# Supplementary material for: Locations and structures of influenza A virus packaging-associated signals and other functional elements via an in silico pipeline for predicting constrained features in RNA viruses
Source: PLoS Comput Biol. 2024 Apr 22;20(4):e1012009. doi: 10.1371/journal.pcbi.1012009 (PMC11034665; doi:10.1371/journal.pcbi.1012009)
Supplement: S1 Appendix — (PDF) [file pcbi.1012009.s001.pdf]

S1 Appendix for

Locations and structures of influenza A virus packaging-associated signals and other functional elements via an *in silico* pipeline for predicting constrained features in RNA viruses

Emma Beniston, Jordan P. Skittrall

E-mail: jps55@cam.ac.uk

1. Additional detail regarding alternative folds of packing-associated regions

In this section, we provide additional detail (figures and text discussion) regarding secondary structure predictions using RNAalifold of regions discussed in the main text. This detail is mostly display and discussion of the different fold predictions from different subtype/host/RNAdescent analysis method combinations. We begin by emphasising the point, made in the Discussion section of the main text, that there are multiple possible reasons for different secondary structure predictions using a minimum free energy algorithm modified by covariation data: some differences may be artefactual, resulting from minor variations in the exact sequences and regions used (i.e. may be regarded as statistical noise). Some differences may be real, but reflecting different local free energy minima between predictions, all of which may be seen in an ensemble of nucleic acids (either at the same time, or at different times during the viral lifecycle). We therefore focus on common features conserved across multiple predictions and with corroborative data from raw RNAdescent output, which are more likely to represent key features of interest.

1.1. PB2 3′ vRNA stem-loops. Fig A presents folds of the PB2 3′ vRNA corresponding to 5′ cRNA regions deemed constrained by RNAdescent for subtype/host/RNAdescent analysis combinations additional to that using H5N8 raw constraint data, i.e. the equivalents of main text Fig 1B.

1.2. PB2 5′ vRNA. Fig B presents folds of the PB2 5′ vRNA corresponding to 3′ cRNA regions deemed constrained by RNAdescent.

Our structural predictions in the vRNA show at least three possible structures in the region just 3′ to the main constrained region mentioned in the main text. There is not a clear delineation between mammalian and avian strains for the predicted formation of these structures, and switching the temperature of the prediction algorithm between mammalian and avian temperatures does not change individual predictions. Inspection of the per-codon output from RNAdescent shows high constraint in all codons where constraint would be required to form the stems of either of these two predicted structures. Of the two structures predicted, only the structure containing (in $-$ sense) an AGUA loop (i.e. with a loop corresponding to PB2 codons 743–744, equivalently NC_007373.1 nucleotides 2254–2259) is fully consistent with the results of the three sets of mutational analyses, i.e. if the structure described is important in packaging, the mutations performed would be consistent with disruption of the structure sufficient to abrogate packaging (see Table A). However, we caution that it is possible for more than one structure to be biologically relevant in a region. This means that although the other predicted structures cannot fully explain the results of the mutational analyses (particularly those of reference (4)), they may still be formed and may still play an important role in viral function.

1.3. PB1 5′ vRNA. Fig C presents folds of the PB1 5′ vRNA corresponding to 3′ cRNA regions deemed constrained by RNAdescent. Folding predictions for the remainder of the highly constrained region (outside the nucleotides of codons 742–747) are more equivocal and include stem-loops and base pairing to form helices with more distant stretches of the PB1 5′ vRNA.

The analysis of Marsh *et al.* (5) notes that disruption of the region we find to be constrained, as well as severely impairing PB1 packaging, moderately impairs packaging of other segments, particularly PB2 and M. These results raise the possibility that our structural predictions for the highly constrained region are only consistent in part of the region because interaction with the vRNA of other segments explains constraint of the second part of the constrained region, and a consistent structure does not occur separately from such an interaction or interactions. It remains possible that, notwithstanding the inconsistent folding predictions, one or more of the structures predicted by RNAalifold covering codons 748–755 is important for packaging. In contrast, the consistent prediction of the stem-loop from codons 742–747, together with the corroboration provided by our individual codon constraint data, leads us to postulate that this consistently predicted structure forms and is important to packaging.

1.4. HA 5′ vRNA. Fig D presents folds of the HA 5′ vRNA corresponding to 3′ cRNA regions deemed constrained by RNAdescent.

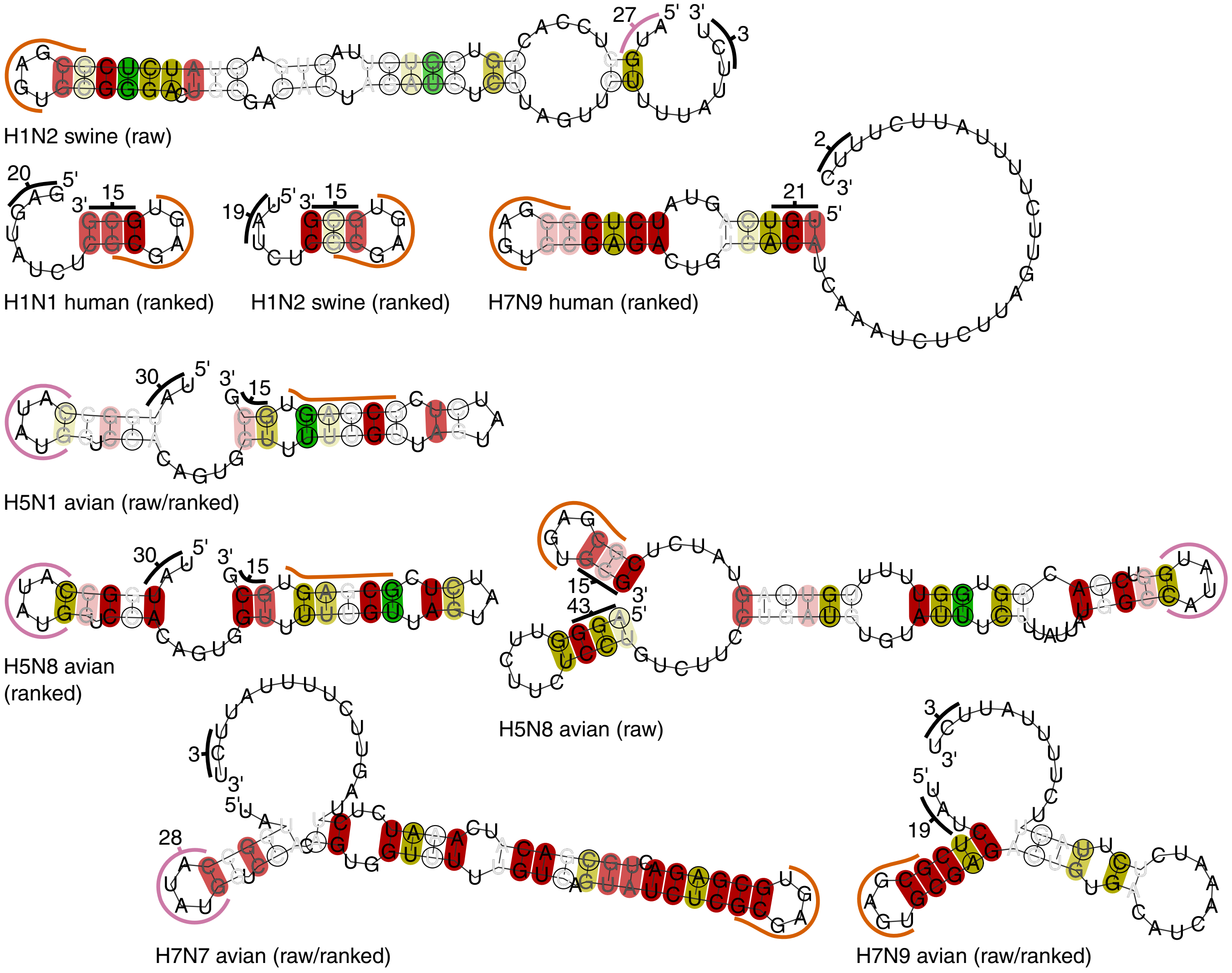

Fig. A. PB2 3′ vRNA regions whose cRNA is deemed constrained by RNAdescent. RNAalifold output from folding the PB2 3′ vRNA corresponding to cRNA regions deemed constrained in RNAdescent analyses. Labels correspond to the influenza A subtype, host, and analysis method (raw constraint data or ranked constraint data) that predicted the constrained region. Folds are of the (− sense) vRNA, produced using RNAalifold (1, 2). The termini of the predicted structures are labelled with the corresponding amino acid number of PB2 over the central nucleotide corresponding to the respective amino acid. Portions of the two predicted stem-loops described in the main text are highlighted: an orange line highlights the nucleotides corresponding to codons 16 and 17 of PB2, and a purple line highlights the nucleotides corresponding to codons 27 and 28 of PB2. In the three cases where RNAalifold does not predict a stem-loop structure consistent with the other predictions for one of these regions, the region deemed constrained by the RNAdescent algorithm does not include the entire region predicted to form the stem-loop. Base pairs are highlighted in deep/mid/light red when all/all but one/all but two sequences are capable of forming the pairs shown. Base pairs are highlighted in deep/mid/light yellow when all/all but one/all but two sequences are capable of forming the pair shown or one other pair (including GU pairs). Base pairs are highlighted in green when all sequences are capable of forming the pair shown or one of two other pairs (including GU pairs). RNAalifold was used with input options disallowing lonely pairs, allowing G-quadruplexes, and with the ribosum scoring matrix enabled. Avian strain folds were produced with the temperature set to 41 ℃.

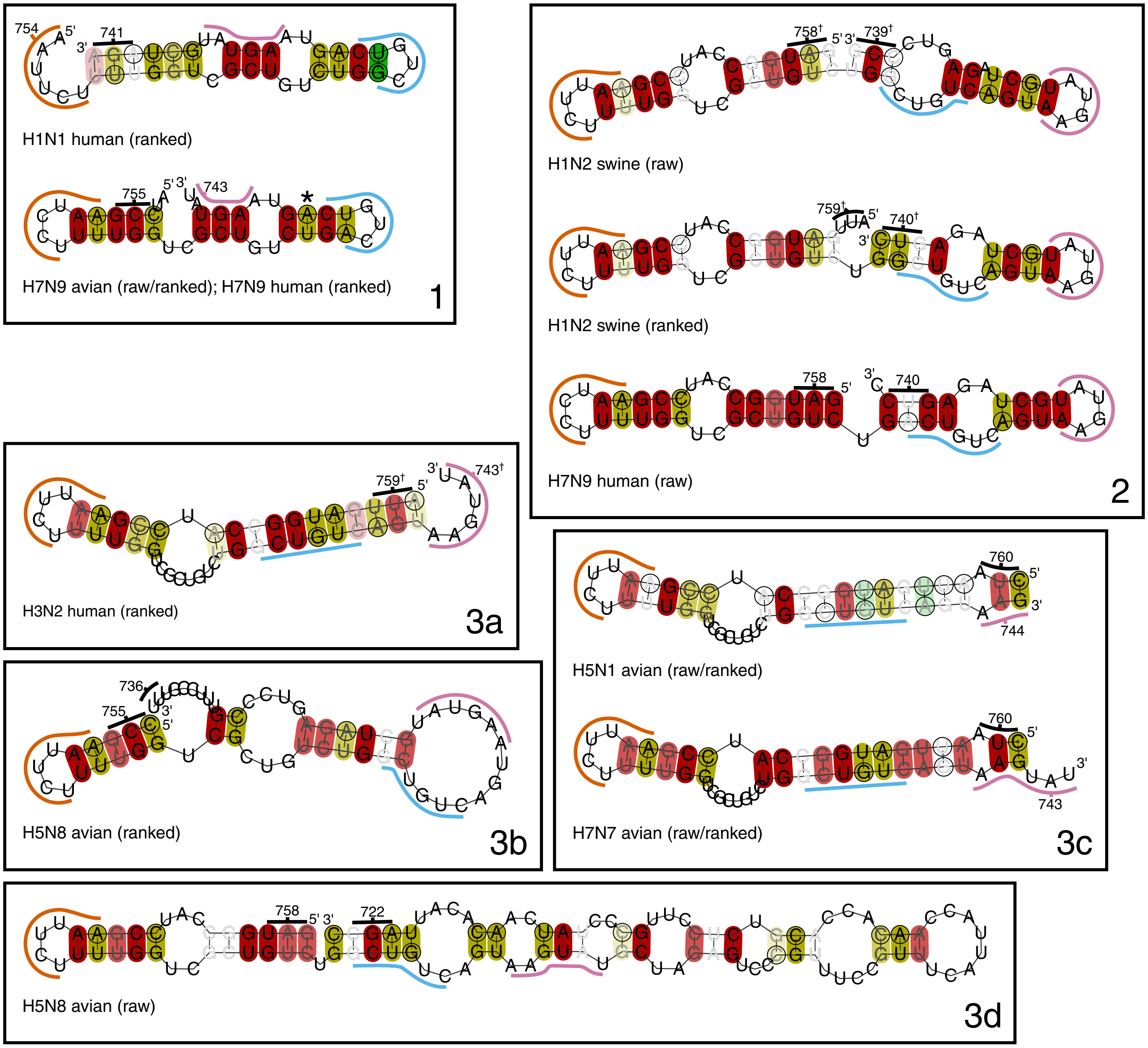

Fig. B. Folds of PB2 5′ vRNA regions whose cRNA is deemed constrained by RNAdescent. Labels correspond to the influenza A subtype, host, and analysis method (raw constraint data or ranked constraint data) that predicted the constrained region. Folds are of the (− sense) vRNA, produced using RNAalifold. The termini of the predicted structures are labelled with the corresponding amino acid number of PB2 over the central nucleotide corresponding to the respective amino acid († indicates cases where the labelled amino acid number differs from the numbering in our analysis output owing to gaps in the alignment used for our analysis). The folds displayed are truncated to the regions containing information on the stem-loops we discuss, using base pair predictions from the folds of the full regions predicted to be constrained; the full region folds may be found in S1 Figures–S8 Figures. Portions of the predicted stem-loops described in the main text are highlighted: an orange line highlights the nucleotides corresponding to codons 753 and 754 of PB2, where there is a highly conserved structure; a purple line highlights the nucleotides corresponding to codons 743 and 744 of PB2, and a light blue line highlights the nucleotides corresponding to codons 746 and 747 of PB2, both of which structures are less well-conserved in predictions. The structural predictions are grouped according to which of the less well-conserved structures is predicted: group 1 contains predictions with a stem-loop around codons 746 and 747 (light blue highlight); group 2 contains predictions with a stem-loop around codons 743 and 744 (purple highlight); group 3 contains structures where neither of these stem-loops is predicted. Group 3 is subdivided into subgroups according to which mutations previously shown to be disruptive to packaging would disrupt the predicted structures (see Table A for more details). The structural predictions in group 3c agree exactly with that of reference (3). See the main text for more discussion of RNAdescent constraint predictions around these predicted structures. Base pairs are highlighted by RNAalifold in deep/mid/light red when all/all but one/all but two sequences are capable of forming the pairs shown. Base pairs are highlighted in deep/mid/light yellow when all/all but one/all but two sequences are capable of forming the pair shown or one other pair (including GU pairs). Base pairs are highlighted in deep/mid/light green when all/all but one/all but two sequences are capable of forming the pair shown or one of two other pairs (including GU pairs). RNAalifold was used with input options disallowing lonely pairs, allowing G-quadruplexes, and with the ribosum scoring matrix enabled. Avian strain folds were produced with the temperature set to 41 °C. * denotes a base-pair that in the H7N9 avian host alignments consists entirely of A-U pairs; the predicted H7N9 avian fold for the region is otherwise identical to the displayed H7N9 human host alignment fold.

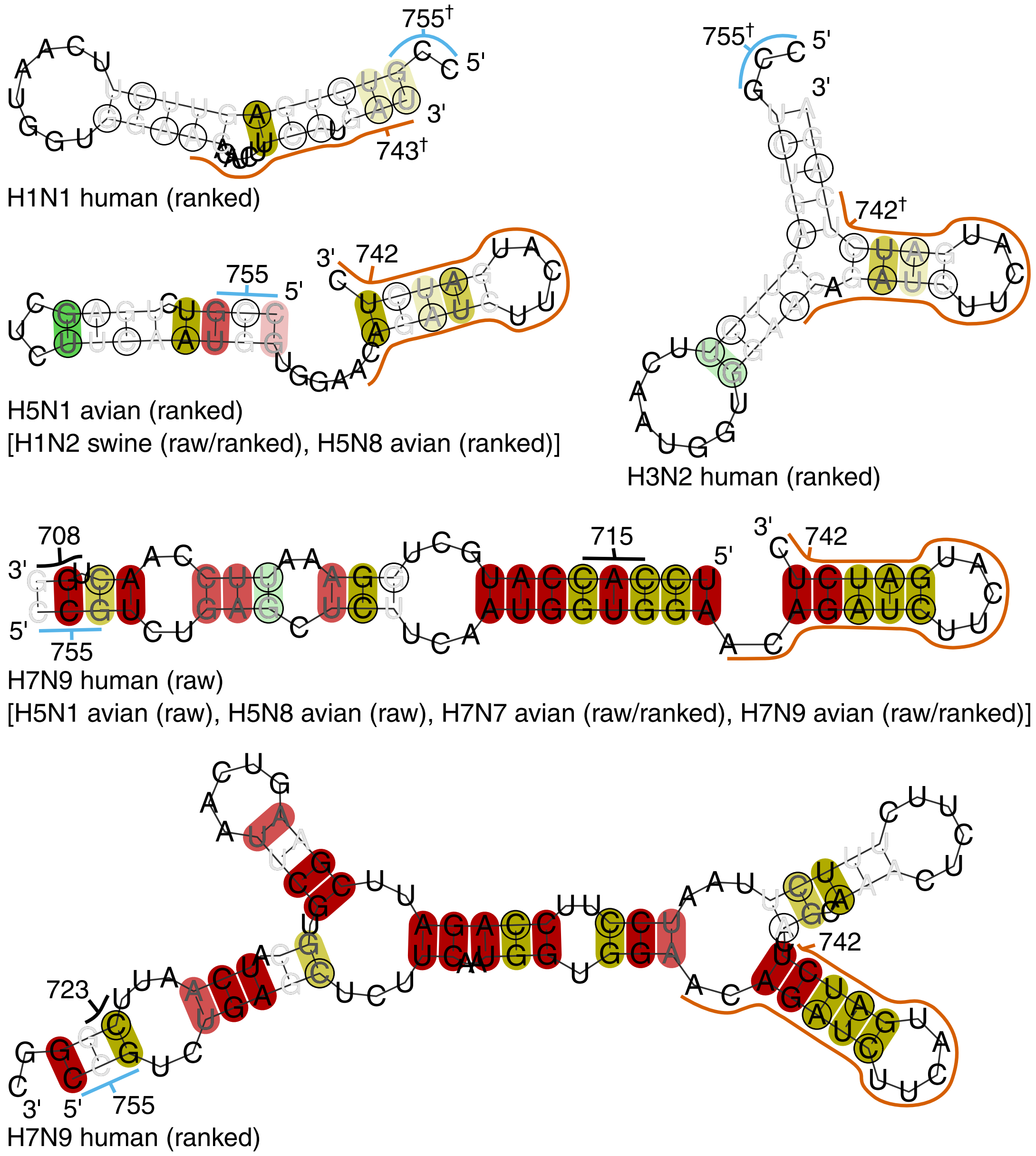

Fig. C. Folds of PB1 5′ vRNA regions whose cRNA is deemed conserved by RNAdescent. Regions corresponding to codons 742–747 of PB1 (NC_007372.1 nucleotides 2248–2265) are marked in orange, and regions corresponding to codon 755 of PB1 (NC_007372.1 nucleotides 2287–2289) are marked in light blue. Codon numbers of non-contiguous regions in folds are labelled († indicates cases where the labelled codon number differs from the numbering in our analysis output owing to gaps in the alignment used for our analysis). In all cases where the fold contains all nucleotides corresponding to codons 742–747 of PB1, a stem-loop is predicted for this region. However, various structures are predicted for the regions corresponding to codons 748–755 of PB1. Labels correspond to the influenza A subtype, host, and analysis method (raw constraint data or ranked constraint data) that predicted the constrained region; subtype/host combinations in square brackets are not displayed, but have similar topology to the displayed fold under which they appear. Folds are of the − sense vRNA, produced using RNAalifold. The folds displayed are truncated to the regions containing the constrained codons we discuss, using base pair predictions from the folds of the full regions predicted to be constrained; the full region folds may be found in S1 Figures–S8 Figures. Base pairs are highlighted by RNAalifold in deep/mid/light red when all/all but one/all but two sequences are capable of forming the pairs shown. Base pairs are highlighted in deep/mid/light yellow when all/all but one/all but two sequences are capable of forming the pair shown or one other pair (including GU pairs). Base pairs are highlighted in deep/mid/light green when all/all but one/all but two sequences are capable of forming the pair shown or one of two other pairs (including GU pairs). RNAalifold was used with input options disallowing lonely pairs, allowing G-quadruplexes, and with the ribosum scoring matrix enabled. Avian strain folds were produced with the temperature set to 41 ℃.

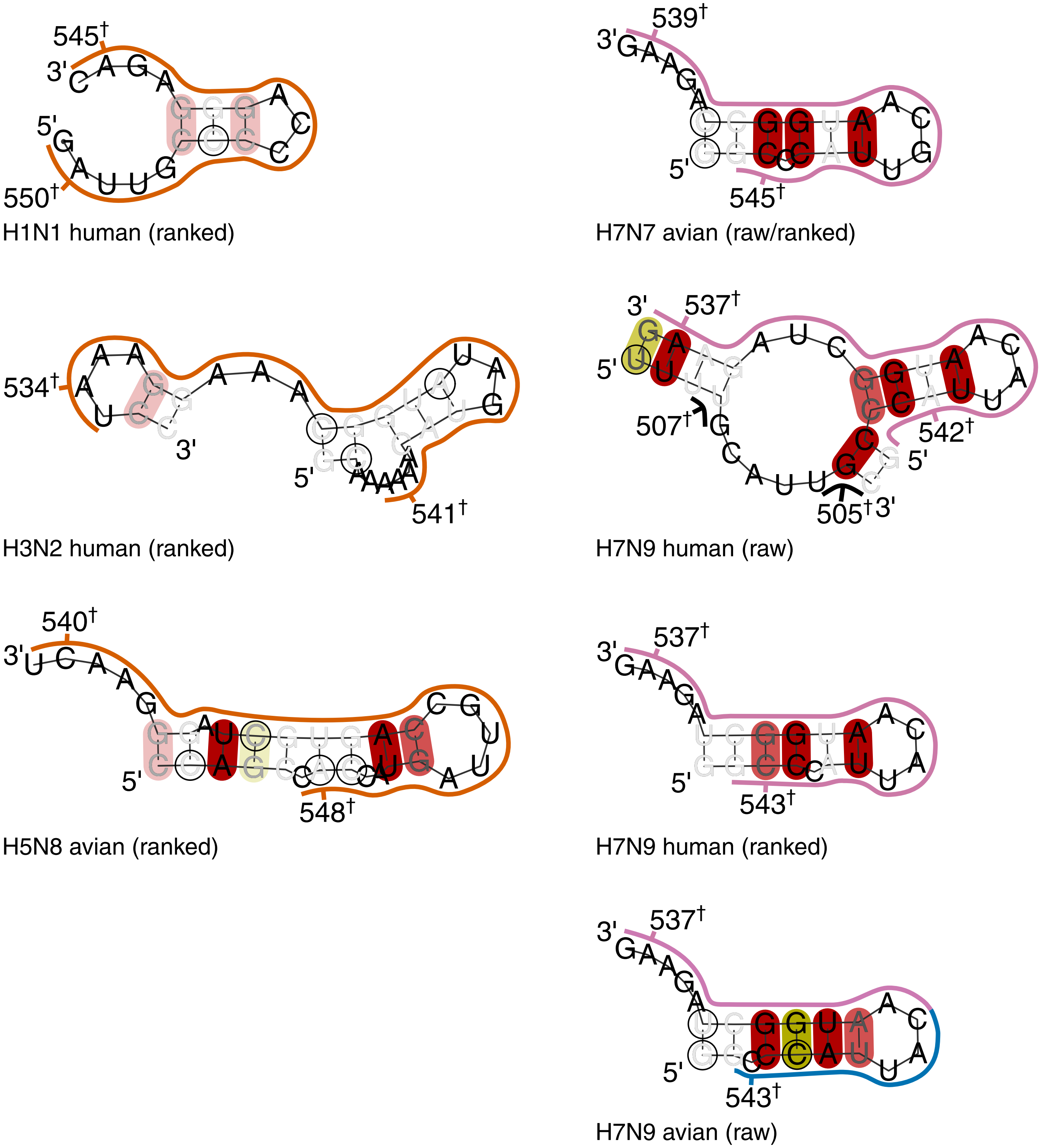

Fig. D. Predicted folds of the comparable constrained sequences in the HA 5′ vRNA packaging-associated region. Folds of regions deemed constrained by RNAdescent and predicted to form small stem-loops are included. Even where secondary structural features are predicted, the agreement on predicted structure between haemagglutinin subtypes is poor. Folds are predicted by applying RNAalifold to the entire regions deemed constrained by the RNAdescent analysis, but then truncating to the intersection of these regions and the comparable constrained regions described in Table 2 of the main text. The constrained subregion that is compared between sequences is highlighted around each fold: blue if deemed constrained just by RNAdescent analysis of raw (unranked) data, orange if deemed constrained just by RNAdescent analysis of ranked data, and purple if deemed constrained by both analyses. Codon numbers are labelled, with † indicating that the labelled codon number differs from the numbering in our analysis output owing to gaps in the alignment used for our analysis. Base pairs are highlighted by RNAalifold in deep/mid/light red when all/all but one/all but two sequences are capable of forming the pairs shown. Base pairs are highlighted in deep/mid/light yellow when all/all but one/all but two sequences are capable of forming the pair shown or one other pair (including GU pairs). RNAalifold was used with input options disallowing lonely pairs, allowing G-quadruplexes, and with the ribosum scoring matrix enabled. Avian strain folds were produced with the temperature set to 41 °C.

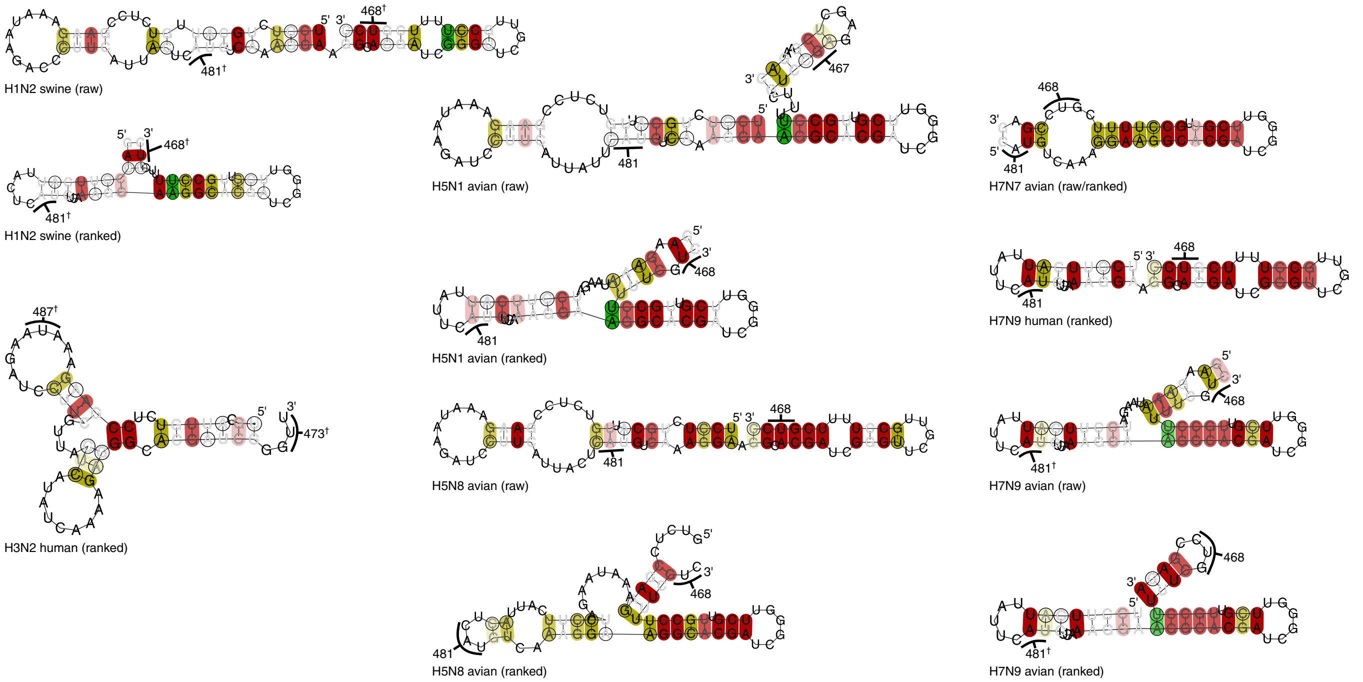

Fig. E. Predicted folds of the comparable constrained sequences in the NP 5′ vRNA packaging-associated region. Truncated folds of regions deemed constrained by RNAdescent, showing just the regions corresponding to codons 468–481 (labels correspond to codon positions in the cRNA, with † indicating that the number differs from the numbering in RNAdescent output owing to gaps in the alignment used for our analysis), plus any additional nucleotides required to show the secondary structure of these regions, are included. A series of highly similar stem-loops is predicted, in all cases except the H3N2 sequences, where the constrained region identified by RNAdescent does not extend to cover codons 468–472. Labels correspond to the influenza A subtype, host, and analysis method (raw constraint data or ranked constraint data) that predicted the constrained region. Base pairs are highlighted by RNAalifold in deep/mid/light red when all/all but one/all but two sequences are capable of forming the pairs shown. Base pairs are highlighted in deep/mid/light yellow when all/all but one/all but two sequences are capable of forming the pair shown or one other pair (including GU pairs). Base pairs are highlighted in deep/mid green when all/all but one sequences are capable of forming the pair shown or one of two other pairs (including GU pairs). RNAalifold was used with input options disallowing lonely pairs, allowing G-quadruplexes, and with the ribosum scoring matrix enabled. Avian strain folds were produced with the temperature set to 41 °C.

Reference	Mutation	Structure number 1	2	3a	3b	3c	3d
Gog *et al.* (4)	744a	✗†	✓	✗	✗	✗	✗
	744b	✓	✓	✗	✗	✓	✗
	745	✓	✓	✓	✗	✓	✗
	748	✗	✗†	✗	✗	✗	✗
Marsh *et al.* (5)	WSN PB2-52		✓				
Liang *et al.* (6)	d1	✓	✓	✓	✗	✓	✓
	m7	✓	✓	✓	✓	✓	✓
	m8	✓	✓†	✓	✓	✓	✓

Table A. Comparison of mutational analyses of the 5′ vRNA (3′ cRNA) packaging-associated region of PB2, and whether mutations that abrogate packaging would be expected to disrupt our predicted structures. Structure 1 has a stem-loop in − sense around PB2 codons 746–747 (CUG in loop). Structure 2 has a stem-loop in − sense around PB2 codons 743–744 (AGUA in loop). Structure subgroups 3 contain neither of these stem-loops. See Fig B for graphical representations of the structures. Mutation nomenclature is taken from the respective references. ✓ indicates that the mutation would be expected to disrupt the structure (i.e. disruption of the predicted structure explains abrogation of packaging). ✗ indicates that the mutation would not be expected to disrupt the structure. † for structures 1 and 2 indicates that the mutation does not affect the four base pairs of the stem closest to the respective predicted loop. A result for the mutation of reference (5) is only included for structure 2 as the mutation is outside the region that would be expected to affect the predicted stem-loops for the other structures.

1.5. NP 5′ vRNA. Fig E presents folds of the NP 5′ vRNA corresponding to 3′ cRNA regions deemed constrained by RNAdescent. These predictions consistently show a stem-loop in the vRNA, with the exception of the fold prediction for the H3N2 sequences: the region folded for H3N2 does not contain the full region predicted in the other subtypes to contain a stem-loop.

1.6. M 3′ vRNA. Fig F presents folds of vRNA corresponding to the three smaller M 3′ cRNA regions deemed constrained by RNAdescent.

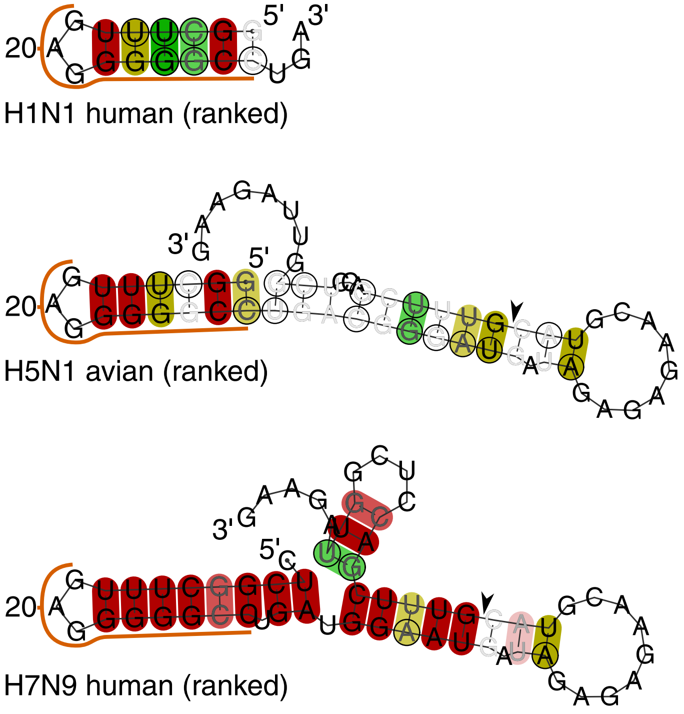

Fig. F. Predicted stem-loop in the M 3′ vRNA packaging-associated region. Three smaller analogous constrained regions identified by RNAdescent in the 5′ cRNA of M1 (3′ vRNA of M) across three different subtypes all have vRNA predicted by RNAalifold to have a highly conserved stem-loop. Synonymous mutations in this region have previously been demonstrated to disrupt packaging (7). Nucleotides corresponding to M1 codons 18–20 are highlighted with orange bars and the central nucleotide corresponding to codon 20 labelled. Positions corresponding to the M2 splice site in cRNA are marked with arrowheads. Labels correspond to the influenza A subtype, host, and analysis method (raw conservation data or ranked conservation data) that predicted the conserved region. Base pairs are highlighted by RNAalifold in deep/mid/light red when all/all but one/all but two sequences are capable of forming the pairs shown. Base pairs are highlighted in deep/mid yellow when all/all but one sequences are capable of forming the pair shown or one other pair (including GU pairs). Base pairs are highlighted in deep/mid green when all/all but one sequences are capable of forming the pair shown or one of two other pairs (including GU pairs). RNAalifold was used with input options disallowing lonely pairs, allowing G-quadruplexes, and with the ribosum scoring matrix enabled. Avian strain folds were produced with the temperature set to 41 ℃.

1.7. The PB1-N92 initiation region. Figs G and H contain predicted folds for the investigated subtype/host combinations, using, respectively, the shorter (found in H5N1 and H5N8) and the longer (found in H7N7) constrained region lengths identified in the avian strains. (The constrained region identified in the H7N9 avian strains has a very short length, and the fold prediction for the strain itself does not predict any base pairing, so we have not produced comparative fold predictions for this length.) Although there are subtle differences in the predicted folds, there is not a clear pattern that dichotomises between the avian and the mammalian strains. In particular, whether the AUG codon that would correspond to PB1-N92 initiation is base paired varies between fold predictions, but not in a manner consistent between the avian and mammalian strains. Although the compatibility of base pairing predictions with the sequences in the alignments appears better for the avian strains, this is likely to be an artefact of the smaller number of sequences (less opportunity for discordance) available from those strains in comparison with mammalian strains (particularly H1N1 and H3N2).

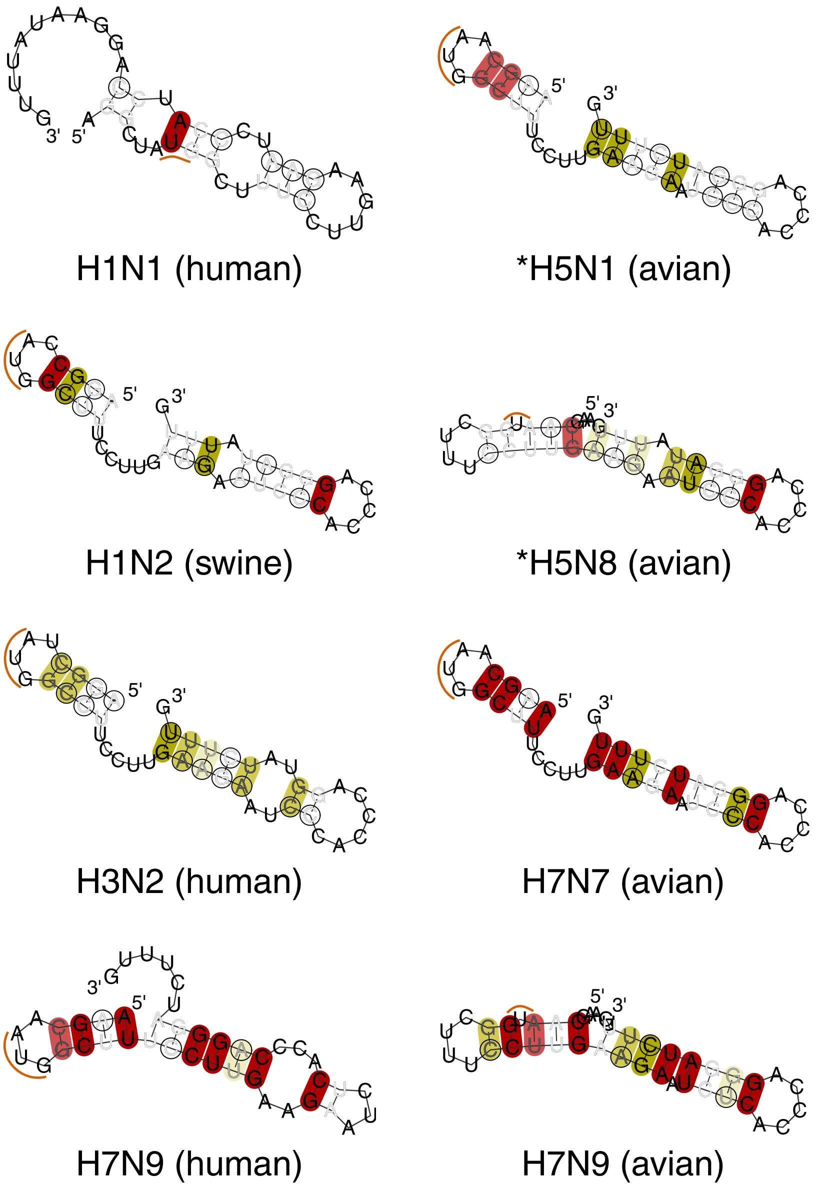

Fig. G. Folds of "short" constrained region cRNA near putative PB1-N92 initiation in investigated mammalian and avian strains. Analogous regions from each of the investigated strains have been folded using RNAalifold. Strains in which this is the region deemed constrained by our algorithm are marked with *. The AUG that would correspond to initiation of the truncated PB1-N92 protein is marked in orange.

2. Additional notes on constraint in packaging-associated regions

In the text that follows, we discuss constrained regions predicted by RNAdescent analyses where the explanations for constraint are less apparent than those for the regions discussed in the main text.

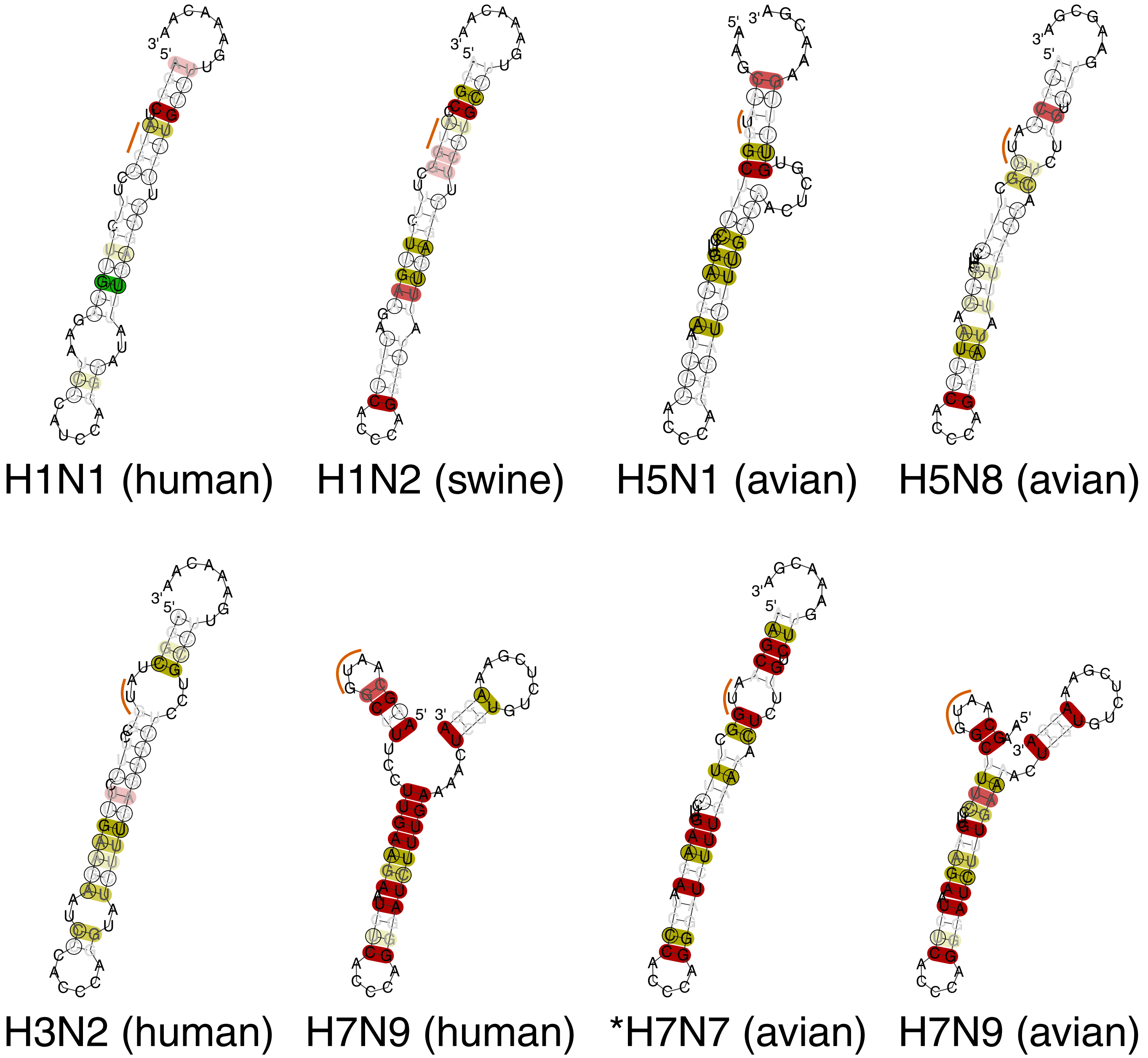

Fig. H. Folds of "long" constrained region cRNA near putative PB1-N92 initiation in investigated mammalian and avian strains. Analogous regions from each of the investigated strains have been folded using RNAalifold. Strains in which this is the region deemed constrained by our algorithm are marked with *. The AUG that would correspond to initiation of the truncated PB1-N92 protein is marked in orange.

2.1. Potential for interaction between constrained PB2 5′ vRNA and constrained HA 3′ cRNA/5′ vRNA. The mutational analysis of Miyamoto *et al.* (8), but not that of Marsh *et al.* (9), predicts an effect on PB2 packaging from mutating the 3′ region of HA in which we find constraint. In Marsh *et al.* the packaging efficiency of PB2 is not impacted at all, whereas in Miyamoto *et al.* a moderate reduction in packaging efficiency is seen, associated with a selection for the mutation C2244A (using the numbering of reference sequence NC_026438.1).

We observe that the highly conserved stem-loop we have already noted in PB2 vRNA (Fig B, orange highlights) has, in most subtypes, a UUCU motif unpaired in the predicted loop. Allowing GU pairs in the possible sequences that will pair with this and considering the reverse complement, any of the sequences UUCU, UCCU, UCCC, UUCC, CCCU in (+ sense) HA cRNA would generate a base-pairing motif. The H7N9 PB2 sequences contain a different, UCCU loop motif, and the corresponding HA sequences do not contain a motif in our region of interest that will generate four base pairs (although they do contain motifs that will generate three pairs).

Both previous mutational analyses use H1 viruses. Although the mutations introduced in HA by Marsh *et al.* (9) would be expected to disrupt the pairing we discuss above by abolishing the UCCCU motif present in the wild-type virus, the mutations also introduce a new UUCU motif immediately 5′ to the motif that has been abolished, and this may rescue our proposed base-pairing. The mutations introduced in HA by Miyamoto *et al.* (8) alter the UCCCU motif to UCACU, which would disrupt base pairing. A compensatory mutation in our proposed PB2 binding site would require an AGA to AGU (arginine to serine) codon change, i.e. changing from an amino acid with a charged R-group to one with a neutral R-group. Therefore, it would be logical to assume that if this proposed interaction occurred, an alternate "rescue" mutation in PB2 may be more evolutionarily advantageous. We note that if we extend the motif in HA cRNA slightly 5′, we obtain in the wild-type virus GUCUCCCU, and the GUCU motif is mutated to GUAU in the experiment. Now, the C2244A mutation observed in PB2 precisely changes a complementary GUCU motif in the PB2 vRNA to GUAU, which is complementary to the mutated HA vRNA.

We are next motivated to consider whether this nearby, but different, interaction between PB2 and HA can explain all the observations (*in silico* and experimental). The mutations of Marsh *et al.* (9) change the GUCU motif to GUAA, which would be expected to disrupt the alternative base pairing possibility, and this is not observed. The motifs are too close to each other in HA to be able to make inferences based on our RNAdescent conservation scores. Whilst sufficiently separate to compare scores, the conservation data for PB2 would be consistent with either of the PB2 motifs needing to be conserved. The structural predictions for PB2 vRNA (Fig B) are more consistent with the UUCU than the GUCU motif being unpaired and available to interact with another segment, but some structural predictions have the second motif mostly available. Neither interaction appears wholly consistent with the H7N9 sequences.

On balance, our analysis provides evidence supportive of either or both of these PB2/HA interactions, with one explanation being that both interactions can occur, one being more common but with packaging rescued by the other when the more common interaction is absent.

2.2. Weak constraint in M 3′ cRNA (5′ vRNA) in a region previously associated with packaging. Overall, few constrained regions are predicted by RNAdescent analysis of M2 codons. This may represent the algorithm finding it harder to distinguish constrained from less-constrained regions in shorter genes. In three subtype/host combinations, constrained regions are predicted with low confidence towards the 3′ end of the M2 coding region in at least one RNAdescent analysis. Notwithstanding the low confidence predictions (with associated increased risk of false positive/artefact and with less precise delineation of the boundaries of constrained regions; see reference (10) for further technical discussion), the predicted regions contain loci associated with packaging (7). Structural prediction for the region using RNAalifold predicts a stem-loop structure in both + and − sense, with the same structures predicted in each of the subtype/host combinations where constraint is predicted (Fig I). There is slightly greater base pairing near the tip of the stem in + sense.

Although a conserved structure in the vRNA (− sense) would yield a more straightforward explanation for the region's role in packaging, whether acting in *cis* or in *trans* (reference (7) notes reduced self-packaging and reduced packaging of the NP segment following synonymous mutation of the region), the possibility of the cRNA acting in *trans* is not excluded – although this would require co-localisation of the cRNA with the vRNA at a stage crucial for packaging. We further note that the synonymous mutations in codons 90–92 undertaken by Hutchinson *et al.* (7) would disrupt only a single base pair in our structural prediction, and the observed disruption to packaging seems disproportionately high for a relatively small structural destabilisation. We therefore caution that our predicted structure either may not form at all, or may not be the only structure critical for function that forms in this region.

2.3. Constraint in NS 3′ cRNA (5′ vRNA) provides evidence to circumscribe earlier results on the region crucial to packaging, and highlights one separate locus with unexplained constraint. Many RNAdescent analyses of NS2 find constraint towards the end of the coding region. Closer inspection of output finds that the five codons prior to the termination codon form a contiguous sub-region of high constraint, and there is isolated high constraint seen in codon 107.

Although a number of similar folds of the analogous constrained regions are predicted by RNAalifold (S1 Figures–S5 Figures; S7 Figures–S8 Figures), the predicted pairings do not correlate well with the individual constraint data. Bearing in mind especially the presence of a contiguous more-constrained region adjacent to the termination codon, this raises the possibility that any conserved structure in the region additionally involves the 3′ UTR.

A marked change in segment incorporation efficiency has previously been described when the final 30 nucleotides of NS2 are included in a recombinant segment compared with the UTR alone (11). Our individual codon data suggest that within these 30, the final 18 nucleotides may be most critical.

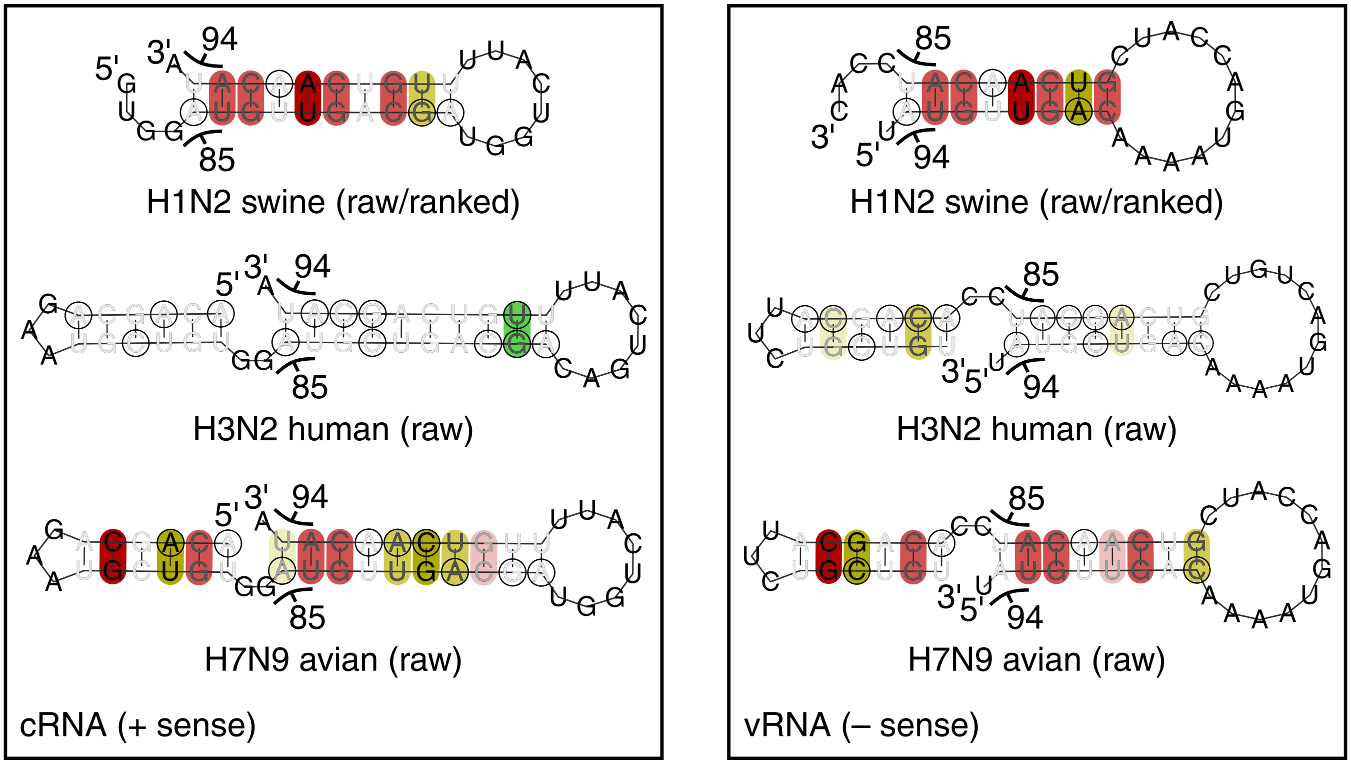

Fig. I. Stem-loops in the 3′ region of M cRNA (/5′ vRNA) associated with packaging. Folds predicted by RNAalifold of all regions in which constraint is found by RNAdescent are shown, with the display truncated in the cases of H3N2 (human) and H7N9 (avian) sequences to the two consistently predicted stem-loops. Folds of both (+ sense) cRNA and (− sense) vRNA are shown. The nucleotides corresponding to M2 codons 85 and 94 are labelled. One stem-loop is consistently predicted in all cases; in the folds of longer regions, a second stem-loop is also consistently predicted. Base pairs are highlighted by RNAalifold in deep/mid/light red when all/all but one/all but two sequences are capable of forming the pairs shown. Base pairs are highlighted in deep/mid/light yellow when all/all but one/all but two sequences are capable of forming the pair shown or one other pair (including GU pairs). Base pairs are highlighted in mid green when all but one sequences are capable of forming the pair shown or one of two other pairs (including GU pairs). RNAalifold was used with input options disallowing lonely pairs, allowing G-quadruplexes, and with the ribosum scoring matrix enabled. Avian strain folds were produced with the temperature set to 41 ℃.

3. Additional notes on constraint associated with alternative protein expression

3.1. The PB1-F2/PB1-N40 initiation regions. The second constrained region described in the RNAdescent analysis of H7N7 (avian host) PB1 ranked constraint data, spanning codons 25–33 (KM922676.1 nucleotides 73–99), encompasses a region near the initiation codon of the PB1-F2 alternative open reading frame. The RNAalifold prediction of the structure of this region (Fig J) contains a stem-loop identical to that predicted in a previous study in association with a pseudoknot (12). Our described constrained region contains the previously described structure 5′ to the initiation codon, and the codons associated with the predicted pseudoknot near the initiation codon, but not the codons previously described as forming the complementary portion of the pseudoknot. The consensus for nucleotide 105 in the H7N7 (avian host) sequences we analyse is A, which will not form the base pair predicted in Priore *et al.* (12), and the constraint of codons in the region corresponding to this earlier predicted helix is less clear. The codon-level output from the RNAdescent analysis shows constraint highly consistent with the predicted base-pairing in the tip of stem-loop. We note further that the codon-level output from the RNAdescent analysis shows relative constraint in two of the three individual codons predicted to form the complement in the pseudoknot region, whilst the third codon encodes a methionine (and is the initiation codon for the PB1-N40 open reading frame), meaning that our analysis does not get any additional nucleotide-level constraint information beyond the amino acid constraint at that locus (it has weight zero).

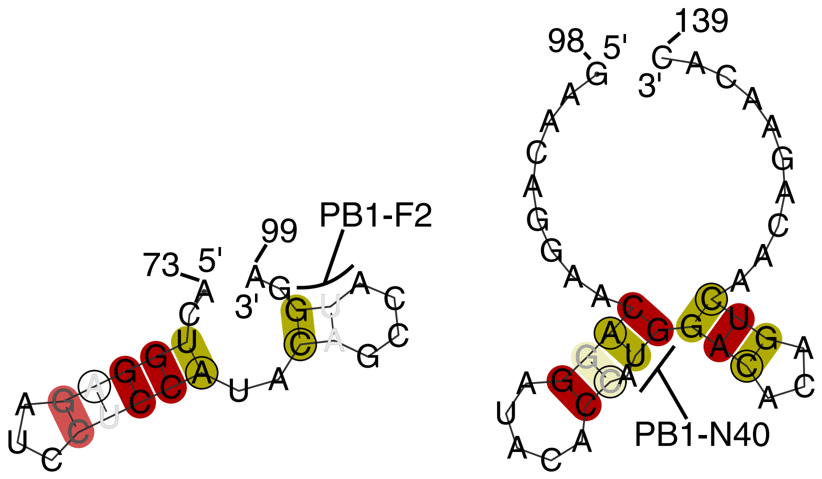

Fig. J. RNAalifold cRNA structure prediction of the constrained regions in H7N7 viruses containing the PB1-F2 and PB1-N40 initiation sites. Left: RNAalifold predicted fold of constrained region found in the RNAdescent analysis of PB1 that contains the PB1-F2 initiation site (marked). Right: fold of constrained region found in the RNAdescent analysis of PB1-F2 that contains the PB1-N40 initiation site (marked). Nucleotide numbering follows GenBank KM922676.1. The larger stem-loop structure predicted in the left hand fold matches that predicted in Figure 1 of Priore *et al.* (12). The corroborative evidence for the formation of the other stem-loops predicted in this figure is weaker. Individual codon constraint is consistent with the pseudoknot formation previously predicted by Priore *et al.*. Base pairs are highlighted by RNAalifold in deep/mid red when all/all but one sequences are capable of forming the pairs shown. Base pairs are highlighted in deep/mid/light yellow when all/all but one/all but two sequences are capable of forming the pair shown or one other pair (including GU pairs). RNAalifold was used with input options disallowing lonely pairs, allowing G-quadruplexes, and with the ribosum scoring matrix enabled. Folds were produced with the temperature set to 41 ℃.

Our RNAdescent analysis of H7N7 (avian host) PB1-F2 non-ranked (raw) constraint data finds a conserved region (PB1-F2 amino acids 2–15, KM922676.1 nucleotides 98–139) that overlaps with the PB1 region containing the PB1-F2 initiation region, and which itself contains the PB1-N40 region (Fig J).

Bearing in mind the predicted pseudoknot, we attempted to fold the contiguous region containing both the noted constrained regions, using RNAPKplex. No pseduoknot was predicted.

Overall, both the earlier analysis by Priore *et al.* (12) and our analysis are supportive of a stem-loop structure for codons 25–29 (KM922676.1 nucleotides 73–87) of PB1 cRNA. Priore *et al.* additionally predict a helix further from the tip of this stem: our analysis has less evidence to support this prediction. With regard to the PB1-N40 initiation region, the observed constraint may simply represent the conserved Kozak sequence, or may represent the constraints on the nucleotides imposed by a pseudoknot. Our analysis is therefore consistent with formation of the previously predicted pseudoknot, but not conclusively supportive of it. We reiterate that formation of a structure at one point in a viral lifecycle does not preclude the formation of other structures.

4. Additional notes on individual structured regions lacking functional explanations

We discuss below regions in which RNAdescent predicts constraint, but for which we do not have a functional explanation, additional to the one discussed in the main text.

4.1. A weakly constrained region in the centre of PB2. RNAdescent constraint analyses of the H5N1 and H7N7 avian host raw (unranked) data detect weak constraint centrally within PB2, approximately located from nucleotide 900 to nucleotide 1100.

Plots of the constraint data suggest the finding is unlikely to be an artefact, and constraint may be present in other subtype/host combinations in this region, although if so is much harder to detect above background noise (S4 Code and S6 Code). Individual codon constraint data are insufficiently clear-cut to corroborate RNAalifold secondary structure predictions (Fig K).

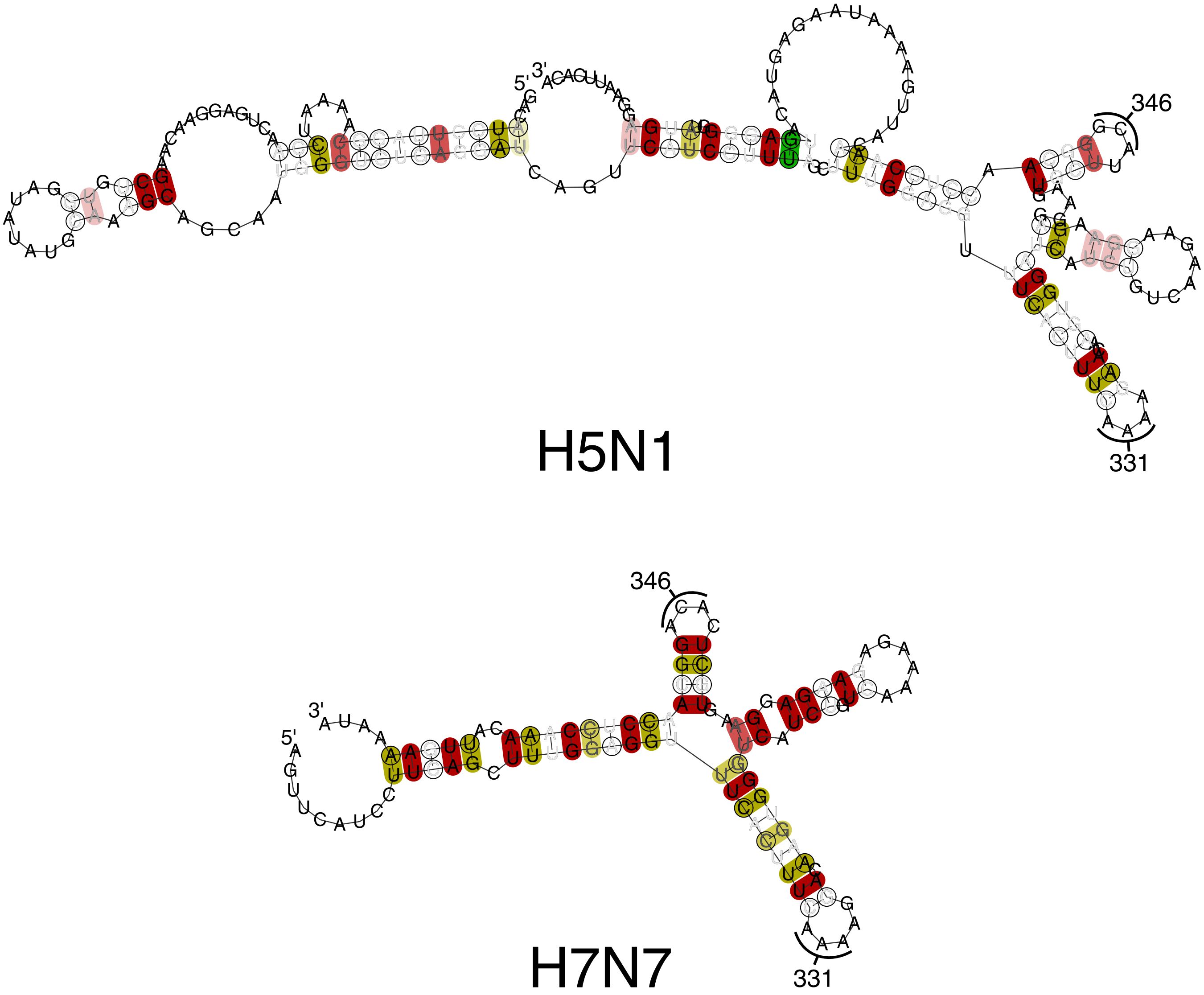

Fig. K. Predicted structures of cRNA in the regions predicted to be constrained in the centre of PB2. Similar structures are seen in the centre of both predicted constrained regions. In both predicted folds, codons 331 and 346 are labelled. Base pairs are highlighted by RNAalifold in deep/mid/light red when all/all but one/all but two sequences are capable of forming the pairs shown. Base pairs are highlighted in deep/mid/light yellow when all/all but one/all but two sequences are capable of forming the pair shown or one other pair (including GU pairs). Base pairs are highlighted in green when all sequences are capable of forming the pair shown or one of two other pairs (including GU pairs). RNAalifold was used with input options disallowing lonely pairs, allowing G-quadruplexes, and with the ribosum scoring matrix enabled. Avian strain folds were produced with the temperature set to 41 ℃.

The regions deemed constrained have termination codons in the -1 frame with a possible preceding open reading frame, but to access this frame would require a -1 frameshift, which has not been described in influenza A. Inspection of the preceding nucleotides does not yield any apparent previously described -1 frameshift motifs (13), although we note that surrounding structure, including pseudoknots (which can be difficult to predict and are not predicted by RNAalifold), are often determinants of such frameshifting.

4.2. Structured cRNA region 3′ to M4 splice junction. Many of our RNAdescent analyses identify constraint in M1 in a region 3′ to the M4 splice junction. The structure of this region has previously been investigated by Jiang *et al.* (14), aiming to distinguish between a single hairpin structure and a previously predicted branched structure (15). The length of the constrained regions our analyses find, and hence the length of the regions for which we attempt structural prediction in our pipeline, are varied, most probably because of the proximity of other constrained features, which are included within single predicted constrained regions in some analyses. The modified minimum free energy structures predicted by RNAalifold for the regions vary (Fig L), and we see represented in the predictions each of the stem-loops previously described by Jiang *et al*, although

we do not see all of the previously predicted branched structure in a single fold prediction. Codon-level constraint data are consistent with either of the previously mentioned structures, and our analysis would be consistent with either, or both, of these previously predicted structures forming.

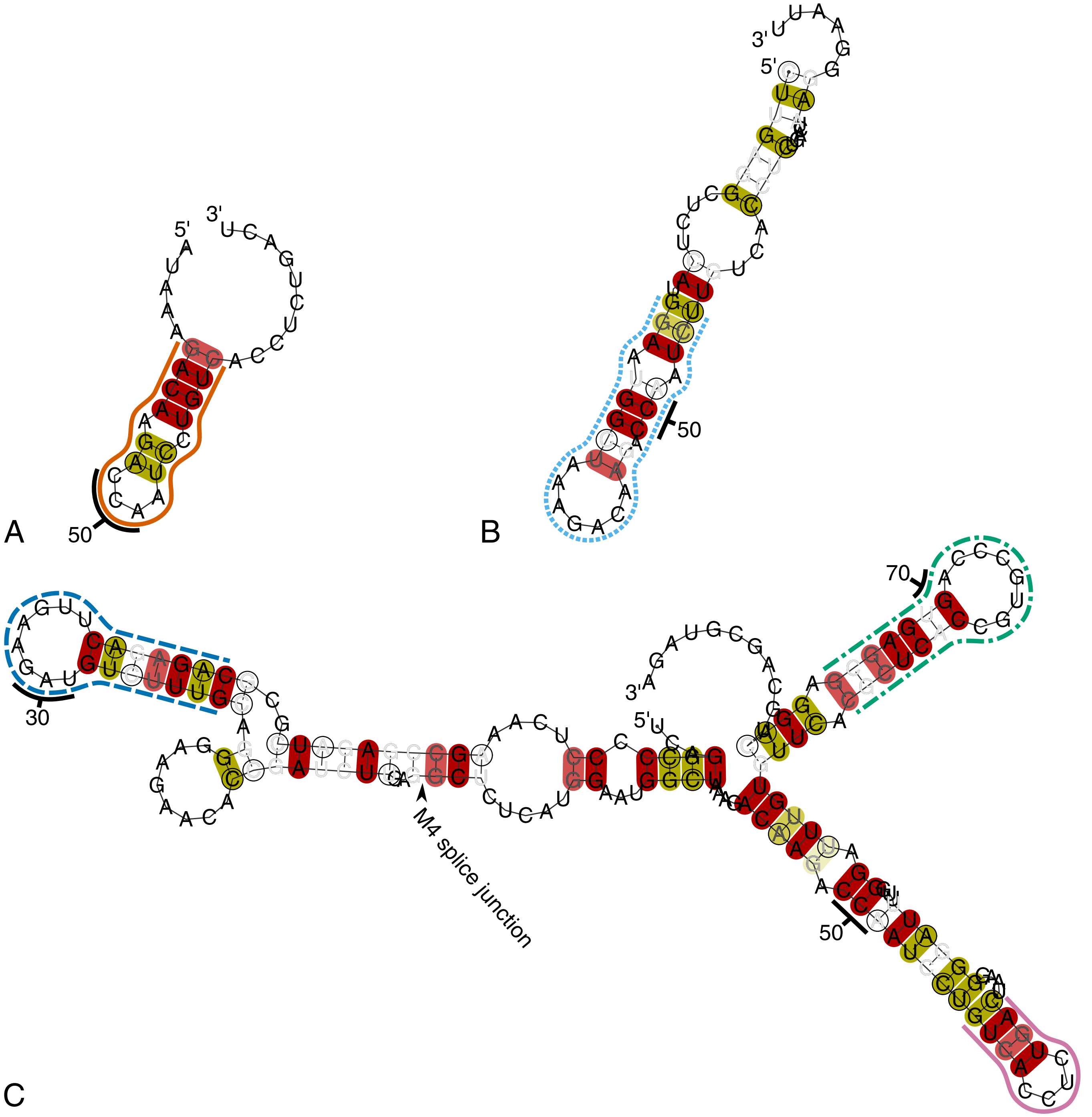

Fig. L. Examples of possible structures 3′ to the M4 splice junction in M cRNA. Folds are annotated with M1 codon references. **A** Fold of the relevant constrained region determined by RNAdescent in analysis of H7N9 (human host) ranked constraint data. The region with the orange solid annotation corresponds to the stem-loop tip in the unbranched model of reference (14) (P3′ by that reference's nomenclature). **B** Fold of the relevant constrained region determined by RNAdescent in analysis of H1N2 (swine host) raw (unranked) constraint data. The region with the light blue dotted annotation corresponds to one of the branched model stem-loops of reference (14) (P2 by that reference's nomenclature). **C** Fold of the relevant constrained region determined by RNAdescent in analysis of H5N8 (avian host) ranked constraint data. The region with the purple solid annotation corresponds to the other of the branched model stem-loops of reference (14) (P3 by that reference's nomenclature). The green dot-dashed annotation corresponds to a region we show to be highly constrained in vRNA (see Fig 5 of main text). The blue dashed annotation corresponds to a stem-loop predicted in a number of the folds of this region. The M4 splice junction is noted with an arrowhead. Base pairs are highlighted by RNAalifold in deep/mid red when all/all but one sequences are capable of forming the pairs shown. Base pairs are highlighted in deep/mid/light yellow when all/all but one/all but two sequences are capable of forming the pair shown or one other pair (including GU pairs). RNAalifold was used with input options disallowing lonely pairs, allowing G-quadruplexes, and with the ribosum scoring matrix enabled. Avian strain folds were produced with the temperature set to 41 °C. Folds of conserved regions from other strain/host/analysis type analyses may be found in S1 Figures–S8 Figures.

Many of the predicted secondary structures also contain a stem-loop whose tip is around 80 nucleotides into the M coding region (Fig L).

4.3. A weakly constrained region in M1 codons 122–132. Three of our RNAdescent constraint analyses find weak constraint in codons 122–131/2 of M1 (one of the analyses extends this constraint to codons 116–133). RNAalifold analyses of the regions predict stem-loop structures, and individual codons that are less-constrained in the RNAdescent analysis are appropriately unpaired in the structural predictions or have a codon usage that would permit alternative canonical/non-canonical base pairing, particularly in − sense (Fig M).

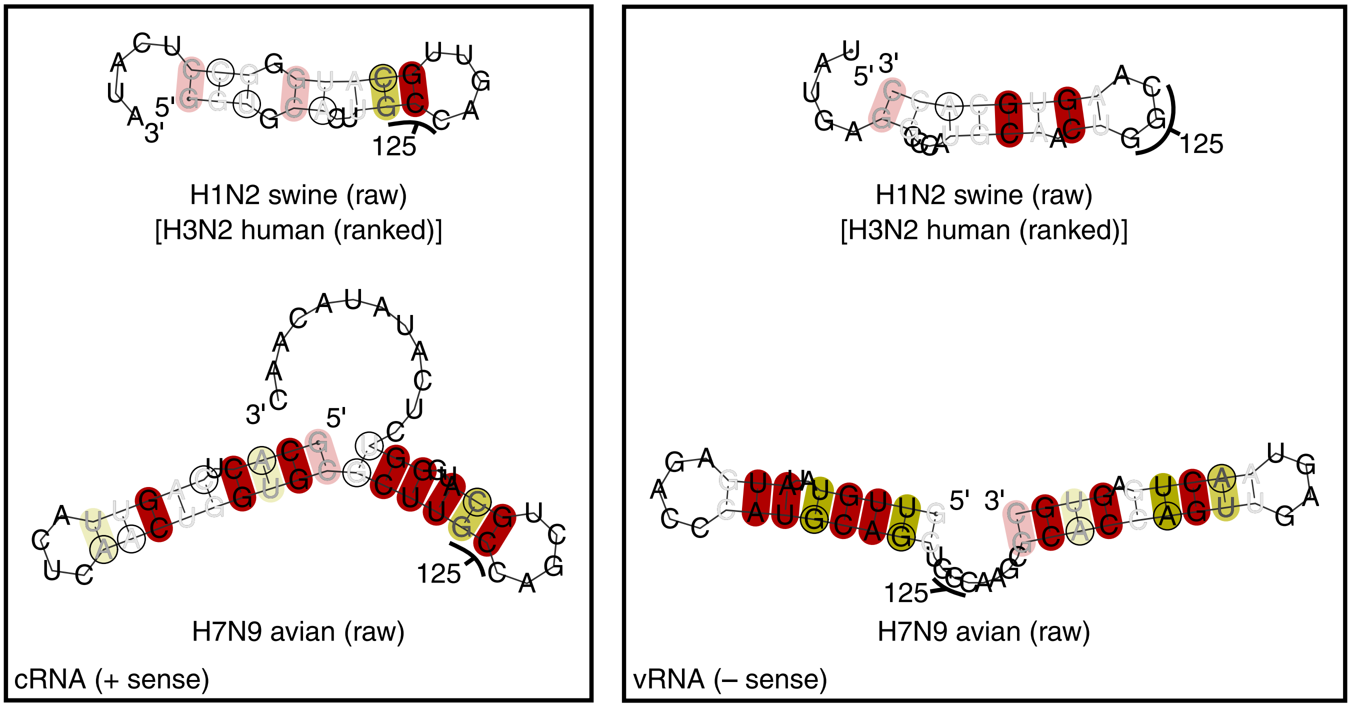

Fig. M. Predicted structures of cRNA and vRNA in the regions of M containing M1 codons 122–131. Predicted folds are labelled according to the subtype/host/data analysed by RNAdescent. The H3N2 predictions are not shown but are similar to the H1N2 predictions. In each case the nucleotides corresponding to M1 codon 125 are labelled. Base pairs are highlighted by RNAalifold in deep/light red when all/all but two sequences are capable of forming the pairs shown. Base pairs are highlighted in deep/mid/light yellow when all/all but one/all but two sequences are capable of forming the pair shown or one other pair (including GU pairs). RNAalifold was used with input options disallowing lonely pairs, allowing G-quadruplexes, and with the ribosum scoring matrix enabled. Avian strain folds were produced with the temperature set to 41 ℃.

We are not aware of any previous specific analysis of this region. There is an initiation codon in-frame with strong Kozak context, but for this to form an alternative initiation site would require the ribosome to initiate almost 300 bases into the segment. Regions this deep into the segment have not been evaluated for packaging potential. We note that although we have found constraint in three of our analyses, covering three subtypes and hosts, nonetheless there was not a sufficiently strong signal for identification in the remainder of the analyses.

References

1. Bernhart SH, Hofacker IL, Will S, Gruber AR, Stadler PF (2008) RNAalifold: improved consensus structure prediction for RNA alignments. *BMC Bioinformatics* 9(1):474.
2. Lorenz R, et al. (2011) ViennaRNA package 2.0. *Algorithms for Molecular Biology* 6(1):26.
3. Mirska B, et al. (2023) In vivo secondary structural analysis of influenza A virus genomic RNA. *Cellular and Molecular Life Sciences* 80:136.
4. Gog JR, et al. (2007) Codon conservation in the influenza A virus genome defines RNA packaging signals. *Nucleic Acids Research* 35(6):1897.
5. Marsh GA, Rabadán R, Levine AJ, Palese P (2008) Highly conserved regions of influenza A virus polymerase gene segments are critical for efficient viral RNA packaging. *Journal of Virology* 82(5):2295–2304.
6. Liang Y, Huang T, Ly H, Parslow TG, Liang Y (2008) Mutational analyses of packaging signals in influenza virus PA, PB1, and PB2 genomic RNA segments. *Journal of Virology* 82(1):229–236.
7. Hutchinson EC, Curran MD, Read EK, Gog JR, Digard P (2008) Mutational analysis of *cis*-acting RNA signals in segment 7 of influenza A virus. *Journal of Virology* 82(23):11869–11879.
8. Miyamoto S, et al. (2022) Contribution of RNA-RNA interactions mediated by the genome packaging signals for the selective genome packaging of influenza A virus. *Journal of Virology* 96(6):e01641–21.
9. Marsh GA, Hatami R, Palese P (2007) Specific residues of the influenza A hemagglutinin viral RNA are important for efficient packaging into budding virions. *Journal of Virology* 81(18):9727–9736.
10. Skittrall JP, Irigoyen N, Brierley I, Gog JR (2023) A novel approach to finding conserved features in low-variability gene alignments characterises RNA motifs in SARS-CoV and SARS-CoV-2. *Scientific Reports* 13:12079.
11. Fujii K, et al. (2005) Importance of both the coding and the segment-specific noncoding regions of the influenza A virus NS segment for its efficient incorporation into virions. *Journal of Virology* 79(6):3766–3774.
12. Priore SF, Kauffmann AD, Baman JR, Turner DH (2015) The influenza A PB1-F2 and N40 start codons are contained within an RNA pseudoknot. *Biochemistry* 54:3413–3415.
13. Atkins JF, Loughran G, Bhatt PR, Firth AE, Baranov PV (2016) Ribosomal frameshifting and transcriptional slippage: from genetic steganography and cryptography to adventitious use. *Nucleic Acids Research* 44(15):7007–7008.
14. Jiang T, Kennedy SD, Moss WN, Kierzek E, Turner DH (2014) Secondary structure of a conserved domain in an intron of influenza A M1 mRNA. *Biochemistry* 53:5236–5248.
15. Moss WN, Priore SF, Turner DH (2011) Identification of potential conserved RNA secondary structure throughout influenza A coding regions. *RNA* 17:991–1011.
16. Ozawa M, et al. (2009) Nucleotide sequence requirements at the 5′ end of the influenza A virus M RNA segment for efficient virus replication. *Journal of Virology* 83(7):3384–3388.
17. Wise HM, et al. (2012) Identification of a novel splice variant form of the influenza A virus M2 ion channel with an antigenically distinct ectodomain. *PLoS Pathogens* 8(11):e1002998.
18. Simon LM, et al. (2019) In vivo analysis of influenza A mRNA secondary structures identifies critical regulatory motifs. *Nucleic Acids Research* 47(13):7003–7017.
19. Fujii K, Ozawa M, Iwatsuki-Horimoto K, Horimoto T, Kawaoka Y (2009) Incorporation of influenza A virus genome segments does not absolutely require wild-type sequences. *Journal of General Virology* 90:1734–1740.
20. Gultyaev AP, Heus HA, Olsthoorn RC (2007) An RNA conformational shift in recent H5N1 influenza A viruses. *Bioinformatics* 23(3):272–276.
21. Liang Y, Hong Y, Parslow TG (2005) *cis*-acting packaging signals in the influenza virus PB1, PB2, and PA genomic RNA segments. *Journal of Virology* 79(16):10348–10355.
22. Muramoto Y, et al. (2006) Hierarchy among viral RNA (vRNA) segments in their role in vRNA incorporation into influenza A virions. *Journal of Virology* 80(5):2318–2325.
23. Duhaut S, Dimmock NJ (2000) Approximately 150 nucleotides from the 5′ end of an influenza A segment 1 defective virion RNA are needed for genome stability during passage of defective virus in infected cells. *Virology* 275:278–285.
24. Dos Santos Afonso E, Escriou N, Leclercq I, van der Werf S, Naffakh N (2005) The generation of recombinant influenza A viruses expressing a PB2 fusion protein requires the conservation of a packaging signal overlapping the coding and noncoding regions at the 5′ end of the PB2 segment. *Virology* 341:34–46.
25. Hagey RJ, et al. (2022) Programmable antivirals targeting critical conserved viral RNA secondary structures from influenza A virus and SARS-CoV-2. *Nature Medicine* 28:1944–1955.
26. Jagger BW, et al. (2012) An overlapping protein-coding region in influenza A virus segment 3 modulates the host response. *Science* 337(6091):199–204.
27. Watanabe T, Watanabe S, Noda T, Fujii Y, Kawaoka Y (2003) Exploitation of nucleic acid packaging signals to generate a novel influenza virus-based vector stably expressing two foreign genes. *Journal of Virology* 77(19):10575–10583.
28. Ozawa M, et al. (2007) Contributions of two nuclear localization signals of influenza A virus nucleoprotein to viral replication. *Journal of Virology* 81(1):30–41.
29. Gultyaev AP, et al. (2014) RNA structural constraints in the evolution of the influenza A virus genome NP segment. *RNA Biology* 11(7):942–952.
30. Hutchinson EC, Wise HM, Kudryavtseva K, Curran MD, Digard P (2009) Characterisation of influenza A viruses with mutations in segment 5 packaging signals. *Vaccine* 27:6270–6275.

31. Takizawa N, Kawaguchi RK (2023) Comprehensive in virio structure probing analysis of the influenza A virus identifies functional RNA structures involved in viral genome replication. *Computational and Structural Biotechnology Journal* 21:5259–5272.
32. Fujii Y, Goto H, Watanabe T, Yoshida T, Kawaoka Y (2003) Selective incorporation of influenza virus RNA segments into virions. *Proceedings of the National Academy of Sciences* 100(4):2002–2007.
33. Seshimo E, Momose F, Morikawa Y (2021) Identification of the 5′-terminal packaging signal of the H1N1 influenza A virus neuraminidase segment at single-nucleotide resolution. *Frontiers in Microbiology* 12:709010.
34. Moss WN, Dela-Moss LI, Priore SF, Turner D (2012) The influenza A segment 7 mRNA 3′ splice site pseudoknot/hairpin family. *RNA Biology* 9(11):1305–1310.
35. Chen JL, Kennedy SD, Turner DH (2015) Structural features of a 3′ splice site in influenza A. *Biochemistry* 54:3269–3285.
36. Peterson JM, O'Leary CA, Moss WN (2022) In silico analysis of local RNA secondary structure in influenza virus A, B and C finds evidence of widespread ordered stability but little evidence of significant covariation. *Scientific Reports* 12:310.
37. Ilyinskii PO, et al. (2009) Importance of mRNA secondary structural elements for the expression of influenza virus genes. *OMICS* 13(5):421–430.
38. Priore SF, et al. (2013) Secondary structure of a conserved domain in the intron of influenza A NS1 mRNA. *PLOS ONE* 8(9):e70615.
39. Gultyaev AP, et al. (2016) Subtype-specific structural constraints in the evolution of influenza A virus hemagglutinin genes. *Scientific Reports* 6:38892.
40. Abolnik C (2017) Evolution of H5 highly pathogenic avian influenza: sequence data indicate stepwise changes in the cleavage site. *Archives of Virology* 162:2219–2230.
41. Gultyaev AP, Richard M, Spronken MI, Olsthoorn RC, Fouchier RA (2019) Conserved structural RNA domains in regions coding for cleavage site motifs in hemagglutinin genes of influenza viruses. *Virus Evolution* 5(2):vez034.
