# Supplementary material for: Locations and structures of influenza A virus packaging-associated signals and other functional elements via an in silico pipeline for predicting constrained features in RNA viruses
Source: PLoS Comput Biol. 2024 Apr 22;20(4):e1012009. doi: 10.1371/journal.pcbi.1012009 (PMC11034665; doi:10.1371/journal.pcbi.1012009)
Supplement: S1 Table — Note that H1N1 sequences contain a non-functional PB1-F2 and so no PB1-F2 analysis was performed for these sequences. (PDF) [file pcbi.1012009.s002.pdf]

Table S1. Number of sequences used in evaluations for each gene/subtype/host combination. Note that H1N1 sequences contain a non-functional PB1-F2 and so no PB1-F2 analysis was performed for these sequences.

	H1N1 (human)	H1N2 (swine)	H3N2 (human)	H5N1 (avian)	H5N8 (avian)	H7N7 (avian)	H7N9 (human)	H7N9 (avian)
PB2	22,232	2,677	40,384	2,687	2,332	572	1,059	898
PB1	20,972	2,626	39,322	2,796	2,350	367	1,094	879
PB1-F2	N/A	1,527	37,993	2,219	875	361	949	664
PA	22,589	2,714	40,665	2,868	2,496	353	1,074	905
PA-X	22,301	2,146	40,345	2,856	2,488	353	1,071	903
HA	39,770	4,525	65,774	4,184	2,718	641	1,120	1,009
NP	23,394	2,748	43,040	2,890	2,479	346	1,146	917
NA	38,907	4,772	58,248	832	2,538	497	1,168	994
M1	31,247	3,948	54,047	3,590	2,676	384	1,296	939
M2	30,867	3,609	53,200	3,202	2,418	339	1,220	918
NS1	24,280	2,835	41,032	3,250	2,635	393	1,262	953
NS2	24,206	2,342	41,097	2,971	1,404	353	1,205	815
