# Supplementary material for: Locations and structures of influenza A virus packaging-associated signals and other functional elements via an in silico pipeline for predicting constrained features in RNA viruses
Source: PLoS Comput Biol. 2024 Apr 22;20(4):e1012009. doi: 10.1371/journal.pcbi.1012009 (PMC11034665; doi:10.1371/journal.pcbi.1012009)
Supplement: S2 Table — Reference sequences used are RefSeq NC_026438.1 (GenBank FJ984387.1), NC_026435.1 (GQ323558.1), NC_026437.1 (FJ969539.1), NC_026433.1 (FJ969540.1), NC_026436.1 (FJ969536.1), NC_026434.1 (FJ984386.1), NC_026431.1 (FJ969537.1), NC_026432.1 (FJ969538.1), for segments 1–8, respectively. Citation details may be found in S1 Appendix. *Denotes a region only found by excluding a potentially interfering signal. Z- and p-values in parentheses denote values prior to removal of the next most significant signal. If parenthetical values are absent, then such a signal was removed in an earlier step only. (PDF) [file pcbi.1012009.s003.pdf]

Table S2. Summary of regions of significant constraint found in H1N1pdm09 (human host) influenza A genes, using weighted and raw (unranked) codon variability values. Reference sequences used are RefSeq NC_026438.1 (GenBank FJ984387.1), NC_026435.1 (GQ323558.1), NC_026437.1 (FJ969539.1), NC_026433.1 (FJ969540.1), NC_026436.1 (FJ969536.1), NC_026434.1 (FJ984386.1), NC_026431.1 (FJ969537.1), NC_026432.1 (FJ969538.1), for segments 1–8, respectively. Citation details may be found in S1 Appendix. *Denotes a region only found by excluding a potentially interfering signal. Z- and p-values in parentheses denote values prior to removal of the next most significant signal. If parenthetical values are absent, then such a signal was removed in an earlier step only.

Gene	Order found	Refseq nt location	Z	p	Comment
PB2	Nil found				
PB1	Nil found				
PB1-F2	Analysis not performed (gene is non-functional)				
PA	Nil found				
PA-X	Nil found				
HA	Nil found				
NP	Nil found				
NA	Nil found				
M1	1*	76–237	0.26 (0.23)	0.0183 (0.0529)	Packaging-associated(16); M42 alternate ORF and m4 splice junction(17); conserved RNA structure(3, 14, 15, 18)
M2	Nil found				
NS1	Nil found				
NS2	5*	7–15	0.59 (0.36)	0.0416 (0.3234)	?Packaging-associated(11, 19)
	1*	19–30, 503–613	0.29 (0.21)	0.0031 (0.1077)	?Packaging-associated(11); splice donor/acceptor; conformationally important region(20); overlapping ORFs
	3*	617–658	0.52 (0.34)	0.015 (0.143)	Overlapping ORFs
	4*	674–718	0.48 (0.39)	0.0367 (0.1108)	?Artefact
	6*	725–742	0.52	0.015	?Artefact
	2*	746–838	0.30	0.0208	Packaging-associated(11)
