# Supplementary material for: Locations and structures of influenza A virus packaging-associated signals and other functional elements via an in silico pipeline for predicting constrained features in RNA viruses
Source: PLoS Comput Biol. 2024 Apr 22;20(4):e1012009. doi: 10.1371/journal.pcbi.1012009 (PMC11034665; doi:10.1371/journal.pcbi.1012009)
Supplement: S3 Table — Reference sequences used are GenBank AB731582.1, AB731583.1, AB731584.1, AB731585.1, AB731586.1, AB731587.1, AB731588.1, AB731589.1, for segments 1–8, respectively. Citation details may be found in S1 Appendix. *Denotes a region only found by excluding a potentially interfering signal. Z- and p-values in parentheses denote values prior to removal of the next most significant signal. If parenthetical values are absent, then such a signal was removed in an earlier step only. (PDF) [file pcbi.1012009.s004.pdf]

Table S3. Summary of regions of significant constraint found in H1N2 (swine host) influenza A genes, using weighted and raw (unranked) codon variability values. Reference sequences used are GenBank AB731582.1, AB731583.1, AB731584.1, AB731585.1, AB731586.1, AB731587.1, AB731588.1, AB731589.1, for segments 1–8, respectively. Citation details may be found in S1 Appendix. *Denotes a region only found by excluding a potentially interfering signal. Z- and p-values in parentheses denote values prior to removal of the next most significant signal. If parenthetical values are absent, then such a signal was removed in an earlier step only.

Gene	Order found	Refseq nt location	Z	p	Comment
PB2	2	7–81	1.19	0.0058	Packaging-associated(21, 22); conserved RNA structure(18)
	1	2212–2277	1.55	<0.0001	Packaging-associated(4–6, 21, 23, 24); conserved RNA structure(3, 25)
PB1	1	2224–2265	1.46	<0.0001	Packaging-associated(5, 6, 21, 22); conserved RNA structure(3, 18)
PB1-F2	Nil found				
PA	3	10–69	1.12	0.0312	Packaging-associated(6, 22)
	2	559–609	1.29	0.0038	Proposed frameshift stimulator (see main text)
	4	640–759	1.12	0.047	Overlap PA-X(26)
	1	2002–2130	1.48	<0.0001	Packaging-associated(5, 6, 21) – but longer than previously described region; conserved cRNA structure(18)
PA-X	2*	10–72	0.99 (0.82)	0.0232 (0.9949)	Packaging-associated(6, 22)
	1	559–572, 574–700	1.12	0.047	Frameshift and overlap PA
HA	1	1612–1692	2.63	<0.0001	Packaging-associated(8, 9, 27)
NP	2	4–57	1.29	0.0056	Packaging-associated(3, 28, 29); conserved cRNA structure(18)
	1	1357–1494	1.62	<0.0001	Packaging-associated(28–31); conserved RNA structure(3, 31)
NA	1	1318–1380	1.40	0.0077	Packaging-associated(32, 33); conserved cRNA structure(18)
M1	2	4–69	0.99	0.0004	Packaging-associated(7, 16); M2 splice donor
	3	115–177	0.89	0.0001	m4 splice junction(17); conserved RNA structure(3, 14, 15)
	1	190–231	1.04	<0.0001	Conserved RNA structure(18)
	4	364–393	0.89	0.0001	Conserved cRNA structure(18)
	5*	682–717	1.01 (0.96)	0.0387 (0.0853)	Structured region 5′ to M2 splice acceptor(34–36)
M2	1*	938–970	0.44 (0.38)	0.0004 (0.1164)	Packaging-associated(7, 16); conserved cRNA structure(18)
NS1	1	490–564	1.25	0.002	Splice acceptor; conformationally important region(20); overlapping ORFs
NS2	3*	19–30, 503–508	1.10 (0.71)	0.0152 (0.0639)	Splice donor/acceptor
	1*	515–613	0.68 (0.49)	0.0002 (0.0728)	Conformationally important region(20); overlapping ORFs
	4*	623–718	0.80	0.0417	?Artefact; some overlapping ORFs
	2*	748–835	0.70	<0.0001	Packaging-associated(11)
