# Supplementary material for: Locations and structures of influenza A virus packaging-associated signals and other functional elements via an in silico pipeline for predicting constrained features in RNA viruses
Source: PLoS Comput Biol. 2024 Apr 22;20(4):e1012009. doi: 10.1371/journal.pcbi.1012009 (PMC11034665; doi:10.1371/journal.pcbi.1012009)
Supplement: S4 Table — Reference sequences used are RefSeq NC_007373.1 (GenBank CY002071.1), NC_007372.1 (CY002070.1), NC_007371.1 (CY002069.1), NC_007366.1 (CY002064.1), NC_007369.1 (CY002067.1), NC_007368.1 (CY002066.1), NC_007367.1 (CY002065.1), NC_007370.1 (CY002068.1), for segments 1–8, respectively. Citation details may be found in S1 Appendix. *Denotes a region only found by excluding a potentially interfering signal. Z- and p-values in parentheses denote values prior to removal of the next most significant signal. If parenthetical values are absent, then such a signal was removed in an earlier step only. (PDF) [file pcbi.1012009.s005.pdf]

Table S4. Summary of regions of significant constraint found in H3N2 (human host) influenza A genes, using weighted and raw (unranked) codon variability values. Reference sequences used are RefSeq NC_007373.1 (GenBank CY002071.1), NC_007372.1 (CY002070.1), NC_007371.1 (CY002069.1), NC_007366.1 (CY002064.1), NC_007369.1 (CY002067.1), NC_007368.1 (CY002066.1), NC_007367.1 (CY002065.1), NC_007370.1 (CY002068.1), for segments 1–8, respectively. Citation details may be found in S1 Appendix. *Denotes a region only found by excluding a potentially interfering signal. Z- and p-values in parentheses denote values prior to removal of the next most significant signal. If parenthetical values are absent, then such a signal was removed in an earlier step only.

Gene	Order found	Refseq nt location	Z	p	Comment
PB2	Nil found				
PB1	Nil found				
PB1-F2	Nil found				
PA	1	553–804	0.32	<0.0001	Proposed frameshift stimulator (see main text); overlap PA-X(26)
	2*	2065–2133	0.30<br>(0.28)	0.0308<br>(0.0528)	Packaging-associated(5, 21) – but 5′ to some previously described regions
PA-X	1	605–772	0.28	<0.0001	Overlap PA
HA	Nil found				
NP	Nil found				
NA	Nil found				
M1	1	38–157	0.22	0.0112	Packaging-associated(16); M42 alternate ORF and m4 splice junction(17); part of conserved RNA structure(3, 14, 15)
M2	1*	924–995	0.19<br>(0.16)	0.015<br>(0.1348)	Conserved RNA structure (3, 18); packaging-associated(7, 16)
NS1	1	465–644	0.31	<0.0001	Splice acceptor; conformationally important region(20); overlapping ORFs
NS2	1	33–56, 529–642	0.20	0.0004	Packaging-associated(11, 19); splice donor/acceptor; conformationally important region(20); overlapping ORFs
