# Supplementary material for: Locations and structures of influenza A virus packaging-associated signals and other functional elements via an in silico pipeline for predicting constrained features in RNA viruses
Source: PLoS Comput Biol. 2024 Apr 22;20(4):e1012009. doi: 10.1371/journal.pcbi.1012009 (PMC11034665; doi:10.1371/journal.pcbi.1012009)
Supplement: S5 Table — Reference sequences used are RefSeq NC_007357.1 (GenBank AF144300.1), NC_007358.1 (AF144301.1), NC_007359.1 (AF144302.1), NC_007362.1 (AF144305.1), NC_007360.1 (AF144303.1), NC_007361.1 (AF144304.1), NC_007363.1 (AF144306.1), NC_007364.1 (AF144307.1), for segments 1–8, respectively. Citation details may be found in S1 Appendix. *Denotes a region only found by excluding a potentially interfering signal. Z- and p-values in parentheses denote values prior to removal of the next most significant signal. If parenthetical values are absent, then such a signal was removed in an earlier step only. (PDF) [file pcbi.1012009.s006.pdf]

Table S5. Summary of regions of significant constraint found in H5N1 (avian host) influenza A genes, using weighted and raw (unranked) codon variability values. Reference sequences used are RefSeq NC_007357.1 (GenBank AF144300.1), NC_007358.1 (AF144301.1), NC_007359.1 (AF144302.1), NC_007362.1 (AF144305.1), NC_007360.1 (AF144303.1), NC_007361.1 (AF144304.1), NC_007363.1 (AF144306.1), NC_007364.1 (AF144307.1), for segments 1–8, respectively. Citation details may be found in S1 Appendix. *Denotes a region only found by excluding a potentially interfering signal. Z- and p-values in parentheses denote values prior to removal of the next most significant signal. If parenthetical values are absent, then such a signal was removed in an earlier step only.

Gene	Order found	Refseq nt location	Z	p	Comment
PB2	2	70–109	1.96	0.0002	Packaging-associated(21, 22); conserved RNA structure(18)
	3*	913–1119	1.64<br>(1.42)	0.0437<br>(0.2075)	Unclear
	1	2152–2307	2.72	<0.0001	Packaging-associated(4–6, 21, 23, 24); conserved RNA structure(3, 25)
PB1	1	2146–2289	1.18	0.0059	Packaging-associated(5, 6, 21, 22) – note region described extends 5′ of previously described regions; conserved RNA structure(3, 18)
PB1-F2	Nil found				
PA	1	589–783	1.29	<0.0001	Proposed frameshift stimulator (see main text); overlap PA-X(26)
	2	1996–2169	1.47	<0.0001	Packaging-associated(5, 6, 21) – but longer than previously described regions; conserved RNA structure(18)
PA-X	2*	589–596; 598–667	0.90<br>(0.80)	0.0271<br>(0.1745)	Proposed frameshift stimulator (see main text); overlap PA
	1	686–775	0.99	<0.0001	Overlap PA
HA	1	1615–1725	1.06	0.0049	Packaging-associated(8, 9, 27)
NP	2	49–108	1.07	0.0011	Packaging-associated(3, 28, 29); conserved cRNA structure(18)
	3*	691–1254	0.92<br>(0.86)	0.0464<br>(0.1094)	?Artefact
	1	1369–1542	1.32	<0.0001	Packaging-associated(28–31) – but longer than previously described region; conserved RNA structure(3, 31)
NA	2*	534–707	0.40<br>(0.38)	0.0155<br>(0.0511)	Unclear
	3*	813–872	0.40<br>(0.37)	0.0496<br>(0.0939)	Unclear
	4*	999–1073	0.40<br>(0.38)	0.0412<br>(0.0772)	Conserved RNA structure(3)
	1	1341–1427	0.45	0.0005	Packaging-associated(4, 31–33); conserved cRNA structure(18)
M1	2	29–67	0.81	0.0005	Packaging-associated(7, 16); M2 splice donor
	1	143–256	0.88	0.0005	m4 splice junction(17); conserved RNA structure(3, 14, 15, 18)
M2	1*	807–845	0.36<br>(0.32)	0.0001<br>(0.3096)	Packaging-associated(16)
NS1	1	18–170	0.60	0.0198	Packaging-associated(11, 19); splice donor; conserved RNA structure(3, 15, 37, 38)
	2	456–590	0.72	0.0201	Splice acceptor; conformationally important region(20); overlapping ORFs
NS2	Nil found				
