# Supplementary material for: Locations and structures of influenza A virus packaging-associated signals and other functional elements via an in silico pipeline for predicting constrained features in RNA viruses
Source: PLoS Comput Biol. 2024 Apr 22;20(4):e1012009. doi: 10.1371/journal.pcbi.1012009 (PMC11034665; doi:10.1371/journal.pcbi.1012009)
Supplement: S6 Table — Reference sequences used are GenBank LC699653.1, LC699654.1, LC699655.1, LC699656.1, LC699657.1, LC699658.1, LC699659.1, LC699660.1, for segments 1–8, respectively. Citation details may be found in S1 Appendix. *Denotes a region only found by excluding a potentially interfering signal. Z- and p-values in parentheses denote values prior to removal of the next most significant signal. If parenthetical values are absent, then such a signal was removed in an earlier step only. (PDF) [file pcbi.1012009.s007.pdf]

Table S6. Summary of regions of significant constraint found in H5N8 (avian host) influenza A genes, using weighted and raw (unranked) codon variability values. Reference sequences used are GenBank LC699653.1, LC699654.1, LC699655.1, LC699656.1, LC699657.1, LC699658.1, LC699659.1, LC699660.1, for segments 1–8, respectively. Citation details may be found in S1 Appendix. *Denotes a region only found by excluding a potentially interfering signal. Z- and p-values in parentheses denote values prior to removal of the next most significant signal. If parenthetical values are absent, then such a signal was removed in an earlier step only.

Gene	Order found	Refseq nt location	Z	p	Comment
PB2	2	66–152	1.27	0.0118	Packaging-associated(21, 22); conserved RNA structure(18)
	1	2106–2303	1.54	<0.0001	Packaging-associated(4–6, 21, 23, 24); conserved RNA structure(3, 25)
PB1	1*	2134–2295	0.78 (0.70)	0.0403 (0.1318)	Packaging-associated(5, 6, 21, 22) – note region described extends 5′ of previously described regions; conserved RNA structure(3, 18)
PB1-F2	Nil found				
PA	1	584–778	0.86	0.0067	Proposed frameshift stimulator (see main text); overlap PA-X(26)
	2	2015–2161	0.86	0.021	Packaging-associated(5, 6, 21) – but longer than previously described regions; conserved cRNA structure(18)
PA-X	1*	603–779	0.70 (0.60)	0.0217 (0.0776)	Proposed frameshift stimulator (see main text); overlap PA
HA	Nil found				
NP	2*	48–296	0.82 (0.71)	0.0176 (0.0947)	Packaging-associated(28–30) – note less conservation in portion that does not overlap with previously described region; conserved RNA structure(3, 18)
	3*	1158–1268	0.86 (0.79)	0.0299 (0.0571)	Unclear; conserved cRNA structure in one subregion(18)
	1	1359–1541	0.83	0.0007	Packaging-associated(28, 29, 31) – but longer than previously described region; conserved RNA structure(3, 31)
NA	2*	263–502	0.60 (0.41)	0.0311 (0.6983)	Unclear
	3*	689–1270	0.48	0.0016	Conserved RNA structure(3)
	1*	1307–1423	0.56 (0.51)	0.0229 (0.0505)	Packaging-associated(31–33); conserved cRNA structure(18)
M1	1	25–255	0.69	0.0003	Packaging-associated(7, 16); M2 splice donor; M42 alternate ORF and m4 splice junction(17); conserved RNA structure(3, 14, 15, 18)
M2	Nil found				
NS1	1	44–136	0.51	0.0004	Packaging-associated(11); splice donor; conserved RNA structure(3, 15, 37, 38)
	2	467–667	0.60	0.0224	Splice acceptor; conformationally important region(20); overlapping ORFs
NS2	Nil found				
