# Supplementary material for: Locations and structures of influenza A virus packaging-associated signals and other functional elements via an in silico pipeline for predicting constrained features in RNA viruses
Source: PLoS Comput Biol. 2024 Apr 22;20(4):e1012009. doi: 10.1371/journal.pcbi.1012009 (PMC11034665; doi:10.1371/journal.pcbi.1012009)
Supplement: S7 Table — Reference sequences used are GenBank KM922674.1, KM922675.1, KM922676.1, KM922677.1, KM922678.1, KM922679.1, KM922680.1, KM922673.1, for segments 1–8, respectively. Citation details may be found in S1 Appendix. *Denotes a region only found by excluding a potentially interfering signal. Z- and p-values in parentheses denote values prior to removal of the next most significant signal. If parenthetical values are absent, then such a signal was removed in an earlier step only. (PDF) [file pcbi.1012009.s008.pdf]

Table S7. Summary of regions of significant constraint found in H7N7 (avian host) influenza A genes, using weighted and raw (un-ranked) codon variability values. Reference sequences used are GenBank KM922674.1, KM922675.1, KM922676.1, KM922677.1, KM922678.1, KM922679.1, KM922680.1, KM922673.1, for segments 1–8, respectively. Citation details may be found in S1 Appendix. *Denotes a region only found by excluding a potentially interfering signal. Z- and p-values in parentheses denote values prior to removal of the next most significant signal. If parenthetical values are absent, then such a signal was removed in an earlier step only.

Gene	Order found	Refseq nt location	Z	p	Comment
PB2	2	7–90	1.55	<0.0001	Packaging-associated(21, 22); conserved RNA structure(18)
	3*	958–1062	1.18<br>(1.12)	0.0373<br>(0.0733)	Unclear
	1	2179–2280	2.19	<0.0001	Packaging-associated(4–6, 21, 23, 24); conserved RNA structure(3, 25)
PB1	1	2125–2265	1.66	<0.0001	Packaging-associated(5, 6, 21, 22) – note region described extends 5′ of previously described regions; conserved RNA structure(3, 18)
PB1-F2	2	98–139	0.51	<0.0001	PB1-N40 initiation region; conserved RNA structure(18)
	1	269–331	0.70	<0.0001	PB1-N92 initiation region (see main text); conserved RNA structure(18)
PA	3	10–78	2.11	<0.0001	Packaging-associated(6, 22) – but longer than one previously described region
	1	565–741	2.55	<0.0001	Proposed frameshift stimulator (see main text); overlap PA-X(26)
	2	1999–2151	2.50	<0.0001	Packaging-associated(5, 6, 21) – but longer than previously described regions; conserved cRNA structure(18)
PA-X	2	10–78	2.19	<0.0001	Packaging-associated(22) – but longer than previously described region
	1	565–570; 572–748	1.97	<0.0001	Proposed frameshift stimulator (see main text); overlap PA
HA	1	1582–1680	1.73	<0.0001	Packaging-associated(8, 9, 27)
NP	2	4–84	1.47	<0.0001	Packaging-associated(28, 29); conserved RNA structure(3, 18)
	1	1363–1494	1.84	<0.0001	Packaging-associated(28–31) – but longer than previously described region; conserved RNA structure(3, 31)
NA	1	1345–1416	2.12	<0.0001	Packaging-associated(31–33); conserved cRNA structure(18)
M1	1	8–235	1.12	<0.0001	Packaging-associated(7, 16); M2 splice donor; M42 alternate ORF and m4 splice junction(17); conserved RNA structure(3, 14, 15, 18)
	2	665–763	0.95	0.0176	Splice acceptor; conformationally important region(34–36); overlapping ORFs
M2	Nil found				
NS1	2	20–169	1.23	0.0015	Packaging-associated(11, 19); splice donor; conserved RNA structure(3, 15, 37, 38)
	1	503–703	1.58	<0.0001	Splice acceptor; conformationally important region(20); overlapping ORFs
NS2	1	38–43; 516–704	0.59	0.0234	Splice donor/acceptor; conformationally important region(20); overlapping ORFs
	2	819–848	0.95	0.0012	Packaging-associated(11)
