# Supplementary material for: Locations and structures of influenza A virus packaging-associated signals and other functional elements via an in silico pipeline for predicting constrained features in RNA viruses
Source: PLoS Comput Biol. 2024 Apr 22;20(4):e1012009. doi: 10.1371/journal.pcbi.1012009 (PMC11034665; doi:10.1371/journal.pcbi.1012009)
Supplement: S8 Table — Reference sequences used are RefSeq NC_026422.1 (GenBank KF021594.1), NC_026423.1 (KF021595.1), NC_026424.1 (KF021596.1), NC_026425.1 (KF021597.1), NC_026426.1 (KF021598.1), NC_026429.1 (KF021599.1), NC_026427.1 (KF021600.1), NC_026428.1 (KF021601.1), for segments 1–8, respectively. Citation details may be found in S1 Appendix. *Denotes a region only found by excluding a potentially interfering signal. Z- and p-values in parentheses denote values prior to removal of the next most significant signal. If parenthetical values are absent, then such a signal was removed in an earlier step only. (PDF) [file pcbi.1012009.s009.pdf]

Table S8. Summary of regions of significant constraint found in H7N9 (human host) influenza A genes, using weighted and raw (unranked) codon variability values. Reference sequences used are RefSeq NC_026422.1 (GenBank KF021594.1), NC_026423.1 (KF021595.1), NC_026424.1 (KF021596.1), NC_026425.1 (KF021597.1), NC_026426.1 (KF021598.1), NC_026429.1 (KF021599.1), NC_026427.1 (KF021600.1), NC_026428.1 (KF021601.1), for segments 1–8, respectively. Citation details may be found in S1 Appendix. *Denotes a region only found by excluding a potentially interfering signal. Z- and p-values in parentheses denote values prior to removal of the next most significant signal. If parenthetical values are absent, then such a signal was removed in an earlier step only.

Gene	Order found	Refseq nt location	Z	p	Comment
PB2	1	2206–2280	0.49	0.0455	Packaging-associated(4–6, 21, 23, 24); conserved RNA structure(3, 25)
PB1	1	2129–2268	0.47	0.0001	Packaging-associated(5, 6, 21, 22) – note region described extends 5′ of previously described regions; conserved RNA structure(3, 18)
PB1-F2	Nil found				
PA	3*	4–159	0.46 (0.46)	0.0001 (0.0041)	Packaging-associated(6, 22) – but longer than previously described regions
	1	553–765	0.49	0.0002	Proposed frameshift stimulator (see main text); overlap PA-X(26)
	2	1504–2151	0.46	0.0041	5′ region: unclear; 3′ region: packaging-associated(5, 6, 21) – but longer than previously described regions; conserved cRNA structure(18)
PA-X	2*	4–159	0.59 (0.38)	0.0031 (0.1941)	Packaging-associated(6, 22) – but longer than previously described region
	3*	349–570; 572–580	0.50	0.0072	5′ region: unclear; 3′ region: frameshift
	1	584–736	0.49	0.0002	Overlap PA
HA	1*	1507–1644	0.29 (0.24)	0.0363 (0.2431)	Packaging-associated(8, 9, 27) – note a single high-variability locus just after this region causes the algorithm not to include the remainder of the gene in the conserved region
NP	Nil found				
NA	Nil found				
M1	1	4–231	0.34	0.0114	Packaging-associated(7, 16); M2 splice donor; M42 alternate ORF and m4 splice junction(17); conserved RNA structure(3, 14, 15, 18)
M2	Nil found				
NS1	2	13–153	0.31	0.0269	Packaging-associated(11, 19); splice donor; conserved RNA structure(3, 15, 37, 38)
	1	472–615	0.31	<0.0001	Splice acceptor; conformationally important region(20); overlapping ORFs
NS2	1	13–30; 503–613	0.22	0.0007	Splice donor/acceptor; conformationally important region(20); overlapping ORFs
	2*	773–838	0.25 (0.23)	0.0281 (0.0549)	Packaging-associated(11)
