# Supplementary material for: Locations and structures of influenza A virus packaging-associated signals and other functional elements via an in silico pipeline for predicting constrained features in RNA viruses
Source: PLoS Comput Biol. 2024 Apr 22;20(4):e1012009. doi: 10.1371/journal.pcbi.1012009 (PMC11034665; doi:10.1371/journal.pcbi.1012009)
Supplement: S9 Table — Reference sequences used are RefSeq NC_026422.1 (GenBank KF021594.1), NC_026423.1 (KF021595.1), NC_026424.1 (KF021596.1), NC_026425.1 (KF021597.1), NC_026426.1 (KF021598.1), NC_026429.1 (KF021599.1), NC_026427.1 (KF021600.1), NC_026428.1 (KF021601.1), for segments 1–8, respectively. Citation details may be found in S1 Appendix. *Denotes a region only found by excluding a potentially interfering signal. Z- and p-values in parentheses denote values prior to removal of the next most significant signal. If parenthetical values are absent, then such a signal was removed in an earlier step only. (PDF) [file pcbi.1012009.s010.pdf]

Table S9. Summary of regions of significant constraint found in H7N9 (avian host) influenza A genes, using weighted and raw (un-ranked) codon variability values. Reference sequences used are RefSeq NC_026422.1 (GenBank KF021594.1), NC_026423.1 (KF021595.1), NC_026424.1 (KF021596.1), NC_026425.1 (KF021597.1), NC_026426.1 (KF021598.1), NC_026429.1 (KF021599.1), NC_026427.1 (KF021600.1), NC_026428.1 (KF021601.1), for segments 1–8, respectively. Citation details may be found in S1 Appendix. *Denotes a region only found by excluding a potentially interfering signal. Z- and p-values in parentheses denote values prior to removal of the next most significant signal. If parenthetical values are absent, then such a signal was removed in an earlier step only.

Gene	Order found	Refseq nt location	Z	p	Comment
PB2	2	7–57	0.95	0.0012	Packaging-associated(21, 22); conserved RNA structure(18)
	1	2116–2277	1.31	<0.0001	Packaging-associated(4–6, 21, 23, 24); conserved RNA structure(3, 25)
PB1	1	2119–2265	1.07	<0.0001	Packaging-associated(5, 6, 21, 22) – note region described extends 5′ of previously described regions; conserved RNA structure(3, 18)
PB1-F2	Nil found				
PA	3*	4–123	0.75 (0.62)	0.0161 (0.1275)	Packaging-associated(6, 22) – but longer than previously described regions
	1	565–756	1.26	<0.0001	Proposed frameshift stimulator (see main text); overlap PA-X(26)
	4*	1600–2010	0.68	0.0365	Unclear; relatively conserved regions visible around nt 1800 and at 3′ end; remainder may be artefact
	2	2014–2142	1.13	<0.0001	Packaging-associated(5, 6, 21) – but longer than previously described regions; conserved cRNA structure(18)
PA-X	2	4–123	0.70	0.0205	Packaging-associated(6, 22) – but longer than previously described regions
	1	565–570; 572–742	1.03	<0.0001	Proposed frameshift stimulator (see main text); overlap PA
HA	1	1582–1680	0.63	0.0027	Packaging-associated(8, 9, 27)
NP	2	4–63	0.69	0.0103	Packaging-associated(28, 29); conserved RNA structure(3, 18)
	1	1372–1482	0.88	<0.0001	Packaging-associated(28–31); conserved RNA structure(3, 31)
NA	2	379–492	0.42	0.0193	Unclear
	1	1306–1398	0.41	0.022	Packaging-associated(4, 31–33); conserved cRNA structure(18)
M1	1	4–231	0.84	<0.0001	Packaging-associated(7, 16); M2 splice donor; M42 alternate ORF and m4 splice junction(17); conserved RNA structure(3, 14, 15, 18)
	2*	346–399	0.74 (0.60)	0.013 (0.1924)	Unclear
	3*	667–759	0.75 (0.67)	0.0387 (0.1097)	Splice acceptor; conformationally important region(34–36); overlapping ORFs
M2	3*	4–26; 715	0.53	0.0157	Packaging-associated(7); M2 splice donor
	4*	719–757	0.65 (0.49)	0.0498 (0.3815)	Conformationally important region(34, 35); overlapping ORFs
	5*	785–799	0.68	0.0423	Unclear
	1*	803–847	0.44 (0.33)	0.037 (0.199)	Unclear
	2*	854–976	0.54	0.0033	Packaging-associated(7, 16); conserved cRNA structure(18)
NS1	2	4–153	0.72	<0.0001	Packaging-associated(11, 19); splice donor; conserved RNA structure(3, 15, 37, 38)
	1	490–615	0.59	<0.0001	Splice acceptor; conformationally important region(20); overlapping ORFs
NS2	1	13–30; 503–613	0.22	0.0383	Splice donor/acceptor; conformationally important region(20); overlapping ORFs
	2	758–838	0.29	0.0079	Packaging-associated(11)
