# Supplementary material for: Locations and structures of influenza A virus packaging-associated signals and other functional elements via an in silico pipeline for predicting constrained features in RNA viruses
Source: PLoS Comput Biol. 2024 Apr 22;20(4):e1012009. doi: 10.1371/journal.pcbi.1012009 (PMC11034665; doi:10.1371/journal.pcbi.1012009)
Supplement: S10 Table — Reference sequences used are RefSeq NC_026438.1 (GenBank FJ984387.1), NC_026435.1 (GQ323558.1), NC_026437.1 (FJ969539.1), NC_026433.1 (FJ969540.1), NC_026436.1 (FJ969536.1), NC_026434.1 (FJ984386.1), NC_026431.1 (FJ969537.1), NC_026432.1 (FJ969538.1), for segments 1–8, respectively. Citation details may be found in S1 Appendix. *Denotes a region only found by excluding a potentially interfering signal. Z- and p-values in parentheses denote values prior to removal of the next most significant signal. If parenthetical values are absent, then such a signal was removed in an earlier step only. (PDF) [file pcbi.1012009.s011.pdf]

Table S10. Summary of regions of significant constraint found in H1N1pdm09 (human host) influenza A genes, using weighted and ranked codon variability values. Reference sequences used are RefSeq NC_026438.1 (GenBank FJ984387.1), NC_026435.1 (GQ323558.1), NC_026437.1 (FJ969539.1), NC_026433.1 (FJ969540.1), NC_026436.1 (FJ969536.1), NC_026434.1 (FJ984386.1), NC_026431.1 (FJ969537.1), NC_026432.1 (FJ969538.1), for segments 1–8, respectively. Citation details may be found in S1 Appendix. *Denotes a region only found by excluding a potentially interfering signal. Z- and p-values in parentheses denote values prior to removal of the next most significant signal. If parenthetical values are absent, then such a signal was removed in an earlier step only.

Gene	Order found	Refseq nt location	Z	p	Comment
PB2	2	43–60	2.37	0.0066	Packaging-associated(21, 22); conserved RNA structure(18)
	1	2221–2262	3.15	<0.0001	Packaging-associated(4–6, 21, 23, 24); conserved RNA structure(3, 25)
PB1	1	2227–2265	2.49	0.0035	Packaging-associated(5, 6, 21, 22); conserved RNA structure(3, 18)
PB1-F2	Analysis not performed (gene is non-functional)				
PA	2	565–615	2.14	0.0426	Proposed frameshift stimulator (see main text)
	3	1258–1296	2.08	0.0364	Unclear
	1	1993–2145	2.24	0.0333	Packaging-associated(5, 6, 21) – but longer than previously described regions; conserved cRNA structure(18)
PA-X	Nil found				
HA	1	1633–1650	2.24	0.0139	Packaging-associated(8, 9, 27)
NP	Nil found				
NA	1*	1375–1392	2.03 (1.95)	0.0184 (0.1196)	Packaging-associated(4, 31–33)
M1	1	49–66	2.14	0.0047	Packaging-associated(7, 16)
M2	Nil found				
NS1	2*	19–57	1.76 (1.72)	0.0256 (0.0686)	Packaging-associated(11)
	1	472–612	2.66	<0.0001	Splice acceptor, conformationally important region(20), overlapping ORFs
NS2	Nil found				
