# Supplementary material for: Locations and structures of influenza A virus packaging-associated signals and other functional elements via an in silico pipeline for predicting constrained features in RNA viruses
Source: PLoS Comput Biol. 2024 Apr 22;20(4):e1012009. doi: 10.1371/journal.pcbi.1012009 (PMC11034665; doi:10.1371/journal.pcbi.1012009)
Supplement: S11 Table — Reference sequences used are GenBank AB731582.1, AB731583.1, AB731584.1, AB731585.1, AB731586.1, AB731587.1, AB731588.1, AB731589.1, for segments 1–8, respectively. Citation details may be found in S1 Appendix. *Denotes a region only found by excluding a potentially interfering signal. Z- and p-values in parentheses denote values prior to removal of the next most significant signal. If parenthetical values are absent, then such a signal was removed in an earlier step only. (PDF) [file pcbi.1012009.s012.pdf]

Table S11. Summary of regions of significant constraint found in H1N2 (swine host) influenza A genes, using weighted and ranked codon variability values. Reference sequences used are GenBank AB731582.1, AB731583.1, AB731584.1, AB731585.1, AB731586.1, AB731587.1, AB731588.1, AB731589.1, for segments 1–8, respectively. Citation details may be found in S1 Appendix. *Denotes a region only found by excluding a potentially interfering signal. Z- and p-values in parentheses denote values prior to removal of the next most significant signal. If parenthetical values are absent, then such a signal was removed in an earlier step only.

Gene	Order found	Refseq nt location	Z	p	Comment
PB2	2	43–57	3.19	<0.0001	Packaging-associated(21, 22); conserved RNA structure(18)
	1	2218–2277	4.32	<0.0001	Packaging-associated(4–6, 21, 23, 24); conserved RNA structure(3, 25)
PB1	1	2224–2265	3.39	<0.0001	Packaging-associated(5, 6, 21, 22); conserved RNA structure(3, 18)
PB1-F2	Nil found				
PA	2	568–591	2.75	0.0012	Frameshift motif/part of proposed stimulator (see main text)
	1	2071–2130	3.51	<0.0001	Packaging-associated(5, 6, 21) – but longer than previously described regions
PA-X	1	565–572, 574–583	2.27	0.0042	Frameshift motif
HA	1	1612–1692	3.16	0.0001	Packaging-associated(8, 9, 27)
NP	2	4–21	2.71	0.0004	Packaging-associated(3, 28); conserved cRNA structure(18)
	1	1381–1479	3.50	<0.0001	Packaging-associated(28–31) conserved RNA structure(3, 31)
NA	1	1318–1380	3.50	<0.0001	Packaging-associated(32, 33); conserved cRNA structure(18)
M1	2	4–69	2.34	0.0002	Packaging-associated(7, 16); M2 splice donor
	1	193–231	3.11	<0.0001	Conserved RNA structure(18)
M2	2*	4–27	1.64	0.0201	M2 splice donor
			(1.49)	(0.2252)	
	1*	938–970	1.77	<0.0001	Packaging-associated(7, 16); conserved cRNA structure(18)
			(1.67)	(0.92)	
NS1	2*	19–57	1.86	0.0229	Packaging-associated(11)
			(1.78)	(0.0934)	
	1	490–546	2.63	<0.0001	Splice acceptor; conformationally important region(20); overlapping ORFs
	3*	574–612	1.67	0.0457	Overlapping ORFs
			(1.59)	(0.1154)	
NS2	1	25–30, 503–547	1.90	0.024	Splice donor/acceptor; conformationally important region(20); overlapping ORFs
	4*	554–571	1.25	0.0192	Overlapping ORFs
	2*	584–613	1.69	0.0039	Overlapping ORFs
			(1.51)	(0.1212)	
	3*	761–835	1.57	0.0226	Packaging-associated(11)
