# Supplementary material for: Locations and structures of influenza A virus packaging-associated signals and other functional elements via an in silico pipeline for predicting constrained features in RNA viruses
Source: PLoS Comput Biol. 2024 Apr 22;20(4):e1012009. doi: 10.1371/journal.pcbi.1012009 (PMC11034665; doi:10.1371/journal.pcbi.1012009)
Supplement: S12 Table — Reference sequences used are RefSeq NC_007373.1 (GenBank CY002071.1), NC_007372.1 (CY002070.1), NC_007371.1 (CY002069.1), NC_007366.1 (CY002064.1), NC_007369.1 (CY002067.1), NC_007368.1 (CY002066.1), NC_007367.1 (CY002065.1), NC_007370.1 (CY002068.1), for segments 1–8, respectively. Citation details may be found in S1 Appendix. *Denotes a region only found by excluding a potentially interfering signal. Z- and p-values in parentheses denote values prior to removal of the next most significant signal. If parenthetical values are absent, then such a signal was removed in an earlier step only. (PDF) [file pcbi.1012009.s013.pdf]

Table S12. Summary of regions of significant constraint found in H3N2 (human host) influenza A genes, using weighted and ranked codon variability values. Reference sequences used are RefSeq NC_007373.1 (GenBank CY002071.1), NC_007372.1 (CY002070.1), NC_007371.1 (CY002069.1), NC_007366.1 (CY002064.1), NC_007369.1 (CY002067.1), NC_007368.1 (CY002066.1), NC_007367.1 (CY002065.1), NC_007370.1 (CY002068.1), for segments 1–8, respectively. Citation details may be found in S1 Appendix. *Denotes a region only found by excluding a potentially interfering signal. Z- and p-values in parentheses denote values prior to removal of the next most significant signal. If parenthetical values are absent, then such a signal was removed in an earlier step only.

Gene	Order found	Refseq nt location	Z	p	Comment
PB2	1	2254–2304	3.37	<0.0001	Packaging-associated(4–6, 21, 23, 24); conserved RNA structure(3, 25)
PB1	1	2245–2289	2.21	0.0447	Packaging-associated(5, 6, 21, 22); conserved RNA structure(3, 18)
PB1-F2	Nil found				
PA	1	589–768	3.59	<0.0001	Proposed frameshift stimulator (see main text); overlap PA-X(26)
	2	2065–2154	3.17	<0.0001	Packaging-associated(5, 6, 21) – but longer than previously described regions
PA-X	2	589–596, 598–643	2.01	0.0048	Proposed frameshift stimulator (see main text)
	1	680–772	3.27	<0.0001	Overlap PA
HA	1	1623–1661	2.17	0.0301	Packaging-associated(8, 9, 27)
NP	1	1462–1524	2.35	0.009	Packaging-associated(28, 29, 31) (found region 5′ of where reference (28) deems critical); conserved RNA structure(3, 31)
NA	2	554–595	2.22	0.0292	Unclear
	1	1367–1399	2.32	0.0201	Packaging-associated(32, 33)
M1	1	29–274	0.22	0.0112	Packaging-associated(16); M42 alternate ORF and m4 splice junction(17); conserved RNA structure(3, 14, 15, 18); unclear for 3′ part of region
	2*	389–421	1.52 (1.42)	0.0009 (0.0656)	Conserved cRNA structure(18)
M2	Nil found				
NS1	2	39–71	1.76	0.0225	Packaging-associated(11); splice donor
	1	507–656	3.14	<0.0001	Splice acceptor; conformationally important region(20); overlapping ORFs
NS2	1	54–56, 529–633	2.17	0.0006	Splice acceptor; conformationally important region(20); overlapping ORFs
	2	844–861	1.38	0.0063	Packaging-associated(11)
