# Supplementary material for: Locations and structures of influenza A virus packaging-associated signals and other functional elements via an in silico pipeline for predicting constrained features in RNA viruses
Source: PLoS Comput Biol. 2024 Apr 22;20(4):e1012009. doi: 10.1371/journal.pcbi.1012009 (PMC11034665; doi:10.1371/journal.pcbi.1012009)
Supplement: S13 Table — Reference sequences used are RefSeq NC_007357.1 (GenBank AF144300.1), NC_007358.1 (AF144301.1), NC_007359.1 (AF144302.1), NC_007362.1 (AF144305.1), NC_007360.1 (AF144303.1), NC_007361.1 (AF144304.1), NC_007363.1 (AF144306.1), NC_007364.1 (AF144307.1), for segments 1–8, respectively. Citation details may be found in S1 Appendix. *Denotes a region only found by excluding a potentially interfering signal. Z- and p-values in parentheses denote values prior to removal of the next most significant signal. If parenthetical values are absent, then such a signal was removed in an earlier step only. (PDF) [file pcbi.1012009.s014.pdf]

Table S13. Summary of regions of significant constraint found in H5N1 (avian host) influenza A genes, using weighted and ranked codon variability values. Reference sequences used are RefSeq NC_007357.1 (GenBank AF144300.1), NC_007358.1 (AF144301.1), NC_007359.1 (AF144302.1), NC_007362.1 (AF144305.1), NC_007360.1 (AF144303.1), NC_007361.1 (AF144304.1), NC_007363.1 (AF144306.1), NC_007364.1 (AF144307.1), for segments 1–8, respectively. Citation details may be found in S1 Appendix. *Denotes a region only found by excluding a potentially interfering signal. Z- and p-values in parentheses denote values prior to removal of the next most significant signal. If parenthetical values are absent, then such a signal was removed in an earlier step only.

Gene	Order found	Refseq nt location	Z	p	Comment
PB2	2	70–109	2.91	0.0006	Packaging-associated(21, 22); conserved RNA structure(18)
	1	2212–2307	4.58	<0.0001	Packaging-associated(4–6, 21, 23, 24); conserved RNA structure(3, 25)
PB1	2*	28–51	1.99 (1.96)	0.0006 (0.2622)	Packaging-associated(6, 21, 22)
	1	2248–2289	3.04	0.0001	Packaging-associated(5, 6, 21, 22); conserved RNA structure(3, 18)
PB1-F2	1	293–334	1.75	0.0372	PB1-N92 initiation region (see main text); conserved RNA structure(18)
PA	4	37–72	1.88	0.036	Packaging-associated(6, 22)
	1	589–780	3.66	<0.0001	Proposed frameshift stimulator (see main text); overlap PA-X(26)
	3	1777–1842	1.92	0.0402	Unclear
	2	2020–2166	3.77	<0.0001	Packaging-associated(5, 6, 21) – but longer than previously described regions; conserved cRNA structure(18)
PA-X	4	37–72	1.66	0.0263	Packaging-associated(6, 22)
	5	136–147	1.14	0.0037	Unclear; may have biological significance but small region raises concern for artefact
	2	589–596; 598–601	1.88	0.0331	Frameshift motif
	3	611–643	1.60	0.0008	Proposed frameshift stimulator (see main text); overlap PA
	1	686–775	2.59	0.0005	Overlap PA
HA	1	1615–1722	2.58	0.0021	Packaging-associated(8, 9, 27)
NP	2	49–108	2.85	0.0001	Packaging-associated(3, 28, 29); conserved cRNA structure(18)
	1	1405–1527	3.77	<0.0001	Packaging-associated(28–31) – mostly 5′ of previously described regions; conserved RNA structure(3, 31)
NA	1	1371–1427	2.66	<0.0001	Packaging-associated(4, 31–33)
M1	2	32–91	2.20	0.0024	Packaging-associated(7, 16); M2 splice donor
	3	164–187	2.04	0.0005	Conserved RNA structure(3, 14, 15)
	1	218–256	3.08	<0.0001	Conserved RNA structure(18)
	4	470–511	1.08	0.0043	Conserved cRNA structure(18)
M2	Nil found				
NS1	1	510–584	2.23	0.009	NS2 splice acceptor; conformationally important region(20); overlapping ORFs
NS2	1	529–582	1.91	0.0153	Conformationally important region(20); overlapping ORFs
	3*	598–621	1.37	0.0012	Overlapping ORFs
	2*	805–849	1.45 (1.32)	0.0277 (0.2838)	Packaging-associated(11)
