# Supplementary material for: Locations and structures of influenza A virus packaging-associated signals and other functional elements via an in silico pipeline for predicting constrained features in RNA viruses
Source: PLoS Comput Biol. 2024 Apr 22;20(4):e1012009. doi: 10.1371/journal.pcbi.1012009 (PMC11034665; doi:10.1371/journal.pcbi.1012009)
Supplement: S14 Table — Reference sequences used are GenBank LC699653.1, LC699654.1, LC699655.1, LC699656.1, LC699657.1, LC699658.1, LC699659.1, LC699660.1, for segments 1–8, respectively. Citation details may be found in S1 Appendix. *Denotes a region only found by excluding a potentially interfering signal. Z- and p-values in parentheses denote values prior to removal of the next most significant signal. If parenthetical values are absent, then such a signal was removed in an earlier step only. (PDF) [file pcbi.1012009.s015.pdf]

Table S14. Summary of regions of significant constraint found in H5N8 (avian host) influenza A genes, using weighted and ranked codon variability values. Reference sequences used are GenBank LC699653.1, LC699654.1, LC699655.1, LC699656.1, LC699657.1, LC699658.1, LC699659.1, LC699660.1, for segments 1–8, respectively. Citation details may be found in S1 Appendix. *Denotes a region only found by excluding a potentially interfering signal. Z- and p-values in parentheses denote values prior to removal of the next most significant signal. If parenthetical values are absent, then such a signal was removed in an earlier step only.

Gene	Order found	Refseq nt location	Z	p	Comment
PB2	2	66–113	2.84	0.0003	Packaging-associated(21, 22); conserved RNA structure(18)
	1	2229–2288	4.22	<0.0001	Packaging-associated(4–6, 21, 23, 24); conserved RNA structure(3, 25)
PB1	1	2248–2289	2.86	0.0009	Packaging-associated(5, 6, 21, 22); conserved RNA structure(3, 18)
PB1-F2	1	293–334	1.93	<0.0001	PB1-N92 initiation region (see main text); conserved RNA structure(18)
PA	2	584–763	2.48	0.0006	Proposed frameshift stimulator (see main text); overlap PA-X(26)
	1	2072–2149	3.19	<0.0001	Packaging-associated(5, 6, 21) – but longer than previously described regions
PA-X	1	744–764	2.48	0.0006	Overlap PA
HA	2*	791–877	1.94 (1.80)	0.0296 (0.2937)	Unclear
	1	1646–1687	2.59	0.0005	Packaging-associated(8, 9, 27)
NP	2	48–68	2.59	0.0022	Packaging-associated(3, 28); conserved cRNA structure(18)
	1	1446–1517	2.75	0.0009	Packaging-associated(28, 29, 31) – but longer than previously described region; conserved RNA structure(3, 31)
NA	2*	287–571	1.98 (1.73)	0.0364 (0.2667)	Unclear; conserved cRNA structure(18)
	1	1331–1420	2.31	0.0061	Packaging-associated(31–33); conserved cRNA structure(18)
M1	1	70–252	2.72	<0.0001	Packaging-associated(7, 16); M2 splice donor; M42 alternate ORF and m4 splice junction(17); conserved RNA structure(3, 14, 15, 18)
M2	Nil found				
NS1	1*	44–178	2.02 (1.67)	0.0004 (0.1496)	Packaging-associated(11); splice donor; conserved RNA structure(3, 15, 37, 38)
	3*	467–520; 599–667	1.77	0.0134	Overlapping ORFs
	2	521–598	2.30	<0.0001	Splice acceptor; conformationally important region(20); overlapping ORFs
NS2	1	44–52; 525–620	2.18	0.0458	Splice donor/acceptor; conformationally important region(20); overlapping ORFs
