# Supplementary material for: Locations and structures of influenza A virus packaging-associated signals and other functional elements via an in silico pipeline for predicting constrained features in RNA viruses
Source: PLoS Comput Biol. 2024 Apr 22;20(4):e1012009. doi: 10.1371/journal.pcbi.1012009 (PMC11034665; doi:10.1371/journal.pcbi.1012009)
Supplement: S16 Table — Reference sequences used are RefSeq NC_026422.1 (GenBank KF021594.1), NC_026423.1 (KF021595.1), NC_026424.1 (KF021596.1), NC_026425.1 (KF021597.1), NC_026426.1 (KF021598.1), NC_026429.1 (KF021599.1), NC_026427.1 (KF021600.1), NC_026428.1 (KF021601.1), for segments 1–8, respectively. Citation details may be found in S1 Appendix. *Denotes a region only found by excluding a potentially interfering signal. Z- and p-values in parentheses denote values prior to removal of the next most significant signal. If parenthetical values are absent, then such a signal was removed in an earlier step only. (PDF) [file pcbi.1012009.s017.pdf]

Table S16. Summary of regions of significant constraint found in H7N9 (human host) influenza A genes, using weighted and ranked codon variability values. Reference sequences used are RefSeq NC_026422.1 (GenBank KF021594.1), NC_026423.1 (KF021595.1), NC_026424.1 (KF021596.1), NC_026425.1 (KF021597.1), NC_026426.1 (KF021598.1), NC_026429.1 (KF021599.1), NC_026427.1 (KF021600.1), NC_026428.1 (KF021601.1), for segments 1–8, respectively. Citation details may be found in S1 Appendix. *Denotes a region only found by excluding a potentially interfering signal. Z- and p-values in parentheses denote values prior to removal of the next most significant signal. If parenthetical values are absent, then such a signal was removed in an earlier step only.

Gene	Order found	Refseq nt location	Z	p	Comment
PB2	2*	4–63	2.08 (2.06)	0.0403 (0.0985)	Packaging-associated(21, 22); conserved RNA structure(18)
	1	2173–2277	3.78	<0.0001	Packaging-associated(4–6, 21, 23, 24); conserved RNA structure(3, 25)
PB1	1	2164–2265	3.33	<0.0001	Packaging-associated(5, 6, 21, 22) – note region described extends 5′ of previously described regions; conserved RNA structure(3, 18)
PB1-F2	Nil found				
PA	1	568–738	3.94	<0.0001	Proposed frameshift stimulator (see main text); overlap PA-X(26)
	2	2044–2142	4.01	<0.0001	Packaging-associated(5, 6, 21) – but longer than previously described regions
PA-X	1	568–570; 572–760	3.73	<0.0001	Proposed frameshift stimulator (see main text); overlap PA
HA	2	1021–1299	2.07	0.045	Conserved stem-loop motifs(39–41)
	1	1561–1680	2.40	0.0046	Packaging-associated(8, 9, 27)
NP	2	4–63	2.72	0.0002	Packaging-associated(28, 29); conserved RNA structure(3, 18)
	1	1381–1479	3.14	<0.0001	Packaging-associated(28–31); conserved RNA structure(3, 31)
NA	Nil found				
M1	2	7–69	2.52	0.0003	Packaging-associated(7, 16); M2 splice donor
	3*	136–168	1.65 (1.62)	0.0316 (0.1105)	Conserved RNA structure(3, 14, 15)
	1	193–231	3.13	<0.0001	Conserved RNA structure(18)
M2	Nil found				
NS1	1	478–612	2.41	<0.0001	Splice acceptor; conformationally important region(20); overlapping ORFs
NS2	1	515–595	2.24	0.0045	Splice acceptor; conformationally important region(20); overlapping ORFs
