# Supplementary material for: Locations and structures of influenza A virus packaging-associated signals and other functional elements via an in silico pipeline for predicting constrained features in RNA viruses
Source: PLoS Comput Biol. 2024 Apr 22;20(4):e1012009. doi: 10.1371/journal.pcbi.1012009 (PMC11034665; doi:10.1371/journal.pcbi.1012009)
Supplement: S17 Table — Reference sequences used are RefSeq NC_026422.1 (GenBank KF021594.1), NC_026423.1 (KF021595.1), NC_026424.1 (KF021596.1), NC_026425.1 (KF021597.1), NC_026426.1 (KF021598.1), NC_026429.1 (KF021599.1), NC_026427.1 (KF021600.1), NC_026428.1 (KF021601.1), for segments 1–8, respectively. Citation details may be found in S1 Appendix. *Denotes a region only found by excluding a potentially interfering signal. Z- and p-values in parentheses denote values prior to removal of the next most significant signal. If parenthetical values are absent, then such a signal was removed in an earlier step only. (PDF) [file pcbi.1012009.s018.pdf]

**Table S17. Summary of regions of significant constraint found in H7N9 (avian host) influenza A genes, using weighted and ranked codon variability values. Reference sequences used are RefSeq NC_026422.1 (GenBank KF021594.1), NC_026423.1 (KF021595.1), NC_026424.1 (KF021596.1), NC_026425.1 (KF021597.1), NC_026426.1 (KF021598.1), NC_026429.1 (KF021599.1), NC_026427.1 (KF021600.1), NC_026428.1 (KF021601.1), for segments 1–8, respectively. Citation details may be found in S1 Appendix. *Denotes a region only found by excluding a potentially interfering signal. $Z$- and $p$-values in parentheses denote values prior to removal of the next most significant signal. If parenthetical values are absent, then such a signal was removed in an earlier step only.**

| Gene | Order found | Refseq nt location | $Z$ | $p$ | Comment |
|---|---|---|---|---|---|
| PB2 | 2 | 7–57 | 2.91 | 0.0003 | Packaging-associated(21, 22); conserved RNA structure(18) |
| | 1 | 2116–2277 | 4.52 | <0.0001 | Packaging-associated(4–6, 21, 23, 24); conserved RNA structure(3, 25) |
| PB1 | 2 | 4–150 | 2.10 | 0.0321 | Packaging-associated(6, 21, 22); conserved RNA structure(18); PB1-F2 initiation and overlap |
| | 1 | 2119–2265 | 3.92 | <0.0001 | Packaging-associated(5, 6, 21, 22) – note region described extends 5′ of previously described regions; conserved RNA structure(3, 18) |
| PB1-F2 | 1 | 281–289 | 1.40 | 0.0001 | PB1-N92 initiation region (see main text); conserved RNA structure(18) |
| PA | 3 | 4–81 | 1.87 | 0.012 | Packaging-associated(6, 22) – but longer than one previously described region |
| | 1 | 565–756 | 5.08 | <0.0001 | Proposed frameshift stimulator (see main text); overlap PA-X(26) |
| | 2 | 1999–2142 | 4.35 | <0.0001 | Packaging-associated(5, 6, 21) – but longer than previously described regions; conserved cRNA structure(18) |
| PA-X | 2 | 4–81 | 1.47 | 0.0097 | Packaging-associated(6, 22) – but longer than one previously described region |
| | 3* | 445–453 | 1.23 (1.22) | 0.0421 (0.0696) | Unclear |
| | 1 | 565–570; 572–742 | 1.03 | <0.0001 | Proposed frameshift stimulator (see main text); overlap PA |
| HA | 1 | 1582–1620 | 2.71 | 0.0002 | Packaging-associated(8, 9, 27) |
| | 2 | 1648–1680 | 2.11 | 0.0191 | Packaging-associated(27) |
| NP | 2 | 4–63 | 2.61 | 0.0003 | Packaging-associated(28, 29); conserved RNA structure(3, 18) |
| | 1 | 1372–1479 | 3.80 | <0.0001 | Packaging-associated(28–31); conserved RNA structure(3, 31) |
| NA | 2 | 379–492 | 1.99 | 0.0391 | Unclear |
| | 1 | 1306–1398 | 2.34 | 0.0059 | Packaging-associated(4, 31–33); conserved cRNA structure(18) |
| M1 | 1 | 7–231 | 3.31 | <0.0001 | Packaging-associated(7, 16); M2 splice donor; M42 alternate ORF and m4 splice junction(17); conserved RNA structure(3, 14, 15, 18) |
| | 2* | 706–714 | 1.47 (1.44) | 0.0079 (0.1511) | Splice acceptor; conformationally important region(34–36) |
| M2 | Nil found | | | | |
| NS1 | 2 | 7–60 | 2.13 | 0.0019 | Packaging-associated(11, 19); splice donor |
| | 3 | 103–153 | 1.68 | 0.0002 | Conserved RNA structure(3, 15, 37, 38) |
| | 1 | 493–615 | 2.68 | <0.0001 | Splice acceptor; conformationally important region(20); overlapping ORFs |
| NS2 | 1 | 13–30; 503–547 | 2.82 | 0.0018 | Splice donor/acceptor; conformationally important region(20); overlapping ORFs |
