# Supplementary material for: Locations and structures of influenza A virus packaging-associated signals and other functional elements via an in silico pipeline for predicting constrained features in RNA viruses
Source: PLoS Comput Biol. 2024 Apr 22;20(4):e1012009. doi: 10.1371/journal.pcbi.1012009 (PMC11034665; doi:10.1371/journal.pcbi.1012009)
Supplement: S2 Code — The content of the notebook follows the same pattern as that in S1 Code. (ZIP) [file pcbi.1012009.s115.zip › S2_code.pdf]

H1N2 swine hosts

PB2

Gene length histogram

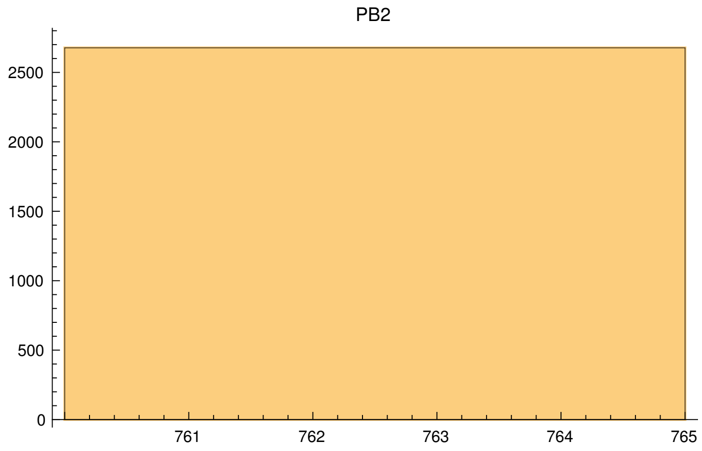

Information vs. nPD

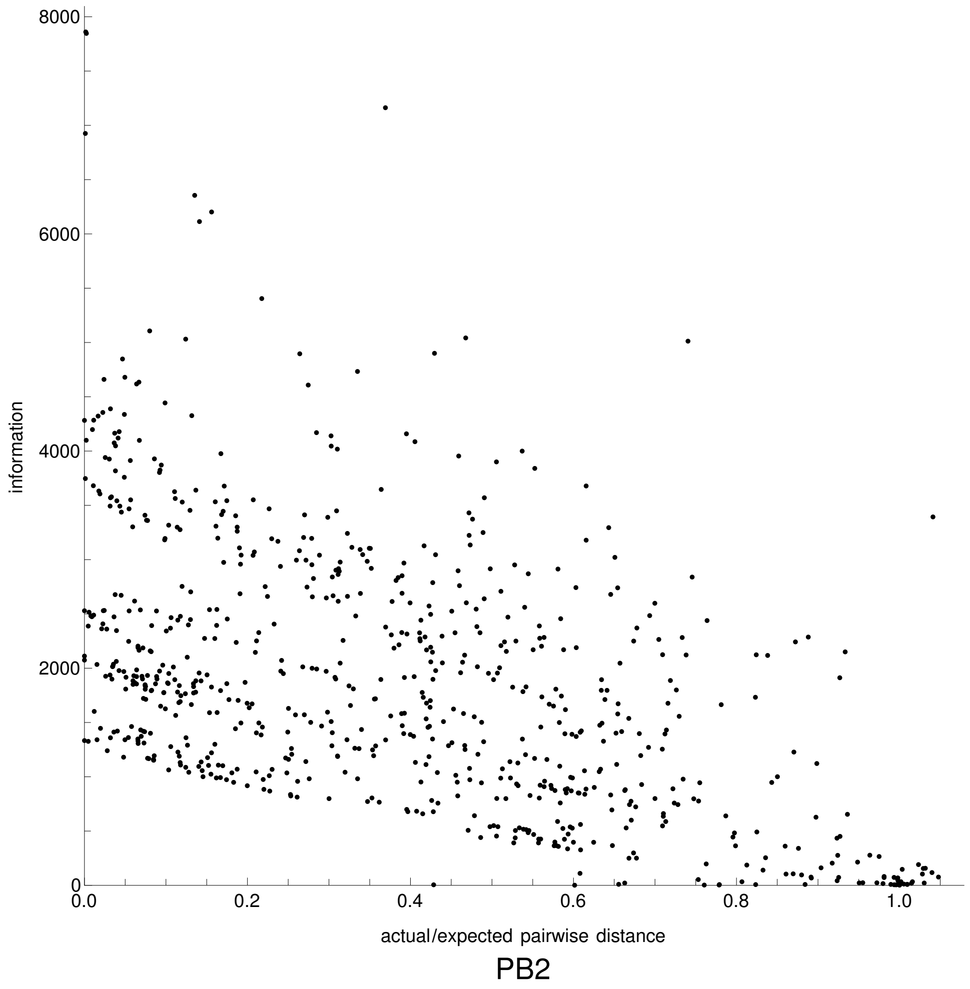

Example sequences highlighted by regions found in analyses to be conserved

Interesting points (by weighted raw PD) highlighted for gene PB2:

ATGGAA
AGAATAAAGGAATTAAGAGATCTAATGTCGCAGTCTCGCACTCGCGAGATACTGA
CAAAAACCACTGTGGACCAT
ATGGCAATAATCAAAAAATACACATCAGGAAGACAAGAGAAGAACCCCGCTCTC --------------
AGAATGAAATGGATGATGGCAATGAAATATCCGATCACAGCAGACAGG -------------------------
AGGATAATGGAGATGATTCCT ---------GAAAGAAATGAACAAGGACAAACACTTTGGAGTAAGACAAATGATGC⋱
TGGATCAGATAGGGTGGTGGTATCACCCCTAGCCGTAACTTGGTGGAATAGGAATGGACCGACAACAGATACGGT⋱
CCACTATCCAAAAGTCTACAAAACATATTTTGAAAAAGTTGAAAGGTTAAAGAATGGGACCTTTGGTCCTGTCCA⋱
TTTTAGAAATCAAATTAAAATACGCCGAAGAGTCGACATAAACCCAGGCCATGCAGATCTCAGTGCCAAGGAAGC⋱
ACAAGATGTTATCATGGAGGTCGTTTTCCCAAATGAAGTGGGAGCCAGAATATTAACATCAGAGTCGCAATTAAC⋱
AATAACAAAAGAGAAGAAAGAGGAGCTCCAGGATTGTAAAATTGCCCCTTTAATGGTGGCATACATGTTGGAAAG⋱
AGAACTGGTTCGCAAAACCAGATTCCTGCCGGTAGCAGGCGGAACAAGCAGTGTGTACATTGAAGTATTGCACTT⋱
GACTCAAGGAACCTGCTGGGAACAGATGTACACTCCAGGTGGAGAAGTGAAGAATGATGATGTCGACCAGAGTTT⋱
AATCATTGCTGCCAGAAACATTGTTAGGAGAGCAACAGTATCGGCAGATCCACTGGCATCACTATTGGAGATGTG⋱
CCACAGCACACAAATTGGTGGGATA ---------AGGATGGTTGACATCCTCAGACAA ------AATCCAACGGAAG⋱
AGCAAGCTGTGGATATATGCAAAGCAGCAATGGGTTTGAGAATCAGCTCCTCTTTTAGCTTCGGAGGTTTCACTT⋱
TCAAAAGAACAAGTGGATCATCTGTTAAAAGGGAAGAAGAAGTG -------------------------
CTTACCGGAAATCTCCAAACA --CTAAAAATA⋱
AGAGTGCATGAGGGGTATGAGGAATTCACAATGGTCGGGAAAAGAGCAACAGCCATATTAAGGAAAGCAACTAGA⋱
AGGCTGGTTCAG ------CTGATAGTAAGTGGAAGAGACGAACAGTCA ---ATTGCTGAGGCAATCATAGTAGCAAT⋱
GGTGTTCTCACAAGAGGATTGCATGATAAAGGCTGTCCGAGGTGATCTGAATTTTGTAAACAGAGCAAATCAACG⋱
GCTGAATCCC ------ATGCATCAACTCCTAAGACACTTCCAGAAGGATGCAAAAGTGCTGATTCAAAAT ---
TGGGGAGTTGAACCTATT ------
GATAATGTCATGGGAATGATAGGCATATTACCTGATATGACTCCAAGCACAGAGATGTCACTAAGA ------------G˙
GGGTAAGGGTCAGTAAAACAGGAGTGGACGAATATTCCAGTACTGAGAGAGTGGTTGTGAGTATTGATCGCTTCT⋱
TGAGAGTTCGAGATCAGCGGGGGAATGTACTCCTATCTCTT --------------------
GAGGAGGTTAGCGAAACACAGGGAACCGAGAAATTAACAATA ---------ACCTATTCATCATCAATGATGTGGGA⋱
GATAAACGGACCTGAGTCAGTGCTCGTTAACACATATCAATGGATCATTAGAAATTGGGAAACTGTGAAGATCCA⋱
ATGGTCTCAAGACCCCACAATGCTATACAACAAGATGGAATTTGAGCCATTTCAGTCTCTCGTACCTAAAGCAGC⋱
CAGAGGC ---CAATACAGTGGATTTGTGAGAACACTATTCCAGCAGATGCGTGATGTGCTGGGGACATTTGATACT⋱
GCCCAAATAATAAAGCTGCTGCCATTTGCAGCAGCGCCACCGGAGCAGAGTAGGATGCAGTTCTCTTCTCTAGCT⋱
GTAAATGTAAGAGGATCAGGAATGAGA -------------------------
ATACTCATAAGAGGTAACTCCCCCGTATTTAACTACAATAAGGCAACTAAAAGG ------
CTAACAGTCCTCGGAAAGGAC --

GCAGGTGTACTAACAGAAGATCCAGATGAGGGAACAGCTGGAGTGGAATCTGCGGTACTGAGAGGGTTCCTAATT --
---------------------CTAGGAAAGGAAAACAAAAGA -----------------TATGGACCCGCATTGAGCA⋱
TCAATGAACTGAGTAATCTTGCAAAAGGGGAGAAAGCCAATGTGTTGATAGGGCAAGGA -------------------
-GACGTGGTGTTGGTAATGAAACGG ---------
AAACGGGACTCTAGCATACTTACTGACAGCCAG-----------------
--
--
--

--------ACAGCGACCAAAAGAATTCGGATGGCCATCAATTAG

Interesting points (by weighted ranked PD) highlighted for gene PB2:

ATGGAAAGAATAAAGGAATTAAGAGATCTAATGTCGCAGTCT
CGCACTCGCGAGATACTGACAAAAACCACTGTGGACCATATGGCAATAATCAAAAAATACACATCAGGAA
GACAAGAGAAGAACCCCGCTCTC --------------
AGAATGAAATGGATGATGGCAATGAAATATCCGATCACAGCAGACAGG -------------------------
AGGATAATGGAGATGATTCCT ---------GAAAGAAATGAACAAGGACAAACACTTTGGAGTAAGACAAATGATGC
TGGATCAGATAGGGTGGTGGTATCACCCCTAGCCGTAACTTGGTGGAATAGGAATGGACCGACAACAGATACGGT
CCACTATCCAAAAGTCTACAAAACATATTTTGAAAAAGTTGAAAGGTTAAAGAATGGGACCTTTGGTCCTGTCCA
TTTTAGAAATCAAATTAAAATACGCCGAAGAGTCGACATAAACCCAGGCCATGCAGATCTCAGTGCCAAGGAAGC
ACAAGATGTTATCATGGAGGTCGTTTTCCCAAATGAAGTGGGAGCCAGAATATTAACATCAGAGTCGCAATTAAC
AATAACAAAAGAGAAGAAAGAGGAGCTCCAGGATTGTAAAATTGCCCCTTTAATGGTGGCATACATGTTGGAAAG
AGAACTGGTTCGCAAAACCAGATTCCTGCCGGTAGCAGGCGGAACAAGCAGTGTGTACATTGAAGTATTGCACTT
GACTCAAGGAACCTGCTGGGAACAGATGTACACTCCAGGTGGAGAAGTGAAGAATGATGATGTCGACCAGAGTTT
AATCATTGCTGCCAGAAACATTGTTAGGAGAGCAACAGTATCGGCAGATCCACTGGCATCACTATTGGAGATGTG
CCACAGCACACAAATTGGTGGGATA ---------AGGATGGTTGACATCCTCAGACAA ------AATCCAACGGAAG
AGCAAGCTGTGGATATATGCAAAGCAGCAATGGGTTTGAGAATCAGCTCCTCTTTTAGCTTCGGAGGTTTCACTT
TCAAAAGAACAAGTGGATCATCTGTTAAAAGGGAAGAAGAAGTG -------------------------
CTTACCGGAAATCTCCAAACA --CTAAAAATA
AGAGTGCATGAGGGGTATGAGGAATTCACAATGGTCGGGAAAAGAGCAACAGCCATATTAAGGAAAGCAACTAGA
AGGCTGGTTCAG ------CTGATAGTAAGTGGAAGAGACGAACAGTCA ---ATTGCTGAGGCAATCATAGTAGCAAT
GGTGTTCTCACAAGAGGATTGCATGATAAAGGCTGTCCGAGGTGATCTGAATTTTGTAAACAGAGCAAATCAACG
GCTGAATCCC ------ATGCATCAACTCCTAAGACACTTCCAGAAGGATGCAAAAGTGCTGATTCAAAAT ---
TGGGGAGTTGAACCTATT ------
GATAATGTCATGGGAATGATAGGCATATTACCTGATATGACTCCAAGCACAGAGATGTCACTAAGA -----------G
GGGTAAGGGTCAGTAAAACAGGAGTGGACGAATATTCCAGTACTGAGAGAGTGGTTGTGAGTATTGATCGCTTCT
TGAGAGTTCGAGATCAGCGGGGGAATGTACTCCTATCTCTT --------------------
GAGGAGGTTAGCGAAACACAGGGAACCGAGAAATTAACAATA ---------ACCTATTCATCATCAATGATGTGGGA
GATAAACGGACCTGAGTCAGTGCTCGTTAACACATATCAATGGATCATTAGAAATTGGGAAACTGTGAAGATCCA
ATGGTCTCAAGACCCCACAATGCTATACAACAAGATGGAATTTGAGCCATTTCAGTCTCTCGTACCTAAAGCAGC
CAGAGGC---CAATACAGTGGATTTGTGAGAACACTATTCCAGCAGATGCGTGATGTGCTGGGGACATTTGATACT
GCCCAAATAATAAAGCTGCTGCCATTTGCAGCAGCGCCACCGGAGCAGAGTAGGATGCAGTTCTCTTCTCTAGCT
GTAAATGTAAGAGGATCAGGAATGAGA -------------------------
ATACTCATAAGAGGTAACTCCCCCGTATTTAACTACAATAAGGCAACTAAAAGG ------
CTAACAGTCCTCGGAAAGGAC --

GCAGGTGTACTAACAGAAGATCCAGATGAGGGAACAGCTGGAGTGGAATCTGCGGTACTGAGAGGGTTCCTAATT --
---------------------CTAGGAAAGGAAAACAAAAGA -----------------TATGGACCCGCATTGAGCA
TCAATGAACTGAGTAATCTTGCAAAAGGGGAGAAAGCCAATGTGTTGATAGGGCAAGGA -------------------
-GACGTGGTGTTGGTAATGAAACGG ---------AAACGG
GACTCTAGCATACTTACTGACAGCCAG----------------------

----ACAGCGACCAAAAGAATTCGGATGGCCATCAATTAG

Per codon conservation report

PB2			
Pos.	1	obs:	exp:
atg	M	2677	2677.00
---	--	------	------
mPD		0	0
		nPD:	1.
		N. weight:	0.
		Sc. PD:	0
		Sc. rank:	0

PB2			
Pos.	2	obs:	exp:
gat	D	3	1.69
gac	D	0	1.31
gaa	E	344	1584.00
gag	E	2330	1090.00
---	--	------	------
mPD		0.23	0.48
		nPD:	0.47
		N. weight:	0.87
		Sc. PD:	0.12
		Sc. rank:	569.1

PB2			
Pos.	3	obs:	exp:
cgt	R	0	84.47
cgc	R	0	141.40
cga	R	3	269.00
cgg	R	0	193.30
aaa	K	1	0.61
aag	K	0	0.39
aga	R	2644	1227.00
agg	R	29	760.60
---	--	------	------
mPD		0.024	0.94
		nPD:	0.03
		N. weight:	1.3
		Sc. PD:	-0.27
		Sc. rank:	-1398.6

PB2			
Pos.	4	obs:	exp:
att	I	0	941.80
atc	I	0	681.00
ata	I	2655	1032.00
atg	M	5	5.00
gtt	V	0	3.77
gtc	V	0	3.21
gta	V	17	4.29
gtg	V	0	5.73
---	--	------	------
mPD		0.016	0.67
		nPD:	0.02
		N. weight:	1.8
		Sc. PD:	-0.36
		Sc. rank:	-1857.1

PB2			
Pos.	5	obs:	exp:
cgt	R	0	0.41
cgc	R	0	0.69
cga	R	0	1.31
cgg	R	0	0.94
aaa	K	2579	1619.00
aag	K	85	1045.00
aga	R	13	5.96
agg	R	0	3.69
---	--	------	------
mPD		0.071	0.49
		nPD:	0.15
		N. weight:	0.69
		Sc. PD:	-0.076
		Sc. rank:	-221.6

PB2			
Pos.	6	obs:	exp:
att	I	0	0.35
atc	I	0	0.26
ata	I	1	0.39
aaa	K	2	1.22
aag	K	0	0.78
gat	D	1	0.56
gac	D	0	0.44
gaa	E	2565	1584.00
gag	E	108	1089.00
---	--	------	------
mPD		0.081	0.49
		nPD:	0.17
		N. weight:	0.69
		Sc. PD:	-0.064
		Sc. rank:	-165.5

PB2			
Pos.	7	obs:	exp:
tta	L	388	291.00
ttg	L	18	502.30
ctt	L	2	540.00
ctc	L	0	389.10
cta	L	1516	466.90
ctg	L	750	484.70
cct	P	0	0.59
ccc	P	0	0.40
cca	P	0	0.79
ccg	P	2	0.22
att	I	0	0.35
atc	I	0	0.26
ata	I	1	0.39
---	--	------	------
mPD		0.67	1.1
		nPD:	0.59
		N. weight:	1.5
		Sc. PD:	0.35
		Sc. rank:	1426.8

PB2			
Pos.	8	obs:	exp:
cgt	R	0	84.41
cgc	R	0	141.30
cga	R	1	268.80
cgg	R	1	193.20
att	I	0	0.35
atc	I	0	0.26
ata	I	1	0.39
aaa	K	2	1.22
aag	K	0	0.78
aga	R	2635	1226.00
agg	R	37	760.00
---	--	------	------
mPD		0.032	0.94
		nPD:	0.03
		N. weight:	1.3
		Sc. PD:	-0.26
		Sc. rank:	-1314.1

PB2			
Pos.	9	obs:	exp:
aat	N	121	69.34
aac	N	1	52.66
gct	A	1	0.25
gcc	A	0	0.23
gca	A	0	0.45
gcg	A	0	0.07
gat	D	2472	1435.00
gac	D	79	1116.00
gaa	E	1	1.19
gag	E	1	0.81
ggt	G	1	0.16
ggc	G	0	0.13
gga	G	0	0.45
ggg	G	0	0.26
---	--	------	------
mPD		0.15	0.58
		nPD:	0.25
		N. weight:	0.84
		Sc. PD:	-0.022
		Sc. rank:	9.7

PB2			
Pos.	10	obs:	exp:
tta	L	155	291.30
ttg	L	9	502.80
ctt	L	3	540.60
ctc	L	0	389.50
cta	L	2236	467.40
ctg	L	274	485.30
---	--	------	------
mPD		0.31	1.1
		nPD:	0.27
		N. weight:	2.2
		Sc. PD:	-0.033
		Sc. rank:	103.2

PB2			
Pos.	11	obs:	exp:
att	I	0	23.06
atc	I	0	16.67
ata	I	65	25.27
atg	M	2611	2611.00
gtt	V	0	0.22
gtc	V	0	0.19
gta	V	1	0.25
gtg	V	0	0.34
---	--	------	------
mPD		0.049	0.049
		nPD:	1.
		N. weight:	0.043
		Sc. PD:	0.024
		Sc. rank:	53.9

PB2			
Pos.	12	obs:	exp:
tta	L	4	1.52
ttg	L	10	2.63
tct	S	9	447.70
tcc	S	0	420.70
tca	S	306	646.40
tcg	S	2347	141.50
ctt	L	0	2.83
ctc	L	0	2.04
cta	L	0	2.44
ctg	L	0	2.54
agt	S	0	495.50
agc	S	0	510.20
gct	A	0	0.25
gcc	A	0	0.23
gca	A	0	0.45
gcg	A	1	0.07
---	--	------	------
mPD		0.22	1.6
		nPD:	0.14
		N. weight:	4.4
		Sc. PD:	-0.52
		Sc. rank:	-1577.3

PB2			
Pos.	13	obs:	exp:
cat	H	9	5.19
cac	H	0	3.81
caa	Q	337	1437.00
cag	Q	2327	1227.00
cgt	R	0	0.03
cgc	R	0	0.05
cga	R	1	0.10
cgg	R	0	0.07
aaa	K	0	1.82
aag	K	3	1.18
aga	R	0	0.46
agg	R	0	0.28
---	--	------	------
mPD		0.23	0.50
		nPD:	0.46
		N. weight:	0.7
		Sc. PD:	0.09
		Sc. rank:	433.9

PB2			
Pos.	14	obs:	exp:
tct	S	542	447.90
tcc	S	2121	420.90
tca	S	0	646.70
tcg	S	0	141.50
cct	P	12	4.13
ccc	P	2	2.82
cca	P	0	5.54
ccg	P	0	1.51
agt	S	0	495.60
agc	S	0	510.40
---	--	------	------
mPD		0.34	1.6
		nPD:	0.21
		N. weight:	2.5
		Sc. PD:	-0.15
		Sc. rank:	-253.1

PB2			
Pos.	15	obs:	exp:
cat	H	0	0.58
cac	H	1	0.42
cgt	R	0	84.47
cgc	R	2675	141.40
cga	R	1	269.00
cgg	R	0	193.30
aga	R	0	1227.00
agg	R	0	760.60
---	--	------	------
mPD		0.0015	0.94
		nPD:	0.
		N. weight:	5.4
		Sc. PD:	-1.2
		Sc. rank:	-6517.4

PB2			
Pos.	16	obs:	exp:
act	T	2608	658.50
acc	T	58	578.90
aca	T	10	1216.00
acg	T	1	223.20
---	--	------	------
mPD		0.050	0.68
		nPD:	0.07
		N. weight:	2.4
		Sc. PD:	-0.39
		Sc. rank:	-1656.1

PB2			
Pos.	17	obs:	exp:
cgt	R	1	84.50
cgc	R	2674	141.50
cga	R	1	269.10
cgg	R	1	193.40
aga	R	0	1228.00
agg	R	0	760.90
---	--	------	------
mPD		0.0022	0.94
		nPD:	0.
		N. weight:	5.4
		Sc. PD:	-1.2
		Sc. rank:	-6389.9

PB2			
Pos.	18	obs:	exp:
gaa	E	2	1586.00
gag	E	2674	1090.00
ggt	G	0	0.16
ggc	G	0	0.13
gga	G	0	0.45
ggg	G	1	0.26
---	--	------	------
mPD		0.0022	0.48
		nPD:	0.
		N. weight:	1.7
		Sc. PD:	-0.36
		Sc. rank:	-1937.5

PB2			
Pos.	19	obs:	exp:
att	I	2	949.60
atc	I	5	686.60
ata	I	2670	1041.00
---	--	------	------
mPD		0.0052	0.66
		nPD:	0.01
		N. weight:	1.7
		Sc. PD:	-0.37
		Sc. rank:	-1986.5

PB2			
Pos.	20	obs:	exp:
ttt	F	0	0.45
ttc	F	1	0.55
tta	L	0	291.20
ttg	L	2	502.60
ctt	L	37	540.40
ctc	L	2206	389.30
cta	L	137	467.30
ctg	L	294	485.10
---	--	------	------
mPD		0.31	1.1
		nPD:	0.27
		N. weight:	2.4
		Sc. PD:	-0.033
		Sc. rank:	131.0

PB2

Pos.	21	obs:	exp:
tct	S	0	0.50
tcc	S	0	0.47
tca	S	0	0.73
tcg	S	0	0.16
att	I	5	4.26
atc	I	3	3.08
ata	I	4	4.67
act	T	2130	651.40
acc	T	81	572.60
aca	T	436	1203.00
acg	T	1	220.80
agt	S	3	0.56
agc	S	0	0.57
gct	A	14	3.43
gcc	A	0	3.21
gca	A	0	6.31
gcg	A	0	1.05
---	--	------	------
mPD		0.35	0.70
		nPD:	0.5
		N. weight:	1.4
		Sc. PD:	0.21
		Sc. rank:	1013.4

,

PB2

Pos.	22	obs:	exp:
caa	Q	1	0.54
cag	Q	0	0.46
cgt	R	0	6.50
cgc	R	0	10.89
cga	R	0	20.71
cgg	R	0	14.88
aat	N	1	1.14
aac	N	1	0.86
aaa	K	485	1499.00
aag	K	1982	967.70
aga	R	139	94.47
agg	R	67	58.55
gaa	E	1	0.59
gag	E	0	0.41
---	--	------	------
mPD		0.50	0.67
		nPD:	0.75
		N. weight:	0.65
		Sc. PD:	0.23
		Sc. rank:	774.3

,

PB2

Pos.	23	obs:	exp:
att	I	0	0.35
atc	I	1	0.26
ata	I	0	0.39
act	T	2	658.20
acc	T	2649	578.70
aca	T	25	1216.00
acg	T	0	223.10
---	--	------	------
mPD		0.021	0.68
		nPD:	0.03
		N. weight:	2.7
		Sc. PD:	-0.54
		Sc. rank:	-2814.8

,

PB2

Pos.	24	obs:	exp:
act	T	2605	658.50
acc	T	5	578.90
aca	T	59	1216.00
acg	T	8	223.20
---	--	------	------
mPD		0.053	0.68
		nPD:	0.08
		N. weight:	2.3
		Sc. PD:	-0.38
		Sc. rank:	-1558.8

,

PB2

Pos.	25	obs:	exp:
gtt	V	8	594.40
gtc	V	0	505.10
gta	V	54	675.80
gtg	V	2615	901.70
---	--	------	------
mPD		0.045	0.74
		nPD:	0.06
		N. weight:	1.8
		Sc. PD:	-0.32
		Sc. rank:	-1441.4

,

PB2

Pos.	26	obs:	exp:
gat	D	79	1506.00
gac	D	2598	1171.00
---	--	------	------
mPD		0.057	0.49
		nPD:	0.12
		N. weight:	1.3
		Sc. PD:	-0.17
		Sc. rank:	-594.0

,

PB2

Pos.	27	obs:	exp:
cat	H	2582	1544.00
cac	H	95	1133.00
---	--	------	------
mPD		0.068	0.49
		nPD:	0.14
		N. weight:	0.76
		Sc. PD:	-0.086
		Sc. rank:	-260.4

,

PB2

Pos.	28	obs:	exp:
atg	M	2677	2677.00
---	--	------	------
mPD		0	0
		nPD:	1.
		N. weight:	0.
		Sc. PD:	0
		Sc. rank:	0

,

PB2

Pos.	29	obs:	exp:
gct	A	80	656.20
gcc	A	2172	613.40
gca	A	418	1207.00
gcg	A	7	200.80
---	--	------	------
mPD		0.32	0.68
		nPD:	0.47
		N. weight:	1.5
		Sc. PD:	0.2
		Sc. rank:	957.8

,

PB2

Pos.	30	obs:	exp:
att	I	2	948.60
atc	I	0	685.90
ata	I	2672	1040.00
gtt	V	0	0.67
gtc	V	0	0.57
gta	V	3	0.76
gtg	V	0	1.01
---	--	------	------
mPD		0.0037	0.66
		nPD:	0.01
		N. weight:	1.7
		Sc. PD:	-0.38
		Sc. rank:	-2029.1

,

PB2

Pos.	31	obs:	exp:
tta	L	5	0.87
ttg	L	0	1.50
ctt	L	0	1.62
ctc	L	3	1.16
cta	L	0	1.40
ctg	L	0	1.45
att	I	39	946.50
atc	I	2300	684.30
ata	I	329	1037.00
gtt	V	0	0.22
gtc	V	1	0.19
gta	V	0	0.25
gtg	V	0	0.34
---	--	------	------
mPD		0.25	0.66
		nPD:	0.38
		N. weight:	1.6
		Sc. PD:	0.11
		Sc. rank:	607.3

,

PB2

Pos.	32	obs:	exp:
cgt	R	0	0.82
cgc	R	0	1.37
cga	R	0	2.61
cgg	R	0	1.88
aaa	K	2593	1611.00
aag	K	58	1040.00
aga	R	25	11.92
agg	R	1	7.39
---	--	------	------
mPD		0.062	0.50
		nPD:	0.12
		N. weight:	0.75
		Sc. PD:	-0.095
		Sc. rank:	-316.4

,

PB2

Pos.	33	obs:	exp:
cgt	R	0	0.60
cgc	R	0	1.00
cga	R	0	1.91
cgg	R	0	1.37
aaa	K	618	1615.00
aag	K	2040	1043.00
aga	R	2	8.71
agg	R	17	5.40
---	--	------	------
mPD		0.37	0.50
		nPD:	0.75
		N. weight:	0.55
		Sc. PD:	0.19
		Sc. rank:	651.6

,

PB2

Pos.	34	obs:	exp:
tat	Y	52	1500.00
tac	Y	2625	1177.00
---	--	------	------
mPD		0.038	0.49
		nPD:	0.08
		N. weight:	1.3
		Sc. PD:	-0.22
		Sc. rank:	-904.8

,

PB2

Pos.	35	obs:	exp:
act	T	24	658.50
acc	T	3	578.90
aca	T	1848	1216.00
acg	T	802	223.20
---	--	------	------
mPD		0.43	0.68
		nPD:	0.64
		N. weight:	1.2
		Sc. PD:	0.32
		Sc. rank:	1225.3

,

PB2

Pos.	36	obs:	exp:
tct	S	4	448.60
tcc	S	0	421.50
tca	S	2606	647.70
tcg	S	57	141.70
act	T	0	2.46
acc	T	0	2.16
aca	T	10	4.54
acg	T	0	0.83
agt	S	0	496.40
agc	S	0	511.10
---	--	------	------
mPD		0.052	1.6
		nPD:	0.03
		N. weight:	2.5
		Sc. PD:	-0.49
		Sc. rank:	-2493.9

,

PB2

Pos.	37	obs:	exp:
ggt	G	0	419.90
ggc	G	0	339.20
gga	G	2464	1213.00
ggg	G	213	705.40
---	--	------	------
mPD		0.15	0.68
		nPD:	0.21
		N. weight:	1.
		Sc. PD:	-0.059
		Sc. rank:	-81.1

,

PB2

Pos.	38	obs:	exp:
cgt	R	0	84.41
cgc	R	0	141.30
cga	R	0	268.80
cgg	R	6	193.20
aaa	K	0	1.82
aag	K	3	1.18
aga	R	628	1226.00
agg	R	2040	760.00
---	--	------	------
mPD		0.37	0.94
		nPD:	0.39
		N. weight:	1.1
		Sc. PD:	0.085
		Sc. rank:	444.3

,

PB2

Pos.	39	obs:	exp:
tta	L	0	0.22
ttg	L	0	0.38
ctt	L	1	0.40
ctc	L	0	0.29
cta	L	0	0.35
ctg	L	1	0.36
caa	Q	1554	1443.00
cag	Q	1121	1232.00
---	--	------	------
mPD		0.49	0.50
		nPD:	0.98
		N. weight:	0.011
		Sc. PD:	0.0059
		Sc. rank:	13.9

,

PB2

Pos.	40	obs:	exp:
gaa	E	209	1586.00
gag	E	2468	1091.00
---	--	------	------
mPD		0.14	0.48
		nPD:	0.3
		N. weight:	1.1
		Sc. PD:	0.0083
		Sc. rank:	149.3

PB2			
Pos.	41	obs:	exp:
cgt	R	0	0.03
cgc	R	0	0.05
cga	R	0	0.10
cgg	R	0	0.07
aaa	K	417	1626.00
aag	K	2259	1050.00
aga	R	0	0.46
agg	R	1	0.28
---	--	------	------
mPD		0.26	0.48
		nPD:	0.55
		N. weight:	0.81
		Sc. PD:	0.16
		Sc. rank:	702.9

PB2			
Pos.	42	obs:	exp:
tct	S	0	0.17
tcc	S	0	0.16
tca	S	0	0.24
tcg	S	0	0.05
aat	N	74	1521.00
aac	N	2602	1155.00
agt	S	0	0.19
agc	S	1	0.19
---	--	------	------
mPD		0.055	0.49
		nPD:	0.11
		N. weight:	1.3
		Sc. PD:	-0.18
		Sc. rank:	-655.3

PB2			
Pos.	43	obs:	exp:
cct	P	214	790.00
ccc	P	2379	539.00
cca	P	82	1060.00
ccg	P	2	288.00
---	--	------	------
mPD		0.20	0.70
		nPD:	0.29
		N. weight:	2.1
		Sc. PD:	-0.00011
		Sc. rank:	255.6

PB2			
Pos.	44	obs:	exp:
tct	S	26	18.50
tcc	S	2	17.38
tca	S	81	26.71
tcg	S	1	5.85
act	T	0	0.25
acc	T	0	0.22
aca	T	1	0.45
acg	T	0	0.08
agt	S	0	20.47
agc	S	0	21.08
gct	A	368	629.00
gcc	A	24	587.90
gca	A	1960	1157.00
gcg	A	214	192.40
---	--	------	------
mPD		0.47	0.79
		nPD:	0.59
		N. weight:	0.62
		Sc. PD:	0.14
		Sc. rank:	592.1

PB2			
Pos.	45	obs:	exp:
tta	L	0	291.20
ttg	L	21	502.60
ctt	L	10	540.40
ctc	L	2626	389.30
cta	L	17	467.30
ctg	L	2	485.10
att	I	0	0.35
atc	I	1	0.26
ata	I	0	0.39
---	--	------	------
mPD		0.053	1.1
		nPD:	0.05
		N. weight:	3.4
		Sc. PD:	-0.62
		Sc. rank:	-2976.2

PB2			
Pos.	46	obs:	exp:
---	-	2677	2677.00
---	--	------	------
mPD		0	0
		nPD:	1.
		N. weight:	0.
		Sc. PD:	0
		Sc. rank:	0

PB2			
Pos.	47	obs:	exp:
---	-	2677	2677.00
---	--	------	------
mPD		0	0
		nPD:	1.
		N. weight:	0.
		Sc. PD:	0
		Sc. rank:	0

PB2			
Pos.	48	obs:	exp:
---	-	2677	2677.00
---	--	------	------
mPD		0	0
		nPD:	1.
		N. weight:	0.
		Sc. PD:	0
		Sc. rank:	0

PB2			
Pos.	49	obs:	exp:
---	-	2677	2677.00
---	--	------	------
mPD		0	0
		nPD:	1.
		N. weight:	0.
		Sc. PD:	0
		Sc. rank:	0

PB2			
Pos.	50	obs:	exp:
---	-	2677	2677.00
---	--	------	------
mPD		0	0
		nPD:	1.
		N. weight:	0.
		Sc. PD:	0
		Sc. rank:	0

PB2			
Pos.	51	obs:	exp:
cgt	R	0	84.50
cgc	R	0	141.50
cga	R	3	269.10
cgg	R	0	193.40
aga	R	2612	1228.00
agg	R	62	760.90
---	--	------	------
mPD		0.048	0.94
		nPD:	0.05
		N. weight:	1.3
		Sc. PD:	-0.23
		Sc. rank:	-1066.9

PB2			
Pos.	52	obs:	exp:
att	I	0	0.71
atc	I	0	0.51
ata	I	2	0.78
atg	M	2675	2675.00
---	--	------	------
mPD		0.0015	0.0015
		nPD:	1.
		N. weight:	0.0013
		Sc. PD:	0.00071
		Sc. rank:	1.6

PB2			
Pos.	53	obs:	exp:
cgt	R	0	0.03
cgc	R	0	0.05
cga	R	0	0.10
cgg	R	0	0.07
aaa	K	628	1626.00
aag	K	2048	1050.00
aga	R	0	0.46
agg	R	1	0.28
---	--	------	------
mPD		0.36	0.48
		nPD:	0.75
		N. weight:	0.54
		Sc. PD:	0.19
		Sc. rank:	635.4

PB2			
Pos.	54	obs:	exp:
tgg	W	2677	2677.00
---	--	------	------
mPD		0	0
		nPD:	1.
		N. weight:	0.
		Sc. PD:	0
		Sc. rank:	0

PB2			
Pos.	55	obs:	exp:
att	I	1	2.13
atc	I	0	1.54
ata	I	5	2.33
atg	M	2668	2668.00
aaa	K	0	0.61
aag	K	1	0.39
gtt	V	0	0.44
gtc	V	0	0.38
gta	V	0	0.50
gtg	V	2	0.67
---	--	------	------
mPD		0.0067	0.0082
		nPD:	0.82
		N. weight:	0.0049
		Sc. PD:	0.002
		Sc. rank:	5.9

PB2			
Pos.	56	obs:	exp:
atg	M	2677	2677.00
---	--	------	------
mPD		0	0
		nPD:	1.
		N. weight:	0.
		Sc. PD:	0
		Sc. rank:	0

PB2			
Pos.	57	obs:	exp:
caa	Q	1	0.54
cag	Q	0	0.46
gct	A	2	656.00
gcc	A	0	613.10
gca	A	2646	1206.00
gcg	A	28	200.70
---	--	------	------
mPD		0.024	0.68
		nPD:	0.03
		N. weight:	1.4
		Sc. PD:	-0.27
		Sc. rank:	-1384.9

PB2			
Pos.	58	obs:	exp:
tct	S	0	0.17
tcc	S	0	0.16
tca	S	0	0.24
tcg	S	1	0.05
atg	M	2676	2676.00
agt	S	0	0.19
agc	S	0	0.19
---	--	------	------
mPD		0.0015	0.0019
		nPD:	0.78
		N. weight:	0.002
		Sc. PD:	0.00076
		Sc. rank:	2.4

PB2			
Pos.	59	obs:	exp:
cgt	R	0	19.85
cgc	R	0	33.25
cga	R	0	63.22
cgg	R	0	45.44
aaa	K	1808	1245.00
aag	K	240	803.40
aga	R	617	288.50
agg	R	12	178.80
---	--	------	------
mPD		0.53	0.97
		nPD:	0.55
		N. weight:	0.57
		Sc. PD:	0.11
		Sc. rank:	493.8

PB2			
Pos.	60	obs:	exp:
ttt	F	0	1.34
ttc	F	3	1.66
tat	Y	455	1496.00
tac	Y	2215	1174.00
cat	H	0	2.31
cac	H	4	1.69
---	--	------	------
mPD		0.29	0.50
		nPD:	0.58
		N. weight:	0.6
		Sc. PD:	0.13
		Sc. rank:	556.6

PB2			
Pos.	61	obs:	exp:
tct	S	0	0.67
tcc	S	0	0.63
tca	S	4	0.97
tcg	S	0	0.21
cct	P	2	788.50
ccc	P	0	538.00
cca	P	2427	1058.00
ccg	P	243	287.40
agt	S	0	0.74
agc	S	0	0.77
gaa	E	1	0.59
gag	E	0	0.41
---	--	------	------
mPD		0.17	0.71
		nPD:	0.24
		N. weight:	1.4
		Sc. PD:	-0.05
		Sc. rank:	-19.9

PB2			
Pos.	62	obs:	exp:
att	I	2417	945.70
atc	I	244	683.80
ata	I	5	1036.00
gtt	V	10	2.22
gtc	V	0	1.89
gta	V	0	2.52
gtg	V	0	3.37
ggt	G	0	0.16
ggc	G	1	0.13
gga	G	0	0.45
ggg	G	0	0.26
---	--	------	------
mPD		0.18	0.67
		nPD:	0.27
		N. weight:	1.4
		Sc. PD:	-0.022
		Sc. rank:	56.8

PB2			
Pos.	63	obs:	exp:
act	T	23	656.80
acc	T	11	577.40
aca	T	2586	1213.00
acg	T	50	222.60
aaa	K	2	1.22
aag	K	0	0.78
gct	A	0	1.23
gcc	A	0	1.15
gca	A	5	2.25
gcg	A	0	0.37
---	--	------	------
mPD		0.067	0.68
		nPD:	0.1
		N. weight:	1.2
		Sc. PD:	-0.18
		Sc. rank:	-702.4

PB2			
Pos.	64	obs:	exp:
gct	A	8	656.00
gcc	A	1	613.10
gca	A	2219	1206.00
gcg	A	448	200.70
gaa	E	1	0.59
gag	E	0	0.41
---	--	------	------
mPD		0.29	0.68
		nPD:	0.42
		N. weight:	1.2
		Sc. PD:	0.12
		Sc. rank:	601.3

PB2			
Pos.	65	obs:	exp:
aat	N	0	7.96
aac	N	14	6.04
aaa	K	2	1.22
aag	K	0	0.78
gat	D	156	1484.00
gac	D	2483	1155.00
gaa	E	19	13.04
gag	E	3	8.96
---	--	------	------
mPD		0.14	0.51
		nPD:	0.27
		N. weight:	1.1
		Sc. PD:	-0.016
		Sc. rank:	53.7

PB2			
Pos.	66	obs:	exp:
cgt	R	0	39.42
cgc	R	0	66.02
cga	R	0	125.50
cgg	R	0	90.23
atg	M	5	5.00
act	T	0	0.98
acc	T	0	0.86
aca	T	0	1.82
acg	T	4	0.33
aaa	K	86	862.40
aag	K	1333	556.60
aga	R	48	572.80
agg	R	1201	355.00
---	--	------	------
mPD		0.60	1.2
		nPD:	0.49
		N. weight:	1.6
		Sc. PD:	0.24
		Sc. rank:	1162.3

,

PB2			
Pos.	67	obs:	exp:
---	-	2677	2677.00
---	--	------	------
mPD		0	0
		nPD:	1.
		N. weight:	0.
		Sc. PD:	0
		Sc. rank:	0

,

PB2			
Pos.	68	obs:	exp:
---	-	2677	2677.00
---	--	------	------
mPD		0	0
		nPD:	1.
		N. weight:	0.
		Sc. PD:	0
		Sc. rank:	0

,

PB2			
Pos.	69	obs:	exp:
---	-	2677	2677.00
---	--	------	------
mPD		0	0
		nPD:	1.
		N. weight:	0.
		Sc. PD:	0
		Sc. rank:	0

,

PB2			
Pos.	70	obs:	exp:
---	-	2677	2677.00
---	--	------	------
mPD		0	0
		nPD:	1.
		N. weight:	0.
		Sc. PD:	0
		Sc. rank:	0

,

PB2			
Pos.	71	obs:	exp:
---	-	2677	2677.00
---	--	------	------
mPD		0	0
		nPD:	1.
		N. weight:	0.
		Sc. PD:	0
		Sc. rank:	0

,

PB2			
Pos.	72	obs:	exp:
---	-	2677	2677.00
---	--	------	------
mPD		0	0
		nPD:	1.
		N. weight:	0.
		Sc. PD:	0
		Sc. rank:	0

,

PB2			
Pos.	73	obs:	exp:
---	-	2677	2677.00
---	--	------	------
mPD		0	0
		nPD:	1.
		N. weight:	0.
		Sc. PD:	0
		Sc. rank:	0

,

PB2			
Pos.	74	obs:	exp:
---	-	2677	2677.00
---	--	------	------
mPD		0	0
		nPD:	1.
		N. weight:	0.
		Sc. PD:	0
		Sc. rank:	0

,

PB2			
Pos.	75	obs:	exp:
---	-	2677	2677.00
---	--	------	------
mPD		0	0
		nPD:	1.
		N. weight:	0.
		Sc. PD:	0
		Sc. rank:	0

,

PB2			
Pos.	76	obs:	exp:
cgt	R	0	80.96
cgc	R	0	135.60
cga	R	0	257.80
cgg	R	0	185.30
aaa	K	88	62.60
aag	K	15	40.40
aga	R	2229	1176.00
agg	R	336	729.00
ggt	G	0	1.41
ggc	G	0	1.14
gga	G	3	4.08
ggg	G	6	2.37
---	--	------	------
mPD		0.31	1.0
		nPD:	0.31
		N. weight:	0.82
		Sc. PD:	0.014
		Sc. rank:	166.9

,

PB2			
Pos.	77	obs:	exp:
att	I	4	941.80
atc	I	5	681.00
ata	I	2646	1032.00
gtt	V	2	4.88
gtc	V	0	4.15
gta	V	20	5.55
gtg	V	0	7.41
---	--	------	------
mPD		0.025	0.68
		nPD:	0.04
		N. weight:	1.7
		Sc. PD:	-0.33
		Sc. rank:	-1680.3

,

PB2			
Pos.	78	obs:	exp:
tta	L	0	0.33
ttg	L	1	0.56
ctt	L	0	0.61
ctc	L	0	0.44
cta	L	0	0.52
ctg	L	2	0.54
cgt	R	0	0.03
cgc	R	0	0.05
cga	R	0	0.10
cgg	R	0	0.07
att	I	23	35.12
atc	I	2	25.39
ata	I	74	38.49
atg	M	2553	2553.00
act	T	2	2.71
acc	T	0	2.38
aca	T	2	5.00
acg	T	7	0.92
aga	R	0	0.46
agg	R	1	0.28
gtt	V	0	2.22
gtc	V	0	1.89
gta	V	0	2.52
gtg	V	10	3.37
---	--	------	------
mPD		0.093	0.10
		nPD:	0.89
		N. weight:	0.047
		Sc. PD:	0.022
		Sc. rank:	57.2

,

PB2			
Pos.	79	obs:	exp:
tct	S	0	0.17
tcc	S	0	0.16
tca	S	0	0.24
tcg	S	0	0.05
aat	N	0	32.97
aac	N	58	25.03
aaa	K	1	0.61
aag	K	0	0.39
agt	S	0	0.19
agc	S	1	0.19
gat	D	21	1196.00
gac	D	2106	930.60
gaa	E	160	277.90
gag	E	309	191.10
ggt	G	0	3.29
ggc	G	18	2.66
gga	G	1	9.51
ggg	G	2	5.53
---	--	------	------
mPD		0.38	0.70
		nPD:	0.54
		N. weight:	1.2
		Sc. PD:	0.24
		Sc. rank:	1039.4

,

PB2			
Pos.	80	obs:	exp:
tta	L	1	0.76
ttg	L	0	1.31
ctt	L	0	1.41
ctc	L	0	1.02
cta	L	0	1.22
ctg	L	6	1.27
att	I	24	30.86
atc	I	3	22.31
ata	I	60	33.82
atg	M	2539	2539.00
act	T	0	6.64
acc	T	0	5.84
aca	T	20	12.27
acg	T	7	2.25
aaa	K	0	0.61
aag	K	1	0.39
gtt	V	0	3.33
gtc	V	0	2.83
gta	V	0	3.79
gtg	V	15	5.05
gct	A	0	0.25
gcc	A	0	0.23
gca	A	0	0.45
gcg	A	1	0.07
---	--	------	------
mPD		0.12	0.13
		nPD:	0.89
		N. weight:	0.053
		Sc. PD:	0.024
		Sc. rank:	64.4

,

PB2			
Pos.	81	obs:	exp:
tta	L	0	0.22
ttg	L	0	0.38
ctt	L	1	0.40
ctc	L	1	0.29
cta	L	0	0.35
ctg	L	0	0.36
cat	H	1	0.58
cac	H	0	0.42
att	I	2304	923.40
atc	I	286	667.60
ata	I	13	1012.00
aat	N	1	0.57
aac	N	0	0.43
gtt	V	59	15.54
gtc	V	11	13.21
gta	V	0	17.67
gtg	V	0	23.58
---	--	------	------
mPD		0.26	0.72
		nPD:	0.36
		N. weight:	1.3
		Sc. PD:	0.076
		Sc. rank:	455.9

,

PB2			
Pos.	82	obs:	exp:
cct	P	353	789.10
ccc	P	22	538.40
cca	P	2276	1059.00
ccg	P	23	287.60
caa	Q	1	0.54
cag	Q	0	0.46
aaa	K	1	0.61
aag	K	0	0.39
gaa	E	1	0.59
gag	E	0	0.41
---	--	------	------
mPD		0.26	0.71
		nPD:	0.37
		N. weight:	0.93
		Sc. PD:	0.058
		Sc. rank:	342.3

,

PB2			
Pos.	83	obs:	exp:
---	-	2677	2677.00
---	--	------	------
mPD		0	0
		nPD:	1.
		N. weight:	0.
		Sc. PD:	0
		Sc. rank:	0

,

PB2			
Pos.	84	obs:	exp:
---	-	2677	2677.00
---	--	------	------
mPD		0	0
		nPD:	1.
		N. weight:	0.
		Sc. PD:	0
		Sc. rank:	0

,

PB2			
Pos.	85	obs:	exp:
---	-	2677	2677.00
---	--	------	------
mPD		0	0
		nPD:	1.
		N. weight:	0.
		Sc. PD:	0
		Sc. rank:	0

,

PB2			
Pos.	86	obs:	exp:
tta	L	0	0.11
ttg	L	0	0.19
ctt	L	0	0.20
ctc	L	0	0.15
cta	L	0	0.17
ctg	L	1	0.18
caa	Q	1	0.54
cag	Q	0	0.46
gtt	V	0	0.44
gtc	V	0	0.38
gta	V	1	0.50
gtg	V	1	0.67
gaa	E	679	1583.00
gag	E	1993	1089.00
ggt	G	0	0.16
ggc	G	0	0.13
gga	G	0	0.45
ggg	G	1	0.26
---	--	------	------
mPD		0.38	0.49
		nPD:	0.79
		N. weight:	0.44
		Sc. PD:	0.17
		Sc. rank:	528.0

,

PB2			
Pos.	87	obs:	exp:
tct	S	0	0.50
tcc	S	0	0.47
tca	S	0	0.73
tcg	S	0	0.16
cgt	R	0	84.28
cgc	R	0	141.10
cga	R	2	268.40
cgg	R	2	192.90
aaa	K	2	2.43
aag	K	2	1.57
agt	S	0	0.56
agc	S	3	0.57
aga	R	451	1224.00
agg	R	2215	758.90
---	--	------	------
mPD		0.29	0.94
		nPD:	0.31
		N. weight:	1.3
		Sc. PD:	0.019
		Sc. rank:	236.3

,

PB2			
Pos.	88	obs:	exp:
tat	Y	1	0.56
tac	Y	0	0.44
act	T	0	0.25
acc	T	1	0.22
aca	T	0	0.45
acg	T	0	0.08
aat	N	2385	1520.00
aac	N	290	1155.00
---	--	------	------
mPD		0.20	0.49
		nPD:	0.4
		N. weight:	0.47
		Sc. PD:	0.039
		Sc. rank:	211.8

,

PB2			
Pos.	89	obs:	exp:
caa	Q	2	1.08
cag	Q	0	0.92
aaa	K	3	1.82
aag	K	0	1.18
gct	A	0	0.25
gcc	A	0	0.23
gca	A	1	0.45
gcg	A	0	0.07
gat	D	0	0.56
gac	D	1	0.44
gaa	E	2599	1581.00
gag	E	70	1088.00
ggt	G	0	0.16
ggc	G	1	0.13
gga	G	0	0.45
ggg	G	0	0.26
---	--	------	------
mPD		0.058	0.49
		nPD:	0.12
		N. weight:	0.77
		Sc. PD:	-0.1
		Sc. rank:	-344.6

,

PB2			
Pos.	90	obs:	exp:
tta	L	0	0.11
ttg	L	0	0.19
ctt	L	0	0.20
ctc	L	0	0.15
cta	L	1	0.17
ctg	L	0	0.18
cct	P	0	0.30
ccc	P	0	0.20
cca	P	1	0.40
ccg	P	0	0.11
cat	H	3	4.61
cac	H	5	3.39
caa	Q	2591	1432.00
cag	Q	64	1223.00
aaa	K	10	6.69
aag	K	1	4.31
gaa	E	1	0.59
gag	E	0	0.41
---	--	------	------
mPD		0.064	0.51
		nPD:	0.12
		N. weight:	0.94
		Sc. PD:	-0.12
		Sc. rank:	-393.5

,

PB2			
Pos.	91	obs:	exp:
cgt	R	0	0.25
cgc	R	0	0.42
cga	R	0	0.80
cgg	R	0	0.58
aga	R	8	3.67
agg	R	0	2.27
gct	A	0	0.25
gcc	A	0	0.23
gca	A	1	0.45
gcg	A	0	0.07
gaa	E	1	0.59
gag	E	0	0.41
ggt	G	1	418.30
ggc	G	4	337.90
gga	G	2321	1208.00
ggg	G	341	702.70
---	--	------	------
mPD		0.23	0.69
		nPD:	0.34
		N. weight:	0.87
		Sc. PD:	0.032
		Sc. rank:	250.0

,

PB2			
Pos.	92	obs:	exp:
cat	H	0	0.58
cac	H	1	0.42
caa	Q	2622	1443.00
cag	Q	54	1233.00
---	--	------	------
mPD		0.040	0.50
		nPD:	0.08
		N. weight:	0.97
		Sc. PD:	-0.15
		Sc. rank:	-630.6

,

PB2			
Pos.	93	obs:	exp:
tct	S	0	0.34
tcc	S	2	0.32
tca	S	0	0.49
tcg	S	0	0.11
cct	P	0	0.30
ccc	P	1	0.20
cca	P	0	0.40
ccg	P	0	0.11
att	I	6	102.50
atc	I	23	74.13
ata	I	260	112.40
atg	M	4	4.00
act	T	211	565.80
acc	T	1962	497.40
aca	T	125	1045.00
acg	T	2	191.80
aat	N	0	0.57
aac	N	1	0.43
aaa	K	2	1.22
aag	K	0	0.78
agt	S	0	0.37
agc	S	0	0.38
gtt	V	0	4.66
gtc	V	1	3.96
gta	V	19	5.30
gtg	V	1	7.07
gct	A	8	13.97
gcc	A	48	13.06
gca	A	1	25.69
gcg	A	0	4.27
---	--	------	------
mPD		0.66	0.95
		nPD:	0.69
		N. weight:	1.7
		Sc. PD:	0.53
		Sc. rank:	1914.4

,

PB2			
Pos.	94	obs:	exp:
ttt	F	0	0.45
ttc	F	1	0.55
tta	L	12	291.20
ttg	L	0	502.60
ctt	L	435	540.40
ctc	L	2144	389.30
cta	L	85	467.30
ctg	L	0	485.10
---	--	------	------
mPD		0.34	1.1
		nPD:	0.3
		N. weight:	2.3
		Sc. PD:	0.018
		Sc. rank:	331.5

,

PB2			
Pos.	95	obs:	exp:
tta	L	0	0.33
ttg	L	3	0.56
tgg	W	2674	2674.00
ctt	L	0	0.61
ctc	L	0	0.44
cta	L	0	0.52
ctg	L	0	0.54
---	--	------	------
mPD		0.0022	0.0052
		nPD:	0.43
		N. weight:	0.0035
		Sc. PD:	0.00037
		Sc. rank:	2.0

,

PB2			
Pos.	96	obs:	exp:
tct	S	0	447.90
tcc	S	0	420.90
tca	S	0	646.70
tcg	S	0	141.50
tgt	C	0	0.43
tgc	C	1	0.57
att	I	0	0.35
atc	I	1	0.26
ata	I	0	0.39
aat	N	2	3.98
aac	N	5	3.02
agt	S	397	495.60
agc	S	2266	510.40
ggt	G	0	0.78
ggc	G	5	0.63
gga	G	0	2.26
ggg	G	0	1.32
---	--	------	------
mPD		0.26	1.6
		nPD:	0.16
		N. weight:	2.3
		Sc. PD:	-0.22
		Sc. rank:	-604.2

,

PB2			
Pos.	97	obs:	exp:
cgt	R	0	32.16
cgc	R	0	53.86
cga	R	0	102.40
cgg	R	0	73.62
act	T	0	1.23
acc	T	0	1.08
aca	T	5	2.27
acg	T	0	0.42
aaa	K	1261	1005.00
aag	K	392	648.40
aga	R	910	467.30
agg	R	109	289.60
---	--	------	------
mPD		0.78	1.2
		nPD:	0.67
		N. weight:	0.42
		Sc. PD:	0.12
		Sc. rank:	452.7

,

PB2			
Pos.	98	obs:	exp:
att	I	0	5.32
atc	I	0	3.85
ata	I	15	5.83
atg	M	13	13.00
act	T	1	648.60
acc	T	1	570.20
aca	T	2603	1198.00
acg	T	32	219.90
aaa	K	1	0.61
aag	K	0	0.39
gtt	V	0	0.22
gtc	V	0	0.19
gta	V	0	0.25
gtg	V	1	0.34
gct	A	0	2.45
gcc	A	0	2.29
gca	A	10	4.51
gcg	A	0	0.75
---	--	------	------
mPD		0.066	0.71
		nPD:	0.09
		N. weight:	1.4
		Sc. PD:	-0.21
		Sc. rank:	-813.3

,

PB2			
Pos.	99	obs:	exp:
tct	S	0	18.17
tcc	S	0	17.07
tca	S	0	26.23
tcg	S	0	5.74
cat	H	1	0.58
cac	H	0	0.42
att	I	0	0.35
atc	I	1	0.26
ata	I	0	0.39
act	T	4	36.90
acc	T	146	32.44
aca	T	0	68.16
acg	T	0	12.51
aat	N	432	1364.00
aac	N	1967	1035.00
aaa	K	8	4.86
aag	K	0	3.14
agt	S	72	20.10
agc	S	36	20.70
gct	A	0	2.45
gcc	A	9	2.29
gca	A	1	4.51
gcg	A	0	0.75
---	--	------	------
mPD		0.51	0.78
		nPD:	0.65
		N. weight:	0.77
		Sc. PD:	0.22
		Sc. rank:	818.4

,

PB2			
Pos.	100	obs:	exp:
gat	D	2564	1505.00
gac	D	112	1171.00
gaa	E	0	0.59
gag	E	1	0.41
---	--	------	------
mPD		0.081	0.49
		nPD:	0.16
		N. weight:	0.77
		Sc. PD:	-0.073
		Sc. rank:	-186.0

,

PB2			
Pos.	101	obs:	exp:
tct	S	1	0.17
tcc	S	0	0.16
tca	S	0	0.24
tcg	S	0	0.05
agt	S	0	0.19
agc	S	0	0.19
gtt	V	3	0.67
gtc	V	0	0.57
gta	V	0	0.76
gtg	V	0	1.01
gct	A	2493	655.20
gcc	A	112	612.40
gca	A	68	1205.00
gcg	A	0	200.50
---	--	------	------
mPD		0.13	0.68
		nPD:	0.19
		N. weight:	2.
		Sc. PD:	-0.15
		Sc. rank:	-280.2

,

PB2			
Pos.	102	obs:	exp:
ggt	G	4	419.90
ggc	G	13	339.20
gga	G	2567	1213.00
ggg	G	93	705.40
---	--	------	------
mPD		0.079	0.68
		nPD:	0.12
		N. weight:	1.2
		Sc. PD:	-0.15
		Sc. rank:	-549.6

,

PB2			
Pos.	103	obs:	exp:
tct	S	7	449.40
tcc	S	1	422.30
tca	S	1269	648.90
tcg	S	1395	142.00
tat	Y	1	0.56
tac	Y	0	0.44
act	T	0	0.98
acc	T	0	0.86
aca	T	1	1.82
acg	T	3	0.33
agt	S	0	497.30
agc	S	0	512.10
---	--	------	------
mPD		0.51	1.6
		nPD:	0.31
		N. weight:	2.8
		Sc. PD:	0.047
		Sc. rank:	556.7

,

PB2			
Pos.	104	obs:	exp:
aat	N	1	13.64
aac	N	23	10.36
gat	D	463	1492.00
gac	D	2189	1160.00
ggt	G	0	0.16
ggc	G	1	0.13
gga	G	0	0.45
ggg	G	0	0.26
---	--	------	------
mPD		0.31	0.51
		nPD:	0.6
		N. weight:	0.6
		Sc. PD:	0.14
		Sc. rank:	582.3

,

PB2			
Pos.	105	obs:	exp:
cgt	R	1064	84.47
cgc	R	440	141.40
cga	R	706	269.00
cgg	R	44	193.30
aaa	K	0	0.61
aag	K	1	0.39
aga	R	54	1227.00
agg	R	368	760.60
---	--	------	------
mPD		0.98	0.94
		nPD:	1.04
		N. weight:	2.3
		Sc. PD:	1.4
		Sc. rank:	2932.3

,

PB2			
Pos.	106	obs:	exp:
tta	L	0	0.22
ttg	L	0	0.38
ctt	L	0	0.40
ctc	L	0	0.29
cta	L	2	0.35
ctg	L	0	0.36
att	I	0	2.48
atc	I	0	1.80
ata	I	7	2.72
atg	M	8	8.00
gtt	V	18	590.60
gtc	V	4	501.90
gta	V	810	671.50
gtg	V	1828	896.00
---	--	------	------
mPD		0.45	0.75
		nPD:	0.6
		N. weight:	0.96
		Sc. PD:	0.23
		Sc. rank:	933.2

,

PB2			
Pos.	107	obs:	exp:
tta	L	0	0.33
ttg	L	2	0.56
ctt	L	0	0.61
ctc	L	0	0.44
cta	L	0	0.52
ctg	L	1	0.54
att	I	2	1.77
atc	I	0	1.28
ata	I	3	1.94
atg	M	2658	2658.00
act	T	0	0.49
acc	T	0	0.43
aca	T	0	0.91
acg	T	2	0.17
gtt	V	0	2.00
gtc	V	0	1.70
gta	V	0	2.27
gtg	V	9	3.03
---	--	------	------
mPD		0.014	0.021
		nPD:	0.66
		N. weight:	0.015
		Sc. PD:	0.0042
		Sc. rank:	15.9

,

PB2			
Pos.	108	obs:	exp:
att	I	0	4.97
atc	I	0	3.59
ata	I	14	5.44
gtt	V	12	591.10
gtc	V	41	502.30
gta	V	2227	672.00
gtg	V	382	896.60
gct	A	0	0.25
gcc	A	0	0.23
gca	A	1	0.45
gcg	A	0	0.07
---	--	------	------
mPD		0.29	0.75
		nPD:	0.39
		N. weight:	1.5
		Sc. PD:	0.11
		Sc. rank:	618.1

,

PB2			
Pos.	109	obs:	exp:
tct	S	0	450.10
tcc	S	5	422.90
tca	S	2654	649.80
tcg	S	17	142.20
caa	Q	1	0.54
cag	Q	0	0.46
agt	S	0	498.10
agc	S	0	512.80
---	--	------	------
mPD		0.018	1.6
		nPD:	0.01
		N. weight:	2.5
		Sc. PD:	-0.54
		Sc. rank:	-2899.1

,

PB2			
Pos.	110	obs:	exp:
ttt	F	0	0.45
ttc	F	1	0.55
tta	L	0	0.11
ttg	L	0	0.19
tct	S	0	0.50
tcc	S	3	0.47
tca	S	0	0.73
tcg	S	0	0.16
ctt	L	0	0.20
ctc	L	1	0.15
cta	L	0	0.17
ctg	L	0	0.18
cct	P	1186	784.40
ccc	P	1450	535.10
cca	P	22	1053.00
ccg	P	0	285.90
act	T	0	2.95
acc	T	12	2.59
aca	T	0	5.45
acg	T	0	1.00
agt	S	0	0.56
agc	S	0	0.57
gct	A	0	0.49
gcc	A	2	0.46
gca	A	0	0.90
gcg	A	0	0.15
---	--	------	------
mPD		0.52	0.72
		nPD:	0.72
		N. weight:	1.3
		Sc. PD:	0.43
		Sc. rank:	1495.6

,

PB2			
Pos.	111	obs:	exp:
tta	L	58	290.00
ttg	L	51	500.60
ctt	L	12	538.20
ctc	L	1	387.70
cta	L	351	465.30
ctg	L	2192	483.10
att	I	0	3.19
atc	I	0	2.31
ata	I	9	3.50
atg	M	2	2.00
gtt	V	0	0.22
gtc	V	0	0.19
gta	V	1	0.25
gtg	V	0	0.34
---	--	------	------
mPD		0.36	1.1
		nPD:	0.31
		N. weight:	2.1
		Sc. PD:	0.04
		Sc. rank:	481.6

,

PB2			
Pos.	112	obs:	exp:
gct	A	115	656.20
gcc	A	2478	613.40
gca	A	83	1207.00
gcg	A	1	200.80
---	--	------	------
mPD		0.14	0.68
		nPD:	0.21
		N. weight:	2.1
		Sc. PD:	-0.13
		Sc. rank:	-230.0

,

PB2			
Pos.	113	obs:	exp:
att	I	0	15.61
atc	I	0	11.29
ata	I	44	17.11
gtt	V	6	584.60
gtc	V	16	496.80
gta	V	1849	664.70
gtg	V	762	886.90
---	--	------	------
mPD		0.45	0.77
		nPD:	0.59
		N. weight:	1.2
		Sc. PD:	0.27
		Sc. rank:	1140.4

,

PB2			
Pos.	114	obs:	exp:
tct	S	0	0.17
tcc	S	0	0.16
tca	S	1	0.24
tcg	S	0	0.05
act	T	331	658.20
acc	T	75	578.70
aca	T	2245	1216.00
acg	T	25	223.10
agt	S	0	0.19
agc	S	0	0.19
---	--	------	------
mPD		0.28	0.68
		nPD:	0.41
		N. weight:	0.66
		Sc. PD:	0.063
		Sc. rank:	324.6

,

PB2			
Pos.	115	obs:	exp:
tgg	W	2677	2677.00
---	--	------	------
mPD		0	0
		nPD:	1.
		N. weight:	0.
		Sc. PD:	0
		Sc. rank:	0

,

PB2

Pos.	116	obs:	exp:
tgg	W	2677	2677.00
---	--	------	------
mPD		0	0
		nPD:	1.
		N. weight:	0.
		Sc. PD:	0
		Sc. rank:	0

,

PB2

Pos.	117	obs:	exp:
aat	N	2573	1522.00
aac	N	104	1155.00
---	--	------	------
mPD		0.075	0.49
		nPD:	0.15
		N. weight:	0.76
		Sc. PD:	-0.08
		Sc. rank:	-235.7

,

PB2

Pos.	118	obs:	exp:
cgt	R	0	84.50
cgc	R	0	141.50
cga	R	0	269.10
cgg	R	1	193.40
aga	R	424	1228.00
agg	R	2252	760.90
---	--	------	------
mPD		0.27	0.94
		nPD:	0.28
		N. weight:	1.4
		Sc. PD:	-0.0037
		Sc. rank:	160.0

,

PB2

Pos.	119	obs:	exp:
tct	S	0	12.95
tcc	S	0	12.17
tca	S	0	18.70
tcg	S	0	4.09
tgt	C	2	0.86
tgc	C	0	1.14
aat	N	2407	1457.00
aac	N	156	1106.00
aaa	K	29	20.06
aag	K	4	12.94
agt	S	76	14.33
agc	S	1	14.76
gat	D	1	0.56
gac	D	0	0.44
gaa	E	1	0.59
gag	E	0	0.41
---	--	------	------
mPD		0.19	0.61
		nPD:	0.32
		N. weight:	0.72
		Sc. PD:	0.017
		Sc. rank:	172.8

,

PB2

Pos.	120	obs:	exp:
ggt	G	180	419.90
ggc	G	2023	339.20
gga	G	436	1213.00
ggg	G	38	705.40
---	--	------	------
mPD		0.40	0.68
		nPD:	0.58
		N. weight:	2.
		Sc. PD:	0.45
		Sc. rank:	1877.0

,

PB2

Pos.	121	obs:	exp:
tta	L	0	0.22
ttg	L	0	0.38
ctt	L	0	0.40
ctc	L	0	0.29
cta	L	2	0.35
ctg	L	0	0.36
cct	P	87	789.10
ccc	P	4	538.40
cca	P	2272	1059.00
ccg	P	311	287.60
caa	Q	1	0.54
cag	Q	0	0.46
---	--	------	------
mPD		0.27	0.71
		nPD:	0.38
		N. weight:	1.1
		Sc. PD:	0.072
		Sc. rank:	406.2

,

PB2

Pos.	122	obs:	exp:
tct	S	0	0.34
tcc	S	0	0.32
tca	S	2	0.49
tcg	S	0	0.11
cgt	R	0	0.06
cgc	R	0	0.11
cga	R	0	0.20
cgg	R	0	0.14
att	I	1	3.19
atc	I	0	2.31
ata	I	8	3.50
act	T	0	645.40
acc	T	3	567.40
aca	T	2580	1192.00
acg	T	41	218.80
aaa	K	10	6.08
aag	K	0	3.92
agt	S	0	0.37
agc	S	0	0.38
aga	R	2	0.92
agg	R	0	0.57
gtt	V	0	0.44
gtc	V	0	0.38
gta	V	1	0.50
gtg	V	1	0.67
gct	A	0	6.86
gcc	A	0	6.42
gca	A	28	12.62
gcg	A	0	2.10
---	--	------	------
mPD		0.075	0.72
		nPD:	0.1
		N. weight:	1.4
		Sc. PD:	-0.19
		Sc. rank:	-708.0

,

PB2

Pos.	123	obs:	exp:
tct	S	1	1.68
tcc	S	0	1.58
tca	S	9	2.43
tcg	S	0	0.53
act	T	14	623.10
acc	T	0	547.70
aca	T	2464	1151.00
acg	T	55	211.20
agt	S	0	1.86
agc	S	0	1.92
gct	A	0	32.85
gcc	A	0	30.70
gca	A	133	60.40
gcg	A	1	10.05
---	--	------	------
mPD		0.15	0.78
		nPD:	0.2
		N. weight:	1.3
		Sc. PD:	-0.091
		Sc. rank:	-162.2

,

PB2

Pos.	124	obs:	exp:
tct	S	0	369.70
tcc	S	0	347.40
tca	S	0	533.80
tcg	S	0	116.80
cgt	R	0	0.03
cgc	R	0	0.05
cga	R	0	0.10
cgg	R	0	0.07
act	T	1	0.25
acc	T	0	0.22
aca	T	0	0.45
acg	T	0	0.08
aat	N	111	87.53
aac	N	43	66.47
agt	S	589	409.10
agc	S	1609	421.20
aga	R	1	0.46
agg	R	0	0.28
gat	D	286	181.70
gac	D	37	141.30
---	--	------	------
mPD		0.97	1.9
		nPD:	0.52
		N. weight:	1.7
		Sc. PD:	0.3
		Sc. rank:	1374.5

,

PB2

Pos.	125	obs:	exp:
att	I	0	0.35
atc	I	0	0.26
ata	I	1	0.39
act	T	11	658.20
acc	T	2	578.70
aca	T	2630	1216.00
acg	T	33	223.10
---	--	------	------
mPD		0.035	0.68
		nPD:	0.05
		N. weight:	1.3
		Sc. PD:	-0.24
		Sc. rank:	-1126.7

,

PB2

Pos.	126	obs:	exp:
ttt	F	3	1.34
ttc	F	0	1.66
att	I	36	23.06
atc	I	29	16.67
ata	I	0	25.27
act	T	2	0.49
acc	T	0	0.43
aca	T	0	0.91
acg	T	0	0.17
gtt	V	2120	578.80
gtc	V	482	491.90
gta	V	3	658.10
gtg	V	2	878.10
---	--	------	------
mPD		0.36	0.79
		nPD:	0.46
		N. weight:	1.9
		Sc. PD:	0.25
		Sc. rank:	1220.5

,

PB2

Pos.	127	obs:	exp:
cat	H	746	1542.00
cac	H	1928	1132.00
caa	Q	2	1.08
cag	Q	0	0.92
aat	N	0	0.57
aac	N	1	0.43
---	--	------	------
mPD		0.40	0.49
		nPD:	0.82
		N. weight:	0.34
		Sc. PD:	0.14
		Sc. rank:	409.5

,

PB2

Pos.	128	obs:	exp:
tat	Y	395	1500.00
tac	Y	2282	1177.00
---	--	------	------
mPD		0.25	0.49
		nPD:	0.51
		N. weight:	0.68
		Sc. PD:	0.12
		Sc. rank:	532.6

,

PB2

Pos.	129	obs:	exp:
tta	L	0	0.11
ttg	L	0	0.19
tct	S	4	0.67
tcc	S	0	0.63
tca	S	0	0.97
tcg	S	0	0.21
ctt	L	0	0.20
ctc	L	1	0.15
cta	L	0	0.17
ctg	L	0	0.18
cct	P	2259	788.50
ccc	P	253	538.00
cca	P	68	1058.00
ccg	P	92	287.40
agt	S	0	0.74
agc	S	0	0.77
---	--	------	------
mPD		0.28	0.71
		nPD:	0.39
		N. weight:	1.3
		Sc. PD:	0.11
		Sc. rank:	574.8

,

PB2

Pos.	130	obs:	exp:
cgt	R	0	0.06
cgc	R	0	0.11
cga	R	0	0.20
cgg	R	0	0.14
act	T	0	0.25
acc	T	0	0.22
aca	T	0	0.45
acg	T	1	0.08
aaa	K	473	1625.00
aag	K	2201	1049.00
aga	R	2	0.92
agg	R	0	0.57
---	--	------	------
mPD		0.29	0.48
		nPD:	0.61
		N. weight:	0.73
		Sc. PD:	0.18
		Sc. rank:	727.3

,

PB2			
Pos.	131	obs:	exp:
att	I	0	1.42
atc	I	1	1.03
ata	I	3	1.56
gtt	V	11	593.50
gtc	V	150	504.40
gta	V	2443	674.80
gtg	V	69	900.30
---	--	------	------
mPD		0.16	0.74
		nPD:	0.22
		N. weight:	1.9
		Sc. PD:	-0.097
		Sc. rank:	-105.3

,

PB2			
Pos.	132	obs:	exp:
tat	Y	1364	1499.00
tac	Y	1312	1177.00
cat	H	0	0.58
cac	H	1	0.42
---	--	------	------
mPD		0.50	0.49
		nPD:	1.01
		N. weight:	0.013
		Sc. PD:	0.0073
		Sc. rank:	16.2

,

PB2			
Pos.	133	obs:	exp:
cgt	R	0	0.95
cgc	R	0	1.59
cga	R	0	3.02
cgg	R	0	2.17
aaa	K	2579	1608.00
aag	K	67	1038.00
aga	R	28	13.76
agg	R	2	8.53
gaa	E	1	0.59
gag	E	0	0.41
---	--	------	------
mPD		0.073	0.51
		nPD:	0.14
		N. weight:	0.73
		Sc. PD:	-0.081
		Sc. rank:	-235.3

,

PB2			
Pos.	134	obs:	exp:
cct	P	3	1.18
ccc	P	0	0.81
cca	P	0	1.58
ccg	P	1	0.43
att	I	4	1.42
atc	I	0	1.03
ata	I	0	1.56
act	T	2219	656.00
acc	T	38	576.70
aca	T	401	1212.00
acg	T	9	222.40
gct	A	0	0.49
gcc	A	0	0.46
gca	A	2	0.90
gcg	A	0	0.15
---	--	------	------
mPD		0.29	0.69
		nPD:	0.43
		N. weight:	1.5
		Sc. PD:	0.16
		Sc. rank:	827.6

,

PB2			
Pos.	135	obs:	exp:
ttt	F	8	3.58
ttc	F	0	4.42
tat	Y	2517	1495.00
tac	Y	152	1174.00
---	--	------	------
mPD		0.11	0.50
		nPD:	0.23
		N. weight:	0.7
		Sc. PD:	-0.033
		Sc. rank:	-29.7

,

PB2			
Pos.	136	obs:	exp:
ttt	F	536	1200.00
ttc	F	2141	1477.00
---	--	------	------
mPD		0.32	0.49
		nPD:	0.65
		N. weight:	0.25
		Sc. PD:	0.07
		Sc. rank:	266.7

,

PB2			
Pos.	137	obs:	exp:
aaa	K	6	3.65
aag	K	0	2.35
gat	D	4	3.94
gac	D	3	3.06
gaa	E	2620	1578.00
gag	E	44	1086.00
---	--	------	------
mPD		0.042	0.49
		nPD:	0.09
		N. weight:	0.83
		Sc. PD:	-0.13
		Sc. rank:	-518.5

,

PB2			
Pos.	138	obs:	exp:
cgt	R	0	0.47
cgc	R	0	0.79
cga	R	0	1.51
cgg	R	0	1.08
aaa	K	1723	1618.00
aag	K	939	1044.00
aga	R	14	6.88
agg	R	1	4.26
---	--	------	------
mPD		0.47	0.49
		nPD:	0.95
		N. weight:	0.016
		Sc. PD:	0.0079
		Sc. rank:	19.2

,

PB2			
Pos.	139	obs:	exp:
ttt	F	0	1.34
ttc	F	3	1.66
att	I	14	13.48
atc	I	24	9.75
ata	I	0	14.77
gtt	V	451	583.50
gtc	V	2162	495.90
gta	V	12	663.40
gtg	V	3	885.20
gct	A	3	1.47
gcc	A	3	1.37
gca	A	0	2.70
gcg	A	0	0.45
ggt	G	0	0.31
ggc	G	2	0.25
gga	G	0	0.91
ggg	G	0	0.53
---	--	------	------
mPD		0.33	0.78
		nPD:	0.43
		N. weight:	2.1
		Sc. PD:	0.23
		Sc. rank:	1229.1

,

PB2			
Pos.	140	obs:	exp:
aaa	K	7	4.25
aag	K	0	2.75
gat	D	0	0.56
gac	D	1	0.44
gaa	E	2572	1581.00
gag	E	97	1088.00
---	--	------	------
mPD		0.076	0.49
		nPD:	0.16
		N. weight:	0.71
		Sc. PD:	-0.072
		Sc. rank:	-212.0

,

PB2			
Pos.	141	obs:	exp:
cgt	R	0	84.50
cgc	R	0	141.50
cga	R	0	269.10
cgg	R	4	193.40
aga	R	506	1228.00
agg	R	2167	760.90
---	--	------	------
mPD		0.31	0.94
		nPD:	0.33
		N. weight:	1.3
		Sc. PD:	0.04
		Sc. rank:	335.2

,

PB2			
Pos.	142	obs:	exp:
tta	L	1600	291.30
ttg	L	995	502.80
ctt	L	0	540.60
ctc	L	0	389.50
cta	L	20	467.40
ctg	L	62	485.30
---	--	------	------
mPD		0.54	1.1
		nPD:	0.47
		N. weight:	2.2
		Sc. PD:	0.31
		Sc. rank:	1525.5

,

PB2			
Pos.	143	obs:	exp:
cgt	R	0	0.76
cgc	R	0	1.27
cga	R	0	2.41
cgg	R	0	1.73
aat	N	0	1.14
aac	N	2	0.86
aaa	K	2210	1611.00
aag	K	441	1040.00
aga	R	19	11.01
agg	R	5	6.82
---	--	------	------
mPD		0.30	0.50
		nPD:	0.59
		N. weight:	0.23
		Sc. PD:	0.054
		Sc. rank:	223.7

,

PB2			
Pos.	144	obs:	exp:
tct	S	0	0.17
tcc	S	0	0.16
tca	S	0	0.24
tcg	S	0	0.05
tat	Y	14	8.40
tac	Y	1	6.60
cat	H	2329	1471.00
cac	H	222	1080.00
caa	Q	1	1.08
cag	Q	1	0.92
cgt	R	1	0.03
cgc	R	0	0.05
cga	R	0	0.10
cgg	R	0	0.07
act	T	2	0.49
acc	T	0	0.43
aca	T	0	0.91
acg	T	0	0.17
aat	N	101	59.68
aac	N	4	45.32
agt	S	1	0.19
agc	S	0	0.19
aga	R	0	0.46
agg	R	0	0.28
---	--	------	------
mPD		0.25	0.58
		nPD:	0.43
		N. weight:	0.54
		Sc. PD:	0.057
		Sc. rank:	299.2

,

PB2			
Pos.	145	obs:	exp:
ggt	G	2137	419.90
ggc	G	77	339.20
gga	G	83	1213.00
ggg	G	380	705.40
---	--	------	------
mPD		0.34	0.68
		nPD:	0.5
		N. weight:	2.
		Sc. PD:	0.32
		Sc. rank:	1525.5

,

PB2

Pos.	146	obs:	exp:
att	I	0	0.71
atc	I	1	0.51
ata	I	1	0.78
act	T	114	655.30
acc	T	2517	576.10
aca	T	33	1211.00
acg	T	0	222.10
gct	A	1	2.70
gcc	A	10	2.52
gca	A	0	4.96
gcg	A	0	0.82
---	--	------	------
mPD		0.12	0.69
		nPD:	0.17
		N. weight:	2.4
		Sc. PD:	-0.22
		Sc. rank:	-530.5

,

PB2

Pos.	147	obs:	exp:
ttt	F	1956	1199.00
ttc	F	720	1477.00
tat	Y	1	0.56
tac	Y	0	0.44
---	--	------	------
mPD		0.39	0.50
		nPD:	0.8
		N. weight:	0.31
		Sc. PD:	0.12
		Sc. rank:	367.2

,

PB2

Pos.	148	obs:	exp:
ggt	G	404	419.90
ggc	G	2239	339.20
gga	G	30	1213.00
ggg	G	4	705.40
---	--	------	------
mPD		0.28	0.68
		nPD:	0.41
		N. weight:	2.8
		Sc. PD:	0.25
		Sc. rank:	1332.5

,

PB2

Pos.	149	obs:	exp:
cct	P	2204	790.00
ccc	P	381	539.00
cca	P	85	1060.00
ccg	P	7	288.00
---	--	------	------
mPD		0.30	0.70
		nPD:	0.43
		N. weight:	1.3
		Sc. PD:	0.14
		Sc. rank:	745.6

,

PB2

Pos.	150	obs:	exp:
tct	S	0	0.17
tcc	S	1	0.16
tca	S	0	0.24
tcg	S	0	0.05
att	I	0	0.35
atc	I	1	0.26
ata	I	0	0.39
agt	S	0	0.19
agc	S	0	0.19
gtt	V	188	593.90
gtc	V	2436	504.80
gta	V	40	675.30
gtg	V	11	901.00
---	--	------	------
mPD		0.17	0.74
		nPD:	0.23
		N. weight:	2.4
		Sc. PD:	-0.11
		Sc. rank:	-106.4

,

PB2

Pos.	151	obs:	exp:
cat	H	1362	1504.00
cac	H	1245	1103.00
caa	Q	1	0.54
cag	Q	0	0.46
cgt	R	8	2.18
cgc	R	61	3.65
cga	R	0	6.94
cgg	R	0	4.98
aga	R	0	31.64
agg	R	0	19.61
---	--	------	------
mPD		0.55	0.60
		nPD:	0.92
		N. weight:	0.14
		Sc. PD:	0.068
		Sc. rank:	173.8

,

PB2

Pos.	152	obs:	exp:
ttt	F	425	1199.00
ttc	F	2251	1477.00
tta	L	0	0.11
ttg	L	0	0.19
ctt	L	1	0.20
ctc	L	0	0.15
cta	L	0	0.17
ctg	L	0	0.18
---	--	------	------
mPD		0.27	0.50
		nPD:	0.54
		N. weight:	0.36
		Sc. PD:	0.069
		Sc. rank:	303.2

,

PB2

Pos.	153	obs:	exp:
caa	Q	9	4.85
cag	Q	0	4.15
cgt	R	0	84.22
cgc	R	0	141.00
cga	R	352	268.20
cgg	R	37	192.70
aga	R	2254	1224.00
agg	R	25	758.30
---	--	------	------
mPD		0.31	0.95
		nPD:	0.32
		N. weight:	0.93
		Sc. PD:	0.024
		Sc. rank:	224.6

,

PB2

Pos.	154	obs:	exp:
tct	S	0	1.68
tcc	S	0	1.58
tca	S	0	2.43
tcg	S	0	0.53
aat	N	2421	1516.00
aac	N	246	1151.00
agt	S	9	1.86
agc	S	1	1.92
---	--	------	------
mPD		0.18	0.50
		nPD:	0.35
		N. weight:	0.53
		Sc. PD:	0.024
		Sc. rank:	166.7

,

PB2

Pos.	155	obs:	exp:
cat	H	0	0.58
cac	H	1	0.42
caa	Q	2638	1443.00
cag	Q	38	1233.00
---	--	------	------
mPD		0.029	0.50
		nPD:	0.06
		N. weight:	1.
		Sc. PD:	-0.18
		Sc. rank:	-826.3

,

PB2

Pos.	156	obs:	exp:
tta	L	0	0.22
ttg	L	0	0.38
ctt	L	2	0.40
ctc	L	0	0.29
cta	L	0	0.35
ctg	L	0	0.36
att	I	57	22.35
atc	I	6	16.16
ata	I	0	24.49
gtt	V	2364	580.00
gtc	V	246	492.90
gta	V	1	659.40
gtg	V	1	879.80
---	--	------	------
mPD		0.22	0.79
		nPD:	0.28
		N. weight:	2.2
		Sc. PD:	-0.016
		Sc. rank:	190.8

,

PB2

Pos.	157	obs:	exp:
cgt	R	0	0.13
cgc	R	0	0.21
cga	R	0	0.40
cgg	R	0	0.29
aaa	K	2609	1624.00
aag	K	64	1049.00
aga	R	4	1.83
agg	R	0	1.14
---	--	------	------
mPD		0.050	0.48
		nPD:	0.1
		N. weight:	0.74
		Sc. PD:	-0.1
		Sc. rank:	-388.4

,

PB2

Pos.	158	obs:	exp:
att	I	1	949.60
atc	I	6	686.60
ata	I	2670	1041.00
---	--	------	------
mPD		0.0052	0.66
		nPD:	0.01
		N. weight:	1.7
		Sc. PD:	-0.37
		Sc. rank:	-1995.2

,

PB2

Pos.	159	obs:	exp:
tct	S	0	0.17
tcc	S	0	0.16
tca	S	0	0.24
tcg	S	0	0.05
cgt	R	22	84.47
cgc	R	349	141.40
cga	R	66	269.00
cgg	R	0	193.30
agt	S	0	0.19
agc	S	1	0.19
aga	R	938	1227.00
agg	R	1301	760.60
---	--	------	------
mPD		0.88	0.94
		nPD:	0.94
		N. weight:	0.45
		Sc. PD:	0.22
		Sc. rank:	560.6

,

PB2

Pos.	160	obs:	exp:
cgt	R	9	83.17
cgc	R	1	139.30
cga	R	319	264.90
cgg	R	109	190.40
aaa	K	0	25.52
aag	K	42	16.48
aga	R	217	1208.00
agg	R	1980	748.90
---	--	------	------
mPD		0.63	0.96
		nPD:	0.65
		N. weight:	1.1
		Sc. PD:	0.31
		Sc. rank:	1168.6

,

PB2

Pos.	161	obs:	exp:
cgt	R	0	84.34
cgc	R	0	141.20
cga	R	1	268.60
cgg	R	1	193.00
aaa	K	3	3.04
aag	K	2	1.96
aga	R	908	1225.00
agg	R	1762	759.50
---	--	------	------
mPD		0.45	0.94
		nPD:	0.48
		N. weight:	0.84
		Sc. PD:	0.12
		Sc. rank:	599.5

,

PB2

Pos.	162	obs:	exp:
gtt	V	2628	594.40
gtc	V	35	505.10
gta	V	12	675.80
gtg	V	2	901.70
---	--	------	------
mPD		0.036	0.74
		nPD:	0.05
		N. weight:	2.6
		Sc. PD:	-0.48
		Sc. rank:	-2275.2

,

PB2

Pos.	163	obs:	exp:
gat	D	717	1505.00
gac	D	1958	1170.00
gaa	E	1	0.59
gag	E	0	0.41
ggt	G	0	0.16
ggc	G	1	0.13
gga	G	0	0.45
ggg	G	0	0.26
---	--	------	------
mPD		0.39	0.49
		nPD:	0.8
		N. weight:	0.33
		Sc. PD:	0.13
		Sc. rank:	399.1

,

PB2

Pos.	164	obs:	exp:
att	I	13	150.40
atc	I	2	108.80
ata	I	409	164.80
atg	M	1	1.00
act	T	1	552.00
acc	T	1	485.20
aca	T	2229	1020.00
acg	T	13	187.10
gtt	V	0	1.78
gtc	V	0	1.51
gta	V	8	2.02
gtg	V	0	2.69
---	--	------	------
mPD		0.30	0.96
		nPD:	0.31
		N. weight:	1.4
		Sc. PD:	0.028
		Sc. rank:	323.5

,

PB2

Pos.	165	obs:	exp:
act	T	0	0.25
acc	T	1	0.22
aca	T	0	0.45
acg	T	0	0.08
aat	N	195	1520.00
aac	N	2480	1155.00
aaa	K	1	0.61
aag	K	0	0.39
---	--	------	------
mPD		0.14	0.49
		nPD:	0.28
		N. weight:	1.
		Sc. PD:	-0.0086
		Sc. rank:	83.7

,

PB2			
Pos.	166	obs:	exp:
cct	P	2123	790.00
ccc	P	59	539.00
cca	P	442	1060.00
ccg	P	53	288.00
---	--	------	------
mPD		0.34	0.70
		nPD:	0.49
		N. weight:	1.
		Sc. PD:	0.16
		Sc. rank:	755.6

,

PB2			
Pos.	167	obs:	exp:
ggt	G	1614	419.90
ggc	G	945	339.20
gga	G	103	1213.00
ggg	G	15	705.40
---	--	------	------
mPD		0.51	0.68
		nPD:	0.75
		N. weight:	2.
		Sc. PD:	0.69
		Sc. rank:	2317.7

,

PB2			
Pos.	168	obs:	exp:
cat	H	2241	1542.00
cac	H	433	1132.00
aat	N	1	1.71
aac	N	2	1.29
---	--	------	------
mPD		0.27	0.49
		nPD:	0.56
		N. weight:	0.29
		Sc. PD:	0.061
		Sc. rank:	262.4

,

PB2			
Pos.	169	obs:	exp:
tct	S	1	2.52
tcc	S	0	2.37
tca	S	14	3.64
tcg	S	0	0.80
act	T	1	0.49
acc	T	0	0.43
aca	T	1	0.91
acg	T	0	0.17
agt	S	0	2.79
agc	S	0	2.87
gtt	V	1	5.55
gtc	V	0	4.72
gta	V	24	6.31
gtg	V	0	8.42
gct	A	10	645.90
gcc	A	0	603.70
gca	A	2546	1188.00
gcg	A	79	197.60
---	--	------	------
mPD		0.098	0.72
		nPD:	0.14
		N. weight:	1.3
		Sc. PD:	-0.15
		Sc. rank:	-459.0

,

PB2			
Pos.	170	obs:	exp:
cat	H	1	0.58
cac	H	0	0.42
gat	D	2204	1503.00
gac	D	469	1170.00
ggt	G	3	0.47
ggc	G	0	0.38
gga	G	0	1.36
ggg	G	0	0.79
---	--	------	------
mPD		0.29	0.50
		nPD:	0.59
		N. weight:	0.29
		Sc. PD:	0.068
		Sc. rank:	279.3

,

PB2			
Pos.	171	obs:	exp:
tta	L	1	291.20
ttg	L	0	502.60
ctt	L	44	540.40
ctc	L	2578	389.30
cta	L	36	467.30
ctg	L	17	485.10
att	I	0	0.35
atc	I	1	0.26
ata	I	0	0.39
---	--	------	------
mPD		0.073	1.1
		nPD:	0.06
		N. weight:	3.2
		Sc. PD:	-0.55
		Sc. rank:	-2483.6

,

PB2			
Pos.	172	obs:	exp:
tct	S	0	445.20
tcc	S	0	418.30
tca	S	0	642.80
tcg	S	0	140.70
aat	N	27	15.35
aac	N	0	11.65
agt	S	2627	492.70
agc	S	20	507.30
ggt	G	3	0.47
ggc	G	0	0.38
gga	G	0	1.36
ggg	G	0	0.79
---	--	------	------
mPD		0.037	1.6
		nPD:	0.02
		N. weight:	3.
		Sc. PD:	-0.61
		Sc. rank:	-3260.4

,

PB2			
Pos.	173	obs:	exp:
tct	S	10	4.88
tcc	S	10	4.58
tca	S	9	7.04
tcg	S	0	1.54
cct	P	4	3.25
ccc	P	4	2.21
cca	P	3	4.36
ccg	P	0	1.18
att	I	0	0.35
atc	I	1	0.26
ata	I	0	0.39
act	T	0	3.94
acc	T	16	3.46
aca	T	0	7.27
acg	T	0	1.33
agt	S	0	5.40
agc	S	0	5.56
gtt	V	0	1.78
gtc	V	8	1.51
gta	V	0	2.02
gtg	V	0	2.69
gct	A	31	639.50
gcc	A	2542	597.80
gca	A	32	1176.00
gcg	A	4	195.70
ggt	G	0	0.47
ggc	G	3	0.38
gga	G	0	1.36
ggg	G	0	0.79
---	--	------	------
mPD		0.12	0.74
		nPD:	0.16
		N. weight:	2.4
		Sc. PD:	-0.24
		Sc. rank:	-659.7

,

PB2			
Pos.	174	obs:	exp:
caa	Q	0	0.54
cag	Q	1	0.46
cgt	R	0	11.11
cgc	R	0	18.61
cga	R	0	35.38
cgg	R	0	25.43
atg	M	2	2.00
aaa	K	338	1411.00
aag	K	1983	910.40
aga	R	39	161.40
agg	R	313	100.00
ggt	G	0	0.16
ggc	G	0	0.13
gga	G	0	0.45
ggg	G	1	0.26
---	--	------	------
mPD		0.47	0.79
		nPD:	0.6
		N. weight:	0.95
		Sc. PD:	0.23
		Sc. rank:	929.7

,

PB2			
Pos.	175	obs:	exp:
gat	D	1	0.56
gac	D	0	0.44
gaa	E	395	1586.00
gag	E	2281	1090.00
---	--	------	------
mPD		0.25	0.48
		nPD:	0.52
		N. weight:	0.79
		Sc. PD:	0.14
		Sc. rank:	635.3

,

PB2			
Pos.	176	obs:	exp:
gct	A	0	656.20
gcc	A	7	613.40
gca	A	2638	1207.00
gcg	A	32	200.80
---	--	------	------
mPD		0.029	0.68
		nPD:	0.04
		N. weight:	1.4
		Sc. PD:	-0.26
		Sc. rank:	-1259.9

,

PB2			
Pos.	177	obs:	exp:
cat	H	2	1.15
cac	H	0	0.85
caa	Q	609	1442.00
cag	Q	2065	1232.00
gaa	E	0	0.59
gag	E	1	0.41
---	--	------	------
mPD		0.35	0.50
		nPD:	0.71
		N. weight:	0.38
		Sc. PD:	0.12
		Sc. rank:	427.0

,

PB2			
Pos.	178	obs:	exp:
gat	D	2628	1506.00
gac	D	49	1171.00
---	--	------	------
mPD		0.036	0.49
		nPD:	0.07
		N. weight:	0.91
		Sc. PD:	-0.15
		Sc. rank:	-644.8

,

PB2			
Pos.	179	obs:	exp:
gtt	V	362	594.40
gtc	V	95	505.10
gta	V	27	675.80
gtg	V	2193	901.70
---	--	------	------
mPD		0.31	0.74
		nPD:	0.42
		N. weight:	1.1
		Sc. PD:	0.11
		Sc. rank:	546.2

,

PB2			
Pos.	180	obs:	exp:
att	I	594	949.60
atc	I	1951	686.60
ata	I	132	1041.00
---	--	------	------
mPD		0.42	0.66
		nPD:	0.63
		N. weight:	1.
		Sc. PD:	0.27
		Sc. rank:	1063.7

,

PB2			
Pos.	181	obs:	exp:
atg	M	2677	2677.00
---	--	------	------
mPD		0	0
		nPD:	1.
		N. weight:	0.
		Sc. PD:	0
		Sc. rank:	0

,

PB2			
Pos.	182	obs:	exp:
gaa	E	2521	1586.00
gag	E	156	1091.00
---	--	------	------
mPD		0.11	0.48
		nPD:	0.23
		N. weight:	0.6
		Sc. PD:	-0.028
		Sc. rank:	-24.6

,

PB2			
Pos.	183	obs:	exp:
att	I	4	1.42
atc	I	0	1.03
ata	I	0	1.56
gtt	V	2553	593.30
gtc	V	110	504.20
gta	V	9	674.50
gtg	V	0	900.00
gct	A	1	0.25
gcc	A	0	0.23
gca	A	0	0.45
gcg	A	0	0.07
---	--	------	------
mPD		0.089	0.74
		nPD:	0.12
		N. weight:	2.4
		Sc. PD:	-0.32
		Sc. rank:	-1062.2

,

PB2			
Pos.	184	obs:	exp:
gtt	V	2639	594.40
gtc	V	32	505.10
gta	V	3	675.80
gtg	V	3	901.70
---	--	------	------
mPD		0.028	0.74
		nPD:	0.04
		N. weight:	2.6
		Sc. PD:	-0.51
		Sc. rank:	-2524.3

,

PB2			
Pos.	185	obs:	exp:
ttt	F	104	1200.00
ttc	F	2573	1477.00
---	--	------	------
mPD		0.075	0.49
		nPD:	0.15
		N. weight:	0.81
		Sc. PD:	-0.086
		Sc. rank:	-252.6

,

PB2			
Pos.	186	obs:	exp:
tct	S	0	0.17
tcc	S	0	0.16
tca	S	1	0.24
tcg	S	0	0.05
cct	P	4	789.70
ccc	P	0	538.80
cca	P	2651	1060.00
ccg	P	21	287.90
agt	S	0	0.19
agc	S	0	0.19
---	--	------	------
mPD		0.019	0.71
		nPD:	0.03
		N. weight:	1.6
		Sc. PD:	-0.33
		Sc. rank:	-1707.2

,

PB2			
Pos.	187	obs:	exp:
aat	N	2633	1521.00
aac	N	43	1155.00
gat	D	1	0.56
gac	D	0	0.44
---	--	------	------
mPD		0.032	0.49
		nPD:	0.07
		N. weight:	0.9
		Sc. PD:	-0.15
		Sc. rank:	-686.0

,

PB2			
Pos.	188	obs:	exp:
gaa	E	2621	1586.00
gag	E	56	1091.00
---	--	------	------
mPD		0.041	0.48
		nPD:	0.08
		N. weight:	0.8
		Sc. PD:	-0.12
		Sc. rank:	-504.2

,

PB2			
Pos.	189	obs:	exp:
atg	M	3	3.00
gtt	V	26	593.70
gtc	V	1	504.60
gta	V	137	675.00
gtg	V	2510	900.70
---	--	------	------
mPD		0.12	0.74
		nPD:	0.16
		N. weight:	1.6
		Sc. PD:	-0.16
		Sc. rank:	-438.1

,

PB2			
Pos.	190	obs:	exp:
ggt	G	44	419.90
ggc	G	1	339.20
gga	G	896	1213.00
ggg	G	1736	705.40
---	--	------	------
mPD		0.47	0.68
		nPD:	0.68
		N. weight:	0.83
		Sc. PD:	0.25
		Sc. rank:	914.1

,

PB2			
Pos.	191	obs:	exp:
gct	A	44	656.20
gcc	A	400	613.40
gca	A	2186	1207.00
gcg	A	47	200.80
---	--	------	------
mPD		0.31	0.68
		nPD:	0.46
		N. weight:	0.66
		Sc. PD:	0.085
		Sc. rank:	407.9

,

PB2			
Pos.	192	obs:	exp:
cgt	R	0	84.18
cgc	R	0	141.00
cga	R	3	268.10
cgg	R	2	192.70
att	I	0	0.35
atc	I	0	0.26
ata	I	1	0.39
aaa	K	9	5.47
aag	K	0	3.53
aga	R	2639	1223.00
agg	R	23	758.00
---	--	------	------
mPD		0.030	0.95
		nPD:	0.03
		N. weight:	1.3
		Sc. PD:	-0.26
		Sc. rank:	-1384.2

,

PB2			
Pos.	193	obs:	exp:
tta	L	0	0.22
ttg	L	0	0.38
ctt	L	0	0.40
ctc	L	0	0.29
cta	L	2	0.35
ctg	L	0	0.36
att	I	1	926.90
atc	I	0	670.20
ata	I	2612	1016.00
act	T	0	3.69
acc	T	0	3.24
aca	T	15	6.82
acg	T	0	1.25
gtt	V	0	10.44
gtc	V	0	8.87
gta	V	46	11.86
gtg	V	1	15.83
---	--	------	------
mPD		0.049	0.71
		nPD:	0.07
		N. weight:	1.8
		Sc. PD:	-0.3
		Sc. rank:	-1289.4

,

PB2			
Pos.	194	obs:	exp:
tta	L	112	291.30
ttg	L	85	502.80
ctt	L	8	540.60
ctc	L	0	389.50
cta	L	385	467.40
ctg	L	2087	485.30
---	--	------	------
mPD		0.44	1.1
		nPD:	0.39
		N. weight:	1.9
		Sc. PD:	0.14
		Sc. rank:	772.4

,

PB2			
Pos.	195	obs:	exp:
att	I	0	0.35
atc	I	0	0.26
ata	I	1	0.39
act	T	7	654.50
acc	T	4	575.40
aca	T	2632	1209.00
acg	T	18	221.90
gct	A	0	3.68
gcc	A	0	3.44
gca	A	15	6.76
gcg	A	0	1.12
---	--	------	------
mPD		0.033	0.69
		nPD:	0.05
		N. weight:	1.4
		Sc. PD:	-0.25
		Sc. rank:	-1201.2

,

PB2			
Pos.	196	obs:	exp:
tta	L	1	0.11
ttg	L	0	0.19
tct	S	6	450.10
tcc	S	4	422.90
tca	S	2635	649.80
tcg	S	31	142.20
ctt	L	0	0.20
ctc	L	0	0.15
cta	L	0	0.17
ctg	L	0	0.18
agt	S	0	498.10
agc	S	0	512.80
---	--	------	------
mPD		0.031	1.6
		nPD:	0.02
		N. weight:	2.5
		Sc. PD:	-0.52
		Sc. rank:	-2735.4

,

PB2			
Pos.	197	obs:	exp:
gat	D	5	3.94
gac	D	2	3.06
gaa	E	399	1582.00
gag	E	2271	1088.00
---	--	------	------
mPD		0.26	0.49
		nPD:	0.53
		N. weight:	0.78
		Sc. PD:	0.15
		Sc. rank:	644.6

,

PB2			
Pos.	198	obs:	exp:
tta	L	0	0.11
ttg	L	1	0.19
tct	S	11	447.60
tcc	S	1	420.60
tca	S	2264	646.20
tcg	S	385	141.40
tgt	C	0	0.43
tgc	C	1	0.57
ctt	L	0	0.20
ctc	L	0	0.15
cta	L	0	0.17
ctg	L	0	0.18
act	T	0	3.44
acc	T	0	3.03
aca	T	14	6.36
acg	T	0	1.17
agt	S	0	495.30
agc	S	0	510.00
---	--	------	------
mPD		0.27	1.6
		nPD:	0.16
		N. weight:	2.2
		Sc. PD:	-0.21
		Sc. rank:	-541.5

,

PB2			
Pos.	199	obs:	exp:
caa	Q	471	1444.00
cag	Q	2206	1233.00
---	--	------	------
mPD		0.29	0.50
		nPD:	0.58
		N. weight:	0.53
		Sc. PD:	0.12
		Sc. rank:	492.5

,

PB2			
Pos.	200	obs:	exp:
ttt	F	0	0.90
ttc	F	2	1.10
tta	L	240	288.50
ttg	L	188	497.90
tct	S	0	0.34
tcc	S	0	0.32
tca	S	2	0.49
tcg	S	0	0.11
ctt	L	10	535.40
ctc	L	0	385.70
cta	L	167	462.90
ctg	L	2046	480.60
caa	Q	0	0.54
cag	Q	1	0.46
atg	M	21	21.00
agt	S	0	0.37
agc	S	0	0.38
---	--	------	------
mPD		0.55	1.1
		nPD:	0.48
		N. weight:	1.8
		Sc. PD:	0.26
		Sc. rank:	1246.0

,

PB2			
Pos .	201	obs :	exp :
tct	S	0	0.50
tcc	S	0	0.47
tca	S	3	0.73
tcg	S	0	0.16
att	I	0	1.06
atc	I	0	0.77
ata	I	3	1.17
atg	M	23	23.00
act	T	3	454.80
acc	T	0	399.80
aca	T	1825	840.20
acg	T	21	154.20
aaa	K	8	4.86
aag	K	0	3.14
agt	S	0	0.56
agc	S	0	0.57
gtt	V	0	0.89
gtc	V	0	0.75
gta	V	4	1.01
gtg	V	0	1.35
gct	A	0	192.70
gcc	A	0	180.10
gca	A	778	354.30
gcg	A	8	58.95
gaa	E	1	0.59
gag	E	0	0.41
---	--	------	------
mPD		0.49	1.1
		nPD :	0.43
		N. weight :	1.4
		Sc. PD :	0.15
		Sc. rank :	803.1

PB2			
Pos .	202	obs :	exp :
tta	L	1	0.22
ttg	L	0	0.38
ctt	L	0	0.40
ctc	L	0	0.29
cta	L	1	0.35
ctg	L	0	0.36
att	I	16	932.30
atc	I	7	674.10
ata	I	2605	1022.00
act	T	0	0.25
acc	T	0	0.22
aca	T	1	0.45
acg	T	0	0.08
gtt	V	1	10.21
gtc	V	2	8.68
gta	V	40	11.61
gtg	V	3	15.49
---	--	------	------
mPD		0.058	0.70
		nPD :	0.08
		N. weight :	1.7
		Sc. PD :	-0.26
		Sc. rank :	-1058.4

PB2			
Pos .	203	obs :	exp :
act	T	23	658.20
acc	T	13	578.70
aca	T	2622	1216.00
acg	T	18	223.10
gct	A	0	0.25
gcc	A	0	0.23
gca	A	1	0.45
gcg	A	0	0.07
---	--	------	------
mPD		0.041	0.68
		nPD :	0.06
		N. weight :	1.3
		Sc. PD :	-0.22
		Sc. rank :	-1029.4

PB2			
Pos .	204	obs :	exp :
caa	Q	2	1.08
cag	Q	0	0.92
cgt	R	0	1.64
cgc	R	0	2.75
cga	R	0	5.23
cgg	R	0	3.76
aaa	K	2453	1593.00
aag	K	169	1029.00
aga	R	52	23.85
agg	R	0	14.78
gaa	E	1	0.59
gag	E	0	0.41
---	--	------	------
mPD		0.16	0.53
		nPD :	0.3
		N. weight :	0.55
		Sc. PD :	0.005
		Sc. rank :	78.9

PB2			
Pos .	205	obs :	exp :
aaa	K	1	1.22
aag	K	1	0.78
gtt	V	0	6.44
gtc	V	0	5.47
gta	V	29	7.32
gtg	V	0	9.77
gct	A	0	2.45
gcc	A	0	2.29
gca	A	0	4.51
gcg	A	10	0.75
gat	D	2	2.25
gac	D	2	1.75
gaa	E	1530	1559.00
gag	E	1101	1072.00
ggt	G	0	0.16
ggc	G	0	0.13
gga	G	1	0.45
ggg	G	0	0.26
---	--	------	------
mPD		0.52	0.52
		nPD :	0.99
		N. weight :	0.051
		Sc. PD :	0.027
		Sc. rank :	62.8

PB2			
Pos .	206	obs :	exp :
aaa	K	83	1626.00
aag	K	2593	1050.00
---	-	1	1.00
---	--	------	------
mPD		0.060	0.48
		nPD :	0.13
		N. weight :	1.5
		Sc. PD :	-0.18
		Sc. rank :	-602.6

PB2			
Pos .	207	obs :	exp :
cgt	R	0	0.44
cgc	R	0	0.74
cga	R	0	1.41
cgg	R	0	1.01
aaa	K	2592	1618.00
aag	K	71	1045.00
aga	R	13	6.42
agg	R	1	3.98
---	--	------	------
mPD		0.063	0.49
		nPD :	0.13
		N. weight :	0.72
		Sc. PD :	-0.089
		Sc. rank :	-292.7

PB2			
Pos .	208	obs :	exp :
tct	S	0	0.17
tcc	S	0	0.16
tca	S	0	0.24
tcg	S	0	0.05
cgt	R	0	0.09
cgc	R	0	0.16
cga	R	0	0.30
cgg	R	0	0.22
aat	N	0	0.57
aac	N	1	0.43
aaa	K	28	20.06
aag	K	5	12.94
agt	S	0	0.19
agc	S	1	0.19
aga	R	1	1.38
agg	R	2	0.85
gat	D	4	27.00
gac	D	44	21.00
gaa	E	2259	1535.00
gag	E	332	1056.00
---	--	------	------
mPD		0.28	0.53
		nPD :	0.53
		N. weight :	0.37
		Sc. PD :	0.069
		Sc. rank :	303.9

PB2			
Pos .	209	obs :	exp :
gtt	V	0	0.22
gtc	V	0	0.19
gta	V	0	0.25
gtg	V	1	0.34
gat	D	4	2.25
gac	D	0	1.75
gaa	E	532	1583.00
gag	E	2140	1089.00
---	--	------	------
mPD		0.32	0.49
		nPD :	0.66
		N. weight :	0.6
		Sc. PD :	0.17
		Sc. rank :	652.9

PB2			
Pos .	210	obs :	exp :
tta	L	2	291.00
ttg	L	0	502.30
ctt	L	116	540.00
ctc	L	2521	389.10
cta	L	35	466.90
ctg	L	0	484.70
att	I	0	1.06
atc	I	3	0.77
ata	I	0	1.17
---	--	------	------
mPD		0.11	1.1
		nPD :	0.1
		N. weight :	3.1
		Sc. PD :	-0.45
		Sc. rank :	-1689.5

PB2			
Pos .	211	obs :	exp :
tta	L	0	4.57
ttg	L	1	7.89
ctt	L	0	8.48
ctc	L	0	6.11
cta	L	1	7.33
ctg	L	40	7.61
cct	P	0	0.30
ccc	P	0	0.20
cca	P	0	0.40
ccg	P	1	0.11
cat	H	27	16.15
cac	H	1	11.85
caa	Q	257	1359.00
cag	Q	2264	1162.00
cgt	R	0	1.61
cgc	R	0	2.70
cga	R	0	5.13
cgg	R	49	3.68
aat	N	1	0.57
aac	N	0	0.43
aaa	K	2	20.06
aag	K	31	12.94
aga	R	0	23.39
agg	R	2	14.50
---	--	------	------
mPD		0.29	0.64
		nPD :	0.45
		N. weight :	0.91
		Sc. PD :	0.11
		Sc. rank :	554.3

PB2			
Pos .	212	obs :	exp :
tct	S	0	0.50
tcc	S	0	0.47
tca	S	0	0.73
tcg	S	0	0.16
tat	Y	1	0.56
tac	Y	0	0.44
aat	N	73	42.63
aac	N	2	32.37
agt	S	3	0.56
agc	S	0	0.57
gat	D	2508	1432.00
gac	D	38	1114.00
gaa	E	3	10.07
gag	E	14	6.93
ggt	G	33	5.49
ggc	G	1	4.43
gga	G	0	15.85
ggg	G	1	9.22
---	--	------	------
mPD		0.13	0.59
		nPD :	0.22
		N. weight :	0.96
		Sc. PD :	-0.053
		Sc. rank :	-72.8

PB2			
Pos .	213	obs :	exp :
tgt	C	2547	1152.00
tgc	C	130	1525.00
---	--	------	------
mPD		0.092	0.49
		nPD :	0.19
		N. weight :	1.2
		Sc. PD :	-0.09
		Sc. rank :	-179.8

PB2			
Pos .	214	obs :	exp :
cat	H	0	0.58
cac	H	1	0.42
caa	Q	7	3.77
cag	Q	0	3.23
cgt	R	0	0.88
cgc	R	0	1.48
cga	R	0	2.81
cgg	R	0	2.02
act	T	0	0.49
acc	T	0	0.43
aca	T	0	0.91
acg	T	2	0.17
aat	N	2	1.71
aac	N	1	1.29
aaa	K	2269	1602.00
aag	K	367	1034.00
aga	R	23	12.84
agg	R	5	7.96
---	--	------	------
mPD		0.27	0.51
		nPD :	0.53
		N. weight :	0.3
		Sc. PD :	0.056
		Sc. rank :	247.5

PB2			
Pos .	215	obs :	exp :
att	I	2000	949.60
atc	I	29	686.60
ata	I	648	1041.00
---	--	------	------
mPD		0.38	0.66
		nPD :	0.58
		N. weight :	0.76
		Sc. PD :	0.17
		Sc. rank :	711.1

PB2			
Pos.	216	obs:	exp:
tct	S	24	5.38
tcc	S	8	5.06
tca	S	0	7.77
tcg	S	0	1.70
act	T	4	0.98
acc	T	0	0.86
aca	T	0	1.82
acg	T	0	0.33
aat	N	2	1.14
aac	N	0	0.86
agt	S	0	5.96
agc	S	0	6.13
gtt	V	1	0.22
gtc	V	0	0.19
gta	V	0	0.25
gtg	V	0	0.34
gct	A	2169	646.70
gcc	A	463	604.40
gca	A	6	1189.00
gcg	A	0	197.80
---	--	------	------
mPD		0.32	0.72
		nPD:	0.45
		N. weight:	1.7
		Sc. PD:	0.22
		Sc. rank:	1061.2

PB2			
Pos.	217	obs:	exp:
cct	P	371	790.00
ccc	P	2258	539.00
cca	P	46	1060.00
ccg	P	2	288.00
---	--	------	------
mPD		0.27	0.70
		nPD:	0.38
		N. weight:	1.9
		Sc. PD:	0.14
		Sc. rank:	765.7

PB2			
Pos.	218	obs:	exp:
tta	L	535	291.30
ttg	L	2075	502.80
ctt	L	0	540.60
ctc	L	0	389.50
cta	L	11	467.40
ctg	L	56	485.30
---	--	------	------
mPD		0.37	1.1
		nPD:	0.33
		N. weight:	2.2
		Sc. PD:	0.066
		Sc. rank:	569.8

PB2			
Pos.	219	obs:	exp:
att	I	0	2.13
atc	I	0	1.54
ata	I	6	2.33
atg	M	2670	2670.00
act	T	0	0.25
acc	T	0	0.22
aca	T	0	0.45
acg	T	1	0.08
---	--	------	------
mPD		0.0052	0.0059
		nPD:	0.88
		N. weight:	0.0056
		Sc. PD:	0.0026
		Sc. rank:	6.9

PB2			
Pos.	220	obs:	exp:
tta	L	0	0.11
ttg	L	1	0.19
ctt	L	0	0.20
ctc	L	0	0.15
cta	L	0	0.17
ctg	L	0	0.18
atg	M	2	2.00
gtt	V	73	593.70
gtc	V	6	504.60
gta	V	70	675.00
gtg	V	2525	900.70
---	--	------	------
mPD		0.11	0.74
		nPD:	0.15
		N. weight:	1.6
		Sc. PD:	-0.17
		Sc. rank:	-497.3

PB2			
Pos.	221	obs:	exp:
gct	A	31	656.20
gcc	A	2	613.40
gca	A	1992	1207.00
gcg	A	652	200.80
---	--	------	------
mPD		0.39	0.68
		nPD:	0.57
		N. weight:	1.2
		Sc. PD:	0.25
		Sc. rank:	1052.7

PB2			
Pos.	222	obs:	exp:
tat	Y	40	1498.00
tac	Y	2634	1176.00
cat	H	0	1.73
cac	H	3	1.27
---	--	------	------
mPD		0.032	0.49
		nPD:	0.06
		N. weight:	1.4
		Sc. PD:	-0.24
		Sc. rank:	-1062.4

PB2			
Pos.	223	obs:	exp:
atg	M	2677	2677.00
---	--	------	------
mPD		0	0
		nPD:	1.
		N. weight:	0.
		Sc. PD:	0
		Sc. rank:	0

PB2			
Pos.	224	obs:	exp:
tta	L	663	291.10
ttg	L	422	502.50
ctt	L	0	540.20
ctc	L	8	389.20
cta	L	1556	467.10
ctg	L	26	484.90
atg	M	2	2.00
---	--	------	------
mPD		0.77	1.1
		nPD:	0.67
		N. weight:	1.6
		Sc. PD:	0.46
		Sc. rank:	1704.0

PB2			
Pos.	225	obs:	exp:
gaa	E	2568	1586.00
gag	E	109	1091.00
---	--	------	------
mPD		0.078	0.48
		nPD:	0.16
		N. weight:	0.68
		Sc. PD:	-0.067
		Sc. rank:	-179.5

PB2			
Pos.	226	obs:	exp:
cgt	R	0	84.37
cgc	R	0	141.30
cga	R	217	268.70
cgg	R	2	193.10
aaa	K	4	2.43
aag	K	0	1.57
aga	R	2394	1226.00
agg	R	60	759.70
---	--	------	------
mPD		0.20	0.94
		nPD:	0.21
		N. weight:	0.97
		Sc. PD:	-0.058
		Sc. rank:	-86.7

PB2			
Pos.	227	obs:	exp:
gaa	E	2230	1586.00
gag	E	447	1091.00
---	--	------	------
mPD		0.28	0.48
		nPD:	0.58
		N. weight:	0.25
		Sc. PD:	0.056
		Sc. rank:	231.9

PB2			
Pos.	228	obs:	exp:
tta	L	100	291.10
ttg	L	2116	502.50
ctt	L	25	540.20
ctc	L	0	389.20
cta	L	83	467.10
ctg	L	351	484.90
atg	M	2	2.00
---	--	------	------
mPD		0.43	1.1
		nPD:	0.38
		N. weight:	1.8
		Sc. PD:	0.12
		Sc. rank:	696.7

PB2			
Pos.	229	obs:	exp:
att	I	9	3.90
atc	I	2	2.82
ata	I	0	4.28
gtt	V	1436	591.90
gtc	V	1228	503.10
gta	V	2	673.00
gtg	V	0	898.00
---	--	------	------
mPD		0.51	0.75
		nPD:	0.68
		N. weight:	1.6
		Sc. PD:	0.49
		Sc. rank:	1805.2

PB2			
Pos.	230	obs:	exp:
tct	S	0	0.17
tcc	S	0	0.16
tca	S	0	0.24
tcg	S	0	0.05
cat	H	1	0.58
cac	H	0	0.42
cgt	R	2149	84.44
cgc	R	402	141.40
cga	R	90	268.90
cgg	R	13	193.30
agt	S	1	0.19
agc	S	0	0.19
aga	R	14	1227.00
agg	R	7	760.30
---	--	------	------
mPD		0.35	0.94
		nPD:	0.37
		N. weight:	5.
		Sc. PD:	0.31
		Sc. rank:	1818.1

PB2			
Pos.	231	obs:	exp:
cgt	R	0	0.66
cgc	R	0	1.11
cga	R	0	2.11
cgg	R	0	1.52
aaa	K	1195	1614.00
aag	K	1461	1042.00
aga	R	21	9.63
agg	R	0	5.97
---	--	------	------
mPD		0.51	0.50
		nPD:	1.03
		N. weight:	0.11
		Sc. PD:	0.061
		Sc. rank:	133.4

PB2			
Pos.	232	obs:	exp:
atg	M	1	1.00
act	T	28	658.20
acc	T	448	578.70
aca	T	715	1216.00
acg	T	1485	223.10
---	--	------	------
mPD		0.59	0.68
		nPD:	0.87
		N. weight:	1.6
		Sc. PD:	0.7
		Sc. rank:	1891.3

PB2			
Pos.	233	obs:	exp:
tct	S	0	0.17
tcc	S	0	0.16
tca	S	0	0.24
tcg	S	0	0.05
cgt	R	0	79.29
cgc	R	0	132.80
cga	R	1	252.50
cgg	R	4	181.50
aaa	K	1	98.45
aag	K	161	63.55
agt	S	0	0.19
agc	S	1	0.19
aga	R	509	1152.00
agg	R	1998	714.00
ggt	G	0	0.31
ggc	G	0	0.25
gga	G	0	0.91
ggg	G	2	0.53
---	--	------	------
mPD		0.43	1.0
		nPD:	0.41
		N. weight:	1.2
		Sc. PD:	0.12
		Sc. rank:	609.8

PB2			
Pos.	234	obs:	exp:
ttt	F	2064	1199.00
ttc	F	612	1477.00
tct	S	1	0.17
tcc	S	0	0.16
tca	S	0	0.24
tcg	S	0	0.05
agt	S	0	0.19
agc	S	0	0.19
---	--	------	------
mPD		0.35	0.50
		nPD:	0.71
		N. weight:	0.41
		Sc. PD:	0.13
		Sc. rank:	462.2

PB2			
Pos.	235	obs:	exp:
tta	L	2	291.30
ttg	L	21	502.80
ctt	L	915	540.60
ctc	L	1326	389.50
cta	L	117	467.40
ctg	L	296	485.30
---	--	------	------
mPD		0.64	1.1
		nPD:	0.56
		N. weight:	1.2
		Sc. PD:	0.25
		Sc. rank:	1080.1

PB2			
Pos.	236	obs:	exp:
cct	P	12	790.00
ccc	P	6	539.00
cca	P	1104	1060.00
ccg	P	1555	288.00
---	--	------	------
mPD		0.49	0.70
		nPD:	0.7
		N. weight:	1.8
		Sc. PD:	0.57
		Sc. rank:	2010.4

,

PB2			
Pos.	237	obs:	exp:
att	I	17	12.06
atc	I	0	8.72
ata	I	17	13.22
gtt	V	24	586.80
gtc	V	6	498.70
gta	V	1076	667.20
gtg	V	1537	890.20
---	--	------	------
mPD		0.53	0.76
		nPD:	0.69
		N. weight:	0.88
		Sc. PD:	0.27
		Sc. rank:	975.5

,

PB2			
Pos.	238	obs:	exp:
tct	S	1	9.42
tcc	S	32	8.85
tca	S	23	13.60
tcg	S	0	2.98
act	T	5	2.21
acc	T	3	1.95
aca	T	1	4.09
acg	T	0	0.75
agt	S	0	10.42
agc	S	0	10.73
gtt	V	8	4.66
gtc	V	2	3.96
gta	V	11	5.30
gtg	V	0	7.07
gct	A	1495	635.10
gcc	A	688	593.70
gca	A	394	1168.00
gcg	A	14	194.30
---	--	------	------
mPD		0.65	0.76
		nPD:	0.85
		N. weight:	0.69
		Sc. PD:	0.3
		Sc. rank:	839.0

,

PB2			
Pos.	239	obs:	exp:
ggt	G	1914	419.90
ggc	G	693	339.20
gga	G	54	1213.00
ggg	G	16	705.40
---	--	------	------
mPD		0.42	0.68
		nPD:	0.62
		N. weight:	2.2
		Sc. PD:	0.55
		Sc. rank:	2224.5

,

PB2			
Pos.	240	obs:	exp:
ggt	G	0	419.90
ggc	G	14	339.20
gga	G	2301	1213.00
ggg	G	362	705.40
---	--	------	------
mPD		0.24	0.68
		nPD:	0.35
		N. weight:	0.83
		Sc. PD:	0.042
		Sc. rank:	276.2

,

PB2			
Pos.	241	obs:	exp:
att	I	0	0.35
atc	I	0	0.26
ata	I	1	0.39
act	T	18	658.20
acc	T	20	578.70
aca	T	2608	1216.00
acg	T	30	223.10
---	--	------	------
mPD		0.051	0.68
		nPD:	0.07
		N. weight:	1.2
		Sc. PD:	-0.21
		Sc. rank:	-865.2

,

PB2			
Pos.	242	obs:	exp:
tct	S	0	275.20
tcc	S	0	258.60
tca	S	0	397.30
tcg	S	0	86.95
agt	S	25	304.50
agc	S	1611	313.50
ggt	G	13	163.30
ggc	G	1021	131.90
gga	G	7	471.50
ggg	G	0	274.30
---	--	------	------
mPD		0.51	1.9
		nPD:	0.27
		N. weight:	3.2
		Sc. PD:	-0.034
		Sc. rank:	242.6

,

PB2			
Pos.	243	obs:	exp:
tct	S	0	449.60
tcc	S	0	422.40
tca	S	0	649.10
tcg	S	0	142.10
act	T	2	0.49
acc	T	0	0.43
aca	T	0	0.91
acg	T	0	0.17
aat	N	2	1.14
aac	N	0	0.86
agt	S	2640	497.50
agc	S	33	512.30
---	--	------	------
mPD		0.027	1.6
		nPD:	0.02
		N. weight:	3.
		Sc. PD:	-0.62
		Sc. rank:	-3317.9

,

PB2			
Pos.	244	obs:	exp:
att	I	29	12.06
atc	I	5	8.72
ata	I	0	13.22
gtt	V	1982	585.30
gtc	V	513	497.40
gta	V	16	665.40
gtg	V	125	887.90
gct	A	5	1.72
gcc	A	1	1.60
gca	A	0	3.16
gcg	A	1	0.52
---	--	------	------
mPD		0.42	0.77
		nPD:	0.55
		N. weight:	1.5
		Sc. PD:	0.3
		Sc. rank:	1300.5

,

PB2			
Pos.	245	obs:	exp:
tat	Y	2265	1500.00
tac	Y	412	1177.00
---	--	------	------
mPD		0.26	0.49
		nPD:	0.53
		N. weight:	0.35
		Sc. PD:	0.064
		Sc. rank:	286.2

,

PB2			
Pos.	246	obs:	exp:
att	I	2477	946.50
atc	I	189	684.30
ata	I	2	1037.00
gtt	V	9	2.00
gtc	V	0	1.70
gta	V	0	2.27
gtg	V	0	3.03
---	--	------	------
mPD		0.14	0.66
		nPD:	0.21
		N. weight:	1.5
		Sc. PD:	-0.09
		Sc. rank:	-139.1

,

PB2			
Pos.	247	obs:	exp:
gaa	E	2553	1586.00
gag	E	124	1091.00
---	--	------	------
mPD		0.088	0.48
		nPD:	0.18
		N. weight:	0.66
		Sc. PD:	-0.053
		Sc. rank:	-119.5

,

PB2			
Pos.	248	obs:	exp:
gtt	V	6	594.40
gtc	V	1	505.10
gta	V	497	675.80
gtg	V	2173	901.70
---	--	------	------
mPD		0.31	0.74
		nPD:	0.42
		N. weight:	1.2
		Sc. PD:	0.12
		Sc. rank:	599.5

,

PB2			
Pos.	249	obs:	exp:
tta	L	119	291.20
ttg	L	1594	502.60
ctt	L	3	540.40
ctc	L	0	389.30
cta	L	306	467.30
ctg	L	654	485.10
atg	M	1	1.00
---	--	------	------
mPD		0.73	1.1
		nPD:	0.64
		N. weight:	1.2
		Sc. PD:	0.34
		Sc. rank:	1289.8

,

PB2			
Pos.	250	obs:	exp:
cat	H	106	1544.00
cac	H	2571	1133.00
---	--	------	------
mPD		0.076	0.49
		nPD:	0.16
		N. weight:	1.3
		Sc. PD:	-0.13
		Sc. rank:	-374.0

,

PB2			
Pos.	251	obs:	exp:
tta	L	2482	291.20
ttg	L	151	502.60
ctt	L	0	540.40
ctc	L	0	389.30
cta	L	40	467.30
ctg	L	3	485.10
atg	M	1	1.00
---	--	------	------
mPD		0.14	1.1
		nPD:	0.12
		N. weight:	3.5
		Sc. PD:	-0.44
		Sc. rank:	-1471.3

,

PB2			
Pos.	252	obs:	exp:
act	T	1915	658.50
acc	T	750	578.90
aca	T	12	1216.00
acg	T	0	223.20
---	--	------	------
mPD		0.41	0.68
		nPD:	0.6
		N. weight:	1.5
		Sc. PD:	0.37
		Sc. rank:	1490.7

,

PB2			
Pos.	253	obs:	exp:
cat	H	4	2.88
cac	H	1	2.12
caa	Q	1123	1441.00
cag	Q	1549	1231.00
---	--	------	------
mPD		0.49	0.50
		nPD:	0.98
		N. weight:	0.056
		Sc. PD:	0.03
		Sc. rank:	69.9

,

PB2			
Pos.	254	obs:	exp:
ggt	G	3	419.90
ggc	G	4	339.20
gga	G	1875	1213.00
ggg	G	795	705.40
---	--	------	------
mPD		0.42	0.68
		nPD:	0.62
		N. weight:	0.61
		Sc. PD:	0.15
		Sc. rank:	613.1

,

PB2			
Pos.	255	obs:	exp:
att	I	0	0.71
atc	I	2	0.51
ata	I	0	0.78
act	T	31	651.60
acc	T	389	572.80
aca	T	1563	1204.00
acg	T	666	220.90
gtt	V	0	0.22
gtc	V	0	0.19
gta	V	0	0.25
gtg	V	1	0.34
gct	A	1	6.13
gcc	A	0	5.73
gca	A	7	11.27
gcg	A	17	1.87
---	--	------	------
mPD		0.59	0.70
		nPD:	0.84
		N. weight:	0.66
		Sc. PD:	0.28
		Sc. rank:	793.4

,

PB2

Pos.	256	obs:	exp:
tct	S	0	0.84
tcc	S	1	0.79
tca	S	0	1.21
tcg	S	0	0.27
tgt	C	93	1150.00
tgc	C	2579	1522.00
agt	S	0	0.93
agc	S	4	0.96
---	--	------	------
mPD		0.071	0.49
		nPD:	0.14
		N. weight:	0.79
		Sc. PD:	-0.088
		Sc. rank:	-254.6

PB2

Pos.	257	obs:	exp:
tgg	W	2677	2677.00
---	--	------	------
mPD		0	0
		nPD:	1.
		N. weight:	0.
		Sc. PD:	0
		Sc. rank:	0

PB2

Pos.	258	obs:	exp:
gaa	E	575	1586.00
gag	E	2101	1090.00
ggt	G	0	0.16
ggc	G	0	0.13
gga	G	1	0.45
ggg	G	0	0.26
---	--	------	------
mPD		0.34	0.48
		nPD:	0.7
		N. weight:	0.55
		Sc. PD:	0.17
		Sc. rank:	619.4

PB2

Pos.	259	obs:	exp:
cat	H	0	0.58
cac	H	1	0.42
caa	Q	1513	1443.00
cag	Q	1163	1233.00
---	--	------	------
mPD		0.49	0.50
		nPD:	0.99
		N. weight:	0.006
		Sc. PD:	0.0032
		Sc. rank:	7.5

PB2

Pos.	260	obs:	exp:
tta	L	1	4.14
ttg	L	4	7.14
ctt	L	0	7.67
ctc	L	0	5.53
cta	L	21	6.64
ctg	L	12	6.89
cgt	R	0	0.09
cgc	R	0	0.16
cga	R	0	0.30
cgg	R	0	0.22
att	I	0	1.77
atc	I	0	1.28
ata	I	5	1.94
atg	M	2619	2619.00
aaa	K	5	7.29
aag	K	7	4.71
aga	R	0	1.38
agg	R	3	0.85
---	--	------	------
mPD		0.063	0.068
		nPD:	0.92
		N. weight:	0.029
		Sc. PD:	0.014
		Sc. rank:	36.1

PB2

Pos.	261	obs:	exp:
tat	Y	261	1500.00
tac	Y	2416	1177.00
---	--	------	------
mPD		0.18	0.49
		nPD:	0.36
		N. weight:	0.89
		Sc. PD:	0.047
		Sc. rank:	304.5

PB2

Pos.	262	obs:	exp:
act	T	2569	655.50
acc	T	82	576.30
aca	T	14	1211.00
acg	T	0	222.20
aat	N	12	6.82
aac	N	0	5.18
---	--	------	------
mPD		0.078	0.69
		nPD:	0.11
		N. weight:	2.3
		Sc. PD:	-0.31
		Sc. rank:	-1106.6

PB2

Pos.	263	obs:	exp:
cct	P	13	790.00
ccc	P	6	539.00
cca	P	2608	1060.00
ccg	P	50	288.00
---	--	------	------
mPD		0.051	0.70
		nPD:	0.07
		N. weight:	1.5
		Sc. PD:	-0.25
		Sc. rank:	-1087.6

PB2

Pos.	264	obs:	exp:
ggt	G	339	419.90
ggc	G	80	339.20
gga	G	1998	1213.00
ggg	G	260	705.40
---	--	------	------
mPD		0.42	0.68
		nPD:	0.61
		N. weight:	0.39
		Sc. PD:	0.095
		Sc. rank:	384.4

PB2

Pos.	265	obs:	exp:
gaa	E	2	1.19
gag	E	0	0.81
ggt	G	7	419.60
ggc	G	2	339.00
gga	G	2460	1212.00
ggg	G	206	704.80
---	--	------	------
mPD		0.15	0.69
		nPD:	0.22
		N. weight:	1.
		Sc. PD:	-0.054
		Sc. rank:	-64.1

PB2

Pos.	266	obs:	exp:
cgt	R	0	0.06
cgc	R	0	0.11
cga	R	0	0.20
cgg	R	0	0.14
aat	N	1	0.57
aac	N	0	0.43
aaa	K	197	133.10
aag	K	22	85.91
aga	R	2	0.92
agg	R	0	0.57
gct	A	0	0.25
gcc	A	0	0.23
gca	A	1	0.45
gcg	A	0	0.07
gat	D	4	5.62
gac	D	6	4.38
gaa	E	2126	1422.00
gag	E	274	978.00
ggt	G	0	6.90
ggc	G	0	5.58
gga	G	39	19.93
ggg	G	5	11.59
---	--	------	------
mPD		0.39	0.68
		nPD:	0.58
		N. weight:	0.41
		Sc. PD:	0.091
		Sc. rank:	376.9

PB2

Pos.	267	obs:	exp:
gtt	V	7	594.40
gtc	V	0	505.10
gta	V	111	675.80
gtg	V	2559	901.70
---	--	------	------
mPD		0.085	0.74
		nPD:	0.11
		N. weight:	1.7
		Sc. PD:	-0.23
		Sc. rank:	-806.7

PB2

Pos.	268	obs:	exp:
cgt	R	0	64.17
cgc	R	0	107.50
cga	R	0	204.30
cgg	R	8	146.90
att	I	0	2.84
atc	I	0	2.05
ata	I	8	3.11
aaa	K	257	385.30
aag	K	377	248.70
aga	R	1955	932.30
agg	R	70	577.80
gaa	E	1	0.59
gag	E	0	0.41
ggt	G	0	0.16
ggc	G	0	0.13
gga	G	0	0.45
ggg	G	1	0.26
---	--	------	------
mPD		0.66	1.2
		nPD:	0.54
		N. weight:	0.93
		Sc. PD:	0.18
		Sc. rank:	781.9

PB2

Pos.	269	obs:	exp:
tct	S	0	0.34
tcc	S	0	0.32
tca	S	0	0.49
tcg	S	0	0.11
aat	N	2598	1520.00
aac	N	77	1155.00
agt	S	2	0.37
agc	S	0	0.38
---	--	------	------
mPD		0.057	0.49
		nPD:	0.12
		N. weight:	0.82
		Sc. PD:	-0.11
		Sc. rank:	-386.9

PB2

Pos.	270	obs:	exp:
gat	D	2653	1506.00
gac	D	24	1171.00
---	--	------	------
mPD		0.018	0.49
		nPD:	0.04
		N. weight:	0.98
		Sc. PD:	-0.19
		Sc. rank:	-963.7

PB2

Pos.	271	obs:	exp:
tat	Y	5	2.80
tac	Y	0	2.20
gat	D	2650	1503.00
gac	D	22	1169.00
---	--	------	------
mPD		0.020	0.50
		nPD:	0.04
		N. weight:	0.98
		Sc. PD:	-0.19
		Sc. rank:	-915.9

PB2

Pos.	272	obs:	exp:
tta	L	12	1.31
ttg	L	0	2.25
ctt	L	0	2.42
ctc	L	0	1.75
cta	L	0	2.10
ctg	L	0	2.18
att	I	118	50.02
atc	I	6	36.16
ata	I	17	54.82
atg	M	86	86.00
gtt	V	2142	541.30
gtc	V	103	460.00
gta	V	184	615.40
gtg	V	9	821.20
---	--	------	------
mPD		0.44	0.90
		nPD:	0.49
		N. weight:	1.8
		Sc. PD:	0.28
		Sc. rank:	1351.6

PB2

Pos.	273	obs:	exp:
aat	N	0	2.84
aac	N	5	2.16
gat	D	116	1502.00
gac	D	2555	1169.00
ggt	G	1	0.16
ggc	G	0	0.13
gga	G	0	0.45
ggg	G	0	0.26
---	--	------	------
mPD		0.088	0.50
		nPD:	0.18
		N. weight:	1.2
		Sc. PD:	-0.1
		Sc. rank:	-219.6

PB2

Pos.	274	obs:	exp:
caa	Q	2196	1444.00
cag	Q	481	1233.00
---	--	------	------
mPD		0.29	0.50
		nPD:	0.59
		N. weight:	0.33
		Sc. PD:	0.076
		Sc. rank:	314.5

PB2

Pos.	275	obs:	exp:
tct	S	0	448.60
tcc	S	0	421.50
tca	S	0	647.70
tcg	S	0	141.70
act	T	10	2.46
acc	T	0	2.16
aca	T	0	4.54
acg	T	0	0.83
agt	S	2594	496.40
agc	S	73	511.10
---	--	------	------
mPD		0.061	1.6
		nPD:	0.04
		N. weight:	2.9
		Sc. PD:	-0.56
		Sc. rank:	-2787.1

PB2			
Pos.	276	obs:	exp:
tta	L	73	291.30
ttg	L	2378	502.80
ctt	L	1	540.60
ctc	L	0	389.50
cta	L	4	467.40
ctg	L	221	485.30
---	--	------	------
mPD		0.21	1.1
		nPD:	0.19
		N. weight:	2.4
		Sc. PD:	-0.19
		Sc. rank:	-408.2

,

PB2			
Pos.	277	obs:	exp:
att	I	2230	938.60
atc	I	403	678.70
ata	I	13	1029.00
gtt	V	26	6.88
gtc	V	3	5.85
gta	V	2	7.83
gtg	V	0	10.44
---	--	------	------
mPD		0.29	0.68
		nPD:	0.42
		N. weight:	1.2
		Sc. PD:	0.12
		Sc. rank:	635.3

,

PB2			
Pos.	278	obs:	exp:
att	I	804	945.00
atc	I	1846	683.30
ata	I	14	1036.00
act	T	0	0.25
acc	T	1	0.22
aca	T	0	0.45
acg	T	0	0.08
gtt	V	0	2.66
gtc	V	12	2.26
gta	V	0	3.03
gtg	V	0	4.04
---	--	------	------
mPD		0.44	0.67
		nPD:	0.65
		N. weight:	1.2
		Sc. PD:	0.33
		Sc. rank:	1241.3

,

PB2			
Pos.	279	obs:	exp:
gct	A	1794	656.20
gcc	A	868	613.40
gca	A	15	1207.00
gcg	A	0	200.80
---	--	------	------
mPD		0.45	0.68
		nPD:	0.66
		N. weight:	1.4
		Sc. PD:	0.4
		Sc. rank:	1524.0

,

PB2			
Pos.	280	obs:	exp:
tct	S	5	5.38
tcc	S	27	5.06
tca	S	0	7.77
tcg	S	0	1.70
agt	S	0	5.96
agc	S	0	6.13
gct	A	2255	648.40
gcc	A	315	606.00
gca	A	69	1192.00
gcg	A	6	198.40
---	--	------	------
mPD		0.29	0.71
		nPD:	0.41
		N. weight:	1.7
		Sc. PD:	0.16
		Sc. rank:	830.0

,

PB2			
Pos.	281	obs:	exp:
cgt	R	0	84.50
cgc	R	0	141.50
cga	R	11	269.10
cgg	R	3	193.40
aga	R	2327	1228.00
agg	R	336	760.90
---	--	------	------
mPD		0.23	0.94
		nPD:	0.25
		N. weight:	0.81
		Sc. PD:	-0.026
		Sc. rank:	-4.2

,

PB2			
Pos.	282	obs:	exp:
tct	S	0	4.54
tcc	S	0	4.27
tca	S	0	6.56
tcg	S	0	1.43
aat	N	157	1506.00
aac	N	2493	1144.00
agt	S	2	5.03
agc	S	25	5.17
---	--	------	------
mPD		0.13	0.53
		nPD:	0.25
		N. weight:	1.1
		Sc. PD:	-0.033
		Sc. rank:	2.3

,

PB2			
Pos.	283	obs:	exp:
att	I	409	949.60
atc	I	6	686.60
ata	I	2262	1041.00
---	--	------	------
mPD		0.26	0.66
		nPD:	0.4
		N. weight:	0.96
		Sc. PD:	0.082
		Sc. rank:	440.2

,

PB2			
Pos.	284	obs:	exp:
att	I	1	0.71
atc	I	0	0.51
ata	I	1	0.78
gtt	V	373	593.90
gtc	V	40	504.80
gta	V	2167	675.30
gtg	V	95	901.00
---	--	------	------
mPD		0.32	0.74
		nPD:	0.44
		N. weight:	1.4
		Sc. PD:	0.16
		Sc. rank:	847.6

,

PB2			
Pos.	285	obs:	exp:
cgt	R	0	84.50
cgc	R	0	141.50
cga	R	1	269.10
cgg	R	0	193.40
aga	R	2546	1228.00
agg	R	130	760.90
---	--	------	------
mPD		0.093	0.94
		nPD:	0.1
		N. weight:	1.1
		Sc. PD:	-0.16
		Sc. rank:	-615.0

,

PB2			
Pos.	286	obs:	exp:
cgt	R	0	84.50
cgc	R	0	141.50
cga	R	2	269.10
cgg	R	0	193.40
aga	R	2583	1228.00
agg	R	92	760.90
---	--	------	------
mPD		0.068	0.94
		nPD:	0.07
		N. weight:	1.2
		Sc. PD:	-0.2
		Sc. rank:	-852.6

,

PB2			
Pos.	287	obs:	exp:
tct	S	0	0.17
tcc	S	0	0.16
tca	S	1	0.24
tcg	S	0	0.05
agt	S	0	0.19
agc	S	0	0.19
gct	A	34	656.00
gcc	A	9	613.10
gca	A	2620	1206.00
gcg	A	13	200.70
---	--	------	------
mPD		0.042	0.68
		nPD:	0.06
		N. weight:	1.3
		Sc. PD:	-0.22
		Sc. rank:	-1023.2

,

PB2			
Pos.	288	obs:	exp:
tct	S	0	1.01
tcc	S	0	0.95
tca	S	6	1.46
tcg	S	0	0.32
att	I	0	108.90
atc	I	0	78.74
ata	I	307	119.40
act	T	0	31.73
acc	T	4	27.90
aca	T	111	58.62
acg	T	14	10.76
agt	S	0	1.12
agc	S	0	1.15
gct	A	6	547.90
gcc	A	1	512.10
gca	A	2177	1007.00
gcg	A	51	167.60
---	--	------	------
mPD		0.54	1.2
		nPD:	0.46
		N. weight:	1.4
		Sc. PD:	0.18
		Sc. rank:	870.7

,

PB2			
Pos.	289	obs:	exp:
gtt	V	5	594.40
gtc	V	2	505.10
gta	V	191	675.80
gtg	V	2479	901.70
---	--	------	------
mPD		0.14	0.74
		nPD:	0.19
		N. weight:	1.5
		Sc. PD:	-0.12
		Sc. rank:	-262.1

,

PB2			
Pos.	290	obs:	exp:
tct	S	26	449.90
tcc	S	20	422.80
tca	S	2543	649.60
tcg	S	86	142.20
agt	S	0	497.90
agc	S	0	512.70
gct	A	0	0.49
gcc	A	0	0.46
gca	A	2	0.90
gcg	A	0	0.15
---	--	------	------
mPD		0.097	1.6
		nPD:	0.06
		N. weight:	2.3
		Sc. PD:	-0.4
		Sc. rank:	-1851.7

,

PB2			
Pos.	291	obs:	exp:
act	T	0	3.94
acc	T	0	3.46
aca	T	16	7.27
acg	T	0	1.33
gct	A	18	652.30
gcc	A	0	609.70
gca	A	2611	1199.00
gcg	A	32	199.60
---	--	------	------
mPD		0.049	0.69
		nPD:	0.07
		N. weight:	1.3
		Sc. PD:	-0.22
		Sc. rank:	-967.4

,

PB2			
Pos.	292	obs:	exp:
gat	D	604	1506.00
gac	D	2073	1171.00
---	--	------	------
mPD		0.35	0.49
		nPD:	0.71
		N. weight:	0.44
		Sc. PD:	0.14
		Sc. rank:	497.9

,

PB2			
Pos.	293	obs:	exp:
cct	P	14	790.00
ccc	P	5	539.00
cca	P	2614	1060.00
ccg	P	44	288.00
---	--	------	------
mPD		0.046	0.70
		nPD:	0.07
		N. weight:	1.5
		Sc. PD:	-0.26
		Sc. rank:	-1169.9

,

PB2			
Pos.	294	obs:	exp:
tta	L	2154	291.30
ttg	L	85	502.80
ctt	L	28	540.60
ctc	L	0	389.50
cta	L	35	467.40
ctg	L	375	485.30
---	--	------	------
mPD		0.58	1.1
		nPD:	0.51
		N. weight:	2.7
		Sc. PD:	0.45
		Sc. rank:	2071.5

,

PB2			
Pos.	295	obs:	exp:
tct	S	0	4.04
tcc	S	0	3.79
tca	S	24	5.83
tcg	S	0	1.28
agt	S	0	4.47
agc	S	0	4.60
gtt	V	0	0.22
gtc	V	0	0.19
gta	V	1	0.25
gtg	V	0	0.34
gct	A	38	650.10
gcc	A	2	607.60
gca	A	2473	1195.00
gcg	A	139	198.90
---	--	------	------
mPD		0.14	0.70
		nPD:	0.21
		N. weight:	1.2
		Sc. PD:	-0.073
		Sc. rank:	-132.8

,

PB2			
Pos.	296	obs:	exp:
ttt	F	1	0.45
ttc	F	0	0.55
tct	S	2187	449.90
tcc	S	72	422.80
tca	S	397	649.60
tcg	S	19	142.20
agt	S	0	497.90
agc	S	0	512.70
gct	A	1	0.25
gcc	A	0	0.23
gca	A	0	0.45
gcg	A	0	0.07
---	--	------	------
mPD		0.31	1.6
		nPD:	0.19
		N. weight:	2.2
		Sc. PD:	-0.16
		Sc. rank:	-316.2

,

PB2			
Pos.	297	obs:	exp:
tta	L	34	291.30
ttg	L	0	502.80
ctt	L	79	540.60
ctc	L	2098	389.50
cta	L	380	467.40
ctg	L	86	485.30
---	--	------	------
mPD		0.39	1.1
		nPD:	0.34
		N. weight:	2.1
		Sc. PD:	0.082
		Sc. rank:	626.0

,

PB2			
Pos.	298	obs:	exp:
tta	L	46	291.20
ttg	L	2461	502.60
ctt	L	0	540.40
ctc	L	0	389.30
cta	L	3	467.30
ctg	L	166	485.10
att	I	0	0.35
atc	I	0	0.26
ata	I	1	0.39
---	--	------	------
mPD		0.16	1.1
		nPD:	0.14
		N. weight:	2.5
		Sc. PD:	-0.29
		Sc. rank:	-870.4

,

PB2			
Pos.	299	obs:	exp:
gaa	E	2126	1586.00
gag	E	551	1091.00
---	--	------	------
mPD		0.33	0.48
		nPD:	0.68
		N. weight:	0.17
		Sc. PD:	0.052
		Sc. rank:	190.0

,

PB2			
Pos.	300	obs:	exp:
tta	L	0	0.11
ttg	L	0	0.19
ctt	L	0	0.20
ctc	L	0	0.15
cta	L	0	0.17
ctg	L	1	0.18
att	I	0	8.16
atc	I	0	5.90
ata	I	23	8.94
atg	M	2653	2653.00
---	--	------	------
mPD		0.018	0.018
		nPD:	0.97
		N. weight:	0.016
		Sc. PD:	0.0085
		Sc. rank:	20.1

,

PB2			
Pos.	301	obs:	exp:
tct	S	0	2.02
tcc	S	12	1.90
tca	S	0	2.91
tcg	S	0	0.64
tgt	C	418	1147.00
tgc	C	2247	1518.00
agt	S	0	2.23
agc	S	0	2.30
---	--	------	------
mPD		0.27	0.50
		nPD:	0.54
		N. weight:	0.34
		Sc. PD:	0.066
		Sc. rank:	287.1

,

PB2			
Pos.	302	obs:	exp:
tat	Y	0	6.16
tac	Y	11	4.84
cat	H	53	1537.00
cac	H	2612	1128.00
aat	N	0	0.57
aac	N	1	0.43
---	--	------	------
mPD		0.048	0.50
		nPD:	0.1
		N. weight:	1.4
		Sc. PD:	-0.21
		Sc. rank:	-807.3

,

PB2			
Pos.	303	obs:	exp:
tct	S	0	399.30
tcc	S	0	375.20
tca	S	0	576.50
tcg	S	0	126.20
aat	N	0	0.57
aac	N	1	0.43
agt	S	19	441.90
agc	S	2355	455.00
ggt	G	17	47.37
ggc	G	277	38.27
gga	G	7	136.80
ggg	G	1	79.57
---	--	------	------
mPD		0.23	1.8
		nPD:	0.13
		N. weight:	3.
		Sc. PD:	-0.36
		Sc. rank:	-1137.1

,

PB2			
Pos.	304	obs:	exp:
act	T	4	658.50
acc	T	3	578.90
aca	T	2547	1216.00
acg	T	123	223.20
---	--	------	------
mPD		0.093	0.68
		nPD:	0.14
		N. weight:	1.2
		Sc. PD:	-0.14
		Sc. rank:	-437.7

,

PB2			
Pos.	305	obs:	exp:
tta	L	0	0.33
ttg	L	0	0.56
ctt	L	0	0.61
ctc	L	0	0.44
cta	L	0	0.52
ctg	L	3	0.54
caa	Q	1332	1431.00
cag	Q	1321	1222.00
cgt	R	0	0.66
cgc	R	0	1.11
cga	R	0	2.11
cgg	R	21	1.52
aga	R	0	9.63
agg	R	0	5.97
---	--	------	------
mPD		0.52	0.53
		nPD:	0.98
		N. weight:	0.05
		Sc. PD:	0.026
		Sc. rank:	61.6

,

PB2			
Pos.	306	obs:	exp:
att	I	2628	949.30
atc	I	39	686.40
ata	I	9	1040.00
gtt	V	1	0.22
gtc	V	0	0.19
gta	V	0	0.25
gtg	V	0	0.34
---	--	------	------
mPD		0.036	0.66
		nPD:	0.05
		N. weight:	1.7
		Sc. PD:	-0.31
		Sc. rank:	-1455.8

,

PB2			
Pos.	307	obs:	exp:
tct	S	0	0.17
tcc	S	0	0.16
tca	S	0	0.24
tcg	S	0	0.05
agt	S	1	0.19
agc	S	0	0.19
ggt	G	436	419.70
ggc	G	5	339.10
gga	G	2157	1212.00
ggg	G	78	705.10
---	--	------	------
mPD		0.32	0.69
		nPD:	0.47
		N. weight:	0.75
		Sc. PD:	0.11
		Sc. rank:	511.3

,

PB2			
Pos.	308	obs:	exp:
cgt	R	0	0.06
cgc	R	0	0.11
cga	R	0	0.20
cgg	R	0	0.14
aga	R	1	0.92
agg	R	1	0.57
ggt	G	4	419.60
ggc	G	0	339.00
gga	G	2329	1212.00
ggg	G	342	704.80
---	--	------	------
mPD		0.23	0.69
		nPD:	0.33
		N. weight:	0.87
		Sc. PD:	0.029
		Sc. rank:	235.9

,

PB2			
Pos.	309	obs:	exp:
tta	L	1	0.33
ttg	L	0	0.56
ctt	L	0	0.61
ctc	L	0	0.44
cta	L	2	0.52
ctg	L	0	0.54
att	I	16	571.10
atc	I	0	412.90
ata	I	1594	625.90
atg	M	175	175.00
act	T	2	31.24
acc	T	0	27.46
aca	T	125	57.71
acg	T	0	10.59
gtt	V	0	160.80
gtc	V	1	136.60
gta	V	644	182.80
gtg	V	79	243.90
gct	A	0	9.07
gcc	A	0	8.48
gca	A	37	16.68
gcg	A	0	2.77
gaa	E	1	0.59
gag	E	0	0.41
---	--	------	------
mPD		0.71	1.3
		nPD:	0.56
		N. weight:	1.6
		Sc. PD:	0.33
		Sc. rank:	1427.4

,

PB2			
Pos.	310	obs:	exp:
---	-	2677	2677.00
---	--	------	------
mPD		0	0
		nPD:	1.
		N. weight:	0.
		Sc. PD:	0
		Sc. rank:	0

,

PB2			
Pos.	311	obs:	exp:
---	-	2677	2677.00
---	--	------	------
mPD		0	0
		nPD:	1.
		N. weight:	0.
		Sc. PD:	0
		Sc. rank:	0

,

PB2			
Pos.	312	obs:	exp:
---	-	2677	2677.00
---	--	------	------
mPD		0	0
		nPD:	1.
		N. weight:	0.
		Sc. PD:	0
		Sc. rank:	0

,

PB2			
Pos.	313	obs:	exp:
tta	L	0	0.33
ttg	L	0	0.56
tgg	W	15	15.00
ctt	L	0	0.61
ctc	L	0	0.44
cta	L	0	0.52
ctg	L	3	0.54
cat	H	0	1.15
cac	H	2	0.85
caa	Q	5	28.58
cag	Q	48	24.42
cgt	R	1	73.20
cgc	R	0	122.60
cga	R	210	233.10
cgg	R	445	167.50
atg	M	1	1.00
aaa	K	10	167.10
aag	K	265	107.90
aga	R	97	1063.00
agg	R	1566	659.10
ggt	G	0	1.41
ggc	G	0	1.14
gga	G	0	4.08
ggg	G	9	2.37
---	--	------	------
mPD		0.84	1.2
		nPD:	0.73
		N. weight:	1.2
		Sc. PD:	0.42
		Sc. rank:	1434.1

,

PB2			
Pos.	314	obs:	exp:
tta	L	7	36.13
ttg	L	325	62.36
ctt	L	0	67.05
ctc	L	0	48.30
cta	L	0	57.97
ctg	L	0	60.18
atg	M	2345	2345.00
---	--	------	------
mPD		0.22	0.37
		nPD:	0.6
		N. weight:	0.36
		Sc. PD:	0.087
		Sc. rank:	353.8

,

PB2			
Pos.	315	obs:	exp:
tta	L	0	0.11
ttg	L	1	0.19
ctt	L	0	0.20
ctc	L	0	0.15
cta	L	0	0.17
ctg	L	0	0.18
att	I	20	11.00
atc	I	0	7.95
ata	I	11	12.05
atg	M	198	198.00
act	T	8	2.21
acc	T	0	1.95
aca	T	0	4.09
acg	T	1	0.75
gtt	V	520	540.20
gtc	V	0	459.10
gta	V	29	614.20
gtg	V	1884	819.50
gct	A	3	1.23
gcc	A	0	1.15
gca	A	0	2.25
gcg	A	2	0.37
---	--	------	------
mPD		0.52	0.90
		nPD:	0.58
		N. weight:	1.
		Sc. PD:	0.23
		Sc. rank:	969.7

,

PB2			
Pos.	316	obs:	exp:
aat	N	0	0.57
aac	N	1	0.43
gat	D	73	1495.00
gac	D	2585	1163.00
gaa	E	18	10.67
gag	E	0	7.33
---	--	------	------
mPD		0.067	0.50
		nPD:	0.13
		N. weight:	1.3
		Sc. PD:	-0.15
		Sc. rank:	-484.0

,

PB2			
Pos.	317	obs:	exp:
tta	L	2	0.33
ttg	L	0	0.56
ctt	L	0	0.61
ctc	L	1	0.44
cta	L	0	0.52
ctg	L	0	0.54
att	I	381	947.50
atc	I	2271	685.10
ata	I	19	1038.00
gtt	V	0	0.67
gtc	V	3	0.57
gta	V	0	0.76
gtg	V	0	1.01
---	--	------	------
mPD		0.26	0.66
		nPD:	0.4
		N. weight:	1.6
		Sc. PD:	0.13
		Sc. rank:	720.3

,

PB2			
Pos.	318	obs:	exp:
tta	L	0	291.00
ttg	L	0	502.30
ctt	L	2387	540.00
ctc	L	283	389.10
cta	L	3	466.90
ctg	L	1	484.70
att	I	3	1.06
atc	I	0	0.77
ata	I	0	1.17
---	--	------	------
mPD		0.19	1.1
		nPD:	0.17
		N. weight:	2.4
		Sc. PD:	-0.22
		Sc. rank:	-515.8

,

PB2			
Pos.	319	obs:	exp:
cgt	R	0	68.21
cgc	R	0	114.20
cga	R	0	217.20
cgg	R	0	156.10
aaa	K	481	313.60
aag	K	35	202.40
aga	R	1914	991.00
agg	R	247	614.20
---	--	------	------
mPD		0.50	1.2
		nPD:	0.42
		N. weight:	0.82
		Sc. PD:	0.085
		Sc. rank:	431.8

,

PB2			
Pos.	320	obs:	exp:
cat	H	4	2.31
cac	H	0	1.69
caa	Q	359	1439.00
cag	Q	2310	1230.00
cgt	R	0	0.09
cgc	R	0	0.16
cga	R	0	0.30
cgg	R	3	0.22
aaa	K	0	0.61
aag	K	1	0.39
aga	R	0	1.38
agg	R	0	0.85
---	--	------	------
mPD		0.24	0.50
		nPD:	0.47
		N. weight:	0.67
		Sc. PD:	0.095
		Sc. rank:	462.8

,

PB2			
Pos.	321	obs:	exp:
---	-	2677	2677.00
---	--	------	------
mPD		0	0
		nPD:	1.
		N. weight:	0.
		Sc. PD:	0
		Sc. rank:	0

,

PB2			
Pos.	322	obs:	exp:
---	-	2677	2677.00
---	--	------	------
mPD		0	0
		nPD:	1.
		N. weight:	0.
		Sc. PD:	0
		Sc. rank:	0

,

PB2			
Pos.	323	obs:	exp:
act	T	0	0.49
acc	T	2	0.43
aca	T	0	0.91
acg	T	0	0.17
aat	N	1466	1520.00
aac	N	1209	1155.00
---	--	------	------
mPD		0.50	0.49
		nPD:	1.01
		N. weight:	0.0066
		Sc. PD:	0.0036
		Sc. rank:	8.1

,

PB2			
Pos.	324	obs:	exp:
tct	S	0	0.67
tcc	S	0	0.63
tca	S	4	0.97
tcg	S	0	0.21
cct	P	33	788.50
ccc	P	1	538.00
cca	P	2599	1058.00
ccg	P	39	287.40
caa	Q	1	0.54
cag	Q	0	0.46
agt	S	0	0.74
agc	S	0	0.77
---	--	------	------
mPD		0.057	0.71
		nPD:	0.08
		N. weight:	1.5
		Sc. PD:	-0.24
		Sc. rank:	-976.0

,

PB2			
Pos.	325	obs:	exp:
atg	M	1	1.00
act	T	742	658.00
acc	T	4	578.40
aca	T	501	1216.00
acg	T	1428	223.10
gct	A	0	0.25
gcc	A	0	0.23
gca	A	1	0.45
gcg	A	0	0.07
---	--	------	------
mPD		0.60	0.68
		nPD:	0.89
		N. weight:	1.6
		Sc. PD:	0.73
		Sc. rank:	1936.5

,

PB2			
Pos.	326	obs:	exp:
gaa	E	469	1586.00
gag	E	2208	1091.00
---	--	------	------
mPD		0.29	0.48
		nPD:	0.6
		N. weight:	0.68
		Sc. PD:	0.16
		Sc. rank:	665.5

,

PB2			
Pos.	327	obs:	exp:
gaa	E	2198	1586.00
gag	E	479	1091.00
---	--	------	------
mPD		0.29	0.48
		nPD:	0.61
		N. weight:	0.23
		Sc. PD:	0.056
		Sc. rank:	224.1

,

PB2			
Pos.	328	obs:	exp:
cct	P	0	0.30
ccc	P	0	0.20
cca	P	1	0.40
ccg	P	0	0.11
caa	Q	2500	1443.00
cag	Q	176	1233.00
---	--	------	------
mPD		0.12	0.50
		nPD:	0.25
		N. weight:	0.72
		Sc. PD:	-0.022
		Sc. rank:	-2.4

,

PB2			
Pos.	329	obs:	exp:
gct	A	381	656.20
gcc	A	2191	613.40
gca	A	102	1207.00
gcg	A	3	200.80
---	--	------	------
mPD		0.31	0.68
		nPD:	0.45
		N. weight:	1.6
		Sc. PD:	0.21
		Sc. rank:	992.8

,

PB2			
Pos.	330	obs:	exp:
att	I	0	15.25
atc	I	0	11.03
ata	I	43	16.72
gtt	V	26	584.80
gtc	V	32	497.00
gta	V	2038	664.90
gtg	V	538	887.20
---	--	------	------
mPD		0.39	0.77
		nPD:	0.5
		N. weight:	1.3
		Sc. PD:	0.21
		Sc. rank:	995.8

,

PB2			
Pos.	331	obs:	exp:
aat	N	4	3.41
aac	N	2	2.59
gat	D	432	1502.00
gac	D	2238	1168.00
gaa	E	1	0.59
gag	E	0	0.41
---	--	------	------
mPD		0.28	0.50
		nPD:	0.56
		N. weight:	0.64
		Sc. PD:	0.13
		Sc. rank:	567.3

PB2			
Pos.	332	obs:	exp:
att	I	16	949.60
atc	I	4	686.60
ata	I	2657	1041.00
---	--	------	------
mPD		0.015	0.66
		nPD:	0.02
		N. weight:	1.7
		Sc. PD:	-0.34
		Sc. rank:	-1795.6

PB2			
Pos.	333	obs:	exp:
tgt	C	33	1152.00
tgc	C	2644	1525.00
---	--	------	------
mPD		0.024	0.49
		nPD:	0.05
		N. weight:	0.93
		Sc. PD:	-0.17
		Sc. rank:	-794.5

PB2			
Pos.	334	obs:	exp:
aaa	K	875	1627.00
aag	K	1802	1050.00
---	--	------	------
mPD		0.44	0.48
		nPD:	0.92
		N. weight:	0.3
		Sc. PD:	0.15
		Sc. rank:	369.4

PB2			
Pos.	335	obs:	exp:
gct	A	41	656.20
gcc	A	9	613.40
gca	A	2618	1207.00
gcg	A	9	200.80
---	--	------	------
mPD		0.043	0.68
		nPD:	0.06
		N. weight:	1.3
		Sc. PD:	-0.22
		Sc. rank:	-1012.6

PB2			
Pos.	336	obs:	exp:
tct	S	0	1.35
tcc	S	0	1.26
tca	S	8	1.94
tcg	S	0	0.43
cct	P	1	0.89
ccc	P	0	0.60
cca	P	2	1.19
ccg	P	0	0.32
agt	S	0	1.49
agc	S	0	1.53
gct	A	7	653.50
gcc	A	30	610.80
gca	A	2570	1202.00
gcg	A	59	199.90
---	--	------	------
mPD		0.079	0.69
		nPD:	0.11
		N. weight:	1.2
		Sc. PD:	-0.16
		Sc. rank:	-593.3

PB2			
Pos.	337	obs:	exp:
tta	L	1	0.33
ttg	L	0	0.56
ctt	L	0	0.61
ctc	L	0	0.44
cta	L	0	0.52
ctg	L	2	0.54
att	I	9	253.30
atc	I	1	183.10
ata	I	704	277.60
atg	M	1960	1960.00
---	--	------	------
mPD		0.40	0.44
		nPD:	0.9
		N. weight:	0.43
		Sc. PD:	0.2
		Sc. rank:	532.0

PB2			
Pos.	338	obs:	exp:
ggt	G	390	419.90
ggc	G	36	339.20
gga	G	127	1213.00
ggg	G	2124	705.40
---	--	------	------
mPD		0.35	0.68
		nPD:	0.51
		N. weight:	1.4
		Sc. PD:	0.23
		Sc. rank:	1046.2

PB2			
Pos.	339	obs:	exp:
tta	L	69	290.60
ttg	L	1085	501.50
ctt	L	16	539.20
ctc	L	0	388.50
cta	L	40	466.20
ctg	L	1460	484.00
atg	M	7	7.00
---	--	------	------
mPD		0.58	1.1
		nPD:	0.51
		N. weight:	1.5
		Sc. PD:	0.26
		Sc. rank:	1204.5

PB2			
Pos.	340	obs:	exp:
cgt	R	0	84.31
cgc	R	0	141.20
cga	R	0	268.50
cgg	R	1	193.00
aaa	K	4	3.65
aag	K	2	2.35
aga	R	638	1225.00
agg	R	2032	759.20
---	--	------	------
mPD		0.37	0.94
		nPD:	0.39
		N. weight:	1.1
		Sc. PD:	0.088
		Sc. rank:	479.2

PB2			
Pos.	341	obs:	exp:
att	I	2027	925.50
atc	I	551	669.20
ata	I	31	1014.00
gtt	V	58	15.10
gtc	V	10	12.83
gta	V	0	17.17
gtg	V	0	22.90
---	--	------	------
mPD		0.40	0.71
		nPD:	0.56
		N. weight:	1.
		Sc. PD:	0.21
		Sc. rank:	900.2

PB2			
Pos.	342	obs:	exp:
tct	S	0	450.30
tcc	S	0	423.10
tca	S	0	650.10
tcg	S	0	142.30
agt	S	129	498.30
agc	S	2548	513.00
---	--	------	------
mPD		0.092	1.6
		nPD:	0.06
		N. weight:	2.7
		Sc. PD:	-0.48
		Sc. rank:	-2234.6

PB2			
Pos.	343	obs:	exp:
tct	S	36	450.30
tcc	S	1800	423.10
tca	S	811	650.10
tcg	S	30	142.30
agt	S	0	498.30
agc	S	0	513.00
---	--	------	------
mPD		0.46	1.6
		nPD:	0.28
		N. weight:	1.8
		Sc. PD:	-0.012
		Sc. rank:	183.7

PB2			
Pos.	344	obs:	exp:
tct	S	2191	450.30
tcc	S	416	423.10
tca	S	61	650.10
tcg	S	9	142.30
agt	S	0	498.30
agc	S	0	513.00
---	--	------	------
mPD		0.31	1.6
		nPD:	0.19
		N. weight:	2.3
		Sc. PD:	-0.18
		Sc. rank:	-368.6

PB2			
Pos.	345	obs:	exp:
ttt	F	430	1200.00
ttc	F	2247	1477.00
---	--	------	------
mPD		0.27	0.49
		nPD:	0.55
		N. weight:	0.35
		Sc. PD:	0.069
		Sc. rank:	300.7

PB2			
Pos.	346	obs:	exp:
tct	S	0	450.30
tcc	S	0	423.10
tca	S	0	650.10
tcg	S	0	142.30
agt	S	895	498.30
agc	S	1782	513.00
---	--	------	------
mPD		0.45	1.6
		nPD:	0.27
		N. weight:	1.9
		Sc. PD:	-0.022
		Sc. rank:	128.9

PB2			
Pos.	347	obs:	exp:
ttt	F	2418	1200.00
ttc	F	259	1477.00
---	--	------	------
mPD		0.17	0.49
		nPD:	0.35
		N. weight:	0.86
		Sc. PD:	0.043
		Sc. rank:	286.7

PB2			
Pos.	348	obs:	exp:
ggt	G	2229	419.90
ggc	G	33	339.20
gga	G	390	1213.00
ggg	G	25	705.40
---	--	------	------
mPD		0.29	0.68
		nPD:	0.42
		N. weight:	2.2
		Sc. PD:	0.21
		Sc. rank:	1094.8

PB2			
Pos.	349	obs:	exp:
cgt	R	0	0.06
cgc	R	0	0.11
cga	R	0	0.20
cgg	R	0	0.14
aga	R	0	0.92
agg	R	2	0.57
gaa	E	0	1.19
gag	E	2	0.81
ggt	G	307	419.30
ggc	G	128	338.70
gga	G	127	1211.00
ggg	G	2111	704.30
---	--	------	------
mPD		0.36	0.69
		nPD:	0.53
		N. weight:	1.3
		Sc. PD:	0.23
		Sc. rank:	1024.6

PB2			
Pos.	350	obs:	exp:
ttt	F	256	1198.00
ttc	F	2418	1476.00
tat	Y	0	1.68
tac	Y	3	1.32
---	--	------	------
mPD		0.18	0.50
		nPD:	0.35
		N. weight:	0.56
		Sc. PD:	0.027
		Sc. rank:	183.7

PB2			
Pos.	351	obs:	exp:
cct	P	1	0.30
ccc	P	0	0.20
cca	P	0	0.40
ccg	P	0	0.11
att	I	0	0.71
atc	I	2	0.51
ata	I	0	0.78
act	T	1112	657.50
acc	T	1453	578.00
aca	T	103	1215.00
acg	T	5	222.90
aat	N	1	0.57
aac	N	0	0.43
---	--	------	------
mPD		0.53	0.68
		nPD:	0.78
		N. weight:	1.2
		Sc. PD:	0.44
		Sc. rank:	1372.6

PB2			
Pos.	352	obs:	exp:
ttt	F	46	1199.00
ttc	F	2630	1477.00
tta	L	1	0.11
ttg	L	0	0.19
ctt	L	0	0.20
ctc	L	0	0.15
cta	L	0	0.17
ctg	L	0	0.18
---	--	------	------
mPD		0.035	0.50
		nPD:	0.07
		N. weight:	0.95
		Sc. PD:	-0.16
		Sc. rank:	-692.9

PB2			
Pos.	353	obs:	exp:
aaa	K	2646	1627.00
aag	K	31	1050.00
---	--	------	------
mPD		0.023	0.48
		nPD:	0.05
		N. weight:	0.82
		Sc. PD:	-0.15
		Sc. rank:	-722.9

PB2			
Pos.	354	obs:	exp:
cgt	R	0	84.15
cgc	R	0	140.90
cga	R	4	268.00
cgg	R	1	192.60
aaa	K	2	6.69
aag	K	9	4.31
aga	R	1020	1223.00
agg	R	1641	757.70
---	--	------	------
mPD		0.48	0.95
		nPD:	0.51
		N. weight:	0.74
		Sc. PD:	0.13
		Sc. rank:	587.0

PB2			
Pos.	355	obs:	exp:
act	T	1	658.50
acc	T	5	578.90
aca	T	2663	1216.00
acg	T	8	223.20
---	--	------	------
mPD		0.010	0.68
		nPD:	0.02
		N. weight:	1.4
		Sc. PD:	-0.3
		Sc. rank:	-1576.1

PB2

Pos.	356	obs:	exp:
tct	S	0	446.60
tcc	S	0	419.60
tca	S	0	644.70
tcg	S	0	141.10
cgt	R	0	0.06
cgc	R	0	0.11
cga	R	0	0.20
cgg	R	0	0.14
aat	N	5	10.23
aac	N	13	7.77
aaa	K	1	1.22
aag	K	1	0.78
agt	S	541	494.20
agc	S	2114	508.80
aga	R	2	0.92
agg	R	0	0.57
---	--	------	------
mPD		0.34	1.6
		nPD:	0.21
		N. weight:	2.1
		Sc. PD:	-0.13
		Sc. rank:	-207.2

,

PB2

Pos.	357	obs:	exp:
ggt	G	26	419.90
ggc	G	23	339.20
gga	G	2360	1213.00
ggg	G	268	705.40
---	--	------	------
mPD		0.21	0.68
		nPD:	0.31
		N. weight:	0.82
		Sc. PD:	0.014
		Sc. rank:	167.9

,

PB2

Pos.	358	obs:	exp:
ttt	F	0	0.45
ttc	F	1	0.55
tct	S	0	449.10
tcc	S	0	422.00
tca	S	2604	648.40
tcg	S	66	141.90
act	T	0	1.23
acc	T	0	1.08
aca	T	5	2.27
acg	T	0	0.42
agt	S	0	497.00
agc	S	0	511.70
gct	A	0	0.25
gcc	A	0	0.23
gca	A	1	0.45
gcg	A	0	0.07
---	--	------	------
mPD		0.054	1.6
		nPD:	0.03
		N. weight:	2.5
		Sc. PD:	-0.49
		Sc. rank:	-2484.0

,

PB2

Pos.	359	obs:	exp:
tct	S	1371	449.90
tcc	S	32	422.80
tca	S	1258	649.60
tcg	S	14	142.20
agt	S	0	497.90
agc	S	0	512.70
gct	A	2	0.49
gcc	A	0	0.46
gca	A	0	0.90
gcg	A	0	0.15
---	--	------	------
mPD		0.52	1.6
		nPD:	0.32
		N. weight:	1.6
		Sc. PD:	0.035
		Sc. rank:	371.2

,

PB2

Pos.	360	obs:	exp:
att	I	125	90.81
atc	I	131	65.66
ata	I	0	99.52
act	T	18	7.87
acc	T	14	6.92
aca	T	0	14.54
acg	T	0	2.67
gtt	V	1599	528.70
gtc	V	762	449.30
gta	V	6	601.10
gtg	V	14	802.00
gct	A	6	1.72
gcc	A	1	1.60
gca	A	0	3.16
gcg	A	0	0.52
gaa	E	1	0.59
gag	E	0	0.41
---	--	------	------
mPD		0.68	0.97
		nPD:	0.7
		N. weight:	1.6
		Sc. PD:	0.5
		Sc. rank:	1761.1

,

PB2

Pos.	361	obs:	exp:
tct	S	0	0.17
tcc	S	0	0.16
tca	S	0	0.24
tcg	S	0	0.05
cgt	R	0	0.38
cgc	R	0	0.63
cga	R	0	1.21
cgg	R	0	0.87
act	T	0	0.25
acc	T	0	0.22
aca	T	1	0.45
acg	T	0	0.08
aat	N	2	1.71
aac	N	1	1.29
aaa	K	541	1617.00
aag	K	2119	1043.00
agt	S	0	0.19
agc	S	1	0.19
aga	R	1	5.50
agg	R	11	3.41
---	--	------	------
mPD		0.34	0.49
		nPD:	0.68
		N. weight:	0.64
		Sc. PD:	0.19
		Sc. rank:	711.1

,

PB2

Pos.	362	obs:	exp:
tct	S	0	0.34
tcc	S	0	0.32
tca	S	0	0.49
tcg	S	0	0.11
cgt	R	0	12.31
cgc	R	0	20.61
cga	R	0	39.20
cgg	R	0	28.18
act	T	0	1.23
acc	T	0	1.08
aca	T	5	2.27
acg	T	0	0.42
aat	N	123	70.48
aac	N	1	53.52
aaa	K	1919	1308.00
aag	K	234	844.50
agt	S	2	0.37
agc	S	0	0.38
aga	R	166	178.90
agg	R	224	110.80
gat	D	3	1.69
gac	D	0	1.31
---	--	------	------
mPD		0.61	0.87
		nPD:	0.71
		N. weight:	0.46
		Sc. PD:	0.15
		Sc. rank:	518.4

,

PB2

Pos.	363	obs:	exp:
aaa	K	1	0.61
aag	K	0	0.39
gat	D	7	7.31
gac	D	6	5.69
gaa	E	2551	1567.00
gag	E	93	1077.00
ggt	G	0	2.98
ggc	G	0	2.41
gga	G	19	8.61
ggg	G	0	5.01
---	--	------	------
mPD		0.091	0.50
		nPD:	0.18
		N. weight:	0.72
		Sc. PD:	-0.059
		Sc. rank:	-131.7

,

PB2

Pos.	364	obs:	exp:
aaa	K	1	0.61
aag	K	0	0.39
gat	D	2	1.12
gac	D	0	0.88
gaa	E	2559	1584.00
gag	E	115	1090.00
---	--	------	------
mPD		0.084	0.48
		nPD:	0.17
		N. weight:	0.67
		Sc. PD:	-0.059
		Sc. rank:	-136.5

,

PB2

Pos.	365	obs:	exp:
gaa	E	2667	1586.00
gag	E	10	1091.00
---	--	------	------
mPD		0.0074	0.48
		nPD:	0.02
		N. weight:	0.93
		Sc. PD:	-0.19
		Sc. rank:	-1036.7

,

PB2

Pos.	366	obs:	exp:
tta	L	0	1.52
ttg	L	13	2.63
ctt	L	0	2.83
ctc	L	0	2.04
cta	L	0	2.44
ctg	L	1	2.54
att	I	0	0.35
atc	I	0	0.26
ata	I	1	0.39
atg	M	218	218.00
gtt	V	3	542.70
gtc	V	0	461.20
gta	V	58	617.00
gtg	V	2383	823.20
---	--	------	------
mPD		0.21	0.88
		nPD:	0.23
		N. weight:	1.7
		Sc. PD:	-0.071
		Sc. rank:	-48.3

,

PB2

Pos.	367	obs:	exp:
---	-	2677	2677.00
---	--	------	------
mPD		0	0
		nPD:	1.
		N. weight:	0.
		Sc. PD:	0
		Sc. rank:	0

,

PB2

Pos.	368	obs:	exp:
---	-	2677	2677.00
---	--	------	------
mPD		0	0
		nPD:	1.
		N. weight:	0.
		Sc. PD:	0
		Sc. rank:	0

,

PB2

Pos.	369	obs:	exp:
---	-	2677	2677.00
---	--	------	------
mPD		0	0
		nPD:	1.
		N. weight:	0.
		Sc. PD:	0
		Sc. rank:	0

,

PB2

Pos.	370	obs:	exp:
---	-	2677	2677.00
---	--	------	------
mPD		0	0
		nPD:	1.
		N. weight:	0.
		Sc. PD:	0
		Sc. rank:	0

,

PB2

Pos.	371	obs:	exp:
---	-	2677	2677.00
---	--	------	------
mPD		0	0
		nPD:	1.
		N. weight:	0.
		Sc. PD:	0
		Sc. rank:	0

,

PB2

Pos.	372	obs:	exp:
---	-	2677	2677.00
---	--	------	------
mPD		0	0
		nPD:	1.
		N. weight:	0.
		Sc. PD:	0
		Sc. rank:	0

,

PB2

Pos.	373	obs:	exp:
---	-	2677	2677.00
---	--	------	------
mPD		0	0
		nPD:	1.
		N. weight:	0.
		Sc. PD:	0
		Sc. rank:	0

,

PB2

Pos.	374	obs:	exp:
---	-	2677	2677.00
---	--	------	------
mPD		0	0
		nPD:	1.
		N. weight:	0.
		Sc. PD:	0
		Sc. rank:	0

,

PB2

Pos.	375	obs:	exp:
---	-	2677	2677.00
---	--	------	------
mPD		0	0
		nPD:	1.
		N. weight:	0.
		Sc. PD:	0
		Sc. rank:	0

,

PB2			
Pos .	376	obs :	exp :
tta	L	13	290.90
ttg	L	0	502.10
ctt	L	329	539.80
ctc	L	1492	388.90
cta	L	826	466.70
ctg	L	13	484.50
att	I	2	1.42
atc	I	2	1.03
ata	I	0	1.56
---	--	------	------
mPD		0.59	1.1
		nPD :	0.52
		N. weight :	1.6
		Sc. PD :	0.27
		Sc. rank :	1232.6

PB2			
Pos .	377	obs :	exp :
act	T	46	658.20
acc	T	4	578.70
aca	T	571	1216.00
acg	T	2055	223.10
gct	A	0	0.25
gcc	A	0	0.23
gca	A	0	0.45
gcg	A	1	0.07
---	--	------	------
mPD		0.37	0.68
		nPD :	0.54
		N. weight :	2.8
		Sc. PD :	0.53
		Sc. rank :	2314.7

PB2			
Pos .	378	obs :	exp :
ggt	G	54	419.90
ggc	G	2602	339.20
gga	G	19	1213.00
ggg	G	2	705.40
---	--	------	------
mPD		0.055	0.68
		nPD :	0.08
		N. weight :	3.5
		Sc. PD :	-0.57
		Sc. rank :	-2321.7

PB2			
Pos .	379	obs :	exp :
tct	S	0	0.17
tcc	S	0	0.16
tca	S	0	0.24
tcg	S	0	0.05
att	I	0	0.35
atc	I	1	0.26
ata	I	0	0.39
aat	N	494	1520.00
aac	N	2181	1155.00
agt	S	0	0.19
agc	S	1	0.19
---	--	------	------
mPD		0.30	0.49
		nPD :	0.61
		N. weight :	0.58
		Sc. PD :	0.14
		Sc. rank :	578.9

PB2			
Pos .	380	obs :	exp :
tta	L	2	291.30
ttg	L	1	502.80
ctt	L	77	540.60
ctc	L	2575	389.50
cta	L	21	467.40
ctg	L	1	485.30
---	--	------	------
mPD		0.076	1.1
		nPD :	0.07
		N. weight :	3.2
		Sc. PD :	-0.55
		Sc. rank :	-2406.3

PB2			
Pos .	381	obs :	exp :
caa	Q	2514	1444.00
cag	Q	163	1233.00
---	--	------	------
mPD		0.11	0.50
		nPD :	0.23
		N. weight :	0.74
		Sc. PD :	-0.033
		Sc. rank :	-24.6

PB2			
Pos .	382	obs :	exp :
tct	S	0	0.17
tcc	S	0	0.16
tca	S	1	0.24
tcg	S	0	0.05
act	T	0	597.20
acc	T	0	525.00
aca	T	2398	1103.00
acg	T	30	202.50
agt	S	0	0.19
agc	S	0	0.19
gtt	V	0	0.22
gtc	V	1	0.19
gta	V	0	0.25
gtg	V	0	0.34
gct	A	0	60.55
gcc	A	18	56.59
gca	A	227	111.30
gcg	A	2	18.52
---	--	------	------
mPD		0.21	0.85
		nPD :	0.24
		N. weight :	1.3
		Sc. PD :	-0.046
		Sc. rank :	-9.9

PB2			
Pos .	383	obs :	exp :
---	-	2677	2677.00
---	--	------	------
mPD		0	0
		nPD :	1.
		N. weight :	0.
		Sc. PD :	0
		Sc. rank :	0

PB2			
Pos .	384	obs :	exp :
---	-	2677	2677.00
---	--	------	------
mPD		0	0
		nPD :	1.
		N. weight :	0.
		Sc. PD :	0
		Sc. rank :	0

PB2			
Pos .	385	obs :	exp :
---	-	2677	2677.00
---	--	------	------
mPD		0	0
		nPD :	1.
		N. weight :	0.
		Sc. PD :	0
		Sc. rank :	0

PB2			
Pos .	386	obs :	exp :
---	-	2677	2677.00
---	--	------	------
mPD		0	0
		nPD :	1.
		N. weight :	0.
		Sc. PD :	0
		Sc. rank :	0

PB2			
Pos .	387	obs :	exp :
---	-	2677	2677.00
---	--	------	------
mPD		0	0
		nPD :	1.
		N. weight :	0.
		Sc. PD :	0
		Sc. rank :	0

PB2			
Pos .	388	obs :	exp :
---	-	2677	2677.00
---	--	------	------
mPD		0	0
		nPD :	1.
		N. weight :	0.
		Sc. PD :	0
		Sc. rank :	0

PB2			
Pos .	389	obs :	exp :
---	-	2677	2677.00
---	--	------	------
mPD		0	0
		nPD :	1.
		N. weight :	0.
		Sc. PD :	0
		Sc. rank :	0

PB2			
Pos .	390	obs :	exp :
---	-	2677	2677.00
---	--	------	------
mPD		0	0
		nPD :	1.
		N. weight :	0.
		Sc. PD :	0
		Sc. rank :	0

PB2			
Pos .	391	obs :	exp :
---	-	2677	2677.00
---	--	------	------
mPD		0	0
		nPD :	1.
		N. weight :	0.
		Sc. PD :	0
		Sc. rank :	0

PB2			
Pos .	392	obs :	exp :
---	-	2677	2677.00
---	--	------	------
mPD		0	0
		nPD :	1.
		N. weight :	0.
		Sc. PD :	0
		Sc. rank :	0

PB2			
Pos .	393	obs :	exp :
---	-	2677	2677.00
---	--	------	------
mPD		0	0
		nPD :	1.
		N. weight :	0.
		Sc. PD :	0
		Sc. rank :	0

PB2			
Pos .	394	obs :	exp :
---	-	2677	2677.00
---	--	------	------
mPD		0	0
		nPD :	1.
		N. weight :	0.
		Sc. PD :	0
		Sc. rank :	0

PB2			
Pos .	395	obs :	exp :
---	-	2677	2677.00
---	--	------	------
mPD		0	0
		nPD :	1.
		N. weight :	0.
		Sc. PD :	0
		Sc. rank :	0

PB2			
Pos .	396	obs :	exp :
---	-	2677	2677.00
---	--	------	------
mPD		0	0
		nPD :	1.
		N. weight :	0.
		Sc. PD :	0
		Sc. rank :	0

PB2			
Pos .	397	obs :	exp :
---	-	2677	2677.00
---	--	------	------
mPD		0	0
		nPD :	1.
		N. weight :	0.
		Sc. PD :	0
		Sc. rank :	0

PB2			
Pos .	398	obs :	exp :
---	-	2677	2677.00
---	--	------	------
mPD		0	0
		nPD :	1.
		N. weight :	0.
		Sc. PD :	0
		Sc. rank :	0

PB2			
Pos .	399	obs :	exp :
---	-	2677	2677.00
---	--	------	------
mPD		0	0
		nPD :	1.
		N. weight :	0.
		Sc. PD :	0
		Sc. rank :	0

PB2			
Pos .	400	obs :	exp :
tta	L	12	291.20
ttg	L	181	502.60
ctt	L	4	540.40
ctc	L	4	389.30
cta	L	162	467.30
ctg	L	2313	485.10
atg	M	1	1.00
---	--	------	------
mPD		0.26	1.1
		nPD :	0.23
		N. weight :	2.2
		Sc. PD :	-0.099
		Sc. rank :	-77.9

PB2			
Pos.	401	obs:	exp:
caa	Q	2	1.08
cag	Q	0	0.92
cgt	R	0	1.14
cgc	R	0	1.90
cga	R	0	3.62
cgg	R	0	2.60
aaa	K	2325	1604.00
aag	K	314	1035.00
aga	R	25	16.51
agg	R	11	10.23
---	--	------	------
mPD		0.24	0.51
		nPD:	0.47
		N. weight:	0.35
		Sc. PD:	0.049
		Sc. rank:	235.7

PB2			
Pos.	402	obs:	exp:
ttt	F	1	0.45
ttc	F	0	0.55
tta	L	82	10.88
ttg	L	11	18.78
ctt	L	0	20.20
ctc	L	0	14.55
cta	L	7	17.46
ctg	L	0	18.13
att	I	1	898.20
atc	I	1	649.40
ata	I	2530	984.40
atg	M	41	41.00
gtt	V	0	0.67
gtc	V	0	0.57
gta	V	3	0.76
gtg	V	0	1.01
---	--	------	------
mPD		0.12	0.75
		nPD:	0.15
		N. weight:	1.8
		Sc. PD:	-0.18
		Sc. rank:	-533.9

PB2			
Pos.	403	obs:	exp:
cgt	R	0	82.83
cgc	R	0	138.70
cga	R	4	263.70
cgg	R	2	189.60
atg	M	2	2.00
act	T	1	0.25
acc	T	0	0.22
aca	T	0	0.45
acg	T	0	0.08
aaa	K	28	28.56
aag	K	19	18.44
aga	R	2335	1203.00
agg	R	283	745.80
ggt	G	0	0.47
ggc	G	0	0.38
gga	G	3	1.36
ggg	G	0	0.79
---	--	------	------
mPD		0.25	0.97
		nPD:	0.25
		N. weight:	0.87
		Sc. PD:	-0.023
		Sc. rank:	8.2

PB2			
Pos.	404	obs:	exp:
att	I	0	8.16
atc	I	0	5.90
ata	I	23	8.94
atg	M	4	4.00
gtt	V	2	588.40
gtc	V	2	500.00
gta	V	1859	669.00
gtg	V	787	892.60
---	--	------	------
mPD		0.44	0.76
		nPD:	0.58
		N. weight:	1.3
		Sc. PD:	0.28
		Sc. rank:	1155.6

PB2			
Pos.	405	obs:	exp:
cat	H	2656	1544.00
cac	H	21	1133.00
---	--	------	------
mPD		0.016	0.49
		nPD:	0.03
		N. weight:	0.94
		Sc. PD:	-0.19
		Sc. rank:	-963.0

PB2			
Pos.	406	obs:	exp:
aaa	K	3	1.82
aag	K	0	1.18
gat	D	0	0.56
gac	D	1	0.44
gaa	E	2226	1584.00
gag	E	447	1089.00
---	--	------	------
mPD		0.28	0.49
		nPD:	0.58
		N. weight:	0.25
		Sc. PD:	0.056
		Sc. rank:	234.1

PB2			
Pos.	407	obs:	exp:
ggt	G	4	419.90
ggc	G	2	339.20
gga	G	1505	1213.00
ggg	G	1166	705.40
---	--	------	------
mPD		0.49	0.68
		nPD:	0.72
		N. weight:	0.62
		Sc. PD:	0.2
		Sc. rank:	706.1

PB2			
Pos.	408	obs:	exp:
ttt	F	2	0.90
ttc	F	0	1.10
tat	Y	2599	1499.00
tac	Y	76	1176.00
---	--	------	------
mPD		0.057	0.49
		nPD:	0.11
		N. weight:	0.85
		Sc. PD:	-0.11
		Sc. rank:	-403.5

PB2			
Pos.	409	obs:	exp:
gaa	E	1436	1586.00
gag	E	1241	1091.00
---	--	------	------
mPD		0.50	0.48
		nPD:	1.03
		N. weight:	0.015
		Sc. PD:	0.0084
		Sc. rank:	18.4

PB2			
Pos.	410	obs:	exp:
gaa	E	2626	1586.00
gag	E	51	1091.00
---	--	------	------
mPD		0.037	0.48
		nPD:	0.08
		N. weight:	0.81
		Sc. PD:	-0.13
		Sc. rank:	-541.3

PB2			
Pos.	411	obs:	exp:
ttt	F	322	1200.00
ttc	F	2355	1477.00
---	--	------	------
mPD		0.21	0.49
		nPD:	0.43
		N. weight:	0.47
		Sc. PD:	0.05
		Sc. rank:	267.0

PB2			
Pos.	412	obs:	exp:
act	T	10	657.70
acc	T	13	578.20
aca	T	2624	1215.00
acg	T	27	223.00
gct	A	0	0.74
gcc	A	0	0.69
gca	A	1	1.35
gcg	A	2	0.22
---	--	------	------
mPD		0.041	0.68
		nPD:	0.06
		N. weight:	1.3
		Sc. PD:	-0.23
		Sc. rank:	-1052.0

PB2			
Pos.	413	obs:	exp:
tta	L	0	1.96
ttg	L	1	3.38
ctt	L	0	3.64
ctc	L	0	2.62
cta	L	0	3.14
ctg	L	17	3.26
att	I	2	94.72
atc	I	0	68.48
ata	I	265	103.80
atg	M	2386	2386.00
gtt	V	0	1.33
gtc	V	0	1.13
gta	V	0	1.51
gtg	V	6	2.02
---	--	------	------
mPD		0.20	0.21
		nPD:	0.92
		N. weight:	0.19
		Sc. PD:	0.093
		Sc. rank:	235.0

PB2			
Pos.	414	obs:	exp:
att	I	1	1.42
atc	I	3	1.03
ata	I	0	1.56
gtt	V	1225	593.10
gtc	V	1380	504.00
gta	V	62	674.30
gtg	V	4	899.70
gct	A	0	0.49
gcc	A	2	0.46
gca	A	0	0.90
gcg	A	0	0.15
---	--	------	------
mPD		0.53	0.74
		nPD:	0.71
		N. weight:	1.5
		Sc. PD:	0.47
		Sc. rank:	1662.3

PB2			
Pos.	415	obs:	exp:
ggt	G	3	419.90
ggc	G	0	339.20
gga	G	469	1213.00
ggg	G	2205	705.40
---	--	------	------
mPD		0.29	0.68
		nPD:	0.42
		N. weight:	1.4
		Sc. PD:	0.15
		Sc. rank:	785.8

PB2			
Pos.	416	obs:	exp:
tct	S	0	0.17
tcc	S	0	0.16
tca	S	0	0.24
tcg	S	0	0.05
cgt	R	0	78.47
cgc	R	0	131.40
cga	R	4	249.90
cgg	R	0	179.60
aaa	K	165	114.90
aag	K	24	74.14
agt	S	1	0.19
agc	S	0	0.19
aga	R	2400	1140.00
agg	R	82	706.60
ggt	G	0	0.16
ggc	G	0	0.13
gga	G	1	0.45
ggg	G	0	0.26
---	--	------	------
mPD		0.21	1.0
		nPD:	0.2
		N. weight:	1.1
		Sc. PD:	-0.075
		Sc. rank:	-133.3

PB2			
Pos.	417	obs:	exp:
cgt	R	0	82.95
cgc	R	0	138.90
cga	R	1	264.10
cgg	R	0	189.90
aaa	K	47	29.78
aag	K	2	19.22
aga	R	2422	1205.00
agg	R	205	746.90
---	--	------	------
mPD		0.18	0.97
		nPD:	0.19
		N. weight:	1.
		Sc. PD:	-0.079
		Sc. rank:	-179.1

PB2			
Pos.	418	obs:	exp:
tct	S	0	1.85
tcc	S	0	1.74
tca	S	9	2.67
tcg	S	2	0.58
agt	S	0	2.05
agc	S	0	2.11
gct	A	4	653.50
gcc	A	3	610.80
gca	A	2646	1202.00
gcg	A	13	199.90
---	--	------	------
mPD		0.025	0.69
		nPD:	0.04
		N. weight:	1.4
		Sc. PD:	-0.27
		Sc. rank:	-1393.7

PB2			
Pos.	419	obs:	exp:
act	T	18	658.50
acc	T	0	578.90
aca	T	2560	1216.00
acg	T	99	223.20
---	--	------	------
mPD		0.084	0.68
		nPD:	0.12
		N. weight:	1.2
		Sc. PD:	-0.15
		Sc. rank:	-527.3

PB2			
Pos.	420	obs:	exp:
tct	S	1	0.17
tcc	S	0	0.16
tca	S	0	0.24
tcg	S	0	0.05
agt	S	0	0.19
agc	S	0	0.19
gct	A	2239	656.00
gcc	A	334	613.10
gca	A	88	1206.00
gcg	A	15	200.70
---	--	------	------
mPD		0.28	0.68
		nPD:	0.42
		N. weight:	1.6
		Sc. PD:	0.16
		Sc. rank:	807.4

PB2			
Pos.	421	obs:	exp:
ttt	F	0	0.45
ttc	F	1	0.55
tta	L	0	1.52
ttg	L	0	2.63
ctt	L	1	2.83
ctc	L	3	2.04
cta	L	10	2.44
ctg	L	0	2.54
att	I	2235	942.60
atc	I	335	681.50
ata	I	87	1033.00
gtt	V	5	1.11
gtc	V	0	0.94
gta	V	0	1.26
gtg	V	0	1.68
---	--	------	------
mPD		0.30	0.67
		nPD:	0.44
		N. weight:	1.
		Sc. PD:	0.12
		Sc. rank:	626.7

,

PB2			
Pos.	422	obs:	exp:
ttt	F	1	5.83
ttc	F	12	7.17
tta	L	341	289.50
ttg	L	57	499.60
tct	S	0	0.17
tcc	S	0	0.16
tca	S	1	0.24
tcg	S	0	0.05
ctt	L	19	537.20
ctc	L	2192	387.00
cta	L	48	464.50
ctg	L	3	482.20
att	I	0	1.06
atc	I	3	0.77
ata	I	0	1.17
agt	S	0	0.19
agc	S	0	0.19
---	--	------	------
mPD		0.56	1.1
		nPD:	0.49
		N. weight:	2.5
		Sc. PD:	0.38
		Sc. rank:	1844.0

,

PB2			
Pos.	423	obs:	exp:
cgt	R	0	84.44
cgc	R	0	141.40
cga	R	205	268.90
cgg	R	0	193.30
aaa	K	1	1.22
aag	K	1	0.78
aga	R	2051	1227.00
agg	R	419	760.30
---	--	------	------
mPD		0.41	0.94
		nPD:	0.43
		N. weight:	0.52
		Sc. PD:	0.058
		Sc. rank:	309.1

,

PB2			
Pos.	424	obs:	exp:
cgt	R	0	0.06
cgc	R	0	0.11
cga	R	0	0.20
cgg	R	0	0.14
aaa	K	1322	1626.00
aag	K	1353	1049.00
aga	R	2	0.92
agg	R	0	0.57
---	--	------	------
mPD		0.50	0.48
		nPD:	1.05
		N. weight:	0.053
		Sc. PD:	0.031
		Sc. rank:	65.9

,

PB2			
Pos.	425	obs:	exp:
gct	A	66	656.00
gcc	A	0	613.10
gca	A	2586	1206.00
gcg	A	24	200.70
ggt	G	0	0.16
ggc	G	0	0.13
gga	G	1	0.45
ggg	G	0	0.26
---	--	------	------
mPD		0.066	0.68
		nPD:	0.1
		N. weight:	1.2
		Sc. PD:	-0.18
		Sc. rank:	-699.1

,

PB2			
Pos.	426	obs:	exp:
act	T	356	658.50
acc	T	2308	578.90
aca	T	10	1216.00
acg	T	3	223.20
---	--	------	------
mPD		0.24	0.68
		nPD:	0.35
		N. weight:	2.
		Sc. PD:	0.098
		Sc. rank:	663.5

,

PB2			
Pos.	427	obs:	exp:
cgt	R	0	84.34
cgc	R	0	141.20
cga	R	0	268.60
cgg	R	2	193.00
aaa	K	0	3.04
aag	K	5	1.96
aga	R	560	1225.00
agg	R	2110	759.50
---	--	------	------
mPD		0.34	0.94
		nPD:	0.36
		N. weight:	1.2
		Sc. PD:	0.063
		Sc. rank:	404.8

,

PB2			
Pos.	428	obs:	exp:
cgt	R	0	84.47
cgc	R	0	141.40
cga	R	12	269.00
cgg	R	1	193.30
atg	M	1	1.00
aga	R	2164	1227.00
agg	R	499	760.60
---	--	------	------
mPD		0.31	0.94
		nPD:	0.34
		N. weight:	0.68
		Sc. PD:	0.024
		Sc. rank:	193.4

,

PB2			
Pos.	429	obs:	exp:
tta	L	60	291.10
ttg	L	2171	502.50
ctt	L	2	540.20
ctc	L	0	389.20
cta	L	47	467.10
ctg	L	395	484.90
atg	M	2	2.00
---	--	------	------
mPD		0.36	1.1
		nPD:	0.31
		N. weight:	2.
		Sc. PD:	0.037
		Sc. rank:	449.9

,

PB2			
Pos.	430	obs:	exp:
att	I	602	881.90
atc	I	1860	637.60
ata	I	24	966.50
gtt	V	41	42.41
gtc	V	150	36.04
gta	V	0	48.22
gtg	V	0	64.33
---	--	------	------
mPD		0.51	0.81
		nPD:	0.63
		N. weight:	1.3
		Sc. PD:	0.35
		Sc. rank:	1345.7

,

PB2			
Pos.	431	obs:	exp:
cat	H	1	0.58
cac	H	0	0.42
caa	Q	219	1443.00
cag	Q	2457	1233.00
---	--	------	------
mPD		0.15	0.50
		nPD:	0.3
		N. weight:	0.89
		Sc. PD:	0.01
		Sc. rank:	144.5

,

PB2			
Pos.	432	obs:	exp:
---	-	2677	2677.00
---	--	------	------
mPD		0	0
		nPD:	1.
		N. weight:	0.
		Sc. PD:	0
		Sc. rank:	0

,

PB2			
Pos.	433	obs:	exp:
---	-	2677	2677.00
---	--	------	------
mPD		0	0
		nPD:	1.
		N. weight:	0.
		Sc. PD:	0
		Sc. rank:	0

,

PB2			
Pos.	434	obs:	exp:
ttt	F	49	24.20
ttc	F	5	29.80
tta	L	1602	285.40
ttg	L	612	492.70
ctt	L	12	529.70
ctc	L	0	381.60
cta	L	20	458.00
ctg	L	377	475.50
---	--	------	------
mPD		0.76	1.2
		nPD:	0.65
		N. weight:	1.9
		Sc. PD:	0.53
		Sc. rank:	2022.9

,

PB2			
Pos.	435	obs:	exp:
tta	L	0	0.11
ttg	L	0	0.19
ctt	L	0	0.20
ctc	L	0	0.15
cta	L	1	0.17
ctg	L	0	0.18
att	I	4	948.20
atc	I	2	685.60
ata	I	2667	1039.00
gtt	V	0	0.67
gtc	V	0	0.57
gta	V	3	0.76
gtg	V	0	1.01
---	--	------	------
mPD		0.0075	0.66
		nPD:	0.01
		N. weight:	1.7
		Sc. PD:	-0.37
		Sc. rank:	-1952.7

,

PB2			
Pos.	436	obs:	exp:
tta	L	0	0.11
ttg	L	1	0.19
ctt	L	0	0.20
ctc	L	0	0.15
cta	L	0	0.17
ctg	L	0	0.18
att	I	0	26.61
atc	I	0	19.24
ata	I	75	29.16
gtt	V	3	577.30
gtc	V	13	490.60
gta	V	2465	656.30
gtg	V	119	875.80
gct	A	0	0.25
gcc	A	0	0.23
gca	A	1	0.45
gcg	A	0	0.07
---	--	------	------
mPD		0.15	0.80
		nPD:	0.19
		N. weight:	2.1
		Sc. PD:	-0.16
		Sc. rank:	-271.0

,

PB2			
Pos.	437	obs:	exp:
tct	S	0	449.40
tcc	S	0	422.30
tca	S	0	648.90
tcg	S	0	142.00
tgt	C	1	0.43
tgc	C	0	0.57
aat	N	1	2.27
aac	N	3	1.73
agt	S	1968	497.30
agc	S	704	512.10
---	--	------	------
mPD		0.39	1.6
		nPD:	0.24
		N. weight:	2.
		Sc. PD:	-0.075
		Sc. rank:	-36.8

,

PB2			
Pos.	438	obs:	exp:
ggt	G	54	419.90
ggc	G	2	339.20
gga	G	703	1213.00
ggg	G	1918	705.40
---	--	------	------
mPD		0.42	0.68
		nPD:	0.61
		N. weight:	0.98
		Sc. PD:	0.24
		Sc. rank:	978.4

,

PB2			
Pos.	439	obs:	exp:
cgt	R	0	77.87
cgc	R	0	130.40
cga	R	13	248.00
cgg	R	0	178.20
aaa	K	187	127.00
aag	K	22	81.98
aga	R	2399	1131.00
agg	R	55	701.20
ggt	G	0	0.16
ggc	G	0	0.13
gga	G	0	0.45
ggg	G	1	0.26
---	--	------	------
mPD		0.21	1.1
		nPD:	0.2
		N. weight:	1.2
		Sc. PD:	-0.079
		Sc. rank:	-142.0

,

PB2			
Pos.	440	obs:	exp:
aat	N	1	3.98
aac	N	6	3.02
gat	D	440	1502.00
gac	D	2230	1168.00
---	--	------	------
mPD		0.28	0.50
		nPD:	0.56
		N. weight:	0.63
		Sc. PD:	0.13
		Sc. rank:	569.3

,

PB2

Pos.	441	obs:	exp:
tat	Y	1	0.56
tac	Y	0	0.44
aat	N	1	1.14
aac	N	1	0.86
gtt	V	0	0.22
gtc	V	0	0.19
gta	V	0	0.25
gtg	V	1	0.34
gat	D	876	591.10
gac	D	175	459.90
gaa	E	501	961.10
gag	E	1121	660.90
---	--	------	------
mPD		0.68	0.73
		nPD:	0.93
		N. weight:	0.31
		Sc. PD:	0.15
		Sc. rank:	384.9

,

PB2

Pos.	442	obs:	exp:
cct	P	0	0.30
ccc	P	0	0.20
cca	P	0	0.40
ccg	P	1	0.11
cat	H	5	4.04
cac	H	2	2.96
caa	Q	1234	1438.00
cag	Q	1433	1229.00
cgt	R	0	0.06
cgc	R	0	0.11
cga	R	0	0.20
cgg	R	2	0.14
aga	R	0	0.92
agg	R	0	0.57
---	--	------	------
mPD		0.50	0.50
		nPD:	1.
		N. weight:	0.031
		Sc. PD:	0.017
		Sc. rank:	38.1

,

PB2

Pos.	443	obs:	exp:
tct	S	0	449.90
tcc	S	2	422.80
tca	S	2552	649.60
tcg	S	121	142.20
agt	S	0	497.90
agc	S	0	512.70
gct	A	0	0.49
gcc	A	0	0.46
gca	A	2	0.90
gcg	A	0	0.15
---	--	------	------
mPD		0.089	1.6
		nPD:	0.05
		N. weight:	2.4
		Sc. PD:	-0.43
		Sc. rank:	-2008.2

,

PB2

Pos.	444	obs:	exp:
---	-	2677	2677.00
---	--	------	------
mPD		0	0
		nPD:	1.
		N. weight:	0.
		Sc. PD:	0
		Sc. rank:	0

,

PB2

Pos.	445	obs:	exp:
tta	L	0	0.33
ttg	L	0	0.56
ctt	L	3	0.61
ctc	L	0	0.44
cta	L	0	0.52
ctg	L	0	0.54
att	I	2536	929.10
atc	I	73	671.70
ata	I	10	1018.00
act	T	2	0.49
acc	T	0	0.43
aca	T	0	0.91
acg	T	0	0.17
gtt	V	37	11.77
gtc	V	2	10.00
gta	V	14	13.38
gtg	V	0	17.85
---	--	------	------
mPD		0.11	0.70
		nPD:	0.16
		N. weight:	1.7
		Sc. PD:	-0.16
		Sc. rank:	-427.2

,

PB2

Pos.	446	obs:	exp:
tct	S	4	0.67
tcc	S	0	0.63
tca	S	0	0.97
tcg	S	0	0.21
act	T	6	1.48
acc	T	0	1.30
aca	T	0	2.73
acg	T	0	0.50
agt	S	0	0.74
agc	S	0	0.77
gtt	V	0	0.22
gtc	V	1	0.19
gta	V	0	0.25
gtg	V	0	0.34
gct	A	2567	653.50
gcc	A	72	610.80
gca	A	22	1202.00
gcg	A	5	199.90
---	--	------	------
mPD		0.081	0.69
		nPD:	0.12
		N. weight:	2.3
		Sc. PD:	-0.3
		Sc. rank:	-1044.4

,

PB2

Pos.	447	obs:	exp:
gat	D	1	0.56
gac	D	0	0.44
gaa	E	466	1586.00
gag	E	2210	1090.00
---	--	------	------
mPD		0.29	0.48
		nPD:	0.6
		N. weight:	0.69
		Sc. PD:	0.16
		Sc. rank:	664.6

,

PB2

Pos.	448	obs:	exp:
att	I	0	0.71
atc	I	0	0.51
ata	I	2	0.78
act	T	0	1.97
acc	T	0	1.73
aca	T	8	3.64
acg	T	0	0.67
gtt	V	0	6.66
gtc	V	0	5.66
gta	V	30	7.57
gtg	V	0	10.10
gct	A	6	646.40
gcc	A	0	604.20
gca	A	2539	1189.00
gcg	A	92	197.80
---	--	------	------
mPD		0.10	0.71
		nPD:	0.14
		N. weight:	1.3
		Sc. PD:	-0.15
		Sc. rank:	-424.5

,

PB2

Pos.	449	obs:	exp:
tta	L	6	1.74
ttg	L	0	3.01
ctt	L	0	3.23
ctc	L	5	2.33
cta	L	5	2.79
ctg	L	0	2.90
att	I	92	936.20
atc	I	300	676.90
ata	I	2247	1026.00
gtt	V	0	4.88
gtc	V	10	4.15
gta	V	12	5.55
gtg	V	0	7.41
---	--	------	------
mPD		0.29	0.69
		nPD:	0.43
		N. weight:	0.93
		Sc. PD:	0.099
		Sc. rank:	521.7

,

PB2

Pos.	450	obs:	exp:
att	I	2235	947.90
atc	I	26	685.30
ata	I	411	1039.00
gtt	V	5	1.11
gtc	V	0	0.94
gta	V	0	1.26
gtg	V	0	1.68
---	--	------	------
mPD		0.28	0.66
		nPD:	0.42
		N. weight:	1.
		Sc. PD:	0.1
		Sc. rank:	540.9

,

PB2

Pos.	451	obs:	exp:
att	I	0	0.35
atc	I	0	0.26
ata	I	1	0.39
gtt	V	272	593.50
gtc	V	57	504.40
gta	V	451	674.80
gtg	V	1893	900.30
gct	A	1	0.74
gcc	A	0	0.69
gca	A	2	1.35
gcg	A	0	0.22
---	--	------	------
mPD		0.46	0.74
		nPD:	0.63
		N. weight:	0.62
		Sc. PD:	0.16
		Sc. rank:	632.5

,

PB2

Pos.	452	obs:	exp:
gct	A	48	656.20
gcc	A	2103	613.40
gca	A	515	1207.00
gcg	A	11	200.80
---	--	------	------
mPD		0.35	0.68
		nPD:	0.51
		N. weight:	1.4
		Sc. PD:	0.24
		Sc. rank:	1078.7

,

PB2

Pos.	453	obs:	exp:
att	I	0	1.42
atc	I	0	1.03
ata	I	4	1.56
atg	M	2673	2673.00
---	--	------	------
mPD		0.0030	0.0030
		nPD:	1.
		N. weight:	0.0026
		Sc. PD:	0.0014
		Sc. rank:	3.2

,

PB2

Pos.	454	obs:	exp:
gtt	V	1	594.20
gtc	V	0	505.00
gta	V	2177	675.50
gtg	V	498	901.40
gct	A	0	0.25
gcc	A	0	0.23
gca	A	1	0.45
gcg	A	0	0.07
---	--	------	------
mPD		0.30	0.74
		nPD:	0.41
		N. weight:	1.6
		Sc. PD:	0.15
		Sc. rank:	758.4

,

PB2

Pos.	455	obs:	exp:
ttt	F	1487	1199.00
ttc	F	1189	1477.00
tta	L	1	0.11
ttg	L	0	0.19
ctt	L	0	0.20
ctc	L	0	0.15
cta	L	0	0.17
ctg	L	0	0.18
---	--	------	------
mPD		0.49	0.50
		nPD:	1.
		N. weight:	0.047
		Sc. PD:	0.026
		Sc. rank:	58.9

,

PB2

Pos.	456	obs:	exp:
tct	S	3	449.90
tcc	S	3	422.80
tca	S	2579	649.60
tcg	S	90	142.20
act	T	0	0.49
acc	T	0	0.43
aca	T	2	0.91
acg	T	0	0.17
agt	S	0	497.90
agc	S	0	512.70
---	--	------	------
mPD		0.071	1.6
		nPD:	0.04
		N. weight:	2.4
		Sc. PD:	-0.45
		Sc. rank:	-2191.6

PB2

Pos.	457	obs:	exp:
caa	Q	2628	1444.00
cag	Q	49	1233.00
---	--	------	------
mPD		0.036	0.50
		nPD:	0.07
		N. weight:	0.98
		Sc. PD:	-0.16
		Sc. rank:	-700.3

PB2

Pos.	458	obs:	exp:
aaa	K	1	0.61
aag	K	0	0.39
gat	D	3	1.69
gac	D	0	1.31
gaa	E	600	1584.00
gag	E	2073	1089.00
---	--	------	------
mPD		0.35	0.48
		nPD:	0.72
		N. weight:	0.52
		Sc. PD:	0.18
		Sc. rank:	602.4

PB2

Pos.	459	obs:	exp:
aat	N	1	0.57
aac	N	0	0.43
gat	D	2547	1503.00
gac	D	125	1169.00
gaa	E	1	2.37
gag	E	3	1.63
---	--	------	------
mPD		0.093	0.49
		nPD:	0.19
		N. weight:	0.74
		Sc. PD:	-0.057
		Sc. rank:	-123.9

PB2

Pos.	460	obs:	exp:
tct	S	0	8.24
tcc	S	0	7.74
tca	S	0	11.90
tcg	S	0	2.60
tgt	C	283	1034.00
tgc	C	2120	1369.00
cat	H	0	0.58
cac	H	1	0.42
cgt	R	18	7.01
cgc	R	200	11.73
cga	R	3	22.31
cgg	R	0	16.04
agt	S	36	9.12
agc	S	13	9.39
aga	R	1	101.80
agg	R	0	63.10
ggt	G	0	0.31
ggc	G	2	0.25
gga	G	0	0.91
ggg	G	0	0.53
---	--	------	------
mPD		0.41	0.76
		nPD:	0.54
		N. weight:	0.83
		Sc. PD:	0.16
		Sc. rank:	707.6

PB2

Pos.	461	obs:	exp:
att	I	0	1.42
atc	I	0	1.03
ata	I	4	1.56
atg	M	2670	2670.00
gtt	V	0	0.67
gtc	V	0	0.57
gta	V	0	0.76
gtg	V	3	1.01
---	--	------	------
mPD		0.0052	0.0067
		nPD:	0.78
		N. weight:	0.0049
		Sc. PD:	0.0018
		Sc. rank:	5.8

PB2

Pos.	462	obs:	exp:
tta	L	0	0.22
ttg	L	0	0.38
ctt	L	0	0.40
ctc	L	1	0.29
cta	L	1	0.35
ctg	L	0	0.36
att	I	193	935.50
atc	I	1966	676.40
ata	I	478	1025.00
atg	M	1	1.00
gtt	V	1	8.22
gtc	V	26	6.98
gta	V	10	9.34
gtg	V	0	12.46
---	--	------	------
mPD		0.44	0.69
		nPD:	0.63
		N. weight:	1.
		Sc. PD:	0.27
		Sc. rank:	1038.7

PB2

Pos.	463	obs:	exp:
cgt	R	0	0.35
cgc	R	0	0.58
cga	R	0	1.11
cgg	R	0	0.79
aaa	K	220	1620.00
aag	K	2446	1046.00
aga	R	0	5.04
agg	R	11	3.13
---	--	------	------
mPD		0.16	0.49
		nPD:	0.33
		N. weight:	1.1
		Sc. PD:	0.033
		Sc. rank:	296.8

PB2

Pos.	464	obs:	exp:
act	T	0	0.25
acc	T	0	0.22
aca	T	1	0.45
acg	T	0	0.08
gct	A	418	656.00
gcc	A	18	613.10
gca	A	2210	1206.00
gcg	A	30	200.70
---	--	------	------
mPD		0.29	0.68
		nPD:	0.43
		N. weight:	0.72
		Sc. PD:	0.08
		Sc. rank:	423.2

PB2

Pos.	465	obs:	exp:
gtt	V	2187	594.40
gtc	V	340	505.10
gta	V	150	675.80
gtg	V	0	901.70
---	--	------	------
mPD		0.31	0.74
		nPD:	0.42
		N. weight:	1.7
		Sc. PD:	0.18
		Sc. rank:	939.7

PB2

Pos.	466	obs:	exp:
cgt	R	0	84.50
cgc	R	0	141.50
cga	R	357	269.10
cgg	R	37	193.40
aga	R	427	1228.00
agg	R	1856	760.90
---	--	------	------
mPD		0.67	0.94
		nPD:	0.71
		N. weight:	0.87
		Sc. PD:	0.28
		Sc. rank:	977.2

PB2

Pos.	467	obs:	exp:
gtt	V	1	0.22
gtc	V	0	0.19
gta	V	0	0.25
gtg	V	0	0.34
ggt	G	477	419.70
ggc	G	2165	339.10
gga	G	31	1212.00
ggg	G	3	705.10
---	--	------	------
mPD		0.31	0.69
		nPD:	0.46
		N. weight:	2.7
		Sc. PD:	0.36
		Sc. rank:	1735.0

PB2

Pos.	468	obs:	exp:
gat	D	2633	1506.00
gac	D	44	1171.00
---	--	------	------
mPD		0.032	0.49
		nPD:	0.07
		N. weight:	0.92
		Sc. PD:	-0.16
		Sc. rank:	-703.9

PB2

Pos.	469	obs:	exp:
tta	L	15	291.20
ttg	L	94	502.60
ctt	L	196	540.40
ctc	L	0	389.30
cta	L	642	467.30
ctg	L	1729	485.10
atg	M	1	1.00
---	--	------	------
mPD		0.55	1.1
		nPD:	0.48
		N. weight:	1.4
		Sc. PD:	0.21
		Sc. rank:	997.1

PB2

Pos.	470	obs:	exp:
aat	N	487	1522.00
aac	N	2190	1155.00
---	--	------	------
mPD		0.30	0.49
		nPD:	0.61
		N. weight:	0.59
		Sc. PD:	0.14
		Sc. rank:	580.2

PB2

Pos.	471	obs:	exp:
ttt	F	2618	1200.00
ttc	F	59	1477.00
---	--	------	------
mPD		0.043	0.49
		nPD:	0.09
		N. weight:	1.3
		Sc. PD:	-0.2
		Sc. rank:	-793.0

PB2

Pos.	472	obs:	exp:
att	I	0	0.35
atc	I	0	0.26
ata	I	1	0.39
gtt	V	124	594.20
gtc	V	2122	505.00
gta	V	416	675.50
gtg	V	14	901.40
---	--	------	------
mPD		0.35	0.74
		nPD:	0.47
		N. weight:	1.8
		Sc. PD:	0.25
		Sc. rank:	1208.5

PB2

Pos.	473	obs:	exp:
aat	N	2118	1522.00
aac	N	559	1155.00
---	--	------	------
mPD		0.33	0.49
		nPD:	0.67
		N. weight:	0.21
		Sc. PD:	0.061
		Sc. rank:	225.3

PB2

Pos.	474	obs:	exp:
cgt	R	0	84.50
cgc	R	0	141.50
cga	R	0	269.10
cgg	R	1	193.40
aga	R	468	1228.00
agg	R	2208	760.90
---	--	------	------
mPD		0.29	0.94
		nPD:	0.31
		N. weight:	1.3
		Sc. PD:	0.02
		Sc. rank:	238.9

PB2

Pos.	475	obs:	exp:
gct	A	5	656.20
gcc	A	3	613.40
gca	A	2621	1207.00
gcg	A	48	200.80
---	--	------	------
mPD		0.041	0.68
		nPD:	0.06
		N. weight:	1.3
		Sc. PD:	-0.23
		Sc. rank:	-1068.9

PB2			
Pos.	476	obs:	exp:
aat	N	562	1522.00
aac	N	2115	1155.00
---	--	------	------
mPD		0.33	0.49
		nPD:	0.68
		N. weight:	0.5
		Sc. PD:	0.15
		Sc. rank:	548.2

,

PB2			
Pos.	477	obs:	exp:
caa	Q	445	1444.00
cag	Q	2232	1233.00
---	--	------	------
mPD		0.28	0.50
		nPD:	0.56
		N. weight:	0.56
		Sc. PD:	0.12
		Sc. rank:	489.0

,

PB2			
Pos.	478	obs:	exp:
cgt	R	13	84.50
cgc	R	0	141.50
cga	R	2043	269.10
cgg	R	341	193.40
aga	R	78	1228.00
agg	R	202	760.90
---	--	------	------
mPD		0.52	0.94
		nPD:	0.55
		N. weight:	2.7
		Sc. PD:	0.54
		Sc. rank:	2327.7

,

PB2			
Pos.	479	obs:	exp:
tta	L	14	291.30
ttg	L	176	502.80
ctt	L	39	540.60
ctc	L	0	389.50
cta	L	185	467.40
ctg	L	2263	485.30
---	--	------	------
mPD		0.30	1.1
		nPD:	0.26
		N. weight:	2.1
		Sc. PD:	-0.045
		Sc. rank:	40.9

,

PB2			
Pos.	480	obs:	exp:
aat	N	2010	1522.00
aac	N	667	1155.00
---	--	------	------
mPD		0.37	0.49
		nPD:	0.76
		N. weight:	0.14
		Sc. PD:	0.05
		Sc. rank:	161.6

,

PB2			
Pos.	481	obs:	exp:
tct	S	0	0.17
tcc	S	1	0.16
tca	S	0	0.24
tcg	S	0	0.05
cct	P	46	789.70
ccc	P	2588	538.80
cca	P	42	1060.00
ccg	P	0	287.90
agt	S	0	0.19
agc	S	0	0.19
---	--	------	------
mPD		0.065	0.71
		nPD:	0.09
		N. weight:	2.6
		Sc. PD:	-0.4
		Sc. rank:	-1576.6

,

PB2			
Pos.	482	obs:	exp:
---	-	2677	2677.00
---	--	------	------
mPD		0	0
		nPD:	1.
		N. weight:	0.
		Sc. PD:	0
		Sc. rank:	0

,

PB2			
Pos.	483	obs:	exp:
---	-	2677	2677.00
---	--	------	------
mPD		0	0
		nPD:	1.
		N. weight:	0.
		Sc. PD:	0
		Sc. rank:	0

,

PB2			
Pos.	484	obs:	exp:
atg	M	2677	2677.00
---	--	------	------
mPD		0	0
		nPD:	1.
		N. weight:	0.
		Sc. PD:	0
		Sc. rank:	0

,

PB2			
Pos.	485	obs:	exp:
cat	H	551	1544.00
cac	H	2125	1132.00
caa	Q	1	0.54
cag	Q	0	0.46
---	--	------	------
mPD		0.33	0.49
		nPD:	0.67
		N. weight:	0.54
		Sc. PD:	0.16
		Sc. rank:	583.6

,

PB2			
Pos.	486	obs:	exp:
caa	Q	2569	1444.00
cag	Q	108	1233.00
---	--	------	------
mPD		0.077	0.50
		nPD:	0.16
		N. weight:	0.84
		Sc. PD:	-0.086
		Sc. rank:	-248.1

,

PB2			
Pos.	487	obs:	exp:
tta	L	14	291.30
ttg	L	0	502.80
ctt	L	135	540.60
ctc	L	2418	389.50
cta	L	84	467.40
ctg	L	26	485.30
---	--	------	------
mPD		0.19	1.1
		nPD:	0.17
		N. weight:	2.8
		Sc. PD:	-0.26
		Sc. rank:	-634.5

,

PB2			
Pos.	488	obs:	exp:
tta	L	44	291.30
ttg	L	2189	502.80
ctt	L	2	540.60
ctc	L	0	389.50
cta	L	63	467.40
ctg	L	379	485.30
---	--	------	------
mPD		0.36	1.1
		nPD:	0.31
		N. weight:	2.
		Sc. PD:	0.036
		Sc. rank:	443.1

,

PB2			
Pos.	489	obs:	exp:
cgt	R	0	84.50
cgc	R	0	141.50
cga	R	0	269.10
cgg	R	12	193.40
aga	R	398	1228.00
agg	R	2267	760.90
---	--	------	------
mPD		0.26	0.94
		nPD:	0.28
		N. weight:	1.4
		Sc. PD:	-0.0097
		Sc. rank:	131.6

,

PB2			
Pos.	490	obs:	exp:
cat	H	2223	1544.00
cac	H	454	1133.00
---	--	------	------
mPD		0.28	0.49
		nPD:	0.58
		N. weight:	0.28
		Sc. PD:	0.061
		Sc. rank:	253.7

,

PB2			
Pos.	491	obs:	exp:
ttt	F	72	1200.00
ttc	F	2605	1477.00
---	--	------	------
mPD		0.052	0.49
		nPD:	0.11
		N. weight:	0.88
		Sc. PD:	-0.12
		Sc. rank:	-457.9

,

PB2			
Pos.	492	obs:	exp:
caa	Q	2308	1444.00
cag	Q	369	1233.00
---	--	------	------
mPD		0.24	0.50
		nPD:	0.48
		N. weight:	0.44
		Sc. PD:	0.065
		Sc. rank:	312.2

,

PB2			
Pos.	493	obs:	exp:
cgt	R	0	0.32
cgc	R	0	0.53
cga	R	0	1.01
cgg	R	0	0.72
aaa	K	1249	1621.00
aag	K	1418	1046.00
aga	R	10	4.59
agg	R	0	2.84
---	--	------	------
mPD		0.51	0.49
		nPD:	1.04
		N. weight:	0.081
		Sc. PD:	0.047
		Sc. rank:	101.2

,

PB2			
Pos.	494	obs:	exp:
aat	N	23	13.07
aac	N	0	9.93
gat	D	2562	1471.00
gac	D	53	1144.00
gaa	E	36	21.33
gag	E	0	14.67
ggt	G	3	0.47
ggc	G	0	0.38
gga	G	0	1.36
ggg	G	0	0.79
---	--	------	------
mPD		0.084	0.53
		nPD:	0.16
		N. weight:	0.9
		Sc. PD:	-0.089
		Sc. rank:	-248.3

,

PB2			
Pos.	495	obs:	exp:
tct	S	0	0.34
tcc	S	1	0.32
tca	S	1	0.49
tcg	S	0	0.11
agt	S	0	0.37
agc	S	0	0.38
gct	A	24	655.70
gcc	A	13	612.90
gca	A	2609	1206.00
gcg	A	29	200.60
---	--	------	------
mPD		0.051	0.68
		nPD:	0.07
		N. weight:	1.3
		Sc. PD:	-0.21
		Sc. rank:	-884.6

,

PB2			
Pos.	496	obs:	exp:
cgt	R	0	0.79
cgc	R	0	1.32
cga	R	0	2.51
cgg	R	0	1.81
aaa	K	2523	1612.00
aag	K	129	1040.00
aga	R	25	11.46
agg	R	0	7.11
---	--	------	------
mPD		0.11	0.50
		nPD:	0.22
		N. weight:	0.61
		Sc. PD:	-0.032
		Sc. rank:	-36.9

,

PB2			
Pos.	497	obs:	exp:
att	I	4	6.39
atc	I	2	4.62
ata	I	12	7.00
gtt	V	10	590.40
gtc	V	13	501.70
gta	V	76	671.20
gtg	V	2560	895.60
---	--	------	------
mPD		0.098	0.75
		nPD:	0.13
		N. weight:	1.7
		Sc. PD:	-0.21
		Sc. rank:	-669.8

,

PB2			
Pos.	498	obs:	exp:
tta	L	1	291.30
ttg	L	16	502.80
ctt	L	1906	540.60
ctc	L	27	389.50
cta	L	79	467.40
ctg	L	648	485.30
---	--	------	------
mPD		0.44	1.1
		nPD:	0.39
		N. weight:	1.6
		Sc. PD:	0.12
		Sc. rank:	660.9

,

PB2			
Pos.	499	obs:	exp:
ttt	F	429	1149.00
ttc	F	2136	1416.00
tta	L	0	0.98
ttg	L	1	1.69
tct	S	0	0.17
tcc	S	1	0.16
tca	S	0	0.24
tcg	S	0	0.05
ctt	L	7	1.82
ctc	L	1	1.31
cta	L	0	1.57
ctg	L	0	1.63
att	I	73	33.70
atc	I	19	24.37
ata	I	3	36.93
agt	S	0	0.19
agc	S	0	0.19
gtt	V	6	1.55
gtc	V	1	1.32
gta	V	0	1.77
gtg	V	0	2.36
---	--	------	------
mPD		0.39	0.59
		nPD:	0.66
		N. weight:	0.37
		Sc. PD:	0.11
		Sc. rank:	396.2

,

PB2			
Pos.	500	obs:	exp:
tta	L	1	0.76
ttg	L	0	1.31
ctt	L	0	1.41
ctc	L	0	1.02
cta	L	0	1.22
ctg	L	6	1.27
cat	H	0	0.58
cac	H	1	0.42
caa	Q	514	1432.00
cag	Q	2142	1224.00
gaa	E	13	7.70
gag	E	0	5.30
---	--	------	------
mPD		0.33	0.51
		nPD:	0.65
		N. weight:	0.48
		Sc. PD:	0.13
		Sc. rank:	505.3

,

PB2			
Pos.	501	obs:	exp:
tct	S	0	0.84
tcc	S	0	0.79
tca	S	0	1.21
tcg	S	0	0.27
act	T	0	0.25
acc	T	1	0.22
aca	T	0	0.45
acg	T	0	0.08
aat	N	451	1518.00
aac	N	2219	1152.00
aaa	K	1	0.61
aag	K	0	0.39
agt	S	0	0.93
agc	S	5	0.96
---	--	------	------
mPD		0.29	0.50
		nPD:	0.57
		N. weight:	0.64
		Sc. PD:	0.14
		Sc. rank:	582.0

,

PB2			
Pos.	502	obs:	exp:
---	-	2677	2677.00
---	--	------	------
mPD		0	0
		nPD:	1.
		N. weight:	0.
		Sc. PD:	0
		Sc. rank:	0

,

PB2			
Pos.	503	obs:	exp:
tgg	W	2677	2677.00
---	--	------	------
mPD		0	0
		nPD:	1.
		N. weight:	0.
		Sc. PD:	0
		Sc. rank:	0

,

PB2			
Pos.	504	obs:	exp:
ggt	G	18	419.90
ggc	G	0	339.20
gga	G	1811	1213.00
ggg	G	848	705.40
---	--	------	------
mPD		0.44	0.68
		nPD:	0.65
		N. weight:	0.58
		Sc. PD:	0.16
		Sc. rank:	602.1

,

PB2			
Pos.	505	obs:	exp:
tta	L	0	4.57
ttg	L	0	7.89
ctt	L	42	8.48
ctc	L	0	6.11
cta	L	0	7.33
ctg	L	0	7.61
att	I	2085	809.20
atc	I	195	585.10
ata	I	1	886.80
atg	M	11	11.00
act	T	216	53.62
acc	T	2	47.14
aca	T	0	99.06
acg	T	0	18.18
aat	N	1	0.57
aac	N	0	0.43
gtt	V	121	27.53
gtc	V	3	23.40
gta	V	0	31.30
gtg	V	0	41.77
---	--	------	------
mPD		0.41	0.95
		nPD:	0.44
		N. weight:	1.6
		Sc. PD:	0.18
		Sc. rank:	943.6

,

PB2			
Pos.	506	obs:	exp:
gaa	E	2502	1586.00
gag	E	175	1091.00
---	--	------	------
mPD		0.12	0.48
		nPD:	0.25
		N. weight:	0.57
		Sc. PD:	-0.015
		Sc. rank:	4.1

,

PB2			
Pos.	507	obs:	exp:
tct	S	24	119.90
tcc	S	676	112.70
tca	S	13	173.10
tcg	S	0	37.89
cct	P	381	570.40
ccc	P	1509	389.20
cca	P	43	765.50
ccg	P	0	207.90
cat	H	1	1.73
cac	H	2	1.27
act	T	4	6.40
acc	T	22	5.62
aca	T	0	11.81
acg	T	0	2.17
agt	S	0	132.70
agc	S	0	136.60
gct	A	0	0.49
gcc	A	2	0.46
gca	A	0	0.90
gcg	A	0	0.15
---	--	------	------
mPD		0.70	1.3
		nPD:	0.53
		N. weight:	2.
		Sc. PD:	0.38
		Sc. rank:	1668.9

,

PB2			
Pos.	508	obs:	exp:
att	I	534	949.60
atc	I	2080	686.60
ata	I	63	1041.00
---	--	------	------
mPD		0.36	0.66
		nPD:	0.54
		N. weight:	1.3
		Sc. PD:	0.25
		Sc. rank:	1079.0

,

PB2			
Pos.	509	obs:	exp:
---	-	2677	2677.00
---	--	------	------
mPD		0	0
		nPD:	1.
		N. weight:	0.
		Sc. PD:	0
		Sc. rank:	0

,

PB2			
Pos.	510	obs:	exp:
---	-	2677	2677.00
---	--	------	------
mPD		0	0
		nPD:	1.
		N. weight:	0.
		Sc. PD:	0
		Sc. rank:	0

,

PB2			
Pos.	511	obs:	exp:
gat	D	472	1505.00
gac	D	2204	1171.00
gaa	E	1	0.59
gag	E	0	0.41
---	--	------	------
mPD		0.29	0.49
		nPD:	0.59
		N. weight:	0.59
		Sc. PD:	0.14
		Sc. rank:	565.1

,

PB2			
Pos.	512	obs:	exp:
tct	S	0	248.90
tcc	S	0	233.90
tca	S	0	359.40
tcg	S	0	78.66
act	T	1	0.25
acc	T	0	0.22
aca	T	0	0.45
acg	T	0	0.08
aat	N	1039	611.60
aac	N	37	464.40
agt	S	1479	275.50
agc	S	1	283.60
gct	A	1	0.25
gcc	A	0	0.23
gca	A	0	0.45
gcg	A	0	0.07
gat	D	110	66.93
gac	D	9	52.07
---	--	------	------
mPD		0.62	1.8
		nPD:	0.35
		N. weight:	2.1
		Sc. PD:	0.093
		Sc. rank:	641.6

,

PB2			
Pos.	513	obs:	exp:
att	I	2	112.10
atc	I	299	81.05
ata	I	15	122.90
gtt	V	24	524.20
gtc	V	91	445.50
gta	V	1047	596.00
gtg	V	1199	795.30
---	--	------	------
mPD		0.83	0.95
		nPD:	0.87
		N. weight:	0.85
		Sc. PD:	0.38
		Sc. rank:	1032.0

,

PB2			
Pos.	514	obs:	exp:
atg	M	2677	2677.00
---	--	------	------
mPD		0	0
		nPD:	1.
		N. weight:	0.
		Sc. PD:	0
		Sc. rank:	0

,

PB2			
Pos.	515	obs:	exp:
ggt	G	3	419.90
ggc	G	14	339.20
gga	G	2489	1213.00
ggg	G	171	705.40
---	--	------	------
mPD		0.13	0.68
		nPD:	0.19
		N. weight:	1.
		Sc. PD:	-0.077
		Sc. rank:	-138.9

,

PB2

Pos.	516	obs:	exp:
atg	M	2677	2677.00
---	--	------	------
mPD		0	0
		nPD:	1.
		N. weight:	0.
		Sc. PD:	0
		Sc. rank:	0

,

PB2

Pos.	517	obs:	exp:
att	I	258	931.90
atc	I	1943	673.80
ata	I	426	1021.00
atg	M	4	4.00
act	T	0	0.25
acc	T	1	0.22
aca	T	0	0.45
acg	T	0	0.08
gtt	V	2	9.77
gtc	V	31	8.30
gta	V	10	11.11
gtg	V	1	14.82
gct	A	0	0.25
gcc	A	0	0.23
gca	A	1	0.45
gcg	A	0	0.07
---	--	------	------
mPD		0.45	0.70
		nPD:	0.65
		N. weight:	0.97
		Sc. PD:	0.27
		Sc. rank:	1028.2

,

PB2

Pos.	518	obs:	exp:
ggt	G	312	419.90
ggc	G	15	339.20
gga	G	1616	1213.00
ggg	G	734	705.40
---	--	------	------
mPD		0.55	0.68
		nPD:	0.8
		N. weight:	0.25
		Sc. PD:	0.099
		Sc. rank:	301.1

,

PB2

Pos.	519	obs:	exp:
tta	L	1	0.22
ttg	L	0	0.38
ctt	L	0	0.40
ctc	L	0	0.29
cta	L	1	0.35
ctg	L	0	0.36
att	I	7	887.90
atc	I	2	642.00
ata	I	2494	973.10
atg	M	3	3.00
gtt	V	0	37.52
gtc	V	0	31.89
gta	V	169	42.66
gtg	V	0	56.92
---	--	------	------
mPD		0.13	0.79
		nPD:	0.16
		N. weight:	1.8
		Sc. PD:	-0.17
		Sc. rank:	-445.4

,

PB2

Pos.	520	obs:	exp:
tta	L	604	290.30
ttg	L	1252	501.10
ctt	L	4	538.80
ctc	L	14	388.20
cta	L	79	465.90
ctg	L	715	483.60
atg	M	9	9.00
---	--	------	------
mPD		0.82	1.1
		nPD:	0.72
		N. weight:	1.2
		Sc. PD:	0.38
		Sc. rank:	1325.5

,

PB2

Pos.	521	obs:	exp:
tct	S	2	0.34
tcc	S	0	0.32
tca	S	0	0.49
tcg	S	0	0.11
cct	P	1797	789.10
ccc	P	855	538.40
cca	P	18	1059.00
ccg	P	4	287.60
cat	H	1	0.58
cac	H	0	0.42
agt	S	0	0.37
agc	S	0	0.38
---	--	------	------
mPD		0.45	0.71
		nPD:	0.63
		N. weight:	1.2
		Sc. PD:	0.33
		Sc. rank:	1271.7

,

PB2

Pos.	522	obs:	exp:
gat	D	1862	1506.00
gac	D	815	1171.00
---	--	------	------
mPD		0.42	0.49
		nPD:	0.86
		N. weight:	0.072
		Sc. PD:	0.032
		Sc. rank:	87.2

,

PB2

Pos.	523	obs:	exp:
tta	L	9	3.16
ttg	L	18	5.45
ctt	L	0	5.86
ctc	L	0	4.22
cta	L	0	5.06
ctg	L	2	5.26
att	I	0	0.35
atc	I	0	0.26
ata	I	1	0.39
atg	M	2647	2647.00
---	--	------	------
mPD		0.029	0.036
		nPD:	0.81
		N. weight:	0.023
		Sc. PD:	0.0091
		Sc. rank:	27.3

,

PB2

Pos.	524	obs:	exp:
tct	S	0	0.50
tcc	S	0	0.47
tca	S	0	0.73
tcg	S	0	0.16
att	I	1	2.84
atc	I	7	2.05
ata	I	0	3.11
act	T	571	642.20
acc	T	2026	564.60
aca	T	9	1186.00
acg	T	5	217.70
agt	S	0	0.56
agc	S	3	0.57
gtt	V	0	0.22
gtc	V	1	0.19
gta	V	0	0.25
gtg	V	0	0.34
gct	A	0	13.24
gcc	A	54	12.37
gca	A	0	24.34
gcg	A	0	4.05
---	--	------	------
mPD		0.39	0.73
		nPD:	0.54
		N. weight:	1.8
		Sc. PD:	0.34
		Sc. rank:	1501.9

,

PB2

Pos.	525	obs:	exp:
tta	L	0	0.11
ttg	L	0	0.19
ctt	L	0	0.20
ctc	L	0	0.15
cta	L	1	0.17
ctg	L	0	0.18
cct	P	11	789.70
ccc	P	18	538.80
cca	P	2614	1060.00
ccg	P	33	287.90
---	--	------	------
mPD		0.046	0.71
		nPD:	0.07
		N. weight:	1.5
		Sc. PD:	-0.26
		Sc. rank:	-1152.8

,

PB2

Pos.	526	obs:	exp:
tct	S	0	449.40
tcc	S	0	422.30
tca	S	0	648.90
tcg	S	0	142.00
aat	N	0	2.84
aac	N	5	2.16
agt	S	77	497.30
agc	S	2595	512.10
---	--	------	------
mPD		0.060	1.6
		nPD:	0.04
		N. weight:	2.8
		Sc. PD:	-0.55
		Sc. rank:	-2748.3

,

PB2

Pos.	527	obs:	exp:
tct	S	0	0.17
tcc	S	0	0.16
tca	S	0	0.24
tcg	S	1	0.05
att	I	1	1.06
atc	I	0	0.77
ata	I	2	1.17
atg	M	194	194.00
act	T	62	607.60
acc	T	2	534.10
aca	T	649	1122.00
acg	T	1757	206.00
agt	S	0	0.19
agc	S	0	0.19
gct	A	0	2.21
gcc	A	0	2.06
gca	A	0	4.06
gcg	A	9	0.67
---	--	------	------
mPD		0.55	0.85
		nPD:	0.64
		N. weight:	2.3
		Sc. PD:	0.62
		Sc. rank:	2379.6

,

PB2

Pos.	528	obs:	exp:
gat	D	5	6.19
gac	D	6	4.81
gaa	E	1584	1579.00
gag	E	1081	1086.00
ggt	G	0	0.16
ggc	G	0	0.13
gga	G	1	0.45
ggg	G	0	0.26
---	--	------	------
mPD		0.49	0.49
		nPD:	1.
		N. weight:	0.0046
		Sc. PD:	0.0025
		Sc. rank:	5.7

,

PB2

Pos.	529	obs:	exp:
cgt	R	0	0.54
cgc	R	0	0.90
cga	R	0	1.71
cgg	R	0	1.23
att	I	0	57.11
atc	I	0	41.29
ata	I	161	62.59
atg	M	2421	2421.00
act	T	0	7.13
acc	T	0	6.27
aca	T	1	13.18
acg	T	28	2.42
aaa	K	6	16.41
aag	K	21	10.59
aga	R	4	7.80
agg	R	13	4.83
gtt	V	0	4.88
gtc	V	0	4.15
gta	V	9	5.55
gtg	V	13	7.41
---	--	------	------
mPD		0.20	0.23
		nPD:	0.84
		N. weight:	0.18
		Sc. PD:	0.074
		Sc. rank:	211.9

,

PB2			
Pos.	530	obs:	exp:
tct	S	4	450.10
tcc	S	4	422.90
tca	S	822	649.80
tcg	S	1846	142.20
cct	P	0	0.30
ccc	P	0	0.20
cca	P	1	0.40
ccg	P	0	0.11
agt	S	0	498.10
agc	S	0	512.80
---	--	------	------
mPD		0.43	1.6
		nPD:	0.26
		N. weight:	3.4
		Sc. PD:	-0.063
		Sc. rank:	126.1

,

PB2			
Pos.	531	obs:	exp:
tta	L	301	284.90
ttg	L	178	491.70
ctt	L	38	528.70
ctc	L	0	380.90
cta	L	178	457.10
ctg	L	1923	474.60
att	I	0	1.42
atc	I	0	1.03
ata	I	4	1.56
atg	M	55	55.00
---	--	------	------
mPD		0.65	1.2
		nPD:	0.56
		N. weight:	1.6
		Sc. PD:	0.33
		Sc. rank:	1397.0

,

PB2			
Pos.	532	obs:	exp:
cgt	R	0	84.50
cgc	R	0	141.50
cga	R	0	269.10
cgg	R	1	193.40
aga	R	1843	1228.00
agg	R	833	760.90
---	--	------	------
mPD		0.43	0.94
		nPD:	0.46
		N. weight:	0.57
		Sc. PD:	0.074
		Sc. rank:	354.6

,

PB2			
Pos.	533	obs:	exp:
---	-	2677	2677.00
---	--	------	------
mPD		0	0
		nPD:	1.
		N. weight:	0.
		Sc. PD:	0
		Sc. rank:	0

,

PB2			
Pos.	534	obs:	exp:
---	-	2677	2677.00
---	--	------	------
mPD		0	0
		nPD:	1.
		N. weight:	0.
		Sc. PD:	0
		Sc. rank:	0

,

PB2			
Pos.	535	obs:	exp:
---	-	2677	2677.00
---	--	------	------
mPD		0	0
		nPD:	1.
		N. weight:	0.
		Sc. PD:	0
		Sc. rank:	0

,

PB2			
Pos.	536	obs:	exp:
---	-	2677	2677.00
---	--	------	------
mPD		0	0
		nPD:	1.
		N. weight:	0.
		Sc. PD:	0
		Sc. rank:	0

,

PB2			
Pos.	537	obs:	exp:
tct	S	0	1.35
tcc	S	0	1.26
tca	S	0	1.94
tcg	S	0	0.43
agt	S	8	1.49
agc	S	0	1.53
ggt	G	1013	418.60
ggc	G	20	338.20
gga	G	397	1209.00
ggg	G	1239	703.20
---	--	------	------
mPD		0.62	0.69
		nPD:	0.9
		N. weight:	0.78
		Sc. PD:	0.36
		Sc. rank:	952.8

,

PB2			
Pos.	538	obs:	exp:
att	I	0	787.50
atc	I	1	569.40
ata	I	2219	863.10
gtt	V	17	101.50
gtc	V	0	86.23
gta	V	48	115.40
gtg	V	392	153.90
---	--	------	------
mPD		0.54	0.98
		nPD:	0.56
		N. weight:	1.7
		Sc. PD:	0.34
		Sc. rank:	1460.0

,

PB2			
Pos.	539	obs:	exp:
cgt	R	0	84.50
cgc	R	0	141.50
cga	R	3	269.10
cgg	R	0	193.40
aga	R	2277	1228.00
agg	R	397	760.90
---	--	------	------
mPD		0.25	0.94
		nPD:	0.27
		N. weight:	0.79
		Sc. PD:	-0.01
		Sc. rank:	45.4

,

PB2			
Pos.	540	obs:	exp:
att	I	14	165.70
atc	I	449	119.80
ata	I	4	181.60
gtt	V	8	490.30
gtc	V	2194	416.60
gta	V	6	557.40
gtg	V	0	743.70
ggt	G	0	0.31
ggc	G	2	0.25
gga	G	0	0.91
ggg	G	0	0.53
---	--	------	------
mPD		0.31	1.0
		nPD:	0.3
		N. weight:	2.9
		Sc. PD:	0.031
		Sc. rank:	430.2

,

PB2			
Pos.	541	obs:	exp:
tct	S	0	450.30
tcc	S	0	423.10
tca	S	0	650.10
tcg	S	0	142.30
agt	S	321	498.30
agc	S	2356	513.00
---	--	------	------
mPD		0.21	1.6
		nPD:	0.13
		N. weight:	2.4
		Sc. PD:	-0.29
		Sc. rank:	-955.6

,

PB2			
Pos.	542	obs:	exp:
cgt	R	0	3.09
cgc	R	0	5.18
cga	R	0	9.85
cgg	R	0	7.08
aat	N	0	0.57
aac	N	1	0.43
aaa	K	2341	1567.00
aag	K	237	1011.00
aga	R	92	44.94
agg	R	6	27.85
---	--	------	------
mPD		0.24	0.57
		nPD:	0.42
		N. weight:	0.46
		Sc. PD:	0.044
		Sc. rank:	226.8

,

PB2			
Pos.	543	obs:	exp:
att	I	2	1.77
atc	I	0	1.28
ata	I	3	1.94
atg	M	2251	2251.00
act	T	2	103.10
acc	T	0	90.61
aca	T	381	190.40
acg	T	36	34.94
gct	A	0	0.49
gcc	A	0	0.46
gca	A	2	0.90
gcg	A	0	0.15
---	--	------	------
mPD		0.52	0.53
		nPD:	0.98
		N. weight:	0.18
		Sc. PD:	0.097
		Sc. rank:	228.0

,

PB2			
Pos.	544	obs:	exp:
ggt	G	14	419.90
ggc	G	1	339.20
gga	G	2529	1213.00
ggg	G	133	705.40
---	--	------	------
mPD		0.11	0.68
		nPD:	0.15
		N. weight:	1.1
		Sc. PD:	-0.11
		Sc. rank:	-331.3

,

PB2			
Pos.	545	obs:	exp:
att	I	0	4.26
atc	I	0	3.08
ata	I	12	4.67
gtt	V	5	591.70
gtc	V	3	502.90
gta	V	2496	672.70
gtg	V	161	897.60
---	--	------	------
mPD		0.13	0.75
		nPD:	0.17
		N. weight:	2.1
		Sc. PD:	-0.19
		Sc. rank:	-434.4

,

PB2			
Pos.	546	obs:	exp:
gat	D	2244	1506.00
gac	D	433	1171.00
---	--	------	------
mPD		0.27	0.49
		nPD:	0.55
		N. weight:	0.32
		Sc. PD:	0.065
		Sc. rank:	281.0

,

PB2			
Pos.	547	obs:	exp:
gaa	E	2496	1586.00
gag	E	180	1090.00
ggt	G	0	0.16
ggc	G	0	0.13
gga	G	1	0.45
ggg	G	0	0.26
---	--	------	------
mPD		0.13	0.48
		nPD:	0.26
		N. weight:	0.56
		Sc. PD:	-0.012
		Sc. rank:	11.9

,

PB2			
Pos.	548	obs:	exp:
ttt	F	3	1.34
ttc	F	0	1.66
tat	Y	1902	1494.00
tac	Y	765	1173.00
cat	H	5	4.04
cac	H	2	2.96
---	--	------	------
mPD		0.42	0.50
		nPD:	0.83
		N. weight:	0.097
		Sc. PD:	0.04
		Sc. rank:	116.7

,

PB2			
Pos.	549	obs:	exp:
ttt	F	0	0.90
ttc	F	2	1.10
tct	S	25	449.30
tcc	S	2624	422.10
tca	S	22	648.60
tcg	S	0	142.00
cct	P	0	0.30
ccc	P	1	0.20
cca	P	0	0.40
ccg	P	0	0.11
act	T	0	0.25
acc	T	1	0.22
aca	T	0	0.45
acg	T	0	0.08
agt	S	0	497.10
agc	S	0	511.90
gct	A	0	0.49
gcc	A	2	0.46
gca	A	0	0.90
gcg	A	0	0.15
---	--	------	------
mPD		0.039	1.6
		nPD:	0.02
		N. weight:	3.2
		Sc. PD:	-0.65
		Sc. rank:	-3432.9

,

PB2			
Pos .	550	obs :	exp :
tct	S	0	447.90
tcc	S	0	420.90
tca	S	0	646.70
tcg	S	0	141.50
tgt	C	1	0.43
tgc	C	0	0.57
cgt	R	0	0.03
cgc	R	0	0.05
cga	R	0	0.10
cgg	R	0	0.07
aat	N	1	1.71
aac	N	2	1.29
agt	S	1288	495.60
agc	S	1375	510.40
aga	R	1	0.46
agg	R	0	0.28
ggt	G	9	1.41
ggc	G	0	1.14
gga	G	0	4.08
ggg	G	0	2.37
---	--	------	------
mPD		0.51	1.6
		nPD :	0.31
		N. weight :	1.8
		Sc. PD:	0.032
		Sc. rank :	388.0

,

PB2			
Pos .	551	obs :	exp :
act	T	422	658.00
acc	T	37	578.40
aca	T	231	1216.00
acg	T	1985	223.10
aat	N	1	0.57
aac	N	0	0.43
gct	A	1	0.25
gcc	A	0	0.23
gca	A	0	0.45
gcg	A	0	0.07
---	--	------	------
mPD		0.42	0.68
		nPD :	0.62
		N. weight :	2.5
		Sc. PD:	0.64
		Sc. rank :	2559.3

,

PB2			
Pos .	552	obs :	exp :
gaa	E	101	1586.00
gag	E	2576	1091.00
---	--	------	------
mPD		0.073	0.48
		nPD :	0.15
		N. weight :	1.3
		Sc. PD:	-0.14
		Sc. rank :	-419.2

,

PB2			
Pos .	553	obs :	exp :
cgt	R	0	84.50
cgc	R	0	141.50
cga	R	1	269.10
cgg	R	0	193.40
aga	R	2579	1228.00
agg	R	97	760.90
---	--	------	------
mPD		0.071	0.94
		nPD :	0.08
		N. weight :	1.2
		Sc. PD:	-0.19
		Sc. rank :	-813.1

,

PB2			
Pos .	554	obs :	exp :
att	I	0	0.71
atc	I	0	0.51
ata	I	2	0.78
gtt	V	11	593.90
gtc	V	1	504.80
gta	V	74	675.30
gtg	V	2589	901.00
---	--	------	------
mPD		0.065	0.74
		nPD :	0.09
		N. weight :	1.7
		Sc. PD:	-0.27
		Sc. rank :	-1071.6

,

PB2			
Pos .	555	obs :	exp :
att	I	24	18.80
atc	I	0	13.59
ata	I	29	20.60
atg	M	2	2.00
act	T	0	0.98
acc	T	0	0.86
aca	T	4	1.82
acg	T	0	0.33
gtt	V	378	524.20
gtc	V	2	445.50
gta	V	1720	596.00
gtg	V	261	795.30
gct	A	29	63.00
gcc	A	0	58.88
gca	A	215	115.80
gcg	A	13	19.27
---	--	------	------
mPD		0.64	0.96
		nPD :	0.67
		N. weight :	1.1
		Sc. PD:	0.31
		Sc. rank :	1155.2

,

PB2			
Pos .	556	obs :	exp :
gtt	V	9	594.40
gtc	V	0	505.10
gta	V	123	675.80
gtg	V	2545	901.70
---	--	------	------
mPD		0.094	0.74
		nPD :	0.13
		N. weight :	1.7
		Sc. PD:	-0.2
		Sc. rank :	-677.0

,

PB2			
Pos .	557	obs :	exp :
tct	S	0	432.80
tcc	S	0	406.60
tca	S	0	624.80
tcg	S	0	136.70
cgt	R	0	0.09
cgc	R	0	0.16
cga	R	0	0.30
cgg	R	0	0.22
act	T	22	6.89
acc	T	3	6.05
aca	T	3	12.72
acg	T	0	2.33
aat	N	60	41.49
aac	N	13	31.51
agt	S	885	478.90
agc	S	1688	493.10
aga	R	3	1.38
agg	R	0	0.85
---	--	------	------
mPD		0.54	1.7
		nPD :	0.32
		N. weight :	1.8
		Sc. PD:	0.048
		Sc. rank :	465.3

,

PB2			
Pos .	558	obs :	exp :
att	I	2586	949.30
atc	I	16	686.40
ata	I	74	1040.00
gtt	V	1	0.22
gtc	V	0	0.19
gta	V	0	0.25
gtg	V	0	0.34
---	--	------	------
mPD		0.066	0.66
		nPD :	0.1
		N. weight :	1.6
		Sc. PD:	-0.23
		Sc. rank :	-879.2

,

PB2			
Pos .	559	obs :	exp :
cat	H	1	0.58
cac	H	0	0.42
gat	D	840	1505.00
gac	D	1836	1171.00
---	--	------	------
mPD		0.43	0.49
		nPD :	0.88
		N. weight :	0.24
		Sc. PD:	0.11
		Sc. rank :	287.5

,

PB2			
Pos .	560	obs :	exp :
tta	L	0	0.11
ttg	L	0	0.19
ctt	L	0	0.20
ctc	L	0	0.15
cta	L	1	0.17
ctg	L	0	0.18
cct	P	0	0.30
ccc	P	1	0.20
cca	P	0	0.40
ccg	P	0	0.11
cgt	R	81	84.37
cgc	R	300	141.30
cga	R	2198	268.70
cgg	R	82	193.10
aaa	K	2	1.22
aag	K	0	0.78
aga	R	12	1226.00
agg	R	0	759.70
---	--	------	------
mPD		0.32	0.94
		nPD :	0.33
		N. weight :	3.3
		Sc. PD:	0.12
		Sc. rank :	926.6

,

PB2			
Pos .	561	obs :	exp :
ttt	F	2265	1195.00
ttc	F	401	1471.00
tat	Y	4	6.16
tac	Y	7	4.84
---	--	------	------
mPD		0.27	0.50
		nPD :	0.53
		N. weight :	0.65
		Sc. PD:	0.12
		Sc. rank :	532.9

,

PB2			
Pos .	562	obs :	exp :
tta	L	948	291.30
ttg	L	1642	502.80
ctt	L	0	540.60
ctc	L	0	389.50
cta	L	7	467.40
ctg	L	80	485.30
---	--	------	------
mPD		0.52	1.1
		nPD :	0.46
		N. weight :	2.
		Sc. PD:	0.26
		Sc. rank :	1257.0

,

PB2			
Pos .	563	obs :	exp :
cgt	R	0	84.50
cgc	R	0	141.50
cga	R	0	269.10
cgg	R	0	193.40
aga	R	1640	1228.00
agg	R	1037	760.90
---	--	------	------
mPD		0.47	0.94
		nPD :	0.51
		N. weight :	0.55
		Sc. PD:	0.092
		Sc. rank :	426.2

,

PB2			
Pos .	564	obs :	exp :
gtt	V	2479	594.20
gtc	V	170	505.00
gta	V	21	675.50
gtg	V	6	901.40
gat	D	1	0.56
gac	D	0	0.44
---	--	------	------
mPD		0.14	0.74
		nPD :	0.19
		N. weight :	2.3
		Sc. PD:	-0.17
		Sc. rank :	-351.2

,

PB2			
Pos.	565	obs:	exp:
caa	Q	0	0.54
cag	Q	1	0.46
cgt	R	5	81.56
cgc	R	0	136.60
cga	R	1054	259.70
cgg	R	790	186.70
aaa	K	76	55.91
aag	K	16	36.09
aga	R	699	1185.00
agg	R	36	734.40
---	--	------	------
mPD		0.93	0.99
		nPD:	0.93
		N. weight:	1.5
		Sc. PD:	0.74
		Sc. rank:	1841.2

,

PB2			
Pos.	566	obs:	exp:
aat	N	3	1.71
aac	N	0	1.29
gat	D	2384	1504.00
gac	D	290	1170.00
---	--	------	------
mPD		0.20	0.49
		nPD:	0.4
		N. weight:	0.48
		Sc. PD:	0.04
		Sc. rank:	215.5

,

PB2			
Pos.	567	obs:	exp:
tta	L	0	0.22
ttg	L	0	0.38
ctt	L	0	0.40
ctc	L	0	0.29
cta	L	2	0.35
ctg	L	0	0.36
cct	P	0	0.89
ccc	P	0	0.60
cca	P	1	1.19
ccg	P	2	0.32
cat	H	7	28.84
cac	H	43	21.16
caa	Q	2181	1413.00
cag	Q	440	1208.00
cgt	R	0	0.03
cgc	R	0	0.05
cga	R	1	0.10
cgg	R	0	0.07
aga	R	0	0.46
agg	R	0	0.28
---	--	------	------
mPD		0.31	0.52
		nPD:	0.6
		N. weight:	0.37
		Sc. PD:	0.088
		Sc. rank:	358.8

,

PB2			
Pos.	568	obs:	exp:
tta	L	0	20.68
ttg	L	0	35.69
tct	S	0	0.17
tcc	S	0	0.16
tca	S	0	0.24
tcg	S	0	0.05
tgg	W	1	1.00
ctt	L	0	38.37
ctc	L	0	27.64
cta	L	190	33.18
ctg	L	0	34.44
caa	Q	49	37.21
cag	Q	20	31.79
cgt	R	4	75.31
cgc	R	0	126.10
cga	R	1232	239.80
cgg	R	307	172.40
att	I	0	0.35
atc	I	0	0.26
ata	I	1	0.39
atg	M	1	1.00
aaa	K	6	16.41
aag	K	21	10.59
agt	S	1	0.19
agc	S	0	0.19
aga	R	775	1094.00
agg	R	68	678.20
ggt	G	0	0.16
ggc	G	0	0.13
gga	G	1	0.45
ggg	G	0	0.26
---	--	------	------
mPD		0.90	1.2
		nPD:	0.74
		N. weight:	1.5
		Sc. PD:	0.51
		Sc. rank:	1711.3

,

PB2			
Pos.	569	obs:	exp:
ggt	G	4	419.90
ggc	G	1	339.20
gga	G	405	1213.00
ggg	G	2267	705.40
---	--	------	------
mPD		0.26	0.68
		nPD:	0.38
		N. weight:	1.5
		Sc. PD:	0.11
		Sc. rank:	588.1

,

PB2			
Pos.	570	obs:	exp:
cat	H	0	1.15
cac	H	2	0.85
aat	N	549	1518.00
aac	N	2122	1153.00
gat	D	1	2.25
gac	D	3	1.75
---	--	------	------
mPD		0.33	0.50
		nPD:	0.67
		N. weight:	0.51
		Sc. PD:	0.15
		Sc. rank:	559.6

,

PB2			
Pos.	571	obs:	exp:
tta	L	2	0.22
ttg	L	0	0.38
ctt	L	0	0.40
ctc	L	0	0.29
cta	L	0	0.35
ctg	L	0	0.36
att	I	0	82.30
atc	I	3	59.51
ata	I	229	90.19
atg	M	1	1.00
gtt	V	0	542.20
gtc	V	16	460.80
gta	V	2338	616.50
gtg	V	88	822.50
---	--	------	------
mPD		0.24	0.90
		nPD:	0.26
		N. weight:	2.1
		Sc. PD:	-0.04
		Sc. rank:	60.6

,

PB2			
Pos.	572	obs:	exp:
tta	L	466	291.20
ttg	L	41	502.60
ctt	L	17	540.40
ctc	L	101	389.30
cta	L	2026	467.30
ctg	L	25	485.10
att	I	0	0.35
atc	I	0	0.26
ata	I	1	0.39
---	--	------	------
mPD		0.44	1.1
		nPD:	0.38
		N. weight:	2.
		Sc. PD:	0.15
		Sc. rank:	783.0

,

PB2			
Pos.	573	obs:	exp:
tta	L	183	291.30
ttg	L	2170	502.80
ctt	L	4	540.60
ctc	L	0	389.50
cta	L	250	467.40
ctg	L	70	485.30
---	--	------	------
mPD		0.49	1.1
		nPD:	0.43
		N. weight:	1.9
		Sc. PD:	0.21
		Sc. rank:	1088.9

,

PB2			
Pos.	574	obs:	exp:
tct	S	1588	446.70
tcc	S	1040	419.80
tca	S	26	645.00
tcg	S	2	141.20
act	T	21	5.17
acc	T	0	4.54
aca	T	0	9.54
acg	T	0	1.75
agt	S	0	494.30
agc	S	0	509.00
---	--	------	------
mPD		0.50	1.6
		nPD:	0.31
		N. weight:	2.
		Sc. PD:	0.031
		Sc. rank:	375.4

,

PB2			
Pos.	575	obs:	exp:
tta	L	0	0.11
ttg	L	0	0.19
ctt	L	1	0.20
ctc	L	0	0.15
cta	L	0	0.17
ctg	L	0	0.18
cct	P	761	789.70
ccc	P	1690	538.80
cca	P	223	1060.00
ccg	P	2	287.90
---	--	------	------
mPD		0.51	0.71
		nPD:	0.73
		N. weight:	1.1
		Sc. PD:	0.36
		Sc. rank:	1245.3

,

PB2			
Pos.	576	obs:	exp:
---	-	2677	2677.00
---	--	------	------
mPD		0	0
		nPD:	1.
		N. weight:	0.
		Sc. PD:	0
		Sc. rank:	0

,

PB2			
Pos.	577	obs:	exp:
---	-	2677	2677.00
---	--	------	------
mPD		0	0
		nPD:	1.
		N. weight:	0.
		Sc. PD:	0
		Sc. rank:	0

,

PB2			
Pos.	578	obs:	exp:
---	-	2677	2677.00
---	--	------	------
mPD		0	0
		nPD:	1.
		N. weight:	0.
		Sc. PD:	0
		Sc. rank:	0

,

PB2			
Pos.	579	obs:	exp:
---	-	2677	2677.00
---	--	------	------
mPD		0	0
		nPD:	1.
		N. weight:	0.
		Sc. PD:	0
		Sc. rank:	0

,

PB2			
Pos.	580	obs:	exp:
---	-	2677	2677.00
---	--	------	------
mPD		0	0
		nPD:	1.
		N. weight:	0.
		Sc. PD:	0
		Sc. rank:	0

,

PB2			
Pos.	581	obs:	exp:
---	-	2677	2677.00
---	--	------	------
mPD		0	0
		nPD:	1.
		N. weight:	0.
		Sc. PD:	0
		Sc. rank:	0

,

PB2			
Pos.	582	obs:	exp:
---	-	2677	2677.00
---	--	------	------
mPD		0	0
		nPD:	1.
		N. weight:	0.
		Sc. PD:	0
		Sc. rank:	0

,

PB2			
Pos.	583	obs:	exp:
gat	D	1	0.56
gac	D	0	0.44
gaa	E	2296	1586.00
gag	E	380	1090.00
---	--	------	------
mPD		0.24	0.48
		nPD:	0.51
		N. weight:	0.31
		Sc. PD:	0.052
		Sc. rank:	241.3

,

PB2			
Pos.	584	obs:	exp:
gaa	E	985	1586.00
gag	E	1692	1091.00
---	--	------	------
mPD		0.47	0.48
		nPD:	0.96
		N. weight:	0.19
		Sc. PD:	0.1
		Sc. rank:	238.1

PB2			
Pos.	585	obs:	exp:
att	I	0	1.42
atc	I	1	1.03
ata	I	3	1.56
gtt	V	332	593.50
gtc	V	2282	504.40
gta	V	55	674.80
gtg	V	4	900.30
---	--	------	------
mPD		0.26	0.74
		nPD:	0.35
		N. weight:	2.1
		Sc. PD:	0.1
		Sc. rank:	694.0

PB2			
Pos.	586	obs:	exp:
tct	S	0	450.30
tcc	S	0	423.10
tca	S	0	650.10
tcg	S	0	142.30
agt	S	1097	498.30
agc	S	1580	513.00
---	--	------	------
mPD		0.48	1.6
		nPD:	0.3
		N. weight:	1.8
		Sc. PD:	0.012
		Sc. rank:	242.5

PB2			
Pos.	587	obs:	exp:
gaa	E	1223	1586.00
gag	E	1454	1091.00
---	--	------	------
mPD		0.50	0.48
		nPD:	1.03
		N. weight:	0.072
		Sc. PD:	0.041
		Sc. rank:	89.4

PB2			
Pos.	588	obs:	exp:
atg	M	1	1.00
act	T	15	658.20
acc	T	4	578.70
aca	T	1975	1216.00
acg	T	682	223.10
---	--	------	------
mPD		0.39	0.68
		nPD:	0.58
		N. weight:	1.1
		Sc. PD:	0.25
		Sc. rank:	1048.9

PB2			
Pos.	589	obs:	exp:
tta	L	0	0.11
ttg	L	0	0.19
ctt	L	0	0.20
ctc	L	0	0.15
cta	L	1	0.17
ctg	L	0	0.18
cat	H	4	2.88
cac	H	1	2.12
caa	Q	1365	1440.00
cag	Q	1306	1231.00
---	--	------	------
mPD		0.50	0.50
		nPD:	1.01
		N. weight:	0.0081
		Sc. PD:	0.0044
		Sc. rank:	10.0

PB2			
Pos.	590	obs:	exp:
ggt	G	3	419.90
ggc	G	0	339.20
gga	G	2425	1213.00
ggg	G	249	705.40
---	--	------	------
mPD		0.17	0.68
		nPD:	0.25
		N. weight:	0.98
		Sc. PD:	-0.029
		Sc. rank:	-0.8

PB2			
Pos.	591	obs:	exp:
att	I	15	7.09
atc	I	0	5.13
ata	I	5	7.78
atg	M	4	4.00
act	T	1875	645.20
acc	T	60	567.20
aca	T	615	1192.00
acg	T	73	218.70
aat	N	8	4.55
aac	N	0	3.45
gtt	V	0	0.22
gtc	V	0	0.19
gta	V	1	0.25
gtg	V	0	0.34
gct	A	12	5.15
gcc	A	0	4.81
gca	A	7	9.47
gcg	A	2	1.57
---	--	------	------
mPD		0.48	0.72
		nPD:	0.66
		N. weight:	0.98
		Sc. PD:	0.28
		Sc. rank:	1057.4

PB2			
Pos.	592	obs:	exp:
gaa	E	329	1586.00
gag	E	2348	1091.00
---	--	------	------
mPD		0.22	0.48
		nPD:	0.45
		N. weight:	0.89
		Sc. PD:	0.11
		Sc. rank:	536.6

PB2			
Pos.	593	obs:	exp:
cgt	R	0	0.85
cgc	R	0	1.43
cga	R	0	2.71
cgg	R	0	1.95
aaa	K	1693	1610.00
aag	K	957	1040.00
aga	R	24	12.38
agg	R	3	7.67
---	--	------	------
mPD		0.48	0.50
		nPD:	0.95
		N. weight:	0.017
		Sc. PD:	0.0085
		Sc. rank:	20.6

PB2			
Pos.	594	obs:	exp:
tta	L	95	291.30
ttg	L	2548	502.80
ctt	L	1	540.60
ctc	L	0	389.50
cta	L	5	467.40
ctg	L	28	485.30
---	--	------	------
mPD		0.098	1.1
		nPD:	0.09
		N. weight:	2.7
		Sc. PD:	-0.42
		Sc. rank:	-1687.1

PB2			
Pos.	595	obs:	exp:
act	T	6	658.50
acc	T	35	578.90
aca	T	2601	1216.00
acg	T	35	223.20
---	--	------	------
mPD		0.056	0.68
		nPD:	0.08
		N. weight:	1.2
		Sc. PD:	-0.2
		Sc. rank:	-798.4

PB2			
Pos.	596	obs:	exp:
att	I	5	949.60
atc	I	3	686.60
ata	I	2669	1041.00
---	--	------	------
mPD		0.0060	0.66
		nPD:	0.01
		N. weight:	1.7
		Sc. PD:	-0.37
		Sc. rank:	-1973.0

PB2			
Pos.	597	obs:	exp:
---	-	2677	2677.00
---	--	------	------
mPD		0	0
		nPD:	1.
		N. weight:	0.
		Sc. PD:	0
		Sc. rank:	0

PB2			
Pos.	598	obs:	exp:
---	-	2677	2677.00
---	--	------	------
mPD		0	0
		nPD:	1.
		N. weight:	0.
		Sc. PD:	0
		Sc. rank:	0

PB2			
Pos.	599	obs:	exp:
---	-	2677	2677.00
---	--	------	------
mPD		0	0
		nPD:	1.
		N. weight:	0.
		Sc. PD:	0
		Sc. rank:	0

PB2			
Pos.	600	obs:	exp:
att	I	1	0.35
atc	I	0	0.26
ata	I	0	0.39
act	T	2246	658.20
acc	T	334	578.70
aca	T	89	1216.00
acg	T	7	223.10
---	--	------	------
mPD		0.28	0.68
		nPD:	0.41
		N. weight:	1.6
		Sc. PD:	0.15
		Sc. rank:	770.4

PB2			
Pos.	601	obs:	exp:
tat	Y	2560	1500.00
tac	Y	117	1177.00
---	--	------	------
mPD		0.084	0.49
		nPD:	0.17
		N. weight:	0.76
		Sc. PD:	-0.069
		Sc. rank:	-169.7

PB2			
Pos.	602	obs:	exp:
tta	L	4	1.41
ttg	L	9	2.44
tct	S	33	447.90
tcc	S	15	420.90
tca	S	527	646.70
tcg	S	2088	141.50
ctt	L	0	2.63
ctc	L	0	1.89
cta	L	0	2.27
ctg	L	0	2.36
act	T	0	0.25
acc	T	0	0.22
aca	T	0	0.45
acg	T	1	0.08
agt	S	0	495.60
agc	S	0	510.40
---	--	------	------
mPD		0.36	1.6
		nPD:	0.22
		N. weight:	3.7
		Sc. PD:	-0.2
		Sc. rank:	-247.6

PB2			
Pos.	603	obs:	exp:
tct	S	10	449.90
tcc	S	0	422.80
tca	S	2500	649.60
tcg	S	165	142.20
act	T	0	0.49
acc	T	0	0.43
aca	T	2	0.91
acg	T	0	0.17
agt	S	0	497.90
agc	S	0	512.70
---	--	------	------
mPD		0.12	1.6
		nPD:	0.08
		N. weight:	2.3
		Sc. PD:	-0.38
		Sc. rank:	-1582.1

,

PB2			
Pos.	604	obs:	exp:
tct	S	34	449.90
tcc	S	0	422.80
tca	S	2607	649.60
tcg	S	34	142.20
agt	S	0	497.90
agc	S	0	512.70
gct	A	0	0.49
gcc	A	0	0.46
gca	A	2	0.90
gcg	A	0	0.15
---	--	------	------
mPD		0.051	1.6
		nPD:	0.03
		N. weight:	2.4
		Sc. PD:	-0.48
		Sc. rank:	-2480.8

,

PB2			
Pos.	605	obs:	exp:
tta	L	0	0.54
ttg	L	0	0.94
ctt	L	0	1.01
ctc	L	0	0.73
cta	L	0	0.87
ctg	L	5	0.91
att	I	0	0.35
atc	I	0	0.26
ata	I	1	0.39
atg	M	2671	2671.00
---	--	------	------
mPD		0.0045	0.0068
		nPD:	0.66
		N. weight:	0.0066
		Sc. PD:	0.0018
		Sc. rank:	7.0

,

PB2			
Pos.	606	obs:	exp:
tta	L	0	7.18
ttg	L	66	12.40
ctt	L	0	13.33
ctc	L	0	9.60
cta	L	0	11.52
ctg	L	0	11.96
atg	M	2611	2611.00
---	--	------	------
mPD		0.048	0.079
		nPD:	0.61
		N. weight:	0.076
		Sc. PD:	0.019
		Sc. rank:	75.4

,

PB2			
Pos.	607	obs:	exp:
tgg	W	2677	2677.00
---	--	------	------
mPD		0	0
		nPD:	1.
		N. weight:	0.
		Sc. PD:	0
		Sc. rank:	0

,

PB2			
Pos.	608	obs:	exp:
gaa	E	293	1586.00
gag	E	2384	1091.00
---	--	------	------
mPD		0.20	0.48
		nPD:	0.4
		N. weight:	0.95
		Sc. PD:	0.084
		Sc. rank:	437.4

,

PB2			
Pos.	609	obs:	exp:
att	I	25	938.30
atc	I	2591	678.40
ata	I	29	1028.00
act	T	0	0.49
acc	T	2	0.43
aca	T	0	0.91
acg	T	0	0.17
gtt	V	10	6.66
gtc	V	20	5.66
gta	V	0	7.57
gtg	V	0	10.10
---	--	------	------
mPD		0.071	0.68
		nPD:	0.1
		N. weight:	2.3
		Sc. PD:	-0.33
		Sc. rank:	-1216.0

,

PB2			
Pos.	610	obs:	exp:
tct	S	0	1.18
tcc	S	0	1.11
tca	S	0	1.70
tcg	S	0	0.37
cat	H	1	0.58
cac	H	0	0.42
aat	N	2294	1517.00
aac	N	375	1152.00
agt	S	3	1.30
agc	S	4	1.34
---	--	------	------
mPD		0.25	0.50
		nPD:	0.5
		N. weight:	0.37
		Sc. PD:	0.06
		Sc. rank:	280.7

,

PB2			
Pos.	611	obs:	exp:
ggt	G	1569	419.90
ggc	G	746	339.20
gga	G	321	1213.00
ggg	G	41	705.40
---	--	------	------
mPD		0.56	0.68
		nPD:	0.82
		N. weight:	1.5
		Sc. PD:	0.6
		Sc. rank:	1767.3

,

PB2			
Pos.	612	obs:	exp:
cct	P	2550	790.00
ccc	P	64	539.00
cca	P	18	1060.00
ccg	P	45	288.00
---	--	------	------
mPD		0.092	0.70
		nPD:	0.13
		N. weight:	1.9
		Sc. PD:	-0.23
		Sc. rank:	-730.6

,

PB2			
Pos.	613	obs:	exp:
gaa	E	283	1586.00
gag	E	2394	1091.00
---	--	------	------
mPD		0.19	0.48
		nPD:	0.39
		N. weight:	0.97
		Sc. PD:	0.077
		Sc. rank:	411.2

,

PB2			
Pos.	614	obs:	exp:
tct	S	2	450.10
tcc	S	2	422.90
tca	S	2638	649.80
tcg	S	34	142.20
agt	S	0	498.10
agc	S	0	512.80
gct	A	0	0.25
gcc	A	0	0.23
gca	A	1	0.45
gcg	A	0	0.07
---	--	------	------
mPD		0.029	1.6
		nPD:	0.02
		N. weight:	2.5
		Sc. PD:	-0.52
		Sc. rank:	-2771.8

,

PB2			
Pos.	615	obs:	exp:
att	I	0	0.71
atc	I	0	0.51
ata	I	2	0.78
atg	M	1	1.00
gtt	V	11	593.70
gtc	V	2	504.60
gta	V	91	675.00
gtg	V	2570	900.70
---	--	------	------
mPD		0.079	0.74
		nPD:	0.11
		N. weight:	1.7
		Sc. PD:	-0.24
		Sc. rank:	-876.0

,

PB2			
Pos.	616	obs:	exp:
tta	L	15	289.00
ttg	L	111	498.90
ctt	L	13	536.40
ctc	L	388	386.40
cta	L	703	463.80
ctg	L	1426	481.50
att	I	0	7.45
atc	I	0	5.39
ata	I	21	8.16
---	--	------	------
mPD		0.68	1.1
		nPD:	0.59
		N. weight:	1.1
		Sc. PD:	0.26
		Sc. rank:	1072.6

,

PB2			
Pos.	617	obs:	exp:
att	I	5	23.77
atc	I	62	17.18
ata	I	0	26.05
gtt	V	533	579.50
gtc	V	2021	492.50
gta	V	32	658.90
gtg	V	24	879.10
---	--	------	------
mPD		0.40	0.79
		nPD:	0.51
		N. weight:	1.9
		Sc. PD:	0.32
		Sc. rank:	1470.1

,

PB2			
Pos.	618	obs:	exp:
aat	N	91	1522.00
aac	N	2586	1155.00
---	--	------	------
mPD		0.066	0.49
		nPD:	0.13
		N. weight:	1.3
		Sc. PD:	-0.15
		Sc. rank:	-468.7

,

PB2			
Pos.	619	obs:	exp:
act	T	1857	658.50
acc	T	384	578.90
aca	T	200	1216.00
acg	T	236	223.20
---	--	------	------
mPD		0.49	0.68
		nPD:	0.71
		N. weight:	0.99
		Sc. PD:	0.32
		Sc. rank:	1127.8

,

PB2			
Pos.	620	obs:	exp:
tat	Y	2374	1500.00
tac	Y	303	1177.00
---	--	------	------
mPD		0.20	0.49
		nPD:	0.41
		N. weight:	0.47
		Sc. PD:	0.043
		Sc. rank:	224.2

,

PB2			
Pos.	621	obs:	exp:
caa	Q	1926	1444.00
cag	Q	751	1233.00
---	--	------	------
mPD		0.40	0.50
		nPD:	0.81
		N. weight:	0.13
		Sc. PD:	0.052
		Sc. rank:	154.5

,

PB2			
Pos.	622	obs:	exp:
tgg	W	2677	2677.00
---	--	------	------
mPD		0	0
		nPD:	1.
		N. weight:	0.
		Sc. PD:	0
		Sc. rank:	0

,

PB2			
Pos.	623	obs:	exp:
att	I	844	942.60
atc	I	1042	681.50
ata	I	771	1033.00
aat	N	1	0.57
aac	N	0	0.43
gtt	V	2	4.22
gtc	V	11	3.59
gta	V	6	4.80
gtg	V	0	6.40
---	--	------	------
mPD		0.68	0.67
		nPD:	1.
		N. weight:	0.1
		Sc. PD:	0.056
		Sc. rank:	126.6

,

PB2			
Pos.	624	obs:	exp:
att	I	419	931.90
atc	I	678	673.80
ata	I	1530	1021.00
act	T	0	1.48
acc	T	0	1.30
aca	T	6	2.73
acg	T	0	0.50
gtt	V	0	9.77
gtc	V	0	8.30
gta	V	44	11.11
gtg	V	0	14.82
---	--	------	------
mPD		0.60	0.70
		nPD:	0.86
		N. weight:	0.25
		Sc. PD:	0.11
		Sc. rank:	302.7

,

PB2			
Pos.	625	obs:	exp:
cgt	R	0	83.90
cgc	R	0	140.50
cga	R	4	267.20
cgg	R	15	192.00
atg	M	1	1.00
aaa	K	8	10.94
aag	K	10	7.06
aga	R	775	1219.00
agg	R	1864	755.50
---	--	------	------
mPD		0.44	0.95
		nPD:	0.47
		N. weight:	0.89
		Sc. PD:	0.12
		Sc. rank:	583.9

,

PB2			
Pos.	626	obs:	exp:
cat	H	0	0.58
cac	H	1	0.42
aat	N	469	1521.00
aac	N	2207	1155.00
---	--	------	------
mPD		0.29	0.49
		nPD:	0.59
		N. weight:	0.61
		Sc. PD:	0.14
		Sc. rank:	582.9

,

PB2			
Pos.	627	obs:	exp:
tgg	W	2677	2677.00
---	--	------	------
mPD		0	0
		nPD:	1.
		N. weight:	0.
		Sc. PD:	0
		Sc. rank:	0

,

PB2			
Pos.	628	obs:	exp:
gaa	E	2625	1586.00
gag	E	52	1091.00
---	--	------	------
mPD		0.038	0.48
		nPD:	0.08
		N. weight:	0.81
		Sc. PD:	-0.13
		Sc. rank:	-538.0

,

PB2			
Pos.	629	obs:	exp:
ttt	F	14	6.27
ttc	F	0	7.73
tta	L	0	0.11
ttg	L	0	0.19
tct	S	0	239.20
tcc	S	0	224.70
tca	S	0	345.30
tcg	S	0	75.58
ctt	L	1	0.20
ctc	L	0	0.15
cta	L	0	0.17
ctg	L	0	0.18
cgt	R	0	0.28
cgc	R	0	0.48
cga	R	0	0.90
cgg	R	0	0.65
att	I	656	234.80
atc	I	6	169.80
ata	I	0	257.40
atg	M	1	1.00
act	T	437	113.90
acc	T	10	100.10
aca	T	3	210.40
acg	T	13	38.61
aat	N	25	25.01
aac	N	19	18.99
agt	S	243	264.70
agc	S	1179	272.50
aga	R	9	4.13
agg	R	0	2.56
gtt	V	36	7.99
gtc	V	0	6.79
gta	V	0	9.09
gtg	V	0	12.13
gct	A	19	4.66
gcc	A	0	4.35
gca	A	0	8.56
gcg	A	0	1.42
ggt	G	2	0.94
ggc	G	4	0.76
gga	G	0	2.72
ggg	G	0	1.58
---	--	------	------
mPD		1.2	1.8
		nPD:	0.65
		N. weight:	2.1
		Sc. PD:	0.58
		Sc. rank:	2210.4

,

PB2			
Pos.	630	obs:	exp:
tta	L	269	160.40
ttg	L	1198	276.90
ctt	L	0	297.70
ctc	L	0	214.50
cta	L	0	257.40
ctg	L	7	267.20
att	I	4	5.32
atc	I	1	3.85
ata	I	10	5.83
atg	M	10	10.00
gtt	V	26	261.60
gtc	V	0	222.30
gta	V	47	297.40
gtg	V	1105	396.80
---	--	------	------
mPD		0.74	1.4
		nPD:	0.54
		N. weight:	2.
		Sc. PD:	0.39
		Sc. rank:	1707.5

,

PB2			
Pos.	631	obs:	exp:
aaa	K	2319	1627.00
aag	K	358	1050.00
---	--	------	------
mPD		0.23	0.48
		nPD:	0.49
		N. weight:	0.3
		Sc. PD:	0.046
		Sc. rank:	220.3

,

PB2			
Pos.	632	obs:	exp:
tta	L	0	0.33
ttg	L	0	0.56
ctt	L	3	0.61
ctc	L	0	0.44
cta	L	0	0.52
ctg	L	0	0.54
att	I	2233	948.20
atc	I	420	685.60
ata	I	20	1039.00
gtt	V	1	0.22
gtc	V	0	0.19
gta	V	0	0.25
gtg	V	0	0.34
---	--	------	------
mPD		0.28	0.66
		nPD:	0.42
		N. weight:	1.1
		Sc. PD:	0.12
		Sc. rank:	609.1

,

PB2			
Pos.	633	obs:	exp:
tta	L	0	0.11
ttg	L	0	0.19
ctt	L	0	0.20
ctc	L	0	0.15
cta	L	1	0.17
ctg	L	0	0.18
caa	Q	2590	1443.00
cag	Q	86	1233.00
---	--	------	------
mPD		0.063	0.50
		nPD:	0.13
		N. weight:	0.89
		Sc. PD:	-0.11
		Sc. rank:	-368.1

,

PB2			
Pos.	634	obs:	exp:
tgg	W	2677	2677.00
---	--	------	------
mPD		0	0
		nPD:	1.
		N. weight:	0.
		Sc. PD:	0
		Sc. rank:	0

,

PB2			
Pos.	635	obs:	exp:
ttt	F	0	0.45
ttc	F	1	0.55
tct	S	147	450.10
tcc	S	257	422.90
tca	S	2244	649.80
tcg	S	28	142.20
agt	S	0	498.10
agc	S	0	512.80
---	--	------	------
mPD		0.29	1.6
		nPD:	0.18
		N. weight:	1.7
		Sc. PD:	-0.15
		Sc. rank:	-320.2

,

PB2			
Pos.	636	obs:	exp:
tta	L	0	0.65
ttg	L	0	1.13
ctt	L	0	1.21
ctc	L	0	0.87
cta	L	0	1.05
ctg	L	6	1.09
cat	H	3	2.31
cac	H	1	1.69
caa	Q	1706	1438.00
cag	Q	960	1228.00
cgt	R	0	0.03
cgc	R	0	0.05
cga	R	1	0.10
cgg	R	0	0.07
aga	R	0	0.46
agg	R	0	0.28
---	--	------	------
mPD		0.47	0.51
		nPD:	0.93
		N. weight:	0.051
		Sc. PD:	0.025
		Sc. rank:	62.2

,

PB2			
Pos.	637	obs:	exp:
aat	N	2	2.84
aac	N	3	2.16
gct	A	0	0.25
gcc	A	1	0.23
gca	A	0	0.45
gcg	A	0	0.07
gat	D	1843	1497.00
gac	D	818	1164.00
gaa	E	2	5.93
gag	E	8	4.07
---	--	------	------
mPD		0.44	0.50
		nPD:	0.87
		N. weight:	0.073
		Sc. PD:	0.033
		Sc. rank:	89.1

,

PB2			
Pos.	638	obs:	exp:
cct	P	76	790.00
ccc	P	2587	539.00
cca	P	14	1060.00
ccg	P	0	288.00
---	--	------	------
mPD		0.065	0.70
		nPD:	0.09
		N. weight:	2.6
		Sc. PD:	-0.4
		Sc. rank:	-1558.3

,

PB2			
Pos.	639	obs:	exp:
tct	S	0	0.34
tcc	S	0	0.32
tca	S	0	0.49
tcg	S	2	0.11
act	T	40	657.70
acc	T	19	578.20
aca	T	1200	1215.00
acg	T	1415	223.00
agt	S	0	0.37
agc	S	0	0.38
gct	A	0	0.25
gcc	A	0	0.23
gca	A	1	0.45
gcg	A	0	0.07
---	--	------	------
mPD		0.52	0.68
		nPD:	0.76
		N. weight:	1.7
		Sc. PD:	0.62
		Sc. rank:	2006.5

,

PB2			
Pos.	640	obs:	exp:
tta	L	0	1.63
ttg	L	2	2.82
ctt	L	0	3.03
ctc	L	0	2.18
cta	L	0	2.62
ctg	L	13	2.72
att	I	3	8.87
atc	I	0	6.41
ata	I	22	9.72
atg	M	2624	2624.00
act	T	0	0.74
acc	T	0	0.65
aca	T	0	1.36
acg	T	3	0.25
gtt	V	0	2.22
gtc	V	0	1.89
gta	V	1	2.52
gtg	V	9	3.37
---	--	------	------
mPD		0.040	0.053
		nPD:	0.75
		N. weight:	0.037
		Sc. PD:	0.013
		Sc. rank:	43.6

,

PB2			
Pos.	641	obs:	exp:
tta	L	2185	291.30
ttg	L	121	502.80
ctt	L	0	540.60
ctc	L	2	389.50
cta	L	342	467.40
ctg	L	27	485.30
---	--	------	------
mPD		0.34	1.1
		nPD:	0.3
		N. weight:	2.8
		Sc. PD:	0.031
		Sc. rank:	448.3

,

PB2			
Pos.	642	obs:	exp:
tat	Y	69	1500.00
tac	Y	2608	1177.00
---	--	------	------
mPD		0.050	0.49
		nPD:	0.1
		N. weight:	1.3
		Sc. PD:	-0.18
		Sc. rank:	-695.6

,

PB2			
Pos.	643	obs:	exp:
aat	N	207	1522.00
aac	N	2470	1155.00
---	--	------	------
mPD		0.14	0.49
		nPD:	0.29
		N. weight:	1.
		Sc. PD:	0.002
		Sc. rank:	126.0

,

PB2			
Pos.	644	obs:	exp:
cgt	R	0	3.22
cgc	R	0	5.39
cga	R	0	10.25
cgg	R	0	7.37
aaa	K	2400	1565.00
aag	K	175	1010.00
aga	R	84	46.78
agg	R	18	28.99
---	--	------	------
mPD		0.21	0.57
		nPD:	0.36
		N. weight:	0.53
		Sc. PD:	0.03
		Sc. rank:	182.3

,

PB2			
Pos.	645	obs:	exp:
tta	L	7	79.55
ttg	L	708	137.30
ctt	L	0	147.60
ctc	L	0	106.40
cta	L	0	127.60
ctg	L	16	132.50
att	I	1	7.80
atc	I	0	5.64
ata	I	21	8.55
atg	M	1899	1899.00
aaa	K	0	1.82
aag	K	3	1.18
gtt	V	0	4.88
gtc	V	0	4.15
gta	V	0	5.55
gtg	V	22	7.41
---	--	------	------
mPD		0.44	0.77
		nPD:	0.57
		N. weight:	0.8
		Sc. PD:	0.17
		Sc. rank:	729.0

,

PB2			
Pos.	646	obs:	exp:
gaa	E	2541	1586.00
gag	E	136	1091.00
---	--	------	------
mPD		0.096	0.48
		nPD:	0.2
		N. weight:	0.63
		Sc. PD:	-0.043
		Sc. rank:	-76.7

,

PB2			
Pos.	647	obs:	exp:
ttt	F	2654	1200.00
ttc	F	23	1477.00
---	--	------	------
mPD		0.017	0.49
		nPD:	0.03
		N. weight:	1.4
		Sc. PD:	-0.27
		Sc. rank:	-1388.6

,

PB2			
Pos.	648	obs:	exp:
gaa	E	2225	1586.00
gag	E	452	1091.00
---	--	------	------
mPD		0.28	0.48
		nPD:	0.58
		N. weight:	0.25
		Sc. PD:	0.056
		Sc. rank:	231.3

,

PB2			
Pos.	649	obs:	exp:
tct	S	0	0.17
tcc	S	0	0.16
tca	S	1	0.24
tcg	S	0	0.05
cct	P	85	789.70
ccc	P	2	538.80
cca	P	2504	1060.00
ccg	P	85	287.90
agt	S	0	0.19
agc	S	0	0.19
---	--	------	------
mPD		0.12	0.71
		nPD:	0.17
		N. weight:	1.3
		Sc. PD:	-0.11
		Sc. rank:	-248.1

,

PB2			
Pos.	650	obs:	exp:
ttt	F	1173	1200.00
ttc	F	1504	1477.00
---	--	------	------
mPD		0.49	0.49
		nPD:	1.
		N. weight:	0.0032
		Sc. PD:	0.0018
		Sc. rank:	4.0

,

PB2			
Pos.	651	obs:	exp:
caa	Q	113	1444.00
cag	Q	2564	1233.00
---	--	------	------
mPD		0.081	0.50
		nPD:	0.16
		N. weight:	1.1
		Sc. PD:	-0.11
		Sc. rank:	-275.9

,

PB2			
Pos.	652	obs:	exp:
tct	S	2566	448.90
tcc	S	102	421.80
tca	S	1	648.10
tcg	S	0	141.90
agt	S	0	496.80
agc	S	0	511.50
gct	A	8	1.96
gcc	A	0	1.83
gca	A	0	3.61
gcg	A	0	0.60
---	--	------	------
mPD		0.080	1.6
		nPD:	0.05
		N. weight:	3.
		Sc. PD:	-0.55
		Sc. rank:	-2602.2

,

PB2			
Pos.	653	obs:	exp:
tta	L	13	291.00
ttg	L	21	502.30
ctt	L	2212	540.00
ctc	L	25	389.10
cta	L	385	466.90
ctg	L	18	484.70
att	I	3	1.06
atc	I	0	0.77
ata	I	0	1.17
---	--	------	------
mPD		0.32	1.1
		nPD:	0.28
		N. weight:	2.
		Sc. PD:	-0.011
		Sc. rank:	205.3

,

PB2			
Pos.	654	obs:	exp:
att	I	2	100.00
atc	I	31	72.33
ata	I	249	109.60
gtt	V	177	531.60
gtc	V	2050	451.70
gta	V	97	604.30
gtg	V	70	806.40
gct	A	0	0.25
gcc	A	1	0.23
gca	A	0	0.45
gcg	A	0	0.07
---	--	------	------
mPD		0.56	0.93
		nPD:	0.6
		N. weight:	1.9
		Sc. PD:	0.46
		Sc. rank:	1855.3

,

PB2			
Pos.	655	obs:	exp:
cct	P	2560	790.00
ccc	P	90	539.00
cca	P	26	1060.00
ccg	P	1	288.00
---	--	------	------
mPD		0.084	0.70
		nPD:	0.12
		N. weight:	1.9
		Sc. PD:	-0.25
		Sc. rank:	-840.5

,

PB2			
Pos.	656	obs:	exp:
cgt	R	0	0.88
cgc	R	0	1.48
cga	R	0	2.81
cgg	R	0	2.02
aat	N	7	3.98
aac	N	0	3.02
aaa	K	475	1606.00
aag	K	2167	1036.00
aga	R	6	12.84
agg	R	22	7.96
---	--	------	------
mPD		0.32	0.51
		nPD:	0.63
		N. weight:	0.72
		Sc. PD:	0.19
		Sc. rank:	735.8

,

PB2			
Pos.	657	obs:	exp:
gct	A	36	656.20
gcc	A	19	613.40
gca	A	2268	1207.00
gcg	A	354	200.80
---	--	------	------
mPD		0.26	0.68
		nPD:	0.39
		N. weight:	1.
		Sc. PD:	0.079
		Sc. rank:	430.1

,

PB2

Pos.	658	obs:	exp:
ttt	F	0	0.45
ttc	F	1	0.55
tct	S	0	2.02
tcc	S	12	1.90
tca	S	0	2.91
tcg	S	0	0.64
att	I	2	42.21
atc	I	117	30.52
ata	I	0	46.26
act	T	19	512.40
acc	T	1990	450.40
aca	T	74	946.50
acg	T	0	173.70
aat	N	0	5.12
aac	N	9	3.88
agt	S	0	2.23
agc	S	0	2.30
gtt	V	7	4.22
gtc	V	12	3.59
gta	V	0	4.80
gtg	V	0	6.40
gct	A	26	106.40
gcc	A	349	99.44
gca	A	59	195.60
gcg	A	0	32.55
---	--	------	------
mPD		0.53	1.1
		nPD:	0.49
		N. weight:	2.2
		Sc. PD:	0.35
		Sc. rank:	1648.1

,

PB2

Pos.	659	obs:	exp:
cgt	R	0	84.41
cgc	R	0	141.30
cga	R	17	268.80
cgg	R	0	193.20
aaa	K	3	1.82
aag	K	0	1.18
aga	R	2530	1226.00
agg	R	127	760.00
---	--	------	------
mPD		0.11	0.94
		nPD:	0.11
		N. weight:	1.1
		Sc. PD:	-0.15
		Sc. rank:	-531.5

,

PB2

Pos.	660	obs:	exp:
tct	S	0	366.70
tcc	S	0	344.50
tca	S	0	529.40
tcg	S	0	115.90
cgt	R	0	0.03
cgc	R	0	0.05
cga	R	0	0.10
cgg	R	0	0.07
aat	N	11	9.66
aac	N	6	7.34
agt	S	1501	405.80
agc	S	679	417.80
aga	R	1	0.46
agg	R	0	0.28
ggt	G	390	75.13
ggc	G	87	60.69
gga	G	2	217.00
ggg	G	0	126.20
---	--	------	------
mPD		0.72	1.8
		nPD:	0.39
		N. weight:	2.1
		Sc. PD:	0.16
		Sc. rank:	884.4

,

PB2

Pos.	661	obs:	exp:
---	-	2677	2677.00
---	--	------	------
mPD		0	0
		nPD:	1.
		N. weight:	0.
		Sc. PD:	0
		Sc. rank:	0

,

PB2

Pos.	662	obs:	exp:
tta	L	0	0.11
ttg	L	0	0.19
ctt	L	0	0.20
ctc	L	0	0.15
cta	L	0	0.17
ctg	L	1	0.18
cat	H	3	2.31
cac	H	1	1.69
caa	Q	408	231.90
cag	Q	22	198.10
cgt	R	1280	69.54
cgc	R	18	116.40
cga	R	83	221.40
cgg	R	794	159.20
aaa	K	11	23.70
aag	K	28	15.30
aga	R	0	1010.00
agg	R	28	626.10
---	--	------	------
mPD		0.97	1.3
		nPD:	0.74
		N. weight:	3.5
		Sc. PD:	1.2
		Sc. rank:	4073.9

,

PB2

Pos.	663	obs:	exp:
tat	Y	60	1500.00
tac	Y	2617	1177.00
---	--	------	------
mPD		0.044	0.49
		nPD:	0.09
		N. weight:	1.3
		Sc. PD:	-0.2
		Sc. rank:	-796.9

,

PB2

Pos.	664	obs:	exp:
tct	S	0	450.30
tcc	S	0	423.10
tca	S	0	650.10
tcg	S	0	142.30
agt	S	2409	498.30
agc	S	268	513.00
---	--	------	------
mPD		0.18	1.6
		nPD:	0.11
		N. weight:	2.5
		Sc. PD:	-0.34
		Sc. rank:	-1262.8

,

PB2

Pos.	665	obs:	exp:
ggt	G	5	419.90
ggc	G	1	339.20
gga	G	2232	1213.00
ggg	G	439	705.40
---	--	------	------
mPD		0.28	0.68
		nPD:	0.41
		N. weight:	0.78
		Sc. PD:	0.071
		Sc. rank:	370.9

,

PB2

Pos.	666	obs:	exp:
ttt	F	511	1200.00
ttc	F	2166	1477.00
---	--	------	------
mPD		0.31	0.49
		nPD:	0.62
		N. weight:	0.27
		Sc. PD:	0.071
		Sc. rank:	277.6

,

PB2

Pos.	667	obs:	exp:
gtt	V	21	594.40
gtc	V	1	505.10
gta	V	815	675.80
gtg	V	1840	901.70
---	--	------	------
mPD		0.43	0.74
		nPD:	0.59
		N. weight:	0.97
		Sc. PD:	0.22
		Sc. rank:	921.1

,

PB2

Pos.	668	obs:	exp:
cgt	R	0	84.50
cgc	R	0	141.50
cga	R	0	269.10
cgg	R	1	193.40
aga	R	500	1228.00
agg	R	2176	760.90
---	--	------	------
mPD		0.30	0.94
		nPD:	0.32
		N. weight:	1.3
		Sc. PD:	0.035
		Sc. rank:	325.3

,

PB2

Pos.	669	obs:	exp:
act	T	7	648.20
acc	T	28	569.80
aca	T	2547	1197.00
acg	T	53	219.70
gct	A	0	10.30
gcc	A	0	9.62
gca	A	42	18.93
gcg	A	0	3.15
---	--	------	------
mPD		0.095	0.71
		nPD:	0.13
		N. weight:	1.2
		Sc. PD:	-0.15
		Sc. rank:	-457.8

,

PB2

Pos.	670	obs:	exp:
tta	L	22	291.30
ttg	L	33	502.80
ctt	L	20	540.60
ctc	L	1	389.50
cta	L	817	467.40
ctg	L	1784	485.30
---	--	------	------
mPD		0.48	1.1
		nPD:	0.42
		N. weight:	1.8
		Sc. PD:	0.18
		Sc. rank:	945.4

,

PB2

Pos.	671	obs:	exp:
ttt	F	186	1200.00
ttc	F	2491	1477.00
---	--	------	------
mPD		0.13	0.49
		nPD:	0.26
		N. weight:	0.66
		Sc. PD:	-0.014
		Sc. rank:	15.2

,

PB2

Pos.	672	obs:	exp:
caa	Q	56	1444.00
cag	Q	2621	1233.00
---	--	------	------
mPD		0.041	0.50
		nPD:	0.08
		N. weight:	1.2
		Sc. PD:	-0.2
		Sc. rank:	-790.8

,

PB2

Pos.	673	obs:	exp:
caa	Q	2479	1444.00
cag	Q	198	1233.00
---	--	------	------
mPD		0.14	0.50
		nPD:	0.28
		N. weight:	0.68
		Sc. PD:	-0.0066
		Sc. rank:	52.9

,

PB2

Pos.	674	obs:	exp:
atg	M	2677	2677.00
---	--	------	------
mPD		0	0
		nPD:	1.
		N. weight:	0.
		Sc. PD:	0
		Sc. rank:	0

,

PB2

Pos.	675	obs:	exp:
cgt	R	201	84.50
cgc	R	212	141.50
cga	R	67	269.10
cgg	R	2096	193.40
aga	R	28	1228.00
agg	R	73	760.90
---	--	------	------
mPD		0.40	0.94
		nPD:	0.43
		N. weight:	3.4
		Sc. PD:	0.37
		Sc. rank:	1959.3

,

PB2			
Pos.	676	obs:	exp:
gat	D	2641	1506.00
gac	D	36	1171.00
---	--	------	------
mPD		0.027	0.49
		nPD:	0.05
		N. weight:	0.94
		Sc. PD:	-0.17
		Sc. rank:	-798.0

PB2			
Pos.	677	obs:	exp:
gtt	V	18	594.40
gtc	V	0	505.10
gta	V	245	675.80
gtg	V	2414	901.70
---	--	------	------
mPD		0.18	0.74
		nPD:	0.24
		N. weight:	1.4
		Sc. PD:	-0.051
		Sc. rank:	-15.5

PB2			
Pos.	678	obs:	exp:
ttt	F	2	0.90
ttc	F	0	1.10
tta	L	1	283.30
ttg	L	22	488.90
ctt	L	2167	525.70
ctc	L	33	378.70
cta	L	19	454.50
ctg	L	361	471.90
att	I	54	19.16
atc	I	0	13.85
ata	I	0	20.99
atg	M	1	1.00
gtt	V	4	3.77
gtc	V	0	3.21
gta	V	0	4.29
gtg	V	13	5.73
---	--	------	------
mPD		0.36	1.2
		nPD:	0.3
		N. weight:	2.
		Sc. PD:	0.024
		Sc. rank:	334.2

PB2			
Pos.	679	obs:	exp:
ggt	G	2	419.90
ggc	G	1	339.20
gga	G	1747	1213.00
ggg	G	927	705.40
---	--	------	------
mPD		0.45	0.68
		nPD:	0.66
		N. weight:	0.61
		Sc. PD:	0.18
		Sc. rank:	659.8

PB2			
Pos.	680	obs:	exp:
act	T	2	658.50
acc	T	18	578.90
aca	T	2608	1216.00
acg	T	49	223.20
---	--	------	------
mPD		0.051	0.68
		nPD:	0.07
		N. weight:	1.3
		Sc. PD:	-0.21
		Sc. rank:	-893.2

PB2			
Pos.	681	obs:	exp:
ttt	F	2629	1200.00
ttc	F	48	1477.00
---	--	------	------
mPD		0.035	0.49
		nPD:	0.07
		N. weight:	1.3
		Sc. PD:	-0.22
		Sc. rank:	-950.7

PB2			
Pos.	682	obs:	exp:
aat	N	1	5.12
aac	N	8	3.88
gat	D	1322	1501.00
gac	D	1346	1167.00
---	--	------	------
mPD		0.51	0.50
		nPD:	1.02
		N. weight:	0.023
		Sc. PD:	0.013
		Sc. rank:	28.7

PB2			
Pos.	683	obs:	exp:
tct	S	2	2.86
tcc	S	13	2.69
tca	S	0	4.13
tcg	S	0	0.90
cct	P	0	0.30
ccc	P	1	0.20
cca	P	0	0.40
ccg	P	0	0.11
act	T	2186	654.10
acc	T	53	575.00
aca	T	402	1208.00
acg	T	18	221.70
agt	S	1	3.16
agc	S	1	3.26
---	--	------	------
mPD		0.32	0.69
		nPD:	0.46
		N. weight:	1.4
		Sc. PD:	0.19
		Sc. rank:	918.9

PB2			
Pos.	684	obs:	exp:
tct	S	1	0.67
tcc	S	3	0.63
tca	S	0	0.97
tcg	S	0	0.21
att	I	5	2.84
atc	I	3	2.05
ata	I	0	3.11
act	T	1	0.74
acc	T	2	0.65
aca	T	0	1.36
acg	T	0	0.25
agt	S	0	0.74
agc	S	0	0.77
gtt	V	126	489.10
gtc	V	2072	415.70
gta	V	4	556.10
gtg	V	1	742.00
gct	A	74	112.50
gcc	A	308	105.20
gca	A	71	206.90
gcg	A	6	34.42
---	--	------	------
mPD		0.50	1.0
		nPD:	0.48
		N. weight:	2.3
		Sc. PD:	0.34
		Sc. rank:	1632.0

PB2			
Pos.	685	obs:	exp:
caa	Q	2627	1444.00
cag	Q	50	1233.00
---	--	------	------
mPD		0.037	0.50
		nPD:	0.07
		N. weight:	0.98
		Sc. PD:	-0.16
		Sc. rank:	-693.6

PB2			
Pos.	686	obs:	exp:
att	I	2	922.00
atc	I	1	666.60
ata	I	2596	1010.00
atg	M	2	2.00
gtt	V	0	16.87
gtc	V	0	14.34
gta	V	41	19.19
gtg	V	35	25.60
---	--	------	------
mPD		0.085	0.72
		nPD:	0.12
		N. weight:	1.7
		Sc. PD:	-0.22
		Sc. rank:	-773.6

PB2			
Pos.	687	obs:	exp:
cgt	R	0	0.03
cgc	R	0	0.05
cga	R	0	0.10
cgg	R	0	0.07
att	I	0	942.60
atc	I	1	681.50
ata	I	2656	1033.00
aga	R	1	0.46
agg	R	0	0.28
gtt	V	0	4.22
gtc	V	0	3.59
gta	V	19	4.80
gtg	V	0	6.40
---	--	------	------
mPD		0.016	0.67
		nPD:	0.02
		N. weight:	1.8
		Sc. PD:	-0.36
		Sc. rank:	-1878.2

PB2			
Pos.	688	obs:	exp:
aaa	K	2283	1627.00
aag	K	394	1050.00
---	--	------	------
mPD		0.25	0.48
		nPD:	0.53
		N. weight:	0.27
		Sc. PD:	0.049
		Sc. rank:	219.7

PB2			
Pos.	689	obs:	exp:
tta	L	77	291.30
ttg	L	11	502.80
ctt	L	2244	540.60
ctc	L	22	389.50
cta	L	209	467.40
ctg	L	114	485.30
---	--	------	------
mPD		0.35	1.1
		nPD:	0.31
		N. weight:	1.8
		Sc. PD:	0.024
		Sc. rank:	322.6

PB2			
Pos.	690	obs:	exp:
ttt	F	0	0.90
ttc	F	2	1.10
tta	L	9	291.10
ttg	L	78	502.50
ctt	L	56	540.20
ctc	L	2145	389.20
cta	L	71	467.10
ctg	L	316	484.90
---	--	------	------
mPD		0.40	1.1
		nPD:	0.35
		N. weight:	2.1
		Sc. PD:	0.1
		Sc. rank:	682.3

PB2			
Pos.	691	obs:	exp:
cct	P	227	789.40
ccc	P	2093	538.60
cca	P	347	1059.00
ccg	P	8	287.70
act	T	0	0.49
acc	T	2	0.43
aca	T	0	0.91
acg	T	0	0.17
---	--	------	------
mPD		0.37	0.71
		nPD:	0.52
		N. weight:	1.5
		Sc. PD:	0.26
		Sc. rank:	1192.3

PB2			
Pos.	692	obs:	exp:
ttt	F	2634	1200.00
ttc	F	43	1477.00
---	--	------	------
mPD		0.032	0.49
		nPD:	0.06
		N. weight:	1.3
		Sc. PD:	-0.23
		Sc. rank:	-1045.0

PB2			
Pos.	693	obs:	exp:
act	T	1	0.25
acc	T	0	0.22
aca	T	0	0.45
acg	T	0	0.08
gct	A	2214	656.00
gcc	A	12	613.10
gca	A	445	1206.00
gcg	A	5	200.70
---	--	------	------
mPD		0.29	0.68
		nPD:	0.42
		N. weight:	1.5
		Sc. PD:	0.16
		Sc. rank:	831.6

PB2			
Pos.	694	obs:	exp:
tct	S	23	4.21
tcc	S	0	3.95
tca	S	1	6.07
tcg	S	1	1.33
agt	S	0	4.65
agc	S	0	4.79
gct	A	2226	650.10
gcc	A	9	607.60
gca	A	402	1195.00
gcg	A	15	198.90
---	--	------	------
mPD		0.29	0.70
		nPD:	0.41
		N. weight:	1.6
		Sc. PD:	0.15
		Sc. rank:	759.0

PB2			
Pos.	695	obs:	exp:
tct	S	1	0.17
tcc	S	0	0.16
tca	S	0	0.24
tcg	S	0	0.05
agt	S	0	0.19
agc	S	0	0.19
gct	A	2343	656.00
gcc	A	218	613.10
gca	A	94	1206.00
gcg	A	21	200.70
---	--	------	------
mPD		0.23	0.68
		nPD:	0.33
		N. weight:	1.7
		Sc. PD:	0.059
		Sc. rank:	471.2

PB2			
Pos.	696	obs:	exp:
cct	P	7	790.00
ccc	P	4	539.00
cca	P	2657	1060.00
ccg	P	9	288.00
---	--	------	------
mPD		0.015	0.70
		nPD:	0.02
		N. weight:	1.6
		Sc. PD:	-0.34
		Sc. rank:	-1781.7

PB2			
Pos.	697	obs:	exp:
cct	P	53	790.00
ccc	P	2	539.00
cca	P	1194	1060.00
ccg	P	1428	288.00
---	--	------	------
mPD		0.52	0.70
		nPD:	0.73
		N. weight:	1.6
		Sc. PD:	0.54
		Sc. rank:	1832.2

PB2			
Pos.	698	obs:	exp:
aaa	K	23	15.19
aag	K	2	9.81
gtt	V	0	1.33
gtc	V	0	1.13
gta	V	0	1.51
gtg	V	6	2.02
gaa	E	2244	1568.00
gag	E	402	1078.00
---	--	------	------
mPD		0.28	0.51
		nPD:	0.56
		N. weight:	0.29
		Sc. PD:	0.061
		Sc. rank:	258.7

PB2			
Pos.	699	obs:	exp:
tta	L	0	0.11
ttg	L	0	0.19
ctt	L	0	0.20
ctc	L	0	0.15
cta	L	0	0.17
ctg	L	1	0.18
cat	H	1	0.58
cac	H	0	0.42
caa	Q	48	1442.00
cag	Q	2626	1232.00
cgt	R	0	0.03
cgc	R	0	0.05
cga	R	0	0.10
cgg	R	1	0.07
aga	R	0	0.46
agg	R	0	0.28
---	--	------	------
mPD		0.037	0.50
		nPD:	0.07
		N. weight:	1.3
		Sc. PD:	-0.21
		Sc. rank:	-873.3

PB2			
Pos.	700	obs:	exp:
tct	S	0	448.80
tcc	S	0	421.70
tca	S	0	647.90
tcg	S	0	141.80
aat	N	8	4.55
aac	N	0	3.45
agt	S	2584	496.60
agc	S	84	511.30
ggt	G	1	0.16
ggc	G	0	0.13
gga	G	0	0.45
ggg	G	0	0.26
---	--	------	------
mPD		0.068	1.6
		nPD:	0.04
		N. weight:	2.9
		Sc. PD:	-0.54
		Sc. rank:	-2634.4

PB2			
Pos.	701	obs:	exp:
tct	S	0	0.17
tcc	S	0	0.16
tca	S	0	0.24
tcg	S	0	0.05
cgt	R	0	84.41
cgc	R	0	141.30
cga	R	26	268.80
cgg	R	1	193.20
aaa	K	0	1.22
aag	K	2	0.78
agt	S	1	0.19
agc	S	0	0.19
aga	R	240	1226.00
agg	R	2407	760.00
---	--	------	------
mPD		0.20	0.94
		nPD:	0.21
		N. weight:	1.6
		Sc. PD:	-0.092
		Sc. rank:	-130.6

PB2			
Pos.	702	obs:	exp:
tta	L	0	0.11
ttg	L	1	0.19
ctt	L	0	0.20
ctc	L	0	0.15
cta	L	0	0.17
ctg	L	0	0.18
att	I	0	0.35
atc	I	0	0.26
ata	I	1	0.39
atg	M	2675	2675.00
---	--	------	------
mPD		0.0015	0.0020
		nPD:	0.76
		N. weight:	0.0018
		Sc. PD:	0.00066
		Sc. rank:	2.1

PB2			
Pos.	703	obs:	exp:
caa	Q	1246	1444.00
cag	Q	1431	1233.00
---	--	------	------
mPD		0.50	0.50
		nPD:	1.
		N. weight:	0.023
		Sc. PD:	0.013
		Sc. rank:	28.7

PB2			
Pos.	704	obs:	exp:
ttt	F	856	1200.00
ttc	F	1821	1477.00
---	--	------	------
mPD		0.44	0.49
		nPD:	0.88
		N. weight:	0.066
		Sc. PD:	0.03
		Sc. rank:	81.0

PB2			
Pos.	705	obs:	exp:
tct	S	433	449.60
tcc	S	2232	422.40
tca	S	4	649.10
tcg	S	4	142.10
cct	P	1	0.30
ccc	P	0	0.20
cca	P	0	0.40
ccg	P	0	0.11
act	T	0	0.74
acc	T	3	0.65
aca	T	0	1.36
acg	T	0	0.25
agt	S	0	497.50
agc	S	0	512.30
---	--	------	------
mPD		0.28	1.6
		nPD:	0.17
		N. weight:	2.5
		Sc. PD:	-0.23
		Sc. rank:	-520.2

PB2			
Pos.	706	obs:	exp:
tct	S	457	450.30
tcc	S	32	423.10
tca	S	1021	650.10
tcg	S	1167	142.30
agt	S	0	498.30
agc	S	0	513.00
---	--	------	------
mPD		0.64	1.6
		nPD:	0.39
		N. weight:	2.
		Sc. PD:	0.15
		Sc. rank:	828.9

PB2			
Pos.	707	obs:	exp:
tta	L	70	291.00
ttg	L	719	502.30
ctt	L	21	540.00
ctc	L	14	389.10
cta	L	599	466.90
ctg	L	1251	484.70
atg	M	3	3.00
---	--	------	------
mPD		0.81	1.1
		nPD:	0.71
		N. weight:	0.96
		Sc. PD:	0.31
		Sc. rank:	1095.8

PB2			
Pos.	708	obs:	exp:
tct	S	0	0.17
tcc	S	0	0.16
tca	S	0	0.24
tcg	S	0	0.05
att	I	12	4.26
atc	I	0	3.08
ata	I	0	4.67
act	T	2625	653.60
acc	T	18	574.60
aca	T	12	1207.00
acg	T	2	221.60
agt	S	1	0.19
agc	S	0	0.19
gct	A	6	1.72
gcc	A	1	1.60
gca	A	0	3.16
gcg	A	0	0.52
---	--	------	------
mPD		0.039	0.69
		nPD:	0.06
		N. weight:	2.5
		Sc. PD:	-0.44
		Sc. rank:	-2012.1

PB2			
Pos.	709	obs:	exp:
tta	L	0	1.85
ttg	L	16	3.19
ctt	L	0	3.43
ctc	L	0	2.47
cta	L	0	2.97
ctg	L	1	3.08
att	I	0	1.77
atc	I	0	1.28
ata	I	5	1.94
gtt	V	42	589.50
gtc	V	2	501.00
gta	V	527	670.20
gtg	V	2084	894.30
---	--	------	------
mPD		0.36	0.75
		nPD:	0.48
		N. weight:	1.1
		Sc. PD:	0.16
		Sc. rank:	754.4

PB2			
Pos.	710	obs:	exp:
tct	S	0	0.17
tcc	S	0	0.16
tca	S	0	0.24
tcg	S	0	0.05
aat	N	2524	1521.00
aac	N	152	1155.00
agt	S	1	0.19
agc	S	0	0.19
---	--	------	------
mPD		0.11	0.49
		nPD:	0.22
		N. weight:	0.67
		Sc. PD:	-0.036
		Sc. rank:	-41.7

PB2			
Pos.	711	obs:	exp:
att	I	0	1.77
atc	I	0	1.28
ata	I	5	1.94
atg	M	2	2.00
gtt	V	21	592.60
gtc	V	2	503.60
gta	V	523	673.80
gtg	V	2123	899.00
ggt	G	0	0.16
ggc	G	0	0.13
gga	G	1	0.45
ggg	G	0	0.26
---	--	------	------
mPD		0.34	0.74
		nPD:	0.45
		N. weight:	1.1
		Sc. PD:	0.14
		Sc. rank:	688.4

PB2			
Pos.	712	obs:	exp:
cgt	R	0	84.50
cgc	R	0	141.50
cga	R	0	269.10
cgg	R	0	193.40
aga	R	2554	1228.00
agg	R	123	760.90
---	--	------	------
mPD		0.088	0.94
		nPD:	0.09
		N. weight:	1.1
		Sc. PD:	-0.17
		Sc. rank:	-668.9

PB2			
Pos.	713	obs:	exp:
ggt	G	8	419.90
ggc	G	1	339.20
gga	G	2565	1213.00
ggg	G	103	705.40
---	--	------	------
mPD		0.080	0.68
		nPD:	0.12
		N. weight:	1.2
		Sc. PD:	-0.15
		Sc. rank:	-533.6

,

PB2			
Pos.	714	obs:	exp:
tct	S	21	446.70
tcc	S	10	419.80
tca	S	2576	645.00
tcg	S	49	141.20
act	T	0	2.46
acc	T	0	2.16
aca	T	10	4.54
acg	T	0	0.83
agt	S	0	494.30
agc	S	0	509.00
gct	A	0	2.70
gcc	A	0	2.52
gca	A	11	4.96
gcg	A	0	0.82
---	--	------	------
mPD		0.074	1.6
		nPD:	0.05
		N. weight:	2.4
		Sc. PD:	-0.44
		Sc. rank:	-2131.1

,

PB2			
Pos.	715	obs:	exp:
ggt	G	8	419.90
ggc	G	4	339.20
gga	G	1255	1213.00
ggg	G	1410	705.40
---	--	------	------
mPD		0.50	0.68
		nPD:	0.73
		N. weight:	0.68
		Sc. PD:	0.23
		Sc. rank:	786.3

,

PB2			
Pos.	716	obs:	exp:
tta	L	17	272.10
ttg	L	1127	469.60
ctt	L	1	504.90
ctc	L	0	363.70
cta	L	7	436.50
ctg	L	1348	453.20
atg	M	177	177.00
---	--	------	------
mPD		0.58	1.2
		nPD:	0.48
		N. weight:	1.6
		Sc. PD:	0.24
		Sc. rank:	1174.2

,

PB2			
Pos.	717	obs:	exp:
cgt	R	0	84.50
cgc	R	0	141.50
cga	R	15	269.10
cgg	R	2	193.40
aga	R	523	1228.00
agg	R	2137	760.90
---	--	------	------
mPD		0.33	0.94
		nPD:	0.36
		N. weight:	1.2
		Sc. PD:	0.061
		Sc. rank:	400.5

,

PB2			
Pos.	718	obs:	exp:
---	-	2677	2677.00
---	--	------	------
mPD		0	0
		nPD:	1.
		N. weight:	0.
		Sc. PD:	0
		Sc. rank:	0

,

PB2			
Pos.	719	obs:	exp:
---	-	2677	2677.00
---	--	------	------
mPD		0	0
		nPD:	1.
		N. weight:	0.
		Sc. PD:	0
		Sc. rank:	0

,

PB2			
Pos.	720	obs:	exp:
---	-	2677	2677.00
---	--	------	------
mPD		0	0
		nPD:	1.
		N. weight:	0.
		Sc. PD:	0
		Sc. rank:	0

,

PB2			
Pos.	721	obs:	exp:
---	-	2677	2677.00
---	--	------	------
mPD		0	0
		nPD:	1.
		N. weight:	0.
		Sc. PD:	0
		Sc. rank:	0

,

PB2			
Pos.	722	obs:	exp:
---	-	2677	2677.00
---	--	------	------
mPD		0	0
		nPD:	1.
		N. weight:	0.
		Sc. PD:	0
		Sc. rank:	0

,

PB2			
Pos.	723	obs:	exp:
---	-	2677	2677.00
---	--	------	------
mPD		0	0
		nPD:	1.
		N. weight:	0.
		Sc. PD:	0
		Sc. rank:	0

,

PB2			
Pos.	724	obs:	exp:
---	-	2677	2677.00
---	--	------	------
mPD		0	0
		nPD:	1.
		N. weight:	0.
		Sc. PD:	0
		Sc. rank:	0

,

PB2			
Pos.	725	obs:	exp:
---	-	2677	2677.00
---	--	------	------
mPD		0	0
		nPD:	1.
		N. weight:	0.
		Sc. PD:	0
		Sc. rank:	0

,

PB2			
Pos.	726	obs:	exp:
---	-	2677	2677.00
---	--	------	------
mPD		0	0
		nPD:	1.
		N. weight:	0.
		Sc. PD:	0
		Sc. rank:	0

,

PB2			
Pos.	727	obs:	exp:
att	I	21	948.60
atc	I	11	685.90
ata	I	2642	1040.00
act	T	0	0.49
acc	T	0	0.43
aca	T	2	0.91
acg	T	0	0.17
gtt	V	0	0.22
gtc	V	0	0.19
gta	V	1	0.25
gtg	V	0	0.34
---	--	------	------
mPD		0.026	0.66
		nPD:	0.04
		N. weight:	1.6
		Sc. PD:	-0.31
		Sc. rank:	-1526.8

,

PB2			
Pos.	728	obs:	exp:
ttt	F	0	2.24
ttc	F	5	2.76
tta	L	1	288.20
ttg	L	64	497.40
ctt	L	52	534.80
ctc	L	386	385.30
cta	L	63	462.40
ctg	L	2082	480.00
atg	M	24	24.00
---	--	------	------
mPD		0.39	1.1
		nPD:	0.34
		N. weight:	1.9
		Sc. PD:	0.072
		Sc. rank:	553.1

,

PB2			
Pos.	729	obs:	exp:
att	I	0	181.60
atc	I	10	131.30
ata	I	502	199.00
aaa	K	1	0.61
aag	K	0	0.39
gtt	V	3	480.50
gtc	V	1	408.30
gta	V	2133	546.30
gtg	V	27	728.90
---	--	------	------
mPD		0.34	1.1
		nPD:	0.32
		N. weight:	2.2
		Sc. PD:	0.059
		Sc. rank:	556.0

,

PB2			
Pos.	730	obs:	exp:
cgt	R	0	84.50
cgc	R	0	141.50
cga	R	17	269.10
cgg	R	0	193.40
aga	R	2319	1228.00
agg	R	341	760.90
---	--	------	------
mPD		0.24	0.94
		nPD:	0.25
		N. weight:	0.8
		Sc. PD:	-0.023
		Sc. rank:	3.3

,

PB2			
Pos.	731	obs:	exp:
gat	D	0	0.56
gac	D	1	0.44
ggt	G	405	419.70
ggc	G	2250	339.10
gga	G	17	1212.00
ggg	G	4	705.10
---	--	------	------
mPD		0.27	0.69
		nPD:	0.4
		N. weight:	2.9
		Sc. PD:	0.24
		Sc. rank:	1278.5

,

PB2			
Pos.	732	obs:	exp:
tct	S	0	0.17
tcc	S	0	0.16
tca	S	0	0.24
tcg	S	0	0.05
aat	N	1460	1521.00
aac	N	1216	1155.00
agt	S	1	0.19
agc	S	0	0.19
---	--	------	------
mPD		0.50	0.49
		nPD:	1.01
		N. weight:	0.006
		Sc. PD:	0.0033
		Sc. rank:	7.4

,

PB2			
Pos.	733	obs:	exp:
ttt	F	1	0.45
ttc	F	0	0.55
tct	S	2219	450.10
tcc	S	450	422.90
tca	S	7	649.80
tcg	S	0	142.20
agt	S	0	498.10
agc	S	0	512.80
---	--	------	------
mPD		0.28	1.6
		nPD:	0.17
		N. weight:	2.5
		Sc. PD:	-0.21
		Sc. rank:	-481.3

,

PB2			
Pos.	734	obs:	exp:
cct	P	55	790.00
ccc	P	283	539.00
cca	P	2317	1060.00
ccg	P	22	288.00
---	--	------	------
mPD		0.24	0.70
		nPD:	0.34
		N. weight:	0.99
		Sc. PD:	0.039
		Sc. rank:	297.8

,

PB2			
Pos.	735	obs:	exp:
tta	L	1	0.44
ttg	L	3	0.75
ctt	L	0	0.81
ctc	L	0	0.58
cta	L	0	0.70
ctg	L	0	0.73
att	I	0	4.61
atc	I	0	3.33
ata	I	13	5.05
gtt	V	4	589.90
gtc	V	2	501.40
gta	V	1193	670.70
gtg	V	1458	895.00
gct	A	0	0.74
gcc	A	0	0.69
gca	A	1	1.35
gcg	A	2	0.22
---	--	------	------
mPD		0.51	0.75
		nPD:	0.68
		N. weight:	0.97
		Sc. PD:	0.29
		Sc. rank:	1066.8

,

PB2

Pos. 736		obs:	exp:
ttt	F	423	1199.00
ttc	F	2253	1477.00
tta	L	1	0.11
ttg	L	0	0.19
ctt	L	0	0.20
ctc	L	0	0.15
cta	L	0	0.17
ctg	L	0	0.18
---	--	------	------
mPD		0.27	0.50
		nPD:	0.54
		N. weight:	0.36
		Sc. PD:	0.069
		Sc. rank:	301.4

PB2

Pos. 737		obs:	exp:
act	T	17	4.43
acc	T	1	3.89
aca	T	0	8.18
acg	T	0	1.50
aat	N	2284	1511.00
aac	N	375	1148.00
---	--	------	------
mPD		0.25	0.51
		nPD:	0.5
		N. weight:	0.38
		Sc. PD:	0.062
		Sc. rank:	289.4

PB2

Pos. 738		obs:	exp:
ttt	F	0	2.69
ttc	F	6	3.31
tat	Y	54	1496.00
tac	Y	2617	1175.00
---	--	------	------
mPD		0.044	0.50
		nPD:	0.09
		N. weight:	1.3
		Sc. PD:	-0.2
		Sc. rank:	-811.1

PB2

Pos. 739		obs:	exp:
aat	N	486	1522.00
aac	N	2191	1155.00
---	--	------	------
mPD		0.30	0.49
		nPD:	0.61
		N. weight:	0.59
		Sc. PD:	0.14
		Sc. rank:	580.5

PB2

Pos. 740		obs:	exp:
cgt	R	0	6.88
cgc	R	0	11.52
cga	R	0	21.91
cgg	R	0	15.75
att	I	0	0.35
atc	I	0	0.26
ata	I	1	0.39
aaa	K	1509	1494.00
aag	K	949	964.20
aga	R	17	99.97
agg	R	201	61.96
---	--	------	------
mPD		0.64	0.68
		nPD:	0.95
		N. weight:	0.15
		Sc. PD:	0.075
		Sc. rank:	183.4

PB2

Pos. 741		obs:	exp:
tct	S	0	1.18
tcc	S	0	1.11
tca	S	4	1.70
tcg	S	0	0.37
att	I	0	0.71
atc	I	0	0.51
ata	I	2	0.78
act	T	0	46.00
acc	T	3	40.44
aca	T	179	84.97
acg	T	5	15.59
aat	N	0	3.98
aac	N	7	3.02
agt	S	0	1.30
agc	S	3	1.34
gtt	V	0	16.43
gtc	V	3	13.96
gta	V	70	18.68
gtg	V	1	24.93
gct	A	17	585.10
gcc	A	4	546.90
gca	A	2349	1076.00
gcg	A	17	179.00
gaa	E	0	0.59
gag	E	1	0.41
ggt	G	0	1.88
ggc	G	12	1.52
gga	G	0	5.44
ggg	G	0	3.16
---	--	------	------
mPD		0.27	0.90
		nPD:	0.3
		N. weight:	1.4
		Sc. PD:	0.0086
		Sc. rank:	174.1

PB2

Pos. 742		obs:	exp:
tct	S	0	0.67
tcc	S	1	0.63
tca	S	1	0.97
tcg	S	0	0.21
act	T	29	653.10
acc	T	2403	574.10
aca	T	222	1206.00
acg	T	1	221.40
aat	N	0	10.23
aac	N	18	7.77
agt	S	0	0.74
agc	S	2	0.77
---	--	------	------
mPD		0.19	0.70
		nPD:	0.27
		N. weight:	2.1
		Sc. PD:	-0.026
		Sc. rank:	130.4

PB2

Pos. 743		obs:	exp:
cgt	R	0	0.60
cgc	R	0	1.00
cga	R	0	1.91
cgg	R	0	1.37
att	I	0	0.35
atc	I	0	0.26
ata	I	1	0.39
aat	N	0	7.39
aac	N	13	5.61
aaa	K	2597	1607.00
aag	K	47	1037.00
aga	R	19	8.71
agg	R	0	5.40
---	--	------	------
mPD		0.059	0.50
		nPD:	0.12
		N. weight:	0.78
		Sc. PD:	-0.1
		Sc. rank:	-358.9

PB2

Pos. 744		obs:	exp:
cgt	R	0	84.44
cgc	R	0	141.40
cga	R	667	268.90
cgg	R	46	193.30
atg	M	1	1.00
aaa	K	0	0.61
aag	K	1	0.39
aga	R	59	1227.00
agg	R	1903	760.30
---	--	------	------
mPD		0.79	0.94
		nPD:	0.84
		N. weight:	1.5
		Sc. PD:	0.62
		Sc. rank:	1770.3

PB2

Pos. 745		obs:	exp:
---	-	2677	2677.00
---	--	------	------
mPD		0	0
		nPD:	1.
		N. weight:	0.
		Sc. PD:	0
		Sc. rank:	0

PB2

Pos. 746		obs:	exp:
---	-	2677	2677.00
---	--	------	------
mPD		0	0
		nPD:	1.
		N. weight:	0.
		Sc. PD:	0
		Sc. rank:	0

PB2

Pos. 747		obs:	exp:
tta	L	8	289.10
ttg	L	1	499.10
ctt	L	2013	536.60
ctc	L	233	386.60
cta	L	377	463.90
ctg	L	25	481.60
att	I	3	6.74
atc	I	1	4.87
ata	I	15	7.39
gat	D	1	0.56
gac	D	0	0.44
---	--	------	------
mPD		0.42	1.1
		nPD:	0.37
		N. weight:	1.6
		Sc. PD:	0.1
		Sc. rank:	614.8

PB2

Pos. 748		obs:	exp:
tct	S	0	0.17
tcc	S	0	0.16
tca	S	1	0.24
tcg	S	0	0.05
att	I	0	1.06
atc	I	0	0.77
ata	I	3	1.17
atg	M	3	3.00
act	T	37	656.80
acc	T	5	577.40
aca	T	2373	1213.00
acg	T	255	222.60
agt	S	0	0.19
agc	S	0	0.19
---	--	------	------
mPD		0.21	0.69
		nPD:	0.3
		N. weight:	1.
		Sc. PD:	0.011
		Sc. rank:	159.6

PB2			
Pos.	749	obs:	exp:
att	I	612	519.30
atc	I	843	375.50
ata	I	9	569.20
gtt	V	797	269.30
gtc	V	378	228.90
gta	V	38	306.20
gtg	V	0	408.60
---	--	------	------
mPD		1.0	1.2
		nPD:	0.82
		N. weight:	1.2
		Sc. PD:	0.49
		Sc. rank:	1437.1

,

PB2			
Pos.	750	obs:	exp:
tta	L	2	288.70
ttg	L	0	498.30
ctt	L	2260	535.80
ctc	L	362	386.00
cta	L	28	463.20
ctg	L	1	480.90
att	I	24	8.51
atc	I	0	6.16
ata	I	0	9.33
---	--	------	------
mPD		0.27	1.2
		nPD:	0.24
		N. weight:	2.2
		Sc. PD:	-0.086
		Sc. rank:	-51.2

,

PB2			
Pos.	751	obs:	exp:
cgt	R	0	0.03
cgc	R	0	0.05
cga	R	0	0.10
cgg	R	0	0.07
aga	R	1	0.46
agg	R	0	0.28
ggt	G	1	419.70
ggc	G	3	339.10
gga	G	2054	1212.00
ggg	G	618	705.10
---	--	------	------
mPD		0.36	0.69
		nPD:	0.52
		N. weight:	0.68
		Sc. PD:	0.12
		Sc. rank:	552.9

,

PB2			
Pos.	752	obs:	exp:
cgt	R	0	0.25
cgc	R	0	0.42
cga	R	0	0.80
cgg	R	0	0.58
aaa	K	1189	1622.00
aag	K	1480	1047.00
aga	R	0	3.67
agg	R	8	2.27
---	--	------	------
mPD		0.50	0.48
		nPD:	1.03
		N. weight:	0.11
		Sc. PD:	0.062
		Sc. rank:	135.2

,

PB2			
Pos.	753	obs:	exp:
tct	S	0	0.17
tcc	S	0	0.16
tca	S	0	0.24
tcg	S	0	0.05
aat	N	1	0.57
aac	N	0	0.43
agt	S	1	0.19
agc	S	0	0.19
gtt	V	3	0.67
gtc	V	0	0.57
gta	V	0	0.76
gtg	V	0	1.01
gat	D	2284	1502.00
gac	D	387	1169.00
gaa	E	1	0.59
gag	E	0	0.41
---	--	------	------
mPD		0.25	0.50
		nPD:	0.51
		N. weight:	0.37
		Sc. PD:	0.063
		Sc. rank:	289.4

,

PB2			
Pos.	754	obs:	exp:
---	-	2677	2677.00
---	--	------	------
mPD		0	0
		nPD:	1.
		N. weight:	0.
		Sc. PD:	0
		Sc. rank:	0

,

PB2			
Pos.	755	obs:	exp:
---	-	2677	2677.00
---	--	------	------
mPD		0	0
		nPD:	1.
		N. weight:	0.
		Sc. PD:	0
		Sc. rank:	0

,

PB2			
Pos.	756	obs:	exp:
---	-	2677	2677.00
---	--	------	------
mPD		0	0
		nPD:	1.
		N. weight:	0.
		Sc. PD:	0
		Sc. rank:	0

,

PB2			
Pos.	757	obs:	exp:
---	-	2677	2677.00
---	--	------	------
mPD		0	0
		nPD:	1.
		N. weight:	0.
		Sc. PD:	0
		Sc. rank:	0

,

PB2			
Pos.	758	obs:	exp:
---	-	2677	2677.00
---	--	------	------
mPD		0	0
		nPD:	1.
		N. weight:	0.
		Sc. PD:	0
		Sc. rank:	0

,

PB2			
Pos.	759	obs:	exp:
---	-	2677	2677.00
---	--	------	------
mPD		0	0
		nPD:	1.
		N. weight:	0.
		Sc. PD:	0
		Sc. rank:	0

,

PB2			
Pos.	760	obs:	exp:
---	-	2677	2677.00
---	--	------	------
mPD		0	0
		nPD:	1.
		N. weight:	0.
		Sc. PD:	0
		Sc. rank:	0

,

PB2			
Pos.	761	obs:	exp:
---	-	2677	2677.00
---	--	------	------
mPD		0	0
		nPD:	1.
		N. weight:	0.
		Sc. PD:	0
		Sc. rank:	0

,

PB2			
Pos.	762	obs:	exp:
---	-	2677	2677.00
---	--	------	------
mPD		0	0
		nPD:	1.
		N. weight:	0.
		Sc. PD:	0
		Sc. rank:	0

,

PB2			
Pos.	763	obs:	exp:
---	-	2677	2677.00
---	--	------	------
mPD		0	0
		nPD:	1.
		N. weight:	0.
		Sc. PD:	0
		Sc. rank:	0

,

PB2			
Pos.	764	obs:	exp:
---	-	2677	2677.00
---	--	------	------
mPD		0	0
		nPD:	1.
		N. weight:	0.
		Sc. PD:	0
		Sc. rank:	0

,

PB2			
Pos.	765	obs:	exp:
---	-	2677	2677.00
---	--	------	------
mPD		0	0
		nPD:	1.
		N. weight:	0.
		Sc. PD:	0
		Sc. rank:	0

,

PB2			
Pos.	766	obs:	exp:
---	-	2677	2677.00
---	--	------	------
mPD		0	0
		nPD:	1.
		N. weight:	0.
		Sc. PD:	0
		Sc. rank:	0

,

PB2			
Pos.	767	obs:	exp:
---	-	2677	2677.00
---	--	------	------
mPD		0	0
		nPD:	1.
		N. weight:	0.
		Sc. PD:	0
		Sc. rank:	0

,

PB2			
Pos.	768	obs:	exp:
---	-	2677	2677.00
---	--	------	------
mPD		0	0
		nPD:	1.
		N. weight:	0.
		Sc. PD:	0
		Sc. rank:	0

,

PB2			
Pos.	769	obs:	exp:
---	-	2677	2677.00
---	--	------	------
mPD		0	0
		nPD:	1.
		N. weight:	0.
		Sc. PD:	0
		Sc. rank:	0

,

PB2			
Pos.	770	obs:	exp:
---	-	2677	2677.00
---	--	------	------
mPD		0	0
		nPD:	1.
		N. weight:	0.
		Sc. PD:	0
		Sc. rank:	0

,

PB2			
Pos.	771	obs:	exp:
---	-	2677	2677.00
---	--	------	------
mPD		0	0
		nPD:	1.
		N. weight:	0.
		Sc. PD:	0
		Sc. rank:	0

,

PB2			
Pos.	772	obs:	exp:
---	-	2677	2677.00
---	--	------	------
mPD		0	0
		nPD:	1.
		N. weight:	0.
		Sc. PD:	0
		Sc. rank:	0

,

PB2			
Pos.	773	obs:	exp:
---	-	2677	2677.00
---	--	------	------
mPD		0	0
		nPD:	1.
		N. weight:	0.
		Sc. PD:	0
		Sc. rank:	0

,

PB2			
Pos.	774	obs:	exp:
---	-	2677	2677.00
---	--	------	------
mPD		0	0
		nPD:	1.
		N. weight:	0.
		Sc. PD:	0
		Sc. rank:	0

,

PB2			
Pos.	775	obs:	exp:
---	-	2677	2677.00
---	--	------	------
mPD		0	0
		nPD:	1.
		N. weight:	0.
		Sc. PD:	0
		Sc. rank:	0

,

PB2			
Pos.	776	obs:	exp:
---	-	2677	2677.00
---	--	------	------
mPD		0	0
		nPD:	1.
		N. weight:	0.
		Sc. PD:	0
		Sc. rank:	0

,

PB2			
Pos.	777	obs:	exp:
---	-	2677	2677.00
---	--	------	------
mPD		0	0
		nPD:	1.
		N. weight:	0.
		Sc. PD:	0
		Sc. rank:	0

,

PB2			
Pos.	778	obs:	exp:
---	-	2677	2677.00
---	--	------	------
mPD		0	0
		nPD:	1.
		N. weight:	0.
		Sc. PD:	0
		Sc. rank:	0

,

PB2			
Pos.	779	obs:	exp:
---	-	2677	2677.00
---	--	------	------
mPD		0	0
		nPD:	1.
		N. weight:	0.
		Sc. PD:	0
		Sc. rank:	0

,

PB2			
Pos.	780	obs:	exp:
---	-	2677	2677.00
---	--	------	------
mPD		0	0
		nPD:	1.
		N. weight:	0.
		Sc. PD:	0
		Sc. rank:	0

,

PB2			
Pos.	781	obs:	exp:
---	-	2677	2677.00
---	--	------	------
mPD		0	0
		nPD:	1.
		N. weight:	0.
		Sc. PD:	0
		Sc. rank:	0

,

PB2			
Pos.	782	obs:	exp:
---	-	2677	2677.00
---	--	------	------
mPD		0	0
		nPD:	1.
		N. weight:	0.
		Sc. PD:	0
		Sc. rank:	0

PB2			
Pos.	783	obs:	exp:
---	-	2677	2677.00
---	--	------	------
mPD		0	0
		nPD:	1.
		N. weight:	0.
		Sc. PD:	0
		Sc. rank:	0

PB2			
Pos.	784	obs:	exp:
---	-	2677	2677.00
---	--	------	------
mPD		0	0
		nPD:	1.
		N. weight:	0.
		Sc. PD:	0
		Sc. rank:	0

PB2			
Pos.	785	obs:	exp:
---	-	2677	2677.00
---	--	------	------
mPD		0	0
		nPD:	1.
		N. weight:	0.
		Sc. PD:	0
		Sc. rank:	0

PB2			
Pos.	786	obs:	exp:
act	T	0	0.25
acc	T	0	0.22
aca	T	1	0.45
acg	T	0	0.08
gct	A	101	656.00
gcc	A	6	613.10
gca	A	2553	1206.00
gcg	A	16	200.70
---	--	------	------
mPD		0.089	0.68
		nPD:	0.13
		N. weight:	1.2
		Sc. PD:	-0.14
		Sc. rank:	-446.6

PB2			
Pos.	787	obs:	exp:
cgt	R	1	0.03
cgc	R	0	0.05
cga	R	0	0.10
cgg	R	0	0.07
aga	R	0	0.46
agg	R	0	0.28
ggt	G	2631	419.70
ggc	G	23	339.10
gga	G	16	1212.00
ggg	G	6	705.10
---	--	------	------
mPD		0.034	0.69
		nPD:	0.05
		N. weight:	3.2
		Sc. PD:	-0.59
		Sc. rank:	-2780.4

PB2			
Pos.	788	obs:	exp:
tct	S	0	2.02
tcc	S	0	1.90
tca	S	12	2.91
tcg	S	0	0.64
act	T	4	9.35
acc	T	2	8.22
aca	T	31	17.27
acg	T	1	3.17
aaa	K	1	0.61
aag	K	0	0.39
agt	S	0	2.23
agc	S	0	2.30
gtt	V	0	2.00
gtc	V	0	1.70
gta	V	5	2.27
gtg	V	4	3.03
gct	A	27	640.80
gcc	A	3	598.90
gca	A	2522	1178.00
gcg	A	62	196.00
gaa	E	3	1.78
gag	E	0	1.22
---	--	------	------
mPD		0.12	0.73
		nPD:	0.17
		N. weight:	1.2
		Sc. PD:	-0.12
		Sc. rank:	-295.1

PB2			
Pos.	789	obs:	exp:
tta	L	533	290.90
ttg	L	2059	502.10
ctt	L	1	539.80
ctc	L	0	388.90
cta	L	18	466.70
ctg	L	62	484.50
atg	M	4	4.00
---	--	------	------
mPD		0.39	1.1
		nPD:	0.34
		N. weight:	2.1
		Sc. PD:	0.086
		Sc. rank:	643.6

PB2			
Pos.	790	obs:	exp:
tta	L	1	0.11
ttg	L	0	0.19
tct	S	2	2.69
tcc	S	0	2.53
tca	S	12	3.89
tcg	S	0	0.85
ctt	L	0	0.20
ctc	L	0	0.15
cta	L	0	0.17
ctg	L	0	0.18
caa	Q	1	0.54
cag	Q	0	0.46
cgt	R	0	0.03
cgc	R	0	0.05
cga	R	0	0.10
cgg	R	0	0.07
att	I	65	26.61
atc	I	0	19.24
ata	I	10	29.16
atg	M	17	17.00
act	T	2023	588.40
acc	T	6	517.20
aca	T	358	1087.00
acg	T	5	199.50
aat	N	114	65.93
aac	N	2	50.07
aaa	K	5	3.04
aag	K	0	1.96
agt	S	2	2.98
agc	S	0	3.07
aga	R	1	0.46
agg	R	0	0.28
gtt	V	1	0.22
gtc	V	0	0.19
gta	V	0	0.25
gtg	V	0	0.34
gct	A	48	12.75
gcc	A	0	11.91
gca	A	4	23.44
gcg	A	0	3.90
---	--	------	------
mPD		0.47	0.89
		nPD:	0.53
		N. weight:	1.6
		Sc. PD:	0.29
		Sc. rank:	1281.2

PB2			
Pos.	791	obs:	exp:
tat	Y	0	0.56
tac	Y	1	0.44
aat	N	0	3.41
aac	N	6	2.59
aaa	K	2	1.22
aag	K	0	0.78
gat	D	12	400.50
gac	D	700	311.50
gaa	E	1916	1154.00
gag	E	32	793.80
ggt	G	0	1.25
ggc	G	1	1.01
gga	G	7	3.62
ggg	G	0	2.11
---	--	------	------
mPD		0.43	0.70
		nPD:	0.61
		N. weight:	0.98
		Sc. PD:	0.24
		Sc. rank:	965.8

PB2			
Pos.	792	obs:	exp:
gat	D	2531	1500.00
gac	D	135	1166.00
gaa	E	3	4.15
gag	E	4	2.85
ggt	G	2	0.63
ggc	G	2	0.51
gga	G	0	1.81
ggg	G	0	1.05
---	--	------	------
mPD		0.11	0.50
		nPD:	0.21
		N. weight:	0.72
		Sc. PD:	-0.043
		Sc. rank:	-66.4

PB2			
Pos.	793	obs:	exp:
tta	L	0	0.22
ttg	L	0	0.38
tct	S	0	0.17
tcc	S	0	0.16
tca	S	1	0.24
tcg	S	0	0.05
ctt	L	0	0.40
ctc	L	0	0.29
cta	L	2	0.35
ctg	L	0	0.36
cct	P	11	788.50
ccc	P	11	538.00
cca	P	2625	1058.00
ccg	P	25	287.40
caa	Q	2	1.08
cag	Q	0	0.92
agt	S	0	0.19
agc	S	0	0.19
---	--	------	------
mPD		0.038	0.71
		nPD:	0.05
		N. weight:	1.6
		Sc. PD:	-0.28
		Sc. rank:	-1311.0

PB2			
Pos.	794	obs:	exp:
aat	N	26	18.76
aac	N	7	14.24
aaa	K	0	0.61
aag	K	1	0.39
gat	D	1680	1375.00
gac	D	765	1070.00
gaa	E	41	113.80
gag	E	151	78.24
ggt	G	6	0.94
ggc	G	0	0.76
gga	G	0	2.72
ggg	G	0	1.58
---	--	------	------
mPD		0.53	0.59
		nPD:	0.9
		N. weight:	0.11
		Sc. PD:	0.053
		Sc. rank:	137.0

PB2			
Pos.	795	obs:	exp:
gaa	E	2249	1586.00
gag	E	427	1090.00
ggt	G	0	0.16
ggc	G	0	0.13
gga	G	0	0.45
ggg	G	1	0.26
---	--	------	------
mPD		0.27	0.48
		nPD:	0.56
		N. weight:	0.27
		Sc. PD:	0.056
		Sc. rank:	237.2

,

PB2			
Pos.	796	obs:	exp:
tct	S	0	12.78
tcc	S	0	12.01
tca	S	0	18.46
tcg	S	0	4.04
tgt	C	0	0.86
tgc	C	2	1.14
cgt	R	0	0.03
cgc	R	0	0.05
cga	R	0	0.10
cgg	R	0	0.07
aat	N	0	0.57
aac	N	1	0.43
agt	S	1	14.15
agc	S	75	14.57
aga	R	1	0.46
agg	R	0	0.28
ggt	G	20	407.30
ggc	G	2098	329.10
gga	G	448	1176.00
ggg	G	31	684.30
---	--	------	------
mPD		0.37	0.78
		nPD:	0.47
		N. weight:	2.4
		Sc. PD:	0.33
		Sc. rank:	1609.9

,

PB2			
Pos.	797	obs:	exp:
tct	S	0	2.02
tcc	S	0	1.90
tca	S	12	2.91
tcg	S	0	0.64
cgt	R	0	0.03
cgc	R	0	0.05
cga	R	0	0.10
cgg	R	0	0.07
att	I	0	2.48
atc	I	0	1.80
ata	I	7	2.72
act	T	23	627.70
acc	T	6	551.80
aca	T	2217	1160.00
acg	T	306	212.80
aat	N	0	0.57
aac	N	1	0.43
aaa	K	7	32.21
aag	K	46	20.79
agt	S	0	2.23
agc	S	0	2.30
aga	R	0	0.46
agg	R	1	0.28
gct	A	0	12.50
gcc	A	0	11.69
gca	A	15	22.99
gcg	A	36	3.82
---	--	------	------
mPD		0.36	0.77
		nPD:	0.47
		N. weight:	1.1
		Sc. PD:	0.15
		Sc. rank:	710.8

,

PB2			
Pos.	798	obs:	exp:
tct	S	729	127.00
tcc	S	8	119.30
tca	S	2	183.30
tcg	S	16	40.13
act	T	32	8.36
acc	T	0	7.35
aca	T	2	15.45
acg	T	0	2.84
agt	S	0	140.50
agc	S	0	144.70
gtt	V	1	0.22
gtc	V	0	0.19
gta	V	0	0.25
gtg	V	0	0.34
gct	A	1821	459.10
gcc	A	15	429.10
gca	A	29	844.30
gcg	A	8	140.50
ggt	G	14	2.20
ggc	G	0	1.77
gga	G	0	6.34
ggg	G	0	3.69
---	--	------	------
mPD		0.49	1.4
		nPD:	0.36
		N. weight:	2.5
		Sc. PD:	0.15
		Sc. rank:	893.3

,

PB2			
Pos.	799	obs:	exp:
ggt	G	17	419.90
ggc	G	0	339.20
gga	G	1969	1213.00
ggg	G	691	705.40
---	--	------	------
mPD		0.39	0.68
		nPD:	0.57
		N. weight:	0.62
		Sc. PD:	0.13
		Sc. rank:	565.0

,

PB2			
Pos.	800	obs:	exp:
tta	L	0	0.22
ttg	L	2	0.38
ctt	L	0	0.40
ctc	L	0	0.29
cta	L	0	0.35
ctg	L	0	0.36
att	I	7	8.87
atc	I	0	6.41
ata	I	18	9.72
gtt	V	18	588.40
gtc	V	0	500.00
gta	V	158	669.00
gtg	V	2474	892.60
---	--	------	------
mPD		0.16	0.76
		nPD:	0.21
		N. weight:	1.6
		Sc. PD:	-0.092
		Sc. rank:	-132.8

,

PB2			
Pos.	801	obs:	exp:
gaa	E	609	1586.00
gag	E	2068	1091.00
---	--	------	------
mPD		0.35	0.48
		nPD:	0.73
		N. weight:	0.51
		Sc. PD:	0.17
		Sc. rank:	593.5

,

PB2			
Pos.	802	obs:	exp:
ttt	F	1	0.45
ttc	F	0	0.55
tct	S	2525	449.80
tcc	S	80	422.60
tca	S	27	649.30
tcg	S	42	142.10
cct	P	0	0.30
ccc	P	1	0.20
cca	P	0	0.40
ccg	P	0	0.11
agt	S	0	497.70
agc	S	0	512.50
gct	A	1	0.25
gcc	A	0	0.23
gca	A	0	0.45
gcg	A	0	0.07
---	--	------	------
mPD		0.11	1.6
		nPD:	0.07
		N. weight:	2.8
		Sc. PD:	-0.48
		Sc. rank:	-2096.0

,

PB2			
Pos.	803	obs:	exp:
gct	A	2237	656.20
gcc	A	25	613.40
gca	A	340	1207.00
gcg	A	75	200.80
---	--	------	------
mPD		0.28	0.68
		nPD:	0.42
		N. weight:	1.5
		Sc. PD:	0.15
		Sc. rank:	782.7

,

PB2			
Pos.	804	obs:	exp:
gtt	V	239	594.40
gtc	V	1995	505.10
gta	V	404	675.80
gtg	V	39	901.70
---	--	------	------
mPD		0.41	0.74
		nPD:	0.56
		N. weight:	1.5
		Sc. PD:	0.32
		Sc. rank:	1365.8

,

PB2			
Pos.	805	obs:	exp:
tta	L	22	291.30
ttg	L	287	502.80
ctt	L	28	540.60
ctc	L	0	389.50
cta	L	110	467.40
ctg	L	2230	485.30
---	--	------	------
mPD		0.32	1.1
		nPD:	0.28
		N. weight:	2.
		Sc. PD:	-0.015
		Sc. rank:	187.1

,

PB2			
Pos.	806	obs:	exp:
cgt	R	0	84.50
cgc	R	0	141.50
cga	R	0	269.10
cgg	R	3	193.40
aga	R	1234	1228.00
agg	R	1440	760.90
---	--	------	------
mPD		0.50	0.94
		nPD:	0.53
		N. weight:	0.64
		Sc. PD:	0.12
		Sc. rank:	526.2

,

PB2			
Pos.	807	obs:	exp:
ggt	G	8	419.90
ggc	G	24	339.20
gga	G	2122	1213.00
ggg	G	523	705.40
---	--	------	------
mPD		0.33	0.68
		nPD:	0.49
		N. weight:	0.65
		Sc. PD:	0.1
		Sc. rank:	473.4

,

PB2			
Pos.	808	obs:	exp:
ttt	F	1161	1200.00
ttc	F	1516	1477.00
---	--	------	------
mPD		0.49	0.49
		nPD:	0.99
		N. weight:	0.0037
		Sc. PD:	0.002
		Sc. rank:	4.5

,

PB2			
Pos.	809	obs:	exp:
tta	L	69	291.30
ttg	L	5	502.80
ctt	L	34	540.60
ctc	L	2203	389.50
cta	L	347	467.40
ctg	L	19	485.30
---	--	------	------
mPD		0.35	1.1
		nPD:	0.31
		N. weight:	2.4
		Sc. PD:	0.038
		Sc. rank:	460.8

,

PB2			
Pos.	810	obs:	exp:
att	I	2581	949.60
atc	I	89	686.60
ata	I	7	1041.00
---	--	------	------
mPD		0.069	0.66
		nPD:	0.11
		N. weight:	1.6
		Sc. PD:	-0.23
		Sc. rank:	-852.5

,

PB2			
Pos.	811	obs:	exp:
---	-	2677	2677.00
---	--	------	------
mPD		0	0
		nPD:	1.
		N. weight:	0.
		Sc. PD:	0
		Sc. rank:	0

,

PB2			
Pos.	812	obs:	exp:
---	-	2677	2677.00
---	--	------	------
mPD		0	0
		nPD:	1.
		N. weight:	0.
		Sc. PD:	0
		Sc. rank:	0

,

PB2			
Pos.	813	obs:	exp:
---	-	2677	2677.00
---	--	------	------
mPD		0	0
		nPD:	1.
		N. weight:	0.
		Sc. PD:	0
		Sc. rank:	0

,

PB2			
Pos.	814	obs:	exp:
---	-	2677	2677.00
---	--	------	------
mPD		0	0
		nPD:	1.
		N. weight:	0.
		Sc. PD:	0
		Sc. rank:	0

,

PB2			
Pos.	815	obs:	exp:
---	-	2677	2677.00
---	--	------	------
mPD		0	0
		nPD:	1.
		N. weight:	0.
		Sc. PD:	0
		Sc. rank:	0

,

PB2			
Pos.	816	obs:	exp:
---	-	2677	2677.00
---	--	------	------
mPD		0	0
		nPD:	1.
		N. weight:	0.
		Sc. PD:	0
		Sc. rank:	0

,

PB2			
Pos.	817	obs:	exp:
---	-	2677	2677.00
---	--	------	------
mPD		0	0
		nPD:	1.
		N. weight:	0.
		Sc. PD:	0
		Sc. rank:	0

,

PB2

Pos.	818	obs:	exp:
---	-	2677	2677.00
---	--	------	------
mPD		0	0
		nPD:	1.
		N. weight:	0.
		Sc. PD:	0
		Sc. rank:	0

PB2

Pos.	819	obs:	exp:
ttt	F	0	0.45
ttc	F	1	0.55
tta	L	314	288.40
ttg	L	1925	497.80
ctt	L	9	535.20
ctc	L	2	385.60
cta	L	370	462.70
ctg	L	30	480.40
att	I	0	9.22
atc	I	0	6.67
ata	I	26	10.11
---	--	------	------
mPD		0.67	1.2
		nPD:	0.58
		N. weight:	1.7
		Sc. PD:	0.39
		Sc. rank:	1601.1

PB2

Pos.	820	obs:	exp:
tct	S	0	0.67
tcc	S	0	0.63
tca	S	0	0.97
tcg	S	0	0.21
agt	S	0	0.74
agc	S	4	0.77
gct	A	0	0.25
gcc	A	0	0.23
gca	A	0	0.45
gcg	A	1	0.07
ggt	G	1081	419.10
ggc	G	1143	338.60
gga	G	117	1210.00
ggg	G	331	704.00
---	--	------	------
mPD		0.64	0.69
		nPD:	0.93
		N. weight:	1.3
		Sc. PD:	0.65
		Sc. rank:	1631.0

PB2

Pos.	821	obs:	exp:
cgt	R	0	0.76
cgc	R	0	1.27
cga	R	0	2.41
cgg	R	0	1.73
aat	N	1	0.57
aac	N	0	0.43
aaa	K	2131	1612.00
aag	K	521	1040.00
aga	R	19	11.01
agg	R	5	6.82
---	--	------	------
mPD		0.33	0.50
		nPD:	0.67
		N. weight:	0.17
		Sc. PD:	0.05
		Sc. rank:	187.8

PB2

Pos.	822	obs:	exp:
aaa	K	1	0.61
aag	K	0	0.39
gaa	E	2621	1585.00
gag	E	54	1090.00
ggt	G	0	0.16
ggc	G	0	0.13
gga	G	1	0.45
ggg	G	0	0.26
---	--	------	------
mPD		0.041	0.48
		nPD:	0.08
		N. weight:	0.8
		Sc. PD:	-0.13
		Sc. rank:	-509.0

PB2

Pos.	823	obs:	exp:
tct	S	0	0.17
tcc	S	0	0.16
tca	S	0	0.24
tcg	S	0	0.05
aat	N	19	226.80
aac	N	380	172.20
agt	S	0	0.19
agc	S	1	0.19
gtt	V	0	0.22
gtc	V	1	0.19
gta	V	0	0.25
gtg	V	0	0.34
gat	D	42	1278.00
gac	D	2230	994.10
gaa	E	4	2.37
gag	E	0	1.63
---	--	------	------
mPD		0.30	0.75
		nPD:	0.4
		N. weight:	1.3
		Sc. PD:	0.12
		Sc. rank:	617.2

PB2

Pos.	824	obs:	exp:
cgt	R	0	2.05
cgc	R	0	3.44
cga	R	0	6.53
cgg	R	0	4.70
atg	M	1	1.00
act	T	0	0.25
acc	T	0	0.22
aca	T	1	0.45
acg	T	0	0.08
aat	N	1	0.57
aac	N	0	0.43
aaa	K	467	1586.00
aag	K	2142	1023.00
aga	R	9	29.81
agg	R	56	18.47
---	--	------	------
mPD		0.34	0.54
		nPD:	0.63
		N. weight:	0.74
		Sc. PD:	0.2
		Sc. rank:	755.0

PB2

Pos.	825	obs:	exp:
tct	S	0	0.17
tcc	S	0	0.16
tca	S	0	0.24
tcg	S	0	0.05
cgt	R	0	81.97
cgc	R	0	137.30
cga	R	0	261.00
cgg	R	0	187.60
att	I	0	0.35
atc	I	0	0.26
ata	I	1	0.39
aaa	K	74	47.40
aag	K	4	30.60
agt	S	0	0.19
agc	S	1	0.19
aga	R	2520	1191.00
agg	R	77	738.10
---	--	------	------
mPD		0.12	0.99
		nPD:	0.12
		N. weight:	1.2
		Sc. PD:	-0.16
		Sc. rank:	-539.2

PB2

Pos.	826	obs:	exp:
---	-	2677	2677.00
---	--	------	------
mPD		0	0
		nPD:	1.
		N. weight:	0.
		Sc. PD:	0
		Sc. rank:	0

PB2

Pos.	827	obs:	exp:
---	-	2677	2677.00
---	--	------	------
mPD		0	0
		nPD:	1.
		N. weight:	0.
		Sc. PD:	0
		Sc. rank:	0

PB2

Pos.	828	obs:	exp:
---	-	2677	2677.00
---	--	------	------
mPD		0	0
		nPD:	1.
		N. weight:	0.
		Sc. PD:	0
		Sc. rank:	0

PB2

Pos.	829	obs:	exp:
---	-	2677	2677.00
---	--	------	------
mPD		0	0
		nPD:	1.
		N. weight:	0.
		Sc. PD:	0
		Sc. rank:	0

PB2

Pos.	830	obs:	exp:
---	-	2677	2677.00
---	--	------	------
mPD		0	0
		nPD:	1.
		N. weight:	0.
		Sc. PD:	0
		Sc. rank:	0

PB2

Pos.	831	obs:	exp:
---	-	2677	2677.00
---	--	------	------
mPD		0	0
		nPD:	1.
		N. weight:	0.
		Sc. PD:	0
		Sc. rank:	0

PB2

Pos.	832	obs:	exp:
ttt	F	1	0.45
ttc	F	0	0.55
tat	Y	2633	1499.00
tac	Y	42	1176.00
cat	H	1	0.58
cac	H	0	0.42
---	--	------	------
mPD		0.032	0.49
		nPD:	0.07
		N. weight:	0.93
		Sc. PD:	-0.16
		Sc. rank:	-719.4

PB2

Pos.	833	obs:	exp:
ggt	G	248	419.90
ggc	G	1929	339.20
gga	G	464	1213.00
ggg	G	36	705.40
---	--	------	------
mPD		0.44	0.68
		nPD:	0.65
		N. weight:	1.9
		Sc. PD:	0.51
		Sc. rank:	1947.2

PB2

Pos.	834	obs:	exp:
tct	S	3	0.67
tcc	S	1	0.63
tca	S	0	0.97
tcg	S	0	0.21
cct	P	15	788.80
ccc	P	21	538.20
cca	P	2602	1059.00
ccg	P	35	287.50
agt	S	0	0.74
agc	S	0	0.77
---	--	------	------
mPD		0.058	0.71
		nPD:	0.08
		N. weight:	1.5
		Sc. PD:	-0.24
		Sc. rank:	-963.3

PB2			
Pos.	835	obs:	exp:
tct	S	0	0.17
tcc	S	0	0.16
tca	S	1	0.24
tcg	S	0	0.05
act	T	0	0.98
acc	T	0	0.86
aca	T	3	1.82
acg	T	1	0.33
agt	S	0	0.19
agc	S	0	0.19
gtt	V	0	2.00
gtc	V	0	1.70
gta	V	9	2.27
gtg	V	0	3.03
gct	A	19	652.80
gcc	A	0	610.20
gca	A	2581	1200.00
gcg	A	63	199.70
---	--	------	------
mPD		0.071	0.69
		nPD:	0.1
		N. weight:	1.3
		Sc. PD:	-0.18
		Sc. rank:	-689.0

,

PB2			
Pos.	836	obs:	exp:
tta	L	2177	291.30
ttg	L	435	502.80
ctt	L	0	540.60
ctc	L	0	389.50
cta	L	56	467.40
ctg	L	9	485.30
---	--	------	------
mPD		0.32	1.1
		nPD:	0.28
		N. weight:	2.9
		Sc. PD:	-0.0082
		Sc. rank:	324.4

,

PB2			
Pos.	837	obs:	exp:
tct	S	0	449.90
tcc	S	0	422.80
tca	S	0	649.60
tcg	S	0	142.20
aat	N	0	0.57
aac	N	1	0.43
agt	S	268	497.90
agc	S	2407	512.70
ggt	G	0	0.16
ggc	G	1	0.13
gga	G	0	0.45
ggg	G	0	0.26
---	--	------	------
mPD		0.18	1.6
		nPD:	0.11
		N. weight:	2.5
		Sc. PD:	-0.33
		Sc. rank:	-1216.8

,

PB2			
Pos.	838	obs:	exp:
att	I	71	949.60
atc	I	2588	686.60
ata	I	18	1041.00
---	--	------	------
mPD		0.065	0.66
		nPD:	0.1
		N. weight:	2.2
		Sc. PD:	-0.32
		Sc. rank:	-1240.1

,

PB2			
Pos.	839	obs:	exp:
tct	S	0	4.21
tcc	S	0	3.95
tca	S	0	6.07
tcg	S	0	1.33
att	I	1	0.35
atc	I	0	0.26
ata	I	0	0.39
aat	N	2628	1505.00
aac	N	20	1143.00
aaa	K	0	0.61
aag	K	1	0.39
agt	S	25	4.65
agc	S	0	4.79
gat	D	2	1.12
gac	D	0	0.88
---	--	------	------
mPD		0.036	0.53
		nPD:	0.07
		N. weight:	0.99
		Sc. PD:	-0.17
		Sc. rank:	-725.0

,

PB2			
Pos.	840	obs:	exp:
gaa	E	2621	1586.00
gag	E	56	1091.00
---	--	------	------
mPD		0.041	0.48
		nPD:	0.08
		N. weight:	0.8
		Sc. PD:	-0.12
		Sc. rank:	-504.2

,

PB2			
Pos.	841	obs:	exp:
tta	L	13	291.30
ttg	L	288	502.80
ctt	L	2	540.60
ctc	L	0	389.50
cta	L	211	467.40
ctg	L	2163	485.30
---	--	------	------
mPD		0.35	1.1
		nPD:	0.31
		N. weight:	2.
		Sc. PD:	0.035
		Sc. rank:	415.4

,

PB2			
Pos.	842	obs:	exp:
tct	S	0	437.00
tcc	S	0	410.60
tca	S	0	630.90
tcg	S	0	138.10
tat	Y	0	6.72
tac	Y	12	5.28
tgt	C	0	18.51
tgc	C	43	24.49
att	I	0	6.74
atc	I	19	4.87
ata	I	0	7.39
agt	S	124	483.50
agc	S	2474	497.90
ggt	G	0	0.78
ggc	G	5	0.63
gga	G	0	2.26
ggg	G	0	1.32
---	--	------	------
mPD		0.16	1.6
		nPD:	0.09
		N. weight:	2.7
		Sc. PD:	-0.4
		Sc. rank:	-1550.6

,

PB2			
Pos.	843	obs:	exp:
tct	S	0	38.85
tcc	S	0	36.51
tca	S	0	56.10
tcg	S	0	12.28
cat	H	11	6.35
cac	H	0	4.65
att	I	4	1.42
atc	I	0	1.03
ata	I	0	1.56
aat	N	2383	1378.00
aac	N	42	1047.00
aaa	K	3	2.43
aag	K	1	1.57
agt	S	230	42.99
agc	S	1	44.27
ggt	G	2	0.31
ggc	G	0	0.25
gga	G	0	0.91
ggg	G	0	0.53
---	--	------	------
mPD		0.21	0.80
		nPD:	0.26
		N. weight:	1.1
		Sc. PD:	-0.025
		Sc. rank:	15.6

,

PB2			
Pos.	844	obs:	exp:
ttt	F	1	0.45
ttc	F	0	0.55
tta	L	0	291.20
ttg	L	0	502.60
ctt	L	2662	540.40
ctc	L	13	389.30
cta	L	0	467.30
ctg	L	1	485.10
---	--	------	------
mPD		0.011	1.1
		nPD:	0.01
		N. weight:	2.9
		Sc. PD:	-0.62
		Sc. rank:	-3325.6

,

PB2			
Pos.	845	obs:	exp:
tct	S	0	5.72
tcc	S	0	5.37
tca	S	34	8.26
tcg	S	0	1.81
act	T	0	11.56
acc	T	0	10.16
aca	T	46	21.36
acg	T	1	3.92
agt	S	0	6.33
agc	S	0	6.52
gtt	V	0	3.77
gtc	V	0	3.21
gta	V	12	4.29
gtg	V	5	5.73
gct	A	1	632.20
gcc	A	26	590.90
gca	A	2537	1162.00
gcg	A	15	193.40
---	--	------	------
mPD		0.11	0.76
		nPD:	0.14
		N. weight:	1.4
		Sc. PD:	-0.15
		Sc. rank:	-460.3

,

PB2			
Pos.	846	obs:	exp:
cgt	R	0	0.98
cgc	R	0	1.64
cga	R	0	3.12
cgg	R	0	2.24
aaa	K	2498	1608.00
aag	K	148	1038.00
aga	R	30	14.22
agg	R	1	8.81
---	--	------	------
mPD		0.13	0.51
		nPD:	0.25
		N. weight:	0.58
		Sc. PD:	-0.016
		Sc. rank:	3.3

,

PB2			
Pos.	847	obs:	exp:
ggt	G	3	419.90
ggc	G	3	339.20
gga	G	2214	1213.00
ggg	G	457	705.40
---	--	------	------
mPD		0.29	0.68
		nPD:	0.42
		N. weight:	0.77
		Sc. PD:	0.077
		Sc. rank:	394.2

,

PB2			
Pos.	848	obs:	exp:
gaa	E	44	1586.00
gag	E	2633	1091.00
---	--	------	------
mPD		0.032	0.48
		nPD:	0.07
		N. weight:	1.5
		Sc. PD:	-0.25
		Sc. rank:	-1118.4

,

PB2			
Pos.	849	obs:	exp:
cgt	R	0	0.06
cgc	R	0	0.11
cga	R	0	0.20
cgg	R	0	0.14
aaa	K	1133	1626.00
aag	K	1542	1049.00
aga	R	0	0.92
agg	R	2	0.57
---	--	------	------
mPD		0.49	0.48
		nPD:	1.02
		N. weight:	0.13
		Sc. PD:	0.075
		Sc. rank:	164.3

,

PB2			
Pos.	850	obs:	exp:
tct	S	5	0.84
tcc	S	0	0.79
tca	S	0	1.21
tcg	S	0	0.27
act	T	4	0.98
acc	T	0	0.86
aca	T	0	1.82
acg	T	0	0.33
agt	S	0	0.93
agc	S	0	0.96
gtt	V	1	0.22
gtc	V	0	0.19
gta	V	0	0.25
gtg	V	0	0.34
gct	A	2243	653.80
gcc	A	419	611.10
gca	A	4	1202.00
gcg	A	1	200.00
---	--	------	------
mPD		0.27	0.69
		nPD:	0.4
		N. weight:	1.8
		Sc. PD:	0.15
		Sc. rank:	820.0

,

PB2			
Pos.	851	obs:	exp:
tat	Y	0	1.68
tac	Y	3	1.32
aat	N	2199	1520.00
aac	N	475	1154.00
---	--	------	------
mPD		0.30	0.49
		nPD:	0.6
		N. weight:	0.27
		Sc. PD:	0.066
		Sc. rank:	267.2

,

PB2			
Pos.	852	obs:	exp:
gtt	V	322	594.40
gtc	V	3	505.10
gta	V	252	675.80
gtg	V	2100	901.70
---	--	------	------
mPD		0.36	0.74
		nPD:	0.49
		N. weight:	0.92
		Sc. PD:	0.14
		Sc. rank:	673.1

,

PB2			
Pos.	853	obs:	exp:
tta	L	36	291.30
ttg	L	386	502.80
ctt	L	0	540.60
ctc	L	0	389.50
cta	L	2204	467.40
ctg	L	51	485.30
---	--	------	------
mPD		0.54	1.1
		nPD:	0.47
		N. weight:	2.2
		Sc. PD:	0.31
		Sc. rank:	1503.9

,

PB2			
Pos.	854	obs:	exp:
att	I	2239	949.60
atc	I	28	686.60
ata	I	410	1041.00
---	--	------	------
mPD		0.28	0.66
		nPD:	0.42
		N. weight:	1.
		Sc. PD:	0.1
		Sc. rank:	528.0

,

PB2			
Pos.	855	obs:	exp:
ggt	G	4	419.90
ggc	G	2	339.20
gga	G	218	1213.00
ggg	G	2453	705.40
---	--	------	------
mPD		0.15	0.68
		nPD:	0.22
		N. weight:	1.8
		Sc. PD:	-0.09
		Sc. rank:	-93.1

,

PB2			
Pos.	856	obs:	exp:
caa	Q	2669	1444.00
cag	Q	8	1233.00
---	--	------	------
mPD		0.0060	0.50
		nPD:	0.01
		N. weight:	1.1
		Sc. PD:	-0.24
		Sc. rank:	-1245.1

,

PB2			
Pos.	857	obs:	exp:
ggt	G	0	419.90
ggc	G	1	339.20
gga	G	1977	1213.00
ggg	G	699	705.40
---	--	------	------
mPD		0.39	0.68
		nPD:	0.56
		N. weight:	0.66
		Sc. PD:	0.14
		Sc. rank:	601.9

,

PB2			
Pos.	858	obs:	exp:
---	-	2677	2677.00
---	--	------	------
mPD		0	0
		nPD:	1.
		N. weight:	0.
		Sc. PD:	0
		Sc. rank:	0

,

PB2			
Pos.	859	obs:	exp:
---	-	2677	2677.00
---	--	------	------
mPD		0	0
		nPD:	1.
		N. weight:	0.
		Sc. PD:	0
		Sc. rank:	0

,

PB2			
Pos.	860	obs:	exp:
---	-	2677	2677.00
---	--	------	------
mPD		0	0
		nPD:	1.
		N. weight:	0.
		Sc. PD:	0
		Sc. rank:	0

,

PB2			
Pos.	861	obs:	exp:
---	-	2677	2677.00
---	--	------	------
mPD		0	0
		nPD:	1.
		N. weight:	0.
		Sc. PD:	0
		Sc. rank:	0

,

PB2			
Pos.	862	obs:	exp:
---	-	2677	2677.00
---	--	------	------
mPD		0	0
		nPD:	1.
		N. weight:	0.
		Sc. PD:	0
		Sc. rank:	0

,

PB2			
Pos.	863	obs:	exp:
---	-	2677	2677.00
---	--	------	------
mPD		0	0
		nPD:	1.
		N. weight:	0.
		Sc. PD:	0
		Sc. rank:	0

,

PB2			
Pos.	864	obs:	exp:
---	-	2677	2677.00
---	--	------	------
mPD		0	0
		nPD:	1.
		N. weight:	0.
		Sc. PD:	0
		Sc. rank:	0

,

PB2			
Pos.	865	obs:	exp:
gat	D	26	1506.00
gac	D	2651	1171.00
---	--	------	------
mPD		0.019	0.49
		nPD:	0.04
		N. weight:	1.4
		Sc. PD:	-0.27
		Sc. rank:	-1348.1

,

PB2			
Pos.	866	obs:	exp:
att	I	0	16.32
atc	I	0	11.80
ata	I	46	17.88
gtt	V	37	584.20
gtc	V	4	496.50
gta	V	821	664.20
gtg	V	1769	886.20
---	--	------	------
mPD		0.49	0.77
		nPD:	0.64
		N. weight:	0.92
		Sc. PD:	0.25
		Sc. rank:	947.5

,

PB2			
Pos.	867	obs:	exp:
gtt	V	19	594.40
gtc	V	1	505.10
gta	V	25	675.80
gtg	V	2632	901.70
---	--	------	------
mPD		0.033	0.74
		nPD:	0.05
		N. weight:	1.8
		Sc. PD:	-0.35
		Sc. rank:	-1667.4

,

PB2			
Pos.	868	obs:	exp:
tta	L	44	291.00
ttg	L	2614	502.30
tct	S	0	0.34
tcc	S	0	0.32
tca	S	0	0.49
tcg	S	2	0.11
ctt	L	0	540.00
ctc	L	0	389.10
cta	L	3	466.90
ctg	L	13	484.70
atg	M	1	1.00
agt	S	0	0.37
agc	S	0	0.38
---	--	------	------
mPD		0.049	1.1
		nPD:	0.04
		N. weight:	2.9
		Sc. PD:	-0.55
		Sc. rank:	-2640.3

,

PB2			
Pos.	869	obs:	exp:
att	I	0	0.71
atc	I	0	0.51
ata	I	2	0.78
gtt	V	10	593.90
gtc	V	5	504.80
gta	V	2576	675.30
gtg	V	84	901.00
---	--	------	------
mPD		0.073	0.74
		nPD:	0.1
		N. weight:	2.2
		Sc. PD:	-0.32
		Sc. rank:	-1232.3

,

PB2			
Pos.	870	obs :	exp :
atg	M	2676	2676.00
gtt	V	0	0.22
gtc	V	0	0.19
gta	V	0	0.25
gtg	V	1	0.34
---	--	------	------
mPD		0.00075	0.0012
		nPD :	0.6
		N. weight :	0.00075
		Sc. PD :	0.00018
		Sc. rank :	0.7

PB2			
Pos.	871	obs :	exp :
cgt	R	0	0.06
cgc	R	0	0.11
cga	R	0	0.20
cgg	R	0	0.14
aaa	K	2674	1626.00
aag	K	1	1049.00
aga	R	2	0.92
agg	R	0	0.57
---	--	------	------
mPD		0.0022	0.48
		nPD :	0.
		N. weight :	0.92
		Sc. PD :	-0.2
		Sc. rank :	-1073.9

PB2			
Pos.	872	obs :	exp :
cgt	R	4	84.50
cgc	R	1	141.50
cga	R	861	269.10
cgg	R	1811	193.40
aga	R	0	1228.00
agg	R	0	760.90
---	--	------	------
mPD		0.44	0.94
		nPD :	0.47
		N. weight :	3.5
		Sc. PD :	0.48
		Sc. rank :	2325.1

PB2			
Pos.	873	obs :	exp :
---	-	2677	2677.00
---	--	------	------
mPD		0	0
		nPD :	1.
		N. weight :	0.
		Sc. PD :	0
		Sc. rank :	0

PB2			
Pos.	874	obs :	exp :
---	-	2677	2677.00
---	--	------	------
mPD		0	0
		nPD :	1.
		N. weight :	0.
		Sc. PD :	0
		Sc. rank :	0

PB2			
Pos.	875	obs :	exp :
---	-	2677	2677.00
---	--	------	------
mPD		0	0
		nPD :	1.
		N. weight :	0.
		Sc. PD :	0
		Sc. rank :	0

PB2			
Pos.	876	obs :	exp :
aaa	K	2659	1626.00
aag	K	17	1050.00
gaa	E	1	0.59
gag	E	0	0.41
---	--	------	------
mPD		0.013	0.48
		nPD :	0.03
		N. weight :	0.86
		Sc. PD :	-0.17
		Sc. rank :	-895.1

PB2			
Pos.	877	obs :	exp :
cgt	R	2	84.50
cgc	R	1	141.50
cga	R	117	269.10
cgg	R	2493	193.40
aga	R	0	1228.00
agg	R	64	760.90
---	--	------	------
mPD		0.13	0.94
		nPD :	0.14
		N. weight :	4.2
		Sc. PD :	-0.48
		Sc. rank :	-1392.5

PB2			
Pos.	878	obs :	exp :
aat	N	0	20.46
aac	N	36	15.54
gat	D	37	1483.00
gac	D	2600	1154.00
gaa	E	4	2.37
gag	E	0	1.63
---	--	------	------
mPD		0.057	0.52
		nPD :	0.11
		N. weight :	1.4
		Sc. PD :	-0.19
		Sc. rank :	-709.4

PB2			
Pos.	879	obs :	exp :
tct	S	2606	450.30
tcc	S	41	423.10
tca	S	27	650.10
tcg	S	3	142.30
agt	S	0	498.30
agc	S	0	513.00
---	--	------	------
mPD		0.052	1.6
		nPD :	0.03
		N. weight :	3.
		Sc. PD :	-0.6
		Sc. rank :	-3085.8

PB2			
Pos.	880	obs :	exp :
tct	S	0	450.30
tcc	S	0	423.10
tca	S	0	650.10
tcg	S	0	142.30
agt	S	25	498.30
agc	S	2652	513.00
---	--	------	------
mPD		0.019	1.6
		nPD :	0.01
		N. weight :	3.
		Sc. PD :	-0.63
		Sc. rank :	-3338.3

PB2			
Pos.	881	obs :	exp :
att	I	0	949.60
atc	I	0	686.60
ata	I	2677	1041.00
---	--	------	------
mPD		0	0.66
		nPD :	0.
		N. weight :	1.7
		Sc. PD :	-0.39
		Sc. rank :	-2186.1

PB2			
Pos.	882	obs :	exp :
tta	L	0	291.30
ttg	L	0	502.80
ctt	L	2677	540.60
ctc	L	0	389.50
cta	L	0	467.40
ctg	L	0	485.30
---	--	------	------
mPD		0	1.1
		nPD :	0.
		N. weight :	3.
		Sc. PD :	-0.66
		Sc. rank :	-3701.6

PB2			
Pos.	883	obs :	exp :
act	T	2676	658.50
acc	T	1	578.90
aca	T	0	1216.00
acg	T	0	223.20
---	--	------	------
mPD		0.00075	0.68
		nPD :	0.
		N. weight :	2.6
		Sc. PD :	-0.57
		Sc. rank :	-3175.4

PB2			
Pos.	884	obs :	exp :
gat	D	218	1506.00
gac	D	2459	1171.00
---	--	------	------
mPD		0.15	0.49
		nPD :	0.3
		N. weight :	0.97
		Sc. PD :	0.012
		Sc. rank :	160.5

PB2			
Pos.	885	obs :	exp :
tct	S	0	450.30
tcc	S	0	423.10
tca	S	0	650.10
tcg	S	0	142.30
agt	S	86	498.30
agc	S	2591	513.00
---	--	------	------
mPD		0.062	1.6
		nPD :	0.04
		N. weight :	2.8
		Sc. PD :	-0.54
		Sc. rank :	-2656.3

PB2			
Pos.	886	obs :	exp :
caa	Q	0	1444.00
cag	Q	2677	1233.00
---	--	------	------
mPD		0	0.50
		nPD :	0.
		N. weight :	1.4
		Sc. PD :	-0.32
		Sc. rank :	-1793.1

PB2			
Pos.	887	obs :	exp :
---	-	2677	2677.00
---	--	------	------
mPD		0	0
		nPD :	1.
		N. weight :	0.
		Sc. PD :	0
		Sc. rank :	0

PB2			
Pos.	888	obs :	exp :
---	-	2677	2677.00
---	--	------	------
mPD		0	0
		nPD :	1.
		N. weight :	0.
		Sc. PD :	0
		Sc. rank :	0

PB2			
Pos.	889	obs :	exp :
---	-	2677	2677.00
---	--	------	------
mPD		0	0
		nPD :	1.
		N. weight :	0.
		Sc. PD :	0
		Sc. rank :	0

PB2			
Pos.	890	obs :	exp :
---	-	2677	2677.00
---	--	------	------
mPD		0	0
		nPD :	1.
		N. weight :	0.
		Sc. PD :	0
		Sc. rank :	0

PB2			
Pos.	891	obs :	exp :
---	-	2677	2677.00
---	--	------	------
mPD		0	0
		nPD :	1.
		N. weight :	0.
		Sc. PD :	0
		Sc. rank :	0

PB2			
Pos.	892	obs :	exp :
---	-	2677	2677.00
---	--	------	------
mPD		0	0
		nPD :	1.
		N. weight :	0.
		Sc. PD :	0
		Sc. rank :	0

PB2			
Pos.	893	obs :	exp :
---	-	2677	2677.00
---	--	------	------
mPD		0	0
		nPD :	1.
		N. weight :	0.
		Sc. PD :	0
		Sc. rank :	0

PB2			
Pos.	894	obs :	exp :
---	-	2677	2677.00
---	--	------	------
mPD		0	0
		nPD :	1.
		N. weight :	0.
		Sc. PD :	0
		Sc. rank :	0

PB2			
Pos.	895	obs :	exp :
---	-	2677	2677.00
---	--	------	------
mPD		0	0
		nPD :	1.
		N. weight :	0.
		Sc. PD :	0
		Sc. rank :	0

PB2			
Pos.	896	obs :	exp :
---	-	2677	2677.00
---	--	------	------
mPD		0	0
		nPD :	1.
		N. weight :	0.
		Sc. PD :	0
		Sc. rank :	0

PB2			
Pos.	897	obs :	exp :
---	-	2677	2677.00
---	--	------	------
mPD		0	0
		nPD :	1.
		N. weight :	0.
		Sc. PD :	0
		Sc. rank :	0

PB2			
Pos.	898	obs :	exp :
---	-	2677	2677.00
---	--	------	------
mPD		0	0
		nPD :	1.
		N. weight :	0.
		Sc. PD :	0
		Sc. rank :	0

PB2			
Pos.	899	obs:	exp:
---	-	2677	2677.00
---	--	------	------
mPD		0	0
		nPD:	1.
		N. weight:	0.
		Sc. PD:	0
		Sc. rank:	0

PB2			
Pos.	900	obs:	exp:
---	-	2677	2677.00
---	--	------	------
mPD		0	0
		nPD:	1.
		N. weight:	0.
		Sc. PD:	0
		Sc. rank:	0

PB2			
Pos.	901	obs:	exp:
---	-	2677	2677.00
---	--	------	------
mPD		0	0
		nPD:	1.
		N. weight:	0.
		Sc. PD:	0
		Sc. rank:	0

PB2			
Pos.	902	obs:	exp:
---	-	2677	2677.00
---	--	------	------
mPD		0	0
		nPD:	1.
		N. weight:	0.
		Sc. PD:	0
		Sc. rank:	0

PB2			
Pos.	903	obs:	exp:
---	-	2677	2677.00
---	--	------	------
mPD		0	0
		nPD:	1.
		N. weight:	0.
		Sc. PD:	0
		Sc. rank:	0

PB2			
Pos.	904	obs:	exp:
---	-	2677	2677.00
---	--	------	------
mPD		0	0
		nPD:	1.
		N. weight:	0.
		Sc. PD:	0
		Sc. rank:	0

PB2			
Pos.	905	obs:	exp:
---	-	2677	2677.00
---	--	------	------
mPD		0	0
		nPD:	1.
		N. weight:	0.
		Sc. PD:	0
		Sc. rank:	0

PB2			
Pos.	906	obs:	exp:
---	-	2677	2677.00
---	--	------	------
mPD		0	0
		nPD:	1.
		N. weight:	0.
		Sc. PD:	0
		Sc. rank:	0

PB2			
Pos.	907	obs:	exp:
---	-	2677	2677.00
---	--	------	------
mPD		0	0
		nPD:	1.
		N. weight:	0.
		Sc. PD:	0
		Sc. rank:	0

PB2			
Pos.	908	obs:	exp:
---	-	2677	2677.00
---	--	------	------
mPD		0	0
		nPD:	1.
		N. weight:	0.
		Sc. PD:	0
		Sc. rank:	0

PB2			
Pos.	909	obs:	exp:
---	-	2677	2677.00
---	--	------	------
mPD		0	0
		nPD:	1.
		N. weight:	0.
		Sc. PD:	0
		Sc. rank:	0

PB2			
Pos.	910	obs:	exp:
---	-	2677	2677.00
---	--	------	------
mPD		0	0
		nPD:	1.
		N. weight:	0.
		Sc. PD:	0
		Sc. rank:	0

PB2			
Pos.	911	obs:	exp:
---	-	2677	2677.00
---	--	------	------
mPD		0	0
		nPD:	1.
		N. weight:	0.
		Sc. PD:	0
		Sc. rank:	0

PB2			
Pos.	912	obs:	exp:
---	-	2677	2677.00
---	--	------	------
mPD		0	0
		nPD:	1.
		N. weight:	0.
		Sc. PD:	0
		Sc. rank:	0

PB2			
Pos.	913	obs:	exp:
---	-	2677	2677.00
---	--	------	------
mPD		0	0
		nPD:	1.
		N. weight:	0.
		Sc. PD:	0
		Sc. rank:	0

PB2			
Pos.	914	obs:	exp:
---	-	2677	2677.00
---	--	------	------
mPD		0	0
		nPD:	1.
		N. weight:	0.
		Sc. PD:	0
		Sc. rank:	0

PB2			
Pos.	915	obs:	exp:
---	-	2677	2677.00
---	--	------	------
mPD		0	0
		nPD:	1.
		N. weight:	0.
		Sc. PD:	0
		Sc. rank:	0

PB2			
Pos.	916	obs:	exp:
---	-	2677	2677.00
---	--	------	------
mPD		0	0
		nPD:	1.
		N. weight:	0.
		Sc. PD:	0
		Sc. rank:	0

PB2			
Pos.	917	obs:	exp:
---	-	2677	2677.00
---	--	------	------
mPD		0	0
		nPD:	1.
		N. weight:	0.
		Sc. PD:	0
		Sc. rank:	0

PB2			
Pos.	918	obs:	exp:
---	-	2677	2677.00
---	--	------	------
mPD		0	0
		nPD:	1.
		N. weight:	0.
		Sc. PD:	0
		Sc. rank:	0

PB2			
Pos.	919	obs:	exp:
---	-	2677	2677.00
---	--	------	------
mPD		0	0
		nPD:	1.
		N. weight:	0.
		Sc. PD:	0
		Sc. rank:	0

PB2			
Pos.	920	obs:	exp:
---	-	2677	2677.00
---	--	------	------
mPD		0	0
		nPD:	1.
		N. weight:	0.
		Sc. PD:	0
		Sc. rank:	0

PB2			
Pos.	921	obs:	exp:
---	-	2677	2677.00
---	--	------	------
mPD		0	0
		nPD:	1.
		N. weight:	0.
		Sc. PD:	0
		Sc. rank:	0

PB2			
Pos.	922	obs:	exp:
---	-	2677	2677.00
---	--	------	------
mPD		0	0
		nPD:	1.
		N. weight:	0.
		Sc. PD:	0
		Sc. rank:	0

PB2			
Pos.	923	obs:	exp:
---	-	2677	2677.00
---	--	------	------
mPD		0	0
		nPD:	1.
		N. weight:	0.
		Sc. PD:	0
		Sc. rank:	0

PB2			
Pos.	924	obs:	exp:
---	-	2677	2677.00
---	--	------	------
mPD		0	0
		nPD:	1.
		N. weight:	0.
		Sc. PD:	0
		Sc. rank:	0

PB2			
Pos.	925	obs:	exp:
---	-	2677	2677.00
---	--	------	------
mPD		0	0
		nPD:	1.
		N. weight:	0.
		Sc. PD:	0
		Sc. rank:	0

PB2			
Pos.	926	obs:	exp:
---	-	2677	2677.00
---	--	------	------
mPD		0	0
		nPD:	1.
		N. weight:	0.
		Sc. PD:	0
		Sc. rank:	0

PB2			
Pos.	927	obs:	exp:
---	-	2677	2677.00
---	--	------	------
mPD		0	0
		nPD:	1.
		N. weight:	0.
		Sc. PD:	0
		Sc. rank:	0

PB2			
Pos.	928	obs:	exp:
---	-	2677	2677.00
---	--	------	------
mPD		0	0
		nPD:	1.
		N. weight:	0.
		Sc. PD:	0
		Sc. rank:	0

PB2			
Pos.	929	obs:	exp:
---	-	2677	2677.00
---	--	------	------
mPD		0	0
		nPD:	1.
		N. weight:	0.
		Sc. PD:	0
		Sc. rank:	0

PB2			
Pos.	930	obs:	exp:
---	-	2677	2677.00
---	--	------	------
mPD		0	0
		nPD:	1.
		N. weight:	0.
		Sc. PD:	0
		Sc. rank:	0

PB2			
Pos.	931	obs:	exp:
---	-	2677	2677.00
---	--	------	------
mPD		0	0
		nPD:	1.
		N. weight:	0.
		Sc. PD:	0
		Sc. rank:	0

PB2			
Pos.	932	obs:	exp:
---	-	2677	2677.00
---	--	------	------
mPD		0	0
		nPD:	1.
		N. weight:	0.
		Sc. PD:	0
		Sc. rank:	0

PB2			
Pos.	933	obs:	exp:
---	-	2677	2677.00
---	--	------	------
mPD		0	0
		nPD:	1.
		N. weight:	0.
		Sc. PD:	0
		Sc. rank:	0

PB2			
Pos.	934	obs:	exp:
---	-	2677	2677.00
---	--	------	------
mPD		0	0
		nPD:	1.
		N. weight:	0.
		Sc. PD:	0
		Sc. rank:	0

PB2			
Pos.	935	obs:	exp:
---	-	2677	2677.00
---	--	------	------
mPD		0	0
		nPD:	1.
		N. weight:	0.
		Sc. PD:	0
		Sc. rank:	0

PB2			
Pos.	936	obs:	exp:
---	-	2677	2677.00
---	--	------	------
mPD		0	0
		nPD:	1.
		N. weight:	0.
		Sc. PD:	0
		Sc. rank:	0

PB2			
Pos.	937	obs:	exp:
---	-	2677	2677.00
---	--	------	------
mPD		0	0
		nPD:	1.
		N. weight:	0.
		Sc. PD:	0
		Sc. rank:	0

PB2			
Pos.	938	obs:	exp:
---	-	2677	2677.00
---	--	------	------
mPD		0	0
		nPD:	1.
		N. weight:	0.
		Sc. PD:	0
		Sc. rank:	0

PB2			
Pos.	939	obs:	exp:
---	-	2677	2677.00
---	--	------	------
mPD		0	0
		nPD:	1.
		N. weight:	0.
		Sc. PD:	0
		Sc. rank:	0

PB2			
Pos.	940	obs:	exp:
---	-	2677	2677.00
---	--	------	------
mPD		0	0
		nPD:	1.
		N. weight:	0.
		Sc. PD:	0
		Sc. rank:	0

PB2			
Pos.	941	obs:	exp:
---	-	2677	2677.00
---	--	------	------
mPD		0	0
		nPD:	1.
		N. weight:	0.
		Sc. PD:	0
		Sc. rank:	0

PB2			
Pos.	942	obs:	exp:
---	-	2677	2677.00
---	--	------	------
mPD		0	0
		nPD:	1.
		N. weight:	0.
		Sc. PD:	0
		Sc. rank:	0

PB2			
Pos.	943	obs:	exp:
---	-	2677	2677.00
---	--	------	------
mPD		0	0
		nPD:	1.
		N. weight:	0.
		Sc. PD:	0
		Sc. rank:	0

PB2			
Pos.	944	obs:	exp:
---	-	2677	2677.00
---	--	------	------
mPD		0	0
		nPD:	1.
		N. weight:	0.
		Sc. PD:	0
		Sc. rank:	0

PB2			
Pos.	945	obs:	exp:
---	-	2677	2677.00
---	--	------	------
mPD		0	0
		nPD:	1.
		N. weight:	0.
		Sc. PD:	0
		Sc. rank:	0

PB2			
Pos.	946	obs:	exp:
---	-	2677	2677.00
---	--	------	------
mPD		0	0
		nPD:	1.
		N. weight:	0.
		Sc. PD:	0
		Sc. rank:	0

PB2			
Pos.	947	obs:	exp:
---	-	2677	2677.00
---	--	------	------
mPD		0	0
		nPD:	1.
		N. weight:	0.
		Sc. PD:	0
		Sc. rank:	0

PB2			
Pos.	948	obs:	exp:
---	-	2677	2677.00
---	--	------	------
mPD		0	0
		nPD:	1.
		N. weight:	0.
		Sc. PD:	0
		Sc. rank:	0

PB2			
Pos.	949	obs:	exp:
---	-	2677	2677.00
---	--	------	------
mPD		0	0
		nPD:	1.
		N. weight:	0.
		Sc. PD:	0
		Sc. rank:	0

PB2			
Pos.	950	obs:	exp:
---	-	2677	2677.00
---	--	------	------
mPD		0	0
		nPD:	1.
		N. weight:	0.
		Sc. PD:	0
		Sc. rank:	0

PB2			
Pos.	951	obs:	exp:
---	-	2677	2677.00
---	--	------	------
mPD		0	0
		nPD:	1.
		N. weight:	0.
		Sc. PD:	0
		Sc. rank:	0

PB2			
Pos.	952	obs:	exp:
---	-	2677	2677.00
---	--	------	------
mPD		0	0
		nPD:	1.
		N. weight:	0.
		Sc. PD:	0
		Sc. rank:	0

PB2			
Pos.	953	obs:	exp:
---	-	2677	2677.00
---	--	------	------
mPD		0	0
		nPD:	1.
		N. weight:	0.
		Sc. PD:	0
		Sc. rank:	0

PB2			
Pos.	954	obs:	exp:
---	-	2677	2677.00
---	--	------	------
mPD		0	0
		nPD:	1.
		N. weight:	0.
		Sc. PD:	0
		Sc. rank:	0

PB2			
Pos.	955	obs:	exp:
act	T	0	658.50
acc	T	0	578.90
aca	T	2677	1216.00
acg	T	0	223.20
---	--	------	------
mPD		0	0.68
		nPD:	0.
		N. weight:	1.5
		Sc. PD:	-0.32
		Sc. rank:	-1825.2

PB2			
Pos.	956	obs:	exp:
gct	A	1	656.20
gcc	A	0	613.40
gca	A	0	1207.00
gcg	A	2676	200.80
---	--	------	------
mPD		0.00075	0.68
		nPD:	0.
		N. weight:	4.8
		Sc. PD:	-1.1
		Sc. rank:	-5809.6

PB2			
Pos.	957	obs:	exp:
act	T	0	658.00
acc	T	2675	578.40
aca	T	0	1216.00
acg	T	0	223.10
gct	A	0	0.49
gcc	A	2	0.46
gca	A	0	0.90
gcg	A	0	0.15
---	--	------	------
mPD		0.0015	0.68
		nPD:	0.
		N. weight:	2.8
		Sc. PD:	-0.62
		Sc. rank:	-3378.1

PB2			
Pos.	958	obs:	exp:
aaa	K	2677	1627.00
aag	K	0	1050.00
---	--	------	------
mPD		0	0.48
		nPD:	0.
		N. weight:	0.92
		Sc. PD:	-0.2
		Sc. rank:	-1152.4

PB2			
Pos.	959	obs:	exp:
cgt	R	0	84.50
cgc	R	0	141.50
cga	R	1	269.10
cgg	R	0	193.40
aga	R	1574	1228.00
agg	R	1102	760.90
---	--	------	------
mPD		0.49	0.94
		nPD:	0.52
		N. weight:	0.55
		Sc. PD:	0.097
		Sc. rank:	439.9

PB2			
Pos.	960	obs:	exp:
tta	L	0	1.74
ttg	L	0	3.01
ctt	L	16	3.23
ctc	L	0	2.33
cta	L	0	2.79
ctg	L	0	2.90
att	I	2643	944.00
atc	I	16	682.50
ata	I	2	1035.00
---	--	------	------
mPD		0.025	0.67
		nPD:	0.04
		N. weight:	1.9
		Sc. PD:	-0.36
		Sc. rank:	-1783.3

PB2			
Pos.	961	obs:	exp:
cgt	R	9	84.50
cgc	R	0	141.50
cga	R	203	269.10
cgg	R	2465	193.40
aga	R	0	1228.00
agg	R	0	760.90
---	--	------	------
mPD		0.15	0.94
		nPD:	0.16
		N. weight:	4.3
		Sc. PD:	-0.44
		Sc. rank:	-1212.8

PB2			
Pos.	962	obs:	exp:
tta	L	0	33.52
ttg	L	308	57.85
ctt	L	0	62.20
ctc	L	0	44.81
cta	L	0	53.78
ctg	L	0	55.83
atg	M	2361	2361.00
gtt	V	0	1.78
gtc	V	0	1.51
gta	V	0	2.02
gtg	V	8	2.69
---	--	------	------
mPD		0.21	0.36
		nPD:	0.59
		N. weight:	0.36
		Sc. PD:	0.083
		Sc. rank:	342.9

PB2			
Pos .	963	obs :	exp :
gtt	V	0	5.11
gtc	V	23	4.34
gta	V	0	5.81
gtg	V	0	7.75
gct	A	0	650.60
gcc	A	2653	608.10
gca	A	1	1196.00
gcg	A	0	199.00
---	--	------	------
mPD		0.018	0.70
		nPD :	0.03
		N. weight :	2.7
		Sc . PD :	-0.55
		Sc . rank :	-2871.2

,

PB2			
Pos .	964	obs :	exp :
att	I	15	942.60
atc	I	2641	681.50
ata	I	1	1033.00
act	T	0	4.67
acc	T	19	4.11
aca	T	0	8.63
acg	T	0	1.58
aat	N	0	0.57
aac	N	1	0.43
---	--	------	------
mPD		0.027	0.67
		nPD :	0.04
		N. weight :	2.5
		Sc . PD :	-0.47
		Sc . rank :	-2289.1

,

PB2			
Pos .	965	obs :	exp :
aat	N	2664	1520.00
aac	N	11	1155.00
aaa	K	1	0.61
aag	K	0	0.39
gat	D	1	0.56
gac	D	0	0.44
---	--	------	------
mPD		0.0097	0.49
		nPD :	0.02
		N. weight :	1.
		Sc . PD :	-0.21
		Sc . rank :	-1094.5

,

PB2			
Pos .	966	obs :	exp :
taa	.	228	1139.00
tag	.	2449	705.20
tga	.	0	832.90
---	--	------	------
mPD		0.16	0.82
		nPD :	0.19
		N. weight :	1.9
		Sc . PD :	-0.14
		Sc . rank :	-264.2

}

Amino acid level conservation per codon

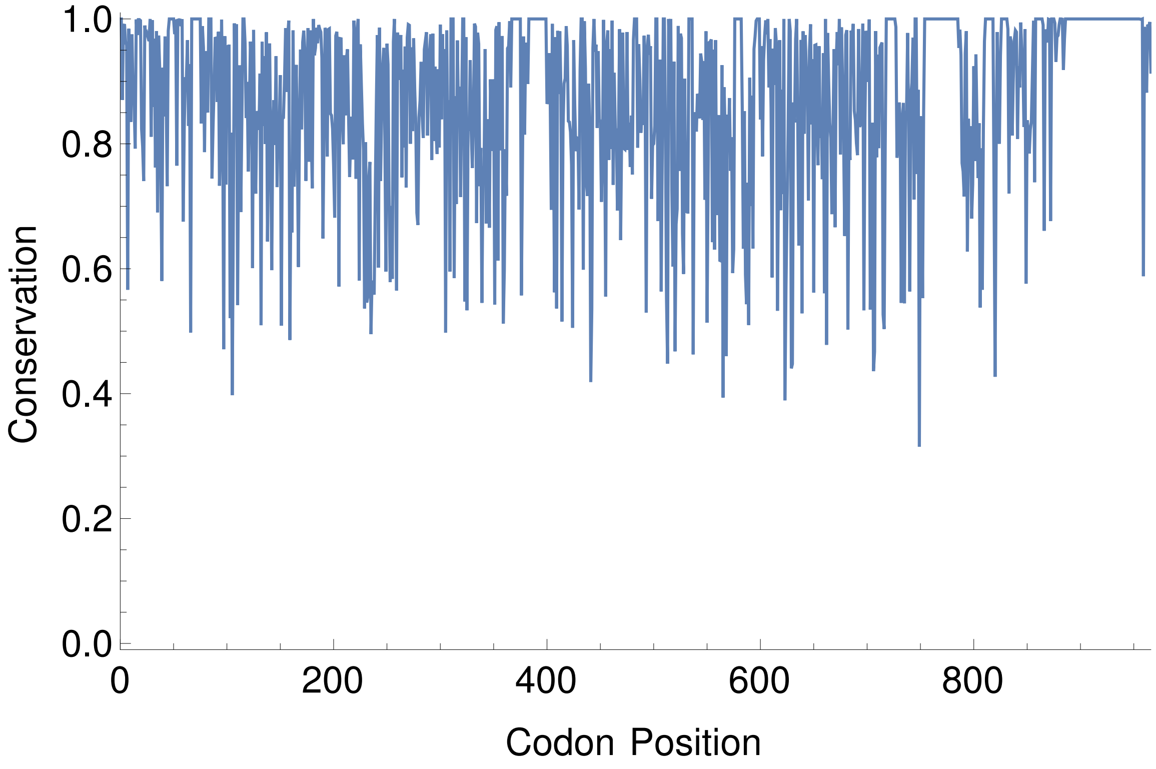

Conservation analysis plots

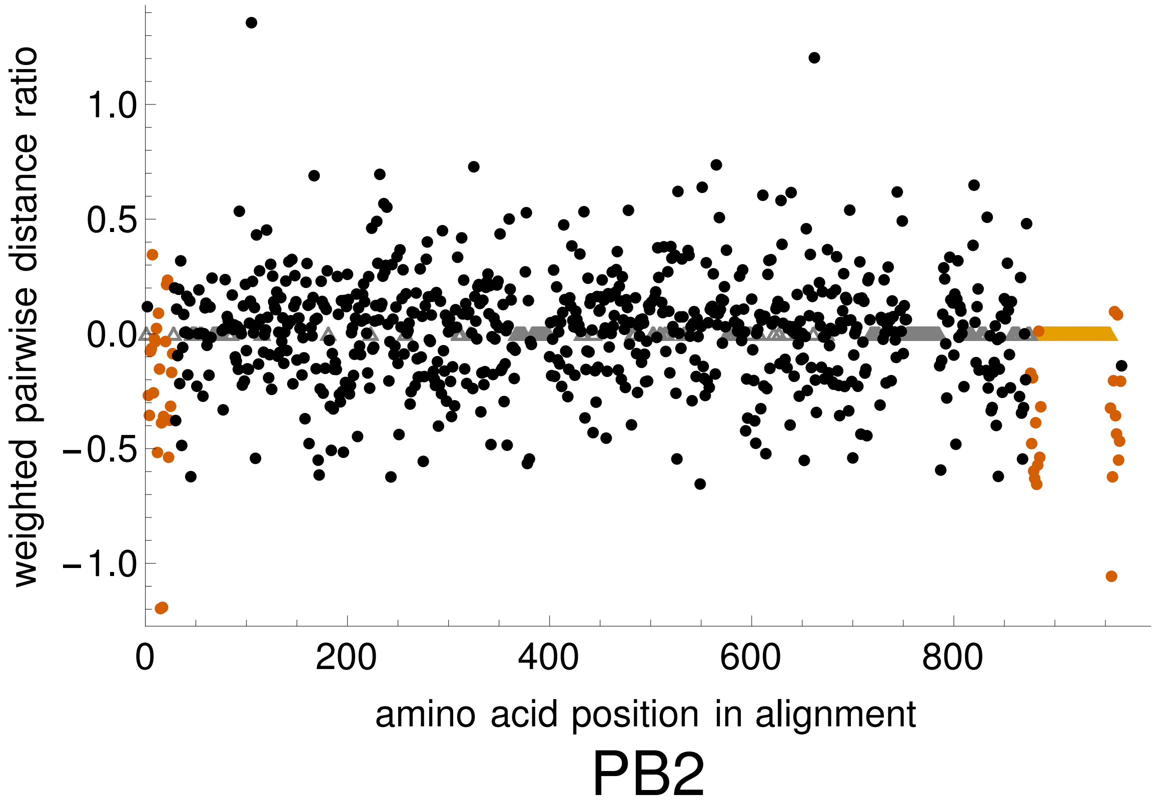

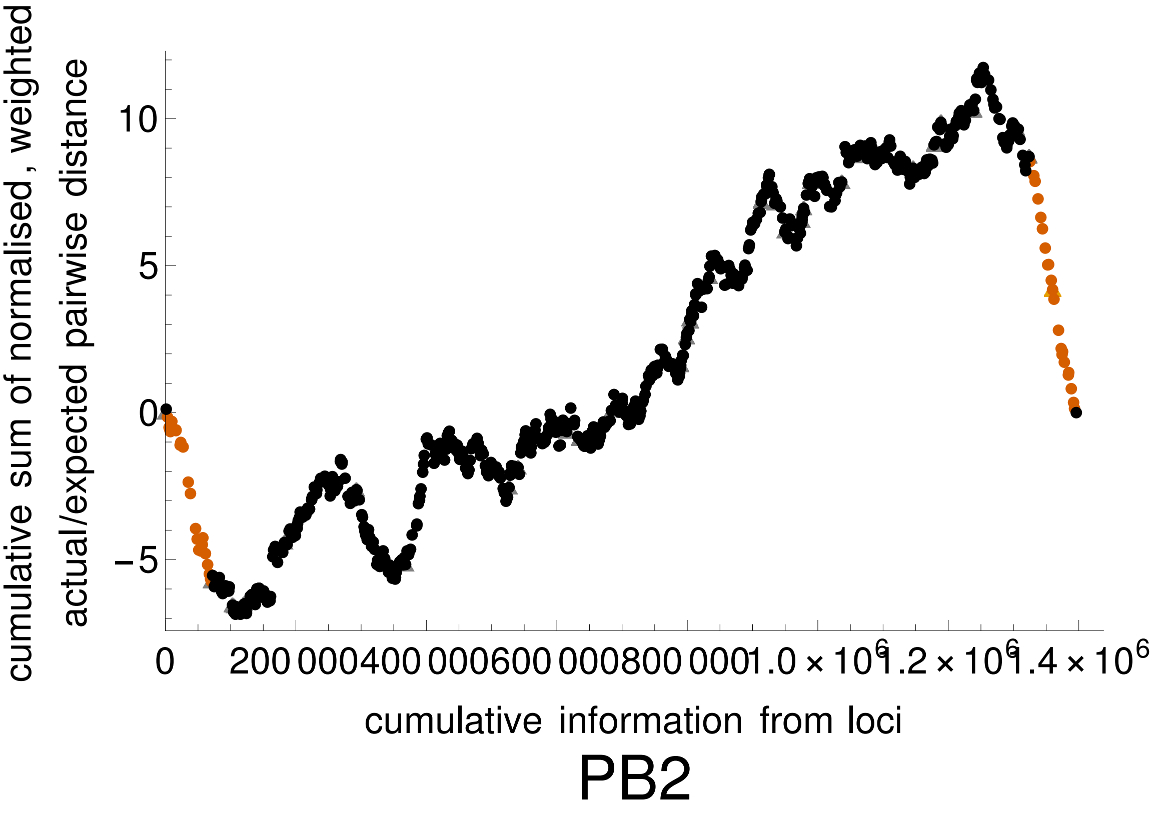

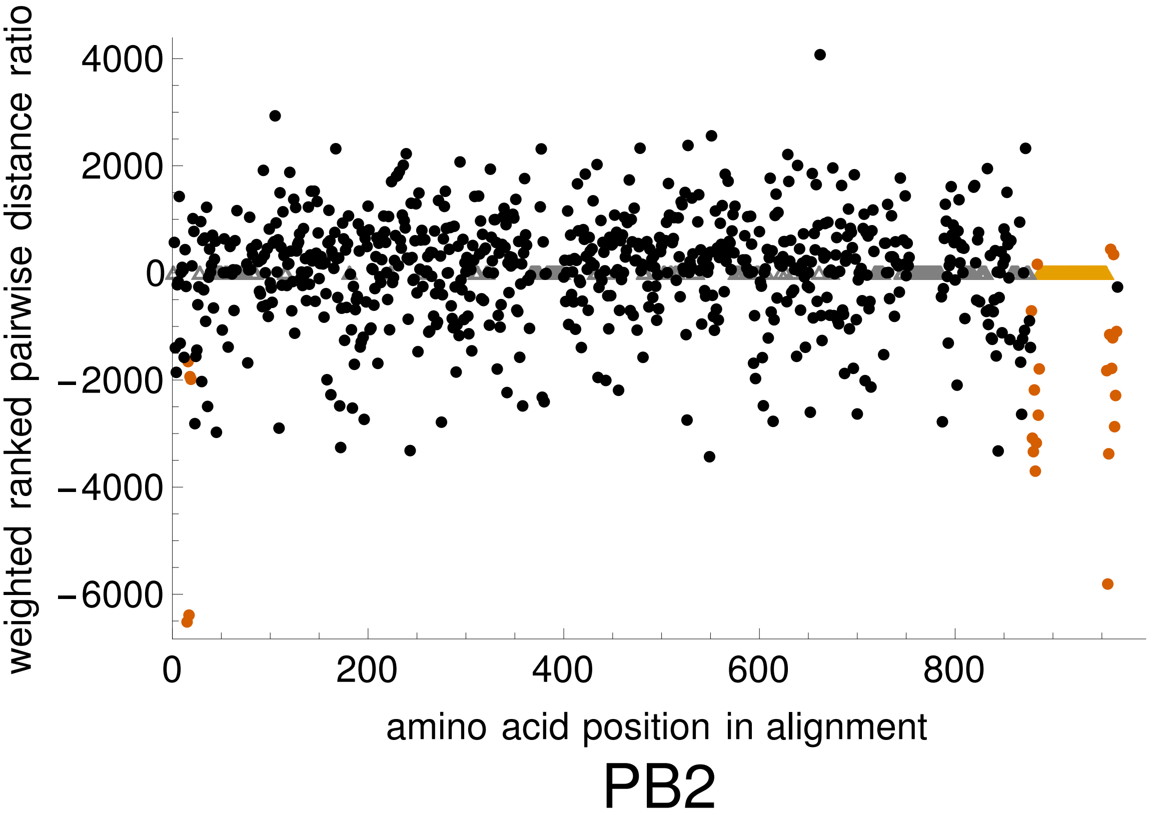

weighted ranked pairwise distance ratio
4000
2000
0
−2000
−4000
−6000
0
200
400
600
800
amino acid position in alignment
PB2

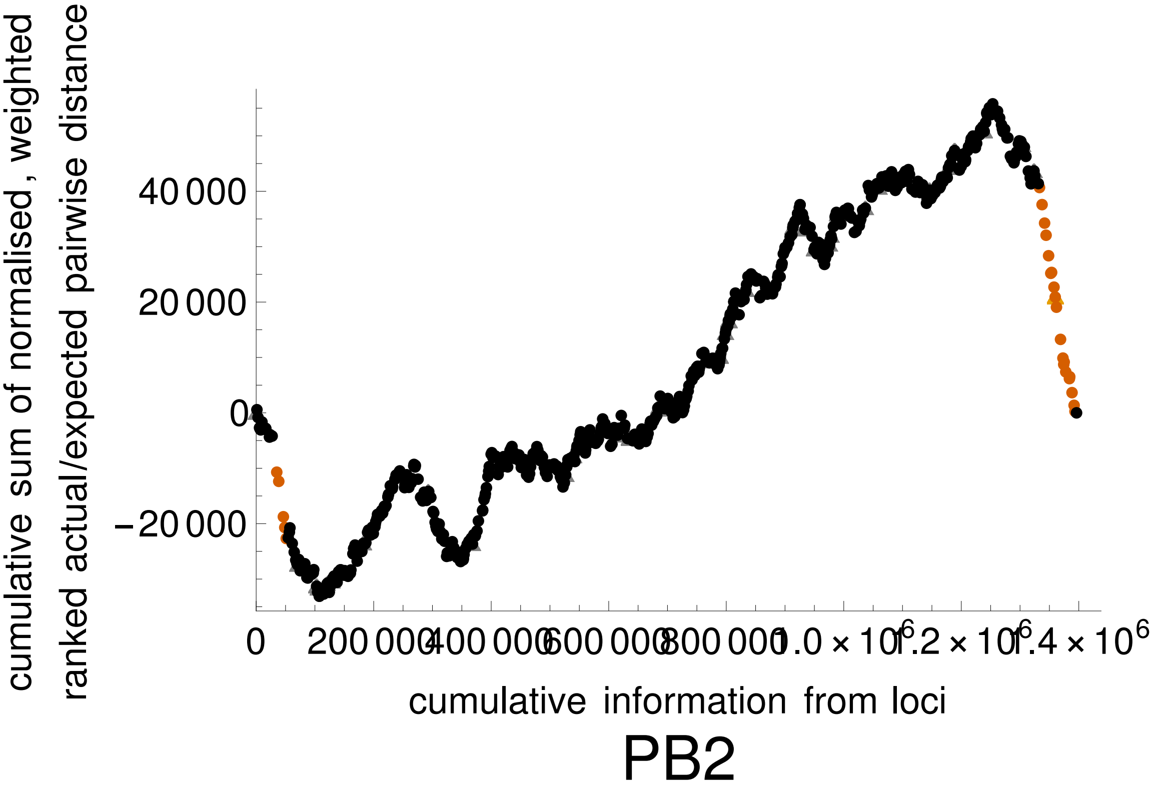

cumulative sum of normalised, weighted ranked actual/expected pairwise distance
40 000
20 000
0
−20 000
0
200 000
400 000
600 000
800 000
1.0 × 10^6
1.2 × 10^6
1.4 × 10^6
cumulative information from loci
PB2

PB1

Gene length histogram

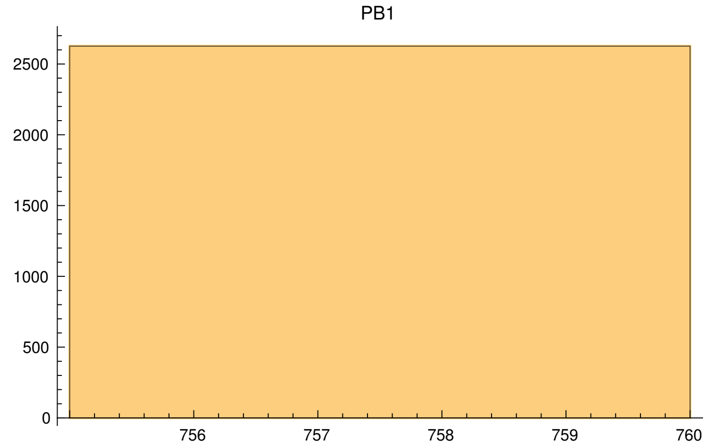

Information vs. nPD

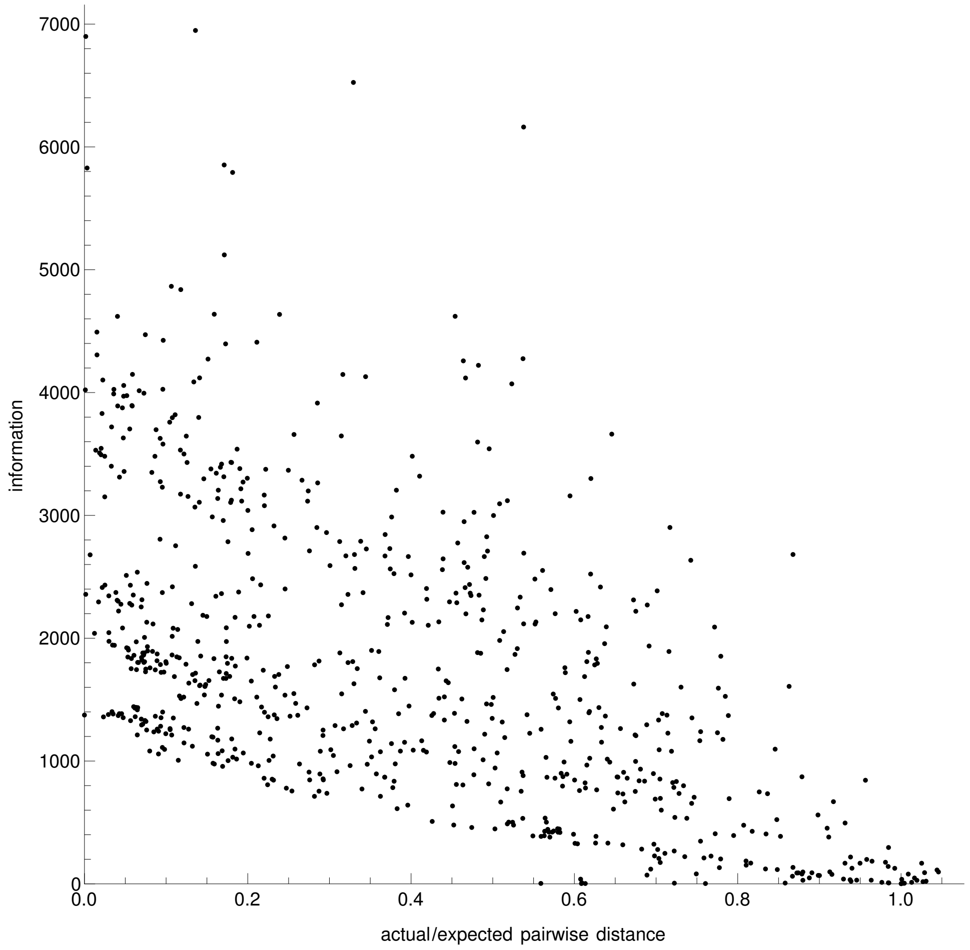

PB1

Example sequences highlighted by regions found in analyses to be conserved

Interesting points (by weighted raw PD) highlighted for gene PB1:

ATGGATGTCAACCCGACTCTACTTTTCTTAAAGGTGCCAGCACAAAATGCTATAAGCACTACATTCCCTTACACTG⋱
GAGATCCCCCATACAGTCATGGAACAGGGACAGGATACACCATGGACACGGTCAACAGAACACATCAATATTCAG⋱
AAAGAGGAAGATGGACAACAAACACAGAAACTGGAGCACCCCAGCTTAACCCGATTGATGGACCACTACCTGAGG⋱
ACAATGAGCCAAGCGGGTATGCTCAAACAGACTGTGTCCTGGAGGCAATGGCTTTCCTTGAGGAATCCCATCCAG⋱
GGATATTTGAAAACTCTTGTCTTGAGACGATGGAGGTTGTCCAACAAACTAGAGTGGACAAATTGACTCAAGGTC⋱
GTCAGACCTATGACTGGACGTTAAATAGAAACCAACCTGCTGCAACTGCTTTGGCCAACACAATAGAGGTCTTCA⋱
GGATGAACAATCTAACAGCCAATGAATCTGGAAGATTAATAGATTTCCTTAAAGATGTGATGGAATCAATGGATA⋱
AAGAAGAGATGGAAATAACAACACATTTCCAAAGGAAAAGAAGAATAAGGGACAACATGACCAAGAAAATGGTAA⋱
CACAAAGGACAATAGGAAAGAAAAAGCAGAAACTGAGCAAGAAAAGTTATCTAATAAGAGCATTGACATTAAACA⋱
CAATGACAAAAGATGCAGAAAGAGGCAAGTTGAAAAGGCGTGCAATTGCAACACCAGGGATGCAGATCAGAGGAT⋱
TTGTGTACTTCGTAGAAACACTGGCGAGGAGCATCTGTGAGAAACTTGAGCAATCTGGGCTCCCAGTTGGGGGAA⋱
ATGAAAAGAAAGCCAAATTGGCAAATGTTGTGAGGAAAATGATGACCAACTCACAAGATACAGAGCTTTCCTTTA⋱
CAATTACTGGAGATAACACCAAATGGAATGAGAATCAAAACCCCAGGATATTTCTGGCGATGATAACGTACATCA⋱
CAAGAAATCAGCCTGAATGGTTCAGAAATGTTTTAAGCATCGCCCCTATAATGTTCTCAAACAAAGTGGCAAGAT⋱
TAGGGAAAGGATACATGTTCGAAAGTAAAAGCATGAAGCTACGAACACAAATACCAGCAGAGATGCTCGCAGATA⋱
TTGACTTGAAATACTTCAATGAATCAACAAGGGAAAAGATTAAGAAGATAAGGCCACTTCTCATAGATGGCACGG⋱
CCTCATTAAGCCCTGGAATGATGATGGGCATGTTTAACATGCTGAGCACAGTTTTAGGAGTCTCAATCCTGAATC⋱
TCGGGCAAAAGAGGTACACTAAAACCACCTACTGGTGGGATGGACTCCAATCTTCTGATGACTTCGCCCTCATAG⋱
TGAATGCACCTAATCATGAAGGAATACAAGCAGGAGTGGATAGGTTCTACAGGACATGCAAACTAGTTGGAATCA⋱
ATATGAGCAAAAAGAAATCTTACATAAATAGGACGGGAACATTTGAATTCACAAGTTTTTTCTACCGCTATGGGT⋱
TTGTAGCCAATTTCAGTATGGAGCTACCCAGCTTTGGGGTATCTGGAGTTAATGAGTCGGCTGACATGAGCATTG⋱
GAGTGACAGTAATAAAGAACAACATGATAAACAATGATCTTGGACCAGCAACAGCCCAAATGGCTCTTCAGCTAT⋱
TCATTAAAGACTACAGATATACATACCGATGCCACAGAAGTGACACGCAAATCCAAACGAAAAGATCATTCGAGC⋱
TAAAAAAGCTATGGGAGCAAACCCACTCGAAGGCAGGGCTACTGGTTTCAGATGGGGGACCAAATCTGTACAATA⋱
TTCGGAATCTCCACATCCCCGAAGTCTGCTTGAAATGGGAGCTAATGGATGTAGATTACCGTGGAAGATTGTGTA⋱
ATCCTCTGAATCCGTTTGTTAACCACAAAGGAATTGAGTCCGTAAACAGTGCTGTAATAATGCCAGCTCATGGTC⋱
CAGCTAAAAGCATGGAATATGACGCTGTTGCAACTACACATTCTTGGATTCCAAAAAGAAATCGTTCCATTCTCA⋱
ATACTAGCCAAAGGGGAATTCTTGAGGATGAACAAATGTACCAGAAGTGCTGCAATCTATTCGAGAAATTCTTCC⋱
CTAGCAGTTCATACAGGAGGCCAGTTGGAATTTCAAGCATGGTGGAGGCCATGGTATCTAGGGCCAGAATTGATG⋱
CACGGATTGATTTCGAGTCTGGAAGGATTAAGAAAGGAGAATTTGCT
GAGATCATGAAGATCTGTTCCACCATTGAAGAGCTCAGACGG---CAA---AAG---TAG

Interesting points (by weighted ranked PD) highlighted for gene PB1:

ATGGATGTCAACCCGACTCTACTTTTCTTAAAGGTGCCAGCACAAAATGCTATAAGCACTACATTCCCTTACACTGGA⋱
GATCCCCCATACAGTCATGGAACAGGGACAGGATACACCATGGACACGGTCAACAGAACACATCAATATTCAGAAAG⋱
AGGAAGATGGACAACAAACACAGAAACTGGAGCACCCCAGCTTAACCCGATTGATGGACCACTACCTGAGGACAATG⋱
AGCCAAGCGGGTATGCTCAAACAGACTGTGTCCTGGAGGCAATGGCTTTCCTTGAGGAATCCCATCCAGGGATATTT⋱
GAAAACTCTTGTCTTGAGACGATGGAGGTTGTCCAACAAACTAGAGTGGACAAATTGACTCAAGGTCGTCAGACCTA⋱
TGACTGGACGTTAAATAGAAACCAACCTGCTGCAACTGCTTTGGCCAACACAATAGAGGTCTTCAGGATGAACAATC⋱
TAACAGCCAATGAATCTGGAAGATTAATAGATTTCCTTAAAGATGTGATGGAATCAATGGATAAAGAAGAGATGGAA⋱
ATAACAACACATTTCCAAAGGAAAAGAAGAATAAGGGACAACATGACCAAGAAAATGGTAACACAAAGGACAATAGG⋱
AAAGAAAAAGCAGAAACTGAGCAAGAAAAGTTATCTAATAAGAGCATTGACATTAAACACAATGACAAAAGATGCAG⋱
AAAGAGGCAAGTTGAAAAGGCGTGCAATTGCAACACCAGGGATGCAGATCAGAGGATTTGTGTACTTCGTAGAAACA⋱

CTGGCGAGGAGCATCTGTGAGAAACTTGAGCAATCTGGGCTCCCAGTTGGGGGAAATGAAAAGAAAGCCAAATTGGC⋱
AAATGTTGTGAGGAAAATGATGACCAACTCACAAGATACAGAGCTTTCCTTTACAATTACTGGAGATAACACCAAAT⋱
GGAATGAGAATCAAAACCCCAGGATATTTCTGGCGATGATAACGTACATCACAAGAAATCAGCCTGAATGGTTCAGA⋱
AATGTTTTAAGCATCGCCCCTATAATGTTCTCAAACAAAGTGGCAAGATTAGGGAAAGGATACATGTTCGAAAGTAA⋱
AAGCATGAAGCTACGAACACAAATACCAGCAGAGATGCTCGCAGATATTGACTTGAAATACTTCAATGAATCAACAA⋱
GGGAAAAGATTAAGAAGATAAGGCCACTTCTCATAGATGGCACGGCCTCATTAAGCCCTGGAATGATGATGGGCATG⋱
TTTAACATGCTGAGCACAGTTTTAGGAGTCTCAATCCTGAATCTCGGGCAAAAGAGGTACACTAAAACCACCTACTG⋱
GTGGGATGGACTCCAATCTTCTGATGACTTCGCCCTCATAGTGAATGCACCTAATCATGAAGGAATACAAGCAGGAG⋱
TGGATAGGTTCTACAGGACATGCAAACTAGTTGGAATCAATATGAGCAAAAAGAAATCTTACATAAATAGGACGGGA⋱
ACATTTGAATTCACAAGTTTTTTCTACCGCTATGGGTTTGTAGCCAATTTCAGTATGGAGCTACCCAGCTTTGGGGT⋱
ATCTGGAGTTAATGAGTCGGCTGACATGAGCATTGGAGTGACAGTAATAAAGAACAACATGATAAACAATGATCTTG⋱
GACCAGCAACAGCCCAAATGGCTCTTCAGCTATTCATTAAAGACTACAGATATACATACCGATGCCACAGAAGTGAC⋱
ACGCAAATCCAAACGAAAAGATCATTCGAGCTAAAAAAGCTATGGGAGCAAACCCACTCGAAGGCAGGGCTACTGGT⋱
TTCAGATGGGGGACCAAATCTGTACAATATTCGGAATCTCCACATCCCCGAAGTCTGCTTGAAATGGGAGCTAATGG⋱
ATGTAGATTACCGTGGAAGATTGTGTAATCCTCTGAATCCGTTTGTTAACCACAAAGGAATTGAGTCCGTAAACAGT⋱
GCTGTAATAATGCCAGCTCATGGTCCAGCTAAAAGCATGGAATATGACGCTGTTGCAACTACACATTCTTGGATTCC⋱
AAAAAGAAATCGTTCCATTCTCAATACTAGCCAAAGGGGAATTCTTGAGGATGAACAAATGTACCAGAAGTGCTGCA⋱
ATCTATTCGAGAAATTCTTCCCTAGCAGTTCATACAGGAGGCCAGTTGGAATTTCAAGCATGGTGGAGGCCATGGTA⋱
TCTAGGGCCAGAATTGATGCACGGATTGATTTCGAGTCTGGAAGGATTAAGAAAGGAGAATTTGCT
GAGATCATGAAGATCTGTTCCACCATTGAAGAGCTCAGACGG---CAA---AAG---TAG

Per codon conservation report

PB1			
Pos.	1	obs:	exp:
atg	M	2626	2626.00
---	--	------	------
mPD		0	0
		nPD:	1.
		N. weight:	0.
		Sc. PD:	0
		Sc. rank:	0

PB1			
Pos.	2	obs:	exp:
gtt	V	0	0.22
gtc	V	0	0.19
gta	V	1	0.25
gtg	V	0	0.34
gat	D	2605	1476.00
gac	D	20	1149.00
---	--	------	------
mPD		0.017	0.49
		nPD:	0.03
		N. weight:	0.83
		Sc. PD:	-0.2
		Sc. rank:	-1050.4

PB1			
Pos.	3	obs:	exp:
att	I	0	1.77
atc	I	5	1.28
ata	I	0	1.94
gtt	V	3	582.00
gtc	V	2611	494.60
gta	V	7	661.60
gtg	V	0	882.80
---	--	------	------
mPD		0.011	0.74
		nPD:	0.02
		N. weight:	2.6
		Sc. PD:	-0.66
		Sc. rank:	-3517.4

PB1			
Pos.	4	obs:	exp:
aat	N	2234	1493.00
aac	N	392	1133.00
---	--	------	------
mPD		0.25	0.49
		nPD:	0.52
		N. weight:	0.29
		Sc. PD:	0.066
		Sc. rank:	272.6

PB1			
Pos.	5	obs:	exp:
cct	P	1	774.90
ccc	P	1	528.70
cca	P	1	1040.00
ccg	P	2623	282.50
---	--	------	------
mPD		0.0023	0.70
		nPD:	0.
		N. weight:	3.5
		Sc. PD:	-0.93
		Sc. rank:	-4894.6

PB1			
Pos.	6	obs:	exp:
att	I	16	8.51
atc	I	8	6.16
ata	I	0	9.33
act	T	2008	639.50
acc	T	590	562.20
aca	T	2	1181.00
acg	T	0	216.80
aat	N	1	0.57
aac	N	0	0.43
gtt	V	1	0.22
gtc	V	0	0.19
gta	V	0	0.25
gtg	V	0	0.34
---	--	------	------
mPD		0.37	0.70
		nPD:	0.53
		N. weight:	1.4
		Sc. PD:	0.34
		Sc. rank:	1356.5

PB1			
Pos.	7	obs:	exp:
tta	L	170	285.80
ttg	L	9	493.30
ctt	L	1	530.30
ctc	L	0	382.10
cta	L	2413	458.50
ctg	L	33	476.00
---	--	------	------
mPD		0.16	1.1
		nPD:	0.14
		N. weight:	2.3
		Sc. PD:	-0.31
		Sc. rank:	-973.2

PB1			
Pos.	8	obs:	exp:
tta	L	0	285.40
ttg	L	0	492.70
ctt	L	2488	529.70
ctc	L	131	381.60
cta	L	4	458.00
ctg	L	0	475.50
att	I	3	1.06
atc	I	0	0.77
ata	I	0	1.17
---	--	------	------
mPD		0.10	1.1
		nPD:	0.09
		N. weight:	2.2
		Sc. PD:	-0.41
		Sc. rank:	-1761.0

PB1			
Pos.	9	obs:	exp:
ttt	F	68	1176.00
ttc	F	2556	1448.00
tta	L	0	0.11
ttg	L	0	0.19
tat	Y	0	0.56
tac	Y	1	0.44
ctt	L	0	0.20
ctc	L	1	0.15
cta	L	0	0.17
ctg	L	0	0.18
---	--	------	------
mPD		0.052	0.50
		nPD:	0.1
		N. weight:	0.75
		Sc. PD:	-0.13
		Sc. rank:	-506.4

PB1			
Pos.	10	obs:	exp:
ttt	F	0	0.45
ttc	F	1	0.55
tta	L	406	285.40
ttg	L	110	492.70
ctt	L	1	529.70
ctc	L	0	381.60
cta	L	2063	458.00
ctg	L	43	475.50
cct	P	0	0.30
ccc	P	0	0.20
cca	P	1	0.40
ccg	P	0	0.11
att	I	0	0.35
atc	I	0	0.26
ata	I	1	0.39
---	--	------	------
mPD		0.43	1.1
		nPD:	0.38
		N. weight:	1.8
		Sc. PD:	0.16
		Sc. rank:	907.9

PB1			
Pos.	11	obs:	exp:
cgt	R	0	0.38
cgc	R	0	0.63
cga	R	0	1.21
cgg	R	0	0.87
aaa	K	1137	1588.00
aag	K	1476	1025.00
aga	R	6	5.50
agg	R	6	3.41
---	-	1	1.00
---	--	------	------
mPD		0.50	0.49
		nPD:	1.03
		N. weight:	0.1
		Sc. PD:	0.071
		Sc. rank:	145.0

PB1			
Pos.	12	obs:	exp:
att	I	749	269.20
atc	I	2	194.70
ata	I	8	295.10
atg	M	1	1.00
gtt	V	1348	414.30
gtc	V	15	352.10
gta	V	20	471.10
gtg	V	483	628.50
---	--	------	------
mPD		0.74	1.2
		nPD:	0.64
		N. weight:	1.2
		Sc. PD:	0.43
		Sc. rank:	1528.1

PB1			
Pos.	13	obs:	exp:
cct	P	12	774.90
ccc	P	0	528.70
cca	P	2455	1040.00
ccg	P	159	282.50
---	--	------	------
mPD		0.12	0.70
		nPD:	0.17
		N. weight:	1.2
		Sc. PD:	-0.12
		Sc. rank:	-199.9

PB1			
Pos.	14	obs:	exp:
act	T	0	0.98
acc	T	0	0.86
aca	T	0	1.82
acg	T	4	0.33
gtt	V	0	15.32
gtc	V	0	13.02
gta	V	5	17.42
gtg	V	64	23.24
gct	A	13	625.80
gcc	A	0	585.00
gca	A	551	1151.00
gcg	A	1989	191.50
---	--	------	------
mPD		0.40	0.74
		nPD:	0.54
		N. weight:	2.5
		Sc. PD:	0.62
		Sc. rank:	2522.0

PB1			
Pos.	15	obs:	exp:
caa	Q	1893	1416.00
cag	Q	733	1210.00
---	--	------	------
mPD		0.40	0.50
		nPD:	0.81
		N. weight:	0.11
		Sc. PD:	0.056
		Sc. rank:	156.5

PB1			
Pos.	16	obs:	exp:
tct	S	0	0.67
tcc	S	0	0.63
tca	S	0	0.97
tcg	S	0	0.21
aat	N	2603	1490.00
aac	N	19	1132.00
agt	S	4	0.74
agc	S	0	0.77
---	--	------	------
mPD		0.017	0.50
		nPD:	0.04
		N. weight:	0.82
		Sc. PD:	-0.2
		Sc. rank:	-1030.1

PB1			
Pos.	17	obs:	exp:
act	T	0	0.25
acc	T	1	0.22
aca	T	0	0.45
acg	T	0	0.08
gct	A	466	643.50
gcc	A	2151	601.50
gca	A	8	1183.00
gcg	A	0	196.90
---	--	------	------
mPD		0.30	0.68
		nPD:	0.44
		N. weight:	1.5
		Sc. PD:	0.23
		Sc. rank:	973.7

PB1			
Pos.	18	obs:	exp:
att	I	43	931.60
atc	I	31	673.50
ata	I	2552	1021.00
---	--	------	------
mPD		0.055	0.66
		nPD:	0.08
		N. weight:	1.3
		Sc. PD:	-0.24
		Sc. rank:	-1040.7

PB1			
Pos.	19	obs:	exp:
tct	S	0	441.70
tcc	S	0	415.00
tca	S	0	637.70
tcg	S	0	139.60
agt	S	225	488.80
agc	S	2401	503.30
---	--	------	------
mPD		0.16	1.6
		nPD:	0.1
		N. weight:	2.1
		Sc. PD:	-0.38
		Sc. rank:	-1517.3

PB1			
Pos.	20	obs:	exp:
tct	S	0	0.17
tcc	S	0	0.16
tca	S	0	0.24
tcg	S	0	0.05
att	I	12	14.54
atc	I	29	10.52
ata	I	0	15.94
act	T	424	634.90
acc	T	2122	558.10
aca	T	32	1173.00
acg	T	3	215.20
agt	S	1	0.19
agc	S	0	0.19
gct	A	3	0.74
gcc	A	0	0.69
gca	A	0	1.35
gcg	A	0	0.22
---	--	------	------
mPD		0.33	0.71
		nPD:	0.47
		N. weight:	1.5
		Sc. PD:	0.28
		Sc. rank:	1184.8

PB1			
Pos.	21	obs:	exp:
act	T	15	645.90
acc	T	25	567.80
aca	T	2488	1193.00
acg	T	98	219.00
---	--	------	------
mPD		0.10	0.68
		nPD:	0.15
		N. weight:	0.96
		Sc. PD:	-0.12
		Sc. rank:	-362.9

,

PB1			
Pos.	22	obs:	exp:
ttt	F	62	1177.00
ttc	F	2564	1449.00
---	--	------	------
mPD		0.046	0.49
		nPD:	0.09
		N. weight:	0.76
		Sc. PD:	-0.14
		Sc. rank:	-576.4

,

PB1			
Pos.	23	obs:	exp:
cct	P	2519	774.90
ccc	P	93	528.70
cca	P	8	1040.00
ccg	P	6	282.50
---	--	------	------
mPD		0.079	0.70
		nPD:	0.11
		N. weight:	1.6
		Sc. PD:	-0.27
		Sc. rank:	-994.5

,

PB1			
Pos.	24	obs:	exp:
tat	Y	2421	1471.00
tac	Y	205	1155.00
---	--	------	------
mPD		0.14	0.49
		nPD:	0.29
		N. weight:	0.51
		Sc. PD:	0.0048
		Sc. rank:	138.1

,

PB1			
Pos.	25	obs:	exp:
tct	S	0	0.17
tcc	S	0	0.16
tca	S	0	0.24
tcg	S	0	0.05
act	T	2495	645.40
acc	T	104	567.40
aca	T	20	1192.00
acg	T	5	218.80
agt	S	1	0.19
agc	S	0	0.19
gct	A	1	0.25
gcc	A	0	0.23
gca	A	0	0.45
gcg	A	0	0.07
---	--	------	------
mPD		0.096	0.68
		nPD:	0.14
		N. weight:	1.8
		Sc. PD:	-0.25
		Sc. rank:	-783.2

,

PB1			
Pos.	26	obs:	exp:
ggt	G	191	411.90
ggc	G	0	332.70
gga	G	2339	1189.00
ggg	G	96	691.90
---	--	------	------
mPD		0.20	0.68
		nPD:	0.29
		N. weight:	0.74
		Sc. PD:	0.007
		Sc. rank:	200.8

,

PB1			
Pos.	27	obs:	exp:
aat	N	0	0.57
aac	N	1	0.43
gat	D	1760	1476.00
gac	D	865	1149.00
---	--	------	------
mPD		0.44	0.49
		nPD:	0.9
		N. weight:	0.041
		Sc. PD:	0.024
		Sc. rank:	59.0

,

PB1			
Pos.	28	obs:	exp:
cct	P	2153	774.60
ccc	P	463	528.50
cca	P	7	1040.00
ccg	P	2	282.40
cgt	R	1	0.03
cgc	R	0	0.05
cga	R	0	0.10
cgg	R	0	0.07
aga	R	0	0.46
agg	R	0	0.28
---	--	------	------
mPD		0.30	0.71
		nPD:	0.42
		N. weight:	1.3
		Sc. PD:	0.17
		Sc. rank:	769.4

,

PB1			
Pos.	29	obs:	exp:
cct	P	23	774.90
ccc	P	8	528.70
cca	P	2587	1040.00
ccg	P	8	282.50
---	--	------	------
mPD		0.029	0.70
		nPD:	0.04
		N. weight:	1.3
		Sc. PD:	-0.31
		Sc. rank:	-1567.0

,

PB1			
Pos.	30	obs:	exp:
tat	Y	190	1471.00
tac	Y	2436	1155.00
---	--	------	------
mPD		0.13	0.49
		nPD:	0.27
		N. weight:	0.85
		Sc. PD:	-0.0081
		Sc. rank:	171.0

,

PB1			
Pos.	31	obs:	exp:
tct	S	0	441.70
tcc	S	0	415.00
tca	S	0	637.70
tcg	S	0	139.60
agt	S	363	488.80
agc	S	2263	503.30
---	--	------	------
mPD		0.24	1.6
		nPD:	0.15
		N. weight:	2.
		Sc. PD:	-0.26
		Sc. rank:	-765.8

,

PB1			
Pos.	32	obs:	exp:
cat	H	2611	1515.00
cac	H	15	1111.00
---	--	------	------
mPD		0.011	0.49
		nPD:	0.02
		N. weight:	0.81
		Sc. PD:	-0.2
		Sc. rank:	-1065.1

,

PB1			
Pos.	33	obs:	exp:
ggt	G	0	411.90
ggc	G	1	332.70
gga	G	2313	1189.00
ggg	G	312	691.90
---	--	------	------
mPD		0.21	0.68
		nPD:	0.31
		N. weight:	0.77
		Sc. PD:	0.018
		Sc. rank:	226.4

,

PB1			
Pos.	34	obs:	exp:
act	T	65	645.90
acc	T	1	567.80
aca	T	2540	1193.00
acg	T	20	219.00
---	--	------	------
mPD		0.064	0.68
		nPD:	0.09
		N. weight:	1.
		Sc. PD:	-0.19
		Sc. rank:	-766.3

,

PB1			
Pos.	35	obs:	exp:
ggt	G	11	411.90
ggc	G	0	332.70
gga	G	2230	1189.00
ggg	G	385	691.90
---	--	------	------
mPD		0.26	0.68
		nPD:	0.38
		N. weight:	0.68
		Sc. PD:	0.061
		Sc. rank:	342.5

,

PB1			
Pos.	36	obs:	exp:
act	T	12	645.90
acc	T	0	567.80
aca	T	2571	1193.00
acg	T	43	219.00
---	--	------	------
mPD		0.041	0.68
		nPD:	0.06
		N. weight:	1.1
		Sc. PD:	-0.24
		Sc. rank:	-1116.5

,

PB1			
Pos.	37	obs:	exp:
ggt	G	1	411.90
ggc	G	0	332.70
gga	G	2486	1189.00
ggg	G	139	691.90
---	--	------	------
mPD		0.10	0.68
		nPD:	0.15
		N. weight:	0.96
		Sc. PD:	-0.12
		Sc. rank:	-366.5

,

PB1			
Pos.	38	obs:	exp:
tat	Y	57	1470.00
tac	Y	2567	1154.00
cat	H	0	1.15
cac	H	2	0.85
---	--	------	------
mPD		0.044	0.49
		nPD:	0.09
		N. weight:	1.1
		Sc. PD:	-0.21
		Sc. rank:	-882.2

,

PB1			
Pos.	39	obs:	exp:
tct	S	0	0.17
tcc	S	0	0.16
tca	S	0	0.24
tcg	S	0	0.05
cct	P	0	0.30
ccc	P	1	0.20
cca	P	0	0.40
ccg	P	0	0.11
act	T	55	645.40
acc	T	2511	567.40
aca	T	57	1192.00
acg	T	1	218.80
agt	S	1	0.19
agc	S	0	0.19
---	--	------	------
mPD		0.086	0.68
		nPD:	0.13
		N. weight:	2.
		Sc. PD:	-0.31
		Sc. rank:	-1057.6

,

PB1			
Pos.	40	obs:	exp:
tta	L	0	1.31
ttg	L	2	2.25
ctt	L	0	2.42
ctc	L	0	1.75
cta	L	0	2.10
ctg	L	10	2.18
att	I	17	78.04
atc	I	0	56.43
ata	I	203	85.53
atg	M	2394	2394.00
---	--	------	------
mPD		0.16	0.17
		nPD:	0.95
		N. weight:	0.1
		Sc. PD:	0.064
		Sc. rank:	144.6

,

PB1

Pos.	41	obs:	exp:
ttt	F	0	0.45
ttc	F	1	0.55
tta	L	0	0.11
ttg	L	0	0.19
ctt	L	0	0.20
ctc	L	1	0.15
cta	L	0	0.17
ctg	L	0	0.18
gat	D	46	1475.00
gac	D	2577	1148.00
gaa	E	0	0.59
gag	E	1	0.41
---	--	------	------
mPD		0.038	0.50
		nPD:	0.08
		N. weight:	1.1
		Sc. PD:	-0.23
		Sc. rank:	-981.4

PB1

Pos.	42	obs:	exp:
tta	L	0	0.11
ttg	L	0	0.19
ctt	L	0	0.20
ctc	L	0	0.15
cta	L	1	0.17
ctg	L	0	0.18
act	T	37	645.70
acc	T	4	567.60
aca	T	2213	1193.00
acg	T	371	218.90
---	--	------	------
mPD		0.27	0.68
		nPD:	0.4
		N. weight:	0.86
		Sc. PD:	0.095
		Sc. rank:	484.7

PB1

Pos.	43	obs:	exp:
att	I	2	1.06
atc	I	1	0.77
ata	I	0	1.17
gtt	V	57	582.40
gtc	V	1820	495.00
gta	V	699	662.10
gtg	V	47	883.50
---	--	------	------
mPD		0.45	0.74
		nPD:	0.61
		N. weight:	1.3
		Sc. PD:	0.4
		Sc. rank:	1458.1

PB1

Pos.	44	obs:	exp:
tct	S	0	1.51
tcc	S	0	1.42
tca	S	0	2.19
tcg	S	0	0.48
aat	N	22	1487.00
aac	N	2595	1130.00
agt	S	0	1.68
agc	S	9	1.72
---	--	------	------
mPD		0.023	0.50
		nPD:	0.05
		N. weight:	1.2
		Sc. PD:	-0.28
		Sc. rank:	-1429.3

PB1

Pos.	45	obs:	exp:
cgt	R	0	82.89
cgc	R	0	138.80
cga	R	2	263.90
cgg	R	0	189.70
aga	R	2444	1204.00
agg	R	180	746.40
---	--	------	------
mPD		0.13	0.94
		nPD:	0.14
		N. weight:	0.87
		Sc. PD:	-0.12
		Sc. rank:	-387.9

PB1

Pos.	46	obs:	exp:
act	T	0	645.90
acc	T	4	567.80
aca	T	2595	1193.00
acg	T	27	219.00
---	--	------	------
mPD		0.023	0.68
		nPD:	0.03
		N. weight:	1.2
		Sc. PD:	-0.28
		Sc. rank:	-1451.4

PB1

Pos.	47	obs:	exp:
tat	Y	0	0.56
tac	Y	1	0.44
cat	H	545	1514.00
cac	H	2080	1111.00
---	--	------	------
mPD		0.33	0.49
		nPD:	0.67
		N. weight:	0.45
		Sc. PD:	0.17
		Sc. rank:	569.6

PB1

Pos.	48	obs:	exp:
tta	L	0	0.11
ttg	L	0	0.19
ctt	L	0	0.20
ctc	L	0	0.15
cta	L	1	0.17
ctg	L	0	0.18
caa	Q	2563	1414.00
cag	Q	60	1209.00
cgt	R	0	0.06
cgc	R	0	0.11
cga	R	2	0.20
cgg	R	0	0.14
aga	R	0	0.92
agg	R	0	0.57
---	--	------	------
mPD		0.047	0.50
		nPD:	0.09
		N. weight:	0.8
		Sc. PD:	-0.15
		Sc. rank:	-605.1

PB1

Pos.	49	obs:	exp:
tat	Y	1845	1471.00
tac	Y	781	1155.00
---	--	------	------
mPD		0.42	0.49
		nPD:	0.85
		N. weight:	0.069
		Sc. PD:	0.038
		Sc. rank:	98.5

PB1

Pos.	50	obs:	exp:
tct	S	5	441.50
tcc	S	0	414.90
tca	S	2410	637.50
tcg	S	210	139.50
agt	S	0	488.60
agc	S	0	503.10
gct	A	0	0.25
gcc	A	0	0.23
gca	A	1	0.45
gcg	A	0	0.07
---	--	------	------
mPD		0.15	1.6
		nPD:	0.09
		N. weight:	1.9
		Sc. PD:	-0.36
		Sc. rank:	-1484.5

PB1

Pos.	51	obs:	exp:
aaa	K	6	3.65
aag	K	0	2.35
gat	D	2	1.12
gac	D	0	0.88
gaa	E	2584	1551.00
gag	E	33	1066.00
ggt	G	0	0.16
ggc	G	0	0.13
gga	G	1	0.45
ggg	G	0	0.26
---	--	------	------
mPD		0.032	0.49
		nPD:	0.06
		N. weight:	0.72
		Sc. PD:	-0.15
		Sc. rank:	-708.4

PB1

Pos.	52	obs:	exp:
cgt	R	0	25.88
cgc	R	0	43.34
cga	R	0	82.42
cgg	R	0	59.24
atg	M	3	3.00
aat	N	0	87.53
aac	N	154	66.47
aaa	K	940	1002.00
aag	K	709	646.80
aga	R	529	376.00
agg	R	291	233.10
---	--	------	------
mPD		0.97	1.1
		nPD:	0.85
		N. weight:	0.23
		Sc. PD:	0.13
		Sc. rank:	327.8

PB1

Pos.	53	obs:	exp:
ggt	G	2	411.90
ggc	G	0	332.70
gga	G	1139	1189.00
ggg	G	1485	691.90
---	--	------	------
mPD		0.49	0.68
		nPD:	0.72
		N. weight:	0.64
		Sc. PD:	0.27
		Sc. rank:	865.1

PB1

Pos.	54	obs:	exp:
cgt	R	0	15.50
cgc	R	0	25.95
cga	R	0	49.35
cgg	R	0	35.47
att	I	0	1.42
atc	I	0	1.03
ata	I	4	1.56
atg	M	2	2.00
aat	N	1	0.57
aac	N	0	0.43
aaa	K	1532	1292.00
aag	K	594	834.00
aga	R	457	225.20
agg	R	34	139.60
gtt	V	0	0.22
gtc	V	0	0.19
gta	V	0	0.25
gtg	V	1	0.34
gaa	E	1	0.59
gag	E	0	0.41
---	--	------	------
mPD		0.68	0.90
		nPD:	0.75
		N. weight:	0.21
		Sc. PD:	0.094
		Sc. rank:	285.1

PB1

Pos.	55	obs:	exp:
tgg	W	2626	2626.00
---	--	------	------
mPD		0	0
		nPD:	1.
		N. weight:	0.
		Sc. PD:	0
		Sc. rank:	0

PB1

Pos.	56	obs:	exp:
tct	S	0	0.17
tcc	S	0	0.16
tca	S	0	0.24
tcg	S	1	0.05
att	I	0	4.61
atc	I	0	3.33
ata	I	13	5.05
atg	M	6	6.00
act	T	15	621.10
acc	T	5	546.00
aca	T	653	1147.00
acg	T	1852	210.60
aaa	K	0	5.47
aag	K	9	3.53
agt	S	0	0.19
agc	S	0	0.19
gtt	V	0	0.67
gtc	V	0	0.57
gta	V	0	0.76
gtg	V	3	1.01
gct	A	0	16.67
gcc	A	0	15.58
gca	A	62	30.65
gcg	A	6	5.10
gaa	E	0	0.59
gag	E	1	0.41
---	--	------	------
mPD		0.49	0.76
		nPD:	0.65
		N. weight:	2.2
		Sc. PD:	0.76
		Sc. rank:	2703.6

,

PB1

Pos.	57	obs:	exp:
att	I	0	40.80
atc	I	0	29.50
ata	I	115	44.71
act	T	40	616.20
acc	T	12	541.70
aca	T	2435	1138.00
acg	T	18	208.90
aaa	K	4	2.43
aag	K	0	1.57
gtt	V	0	0.22
gtc	V	0	0.19
gta	V	1	0.25
gtg	V	0	0.34
gct	A	1	0.25
gcc	A	0	0.23
gca	A	0	0.45
gcg	A	0	0.07
---	--	------	------
mPD		0.14	0.77
		nPD:	0.18
		N. weight:	1.1
		Sc. PD:	-0.099
		Sc. rank:	-91.5

,

PB1

Pos.	58	obs:	exp:
tgt	C	0	0.43
tgc	C	1	0.57
aat	N	990	1491.00
aac	N	1634	1133.00
gat	D	0	0.56
gac	D	1	0.44
---	--	------	------
mPD		0.47	0.49
		nPD:	0.96
		N. weight:	0.12
		Sc. PD:	0.077
		Sc. rank:	171.3

,

PB1

Pos.	59	obs:	exp:
cct	P	0	0.30
ccc	P	0	0.20
cca	P	1	0.40
ccg	P	0	0.11
att	I	0	1.77
atc	I	0	1.28
ata	I	5	1.94
atg	M	1	1.00
act	T	24	643.20
acc	T	64	565.50
aca	T	2458	1188.00
acg	T	69	218.10
aaa	K	0	2.43
aag	K	4	1.57
---	--	------	------
mPD		0.13	0.69
		nPD:	0.18
		N. weight:	0.9
		Sc. PD:	-0.085
		Sc. rank:	-88.1

,

PB1

Pos.	60	obs:	exp:
gaa	E	515	1556.00
gag	E	2111	1070.00
---	--	------	------
mPD		0.32	0.48
		nPD:	0.65
		N. weight:	0.52
		Sc. PD:	0.18
		Sc. rank:	645.1

,

PB1

Pos.	61	obs:	exp:
act	T	2518	645.90
acc	T	55	567.80
aca	T	15	1193.00
acg	T	38	219.00
---	--	------	------
mPD		0.080	0.68
		nPD:	0.12
		N. weight:	1.9
		Sc. PD:	-0.3
		Sc. rank:	-1081.9

,

PB1

Pos.	62	obs:	exp:
tgg	W	1	1.00
cgt	R	0	0.03
cgc	R	0	0.05
cga	R	0	0.10
cgg	R	0	0.07
aga	R	1	0.46
agg	R	0	0.28
gat	D	42	23.62
gac	D	0	18.38
gaa	E	1	0.59
gag	E	0	0.41
ggt	G	595	404.80
ggc	G	45	327.00
gga	G	613	1169.00
ggg	G	1328	680.10
---	--	------	------
mPD		0.66	0.72
		nPD:	0.92
		N. weight:	0.4
		Sc. PD:	0.24
		Sc. rank:	574.4

,

PB1

Pos.	63	obs:	exp:
tct	S	0	0.17
tcc	S	0	0.16
tca	S	1	0.24
tcg	S	0	0.05
agt	S	0	0.19
agc	S	0	0.19
gct	A	7	643.50
gcc	A	0	601.50
gca	A	2599	1183.00
gcg	A	19	196.90
---	--	------	------
mPD		0.020	0.68
		nPD:	0.03
		N. weight:	1.2
		Sc. PD:	-0.29
		Sc. rank:	-1502.4

,

PB1

Pos.	64	obs:	exp:
tta	L	0	4.68
ttg	L	0	8.08
tct	S	1	1.51
tcc	S	6	1.42
tca	S	2	2.19
tcg	S	0	0.48
ctt	L	1	8.68
ctc	L	42	6.26
cta	L	0	7.51
ctg	L	0	7.79
cct	P	161	754.30
ccc	P	2364	514.60
cca	P	30	1012.00
ccg	P	1	274.90
cat	H	0	0.58
cac	H	1	0.42
act	T	0	3.44
acc	T	14	3.03
aca	T	0	6.36
acg	T	0	1.17
agt	S	0	1.68
agc	S	0	1.72
gct	A	0	0.74
gcc	A	3	0.69
gca	A	0	1.35
gcg	A	0	0.22
---	--	------	------
mPD		0.19	0.77
		nPD:	0.25
		N. weight:	2.
		Sc. PD:	-0.063
		Sc. rank:	329.6

,

PB1

Pos.	65	obs:	exp:
caa	Q	150	1416.00
cag	Q	2476	1210.00
---	--	------	------
mPD		0.11	0.50
		nPD:	0.22
		N. weight:	0.86
		Sc. PD:	-0.054
		Sc. rank:	41.9

,

PB1

Pos.	66	obs:	exp:
ttt	F	0	0.45
ttc	F	1	0.55
tta	L	14	283.30
ttg	L	0	488.90
ctt	L	378	525.70
ctc	L	2193	378.70
cta	L	15	454.50
ctg	L	3	471.90
att	I	0	7.80
atc	I	22	5.64
ata	I	0	8.55
---	--	------	------
mPD		0.30	1.1
		nPD:	0.26
		N. weight:	2.2
		Sc. PD:	-0.054
		Sc. rank:	394.3

,

PB1

Pos.	67	obs:	exp:
aat	N	1069	1493.00
aac	N	1557	1133.00
---	--	------	------
mPD		0.48	0.49
		nPD:	0.98
		N. weight:	0.084
		Sc. PD:	0.057
		Sc. rank:	122.2

,

PB1

Pos.	68	obs:	exp:
cct	P	148	774.90
ccc	P	14	528.70
cca	P	191	1040.00
ccg	P	2273	282.50
---	--	------	------
mPD		0.24	0.70
		nPD:	0.34
		N. weight:	2.5
		Sc. PD:	0.15
		Sc. rank:	1045.4

,

PB1

Pos.	69	obs:	exp:
att	I	2528	923.00
atc	I	73	667.40
ata	I	1	1012.00
act	T	1	0.25
acc	T	0	0.22
aca	T	0	0.45
acg	T	0	0.08
gtt	V	23	5.11
gtc	V	0	4.34
gta	V	0	5.81
gtg	V	0	7.75
---	--	------	------
mPD		0.073	0.68
		nPD:	0.11
		N. weight:	1.4
		Sc. PD:	-0.24
		Sc. rank:	-912.0

,

PB1

Pos.	70	obs:	exp:
gat	D	2421	1477.00
gac	D	205	1149.00
---	--	------	------
mPD		0.14	0.49
		nPD:	0.29
		N. weight:	0.5
		Sc. PD:	0.0049
		Sc. rank:	137.7

,

PB1			
Pos.	71	obs:	exp:
ggt	G	3	411.90
ggc	G	0	332.70
gga	G	2301	1189.00
ggg	G	322	691.90
---	--	------	------
mPD		0.22	0.68
		nPD:	0.32
		N. weight:	0.75
		Sc. PD:	0.025
		Sc. rank:	253.3

,

PB1			
Pos.	72	obs:	exp:
tct	S	0	0.34
tcc	S	0	0.32
tca	S	1	0.49
tcg	S	1	0.11
cct	P	33	774.30
ccc	P	2	528.30
cca	P	2493	1039.00
ccg	P	96	282.30
agt	S	0	0.37
agc	S	0	0.38
---	--	------	------
mPD		0.098	0.71
		nPD:	0.14
		N. weight:	1.2
		Sc. PD:	-0.16
		Sc. rank:	-516.0

,

PB1			
Pos.	73	obs:	exp:
ttt	F	0	0.45
ttc	F	1	0.55
tta	L	93	285.70
ttg	L	2	493.10
ctt	L	13	530.10
ctc	L	23	381.90
cta	L	2458	458.40
ctg	L	36	475.80
---	--	------	------
mPD		0.13	1.1
		nPD:	0.11
		N. weight:	2.3
		Sc. PD:	-0.37
		Sc. rank:	-1394.0

,

PB1			
Pos.	74	obs:	exp:
tct	S	0	0.17
tcc	S	0	0.16
tca	S	1	0.24
tcg	S	0	0.05
cct	P	2539	774.60
ccc	P	68	528.50
cca	P	13	1040.00
ccg	P	5	282.40
agt	S	0	0.19
agc	S	0	0.19
---	--	------	------
mPD		0.065	0.71
		nPD:	0.09
		N. weight:	1.7
		Sc. PD:	-0.3
		Sc. rank:	-1298.2

,

PB1			
Pos.	75	obs:	exp:
act	T	0	0.25
acc	T	0	0.22
aca	T	1	0.45
acg	T	0	0.08
aat	N	1	0.57
aac	N	0	0.43
aaa	K	2	3.04
aag	K	3	1.96
gtt	V	0	1.55
gtc	V	0	1.32
gta	V	0	1.77
gtg	V	7	2.36
gct	A	0	0.49
gcc	A	0	0.46
gca	A	2	0.90
gcg	A	0	0.15
gat	D	26	14.62
gac	D	0	11.38
gaa	E	1260	1530.00
gag	E	1322	1052.00
ggt	G	0	0.31
ggc	G	0	0.25
gga	G	0	0.91
ggg	G	2	0.53
---	--	------	------
mPD		0.52	0.51
		nPD:	1.03
		N. weight:	0.054
		Sc. PD:	0.039
		Sc. rank:	78.7

,

PB1			
Pos.	76	obs:	exp:
caa	Q	1	0.54
cag	Q	0	0.46
aat	N	29	22.17
aac	N	10	16.83
gtt	V	1	0.22
gtc	V	0	0.19
gta	V	0	0.25
gtg	V	0	0.34
gat	D	2029	1446.00
gac	D	542	1125.00
gaa	E	2	1.19
gag	E	0	0.81
ggt	G	11	1.88
ggc	G	1	1.52
gga	G	0	5.44
ggg	G	0	3.16
---	--	------	------
mPD		0.37	0.54
		nPD:	0.7
		N. weight:	0.19
		Sc. PD:	0.077
		Sc. rank:	250.7

,

PB1			
Pos.	77	obs:	exp:
tct	S	0	0.17
tcc	S	0	0.16
tca	S	0	0.24
tcg	S	0	0.05
cat	H	1	0.58
cac	H	0	0.42
aat	N	2600	1488.00
aac	N	18	1130.00
agt	S	1	0.19
agc	S	0	0.19
gct	A	0	0.25
gcc	A	1	0.23
gca	A	0	0.45
gcg	A	0	0.07
gat	D	3	2.25
gac	D	1	1.75
ggt	G	1	0.16
ggc	G	0	0.13
gga	G	0	0.45
ggg	G	0	0.26
---	--	------	------
mPD		0.023	0.50
		nPD:	0.05
		N. weight:	0.82
		Sc. PD:	-0.19
		Sc. rank:	-961.8

,

PB1			
Pos.	78	obs:	exp:
aaa	K	1	0.61
aag	K	0	0.39
gat	D	4	7.87
gac	D	10	6.13
gaa	E	2190	1546.00
gag	E	420	1064.00
ggt	G	0	0.16
ggc	G	0	0.13
gga	G	1	0.45
ggg	G	0	0.26
---	--	------	------
mPD		0.28	0.49
		nPD:	0.57
		N. weight:	0.23
		Sc. PD:	0.063
		Sc. rank:	240.3

,

PB1			
Pos.	79	obs:	exp:
cct	P	37	774.60
ccc	P	7	528.50
cca	P	2432	1040.00
ccg	P	149	282.40
ggt	G	0	0.16
ggc	G	0	0.13
gga	G	0	0.45
ggg	G	1	0.26
---	--	------	------
mPD		0.14	0.71
		nPD:	0.2
		N. weight:	1.1
		Sc. PD:	-0.088
		Sc. rank:	-34.5

,

PB1			
Pos.	80	obs:	exp:
tct	S	0	436.50
tcc	S	0	410.10
tca	S	0	630.20
tcg	S	0	137.90
tgt	C	5	2.15
tgc	C	0	2.85
att	I	1	0.35
atc	I	0	0.26
ata	I	0	0.39
act	T	24	5.90
acc	T	0	5.19
aca	T	0	10.91
acg	T	0	2.00
agt	S	2231	483.00
agc	S	364	497.30
gaa	E	1	0.59
gag	E	0	0.41
---	--	------	------
mPD		0.26	1.6
		nPD:	0.16
		N. weight:	2.
		Sc. PD:	-0.23
		Sc. rank:	-602.7

,

PB1			
Pos.	81	obs:	exp:
ttt	F	0	0.45
ttc	F	1	0.55
ggt	G	31	411.70
ggc	G	7	332.60
gga	G	1532	1189.00
ggg	G	1055	691.70
---	--	------	------
mPD		0.50	0.69
		nPD:	0.73
		N. weight:	0.44
		Sc. PD:	0.19
		Sc. rank:	593.6

,

PB1			
Pos.	82	obs:	exp:
tta	L	0	0.11
ttg	L	1	0.19
tat	Y	2599	1471.00
tac	Y	26	1154.00
ctt	L	0	0.20
ctc	L	0	0.15
cta	L	0	0.17
ctg	L	0	0.18
---	--	------	------
mPD		0.021	0.49
		nPD:	0.04
		N. weight:	0.82
		Sc. PD:	-0.19
		Sc. rank:	-974.9

,

PB1			
Pos.	83	obs:	exp:
tct	S	0	0.50
tcc	S	0	0.47
tca	S	3	0.73
tcg	S	0	0.16
aaa	K	0	0.61
aag	K	1	0.39
agt	S	0	0.56
agc	S	0	0.57
gtt	V	0	0.22
gtc	V	0	0.19
gta	V	1	0.25
gtg	V	0	0.34
gct	A	309	642.50
gcc	A	36	600.50
gca	A	2273	1181.00
gcg	A	3	196.60
---	--	------	------
mPD		0.24	0.68
		nPD:	0.35
		N. weight:	0.69
		Sc. PD:	0.044
		Sc. rank:	301.8

,

PB1			
Pos.	84	obs:	exp:
caa	Q	2587	1416.00
cag	Q	38	1209.00
atg	M	1	1.00
---	--	------	------
mPD		0.031	0.50
		nPD:	0.06
		N. weight:	0.85
		Sc. PD:	-0.18
		Sc. rank:	-856.0

,

PB1			
Pos.	85	obs:	exp:
cgt	R	0	0.03
cgc	R	0	0.05
cga	R	0	0.10
cgg	R	0	0.07
act	T	4	645.70
acc	T	1	567.60
aca	T	2534	1193.00
acg	T	86	218.90
aga	R	1	0.46
agg	R	0	0.28
---	--	------	------
mPD		0.068	0.68
		nPD:	0.1
		N. weight:	1.1
		Sc. PD:	-0.19
		Sc. rank:	-741.4

,

PB1			
Pos. 86		obs:	exp:
aat	N	0	0.57
aac	N	1	0.43
gat	D	82	1476.00
gac	D	2543	1149.00
---	--	------	------
mPD		0.061	0.49
		nPD:	0.12
		N. weight:	1.1
		Sc. PD:	-0.16
		Sc. rank:	-568.4

,

PB1			
Pos. 87		obs:	exp:
tgt	C	2515	1130.00
tgc	C	111	1496.00
---	--	------	------
mPD		0.081	0.49
		nPD:	0.17
		N. weight:	1.
		Sc. PD:	-0.12
		Sc. rank:	-280.8

,

PB1			
Pos. 88		obs:	exp:
gtt	V	925	583.10
gtc	V	1681	495.50
gta	V	20	662.90
gtg	V	0	884.50
---	--	------	------
mPD		0.47	0.74
		nPD:	0.63
		N. weight:	1.4
		Sc. PD:	0.48
		Sc. rank:	1739.2

,

PB1			
Pos. 89		obs:	exp:
tta	L	43	285.80
ttg	L	54	493.30
ctt	L	13	530.30
ctc	L	32	382.10
cta	L	760	458.50
ctg	L	1724	476.00
---	--	------	------
mPD		0.52	1.1
		nPD:	0.46
		N. weight:	1.4
		Sc. PD:	0.23
		Sc. rank:	963.4

,

PB1			
Pos. 90		obs:	exp:
ttt	F	1	0.45
ttc	F	0	0.55
gaa	E	158	1555.00
gag	E	2467	1070.00
---	--	------	------
mPD		0.12	0.48
		nPD:	0.24
		N. weight:	1.
		Sc. PD:	-0.043
		Sc. rank:	126.9

,

PB1			
Pos. 91		obs:	exp:
gct	A	743	643.70
gcc	A	1437	601.70
gca	A	409	1184.00
gcg	A	37	196.90
---	--	------	------
mPD		0.60	0.68
		nPD:	0.88
		N. weight:	0.52
		Sc. PD:	0.3
		Sc. rank:	745.3

,

PB1			
Pos. 92		obs:	exp:
tta	L	0	0.22
ttg	L	1	0.38
ctt	L	0	0.40
ctc	L	0	0.29
cta	L	0	0.35
ctg	L	1	0.36
att	I	0	0.71
atc	I	0	0.51
ata	I	2	0.78
atg	M	2621	2621.00
gtt	V	0	0.22
gtc	V	0	0.19
gta	V	0	0.25
gtg	V	1	0.34
---	--	------	------
mPD		0.0038	0.0053
		nPD:	0.72
		N. weight:	0.0034
		Sc. PD:	0.0014
		Sc. rank:	4.6

,

PB1			
Pos. 93		obs:	exp:
gtt	V	1	0.22
gtc	V	0	0.19
gta	V	0	0.25
gtg	V	0	0.34
gct	A	2509	643.50
gcc	A	81	601.50
gca	A	13	1183.00
gcg	A	22	196.90
---	--	------	------
mPD		0.086	0.68
		nPD:	0.13
		N. weight:	1.9
		Sc. PD:	-0.28
		Sc. rank:	-958.9

,

PB1			
Pos. 94		obs:	exp:
ttt	F	92	1147.00
ttc	F	2467	1412.00
tta	L	24	3.37
ttg	L	1	5.82
tct	S	0	0.50
tcc	S	3	0.47
tca	S	0	0.73
tcg	S	0	0.16
tat	Y	0	18.49
tac	Y	33	14.51
ctt	L	0	6.26
ctc	L	6	4.51
cta	L	0	5.41
ctg	L	0	5.62
agt	S	0	0.56
agc	S	0	0.57
---	--	------	------
mPD		0.12	0.55
		nPD:	0.21
		N. weight:	0.73
		Sc. PD:	-0.048
		Sc. rank:	29.4

,

PB1			
Pos. 95		obs:	exp:
tta	L	0	285.40
ttg	L	0	492.70
ctt	L	2557	529.70
ctc	L	54	381.60
cta	L	7	458.00
ctg	L	5	475.50
atg	M	2	2.00
gtt	V	1	0.22
gtc	V	0	0.19
gta	V	0	0.25
gtg	V	0	0.34
---	--	------	------
mPD		0.053	1.1
		nPD:	0.05
		N. weight:	2.3
		Sc. PD:	-0.52
		Sc. rank:	-2670.1

,

PB1			
Pos. 96		obs:	exp:
gct	A	0	0.25
gcc	A	0	0.23
gca	A	1	0.45
gcg	A	0	0.07
gat	D	2	2.25
gac	D	2	1.75
gaa	E	2061	1553.00
gag	E	560	1068.00
---	--	------	------
mPD		0.34	0.49
		nPD:	0.7
		N. weight:	0.14
		Sc. PD:	0.054
		Sc. rank:	176.7

,

PB1			
Pos. 97		obs:	exp:
cgt	R	0	0.13
cgc	R	0	0.21
cga	R	0	0.40
cgg	R	0	0.29
aat	N	2	1.71
aac	N	1	1.29
aaa	K	52	32.82
aag	K	2	21.18
aga	R	2	1.83
agg	R	2	1.14
gct	A	0	0.49
gcc	A	0	0.46
gca	A	2	0.90
gcg	A	0	0.15
gat	D	0	6.19
gac	D	11	4.81
gaa	E	2443	1510.00
gag	E	105	1038.00
ggt	G	0	0.63
ggc	G	0	0.51
gga	G	4	1.81
ggg	G	0	1.05
---	--	------	------
mPD		0.14	0.54
		nPD:	0.26
		N. weight:	0.58
		Sc. PD:	-0.011
		Sc. rank:	110.2

,

PB1			
Pos. 98		obs:	exp:
tct	S	176	439.80
tcc	S	2406	413.30
tca	S	7	635.00
tcg	S	26	139.00
cct	P	0	0.30
ccc	P	1	0.20
cca	P	0	0.40
ccg	P	0	0.11
act	T	0	2.21
acc	T	9	1.95
aca	T	0	4.09
acg	T	0	0.75
agt	S	0	486.70
agc	S	0	501.20
gct	A	0	0.25
gcc	A	1	0.23
gca	A	0	0.45
gcg	A	0	0.07
---	--	------	------
mPD		0.16	1.6
		nPD:	0.1
		N. weight:	2.4
		Sc. PD:	-0.43
		Sc. rank:	-1725.9

,

PB1			
Pos. 99		obs:	exp:
cct	P	0	0.30
ccc	P	1	0.20
cca	P	0	0.40
ccg	P	0	0.11
cat	H	377	1514.00
cac	H	2247	1110.00
caa	Q	1	0.54
cag	Q	0	0.46
---	--	------	------
mPD		0.25	0.49
		nPD:	0.51
		N. weight:	0.63
		Sc. PD:	0.14
		Sc. rank:	575.6

,

PB1			
Pos. 100		obs:	exp:
tct	S	0	0.17
tcc	S	0	0.16
tca	S	1	0.24
tcg	S	0	0.05
cct	P	72	774.30
ccc	P	17	528.30
cca	P	2474	1039.00
ccg	P	61	282.30
caa	Q	0	0.54
cag	Q	1	0.46
agt	S	0	0.19
agc	S	0	0.19
---	--	------	------
mPD		0.11	0.71
		nPD:	0.16
		N. weight:	1.1
		Sc. PD:	-0.13
		Sc. rank:	-358.6

,

PB1			
Pos. 101		obs:	exp:
gaa	E	0	0.59
gag	E	1	0.41
ggt	G	13	411.70
ggc	G	22	332.60
gga	G	965	1189.00
ggg	G	1625	691.70
---	--	------	------
mPD		0.48	0.69
		nPD:	0.7
		N. weight:	0.65
		Sc. PD:	0.26
		Sc. rank:	856.5

,

PB1			
Pos. 102		obs:	exp:
tta	L	1	0.22
ttg	L	0	0.38
ctt	L	0	0.40
ctc	L	1	0.29
cta	L	0	0.35
ctg	L	0	0.36
att	I	14	930.50
atc	I	18	672.80
ata	I	2591	1020.00
gtt	V	0	0.22
gtc	V	0	0.19
gta	V	1	0.25
gtg	V	0	0.34
---	--	------	------
mPD		0.027	0.66
		nPD:	0.04
		N. weight:	1.4
		Sc. PD:	-0.32
		Sc. rank:	-1635.0

,

PB1			
Pos. 103		obs:	exp:
ttt	F	2567	1177.00
ttc	F	59	1449.00
---	--	------	------
mPD		0.044	0.49
		nPD:	0.09
		N. weight:	1.1
		Sc. PD:	-0.2
		Sc. rank:	-860.6

,

PB1			
Pos. 104		obs:	exp:
aaa	K	1	0.61
aag	K	0	0.39
gat	D	3	1.69
gac	D	0	1.31
gaa	E	613	1552.00
gag	E	2007	1068.00
ggt	G	0	0.31
ggc	G	0	0.25
gga	G	2	0.91
ggg	G	0	0.53
---	--	------	------
mPD		0.36	0.49
		nPD:	0.75
		N. weight:	0.42
		Sc. PD:	0.19
		Sc. rank:	577.0

,

PB1			
Pos. 105		obs:	exp:
tct	S	0	1.01
tcc	S	0	0.95
tca	S	0	1.46
tcg	S	0	0.32
att	I	1	0.35
atc	I	0	0.26
ata	I	0	0.39
act	T	1	0.49
acc	T	1	0.43
aca	T	0	0.91
acg	T	0	0.17
aat	N	2171	1487.00
aac	N	446	1130.00
agt	S	3	1.12
agc	S	3	1.15
---	--	------	------
mPD		0.29	0.50
		nPD:	0.58
		N. weight:	0.25
		Sc. PD:	0.072
		Sc. rank:	271.4

,

PB1

Pos.	106	obs:	exp:
tct	S	288	441.40
tcc	S	7	414.70
tca	S	2110	637.20
tcg	S	219	139.50
agt	S	0	488.40
agc	S	0	502.90
gct	A	0	0.49
gcc	A	0	0.46
gca	A	2	0.90
gcg	A	0	0.15
---	--	------	------
mPD		0.34	1.6
		nPD:	0.21
		N. weight:	1.5
		Sc. PD:	-0.11
		Sc. rank:	17.7

,

PB1

Pos.	107	obs:	exp:
tgt	C	619	1130.00
tgc	C	2007	1496.00
---	--	------	------
mPD		0.36	0.49
		nPD:	0.74
		N. weight:	0.13
		Sc. PD:	0.057
		Sc. rank:	179.4

,

PB1

Pos.	108	obs:	exp:
ttt	F	2	0.90
ttc	F	0	1.10
tta	L	0	277.10
ttg	L	0	478.20
ctt	L	2521	514.20
ctc	L	11	370.40
cta	L	9	444.60
ctg	L	5	461.50
cgt	R	6	0.19
cgc	R	0	0.32
cga	R	0	0.60
cgg	R	0	0.43
att	I	70	24.83
atc	I	0	17.95
ata	I	0	27.21
act	T	1	0.25
acc	T	0	0.22
aca	T	0	0.45
acg	T	0	0.08
aga	R	0	2.75
agg	R	0	1.71
gtt	V	1	0.22
gtc	V	0	0.19
gta	V	0	0.25
gtg	V	0	0.34
---	--	------	------
mPD		0.079	1.2
		nPD:	0.07
		N. weight:	2.4
		Sc. PD:	-0.49
		Sc. rank:	-2289.0

,

PB1

Pos.	109	obs:	exp:
gaa	E	2522	1555.00
gag	E	103	1070.00
ggt	G	0	0.16
ggc	G	0	0.13
gga	G	1	0.45
ggg	G	0	0.26
---	--	------	------
mPD		0.076	0.48
		nPD:	0.16
		N. weight:	0.58
		Sc. PD:	-0.07
		Sc. rank:	-194.5

,

PB1

Pos.	110	obs:	exp:
act	T	4	634.60
acc	T	6	557.90
aca	T	2228	1172.00
acg	T	342	215.10
gct	A	0	11.28
gcc	A	0	10.54
gca	A	40	20.73
gcg	A	6	3.45
---	--	------	------
mPD		0.27	0.71
		nPD:	0.38
		N. weight:	0.94
		Sc. PD:	0.088
		Sc. rank:	492.6

,

PB1

Pos.	111	obs:	exp:
tta	L	0	0.33
ttg	L	2	0.56
ctt	L	0	0.61
ctc	L	0	0.44
cta	L	0	0.52
ctg	L	1	0.54
att	I	6	51.08
atc	I	0	36.93
ata	I	138	55.98
atg	M	2470	2470.00
gtt	V	0	2.00
gtc	V	0	1.70
gta	V	0	2.27
gtg	V	9	3.03
---	--	------	------
mPD		0.11	0.12
		nPD:	0.94
		N. weight:	0.076
		Sc. PD:	0.048
		Sc. rank:	109.4

,

PB1

Pos.	112	obs:	exp:
gaa	E	2213	1556.00
gag	E	413	1070.00
---	--	------	------
mPD		0.27	0.48
		nPD:	0.55
		N. weight:	0.23
		Sc. PD:	0.06
		Sc. rank:	238.0

,

PB1

Pos.	113	obs:	exp:
ttt	F	10	4.48
ttc	F	0	5.52
tta	L	0	0.87
ttg	L	0	1.50
ctt	L	8	1.62
ctc	L	0	1.16
cta	L	0	1.40
ctg	L	0	1.45
att	I	1314	533.20
atc	I	182	385.50
ata	I	7	584.30
atg	M	9	9.00
act	T	3	0.74
acc	T	0	0.65
aca	T	0	1.36
acg	T	0	0.25
aat	N	2	1.14
aac	N	0	0.86
gtt	V	1023	238.70
gtc	V	49	202.80
gta	V	3	271.40
gtg	V	0	362.10
gct	A	16	3.92
gcc	A	0	3.67
gca	A	0	7.21
gcg	A	0	1.20
---	--	------	------
mPD		0.68	1.2
		nPD:	0.55
		N. weight:	1.5
		Sc. PD:	0.38
		Sc. rank:	1521.9

,

PB1

Pos.	114	obs:	exp:
att	I	18	14.19
atc	I	22	10.26
ata	I	0	15.55
gtt	V	849	573.30
gtc	V	1727	487.20
gta	V	4	651.80
gtg	V	2	869.70
gct	A	0	0.98
gcc	A	4	0.92
gca	A	0	1.80
gcg	A	0	0.30
---	--	------	------
mPD		0.48	0.77
		nPD:	0.62
		N. weight:	1.5
		Sc. PD:	0.49
		Sc. rank:	1767.2

,

PB1

Pos.	115	obs:	exp:
caa	Q	2540	1416.00
cag	Q	86	1210.00
---	--	------	------
mPD		0.063	0.50
		nPD:	0.13
		N. weight:	0.75
		Sc. PD:	-0.11
		Sc. rank:	-380.8

,

PB1

Pos.	116	obs:	exp:
caa	Q	2569	1416.00
cag	Q	56	1209.00
cgt	R	0	0.03
cgc	R	0	0.05
cga	R	0	0.10
cgg	R	1	0.07
aga	R	0	0.46
agg	R	0	0.28
---	--	------	------
mPD		0.043	0.50
		nPD:	0.09
		N. weight:	0.81
		Sc. PD:	-0.15
		Sc. rank:	-656.7

,

PB1

Pos.	117	obs:	exp:
tct	S	0	0.17
tcc	S	0	0.16
tca	S	1	0.24
tcg	S	0	0.05
act	T	7	644.50
acc	T	0	566.60
aca	T	2577	1191.00
acg	T	36	218.50
agt	S	0	0.19
agc	S	0	0.19
gct	A	0	1.23
gcc	A	0	1.15
gca	A	5	2.25
gcg	A	0	0.37
---	--	------	------
mPD		0.037	0.68
		nPD:	0.05
		N. weight:	1.1
		Sc. PD:	-0.25
		Sc. rank:	-1227.7

,

PB1

Pos.	118	obs:	exp:
cgt	R	0	82.89
cgc	R	0	138.80
cga	R	10	263.90
cgg	R	14	189.70
aga	R	668	1204.00
agg	R	1934	746.40
---	--	------	------
mPD		0.40	0.94
		nPD:	0.43
		N. weight:	0.82
		Sc. PD:	0.12
		Sc. rank:	513.0

,

PB1

Pos.	119	obs:	exp:
tta	L	0	0.33
ttg	L	3	0.56
ctt	L	0	0.61
ctc	L	0	0.44
cta	L	0	0.52
ctg	L	0	0.54
att	I	0	1.77
atc	I	0	1.28
ata	I	5	1.94
atg	M	25	25.00
gtt	V	18	575.70
gtc	V	3	489.30
gta	V	574	654.60
gtg	V	1998	873.40
---	--	------	------
mPD		0.38	0.76
		nPD:	0.5
		N. weight:	0.9
		Sc. PD:	0.19
		Sc. rank:	808.5

,

PB1

Pos.	120	obs:	exp:
gat	D	1904	1477.00
gac	D	722	1149.00
---	--	------	------
mPD		0.40	0.49
		nPD:	0.81
		N. weight:	0.09
		Sc. PD:	0.046
		Sc. rank:	127.9

,

PB1

Pos.	121	obs:	exp:
cgt	R	0	0.03
cgc	R	0	0.05
cga	R	0	0.10
cgg	R	0	0.07
aaa	K	2477	1595.00
aag	K	148	1030.00
aga	R	1	0.46
agg	R	0	0.28
---	--	------	------
mPD		0.11	0.48
		nPD:	0.22
		N. weight:	0.48
		Sc. PD:	-0.027
		Sc. rank:	39.3

PB1

Pos.	122	obs:	exp:
tta	L	35	285.70
ttg	L	127	493.10
ctt	L	5	530.10
ctc	L	0	381.90
cta	L	2104	458.40
ctg	L	354	475.80
atg	M	1	1.00
---	--	------	------
mPD		0.42	1.1
		nPD:	0.37
		N. weight:	1.7
		Sc. PD:	0.14
		Sc. rank:	811.9

PB1

Pos.	123	obs:	exp:
act	T	1681	645.20
acc	T	910	567.20
aca	T	19	1192.00
acg	T	13	218.70
gct	A	2	0.74
gcc	A	1	0.69
gca	A	0	1.35
gcg	A	0	0.22
---	--	------	------
mPD		0.47	0.68
		nPD:	0.69
		N. weight:	1.2
		Sc. PD:	0.45
		Sc. rank:	1499.5

PB1

Pos.	124	obs:	exp:
cat	H	1	5.19
cac	H	8	3.81
caa	Q	1705	1411.00
cag	Q	912	1206.00
---	--	------	------
mPD		0.46	0.50
		nPD:	0.92
		N. weight:	0.046
		Sc. PD:	0.028
		Sc. rank:	66.1

PB1

Pos.	125	obs:	exp:
ggt	G	2483	411.90
ggc	G	127	332.70
gga	G	15	1189.00
ggg	G	1	691.90
---	--	------	------
mPD		0.10	0.68
		nPD:	0.15
		N. weight:	2.5
		Sc. PD:	-0.32
		Sc. rank:	-912.0

PB1

Pos.	126	obs:	exp:
cat	H	0	1.15
cac	H	2	0.85
cgt	R	454	82.83
cgc	R	2134	138.70
cga	R	19	263.70
cgg	R	17	189.60
aga	R	0	1203.00
agg	R	0	745.80
---	--	------	------
mPD		0.31	0.94
		nPD:	0.33
		N. weight:	3.9
		Sc. PD:	0.18
		Sc. rank:	1462.9

PB1

Pos.	127	obs:	exp:
caa	Q	176	1416.00
cag	Q	2450	1210.00
---	--	------	------
mPD		0.13	0.50
		nPD:	0.25
		N. weight:	0.81
		Sc. PD:	-0.024
		Sc. rank:	136.1

PB1

Pos.	128	obs:	exp:
act	T	1357	645.90
acc	T	456	567.80
aca	T	800	1193.00
acg	T	13	219.00
---	--	------	------
mPD		0.61	0.68
		nPD:	0.9
		N. weight:	0.33
		Sc. PD:	0.2
		Sc. rank:	480.1

PB1

Pos.	129	obs:	exp:
ttt	F	13	9.86
ttc	F	9	12.14
tat	Y	2591	1459.00
tac	Y	13	1145.00
---	--	------	------
mPD		0.033	0.51
		nPD:	0.07
		N. weight:	0.85
		Sc. PD:	-0.18
		Sc. rank:	-829.3

PB1

Pos.	130	obs:	exp:
gtt	V	1	0.22
gtc	V	0	0.19
gta	V	0	0.25
gtg	V	0	0.34
gat	D	2178	1476.00
gac	D	447	1149.00
---	--	------	------
mPD		0.28	0.49
		nPD:	0.57
		N. weight:	0.26
		Sc. PD:	0.072
		Sc. rank:	274.5

PB1

Pos.	131	obs:	exp:
tgg	W	2625	2625.00
cgt	R	0	0.03
cgc	R	0	0.05
cga	R	0	0.10
cgg	R	1	0.07
aga	R	0	0.46
agg	R	0	0.28
---	--	------	------
mPD		0.00076	0.0013
		nPD:	0.61
		N. weight:	0.0016
		Sc. PD:	0.00049
		Sc. rank:	1.8

PB1

Pos.	132	obs:	exp:
act	T	33	645.70
acc	T	5	567.60
aca	T	2566	1193.00
acg	T	21	218.90
aaa	K	1	0.61
aag	K	0	0.39
---	--	------	------
mPD		0.045	0.68
		nPD:	0.07
		N. weight:	1.1
		Sc. PD:	-0.22
		Sc. rank:	-1037.1

PB1

Pos.	133	obs:	exp:
ttt	F	0	0.45
ttc	F	1	0.55
tta	L	2099	269.70
ttg	L	263	465.50
ctt	L	0	500.50
ctc	L	0	360.50
cta	L	94	432.70
ctg	L	22	449.20
att	I	0	9.93
atc	I	0	7.18
ata	I	28	10.89
gtt	V	0	26.42
gtc	V	0	22.46
gta	V	119	30.04
gtg	V	0	40.08
---	--	------	------
mPD		0.38	1.2
		nPD:	0.32
		N. weight:	2.5
		Sc. PD:	0.081
		Sc. rank:	825.1

PB1

Pos.	134	obs:	exp:
tct	S	0	0.17
tcc	S	0	0.16
tca	S	0	0.24
tcg	S	0	0.05
cct	P	1	0.30
ccc	P	0	0.20
cca	P	0	0.40
ccg	P	0	0.11
aat	N	1263	1489.00
aac	N	1357	1131.00
agt	S	1	0.19
agc	S	0	0.19
gat	D	3	2.25
gac	D	1	1.75
---	--	------	------
mPD		0.50	0.50
		nPD:	1.02
		N. weight:	0.028
		Sc. PD:	0.02
		Sc. rank:	41.1

PB1

Pos.	135	obs:	exp:
cgt	R	0	82.83
cgc	R	0	138.70
cga	R	3	263.70
cgg	R	0	189.60
aaa	K	2	1.22
aag	K	0	0.78
aga	R	2467	1203.00
agg	R	154	745.80
---	--	------	------
mPD		0.11	0.94
		nPD:	0.12
		N. weight:	0.9
		Sc. PD:	-0.14
		Sc. rank:	-499.2

PB1

Pos.	136	obs:	exp:
aat	N	2113	1493.00
aac	N	513	1133.00
---	--	------	------
mPD		0.31	0.49
		nPD:	0.64
		N. weight:	0.2
		Sc. PD:	0.068
		Sc. rank:	242.9

PB1

Pos.	137	obs:	exp:
caa	Q	2544	1416.00
cag	Q	82	1210.00
---	--	------	------
mPD		0.061	0.50
		nPD:	0.12
		N. weight:	0.76
		Sc. PD:	-0.12
		Sc. rank:	-415.6

PB1

Pos.	138	obs:	exp:
cct	P	128	774.30
ccc	P	1	528.30
cca	P	106	1039.00
ccg	P	2389	282.30
act	T	0	0.49
acc	T	0	0.43
aca	T	0	0.91
acg	T	2	0.17
---	--	------	------
mPD		0.17	0.71
		nPD:	0.24
		N. weight:	2.8
		Sc. PD:	-0.12
		Sc. rank:	374.3

PB1

Pos.	139	obs:	exp:
gct	A	433	643.70
gcc	A	36	601.70
gca	A	2148	1184.00
gcg	A	9	196.90
---	--	------	------
mPD		0.30	0.68
		nPD:	0.45
		N. weight:	0.59
		Sc. PD:	0.093
		Sc. rank:	396.2

PB1

Pos.	140	obs:	exp:
tct	S	0	0.34
tcc	S	0	0.32
tca	S	2	0.49
tcg	S	0	0.11
agt	S	0	0.37
agc	S	0	0.38
gct	A	3	643.20
gcc	A	3	601.20
gca	A	2559	1183.00
gcg	A	59	196.80
---	--	------	------
mPD		0.050	0.68
		nPD:	0.07
		N. weight:	1.1
		Sc. PD:	-0.22
		Sc. rank:	-1012.1

PB1

Pos.	141	obs:	exp:
act	T	2500	645.90
acc	T	10	567.80
aca	T	111	1193.00
acg	T	5	219.00
---	--	------	------
mPD		0.092	0.68
		nPD:	0.14
		N. weight:	1.8
		Sc. PD:	-0.26
		Sc. rank:	-869.7

PB1

Pos.	142	obs:	exp:
gct	A	393	643.70
gcc	A	20	601.70
gca	A	2204	1184.00
gcg	A	9	196.90
---	--	------	------
mPD		0.27	0.68
		nPD:	0.4
		N. weight:	0.65
		Sc. PD:	0.075
		Sc. rank:	378.0

PB1

Pos.	143	obs:	exp:
tta	L	117	285.80
ttg	L	2464	493.30
ctt	L	0	530.30
ctc	L	0	382.10
cta	L	0	458.50
ctg	L	45	476.00
---	--	------	------
mPD		0.12	1.1
		nPD:	0.1
		N. weight:	2.2
		Sc. PD:	-0.38
		Sc. rank:	-1512.4

PB1

Pos.	144	obs:	exp:
tct	S	0	0.17
tcc	S	1	0.16
tca	S	0	0.24
tcg	S	0	0.05
act	T	0	1.48
acc	T	6	1.30
aca	T	0	2.73
acg	T	0	0.50
agt	S	0	0.19
agc	S	0	0.19
gct	A	14	642.00
gcc	A	2596	600.10
gca	A	9	1181.00
gcg	A	0	196.40
---	--	------	------
mPD		0.023	0.68
		nPD:	0.03
		N. weight:	2.2
		Sc. PD:	-0.53
		Sc. rank:	-2794.5

PB1

Pos.	145	obs:	exp:
aat	N	71	1493.00
aac	N	2555	1133.00
---	--	------	------
mPD		0.053	0.49
		nPD:	0.11
		N. weight:	1.1
		Sc. PD:	-0.19
		Sc. rank:	-723.4

PB1

Pos.	146	obs:	exp:
tct	S	0	0.17
tcc	S	0	0.16
tca	S	0	0.24
tcg	S	0	0.05
act	T	110	645.70
acc	T	2133	567.60
aca	T	381	1193.00
acg	T	1	218.90
agt	S	0	0.19
agc	S	1	0.19
---	--	------	------
mPD		0.32	0.68
		nPD:	0.47
		N. weight:	1.3
		Sc. PD:	0.23
		Sc. rank:	998.9

PB1

Pos.	147	obs:	exp:
att	I	10	930.80
atc	I	85	673.00
ata	I	2529	1020.00
atg	M	1	1.00
gtt	V	0	0.22
gtc	V	0	0.19
gta	V	1	0.25
gtg	V	0	0.34
---	--	------	------
mPD		0.072	0.66
		nPD:	0.11
		N. weight:	1.2
		Sc. PD:	-0.21
		Sc. rank:	-779.0

PB1

Pos.	148	obs:	exp:
gaa	E	2255	1556.00
gag	E	371	1070.00
---	--	------	------
mPD		0.24	0.48
		nPD:	0.5
		N. weight:	0.27
		Sc. PD:	0.056
		Sc. rank:	240.5

PB1

Pos.	149	obs:	exp:
att	I	5	17.03
atc	I	43	12.31
ata	I	0	18.66
gtt	V	1339	572.40
gtc	V	1010	486.50
gta	V	48	650.80
gtg	V	181	868.30
---	--	------	------
mPD		0.61	0.78
		nPD:	0.78
		N. weight:	0.91
		Sc. PD:	0.44
		Sc. rank:	1277.0

PB1

Pos.	150	obs:	exp:
ttt	F	1996	1177.00
ttc	F	630	1449.00
---	--	------	------
mPD		0.36	0.49
		nPD:	0.74
		N. weight:	0.32
		Sc. PD:	0.14
		Sc. rank:	433.1

PB1

Pos.	151	obs:	exp:
tct	S	0	0.17
tcc	S	0	0.16
tca	S	0	0.24
tcg	S	0	0.05
cgt	R	0	81.15
cgc	R	0	135.90
cga	R	1	258.40
cgg	R	0	185.70
aaa	K	16	26.74
aag	K	28	17.26
agt	S	1	0.19
agc	S	0	0.19
aga	R	2057	1179.00
agg	R	513	730.70
ggt	G	0	1.57
ggc	G	0	1.27
gga	G	8	4.53
ggg	G	2	2.63
---	--	------	------
mPD		0.37	0.97
		nPD:	0.38
		N. weight:	0.58
		Sc. PD:	0.055
		Sc. rank:	305.8

PB1

Pos.	152	obs:	exp:
ttt	F	2	0.90
ttc	F	0	1.10
tta	L	28	14.26
ttg	L	96	24.61
tct	S	65	366.20
tcc	S	0	344.10
tca	S	1456	528.70
tcg	S	656	115.70
ctt	L	0	26.46
ctc	L	0	19.06
cta	L	0	22.87
ctg	L	7	23.75
att	I	0	0.71
atc	I	0	0.51
ata	I	2	0.78
atg	M	283	283.00
act	T	0	0.74
acc	T	0	0.65
aca	T	1	1.36
acg	T	2	0.25
aaa	K	1	10.33
aag	K	16	6.67
agt	S	0	405.20
agc	S	0	417.20
gtt	V	0	0.44
gtc	V	0	0.38
gta	V	0	0.50
gtg	V	2	0.67
gct	A	0	2.21
gcc	A	0	2.06
gca	A	2	4.06
gcg	A	7	0.67
---	--	------	------
mPD		1.0	1.9
		nPD:	0.54
		N. weight:	1.6
		Sc. PD:	0.39
		Sc. rank:	1631.2

PB1

Pos.	153	obs:	exp:
aat	N	2186	1493.00
aac	N	440	1133.00
---	--	------	------
mPD		0.28	0.49
		nPD:	0.57
		N. weight:	0.25
		Sc. PD:	0.069
		Sc. rank:	266.0

PB1

Pos.	154	obs:	exp:
tct	S	0	39.02
tcc	S	0	36.67
tca	S	0	56.34
tcg	S	0	12.33
tgt	C	22	11.19
tgc	C	4	14.81
cgt	R	1	0.03
cgc	R	0	0.05
cga	R	0	0.10
cgg	R	0	0.07
aat	N	161	92.65
aac	N	2	70.35
agt	S	216	43.18
agc	S	16	44.46
aga	R	0	0.46
agg	R	0	0.28
gat	D	36	46.68
gac	D	47	36.32
ggt	G	1610	332.70
ggc	G	510	268.80
gga	G	1	960.70
ggg	G	0	558.90
---	--	------	------
mPD		0.79	1.3
		nPD:	0.62
		N. weight:	2.
		Sc. PD:	0.64
		Sc. rank:	2326.8

PB1

Pos.	155	obs:	exp:
tta	L	84	285.70
ttg	L	7	493.10
ctt	L	1	530.10
ctc	L	100	381.90
cta	L	2333	458.40
ctg	L	100	475.80
att	I	0	0.35
atc	I	0	0.26
ata	I	1	0.39
---	--	------	------
mPD		0.22	1.1
		nPD:	0.19
		N. weight:	2.
		Sc. PD:	-0.18
		Sc. rank:	-127.4

PB1			
Pos.	156	obs:	exp:
cgt	R	0	0.09
cgc	R	0	0.16
cga	R	0	0.30
cgg	R	0	0.22
att	I	1	3.90
atc	I	0	2.82
ata	I	10	4.28
act	T	15	640.80
acc	T	2	563.30
aca	T	2563	1184.00
acg	T	25	217.20
aaa	K	3	1.82
aag	K	0	1.18
aga	R	3	1.38
agg	R	0	0.85
gtt	V	0	0.22
gtc	V	0	0.19
gta	V	1	0.25
gtg	V	0	0.34
gct	A	0	0.49
gcc	A	0	0.46
gca	A	2	0.90
gcg	A	0	0.15
gaa	E	1	0.59
gag	E	0	0.41
---	--	------	------
mPD		0.050	0.70
		nPD:	0.07
		N. weight:	1.1
		Sc. PD:	-0.23
		Sc. rank:	-1036.0

,

PB1			
Pos.	157	obs:	exp:
tct	S	1	0.50
tcc	S	2	0.47
tca	S	0	0.73
tcg	S	0	0.16
att	I	1	0.35
atc	I	0	0.26
ata	I	0	0.39
act	T	204	54.36
acc	T	17	47.79
aca	T	0	100.40
acg	T	0	18.43
aat	N	3	2.27
aac	N	1	1.73
agt	S	0	0.56
agc	S	0	0.57
gtt	V	0	0.22
gtc	V	1	0.19
gta	V	0	0.25
gtg	V	0	0.34
gct	A	1898	585.40
gcc	A	479	547.10
gca	A	6	1076.00
gcg	A	5	179.10
gat	D	7	3.94
gac	D	0	3.06
ggt	G	0	0.16
ggc	G	1	0.13
gga	G	0	0.45
ggg	G	0	0.26
---	--	------	------
mPD		0.49	0.85
		nPD:	0.57
		N. weight:	1.4
		Sc. PD:	0.4
		Sc. rank:	1520.8

,

PB1			
Pos.	158	obs:	exp:
tct	S	0	20.18
tcc	S	0	18.97
tca	S	0	29.14
tcg	S	0	6.38
tat	Y	1	0.56
tac	Y	0	0.44
aat	N	2374	1424.00
aac	N	131	1081.00
agt	S	101	22.33
agc	S	19	23.00
---	--	------	------
mPD		0.20	0.65
		nPD:	0.3
		N. weight:	0.65
		Sc. PD:	0.012
		Sc. rank:	188.4

,

PB1			
Pos.	159	obs:	exp:
gat	D	4	7.87
gac	D	10	6.13
gaa	E	737	1543.00
gag	E	1867	1061.00
ggt	G	0	1.25
ggc	G	0	1.01
gga	G	7	3.62
ggg	G	1	2.11
---	--	------	------
mPD		0.42	0.50
		nPD:	0.85
		N. weight:	0.31
		Sc. PD:	0.17
		Sc. rank:	442.7

,

PB1			
Pos.	160	obs:	exp:
tct	S	124	441.50
tcc	S	3	414.90
tca	S	2194	637.50
tcg	S	304	139.50
act	T	0	0.25
acc	T	0	0.22
aca	T	1	0.45
acg	T	0	0.08
agt	S	0	488.60
agc	S	0	503.10
---	--	------	------
mPD		0.29	1.6
		nPD:	0.18
		N. weight:	1.7
		Sc. PD:	-0.17
		Sc. rank:	-265.0

,

PB1			
Pos.	161	obs:	exp:
ggt	G	4	411.90
ggc	G	17	332.70
gga	G	2412	1189.00
ggg	G	193	691.90
---	--	------	------
mPD		0.15	0.68
		nPD:	0.22
		N. weight:	0.83
		Sc. PD:	-0.05
		Sc. rank:	60.1

,

PB1			
Pos.	162	obs:	exp:
tct	S	0	0.17
tcc	S	0	0.16
tca	S	0	0.24
tcg	S	0	0.05
cgt	R	0	82.86
cgc	R	0	138.70
cga	R	0	263.80
cgg	R	12	189.60
agt	S	0	0.19
agc	S	1	0.19
aga	R	491	1204.00
agg	R	2122	746.10
---	--	------	------
mPD		0.31	0.94
		nPD:	0.33
		N. weight:	1.
		Sc. PD:	0.052
		Sc. rank:	416.4

,

PB1			
Pos.	163	obs:	exp:
tta	L	320	285.80
ttg	L	46	493.30
ctt	L	9	530.30
ctc	L	23	382.10
cta	L	1341	458.50
ctg	L	887	476.00
---	--	------	------
mPD		0.71	1.1
		nPD:	0.63
		N. weight:	1.1
		Sc. PD:	0.36
		Sc. rank:	1302.5

,

PB1			
Pos.	164	obs:	exp:
tta	L	1	0.22
ttg	L	0	0.38
ctt	L	0	0.40
ctc	L	0	0.29
cta	L	1	0.35
ctg	L	0	0.36
att	I	7	926.60
atc	I	0	669.90
ata	I	2605	1015.00
atg	M	5	5.00
gtt	V	0	1.55
gtc	V	0	1.32
gta	V	6	1.77
gtg	V	1	2.36
---	--	------	------
mPD		0.017	0.67
		nPD:	0.03
		N. weight:	1.4
		Sc. PD:	-0.36
		Sc. rank:	-1876.1

,

PB1			
Pos.	165	obs:	exp:
gat	D	2580	1477.00
gac	D	46	1149.00
---	--	------	------
mPD		0.034	0.49
		nPD:	0.07
		N. weight:	0.77
		Sc. PD:	-0.16
		Sc. rank:	-725.8

,

PB1			
Pos.	166	obs:	exp:
ttt	F	123	1175.00
ttc	F	2500	1448.00
tta	L	0	0.33
ttg	L	0	0.56
ctt	L	0	0.61
ctc	L	3	0.44
cta	L	0	0.52
ctg	L	0	0.54
---	--	------	------
mPD		0.092	0.50
		nPD:	0.18
		N. weight:	0.65
		Sc. PD:	-0.062
		Sc. rank:	-62.5

,

PB1			
Pos.	167	obs:	exp:
tta	L	1532	285.80
ttg	L	57	493.30
ctt	L	404	530.30
ctc	L	14	382.10
cta	L	593	458.50
ctg	L	26	476.00
---	--	------	------
mPD		0.80	1.1
		nPD:	0.7
		N. weight:	1.4
		Sc. PD:	0.57
		Sc. rank:	1862.3

,

PB1			
Pos.	168	obs:	exp:
cgt	R	0	0.06
cgc	R	0	0.11
cga	R	0	0.20
cgg	R	0	0.14
aaa	K	1254	1595.00
aag	K	1370	1029.00
aga	R	0	0.92
agg	R	2	0.57
---	--	------	------
mPD		0.50	0.48
		nPD:	1.05
		N. weight:	0.058
		Sc. PD:	0.042
		Sc. rank:	83.8

,

PB1			
Pos.	169	obs:	exp:
gat	D	2566	1477.00
gac	D	60	1149.00
---	--	------	------
mPD		0.045	0.49
		nPD:	0.09
		N. weight:	0.74
		Sc. PD:	-0.14
		Sc. rank:	-579.8

,

PB1			
Pos.	170	obs:	exp:
att	I	0	0.35
atc	I	0	0.26
ata	I	1	0.39
gtt	V	24	582.80
gtc	V	13	495.30
gta	V	2161	662.70
gtg	V	427	884.20
---	--	------	------
mPD		0.30	0.74
		nPD:	0.4
		N. weight:	1.3
		Sc. PD:	0.14
		Sc. rank:	726.2

,

PB1			
Pos.	171	obs:	exp:
tta	L	0	1.41
ttg	L	1	2.44
ctt	L	0	2.63
ctc	L	0	1.89
cta	L	2	2.27
ctg	L	10	2.36
att	I	1	9.93
atc	I	0	7.18
ata	I	27	10.89
atg	M	2545	2545.00
act	T	0	1.97
acc	T	0	1.73
aca	T	0	3.64
acg	T	8	0.67
gtt	V	1	7.11
gtc	V	0	6.04
gta	V	4	8.08
gtg	V	27	10.78
---	--	------	------
mPD		0.066	0.088
		nPD:	0.75
		N. weight:	0.048
		Sc. PD:	0.022
		Sc. rank:	66.4

,

PB1			
Pos.	172	obs:	exp:
cgt	R	0	0.41
cgc	R	0	0.69
cga	R	0	1.31
cgg	R	0	0.94
aat	N	1	0.57
aac	N	0	0.43
aaa	K	15	9.12
aag	K	0	5.88
aga	R	13	5.96
agg	R	0	3.69
gat	D	26	20.81
gac	D	11	16.19
gaa	E	2473	1513.00
gag	E	80	1040.00
ggt	G	0	1.10
ggc	G	0	0.89
gga	G	7	3.17
ggg	G	0	1.84
---	--	------	------
mPD		0.12	0.54
		nPD:	0.23
		N. weight:	0.62
		Sc. PD:	-0.031
		Sc. rank:	61.3

,

PB1			
Pos.	173	obs:	exp:
tct	S	48	441.70
tcc	S	0	415.00
tca	S	2554	637.70
tcg	S	24	139.60
agt	S	0	488.80
agc	S	0	503.30
---	--	------	------
mPD		0.054	1.6
		nPD:	0.03
		N. weight:	2.
		Sc. PD:	-0.49
		Sc. rank:	-2571.4

,

PB1			
Pos.	174	obs:	exp:
tta	L	0	0.22
ttg	L	1	0.38
ctt	L	0	0.40
ctc	L	0	0.29
cta	L	0	0.35
ctg	L	1	0.36
atg	M	2431	2431.00
act	T	0	7.38
acc	T	0	6.49
aca	T	29	13.63
acg	T	1	2.50
gtt	V	0	35.75
gtc	V	0	30.38
gta	V	8	40.64
gtg	V	153	54.23
gct	A	0	0.49
gcc	A	0	0.46
gca	A	2	0.90
gcg	A	0	0.15
---	--	------	------
mPD		0.17	0.24
		nPD:	0.71
		N. weight:	0.1
		Sc. PD:	0.042
		Sc. rank:	136.7

,

PB1			
Pos.	175	obs:	exp:
aat	N	30	380.80
aac	N	640	289.20
gat	D	1683	1100.00
gac	D	272	855.40
gaa	E	1	0.59
gag	E	0	0.41
---	--	------	------
mPD		0.83	0.87
		nPD:	0.96
		N. weight:	0.5
		Sc. PD:	0.33
		Sc. rank:	726.7

,

PB1			
Pos.	176	obs:	exp:
caa	Q	2	1.08
cag	Q	0	0.92
cgt	R	0	6.06
cgc	R	0	10.15
cga	R	0	19.30
cgg	R	0	13.87
aat	N	2	1.14
aac	N	0	0.86
aaa	K	2366	1477.00
aag	K	64	953.20
aga	R	172	88.05
agg	R	20	54.57
---	--	------	------
mPD		0.20	0.66
		nPD:	0.3
		N. weight:	0.62
		Sc. PD:	0.013
		Sc. rank:	181.8

,

PB1			
Pos.	177	obs:	exp:
aaa	K	6	4.25
aag	K	1	2.75
gtt	V	0	0.22
gtc	V	0	0.19
gta	V	0	0.25
gtg	V	1	0.34
gat	D	20	12.37
gac	D	2	9.63
gaa	E	489	1532.00
gag	E	2096	1053.00
ggt	G	0	1.73
ggc	G	0	1.39
gga	G	2	4.98
ggg	G	9	2.90
---	--	------	------
mPD		0.33	0.51
		nPD:	0.66
		N. weight:	0.54
		Sc. PD:	0.2
		Sc. rank:	678.3

,

PB1			
Pos.	178	obs:	exp:
act	T	0	1.48
acc	T	0	1.30
aca	T	0	2.73
acg	T	6	0.50
aaa	K	1	3.65
aag	K	5	2.35
gat	D	11	6.75
gac	D	1	5.25
gaa	E	2162	1529.00
gag	E	419	1052.00
ggt	G	0	3.29
ggc	G	0	2.66
gga	G	20	9.51
ggg	G	1	5.53
---	--	------	------
mPD		0.31	0.52
		nPD:	0.6
		N. weight:	0.24
		Sc. PD:	0.073
		Sc. rank:	269.5

,

PB1			
Pos.	179	obs:	exp:
tta	L	1	0.87
ttg	L	0	1.50
ctt	L	0	1.62
ctc	L	0	1.16
cta	L	7	1.40
ctg	L	0	1.45
att	I	10	724.70
atc	I	2	524.00
ata	I	2031	794.30
atg	M	562	562.00
gtt	V	0	2.89
gtc	V	0	2.45
gta	V	0	3.28
gtg	V	13	4.38
---	--	------	------
mPD		0.37	0.76
		nPD:	0.48
		N. weight:	1.1
		Sc. PD:	0.21
		Sc. rank:	902.3

,

PB1			
Pos.	180	obs:	exp:
aaa	K	2	3.04
aag	K	3	1.96
gtt	V	0	0.44
gtc	V	0	0.38
gta	V	2	0.50
gtg	V	0	0.67
gat	D	1	0.56
gac	D	0	0.44
gaa	E	532	1549.00
gag	E	2082	1065.00
ggt	G	0	0.63
ggc	G	0	0.51
gga	G	1	1.81
ggg	G	3	1.05
---	--	------	------
mPD		0.33	0.49
		nPD:	0.68
		N. weight:	0.5
		Sc. PD:	0.19
		Sc. rank:	641.1

,

PB1			
Pos.	181	obs:	exp:
tta	L	0	0.11
ttg	L	0	0.19
ctt	L	0	0.20
ctc	L	0	0.15
cta	L	1	0.17
ctg	L	0	0.18
att	I	14	919.10
atc	I	11	664.60
ata	I	2566	1007.00
atg	M	21	21.00
gtt	V	0	2.89
gtc	V	0	2.45
gta	V	10	3.28
gtg	V	3	4.38
---	--	------	------
mPD		0.047	0.67
		nPD:	0.07
		N. weight:	1.4
		Sc. PD:	-0.28
		Sc. rank:	-1284.7

,

PB1			
Pos.	182	obs:	exp:
cgt	R	0	0.03
cgc	R	0	0.05
cga	R	1	0.10
cgg	R	0	0.07
att	I	1	9.58
atc	I	0	6.93
ata	I	26	10.50
act	T	8	633.40
acc	T	2	556.80
aca	T	2512	1170.00
acg	T	53	214.70
aga	R	0	0.46
agg	R	0	0.28
gtt	V	0	4.88
gtc	V	0	4.15
gta	V	20	5.55
gtg	V	2	7.41
gct	A	0	0.25
gcc	A	0	0.23
gca	A	1	0.45
gcg	A	0	0.07
---	--	------	------
mPD		0.10	0.74
		nPD:	0.14
		N. weight:	1.1
		Sc. PD:	-0.15
		Sc. rank:	-444.4

,

PB1			
Pos.	183	obs:	exp:
att	I	0	0.35
atc	I	0	0.26
ata	I	1	0.39
act	T	161	645.20
acc	T	581	567.20
aca	T	1869	1192.00
acg	T	12	218.70
gct	A	0	0.49
gcc	A	0	0.46
gca	A	2	0.90
gcg	A	0	0.15
---	--	------	------
mPD		0.44	0.68
		nPD:	0.65
		N. weight:	0.36
		Sc. PD:	0.13
		Sc. rank:	449.8

,

PB1			
Pos.	184	obs:	exp:
cat	H	580	1515.00
cac	H	2046	1111.00
---	--	------	------
mPD		0.34	0.49
		nPD:	0.71
		N. weight:	0.41
		Sc. PD:	0.17
		Sc. rank:	547.7

,

PB1			
Pos.	185	obs:	exp:
ttt	F	2084	1176.00
ttc	F	540	1448.00
tta	L	0	0.22
ttg	L	0	0.38
ctt	L	2	0.40
ctc	L	0	0.29
cta	L	0	0.35
ctg	L	0	0.36
---	--	------	------
mPD		0.33	0.50
		nPD:	0.66
		N. weight:	0.4
		Sc. PD:	0.14
		Sc. rank:	498.9

,

PB1			
Pos.	186	obs:	exp:
tta	L	0	0.11
ttg	L	0	0.19
ctt	L	0	0.20
ctc	L	0	0.15
cta	L	1	0.17
ctg	L	0	0.18
caa	Q	2583	1416.00
cag	Q	42	1209.00
---	--	------	------
mPD		0.032	0.50
		nPD:	0.06
		N. weight:	0.84
		Sc. PD:	-0.18
		Sc. rank:	-825.1

,

PB1			
Pos.	187	obs:	exp:
cgt	R	0	82.61
cgc	R	0	138.30
cga	R	67	263.00
cgg	R	1	189.10
aaa	K	2	5.47
aag	K	7	3.53
aga	R	2073	1200.00
agg	R	476	743.80
---	--	------	------
mPD		0.36	0.94
		nPD:	0.38
		N. weight:	0.5
		Sc. PD:	0.046
		Sc. rank:	258.7

,

PB1			
Pos.	188	obs:	exp:
cgt	R	0	0.03
cgc	R	0	0.05
cga	R	0	0.10
cgg	R	0	0.07
aaa	K	1981	1595.00
aag	K	644	1030.00
aga	R	0	0.46
agg	R	1	0.28
---	--	------	------
mPD		0.37	0.48
		nPD:	0.78
		N. weight:	0.078
		Sc. PD:	0.037
		Sc. rank:	109.6

,

PB1			
Pos.	189	obs:	exp:
cgt	R	0	80.37
cgc	R	0	134.60
cga	R	3	255.90
cgg	R	7	183.90
aaa	K	3	48.62
aag	K	77	31.38
aga	R	617	1168.00
agg	R	1919	723.60
---	--	------	------
mPD		0.43	0.99
		nPD:	0.43
		N. weight:	0.9
		Sc. PD:	0.13
		Sc. rank:	570.1

,

PB1			
Pos.	190	obs:	exp:
cgt	R	0	82.70
cgc	R	0	138.50
cga	R	0	263.30
cgg	R	0	189.30
aaa	K	6	3.65
aag	K	0	2.35
aga	R	2545	1202.00
agg	R	75	744.70
---	--	------	------
mPD		0.060	0.94
		nPD:	0.06
		N. weight:	1.
		Sc. PD:	-0.22
		Sc. rank:	-1027.8

,

PB1			
Pos.	191	obs:	exp:
tta	L	7	0.76
ttg	L	0	1.31
ctt	L	0	1.41
ctc	L	0	1.02
cta	L	0	1.22
ctg	L	0	1.27
att	I	13	198.70
atc	I	1	143.60
ata	I	546	217.70
atg	M	1	1.00
act	T	0	0.74
acc	T	0	0.65
aca	T	3	1.36
acg	T	0	0.25
gtt	V	15	456.10
gtc	V	0	387.60
gta	V	1191	518.50
gtg	V	848	691.80
gct	A	0	0.25
gcc	A	0	0.23
gca	A	1	0.45
gcg	A	0	0.07
---	--	------	------
mPD		0.80	1.1
		nPD:	0.73
		N. weight:	0.95
		Sc. PD:	0.41
		Sc. rank:	1295.6

PB1			
Pos.	192	obs:	exp:
cgt	R	0	82.73
cgc	R	0	138.50
cga	R	0	263.40
cgg	R	1	189.40
atg	M	1	1.00
aaa	K	2	2.43
aag	K	2	1.57
aga	R	1796	1202.00
agg	R	824	745.00
---	--	------	------
mPD		0.44	0.94
		nPD:	0.46
		N. weight:	0.48
		Sc. PD:	0.083
		Sc. rank:	345.7

PB1			
Pos.	193	obs:	exp:
gat	D	949	1477.00
gac	D	1677	1149.00
---	--	------	------
mPD		0.46	0.49
		nPD:	0.94
		N. weight:	0.13
		Sc. PD:	0.082
		Sc. rank:	187.8

PB1			
Pos.	194	obs:	exp:
tct	S	0	2.02
tcc	S	0	1.90
tca	S	0	2.91
tcg	S	0	0.64
tat	Y	1	6.72
tac	Y	11	5.28
cat	H	2	2.31
cac	H	2	1.69
act	T	0	0.25
acc	T	1	0.22
aca	T	0	0.45
acg	T	0	0.08
aat	N	91	1476.00
aac	N	2506	1121.00
agt	S	0	2.23
agc	S	12	2.30
---	--	------	------
mPD		0.091	0.52
		nPD:	0.18
		N. weight:	1.1
		Sc. PD:	-0.11
		Sc. rank:	-177.6

PB1			
Pos.	195	obs:	exp:
tta	L	0	0.44
ttg	L	3	0.75
tgg	W	2	2.00
ctt	L	0	0.81
ctc	L	0	0.58
cta	L	0	0.70
ctg	L	1	0.73
att	I	5	24.12
atc	I	1	17.44
ata	I	62	26.44
atg	M	2509	2509.00
act	T	0	6.64
acc	T	0	5.84
aca	T	18	12.27
acg	T	9	2.25
gtt	V	0	3.55
gtc	V	0	3.02
gta	V	0	4.04
gtg	V	16	5.39
---	--	------	------
mPD		0.10	0.12
		nPD:	0.87
		N. weight:	0.053
		Sc. PD:	0.03
		Sc. rank:	75.8

PB1			
Pos.	196	obs:	exp:
tct	S	1	2.19
tcc	S	2	2.05
tca	S	0	3.16
tcg	S	0	0.69
act	T	78	642.70
acc	T	2482	565.00
aca	T	25	1187.00
acg	T	28	217.90
agt	S	0	2.42
agc	S	10	2.49
---	--	------	------
mPD		0.11	0.69
		nPD:	0.15
		N. weight:	2.
		Sc. PD:	-0.25
		Sc. rank:	-698.0

PB1			
Pos.	197	obs:	exp:
cgt	R	0	0.51
cgc	R	0	0.85
cga	R	0	1.61
cgg	R	0	1.16
aaa	K	835	1586.00
aag	K	1774	1023.00
aga	R	7	7.34
agg	R	9	4.55
ggt	G	0	0.16
ggc	G	0	0.13
gga	G	0	0.45
ggg	G	1	0.26
---	--	------	------
mPD		0.45	0.49
		nPD:	0.91
		N. weight:	0.27
		Sc. PD:	0.16
		Sc. rank:	388.4

PB1			
Pos.	198	obs:	exp:
cgt	R	0	0.54
cgc	R	0	0.90
cga	R	0	1.71
cgg	R	0	1.23
aaa	K	483	1586.00
aag	K	2126	1023.00
aga	R	9	7.80
agg	R	8	4.83
---	--	------	------
mPD		0.32	0.49
		nPD:	0.64
		N. weight:	0.59
		Sc. PD:	0.2
		Sc. rank:	726.8

PB1			
Pos.	199	obs:	exp:
tta	L	0	0.44
ttg	L	4	0.75
ctt	L	0	0.81
ctc	L	0	0.58
cta	L	0	0.70
ctg	L	0	0.73
atg	M	2620	2620.00
gtt	V	0	0.44
gtc	V	0	0.38
gta	V	0	0.50
gtg	V	2	0.67
---	--	------	------
mPD		0.0046	0.0075
		nPD:	0.61
		N. weight:	0.0053
		Sc. PD:	0.0017
		Sc. rank:	6.0

PB1			
Pos.	200	obs:	exp:
ttt	F	0	0.45
ttc	F	1	0.55
att	I	0	49.66
atc	I	61	35.91
ata	I	79	54.43
gtt	V	16	551.80
gtc	V	1938	468.90
gta	V	500	627.30
gtg	V	31	837.00
---	--	------	------
mPD		0.47	0.84
		nPD:	0.56
		N. weight:	1.5
		Sc. PD:	0.41
		Sc. rank:	1594.0

PB1			
Pos.	201	obs:	exp:
att	I	0	0.35
atc	I	0	0.26
ata	I	1	0.39
atg	M	1	1.00
act	T	4	645.20
acc	T	1	567.20
aca	T	1905	1192.00
acg	T	713	218.70
gct	A	0	0.25
gcc	A	0	0.23
gca	A	0	0.45
gcg	A	1	0.07
---	--	------	------
mPD		0.40	0.68
		nPD:	0.59
		N. weight:	1.
		Sc. PD:	0.3
		Sc. rank:	1131.2

PB1			
Pos.	202	obs:	exp:
cat	H	1	1.73
cac	H	2	1.27
caa	Q	2584	1413.00
cag	Q	37	1208.00
cgt	R	0	0.03
cgc	R	0	0.05
cga	R	1	0.10
cgg	R	0	0.07
aaa	K	1	0.61
aag	K	0	0.39
aga	R	0	0.46
agg	R	0	0.28
---	--	------	------
mPD		0.032	0.50
		nPD:	0.06
		N. weight:	0.85
		Sc. PD:	-0.18
		Sc. rank:	-853.9

PB1			
Pos.	203	obs:	exp:
cgt	R	0	82.89
cgc	R	0	138.80
cga	R	28	263.90
cgg	R	3	189.70
aga	R	2069	1204.00
agg	R	526	746.40
---	--	------	------
mPD		0.35	0.94
		nPD:	0.37
		N. weight:	0.52
		Sc. PD:	0.042
		Sc. rank:	241.4

PB1			
Pos.	204	obs:	exp:
atg	M	1	1.00
act	T	24	645.40
acc	T	0	567.40
aca	T	2563	1192.00
acg	T	37	218.80
gct	A	0	0.25
gcc	A	0	0.23
gca	A	1	0.45
gcg	A	0	0.07
---	--	------	------
mPD		0.048	0.68
		nPD:	0.07
		N. weight:	1.1
		Sc. PD:	-0.22
		Sc. rank:	-1018.0

PB1			
Pos.	205	obs:	exp:
tta	L	1	0.11
ttg	L	0	0.19
ctt	L	0	0.20
ctc	L	0	0.15
cta	L	0	0.17
ctg	L	0	0.18
att	I	6	928.40
atc	I	4	671.20
ata	I	2607	1017.00
atg	M	4	4.00
act	T	0	0.25
acc	T	0	0.22
aca	T	1	0.45
acg	T	0	0.08
gtt	V	0	0.67
gtc	V	0	0.57
gta	V	3	0.76
gtg	V	0	1.01
---	--	------	------
mPD		0.014	0.66
		nPD:	0.02
		N. weight:	1.4
		Sc. PD:	-0.36
		Sc. rank:	-1908.4

PB1			
Pos.	206	obs:	exp:
ggt	G	16	411.90
ggc	G	13	332.70
gga	G	666	1189.00
ggg	G	1931	691.90
---	--	------	------
mPD		0.40	0.68
		nPD:	0.58
		N. weight:	0.9
		Sc. PD:	0.25
		Sc. rank:	972.6

PB1			
Pos.	207	obs:	exp:
cgt	R	0	0.13
cgc	R	0	0.21
cga	R	0	0.40
cgg	R	0	0.29
aaa	K	33	1593.00
aag	K	2589	1029.00
aga	R	0	1.83
agg	R	4	1.14
---	--	------	------
mPD		0.028	0.48
		nPD:	0.06
		N. weight:	1.3
		Sc. PD:	-0.29
		Sc. rank:	-1416.0

PB1			
Pos.	208	obs:	exp:
cgt	R	0	0.13
cgc	R	0	0.21
cga	R	0	0.40
cgg	R	0	0.29
aaa	K	2462	1593.00
aag	K	160	1029.00
aga	R	2	1.83
agg	R	2	1.14
---	--	------	------
mPD		0.12	0.48
		nPD:	0.25
		N. weight:	0.46
		Sc. PD:	-0.016
		Sc. rank:	70.8

PB1			
Pos.	209	obs:	exp:
cgt	R	0	0.19
cgc	R	0	0.32
cga	R	0	0.60
cgg	R	0	0.43
aaa	K	2286	1592.00
aag	K	334	1028.00
aga	R	2	2.75
agg	R	4	1.71
---	--	------	------
mPD		0.23	0.48
		nPD:	0.47
		N. weight:	0.27
		Sc. PD:	0.05
		Sc. rank:	215.7

PB1			
Pos.	210	obs:	exp:
tta	L	0	0.65
ttg	L	0	1.13
ctt	L	0	1.21
ctc	L	0	0.87
cta	L	2	1.05
ctg	L	4	1.09
cat	H	15	9.81
cac	H	2	7.19
caa	Q	2180	1404.00
cag	Q	423	1199.00
---	--	------	------
mPD		0.29	0.51
		nPD:	0.56
		N. weight:	0.32
		Sc. PD:	0.086
		Sc. rank:	335.4

PB1			
Pos.	211	obs:	exp:
cgt	R	0	59.41
cgc	R	0	99.47
cga	R	5	189.20
cgg	R	0	136.00
act	T	0	0.25
acc	T	0	0.22
aca	T	1	0.45
acg	T	0	0.08
aat	N	0	0.57
aac	N	1	0.43
aaa	K	623	450.90
aag	K	119	291.10
aga	R	1730	863.10
agg	R	147	534.90
---	--	------	------
mPD		0.59	1.2
		nPD:	0.48
		N. weight:	0.65
		Sc. PD:	0.12
		Sc. rank:	524.3

,

PB1			
Pos.	212	obs:	exp:
tta	L	25	124.80
ttg	L	65	215.40
ctt	L	4	231.60
ctc	L	0	166.90
cta	L	45	200.30
ctg	L	1008	207.90
att	I	12	4.26
atc	I	0	3.08
ata	I	0	4.67
atg	M	760	760.00
gtt	V	0	157.00
gtc	V	0	133.40
gta	V	15	178.50
gtg	V	692	238.10
---	--	------	------
mPD		0.75	1.4
		nPD:	0.55
		N. weight:	1.3
		Sc. PD:	0.33
		Sc. rank:	1303.3

,

PB1			
Pos.	213	obs:	exp:
tct	S	0	27.92
tcc	S	0	26.24
tca	S	0	40.31
tcg	S	0	8.82
tat	Y	0	0.56
tac	Y	1	0.44
cat	H	1	0.58
cac	H	0	0.42
act	T	14	3.69
acc	T	0	3.24
aca	T	1	6.82
acg	T	0	1.25
aat	N	1806	1296.00
aac	N	475	984.50
aaa	K	5	13.98
aag	K	18	9.02
agt	S	49	30.90
agc	S	117	31.81
gtt	V	1	0.22
gtc	V	0	0.19
gta	V	0	0.25
gtg	V	0	0.34
gat	D	133	75.37
gac	D	1	58.63
gaa	E	2	1.19
gag	E	0	0.81
ggt	G	0	0.31
ggc	G	2	0.25
gga	G	0	0.91
ggg	G	0	0.53
---	--	------	------
mPD		0.60	0.83
		nPD:	0.72
		N. weight:	0.32
		Sc. PD:	0.14
		Sc. rank:	435.4

,

PB1			
Pos.	214	obs:	exp:
cgt	R	0	5.27
cgc	R	0	8.83
cga	R	0	16.79
cgg	R	0	12.06
aat	N	1	0.57
aac	N	0	0.43
aaa	K	127	1494.00
aag	K	2331	964.20
aga	R	16	76.59
agg	R	151	47.47
---	--	------	------
mPD		0.22	0.63
		nPD:	0.35
		N. weight:	1.1
		Sc. PD:	0.075
		Sc. rank:	498.5

,

PB1			
Pos.	215	obs:	exp:
cgt	R	0	74.27
cgc	R	0	124.40
cga	R	0	236.50
cgg	R	0	170.00
aaa	K	89	165.90
aag	K	184	107.10
aga	R	1324	1079.00
agg	R	1029	668.80
---	--	------	------
mPD		0.68	1.1
		nPD:	0.63
		N. weight:	0.45
		Sc. PD:	0.15
		Sc. rank:	545.0

,

PB1			
Pos.	216	obs:	exp:
tct	S	0	299.60
tcc	S	0	281.50
tca	S	0	432.50
tcg	S	0	94.66
tgt	C	8	5.60
tgc	C	5	7.40
cat	H	1	0.58
cac	H	0	0.42
cgt	R	0	0.03
cgc	R	1	0.05
cga	R	0	0.10
cgg	R	0	0.07
att	I	2	0.71
atc	I	0	0.51
ata	I	0	0.78
aat	N	87	68.77
aac	N	34	52.23
aaa	K	12	7.29
aag	K	0	4.71
agt	S	1653	331.50
agc	S	128	341.30
aga	R	0	0.46
agg	R	0	0.28
gct	A	0	1.47
gcc	A	6	1.37
gca	A	0	2.70
gcg	A	0	0.45
gat	D	1	11.81
gac	D	20	9.19
ggt	G	122	104.80
ggc	G	543	84.64
gga	G	2	302.60
ggg	G	1	176.00
---	--	------	------
mPD		0.93	1.9
		nPD:	0.48
		N. weight:	2.1
		Sc. PD:	0.41
		Sc. rank:	1741.4

,

PB1			
Pos.	217	obs:	exp:
tat	Y	2512	1471.00
tac	Y	114	1155.00
---	--	------	------
mPD		0.083	0.49
		nPD:	0.17
		N. weight:	0.64
		Sc. PD:	-0.071
		Sc. rank:	-158.9

,

PB1			
Pos.	218	obs:	exp:
tta	L	900	285.00
ttg	L	11	491.90
ctt	L	0	528.90
ctc	L	3	381.10
cta	L	1659	457.30
ctg	L	46	474.80
att	I	0	2.48
atc	I	0	1.80
ata	I	7	2.72
---	--	------	------
mPD		0.50	1.1
		nPD:	0.44
		N. weight:	1.8
		Sc. PD:	0.27
		Sc. rank:	1164.2

,

PB1			
Pos.	219	obs:	exp:
tta	L	0	0.22
ttg	L	0	0.38
ctt	L	0	0.40
ctc	L	0	0.29
cta	L	2	0.35
ctg	L	0	0.36
att	I	31	927.70
atc	I	6	670.70
ata	I	2578	1017.00
act	T	0	0.25
acc	T	0	0.22
aca	T	1	0.45
acg	T	0	0.08
gtt	V	0	1.78
gtc	V	1	1.51
gta	V	7	2.02
gtg	V	0	2.69
---	--	------	------
mPD		0.037	0.67
		nPD:	0.06
		N. weight:	1.4
		Sc. PD:	-0.3
		Sc. rank:	-1465.5

,

PB1			
Pos.	220	obs:	exp:
tct	S	0	1.51
tcc	S	0	1.42
tca	S	0	2.19
tcg	S	0	0.48
cgt	R	1	82.07
cgc	R	0	137.40
cga	R	12	261.30
cgg	R	0	187.80
aaa	K	17	10.33
aag	K	0	6.67
agt	S	9	1.68
agc	S	0	1.72
aga	R	2531	1192.00
agg	R	56	739.00
---	--	------	------
mPD		0.072	0.96
		nPD:	0.08
		N. weight:	1.
		Sc. PD:	-0.21
		Sc. rank:	-910.3

,

PB1			
Pos.	221	obs:	exp:
tct	S	0	2.52
tcc	S	0	2.37
tca	S	15	3.64
tcg	S	0	0.80
act	T	0	0.25
acc	T	0	0.22
aca	T	1	0.45
acg	T	0	0.08
agt	S	0	2.79
agc	S	0	2.87
gct	A	5	639.80
gcc	A	31	598.00
gca	A	2559	1176.00
gcg	A	15	195.70
---	--	------	------
mPD		0.050	0.70
		nPD:	0.07
		N. weight:	1.1
		Sc. PD:	-0.22
		Sc. rank:	-1021.8

PB1			
Pos.	222	obs:	exp:
tta	L	44	285.80
ttg	L	551	493.30
ctt	L	43	530.30
ctc	L	6	382.10
cta	L	970	458.50
ctg	L	1012	476.00
---	--	------	------
mPD		0.85	1.1
		nPD:	0.74
		N. weight:	0.8
		Sc. PD:	0.36
		Sc. rank:	1104.4

PB1			
Pos.	223	obs:	exp:
act	T	32	645.90
acc	T	7	567.80
aca	T	2553	1193.00
acg	T	34	219.00
---	--	------	------
mPD		0.055	0.68
		nPD:	0.08
		N. weight:	1.
		Sc. PD:	-0.2
		Sc. rank:	-882.5

PB1			
Pos.	224	obs:	exp:
ttt	F	1	0.45
ttc	F	0	0.55
tta	L	1122	285.70
ttg	L	1416	493.10
ctt	L	4	530.10
ctc	L	0	381.90
cta	L	56	458.40
ctg	L	27	475.80
---	--	------	------
mPD		0.56	1.1
		nPD:	0.49
		N. weight:	1.7
		Sc. PD:	0.34
		Sc. rank:	1461.8

PB1			
Pos.	225	obs:	exp:
aat	N	2068	1493.00
aac	N	558	1133.00
---	--	------	------
mPD		0.33	0.49
		nPD:	0.68
		N. weight:	0.17
		Sc. PD:	0.065
		Sc. rank:	216.6

PB1			
Pos.	226	obs:	exp:
act	T	17	645.90
acc	T	1	567.80
aca	T	518	1193.00
acg	T	2090	219.00
---	--	------	------
mPD		0.33	0.68
		nPD:	0.48
		N. weight:	2.5
		Sc. PD:	0.48
		Sc. rank:	2067.9

PB1			
Pos.	227	obs:	exp:
atg	M	2626	2626.00
---	--	------	------
mPD		0	0
		nPD:	1.
		N. weight:	0.
		Sc. PD:	0
		Sc. rank:	0

PB1			
Pos.	228	obs:	exp:
act	T	49	645.70
acc	T	2149	567.60
aca	T	425	1193.00
acg	T	2	218.90
gct	A	0	0.25
gcc	A	1	0.23
gca	A	0	0.45
gcg	A	0	0.07
---	--	------	------
mPD		0.30	0.68
		nPD:	0.45
		N. weight:	1.4
		Sc. PD:	0.22
		Sc. rank:	917.8

PB1			
Pos.	229	obs:	exp:
aaa	K	2436	1596.00
aag	K	190	1030.00
---	--	------	------
mPD		0.13	0.48
		nPD:	0.28
		N. weight:	0.42
		Sc. PD:	-0.00032
		Sc. rank:	96.1

PB1			
Pos.	230	obs:	exp:
aat	N	0	0.57
aac	N	1	0.43
gat	D	2385	1476.00
gac	D	240	1149.00
---	--	------	------
mPD		0.17	0.49
		nPD:	0.34
		N. weight:	0.46
		Sc. PD:	0.025
		Sc. rank:	187.3

PB1			
Pos.	231	obs:	exp:
gct	A	88	642.50
gcc	A	7	600.50
gca	A	2495	1181.00
gcg	A	31	196.60
ggt	G	0	0.78
ggc	G	0	0.63
gga	G	5	2.26
ggg	G	0	1.32
---	--	------	------
mPD		0.096	0.68
		nPD:	0.14
		N. weight:	0.96
		Sc. PD:	-0.13
		Sc. rank:	-403.2

PB1			
Pos.	232	obs:	exp:
gaa	E	514	1556.00
gag	E	2112	1070.00
---	--	------	------
mPD		0.31	0.48
		nPD:	0.65
		N. weight:	0.52
		Sc. PD:	0.18
		Sc. rank:	644.6

PB1			
Pos.	233	obs:	exp:
cgt	R	0	82.86
cgc	R	0	138.70
cga	R	9	263.80
cgg	R	0	189.60
aga	R	2477	1204.00
agg	R	139	746.10
gaa	E	0	0.59
gag	E	1	0.41
---	--	------	------
mPD		0.11	0.94
		nPD:	0.12
		N. weight:	0.91
		Sc. PD:	-0.15
		Sc. rank:	-535.4

PB1			
Pos.	234	obs:	exp:
cgt	R	0	0.03
cgc	R	0	0.05
cga	R	0	0.10
cgg	R	0	0.07
aga	R	0	0.46
agg	R	1	0.28
ggt	G	95	411.70
ggc	G	2426	332.60
gga	G	94	1189.00
ggg	G	10	691.70
---	--	------	------
mPD		0.14	0.69
		nPD:	0.21
		N. weight:	2.6
		Sc. PD:	-0.18
		Sc. rank:	79.8

PB1			
Pos.	235	obs:	exp:
caa	Q	1	0.54
cag	Q	0	0.46
aaa	K	1738	1595.00
aag	K	887	1030.00
---	--	------	------
mPD		0.45	0.48
		nPD:	0.94
		N. weight:	0.013
		Sc. PD:	0.008
		Sc. rank:	18.3

PB1			
Pos.	236	obs:	exp:
tta	L	1314	285.80
ttg	L	1254	493.30
ctt	L	0	530.30
ctc	L	0	382.10
cta	L	21	458.50
ctg	L	37	476.00
---	--	------	------
mPD		0.54	1.1
		nPD:	0.48
		N. weight:	1.8
		Sc. PD:	0.34
		Sc. rank:	1437.4

PB1			
Pos.	237	obs:	exp:
cgt	R	0	0.38
cgc	R	0	0.63
cga	R	0	1.21
cgg	R	0	0.87
aaa	K	2567	1589.00
aag	K	47	1025.00
aga	R	7	5.50
agg	R	5	3.41
---	--	------	------
mPD		0.048	0.49
		nPD:	0.1
		N. weight:	0.65
		Sc. PD:	-0.12
		Sc. rank:	-453.2

PB1			
Pos.	238	obs:	exp:
cgt	R	0	82.89
cgc	R	0	138.80
cga	R	0	263.90
cgg	R	3	189.70
aga	R	1552	1204.00
agg	R	1071	746.40
---	--	------	------
mPD		0.49	0.94
		nPD:	0.52
		N. weight:	0.46
		Sc. PD:	0.1
		Sc. rank:	431.2

PB1			
Pos.	239	obs:	exp:
cgt	R	409	82.89
cgc	R	1	138.80
cga	R	2	263.90
cgg	R	2	189.70
aga	R	377	1204.00
agg	R	1835	746.40
---	--	------	------
mPD		0.73	0.94
		nPD:	0.78
		N. weight:	1.1
		Sc. PD:	0.53
		Sc. rank:	1543.4

PB1			
Pos.	240	obs:	exp:
tct	S	0	0.17
tcc	S	0	0.16
tca	S	1	0.24
tcg	S	0	0.05
agt	S	0	0.19
agc	S	0	0.19
gct	A	1920	643.50
gcc	A	96	601.50
gca	A	601	1183.00
gcg	A	8	196.90
---	--	------	------
mPD		0.41	0.68
		nPD:	0.61
		N. weight:	0.89
		Sc. PD:	0.28
		Sc. rank:	1013.5

PB1			
Pos.	241	obs:	exp:
att	I	1525	931.60
atc	I	1075	673.50
ata	I	26	1021.00
---	--	------	------
mPD		0.50	0.66
		nPD:	0.75
		N. weight:	0.69
		Sc. PD:	0.31
		Sc. rank:	955.7

PB1			
Pos.	242	obs:	exp:
gct	A	4	643.70
gcc	A	18	601.70
gca	A	2431	1184.00
gcg	A	173	196.90
---	--	------	------
mPD		0.14	0.68
		nPD:	0.2
		N. weight:	0.98
		Sc. PD:	-0.074
		Sc. rank:	-2.4

PB1			
Pos.	243	obs:	exp:
act	T	11	645.90
acc	T	21	567.80
aca	T	2567	1193.00
acg	T	27	219.00
---	--	------	------
mPD		0.044	0.68
		nPD:	0.07
		N. weight:	1.1
		Sc. PD:	-0.22
		Sc. rank:	-1045.6

PB1			
Pos.	244	obs:	exp:
cct	P	2130	774.60
ccc	P	38	528.50
cca	P	440	1040.00
ccg	P	17	282.40
cat	H	1	0.58
cac	H	0	0.42
---	--	------	------
mPD		0.31	0.70
		nPD:	0.45
		N. weight:	0.97
		Sc. PD:	0.15
		Sc. rank:	649.6

PB1

Pos.	245	obs:	exp:
ggt	G	6	411.90
ggc	G	0	332.70
gga	G	188	1189.00
ggg	G	2432	691.90
---	--	------	------
mPD		0.14	0.68
		nPD:	0.2
		N. weight:	1.6
		Sc. PD:	-0.13
		Sc. rank:	-17.6

PB1

Pos.	246	obs:	exp:
att	I	2	0.71
atc	I	0	0.51
ata	I	0	0.78
atg	M	2624	2624.00
---	--	------	------
mPD		0.0015	0.0015
		nPD:	1.
		N. weight:	0.0012
		Sc. PD:	0.00085
		Sc. rank:	1.8

PB1

Pos.	247	obs:	exp:
cat	H	0	2.31
cac	H	4	1.69
caa	Q	1583	1414.00
cag	Q	1039	1208.00
---	--	------	------
mPD		0.48	0.50
		nPD:	0.96
		N. weight:	0.018
		Sc. PD:	0.012
		Sc. rank:	25.6

PB1

Pos.	248	obs:	exp:
tta	L	0	0.11
ttg	L	0	0.19
ctt	L	1	0.20
ctc	L	0	0.15
cta	L	0	0.17
ctg	L	0	0.18
att	I	2030	930.80
atc	I	561	673.00
ata	I	33	1020.00
act	T	1	0.25
acc	T	0	0.22
aca	T	0	0.45
acg	T	0	0.08
---	--	------	------
mPD		0.36	0.66
		nPD:	0.54
		N. weight:	0.82
		Sc. PD:	0.2
		Sc. rank:	836.9

PB1

Pos.	249	obs:	exp:
cgt	R	0	82.89
cgc	R	0	138.80
cga	R	4	263.90
cgg	R	5	189.70
aga	R	2559	1204.00
agg	R	58	746.40
---	--	------	------
mPD		0.054	0.94
		nPD:	0.06
		N. weight:	1.
		Sc. PD:	-0.23
		Sc. rank:	-1102.7

PB1

Pos.	250	obs:	exp:
ggt	G	743	411.90
ggc	G	19	332.70
gga	G	378	1189.00
ggg	G	1486	691.90
---	--	------	------
mPD		0.58	0.68
		nPD:	0.85
		N. weight:	0.65
		Sc. PD:	0.35
		Sc. rank:	927.8

PB1

Pos.	251	obs:	exp:
ttt	F	611	1177.00
ttc	F	2015	1449.00
---	--	------	------
mPD		0.36	0.49
		nPD:	0.72
		N. weight:	0.16
		Sc. PD:	0.067
		Sc. rank:	215.1

PB1

Pos.	252	obs:	exp:
gtt	V	20	583.10
gtc	V	12	495.50
gta	V	793	662.90
gtg	V	1801	884.50
---	--	------	------
mPD		0.44	0.74
		nPD:	0.59
		N. weight:	0.78
		Sc. PD:	0.24
		Sc. rank:	871.9

PB1

Pos.	253	obs:	exp:
ttt	F	1	0.90
ttc	F	1	1.10
tta	L	0	0.11
ttg	L	0	0.19
tat	Y	296	1469.00
tac	Y	2326	1153.00
ctt	L	1	0.20
ctc	L	0	0.15
cta	L	0	0.17
ctg	L	0	0.18
cat	H	0	0.58
cac	H	1	0.42
---	--	------	------
mPD		0.21	0.50
		nPD:	0.41
		N. weight:	0.69
		Sc. PD:	0.087
		Sc. rank:	411.6

PB1

Pos.	254	obs:	exp:
ttt	F	2215	1173.00
ttc	F	402	1444.00
tta	L	0	0.22
ttg	L	0	0.38
tct	S	0	0.17
tcc	S	1	0.16
tca	S	0	0.24
tcg	S	0	0.05
tgt	C	1	0.86
tgc	C	1	1.14
ctt	L	1	0.40
ctc	L	1	0.29
cta	L	0	0.35
ctg	L	0	0.36
att	I	1	0.35
atc	I	0	0.26
ata	I	0	0.39
agt	S	0	0.19
agc	S	0	0.19
gtt	V	0	0.67
gtc	V	3	0.57
gta	V	0	0.76
gtg	V	0	1.01
---	--	------	------
mPD		0.27	0.50
		nPD:	0.54
		N. weight:	0.54
		Sc. PD:	0.13
		Sc. rank:	530.7

PB1

Pos.	255	obs:	exp:
gtt	V	2032	582.80
gtc	V	13	495.30
gta	V	527	662.70
gtg	V	53	884.20
gct	A	1	0.25
gcc	A	0	0.23
gca	A	0	0.45
gcg	A	0	0.07
---	--	------	------
mPD		0.36	0.74
		nPD:	0.49
		N. weight:	1.3
		Sc. PD:	0.26
		Sc. rank:	1121.8

PB1

Pos.	256	obs:	exp:
gaa	E	1406	1556.00
gag	E	1220	1070.00
---	--	------	------
mPD		0.50	0.48
		nPD:	1.03
		N. weight:	0.013
		Sc. PD:	0.0093
		Sc. rank:	18.7

PB1

Pos.	257	obs:	exp:
tct	S	32	5.55
tcc	S	0	5.22
tca	S	0	8.01
tcg	S	0	1.75
att	I	23	8.87
atc	I	1	6.41
ata	I	1	9.72
atg	M	2	2.00
act	T	2012	592.10
acc	T	13	520.50
aca	T	361	1094.00
acg	T	21	200.70
aat	N	3	1.71
aac	N	0	1.29
agt	S	1	6.14
agc	S	0	6.32
gtt	V	1	0.22
gtc	V	0	0.19
gta	V	0	0.25
gtg	V	0	0.34
gct	A	97	38.00
gcc	A	6	35.51
gca	A	49	69.87
gcg	A	3	11.62
---	--	------	------
mPD		0.45	0.84
		nPD:	0.54
		N. weight:	1.3
		Sc. PD:	0.31
		Sc. rank:	1274.9

PB1

Pos.	258	obs:	exp:
tta	L	1239	285.80
ttg	L	61	493.30
ctt	L	1	530.30
ctc	L	2	382.10
cta	L	974	458.50
ctg	L	349	476.00
---	--	------	------
mPD		0.77	1.1
		nPD:	0.67
		N. weight:	1.4
		Sc. PD:	0.52
		Sc. rank:	1738.6

PB1

Pos.	259	obs:	exp:
gct	A	2122	643.70
gcc	A	59	601.70
gca	A	100	1184.00
gcg	A	345	196.90
---	--	------	------
mPD		0.33	0.68
		nPD:	0.48
		N. weight:	1.4
		Sc. PD:	0.27
		Sc. rank:	1158.8

PB1

Pos.	260	obs:	exp:
cgt	R	0	82.20
cgc	R	0	137.60
cga	R	4	261.70
cgg	R	3	188.10
aaa	K	14	12.76
aag	K	7	8.24
aga	R	1504	1194.00
agg	R	1093	740.10
ggt	G	0	0.16
ggc	G	0	0.13
gga	G	1	0.45
ggg	G	0	0.26
---	--	------	------
mPD		0.51	0.95
		nPD:	0.53
		N. weight:	0.45
		Sc. PD:	0.11
		Sc. rank:	438.8

PB1

Pos.	261	obs:	exp:
tct	S	0	432.40
tcc	S	0	406.30
tca	S	0	624.30
tcg	S	0	136.60
tgt	C	0	1.72
tgc	C	4	2.28
cgt	R	0	0.38
cgc	R	5	0.63
cga	R	0	1.21
cgg	R	0	0.87
att	I	0	3.55
atc	I	10	2.56
ata	I	0	3.89
aat	N	1	15.35
aac	N	26	11.65
agt	S	50	478.50
agc	S	2521	492.70
aga	R	7	5.50
agg	R	0	3.41
ggt	G	0	0.31
ggc	G	2	0.25
gga	G	0	0.91
ggg	G	0	0.53
---	--	------	------
mPD		0.080	1.7
		nPD:	0.05
		N. weight:	2.4
		Sc. PD:	-0.54
		Sc. rank:	-2712.9

PB1

Pos.	262	obs:	exp:
att	I	2174	931.60
atc	I	414	673.50
ata	I	38	1021.00
---	--	------	------
mPD		0.29	0.66
		nPD:	0.44
		N. weight:	0.9
		Sc. PD:	0.14
		Sc. rank:	597.1

PB1

Pos.	263	obs:	exp:
tgt	C	590	1130.00
tgc	C	2036	1496.00
---	--	------	------
mPD		0.35	0.49
		nPD:	0.71
		N. weight:	0.15
		Sc. PD:	0.061
		Sc. rank:	196.2

PB1

Pos.	264	obs:	exp:
gat	D	2	1.12
gac	D	0	0.88
gaa	E	1431	1554.00
gag	E	1192	1069.00
ggt	G	0	0.16
ggc	G	0	0.13
gga	G	1	0.45
ggg	G	0	0.26
---	--	------	------
mPD		0.50	0.48
		nPD:	1.03
		N. weight:	0.011
		Sc. PD:	0.0076
		Sc. rank:	15.5

PB1

Pos.	265	obs:	exp:
cgt	R	0	1.39
cgc	R	0	2.33
cga	R	0	4.42
cgg	R	0	3.18
aaa	K	525	1569.00
aag	K	2057	1013.00
aga	R	2	20.18
agg	R	42	12.51
---	--	------	------
mPD		0.35	0.52
		nPD:	0.68
		N. weight:	0.56
		Sc. PD:	0.21
		Sc. rank:	714.8

PB1

Pos.	266	obs:	exp:
tta	L	0	285.70
ttg	L	0	493.10
ctt	L	2564	530.10
ctc	L	45	381.90
cta	L	13	458.40
ctg	L	3	475.80
att	I	1	0.35
atc	I	0	0.26
ata	I	0	0.39
---	--	------	------
mPD		0.046	1.1
		nPD:	0.04
		N. weight:	2.3
		Sc. PD:	-0.54
		Sc. rank:	-2776.2

PB1

Pos.	267	obs:	exp:
gaa	E	2164	1556.00
gag	E	462	1070.00
---	--	------	------
mPD		0.29	0.48
		nPD:	0.6
		N. weight:	0.2
		Sc. PD:	0.06
		Sc. rank:	220.5

PB1

Pos.	268	obs:	exp:
caa	Q	1379	1416.00
cag	Q	1247	1210.00
---	--	------	------
mPD		0.50	0.50
		nPD:	1.
		N. weight:	0.0031
		Sc. PD:	0.0022
		Sc. rank:	4.5

PB1

Pos.	269	obs:	exp:
tct	S	1175	441.70
tcc	S	1290	415.00
tca	S	123	637.70
tcg	S	38	139.60
agt	S	0	488.80
agc	S	0	503.30
---	--	------	------
mPD		0.56	1.6
		nPD:	0.34
		N. weight:	1.4
		Sc. PD:	0.08
		Sc. rank:	582.5

PB1

Pos.	270	obs:	exp:
ggt	G	1	411.90
ggc	G	5	332.70
gga	G	1572	1189.00
ggg	G	1048	691.90
---	--	------	------
mPD		0.48	0.68
		nPD:	0.7
		N. weight:	0.51
		Sc. PD:	0.21
		Sc. rank:	670.5

PB1

Pos.	271	obs:	exp:
tta	L	17	285.80
ttg	L	14	493.30
ctt	L	110	530.30
ctc	L	2436	382.10
cta	L	39	458.50
ctg	L	10	476.00
---	--	------	------
mPD		0.16	1.1
		nPD:	0.14
		N. weight:	2.4
		Sc. PD:	-0.33
		Sc. rank:	-1006.6

PB1

Pos.	272	obs:	exp:
cct	P	10	774.90
ccc	P	4	528.70
cca	P	1867	1040.00
ccg	P	745	282.50
---	--	------	------
mPD		0.41	0.70
		nPD:	0.59
		N. weight:	1.
		Sc. PD:	0.31
		Sc. rank:	1153.7

PB1

Pos.	273	obs:	exp:
att	I	0	0.35
atc	I	0	0.26
ata	I	1	0.39
gtt	V	431	582.80
gtc	V	28	495.30
gta	V	2121	662.70
gtg	V	45	884.20
---	--	------	------
mPD		0.32	0.74
		nPD:	0.43
		N. weight:	1.3
		Sc. PD:	0.18
		Sc. rank:	800.2

PB1

Pos.	274	obs:	exp:
cgt	R	0	0.03
cgc	R	0	0.05
cga	R	0	0.10
cgg	R	0	0.07
aga	R	0	0.46
agg	R	1	0.28
ggt	G	81	411.70
ggc	G	0	332.60
gga	G	297	1189.00
ggg	G	2247	691.70
---	--	------	------
mPD		0.25	0.69
		nPD:	0.37
		N. weight:	1.3
		Sc. PD:	0.11
		Sc. rank:	608.9

PB1

Pos.	275	obs:	exp:
ggt	G	48	411.90
ggc	G	2091	332.70
gga	G	283	1189.00
ggg	G	204	691.90
---	--	------	------
mPD		0.35	0.68
		nPD:	0.51
		N. weight:	1.8
		Sc. PD:	0.4
		Sc. rank:	1684.2

PB1

Pos.	276	obs:	exp:
aat	N	2576	1493.00
aac	N	50	1133.00
---	--	------	------
mPD		0.037	0.49
		nPD:	0.08
		N. weight:	0.74
		Sc. PD:	-0.15
		Sc. rank:	-647.6

PB1

Pos.	277	obs:	exp:
gaa	E	2521	1556.00
gag	E	105	1070.00
---	--	------	------
mPD		0.077	0.48
		nPD:	0.16
		N. weight:	0.58
		Sc. PD:	-0.069
		Sc. rank:	-183.4

PB1

Pos.	278	obs:	exp:
aaa	K	166	1596.00
aag	K	2460	1030.00
---	--	------	------
mPD		0.12	0.48
		nPD:	0.25
		N. weight:	1.1
		Sc. PD:	-0.034
		Sc. rank:	165.1

PB1

Pos.	279	obs:	exp:
aaa	K	1860	1596.00
aag	K	766	1030.00
---	--	------	------
mPD		0.41	0.48
		nPD:	0.87
		N. weight:	0.037
		Sc. PD:	0.021
		Sc. rank:	52.7

PB1

Pos.	280	obs:	exp:
cct	P	0	0.30
ccc	P	1	0.20
cca	P	0	0.40
ccg	P	0	0.11
gct	A	80	643.50
gcc	A	2474	601.50
gca	A	70	1183.00
gcg	A	1	196.90
---	--	------	------
mPD		0.11	0.68
		nPD:	0.16
		N. weight:	1.9
		Sc. PD:	-0.21
		Sc. rank:	-546.9

PB1

Pos.	281	obs:	exp:
aaa	K	2568	1596.00
aag	K	58	1030.00
---	--	------	------
mPD		0.043	0.48
		nPD:	0.09
		N. weight:	0.63
		Sc. PD:	-0.12
		Sc. rank:	-495.1

PB1

Pos.	282	obs:	exp:
tta	L	14	285.80
ttg	L	1859	493.30
ctt	L	6	530.30
ctc	L	0	382.10
cta	L	2	458.50
ctg	L	745	476.00
---	--	------	------
mPD		0.43	1.1
		nPD:	0.37
		N. weight:	1.6
		Sc. PD:	0.14
		Sc. rank:	805.1

PB1

Pos.	283	obs:	exp:
gct	A	0	643.70
gcc	A	1	601.70
gca	A	2615	1184.00
gcg	A	10	196.90
---	--	------	------
mPD		0.0083	0.68
		nPD:	0.01
		N. weight:	1.2
		Sc. PD:	-0.32
		Sc. rank:	-1699.9

PB1

Pos.	284	obs:	exp:
aat	N	1703	1493.00
aac	N	923	1133.00
---	--	------	------
mPD		0.46	0.49
		nPD:	0.93
		N. weight:	0.023
		Sc. PD:	0.014
		Sc. rank:	33.6

PB1

Pos. 285		obs:	exp:
gtt	V	2605	583.10
gtc	V	19	495.50
gta	V	2	662.90
gtg	V	0	884.50
---	--	------	------
mPD		0.016	0.74
		nPD:	0.02
		N. weight:	2.3
		Sc. PD:	-0.57
		Sc. rank:	-3041.3

PB1

Pos. 286		obs:	exp:
att	I	1	0.35
atc	I	0	0.26
ata	I	0	0.39
gtt	V	24	582.80
gtc	V	0	495.30
gta	V	165	662.70
gtg	V	2436	884.20
---	--	------	------
mPD		0.14	0.74
		nPD:	0.18
		N. weight:	1.3
		Sc. PD:	-0.12
		Sc. rank:	-117.2

PB1

Pos. 287		obs:	exp:
cgt	R	0	82.80
cgc	R	0	138.60
cga	R	24	263.60
cgg	R	2	189.50
atg	M	1	1.00
aaa	K	1	1.22
aag	K	1	0.78
aga	R	2151	1203.00
agg	R	446	745.50
---	--	------	------
mPD		0.31	0.94
		nPD:	0.33
		N. weight:	0.57
		Sc. PD:	0.024
		Sc. rank:	203.3

PB1

Pos. 288		obs:	exp:
aaa	K	578	1596.00
aag	K	2048	1030.00
---	--	------	------
mPD		0.34	0.48
		nPD:	0.72
		N. weight:	0.49
		Sc. PD:	0.21
		Sc. rank:	661.6

PB1

Pos. 289		obs:	exp:
atg	M	2626	2626.00
---	--	------	------
mPD		0	0
		nPD:	1.
		N. weight:	0.
		Sc. PD:	0
		Sc. rank:	0

PB1

Pos. 290		obs:	exp:
att	I	1	0.35
atc	I	0	0.26
ata	I	0	0.39
atg	M	2625	2625.00
---	--	------	------
mPD		0.00076	0.00076
		nPD:	1.
		N. weight:	0.00062
		Sc. PD:	0.00043
		Sc. rank:	0.9

PB1

Pos. 291		obs:	exp:
att	I	3	3.19
atc	I	6	2.31
ata	I	0	3.50
act	T	2139	642.20
acc	T	441	564.60
aca	T	29	1186.00
acg	T	2	217.70
aat	N	1	1.14
aac	N	1	0.86
gct	A	3	0.98
gcc	A	1	0.92
gca	A	0	1.80
gcg	A	0	0.30
---	--	------	------
mPD		0.31	0.69
		nPD:	0.45
		N. weight:	1.4
		Sc. PD:	0.23
		Sc. rank:	986.5

PB1

Pos. 292		obs:	exp:
tct	S	0	2.52
tcc	S	0	2.37
tca	S	0	3.64
tcg	S	0	0.80
aat	N	2036	1484.00
aac	N	575	1127.00
agt	S	3	2.79
agc	S	12	2.87
---	--	------	------
mPD		0.36	0.51
		nPD:	0.7
		N. weight:	0.17
		Sc. PD:	0.067
		Sc. rank:	218.4

PB1

Pos. 293		obs:	exp:
tct	S	10	441.50
tcc	S	16	414.90
tca	S	2582	637.50
tcg	S	17	139.50
agt	S	0	488.60
agc	S	0	503.10
gct	A	0	0.25
gcc	A	0	0.23
gca	A	1	0.45
gcg	A	0	0.07
---	--	------	------
mPD		0.033	1.6
		nPD:	0.02
		N. weight:	2.1
		Sc. PD:	-0.52
		Sc. rank:	-2809.4

PB1

Pos. 294		obs:	exp:
cat	H	0	1.15
cac	H	2	0.85
caa	Q	2587	1414.00
cag	Q	36	1209.00
cgt	R	0	0.03
cgc	R	0	0.05
cga	R	0	0.10
cgg	R	1	0.07
aga	R	0	0.46
agg	R	0	0.28
---	--	------	------
mPD		0.030	0.50
		nPD:	0.06
		N. weight:	0.86
		Sc. PD:	-0.18
		Sc. rank:	-869.2

PB1

Pos. 295		obs:	exp:
gat	D	449	1477.00
gac	D	2177	1149.00
---	--	------	------
mPD		0.28	0.49
		nPD:	0.58
		N. weight:	0.51
		Sc. PD:	0.14
		Sc. rank:	549.9

PB1

Pos. 296		obs:	exp:
tct	S	0	0.17
tcc	S	0	0.16
tca	S	1	0.24
tcg	S	0	0.05
act	T	23	645.70
acc	T	1	567.60
aca	T	2559	1193.00
acg	T	42	218.90
agt	S	0	0.19
agc	S	0	0.19
---	--	------	------
mPD		0.050	0.68
		nPD:	0.07
		N. weight:	1.1
		Sc. PD:	-0.22
		Sc. rank:	-964.1

PB1

Pos. 297		obs:	exp:
gat	D	1	0.56
gac	D	0	0.44
gaa	E	69	1555.00
gag	E	2556	1070.00
---	--	------	------
mPD		0.052	0.48
		nPD:	0.11
		N. weight:	1.2
		Sc. PD:	-0.2
		Sc. rank:	-776.8

PB1

Pos. 298		obs:	exp:
tta	L	0	207.60
ttg	L	0	358.40
ctt	L	1769	385.30
ctc	L	132	277.60
cta	L	3	333.20
ctg	L	4	345.90
att	I	684	251.50
atc	I	18	181.90
ata	I	7	275.60
atg	M	1	1.00
act	T	5	1.23
acc	T	0	1.08
aca	T	0	2.27
acg	T	0	0.42
gtt	V	3	0.67
gtc	V	0	0.57
gta	V	0	0.76
gtg	V	0	1.01
---	--	------	------
mPD		0.52	1.4
		nPD:	0.38
		N. weight:	1.9
		Sc. PD:	0.18
		Sc. rank:	1017.3

PB1

Pos. 299		obs:	exp:
tct	S	1349	441.70
tcc	S	1214	415.00
tca	S	63	637.70
tcg	S	0	139.60
agt	S	0	488.80
agc	S	0	503.30
---	--	------	------
mPD		0.52	1.6
		nPD:	0.32
		N. weight:	1.6
		Sc. PD:	0.058
		Sc. rank:	545.5

PB1

Pos. 300		obs:	exp:
ttt	F	455	1177.00
ttc	F	2171	1449.00
---	--	------	------
mPD		0.29	0.49
		nPD:	0.58
		N. weight:	0.27
		Sc. PD:	0.076
		Sc. rank:	289.4

PB1

Pos. 301		obs:	exp:
act	T	9	645.90
acc	T	35	567.80
aca	T	2525	1193.00
acg	T	57	219.00
---	--	------	------
mPD		0.075	0.68
		nPD:	0.11
		N. weight:	1.
		Sc. PD:	-0.17
		Sc. rank:	-624.7

PB1

Pos. 302		obs:	exp:
att	I	1183	929.80
atc	I	1394	672.30
ata	I	44	1019.00
gtt	V	4	1.11
gtc	V	1	0.94
gta	V	0	1.26
gtg	V	0	1.68
---	--	------	------
mPD		0.52	0.66
		nPD:	0.78
		N. weight:	0.7
		Sc. PD:	0.34
		Sc. rank:	981.6

PB1

Pos. 303		obs:	exp:
act	T	2539	645.70
acc	T	31	567.60
aca	T	31	1193.00
acg	T	24	218.90
gct	A	1	0.25
gcc	A	0	0.23
gca	A	0	0.45
gcg	A	0	0.07
---	--	------	------
mPD		0.065	0.68
		nPD:	0.1
		N. weight:	1.9
		Sc. PD:	-0.35
		Sc. rank:	-1422.9

PB1

Pos. 304		obs:	exp:
ggt	G	32	411.90
ggc	G	1	332.70
gga	G	741	1189.00
ggg	G	1852	691.90
---	--	------	------
mPD		0.42	0.68
		nPD:	0.62
		N. weight:	0.83
		Sc. PD:	0.27
		Sc. rank:	966.6

PB1

Pos. 305		obs:	exp:
gat	D	340	1477.00
gac	D	2286	1149.00
---	--	------	------
mPD		0.23	0.49
		nPD:	0.46
		N. weight:	0.64
		Sc. PD:	0.11
		Sc. rank:	458.9

PB1

Pos. 306		obs:	exp:
aat	N	714	1493.00
aac	N	1912	1133.00
---	--	------	------
mPD		0.40	0.49
		nPD:	0.81
		N. weight:	0.28
		Sc. PD:	0.14
		Sc. rank:	401.2

PB1

Pos. 307		obs:	exp:
act	T	2104	645.90
acc	T	502	567.80
aca	T	20	1193.00
acg	T	0	219.00
---	--	------	------
mPD		0.32	0.68
		nPD:	0.47
		N. weight:	1.4
		Sc. PD:	0.26
		Sc. rank:	1101.6

PB1

Pos. 308		obs:	exp:
aaa	K	1828	1596.00
aag	K	798	1030.00
---	--	------	------
mPD		0.42	0.48
		nPD:	0.89
		N. weight:	0.029
		Sc. PD:	0.017
		Sc. rank:	41.5

PB1

Pos. 309		obs:	exp:
tgg	W	2626	2626.00
---	--	------	------
mPD		0	0
		nPD:	1.
		N. weight:	0.
		Sc. PD:	0
		Sc. rank:	0

PB1			
Pos.	310	obs:	exp:
aat	N	2607	1493.00
aac	N	19	1133.00
---	--	------	------
mPD		0.014	0.49
		nPD:	0.03
		N. weight:	0.82
		Sc. PD:	-0.2
		Sc. rank:	-1060.7

,

PB1			
Pos.	311	obs:	exp:
gaa	E	2161	1556.00
gag	E	465	1070.00
---	--	------	------
mPD		0.29	0.48
		nPD:	0.6
		N. weight:	0.19
		Sc. PD:	0.06
		Sc. rank:	219.3

,

PB1			
Pos.	312	obs:	exp:
aat	N	1179	1493.00
aac	N	1447	1133.00
---	--	------	------
mPD		0.49	0.49
		nPD:	1.01
		N. weight:	0.047
		Sc. PD:	0.033
		Sc. rank:	68.7

,

PB1			
Pos.	313	obs:	exp:
caa	Q	2519	1416.00
cag	Q	107	1210.00
---	--	------	------
mPD		0.078	0.50
		nPD:	0.16
		N. weight:	0.71
		Sc. PD:	-0.085
		Sc. rank:	-238.1

,

PB1			
Pos.	314	obs:	exp:
tct	S	0	0.50
tcc	S	0	0.47
tca	S	0	0.73
tcg	S	0	0.16
aat	N	2181	1491.00
aac	N	442	1132.00
agt	S	1	0.56
agc	S	2	0.57
---	--	------	------
mPD		0.28	0.49
		nPD:	0.57
		N. weight:	0.25
		Sc. PD:	0.07
		Sc. rank:	267.9

,

PB1			
Pos.	315	obs:	exp:
tta	L	0	0.11
ttg	L	0	0.19
ctt	L	0	0.20
ctc	L	1	0.15
cta	L	0	0.17
ctg	L	0	0.18
cct	P	1993	774.60
ccc	P	604	528.50
cca	P	26	1040.00
ccg	P	2	282.40
---	--	------	------
mPD		0.37	0.71
		nPD:	0.53
		N. weight:	1.1
		Sc. PD:	0.26
		Sc. rank:	1069.1

,

PB1			
Pos.	316	obs:	exp:
cgt	R	9	82.86
cgc	R	3	138.70
cga	R	2154	263.80
cgg	R	145	189.60
aaa	K	0	0.61
aag	K	1	0.39
aga	R	34	1204.00
agg	R	280	746.10
---	--	------	------
mPD		0.49	0.94
		nPD:	0.52
		N. weight:	2.4
		Sc. PD:	0.56
		Sc. rank:	2314.0

,

PB1			
Pos.	317	obs:	exp:
att	I	34	86.91
atc	I	0	62.84
ata	I	211	95.25
atg	M	2070	2070.00
act	T	0	2.95
acc	T	0	2.59
aca	T	1	5.45
acg	T	11	1.00
gtt	V	1	66.39
gtc	V	0	56.42
gta	V	25	75.48
gtg	V	273	100.70
---	--	------	------
mPD		0.40	0.52
		nPD:	0.77
		N. weight:	0.24
		Sc. PD:	0.11
		Sc. rank:	337.1

,

PB1			
Pos.	318	obs:	exp:
ttt	F	503	1177.00
ttc	F	2123	1449.00
---	--	------	------
mPD		0.31	0.49
		nPD:	0.63
		N. weight:	0.23
		Sc. PD:	0.076
		Sc. rank:	274.1

,

PB1			
Pos.	319	obs:	exp:
tta	L	22	285.70
ttg	L	79	493.10
ctt	L	39	530.10
ctc	L	0	381.90
cta	L	145	458.40
ctg	L	2340	475.80
cct	P	0	0.30
ccc	P	0	0.20
cca	P	0	0.40
ccg	P	1	0.11
---	--	------	------
mPD		0.22	1.1
		nPD:	0.19
		N. weight:	1.9
		Sc. PD:	-0.17
		Sc. rank:	-74.2

,

PB1			
Pos.	320	obs:	exp:
act	T	3	9.35
acc	T	0	8.22
aca	T	7	17.27
acg	T	28	3.17
gtt	V	0	0.44
gtc	V	0	0.38
gta	V	0	0.50
gtg	V	2	0.67
gct	A	83	633.90
gcc	A	6	592.50
gca	A	347	1166.00
gcg	A	2150	193.90
---	--	------	------
mPD		0.32	0.71
		nPD:	0.45
		N. weight:	2.7
		Sc. PD:	0.45
		Sc. rank:	1910.9

,

PB1			
Pos.	321	obs:	exp:
atg	M	2626	2626.00
---	--	------	------
mPD		0	0
		nPD:	1.
		N. weight:	0.
		Sc. PD:	0
		Sc. rank:	0

,

PB1			
Pos.	322	obs:	exp:
att	I	2076	902.10
atc	I	58	652.30
ata	I	409	988.60
atg	M	1	1.00
gtt	V	79	18.21
gtc	V	0	15.47
gta	V	3	20.70
gtg	V	0	27.62
---	--	------	------
mPD		0.36	0.73
		nPD:	0.5
		N. weight:	0.8
		Sc. PD:	0.17
		Sc. rank:	714.9

,

PB1			
Pos.	323	obs:	exp:
atg	M	2	2.00
act	T	60	645.40
acc	T	2	567.40
aca	T	2282	1192.00
acg	T	280	218.80
---	--	------	------
mPD		0.23	0.68
		nPD:	0.34
		N. weight:	0.83
		Sc. PD:	0.05
		Sc. rank:	347.7

,

PB1			
Pos.	324	obs:	exp:
tat	Y	1373	1471.00
tac	Y	1252	1154.00
cat	H	0	0.58
cac	H	1	0.42
---	--	------	------
mPD		0.50	0.49
		nPD:	1.01
		N. weight:	0.0073
		Sc. PD:	0.0052
		Sc. rank:	10.7

,

PB1			
Pos.	325	obs:	exp:
att	I	215	930.80
atc	I	2268	673.00
ata	I	141	1020.00
atg	M	2	2.00
---	--	------	------
mPD		0.24	0.66
		nPD:	0.37
		N. weight:	1.3
		Sc. PD:	0.11
		Sc. rank:	631.5

,

PB1			
Pos.	326	obs:	exp:
act	T	82	645.90
acc	T	2063	567.80
aca	T	479	1193.00
acg	T	2	219.00
---	--	------	------
mPD		0.35	0.68
		nPD:	0.51
		N. weight:	1.2
		Sc. PD:	0.27
		Sc. rank:	1134.5

,

PB1			
Pos.	327	obs:	exp:
cgt	R	0	82.20
cgc	R	0	137.60
cga	R	17	261.70
cgg	R	0	188.10
act	T	0	0.25
acc	T	0	0.22
aca	T	1	0.45
acg	T	0	0.08
aaa	K	21	12.76
aag	K	0	8.24
aga	R	2531	1194.00
agg	R	56	740.10
---	--	------	------
mPD		0.071	0.95
		nPD:	0.07
		N. weight:	1.
		Sc. PD:	-0.21
		Sc. rank:	-902.8

,

PB1			
Pos.	328	obs:	exp:
tct	S	0	2.35
tcc	S	0	2.21
tca	S	0	3.40
tcg	S	0	0.74
act	T	1	0.25
acc	T	0	0.22
aca	T	0	0.45
acg	T	0	0.08
aat	N	1669	1462.00
aac	N	903	1110.00
aaa	K	8	4.86
aag	K	0	3.14
agt	S	13	2.61
agc	S	1	2.68
gat	D	11	6.75
gac	D	1	5.25
ggt	G	6	2.98
ggc	G	13	2.41
gga	G	0	8.61
ggg	G	0	5.01
---	--	------	------
mPD		0.51	0.56
		nPD:	0.91
		N. weight:	0.058
		Sc. PD:	0.035
		Sc. rank:	83.6

,

PB1			
Pos.	329	obs:	exp:
caa	Q	2182	1416.00
cag	Q	444	1210.00
---	--	------	------
mPD		0.28	0.50
		nPD:	0.57
		N. weight:	0.3
		Sc. PD:	0.081
		Sc. rank:	315.6

,

PB1			
Pos.	330	obs:	exp:
cct	P	520	774.90
ccc	P	2011	528.70
cca	P	89	1040.00
ccg	P	6	282.50
---	--	------	------
mPD		0.37	0.70
		nPD:	0.53
		N. weight:	1.3
		Sc. PD:	0.32
		Sc. rank:	1298.1

PB1			
Pos.	331	obs:	exp:
gtt	V	0	0.22
gtc	V	0	0.19
gta	V	0	0.25
gtg	V	1	0.34
gat	D	21	12.94
gac	D	2	10.06
gaa	E	458	1542.00
gag	E	2144	1060.00
---	--	------	------
mPD		0.30	0.49
		nPD:	0.62
		N. weight:	0.57
		Sc. PD:	0.18
		Sc. rank:	663.9

PB1			
Pos.	332	obs:	exp:
tgg	W	2626	2626.00
---	--	------	------
mPD		0	0
		nPD:	1.
		N. weight:	0.
		Sc. PD:	0
		Sc. rank:	0

PB1			
Pos.	333	obs:	exp:
ttt	F	100	1172.00
ttc	F	2515	1443.00
tta	L	7	1.20
ttg	L	0	2.07
ctt	L	0	2.22
ctc	L	3	1.60
cta	L	1	1.92
ctg	L	0	1.99
---	--	------	------
mPD		0.082	0.50
		nPD:	0.16
		N. weight:	0.7
		Sc. PD:	-0.08
		Sc. rank:	-202.0

PB1			
Pos.	334	obs:	exp:
cgt	R	0	82.89
cgc	R	0	138.80
cga	R	15	263.90
cgg	R	0	189.70
aga	R	2473	1204.00
agg	R	138	746.40
---	--	------	------
mPD		0.11	0.94
		nPD:	0.12
		N. weight:	0.9
		Sc. PD:	-0.14
		Sc. rank:	-498.5

PB1			
Pos.	335	obs:	exp:
aat	N	484	1493.00
aac	N	2142	1133.00
---	--	------	------
mPD		0.30	0.49
		nPD:	0.61
		N. weight:	0.49
		Sc. PD:	0.16
		Sc. rank:	560.6

PB1			
Pos.	336	obs:	exp:
att	I	89	775.10
atc	I	1786	560.40
ata	I	310	849.50
act	T	0	0.25
acc	T	0	0.22
aca	T	1	0.45
acg	T	0	0.08
gtt	V	399	97.70
gtc	V	35	83.03
gta	V	6	111.10
gtg	V	0	148.20
---	--	------	------
mPD		0.75	0.97
		nPD:	0.77
		N. weight:	1.2
		Sc. PD:	0.59
		Sc. rank:	1726.0

PB1			
Pos.	337	obs:	exp:
tta	L	325	284.80
ttg	L	76	491.60
ctt	L	1	528.50
ctc	L	0	380.80
cta	L	89	457.00
ctg	L	2126	474.40
atg	M	9	9.00
---	--	------	------
mPD		0.53	1.1
		nPD:	0.46
		N. weight:	1.8
		Sc. PD:	0.31
		Sc. rank:	1294.7

PB1			
Pos.	338	obs:	exp:
tct	S	0	441.70
tcc	S	0	415.00
tca	S	0	637.70
tcg	S	0	139.60
agt	S	469	488.80
agc	S	2157	503.30
---	--	------	------
mPD		0.29	1.6
		nPD:	0.18
		N. weight:	1.9
		Sc. PD:	-0.18
		Sc. rank:	-245.0

PB1			
Pos.	339	obs:	exp:
tta	L	0	0.22
ttg	L	0	0.38
ctt	L	0	0.40
ctc	L	0	0.29
cta	L	2	0.35
ctg	L	0	0.36
att	I	62	150.40
atc	I	333	108.80
ata	I	29	164.80
atg	M	2175	2175.00
gtt	V	18	5.55
gtc	V	2	4.72
gta	V	0	6.31
gtg	V	5	8.42
---	--	------	------
mPD		0.31	0.32
		nPD:	0.98
		N. weight:	0.18
		Sc. PD:	0.12
		Sc. rank:	255.4

PB1			
Pos.	340	obs:	exp:
gct	A	45	643.70
gcc	A	389	601.70
gca	A	2123	1184.00
gcg	A	69	196.90
---	--	------	------
mPD		0.32	0.68
		nPD:	0.48
		N. weight:	0.53
		Sc. PD:	0.099
		Sc. rank:	418.4

PB1			
Pos.	341	obs:	exp:
cct	P	465	774.90
ccc	P	1886	528.70
cca	P	273	1040.00
ccg	P	2	282.50
---	--	------	------
mPD		0.44	0.70
		nPD:	0.63
		N. weight:	1.1
		Sc. PD:	0.35
		Sc. rank:	1282.4

PB1			
Pos.	342	obs:	exp:
att	I	3	926.60
atc	I	17	669.90
ata	I	2592	1015.00
gtt	V	0	3.11
gtc	V	0	2.64
gta	V	14	3.53
gtg	V	0	4.72
---	--	------	------
mPD		0.026	0.67
		nPD:	0.04
		N. weight:	1.4
		Sc. PD:	-0.33
		Sc. rank:	-1727.3

PB1			
Pos.	343	obs:	exp:
atg	M	2626	2626.00
---	--	------	------
mPD		0	0
		nPD:	1.
		N. weight:	0.
		Sc. PD:	0
		Sc. rank:	0

PB1			
Pos.	344	obs:	exp:
ttt	F	133	1177.00
ttc	F	2493	1449.00
---	--	------	------
mPD		0.096	0.49
		nPD:	0.19
		N. weight:	0.63
		Sc. PD:	-0.054
		Sc. rank:	-22.6

PB1			
Pos.	345	obs:	exp:
tct	S	11	441.70
tcc	S	5	415.00
tca	S	2572	637.70
tcg	S	38	139.60
agt	S	0	488.80
agc	S	0	503.30
---	--	------	------
mPD		0.040	1.6
		nPD:	0.02
		N. weight:	2.1
		Sc. PD:	-0.51
		Sc. rank:	-2711.3

PB1			
Pos.	346	obs:	exp:
aat	N	169	1493.00
aac	N	2457	1133.00
---	--	------	------
mPD		0.12	0.49
		nPD:	0.25
		N. weight:	0.91
		Sc. PD:	-0.032
		Sc. rank:	133.1

PB1			
Pos.	347	obs:	exp:
aaa	K	2575	1596.00
aag	K	51	1030.00
---	--	------	------
mPD		0.038	0.48
		nPD:	0.08
		N. weight:	0.64
		Sc. PD:	-0.13
		Sc. rank:	-545.2

PB1			
Pos.	348	obs:	exp:
tta	L	0	1.96
ttg	L	16	3.38
ctt	L	0	3.64
ctc	L	0	2.62
cta	L	0	3.14
ctg	L	2	3.26
atg	M	2596	2596.00
gtt	V	0	2.66
gtc	V	0	2.26
gta	V	0	3.03
gtg	V	12	4.04
---	--	------	------
mPD		0.023	0.037
		nPD:	0.61
		N. weight:	0.023
		Sc. PD:	0.0071
		Sc. rank:	25.8

PB1			
Pos.	349	obs:	exp:
act	T	0	0.25
acc	T	0	0.22
aca	T	1	0.45
acg	T	0	0.08
gct	A	33	643.50
gcc	A	7	601.50
gca	A	2560	1183.00
gcg	A	25	196.90
---	--	------	------
mPD		0.049	0.68
		nPD:	0.07
		N. weight:	1.1
		Sc. PD:	-0.22
		Sc. rank:	-984.6

PB1			
Pos.	350	obs:	exp:
cgt	R	0	81.97
cgc	R	0	137.30
cga	R	1	261.00
cgg	R	1	187.60
aaa	K	24	17.62
aag	K	5	11.38
aga	R	2412	1191.00
agg	R	183	738.10
---	--	------	------
mPD		0.16	0.96
		nPD:	0.16
		N. weight:	0.86
		Sc. PD:	-0.098
		Sc. rank:	-238.7

PB1			
Pos.	351	obs:	exp:
tta	L	419	285.60
ttg	L	46	492.90
ctt	L	70	529.90
ctc	L	0	381.80
cta	L	1981	458.20
ctg	L	108	475.70
att	I	0	0.71
atc	I	0	0.51
ata	I	2	0.78
---	--	------	------
mPD		0.45	1.1
		nPD:	0.4
		N. weight:	1.6
		Sc. PD:	0.17
		Sc. rank:	887.0

PB1			
Pos.	352	obs:	exp:
ggt	G	30	411.90
ggc	G	1	332.70
gga	G	882	1189.00
ggg	G	1713	691.90
---	--	------	------
mPD		0.46	0.68
		nPD:	0.67
		N. weight:	0.72
		Sc. PD:	0.27
		Sc. rank:	916.5

PB1			
Pos.	353	obs:	exp:
cgt	R	0	3.00
cgc	R	0	5.02
cga	R	0	9.55
cgg	R	0	6.86
aat	N	1	0.57
aac	N	0	0.43
aaa	K	2415	1538.00
aag	K	115	992.40
aga	R	92	43.57
agg	R	3	27.00
---	--	------	------
mPD		0.16	0.57
		nPD:	0.28
		N. weight:	0.54
		Sc. PD:	-0.0039
		Sc. rank:	117.5

PB1			
Pos.	354	obs:	exp:
ggt	G	89	411.90
ggc	G	19	332.70
gga	G	655	1189.00
ggg	G	1863	691.90
---	--	------	------
mPD		0.43	0.68
		nPD:	0.63
		N. weight:	0.76
		Sc. PD:	0.26
		Sc. rank:	918.5

PB1			
Pos.	355	obs:	exp:
ttt	F	0	3.14
ttc	F	7	3.86
tat	Y	111	1467.00
tac	Y	2508	1152.00
---	--	------	------
mPD		0.086	0.50
		nPD:	0.17
		N. weight:	0.99
		Sc. PD:	-0.1
		Sc. rank:	-186.7

,

PB1			
Pos.	356	obs:	exp:
atg	M	2626	2626.00
---	--	------	------
mPD		0	0
		nPD:	1.
		N. weight:	0.
		Sc. PD:	0
		Sc. rank:	0

,

PB1			
Pos.	357	obs:	exp:
ttt	F	156	1177.00
ttc	F	2470	1449.00
---	--	------	------
mPD		0.11	0.49
		nPD:	0.23
		N. weight:	0.6
		Sc. PD:	-0.032
		Sc. rank:	55.1

,

PB1			
Pos.	358	obs:	exp:
gaa	E	449	1556.00
gag	E	2177	1070.00
---	--	------	------
mPD		0.28	0.48
		nPD:	0.59
		N. weight:	0.59
		Sc. PD:	0.17
		Sc. rank:	648.0

,

PB1			
Pos.	359	obs:	exp:
tct	S	0	441.70
tcc	S	0	415.00
tca	S	0	637.70
tcg	S	0	139.60
agt	S	2495	488.80
agc	S	131	503.30
---	--	------	------
mPD		0.095	1.6
		nPD:	0.06
		N. weight:	2.3
		Sc. PD:	-0.5
		Sc. rank:	-2409.0

,

PB1			
Pos.	360	obs:	exp:
cgt	R	0	1.20
cgc	R	0	2.01
cga	R	0	3.82
cgg	R	0	2.75
aaa	K	2203	1573.00
aag	K	385	1015.00
aga	R	22	17.43
agg	R	16	10.80
---	--	------	------
mPD		0.29	0.51
		nPD:	0.56
		N. weight:	0.23
		Sc. PD:	0.061
		Sc. rank:	239.3

,

PB1			
Pos.	361	obs:	exp:
tct	S	0	68.63
tcc	S	0	64.48
tca	S	0	99.08
tcg	S	0	21.68
cgt	R	0	60.95
cgc	R	0	102.10
cga	R	4	194.10
cgg	R	3	139.50
att	I	0	7.80
atc	I	0	5.64
ata	I	22	8.55
atg	M	8	8.00
act	T	0	0.49
acc	T	2	0.43
aca	T	0	0.91
acg	T	0	0.17
aat	N	0	12.50
aac	N	22	9.50
aaa	K	206	138.00
aag	K	21	89.04
agt	S	15	75.94
agc	S	393	78.19
aga	R	1190	885.60
agg	R	734	548.80
ggt	G	1	0.94
ggc	G	0	0.76
gga	G	5	2.72
ggg	G	0	1.58
---	--	------	------
mPD		0.80	1.4
		nPD:	0.56
		N. weight:	0.75
		Sc. PD:	0.2
		Sc. rank:	781.9

,

PB1			
Pos.	362	obs:	exp:
tta	L	0	0.22
ttg	L	1	0.38
ctt	L	0	0.40
ctc	L	0	0.29
cta	L	0	0.35
ctg	L	1	0.36
atg	M	2624	2624.00
---	--	------	------
mPD		0.0015	0.0025
		nPD:	0.61
		N. weight:	0.0016
		Sc. PD:	0.00051
		Sc. rank:	1.8

,

PB1			
Pos.	363	obs:	exp:
cgt	R	0	1.23
cgc	R	0	2.06
cga	R	0	3.92
cgg	R	0	2.82
aaa	K	544	1572.00
aag	K	2043	1015.00
aga	R	2	17.89
agg	R	37	11.08
---	--	------	------
mPD		0.36	0.51
		nPD:	0.7
		N. weight:	0.53
		Sc. PD:	0.21
		Sc. rank:	696.4

,

PB1			
Pos.	364	obs:	exp:
tta	L	17	201.20
ttg	L	0	347.30
ctt	L	66	373.40
ctc	L	1396	269.00
cta	L	358	322.90
ctg	L	12	335.20
att	I	652	266.10
atc	I	67	192.40
ata	I	31	291.60
act	T	1	0.25
acc	T	0	0.22
aca	T	0	0.45
acg	T	0	0.08
gtt	V	24	5.77
gtc	V	1	4.91
gta	V	1	6.56
gtg	V	0	8.76
---	--	------	------
mPD		1.0	1.4
		nPD:	0.74
		N. weight:	1.6
		Sc. PD:	0.69
		Sc. rank:	2146.1

,

PB1			
Pos.	365	obs:	exp:
cgt	R	0	82.89
cgc	R	0	138.80
cga	R	2425	263.90
cgg	R	136	189.70
aga	R	44	1204.00
agg	R	21	746.40
---	--	------	------
mPD		0.16	0.94
		nPD:	0.17
		N. weight:	3.
		Sc. PD:	-0.33
		Sc. rank:	-613.7

,

PB1			
Pos.	366	obs:	exp:
act	T	24	643.00
acc	T	1	565.30
aca	T	2562	1188.00
acg	T	27	218.00
gct	A	0	2.94
gcc	A	0	2.75
gca	A	12	5.41
gcg	A	0	0.90
---	--	------	------
mPD		0.048	0.69
		nPD:	0.07
		N. weight:	1.1
		Sc. PD:	-0.22
		Sc. rank:	-1037.3

,

PB1			
Pos.	367	obs:	exp:
caa	Q	1389	1416.00
cag	Q	1237	1210.00
---	--	------	------
mPD		0.50	0.50
		nPD:	1.
		N. weight:	0.0028
		Sc. PD:	0.0019
		Sc. rank:	4.1

,

PB1			
Pos.	368	obs:	exp:
tta	L	28	4.24
ttg	L	1	7.33
ctt	L	0	7.88
ctc	L	0	5.67
cta	L	10	6.81
ctg	L	0	7.07
att	I	3	882.20
atc	I	31	637.90
ata	I	2453	966.90
gtt	V	0	22.20
gtc	V	0	18.87
gta	V	98	25.24
gtg	V	2	33.68
---	--	------	------
mPD		0.13	0.77
		nPD:	0.17
		N. weight:	1.4
		Sc. PD:	-0.15
		Sc. rank:	-366.0

,

PB1			
Pos.	369	obs:	exp:
tct	S	1	3.03
tcc	S	4	2.84
tca	S	13	4.37
tcg	S	0	0.96
cct	P	22	769.00
ccc	P	9	524.70
cca	P	2520	1032.00
ccg	P	55	280.30
caa	Q	1	0.54
cag	Q	0	0.46
act	T	0	0.25
acc	T	0	0.22
aca	T	1	0.45
acg	T	0	0.08
agt	S	0	3.35
agc	S	0	3.45
---	--	------	------
mPD		0.083	0.72
		nPD:	0.11
		N. weight:	1.2
		Sc. PD:	-0.2
		Sc. rank:	-737.2

,

PB1			
Pos.	370	obs:	exp:
tct	S	0	3.53
tcc	S	1	3.32
tca	S	20	5.10
tcg	S	0	1.12
act	T	0	1.48
acc	T	0	1.30
aca	T	6	2.73
acg	T	0	0.50
agt	S	0	3.91
agc	S	0	4.02
gct	A	18	637.10
gcc	A	26	595.50
gca	A	2498	1171.00
gcg	A	57	194.90
---	--	------	------
mPD		0.096	0.71
		nPD:	0.14
		N. weight:	1.
		Sc. PD:	-0.14
		Sc. rank:	-456.4

,

PB1			
Pos.	371	obs:	exp:
gaa	E	2429	1555.00
gag	E	196	1070.00
ggt	G	0	0.16
ggc	G	0	0.13
gga	G	1	0.45
ggg	G	0	0.26
---	--	------	------
mPD		0.14	0.48
		nPD:	0.29
		N. weight:	0.45
		Sc. PD:	0.0021
		Sc. rank:	114.6

,

PB1			
Pos.	372	obs:	exp:
tta	L	0	1.09
ttg	L	0	1.88
ctt	L	1	2.02
ctc	L	0	1.45
cta	L	0	1.75
ctg	L	9	1.81
att	I	0	4.97
atc	I	0	3.59
ata	I	14	5.44
atg	M	2562	2562.00
act	T	0	0.74
acc	T	0	0.65
aca	T	0	1.36
acg	T	3	0.25
gtt	V	0	7.99
gtc	V	0	6.79
gta	V	2	9.09
gtg	V	34	12.13
gct	A	0	0.25
gcc	A	0	0.23
gca	A	0	0.45
gcg	A	1	0.07
---	--	------	------
mPD		0.051	0.074
		nPD:	0.69
		N. weight:	0.042
		Sc. PD:	0.017
		Sc. rank:	54.8

,

PB1			
Pos.	373	obs:	exp:
tta	L	89	281.20
ttg	L	1	485.40
ctt	L	63	521.90
ctc	L	305	376.00
cta	L	2064	451.20
ctg	L	62	468.40
att	I	13	14.90
atc	I	16	10.77
ata	I	13	16.33
---	--	------	------
mPD		0.40	1.2
		nPD:	0.35
		N. weight:	1.6
		Sc. PD:	0.098
		Sc. rank:	700.3

,

PB1			
Pos.	374	obs:	exp:
tta	L	2	0.22
ttg	L	0	0.38
tct	S	1	148.20
tcc	S	25	139.20
tca	S	855	213.90
tcg	S	0	46.82
ctt	L	0	0.40
ctc	L	0	0.29
cta	L	0	0.35
ctg	L	0	0.36
cct	P	0	0.30
ccc	P	0	0.20
cca	P	1	0.40
ccg	P	0	0.11
att	I	0	1.42
atc	I	0	1.03
ata	I	4	1.56
act	T	0	8.12
acc	T	0	7.14
aca	T	33	14.99
acg	T	0	2.75
agt	S	0	164.00
agc	S	0	168.80
gtt	V	0	33.97
gtc	V	0	28.87
gta	V	152	38.62
gtg	V	1	51.53
gct	A	3	380.20
gcc	A	6	355.40
gca	A	1529	699.10
gcg	A	13	116.30
gaa	E	1	0.59
gag	E	0	0.41
---	--	------	------
mPD		0.62	1.5
		nPD:	0.4
		N. weight:	1.5
		Sc. PD:	0.17
		Sc. rank:	850.8

,

PB1			
Pos.	375	obs:	exp:
tct	S	2	359.90
tcc	S	0	338.20
tca	S	0	519.70
tcg	S	0	113.70
tat	Y	47	26.33
tac	Y	0	20.67
tgt	C	2	0.86
tgc	C	0	1.14
cat	H	2	1.15
cac	H	0	0.85
cgt	R	0	0.06
cgc	R	1	0.11
cga	R	0	0.20
cgg	R	0	0.14
act	T	0	1.23
acc	T	5	1.08
aca	T	0	2.27
acg	T	0	0.42
aat	N	21	92.08
aac	N	141	69.92
aaa	K	1	0.61
aag	K	0	0.39
agt	S	85	398.30
agc	S	2053	410.10
aga	R	1	0.92
agg	R	0	0.57
gat	D	209	146.80
gac	D	52	114.20
gaa	E	1	0.59
gag	E	0	0.41
ggt	G	1	0.47
ggc	G	0	0.38
gga	G	2	1.36
ggg	G	0	0.79
---	--	------	------
mPD		0.76	1.9
		nPD:	0.41
		N. weight:	2.
		Sc. PD:	0.24
		Sc. rank:	1167.5

,

PB1			
Pos.	376	obs:	exp:
att	I	2361	931.60
atc	I	202	673.50
ata	I	63	1021.00
---	--	------	------
mPD		0.19	0.66
		nPD:	0.28
		N. weight:	1.1
		Sc. PD:	-0.00064
		Sc. rank:	244.8

,

PB1			
Pos.	377	obs:	exp:
aat	N	3	16.48
aac	N	26	12.52
gat	D	104	1443.00
gac	D	2462	1123.00
gaa	E	29	18.37
gag	E	2	12.63
---	--	------	------
mPD		0.12	0.53
		nPD:	0.23
		N. weight:	1.
		Sc. PD:	-0.048
		Sc. rank:	115.1

,

PB1			
Pos.	378	obs:	exp:
tta	L	22	285.70
ttg	L	374	493.10
ctt	L	35	530.10
ctc	L	0	381.90
cta	L	109	458.40
ctg	L	2085	475.80
atg	M	1	1.00
---	--	------	------
mPD		0.38	1.1
		nPD:	0.33
		N. weight:	1.6
		Sc. PD:	0.074
		Sc. rank:	616.8

,

PB1			
Pos.	379	obs:	exp:
cgt	R	0	0.98
cgc	R	0	1.64
cga	R	0	3.12
cgg	R	0	2.24
aaa	K	576	1577.00
aag	K	2019	1018.00
aga	R	4	14.22
agg	R	27	8.81
---	--	------	------
mPD		0.37	0.51
		nPD:	0.72
		N. weight:	0.5
		Sc. PD:	0.21
		Sc. rank:	671.4

,

PB1			
Pos.	380	obs:	exp:
tat	Y	1539	1471.00
tac	Y	1087	1155.00
---	--	------	------
mPD		0.49	0.49
		nPD:	0.99
		N. weight:	0.0046
		Sc. PD:	0.0031
		Sc. rank:	6.7

,

PB1			
Pos.	381	obs:	exp:
ttt	F	46	1177.00
ttc	F	2580	1449.00
---	--	------	------
mPD		0.034	0.49
		nPD:	0.07
		N. weight:	0.8
		Sc. PD:	-0.16
		Sc. rank:	-758.3

,

PB1			
Pos.	382	obs:	exp:
aat	N	2545	1493.00
aac	N	81	1133.00
---	--	------	------
mPD		0.060	0.49
		nPD:	0.12
		N. weight:	0.68
		Sc. PD:	-0.11
		Sc. rank:	-367.5

,

PB1			
Pos.	383	obs:	exp:
aaa	K	3	1.82
aag	K	0	1.18
gct	A	0	0.74
gcc	A	0	0.69
gca	A	3	1.35
gcg	A	0	0.22
gat	D	17	20.25
gac	D	19	15.75
gaa	E	2433	1530.00
gag	E	150	1053.00
ggt	G	0	0.16
ggc	G	0	0.13
gga	G	1	0.45
ggg	G	0	0.26
---	--	------	------
mPD		0.14	0.50
		nPD:	0.28
		N. weight:	0.5
		Sc. PD:	-0.0032
		Sc. rank:	113.3

,

PB1			
Pos.	384	obs:	exp:
tta	L	5	0.65
ttg	L	1	1.13
tct	S	8	428.60
tcc	S	5	402.70
tca	S	2446	618.80
tcg	S	89	135.40
ctt	L	0	1.21
ctc	L	0	0.87
cta	L	0	1.05
ctg	L	0	1.09
cct	P	0	10.33
ccc	P	0	7.05
cca	P	35	13.86
ccg	P	0	3.76
caa	Q	3	1.62
cag	Q	0	1.38
act	T	0	8.12
acc	T	0	7.14
aca	T	33	14.99
acg	T	0	2.75
agt	S	0	474.20
agc	S	0	488.30
gct	A	0	0.25
gcc	A	0	0.23
gca	A	1	0.45
gcg	A	0	0.07
---	--	------	------
mPD		0.14	1.7
		nPD:	0.08
		N. weight:	2.
		Sc. PD:	-0.38
		Sc. rank:	-1665.7

,

PB1			
Pos.	385	obs:	exp:
act	T	10	645.90
acc	T	14	567.80
aca	T	2574	1193.00
acg	T	28	219.00
---	--	------	------
mPD		0.039	0.68
		nPD:	0.06
		N. weight:	1.1
		Sc. PD:	-0.24
		Sc. rank:	-1150.2

,

PB1			
Pos.	386	obs:	exp:
cgt	R	0	59.12
cgc	R	0	99.00
cga	R	0	188.30
cgg	R	0	135.30
aaa	K	55	457.60
aag	K	698	295.40
aga	R	101	859.00
agg	R	1772	532.40
---	--	------	------
mPD		0.52	1.2
		nPD:	0.42
		N. weight:	1.4
		Sc. PD:	0.19
		Sc. rank:	864.4

,

PB1			
Pos.	387	obs:	exp:
caa	Q	0	1.08
cag	Q	2	0.92
cgt	R	0	0.54
cgc	R	0	0.90
cga	R	0	1.71
cgg	R	0	1.23
atg	M	1	1.00
act	T	0	0.25
acc	T	0	0.22
aca	T	0	0.45
acg	T	1	0.08
aat	N	3	1.71
aac	N	0	1.29
aaa	K	250	1485.00
aag	K	2194	958.70
aga	R	1	7.80
agg	R	16	4.83
gaa	E	137	91.84
gag	E	18	63.16
ggt	G	0	0.47
ggc	G	0	0.38
gga	G	3	1.36
ggg	G	0	0.79
---	--	------	------
mPD		0.39	0.61
		nPD:	0.63
		N. weight:	0.85
		Sc. PD:	0.29
		Sc. rank:	1028.2

,

PB1			
Pos.	388	obs:	exp:
cgt	R	0	0.09
cgc	R	0	0.16
cga	R	0	0.30
cgg	R	0	0.22
aaa	K	2303	1594.00
aag	K	320	1029.00
aga	R	2	1.38
agg	R	1	0.85
---	--	------	------
mPD		0.22	0.48
		nPD:	0.45
		N. weight:	0.28
		Sc. PD:	0.047
		Sc. rank:	192.8

,

PB1			
Pos.	389	obs:	exp:
att	I	2144	930.80
atc	I	448	673.00
ata	I	32	1020.00
gtt	V	2	0.44
gtc	V	0	0.38
gta	V	0	0.50
gtg	V	0	0.67
---	--	------	------
mPD		0.30	0.66
		nPD:	0.46
		N. weight:	0.89
		Sc. PD:	0.15
		Sc. rank:	644.5

,

PB1			
Pos.	390	obs:	exp:
aaa	K	1	69.89
aag	K	114	45.11
gaa	E	305	1488.00
gag	E	2206	1023.00
---	--	------	------
mPD		0.29	0.57
		nPD:	0.51
		N. weight:	0.78
		Sc. PD:	0.17
		Sc. rank:	724.1

,

PB1			
Pos.	391	obs:	exp:
cgt	R	0	1.74
cgc	R	0	2.91
cga	R	0	5.53
cgg	R	0	3.97
atg	M	1	1.00
act	T	0	0.25
acc	T	0	0.22
aca	T	1	0.45
acg	T	0	0.08
aaa	K	2178	1561.00
aag	K	390	1007.00
aga	R	50	25.22
agg	R	5	15.63
gaa	E	0	0.59
gag	E	1	0.41
---	--	------	------
mPD		0.30	0.53
		nPD:	0.56
		N. weight:	0.23
		Sc. PD:	0.063
		Sc. rank:	244.8

,

PB1			
Pos.	392	obs:	exp:
tta	L	1	0.33
ttg	L	0	0.56
ctt	L	0	0.61
ctc	L	0	0.44
cta	L	2	0.52
ctg	L	0	0.54
att	I	23	924.80
atc	I	20	668.70
ata	I	2564	1014.00
atg	M	1	1.00
gtt	V	0	3.33
gtc	V	0	2.83
gta	V	15	3.79
gtg	V	0	5.05
---	--	------	------
mPD		0.047	0.67
		nPD:	0.07
		N. weight:	1.3
		Sc. PD:	-0.27
		Sc. rank:	-1278.5

,

PB1			
Pos.	393	obs:	exp:
cgt	R	0	82.64
cgc	R	0	138.40
cga	R	17	263.10
cgg	R	5	189.10
aaa	K	0	4.86
aag	K	8	3.14
aga	R	206	1201.00
agg	R	2390	744.10
---	--	------	------
mPD		0.18	0.94
		nPD:	0.19
		N. weight:	1.4
		Sc. PD:	-0.13
		Sc. rank:	-100.4

,

PB1

Pos.	394	obs:	exp:
tta	L	0	0.11
ttg	L	0	0.19
tct	S	1	0.34
tcc	S	0	0.32
tca	S	0	0.49
tcg	S	1	0.11
ctt	L	1	0.20
ctc	L	0	0.15
cta	L	0	0.17
ctg	L	0	0.18
cct	P	1945	774.00
ccc	P	244	528.10
cca	P	395	1039.00
ccg	P	39	282.20
agt	S	0	0.37
agc	S	0	0.38
---	--	------	------
mPD		0.42	0.71
		nPD:	0.6
		N. weight:	0.69
		Sc. PD:	0.21
		Sc. rank:	773.7

,

PB1

Pos.	395	obs:	exp:
tta	L	1	285.80
ttg	L	0	493.30
ctt	L	864	530.30
ctc	L	1641	382.10
cta	L	118	458.50
ctg	L	2	476.00
---	--	------	------
mPD		0.50	1.1
		nPD:	0.44
		N. weight:	1.6
		Sc. PD:	0.24
		Sc. rank:	1027.6

,

PB1

Pos.	396	obs:	exp:
tta	L	190	285.10
ttg	L	3	492.10
ctt	L	10	529.10
ctc	L	280	381.20
cta	L	2069	457.50
ctg	L	68	474.90
att	I	0	2.13
atc	I	3	1.54
ata	I	3	2.33
---	--	------	------
mPD		0.39	1.1
		nPD:	0.34
		N. weight:	1.7
		Sc. PD:	0.089
		Sc. rank:	673.4

,

PB1

Pos.	397	obs:	exp:
att	I	22	897.50
atc	I	7	648.90
ata	I	2501	983.60
atg	M	26	26.00
act	T	0	15.74
acc	T	0	13.84
aca	T	63	29.08
acg	T	1	5.34
gtt	V	0	1.33
gtc	V	0	1.13
gta	V	6	1.51
gtg	V	0	2.02
---	--	------	------
mPD		0.094	0.72
		nPD:	0.13
		N. weight:	1.4
		Sc. PD:	-0.2
		Sc. rank:	-677.6

,

PB1

Pos.	398	obs:	exp:
tct	S	0	0.34
tcc	S	0	0.32
tca	S	0	0.49
tcg	S	0	0.11
aat	N	10	8.53
aac	N	5	6.47
agt	S	0	0.37
agc	S	2	0.38
gct	A	15	6.62
gcc	A	6	6.19
gca	A	6	12.17
gcg	A	0	2.02
gat	D	2375	1348.00
gac	D	21	1048.00
gaa	E	113	109.00
gag	E	71	74.98
ggt	G	2	0.31
ggc	G	0	0.25
gga	G	0	0.91
ggg	G	0	0.53
---	--	------	------
mPD		0.20	0.60
		nPD:	0.33
		N. weight:	0.77
		Sc. PD:	0.033
		Sc. rank:	274.5

,

PB1

Pos.	399	obs:	exp:
tct	S	0	0.50
tcc	S	0	0.47
tca	S	0	0.73
tcg	S	0	0.16
tgt	C	0	0.43
tgc	C	1	0.57
agt	S	0	0.56
agc	S	3	0.57
gat	D	0	1.69
gac	D	3	1.31
ggt	G	33	410.80
ggc	G	2527	331.90
gga	G	50	1186.00
ggg	G	9	690.10
---	--	------	------
mPD		0.074	0.69
		nPD:	0.11
		N. weight:	2.9
		Sc. PD:	-0.49
		Sc. rank:	-1908.8

,

PB1

Pos.	400	obs:	exp:
tct	S	1	0.17
tcc	S	0	0.16
tca	S	0	0.24
tcg	S	0	0.05
atg	M	1	1.00
act	T	59	639.80
acc	T	9	562.40
aca	T	2203	1182.00
acg	T	330	216.90
aaa	K	1	0.61
aag	K	0	0.39
agt	S	0	0.19
agc	S	0	0.19
gct	A	21	5.39
gcc	A	0	5.04
gca	A	1	9.92
gcg	A	0	1.65
---	--	------	------
mPD		0.30	0.70
		nPD:	0.43
		N. weight:	0.81
		Sc. PD:	0.11
		Sc. rank:	503.5

,

PB1

Pos.	401	obs:	exp:
gtt	V	0	3.11
gtc	V	6	2.64
gta	V	8	3.53
gtg	V	0	4.72
gct	A	18	640.30
gcc	A	361	598.50
gca	A	2192	1177.00
gcg	A	41	195.90
---	--	------	------
mPD		0.29	0.69
		nPD:	0.42
		N. weight:	0.64
		Sc. PD:	0.084
		Sc. rank:	382.4

,

PB1

Pos.	402	obs:	exp:
tct	S	9	441.70
tcc	S	14	415.00
tca	S	2586	637.70
tcg	S	17	139.60
agt	S	0	488.80
agc	S	0	503.30
---	--	------	------
mPD		0.030	1.6
		nPD:	0.02
		N. weight:	2.1
		Sc. PD:	-0.53
		Sc. rank:	-2838.2

,

PB1

Pos.	403	obs:	exp:
tta	L	188	285.60
ttg	L	847	492.90
ctt	L	1	529.90
ctc	L	0	381.80
cta	L	163	458.20
ctg	L	1425	475.70
att	I	0	0.71
atc	I	0	0.51
ata	I	2	0.78
---	--	------	------
mPD		0.71	1.1
		nPD:	0.62
		N. weight:	1.1
		Sc. PD:	0.35
		Sc. rank:	1261.3

,

PB1

Pos.	404	obs:	exp:
tct	S	0	441.70
tcc	S	0	415.00
tca	S	0	637.70
tcg	S	0	139.60
agt	S	726	488.80
agc	S	1900	503.30
---	--	------	------
mPD		0.40	1.6
		nPD:	0.25
		N. weight:	1.7
		Sc. PD:	-0.06
		Sc. rank:	238.0

,

PB1

Pos.	405	obs:	exp:
cct	P	2496	774.90
ccc	P	50	528.70
cca	P	64	1040.00
ccg	P	16	282.50
---	--	------	------
mPD		0.096	0.70
		nPD:	0.14
		N. weight:	1.5
		Sc. PD:	-0.22
		Sc. rank:	-718.2

,

PB1

Pos.	406	obs:	exp:
ggt	G	5	411.90
ggc	G	0	332.70
gga	G	597	1189.00
ggg	G	2024	691.90
---	--	------	------
mPD		0.35	0.68
		nPD:	0.52
		N. weight:	1.
		Sc. PD:	0.23
		Sc. rank:	970.6

,

PB1

Pos.	407	obs:	exp:
atg	M	2626	2626.00
---	--	------	------
mPD		0	0
		nPD:	1.
		N. weight:	0.
		Sc. PD:	0
		Sc. rank:	0

,

PB1

Pos.	408	obs:	exp:
atg	M	2626	2626.00
---	--	------	------
mPD		0	0
		nPD:	1.
		N. weight:	0.
		Sc. PD:	0
		Sc. rank:	0

,

PB1

Pos.	409	obs:	exp:
atg	M	2626	2626.00
---	--	------	------
mPD		0	0
		nPD:	1.
		N. weight:	0.
		Sc. PD:	0
		Sc. rank:	0

,

PB1

Pos.	410	obs:	exp:
ggt	G	88	411.90
ggc	G	2477	332.70
gga	G	46	1189.00
ggg	G	15	691.90
---	--	------	------
mPD		0.11	0.68
		nPD:	0.16
		N. weight:	2.8
		Sc. PD:	-0.33
		Sc. rank:	-877.8

,

PB1

Pos.	411	obs:	exp:
atg	M	2626	2626.00
---	--	------	------
mPD		0	0
		nPD:	1.
		N. weight:	0.
		Sc. PD:	0
		Sc. rank:	0

,

PB1

Pos.	412	obs:	exp:
ttt	F	457	1177.00
ttc	F	2169	1449.00
---	--	------	------
mPD		0.29	0.49
		nPD:	0.58
		N. weight:	0.27
		Sc. PD:	0.076
		Sc. rank:	289.0

,

PB1

Pos.	413	obs:	exp:
aat	N	120	1493.00
aac	N	2506	1133.00
---	--	------	------
mPD		0.087	0.49
		nPD:	0.18
		N. weight:	1.
		Sc. PD:	-0.1
		Sc. rank:	-154.5

,

PB1			
Pos.	414	obs :	exp :
atg	M	2626	2626.00
---	--	------	------
mPD		0	0
		nPD :	1.
		N. weight :	0.
		Sc. PD :	0
		Sc. rank :	0

PB1			
Pos.	415	obs :	exp :
tta	L	47	285.80
ttg	L	22	493.30
ctt	L	9	530.30
ctc	L	1	382.10
cta	L	2178	458.50
ctg	L	369	476.00
---	--	------	------
mPD		0.31	1.1
		nPD :	0.27
		N. weight :	1.9
		Sc. PD :	-0.016
		Sc. rank :	384.9

PB1			
Pos.	416	obs :	exp :
tct	S	0	441.50
tcc	S	0	414.90
tca	S	0	637.50
tcg	S	0	139.50
cgt	R	0	0.03
cgc	R	0	0.05
cga	R	0	0.10
cgg	R	0	0.07
agt	S	1961	488.60
agc	S	664	503.10
aga	R	1	0.46
agg	R	0	0.28
---	--	------	------
mPD		0.38	1.6
		nPD :	0.23
		N. weight :	1.7
		Sc. PD :	-0.084
		Sc. rank :	186.6

PB1			
Pos.	417	obs :	exp :
act	T	60	645.90
acc	T	5	567.80
aca	T	923	1193.00
acg	T	1638	219.00
---	--	------	------
mPD		0.49	0.68
		nPD :	0.72
		N. weight :	1.7
		Sc. PD :	0.72
		Sc. rank :	2321.1

PB1			
Pos.	418	obs :	exp :
att	I	2	0.71
atc	I	0	0.51
ata	I	0	0.78
gtt	V	1761	582.40
gtc	V	807	495.00
gta	V	34	662.10
gtg	V	21	883.50
ggt	G	1	0.16
ggc	G	0	0.13
gga	G	0	0.45
ggg	G	0	0.26
---	--	------	------
mPD		0.46	0.74
		nPD :	0.62
		N. weight :	1.3
		Sc. PD :	0.42
		Sc. rank :	1500.8

PB1			
Pos.	419	obs :	exp :
tta	L	269	285.80
ttg	L	2045	493.30
ctt	L	2	530.30
ctc	L	1	382.10
cta	L	252	458.50
ctg	L	57	476.00
---	--	------	------
mPD		0.53	1.1
		nPD :	0.46
		N. weight :	1.6
		Sc. PD :	0.27
		Sc. rank :	1157.5

PB1			
Pos.	420	obs :	exp :
ggt	G	38	411.90
ggc	G	1	332.70
gga	G	2397	1189.00
ggg	G	190	691.90
---	--	------	------
mPD		0.16	0.68
		nPD :	0.24
		N. weight :	0.8
		Sc. PD :	-0.036
		Sc. rank :	97.1

PB1			
Pos.	421	obs :	exp :
gtt	V	135	583.10
gtc	V	2396	495.50
gta	V	88	662.90
gtg	V	7	884.50
---	--	------	------
mPD		0.16	0.74
		nPD :	0.22
		N. weight :	2.
		Sc. PD :	-0.12
		Sc. rank :	160.5

PB1			
Pos.	422	obs :	exp :
tct	S	14	441.70
tcc	S	21	415.00
tca	S	1333	637.70
tcg	S	1258	139.60
agt	S	0	488.80
agc	S	0	503.30
---	--	------	------
mPD		0.51	1.6
		nPD :	0.31
		N. weight :	2.2
		Sc. PD :	0.067
		Sc. rank :	686.2

PB1			
Pos.	423	obs :	exp :
att	I	40	913.10
atc	I	363	660.20
ata	I	2171	1001.00
act	T	0	0.25
acc	T	1	0.22
aca	T	0	0.45
acg	T	0	0.08
gtt	V	1	11.32
gtc	V	25	9.62
gta	V	25	12.87
gtg	V	0	17.18
---	--	------	------
mPD		0.32	0.70
		nPD :	0.45
		N. weight :	0.83
		Sc. PD :	0.14
		Sc. rank :	561.6

PB1			
Pos.	424	obs :	exp :
tta	L	6	285.80
ttg	L	396	493.30
ctt	L	30	530.30
ctc	L	0	382.10
cta	L	70	458.50
ctg	L	2124	476.00
---	--	------	------
mPD		0.34	1.1
		nPD :	0.3
		N. weight :	1.7
		Sc. PD :	0.023
		Sc. rank :	476.4

PB1			
Pos.	425	obs :	exp :
aat	N	2191	1493.00
aac	N	435	1133.00
---	--	------	------
mPD		0.28	0.49
		nPD :	0.56
		N. weight :	0.25
		Sc. PD :	0.069
		Sc. rank :	268.0

PB1			
Pos.	426	obs :	exp :
tta	L	0	282.50
ttg	L	0	487.60
ctt	L	2349	524.30
ctc	L	239	377.70
cta	L	8	453.30
ctg	L	0	470.60
att	I	30	10.64
atc	I	0	7.69
ata	I	0	11.66
---	--	------	------
mPD		0.19	1.2
		nPD :	0.17
		N. weight :	2.
		Sc. PD :	-0.22
		Sc. rank :	-506.5

PB1			
Pos.	427	obs :	exp :
ggt	G	2	411.90
ggc	G	7	332.70
gga	G	2178	1189.00
ggg	G	439	691.90
---	--	------	------
mPD		0.28	0.68
		nPD :	0.42
		N. weight :	0.65
		Sc. PD :	0.083
		Sc. rank :	385.7

PB1			
Pos.	428	obs :	exp :
caa	Q	2552	1408.00
cag	Q	59	1203.00
aaa	K	15	9.12
aag	K	0	5.88
---	--	------	------
mPD		0.055	0.51
		nPD :	0.11
		N. weight :	0.8
		Sc. PD :	-0.13
		Sc. rank :	-502.9

PB1			
Pos.	429	obs :	exp :
caa	Q	0	0.54
cag	Q	1	0.46
cgt	R	0	1.48
cgc	R	0	2.48
cga	R	0	4.72
cgg	R	0	3.40
aaa	K	57	1567.00
aag	K	2521	1011.00
aga	R	0	21.55
agg	R	47	13.36
---	--	------	------
mPD		0.078	0.52
		nPD :	0.15
		N. weight :	1.3
		Sc. PD :	-0.17
		Sc. rank :	-480.3

PB1			
Pos.	430	obs :	exp :
tct	S	0	0.17
tcc	S	0	0.16
tca	S	0	0.24
tcg	S	0	0.05
cgt	R	0	11.08
cgc	R	0	18.55
cga	R	0	35.28
cgg	R	0	25.36
aaa	K	2150	1371.00
aag	K	106	884.90
agt	S	1	0.19
agc	S	0	0.19
aga	R	142	161.00
agg	R	209	99.76
gaa	E	1	10.67
gag	E	17	7.33
---	--	------	------
mPD		0.47	0.80
		nPD :	0.58
		N. weight :	0.53
		Sc. PD :	0.16
		Sc. rank :	584.7

PB1			
Pos.	431	obs :	exp :
tat	Y	34	1467.00
tac	Y	2584	1151.00
cat	H	0	4.61
cac	H	8	3.39
---	--	------	------
mPD		0.032	0.50
		nPD :	0.06
		N. weight :	1.2
		Sc. PD :	-0.25
		Sc. rank :	-1166.0

PB1			
Pos.	432	obs :	exp :
act	T	407	645.90
acc	T	2124	567.80
aca	T	81	1193.00
acg	T	14	219.00
---	--	------	------
mPD		0.32	0.68
		nPD :	0.47
		N. weight :	1.4
		Sc. PD :	0.26
		Sc. rank :	1103.2

PB1			
Pos.	433	obs :	exp :
cgt	R	0	47.25
cgc	R	0	79.12
cga	R	0	150.50
cgg	R	2	108.10
aat	N	1	0.57
aac	N	0	0.43
aaa	K	413	685.50
aag	K	715	442.50
aga	R	40	686.50
agg	R	1455	425.50
---	--	------	------
mPD		0.78	1.3
		nPD :	0.62
		N. weight :	1.1
		Sc. PD :	0.34
		Sc. rank :	1239.9

PB1			
Pos.	434	obs:	exp:
act	T	28	645.40
acc	T	408	567.40
aca	T	2146	1192.00
acg	T	42	218.80
aat	N	0	1.14
aac	N	2	0.86
---	--	------	------
mPD		0.31	0.68
		nPD:	0.45
		N. weight:	0.58
		Sc. PD:	0.096
		Sc. rank:	399.3

,

PB1			
Pos.	435	obs:	exp:
tct	S	18	3.03
tcc	S	0	2.84
tca	S	0	4.37
tcg	S	0	0.96
cct	P	0	0.30
ccc	P	1	0.20
cca	P	0	0.40
ccg	P	0	0.11
att	I	0	168.50
atc	I	1	121.80
ata	I	474	184.70
act	T	12	473.50
acc	T	271	416.30
aca	T	1247	874.70
acg	T	395	160.50
agt	S	0	3.35
agc	S	0	3.45
gtt	V	1	39.74
gtc	V	0	33.78
gta	V	176	45.19
gtg	V	2	60.29
gct	A	2	6.86
gcc	A	0	6.42
gca	A	24	12.62
gcg	A	2	2.10
---	--	------	------
mPD		0.96	1.2
		nPD:	0.79
		N. weight:	0.81
		Sc. PD:	0.4
		Sc. rank:	1148.8

,

PB1			
Pos.	436	obs:	exp:
ttt	F	0	0.45
ttc	F	1	0.55
tat	Y	177	1470.00
tac	Y	2446	1153.00
tgt	C	0	0.43
tgc	C	1	0.57
cat	H	0	0.58
cac	H	1	0.42
---	--	------	------
mPD		0.13	0.50
		nPD:	0.26
		N. weight:	0.87
		Sc. PD:	-0.02
		Sc. rank:	161.3

,

PB1			
Pos.	437	obs:	exp:
tgg	W	2626	2626.00
---	--	------	------
mPD		0	0
		nPD:	1.
		N. weight:	0.
		Sc. PD:	0
		Sc. rank:	0

,

PB1			
Pos.	438	obs:	exp:
tgg	W	2626	2626.00
---	--	------	------
mPD		0	0
		nPD:	1.
		N. weight:	0.
		Sc. PD:	0
		Sc. rank:	0

,

PB1			
Pos.	439	obs:	exp:
aat	N	2	1.14
aac	N	0	0.86
gat	D	1571	1476.00
gac	D	1053	1148.00
---	--	------	------
mPD		0.48	0.49
		nPD:	0.98
		N. weight:	0.0073
		Sc. PD:	0.0049
		Sc. rank:	10.6

,

PB1			
Pos.	440	obs:	exp:
ggt	G	20	411.90
ggc	G	13	332.70
gga	G	1861	1189.00
ggg	G	732	691.90
---	--	------	------
mPD		0.42	0.68
		nPD:	0.61
		N. weight:	0.46
		Sc. PD:	0.15
		Sc. rank:	532.8

,

PB1			
Pos.	441	obs:	exp:
tta	L	2	285.80
ttg	L	0	493.30
ctt	L	61	530.30
ctc	L	2513	382.10
cta	L	44	458.50
ctg	L	6	476.00
---	--	------	------
mPD		0.085	1.1
		nPD:	0.07
		N. weight:	2.7
		Sc. PD:	-0.53
		Sc. rank:	-2348.2

,

PB1			
Pos.	442	obs:	exp:
caa	Q	2520	1416.00
cag	Q	106	1210.00
---	--	------	------
mPD		0.078	0.50
		nPD:	0.16
		N. weight:	0.71
		Sc. PD:	-0.087
		Sc. rank:	-245.5

,

PB1			
Pos.	443	obs:	exp:
tct	S	440	441.70
tcc	S	1475	415.00
tca	S	689	637.70
tcg	S	22	139.60
agt	S	0	488.80
agc	S	0	503.30
---	--	------	------
mPD		0.59	1.6
		nPD:	0.36
		N. weight:	1.1
		Sc. PD:	0.084
		Sc. rank:	512.6

,

PB1			
Pos.	444	obs:	exp:
tct	S	517	441.70
tcc	S	2037	415.00
tca	S	69	637.70
tcg	S	3	139.60
agt	S	0	488.80
agc	S	0	503.30
---	--	------	------
mPD		0.36	1.6
		nPD:	0.22
		N. weight:	1.9
		Sc. PD:	-0.11
		Sc. rank:	116.9

,

PB1			
Pos.	445	obs:	exp:
gat	D	758	1477.00
gac	D	1868	1149.00
---	--	------	------
mPD		0.41	0.49
		nPD:	0.83
		N. weight:	0.24
		Sc. PD:	0.13
		Sc. rank:	342.7

,

PB1			
Pos.	446	obs:	exp:
gat	D	2171	1477.00
gac	D	455	1149.00
---	--	------	------
mPD		0.29	0.49
		nPD:	0.58
		N. weight:	0.25
		Sc. PD:	0.072
		Sc. rank:	271.4

,

PB1			
Pos.	447	obs:	exp:
ttt	F	2244	1177.00
ttc	F	382	1449.00
---	--	------	------
mPD		0.25	0.49
		nPD:	0.5
		N. weight:	0.56
		Sc. PD:	0.12
		Sc. rank:	507.9

,

PB1			
Pos.	448	obs:	exp:
gtt	V	11	2.89
gtc	V	1	2.45
gta	V	0	3.28
gtg	V	1	4.38
gct	A	707	640.00
gcc	A	1892	598.20
gca	A	9	1177.00
gcg	A	3	195.80
ggt	G	0	0.31
ggc	G	2	0.25
gga	G	0	0.91
ggg	G	0	0.53
---	--	------	------
mPD		0.42	0.69
		nPD:	0.6
		N. weight:	1.3
		Sc. PD:	0.41
		Sc. rank:	1490.5

,

PB1			
Pos.	449	obs:	exp:
ttt	F	1	0.45
ttc	F	0	0.55
tta	L	1	285.70
ttg	L	1	493.10
ctt	L	97	530.10
ctc	L	2417	381.90
cta	L	90	458.40
ctg	L	19	475.80
---	--	------	------
mPD		0.15	1.1
		nPD:	0.13
		N. weight:	2.4
		Sc. PD:	-0.35
		Sc. rank:	-1175.6

,

PB1			
Pos.	450	obs:	exp:
att	I	11	931.20
atc	I	27	673.30
ata	I	2587	1021.00
gtt	V	0	0.22
gtc	V	0	0.19
gta	V	1	0.25
gtg	V	0	0.34
---	--	------	------
mPD		0.029	0.66
		nPD:	0.04
		N. weight:	1.4
		Sc. PD:	-0.31
		Sc. rank:	-1584.8

,

PB1			
Pos.	451	obs:	exp:
att	I	2	19.87
atc	I	0	14.36
ata	I	54	21.77
atg	M	1	1.00
gtt	V	33	570.40
gtc	V	1	484.80
gta	V	1390	648.50
gtg	V	1145	865.30
---	--	------	------
mPD		0.55	0.78
		nPD:	0.7
		N. weight:	0.79
		Sc. PD:	0.32
		Sc. rank:	1044.1

,

PB1			
Pos.	452	obs:	exp:
aat	N	1801	1493.00
aac	N	825	1133.00
---	--	------	------
mPD		0.43	0.49
		nPD:	0.88
		N. weight:	0.048
		Sc. PD:	0.027
		Sc. rank:	68.6

,

PB1			
Pos.	453	obs:	exp:
tct	S	0	0.17
tcc	S	0	0.16
tca	S	1	0.24
tcg	S	0	0.05
agt	S	0	0.19
agc	S	0	0.19
gct	A	2	643.50
gcc	A	16	601.50
gca	A	2534	1183.00
gcg	A	73	196.90
---	--	------	------
mPD		0.068	0.68
		nPD:	0.1
		N. weight:	1.1
		Sc. PD:	-0.19
		Sc. rank:	-731.7

,

PB1

Pos.	454	obs:	exp:
tta	L	0	0.33
ttg	L	0	0.56
tct	S	10	4.37
tcc	S	1	4.11
tca	S	15	6.31
tcg	S	0	1.38
ctt	L	0	0.61
ctc	L	0	0.44
cta	L	3	0.52
ctg	L	0	0.54
cct	P	354	720.30
ccc	P	53	491.40
cca	P	2022	966.60
ccg	P	12	262.60
caa	Q	151	81.43
cag	Q	0	69.57
act	T	2	1.23
acc	T	0	1.08
aca	T	3	2.27
acg	T	0	0.42
agt	S	0	4.84
agc	S	0	4.98
---	--	------	------
mPD		0.42	0.85
		nPD:	0.49
		N. weight:	0.72
		Sc. PD:	0.14
		Sc. rank:	619.1

PB1

Pos.	455	obs:	exp:
tct	S	0	2.02
tcc	S	0	1.90
tca	S	0	2.91
tcg	S	0	0.64
act	T	2	0.98
acc	T	2	0.86
aca	T	0	1.82
acg	T	0	0.33
aat	N	1863	1459.00
aac	N	704	1108.00
agt	S	6	2.23
agc	S	6	2.30
gat	D	32	22.50
gac	D	8	17.50
ggt	G	1	0.47
ggc	G	2	0.38
gga	G	0	1.36
ggg	G	0	0.79
---	--	------	------
mPD		0.45	0.55
		nPD:	0.82
		N. weight:	0.1
		Sc. PD:	0.052
		Sc. rank:	142.2

PB1

Pos.	456	obs:	exp:
tct	S	0	0.17
tcc	S	0	0.16
tca	S	0	0.24
tcg	S	0	0.05
tat	Y	55	30.81
tac	Y	0	24.19
cat	H	2337	1477.00
cac	H	223	1083.00
caa	Q	2	1.08
cag	Q	0	0.92
cgt	R	7	0.22
cgc	R	0	0.37
cga	R	0	0.70
cgg	R	0	0.51
aat	N	1	0.57
aac	N	0	0.43
agt	S	1	0.19
agc	S	0	0.19
aga	R	0	3.21
agg	R	0	1.99
---	--	------	------
mPD		0.21	0.54
		nPD:	0.38
		N. weight:	0.47
		Sc. PD:	0.043
		Sc. rank:	239.0

PB1

Pos.	457	obs:	exp:
aaa	K	1	1.22
aag	K	1	0.78
gct	A	0	0.25
gcc	A	0	0.23
gca	A	0	0.45
gcg	A	1	0.07
gat	D	30	19.12
gac	D	4	14.88
gaa	E	558	1528.00
gag	E	2021	1051.00
ggt	G	0	1.57
ggc	G	0	1.27
gga	G	2	4.53
ggg	G	8	2.63
---	--	------	------
mPD		0.37	0.51
		nPD:	0.72
		N. weight:	0.47
		Sc. PD:	0.2
		Sc. rank:	630.8

PB1

Pos.	458	obs:	exp:
ggt	G	1	411.90
ggc	G	0	332.70
gga	G	2486	1189.00
ggg	G	139	691.90
---	--	------	------
mPD		0.10	0.68
		nPD:	0.15
		N. weight:	0.96
		Sc. PD:	-0.12
		Sc. rank:	-366.5

PB1

Pos.	459	obs:	exp:
att	I	52	926.60
atc	I	766	669.90
ata	I	1794	1015.00
aaa	K	1	0.61
aag	K	0	0.39
gtt	V	0	2.89
gtc	V	1	2.45
gta	V	12	3.28
gtg	V	0	4.38
---	--	------	------
mPD		0.45	0.67
		nPD:	0.67
		N. weight:	0.59
		Sc. PD:	0.22
		Sc. rank:	755.9

PB1

Pos.	460	obs:	exp:
caa	Q	2405	1413.00
cag	Q	216	1208.00
gaa	E	5	2.96
gag	E	0	2.04
---	--	------	------
mPD		0.15	0.50
		nPD:	0.31
		N. weight:	0.54
		Sc. PD:	0.014
		Sc. rank:	161.5

PB1

Pos.	461	obs:	exp:
tct	S	0	0.17
tcc	S	0	0.16
tca	S	1	0.24
tcg	S	0	0.05
act	T	0	0.98
acc	T	0	0.86
aca	T	4	1.82
acg	T	0	0.33
agt	S	0	0.19
agc	S	0	0.19
gct	A	202	642.50
gcc	A	42	600.50
gca	A	2341	1181.00
gcg	A	36	196.60
---	--	------	------
mPD		0.20	0.68
		nPD:	0.29
		N. weight:	0.72
		Sc. PD:	0.0066
		Sc. rank:	191.7

PB1

Pos.	462	obs:	exp:
ggt	G	10	411.90
ggc	G	0	332.70
gga	G	2466	1189.00
ggg	G	150	691.90
---	--	------	------
mPD		0.11	0.68
		nPD:	0.17
		N. weight:	0.91
		Sc. PD:	-0.1
		Sc. rank:	-234.9

PB1

Pos.	463	obs:	exp:
atg	M	1	1.00
gtt	V	33	582.80
gtc	V	2	495.30
gta	V	174	662.70
gtg	V	2416	884.20
---	--	------	------
mPD		0.15	0.74
		nPD:	0.2
		N. weight:	1.2
		Sc. PD:	-0.097
		Sc. rank:	-7.8

PB1

Pos.	464	obs:	exp:
aat	N	112	71.05
aac	N	13	53.95
gat	D	1743	1407.00
gac	D	758	1094.00
---	--	------	------
mPD		0.51	0.58
		nPD:	0.87
		N. weight:	0.08
		Sc. PD:	0.045
		Sc. rank:	113.9

PB1

Pos.	465	obs:	exp:
cgt	R	0	81.34
cgc	R	0	136.20
cga	R	19	259.00
cgg	R	0	186.20
aaa	K	44	29.78
aag	K	5	19.22
aga	R	2112	1182.00
agg	R	446	732.40
---	--	------	------
mPD		0.34	0.97
		nPD:	0.35
		N. weight:	0.58
		Sc. PD:	0.036
		Sc. rank:	251.4

PB1

Pos.	466	obs:	exp:
ttt	F	202	1176.00
ttc	F	2423	1449.00
tat	Y	0	0.56
tac	Y	1	0.44
---	--	------	------
mPD		0.14	0.50
		nPD:	0.29
		N. weight:	0.53
		Sc. PD:	0.003
		Sc. rank:	139.2

PB1

Pos.	467	obs:	exp:
tat	Y	440	1471.00
tac	Y	2186	1155.00
---	--	------	------
mPD		0.28	0.49
		nPD:	0.57
		N. weight:	0.52
		Sc. PD:	0.14
		Sc. rank:	546.5

PB1

Pos.	468	obs:	exp:
cgt	R	0	82.89
cgc	R	0	138.80
cga	R	11	263.90
cgg	R	15	189.70
aga	R	271	1204.00
agg	R	2329	746.40
---	--	------	------
mPD		0.21	0.94
		nPD:	0.23
		N. weight:	1.3
		Sc. PD:	-0.071
		Sc. rank:	116.5

PB1

Pos.	469	obs:	exp:
att	I	0	8.16
atc	I	22	5.90
ata	I	1	8.94
act	T	83	638.80
acc	T	2347	561.60
aca	T	167	1180.00
acg	T	0	216.60
gct	A	0	1.47
gcc	A	5	1.37
gca	A	1	2.70
gcg	A	0	0.45
---	--	------	------
mPD		0.20	0.70
		nPD:	0.28
		N. weight:	1.7
		Sc. PD:	0.0036
		Sc. rank:	409.7

PB1

Pos.	470	obs:	exp:
tgt	C	88	1130.00
tgc	C	2538	1496.00
---	--	------	------
mPD		0.065	0.49
		nPD:	0.13
		N. weight:	0.67
		Sc. PD:	-0.096
		Sc. rank:	-331.1

PB1

Pos.	471	obs:	exp:
aaa	K	1222	1596.00
aag	K	1404	1030.00
---	--	------	------
mPD		0.50	0.48
		nPD:	1.04
		N. weight:	0.067
		Sc. PD:	0.049
		Sc. rank:	97.4

PB1

Pos.	472	obs:	exp:
tta	L	1908	285.60
ttg	L	284	492.90
ctt	L	6	529.90
ctc	L	0	381.80
cta	L	207	458.20
ctg	L	219	475.70
att	I	0	0.35
atc	I	0	0.26
ata	I	1	0.39
atg	M	1	1.00
---	--	------	------
mPD		0.59	1.1
		nPD:	0.52
		N. weight:	1.9
		Sc. PD:	0.42
		Sc. rank:	1754.1

PB1

Pos.	473	obs:	exp:
tta	L	0	2.39
ttg	L	0	4.13
ctt	L	16	4.44
ctc	L	5	3.20
cta	L	1	3.84
ctg	L	0	3.99
att	I	1	1.06
atc	I	0	0.77
ata	I	2	1.17
atg	M	3	3.00
gtt	V	398	576.80
gtc	V	33	490.20
gta	V	1387	655.80
gtg	V	780	875.10
---	--	------	------
mPD		0.63	0.76
		nPD:	0.83
		N. weight:	0.45
		Sc. PD:	0.23
		Sc. rank:	631.0

PB1			
Pos.	474	obs:	exp:
ggt	G	3	411.90
ggc	G	3	332.70
gga	G	1891	1189.00
ggg	G	729	691.90
---	--	------	------
mPD		0.40	0.68
		nPD:	0.59
		N. weight:	0.53
		Sc. PD:	0.16
		Sc. rank:	588.5

PB1			
Pos.	475	obs:	exp:
att	I	71	929.80
atc	I	2318	672.30
ata	I	232	1019.00
gtt	V	1	1.11
gtc	V	4	0.94
gta	V	0	1.26
gtg	V	0	1.68
---	--	------	------
mPD		0.21	0.66
		nPD:	0.32
		N. weight:	1.4
		Sc. PD:	0.054
		Sc. rank:	488.9

PB1			
Pos.	476	obs:	exp:
aat	N	471	1493.00
aac	N	2155	1133.00
---	--	------	------
mPD		0.29	0.49
		nPD:	0.6
		N. weight:	0.5
		Sc. PD:	0.15
		Sc. rank:	565.1

PB1			
Pos.	477	obs:	exp:
atg	M	2626	2626.00
---	--	------	------
mPD		0	0
		nPD:	1.
		N. weight:	0.
		Sc. PD:	0
		Sc. rank:	0

PB1			
Pos.	478	obs:	exp:
tct	S	0	441.50
tcc	S	0	414.90
tca	S	0	637.50
tcg	S	0	139.50
aat	N	0	0.57
aac	N	1	0.43
agt	S	48	488.60
agc	S	2577	503.10
---	--	------	------
mPD		0.037	1.6
		nPD:	0.02
		N. weight:	2.4
		Sc. PD:	-0.61
		Sc. rank:	-3221.8

PB1			
Pos.	479	obs:	exp:
aaa	K	2552	1596.00
aag	K	74	1030.00
---	--	------	------
mPD		0.055	0.48
		nPD:	0.11
		N. weight:	0.6
		Sc. PD:	-0.096
		Sc. rank:	-356.8

PB1			
Pos.	480	obs:	exp:
cgt	R	0	3.16
cgc	R	0	5.29
cga	R	0	10.05
cgg	R	0	7.22
aaa	K	23	1535.00
aag	K	2503	990.90
aga	R	2	45.86
agg	R	98	28.42
---	--	------	------
mPD		0.092	0.57
		nPD:	0.16
		N. weight:	1.4
		Sc. PD:	-0.16
		Sc. rank:	-432.7

PB1			
Pos.	481	obs:	exp:
aaa	K	593	1596.00
aag	K	2033	1030.00
---	--	------	------
mPD		0.35	0.48
		nPD:	0.73
		N. weight:	0.47
		Sc. PD:	0.21
		Sc. rank:	647.3

PB1			
Pos.	482	obs:	exp:
tct	S	460	441.70
tcc	S	2107	415.00
tca	S	56	637.70
tcg	S	3	139.60
agt	S	0	488.80
agc	S	0	503.30
---	--	------	------
mPD		0.33	1.6
		nPD:	0.2
		N. weight:	2.
		Sc. PD:	-0.16
		Sc. rank:	-47.2

PB1			
Pos.	483	obs:	exp:
tat	Y	1973	1471.00
tac	Y	653	1155.00
---	--	------	------
mPD		0.37	0.49
		nPD:	0.76
		N. weight:	0.13
		Sc. PD:	0.057
		Sc. rank:	173.3

PB1			
Pos.	484	obs:	exp:
att	I	18	930.50
atc	I	5	672.80
ata	I	2600	1020.00
atg	M	1	1.00
gtt	V	0	0.44
gtc	V	0	0.38
gta	V	2	0.50
gtg	V	0	0.67
---	--	------	------
mPD		0.020	0.66
		nPD:	0.03
		N. weight:	1.4
		Sc. PD:	-0.34
		Sc. rank:	-1796.7

PB1			
Pos.	485	obs:	exp:
aat	N	2516	1493.00
aac	N	110	1133.00
---	--	------	------
mPD		0.080	0.49
		nPD:	0.16
		N. weight:	0.63
		Sc. PD:	-0.072
		Sc. rank:	-181.6

PB1			
Pos.	486	obs:	exp:
cgt	R	0	14.84
cgc	R	0	24.84
cga	R	0	47.24
cgg	R	3	33.95
aaa	K	205	1310.00
aag	K	1951	845.70
aga	R	100	215.50
agg	R	367	133.60
---	--	------	------
mPD		0.50	0.87
		nPD:	0.57
		N. weight:	0.92
		Sc. PD:	0.26
		Sc. rank:	985.0

PB1			
Pos.	487	obs:	exp:
act	T	719	645.90
acc	T	7	567.80
aca	T	1812	1193.00
acg	T	88	219.00
---	--	------	------
mPD		0.45	0.68
		nPD:	0.66
		N. weight:	0.44
		Sc. PD:	0.16
		Sc. rank:	545.9

PB1			
Pos.	488	obs:	exp:
ggt	G	93	411.90
ggc	G	20	332.70
gga	G	553	1189.00
ggg	G	1960	691.90
---	--	------	------
mPD		0.40	0.68
		nPD:	0.58
		N. weight:	0.85
		Sc. PD:	0.24
		Sc. rank:	927.0

PB1			
Pos.	489	obs:	exp:
act	T	26	645.90
acc	T	11	567.80
aca	T	2547	1193.00
acg	T	42	219.00
---	--	------	------
mPD		0.059	0.68
		nPD:	0.09
		N. weight:	1.
		Sc. PD:	-0.2
		Sc. rank:	-841.8

PB1			
Pos.	490	obs:	exp:
ttt	F	2592	1177.00
ttc	F	34	1449.00
---	--	------	------
mPD		0.026	0.49
		nPD:	0.05
		N. weight:	1.1
		Sc. PD:	-0.25
		Sc. rank:	-1264.7

PB1			
Pos.	491	obs:	exp:
gaa	E	2470	1556.00
gag	E	156	1070.00
---	--	------	------
mPD		0.11	0.48
		nPD:	0.23
		N. weight:	0.5
		Sc. PD:	-0.025
		Sc. rank:	50.7

PB1			
Pos.	492	obs:	exp:
ttt	F	41	1177.00
ttc	F	2585	1449.00
---	--	------	------
mPD		0.031	0.49
		nPD:	0.06
		N. weight:	0.81
		Sc. PD:	-0.17
		Sc. rank:	-811.5

PB1			
Pos.	493	obs:	exp:
act	T	26	645.90
acc	T	3	567.80
aca	T	2578	1193.00
acg	T	19	219.00
---	--	------	------
mPD		0.036	0.68
		nPD:	0.05
		N. weight:	1.1
		Sc. PD:	-0.24
		Sc. rank:	-1200.6

PB1			
Pos.	494	obs:	exp:
tct	S	0	441.70
tcc	S	0	415.00
tca	S	0	637.70
tcg	S	0	139.60
agt	S	79	488.80
agc	S	2547	503.30
---	--	------	------
mPD		0.058	1.6
		nPD:	0.04
		N. weight:	2.4
		Sc. PD:	-0.56
		Sc. rank:	-2949.5

PB1			
Pos.	495	obs:	exp:
ttt	F	2481	1177.00
ttc	F	145	1449.00
---	--	------	------
mPD		0.10	0.49
		nPD:	0.21
		N. weight:	0.9
		Sc. PD:	-0.062
		Sc. rank:	18.4

PB1			
Pos.	496	obs:	exp:
ttt	F	2212	1177.00
ttc	F	414	1449.00
---	--	------	------
mPD		0.27	0.49
		nPD:	0.54
		N. weight:	0.52
		Sc. PD:	0.13
		Sc. rank:	520.9

PB1			
Pos.	497	obs:	exp:
tat	Y	1755	1471.00
tac	Y	871	1155.00
---	--	------	------
mPD		0.44	0.49
		nPD:	0.9
		N. weight:	0.04
		Sc. PD:	0.024
		Sc. rank:	58.2

PB1			
Pos.	498	obs:	exp:
cgt	R	103	82.89
cgc	R	2452	138.80
cga	R	62	263.90
cgg	R	7	189.70
aga	R	0	1204.00
agg	R	2	746.40
---	--	------	------
mPD		0.13	0.94
		nPD:	0.14
		N. weight:	4.1
		Sc. PD:	-0.58
		Sc. rank:	-1864.9

PB1			
Pos.	499	obs:	exp:
tat	Y	2579	1471.00
tac	Y	47	1155.00
---	--	------	------
mPD		0.035	0.49
		nPD:	0.07
		N. weight:	0.77
		Sc. PD:	-0.16
		Sc. rank:	-719.7

PB1			
Pos.	500	obs:	exp:
ggt	G	24	411.90
ggc	G	4	332.70
gga	G	891	1189.00
ggg	G	1707	691.90
---	--	------	------
mPD		0.46	0.68
		nPD:	0.68
		N. weight:	0.72
		Sc. PD:	0.27
		Sc. rank:	920.4

PB1			
Pos.	501	obs:	exp:
ttt	F	2523	1177.00
ttc	F	103	1449.00
---	--	------	------
mPD		0.075	0.49
		nPD:	0.15
		N. weight:	0.98
		Sc. PD:	-0.12
		Sc. rank:	-349.6

PB1			
Pos.	502	obs:	exp:
gtt	V	174	583.10
gtc	V	1	495.50
gta	V	1781	662.90
gtg	V	670	884.50
---	--	------	------
mPD		0.47	0.74
		nPD:	0.64
		N. weight:	0.81
		Sc. PD:	0.28
		Sc. rank:	993.6

PB1			
Pos.	503	obs:	exp:
gct	A	2232	643.70
gcc	A	375	601.70
gca	A	19	1184.00
gcg	A	0	196.90
---	--	------	------
mPD		0.26	0.68
		nPD:	0.38
		N. weight:	1.5
		Sc. PD:	0.14
		Sc. rank:	779.1

PB1			
Pos.	504	obs:	exp:
aat	N	2227	1493.00
aac	N	399	1133.00
---	--	------	------
mPD		0.26	0.49
		nPD:	0.53
		N. weight:	0.28
		Sc. PD:	0.067
		Sc. rank:	272.6

,

PB1			
Pos.	505	obs:	exp:
ttt	F	2144	1177.00
ttc	F	482	1449.00
---	--	------	------
mPD		0.30	0.49
		nPD:	0.61
		N. weight:	0.45
		Sc. PD:	0.14
		Sc. rank:	511.5

,

PB1			
Pos.	506	obs:	exp:
tct	S	0	441.70
tcc	S	0	415.00
tca	S	0	637.70
tcg	S	0	139.60
agt	S	417	488.80
agc	S	2209	503.30
---	--	------	------
mPD		0.27	1.6
		nPD:	0.16
		N. weight:	1.9
		Sc. PD:	-0.22
		Sc. rank:	-535.1

,

PB1			
Pos.	507	obs:	exp:
atg	M	2624	2624.00
act	T	0	0.25
acc	T	0	0.22
aca	T	0	0.45
acg	T	1	0.08
gtt	V	0	0.22
gtc	V	0	0.19
gta	V	0	0.25
gtg	V	1	0.34
---	--	------	------
mPD		0.0015	0.0027
		nPD:	0.56
		N. weight:	0.0021
		Sc. PD:	0.00056
		Sc. rank:	2.2

,

PB1			
Pos.	508	obs:	exp:
gat	D	1	0.56
gac	D	0	0.44
gaa	E	114	1555.00
gag	E	2511	1070.00
---	--	------	------
mPD		0.084	0.48
		nPD:	0.17
		N. weight:	1.1
		Sc. PD:	-0.12
		Sc. rank:	-201.7

,

PB1			
Pos.	509	obs:	exp:
tta	L	28	285.80
ttg	L	70	493.30
ctt	L	12	530.30
ctc	L	50	382.10
cta	L	879	458.50
ctg	L	1587	476.00
---	--	------	------
mPD		0.55	1.1
		nPD:	0.49
		N. weight:	1.3
		Sc. PD:	0.25
		Sc. rank:	1071.1

,

PB1			
Pos.	510	obs:	exp:
cct	P	41	774.90
ccc	P	2571	528.70
cca	P	14	1040.00
ccg	P	0	282.50
---	--	------	------
mPD		0.041	0.70
		nPD:	0.06
		N. weight:	2.3
		Sc. PD:	-0.5
		Sc. rank:	-2386.2

,

PB1			
Pos.	511	obs:	exp:
tct	S	0	441.40
tcc	S	0	414.70
tca	S	0	637.20
tcg	S	0	139.50
aat	N	2	1.14
aac	N	0	0.86
agt	S	1735	488.40
agc	S	889	502.90
---	--	------	------
mPD		0.45	1.6
		nPD:	0.28
		N. weight:	1.6
		Sc. PD:	-0.011
		Sc. rank:	359.9

,

PB1			
Pos.	512	obs:	exp:
ttt	F	2591	1177.00
ttc	F	35	1449.00
---	--	------	------
mPD		0.026	0.49
		nPD:	0.05
		N. weight:	1.1
		Sc. PD:	-0.25
		Sc. rank:	-1241.7

,

PB1			
Pos.	513	obs:	exp:
ggt	G	0	411.90
ggc	G	1	332.70
gga	G	2207	1189.00
ggg	G	418	691.90
---	--	------	------
mPD		0.27	0.68
		nPD:	0.39
		N. weight:	0.69
		Sc. PD:	0.072
		Sc. rank:	372.4

,

PB1			
Pos.	514	obs:	exp:
att	I	0	0.35
atc	I	0	0.26
ata	I	1	0.39
gtt	V	24	582.80
gtc	V	5	495.30
gta	V	423	662.70
gtg	V	2173	884.20
---	--	------	------
mPD		0.29	0.74
		nPD:	0.39
		N. weight:	0.99
		Sc. PD:	0.11
		Sc. rank:	549.5

,

PB1			
Pos.	515	obs:	exp:
tct	S	2464	439.00
tcc	S	68	412.50
tca	S	42	633.80
tcg	S	36	138.70
cct	P	8	2.36
ccc	P	0	1.61
cca	P	0	3.17
ccg	P	0	0.86
act	T	1	0.25
acc	T	0	0.22
aca	T	0	0.45
acg	T	0	0.08
agt	S	0	485.80
agc	S	0	500.20
gct	A	7	1.72
gcc	A	0	1.60
gca	A	0	3.16
gcg	A	0	0.52
---	--	------	------
mPD		0.12	1.6
		nPD:	0.07
		N. weight:	2.4
		Sc. PD:	-0.48
		Sc. rank:	-2160.1

,

PB1			
Pos.	516	obs:	exp:
ggt	G	12	411.90
ggc	G	0	332.70
gga	G	2443	1189.00
ggg	G	171	691.90
---	--	------	------
mPD		0.13	0.68
		nPD:	0.19
		N. weight:	0.88
		Sc. PD:	-0.078
		Sc. rank:	-52.9

,

PB1			
Pos.	517	obs:	exp:
att	I	41	554.80
atc	I	2	401.10
ata	I	1521	608.00
act	T	0	1.48
acc	T	0	1.30
aca	T	5	2.73
acg	T	1	0.50
gtt	V	368	234.50
gtc	V	20	199.30
gta	V	654	266.60
gtg	V	14	355.70
---	--	------	------
mPD		0.77	1.2
		nPD:	0.64
		N. weight:	1.2
		Sc. PD:	0.4
		Sc. rank:	1419.0

,

PB1			
Pos.	518	obs:	exp:
aat	N	1314	1493.00
aac	N	1312	1133.00
---	--	------	------
mPD		0.50	0.49
		nPD:	1.02
		N. weight:	0.017
		Sc. PD:	0.012
		Sc. rank:	24.8

,

PB1			
Pos.	519	obs:	exp:
gaa	E	2422	1556.00
gag	E	204	1070.00
---	--	------	------
mPD		0.14	0.48
		nPD:	0.3
		N. weight:	0.44
		Sc. PD:	0.0061
		Sc. rank:	123.5

,

PB1			
Pos.	520	obs:	exp:
tct	S	30	435.00
tcc	S	0	408.70
tca	S	2117	628.00
tcg	S	439	137.40
act	T	0	7.13
acc	T	0	6.27
aca	T	29	13.18
acg	T	0	2.42
agt	S	0	481.30
agc	S	0	495.60
gct	A	0	2.70
gcc	A	0	2.52
gca	A	11	4.96
gcg	A	0	0.82
---	--	------	------
mPD		0.33	1.6
		nPD:	0.2
		N. weight:	1.8
		Sc. PD:	-0.14
		Sc. rank:	-30.9

,

PB1			
Pos.	521	obs:	exp:
gct	A	2480	643.70
gcc	A	37	601.70
gca	A	109	1184.00
gcg	A	0	196.90
---	--	------	------
mPD		0.11	0.68
		nPD:	0.16
		N. weight:	1.8
		Sc. PD:	-0.22
		Sc. rank:	-600.4

,

PB1			
Pos.	522	obs:	exp:
gat	D	1659	1477.00
gac	D	967	1149.00
---	--	------	------
mPD		0.47	0.49
		nPD:	0.95
		N. weight:	0.018
		Sc. PD:	0.011
		Sc. rank:	25.9

,

PB1			
Pos.	523	obs:	exp:
atg	M	2626	2626.00
---	--	------	------
mPD		0	0
		nPD:	1.
		N. weight:	0.
		Sc. PD:	0
		Sc. rank:	0

,

PB1			
Pos.	524	obs:	exp:
tct	S	0	441.40
tcc	S	0	414.70
tca	S	0	637.20
tcg	S	0	139.50
att	I	0	0.35
atc	I	1	0.26
ata	I	0	0.39
agt	S	2117	488.40
agc	S	507	502.90
ggt	G	0	0.16
ggc	G	1	0.13
gga	G	0	0.45
ggg	G	0	0.26
---	--	------	------
mPD		0.31	1.6
		nPD:	0.19
		N. weight:	1.9
		Sc. PD:	-0.16
		Sc. rank:	-84.5

PB1			
Pos.	525	obs:	exp:
att	I	2558	929.10
atc	I	58	671.70
ata	I	3	1018.00
gtt	V	7	1.55
gtc	V	0	1.32
gta	V	0	1.77
gtg	V	0	2.36
---	--	------	------
mPD		0.051	0.66
		nPD:	0.08
		N. weight:	1.5
		Sc. PD:	-0.29
		Sc. rank:	-1249.5

PB1			
Pos.	526	obs:	exp:
ggt	G	41	411.90
ggc	G	2	332.70
gga	G	1779	1189.00
ggg	G	804	691.90
---	--	------	------
mPD		0.45	0.68
		nPD:	0.65
		N. weight:	0.44
		Sc. PD:	0.16
		Sc. rank:	548.7

PB1			
Pos.	527	obs:	exp:
tta	L	4	0.76
ttg	L	2	1.31
ctt	L	0	1.41
ctc	L	0	1.02
cta	L	0	1.22
ctg	L	1	1.27
att	I	11	21.99
atc	I	1	15.90
ata	I	50	24.10
atg	M	22	22.00
act	T	2	0.49
acc	T	0	0.43
aca	T	0	0.91
acg	T	0	0.17
gtt	V	17	562.00
gtc	V	9	477.60
gta	V	1798	638.90
gtg	V	707	852.50
gct	A	0	0.49
gcc	A	0	0.46
gca	A	0	0.90
gcg	A	2	0.15
---	--	------	------
mPD		0.50	0.81
		nPD:	0.61
		N. weight:	1.
		Sc. PD:	0.32
		Sc. rank:	1148.6

PB1			
Pos.	528	obs:	exp:
tct	S	0	0.34
tcc	S	0	0.32
tca	S	2	0.49
tcg	S	0	0.11
act	T	29	641.50
acc	T	4	564.00
aca	T	2540	1185.00
acg	T	35	217.50
agt	S	0	0.37
agc	S	0	0.38
gct	A	0	3.92
gcc	A	0	3.67
gca	A	16	7.21
gcg	A	0	1.20
---	--	------	------
mPD		0.064	0.69
		nPD:	0.09
		N. weight:	1.1
		Sc. PD:	-0.19
		Sc. rank:	-805.0

PB1			
Pos.	529	obs:	exp:
atg	M	1	1.00
gtt	V	27	582.80
gtc	V	23	495.30
gta	V	355	662.70
gtg	V	2220	884.20
---	--	------	------
mPD		0.27	0.74
		nPD:	0.36
		N. weight:	1.
		Sc. PD:	0.076
		Sc. rank:	458.3

PB1			
Pos.	530	obs:	exp:
att	I	4	930.80
atc	I	29	673.00
ata	I	2591	1020.00
gtt	V	0	0.44
gtc	V	0	0.38
gta	V	2	0.50
gtg	V	0	0.67
---	--	------	------
mPD		0.026	0.66
		nPD:	0.04
		N. weight:	1.4
		Sc. PD:	-0.32
		Sc. rank:	-1674.0

PB1			
Pos.	531	obs:	exp:
cgt	R	0	1.61
cgc	R	0	2.70
cga	R	0	5.13
cgg	R	0	3.68
aaa	K	158	1565.00
aag	K	2417	1010.00
aga	R	0	23.39
agg	R	51	14.50
---	--	------	------
mPD		0.15	0.53
		nPD:	0.29
		N. weight:	1.1
		Sc. PD:	0.005
		Sc. rank:	280.1

PB1			
Pos.	532	obs:	exp:
aat	N	75	1493.00
aac	N	2551	1133.00
---	--	------	------
mPD		0.056	0.49
		nPD:	0.11
		N. weight:	1.1
		Sc. PD:	-0.18
		Sc. rank:	-663.1

PB1			
Pos.	533	obs:	exp:
tct	S	0	0.67
tcc	S	0	0.63
tca	S	0	0.97
tcg	S	0	0.21
aat	N	114	1490.00
aac	N	2508	1132.00
agt	S	0	0.74
agc	S	4	0.77
---	--	------	------
mPD		0.086	0.50
		nPD:	0.17
		N. weight:	1.
		Sc. PD:	-0.11
		Sc. rank:	-182.9

PB1			
Pos.	534	obs:	exp:
atg	M	2626	2626.00
---	--	------	------
mPD		0	0
		nPD:	1.
		N. weight:	0.
		Sc. PD:	0
		Sc. rank:	0

PB1			
Pos.	535	obs:	exp:
att	I	21	921.30
atc	I	3	666.10
ata	I	2573	1010.00
gtt	V	0	6.44
gtc	V	0	5.47
gta	V	28	7.32
gtg	V	1	9.77
---	--	------	------
mPD		0.041	0.68
		nPD:	0.06
		N. weight:	1.4
		Sc. PD:	-0.3
		Sc. rank:	-1421.6

PB1			
Pos.	536	obs:	exp:
tat	Y	0	0.56
tac	Y	1	0.44
aat	N	334	1492.00
aac	N	2291	1133.00
---	--	------	------
mPD		0.22	0.49
		nPD:	0.45
		N. weight:	0.66
		Sc. PD:	0.11
		Sc. rank:	453.8

PB1			
Pos.	537	obs:	exp:
tct	S	0	2.35
tcc	S	0	2.21
tca	S	0	3.40
tcg	S	0	0.74
aat	N	2575	1484.00
aac	N	36	1127.00
aaa	K	0	0.61
aag	K	1	0.39
agt	S	14	2.61
agc	S	0	2.68
---	--	------	------
mPD		0.038	0.51
		nPD:	0.08
		N. weight:	0.79
		Sc. PD:	-0.16
		Sc. rank:	-685.8

PB1			
Pos.	538	obs:	exp:
gat	D	1804	1477.00
gac	D	822	1149.00
---	--	------	------
mPD		0.43	0.49
		nPD:	0.87
		N. weight:	0.053
		Sc. PD:	0.03
		Sc. rank:	76.5

PB1			
Pos.	539	obs:	exp:
ttt	F	0	0.90
ttc	F	2	1.10
tta	L	0	285.60
ttg	L	0	492.90
ctt	L	2306	529.90
ctc	L	288	381.80
cta	L	12	458.20
ctg	L	18	475.70
---	--	------	------
mPD		0.22	1.1
		nPD:	0.19
		N. weight:	1.9
		Sc. PD:	-0.17
		Sc. rank:	-100.7

PB1			
Pos.	540	obs:	exp:
ggt	G	13	411.90
ggc	G	38	332.70
gga	G	2254	1189.00
ggg	G	321	691.90
---	--	------	------
mPD		0.25	0.68
		nPD:	0.36
		N. weight:	0.64
		Sc. PD:	0.049
		Sc. rank:	295.4

PB1			
Pos.	541	obs:	exp:
cct	P	2133	774.90
ccc	P	24	528.70
cca	P	442	1040.00
ccg	P	27	282.50
---	--	------	------
mPD		0.31	0.70
		nPD:	0.44
		N. weight:	0.98
		Sc. PD:	0.15
		Sc. rank:	651.9

PB1			
Pos.	542	obs:	exp:
gct	A	7	643.70
gcc	A	3	601.70
gca	A	2593	1184.00
gcg	A	23	196.90
---	--	------	------
mPD		0.025	0.68
		nPD:	0.04
		N. weight:	1.2
		Sc. PD:	-0.27
		Sc. rank:	-1420.5

PB1			
Pos.	543	obs:	exp:
act	T	129	645.90
acc	T	9	567.80
aca	T	1840	1193.00
acg	T	648	219.00
---	--	------	------
mPD		0.45	0.68
		nPD:	0.66
		N. weight:	0.75
		Sc. PD:	0.27
		Sc. rank:	940.8

PB1			
Pos.	544	obs:	exp:
tct	S	1	0.17
tcc	S	0	0.16
tca	S	0	0.24
tcg	S	0	0.05
agt	S	0	0.19
agc	S	0	0.19
gct	A	997	643.50
gcc	A	1597	601.50
gca	A	31	1183.00
gcg	A	0	196.90
---	--	------	------
mPD		0.49	0.68
		nPD:	0.72
		N. weight:	1.1
		Sc. PD:	0.47
		Sc. rank:	1505.8

PB1			
Pos.	545	obs:	exp:
caa	Q	711	1416.00
cag	Q	1915	1210.00
---	--	------	------
mPD		0.40	0.50
		nPD:	0.79
		N. weight:	0.23
		Sc. PD:	0.12
		Sc. rank:	330.5

PB1			
Pos.	546	obs:	exp:
atg	M	2626	2626.00
---	--	------	------
mPD		0	0
		nPD:	1.
		N. weight:	0.
		Sc. PD:	0
		Sc. rank:	0

PB1			
Pos.	547	obs:	exp:
gct	A	1107	643.70
gcc	A	1425	601.70
gca	A	92	1184.00
gcg	A	2	196.90
---	--	------	------
mPD		0.53	0.68
		nPD:	0.78
		N. weight:	0.95
		Sc. PD:	0.45
		Sc. rank:	1322.2

PB1			
Pos.	548	obs:	exp:
tta	L	1	285.70
ttg	L	0	493.10
ctt	L	2432	530.10
ctc	L	172	381.90
cta	L	17	458.40
ctg	L	3	475.80
att	I	1	0.35
atc	I	0	0.26
ata	I	0	0.39
---	--	------	------
mPD		0.14	1.1
		nPD:	0.12
		N. weight:	2.1
		Sc. PD:	-0.32
		Sc. rank:	-1126.4

PB1			
Pos.	549	obs:	exp:
caa	Q	954	1416.00
cag	Q	1672	1210.00
---	--	------	------
mPD		0.46	0.50
		nPD:	0.93
		N. weight:	0.1
		Sc. PD:	0.062
		Sc. rank:	144.4

PB1			
Pos.	550	obs:	exp:
ttt	F	3	1.34
ttc	F	0	1.66
tta	L	205	285.40
ttg	L	1896	492.70
ctt	L	0	529.70
ctc	L	8	381.60
cta	L	346	458.00
ctg	L	168	475.50
---	--	------	------
mPD		0.66	1.1
		nPD:	0.58
		N. weight:	1.3
		Sc. PD:	0.37
		Sc. rank:	1412.3

PB1			
Pos.	551	obs:	exp:
ttt	F	49	1177.00
ttc	F	2577	1449.00
---	--	------	------
mPD		0.037	0.49
		nPD:	0.07
		N. weight:	0.79
		Sc. PD:	-0.16
		Sc. rank:	-706.7

PB1			
Pos.	552	obs:	exp:
tta	L	0	0.22
ttg	L	0	0.38
ctt	L	0	0.40
ctc	L	2	0.29
cta	L	0	0.35
ctg	L	0	0.36
att	I	342	926.20
atc	I	2130	669.70
ata	I	139	1015.00
act	T	0	0.25
acc	T	1	0.22
aca	T	0	0.45
acg	T	0	0.08
gtt	V	1	2.66
gtc	V	11	2.26
gta	V	0	3.03
gtg	V	0	4.04
---	--	------	------
mPD		0.33	0.67
		nPD:	0.49
		N. weight:	1.1
		Sc. PD:	0.22
		Sc. rank:	930.3

PB1			
Pos.	553	obs:	exp:
aaa	K	2325	1596.00
aag	K	301	1030.00
---	--	------	------
mPD		0.20	0.48
		nPD:	0.43
		N. weight:	0.3
		Sc. PD:	0.042
		Sc. rank:	187.0

PB1			
Pos.	554	obs:	exp:
gat	D	115	1477.00
gac	D	2511	1149.00
---	--	------	------
mPD		0.084	0.49
		nPD:	0.17
		N. weight:	0.99
		Sc. PD:	-0.11
		Sc. rank:	-232.8

PB1			
Pos.	555	obs:	exp:
tat	Y	51	1471.00
tac	Y	2575	1155.00
---	--	------	------
mPD		0.038	0.49
		nPD:	0.08
		N. weight:	1.1
		Sc. PD:	-0.22
		Sc. rank:	-958.8

PB1			
Pos.	556	obs:	exp:
cgt	R	0	82.89
cgc	R	1	138.80
cga	R	26	263.90
cgg	R	3	189.70
aga	R	2070	1204.00
agg	R	526	746.40
---	--	------	------
mPD		0.35	0.94
		nPD:	0.37
		N. weight:	0.52
		Sc. PD:	0.042
		Sc. rank:	240.4

PB1			
Pos.	557	obs:	exp:
tat	Y	367	1471.00
tac	Y	2259	1155.00
---	--	------	------
mPD		0.24	0.49
		nPD:	0.49
		N. weight:	0.6
		Sc. PD:	0.12
		Sc. rank:	504.8

PB1			
Pos.	558	obs:	exp:
act	T	15	645.90
acc	T	799	567.80
aca	T	1773	1193.00
acg	T	39	219.00
---	--	------	------
mPD		0.45	0.68
		nPD:	0.66
		N. weight:	0.51
		Sc. PD:	0.19
		Sc. rank:	644.4

PB1			
Pos.	559	obs:	exp:
tat	Y	2185	1471.00
tac	Y	441	1155.00
---	--	------	------
mPD		0.28	0.49
		nPD:	0.57
		N. weight:	0.26
		Sc. PD:	0.072
		Sc. rank:	279.6

PB1			
Pos.	560	obs:	exp:
cgt	R	15	82.86
cgc	R	9	138.70
cga	R	289	263.80
cgg	R	114	189.60
aaa	K	0	0.61
aag	K	1	0.39
aga	R	1430	1204.00
agg	R	768	746.10
---	--	------	------
mPD		0.73	0.94
		nPD:	0.78
		N. weight:	0.12
		Sc. PD:	0.058
		Sc. rank:	168.3

PB1			
Pos.	561	obs:	exp:
tct	S	0	0.50
tcc	S	0	0.47
tca	S	0	0.73
tcg	S	0	0.16
tgt	C	120	1129.00
tgc	C	2503	1494.00
agt	S	0	0.56
agc	S	3	0.57
---	--	------	------
mPD		0.090	0.49
		nPD:	0.18
		N. weight:	0.61
		Sc. PD:	-0.059
		Sc. rank:	-62.3

PB1			
Pos.	562	obs:	exp:
cat	H	2132	1515.00
cac	H	494	1111.00
---	--	------	------
mPD		0.31	0.49
		nPD:	0.63
		N. weight:	0.2
		Sc. PD:	0.065
		Sc. rank:	235.2

PB1			
Pos.	563	obs:	exp:
cgt	R	0	81.75
cgc	R	0	136.90
cga	R	5	260.30
cgg	R	0	187.10
aaa	K	31	21.88
aag	K	5	14.12
aga	R	1844	1188.00
agg	R	741	736.10
---	--	------	------
mPD		0.44	0.96
		nPD:	0.46
		N. weight:	0.48
		Sc. PD:	0.08
		Sc. rank:	338.1

PB1			
Pos.	564	obs:	exp:
tct	S	0	12.45
tcc	S	0	11.70
tca	S	0	17.97
tcg	S	0	3.93
aat	N	1	0.57
aac	N	0	0.43
agt	S	74	13.77
agc	S	0	14.18
ggt	G	310	400.10
ggc	G	37	323.20
gga	G	784	1155.00
ggg	G	1420	672.20
---	--	------	------
mPD		0.65	0.78
		nPD:	0.84
		N. weight:	0.44
		Sc. PD:	0.23
		Sc. rank:	620.0

,

PB1			
Pos.	565	obs:	exp:
gat	D	55	1477.00
gac	D	2571	1149.00
---	--	------	------
mPD		0.041	0.49
		nPD:	0.08
		N. weight:	1.1
		Sc. PD:	-0.22
		Sc. rank:	-936.3

,

PB1			
Pos.	566	obs:	exp:
tct	S	0	0.67
tcc	S	0	0.63
tca	S	1	0.97
tcg	S	3	0.21
atg	M	26	26.00
act	T	68	636.30
acc	T	6	559.40
aca	T	2186	1176.00
acg	T	327	215.70
aaa	K	3	4.86
aag	K	5	3.14
agt	S	0	0.74
agc	S	0	0.77
gct	A	1	0.25
gcc	A	0	0.23
gca	A	0	0.45
gcg	A	0	0.07
---	--	------	------
mPD		0.31	0.71
		nPD:	0.44
		N. weight:	0.79
		Sc. PD:	0.12
		Sc. rank:	520.3

,

PB1			
Pos.	567	obs:	exp:
tta	L	0	5.11
ttg	L	0	8.83
ctt	L	41	9.49
ctc	L	0	6.84
cta	L	6	8.21
ctg	L	0	8.52
cat	H	1	1.73
cac	H	2	1.27
caa	Q	2396	1389.00
cag	Q	179	1186.00
aaa	K	1	0.61
aag	K	0	0.39
---	--	------	------
mPD		0.19	0.55
		nPD:	0.35
		N. weight:	0.61
		Sc. PD:	0.041
		Sc. rank:	272.5

,

PB1			
Pos.	568	obs:	exp:
att	I	2103	924.80
atc	I	465	668.70
ata	I	39	1014.00
act	T	0	1.48
acc	T	6	1.30
aca	T	0	2.73
acg	T	0	0.50
gtt	V	8	2.89
gtc	V	5	2.45
gta	V	0	3.28
gtg	V	0	4.38
---	--	------	------
mPD		0.34	0.67
		nPD:	0.5
		N. weight:	0.87
		Sc. PD:	0.18
		Sc. rank:	772.4

,

PB1			
Pos.	569	obs:	exp:
tta	L	0	0.11
ttg	L	0	0.19
ctt	L	0	0.20
ctc	L	0	0.15
cta	L	0	0.17
ctg	L	1	0.18
cat	H	1	0.58
cac	H	0	0.42
caa	Q	1218	1412.00
cag	Q	1401	1207.00
atg	M	2	2.00
aaa	K	1	0.61
aag	K	0	0.39
gaa	E	2	1.19
gag	E	0	0.81
---	--	------	------
mPD		0.50	0.50
		nPD:	1.
		N. weight:	0.022
		Sc. PD:	0.015
		Sc. rank:	31.8

,

PB1			
Pos.	570	obs:	exp:
act	T	70	645.90
acc	T	0	567.80
aca	T	426	1193.00
acg	T	2130	219.00
---	--	------	------
mPD		0.32	0.68
		nPD:	0.46
		N. weight:	2.5
		Sc. PD:	0.44
		Sc. rank:	1852.4

,

PB1			
Pos.	571	obs:	exp:
cgt	R	0	68.02
cgc	R	0	113.90
cga	R	16	216.60
cgg	R	6	155.70
aaa	K	435	286.20
aag	K	36	184.80
aga	R	2097	988.30
agg	R	36	612.50
---	--	------	------
mPD		0.37	1.2
		nPD:	0.31
		N. weight:	0.92
		Sc. PD:	0.029
		Sc. rank:	299.1

,

PB1			
Pos.	572	obs:	exp:
cgt	R	0	82.89
cgc	R	0	138.80
cga	R	12	263.90
cgg	R	0	189.70
aga	R	2473	1204.00
agg	R	141	746.40
---	--	------	------
mPD		0.11	0.94
		nPD:	0.12
		N. weight:	0.9
		Sc. PD:	-0.14
		Sc. rank:	-502.9

,

PB1			
Pos.	573	obs:	exp:
tct	S	43	437.00
tcc	S	3	410.60
tca	S	2520	630.90
tcg	S	32	138.10
act	T	0	6.89
acc	T	0	6.05
aca	T	28	12.72
acg	T	0	2.33
agt	S	0	483.50
agc	S	0	497.90
---	--	------	------
mPD		0.079	1.6
		nPD:	0.05
		N. weight:	2.
		Sc. PD:	-0.45
		Sc. rank:	-2229.5

,

PB1			
Pos.	574	obs:	exp:
ttt	F	857	1174.00
ttc	F	1762	1445.00
tta	L	0	0.11
ttg	L	0	0.19
tct	S	0	0.17
tcc	S	1	0.16
tca	S	0	0.24
tcg	S	0	0.05
tat	Y	1	2.24
tac	Y	3	1.76
ctt	L	1	0.20
ctc	L	0	0.15
cta	L	0	0.17
ctg	L	0	0.18
agt	S	0	0.19
agc	S	0	0.19
gtt	V	1	0.22
gtc	V	0	0.19
gta	V	0	0.25
gtg	V	0	0.34
---	--	------	------
mPD		0.45	0.50
		nPD:	0.89
		N. weight:	0.054
		Sc. PD:	0.031
		Sc. rank:	77.2

,

PB1			
Pos.	575	obs:	exp:
gaa	E	147	1556.00
gag	E	2479	1070.00
---	--	------	------
mPD		0.11	0.48
		nPD:	0.22
		N. weight:	1.
		Sc. PD:	-0.063
		Sc. rank:	54.8

,

PB1			
Pos.	576	obs:	exp:
tta	L	715	279.10
ttg	L	53	481.80
ctt	L	0	518.00
ctc	L	0	373.20
cta	L	1676	447.90
ctg	L	121	465.00
att	I	0	21.28
atc	I	2	15.39
ata	I	58	23.33
atg	M	1	1.00
---	--	------	------
mPD		0.57	1.2
		nPD:	0.49
		N. weight:	1.6
		Sc. PD:	0.32
		Sc. rank:	1349.4

,

PB1			
Pos.	577	obs:	exp:
caa	Q	1	0.54
cag	Q	0	0.46
cgt	R	0	2.02
cgc	R	0	3.38
cga	R	0	6.43
cgg	R	31	4.62
atg	M	1	1.00
aat	N	2	1.14
aac	N	0	0.86
aaa	K	410	1554.00
aag	K	2147	1003.00
aga	R	9	29.35
agg	R	24	18.19
gaa	E	0	0.59
gag	E	1	0.41
---	--	------	------
mPD		0.34	0.54
		nPD:	0.63
		N. weight:	0.69
		Sc. PD:	0.23
		Sc. rank:	833.8

,

PB1			
Pos.	578	obs:	exp:
tct	S	0	39.53
tcc	S	0	37.14
tca	S	0	57.07
tcg	S	0	12.49
cgt	R	0	8.24
cgc	R	0	13.80
cga	R	0	26.23
cgg	R	0	18.86
aat	N	2	3.98
aac	N	5	3.02
aaa	K	401	1287.00
aag	K	1717	830.80
agt	S	1	43.74
agc	S	234	45.04
aga	R	34	119.70
agg	R	227	74.18
gaa	E	0	2.37
gag	E	4	1.63
ggt	G	0	0.16
ggc	G	0	0.13
gga	G	0	0.45
ggg	G	1	0.26
- - -	- -	- - - - - -	- - - - - -
mPD		0.72	1.0
		nPD:	0.71
		N. weight:	0.82
		Sc. PD:	0.34
		Sc. rank:	1094.6

,

PB1			
Pos.	579	obs:	exp:
tta	L	37	285.20
ttg	L	22	492.30
ctt	L	31	529.30
ctc	L	4	381.30
cta	L	561	457.70
ctg	L	1966	475.10
atg	M	5	5.00
- - -	- -	- - - - - -	- - - - - -
mPD		0.42	1.1
		nPD:	0.37
		N. weight:	1.6
		Sc. PD:	0.13
		Sc. rank:	751.8

,

PB1			
Pos.	580	obs:	exp:
tgg	W	2626	2626.00
- - -	- -	- - - - - -	- - - - - -
mPD		0	0
		nPD:	1.
		N. weight:	0.
		Sc. PD:	0
		Sc. rank:	0

,

PB1			
Pos.	581	obs:	exp:
tta	L	0	0.44
ttg	L	0	0.75
tat	Y	6	3.36
tac	Y	0	2.64
ctt	L	0	0.81
ctc	L	0	0.58
cta	L	0	0.70
ctg	L	4	0.73
aat	N	89	51.15
aac	N	1	38.85
aaa	K	0	0.61
aag	K	1	0.39
gct	A	1	0.25
gcc	A	0	0.23
gca	A	0	0.45
gcg	A	0	0.07
gat	D	1963	1181.00
gac	D	137	918.80
gaa	E	47	248.30
gag	E	372	170.70
ggt	G	5	0.78
ggc	G	0	0.63
gga	G	0	2.26
ggg	G	0	1.32
- - -	- -	- - - - - -	- - - - - -
mPD		0.44	0.71
		nPD:	0.62
		N. weight:	0.61
		Sc. PD:	0.2
		Sc. rank:	713.0

,

PB1			
Pos.	582	obs:	exp:
caa	Q	2565	1408.00
cag	Q	46	1203.00
aaa	K	11	6.69
aag	K	0	4.31
gat	D	4	2.25
gac	D	0	1.75
- - -	- -	- - - - - -	- - - - - -
mPD		0.049	0.51
		nPD:	0.1
		N. weight:	0.83
		Sc. PD:	-0.15
		Sc. rank:	-607.2

,

PB1			
Pos.	583	obs:	exp:
act	T	102	645.90
acc	T	2517	567.80
aca	T	3	1193.00
acg	T	4	219.00
- - -	- -	- - - - - -	- - - - - -
mPD		0.080	0.68
		nPD:	0.12
		N. weight:	2.1
		Sc. PD:	-0.33
		Sc. rank:	-1219.4

,

PB1			
Pos.	584	obs:	exp:
tta	L	0	0.33
ttg	L	0	0.56
tat	Y	0	0.56
tac	Y	1	0.44
tgt	C	1	0.43
tgc	C	0	0.57
ctt	L	3	0.61
ctc	L	0	0.44
cta	L	0	0.52
ctg	L	0	0.54
cct	P	0	1.18
ccc	P	0	0.81
cca	P	4	1.58
ccg	P	0	0.43
cat	H	251	218.00
cac	H	127	160.00
caa	Q	2067	1152.00
cag	Q	70	984.60
cgt	R	40	2.78
cgc	R	2	4.65
cga	R	42	8.85
cgg	R	4	6.36
aat	N	2	1.14
aac	N	0	0.86
aaa	K	12	7.29
aag	K	0	4.71
aga	R	0	40.36
agg	R	0	25.01
- - -	- -	- - - - - -	- - - - - -
mPD		0.41	0.76
		nPD:	0.55
		N. weight:	0.73
		Sc. PD:	0.18
		Sc. rank:	747.1

,

PB1			
Pos.	585	obs:	exp:
tct	S	17	441.00
tcc	S	38	414.40
tca	S	2532	636.70
tcg	S	35	139.40
agt	S	0	488.00
agc	S	0	502.50
gct	A	0	0.98
gcc	A	0	0.92
gca	A	4	1.80
gcg	A	0	0.30
- - -	- -	- - - - - -	- - - - - -
mPD		0.070	1.6
		nPD:	0.04
		N. weight:	2.
		Sc. PD:	-0.45
		Sc. rank:	-2315.4

,

PB1			
Pos.	586	obs:	exp:
cgt	R	0	1.26
cgc	R	0	2.11
cga	R	0	4.02
cgg	R	0	2.89
aaa	K	482	1572.00
aag	K	2104	1014.00
aga	R	1	18.34
agg	R	39	11.37
- - -	- -	- - - - - -	- - - - - -
mPD		0.33	0.52
		nPD:	0.64
		N. weight:	0.6
		Sc. PD:	0.21
		Sc. rank:	742.8

,

PB1			
Pos.	587	obs:	exp:
tta	L	2	0.22
ttg	L	0	0.38
tct	S	0	2.02
tcc	S	0	1.90
tca	S	12	2.91
tcg	S	0	0.64
ctt	L	0	0.40
ctc	L	0	0.29
cta	L	0	0.35
ctg	L	0	0.36
cct	P	0	0.30
ccc	P	0	0.20
cca	P	1	0.40
ccg	P	0	0.11
att	I	0	6.03
atc	I	0	4.36
ata	I	17	6.61
act	T	40	45.01
acc	T	1	39.57
aca	T	140	83.15
acg	T	2	15.26
agt	S	0	2.23
agc	S	0	2.30
gtt	V	0	139.00
gtc	V	0	118.10
gta	V	580	158.00
gtg	V	46	210.90
gct	A	45	437.10
gcc	A	1	408.50
gca	A	1726	803.70
gcg	A	11	133.70
ggt	G	0	0.31
ggc	G	0	0.25
gga	G	2	0.91
ggg	G	0	0.53
- - -	- -	- - - - - -	- - - - - -
mPD		0.63	1.2
		nPD:	0.51
		N. weight:	1.2
		Sc. PD:	0.26
		Sc. rank:	1083.8

,

PB1			
Pos.	588	obs:	exp:
ggt	G	5	411.90
ggc	G	3	332.70
gga	G	1649	1189.00
ggg	G	969	691.90
- - -	- -	- - - - - -	- - - - - -
mPD		0.47	0.68
		nPD:	0.69
		N. weight:	0.5
		Sc. PD:	0.19
		Sc. rank:	641.2

,

PB1			
Pos.	589	obs:	exp:
tta	L	1381	285.60
ttg	L	49	492.90
ctt	L	1	529.90
ctc	L	0	381.80
cta	L	1142	458.20
ctg	L	51	475.70
att	I	0	0.71
atc	I	0	0.51
ata	I	2	0.78
- - -	- -	- - - - - -	- - - - - -
mPD		0.57	1.1
		nPD:	0.5
		N. weight:	1.8
		Sc. PD:	0.37
		Sc. rank:	1609.6

,

PB1			
Pos.	590	obs:	exp:
tta	L	1321	285.80
ttg	L	91	493.30
ctt	L	4	530.30
ctc	L	3	382.10
cta	L	850	458.50
ctg	L	357	476.00
- - -	- -	- - - - - -	- - - - - -
mPD		0.78	1.1
		nPD:	0.69
		N. weight:	1.3
		Sc. PD:	0.53
		Sc. rank:	1752.3

,

PB1			
Pos.	591	obs:	exp:
att	I	67	33.35
atc	I	5	24.11
ata	I	22	36.54
gtt	V	344	562.20
gtc	V	20	477.80
gta	V	2034	639.20
gtg	V	134	852.80
- - -	- -	- - - - - -	- - - - - -
mPD		0.43	0.81
		nPD:	0.53
		N. weight:	1.1
		Sc. PD:	0.27
		Sc. rank:	1101.1

,

PB1			
Pos.	592	obs:	exp:
tct	S	1487	440.20
tcc	S	40	413.60
tca	S	1077	635.50
tcg	S	13	139.10
tgt	C	1	0.43
tgc	C	0	0.57
agt	S	0	487.10
agc	S	0	501.50
gct	A	7	1.96
gcc	A	0	1.83
gca	A	1	3.61
gcg	A	0	0.60
- - -	- -	- - - - - -	- - - - - -
mPD		0.51	1.6
		nPD:	0.31
		N. weight:	1.4
		Sc. PD:	0.042
		Sc. rank:	434.6

,

PB1			
Pos.	593	obs:	exp:
gat	D	2589	1477.00
gac	D	37	1149.00
---	--	------	------
mPD		0.028	0.49
		nPD:	0.06
		N. weight:	0.79
		Sc. PD:	-0.17
		Sc. rank:	-839.4

,

PB1			
Pos.	594	obs:	exp:
ggt	G	17	411.90
ggc	G	2	332.70
gga	G	2399	1189.00
ggg	G	208	691.90
---	--	------	------
mPD		0.16	0.68
		nPD:	0.23
		N. weight:	0.82
		Sc. PD:	-0.039
		Sc. rank:	90.7

,

PB1			
Pos.	595	obs:	exp:
ggt	G	21	411.90
ggc	G	5	332.70
gga	G	2219	1189.00
ggg	G	381	691.90
---	--	------	------
mPD		0.26	0.68
		nPD:	0.39
		N. weight:	0.64
		Sc. PD:	0.065
		Sc. rank:	347.6

,

PB1			
Pos.	596	obs:	exp:
cct	P	6	774.90
ccc	P	16	528.70
cca	P	2554	1040.00
ccg	P	50	282.50
---	--	------	------
mPD		0.054	0.70
		nPD:	0.08
		N. weight:	1.3
		Sc. PD:	-0.25
		Sc. rank:	-1095.1

,

PB1			
Pos.	597	obs:	exp:
aat	N	1685	1493.00
aac	N	941	1133.00
---	--	------	------
mPD		0.46	0.49
		nPD:	0.94
		N. weight:	0.02
		Sc. PD:	0.013
		Sc. rank:	28.7

,

PB1			
Pos.	598	obs:	exp:
tta	L	2042	284.90
ttg	L	160	491.70
tct	S	0	0.34
tcc	S	0	0.32
tca	S	2	0.49
tcg	S	0	0.11
ctt	L	3	528.70
ctc	L	2	380.90
cta	L	109	457.10
ctg	L	302	474.60
att	I	0	1.06
atc	I	0	0.77
ata	I	3	1.17
atg	M	3	3.00
agt	S	0	0.37
agc	S	0	0.38
---	--	------	------
mPD		0.57	1.1
		nPD:	0.5
		N. weight:	2.1
		Sc. PD:	0.43
		Sc. rank:	1866.0

,

PB1			
Pos.	599	obs:	exp:
tat	Y	255	1471.00
tac	Y	2371	1155.00
---	--	------	------
mPD		0.18	0.49
		nPD:	0.36
		N. weight:	0.75
		Sc. PD:	0.053
		Sc. rank:	337.3

,

PB1			
Pos.	600	obs:	exp:
aat	N	1873	1493.00
aac	N	753	1133.00
---	--	------	------
mPD		0.41	0.49
		nPD:	0.83
		N. weight:	0.072
		Sc. PD:	0.038
		Sc. rank:	102.3

,

PB1			
Pos.	601	obs:	exp:
att	I	112	931.20
atc	I	1807	673.30
ata	I	706	1021.00
gtt	V	0	0.22
gtc	V	0	0.19
gta	V	1	0.25
gtg	V	0	0.34
---	--	------	------
mPD		0.45	0.66
		nPD:	0.69
		N. weight:	0.77
		Sc. PD:	0.3
		Sc. rank:	995.5

,

PB1			
Pos.	602	obs:	exp:
cgt	R	8	82.89
cgc	R	0	138.80
cga	R	152	263.90
cgg	R	2392	189.70
aga	R	1	1204.00
agg	R	73	746.40
---	--	------	------
mPD		0.17	0.94
		nPD:	0.18
		N. weight:	3.4
		Sc. PD:	-0.33
		Sc. rank:	-358.4

,

PB1			
Pos.	603	obs:	exp:
aat	N	2500	1493.00
aac	N	126	1133.00
---	--	------	------
mPD		0.091	0.49
		nPD:	0.19
		N. weight:	0.6
		Sc. PD:	-0.056
		Sc. rank:	-51.0

,

PB1			
Pos.	604	obs:	exp:
ttt	F	3	1.34
ttc	F	0	1.66
tta	L	0	285.10
ttg	L	0	492.10
ctt	L	2047	529.10
ctc	L	502	381.20
cta	L	71	457.50
ctg	L	0	474.90
att	I	2	1.06
atc	I	1	0.77
ata	I	0	1.17
---	--	------	------
mPD		0.36	1.1
		nPD:	0.31
		N. weight:	1.7
		Sc. PD:	0.048
		Sc. rank:	503.6

,

PB1			
Pos.	605	obs:	exp:
cat	H	48	1515.00
cac	H	2578	1111.00
---	--	------	------
mPD		0.036	0.49
		nPD:	0.07
		N. weight:	1.2
		Sc. PD:	-0.24
		Sc. rank:	-1074.5

,

PB1			
Pos.	606	obs:	exp:
tta	L	0	0.33
ttg	L	0	0.56
ctt	L	3	0.61
ctc	L	0	0.44
cta	L	0	0.52
ctg	L	0	0.54
att	I	2305	930.10
atc	I	305	672.50
ata	I	12	1019.00
gtt	V	0	0.22
gtc	V	1	0.19
gta	V	0	0.25
gtg	V	0	0.34
---	--	------	------
mPD		0.22	0.66
		nPD:	0.33
		N. weight:	1.1
		Sc. PD:	0.048
		Sc. rank:	389.8

,

PB1			
Pos.	607	obs:	exp:
cct	P	2172	774.90
ccc	P	105	528.70
cca	P	132	1040.00
ccg	P	217	282.50
---	--	------	------
mPD		0.31	0.70
		nPD:	0.43
		N. weight:	1.
		Sc. PD:	0.15
		Sc. rank:	651.2

,

PB1			
Pos.	608	obs:	exp:
gaa	E	2564	1555.00
gag	E	61	1070.00
ggt	G	0	0.16
ggc	G	0	0.13
gga	G	1	0.45
ggg	G	0	0.26
---	--	------	------
mPD		0.046	0.48
		nPD:	0.1
		N. weight:	0.66
		Sc. PD:	-0.12
		Sc. rank:	-484.2

,

PB1			
Pos.	609	obs:	exp:
gtt	V	265	582.40
gtc	V	2317	495.00
gta	V	41	662.10
gtg	V	0	883.50
gct	A	0	0.74
gcc	A	3	0.69
gca	A	0	1.35
gcg	A	0	0.22
---	--	------	------
mPD		0.21	0.74
		nPD:	0.29
		N. weight:	1.9
		Sc. PD:	0.0059
		Sc. rank:	492.7

,

PB1			
Pos.	610	obs:	exp:
tgt	C	145	1130.00
tgc	C	2481	1496.00
---	--	------	------
mPD		0.10	0.49
		nPD:	0.21
		N. weight:	0.57
		Sc. PD:	-0.038
		Sc. rank:	18.6

,

PB1			
Pos.	611	obs:	exp:
tta	L	1985	285.60
ttg	L	397	492.90
ctt	L	0	529.90
ctc	L	0	381.80
cta	L	182	458.20
ctg	L	60	475.70
caa	Q	1	0.54
cag	Q	0	0.46
atg	M	1	1.00
---	--	------	------
mPD		0.46	1.1
		nPD:	0.4
		N. weight:	2.1
		Sc. PD:	0.24
		Sc. rank:	1203.8

,

PB1			
Pos.	612	obs:	exp:
aaa	K	2456	1596.00
aag	K	170	1030.00
---	--	------	------
mPD		0.12	0.48
		nPD:	0.25
		N. weight:	0.45
		Sc. PD:	-0.012
		Sc. rank:	76.2

,

PB1			
Pos.	613	obs:	exp:
tgg	W	2626	2626.00
---	--	------	------
mPD		0	0
		nPD:	1.
		N. weight:	0.
		Sc. PD:	0
		Sc. rank:	0

,

PB1			
Pos.	614	obs:	exp:
gat	D	46	42.18
gac	D	29	32.82
gaa	E	163	1512.00
gag	E	2388	1039.00
---	--	------	------
mPD		0.17	0.51
		nPD:	0.33
		N. weight:	0.97
		Sc. PD:	0.044
		Sc. rank:	368.9

,

PB1			
Pos.	615	obs:	exp:
tta	L	36	285.60
ttg	L	3	492.90
ctt	L	32	529.90
ctc	L	13	381.80
cta	L	2464	458.20
ctg	L	76	475.70
atg	M	1	1.00
gtt	V	0	0.22
gtc	V	0	0.19
gta	V	1	0.25
gtg	V	0	0.34
---	--	------	------
mPD		0.12	1.1
		nPD:	0.11
		N. weight:	2.3
		Sc. PD:	-0.38
		Sc. rank:	-1443.6

,

PB1			
Pos.	616	obs:	exp:
atg	M	2626	2626.00
---	--	------	------
mPD		0	0
		nPD:	1.
		N. weight:	0.
		Sc. PD:	0
		Sc. rank:	0

,

PB1			
Pos.	617	obs:	exp:
gat	D	2599	1477.00
gac	D	27	1149.00
---	--	------	------
mPD		0.020	0.49
		nPD:	0.04
		N. weight:	0.81
		Sc. PD:	-0.19
		Sc. rank:	-970.6

PB1			
Pos.	618	obs:	exp:
att	I	0	6.39
atc	I	0	4.62
ata	I	18	7.00
act	T	0	0.25
acc	T	0	0.22
aca	T	1	0.45
acg	T	0	0.08
aaa	K	9	20.66
aag	K	25	13.34
gtt	V	0	67.50
gtc	V	0	57.36
gta	V	261	76.74
gtg	V	43	102.40
gct	A	0	0.25
gcc	A	0	0.23
gca	A	1	0.45
gcg	A	0	0.07
gat	D	762	445.50
gac	D	30	346.50
gaa	E	90	874.00
gag	E	1385	601.00
ggt	G	0	0.16
ggc	G	0	0.13
gga	G	0	0.45
ggg	G	1	0.26
---	--	------	------
mPD		0.85	0.98
		nPD:	0.86
		N. weight:	0.96
		Sc. PD:	0.53
		Sc. rank:	1366.0

PB1			
Pos.	619	obs:	exp:
aat	N	21	17.05
aac	N	9	12.95
gat	D	2520	1446.00
gac	D	51	1125.00
gaa	E	9	8.30
gag	E	5	5.70
ggt	G	10	1.73
ggc	G	1	1.39
gga	G	0	4.98
ggg	G	0	2.90
---	--	------	------
mPD		0.087	0.53
		nPD:	0.16
		N. weight:	0.75
		Sc. PD:	-0.086
		Sc. rank:	-226.3

PB1			
Pos.	620	obs:	exp:
tat	Y	2226	1471.00
tac	Y	400	1155.00
---	--	------	------
mPD		0.26	0.49
		nPD:	0.52
		N. weight:	0.3
		Sc. PD:	0.069
		Sc. rank:	285.5

PB1			
Pos.	621	obs:	exp:
caa	Q	0	15.64
cag	Q	29	13.36
cgt	R	8	81.88
cgc	R	0	137.10
cga	R	79	260.70
cgg	R	2408	187.40
aaa	K	0	1.82
aag	K	3	1.18
aga	R	4	1190.00
agg	R	95	737.30
---	--	------	------
mPD		0.17	0.97
		nPD:	0.17
		N. weight:	3.5
		Sc. PD:	-0.37
		Sc. rank:	-742.0

PB1			
Pos.	622	obs:	exp:
ggt	G	67	411.90
ggc	G	16	332.70
gga	G	2489	1189.00
ggg	G	54	691.90
---	--	------	------
mPD		0.10	0.68
		nPD:	0.15
		N. weight:	0.91
		Sc. PD:	-0.12
		Sc. rank:	-354.0

PB1			
Pos.	623	obs:	exp:
cgt	R	0	82.89
cgc	R	0	138.80
cga	R	24	263.90
cgg	R	2	189.70
aga	R	2086	1204.00
agg	R	514	746.40
---	--	------	------
mPD		0.34	0.94
		nPD:	0.36
		N. weight:	0.53
		Sc. PD:	0.038
		Sc. rank:	240.3

PB1			
Pos.	624	obs:	exp:
tta	L	25	285.30
ttg	L	380	492.50
ctt	L	2153	529.50
ctc	L	17	381.50
cta	L	1	457.80
ctg	L	46	475.30
att	I	3	1.06
atc	I	0	0.77
ata	I	0	1.17
atg	M	1	1.00
---	--	------	------
mPD		0.56	1.1
		nPD:	0.49
		N. weight:	1.6
		Sc. PD:	0.33
		Sc. rank:	1411.4

PB1			
Pos.	625	obs:	exp:
tgt	C	1530	1130.00
tgc	C	1096	1496.00
---	--	------	------
mPD		0.49	0.49
		nPD:	0.99
		N. weight:	0.075
		Sc. PD:	0.051
		Sc. rank:	109.3

PB1			
Pos.	626	obs:	exp:
cat	H	14	8.08
cac	H	0	5.92
aat	N	2228	1485.00
aac	N	384	1127.00
---	--	------	------
mPD		0.26	0.50
		nPD:	0.52
		N. weight:	0.3
		Sc. PD:	0.068
		Sc. rank:	282.8

PB1			
Pos.	627	obs:	exp:
tct	S	0	0.17
tcc	S	1	0.16
tca	S	0	0.24
tcg	S	0	0.05
cct	P	541	774.60
ccc	P	2053	528.50
cca	P	22	1040.00
ccg	P	9	282.40
agt	S	0	0.19
agc	S	0	0.19
---	--	------	------
mPD		0.35	0.71
		nPD:	0.49
		N. weight:	1.5
		Sc. PD:	0.3
		Sc. rank:	1271.6

PB1			
Pos.	628	obs:	exp:
tta	L	15	281.40
ttg	L	31	485.70
ctt	L	27	522.30
ctc	L	5	376.30
cta	L	155	451.50
ctg	L	2353	468.80
caa	Q	0	14.02
cag	Q	26	11.98
atg	M	14	14.00
---	--	------	------
mPD		0.21	1.2
		nPD:	0.18
		N. weight:	2.
		Sc. PD:	-0.2
		Sc. rank:	-283.7

PB1			
Pos.	629	obs:	exp:
aat	N	2487	1493.00
aac	N	139	1133.00
---	--	------	------
mPD		0.10	0.49
		nPD:	0.2
		N. weight:	0.58
		Sc. PD:	-0.044
		Sc. rank:	-0.1

PB1			
Pos.	630	obs:	exp:
cct	P	69	774.90
ccc	P	2123	528.70
cca	P	189	1040.00
ccg	P	245	282.50
---	--	------	------
mPD		0.33	0.70
		nPD:	0.47
		N. weight:	1.4
		Sc. PD:	0.26
		Sc. rank:	1128.3

PB1			
Pos.	631	obs:	exp:
ttt	F	2537	1177.00
ttc	F	89	1449.00
---	--	------	------
mPD		0.066	0.49
		nPD:	0.13
		N. weight:	1.
		Sc. PD:	-0.15
		Sc. rank:	-499.6

PB1

Pos.	632	obs:	exp:
tta	L	0	0.11
ttg	L	0	0.19
ctt	L	1	0.20
ctc	L	0	0.15
cta	L	0	0.17
ctg	L	0	0.18
gtt	V	122	582.60
gtc	V	2436	495.10
gta	V	64	662.40
gtg	V	2	883.80
gct	A	1	0.25
gcc	A	0	0.23
gca	A	0	0.45
gcg	A	0	0.07
---	--	------	------
mPD		0.14	0.74
		nPD:	0.19
		N. weight:	2.1
		Sc. PD:	-0.19
		Sc. rank:	-160.9

,

PB1

Pos.	633	obs:	exp:
tct	S	0	381.50
tcc	S	0	358.40
tca	S	0	550.80
tcg	S	0	120.50
cgt	R	0	0.03
cgc	R	1	0.05
cga	R	0	0.10
cgg	R	0	0.07
att	I	0	0.35
atc	I	1	0.26
ata	I	0	0.39
act	T	1	3.20
acc	T	12	2.81
aca	T	0	5.91
acg	T	0	1.08
aat	N	23	188.10
aac	N	308	142.90
agt	S	752	422.10
agc	S	1516	434.70
aga	R	0	0.46
agg	R	0	0.28
ggt	G	11	1.88
ggc	G	1	1.52
gga	G	0	5.44
ggg	G	0	3.16
---	--	------	------
mPD		0.66	1.8
		nPD:	0.37
		N. weight:	1.5
		Sc. PD:	0.13
		Sc. rank:	765.2

,

PB1

Pos.	634	obs:	exp:
tat	Y	1	0.56
tac	Y	0	0.44
cat	H	2341	1513.00
cac	H	282	1110.00
caa	Q	2	1.08
cag	Q	0	0.92
---	--	------	------
mPD		0.19	0.49
		nPD:	0.4
		N. weight:	0.38
		Sc. PD:	0.042
		Sc. rank:	211.0

,

PB1

Pos.	635	obs:	exp:
cgt	R	0	0.57
cgc	R	0	0.95
cga	R	0	1.81
cgg	R	0	1.30
aat	N	1	0.57
aac	N	0	0.43
aaa	K	2053	1584.00
aag	K	554	1023.00
aga	R	16	8.25
agg	R	2	5.12
---	--	------	------
mPD		0.35	0.49
		nPD:	0.7
		N. weight:	0.12
		Sc. PD:	0.05
		Sc. rank:	162.0

,

PB1

Pos.	636	obs:	exp:
cgt	R	0	0.03
cgc	R	0	0.05
cga	R	0	0.10
cgg	R	0	0.07
aga	R	1	0.46
agg	R	0	0.28
gct	A	0	0.25
gcc	A	0	0.23
gca	A	0	0.45
gcg	A	1	0.07
gat	D	3	1.69
gac	D	0	1.31
gaa	E	301	1360.00
gag	E	1994	935.20
ggt	G	0	51.13
ggc	G	1	41.31
gga	G	291	147.70
ggg	G	34	85.90
---	--	------	------
mPD		0.57	0.74
		nPD:	0.78
		N. weight:	0.73
		Sc. PD:	0.35
		Sc. rank:	1018.6

,

PB1

Pos.	637	obs:	exp:
ttt	F	2	0.90
ttc	F	0	1.10
tta	L	0	0.11
ttg	L	0	0.19
ctt	L	1	0.20
ctc	L	0	0.15
cta	L	0	0.17
ctg	L	0	0.18
att	I	2568	923.40
atc	I	26	667.60
ata	I	9	1012.00
act	T	1	0.25
acc	T	0	0.22
aca	T	0	0.45
acg	T	0	0.08
gtt	V	19	4.22
gtc	V	0	3.59
gta	V	0	4.80
gtg	V	0	6.40
---	--	------	------
mPD		0.044	0.68
		nPD:	0.06
		N. weight:	1.5
		Sc. PD:	-0.32
		Sc. rank:	-1486.7

,

PB1

Pos.	638	obs:	exp:
aat	N	2	1.14
aac	N	0	0.86
gat	D	2062	1227.00
gac	D	120	954.70
gaa	E	64	256.00
gag	E	368	176.00
ggt	G	10	1.57
ggc	G	0	1.27
gga	G	0	4.53
ggg	G	0	2.63
---	--	------	------
mPD		0.36	0.64
		nPD:	0.57
		N. weight:	0.61
		Sc. PD:	0.17
		Sc. rank:	646.5

,

PB1

Pos.	639	obs:	exp:
ttt	F	1	1.34
ttc	F	2	1.66
tct	S	2215	440.20
tcc	S	311	413.60
tca	S	70	635.50
tcg	S	21	139.10
tat	Y	1	0.56
tac	Y	0	0.44
cct	P	0	0.30
ccc	P	1	0.20
cca	P	0	0.40
ccg	P	0	0.11
act	T	0	0.25
acc	T	1	0.22
aca	T	0	0.45
acg	T	0	0.08
agt	S	0	487.10
agc	S	0	501.50
gct	A	0	0.74
gcc	A	3	0.69
gca	A	0	1.35
gcg	A	0	0.22
---	--	------	------
mPD		0.28	1.6
		nPD:	0.17
		N. weight:	2.
		Sc. PD:	-0.21
		Sc. rank:	-446.4

,

PB1

Pos.	640	obs:	exp:
att	I	0	11.00
atc	I	0	7.95
ata	I	31	12.05
gtt	V	13	575.50
gtc	V	3	489.10
gta	V	2448	654.30
gtg	V	128	873.10
gct	A	0	0.74
gcc	A	0	0.69
gca	A	3	1.35
gcg	A	0	0.22
---	--	------	------
mPD		0.13	0.76
		nPD:	0.17
		N. weight:	1.8
		Sc. PD:	-0.19
		Sc. rank:	-418.4

,

PB1

Pos.	641	obs:	exp:
aat	N	77	1493.00
aac	N	2549	1133.00
---	--	------	------
mPD		0.057	0.49
		nPD:	0.12
		N. weight:	1.1
		Sc. PD:	-0.18
		Sc. rank:	-648.1

,

PB1

Pos.	642	obs:	exp:
tct	S	0	285.60
tcc	S	0	268.40
tca	S	0	412.30
tcg	S	0	90.24
tat	Y	3	1.68
tac	Y	0	1.32
tgt	C	1	0.43
tgc	C	0	0.57
aat	N	916	524.10
aac	N	6	397.90
aaa	K	1	0.61
aag	K	0	0.39
agt	S	1668	316.00
agc	S	30	325.40
ggt	G	1	0.16
ggc	G	0	0.13
gga	G	0	0.45
ggg	G	0	0.26
---	--	------	------
mPD		0.49	1.8
		nPD:	0.27
		N. weight:	1.9
		Sc. PD:	-0.015
		Sc. rank:	409.0

,

PB1

Pos.	643	obs:	exp:
tct	S	1	0.50
tcc	S	2	0.47
tca	S	0	0.73
tcg	S	0	0.16
act	T	3	1.23
acc	T	2	1.08
aca	T	0	2.27
acg	T	0	0.42
agt	S	0	0.56
agc	S	0	0.57
gct	A	2297	641.80
gcc	A	272	599.80
gca	A	42	1180.00
gcg	A	7	196.30
---	--	------	------
mPD		0.23	0.69
		nPD:	0.33
		N. weight:	1.5
		Sc. PD:	0.072
		Sc. rank:	599.7

,

PB1

Pos.	644	obs:	exp:
ttt	F	1	0.45
ttc	F	0	0.55
att	I	1	1.77
atc	I	0	1.28
ata	I	4	1.94
atg	M	27	27.00
gtt	V	19	575.30
gtc	V	0	488.90
gta	V	160	654.10
gtg	V	2412	872.70
gct	A	0	0.49
gcc	A	0	0.46
gca	A	0	0.90
gcg	A	2	0.15
---	--	------	------
mPD		0.16	0.76
		nPD:	0.21
		N. weight:	1.3
		Sc. PD:	-0.093
		Sc. rank:	21.9

,

PB1

Pos.	645	obs:	exp:
att	I	0	50.37
atc	I	0	36.42
ata	I	142	55.21
atg	M	4	4.00
gtt	V	16	550.60
gtc	V	0	468.00
gta	V	1001	626.00
gtg	V	1463	835.30
---	--	------	------
mPD		0.60	0.85
		nPD:	0.71
		N. weight:	0.82
		Sc. PD:	0.34
		Sc. rank:	1087.6

,

PB1

Pos.	646	obs:	exp:
tta	L	0	1.96
ttg	L	17	3.38
ctt	L	0	3.64
ctc	L	0	2.62
cta	L	0	3.14
ctg	L	1	3.26
att	I	1	9.22
atc	I	0	6.67
ata	I	25	10.11
atg	M	2516	2516.00
act	T	0	4.18
acc	T	0	3.68
aca	T	3	7.72
acg	T	14	1.42
gtt	V	2	10.88
gtc	V	0	9.25
gta	V	2	12.37
gtg	V	45	16.50
---	--	------	------
mPD		0.088	0.13
		nPD:	0.69
		N. weight:	0.071
		Sc. PD:	0.028
		Sc. rank:	92.9

PB1			
Pos.	647	obs:	exp:
tct	S	0	0.17
tcc	S	0	0.16
tca	S	1	0.24
tcg	S	0	0.05
cct	P	151	772.00
ccc	P	57	526.70
cca	P	2393	1036.00
ccg	P	15	281.40
caa	Q	7	3.77
cag	Q	0	3.23
act	T	0	0.49
acc	T	0	0.43
aca	T	2	0.91
acg	T	0	0.17
agt	S	0	0.19
agc	S	0	0.19
---	--	------	------
mPD		0.17	0.71
		nPD:	0.23
		N. weight:	0.95
		Sc. PD:	-0.045
		Sc. rank:	112.7

,

PB1			
Pos.	648	obs:	exp:
tct	S	22	13.96
tcc	S	38	13.12
tca	S	23	20.16
tcg	S	0	4.41
cct	P	0	0.59
ccc	P	2	0.40
cca	P	0	0.79
ccg	P	0	0.22
act	T	0	7.63
acc	T	15	6.70
aca	T	16	14.09
acg	T	0	2.59
agt	S	0	15.45
agc	S	0	15.91
gtt	V	0	0.67
gtc	V	3	0.57
gta	V	0	0.76
gtg	V	0	1.01
gct	A	347	614.50
gcc	A	2018	574.40
gca	A	142	1130.00
gcg	A	0	188.00
---	--	------	------
mPD		0.44	0.79
		nPD:	0.55
		N. weight:	1.3
		Sc. PD:	0.33
		Sc. rank:	1319.9

,

PB1			
Pos.	649	obs:	exp:
cat	H	2508	1515.00
cac	H	118	1111.00
---	--	------	------
mPD		0.086	0.49
		nPD:	0.18
		N. weight:	0.6
		Sc. PD:	-0.061
		Sc. rank:	-94.1

,

PB1			
Pos.	650	obs:	exp:
ggt	G	2341	411.90
ggc	G	262	332.70
gga	G	20	1189.00
ggg	G	3	691.90
---	--	------	------
mPD		0.20	0.68
		nPD:	0.29
		N. weight:	2.3
		Sc. PD:	0.0063
		Sc. rank:	573.5

,

PB1			
Pos.	651	obs:	exp:
cct	P	189	774.60
ccc	P	17	528.50
cca	P	2369	1040.00
ccg	P	50	282.40
act	T	0	0.25
acc	T	0	0.22
aca	T	1	0.45
acg	T	0	0.08
---	--	------	------
mPD		0.18	0.70
		nPD:	0.26
		N. weight:	0.92
		Sc. PD:	-0.023
		Sc. rank:	159.3

,

PB1			
Pos.	652	obs:	exp:
tct	S	24	6.06
tcc	S	12	5.69
tca	S	0	8.74
tcg	S	0	1.91
att	I	0	2.48
atc	I	7	1.80
ata	I	0	2.72
act	T	31	12.30
acc	T	19	10.81
aca	T	0	22.72
acg	T	0	4.17
agt	S	0	6.70
agc	S	0	6.90
gtt	V	27	11.55
gtc	V	25	9.81
gta	V	0	13.13
gtg	V	0	17.52
gct	A	289	607.90
gcc	A	2173	568.20
gca	A	17	1118.00
gcg	A	1	186.00
ggt	G	0	0.16
ggc	G	0	0.13
gga	G	0	0.45
ggg	G	1	0.26
---	--	------	------
mPD		0.37	0.81
		nPD:	0.46
		N. weight:	1.6
		Sc. PD:	0.28
		Sc. rank:	1178.6

,

PB1			
Pos.	653	obs:	exp:
cgt	R	0	0.57
cgc	R	0	0.95
cga	R	0	1.81
cgg	R	0	1.30
aat	N	0	0.57
aac	N	1	0.43
aaa	K	2364	1584.00
aag	K	242	1022.00
aga	R	7	8.25
agg	R	11	5.12
gat	D	1	0.56
gac	D	0	0.44
---	--	------	------
mPD		0.19	0.50
		nPD:	0.38
		N. weight:	0.36
		Sc. PD:	0.035
		Sc. rank:	194.8

,

PB1			
Pos.	654	obs:	exp:
tct	S	0	407.40
tcc	S	0	382.80
tca	S	0	588.20
tcg	S	0	128.70
cgt	R	0	0.06
cgc	R	0	0.11
cga	R	0	0.20
cgg	R	0	0.14
att	I	1	7.45
atc	I	20	5.39
ata	I	0	8.16
act	T	8	3.69
acc	T	7	3.24
aca	T	0	6.82
acg	T	0	1.25
aat	N	26	92.08
aac	N	136	69.92
agt	S	199	450.80
agc	S	2223	464.20
aga	R	0	0.92
agg	R	2	0.57
ggt	G	1	0.63
ggc	G	3	0.51
gga	G	0	1.81
ggg	G	0	1.05
---	--	------	------
mPD		0.31	1.7
		nPD:	0.18
		N. weight:	2.
		Sc. PD:	-0.2
		Sc. rank:	-253.1

,

PB1			
Pos.	655	obs:	exp:
tta	L	0	0.11
ttg	L	1	0.19
ctt	L	0	0.20
ctc	L	0	0.15
cta	L	0	0.17
ctg	L	0	0.18
att	I	10	12.77
atc	I	0	9.23
ata	I	26	14.00
atg	M	2582	2582.00
act	T	0	0.74
acc	T	0	0.65
aca	T	0	1.36
acg	T	3	0.25
gtt	V	0	0.89
gtc	V	0	0.75
gta	V	0	1.01
gtg	V	4	1.35
---	--	------	------
mPD		0.033	0.038
		nPD:	0.88
		N. weight:	0.017
		Sc. PD:	0.0099
		Sc. rank:	24.8

,

PB1			
Pos.	656	obs:	exp:
cat	H	0	0.58
cac	H	1	0.42
gaa	E	2472	1555.00
gag	E	153	1070.00
---	--	------	------
mPD		0.11	0.48
		nPD:	0.23
		N. weight:	0.51
		Sc. PD:	-0.026
		Sc. rank:	49.0

,

PB1			
Pos.	657	obs:	exp:
tat	Y	2567	1471.00
tac	Y	58	1154.00
cat	H	1	0.58
cac	H	0	0.42
---	--	------	------
mPD		0.044	0.49
		nPD:	0.09
		N. weight:	0.75
		Sc. PD:	-0.14
		Sc. rank:	-591.6

,

PB1			
Pos.	658	obs:	exp:
gat	D	2555	1476.00
gac	D	69	1148.00
gaa	E	1	1.19
gag	E	1	0.81
---	--	------	------
mPD		0.053	0.49
		nPD:	0.11
		N. weight:	0.72
		Sc. PD:	-0.12
		Sc. rank:	-473.5

,

PB1			
Pos.	659	obs:	exp:
tct	S	1	0.34
tcc	S	1	0.32
tca	S	0	0.49
tcg	S	0	0.11
agt	S	0	0.37
agc	S	0	0.38
gct	A	472	643.20
gcc	A	2119	601.20
gca	A	26	1183.00
gcg	A	7	196.80
---	--	------	------
mPD		0.32	0.68
		nPD:	0.47
		N. weight:	1.4
		Sc. PD:	0.25
		Sc. rank:	1075.3

,

PB1			
Pos.	660	obs:	exp:
att	I	12	4.26
atc	I	0	3.08
ata	I	0	4.67
gtt	V	2571	580.40
gtc	V	30	493.30
gta	V	11	659.90
gtg	V	2	880.50
---	--	------	------
mPD		0.041	0.75
		nPD:	0.06
		N. weight:	2.2
		Sc. PD:	-0.48
		Sc. rank:	-2358.8

PB1			
Pos.	661	obs:	exp:
tct	S	0	0.34
tcc	S	0	0.32
tca	S	2	0.49
tcg	S	0	0.11
agt	S	0	0.37
agc	S	0	0.38
gct	A	146	643.20
gcc	A	32	601.20
gca	A	2412	1183.00
gcg	A	34	196.80
---	--	------	------
mPD		0.15	0.68
		nPD:	0.22
		N. weight:	0.81
		Sc. PD:	-0.045
		Sc. rank:	68.7

PB1			
Pos.	662	obs:	exp:
act	T	2431	645.40
acc	T	148	567.40
aca	T	30	1192.00
acg	T	15	218.80
aat	N	2	1.14
aac	N	0	0.86
---	--	------	------
mPD		0.14	0.68
		nPD:	0.21
		N. weight:	1.7
		Sc. PD:	-0.13
		Sc. rank:	10.8

PB1			
Pos.	663	obs:	exp:
att	I	0	0.35
atc	I	0	0.26
ata	I	1	0.39
act	T	35	645.70
acc	T	5	567.60
aca	T	2537	1193.00
acg	T	48	218.90
---	--	------	------
mPD		0.066	0.68
		nPD:	0.1
		N. weight:	1.
		Sc. PD:	-0.18
		Sc. rank:	-716.4

PB1			
Pos.	664	obs:	exp:
cat	H	1042	1515.00
cac	H	1584	1111.00
---	--	------	------
mPD		0.48	0.49
		nPD:	0.98
		N. weight:	0.1
		Sc. PD:	0.07
		Sc. rank:	151.6

PB1			
Pos.	665	obs:	exp:
tct	S	456	441.70
tcc	S	2050	415.00
tca	S	85	637.70
tcg	S	35	139.60
agt	S	0	488.80
agc	S	0	503.30
---	--	------	------
mPD		0.36	1.6
		nPD:	0.22
		N. weight:	1.8
		Sc. PD:	-0.11
		Sc. rank:	126.5

PB1			
Pos.	666	obs:	exp:
tgg	W	2626	2626.00
---	--	------	------
mPD		0	0
		nPD:	1.
		N. weight:	0.
		Sc. PD:	0
		Sc. rank:	0

PB1			
Pos.	667	obs:	exp:
ttt	F	0	0.45
ttc	F	1	0.55
tta	L	0	0.11
ttg	L	0	0.19
tct	S	0	0.34
tcc	S	0	0.32
tca	S	0	0.49
tcg	S	0	0.11
ctt	L	1	0.20
ctc	L	0	0.15
cta	L	0	0.17
ctg	L	0	0.18
att	I	1515	865.60
atc	I	890	625.80
ata	I	35	948.60
act	T	19	5.41
acc	T	3	4.76
aca	T	0	10.00
acg	T	0	1.83
aat	N	2	1.14
aac	N	0	0.86
agt	S	2	0.37
agc	S	0	0.38
gtt	V	128	35.08
gtc	V	26	29.81
gta	V	0	39.89
gtg	V	4	53.22
---	--	------	------
mPD		0.61	0.81
		nPD:	0.75
		N. weight:	0.74
		Sc. PD:	0.33
		Sc. rank:	1018.8

PB1			
Pos.	668	obs:	exp:
tct	S	0	0.17
tcc	S	1	0.16
tca	S	0	0.24
tcg	S	0	0.05
cct	P	482	774.30
ccc	P	1814	528.30
cca	P	324	1039.00
ccg	P	4	282.30
agt	S	0	0.19
agc	S	0	0.19
gat	D	0	0.56
gac	D	1	0.44
---	--	------	------
mPD		0.48	0.71
		nPD:	0.67
		N. weight:	0.97
		Sc. PD:	0.36
		Sc. rank:	1227.0

PB1			
Pos.	669	obs:	exp:
cgt	R	0	0.09
cgc	R	0	0.16
cga	R	0	0.30
cgg	R	0	0.22
aaa	K	659	1594.00
aag	K	1964	1029.00
aga	R	1	1.38
agg	R	2	0.85
---	--	------	------
mPD		0.38	0.48
		nPD:	0.79
		N. weight:	0.41
		Sc. PD:	0.2
		Sc. rank:	582.3

PB1			
Pos.	670	obs:	exp:
cgt	R	0	82.89
cgc	R	0	138.80
cga	R	3	263.90
cgg	R	3	189.70
aga	R	476	1204.00
agg	R	2144	746.40
---	--	------	------
mPD		0.30	0.94
		nPD:	0.32
		N. weight:	1.1
		Sc. PD:	0.042
		Sc. rank:	378.1

PB1			
Pos.	671	obs:	exp:
aat	N	1008	1493.00
aac	N	1618	1133.00
---	--	------	------
mPD		0.47	0.49
		nPD:	0.96
		N. weight:	0.11
		Sc. PD:	0.072
		Sc. rank:	158.9

PB1			
Pos.	672	obs:	exp:
cat	H	0	0.58
cac	H	1	0.42
cgt	R	1022	82.86
cgc	R	1540	138.70
cga	R	50	263.80
cgg	R	12	189.60
aga	R	1	1204.00
agg	R	0	746.10
---	--	------	------
mPD		0.51	0.94
		nPD:	0.54
		N. weight:	3.7
		Sc. PD:	0.9
		Sc. rank:	3693.1

PB1			
Pos.	673	obs:	exp:
tct	S	2211	441.70
tcc	S	366	415.00
tca	S	47	637.70
tcg	S	2	139.60
agt	S	0	488.80
agc	S	0	503.30
---	--	------	------
mPD		0.27	1.6
		nPD:	0.17
		N. weight:	2.
		Sc. PD:	-0.23
		Sc. rank:	-536.2

PB1			
Pos.	674	obs:	exp:
att	I	2468	928.70
atc	I	96	671.50
ata	I	54	1018.00
gtt	V	8	1.78
gtc	V	0	1.51
gta	V	0	2.02
gtg	V	0	2.69
---	--	------	------
mPD		0.12	0.66
		nPD:	0.17
		N. weight:	1.2
		Sc. PD:	-0.13
		Sc. rank:	-216.8

PB1			
Pos.	675	obs:	exp:
ttt	F	0	0.90
ttc	F	2	1.10
tta	L	7	285.40
ttg	L	16	492.70
ctt	L	20	529.70
ctc	L	2500	381.60
cta	L	80	458.00
ctg	L	0	475.50
att	I	0	0.35
atc	I	1	0.26
ata	I	0	0.39
---	--	------	------
mPD		0.11	1.1
		nPD:	0.1
		N. weight:	2.6
		Sc. PD:	-0.47
		Sc. rank:	-1848.8

PB1			
Pos.	676	obs:	exp:
aat	N	456	1492.00
aac	N	2169	1133.00
aaa	K	0	0.61
aag	K	1	0.39
---	--	------	------
mPD		0.29	0.49
		nPD:	0.59
		N. weight:	0.52
		Sc. PD:	0.15
		Sc. rank:	569.0

PB1			
Pos.	677	obs:	exp:
act	T	127	645.40
acc	T	290	567.40
aca	T	1830	1192.00
acg	T	377	218.80
aat	N	1	0.57
aac	N	0	0.43
gat	D	0	0.56
gac	D	1	0.44
---	--	------	------
mPD		0.48	0.68
		nPD:	0.71
		N. weight:	0.36
		Sc. PD:	0.15
		Sc. rank:	472.8

PB1			
Pos.	678	obs:	exp:
tct	S	0	434.80
tcc	S	0	408.50
tca	S	0	627.70
tcg	S	0	137.40
cgt	R	0	0.03
cgc	R	0	0.05
cga	R	0	0.10
cgg	R	0	0.07
act	T	0	0.25
acc	T	1	0.22
aca	T	0	0.45
acg	T	0	0.08
aat	N	0	22.17
aac	N	39	16.83
agt	S	74	481.10
agc	S	2511	495.40
aga	R	1	0.46
agg	R	0	0.28
---	--	------	------
mPD		0.086	1.6
		nPD:	0.05
		N. weight:	2.4
		Sc. PD:	-0.52
		Sc. rank:	-2594.2

PB1			
Pos.	679	obs:	exp:
cat	H	0	6.35
cac	H	11	4.65
caa	Q	2502	1410.00
cag	Q	113	1205.00
---	--	------	------
mPD		0.090	0.50
		nPD:	0.18
		N. weight:	0.7
		Sc. PD:	-0.069
		Sc. rank:	-82.2

,

PB1			
Pos.	680	obs:	exp:
cgt	R	0	80.18
cgc	R	0	134.30
cga	R	0	255.30
cgg	R	1	183.50
aaa	K	1	52.27
aag	K	85	33.73
aga	R	213	1165.00
agg	R	2326	721.90
---	--	------	------
mPD		0.21	0.99
		nPD:	0.22
		N. weight:	1.4
		Sc. PD:	-0.093
		Sc. rank:	66.2

,

PB1			
Pos.	681	obs:	exp:
ggt	G	2	411.90
ggc	G	0	332.70
gga	G	2492	1189.00
ggg	G	132	691.90
---	--	------	------
mPD		0.097	0.68
		nPD:	0.14
		N. weight:	0.96
		Sc. PD:	-0.13
		Sc. rank:	-391.9

,

PB1			
Pos.	682	obs:	exp:
att	I	2496	928.70
atc	I	86	671.50
ata	I	36	1018.00
gtt	V	7	1.78
gtc	V	1	1.51
gta	V	0	2.02
gtg	V	0	2.69
---	--	------	------
mPD		0.096	0.66
		nPD:	0.15
		N. weight:	1.3
		Sc. PD:	-0.17
		Sc. rank:	-517.5

,

PB1			
Pos.	683	obs:	exp:
ttt	F	1	0.45
ttc	F	0	0.55
tta	L	1	285.20
ttg	L	2	492.30
ctt	L	2484	529.30
ctc	L	115	381.30
cta	L	12	457.70
ctg	L	7	475.10
att	I	4	1.42
atc	I	0	1.03
ata	I	0	1.56
---	--	------	------
mPD		0.11	1.1
		nPD:	0.09
		N. weight:	2.2
		Sc. PD:	-0.39
		Sc. rank:	-1660.3

,

PB1			
Pos.	684	obs:	exp:
gaa	E	272	1556.00
gag	E	2354	1070.00
---	--	------	------
mPD		0.19	0.48
		nPD:	0.38
		N. weight:	0.82
		Sc. PD:	0.081
		Sc. rank:	443.4

,

PB1			
Pos.	685	obs:	exp:
gat	D	2560	1477.00
gac	D	66	1149.00
---	--	------	------
mPD		0.049	0.49
		nPD:	0.1
		N. weight:	0.73
		Sc. PD:	-0.13
		Sc. rank:	-504.0

,

PB1			
Pos.	686	obs:	exp:
gaa	E	2514	1556.00
gag	E	112	1070.00
---	--	------	------
mPD		0.082	0.48
		nPD:	0.17
		N. weight:	0.57
		Sc. PD:	-0.062
		Sc. rank:	-139.1

,

PB1			
Pos.	687	obs:	exp:
tta	L	0	0.11
ttg	L	0	0.19
tgg	W	1	1.00
ctt	L	0	0.20
ctc	L	0	0.15
cta	L	0	0.17
ctg	L	1	0.18
cat	H	92	91.14
cac	H	66	66.86
caa	Q	475	1276.00
cag	Q	1891	1090.00
cgt	R	0	1.14
cgc	R	0	1.90
cga	R	18	3.62
cgg	R	17	2.60
aaa	K	20	38.90
aag	K	44	25.10
aga	R	1	16.51
agg	R	0	10.23
---	--	------	------
mPD		0.48	0.65
		nPD:	0.74
		N. weight:	0.39
		Sc. PD:	0.17
		Sc. rank:	534.4

,

PB1			
Pos.	688	obs:	exp:
att	I	5	2.13
atc	I	0	1.54
ata	I	1	2.33
atg	M	2617	2617.00
gtt	V	0	0.44
gtc	V	0	0.38
gta	V	0	0.50
gtg	V	2	0.67
---	-	1	1.00
---	--	------	------
mPD		0.0061	0.0071
		nPD:	0.86
		N. weight:	0.0039
		Sc. PD:	0.0021
		Sc. rank:	5.5

,

PB1			
Pos.	689	obs:	exp:
tat	Y	236	1470.00
tac	Y	2388	1154.00
cat	H	0	0.58
cac	H	1	0.42
---	-	1	1.00
---	--	------	------
mPD		0.16	0.49
		nPD:	0.33
		N. weight:	0.78
		Sc. PD:	0.038
		Sc. rank:	308.1

,

PB1			
Pos.	690	obs:	exp:
cat	H	2	1.15
cac	H	0	0.85
caa	Q	175	1411.00
cag	Q	2442	1206.00
cgt	R	0	0.09
cgc	R	0	0.16
cga	R	0	0.30
cgg	R	3	0.22
aaa	K	1	2.43
aag	K	3	1.57
aga	R	0	1.38
agg	R	0	0.85
---	--	------	------
mPD		0.13	0.50
		nPD:	0.26
		N. weight:	0.82
		Sc. PD:	-0.017
		Sc. rank:	153.2

,

PB1			
Pos.	691	obs:	exp:
cgt	R	0	0.25
cgc	R	0	0.42
cga	R	0	0.80
cgg	R	0	0.58
aaa	K	331	1591.00
aag	K	2287	1027.00
aga	R	1	3.67
agg	R	7	2.27
---	--	------	------
mPD		0.23	0.48
		nPD:	0.47
		N. weight:	0.79
		Sc. PD:	0.14
		Sc. rank:	603.3

,

PB1			
Pos.	692	obs:	exp:
tgt	C	257	1130.00
tgc	C	2368	1495.00
ggt	G	0	0.16
ggc	G	0	0.13
gga	G	1	0.45
ggg	G	0	0.26
---	--	------	------
mPD		0.18	0.49
		nPD:	0.36
		N. weight:	0.42
		Sc. PD:	0.033
		Sc. rank:	196.5

,

PB1			
Pos.	693	obs:	exp:
tat	Y	0	1.68
tac	Y	3	1.32
tgt	C	53	1129.00
tgc	C	2570	1494.00
---	--	------	------
mPD		0.042	0.49
		nPD:	0.08
		N. weight:	0.74
		Sc. PD:	-0.14
		Sc. rank:	-606.5

,

PB1			
Pos.	694	obs:	exp:
tct	S	0	5.55
tcc	S	0	5.22
tca	S	0	8.01
tcg	S	0	1.75
tat	Y	8	4.48
tac	Y	0	3.52
att	I	0	0.35
atc	I	1	0.26
ata	I	0	0.39
act	T	2	2.21
acc	T	7	1.95
aca	T	0	4.09
acg	T	0	0.75
aat	N	1914	1464.00
aac	N	661	1111.00
agt	S	22	6.14
agc	S	11	6.32
---	--	------	------
mPD		0.42	0.55
		nPD:	0.77
		N. weight:	0.13
		Sc. PD:	0.063
		Sc. rank:	186.0

,

PB1			
Pos.	695	obs:	exp:
tta	L	213	281.10
ttg	L	61	485.20
ctt	L	17	521.70
ctc	L	6	375.80
cta	L	878	451.00
ctg	L	1408	468.20
att	I	0	1.77
atc	I	1	1.28
ata	I	4	1.94
atg	M	38	38.00
---	--	------	------
mPD		0.71	1.2
		nPD:	0.62
		N. weight:	1.1
		Sc. PD:	0.36
		Sc. rank:	1304.3

,

PB1			
Pos.	696	obs:	exp:
ttt	F	1099	1177.00
ttc	F	1527	1449.00
---	--	------	------
mPD		0.49	0.49
		nPD:	0.98
		N. weight:	0.0052
		Sc. PD:	0.0035
		Sc. rank:	7.6

PB1			
Pos.	697	obs:	exp:
gaa	E	118	1555.00
gag	E	2506	1069.00
ggt	G	0	0.31
ggc	G	0	0.25
gga	G	0	0.91
ggg	G	2	0.53
---	--	------	------
mPD		0.087	0.48
		nPD:	0.18
		N. weight:	1.1
		Sc. PD:	-0.11
		Sc. rank:	-130.9

PB1			
Pos.	698	obs:	exp:
cgt	R	0	0.63
cgc	R	0	1.06
cga	R	0	2.01
cgg	R	0	1.44
aat	N	1	0.57
aac	N	0	0.43
aaa	K	2477	1583.00
aag	K	128	1022.00
aga	R	19	9.17
agg	R	1	5.68
---	--	------	------
mPD		0.11	0.50
		nPD:	0.22
		N. weight:	0.51
		Sc. PD:	-0.031
		Sc. rank:	28.1

PB1			
Pos.	699	obs:	exp:
ttt	F	2165	1177.00
ttc	F	461	1449.00
---	--	------	------
mPD		0.29	0.49
		nPD:	0.59
		N. weight:	0.47
		Sc. PD:	0.14
		Sc. rank:	517.9

PB1			
Pos.	700	obs:	exp:
ttt	F	70	1177.00
ttc	F	2556	1449.00
---	--	------	------
mPD		0.052	0.49
		nPD:	0.1
		N. weight:	0.75
		Sc. PD:	-0.13
		Sc. rank:	-500.9

PB1			
Pos.	701	obs:	exp:
cct	P	2377	774.60
ccc	P	229	528.50
cca	P	15	1040.00
ccg	P	4	282.40
cat	H	1	0.58
cac	H	0	0.42
---	--	------	------
mPD		0.17	0.70
		nPD:	0.25
		N. weight:	1.4
		Sc. PD:	-0.051
		Sc. rank:	215.8

PB1			
Pos.	702	obs:	exp:
tct	S	0	441.50
tcc	S	0	414.90
tca	S	0	637.50
tcg	S	0	139.50
agt	S	1497	488.60
agc	S	1128	503.10
---	-	1	1.00
---	--	------	------
mPD		0.49	1.6
		nPD:	0.3
		N. weight:	1.5
		Sc. PD:	0.027
		Sc. rank:	443.2

PB1			
Pos.	703	obs:	exp:
tct	S	0	441.50
tcc	S	0	414.90
tca	S	0	637.50
tcg	S	0	139.50
cgt	R	0	0.03
cgc	R	0	0.05
cga	R	0	0.10
cgg	R	0	0.07
agt	S	2519	488.60
agc	S	106	503.10
aga	R	1	0.46
agg	R	0	0.28
---	--	------	------
mPD		0.078	1.6
		nPD:	0.05
		N. weight:	2.4
		Sc. PD:	-0.53
		Sc. rank:	-2676.1

PB1			
Pos.	704	obs:	exp:
tta	L	0	0.11
ttg	L	1	0.19
tct	S	6	441.50
tcc	S	2	414.90
tca	S	1798	637.50
tcg	S	819	139.50
ctt	L	0	0.20
ctc	L	0	0.15
cta	L	0	0.17
ctg	L	0	0.18
agt	S	0	488.60
agc	S	0	503.10
---	--	------	------
mPD		0.43	1.6
		nPD:	0.27
		N. weight:	2.
		Sc. PD:	-0.03
		Sc. rank:	385.8

PB1			
Pos.	705	obs:	exp:
tat	Y	734	1471.00
tac	Y	1892	1155.00
---	--	------	------
mPD		0.40	0.49
		nPD:	0.82
		N. weight:	0.25
		Sc. PD:	0.13
		Sc. rank:	360.1

PB1			
Pos.	706	obs:	exp:
cgt	R	0	82.39
cgc	R	0	138.00
cga	R	1	262.30
cgg	R	1	188.60
aaa	K	0	9.72
aag	K	16	6.28
aga	R	447	1197.00
agg	R	2161	741.80
---	--	------	------
mPD		0.30	0.95
		nPD:	0.31
		N. weight:	1.1
		Sc. PD:	0.033
		Sc. rank:	344.5

PB1			
Pos.	707	obs:	exp:
cgt	R	0	82.70
cgc	R	0	138.50
cga	R	6	263.30
cgg	R	0	189.30
aaa	K	6	3.65
aag	K	0	2.35
aga	R	2205	1202.00
agg	R	409	744.70
---	--	------	------
mPD		0.27	0.94
		nPD:	0.29
		N. weight:	0.64
		Sc. PD:	0.0039
		Sc. rank:	169.6

PB1			
Pos.	708	obs:	exp:
tct	S	1	0.17
tcc	S	0	0.16
tca	S	0	0.24
tcg	S	0	0.05
cct	P	26	774.60
ccc	P	6	528.50
cca	P	727	1040.00
ccg	P	1866	282.40
agt	S	0	0.19
agc	S	0	0.19
---	--	------	------
mPD		0.42	0.71
		nPD:	0.59
		N. weight:	1.9
		Sc. PD:	0.56
		Sc. rank:	2101.1

PB1			
Pos.	709	obs:	exp:
att	I	78	27.67
atc	I	0	20.01
ata	I	0	30.32
gtt	V	2492	565.70
gtc	V	53	480.80
gta	V	1	643.20
gtg	V	2	858.20
---	--	------	------
mPD		0.099	0.80
		nPD:	0.12
		N. weight:	2.2
		Sc. PD:	-0.33
		Sc. rank:	-1140.8

PB1			
Pos.	710	obs:	exp:
ggt	G	6	411.90
ggc	G	2	332.70
gga	G	2506	1189.00
ggg	G	112	691.90
---	--	------	------
mPD		0.088	0.68
		nPD:	0.13
		N. weight:	0.97
		Sc. PD:	-0.14
		Sc. rank:	-490.5

PB1			
Pos.	711	obs:	exp:
tta	L	0	0.11
ttg	L	0	0.19
ctt	L	1	0.20
ctc	L	0	0.15
cta	L	0	0.17
ctg	L	0	0.18
att	I	2541	931.20
atc	I	82	673.30
ata	I	2	1021.00
---	--	------	------
mPD		0.063	0.66
		nPD:	0.1
		N. weight:	1.4
		Sc. PD:	-0.25
		Sc. rank:	-1037.4

PB1			
Pos.	712	obs:	exp:
tct	S	2186	441.70
tcc	S	143	415.00
tca	S	291	637.70
tcg	S	6	139.60
agt	S	0	488.80
agc	S	0	503.30
---	--	------	------
mPD		0.29	1.6
		nPD:	0.18
		N. weight:	1.8
		Sc. PD:	-0.18
		Sc. rank:	-271.0

PB1			
Pos.	713	obs:	exp:
tct	S	0	439.80
tcc	S	0	413.30
tca	S	0	635.00
tcg	S	0	139.00
act	T	0	2.46
acc	T	10	2.16
aca	T	0	4.54
acg	T	0	0.83
aat	N	0	0.57
aac	N	1	0.43
agt	S	68	486.70
agc	S	2547	501.20
---	--	------	------
mPD		0.059	1.6
		nPD:	0.04
		N. weight:	2.4
		Sc. PD:	-0.57
		Sc. rank:	-2954.9

PB1			
Pos.	714	obs:	exp:
tta	L	0	0.11
ttg	L	1	0.19
ctt	L	0	0.20
ctc	L	0	0.15
cta	L	0	0.17
ctg	L	0	0.18
att	I	0	0.35
atc	I	0	0.26
ata	I	1	0.39
atg	M	2624	2624.00
---	--	------	------
mPD		0.0015	0.0020
		nPD:	0.76
		N. weight:	0.0016
		Sc. PD:	0.00072
		Sc. rank:	2.2

PB1			
Pos.	715	obs:	exp:
ttt	F	1	0.45
ttc	F	0	0.55
tta	L	0	0.11
ttg	L	1	0.19
ctt	L	0	0.20
ctc	L	0	0.15
cta	L	0	0.17
ctg	L	0	0.18
atg	M	30	30.00
gtt	V	4	575.50
gtc	V	0	489.10
gta	V	869	654.30
gtg	V	1719	873.10
gct	A	0	0.25
gcc	A	0	0.23
gca	A	0	0.45
gcg	A	1	0.07
ggt	G	0	0.16
ggc	G	0	0.13
gga	G	0	0.45
ggg	G	1	0.26
---	--	------	------
mPD		0.47	0.76
		nPD:	0.62
		N. weight:	0.83
		Sc. PD:	0.27
		Sc. rank:	976.6

PB1			
Pos.	716	obs:	exp:
gat	D	2	1.12
gac	D	0	0.88
gaa	E	9	1555.00
gag	E	2615	1069.00
---	--	------	------
mPD		0.0084	0.48
		nPD:	0.02
		N. weight:	1.4
		Sc. PD:	-0.35
		Sc. rank:	-1867.7

PB1			
Pos.	717	obs:	exp:
gct	A	37	643.70
gcc	A	2583	601.70
gca	A	6	1184.00
gcg	A	0	196.90
---	--	------	------
mPD		0.032	0.68
		nPD:	0.05
		N. weight:	2.2
		Sc. PD:	-0.49
		Sc. rank:	-2466.6

PB1			
Pos.	718	obs:	exp:
atg	M	2626	2626.00
---	--	------	------
mPD		0	0
		nPD:	1.
		N. weight:	0.
		Sc. PD:	0
		Sc. rank:	0

PB1			
Pos.	719	obs:	exp:
tta	L	2	1.52
ttg	L	12	2.63
ctt	L	0	2.83
ctc	L	0	2.04
cta	L	0	2.44
ctg	L	0	2.54
att	I	0	7.45
atc	I	0	5.39
ata	I	21	8.16
atg	M	18	18.00
gtt	V	79	570.90
gtc	V	4	485.10
gta	V	442	649.00
gtg	V	2046	866.00
gct	A	0	0.49
gcc	A	0	0.46
gca	A	1	0.90
gcg	A	1	0.15
---	--	------	------
mPD		0.38	0.78
		nPD:	0.49
		N. weight:	0.87
		Sc. PD:	0.18
		Sc. rank:	752.2

PB1			
Pos.	720	obs:	exp:
ttt	F	1	0.45
ttc	F	0	0.55
tct	S	2497	439.20
tcc	S	69	412.60
tca	S	12	634.10
tcg	S	33	138.80
agt	S	0	486.00
agc	S	0	500.40
gct	A	13	3.43
gcc	A	0	3.21
gca	A	0	6.31
gcg	A	1	1.05
---	--	------	------
mPD		0.096	1.6
		nPD:	0.06
		N. weight:	2.5
		Sc. PD:	-0.53
		Sc. rank:	-2520.9

PB1			
Pos.	721	obs:	exp:
cgt	R	0	82.70
cgc	R	0	138.50
cga	R	0	263.30
cgg	R	0	189.30
aaa	K	0	3.65
aag	K	6	2.35
aga	R	25	1202.00
agg	R	2595	744.70
---	--	------	------
mPD		0.023	0.94
		nPD:	0.02
		N. weight:	1.9
		Sc. PD:	-0.46
		Sc. rank:	-2440.6

PB1			
Pos.	722	obs:	exp:
gct	A	70	643.70
gcc	A	2547	601.70
gca	A	9	1184.00
gcg	A	0	196.90
---	--	------	------
mPD		0.059	0.68
		nPD:	0.09
		N. weight:	2.1
		Sc. PD:	-0.39
		Sc. rank:	-1689.9

PB1			
Pos.	723	obs:	exp:
tta	L	0	0.11
ttg	L	0	0.19
ctt	L	0	0.20
ctc	L	0	0.15
cta	L	0	0.17
ctg	L	1	0.18
caa	Q	4	4.31
cag	Q	4	3.69
cgt	R	104	82.45
cgc	R	0	138.10
cga	R	845	262.50
cgg	R	1226	188.70
aaa	K	2	3.04
aag	K	3	1.96
aga	R	310	1198.00
agg	R	127	742.40
---	--	------	------
mPD		0.83	0.95
		nPD:	0.87
		N. weight:	1.6
		Sc. PD:	0.9
		Sc. rank:	2288.7

PB1			
Pos.	724	obs:	exp:
att	I	2581	931.60
atc	I	45	673.50
ata	I	0	1021.00
---	--	------	------
mPD		0.034	0.66
		nPD:	0.05
		N. weight:	1.5
		Sc. PD:	-0.33
		Sc. rank:	-1658.9

PB1			
Pos.	725	obs:	exp:
gat	D	2595	1477.00
gac	D	31	1149.00
---	--	------	------
mPD		0.023	0.49
		nPD:	0.05
		N. weight:	0.8
		Sc. PD:	-0.18
		Sc. rank:	-925.9

PB1			
Pos.	726	obs:	exp:
gct	A	9	643.70
gcc	A	2179	601.70
gca	A	432	1184.00
gcg	A	6	196.90
---	--	------	------
mPD		0.28	0.68
		nPD:	0.42
		N. weight:	1.4
		Sc. PD:	0.18
		Sc. rank:	840.2

PB1			
Pos.	727	obs:	exp:
cgt	R	0	82.89
cgc	R	0	138.80
cga	R	69	263.90
cgg	R	358	189.70
aga	R	63	1204.00
agg	R	2136	746.40
---	--	------	------
mPD		0.37	0.94
		nPD:	0.39
		N. weight:	1.3
		Sc. PD:	0.14
		Sc. rank:	718.9

PB1			
Pos.	728	obs:	exp:
att	I	1763	663.40
atc	I	106	479.60
ata	I	1	727.00
gtt	V	281	167.90
gtc	V	462	142.70
gta	V	13	190.80
gtg	V	0	254.60
---	--	------	------
mPD		0.76	1.1
		nPD:	0.68
		N. weight:	1.3
		Sc. PD:	0.5
		Sc. rank:	1689.5

PB1			
Pos.	729	obs:	exp:
gat	D	545	1451.00
gac	D	2035	1129.00
gaa	E	45	27.26
gag	E	1	18.74
---	--	------	------
mPD		0.36	0.51
		nPD:	0.7
		N. weight:	0.41
		Sc. PD:	0.16
		Sc. rank:	536.5

PB1			
Pos.	730	obs:	exp:
ttt	F	334	1176.00
ttc	F	2290	1448.00
tat	Y	0	1.12
tac	Y	2	0.88
---	--	------	------
mPD		0.22	0.50
		nPD:	0.45
		N. weight:	0.38
		Sc. PD:	0.061
		Sc. rank:	255.0

PB1			
Pos.	731	obs:	exp:
gaa	E	858	1556.00
gag	E	1768	1070.00
---	--	------	------
mPD		0.44	0.48
		nPD:	0.91
		N. weight:	0.23
		Sc. PD:	0.14
		Sc. rank:	326.8

PB1			
Pos.	732	obs:	exp:
tct	S	2593	441.70
tcc	S	27	415.00
tca	S	4	637.70
tcg	S	2	139.60
agt	S	0	488.80
agc	S	0	503.30
---	--	------	------
mPD		0.025	1.6
		nPD:	0.02
		N. weight:	2.7
		Sc. PD:	-0.69
		Sc. rank:	-3695.3

PB1

Pos.	733	obs:	exp:
cgt	R	0	0.19
cgc	R	0	0.32
cga	R	0	0.60
cgg	R	0	0.43
aga	R	3	2.75
agg	R	3	1.71
gaa	E	2	1.19
gag	E	0	0.81
ggt	G	2	410.60
ggc	G	1	331.70
gga	G	2501	1186.00
ggg	G	114	689.80
---	--	------	------
mPD		0.093	0.69
		nPD:	0.14
		N. weight:	0.98
		Sc. PD:	-0.14
		Sc. rank:	-465.4

,

PB1

Pos.	734	obs:	exp:
cgt	R	6	82.80
cgc	R	0	138.60
cga	R	47	263.60
cgg	R	1897	189.50
aaa	K	0	1.82
aag	K	3	1.18
aga	R	20	1203.00
agg	R	653	745.50
---	--	------	------
mPD		0.44	0.94
		nPD:	0.47
		N. weight:	2.4
		Sc. PD:	0.43
		Sc. rank:	1859.1

,

PB1

Pos.	735	obs:	exp:
tta	L	0	0.33
ttg	L	0	0.56
tct	S	0	0.17
tcc	S	0	0.16
tca	S	0	0.24
tcg	S	0	0.05
ctt	L	0	0.61
ctc	L	2	0.44
cta	L	0	0.52
ctg	L	1	0.54
att	I	1689	910.60
atc	I	859	658.40
ata	I	19	998.00
atg	M	36	36.00
act	T	2	1.23
acc	T	1	1.08
aca	T	2	2.27
acg	T	0	0.42
agt	S	1	0.19
agc	S	0	0.19
gtt	V	5	2.89
gtc	V	8	2.45
gta	V	0	3.28
gtg	V	0	4.38
gct	A	0	0.25
gcc	A	1	0.23
gca	A	0	0.45
gcg	A	0	0.07
---	--	------	------
mPD		0.49	0.69
		nPD:	0.71
		N. weight:	0.73
		Sc. PD:	0.3
		Sc. rank:	973.5

,

PB1

Pos.	736	obs:	exp:
tct	S	0	0.34
tcc	S	0	0.32
tca	S	0	0.49
tcg	S	0	0.11
caa	Q	0	3.24
cag	Q	6	2.76
cgt	R	0	0.92
cgc	R	0	1.53
cga	R	0	2.91
cgg	R	0	2.10
act	T	0	0.74
acc	T	0	0.65
aca	T	0	1.36
acg	T	3	0.25
aat	N	16	9.66
aac	N	1	7.34
aaa	K	69	1554.00
aag	K	2488	1003.00
agt	S	2	0.37
agc	S	0	0.38
aga	R	6	13.30
agg	R	23	8.24
gct	A	0	0.25
gcc	A	0	0.23
gca	A	1	0.45
gcg	A	0	0.07
gaa	E	0	2.37
gag	E	4	1.63
ggt	G	0	1.10
ggc	G	0	0.89
gga	G	0	3.17
ggg	G	7	1.84
---	--	------	------
mPD		0.12	0.54
		nPD:	0.21
		N. weight:	1.3
		Sc. PD:	-0.082
		Sc. rank:	46.8

,

PB1

Pos.	737	obs:	exp:
cgt	R	0	11.05
cgc	R	0	18.50
cga	R	0	35.18
cgg	R	0	25.29
act	T	0	2.21
acc	T	0	1.95
aca	T	9	4.09
acg	T	0	0.75
aat	N	13	9.09
aac	N	3	6.91
aaa	K	2037	1362.00
aag	K	204	879.10
aga	R	350	160.50
agg	R	0	99.48
gaa	E	10	5.93
gag	E	0	4.07
---	--	------	------
mPD		0.40	0.81
		nPD:	0.5
		N. weight:	0.48
		Sc. PD:	0.099
		Sc. rank:	425.1

,

PB1

Pos.	738	obs:	exp:
gat	D	6	4.50
gac	D	2	3.50
gaa	E	2529	1544.00
gag	E	77	1062.00
ggt	G	0	1.88
ggc	G	0	1.52
gga	G	8	5.44
ggg	G	4	3.16
---	--	------	------
mPD		0.075	0.50
		nPD:	0.15
		N. weight:	0.63
		Sc. PD:	-0.08
		Sc. rank:	-231.5

,

PB1

Pos.	739	obs:	exp:
aaa	K	0	0.61
aag	K	1	0.39
gat	D	31	24.75
gac	D	13	19.25
gaa	E	342	1526.00
gag	E	2234	1050.00
ggt	G	0	0.78
ggc	G	0	0.63
gga	G	0	2.26
ggg	G	5	1.32
---	--	------	------
mPD		0.26	0.51
		nPD:	0.51
		N. weight:	0.71
		Sc. PD:	0.16
		Sc. rank:	659.8

,

PB1

Pos.	740	obs:	exp:
ttt	F	399	1172.00
ttc	F	2217	1444.00
tta	L	0	0.22
ttg	L	0	0.38
tat	Y	8	4.48
tac	Y	0	3.52
ctt	L	0	0.40
ctc	L	2	0.29
cta	L	0	0.35
ctg	L	0	0.36
---	--	------	------
mPD		0.27	0.50
		nPD:	0.54
		N. weight:	0.32
		Sc. PD:	0.078
		Sc. rank:	311.4

,

PB1

Pos.	741	obs:	exp:
ttt	F	4	1.79
ttc	F	0	2.21
tct	S	2176	368.00
tcc	S	1	345.80
tca	S	11	531.30
tcg	S	0	116.30
tat	Y	11	6.16
tac	Y	0	4.84
act	T	11	2.71
acc	T	0	2.38
aca	T	0	5.00
acg	T	0	0.92
agt	S	0	407.20
agc	S	0	419.30
gtt	V	4	0.89
gtc	V	0	0.75
gta	V	0	1.01
gtg	V	0	1.35
gct	A	401	99.77
gcc	A	6	93.25
gca	A	0	183.50
gcg	A	0	30.52
gat	D	1	0.56
gac	D	0	0.44
---	--	------	------
mPD		0.30	1.7
		nPD:	0.17
		N. weight:	2.6
		Sc. PD:	-0.28
		Sc. rank:	-500.7

,

PB1			
Pos.	742	obs:	exp:
gat	D	1	0.56
gac	D	0	0.44
gaa	E	0	1555.00
gag	E	2625	1070.00
---	--	------	------
mPD		0.00076	0.48
		nPD:	0.
		N. weight:	1.4
		Sc. PD:	-0.38
		Sc. rank:	-2020.1

,

PB1			
Pos.	743	obs:	exp:
ttt	F	0	0.45
ttc	F	1	0.55
att	I	6	929.40
atc	I	2614	672.00
ata	I	0	1019.00
act	T	0	1.23
acc	T	5	1.08
aca	T	0	2.27
acg	T	0	0.42
---	--	------	------
mPD		0.0091	0.66
		nPD:	0.01
		N. weight:	2.1
		Sc. PD:	-0.54
		Sc. rank:	-2925.6

,

PB1			
Pos.	744	obs:	exp:
tta	L	2	1.31
ttg	L	8	2.25
ctt	L	0	2.42
ctc	L	0	1.75
cta	L	0	2.10
ctg	L	2	2.18
att	I	49	45.41
atc	I	0	32.83
ata	I	79	49.76
atg	M	2464	2464.00
act	T	0	3.69
acc	T	0	3.24
aca	T	1	6.82
acg	T	14	1.25
gtt	V	0	1.55
gtc	V	0	1.32
gta	V	0	1.77
gtg	V	7	2.36
---	--	------	------
mPD		0.12	0.14
		nPD:	0.88
		N. weight:	0.057
		Sc. PD:	0.033
		Sc. rank:	82.6

,

PB1			
Pos.	745	obs:	exp:
cgt	R	0	1.04
cgc	R	0	1.74
cga	R	0	3.32
cgg	R	0	2.38
aaa	K	5	1576.00
aag	K	2588	1017.00
aga	R	0	15.13
agg	R	33	9.38
---	--	------	------
mPD		0.029	0.51
		nPD:	0.06
		N. weight:	1.4
		Sc. PD:	-0.31
		Sc. rank:	-1540.5

,

PB1			
Pos.	746	obs:	exp:
att	I	4	926.60
atc	I	2608	669.90
ata	I	0	1015.00
act	T	0	3.44
acc	T	14	3.03
aca	T	0	6.36
acg	T	0	1.17
---	--	------	------
mPD		0.014	0.67
		nPD:	0.02
		N. weight:	2.1
		Sc. PD:	-0.53
		Sc. rank:	-2834.5

,

PB1			
Pos.	747	obs:	exp:
tgt	C	2375	1130.00
tgc	C	251	1496.00
---	--	------	------
mPD		0.17	0.49
		nPD:	0.35
		N. weight:	0.78
		Sc. PD:	0.053
		Sc. rank:	350.2

,

PB1			
Pos.	748	obs:	exp:
ttt	F	0	5.83
ttc	F	13	7.17
tct	S	5	432.30
tcc	S	2538	406.20
tca	S	27	624.10
tcg	S	0	136.60
tat	Y	0	0.56
tac	Y	1	0.44
act	T	0	9.10
acc	T	37	8.00
aca	T	0	16.81
acg	T	0	3.09
agt	S	0	478.30
agc	S	0	492.50
gct	A	0	1.23
gcc	A	5	1.15
gca	A	0	2.25
gcg	A	0	0.37
---	--	------	------
mPD		0.066	1.6
		nPD:	0.04
		N. weight:	2.7
		Sc. PD:	-0.64
		Sc. rank:	-3320.8

,

PB1			
Pos.	749	obs:	exp:
tct	S	0	0.17
tcc	S	1	0.16
tca	S	0	0.24
tcg	S	0	0.05
act	T	0	645.70
acc	T	2625	567.60
aca	T	0	1193.00
acg	T	0	218.90
agt	S	0	0.19
agc	S	0	0.19
---	--	------	------
mPD		0.00076	0.68
		nPD:	0.
		N. weight:	2.4
		Sc. PD:	-0.65
		Sc. rank:	-3459.4

,

PB1			
Pos.	750	obs:	exp:
att	I	2620	931.60
atc	I	1	673.50
ata	I	5	1021.00
---	--	------	------
mPD		0.0046	0.66
		nPD:	0.01
		N. weight:	1.6
		Sc. PD:	-0.42
		Sc. rank:	-2240.0

,

PB1			
Pos.	751	obs:	exp:
gaa	E	2626	1556.00
gag	E	0	1070.00
---	--	------	------
mPD		0	0.48
		nPD:	0.
		N. weight:	0.82
		Sc. PD:	-0.22
		Sc. rank:	-1189.7

,

PB1			
Pos.	752	obs:	exp:
aat	N	1	0.57
aac	N	0	0.43
gat	D	29	18.00
gac	D	3	14.00
gaa	E	2081	1517.00
gag	E	480	1044.00
ggt	G	0	5.02
ggc	G	0	4.05
gga	G	29	14.49
ggg	G	3	8.43
---	--	------	------
mPD		0.35	0.52
		nPD:	0.66
		N. weight:	0.19
		Sc. PD:	0.069
		Sc. rank:	236.8

,

PB1			
Pos.	753	obs:	exp:
ttt	F	1	60.94
ttc	F	135	75.06
tta	L	0	266.70
ttg	L	0	460.40
tct	S	0	3.03
tcc	S	18	2.84
tca	S	0	4.37
tcg	S	0	0.96
ctt	L	3	495.00
ctc	L	2448	356.60
cta	L	0	428.00
ctg	L	0	444.30
cat	H	0	1.73
cac	H	3	1.27
att	I	0	6.39
atc	I	18	4.62
ata	I	0	7.00
agt	S	0	3.35
agc	S	0	3.45
---	--	------	------
mPD		0.14	1.2
		nPD:	0.12
		N. weight:	2.9
		Sc. PD:	-0.46
		Sc. rank:	-1618.1

,

PB1			
Pos.	754	obs :	exp :
cgt	R	0	81.69
cgc	R	0	136.80
cga	R	0	260.10
cgg	R	0	187.00
aaa	K	8	4.86
aag	K	0	3.14
aga	R	2588	1187.00
agg	R	0	735.60
ggt	G	0	4.71
ggc	G	0	3.80
gga	G	30	13.59
ggg	G	0	7.90
---	--	------	------
mPD		0.029	0.96
		nPD :	0.03
		N. weight :	1.2
		Sc. PD :	-0.3
		Sc. rank :	-1561.5

,

PB1			
Pos.	755	obs :	exp :
cct	P	0	0.30
ccc	P	1	0.20
cca	P	0	0.40
ccg	P	0	0.11
cgt	R	0	82.86
cgc	R	0	138.70
cga	R	0	263.80
cgg	R	2625	189.60
aga	R	0	1204.00
agg	R	0	746.10
---	--	------	------
mPD		0.0015	0.94
		nPD :	0.
		N. weight :	4.1
		Sc. PD :	-1.1
		Sc. rank :	-5848.4

,

PB1			
Pos.	756	obs :	exp :
caa	Q	27	14.56
cag	Q	0	12.44
---	-	2599	2599.00
---	--	------	------
mPD		0	0.000053
		nPD :	0.
		N. weight :	0.
		Sc. PD :	0
		Sc. rank :	0

,

PB1			
Pos.	757	obs :	exp :
tta	L	0	0.11
ttg	L	0	0.19
ctt	L	0	0.20
ctc	L	0	0.15
cta	L	1	0.17
ctg	L	0	0.18
cct	P	0	0.59
ccc	P	0	0.40
cca	P	2	0.79
ccg	P	0	0.22
cat	H	1	2.88
cac	H	4	2.12
caa	Q	2459	1396.00
cag	Q	129	1192.00
cgt	R	0	0.95
cgc	R	0	1.59
cga	R	28	3.02
cgg	R	2	2.17
aga	R	0	13.76
agg	R	0	8.53
---	--	------	------
mPD		0.12	0.54
		nPD :	0.23
		N. weight :	0.7
		Sc. PD :	-0.037
		Sc. rank :	66.5

,

PB1			
Pos.	758	obs :	exp :
aaa	K	1	0.61
aag	K	0	0.39
---	-	2625	2625.00
---	--	------	------
mPD		0	6.9×10^{-8}
		nPD :	0.
		N. weight :	0.
		Sc. PD :	0
		Sc. rank :	0

,

PB1			
Pos.	759	obs :	exp :
caa	Q	61	33.43
cag	Q	1	28.57
cgt	R	0	0.85
cgc	R	0	1.43
cga	R	0	2.71
cgg	R	0	1.95
aat	N	13	15.35
aac	N	14	11.65
aaa	K	787	1522.00
aag	K	1718	982.60
aga	R	16	12.38
agg	R	11	7.67
gtt	V	0	0.44
gtc	V	0	0.38
gta	V	0	0.50
gtg	V	2	0.67
gaa	E	1	1.19
gag	E	1	0.81
ggt	G	0	0.16
ggc	G	0	0.13
gga	G	0	0.45
ggg	G	1	0.26
---	--	------	------
mPD		0.53	0.57
		nPD :	0.93
		N. weight :	0.29
		Sc. PD :	0.18
		Sc. rank :	425.8

,

PB1			
Pos.	760	obs :	exp :
tta	L	1	0.11
ttg	L	0	0.19
tat	Y	2	1.12
tac	Y	0	0.88
tgg	W	1	1.00
ctt	L	0	0.20
ctc	L	0	0.15
cta	L	0	0.17
ctg	L	0	0.18
caa	Q	20	12.40
cag	Q	3	10.60
aaa	K	0	0.61
aag	K	1	0.39
gaa	E	3	1.78
gag	E	0	1.22
---	-	2595	2595.00
---	--	------	------
mPD		0.00013	0.00015
		nPD :	0.84
		N. weight :	0.
		Sc. PD :	0
		Sc. rank :	0

,

PB1			
Pos.	761	obs :	exp :
taa	.	1997	1117.00
tag	.	412	691.80
tga	.	217	817.00
---	--	------	------
mPD		0.42	0.82
		nPD :	0.51
		N. weight :	0.4
		Sc. PD :	0.087
		Sc. rank :	364.9

}

Amino acid level conservation per codon

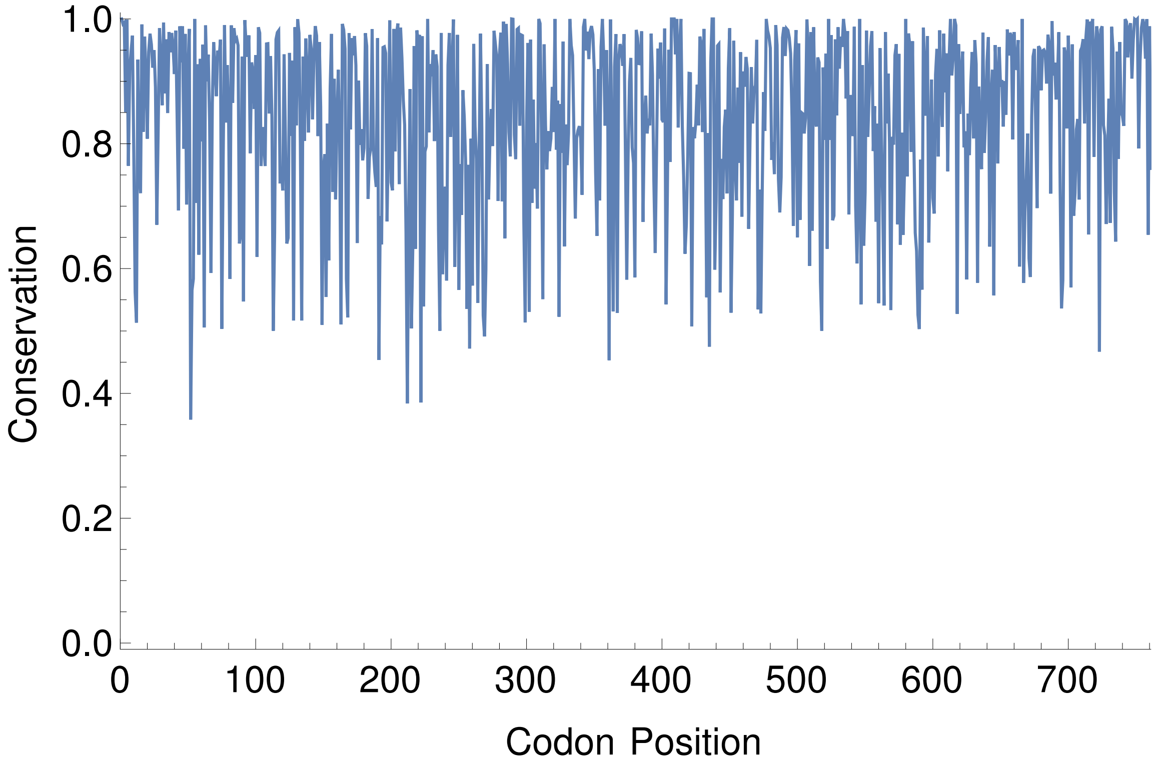

Conservation analysis plots

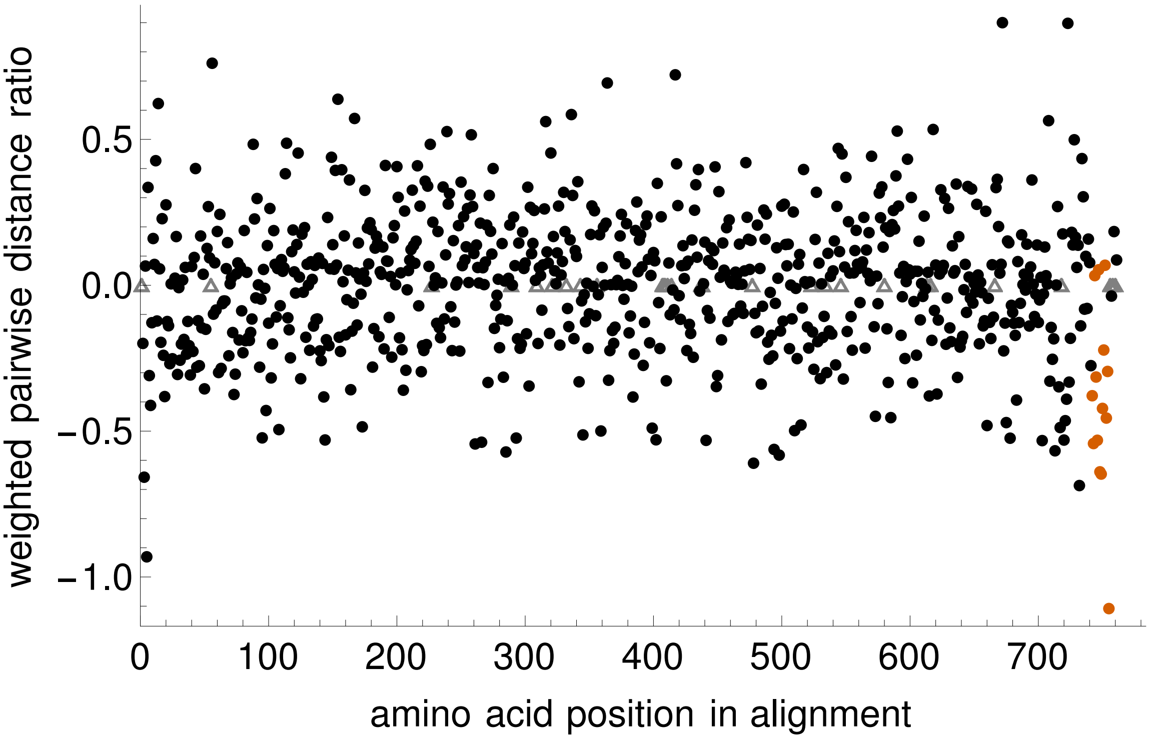

PB1

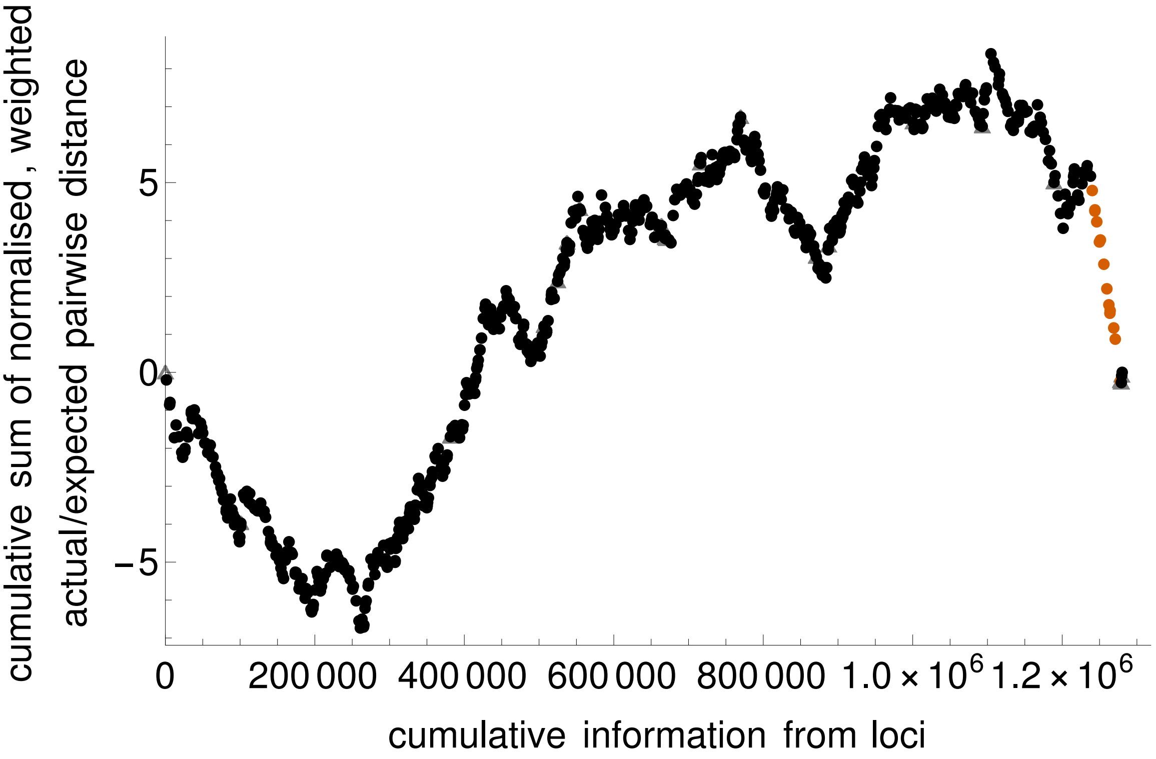

PB1

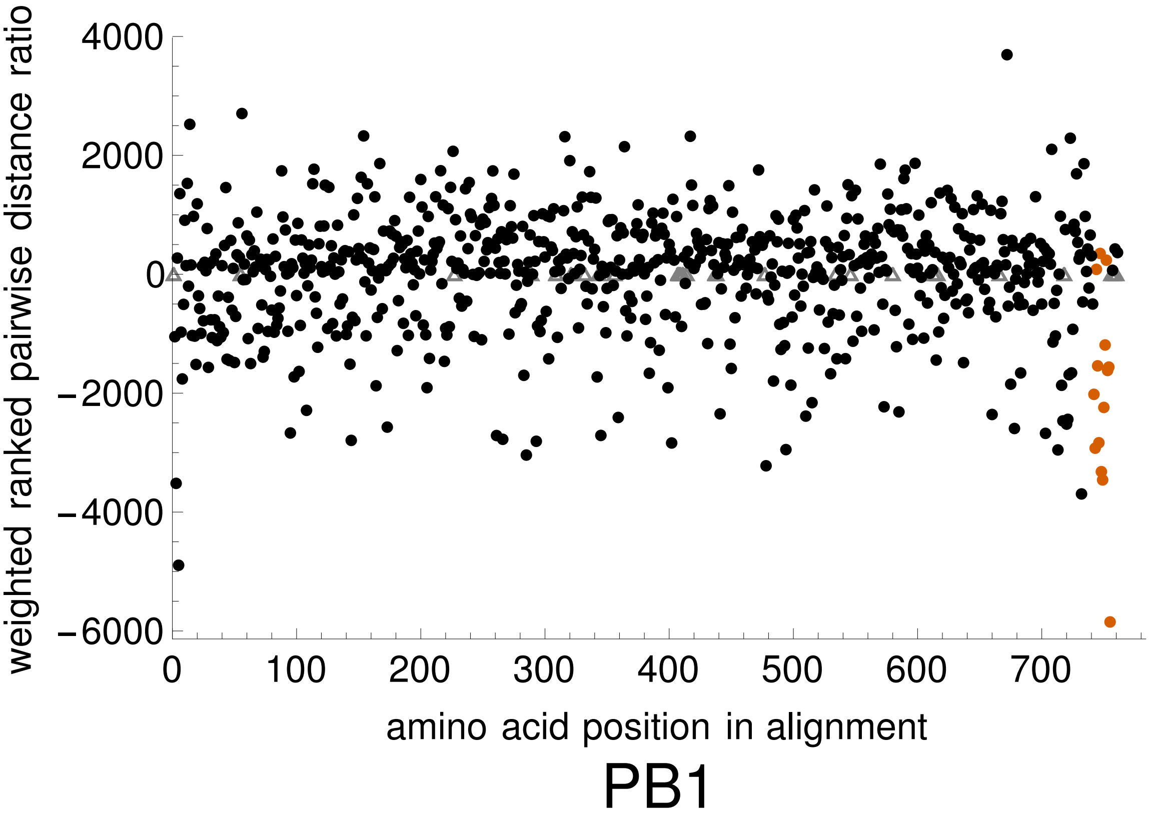
weighted ranked pairwise distance ratio
4000
2000
0
−2000
−4000
−6000
0
100
200
300
400
500
600
700
amino acid position in alignment
PB1

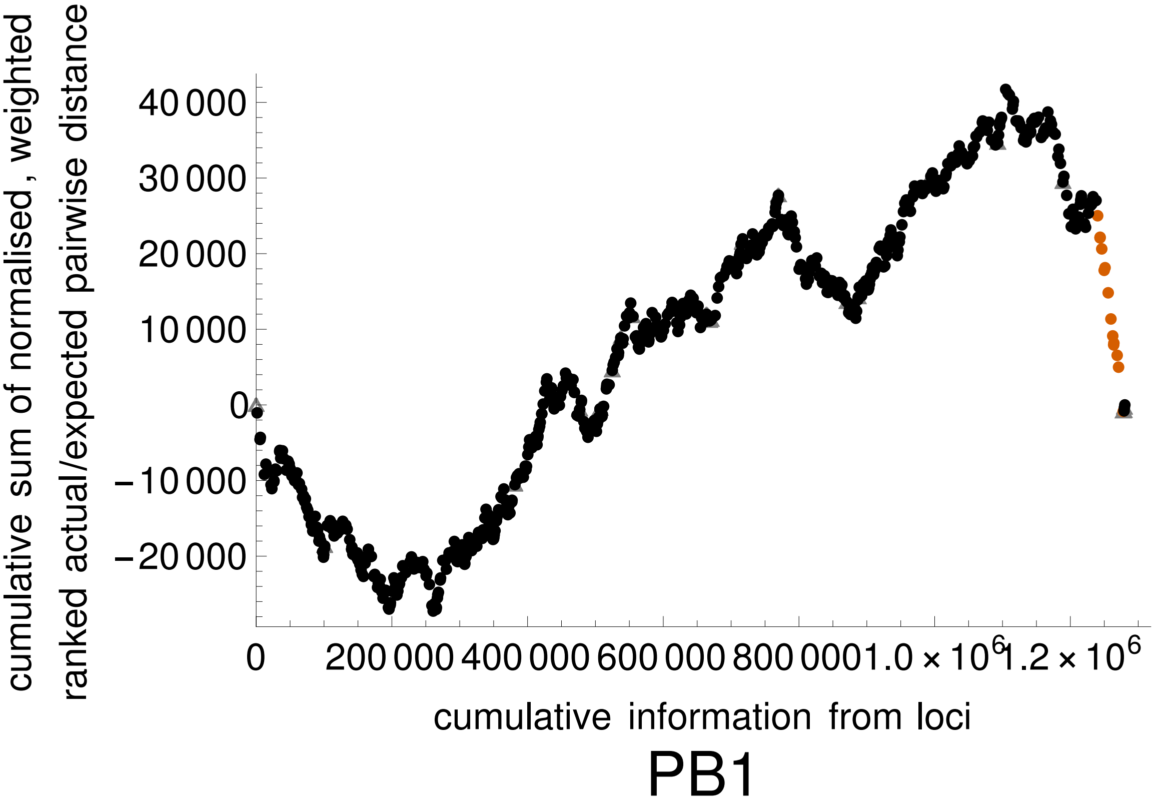
cumulative sum of normalised, weighted ranked actual/expected pairwise distance
40 000
30 000
20 000
10 000
0
−10 000
−20 000
0
200 000
400 000
600 000
800 000
1.0 × 10^6
1.2 × 10^6
cumulative information from loci
PB1

PB1-F2

Gene length histogram

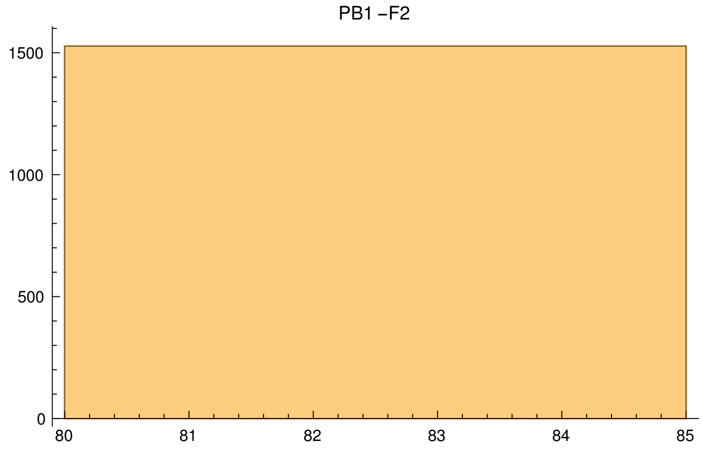

Information vs. nPD

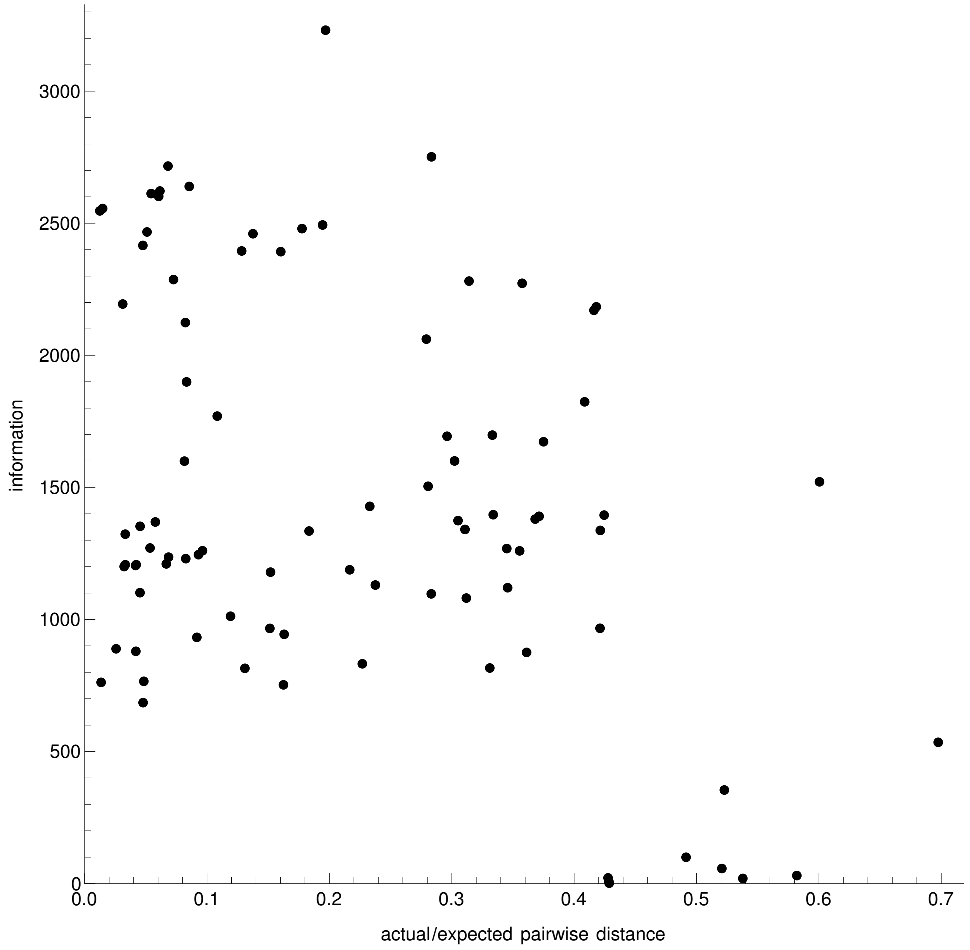

PB1–F2

Example sequences highlighted by regions found in analyses to be conserved

```
Interesting points (by weighted raw PD) highlighted for gene PB1-F2:
ATGGAACAGGGACAGGATACACCATGGACACGGTCAACAGAACACATCAATATTCAGAAAGGGGAAGATGGACGACAA⋱.
    ACACAGAAACTGGAGCACCCCAGCTTAACCCGATTGATGGACCACTACCTGAGGACAATGAGCCAAGCGGGTATGCT⋱.
    CAAACAGACTGTGTCCTGGAGGCAATGGCTTTCCTTGAGGAATCCCACCCAGGGATATTTGAAAACTCGTGTCTTGA⋱.
    AACGA-------------------------------TAG
```

```
Interesting points (by weighted ranked PD) highlighted for gene PB1-F2:
ATGGAACAGGGACAGGATACACCATGGACACGGTCAACAGAACACATCAATATTCAGAAAGGGGAAGATGGACGACAA⋱.
    ACACAGAAACTGGAGCACCCCAGCTTAACCCGATTGATGGACCACTACCTGAGGACAATGAGCCAAGCGGGTATGCT⋱.
    CAAACAGACTGTGTCCTGGAGGCAATGGCTTTCCTTGAGGAATCCCACCCAGGGATATTTGAAAACTCGTGTCTTGA⋱.
    AACGA-------------------------------TAG
```

Per codon conservation report

PB1-F2

Pos.	1	obs:	exp:
atg	M	1527	1527.00
---	--	------	------
mPD		0	0
		nPD:	1.
		N. weight:	0.
		Sc. PD:	0
		Sc. rank:	0

PB1-F2

Pos.	2	obs:	exp:
gct	A	0	0.25
gcc	A	0	0.23
gca	A	1	0.45
gcg	A	0	0.07
gaa	E	1468	869.80
gag	E	0	598.20
ggt	G	0	9.10
ggc	G	0	7.35
gga	G	58	26.27
ggg	G	0	15.28
---	--	------	------
mPD		0.074	0.57
		nPD:	0.13
		N. weight:	0.58
		Sc. PD:	-0.031
		Sc. rank:	-41.0

PB1-F2

Pos.	3	obs:	exp:
tta	L	0	1.20
ttg	L	0	2.07
ctt	L	0	2.22
ctc	L	0	1.60
cta	L	0	1.92
ctg	L	11	1.99
cct	P	0	0.30
ccc	P	0	0.20
cca	P	0	0.40
ccg	P	1	0.11
caa	Q	0	814.30
cag	Q	1510	695.70
cgt	R	0	0.16
cgc	R	0	0.26
cga	R	0	0.50
cgg	R	5	0.36
aga	R	0	2.29
agg	R	0	1.42
---	--	------	------
mPD		0.022	0.53
		nPD:	0.04
		N. weight:	0.85
		Sc. PD:	-0.12
		Sc. rank:	-832.6

PB1-F2

Pos.	4	obs:	exp:
gtt	V	0	0.22
gtc	V	0	0.19
gta	V	1	0.25
gtg	V	0	0.34
gaa	E	1246	738.30
gag	E	0	507.70
ggt	G	0	43.92
ggc	G	0	35.48
gga	G	280	126.80
ggg	G	0	73.78
---	--	------	------
mPD		0.30	0.83
		nPD:	0.36
		N. weight:	0.62
		Sc. PD:	0.1
		Sc. rank:	547.3

PB1-F2

Pos.	5	obs:	exp:
tta	L	0	0.76
ttg	L	0	1.31
ctt	L	0	1.41
ctc	L	0	1.02
cta	L	0	1.22
ctg	L	7	1.27
caa	Q	0	799.20
cag	Q	1482	682.80
cgt	R	0	1.20
cgc	R	0	2.01
cga	R	0	3.82
cgg	R	38	2.75
aga	R	0	17.43
agg	R	0	10.80
---	--	------	------
mPD		0.057	0.60
		nPD:	0.1
		N. weight:	0.89
		Sc. PD:	-0.078
		Sc. rank:	-164.9

PB1-F2

Pos.	6	obs:	exp:
gat	D	1452	817.30
gac	D	1	635.70
ggt	G	74	11.61
ggc	G	0	9.38
gga	G	0	33.52
ggg	G	0	19.50
---	--	------	------
mPD		0.094	0.62
		nPD:	0.15
		N. weight:	0.68
		Sc. PD:	-0.023
		Sc. rank:	-4.2

PB1-F2

Pos.	7	obs:	exp:
att	I	0	13.48
atc	I	0	9.75
ata	I	38	14.77
act	T	0	366.30
acc	T	0	322.00
aca	T	1489	676.60
acg	T	0	124.20
---	--	------	------
mPD		0.049	0.73
		nPD:	0.07
		N. weight:	0.85
		Sc. PD:	-0.099
		Sc. rank:	-470.0

PB1-F2

Pos.	8	obs:	exp:
tta	L	0	1.52
ttg	L	0	2.63
ctt	L	0	2.83
ctc	L	0	2.04
cta	L	14	2.44
ctg	L	0	2.54
cct	P	2	443.80
ccc	P	8	302.80
cca	P	1494	595.60
ccg	P	0	161.80
caa	Q	8	4.31
cag	Q	0	3.69
cgt	R	0	0.03
cgc	R	0	0.05
cga	R	1	0.10
cgg	R	0	0.07
aga	R	0	0.46
agg	R	0	0.28
---	--	------	------
mPD		0.043	0.74
		nPD:	0.06
		N. weight:	0.97
		Sc. PD:	-0.12
		Sc. rank:	-650.0

PB1-F2

Pos.	9	obs:	exp:
tta	L	0	0.11
ttg	L	1	0.19
tgg	W	1526	1526.00
ctt	L	0	0.20
ctc	L	0	0.15
cta	L	0	0.17
ctg	L	0	0.18
---	--	------	------
mPD		0.0013	0.0031
		nPD:	0.43
		N. weight:	0.0012
		Sc. PD:	0.00028
		Sc. rank:	1.4

PB1-F2

Pos.	10	obs:	exp:
cgt	R	0	0.03
cgc	R	0	0.05
cga	R	0	0.10
cgg	R	0	0.07
att	I	0	6.03
atc	I	0	4.36
ata	I	17	6.61
act	T	0	371.20
acc	T	0	326.30
aca	T	1509	685.70
acg	T	0	125.80
aga	R	1	0.46
agg	R	0	0.28
---	--	------	------
mPD		0.023	0.70
		nPD:	0.03
		N. weight:	0.85
		Sc. PD:	-0.13
		Sc. rank:	-854.1

PB1-F2

Pos.	11	obs:	exp:
tta	L	0	0.98
ttg	L	0	1.69
ctt	L	0	1.82
ctc	L	0	1.31
cta	L	0	1.57
ctg	L	9	1.63
cct	P	0	0.59
ccc	P	0	0.40
cca	P	0	0.79
ccg	P	2	0.22
caa	Q	0	667.60
cag	Q	1238	570.40
cgt	R	0	8.78
cgc	R	0	14.69
cga	R	3	27.94
cgg	R	275	20.08
aga	R	0	127.50
agg	R	0	79.01
---	--	------	------
mPD		0.31	1.1
		nPD:	0.3
		N. weight:	1.2
		Sc. PD:	0.13
		Sc. rank:	640.8

PB1-F2

Pos.	12	obs:	exp:
tta	L	41	4.46
ttg	L	0	7.70
tct	S	0	249.90
tcc	S	0	234.90
tca	S	1486	360.90
tcg	S	0	78.98
ctt	L	0	8.28
ctc	L	0	5.97
cta	L	0	7.16
ctg	L	0	7.43
agt	S	0	276.60
agc	S	0	284.80
---	--	------	------
mPD		0.052	1.7
		nPD:	0.03
		N. weight:	1.5
		Sc. PD:	-0.23
		Sc. rank:	-1662.4

PB1-F2

Pos.	13	obs:	exp:
att	I	0	6.03
atc	I	0	4.36
ata	I	17	6.61
act	T	0	370.00
acc	T	0	325.20
aca	T	1504	683.40
acg	T	0	125.40
gct	A	0	1.47
gcc	A	0	1.37
gca	A	6	2.70
gcg	A	0	0.45
---	--	------	------
mPD		0.030	0.71
		nPD:	0.04
		N. weight:	0.85
		Sc. PD:	-0.12
		Sc. rank:	-800.8

PB1-F2

Pos.	14	obs:	exp:
gaa	E	1404	831.90
gag	E	0	572.10
ggt	G	0	19.29
ggc	G	0	15.59
gga	G	123	55.71
ggg	G	0	32.41
---	--	------	------
mPD		0.15	0.65
		nPD:	0.23
		N. weight:	0.59
		Sc. PD:	0.023
		Sc. rank:	184.5

PB1-F2

Pos.	15	obs:	exp:
cat	H	0	869.80
cac	H	1508	638.20
cgt	R	0	0.60
cgc	R	19	1.00
cga	R	0	1.91
cgg	R	0	1.37
aga	R	0	8.71
agg	R	0	5.40
mPD		0.025	0.54
		nPD:	0.05
		N. weight:	0.96
		Sc. PD:	-0.13
		Sc. rank:	-852.7

PB1-F2

Pos.	16	obs:	exp:
att	I	0	136.60
atc	I	385	98.75
ata	I	0	149.70
act	T	0	280.90
acc	T	1142	246.90
aca	T	0	518.90
acg	T	0	95.23
mPD		0.38	1.1
		nPD:	0.36
		N. weight:	1.6
		Sc. PD:	0.27
		Sc. rank:	1394.4

PB1-F2

Pos.	17	obs:	exp:
tct	S	0	2.52
tcc	S	0	2.37
tca	S	0	3.64
tcg	S	0	0.80
aat	N	1510	858.30
aac	N	0	651.70
agt	S	15	2.79
agc	S	0	2.87
gat	D	2	1.12
gac	D	0	0.88
mPD		0.022	0.53
		nPD:	0.04
		N. weight:	0.62
		Sc. PD:	-0.087
		Sc. rank:	-597.6

PB1-F2

Pos.	18	obs:	exp:
att	I	1481	525.40
atc	I	0	379.90
ata	I	0	575.80
act	T	46	11.32
acc	T	0	9.95
aca	T	0	20.90
acg	T	0	3.84
mPD		0.058	0.72
		nPD:	0.08
		N. weight:	1.1
		Sc. PD:	-0.12
		Sc. rank:	-452.7

PB1-F2

Pos.	19	obs:	exp:
tta	L	0	0.44
ttg	L	0	0.75
ctt	L	0	0.81
ctc	L	0	0.58
cta	L	0	0.70
ctg	L	4	0.73
caa	Q	2	804.60
cag	Q	1490	687.40
cgt	R	0	0.98
cgc	R	0	1.64
cga	R	0	3.12
cgg	R	31	2.24
aga	R	0	14.22
agg	R	0	8.81
mPD		0.048	0.58
		nPD:	0.08
		N. weight:	0.87
		Sc. PD:	-0.088
		Sc. rank:	-292.8

PB1-F2

Pos.	20	obs:	exp:
cgt	R	0	0.16
cgc	R	0	0.26
cga	R	0	0.50
cgg	R	0	0.36
aaa	K	1522	925.00
aag	K	0	597.00
aga	R	5	2.29
agg	R	0	1.42
mPD		0.0065	0.49
		nPD:	0.01
		N. weight:	0.54
		Sc. PD:	-0.09
		Sc. rank:	-634.9

PB1-F2

Pos.	21	obs:	exp:
tgg	W	2	2.00
cgt	R	0	2.71
cgc	R	0	4.55
cga	R	0	8.64
cgg	R	0	6.21
aaa	K	0	472.20
aag	K	777	304.80
aga	R	0	39.44
agg	R	86	24.44
gaa	E	0	267.80
gag	E	452	184.20
ggt	G	0	32.94
ggc	G	0	26.61
gga	G	0	95.12
ggg	G	210	55.33
mPD		0.81	1.3
		nPD:	0.6
		N. weight:	1.1
		Sc. PD:	0.43
		Sc. rank:	1287.0

PB1-F2

Pos.	22	obs:	exp:
gtt	V	0	0.44
gtc	V	0	0.38
gta	V	2	0.50
gtg	V	0	0.67
gaa	E	336	199.10
gag	E	0	136.90
ggt	G	0	186.50
ggc	G	0	150.70
gga	G	1189	538.60
ggg	G	0	313.30
mPD		0.35	1.0
		nPD:	0.35
		N. weight:	0.79
		Sc. PD:	0.12
		Sc. rank:	634.2

PB1-F2

Pos.	23	obs:	exp:
tct	S	0	12.45
tcc	S	0	11.70
tca	S	0	17.97
tcg	S	0	3.93
tat	Y	2	1.12
tac	Y	0	0.88
att	I	1	0.35
atc	I	0	0.26
ata	I	0	0.39
aat	N	1174	667.30
aac	N	0	506.70
agt	S	74	13.77
agc	S	0	14.18
gat	D	262	147.40
gac	D	0	114.60
ggt	G	14	2.20
ggc	G	0	1.77
gga	G	0	6.34
ggg	G	0	3.69
mPD		0.41	0.97
		nPD:	0.42
		N. weight:	0.68
		Sc. PD:	0.16
		Sc. rank:	754.9

PB1-F2

Pos.	24	obs:	exp:
ggt	G	0	239.50
ggc	G	0	193.50
gga	G	1503	691.70
ggg	G	24	402.30
mPD		0.031	0.68
		nPD:	0.05
		N. weight:	0.78
		Sc. PD:	-0.11
		Sc. rank:	-714.3

PB1-F2

Pos.	25	obs:	exp:
tta	L	0	0.22
ttg	L	0	0.38
ctt	L	0	0.40
ctc	L	0	0.29
cta	L	2	0.35
ctg	L	0	0.36
caa	Q	404	217.90
cag	Q	0	186.10
cgt	R	0	35.38
cgc	R	0	59.25
cga	R	1110	112.70
cgg	R	1	80.99
aga	R	10	514.10
agg	R	0	318.60
mPD		0.41	1.4
		nPD:	0.28
		N. weight:	1.9
		Sc. PD:	0.18
		Sc. rank:	978.5

PB1-F2

Pos.	26	obs:	exp:
tta	L	0	4.35
ttg	L	0	7.51
ctt	L	0	8.08
ctc	L	0	5.82
cta	L	40	6.98
ctg	L	0	7.25
cct	P	0	0.59
ccc	P	0	0.40
cca	P	2	0.79
ccg	P	0	0.22
caa	Q	1470	792.70
cag	Q	0	677.30
cgt	R	0	0.44
cgc	R	0	0.74
cga	R	14	1.41
cgg	R	0	1.01
aaa	K	1	0.61
aag	K	0	0.39
aga	R	0	6.42
agg	R	0	3.98
mPD		0.073	0.61
		nPD:	0.12
		N. weight:	0.71
		Sc. PD:	-0.047
		Sc. rank:	-94.6

PB1-F2

Pos.	27	obs:	exp:
att	I	0	314.70
atc	I	0	227.50
ata	I	887	344.80
act	T	0	157.40
acc	T	1	138.40
aca	T	639	290.80
acg	T	0	53.37
mPD		0.49	1.2
		nPD:	0.42
		N. weight:	0.94
		Sc. PD:	0.22
		Sc. rank:	1068.3

PB1-F2

Pos.	28	obs:	exp:
tta	L	0	1.96
ttg	L	0	3.38
tgg	W	1	1.00
ctt	L	0	3.64
ctc	L	0	2.62
cta	L	0	3.14
ctg	L	18	3.26
cct	P	0	10.92
ccc	P	0	7.45
cca	P	0	14.65
ccg	P	37	3.98
caa	Q	0	769.50
cag	Q	1427	657.50
cgt	R	0	1.39
cgc	R	0	2.33
cga	R	0	4.42
cgg	R	44	3.18
aga	R	0	20.18
agg	R	0	12.51
mPD		0.13	0.69
		nPD:	0.18
		N. weight:	0.94
		Sc. PD:	-0.0028
		Sc. rank:	157.0

PB1-F2

Pos.	29	obs:	exp:
cgt	R	0	37.72
cgc	R	0	63.16
cga	R	0	120.10
cgg	R	0	86.33
aaa	K	332	201.80
aag	K	0	130.20
aga	R	1195	548.00
agg	R	0	339.60
---	--	------	------
mPD		0.34	1.2
		nPD:	0.28
		N. weight:	0.77
		Sc. PD:	0.073
		Sc. rank:	349.6

PB1-F2

Pos.	30	obs:	exp:
tta	L	0	157.00
ttg	L	0	271.00
ctt	L	0	291.40
ctc	L	0	210.00
cta	L	1	252.00
ctg	L	1442	261.60
cct	P	0	10.62
ccc	P	0	7.25
cca	P	0	14.26
ccg	P	36	3.87
caa	Q	0	7.01
cag	Q	13	5.99
cgt	R	0	1.10
cgc	R	0	1.85
cga	R	0	3.52
cgg	R	35	2.53
aga	R	0	16.05
agg	R	0	9.95
---	--	------	------
mPD		0.11	1.3
		nPD:	0.09
		N. weight:	1.9
		Sc. PD:	-0.18
		Sc. rank:	-467.2

PB1-F2

Pos.	31	obs:	exp:
gtt	V	0	0.22
gtc	V	0	0.19
gta	V	0	0.25
gtg	V	1	0.34
gct	A	0	0.49
gcc	A	0	0.46
gca	A	0	0.90
gcg	A	2	0.15
gaa	E	0	292.10
gag	E	493	200.90
ggt	G	0	161.70
ggc	G	0	130.60
gga	G	0	467.00
ggg	G	1031	271.70
---	--	------	------
mPD		0.44	1.1
		nPD:	0.41
		N. weight:	1.3
		Sc. PD:	0.28
		Sc. rank:	1294.1

PB1-F2

Pos.	32	obs:	exp:
tta	L	0	0.76
ttg	L	0	1.31
ctt	L	0	1.41
ctc	L	7	1.02
cta	L	0	1.22
ctg	L	0	1.27
cat	H	0	873.30
cac	H	1514	640.70
caa	Q	1	2.16
cag	Q	3	1.84
cgt	R	0	0.06
cgc	R	2	0.11
cga	R	0	0.20
cgg	R	0	0.14
aga	R	0	0.92
agg	R	0	0.57
---	--	------	------
mPD		0.017	0.51
		nPD:	0.03
		N. weight:	0.93
		Sc. PD:	-0.14
		Sc. rank:	-957.5

PB1-F2

Pos.	33	obs:	exp:
tta	L	0	13.28
ttg	L	0	22.92
tct	S	0	0.50
tcc	S	3	0.47
tca	S	0	0.73
tcg	S	0	0.16
ctt	L	0	24.64
ctc	L	122	17.75
cta	L	0	21.30
ctg	L	0	22.12
cct	P	0	410.50
ccc	P	1391	280.10
cca	P	0	550.80
ccg	P	0	149.60
cat	H	0	6.35
cac	H	11	4.65
agt	S	0	0.56
agc	S	0	0.57
---	--	------	------
mPD		0.16	0.92
		nPD:	0.18
		N. weight:	1.8
		Sc. PD:	-0.015
		Sc. rank:	247.3

PB1-F2

Pos.	34	obs:	exp:
tct	S	0	245.60
tcc	S	0	230.70
tca	S	0	354.50
tcg	S	0	77.60
cgt	R	0	0.69
cgc	R	0	1.16
cga	R	0	2.21
cgg	R	0	1.59
aat	N	0	25.58
aac	N	45	19.42
agt	S	0	271.70
agc	S	1460	279.80
aga	R	22	10.09
agg	R	0	6.25
---	--	------	------
mPD		0.086	1.7
		nPD:	0.05
		N. weight:	1.7
		Sc. PD:	-0.23
		Sc. rank:	-1345.3

PB1-F2

Pos.	35	obs:	exp:
tta	L	288	31.34
ttg	L	0	54.10
tct	S	0	208.40
tcc	S	0	195.80
tca	S	1239	300.90
tcg	S	0	65.85
ctt	L	0	58.16
ctc	L	0	41.90
cta	L	0	50.29
ctg	L	0	52.21
agt	S	0	230.60
agc	S	0	237.50
---	--	------	------
mPD		0.31	1.9
		nPD:	0.16
		N. weight:	1.7
		Sc. PD:	-0.043
		Sc. rank:	104.4

PB1-F2

Pos.	36	obs:	exp:
att	I	0	321.80
atc	I	907	232.60
ata	I	0	352.60
act	T	0	152.50
acc	T	620	134.10
aca	T	0	281.70
acg	T	0	51.70
---	--	------	------
mPD		0.48	1.2
		nPD:	0.42
		N. weight:	1.5
		Sc. PD:	0.35
		Sc. rank:	1676.9

PB1-F2

Pos.	37	obs:	exp:
tta	L	0	11.21
ttg	L	0	19.35
ctt	L	0	20.80
ctc	L	0	14.99
cta	L	103	17.99
ctg	L	0	18.67
cct	P	0	0.89
ccc	P	0	0.60
cca	P	3	1.19
ccg	P	0	0.32
caa	Q	97	52.31
cag	Q	0	44.69
cgt	R	0	41.79
cgc	R	0	69.98
cga	R	1314	133.10
cgg	R	10	95.65
aga	R	0	607.20
agg	R	0	376.30
---	--	------	------
mPD		0.25	1.3
		nPD:	0.2
		N. weight:	2.3
		Sc. PD:	0.023
		Sc. rank:	628.5

PB1-F2

Pos.	38	obs:	exp:
tta	L	0	159.60
ttg	L	1466	275.60
tct	S	0	10.09
tcc	S	0	9.48
tca	S	0	14.57
tcg	S	60	3.19
ctt	L	0	296.30
ctc	L	0	213.40
cta	L	0	256.20
ctg	L	1	265.90
agt	S	0	11.17
agc	S	0	11.50
---	--	------	------
mPD		0.077	1.3
		nPD:	0.06
		N. weight:	1.9
		Sc. PD:	-0.22
		Sc. rank:	-1060.8

PB1-F2

Pos.	39	obs:	exp:
atg	M	1504	1504.00
act	T	0	5.66
acc	T	0	4.97
aca	T	0	10.45
acg	T	23	1.92
---	--	------	------
mPD		0.030	0.057
		nPD:	0.52
		N. weight:	0.04
		Sc. PD:	0.013
		Sc. rank:	46.9

PB1-F2

Pos.	40	obs:	exp:
gat	D	1	780.10
gac	D	1386	606.90
ggt	G	0	21.96
ggc	G	140	17.74
gga	G	0	63.41
ggg	G	0	36.89
---	--	------	------
mPD		0.17	0.72
		nPD:	0.23
		N. weight:	1.
		Sc. PD:	0.046
		Sc. rank:	344.0

PB1-F2

Pos.	41	obs:	exp:
tta	L	0	2.07
ttg	L	0	3.57
ctt	L	0	3.84
ctc	L	19	2.76
cta	L	0	3.32
ctg	L	0	3.44
cct	P	0	0.30
ccc	P	1	0.20
cca	P	0	0.40
ccg	P	0	0.11
cat	H	63	849.10
cac	H	1409	622.90
cgt	R	0	1.10
cgc	R	35	1.85
cga	R	0	3.52
cgg	R	0	2.53
aga	R	0	16.05
agg	R	0	9.95
---	--	------	------
mPD		0.15	0.63
		nPD:	0.24
		N. weight:	0.8
		Sc. PD:	0.04
		Sc. rank:	289.4

PB1-F2

Pos.	42	obs:	exp:
tct	S	0	2.86
tcc	S	17	2.69
tca	S	0	4.13
tcg	S	0	0.90
tat	Y	0	843.20
tac	Y	1505	661.80
tgt	C	0	2.15
tgc	C	5	2.85
agt	S	0	3.16
agc	S	0	3.26
---	--	------	------
mPD		0.028	0.53
		nPD:	0.05
		N. weight:	0.9
		Sc. PD:	-0.12
		Sc. rank:	-671.2

,

PB1-F2

Pos.	43	obs:	exp:
tta	L	0	160.30
ttg	L	0	276.70
ctt	L	0	297.50
ctc	L	0	214.30
cta	L	2	257.20
ctg	L	1471	267.00
cct	P	0	10.92
ccc	P	0	7.45
cca	P	1	14.65
ccg	P	36	3.98
caa	Q	0	6.47
cag	Q	12	5.53
cgt	R	0	0.16
cgc	R	0	0.26
cga	R	0	0.50
cgg	R	5	0.36
aga	R	0	2.29
agg	R	0	1.42
---	--	------	------
mPD		0.073	1.2
		nPD:	0.06
		N. weight:	1.8
		Sc. PD:	-0.22
		Sc. rank:	-1144.3

,

PB1-F2

Pos.	44	obs:	exp:
caa	Q	0	0.54
cag	Q	1	0.46
cgt	R	0	12.15
cgc	R	0	20.35
cga	R	0	38.70
cgg	R	0	27.81
att	I	0	0.35
atc	I	0	0.26
ata	I	1	0.39
atg	M	10	10.00
aaa	K	27	686.10
aag	K	1102	442.90
aga	R	2	176.60
agg	R	383	109.40
ggt	G	0	0.16
ggc	G	0	0.13
gga	G	0	0.45
ggg	G	1	0.26
---	--	------	------
mPD		0.43	1.0
		nPD:	0.42
		N. weight:	0.99
		Sc. PD:	0.23
		Sc. rank:	1140.7

,

PB1-F2

Pos.	45	obs:	exp:
ttt	F	0	0.45
ttc	F	1	0.55
att	I	0	403.00
atc	I	0	291.40
ata	I	1136	441.60
atg	M	2	2.00
act	T	0	94.46
acc	T	0	83.04
aca	T	384	174.50
acg	T	0	32.02
aaa	K	2	1.22
aag	K	0	0.78
gtt	V	0	0.22
gtc	V	0	0.19
gta	V	1	0.25
gtg	V	0	0.34
gct	A	0	0.25
gcc	A	0	0.23
gca	A	1	0.45
gcg	A	0	0.07
---	--	------	------
mPD		0.39	1.1
		nPD:	0.37
		N. weight:	0.97
		Sc. PD:	0.17
		Sc. rank:	888.5

,

PB1-F2

Pos.	46	obs:	exp:
atg	M	1518	1518.00
act	T	0	1.72
acc	T	0	1.51
aca	T	0	3.18
acg	T	7	0.58
gtt	V	0	0.44
gtc	V	0	0.38
gta	V	0	0.50
gtg	V	2	0.67
---	--	------	------
mPD		0.012	0.022
		nPD:	0.54
		N. weight:	0.014
		Sc. PD:	0.0047
		Sc. rank:	16.1

,

PB1-F2

Pos.	47	obs:	exp:
tct	S	0	45.58
tcc	S	0	42.83
tca	S	0	65.81
tcg	S	0	14.40
att	I	0	0.35
atc	I	1	0.26
ata	I	0	0.39
act	T	0	0.25
acc	T	1	0.22
aca	T	0	0.45
acg	T	0	0.08
aat	N	0	712.80
aac	N	1254	541.20
agt	S	0	50.44
agc	S	271	51.94
---	--	------	------
mPD		0.29	1.0
		nPD:	0.28
		N. weight:	1.1
		Sc. PD:	0.097
		Sc. rank:	457.2

,

PB1-F2

Pos.	48	obs:	exp:
tta	L	0	0.44
ttg	L	0	0.75
ctt	L	0	0.81
ctc	L	0	0.58
cta	L	4	0.70
ctg	L	0	0.73
cct	P	0	0.30
ccc	P	0	0.20
cca	P	1	0.40
ccg	P	0	0.11
caa	Q	1379	743.60
cag	Q	0	635.40
cgt	R	0	4.51
cgc	R	0	7.56
cga	R	143	14.37
cgg	R	0	10.33
aga	R	0	65.58
agg	R	0	40.64
---	--	------	------
mPD		0.18	0.81
		nPD:	0.22
		N. weight:	0.84
		Sc. PD:	0.025
		Sc. rank:	250.1

,

PB1-F2

Pos.	49	obs:	exp:
gtt	V	0	280.00
gtc	V	0	237.90
gta	V	0	318.30
gtg	V	1261	424.70
gct	A	0	65.21
gcc	A	0	60.95
gca	A	0	119.90
gcg	A	266	19.95
---	--	------	------
mPD		0.29	1.0
		nPD:	0.28
		N. weight:	1.5
		Sc. PD:	0.13
		Sc. rank:	584.8

,

PB1-F2

Pos.	50	obs:	exp:
gtt	V	4	0.89
gtc	V	0	0.75
gta	V	0	1.01
gtg	V	0	1.35
gct	A	5	1.23
gcc	A	0	1.15
gca	A	0	2.25
gcg	A	0	0.37
gat	D	1219	685.60
gac	D	0	533.40
ggt	G	299	46.90
ggc	G	0	37.89
gga	G	0	135.40
ggg	G	0	78.78
---	--	------	------
mPD		0.32	0.94
		nPD:	0.34
		N. weight:	0.9
		Sc. PD:	0.14
		Sc. rank:	699.0

,

PB1-F2

Pos.	51	obs:	exp:
att	I	3	1.06
atc	I	0	0.77
ata	I	0	1.17
atg	M	1513	1513.00
act	T	0	2.71
acc	T	0	2.38
aca	T	0	5.00
acg	T	11	0.92
---	--	------	------
mPD		0.018	0.031
		nPD:	0.58
		N. weight:	0.021
		Sc. PD:	0.0083
		Sc. rank:	25.1

,

PB1-F2

Pos.	52	obs:	exp:
tta	L	0	27.75
ttg	L	0	47.90
ctt	L	0	51.50
ctc	L	255	37.10
cta	L	0	44.53
ctg	L	0	46.22
cct	P	0	5.02
ccc	P	17	3.42
cca	P	0	6.73
ccg	P	0	1.83
cat	H	0	723.30
cac	H	1254	530.70
cgt	R	0	0.03
cgc	R	1	0.05
cga	R	0	0.10
cgg	R	0	0.07
aga	R	0	0.46
agg	R	0	0.28
---	--	------	------
mPD		0.30	0.99
		nPD:	0.3
		N. weight:	1.1
		Sc. PD:	0.13
		Sc. rank:	639.8

,

PB1-F2

Pos.	53	obs:	exp:
cgt	R	0	0.60
cgc	R	0	1.00
cga	R	0	1.91
cgg	R	0	1.37
aaa	K	1508	916.50
aag	K	0	591.50
aga	R	19	8.71
agg	R	0	5.40
---	--	------	------
mPD		0.025	0.51
		nPD:	0.05
		N. weight:	0.54
		Sc. PD:	-0.073
		Sc. rank:	-443.0

,

PB1-F2

Pos.	54	obs:	exp:
tta	L	0	0.11
ttg	L	0	0.19
ctt	L	0	0.20
ctc	L	0	0.15
cta	L	0	0.17
ctg	L	1	0.18
cct	P	0	0.30
ccc	P	0	0.20
cca	P	0	0.40
ccg	P	1	0.11
caa	Q	0	807.30
cag	Q	1497	689.70
cgt	R	0	0.85
cgc	R	0	1.43
cga	R	0	2.71
cgg	R	27	1.95
aga	R	0	12.38
agg	R	0	7.67
gaa	E	0	0.59
gag	E	1	0.41
---	--	------	------
mPD		0.039	0.56
		nPD:	0.07
		N. weight:	0.87
		Sc. PD:	-0.1
		Sc. rank:	-414.3

,

PB1-F2

Pos.	55	obs:	exp:
att	I	48	17.03
atc	I	0	12.31
ata	I	0	18.66
act	T	1479	363.80
acc	T	0	319.80
aca	T	0	672.00
acg	T	0	123.30
---	--	------	------
mPD		0.061	0.74
		nPD:	0.08
		N. weight:	1.5
		Sc. PD:	-0.15
		Sc. rank:	-540.6

,

PB1-F2

Pos.	56	obs:	exp:
gtt	V	0	323.10
gtc	V	0	274.60
gta	V	0	367.30
gtg	V	1455	490.10
gct	A	0	17.65
gcc	A	0	16.50
gca	A	0	32.45
gcg	A	72	5.40
---	--	------	------
mPD		0.090	0.83
		nPD:	0.11
		N. weight:	1.2
		Sc. PD:	-0.095
		Sc. rank:	-189.5

,

PB1-F2

Pos.	57	obs:	exp:
ttt	F	3	66.32
ttc	F	145	81.68
tct	S	30	230.10
tcc	S	1338	216.20
tca	S	0	332.20
tcg	S	0	72.71
tat	Y	2	6.16
tac	Y	9	4.84
agt	S	0	254.60
agc	S	0	262.20
---	--	------	------
mPD		0.23	1.7
		nPD:	0.14
		N. weight:	1.7
		Sc. PD:	-0.083
		Sc. rank:	-42.6

,

PB1-F2

Pos.	58	obs:	exp:
tta	L	0	0.76
ttg	L	7	1.31
tct	S	0	5.05
tcc	S	0	4.74
tca	S	0	7.29
tcg	S	30	1.59
tgg	W	1490	1490.00
ctt	L	0	1.41
ctc	L	0	1.02
cta	L	0	1.22
ctg	L	0	1.27
agt	S	0	5.58
agc	S	0	5.75
---	--	------	------
mPD		0.047	0.097
		nPD:	0.49
		N. weight:	0.07
		Sc. PD:	0.021
		Sc. rank:	81.7

,

PB1-F2

Pos.	59	obs:	exp:
cgt	R	0	46.09
cgc	R	0	77.17
cga	R	0	146.70
cgg	R	0	105.50
aaa	K	0	40.72
aag	K	67	26.28
aga	R	0	669.60
agg	R	1460	415.00
---	--	------	------
mPD		0.084	1.0
		nPD:	0.08
		N. weight:	1.3
		Sc. PD:	-0.13
		Sc. rank:	-403.6

,

PB1-F2

Pos.	60	obs:	exp:
tta	L	0	5.01
ttg	L	0	8.64
ctt	L	0	9.29
ctc	L	0	6.69
cta	L	46	8.03
ctg	L	0	8.34
cct	P	1	346.70
ccc	P	1	236.60
cca	P	1173	465.30
ccg	P	0	126.40
caa	Q	275	148.30
cag	Q	0	126.70
cgt	R	0	0.98
cgc	R	0	1.64
cga	R	31	3.12
cgg	R	0	2.24
aga	R	0	14.22
agg	R	0	8.81
---	--	------	------
mPD		0.38	1.1
		nPD:	0.33
		N. weight:	0.99
		Sc. PD:	0.14
		Sc. rank:	745.8

,

PB1-F2

Pos.	61	obs:	exp:
tgg	W	1527	1527.00
---	--	------	------
mPD		0	0
		nPD:	1.
		N. weight:	0.
		Sc. PD:	0
		Sc. rank:	0

,

PB1-F2

Pos.	62	obs:	exp:
ttt	F	1	0.45
ttc	F	0	0.55
tta	L	0	161.70
ttg	L	0	279.10
ctt	L	1484	300.10
ctc	L	2	216.20
cta	L	0	259.50
ctg	L	0	269.40
cct	P	30	9.15
ccc	P	1	6.24
cca	P	0	12.28
ccg	P	0	3.33
cat	H	5	2.88
cac	H	0	2.12
cgt	R	4	0.13
cgc	R	0	0.21
cga	R	0	0.40
cgg	R	0	0.29
aga	R	0	1.83
agg	R	0	1.14
---	--	------	------
mPD		0.057	1.2
		nPD:	0.05
		N. weight:	1.7
		Sc. PD:	-0.23
		Sc. rank:	-1444.6

,

PB1-F2

Pos.	63	obs:	exp:
ttt	F	0	25.09
ttc	F	56	30.91
tct	S	0	242.40
tcc	S	1438	227.70
tca	S	2	349.90
tcg	S	1	76.59
tat	Y	0	0.56
tac	Y	1	0.44
cct	P	0	0.89
ccc	P	3	0.60
cca	P	0	1.19
ccg	P	0	0.32
act	T	0	6.40
acc	T	26	5.62
aca	T	0	11.81
acg	T	0	2.17
agt	S	0	268.20
agc	S	0	276.20
---	--	------	------
mPD		0.11	1.7
		nPD:	0.07
		N. weight:	1.9
		Sc. PD:	-0.22
		Sc. rank:	-955.7

,

PB1-F2

Pos.	64	obs:	exp:
tta	L	0	165.00
ttg	L	1516	284.80
tct	S	0	0.67
tcc	S	0	0.63
tca	S	0	0.97
tcg	S	4	0.21
tgg	W	7	7.00
ctt	L	0	306.20
ctc	L	0	220.60
cta	L	0	264.70
ctg	L	0	274.80
agt	S	0	0.74
agc	S	0	0.77
---	--	------	------
mPD		0.014	1.2
		nPD:	0.01
		N. weight:	1.8
		Sc. PD:	-0.3
		Sc. rank:	-2148.5

,

PB1-F2

Pos.	65	obs:	exp:
caa	Q	0	0.54
cag	Q	1	0.46
cgt	R	0	8.43
cgc	R	0	14.11
cga	R	0	26.84
cgg	R	0	19.29
atg	M	2	2.00
act	T	0	0.49
acc	T	0	0.43
aca	T	0	0.91
acg	T	2	0.17
aaa	K	19	762.70
aag	K	1236	492.30
aga	R	4	122.40
agg	R	263	75.89
---	--	------	------
mPD		0.32	0.87
		nPD:	0.37
		N. weight:	0.98
		Sc. PD:	0.18
		Sc. rank:	921.3

,

PB1-F2

Pos.	66	obs:	exp:
tct	S	0	11.27
tcc	S	0	10.59
tca	S	0	16.27
tcg	S	0	3.56
act	T	8	1.97
acc	T	0	1.73
aca	T	0	3.64
acg	T	0	0.67
aat	N	1441	819.00
aac	N	0	622.00
aaa	K	7	4.25
aag	K	0	2.75
agt	S	67	12.47
agc	S	0	12.84
gat	D	4	2.25
gac	D	0	1.75
---	--	------	------
mPD		0.11	0.66
		nPD:	0.16
		N. weight:	0.67
		Sc. PD:	-0.015
		Sc. rank:	62.7

,

PB1-F2

Pos.	67	obs:	exp:
tta	L	0	16.65
ttg	L	0	28.74
ctt	L	0	30.90
ctc	L	153	22.26
cta	L	0	26.72
ctg	L	0	27.73
cct	P	0	404.00
ccc	P	1369	275.60
cca	P	0	542.10
ccg	P	0	147.30
cat	H	0	2.88
cac	H	5	2.12
---	--	------	------
mPD		0.19	0.96
		nPD:	0.19
		N. weight:	1.8
		Sc. PD:	0.014
		Sc. rank:	376.9

,

PB1-F2

Pos.	68	obs:	exp:
att	I	0	102.90
atc	I	290	74.38
ata	I	0	112.70
act	T	1	304.00
acc	T	1235	267.30
aca	T	0	561.60
acg	T	0	103.10
aat	N	0	0.57
aac	N	1	0.43
---	--	------	------
mPD		0.31	0.99
		nPD:	0.31
		N. weight:	1.6
		Sc. PD:	0.2
		Sc. rank:	1098.3

,

PB1-F2

Pos.	69	obs:	exp:
tta	L	0	0.54
ttg	L	0	0.94
ctt	L	0	1.01
ctc	L	0	0.73
cta	L	0	0.87
ctg	L	5	0.91
cct	P	0	3.54
ccc	P	0	2.42
cca	P	0	4.75
ccg	P	12	1.29
caa	Q	0	801.30
cag	Q	1486	684.70
cgt	R	0	0.76
cgc	R	0	1.27
cga	R	0	2.41
cgg	R	23	1.73
aga	R	0	11.01
agg	R	1	6.82
---	--	------	------
mPD		0.054	0.58
		nPD:	0.09
		N. weight:	0.88
		Sc. PD:	-0.08
		Sc. rank:	-184.0

,

PB1-F2

Pos.	70	obs:	exp:
cgt	R	0	0.03
cgc	R	0	0.05
cga	R	0	0.10
cgg	R	0	0.07
aga	R	1	0.46
agg	R	0	0.28
gtt	V	0	0.44
gtc	V	0	0.38
gta	V	2	0.50
gtg	V	0	0.67
gat	D	0	0.56
gac	D	1	0.44
gaa	E	92	54.51
gag	E	0	37.49
ggt	G	0	224.50
ggc	G	0	181.30
gga	G	1430	648.20
ggg	G	1	377.00
---	--	------	------
mPD		0.12	0.80
		nPD:	0.15
		N. weight:	0.83
		Sc. PD:	-0.028
		Sc. rank:	13.6

,

PB1-F2

Pos.	71	obs:	exp:
ttt	F	8	3.58
ttc	F	0	4.42
tct	S	2	0.34
tcc	S	0	0.32
tca	S	0	0.49
tcg	S	0	0.11
tat	Y	1517	849.90
tac	Y	0	667.10
agt	S	0	0.37
agc	S	0	0.38
---	--	------	------
mPD		0.013	0.51
		nPD:	0.03
		N. weight:	0.63
		Sc. PD:	-0.098
		Sc. rank:	-699.7

,

PB1-F2

Pos.	72	obs:	exp:
tta	L	1	160.50
ttg	L	1474	277.10
tct	S	0	8.75
tcc	S	0	8.22
tca	S	0	12.63
tcg	S	52	2.76
ctt	L	0	297.90
ctc	L	0	214.60
cta	L	0	257.60
ctg	L	0	267.40
agt	S	0	9.68
agc	S	0	9.97
---	--	------	------
mPD		0.067	1.2
		nPD:	0.05
		N. weight:	1.8
		Sc. PD:	-0.24
		Sc. rank:	-1288.3

,

PB1-F2

Pos.	73	obs:	exp:
cgt	R	0	35.83
cgc	R	0	59.99
cga	R	0	114.10
cgg	R	0	82.00
att	I	0	0.71
atc	I	0	0.51
ata	I	2	0.78
aaa	K	389	236.40
aag	K	0	152.60
aga	R	1135	520.50
agg	R	0	322.60
gaa	E	1	0.59
gag	E	0	0.41
---	--	------	------
mPD		0.38	1.2
		nPD:	0.31
		N. weight:	0.76
		Sc. PD:	0.093
		Sc. rank:	487.4

,

PB1-F2

Pos.	74	obs:	exp:
cct	P	1	0.30
ccc	P	0	0.20
cca	P	0	0.40
ccg	P	0	0.11
att	I	1212	429.90
atc	I	0	310.90
ata	I	0	471.20
act	T	310	76.25
acc	T	0	67.03
aca	T	0	140.90
acg	T	0	25.85
gtt	V	2	0.44
gtc	V	0	0.38
gta	V	0	0.50
gtg	V	0	0.67
gct	A	2	0.49
gcc	A	0	0.46
gca	A	0	0.90
gcg	A	0	0.15
---	--	------	------
mPD		0.33	1.0
		nPD:	0.33
		N. weight:	1.2
		Sc. PD:	0.17
		Sc. rank:	874.9

,

PB1-F2

Pos.	75	obs:	exp:
tta	L	0	25.79
ttg	L	0	44.52
ctt	L	237	47.86
ctc	L	0	34.48
cta	L	0	41.38
ctg	L	0	42.96
cct	P	3	0.89
ccc	P	0	0.60
cca	P	0	1.19
ccg	P	0	0.32
cat	H	1201	692.80
cac	H	0	508.20
cgt	R	86	2.71
cgc	R	0	4.55
cga	R	0	8.64
cgg	R	0	6.21
aga	R	0	39.44
agg	R	0	24.44
---	--	------	------
mPD		0.35	1.1
		nPD:	0.31
		N. weight:	0.95
		Sc. PD:	0.11
		Sc. rank:	585.0

,

PB1-F2

Pos.	76	obs:	exp:
gtt	V	0	106.10
gtc	V	448	90.20
gta	V	29	120.70
gtg	V	1	161.00
gct	A	0	257.10
gcc	A	1041	240.40
gca	A	8	472.80
gcg	A	0	78.67
---	--	------	------
mPD		0.48	1.2
		nPD:	0.42
		N. weight:	1.5
		Sc. PD:	0.34
		Sc. rank:	1603.2

,

PB1-F2

Pos.	77	obs:	exp:
tta	L	0	164.80
ttg	L	1514	284.40
tct	S	0	1.01
tcc	S	0	0.95
tca	S	0	1.46
tcg	S	6	0.32
tgg	W	1	1.00
ctt	L	0	305.80
ctc	L	0	220.30
cta	L	0	264.40
ctg	L	0	274.40
agt	S	0	1.12
agc	S	0	1.15
gtt	V	0	1.33
gtc	V	0	1.13
gta	V	0	1.51
gtg	V	6	2.02
---	--	------	------
mPD		0.017	1.2
		nPD:	0.01
		N. weight:	1.8
		Sc. PD:	-0.3
		Sc. rank:	-2042.3

,

PB1-F2

Pos.	78	obs:	exp:
cgt	R	0	2.24
cgc	R	0	3.75
cga	R	0	7.14
cgg	R	0	5.13
aaa	K	1451	884.30
aag	K	4	570.70
aga	R	70	32.56
agg	R	1	20.18
gaa	E	1	0.59
gag	E	0	0.41
---	--	------	------
mPD		0.097	0.59
		nPD:	0.16
		N. weight:	0.53
		Sc. PD:	-0.012
		Sc. rank:	40.4

,

PB1-F2

Pos.	79	obs:	exp:
tta	L	0	0.44
ttg	L	0	0.75
ctt	L	0	0.81
ctc	L	0	0.58
cta	L	4	0.70
ctg	L	0	0.73
cct	P	0	1.48
ccc	P	0	1.01
cca	P	5	1.98
ccg	P	0	0.54
cat	H	0	0.58
cac	H	1	0.42
caa	Q	1229	665.40
cag	Q	5	568.60
cgt	R	2	8.90
cgc	R	0	14.91
cga	R	278	28.34
cgg	R	2	20.37
aga	R	0	129.30
agg	R	0	80.15
---	-	1	1.00
---	--	------	------
mPD		0.32	1.1
		nPD:	0.31
		N. weight:	0.97
		Sc. PD:	0.11
		Sc. rank:	574.9

,

PB1-F2

Pos.	80	obs:	exp:
tta	L	0	0.54
ttg	L	5	0.94
tgg	W	1413	1413.00
ctt	L	0	1.01
ctc	L	0	0.73
cta	L	0	0.87
ctg	L	0	0.91
---	-	109	109.00
---	--	------	------
mPD		0.0061	0.014
		nPD:	0.43
		N. weight:	0.0059
		Sc. PD:	0.0014
		Sc. rank:	6.8

,

PB1-F2

Pos.	81	obs:	exp:
tct	S	0	0.34
tcc	S	0	0.32
tca	S	0	0.49
tcg	S	0	0.11
cgt	R	0	8.40
cgc	R	0	14.06
cga	R	0	26.74
cgg	R	0	19.22
aat	N	0	2.27
aac	N	4	1.73
aaa	K	1081	695.90
aag	K	64	449.10
agt	S	2	0.37
agc	S	0	0.38
aga	R	56	122.00
agg	R	210	75.60
---	-	110	110.00
---	--	------	------
mPD		0.54	0.77
		nPD:	0.7
		N. weight:	0.38
		Sc. PD:	0.19
		Sc. rank:	456.4

,

PB1-F2

Pos.	82	obs:	exp:
tta	L	13	149.40
ttg	L	1353	257.90
tct	S	0	5.55
tcc	S	0	5.22
tca	S	0	8.01
tcg	S	33	1.75
tgg	W	9	9.00
ctt	L	0	277.30
ctc	L	0	199.80
cta	L	0	239.70
ctg	L	7	248.90
atg	M	2	2.00
agt	S	0	6.14
agc	S	0	6.32
---	-	110	110.00
---	--	------	------
mPD		0.076	1.1
		nPD:	0.07
		N. weight:	1.6
		Sc. PD:	-0.18
		Sc. rank:	-696.4

,

PB1-F2

Pos.	83	obs:	exp:
ttt	F	0	78.41
ttc	F	175	96.59
tct	S	0	208.70
tcc	S	1241	196.10
tca	S	0	301.40
tcg	S	0	65.96
cct	P	0	0.30
ccc	P	1	0.20
cca	P	0	0.40
ccg	P	0	0.11
agt	S	0	231.00
agc	S	0	237.80
---	-	110	110.00
---	--	------	------
mPD		0.19	1.5
		nPD:	0.13
		N. weight:	1.7
		Sc. PD:	-0.095
		Sc. rank:	-146.8

PB1-F2

Pos.	84	obs:	exp:
tct	S	0	2.02
tcc	S	0	1.90
tca	S	0	2.91
tcg	S	0	0.64
aat	N	0	798.60
aac	N	1405	606.40
agt	S	0	2.23
agc	S	12	2.30
---	-	110	110.00
---	--	------	------
mPD		0.014	0.45
		nPD:	0.03
		N. weight:	0.85
		Sc. PD:	-0.13
		Sc. rank:	-890.1

PB1-F2

Pos.	85	obs:	exp:
cgt	R	0	0.38
cgc	R	0	0.63
cga	R	0	1.21
cgg	R	0	0.87
aat	N	1	0.57
aac	N	0	0.43
aaa	K	1400	853.30
aag	K	4	550.70
aga	R	12	5.50
agg	R	0	3.41
---	-	110	110.00
---	--	------	------
mPD		0.021	0.43
		nPD:	0.05
		N. weight:	0.48
		Sc. PD:	-0.065
		Sc. rank:	-403.3

PB1-F2

Pos.	86	obs:	exp:
tta	L	0	0.76
ttg	L	0	1.31
ctt	L	0	1.41
ctc	L	0	1.02
cta	L	7	1.22
ctg	L	0	1.27
caa	Q	1379	743.60
cag	Q	0	635.40
cgt	R	0	0.98
cgc	R	1	1.64
cga	R	30	3.12
cgg	R	0	2.24
aga	R	0	14.22
agg	R	0	8.81
---	-	110	110.00
---	--	------	------
mPD		0.046	0.51
		nPD:	0.09
		N. weight:	0.66
		Sc. PD:	-0.061
		Sc. rank:	-153.4

PB1-F2

Pos.	87	obs:	exp:
cgt	R	0	0.03
cgc	R	0	0.05
cga	R	1	0.10
cgg	R	0	0.07
aga	R	0	0.46
agg	R	0	0.28
gaa	E	10	194.30
gag	E	318	133.70
ggt	G	1	170.50
ggc	G	0	137.70
gga	G	3	492.40
ggg	G	1083	286.40
---	-	111	111.00
---	--	------	------
mPD		0.33	0.87
		nPD:	0.37
		N. weight:	1.2
		Sc. PD:	0.22
		Sc. rank:	1145.9

PB1-F2

Pos.	88	obs:	exp:
tta	L	0	1.41
ttg	L	13	2.44
tgg	W	1376	1376.00
ctt	L	0	2.63
ctc	L	0	1.89
cta	L	0	2.27
ctg	L	0	2.36
---	-	138	138.00
---	--	------	------
mPD		0.015	0.036
		nPD:	0.43
		N. weight:	0.015
		Sc. PD:	0.0036
		Sc. rank:	17.8

PB1-F2

Pos.	89	obs:	exp:
att	I	0	373.20
atc	I	0	269.80
ata	I	1052	409.00
act	T	0	82.89
acc	T	0	72.87
aca	T	337	153.10
acg	T	0	28.10
---	-	138	138.00
---	--	------	------
mPD		0.30	0.86
		nPD:	0.36
		N. weight:	0.89
		Sc. PD:	0.15
		Sc. rank:	734.5

PB1-F2

Pos.	90	obs:	exp:
tct	S	0	6.39
tcc	S	0	6.01
tca	S	0	9.23
tcg	S	0	2.02
aat	N	100	767.90
aac	N	1251	583.10
aaa	K	1	0.61
aag	K	0	0.39
agt	S	1	7.07
agc	S	37	7.28
gaa	E	0	0.59
gag	E	1	0.41
ggt	G	0	0.16
ggc	G	0	0.13
gga	G	0	0.45
ggg	G	1	0.26
---	-	135	135.00
---	--	------	------
mPD		0.16	0.49
		nPD:	0.33
		N. weight:	0.58
		Sc. PD:	0.081
		Sc. rank:	401.9

PB1-F2

Pos.	91	obs:	exp:
taa	.	1152	649.70
tag	.	138	402.30
tga	.	237	475.10
---	--	------	------
mPD		0.43	0.82
		nPD:	0.52
		N. weight:	0.25
		Sc. PD:	0.082
		Sc. rank:	292.3

Amino acid level conservation per codon

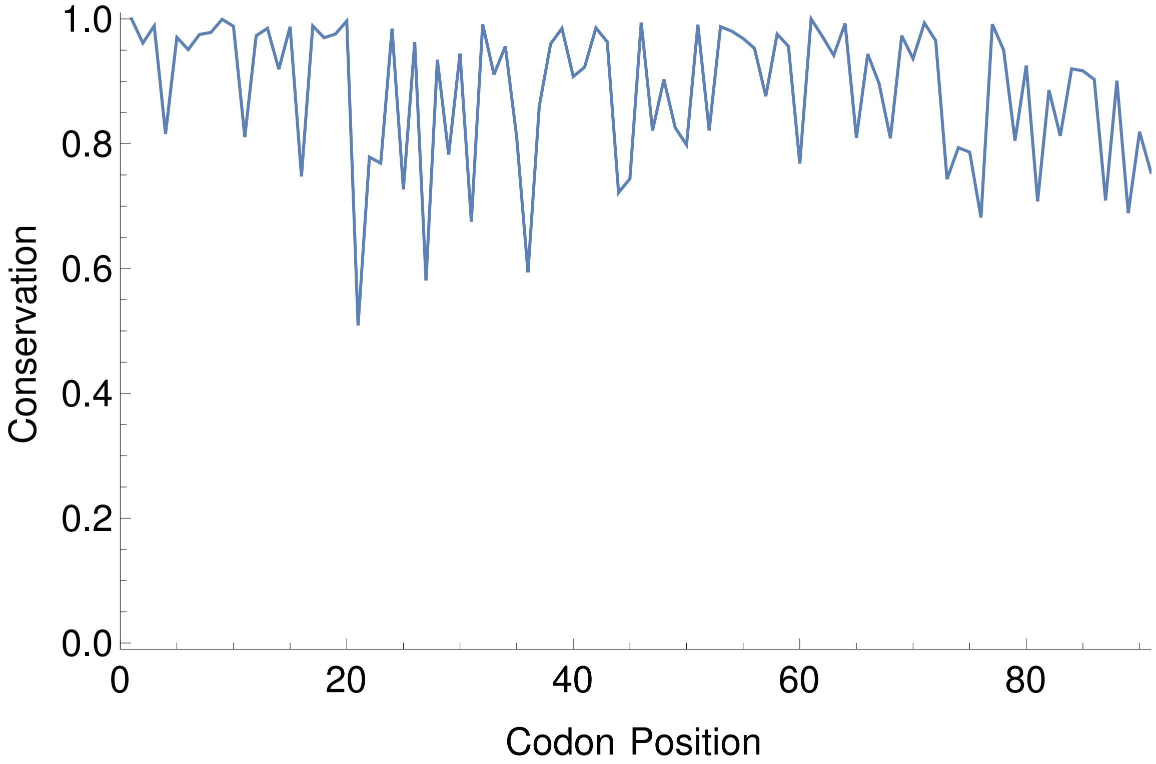

Conservation analysis plots

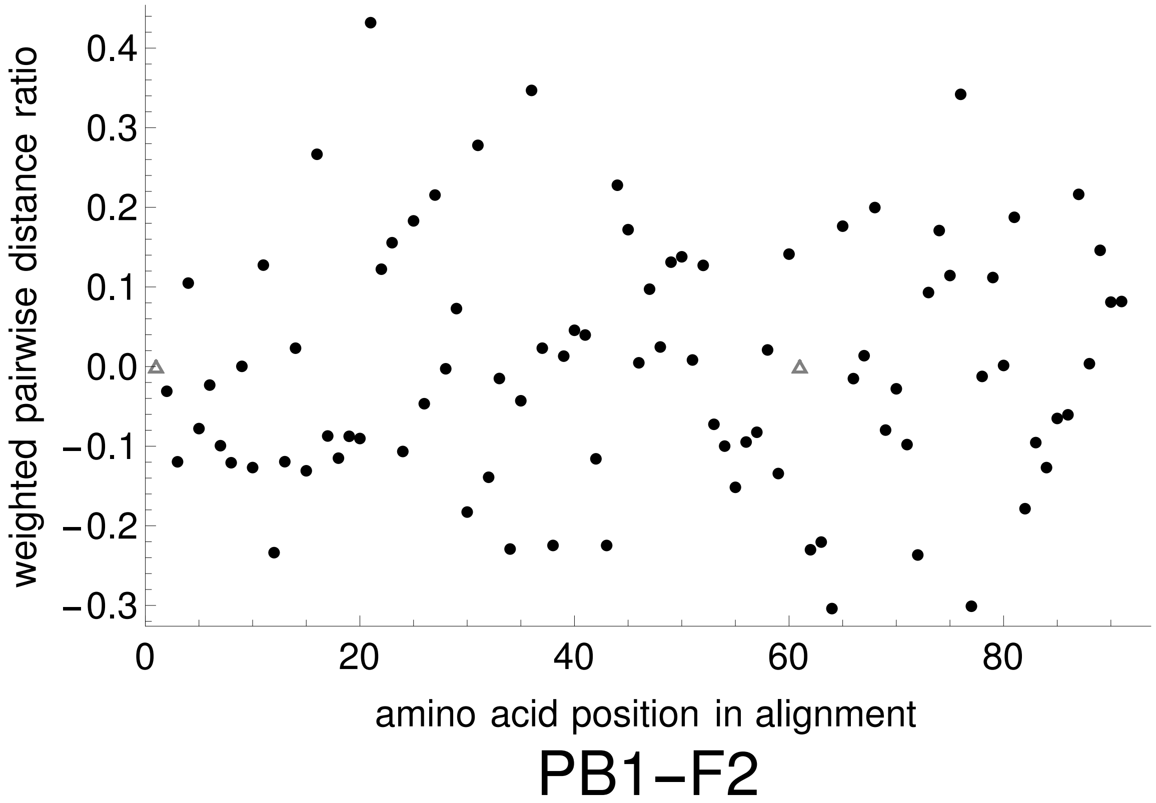

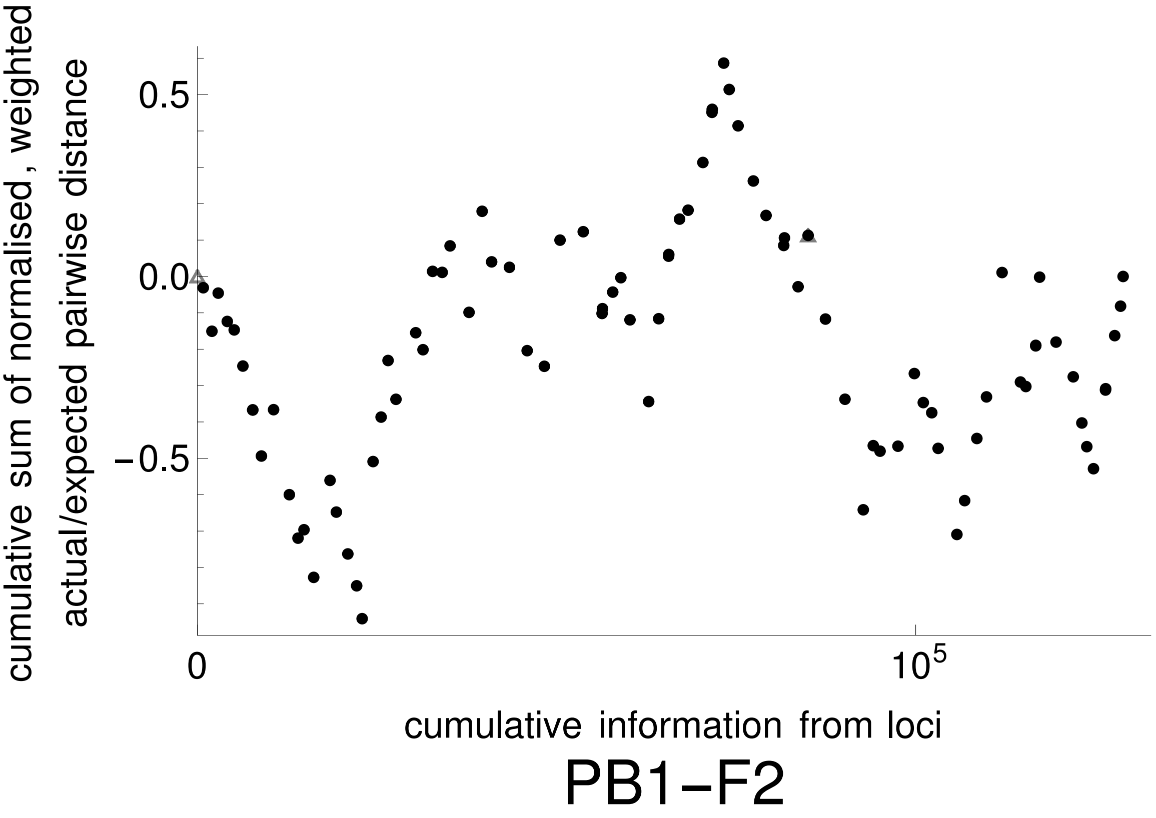

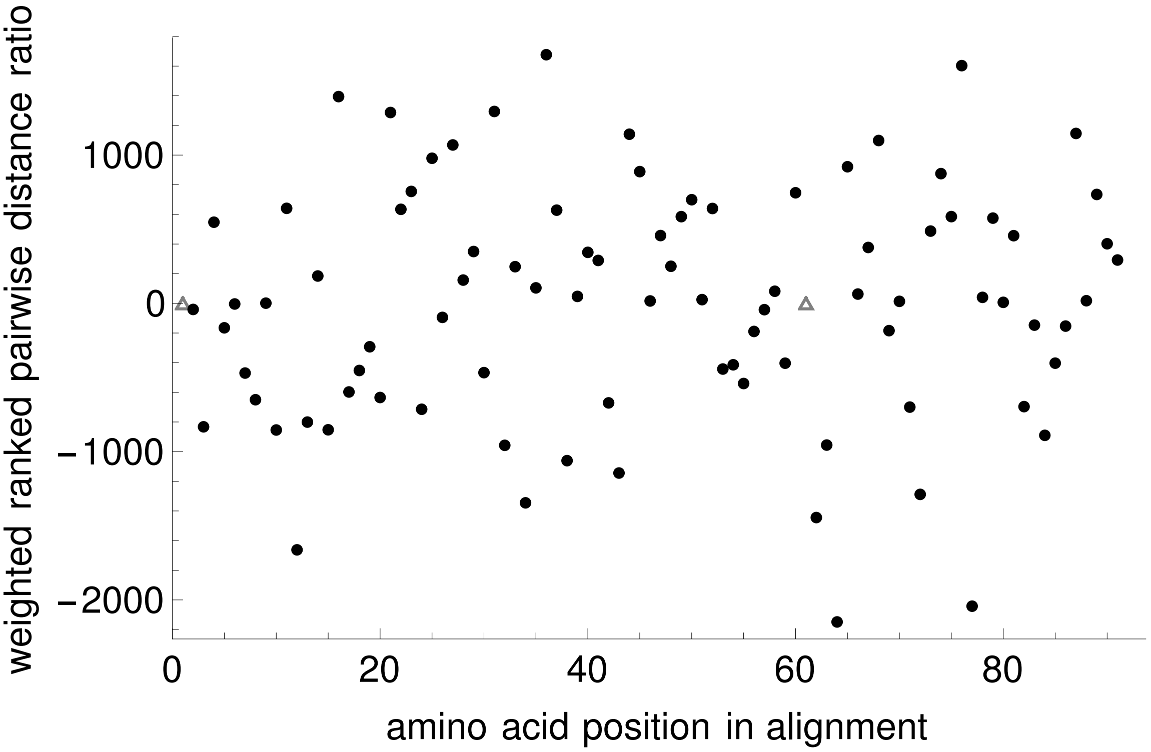
weighted ranked pairwise distance ratio
1000
0
−1000
−2000
0
20
40
60
80
amino acid position in alignment

PB1−F2

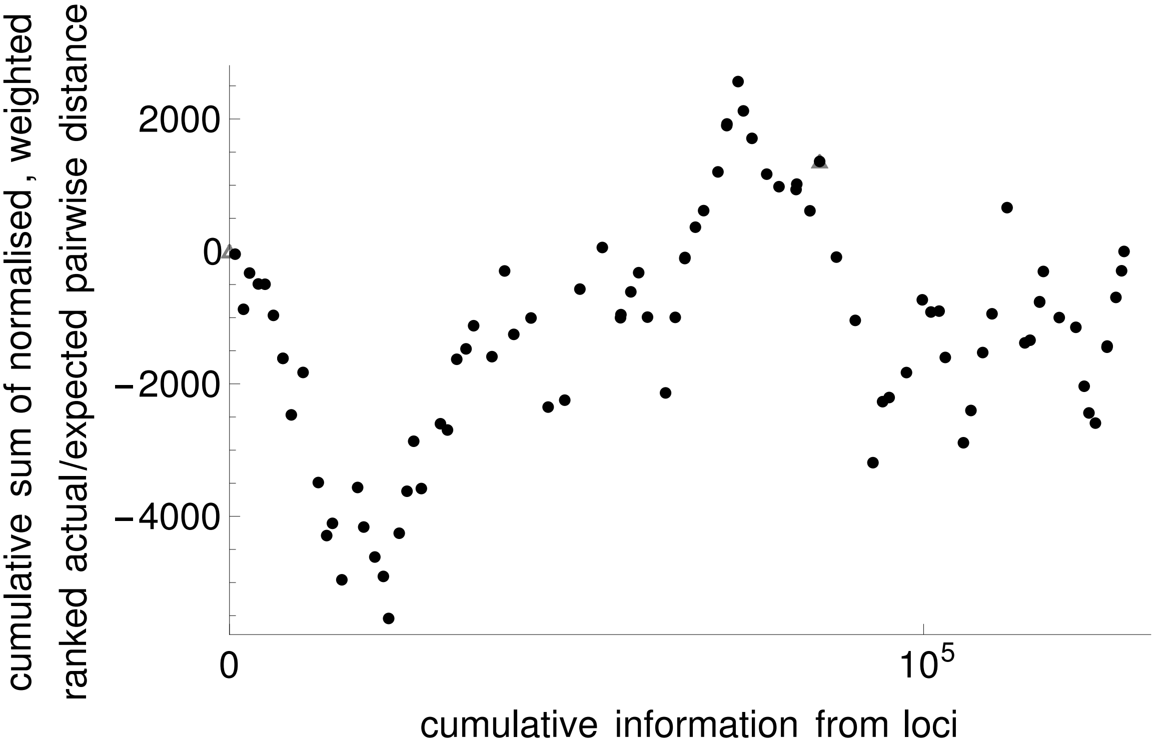
cumulative sum of normalised, weighted
ranked actual/expected pairwise distance
2000
0
−2000
−4000
0
10^5
cumulative information from loci

PB1−F2

PA

Gene length histogram

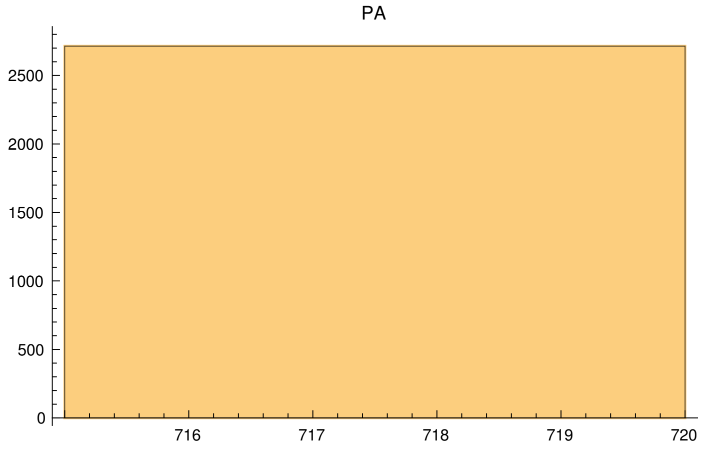

Information vs. nPD

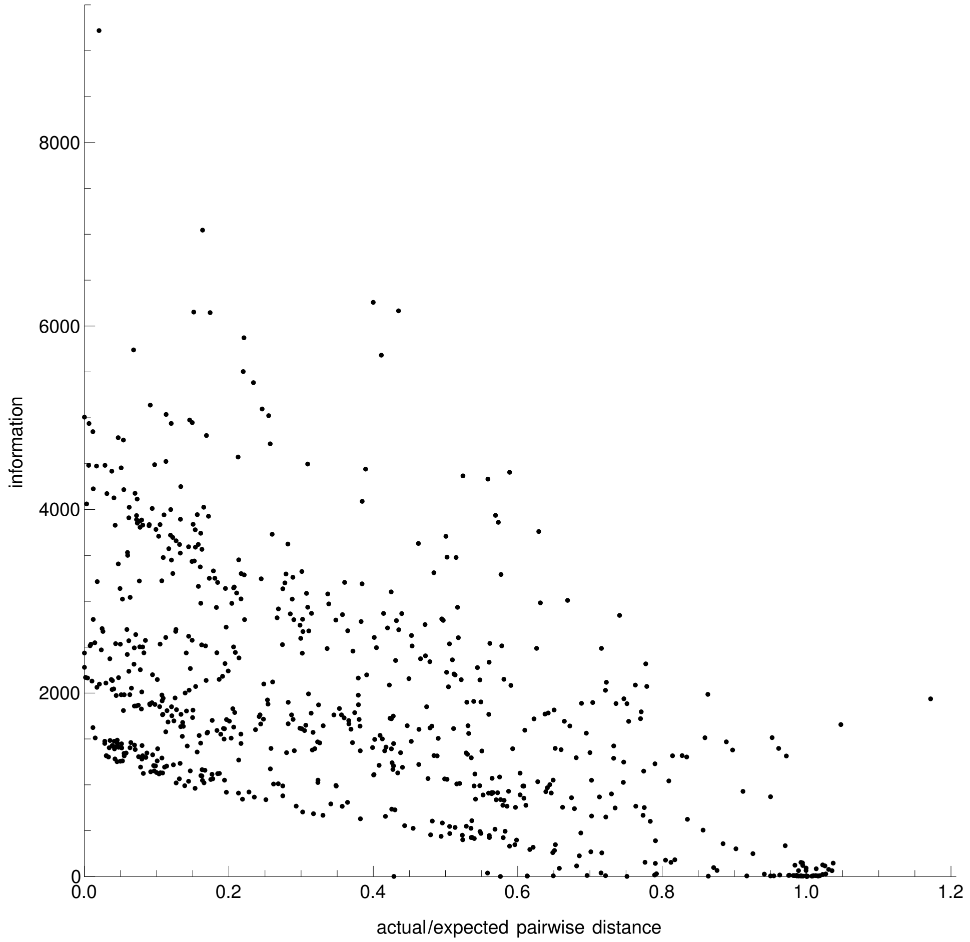

PA

Example sequences highlighted by regions found in analyses to be conserved

Interesting points (by weighted raw PD) highlighted for gene PA:

ATGGAAGAC
TTTGTGCGGCAATGCTTCAATCCAATGATCGTCGAGCTTGCGGAAAAAACAATGA
AAGAACATGGAGAAGACCCGAAAATTGAAACAAACAAATTTGCTGCAATATGTACACACCTGGAAGTCT⋱
GTTTCATGTATTCGGATTTCCACTTCATTGATGAACAAGGTGAATCAATAGTCGTAGAATCTGGTGATCC⋱
AAATTTACTACTAAAACACAGATTTGAAATAATTGAGGGAAGGGATCGCACAATGGCCTGGACAGTGGTG⋱
AACAGCATCTGCAATACCACAGAGGTTGAGAAGCCCAAGTTTCTTCCGGATCTATATGACTATAAGGAGA⋱
ACCGATTCATTGAAATCGGTGTAACAAGAAGAGAAGTCCACATATACTACCTAGAAAAAGCCAACAAGAT⋱
AAAATCAGAGAAGACACACATCCACATTTTCTCGTTCACTGGGGAAGAAATGGCCACTAAGGCAGATTAC⋱
ACTCTTGACGAGGAGAGCAGAGCAAGAATCAAGACCAGACTATTCACCATCAGGCAGGAAATGGCCATTA⋱
GGGGC
CTATGGGACTCCTTTCGTCAGTCCGAAAGAGGTGAAGAGACAATTGAAGAA
AGGTTTGAAATCAAGGGAACCATCCGTAGG
CTTGCCAACCAAAGTCTCCCACCGAACTTCTCCAGCCTTGAAAACTTTAGAGCCT
ATGTGGATGGATTCGAACCGAACGGCTGCATTGAGGGCAAGCTTTCTCAAAT
GTCCAAAGAAGTGAATGCCAGGATTGACCCATTCTTGAGGGAAACACCACGCCCTCTCAGATTACC⋱
TGATGGACCTCCCTGTTCTCAACGGTCAAAATTCCTGCTGATGGATGCCTTAAAATTAAGCATTGAGGAT⋱
CCGAGTCATGAGGGGGAGGGAATACCGCTATATGATGCAATCAAGTGCATGAAGACATTTTTCGGGTGGA⋱
AAGAGCCCAACATTGTCAAACCACATGAGAAAGGCATAAACTCCAATTATCTCCTAGCTTGGAAACAGGT⋱
GCTAGCAGAGATACAGGACATTGAAAATGAGGAGAAAATTCAAAAGACAAAAAACATGAAGAAAACAAGT⋱
CAGCTAAAGTGGGCACTTGGTGAAAACATGGCACCTGAGAAAGTGGATTTTGAAGATTGCAAAGATATTG⋱
GTGATTTAAAACAGTATGACAGTGATGAGCCAAAAATGAGATCACTAGCAAGCTGGATTCAGAGTGAATT⋱
CAATAAGGCATGTGAGCTGACTGATTCTAGTTGGATAGAGCTTGACGAAATAGGGGAGGATGTCGCCCCA⋱
ATCGAGCATATTGCAAGTATGAGGAGGAACTACTTCACAGCAGAAGTGTCCCACTGCAGGGCCACTGAGT⋱
ACATAATGAAGGGAGTGTACATAAACACAGCCTTACTCAATGCATCTTGTGCAGCCATGGATGACTTCCA⋱
GTTAATCCCAATGATAAGCAAGTGCAGGACGAAAGAAGGAAGGCGGAAGACAAATCTATATGGTTTCATT⋱
GTAAAAGGGAGATCTCATTTAAGGAACGATACTGACGTGGTAAATTTTGTGAGTATGGAGTTCTCACTTA⋱
CTGACCCGAGGCTAGAGCCACACAAATGGGAGAAGTATTGTGTTCTTGAAATAGGAGACATGCTTCTAAG⋱
AACTTCAATTGGCCAAGTGTCGAGGCCAATGTTTCTGTATGTGAGAACCAATGGAACCTCCAAAATCAAA⋱
ATGAAGTGGGGTATGGAAATGAGACGTTGCTTTCTTCAATCGCTCCAACAGATTGAGAGCATGATCGAGG⋱
CGGAATCCTCTGTTAAAGAAAAGGACATGACCAAAGAATTTTTTGAAAACAGATCTGAAACATGGCCCAT⋱
TGGGGAATCACCCAAAGGAGTGGAAGAAGGCTCCATTGGGAAAGTATGCAGGACCTTACTGGCAAAGTCT⋱
GTATTCAATAGCCTGTACGCATCCCCACAACTCGAGGGATTCTCAGCTGAATCAAGGAAGTTACTTCTA
ATTGTTCAGGCACTTAGGGACAACCTGGAACCTGGGACCTTTGATCTTGAAGGGC
TATATGATGCAATTGAGGAGTGCCTGATTAACGATCCCTGGGTTTTGCTTAA
TGCGTCTTGGTTCAACTCCTTCCTTGTGCATGCACTGAAATAG

Interesting points (by weighted ranked PD) highlighted for gene PA:

ATGGAAGACTTTGTGCGGCAATGCTTCAATCCAATGATCGTCGAGCTTGCGGAAAAAACAATGAAAGAACATGGAGAA⋱
GACCCGAAAATTGAAACAAACAAATTTGCTGCAATATGTACACACCTGGAAGTCTGTTTCATGTATTCGGATTTCCA⋱
CTTCATTGATGAACAAGGTGAATCAATAGTCGTAGAATCTGGTGATCCAAATTTACTACTAAAACACAGATTTGAAA⋱
TAATTGAGGGAAGGGATCGCACAATGGCCTGGACAGTGGTGAACAGCATCTGCAATACCACAGAGGTTGAGAAGCCC⋱
AAGTTTCTTCCGGATCTATATGACTATAAGGAGAACCGATTCATTGAAATCGGTGTAACAAGAAGAGAAGTCCACAT⋱
ATACTACCTAGAAAAAGCCAACAAGATAAAATCAGAGAAGACACACATCCACATTTTCTCGTTCACTGGGGAAGAAA⋱

TGGCCACTAAGGCAGATTACACTCTTGACGAGGAGAGCAGAGCAAGAATCAAGACCAGACTATTCACCATCAGGCAG⋱
GAAATGGCCATTAGGGGCCTATGGGAC
TCCTTTCGTCAGTCCGAAAGAGGTGAAGAGACAATTGAAGAAAGGTTTGAAATCAAGGGAACCATC⋱
CGTAGGCTTGCCAACCAAAGTCTCCCACCGAACTTCTCCAGCCTTGAAAACTTTAGAGCCTATGTGGATG⋱
GATTCGAACCGAACGGCTGCATTGAGGGCAAGCTTTCTCAAATGTCCAAAGAAGTGAATGCCAGGATTGA⋱
CCCATTCTTGAGGGAAACACCACGCCCTCTCAGATTACCTGATGGACCTCCCTGTTCTCAACGGTCAAAA⋱
TTCCTGCTGATGGATGCCTTAAAATTAAGCATTGAGGATCCGAGTCATGAGGGGGAGGGAATACCGCTAT⋱
ATGATGCAATCAAGTGCATGAAGACATTTTTCGGGTGGAAAGAGCCCAACATTGTCAAACCACATGAGAA⋱
AGGCATAAACTCCAATTATCTCCTAGCTTGGAAACAGGTGCTAGCAGAGATACAGGACATTGAAAATGAG⋱
GAGAAAATTCAAAAGACAAAAAACATGAAGAAAACAAGTCAGCTAAAGTGGGCACTTGGTGAAAACATGG⋱
CACCTGAGAAAGTGGATTTTGAAGATTGCAAAGATATTGGTGATTTAAAACAGTATGACAGTGATGAGCC⋱
AAAAATGAGATCACTAGCAAGCTGGATTCAGAGTGAATTCAATAAGGCATGTGAGCTGACTGATTCTAGT⋱
TGGATAGAGCTTGACGAAATAGGGGAGGATGTCGCCCCAATCGAGCATATTGCAAGTATGAGGAGGAACT⋱
ACTTCACAGCAGAAGTGTCCCACTGCAGGGCCACTGAGTACATAATGAAGGGAGTGTACATAAACACAGC⋱
CTTACTCAATGCATCTTGTGCAGCCATGGATGACTTCCAGTTAATCCCAATGATAAGCAAGTGCAGGACG⋱
AAAGAAGGAAGGCGGAAGACAAATCTATATGGTTTCATTGTAAAAGGGAGATCTCATTTAAGGAACGATA⋱
CTGACGTGGTAAATTTTGTGAGTATGGAGTTCTCACTTACTGACCCGAGGCTAGAGCCACACAAATGGGA⋱
GAAGTATTGTGTTCTTGAAATAGGAGACATGCTTCTAAGAACTTCAATTGGCCAAGTGTCGAGGCCAATG⋱
TTTCTGTATGTGAGAACCAATGGAACCTCCAAAATCAAAATGAAGTGGGGTATGGAAATGAGACGTTGCT⋱
TTCTTCAATCGCTCCAACAGATTGAGAGCATGATCGAGGCGGAATCCTCTGTTAAAGAAAAGGACATGAC⋱
CAAAGAATTTTTTGAAAACAGATCTGAAACATGGCCCATTGGGGAATCACCCAAAGGAGTGGAAGAAGGC⋱
TCCATTGGGAAAGTATGCAGGACCTTACTGGCAAAGTCTGTATTCAATAGCCTGTACGCATCCCCACAAC⋱
TCGAGGGATTCTCAGCTGAATCAAGGAAGTTACTTCTAATTGTTCAGGCACTTAGGGACAACCTGGAACC⋱
TGGGACCTTTGATCTTGAAGGGCTATATGATGCAATT
GAGGAGTGCCTGATTAACGATCCCTGGGTTTTGCTTAATGCGTCTTGGTTCAACT
CCTTCCTTGTGCATGCACTGAAATAG

Per codon conservation report

PA

Pos.	1	obs:	exp:
atg	M	2714	2714.00
mPD		0	0
		nPD:	1.
		N. weight:	0.
		Sc. PD:	0
		Sc. rank:	0

PA

Pos.	2	obs:	exp:
gat	D	1	0.56
gac	D	0	0.44
gaa	E	2694	1608.00
gag	E	19	1105.00
mPD		0.015	0.48
		nPD:	0.03
		N. weight:	0.71
		Sc. PD:	-0.17
		Sc. rank:	-985.1

PA

Pos.	3	obs:	exp:
cat	H	0	0.58
cac	H	1	0.42
aat	N	0	9.66
aac	N	17	7.34
gct	A	0	0.49
gcc	A	2	0.46
gca	A	0	0.90
gcg	A	0	0.15
gat	D	468	1506.00
gac	D	2210	1172.00
gaa	E	1	7.70
gag	E	12	5.30
ggt	G	1	0.47
ggc	G	2	0.38
gga	G	0	1.36
ggg	G	0	0.79
mPD		0.31	0.52
		nPD:	0.6
		N. weight:	0.48
		Sc. PD:	0.16
		Sc. rank:	631.8

PA

Pos.	4	obs:	exp:
ttt	F	2609	1214.00
ttc	F	101	1496.00
tta	L	2	0.33
ttg	L	0	0.56
tct	S	1	0.17
tcc	S	0	0.16
tca	S	0	0.24
tcg	S	0	0.05
ctt	L	1	0.61
ctc	L	0	0.44
cta	L	0	0.52
ctg	L	0	0.54
agt	S	0	0.19
agc	S	0	0.19
mPD		0.075	0.50
		nPD:	0.15
		N. weight:	0.93
		Sc. PD:	-0.11
		Sc. rank:	-317.6

PA

Pos.	5	obs:	exp:
att	I	0	0.35
atc	I	0	0.26
ata	I	1	0.39
atg	M	1	1.00
gtt	V	16	601.50
gtc	V	1	511.20
gta	V	64	683.90
gtg	V	2628	912.50
gaa	E	0	1.78
gag	E	3	1.22
mPD		0.063	0.74
		nPD:	0.08
		N. weight:	1.4
		Sc. PD:	-0.25
		Sc. rank:	-1198.4

PA

Pos.	6	obs:	exp:
cgt	R	3	85.67
cgc	R	2	143.50
cga	R	2626	272.80
cgg	R	46	196.10
aga	R	37	1245.00
agg	R	0	771.40
mPD		0.064	0.94
		nPD:	0.07
		N. weight:	3.1
		Sc. PD:	-0.61
		Sc. rank:	-3254.6

PA

Pos.	7	obs:	exp:
caa	Q	2388	1464.00
cag	Q	326	1250.00
mPD		0.21	0.50
		nPD:	0.43
		N. weight:	0.39
		Sc. PD:	0.06
		Sc. rank:	328.8

PA

Pos.	8	obs:	exp:
ttt	F	1	0.45
ttc	F	0	0.55
tct	S	0	2.19
tcc	S	1	2.05
tca	S	0	3.16
tcg	S	0	0.69
tat	Y	0	2.80
tac	Y	5	2.20
tgt	C	40	1160.00
tgc	C	2655	1535.00
agt	S	0	2.42
agc	S	12	2.49
mPD		0.044	0.51
		nPD:	0.09
		N. weight:	0.72
		Sc. PD:	-0.13
		Sc. rank:	-622.4

PA

Pos.	9	obs:	exp:
ttt	F	31	1216.00
ttc	F	2683	1498.00
mPD		0.023	0.49
		nPD:	0.05
		N. weight:	0.78
		Sc. PD:	-0.17
		Sc. rank:	-995.4

PA

Pos.	10	obs:	exp:
tct	S	0	0.17
tcc	S	0	0.16
tca	S	0	0.24
tcg	S	0	0.05
act	T	1	0.25
acc	T	0	0.22
aca	T	0	0.45
acg	T	0	0.08
aat	N	2663	1541.00
aac	N	49	1171.00
agt	S	1	0.19
agc	S	0	0.19
mPD		0.037	0.49
		nPD:	0.07
		N. weight:	0.7
		Sc. PD:	-0.13
		Sc. rank:	-677.8

PA

Pos.	11	obs:	exp:
cct	P	1	800.90
ccc	P	0	546.40
cca	P	2680	1075.00
ccg	P	33	291.90
mPD		0.025	0.70
		nPD:	0.04
		N. weight:	1.3
		Sc. PD:	-0.29
		Sc. rank:	-1743.3

PA

Pos.	12	obs:	exp:
tta	L	0	0.11
ttg	L	0	0.19
ctt	L	0	0.20
ctc	L	0	0.15
cta	L	0	0.17
ctg	L	1	0.18
att	I	3	3.90
atc	I	0	2.82
ata	I	8	4.28
atg	M	2702	2702.00
mPD		0.0088	0.0093
		nPD:	0.95
		N. weight:	0.0039
		Sc. PD:	0.0026
		Sc. rank:	6.2

PA

Pos.	13	obs:	exp:
ttt	F	1	0.90
ttc	F	1	1.10
att	I	104	898.20
atc	I	2414	649.40
ata	I	14	984.40
gtt	V	11	39.97
gtc	V	166	33.97
gta	V	3	45.44
gtg	V	0	60.63
mPD		0.22	0.80
		nPD:	0.27
		N. weight:	1.7
		Sc. PD:	0.0089
		Sc. rank:	562.0

PA

Pos.	14	obs:	exp:
att	I	1	14.90
atc	I	41	10.77
ata	I	0	16.33
gtt	V	141	592.60
gtc	V	2527	503.60
gta	V	1	673.80
gtg	V	0	899.00
gat	D	0	1.69
gac	D	3	1.31
mPD		0.13	0.77
		nPD:	0.17
		N. weight:	2.1
		Sc. PD:	-0.2
		Sc. rank:	-255.5

PA

Pos.	15	obs:	exp:
gat	D	6	3.37
gac	D	0	2.63
gaa	E	35	1605.00
gag	E	2673	1103.00
mPD		0.030	0.49
		nPD:	0.06
		N. weight:	1.2
		Sc. PD:	-0.24
		Sc. rank:	-1325.8

PA

Pos.	16	obs:	exp:
tta	L	0	295.30
ttg	L	0	509.80
ctt	L	2695	548.10
ctc	L	2	394.90
cta	L	16	473.90
ctg	L	1	492.00
mPD		0.014	1.1
		nPD:	0.01
		N. weight:	2.3
		Sc. PD:	-0.57
		Sc. rank:	-3401.6

PA

Pos.	17	obs:	exp:
gct	A	27	665.30
gcc	A	0	621.80
gca	A	119	1223.00
gcg	A	2568	203.50
mPD		0.10	0.68
		nPD:	0.15
		N. weight:	3.3
		Sc. PD:	-0.38
		Sc. rank:	-1033.3

PA

Pos.	18	obs:	exp:
gaa	E	2686	1608.00
gag	E	27	1105.00
ggt	G	0	0.16
ggc	G	0	0.13
gga	G	1	0.45
ggg	G	0	0.26
mPD		0.020	0.48
		nPD:	0.04
		N. weight:	0.69
		Sc. PD:	-0.15
		Sc. rank:	-899.3

PA

Pos.	19	obs:	exp:
cgt	R	0	1.17
cgc	R	0	1.96
cga	R	0	3.72
cgg	R	0	2.67
aat	N	4	2.27
aac	N	0	1.73
aaa	K	185	1624.00
aag	K	2488	1049.00
aga	R	7	16.97
agg	R	30	10.52
mPD		0.16	0.51
		nPD:	0.31
		N. weight:	0.96
		Sc. PD:	0.042
		Sc. rank:	488.5

PA

Pos.	20	obs:	exp:
tct	S	0	3.20
tcc	S	0	3.00
tca	S	19	4.61
tcg	S	0	1.01
act	T	0	48.46
acc	T	0	42.60
aca	T	197	89.52
acg	T	0	16.43
aaa	K	1	0.61
aag	K	0	0.39
agt	S	0	3.54
agc	S	0	3.64
gtt	V	0	0.89
gtc	V	0	0.75
gta	V	4	1.01
gtg	V	0	1.35
gct	A	2	610.60
gcc	A	0	570.70
gca	A	2487	1123.00
gcg	A	2	186.80
gaa	E	2	1.19
gag	E	0	0.81
mPD		0.16	0.84
		nPD:	0.19
		N. weight:	1.2
		Sc. PD:	-0.093
		Sc. rank:	-44.0

PA

Pos.	21	obs:	exp:
atg	M	2714	2714.00
mPD		0	0
		nPD:	1.
		N. weight:	0.
		Sc. PD:	0
		Sc. rank:	0

PA

Pos.	22	obs:	exp:
cgt	R	0	0.32
cgc	R	0	0.53
cga	R	0	1.01
cgg	R	0	0.72
act	T	0	0.25
acc	T	0	0.22
aca	T	1	0.45
acg	T	0	0.08
aaa	K	2684	1643.00
aag	K	19	1060.00
aga	R	10	4.59
agg	R	0	2.84
mPD		0.022	0.49
		nPD:	0.05
		N. weight:	0.67
		Sc. PD:	-0.15
		Sc. rank:	-862.8

PA

Pos.	23	obs:	exp:
cgt	R	0	0.03
cgc	R	0	0.05
cga	R	0	0.10
cgg	R	0	0.07
aga	R	1	0.46
agg	R	0	0.28
gaa	E	2650	1607.00
gag	E	62	1105.00
ggt	G	0	0.16
ggc	G	0	0.13
gga	G	1	0.45
ggg	G	0	0.26
mPD		0.047	0.49
		nPD:	0.1
		N. weight:	0.62
		Sc. PD:	-0.1
		Sc. rank:	-488.1

PA

Pos.	24	obs:	exp:
ttt	F	1	0.90
ttc	F	1	1.10
tat	Y	2283	1315.00
tac	Y	64	1032.00
cat	H	343	210.50
cac	H	22	154.50
mPD		0.30	0.73
		nPD:	0.41
		N. weight:	0.65
		Sc. PD:	0.088
		Sc. rank:	497.8

PA

Pos.	25	obs:	exp:
ggt	G	0	425.70
ggc	G	0	343.90
gga	G	912	1229.00
ggg	G	1802	715.10
mPD		0.45	0.68
		nPD:	0.65
		N. weight:	0.75
		Sc. PD:	0.28
		Sc. rank:	1047.1

PA

Pos.	26	obs:	exp:
gaa	E	2647	1608.00
gag	E	67	1106.00
mPD		0.048	0.48
		nPD:	0.1
		N. weight:	0.61
		Sc. PD:	-0.1
		Sc. rank:	-462.4

PA

Pos.	27	obs:	exp:
tct	S	0	0.34
tcc	S	0	0.32
tca	S	0	0.49
tcg	S	0	0.11
tat	Y	1	0.56
tac	Y	0	0.44
cat	H	1	0.58
cac	H	0	0.42
act	T	0	0.25
acc	T	1	0.22
aca	T	0	0.45
acg	T	0	0.08
aat	N	99	111.40
aac	N	97	84.60
agt	S	2	0.37
agc	S	0	0.38
gat	D	2123	1413.00
gac	D	389	1099.00
gaa	E	1	0.59
gag	E	0	0.41
mPD		0.43	0.63
		nPD:	0.69
		N. weight:	0.26
		Sc. PD:	0.1
		Sc. rank:	363.9

PA

Pos.	28	obs:	exp:
tta	L	0	0.22
ttg	L	0	0.38
tct	S	20	156.30
tcc	S	0	146.80
tca	S	30	225.60
tcg	S	879	49.37
ctt	L	0	0.40
ctc	L	0	0.29
cta	L	1	0.35
ctg	L	1	0.36
cct	P	3	523.50
ccc	P	4	357.20
cca	P	143	702.50
ccg	P	1624	190.80
act	T	0	2.21
acc	T	0	1.95
aca	T	0	4.09
acg	T	9	0.75
agt	S	0	172.90
agc	S	0	178.00
mPD		0.59	1.4
		nPD:	0.41
		N. weight:	3.1
		Sc. PD:	0.42
		Sc. rank:	2386.3

PA

Pos.	29	obs:	exp:
cgt	R	0	0.38
cgc	R	0	0.63
cga	R	0	1.21
cgg	R	0	0.87
aaa	K	2582	1641.00
aag	K	119	1060.00
aga	R	9	5.50
agg	R	3	3.41
gaa	E	0	0.59
gag	E	1	0.41
mPD		0.096	0.49
		nPD:	0.2
		N. weight:	0.49
		Sc. PD:	-0.035
		Sc. rank:	4.7

PA

Pos.	30	obs:	exp:
tta	L	0	0.33
ttg	L	0	0.56
ctt	L	0	0.61
ctc	L	0	0.44
cta	L	3	0.52
ctg	L	0	0.54
att	I	498	887.20
atc	I	1964	641.50
ata	I	39	972.30
atg	M	1	1.00
act	T	2	38.62
acc	T	155	33.95
aca	T	0	71.34
acg	T	0	13.09
gtt	V	11	11.55
gtc	V	40	9.81
gta	V	1	13.13
gtg	V	0	17.52
mPD		0.48	0.81
		nPD:	0.59
		N. weight:	1.1
		Sc. PD:	0.35
		Sc. rank:	1464.3

PA			
Pos.	31	obs:	exp:
gaa	E	2692	1608.00
gag	E	22	1106.00
---	--	------	------
mPD		0.016	0.48
		nPD:	0.03
		N. weight:	0.7
		Sc. PD:	-0.16
		Sc. rank:	-963.5

,

PA			
Pos.	32	obs:	exp:
tta	L	0	0.11
ttg	L	0	0.19
ctt	L	0	0.20
ctc	L	1	0.15
cta	L	0	0.17
ctg	L	0	0.18
att	I	5	2.48
atc	I	2	1.80
ata	I	0	2.72
act	T	960	664.60
acc	T	1288	584.30
aca	T	452	1228.00
acg	T	2	225.30
aat	N	3	1.71
aac	N	0	1.29
gct	A	0	0.25
gcc	A	0	0.23
gca	A	1	0.45
gcg	A	0	0.07
---	--	------	------
mPD		0.63	0.69
		nPD:	0.91
		N. weight:	0.5
		Sc. PD:	0.31
		Sc. rank:	789.0

,

PA			
Pos.	33	obs:	exp:
aat	N	104	1543.00
aac	N	2610	1171.00
---	--	------	------
mPD		0.074	0.49
		nPD:	0.15
		N. weight:	0.97
		Sc. PD:	-0.11
		Sc. rank:	-328.2

,

PA			
Pos.	34	obs:	exp:
aaa	K	744	1649.00
aag	K	1970	1065.00
---	--	------	------
mPD		0.40	0.48
		nPD:	0.83
		N. weight:	0.34
		Sc. PD:	0.19
		Sc. rank:	523.4

,

PA			
Pos.	35	obs:	exp:
ttt	F	1491	1214.00
ttc	F	1219	1496.00
tta	L	2	0.33
ttg	L	0	0.56
tat	Y	1	0.56
tac	Y	0	0.44
ctt	L	1	0.61
ctc	L	0	0.44
cta	L	0	0.52
ctg	L	0	0.54
---	--	------	------
mPD		0.50	0.50
		nPD:	1.
		N. weight:	0.036
		Sc. PD:	0.025
		Sc. rank:	57.0

,

PA			
Pos.	36	obs:	exp:
tct	S	5	0.84
tcc	S	0	0.79
tca	S	0	1.21
tcg	S	0	0.27
act	T	9	2.21
acc	T	0	1.95
aca	T	0	4.09
acg	T	0	0.75
agt	S	0	0.93
agc	S	0	0.96
gct	A	2561	661.60
gcc	A	80	618.40
gca	A	33	1217.00
gcg	A	25	202.40
ggt	G	1	0.16
ggc	G	0	0.13
gga	G	0	0.45
ggg	G	0	0.26
---	--	------	------
mPD		0.11	0.69
		nPD:	0.16
		N. weight:	1.7
		Sc. PD:	-0.19
		Sc. rank:	-424.9

,

PA			
Pos.	37	obs:	exp:
tct	S	0	0.34
tcc	S	0	0.32
tca	S	2	0.49
tcg	S	0	0.11
agt	S	0	0.37
agc	S	0	0.38
gtt	V	0	0.22
gtc	V	0	0.19
gta	V	1	0.25
gtg	V	0	0.34
gct	A	2	663.60
gcc	A	0	620.20
gca	A	2676	1220.00
gcg	A	29	203.00
ggt	G	0	0.63
ggc	G	0	0.51
gga	G	4	1.81
ggg	G	0	1.05
---	--	------	------
mPD		0.028	0.68
		nPD:	0.04
		N. weight:	1.1
		Sc. PD:	-0.25
		Sc. rank:	-1460.0

,

PA			
Pos.	38	obs:	exp:
tta	L	0	0.11
ttg	L	0	0.19
ctt	L	0	0.20
ctc	L	0	0.15
cta	L	1	0.17
ctg	L	0	0.18
att	I	5	948.90
atc	I	1	686.10
ata	I	2669	1040.00
gtt	V	0	8.44
gtc	V	0	7.17
gta	V	36	9.59
gtg	V	2	12.80
---	--	------	------
mPD		0.034	0.69
		nPD:	0.05
		N. weight:	1.4
		Sc. PD:	-0.29
		Sc. rank:	-1673.2

,

PA			
Pos.	39	obs:	exp:
tct	S	0	0.17
tcc	S	0	0.16
tca	S	0	0.24
tcg	S	0	0.05
tgt	C	123	1168.00
tgc	C	2590	1545.00
agt	S	0	0.19
agc	S	1	0.19
---	--	------	------
mPD		0.087	0.49
		nPD:	0.18
		N. weight:	0.57
		Sc. PD:	-0.051
		Sc. rank:	-49.5

,

PA			
Pos.	40	obs:	exp:
att	I	1	0.35
atc	I	0	0.26
ata	I	0	0.39
act	T	10	667.30
acc	T	9	586.70
aca	T	1860	1233.00
acg	T	834	226.20
---	--	------	------
mPD		0.44	0.68
		nPD:	0.64
		N. weight:	0.96
		Sc. PD:	0.35
		Sc. rank:	1320.5

,

PA			
Pos.	41	obs:	exp:
cat	H	1116	1565.00
cac	H	1598	1149.00
---	--	------	------
mPD		0.48	0.49
		nPD:	0.99
		N. weight:	0.083
		Sc. PD:	0.059
		Sc. rank:	132.3

,

PA			
Pos.	42	obs:	exp:
tta	L	45	263.60
ttg	L	2207	454.90
ctt	L	5	489.10
ctc	L	0	352.40
cta	L	8	422.90
ctg	L	157	439.00
att	I	0	0.35
atc	I	0	0.26
ata	I	1	0.39
atg	M	291	291.00
---	--	------	------
mPD		0.34	1.2
		nPD:	0.28
		N. weight:	1.7
		Sc. PD:	0.014
		Sc. rank:	586.8

,

PA			
Pos.	43	obs:	exp:
gaa	E	2538	1608.00
gag	E	176	1106.00
---	--	------	------
mPD		0.12	0.48
		nPD:	0.25
		N. weight:	0.45
		Sc. PD:	-0.0079
		Sc. rank:	109.7

,

PA			
Pos.	44	obs:	exp:
tta	L	0	0.11
ttg	L	0	0.19
ctt	L	0	0.20
ctc	L	1	0.15
cta	L	0	0.17
ctg	L	0	0.18
att	I	63	109.60
atc	I	243	79.26
ata	I	3	120.10
act	T	0	0.49
acc	T	2	0.43
aca	T	0	0.91
acg	T	0	0.17
gtt	V	993	528.70
gtc	V	1314	449.30
gta	V	55	601.10
gtg	V	19	802.00
gct	A	1	5.15
gcc	A	4	4.81
gca	A	16	9.47
gcg	A	0	1.57
---	--	------	------
mPD		0.74	0.96
		nPD:	0.76
		N. weight:	1.1
		Sc. PD:	0.54
		Sc. rank:	1696.1

,

PA			
Pos.	45	obs:	exp:
tct	S	1	0.17
tcc	S	0	0.16
tca	S	0	0.24
tcg	S	0	0.05
tgt	C	2576	1168.00
tgc	C	137	1545.00
agt	S	0	0.19
agc	S	0	0.19
---	--	------	------
mPD		0.097	0.49
		nPD:	0.2
		N. weight:	0.92
		Sc. PD:	-0.065
		Sc. rank:	12.6

,

PA			
Pos.	46	obs:	exp:
ttt	F	48	1216.00
ttc	F	2666	1498.00
---	--	------	------
mPD		0.035	0.49
		nPD:	0.07
		N. weight:	0.74
		Sc. PD:	-0.14
		Sc. rank:	-760.6

,

PA			
Pos.	47	obs:	exp:
atg	M	2714	2714.00
---	--	------	------
mPD		0	0
		nPD:	1.
		N. weight:	0.
		Sc. PD:	0
		Sc. rank:	0

,

PA			
Pos.	48	obs:	exp:
tat	Y	2682	1520.00
tac	Y	32	1194.00
---	--	------	------
mPD		0.023	0.49
		nPD:	0.05
		N. weight:	0.76
		Sc. PD:	-0.16
		Sc. rank:	-941.3

,

PA			
Pos.	49	obs:	exp:
tct	S	480	456.50
tcc	S	17	428.90
tca	S	210	659.10
tcg	S	2007	144.20
agt	S	0	505.10
agc	S	0	520.10
---	--	------	------
mPD		0.42	1.6
		nPD:	0.26
		N. weight:	2.7
		Sc. PD:	-0.037
		Sc. rank:	714.7

,

PA			
Pos.	50	obs:	exp:
gat	D	1965	1527.00
gac	D	749	1187.00
---	--	------	------
mPD		0.40	0.49
		nPD:	0.81
		N. weight:	0.083
		Sc. PD:	0.044
		Sc. rank:	129.1

,

PA			
Pos.	51	obs:	exp:
ttt	F	130	1215.00
ttc	F	2582	1497.00
tta	L	0	0.22
ttg	L	0	0.38
ctt	L	0	0.40
ctc	L	2	0.29
cta	L	0	0.35
ctg	L	0	0.36
---	--	------	------
mPD		0.093	0.50
		nPD:	0.19
		N. weight:	0.6
		Sc. PD:	-0.049
		Sc. rank:	-21.4

,

PA			
Pos.	52	obs:	exp:
cat	H	2270	1565.00
cac	H	444	1149.00
---	--	------	------
mPD		0.27	0.49
		nPD:	0.56
		N. weight:	0.23
		Sc. PD:	0.065
		Sc. rank:	278.4

,

PA			
Pos.	53	obs:	exp:
ttt	F	26	1216.00
ttc	F	2687	1497.00
tat	Y	1	0.56
tac	Y	0	0.44
---	--	------	------
mPD		0.020	0.50
		nPD:	0.04
		N. weight:	0.79
		Sc. PD:	-0.18
		Sc. rank:	-1042.0

,

PA			
Pos.	54	obs:	exp:
att	I	1240	962.10
atc	I	1379	695.60
ata	I	93	1054.00
aaa	K	0	0.61
aag	K	1	0.39
gtt	V	0	0.22
gtc	V	1	0.19
gta	V	0	0.25
gtg	V	0	0.34
---	--	------	------
mPD		0.53	0.66
		nPD:	0.81
		N. weight:	0.56
		Sc. PD:	0.3
		Sc. rank:	867.6

,

PA			
Pos.	55	obs:	exp:
aat	N	54	113.10
aac	N	145	85.89
gat	D	375	1414.00
gac	D	2139	1100.00
gaa	E	0	0.59
gag	E	1	0.41
---	--	------	------
mPD		0.40	0.63
		nPD:	0.64
		N. weight:	0.52
		Sc. PD:	0.19
		Sc. rank:	714.9

,

PA			
Pos.	56	obs:	exp:
cgt	R	0	0.03
cgc	R	0	0.05
cga	R	0	0.10
cgg	R	1	0.07
aat	N	0	0.57
aac	N	1	0.43
aga	R	0	0.46
agg	R	0	0.28
gat	D	0	1.12
gac	D	2	0.88
gaa	E	2385	1606.00
gag	E	325	1104.00
---	--	------	------
mPD		0.22	0.49
		nPD:	0.44
		N. weight:	0.3
		Sc. PD:	0.051
		Sc. rank:	269.0

,

PA			
Pos.	57	obs:	exp:
tta	L	0	0.33
ttg	L	0	0.56
tgg	W	13	13.00
ctt	L	0	0.61
ctc	L	0	0.44
cta	L	0	0.52
ctg	L	3	0.54
cat	H	2	1.15
cac	H	0	0.85
caa	Q	62	513.90
cag	Q	891	439.10
cgt	R	0	54.13
cgc	R	0	90.65
cga	R	462	172.40
cgg	R	1050	123.90
act	T	0	0.25
acc	T	0	0.22
aca	T	0	0.45
acg	T	1	0.08
aaa	K	6	15.80
aag	K	20	10.20
aga	R	18	786.50
agg	R	185	487.40
ggt	G	0	0.16
ggc	G	0	0.13
gga	G	1	0.45
ggg	G	0	0.26
---	--	------	------
mPD		0.95	1.5
		nPD:	0.63
		N. weight:	1.6
		Sc. PD:	0.57
		Sc. rank:	2188.4

,

PA			
Pos.	58	obs:	exp:
tct	S	0	0.17
tcc	S	0	0.16
tca	S	0	0.24
tcg	S	0	0.05
tgg	W	1	1.00
agt	S	1	0.19
agc	S	0	0.19
gat	D	1	0.56
gac	D	0	0.44
ggt	G	2664	425.20
ggc	G	34	343.50
gga	G	3	1228.00
ggg	G	10	714.30
---	--	------	------
mPD		0.037	0.69
		nPD:	0.05
		N. weight:	2.6
		Sc. PD:	-0.54
		Sc. rank:	-3011.2

,

PA			
Pos.	59	obs:	exp:
aaa	K	5	3.04
aag	K	0	1.96
gct	A	0	0.49
gcc	A	0	0.46
gca	A	2	0.90
gcg	A	0	0.15
gat	D	3	4.50
gac	D	5	3.50
gaa	E	1989	1577.00
gag	E	672	1084.00
ggt	G	0	5.96
ggc	G	0	4.82
gga	G	8	17.21
ggg	G	30	10.01
---	--	------	------
mPD		0.42	0.52
		nPD:	0.8
		N. weight:	0.096
		Sc. PD:	0.05
		Sc. rank:	148.8

,

PA			
Pos.	60	obs:	exp:
tct	S	5	455.30
tcc	S	5	427.80
tca	S	2415	657.40
tcg	S	282	143.90
cct	P	0	0.89
ccc	P	0	0.60
cca	P	1	1.19
ccg	P	2	0.32
act	T	0	0.49
acc	T	0	0.43
aca	T	1	0.91
acg	T	1	0.17
agt	S	0	503.80
agc	S	0	518.80
gct	A	0	0.49
gcc	A	0	0.46
gca	A	2	0.90
gcg	A	0	0.15
---	--	------	------
mPD		0.20	1.6
		nPD:	0.12
		N. weight:	1.8
		Sc. PD:	-0.25
		Sc. rank:	-955.1

,

PA			
Pos.	61	obs:	exp:
ttt	F	0	1.79
ttc	F	4	2.21
cgt	R	0	0.03
cgc	R	0	0.05
cga	R	0	0.10
cgg	R	0	0.07
att	I	19	794.60
atc	I	1068	574.50
ata	I	1153	870.80
atg	M	9	9.00
act	T	0	70.84
acc	T	6	62.28
aca	T	279	130.90
acg	T	3	24.02
aaa	K	4	2.43
aag	K	0	1.57
aga	R	1	0.46
agg	R	0	0.28
gtt	V	0	37.30
gtc	V	11	31.70
gta	V	157	42.41
gtg	V	0	56.59
---	--	------	------
mPD		0.81	0.99
		nPD:	0.81
		N. weight:	0.71
		Sc. PD:	0.38
		Sc. rank:	1098.2

,

PA			
Pos.	62	obs:	exp:
att	I	2108	909.60
atc	I	183	657.60
ata	I	273	996.80
act	T	2	0.49
acc	T	0	0.43
aca	T	0	0.91
acg	T	0	0.17
gtt	V	127	32.86
gtc	V	1	27.93
gta	V	18	37.36
gtg	V	2	49.85
---	--	------	------
mPD		0.41	0.77
		nPD:	0.53
		N. weight:	0.72
		Sc. PD:	0.18
		Sc. rank:	818.3

,

PA			
Pos.	63	obs:	exp:
tta	L	1	0.22
ttg	L	0	0.38
ctt	L	0	0.40
ctc	L	0	0.29
cta	L	1	0.35
ctg	L	0	0.36
att	I	0	30.15
atc	I	0	21.80
ata	I	85	33.05
atg	M	8	8.00
act	T	0	0.25
acc	T	0	0.22
aca	T	1	0.45
acg	T	0	0.08
gtt	V	10	580.80
gtc	V	0	493.60
gta	V	2481	660.40
gtg	V	125	881.10
gct	A	0	0.49
gcc	A	0	0.46
gca	A	2	0.90
gcg	A	0	0.15
---	--	------	------
mPD		0.17	0.81
		nPD:	0.21
		N. weight:	1.7
		Sc. PD:	-0.095
		Sc. rank:	135.0

,

PA			
Pos.	64	obs:	exp:
gat	D	0	2.25
gac	D	4	1.75
gaa	E	2641	1606.00
gag	E	69	1104.00
---	--	------	------
mPD		0.052	0.48
		nPD:	0.11
		N. weight:	0.61
		Sc. PD:	-0.095
		Sc. rank:	-415.9

,

PA			
Pos.	65	obs:	exp:
tta	L	0	0.22
ttg	L	0	0.38
tct	S	2396	449.10
tcc	S	264	422.00
tca	S	6	648.40
tcg	S	4	141.90
tat	Y	4	3.36
tac	Y	2	2.64
ctt	L	2	0.40
ctc	L	0	0.29
cta	L	0	0.35
ctg	L	0	0.36
cct	P	30	9.74
ccc	P	3	6.64
cca	P	0	13.07
ccg	P	0	3.55
agt	S	0	497.00
agc	S	0	511.70
gct	A	3	0.74
gcc	A	0	0.69
gca	A	0	1.35
gcg	A	0	0.22
---	--	------	------
mPD		0.22	1.6
		nPD:	0.13
		N. weight:	2.1
		Sc. PD:	-0.28
		Sc. rank:	-974.1

,

PA			
Pos.	66	obs:	exp:
tct	S	0	41.55
tcc	S	0	39.04
tca	S	0	59.98
tcg	S	0	13.13
aat	N	10	5.68
aac	N	0	4.32
agt	S	232	45.97
agc	S	15	47.34
gat	D	219	123.70
gac	D	1	96.26
ggt	G	2214	350.90
ggc	G	19	283.50
gga	G	2	1013.00
ggg	G	2	589.40
---	--	------	------
mPD		0.36	1.1
		nPD:	0.31
		N. weight:	2.4
		Sc. PD:	0.094
		Sc. rank:	1178.5

,

PA			
Pos.	67	obs:	exp:
aat	N	2	5.12
aac	N	7	3.88
gat	D	1564	1521.00
gac	D	1141	1184.00
---	--	------	------
mPD		0.49	0.50
		nPD:	0.99
		N. weight:	0.0048
		Sc. PD:	0.0034
		Sc. rank:	7.7

,

PA			
Pos.	68	obs:	exp:
tct	S	18	4.88
tcc	S	0	4.58
tca	S	9	7.04
tcg	S	2	1.54
cct	P	21	792.00
ccc	P	8	540.40
cca	P	1675	1063.00
ccg	P	980	288.70
act	T	0	0.25
acc	T	0	0.22
aca	T	1	0.45
acg	T	0	0.08
agt	S	0	5.40
agc	S	0	5.56
---	--	------	------
mPD		0.51	0.73
		nPD:	0.69
		N. weight:	1.
		Sc. PD:	0.42
		Sc. rank:	1450.6

,

PA			
Pos.	69	obs:	exp:
tat	Y	1	0.56
tac	Y	0	0.44
aat	N	2675	1542.00
aac	N	38	1171.00
---	--	------	------
mPD		0.028	0.49
		nPD:	0.06
		N. weight:	0.72
		Sc. PD:	-0.15
		Sc. rank:	-834.5

,

PA			
Pos.	70	obs:	exp:
tta	L	1	0.11
ttg	L	0	0.19
ctt	L	0	0.20
ctc	L	0	0.15
cta	L	0	0.17
ctg	L	0	0.18
act	T	0	1.23
acc	T	0	1.08
aca	T	5	2.27
acg	T	0	0.42
gtt	V	0	1.78
gtc	V	0	1.51
gta	V	8	2.02
gtg	V	0	2.69
gct	A	9	661.90
gcc	A	17	618.60
gca	A	2629	1217.00
gcg	A	45	202.50
---	--	------	------
mPD		0.062	0.69
		nPD:	0.09
		N. weight:	1.
		Sc. PD:	-0.18
		Sc. rank:	-846.3

,

PA			
Pos.	71	obs:	exp:
tta	L	142	292.40
ttg	L	1034	504.70
ctt	L	14	542.70
ctc	L	389	390.90
cta	L	1037	469.20
ctg	L	71	487.10
cct	P	0	0.30
ccc	P	1	0.20
cca	P	0	0.40
ccg	P	0	0.11
att	I	0	9.22
atc	I	0	6.67
ata	I	26	10.11
---	--	------	------
mPD		1.1	1.2
		nPD:	0.97
		N. weight:	0.71
		Sc. PD:	0.48
		Sc. rank:	1126.6

,

PA			
Pos.	72	obs:	exp:
tta	L	236	293.40
ttg	L	960	506.40
ctt	L	6	544.50
ctc	L	1	392.30
cta	L	340	470.80
ctg	L	1153	488.70
att	I	0	0.35
atc	I	0	0.26
ata	I	1	0.39
atg	M	17	17.00
---	--	------	------
mPD		0.84	1.1
		nPD:	0.73
		N. weight:	0.77
		Sc. PD:	0.35
		Sc. rank:	1128.8

,

PA			
Pos.	73	obs:	exp:
cgt	R	0	0.47
cgc	R	0	0.79
cga	R	0	1.51
cgg	R	0	1.08
aaa	K	664	1640.00
aag	K	2035	1059.00
aga	R	0	6.88
agg	R	15	4.26
---	--	------	------
mPD		0.38	0.49
		nPD:	0.78
		N. weight:	0.4
		Sc. PD:	0.2
		Sc. rank:	617.9

,

PA			
Pos.	74	obs:	exp:
cat	H	234	1565.00
cac	H	2480	1149.00
---	--	------	------
mPD		0.16	0.49
		nPD:	0.32
		N. weight:	0.79
		Sc. PD:	0.041
		Sc. rank:	420.5

,

PA			
Pos.	75	obs:	exp:
cgt	R	23	85.67
cgc	R	1	143.50
cga	R	2059	272.80
cgg	R	176	196.10
aga	R	366	1245.00
agg	R	89	771.40
---	--	------	------
mPD		0.47	0.94
		nPD:	0.5
		N. weight:	1.9
		Sc. PD:	0.43
		Sc. rank:	1959.1

,

PA			
Pos.	76	obs:	exp:
ttt	F	2707	1216.00
ttc	F	7	1498.00
---	--	------	------
mPD		0.0051	0.49
		nPD:	0.01
		N. weight:	1.1
		Sc. PD:	-0.29
		Sc. rank:	-1752.2

,

PA			
Pos.	77	obs:	exp:
gct	A	0	0.25
gcc	A	0	0.23
gca	A	1	0.45
gcg	A	0	0.07
gaa	E	484	1608.00
gag	E	2229	1105.00
---	--	------	------
mPD		0.29	0.48
		nPD:	0.61
		N. weight:	0.53
		Sc. PD:	0.18
		Sc. rank:	703.7

,

PA			
Pos.	78	obs:	exp:
tta	L	2	0.33
ttg	L	0	0.56
ctt	L	0	0.61
ctc	L	0	0.44
cta	L	1	0.52
ctg	L	0	0.54
att	I	1	961.70
atc	I	4	695.30
ata	I	2706	1054.00
---	--	------	------
mPD		0.0059	0.66
		nPD:	0.01
		N. weight:	1.4
		Sc. PD:	-0.35
		Sc. rank:	-2089.3

,

PA			
Pos.	79	obs:	exp:
tta	L	0	0.44
ttg	L	0	0.75
ctt	L	4	0.81
ctc	L	0	0.58
cta	L	0	0.70
ctg	L	0	0.73
att	I	2640	960.60
atc	I	62	694.60
ata	I	6	1053.00
gtt	V	2	0.44
gtc	V	0	0.38
gta	V	0	0.50
gtg	V	0	0.67
---	--	------	------
mPD		0.053	0.66
		nPD:	0.08
		N. weight:	1.3
		Sc. PD:	-0.25
		Sc. rank:	-1197.4

,

PA			
Pos.	80	obs:	exp:
aaa	K	0	0.61
aag	K	1	0.39
gaa	E	2216	1608.00
gag	E	497	1105.00
---	--	------	------
mPD		0.30	0.48
		nPD:	0.62
		N. weight:	0.17
		Sc. PD:	0.059
		Sc. rank:	229.0

,

PA			
Pos.	81	obs:	exp:
gct	A	0	0.25
gcc	A	0	0.23
gca	A	1	0.45
gcg	A	0	0.07
ggt	G	0	425.50
ggc	G	0	343.80
gga	G	2609	1229.00
ggg	G	104	714.80
---	--	------	------
mPD		0.074	0.69
		nPD:	0.11
		N. weight:	0.95
		Sc. PD:	-0.15
		Sc. rank:	-647.2

,

PA			
Pos.	82	obs:	exp:
cgt	R	0	85.67
cgc	R	0	143.50
cga	R	16	272.80
cgg	R	1	196.10
aga	R	2413	1245.00
agg	R	284	771.40
---	--	------	------
mPD		0.20	0.94
		nPD:	0.21
		N. weight:	0.68
		Sc. PD:	-0.037
		Sc. rank:	66.6

,

PA			
Pos.	83	obs:	exp:
cat	H	0	0.58
cac	H	1	0.42
gat	D	402	1525.00
gac	D	2309	1186.00
ggt	G	1	0.31
ggc	G	1	0.25
gga	G	0	0.91
ggg	G	0	0.53
---	--	------	------
mPD		0.26	0.49
		nPD:	0.52
		N. weight:	0.54
		Sc. PD:	0.13
		Sc. rank:	593.3

,

PA			
Pos.	84	obs:	exp:
cct	P	0	0.30
ccc	P	0	0.20
cca	P	1	0.40
ccg	P	0	0.11
cgt	R	81	85.64
cgc	R	299	143.40
cga	R	1148	272.70
cgg	R	75	196.00
aga	R	65	1244.00
agg	R	1045	771.10
---	--	------	------
mPD		1.1	0.94
		nPD:	1.17
		N. weight:	1.
		Sc. PD:	0.92
		Sc. rank:	1673.4

,

PA			
Pos.	85	obs:	exp:
tct	S	0	1.35
tcc	S	0	1.26
tca	S	0	1.94
tcg	S	0	0.43
cct	P	0	0.30
ccc	P	1	0.20
cca	P	0	0.40
ccg	P	0	0.11
att	I	52	378.50
atc	I	980	273.70
ata	I	35	414.80
act	T	826	362.60
acc	T	262	318.70
aca	T	358	669.80
acg	T	28	122.90
aat	N	22	19.89
aac	N	13	15.11
agt	S	6	1.49
agc	S	2	1.53
gtt	V	0	5.33
gtc	V	24	4.53
gta	V	0	6.06
gtg	V	0	8.08
gct	A	18	25.25
gcc	A	41	23.60
gca	A	44	46.43
gcg	A	0	7.72
gat	D	0	0.56
gac	D	1	0.44
ggt	G	1	0.16
ggc	G	0	0.13
gga	G	0	0.45
ggg	G	0	0.26
---	--	------	------
mPD		1.2	1.3
		nPD:	0.95
		N. weight:	0.81
		Sc. PD:	0.54
		Sc. rank:	1292.0

,

PA			
Pos.	86	obs:	exp:
att	I	9	12.77
atc	I	0	9.23
ata	I	27	14.00
atg	M	2584	2584.00
act	T	0	0.25
acc	T	0	0.22
aca	T	0	0.45
acg	T	1	0.08
gtt	V	1	20.65
gtc	V	0	17.55
gta	V	0	23.48
gtg	V	92	31.33
---	--	------	------
mPD		0.094	0.14
		nPD:	0.68
		N. weight:	0.062
		Sc. PD:	0.025
		Sc. rank:	88.8

,

PA			
Pos.	87	obs:	exp:
gct	A	442	665.30
gcc	A	2157	621.80
gca	A	109	1223.00
gcg	A	6	203.50
---	--	------	------
mPD		0.34	0.68
		nPD:	0.5
		N. weight:	1.2
		Sc. PD:	0.27
		Sc. rank:	1239.7

,

PA			
Pos.	88	obs:	exp:
tgg	W	2714	2714.00
---	--	------	------
mPD		0	0
		nPD:	1.
		N. weight:	0.
		Sc. PD:	0
		Sc. rank:	0

,

PA			
Pos.	89	obs:	exp:
act	T	8	667.10
acc	T	1	586.40
aca	T	2628	1232.00
acg	T	75	226.10
gct	A	0	0.49
gcc	A	0	0.46
gca	A	2	0.90
gcg	A	0	0.15
---	--	------	------
mPD		0.062	0.68
		nPD:	0.09
		N. weight:	1.
		Sc. PD:	-0.18
		Sc. rank:	-838.0

,

PA			
Pos.	90	obs:	exp:
tta	L	0	1.09
ttg	L	10	1.88
ctt	L	0	2.02
ctc	L	0	1.45
cta	L	0	1.75
ctg	L	0	1.81
att	I	1	18.45
atc	I	1	13.34
ata	I	50	20.22
atg	M	10	10.00
gtt	V	22	586.00
gtc	V	1	498.00
gta	V	1110	666.20
gtg	V	1506	888.90
gct	A	2	0.74
gcc	A	0	0.69
gca	A	1	1.35
gcg	A	0	0.22
---	--	------	------
mPD		0.56	0.79
		nPD:	0.7
		N. weight:	0.73
		Sc. PD:	0.31
		Sc. rank:	1043.0

,

PA

Pos.	91	obs:	exp:
gtt	V	7	602.60
gtc	V	0	512.10
gta	V	616	685.10
gtg	V	2091	914.20
---	--	------	------
mPD		0.36	0.74
		nPD:	0.48
		N. weight:	0.88
		Sc. PD:	0.18
		Sc. rank:	866.7

,

PA

Pos.	92	obs:	exp:
tat	Y	0	0.56
tac	Y	1	0.44
att	I	0	0.35
atc	I	1	0.26
ata	I	0	0.39
aat	N	52	1541.00
aac	N	2660	1171.00
---	--	------	------
mPD		0.039	0.49
		nPD:	0.08
		N. weight:	1.1
		Sc. PD:	-0.2
		Sc. rank:	-980.0

,

PA

Pos.	93	obs:	exp:
tct	S	0	456.00
tcc	S	0	428.50
tca	S	0	658.30
tcg	S	0	144.10
cgt	R	0	0.03
cgc	R	0	0.05
cga	R	0	0.10
cgg	R	0	0.07
act	T	2	0.49
acc	T	0	0.43
aca	T	0	0.91
acg	T	0	0.17
agt	S	2134	504.60
agc	S	577	519.60
aga	R	0	0.46
agg	R	1	0.28
---	--	------	------
mPD		0.34	1.6
		nPD:	0.21
		N. weight:	1.7
		Sc. PD:	-0.1
		Sc. rank:	88.0

,

PA

Pos.	94	obs:	exp:
att	I	57	957.80
atc	I	1548	692.50
ata	I	1095	1050.00
gtt	V	4	3.11
gtc	V	8	2.64
gta	V	2	3.53
gtg	V	0	4.72
---	--	------	------
mPD		0.52	0.67
		nPD:	0.77
		N. weight:	0.62
		Sc. PD:	0.3
		Sc. rank:	943.0

,

PA

Pos.	95	obs:	exp:
tgt	C	1120	1168.00
tgc	C	1593	1545.00
---	-	1	1.00
---	--	------	------
mPD		0.48	0.49
		nPD:	0.99
		N. weight:	0.0032
		Sc. PD:	0.0022
		Sc. rank:	5.0

,

PA

Pos.	96	obs:	exp:
tct	S	0	0.17
tcc	S	0	0.16
tca	S	0	0.24
tcg	S	0	0.05
aat	N	390	1541.00
aac	N	2322	1171.00
agt	S	0	0.19
agc	S	1	0.19
---	-	1	1.00
---	--	------	------
mPD		0.25	0.49
		nPD:	0.5
		N. weight:	0.57
		Sc. PD:	0.13
		Sc. rank:	591.7

,

PA

Pos.	97	obs:	exp:
att	I	0	0.35
atc	I	0	0.26
ata	I	1	0.39
act	T	342	653.80
acc	T	610	574.80
aca	T	1697	1208.00
acg	T	9	221.60
aat	N	7	3.98
aac	N	0	3.02
gtt	V	0	0.22
gtc	V	0	0.19
gta	V	1	0.25
gtg	V	0	0.34
gct	A	21	11.28
gcc	A	6	10.54
gca	A	19	20.73
gcg	A	0	3.45
---	-	1	1.00
---	--	------	------
mPD		0.57	0.72
		nPD:	0.79
		N. weight:	0.21
		Sc. PD:	0.11
		Sc. rank:	324.6

,

PA

Pos.	98	obs:	exp:
att	I	0	1.06
atc	I	0	0.77
ata	I	3	1.17
act	T	15	666.40
acc	T	24	585.80
aca	T	2649	1231.00
acg	T	21	225.90
gct	A	0	0.49
gcc	A	0	0.46
gca	A	2	0.90
gcg	A	0	0.15
---	--	------	------
mPD		0.047	0.68
		nPD:	0.07
		N. weight:	1.
		Sc. PD:	-0.2
		Sc. rank:	-1037.7

,

PA

Pos.	99	obs:	exp:
tct	S	0	0.17
tcc	S	0	0.16
tca	S	0	0.24
tcg	S	0	0.05
cgt	R	0	1.01
cgc	R	0	1.69
cga	R	0	3.22
cgg	R	0	2.31
aaa	K	2	6.69
aag	K	9	4.31
agt	S	1	0.19
agc	S	0	0.19
aga	R	0	14.68
agg	R	32	9.10
gat	D	0	0.56
gac	D	1	0.44
gaa	E	10	60.44
gag	E	92	41.56
ggt	G	3	402.60
ggc	G	0	325.30
gga	G	167	1163.00
ggg	G	2397	676.40
---	--	------	------
mPD		0.24	0.79
		nPD:	0.3
		N. weight:	1.5
		Sc. PD:	0.048
		Sc. rank:	683.4

,

PA

Pos.	100	obs:	exp:
tta	L	2	0.44
ttg	L	0	0.75
ctt	L	0	0.81
ctc	L	0	0.58
cta	L	2	0.70
ctg	L	0	0.73
att	I	32	63.85
atc	I	1	46.17
ata	I	147	69.98
act	T	1	0.25
acc	T	0	0.22
aca	T	0	0.45
acg	T	0	0.08
gtt	V	391	554.40
gtc	V	10	471.20
gta	V	2043	630.30
gtg	V	53	841.10
gct	A	10	7.84
gcc	A	0	7.33
gca	A	22	14.42
gcg	A	0	2.40
---	--	------	------
mPD		0.46	0.89
		nPD:	0.51
		N. weight:	1.2
		Sc. PD:	0.28
		Sc. rank:	1278.1

,

PA

Pos.	101	obs:	exp:
aaa	K	0	1.22
aag	K	2	0.78
gat	D	45	25.87
gac	D	1	20.13
gaa	E	214	1569.00
gag	E	2434	1079.00
ggt	G	0	2.82
ggc	G	0	2.28
gga	G	0	8.15
ggg	G	18	4.74
---	--	------	------
mPD		0.19	0.52
		nPD:	0.37
		N. weight:	0.86
		Sc. PD:	0.084
		Sc. rank:	552.2

,

PA

Pos.	102	obs:	exp:
aaa	K	234	1649.00
aag	K	2480	1065.00
---	--	------	------
mPD		0.16	0.48
		nPD:	0.33
		N. weight:	0.88
		Sc. PD:	0.053
		Sc. rank:	487.4

,

PA

Pos.	103	obs:	exp:
cct	P	2166	800.90
ccc	P	459	546.40
cca	P	85	1075.00
ccg	P	4	291.90
---	--	------	------
mPD		0.33	0.70
		nPD:	0.47
		N. weight:	0.99
		Sc. PD:	0.2
		Sc. rank:	961.4

,

PA

Pos.	104	obs:	exp:
caa	Q	3	1.62
cag	Q	0	1.38
cgt	R	0	1.26
cgc	R	0	2.11
cga	R	0	4.02
cgg	R	1	2.89
att	I	0	0.35
atc	I	0	0.26
ata	I	1	0.39
atg	M	1	1.00
aat	N	38	25.58
aac	N	7	19.42
aaa	K	2249	1595.00
aag	K	375	1029.00
aga	R	20	18.34
agg	R	19	11.37
---	--	------	------
mPD		0.31	0.54
		nPD:	0.58
		N. weight:	0.23
		Sc. PD:	0.069
		Sc. rank:	291.7

,

PA

Pos.	105	obs:	exp:
ttt	F	2329	1202.00
ttc	F	354	1481.00
tta	L	0	1.31
ttg	L	0	2.25
tat	Y	3	10.64
tac	Y	16	8.36
ctt	L	12	2.42
ctc	L	0	1.75
cta	L	0	2.10
ctg	L	0	2.18
---	--	------	------
mPD		0.26	0.52
		nPD:	0.5
		N. weight:	0.57
		Sc. PD:	0.13
		Sc. rank:	584.6

,

PA

Pos.	106	obs:	exp:
tta	L	0	294.80
ttg	L	0	508.80
ctt	L	2590	547.10
ctc	L	115	394.10
cta	L	1	473.00
ctg	L	3	491.10
att	I	5	1.77
atc	I	0	1.28
ata	I	0	1.94
---	--	------	------
mPD		0.088	1.1
		nPD:	0.08
		N. weight:	2.1
		Sc. PD:	-0.39
		Sc. rank:	-1962.2

,

PA

Pos.	107	obs:	exp:
tta	L	0	0.11
ttg	L	0	0.19
ctt	L	1	0.20
ctc	L	0	0.15
cta	L	0	0.17
ctg	L	0	0.18
cct	P	1141	800.60
ccc	P	18	546.20
cca	P	135	1074.00
ccg	P	1419	291.80
---	--	------	------
mPD		0.55	0.70
		nPD:	0.78
		N. weight:	1.2
		Sc. PD:	0.62
		Sc. rank:	1912.2

,

PA

Pos.	108	obs:	exp:
gat	D	2645	1527.00
gac	D	69	1187.00
---	--	------	------
mPD		0.050	0.49
		nPD:	0.1
		N. weight:	0.68
		Sc. PD:	-0.11
		Sc. rank:	-511.8

,

PA

Pos.	109	obs:	exp:
tta	L	914	295.30
ttg	L	1219	509.80
ctt	L	3	548.10
ctc	L	1	394.90
cta	L	445	473.90
ctg	L	132	492.00
---	--	------	------
mPD		0.84	1.1
		nPD:	0.74
		N. weight:	1.
		Sc. PD:	0.46
		Sc. rank:	1501.4

,

PA

Pos.	110	obs:	exp:
ttt	F	0	0.45
ttc	F	1	0.55
tat	Y	1594	1519.00
tac	Y	1117	1192.00
tgt	C	2	0.86
tgc	C	0	1.14
---	--	------	------
mPD		0.49	0.49
		nPD:	0.98
		N. weight:	0.0057
		Sc. PD:	0.004
		Sc. rank:	9.2

,

PA

Pos.	111	obs:	exp:
gat	D	952	1527.00
gac	D	1762	1187.00
---	--	------	------
mPD		0.46	0.49
		nPD:	0.93
		N. weight:	0.13
		Sc. PD:	0.086
		Sc. rank:	212.5

,

PA

Pos.	112	obs:	exp:
tat	Y	448	1520.00
tac	Y	2266	1194.00
---	--	------	------
mPD		0.28	0.49
		nPD:	0.56
		N. weight:	0.49
		Sc. PD:	0.14
		Sc. rank:	589.1

,

PA

Pos.	113	obs:	exp:
cgt	R	0	0.16
cgc	R	0	0.26
cga	R	0	0.50
cgg	R	0	0.36
aaa	K	2155	1646.00
aag	K	554	1063.00
aga	R	2	2.29
agg	R	3	1.42
---	--	------	------
mPD		0.33	0.48
		nPD:	0.69
		N. weight:	0.12
		Sc. PD:	0.049
		Sc. rank:	173.2

,

PA

Pos.	114	obs:	exp:
aaa	K	0	2.43
aag	K	4	1.57
gaa	E	623	1605.00
gag	E	2085	1103.00
ggt	G	0	0.31
ggc	G	0	0.25
gga	G	0	0.91
ggg	G	2	0.53
---	--	------	------
mPD		0.36	0.49
		nPD:	0.73
		N. weight:	0.4
		Sc. PD:	0.18
		Sc. rank:	593.5

,

PA

Pos.	115	obs:	exp:
tct	S	0	3.03
tcc	S	0	2.84
tca	S	0	4.37
tcg	S	0	0.96
aat	N	870	1519.00
aac	N	1802	1153.00
aaa	K	2	1.22
aag	K	0	0.78
agt	S	0	3.35
agc	S	18	3.45
gat	D	14	11.81
gac	D	7	9.19
ggt	G	0	0.16
ggc	G	1	0.13
gga	G	0	0.45
ggg	G	0	0.26
---	--	------	------
mPD		0.47	0.53
		nPD:	0.88
		N. weight:	0.19
		Sc. PD:	0.12
		Sc. rank:	302.8

,

PA

Pos.	116	obs:	exp:
cgt	R	7	85.67
cgc	R	0	143.50
cga	R	1615	272.80
cgg	R	1022	196.10
aga	R	63	1245.00
agg	R	7	771.40
---	--	------	------
mPD		0.52	0.94
		nPD:	0.56
		N. weight:	2.3
		Sc. PD:	0.66
		Sc. rank:	2801.7

,

PA

Pos.	117	obs:	exp:
ttt	F	64	1216.00
ttc	F	2649	1497.00
att	I	0	0.35
atc	I	1	0.26
ata	I	0	0.39
---	--	------	------
mPD		0.047	0.50
		nPD:	0.09
		N. weight:	0.71
		Sc. PD:	-0.12
		Sc. rank:	-566.6

,

PA

Pos.	118	obs:	exp:
tta	L	0	0.44
ttg	L	0	0.75
ctt	L	4	0.81
ctc	L	0	0.58
cta	L	0	0.70
ctg	L	0	0.73
att	I	1606	581.40
atc	I	32	420.40
ata	I	1	637.20
gtt	V	1059	237.60
gtc	V	9	201.90
gta	V	2	270.10
gtg	V	0	360.40
gct	A	1	0.25
gcc	A	0	0.23
gca	A	0	0.45
gcg	A	0	0.07
---	--	------	------
mPD		0.51	1.2
		nPD:	0.42
		N. weight:	1.7
		Sc. PD:	0.25
		Sc. rank:	1373.1

,

PA

Pos.	119	obs:	exp:
gaa	E	2296	1608.00
gag	E	418	1106.00
---	--	------	------
mPD		0.26	0.48
		nPD:	0.54
		N. weight:	0.22
		Sc. PD:	0.059
		Sc. rank:	259.2

,

PA

Pos.	120	obs:	exp:
att	I	2385	955.30
atc	I	282	690.70
ata	I	26	1047.00
gtt	V	21	4.66
gtc	V	0	3.96
gta	V	0	5.30
gtg	V	0	7.07
---	--	------	------
mPD		0.22	0.67
		nPD:	0.32
		N. weight:	1.
		Sc. PD:	0.054
		Sc. rank:	545.6

,

PA

Pos.	121	obs:	exp:
ggt	G	424	425.70
ggc	G	11	343.90
gga	G	2242	1229.00
ggg	G	37	715.10
---	--	------	------
mPD		0.29	0.68
		nPD:	0.43
		N. weight:	0.65
		Sc. PD:	0.1
		Sc. rank:	548.5

,

PA

Pos.	122	obs:	exp:
att	I	0	1.42
atc	I	0	1.03
ata	I	4	1.56
gtt	V	20	601.70
gtc	V	129	511.40
gta	V	1347	684.10
gtg	V	1214	912.80
---	--	------	------
mPD		0.55	0.74
		nPD:	0.75
		N. weight:	0.55
		Sc. PD:	0.26
		Sc. rank:	822.6

,

PA

Pos.	123	obs:	exp:
act	T	23	667.60
acc	T	70	586.90
aca	T	2587	1233.00
acg	T	34	226.30
---	--	------	------
mPD		0.091	0.68
		nPD:	0.13
		N. weight:	0.88
		Sc. PD:	-0.12
		Sc. rank:	-405.3

,

PA

Pos.	124	obs:	exp:
cgt	R	6	85.67
cgc	R	0	143.50
cga	R	508	272.80
cgg	R	1068	196.10
aga	R	990	1245.00
agg	R	142	771.40
---	--	------	------
mPD		0.98	0.94
		nPD:	1.05
		N. weight:	0.89
		Sc. PD:	0.68
		Sc. rank:	1426.9

,

PA

Pos.	125	obs:	exp:
cgt	R	0	85.67
cgc	R	0	143.50
cga	R	3	272.80
cgg	R	4	196.10
aga	R	214	1245.00
agg	R	2493	771.40
---	--	------	------
mPD		0.15	0.94
		nPD:	0.16
		N. weight:	1.4
		Sc. PD:	-0.14
		Sc. rank:	-284.4

,

PA			
Pos.	126	obs:	exp:
aaa	K	0	1.22
aag	K	2	0.78
gat	D	3	2.25
gac	D	1	1.75
gaa	E	1665	1604.00
gag	E	1042	1103.00
ggt	G	0	0.16
ggc	G	0	0.13
gga	G	0	0.45
ggg	G	1	0.26
---	--	------	------
mPD		0.48	0.49
		nPD:	0.98
		N. weight:	0.0061
		Sc. PD:	0.0043
		Sc. rank:	9.8

,

PA			
Pos.	127	obs:	exp:
att	I	1	12.06
atc	I	33	8.72
ata	I	0	13.22
act	T	0	0.25
acc	T	1	0.22
aca	T	0	0.45
acg	T	0	0.08
gtt	V	329	592.60
gtc	V	2291	503.60
gta	V	47	673.80
gtg	V	2	899.00
gct	A	0	2.45
gcc	A	10	2.29
gca	A	0	4.51
gcg	A	0	0.75
---	--	------	------
mPD		0.28	0.77
		nPD:	0.36
		N. weight:	1.7
		Sc. PD:	0.15
		Sc. rank:	1067.1

,

PA			
Pos.	128	obs:	exp:
cat	H	73	1565.00
cac	H	2641	1149.00
---	--	------	------
mPD		0.052	0.49
		nPD:	0.11
		N. weight:	1.1
		Sc. PD:	-0.17
		Sc. rank:	-744.5

,

PA			
Pos.	129	obs:	exp:
tta	L	3	0.87
ttg	L	0	1.50
ctt	L	0	1.62
ctc	L	0	1.16
cta	L	5	1.40
ctg	L	0	1.45
att	I	0	950.00
atc	I	3	686.90
ata	I	2675	1041.00
atg	M	11	11.00
act	T	0	1.23
acc	T	0	1.08
aca	T	5	2.27
acg	T	0	0.42
gtt	V	0	2.66
gtc	V	0	2.26
gta	V	11	3.03
gtg	V	1	4.04
---	--	------	------
mPD		0.029	0.68
		nPD:	0.04
		N. weight:	1.4
		Sc. PD:	-0.3
		Sc. rank:	-1759.6

,

PA			
Pos.	130	obs:	exp:
ttt	F	0	0.45
ttc	F	1	0.55
tat	Y	1119	1520.00
tac	Y	1594	1193.00
---	--	------	------
mPD		0.49	0.49
		nPD:	0.98
		N. weight:	0.067
		Sc. PD:	0.046
		Sc. rank:	106.2

,

PA			
Pos.	131	obs:	exp:
tat	Y	123	1520.00
tac	Y	2590	1193.00
tgt	C	0	0.43
tgc	C	1	0.57
---	--	------	------
mPD		0.087	0.49
		nPD:	0.18
		N. weight:	0.91
		Sc. PD:	-0.083
		Sc. rank:	-81.5

,

PA			
Pos.	132	obs:	exp:
tta	L	134	294.90
ttg	L	20	509.00
ctt	L	3	547.30
ctc	L	1	394.30
cta	L	2200	473.20
ctg	L	352	491.30
att	I	0	0.71
atc	I	0	0.51
ata	I	2	0.78
atg	M	1	1.00
act	T	1	0.25
acc	T	0	0.22
aca	T	0	0.45
acg	T	0	0.08
---	--	------	------
mPD		0.35	1.1
		nPD:	0.31
		N. weight:	1.7
		Sc. PD:	0.062
		Sc. rank:	791.1

,

PA			
Pos.	133	obs:	exp:
aaa	K	0	0.61
aag	K	1	0.39
gaa	E	441	1608.00
gag	E	2272	1105.00
---	--	------	------
mPD		0.27	0.48
		nPD:	0.56
		N. weight:	0.58
		Sc. PD:	0.17
		Sc. rank:	708.5

,

PA			
Pos.	134	obs:	exp:
aaa	K	2621	1649.00
aag	K	93	1065.00
---	--	------	------
mPD		0.066	0.48
		nPD:	0.14
		N. weight:	0.53
		Sc. PD:	-0.068
		Sc. rank:	-230.4

,

PA			
Pos.	135	obs:	exp:
gct	A	92	665.30
gcc	A	2599	621.80
gca	A	23	1223.00
gcg	A	0	203.50
---	--	------	------
mPD		0.082	0.68
		nPD:	0.12
		N. weight:	1.9
		Sc. PD:	-0.27
		Sc. rank:	-1044.8

,

PA			
Pos.	136	obs:	exp:
aat	N	140	1542.00
aac	N	2573	1171.00
gat	D	0	0.56
gac	D	1	0.44
---	--	------	------
mPD		0.099	0.49
		nPD:	0.2
		N. weight:	0.91
		Sc. PD:	-0.061
		Sc. rank:	21.1

,

PA			
Pos.	137	obs:	exp:
cgt	R	0	0.06
cgc	R	0	0.11
cga	R	0	0.20
cgg	R	0	0.14
aaa	K	2629	1648.00
aag	K	83	1064.00
aga	R	2	0.92
agg	R	0	0.57
---	--	------	------
mPD		0.061	0.48
		nPD:	0.13
		N. weight:	0.55
		Sc. PD:	-0.076
		Sc. rank:	-275.3

,

PA			
Pos.	138	obs:	exp:
att	I	3	962.80
atc	I	4	696.10
ata	I	2707	1055.00
---	--	------	------
mPD		0.0052	0.66
		nPD:	0.01
		N. weight:	1.4
		Sc. PD:	-0.35
		Sc. rank:	-2084.3

,

PA			
Pos.	139	obs:	exp:
act	T	0	0.25
acc	T	0	0.22
aca	T	1	0.45
acg	T	0	0.08
aaa	K	2564	1649.00
aag	K	149	1064.00
---	--	------	------
mPD		0.10	0.48
		nPD:	0.22
		N. weight:	0.45
		Sc. PD:	-0.022
		Sc. rank:	61.7

,

PA			
Pos.	140	obs:	exp:
tct	S	1241	456.50
tcc	S	1047	428.90
tca	S	420	659.10
tcg	S	6	144.20
agt	S	0	505.10
agc	S	0	520.10
---	--	------	------
mPD		0.62	1.6
		nPD:	0.38
		N. weight:	1.1
		Sc. PD:	0.11
		Sc. rank:	704.9

,

PA			
Pos.	141	obs:	exp:
aaa	K	0	0.61
aag	K	1	0.39
gat	D	18	10.12
gac	D	0	7.88
gaa	E	288	1594.00
gag	E	2402	1096.00
ggt	G	0	0.78
ggc	G	0	0.63
gga	G	1	2.26
ggg	G	4	1.32
---	--	------	------
mPD		0.21	0.49
		nPD:	0.42
		N. weight:	0.76
		Sc. PD:	0.11
		Sc. rank:	606.9

,

PA			
Pos.	142	obs:	exp:
cgt	R	0	1.23
cgc	R	0	2.06
cga	R	2	3.92
cgg	R	1	2.82
act	T	0	0.49
acc	T	0	0.43
aca	T	0	0.91
acg	T	2	0.17
aat	N	24	14.78
aac	N	2	11.22
aaa	K	168	1597.00
aag	K	2460	1031.00
aga	R	1	17.89
agg	R	35	11.08
gat	D	8	5.06
gac	D	1	3.94
gaa	E	0	3.56
gag	E	6	2.44
ggt	G	3	0.63
ggc	G	0	0.51
gga	G	0	1.81
ggg	G	1	1.05
---	--	------	------
mPD		0.19	0.55
		nPD:	0.35
		N. weight:	0.98
		Sc. PD:	0.081
		Sc. rank:	585.1

,

PA			
Pos.	143	obs:	exp:
att	I	0	0.35
atc	I	0	0.26
ata	I	1	0.39
act	T	2	667.30
acc	T	0	586.70
aca	T	2625	1233.00
acg	T	86	226.20
---	--	------	------
mPD		0.064	0.68
		nPD:	0.09
		N. weight:	1.
		Sc. PD:	-0.17
		Sc. rank:	-827.4

,

PA			
Pos.	144	obs:	exp:
cat	H	81	1563.00
cac	H	2628	1146.00
caa	Q	5	2.70
cag	Q	0	2.30
---	--	------	------
mPD		0.061	0.49
		nPD:	0.13
		N. weight:	1.
		Sc. PD:	-0.15
		Sc. rank:	-550.6

,

PA			
Pos.	145	obs:	exp:
tta	L	0	0.22
ttg	L	0	0.38
ctt	L	2	0.40
ctc	L	0	0.29
cta	L	0	0.35
ctg	L	0	0.36
att	I	2259	961.70
atc	I	448	695.30
ata	I	4	1054.00
gtt	V	1	0.22
gtc	V	0	0.19
gta	V	0	0.25
gtg	V	0	0.34
---	--	------	------
mPD		0.28	0.66
		nPD:	0.43
		N. weight:	0.92
		Sc. PD:	0.14
		Sc. rank:	768.2

,

PA			
Pos.	146	obs:	exp:
cat	H	1124	1565.00
cac	H	1590	1149.00
---	--	------	------
mPD		0.49	0.49
		nPD:	0.99
		N. weight:	0.08
		Sc. PD:	0.057
		Sc. rank:	127.8

PA			
Pos.	147	obs:	exp:
att	I	1636	962.80
atc	I	1069	696.10
ata	I	9	1055.00
---	--	------	------
mPD		0.48	0.66
		nPD:	0.73
		N. weight:	0.69
		Sc. PD:	0.31
		Sc. rank:	1019.7

PA			
Pos.	148	obs:	exp:
ttt	F	2242	1216.00
ttc	F	472	1498.00
---	--	------	------
mPD		0.29	0.49
		nPD:	0.58
		N. weight:	0.45
		Sc. PD:	0.14
		Sc. rank:	573.9

PA			
Pos.	149	obs:	exp:
tct	S	94	456.50
tcc	S	2	428.90
tca	S	2308	659.10
tcg	S	310	144.20
agt	S	0	505.10
agc	S	0	520.10
---	--	------	------
mPD		0.26	1.6
		nPD:	0.16
		N. weight:	1.6
		Sc. PD:	-0.17
		Sc. rank:	-349.5

PA			
Pos.	150	obs:	exp:
ttt	F	92	1216.00
ttc	F	2622	1498.00
---	--	------	------
mPD		0.066	0.49
		nPD:	0.13
		N. weight:	0.66
		Sc. PD:	-0.089
		Sc. rank:	-320.5

PA			
Pos.	151	obs:	exp:
att	I	41	15.25
atc	I	2	11.03
ata	I	0	16.72
act	T	2398	646.20
acc	T	94	568.10
aca	T	128	1194.00
acg	T	7	219.10
aat	N	41	23.30
aac	N	0	17.70
gct	A	3	0.74
gcc	A	0	0.69
gca	A	0	1.35
gcg	A	0	0.22
---	--	------	------
mPD		0.22	0.74
		nPD:	0.3
		N. weight:	1.5
		Sc. PD:	0.042
		Sc. rank:	622.3

PA			
Pos.	152	obs:	exp:
ggt	G	15	425.70
ggc	G	0	343.90
gga	G	2546	1229.00
ggg	G	153	715.10
---	--	------	------
mPD		0.12	0.68
		nPD:	0.17
		N. weight:	0.85
		Sc. PD:	-0.082
		Sc. rank:	-110.4

PA			
Pos.	153	obs:	exp:
gaa	E	484	1608.00
gag	E	2230	1106.00
---	--	------	------
mPD		0.29	0.48
		nPD:	0.61
		N. weight:	0.53
		Sc. PD:	0.17
		Sc. rank:	701.1

PA			
Pos.	154	obs:	exp:
gaa	E	517	1608.00
gag	E	2197	1106.00
---	--	------	------
mPD		0.31	0.48
		nPD:	0.64
		N. weight:	0.5
		Sc. PD:	0.18
		Sc. rank:	682.1

PA			
Pos.	155	obs:	exp:
att	I	0	0.35
atc	I	0	0.26
ata	I	1	0.39
atg	M	2712	2712.00
gtt	V	0	0.22
gtc	V	0	0.19
gta	V	0	0.25
gtg	V	1	0.34
---	--	------	------
mPD		0.0015	0.0020
		nPD:	0.75
		N. weight:	0.0011
		Sc. PD:	0.00051
		Sc. rank:	1.6

PA			
Pos.	156	obs:	exp:
gct	A	214	665.30
gcc	A	2427	621.80
gca	A	73	1223.00
gcg	A	0	203.50
---	--	------	------
mPD		0.19	0.68
		nPD:	0.29
		N. weight:	1.5
		Sc. PD:	0.024
		Sc. rank:	579.5

PA			
Pos.	157	obs:	exp:
tgt	C	1	0.43
tgc	C	0	0.57
act	T	459	667.30
acc	T	2212	586.70
aca	T	41	1233.00
acg	T	1	226.20
---	--	------	------
mPD		0.31	0.68
		nPD:	0.45
		N. weight:	1.4
		Sc. PD:	0.25
		Sc. rank:	1300.6

PA			
Pos.	158	obs:	exp:
cgt	R	0	0.92
cgc	R	0	1.53
cga	R	0	2.91
cgg	R	0	2.10
aat	N	10	6.82
aac	N	2	5.18
aaa	K	2295	1624.00
aag	K	377	1048.00
aga	R	21	13.30
agg	R	8	8.24
gaa	E	0	0.59
gag	E	1	0.41
---	--	------	------
mPD		0.27	0.51
		nPD:	0.54
		N. weight:	0.23
		Sc. PD:	0.06
		Sc. rank:	265.2

PA			
Pos.	159	obs:	exp:
tct	S	0	0.50
tcc	S	0	0.47
tca	S	0	0.73
tcg	S	3	0.16
aat	N	0	0.57
aac	N	1	0.43
agt	S	0	0.56
agc	S	0	0.57
gct	A	46	664.30
gcc	A	23	620.90
gca	A	718	1222.00
gcg	A	1923	203.20
---	--	------	------
mPD		0.43	0.68
		nPD:	0.63
		N. weight:	2.
		Sc. PD:	0.71
		Sc. rank:	2744.0

PA			
Pos.	160	obs:	exp:
cat	H	0	0.58
cac	H	1	0.42
aat	N	0	1.14
aac	N	2	0.86
gat	D	398	1519.00
gac	D	2303	1182.00
gaa	E	10	5.93
gag	E	0	4.07
---	--	------	------
mPD		0.26	0.50
		nPD:	0.52
		N. weight:	0.55
		Sc. PD:	0.13
		Sc. rank:	604.9

PA			
Pos.	161	obs:	exp:
tat	Y	20	1520.00
tac	Y	2694	1194.00
---	--	------	------
mPD		0.015	0.49
		nPD:	0.03
		N. weight:	1.1
		Sc. PD:	-0.27
		Sc. rank:	-1581.4

PA			
Pos.	162	obs:	exp:
tct	S	0	0.17
tcc	S	1	0.16
tca	S	0	0.24
tcg	S	0	0.05
att	I	11	31.57
atc	I	78	22.83
ata	I	0	34.60
act	T	480	645.40
acc	T	2047	567.40
aca	T	97	1192.00
acg	T	0	218.80
agt	S	0	0.19
agc	S	0	0.19
---	--	------	------
mPD		0.42	0.74
		nPD:	0.56
		N. weight:	1.3
		Sc. PD:	0.36
		Sc. rank:	1526.1

PA			
Pos.	163	obs:	exp:
tta	L	11	294.90
ttg	L	3	509.00
ctt	L	2606	547.30
ctc	L	55	394.30
cta	L	20	473.20
ctg	L	15	491.30
att	I	4	1.42
atc	I	0	1.03
ata	I	0	1.56
---	--	------	------
mPD		0.088	1.1
		nPD:	0.08
		N. weight:	2.
		Sc. PD:	-0.38
		Sc. rank:	-1909.0

PA			
Pos.	164	obs:	exp:
gat	D	602	1526.00
gac	D	2111	1187.00
gaa	E	1	0.59
gag	E	0	0.41
---	--	------	------
mPD		0.35	0.49
		nPD:	0.7
		N. weight:	0.35
		Sc. PD:	0.15
		Sc. rank:	511.1

PA			
Pos.	165	obs:	exp:
gat	D	66	37.12
gac	D	0	28.88
gaa	E	2275	1569.00
gag	E	373	1079.00
---	--	------	------
mPD		0.28	0.51
		nPD:	0.55
		N. weight:	0.26
		Sc. PD:	0.072
		Sc. rank:	311.7

PA			
Pos.	166	obs:	exp:
gaa	E	485	1608.00
gag	E	2229	1106.00
---	--	------	------
mPD		0.29	0.48
		nPD:	0.61
		N. weight:	0.53
		Sc. PD:	0.18
		Sc. rank:	701.0

PA			
Pos.	167	obs:	exp:
tct	S	0	456.30
tcc	S	0	428.80
tca	S	0	658.80
tcg	S	0	144.20
agt	S	172	505.00
agc	S	2541	519.90
ggt	G	0	0.16
ggc	G	1	0.13
gga	G	0	0.45
ggg	G	0	0.26
---	--	------	------
mPD		0.12	1.6
		nPD:	0.07
		N. weight:	2.1
		Sc. PD:	-0.4
		Sc. rank:	-2014.5

PA			
Pos.	168	obs:	exp:
cgt	R	0	85.64
cgc	R	0	143.40
cga	R	3	272.70
cgg	R	0	196.00
aaa	K	0	0.61
aag	K	1	0.39
aga	R	588	1244.00
agg	R	2122	771.10
---	--	------	------
mPD		0.34	0.94
		nPD:	0.37
		N. weight:	0.91
		Sc. PD:	0.086
		Sc. rank:	577.4

PA			
Pos.	169	obs:	exp:
gct	A	14	665.30
gcc	A	26	621.80
gca	A	2647	1223.00
gcg	A	27	203.50
---	--	------	------
mPD		0.049	0.68
		nPD:	0.07
		N. weight:	1.
		Sc. PD:	-0.19
		Sc. rank:	-1010.5

PA			
Pos.	170	obs:	exp:
cgt	R	0	85.67
cgc	R	0	143.50
cga	R	12	272.80
cgg	R	0	196.10
aga	R	2644	1245.00
agg	R	58	771.40
---	--	------	------
mPD		0.051	0.94
		nPD:	0.05
		N. weight:	0.97
		Sc. PD:	-0.2
		Sc. rank:	-1156.6

PA

Pos.	171	obs:	exp:
att	I	21	962.40
atc	I	2501	695.90
ata	I	191	1055.00
gtt	V	0	0.22
gtc	V	1	0.19
gta	V	0	0.25
gtg	V	0	0.34
---	--	------	------
mPD		0.15	0.66
		nPD:	0.22
		N. weight:	1.5
		Sc. PD:	-0.07
		Sc. rank:	268.5

PA

Pos.	172	obs:	exp:
aaa	K	1597	1649.00
aag	K	1117	1065.00
---	--	------	------
mPD		0.48	0.48
		nPD:	1.02
		N. weight:	0.0034
		Sc. PD:	0.0025
		Sc. rank:	5.4

PA

Pos.	173	obs:	exp:
act	T	1468	667.60
acc	T	1214	586.90
aca	T	15	1233.00
acg	T	17	226.30
---	--	------	------
mPD		0.51	0.68
		nPD:	0.75
		N. weight:	1.
		Sc. PD:	0.49
		Sc. rank:	1560.6

PA

Pos.	174	obs:	exp:
cgt	R	0	85.67
cgc	R	0	143.50
cga	R	2	272.80
cgg	R	12	196.10
aga	R	527	1245.00
agg	R	2173	771.40
---	--	------	------
mPD		0.32	0.94
		nPD:	0.35
		N. weight:	0.95
		Sc. PD:	0.07
		Sc. rank:	547.7

PA

Pos.	175	obs:	exp:
tta	L	27	295.30
ttg	L	18	509.80
ctt	L	1402	548.10
ctc	L	895	394.90
cta	L	351	473.90
ctg	L	21	492.00
---	--	------	------
mPD		0.64	1.1
		nPD:	0.56
		N. weight:	0.95
		Sc. PD:	0.27
		Sc. rank:	1149.5

PA

Pos.	176	obs:	exp:
ttt	F	58	1191.00
ttc	F	2599	1466.00
tat	Y	1	31.93
tac	Y	56	25.07
---	--	------	------
mPD		0.084	0.54
		nPD:	0.16
		N. weight:	0.73
		Sc. PD:	-0.081
		Sc. rank:	-201.3

PA

Pos.	177	obs:	exp:
tct	S	1	0.34
tcc	S	1	0.32
tca	S	0	0.49
tcg	S	0	0.11
att	I	0	0.35
atc	I	1	0.26
ata	I	0	0.39
act	T	1134	666.60
acc	T	1568	586.00
aca	T	8	1231.00
acg	T	0	226.00
agt	S	0	0.37
agc	S	0	0.38
gct	A	0	0.25
gcc	A	1	0.23
gca	A	0	0.45
gcg	A	0	0.07
---	--	------	------
mPD		0.49	0.68
		nPD:	0.72
		N. weight:	1.1
		Sc. PD:	0.5
		Sc. rank:	1668.6

PA

Pos.	178	obs:	exp:
tta	L	0	0.11
ttg	L	0	0.19
ctt	L	0	0.20
ctc	L	0	0.15
cta	L	1	0.17
ctg	L	0	0.18
att	I	62	960.60
atc	I	343	694.60
ata	I	2303	1053.00
gtt	V	0	1.11
gtc	V	0	0.94
gta	V	5	1.26
gtg	V	0	1.68
---	--	------	------
mPD		0.26	0.66
		nPD:	0.4
		N. weight:	0.76
		Sc. PD:	0.096
		Sc. rank:	547.6

PA

Pos.	179	obs:	exp:
cgt	R	0	85.67
cgc	R	0	143.50
cga	R	9	272.80
cgg	R	0	196.10
aga	R	2336	1245.00
agg	R	369	771.40
---	--	------	------
mPD		0.24	0.94
		nPD:	0.26
		N. weight:	0.63
		Sc. PD:	-0.0073
		Sc. rank:	176.0

PA

Pos.	180	obs:	exp:
cat	H	1	0.58
cac	H	0	0.42
caa	Q	2252	1463.00
cag	Q	461	1250.00
---	--	------	------
mPD		0.28	0.50
		nPD:	0.57
		N. weight:	0.28
		Sc. PD:	0.081
		Sc. rank:	342.2

PA

Pos.	181	obs:	exp:
gat	D	0	2.25
gac	D	4	1.75
gaa	E	2016	1606.00
gag	E	694	1104.00
---	--	------	------
mPD		0.38	0.48
		nPD:	0.79
		N. weight:	0.077
		Sc. PD:	0.039
		Sc. rank:	119.3

PA

Pos.	182	obs:	exp:
tta	L	0	0.65
ttg	L	2	1.13
ctt	L	0	1.21
ctc	L	0	0.87
cta	L	0	1.05
ctg	L	4	1.09
att	I	0	0.35
atc	I	0	0.26
ata	I	1	0.39
atg	M	2707	2707.00
---	--	------	------
mPD		0.0052	0.0079
		nPD:	0.65
		N. weight:	0.0045
		Sc. PD:	0.0017
		Sc. rank:	6.3

PA

Pos.	183	obs:	exp:
act	T	0	0.25
acc	T	1	0.22
aca	T	0	0.45
acg	T	0	0.08
gtt	V	0	1.11
gtc	V	5	0.94
gta	V	0	1.26
gtg	V	0	1.68
gct	A	26	663.80
gcc	A	2610	620.50
gca	A	72	1221.00
gcg	A	0	203.10
---	--	------	------
mPD		0.075	0.68
		nPD:	0.11
		N. weight:	1.9
		Sc. PD:	-0.29
		Sc. rank:	-1248.4

PA

Pos.	184	obs:	exp:
tta	L	0	0.65
ttg	L	0	1.13
tct	S	0	366.80
tcc	S	0	344.70
tca	S	0	529.60
tcg	S	0	115.90
tgt	C	1	0.43
tgc	C	0	0.57
ctt	L	6	1.21
ctc	L	0	0.87
cta	L	0	1.05
ctg	L	0	1.09
cat	H	2	1.15
cac	H	0	0.85
cgt	R	1	0.03
cgc	R	0	0.05
cga	R	0	0.10
cgg	R	0	0.07
att	I	365	141.20
atc	I	27	102.10
ata	I	6	154.70
atg	M	1	1.00
act	T	7	2.21
acc	T	0	1.95
aca	T	2	4.09
acg	T	0	0.75
aat	N	22	18.19
aac	N	10	13.81
agt	S	1913	405.90
agc	S	268	418.00
aga	R	0	0.46
agg	R	0	0.28
gct	A	1	0.25
gcc	A	0	0.23
gca	A	0	0.45
gcg	A	0	0.07
ggt	G	82	12.86
ggc	G	0	10.39
gga	G	0	37.14
ggg	G	0	21.61
---	--	------	------
mPD		0.55	1.8
		nPD:	0.3
		N. weight:	1.8
		Sc. PD:	0.055
		Sc. rank:	787.7

PA

Pos.	185	obs:	exp:
cgt	R	0	85.42
cgc	R	0	143.00
cga	R	2	272.00
cgg	R	31	195.50
atg	M	3	3.00
aaa	K	0	3.04
aag	K	5	1.96
aga	R	185	1241.00
agg	R	2488	769.10
---	--	------	------
mPD		0.16	0.94
		nPD:	0.17
		N. weight:	1.4
		Sc. PD:	-0.13
		Sc. rank:	-209.3

PA

Pos.	186	obs:	exp:
tct	S	0	175.80
tcc	S	0	165.20
tca	S	0	253.80
tcg	S	0	55.54
tgt	C	0	0.43
tgc	C	1	0.57
aat	N	44	28.42
aac	N	6	21.58
agt	S	1011	194.50
agc	S	34	200.30
gct	A	6	69.13
gcc	A	275	64.61
gca	A	1	127.10
gcg	A	0	21.15
ggt	G	1151	209.60
ggc	G	176	169.30
gga	G	7	605.10
ggg	G	2	352.00
---	--	------	------
mPD		1.0	1.8
		nPD:	0.57
		N. weight:	2.1
		Sc. PD:	0.62
		Sc. rank:	2631.4

,

PA

Pos.	187	obs:	exp:
tta	L	28	294.70
ttg	L	2	508.70
ctt	L	22	546.90
ctc	L	9	394.00
cta	L	2540	472.90
ctg	L	107	490.90
att	I	0	1.77
atc	I	1	1.28
ata	I	4	1.94
gtt	V	0	0.22
gtc	V	0	0.19
gta	V	1	0.25
gtg	V	0	0.34
---	--	------	------
mPD		0.13	1.1
		nPD:	0.11
		N. weight:	2.1
		Sc. PD:	-0.33
		Sc. rank:	-1395.2

,

PA

Pos.	188	obs:	exp:
tgg	W	2714	2714.00
---	--	------	------
mPD		0	0
		nPD:	1.
		N. weight:	0.
		Sc. PD:	0
		Sc. rank:	0

,

PA

Pos.	189	obs:	exp:
gtt	V	1	0.22
gtc	V	0	0.19
gta	V	0	0.25
gtg	V	0	0.34
gct	A	1	0.25
gcc	A	0	0.23
gca	A	0	0.45
gcg	A	0	0.07
gat	D	2310	1523.00
gac	D	398	1185.00
gaa	E	2	2.37
gag	E	2	1.63
---	--	------	------
mPD		0.25	0.50
		nPD:	0.51
		N. weight:	0.29
		Sc. PD:	0.069
		Sc. rank:	310.0

,

PA

Pos.	190	obs:	exp:
ttt	F	0	0.45
ttc	F	1	0.55
tct	S	3	455.70
tcc	S	2700	428.10
tca	S	6	657.80
tcg	S	0	144.00
tat	Y	0	1.12
tac	Y	2	0.88
cct	P	0	0.59
ccc	P	2	0.40
cca	P	0	0.79
ccg	P	0	0.22
agt	S	0	504.20
agc	S	0	519.20
---	--	------	------
mPD		0.010	1.6
		nPD:	0.01
		N. weight:	2.7
		Sc. PD:	-0.68
		Sc. rank:	-4107.1

,

PA

Pos.	191	obs:	exp:
ttt	F	2713	1216.00
ttc	F	1	1498.00
---	--	------	------
mPD		0.00074	0.49
		nPD:	0.
		N. weight:	1.2
		Sc. PD:	-0.31
		Sc. rank:	-1849.5

,

PA

Pos.	192	obs:	exp:
tgt	C	2	0.86
tgc	C	0	1.14
cat	H	2	1.15
cac	H	0	0.85
cgt	R	2688	85.54
cgc	R	18	143.20
cga	R	4	272.40
cgg	R	0	195.80
aga	R	0	1243.00
agg	R	0	770.30
---	--	------	------
mPD		0.019	0.94
		nPD:	0.02
		N. weight:	5.
		Sc. PD:	-1.2
		Sc. rank:	-7145.7

,

PA

Pos.	193	obs:	exp:
tta	L	0	0.33
ttg	L	0	0.56
ctt	L	0	0.61
ctc	L	0	0.44
cta	L	0	0.52
ctg	L	3	0.54
cat	H	2	1.15
cac	H	0	0.85
caa	Q	76	1459.00
cag	Q	2629	1246.00
cgt	R	0	0.03
cgc	R	0	0.05
cga	R	0	0.10
cgg	R	1	0.07
aaa	K	0	1.82
aag	K	3	1.18
aga	R	0	0.46
agg	R	0	0.28
---	--	------	------
mPD		0.061	0.50
		nPD:	0.12
		N. weight:	0.94
		Sc. PD:	-0.14
		Sc. rank:	-522.7

,

PA

Pos.	194	obs:	exp:
ttt	F	0	0.45
ttc	F	1	0.55
tct	S	7	456.20
tcc	S	2688	428.60
tca	S	16	658.60
tcg	S	1	144.10
agt	S	0	504.80
agc	S	0	519.70
gct	A	0	0.25
gcc	A	1	0.23
gca	A	0	0.45
gcg	A	0	0.07
---	--	------	------
mPD		0.019	1.6
		nPD:	0.01
		N. weight:	2.6
		Sc. PD:	-0.66
		Sc. rank:	-3957.4

,

PA

Pos.	195	obs:	exp:
gaa	E	1547	1608.00
gag	E	1167	1106.00
---	--	------	------
mPD		0.49	0.48
		nPD:	1.02
		N. weight:	0.0038
		Sc. PD:	0.0027
		Sc. rank:	6.0

,

PA

Pos.	196	obs:	exp:
tct	S	0	0.17
tcc	S	0	0.16
tca	S	0	0.24
tcg	S	0	0.05
cgt	R	0	84.25
cgc	R	0	141.10
cga	R	2	268.30
cgg	R	0	192.80
aaa	K	39	26.74
aag	K	5	17.26
agt	S	1	0.19
agc	S	0	0.19
aga	R	2499	1224.00
agg	R	168	758.60
---	--	------	------
mPD		0.15	0.97
		nPD:	0.16
		N. weight:	0.83
		Sc. PD:	-0.089
		Sc. rank:	-203.4

,

PA

Pos.	197	obs:	exp:
tgt	C	0	0.43
tgc	C	1	0.57
ggt	G	67	425.50
ggc	G	2627	343.80
gga	G	12	1229.00
ggg	G	7	714.80
---	--	------	------
mPD		0.062	0.69
		nPD:	0.09
		N. weight:	2.8
		Sc. PD:	-0.48
		Sc. rank:	-2264.2

,

PA

Pos.	198	obs:	exp:
gaa	E	2616	1608.00
gag	E	98	1106.00
---	--	------	------
mPD		0.070	0.48
		nPD:	0.14
		N. weight:	0.56
		Sc. PD:	-0.068
		Sc. rank:	-225.0

,

PA

Pos.	199	obs:	exp:
gat	D	23	14.62
gac	D	3	11.38
gaa	E	292	1592.00
gag	E	2395	1095.00
ggt	G	0	0.16
ggc	G	0	0.13
gga	G	0	0.45
ggg	G	1	0.26
---	--	------	------
mPD		0.21	0.49
		nPD:	0.43
		N. weight:	0.75
		Sc. PD:	0.11
		Sc. rank:	618.7

,

PA

Pos.	200	obs:	exp:
act	T	0	667.60
acc	T	2	586.90
aca	T	2598	1233.00
acg	T	114	226.30
---	--	------	------
mPD		0.082	0.68
		nPD:	0.12
		N. weight:	0.99
		Sc. PD:	-0.14
		Sc. rank:	-556.2

,

PA

Pos.	201	obs:	exp:
att	I	2594	930.80
atc	I	30	673.00
ata	I	0	1020.00
atg	M	13	13.00
act	T	7	2.95
acc	T	5	2.59
aca	T	0	5.45
acg	T	0	1.00
gtt	V	64	14.43
gtc	V	1	12.27
gta	V	0	16.41
gtg	V	0	21.89
---	--	------	------
mPD		0.091	0.72
		nPD:	0.13
		N. weight:	1.4
		Sc. PD:	-0.2
		Sc. rank:	-746.6

,

PA

Pos.	202	obs:	exp:
gaa	E	2550	1608.00
gag	E	164	1106.00
---	--	------	------
mPD		0.11	0.48
		nPD:	0.24
		N. weight:	0.47
		Sc. PD:	-0.015
		Sc. rank:	91.1

,

PA

Pos.	203	obs:	exp:
gat	D	0	0.56
gac	D	1	0.44
gaa	E	2694	1608.00
gag	E	19	1105.00
---	--	------	------
mPD		0.015	0.48
		nPD:	0.03
		N. weight:	0.71
		Sc. PD:	-0.17
		Sc. rank:	-985.3

,

PA

Pos.	204	obs:	exp:
cgt	R	0	47.06
cgc	R	0	78.81
cga	R	7	149.90
cgg	R	0	107.70
att	I	0	0.35
atc	I	0	0.26
ata	I	1	0.39
aaa	K	1119	742.70
aag	K	103	479.30
aga	R	1259	683.80
agg	R	225	423.80
---	--	------	------
mPD		0.71	1.3
		nPD:	0.57
		N. weight:	0.49
		Sc. PD:	0.14
		Sc. rank:	606.0

,

PA

Pos.	205	obs:	exp:
ttt	F	2577	1216.00
ttc	F	137	1498.00
---	--	------	------
mPD		0.096	0.49
		nPD:	0.19
		N. weight:	0.87
		Sc. PD:	-0.064
		Sc. rank:	-13.0

,

PA

Pos. 206		obs:	exp:
aaa	K	0	0.61
aag	K	1	0.39
gct	A	0	0.25
gcc	A	0	0.23
gca	A	1	0.45
gcg	A	0	0.07
gat	D	4	3.94
gac	D	3	3.06
gaa	E	1762	1603.00
gag	E	943	1102.00
---	--	------	------
mPD		0.46	0.49
		nPD:	0.94
		N. weight:	0.014
		Sc. PD:	0.0095
		Sc. rank:	22.8

PA

Pos. 207		obs:	exp:
tta	L	0	0.11
ttg	L	0	0.19
ctt	L	1	0.20
ctc	L	0	0.15
cta	L	0	0.17
ctg	L	0	0.18
att	I	2187	961.40
atc	I	516	695.10
ata	I	7	1054.00
gtt	V	1	0.67
gtc	V	2	0.57
gta	V	0	0.76
gtg	V	0	1.01
---	--	------	------
mPD		0.32	0.66
		nPD:	0.48
		N. weight:	0.87
		Sc. PD:	0.18
		Sc. rank:	852.1

PA

Pos. 208		obs:	exp:
tct	S	0	1.85
tcc	S	0	1.74
tca	S	11	2.67
tcg	S	0	0.58
caa	Q	1	0.54
cag	Q	0	0.46
cgt	R	0	0.41
cgc	R	0	0.69
cga	R	0	1.31
cgg	R	0	0.94
att	I	0	0.35
atc	I	0	0.26
ata	I	1	0.39
act	T	4	535.00
acc	T	115	470.30
aca	T	2015	988.30
acg	T	41	181.40
aaa	K	123	302.00
aag	K	374	195.00
agt	S	0	2.05
agc	S	0	2.11
aga	R	0	5.96
agg	R	13	3.69
gct	A	0	3.92
gcc	A	0	3.67
gca	A	16	7.21
gcg	A	0	1.20
---	--	------	------
mPD		0.66	1.0
		nPD:	0.66
		N. weight:	0.74
		Sc. PD:	0.28
		Sc. rank:	1039.8

PA

Pos. 209		obs:	exp:
cgt	R	0	0.13
cgc	R	0	0.21
cga	R	0	0.40
cgg	R	0	0.29
aga	R	4	1.83
agg	R	0	1.14
gaa	E	1	0.59
gag	E	0	0.41
ggt	G	0	424.90
ggc	G	0	343.30
gga	G	2531	1227.00
ggg	G	178	713.80
---	--	------	------
mPD		0.13	0.69
		nPD:	0.18
		N. weight:	0.86
		Sc. PD:	-0.072
		Sc. rank:	-46.0

PA

Pos. 210		obs:	exp:
att	I	1	0.35
atc	I	0	0.26
ata	I	0	0.39
act	T	996	662.20
acc	T	1610	582.10
aca	T	31	1223.00
acg	T	55	224.50
aat	N	1	0.57
aac	N	0	0.43
gct	A	16	4.90
gcc	A	4	4.58
gca	A	0	9.01
gcg	A	0	1.50
---	--	------	------
mPD		0.52	0.70
		nPD:	0.75
		N. weight:	1.
		Sc. PD:	0.48
		Sc. rank:	1522.1

PA

Pos. 211		obs:	exp:
att	I	3	1.77
atc	I	1	1.28
ata	I	1	1.94
atg	M	2702	2702.00
gtt	V	0	1.55
gtc	V	0	1.32
gta	V	0	1.77
gtg	V	7	2.36
---	--	------	------
mPD		0.0088	0.012
		nPD:	0.72
		N. weight:	0.0054
		Sc. PD:	0.0024
		Sc. rank:	7.9

PA

Pos. 212		obs:	exp:
tta	L	0	4.46
ttg	L	0	7.70
tct	S	0	0.17
tcc	S	0	0.16
tca	S	0	0.24
tcg	S	0	0.05
tat	Y	4	2.24
tac	Y	0	1.76
tgt	C	0	0.43
tgc	C	1	0.57
ctt	L	16	8.28
ctc	L	24	5.97
cta	L	1	7.16
ctg	L	0	7.43
cat	H	254	169.00
cac	H	39	124.00
caa	Q	2	2.16
cag	Q	2	1.84
cgt	R	273	74.81
cgc	R	2064	125.30
cga	R	26	238.20
cgg	R	3	171.20
agt	S	0	0.19
agc	S	1	0.19
aga	R	4	1087.00
agg	R	0	673.60
---	--	------	------
mPD		0.58	1.3
		nPD:	0.43
		N. weight:	3.3
		Sc. PD:	0.54
		Sc. rank:	2919.3

PA

Pos. 213		obs:	exp:
tct	S	0	0.17
tcc	S	0	0.16
tca	S	0	0.24
tcg	S	0	0.05
caa	Q	0	0.54
cag	Q	1	0.46
cgt	R	0	50.22
cgc	R	0	84.09
cga	R	5	159.90
cgg	R	11	114.90
atg	M	1	1.00
aat	N	3	1.71
aac	N	0	1.29
aaa	K	49	678.80
aag	K	1068	438.20
agt	S	1	0.19
agc	S	0	0.19
aga	R	99	729.60
agg	R	1476	452.20
---	--	------	------
mPD		0.61	1.3
		nPD:	0.48
		N. weight:	1.3
		Sc. PD:	0.26
		Sc. rank:	1226.9

PA

Pos. 214		obs:	exp:
tta	L	0	294.40
ttg	L	0	508.10
ctt	L	2586	546.30
ctc	L	118	393.60
cta	L	1	472.30
ctg	L	0	490.30
att	I	9	3.19
atc	I	0	2.31
ata	I	0	3.50
---	--	------	------
mPD		0.091	1.1
		nPD:	0.08
		N. weight:	2.1
		Sc. PD:	-0.39
		Sc. rank:	-1902.4

PA

Pos. 215		obs:	exp:
gct	A	125	665.30
gcc	A	2524	621.80
gca	A	64	1223.00
gcg	A	1	203.50
---	--	------	------
mPD		0.13	0.68
		nPD:	0.2
		N. weight:	1.7
		Sc. PD:	-0.12
		Sc. rank:	1.3

PA

Pos. 216		obs:	exp:
aat	N	46	260.30
aac	N	412	197.70
aaa	K	2	1.22
aag	K	0	0.78
gct	A	0	0.25
gcc	A	1	0.23
gca	A	0	0.45
gcg	A	0	0.07
gat	D	114	1266.00
gac	D	2136	984.50
gaa	E	3	1.78
gag	E	0	1.22
---	--	------	------
mPD		0.40	0.78
		nPD:	0.51
		N. weight:	0.87
		Sc. PD:	0.2
		Sc. rank:	927.8

PA

Pos. 217		obs:	exp:
cat	H	0	0.58
cac	H	1	0.42
caa	Q	2519	1463.00
cag	Q	194	1250.00
---	--	------	------
mPD		0.13	0.50
		nPD:	0.27
		N. weight:	0.54
		Sc. PD:	-0.00048
		Sc. rank:	167.6

PA

Pos. 218		obs:	exp:
tct	S	0	456.30
tcc	S	0	428.80
tca	S	0	658.80
tcg	S	0	144.20
agt	S	2546	505.00
agc	S	167	519.90
gct	A	0	0.25
gcc	A	1	0.23
gca	A	0	0.45
gcg	A	0	0.07
---	--	------	------
mPD		0.12	1.6
		nPD:	0.07
		N. weight:	2.1
		Sc. PD:	-0.41
		Sc. rank:	-2098.2

PA

Pos. 219		obs:	exp:
tta	L	9	295.30
ttg	L	0	509.80
ctt	L	60	548.10
ctc	L	2567	394.90
cta	L	72	473.90
ctg	L	6	492.00
---	--	------	------
mPD		0.11	1.1
		nPD:	0.1
		N. weight:	2.4
		Sc. PD:	-0.41
		Sc. rank:	-1876.6

PA

Pos. 220		obs:	exp:
cct	P	29	800.90
ccc	P	28	546.40
cca	P	2175	1075.00
ccg	P	482	291.90
---	--	------	------
mPD		0.33	0.70
		nPD:	0.46
		N. weight:	0.86
		Sc. PD:	0.16
		Sc. rank:	814.0

PA

Pos.	221	obs:	exp:
cct	P	29	800.90
ccc	P	9	546.40
cca	P	231	1075.00
ccg	P	2445	291.90
---	--	------	------
mPD		0.18	0.70
		nPD:	0.26
		N. weight:	2.5
		Sc. PD:	-0.03
		Sc. rank:	699.9

,

PA

Pos.	222	obs:	exp:
tct	S	0	0.17
tcc	S	0	0.16
tca	S	0	0.24
tcg	S	0	0.05
cat	H	0	0.58
cac	H	1	0.42
act	T	0	0.25
acc	T	1	0.22
aca	T	0	0.45
acg	T	0	0.08
aat	N	69	1539.00
aac	N	2639	1169.00
aaa	K	3	1.82
aag	K	0	1.18
agt	S	0	0.19
agc	S	1	0.19
---	--	------	------
mPD		0.054	0.49
		nPD:	0.11
		N. weight:	1.
		Sc. PD:	-0.16
		Sc. rank:	-706.6

,

PA

Pos.	223	obs:	exp:
ttt	F	43	1216.00
ttc	F	2671	1498.00
---	--	------	------
mPD		0.031	0.49
		nPD:	0.06
		N. weight:	0.75
		Sc. PD:	-0.15
		Sc. rank:	-818.5

,

PA

Pos.	224	obs:	exp:
tct	S	36	455.10
tcc	S	2598	427.70
tca	S	72	657.10
tcg	S	0	143.80
tat	Y	0	0.56
tac	Y	1	0.44
cct	P	0	1.18
ccc	P	4	0.81
cca	P	0	1.58
ccg	P	0	0.43
act	T	0	0.74
acc	T	3	0.65
aca	T	0	1.36
acg	T	0	0.25
agt	S	0	503.70
agc	S	0	518.60
---	--	------	------
mPD		0.083	1.6
		nPD:	0.05
		N. weight:	2.4
		Sc. PD:	-0.51
		Sc. rank:	-2909.9

,

PA

Pos.	225	obs:	exp:
tct	S	0	438.80
tcc	S	0	412.30
tca	S	0	633.60
tcg	S	0	138.70
tgt	C	0	19.37
tgc	C	45	25.63
act	T	0	0.25
acc	T	1	0.22
aca	T	0	0.45
acg	T	0	0.08
aat	N	0	32.40
aac	N	57	24.60
agt	S	241	485.60
agc	S	2368	500.00
ggt	G	1	0.31
ggc	G	0	0.25
gga	G	1	0.91
ggg	G	0	0.53
---	--	------	------
mPD		0.24	1.7
		nPD:	0.14
		N. weight:	1.9
		Sc. PD:	-0.24
		Sc. rank:	-783.6

,

PA

Pos.	226	obs:	exp:
ttt	F	118	58.25
ttc	F	12	71.75
tta	L	0	278.80
ttg	L	0	481.20
ctt	L	2526	517.40
ctc	L	33	372.80
cta	L	2	447.40
ctg	L	1	464.40
att	I	22	7.80
atc	I	0	5.64
ata	I	0	8.55
---	--	------	------
mPD		0.14	1.2
		nPD:	0.12
		N. weight:	2.1
		Sc. PD:	-0.31
		Sc. rank:	-1255.1

,

PA

Pos.	227	obs:	exp:
aaa	K	3	1.82
aag	K	0	1.18
gct	A	0	0.25
gcc	A	0	0.23
gca	A	1	0.45
gcg	A	0	0.07
gat	D	18	12.94
gac	D	5	10.06
gaa	E	2638	1592.00
gag	E	48	1094.00
ggt	G	0	0.16
ggc	G	0	0.13
gga	G	1	0.45
ggg	G	0	0.26
---	--	------	------
mPD		0.055	0.50
		nPD:	0.11
		N. weight:	0.64
		Sc. PD:	-0.099
		Sc. rank:	-420.4

,

PA

Pos.	228	obs:	exp:
tct	S	0	2.35
tcc	S	0	2.21
tca	S	0	3.40
tcg	S	0	0.74
cat	H	0	2.88
cac	H	5	2.12
act	T	1	3.94
acc	T	15	3.46
aca	T	0	7.27
acg	T	0	1.33
aat	N	54	1516.00
aac	N	2614	1152.00
aaa	K	4	2.43
aag	K	0	1.57
agt	S	0	2.61
agc	S	14	2.68
ggt	G	0	1.10
ggc	G	7	0.89
gga	G	0	3.17
ggg	G	0	1.84
---	--	------	------
mPD		0.078	0.54
		nPD:	0.14
		N. weight:	1.1
		Sc. PD:	-0.13
		Sc. rank:	-427.9

,

PA

Pos.	229	obs:	exp:
ttt	F	2635	1216.00
ttc	F	79	1498.00
---	--	------	------
mPD		0.057	0.49
		nPD:	0.11
		N. weight:	0.97
		Sc. PD:	-0.15
		Sc. rank:	-597.8

,

PA

Pos.	230	obs:	exp:
cgt	R	0	85.64
cgc	R	0	143.40
cga	R	29	272.70
cgg	R	0	196.00
aaa	K	0	0.61
aag	K	1	0.39
aga	R	2566	1244.00
agg	R	118	771.10
---	--	------	------
mPD		0.11	0.94
		nPD:	0.11
		N. weight:	0.85
		Sc. PD:	-0.13
		Sc. rank:	-547.4

,

PA

Pos.	231	obs:	exp:
ttt	F	0	0.90
ttc	F	2	1.10
tct	S	0	0.34
tcc	S	2	0.32
tca	S	0	0.49
tcg	S	0	0.11
act	T	0	1.23
acc	T	5	1.08
aca	T	0	2.27
acg	T	0	0.42
agt	S	0	0.37
agc	S	0	0.38
gtt	V	0	0.44
gtc	V	2	0.38
gta	V	0	0.50
gtg	V	0	0.67
gct	A	19	662.60
gcc	A	2676	619.30
gca	A	8	1218.00
gcg	A	0	202.70
---	--	------	------
mPD		0.029	0.69
		nPD:	0.04
		N. weight:	2.1
		Sc. PD:	-0.46
		Sc. rank:	-2666.6

,

PA

Pos.	232	obs:	exp:
tat	Y	2629	1520.00
tac	Y	85	1194.00
---	--	------	------
mPD		0.061	0.49
		nPD:	0.12
		N. weight:	0.65
		Sc. PD:	-0.093
		Sc. rank:	-350.3

,

PA

Pos.	233	obs:	exp:
att	I	0	2.84
atc	I	0	2.05
ata	I	8	3.11
gtt	V	3	600.80
gtc	V	5	510.60
gta	V	2147	683.10
gtg	V	551	911.50
---	--	------	------
mPD		0.33	0.74
		nPD:	0.45
		N. weight:	1.2
		Sc. PD:	0.2
		Sc. rank:	1056.3

,

PA

Pos.	234	obs:	exp:
aat	N	4	2.27
aac	N	0	1.73
gat	D	2695	1524.00
gac	D	15	1186.00
---	--	------	------
mPD		0.014	0.50
		nPD:	0.03
		N. weight:	0.79
		Sc. PD:	-0.19
		Sc. rank:	-1118.4

,

PA

Pos.	235	obs:	exp:
cgt	R	0	0.19
cgc	R	0	0.32
cga	R	0	0.60
cgg	R	0	0.43
aga	R	3	2.75
agg	R	3	1.71
ggt	G	28	424.80
ggc	G	0	343.10
gga	G	2277	1227.00
ggg	G	403	713.50
---	--	------	------
mPD		0.28	0.69
		nPD:	0.4
		N. weight:	0.6
		Sc. PD:	0.077
		Sc. rank:	445.2

,

PA

Pos.	236	obs:	exp:
ttt	F	375	1216.00
ttc	F	2339	1498.00
---	--	------	------
mPD		0.24	0.49
		nPD:	0.48
		N. weight:	0.33
		Sc. PD:	0.067
		Sc. rank:	320.4

,

PA

Pos.	237	obs:	exp:
aaa	K	20	19.45
aag	K	12	12.55
gtt	V	0	0.44
gtc	V	0	0.38
gta	V	2	0.50
gtg	V	0	0.67
gaa	E	1588	1588.00
gag	E	1092	1092.00
---	--	------	------
mPD		0.51	0.51
		nPD:	1.
		N. weight:	0.0048
		Sc. PD:	0.0034
		Sc. rank:	7.6

,

PA

Pos.	238	obs:	exp:
cct	P	25	799.70
ccc	P	2	545.60
cca	P	1184	1073.00
ccg	P	1499	291.50
caa	Q	0	0.54
cag	Q	1	0.46
act	T	0	0.25
acc	T	0	0.22
aca	T	0	0.45
acg	T	1	0.08
gct	A	0	0.49
gcc	A	0	0.46
gca	A	1	0.90
gcg	A	1	0.15
---	--	------	------
mPD		0.51	0.71
		nPD:	0.72
		N. weight:	1.3
		Sc. PD:	0.58
		Sc. rank:	1943.6

,

PA

Pos.	239	obs:	exp:
tct	S	0	0.84
tcc	S	0	0.79
tca	S	0	1.21
tcg	S	0	0.27
att	I	0	1.42
atc	I	4	1.03
ata	I	0	1.56
atg	M	2	2.00
act	T	3	2.71
acc	T	8	2.38
aca	T	0	5.00
acg	T	0	0.92
aat	N	462	1530.00
aac	N	2230	1162.00
agt	S	0	0.93
agc	S	5	0.96
---	--	------	------
mPD		0.30	0.51
		nPD:	0.59
		N. weight:	0.5
		Sc. PD:	0.16
		Sc. rank:	645.1

,

PA

Pos.	240	obs:	exp:
tct	S	0	0.17
tcc	S	0	0.16
tca	S	0	0.24
tcg	S	0	0.05
agt	S	0	0.19
agc	S	1	0.19
ggt	G	108	425.50
ggc	G	2504	343.80
gga	G	99	1229.00
ggg	G	2	714.80
---	--	------	------
mPD		0.15	0.69
		nPD:	0.21
		N. weight:	2.5
		Sc. PD:	-0.14
		Sc. rank:	226.9

,

PA

Pos.	241	obs:	exp:
ttt	F	0	2.69
ttc	F	6	3.31
tct	S	0	0.50
tcc	S	0	0.47
tca	S	0	0.73
tcg	S	0	0.16
tat	Y	13	121.60
tac	Y	204	95.43
tgt	C	34	1070.00
tgc	C	2453	1417.00
cgt	R	0	0.03
cgc	R	1	0.05
cga	R	0	0.10
cgg	R	0	0.07
agt	S	0	0.56
agc	S	3	0.57
aga	R	0	0.46
agg	R	0	0.28
---	--	------	------
mPD		0.19	0.65
		nPD:	0.29
		N. weight:	0.74
		Sc. PD:	0.015
		Sc. rank:	302.5

,

PA

Pos.	242	obs:	exp:
att	I	2691	962.80
atc	I	13	696.10
ata	I	10	1055.00
---	--	------	------
mPD		0.017	0.66
		nPD:	0.03
		N. weight:	1.4
		Sc. PD:	-0.34
		Sc. rank:	-2036.4

,

PA

Pos.	243	obs:	exp:
gat	D	4	2.25
gac	D	0	1.75
gaa	E	195	1605.00
gag	E	2514	1104.00
ggt	G	0	0.16
ggc	G	0	0.13
gga	G	0	0.45
ggg	G	1	0.26
---	--	------	------
mPD		0.14	0.49
		nPD:	0.28
		N. weight:	0.89
		Sc. PD:	0.011
		Sc. rank:	326.8

,

PA

Pos.	244	obs:	exp:
tct	S	0	0.67
tcc	S	0	0.63
tca	S	0	0.97
tcg	S	0	0.21
agt	S	0	0.74
agc	S	4	0.77
gct	A	0	0.25
gcc	A	1	0.23
gca	A	0	0.45
gcg	A	0	0.07
ggt	G	71	424.90
ggc	G	2605	343.30
gga	G	27	1227.00
ggg	G	6	713.80
---	--	------	------
mPD		0.078	0.69
		nPD:	0.11
		N. weight:	2.7
		Sc. PD:	-0.41
		Sc. rank:	-1684.9

,

PA

Pos.	245	obs:	exp:
cgt	R	0	0.09
cgc	R	0	0.16
cga	R	0	0.30
cgg	R	0	0.22
aat	N	1	0.57
aac	N	0	0.43
aaa	K	100	1647.00
aag	K	2610	1063.00
aga	R	0	1.38
agg	R	3	0.85
---	--	------	------
mPD		0.074	0.48
		nPD:	0.15
		N. weight:	1.1
		Sc. PD:	-0.13
		Sc. rank:	-310.8

,

PA

Pos.	246	obs:	exp:
tta	L	0	295.30
ttg	L	0	509.80
ctt	L	2616	548.10
ctc	L	84	394.90
cta	L	12	473.90
ctg	L	2	492.00
---	--	------	------
mPD		0.070	1.1
		nPD:	0.06
		N. weight:	2.1
		Sc. PD:	-0.43
		Sc. rank:	-2328.5

,

PA

Pos.	247	obs:	exp:
tct	S	1720	456.50
tcc	S	976	428.90
tca	S	16	659.10
tcg	S	2	144.20
agt	S	0	505.10
agc	S	0	520.10
---	--	------	------
mPD		0.47	1.6
		nPD:	0.29
		N. weight:	1.6
		Sc. PD:	0.029
		Sc. rank:	630.8

,

PA

Pos.	248	obs:	exp:
caa	Q	2670	1464.00
cag	Q	44	1250.00
---	--	------	------
mPD		0.032	0.50
		nPD:	0.06
		N. weight:	0.78
		Sc. PD:	-0.16
		Sc. rank:	-843.1

,

PA

Pos.	249	obs:	exp:
tta	L	0	0.54
ttg	L	1	0.94
ctt	L	0	1.01
ctc	L	0	0.73
cta	L	0	0.87
ctg	L	4	0.91
atg	M	2709	2709.00
---	--	------	------
mPD		0.0037	0.0060
		nPD:	0.61
		N. weight:	0.0037
		Sc. PD:	0.0012
		Sc. rank:	4.9

,

PA

Pos.	250	obs:	exp:
tct	S	58	452.60
tcc	S	414	425.30
tca	S	2167	653.50
tcg	S	52	143.00
cct	P	0	1.48
ccc	P	1	1.01
cca	P	4	1.98
ccg	P	0	0.54
act	T	0	0.49
acc	T	0	0.43
aca	T	2	0.91
acg	T	0	0.17
agt	S	0	500.90
agc	S	0	515.70
gct	A	0	3.92
gcc	A	0	3.67
gca	A	16	7.21
gcg	A	0	1.20
---	--	------	------
mPD		0.34	1.6
		nPD:	0.21
		N. weight:	1.3
		Sc. PD:	-0.077
		Sc. rank:	96.9

,

PA

Pos.	251	obs:	exp:
cgt	R	0	1.29
cgc	R	0	2.17
cga	R	0	4.12
cgg	R	0	2.96
aaa	K	2551	1623.00
aag	K	120	1048.00
aga	R	38	18.80
agg	R	3	11.65
gaa	E	2	1.19
gag	E	0	0.81
---	--	------	------
mPD		0.12	0.52
		nPD:	0.23
		N. weight:	0.5
		Sc. PD:	-0.02
		Sc. rank:	89.5

,

PA

Pos.	252	obs:	exp:
caa	Q	2	1.08
cag	Q	0	0.92
aaa	K	10	6.08
aag	K	0	3.92
gat	D	8	5.06
gac	D	1	3.94
gaa	E	2591	1590.00
gag	E	92	1093.00
ggt	G	0	1.57
ggc	G	0	1.27
gga	G	10	4.53
ggg	G	0	2.63
---	--	------	------
mPD		0.088	0.50
		nPD:	0.17
		N. weight:	0.57
		Sc. PD:	-0.052
		Sc. rank:	-54.5

,

PA

Pos.	253	obs:	exp:
gtt	V	6	602.60
gtc	V	0	512.10
gta	V	210	685.10
gtg	V	2498	914.20
---	--	------	------
mPD		0.15	0.74
		nPD:	0.2
		N. weight:	1.2
		Sc. PD:	-0.083
		Sc. rank:	23.0

,

PA

Pos.	254	obs:	exp:
tct	S	0	184.70
tcc	S	0	173.50
tca	S	0	266.60
tcg	S	0	58.36
tgt	C	4	1.72
tgc	C	0	2.28
cat	H	3	1.73
cac	H	0	1.27
cgt	R	0	0.03
cgc	R	0	0.05
cga	R	0	0.10
cgg	R	0	0.07
att	I	1	0.35
atc	I	0	0.26
ata	I	0	0.39
act	T	0	4.43
acc	T	18	3.89
aca	T	0	8.18
acg	T	0	1.50
aat	N	553	891.20
aac	N	1015	676.80
agt	S	1016	204.40
agc	S	82	210.40
aga	R	1	0.46
agg	R	0	0.28
gat	D	0	3.37
gac	D	6	2.63
ggt	G	14	2.35
ggc	G	1	1.90
gga	G	0	6.79
ggg	G	0	3.95
---	--	------	------
mPD		1.0	1.6
		nPD:	0.64
		N. weight:	0.95
		Sc. PD:	0.34
		Sc. rank:	1303.4

,

PA

Pos.	255	obs:	exp:
gct	A	92	665.30
gcc	A	2502	621.80
gca	A	119	1223.00
gcg	A	1	203.50
---	--	------	------
mPD		0.15	0.68
		nPD:	0.22
		N. weight:	1.6
		Sc. PD:	-0.083
		Sc. rank:	199.6

,

PA

Pos.	256	obs:	exp:
cat	H	3	1.73
cac	H	0	1.27
caa	Q	1063	580.80
cag	Q	14	496.20
cgt	R	0	15.44
cgc	R	0	25.85
cga	R	21	49.15
cgg	R	8	35.33
atg	M	1	1.00
act	T	0	0.74
acc	T	0	0.65
aca	T	3	1.36
acg	T	0	0.25
aat	N	1	0.57
aac	N	0	0.43
aaa	K	1109	692.20
aag	K	30	446.80
aga	R	136	224.30
agg	R	324	139.00
ggt	G	0	0.16
ggc	G	0	0.13
gga	G	0	0.45
ggg	G	1	0.26
---	--	------	------
mPD		1.0	1.3
		nPD:	0.79
		N. weight:	0.66
		Sc. PD:	0.34
		Sc. rank:	1020.0

,

PA

Pos.	257	obs:	exp:
att	I	2639	962.40
atc	I	41	695.90
ata	I	33	1055.00
gtt	V	1	0.22
gtc	V	0	0.19
gta	V	0	0.25
gtg	V	0	0.34
---	--	------	------
mPD		0.054	0.66
		nPD:	0.08
		N. weight:	1.3
		Sc. PD:	-0.24
		Sc. rank:	-1143.5

,

PA

Pos.	258	obs:	exp:
tct	S	0	0.67
tcc	S	0	0.63
tca	S	0	0.97
tcg	S	0	0.21
tat	Y	2	2.24
tac	Y	2	1.76
cat	H	0	0.58
cac	H	1	0.42
caa	Q	1	0.54
cag	Q	0	0.46
act	T	0	0.98
acc	T	4	0.86
aca	T	0	1.82
acg	T	0	0.33
aat	N	0	2.27
aac	N	4	1.73
aaa	K	2	1.22
aag	K	0	0.78
agt	S	0	0.74
agc	S	4	0.77
gct	A	0	0.25
gcc	A	0	0.23
gca	A	0	0.45
gcg	A	1	0.07
gat	D	28	68.06
gac	D	93	52.94
gaa	E	2484	1524.00
gag	E	88	1048.00
---	--	------	------
mPD		0.18	0.55
		nPD:	0.32
		N. weight:	0.56
		Sc. PD:	0.03
		Sc. rank:	303.9

,

PA

Pos.	259	obs:	exp:
tta	L	0	0.11
ttg	L	0	0.19
tct	S	0	0.67
tcc	S	0	0.63
tca	S	4	0.97
tcg	S	0	0.21
tat	Y	1	0.56
tac	Y	0	0.44
ctt	L	0	0.20
ctc	L	0	0.15
cta	L	1	0.17
ctg	L	0	0.18
cct	P	29	797.10
ccc	P	9	543.80
cca	P	2618	1070.00
ccg	P	45	290.50
caa	Q	1	0.54
cag	Q	0	0.46
cgt	R	0	0.06
cgc	R	0	0.11
cga	R	0	0.20
cgg	R	0	0.14
act	T	0	0.98
acc	T	0	0.86
aca	T	4	1.82
acg	T	0	0.33
agt	S	0	0.74
agc	S	0	0.77
aga	R	2	0.92
agg	R	0	0.57
---	--	------	------
mPD		0.072	0.72
		nPD:	0.1
		N. weight:	1.2
		Sc. PD:	-0.19
		Sc. rank:	-865.9

,

PA

Pos.	260	obs:	exp:
ttt	F	117	1216.00
ttc	F	2597	1498.00
---	--	------	------
mPD		0.083	0.49
		nPD:	0.17
		N. weight:	0.62
		Sc. PD:	-0.062
		Sc. rank:	-100.2

,

PA			
Pos.	261	obs:	exp:
ttt	F	3	1.34
ttc	F	0	1.66
tta	L	46	283.40
ttg	L	2489	489.10
tct	S	0	1.35
tcc	S	0	1.26
tca	S	0	1.94
tcg	S	8	0.43
tgg	W	23	23.00
ctt	L	0	525.90
ctc	L	0	378.90
cta	L	0	454.70
ctg	L	69	472.00
atg	M	3	3.00
act	T	0	15.25
acc	T	0	13.41
aca	T	1	28.17
acg	T	61	5.17
agt	S	0	1.49
agc	S	0	1.53
gtt	V	0	2.44
gtc	V	0	2.08
gta	V	0	2.78
gtg	V	11	3.71
---	--	------	------
mPD		0.21	1.2
		nPD:	0.17
		N. weight:	2.2
		Sc. PD:	-0.22
		Sc. rank:	-359.6

,

PA			
Pos.	262	obs:	exp:
tct	S	0	0.50
tcc	S	0	0.47
tca	S	0	0.73
tcg	S	0	0.16
cgt	R	0	39.96
cgc	R	0	66.92
cga	R	0	127.20
cgg	R	1	91.46
aaa	K	134	876.40
aag	K	1308	565.60
agt	S	3	0.56
agc	S	0	0.57
aga	R	69	580.60
agg	R	1196	359.80
gaa	E	0	0.59
gag	E	1	0.41
ggt	G	0	0.31
ggc	G	0	0.25
gga	G	0	0.91
ggg	G	2	0.53
---	--	------	------
mPD		0.64	1.2
		nPD:	0.52
		N. weight:	1.2
		Sc. PD:	0.28
		Sc. rank:	1284.7

,

PA			
Pos.	263	obs:	exp:
tct	S	0	0.67
tcc	S	0	0.63
tca	S	3	0.97
tcg	S	0	0.21
cgt	R	0	0.09
cgc	R	0	0.16
cga	R	0	0.30
cgg	R	0	0.22
att	I	0	0.35
atc	I	0	0.26
ata	I	1	0.39
atg	M	22	22.00
act	T	108	458.70
acc	T	32	403.30
aca	T	981	847.40
acg	T	744	155.50
aat	N	89	50.59
aac	N	0	38.41
aaa	K	213	160.40
aag	K	51	103.60
agt	S	1	0.74
agc	S	0	0.77
aga	R	3	1.38
agg	R	0	0.85
gtt	V	0	0.67
gtc	V	0	0.57
gta	V	0	0.76
gtg	V	3	1.01
gct	A	1	11.77
gcc	A	0	11.00
gca	A	14	21.64
gcg	A	33	3.60
gat	D	6	3.94
gac	D	1	3.06
gaa	E	372	234.00
gag	E	23	161.00
ggt	G	0	2.04
ggc	G	0	1.65
gga	G	13	5.89
ggg	G	0	3.43
---	--	------	------
mPD		1.3	1.4
		nPD:	0.9
		N. weight:	0.74
		Sc. PD:	0.45
		Sc. rank:	1169.9

,

PA			
Pos.	264	obs:	exp:
act	T	3	667.60
acc	T	1	586.90
aca	T	2621	1233.00
acg	T	89	226.30
---	--	------	------
mPD		0.066	0.68
		nPD:	0.1
		N. weight:	1.
		Sc. PD:	-0.17
		Sc. rank:	-779.7

,

PA			
Pos.	265	obs:	exp:
tct	S	0	0.34
tcc	S	0	0.32
tca	S	2	0.49
tcg	S	0	0.11
cct	P	4	800.00
ccc	P	1	545.80
cca	P	2638	1074.00
ccg	P	68	291.60
agt	S	0	0.37
agc	S	0	0.38
gct	A	0	0.25
gcc	A	0	0.23
gca	A	1	0.45
gcg	A	0	0.07
---	--	------	------
mPD		0.055	0.71
		nPD:	0.08
		N. weight:	1.2
		Sc. PD:	-0.23
		Sc. rank:	-1124.0

,

PA			
Pos.	266	obs:	exp:
cat	H	0	4.61
cac	H	8	3.39
cgt	R	122	85.42
cgc	R	2492	143.00
cga	R	80	272.00
cgg	R	11	195.50
aga	R	1	1241.00
agg	R	0	769.10
---	--	------	------
mPD		0.16	0.95
		nPD:	0.16
		N. weight:	3.8
		Sc. PD:	-0.39
		Sc. rank:	-666.2

,

PA			
Pos.	267	obs:	exp:
tta	L	0	13.82
ttg	L	0	23.85
tct	S	124	31.96
tcc	S	66	30.03
tca	S	0	46.14
tcg	S	0	10.10
tat	Y	1	0.56
tac	Y	0	0.44
ctt	L	93	25.65
ctc	L	11	18.48
cta	L	23	22.18
ctg	L	0	23.02
cct	P	1186	703.50
ccc	P	1107	480.00
cca	P	91	944.10
ccg	P	0	256.40
cat	H	2	4.04
cac	H	5	2.96
act	T	0	0.98
acc	T	4	0.86
aca	T	0	1.82
acg	T	0	0.33
agt	S	0	35.36
agc	S	0	36.41
gct	A	0	0.25
gcc	A	1	0.23
gca	A	0	0.45
gcg	A	0	0.07
---	--	------	------
mPD		0.77	1.0
		nPD:	0.75
		N. weight:	0.91
		Sc. PD:	0.43
		Sc. rank:	1370.2

,

PA			
Pos.	268	obs:	exp:
ttt	F	1	3.58
ttc	F	7	4.42
tta	L	0	290.60
ttg	L	2	501.50
ctt	L	79	539.20
ctc	L	2538	388.50
cta	L	48	466.20
ctg	L	3	484.00
att	I	3	12.06
atc	I	30	8.72
ata	I	1	13.22
gtt	V	0	0.44
gtc	V	2	0.38
gta	V	0	0.50
gtg	V	0	0.67
---	--	------	------
mPD		0.13	1.2
		nPD:	0.11
		N. weight:	2.4
		Sc. PD:	-0.37
		Sc. rank:	-1543.1

,

PA			
Pos.	269	obs:	exp:
cgt	R	0	76.80
cgc	R	0	128.60
cga	R	15	244.50
cgg	R	0	175.80
att	I	0	1.06
atc	I	0	0.77
ata	I	3	1.17
aaa	K	273	167.10
aag	K	2	107.90
aga	R	2380	1116.00
agg	R	38	691.50
gaa	E	1	0.59
gag	E	0	0.41
ggt	G	0	0.31
ggc	G	0	0.25
gga	G	2	0.91
ggg	G	0	0.53
---	--	------	------
mPD		0.23	1.1
		nPD:	0.21
		N. weight:	0.96
		Sc. PD:	-0.057
		Sc. rank:	65.3

,

PA			
Pos.	270	obs:	exp:
tta	L	444	294.50
ttg	L	2205	508.30
ctt	L	4	546.50
ctc	L	0	393.70
cta	L	13	472.50
ctg	L	40	490.50
atg	M	8	8.00
---	--	------	------
mPD		0.33	1.1
		nPD:	0.29
		N. weight:	1.8
		Sc. PD:	0.033
		Sc. rank:	694.3

,

PA

Pos.	271	obs:	exp:
cct	P	2663	800.90
ccc	P	8	546.40
cca	P	35	1075.00
ccg	P	8	291.90
---	--	------	------
mPD		0.037	0.70
		nPD:	0.05
		N. weight:	1.6
		Sc. PD:	-0.34
		Sc. rank:	-1944.6

,

PA

Pos.	272	obs:	exp:
tct	S	1	2.35
tcc	S	0	2.21
tca	S	0	3.40
tcg	S	0	0.74
tat	Y	2	1.12
tac	Y	0	0.88
tgt	C	8	3.44
tgc	C	0	4.56
aat	N	119	68.77
aac	N	2	52.23
agt	S	13	2.61
agc	S	0	2.68
gtt	V	3	0.67
gtc	V	0	0.57
gta	V	0	0.76
gtg	V	0	1.01
gct	A	1	0.25
gcc	A	0	0.23
gca	A	0	0.45
gcg	A	0	0.07
gat	D	2449	1402.00
gac	D	44	1091.00
gaa	E	19	20.74
gag	E	16	14.26
ggt	G	31	5.80
ggc	G	6	4.69
gga	G	0	16.76
ggg	G	0	9.75
---	--	------	------
mPD		0.21	0.66
		nPD:	0.32
		N. weight:	0.74
		Sc. PD:	0.034
		Sc. rank:	386.0

,

PA

Pos.	273	obs:	exp:
ggt	G	36	425.70
ggc	G	39	343.90
gga	G	544	1229.00
ggg	G	2095	715.10
---	--	------	------
mPD		0.36	0.68
		nPD:	0.53
		N. weight:	0.88
		Sc. PD:	0.23
		Sc. rank:	1009.9

,

PA

Pos.	274	obs:	exp:
tta	L	0	0.44
ttg	L	0	0.75
tct	S	3	0.50
tcc	S	0	0.47
tca	S	0	0.73
tcg	S	0	0.16
ctt	L	4	0.81
ctc	L	0	0.58
cta	L	0	0.70
ctg	L	0	0.73
cct	P	1774	797.90
ccc	P	908	544.40
cca	P	21	1071.00
ccg	P	1	290.90
cat	H	1	0.58
cac	H	0	0.42
caa	Q	1	0.54
cag	Q	0	0.46
agt	S	0	0.56
agc	S	0	0.57
gct	A	1	0.25
gcc	A	0	0.23
gca	A	0	0.45
gcg	A	0	0.07
---	--	------	------
mPD		0.46	0.71
		nPD:	0.65
		N. weight:	0.98
		Sc. PD:	0.36
		Sc. rank:	1356.6

,

PA

Pos.	275	obs:	exp:
ttt	F	1	0.90
ttc	F	1	1.10
tta	L	0	129.70
ttg	L	0	223.90
tct	S	0	0.67
tcc	S	4	0.63
tca	S	0	0.97
tcg	S	0	0.21
ctt	L	1165	240.70
ctc	L	24	173.40
cta	L	3	208.10
ctg	L	0	216.10
cct	P	1138	445.30
ccc	P	370	303.80
cca	P	1	597.60
ccg	P	0	162.30
cat	H	2	1.15
cac	H	0	0.85
att	I	4	1.42
atc	I	0	1.03
ata	I	0	1.56
agt	S	0	0.74
agc	S	0	0.77
gct	A	1	0.25
gcc	A	0	0.23
gca	A	0	0.45
gcg	A	0	0.07
---	--	------	------
mPD		0.76	1.5
		nPD:	0.52
		N. weight:	1.6
		Sc. PD:	0.38
		Sc. rank:	1735.0

,

PA

Pos.	276	obs:	exp:
tct	S	0	0.17
tcc	S	0	0.16
tca	S	0	0.24
tcg	S	0	0.05
tgt	C	597	1168.00
tgc	C	2116	1545.00
agt	S	1	0.19
agc	S	0	0.19
---	--	------	------
mPD		0.34	0.49
		nPD:	0.7
		N. weight:	0.15
		Sc. PD:	0.061
		Sc. rank:	208.8

,

PA

Pos.	277	obs:	exp:
ttt	F	30	17.48
ttc	F	9	21.52
tct	S	1226	250.80
tcc	S	254	235.60
tca	S	10	362.10
tcg	S	1	79.24
tat	Y	48	28.01
tac	Y	2	21.99
cct	P	3	1.48
ccc	P	0	1.01
cca	P	2	1.98
ccg	P	0	0.54
cat	H	1020	612.00
cac	H	41	449.00
cgt	R	1	0.03
cgc	R	0	0.05
cga	R	0	0.10
cgg	R	0	0.07
act	T	61	15.00
acc	T	0	13.19
aca	T	0	27.72
acg	T	0	5.09
aat	N	3	1.71
aac	N	0	1.29
agt	S	0	277.50
agc	S	0	285.70
aga	R	0	0.46
agg	R	0	0.28
gct	A	3	0.74
gcc	A	0	0.69
gca	A	0	1.35
gcg	A	0	0.22
---	--	------	------
mPD		1.2	1.9
		nPD:	0.63
		N. weight:	1.3
		Sc. PD:	0.47
		Sc. rank:	1799.7

,

PA

Pos.	278	obs:	exp:
caa	Q	473	1464.00
cag	Q	2241	1250.00
---	--	------	------
mPD		0.29	0.50
		nPD:	0.58
		N. weight:	0.42
		Sc. PD:	0.13
		Sc. rank:	533.6

,

PA

Pos.	279	obs:	exp:
cgt	R	9	85.51
cgc	R	0	143.20
cga	R	208	272.30
cgg	R	2432	195.70
aaa	K	0	3.04
aag	K	5	1.96
aga	R	16	1242.00
agg	R	44	770.00
---	--	------	------
mPD		0.21	0.94
		nPD:	0.22
		N. weight:	3.2
		Sc. PD:	-0.15
		Sc. rank:	516.6

,

PA

Pos.	280	obs:	exp:
tct	S	8	456.20
tcc	S	165	428.60
tca	S	2535	658.60
tcg	S	4	144.10
act	T	0	0.49
acc	T	0	0.43
aca	T	2	0.91
acg	T	0	0.17
agt	S	0	504.80
agc	S	0	519.70
---	--	------	------
mPD		0.12	1.6
		nPD:	0.08
		N. weight:	1.7
		Sc. PD:	-0.33
		Sc. rank:	-1658.0

,

PA

Pos.	281	obs:	exp:
aaa	K	649	1649.00
aag	K	2065	1065.00
---	--	------	------
mPD		0.36	0.48
		nPD:	0.76
		N. weight:	0.41
		Sc. PD:	0.2
		Sc. rank:	623.9

,

PA

Pos.	282	obs:	exp:
ttt	F	130	1216.00
ttc	F	2584	1498.00
---	--	------	------
mPD		0.091	0.49
		nPD:	0.18
		N. weight:	0.6
		Sc. PD:	-0.05
		Sc. rank:	-26.1

,

PA

Pos.	283	obs:	exp:
tta	L	23	295.30
ttg	L	1284	509.80
ctt	L	32	548.10
ctc	L	1	394.90
cta	L	61	473.90
ctg	L	1313	492.00
---	--	------	------
mPD		0.58	1.1
		nPD:	0.51
		N. weight:	1.2
		Sc. PD:	0.28
		Sc. rank:	1276.1

,

PA

Pos.	284	obs:	exp:
tta	L	6	295.00
ttg	L	28	509.20
ctt	L	23	547.50
ctc	L	0	394.40
cta	L	195	473.40
ctg	L	2459	491.40
atg	M	3	3.00
---	--	------	------
mPD		0.18	1.1
		nPD:	0.16
		N. weight:	1.9
		Sc. PD:	-0.21
		Sc. rank:	-501.0

,

PA

Pos.	285	obs:	exp:
tta	L	0	0.44
ttg	L	3	0.75
ctt	L	0	0.81
ctc	L	0	0.58
cta	L	0	0.70
ctg	L	1	0.73
atg	M	2710	2710.00
---	--	------	------
mPD		0.0029	0.0048
		nPD:	0.61
		N. weight:	0.0029
		Sc. PD:	0.00096
		Sc. rank:	3.8

,

PA

Pos.	286	obs:	exp:
gat	D	2450	1527.00
gac	D	264	1187.00
---	--	------	------
mPD		0.18	0.49
		nPD:	0.36
		N. weight:	0.41
		Sc. PD:	0.035
		Sc. rank:	247.1

,

PA

Pos.	287	obs:	exp:
tct	S	4	1.51
tcc	S	5	1.42
tca	S	0	2.19
tcg	S	0	0.48
agt	S	0	1.68
agc	S	0	1.72
gct	A	2280	663.10
gcc	A	379	619.80
gca	A	45	1219.00
gcg	A	1	202.90
---	--	------	------
mPD		0.28	0.69
		nPD:	0.4
		N. weight:	1.3
		Sc. PD:	0.18
		Sc. rank:	1020.4

,

PA

Pos.	288	obs:	exp:
tta	L	391	293.20
ttg	L	183	506.00
ctt	L	2	544.10
ctc	L	1	392.00
cta	L	141	470.40
ctg	L	1976	488.30
atg	M	20	20.00
---	--	------	------
mPD		0.66	1.1
		nPD:	0.58
		N. weight:	1.4
		Sc. PD:	0.41
		Sc. rank:	1724.2

,

PA

Pos.	289	obs:	exp:
aaa	K	2611	1649.00
aag	K	103	1065.00
---	--	------	------
mPD		0.073	0.48
		nPD:	0.15
		N. weight:	0.52
		Sc. PD:	-0.059
		Sc. rank:	-156.1

,

PA

Pos.	290	obs:	exp:
tta	L	1044	295.30
ttg	L	426	509.80
ctt	L	0	548.10
ctc	L	2	394.90
cta	L	1117	473.90
ctg	L	125	492.00
---	--	------	------
mPD		0.82	1.1
		nPD:	0.72
		N. weight:	1.1
		Sc. PD:	0.48
		Sc. rank:	1592.6

,

PA

Pos.	291	obs:	exp:
tct	S	0	454.80
tcc	S	0	427.30
tca	S	0	656.60
tcg	S	0	143.70
aat	N	3	1.71
aac	N	0	1.29
agt	S	2134	503.30
agc	S	570	518.20
ggt	G	7	1.10
ggc	G	0	0.89
gga	G	0	3.17
ggg	G	0	1.84
---	--	------	------
mPD		0.34	1.6
		nPD:	0.21
		N. weight:	1.7
		Sc. PD:	-0.1
		Sc. rank:	107.9

,

PA

Pos.	292	obs:	exp:
att	I	2660	951.40
atc	I	16	687.90
ata	I	6	1043.00
gtt	V	31	7.11
gtc	V	1	6.04
gta	V	0	8.08
gtg	V	0	10.78
---	--	------	------
mPD		0.040	0.68
		nPD:	0.06
		N. weight:	1.4
		Sc. PD:	-0.3
		Sc. rank:	-1659.2

,

PA

Pos.	293	obs:	exp:
gtt	V	0	0.44
gtc	V	0	0.38
gta	V	0	0.50
gtg	V	2	0.67
gat	D	1	2.25
gac	D	3	1.75
gaa	E	2328	1605.00
gag	E	380	1103.00
---	--	------	------
mPD		0.25	0.49
		nPD:	0.51
		N. weight:	0.25
		Sc. PD:	0.058
		Sc. rank:	267.3

,

PA

Pos.	294	obs:	exp:
aat	N	3	3.41
aac	N	3	2.59
gat	D	449	1520.00
gac	D	2254	1183.00
gaa	E	5	2.96
gag	E	0	2.04
---	--	------	------
mPD		0.29	0.50
		nPD:	0.57
		N. weight:	0.49
		Sc. PD:	0.15
		Sc. rank:	612.8

,

PA

Pos.	295	obs:	exp:
cct	P	188	800.90
ccc	P	33	546.40
cca	P	365	1075.00
ccg	P	2128	291.90
---	--	------	------
mPD		0.36	0.70
		nPD:	0.51
		N. weight:	1.9
		Sc. PD:	0.45
		Sc. rank:	2038.3

,

PA

Pos.	296	obs:	exp:
tct	S	0	449.90
tcc	S	0	422.80
tca	S	0	649.60
tcg	S	0	142.20
aat	N	24	18.19
aac	N	8	13.81
agt	S	2399	497.90
agc	S	276	512.70
gat	D	1	0.56
gac	D	0	0.44
ggt	G	5	0.94
ggc	G	1	0.76
gga	G	0	2.72
ggg	G	0	1.58
---	--	------	------
mPD		0.22	1.6
		nPD:	0.13
		N. weight:	1.9
		Sc. PD:	-0.26
		Sc. rank:	-946.0

,

PA

Pos.	297	obs:	exp:
tat	Y	4	3.92
tac	Y	3	3.08
cat	H	1687	1561.00
cac	H	1020	1146.00
---	--	------	------
mPD		0.48	0.49
		nPD:	0.96
		N. weight:	0.0094
		Sc. PD:	0.0063
		Sc. rank:	14.9

,

PA

Pos.	298	obs:	exp:
aaa	K	0	0.61
aag	K	1	0.39
gaa	E	1326	1608.00
gag	E	1387	1105.00
---	--	------	------
mPD		0.50	0.48
		nPD:	1.04
		N. weight:	0.035
		Sc. PD:	0.026
		Sc. rank:	55.4

,

PA

Pos.	299	obs:	exp:
ggt	G	12	425.70
ggc	G	0	343.90
gga	G	271	1229.00
ggg	G	2431	715.10
---	--	------	------
mPD		0.19	0.68
		nPD:	0.27
		N. weight:	1.4
		Sc. PD:	0.0065
		Sc. rank:	436.5

,

PA

Pos.	300	obs:	exp:
gat	D	2	1.12
gac	D	0	0.88
gaa	E	1221	1607.00
gag	E	1491	1105.00
---	--	------	------
mPD		0.50	0.48
		nPD:	1.03
		N. weight:	0.063
		Sc. PD:	0.046
		Sc. rank:	100.1

,

PA

Pos.	301	obs:	exp:
ggt	G	2	425.70
ggc	G	0	343.90
gga	G	2159	1229.00
ggg	G	553	715.10
---	--	------	------
mPD		0.33	0.68
		nPD:	0.48
		N. weight:	0.57
		Sc. PD:	0.12
		Sc. rank:	556.5

,

PA

Pos.	302	obs:	exp:
tta	L	1	0.11
ttg	L	0	0.19
ctt	L	0	0.20
ctc	L	0	0.15
cta	L	0	0.17
ctg	L	0	0.18
att	I	12	961.40
atc	I	3	695.10
ata	I	2695	1054.00
gtt	V	0	0.67
gtc	V	1	0.57
gta	V	1	0.76
gtg	V	1	1.01
---	--	------	------
mPD		0.015	0.66
		nPD:	0.02
		N. weight:	1.3
		Sc. PD:	-0.32
		Sc. rank:	-1900.1

,

PA

Pos.	303	obs:	exp:
cct	P	3	800.90
ccc	P	0	546.40
cca	P	2284	1075.00
ccg	P	427	291.90
---	--	------	------
mPD		0.27	0.70
		nPD:	0.38
		N. weight:	1.
		Sc. PD:	0.11
		Sc. rank:	672.3

,

PA

Pos.	304	obs:	exp:
tta	L	146	294.20
ttg	L	0	507.70
ctt	L	61	545.90
ctc	L	2	393.30
cta	L	2469	472.00
ctg	L	25	490.00
att	I	4	3.90
atc	I	0	2.82
ata	I	7	4.28
---	--	------	------
mPD		0.18	1.1
		nPD:	0.15
		N. weight:	2.
		Sc. PD:	-0.23
		Sc. rank:	-594.7

,

PA

Pos.	305	obs:	exp:
ttt	F	12	5.38
ttc	F	0	6.62
tat	Y	2642	1501.00
tac	Y	37	1178.00
tgt	C	8	3.44
tgc	C	0	4.56
cat	H	15	8.65
cac	H	0	6.35
---	--	------	------
mPD		0.053	0.52
		nPD:	0.1
		N. weight:	0.75
		Sc. PD:	-0.12
		Sc. rank:	-559.3

,

PA			
Pos.	306	obs:	exp:
gat	D	2656	1527.00
gac	D	58	1187.00
---	--	------	------
mPD		0.042	0.49
		nPD:	0.09
		N. weight:	0.7
		Sc. PD:	-0.13
		Sc. rank:	-602.5

PA			
Pos.	307	obs:	exp:
gct	A	29	665.30
gcc	A	5	621.80
gca	A	2644	1223.00
gcg	A	36	203.50
---	--	------	------
mPD		0.051	0.68
		nPD:	0.07
		N. weight:	1.
		Sc. PD:	-0.19
		Sc. rank:	-972.2

PA			
Pos.	308	obs:	exp:
tta	L	0	0.22
ttg	L	0	0.38
ctt	L	0	0.40
ctc	L	1	0.29
cta	L	1	0.35
ctg	L	0	0.36
att	I	49	930.10
atc	I	2566	672.50
ata	I	7	1019.00
act	T	0	6.40
acc	T	26	5.62
aca	T	0	11.81
acg	T	0	2.17
gtt	V	1	14.21
gtc	V	51	12.08
gta	V	12	16.16
gtg	V	0	21.56
---	--	------	------
mPD		0.12	0.73
		nPD:	0.16
		N. weight:	1.8
		Sc. PD:	-0.19
		Sc. rank:	-431.8

PA			
Pos.	309	obs:	exp:
cgt	R	0	1.07
cgc	R	0	1.80
cga	R	0	3.42
cgg	R	0	2.46
atg	M	2	2.00
aat	N	1	1.14
aac	N	1	0.86
aaa	K	1344	1626.00
aag	K	1332	1050.00
aga	R	20	15.59
agg	R	14	9.66
---	--	------	------
mPD		0.53	0.51
		nPD:	1.03
		N. weight:	0.042
		Sc. PD:	0.031
		Sc. rank:	67.0

PA			
Pos.	310	obs:	exp:
tgt	C	54	1168.00
tgc	C	2660	1546.00
---	--	------	------
mPD		0.039	0.49
		nPD:	0.08
		N. weight:	0.69
		Sc. PD:	-0.13
		Sc. rank:	-621.5

PA			
Pos.	311	obs:	exp:
tta	L	0	0.11
ttg	L	1	0.19
ctt	L	0	0.20
ctc	L	0	0.15
cta	L	0	0.17
ctg	L	0	0.18
att	I	0	2.13
atc	I	0	1.54
ata	I	6	2.33
atg	M	2707	2707.00
---	--	------	------
mPD		0.0052	0.0056
		nPD:	0.92
		N. weight:	0.0039
		Sc. PD:	0.0025
		Sc. rank:	6.2

PA			
Pos.	312	obs:	exp:
cgt	R	0	0.79
cgc	R	0	1.32
cga	R	0	2.51
cgg	R	0	1.81
aat	N	43	25.01
aac	N	1	18.99
aaa	K	67	1607.00
aag	K	2578	1038.00
aga	R	2	11.46
agg	R	23	7.11
---	--	------	------
mPD		0.099	0.52
		nPD:	0.19
		N. weight:	1.2
		Sc. PD:	-0.089
		Sc. rank:	-30.9

PA			
Pos.	313	obs:	exp:
tct	S	0	0.17
tcc	S	0	0.16
tca	S	1	0.24
tcg	S	0	0.05
act	T	13	667.30
acc	T	2	586.70
aca	T	2468	1233.00
acg	T	230	226.20
agt	S	0	0.19
agc	S	0	0.19
---	--	------	------
mPD		0.17	0.68
		nPD:	0.24
		N. weight:	0.89
		Sc. PD:	-0.022
		Sc. rank:	189.5

PA			
Pos.	314	obs:	exp:
ttt	F	1185	1216.00
ttc	F	1529	1498.00
---	--	------	------
mPD		0.49	0.49
		nPD:	1.
		N. weight:	0.0026
		Sc. PD:	0.0019
		Sc. rank:	4.2

PA			
Pos.	315	obs:	exp:
ttt	F	2239	1202.00
ttc	F	443	1480.00
tta	L	0	3.37
ttg	L	31	5.82
ctt	L	0	6.26
ctc	L	0	4.51
cta	L	0	5.41
ctg	L	0	5.62
gtt	V	1	0.22
gtc	V	0	0.19
gta	V	0	0.25
gtg	V	0	0.34
---	--	------	------
mPD		0.29	0.52
		nPD:	0.56
		N. weight:	0.49
		Sc. PD:	0.14
		Sc. rank:	604.6

PA			
Pos.	316	obs:	exp:
ggt	G	41	425.70
ggc	G	2178	343.90
gga	G	418	1229.00
ggg	G	77	715.10
---	--	------	------
mPD		0.33	0.68
		nPD:	0.48
		N. weight:	1.8
		Sc. PD:	0.37
		Sc. rank:	1767.7

PA			
Pos.	317	obs:	exp:
tgg	W	2714	2714.00
---	--	------	------
mPD		0	0
		nPD:	1.
		N. weight:	0.
		Sc. PD:	0
		Sc. rank:	0

PA			
Pos.	318	obs:	exp:
cgt	R	0	0.03
cgc	R	0	0.05
cga	R	0	0.10
cgg	R	0	0.07
act	T	0	0.25
acc	T	0	0.22
aca	T	1	0.45
acg	T	0	0.08
aaa	K	2660	1648.00
aag	K	51	1063.00
aga	R	1	0.46
agg	R	0	0.28
gaa	E	1	0.59
gag	E	0	0.41
---	--	------	------
mPD		0.039	0.48
		nPD:	0.08
		N. weight:	0.6
		Sc. PD:	-0.11
		Sc. rank:	-530.7

PA			
Pos.	319	obs:	exp:
aaa	K	0	1.22
aag	K	2	0.78
gat	D	19	12.94
gac	D	4	10.06
gaa	E	196	1593.00
gag	E	2493	1096.00
---	--	------	------
mPD		0.15	0.49
		nPD:	0.31
		N. weight:	0.89
		Sc. PD:	0.032
		Sc. rank:	416.1

PA			
Pos.	320	obs:	exp:
cct	P	2271	800.90
ccc	P	427	546.40
cca	P	13	1075.00
ccg	P	3	291.90
---	--	------	------
mPD		0.28	0.70
		nPD:	0.39
		N. weight:	1.2
		Sc. PD:	0.14
		Sc. rank:	852.0

PA			
Pos.	321	obs:	exp:
tta	L	0	0.11
ttg	L	0	0.19
tct	S	0	13.62
tcc	S	0	12.80
tca	S	0	19.67
tcg	S	0	4.30
tat	Y	2	2.80
tac	Y	3	2.20
ctt	L	0	0.20
ctc	L	1	0.15
cta	L	0	0.17
ctg	L	0	0.18
cat	H	5	159.20
cac	H	271	116.80
caa	Q	45	26.96
cag	Q	5	23.04
cgt	R	0	0.09
cgc	R	1	0.16
cga	R	0	0.30
cgg	R	0	0.22
att	I	2	1.42
atc	I	2	1.03
ata	I	0	1.56
act	T	11	5.90
acc	T	12	5.19
aca	T	0	10.91
acg	T	1	2.00
aat	N	176	1217.00
aac	N	1965	924.10
aaa	K	123	75.97
aag	K	2	49.03
agt	S	0	15.08
agc	S	81	15.52
aga	R	2	1.38
agg	R	0	0.85
gat	D	0	2.25
gac	D	4	1.75
---	--	------	------
mPD		0.55	0.90
		nPD:	0.61
		N. weight:	0.86
		Sc. PD:	0.29
		Sc. rank:	1142.5

,

PA			
Pos.	322	obs:	exp:
att	I	434	956.00
atc	I	44	691.20
ata	I	2217	1048.00
gtt	V	3	4.22
gtc	V	1	3.59
gta	V	15	4.80
gtg	V	0	6.40
---	--	------	------
mPD		0.31	0.67
		nPD:	0.46
		N. weight:	0.66
		Sc. PD:	0.12
		Sc. rank:	617.2

,

PA			
Pos.	323	obs:	exp:
att	I	23	430.30
atc	I	1179	311.10
ata	I	11	471.60
act	T	0	1.72
acc	T	7	1.51
aca	T	0	3.18
acg	T	0	0.58
gtt	V	37	331.30
gtc	V	1425	281.50
gta	V	27	376.60
gtg	V	3	502.50
gct	A	0	0.49
gcc	A	2	0.46
gca	A	0	0.90
gcg	A	0	0.15
---	--	------	------
mPD		0.57	1.2
		nPD:	0.46
		N. weight:	2.
		Sc. PD:	0.37
		Sc. rank:	1828.3

,

PA			
Pos.	324	obs:	exp:
aaa	K	2357	1649.00
aag	K	357	1065.00
---	--	------	------
mPD		0.23	0.48
		nPD:	0.48
		N. weight:	0.24
		Sc. PD:	0.05
		Sc. rank:	239.7

,

PA			
Pos.	325	obs:	exp:
tct	S	1	2.86
tcc	S	0	2.69
tca	S	16	4.13
tcg	S	0	0.90
cct	P	802	795.00
ccc	P	19	542.40
cca	P	1584	1067.00
ccg	P	289	289.80
caa	Q	3	1.62
cag	Q	0	1.38
agt	S	0	3.16
agc	S	0	3.26
---	--	------	------
mPD		0.57	0.72
		nPD:	0.78
		N. weight:	0.32
		Sc. PD:	0.16
		Sc. rank:	498.8

,

PA			
Pos.	326	obs:	exp:
cat	H	2677	1565.00
cac	H	36	1148.00
aat	N	1	0.57
aac	N	0	0.43
---	--	------	------
mPD		0.027	0.49
		nPD:	0.06
		N. weight:	0.71
		Sc. PD:	-0.15
		Sc. rank:	-817.7

,

PA			
Pos.	327	obs:	exp:
aaa	K	6	13.37
aag	K	16	8.63
gtt	V	0	1.11
gtc	V	0	0.94
gta	V	0	1.26
gtg	V	5	1.68
gat	D	5	5.06
gac	D	4	3.94
gaa	E	254	1571.00
gag	E	2398	1081.00
ggt	G	0	4.08
ggc	G	0	3.29
gga	G	0	11.78
ggg	G	26	6.85
---	--	------	------
mPD		0.22	0.53
		nPD:	0.41
		N. weight:	0.81
		Sc. PD:	0.11
		Sc. rank:	633.0

,

PA			
Pos.	328	obs:	exp:
cgt	R	0	4.14
cgc	R	0	6.92
cga	R	0	13.17
cgg	R	0	9.46
aat	N	0	0.57
aac	N	1	0.43
aaa	K	2403	1569.00
aag	K	179	1013.00
aga	R	128	60.08
agg	R	3	37.23
---	--	------	------
mPD		0.22	0.60
		nPD:	0.36
		N. weight:	0.43
		Sc. PD:	0.04
		Sc. rank:	270.1

,

PA			
Pos.	329	obs:	exp:
tgt	C	0	0.43
tgc	C	1	0.57
ggt	G	48	425.50
ggc	G	2599	343.80
gga	G	43	1229.00
ggg	G	23	714.80
---	--	------	------
mPD		0.082	0.69
		nPD:	0.12
		N. weight:	2.7
		Sc. PD:	-0.39
		Sc. rank:	-1518.0

,

PA			
Pos.	330	obs:	exp:
att	I	4	908.90
atc	I	17	657.10
ata	I	2541	996.00
atg	M	13	13.00
act	T	0	5.41
acc	T	0	4.76
aca	T	22	10.00
acg	T	0	1.83
gtt	V	0	25.98
gtc	V	0	22.08
gta	V	59	29.54
gtg	V	58	39.41
---	--	------	------
mPD		0.16	0.77
		nPD:	0.21
		N. weight:	1.3
		Sc. PD:	-0.069
		Sc. rank:	143.0

,

PA

Pos.	331	obs:	exp:
tct	S	0	1.18
tcc	S	0	1.11
tca	S	0	1.70
tcg	S	0	0.37
aat	N	1568	1539.00
aac	N	1139	1168.00
agt	S	2	1.30
agc	S	5	1.34
---	--	------	------
mPD		0.49	0.50
		nPD:	0.99
		N. weight:	0.0072
		Sc. PD:	0.005
		Sc. rank:	11.5

,

PA

Pos.	332	obs:	exp:
tta	L	0	0.98
ttg	L	0	1.69
tct	S	2	8.58
tcc	S	23	8.06
tca	S	26	12.38
tcg	S	0	2.71
ctt	L	0	1.82
ctc	L	9	1.31
cta	L	0	1.57
ctg	L	0	1.63
cct	P	79	779.30
ccc	P	2230	531.70
cca	P	325	1046.00
ccg	P	7	284.10
cat	H	0	2.31
cac	H	4	1.69
act	T	0	1.48
acc	T	1	1.30
aca	T	5	2.73
acg	T	0	0.50
agt	S	0	9.49
agc	S	0	9.77
gct	A	0	0.74
gcc	A	0	0.69
gca	A	3	1.35
gcg	A	0	0.22
---	--	------	------
mPD		0.34	0.77
		nPD:	0.44
		N. weight:	1.4
		Sc. PD:	0.23
		Sc. rank:	1283.1

,

PA

Pos.	333	obs:	exp:
cat	H	1	0.58
cac	H	0	0.42
aat	N	2267	1542.00
aac	N	446	1171.00
---	--	------	------
mPD		0.28	0.49
		nPD:	0.56
		N. weight:	0.24
		Sc. PD:	0.068
		Sc. rank:	292.6

,

PA

Pos.	334	obs:	exp:
tat	Y	1514	1520.00
tac	Y	1200	1194.00
---	--	------	------
mPD		0.49	0.49
		nPD:	1.
		N. weight:	0.0023
		Sc. PD:	0.0016
		Sc. rank:	3.6

,

PA

Pos.	335	obs:	exp:
tta	L	45	294.80
ttg	L	0	508.80
ctt	L	85	547.10
ctc	L	1497	394.10
cta	L	995	473.00
ctg	L	87	491.10
att	I	0	1.77
atc	I	4	1.28
ata	I	1	1.94
---	--	------	------
mPD		0.58	1.1
		nPD:	0.51
		N. weight:	1.3
		Sc. PD:	0.3
		Sc. rank:	1353.8

,

PA

Pos.	336	obs:	exp:
tta	L	20	174.20
ttg	L	43	300.70
ctt	L	30	323.30
ctc	L	1	232.90
cta	L	114	279.60
ctg	L	1393	290.20
caa	Q	0	1.62
cag	Q	3	1.38
cgt	R	0	0.03
cgc	R	0	0.05
cga	R	0	0.10
cgg	R	0	0.07
att	I	0	0.71
atc	I	0	0.51
ata	I	2	0.78
atg	M	1105	1105.00
act	T	0	0.25
acc	T	0	0.22
aca	T	0	0.45
acg	T	1	0.08
aaa	K	0	0.61
aag	K	1	0.39
aga	R	0	0.46
agg	R	1	0.28
---	--	------	------
mPD		0.63	1.2
		nPD:	0.53
		N. weight:	1.
		Sc. PD:	0.26
		Sc. rank:	1159.8

,

PA

Pos.	337	obs:	exp:
tct	S	3	1.51
tcc	S	1	1.42
tca	S	3	2.19
tcg	S	2	0.48
act	T	43	11.07
acc	T	1	9.73
aca	T	1	20.45
acg	T	0	3.75
agt	S	0	1.68
agc	S	0	1.72
gtt	V	4	0.89
gtc	V	0	0.75
gta	V	0	1.01
gtg	V	0	1.35
gct	A	2548	651.10
gcc	A	40	608.60
gca	A	43	1197.00
gcg	A	25	199.20
---	--	------	------
mPD		0.13	0.72
		nPD:	0.17
		N. weight:	1.7
		Sc. PD:	-0.16
		Sc. rank:	-197.8

,

PA

Pos.	338	obs:	exp:
tgg	W	2714	2714.00
---	--	------	------
mPD		0	0
		nPD:	1.
		N. weight:	0.
		Sc. PD:	0
		Sc. rank:	0

,

PA

Pos.	339	obs:	exp:
cgt	R	0	0.03
cgc	R	0	0.05
cga	R	0	0.10
cgg	R	0	0.07
act	T	0	0.25
acc	T	0	0.22
aca	T	0	0.45
acg	T	1	0.08
aaa	K	986	1648.00
aag	K	1725	1063.00
aga	R	0	0.46
agg	R	1	0.28
gaa	E	1	0.59
gag	E	0	0.41
---	--	------	------
mPD		0.47	0.48
		nPD:	0.97
		N. weight:	0.18
		Sc. PD:	0.12
		Sc. rank:	288.0

,

PA

Pos.	340	obs:	exp:
caa	Q	1177	1464.00
cag	Q	1537	1250.00
---	--	------	------
mPD		0.49	0.50
		nPD:	0.99
		N. weight:	0.035
		Sc. PD:	0.024
		Sc. rank:	55.6

,

PA

Pos.	341	obs:	exp:
tta	L	1	0.44
ttg	L	3	0.75
ctt	L	0	0.81
ctc	L	0	0.58
cta	L	0	0.70
ctg	L	0	0.73
att	I	1	2.84
atc	I	0	2.05
ata	I	7	3.11
atg	M	1	1.00
act	T	0	0.25
acc	T	0	0.22
aca	T	0	0.45
acg	T	1	0.08
gtt	V	25	599.50
gtc	V	6	509.50
gta	V	795	681.60
gtg	V	1874	909.40
---	--	------	------
mPD		0.44	0.75
		nPD:	0.59
		N. weight:	0.75
		Sc. PD:	0.24
		Sc. rank:	982.0

PA

Pos.	342	obs:	exp:
tta	L	110	292.50
ttg	L	5	504.90
ctt	L	8	542.90
ctc	L	14	391.10
cta	L	2459	469.40
ctg	L	92	487.30
caa	Q	4	2.16
cag	Q	0	1.84
att	I	0	7.09
atc	I	0	5.13
ata	I	20	7.78
atg	M	2	2.00
---	--	------	------
mPD		0.19	1.2
		nPD:	0.16
		N. weight:	2.
		Sc. PD:	-0.21
		Sc. rank:	-453.5

PA

Pos.	343	obs:	exp:
tct	S	16	5.55
tcc	S	0	5.22
tca	S	17	8.01
tcg	S	0	1.75
act	T	48	34.19
acc	T	0	30.06
aca	T	91	63.16
acg	T	0	11.59
aat	N	1	0.57
aac	N	0	0.43
agt	S	0	6.14
agc	S	0	6.32
gtt	V	1	1.11
gtc	V	0	0.94
gta	V	4	1.26
gtg	V	0	1.68
gct	A	278	620.70
gcc	A	3	580.10
gca	A	2243	1141.00
gcg	A	8	189.90
gat	D	2	1.12
gac	D	0	0.88
gaa	E	2	1.19
gag	E	0	0.81
---	--	------	------
mPD		0.36	0.82
		nPD:	0.44
		N. weight:	0.73
		Sc. PD:	0.12
		Sc. rank:	646.4

PA

Pos.	344	obs:	exp:
gat	D	1	0.56
gac	D	0	0.44
gaa	E	418	1608.00
gag	E	2295	1105.00
---	--	------	------
mPD		0.26	0.48
		nPD:	0.54
		N. weight:	0.6
		Sc. PD:	0.16
		Sc. rank:	700.1

PA

Pos.	345	obs:	exp:
tta	L	87	290.80
ttg	L	2	501.90
ctt	L	21	539.60
ctc	L	1105	388.80
cta	L	1347	466.60
ctg	L	110	484.40
att	I	0	14.90
atc	I	5	10.77
ata	I	37	16.33
---	--	------	------
mPD		0.63	1.2
		nPD:	0.54
		N. weight:	1.2
		Sc. PD:	0.33
		Sc. rank:	1435.7

PA

Pos.	346	obs:	exp:
tta	L	0	0.11
ttg	L	0	0.19
ctt	L	0	0.20
ctc	L	0	0.15
cta	L	0	0.17
ctg	L	1	0.18
cct	P	0	0.30
ccc	P	0	0.20
cca	P	0	0.40
ccg	P	1	0.11
cat	H	53	30.57
cac	H	0	22.43
caa	Q	117	1432.00
cag	Q	2538	1223.00
cgt	R	0	0.03
cgc	R	0	0.05
cga	R	0	0.10
cgg	R	1	0.07
aaa	K	0	1.22
aag	K	2	0.78
aga	R	0	0.46
agg	R	0	0.28
gaa	E	0	0.59
gag	E	1	0.41
---	--	------	------
mPD		0.12	0.52
		nPD:	0.24
		N. weight:	0.86
		Sc. PD:	-0.027
		Sc. rank:	171.7

PA

Pos.	347	obs:	exp:
tct	S	0	0.67
tcc	S	0	0.63
tca	S	0	0.97
tcg	S	0	0.21
aat	N	0	1.14
aac	N	2	0.86
agt	S	0	0.74
agc	S	4	0.77
gat	D	256	1404.00
gac	D	2241	1093.00
gaa	E	208	123.80
gag	E	1	85.16
ggt	G	0	0.31
ggc	G	2	0.25
gga	G	0	0.91
ggg	G	0	0.53
---	--	------	------
mPD		0.31	0.57
		nPD:	0.54
		N. weight:	0.7
		Sc. PD:	0.18
		Sc. rank:	801.7

PA

Pos.	348	obs:	exp:
ttt	F	27	12.55
ttc	F	1	15.45
tta	L	2	0.33
ttg	L	0	0.56
ctt	L	1	0.61
ctc	L	0	0.44
cta	L	0	0.52
ctg	L	0	0.54
att	I	2607	939.00
atc	I	31	678.90
ata	I	9	1029.00
act	T	5	1.23
acc	T	0	1.08
aca	T	0	2.27
acg	T	0	0.42
gtt	V	31	6.88
gtc	V	0	5.85
gta	V	0	7.83
gtg	V	0	10.44
---	--	------	------
mPD		0.080	0.71
		nPD:	0.11
		N. weight:	1.4
		Sc. PD:	-0.21
		Sc. rank:	-909.5

PA

Pos.	349	obs:	exp:
aaa	K	2	1.22
aag	K	0	0.78
gaa	E	2643	1606.00
gag	E	68	1105.00
ggt	G	0	0.16
ggc	G	0	0.13
gga	G	1	0.45
ggg	G	0	0.26
---	--	------	------
mPD		0.051	0.49
		nPD:	0.11
		N. weight:	0.61
		Sc. PD:	-0.097
		Sc. rank:	-433.4

PA

Pos.	350	obs:	exp:
tct	S	0	4.54
tcc	S	0	4.27
tca	S	0	6.56
tcg	S	0	1.43
cat	H	7	4.04
cac	H	0	2.96
att	I	4	1.42
atc	I	0	1.03
ata	I	0	1.56
act	T	6	2.21
acc	T	3	1.95
aca	T	0	4.09
acg	T	0	0.75
aat	N	2608	1498.00
aac	N	27	1137.00
aaa	K	5	4.25
aag	K	2	2.75
agt	S	27	5.03
agc	S	0	5.17
gat	D	24	14.06
gac	D	1	10.94
---	--	------	------
mPD		0.080	0.56
		nPD:	0.14
		N. weight:	0.76
		Sc. PD:	-0.095
		Sc. rank:	-316.8

PA

Pos.	351	obs:	exp:
gat	D	7	4.50
gac	D	1	3.50
gaa	E	2308	1600.00
gag	E	393	1101.00
ggt	G	0	0.78
ggc	G	0	0.63
gga	G	5	2.26
ggg	G	0	1.32
---	--	------	------
mPD		0.26	0.49
		nPD:	0.52
		N. weight:	0.24
		Sc. PD:	0.06
		Sc. rank:	270.2

PA

Pos.	352	obs:	exp:
aaa	K	15	17.02
aag	K	13	10.98
gat	D	36	20.81
gac	D	1	16.19
gaa	E	104	1568.00
gag	E	2543	1079.00
ggt	G	0	0.31
ggc	G	0	0.25
gga	G	0	0.91
ggg	G	2	0.53
---	--	------	------
mPD		0.13	0.52
		nPD:	0.25
		N. weight:	1.
		Sc. PD:	-0.016
		Sc. rank:	256.6

PA

Pos.	353	obs:	exp:
cgt	R	0	0.88
cgc	R	0	1.48
cga	R	0	2.81
cgg	R	0	2.02
act	T	0	0.98
acc	T	0	0.86
aca	T	0	1.82
acg	T	4	0.33
aat	N	11	6.25
aac	N	0	4.75
aaa	K	486	1622.00
aag	K	2183	1047.00
aga	R	13	12.84
agg	R	15	7.96
gaa	E	1	0.59
gag	E	0	0.41
ggt	G	0	0.16
ggc	G	0	0.13
gga	G	1	0.45
ggg	G	0	0.26
---	--	------	------
mPD		0.33	0.51
		nPD:	0.65
		N. weight:	0.56
		Sc. PD:	0.21
		Sc. rank:	783.1

PA

Pos.	354	obs:	exp:
ttt	F	1	0.45
ttc	F	0	0.55
tta	L	0	2.83
ttg	L	0	4.88
ctt	L	6	5.25
ctc	L	20	3.78
cta	L	0	4.54
ctg	L	0	4.71
att	I	548	927.30
atc	I	2060	670.50
ata	I	6	1016.00
atg	M	1	1.00
act	T	0	11.81
acc	T	48	10.38
aca	T	0	21.81
acg	T	0	4.00
gtt	V	2	5.33
gtc	V	22	4.53
gta	V	0	6.06
gtg	V	0	8.08
---	--	------	------
mPD		0.40	0.73
		nPD:	0.55
		N. weight:	1.2
		Sc. PD:	0.31
		Sc. rank:	1359.8

PA

Pos.	355	obs:	exp:
tta	L	0	0.11
ttg	L	0	0.19
tct	S	0	0.17
tcc	S	1	0.16
tca	S	0	0.24
tcg	S	0	0.05
ctt	L	0	0.20
ctc	L	0	0.15
cta	L	1	0.17
ctg	L	0	0.18
cct	P	43	797.10
ccc	P	276	543.80
cca	P	2374	1070.00
ccg	P	8	290.50
caa	Q	2	1.08
cag	Q	0	0.92
act	T	0	2.21
acc	T	0	1.95
aca	T	9	4.09
acg	T	0	0.75
agt	S	0	0.19
agc	S	0	0.19
---	--	------	------
mPD		0.23	0.71
		nPD:	0.32
		N. weight:	0.84
		Sc. PD:	0.039
		Sc. rank:	443.0

PA

Pos.	356	obs:	exp:
cgt	R	0	34.94
cgc	R	0	58.51
cga	R	2	111.30
cgg	R	0	79.97
aaa	K	287	976.60
aag	K	1320	630.40
aga	R	254	507.70
agg	R	851	314.60
---	--	------	------
mPD		0.80	1.2
		nPD:	0.68
		N. weight:	0.7
		Sc. PD:	0.28
		Sc. rank:	990.7

PA

Pos.	357	obs:	exp:
att	I	8	35.47
atc	I	4	25.65
ata	I	88	38.88
act	T	394	636.60
acc	T	149	559.60
aca	T	2022	1176.00
acg	T	23	215.80
aat	N	2	1.14
aac	N	0	0.86
aaa	K	2	1.22
aag	K	0	0.78
gtt	V	17	3.77
gtc	V	0	3.21
gta	V	0	4.29
gtg	V	0	5.73
gct	A	2	1.23
gcc	A	0	1.15
gca	A	3	2.25
gcg	A	0	0.37
---	--	------	------
mPD		0.47	0.78
		nPD:	0.6
		N. weight:	0.41
		Sc. PD:	0.13
		Sc. rank:	532.8

PA

Pos.	358	obs:	exp:
cgt	R	0	0.03
cgc	R	0	0.05
cga	R	0	0.10
cgg	R	0	0.07
aaa	K	588	1649.00
aag	K	2125	1064.00
aga	R	1	0.46
agg	R	0	0.28
---	--	------	------
mPD		0.34	0.48
		nPD:	0.71
		N. weight:	0.47
		Sc. PD:	0.2
		Sc. rank:	675.5

PA

Pos.	359	obs:	exp:
att	I	5	1.77
atc	I	0	1.28
ata	I	0	1.94
act	T	0	1.48
acc	T	6	1.30
aca	T	0	2.73
acg	T	0	0.50
aat	N	223	1535.00
aac	N	2478	1166.00
aaa	K	1	1.22
aag	K	1	0.78
---	--	------	------
mPD		0.16	0.50
		nPD:	0.33
		N. weight:	0.78
		Sc. PD:	0.043
		Sc. rank:	427.6

PA

Pos.	360	obs:	exp:
tta	L	0	0.76
ttg	L	4	1.31
ctt	L	0	1.41
ctc	L	0	1.02
cta	L	0	1.22
ctg	L	3	1.27
att	I	1	3.90
atc	I	0	2.82
ata	I	10	4.28
atg	M	2695	2695.00
gtt	V	0	0.22
gtc	V	0	0.19
gta	V	0	0.25
gtg	V	1	0.34
---	--	------	------
mPD		0.014	0.018
		nPD:	0.79
		N. weight:	0.0094
		Sc. PD:	0.0048
		Sc. rank:	14.4

PA

Pos.	361	obs:	exp:
cgt	R	0	0.51
cgc	R	0	0.85
cga	R	0	1.61
cgg	R	0	1.16
act	T	0	0.49
acc	T	0	0.43
aca	T	0	0.91
acg	T	2	0.17
aat	N	2	1.14
aac	N	0	0.86
aaa	K	449	1635.00
aag	K	2242	1056.00
aga	R	13	7.34
agg	R	3	4.55
gaa	E	1	1.78
gag	E	2	1.22
---	--	------	------
mPD		0.30	0.50
		nPD:	0.6
		N. weight:	0.61
		Sc. PD:	0.2
		Sc. rank:	798.1

PA

Pos.	362	obs:	exp:
tct	S	0	0.17
tcc	S	0	0.16
tca	S	0	0.24
tcg	S	0	0.05
cgt	R	0	60.19
cgc	R	0	100.80
cga	R	0	191.70
cgg	R	0	137.80
act	T	0	0.49
acc	T	0	0.43
aca	T	2	0.91
acg	T	0	0.17
aaa	K	786	486.20
aag	K	14	313.80
agt	S	0	0.19
agc	S	1	0.19
aga	R	1869	874.50
agg	R	38	542.00
ggt	G	0	0.63
ggc	G	0	0.51
gga	G	4	1.81
ggg	G	0	1.05
---	--	------	------
mPD		0.46	1.3
		nPD:	0.37
		N. weight:	0.89
		Sc. PD:	0.085
		Sc. rank:	568.6

PA

Pos.	363	obs:	exp:
act	T	3	667.30
acc	T	1	586.70
aca	T	2698	1233.00
acg	T	11	226.20
gct	A	0	0.25
gcc	A	0	0.23
gca	A	1	0.45
gcg	A	0	0.07
---	--	------	------
mPD		0.012	0.68
		nPD:	0.02
		N. weight:	1.1
		Sc. PD:	-0.27
		Sc. rank:	-1632.0

PA

Pos.	364	obs:	exp:
tct	S	0	441.70
tcc	S	0	415.00
tca	S	0	637.70
tcg	S	0	139.60
att	I	1	4.61
atc	I	12	3.33
ata	I	0	5.05
aat	N	23	35.81
aac	N	40	27.19
agt	S	790	488.80
agc	S	1836	503.30
ggt	G	4	1.88
ggc	G	8	1.52
gga	G	0	5.44
ggg	G	0	3.16
---	--	------	------
mPD		0.48	1.7
		nPD:	0.29
		N. weight:	1.5
		Sc. PD:	0.03
		Sc. rank:	612.3

PA

Pos.	365	obs:	exp:
tct	S	0	1.51
tcc	S	0	1.42
tca	S	9	2.19
tcg	S	0	0.48
cct	P	0	4.72
ccc	P	0	3.22
cca	P	16	6.34
ccg	P	0	1.72
cat	H	3	2.31
cac	H	1	1.69
caa	Q	2276	1448.00
cag	Q	409	1237.00
agt	S	0	1.68
agc	S	0	1.72
---	--	------	------
mPD		0.28	0.53
		nPD:	0.54
		N. weight:	0.33
		Sc. PD:	0.085
		Sc. rank:	377.8

PA

Pos.	366	obs:	exp:
ttt	F	0	1.34
ttc	F	3	1.66
tta	L	493	295.00
ttg	L	2124	509.20
ctt	L	0	547.50
ctc	L	0	394.40
cta	L	39	473.40
ctg	L	55	491.40
---	--	------	------
mPD		0.38	1.1
		nPD:	0.34
		N. weight:	1.7
		Sc. PD:	0.11
		Sc. rank:	935.4

PA

Pos.	367	obs:	exp:
caa	Q	0	1.08
cag	Q	2	0.92
cgt	R	0	1.20
cgc	R	0	2.01
cga	R	0	3.82
cgg	R	0	2.75
atg	M	1	1.00
aaa	K	143	1624.00
aag	K	2530	1049.00
aga	R	24	17.43
agg	R	14	10.80
---	--	------	------
mPD		0.15	0.52
		nPD:	0.28
		N. weight:	1.
		Sc. PD:	0.013
		Sc. rank:	377.4

PA

Pos.	368	obs:	exp:
tgg	W	2714	2714.00
---	--	------	------
mPD		0	0
		nPD:	1.
		N. weight:	0.
		Sc. PD:	0
		Sc. rank:	0

PA

Pos.	369	obs:	exp:
tct	S	0	2.35
tcc	S	0	2.21
tca	S	14	3.40
tcg	S	0	0.74
act	T	0	0.98
acc	T	0	0.86
aca	T	4	1.82
acg	T	0	0.33
agt	S	0	2.61
agc	S	0	2.68
gtt	V	0	5.77
gtc	V	0	4.91
gta	V	25	6.56
gtg	V	1	8.76
gct	A	20	654.50
gcc	A	43	611.80
gca	A	2589	1204.00
gcg	A	18	200.20
---	--	------	------
mPD		0.091	0.72
		nPD:	0.13
		N. weight:	0.98
		Sc. PD:	-0.14
		Sc. rank:	-486.4

PA

Pos.	370	obs:	exp:
tta	L	0	295.20
ttg	L	0	509.60
ctt	L	1575	547.90
ctc	L	1124	394.70
cta	L	9	473.70
ctg	L	5	491.80
att	I	1	0.35
atc	I	0	0.26
ata	I	0	0.39
---	--	------	------
mPD		0.49	1.1
		nPD:	0.43
		N. weight:	1.5
		Sc. PD:	0.24
		Sc. rank:	1294.5

PA

Pos.	371	obs:	exp:
ggt	G	2670	425.70
ggc	G	35	343.90
gga	G	2	1229.00
ggg	G	7	715.10
---	--	------	------
mPD		0.032	0.68
		nPD:	0.05
		N. weight:	2.6
		Sc. PD:	-0.56
		Sc. rank:	-3238.7

PA

Pos.	372	obs:	exp:
gat	D	2	1.12
gac	D	0	0.88
gaa	E	1532	1607.00
gag	E	1180	1105.00
---	--	------	------
mPD		0.49	0.48
		nPD:	1.02
		N. weight:	0.0051
		Sc. PD:	0.0038
		Sc. rank:	8.2

PA

Pos.	373	obs:	exp:
tct	S	0	1.18
tcc	S	0	1.11
tca	S	0	1.70
tcg	S	0	0.37
aat	N	2273	1539.00
aac	N	434	1168.00
agt	S	6	1.30
agc	S	1	1.34
---	--	------	------
mPD		0.27	0.50
		nPD:	0.55
		N. weight:	0.25
		Sc. PD:	0.069
		Sc. rank:	300.4

PA

Pos.	374	obs:	exp:
tta	L	0	0.65
ttg	L	6	1.13
ctt	L	0	1.21
ctc	L	0	0.87
cta	L	0	1.05
ctg	L	0	1.09
cgt	R	0	0.03
cgc	R	0	0.05
cga	R	0	0.10
cgg	R	0	0.07
att	I	15	6.74
atc	I	0	4.87
ata	I	4	7.39
atg	M	2680	2680.00
act	T	0	0.25
acc	T	0	0.22
aca	T	0	0.45
acg	T	1	0.08
aga	R	0	0.46
agg	R	1	0.28
gtt	V	0	1.55
gtc	V	0	1.32
gta	V	1	1.77
gtg	V	6	2.36
---	--	------	------
mPD		0.026	0.032
		nPD:	0.79
		N. weight:	0.017
		Sc. PD:	0.0085
		Sc. rank:	25.6

PA

Pos.	375	obs:	exp:
gct	A	152	665.30
gcc	A	27	621.80
gca	A	2525	1223.00
gcg	A	10	203.50
---	--	------	------
mPD		0.13	0.68
		nPD:	0.19
		N. weight:	0.81
		Sc. PD:	-0.06
		Sc. rank:	-15.2

PA

Pos.	376	obs:	exp:
tct	S	0	0.17
tcc	S	0	0.16
tca	S	1	0.24
tcg	S	0	0.05
cct	P	403	800.60
ccc	P	12	546.20
cca	P	2285	1074.00
ccg	P	13	291.80
agt	S	0	0.19
agc	S	0	0.19
---	--	------	------
mPD		0.27	0.71
		nPD:	0.38
		N. weight:	0.74
		Sc. PD:	0.081
		Sc. rank:	500.6

PA

Pos.	377	obs:	exp:
gaa	E	1202	1608.00
gag	E	1512	1106.00
---	--	------	------
mPD		0.49	0.48
		nPD:	1.02
		N. weight:	0.068
		Sc. PD:	0.05
		Sc. rank:	109.2

PA

Pos.	378	obs:	exp:
atg	M	1	1.00
act	T	0	0.25
acc	T	0	0.22
aca	T	1	0.45
acg	T	0	0.08
aaa	K	2228	1648.00
aag	K	484	1064.00
---	--	------	------
mPD		0.30	0.48
		nPD:	0.62
		N. weight:	0.16
		Sc. PD:	0.054
		Sc. rank:	211.8

PA

Pos.	379	obs:	exp:
tgg	W	1	1.00
att	I	0	15.61
atc	I	0	11.29
ata	I	44	17.11
atg	M	5	5.00
gtt	V	3	591.30
gtc	V	3	502.50
gta	V	2271	672.20
gtg	V	386	897.00
gct	A	0	0.25
gcc	A	0	0.23
gca	A	1	0.45
gcg	A	0	0.07
---	--	------	------
mPD		0.29	0.78
		nPD:	0.37
		N. weight:	1.3
		Sc. PD:	0.13
		Sc. rank:	857.3

PA

Pos.	380	obs:	exp:
gat	D	1476	1526.00
gac	D	1237	1187.00
gaa	E	1	0.59
gag	E	0	0.41
---	--	------	------
mPD		0.50	0.49
		nPD:	1.01
		N. weight:	0.0035
		Sc. PD:	0.0025
		Sc. rank:	5.6

PA

Pos.	381	obs:	exp:
ttt	F	2700	1216.00
ttc	F	13	1497.00
tta	L	1	0.11
ttg	L	0	0.19
ctt	L	0	0.20
ctc	L	0	0.15
cta	L	0	0.17
ctg	L	0	0.18
---	--	------	------
mPD		0.010	0.50
		nPD:	0.02
		N. weight:	1.1
		Sc. PD:	-0.27
		Sc. rank:	-1619.8

PA

Pos.	382	obs:	exp:
aat	N	5	2.84
aac	N	0	2.16
gat	D	2287	1291.00
gac	D	8	1004.00
gaa	E	404	245.30
gag	E	10	168.70
---	--	------	------
mPD		0.27	0.63
		nPD:	0.43
		N. weight:	0.78
		Sc. PD:	0.12
		Sc. rank:	661.6

PA

Pos.	383	obs:	exp:
tat	Y	1	0.56
tac	Y	0	0.44
aat	N	0	0.57
aac	N	1	0.43
gat	D	477	1512.00
gac	D	2212	1177.00
gaa	E	17	13.63
gag	E	6	9.37
---	--	------	------
mPD		0.31	0.50
		nPD:	0.61
		N. weight:	0.46
		Sc. PD:	0.15
		Sc. rank:	610.0

PA

Pos.	384	obs:	exp:
tct	S	0	0.17
tcc	S	1	0.16
tca	S	0	0.24
tcg	S	0	0.05
tat	Y	0	0.56
tac	Y	1	0.44
tgt	C	95	1166.00
tgc	C	2614	1543.00
cgt	R	0	0.03
cgc	R	1	0.05
cga	R	0	0.10
cgg	R	0	0.07
act	T	0	0.49
acc	T	2	0.43
aca	T	0	0.91
acg	T	0	0.17
agt	S	0	0.19
agc	S	0	0.19
aga	R	0	0.46
agg	R	0	0.28
---	--	------	------
mPD		0.073	0.50
		nPD:	0.15
		N. weight:	0.62
		Sc. PD:	-0.074
		Sc. rank:	-233.4

PA

Pos.	385	obs:	exp:
cgt	R	0	0.47
cgc	R	0	0.79
cga	R	0	1.51
cgg	R	0	1.08
aaa	K	2652	1640.00
aag	K	47	1059.00
aga	R	15	6.88
agg	R	0	4.26
---	--	------	------
mPD		0.045	0.49
		nPD:	0.09
		N. weight:	0.61
		Sc. PD:	-0.11
		Sc. rank:	-501.7

PA			
Pos.	386	obs:	exp:
tct	S	0	0.17
tcc	S	0	0.16
tca	S	0	0.24
tcg	S	0	0.05
aat	N	9	5.68
aac	N	1	4.32
agt	S	1	0.19
agc	S	0	0.19
gat	D	2524	1506.00
gac	D	154	1172.00
gaa	E	22	14.22
gag	E	2	9.78
ggt	G	1	0.16
ggc	G	0	0.13
gga	G	0	0.45
ggg	G	0	0.26
---	--	------	------
mPD		0.13	0.51
		nPD:	0.26
		N. weight:	0.54
		Sc. PD:	-0.0039
		Sc. rank:	162.0

,

PA			
Pos.	387	obs:	exp:
att	I	224	185.90
atc	I	297	134.40
ata	I	3	203.70
act	T	0	0.49
acc	T	2	0.43
aca	T	0	0.91
acg	T	0	0.17
gtt	V	1121	485.60
gtc	V	1038	412.70
gta	V	7	552.10
gtg	V	21	736.60
gct	A	0	0.25
gcc	A	1	0.23
gca	A	0	0.45
gcg	A	0	0.07
---	--	------	------
mPD		0.83	1.1
		nPD:	0.78
		N. weight:	1.1
		Sc. PD:	0.55
		Sc. rank:	1714.8

,

PA			
Pos.	388	obs:	exp:
tct	S	0	49.45
tcc	S	0	46.46
tca	S	0	71.39
tcg	S	0	15.63
tgt	C	8	3.44
tgc	C	0	4.56
cgt	R	0	0.06
cgc	R	0	0.11
cga	R	0	0.20
cgg	R	0	0.14
act	T	1	0.25
acc	T	0	0.22
aca	T	0	0.45
acg	T	0	0.08
aat	N	43	24.44
aac	N	0	18.56
agt	S	289	54.72
agc	S	5	56.34
aga	R	2	0.92
agg	R	0	0.57
gct	A	2	0.49
gcc	A	0	0.46
gca	A	0	0.90
gcg	A	0	0.15
gat	D	15	9.00
gac	D	1	7.00
gaa	E	35	21.33
gag	E	1	14.67
ggt	G	1234	362.60
ggc	G	6	293.00
gga	G	1030	1047.00
ggg	G	42	609.20
---	--	------	------
mPD		0.80	1.1
		nPD:	0.7
		N. weight:	1.
		Sc. PD:	0.43
		Sc. rank:	1474.0

,

PA			
Pos.	389	obs:	exp:
gat	D	1589	1527.00
gac	D	1125	1187.00
---	--	------	------
mPD		0.49	0.49
		nPD:	0.99
		N. weight:	0.0038
		Sc. PD:	0.0027
		Sc. rank:	6.1

,

PA			
Pos.	390	obs:	exp:
tta	L	345	295.30
ttg	L	62	509.80
ctt	L	1321	548.10
ctc	L	945	394.90
cta	L	21	473.90
ctg	L	20	492.00
---	--	------	------
mPD		0.88	1.1
		nPD:	0.77
		N. weight:	0.97
		Sc. PD:	0.47
		Sc. rank:	1469.4

,

PA			
Pos.	391	obs:	exp:
caa	Q	2	1.08
cag	Q	0	0.92
cgt	R	0	0.41
cgc	R	0	0.69
cga	R	0	1.31
cgg	R	0	0.94
act	T	0	0.25
acc	T	0	0.22
aca	T	1	0.45
acg	T	0	0.08
aaa	K	2645	1640.00
aag	K	53	1058.00
aga	R	12	5.96
agg	R	1	3.69
---	--	------	------
mPD		0.051	0.49
		nPD:	0.1
		N. weight:	0.6
		Sc. PD:	-0.098
		Sc. rank:	-442.2

,

PA			
Pos.	392	obs:	exp:
caa	Q	204	1464.00
cag	Q	2510	1250.00
---	--	------	------
mPD		0.14	0.50
		nPD:	0.28
		N. weight:	0.73
		Sc. PD:	0.0075
		Sc. rank:	255.7

,

PA			
Pos.	393	obs:	exp:
tat	Y	2683	1520.00
tac	Y	30	1193.00
cat	H	1	0.58
cac	H	0	0.42
---	--	------	------
mPD		0.023	0.49
		nPD:	0.05
		N. weight:	0.76
		Sc. PD:	-0.17
		Sc. rank:	-968.5

,

PA			
Pos.	394	obs:	exp:
tct	S	0	0.17
tcc	S	0	0.16
tca	S	0	0.24
tcg	S	0	0.05
aat	N	11	76.73
aac	N	124	58.27
agt	S	0	0.19
agc	S	1	0.19
gtt	V	0	0.44
gtc	V	2	0.38
gta	V	0	0.50
gtg	V	0	0.67
gat	D	361	1440.00
gac	D	2199	1120.00
gaa	E	5	3.56
gag	E	1	2.44
ggt	G	2	1.57
ggc	G	8	1.27
gga	G	0	4.53
ggg	G	0	2.63
---	--	------	------
mPD		0.35	0.60
		nPD:	0.57
		N. weight:	0.58
		Sc. PD:	0.17
		Sc. rank:	734.8

,

PA			
Pos.	395	obs:	exp:
tct	S	0	450.10
tcc	S	0	422.90
tca	S	0	649.80
tcg	S	0	142.20
tgt	C	0	0.43
tgc	C	1	0.57
aat	N	26	16.48
aac	N	3	12.52
agt	S	1565	498.10
agc	S	1111	512.80
ggt	G	7	1.25
ggc	G	1	1.01
gga	G	0	3.62
ggg	G	0	2.11
---	--	------	------
mPD		0.51	1.6
		nPD:	0.31
		N. weight:	1.4
		Sc. PD:	0.058
		Sc. rank:	728.2

,

PA

Pos.	396	obs:	exp:
aat	N	16	9.66
aac	N	1	7.34
gtt	V	1	5.33
gtc	V	23	4.53
gta	V	0	6.06
gtg	V	0	8.08
gat	D	2487	1488.00
gac	D	158	1157.00
gaa	E	17	12.44
gag	E	4	8.56
ggt	G	6	1.10
ggc	G	1	0.89
gga	G	0	3.17
ggg	G	0	1.84
---	--	------	------
mPD		0.18	0.54
		nPD:	0.32
		N. weight:	0.55
		Sc. PD:	0.029
		Sc. rank:	295.1

,

PA

Pos.	397	obs:	exp:
aaa	K	0	1.22
aag	K	2	0.78
gtt	V	0	0.22
gtc	V	0	0.19
gta	V	0	0.25
gtg	V	1	0.34
gat	D	1	0.56
gac	D	0	0.44
gaa	E	163	1604.00
gag	E	2544	1103.00
ggt	G	0	0.47
ggc	G	0	0.38
gga	G	1	1.36
ggg	G	2	0.79
---	--	------	------
mPD		0.12	0.49
		nPD:	0.24
		N. weight:	0.95
		Sc. PD:	-0.024
		Sc. rank:	197.1

,

PA

Pos.	398	obs:	exp:
cct	P	9	800.90
ccc	P	70	546.40
cca	P	2411	1075.00
ccg	P	224	291.90
---	--	------	------
mPD		0.20	0.70
		nPD:	0.29
		N. weight:	0.92
		Sc. PD:	0.018
		Sc. rank:	368.4

,

PA

Pos.	399	obs:	exp:
cgt	R	0	0.03
cgc	R	0	0.05
cga	R	0	0.10
cgg	R	0	0.07
att	I	0	1.77
atc	I	0	1.28
ata	I	5	1.94
act	T	0	1.72
acc	T	0	1.51
aca	T	7	3.18
acg	T	0	0.58
aat	N	6	3.41
aac	N	0	2.59
aaa	K	358	235.80
aag	K	30	152.20
aga	R	1	0.46
agg	R	0	0.28
gat	D	15	8.44
gac	D	0	6.56
gaa	E	251	1356.00
gag	E	2038	932.70
ggt	G	2	0.47
ggc	G	0	0.38
gga	G	0	1.36
ggg	G	1	0.79
---	--	------	------
mPD		0.63	0.76
		nPD:	0.83
		N. weight:	0.7
		Sc. PD:	0.39
		Sc. rank:	1091.9

,

PA

Pos.	400	obs:	exp:
ttt	F	8	4.03
ttc	F	1	4.97
tta	L	13	8.16
ttg	L	3	14.09
tct	S	8	8.58
tcc	S	32	8.06
tca	S	1	12.38
tcg	S	0	2.71
ctt	L	13	15.15
ctc	L	45	10.91
cta	L	0	13.10
ctg	L	1	13.60
cct	P	902	643.60
ccc	P	1273	439.10
cca	P	6	863.70
ccg	P	0	234.60
cat	H	2	3.46
cac	H	4	2.54
cgt	R	0	0.13
cgc	R	0	0.21
cga	R	0	0.40
cgg	R	0	0.29
att	I	206	79.46
atc	I	15	57.45
ata	I	3	87.08
atg	M	86	86.00
act	T	21	8.86
acc	T	10	7.78
aca	T	0	16.36
acg	T	5	3.00
aat	N	15	8.53
aac	N	0	6.47
aaa	K	0	0.61
aag	K	1	0.39
agt	S	10	9.49
agc	S	0	9.77
aga	R	0	1.83
agg	R	4	1.14
gtt	V	5	5.77
gtc	V	0	4.91
gta	V	0	6.56
gtg	V	21	8.76
---	--	------	------
mPD		1.1	1.3
		nPD:	0.86
		N. weight:	1.1
		Sc. PD:	0.62
		Sc. rank:	1675.6

,

PA

Pos.	401	obs:	exp:
tct	S	0	0.17
tcc	S	0	0.16
tca	S	0	0.24
tcg	S	0	0.05
cgt	R	0	84.37
cgc	R	0	141.30
cga	R	5	268.70
cgg	R	0	193.10
aaa	K	40	24.31
aag	K	0	15.69
agt	S	0	0.19
agc	S	1	0.19
aga	R	2609	1226.00
agg	R	59	759.70
---	--	------	------
mPD		0.076	0.96
		nPD:	0.08
		N. weight:	0.98
		Sc. PD:	-0.18
		Sc. rank:	-903.3

,

PA

Pos.	402	obs:	exp:
tct	S	2245	456.50
tcc	S	29	428.90
tca	S	393	659.10
tcg	S	47	144.20
agt	S	0	505.10
agc	S	0	520.10
---	--	------	------
mPD		0.29	1.6
		nPD:	0.18
		N. weight:	1.7
		Sc. PD:	-0.15
		Sc. rank:	-123.3

,

PA

Pos.	403	obs:	exp:
tta	L	43	221.20
ttg	L	155	381.90
ctt	L	27	410.60
ctc	L	1	295.80
cta	L	1542	355.00
ctg	L	265	368.50
cct	P	0	1.18
ccc	P	0	0.81
cca	P	2	1.58
ccg	P	2	0.43
att	I	0	238.00
atc	I	0	172.10
ata	I	671	260.90
atg	M	5	5.00
gtt	V	0	0.22
gtc	V	0	0.19
gta	V	1	0.25
gtg	V	0	0.34
---	--	------	------
mPD		0.76	1.4
		nPD:	0.56
		N. weight:	1.4
		Sc. PD:	0.39
		Sc. rank:	1671.4

,

PA

Pos.	404	obs:	exp:
tct	S	0	9.42
tcc	S	0	8.85
tca	S	55	13.60
tcg	S	1	2.98
act	T	0	6.64
acc	T	0	5.84
aca	T	27	12.27
acg	T	0	2.25
agt	S	0	10.42
agc	S	0	10.73
gtt	V	0	1.55
gtc	V	0	1.32
gta	V	7	1.77
gtg	V	0	2.36
gct	A	8	643.20
gcc	A	0	601.20
gca	A	2568	1183.00
gcg	A	48	196.80
---	--	------	------
mPD		0.11	0.76
		nPD:	0.14
		N. weight:	1.1
		Sc. PD:	-0.14
		Sc. rank:	-460.2

,

PA

Pos.	405	obs:	exp:
tct	S	0	445.90
tcc	S	0	419.00
tca	S	0	643.80
tcg	S	0	140.90
agt	S	63	493.40
agc	S	2588	508.10
ggt	G	0	9.88
ggc	G	63	7.98
gga	G	0	28.54
ggg	G	0	16.60
---	--	------	------
mPD		0.091	1.7
		nPD:	0.05
		N. weight:	2.3
		Sc. PD:	-0.48
		Sc. rank:	-2645.9

,

PA

Pos.	406	obs:	exp:
tgg	W	2714	2714.00
---	--	------	------
mPD		0	0
		nPD:	1.
		N. weight:	0.
		Sc. PD:	0
		Sc. rank:	0

,

PA

Pos.	407	obs:	exp:
att	I	340	166.40
atc	I	109	120.30
ata	I	20	182.30
atg	M	3	3.00
gtt	V	106	497.80
gtc	V	2103	423.10
gta	V	6	566.00
gtg	V	27	755.20
---	--	------	------
mPD		0.60	1.0
		nPD:	0.58
		N. weight:	1.8
		Sc. PD:	0.53
		Sc. rank:	2246.9

,

PA

Pos.	408	obs:	exp:
caa	Q	2384	1464.00
cag	Q	330	1250.00
---	--	------	------
mPD		0.21	0.50
		nPD:	0.43
		N. weight:	0.39
		Sc. PD:	0.061
		Sc. rank:	332.6

,

PA

Pos.	409	obs:	exp:
tct	S	0	62.57
tcc	S	0	58.79
tca	S	0	90.34
tcg	S	0	19.77
tgt	C	1	0.43
tgc	C	0	0.57
aat	N	2303	1329.00
aac	N	36	1010.00
agt	S	369	69.24
agc	S	3	71.29
ggt	G	2	0.31
ggc	G	0	0.25
gga	G	0	0.91
ggg	G	0	0.53
---	--	------	------
mPD		0.27	0.94
		nPD:	0.29
		N. weight:	0.95
		Sc. PD:	0.016
		Sc. rank:	360.7

,

PA

Pos.	410	obs:	exp:
gaa	E	1890	1608.00
gag	E	824	1106.00
---	--	------	------
mPD		0.42	0.48
		nPD:	0.88
		N. weight:	0.036
		Sc. PD:	0.021
		Sc. rank:	56.5

,

PA

Pos.	411	obs:	exp:
ttt	F	25	1213.00
ttc	F	2683	1495.00
tta	L	0	0.65
ttg	L	0	1.13
ctt	L	0	1.21
ctc	L	6	0.87
cta	L	0	1.05
ctg	L	0	1.09
---	--	------	------
mPD		0.023	0.50
		nPD:	0.05
		N. weight:	0.8
		Sc. PD:	-0.17
		Sc. rank:	-1021.5

,

PA

Pos.	412	obs:	exp:
aat	N	1146	1543.00
aac	N	1568	1171.00
---	--	------	------
mPD		0.49	0.49
		nPD:	0.99
		N. weight:	0.065
		Sc. PD:	0.046
		Sc. rank:	103.7

,

PA

Pos.	413	obs:	exp:
cgt	R	0	0.06
cgc	R	0	0.11
cga	R	0	0.20
cgg	R	0	0.14
aaa	K	290	1648.00
aag	K	2422	1064.00
aga	R	0	0.92
agg	R	2	0.57
---	--	------	------
mPD		0.19	0.48
		nPD:	0.4
		N. weight:	0.8
		Sc. PD:	0.1
		Sc. rank:	606.6

,

PA

Pos.	414	obs:	exp:
tct	S	0	0.17
tcc	S	0	0.16
tca	S	1	0.24
tcg	S	0	0.05
act	T	0	0.49
acc	T	0	0.43
aca	T	2	0.91
acg	T	0	0.17
agt	S	0	0.19
agc	S	0	0.19
gct	A	165	664.60
gcc	A	8	621.20
gca	A	2514	1222.00
gcg	A	24	203.30
---	--	------	------
mPD		0.14	0.68
		nPD:	0.2
		N. weight:	0.81
		Sc. PD:	-0.052
		Sc. rank:	21.7

,

PA

Pos.	415	obs:	exp:
tct	S	0	0.17
tcc	S	0	0.16
tca	S	0	0.24
tcg	S	0	0.05
tgt	C	2689	1168.00
tgc	C	24	1545.00
agt	S	1	0.19
agc	S	0	0.19
---	--	------	------
mPD		0.018	0.49
		nPD:	0.04
		N. weight:	1.2
		Sc. PD:	-0.26
		Sc. rank:	-1566.6

,

PA

Pos.	416	obs:	exp:
gaa	E	2373	1608.00
gag	E	341	1106.00
---	--	------	------
mPD		0.22	0.48
		nPD:	0.46
		N. weight:	0.28
		Sc. PD:	0.051
		Sc. rank:	262.4

,

PA

Pos.	417	obs:	exp:
tta	L	779	295.10
ttg	L	1719	509.40
ctt	L	24	547.70
ctc	L	0	394.60
cta	L	23	473.50
ctg	L	167	491.60
att	I	0	0.71
atc	I	0	0.51
ata	I	2	0.78
---	--	------	------
mPD		0.58	1.1
		nPD:	0.51
		N. weight:	1.4
		Sc. PD:	0.31
		Sc. rank:	1442.4

,

PA

Pos.	418	obs:	exp:
tct	S	0	0.34
tcc	S	0	0.32
tca	S	0	0.49
tcg	S	1	0.11
act	T	1220	667.10
acc	T	1159	586.40
aca	T	43	1232.00
acg	T	290	226.10
agt	S	0	0.37
agc	S	1	0.38
---	--	------	------
mPD		0.61	0.68
		nPD:	0.89
		N. weight:	0.79
		Sc. PD:	0.48
		Sc. rank:	1242.7

,

PA

Pos.	419	obs:	exp:
aat	N	15	8.53
aac	N	0	6.47
gat	D	2599	1518.00
gac	D	99	1180.00
gaa	E	1	0.59
gag	E	0	0.41
---	--	------	------
mPD		0.082	0.50
		nPD:	0.16
		N. weight:	0.63
		Sc. PD:	-0.065
		Sc. rank:	-122.4

,

PA

Pos.	420	obs:	exp:
tct	S	135	456.30
tcc	S	279	428.80
tca	S	2251	658.80
tcg	S	48	144.20
act	T	0	0.25
acc	T	0	0.22
aca	T	1	0.45
acg	T	0	0.08
agt	S	0	505.00
agc	S	0	519.90
---	--	------	------
mPD		0.30	1.6
		nPD:	0.18
		N. weight:	1.3
		Sc. PD:	-0.11
		Sc. rank:	-75.5

,

PA

Pos.	421	obs:	exp:
tct	S	0	451.40
tcc	S	0	424.20
tca	S	0	651.80
tcg	S	0	142.60
att	I	1	2.84
atc	I	7	2.05
ata	I	0	3.11
aat	N	4	2.27
aac	N	0	1.73
agt	S	178	499.60
agc	S	2506	514.40
ggt	G	0	2.82
ggc	G	18	2.28
gga	G	0	8.15
ggg	G	0	4.74
---	--	------	------
mPD		0.15	1.6
		nPD:	0.09
		N. weight:	2.1
		Sc. PD:	-0.36
		Sc. rank:	-1734.6

,

PA

Pos.	422	obs:	exp:
tgg	W	2714	2714.00
---	--	------	------
mPD		0	0
		nPD:	1.
		N. weight:	0.
		Sc. PD:	0
		Sc. rank:	0

,

PA

Pos.	423	obs:	exp:
tta	L	23	2.50
ttg	L	0	4.32
ctt	L	0	4.65
ctc	L	0	3.35
cta	L	0	4.02
ctg	L	0	4.17
att	I	10	944.70
atc	I	3	683.00
ata	I	2650	1035.00
atg	M	19	19.00
act	T	0	0.49
acc	T	0	0.43
aca	T	2	0.91
acg	T	0	0.17
gtt	V	0	1.55
gtc	V	0	1.32
gta	V	6	1.77
gtg	V	1	2.36
---	--	------	------
mPD		0.047	0.69
		nPD:	0.07
		N. weight:	1.3
		Sc. PD:	-0.26
		Sc. rank:	-1397.8

,

PA

Pos.	424	obs:	exp:
gat	D	0	0.56
gac	D	1	0.44
gaa	E	2184	1608.00
gag	E	529	1105.00
---	--	------	------
mPD		0.31	0.48
		nPD:	0.65
		N. weight:	0.15
		Sc. PD:	0.057
		Sc. rank:	212.5

,

PA

Pos.	425	obs:	exp:
ttt	F	27	12.10
ttc	F	0	14.90
tta	L	0	292.30
ttg	L	0	504.50
ctt	L	2554	542.50
ctc	L	114	390.80
cta	L	17	469.00
ctg	L	1	486.90
cct	P	1	0.30
ccc	P	0	0.20
cca	P	0	0.40
ccg	P	0	0.11
---	--	------	------
mPD		0.11	1.1
		nPD:	0.1
		N. weight:	2.
		Sc. PD:	-0.34
		Sc. rank:	-1547.9

,

PA			
Pos.	426	obs:	exp:
aat	N	1	0.57
aac	N	0	0.43
gat	D	2233	1498.00
gac	D	430	1165.00
gaa	E	50	29.63
gag	E	0	20.37
---	--	------	------
mPD		0.30	0.51
		nPD:	0.58
		N. weight:	0.27
		Sc. PD:	0.081
		Sc. rank:	341.5

,

PA			
Pos.	427	obs:	exp:
gaa	E	1582	1608.00
gag	E	1132	1106.00
---	--	------	------
mPD		0.49	0.48
		nPD:	1.01
		N. weight:	0.0025
		Sc. PD:	0.0018
		Sc. rank:	4.0

,

PA			
Pos.	428	obs:	exp:
tta	L	1	0.87
ttg	L	0	1.50
ctt	L	0	1.62
ctc	L	0	1.16
cta	L	7	1.40
ctg	L	0	1.45
att	I	41	957.80
atc	I	8	692.50
ata	I	2651	1050.00
atg	M	1	1.00
act	T	0	0.25
acc	T	0	0.22
aca	T	1	0.45
acg	T	0	0.08
gtt	V	0	0.89
gtc	V	0	0.75
gta	V	4	1.01
gtg	V	0	1.35
---	--	------	------
mPD		0.046	0.67
		nPD:	0.07
		N. weight:	1.2
		Sc. PD:	-0.24
		Sc. rank:	-1305.5

,

PA			
Pos.	429	obs:	exp:
ggt	G	23	425.70
ggc	G	1	343.90
gga	G	2497	1229.00
ggg	G	193	715.10
---	--	------	------
mPD		0.15	0.68
		nPD:	0.22
		N. weight:	0.78
		Sc. PD:	-0.04
		Sc. rank:	98.4

,

PA			
Pos.	430	obs:	exp:
aaa	K	1	0.61
aag	K	0	0.39
gat	D	0	1.12
gac	D	2	0.88
gaa	E	2299	1606.00
gag	E	411	1104.00
ggt	G	0	0.16
ggc	G	0	0.13
gga	G	1	0.45
ggg	G	0	0.26
---	--	------	------
mPD		0.26	0.49
		nPD:	0.54
		N. weight:	0.23
		Sc. PD:	0.059
		Sc. rank:	262.9

,

PA			
Pos.	431	obs:	exp:
aat	N	1	0.57
aac	N	0	0.43
gat	D	2688	1526.00
gac	D	25	1187.00
---	--	------	------
mPD		0.019	0.49
		nPD:	0.04
		N. weight:	0.77
		Sc. PD:	-0.17
		Sc. rank:	-1027.7

,

PA			
Pos.	432	obs:	exp:
tta	L	0	0.76
ttg	L	0	1.31
ctt	L	6	1.41
ctc	L	1	1.02
cta	L	0	1.22
ctg	L	0	1.27
att	I	34	13.83
atc	I	3	10.00
ata	I	2	15.16
gtt	V	2264	592.40
gtc	V	345	503.40
gta	V	51	673.50
gtg	V	8	898.70
---	--	------	------
mPD		0.30	0.77
		nPD:	0.38
		N. weight:	1.5
		Sc. PD:	0.17
		Sc. rank:	1027.2

,

PA			
Pos.	433	obs:	exp:
gct	A	397	665.30
gcc	A	1788	621.80
gca	A	529	1223.00
gcg	A	0	203.50
---	--	------	------
mPD		0.51	0.68
		nPD:	0.75
		N. weight:	0.67
		Sc. PD:	0.31
		Sc. rank:	1001.7

,

PA			
Pos.	434	obs:	exp:
cct	P	32	800.90
ccc	P	0	546.40
cca	P	575	1075.00
ccg	P	2107	291.90
---	--	------	------
mPD		0.35	0.70
		nPD:	0.5
		N. weight:	2.
		Sc. PD:	0.45
		Sc. rank:	2054.3

,

PA			
Pos.	435	obs:	exp:
ttt	F	1	0.45
ttc	F	0	0.55
att	I	2130	962.40
atc	I	504	695.90
ata	I	79	1055.00
---	--	------	------
mPD		0.35	0.66
		nPD:	0.53
		N. weight:	0.72
		Sc. PD:	0.18
		Sc. rank:	815.8

,

PA			
Pos.	436	obs:	exp:
gat	D	0	0.56
gac	D	1	0.44
gaa	E	2241	1608.00
gag	E	472	1105.00
---	--	------	------
mPD		0.29	0.48
		nPD:	0.6
		N. weight:	0.19
		Sc. PD:	0.06
		Sc. rank:	245.1

,

PA			
Pos.	437	obs:	exp:
tat	Y	23	19.61
tac	Y	12	15.39
cat	H	1475	1545.00
cac	H	1204	1134.00
---	--	------	------
mPD		0.52	0.51
		nPD:	1.01
		N. weight:	0.0057
		Sc. PD:	0.0041
		Sc. rank:	9.1

,

PA			
Pos.	438	obs:	exp:
att	I	502	934.70
atc	I	2019	675.80
ata	I	114	1024.00
act	T	3	0.98
acc	T	1	0.86
aca	T	0	1.82
acg	T	0	0.33
gtt	V	52	16.65
gtc	V	19	14.15
gta	V	2	18.93
gtg	V	2	25.26
---	--	------	------
mPD		0.45	0.72
		nPD:	0.62
		N. weight:	0.92
		Sc. PD:	0.32
		Sc. rank:	1238.0

,

PA			
Pos.	439	obs:	exp:
gct	A	71	665.30
gcc	A	1	621.80
gca	A	2218	1223.00
gcg	A	424	203.50
---	--	------	------
mPD		0.31	0.68
		nPD:	0.45
		N. weight:	0.79
		Sc. PD:	0.14
		Sc. rank:	724.3

,

PA			
Pos.	440	obs:	exp:
tct	S	0	456.50
tcc	S	0	428.90
tca	S	0	659.10
tcg	S	0	144.20
agt	S	500	505.10
agc	S	2214	520.10
---	--	------	------
mPD		0.30	1.6
		nPD:	0.18
		N. weight:	1.7
		Sc. PD:	-0.14
		Sc. rank:	-79.3

,

PA			
Pos.	441	obs:	exp:
tta	L	0	2.39
ttg	L	22	4.13
ctt	L	0	4.44
ctc	L	0	3.20
cta	L	0	3.84
ctg	L	0	3.99
cgt	R	0	0.09
cgc	R	0	0.16
cga	R	0	0.30
cgg	R	0	0.22
att	I	1	6.03
atc	I	0	4.36
ata	I	16	6.61
atg	M	2654	2654.00
act	T	0	2.95
acc	T	0	2.59
aca	T	0	5.45
acg	T	12	1.00
aaa	K	0	0.61
aag	K	1	0.39
aga	R	0	1.38
agg	R	3	0.85
gtt	V	0	1.11
gtc	V	0	0.94
gta	V	0	1.26
gtg	V	5	1.68
---	--	------	------
mPD		0.044	0.067
		nPD:	0.66
		N. weight:	0.048
		Sc. PD:	0.018
		Sc. rank:	67.5

,

PA			
Pos.	442	obs:	exp:
cgt	R	0	85.64
cgc	R	0	143.40
cga	R	1	272.70
cgg	R	0	196.00
act	T	0	0.25
acc	T	0	0.22
aca	T	1	0.45
acg	T	0	0.08
aga	R	194	1244.00
agg	R	2518	771.10
---	--	------	------
mPD		0.14	0.94
		nPD:	0.14
		N. weight:	1.4
		Sc. PD:	-0.17
		Sc. rank:	-558.6

,

PA			
Pos.	443	obs:	exp:
cgt	R	0	85.67
cgc	R	0	143.50
cga	R	0	272.80
cgg	R	0	196.10
aga	R	387	1245.00
agg	R	2327	771.40
---	--	------	------
mPD		0.24	0.94
		nPD:	0.26
		N. weight:	1.1
		Sc. PD:	-0.0097
		Sc. rank:	338.0

,

PA			
Pos.	444	obs:	exp:
tct	S	0	0.17
tcc	S	0	0.16
tca	S	0	0.24
tcg	S	0	0.05
act	T	0	0.49
acc	T	2	0.43
aca	T	0	0.91
acg	T	0	0.17
aat	N	308	1532.00
aac	N	2388	1164.00
agt	S	0	0.19
agc	S	1	0.19
gat	D	1	8.44
gac	D	14	6.56
---	--	------	------
mPD		0.22	0.50
		nPD:	0.43
		N. weight:	0.67
		Sc. PD:	0.1
		Sc. rank:	557.2

,

PA			
Pos.	445	obs:	exp:
ttt	F	1	0.45
ttc	F	0	0.55
tat	Y	2318	1520.00
tac	Y	395	1193.00
---	--	------	------
mPD		0.25	0.49
		nPD:	0.51
		N. weight:	0.29
		Sc. PD:	0.068
		Sc. rank:	311.1

,

PA			
Pos.	446	obs:	exp:
ttt	F	2247	1216.00
ttc	F	467	1498.00
---	--	------	------
mPD		0.29	0.49
		nPD:	0.58
		N. weight:	0.45
		Sc. PD:	0.14
		Sc. rank:	569.1

,

PA			
Pos.	447	obs:	exp:
tct	S	1	2.35
tcc	S	0	2.21
tca	S	13	3.40
tcg	S	0	0.74
act	T	2	661.40
acc	T	15	581.50
aca	T	2655	1222.00
acg	T	17	224.20
agt	S	0	2.61
agc	S	0	2.68
gct	A	0	2.70
gcc	A	0	2.52
gca	A	11	4.96
gcg	A	0	0.82
---	--	------	------
mPD		0.044	0.70
		nPD:	0.06
		N. weight:	1.1
		Sc. PD:	-0.21
		Sc. rank:	-1159.3

,

PA			
Pos.	448	obs:	exp:
tct	S	0	0.67
tcc	S	1	0.63
tca	S	3	0.97
tcg	S	0	0.21
act	T	0	10.09
acc	T	0	8.87
aca	T	41	18.63
acg	T	0	3.42
agt	S	0	0.74
agc	S	0	0.77
gct	A	34	653.30
gcc	A	1	610.60
gca	A	2609	1201.00
gcg	A	21	199.90
gaa	E	2	1.19
gag	E	0	0.81
ggt	G	0	0.31
ggc	G	0	0.25
gga	G	2	0.91
ggg	G	0	0.53
---	--	------	------
mPD		0.077	0.72
		nPD:	0.11
		N. weight:	1.
		Sc. PD:	-0.16
		Sc. rank:	-712.7

,

PA			
Pos.	449	obs:	exp:
aaa	K	2	1.22
aag	K	0	0.78
gaa	E	2566	1607.00
gag	E	146	1105.00
---	--	------	------
mPD		0.10	0.48
		nPD:	0.21
		N. weight:	0.49
		Sc. PD:	-0.027
		Sc. rank:	46.2

,

PA			
Pos.	450	obs:	exp:
att	I	3	2.48
atc	I	0	1.80
ata	I	4	2.72
gtt	V	11	601.10
gtc	V	1	510.80
gta	V	83	683.40
gtg	V	2612	911.80
---	--	------	------
mPD		0.078	0.74
		nPD:	0.1
		N. weight:	1.4
		Sc. PD:	-0.22
		Sc. rank:	-974.1

,

PA			
Pos.	451	obs:	exp:
tct	S	373	456.20
tcc	S	2330	428.60
tca	S	9	658.60
tcg	S	0	144.10
tgt	C	1	0.43
tgc	C	0	0.57
agt	S	0	504.80
agc	S	0	519.70
gct	A	1	0.25
gcc	A	0	0.23
gca	A	0	0.45
gcg	A	0	0.07
---	--	------	------
mPD		0.25	1.6
		nPD:	0.15
		N. weight:	2.1
		Sc. PD:	-0.24
		Sc. rank:	-675.5

,

PA			
Pos.	452	obs:	exp:
tat	Y	3	6.16
tac	Y	8	4.84
cat	H	1412	1559.00
cac	H	1291	1144.00
---	--	------	------
mPD		0.51	0.50
		nPD:	1.02
		N. weight:	0.013
		Sc. PD:	0.0093
		Sc. rank:	20.3

,

PA			
Pos.	453	obs:	exp:
tgt	C	197	1168.00
tgc	C	2517	1546.00
---	--	------	------
mPD		0.13	0.49
		nPD:	0.27
		N. weight:	0.47
		Sc. PD:	0.0025
		Sc. rank:	154.1

,

PA			
Pos.	454	obs:	exp:
cgt	R	0	85.64
cgc	R	0	143.40
cga	R	0	272.70
cgg	R	2	196.00
act	T	0	0.25
acc	T	0	0.22
aca	T	0	0.45
acg	T	1	0.08
aga	R	169	1244.00
agg	R	2542	771.10
---	--	------	------
mPD		0.12	0.94
		nPD:	0.13
		N. weight:	1.4
		Sc. PD:	-0.2
		Sc. rank:	-742.9

,

PA			
Pos.	455	obs:	exp:
gct	A	1999	665.30
gcc	A	683	621.80
gca	A	29	1223.00
gcg	A	3	203.50
---	--	------	------
mPD		0.39	0.68
		nPD:	0.58
		N. weight:	1.2
		Sc. PD:	0.35
		Sc. rank:	1483.8

,

PA

Pos.	456	obs:	exp:
act	T	2337	667.30
acc	T	9	586.70
aca	T	106	1233.00
acg	T	261	226.20
gct	A	1	0.25
gcc	A	0	0.23
gca	A	0	0.45
gcg	A	0	0.07
---	--	------	------
mPD		0.25	0.68
		nPD:	0.36
		N. weight:	1.4
		Sc. PD:	0.13
		Sc. rank:	903.8

PA

Pos.	457	obs:	exp:
gaa	E	2506	1608.00
gag	E	208	1106.00
---	--	------	------
mPD		0.14	0.48
		nPD:	0.29
		N. weight:	0.41
		Sc. PD:	0.0096
		Sc. rank:	170.0

PA

Pos.	458	obs:	exp:
tat	Y	146	1520.00
tac	Y	2568	1194.00
---	--	------	------
mPD		0.10	0.49
		nPD:	0.21
		N. weight:	0.88
		Sc. PD:	-0.054
		Sc. rank:	49.0

PA

Pos.	459	obs:	exp:
tta	L	1	0.11
ttg	L	0	0.19
ctt	L	0	0.20
ctc	L	0	0.15
cta	L	0	0.17
ctg	L	0	0.18
att	I	1	958.90
atc	I	1	693.30
ata	I	2701	1051.00
atg	M	4	4.00
act	T	0	0.25
acc	T	0	0.22
aca	T	1	0.45
acg	T	0	0.08
gtt	V	0	1.11
gtc	V	0	0.94
gta	V	5	1.26
gtg	V	0	1.68
---	--	------	------
mPD		0.0096	0.66
		nPD:	0.01
		N. weight:	1.4
		Sc. PD:	-0.34
		Sc. rank:	-2042.8

PA

Pos.	460	obs:	exp:
att	I	20	9.58
atc	I	0	6.93
ata	I	7	10.50
atg	M	2686	2686.00
gtt	V	0	0.22
gtc	V	0	0.19
gta	V	0	0.25
gtg	V	1	0.34
---	--	------	------
mPD		0.020	0.021
		nPD:	0.98
		N. weight:	0.0079
		Sc. PD:	0.0055
		Sc. rank:	12.6

PA

Pos.	461	obs:	exp:
cgt	R	0	0.06
cgc	R	0	0.11
cga	R	0	0.20
cgg	R	0	0.14
aaa	K	364	1648.00
aag	K	2348	1064.00
aga	R	0	0.92
agg	R	2	0.57
---	--	------	------
mPD		0.23	0.48
		nPD:	0.49
		N. weight:	0.71
		Sc. PD:	0.15
		Sc. rank:	706.7

PA

Pos.	462	obs:	exp:
ggt	G	22	425.70
ggc	G	11	343.90
gga	G	2603	1229.00
ggg	G	78	715.10
---	--	------	------
mPD		0.079	0.68
		nPD:	0.12
		N. weight:	0.91
		Sc. PD:	-0.14
		Sc. rank:	-553.3

PA

Pos.	463	obs:	exp:
atg	M	1	1.00
gtt	V	233	602.40
gtc	V	2	511.90
gta	V	235	684.90
gtg	V	2243	913.80
---	--	------	------
mPD		0.30	0.74
		nPD:	0.41
		N. weight:	0.83
		Sc. PD:	0.11
		Sc. rank:	634.9

PA

Pos.	464	obs:	exp:
tat	Y	322	1519.00
tac	Y	2389	1192.00
cat	H	0	1.73
cac	H	3	1.27
---	--	------	------
mPD		0.21	0.49
		nPD:	0.43
		N. weight:	0.63
		Sc. PD:	0.097
		Sc. rank:	526.1

PA

Pos.	465	obs:	exp:
tta	L	0	0.33
ttg	L	0	0.56
ctt	L	0	0.61
ctc	L	0	0.44
cta	L	3	0.52
ctg	L	0	0.54
att	I	9	931.90
atc	I	42	673.80
ata	I	2576	1021.00
atg	M	50	50.00
act	T	0	5.17
acc	T	0	4.54
aca	T	17	9.54
acg	T	4	1.75
gtt	V	0	2.89
gtc	V	0	2.45
gta	V	13	3.28
gtg	V	0	4.38
---	--	------	------
mPD		0.10	0.70
		nPD:	0.15
		N. weight:	1.2
		Sc. PD:	-0.15
		Sc. rank:	-452.9

PA

Pos.	466	obs:	exp:
tct	S	0	0.17
tcc	S	0	0.16
tca	S	0	0.24
tcg	S	0	0.05
aat	N	2228	1542.00
aac	N	485	1171.00
agt	S	1	0.19
agc	S	0	0.19
---	--	------	------
mPD		0.29	0.49
		nPD:	0.6
		N. weight:	0.21
		Sc. PD:	0.069
		Sc. rank:	281.2

PA

Pos.	467	obs:	exp:
act	T	176	667.30
acc	T	2	586.70
aca	T	475	1233.00
acg	T	2060	226.20
gct	A	0	0.25
gcc	A	0	0.23
gca	A	1	0.45
gcg	A	0	0.07
---	--	------	------
mPD		0.39	0.68
		nPD:	0.57
		N. weight:	2.1
		Sc. PD:	0.62
		Sc. rank:	2608.7

PA

Pos.	468	obs:	exp:
gct	A	1227	665.30
gcc	A	1417	621.80
gca	A	68	1223.00
gcg	A	2	203.50
---	--	------	------
mPD		0.52	0.68
		nPD:	0.77
		N. weight:	0.92
		Sc. PD:	0.45
		Sc. rank:	1404.0

PA

Pos.	469	obs:	exp:
tta	L	473	295.30
ttg	L	1298	509.80
ctt	L	60	548.10
ctc	L	655	394.90
cta	L	199	473.90
ctg	L	29	492.00
---	--	------	------
mPD		1.1	1.1
		nPD:	0.96
		N. weight:	0.75
		Sc. PD:	0.51
		Sc. rank:	1194.3

PA

Pos.	470	obs:	exp:
tta	L	0	295.20
ttg	L	0	509.60
ctt	L	1154	547.90
ctc	L	1533	394.70
cta	L	24	473.70
ctg	L	2	491.80
att	I	0	0.35
atc	I	1	0.26
ata	I	0	0.39
---	--	------	------
mPD		0.50	1.1
		nPD:	0.44
		N. weight:	1.5
		Sc. PD:	0.26
		Sc. rank:	1382.9

PA

Pos.	471	obs:	exp:
aat	N	2636	1543.00
aac	N	78	1171.00
---	--	------	------
mPD		0.056	0.49
		nPD:	0.11
		N. weight:	0.65
		Sc. PD:	-0.098
		Sc. rank:	-400.9

PA

Pos.	472	obs:	exp:
tct	S	0	1.68
tcc	S	0	1.58
tca	S	10	2.43
tcg	S	0	0.53
agt	S	0	1.86
agc	S	0	1.92
gct	A	14	662.80
gcc	A	6	619.60
gca	A	2666	1219.00
gcg	A	18	202.80
---	--	------	------
mPD		0.035	0.69
		nPD:	0.05
		N. weight:	1.1
		Sc. PD:	-0.23
		Sc. rank:	-1289.6

PA

Pos.	473	obs:	exp:
tct	S	1443	456.50
tcc	S	1105	428.90
tca	S	166	659.10
tcg	S	0	144.20
agt	S	0	505.10
agc	S	0	520.10
---	--	------	------
mPD		0.55	1.6
		nPD:	0.34
		N. weight:	1.3
		Sc. PD:	0.087
		Sc. rank:	744.8

PA

Pos.	474	obs:	exp:
tgt	C	2688	1168.00
tgc	C	26	1546.00
---	--	------	------
mPD		0.019	0.49
		nPD:	0.04
		N. weight:	1.1
		Sc. PD:	-0.26
		Sc. rank:	-1531.0

PA

Pos.	475	obs:	exp:
tct	S	0	0.84
tcc	S	0	0.79
tca	S	5	1.21
tcg	S	0	0.27
act	T	0	1.72
acc	T	0	1.51
aca	T	7	3.18
acg	T	0	0.58
agt	S	0	0.93
agc	S	0	0.96
gtt	V	0	0.67
gtc	V	0	0.57
gta	V	3	0.76
gtg	V	0	1.01
gct	A	14	661.60
gcc	A	7	618.40
gca	A	2662	1217.00
gcg	A	16	202.40
---	--	------	------
mPD		0.038	0.69
		nPD:	0.06
		N. weight:	1.1
		Sc. PD:	-0.22
		Sc. rank:	-1234.9

PA			
Pos.	476	obs:	exp:
tct	S	0	0.34
tcc	S	2	0.32
tca	S	0	0.49
tcg	S	0	0.11
act	T	0	2.46
acc	T	0	2.16
aca	T	10	4.54
acg	T	0	0.83
aat	N	0	1.71
aac	N	3	1.29
agt	S	0	0.37
agc	S	0	0.38
gct	A	48	661.60
gcc	A	2253	618.40
gca	A	396	1217.00
gcg	A	2	202.40
---	--	------	------
mPD		0.30	0.69
		nPD:	0.43
		N. weight:	1.3
		Sc. PD:	0.2
		Sc. rank:	1084.4

,

PA			
Pos.	477	obs:	exp:
atg	M	2714	2714.00
---	--	------	------
mPD		0	0
		nPD:	1.
		N. weight:	0.
		Sc. PD:	0
		Sc. rank:	0

,

PA			
Pos.	478	obs:	exp:
aat	N	10	5.68
aac	N	0	4.32
gat	D	2666	1513.00
gac	D	24	1177.00
gaa	E	11	7.11
gag	E	1	4.89
ggt	G	2	0.31
ggc	G	0	0.25
gga	G	0	0.91
ggg	G	0	0.53
---	--	------	------
mPD		0.035	0.51
		nPD:	0.07
		N. weight:	0.77
		Sc. PD:	-0.15
		Sc. rank:	-797.4

,

PA			
Pos.	479	obs:	exp:
aat	N	1	0.57
aac	N	0	0.43
gat	D	539	1513.00
gac	D	2151	1177.00
gaa	E	10	11.26
gag	E	9	7.74
ggt	G	0	0.63
ggc	G	4	0.51
gga	G	0	1.81
ggg	G	0	1.05
---	--	------	------
mPD		0.33	0.50
		nPD:	0.66
		N. weight:	0.41
		Sc. PD:	0.16
		Sc. rank:	568.6

,

PA			
Pos.	480	obs:	exp:
ttt	F	2271	1215.00
ttc	F	440	1496.00
tct	S	0	0.17
tcc	S	1	0.16
tca	S	0	0.24
tcg	S	0	0.05
tat	Y	2	1.12
tac	Y	0	0.88
agt	S	0	0.19
agc	S	0	0.19
---	--	------	------
mPD		0.27	0.50
		nPD:	0.55
		N. weight:	0.48
		Sc. PD:	0.13
		Sc. rank:	570.1

,

PA			
Pos.	481	obs:	exp:
caa	Q	187	1464.00
cag	Q	2527	1250.00
---	--	------	------
mPD		0.13	0.50
		nPD:	0.26
		N. weight:	0.75
		Sc. PD:	-0.0081
		Sc. rank:	211.9

,

PA			
Pos.	482	obs:	exp:
tta	L	340	295.10
ttg	L	117	509.40
ctt	L	8	547.70
ctc	L	10	394.60
cta	L	104	473.50
ctg	L	2133	491.60
atg	M	2	2.00
---	--	------	------
mPD		0.57	1.1
		nPD:	0.5
		N. weight:	1.5
		Sc. PD:	0.33
		Sc. rank:	1529.6

,

PA			
Pos.	483	obs:	exp:
att	I	279	962.40
atc	I	2416	695.90
ata	I	18	1055.00
gtt	V	0	0.22
gtc	V	1	0.19
gta	V	0	0.25
gtg	V	0	0.34
---	--	------	------
mPD		0.20	0.66
		nPD:	0.3
		N. weight:	1.4
		Sc. PD:	0.041
		Sc. rank:	598.4

,

PA			
Pos.	484	obs:	exp:
tct	S	0	0.17
tcc	S	0	0.16
tca	S	1	0.24
tcg	S	0	0.05
cct	P	4	800.60
ccc	P	4	546.20
cca	P	2621	1074.00
ccg	P	84	291.80
agt	S	0	0.19
agc	S	0	0.19
---	--	------	------
mPD		0.066	0.71
		nPD:	0.09
		N. weight:	1.2
		Sc. PD:	-0.2
		Sc. rank:	-943.0

,

PA			
Pos.	485	obs:	exp:
att	I	0	0.35
atc	I	0	0.26
ata	I	1	0.39
atg	M	2713	2713.00
---	--	------	------
mPD		0.00074	0.00074
		nPD:	1.
		N. weight:	0.00051
		Sc. PD:	0.00036
		Sc. rank:	0.8

,

PA			
Pos.	486	obs:	exp:
tta	L	1	2.29
ttg	L	0	3.94
ctt	L	0	4.24
ctc	L	0	3.06
cta	L	20	3.67
ctg	L	0	3.81
att	I	10	952.80
atc	I	17	688.90
ata	I	2659	1044.00
atg	M	3	3.00
gtt	V	0	0.89
gtc	V	0	0.75
gta	V	4	1.01
gtg	V	0	1.35
---	--	------	------
mPD		0.040	0.68
		nPD:	0.06
		N. weight:	1.3
		Sc. PD:	-0.27
		Sc. rank:	-1484.1

,

PA			
Pos.	487	obs:	exp:
tct	S	0	453.80
tcc	S	0	426.40
tca	S	0	655.20
tcg	S	0	143.40
act	T	0	0.25
acc	T	1	0.22
aca	T	0	0.45
acg	T	0	0.08
aat	N	0	8.53
aac	N	15	6.47
agt	S	77	502.20
agc	S	2621	517.10
---	--	------	------
mPD		0.067	1.6
		nPD:	0.04
		N. weight:	2.2
		Sc. PD:	-0.49
		Sc. rank:	-2924.7

,

PA			
Pos.	488	obs:	exp:
aaa	K	2502	1649.00
aag	K	212	1065.00
---	--	------	------
mPD		0.14	0.48
		nPD:	0.3
		N. weight:	0.38
		Sc. PD:	0.012
		Sc. rank:	172.2

,

PA			
Pos.	489	obs:	exp:
tct	S	0	0.34
tcc	S	1	0.32
tca	S	0	0.49
tcg	S	0	0.11
tgt	C	2143	1167.00
tgc	C	569	1545.00
agt	S	0	0.37
agc	S	1	0.38
---	--	------	------
mPD		0.33	0.49
		nPD:	0.68
		N. weight:	0.4
		Sc. PD:	0.16
		Sc. rank:	564.9

,

PA			
Pos.	490	obs:	exp:
cgt	R	0	85.57
cgc	R	0	143.30
cga	R	1	272.50
cgg	R	1	195.90
aaa	K	2	1.82
aag	K	1	1.18
aga	R	573	1243.00
agg	R	2136	770.50
---	--	------	------
mPD		0.34	0.94
		nPD:	0.36
		N. weight:	0.93
		Sc. PD:	0.082
		Sc. rank:	568.6

,

PA			
Pos.	491	obs:	exp:
act	T	51	667.30
acc	T	2204	586.70
aca	T	88	1233.00
acg	T	370	226.20
aat	N	0	0.57
aac	N	1	0.43
---	--	------	------
mPD		0.32	0.68
		nPD:	0.47
		N. weight:	1.5
		Sc. PD:	0.29
		Sc. rank:	1411.5

,

PA			
Pos.	492	obs:	exp:
cgt	R	0	1.29
cgc	R	0	2.17
cga	R	0	4.12
cgg	R	0	2.96
aaa	K	2008	1624.00
aag	K	665	1049.00
aga	R	40	18.80
agg	R	1	11.65
---	--	------	------
mPD		0.40	0.52
		nPD:	0.78
		N. weight:	0.084
		Sc. PD:	0.041
		Sc. rank:	127.8

,

PA			
Pos.	493	obs:	exp:
aaa	K	1	0.61
aag	K	0	0.39
gaa	E	2662	1607.00
gag	E	50	1105.00
ggt	G	0	0.16
ggc	G	0	0.13
gga	G	1	0.45
ggg	G	0	0.26
---	--	------	------
mPD		0.038	0.48
		nPD:	0.08
		N. weight:	0.64
		Sc. PD:	-0.12
		Sc. rank:	-593.0

,

PA			
Pos.	494	obs:	exp:
gaa	E	1	0.59
gag	E	0	0.41
ggt	G	12	425.50
ggc	G	6	343.80
gga	G	2547	1229.00
ggg	G	148	714.80
---	--	------	------
mPD		0.12	0.69
		nPD:	0.17
		N. weight:	0.84
		Sc. PD:	-0.082
		Sc. rank:	-112.9

,

PA			
Pos.	495	obs:	exp:
cgt	R	0	85.57
cgc	R	0	143.30
cga	R	0	272.50
cgg	R	21	195.90
aaa	K	1	1.82
aag	K	2	1.18
aga	R	2192	1243.00
agg	R	498	770.50
---	--	------	------
mPD		0.33	0.94
		nPD:	0.35
		N. weight:	0.53
		Sc. PD:	0.041
		Sc. rank:	312.8

,

PA			
Pos.	496	obs:	exp:
cct	P	0	0.30
ccc	P	0	0.20
cca	P	0	0.40
ccg	P	1	0.11
caa	Q	0	1.08
cag	Q	2	0.92
cgt	R	4	85.54
cgc	R	0	143.20
cga	R	1160	272.40
cgg	R	1464	195.80
aaa	K	0	0.61
aag	K	1	0.39
aga	R	12	1243.00
agg	R	70	770.30
---	--	------	------
mPD		0.55	0.94
		nPD:	0.59
		N. weight:	2.4
		Sc. PD:	0.74
		Sc. rank:	3084.9

,

PA			
Pos.	497	obs:	exp:
cgt	R	0	25.54
cgc	R	0	42.76
cga	R	0	81.31
cgg	R	0	58.45
aaa	K	1555	1158.00
aag	K	350	747.30
aga	R	798	371.00
agg	R	11	229.90
---	--	------	------
mPD		0.65	1.1
		nPD:	0.61
		N. weight:	0.42
		Sc. PD:	0.14
		Sc. rank:	557.2

,

PA			
Pos.	498	obs:	exp:
tct	S	0	0.17
tcc	S	0	0.16
tca	S	1	0.24
tcg	S	0	0.05
act	T	29	667.30
acc	T	25	586.70
aca	T	2501	1233.00
acg	T	158	226.20
agt	S	0	0.19
agc	S	0	0.19
---	--	------	------
mPD		0.15	0.68
		nPD:	0.22
		N. weight:	0.83
		Sc. PD:	-0.043
		Sc. rank:	96.3

,

PA			
Pos.	499	obs:	exp:
tct	S	0	0.17
tcc	S	0	0.16
tca	S	0	0.24
tcg	S	0	0.05
aat	N	1635	1542.00
aac	N	1078	1171.00
agt	S	1	0.19
agc	S	0	0.19
---	--	------	------
mPD		0.48	0.49
		nPD:	0.98
		N. weight:	0.0067
		Sc. PD:	0.0046
		Sc. rank:	10.6

,

PA			
Pos.	500	obs:	exp:
tta	L	50	295.30
ttg	L	79	509.80
ctt	L	4	548.10
ctc	L	14	394.90
cta	L	438	473.90
ctg	L	2129	492.00
---	--	------	------
mPD		0.40	1.1
		nPD:	0.35
		N. weight:	1.5
		Sc. PD:	0.12
		Sc. rank:	879.2

,

PA			
Pos.	501	obs:	exp:
ttt	F	1	0.45
ttc	F	0	0.55
tat	Y	1867	1520.00
tac	Y	846	1193.00
---	--	------	------
mPD		0.43	0.49
		nPD:	0.87
		N. weight:	0.053
		Sc. PD:	0.031
		Sc. rank:	82.8

,

PA			
Pos.	502	obs:	exp:
tgg	W	1	1.00
ggt	G	310	425.50
ggc	G	816	343.80
gga	G	83	1229.00
ggg	G	1504	714.80
---	--	------	------
mPD		0.59	0.69
		nPD:	0.86
		N. weight:	0.81
		Sc. PD:	0.47
		Sc. rank:	1272.8

,

PA			
Pos.	503	obs:	exp:
ttt	F	74	1216.00
ttc	F	2640	1498.00
---	--	------	------
mPD		0.053	0.49
		nPD:	0.11
		N. weight:	0.69
		Sc. PD:	-0.11
		Sc. rank:	-484.0

,

PA			
Pos.	504	obs:	exp:
tta	L	0	0.22
ttg	L	0	0.38
ctt	L	2	0.40
ctc	L	0	0.29
cta	L	0	0.35
ctg	L	0	0.36
att	I	2469	957.10
atc	I	226	692.00
ata	I	3	1049.00
gtt	V	14	3.11
gtc	V	0	2.64
gta	V	0	3.53
gtg	V	0	4.72
---	--	------	------
mPD		0.17	0.67
		nPD:	0.25
		N. weight:	1.1
		Sc. PD:	-0.023
		Sc. rank:	272.3

,

PA			
Pos.	505	obs:	exp:
att	I	0	707.70
atc	I	4	511.70
ata	I	1991	775.60
gtt	V	1	159.60
gtc	V	0	135.70
gta	V	652	181.50
gtg	V	66	242.20
---	--	------	------
mPD		0.44	1.1
		nPD:	0.4
		N. weight:	1.4
		Sc. PD:	0.18
		Sc. rank:	1052.4

,

PA			
Pos.	506	obs:	exp:
aat	N	0	0.57
aac	N	1	0.43
aaa	K	2344	1649.00
aag	K	369	1064.00
---	--	------	------
mPD		0.24	0.48
		nPD:	0.49
		N. weight:	0.24
		Sc. PD:	0.052
		Sc. rank:	236.7

,

PA			
Pos.	507	obs:	exp:
cgt	R	0	0.03
cgc	R	0	0.05
cga	R	0	0.10
cgg	R	0	0.07
aga	R	1	0.46
agg	R	0	0.28
ggt	G	1	425.50
ggc	G	5	343.80
gga	G	2526	1229.00
ggg	G	181	714.80
---	--	------	------
mPD		0.13	0.69
		nPD:	0.19
		N. weight:	0.83
		Sc. PD:	-0.066
		Sc. rank:	-26.4

,

PA			
Pos.	508	obs:	exp:
cgt	R	0	85.42
cgc	R	0	143.00
cga	R	19	272.00
cgg	R	1	195.50
aaa	K	2	4.86
aag	K	6	3.14
aga	R	511	1241.00
agg	R	2175	769.10
---	--	------	------
mPD		0.34	0.94
		nPD:	0.36
		N. weight:	0.95
		Sc. PD:	0.08
		Sc. rank:	567.0

,

PA			
Pos.	509	obs:	exp:
tct	S	1458	456.50
tcc	S	1236	428.90
tca	S	17	659.10
tcg	S	3	144.20
agt	S	0	505.10
agc	S	0	520.10
---	--	------	------
mPD		0.50	1.6
		nPD:	0.31
		N. weight:	1.6
		Sc. PD:	0.061
		Sc. rank:	780.7

,

PA			
Pos.	510	obs:	exp:
cat	H	2649	1565.00
cac	H	65	1149.00
---	--	------	------
mPD		0.047	0.49
		nPD:	0.1
		N. weight:	0.65
		Sc. PD:	-0.11
		Sc. rank:	-514.6

,

PA			
Pos.	511	obs:	exp:
tta	L	105	295.30
ttg	L	1253	509.80
ctt	L	0	548.10
ctc	L	0	394.90
cta	L	305	473.90
ctg	L	1051	492.00
---	--	------	------
mPD		0.76	1.1
		nPD:	0.66
		N. weight:	0.91
		Sc. PD:	0.35
		Sc. rank:	1277.9

,

PA			
Pos.	512	obs:	exp:
cgt	R	0	85.54
cgc	R	0	143.20
cga	R	4	272.40
cgg	R	1	195.80
aaa	K	3	1.82
aag	K	0	1.18
aga	R	2161	1243.00
agg	R	544	770.30
ggt	G	0	0.16
ggc	G	0	0.13
gga	G	1	0.45
ggg	G	0	0.26
---	--	------	------
mPD		0.33	0.94
		nPD:	0.35
		N. weight:	0.53
		Sc. PD:	0.041
		Sc. rank:	313.6

,

PA			
Pos.	513	obs:	exp:
tct	S	0	0.34
tcc	S	0	0.32
tca	S	0	0.49
tcg	S	0	0.11
aat	N	2400	1541.00
aac	N	312	1171.00
agt	S	1	0.37
agc	S	1	0.38
---	--	------	------
mPD		0.21	0.49
		nPD:	0.42
		N. weight:	0.35
		Sc. PD:	0.051
		Sc. rank:	281.1

,

PA			
Pos.	514	obs:	exp:
gat	D	2463	1527.00
gac	D	251	1187.00
---	--	------	------
mPD		0.17	0.49
		nPD:	0.34
		N. weight:	0.43
		Sc. PD:	0.03
		Sc. rank:	244.3

,

PA			
Pos.	515	obs:	exp:
act	T	2515	667.30
acc	T	130	586.70
aca	T	27	1233.00
acg	T	41	226.20
gct	A	1	0.25
gcc	A	0	0.23
gca	A	0	0.45
gcg	A	0	0.07
---	--	------	------
mPD		0.14	0.68
		nPD:	0.2
		N. weight:	1.6
		Sc. PD:	-0.1
		Sc. rank:	54.4

,

PA

Pos.	516	obs:	exp:
gat	D	1182	1526.00
gac	D	1531	1187.00
gaa	E	1	0.59
gag	E	0	0.41
---	--	------	------
mPD		0.49	0.49
		nPD:	1.
		N. weight:	0.05
		Sc. PD:	0.035
		Sc. rank:	79.3

PA

Pos.	517	obs:	exp:
tta	L	0	0.11
ttg	L	0	0.19
ctt	L	0	0.20
ctc	L	0	0.15
cta	L	1	0.17
ctg	L	0	0.18
gtt	V	2	602.40
gtc	V	1	511.90
gta	V	68	684.90
gtg	V	2642	913.80
---	--	------	------
mPD		0.052	0.74
		nPD:	0.07
		N. weight:	1.4
		Sc. PD:	-0.27
		Sc. rank:	-1439.7

PA

Pos.	518	obs:	exp:
gtt	V	15	601.70
gtc	V	0	511.40
gta	V	499	684.10
gtg	V	2196	912.80
gct	A	0	0.74
gcc	A	0	0.69
gca	A	3	1.35
gcg	A	0	0.22
ggt	G	0	0.16
ggc	G	0	0.13
gga	G	0	0.45
ggg	G	1	0.26
---	--	------	------
mPD		0.31	0.74
		nPD:	0.42
		N. weight:	0.93
		Sc. PD:	0.14
		Sc. rank:	756.5

PA

Pos.	519	obs:	exp:
aat	N	624	1543.00
aac	N	2090	1171.00
---	--	------	------
mPD		0.35	0.49
		nPD:	0.72
		N. weight:	0.35
		Sc. PD:	0.15
		Sc. rank:	509.9

PA

Pos.	520	obs:	exp:
ttt	F	2688	1215.00
ttc	F	24	1497.00
tat	Y	2	1.12
tac	Y	0	0.88
---	--	------	------
mPD		0.019	0.50
		nPD:	0.04
		N. weight:	1.1
		Sc. PD:	-0.25
		Sc. rank:	-1470.3

PA

Pos.	521	obs:	exp:
tta	L	110	13.28
ttg	L	2	22.92
ctt	L	0	24.64
ctc	L	0	17.75
cta	L	3	21.30
ctg	L	7	22.12
att	I	0	6.74
atc	I	0	4.87
ata	I	19	7.39
atg	M	2	2.00
gtt	V	9	570.90
gtc	V	5	485.10
gta	V	2109	649.00
gtg	V	448	866.00
---	--	------	------
mPD		0.39	0.84
		nPD:	0.47
		N. weight:	1.3
		Sc. PD:	0.24
		Sc. rank:	1211.7

PA

Pos.	522	obs:	exp:
tct	S	0	456.30
tcc	S	0	428.80
tca	S	0	658.80
tcg	S	0	144.20
cgt	R	0	0.03
cgc	R	0	0.05
cga	R	0	0.10
cgg	R	0	0.07
agt	S	1518	505.00
agc	S	1195	519.90
aga	R	1	0.46
agg	R	0	0.28
---	--	------	------
mPD		0.49	1.6
		nPD:	0.3
		N. weight:	1.4
		Sc. PD:	0.047
		Sc. rank:	662.5

PA

Pos.	523	obs:	exp:
atg	M	2714	2714.00
---	--	------	------
mPD		0	0
		nPD:	1.
		N. weight:	0.
		Sc. PD:	0
		Sc. rank:	0

PA

Pos.	524	obs:	exp:
gaa	E	214	1608.00
gag	E	2500	1106.00
---	--	------	------
mPD		0.15	0.48
		nPD:	0.3
		N. weight:	0.87
		Sc. PD:	0.027
		Sc. rank:	374.7

PA

Pos.	525	obs:	exp:
ttt	F	426	1216.00
ttc	F	2288	1498.00
---	--	------	------
mPD		0.26	0.49
		nPD:	0.54
		N. weight:	0.28
		Sc. PD:	0.073
		Sc. rank:	324.7

PA

Pos.	526	obs:	exp:
tct	S	420	456.50
tcc	S	1135	428.90
tca	S	1155	659.10
tcg	S	4	144.20
agt	S	0	505.10
agc	S	0	520.10
---	--	------	------
mPD		0.62	1.6
		nPD:	0.38
		N. weight:	0.92
		Sc. PD:	0.1
		Sc. rank:	618.7

PA

Pos.	527	obs:	exp:
tta	L	2	295.20
ttg	L	0	509.60
ctt	L	129	547.90
ctc	L	2497	394.70
cta	L	78	473.70
ctg	L	7	491.80
att	I	0	0.35
atc	I	1	0.26
ata	I	0	0.39
---	--	------	------
mPD		0.15	1.1
		nPD:	0.13
		N. weight:	2.3
		Sc. PD:	-0.3
		Sc. rank:	-1030.6

PA

Pos.	528	obs:	exp:
tct	S	0	0.84
tcc	S	0	0.79
tca	S	0	1.21
tcg	S	0	0.27
att	I	6	4.97
atc	I	8	3.59
ata	I	0	5.44
act	T	2164	628.20
acc	T	345	552.30
aca	T	45	1161.00
acg	T	0	213.00
aat	N	0	0.57
aac	N	1	0.43
agt	S	5	0.93
agc	S	0	0.96
gtt	V	140	31.08
gtc	V	0	26.42
gta	V	0	35.34
gtg	V	0	47.16
---	--	------	------
mPD		0.46	0.90
		nPD:	0.52
		N. weight:	1.4
		Sc. PD:	0.34
		Sc. rank:	1548.1

PA

Pos.	529	obs:	exp:
aat	N	8	7.39
aac	N	5	5.61
gat	D	890	1519.00
gac	D	1810	1181.00
gaa	E	1	0.59
gag	E	0	0.41
---	--	------	------
mPD		0.45	0.50
		nPD:	0.9
		N. weight:	0.16
		Sc. PD:	0.1
		Sc. rank:	257.0

PA

Pos.	530	obs:	exp:
cct	P	173	800.90
ccc	P	13	546.40
cca	P	228	1075.00
ccg	P	2300	291.90
---	--	------	------
mPD		0.27	0.70
		nPD:	0.38
		N. weight:	2.2
		Sc. PD:	0.25
		Sc. rank:	1550.0

PA			
Pos.	531	obs:	exp:
tct	S	0	0.84
tcc	S	0	0.79
tca	S	0	1.21
tcg	S	0	0.27
cgt	R	0	81.44
cgc	R	0	136.40
cga	R	0	259.30
cgg	R	0	186.40
att	I	0	0.35
atc	I	0	0.26
ata	I	1	0.39
atg	M	9	9.00
aaa	K	113	72.32
aag	K	6	46.68
agt	S	5	0.93
agc	S	0	0.96
aga	R	1449	1183.00
agg	R	1131	733.30
---	--	------	------
mPD		0.58	1.0
		nPD:	0.57
		N. weight:	0.45
		Sc. PD:	0.13
		Sc. rank:	560.5

PA			
Pos.	532	obs:	exp:
ttt	F	1	0.45
ttc	F	0	0.55
tta	L	0	294.30
ttg	L	146	507.90
ctt	L	27	546.10
ctc	L	4	393.40
cta	L	61	472.20
ctg	L	2466	490.20
caa	Q	0	0.54
cag	Q	1	0.46
atg	M	8	8.00
---	--	------	------
mPD		0.18	1.1
		nPD:	0.15
		N. weight:	1.9
		Sc. PD:	-0.22
		Sc. rank:	-548.1

PA			
Pos.	533	obs:	exp:
gaa	E	270	1608.00
gag	E	2444	1106.00
---	--	------	------
mPD		0.18	0.48
		nPD:	0.37
		N. weight:	0.78
		Sc. PD:	0.078
		Sc. rank:	504.7

PA			
Pos.	534	obs:	exp:
tta	L	0	3.70
ttg	L	3	6.39
tct	S	0	2.35
tcc	S	0	2.21
tca	S	14	3.40
tcg	S	0	0.74
ctt	L	0	6.87
ctc	L	4	4.95
cta	L	12	5.94
ctg	L	15	6.16
cct	P	15	782.60
ccc	P	23	533.90
cca	P	2568	1050.00
ccg	P	46	285.30
caa	Q	5	3.24
cag	Q	1	2.76
att	I	0	0.35
atc	I	0	0.26
ata	I	1	0.39
atg	M	3	3.00
act	T	0	0.74
acc	T	0	0.65
aca	T	3	1.36
acg	T	0	0.25
agt	S	0	2.61
agc	S	0	2.68
gct	A	0	0.25
gcc	A	0	0.23
gca	A	1	0.45
gcg	A	0	0.07
---	--	------	------
mPD		0.13	0.77
		nPD:	0.17
		N. weight:	1.1
		Sc. PD:	-0.11
		Sc. rank:	-172.0

PA			
Pos.	535	obs:	exp:
ttt	F	0	0.45
ttc	F	1	0.55
tat	Y	1	7.84
tac	Y	13	6.16
cat	H	186	1548.00
cac	H	2497	1135.00
caa	Q	6	3.24
cag	Q	0	2.76
aat	N	6	5.68
aac	N	4	4.32
---	--	------	------
mPD		0.16	0.51
		nPD:	0.3
		N. weight:	0.86
		Sc. PD:	0.03
		Sc. rank:	398.2

PA			
Pos.	536	obs:	exp:
aat	N	1	0.57
aac	N	0	0.43
aaa	K	2317	1649.00
aag	K	396	1064.00
---	--	------	------
mPD		0.25	0.48
		nPD:	0.52
		N. weight:	0.22
		Sc. PD:	0.054
		Sc. rank:	240.7

PA			
Pos.	537	obs:	exp:
tgg	W	2714	2714.00
---	--	------	------
mPD		0	0
		nPD:	1.
		N. weight:	0.
		Sc. PD:	0
		Sc. rank:	0

PA			
Pos.	538	obs:	exp:
cgt	R	0	0.03
cgc	R	0	0.05
cga	R	0	0.10
cgg	R	0	0.07
aaa	K	11	6.69
aag	K	0	4.31
aga	R	1	0.46
agg	R	0	0.28
gtt	V	0	0.22
gtc	V	0	0.19
gta	V	0	0.25
gtg	V	1	0.34
gaa	E	2073	1571.00
gag	E	578	1080.00
ggt	G	0	7.84
ggc	G	0	6.34
gga	G	48	22.65
ggg	G	2	13.17
---	--	------	------
mPD		0.38	0.53
		nPD:	0.72
		N. weight:	0.14
		Sc. PD:	0.061
		Sc. rank:	202.1

PA			
Pos.	539	obs:	exp:
cgt	R	0	0.06
cgc	R	0	0.11
cga	R	0	0.20
cgg	R	0	0.14
aat	N	1	0.57
aac	N	0	0.43
aaa	K	1217	1646.00
aag	K	1492	1063.00
aga	R	0	0.92
agg	R	2	0.57
gaa	E	1	1.19
gag	E	1	0.81
---	--	------	------
mPD		0.50	0.48
		nPD:	1.04
		N. weight:	0.079
		Sc. PD:	0.059
		Sc. rank:	125.9

PA			
Pos.	540	obs:	exp:
tat	Y	451	1519.00
tac	Y	2261	1193.00
cat	H	0	1.15
cac	H	2	0.85
---	--	------	------
mPD		0.28	0.49
		nPD:	0.56
		N. weight:	0.49
		Sc. PD:	0.14
		Sc. rank:	595.7

PA			
Pos.	541	obs:	exp:
tgt	C	2670	1168.00
tgc	C	44	1546.00
---	--	------	------
mPD		0.032	0.49
		nPD:	0.07
		N. weight:	1.1
		Sc. PD:	-0.22
		Sc. rank:	-1179.6

PA			
Pos.	542	obs:	exp:
att	I	10	10.29
atc	I	19	7.44
ata	I	0	11.27
gtt	V	2603	596.20
gtc	V	79	506.70
gta	V	2	677.80
gtg	V	1	904.40
---	--	------	------
mPD		0.093	0.76
		nPD:	0.12
		N. weight:	2.
		Sc. PD:	-0.29
		Sc. rank:	-1084.6

PA			
Pos.	543	obs:	exp:
tta	L	1	293.00
ttg	L	0	505.60
ctt	L	2601	543.70
ctc	L	56	391.70
cta	L	33	470.10
ctg	L	1	488.00
att	I	21	7.80
atc	I	1	5.64
ata	I	0	8.55
---	--	------	------
mPD		0.083	1.1
		nPD:	0.07
		N. weight:	2.1
		Sc. PD:	-0.4
		Sc. rank:	-2094.8

PA			
Pos.	544	obs:	exp:
gaa	E	2625	1608.00
gag	E	89	1106.00
---	--	------	------
mPD		0.063	0.48
		nPD:	0.13
		N. weight:	0.57
		Sc. PD:	-0.077
		Sc. rank:	-283.4

PA			
Pos.	545	obs:	exp:
att	I	7	943.60
atc	I	1	682.30
ata	I	2652	1034.00
gtt	V	0	11.99
gtc	V	0	10.19
gta	V	42	13.63
gtg	V	12	18.19
---	--	------	------
mPD		0.054	0.70
		nPD:	0.08
		N. weight:	1.3
		Sc. PD:	-0.25
		Sc. rank:	-1280.6

,

PA			
Pos.	546	obs:	exp:
ggt	G	64	425.70
ggc	G	1	343.90
gga	G	2582	1229.00
ggg	G	67	715.10
---	--	------	------
mPD		0.094	0.68
		nPD:	0.14
		N. weight:	0.88
		Sc. PD:	-0.11
		Sc. rank:	-383.0

,

PA			
Pos.	547	obs:	exp:
aat	N	0	17.62
aac	N	31	13.38
gat	D	119	1489.00
gac	D	2529	1159.00
gaa	E	33	20.74
gag	E	2	14.26
---	--	------	------
mPD		0.13	0.53
		nPD:	0.25
		N. weight:	0.92
		Sc. PD:	-0.019
		Sc. rank:	217.9

,

PA			
Pos.	548	obs:	exp:
att	I	2	1.42
atc	I	0	1.03
ata	I	2	1.56
atg	M	2710	2710.00
---	--	------	------
mPD		0.0029	0.0029
		nPD:	1.
		N. weight:	0.0012
		Sc. PD:	0.00083
		Sc. rank:	1.9

,

PA			
Pos.	549	obs:	exp:
ttt	F	0	1.79
ttc	F	4	2.21
tta	L	1	250.00
ttg	L	0	431.50
ctt	L	22	463.90
ctc	L	2256	334.20
cta	L	18	401.10
ctg	L	0	416.40
att	I	322	142.30
atc	I	77	102.90
ata	I	2	155.90
atg	M	1	1.00
act	T	1	0.25
acc	T	0	0.22
aca	T	0	0.45
acg	T	0	0.08
gtt	V	10	2.22
gtc	V	0	1.89
gta	V	0	2.52
gtg	V	0	3.37
---	--	------	------
mPD		0.50	1.3
		nPD:	0.39
		N. weight:	2.4
		Sc. PD:	0.28
		Sc. rank:	1708.6

,

PA			
Pos.	550	obs:	exp:
tta	L	50	290.20
ttg	L	1053	501.00
ctt	L	5	538.60
ctc	L	19	388.00
cta	L	409	465.70
ctg	L	1131	483.50
att	I	1	4.61
atc	I	0	3.33
ata	I	12	5.05
atg	M	19	19.00
gtt	V	0	3.33
gtc	V	0	2.83
gta	V	0	3.79
gtg	V	15	5.05
---	--	------	------
mPD		0.80	1.2
		nPD:	0.69
		N. weight:	0.84
		Sc. PD:	0.35
		Sc. rank:	1203.8

,

PA			
Pos.	551	obs:	exp:
caa	Q	1	0.54
cag	Q	0	0.46
cgt	R	1	83.58
cgc	R	1	140.00
cga	R	360	266.20
cgg	R	40	191.30
atg	M	1	1.00
aaa	K	32	38.90
aag	K	32	25.10
aga	R	236	1214.00
agg	R	2010	752.60
---	--	------	------
mPD		0.66	0.98
		nPD:	0.67
		N. weight:	0.88
		Sc. PD:	0.35
		Sc. rank:	1248.9

,

PA			
Pos.	552	obs:	exp:
tct	S	0	0.34
tcc	S	0	0.32
tca	S	0	0.49
tcg	S	0	0.11
act	T	2658	666.60
acc	T	40	586.00
aca	T	4	1231.00
acg	T	8	226.00
agt	S	2	0.37
agc	S	0	0.38
gct	A	2	0.49
gcc	A	0	0.46
gca	A	0	0.90
gcg	A	0	0.15
---	--	------	------
mPD		0.041	0.68
		nPD:	0.06
		N. weight:	1.9
		Sc. PD:	-0.39
		Sc. rank:	-2149.1

,

PA			
Pos.	553	obs:	exp:
tct	S	3	71.99
tcc	S	11	67.64
tca	S	405	103.90
tcg	S	9	22.75
agt	S	0	79.66
agc	S	0	82.03
gct	A	79	560.40
gcc	A	3	523.80
gca	A	916	1030.00
gcg	A	1288	171.40
---	--	------	------
mPD		0.80	1.1
		nPD:	0.74
		N. weight:	1.5
		Sc. PD:	0.7
		Sc. rank:	2276.9

,

PA			
Pos.	554	obs:	exp:
ttt	F	1	0.45
ttc	F	0	0.55
att	I	401	937.20
atc	I	32	677.60
ata	I	2209	1027.00
atg	M	1	1.00
gtt	V	44	15.54
gtc	V	0	13.21
gta	V	26	17.67
gtg	V	0	23.58
---	--	------	------
mPD		0.35	0.71
		nPD:	0.48
		N. weight:	0.71
		Sc. PD:	0.15
		Sc. rank:	706.3

,

PA			
Pos.	555	obs:	exp:
ggt	G	125	425.70
ggc	G	2549	343.90
gga	G	30	1229.00
ggg	G	10	715.10
---	--	------	------
mPD		0.12	0.68
		nPD:	0.17
		N. weight:	2.6
		Sc. PD:	-0.25
		Sc. rank:	-356.9

,

PA			
Pos.	556	obs:	exp:
cat	H	0	0.58
cac	H	1	0.42
caa	Q	2659	1462.00
cag	Q	53	1250.00
cgt	R	0	0.03
cgc	R	0	0.05
cga	R	1	0.10
cgg	R	0	0.07
aga	R	0	0.46
agg	R	0	0.28
---	--	------	------
mPD		0.040	0.50
		nPD:	0.08
		N. weight:	0.77
		Sc. PD:	-0.14
		Sc. rank:	-690.5

,

PA			
Pos.	557	obs:	exp:
tta	L	0	0.11
ttg	L	1	0.19
ctt	L	0	0.20
ctc	L	0	0.15
cta	L	0	0.17
ctg	L	0	0.18
att	I	4	3.90
atc	I	0	2.82
ata	I	7	4.28
atg	M	3	3.00
gtt	V	20	599.10
gtc	V	0	509.10
gta	V	102	681.10
gtg	V	2576	908.80
gct	A	0	0.25
gcc	A	0	0.23
gca	A	0	0.45
gcg	A	1	0.07
---	--	------	------
mPD		0.11	0.75
		nPD:	0.14
		N. weight:	1.3
		Sc. PD:	-0.16
		Sc. rank:	-552.9

,

PA			
Pos.	558	obs:	exp:
tta	L	1	1.31
ttg	L	11	2.25
tct	S	17	449.80
tcc	S	0	422.60
tca	S	367	649.30
tcg	S	2290	142.10
ctt	L	0	2.42
ctc	L	0	1.75
cta	L	0	2.10
ctg	L	0	2.18
cct	P	0	0.30
ccc	P	0	0.20
cca	P	1	0.40
ccg	P	0	0.11
act	T	0	4.18
acc	T	0	3.68
aca	T	13	7.72
acg	T	4	1.42
agt	S	0	497.70
agc	S	0	512.50
gct	A	0	2.45
gcc	A	0	2.29
gca	A	8	4.51
gcg	A	2	0.75
---	--	------	------
mPD		0.29	1.6
		nPD:	0.17
		N. weight:	3.3
		Sc. PD:	-0.31
		Sc. rank:	-325.0

,

PA			
Pos.	559	obs:	exp:
cgt	R	0	85.32
cgc	R	0	142.90
cga	R	0	271.70
cgg	R	2	195.30
aaa	K	0	6.69
aag	K	11	4.31
aga	R	70	1240.00
agg	R	2631	768.30
---	--	------	------
mPD		0.060	0.95
		nPD:	0.06
		N. weight:	1.6
		Sc. PD:	-0.33
		Sc. rank:	-1762.1

,

PA

Pos.	560	obs:	exp:
tta	L	0	0.11
ttg	L	0	0.19
tct	S	0	1.18
tcc	S	6	1.11
tca	S	1	1.70
tcg	S	0	0.37
ctt	L	1	0.20
ctc	L	0	0.15
cta	L	0	0.17
ctg	L	0	0.18
cct	P	67	798.20
ccc	P	2167	544.60
cca	P	451	1071.00
ccg	P	20	291.00
agt	S	0	1.30
agc	S	0	1.34
gct	A	0	0.25
gcc	A	1	0.23
gca	A	0	0.45
gcg	A	0	0.07
---	--	------	------
mPD		0.34	0.71
		nPD:	0.47
		N. weight:	1.3
		Sc. PD:	0.26
		Sc. rank:	1244.1

,

PA

Pos.	561	obs:	exp:
tta	L	0	2.07
ttg	L	4	3.57
ctt	L	0	3.84
ctc	L	0	2.76
cta	L	0	3.32
ctg	L	15	3.44
att	I	0	4.61
atc	I	0	3.33
ata	I	13	5.05
atg	M	2679	2679.00
gtt	V	0	0.67
gtc	V	0	0.57
gta	V	0	0.76
gtg	V	3	1.01
---	--	------	------
mPD		0.026	0.036
		nPD:	0.72
		N. weight:	0.021
		Sc. PD:	0.0093
		Sc. rank:	30.8

,

PA

Pos.	562	obs:	exp:
ttt	F	410	1212.00
ttc	F	2294	1492.00
tat	Y	3	5.60
tac	Y	7	4.40
---	--	------	------
mPD		0.27	0.50
		nPD:	0.53
		N. weight:	0.29
		Sc. PD:	0.075
		Sc. rank:	333.5

,

PA

Pos.	563	obs:	exp:
tta	L	193	295.30
ttg	L	16	509.80
ctt	L	5	548.10
ctc	L	8	394.90
cta	L	2098	473.90
ctg	L	394	492.00
---	--	------	------
mPD		0.41	1.1
		nPD:	0.36
		N. weight:	1.5
		Sc. PD:	0.13
		Sc. rank:	931.9

,

PA

Pos.	564	obs:	exp:
tct	S	1	0.17
tcc	S	0	0.16
tca	S	0	0.24
tcg	S	0	0.05
tat	Y	2679	1520.00
tac	Y	34	1193.00
agt	S	0	0.19
agc	S	0	0.19
---	--	------	------
mPD		0.025	0.49
		nPD:	0.05
		N. weight:	0.75
		Sc. PD:	-0.16
		Sc. rank:	-907.1

,

PA

Pos.	565	obs:	exp:
att	I	0	1.06
atc	I	0	0.77
ata	I	3	1.17
atg	M	68	68.00
gtt	V	20	586.60
gtc	V	0	498.50
gta	V	121	666.90
gtg	V	2501	889.90
gct	A	0	0.25
gcc	A	0	0.23
gca	A	0	0.45
gcg	A	1	0.07
---	--	------	------
mPD		0.15	0.79
		nPD:	0.19
		N. weight:	1.2
		Sc. PD:	-0.091
		Sc. rank:	-8.5

,

PA

Pos.	566	obs:	exp:
cgt	R	0	85.60
cgc	R	0	143.30
cga	R	1	272.60
cgg	R	0	195.90
aaa	K	2	1.22
aag	K	0	0.78
aga	R	2491	1244.00
agg	R	220	770.80
---	--	------	------
mPD		0.15	0.94
		nPD:	0.16
		N. weight:	0.78
		Sc. PD:	-0.083
		Sc. rank:	-183.3

,

PA

Pos.	567	obs:	exp:
act	T	59	667.60
acc	T	2603	586.90
aca	T	51	1233.00
acg	T	1	226.30
---	--	------	------
mPD		0.079	0.68
		nPD:	0.12
		N. weight:	1.9
		Sc. PD:	-0.29
		Sc. rank:	-1157.1

,

PA

Pos.	568	obs:	exp:
cat	H	1	0.58
cac	H	0	0.42
aat	N	2686	1542.00
aac	N	27	1171.00
---	--	------	------
mPD		0.020	0.49
		nPD:	0.04
		N. weight:	0.75
		Sc. PD:	-0.17
		Sc. rank:	-978.7

,

PA

Pos.	569	obs:	exp:
ggt	G	5	425.70
ggc	G	4	343.90
gga	G	2594	1229.00
ggg	G	111	715.10
---	--	------	------
mPD		0.085	0.68
		nPD:	0.12
		N. weight:	0.91
		Sc. PD:	-0.13
		Sc. rank:	-484.2

,

PA

Pos.	570	obs:	exp:
tct	S	0	0.34
tcc	S	1	0.32
tca	S	0	0.49
tcg	S	0	0.11
cct	P	0	0.30
ccc	P	1	0.20
cca	P	0	0.40
ccg	P	0	0.11
att	I	0	0.35
atc	I	1	0.26
ata	I	0	0.39
act	T	28	666.60
acc	T	2568	586.00
aca	T	77	1231.00
acg	T	37	226.00
agt	S	0	0.37
agc	S	1	0.38
---	--	------	------
mPD		0.10	0.68
		nPD:	0.15
		N. weight:	1.8
		Sc. PD:	-0.21
		Sc. rank:	-562.3

,

PA

Pos.	571	obs:	exp:
tct	S	28	456.30
tcc	S	2295	428.80
tca	S	388	658.80
tcg	S	2	144.20
aat	N	0	0.57
aac	N	1	0.43
agt	S	0	505.00
agc	S	0	519.90
---	--	------	------
mPD		0.27	1.6
		nPD:	0.16
		N. weight:	1.9
		Sc. PD:	-0.2
		Sc. rank:	-380.1

,

PA

Pos.	572	obs:	exp:
aaa	K	497	1649.00
aag	K	2217	1065.00
---	--	------	------
mPD		0.30	0.48
		nPD:	0.63
		N. weight:	0.56
		Sc. PD:	0.19
		Sc. rank:	749.5

,

PA

Pos.	573	obs:	exp:
att	I	71	959.90
atc	I	2615	694.10
ata	I	20	1052.00
gtt	V	0	1.78
gtc	V	8	1.51
gta	V	0	2.02
gtg	V	0	2.69
---	--	------	------
mPD		0.071	0.66
		nPD:	0.11
		N. weight:	1.7
		Sc. PD:	-0.27
		Sc. rank:	-1221.2

,

PA

Pos.	574	obs:	exp:
cgt	R	0	0.03
cgc	R	0	0.05
cga	R	0	0.10
cgg	R	0	0.07
aaa	K	514	1649.00
aag	K	2199	1064.00
aga	R	0	0.46
agg	R	1	0.28
---	--	------	------
mPD		0.31	0.48
		nPD:	0.64
		N. weight:	0.54
		Sc. PD:	0.2
		Sc. rank:	743.5

,

PA

Pos.	575	obs:	exp:
atg	M	2714	2714.00
---	--	------	------
mPD		0	0
		nPD:	1.
		N. weight:	0.
		Sc. PD:	0
		Sc. rank:	0

,

PA

Pos.	576	obs:	exp:
cgt	R	0	0.13
cgc	R	0	0.21
cga	R	0	0.40
cgg	R	0	0.29
aaa	K	2479	1647.00
aag	K	231	1063.00
aga	R	4	1.83
agg	R	0	1.14
---	--	------	------
mPD		0.16	0.48
		nPD:	0.33
		N. weight:	0.36
		Sc. PD:	0.021
		Sc. rank:	196.6

,

PA

Pos.	577	obs:	exp:
tgg	W	2714	2714.00
---	--	------	------
mPD		0	0
		nPD:	1.
		N. weight:	0.
		Sc. PD:	0
		Sc. rank:	0

,

PA

Pos.	578	obs:	exp:
tct	S	0	1.01
tcc	S	0	0.95
tca	S	0	1.46
tcg	S	0	0.32
agt	S	6	1.12
agc	S	0	1.15
ggt	G	585	424.80
ggc	G	1896	343.10
gga	G	208	1227.00
ggg	G	19	713.50
---	--	------	------
mPD		0.46	0.69
		nPD:	0.67
		N. weight:	1.6
		Sc. PD:	0.63
		Sc. rank:	2284.3

,

PA

Pos.	579	obs:	exp:
att	I	3	4.61
atc	I	0	3.33
ata	I	10	5.05
atg	M	2700	2700.00
gtt	V	0	0.22
gtc	V	0	0.19
gta	V	0	0.25
gtg	V	1	0.34
---	--	------	------
mPD		0.010	0.011
		nPD:	0.95
		N. weight:	0.0043
		Sc. PD:	0.0029
		Sc. rank:	6.8

,

PA			
Pos.	580	obs:	exp:
gaa	E	2435	1608.00
gag	E	279	1106.00
---	--	------	------
mPD		0.18	0.48
		nPD:	0.38
		N. weight:	0.34
		Sc. PD:	0.037
		Sc. rank:	230.4

,

PA			
Pos.	581	obs:	exp:
tta	L	0	0.11
ttg	L	0	0.19
ctt	L	0	0.20
ctc	L	0	0.15
cta	L	0	0.17
ctg	L	1	0.18
att	I	2	0.71
atc	I	0	0.51
ata	I	0	0.78
atg	M	2697	2697.00
act	T	0	3.44
acc	T	0	3.03
aca	T	0	6.36
acg	T	14	1.17
---	--	------	------
mPD		0.012	0.022
		nPD:	0.56
		N. weight:	0.021
		Sc. PD:	0.0058
		Sc. rank:	24.7

,

PA			
Pos.	582	obs:	exp:
cgt	R	0	85.67
cgc	R	0	143.50
cga	R	0	272.80
cgg	R	0	196.10
aga	R	634	1245.00
agg	R	2080	771.40
---	--	------	------
mPD		0.36	0.94
		nPD:	0.38
		N. weight:	0.88
		Sc. PD:	0.097
		Sc. rank:	595.8

,

PA			
Pos.	583	obs:	exp:
tta	L	0	0.22
ttg	L	0	0.38
ctt	L	0	0.40
ctc	L	2	0.29
cta	L	0	0.35
ctg	L	0	0.36
cat	H	0	0.58
cac	H	1	0.42
cgt	R	470	85.57
cgc	R	2103	143.30
cga	R	94	272.50
cgg	R	33	195.90
aga	R	1	1243.00
agg	R	10	770.50
---	--	------	------
mPD		0.38	0.94
		nPD:	0.4
		N. weight:	3.4
		Sc. PD:	0.43
		Sc. rank:	2488.0

,

PA			
Pos.	584	obs:	exp:
tgt	C	111	1168.00
tgc	C	2602	1545.00
ggt	G	0	0.16
ggc	G	1	0.13
gga	G	0	0.45
ggg	G	0	0.26
---	--	------	------
mPD		0.079	0.49
		nPD:	0.16
		N. weight:	0.59
		Sc. PD:	-0.062
		Sc. rank:	-127.5

,

PA			
Pos.	585	obs:	exp:
ttt	F	6	2.69
ttc	F	0	3.31
tta	L	0	294.70
ttg	L	0	508.70
ctt	L	2584	546.90
ctc	L	110	394.00
cta	L	13	472.90
ctg	L	1	490.90
---	--	------	------
mPD		0.092	1.1
		nPD:	0.08
		N. weight:	2.1
		Sc. PD:	-0.38
		Sc. rank:	-1813.9

,

PA			
Pos.	586	obs:	exp:
tta	L	0	295.20
ttg	L	0	509.60
ctt	L	1686	547.90
ctc	L	1025	394.70
cta	L	1	473.70
ctg	L	1	491.80
att	I	1	0.35
atc	I	0	0.26
ata	I	0	0.39
---	--	------	------
mPD		0.47	1.1
		nPD:	0.41
		N. weight:	1.5
		Sc. PD:	0.22
		Sc. rank:	1220.7

,

PA			
Pos.	587	obs:	exp:
caa	Q	304	1464.00
cag	Q	2410	1250.00
---	--	------	------
mPD		0.20	0.50
		nPD:	0.4
		N. weight:	0.59
		Sc. PD:	0.076
		Sc. rank:	441.8

,

PA			
Pos.	588	obs:	exp:
tct	S	2491	455.00
tcc	S	188	427.50
tca	S	23	656.90
tcg	S	3	143.80
cct	P	2	0.59
ccc	P	0	0.40
cca	P	0	0.79
ccg	P	0	0.22
agt	S	0	503.50
agc	S	0	518.40
gct	A	7	1.72
gcc	A	0	1.60
gca	A	0	3.16
gcg	A	0	0.52
---	--	------	------
mPD		0.15	1.6
		nPD:	0.09
		N. weight:	2.2
		Sc. PD:	-0.37
		Sc. rank:	-1731.2

,

PA			
Pos.	589	obs:	exp:
tta	L	3	294.60
ttg	L	0	508.50
ctt	L	2550	546.70
ctc	L	130	393.90
cta	L	23	472.70
ctg	L	1	490.70
att	I	7	2.48
atc	I	0	1.80
ata	I	0	2.72
---	--	------	------
mPD		0.12	1.1
		nPD:	0.1
		N. weight:	2.
		Sc. PD:	-0.32
		Sc. rank:	-1471.0

,

PA			
Pos.	590	obs:	exp:
caa	Q	479	1464.00
cag	Q	2235	1250.00
---	--	------	------
mPD		0.29	0.50
		nPD:	0.59
		N. weight:	0.41
		Sc. PD:	0.13
		Sc. rank:	531.3

,

PA			
Pos.	591	obs:	exp:
caa	Q	95	1464.00
cag	Q	2619	1250.00
---	--	------	------
mPD		0.068	0.50
		nPD:	0.14
		N. weight:	0.9
		Sc. PD:	-0.12
		Sc. rank:	-396.5

,

PA			
Pos.	592	obs:	exp:
att	I	2658	961.00
atc	I	48	694.80
ata	I	3	1053.00
gtt	V	5	1.11
gtc	V	0	0.94
gta	V	0	1.26
gtg	V	0	1.68
---	--	------	------
mPD		0.041	0.66
		nPD:	0.06
		N. weight:	1.4
		Sc. PD:	-0.28
		Sc. rank:	-1543.6

,

PA			
Pos.	593	obs:	exp:
gaa	E	142	1608.00
gag	E	2572	1106.00
---	--	------	------
mPD		0.099	0.48
		nPD:	0.21
		N. weight:	0.98
		Sc. PD:	-0.061
		Sc. rank:	37.8

,

PA			
Pos.	594	obs:	exp:
tct	S	0	455.70
tcc	S	0	428.10
tca	S	0	657.80
tcg	S	0	144.00
aat	N	0	2.27
aac	N	4	1.73
agt	S	856	504.20
agc	S	1853	519.20
ggt	G	0	0.16
ggc	G	1	0.13
gga	G	0	0.45
ggg	G	0	0.26
---	--	------	------
mPD		0.44	1.6
		nPD:	0.27
		N. weight:	1.5
		Sc. PD:	-0.0037
		Sc. rank:	463.4

,

PA			
Pos.	595	obs:	exp:
tta	L	0	0.11
ttg	L	0	0.19
ctt	L	0	0.20
ctc	L	0	0.15
cta	L	0	0.17
ctg	L	1	0.18
att	I	0	1.06
atc	I	0	0.77
ata	I	3	1.17
atg	M	2710	2710.00
---	--	------	------
mPD		0.0029	0.0034
		nPD:	0.86
		N. weight:	0.0024
		Sc. PD:	0.0014
		Sc. rank:	3.8

,

PA			
Pos.	596	obs:	exp:
att	I	2247	944.70
atc	I	401	683.00
ata	I	15	1035.00
gtt	V	50	11.32
gtc	V	1	9.62
gta	V	0	12.87
gtg	V	0	17.18
---	--	------	------
mPD		0.30	0.70
		nPD:	0.43
		N. weight:	0.94
		Sc. PD:	0.15
		Sc. rank:	792.3

,

PA			
Pos.	597	obs:	exp:
gat	D	2	1.12
gac	D	0	0.88
gaa	E	554	1607.00
gag	E	2158	1105.00
---	--	------	------
mPD		0.33	0.48
		nPD:	0.67
		N. weight:	0.46
		Sc. PD:	0.18
		Sc. rank:	655.0

,

PA			
Pos.	598	obs:	exp:
tct	S	3	3.70
tcc	S	0	3.48
tca	S	18	5.34
tcg	S	1	1.17
act	T	11	5.17
acc	T	1	4.54
aca	T	4	9.54
acg	T	5	1.75
agt	S	0	4.09
agc	S	0	4.22
gtt	V	7	3.33
gtc	V	2	2.83
gta	V	3	3.79
gtg	V	3	5.05
gct	A	1341	651.10
gcc	A	726	608.60
gca	A	285	1197.00
gcg	A	304	199.20
---	--	------	------
mPD		0.69	0.73
		nPD:	0.95
		N. weight:	0.47
		Sc. PD:	0.31
		Sc. rank:	740.7

,

PA

Pos. 599		obs:	exp:
gat	D	1	0.56
gac	D	0	0.44
gaa	E	792	1607.00
gag	E	1920	1105.00
ggt	G	0	0.16
ggc	G	0	0.13
gga	G	0	0.45
ggg	G	1	0.26
---	--	------	------
mPD		0.41	0.48
		nPD:	0.86
		N. weight:	0.27
		Sc. PD:	0.16
		Sc. rank:	424.8

,

PA

Pos. 600		obs:	exp:
tct	S	2544	456.30
tcc	S	130	428.80
tca	S	22	658.80
tcg	S	17	144.20
tgt	C	1	0.43
tgc	C	0	0.57
agt	S	0	505.00
agc	S	0	519.90
---	--	------	------
mPD		0.12	1.6
		nPD:	0.07
		N. weight:	2.2
		Sc. PD:	-0.42
		Sc. rank:	-2172.2

,

PA

Pos. 601		obs:	exp:
tct	S	2407	456.00
tcc	S	205	428.50
tca	S	83	658.30
tcg	S	16	144.10
agt	S	0	504.60
agc	S	0	519.60
gct	A	3	0.74
gcc	A	0	0.69
gca	A	0	1.35
gcg	A	0	0.22
---	--	------	------
mPD		0.21	1.6
		nPD:	0.13
		N. weight:	2.
		Sc. PD:	-0.27
		Sc. rank:	-992.2

,

PA

Pos. 602		obs:	exp:
att	I	47	31.57
atc	I	38	22.83
ata	I	4	34.60
act	T	0	0.25
acc	T	1	0.22
aca	T	0	0.45
acg	T	0	0.08
gtt	V	395	582.60
gtc	V	2130	495.10
gta	V	99	662.40
gtg	V	0	883.80
---	--	------	------
mPD		0.40	0.81
		nPD:	0.49
		N. weight:	1.5
		Sc. PD:	0.33
		Sc. rank:	1525.9

,

PA

Pos. 603		obs:	exp:
cgt	R	0	5.27
cgc	R	0	8.83
cga	R	0	16.79
cgg	R	2	12.06
act	T	0	0.49
acc	T	0	0.43
aca	T	0	0.91
acg	T	2	0.17
aat	N	15	40.92
aac	N	57	31.08
aaa	K	2434	1503.00
aag	K	39	970.10
aga	R	30	76.59
agg	R	135	47.47
---	--	------	------
mPD		0.29	0.66
		nPD:	0.44
		N. weight:	0.64
		Sc. PD:	0.11
		Sc. rank:	576.3

,

PA

Pos. 604		obs:	exp:
gaa	E	674	1608.00
gag	E	2039	1105.00
ggt	G	0	0.16
ggc	G	0	0.13
gga	G	0	0.45
ggg	G	1	0.26
---	--	------	------
mPD		0.37	0.48
		nPD:	0.77
		N. weight:	0.36
		Sc. PD:	0.18
		Sc. rank:	546.9

,

PA

Pos. 605		obs:	exp:
cgt	R	0	0.09
cgc	R	0	0.16
cga	R	0	0.30
cgg	R	0	0.22
aaa	K	2257	1648.00
aag	K	454	1063.00
aga	R	0	1.38
agg	R	3	0.85
---	--	------	------
mPD		0.28	0.48
		nPD:	0.59
		N. weight:	0.18
		Sc. PD:	0.056
		Sc. rank:	230.3

,

PA

Pos. 606		obs:	exp:
gat	D	233	1527.00
gac	D	2481	1187.00
---	--	------	------
mPD		0.16	0.49
		nPD:	0.32
		N. weight:	0.75
		Sc. PD:	0.036
		Sc. rank:	396.6

,

PA

Pos. 607		obs:	exp:
tta	L	0	0.22
ttg	L	1	0.38
ctt	L	0	0.40
ctc	L	0	0.29
cta	L	0	0.35
ctg	L	1	0.36
cgt	R	0	0.03
cgc	R	0	0.05
cga	R	0	0.10
cgg	R	0	0.07
att	I	0	1.42
atc	I	0	1.03
ata	I	4	1.56
atg	M	2703	2703.00
aga	R	0	0.46
agg	R	1	0.28
gtt	V	0	0.89
gtc	V	0	0.75
gta	V	0	1.01
gtg	V	4	1.35
---	--	------	------
mPD		0.0081	0.012
		nPD:	0.7
		N. weight:	0.0065
		Sc. PD:	0.0027
		Sc. rank:	9.3

,

PA

Pos. 608		obs:	exp:
act	T	53	667.60
acc	T	2571	586.90
aca	T	90	1233.00
acg	T	0	226.30
---	--	------	------
mPD		0.10	0.68
		nPD:	0.15
		N. weight:	1.8
		Sc. PD:	-0.22
		Sc. rank:	-670.7

,

PA

Pos. 609		obs:	exp:
cgt	R	0	9.06
cgc	R	0	15.17
cga	R	0	28.85
cgg	R	0	20.73
aaa	K	522	1475.00
aag	K	1905	952.00
aga	R	25	131.60
agg	R	262	81.57
---	--	------	------
mPD		0.51	0.73
		nPD:	0.7
		N. weight:	0.56
		Sc. PD:	0.24
		Sc. rank:	812.3

,

PA

Pos. 610		obs:	exp:
aaa	K	4	2.43
aag	K	0	1.57
gat	D	10	8.44
gac	D	5	6.56
gaa	E	2647	1595.00
gag	E	45	1097.00
ggt	G	0	0.47
ggc	G	0	0.38
gga	G	3	1.36
ggg	G	0	0.79
---	--	------	------
mPD		0.049	0.49
		nPD:	0.1
		N. weight:	0.65
		Sc. PD:	-0.11
		Sc. rank:	-499.4

,

PA

Pos. 611		obs:	exp:
ttt	F	388	1216.00
ttc	F	2326	1498.00
---	--	------	------
mPD		0.25	0.49
		nPD:	0.5
		N. weight:	0.31
		Sc. PD:	0.069
		Sc. rank:	317.9

,

PA

Pos. 612		obs:	exp:
ttt	F	2578	1216.00
ttc	F	136	1498.00
---	--	------	------
mPD		0.095	0.49
		nPD:	0.19
		N. weight:	0.87
		Sc. PD:	-0.065
		Sc. rank:	-19.5

,

PA

Pos.	613	obs:	exp:
aaa	K	5	3.04
aag	K	0	1.96
gtt	V	0	0.22
gtc	V	0	0.19
gta	V	1	0.25
gtg	V	0	0.34
gct	A	0	0.25
gcc	A	0	0.23
gca	A	1	0.45
gcg	A	0	0.07
gat	D	4	10.12
gac	D	14	7.88
gaa	E	2587	1577.00
gag	E	75	1085.00
ggt	G	0	4.24
ggc	G	0	3.42
gga	G	27	12.23
ggg	G	0	7.11
---	--	------	------
mPD		0.091	0.52
		nPD:	0.18
		N. weight:	0.6
		Sc. PD:	-0.054
		Sc. rank:	-55.8

,

PA

Pos.	614	obs:	exp:
tct	S	0	6.39
tcc	S	0	6.01
tca	S	0	9.23
tcg	S	0	2.02
act	T	1	3.44
acc	T	13	3.03
aca	T	0	6.36
acg	T	0	1.17
aat	N	94	1478.00
aac	N	2507	1123.00
aaa	K	20	12.15
aag	K	0	7.85
agt	S	0	7.07
agc	S	38	7.28
gat	D	1	23.06
gac	D	40	17.94
---	--	------	------
mPD		0.15	0.59
		nPD:	0.25
		N. weight:	1.
		Sc. PD:	-0.015
		Sc. rank:	255.2

,

PA

Pos.	615	obs:	exp:
cgt	R	0	3.82
cgc	R	0	6.40
cga	R	1	12.16
cgg	R	0	8.74
aat	N	0	0.57
aac	N	1	0.43
aaa	K	2487	1575.00
aag	K	105	1017.00
aga	R	119	55.49
agg	R	1	34.39
---	--	------	------
mPD		0.16	0.59
		nPD:	0.27
		N. weight:	0.53
		Sc. PD:	0.0028
		Sc. rank:	171.9

,

PA

Pos.	616	obs:	exp:
tta	L	0	1.96
ttg	L	18	3.38
tct	S	417	451.80
tcc	S	14	424.50
tca	S	157	652.30
tcg	S	2098	142.80
ctt	L	0	3.64
ctc	L	0	2.62
cta	L	0	3.14
ctg	L	0	3.26
cct	P	0	0.30
ccc	P	0	0.20
cca	P	0	0.40
ccg	P	1	0.11
act	T	0	2.21
acc	T	0	1.95
aca	T	9	4.09
acg	T	0	0.75
agt	S	0	499.90
agc	S	0	514.80
---	--	------	------
mPD		0.38	1.6
		nPD:	0.23
		N. weight:	2.9
		Sc. PD:	-0.1
		Sc. rank:	560.1

,

PA

Pos.	617	obs:	exp:
caa	Q	2	1.08
cag	Q	0	0.92
aaa	K	1	0.61
aag	K	0	0.39
gtt	V	0	0.44
gtc	V	0	0.38
gta	V	2	0.50
gtg	V	0	0.67
gat	D	0	1.69
gac	D	3	1.31
gaa	E	2600	1603.00
gag	E	105	1102.00
ggt	G	0	0.16
ggc	G	0	0.13
gga	G	1	0.45
ggg	G	0	0.26
---	--	------	------
mPD		0.081	0.49
		nPD:	0.17
		N. weight:	0.55
		Sc. PD:	-0.056
		Sc. rank:	-89.9

,

PA

Pos.	618	obs:	exp:
tct	S	0	0.34
tcc	S	0	0.32
tca	S	2	0.49
tcg	S	0	0.11
cgt	R	0	0.06
cgc	R	0	0.11
cga	R	0	0.20
cgg	R	0	0.14
atg	M	2	2.00
act	T	155	543.40
acc	T	0	477.70
aca	T	2027	1004.00
acg	T	27	184.20
aaa	K	195	119.10
aag	K	1	76.88
agt	S	0	0.37
agc	S	0	0.38
aga	R	2	0.92
agg	R	0	0.57
gtt	V	3	48.18
gtc	V	0	40.95
gta	V	202	54.78
gtg	V	12	73.09
gct	A	12	20.35
gcc	A	0	19.02
gca	A	70	37.41
gcg	A	1	6.22
ggt	G	0	0.47
ggc	G	0	0.38
gga	G	3	1.36
ggg	G	0	0.79
---	--	------	------
mPD		0.62	1.2
		nPD:	0.53
		N. weight:	0.84
		Sc. PD:	0.22
		Sc. rank:	961.3

,

PA

Pos.	619	obs:	exp:
tgg	W	2713	2713.00
ggt	G	0	0.16
ggc	G	0	0.13
gga	G	0	0.45
ggg	G	1	0.26
---	--	------	------
mPD		0.00074	0.0013
		nPD:	0.58
		N. weight:	0.00072
		Sc. PD:	0.00021
		Sc. rank:	0.9

,

PA

Pos.	620	obs:	exp:
cct	P	32	800.90
ccc	P	405	546.40
cca	P	2246	1075.00
ccg	P	31	291.90
---	--	------	------
mPD		0.29	0.70
		nPD:	0.42
		N. weight:	0.74
		Sc. PD:	0.11
		Sc. rank:	585.8

,

PA

Pos.	621	obs:	exp:
att	I	529	958.90
atc	I	2155	693.30
ata	I	19	1051.00
gtt	V	2	2.44
gtc	V	6	2.08
gta	V	3	2.78
gtg	V	0	3.71
---	--	------	------
mPD		0.34	0.67
		nPD:	0.5
		N. weight:	1.1
		Sc. PD:	0.25
		Sc. rank:	1169.4

,

PA

Pos.	622	obs:	exp:
ggt	G	1	425.70
ggc	G	0	343.90
gga	G	2222	1229.00
ggg	G	491	715.10
---	--	------	------
mPD		0.30	0.68
		nPD:	0.43
		N. weight:	0.61
		Sc. PD:	0.098
		Sc. rank:	525.8

,

PA

Pos.	623	obs:	exp:
gat	D	1	0.56
gac	D	0	0.44
gaa	E	1634	1608.00
gag	E	1079	1105.00
---	--	------	------
mPD		0.48	0.48
		nPD:	0.99
		N. weight:	0.0028
		Sc. PD:	0.002
		Sc. rank:	4.5

,

PA

Pos.	624	obs:	exp:
tct	S	1	456.50
tcc	S	74	428.90
tca	S	2607	659.10
tcg	S	32	144.20
agt	S	0	505.10
agc	S	0	520.10
---	--	------	------
mPD		0.076	1.6
		nPD:	0.05
		N. weight:	1.8
		Sc. PD:	-0.4
		Sc. rank:	-2292.1

,

PA

Pos.	625	obs:	exp:
cct	P	208	800.90
ccc	P	2493	546.40
cca	P	13	1075.00
ccg	P	0	291.90
---	--	------	------
mPD		0.15	0.70
		nPD:	0.21
		N. weight:	1.9
		Sc. PD:	-0.1
		Sc. rank:	196.5

,

PA

Pos.	626	obs:	exp:
tct	S	0	0.17
tcc	S	0	0.16
tca	S	0	0.24
tcg	S	0	0.05
caa	Q	2	1.08
cag	Q	0	0.92
cgt	R	0	34.47
cgc	R	0	57.72
cga	R	0	109.80
cgg	R	0	78.89
aat	N	1	2.27
aac	N	3	1.73
aaa	K	1430	970.60
aag	K	167	626.40
agt	S	1	0.19
agc	S	0	0.19
aga	R	112	500.80
agg	R	980	310.40
gaa	E	5	4.74
gag	E	3	3.26
ggt	G	0	1.57
ggc	G	0	1.27
gga	G	0	4.53
ggg	G	10	2.63
---	--	------	------
mPD		0.99	1.2
		nPD:	0.83
		N. weight:	0.71
		Sc. PD:	0.39
		Sc. rank:	1102.2

,

PA

Pos.	627	obs:	exp:
cgt	R	0	0.16
cgc	R	0	0.26
cga	R	0	0.50
cgg	R	0	0.36
aga	R	4	2.29
agg	R	1	1.42
ggt	G	68	424.90
ggc	G	0	343.30
gga	G	2075	1227.00
ggg	G	566	713.80
---	--	------	------
mPD		0.37	0.69
		nPD:	0.54
		N. weight:	0.45
		Sc. PD:	0.12
		Sc. rank:	529.5

,

PA

Pos.	628	obs:	exp:
tta	L	1	0.65
ttg	L	5	1.13
ctt	L	0	1.21
ctc	L	0	0.87
cta	L	0	1.05
ctg	L	0	1.09
att	I	0	1.77
atc	I	1	1.28
ata	I	4	1.94
atg	M	12	12.00
gtt	V	14	597.30
gtc	V	0	507.60
gta	V	312	679.10
gtg	V	2364	906.10
gct	A	0	0.25
gcc	A	0	0.23
gca	A	1	0.45
gcg	A	0	0.07
---	--	------	------
mPD		0.23	0.75
		nPD:	0.31
		N. weight:	1.1
		Sc. PD:	0.043
		Sc. rank:	534.6

,

PA

Pos.	629	obs:	exp:
gaa	E	525	1608.00
gag	E	2189	1106.00
---	--	------	------
mPD		0.31	0.48
		nPD:	0.65
		N. weight:	0.49
		Sc. PD:	0.18
		Sc. rank:	676.9

,

PA

Pos.	630	obs:	exp:
cat	H	1	0.58
cac	H	0	0.42
aaa	K	1	0.61
aag	K	0	0.39
gtt	V	0	0.22
gtc	V	0	0.19
gta	V	1	0.25
gtg	V	0	0.34
gat	D	6	6.75
gac	D	6	5.25
gaa	E	2602	1597.00
gag	E	94	1099.00
ggt	G	0	0.47
ggc	G	0	0.38
gga	G	3	1.36
ggg	G	0	0.79
---	--	------	------
mPD		0.081	0.49
		nPD:	0.16
		N. weight:	0.56
		Sc. PD:	-0.058
		Sc. rank:	-108.7

,

PA

Pos.	631	obs:	exp:
tct	S	0	5.05
tcc	S	0	4.74
tca	S	0	7.29
tcg	S	0	1.59
agt	S	0	5.58
agc	S	30	5.75
gct	A	0	0.25
gcc	A	0	0.23
gca	A	1	0.45
gcg	A	0	0.07
ggt	G	79	420.80
ggc	G	2568	340.00
gga	G	25	1215.00
ggg	G	11	706.90
---	--	------	------
mPD		0.11	0.72
		nPD:	0.15
		N. weight:	2.7
		Sc. PD:	-0.32
		Sc. rank:	-1016.0

,

PA

Pos.	632	obs:	exp:
ttt	F	2	0.90
ttc	F	0	1.10
tta	L	0	0.11
ttg	L	1	0.19
tct	S	1982	455.70
tcc	S	715	428.10
tca	S	12	657.80
tcg	S	0	144.00
ctt	L	0	0.20
ctc	L	0	0.15
cta	L	0	0.17
ctg	L	0	0.18
cct	P	0	0.59
ccc	P	2	0.40
cca	P	0	0.79
ccg	P	0	0.22
agt	S	0	504.20
agc	S	0	519.20
---	--	------	------
mPD		0.40	1.6
		nPD:	0.24
		N. weight:	1.7
		Sc. PD:	-0.042
		Sc. rank:	383.5

,

PA

Pos.	633	obs:	exp:
att	I	2326	962.40
atc	I	378	695.90
ata	I	9	1055.00
gtt	V	1	0.22
gtc	V	0	0.19
gta	V	0	0.25
gtg	V	0	0.34
---	--	------	------
mPD		0.25	0.66
		nPD:	0.37
		N. weight:	0.96
		Sc. PD:	0.098
		Sc. rank:	627.4

,

PA

Pos.	634	obs:	exp:
ggt	G	40	425.70
ggc	G	1	343.90
gga	G	367	1229.00
ggg	G	2306	715.10
---	--	------	------
mPD		0.26	0.68
		nPD:	0.38
		N. weight:	1.2
		Sc. PD:	0.12
		Sc. rank:	765.9

,

PA

Pos.	635	obs:	exp:
tct	S	0	0.17
tcc	S	0	0.16
tca	S	0	0.24
tcg	S	0	0.05
aaa	K	2491	1649.00
aag	K	222	1064.00
agt	S	0	0.19
agc	S	1	0.19
---	--	------	------
mPD		0.15	0.48
		nPD:	0.32
		N. weight:	0.37
		Sc. PD:	0.017
		Sc. rank:	193.7

,

PA

Pos.	636	obs:	exp:
att	I	0	0.71
atc	I	0	0.51
ata	I	2	0.78
aaa	K	1	0.61
aag	K	0	0.39
gtt	V	23	601.70
gtc	V	21	511.40
gta	V	572	684.10
gtg	V	2094	912.80
gct	A	0	0.25
gcc	A	0	0.23
gca	A	1	0.45
gcg	A	0	0.07
---	--	------	------
mPD		0.36	0.74
		nPD:	0.49
		N. weight:	0.81
		Sc. PD:	0.17
		Sc. rank:	812.1

,

PA

Pos.	637	obs:	exp:
tct	S	0	0.17
tcc	S	0	0.16
tca	S	0	0.24
tcg	S	0	0.05
tgt	C	112	1168.00
tgc	C	2601	1545.00
agt	S	0	0.19
agc	S	1	0.19
---	--	------	------
mPD		0.080	0.49
		nPD:	0.16
		N. weight:	0.59
		Sc. PD:	-0.061
		Sc. rank:	-122.0

,

PA

Pos.	638	obs:	exp:
cgt	R	0	85.67
cgc	R	0	143.50
cga	R	0	272.80
cgg	R	15	196.10
aga	R	188	1245.00
agg	R	2511	771.40
---	--	------	------
mPD		0.14	0.94
		nPD:	0.15
		N. weight:	1.4
		Sc. PD:	-0.16
		Sc. rank:	-494.4

,

PA

Pos.	639	obs:	exp:
act	T	50	594.30
acc	T	2252	522.40
aca	T	106	1098.00
acg	T	8	201.50
gct	A	20	73.05
gcc	A	260	68.28
gca	A	14	134.30
gcg	A	4	22.35
---	--	------	------
mPD		0.34	0.87
		nPD:	0.38
		N. weight:	1.7
		Sc. PD:	0.19
		Sc. rank:	1192.3

,

PA

Pos.	640	obs:	exp:
ttt	F	2	0.90
ttc	F	0	1.10
tta	L	2476	294.80
ttg	L	169	508.80
ctt	L	0	547.10
ctc	L	0	394.10
cta	L	58	473.00
ctg	L	6	491.10
att	I	0	1.06
atc	I	0	0.77
ata	I	3	1.17
---	--	------	------
mPD		0.17	1.1
		nPD:	0.15
		N. weight:	2.7
		Sc. PD:	-0.31
		Sc. rank:	-917.9

,

PA

Pos.	641	obs:	exp:
tta	L	21	295.20
ttg	L	166	509.60
ctt	L	8	547.90
ctc	L	0	394.70
cta	L	149	473.70
ctg	L	2369	491.80
atg	M	1	1.00
---	--	------	------
mPD		0.25	1.1
		nPD:	0.22
		N. weight:	1.8
		Sc. PD:	-0.083
		Sc. rank:	303.3

,

PA

Pos.	642	obs:	exp:
gct	A	15	665.00
gcc	A	2	621.60
gca	A	2615	1223.00
gcg	A	81	203.50
gaa	E	1	0.59
gag	E	0	0.41
---	--	------	------
mPD		0.071	0.68
		nPD:	0.1
		N. weight:	1.
		Sc. PD:	-0.16
		Sc. rank:	-727.3

,

PA

Pos.	643	obs:	exp:
cgt	R	0	0.06
cgc	R	0	0.11
cga	R	0	0.20
cgg	R	0	0.14
aaa	K	2195	1648.00
aag	K	517	1064.00
aga	R	1	0.92
agg	R	1	0.57
---	--	------	------
mPD		0.31	0.48
		nPD:	0.65
		N. weight:	0.14
		Sc. PD:	0.052
		Sc. rank:	194.1

,

PA

Pos.	644	obs:	exp:
tct	S	2650	456.50
tcc	S	29	428.90
tca	S	33	659.10
tcg	S	2	144.20
agt	S	0	505.10
agc	S	0	520.10
---	--	------	------
mPD		0.046	1.6
		nPD:	0.03
		N. weight:	2.4
		Sc. PD:	-0.57
		Sc. rank:	-3372.7

,

PA

Pos.	645	obs:	exp:
gtt	V	17	602.40
gtc	V	0	511.90
gta	V	2518	684.90
gtg	V	178	913.80
gct	A	0	0.25
gcc	A	0	0.23
gca	A	1	0.45
gcg	A	0	0.07
---	--	------	------
mPD		0.13	0.74
		nPD:	0.18
		N. weight:	1.6
		Sc. PD:	-0.13
		Sc. rank:	-100.1

,

PA

Pos.	646	obs:	exp:
ttt	F	33	1215.00
ttc	F	2679	1497.00
tta	L	0	0.11
ttg	L	0	0.19
tct	S	0	0.17
tcc	S	1	0.16
tca	S	0	0.24
tcg	S	0	0.05
ctt	L	0	0.20
ctc	L	1	0.15
cta	L	0	0.17
ctg	L	0	0.18
agt	S	0	0.19
agc	S	0	0.19
---	--	------	------
mPD		0.026	0.50
		nPD:	0.05
		N. weight:	0.78
		Sc. PD:	-0.17
		Sc. rank:	-938.8

,

PA

Pos.	647	obs:	exp:
aat	N	426	1543.00
aac	N	2288	1171.00
---	--	------	------
mPD		0.26	0.49
		nPD:	0.54
		N. weight:	0.53
		Sc. PD:	0.14
		Sc. rank:	616.5

,

PA

Pos.	648	obs:	exp:
tct	S	0	452.50
tcc	S	0	425.10
tca	S	0	653.20
tcg	S	0	143.00
tgt	C	11	9.90
tgc	C	12	13.10
aat	N	1	0.57
aac	N	0	0.43
agt	S	2258	500.70
agc	S	432	515.50
---	--	------	------
mPD		0.29	1.6
		nPD:	0.18
		N. weight:	1.8
		Sc. PD:	-0.16
		Sc. rank:	-140.4

,

PA

Pos.	649	obs:	exp:
tta	L	74	293.80
ttg	L	31	507.10
ctt	L	7	545.30
ctc	L	1	392.80
cta	L	2125	471.50
ctg	L	462	489.40
att	I	0	4.26
atc	I	0	3.08
ata	I	12	4.67
atg	M	2	2.00
---	--	------	------
mPD		0.39	1.1
		nPD:	0.34
		N. weight:	1.6
		Sc. PD:	0.11
		Sc. rank:	914.0

,

PA

Pos.	650	obs:	exp:
ttt	F	1	0.45
ttc	F	0	0.55
tat	Y	2168	1520.00
tac	Y	545	1193.00
---	--	------	------
mPD		0.32	0.49
		nPD:	0.65
		N. weight:	0.19
		Sc. PD:	0.07
		Sc. rank:	260.7

,

PA

Pos.	651	obs:	exp:
tct	S	0	0.34
tcc	S	0	0.32
tca	S	2	0.49
tcg	S	0	0.11
act	T	0	0.25
acc	T	0	0.22
aca	T	0	0.45
acg	T	1	0.08
agt	S	0	0.37
agc	S	0	0.38
gct	A	46	664.60
gcc	A	1	621.20
gca	A	559	1222.00
gcg	A	2105	203.30
---	--	------	------
mPD		0.36	0.68
		nPD:	0.52
		N. weight:	2.3
		Sc. PD:	0.58
		Sc. rank:	2644.0

,

PA

Pos.	652	obs:	exp:
tct	S	2550	456.50
tcc	S	149	428.90
tca	S	14	659.10
tcg	S	1	144.20
agt	S	0	505.10
agc	S	0	520.10
---	--	------	------
mPD		0.11	1.6
		nPD:	0.07
		N. weight:	2.2
		Sc. PD:	-0.44
		Sc. rank:	-2302.0

,

PA

Pos.	653	obs:	exp:
tct	S	0	44.07
tcc	S	0	41.41
tca	S	262	63.62
tcg	S	0	13.92
cct	P	0	723.00
ccc	P	7	493.30
cca	P	2420	970.20
ccg	P	23	263.50
agt	S	0	48.76
agc	S	0	50.21
gct	A	0	0.49
gcc	A	0	0.46
gca	A	2	0.90
gcg	A	0	0.15
---	--	------	------
mPD		0.20	0.96
		nPD:	0.21
		N. weight:	1.3
		Sc. PD:	-0.083
		Sc. rank:	59.8

,

PA

Pos.	654	obs:	exp:
caa	Q	1945	1464.00
cag	Q	769	1250.00
---	--	------	------
mPD		0.41	0.50
		nPD:	0.82
		N. weight:	0.099
		Sc. PD:	0.053
		Sc. rank:	153.0

,

PA

Pos.	655	obs:	exp:
tta	L	13	295.10
ttg	L	14	509.40
ctt	L	2268	547.70
ctc	L	321	394.60
cta	L	94	473.50
ctg	L	2	491.60
att	I	2	0.71
atc	I	0	0.51
ata	I	0	0.78
---	--	------	------
mPD		0.31	1.1
		nPD:	0.27
		N. weight:	1.6
		Sc. PD:	-0.0011
		Sc. rank:	494.2

,

PA

Pos.	656	obs:	exp:
gaa	E	169	1608.00
gag	E	2545	1106.00
---	--	------	------
mPD		0.12	0.48
		nPD:	0.24
		N. weight:	0.94
		Sc. PD:	-0.025
		Sc. rank:	191.0

,

PA

Pos.	657	obs:	exp:
cgt	R	0	0.03
cgc	R	0	0.05
cga	R	0	0.10
cgg	R	0	0.07
aga	R	1	0.46
agg	R	0	0.28
ggt	G	4	425.50
ggc	G	2	343.80
gga	G	466	1229.00
ggg	G	2241	714.80
---	--	------	------
mPD		0.29	0.69
		nPD:	0.42
		N. weight:	1.1
		Sc. PD:	0.17
		Sc. rank:	910.8

PA

Pos.	658	obs:	exp:
ttt	F	2623	1216.00
ttc	F	90	1497.00
cat	H	0	0.58
cac	H	1	0.42
---	--	------	------
mPD		0.066	0.50
		nPD:	0.13
		N. weight:	0.95
		Sc. PD:	-0.13
		Sc. rank:	-424.7

PA

Pos.	659	obs:	exp:
tta	L	0	0.54
ttg	L	5	0.94
tct	S	95	455.70
tcc	S	4	428.10
tca	S	599	657.80
tcg	S	2011	144.00
ctt	L	0	1.01
ctc	L	0	0.73
cta	L	0	0.87
ctg	L	0	0.91
agt	S	0	504.20
agc	S	0	519.20
---	--	------	------
mPD		0.40	1.6
		nPD:	0.25
		N. weight:	2.7
		Sc. PD:	-0.062
		Sc. rank:	635.9

PA

Pos.	660	obs:	exp:
tct	S	2	0.34
tcc	S	0	0.32
tca	S	0	0.49
tcg	S	0	0.11
agt	S	0	0.37
agc	S	0	0.38
gct	A	2658	664.80
gcc	A	24	621.40
gca	A	23	1222.00
gcg	A	7	203.40
---	--	------	------
mPD		0.041	0.68
		nPD:	0.06
		N. weight:	1.9
		Sc. PD:	-0.38
		Sc. rank:	-2114.4

PA

Pos.	661	obs:	exp:
gaa	E	2681	1608.00
gag	E	33	1106.00
---	--	------	------
mPD		0.024	0.48
		nPD:	0.05
		N. weight:	0.68
		Sc. PD:	-0.14
		Sc. rank:	-828.5

PA

Pos.	662	obs:	exp:
tct	S	3	456.30
tcc	S	4	428.80
tca	S	623	658.80
tcg	S	2083	144.20
act	T	0	0.25
acc	T	0	0.22
aca	T	0	0.45
acg	T	1	0.08
agt	S	0	505.00
agc	S	0	519.90
---	--	------	------
mPD		0.36	1.6
		nPD:	0.22
		N. weight:	3.
		Sc. PD:	-0.14
		Sc. rank:	442.3

PA

Pos.	663	obs:	exp:
cgt	R	0	85.67
cgc	R	0	143.50
cga	R	1	272.80
cgg	R	0	196.10
aga	R	2529	1245.00
agg	R	184	771.40
---	--	------	------
mPD		0.13	0.94
		nPD:	0.14
		N. weight:	0.82
		Sc. PD:	-0.11
		Sc. rank:	-364.1

PA

Pos.	664	obs:	exp:
cgt	R	0	0.57
cgc	R	0	0.95
cga	R	0	1.81
cgg	R	0	1.30
aaa	K	1494	1638.00
aag	K	1202	1058.00
aga	R	15	8.25
agg	R	3	5.12
---	--	------	------
mPD		0.51	0.49
		nPD:	1.03
		N. weight:	0.016
		Sc. PD:	0.012
		Sc. rank:	24.9

PA

Pos.	665	obs:	exp:
ttt	F	8	3.58
ttc	F	0	4.42
tta	L	378	289.10
ttg	L	2223	499.10
ctt	L	0	536.60
ctc	L	0	386.60
cta	L	6	463.90
ctg	L	50	481.60
atg	M	49	49.00
---	--	------	------
mPD		0.32	1.2
		nPD:	0.28
		N. weight:	1.8
		Sc. PD:	0.017
		Sc. rank:	618.7

PA

Pos.	666	obs:	exp:
tta	L	1	295.20
ttg	L	0	509.60
ctt	L	1708	547.90
ctc	L	966	394.70
cta	L	37	473.70
ctg	L	1	491.80
cat	H	1	0.58
cac	H	0	0.42
---	--	------	------
mPD		0.48	1.1
		nPD:	0.42
		N. weight:	1.5
		Sc. PD:	0.21
		Sc. rank:	1174.9

PA

Pos.	667	obs:	exp:
tta	L	20	295.20
ttg	L	0	509.60
ctt	L	47	547.90
ctc	L	2217	394.70
cta	L	429	473.70
ctg	L	0	491.80
ggt	G	0	0.16
ggc	G	0	0.13
gga	G	1	0.45
ggg	G	0	0.26
---	--	------	------
mPD		0.32	1.1
		nPD:	0.28
		N. weight:	1.9
		Sc. PD:	0.024
		Sc. rank:	703.3

PA

Pos.	668	obs:	exp:
att	I	2692	961.70
atc	I	17	695.30
ata	I	2	1054.00
aat	N	1	0.57
aac	N	0	0.43
gtt	V	2	0.44
gtc	V	0	0.38
gta	V	0	0.50
gtg	V	0	0.67
---	--	------	------
mPD		0.016	0.66
		nPD:	0.02
		N. weight:	1.5
		Sc. PD:	-0.35
		Sc. rank:	-2069.1

PA

Pos.	669	obs:	exp:
tta	L	0	0.11
ttg	L	0	0.19
ctt	L	0	0.20
ctc	L	0	0.15
cta	L	0	0.17
ctg	L	1	0.18
att	I	35	14.90
atc	I	7	10.77
ata	I	0	16.33
gtt	V	2594	592.20
gtc	V	71	503.30
gta	V	1	673.30
gtg	V	1	898.30
gct	A	4	0.98
gcc	A	0	0.92
gca	A	0	1.80
gcg	A	0	0.30
---	--	------	------
mPD		0.092	0.77
		nPD:	0.12
		N. weight:	2.
		Sc. PD:	-0.29
		Sc. rank:	-1186.8

PA

Pos.	670	obs:	exp:
tta	L	1	0.11
ttg	L	0	0.19
ctt	L	0	0.20
ctc	L	0	0.15
cta	L	0	0.17
ctg	L	0	0.18
caa	Q	72	1463.00
cag	Q	2641	1250.00
---	--	------	------
mPD		0.054	0.50
		nPD:	0.11
		N. weight:	0.95
		Sc. PD:	-0.15
		Sc. rank:	-652.4

PA

Pos.	671	obs:	exp:
gtt	V	0	0.22
gtc	V	0	0.19
gta	V	1	0.25
gtg	V	0	0.34
gct	A	31	665.00
gcc	A	0	621.60
gca	A	2673	1223.00
gcg	A	9	203.50
---	--	------	------
mPD		0.030	0.68
		nPD:	0.04
		N. weight:	1.1
		Sc. PD:	-0.23
		Sc. rank:	-1362.7

PA

Pos.	672	obs:	exp:
ttt	F	40	18.37
ttc	F	1	22.63
tta	L	0	290.80
ttg	L	1	501.90
ctt	L	2546	539.60
ctc	L	125	388.80
cta	L	0	466.60
ctg	L	0	484.40
att	I	1	0.35
atc	I	0	0.26
ata	I	0	0.39
---	--	------	------
mPD		0.12	1.2
		nPD:	0.1
		N. weight:	2.1
		Sc. PD:	-0.33
		Sc. rank:	-1487.6

PA

Pos.	673	obs:	exp:
cgt	R	0	85.26
cgc	R	0	142.80
cga	R	3	271.50
cgg	R	5	195.10
aaa	K	0	7.29
aag	K	12	4.71
aga	R	40	1239.00
agg	R	2653	767.70
ggt	G	0	0.16
ggc	G	0	0.13
gga	G	0	0.45
ggg	G	1	0.26
---	--	------	------
mPD		0.047	0.95
		nPD:	0.05
		N. weight:	1.7
		Sc. PD:	-0.36
		Sc. rank:	-2084.4

PA

Pos.	674	obs:	exp:
gct	A	0	0.25
gcc	A	1	0.23
gca	A	0	0.45
gcg	A	0	0.07
gat	D	97	1526.00
gac	D	2616	1187.00
---	--	------	------
mPD		0.070	0.49
		nPD:	0.14
		N. weight:	0.97
		Sc. PD:	-0.12
		Sc. rank:	-405.2

PA

Pos.	675	obs:	exp:
tct	S	0	3.03
tcc	S	0	2.84
tca	S	0	4.37
tcg	S	0	0.96
aat	N	431	1532.00
aac	N	2265	1164.00
agt	S	2	3.35
agc	S	16	3.45
---	--	------	------
mPD		0.28	0.51
		nPD:	0.55
		N. weight:	0.53
		Sc. PD:	0.14
		Sc. rank:	625.6

PA

Pos.	676	obs:	exp:
tta	L	2	295.30
ttg	L	1080	509.80
ctt	L	35	548.10
ctc	L	1	394.90
cta	L	13	473.90
ctg	L	1583	492.00
---	--	------	------
mPD		0.52	1.1
		nPD:	0.45
		N. weight:	1.4
		Sc. PD:	0.24
		Sc. rank:	1252.1

PA			
Pos.	677	obs:	exp:
gat	D	2	1.69
gac	D	1	1.31
gaa	E	2679	1606.00
gag	E	32	1105.00
---	--	------	------
mPD		0.025	0.48
		nPD:	0.05
		N. weight:	0.68
		Sc. PD:	-0.14
		Sc. rank:	-808.6

PA			
Pos.	678	obs:	exp:
cct	P	2697	800.90
ccc	P	15	546.40
cca	P	0	1075.00
ccg	P	2	291.90
---	--	------	------
mPD		0.012	0.70
		nPD:	0.02
		N. weight:	1.7
		Sc. PD:	-0.42
		Sc. rank:	-2529.7

PA			
Pos.	679	obs:	exp:
ggt	G	9	425.70
ggc	G	1	343.90
gga	G	1475	1229.00
ggg	G	1229	715.10
---	--	------	------
mPD		0.50	0.68
		nPD:	0.73
		N. weight:	0.48
		Sc. PD:	0.22
		Sc. rank:	711.1

PA			
Pos.	680	obs:	exp:
tct	S	0	0.34
tcc	S	0	0.32
tca	S	0	0.49
tcg	S	0	0.11
act	T	71	666.80
acc	T	2587	586.20
aca	T	53	1232.00
acg	T	0	226.10
agt	S	0	0.37
agc	S	2	0.38
gct	A	0	0.25
gcc	A	1	0.23
gca	A	0	0.45
gcg	A	0	0.07
---	--	------	------
mPD		0.090	0.68
		nPD:	0.13
		N. weight:	1.9
		Sc. PD:	-0.25
		Sc. rank:	-899.7

PA			
Pos.	681	obs:	exp:
ttt	F	1304	1216.00
ttc	F	1410	1498.00
---	--	------	------
mPD		0.50	0.49
		nPD:	1.01
		N. weight:	0.0053
		Sc. PD:	0.0039
		Sc. rank:	8.5

PA			
Pos.	682	obs:	exp:
aat	N	2	1.14
aac	N	0	0.86
gat	D	2659	1525.00
gac	D	53	1187.00
---	--	------	------
mPD		0.040	0.49
		nPD:	0.08
		N. weight:	0.71
		Sc. PD:	-0.13
		Sc. rank:	-634.4

PA			
Pos.	683	obs:	exp:
tta	L	0	291.60
ttg	L	0	503.40
ctt	L	2616	541.30
ctc	L	59	389.90
cta	L	4	468.00
ctg	L	1	485.80
cgt	R	1	0.03
cgc	R	0	0.05
cga	R	0	0.10
cgg	R	0	0.07
att	I	32	11.71
atc	I	1	8.46
ata	I	0	12.83
aga	R	0	0.46
agg	R	0	0.28
---	--	------	------
mPD		0.072	1.2
		nPD:	0.06
		N. weight:	2.2
		Sc. PD:	-0.44
		Sc. rank:	-2364.3

PA			
Pos.	684	obs:	exp:
tgg	W	2	2.00
cgt	R	0	0.06
cgc	R	0	0.11
cga	R	0	0.20
cgg	R	0	0.14
aga	R	0	0.92
agg	R	2	0.57
gat	D	1	0.56
gac	D	0	0.44
gaa	E	51	122.70
gag	E	156	84.35
ggt	G	1	392.40
ggc	G	4	317.00
gga	G	106	1133.00
ggg	G	2391	659.20
---	--	------	------
mPD		0.26	0.82
		nPD:	0.31
		N. weight:	1.5
		Sc. PD:	0.068
		Sc. rank:	797.6

PA			
Pos.	685	obs:	exp:
ggt	G	16	425.70
ggc	G	0	343.90
gga	G	299	1229.00
ggg	G	2399	715.10
---	--	------	------
mPD		0.21	0.68
		nPD:	0.3
		N. weight:	1.3
		Sc. PD:	0.041
		Sc. rank:	584.5

PA			
Pos.	686	obs:	exp:
tta	L	253	295.30
ttg	L	0	509.80
ctt	L	0	548.10
ctc	L	0	394.90
cta	L	2449	473.90
ctg	L	12	492.00
---	--	------	------
mPD		0.18	1.1
		nPD:	0.16
		N. weight:	2.1
		Sc. PD:	-0.23
		Sc. rank:	-564.5

PA			
Pos.	687	obs:	exp:
tat	Y	2704	1520.00
tac	Y	10	1194.00
---	--	------	------
mPD		0.0073	0.49
		nPD:	0.01
		N. weight:	0.81
		Sc. PD:	-0.2
		Sc. rank:	-1208.3

PA			
Pos.	688	obs:	exp:
tct	S	0	0.67
tcc	S	0	0.63
tca	S	0	0.97
tcg	S	0	0.21
aat	N	83	47.18
aac	N	0	35.82
agt	S	4	0.74
agc	S	0	0.77
gtt	V	0	4.00
gtc	V	0	3.40
gta	V	18	4.54
gtg	V	0	6.06
gat	D	264	149.60
gac	D	2	116.40
gaa	E	2230	1334.00
gag	E	21	917.20
ggt	G	59	14.43
ggc	G	0	11.66
gga	G	33	41.67
ggg	G	0	24.24
---	--	------	------
mPD		0.41	0.75
		nPD:	0.55
		N. weight:	0.74
		Sc. PD:	0.2
		Sc. rank:	875.8

PA			
Pos.	689	obs:	exp:
tct	S	0	7.23
tcc	S	0	6.80
tca	S	43	10.44
tcg	S	0	2.29
agt	S	0	8.00
agc	S	0	8.24
gct	A	0	654.70
gcc	A	0	612.00
gca	A	2667	1204.00
gcg	A	4	200.30
---	--	------	------
mPD		0.034	0.72
		nPD:	0.05
		N. weight:	1.2
		Sc. PD:	-0.25
		Sc. rank:	-1452.3

PA			
Pos.	690	obs:	exp:
att	I	576	959.60
atc	I	2091	693.80
ata	I	38	1052.00
act	T	0	0.25
acc	T	1	0.22
aca	T	0	0.45
acg	T	0	0.08
gtt	V	5	1.78
gtc	V	3	1.51
gta	V	0	2.02
gtg	V	0	2.69
---	--	------	------
mPD		0.36	0.66
		nPD:	0.55
		N. weight:	1.
		Sc. PD:	0.28
		Sc. rank:	1213.5

PA			
Pos.	691	obs:	exp:
gaa	E	0	1608.00
gag	E	2714	1106.00
---	--	------	------
mPD		0	0.48
		nPD:	0.
		N. weight:	1.3
		Sc. PD:	-0.34
		Sc. rank:	-2103.5

PA			
Pos.	692	obs:	exp:
gaa	E	212	1608.00
gag	E	2502	1106.00
---	--	------	------
mPD		0.14	0.48
		nPD:	0.3
		N. weight:	0.87
		Sc. PD:	0.025
		Sc. rank:	361.5

PA			
Pos.	693	obs:	exp:
tgt	C	23	1168.00
tgc	C	2691	1546.00
---	--	------	------
mPD		0.017	0.49
		nPD:	0.03
		N. weight:	0.75
		Sc. PD:	-0.17
		Sc. rank:	-1035.4

PA			
Pos.	694	obs:	exp:
tta	L	0	295.30
ttg	L	4	509.80
ctt	L	3	548.10
ctc	L	0	394.90
cta	L	19	473.90
ctg	L	2688	492.00
---	--	------	------
mPD		0.019	1.1
		nPD:	0.02
		N. weight:	2.4
		Sc. PD:	-0.59
		Sc. rank:	-3551.7

PA			
Pos.	695	obs:	exp:
att	I	2703	959.60
atc	I	2	693.80
ata	I	0	1052.00
gtt	V	9	2.00
gtc	V	0	1.70
gta	V	0	2.27
gtg	V	0	3.03
---	--	------	------
mPD		0.0081	0.66
		nPD:	0.01
		N. weight:	1.5
		Sc. PD:	-0.38
		Sc. rank:	-2271.2

,

PA			
Pos.	696	obs:	exp:
tct	S	0	0.67
tcc	S	0	0.63
tca	S	0	0.97
tcg	S	0	0.21
aat	N	2695	1540.00
aac	N	15	1170.00
agt	S	4	0.74
agc	S	0	0.77
---	--	------	------
mPD		0.014	0.50
		nPD:	0.03
		N. weight:	0.78
		Sc. PD:	-0.18
		Sc. rank:	-1103.3

,

PA			
Pos.	697	obs:	exp:
gat	D	2686	1527.00
gac	D	28	1187.00
---	--	------	------
mPD		0.020	0.49
		nPD:	0.04
		N. weight:	0.76
		Sc. PD:	-0.17
		Sc. rank:	-997.7

,

PA			
Pos.	698	obs:	exp:
cct	P	29	800.90
ccc	P	2684	546.40
cca	P	0	1075.00
ccg	P	1	291.90
---	--	------	------
mPD		0.022	0.70
		nPD:	0.03
		N. weight:	2.2
		Sc. PD:	-0.52
		Sc. rank:	-3092.7

,

PA			
Pos.	699	obs:	exp:
tgg	W	2714	2714.00
---	--	------	------
mPD		0	0
		nPD:	1.
		N. weight:	0.
		Sc. PD:	0
		Sc. rank:	0

,

PA			
Pos.	700	obs:	exp:
gtt	V	2711	602.60
gtc	V	1	512.10
gta	V	2	685.10
gtg	V	0	914.20
---	--	------	------
mPD		0.0022	0.74
		nPD:	0.
		N. weight:	2.2
		Sc. PD:	-0.57
		Sc. rank:	-3439.1

,

PA			
Pos.	701	obs:	exp:
tta	L	3	295.30
ttg	L	2706	509.80
ctt	L	0	548.10
ctc	L	0	394.90
cta	L	1	473.90
ctg	L	4	492.00
---	--	------	------
mPD		0.0066	1.1
		nPD:	0.01
		N. weight:	2.4
		Sc. PD:	-0.62
		Sc. rank:	-3756.7

,

PA			
Pos.	702	obs:	exp:
tta	L	0	295.30
ttg	L	0	509.80
ctt	L	2568	548.10
ctc	L	146	394.90
cta	L	0	473.90
ctg	L	0	492.00
---	--	------	------
mPD		0.10	1.1
		nPD:	0.09
		N. weight:	2.1
		Sc. PD:	-0.36
		Sc. rank:	-1748.3

,

PA			
Pos.	703	obs:	exp:
tct	S	0	0.67
tcc	S	0	0.63
tca	S	0	0.97
tcg	S	0	0.21
aat	N	2579	1540.00
aac	N	131	1170.00
agt	S	2	0.74
agc	S	2	0.77
---	--	------	------
mPD		0.096	0.50
		nPD:	0.19
		N. weight:	0.56
		Sc. PD:	-0.042
		Sc. rank:	-7.0

,

PA			
Pos.	704	obs:	exp:
gct	A	24	665.30
gcc	A	0	621.80
gca	A	2215	1223.00
gcg	A	475	203.50
---	--	------	------
mPD		0.30	0.68
		nPD:	0.45
		N. weight:	0.88
		Sc. PD:	0.15
		Sc. rank:	800.5

,

PA			
Pos.	705	obs:	exp:
tct	S	2628	456.50
tcc	S	69	428.90
tca	S	10	659.10
tcg	S	7	144.20
agt	S	0	505.10
agc	S	0	520.10
---	--	------	------
mPD		0.062	1.6
		nPD:	0.04
		N. weight:	2.4
		Sc. PD:	-0.54
		Sc. rank:	-3198.3

,

PA			
Pos.	706	obs:	exp:
tta	L	0	0.11
ttg	L	1	0.19
tgg	W	2713	2713.00
ctt	L	0	0.20
ctc	L	0	0.15
cta	L	0	0.17
ctg	L	0	0.18
---	--	------	------
mPD		0.00074	0.0017
		nPD:	0.43
		N. weight:	0.0009
		Sc. PD:	0.00014
		Sc. rank:	0.8

,

PA			
Pos.	707	obs:	exp:
ttt	F	24	1216.00
ttc	F	2690	1498.00
---	--	------	------
mPD		0.018	0.49
		nPD:	0.04
		N. weight:	0.8
		Sc. PD:	-0.18
		Sc. rank:	-1086.4

,

PA			
Pos.	708	obs:	exp:
aat	N	0	1543.00
aac	N	2714	1171.00
---	--	------	------
mPD		0	0.49
		nPD:	0.
		N. weight:	1.2
		Sc. PD:	-0.32
		Sc. rank:	-1968.7

,

PA			
Pos.	709	obs:	exp:
tct	S	0	456.50
tcc	S	2714	428.90
tca	S	0	659.10
tcg	S	0	144.20
agt	S	0	505.10
agc	S	0	520.10
---	--	------	------
mPD		0	1.6
		nPD:	0.
		N. weight:	2.7
		Sc. PD:	-0.71
		Sc. rank:	-4322.6

,

PA			
Pos.	710	obs:	exp:
ttt	F	0	1213.00
ttc	F	2706	1493.00
tta	L	0	0.87
ttg	L	0	1.50
ctt	L	0	1.62
ctc	L	8	1.16
cta	L	0	1.40
ctg	L	0	1.45
---	--	------	------
mPD		0.0059	0.50
		nPD:	0.01
		N. weight:	0.87
		Sc. PD:	-0.22
		Sc. rank:	-1321.8

,

PA			
Pos.	711	obs:	exp:
tta	L	0	293.90
ttg	L	1	507.30
ctt	L	439	545.50
ctc	L	2239	393.00
cta	L	13	471.60
ctg	L	9	489.60
att	I	4	4.61
atc	I	9	3.33
ata	I	0	5.05
---	--	------	------
mPD		0.30	1.1
		nPD:	0.26
		N. weight:	2.
		Sc. PD:	-0.018
		Sc. rank:	584.2

,

PA			
Pos.	712	obs:	exp:
att	I	0	38.31
atc	I	0	27.70
ata	I	108	41.99
atg	M	160	160.00
act	T	1	562.30
acc	T	0	494.30
aca	T	2250	1039.00
acg	T	35	190.60
aaa	K	6	3.65
aag	K	0	2.35
gtt	V	0	32.20
gtc	V	0	27.36
gta	V	13	36.60
gtg	V	132	48.84
gct	A	0	2.21
gcc	A	0	2.06
gca	A	9	4.06
gcg	A	0	0.67
---	--	------	------
mPD		0.58	1.1
		nPD:	0.54
		N. weight:	1.
		Sc. PD:	0.27
		Sc. rank:	1189.5

,

PA			
Pos.	713	obs:	exp:
cat	H	2677	1561.00
cac	H	30	1146.00
caa	Q	1	3.24
cag	Q	5	2.76
cgt	R	1	0.03
cgc	R	0	0.05
cga	R	0	0.10
cgg	R	0	0.07
aga	R	0	0.46
agg	R	0	0.28
---	--	------	------
mPD		0.027	0.49
		nPD:	0.05
		N. weight:	0.72
		Sc. PD:	-0.15
		Sc. rank:	-840.1

,

PA			
Pos.	714	obs:	exp:
act	T	0	0.49
acc	T	0	0.43
aca	T	2	0.91
acg	T	0	0.17
gtt	V	0	0.44
gtc	V	0	0.38
gta	V	2	0.50
gtg	V	0	0.67
gct	A	0	664.30
gcc	A	0	620.90
gca	A	2710	1222.00
gcg	A	0	203.20
---	--	------	------
mPD		0.0029	0.68
		nPD:	0.
		N. weight:	1.2
		Sc. PD:	-0.3
		Sc. rank:	-1826.2

,

PA			
Pos.	715	obs:	exp:
tta	L	3	295.30
ttg	L	74	509.80
ctt	L	5	548.10
ctc	L	17	394.90
cta	L	264	473.90
ctg	L	2351	492.00
---	--	------	------
mPD		0.25	1.1
		nPD:	0.22
		N. weight:	1.8
		Sc. PD:	-0.091
		Sc. rank:	238.1

,

PA			
Pos.	716	obs:	exp:
caa	Q	0	4.31
cag	Q	8	3.69
cgt	R	0	1.48
cgc	R	0	2.48
cga	R	0	4.72
cgg	R	0	3.40
aat	N	16	10.80
aac	N	3	8.20
aaa	K	1411	1585.00
aag	K	1197	1023.00
aga	R	47	21.55
agg	R	0	13.36
gat	D	1	0.56
gac	D	0	0.44
gaa	E	28	18.37
gag	E	3	12.63
---	--	------	------
mPD		0.57	0.56
		nPD:	1.01
		N. weight:	0.046
		Sc. PD:	0.033
		Sc. rank:	73.1

,

PA			
Pos.	717	obs:	exp:
taa	.	188	1155.00
tag	.	2503	714.90
tga	.	23	844.40
---	--	------	------
mPD		0.16	0.82
		nPD:	0.2
		N. weight:	1.5
		Sc. PD:	-0.1
		Sc. rank:	10.7

}

Amino acid level conservation per codon

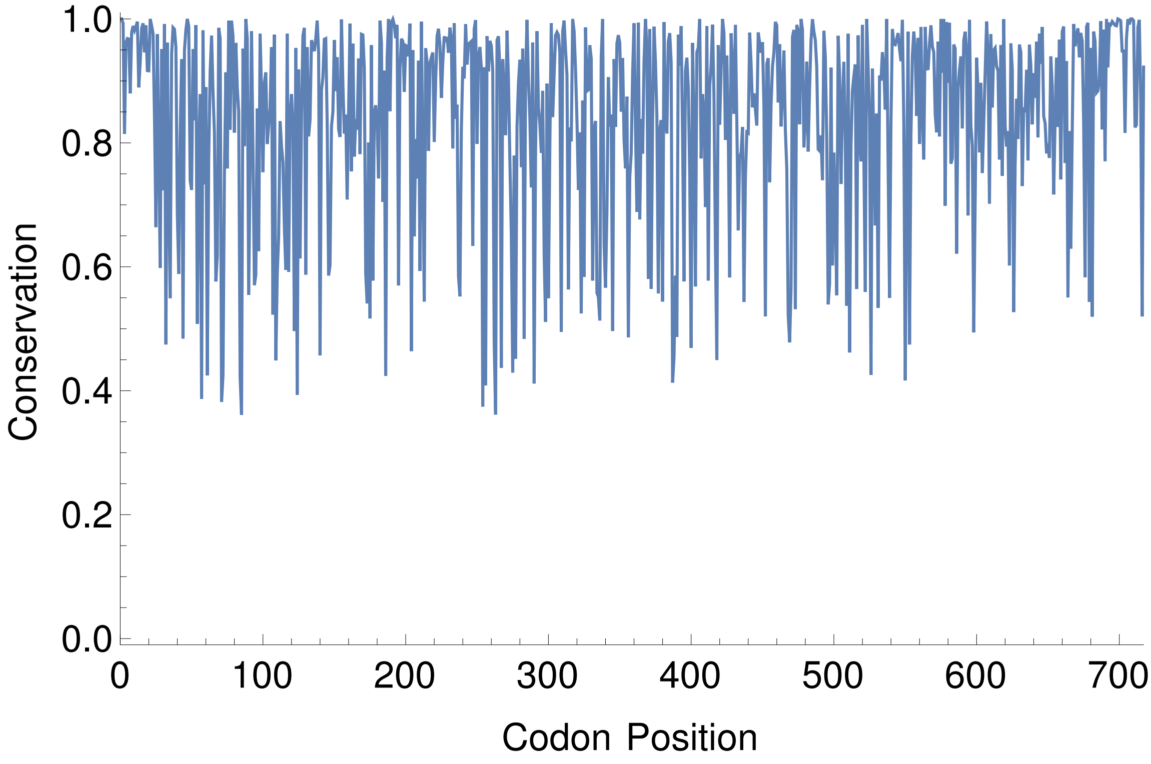

Conservation analysis plots

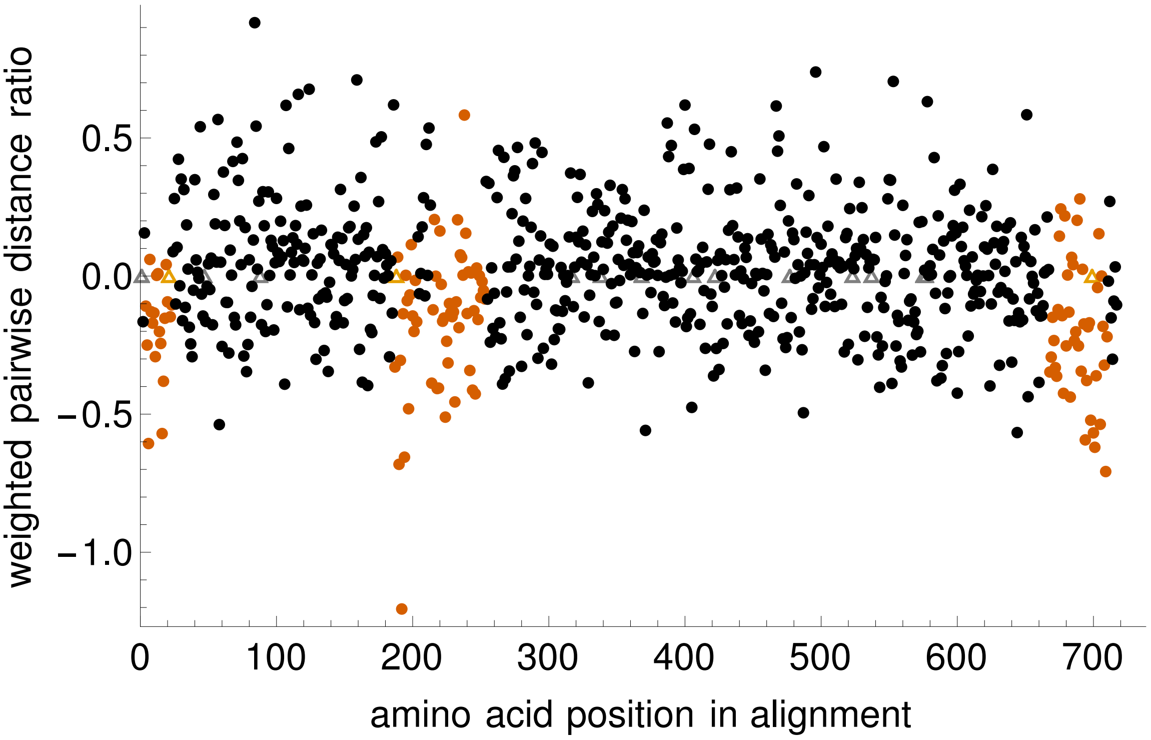

PA

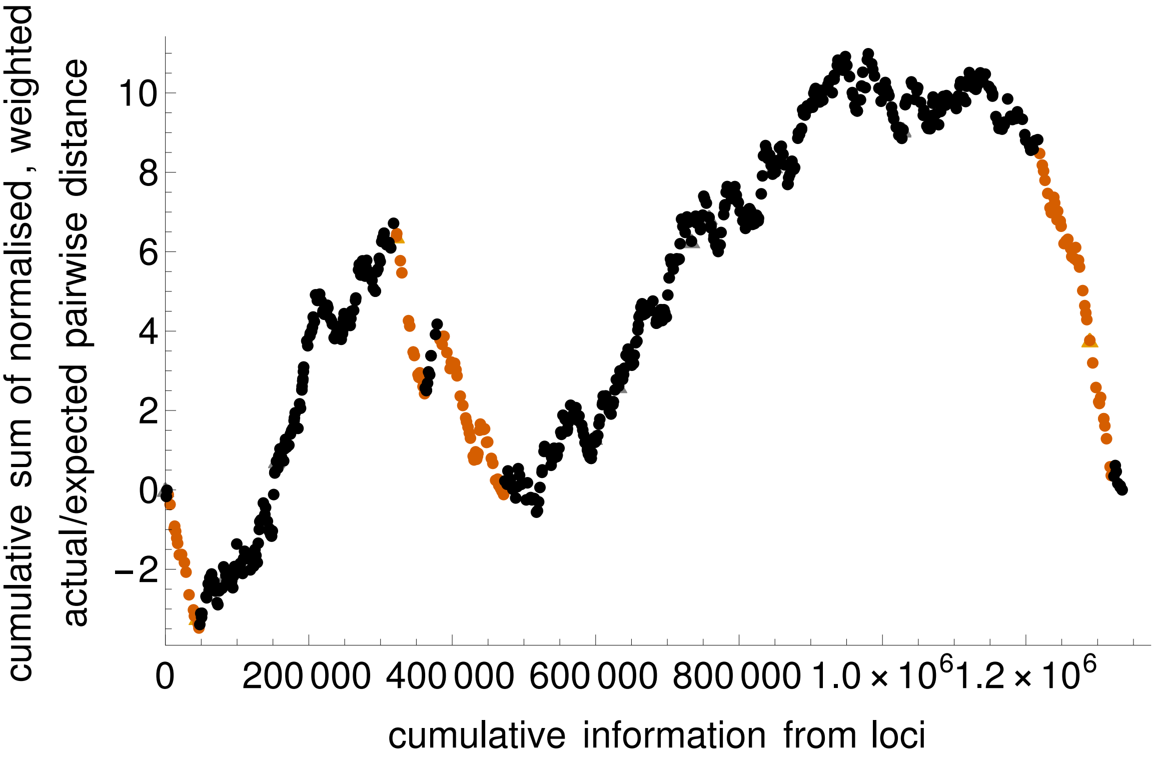

PA

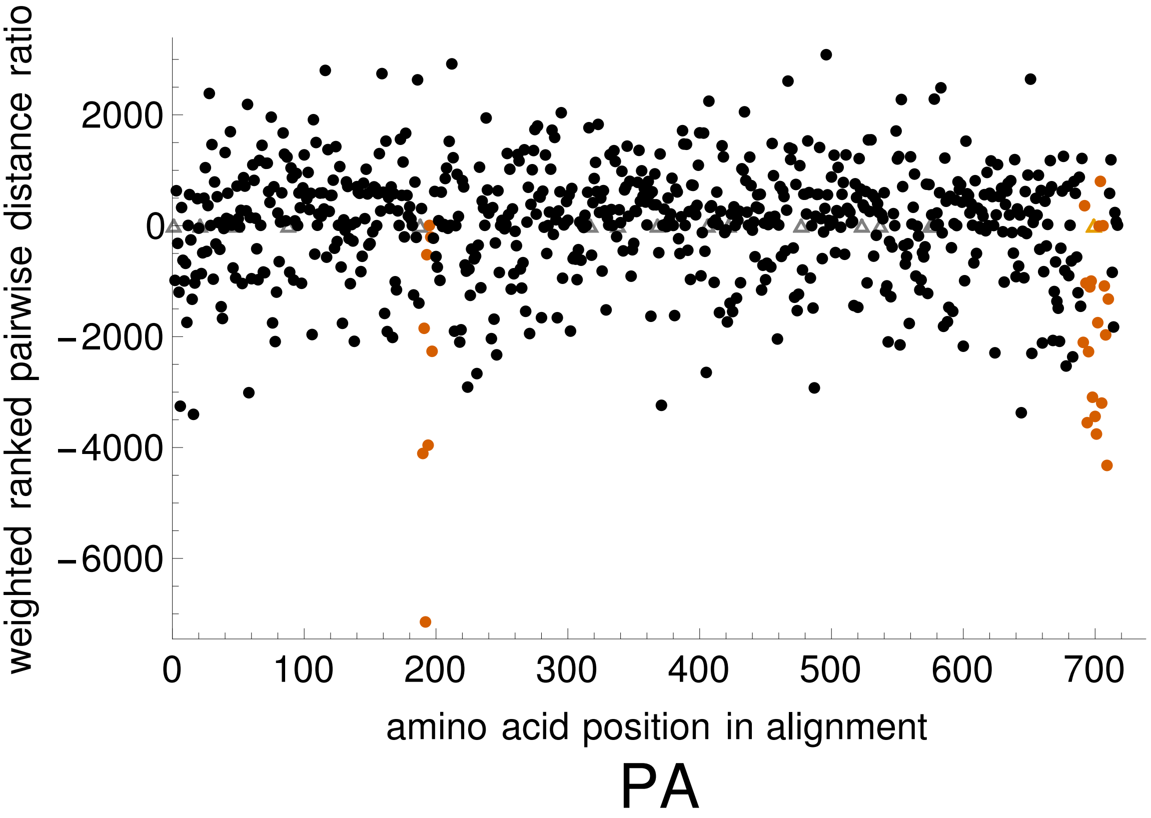
weighted ranked pairwise distance ratio
2000
0
−2000
−4000
−6000
0
100
200
300
400
500
600
700
amino acid position in alignment
PA

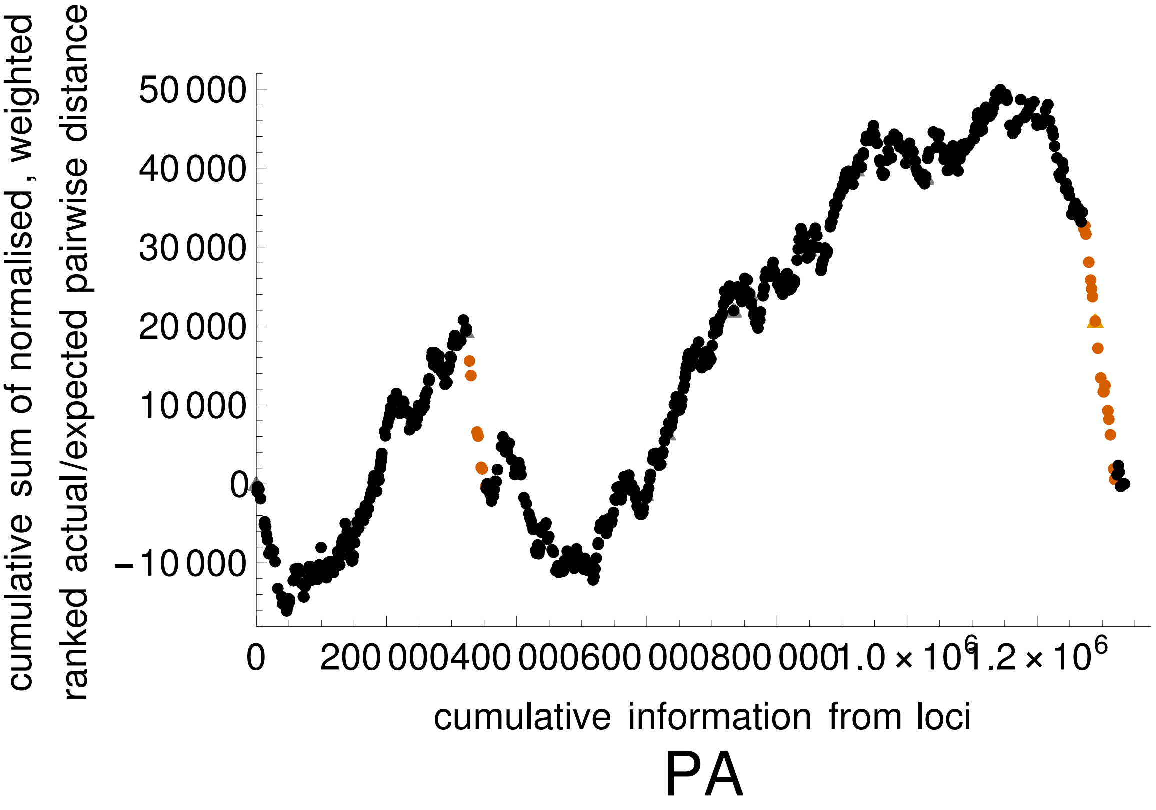
cumulative sum of normalised, weighted ranked actual/expected pairwise distance
50 000
40 000
30 000
20 000
10 000
0
−10 000
0
200 000
400 000
600 000
800 000
1.0 × 10^6
1.2 × 10^6
cumulative information from loci
PA

PA-X

Gene length histogram

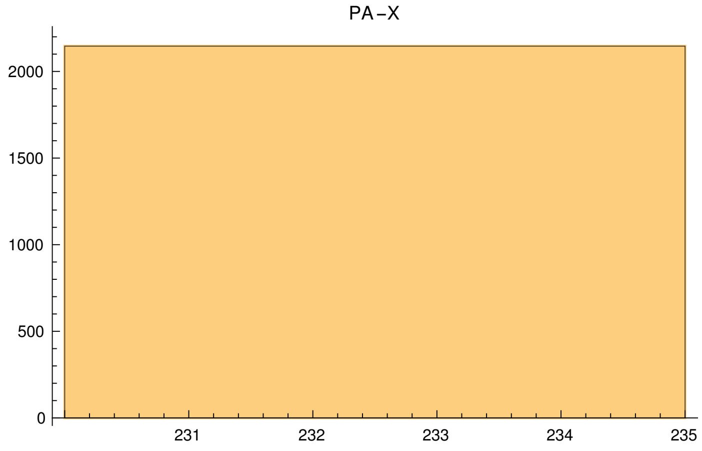

Information vs. nPD

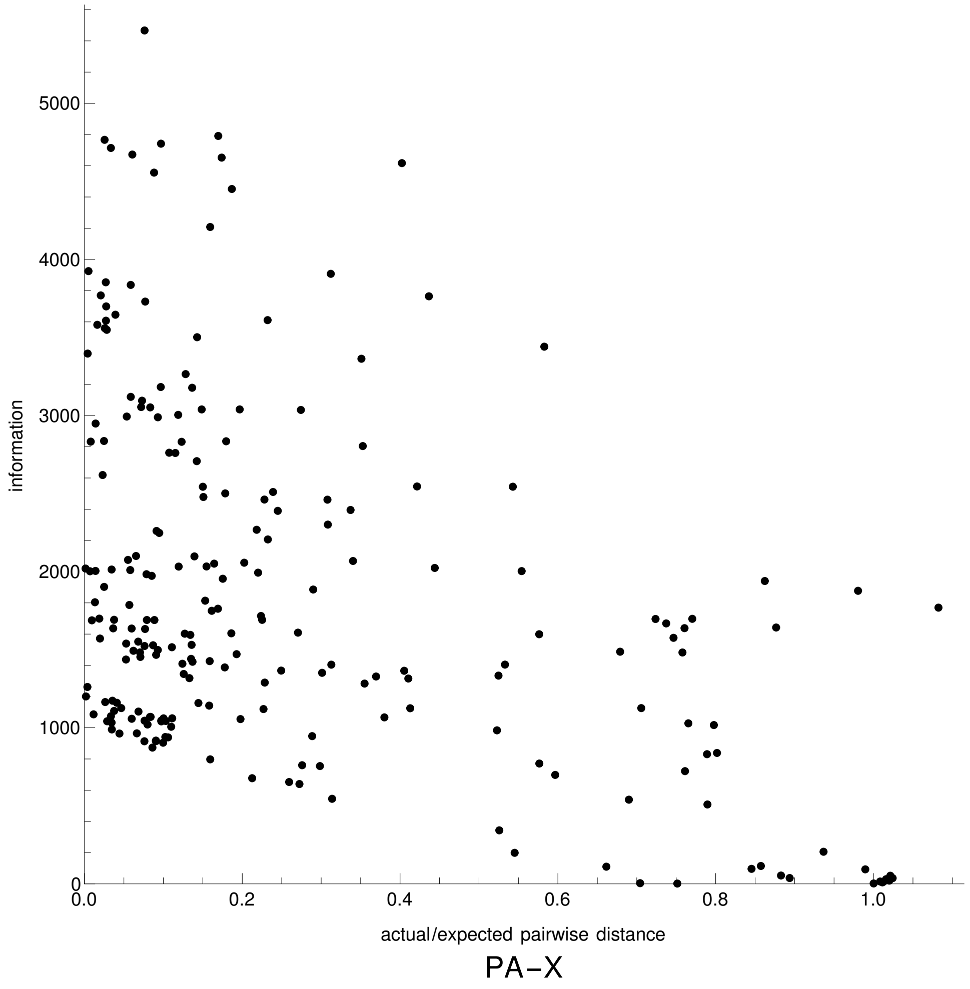

Example sequences highlighted by regions found in analyses to be conserved

Interesting points (by weighted raw PD) highlighted for gene PA-X:

ATGGAAGAC
TTTGTGCGACAATGCTTCAATCCAATGATCGTCGAGCTTGCGGAAAAGGCAATGA AAGAATATGGGGAAGATCCGAAAATCGAAACTAACAAGTTTGCTGCAATATGCACACATTTGGAAGTT⋱
TGTTTCATGTATTCGGATTTCCATTTCATCGACGAACGGGGTGAATCAATAATTGTAGAATCTGGTGACC⋱
CAAATGCACTATTGAAGCACCGATTTGAGATAATTGAAGGAAGAGACCGAATCATGGCATGGACAGTGGT⋱
GAACAGTATATGTAACACAACAGGGGTAGAGAAGCCTAAATTTCTTCCTGATTTGTATGATTACAAAGAG⋱
AACCGGTTCATCGAAATTGGAGTAACACGGAGGGAAGTCCACATATATTACCTAGAGAAAGCCAACAAAA⋱
TAAAATCCGAGAAAACACACATTCACATCTTTTCATTCACTGGAGAGGAGATGGCCACCAAAGCGGACTA⋱
CACCCTTGACGAAGAGAGCAGGGCAAGAATCAAAACTAGGCTTTTCACTATAAGACAAGAAATGGTCAGT⋱
AGGAGT
CTATGGGATTCCTTCGTCAGTCCGAAAGAGGCGAAGAGACAATTGAAGAAAAATT TGAGATTACAGGAACTATGCGCAAGCTTGCCGACCAAAGTCTCCCACCGAAC TTCTCCAGCCTTGAAAACTTTAGAGCCTATGTAG

Interesting points (by weighted ranked PD) highlighted for gene PA-X:

ATGGAAGACTTTGTGCGACAATGCTTCAATCCAATGATCGTCGAGCTTGCGGAAAAGGCAATGAAAGAATATGGGGAA⋱
GATCCGAAAATCGAAACTAACAAGTTTGCTGCAATATGCACACATTTGGAAGTTTGTTTCATGTATTCGGATTTCCA⋱
TTTCATCGACGAACGGGGTGAATCAATAATTGTAGAATCTGGTGACCCAAATGCACTATTGAAGCACCGATTTGAGA⋱
TAATTGAAGGAAGAGACCGAATCATGGCATGGACAGTGGTGAACAGTATATGTAACACAACAGGGGTAGAGAAGCCT⋱
AAATTTCTTCCTGATTTGTATGATTACAAAGAGAACCGGTTCATCGAAATTGGAGTAACACGGAGGGAAGTCCACAT⋱
ATATTACCTAGAGAAAGCCAACAAAATAAAATCCGAGAAAACACACATTCACATCTTTTCATTCACTGGAGAGGAGA⋱
TGGCCACCAAAGCGGACTACACCCTTGACGAAGAGAGCAGGGCAAGAATCAAAACTAGGCTTTTCACTATAAGACAA⋱
GAAATGGTCAGTAGGAGTCTATGG
GATTCCTTCGTCAGTCCGAAAGAGGCGAAGAGACAATTGAAGAAAAATTTGAGATTACAGGAACTATGC⋱
GCAAGCTTGCCGACCAAAGTCTCCCACCGAACTTCTCCAGCCTTGAAAACTTTAGAGCCTATGTAG

Per codon conservation report

PA-X

Pos. 1		obs:	exp:
atg	M	2146	2146.00
mPD		0	0
		nPD:	1.
		N. weight:	0.
		Sc. PD:	0
		Sc. rank:	0

PA-X

Pos. 2		obs:	exp:
gaa	E	2131	1272.00
gag	E	15	874.50
mPD		0.014	0.48
		nPD:	0.03
		N. weight:	0.58
		Sc. PD:	-0.092
		Sc. rank:	-607.3

PA-X

Pos. 3		obs:	exp:
cat	H	0	0.58
cac	H	1	0.42
aat	N	0	5.68
aac	N	10	4.32
gct	A	0	0.49
gcc	A	2	0.46
gca	A	0	0.90
gcg	A	0	0.15
gat	D	374	1198.00
gac	D	1756	932.00
ggt	G	1	0.47
ggc	G	2	0.38
gga	G	0	1.36
ggg	G	0	0.79
mPD		0.30	0.51
		nPD:	0.6
		N. weight:	0.39
		Sc. PD:	0.15
		Sc. rank:	525.0

PA-X

Pos. 4		obs:	exp:
ttt	F	2109	959.80
ttc	F	33	1182.00
tta	L	2	0.33
ttg	L	0	0.56
tct	S	1	0.17
tcc	S	0	0.16
tca	S	0	0.24
tcg	S	0	0.05
ctt	L	1	0.61
ctc	L	0	0.44
cta	L	0	0.52
ctg	L	0	0.54
agt	S	0	0.19
agc	S	0	0.19
mPD		0.034	0.50
		nPD:	0.07
		N. weight:	0.87
		Sc. PD:	-0.1
		Sc. rank:	-553.4

PA-X

Pos. 5		obs:	exp:
atg	M	1	1.00
gtt	V	2	476.30
gtc	V	0	404.80
gta	V	50	541.50
gtg	V	2093	722.50
mPD		0.048	0.74
		nPD:	0.07
		N. weight:	1.2
		Sc. PD:	-0.14
		Sc. rank:	-771.0

PA-X

Pos. 6		obs:	exp:
cgt	R	0	67.74
cgc	R	1	113.40
cga	R	2112	215.70
cgg	R	16	155.00
aga	R	17	984.10
agg	R	0	609.90
mPD		0.031	0.94
		nPD:	0.03
		N. weight:	2.6
		Sc. PD:	-0.4
		Sc. rank:	-2656.1

PA-X

Pos. 7		obs:	exp:
caa	Q	1973	1157.00
cag	Q	173	988.70
mPD		0.15	0.50
		nPD:	0.3
		N. weight:	0.42
		Sc. PD:	0.044
		Sc. rank:	385.1

PA-X

Pos. 8		obs:	exp:
tct	S	0	1.68
tcc	S	0	1.58
tca	S	0	2.43
tcg	S	0	0.53
tat	Y	0	2.80
tac	Y	5	2.20
tgt	C	31	917.30
tgc	C	2100	1214.00
agt	S	0	1.86
agc	S	10	1.92
mPD		0.042	0.51
		nPD:	0.08
		N. weight:	0.6
		Sc. PD:	-0.062
		Sc. rank:	-229.0

PA-X

Pos. 9		obs:	exp:
ttt	F	22	961.60
ttc	F	2124	1184.00
mPD		0.020	0.49
		nPD:	0.04
		N. weight:	0.65
		Sc. PD:	-0.095
		Sc. rank:	-578.9

PA-X

Pos. 10		obs:	exp:
act	T	1	0.25
acc	T	0	0.22
aca	T	0	0.45
acg	T	0	0.08
aat	N	2103	1219.00
aac	N	42	925.80
mPD		0.039	0.49
		nPD:	0.08
		N. weight:	0.57
		Sc. PD:	-0.062
		Sc. rank:	-240.5

PA-X

Pos. 11		obs:	exp:
cct	P	1	633.30
ccc	P	0	432.10
cca	P	2127	849.80
ccg	P	18	230.80
mPD		0.018	0.70
		nPD:	0.02
		N. weight:	1.1
		Sc. PD:	-0.17
		Sc. rank:	-1308.1

PA-X

Pos. 12		obs:	exp:
att	I	3	1.42
atc	I	0	1.03
ata	I	1	1.56
atg	M	2142	2142.00
mPD		0.0037	0.0037
		nPD:	1.
		N. weight:	0.0015
		Sc. PD:	0.0012
		Sc. rank:	2.3

PA-X

Pos. 13		obs:	exp:
ttt	F	1	0.45
ttc	F	0	0.55
att	I	36	736.80
atc	I	2038	532.70
ata	I	3	807.50
gtt	V	3	15.10
gtc	V	63	12.83
gta	V	2	17.17
gtg	V	0	22.90
mPD		0.10	0.73
		nPD:	0.14
		N. weight:	1.5
		Sc. PD:	-0.071
		Sc. rank:	292.3

PA-X

Pos. 14		obs:	exp:
att	I	1	11.35
atc	I	31	8.21
ata	I	0	12.44
gtt	V	136	468.70
gtc	V	1975	398.30
gta	V	0	532.90
gtg	V	0	711.00
gat	D	0	1.69
gac	D	3	1.31
mPD		0.15	0.77
		nPD:	0.2
		N. weight:	1.7
		Sc. PD:	0.01
		Sc. rank:	1063.7

PA-X

Pos. 15		obs:	exp:
gat	D	6	3.37
gac	D	0	2.63
gaa	E	24	1268.00
gag	E	2116	872.00
mPD		0.028	0.49
		nPD:	0.06
		N. weight:	1.
		Sc. PD:	-0.13
		Sc. rank:	-803.4

PA-X

Pos. 16		obs:	exp:
tta	L	0	233.50
ttg	L	0	403.10
ctt	L	2141	433.40
ctc	L	2	312.20
cta	L	2	374.70
ctg	L	1	389.00
mPD		0.0047	1.1
		nPD:	0.
		N. weight:	1.9
		Sc. PD:	-0.35
		Sc. rank:	-2846.5

PA-X

Pos. 17		obs:	exp:
gct	A	27	526.10
gcc	A	0	491.70
gca	A	103	967.30
gcg	A	2016	160.90
mPD		0.12	0.68
		nPD:	0.17
		N. weight:	2.7
		Sc. PD:	-0.055
		Sc. rank:	1202.2

PA-X

Pos. 18		obs:	exp:
gaa	E	2128	1271.00
gag	E	17	874.00
ggt	G	0	0.16
ggc	G	0	0.13
gga	G	1	0.45
ggg	G	0	0.26
mPD		0.017	0.48
		nPD:	0.03
		N. weight:	0.58
		Sc. PD:	-0.088
		Sc. rank:	-573.0

PA-X

Pos. 19		obs:	exp:
cgt	R	0	1.14
cgc	R	0	1.90
cga	R	0	3.62
cgg	R	0	2.60
aaa	K	49	1282.00
aag	K	2061	827.70
aga	R	7	16.51
agg	R	29	10.23
mPD		0.084	0.52
		nPD:	0.16
		N. weight:	0.98
		Sc. PD:	-0.028
		Sc. rank:	376.5

PA-X

Pos. 20		obs:	exp:
act	T	0	15.00
acc	T	0	13.19
aca	T	61	27.72
acg	T	0	5.09
gct	A	2	511.10
gcc	A	0	477.70
gca	A	2082	939.80
gcg	A	1	156.40
mPD		0.058	0.73
		nPD:	0.08
		N. weight:	0.94
		Sc. PD:	-0.1
		Sc. rank:	-405.5

PA-X

Pos. 21		obs:	exp:
atg	M	2146	2146.00
mPD		0	0
		nPD:	1.
		N. weight:	0.
		Sc. PD:	0
		Sc. rank:	0

PA-X

Pos. 22		obs:	exp:
cgt	R	0	0.09
cgc	R	0	0.16
cga	R	0	0.30
cgg	R	0	0.22
aaa	K	2128	1302.00
aag	K	15	840.60
aga	R	3	1.38
agg	R	0	0.85
mPD		0.017	0.48
		nPD:	0.03
		N. weight:	0.55
		Sc. PD:	-0.084
		Sc. rank:	-536.3

PA-X

Pos. 23		obs:	exp:
gaa	E	2111	1271.00
gag	E	34	874.00
ggt	G	0	0.16
ggc	G	0	0.13
gga	G	1	0.45
ggg	G	0	0.26
mPD		0.032	0.48
		nPD:	0.07
		N. weight:	0.54
		Sc. PD:	-0.065
		Sc. rank:	-350.1

PA-X

Pos. 24		obs:	exp:
ttt	F	1	0.90
ttc	F	1	1.10
tat	Y	2088	1200.00
tac	Y	54	942.00
cat	H	1	1.15
cac	H	1	0.85
mPD		0.055	0.50
		nPD:	0.11
		N. weight:	0.56
		Sc. PD:	-0.044
		Sc. rank:	-42.0

PA-X

Pos. 25		obs:	exp:
ggt	G	0	336.60
ggc	G	0	271.90
gga	G	515	972.00
ggg	G	1631	565.40
mPD		0.36	0.68
		nPD:	0.53
		N. weight:	0.78
		Sc. PD:	0.26
		Sc. rank:	991.1

PA-X

Pos.	26	obs:	exp:
gaa	E	2098	1272.00
gag	E	48	874.50
---	--	------	------
mPD		0.044	0.48
		nPD:	0.09
		N. weight:	0.51
		Sc. PD:	-0.05
		Sc. rank:	-148.6

PA-X

Pos.	27	obs:	exp:
tct	S	0	0.34
tcc	S	0	0.32
tca	S	0	0.49
tcg	S	0	0.11
tat	Y	1	0.56
tac	Y	0	0.44
cat	H	1	0.58
cac	H	0	0.42
aat	N	93	62.52
aac	N	17	47.48
agt	S	2	0.37
agc	S	0	0.38
gat	D	1972	1142.00
gac	D	59	888.60
gaa	E	1	0.59
gag	E	0	0.41
---	--	------	------
mPD		0.17	0.60
		nPD:	0.29
		N. weight:	0.53
		Sc. PD:	0.05
		Sc. rank:	472.6

PA-X

Pos.	28	obs:	exp:
tct	S	20	142.30
tcc	S	0	133.70
tca	S	29	205.40
tcg	S	797	44.96
cct	P	1	381.00
ccc	P	4	259.90
cca	P	92	511.20
ccg	P	1194	138.90
act	T	0	2.21
acc	T	0	1.95
aca	T	0	4.09
acg	T	9	0.75
agt	S	0	157.50
agc	S	0	162.10
---	--	------	------
mPD		0.61	1.5
		nPD:	0.4
		N. weight:	2.6
		Sc. PD:	0.53
		Sc. rank:	2947.7

PA-X

Pos.	29	obs:	exp:
cgt	R	0	0.19
cgc	R	0	0.32
cga	R	0	0.60
cgg	R	0	0.43
aaa	K	2106	1301.00
aag	K	34	839.40
aga	R	6	2.75
agg	R	0	1.71
---	--	------	------
mPD		0.037	0.48
		nPD:	0.08
		N. weight:	0.51
		Sc. PD:	-0.057
		Sc. rank:	-283.7

PA-X

Pos.	30	obs:	exp:
tta	L	0	0.33
ttg	L	0	0.56
ctt	L	0	0.61
ctc	L	0	0.44
cta	L	3	0.52
ctg	L	0	0.54
att	I	84	691.70
atc	I	1830	500.20
ata	I	36	758.10
act	T	0	37.88
acc	T	154	33.30
aca	T	0	69.98
acg	T	0	12.84
gtt	V	0	8.66
gtc	V	38	7.36
gta	V	1	9.85
gtg	V	0	13.14
---	--	------	------
mPD		0.28	0.84
		nPD:	0.34
		N. weight:	1.3
		Sc. PD:	0.19
		Sc. rank:	1364.9

PA-X

Pos.	31	obs:	exp:
gaa	E	2140	1272.00
gag	E	6	874.50
---	--	------	------
mPD		0.0056	0.48
		nPD:	0.01
		N. weight:	0.61
		Sc. PD:	-0.11
		Sc. rank:	-858.1

PA-X

Pos.	32	obs:	exp:
tta	L	0	0.11
ttg	L	0	0.19
ctt	L	0	0.20
ctc	L	1	0.15
cta	L	0	0.17
ctg	L	0	0.18
att	I	5	2.48
atc	I	2	1.80
ata	I	0	2.72
act	T	901	525.20
acc	T	1188	461.70
aca	T	45	970.10
acg	T	1	178.00
aat	N	3	1.71
aac	N	0	1.29
---	--	------	------
mPD		0.52	0.69
		nPD:	0.76
		N. weight:	0.83
		Sc. PD:	0.46
		Sc. rank:	1174.5

PA-X

Pos.	33	obs:	exp:
aat	N	60	1220.00
aac	N	2086	926.20
---	--	------	------
mPD		0.054	0.49
		nPD:	0.11
		N. weight:	0.85
		Sc. PD:	-0.066
		Sc. rank:	-53.8

PA-X

Pos.	34	obs:	exp:
aaa	K	313	1304.00
aag	K	1833	841.80
---	--	------	------
mPD		0.25	0.48
		nPD:	0.52
		N. weight:	0.55
		Sc. PD:	0.18
		Sc. rank:	681.3

PA-X

Pos.	35	obs:	exp:
ttt	F	1041	960.20
ttc	F	1102	1183.00
tta	L	2	0.33
ttg	L	0	0.56
ctt	L	1	0.61
ctc	L	0	0.44
cta	L	0	0.52
ctg	L	0	0.54
---	--	------	------
mPD		0.50	0.50
		nPD:	1.01
		N. weight:	0.0084
		Sc. PD:	0.0067
		Sc. rank:	12.9

PA-X

Pos.	36	obs:	exp:
tct	S	5	0.84
tcc	S	0	0.79
tca	S	0	1.21
tcg	S	0	0.27
act	T	6	1.48
acc	T	0	1.30
aca	T	0	2.73
acg	T	0	0.50
agt	S	0	0.93
agc	S	0	0.96
gct	A	2031	523.10
gcc	A	65	489.00
gca	A	15	961.90
gcg	A	23	160.00
ggt	G	1	0.16
ggc	G	0	0.13
gga	G	0	0.45
ggg	G	0	0.26
---	--	------	------
mPD		0.10	0.69
		nPD:	0.15
		N. weight:	1.4
		Sc. PD:	-0.056
		Sc. rank:	382.6

PA-X

Pos.	37	obs:	exp:
tct	S	0	0.34
tcc	S	0	0.32
tca	S	2	0.49
tcg	S	0	0.11
agt	S	0	0.37
agc	S	0	0.38
gtt	V	0	0.22
gtc	V	0	0.19
gta	V	1	0.25
gtg	V	0	0.34
gct	A	0	524.30
gcc	A	0	490.10
gca	A	2119	964.20
gcg	A	20	160.40
ggt	G	0	0.63
ggc	G	0	0.51
gga	G	4	1.81
ggg	G	0	1.05
---	--	------	------
mPD		0.025	0.69
		nPD:	0.04
		N. weight:	0.91
		Sc. PD:	-0.14
		Sc. rank:	-868.5

PA-X

Pos.	38	obs:	exp:
tta	L	0	0.11
ttg	L	0	0.19
ctt	L	0	0.20
ctc	L	0	0.15
cta	L	1	0.17
ctg	L	0	0.18
att	I	4	747.80
atc	I	0	540.70
ata	I	2104	819.50
gtt	V	0	8.22
gtc	V	0	6.98
gta	V	35	9.34
gtg	V	2	12.46
---	--	------	------
mPD		0.040	0.70
		nPD:	0.06
		N. weight:	1.1
		Sc. PD:	-0.15
		Sc. rank:	-887.2

PA-X

Pos.	39	obs:	exp:
tct	S	0	0.17
tcc	S	0	0.16
tca	S	0	0.24
tcg	S	0	0.05
tgt	C	19	923.30
tgc	C	2126	1222.00
agt	S	0	0.19
agc	S	1	0.19
---	--	------	------
mPD		0.018	0.49
		nPD:	0.04
		N. weight:	0.62
		Sc. PD:	-0.092
		Sc. rank:	-575.1

PA-X

Pos.	40	obs:	exp:
att	I	1	0.35
atc	I	0	0.26
ata	I	0	0.39
act	T	8	527.60
acc	T	7	463.80
aca	T	1393	974.70
acg	T	737	178.90
---	--	------	------
mPD		0.46	0.68
		nPD:	0.68
		N. weight:	0.83
		Sc. PD:	0.4
		Sc. rank:	1128.5

PA-X

Pos. 41		obs:	exp:
cat	H	1052	1238.00
cac	H	1094	908.10
---	--	------	------
mPD		0.50	0.49
		nPD:	1.02
		N. weight:	0.02
		Sc. PD:	0.017
		Sc. rank:	31.2

PA-X

Pos. 42		obs:	exp:
tta	L	38	231.90
ttg	L	1994	400.30
ctt	L	0	430.40
ctc	L	0	310.10
cta	L	0	372.10
ctg	L	99	386.30
atg	M	15	15.00
---	--	------	------
mPD		0.14	1.1
		nPD:	0.12
		N. weight:	1.7
		Sc. PD:	-0.12
		Sc. rank:	-21.6

PA-X

Pos. 43		obs:	exp:
gaa	E	2002	1272.00
gag	E	144	874.50
---	--	------	------
mPD		0.13	0.48
		nPD:	0.26
		N. weight:	0.36
		Sc. PD:	0.024
		Sc. rank:	306.9

PA-X

Pos. 44		obs:	exp:
tta	L	0	0.11
ttg	L	0	0.19
ctt	L	0	0.20
ctc	L	1	0.15
cta	L	0	0.17
ctg	L	0	0.18
att	I	63	73.43
atc	I	144	53.09
ata	I	0	80.48
act	T	0	0.49
acc	T	2	0.43
aca	T	0	0.91
acg	T	0	0.17
gtt	V	950	429.20
gtc	V	934	364.80
gta	V	32	488.00
gtg	V	17	651.10
gct	A	1	0.74
gcc	A	2	0.69
gca	A	0	1.35
gcg	A	0	0.22
---	--	------	------
mPD		0.70	0.92
		nPD:	0.76
		N. weight:	0.91
		Sc. PD:	0.51
		Sc. rank:	1309.1

PA-X

Pos. 45		obs:	exp:
tgt	C	2048	923.70
tgc	C	98	1222.00
---	--	------	------
mPD		0.087	0.49
		nPD:	0.18
		N. weight:	0.77
		Sc. PD:	-0.0097
		Sc. rank:	393.9

PA-X

Pos. 46		obs:	exp:
ttt	F	45	961.60
ttc	F	2101	1184.00
---	--	------	------
mPD		0.041	0.49
		nPD:	0.08
		N. weight:	0.6
		Sc. PD:	-0.063
		Sc. rank:	-247.2

PA-X

Pos. 47		obs:	exp:
atg	M	2146	2146.00
---	--	------	------
mPD		0	0
		nPD:	1.
		N. weight:	0.
		Sc. PD:	0
		Sc. rank:	0

PA-X

Pos. 48		obs:	exp:
tat	Y	2132	1202.00
tac	Y	14	943.70
---	--	------	------
mPD		0.013	0.49
		nPD:	0.03
		N. weight:	0.65
		Sc. PD:	-0.1
		Sc. rank:	-755.0

PA-X

Pos. 49		obs:	exp:
tct	S	165	361.00
tcc	S	0	339.20
tca	S	181	521.10
tcg	S	1800	114.10
agt	S	0	399.40
agc	S	0	411.30
---	--	------	------
mPD		0.28	1.6
		nPD:	0.17
		N. weight:	2.6
		Sc. PD:	-0.043
		Sc. rank:	1257.1

PA-X

Pos. 50		obs:	exp:
gat	D	1513	1207.00
gac	D	633	939.00
---	--	------	------
mPD		0.42	0.49
		nPD:	0.85
		N. weight:	0.054
		Sc. PD:	0.034
		Sc. rank:	79.6

PA-X

Pos. 51		obs:	exp:
ttt	F	52	961.10
ttc	F	2093	1184.00
tta	L	0	0.11
ttg	L	0	0.19
ctt	L	0	0.20
ctc	L	1	0.15
cta	L	0	0.17
ctg	L	0	0.18
---	--	------	------
mPD		0.048	0.50
		nPD:	0.1
		N. weight:	0.58
		Sc. PD:	-0.053
		Sc. rank:	-85.7

PA-X

Pos. 52		obs:	exp:
cat	H	2089	1238.00
cac	H	57	908.10
---	--	------	------
mPD		0.052	0.49
		nPD:	0.11
		N. weight:	0.52
		Sc. PD:	-0.043
		Sc. rank:	-53.9

PA-X

Pos. 53		obs:	exp:
ttt	F	19	961.60
ttc	F	2127	1184.00
---	--	------	------
mPD		0.018	0.49
		nPD:	0.04
		N. weight:	0.65
		Sc. PD:	-0.099
		Sc. rank:	-630.4

PA-X

Pos. 54		obs:	exp:
att	I	1061	761.30
atc	I	1025	550.40
ata	I	60	834.30
---	--	------	------
mPD		0.53	0.66
		nPD:	0.8
		N. weight:	0.47
		Sc. PD:	0.28
		Sc. rank:	693.3

PA-X

Pos. 55		obs:	exp:
aat	N	21	88.10
aac	N	134	66.90
gat	D	89	1120.00
gac	D	1902	871.10
---	--	------	------
mPD		0.23	0.63
		nPD:	0.37
		N. weight:	0.74
		Sc. PD:	0.13
		Sc. rank:	816.7

PA-X

Pos. 56		obs:	exp:
gaa	E	2060	1272.00
gag	E	86	874.50
---	--	------	------
mPD		0.077	0.48
		nPD:	0.16
		N. weight:	0.45
		Sc. PD:	-0.014
		Sc. rank:	165.9

PA-X

Pos. 57		obs:	exp:
tta	L	0	0.33
ttg	L	0	0.56
tgg	W	13	13.00
ctt	L	0	0.61
ctc	L	0	0.44
cta	L	0	0.52
ctg	L	3	0.54
cat	H	1	0.58
cac	H	0	0.42
caa	Q	54	465.40
cag	Q	809	397.60
cgt	R	0	39.24
cgc	R	0	65.70
cga	R	114	124.90
cgg	R	940	89.80
act	T	0	0.25
acc	T	0	0.22
aca	T	0	0.45
acg	T	1	0.08
aaa	K	1	12.76
aag	K	20	8.24
aga	R	6	570.00
agg	R	183	353.30
ggt	G	0	0.16
ggc	G	0	0.13
gga	G	1	0.45
ggg	G	0	0.26
---	--	------	------
mPD		0.83	1.5
		nPD:	0.54
		N. weight:	1.4
		Sc. PD:	0.49
		Sc. rank:	1822.0

PA-X

Pos. 58		obs:	exp:
tgg	W	1	1.00
ggt	G	2127	336.40
ggc	G	9	271.80
gga	G	2	971.60
ggg	G	7	565.20
---	--	------	------
mPD		0.019	0.69
		nPD:	0.03
		N. weight:	2.2
		Sc. PD:	-0.34
		Sc. rank:	-2436.7

PA-X

Pos. 59		obs:	exp:
aaa	K	5	3.04
aag	K	0	1.96
gct	A	0	0.25
gcc	A	0	0.23
gca	A	1	0.45
gcg	A	0	0.07
gat	D	3	4.50
gac	D	5	3.50
gaa	E	1509	1243.00
gag	E	589	854.90
ggt	G	0	5.33
ggc	G	0	4.31
gga	G	4	15.40
ggg	G	30	8.96
---	--	------	------
mPD		0.45	0.53
		nPD:	0.86
		N. weight:	0.064
		Sc. PD:	0.042
		Sc. rank:	94.8

PA-X

Pos. 60		obs:	exp:
tct	S	0	360.50
tcc	S	0	338.70
tca	S	2121	520.40
tcg	S	22	113.90
act	T	0	0.25
acc	T	0	0.22
aca	T	1	0.45
acg	T	0	0.08
agt	S	0	398.90
agc	S	0	410.70
gct	A	0	0.49
gcc	A	0	0.46
gca	A	2	0.90
gcg	A	0	0.15
---	--	------	------
mPD		0.023	1.6
		nPD:	0.01
		N. weight:	1.6
		Sc. PD:	-0.28
		Sc. rank:	-2247.3

PA-X

Pos.	61	obs:	exp:
ttt	F	0	1.79
ttc	F	4	2.21
cgt	R	0	0.03
cgc	R	0	0.05
cga	R	0	0.10
cgg	R	0	0.07
att	I	15	697.40
atc	I	977	504.30
ata	I	974	764.30
atg	M	9	9.00
act	T	0	3.20
acc	T	4	2.81
aca	T	9	5.91
acg	T	0	1.08
aga	R	1	0.46
agg	R	0	0.28
gtt	V	0	33.97
gtc	V	11	28.87
gta	V	142	38.62
gtg	V	0	51.53
---	--	------	------
mPD		0.66	0.83
		nPD:	0.8
		N. weight:	0.57
		Sc. PD:	0.34
		Sc. rank:	837.4

,

PA-X

Pos.	62	obs:	exp:
att	I	1932	752.10
atc	I	173	543.80
ata	I	15	824.20
act	T	2	0.49
acc	T	0	0.43
aca	T	0	0.91
acg	T	0	0.17
gtt	V	24	5.33
gtc	V	0	4.53
gta	V	0	6.06
gtg	V	0	8.08
---	--	------	------
mPD		0.19	0.68
		nPD:	0.27
		N. weight:	0.9
		Sc. PD:	0.07
		Sc. rank:	767.8

,

PA-X

Pos.	63	obs:	exp:
tta	L	1	0.22
ttg	L	0	0.38
ctt	L	0	0.40
ctc	L	0	0.29
cta	L	1	0.35
ctg	L	0	0.36
att	I	0	15.96
atc	I	0	11.54
ata	I	45	17.49
atg	M	7	7.00
gtt	V	5	464.10
gtc	V	0	394.40
gta	V	1996	527.60
gtg	V	89	704.00
gct	A	0	0.49
gcc	A	0	0.46
gca	A	2	0.90
gcg	A	0	0.15
---	--	------	------
mPD		0.14	0.79
		nPD:	0.18
		N. weight:	1.4
		Sc. PD:	-0.017
		Sc. rank:	736.5

,

PA-X

Pos.	64	obs:	exp:
gat	D	0	0.56
gac	D	1	0.44
gaa	E	2098	1271.00
gag	E	47	874.00
---	--	------	------
mPD		0.044	0.48
		nPD:	0.09
		N. weight:	0.51
		Sc. PD:	-0.05
		Sc. rank:	-152.6

,

PA-X

Pos.	65	obs:	exp:
tct	S	2099	355.70
tcc	S	10	334.30
tca	S	4	513.60
tcg	S	2	112.40
tat	Y	4	2.24
tac	Y	0	1.76
cct	P	22	7.08
ccc	P	2	4.83
cca	P	0	9.50
ccg	P	0	2.58
agt	S	0	393.70
agc	S	0	405.30
gct	A	3	0.74
gcc	A	0	0.69
gca	A	0	1.35
gcg	A	0	0.22
---	--	------	------
mPD		0.045	1.6
		nPD:	0.03
		N. weight:	2.1
		Sc. PD:	-0.33
		Sc. rank:	-2226.9

,

PA-X

Pos.	66	obs:	exp:
tct	S	0	6.06
tcc	S	0	5.69
tca	S	0	8.74
tcg	S	0	1.91
aat	N	3	1.71
aac	N	0	1.29
agt	S	36	6.70
agc	S	0	6.90
gat	D	187	105.20
gac	D	0	81.82
ggt	G	1900	301.20
ggc	G	17	243.30
gga	G	1	869.70
ggg	G	2	505.90
---	--	------	------
mPD		0.22	0.93
		nPD:	0.23
		N. weight:	2.
		Sc. PD:	0.081
		Sc. rank:	1562.3

,

PA-X

Pos.	67	obs:	exp:
aat	N	0	3.98
aac	N	7	3.02
gat	D	1058	1203.00
gac	D	1081	935.90
---	--	------	------
mPD		0.51	0.50
		nPD:	1.02
		N. weight:	0.017
		Sc. PD:	0.013
		Sc. rank:	25.4

,

PA-X

Pos.	68	obs:	exp:
tct	S	13	2.52
tcc	S	0	2.37
tca	S	0	3.64
tcg	S	2	0.80
cct	P	14	628.80
ccc	P	3	429.00
cca	P	1157	843.90
ccg	P	957	229.20
agt	S	0	2.79
agc	S	0	2.87
---	--	------	------
mPD		0.52	0.72
		nPD:	0.72
		N. weight:	0.95
		Sc. PD:	0.49
		Sc. rank:	1311.3

,

PA-X

Pos.	69	obs:	exp:
aat	N	2114	1220.00
aac	N	32	926.20
---	--	------	------
mPD		0.029	0.49
		nPD:	0.06
		N. weight:	0.59
		Sc. PD:	-0.075
		Sc. rank:	-424.4

,

PA-X

Pos.	70	obs:	exp:
gtt	V	0	1.11
gtc	V	0	0.94
gta	V	5	1.26
gtg	V	0	1.68
gct	A	7	524.80
gcc	A	16	490.60
gca	A	2078	965.10
gcg	A	40	160.60
---	--	------	------
mPD		0.062	0.68
		nPD:	0.09
		N. weight:	0.82
		Sc. PD:	-0.08
		Sc. rank:	-229.5

,

PA-X

Pos.	71	obs:	exp:
tta	L	111	231.00
ttg	L	946	398.80
ctt	L	0	428.80
ctc	L	2	308.90
cta	L	1002	370.70
ctg	L	62	384.80
att	I	0	8.16
atc	I	0	5.90
ata	I	23	8.94
---	--	------	------
mPD		1.0	1.2
		nPD:	0.88
		N. weight:	0.92
		Sc. PD:	0.61
		Sc. rank:	1383.5

,

PA-X

Pos.	72	obs:	exp:
tta	L	128	231.60
ttg	L	921	399.70
ctt	L	6	429.80
ctc	L	0	309.60
cta	L	43	371.60
ctg	L	1030	385.70
att	I	0	0.35
atc	I	0	0.26
ata	I	1	0.39
atg	M	17	17.00
---	--	------	------
mPD		0.66	1.1
		nPD:	0.58
		N. weight:	0.89
		Sc. PD:	0.34
		Sc. rank:	1170.3

,

PA-X

Pos.	73	obs:	exp:
cgt	R	0	0.35
cgc	R	0	0.58
cga	R	0	1.11
cgg	R	0	0.79
aaa	K	193	1298.00
aag	K	1942	837.50
aga	R	0	5.04
agg	R	11	3.13
---	--	------	------
mPD		0.17	0.49
		nPD:	0.36
		N. weight:	0.72
		Sc. PD:	0.11
		Sc. rank:	781.8

,

PA-X

Pos.	74	obs:	exp:
cat	H	222	1238.00
cac	H	1924	908.10
---	--	------	------
mPD		0.19	0.49
		nPD:	0.38
		N. weight:	0.6
		Sc. PD:	0.11
		Sc. rank:	660.5

,

PA-X

Pos.	75	obs:	exp:
cgt	R	23	67.74
cgc	R	1	113.40
cga	R	1978	215.70
cgg	R	109	155.00
aga	R	35	984.10
agg	R	0	609.90
---	--	------	------
mPD		0.15	0.94
		nPD:	0.16
		N. weight:	2.3
		Sc. PD:	-0.072
		Sc. rank:	861.3

,

PA-X

Pos.	76	obs:	exp:
ttt	F	2141	961.60
ttc	F	5	1184.00
---	--	------	------
mPD		0.0047	0.49
		nPD:	0.01
		N. weight:	0.94
		Sc. PD:	-0.17
		Sc. rank:	-1341.5

,

PA-X

Pos.	77	obs:	exp:
gct	A	0	0.25
gcc	A	0	0.23
gca	A	1	0.45
gcg	A	0	0.07
gaa	E	70	1271.00
gag	E	2075	874.00
---	--	------	------
mPD		0.065	0.48
		nPD:	0.13
		N. weight:	0.89
		Sc. PD:	-0.049
		Sc. rank:	90.3

,

PA-X

Pos.	78	obs:	exp:
tta	L	0	0.11
ttg	L	0	0.19
ctt	L	0	0.20
ctc	L	0	0.15
cta	L	1	0.17
ctg	L	0	0.18
att	I	0	760.90
atc	I	4	550.20
ata	I	2141	833.90
---	--	------	------
mPD		0.0047	0.66
		nPD:	0.01
		N. weight:	1.1
		Sc. PD:	-0.2
		Sc. rank:	-1628.8

,

PA-X

Pos.	79	obs:	exp:
tta	L	0	0.22
ttg	L	0	0.38
ctt	L	2	0.40
ctc	L	0	0.29
cta	L	0	0.35
ctg	L	0	0.36
att	I	2089	759.90
atc	I	49	549.40
ata	I	4	832.70
gtt	V	2	0.44
gtc	V	0	0.38
gta	V	0	0.50
gtg	V	0	0.67
---	--	------	------
mPD		0.052	0.66
		nPD:	0.08
		N. weight:	1.1
		Sc. PD:	-0.12
		Sc. rank:	-489.7

,

PA-X

Pos.	80	obs:	exp:
aaa	K	0	0.61
aag	K	1	0.39
gaa	E	1995	1271.00
gag	E	150	874.00
---	--	------	------
mPD		0.13	0.48
		nPD:	0.27
		N. weight:	0.36
		Sc. PD:	0.028
		Sc. rank:	306.8

,

PA-X			
Pos.	81	obs:	exp:
ggt	G	0	336.60
ggc	G	0	271.90
gga	G	2093	972.00
ggg	G	53	565.40
---	--	------	------
mPD		0.048	0.68
		nPD:	0.07
		N. weight:	0.83
		Sc. PD:	-0.097
		Sc. rank:	-512.9

PA-X			
Pos.	82	obs:	exp:
cgt	R	0	67.74
cgc	R	0	113.40
cga	R	6	215.70
cgg	R	0	155.00
aga	R	1974	984.10
agg	R	166	609.90
---	--	------	------
mPD		0.15	0.94
		nPD:	0.16
		N. weight:	0.64
		Sc. PD:	-0.02
		Sc. rank:	207.0

PA-X			
Pos.	83	obs:	exp:
gat	D	73	1206.00
gac	D	2072	938.50
ggt	G	0	0.16
ggc	G	1	0.13
gga	G	0	0.45
ggg	G	0	0.26
---	--	------	------
mPD		0.067	0.49
		nPD:	0.14
		N. weight:	0.8
		Sc. PD:	-0.044
		Sc. rank:	90.2

PA-X			
Pos.	84	obs:	exp:
cgt	R	21	67.74
cgc	R	16	113.40
cga	R	1024	215.70
cgg	R	64	155.00
aga	R	59	984.10
agg	R	962	609.90
---	--	------	------
mPD		1.0	0.94
		nPD:	1.08
		N. weight:	0.99
		Sc. PD:	0.86
		Sc. rank:	1521.7

PA-X			
Pos.	85	obs:	exp:
tct	S	0	1.35
tcc	S	0	1.26
tca	S	0	1.94
tcg	S	0	0.43
cct	P	0	0.30
ccc	P	1	0.20
cca	P	0	0.40
ccg	P	0	0.11
att	I	52	365.70
atc	I	946	264.40
ata	I	33	400.80
act	T	746	245.50
acc	T	246	215.80
aca	T	5	453.50
acg	T	1	83.22
aat	N	13	14.21
aac	N	12	10.79
agt	S	6	1.49
agc	S	2	1.53
gtt	V	0	5.11
gtc	V	23	4.34
gta	V	0	5.81
gtg	V	0	7.75
gct	A	18	14.22
gcc	A	26	13.29
gca	A	14	26.14
gcg	A	0	4.35
gat	D	0	0.56
gac	D	1	0.44
ggt	G	1	0.16
ggc	G	0	0.13
gga	G	0	0.45
ggg	G	0	0.26
---	--	------	------
mPD		1.1	1.3
		nPD:	0.86
		N. weight:	1.1
		Sc. PD:	0.71
		Sc. rank:	1621.2

PA-X			
Pos.	86	obs:	exp:
att	I	5	9.22
atc	I	0	6.67
ata	I	21	10.11
atg	M	2033	2033.00
act	T	0	0.25
acc	T	0	0.22
aca	T	0	0.45
acg	T	1	0.08
gtt	V	0	19.10
gtc	V	0	16.23
gta	V	0	21.71
gtg	V	86	28.97
---	--	------	------
mPD		0.10	0.15
		nPD:	0.66
		N. weight:	0.061
		Sc. PD:	0.028
		Sc. rank:	82.8

PA-X			
Pos.	87	obs:	exp:
gct	A	164	526.10
gcc	A	1872	491.70
gca	A	104	967.30
gcg	A	6	160.90
---	--	------	------
mPD		0.23	0.68
		nPD:	0.34
		N. weight:	1.2
		Sc. PD:	0.17
		Sc. rank:	1196.5

PA-X			
Pos.	88	obs:	exp:
tgg	W	2146	2146.00
---	--	------	------
mPD		0	0
		nPD:	1.
		N. weight:	0.
		Sc. PD:	0
		Sc. rank:	0

PA-X			
Pos.	89	obs:	exp:
act	T	2	527.40
acc	T	0	463.60
aca	T	2089	974.20
acg	T	53	178.80
gct	A	0	0.49
gcc	A	0	0.46
gca	A	2	0.90
gcg	A	0	0.15
---	--	------	------
mPD		0.052	0.68
		nPD:	0.08
		N. weight:	0.85
		Sc. PD:	-0.095
		Sc. rank:	-463.7

PA-X			
Pos.	90	obs:	exp:
tta	L	0	0.76
ttg	L	7	1.31
ctt	L	0	1.41
ctc	L	0	1.02
cta	L	0	1.22
ctg	L	0	1.27
att	I	0	16.67
atc	I	0	12.05
ata	I	47	18.27
atg	M	9	9.00
gtt	V	8	462.30
gtc	V	1	392.90
gta	V	1003	525.60
gtg	V	1070	701.30
gct	A	0	0.25
gcc	A	0	0.23
gca	A	1	0.45
gcg	A	0	0.07
---	--	------	------
mPD		0.56	0.80
		nPD:	0.71
		N. weight:	0.63
		Sc. PD:	0.32
		Sc. rank:	862.1

PA-X			
Pos.	91	obs:	exp:
gtt	V	7	476.50
gtc	V	0	404.90
gta	V	187	541.70
gtg	V	1952	722.80
---	--	------	------
mPD		0.17	0.74
		nPD:	0.22
		N. weight:	0.96
		Sc. PD:	0.031
		Sc. rank:	670.1

PA-X			
Pos.	92	obs:	exp:
att	I	0	0.35
atc	I	1	0.26
ata	I	0	0.39
aat	N	31	1219.00
aac	N	2114	925.80
---	--	------	------
mPD		0.029	0.49
		nPD:	0.06
		N. weight:	0.91
		Sc. PD:	-0.12
		Sc. rank:	-663.6

PA-X			
Pos.	93	obs:	exp:
tct	S	0	360.50
tcc	S	0	338.70
tca	S	0	520.40
tcg	S	0	113.90
cgt	R	0	0.03
cgc	R	0	0.05
cga	R	0	0.10
cgg	R	0	0.07
act	T	2	0.49
acc	T	0	0.43
aca	T	0	0.91
acg	T	0	0.17
agt	S	1989	398.90
agc	S	154	410.70
aga	R	0	0.46
agg	R	1	0.28
---	--	------	------
mPD		0.14	1.6
		nPD:	0.08
		N. weight:	1.7
		Sc. PD:	-0.18
		Sc. rank:	-667.1

PA-X			
Pos.	94	obs:	exp:
att	I	38	757.70
atc	I	1046	547.90
ata	I	1052	830.40
gtt	V	0	2.22
gtc	V	8	1.89
gta	V	2	2.52
gtg	V	0	3.37
---	--	------	------
mPD		0.53	0.67
		nPD:	0.79
		N. weight:	0.46
		Sc. PD:	0.27
		Sc. rank:	678.6

PA-X			
Pos.	95	obs:	exp:
tgt	C	1057	923.30
tgc	C	1088	1222.00
---	-	1	1.00
---	--	------	------
mPD		0.50	0.49
		nPD:	1.02
		N. weight:	0.012
		Sc. PD:	0.0094
		Sc. rank:	17.8

PA-X

Pos. 96		obs:	exp:
tct	S	0	0.17
tcc	S	0	0.16
tca	S	0	0.24
tcg	S	0	0.05
aat	N	19	1219.00
aac	N	2125	925.40
agt	S	0	0.19
agc	S	1	0.19
---	-	1	1.00
---	--	------	------
mPD		0.018	0.49
		nPD:	0.04
		N. weight:	0.94
		Sc. PD:	-0.14
		Sc. rank:	-885.7

,

PA-X

Pos. 97		obs:	exp:
att	I	0	0.35
atc	I	0	0.26
ata	I	1	0.39
act	T	27	521.70
acc	T	503	458.60
aca	T	1588	963.80
acg	T	3	176.90
aat	N	1	0.57
aac	N	0	0.43
gtt	V	0	0.22
gtc	V	0	0.19
gta	V	1	0.25
gtg	V	0	0.34
gct	A	0	5.15
gcc	A	2	4.81
gca	A	19	9.47
gcg	A	0	1.57
---	-	1	1.00
---	--	------	------
mPD		0.40	0.70
		nPD:	0.58
		N. weight:	0.43
		Sc. PD:	0.16
		Sc. rank:	566.6

,

PA-X

Pos. 98		obs:	exp:
act	T	4	527.40
acc	T	22	463.60
aca	T	2100	974.20
acg	T	18	178.80
gct	A	0	0.49
gcc	A	0	0.46
gca	A	2	0.90
gcg	A	0	0.15
---	--	------	------
mPD		0.042	0.68
		nPD:	0.06
		N. weight:	0.83
		Sc. PD:	-0.1
		Sc. rank:	-561.1

,

PA-X

Pos. 99		obs:	exp:
tct	S	0	0.17
tcc	S	0	0.16
tca	S	0	0.24
tcg	S	0	0.05
cgt	R	0	1.01
cgc	R	0	1.69
cga	R	0	3.22
cgg	R	0	2.31
aaa	K	1	1.82
aag	K	2	1.18
agt	S	1	0.19
agc	S	0	0.19
aga	R	0	14.68
agg	R	32	9.10
gaa	E	0	0.59
gag	E	1	0.41
ggt	G	2	330.80
ggc	G	0	267.20
gga	G	76	955.30
ggg	G	2031	555.70
---	--	------	------
mPD		0.11	0.72
		nPD:	0.15
		N. weight:	1.4
		Sc. PD:	-0.054
		Sc. rank:	398.2

,

PA-X

Pos. 100		obs:	exp:
tta	L	2	0.22
ttg	L	0	0.38
ctt	L	0	0.40
ctc	L	0	0.29
cta	L	0	0.35
ctg	L	0	0.36
att	I	0	50.73
atc	I	0	36.68
ata	I	143	55.59
gtt	V	25	440.10
gtc	V	0	374.00
gta	V	1907	500.30
gtg	V	50	667.60
gct	A	0	4.66
gcc	A	0	4.35
gca	A	19	8.56
gcg	A	0	1.42
---	--	------	------
mPD		0.21	0.89
		nPD:	0.24
		N. weight:	1.4
		Sc. PD:	0.066
		Sc. rank:	1135.1

,

PA-X

Pos. 101		obs:	exp:
aaa	K	0	1.22
aag	K	2	0.78
gat	D	42	23.62
gac	D	0	18.38
gaa	E	125	1235.00
gag	E	1960	849.60
ggt	G	0	2.67
ggc	G	0	2.15
gga	G	0	7.70
ggg	G	17	4.48
---	--	------	------
mPD		0.16	0.52
		nPD:	0.31
		N. weight:	0.78
		Sc. PD:	0.093
		Sc. rank:	783.1

,

PA-X

Pos. 102		obs:	exp:
aaa	K	167	1304.00
aag	K	1979	841.80
---	--	------	------
mPD		0.14	0.48
		nPD:	0.3
		N. weight:	0.75
		Sc. PD:	0.081
		Sc. rank:	697.5

,

PA-X

Pos. 103		obs:	exp:
cct	P	2016	633.30
ccc	P	74	432.10
cca	P	53	849.80
ccg	P	3	230.80
---	--	------	------
mPD		0.12	0.70
		nPD:	0.16
		N. weight:	1.1
		Sc. PD:	-0.029
		Sc. rank:	459.0

,

PA-X

Pos. 104		obs:	exp:
caa	Q	3	1.62
cag	Q	0	1.38
cgt	R	0	0.76
cgc	R	0	1.27
cga	R	0	2.41
cgg	R	0	1.73
att	I	0	0.35
atc	I	0	0.26
ata	I	1	0.39
aat	N	38	25.58
aac	N	7	19.42
aaa	K	1995	1260.00
aag	K	78	813.20
aga	R	11	11.01
agg	R	13	6.82
---	--	------	------
mPD		0.15	0.53
		nPD:	0.28
		N. weight:	0.42
		Sc. PD:	0.035
		Sc. rank:	376.6

,

PA-X

Pos. 105		obs:	exp:
ttt	F	2082	949.90
ttc	F	38	1170.00
tta	L	0	1.20
ttg	L	0	2.07
tat	Y	2	8.40
tac	Y	13	6.60
ctt	L	11	2.22
ctc	L	0	1.60
cta	L	0	1.92
ctg	L	0	1.99
---	--	------	------
mPD		0.071	0.52
		nPD:	0.14
		N. weight:	0.85
		Sc. PD:	-0.046
		Sc. rank:	105.6

,

PA-X

Pos. 106		obs:	exp:
tta	L	0	233.00
ttg	L	0	402.20
ctt	L	2053	432.40
ctc	L	88	311.50
cta	L	0	373.80
ctg	L	0	388.10
att	I	5	1.77
atc	I	0	1.28
ata	I	0	1.94
---	--	------	------
mPD		0.083	1.1
		nPD:	0.07
		N. weight:	1.7
		Sc. PD:	-0.2
		Sc. rank:	-973.2

,

PA-X

Pos. 107		obs:	exp:
tta	L	0	0.11
ttg	L	0	0.19
ctt	L	1	0.20
ctc	L	0	0.15
cta	L	0	0.17
ctg	L	0	0.18
cct	P	1105	633.00
ccc	P	7	431.90
cca	P	97	849.40
ccg	P	936	230.70
---	--	------	------
mPD		0.54	0.71
		nPD:	0.77
		N. weight:	0.95
		Sc. PD:	0.54
		Sc. rank:	1381.2

,

PA-X

Pos. 108		obs:	exp:
gat	D	2105	1207.00
gac	D	41	939.00
---	--	------	------
mPD		0.037	0.49
		nPD:	0.08
		N. weight:	0.58
		Sc. PD:	-0.065
		Sc. rank:	-313.5

,

PA-X

Pos. 109		obs:	exp:
tta	L	815	233.50
ttg	L	1161	403.10
ctt	L	0	433.40
ctc	L	0	312.20
cta	L	71	374.70
ctg	L	99	389.00
---	--	------	------
mPD		0.63	1.1
		nPD:	0.55
		N. weight:	1.1
		Sc. PD:	0.4
		Sc. rank:	1453.1

,

PA-X

Pos. 110		obs:	exp:
ttt	F	0	0.45
ttc	F	1	0.55
tat	Y	1132	1201.00
tac	Y	1011	942.40
tgt	C	2	0.86
tgc	C	0	1.14
---	--	------	------
mPD		0.50	0.50
		nPD:	1.01
		N. weight:	0.006
		Sc. PD:	0.0048
		Sc. rank:	9.2

,

PA-X

Pos. 111		obs:	exp:
gat	D	899	1207.00
gac	D	1247	939.00
---	--	------	------
mPD		0.49	0.49
		nPD:	0.99
		N. weight:	0.052
		Sc. PD:	0.04
		Sc. rank:	79.2

,

PA-X

Pos. 112		obs:	exp:
tat	Y	47	1202.00
tac	Y	2099	943.70
---	--	------	------
mPD		0.043	0.49
		nPD:	0.09
		N. weight:	0.85
		Sc. PD:	-0.086
		Sc. rank:	-299.6

,

PA-X

Pos. 113		obs:	exp:
cgt	R	0	0.03
cgc	R	0	0.05
cga	R	0	0.10
cgg	R	0	0.07
aaa	K	1971	1304.00
aag	K	174	841.40
aga	R	1	0.46
agg	R	0	0.28
---	--	------	------
mPD		0.15	0.48
		nPD:	0.31
		N. weight:	0.3
		Sc. PD:	0.037
		Sc. rank:	305.1

,

PA-X

Pos. 114		obs:	exp:
aaa	K	0	1.82
aag	K	3	1.18
gaa	E	548	1269.00
gag	E	1593	872.40
ggt	G	0	0.31
ggc	G	0	0.25
gga	G	0	0.91
ggg	G	2	0.53
---	--	------	------
mPD		0.39	0.49
		nPD:	0.79
		N. weight:	0.28
		Sc. PD:	0.17
		Sc. rank:	416.6

,

PA-X

Pos.	115	obs:	exp:
tct	S	0	0.84
tcc	S	0	0.79
tca	S	0	1.21
tcg	S	0	0.27
aat	N	760	1204.00
aac	N	1358	914.20
aaa	K	2	1.22
aag	K	0	0.78
agt	S	0	0.93
agc	S	5	0.96
gat	D	14	11.25
gac	D	6	8.75
ggt	G	0	0.16
ggc	G	1	0.13
gga	G	0	0.45
ggg	G	0	0.26
mPD		0.49	0.52
		nPD:	0.94
		N. weight:	0.11
		Sc. PD:	0.084
		Sc. rank:	173.5

PA-X

Pos.	116	obs:	exp:
cgt	R	7	67.74
cgc	R	0	113.40
cga	R	1123	215.70
cgg	R	963	155.00
aga	R	50	984.10
agg	R	3	609.90
mPD		0.55	0.94
		nPD:	0.58
		N. weight:	1.9
		Sc. PD:	0.73
		Sc. rank:	2578.7

PA-X

Pos.	117	obs:	exp:
ttt	F	36	961.10
ttc	F	2109	1184.00
att	I	0	0.35
atc	I	1	0.26
ata	I	0	0.39
mPD		0.034	0.50
		nPD:	0.07
		N. weight:	0.62
		Sc. PD:	-0.073
		Sc. rank:	-388.6

PA-X

Pos.	118	obs:	exp:
tta	L	0	0.44
ttg	L	0	0.75
ctt	L	4	0.81
ctc	L	0	0.58
cta	L	0	0.70
ctg	L	0	0.73
att	I	1188	426.40
atc	I	14	308.30
ata	I	0	467.30
gtt	V	930	208.50
gtc	V	7	177.20
gta	V	2	237.00
gtg	V	0	316.30
gct	A	1	0.25
gcc	A	0	0.23
gca	A	0	0.45
gcg	A	0	0.07
mPD		0.52	1.2
		nPD:	0.42
		N. weight:	1.4
		Sc. PD:	0.32
		Sc. rank:	1692.3

PA-X

Pos.	119	obs:	exp:
gaa	E	1826	1272.00
gag	E	320	874.50
mPD		0.25	0.48
		nPD:	0.53
		N. weight:	0.19
		Sc. PD:	0.063
		Sc. rank:	239.9

PA-X

Pos.	120	obs:	exp:
att	I	2106	757.00
atc	I	21	547.30
ata	I	7	829.60
gtt	V	12	2.66
gtc	V	0	2.26
gta	V	0	3.03
gtg	V	0	4.04
mPD		0.037	0.67
		nPD:	0.06
		N. weight:	1.2
		Sc. PD:	-0.15
		Sc. rank:	-948.6

PA-X

Pos.	121	obs:	exp:
ggt	G	19	336.60
ggc	G	3	271.90
gga	G	2093	972.00
ggg	G	31	565.40
mPD		0.049	0.68
		nPD:	0.07
		N. weight:	0.81
		Sc. PD:	-0.095
		Sc. rank:	-494.1

PA-X

Pos.	122	obs:	exp:
gtt	V	19	476.50
gtc	V	129	404.90
gta	V	940	541.70
gtg	V	1058	722.80
mPD		0.56	0.74
		nPD:	0.76
		N. weight:	0.4
		Sc. PD:	0.22
		Sc. rank:	579.2

PA-X

Pos.	123	obs:	exp:
act	T	1	527.90
acc	T	67	464.10
aca	T	2051	975.10
acg	T	27	178.90
mPD		0.085	0.68
		nPD:	0.13
		N. weight:	0.75
		Sc. PD:	-0.047
		Sc. rank:	32.8

PA-X

Pos.	124	obs:	exp:
cgt	R	6	67.74
cgc	R	0	113.40
cga	R	492	215.70
cgg	R	1012	155.00
aga	R	588	984.10
agg	R	48	609.90
mPD		0.92	0.94
		nPD:	0.98
		N. weight:	1.
		Sc. PD:	0.81
		Sc. rank:	1598.5

PA-X

Pos.	125	obs:	exp:
cgt	R	0	67.74
cgc	R	0	113.40
cga	R	0	215.70
cgg	R	1	155.00
aga	R	99	984.10
agg	R	2046	609.90
mPD		0.089	0.94
		nPD:	0.09
		N. weight:	1.3
		Sc. PD:	-0.12
		Sc. rank:	-267.8

PA-X

Pos.	126	obs:	exp:
aaa	K	0	1.22
aag	K	2	0.78
gat	D	3	1.69
gac	D	0	1.31
gaa	E	1236	1268.00
gag	E	904	872.00
ggt	G	0	0.16
ggc	G	0	0.13
gga	G	0	0.45
ggg	G	1	0.26
mPD		0.49	0.49
		nPD:	1.01
		N. weight:	0.0056
		Sc. PD:	0.0045
		Sc. rank:	8.5

PA-X

Pos.	127	obs:	exp:
att	I	0	10.29
atc	I	29	7.44
ata	I	0	11.27
act	T	0	0.25
acc	T	1	0.22
aca	T	0	0.45
acg	T	0	0.08
gtt	V	60	468.50
gtc	V	2031	398.20
gta	V	19	532.60
gtg	V	0	710.70
gct	A	0	1.47
gcc	A	6	1.37
gca	A	0	2.70
gcg	A	0	0.45
mPD		0.11	0.77
		nPD:	0.14
		N. weight:	1.8
		Sc. PD:	-0.094
		Sc. rank:	261.0

PA-X

Pos.	128	obs:	exp:
cat	H	41	1238.00
cac	H	2105	908.10
mPD		0.037	0.49
		nPD:	0.08
		N. weight:	0.91
		Sc. PD:	-0.1
		Sc. rank:	-441.7

PA-X

Pos.	129	obs:	exp:
tta	L	2	0.54
ttg	L	0	0.94
ctt	L	0	1.01
ctc	L	0	0.73
cta	L	3	0.87
ctg	L	0	0.91
att	I	0	752.80
atc	I	1	544.30
ata	I	2121	825.00
atg	M	11	11.00
act	T	0	0.98
acc	T	0	0.86
aca	T	4	1.82
acg	T	0	0.33
gtt	V	0	0.89
gtc	V	0	0.75
gta	V	4	1.01
gtg	V	0	1.35
mPD		0.023	0.67
		nPD:	0.03
		N. weight:	1.1
		Sc. PD:	-0.17
		Sc. rank:	-1100.1

PA-X

Pos.	130	obs:	exp:
tat	Y	1066	1202.00
tac	Y	1080	943.70
mPD		0.50	0.49
		nPD:	1.02
		N. weight:	0.012
		Sc. PD:	0.0096
		Sc. rank:	18.3

PA-X

Pos.	131	obs:	exp:
tat	Y	75	1202.00
tac	Y	2071	943.70
mPD		0.067	0.49
		nPD:	0.14
		N. weight:	0.79
		Sc. PD:	-0.042
		Sc. rank:	125.2

PA-X

Pos.	132	obs:	exp:
tta	L	126	233.30
ttg	L	0	402.70
ctt	L	1	433.00
ctc	L	1	311.90
cta	L	1955	374.40
ctg	L	61	388.60
att	I	0	0.71
atc	I	0	0.51
ata	I	2	0.78
mPD		0.17	1.1
		nPD:	0.15
		N. weight:	1.7
		Sc. PD:	-0.07
		Sc. rank:	425.0

PA-X

Pos.	133	obs:	exp:
aaa	K	0	0.61
aag	K	1	0.39
gaa	E	46	1271.00
gag	E	2099	874.00
mPD		0.043	0.48
		nPD:	0.09
		N. weight:	0.94
		Sc. PD:	-0.094
		Sc. rank:	-287.5

PA-X

Pos.	134	obs:	exp:
aaa	K	2101	1304.00
aag	K	45	841.80
mPD		0.041	0.48
		nPD:	0.09
		N. weight:	0.49
		Sc. PD:	-0.05
		Sc. rank:	-176.6

PA-X			
Pos.	135	obs:	exp:
gct	A	77	526.10
gcc	A	2059	491.70
gca	A	10	967.30
gcg	A	0	160.90
---	--	------	------
mPD		0.078	0.68
		nPD:	0.12
		N. weight:	1.5
		Sc. PD:	-0.11
		Sc. rank:	-54.3

PA-X			
Pos.	136	obs:	exp:
aat	N	127	1219.00
aac	N	2018	925.80
gat	D	0	0.56
gac	D	1	0.44
---	--	------	------
mPD		0.11	0.49
		nPD:	0.23
		N. weight:	0.72
		Sc. PD:	0.027
		Sc. rank:	538.5

PA-X			
Pos.	137	obs:	exp:
cgt	R	0	0.06
cgc	R	0	0.11
cga	R	0	0.20
cgg	R	0	0.14
aaa	K	2123	1303.00
aag	K	21	841.00
aga	R	2	0.92
agg	R	0	0.57
---	--	------	------
mPD		0.021	0.48
		nPD:	0.04
		N. weight:	0.54
		Sc. PD:	-0.077
		Sc. rank:	-476.7

PA-X			
Pos.	138	obs:	exp:
att	I	1	761.30
atc	I	0	550.40
ata	I	2145	834.30
---	--	------	------
mPD		0.00093	0.66
		nPD:	0.
		N. weight:	1.1
		Sc. PD:	-0.21
		Sc. rank:	-1741.6

PA-X			
Pos.	139	obs:	exp:
act	T	0	0.25
acc	T	0	0.22
aca	T	1	0.45
acg	T	0	0.08
aaa	K	2031	1304.00
aag	K	114	841.40
---	--	------	------
mPD		0.10	0.48
		nPD:	0.21
		N. weight:	0.38
		Sc. PD:	0.0081
		Sc. rank:	247.5

PA-X			
Pos.	140	obs:	exp:
tct	S	1192	361.00
tcc	S	932	339.20
tca	S	21	521.10
tcg	S	1	114.10
agt	S	0	399.40
agc	S	0	411.30
---	--	------	------
mPD		0.50	1.6
		nPD:	0.31
		N. weight:	1.3
		Sc. PD:	0.15
		Sc. rank:	1232.9

PA-X			
Pos.	141	obs:	exp:
gat	D	16	9.00
gac	D	0	7.00
gaa	E	224	1260.00
gag	E	1903	866.70
ggt	G	0	0.47
ggc	G	0	0.38
gga	G	1	1.36
ggg	G	2	0.79
---	--	------	------
mPD		0.20	0.49
		nPD:	0.41
		N. weight:	0.63
		Sc. PD:	0.14
		Sc. rank:	735.9

PA-X			
Pos.	142	obs:	exp:
cgt	R	0	0.92
cgc	R	0	1.53
cga	R	0	2.91
cgg	R	0	2.10
act	T	0	0.25
acc	T	0	0.22
aca	T	0	0.45
acg	T	1	0.08
aat	N	7	3.98
aac	N	0	3.02
aaa	K	66	1273.00
aag	K	2029	821.80
aga	R	1	13.30
agg	R	28	8.24
gat	D	8	5.06
gac	D	1	3.94
gaa	E	0	1.19
gag	E	2	0.81
ggt	G	3	0.47
ggc	G	0	0.38
gga	G	0	1.36
ggg	G	0	0.79
---	--	------	------
mPD		0.12	0.54
		nPD:	0.23
		N. weight:	0.94
		Sc. PD:	0.032
		Sc. rank:	672.2

PA-X			
Pos.	143	obs:	exp:
att	I	0	0.35
atc	I	0	0.26
ata	I	1	0.39
act	T	2	527.60
acc	T	0	463.80
aca	T	2076	974.70
acg	T	67	178.90
---	--	------	------
mPD		0.063	0.68
		nPD:	0.09
		N. weight:	0.84
		Sc. PD:	-0.08
		Sc. rank:	-211.0

PA-X			
Pos.	144	obs:	exp:
cat	H	6	1237.00
cac	H	2139	907.70
caa	Q	1	0.54
cag	Q	0	0.46
---	--	------	------
mPD		0.0065	0.49
		nPD:	0.01
		N. weight:	1.
		Sc. PD:	-0.17
		Sc. rank:	-1411.9

PA-X			
Pos.	145	obs:	exp:
tta	L	0	0.22
ttg	L	0	0.38
ctt	L	2	0.40
ctc	L	0	0.29
cta	L	0	0.35
ctg	L	0	0.36
att	I	2084	760.20
atc	I	58	549.70
ata	I	1	833.10
gtt	V	1	0.22
gtc	V	0	0.19
gta	V	0	0.25
gtg	V	0	0.34
---	--	------	------
mPD		0.056	0.66
		nPD:	0.09
		N. weight:	1.1
		Sc. PD:	-0.11
		Sc. rank:	-406.4

PA-X			
Pos.	146	obs:	exp:
cat	H	1012	1238.00
cac	H	1134	908.10
---	--	------	------
mPD		0.50	0.49
		nPD:	1.02
		N. weight:	0.029
		Sc. PD:	0.024
		Sc. rank:	44.4

PA-X			
Pos.	147	obs:	exp:
att	I	1115	761.30
atc	I	1023	550.40
ata	I	8	834.30
---	--	------	------
mPD		0.50	0.66
		nPD:	0.77
		N. weight:	0.57
		Sc. PD:	0.32
		Sc. rank:	829.4

PA-X			
Pos.	148	obs:	exp:
ttt	F	2078	961.60
ttc	F	68	1184.00
---	--	------	------
mPD		0.061	0.49
		nPD:	0.12
		N. weight:	0.79
		Sc. PD:	-0.051
		Sc. rank:	26.5

PA-X			
Pos.	149	obs:	exp:
tct	S	20	361.00
tcc	S	1	339.20
tca	S	2102	521.10
tcg	S	23	114.10
agt	S	0	399.40
agc	S	0	411.30
---	--	------	------
mPD		0.040	1.6
		nPD:	0.02
		N. weight:	1.6
		Sc. PD:	-0.26
		Sc. rank:	-1973.1

PA-X			
Pos.	150	obs:	exp:
ttt	F	53	961.60
ttc	F	2093	1184.00
---	--	------	------
mPD		0.048	0.49
		nPD:	0.1
		N. weight:	0.58
		Sc. PD:	-0.053
		Sc. rank:	-80.8

PA-X			
Pos.	151	obs:	exp:
att	I	40	14.90
atc	I	2	10.77
ata	I	0	16.33
act	T	1958	509.90
acc	T	91	448.30
aca	T	20	942.00
acg	T	4	172.90
aat	N	28	15.91
aac	N	0	12.09
gct	A	3	0.74
gcc	A	0	0.69
gca	A	0	1.35
gcg	A	0	0.22
---	--	------	------
mPD		0.17	0.75
		nPD:	0.23
		N. weight:	1.4
		Sc. PD:	0.05
		Sc. rank:	1015.0

PA-X			
Pos.	152	obs:	exp:
ggt	G	13	336.60
ggc	G	0	271.90
gga	G	2044	972.00
ggg	G	89	565.40
---	--	------	------
mPD		0.091	0.68
		nPD:	0.13
		N. weight:	0.74
		Sc. PD:	-0.041
		Sc. rank:	65.9

PA-X			
Pos.	153	obs:	exp:
gaa	E	68	1272.00
gag	E	2078	874.50
---	--	------	------
mPD		0.061	0.48
		nPD:	0.13
		N. weight:	0.89
		Sc. PD:	-0.055
		Sc. rank:	49.7

PA-X			
Pos.	154	obs:	exp:
gaa	E	105	1272.00
gag	E	2041	874.50
---	--	------	------
mPD		0.093	0.48
		nPD:	0.19
		N. weight:	0.82
		Sc. PD:	0.0017
		Sc. rank:	496.4

PA-X			
Pos.	155	obs:	exp:
att	I	0	0.35
atc	I	0	0.26
ata	I	1	0.39
atg	M	2144	2144.00
gtt	V	0	0.22
gtc	V	0	0.19
gta	V	0	0.25
gtg	V	1	0.34
---	--	------	------
mPD		0.0019	0.0025
		nPD:	0.75
		N. weight:	0.0011
		Sc. PD:	0.00062
		Sc. rank:	1.6

,

PA-X			
Pos.	156	obs:	exp:
gct	A	156	526.10
gcc	A	1953	491.70
gca	A	37	967.30
gcg	A	0	160.90
---	--	------	------
mPD		0.17	0.68
		nPD:	0.25
		N. weight:	1.3
		Sc. PD:	0.071
		Sc. rank:	1104.1

,

PA-X			
Pos.	157	obs:	exp:
tgt	C	1	0.43
tgc	C	0	0.57
act	T	61	527.60
acc	T	2055	463.80
aca	T	29	974.70
acg	T	0	178.90
---	--	------	------
mPD		0.084	0.68
		nPD:	0.12
		N. weight:	1.6
		Sc. PD:	-0.1
		Sc. rank:	36.7

,

PA-X			
Pos.	158	obs:	exp:
cgt	R	0	0.57
cgc	R	0	0.95
cga	R	0	1.81
cgg	R	0	1.30
aat	N	8	4.55
aac	N	0	3.45
aaa	K	2091	1288.00
aag	K	28	831.20
aga	R	18	8.25
agg	R	0	5.12
gaa	E	0	0.59
gag	E	1	0.41
---	--	------	------
mPD		0.052	0.50
		nPD:	0.1
		N. weight:	0.52
		Sc. PD:	-0.045
		Sc. rank:	-61.7

,

PA-X			
Pos.	159	obs:	exp:
tct	S	0	0.50
tcc	S	0	0.47
tca	S	0	0.73
tcg	S	3	0.16
aat	N	0	0.57
aac	N	1	0.43
agt	S	0	0.56
agc	S	0	0.57
gct	A	42	525.10
gcc	A	14	490.80
gca	A	314	965.50
gcg	A	1772	160.60
---	--	------	------
mPD		0.30	0.68
		nPD:	0.44
		N. weight:	2.1
		Sc. PD:	0.5
		Sc. rank:	2560.8

,

PA-X			
Pos.	160	obs:	exp:
cat	H	0	0.58
cac	H	1	0.42
aat	N	0	0.57
aac	N	1	0.43
gat	D	76	1200.00
gac	D	2058	933.70
gaa	E	10	5.93
gag	E	0	4.07
---	--	------	------
mPD		0.079	0.50
		nPD:	0.16
		N. weight:	0.8
		Sc. PD:	-0.025
		Sc. rank:	267.2

,

PA-X			
Pos.	161	obs:	exp:
tat	Y	10	1202.00
tac	Y	2136	943.70
---	--	------	------
mPD		0.0093	0.49
		nPD:	0.02
		N. weight:	0.95
		Sc. PD:	-0.16
		Sc. rank:	-1257.7

,

PA-X			
Pos.	162	obs:	exp:
tct	S	0	0.17
tcc	S	1	0.16
tca	S	0	0.24
tcg	S	0	0.05
att	I	2	28.38
atc	I	78	20.52
ata	I	0	31.10
act	T	97	507.90
acc	T	1885	446.50
aca	T	83	938.30
acg	T	0	172.20
agt	S	0	0.19
agc	S	0	0.19
---	--	------	------
mPD		0.23	0.75
		nPD:	0.31
		N. weight:	1.4
		Sc. PD:	0.16
		Sc. rank:	1295.0

,

PA-X			
Pos.	163	obs:	exp:
tta	L	9	233.40
ttg	L	3	402.90
ctt	L	2085	433.20
ctc	L	36	312.10
cta	L	1	374.50
ctg	L	11	388.80
att	I	1	0.35
atc	I	0	0.26
ata	I	0	0.39
---	--	------	------
mPD		0.067	1.1
		nPD:	0.06
		N. weight:	1.7
		Sc. PD:	-0.22
		Sc. rank:	-1286.9

,

PA-X			
Pos.	164	obs:	exp:
gat	D	465	1207.00
gac	D	1681	939.00
---	--	------	------
mPD		0.34	0.49
		nPD:	0.69
		N. weight:	0.3
		Sc. PD:	0.15
		Sc. rank:	410.4

,

PA-X			
Pos.	165	obs:	exp:
gat	D	2	1.12
gac	D	0	0.88
gaa	E	2093	1270.00
gag	E	51	873.60
---	--	------	------
mPD		0.048	0.48
		nPD:	0.1
		N. weight:	0.5
		Sc. PD:	-0.045
		Sc. rank:	-66.8

,

PA-X			
Pos.	166	obs:	exp:
gaa	E	138	1272.00
gag	E	2008	874.50
---	--	------	------
mPD		0.12	0.48
		nPD:	0.25
		N. weight:	0.76
		Sc. PD:	0.044
		Sc. rank:	639.0

,

PA-X			
Pos.	167	obs:	exp:
tct	S	0	360.80
tcc	S	0	339.00
tca	S	0	520.90
tcg	S	0	114.00
agt	S	133	399.20
agc	S	2012	411.10
ggt	G	0	0.16
ggc	G	1	0.13
gga	G	0	0.45
ggg	G	0	0.26
---	--	------	------
mPD		0.12	1.6
		nPD:	0.07
		N. weight:	1.7
		Sc. PD:	-0.2
		Sc. rank:	-999.6

,

PA-X			
Pos.	168	obs:	exp:
cgt	R	0	67.71
cgc	R	0	113.40
cga	R	0	215.60
cgg	R	0	155.00
aaa	K	0	0.61
aag	K	1	0.39
aga	R	168	983.70
agg	R	1977	609.70
---	--	------	------
mPD		0.15	0.94
		nPD:	0.15
		N. weight:	1.1
		Sc. PD:	-0.04
		Sc. rank:	359.1

,

PA-X			
Pos.	169	obs:	exp:
gct	A	2	526.10
gcc	A	15	491.70
gca	A	2107	967.30
gcg	A	22	160.90
---	--	------	------
mPD		0.036	0.68
		nPD:	0.05
		N. weight:	0.86
		Sc. PD:	-0.12
		Sc. rank:	-735.8

,

PA-X			
Pos.	170	obs:	exp:
cgt	R	0	67.74
cgc	R	0	113.40
cga	R	0	215.70
cgg	R	0	155.00
aga	R	2126	984.10
agg	R	20	609.90
---	--	------	------
mPD		0.018	0.94
		nPD:	0.02
		N. weight:	0.88
		Sc. PD:	-0.15
		Sc. rank:	-1153.1

,

PA-X			
Pos.	171	obs:	exp:
att	I	9	760.90
atc	I	1968	550.20
ata	I	168	833.90
gtt	V	0	0.22
gtc	V	1	0.19
gta	V	0	0.25
gtg	V	0	0.34
---	--	------	------
mPD		0.15	0.66
		nPD:	0.23
		N. weight:	1.2
		Sc. PD:	0.05
		Sc. rank:	974.7

,

PA-X			
Pos.	172	obs:	exp:
aaa	K	1486	1304.00
aag	K	660	841.80
---	--	------	------
mPD		0.43	0.48
		nPD:	0.89
		N. weight:	0.021
		Sc. PD:	0.014
		Sc. rank:	31.5

,

PA-X			
Pos.	173	obs:	exp:
act	T	1075	527.90
acc	T	1056	464.10
aca	T	11	975.10
acg	T	4	178.90
---	--	------	------
mPD		0.51	0.68
		nPD:	0.75
		N. weight:	0.88
		Sc. PD:	0.48
		Sc. rank:	1239.3

,

PA-X			
Pos.	174	obs:	exp:
cgt	R	0	67.74
cgc	R	0	113.40
cga	R	1	215.70
cgg	R	2	155.00
aga	R	92	984.10
agg	R	2051	609.90
---	--	------	------
mPD		0.086	0.94
		nPD:	0.09
		N. weight:	1.3
		Sc. PD:	-0.12
		Sc. rank:	-332.4

,

PA-X			
Pos.	175	obs:	exp:
tta	L	0	233.50
ttg	L	1	403.10
ctt	L	1274	433.40
ctc	L	810	312.20
cta	L	57	374.70
ctg	L	4	389.00
---	--	------	------
mPD		0.51	1.1
		nPD:	0.44
		N. weight:	1.1
		Sc. PD:	0.28
		Sc. rank:	1393.9

PA-X			
Pos.	176	obs:	exp:
ttt	F	48	958.50
ttc	F	2091	1181.00
tat	Y	0	3.92
tac	Y	7	3.08
---	--	------	------
mPD		0.050	0.50
		nPD:	0.1
		N. weight:	0.59
		Sc. PD:	-0.052
		Sc. rank:	-73.7

PA-X			
Pos.	177	obs:	exp:
tct	S	1	0.34
tcc	S	1	0.32
tca	S	0	0.49
tcg	S	0	0.11
act	T	1060	527.10
acc	T	1083	463.40
aca	T	0	973.80
acg	T	0	178.70
agt	S	0	0.37
agc	S	0	0.38
gct	A	0	0.25
gcc	A	1	0.23
gca	A	0	0.45
gcg	A	0	0.07
---	--	------	------
mPD		0.50	0.68
		nPD:	0.74
		N. weight:	0.93
		Sc. PD:	0.5
		Sc. rank:	1300.9

PA-X			
Pos.	178	obs:	exp:
tta	L	0	0.11
ttg	L	0	0.19
ctt	L	0	0.20
ctc	L	0	0.15
cta	L	1	0.17
ctg	L	0	0.18
att	I	0	759.10
atc	I	4	548.90
ata	I	2136	832.00
gtt	V	0	1.11
gtc	V	0	0.94
gta	V	5	1.26
gtg	V	0	1.68
---	--	------	------
mPD		0.0093	0.66
		nPD:	0.01
		N. weight:	1.1
		Sc. PD:	-0.19
		Sc. rank:	-1552.3

PA-X			
Pos.	179	obs:	exp:
cgt	R	0	67.74
cgc	R	0	113.40
cga	R	8	215.70
cgg	R	0	155.00
aga	R	2092	984.10
agg	R	46	609.90
---	--	------	------
mPD		0.049	0.94
		nPD:	0.05
		N. weight:	0.8
		Sc. PD:	-0.11
		Sc. rank:	-696.2

PA-X			
Pos.	180	obs:	exp:
caa	Q	2085	1157.00
cag	Q	61	988.70
---	--	------	------
mPD		0.055	0.50
		nPD:	0.11
		N. weight:	0.59
		Sc. PD:	-0.046
		Sc. rank:	-33.0

PA-X			
Pos.	181	obs:	exp:
gat	D	0	2.25
gac	D	4	1.75
gaa	E	1484	1269.00
gag	E	658	872.80
---	--	------	------
mPD		0.43	0.48
		nPD:	0.88
		N. weight:	0.03
		Sc. PD:	0.02
		Sc. rank:	45.0

PA-X			
Pos.	182	obs:	exp:
tta	L	0	0.22
ttg	L	0	0.38
ctt	L	0	0.40
ctc	L	0	0.29
cta	L	0	0.35
ctg	L	2	0.36
att	I	0	0.35
atc	I	0	0.26
ata	I	1	0.39
atg	M	2143	2143.00
---	--	------	------
mPD		0.0028	0.0040
		nPD:	0.7
		N. weight:	0.0024
		Sc. PD:	0.0012
		Sc. rank:	3.3

PA-X			
Pos.	183	obs:	exp:
act	T	0	0.25
acc	T	1	0.22
aca	T	0	0.45
acg	T	0	0.08
gtt	V	0	1.11
gtc	V	5	0.94
gta	V	0	1.26
gtg	V	0	1.68
gct	A	19	524.60
gcc	A	2065	490.30
gca	A	56	964.60
gcg	A	0	160.50
---	--	------	------
mPD		0.074	0.68
		nPD:	0.11
		N. weight:	1.5
		Sc. PD:	-0.13
		Sc. rank:	-126.9

PA-X			
Pos.	184	obs:	exp:
tct	S	0	342.60
tcc	S	0	321.90
tca	S	0	494.70
tcg	S	0	108.30
tgt	C	1	0.43
tgc	C	0	0.57
cat	H	2	1.15
cac	H	0	0.85
cgt	R	1	0.03
cgc	R	0	0.05
cga	R	0	0.10
cgg	R	0	0.07
att	I	2	1.06
atc	I	1	0.77
ata	I	0	1.17
aat	N	22	12.50
aac	N	0	9.50
agt	S	1789	379.10
agc	S	248	390.40
aga	R	0	0.46
agg	R	0	0.28
ggt	G	80	12.55
ggc	G	0	10.14
gga	G	0	36.24
ggg	G	0	21.08
---	--	------	------
mPD		0.31	1.7
		nPD:	0.18
		N. weight:	1.6
		Sc. PD:	-0.017
		Sc. rank:	868.1

PA-X			
Pos.	185	obs:	exp:
cgt	R	0	67.55
cgc	R	0	113.10
cga	R	2	215.10
cgg	R	31	154.60
atg	M	3	3.00
aaa	K	0	1.82
aag	K	3	1.18
aga	R	106	981.40
agg	R	2001	608.20
---	--	------	------
mPD		0.13	0.94
		nPD:	0.14
		N. weight:	1.2
		Sc. PD:	-0.059
		Sc. rank:	202.9

PA-X			
Pos.	186	obs:	exp:
tct	S	0	170.60
tcc	S	0	160.30
tca	S	0	246.20
tcg	S	0	53.89
aat	N	44	28.42
aac	N	6	21.58
agt	S	980	188.70
agc	S	34	194.30
gct	A	1	0.74
gcc	A	2	0.69
gca	A	0	1.35
gcg	A	0	0.22
ggt	G	1028	169.20
ggc	G	47	136.70
gga	G	4	488.70
ggg	G	0	284.30
---	--	------	------
mPD		0.63	1.8
		nPD:	0.35
		N. weight:	1.9
		Sc. PD:	0.29
		Sc. rank:	1992.9

PA-X			
Pos.	187	obs:	exp:
tta	L	11	233.30
ttg	L	0	402.70
ctt	L	14	433.00
ctc	L	8	311.90
cta	L	2023	374.40
ctg	L	88	388.60
att	I	0	0.71
atc	I	0	0.51
ata	I	2	0.78
---	--	------	------
mPD		0.11	1.1
		nPD:	0.1
		N. weight:	1.8
		Sc. PD:	-0.16
		Sc. rank:	-337.2

PA-X			
Pos.	188	obs:	exp:
tgg	W	2146	2146.00
---	--	------	------
mPD		0	0
		nPD:	1.
		N. weight:	0.
		Sc. PD:	0
		Sc. rank:	0

PA-X			
Pos.	189	obs:	exp:
gtt	V	1	0.22
gtc	V	0	0.19
gta	V	0	0.25
gtg	V	0	0.34
gct	A	1	0.25
gcc	A	0	0.23
gca	A	0	0.45
gcg	A	0	0.07
gat	D	2121	1205.00
gac	D	21	937.20
gaa	E	1	1.19
gag	E	1	0.81
---	--	------	------
mPD		0.023	0.50
		nPD:	0.05
		N. weight:	0.63
		Sc. PD:	-0.088
		Sc. rank:	-552.2

PA-X			
Pos.	190	obs:	exp:
ttt	F	0	0.45
ttc	F	1	0.55
tct	S	1	360.10
tcc	S	2137	338.40
tca	S	3	519.90
tcg	S	0	113.80
tat	Y	0	1.12
tac	Y	2	0.88
cct	P	0	0.59
ccc	P	2	0.40
cca	P	0	0.79
ccg	P	0	0.22
agt	S	0	398.50
agc	S	0	410.30
---	--	------	------
mPD		0.0084	1.6
		nPD:	0.01
		N. weight:	2.2
		Sc. PD:	-0.4
		Sc. rank:	-3225.2

PA-X			
Pos.	191	obs:	exp:
ttt	F	2	961.60
ttc	F	2144	1184.00
---	--	------	------
mPD		0.0019	0.49
		nPD:	0.
		N. weight:	0.7
		Sc. PD:	-0.13
		Sc. rank:	-1074.6

PA-X			
Pos.	192	obs:	exp:
cgt	R	1	0.03
cgc	R	0	0.05
cga	R	0	0.10
cgg	R	0	0.07
att	I	0	0.35
atc	I	1	0.26
ata	I	0	0.39
aga	R	0	0.46
agg	R	0	0.28
gtt	V	0	472.70
gtc	V	2127	401.70
gta	V	2	537.40
gtg	V	0	717.10
gct	A	0	2.70
gcc	A	11	2.52
gca	A	0	4.96
gcg	A	0	0.82
gat	D	0	2.25
gac	D	4	1.75
---	--	------	------
mPD		0.019	0.76
		nPD:	0.03
		N. weight:	2.
		Sc. PD:	-0.32
		Sc. rank:	-2324.7

PA-X			
Pos.	193	obs:	exp:
tct	S	0	356.40
tcc	S	0	334.90
tca	S	0	514.60
tcg	S	0	112.60
tgt	C	3	1.29
tgc	C	0	1.71
caa	Q	0	0.54
cag	Q	1	0.46
att	I	2	0.71
atc	I	0	0.51
ata	I	0	0.78
aat	N	20	11.37
aac	N	0	8.63
agt	S	2119	394.40
agc	S	0	406.10
ggt	G	1	0.16
ggc	G	0	0.13
gga	G	0	0.45
ggg	G	0	0.26
---	--	------	------
mPD		0.027	1.6
		nPD:	0.02
		N. weight:	2.
		Sc. PD:	-0.34
		Sc. rank:	-2676.3

PA-X			
Pos.	194	obs:	exp:
tta	L	0	0.76
ttg	L	0	1.31
tct	S	0	0.17
tcc	S	0	0.16
tca	S	0	0.24
tcg	S	1	0.05
ctt	L	0	1.41
ctc	L	0	1.02
cta	L	0	1.22
ctg	L	7	1.27
cct	P	0	627.40
ccc	P	0	428.00
cca	P	0	841.90
ccg	P	2126	228.70
caa	Q	0	5.93
cag	Q	11	5.07
cgt	R	0	0.03
cgc	R	0	0.05
cga	R	0	0.10
cgg	R	1	0.07
agt	S	0	0.19
agc	S	0	0.19
aga	R	0	0.46
agg	R	0	0.28
---	--	------	------
mPD		0.019	0.73
		nPD:	0.03
		N. weight:	2.7
		Sc. PD:	-0.43
		Sc. rank:	-3183.1

PA-X			
Pos.	195	obs:	exp:
tct	S	0	0.17
tcc	S	0	0.16
tca	S	0	0.24
tcg	S	0	0.05
cgt	R	0	33.05
cgc	R	0	55.34
cga	R	0	105.20
cgg	R	0	75.64
aat	N	0	0.57
aac	N	1	0.43
aaa	K	1097	666.70
aag	K	0	430.30
agt	S	0	0.19
agc	S	1	0.19
aga	R	1047	480.20
agg	R	0	297.60
---	--	------	------
mPD		0.50	1.2
		nPD:	0.41
		N. weight:	0.76
		Sc. PD:	0.16
		Sc. rank:	879.2

PA-X			
Pos.	196	obs:	exp:
aat	N	1	0.57
aac	N	0	0.43
aaa	K	0	4.25
aag	K	7	2.75
gtt	V	0	0.22
gtc	V	0	0.19
gta	V	0	0.25
gtg	V	1	0.34
gaa	E	0	1176.00
gag	E	1984	808.40
ggt	G	0	24.00
ggc	G	0	19.39
gga	G	0	69.30
ggg	G	153	40.31
---	--	------	------
mPD		0.14	0.64
		nPD:	0.22
		N. weight:	1.1
		Sc. PD:	0.032
		Sc. rank:	764.2

PA-X			
Pos.	197	obs:	exp:
gtt	V	0	11.99
gtc	V	0	10.19
gta	V	0	13.63
gtg	V	54	18.19
gct	A	0	510.90
gcc	A	0	477.50
gca	A	0	939.40
gcg	A	2084	156.30
gaa	E	0	1.19
gag	E	2	0.81
ggt	G	0	0.94
ggc	G	0	0.76
gga	G	0	2.72
ggg	G	6	1.58
---	--	------	------
mPD		0.056	0.74
		nPD:	0.08
		N. weight:	3.1
		Sc. PD:	-0.34
		Sc. rank:	-1516.1

PA-X			
Pos.	198	obs:	exp:
cgt	R	0	2.37
cgc	R	0	3.96
cga	R	0	7.54
cgg	R	0	5.42
aaa	K	0	1259.00
aag	K	2071	812.40
aga	R	0	34.39
agg	R	75	21.32
---	--	------	------
mPD		0.067	0.57
		nPD:	0.12
		N. weight:	1.1
		Sc. PD:	-0.079
		Sc. rank:	2.5

PA-X			
Pos.	199	obs:	exp:
cgt	R	0	59.88
cgc	R	0	100.30
cga	R	0	190.70
cgg	R	0	137.00
att	I	0	1.42
atc	I	0	1.03
ata	I	4	1.56
aaa	K	244	148.30
aag	K	0	95.71
aga	R	1897	870.00
agg	R	0	539.20
ggt	G	0	0.16
ggc	G	0	0.13
gga	G	1	0.45
ggg	G	0	0.26
---	--	------	------
mPD		0.21	1.1
		nPD:	0.19
		N. weight:	0.9
		Sc. PD:	-0.004
		Sc. rank:	502.1

PA-X			
Pos.	200	obs:	exp:
cct	P	0	0.59
ccc	P	0	0.40
cca	P	2	0.79
ccg	P	0	0.22
caa	Q	2061	1137.00
cag	Q	48	971.70
cgt	R	0	1.10
cgc	R	0	1.85
cga	R	32	3.52
cgg	R	3	2.53
aga	R	0	16.05
agg	R	0	9.95
---	--	------	------
mPD		0.080	0.56
		nPD:	0.14
		N. weight:	0.65
		Sc. PD:	-0.029
		Sc. rank:	147.4

PA-X			
Pos.	201	obs:	exp:
tta	L	0	230.30
ttg	L	2110	397.50
tct	S	0	2.86
tcc	S	0	2.69
tca	S	0	4.13
tcg	S	17	0.90
tgg	W	13	13.00
ctt	L	0	427.30
ctc	L	0	307.90
cta	L	0	369.50
ctg	L	6	383.60
agt	S	0	3.16
agc	S	0	3.26
---	--	------	------
mPD		0.033	1.2
		nPD:	0.03
		N. weight:	2.
		Sc. PD:	-0.31
		Sc. rank:	-2084.4

PA-X			
Pos.	202	obs:	exp:
cgt	R	0	4.80
cgc	R	0	8.03
cga	R	0	15.28
cgg	R	0	10.98
aaa	K	0	1212.00
aag	K	1994	782.20
aga	R	0	69.71
agg	R	152	43.20
---	--	------	------
mPD		0.13	0.65
		nPD:	0.2
		N. weight:	1.1
		Sc. PD:	0.013
		Sc. rank:	746.5

PA-X			
Pos.	203	obs:	exp:
cgt	R	0	0.47
cgc	R	0	0.79
cga	R	0	1.51
cgg	R	0	1.08
act	T	0	0.25
acc	T	0	0.22
aca	T	1	0.45
acg	T	0	0.08
aat	N	0	1.14
aac	N	2	0.86
aaa	K	2128	1293.00
aag	K	0	834.70
aga	R	15	6.88
agg	R	0	4.26
---	--	------	------
mPD		0.017	0.50
		nPD:	0.03
		N. weight:	0.6
		Sc. PD:	-0.092
		Sc. rank:	-600.3

PA-X

Pos.	204	obs:	exp:
tct	S	0	12.78
tcc	S	0	12.01
tca	S	0	18.46
tcg	S	0	4.04
tat	Y	1	0.56
tac	Y	0	0.44
aat	N	1005	571.20
aac	N	0	433.80
agt	S	76	14.15
agc	S	0	14.57
gat	D	1044	587.20
gac	D	0	456.80
ggt	G	20	3.14
ggc	G	0	2.53
gga	G	0	9.06
ggg	G	0	5.27
---	--	------	------
mPD		0.59	1.1
		nPD:	0.52
		N. weight:	0.74
		Sc. PD:	0.24
		Sc. rank:	931.6

,

PA-X

Pos.	205	obs:	exp:
tta	L	1	228.00
ttg	L	2094	393.50
tct	S	0	8.58
tcc	S	0	8.06
tca	S	0	12.38
tcg	S	51	2.71
ctt	L	0	423.10
ctc	L	0	304.80
cta	L	0	365.80
ctg	L	0	379.80
agt	S	0	9.49
agc	S	0	9.77
---	--	------	------
mPD		0.047	1.2
		nPD:	0.04
		N. weight:	2.
		Sc. PD:	-0.3
		Sc. rank:	-1837.1

,

PA-X

Pos.	206	obs:	exp:
cgt	R	0	28.19
cgc	R	0	47.20
cga	R	0	89.76
cgg	R	0	64.51
att	I	0	1.42
atc	I	0	1.03
ata	I	4	1.56
aaa	K	1248	759.10
aag	K	1	489.90
aga	R	893	409.50
agg	R	0	253.80
---	--	------	------
mPD		0.49	1.2
		nPD:	0.41
		N. weight:	0.73
		Sc. PD:	0.16
		Sc. rank:	854.3

,

PA-X

Pos.	207	obs:	exp:
ttt	F	4	1.79
ttc	F	0	2.21
tta	L	2027	222.30
ttg	L	16	383.70
tct	S	6	16.65
tcc	S	0	15.65
tca	S	93	24.04
tcg	S	0	5.26
ctt	L	0	412.60
ctc	L	0	297.20
cta	L	0	356.70
ctg	L	0	370.30
agt	S	0	18.43
agc	S	0	18.97
---	--	------	------
mPD		0.11	1.3
		nPD:	0.09
		N. weight:	2.5
		Sc. PD:	-0.25
		Sc. rank:	-806.9

,

PA-X

Pos.	208	obs:	exp:
tta	L	0	0.44
ttg	L	0	0.75
ctt	L	0	0.81
ctc	L	0	0.58
cta	L	0	0.70
ctg	L	4	0.73
cct	P	0	33.94
ccc	P	0	23.15
cca	P	1	45.54
ccg	P	114	12.37
caa	Q	3	1026.00
cag	Q	1899	876.30
cgt	R	0	1.42
cgc	R	0	2.38
cga	R	0	4.52
cgg	R	41	3.25
aaa	K	0	48.62
aag	K	80	31.38
aga	R	0	20.64
agg	R	4	12.79
---	--	------	------
mPD		0.22	0.77
		nPD:	0.29
		N. weight:	1.1
		Sc. PD:	0.1
		Sc. rank:	956.4

,

PA-X

Pos.	209	obs:	exp:
aaa	K	1	0.61
aag	K	0	0.39
gaa	E	2014	1204.00
gag	E	18	828.00
ggt	G	0	17.72
ggc	G	0	14.32
gga	G	112	51.18
ggg	G	1	29.77
---	--	------	------
mPD		0.12	0.60
		nPD:	0.2
		N. weight:	0.59
		Sc. PD:	0.0041
		Sc. rank:	374.0

,

PA-X

Pos.	210	obs:	exp:
tta	L	1	105.80
ttg	L	0	182.60
ctt	L	0	196.30
ctc	L	0	141.40
cta	L	964	169.70
ctg	L	7	176.20
cct	P	0	324.90
ccc	P	0	221.70
cca	P	1101	436.00
ccg	P	0	118.40
caa	Q	19	10.25
cag	Q	0	8.75
cgt	R	0	1.67
cgc	R	0	2.80
cga	R	53	5.33
cgg	R	0	3.83
att	I	0	0.35
atc	I	0	0.26
ata	I	1	0.39
aga	R	0	24.31
agg	R	0	15.06
---	--	------	------
mPD		0.54	1.5
		nPD:	0.35
		N. weight:	1.6
		Sc. PD:	0.25
		Sc. rank:	1694.0

,

PA-X

Pos.	211	obs:	exp:
tgt	C	1	923.70
tgc	C	2145	1222.00
---	--	------	------
mPD		0.00093	0.49
		nPD:	0.
		N. weight:	0.67
		Sc. PD:	-0.12
		Sc. rank:	-1029.2

,

PA-X

Pos.	212	obs:	exp:
tta	L	1	0.11
ttg	L	0	0.19
tct	S	0	3.53
tcc	S	0	3.32
tca	S	21	5.10
tcg	S	0	1.12
ctt	L	0	0.20
ctc	L	0	0.15
cta	L	0	0.17
ctg	L	0	0.18
att	I	0	0.71
atc	I	0	0.51
ata	I	2	0.78
act	T	0	4.18
acc	T	0	3.68
aca	T	17	7.72
acg	T	0	1.42
agt	S	0	3.91
agc	S	0	4.02
gtt	V	0	19.54
gtc	V	0	16.61
gta	V	88	22.21
gtg	V	0	29.64
gct	A	0	488.50
gcc	A	1	456.60
gca	A	1992	898.30
gcg	A	0	149.50
gaa	E	23	13.63
gag	E	0	9.37
ggt	G	0	0.16
ggc	G	0	0.13
gga	G	1	0.45
ggg	G	0	0.26
---	--	------	------
mPD		0.14	0.83
		nPD:	0.17
		N. weight:	0.98
		Sc. PD:	-0.021
		Sc. rank:	407.2

,

PA-X

Pos.	213	obs:	exp:
tct	S	0	170.70
tcc	S	0	160.40
tca	S	0	246.50
tcg	S	0	53.94
tgt	C	0	0.43
tgc	C	1	0.57
cgt	R	0	0.19
cgc	R	0	0.32
cga	R	0	0.60
cgg	R	0	0.43
att	I	0	1.06
atc	I	3	0.77
ata	I	0	1.17
aat	N	0	26.71
aac	N	47	20.29
agt	S	0	188.90
agc	S	1015	194.50
aga	R	6	2.75
agg	R	0	1.71
gat	D	0	6.19
gac	D	11	4.81
ggt	G	0	166.70
ggc	G	1060	134.70
gga	G	3	481.50
ggg	G	0	280.10
---	--	------	------
mPD		0.56	1.8
		nPD:	0.31
		N. weight:	2.2
		Sc. PD:	0.26
		Sc. rank:	2156.8

,

PA-X

Pos.	214	obs:	exp:
tta	L	0	221.30
ttg	L	2034	382.10
tct	S	0	18.84
tcc	S	0	17.70
tca	S	0	27.20
tcg	S	112	5.95
ctt	L	0	410.80
ctc	L	0	295.90
cta	L	0	355.20
ctg	L	0	368.70
agt	S	0	20.85
agc	S	0	21.46
---	--	------	------
mPD		0.099	1.3
		nPD:	0.08
		N. weight:	2.1
		Sc. PD:	-0.23
		Sc. rank:	-951.5

,

PA-X

Pos.	215	obs:	exp:
tta	L	0	11.97
ttg	L	0	20.66
ctt	L	0	22.22
ctc	L	0	16.00
cta	L	1	19.21
ctg	L	109	19.94
cct	P	0	589.90
ccc	P	0	402.50
cca	P	34	791.60
ccg	P	1965	215.00
caa	Q	1	19.95
cag	Q	36	17.05
---	--	------	------
mPD		0.16	0.87
		nPD:	0.19
		N. weight:	2.5
		Sc. PD:	-0.0094
		Sc. rank:	1474.6

,

PA-X

Pos.	216	obs:	exp:
att	I	0	39.02
atc	I	110	28.21
ata	I	0	42.76
act	T	0	500.10
acc	T	2033	439.60
aca	T	0	923.80
acg	T	0	169.50
aat	N	0	1.71
aac	N	3	1.29
---	--	------	------
mPD		0.10	0.78
		nPD:	0.13
		N. weight:	1.8
		Sc. PD:	-0.11
		Sc. rank:	145.5

,

PA-X

Pos.	217	obs:	exp:
cgt	R	0	5.71
cgc	R	0	9.57
cga	R	0	18.19
cgg	R	0	13.08
aaa	K	1965	1194.00
aag	K	0	770.80
aga	R	181	83.01
agg	R	0	51.44
---	--	------	------
mPD		0.15	0.68
		nPD:	0.23
		N. weight:	0.62
		Sc. PD:	0.022
		Sc. rank:	450.2

,

PA-X

Pos.	218	obs:	exp:
gtt	V	8	447.40
gtc	V	2007	380.20
gta	V	0	508.70
gtg	V	0	678.70
gct	A	0	32.11
gcc	A	131	30.02
gca	A	0	59.05
gcg	A	0	9.82
---	--	------	------
mPD		0.12	0.86
		nPD:	0.14
		N. weight:	2.
		Sc. PD:	-0.091
		Sc. rank:	428.8

,

PA-X

Pos.	219	obs:	exp:
ttt	F	0	20.16
ttc	F	45	24.84
tct	S	0	342.60
tcc	S	2037	321.90
tca	S	0	494.70
tcg	S	0	108.30
tat	Y	0	34.73
tac	Y	62	27.27
tgt	C	0	0.86
tgc	C	2	1.14
agt	S	0	379.10
agc	S	0	390.40
---	--	------	------
mPD		0.098	1.7
		nPD:	0.06
		N. weight:	2.1
		Sc. PD:	-0.28
		Sc. rank:	-1632.4

,

PA-X

Pos.	220	obs:	exp:
tta	L	0	1.74
ttg	L	0	3.01
ctt	L	0	3.23
ctc	L	16	2.33
cta	L	0	2.79
ctg	L	0	2.90
cct	P	0	8.26
ccc	P	28	5.64
cca	P	0	11.09
ccg	P	0	3.01
cat	H	0	1106.00
cac	H	1917	811.20
cgt	R	0	5.84
cgc	R	185	9.78
cga	R	0	18.59
cgg	R	0	13.37
aga	R	0	84.84
agg	R	0	52.58
---	--	------	------
mPD		0.19	0.89
		nPD:	0.22
		N. weight:	1.3
		Sc. PD:	0.034
		Sc. rank:	850.8

,

PA-X

Pos.	221	obs:	exp:
tta	L	0	1.41
ttg	L	0	2.44
ctt	L	0	2.63
ctc	L	0	1.89
cta	L	13	2.27
ctg	L	0	2.36
caa	Q	104	56.08
cag	Q	0	47.92
cgt	R	0	64.05
cgc	R	1	107.20
cga	R	2028	203.90
cgg	R	0	146.60
aga	R	0	930.50
agg	R	0	576.70
---	--	------	------
mPD		0.10	1.1
		nPD:	0.1
		N. weight:	2.6
		Sc. PD:	-0.24
		Sc. rank:	-409.2

,

PA-X

Pos.	222	obs:	exp:
cct	P	1	0.30
ccc	P	0	0.20
cca	P	0	0.40
ccg	P	0	0.11
att	I	37	13.13
atc	I	0	9.49
ata	I	0	14.38
act	T	2104	517.50
acc	T	0	455.00
aca	T	0	956.00
acg	T	0	175.40
aat	N	3	1.71
aac	N	0	1.29
gct	A	1	0.25
gcc	A	0	0.23
gca	A	0	0.45
gcg	A	0	0.07
---	--	------	------
mPD		0.039	0.72
		nPD:	0.05
		N. weight:	1.7
		Sc. PD:	-0.22
		Sc. rank:	-1394.1

,

PA-X

Pos.	223	obs:	exp:
ttt	F	34	15.23
ttc	F	0	18.77
tct	S	2109	355.20
tcc	S	2	333.80
tca	S	1	512.90
tcg	S	0	112.20
agt	S	0	393.10
agc	S	0	404.80
---	--	------	------
mPD		0.034	1.6
		nPD:	0.02
		N. weight:	2.1
		Sc. PD:	-0.35
		Sc. rank:	-2707.0

,

PA-X

Pos.	224	obs:	exp:
tta	L	0	3.05
ttg	L	0	5.26
ctt	L	0	5.65
ctc	L	0	4.07
cta	L	28	4.89
ctg	L	0	5.08
cct	P	38	604.40
ccc	P	0	412.30
cca	P	2010	811.00
ccg	P	0	220.30
caa	Q	70	37.75
cag	Q	0	32.25
---	--	------	------
mPD		0.12	0.80
		nPD:	0.15
		N. weight:	1.
		Sc. PD:	-0.037
		Sc. rank:	305.1

,

PA-X

Pos.	225	obs:	exp:
act	T	32	11.81
acc	T	15	10.38
aca	T	1	21.81
acg	T	0	4.00
gtt	V	15	37.08
gtc	V	152	31.51
gta	V	0	42.16
gtg	V	0	56.25
gct	A	7	473.30
gcc	A	1920	442.40
gca	A	4	870.40
gcg	A	0	144.80
---	--	------	------
mPD		0.24	0.88
		nPD:	0.27
		N. weight:	1.7
		Sc. PD:	0.14
		Sc. rank:	1494.7

,

PA-X

Pos.	226	obs:	exp:
tta	L	2	230.10
ttg	L	2112	397.10
tct	S	0	5.21
tcc	S	0	4.90
tca	S	1	7.53
tcg	S	30	1.65
tgg	W	1	1.00
ctt	L	0	426.90
ctc	L	0	307.60
cta	L	0	369.10
ctg	L	0	383.20
agt	S	0	5.77
agc	S	0	5.94
---	--	------	------
mPD		0.032	1.2
		nPD:	0.03
		N. weight:	2.
		Sc. PD:	-0.32
		Sc. rank:	-2227.1

,

PA-X

Pos.	227	obs:	exp:
caa	Q	1	0.54
cag	Q	0	0.46
cgt	R	0	1.01
cgc	R	0	1.69
cga	R	0	3.22
cgg	R	0	2.31
att	I	0	3.55
atc	I	0	2.56
ata	I	10	3.89
act	T	0	0.98
acc	T	0	0.86
aca	T	4	1.82
acg	T	0	0.33
aat	N	0	2.84
aac	N	5	2.16
aaa	K	2086	1272.00
aag	K	7	821.00
aga	R	32	14.68
agg	R	0	9.10
gaa	E	1	0.59
gag	E	0	0.41
---	--	------	------
mPD		0.055	0.54
		nPD:	0.1
		N. weight:	0.58
		Sc. PD:	-0.05
		Sc. rank:	-63.9

,

PA-X

Pos.	228	obs:	exp:
cct	P	12	3.54
ccc	P	0	2.42
cca	P	0	4.75
ccg	P	0	1.29
att	I	42	14.90
atc	I	0	10.77
ata	I	0	16.33
act	T	2070	509.20
acc	T	0	447.60
aca	T	0	940.60
acg	T	0	172.60
aat	N	4	2.27
aac	N	0	1.73
gct	A	18	4.41
gcc	A	0	4.12
gca	A	0	8.11
gcg	A	0	1.35
---	--	------	------
mPD		0.070	0.75
		nPD:	0.09
		N. weight:	1.7
		Sc. PD:	-0.16
		Sc. rank:	-384.0

,

PA-X

Pos.	229	obs:	exp:
ttt	F	0	12.55
ttc	F	28	15.45
tta	L	2065	224.70
ttg	L	0	387.90
tct	S	0	8.91
tcc	S	0	8.38
tca	S	53	12.87
tcg	S	0	2.82
ctt	L	0	417.00
ctc	L	0	300.50
cta	L	0	360.60
ctg	L	0	374.30
agt	S	0	9.86
agc	S	0	10.16
---	--	------	------
mPD		0.074	1.2
		nPD:	0.06
		N. weight:	2.6
		Sc. PD:	-0.33
		Sc. rank:	-1784.6

,

PA-X

Pos.	230	obs:	exp:
cgt	R	0	0.03
cgc	R	0	0.05
cga	R	0	0.10
cgg	R	0	0.07
aga	R	0	0.46
agg	R	1	0.28
gaa	E	3	1203.00
gag	E	2028	827.60
ggt	G	0	17.88
ggc	G	0	14.45
gga	G	0	51.64
ggg	G	114	30.04
---	--	------	------
mPD		0.11	0.60
		nPD:	0.18
		N. weight:	1.1
		Sc. PD:	-0.016
		Sc. rank:	544.0

,

PA-X

Pos.	231	obs:	exp:
tta	L	0	0.98
ttg	L	0	1.69
tct	S	2	0.34
tcc	S	0	0.32
tca	S	0	0.49
tcg	S	0	0.11
ctt	L	9	1.82
ctc	L	0	1.31
cta	L	0	1.57
ctg	L	0	1.63
cct	P	2128	628.00
ccc	P	0	428.40
cca	P	0	842.70
ccg	P	0	228.90
cat	H	7	4.04
cac	H	0	2.96
agt	S	0	0.37
agc	S	0	0.38
---	--	------	------
mPD		0.017	0.72
		nPD:	0.02
		N. weight:	1.5
		Sc. PD:	-0.24
		Sc. rank:	-1852.3

,

PA-X

Pos.	232	obs:	exp:
att	I	0	2.84
atc	I	0	2.05
ata	I	8	3.11
atg	M	2061	2061.00
act	T	0	18.94
acc	T	0	16.65
aca	T	0	34.99
acg	T	77	6.42
---	--	------	------
mPD		0.077	0.14
		nPD:	0.55
		N. weight:	0.11
		Sc. PD:	0.038
		Sc. rank:	142.6

,

PA-X

Pos.	233	obs:	exp:
taa	.	1	913.00
tag	.	2142	565.30
tga	.	3	667.70
---	--	------	------
mPD		0.0065	0.82
		nPD:	0.01
		N. weight:	1.6
		Sc. PD:	-0.28
		Sc. rank:	-2270.9

}

Amino acid level conservation per codon

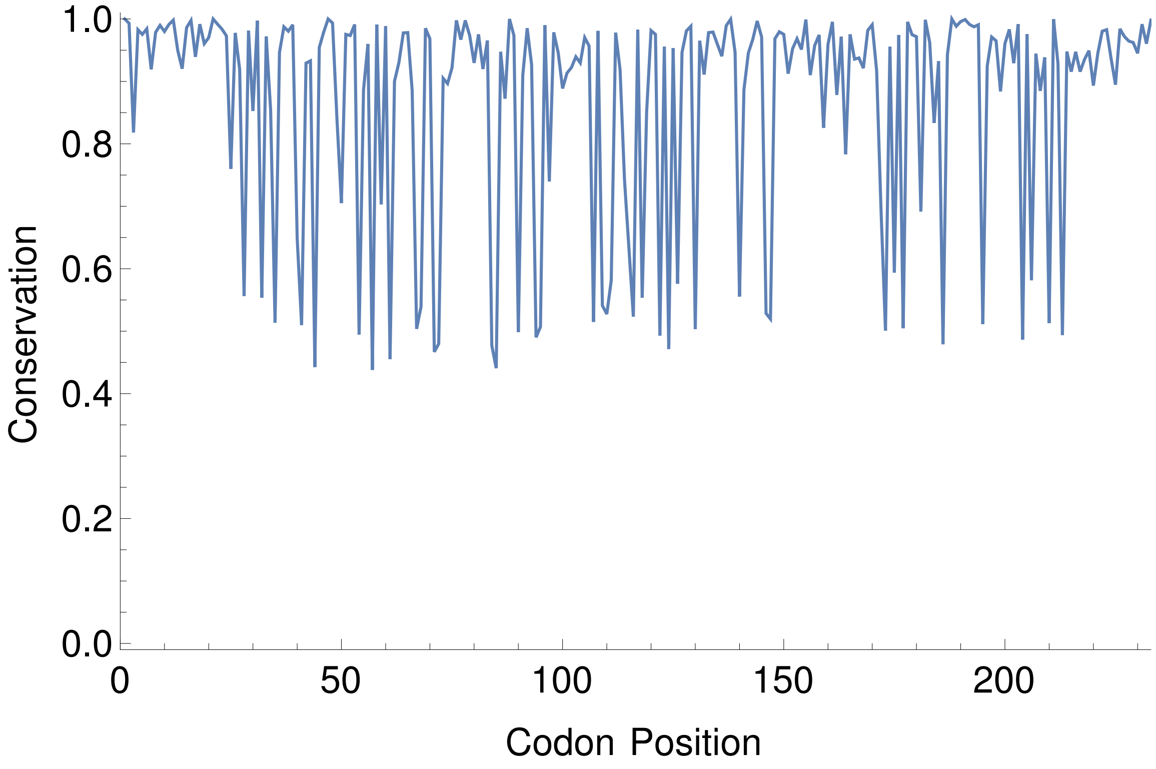

Conservation analysis plots

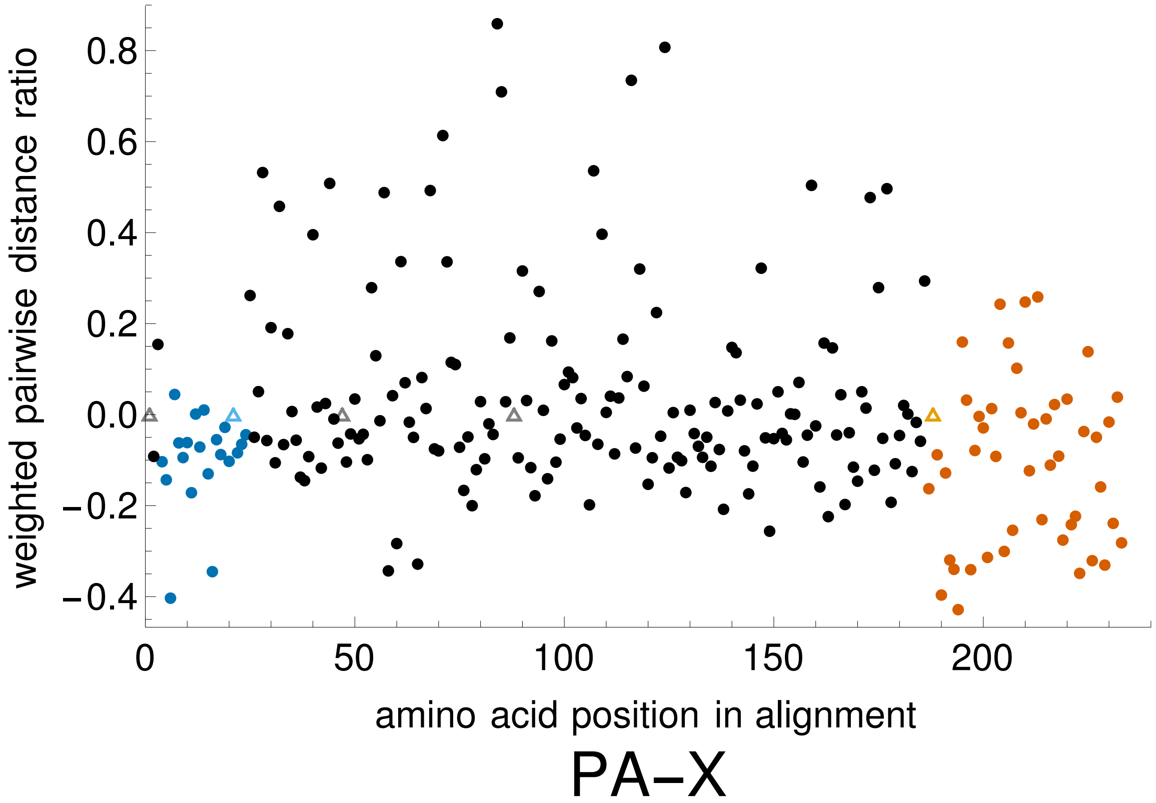

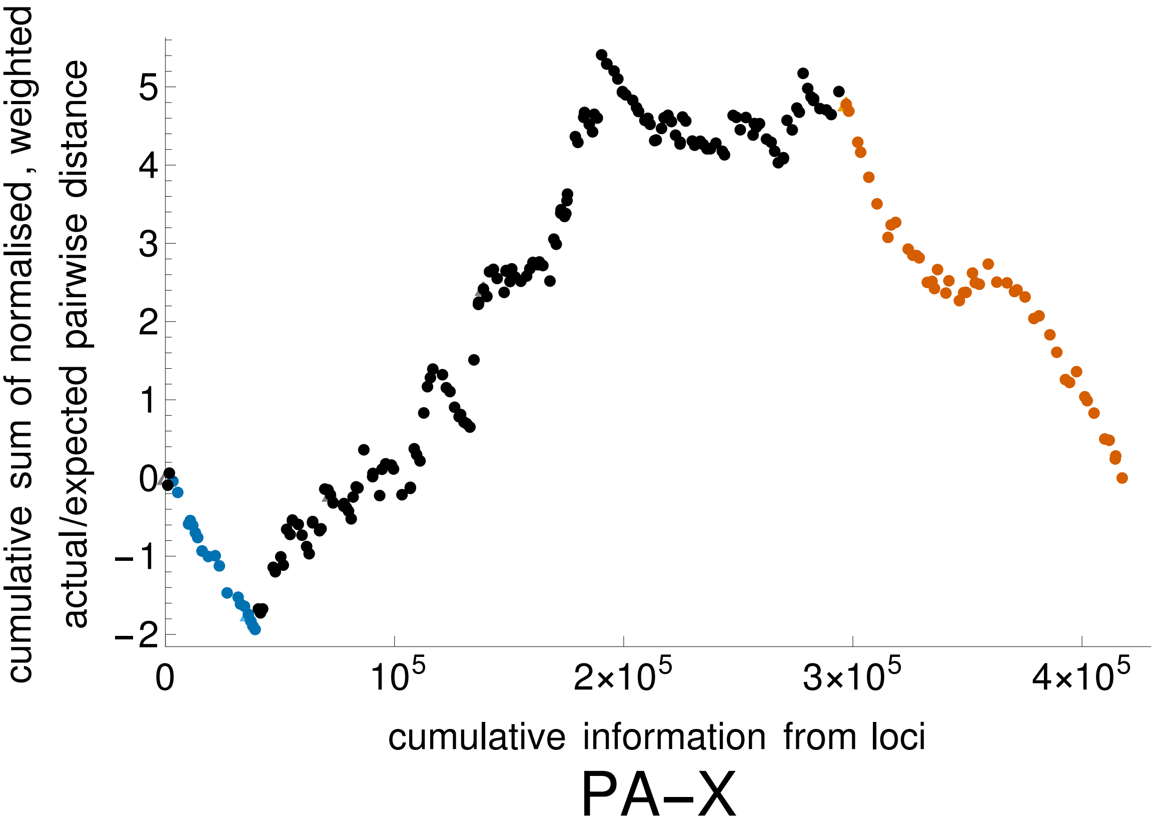

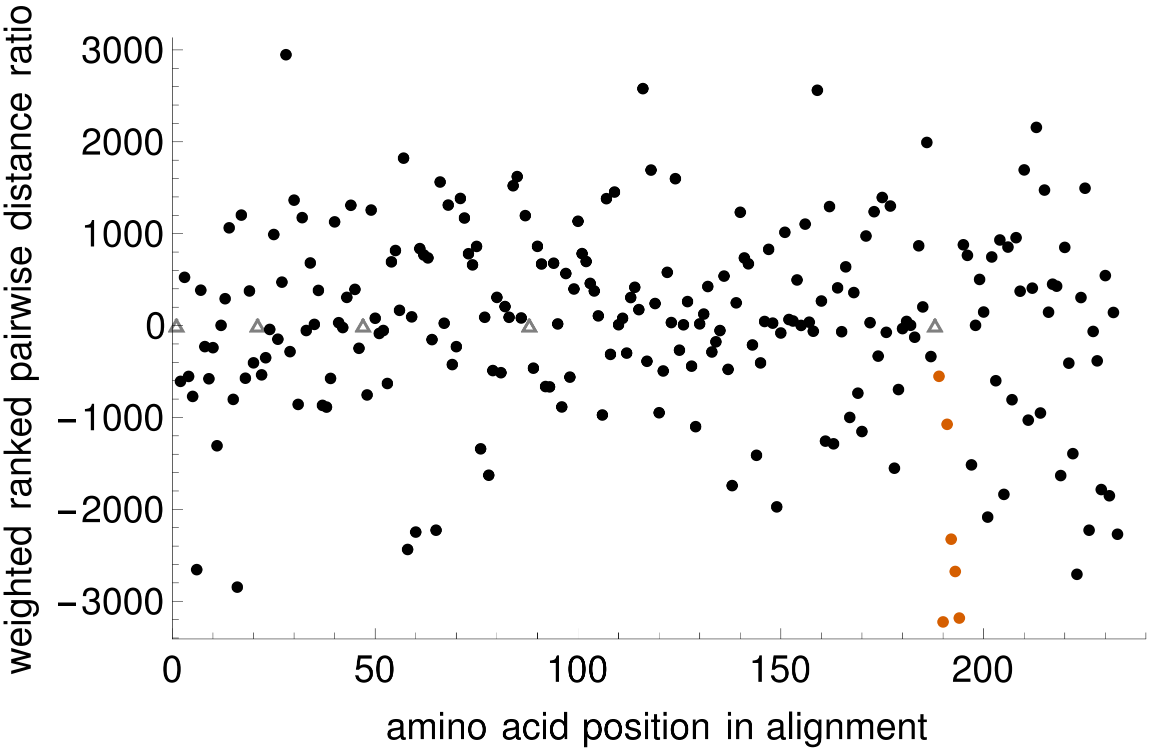

weighted ranked pairwise distance ratio
3000
2000
1000
0
−1000
−2000
−3000
0
50
100
150
200
amino acid position in alignment

PA–X

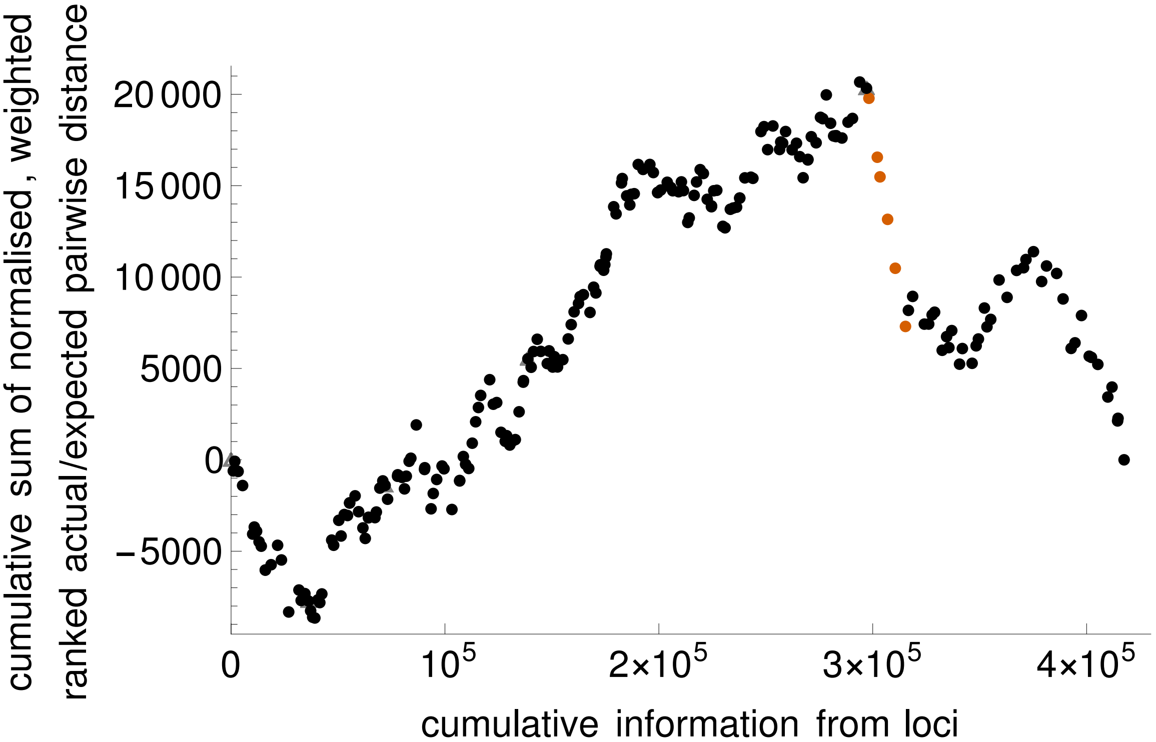

cumulative sum of normalised, weighted ranked actual/expected pairwise distance
20 000
15 000
10 000
5000
0
−5000
0
10^5
2×10^5
3×10^5
4×10^5
cumulative information from loci

PA–X

HA

Gene length histogram

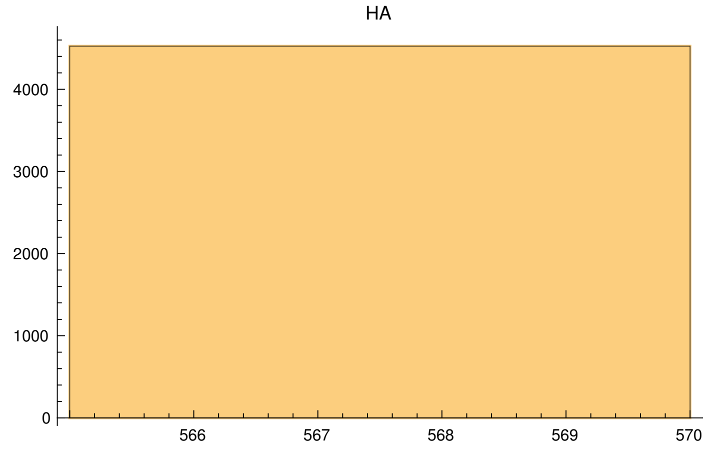

Information vs. nPD

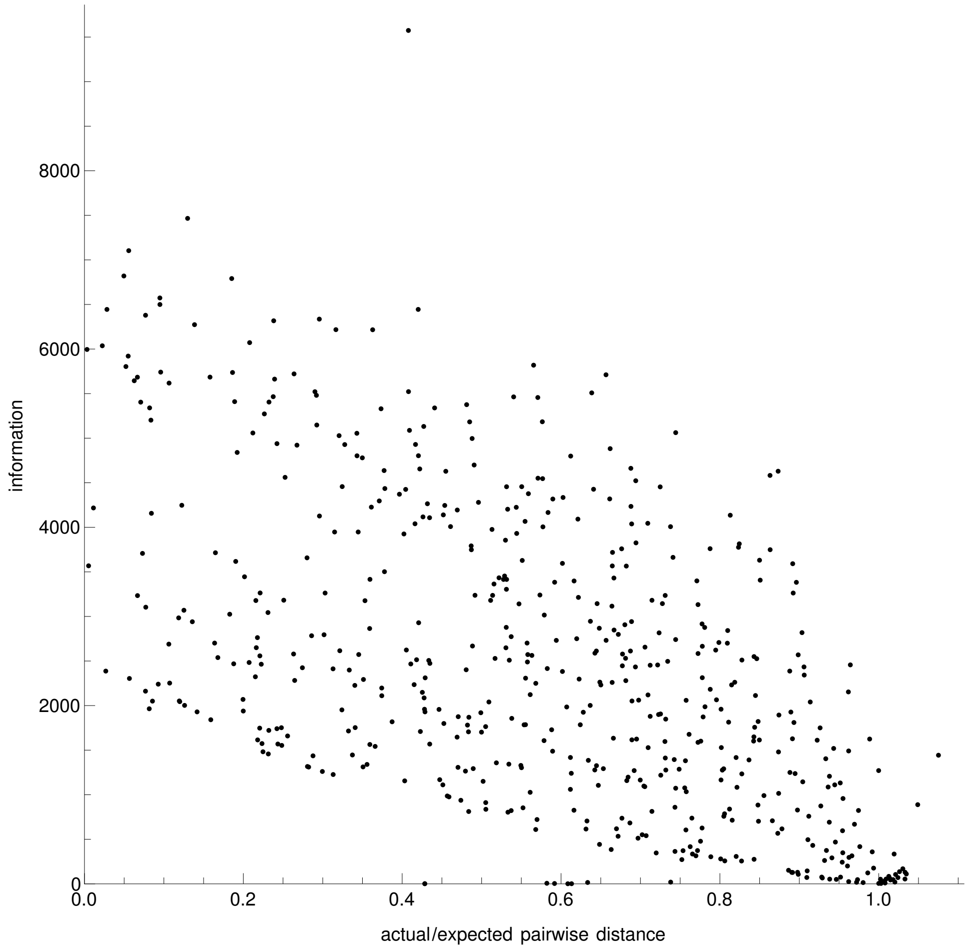

HA

Example sequences highlighted by regions found in analyses to be conserved

Interesting points (by weighted raw PD) highlighted for gene HA:

-----------------ATGGAA---GCAAAACTGTTTGTATTATTCTGTGCATTCACTGCACTGAAAGCTGAC ----

--------------------------ACCATTTGTGTAGGCTACCATGCTAACAATTCCACAGACACTGTCGACACAA⋱

TACTGGAGAAGAATGTGACTGTTACCCATTCAGTTAATTTACTAGAAAACAACCATAATGGAAAACTCTGCAGCC⋱

TGAATGGAAAGGCCCCTTTACAACTG ---------GGGGACTGCAACGTAGCAGGATGGATCCTTGGCAACCCAGAA⋱

TGTGACCTGCTGCTCACAGCAAATTCGTGGTCTTACATTATAGAGACTTCAAATTCAAAAAATGGAGCATGCTAC⋱

CCTGGAGAATTCGCTGATTATGAGGAATTAAGGGAGCAGCTGAATGCAGTTTCTTCATTTGAAAGATTTGAAATT⋱

TTCCCAAAAGCAACC ---TCATGGCCAAATCATGAGACAACCAGAGGC ---ACCACAGTTGCATGCTCC ---

CACTCTGGAGCCAACAGTTTTTATCGGAACTTGCTATGGATAGTA ---AAG------AAGGGAAACTCCTATCCTAA⋱

GCTCAACAAGTCATACACAAACAACAAAGGAAAAGAAGTGCTTGTAATCTGGGGAGTGCACCACCCTCCGACTGA⋱

CAGTGACCAACAAACCCTCTATCAGAATAATCATACATATGTTTCAGTTGGATCATCAAAATACTACCGAAGGTT⋱

CACACCAGAAATAGTAGTCAGACCTAAAGTCAGAGGACAAGCAGGCAGAATGAATTATTATTGGACGCTGTTAGA⋱

TCAAGGAGACACCATAACCTTTGAGGCCACTGGGAACTTAATAGCACCATGGCACGCATTTGCATTGAATAAGGG⋱

CTCTAGTTCTGGAATTATAATGTCGGATGCCCATGTTCACAATTGCACTACAAAGTGCCAAACTCCTCATGGGGC⋱

CTTGAAAAGCAATCTTCCTTTTCAGAACGTACATCCCATCACTATTGGAGAATGCCCCAAATATGTTAAAAGCAC⋱

CCAACTAAGAATGGCAACAGGATTAAGAAACGTCCCCTCTATTCAATCCAGAGGACTGTTTGGGGCAATTGCCGG⋱

ATTCATTGAAGGAGGATGGACAGGAATGATAGATGGATGGTATGGATATCACCATCAAAATGAGCAGGGATCTGG⋱

TTACGCAGCAGATCAGAAAAGCACACAAATCGCAATTGACGGGATCAGCAACAAAGTGAACTCAGTAATTGAAAA⋱

AATGAACATTCAATTTACTTCAGTGGGCAAGGAGTTCAATGATCTAGAGAAAAGGATTGAGAATTTGAATAAGAA⋱

GGTCGATGATGGGTTTTTGGATGTATGGACATATAACGCTGAGTTGCTCATTTTGCTCGAGAACGAAAGGACTCT⋱

AGATTTCCATGACTTCAACGTAAAAAATTTATATGAAAAGGTCAAATCACAACTGAGGAATAATGCCAAGGAAAT⋱

CGGAAATGGCTGTTTTGAGTTCTATCACAAATGTGATAATGAATGCATGGAGAGCGTAAAAAATGGCACATATAA⋱

TTATCCCAAATATTCAGAAGAATCTAAATTGAATAGAGAGGAAATAGACGGTGTGAAACTAGAATCAATGGGAGT⋱

TCACCAGATTTTGGCGATCTACTCCACAGTC

GCCAGTTCCCTGGTCTTGTTAGTCTCCCTGGGGGCAATCAGCTTCTGGATGTGTT

CTAATGGGTCATTGCAATGCAGAGTATGCATTTAA

Interesting points (by weighted ranked PD) highlighted for gene HA:

-----------------ATGGAA---GCAAAACTGTTTGTATTATTCTGTGCATTCACTGCACTGAAAGCTGAC ------

------------------------ACCATTTGTGTAGGCTACCATGCTAACAATTCCACAGACACTGTCGACACAATA⋱

CTGGAGAAGAATGTGACTGTTACCCATTCAGTTAATTTACTAGAAAACAACCATAATGGAAAACTCTGCAGCCTG⋱

AATGGAAAGGCCCCTTTACAACTG ---------GGGGACTGCAACGTAGCAGGATGGATCCTTGGCAACCCAGAATG⋱

TGACCTGCTGCTCACAGCAAATTCGTGGTCTTACATTATAGAGACTTCAAATTCAAAAAATGGAGCATGCTACCC⋱

TGGAGAATTCGCTGATTATGAGGAATTAAGGGAGCAGCTGAATGCAGTTTCTTCATTTGAAAGATTTGAAATTTT⋱

CCCAAAAGCAACC ---TCATGGCCAAATCATGAGACAACCAGAGGC ---ACCACAGTTGCATGCTCC ---

CACTCTGGAGCCAACAGTTTTTATCGGAACTTGCTATGGATAGTA ---AAG------AAGGGAAACTCCTATCCTAA⋱

GCTCAACAAGTCATACACAAACAACAAAGGAAAAGAAGTGCTTGTAATCTGGGGAGTGCACCACCCTCCGACTGA⋱

CAGTGACCAACAAACCCTCTATCAGAATAATCATACATATGTTTCAGTTGGATCATCAAAATACTACCGAAGGTT⋱

CACACCAGAAATAGTAGTCAGACCTAAAGTCAGAGGACAAGCAGGCAGAATGAATTATTATTGGACGCTGTTAGA⋱

TCAAGGAGACACCATAACCTTTGAGGCCACTGGGAACTTAATAGCACCATGGCACGCATTTGCATTGAATAAGGG⋱

CTCTAGTTCTGGAATTATAATGTCGGATGCCCATGTTCACAATTGCACTACAAAGTGCCAAACTCCTCATGGGGC⋱

CTTGAAAAGCAATCTTCCTTTTCAGAACGTACATCCCATCACTATTGGAGAATGCCCCAAATATGTTAAAAGCAC⋱

CCAACTAAGAATGGCAACAGGATTAAGAAACGTCCCCTCTATTCAATCCAGAGGACTGTTTGGGGCAATTGCCGG⋱

ATTCATTGAAGGAGGATGGACAGGAATGATAGATGGATGGTATGGATATCACCATCAAAATGAGCAGGGATCTGG⋱

TTACGCAGCAGATCAGAAAAGCACACAAATCGCAATTGACGGGATCAGCAACAAAGTGAACTCAGTAATTGAAAA∵.
AATGAACATTCAATTTACTTCAGTGGGCAAGGAGTTCAATGATCTAGAGAAAAGGATTGAGAATTTGAATAAGAA∵.
GGTCGATGATGGGTTTTTGGATGTATGGACATATAACGCTGAGTTGCTCATTTTGCTCGAGAACGAAAGGACTCT∵.
AGATTTCCATGACTTCAACGTAAAAAATTTATATGAAAAGGTCAAATCACAACTGAGGAATAATGCCAAGGAAAT∵.
CGGAAATGGCTGTTTTGAGTTCTATCACAAATGTGATAATGAATGCATGGAGAGCGTAAAAAATGGCACATATAA∵.
TTATCCCAAATATTCAGAAGAATCTAAATTGAATAGAGAGGAAATAGACGGTGTGAAACTAGAATCAATGGGAGT∵.
TCACCAGATTTTGGCGATCTACTCCACAGTC
GCCAGTTCCCTGGTCTTGTTAGTCTCCCTGGGGGCAATCAGCTTCTGGATGTGTT
CTAATGGGTCATTGCAATGCAGAGTATGCATTTAA

Per codon conservation report

{

HA

Pos.	1	obs:	exp:
atg	M	10	10.00
---	-	4515	4515.00
---	--	------	------
mPD		0	0
		nPD:	1.
		N. weight:	0.
		Sc. PD:	0
		Sc. rank:	0

,

HA

Pos.	2	obs:	exp:
att	I	0	1.06
atc	I	3	0.77
ata	I	0	1.17
---	-	4522	4522.00
---	--	------	------
mPD		0	2.9×10^{-7}
		nPD:	0.
		N. weight:	0.
		Sc. PD:	0
		Sc. rank:	0

,

HA

Pos.	3	obs:	exp:
aaa	K	10	6.08
aag	K	0	3.92
---	-	4515	4515.00
---	--	------	------
mPD		0	2.3×10^{-6}
		nPD:	0.
		N. weight:	0.
		Sc. PD:	0
		Sc. rank:	0

,

HA

Pos.	4	obs:	exp:
act	T	0	2.46
acc	T	0	2.16
aca	T	10	4.54
acg	T	0	0.83
---	-	4515	4515.00
---	--	------	------
mPD		0	3.3×10^{-6}
		nPD:	0.
		N. weight:	0.
		Sc. PD:	0
		Sc. rank:	0

,

HA

Pos.	5	obs:	exp:
att	I	0	0.35
atc	I	1	0.26
ata	I	0	0.39
act	T	0	2.21
acc	T	9	1.95
aca	T	0	4.09
acg	T	0	0.75
---	-	4515	4515.00
---	--	------	------
mPD		8.8×10^{-7}	4.2×10^{-6}
		nPD:	0.21
		N. weight:	0.
		Sc. PD:	0
		Sc. rank:	0

,

HA

Pos.	6	obs:	exp:
aaa	K	7	6.08
aag	K	3	3.92
---	-	4515	4515.00
---	--	------	------
mPD		2.1×10^{-6}	2.3×10^{-6}
		nPD:	0.88
		N. weight:	0.
		Sc. PD:	0
		Sc. rank:	0

,

HA

Pos.	7	obs:	exp:
atg	M	4525	4525.00
---	--	------	------
mPD		0	0
		nPD:	1.
		N. weight:	0.
		Sc. PD:	0
		Sc. rank:	0

,

HA

Pos.	8	obs:	exp:
cgt	R	0	3.54
cgc	R	0	5.92
cga	R	0	11.26
cgg	R	0	8.09
att	I	0	0.35
atc	I	0	0.26
ata	I	1	0.39
act	T	0	0.98
acc	T	0	0.86
aca	T	1	1.82
acg	T	3	0.33
aat	N	0	0.57
aac	N	1	0.43
aaa	K	3343	2531.00
aag	K	822	1634.00
aga	R	112	51.36
agg	R	0	31.83
gtt	V	0	0.22
gtc	V	0	0.19
gta	V	1	0.25
gtg	V	0	0.34
gaa	E	223	142.20
gag	E	17	97.80
ggt	G	0	0.16
ggc	G	0	0.13
gga	G	1	0.45
ggg	G	0	0.26
---	--	------	------
mPD		0.46	0.64
		nPD:	0.71
		N. weight:	0.23
		Sc. PD:	0.051
		Sc. rank:	277.4

,

HA

Pos.	9	obs:	exp:
gtt	V	0	0.67
gtc	V	0	0.57
gta	V	3	0.76
gtg	V	0	1.01
---	-	4522	4522.00
---	--	------	------
mPD		0	3.2×10^{-7}
		nPD:	0.
		N. weight:	0.
		Sc. PD:	0
		Sc. rank:	0

,

HA

Pos.	10	obs:	exp:
tta	L	6	1.74
ttg	L	0	3.01
tct	S	0	1.18
tcc	S	0	1.11
tca	S	3	1.70
tcg	S	0	0.37
ctt	L	0	3.23
ctc	L	0	2.33
cta	L	10	2.79
ctg	L	0	2.90
cct	P	0	31.87
ccc	P	0	21.74
cca	P	108	42.77
ccg	P	0	11.62
att	I	0	18.45
atc	I	0	13.34
ata	I	52	20.22
act	T	21	48.46
acc	T	2	42.60
aca	T	172	89.52
acg	T	2	16.43
agt	S	4	1.30
agc	S	0	1.34
gtt	V	2	581.50
gtc	V	0	494.20
gta	V	2604	661.10
gtg	V	13	882.20
gct	A	248	372.60
gcc	A	10	348.30
gca	A	1251	685.10
gcg	A	11	114.00
gat	D	2	1.12
gac	D	0	0.88
gaa	E	3	1.78
gag	E	0	1.22
ggt	G	0	0.16
ggc	G	0	0.13
gga	G	1	0.45
ggg	G	0	0.26
---	--	------	------
mPD		0.77	1.4
		nPD:	0.56
		N. weight:	1.9
		Sc. PD:	0.15
		Sc. rank:	810.9

,

HA

Pos .	11	obs :	exp :
tta	L	0	0.11
ttg	L	0	0.19
ctt	L	0	0.20
ctc	L	0	0.15
cta	L	1	0.17
ctg	L	0	0.18
cgt	R	0	2.37
cgc	R	0	3.96
cga	R	0	7.54
cgg	R	0	5.42
att	I	0	295.50
atc	I	0	213.70
ata	I	833	323.80
atg	M	6	6.00
act	T	0	44.52
acc	T	0	39.14
aca	T	162	82.25
acg	T	19	15.09
aaa	K	3182	1952.00
aag	K	30	1260.00
aga	R	74	34.39
agg	R	1	21.32
gtt	V	0	35.97
gtc	V	0	30.57
gta	V	162	40.90
gtg	V	0	54.57
gct	A	0	10.05
gcc	A	0	9.39
gca	A	41	18.48
gcg	A	0	3.07
gaa	E	14	8.30
gag	E	0	5.70
---	--	------	------
mPD		0.56	1.1
		nPD :	0.49
		N. weight :	1.2
		Sc. PD :	0.013
		Sc. rank :	30.6

,

HA

Pos .	12	obs :	exp :
tta	L	30	481.30
ttg	L	343	830.80
ctt	L	8	893.30
ctc	L	1	643.50
cta	L	3760	772.30
ctg	L	281	801.80
cct	P	0	3.54
ccc	P	0	2.42
cca	P	12	4.75
ccg	P	0	1.29
caa	Q	1	0.54
cag	Q	0	0.46
att	I	0	28.73
atc	I	0	20.78
ata	I	81	31.49
atg	M	6	6.00
act	T	1	0.25
acc	T	0	0.22
aca	T	0	0.45
acg	T	0	0.08
gtt	V	0	0.22
gtc	V	0	0.19
gta	V	1	0.25
gtg	V	0	0.34
---	--	------	------
mPD		0.44	1.2
		nPD :	0.37
		N. weight :	2.3
		Sc. PD :	-0.22
		Sc. rank :	-1308.1

,

HA

Pos .	13	obs :	exp :
ttt	F	222	103.10
ttc	F	8	126.90
tta	L	229	303.20
ttg	L	594	523.30
ctt	L	11	562.70
ctc	L	0	405.40
cta	L	295	486.50
ctg	L	1657	505.00
att	I	188	132.30
atc	I	0	95.67
ata	I	185	145.00
atg	M	744	744.00
act	T	5	1.48
acc	T	0	1.30
aca	T	0	2.73
acg	T	1	0.50
aaa	K	0	0.61
aag	K	1	0.39
gtt	V	8	83.71
gtc	V	0	71.14
gta	V	361	95.17
gtg	V	8	127.00
gct	A	0	1.96
gcc	A	0	1.83
gca	A	8	3.61
gcg	A	0	0.60
---	--	------	------
mPD		1.2	1.4
		nPD :	0.85
		N. weight :	1.1
		Sc. PD :	0.38
		Sc. rank :	1854.0

,

HA

Pos .	14	obs :	exp :
ttt	F	3	1.79
ttc	F	1	2.21
tta	L	3	0.65
ttg	L	0	1.13
ctt	L	0	1.21
ctc	L	2	0.87
cta	L	1	1.05
ctg	L	0	1.09
att	I	86	627.90
atc	I	1664	454.00
ata	I	20	688.10
act	T	2	73.06
acc	T	295	64.22
aca	T	0	135.00
acg	T	0	24.77
gtt	V	1265	541.80
gtc	V	920	460.40
gta	V	239	615.90
gtg	V	16	821.90
gct	A	1	1.47
gcc	A	3	1.37
gca	A	2	2.70
gcg	A	0	0.45
gat	D	1	0.56
gac	D	0	0.44
ggt	G	1	0.16
ggc	G	0	0.13
gga	G	0	0.45
ggg	G	0	0.26
---	--	------	------
mPD		1.1	1.4
		nPD :	0.82
		N. weight :	1.7
		Sc. PD :	0.54
		Sc. rank :	2713.4

,

HA

Pos .	15	obs :	exp :
ttt	F	5	2.24
ttc	F	0	2.76
tta	L	507	489.50
ttg	L	554	844.90
tct	S	0	0.34
tcc	S	0	0.32
tca	S	0	0.49
tcg	S	2	0.11
ctt	L	13	908.40
ctc	L	1	654.40
cta	L	104	785.40
ctg	L	3319	815.40
cct	P	1	0.89
ccc	P	0	0.60
cca	P	0	1.19
ccg	P	2	0.32
caa	Q	0	0.54
cag	Q	1	0.46
att	I	0	0.71
atc	I	0	0.51
ata	I	2	0.78
atg	M	14	14.00
agt	S	0	0.37
agc	S	0	0.38
---	--	------	------
mPD		0.61	1.1
		nPD :	0.53
		N. weight :	1.8
		Sc. PD :	0.097
		Sc. rank :	456.1

,

HA

Pos .	16	obs :	exp :
ttt	F	563	354.90
ttc	F	229	437.10
tta	L	2075	371.20
ttg	L	210	640.70
tct	S	0	43.40
tcc	S	0	40.77
tca	S	258	62.65
tcg	S	0	13.71
ctt	L	2	688.90
ctc	L	4	496.30
cta	L	1090	595.60
ctg	L	30	618.30
caa	Q	2	1.08
cag	Q	0	0.92
att	I	0	21.28
atc	I	0	15.39
ata	I	60	23.33
atg	M	1	1.00
agt	S	0	48.02
agc	S	0	49.45
gct	A	0	0.25
gcc	A	1	0.23
gca	A	0	0.45
gcg	A	0	0.07
---	--	------	------
mPD		0.89	1.4
		nPD :	0.64
		N. weight :	1.9
		Sc. PD :	0.3
		Sc. rank :	1525.4

,

HA

Pos .	17	obs :	exp :
ttt	F	28	13.44
ttc	F	2	16.56
tct	S	0	10.76
tcc	S	0	10.11
tca	S	0	15.54
tcg	S	0	3.40
tat	Y	667	395.00
tac	Y	38	310.00
tgt	C	3023	1430.00
tgc	C	300	1893.00
cat	H	261	229.60
cac	H	137	168.40
caa	Q	0	0.54
cag	Q	1	0.46
aat	N	3	1.71
aac	N	0	1.29
agt	S	63	11.91
agc	S	1	12.27
ggt	G	1	0.16
ggc	G	0	0.13
gga	G	0	0.45
ggg	G	0	0.26
---	--	------	------
mPD		0.76	1.1
		nPD :	0.71
		N. weight :	0.92
		Sc. PD :	0.2
		Sc. rank :	1104.5

,

HA

Pos .	18	obs :	exp :
tta	L	0	0.11
ttg	L	0	0.19
tct	S	0	1.51
tcc	S	0	1.42
tca	S	9	2.19
tcg	S	0	0.48
ctt	L	0	0.20
ctc	L	0	0.15
cta	L	1	0.17
ctg	L	0	0.18
cgt	R	0	0.03
cgc	R	0	0.05
cga	R	0	0.10
cgg	R	0	0.07
att	I	0	15.25
atc	I	0	11.03
ata	I	43	16.72
atg	M	2	2.00
act	T	2	954.40
acc	T	2	839.00
aca	T	3851	1763.00
acg	T	25	323.50
aaa	K	17	10.33
aag	K	0	6.67
agt	S	0	1.68
agc	S	0	1.72
aga	R	1	0.46
agg	R	0	0.28
gtt	V	0	22.65
gtc	V	0	19.25
gta	V	97	25.75
gtg	V	5	34.36
gct	A	1	115.20
gcc	A	0	107.70
gca	A	375	211.90
gcg	A	94	35.25
---	--	------	------
mPD		0.35	0.98
		nPD :	0.36
		N. weight :	1.5
		Sc. PD :	-0.16
		Sc. rank :	-932.1

,

HA

Pos .	19	obs :	exp :
ttt	F	3656	1808.00
ttc	F	380	2228.00
tta	L	2	43.09
ttg	L	1	74.38
tgt	C	2	1.29
tgc	C	1	1.71
ctt	L	358	79.98
ctc	L	32	57.62
cta	L	2	69.15
ctg	L	1	71.78
att	I	32	11.71
atc	I	0	8.46
ata	I	1	12.83
gtt	V	10	12.66
gtc	V	47	10.76
gta	V	0	14.39
gtg	V	0	19.20
---	--	------	------
mPD		0.38	0.71
		nPD :	0.53
		N. weight :	1.1
		Sc. PD :	0.06
		Sc. rank :	287.1

,

HA

Pos .	20	obs :	exp :
tta	L	1	0.11
ttg	L	0	0.19
tct	S	7	51.47
tcc	S	4	48.36
tca	S	294	74.31
tcg	S	0	16.26
ctt	L	0	0.20
ctc	L	0	0.15
cta	L	0	0.17
ctg	L	0	0.18
cgt	R	0	0.03
cgc	R	0	0.05
cga	R	0	0.10
cgg	R	0	0.07
att	I	0	5.32
atc	I	0	3.85
ata	I	15	5.83
act	T	220	789.60
acc	T	6	694.10
aca	T	2971	1459.00
acg	T	13	267.70
aat	N	40	23.87
aac	N	2	18.13
aaa	K	6	3.65
aag	K	0	2.35
agt	S	1	56.95
agc	S	0	58.64
aga	R	1	0.46
agg	R	0	0.28
gtt	V	0	2.22
gtc	V	0	1.89
gta	V	10	2.52
gtg	V	0	3.37
gct	A	4	229.00
gcc	A	4	214.00
gca	A	832	421.00
gcg	A	94	70.04
---	--	------	------
mPD		0.62	1.2
		nPD :	0.54
		N. weight :	1.2
		Sc. PD :	0.069
		Sc. rank :	330.0

,

HA

Pos.	21	obs:	exp:
tct	S	0	3.36
tcc	S	5	3.16
tca	S	11	4.86
tcg	S	4	1.06
cct	P	0	1.77
ccc	P	6	1.21
cca	P	0	2.38
ccg	P	0	0.65
cgt	R	0	0.66
cgc	R	0	1.11
cga	R	0	2.11
cgg	R	0	1.52
att	I	1	12.06
atc	I	31	8.72
ata	I	2	13.22
act	T	139	137.30
acc	T	384	120.70
aca	T	34	253.60
acg	T	1	46.53
aat	N	0	0.57
aac	N	1	0.43
agt	S	0	3.72
agc	S	0	3.83
aga	R	1	9.63
agg	R	20	5.97
gtt	V	59	35.75
gtc	V	70	30.38
gta	V	31	40.64
gtg	V	1	54.23
gct	A	2953	912.90
gcc	A	595	853.30
gca	A	169	1679.00
gcg	A	7	279.30
---	--	------	------
mPD		0.79	1.0
		nPD:	0.77
		N. weight:	1.5
		Sc. PD:	0.41
		Sc. rank:	2090.5

,

HA

Pos.	22	obs:	exp:
tta	L	22	25.90
ttg	L	20	44.70
tct	S	0	1.18
tcc	S	0	1.11
tca	S	7	1.70
tcg	S	0	0.37
ctt	L	1	48.07
ctc	L	1	34.63
cta	L	60	41.56
ctg	L	134	43.14
att	I	0	99.33
atc	I	0	71.82
ata	I	280	108.90
atg	M	1	1.00
act	T	5	604.40
acc	T	0	531.30
aca	T	1853	1116.00
acg	T	599	204.90
agt	S	0	1.30
agc	S	0	1.34
gtt	V	0	1.33
gtc	V	0	1.13
gta	V	3	1.51
gtg	V	3	2.02
gct	A	0	371.40
gcc	A	3	347.10
gca	A	1507	682.90
gcg	A	5	113.60
ggt	G	0	3.29
ggc	G	0	2.66
gga	G	21	9.51
ggg	G	0	5.53
---	--	------	------
mPD		1.0	1.4
		nPD:	0.71
		N. weight:	1.4
		Sc. PD:	0.31
		Sc. rank:	1697.5

,

HA

Pos.	23	obs:	exp:
ttt	F	6	2.69
ttc	F	0	3.31
tct	S	5	4.54
tcc	S	0	4.27
tca	S	0	6.56
tcg	S	0	1.43
tat	Y	2638	1498.00
tac	Y	35	1175.00
tgt	C	4	1.72
tgc	C	0	2.28
cat	H	34	25.38
cac	H	10	18.62
cgt	R	0	0.09
cgc	R	0	0.16
cga	R	0	0.30
cgg	R	0	0.22
att	I	3	1.06
atc	I	0	0.77
ata	I	0	1.17
act	T	11	2.71
acc	T	0	2.38
aca	T	0	5.00
acg	T	0	0.92
aat	N	1133	653.60
aac	N	17	496.40
aaa	K	221	141.00
aag	K	11	91.01
agt	S	22	5.03
agc	S	0	5.17
aga	R	3	1.38
agg	R	0	0.85
gat	D	369	208.10
gac	D	1	161.90
gaa	E	2	1.19
gag	E	0	0.81
---	--	------	------
mPD		0.69	1.1
		nPD:	0.62
		N. weight:	1.
		Sc. PD:	0.14
		Sc. rank:	729.5

,

HA

Pos.	24	obs:	exp:
act	T	1	0.98
acc	T	0	0.86
aca	T	3	1.82
acg	T	0	0.33
gct	A	316	1108.00
gcc	A	77	1035.00
gca	A	2478	2037.00
gcg	A	1648	338.90
ggt	G	0	0.31
ggc	G	0	0.25
gga	G	1	0.91
ggg	G	1	0.53
---	--	------	------
mPD		0.56	0.68
		nPD:	0.83
		N. weight:	1.1
		Sc. PD:	0.36
		Sc. rank:	1798.6

,

HA

Pos.	25	obs:	exp:
gat	D	144	2545.00
gac	D	4381	1980.00
---	--	------	------
mPD		0.062	0.49
		nPD:	0.13
		N. weight:	1.3
		Sc. PD:	-0.44
		Sc. rank:	-2054.6

,

HA

Pos.	26	obs:	exp:
---	-	4525	4525.00
---	--	------	------
mPD		0	0
		nPD:	1.
		N. weight:	0.
		Sc. PD:	0
		Sc. rank:	0

,

HA

Pos.	27	obs:	exp:
---	-	4525	4525.00
---	--	------	------
mPD		0	0
		nPD:	1.
		N. weight:	0.
		Sc. PD:	0
		Sc. rank:	0

,

HA

Pos.	28	obs:	exp:
---	-	4525	4525.00
---	--	------	------
mPD		0	0
		nPD:	1.
		N. weight:	0.
		Sc. PD:	0
		Sc. rank:	0

,

HA

Pos.	29	obs:	exp:
---	-	4525	4525.00
---	--	------	------
mPD		0	0
		nPD:	1.
		N. weight:	0.
		Sc. PD:	0
		Sc. rank:	0

,

HA

Pos.	30	obs:	exp:
---	-	4525	4525.00
---	--	------	------
mPD		0	0
		nPD:	1.
		N. weight:	0.
		Sc. PD:	0
		Sc. rank:	0

,

HA

Pos.	31	obs:	exp:
---	-	4525	4525.00
---	--	------	------
mPD		0	0
		nPD:	1.
		N. weight:	0.
		Sc. PD:	0
		Sc. rank:	0

,

HA

Pos.	32	obs:	exp:
---	-	4525	4525.00
---	--	------	------
mPD		0	0
		nPD:	1.
		N. weight:	0.
		Sc. PD:	0
		Sc. rank:	0

,

HA

Pos.	33	obs:	exp:
---	-	4525	4525.00
---	--	------	------
mPD		0	0
		nPD:	1.
		N. weight:	0.
		Sc. PD:	0
		Sc. rank:	0

,

HA

Pos.	34	obs:	exp:
---	-	4525	4525.00
---	--	------	------
mPD		0	0
		nPD:	1.
		N. weight:	0.
		Sc. PD:	0
		Sc. rank:	0

,

HA

Pos.	35	obs:	exp:
---	-	4525	4525.00
---	--	------	------
mPD		0	0
		nPD:	1.
		N. weight:	0.
		Sc. PD:	0
		Sc. rank:	0

,

HA

Pos.	36	obs:	exp:
tct	S	0	0.34
tcc	S	0	0.32
tca	S	0	0.49
tcg	S	0	0.11
cct	P	0	0.30
ccc	P	0	0.20
cca	P	1	0.40
ccg	P	0	0.11
att	I	0	15.61
atc	I	0	11.29
ata	I	44	17.11
atg	M	170	170.00
act	T	15	1055.00
acc	T	219	927.90
aca	T	3886	1950.00
acg	T	171	357.80
aaa	K	16	10.33
aag	K	1	6.67
agt	S	0	0.37
agc	S	2	0.38
---	--	------	------
mPD		0.33	0.79
		nPD:	0.42
		N. weight:	0.97
		Sc. PD:	-0.056
		Sc. rank:	-378.3

,

HA

Pos.	37	obs:	exp:
tta	L	358	87.49
ttg	L	17	151.00
ctt	L	0	162.40
ctc	L	0	117.00
cta	L	214	140.40
ctg	L	215	145.70
att	I	257	1320.00
atc	I	13	954.40
ata	I	3451	1447.00
---	--	------	------
mPD		0.51	1.0
		nPD:	0.51
		N. weight:	1.4
		Sc. PD:	0.046
		Sc. rank:	145.4

,

HA

Pos.	38	obs:	exp:
tta	L	0	0.11
ttg	L	0	0.19
tct	S	0	0.17
tcc	S	0	0.16
tca	S	0	0.24
tcg	S	0	0.05
tgt	C	3532	1947.00
tgc	C	991	2576.00
ctt	L	0	0.20
ctc	L	0	0.15
cta	L	0	0.17
ctg	L	1	0.18
agt	S	0	0.19
agc	S	1	0.19
---	--	------	------
mPD		0.34	0.49
		nPD:	0.7
		N. weight:	0.51
		Sc. PD:	0.11
		Sc. rank:	589.0

,

HA

Pos.	39	obs:	exp:
tta	L	2	0.22
ttg	L	0	0.38
ctt	L	0	0.40
ctc	L	0	0.29
cta	L	0	0.35
ctg	L	0	0.36
cgt	R	0	0.03
cgc	R	0	0.05
cga	R	0	0.10
cgg	R	0	0.07
att	I	9	1178.00
atc	I	1	851.80
ata	I	3311	1291.00
aga	R	0	0.46
agg	R	1	0.28
gtt	V	2	266.70
gtc	V	0	226.60
gta	V	1108	303.20
gtg	V	91	404.50
---	--	------	------
mPD		0.44	1.1
		nPD:	0.4
		N. weight:	1.9
		Sc. PD:	-0.14
		Sc. rank:	-945.0

,

HA

Pos.	40	obs:	exp:
tgt	C	1	0.43
tgc	C	0	0.57
gct	A	0	0.25
gcc	A	1	0.23
gca	A	0	0.45
gcg	A	0	0.07
ggt	G	1216	709.40
ggc	G	1573	573.10
gga	G	1627	2049.00
ggg	G	107	1192.00
---	--	------	------
mPD		0.68	0.69
		nPD:	0.99
		N. weight:	0.71
		Sc. PD:	0.34
		Sc. rank:	1391.0

,

HA

Pos.	41	obs:	exp:
tat	Y	1416	2535.00
tac	Y	3109	1990.00
mPD		0.43	0.49
		nPD:	0.87
		N. weight:	0.25
		Sc. PD:	0.092
		Sc. rank:	432.4

,

HA

Pos.	42	obs:	exp:
cat	H	4263	2610.00
cac	H	262	1915.00
mPD		0.11	0.49
		nPD:	0.22
		N. weight:	0.68
		Sc. PD:	-0.16
		Sc. rank:	-825.7

,

HA

Pos.	43	obs:	exp:
tct	S	0	6.73
tcc	S	0	6.32
tca	S	40	9.71
tcg	S	0	2.13
agt	S	0	7.44
agc	S	0	7.67
gct	A	259	1099.00
gcc	A	2716	1027.00
gca	A	583	2021.00
gcg	A	926	336.30
ggt	G	0	0.16
ggc	G	1	0.13
gga	G	0	0.45
ggg	G	0	0.26
mPD		0.59	0.70
		nPD:	0.84
		N. weight:	1.1
		Sc. PD:	0.38
		Sc. rank:	1849.7

,

HA

Pos.	44	obs:	exp:
aat	N	832	2572.00
aac	N	3693	1953.00
mPD		0.30	0.49
		nPD:	0.61
		N. weight:	0.62
		Sc. PD:	0.079
		Sc. rank:	409.6

,

HA

Pos.	45	obs:	exp:
aat	N	1693	2572.00
aac	N	2832	1953.00
mPD		0.47	0.49
		nPD:	0.95
		N. weight:	0.15
		Sc. PD:	0.068
		Sc. rank:	293.5

,

HA

Pos.	46	obs:	exp:
tct	S	4	761.10
tcc	S	237	715.10
tca	S	4249	1099.00
tcg	S	35	240.50
agt	S	0	842.20
agc	S	0	867.20
mPD		0.12	1.6
		nPD:	0.07
		N. weight:	2.3
		Sc. PD:	-0.9
		Sc. rank:	-4166.2

,

HA

Pos.	47	obs:	exp:
tct	S	1	0.34
tcc	S	0	0.32
tca	S	0	0.49
tcg	S	0	0.11
cct	P	0	0.30
ccc	P	1	0.20
cca	P	0	0.40
ccg	P	0	0.11
act	T	2282	1112.00
acc	T	1831	977.80
aca	T	399	2055.00
acg	T	10	377.10
agt	S	0	0.37
agc	S	1	0.38
mPD		0.58	0.68
		nPD:	0.84
		N. weight:	0.92
		Sc. PD:	0.32
		Sc. rank:	1543.9

,

HA

Pos.	48	obs:	exp:
aat	N	0	13.64
aac	N	24	10.36
gat	D	102	2450.00
gac	D	4254	1906.00
gaa	E	142	85.92
gag	E	3	59.08
mPD		0.12	0.53
		nPD:	0.22
		N. weight:	1.4
		Sc. PD:	-0.34
		Sc. rank:	-1742.6

,

HA

Pos.	49	obs:	exp:
tct	S	0	0.17
tcc	S	0	0.16
tca	S	0	0.24
tcg	S	0	0.05
att	I	39	13.83
atc	I	0	10.00
ata	I	0	15.16
act	T	4371	1103.00
acc	T	88	969.80
aca	T	26	2038.00
acg	T	0	374.00
agt	S	1	0.19
agc	S	0	0.19
mPD		0.067	0.70
		nPD:	0.1
		N. weight:	2.5
		Sc. PD:	-0.89
		Sc. rank:	-4024.7

,

HA

Pos.	50	obs:	exp:
att	I	100	37.25
atc	I	3	26.93
ata	I	2	40.82
gtt	V	3661	981.00
gtc	V	352	833.70
gta	V	380	1115.00
gtg	V	25	1488.00
gct	A	1	0.25
gcc	A	0	0.23
gca	A	0	0.45
gcg	A	0	0.07
gat	D	1	0.56
gac	D	0	0.44
mPD		0.34	0.79
		nPD:	0.43
		N. weight:	1.8
		Sc. PD:	-0.07
		Sc. rank:	-369.3

,

HA

Pos.	51	obs:	exp:
cat	H	0	0.58
cac	H	1	0.42
gat	D	890	2545.00
gac	D	3634	1979.00
mPD		0.32	0.49
		nPD:	0.64
		N. weight:	0.55
		Sc. PD:	0.087
		Sc. rank:	442.4

,

HA

Pos.	52	obs:	exp:
tta	L	1	0.11
ttg	L	0	0.19
ctt	L	0	0.20
ctc	L	0	0.15
cta	L	0	0.17
ctg	L	0	0.18
act	T	92	1113.00
acc	T	94	978.10
aca	T	4257	2055.00
acg	T	80	377.20
aaa	K	1	0.61
aag	K	0	0.39
mPD		0.11	0.68
		nPD:	0.17
		N. weight:	1.1
		Sc. PD:	-0.32
		Sc. rank:	-1579.9

,

HA

Pos.	53	obs:	exp:
tta	L	28	4.35
ttg	L	0	7.51
ctt	L	0	8.08
ctc	L	0	5.82
cta	L	11	6.98
ctg	L	1	7.25
cct	P	0	0.30
ccc	P	0	0.20
cca	P	1	0.40
ccg	P	0	0.11
att	I	6	172.80
atc	I	16	124.90
ata	I	465	189.30
gtt	V	58	887.00
gtc	V	234	753.80
gta	V	3105	1008.00
gtg	V	598	1346.00
gct	A	0	0.25
gcc	A	1	0.23
gca	A	0	0.45
gcg	A	0	0.07
ggt	G	0	0.16
ggc	G	0	0.13
gga	G	1	0.45
ggg	G	0	0.26
mPD		0.55	0.95
		nPD:	0.58
		N. weight:	1.3
		Sc. PD:	0.13
		Sc. rank:	737.3

,

HA

Pos.	54	obs:	exp:
ttt	F	6	2.69
ttc	F	0	3.31
tta	L	348	485.90
ttg	L	77	838.70
ctt	L	2800	901.80
ctc	L	259	649.60
cta	L	592	779.60
ctg	L	389	809.40
cct	P	0	0.59
ccc	P	2	0.40
cca	P	0	0.79
ccg	P	0	0.22
cgt	R	0	0.03
cgc	R	0	0.05
cga	R	1	0.10
cgg	R	0	0.07
atg	M	39	39.00
aga	R	0	0.46
agg	R	0	0.28
gtt	V	0	2.66
gtc	V	0	2.26
gta	V	12	3.03
gtg	V	0	4.04
mPD		0.75	1.2
		nPD:	0.65
		N. weight:	0.97
		Sc. PD:	0.16
		Sc. rank:	825.1

,

HA

Pos.	55	obs:	exp:
cct	P	0	0.30
ccc	P	0	0.20
cca	P	0	0.40
ccg	P	1	0.11
aaa	K	3	1.82
aag	K	0	1.18
gct	A	0	0.25
gcc	A	1	0.23
gca	A	0	0.45
gcg	A	0	0.07
gaa	E	2366	2678.00
gag	E	2153	1841.00
ggt	G	0	0.16
ggc	G	0	0.13
gga	G	1	0.45
ggg	G	0	0.26
mPD		0.50	0.49
		nPD:	1.03
		N. weight:	0.024
		Sc. PD:	0.012
		Sc. rank:	46.7

,

HA

Pos.	56	obs:	exp:
tta	L	0	0.11
ttg	L	0	0.19
ctt	L	0	0.20
ctc	L	0	0.15
cta	L	0	0.17
ctg	L	1	0.18
caa	Q	0	0.54
cag	Q	1	0.46
cgt	R	0	1.04
cgc	R	0	1.74
cga	R	0	3.32
cgg	R	0	2.38
aaa	K	219	2729.00
aag	K	4271	1761.00
aga	R	5	15.13
agg	R	28	9.38
---	--	------	------
mPD		0.11	0.50
		nPD:	0.22
		N. weight:	1.4
		Sc. PD:	-0.34
		Sc. rank:	-1728.9

HA

Pos.	57	obs:	exp:
aat	N	4230	2571.00
aac	N	294	1953.00
aaa	K	1	0.61
aag	K	0	0.39
---	--	------	------
mPD		0.12	0.49
		nPD:	0.25
		N. weight:	0.67
		Sc. PD:	-0.14
		Sc. rank:	-721.5

HA

Pos.	58	obs:	exp:
att	I	0	1.77
atc	I	0	1.28
ata	I	5	1.94
gtt	V	98	1004.00
gtc	V	4	852.90
gta	V	1177	1141.00
gtg	V	3241	1522.00
---	--	------	------
mPD		0.42	0.74
		nPD:	0.57
		N. weight:	0.98
		Sc. PD:	0.085
		Sc. rank:	457.8

HA

Pos.	59	obs:	exp:
act	T	245	1095.00
acc	T	4	962.90
aca	T	4152	2023.00
acg	T	52	371.30
aaa	K	4	2.43
aag	K	0	1.57
gct	A	0	16.67
gcc	A	0	15.58
gca	A	68	30.65
gcg	A	0	5.10
---	--	------	------
mPD		0.16	0.71
		nPD:	0.22
		N. weight:	1.1
		Sc. PD:	-0.27
		Sc. rank:	-1365.5

HA

Pos.	60	obs:	exp:
gtt	V	225	1005.00
gtc	V	27	853.90
gta	V	1779	1142.00
gtg	V	2494	1524.00
---	--	------	------
mPD		0.54	0.74
		nPD:	0.73
		N. weight:	0.69
		Sc. PD:	0.17
		Sc. rank:	892.3

HA

Pos.	61	obs:	exp:
act	T	83	1113.00
acc	T	765	978.50
aca	T	3655	2056.00
acg	T	22	377.30
---	--	------	------
mPD		0.32	0.68
		nPD:	0.47
		N. weight:	0.71
		Sc. PD:	-0.0048
		Sc. rank:	-72.8

HA

Pos.	62	obs:	exp:
cct	P	0	0.30
ccc	P	1	0.20
cca	P	0	0.40
ccg	P	0	0.11
cat	H	535	2610.00
cac	H	3989	1914.00
---	--	------	------
mPD		0.21	0.49
		nPD:	0.43
		N. weight:	0.91
		Sc. PD:	-0.042
		Sc. rank:	-232.7

HA

Pos.	63	obs:	exp:
tct	S	4151	759.90
tcc	S	62	714.00
tca	S	299	1097.00
tcg	S	6	240.10
tat	Y	5	2.80
tac	Y	0	2.20
cgt	R	0	0.03
cgc	R	0	0.05
cga	R	0	0.10
cgg	R	0	0.07
agt	S	0	840.90
agc	S	0	865.90
aga	R	0	0.46
agg	R	1	0.28
gct	A	1	0.25
gcc	A	0	0.23
gca	A	0	0.45
gcg	A	0	0.07
---	--	------	------
mPD		0.16	1.6
		nPD:	0.1
		N. weight:	2.8
		Sc. PD:	-1.
		Sc. rank:	-4603.8

HA

Pos.	64	obs:	exp:
att	I	17	24.12
atc	I	51	17.44
ata	I	0	26.44
gtt	V	773	989.40
gtc	V	3656	840.80
gta	V	24	1125.00
gtg	V	3	1501.00
gaa	E	0	0.59
gag	E	1	0.41
---	--	------	------
mPD		0.33	0.77
		nPD:	0.43
		N. weight:	2.2
		Sc. PD:	-0.1
		Sc. rank:	-586.0

HA

Pos.	65	obs:	exp:
tct	S	0	1.35
tcc	S	0	1.26
tca	S	0	1.94
tcg	S	0	0.43
act	T	0	0.25
acc	T	1	0.22
aca	T	0	0.45
acg	T	0	0.08
aat	N	602	2561.00
aac	N	3903	1944.00
agt	S	1	1.49
agc	S	7	1.53
gat	D	10	6.19
gac	D	1	4.81
---	--	------	------
mPD		0.24	0.50
		nPD:	0.48
		N. weight:	0.81
		Sc. PD:	0.0059
		Sc. rank:	-26.9

HA

Pos.	66	obs:	exp:
tta	L	224	492.30
ttg	L	33	849.80
ctt	L	979	913.70
ctc	L	400	658.20
cta	L	2870	789.90
ctg	L	18	820.10
att	I	1	0.35
atc	I	0	0.26
ata	I	0	0.39
---	--	------	------
mPD		0.59	1.1
		nPD:	0.51
		N. weight:	1.4
		Sc. PD:	0.05
		Sc. rank:	177.6

HA

Pos.	67	obs:	exp:
tta	L	83	492.30
ttg	L	0	849.80
ctt	L	3376	913.70
ctc	L	19	658.20
cta	L	994	789.90
ctg	L	52	820.10
att	I	1	0.35
atc	I	0	0.26
ata	I	0	0.39
---	--	------	------
mPD		0.42	1.1
		nPD:	0.37
		N. weight:	1.9
		Sc. PD:	-0.18
		Sc. rank:	-1083.4

HA

Pos.	68	obs:	exp:
tta	L	0	0.11
ttg	L	1	0.19
ctt	L	0	0.20
ctc	L	0	0.15
cta	L	0	0.17
ctg	L	0	0.18
aaa	K	1	0.61
aag	K	0	0.39
gaa	E	2156	2680.00
gag	E	2367	1843.00
---	--	------	------
mPD		0.50	0.48
		nPD:	1.03
		N. weight:	0.056
		Sc. PD:	0.03
		Sc. rank:	111.7

HA

Pos.	69	obs:	exp:
tct	S	0	3.36
tcc	S	0	3.16
tca	S	0	4.86
tcg	S	0	1.06
tat	Y	0	1.12
tac	Y	2	0.88
act	T	0	77.48
acc	T	315	68.12
aca	T	0	143.10
acg	T	0	26.27
aat	N	7	251.20
aac	N	435	190.80
agt	S	1	3.72
agc	S	19	3.83
gat	D	30	2064.00
gac	D	3640	1606.00
gaa	E	73	43.25
gag	E	0	29.75
ggt	G	0	0.47
ggc	G	3	0.38
gga	G	0	1.36
ggg	G	0	0.79
---	--	------	------
mPD		0.47	0.97
		nPD:	0.49
		N. weight:	1.6
		Sc. PD:	0.017
		Sc. rank:	6.6

HA

Pos.	70	obs:	exp:
tct	S	0	524.10
tcc	S	0	492.50
tca	S	0	756.70
tcg	S	0	165.60
tgg	W	1	1.00
caa	Q	1	0.54
cag	Q	0	0.46
cgt	R	5	7.23
cgc	R	0	12.10
cga	R	0	23.02
cgg	R	0	16.54
att	I	1	1.06
atc	I	2	0.77
ata	I	0	1.17
act	T	46	79.94
acc	T	9	70.28
aca	T	251	147.70
acg	T	19	27.10
aat	N	56	63.09
aac	N	55	47.91
aaa	K	593	444.90
aag	K	139	287.10
agt	S	1867	580.00
agc	S	1249	597.20
aga	R	79	105.00
agg	R	145	65.09
gct	A	0	0.25
gcc	A	0	0.23
gca	A	1	0.45
gcg	A	0	0.07
ggt	G	3	0.94
ggc	G	3	0.76
gga	G	0	2.72
ggg	G	0	1.58
---	--	------	------
mPD		1.1	1.9
		nPD:	0.59
		N. weight:	1.5
		Sc. PD:	0.16
		Sc. rank:	902.4

HA

Pos.	71	obs:	exp:
tat	Y	2	1.68
tac	Y	1	1.32
cat	H	1364	2599.00
cac	H	3142	1907.00
caa	Q	2	1.08
cag	Q	0	0.92
aat	N	0	7.96
aac	N	14	6.04
---	--	------	------
mPD		0.43	0.50
		nPD:	0.87
		N. weight:	0.31
		Sc. PD:	0.11
		Sc. rank:	539.4

,

HA

Pos.	72	obs:	exp:
ttt	F	0	0.45
ttc	F	1	0.55
tct	S	0	0.17
tcc	S	0	0.16
tca	S	0	0.24
tcg	S	0	0.05
aat	N	3976	2550.00
aac	N	510	1936.00
agt	S	0	0.19
agc	S	1	0.19
gat	D	4	20.81
gac	D	33	16.19
---	--	------	------
mPD		0.23	0.51
		nPD:	0.45
		N. weight:	0.48
		Sc. PD:	-0.011
		Sc. rank:	-80.7

,

HA

Pos.	73	obs:	exp:
cgt	R	0	0.13
cgc	R	0	0.21
cga	R	0	0.40
cgg	R	0	0.29
aaa	K	1	0.61
aag	K	0	0.39
aga	R	1	1.83
agg	R	3	1.14
gaa	E	64	41.48
gag	E	6	28.52
ggt	G	0	698.00
ggc	G	2	563.90
gga	G	1511	2016.00
ggg	G	2937	1173.00
---	--	------	------
mPD		0.49	0.72
		nPD:	0.68
		N. weight:	0.99
		Sc. PD:	0.19
		Sc. rank:	1019.5

,

HA

Pos.	74	obs:	exp:
caa	Q	6	3.24
cag	Q	0	2.76
cgt	R	0	2.34
cgc	R	0	3.91
cga	R	0	7.44
cgg	R	0	5.35
atg	M	1	1.00
aaa	K	2952	2700.00
aag	K	1490	1742.00
aga	R	25	33.94
agg	R	49	21.03
gaa	E	2	1.19
gag	E	0	0.81
---	--	------	------
mPD		0.49	0.52
		nPD:	0.93
		N. weight:	0.033
		Sc. PD:	0.014
		Sc. rank:	62.8

,

HA

Pos.	75	obs:	exp:
tta	L	499	492.20
ttg	L	27	849.60
ctt	L	10	913.50
ctc	L	221	658.10
cta	L	2042	789.80
ctg	L	1724	819.90
cgt	R	0	0.03
cgc	R	0	0.05
cga	R	0	0.10
cgg	R	1	0.07
atg	M	1	1.00
aga	R	0	0.46
agg	R	0	0.28
---	--	------	------
mPD		0.74	1.1
		nPD:	0.65
		N. weight:	1.2
		Sc. PD:	0.2
		Sc. rank:	1042.4

,

HA

Pos.	76	obs:	exp:
tct	S	0	0.17
tcc	S	0	0.16
tca	S	0	0.24
tcg	S	0	0.05
tgt	C	1586	1947.00
tgc	C	2938	2577.00
agt	S	1	0.19
agc	S	0	0.19
---	--	------	------
mPD		0.46	0.49
		nPD:	0.93
		N. weight:	0.029
		Sc. PD:	0.012
		Sc. rank:	54.7

,

HA

Pos.	77	obs:	exp:
tta	L	26	288.80
ttg	L	0	498.50
tct	S	0	38.69
tcc	S	0	36.35
tca	S	7	55.85
tcg	S	0	12.22
ctt	L	1	536.00
ctc	L	0	386.10
cta	L	2579	463.40
ctg	L	48	481.10
caa	Q	15	8.09
cag	Q	0	6.91
cgt	R	0	9.44
cgc	R	0	15.80
cga	R	66	30.05
cgg	R	0	21.60
aat	N	23	21.60
aac	N	15	16.40
aaa	K	1087	778.50
aag	K	194	502.50
agt	S	29	42.81
agc	S	194	44.08
aga	R	230	137.10
agg	R	3	84.98
ggt	G	0	1.25
ggc	G	2	1.01
gga	G	5	3.62
ggg	G	1	2.11
---	--	------	------
mPD		1.3	1.9
		nPD:	0.66
		N. weight:	2.1
		Sc. PD:	0.37
		Sc. rank:	1922.6

,

HA

Pos.	78	obs:	exp:
tta	L	968	489.80
ttg	L	111	845.40
ctt	L	1	909.00
ctc	L	2	654.90
cta	L	3050	785.90
ctg	L	369	815.90
att	I	0	2.84
atc	I	0	2.05
ata	I	8	3.11
atg	M	16	16.00
---	--	------	------
mPD		0.57	1.1
		nPD:	0.5
		N. weight:	1.9
		Sc. PD:	0.035
		Sc. rank:	122.4

,

HA

Pos.	79	obs:	exp:
tct	S	0	0.50
tcc	S	0	0.47
tca	S	1	0.73
tcg	S	0	0.16
tgg	W	1	1.00
caa	Q	12	6.47
cag	Q	0	5.53
cgt	R	0	18.02
cgc	R	0	30.18
cga	R	69	57.39
cgg	R	0	41.25
aat	N	268	156.90
aac	N	8	119.10
aaa	K	1231	1819.00
aag	K	1762	1174.00
agt	S	1	0.56
agc	S	1	0.57
aga	R	373	261.90
agg	R	129	162.30
gat	D	2	1.12
gac	D	0	0.88
gaa	E	12	91.84
gag	E	143	63.16
ggt	G	0	80.31
ggc	G	0	64.88
gga	G	34	231.90
ggg	G	478	134.90
---	--	------	------
mPD		1.2	1.3
		nPD:	0.95
		N. weight:	0.49
		Sc. PD:	0.22
		Sc. rank:	950.8

,

HA

Pos.	80	obs:	exp:
aaa	K	1	0.61
aag	K	0	0.39
gtt	V	0	0.22
gtc	V	0	0.19
gta	V	1	0.25
gtg	V	0	0.34
gct	A	0	0.25
gcc	A	0	0.23
gca	A	1	0.45
gcg	A	0	0.07
gaa	E	8	4.74
gag	E	0	3.26
ggt	G	1	708.00
ggc	G	0	572.00
gga	G	3295	2045.00
ggg	G	1218	1189.00
---	--	------	------
mPD		0.40	0.69
		nPD:	0.58
		N. weight:	0.7
		Sc. PD:	0.067
		Sc. rank:	386.7

,

HA

Pos.	81	obs:	exp:
tta	L	0	0.33
ttg	L	1	0.56
ctt	L	0	0.61
ctc	L	0	0.44
cta	L	1	0.52
ctg	L	1	0.54
cgt	R	0	1.29
cgc	R	0	2.17
cga	R	1	4.12
cgg	R	0	2.96
att	I	16	1024.00
atc	I	0	740.20
ata	I	2870	1122.00
atg	M	385	385.00
act	T	0	3.69
acc	T	0	3.24
aca	T	6	6.82
acg	T	9	1.25
aat	N	1	0.57
aac	N	0	0.43
aaa	K	453	391.40
aag	K	191	252.60
aga	R	0	18.80
agg	R	40	11.65
gtt	V	1	119.00
gtc	V	0	101.10
gta	V	456	135.30
gtg	V	79	180.50
gaa	E	5	4.15
gag	E	2	2.85
ggt	G	0	1.10
ggc	G	0	0.89
gga	G	7	3.17
ggg	G	0	1.84
---	--	------	------
mPD		0.76	1.2
		nPD:	0.62
		N. weight:	1.4
		Sc. PD:	0.19
		Sc. rank:	1011.2

,

HA

Pos.	82	obs:	exp:
tct	S	1	19.01
tcc	S	112	17.86
tca	S	0	27.44
tcg	S	0	6.01
tgt	C	0	0.43
tgc	C	1	0.57
cct	P	1	26.26
ccc	P	88	17.92
cca	P	0	35.24
ccg	P	0	9.57
att	I	0	3.19
atc	I	9	2.31
ata	I	0	3.50
act	T	2	18.45
acc	T	73	16.22
aca	T	0	34.08
acg	T	0	6.25
aat	N	0	1.71
aac	N	3	1.29
agt	S	0	21.03
agc	S	0	21.66
gtt	V	0	1.55
gtc	V	7	1.32
gta	V	0	1.77
gtg	V	0	2.36
gct	A	1163	1036.00
gcc	A	3058	968.70
gca	A	5	1906.00
gcg	A	2	317.10
---	--	------	------
mPD		0.52	0.83
		nPD:	0.62
		N. weight:	1.8
		Sc. PD:	0.24
		Sc. rank:	1270.8

,

HA

Pos.	83	obs:	exp:
cct	P	153	1335.00
ccc	P	1678	911.00
cca	P	2367	1792.00
ccg	P	327	486.80
---	--	------	------
mPD		0.58	0.70
		nPD:	0.83
		N. weight:	0.54
		Sc. PD:	0.18
		Sc. rank:	879.9

,

HA

Pos.	84	obs:	exp:
ttt	F	0	0.45
ttc	F	1	0.55
tta	L	359	487.60
ttg	L	871	841.70
tct	S	0	0.34
tcc	S	0	0.32
tca	S	0	0.49
tcg	S	2	0.11
ctt	L	1	905.00
ctc	L	0	652.00
cta	L	2730	782.40
ctg	L	520	812.30
caa	Q	1	0.54
cag	Q	0	0.46
att	I	2	14.19
atc	I	0	10.26
ata	I	38	15.55
agt	S	0	0.37
agc	S	0	0.38
---	--	------	------
mPD		0.84	1.2
		nPD:	0.73
		N. weight:	1.4
		Sc. PD:	0.32
		Sc. rank:	1750.3

,

HA

Pos.	85	obs:	exp:
tat	Y	6	233.10
tac	Y	410	182.90
cat	H	796	478.80
cac	H	34	351.20
caa	Q	3230	1764.00
cag	Q	42	1508.00
cgt	R	0	0.06
cgc	R	0	0.11
cga	R	2	0.20
cgg	R	0	0.14
aat	N	0	2.27
aac	N	4	1.73
aaa	K	1	0.61
aag	K	0	0.39
aga	R	0	0.92
agg	R	0	0.57
---	--	------	------
mPD		0.62	0.87
		nPD:	0.71
		N. weight:	1.1
		Sc. PD:	0.24
		Sc. rank:	1290.4

,

HA

Pos.	86	obs:	exp:
tta	L	303	492.20
ttg	L	3563	849.60
tct	S	0	0.17
tcc	S	0	0.16
tca	S	0	0.24
tcg	S	1	0.05
ctt	L	14	913.50
ctc	L	0	658.10
cta	L	138	789.80
ctg	L	505	819.90
agt	S	0	0.19
agc	S	0	0.19
gtt	V	0	0.22
gtc	V	1	0.19
gta	V	0	0.25
gtg	V	0	0.34
---	--	------	------
mPD		0.43	1.1
		nPD:	0.38
		N. weight:	1.9
		Sc. PD:	-0.18
		Sc. rank:	-993.6

,

HA

Pos.	87	obs:	exp:
---	-	4525	4525.00
---	--	------	------
mPD		0	0
		nPD:	1.
		N. weight:	0.
		Sc. PD:	0
		Sc. rank:	0

,

HA

Pos.	88	obs:	exp:
---	-	4525	4525.00
---	--	------	------
mPD		0	0
		nPD:	1.
		N. weight:	0.
		Sc. PD:	0
		Sc. rank:	0

,

HA

Pos.	89	obs:	exp:
---	-	4525	4525.00
---	--	------	------
mPD		0	0
		nPD:	1.
		N. weight:	0.
		Sc. PD:	0
		Sc. rank:	0

,

HA

Pos.	90	obs:	exp:
tct	S	0	0.84
tcc	S	0	0.79
tca	S	0	1.21
tcg	S	0	0.27
cgt	R	0	0.35
cgc	R	0	0.58
cga	R	0	1.11
cgg	R	0	0.79
aaa	K	1	0.61
aag	K	0	0.39
agt	S	5	0.93
agc	S	0	0.96
aga	R	1	5.04
agg	R	10	3.13
gct	A	0	0.25
gcc	A	1	0.23
gca	A	0	0.45
gcg	A	0	0.07
gat	D	3	1.69
gac	D	0	1.31
gaa	E	1	1.78
gag	E	2	1.22
ggt	G	3819	706.00
ggc	G	131	570.30
gga	G	115	2039.00
ggg	G	436	1186.00
---	--	------	------
mPD		0.28	0.70
		nPD:	0.41
		N. weight:	2.4
		Sc. PD:	-0.15
		Sc. rank:	-1003.2

,

HA			
Pos.	91	obs:	exp:
tct	S	0	167.50
tcc	S	0	157.40
tca	S	0	241.90
tcg	S	0	52.94
cat	H	1	1.73
cac	H	2	1.27
cgt	R	0	4.26
cgc	R	0	7.14
cga	R	0	13.57
cgg	R	0	9.75
act	T	1	3.94
acc	T	0	3.46
aca	T	15	7.27
acg	T	0	1.33
aat	N	1835	1118.00
aac	N	132	849.00
aaa	K	1340	827.10
aag	K	21	533.90
agt	S	975	185.40
agc	S	21	190.90
aga	R	134	61.91
agg	R	1	38.37
gat	D	27	22.50
gac	D	13	17.50
gaa	E	4	2.37
gag	E	0	1.63
ggt	G	3	0.47
ggc	G	0	0.38
gga	G	0	1.36
ggg	G	0	0.79
---	--	------	------
mPD		0.90	1.4
		nPD:	0.64
		N. weight:	1.3
		Sc. PD:	0.19
		Sc. rank:	977.9

HA			
Pos.	92	obs:	exp:
tgt	C	1142	1948.00
tgc	C	3383	2577.00
---	--	------	------
mPD		0.38	0.49
		nPD:	0.77
		N. weight:	0.14
		Sc. PD:	0.038
		Sc. rank:	192.3

HA			
Pos.	93	obs:	exp:
tct	S	0	511.30
tcc	S	0	480.40
tca	S	0	738.20
tcg	S	0	161.60
att	I	0	0.35
atc	I	1	0.26
ata	I	0	0.39
act	T	0	1.23
acc	T	4	1.08
aca	T	1	2.27
acg	T	0	0.42
aat	N	90	840.10
aac	N	1388	637.90
agt	S	99	565.80
agc	S	2941	582.60
gat	D	0	0.56
gac	D	1	0.44
---	--	------	------
mPD		0.52	1.8
		nPD:	0.29
		N. weight:	2.4
		Sc. PD:	-0.41
		Sc. rank:	-2206.7

HA			
Pos.	94	obs:	exp:
tta	L	0	0.11
ttg	L	0	0.19
ctt	L	1	0.20
ctc	L	0	0.15
cta	L	0	0.17
ctg	L	0	0.18
att	I	1400	569.00
atc	I	159	411.40
ata	I	45	623.60
aat	N	1	0.57
aac	N	0	0.43
gtt	V	2684	647.50
gtc	V	13	550.20
gta	V	204	736.10
gtg	V	15	982.20
gct	A	0	0.74
gcc	A	3	0.69
gca	A	0	1.35
gcg	A	0	0.22
---	--	------	------
mPD		0.64	1.2
		nPD:	0.53
		N. weight:	1.9
		Sc. PD:	0.1
		Sc. rank:	440.4

HA			
Pos.	95	obs:	exp:
tct	S	0	0.17
tcc	S	0	0.16
tca	S	1	0.24
tcg	S	0	0.05
act	T	1	0.49
acc	T	1	0.43
aca	T	0	0.91
acg	T	0	0.17
agt	S	0	0.19
agc	S	0	0.19
gtt	V	0	0.67
gtc	V	3	0.57
gta	V	0	0.76
gtg	V	0	1.01
gct	A	1114	1108.00
gcc	A	3051	1035.00
gca	A	326	2037.00
gcg	A	28	338.90
---	--	------	------
mPD		0.48	0.68
		nPD:	0.71
		N. weight:	1.2
		Sc. PD:	0.25
		Sc. rank:	1361.2

HA			
Pos.	96	obs:	exp:
ggt	G	9	709.80
ggc	G	365	573.40
gga	G	3850	2050.00
ggg	G	301	1192.00
---	--	------	------
mPD		0.27	0.68
		nPD:	0.39
		N. weight:	0.79
		Sc. PD:	-0.066
		Sc. rank:	-403.2

HA			
Pos.	97	obs:	exp:
tgg	W	4524	4524.00
cgt	R	0	0.03
cgc	R	0	0.05
cga	R	0	0.10
cgg	R	0	0.07
aga	R	0	0.46
agg	R	1	0.28
---	--	------	------
mPD		0.00044	0.00073
		nPD:	0.61
		N. weight:	0.00055
		Sc. PD:	0.000068
		Sc. rank:	0.4

HA			
Pos.	98	obs:	exp:
tta	L	0	79.33
ttg	L	0	136.90
ctt	L	519	147.20
ctc	L	208	106.10
cta	L	2	127.30
ctg	L	0	132.10
att	I	258	1327.00
atc	I	1692	959.30
ata	I	1790	1454.00
atg	M	2	2.00
gtt	V	39	11.99
gtc	V	9	10.19
gta	V	5	13.63
gtg	V	1	18.19
---	--	------	------
mPD		0.92	1.0
		nPD:	0.93
		N. weight:	0.76
		Sc. PD:	0.32
		Sc. rank:	1445.8

HA			
Pos.	99	obs:	exp:
tta	L	2879	492.30
ttg	L	782	849.80
tct	S	0	0.17
tcc	S	1	0.16
tca	S	0	0.24
tcg	S	0	0.05
ctt	L	225	913.70
ctc	L	2	658.20
cta	L	128	789.90
ctg	L	508	820.10
agt	S	0	0.19
agc	S	0	0.19
---	--	------	------
mPD		0.78	1.1
		nPD:	0.69
		N. weight:	1.8
		Sc. PD:	0.37
		Sc. rank:	1991.0

HA			
Pos.	100	obs:	exp:
cgt	R	0	0.06
cgc	R	0	0.11
cga	R	0	0.20
cgg	R	0	0.14
aga	R	1	0.92
agg	R	1	0.57
ggt	G	42	709.40
ggc	G	209	573.10
gga	G	3304	2049.00
ggg	G	968	1192.00
---	--	------	------
mPD		0.42	0.69
		nPD:	0.61
		N. weight:	0.46
		Sc. PD:	0.059
		Sc. rank:	304.4

HA			
Pos.	101	obs:	exp:
tct	S	0	0.34
tcc	S	0	0.32
tca	S	0	0.49
tcg	S	0	0.11
act	T	0	0.25
acc	T	1	0.22
aca	T	0	0.45
acg	T	0	0.08
aat	N	907	2570.00
aac	N	3615	1952.00
agt	S	0	0.37
agc	S	2	0.38
---	--	------	------
mPD		0.32	0.49
		nPD:	0.65
		N. weight:	0.56
		Sc. PD:	0.094
		Sc. rank:	480.0

HA			
Pos.	102	obs:	exp:
tct	S	0	0.17
tcc	S	0	0.16
tca	S	1	0.24
tcg	S	0	0.05
cct	P	24	1335.00
ccc	P	3	910.80
cca	P	4406	1792.00
ccg	P	91	486.60
agt	S	0	0.19
agc	S	0	0.19
---	--	------	------
mPD		0.051	0.70
		nPD:	0.07
		N. weight:	1.6
		Sc. PD:	-0.61
		Sc. rank:	-2840.6

HA			
Pos.	103	obs:	exp:
cgt	R	0	0.03
cgc	R	0	0.05
cga	R	0	0.10
cgg	R	0	0.07
aat	N	9	5.68
aac	N	1	4.32
aaa	K	67	46.19
aag	K	9	29.81
aga	R	1	0.46
agg	R	0	0.28
gtt	V	0	0.44
gtc	V	0	0.38
gta	V	1	0.50
gtg	V	1	0.67
gct	A	0	2.21
gcc	A	0	2.06
gca	A	5	4.06
gcg	A	4	0.67
gat	D	26	15.19
gac	D	1	11.81
gaa	E	2422	2582.00
gag	E	1936	1776.00
ggt	G	0	6.59
ggc	G	2	5.32
gga	G	11	19.02
ggg	G	29	11.07
---	--	------	------
mPD		0.56	0.56
		nPD:	1.01
		N. weight:	0.036
		Sc. PD:	0.018
		Sc. rank:	72.1

HA			
Pos.	104	obs:	exp:
tgt	C	1622	1948.00
tgc	C	2903	2577.00
---	--	------	------
mPD		0.46	0.49
		nPD:	0.94
		N. weight:	0.023
		Sc. PD:	0.01
		Sc. rank:	44.2

HA			
Pos.	105	obs:	exp:
aat	N	0	7.96
aac	N	14	6.04
aaa	K	11	6.69
aag	K	0	4.31
gat	D	52	160.90
gac	D	234	125.10
gaa	E	4092	2473.00
gag	E	82	1701.00
ggt	G	1	6.27
ggc	G	0	5.07
gga	G	39	18.12
ggg	G	0	10.54
---	--	------	------
mPD		0.19	0.58
		nPD:	0.32
		N. weight:	0.85
		Sc. PD:	-0.12
		Sc. rank:	-671.2

HA			
Pos.	106	obs:	exp:
ttt	F	35	16.13
ttc	F	1	19.87
tta	L	118	124.20
ttg	L	752	214.30
tct	S	5	477.90
tcc	S	8	449.00
tca	S	2800	689.90
tcg	S	28	151.00
ctt	L	0	230.40
ctc	L	0	166.00
cta	L	5	199.20
ctg	L	266	206.80
cct	P	0	18.89
ccc	P	0	12.89
cca	P	57	25.34
ccg	P	7	6.88
caa	Q	0	1.08
cag	Q	2	0.92
cgt	R	0	0.19
cgc	R	0	0.32
cga	R	0	0.60
cgg	R	6	0.43
att	I	0	88.69
atc	I	0	64.12
ata	I	250	97.19
atg	M	145	145.00
act	T	0	7.38
acc	T	0	6.49
aca	T	30	13.63
acg	T	0	2.50
aaa	K	1	0.61
aag	K	0	0.39
agt	S	0	528.80
agc	S	0	544.50
aga	R	0	2.75
agg	R	0	1.71
gtt	V	0	1.78
gtc	V	0	1.51
gta	V	8	2.02
gtg	V	0	2.69
gct	A	0	0.25
gcc	A	0	0.23
gca	A	1	0.45
gcg	A	0	0.07
---	--	------	------
mPD		1.2	2.0
		nPD:	0.58
		N. weight:	2.3
		Sc. PD:	0.21
		Sc. rank:	1180.8

,

HA			
Pos.	107	obs:	exp:
tta	L	17	443.70
ttg	L	51	765.80
tct	S	0	1.18
tcc	S	0	1.11
tca	S	0	1.70
tcg	S	7	0.37
ctt	L	16	823.40
ctc	L	386	593.20
cta	L	645	711.90
ctg	L	2962	739.10
cct	P	0	0.89
ccc	P	0	0.60
cca	P	2	1.19
ccg	P	1	0.32
caa	Q	0	11.86
cag	Q	22	10.14
cgt	R	0	0.03
cgc	R	0	0.05
cga	R	0	0.10
cgg	R	1	0.07
att	I	1	140.80
atc	I	1	101.80
ata	I	395	154.30
act	T	0	2.71
acc	T	0	2.38
aca	T	11	5.00
acg	T	0	0.92
agt	S	0	1.30
agc	S	0	1.34
aga	R	0	0.46
agg	R	0	0.28
gtt	V	0	1.55
gtc	V	0	1.32
gta	V	7	1.77
gtg	V	0	2.36
---	--	------	------
mPD		0.70	1.3
		nPD:	0.55
		N. weight:	1.8
		Sc. PD:	0.13
		Sc. rank:	669.6

,

HA			
Pos.	108	obs:	exp:
ttt	F	283	237.90
ttc	F	248	293.10
tta	L	4	73.24
ttg	L	3	126.40
tct	S	9	98.90
tcc	S	561	92.93
tca	S	11	142.80
tcg	S	0	31.25
tat	Y	0	1.68
tac	Y	3	1.32
ctt	L	74	135.90
ctc	L	590	97.92
cta	L	2	117.50
ctg	L	0	122.00
cct	P	1	13.57
ccc	P	45	9.26
cca	P	0	18.22
ccg	P	0	4.95
att	I	2075	756.30
atc	I	55	546.80
ata	I	2	828.90
act	T	367	92.00
acc	T	4	80.87
aca	T	0	169.90
acg	T	3	31.19
aat	N	160	90.94
aac	N	0	69.06
aaa	K	2	1.22
aag	K	0	0.78
agt	S	7	109.40
agc	S	0	112.70
gtt	V	8	2.22
gtc	V	2	1.89
gta	V	0	2.52
gtg	V	0	3.37
gct	A	5	1.23
gcc	A	0	1.15
gca	A	0	2.25
gcg	A	0	0.37
gat	D	1	0.56
gac	D	0	0.44
---	--	------	------
mPD		1.4	1.6
		nPD:	0.86
		N. weight:	2.
		Sc. PD:	0.73
		Sc. rank:	3465.4

,

HA			
Pos.	109	obs:	exp:
ttt	F	3	5.38
ttc	F	9	6.62
tct	S	298	499.90
tcc	S	2657	469.70
tca	S	15	721.70
tcg	S	0	158.00
tat	Y	0	1.12
tac	Y	2	0.88
cct	P	4	24.20
ccc	P	77	16.51
cca	P	1	32.47
ccg	P	0	8.82
act	T	2	338.50
acc	T	6	297.50
aca	T	1352	625.20
acg	T	16	114.70
aat	N	28	15.91
aac	N	0	12.09
aaa	K	26	28.56
aag	K	21	18.44
agt	S	2	553.20
agc	S	0	569.60
gct	A	0	1.47
gcc	A	0	1.37
gca	A	6	2.70
gcg	A	0	0.45
---	--	------	------
mPD		1.0	1.6
		nPD:	0.64
		N. weight:	2.4
		Sc. PD:	0.37
		Sc. rank:	1867.2

,

HA			
Pos.	110	obs:	exp:
tct	S	0	5.55
tcc	S	0	5.22
tca	S	28	8.01
tcg	S	4	1.75
caa	Q	0	4.31
cag	Q	8	3.69
cgt	R	0	2.81
cgc	R	0	4.70
cga	R	0	8.95
cgg	R	0	6.43
att	I	0	0.35
atc	I	0	0.26
ata	I	1	0.39
act	T	0	4.92
acc	T	0	4.32
aca	T	13	9.09
acg	T	7	1.67
aat	N	4	2.27
aac	N	0	1.73
aaa	K	129	1801.00
aag	K	2835	1163.00
agt	S	1	6.14
agc	S	0	6.32
aga	R	10	40.82
agg	R	79	25.30
gtt	V	0	161.20
gtc	V	0	137.00
gta	V	687	183.30
gtg	V	39	244.50
gct	A	2	164.50
gcc	A	2	153.70
gca	A	590	302.50
gcg	A	77	50.32
gaa	E	9	5.33
gag	E	0	3.67
---	--	------	------
mPD		1.4	1.6
		nPD:	0.89
		N. weight:	1.6
		Sc. PD:	0.61
		Sc. rank:	2834.0

,

HA			
Pos.	111	obs:	exp:
tct	S	0	165.30
tcc	S	0	155.40
tca	S	0	238.70
tcg	S	0	52.24
caa	Q	1	0.54
cag	Q	0	0.46
cgt	R	0	1.20
cgc	R	0	2.01
cga	R	0	3.82
cgg	R	0	2.75
att	I	0	0.35
atc	I	1	0.26
ata	I	0	0.39
act	T	5	2.21
acc	T	0	1.95
aca	T	3	4.09
acg	T	1	0.75
aat	N	215	241.00
aac	N	209	183.00
aaa	K	1494	922.50
aag	K	24	595.50
agt	S	36	183.00
agc	S	947	188.40
aga	R	38	17.43
agg	R	0	10.80
gtt	V	0	2.66
gtc	V	0	2.26
gta	V	12	3.03
gtg	V	0	4.04
gat	D	5	6.75
gac	D	7	5.25
gaa	E	1510	902.40
gag	E	13	620.60
ggt	G	0	0.63
ggc	G	3	0.51
gga	G	1	1.81
ggg	G	0	1.05
---	--	------	------
mPD		1.3	1.6
		nPD:	0.78
		N. weight:	1.3
		Sc. PD:	0.36
		Sc. rank:	1840.4

,

HA			
Pos.	112	obs:	exp:
tta	L	1	0.11
ttg	L	0	0.19
tct	S	21	759.90
tcc	S	2	714.00
tca	S	4326	1097.00
tcg	S	169	240.10
ctt	L	0	0.20
ctc	L	0	0.15
cta	L	0	0.17
ctg	L	0	0.18
cct	P	0	0.59
ccc	P	0	0.40
cca	P	1	0.79
ccg	P	1	0.22
act	T	0	0.98
acc	T	0	0.86
aca	T	4	1.82
acg	T	0	0.33
agt	S	0	840.90
agc	S	0	865.90
---	--	------	------
mPD		0.085	1.6
		nPD:	0.05
		N. weight:	2.5
		Sc. PD:	-1.
		Sc. rank:	-4733.8

,

HA			
Pos.	113	obs:	exp:
tgg	W	4525	4525.00
---	--	------	------
mPD		0	0
		nPD:	1.
		N. weight:	0.
		Sc. PD:	0
		Sc. rank:	0

,

HA			
Pos.	114	obs:	exp:
tct	S	1102	760.60
tcc	S	3366	714.70
tca	S	47	1098.00
tcg	S	7	240.30
cct	P	0	0.59
ccc	P	2	0.40
cca	P	0	0.79
ccg	P	0	0.22
agt	S	0	841.70
agc	S	0	866.60
gct	A	0	0.25
gcc	A	1	0.23
gca	A	0	0.45
gcg	A	0	0.07
---	--	------	------
mPD		0.39	1.6
		nPD:	0.24
		N. weight:	2.4
		Sc. PD:	-0.53
		Sc. rank:	-2702.1

,

HA			
Pos.	115	obs:	exp:
tat	Y	650	2535.00
tac	Y	3874	1989.00
aat	N	0	0.57
aac	N	1	0.43
---	--	------	------
mPD		0.25	0.49
		nPD:	0.5
		N. weight:	0.74
		Sc. PD:	0.016
		Sc. rank:	56.5

,

HA

Pos.	116	obs:	exp:
ttt	F	1	0.45
ttc	F	0	0.55
tta	L	0	0.22
ttg	L	0	0.38
ctt	L	2	0.40
ctc	L	0	0.29
cta	L	0	0.35
ctg	L	0	0.36
att	I	4204	1602.00
atc	I	31	1158.00
ata	I	281	1756.00
atg	M	2	2.00
act	T	1	0.25
acc	T	0	0.22
aca	T	0	0.45
acg	T	0	0.08
gtt	V	3	0.67
gtc	V	0	0.57
gta	V	0	0.76
gtg	V	0	1.01
---	--	------	------
mPD		0.13	0.66
		nPD :	0.2
		N. weight :	1.5
		Sc. PD :	-0.39
		Sc. rank :	-1972.2

,

HA

Pos.	117	obs:	exp:
att	I	0	84.78
atc	I	0	61.30
ata	I	239	92.92
act	T	0	1.23
acc	T	0	1.08
aca	T	5	2.27
acg	T	0	0.42
gtt	V	5	866.60
gtc	V	4	736.50
gta	V	2546	985.30
gtg	V	1348	1315.00
gct	A	8	92.66
gcc	A	0	86.61
gca	A	369	170.40
gcg	A	1	28.35
---	--	------	------
mPD		0.68	1.0
		nPD :	0.68
		N. weight :	1.3
		Sc. PD :	0.24
		Sc. rank :	1282.2

,

HA

Pos.	118	obs:	exp:
aaa	K	2	1.22
aag	K	0	0.78
gaa	E	3511	2680.00
gag	E	1012	1843.00
---	--	------	------
mPD		0.35	0.48
		nPD :	0.72
		N. weight :	0.15
		Sc. PD :	0.035
		Sc. rank :	185.2

,

HA

Pos.	119	obs:	exp:
tct	S	0	0.50
tcc	S	0	0.47
tca	S	3	0.73
tcg	S	0	0.16
cct	P	0	4.72
ccc	P	0	3.22
cca	P	16	6.34
ccg	P	0	1.72
att	I	0	4.97
atc	I	0	3.59
ata	I	14	5.44
act	T	206	892.40
acc	T	28	784.50
aca	T	3318	1649.00
acg	T	76	302.50
aat	N	8	6.25
aac	N	3	4.75
aaa	K	65	39.50
aag	K	0	25.50
agt	S	0	0.56
agc	S	0	0.57
gtt	V	0	3.33
gtc	V	0	2.83
gta	V	15	3.79
gtg	V	0	5.05
gct	A	11	188.80
gcc	A	4	176.40
gca	A	731	347.10
gcg	A	24	57.75
gaa	E	3	1.78
gag	E	0	1.22
---	--	------	------
mPD		0.49	1.0
		nPD :	0.48
		N. weight :	1.
		Sc. PD :	0.0043
		Sc. rank :	-69.7

,

HA

Pos.	120	obs:	exp:
ttt	F	2	0.90
ttc	F	0	1.10
tta	L	0	0.11
ttg	L	0	0.19
tct	S	810	295.20
tcc	S	22	277.40
tca	S	850	426.20
tcg	S	73	93.27
tat	Y	1	0.56
tac	Y	0	0.44
ctt	L	0	0.20
ctc	L	1	0.15
cta	L	0	0.17
ctg	L	0	0.18
cct	P	213	781.10
ccc	P	583	532.90
cca	P	1785	1048.00
ccg	P	66	284.70
caa	Q	31	16.72
cag	Q	0	14.28
act	T	4	19.43
acc	T	0	17.08
aca	T	75	35.90
acg	T	0	6.59
agt	S	0	326.60
agc	S	0	336.30
gct	A	0	2.21
gcc	A	0	2.06
gca	A	9	4.06
gcg	A	0	0.67
---	--	------	------
mPD		1.1	1.5
		nPD :	0.7
		N. weight :	0.89
		Sc. PD :	0.19
		Sc. rank :	1039.3

,

HA

Pos.	121	obs:	exp:
tct	S	0	42.22
tcc	S	0	39.67
tca	S	0	60.95
tcg	S	0	13.34
tat	Y	1	2.24
tac	Y	3	1.76
cat	H	1	0.58
cac	H	0	0.42
cgt	R	0	1.64
cgc	R	0	2.75
cga	R	0	5.23
cgg	R	0	3.76
att	I	10	3.90
atc	I	1	2.82
ata	I	0	4.28
act	T	5	11.81
acc	T	43	10.38
aca	T	0	21.81
acg	T	0	4.00
aat	N	3334	2321.00
aac	N	750	1763.00
aaa	K	10	9.72
aag	K	6	6.28
agt	S	240	46.72
agc	S	11	48.10
aga	R	0	23.85
agg	R	52	14.78
gtt	V	1	0.22
gtc	V	0	0.19
gta	V	0	0.25
gtg	V	0	0.34
gct	A	2	0.49
gcc	A	0	0.46
gca	A	0	0.90
gcg	A	0	0.15
gat	D	20	23.62
gac	D	22	18.38
gaa	E	9	5.33
gag	E	0	3.67
ggt	G	4	0.63
ggc	G	0	0.51
gga	G	0	1.81
ggg	G	0	1.05
---	--	------	------
mPD		0.51	0.78
		nPD :	0.65
		N. weight :	0.48
		Sc. PD :	0.077
		Sc. rank :	397.7

,

HA

Pos.	122	obs:	exp:
tct	S	910	391.10
tcc	S	9	367.40
tca	S	1393	564.60
tcg	S	13	123.60
tat	Y	1	0.56
tac	Y	0	0.44
cct	P	2023	628.30
ccc	P	28	428.60
cca	P	70	843.10
ccg	P	8	229.00
cat	H	1	0.58
cac	H	0	0.42
agt	S	0	432.70
agc	S	0	445.60
gct	A	69	16.91
gcc	A	0	15.81
gca	A	0	31.10
gcg	A	0	5.17
---	--	------	------
mPD		0.97	1.7
		nPD :	0.58
		N. weight :	1.8
		Sc. PD :	0.18
		Sc. rank :	1053.4

,

HA

Pos.	123	obs:	exp:
tat	Y	0	6.16
tac	Y	11	4.84
cgt	R	0	0.28
cgc	R	0	0.48
cga	R	0	0.90
cgg	R	0	0.65
act	T	0	3.44
acc	T	0	3.03
aca	T	14	6.36
acg	T	0	1.17
aat	N	4	6.25
aac	N	7	4.75
aaa	K	119	91.16
aag	K	31	58.84
aga	R	9	4.13
agg	R	0	2.56
gtt	V	6	2.22
gtc	V	0	1.89
gta	V	0	2.52
gtg	V	4	3.37
gat	D	744	1060.00
gac	D	1141	824.80
gaa	E	496	1424.00
gag	E	1907	979.20
ggt	G	1	5.02
ggc	G	26	4.05
gga	G	3	14.49
ggg	G	2	8.43
---	--	------	------
mPD		0.81	0.85
		nPD :	0.95
		N. weight :	0.42
		Sc. PD :	0.19
		Sc. rank :	806.8

,

HA

Pos.	124	obs:	exp:
tct	S	0	0.50
tcc	S	0	0.47
tca	S	0	0.73
tcg	S	0	0.16
tat	Y	101	57.14
tac	Y	1	44.86
cat	H	7	4.04
cac	H	0	2.96
att	I	1	0.35
atc	I	0	0.26
ata	I	0	0.39
aat	N	4272	2490.00
aac	N	109	1891.00
aaa	K	15	13.98
aag	K	8	9.02
agt	S	3	0.56
agc	S	0	0.57
gat	D	8	4.50
gac	D	0	3.50
---	--	------	------
mPD		0.11	0.55
		nPD :	0.2
		N. weight :	0.9
		Sc. PD :	-0.23
		Sc. rank :	-1198.7

,

HA

Pos.	125	obs:	exp:
tgt	C	1	0.43
tgc	C	0	0.57
gat	D	2	1.12
gac	D	0	0.88
ggt	G	597	709.30
ggc	G	3	573.00
gga	G	2885	2048.00
ggg	G	1037	1191.00
---	--	------	------
mPD		0.52	0.69
		nPD :	0.76
		N. weight :	0.32
		Sc. PD :	0.087
		Sc. rank :	449.7

,

HA			
Pos.	126	obs:	exp:
tct	S	0	1.68
tcc	S	0	1.58
tca	S	9	2.43
tcg	S	1	0.53
cct	P	0	0.89
ccc	P	0	0.60
cca	P	3	1.19
ccg	P	0	0.32
att	I	4	123.10
atc	I	3	89.00
ata	I	340	134.90
atg	M	10	10.00
act	T	165	578.80
acc	T	4	508.80
aca	T	1895	1069.00
acg	T	289	196.20
aaa	K	49	29.78
aag	K	0	19.22
agt	S	0	1.86
agc	S	0	1.92
gtt	V	0	6.22
gtc	V	0	5.28
gta	V	28	7.07
gtg	V	0	9.43
gct	A	6	422.90
gcc	A	8	395.20
gca	A	1695	777.50
gcg	A	16	129.40
---	--	------	------
mPD		0.86	1.3
		nPD:	0.64
		N. weight:	1.1
		Sc. PD:	0.18
		Sc. rank:	904.5

,

HA			
Pos.	127	obs:	exp:
ttt	F	1	0.45
ttc	F	0	0.55
tgt	C	4146	1947.00
tgc	C	378	2577.00
---	--	------	------
mPD		0.15	0.49
		nPD:	0.31
		N. weight:	1.
		Sc. PD:	-0.16
		Sc. rank:	-893.8

,

HA			
Pos.	128	obs:	exp:
tat	Y	543	2535.00
tac	Y	3982	1990.00
---	--	------	------
mPD		0.21	0.49
		nPD:	0.43
		N. weight:	0.84
		Sc. PD:	-0.038
		Sc. rank:	-205.7

,

HA			
Pos.	129	obs:	exp:
cct	P	551	1335.00
ccc	P	64	910.80
cca	P	3886	1792.00
ccg	P	23	486.60
cgt	R	0	0.03
cgc	R	0	0.05
cga	R	1	0.10
cgg	R	0	0.07
aga	R	0	0.46
agg	R	0	0.28
---	--	------	------
mPD		0.25	0.70
		nPD:	0.35
		N. weight:	1.
		Sc. PD:	-0.12
		Sc. rank:	-661.5

,

HA			
Pos.	130	obs:	exp:
tgg	W	1	1.00
cgt	R	0	0.03
cgc	R	0	0.05
cga	R	0	0.10
cgg	R	0	0.07
aga	R	0	0.46
agg	R	1	0.28
ggt	G	11	709.40
ggc	G	3	573.10
gga	G	1688	2049.00
ggg	G	2821	1192.00
---	--	------	------
mPD		0.47	0.69
		nPD:	0.69
		N. weight:	0.89
		Sc. PD:	0.18
		Sc. rank:	992.3

,

HA			
Pos.	131	obs:	exp:
tta	L	2	0.22
ttg	L	0	0.38
tct	S	0	66.44
tcc	S	0	62.43
tca	S	0	95.92
tcg	S	0	20.99
tat	Y	1283	737.80
tac	Y	34	579.20
tgt	C	1	0.43
tgc	C	0	0.57
ctt	L	0	0.40
ctc	L	0	0.29
cta	L	0	0.35
ctg	L	0	0.36
cat	H	77	86.52
cac	H	73	63.48
caa	Q	35	395.80
cag	Q	699	338.20
att	I	4	1.42
atc	I	0	1.03
ata	I	0	1.56
act	T	1	0.25
acc	T	0	0.22
aca	T	0	0.45
acg	T	0	0.08
aat	N	12	6.82
aac	N	0	5.18
aaa	K	7	12.76
aag	K	14	8.24
agt	S	392	73.52
agc	S	3	75.70
gtt	V	0	0.67
gtc	V	0	0.57
gta	V	2	0.76
gtg	V	1	1.01
gct	A	1	0.25
gcc	A	0	0.23
gca	A	0	0.45
gcg	A	0	0.07
gat	D	984	583.30
gac	D	53	453.70
gaa	E	177	501.90
gag	E	670	345.10
---	--	------	------
mPD		1.4	1.6
		nPD:	0.89
		N. weight:	1.
		Sc. PD:	0.4
		Sc. rank:	1857.2

,

HA			
Pos.	132	obs:	exp:
ttt	F	100	2024.00
ttc	F	4418	2494.00
tct	S	0	0.17
tcc	S	1	0.16
tca	S	0	0.24
tcg	S	0	0.05
att	I	0	1.06
atc	I	3	0.77
ata	I	0	1.17
agt	S	0	0.19
agc	S	0	0.19
gtt	V	0	0.67
gtc	V	3	0.57
gta	V	0	0.76
gtg	V	0	1.01
---	--	------	------
mPD		0.046	0.50
		nPD:	0.09
		N. weight:	0.97
		Sc. PD:	-0.35
		Sc. rank:	-1624.0

,

HA			
Pos.	133	obs:	exp:
ttt	F	0	2.24
ttc	F	5	2.76
tct	S	56	72.66
tcc	S	264	68.27
tca	S	44	104.90
tcg	S	0	22.96
att	I	13	164.20
atc	I	441	118.80
ata	I	9	180.00
atg	M	1	1.00
act	T	57	236.60
acc	T	657	208.00
aca	T	248	437.10
acg	T	0	80.22
aat	N	2	2.84
aac	N	3	2.16
agt	S	0	80.41
agc	S	68	82.79
gtt	V	0	10.66
gtc	V	48	9.06
gta	V	0	12.12
gtg	V	0	16.17
gct	A	259	454.70
gcc	A	1589	425.00
gca	A	7	836.10
gcg	A	0	139.10
gat	D	10	7.31
gac	D	3	5.69
gaa	E	738	438.50
gag	E	2	301.50
ggt	G	0	0.16
ggc	G	1	0.13
gga	G	0	0.45
ggg	G	0	0.26
---	--	------	------
mPD		1.5	1.7
		nPD:	0.86
		N. weight:	1.6
		Sc. PD:	0.59
		Sc. rank:	2852.9

,

HA			
Pos.	134	obs:	exp:
tct	S	0	2.35
tcc	S	0	2.21
tca	S	0	3.40
tcg	S	0	0.74
tat	Y	0	0.56
tac	Y	1	0.44
aat	N	687	579.20
aac	N	332	439.80
agt	S	4	2.61
agc	S	10	2.68
gat	D	625	1960.00
gac	D	2859	1524.00
ggt	G	1	1.10
ggc	G	6	0.89
gga	G	0	3.17
ggg	G	0	1.84
---	--	------	------
mPD		0.77	0.86
		nPD:	0.9
		N. weight:	0.5
		Sc. PD:	0.2
		Sc. rank:	928.4

,

HA			
Pos.	135	obs:	exp:
tat	Y	4387	2535.00
tac	Y	137	1989.00
aat	N	1	0.57
aac	N	0	0.43
---	--	------	------
mPD		0.059	0.49
		nPD:	0.12
		N. weight:	0.89
		Sc. PD:	-0.3
		Sc. rank:	-1387.4

,

HA			
Pos.	136	obs:	exp:
gaa	E	2194	2681.00
gag	E	2331	1844.00
---	--	------	------
mPD		0.50	0.48
		nPD:	1.03
		N. weight:	0.048
		Sc. PD:	0.025
		Sc. rank:	95.5

,

HA			
Pos.	137	obs:	exp:
gaa	E	1720	2681.00
gag	E	2805	1844.00
---	--	------	------
mPD		0.47	0.48
		nPD:	0.98
		N. weight:	0.18
		Sc. PD:	0.086
		Sc. rank:	356.7

,

HA			
Pos.	138	obs:	exp:
tta	L	211	492.20
ttg	L	91	849.60
ctt	L	10	913.50
ctc	L	1	658.10
cta	L	2072	789.80
ctg	L	2138	819.90
atg	M	2	2.00
---	--	------	------
mPD		0.63	1.1
		nPD:	0.55
		N. weight:	1.6
		Sc. PD:	0.11
		Sc. rank:	566.6

,

HA			
Pos.	139	obs:	exp:
cgt	R	0	140.70
cgc	R	0	235.60
cga	R	2	448.00
cgg	R	6	322.00
act	T	0	0.25
acc	T	0	0.22
aca	T	0	0.45
acg	T	1	0.08
aaa	K	12	40.72
aag	K	55	26.28
aga	R	1167	2044.00
agg	R	3282	1267.00
---	--	------	------
mPD		0.42	0.96
		nPD:	0.43
		N. weight:	1.1
		Sc. PD:	-0.042
		Sc. rank:	-209.7

,

HA			
Pos.	140	obs:	exp:
gct	A	0	0.25
gcc	A	0	0.23
gca	A	0	0.45
gcg	A	1	0.07
gat	D	0	0.56
gac	D	1	0.44
gaa	E	382	2680.00
gag	E	4141	1843.00
---	--	------	------
mPD		0.16	0.48
		nPD:	0.32
		N. weight:	1.1
		Sc. PD:	-0.17
		Sc. rank:	-905.9

,

HA			
Pos.	141	obs:	exp:
tta	L	2	5.66
ttg	L	8	9.77
ctt	L	0	10.50
ctc	L	0	7.57
cta	L	37	9.08
ctg	L	5	9.43
cat	H	57	35.19
cac	H	4	25.81
caa	Q	3898	2377.00
cag	Q	510	2031.00
cgt	R	0	0.13
cgc	R	0	0.21
cga	R	3	0.40
cgg	R	1	0.29
aga	R	0	1.83
agg	R	0	1.14
---	--	------	------
mPD		0.26	0.55
		nPD:	0.47
		N. weight:	0.57
		Sc. PD:	-0.0032
		Sc. rank:	-48.6

,

HA			
Pos.	142	obs:	exp:
tta	L	183	491.70
ttg	L	4040	848.60
tgg	W	1	1.00
ctt	L	4	912.50
ctc	L	0	657.40
cta	L	21	788.90
ctg	L	270	819.00
atg	M	6	6.00
---	--	------	------
mPD		0.21	1.1
		nPD:	0.19
		N. weight:	2.5
		Sc. PD:	-0.68
		Sc. rank:	-3457.3

,

HA			
Pos.	143	obs:	exp:
tct	S	0	743.40
tcc	S	0	698.50
tca	S	0	1073.00
tcg	S	0	234.90
att	I	2	0.71
atc	I	0	0.51
ata	I	0	0.78
aat	N	13	20.46
aac	N	23	15.54
agt	S	3198	822.70
agc	S	1222	847.10
ggt	G	66	10.51
ggc	G	1	8.49
gga	G	0	30.35
ggg	G	0	17.65
---	--	------	------
mPD		0.45	1.7
		nPD:	0.27
		N. weight:	2.1
		Sc. PD:	-0.42
		Sc. rank:	-2130.8

,

HA			
Pos.	144	obs:	exp:
tct	S	50	721.70
tcc	S	3	678.20
tca	S	4130	1042.00
tcg	S	108	228.10
att	I	0	1.42
atc	I	0	1.03
ata	I	4	1.56
atg	M	1	1.00
act	T	4	52.15
acc	T	0	45.84
aca	T	202	96.33
acg	T	6	17.68
agt	S	0	798.70
agc	S	0	822.40
gct	A	0	3.92
gcc	A	0	3.67
gca	A	16	7.21
gcg	A	0	1.20
gaa	E	1	0.59
gag	E	0	0.41
---	--	------	------
mPD		0.17	1.6
		nPD:	0.11
		N. weight:	2.4
		Sc. PD:	-0.85
		Sc. rank:	-3879.2

,

HA			
Pos.	145	obs:	exp:
tta	L	1	0.11
ttg	L	0	0.19
ctt	L	0	0.20
ctc	L	0	0.15
cta	L	0	0.17
ctg	L	0	0.18
att	I	11	17.74
atc	I	2	12.82
ata	I	37	19.44
atg	M	36	36.00
act	T	0	0.25
acc	T	0	0.22
aca	T	1	0.45
acg	T	0	0.08
gtt	V	339	983.60
gtc	V	117	835.90
gta	V	2262	1118.00
gtg	V	1712	1492.00
gct	A	0	1.47
gcc	A	0	1.37
gca	A	6	2.70
gcg	A	0	0.45
ggt	G	0	0.16
ggc	G	0	0.13
gga	G	0	0.45
ggg	G	1	0.26
---	--	------	------
mPD		0.63	0.78
		nPD:	0.8
		N. weight:	0.55
		Sc. PD:	0.17
		Sc. rank:	857.6

,

HA			
Pos.	146	obs:	exp:
tta	L	1	0.22
ttg	L	1	0.38
tct	S	2867	759.90
tcc	S	83	714.00
tca	S	1547	1097.00
tcg	S	21	240.10
tat	Y	2	1.12
tac	Y	0	0.88
ctt	L	0	0.40
ctc	L	0	0.29
cta	L	0	0.35
ctg	L	0	0.36
cct	P	2	0.59
ccc	P	0	0.40
cca	P	0	0.79
ccg	P	0	0.22
agt	S	0	840.90
agc	S	0	865.90
gct	A	1	0.25
gcc	A	0	0.23
gca	A	0	0.45
gcg	A	0	0.07
---	--	------	------
mPD		0.48	1.6
		nPD:	0.3
		N. weight:	1.8
		Sc. PD:	-0.3
		Sc. rank:	-1579.9

,

HA			
Pos.	147	obs:	exp:
tta	L	1	0.11
ttg	L	0	0.19
tct	S	25	760.40
tcc	S	3	714.50
tca	S	4441	1098.00
tcg	S	52	240.30
ctt	L	0	0.20
ctc	L	0	0.15
cta	L	0	0.17
ctg	L	0	0.18
cct	P	0	0.30
ccc	P	0	0.20
cca	P	1	0.40
ccg	P	0	0.11
act	T	0	0.25
acc	T	0	0.22
aca	T	1	0.45
acg	T	0	0.08
agt	S	0	841.50
agc	S	0	866.40
gct	A	0	0.25
gcc	A	0	0.23
gca	A	1	0.45
gcg	A	0	0.07
---	--	------	------
mPD		0.037	1.6
		nPD:	0.02
		N. weight:	2.6
		Sc. PD:	-1.1
		Sc. rank:	-5088.0

,

HA			
Pos.	148	obs:	exp:
ttt	F	3986	2012.00
ttc	F	504	2478.00
tta	L	1	2.18
ttg	L	4	3.76
ctt	L	8	4.04
ctc	L	7	2.91
cta	L	0	3.49
ctg	L	0	3.63
att	I	0	0.71
atc	I	2	0.51
ata	I	0	0.78
gtt	V	4	2.89
gtc	V	9	2.45
gta	V	0	3.28
gtg	V	0	4.38
---	--	------	------
mPD		0.22	0.51
		nPD:	0.43
		N. weight:	0.85
		Sc. PD:	-0.039
		Sc. rank:	-213.7

,

HA			
Pos.	149	obs:	exp:
cgt	R	0	0.03
cgc	R	0	0.05
cga	R	0	0.10
cgg	R	0	0.07
aat	N	4	2.27
aac	N	0	1.73
aaa	K	200	647.80
aag	K	866	418.20
aga	R	0	0.46
agg	R	1	0.28
gat	D	3	9.56
gac	D	14	7.44
gaa	E	1378	2036.00
gag	E	2059	1401.00
---	--	------	------
mPD		0.82	0.85
		nPD:	0.97
		N. weight:	0.29
		Sc. PD:	0.14
		Sc. rank:	569.9

,

HA			
Pos.	150	obs:	exp:
caa	Q	1	44.22
cag	Q	81	37.78
cgt	R	146	111.10
cgc	R	0	186.10
cga	R	13	353.90
cgg	R	15	254.40
act	T	0	0.25
acc	T	0	0.22
aca	T	1	0.45
acg	T	0	0.08
aaa	K	436	558.50
aag	K	483	360.50
aga	R	2584	1615.00
agg	R	763	1001.00
gaa	E	0	1.19
gag	E	2	0.81
---	--	------	------
mPD		0.91	1.2
		nPD:	0.74
		N. weight:	0.47
		Sc. PD:	0.12
		Sc. rank:	632.6

,

HA			
Pos.	151	obs:	exp:
ttt	F	1158	2024.00
ttc	F	3358	2492.00
tta	L	0	0.54
ttg	L	0	0.94
ctt	L	0	1.01
ctc	L	5	0.73
cta	L	0	0.87
ctg	L	0	0.91
cct	P	0	0.30
ccc	P	1	0.20
cca	P	0	0.40
ccg	P	0	0.11
att	I	1	0.71
atc	I	1	0.51
ata	I	0	0.78
gtt	V	0	0.22
gtc	V	1	0.19
gta	V	0	0.25
gtg	V	0	0.34
---	--	------	------
mPD		0.39	0.50
		nPD:	0.77
		N. weight:	0.16
		Sc. PD:	0.045
		Sc. rank:	229.9

,

HA			
Pos.	152	obs:	exp:
caa	Q	1	0.54
cag	Q	0	0.46
aaa	K	4	3.65
aag	K	2	2.35
gaa	E	2528	2675.00
gag	E	1987	1840.00
ggt	G	0	0.47
ggc	G	0	0.38
gga	G	0	1.36
ggg	G	3	0.79
---	--	------	------
mPD		0.50	0.49
		nPD:	1.02
		N. weight:	0.0087
		Sc. PD:	0.0045
		Sc. rank:	17.2

,

HA			
Pos.	153	obs:	exp:
tta	L	0	0.11
ttg	L	0	0.19
ctt	L	1	0.20
ctc	L	0	0.15
cta	L	0	0.17
ctg	L	0	0.18
att	I	462	1575.00
atc	I	37	1139.00
ata	I	3942	1727.00
atg	M	80	80.00
gtt	V	0	0.67
gtc	V	2	0.57
gta	V	1	0.76
gtg	V	0	1.01
---	--	------	------
mPD		0.23	0.67
		nPD:	0.35
		N. weight:	1.1
		Sc. PD:	-0.14
		Sc. rank:	-769.6

,

HA			
Pos.	154	obs:	exp:
ttt	F	273	2026.00
ttc	F	4249	2496.00
tta	L	0	0.33
ttg	L	0	0.56
ctt	L	1	0.61
ctc	L	2	0.44
cta	L	0	0.52
ctg	L	0	0.54
---	--	------	------
mPD		0.12	0.50
		nPD:	0.23
		N. weight:	0.75
		Sc. PD:	-0.17
		Sc. rank:	-874.9

,

HA			
Pos.	155	obs:	exp:
tct	S	0	1.18
tcc	S	7	1.11
tca	S	0	1.70
tcg	S	0	0.37
cct	P	477	1332.00
ccc	P	3666	909.00
cca	P	350	1788.00
ccg	P	22	485.70
act	T	0	0.25
acc	T	1	0.22
aca	T	0	0.45
acg	T	0	0.08
agt	S	0	1.30
agc	S	0	1.34
---	-	2	2.00
---	--	------	------
mPD		0.33	0.71
		nPD:	0.46
		N. weight:	1.7
		Sc. PD:	-0.025
		Sc. rank:	-185.5

,

HA			
Pos.	156	obs:	exp:
tct	S	0	0.67
tcc	S	0	0.63
tca	S	3	0.97
tcg	S	0	0.21
cgt	R	0	21.84
cgc	R	0	36.58
cga	R	1	69.55
cgg	R	0	49.99
att	I	2	114.20
atc	I	0	82.59
ata	I	320	125.20
atg	M	73	73.00
act	T	0	1.72
acc	T	0	1.51
aca	T	4	3.18
acg	T	3	0.58
aat	N	123	71.62
aac	N	3	54.38
aaa	K	2324	1893.00
aag	K	791	1222.00
agt	S	1	0.74
agc	S	0	0.77
aga	R	687	317.30
agg	R	4	196.70
gtt	V	0	0.22
gtc	V	0	0.19
gta	V	0	0.25
gtg	V	1	0.34
gct	A	0	0.25
gcc	A	0	0.23
gca	A	1	0.45
gcg	A	0	0.07
gaa	E	153	98.36
gag	E	13	67.64
ggt	G	0	2.82
ggc	G	0	2.28
gga	G	17	8.15
ggg	G	1	4.74
---	--	------	------
mPD		0.84	1.1
		nPD:	0.76
		N. weight:	0.47
		Sc. PD:	0.12
		Sc. rank:	645.9

,

HA			
Pos.	157	obs:	exp:
tct	S	0	27.25
tcc	S	0	25.60
tca	S	161	39.34
tcg	S	0	8.61
cgt	R	0	1.17
cgc	R	0	1.96
cga	R	0	3.72
cgg	R	0	2.67
att	I	0	1.77
atc	I	0	1.28
ata	I	5	1.94
atg	M	38	38.00
act	T	5	91.75
acc	T	0	80.66
aca	T	365	169.50
acg	T	3	31.10
aat	N	3	1.71
aac	N	0	1.29
aaa	K	33	383.50
aag	K	598	247.50
agt	S	1	30.15
agc	S	0	31.05
aga	R	8	16.97
agg	R	29	10.52
gtt	V	0	0.22
gtc	V	0	0.19
gta	V	1	0.25
gtg	V	0	0.34
gct	A	1	105.90
gcc	A	0	98.98
gca	A	430	194.70
gcg	A	1	32.40
gat	D	45	29.25
gac	D	7	22.75
gaa	E	2375	1629.00
gag	E	374	1120.00
ggt	G	0	5.02
ggc	G	1	4.05
gga	G	28	14.49
ggg	G	3	8.43
---	-	10	10.00
---	--	------	------
mPD		1.2	1.4
		nPD:	0.85
		N. weight:	0.79
		Sc. PD:	0.28
		Sc. rank:	1343.0

,

HA			
Pos.	158	obs:	exp:
tct	S	0	662.50
tcc	S	0	622.50
tca	S	0	956.50
tcg	S	0	209.30
cat	H	0	2.88
cac	H	5	2.12
cgt	R	0	0.88
cgc	R	10	1.48
cga	R	0	2.81
cgg	R	0	2.02
att	I	0	2.48
atc	I	7	1.80
ata	I	0	2.72
act	T	4	57.56
acc	T	162	50.60
aca	T	68	106.30
acg	T	0	19.51
aat	N	47	93.22
aac	N	117	70.78
aaa	K	1	0.61
aag	K	0	0.39
agt	S	1067	733.10
agc	S	2872	754.90
aga	R	18	12.84
agg	R	0	7.96
gat	D	4	9.56
gac	D	13	7.44
ggt	G	36	20.39
ggc	G	90	16.47
gga	G	4	58.88
ggg	G	0	34.25
---	--	------	------
mPD		0.66	1.7
		nPD:	0.38
		N. weight:	2.
		Sc. PD:	-0.19
		Sc. rank:	-1085.4

,

HA			
Pos.	159	obs:	exp:
---	-	4525	4525.00
---	--	------	------
mPD		0	0
		nPD:	1.
		N. weight:	0.
		Sc. PD:	0
		Sc. rank:	0

,

HA			
Pos.	160	obs:	exp:
ttt	F	1	0.45
ttc	F	0	0.55
tta	L	2	0.22
ttg	L	0	0.38
tct	S	51	755.40
tcc	S	13	709.80
tca	S	4291	1091.00
tcg	S	136	238.70
ctt	L	0	0.40
ctc	L	0	0.29
cta	L	0	0.35
ctg	L	0	0.36
cct	P	0	1.18
ccc	P	0	0.81
cca	P	4	1.58
ccg	P	0	0.43
act	T	0	5.90
acc	T	2	5.19
aca	T	20	10.91
acg	T	2	2.00
agt	S	0	835.90
agc	S	0	860.70
gct	A	0	0.74
gcc	A	0	0.69
gca	A	3	1.35
gcg	A	0	0.22
---	--	------	------
mPD		0.10	1.6
		nPD:	0.06
		N. weight:	2.5
		Sc. PD:	-0.96
		Sc. rank:	-4453.8

,

HA			
Pos.	161	obs:	exp:
tta	L	1	0.11
ttg	L	0	0.19
tgg	W	4517	4517.00
ctt	L	0	0.20
ctc	L	0	0.15
cta	L	0	0.17
ctg	L	0	0.18
cgt	R	0	0.09
cgc	R	0	0.16
cga	R	0	0.30
cgg	R	3	0.22
aga	R	0	1.38
agg	R	0	0.85
ggt	G	0	0.63
ggc	G	0	0.51
gga	G	0	1.81
ggg	G	4	1.05
---	--	------	------
mPD		0.0040	0.0063
		nPD:	0.63
		N. weight:	0.0067
		Sc. PD:	0.00099
		Sc. rank:	5.0

,

HA			
Pos.	162	obs:	exp:
ttt	F	0	0.90
ttc	F	2	1.10
tta	L	0	1.74
ttg	L	0	3.01
tct	S	1	11.94
tcc	S	63	11.22
tca	S	4	17.24
tcg	S	3	3.77
ctt	L	5	3.23
ctc	L	3	2.33
cta	L	8	2.79
ctg	L	0	2.90
cct	P	127	1295.00
ccc	P	4029	883.60
cca	P	231	1738.00
ccg	P	2	472.10
cat	H	3	1.73
cac	H	0	1.27
caa	Q	7	3.77
cag	Q	0	3.23
act	T	1	7.63
acc	T	30	6.70
aca	T	0	14.09
acg	T	0	2.59
agt	S	0	13.21
agc	S	0	13.61
gaa	E	6	3.56
gag	E	0	2.44
---	--	------	------
mPD		0.23	0.78
		nPD:	0.29
		N. weight:	2.4
		Sc. PD:	-0.42
		Sc. rank:	-2261.4

,

HA			
Pos.	163	obs:	exp:
tta	L	0	19.04
ttg	L	0	32.87
tct	S	0	4.88
tcc	S	0	4.58
tca	S	0	7.04
tcg	S	0	1.54
tat	Y	7	4.48
tac	Y	1	3.52
ctt	L	174	35.34
ctc	L	1	25.46
cta	L	0	30.56
ctg	L	0	31.72
cat	H	7	51.91
cac	H	83	38.09
caa	Q	7	3.77
cag	Q	0	3.23
cgt	R	0	0.03
cgc	R	0	0.05
cga	R	0	0.10
cgg	R	0	0.07
aat	N	1134	2063.00
aac	N	2496	1567.00
aaa	K	299	186.60
aag	K	8	120.40
agt	S	10	5.40
agc	S	19	5.56
aga	R	1	0.46
agg	R	0	0.28
gat	D	177	120.90
gac	D	38	94.07
gaa	E	25	14.81
gag	E	0	10.19
ggt	G	36	5.96
ggc	G	0	4.82
gga	G	2	17.21
ggg	G	0	10.01
---	--	------	------
mPD		0.87	0.92
		nPD:	0.94
		N. weight:	0.47
		Sc. PD:	0.2
		Sc. rank:	903.4

,

HA			
Pos.	164	obs:	exp:
tat	Y	165	99.16
tac	Y	12	77.84
cat	H	1291	2500.00
cac	H	3043	1834.00
aat	N	10	7.39
aac	N	3	5.61
gaa	E	1	0.59
gag	E	0	0.41
---	--	------	------
mPD		0.52	0.57
		nPD:	0.91
		N. weight:	0.33
		Sc. PD:	0.14
		Sc. rank:	621.2

,

HA			
Pos.	165	obs:	exp:
tct	S	0	45.92
tcc	S	0	43.15
tca	S	1	66.29
tcg	S	0	14.51
caa	Q	0	2.70
cag	Q	5	2.30
act	T	1406	614.20
acc	T	1059	540.00
aca	T	32	1135.00
acg	T	0	208.20
aat	N	219	175.60
aac	N	90	133.40
aaa	K	383	238.20
aag	K	9	153.80
agt	S	30	50.81
agc	S	242	52.32
gtt	V	0	0.44
gtc	V	1	0.38
gta	V	0	0.50
gtg	V	1	0.67
gct	A	6	1.72
gcc	A	1	1.60
gca	A	0	3.16
gcg	A	0	0.52
gat	D	86	205.90
gac	D	280	160.10
gaa	E	511	389.90
gag	E	147	268.10
ggt	G	3	0.94
ggc	G	2	0.76
gga	G	1	2.72
ggg	G	0	1.58
---	-	10	10.00
---	--	------	------
mPD		1.6	1.6
		nPD:	0.96
		N. weight:	1.1
		Sc. PD:	0.49
		Sc. rank:	2090.7

,

HA			
Pos.	166	obs:	exp:
tta	L	0	1.20
ttg	L	10	2.07
tct	S	1	46.93
tcc	S	0	44.09
tca	S	71	67.75
tcg	S	207	14.83
tgg	W	1	1.00
ctt	L	0	2.22
ctc	L	0	1.60
cta	L	1	1.92
ctg	L	0	1.99
cct	P	0	7.08
ccc	P	0	4.83
cca	P	18	9.50
ccg	P	6	2.58
att	I	0	88.33
atc	I	4	63.87
ata	I	245	96.80
act	T	10	300.10
acc	T	6	263.80
aca	T	994	554.40
acg	T	210	101.70
aaa	K	5	3.04
aag	K	0	1.96
agt	S	0	51.93
agc	S	0	53.47
gtt	V	0	429.00
gtc	V	6	364.60
gta	V	1888	487.70
gtg	V	38	650.80
gct	A	1	193.20
gcc	A	0	180.60
gca	A	782	355.20
gcg	A	5	59.10
gaa	E	0	3.56
gag	E	6	2.44
---	-	10	10.00
---	--	------	------
mPD		1.2	1.8
		nPD:	0.69
		N. weight:	2.
		Sc. PD:	0.4
		Sc. rank:	2234.5

,

HA			
Pos.	167	obs:	exp:
tct	S	0	21.70
tcc	S	6	20.39
tca	S	0	31.33
tcg	S	0	6.86
cct	P	0	0.30
ccc	P	1	0.20
cca	P	0	0.40
ccg	P	0	0.11
att	I	0	1.42
atc	I	4	1.03
ata	I	0	1.56
act	T	81	664.10
acc	T	2566	583.90
aca	T	52	1227.00
acg	T	1	225.10
aat	N	321	529.70
aac	N	611	402.30
aaa	K	9	6.08
aag	K	1	3.92
agt	S	2	24.01
agc	S	121	24.72
gct	A	10	29.17
gcc	A	108	27.27
gca	A	0	53.64
gcg	A	1	8.92
gat	D	43	61.87
gac	D	67	48.13
gaa	E	3	1.78
gag	E	0	1.22
ggt	G	0	0.16
ggc	G	0	0.13
gga	G	1	0.45
ggg	G	0	0.26
---	-	516	516.00
---	--	------	------
mPD		0.61	0.98
		nPD:	0.62
		N. weight:	0.
		Sc. PD:	0
		Sc. rank:	0

,

HA			
Pos.	168	obs:	exp:
tct	S	0	0.50
tcc	S	0	0.47
tca	S	0	0.73
tcg	S	0	0.16
caa	Q	10	5.39
cag	Q	0	4.61
cgt	R	0	4.58
cgc	R	0	7.66
cga	R	0	14.57
cgg	R	0	10.48
att	I	0	2.48
atc	I	0	1.80
ata	I	7	2.72
atg	M	2	2.00
act	T	0	10.09
acc	T	0	8.87
aca	T	41	18.63
acg	T	0	3.42
aat	N	25	18.19
aac	N	7	13.81
aaa	K	914	596.80
aag	K	68	385.20
agt	S	1	0.56
agc	S	2	0.57
aga	R	140	66.50
agg	R	5	41.21
gat	D	0	1.12
gac	D	2	0.88
gaa	E	14	8.30
gag	E	0	5.70
ggt	G	0	2.51
ggc	G	0	2.03
gga	G	15	7.25
ggg	G	1	4.22
---	-	3271	3271.00
---	--	------	------
mPD		0.040	0.073
		nPD:	0.55
		N. weight:	0.
		Sc. PD:	0
		Sc. rank:	0

,

HA			
Pos.	169	obs:	exp:
tct	S	0	0.67
tcc	S	0	0.63
tca	S	0	0.97
tcg	S	0	0.21
cgt	R	0	0.06
cgc	R	0	0.11
cga	R	2	0.20
cgg	R	0	0.14
agt	S	4	0.74
agc	S	0	0.77
aga	R	0	0.92
agg	R	0	0.57
ggt	G	1344	708.70
ggc	G	35	572.50
gga	G	2993	2046.00
ggg	G	146	1190.00
---	-	1	1.00
---	--	------	------
mPD		0.47	0.69
		nPD:	0.69
		N. weight:	0.7
		Sc. PD:	0.14
		Sc. rank:	777.2

,

HA

Pos.	170	obs:	exp:
gct	A	0	0.25
gcc	A	0	0.23
gca	A	0	0.45
gcg	A	1	0.07
---	-	4524	4524.00
---	--	------	------
mPD		0	3.3×10^{-8}
		nPD:	0.
		N. weight:	0.
		Sc. PD:	0
		Sc. rank:	0

,

HA

Pos.	171	obs:	exp:
tta	L	0	0.11
ttg	L	1	0.19
tct	S	0	1.18
tcc	S	2	1.11
tca	S	5	1.70
tcg	S	0	0.37
ctt	L	0	0.20
ctc	L	0	0.15
cta	L	0	0.17
ctg	L	0	0.18
cgt	R	0	0.09
cgc	R	0	0.16
cga	R	1	0.30
cgg	R	0	0.22
att	I	0	1.77
atc	I	0	1.28
ata	I	5	1.94
atg	M	59	59.00
act	T	8	57.56
acc	T	205	50.60
aca	T	17	106.30
acg	T	4	19.51
aat	N	2	1.14
aac	N	0	0.86
aaa	K	50	161.00
aag	K	215	104.00
agt	S	0	1.30
agc	S	0	1.34
aga	R	0	1.38
agg	R	2	0.85
gtt	V	685	775.80
gtc	V	4	659.30
gta	V	758	882.00
gtg	V	2047	1177.00
gct	A	0	44.12
gcc	A	7	41.24
gca	A	122	81.13
gcg	A	51	13.50
gat	D	4	2.25
gac	D	0	1.75
gaa	E	9	68.73
gag	E	107	47.27
ggt	G	6	24.31
ggc	G	0	19.64
gga	G	5	70.21
ggg	G	144	40.84
---	--	------	------
mPD		1.2	1.3
		nPD:	0.89
		N. weight:	0.71
		Sc. PD:	0.28
		Sc. rank:	1276.7

,

HA

Pos.	172	obs:	exp:
tct	S	0	457.70
tcc	S	3	430.00
tca	S	2611	660.80
tcg	S	106	144.60
act	T	273	443.50
acc	T	5	389.90
aca	T	782	819.30
acg	T	743	150.30
agt	S	1	506.40
agc	S	0	521.50
gct	A	0	0.25
gcc	A	0	0.23
gca	A	1	0.45
gcg	A	0	0.07
---	--	------	------
mPD		0.88	1.5
		nPD:	0.58
		N. weight:	2.
		Sc. PD:	0.19
		Sc. rank:	1061.7

,

HA

Pos.	173	obs:	exp:
tct	S	1	169.70
tcc	S	9	159.50
tca	S	999	245.00
tcg	S	0	53.63
cgt	R	0	0.03
cgc	R	0	0.05
cga	R	0	0.10
cgg	R	0	0.07
att	I	43	20.58
atc	I	1	14.88
ata	I	14	22.55
atg	M	22	22.00
act	T	1	9.59
acc	T	3	8.43
aca	T	35	17.72
acg	T	0	3.25
aaa	K	23	13.98
aag	K	0	9.02
agt	S	0	187.80
agc	S	0	193.40
aga	R	1	0.46
agg	R	0	0.28
gtt	V	129	61.73
gtc	V	23	52.46
gta	V	120	70.18
gtg	V	6	93.64
gct	A	38	748.40
gcc	A	503	699.50
gca	A	2486	1376.00
gcg	A	26	229.00
gaa	E	8	7.70
gag	E	5	5.30
ggt	G	25	4.55
ggc	G	1	3.67
gga	G	3	13.14
ggg	G	0	7.64
---	--	------	------
mPD		0.88	1.4
		nPD:	0.62
		N. weight:	1.2
		Sc. PD:	0.16
		Sc. rank:	839.9

,

HA

Pos.	174	obs:	exp:
tct	S	13	599.30
tcc	S	5	563.10
tca	S	3494	865.20
tcg	S	51	189.40
agt	S	0	663.20
agc	S	0	682.80
gct	A	18	235.80
gcc	A	21	220.40
gca	A	892	433.60
gcg	A	31	72.14
---	--	------	------
mPD		0.39	1.7
		nPD:	0.23
		N. weight:	2.3
		Sc. PD:	-0.54
		Sc. rank:	-2720.9

,

HA

Pos.	175	obs:	exp:
tgt	C	584	1947.00
tgc	C	3940	2577.00
ggt	G	0	0.16
ggc	G	1	0.13
gga	G	0	0.45
ggg	G	0	0.26
---	--	------	------
mPD		0.23	0.49
		nPD:	0.46
		N. weight:	0.42
		Sc. PD:	-0.0069
		Sc. rank:	-50.1

,

HA

Pos.	176	obs:	exp:
ttt	F	0	3.14
ttc	F	7	3.86
tta	L	1	0.22
ttg	L	0	0.38
tct	S	121	574.10
tcc	S	3262	539.40
tca	S	28	828.80
tcg	S	2	181.40
tat	Y	1	1.68
tac	Y	2	1.32
ctt	L	0	0.40
ctc	L	1	0.29
cta	L	0	0.35
ctg	L	0	0.36
cct	P	770	297.20
ccc	P	231	202.70
cca	P	6	398.80
ccg	P	0	108.30
caa	Q	2	1.08
cag	Q	0	0.92
act	T	83	22.14
acc	T	7	19.46
aca	T	0	40.90
acg	T	0	7.50
agt	S	0	635.20
agc	S	0	654.10
gct	A	1	0.25
gcc	A	0	0.23
gca	A	0	0.45
gcg	A	0	0.07
---	--	------	------
mPD		0.74	1.7
		nPD:	0.42
		N. weight:	2.8
		Sc. PD:	-0.15
		Sc. rank:	-944.3

,

HA

Pos.	177	obs:	exp:
aat	N	1	0.57
aac	N	0	0.43
---	-	4524	4524.00
---	--	------	------
mPD		0	2.4×10^{-8}
		nPD:	0.
		N. weight:	0.
		Sc. PD:	0
		Sc. rank:	0

,

HA

Pos.	178	obs:	exp:
ttt	F	2	0.90
ttc	F	0	1.10
tta	L	0	1.09
ttg	L	0	1.88
tct	S	1	0.50
tcc	S	1	0.47
tca	S	0	0.73
tcg	S	0	0.16
tat	Y	363	219.60
tac	Y	29	172.40
ctt	L	10	2.02
ctc	L	0	1.45
cta	L	0	1.75
ctg	L	0	1.81
cat	H	3481	2157.00
cac	H	259	1583.00
caa	Q	25	16.18
cag	Q	5	13.82
cgt	R	6	0.41
cgc	R	6	0.69
cga	R	1	1.31
cgg	R	0	0.94
aat	N	34	25.01
aac	N	10	18.99
aaa	K	97	76.57
aag	K	29	49.43
agt	S	1	0.56
agc	S	0	0.57
aga	R	0	5.96
agg	R	0	3.69
gat	D	160	92.24
gac	D	4	71.76
gaa	E	1	0.59
gag	E	0	0.41
---	--	------	------
mPD		0.49	0.83
		nPD:	0.59
		N. weight:	0.65
		Sc. PD:	0.069
		Sc. rank:	382.3

,

HA

Pos.	179	obs:	exp:
ttt	F	3	1.34
ttc	F	0	1.66
tta	L	0	0.22
ttg	L	2	0.38
tct	S	214	50.29
tcc	S	25	47.25
tca	S	4	72.61
tcg	S	2	15.89
tat	Y	1	0.56
tac	Y	0	0.44
ctt	L	0	0.40
ctc	L	0	0.29
cta	L	0	0.35
ctg	L	0	0.36
cct	P	1	0.30
ccc	P	0	0.20
cca	P	0	0.40
ccg	P	0	0.11
cat	H	0	0.58
cac	H	1	0.42
caa	Q	50	37.75
cag	Q	20	32.25
cgt	R	0	0.13
cgc	R	0	0.21
cga	R	0	0.40
cgg	R	0	0.29
act	T	1	0.25
acc	T	0	0.22
aca	T	0	0.45
acg	T	0	0.08
aat	N	1753	1502.00
aac	N	890	1141.00
aaa	K	140	201.20
aag	K	191	129.80
agt	S	50	55.65
agc	S	4	57.30
aga	R	1	1.83
agg	R	3	1.14
gtt	V	3	0.67
gtc	V	0	0.57
gta	V	0	0.76
gtg	V	0	1.01
gct	A	1100	272.60
gcc	A	3	254.80
gca	A	4	501.20
gcg	A	5	83.39
gat	D	41	23.62
gac	D	1	18.38
gaa	E	3	7.11
gag	E	9	4.89
---	--	------	------
mPD		1.4	1.6
		nPD:	0.87
		N. weight:	0.82
		Sc. PD:	0.31
		Sc. rank:	1466.0

,

HA			
Pos.	180	obs:	exp:
cgt	R	0	0.28
cgc	R	0	0.48
cga	R	0	0.90
cgg	R	0	0.65
atg	M	5	5.00
aga	R	5	4.13
agg	R	4	2.56
gaa	E	3	5.33
gag	E	6	3.67
ggt	G	7	706.20
ggc	G	0	570.50
gga	G	1528	2039.00
ggg	G	2967	1186.00
---	--	------	------
mPD		0.46	0.70
		nPD:	0.66
		N. weight:	0.98
		Sc. PD:	0.17
		Sc. rank:	906.3

,

HA			
Pos.	181	obs:	exp:
tct	S	1	3.20
tcc	S	8	3.00
tca	S	1	4.61
tcg	S	0	1.01
caa	Q	14	7.55
cag	Q	0	6.45
cgt	R	0	3.50
cgc	R	0	5.87
cga	R	0	11.16
cgg	R	0	8.02
att	I	0	0.71
atc	I	1	0.51
ata	I	1	0.78
act	T	0	75.02
acc	T	21	65.95
aca	T	279	138.60
acg	T	5	25.43
aat	N	33	525.20
aac	N	891	398.80
aaa	K	1161	711.10
aag	K	9	458.90
agt	S	1	3.54
agc	S	8	3.64
aga	R	107	50.90
agg	R	4	31.55
gtt	V	0	5.99
gtc	V	11	5.09
gta	V	16	6.82
gtg	V	0	9.09
gct	A	34	208.60
gcc	A	166	195.00
gca	A	651	383.60
gcg	A	0	63.82
gat	D	0	1.69
gac	D	3	1.31
gaa	E	1041	625.70
gag	E	15	430.30
ggt	G	0	6.59
ggc	G	0	5.32
gga	G	42	19.02
ggg	G	0	11.07
---	-	1	1.00
---	--	------	------
mPD		1.3	1.6
		nPD:	0.81
		N. weight:	0.97
		Sc. PD:	0.31
		Sc. rank:	1549.7

,

HA			
Pos.	182	obs:	exp:
tct	S	0	330.20
tcc	S	0	310.20
tca	S	4	476.70
tcg	S	0	104.30
cct	P	1	1.77
ccc	P	0	1.21
cca	P	5	2.38
ccg	P	0	0.65
cat	H	1	0.58
cac	H	0	0.42
caa	Q	1	6.47
cag	Q	11	5.53
cgt	R	10	32.83
cgc	R	11	54.97
cga	R	8	104.50
cgg	R	0	75.13
att	I	0	1.42
atc	I	0	1.03
ata	I	4	1.56
atg	M	3	3.00
act	T	1	0.25
acc	T	0	0.22
aca	T	0	0.45
acg	T	0	0.08
aat	N	57	289.90
aac	N	453	220.10
aaa	K	346	250.40
aag	K	66	161.60
agt	S	253	365.40
agc	S	1706	376.20
aga	R	664	476.90
agg	R	347	295.60
gtt	V	0	0.44
gtc	V	0	0.38
gta	V	1	0.50
gtg	V	1	0.67
gct	A	0	2.45
gcc	A	0	2.29
gca	A	10	4.51
gcg	A	0	0.75
gaa	E	1	0.59
gag	E	0	0.41
ggt	G	1	86.74
ggc	G	0	70.07
gga	G	536	250.50
ggg	G	16	145.70
---	-	7	7.00
---	--	------	------
mPD		1.2	2.0
		nPD:	0.62
		N. weight:	1.5
		Sc. PD:	0.2
		Sc. rank:	1025.9

,

HA			
Pos.	183	obs:	exp:
tct	S	0	740.10
tcc	S	0	695.40
tca	S	0	1068.00
tcg	S	0	233.90
cgt	R	0	0.06
cgc	R	0	0.11
cga	R	0	0.20
cgg	R	0	0.14
aat	N	72	40.92
aac	N	0	31.08
aaa	K	0	3.04
aag	K	5	1.96
agt	S	2550	818.90
agc	S	1850	843.20
aga	R	0	0.92
agg	R	2	0.57
gat	D	2	2.25
gac	D	2	1.75
ggt	G	11	5.80
ggc	G	26	4.69
gga	G	0	16.76
ggg	G	0	9.75
---	-	5	5.00
---	--	------	------
mPD		0.54	1.7
		nPD:	0.32
		N. weight:	1.9
		Sc. PD:	-0.28
		Sc. rank:	-1507.4

,

HA			
Pos.	184	obs:	exp:
ttt	F	2361	2028.00
ttc	F	2164	2497.00
---	--	------	------
mPD		0.50	0.49
		nPD:	1.01
		N. weight:	0.023
		Sc. PD:	0.012
		Sc. rank:	46.2

,

HA			
Pos.	185	obs:	exp:
tat	Y	418	2535.00
tac	Y	4107	1990.00
---	--	------	------
mPD		0.17	0.49
		nPD:	0.34
		N. weight:	0.97
		Sc. PD:	-0.12
		Sc. rank:	-717.2

,

HA			
Pos.	186	obs:	exp:
caa	Q	7	8.09
cag	Q	8	6.91
cgt	R	6	125.50
cgc	R	0	210.10
cga	R	34	399.50
cgg	R	188	287.20
act	T	0	12.05
acc	T	0	10.60
aca	T	49	22.27
acg	T	0	4.09
aaa	K	370	292.30
aag	K	111	188.70
aga	R	3485	1823.00
agg	R	262	1130.00
gct	A	0	0.25
gcc	A	0	0.23
gca	A	1	0.45
gcg	A	0	0.07
gaa	E	1	0.59
gag	E	0	0.41
ggt	G	0	0.47
ggc	G	0	0.38
gga	G	3	1.36
ggg	G	0	0.79
---	--	------	------
mPD		0.54	1.1
		nPD:	0.48
		N. weight:	0.77
		Sc. PD:	0.0044
		Sc. rank:	-35.5

,

HA			
Pos.	187	obs:	exp:
att	I	1	0.35
atc	I	0	0.26
ata	I	0	0.39
aat	N	4179	2571.00
aac	N	345	1953.00
---	--	------	------
mPD		0.14	0.49
		nPD:	0.29
		N. weight:	0.62
		Sc. PD:	-0.11
		Sc. rank:	-598.3

,

HA			
Pos.	188	obs:	exp:
tta	L	1210	492.30
ttg	L	1544	849.80
ctt	L	2	913.70
ctc	L	0	658.20
cta	L	53	789.90
ctg	L	1715	820.10
atg	M	1	1.00
---	--	------	------
mPD		0.88	1.1
		nPD:	0.77
		N. weight:	1.4
		Sc. PD:	0.38
		Sc. rank:	1947.0

,

HA			
Pos.	189	obs:	exp:
tta	L	123	181.10
ttg	L	4	312.60
tct	S	0	0.50
tcc	S	0	0.47
tca	S	3	0.73
tcg	S	0	0.16
ctt	L	66	336.10
ctc	L	3	242.10
cta	L	1409	290.60
ctg	L	59	301.60
caa	Q	1	0.54
cag	Q	0	0.46
cgt	R	0	0.03
cgc	R	0	0.05
cga	R	1	0.10
cgg	R	0	0.07
att	I	14	762.30
atc	I	616	551.20
ata	I	1519	835.50
atg	M	342	342.00
agt	S	0	0.56
agc	S	0	0.57
aga	R	0	0.46
agg	R	0	0.28
gtt	V	0	80.82
gtc	V	39	68.69
gta	V	315	91.89
gtg	V	10	122.60
gct	A	0	0.25
gcc	A	0	0.23
gca	A	1	0.45
gcg	A	0	0.07
---	--	------	------
mPD		0.99	1.4
		nPD:	0.73
		N. weight:	1.4
		Sc. PD:	0.34
		Sc. rank:	1821.1

,

HA			
Pos.	190	obs:	exp:
tgg	W	4525	4525.00
---	--	------	------
mPD		0	0
		nPD:	1.
		N. weight:	0.
		Sc. PD:	0
		Sc. rank:	0

,

HA			
Pos.	191	obs:	exp:
tta	L	16	464.10
ttg	L	56	801.10
ctt	L	98	861.40
ctc	L	6	620.50
cta	L	684	744.70
ctg	L	3405	773.10
cct	P	0	0.59
ccc	P	1	0.40
cca	P	0	0.79
ccg	P	1	0.22
att	I	0	90.81
atc	I	1	65.66
ata	I	255	99.52
gtt	V	0	0.44
gtc	V	0	0.38
gta	V	2	0.50
gtg	V	0	0.67
---	--	------	------
mPD		0.51	1.2
		nPD:	0.42
		N. weight:	2.1
		Sc. PD:	-0.11
		Sc. rank:	-674.5

,

HA			
Pos.	192	obs:	exp:
tta	L	59	8.49
ttg	L	18	14.65
ctt	L	0	15.75
ctc	L	0	11.35
cta	L	1	13.62
ctg	L	0	14.14
cgt	R	0	0.03
cgc	R	0	0.05
cga	R	0	0.10
cgg	R	0	0.07
att	I	8	15.96
atc	I	0	11.54
ata	I	37	17.49
act	T	74	749.50
acc	T	7	658.90
aca	T	2261	1385.00
acg	T	705	254.10
aaa	K	29	18.23
aag	K	1	11.77
aga	R	1	0.46
agg	R	0	0.28
gtt	V	212	294.00
gtc	V	109	249.80
gta	V	301	334.20
gtg	V	702	446.00
---	--	------	------
mPD		1.4	1.6
		nPD:	0.89
		N. weight:	0.84
		Sc. PD:	0.33
		Sc. rank:	1507.4

,

HA			
Pos.	193	obs:	exp:
aaa	K	0	0.61
aag	K	1	0.39
---	-	4524	4524.00
---	--	------	------
mPD		0	2.3×10^{-8}
		nPD:	0.
		N. weight:	0.
		Sc. PD:	0
		Sc. rank:	0

,

HA			
Pos.	194	obs:	exp:
tta	L	1	0.11
ttg	L	0	0.19
tct	S	0	4.54
tcc	S	0	4.27
tca	S	0	6.56
tcg	S	0	1.43
ctt	L	0	0.20
ctc	L	0	0.15
cta	L	0	0.17
ctg	L	0	0.18
caa	Q	30	18.87
cag	Q	5	16.13
cgt	R	0	0.95
cgc	R	0	1.59
cga	R	2	3.02
cgg	R	0	2.17
atg	M	61	61.00
act	T	0	4.43
acc	T	0	3.89
aca	T	0	8.18
acg	T	18	1.50
aat	N	1	1.14
aac	N	1	0.86
aaa	K	1049	834.40
aag	K	324	538.60
agt	S	25	5.03
agc	S	2	5.17
aga	R	1	13.76
agg	R	27	8.53
gtt	V	7	498.70
gtc	V	0	423.80
gta	V	680	567.00
gtg	V	1559	756.50
gct	A	0	10.05
gcc	A	0	9.39
gca	A	13	18.48
gcg	A	28	3.07
gaa	E	89	349.00
gag	E	500	240.00
ggt	G	1	16.00
ggc	G	0	12.92
gga	G	22	46.20
ggg	G	79	26.88
---	--	------	------
mPD		1.5	1.7
		nPD:	0.89
		N. weight:	0.79
		Sc. PD:	0.31
		Sc. rank:	1439.8

,

HA			
Pos.	195	obs:	exp:
aaa	K	4	2.43
aag	K	0	1.57
---	-	4521	4521.00
---	--	------	------
mPD		0	3.7×10^{-7}
		nPD:	0.
		N. weight:	0.
		Sc. PD:	0
		Sc. rank:	0

,

HA			
Pos.	196	obs:	exp:
gaa	E	4	2.37
gag	E	0	1.63
---	-	4521	4521.00
---	--	------	------
mPD		0	3.8×10^{-7}
		nPD:	0.
		N. weight:	0.
		Sc. PD:	0
		Sc. rank:	0

,

HA			
Pos.	197	obs:	exp:
cgt	R	0	0.66
cgc	R	0	1.11
cga	R	0	2.11
cgg	R	0	1.52
att	I	0	0.35
atc	I	0	0.26
ata	I	1	0.39
act	T	0	0.49
acc	T	0	0.43
aca	T	2	0.91
acg	T	0	0.17
aaa	K	3786	2731.00
aag	K	708	1763.00
aga	R	17	9.63
agg	R	4	5.97
gaa	E	2	2.96
gag	E	3	2.04
ggt	G	0	0.31
ggc	G	0	0.25
gga	G	1	0.91
ggg	G	1	0.53
---	--	------	------
mPD		0.28	0.49
		nPD:	0.57
		N. weight:	0.26
		Sc. PD:	0.023
		Sc. rank:	122.3

,

HA			
Pos.	198	obs:	exp:
tct	S	0	3.70
tcc	S	0	3.48
tca	S	0	5.34
tcg	S	0	1.17
tat	Y	1	0.56
tac	Y	0	0.44
cat	H	6	3.46
cac	H	0	2.54
caa	Q	3	1.62
cag	Q	0	1.38
cgt	R	0	0.82
cgc	R	0	1.37
cga	R	0	2.61
cgg	R	0	1.88
att	I	0	0.35
atc	I	0	0.26
ata	I	1	0.39
act	T	0	3.69
acc	T	1	3.24
aca	T	3	6.82
acg	T	11	1.25
aat	N	1982	1663.00
aac	N	943	1262.00
aaa	K	21	16.41
aag	K	6	10.59
agt	S	17	4.09
agc	S	5	4.22
aga	R	24	11.92
agg	R	2	7.39
gtt	V	1	0.67
gtc	V	0	0.57
gta	V	2	0.76
gtg	V	0	1.01
gct	A	15	4.41
gcc	A	0	4.12
gca	A	3	8.11
gcg	A	0	1.35
gat	D	279	173.80
gac	D	30	135.20
gaa	E	292	178.90
gag	E	10	123.10
ggt	G	186	134.70
ggc	G	4	108.80
gga	G	588	389.10
ggg	G	81	226.30
---	-	8	8.00
---	--	------	------
mPD		1.4	1.4
		nPD:	0.95
		N. weight:	0.26
		Sc. PD:	0.12
		Sc. rank:	501.2

,

HA

Pos.	199	obs:	exp:
tct	S	0	9.59
tcc	S	0	9.01
tca	S	0	13.84
tcg	S	0	3.03
tat	Y	63	35.30
tac	Y	0	27.70
caa	Q	1	0.54
cag	Q	0	0.46
cgt	R	0	0.13
cgc	R	0	0.21
cga	R	0	0.40
cgg	R	0	0.29
act	T	146	37.63
acc	T	4	33.08
aca	T	1	69.52
acg	T	2	12.76
aat	N	1295	871.30
aac	N	238	661.70
aaa	K	3	3.04
aag	K	2	1.96
agt	S	21	10.61
agc	S	36	10.92
aga	R	4	1.83
agg	R	0	1.14
gct	A	0	0.74
gcc	A	3	0.69
gca	A	0	1.35
gcg	A	0	0.22
gat	D	400	239.60
gac	D	26	186.40
gaa	E	1	1.19
gag	E	1	0.81
ggt	G	1842	357.20
ggc	G	368	288.50
gga	G	9	1031.00
ggg	G	58	600.00
---	-	1	1.00
---	--	------	------
mPD		1.3	1.8
		nPD:	0.74
		N. weight:	1.6
		Sc. PD:	0.4
		Sc. rank:	2129.9

,

HA

Pos.	200	obs:	exp:
ttt	F	1	1.34
ttc	F	2	1.66
tta	L	42	299.80
ttg	L	2670	517.50
tct	S	222	274.80
tcc	S	190	258.20
tca	S	1174	396.80
tcg	S	48	86.84
ctt	L	0	556.40
ctc	L	0	400.80
cta	L	0	481.10
ctg	L	43	499.40
cct	P	1	1.18
ccc	P	0	0.81
cca	P	3	1.58
ccg	P	0	0.43
cat	H	0	1.73
cac	H	3	1.27
caa	Q	0	0.54
cag	Q	1	0.46
cgt	R	0	0.06
cgc	R	0	0.11
cga	R	0	0.20
cgg	R	2	0.14
att	I	41	14.90
atc	I	1	10.77
ata	I	0	16.33
act	T	42	13.04
acc	T	1	11.46
aca	T	10	24.08
acg	T	0	4.42
aat	N	3	1.71
aac	N	0	1.29
agt	S	0	304.10
agc	S	0	313.20
aga	R	0	0.92
agg	R	0	0.57
gtt	V	0	4.66
gtc	V	6	3.96
gta	V	5	5.30
gtg	V	10	7.07
gct	A	3	0.74
gcc	A	0	0.69
gca	A	0	1.35
gcg	A	0	0.22
gaa	E	1	0.59
gag	E	0	0.41
---	--	------	------
mPD		1.1	1.9
		nPD:	0.57
		N. weight:	2.4
		Sc. PD:	0.21
		Sc. rank:	1153.1

,

HA

Pos.	201	obs:	exp:
tat	Y	465	2535.00
tac	Y	4060	1990.00
---	--	------	------
mPD		0.18	0.49
		nPD:	0.37
		N. weight:	0.92
		Sc. PD:	-0.088
		Sc. rank:	-506.5

,

HA

Pos.	202	obs:	exp:
tta	L	0	0.11
ttg	L	0	0.19
tct	S	1	0.34
tcc	S	0	0.32
tca	S	1	0.49
tcg	S	0	0.11
ctt	L	0	0.20
ctc	L	0	0.15
cta	L	1	0.17
ctg	L	0	0.18
cct	P	149	1330.00
ccc	P	37	907.20
cca	P	3777	1784.00
ccg	P	543	484.70
caa	Q	5	8.63
cag	Q	11	7.37
agt	S	0	0.37
agc	S	0	0.38
---	--	------	------
mPD		0.29	0.71
		nPD:	0.41
		N. weight:	1.1
		Sc. PD:	-0.066
		Sc. rank:	-424.5

,

HA

Pos.	203	obs:	exp:
tct	S	0	1.35
tcc	S	0	1.26
tca	S	0	1.94
tcg	S	0	0.43
tat	Y	0	0.56
tac	Y	1	0.44
cat	H	0	0.58
cac	H	1	0.42
cgt	R	0	12.18
cgc	R	0	20.40
cga	R	0	38.80
cgg	R	0	27.89
att	I	0	1.06
atc	I	0	0.77
ata	I	3	1.17
atg	M	56	56.00
act	T	0	6.40
acc	T	19	5.62
aca	T	3	11.81
acg	T	4	2.17
aat	N	346	1714.00
aac	N	2670	1302.00
aaa	K	242	623.50
aag	K	784	402.50
agt	S	0	1.49
agc	S	8	1.53
aga	R	82	177.00
agg	R	304	109.70
gtt	V	0	0.22
gtc	V	0	0.19
gta	V	0	0.25
gtg	V	1	0.34
gat	D	0	0.56
gac	D	1	0.44
---	--	------	------
mPD		0.76	0.95
		nPD:	0.8
		N. weight:	0.85
		Sc. PD:	0.26
		Sc. rank:	1309.5

,

HA

Pos.	204	obs:	exp:
ttt	F	4	22.40
ttc	F	46	27.60
tta	L	5	454.70
ttg	L	117	784.80
ctt	L	17	843.80
ctc	L	1135	607.90
cta	L	87	729.50
ctg	L	2817	757.40
att	I	10	95.78
atc	I	245	69.25
ata	I	15	105.00
gtt	V	1	5.99
gtc	V	9	5.09
gta	V	0	6.82
gtg	V	17	9.09
---	--	------	------
mPD		0.67	1.2
		nPD:	0.54
		N. weight:	1.8
		Sc. PD:	0.12
		Sc. rank:	564.4

,

HA

Pos.	205	obs:	exp:
tct	S	0	612.80
tcc	S	0	575.70
tca	S	0	884.70
tcg	S	0	193.60
tat	Y	0	1.12
tac	Y	2	0.88
tgt	C	0	0.43
tgc	C	1	0.57
cgt	R	0	0.60
cgc	R	1	1.00
cga	R	0	1.91
cgg	R	0	1.37
att	I	2	2.13
atc	I	4	1.54
ata	I	0	2.33
act	T	0	18.94
acc	T	77	16.65
aca	T	0	34.99
acg	T	0	6.42
aat	N	199	430.30
aac	N	558	326.70
aaa	K	14	12.15
aag	K	6	7.85
agt	S	139	678.00
agc	S	3504	698.20
aga	R	16	8.71
agg	R	2	5.40
---	--	------	------
mPD		0.47	1.8
		nPD:	0.26
		N. weight:	2.5
		Sc. PD:	-0.5
		Sc. rank:	-2529.4

,

HA

Pos.	206	obs:	exp:
caa	Q	18	12.94
cag	Q	6	11.06
cgt	R	0	0.98
cgc	R	0	1.64
cga	R	0	3.12
cgg	R	0	2.24
att	I	5	154.70
atc	I	4	111.80
ata	I	427	169.50
atg	M	117	117.00
act	T	0	0.74
acc	T	0	0.65
aca	T	2	1.36
acg	T	1	0.25
aat	N	54	32.97
aac	N	4	25.03
aaa	K	943	2326.00
aag	K	2885	1502.00
aga	R	4	14.22
agg	R	27	8.81
gtt	V	0	1.11
gtc	V	0	0.94
gta	V	1	1.26
gtg	V	4	1.68
gct	A	0	0.25
gcc	A	0	0.23
gca	A	1	0.45
gcg	A	0	0.07
gaa	E	1	1.19
gag	E	1	0.81
ggt	G	0	3.14
ggc	G	0	2.53
gga	G	20	9.06
ggg	G	0	5.27
---	--	------	------
mPD		0.71	0.81
		nPD:	0.87
		N. weight:	0.64
		Sc. PD:	0.24
		Sc. rank:	1140.2

,

HA

Pos.	207	obs:	exp:
ttt	F	1	1.34
ttc	F	2	1.66
tta	L	1	0.11
ttg	L	0	0.19
tct	S	232	754.00
tcc	S	3985	708.50
tca	S	263	1089.00
tcg	S	3	238.30
tat	Y	0	1.68
tac	Y	3	1.32
ctt	L	0	0.20
ctc	L	0	0.15
cta	L	0	0.17
ctg	L	0	0.18
cct	P	0	1.48
ccc	P	5	1.01
cca	P	0	1.98
ccg	P	0	0.54
cat	H	0	3.46
cac	H	6	2.54
act	T	1	4.92
acc	T	16	4.32
aca	T	3	9.09
acg	T	0	1.67
agt	S	0	834.40
agc	S	0	859.20
gtt	V	1	0.22
gtc	V	0	0.19
gta	V	0	0.25
gtg	V	0	0.34
gct	A	0	0.49
gcc	A	2	0.46
gca	A	0	0.90
gcg	A	0	0.15
gat	D	1	0.56
gac	D	0	0.44
---	--	------	------
mPD		0.23	1.6
		nPD:	0.14
		N. weight:	2.7
		Sc. PD:	-0.87
		Sc. rank:	-4050.8

,

HA

Pos.	208	obs:	exp:
ttt	F	1	0.45
ttc	F	0	0.55
tat	Y	3803	2523.00
tac	Y	701	1981.00
tgg	W	1	1.00
cat	H	0	10.96
cac	H	19	8.04
---	--	------	------
mPD		0.28	0.50
		nPD:	0.55
		N. weight:	0.37
		Sc. PD:	0.027
		Sc. rank:	134.2

,

HA

Pos.	209	obs:	exp:
ttt	F	2	10.31
ttc	F	21	12.69
tta	L	0	0.11
ttg	L	0	0.19
tat	Y	1	1.12
tac	Y	1	0.88
ctt	L	0	0.20
ctc	L	1	0.15
cta	L	0	0.17
ctg	L	0	0.18
caa	Q	1	0.54
cag	Q	0	0.46
cgt	R	0	0.22
cgc	R	0	0.37
cga	R	0	0.70
cgg	R	0	0.51
att	I	243	101.10
atc	I	28	73.10
ata	I	14	110.80
atg	M	76	76.00
act	T	30	310.90
acc	T	7	273.30
aca	T	1212	574.40
acg	T	15	105.40
aaa	K	338	359.80
aag	K	254	232.20
aga	R	5	3.21
agg	R	2	1.99
gtt	V	736	259.60
gtc	V	155	220.60
gta	V	130	295.10
gtg	V	148	393.80
gct	A	38	22.55
gcc	A	0	21.08
gca	A	51	41.47
gcg	A	3	6.90
gaa	E	686	600.20
gag	E	327	412.80
---	--	------	------
mPD		1.8	1.9
		nPD:	0.96
		N. weight:	0.65
		Sc. PD:	0.3
		Sc. rank:	1263.6

,

HA

Pos.	210	obs:	exp:
aat	N	314	2572.00
aac	N	4211	1953.00
---	--	------	------
mPD		0.13	0.49
		nPD:	0.26
		N. weight:	1.1
		Sc. PD:	-0.22
		Sc. rank:	-1158.8

,

HA

Pos.	211	obs:	exp:
tct	S	0	6.56
tcc	S	0	6.16
tca	S	0	9.47
tcg	S	0	2.07
cgt	R	0	0.35
cgc	R	0	0.58
cga	R	0	1.11
cgg	R	0	0.79
act	T	0	2.21
acc	T	1	1.95
aca	T	8	4.09
acg	T	0	0.75
aat	N	616	786.10
aac	N	767	596.90
aaa	K	950	578.00
aag	K	1	373.00
agt	S	20	7.26
agc	S	19	7.47
aga	R	11	5.04
agg	R	0	3.13
gtt	V	0	0.89
gtc	V	3	0.75
gta	V	1	1.01
gtg	V	0	1.35
gct	A	0	0.49
gcc	A	2	0.46
gca	A	0	0.90
gcg	A	0	0.15
gat	D	393	1101.00
gac	D	1565	856.70
gaa	E	164	97.17
gag	E	0	66.83
ggt	G	2	0.63
ggc	G	1	0.51
gga	G	1	1.81
ggg	G	0	1.05
---	--	------	------
mPD		1.1	1.2
		nPD:	0.94
		N. weight:	0.52
		Sc. PD:	0.23
		Sc. rank:	1006.6

,

HA

Pos.	212	obs:	exp:
tgg	W	1	1.00
caa	Q	3	1.62
cag	Q	0	1.38
cgt	R	0	6.12
cgc	R	0	10.25
cga	R	1	19.50
cgg	R	0	14.02
aat	N	3	3.41
aac	N	3	2.59
aaa	K	2724	2372.00
aag	K	1179	1531.00
aga	R	161	88.97
agg	R	32	55.14
gat	D	1	0.56
gac	D	0	0.44
gaa	E	14	242.90
gag	E	396	167.10
ggt	G	0	1.10
ggc	G	0	0.89
gga	G	0	3.17
ggg	G	7	1.84
---	--	------	------
mPD		0.72	0.76
		nPD:	0.95
		N. weight:	0.2
		Sc. PD:	0.09
		Sc. rank:	393.1

,

HA

Pos.	213	obs:	exp:
cat	H	0	0.58
cac	H	1	0.42
cgt	R	0	8.11
cgc	R	0	13.58
cga	R	0	25.83
cgg	R	1	18.57
atg	M	3	3.00
aat	N	7	3.98
aac	N	0	3.02
aaa	K	25	68.67
aag	K	88	44.33
aga	R	8	117.90
agg	R	248	73.05
gat	D	80	45.56
gac	D	1	35.44
gaa	E	194	1135.00
gag	E	1722	780.70
ggt	G	20	336.80
ggc	G	0	272.00
gga	G	139	972.50
ggg	G	1988	565.70
---	--	------	------
mPD		0.84	1.3
		nPD:	0.66
		N. weight:	1.5
		Sc. PD:	0.28
		Sc. rank:	1446.6

,

HA			
Pos.	214	obs:	exp:
cgt	R	0	2.84
cgc	R	0	4.76
cga	R	0	9.05
cgg	R	0	6.50
atg	M	1	1.00
act	T	0	0.74
acc	T	0	0.65
aca	T	2	1.36
acg	T	1	0.25
aat	N	2	1.14
aac	N	0	0.86
aaa	K	4070	2621.00
aag	K	242	1691.00
aga	R	88	41.27
agg	R	2	25.58
gtt	V	0	0.22
gtc	V	0	0.19
gta	V	1	0.25
gtg	V	0	0.34
gaa	E	114	68.73
gag	E	2	47.27
---	--	------	------
mPD		0.20	0.58
		nPD:	0.34
		N. weight:	0.63
		Sc. PD:	-0.082
		Sc. rank:	-469.8

,

HA			
Pos.	215	obs:	exp:
atg	M	1	1.00
aaa	K	18	10.94
aag	K	0	7.06
gct	A	0	0.25
gcc	A	0	0.23
gca	A	1	0.45
gcg	A	0	0.07
gat	D	2	4.50
gac	D	6	3.50
gaa	E	4430	2664.00
gag	E	66	1832.00
ggt	G	0	0.16
ggc	G	0	0.13
gga	G	1	0.45
ggg	G	0	0.26
---	--	------	------
mPD		0.042	0.49
		nPD:	0.09
		N. weight:	0.89
		Sc. PD:	-0.33
		Sc. rank:	-1491.8

,

HA			
Pos.	216	obs:	exp:
ttt	F	0	0.45
ttc	F	1	0.55
att	I	25	223.10
atc	I	600	161.30
ata	I	4	244.50
gtt	V	489	864.80
gtc	V	3137	735.00
gta	V	58	983.20
gtg	V	211	1312.00
---	--	------	------
mPD		0.54	0.98
		nPD:	0.55
		N. weight:	1.9
		Sc. PD:	0.14
		Sc. rank:	675.4

,

HA			
Pos.	217	obs:	exp:
ttt	F	1	0.45
ttc	F	0	0.55
tta	L	1	492.10
ttg	L	0	849.40
ctt	L	3988	913.30
ctc	L	393	657.90
cta	L	132	789.60
ctg	L	8	819.70
cct	P	2	0.59
ccc	P	0	0.40
cca	P	0	0.79
ccg	P	0	0.22
---	--	------	------
mPD		0.22	1.1
		nPD:	0.19
		N. weight:	2.3
		Sc. PD:	-0.64
		Sc. rank:	-3206.2

,

HA			
Pos.	218	obs:	exp:
tta	L	1	0.87
ttg	L	2	1.50
ctt	L	0	1.62
ctc	L	0	1.16
cta	L	5	1.40
ctg	L	0	1.45
att	I	6	619.70
atc	I	6	448.10
ata	I	1735	679.20
gtt	V	68	615.00
gtc	V	277	522.70
gta	V	1352	699.30
gtg	V	1073	933.00
---	--	------	------
mPD		0.95	1.2
		nPD:	0.78
		N. weight:	1.
		Sc. PD:	0.29
		Sc. rank:	1468.3

,

HA			
Pos.	219	obs:	exp:
ttt	F	1	0.45
ttc	F	0	0.55
tta	L	924	446.50
ttg	L	52	770.70
ctt	L	6	828.60
ctc	L	4	597.00
cta	L	2361	716.40
ctg	L	756	743.80
att	I	31	144.70
atc	I	149	104.60
ata	I	228	158.60
atg	M	1	1.00
gtt	V	0	2.66
gtc	V	3	2.26
gta	V	9	3.03
gtg	V	0	4.04
---	--	------	------
mPD		0.83	1.2
		nPD:	0.67
		N. weight:	1.5
		Sc. PD:	0.27
		Sc. rank:	1429.8

,

HA			
Pos.	220	obs:	exp:
tgg	W	4525	4525.00
---	--	------	------
mPD		0	0
		nPD:	1.
		N. weight:	0.
		Sc. PD:	0
		Sc. rank:	0

,

HA			
Pos.	221	obs:	exp:
gct	A	1	16.18
gcc	A	65	15.12
gca	A	0	29.75
gcg	A	0	4.95
ggt	G	3006	699.40
ggc	G	1081	565.00
gga	G	332	2020.00
ggg	G	40	1175.00
---	--	------	------
mPD		0.52	0.71
		nPD:	0.72
		N. weight:	1.9
		Sc. PD:	0.45
		Sc. rank:	2444.0

,

HA			
Pos.	222	obs:	exp:
att	I	982	473.90
atc	I	171	342.70
ata	I	183	519.40
gtt	V	2603	708.10
gtc	V	321	601.80
gta	V	59	805.00
gtg	V	206	1074.00
---	--	------	------
mPD		0.77	1.2
		nPD:	0.66
		N. weight:	1.4
		Sc. PD:	0.24
		Sc. rank:	1240.6

,

HA			
Pos.	223	obs:	exp:
cct	P	1	0.30
ccc	P	0	0.20
cca	P	0	0.40
ccg	P	0	0.11
cat	H	2772	2610.00
cac	H	1752	1914.00
---	--	------	------
mPD		0.48	0.49
		nPD:	0.97
		N. weight:	0.0077
		Sc. PD:	0.0036
		Sc. rank:	15.1

,

HA			
Pos.	224	obs:	exp:
tta	L	0	0.11
ttg	L	0	0.19
tat	Y	2	2.24
tac	Y	2	1.76
ctt	L	1	0.20
ctc	L	0	0.15
cta	L	0	0.17
ctg	L	0	0.18
cat	H	2573	2595.00
cac	H	1925	1903.00
caa	Q	0	0.54
cag	Q	1	0.46
cgt	R	0	0.03
cgc	R	1	0.05
cga	R	0	0.10
cgg	R	0	0.07
aat	N	4	11.37
aac	N	16	8.63
aga	R	0	0.46
agg	R	0	0.28
---	--	------	------
mPD		0.50	0.50
		nPD:	1.
		N. weight:	0.0079
		Sc. PD:	0.0039
		Sc. rank:	15.6

,

HA			
Pos.	225	obs:	exp:
cct	P	240	1335.00
ccc	P	15	911.00
cca	P	2290	1792.00
ccg	P	1980	486.80
---	--	------	------
mPD		0.55	0.70
		nPD:	0.78
		N. weight:	1.2
		Sc. PD:	0.36
		Sc. rank:	1853.2

,

HA			
Pos.	226	obs:	exp:
tct	S	2151	400.70
tcc	S	182	376.50
tca	S	49	578.40
tcg	S	0	126.60
tat	Y	2	1.12
tac	Y	0	0.88
cct	P	1726	628.80
ccc	P	164	429.00
cca	P	156	843.90
ccg	P	85	229.20
act	T	2	0.49
acc	T	0	0.43
aca	T	0	0.91
acg	T	0	0.17
agt	S	0	443.30
agc	S	0	456.50
gct	A	7	1.96
gcc	A	1	1.83
gca	A	0	3.61
gcg	A	0	0.60
---	--	------	------
mPD		0.76	1.7
		nPD:	0.45
		N. weight:	2.
		Sc. PD:	-0.04
		Sc. rank:	-249.3

,

HA			
Pos.	227	obs:	exp:
tct	S	1	2.86
tcc	S	0	2.69
tca	S	0	4.13
tcg	S	0	0.90
cct	P	0	0.30
ccc	P	1	0.20
cca	P	0	0.40
ccg	P	0	0.11
cgt	R	0	0.03
cgc	R	0	0.05
cga	R	0	0.10
cgg	R	0	0.07
act	T	837	354.00
acc	T	589	311.20
aca	T	13	653.90
acg	T	0	120.00
aat	N	267	1631.00
aac	N	2602	1238.00
aaa	K	3	2.43
aag	K	1	1.57
agt	S	3	3.16
agc	S	13	3.26
aga	R	1	0.46
agg	R	0	0.28
gat	D	5	108.60
gac	D	188	84.45
ggt	G	0	0.16
ggc	G	1	0.13
gga	G	0	0.45
ggg	G	0	0.26
---	--	------	------
mPD		0.90	1.2
		nPD:	0.78
		N. weight:	1.2
		Sc. PD:	0.33
		Sc. rank:	1701.1

,

HA			
Pos.	228	obs:	exp:
tta	L	1	0.33
ttg	L	0	0.56
tct	S	0	93.86
tcc	S	0	88.19
tca	S	0	135.50
tcg	S	0	29.66
tat	Y	2	67.79
tac	Y	119	53.21
ctt	L	0	0.61
ctc	L	0	0.44
cta	L	2	0.52
ctg	L	0	0.54
cat	H	3	1.73
cac	H	0	1.27
cgt	R	0	0.82
cgc	R	0	1.37
cga	R	0	2.61
cgg	R	0	1.88
att	I	35	814.80
atc	I	5	589.20
ata	I	2257	893.00
atg	M	778	778.00
act	T	13	3.44
acc	T	0	3.03
aca	T	0	6.36
acg	T	1	1.17
aat	N	167	104.60
aac	N	17	79.42
aaa	K	17	10.94
aag	K	1	7.06
agt	S	555	103.90
agc	S	3	106.90
aga	R	7	11.92
agg	R	19	7.39
gtt	V	0	2.66
gtc	V	2	2.26
gta	V	9	3.03
gtg	V	1	4.04
gct	A	10	2.70
gcc	A	0	2.52
gca	A	1	4.96
gcg	A	0	0.82
gat	D	66	82.68
gac	D	81	64.32
gaa	E	22	13.04
gag	E	0	8.96
ggt	G	300	51.92
ggc	G	30	41.94
gga	G	1	149.90
ggg	G	0	87.21
---	--	------	------
mPD		1.4	1.6
		nPD:	0.85
		N. weight:	1.6
		Sc. PD:	0.56
		Sc. rank:	2704.5

,

HA			
Pos.	229	obs:	exp:
tct	S	1	30.44
tcc	S	0	28.61
tca	S	0	43.95
tcg	S	0	9.62
caa	Q	1	2.70
cag	Q	4	2.30
cgt	R	2	3.47
cgc	R	0	5.81
cga	R	0	11.06
cgg	R	0	7.95
att	I	4	1.42
atc	I	0	1.03
ata	I	0	1.56
act	T	310	80.19
acc	T	14	70.49
aca	T	2	148.10
acg	T	0	27.18
aat	N	460	263.20
aac	N	3	199.80
aaa	K	4	23.09
aag	K	34	14.91
agt	S	146	33.69
agc	S	34	34.69
aga	R	12	50.45
agg	R	96	31.26
gtt	V	7	2.00
gtc	V	0	1.70
gta	V	0	2.27
gtg	V	2	3.03
gct	A	198	69.62
gcc	A	82	65.07
gca	A	2	128.00
gcg	A	2	21.30
gat	D	74	88.31
gac	D	83	68.69
gaa	E	187	1211.00
gag	E	1857	832.90
ggt	G	18	141.80
ggc	G	6	114.50
gga	G	41	409.50
ggg	G	839	238.20
---	--	------	------
mPD		1.5	1.6
		nPD:	0.89
		N. weight:	1.4
		Sc. PD:	0.56
		Sc. rank:	2587.9

,

HA			
Pos.	230	obs:	exp:
tct	S	0	24.39
tcc	S	2	22.92
tca	S	0	35.21
tcg	S	0	7.71
att	I	0	0.71
atc	I	2	0.51
ata	I	0	0.78
act	T	0	2.95
acc	T	12	2.59
aca	T	0	5.45
acg	T	0	1.00
aat	N	26	494.50
aac	N	844	375.50
agt	S	29	26.99
agc	S	114	27.79
gtt	V	23	5.77
gtc	V	3	4.91
gta	V	0	6.56
gtg	V	0	8.76
gct	A	0	2.21
gcc	A	9	2.06
gca	A	0	4.06
gcg	A	0	0.67
gat	D	986	1942.00
gac	D	2466	1510.00
gaa	E	7	4.15
gag	E	0	2.85
ggt	G	0	0.31
ggc	G	2	0.25
gga	G	0	0.91
ggg	G	0	0.53
---	--	------	------
mPD		0.80	0.95
		nPD:	0.84
		N. weight:	0.6
		Sc. PD:	0.21
		Sc. rank:	998.4

,

HA			
Pos.	231	obs:	exp:
tta	L	0	0.11
ttg	L	0	0.19
ctt	L	0	0.20
ctc	L	0	0.15
cta	L	1	0.17
ctg	L	0	0.18
caa	Q	4226	2439.00
cag	Q	297	2084.00
cgt	R	0	0.03
cgc	R	0	0.05
cga	R	1	0.10
cgg	R	0	0.07
aga	R	0	0.46
agg	R	0	0.28
---	--	------	------
mPD		0.12	0.50
		nPD:	0.25
		N. weight:	0.76
		Sc. PD:	-0.16
		Sc. rank:	-817.3

,

HA			
Pos.	232	obs:	exp:
tta	L	0	2.39
ttg	L	0	4.13
tat	Y	0	7.84
tac	Y	14	6.16
tgg	W	5	5.00
ctt	L	0	4.44
ctc	L	0	3.20
cta	L	22	3.84
ctg	L	0	3.99
cat	H	0	1.15
cac	H	2	0.85
caa	Q	1138	642.30
cag	Q	53	548.70
cgt	R	0	93.78
cgc	R	0	157.00
cga	R	177	298.60
cgg	R	56	214.60
att	I	3	2.13
atc	I	0	1.54
ata	I	3	2.33
atg	M	18	18.00
aaa	K	65	178.10
aag	K	228	114.90
aga	R	70	1362.00
agg	R	2668	844.40
gaa	E	3	1.78
gag	E	0	1.22
---	--	------	------
mPD		1.3	1.5
		nPD:	0.9
		N. weight:	1.5
		Sc. PD:	0.58
		Sc. rank:	2710.8

,

HA			
Pos.	233	obs:	exp:
tct	S	0	94.36
tcc	S	7	88.66
tca	S	0	136.20
tcg	S	0	29.82
tat	Y	1	0.56
tac	Y	0	0.44
tgg	W	451	451.00
caa	Q	1	11.32
cag	Q	20	9.68
cgt	R	2	0.85
cgc	R	0	1.43
cga	R	0	2.71
cgg	R	12	1.95
att	I	4	1.77
atc	I	1	1.28
ata	I	0	1.94
act	T	157	455.80
acc	T	1640	400.70
aca	T	55	842.00
acg	T	1	154.50
aat	N	1	1.14
aac	N	1	0.86
aaa	K	3	10.33
aag	K	14	6.67
agt	S	527	104.40
agc	S	27	107.50
aga	R	9	12.38
agg	R	4	7.67
gtt	V	2	1.33
gtc	V	4	1.13
gta	V	0	1.51
gtg	V	0	2.02
gct	A	41	386.30
gcc	A	1457	361.10
gca	A	77	710.40
gcg	A	1	118.20
gat	D	1	0.56
gac	D	0	0.44
gaa	E	2	1.78
gag	E	1	1.22
ggt	G	1	0.16
ggc	G	0	0.13
gga	G	0	0.45
ggg	G	0	0.26
---	--	------	------
mPD		1.4	1.6
		nPD:	0.87
		N. weight:	2.
		Sc. PD:	0.75
		Sc. rank:	3557.6

,

HA

Pos.	234	obs:	exp:
tta	L	9	449.10
ttg	L	0	775.20
ctt	L	230	833.50
ctc	L	3607	600.50
cta	L	279	720.60
ctg	L	2	748.10
att	I	22	140.80
atc	I	349	101.80
ata	I	26	154.30
atg	M	1	1.00
---	--	------	------
mPD		0.39	1.2
		nPD:	0.32
		N. weight:	2.7
		Sc. PD:	-0.41
		Sc. rank:	-2213.8

,

HA

Pos.	235	obs:	exp:
tta	L	1	0.11
ttg	L	0	0.19
tat	Y	3410	2535.00
tac	Y	1114	1989.00
ctt	L	0	0.20
ctc	L	0	0.15
cta	L	0	0.17
ctg	L	0	0.18
---	--	------	------
mPD		0.37	0.49
		nPD:	0.75
		N. weight:	0.16
		Sc. PD:	0.042
		Sc. rank:	222.8

,

HA

Pos.	236	obs:	exp:
tta	L	0	0.33
ttg	L	0	0.56
tct	S	0	1.01
tcc	S	0	0.95
tca	S	0	1.46
tcg	S	0	0.32
tat	Y	1	0.56
tac	Y	0	0.44
tgt	C	1	0.43
tgc	C	0	0.57
ctt	L	0	0.61
ctc	L	1	0.44
cta	L	2	0.52
ctg	L	0	0.54
cct	P	0	0.30
ccc	P	1	0.20
cca	P	0	0.40
ccg	P	0	0.11
cat	H	937	1321.00
cac	H	1354	969.50
caa	Q	146	790.60
cag	Q	1320	675.40
cgt	R	316	18.91
cgc	R	271	31.66
cga	R	10	60.21
cgg	R	2	43.27
att	I	0	1.06
atc	I	0	0.77
ata	I	3	1.17
aat	N	70	60.25
aac	N	36	45.75
aaa	K	18	27.35
aag	K	27	17.65
agt	S	0	1.12
agc	S	6	1.15
aga	R	0	274.70
agg	R	0	170.30
gat	D	1	0.56
gac	D	0	0.44
gaa	E	1	1.19
gag	E	1	0.81
---	--	------	------
mPD		0.99	1.2
		nPD:	0.82
		N. weight:	0.98
		Sc. PD:	0.32
		Sc. rank:	1578.8

,

HA

Pos.	237	obs:	exp:
tct	S	0	2.19
tcc	S	0	2.05
tca	S	1	3.16
tcg	S	2	0.69
cct	P	0	0.30
ccc	P	0	0.20
cca	P	1	0.40
ccg	P	0	0.11
cat	H	11	6.35
cac	H	0	4.65
cgt	R	0	0.09
cgc	R	0	0.16
cga	R	0	0.30
cgg	R	0	0.22
att	I	1	0.71
atc	I	0	0.51
ata	I	1	0.78
atg	M	2	2.00
act	T	39	601.90
acc	T	0	529.10
aca	T	1777	1112.00
acg	T	631	204.00
aat	N	1405	815.10
aac	N	29	618.90
aaa	K	573	356.10
aag	K	13	229.90
agt	S	10	2.42
agc	S	0	2.49
aga	R	3	1.38
agg	R	0	0.85
gct	A	0	4.66
gcc	A	0	4.35
gca	A	2	8.56
gcg	A	17	1.42
gat	D	4	2.25
gac	D	0	1.75
gaa	E	1	1.78
gag	E	2	1.22
---	--	------	------
mPD		1.1	1.2
		nPD:	0.91
		N. weight:	1.1
		Sc. PD:	0.43
		Sc. rank:	1981.2

,

HA

Pos.	238	obs:	exp:
tta	L	1	0.11
ttg	L	0	0.19
tct	S	0	1.51
tcc	S	0	1.42
tca	S	1	2.19
tcg	S	0	0.48
ctt	L	0	0.20
ctc	L	0	0.15
cta	L	0	0.17
ctg	L	0	0.18
cct	P	0	4.72
ccc	P	0	3.22
cca	P	16	6.34
ccg	P	0	1.72
act	T	1	10.58
acc	T	2	9.30
aca	T	40	19.54
acg	T	0	3.59
aat	N	429	270.00
aac	N	46	205.00
aaa	K	1	1.22
aag	K	1	0.78
agt	S	6	1.68
agc	S	2	1.72
gtt	V	0	2.22
gtc	V	0	1.89
gta	V	10	2.52
gtg	V	0	3.37
gct	A	8	199.80
gcc	A	0	186.70
gca	A	806	367.40
gcg	A	1	61.12
gat	D	50	31.50
gac	D	6	24.50
gaa	E	3048	1828.00
gag	E	37	1257.00
ggt	G	0	2.04
ggc	G	0	1.65
gga	G	13	5.89
ggg	G	0	3.43
---	--	------	------
mPD		0.77	1.2
		nPD:	0.65
		N. weight:	0.98
		Sc. PD:	0.16
		Sc. rank:	830.3

,

HA

Pos.	239	obs:	exp:
tct	S	0	4.21
tcc	S	0	3.95
tca	S	0	6.07
tcg	S	0	1.33
tat	Y	4	2.80
tac	Y	1	2.20
cct	P	1	0.30
ccc	P	0	0.20
cca	P	0	0.40
ccg	P	0	0.11
cat	H	168	122.90
cac	H	45	90.14
caa	Q	5	2.70
cag	Q	0	2.30
cgt	R	1	0.06
cgc	R	1	0.11
cga	R	0	0.20
cgg	R	0	0.14
att	I	1	0.35
atc	I	0	0.26
ata	I	0	0.39
act	T	251	75.76
acc	T	6	66.60
aca	T	50	140.00
acg	T	1	25.68
aat	N	3123	1800.00
aac	N	44	1367.00
agt	S	25	4.65
agc	S	0	4.79
aga	R	0	0.92
agg	R	0	0.57
gct	A	4	1.47
gcc	A	0	1.37
gca	A	2	2.70
gcg	A	0	0.45
gat	D	782	444.30
gac	D	8	345.70
gaa	E	2	1.19
gag	E	0	0.81
---	--	------	------
mPD		0.58	1.0
		nPD:	0.56
		N. weight:	1.
		Sc. PD:	0.075
		Sc. rank:	391.9

,

HA

Pos.	240	obs:	exp:
tct	S	0	10.76
tcc	S	62	10.11
tca	S	2	15.54
tcg	S	0	3.40
cct	P	0	25.08
ccc	P	85	17.11
cca	P	0	33.66
ccg	P	0	9.14
act	T	210	152.80
acc	T	189	134.30
aca	T	212	282.20
acg	T	10	51.78
agt	S	0	11.91
agc	S	0	12.27
gtt	V	1	0.22
gtc	V	0	0.19
gta	V	0	0.25
gtg	V	0	0.34
gct	A	2834	920.20
gcc	A	593	860.10
gca	A	303	1692.00
gcg	A	24	281.50
---	--	------	------
mPD		0.78	0.98
		nPD:	0.8
		N. weight:	1.2
		Sc. PD:	0.36
		Sc. rank:	1798.0

,

HA

Pos.	241	obs:	exp:
ttt	F	6	2.69
ttc	F	0	3.31
tct	S	370	66.94
tcc	S	1	62.90
tca	S	19	96.65
tcg	S	8	21.15
tat	Y	3969	2243.00
tac	Y	35	1761.00
cat	H	117	67.49
cac	H	0	49.51
agt	S	0	74.08
agc	S	0	76.28
---	--	------	------
mPD		0.24	0.80
		nPD:	0.3
		N. weight:	1.2
		Sc. PD:	-0.2
		Sc. rank:	-1055.5

,

HA

Pos.	242	obs:	exp:
att	I	46	30.15
atc	I	39	21.80
ata	I	0	33.05
gtt	V	1560	985.40
gtc	V	2772	837.40
gta	V	28	1120.00
gtg	V	78	1495.00
gct	A	1	0.49
gcc	A	0	0.46
gca	A	0	0.90
gcg	A	1	0.15
---	--	------	------
mPD		0.53	0.78
		nPD:	0.68
		N. weight:	1.6
		Sc. PD:	0.31
		Sc. rank:	1616.8

,

HA

Pos.	243	obs:	exp:
ttt	F	1142	547.60
ttc	F	80	674.40
tct	S	2924	554.40
tcc	S	110	520.90
tca	S	251	800.40
tcg	S	11	175.20
tat	Y	1	0.56
tac	Y	0	0.44
act	T	0	1.48
acc	T	1	1.30
aca	T	5	2.73
acg	T	0	0.50
agt	S	0	613.50
agc	S	0	631.70
---	--	------	------
mPD		0.58	1.7
		nPD:	0.34
		N. weight:	2.2
		Sc. PD:	-0.28
		Sc. rank:	-1584.7

,

HA			
Pos.	244	obs:	exp:
gtt	V	250	1005.00
gtc	V	6	853.90
gta	V	3415	1142.00
gtg	V	854	1524.00
---	--	------	------
mPD		0.39	0.74
		nPD:	0.53
		N. weight:	1.2
		Sc. PD:	0.065
		Sc. rank:	256.1

,

HA			
Pos.	245	obs:	exp:
tta	L	1	0.33
ttg	L	2	0.56
tct	S	0	1.18
tcc	S	0	1.11
tca	S	2	1.70
tcg	S	1	0.37
ctt	L	0	0.61
ctc	L	0	0.44
cta	L	0	0.52
ctg	L	0	0.54
att	I	2	12.77
atc	I	0	9.23
ata	I	34	14.00
atg	M	675	675.00
act	T	0	9.84
acc	T	0	8.65
aca	T	6	18.18
acg	T	34	3.34
agt	S	2	1.30
agc	S	2	1.34
gtt	V	16	485.60
gtc	V	1	412.70
gta	V	103	552.10
gtg	V	2067	736.60
gct	A	1	44.86
gcc	A	0	41.93
gca	A	173	82.49
gcg	A	9	13.72
gaa	E	70	48.59
gag	E	12	33.41
ggt	G	3	205.80
ggc	G	1	166.20
gga	G	504	594.30
ggg	G	804	345.70
---	--	------	------
mPD		1.1	1.5
		nPD:	0.74
		N. weight:	1.2
		Sc. PD:	0.3
		Sc. rank:	1612.4

,

HA			
Pos.	246	obs:	exp:
ttt	F	1	0.45
ttc	F	0	0.55
tct	S	2590	596.30
tcc	S	275	560.30
tca	S	648	860.90
tcg	S	32	188.40
act	T	47	218.20
acc	T	0	191.80
aca	T	826	403.00
acg	T	14	73.96
agt	S	0	659.80
agc	S	0	679.40
gct	A	1	22.55
gcc	A	57	21.08
gca	A	34	41.47
gcg	A	0	6.90
---	--	------	------
mPD		0.89	1.6
		nPD:	0.54
		N. weight:	1.7
		Sc. PD:	0.11
		Sc. rank:	544.9

,

HA			
Pos.	247	obs:	exp:
tta	L	2	0.22
ttg	L	0	0.38
tct	S	13	756.90
tcc	S	22	711.20
tca	S	4273	1093.00
tcg	S	192	239.20
ctt	L	0	0.40
ctc	L	0	0.29
cta	L	0	0.35
ctg	L	0	0.36
cct	P	0	4.72
ccc	P	1	3.22
cca	P	11	6.34
ccg	P	4	1.72
act	T	0	1.23
acc	T	0	1.08
aca	T	5	2.27
acg	T	0	0.42
agt	S	0	837.60
agc	S	0	862.40
gct	A	0	0.49
gcc	A	0	0.46
gca	A	2	0.90
gcg	A	0	0.15
---	--	------	------
mPD		0.11	1.6
		nPD:	0.07
		N. weight:	2.5
		Sc. PD:	-0.95
		Sc. rank:	-4444.2

,

HA			
Pos.	248	obs:	exp:
tta	L	0	0.22
ttg	L	0	0.38
tct	S	0	0.17
tcc	S	0	0.16
tca	S	0	0.24
tcg	S	0	0.05
tat	Y	9	5.04
tac	Y	0	3.96
ctt	L	2	0.40
ctc	L	0	0.29
cta	L	0	0.35
ctg	L	0	0.36
cct	P	1	0.30
ccc	P	0	0.20
cca	P	0	0.40
ccg	P	0	0.11
cat	H	2836	1755.00
cac	H	206	1287.00
caa	Q	1	1.08
cag	Q	1	0.92
cgt	R	5	9.03
cgc	R	0	15.12
cga	R	1	28.75
cgg	R	0	20.66
act	T	2	183.50
acc	T	0	161.30
aca	T	381	339.00
acg	T	363	62.21
aat	N	4	2.84
aac	N	1	2.16
aaa	K	414	260.10
aag	K	14	167.90
agt	S	0	0.19
agc	S	1	0.19
aga	R	277	131.20
agg	R	3	81.29
gct	A	0	0.25
gcc	A	0	0.23
gca	A	0	0.45
gcg	A	1	0.07
gaa	E	2	1.19
gag	E	0	0.81
---	--	------	------
mPD		1.4	1.5
		nPD:	0.91
		N. weight:	0.89
		Sc. PD:	0.37
		Sc. rank:	1676.1

,

HA			
Pos.	249	obs:	exp:
ttt	F	48	21.96
ttc	F	1	27.04
tat	Y	2956	2500.00
tac	Y	1506	1962.00
cat	H	1	7.50
cac	H	12	5.50
gat	D	1	0.56
gac	D	0	0.44
---	--	------	------
mPD		0.47	0.52
		nPD:	0.91
		N. weight:	0.063
		Sc. PD:	0.026
		Sc. rank:	118.9

,

HA			
Pos.	250	obs:	exp:
tct	S	0	646.40
tcc	S	0	607.40
tca	S	0	933.20
tcg	S	0	204.20
tat	Y	17	133.90
tac	Y	222	105.10
cat	H	0	3.46
cac	H	6	2.54
aat	N	36	246.70
aac	N	398	187.30
agt	S	87	715.30
agc	S	3756	736.50
ggt	G	0	0.47
ggc	G	3	0.38
gga	G	0	1.36
ggg	G	0	0.79
---	--	------	------
mPD		0.42	1.8
		nPD:	0.24
		N. weight:	2.7
		Sc. PD:	-0.62
		Sc. rank:	-3072.5

,

HA			
Pos.	251	obs:	exp:
cat	H	13	7.50
cac	H	0	5.50
caa	Q	124	284.70
cag	Q	404	243.30
cgt	R	0	78.06
cgc	R	0	130.70
cga	R	137	248.60
cgg	R	3	178.70
atg	M	7	7.00
aaa	K	166	411.40
aag	K	511	265.60
aga	R	1968	1134.00
agg	R	365	702.90
gaa	E	47	27.85
gag	E	0	19.15
ggt	G	2	122.30
ggc	G	0	98.83
gga	G	769	353.30
ggg	G	9	205.50
---	--	------	------
mPD		1.3	1.6
		nPD:	0.84
		N. weight:	0.72
		Sc. PD:	0.25
		Sc. rank:	1191.1

,

HA			
Pos.	252	obs:	exp:
cct	P	0	0.30
ccc	P	0	0.20
cca	P	1	0.40
ccg	P	0	0.11
cgt	R	0	98.42
cgc	R	0	164.80
cga	R	5	313.40
cgg	R	7	225.30
att	I	0	0.35
atc	I	0	0.26
ata	I	1	0.39
atg	M	1	1.00
act	T	0	5.41
acc	T	0	4.76
aca	T	20	10.00
acg	T	2	1.83
aat	N	2	1.14
aac	N	0	0.86
aaa	K	775	705.60
aag	K	386	455.40
aga	R	2692	1430.00
agg	R	414	886.20
gaa	E	136	129.20
gag	E	82	88.83
ggt	G	0	0.16
ggc	G	0	0.13
gga	G	1	0.45
ggg	G	0	0.26
---	--	------	------
mPD		0.85	1.3
		nPD:	0.63
		N. weight:	0.6
		Sc. PD:	0.09
		Sc. rank:	451.3

,

HA			
Pos.	253	obs:	exp:
ttt	F	200	2010.00
ttc	F	4286	2476.00
tta	L	4	0.76
ttg	L	0	1.31
tat	Y	1	3.92
tac	Y	6	3.08
ctt	L	0	1.41
ctc	L	3	1.02
cta	L	0	1.22
ctg	L	0	1.27
att	I	0	8.16
atc	I	22	5.90
ata	I	1	8.94
gtt	V	1	0.44
gtc	V	1	0.38
gta	V	0	0.50
gtg	V	0	0.67
---	--	------	------
mPD		0.10	0.51
		nPD:	0.2
		N. weight:	0.84
		Sc. PD:	-0.22
		Sc. rank:	-1118.4

,

HA

Pos.	254	obs:	exp:
tct	S	0	0.50
tcc	S	2	0.47
tca	S	0	0.73
tcg	S	0	0.16
att	I	18	47.18
atc	I	103	34.11
ata	I	12	51.71
atg	M	56	56.00
act	T	51	839.00
acc	T	2845	737.60
aca	T	235	1550.00
acg	T	280	284.40
aat	N	0	7.96
aac	N	14	6.04
aaa	K	499	486.80
aag	K	302	314.20
agt	S	0	0.56
agc	S	1	0.57
gtt	V	1	4.22
gtc	V	1	3.59
gta	V	1	4.80
gtg	V	16	6.40
gct	A	0	1.23
gcc	A	4	1.15
gca	A	0	2.25
gcg	A	1	0.37
gaa	E	20	49.18
gag	E	63	33.82
---	--	------	------
mPD		0.95	1.1
		nPD:	0.85
		N. weight:	1.5
		Sc. PD:	0.52
		Sc. rank:	2552.6

,

HA

Pos.	255	obs:	exp:
tct	S	0	1.68
tcc	S	0	1.58
tca	S	9	2.43
tcg	S	1	0.53
cct	P	18	1289.00
ccc	P	52	879.40
cca	P	4075	1730.00
ccg	P	223	469.90
att	I	0	51.79
atc	I	0	37.45
ata	I	146	56.76
act	T	0	0.25
acc	T	0	0.22
aca	T	1	0.45
acg	T	0	0.08
agt	S	0	1.86
agc	S	0	1.92
---	--	------	------
mPD		0.25	0.83
		nPD:	0.3
		N. weight:	1.4
		Sc. PD:	-0.23
		Sc. rank:	-1218.8

,

HA

Pos.	256	obs:	exp:
caa	Q	1	0.54
cag	Q	0	0.46
aaa	K	22	13.37
aag	K	0	8.63
gtt	V	0	2.00
gtc	V	0	1.70
gta	V	9	2.27
gtg	V	0	3.03
gat	D	0	9.00
gac	D	16	7.00
gaa	E	4267	2653.00
gag	E	210	1824.00
---	--	------	------
mPD		0.11	0.50
		nPD:	0.22
		N. weight:	0.7
		Sc. PD:	-0.17
		Sc. rank:	-871.0

,

HA

Pos.	257	obs:	exp:
tta	L	11	1.20
ttg	L	0	2.07
ctt	L	0	2.22
ctc	L	0	1.60
cta	L	0	1.92
ctg	L	0	1.99
att	I	333	1596.00
atc	I	15	1154.00
ata	I	4152	1749.00
gtt	V	1	3.11
gtc	V	0	2.64
gta	V	11	3.53
gtg	V	2	4.72
---	--	------	------
mPD		0.15	0.67
		nPD:	0.23
		N. weight:	1.3
		Sc. PD:	-0.31
		Sc. rank:	-1559.0

,

HA

Pos.	258	obs:	exp:
tct	S	0	4.04
tcc	S	14	3.79
tca	S	4	5.83
tcg	S	0	1.28
att	I	0	26.61
atc	I	48	19.24
ata	I	27	29.16
atg	M	1	1.00
act	T	13	384.20
acc	T	1524	337.80
aca	T	25	709.80
acg	T	0	130.30
aat	N	0	8.53
aac	N	15	6.47
agt	S	0	4.47
agc	S	6	4.60
gtt	V	0	67.50
gtc	V	93	57.36
gta	V	183	76.74
gtg	V	28	102.40
gct	A	278	619.90
gcc	A	1039	579.50
gca	A	843	1140.00
gcg	A	369	189.70
gaa	E	7	4.15
gag	E	0	2.85
ggt	G	0	1.25
ggc	G	1	1.01
gga	G	5	3.62
ggg	G	2	2.11
---	--	------	------
mPD		1.2	1.3
		nPD:	0.9
		N. weight:	1.2
		Sc. PD:	0.49
		Sc. rank:	2281.6

,

HA

Pos.	259	obs:	exp:
tct	S	1	3.87
tcc	S	11	3.63
tca	S	11	5.59
tcg	S	0	1.22
cct	P	0	0.30
ccc	P	1	0.20
cca	P	0	0.40
ccg	P	0	0.11
caa	Q	2	1.62
cag	Q	1	1.38
cgt	R	0	13.29
cgc	R	0	22.25
cga	R	3	42.32
cgg	R	1	30.41
att	I	0	77.69
atc	I	43	56.17
ata	I	176	85.14
act	T	4	152.50
acc	T	100	134.10
aca	T	515	281.70
acg	T	1	51.70
aat	N	0	0.57
aac	N	1	0.43
aaa	K	2039	1646.00
aag	K	670	1063.00
agt	S	0	4.28
agc	S	0	4.41
aga	R	171	193.10
agg	R	246	119.70
gtt	V	0	5.99
gtc	V	11	5.09
gta	V	16	6.82
gtg	V	0	9.09
gct	A	18	102.70
gcc	A	96	96.00
gca	A	305	188.90
gcg	A	0	31.42
gat	D	0	5.06
gac	D	9	3.94
gaa	E	72	42.66
gag	E	0	29.34
ggt	G	0	0.16
ggc	G	0	0.13
gga	G	1	0.45
ggg	G	0	0.26
---	--	------	------
mPD		1.2	1.4
		nPD:	0.85
		N. weight:	0.38
		Sc. PD:	0.13
		Sc. rank:	649.9

,

HA

Pos.	260	obs:	exp:
cgt	R	0	142.80
cgc	R	1	239.20
cga	R	6	454.80
cgg	R	4	326.90
aga	R	2450	2075.00
agg	R	2064	1286.00
---	--	------	------
mPD		0.50	0.94
		nPD:	0.53
		N. weight:	0.58
		Sc. PD:	0.032
		Sc. rank:	149.4

,

HA

Pos.	261	obs:	exp:
tct	S	0	0.34
tcc	S	2	0.32
tca	S	0	0.49
tcg	S	0	0.11
cct	P	343	1326.00
ccc	P	3977	905.00
cca	P	166	1780.00
ccg	P	9	483.50
caa	Q	19	10.25
cag	Q	0	8.75
act	T	2	1.72
acc	T	5	1.51
aca	T	0	3.18
acg	T	0	0.58
aat	N	0	0.57
aac	N	1	0.43
agt	S	0	0.37
agc	S	0	0.38
gct	A	0	0.25
gcc	A	1	0.23
gca	A	0	0.45
gcg	A	0	0.07
---	--	------	------
mPD		0.23	0.72
		nPD:	0.32
		N. weight:	2.2
		Sc. PD:	-0.32
		Sc. rank:	-1758.3

,

HA

Pos.	262	obs:	exp:
tgg	W	1	1.00
cgt	R	0	0.76
cgc	R	0	1.27
cga	R	0	2.41
cgg	R	0	1.73
atg	M	1	1.00
aaa	K	2864	2723.00
aag	K	1616	1757.00
aga	R	16	11.01
agg	R	8	6.82
gaa	E	14	11.26
gag	E	5	7.74
---	--	------	------
mPD		0.48	0.50
		nPD:	0.96
		N. weight:	0.011
		Sc. PD:	0.005
		Sc. rank:	21.5

,

HA

Pos.	263	obs:	exp:
tta	L	3	0.54
ttg	L	1	0.94
ctt	L	0	1.01
ctc	L	0	0.73
cta	L	0	0.87
ctg	L	1	0.91
att	I	4	124.90
atc	I	142	90.28
ata	I	206	136.80
atg	M	3	3.00
gtt	V	13	924.60
gtc	V	79	785.70
gta	V	1834	1051.00
gtg	V	2238	1403.00
gct	A	0	0.25
gcc	A	0	0.23
gca	A	1	0.45
gcg	A	0	0.07
---	--	------	------
mPD		0.70	0.89
		nPD:	0.78
		N. weight:	0.86
		Sc. PD:	0.25
		Sc. rank:	1285.0

,

HA

Pos.	264	obs:	exp:
tct	S	0	0.17
tcc	S	0	0.16
tca	S	0	0.24
tcg	S	0	0.05
cgt	R	0	141.90
cgc	R	0	237.50
cga	R	460	451.70
cgg	R	1	324.70
atg	M	1	1.00
aat	N	4	2.27
aac	N	0	1.73
aaa	K	16	14.59
aag	K	8	9.41
agt	S	1	0.19
agc	S	0	0.19
aga	R	3285	2061.00
agg	R	748	1277.00
ggt	G	0	0.16
ggc	G	0	0.13
gga	G	0	0.45
ggg	G	1	0.26
---	--	------	------
mPD		0.48	0.95
		nPD:	0.5
		N. weight:	0.5
		Sc. PD:	0.012
		Sc. rank:	39.9

,

HA

Pos.	265	obs:	exp:
tat	Y	0	1.12
tac	Y	2	0.88
act	T	1	3.20
acc	T	0	2.81
aca	T	12	5.91
acg	T	0	1.08
aat	N	1596	936.10
aac	N	51	710.90
aaa	K	14	10.94
aag	K	4	7.06
gat	D	1830	1246.00
gac	D	385	969.20
gaa	E	139	112.00
gag	E	50	77.01
ggt	G	430	69.17
ggc	G	2	55.88
gga	G	4	199.80
ggg	G	5	116.20
---	--	------	------
mPD		0.91	1.2
		nPD:	0.73
		N. weight:	0.8
		Sc. PD:	0.19
		Sc. rank:	1047.8

,

HA

Pos.	266	obs:	exp:
cat	H	0	0.58
cac	H	1	0.42
caa	Q	1784	2439.00
cag	Q	2738	2083.00
cgt	R	0	0.06
cgc	R	0	0.11
cga	R	2	0.20
cgg	R	0	0.14
aga	R	0	0.92
agg	R	0	0.57
---	--	------	------
mPD		0.48	0.50
		nPD:	0.96
		N. weight:	0.087
		Sc. PD:	0.04
		Sc. rank:	168.8

,

HA

Pos.	267	obs:	exp:
tta	L	1	0.11
ttg	L	0	0.19
tct	S	0	2.86
tcc	S	0	2.69
tca	S	17	4.13
tcg	S	0	0.90
ctt	L	0	0.20
ctc	L	0	0.15
cta	L	0	0.17
ctg	L	0	0.18
caa	Q	2	1.08
cag	Q	0	0.92
cgt	R	0	0.38
cgc	R	0	0.63
cga	R	0	1.21
cgg	R	0	0.87
act	T	0	62.23
acc	T	0	54.71
aca	T	251	115.00
acg	T	2	21.10
aaa	K	28	20.66
aag	K	6	13.34
agt	S	0	3.16
agc	S	0	3.26
aga	R	10	5.50
agg	R	2	3.41
gct	A	10	224.30
gcc	A	0	209.60
gca	A	839	412.40
gcg	A	66	68.62
gat	D	4	2.25
gac	D	0	1.75
gaa	E	3063	1876.00
gag	E	103	1290.00
ggt	G	0	18.98
ggc	G	0	15.33
gga	G	115	54.81
ggg	G	6	31.88
---	--	------	------
mPD		0.64	1.1
		nPD:	0.56
		N. weight:	0.92
		Sc. PD:	0.074
		Sc. rank:	401.7

,

HA

Pos.	268	obs:	exp:
ggt	G	73	709.80
ggc	G	152	573.40
gga	G	2775	2050.00
ggg	G	1525	1192.00
---	--	------	------
mPD		0.51	0.68
		nPD:	0.74
		N. weight:	0.37
		Sc. PD:	0.094
		Sc. rank:	502.1

,

HA

Pos.	269	obs:	exp:
cgt	R	0	142.80
cgc	R	0	239.20
cga	R	2	454.80
cgg	R	1	326.90
aga	R	3653	2075.00
agg	R	869	1286.00
---	--	------	------
mPD		0.31	0.94
		nPD:	0.33
		N. weight:	0.74
		Sc. PD:	-0.1
		Sc. rank:	-565.6

,

HA

Pos.	270	obs:	exp:
att	I	264	1109.00
atc	I	2058	802.00
ata	I	805	1216.00
atg	M	1397	1397.00
gtt	V	0	0.22
gtc	V	0	0.19
gta	V	0	0.25
gtg	V	1	0.34
---	--	------	------
mPD		0.66	0.74
		nPD:	0.89
		N. weight:	0.54
		Sc. PD:	0.21
		Sc. rank:	986.2

,

HA

Pos.	271	obs:	exp:
tct	S	0	1.01
tcc	S	0	0.95
tca	S	0	1.46
tcg	S	0	0.32
att	I	0	0.35
atc	I	1	0.26
ata	I	0	0.39
act	T	1	0.25
acc	T	0	0.22
aca	T	0	0.45
acg	T	0	0.08
aat	N	609	2490.00
aac	N	3772	1891.00
aaa	K	46	27.96
aag	K	0	18.04
agt	S	0	1.12
agc	S	6	1.15
gat	D	1	50.62
gac	D	89	39.38
---	--	------	------
mPD		0.29	0.55
		nPD:	0.54
		N. weight:	0.81
		Sc. PD:	0.047
		Sc. rank:	225.2

,

HA

Pos.	272	obs:	exp:
tat	Y	2238	2535.00
tac	Y	2287	1990.00
---	--	------	------
mPD		0.50	0.49
		nPD:	1.01
		N. weight:	0.019
		Sc. PD:	0.0096
		Sc. rank:	37.5

,

HA

Pos.	273	obs:	exp:
ttt	F	0	0.45
ttc	F	1	0.55
tat	Y	989	2521.00
tac	Y	3510	1978.00
cat	H	8	14.42
cac	H	17	10.58
---	--	------	------
mPD		0.36	0.50
		nPD:	0.7
		N. weight:	0.48
		Sc. PD:	0.1
		Sc. rank:	557.1

,

HA			
Pos.	274	obs:	exp:
tgg	W	4523	4523.00
cgt	R	0	0.03
cgc	R	0	0.05
cga	R	0	0.10
cgg	R	1	0.07
aga	R	0	0.46
agg	R	0	0.28
ggt	G	0	0.16
ggc	G	0	0.13
gga	G	0	0.45
ggg	G	1	0.26
---	--	------	------
mPD		0.00088	0.0015
		nPD :	0.59
		N. weight :	0.0017
		Sc. PD:	0.00019
		Sc. rank :	1.0

,

HA			
Pos.	275	obs:	exp:
att	I	25	14.19
atc	I	1	10.26
ata	I	14	15.55
act	T	3002	1093.00
acc	T	22	961.00
aca	T	1408	2019.00
acg	T	12	370.60
gct	A	2	10.05
gcc	A	0	9.39
gca	A	33	18.48
gcg	A	6	3.07
---	--	------	------
mPD		0.48	0.71
		nPD :	0.68
		N. weight :	1.1
		Sc. PD:	0.2
		Sc. rank :	1062.0

,

HA			
Pos.	276	obs:	exp:
tta	L	40	470.40
ttg	L	319	812.00
ctt	L	13	873.10
ctc	L	6	629.00
cta	L	1034	754.80
ctg	L	2911	783.60
caa	Q	0	1.08
cag	Q	2	0.92
att	I	0	63.85
atc	I	0	46.17
ata	I	180	69.98
atg	M	18	18.00
gtt	V	0	0.44
gtc	V	0	0.38
gta	V	2	0.50
gtg	V	0	0.67
---	--	------	------
mPD		0.63	1.2
		nPD :	0.53
		N. weight :	1.7
		Sc. PD:	0.085
		Sc. rank :	316.2

,

HA			
Pos.	277	obs:	exp:
tta	L	266	361.60
ttg	L	59	624.20
ctt	L	22	671.10
ctc	L	0	483.50
cta	L	1024	580.20
ctg	L	1952	602.40
att	I	0	68.47
atc	I	0	49.50
ata	I	193	75.03
atg	M	4	4.00
gtt	V	7	223.10
gtc	V	0	189.60
gta	V	975	253.70
gtg	V	23	338.50
---	--	------	------
mPD		1.0	1.4
		nPD :	0.74
		N. weight :	1.7
		Sc. PD:	0.43
		Sc. rank :	2311.5

,

HA			
Pos.	278	obs:	exp:
caa	Q	15	8.09
cag	Q	0	6.91
cgt	R	0	0.06
cgc	R	0	0.11
cga	R	0	0.20
cgg	R	0	0.14
aat	N	8	5.12
aac	N	1	3.88
aaa	K	84	54.09
aag	K	5	34.91
aga	R	2	0.92
agg	R	0	0.57
gct	A	0	9.56
gcc	A	0	8.94
gca	A	39	17.58
gcg	A	0	2.92
gat	D	127	135.00
gac	D	113	105.00
gaa	E	3157	2388.00
gag	E	873	1642.00
ggt	G	1	15.84
ggc	G	2	12.80
gga	G	97	45.75
ggg	G	1	26.61
---	--	------	------
mPD		0.51	0.66
		nPD :	0.78
		N. weight :	0.21
		Sc. PD:	0.059
		Sc. rank :	299.0

,

HA			
Pos.	279	obs:	exp:
tct	S	0	1.68
tcc	S	7	1.58
tca	S	3	2.43
tcg	S	0	0.53
cct	P	655	1251.00
ccc	P	3291	853.40
cca	P	115	1679.00
ccg	P	178	456.00
cat	H	1	1.15
cac	H	1	0.85
caa	Q	200	115.40
cag	Q	14	98.60
cgt	R	0	0.03
cgc	R	0	0.05
cga	R	0	0.10
cgg	R	1	0.07
act	T	0	0.25
acc	T	1	0.22
aca	T	0	0.45
acg	T	0	0.08
aaa	K	19	12.15
aag	K	1	7.85
agt	S	0	1.86
agc	S	0	1.92
aga	R	0	0.46
agg	R	0	0.28
gct	A	0	9.32
gcc	A	38	8.71
gca	A	0	17.13
gcg	A	0	2.85
---	--	------	------
mPD		0.56	0.84
		nPD :	0.66
		N. weight :	1.6
		Sc. PD:	0.29
		Sc. rank :	1525.8

,

HA			
Pos.	280	obs:	exp:
tct	S	0	1.01
tcc	S	0	0.95
tca	S	0	1.46
tcg	S	0	0.32
caa	Q	0	0.54
cag	Q	1	0.46
cgt	R	0	7.99
cgc	R	0	13.37
cga	R	0	25.43
cgg	R	11	18.28
atg	M	2	2.00
aat	N	13	7.96
aac	N	1	6.04
aaa	K	7	22.49
aag	K	30	14.51
agt	S	6	1.12
agc	S	0	1.15
aga	R	33	116.00
agg	R	209	71.91
gat	D	3	1.69
gac	D	0	1.31
gaa	E	29	74.06
gag	E	96	50.94
ggt	G	74	640.60
ggc	G	0	517.50
gga	G	2210	1850.00
ggg	G	1800	1076.00
---	--	------	------
mPD		0.73	0.89
		nPD :	0.82
		N. weight :	0.62
		Sc. PD:	0.2
		Sc. rank :	991.9

,

HA			
Pos.	281	obs:	exp:
aat	N	2	1.14
aac	N	0	0.86
gat	D	2196	2502.00
gac	D	2252	1946.00
gaa	E	55	42.66
gag	E	17	29.34
ggt	G	0	0.47
ggc	G	3	0.38
gga	G	0	1.36
ggg	G	0	0.79
---	--	------	------
mPD		0.52	0.51
		nPD :	1.01
		N. weight :	0.026
		Sc. PD:	0.013
		Sc. rank :	52.4

,

HA			
Pos.	282	obs:	exp:
tct	S	0	0.50
tcc	S	0	0.47
tca	S	1	0.73
tcg	S	0	0.16
cgt	R	0	0.03
cgc	R	0	0.05
cga	R	0	0.10
cgg	R	0	0.07
att	I	0	2.48
atc	I	1	1.80
ata	I	6	2.72
act	T	11	1019.00
acc	T	217	895.70
aca	T	3729	1882.00
acg	T	185	345.40
aat	N	1	7.39
aac	N	12	5.61
aaa	K	295	217.00
aag	K	62	140.00
agt	S	0	0.56
agc	S	2	0.57
aga	R	1	0.46
agg	R	0	0.28
gtt	V	0	0.22
gtc	V	0	0.19
gta	V	1	0.25
gtg	V	0	0.34
gaa	E	1	0.59
gag	E	0	0.41
---	--	------	------
mPD		0.36	0.84
		nPD :	0.43
		N. weight :	0.93
		Sc. PD:	-0.045
		Sc. rank :	-270.5

,

HA			
Pos.	283	obs:	exp:
tta	L	1	0.11
ttg	L	0	0.19
ctt	L	0	0.20
ctc	L	0	0.15
cta	L	0	0.17
ctg	L	0	0.18
att	I	9	1602.00
atc	I	0	1159.00
ata	I	4508	1756.00
gtt	V	0	1.55
gtc	V	0	1.32
gta	V	7	1.77
gtg	V	0	2.36
---	--	------	------
mPD		0.0075	0.66
		nPD :	0.01
		N. weight :	1.8
		Sc. PD:	-0.8
		Sc. rank :	-3586.9

,

HA			
Pos.	284	obs:	exp:
cgt	R	0	0.06
cgc	R	0	0.11
cga	R	0	0.20
cgg	R	0	0.14
att	I	1	1027.00
atc	I	1	742.80
ata	I	2894	1126.00
atg	M	123	123.00
act	T	146	367.00
acc	T	85	322.60
aca	T	1204	678.00
acg	T	57	124.40
aaa	K	2	3.04
aag	K	3	1.96
aga	R	1	0.92
agg	R	1	0.57
gtt	V	0	1.33
gtc	V	0	1.13
gta	V	6	1.51
gtg	V	0	2.02
gct	A	0	0.25
gcc	A	0	0.23
gca	A	1	0.45
gcg	A	0	0.07
---	--	------	------
mPD		0.62	1.1
		nPD:	0.55
		N. weight:	1.4
		Sc. PD:	0.091
		Sc. rank:	447.8

,

HA			
Pos.	285	obs:	exp:
ttt	F	3941	2023.00
ttc	F	574	2492.00
tta	L	1	0.54
ttg	L	1	0.94
tat	Y	1	0.56
tac	Y	0	0.44
tgt	C	3	1.29
tgc	C	0	1.71
ctt	L	3	1.01
ctc	L	0	0.73
cta	L	0	0.87
ctg	L	0	0.91
att	I	1	0.35
atc	I	0	0.26
ata	I	0	0.39
---	--	------	------
mPD		0.23	0.50
		nPD:	0.45
		N. weight:	0.78
		Sc. PD:	-0.017
		Sc. rank:	-117.1

,

HA			
Pos.	286	obs:	exp:
aat	N	1	0.57
aac	N	0	0.43
aaa	K	2	24.92
aag	K	39	16.08
gct	A	0	0.25
gcc	A	0	0.23
gca	A	0	0.45
gcg	A	1	0.07
gaa	E	2118	2656.00
gag	E	2364	1826.00
---	--	------	------
mPD		0.52	0.50
		nPD:	1.03
		N. weight:	0.073
		Sc. PD:	0.038
		Sc. rank:	145.6

,

HA			
Pos.	287	obs:	exp:
tct	S	0	0.50
tcc	S	1	0.47
tca	S	2	0.73
tcg	S	0	0.16
act	T	0	173.70
acc	T	5	152.70
aca	T	701	320.80
acg	T	0	58.87
agt	S	0	0.56
agc	S	0	0.57
gct	A	55	935.40
gcc	A	197	874.30
gca	A	3512	1720.00
gcg	A	52	286.20
---	--	------	------
mPD		0.39	0.94
		nPD:	0.42
		N. weight:	1.1
		Sc. PD:	-0.06
		Sc. rank:	-389.0

,

HA			
Pos.	288	obs:	exp:
tct	S	0	133.90
tcc	S	0	125.80
tca	S	0	193.30
tcg	S	0	42.31
act	T	1396	365.00
acc	T	33	320.90
aca	T	13	674.30
acg	T	42	123.70
aat	N	2225	1275.00
aac	N	18	968.10
agt	S	792	148.20
agc	S	4	152.60
gat	D	2	1.12
gac	D	0	0.88
---	--	------	------
mPD		0.66	1.4
		nPD:	0.47
		N. weight:	1.8
		Sc. PD:	-0.012
		Sc. rank:	-163.1

,

HA			
Pos.	289	obs:	exp:
ggt	G	170	709.80
ggc	G	148	573.40
gga	G	3661	2050.00
ggg	G	546	1192.00
---	--	------	------
mPD		0.33	0.68
		nPD:	0.48
		N. weight:	0.55
		Sc. PD:	0.0018
		Sc. rank:	-40.5

,

HA			
Pos.	290	obs:	exp:
aat	N	3765	2572.00
aac	N	760	1953.00
---	--	------	------
mPD		0.28	0.49
		nPD:	0.57
		N. weight:	0.31
		Sc. PD:	0.028
		Sc. rank:	147.5

,

HA			
Pos.	291	obs:	exp:
ttt	F	41	18.37
ttc	F	0	22.63
tta	L	563	487.70
ttg	L	880	841.90
ctt	L	2	905.20
ctc	L	1	652.10
cta	L	2937	782.60
ctg	L	99	812.50
att	I	0	0.35
atc	I	0	0.26
ata	I	1	0.39
gtt	V	0	0.22
gtc	V	0	0.19
gta	V	0	0.25
gtg	V	1	0.34
---	--	------	------
mPD		0.80	1.1
		nPD:	0.69
		N. weight:	1.7
		Sc. PD:	0.34
		Sc. rank:	1908.7

,

HA			
Pos.	292	obs:	exp:
tta	L	1	0.11
ttg	L	0	0.19
ctt	L	0	0.20
ctc	L	0	0.15
cta	L	0	0.17
ctg	L	0	0.18
att	I	7	1161.00
atc	I	3	839.70
ata	I	3264	1273.00
atg	M	2	2.00
aaa	K	1	0.61
aag	K	0	0.39
gtt	V	3	276.90
gtc	V	9	235.30
gta	V	82	314.80
gtg	V	1153	420.00
---	--	------	------
mPD		0.79	1.1
		nPD:	0.71
		N. weight:	1.8
		Sc. PD:	0.39
		Sc. rank:	2096.8

,

HA			
Pos.	293	obs:	exp:
tta	L	25	2.72
ttg	L	0	4.70
ctt	L	0	5.05
ctc	L	0	3.64
cta	L	0	4.37
ctg	L	0	4.53
att	I	0	2.13
atc	I	0	1.54
ata	I	6	2.33
gtt	V	2	234.70
gtc	V	0	199.50
gta	V	813	266.80
gtg	V	242	356.00
gct	A	761	842.50
gcc	A	36	787.50
gca	A	1497	1549.00
gcg	A	1143	257.80
---	--	------	------
mPD		0.99	1.1
		nPD:	0.91
		N. weight:	1.
		Sc. PD:	0.41
		Sc. rank:	1913.7

,

HA			
Pos.	294	obs:	exp:
tct	S	0	0.34
tcc	S	0	0.32
tca	S	2	0.49
tcg	S	0	0.11
cct	P	16	1335.00
ccc	P	13	910.60
cca	P	4085	1791.00
ccg	P	409	486.50
agt	S	0	0.37
agc	S	0	0.38
---	--	------	------
mPD		0.18	0.71
		nPD:	0.25
		N. weight:	1.4
		Sc. PD:	-0.29
		Sc. rank:	-1465.4

,

HA			
Pos.	295	obs:	exp:
tta	L	11	1.41
ttg	L	2	2.44
tct	S	0	0.17
tcc	S	0	0.16
tca	S	1	0.24
tcg	S	0	0.05
tgg	W	2131	2131.00
ctt	L	0	2.63
ctc	L	0	1.89
cta	L	0	2.27
ctg	L	0	2.36
caa	Q	5	2.70
cag	Q	0	2.30
cgt	R	3	69.22
cgc	R	0	115.90
cga	R	44	220.40
cgg	R	24	158.40
aaa	K	27	110.00
aag	K	154	71.00
agt	S	0	0.19
agc	S	0	0.19
aga	R	909	1006.00
agg	R	1213	623.30
ggt	G	0	0.16
ggc	G	0	0.13
gga	G	0	0.45
ggg	G	1	0.26
---	--	------	------
mPD		0.95	1.2
		nPD:	0.82
		N. weight:	0.31
		Sc. PD:	0.099
		Sc. rank:	496.4

,

HA			
Pos.	296	obs:	exp:
ttt	F	10	4.48
ttc	F	0	5.52
tat	Y	4057	2440.00
tac	Y	299	1916.00
cat	H	130	91.71
cac	H	29	67.29
---	--	------	------
mPD		0.21	0.56
		nPD:	0.37
		N. weight:	0.67
		Sc. PD:	-0.07
		Sc. rank:	-397.3

,

HA			
Pos.	297	obs:	exp:
tct	S	0	0.17
tcc	S	0	0.16
tca	S	1	0.24
tcg	S	0	0.05
agt	S	0	0.19
agc	S	0	0.19
gct	A	2592	1108.00
gcc	A	1248	1035.00
gca	A	602	2036.00
gcg	A	76	338.80
ggt	G	6	0.94
ggc	G	0	0.76
gga	G	0	2.72
ggg	G	0	1.58
---	--	------	------
mPD		0.58	0.68
		nPD:	0.85
		N. weight:	0.7
		Sc. PD:	0.25
		Sc. rank:	1194.8

,

HA			
Pos.	298	obs:	exp:
ttt	F	1297	2026.00
ttc	F	3225	2496.00
tta	L	0	0.22
ttg	L	2	0.38
tat	Y	1	0.56
tac	Y	0	0.44
ctt	L	0	0.40
ctc	L	0	0.29
cta	L	0	0.35
ctg	L	0	0.36
---	--	------	------
mPD		0.41	0.50
		nPD:	0.83
		N. weight:	0.11
		Sc. PD:	0.037
		Sc. rank:	182.4

,

HA

Pos.	299	obs:	exp:
tct	S	0	2.35
tcc	S	0	2.21
tca	S	14	3.40
tcg	S	0	0.74
cct	P	0	0.30
ccc	P	0	0.20
cca	P	1	0.40
ccg	P	0	0.11
caa	Q	2	1.08
cag	Q	0	0.92
att	I	0	23.77
atc	I	0	17.18
ata	I	67	26.05
atg	M	1	1.00
act	T	0	195.60
acc	T	0	171.90
aca	T	793	361.20
acg	T	2	66.29
aaa	K	47	28.56
aag	K	0	18.44
agt	S	0	2.61
agc	S	0	2.68
gtt	V	0	2.44
gtc	V	0	2.08
gta	V	11	2.78
gtg	V	0	3.71
gct	A	64	644.70
gcc	A	37	602.60
gca	A	2502	1185.00
gcg	A	27	197.20
gaa	E	929	567.00
gag	E	28	390.00
---	--	------	------
mPD		0.77	1.4
		nPD:	0.56
		N. weight:	1.2
		Sc. PD:	0.09
		Sc. rank:	468.1

,

HA

Pos.	300	obs:	exp:
tta	L	28	368.40
ttg	L	1011	635.80
ctt	L	13	683.60
ctc	L	0	492.50
cta	L	50	591.10
ctg	L	2283	613.60
att	I	7	11.00
atc	I	1	7.95
ata	I	23	12.05
atg	M	1056	1056.00
act	T	0	0.25
acc	T	0	0.22
aca	T	0	0.45
acg	T	1	0.08
gtt	V	0	11.55
gtc	V	0	9.81
gta	V	0	13.13
gtg	V	52	17.52
---	--	------	------
mPD		0.67	1.3
		nPD:	0.53
		N. weight:	1.4
		Sc. PD:	0.074
		Sc. rank:	308.0

,

HA

Pos.	301	obs:	exp:
tct	S	0	483.60
tcc	S	0	454.40
tca	S	0	698.20
tcg	S	0	152.80
cgt	R	1	0.28
cgc	R	0	0.48
cga	R	0	0.90
cgg	R	0	0.65
att	I	16	7.09
atc	I	0	5.13
ata	I	4	7.78
act	T	91	22.63
acc	T	0	19.89
aca	T	1	41.80
acg	T	0	7.67
aat	N	807	466.10
aac	N	13	353.90
aaa	K	263	168.30
aag	K	14	108.70
agt	S	2727	535.10
agc	S	148	551.00
aga	R	8	4.13
agg	R	0	2.56
gct	A	0	0.25
gcc	A	1	0.23
gca	A	0	0.45
gcg	A	0	0.07
gat	D	95	88.87
gac	D	63	69.13
gaa	E	231	149.90
gag	E	22	103.10
ggt	G	20	3.14
ggc	G	0	2.53
gga	G	0	9.06
ggg	G	0	5.27
---	--	------	------
mPD		0.95	1.9
		nPD:	0.49
		N. weight:	2.2
		Sc. PD:	0.024
		Sc. rank:	40.4

,

HA

Pos.	302	obs:	exp:
cgt	R	0	104.20
cgc	R	0	174.40
cga	R	8	331.70
cgg	R	0	238.40
att	I	0	0.35
atc	I	0	0.26
ata	I	1	0.39
atg	M	10	10.00
act	T	0	0.74
acc	T	0	0.65
aca	T	2	1.36
acg	T	1	0.25
aat	N	6	3.41
aac	N	0	2.59
aaa	K	139	725.60
aag	K	1055	468.40
aga	R	3130	1513.00
agg	R	162	937.90
gaa	E	0	0.59
gag	E	1	0.41
ggt	G	0	1.57
ggc	G	0	1.27
gga	G	10	4.53
ggg	G	0	2.63
---	--	------	------
mPD		0.80	1.2
		nPD:	0.64
		N. weight:	1.1
		Sc. PD:	0.18
		Sc. rank:	921.5

,

HA

Pos.	303	obs:	exp:
tct	S	0	61.06
tcc	S	0	57.37
tca	S	0	88.15
tcg	S	0	19.29
tat	Y	0	0.56
tac	Y	1	0.44
cat	H	2	1.15
cac	H	0	0.85
cgt	R	0	0.03
cgc	R	0	0.05
cga	R	0	0.10
cgg	R	0	0.07
aat	N	360	214.30
aac	N	17	162.70
aaa	K	1	0.61
aag	K	0	0.39
agt	S	317	67.56
agc	S	46	69.57
aga	R	1	0.46
agg	R	0	0.28
gct	A	1	1.47
gcc	A	5	1.37
gca	A	0	2.70
gcg	A	0	0.45
gat	D	26	18.56
gac	D	7	14.44
gaa	E	7	7.11
gag	E	5	4.89
ggt	G	1132	584.90
ggc	G	2104	472.50
gga	G	426	1689.00
ggg	G	67	982.50
---	--	------	------
mPD		1.0	1.3
		nPD:	0.82
		N. weight:	1.6
		Sc. PD:	0.54
		Sc. rank:	2667.4

,

HA

Pos.	304	obs:	exp:
ttt	F	1317	596.40
ttc	F	14	734.60
tta	L	10	179.60
ttg	L	17	309.90
tct	S	1206	209.40
tcc	S	35	196.80
tca	S	4	302.30
tcg	S	0	66.17
tat	Y	4	2.24
tac	Y	0	1.76
ctt	L	1614	333.20
ctc	L	2	240.10
cta	L	0	288.10
ctg	L	7	299.10
cct	P	26	8.85
ccc	P	2	6.04
cca	P	1	11.88
ccg	P	1	3.23
cat	H	9	5.19
cac	H	0	3.81
att	I	32	11.35
atc	I	0	8.21
ata	I	0	12.44
act	T	19	4.67
acc	T	0	4.11
aca	T	0	8.63
acg	T	0	1.58
agt	S	0	231.70
agc	S	0	238.60
gtt	V	7	1.55
gtc	V	0	1.32
gta	V	0	1.77
gtg	V	0	2.36
gct	A	191	48.29
gcc	A	3	45.14
gca	A	3	88.80
gcg	A	0	14.77
gat	D	1	0.56
gac	D	0	0.44
---	--	------	------
mPD		1.0	1.8
		nPD:	0.57
		N. weight:	2.5
		Sc. PD:	0.21
		Sc. rank:	1163.0

,

HA

Pos.	305	obs:	exp:
tct	S	0	28.43
tcc	S	0	26.71
tca	S	0	41.04
tcg	S	0	8.98
cgt	R	6	1.96
cgc	R	0	3.28
cga	R	0	6.23
cgg	R	0	4.48
att	I	11	3.90
atc	I	0	2.82
ata	I	0	4.28
act	T	1	0.25
acc	T	0	0.22
aca	T	0	0.45
acg	T	0	0.08
aat	N	38	24.44
aac	N	5	18.56
aaa	K	94	61.38
aag	K	7	39.62
agt	S	165	31.45
agc	S	4	32.39
aga	R	55	28.43
agg	R	1	17.62
gat	D	4	2.25
gac	D	0	1.75
gaa	E	93	136.90
gag	E	138	94.13
ggt	G	45	612.20
ggc	G	6	494.60
gga	G	3683	1768.00
ggg	G	169	1028.00
---	--	------	------
mPD		0.55	1.0
		nPD:	0.53
		N. weight:	1.2
		Sc. PD:	0.059
		Sc. rank:	226.1

,

HA

Pos.	306	obs:	exp:
ttt	F	1	0.45
ttc	F	0	0.55
tta	L	2	0.22
ttg	L	0	0.38
tct	S	1331	760.10
tcc	S	121	714.20
tca	S	3059	1097.00
tcg	S	8	240.20
tat	Y	1	0.56
tac	Y	0	0.44
ctt	L	0	0.40
ctc	L	0	0.29
cta	L	0	0.35
ctg	L	0	0.36
cct	P	1	0.30
ccc	P	0	0.20
cca	P	0	0.40
ccg	P	0	0.11
agt	S	0	841.10
agc	S	0	866.10
gct	A	1	0.25
gcc	A	0	0.23
gca	A	0	0.45
gcg	A	0	0.07
---	--	------	------
mPD		0.46	1.6
		nPD:	0.28
		N. weight:	1.6
		Sc. PD:	-0.29
		Sc. rank:	-1555.5

,

HA

Pos.	307	obs:	exp:
tct	S	0	11.44
tcc	S	0	10.75
tca	S	0	16.51
tcg	S	0	3.61
cgt	R	0	0.06
cgc	R	0	0.11
cga	R	1	0.20
cgg	R	0	0.14
agt	S	68	12.66
agc	S	0	13.03
aga	R	1	0.92
agg	R	0	0.57
ggt	G	1144	698.80
ggc	G	9	564.50
gga	G	3234	2018.00
ggg	G	68	1174.00
---	--	------	------
mPD		0.45	0.74
		nPD:	0.61
		N. weight:	0.86
		Sc. PD:	0.11
		Sc. rank:	567.0

,

HA

Pos.	308	obs:	exp:
att	I	1368	1603.00
atc	I	3069	1159.00
ata	I	83	1757.00
gtt	V	2	1.11
gtc	V	3	0.94
gta	V	0	1.26
gtg	V	0	1.68
---	--	------	------
mPD		0.45	0.66
		nPD:	0.68
		N. weight:	1.1
		Sc. PD:	0.21
		Sc. rank:	1124.2

,

HA

Pos.	309	obs:	exp:
tta	L	1	1.09
ttg	L	4	1.88
ctt	L	0	2.02
ctc	L	3	1.45
cta	L	2	1.75
ctg	L	0	1.81
att	I	225	1505.00
atc	I	3140	1088.00
ata	I	878	1650.00
atg	M	210	210.00
gtt	V	0	13.77
gtc	V	5	11.70
gta	V	57	15.65
gtg	V	0	20.88
---	--	------	------
mPD		0.50	0.72
		nPD:	0.69
		N. weight:	1.1
		Sc. PD:	0.22
		Sc. rank:	1188.5

,

HA

Pos.	310	obs:	exp:
tct	S	0	0.17
tcc	S	1	0.16
tca	S	0	0.24
tcg	S	0	0.05
att	I	721	319.30
atc	I	131	230.80
ata	I	48	349.90
atg	M	133	133.00
act	T	231	666.80
acc	T	1648	586.20
aca	T	826	1232.00
acg	T	6	226.10
aat	N	43	33.53
aac	N	16	25.47
aaa	K	41	30.39
aag	K	9	19.61
agt	S	0	0.19
agc	S	0	0.19
gtt	V	451	149.00
gtc	V	215	126.60
gta	V	4	169.40
gtg	V	1	226.00
---	--	------	------
mPD		1.4	1.5
		nPD:	0.96
		N. weight:	0.94
		Sc. PD:	0.43
		Sc. rank:	1820.9

,

HA

Pos.	311	obs:	exp:
tct	S	1427	760.60
tcc	S	11	714.70
tca	S	2279	1098.00
tcg	S	805	240.30
act	T	0	0.49
acc	T	0	0.43
aca	T	2	0.91
acg	T	0	0.17
agt	S	0	841.70
agc	S	0	866.60
gct	A	0	0.25
gcc	A	0	0.23
gca	A	1	0.45
gcg	A	0	0.07
---	--	------	------
mPD		0.62	1.6
		nPD:	0.38
		N. weight:	1.5
		Sc. PD:	-0.14
		Sc. rank:	-804.3

,

HA

Pos.	312	obs:	exp:
tct	S	0	2.52
tcc	S	0	2.37
tca	S	0	3.64
tcg	S	0	0.80
cat	H	1	0.58
cac	H	0	0.42
caa	Q	1	0.54
cag	Q	0	0.46
cgt	R	0	0.13
cgc	R	0	0.21
cga	R	0	0.40
cgg	R	0	0.29
act	T	1	0.25
acc	T	0	0.22
aca	T	0	0.45
acg	T	0	0.08
aat	N	2214	1304.00
aac	N	81	990.60
aaa	K	686	429.10
aag	K	20	276.90
agt	S	15	2.79
agc	S	0	2.87
aga	R	4	1.83
agg	R	0	1.14
gat	D	1353	793.10
gac	D	57	616.90
gaa	E	89	53.92
gag	E	2	37.08
ggt	G	1	0.16
ggc	G	0	0.13
gga	G	0	0.45
ggg	G	0	0.26
---	--	------	------
mPD		0.80	1.1
		nPD:	0.73
		N. weight:	0.83
		Sc. PD:	0.19
		Sc. rank:	1050.6

,

HA

Pos.	313	obs:	exp:
tct	S	6	3.03
tcc	S	1	2.84
tca	S	11	4.37
tcg	S	0	0.96
att	I	1	6.03
atc	I	0	4.36
ata	I	16	6.61
atg	M	96	96.00
act	T	27	100.40
acc	T	5	88.23
aca	T	371	185.40
acg	T	5	34.02
aaa	K	29	18.23
aag	K	1	11.77
agt	S	0	3.35
agc	S	0	3.45
gtt	V	24	63.95
gtc	V	2	54.34
gta	V	97	72.70
gtg	V	165	97.01
gct	A	219	898.90
gcc	A	379	840.20
gca	A	2612	1653.00
gcg	A	457	275.00
gat	D	1	0.56
gac	D	0	0.44
---	--	------	------
mPD		0.88	1.1
		nPD:	0.8
		N. weight:	0.51
		Sc. PD:	0.16
		Sc. rank:	776.1

,

HA

Pos.	314	obs:	exp:
ttt	F	1	0.45
ttc	F	0	0.55
tta	L	1	0.33
ttg	L	0	0.56
tct	S	43	97.72
tcc	S	0	91.82
tca	S	327	141.10
tcg	S	211	30.88
tat	Y	6	3.36
tac	Y	0	2.64
ctt	L	2	0.61
ctc	L	0	0.44
cta	L	0	0.52
ctg	L	0	0.54
cct	P	140	1087.00
ccc	P	22	741.30
cca	P	3506	1458.00
ccg	P	14	396.10
cat	H	196	117.10
cac	H	7	85.91
caa	Q	5	10.25
cag	Q	14	8.75
cgt	R	1	0.03
cgc	R	0	0.05
cga	R	0	0.10
cgg	R	0	0.07
act	T	0	4.92
acc	T	0	4.32
aca	T	9	9.09
acg	T	11	1.67
aat	N	9	5.12
aac	N	0	3.88
agt	S	0	108.10
agc	S	0	111.30
aga	R	0	0.46
agg	R	0	0.28
---	--	------	------
mPD		0.61	1.1
		nPD:	0.53
		N. weight:	1.5
		Sc. PD:	0.075
		Sc. rank:	266.4

,

HA

Pos.	315	obs:	exp:
ttt	F	10	4.93
ttc	F	1	6.07
tta	L	0	37.98
ttg	L	0	65.55
ctt	L	320	70.48
ctc	L	1	50.78
cta	L	4	60.94
ctg	L	24	63.26
cgt	R	0	0.13
cgc	R	0	0.21
cga	R	0	0.40
cgg	R	0	0.29
att	I	367	165.70
atc	I	43	119.80
ata	I	57	181.60
atg	M	2814	2814.00
act	T	0	7.38
acc	T	1	6.49
aca	T	14	13.63
acg	T	15	2.50
aga	R	0	1.83
agg	R	4	1.14
gtt	V	262	187.80
gtc	V	356	159.60
gta	V	94	213.60
gtg	V	134	285.00
gct	A	0	0.98
gcc	A	4	0.92
gca	A	0	1.80
gcg	A	0	0.30
---	--	------	------
mPD		0.95	0.90
		nPD:	1.05
		N. weight:	0.39
		Sc. PD:	0.21
		Sc. rank:	765.3

,

HA

Pos.	316	obs:	exp:
tct	S	0	1.68
tcc	S	0	1.58
tca	S	0	2.43
tcg	S	0	0.53
tat	Y	29	22.41
tac	Y	11	17.59
cat	H	418	804.10
cac	H	976	589.90
caa	Q	18	10.25
cag	Q	1	8.75
cgt	R	0	0.13
cgc	R	3	0.21
cga	R	1	0.40
cgg	R	0	0.29
aat	N	17	17.05
aac	N	13	12.95
agt	S	10	1.86
agc	S	0	1.92
aga	R	0	1.83
agg	R	0	1.14
gct	A	2	0.74
gcc	A	1	0.69
gca	A	0	1.35
gcg	A	0	0.22
gat	D	347	1005.00
gac	D	1440	781.90
gaa	E	3	27.85
gag	E	44	19.15
ggt	G	1048	186.80
ggc	G	134	150.90
gga	G	5	539.50
ggg	G	4	313.80
---	--	------	------
mPD		1.4	1.5
		nPD:	0.9
		N. weight:	1.1
		Sc. PD:	0.44
		Sc. rank:	2070.3

,

HA

Pos.	317	obs:	exp:
caa	Q	2	1.08
cag	Q	0	0.92
cgt	R	0	0.16
cgc	R	0	0.26
cga	R	0	0.50
cgg	R	0	0.36
aat	N	365	218.80
aac	N	20	166.20
aaa	K	262	234.60
aag	K	124	151.40
aga	R	0	2.29
agg	R	5	1.42
gtt	V	0	0.22
gtc	V	0	0.19
gta	V	1	0.25
gtg	V	0	0.34
gct	A	0	0.49
gcc	A	0	0.46
gca	A	2	0.90
gcg	A	0	0.15
gat	D	1088	613.60
gac	D	3	477.40
gaa	E	1779	1513.00
gag	E	774	1040.00
ggt	G	0	15.69
ggc	G	0	12.67
gga	G	61	45.29
ggg	G	39	26.35
---	--	------	------
mPD		0.97	1.0
		nPD:	0.93
		N. weight:	0.38
		Sc. PD:	0.16
		Sc. rank:	722.8

,

HA

Pos.	318	obs:	exp:
tct	S	0	0.17
tcc	S	0	0.16
tca	S	0	0.24
tcg	S	0	0.05
tgt	C	3190	1947.00
tgc	C	1334	2577.00
agt	S	0	0.19
agc	S	1	0.19
---	--	------	------
mPD		0.42	0.49
		nPD:	0.85
		N. weight:	0.3
		Sc. PD:	0.11
		Sc. rank:	518.5

,

HA

Pos.	319	obs:	exp:
tct	S	12	5.72
tcc	S	0	5.37
tca	S	0	8.26
tcg	S	0	1.81
tat	Y	1	1.12
tac	Y	1	0.88
tgt	C	1	0.43
tgc	C	0	0.57
act	T	295	101.30
acc	T	116	89.09
aca	T	1	187.20
acg	T	0	34.36
aat	N	1850	1161.00
aac	N	192	881.40
agt	S	21	6.33
agc	S	1	6.52
gat	D	1747	1136.00
gac	D	273	883.80
gaa	E	13	8.30
gag	E	1	5.70
---	--	------	------
mPD		0.91	1.2
		nPD:	0.74
		N. weight:	0.61
		Sc. PD:	0.15
		Sc. rank:	813.3

HA

Pos.	320	obs:	exp:
tct	S	1	88.98
tcc	S	0	83.60
tca	S	10	128.50
tcg	S	518	28.12
act	T	6	587.40
acc	T	5	516.40
aca	T	1496	1085.00
acg	T	881	199.10
agt	S	0	98.46
agc	S	0	101.40
gtt	V	0	1.33
gtc	V	0	1.13
gta	V	4	1.51
gtg	V	2	2.02
gct	A	41	392.70
gcc	A	0	367.10
gca	A	1238	722.10
gcg	A	323	120.10
---	--	------	------
mPD		1.1	1.3
		nPD:	0.81
		N. weight:	1.8
		Sc. PD:	0.57
		Sc. rank:	2859.6

HA

Pos.	321	obs:	exp:
cct	P	1	0.30
ccc	P	0	0.20
cca	P	0	0.40
ccg	P	0	0.11
caa	Q	1	0.54
cag	Q	0	0.46
cgt	R	0	0.82
cgc	R	0	1.37
cga	R	0	2.61
cgg	R	0	1.88
att	I	10	3.90
atc	I	1	2.82
ata	I	0	4.28
atg	M	7	7.00
act	T	813	205.40
acc	T	11	180.60
aca	T	8	379.40
acg	T	3	69.63
aat	N	44	25.01
aac	N	0	18.99
aaa	K	1448	2142.00
aag	K	2076	1382.00
aga	R	4	11.92
agg	R	22	7.39
gct	A	17	4.17
gcc	A	0	3.90
gca	A	0	7.66
gcg	A	0	1.27
gaa	E	58	34.96
gag	E	1	24.04
---	--	------	------
mPD		0.99	0.92
		nPD:	1.08
		N. weight:	0.63
		Sc. PD:	0.35
		Sc. rank:	1245.9

HA

Pos.	322	obs:	exp:
tgt	C	3923	1948.00
tgc	C	602	2577.00
---	--	------	------
mPD		0.23	0.49
		nPD:	0.47
		N. weight:	0.81
		Sc. PD:	-0.0043
		Sc. rank:	-65.4

HA

Pos.	323	obs:	exp:
caa	Q	3571	2440.00
cag	Q	954	2085.00
---	--	------	------
mPD		0.33	0.50
		nPD:	0.67
		N. weight:	0.27
		Sc. PD:	0.049
		Sc. rank:	260.3

HA

Pos.	324	obs:	exp:
act	T	232	1113.00
acc	T	61	978.50
aca	T	4174	2056.00
acg	T	58	377.30
---	--	------	------
mPD		0.15	0.68
		nPD:	0.22
		N. weight:	1.
		Sc. PD:	-0.25
		Sc. rank:	-1283.1

HA

Pos.	325	obs:	exp:
tta	L	0	0.11
ttg	L	0	0.19
ctt	L	0	0.20
ctc	L	1	0.15
cta	L	0	0.17
ctg	L	0	0.18
cct	P	2808	1335.00
ccc	P	1130	910.80
cca	P	584	1792.00
ccg	P	2	486.60
---	--	------	------
mPD		0.54	0.70
		nPD:	0.76
		N. weight:	0.73
		Sc. PD:	0.2
		Sc. rank:	1020.6

HA

Pos.	326	obs:	exp:
tta	L	0	3.26
ttg	L	0	5.63
tat	Y	14	7.84
tac	Y	0	6.16
ctt	L	5	6.06
ctc	L	0	4.36
cta	L	3	5.24
ctg	L	22	5.44
cct	P	0	0.30
ccc	P	0	0.20
cca	P	1	0.40
ccg	P	0	0.11
cat	H	269	158.00
cac	H	5	116.00
caa	Q	556	1094.00
cag	Q	1473	934.80
cgt	R	12	5.65
cgc	R	0	9.46
cga	R	31	17.99
cgg	R	117	12.93
aat	N	63	38.08
aac	N	4	28.92
aaa	K	931	1160.00
aag	K	977	748.40
aga	R	16	82.09
agg	R	3	50.88
gat	D	2	1.12
gac	D	0	0.88
gaa	E	2	12.44
gag	E	19	8.56
---	--	------	------
mPD		1.1	1.2
		nPD:	0.97
		N. weight:	0.36
		Sc. PD:	0.17
		Sc. rank:	702.5

HA

Pos.	327	obs:	exp:
ggt	G	1201	709.80
ggc	G	6	573.40
gga	G	2097	2050.00
ggg	G	1221	1192.00
---	--	------	------
mPD		0.64	0.68
		nPD:	0.94
		N. weight:	0.3
		Sc. PD:	0.13
		Sc. rank:	575.8

HA

Pos.	328	obs:	exp:
tct	S	1	0.34
tcc	S	1	0.32
tca	S	0	0.49
tcg	S	0	0.11
agt	S	0	0.37
agc	S	0	0.38
gct	A	3228	1106.00
gcc	A	901	1034.00
gca	A	238	2034.00
gcg	A	146	338.50
ggt	G	10	1.57
ggc	G	0	1.27
gga	G	0	4.53
ggg	G	0	2.63
---	--	------	------
mPD		0.45	0.68
		nPD:	0.66
		N. weight:	1.2
		Sc. PD:	0.2
		Sc. rank:	1043.9

HA			
Pos.	329	obs:	exp:
tta	L	334	98.16
ttg	L	221	169.40
ctt	L	0	182.20
ctc	L	0	131.20
cta	L	338	157.50
ctg	L	9	163.50
att	I	0	1278.00
atc	I	0	924.10
ata	I	3603	1401.00
atg	M	17	17.00
gtt	V	0	0.67
gtc	V	0	0.57
gta	V	3	0.76
gtg	V	0	1.01
---	--	------	------
mPD		0.44	1.0
		nPD:	0.43
		N. weight:	1.8
		Sc. PD:	-0.084
		Sc. rank:	-496.9

,

HA			
Pos.	330	obs:	exp:
tct	S	0	0.17
tcc	S	0	0.16
tca	S	0	0.24
tcg	S	0	0.05
cat	H	0	1.15
cac	H	2	0.85
cgt	R	0	0.85
cgc	R	0	1.43
cga	R	0	2.71
cgg	R	0	1.95
att	I	0	0.35
atc	I	1	0.26
ata	I	0	0.39
act	T	0	1.97
acc	T	7	1.73
aca	T	1	3.64
acg	T	0	0.67
aat	N	319	2432.00
aac	N	3959	1846.00
aaa	K	191	117.30
aag	K	2	75.71
agt	S	0	0.19
agc	S	1	0.19
aga	R	27	12.38
agg	R	0	7.67
gat	D	0	0.56
gac	D	1	0.44
gaa	E	13	7.70
gag	E	0	5.30
ggt	G	0	0.16
ggc	G	0	0.13
gga	G	1	0.45
ggg	G	0	0.26
---	--	------	------
mPD		0.25	0.57
		nPD:	0.43
		N. weight:	1.1
		Sc. PD:	-0.044
		Sc. rank:	-238.4

,

HA			
Pos.	331	obs:	exp:
tct	S	0	526.30
tcc	S	2	494.50
tca	S	0	759.80
tcg	S	0	166.30
cgt	R	0	0.03
cgc	R	1	0.05
cga	R	0	0.10
cgg	R	0	0.07
act	T	44	300.30
acc	T	1173	264.00
aca	T	4	554.80
acg	T	0	101.80
aat	N	71	50.02
aac	N	17	37.98
agt	S	198	582.40
agc	S	2929	599.70
aga	R	0	0.46
agg	R	0	0.28
gtt	V	1	0.22
gtc	V	0	0.19
gta	V	0	0.25
gtg	V	0	0.34
ggt	G	1	13.33
ggc	G	72	10.77
gga	G	12	38.50
ggg	G	0	22.40
---	--	------	------
mPD		0.60	1.6
		nPD:	0.36
		N. weight:	2.7
		Sc. PD:	-0.29
		Sc. rank:	-1613.7

,

HA			
Pos.	332	obs:	exp:
tct	S	0	575.90
tcc	S	0	541.10
tca	S	0	831.50
tcg	S	0	182.00
cat	H	1	0.58
cac	H	0	0.42
cgt	R	2	0.09
cgc	R	0	0.16
cga	R	0	0.30
cgg	R	0	0.22
att	I	1	0.35
atc	I	0	0.26
ata	I	0	0.39
act	T	15	3.94
acc	T	1	3.46
aca	T	0	7.27
acg	T	0	1.33
aat	N	980	588.80
aac	N	56	447.20
aaa	K	5	3.04
aag	K	0	1.96
agt	S	1524	637.30
agc	S	1900	656.20
aga	R	1	1.38
agg	R	0	0.85
gat	D	39	21.94
gac	D	0	17.06
---	--	------	------
mPD		0.88	1.8
		nPD:	0.49
		N. weight:	1.6
		Sc. PD:	0.017
		Sc. rank:	-11.3

,

HA			
Pos.	333	obs:	exp:
ttt	F	1	0.45
ttc	F	0	0.55
tta	L	0	487.50
ttg	L	1	841.50
ctt	L	3438	904.80
ctc	L	975	651.80
cta	L	17	782.30
ctg	L	49	812.10
cgt	R	42	1.36
cgc	R	1	2.27
cga	R	0	4.32
cgg	R	0	3.11
atg	M	1	1.00
aga	R	0	19.72
agg	R	0	12.22
---	--	------	------
mPD		0.38	1.2
		nPD:	0.33
		N. weight:	2.1
		Sc. PD:	-0.3
		Sc. rank:	-1636.2

,

HA			
Pos.	334	obs:	exp:
tta	L	0	0.11
ttg	L	0	0.19
ctt	L	0	0.20
ctc	L	0	0.15
cta	L	1	0.17
ctg	L	0	0.18
cct	P	2429	1335.00
ccc	P	713	910.80
cca	P	1167	1792.00
ccg	P	215	486.60
---	--	------	------
mPD		0.62	0.70
		nPD:	0.88
		N. weight:	0.27
		Sc. PD:	0.1
		Sc. rank:	478.2

,

HA			
Pos.	335	obs:	exp:
ttt	F	1505	2028.00
ttc	F	3020	2497.00
---	--	------	------
mPD		0.44	0.49
		nPD:	0.9
		N. weight:	0.056
		Sc. PD:	0.022
		Sc. rank:	104.0

,

HA			
Pos.	336	obs:	exp:
cat	H	2	1.73
cac	H	1	1.27
caa	Q	263	2439.00
cag	Q	4259	2083.00
---	--	------	------
mPD		0.11	0.50
		nPD:	0.22
		N. weight:	1.1
		Sc. PD:	-0.26
		Sc. rank:	-1297.6

,

HA			
Pos.	337	obs:	exp:
tct	S	0	0.34
tcc	S	0	0.32
tca	S	0	0.49
tcg	S	0	0.11
cat	H	1	0.58
cac	H	0	0.42
act	T	0	0.25
acc	T	1	0.22
aca	T	0	0.45
acg	T	0	0.08
aat	N	4362	2567.00
aac	N	155	1950.00
aaa	K	3	2.43
aag	K	1	1.57
agt	S	2	0.37
agc	S	0	0.38
---	--	------	------
mPD		0.070	0.49
		nPD:	0.14
		N. weight:	0.84
		Sc. PD:	-0.26
		Sc. rank:	-1241.2

,

HA			
Pos.	338	obs:	exp:
att	I	4	180.60
atc	I	21	130.60
ata	I	484	197.90
gtt	V	9	891.30
gtc	V	1	757.40
gta	V	3742	1013.00
gtg	V	262	1352.00
gct	A	0	0.49
gcc	A	0	0.46
gca	A	2	0.90
gcg	A	0	0.15
---	--	------	------
mPD		0.32	0.94
		nPD:	0.34
		N. weight:	2.1
		Sc. PD:	-0.26
		Sc. rank:	-1476.3

,

HA			
Pos.	339	obs:	exp:
cct	P	0	0.30
ccc	P	1	0.20
cca	P	0	0.40
ccg	P	0	0.11
cat	H	2141	2605.00
cac	H	2376	1912.00
caa	Q	0	0.54
cag	Q	1	0.46
aat	N	2	3.41
aac	N	4	2.59
---	--	------	------
mPD		0.50	0.49
		nPD:	1.02
		N. weight:	0.046
		Sc. PD:	0.023
		Sc. rank:	90.3

,

HA			
Pos.	340	obs:	exp:
tct	S	0	0.84
tcc	S	0	0.79
tca	S	5	1.21
tcg	S	0	0.27
cct	P	184	1333.00
ccc	P	236	909.60
cca	P	3712	1789.00
ccg	P	386	486.00
caa	Q	1	0.54
cag	Q	0	0.46
agt	S	0	0.93
agc	S	0	0.96
gct	A	0	0.25
gcc	A	0	0.23
gca	A	1	0.45
gcg	A	0	0.07
---	--	------	------
mPD		0.32	0.71
		nPD:	0.45
		N. weight:	0.85
		Sc. PD:	-0.024
		Sc. rank:	-147.8

,

HA			
Pos.	341	obs:	exp:
ttt	F	3	4.48
ttc	F	7	5.52
tta	L	0	0.44
ttg	L	0	0.75
ctt	L	2	0.81
ctc	L	1	0.58
cta	L	1	0.70
ctg	L	0	0.73
att	I	263	239.10
atc	I	386	172.90
ata	I	25	262.00
act	T	0	0.49
acc	T	2	0.43
aca	T	0	0.91
acg	T	0	0.17
gtt	V	648	849.70
gtc	V	2359	722.10
gta	V	802	966.10
gtg	V	18	1289.00
gct	A	0	1.96
gcc	A	6	1.83
gca	A	2	3.61
gcg	A	0	0.60
---	--	------	------
mPD		0.82	1.0
		nPD:	0.81
		N. weight:	1.2
		Sc. PD:	0.37
		Sc. rank:	1834.0

,

HA			
Pos.	342	obs:	exp:
tct	S	1	0.67
tcc	S	1	0.63
tca	S	2	0.97
tcg	S	0	0.21
act	T	268	1110.00
acc	T	20	975.50
aca	T	4192	2050.00
acg	T	31	376.20
agt	S	0	0.74
agc	S	0	0.77
gct	A	7	2.45
gcc	A	0	2.29
gca	A	3	4.51
gcg	A	0	0.75
---	--	------	------
mPD		0.14	0.69
		nPD:	0.21
		N. weight:	1.1
		Sc. PD:	-0.27
		Sc. rank:	-1413.9

,

HA			
Pos.	343	obs:	exp:
tta	L	5	0.76
ttg	L	0	1.31
ctt	L	0	1.41
ctc	L	0	1.02
cta	L	2	1.22
ctg	L	0	1.27
att	I	1406	1600.00
atc	I	82	1157.00
ata	I	3023	1754.00
atg	M	1	1.00
gtt	V	2	1.33
gtc	V	1	1.13
gta	V	3	1.51
gtg	V	0	2.02
---	--	------	------
mPD		0.46	0.66
		nPD:	0.69
		N. weight:	0.55
		Sc. PD:	0.11
		Sc. rank:	616.7

,

HA			
Pos.	344	obs:	exp:
ggt	G	24	709.80
ggc	G	0	573.40
gga	G	4072	2050.00
ggg	G	429	1192.00
---	--	------	------
mPD		0.18	0.68
		nPD:	0.26
		N. weight:	0.99
		Sc. PD:	-0.2
		Sc. rank:	-1002.3

,

HA			
Pos.	345	obs:	exp:
caa	Q	11	6.47
cag	Q	1	5.53
aaa	K	256	157.40
aag	K	3	101.60
gct	A	0	0.25
gcc	A	0	0.23
gca	A	1	0.45
gcg	A	0	0.07
gat	D	0	0.56
gac	D	1	0.44
gaa	E	3060	2519.00
gag	E	1192	1733.00
---	--	------	------
mPD		0.50	0.60
		nPD:	0.84
		N. weight:	0.12
		Sc. PD:	0.041
		Sc. rank:	200.0

,

HA			
Pos.	346	obs:	exp:
tgt	C	3870	1948.00
tgc	C	655	2577.00
---	--	------	------
mPD		0.25	0.49
		nPD:	0.51
		N. weight:	0.77
		Sc. PD:	0.021
		Sc. rank:	65.1

,

HA			
Pos.	347	obs:	exp:
cct	P	74	1335.00
ccc	P	397	910.80
cca	P	3936	1792.00
ccg	P	117	486.60
gct	A	0	0.25
gcc	A	0	0.23
gca	A	1	0.45
gcg	A	0	0.07
---	--	------	------
mPD		0.23	0.70
		nPD:	0.33
		N. weight:	1.
		Sc. PD:	-0.14
		Sc. rank:	-783.8

,

HA			
Pos.	348	obs:	exp:
cgt	R	0	0.57
cgc	R	0	0.95
cga	R	0	1.81
cgg	R	0	1.30
act	T	1	0.25
acc	T	0	0.22
aca	T	0	0.45
acg	T	0	0.08
aaa	K	1900	2738.00
aag	K	2606	1768.00
aga	R	17	8.25
agg	R	1	5.12
---	--	------	------
mPD		0.50	0.49
		nPD:	1.02
		N. weight:	0.15
		Sc. PD:	0.074
		Sc. rank:	287.6

,

HA			
Pos.	349	obs:	exp:
ttt	F	3	1.34
ttc	F	0	1.66
tat	Y	3865	2533.00
tac	Y	657	1989.00
---	--	------	------
mPD		0.25	0.49
		nPD:	0.51
		N. weight:	0.4
		Sc. PD:	0.011
		Sc. rank:	34.7

,

HA			
Pos.	350	obs:	exp:
att	I	12	10.29
atc	I	17	7.44
ata	I	0	11.27
gtt	V	297	998.30
gtc	V	3716	848.40
gta	V	463	1135.00
gtg	V	20	1514.00
---	--	------	------
mPD		0.32	0.75
		nPD:	0.42
		N. weight:	2.
		Sc. PD:	-0.1
		Sc. rank:	-608.9

,

HA			
Pos.	351	obs:	exp:
cgt	R	0	91.67
cgc	R	0	153.50
cga	R	0	291.90
cgg	R	4	209.80
aaa	K	1303	983.30
aag	K	315	634.70
aga	R	372	1332.00
agg	R	2528	825.40
gaa	E	3	1.78
gag	E	0	1.22
---	--	------	------
mPD		0.93	1.3
		nPD:	0.73
		N. weight:	1.1
		Sc. PD:	0.26
		Sc. rank:	1427.2

,

HA			
Pos.	352	obs:	exp:
ttt	F	1	0.45
ttc	F	0	0.55
tct	S	0	760.80
tcc	S	0	714.80
tca	S	0	1098.00
tcg	S	0	240.40
act	T	1	0.25
acc	T	0	0.22
aca	T	0	0.45
acg	T	0	0.08
agt	S	3303	841.80
agc	S	1220	866.80
---	--	------	------
mPD		0.40	1.6
		nPD:	0.24
		N. weight:	2.1
		Sc. PD:	-0.47
		Sc. rank:	-2324.8

,

HA			
Pos.	353	obs:	exp:
tct	S	1	4.04
tcc	S	1	3.79
tca	S	22	5.83
tcg	S	0	1.28
cct	P	0	0.30
ccc	P	0	0.20
cca	P	1	0.40
ccg	P	0	0.11
cgt	R	0	3.57
cgc	R	0	5.97
cga	R	0	11.36
cgg	R	0	8.16
att	I	0	1.42
atc	I	3	1.03
ata	I	1	1.56
act	T	87	592.60
acc	T	317	520.90
aca	T	1995	1095.00
acg	T	10	200.90
aat	N	1	1.14
aac	N	1	0.86
aaa	K	76	47.40
aag	K	2	30.60
agt	S	0	4.47
agc	S	0	4.60
aga	R	113	51.82
agg	R	0	32.12
gtt	V	1	5.33
gtc	V	14	4.53
gta	V	9	6.06
gtg	V	0	8.08
gct	A	165	454.70
gcc	A	233	425.00
gca	A	1454	836.10
gcg	A	3	139.10
gaa	E	14	8.30
gag	E	0	5.70
ggt	G	0	0.16
ggc	G	0	0.13
gga	G	1	0.45
ggg	G	0	0.26
---	--	------	------
mPD		0.91	1.3
		nPD:	0.71
		N. weight:	0.66
		Sc. PD:	0.15
		Sc. rank:	799.3

,

HA

Pos.	354	obs:	exp:
tct	S	0	0.17
tcc	S	0	0.16
tca	S	0	0.24
tcg	S	0	0.05
cat	H	1	0.58
cac	H	0	0.42
caa	Q	223	124.00
cag	Q	7	106.00
cgt	R	1	9.88
cgc	R	1	16.54
cga	R	2	31.46
cgg	R	0	22.61
aat	N	4	2.84
aac	N	1	2.16
aaa	K	3825	2404.00
aag	K	130	1551.00
agt	S	1	0.19
agc	S	0	0.19
aga	R	262	143.50
agg	R	47	88.96
gaa	E	20	11.85
gag	E	0	8.15
---	--	------	------
mPD		0.32	0.75
		nPD:	0.42
		N. weight:	0.74
		Sc. PD:	-0.037
		Sc. rank:	-219.9

,

HA

Pos.	355	obs:	exp:
tta	L	3297	492.40
ttg	L	500	849.90
ctt	L	1	913.90
ctc	L	3	658.40
cta	L	226	790.10
ctg	L	498	820.30
---	--	------	------
mPD		0.62	1.1
		nPD:	0.54
		N. weight:	2.4
		Sc. PD:	0.14
		Sc. rank:	700.7

,

HA

Pos.	356	obs:	exp:
cgt	R	0	126.70
cgc	R	0	212.10
cga	R	65	403.40
cgg	R	1	289.90
aaa	K	428	311.20
aag	K	84	200.80
aga	R	1145	1840.00
agg	R	2802	1141.00
---	--	------	------
mPD		0.69	1.1
		nPD:	0.63
		N. weight:	0.84
		Sc. PD:	0.12
		Sc. rank:	620.4

,

HA

Pos.	357	obs:	exp:
tta	L	0	26.01
ttg	L	11	44.89
ctt	L	0	48.27
ctc	L	0	34.77
cta	L	6	41.73
ctg	L	222	43.32
cgt	R	0	0.03
cgc	R	0	0.05
cga	R	0	0.10
cgg	R	0	0.07
att	I	0	16.67
atc	I	0	12.05
ata	I	47	18.27
atg	M	4238	4238.00
aga	R	0	0.46
agg	R	1	0.28
---	--	------	------
mPD		0.12	0.19
		nPD:	0.66
		N. weight:	0.17
		Sc. PD:	0.029
		Sc. rank:	151.9

,

HA

Pos.	358	obs:	exp:
gtt	V	2116	637.50
gtc	V	84	541.80
gta	V	667	724.80
gtg	V	4	967.00
gct	A	843	405.40
gcc	A	552	379.00
gca	A	242	745.50
gcg	A	17	124.00
---	--	------	------
mPD		0.98	1.2
		nPD:	0.81
		N. weight:	1.2
		Sc. PD:	0.39
		Sc. rank:	1940.7

,

HA

Pos.	359	obs:	exp:
cct	P	0	0.30
ccc	P	0	0.20
cca	P	1	0.40
ccg	P	0	0.11
act	T	35	1113.00
acc	T	0	978.30
aca	T	4336	2056.00
acg	T	153	377.20
---	--	------	------
mPD		0.081	0.68
		nPD:	0.12
		N. weight:	1.3
		Sc. PD:	-0.44
		Sc. rank:	-2040.7

,

HA

Pos.	360	obs:	exp:
atg	M	1	1.00
gaa	E	1	0.59
gag	E	0	0.41
ggt	G	102	709.40
ggc	G	11	573.10
gga	G	4220	2049.00
ggg	G	190	1192.00
---	--	------	------
mPD		0.13	0.69
		nPD:	0.19
		N. weight:	1.1
		Sc. PD:	-0.29
		Sc. rank:	-1480.0

,

HA

Pos.	361	obs:	exp:
tta	L	1050	492.20
ttg	L	427	849.60
ctt	L	67	913.50
ctc	L	3	658.10
cta	L	2922	789.80
ctg	L	54	819.90
cgt	R	0	0.03
cgc	R	0	0.05
cga	R	1	0.10
cgg	R	0	0.07
att	I	0	0.35
atc	I	0	0.26
ata	I	1	0.39
aga	R	0	0.46
agg	R	0	0.28
---	--	------	------
mPD		0.66	1.1
		nPD:	0.58
		N. weight:	1.7
		Sc. PD:	0.17
		Sc. rank:	955.7

,

HA

Pos.	362	obs:	exp:
cgt	R	0	142.80
cgc	R	0	239.20
cga	R	0	454.80
cgg	R	6	326.90
aga	R	960	2075.00
agg	R	3559	1286.00
---	--	------	------
mPD		0.34	0.94
		nPD:	0.36
		N. weight:	1.2
		Sc. PD:	-0.14
		Sc. rank:	-799.6

,

HA

Pos.	363	obs:	exp:
tct	S	0	0.17
tcc	S	0	0.16
tca	S	0	0.24
tcg	S	0	0.05
cat	H	0	0.58
cac	H	1	0.42
aat	N	1526	2571.00
aac	N	2997	1952.00
agt	S	0	0.19
agc	S	1	0.19
---	--	------	------
mPD		0.45	0.49
		nPD:	0.91
		N. weight:	0.22
		Sc. PD:	0.088
		Sc. rank:	405.4

,

HA

Pos.	364	obs:	exp:
ttt	F	0	0.45
ttc	F	1	0.55
att	I	510	1016.00
atc	I	2332	734.60
ata	I	22	1113.00
act	T	129	277.00
acc	T	995	243.50
aca	T	2	511.60
acg	T	0	93.89
aat	N	0	30.69
aac	N	54	23.31
aaa	K	0	1.22
aag	K	2	0.78
gtt	V	103	103.70
gtc	V	340	88.12
gta	V	24	117.90
gtg	V	0	157.30
gat	D	1	6.19
gac	D	10	4.81
---	--	------	------
mPD		0.88	1.3
		nPD:	0.69
		N. weight:	1.8
		Sc. PD:	0.35
		Sc. rank:	1934.3

,

HA

Pos.	365	obs:	exp:
tta	L	0	0.33
ttg	L	0	0.56
ctt	L	0	0.61
ctc	L	0	0.44
cta	L	2	0.52
ctg	L	1	0.54
cct	P	127	1331.00
ccc	P	193	907.80
cca	P	3557	1786.00
ccg	P	632	485.00
caa	Q	11	7.01
cag	Q	2	5.99
---	--	------	------
mPD		0.36	0.71
		nPD:	0.51
		N. weight:	0.89
		Sc. PD:	0.028
		Sc. rank:	85.1

,

HA

Pos.	366	obs:	exp:
tct	S	1667	759.40
tcc	S	2472	713.60
tca	S	352	1096.00
tcg	S	24	240.00
cct	P	1	0.59
ccc	P	1	0.40
cca	P	0	0.79
ccg	P	0	0.22
act	T	3	1.97
acc	T	5	1.73
aca	T	0	3.64
acg	T	0	0.67
agt	S	0	840.30
agc	S	0	865.30
---	--	------	------
mPD		0.56	1.6
		nPD:	0.34
		N. weight:	1.7
		Sc. PD:	-0.21
		Sc. rank:	-1193.7

,

HA

Pos.	367	obs:	exp:
tta	L	0	0.33
ttg	L	0	0.56
ctt	L	3	0.61
ctc	L	0	0.44
cta	L	0	0.52
ctg	L	0	0.54
att	I	3443	1594.00
atc	I	1032	1153.00
ata	I	19	1747.00
act	T	1	0.49
acc	T	0	0.43
aca	T	1	0.91
acg	T	0	0.17
gtt	V	16	5.77
gtc	V	10	4.91
gta	V	0	6.56
gtg	V	0	8.76
---	--	------	------
mPD		0.37	0.67
		nPD:	0.56
		N. weight:	1.1
		Sc. PD:	0.083
		Sc. rank:	439.2

,

HA

Pos.	368	obs:	exp:
cat	H	1	0.58
cac	H	0	0.42
caa	Q	4401	2440.00
cag	Q	123	2084.00
---	--	------	------
mPD		0.053	0.50
		nPD:	0.11
		N. weight:	0.98
		Sc. PD:	-0.34
		Sc. rank:	-1548.8

,

HA			
Pos.	369	obs:	exp:
ttt	F	0	7.62
ttc	F	17	9.38
tct	S	820	741.40
tcc	S	3500	696.70
tca	S	88	1070.00
tcg	S	0	234.30
tat	Y	1	0.56
tac	Y	0	0.44
act	T	54	14.02
acc	T	3	12.33
aca	T	0	25.90
acg	T	0	4.75
agt	S	0	820.40
agc	S	0	844.80
gct	A	0	10.30
gcc	A	42	9.62
gca	A	0	18.93
gcg	A	0	3.15
---	--	------	------
mPD		0.39	1.6
		nPD:	0.24
		N. weight:	2.5
		Sc. PD:	-0.55
		Sc. rank:	-2713.4

HA			
Pos.	370	obs:	exp:
cgt	R	0	142.60
cgc	R	0	238.80
cga	R	19	454.10
cgg	R	0	326.40
aaa	K	7	4.25
aag	K	0	2.75
aga	R	4289	2072.00
agg	R	210	1284.00
---	--	------	------
mPD		0.10	0.94
		nPD:	0.11
		N. weight:	1.2
		Sc. PD:	-0.41
		Sc. rank:	-1876.2

HA			
Pos.	371	obs:	exp:
cgt	R	0	0.03
cgc	R	0	0.05
cga	R	0	0.10
cgg	R	0	0.07
aga	R	0	0.46
agg	R	1	0.28
ggt	G	3225	709.60
ggc	G	837	573.20
gga	G	254	2049.00
ggg	G	208	1192.00
---	--	------	------
mPD		0.45	0.69
		nPD:	0.66
		N. weight:	1.9
		Sc. PD:	0.33
		Sc. rank:	1673.8

HA			
Pos.	372	obs:	exp:
ttt	F	1	0.45
ttc	F	0	0.55
tta	L	49	489.70
ttg	L	2736	845.20
tgg	W	1	1.00
ctt	L	284	908.80
ctc	L	15	654.70
cta	L	791	785.80
ctg	L	625	815.70
att	I	4	7.80
atc	I	0	5.64
ata	I	18	8.55
gtt	V	0	0.22
gtc	V	0	0.19
gta	V	0	0.25
gtg	V	1	0.34
---	--	------	------
mPD		0.89	1.1
		nPD:	0.77
		N. weight:	1.1
		Sc. PD:	0.31
		Sc. rank:	1614.4

HA			
Pos.	373	obs:	exp:
ttt	F	4367	2025.00
ttc	F	152	2494.00
tta	L	1	0.65
ttg	L	2	1.13
ctt	L	3	1.21
ctc	L	0	0.87
cta	L	0	1.05
ctg	L	0	1.09
---	--	------	------
mPD		0.068	0.50
		nPD:	0.14
		N. weight:	1.3
		Sc. PD:	-0.41
		Sc. rank:	-1922.2

HA			
Pos.	374	obs:	exp:
gaa	E	0	0.59
gag	E	1	0.41
ggt	G	80	709.60
ggc	G	3	573.20
gga	G	2217	2049.00
ggg	G	2224	1192.00
---	--	------	------
mPD		0.52	0.69
		nPD:	0.76
		N. weight:	0.6
		Sc. PD:	0.16
		Sc. rank:	830.9

HA			
Pos.	375	obs:	exp:
gct	A	771	1109.00
gcc	A	3438	1037.00
gca	A	309	2040.00
gcg	A	7	339.40
---	--	------	------
mPD		0.39	0.68
		nPD:	0.57
		N. weight:	1.4
		Sc. PD:	0.13
		Sc. rank:	716.8

HA			
Pos.	376	obs:	exp:
ttt	F	1	0.45
ttc	F	0	0.55
tta	L	0	0.11
ttg	L	0	0.19
ctt	L	0	0.20
ctc	L	0	0.15
cta	L	1	0.17
ctg	L	0	0.18
att	I	3541	1603.00
atc	I	519	1159.00
ata	I	460	1757.00
atg	M	1	1.00
aat	N	1	0.57
aac	N	0	0.43
gtt	V	1	0.22
gtc	V	0	0.19
gta	V	0	0.25
gtg	V	0	0.34
---	--	------	------
mPD		0.36	0.66
		nPD:	0.55
		N. weight:	0.77
		Sc. PD:	0.056
		Sc. rank:	284.6

HA			
Pos.	377	obs:	exp:
cct	P	0	0.30
ccc	P	1	0.20
cca	P	0	0.40
ccg	P	0	0.11
gct	A	1127	1109.00
gcc	A	3193	1037.00
gca	A	202	2039.00
gcg	A	2	339.30
---	--	------	------
mPD		0.44	0.68
		nPD:	0.65
		N. weight:	1.4
		Sc. PD:	0.22
		Sc. rank:	1127.0

HA			
Pos.	378	obs:	exp:
gtt	V	0	0.22
gtc	V	0	0.19
gta	V	0	0.25
gtg	V	1	0.34
ggt	G	3652	709.60
ggc	G	529	573.20
gga	G	227	2049.00
ggg	G	116	1192.00
---	--	------	------
mPD		0.33	0.69
		nPD:	0.48
		N. weight:	2.3
		Sc. PD:	0.019
		Sc. rank:	-40.4

HA			
Pos.	379	obs:	exp:
ttt	F	1469	2026.00
ttc	F	3053	2496.00
tta	L	2	0.22
ttg	L	0	0.38
tgt	C	0	0.43
tgc	C	1	0.57
ctt	L	0	0.40
ctc	L	0	0.29
cta	L	0	0.35
ctg	L	0	0.36
---	--	------	------
mPD		0.44	0.50
		nPD:	0.89
		N. weight:	0.066
		Sc. PD:	0.026
		Sc. rank:	118.0

HA			
Pos.	380	obs:	exp:
ttt	F	1	0.45
ttc	F	0	0.55
tta	L	1	0.44
ttg	L	0	0.75
ctt	L	3	0.81
ctc	L	0	0.58
cta	L	0	0.70
ctg	L	0	0.73
att	I	4397	1601.00
atc	I	107	1157.00
ata	I	8	1754.00
atg	M	8	8.00
---	--	------	------
mPD		0.056	0.66
		nPD:	0.08
		N. weight:	1.8
		Sc. PD:	-0.67
		Sc. rank:	-3036.1

HA			
Pos.	381	obs:	exp:
cat	H	1	0.58
cac	H	0	0.42
aaa	K	1	0.61
aag	K	0	0.39
gct	A	0	0.25
gcc	A	0	0.23
gca	A	1	0.45
gcg	A	0	0.07
gaa	E	4265	2679.00
gag	E	257	1843.00
---	--	------	------
mPD		0.11	0.48
		nPD:	0.22
		N. weight:	0.64
		Sc. PD:	-0.15
		Sc. rank:	-774.6

HA			
Pos.	382	obs:	exp:
cgt	R	1	0.03
cgc	R	0	0.05
cga	R	0	0.10
cgg	R	0	0.07
aga	R	0	0.46
agg	R	0	0.28
gct	A	0	0.25
gcc	A	0	0.23
gca	A	0	0.45
gcg	A	1	0.07
ggt	G	18	709.40
ggc	G	5	573.10
gga	G	1822	2049.00
ggg	G	2678	1192.00
---	--	------	------
mPD		0.49	0.69
		nPD:	0.71
		N. weight:	0.82
		Sc. PD:	0.18
		Sc. rank:	993.2

HA			
Pos.	383	obs:	exp:
cgt	R	0	0.09
cgc	R	0	0.16
cga	R	1	0.30
cgg	R	0	0.22
aga	R	2	1.38
agg	R	0	0.85
gat	D	1	0.56
gac	D	0	0.44
ggt	G	106	709.10
ggc	G	20	572.90
gga	G	2872	2048.00
ggg	G	1523	1191.00
---	--	------	------
mPD		0.48	0.69
		nPD:	0.71
		N. weight:	0.47
		Sc. PD:	0.1
		Sc. rank:	555.3

HA			
Pos.	384	obs:	exp:
tgg	W	4520	4520.00
cgt	R	0	0.03
cgc	R	0	0.05
cga	R	0	0.10
cgg	R	0	0.07
aga	R	0	0.46
agg	R	1	0.28
ggt	G	0	0.63
ggc	G	0	0.51
gga	G	0	1.81
ggg	G	4	1.05
---	--	------	------
mPD		0.0022	0.0038
		nPD:	0.58
		N. weight:	0.0029
		Sc. PD:	0.00029
		Sc. rank:	1.6

,

HA			
Pos.	385	obs:	exp:
tct	S	1	0.17
tcc	S	0	0.16
tca	S	0	0.24
tcg	S	0	0.05
att	I	1	0.35
atc	I	0	0.26
ata	I	0	0.39
act	T	2722	1111.00
acc	T	334	976.80
aca	T	1461	2052.00
acg	T	0	376.70
aat	N	3	1.71
aac	N	0	1.29
agt	S	0	0.19
agc	S	0	0.19
gct	A	3	0.74
gcc	A	0	0.69
gca	A	0	1.35
gcg	A	0	0.22
---	--	------	------
mPD		0.53	0.68
		nPD:	0.78
		N. weight:	0.7
		Sc. PD:	0.2
		Sc. rank:	1004.4

,

HA			
Pos.	386	obs:	exp:
gct	A	0	0.25
gcc	A	0	0.23
gca	A	1	0.45
gcg	A	0	0.07
gaa	E	15	8.89
gag	E	0	6.11
ggt	G	7	707.30
ggc	G	4	571.30
gga	G	4255	2042.00
ggg	G	243	1188.00
---	--	------	------
mPD		0.11	0.69
		nPD:	0.16
		N. weight:	1.2
		Sc. PD:	-0.35
		Sc. rank:	-1702.5

,

HA			
Pos.	387	obs:	exp:
tta	L	0	0.33
ttg	L	1	0.56
ctt	L	0	0.61
ctc	L	0	0.44
cta	L	0	0.52
ctg	L	2	0.54
atg	M	4505	4505.00
aaa	K	0	1.22
aag	K	2	0.78
gtt	V	0	0.22
gtc	V	0	0.19
gta	V	0	0.25
gtg	V	1	0.34
gaa	E	0	8.30
gag	E	14	5.70
---	--	------	------
mPD		0.015	0.020
		nPD:	0.74
		N. weight:	0.0085
		Sc. PD:	0.0021
		Sc. rank:	11.3

,

HA			
Pos.	388	obs:	exp:
tta	L	1	0.11
ttg	L	0	0.19
ctt	L	0	0.20
ctc	L	0	0.15
cta	L	0	0.17
ctg	L	0	0.18
att	I	8	546.70
atc	I	5	395.20
ata	I	1528	599.10
atg	M	1	1.00
gtt	V	9	662.10
gtc	V	1	562.70
gta	V	2906	752.80
gtg	V	66	1004.00
---	--	------	------
mPD		0.49	1.2
		nPD:	0.41
		N. weight:	2.2
		Sc. PD:	-0.14
		Sc. rank:	-891.4

,

HA			
Pos.	389	obs:	exp:
tat	Y	2	1.12
tac	Y	0	0.88
cat	H	1	0.58
cac	H	0	0.42
aat	N	41	23.30
aac	N	0	17.70
gat	D	4229	2510.00
gac	D	234	1953.00
gaa	E	7	10.67
gag	E	11	7.33
---	--	------	------
mPD		0.12	0.52
		nPD:	0.24
		N. weight:	0.76
		Sc. PD:	-0.17
		Sc. rank:	-830.4

,

HA			
Pos.	390	obs:	exp:
ggt	G	316	709.80
ggc	G	9	573.40
gga	G	2393	2050.00
ggg	G	1807	1192.00
---	--	------	------
mPD		0.56	0.68
		nPD:	0.81
		N. weight:	0.36
		Sc. PD:	0.12
		Sc. rank:	575.8

,

HA			
Pos.	391	obs:	exp:
tta	L	0	0.11
ttg	L	1	0.19
tgg	W	4524	4524.00
ctt	L	0	0.20
ctc	L	0	0.15
cta	L	0	0.17
ctg	L	0	0.18
---	--	------	------
mPD		0.00044	0.0010
		nPD:	0.43
		N. weight:	0.00073
		Sc. PD:	-0.000033
		Sc. rank:	-0.2

,

HA			
Pos.	392	obs:	exp:
tat	Y	3578	2535.00
tac	Y	947	1990.00
---	--	------	------
mPD		0.33	0.49
		nPD:	0.67
		N. weight:	0.23
		Sc. PD:	0.043
		Sc. rank:	225.2

,

HA			
Pos.	393	obs:	exp:
tct	S	0	0.17
tcc	S	0	0.16
tca	S	0	0.24
tcg	S	0	0.05
agt	S	1	0.19
agc	S	0	0.19
ggt	G	4222	709.60
ggc	G	27	573.20
gga	G	211	2049.00
ggg	G	64	1192.00
---	--	------	------
mPD		0.13	0.69
		nPD:	0.19
		N. weight:	2.9
		Sc. PD:	-0.81
		Sc. rank:	-4141.8

,

HA			
Pos.	394	obs:	exp:
ttt	F	4	1.79
ttc	F	0	2.21
tat	Y	4092	2533.00
tac	Y	429	1988.00
---	--	------	------
mPD		0.17	0.49
		nPD:	0.35
		N. weight:	0.57
		Sc. PD:	-0.067
		Sc. rank:	-382.4

,

HA			
Pos.	395	obs:	exp:
tta	L	0	0.11
ttg	L	0	0.19
tat	Y	49	28.01
tac	Y	1	21.99
tgt	C	1	0.43
tgc	C	0	0.57
ctt	L	1	0.20
ctc	L	0	0.15
cta	L	0	0.17
ctg	L	0	0.18
cat	H	3638	2577.00
cac	H	829	1890.00
caa	Q	1	1.08
cag	Q	1	0.92
cgt	R	2	0.13
cgc	R	2	0.21
cga	R	0	0.40
cgg	R	0	0.29
aga	R	0	1.83
agg	R	0	1.14
---	--	------	------
mPD		0.33	0.52
		nPD:	0.63
		N. weight:	0.27
		Sc. PD:	0.039
		Sc. rank:	198.7

,

HA			
Pos.	396	obs:	exp:
tta	L	0	0.11
ttg	L	0	0.19
tat	Y	4	2.24
tac	Y	0	1.76
ctt	L	1	0.20
ctc	L	0	0.15
cta	L	0	0.17
ctg	L	0	0.18
cat	H	3871	2607.00
cac	H	649	1913.00
---	--	------	------
mPD		0.25	0.49
		nPD:	0.51
		N. weight:	0.36
		Sc. PD:	0.01
		Sc. rank:	32.7

,

HA			
Pos.	397	obs:	exp:
tta	L	0	2.39
ttg	L	0	4.13
ctt	L	0	4.44
ctc	L	0	3.20
cta	L	0	3.84
ctg	L	22	3.99
cct	P	0	0.30
ccc	P	1	0.20
cca	P	0	0.40
ccg	P	0	0.11
cat	H	20	13.27
cac	H	3	9.73
caa	Q	1785	2380.00
cag	Q	2628	2033.00
cgt	R	0	1.67
cgc	R	0	2.80
cga	R	4	5.33
cgg	R	35	3.83
aaa	K	7	7.29
aag	K	5	4.71
aga	R	0	24.31
agg	R	14	15.06
gaa	E	1	0.59
gag	E	0	0.41
---	--	------	------
mPD		0.53	0.56
		nPD:	0.94
		N. weight:	0.13
		Sc. PD:	0.056
		Sc. rank:	243.3

,

HA

Pos.	398	obs:	exp:
aat	N	4341	2572.00
aac	N	184	1953.00
---	--	------	------
mPD		0.078	0.49
		nPD:	0.16
		N. weight:	0.8
		Sc. PD:	-0.24
		Sc. rank:	-1166.7

HA

Pos.	399	obs:	exp:
aat	N	0	0.57
aac	N	1	0.43
aaa	K	0	0.61
aag	K	1	0.39
gat	D	80	217.70
gac	D	307	169.30
gaa	E	912	2426.00
gag	E	3183	1669.00
ggt	G	0	6.43
ggc	G	0	5.20
gga	G	24	18.57
ggg	G	17	10.80
---	--	------	------
mPD		0.47	0.58
		nPD:	0.8
		N. weight:	0.56
		Sc. PD:	0.17
		Sc. rank:	869.4

HA

Pos.	400	obs:	exp:
cct	P	0	0.30
ccc	P	0	0.20
cca	P	1	0.40
ccg	P	0	0.11
caa	Q	2117	2439.00
cag	Q	2406	2084.00
aaa	K	1	0.61
aag	K	0	0.39
---	--	------	------
mPD		0.50	0.50
		nPD:	1.
		N. weight:	0.023
		Sc. PD:	0.011
		Sc. rank:	44.5

HA

Pos.	401	obs:	exp:
cgt	R	0	0.03
cgc	R	0	0.05
cga	R	0	0.10
cgg	R	0	0.07
aga	R	0	0.46
agg	R	1	0.28
ggt	G	77	709.60
ggc	G	23	573.20
gga	G	3235	2049.00
ggg	G	1189	1192.00
---	--	------	------
mPD		0.42	0.69
		nPD:	0.61
		N. weight:	0.54
		Sc. PD:	0.069
		Sc. rank:	367.8

HA

Pos.	402	obs:	exp:
tct	S	2703	673.80
tcc	S	50	633.10
tca	S	864	972.80
tcg	S	387	212.90
tat	Y	12	6.72
tac	Y	0	5.28
cat	H	5	2.88
cac	H	0	2.12
act	T	478	119.10
acc	T	5	104.70
aca	T	0	219.90
acg	T	1	40.36
aat	N	14	7.96
aac	N	0	6.04
agt	S	2	745.60
agc	S	0	767.70
gct	A	1	0.98
gcc	A	0	0.92
gca	A	3	1.80
gcg	A	0	0.30
---	--	------	------
mPD		0.67	1.6
		nPD:	0.4
		N. weight:	1.9
		Sc. PD:	-0.13
		Sc. rank:	-903.4

HA

Pos.	403	obs:	exp:
ggt	G	289	709.80
ggc	G	1891	573.40
gga	G	1874	2050.00
ggg	G	471	1192.00
---	--	------	------
mPD		0.64	0.68
		nPD:	0.93
		N. weight:	0.61
		Sc. PD:	0.26
		Sc. rank:	1164.6

HA

Pos.	404	obs:	exp:
ttt	F	29	15.23
ttc	F	5	18.77
tat	Y	4219	2516.00
tac	Y	272	1975.00
---	--	------	------
mPD		0.13	0.51
		nPD:	0.26
		N. weight:	0.72
		Sc. PD:	-0.15
		Sc. rank:	-750.9

HA

Pos.	405	obs:	exp:
tct	S	1	0.17
tcc	S	0	0.16
tca	S	0	0.24
tcg	S	0	0.05
agt	S	0	0.19
agc	S	0	0.19
gtt	V	1	0.22
gtc	V	0	0.19
gta	V	0	0.25
gtg	V	0	0.34
gct	A	3039	1108.00
gcc	A	448	1036.00
gca	A	991	2038.00
gcg	A	44	339.10
gat	D	1	0.56
gac	D	0	0.44
---	--	------	------
mPD		0.49	0.68
		nPD:	0.72
		N. weight:	0.82
		Sc. PD:	0.19
		Sc. rank:	1024.9

HA

Pos.	406	obs:	exp:
gct	A	540	1109.00
gcc	A	461	1037.00
gca	A	3215	2040.00
gcg	A	309	339.40
---	--	------	------
mPD		0.47	0.68
		nPD:	0.69
		N. weight:	0.3
		Sc. PD:	0.059
		Sc. rank:	311.3

HA

Pos.	407	obs:	exp:
aat	N	0	1.14
aac	N	2	0.86
gat	D	3304	2543.00
gac	D	1218	1979.00
gaa	E	1	0.59
gag	E	0	0.41
---	--	------	------
mPD		0.40	0.49
		nPD:	0.8
		N. weight:	0.12
		Sc. PD:	0.037
		Sc. rank:	186.0

HA

Pos.	408	obs:	exp:
tta	L	2	52.78
ttg	L	30	91.10
ctt	L	0	97.95
ctc	L	0	70.57
cta	L	78	84.69
ctg	L	375	87.92
cat	H	0	5.77
cac	H	10	4.23
caa	Q	3193	2092.00
cag	Q	687	1788.00
cgt	R	1	3.47
cgc	R	0	5.81
cga	R	81	11.06
cgg	R	28	7.95
atg	M	1	1.00
aaa	K	19	23.70
aag	K	20	15.30
aga	R	0	50.45
agg	R	0	31.26
---	--	------	------
mPD		0.65	0.88
		nPD:	0.73
		N. weight:	0.61
		Sc. PD:	0.15
		Sc. rank:	797.2

HA

Pos.	409	obs:	exp:
tta	L	0	0.11
ttg	L	0	0.19
tct	S	0	0.17
tcc	S	0	0.16
tca	S	0	0.24
tcg	S	0	0.05
ctt	L	0	0.20
ctc	L	0	0.15
cta	L	1	0.17
ctg	L	0	0.18
cat	H	1	0.58
cac	H	0	0.42
caa	Q	955	524.20
cag	Q	17	447.80
cgt	R	0	0.60
cgc	R	0	1.00
cga	R	5	1.91
cgg	R	0	1.37
aaa	K	695	1155.00
aag	K	1205	745.30
agt	S	0	0.19
agc	S	1	0.19
aga	R	2	8.71
agg	R	12	5.40
gaa	E	1594	946.30
gag	E	3	650.70
ggt	G	0	5.33
ggc	G	0	4.31
gga	G	34	15.40
ggg	G	0	8.96
---	--	------	------
mPD		1.1	1.2
		nPD:	0.92
		N. weight:	0.7
		Sc. PD:	0.29
		Sc. rank:	1328.0

HA

Pos.	410	obs:	exp:
tct	S	0	760.90
tcc	S	0	715.00
tca	S	0	1099.00
tcg	S	0	240.40
act	T	1	0.25
acc	T	0	0.22
aca	T	0	0.45
acg	T	0	0.08
agt	S	302	842.00
agc	S	4222	867.00
---	--	------	------
mPD		0.13	1.6
		nPD:	0.08
		N. weight:	2.8
		Sc. PD:	-1.
		Sc. rank:	-4818.6

HA

Pos.	411	obs:	exp:
cct	P	0	0.30
ccc	P	0	0.20
cca	P	1	0.40
ccg	P	0	0.11
act	T	37	1113.00
acc	T	30	978.30
aca	T	4169	2056.00
acg	T	288	377.20
---	--	------	------
mPD		0.15	0.68
		nPD:	0.22
		N. weight:	1.2
		Sc. PD:	-0.28
		Sc. rank:	-1443.7

HA

Pos.	412	obs:	exp:
cct	P	0	0.59
ccc	P	0	0.40
cca	P	2	0.79
ccg	P	0	0.22
caa	Q	3640	2439.00
cag	Q	883	2084.00
---	--	------	------
mPD		0.32	0.50
		nPD:	0.63
		N. weight:	0.31
		Sc. PD:	0.045
		Sc. rank:	228.4

HA

Pos.	413	obs:	exp:
tta	L	0	0.98
ttg	L	0	1.69
tct	S	0	5.05
tcc	S	0	4.74
tca	S	0	7.29
tcg	S	0	1.59
tat	Y	1	0.56
tac	Y	0	0.44
ctt	L	2	1.82
ctc	L	6	1.31
cta	L	0	1.57
ctg	L	1	1.63
cat	H	6	3.46
cac	H	0	2.54
cgt	R	0	12.97
cgc	R	0	21.72
cga	R	2	41.31
cgg	R	0	29.69
att	I	322	171.00
atc	I	139	123.60
ata	I	21	187.40
atg	M	3	3.00
act	T	26	7.63
acc	T	5	6.70
aca	T	0	14.09
acg	T	0	2.59
aat	N	3383	1987.00
aac	N	112	1508.00
aaa	K	8	4.86
aag	K	0	3.14
agt	S	30	5.58
agc	S	0	5.75
aga	R	351	188.50
agg	R	58	116.80
gtt	V	3	1.11
gtc	V	2	0.94
gta	V	0	1.26
gtg	V	0	1.68
gct	A	0	0.25
gcc	A	1	0.23
gca	A	0	0.45
gcg	A	0	0.07
gat	D	42	23.62
gac	D	0	18.38
gaa	E	1	0.59
gag	E	0	0.41
---	--	------	------
mPD		0.67	1.1
		nPD:	0.64
		N. weight:	0.87
		Sc. PD:	0.13
		Sc. rank:	657.0

,

HA

Pos.	414	obs:	exp:
gct	A	111	1109.00
gcc	A	4179	1037.00
gca	A	229	2039.00
gcg	A	5	339.30
gat	D	0	0.56
gac	D	1	0.44
---	--	------	------
mPD		0.14	0.68
		nPD:	0.21
		N. weight:	2.2
		Sc. PD:	-0.55
		Sc. rank:	-2808.5

,

HA

Pos.	415	obs:	exp:
ttt	F	1	0.45
ttc	F	0	0.55
tta	L	0	0.22
ttg	L	0	0.38
ctt	L	2	0.40
ctc	L	0	0.29
cta	L	0	0.35
ctg	L	0	0.36
att	I	4265	1591.00
atc	I	119	1150.00
ata	I	100	1743.00
gtt	V	37	8.44
gtc	V	0	7.17
gta	V	1	9.59
gtg	V	0	12.80
---	--	------	------
mPD		0.11	0.68
		nPD:	0.16
		N. weight:	1.6
		Sc. PD:	-0.47
		Sc. rank:	-2323.8

,

HA

Pos.	416	obs:	exp:
tct	S	0	0.34
tcc	S	0	0.32
tca	S	0	0.49
tcg	S	0	0.11
cat	H	1	0.58
cac	H	0	0.42
aat	N	1549	1724.00
aac	N	1485	1310.00
aaa	K	1	0.61
aag	K	0	0.39
agt	S	1	0.37
agc	S	1	0.38
gat	D	840	836.40
gac	D	647	650.60
---	--	------	------
mPD		0.94	0.93
		nPD:	1.01
		N. weight:	0.014
		Sc. PD:	0.007
		Sc. rank:	27.8

,

HA

Pos.	417	obs:	exp:
tct	S	0	4.21
tcc	S	0	3.95
tca	S	0	6.07
tcg	S	0	1.33
cgt	R	0	10.45
cgc	R	0	17.50
cga	R	0	33.27
cgg	R	0	23.91
atg	M	1	1.00
aaa	K	16	31.60
aag	K	36	20.40
agt	S	25	4.65
agc	S	0	4.79
aga	R	320	151.80
agg	R	11	94.08
gaa	E	3	92.43
gag	E	153	63.57
ggt	G	56	621.10
ggc	G	0	501.80
gga	G	905	1794.00
ggg	G	2999	1043.00
---	--	------	------
mPD		0.68	0.94
		nPD:	0.72
		N. weight:	1.2
		Sc. PD:	0.29
		Sc. rank:	1529.4

,

HA

Pos.	418	obs:	exp:
tta	L	0	0.11
ttg	L	0	0.19
ctt	L	0	0.20
ctc	L	1	0.15
cta	L	0	0.17
ctg	L	0	0.18
att	I	2600	1585.00
atc	I	1829	1146.00
ata	I	38	1737.00
atg	M	1	1.00
gtt	V	28	12.43
gtc	V	28	10.57
gta	V	0	14.14
gtg	V	0	18.86
---	--	------	------
mPD		0.52	0.69
		nPD:	0.76
		N. weight:	0.89
		Sc. PD:	0.24
		Sc. rank:	1245.7

,

HA

Pos.	419	obs:	exp:
tct	S	0	33.14
tcc	S	0	31.13
tca	S	0	47.84
tcg	S	0	10.47
cgt	R	0	0.47
cgc	R	5	0.79
cga	R	0	1.51
cgg	R	0	1.08
act	T	862	1055.00
acc	T	101	927.00
aca	T	3302	1948.00
acg	T	22	357.50
aat	N	0	14.78
aac	N	26	11.22
agt	S	5	36.67
agc	S	192	37.75
aga	R	10	6.88
agg	R	0	4.26
---	--	------	------
mPD		0.52	0.79
		nPD:	0.67
		N. weight:	0.71
		Sc. PD:	0.13
		Sc. rank:	673.8

,

HA

Pos.	420	obs:	exp:
tct	S	0	0.84
tcc	S	0	0.79
tca	S	0	1.21
tcg	S	0	0.27
act	T	0	8.36
acc	T	34	7.35
aca	T	0	15.45
acg	T	0	2.84
aat	N	181	2550.00
aac	N	4305	1936.00
agt	S	0	0.93
agc	S	5	0.96
---	--	------	------
mPD		0.094	0.51
		nPD:	0.18
		N. weight:	1.3
		Sc. PD:	-0.36
		Sc. rank:	-1871.0

,

HA

Pos.	421	obs:	exp:
aaa	K	1209	2750.00
aag	K	3316	1775.00
---	--	------	------
mPD		0.39	0.48
		nPD:	0.82
		N. weight:	0.47
		Sc. PD:	0.15
		Sc. rank:	760.0

,

HA

Pos.	422	obs:	exp:
att	I	0	5.68
atc	I	0	4.10
ata	I	16	6.22
atg	M	2	2.00
gtt	V	2	1000.00
gtc	V	3	850.30
gta	V	1289	1137.00
gtg	V	3212	1518.00
gaa	E	0	0.59
gag	E	1	0.41
---	--	------	------
mPD		0.42	0.75
		nPD:	0.56
		N. weight:	1.1
		Sc. PD:	0.091
		Sc. rank:	493.3

,

HA			
Pos.	423	obs:	exp:
cat	H	1	0.58
cac	H	0	0.42
aat	N	3943	2571.00
aac	N	581	1953.00
---	--	------	------
mPD		0.22	0.49
		nPD:	0.46
		N. weight:	0.43
		Sc. PD:	-0.0078
		Sc. rank:	-51.9

,

HA			
Pos.	424	obs:	exp:
ttt	F	2	1.34
ttc	F	1	1.66
tct	S	3388	759.40
tcc	S	662	713.60
tca	S	423	1096.00
tcg	S	42	240.00
act	T	0	0.25
acc	T	0	0.22
aca	T	1	0.45
acg	T	0	0.08
agt	S	0	840.30
agc	S	0	865.30
gtt	V	3	0.67
gtc	V	0	0.57
gta	V	0	0.76
gtg	V	0	1.01
gct	A	2	0.74
gcc	A	0	0.69
gca	A	1	1.35
gcg	A	0	0.22
---	--	------	------
mPD		0.41	1.6
		nPD:	0.25
		N. weight:	2.
		Sc. PD:	-0.42
		Sc. rank:	-2073.8

,

HA			
Pos.	425	obs:	exp:
tta	L	5	0.54
ttg	L	0	0.94
ctt	L	0	1.01
ctc	L	0	0.73
cta	L	0	0.87
ctg	L	0	0.91
att	I	94	158.60
atc	I	9	114.70
ata	I	344	173.80
gtt	V	1170	904.40
gtc	V	17	768.60
gta	V	2626	1028.00
gtg	V	260	1372.00
---	--	------	------
mPD		0.67	0.92
		nPD:	0.72
		N. weight:	1.1
		Sc. PD:	0.25
		Sc. rank:	1319.0

,

HA			
Pos.	426	obs:	exp:
tta	L	0	0.11
ttg	L	0	0.19
ctt	L	1	0.20
ctc	L	0	0.15
cta	L	0	0.17
ctg	L	0	0.18
att	I	4223	1599.00
atc	I	253	1156.00
ata	I	32	1753.00
gtt	V	15	3.55
gtc	V	0	3.02
gta	V	1	4.04
gtg	V	0	5.39
---	--	------	------
mPD		0.13	0.67
		nPD:	0.19
		N. weight:	1.6
		Sc. PD:	-0.42
		Sc. rank:	-2127.2

,

HA			
Pos.	427	obs:	exp:
caa	Q	3	2.16
cag	Q	1	1.84
aat	N	4	2.27
aac	N	0	1.73
aaa	K	1	1.82
aag	K	2	1.18
gct	A	0	0.49
gcc	A	0	0.46
gca	A	0	0.90
gcg	A	2	0.15
gat	D	114	88.87
gac	D	44	69.13
gaa	E	2420	2575.00
gag	E	1926	1771.00
ggt	G	0	1.25
ggc	G	0	1.01
gga	G	0	3.62
ggg	G	8	2.11
---	--	------	------
mPD		0.54	0.53
		nPD:	1.02
		N. weight:	0.021
		Sc. PD:	0.011
		Sc. rank:	40.9

,

HA			
Pos.	428	obs:	exp:
cgt	R	0	0.69
cgc	R	0	1.16
cga	R	0	2.21
cgg	R	0	1.59
aaa	K	3649	2737.00
aag	K	854	1766.00
aga	R	17	10.09
agg	R	5	6.25
---	--	------	------
mPD		0.32	0.49
		nPD:	0.65
		N. weight:	0.19
		Sc. PD:	0.031
		Sc. rank:	161.2

,

HA			
Pos.	429	obs:	exp:
tta	L	0	0.11
ttg	L	0	0.19
ctt	L	0	0.20
ctc	L	0	0.15
cta	L	0	0.17
ctg	L	1	0.18
att	I	0	18.09
atc	I	1	13.08
ata	I	50	19.83
atg	M	4472	4472.00
gtt	V	0	0.22
gtc	V	0	0.19
gta	V	0	0.25
gtg	V	1	0.34
---	--	------	------
mPD		0.023	0.024
		nPD:	0.97
		N. weight:	0.021
		Sc. PD:	0.0097
		Sc. rank:	40.5

,

HA			
Pos.	430	obs:	exp:
tct	S	0	0.17
tcc	S	0	0.16
tca	S	0	0.24
tcg	S	0	0.05
aat	N	965	2570.00
aac	N	3557	1952.00
agt	S	0	0.19
agc	S	1	0.19
gat	D	0	1.12
gac	D	2	0.88
---	--	------	------
mPD		0.34	0.49
		nPD:	0.68
		N. weight:	0.52
		Sc. PD:	0.1
		Sc. rank:	544.3

,

HA			
Pos.	431	obs:	exp:
ttt	F	4	1.79
ttc	F	0	2.21
tct	S	1	1.85
tcc	S	0	1.74
tca	S	10	2.67
tcg	S	0	0.58
cct	P	1	0.30
ccc	P	0	0.20
cca	P	0	0.40
ccg	P	0	0.11
att	I	200	84.78
atc	I	32	61.30
ata	I	7	92.92
atg	M	1	1.00
act	T	3152	1041.00
acc	T	146	915.60
aca	T	525	1924.00
acg	T	411	353.10
aat	N	14	7.96
aac	N	0	6.04
agt	S	0	2.05
agc	S	0	2.11
gtt	V	12	2.89
gtc	V	1	2.45
gta	V	0	3.28
gtg	V	0	4.38
gct	A	4	1.96
gcc	A	2	1.83
gca	A	2	3.61
gcg	A	0	0.60
---	--	------	------
mPD		0.54	0.81
		nPD:	0.67
		N. weight:	1.2
		Sc. PD:	0.22
		Sc. rank:	1190.4

,

HA			
Pos.	432	obs:	exp:
tta	L	1	0.11
ttg	L	0	0.19
ctt	L	0	0.20
ctc	L	0	0.15
cta	L	0	0.17
ctg	L	0	0.18
cat	H	0	0.58
cac	H	1	0.42
caa	Q	3507	2439.00
cag	Q	1016	2084.00
---	--	------	------
mPD		0.35	0.50
		nPD:	0.7
		N. weight:	0.24
		Sc. PD:	0.051
		Sc. rank:	279.0

,

HA

Pos.	433	obs:	exp:
ttt	F	441	2028.00
ttc	F	4084	2497.00
---	--	------	------
mPD		0.18	0.49
		nPD:	0.36
		N. weight:	0.58
		Sc. PD:	-0.066
		Sc. rank:	-378.8

,

HA

Pos.	434	obs:	exp:
tct	S	1	17.49
tcc	S	0	16.44
tca	S	101	25.26
tcg	S	0	5.53
att	I	0	1.77
atc	I	0	1.28
ata	I	5	1.94
act	T	159	1075.00
acc	T	105	945.40
aca	T	4054	1987.00
acg	T	54	364.60
aat	N	3	1.71
aac	N	0	1.29
aaa	K	1	0.61
aag	K	0	0.39
agt	S	1	19.36
agc	S	1	19.93
gtt	V	0	0.22
gtc	V	0	0.19
gta	V	1	0.25
gtg	V	0	0.34
gct	A	7	9.56
gcc	A	0	8.94
gca	A	32	17.58
gcg	A	0	2.92
---	--	------	------
mPD		0.21	0.75
		nPD:	0.27
		N. weight:	1.1
		Sc. PD:	-0.2
		Sc. rank:	-1042.3

,

HA

Pos.	435	obs:	exp:
ttt	F	6	2.69
ttc	F	0	3.31
tct	S	45	50.12
tcc	S	2	47.10
tca	S	248	72.37
tcg	S	3	15.84
act	T	12	3.20
acc	T	0	2.81
aca	T	1	5.91
acg	T	0	1.08
agt	S	0	55.46
agc	S	0	57.11
gtt	V	3	0.67
gtc	V	0	0.57
gta	V	0	0.76
gtg	V	0	1.01
gct	A	2975	1031.00
gcc	A	17	963.50
gca	A	1208	1895.00
gcg	A	5	315.40
---	--	------	------
mPD		0.58	0.87
		nPD:	0.67
		N. weight:	1.2
		Sc. PD:	0.22
		Sc. rank:	1196.9

,

HA

Pos.	436	obs:	exp:
tta	L	0	0.11
ttg	L	1	0.19
ctt	L	0	0.20
ctc	L	0	0.15
cta	L	0	0.17
ctg	L	0	0.18
att	I	0	3.90
atc	I	0	2.82
ata	I	11	4.28
atg	M	1	1.00
gtt	V	8	999.60
gtc	V	44	849.50
gta	V	1042	1136.00
gtg	V	3408	1516.00
gct	A	0	2.45
gcc	A	0	2.29
gca	A	9	4.51
gcg	A	1	0.75
---	--	------	------
mPD		0.39	0.75
		nPD:	0.52
		N. weight:	1.1
		Sc. PD:	0.042
		Sc. rank:	157.7

,

HA

Pos.	437	obs:	exp:
tct	S	0	3.36
tcc	S	0	3.16
tca	S	0	4.86
tcg	S	0	1.06
agt	S	9	3.72
agc	S	11	3.83
gat	D	3	15.75
gac	D	25	12.25
ggt	G	1349	702.20
ggc	G	3038	567.30
gga	G	75	2028.00
ggg	G	15	1180.00
---	--	------	------
mPD		0.47	0.71
		nPD:	0.66
		N. weight:	2.5
		Sc. PD:	0.42
		Sc. rank:	2162.5

,

HA

Pos.	438	obs:	exp:
cgt	R	0	0.09
cgc	R	0	0.16
cga	R	0	0.30
cgg	R	0	0.22
act	T	0	0.49
acc	T	0	0.43
aca	T	1	0.91
acg	T	1	0.17
aat	N	5	2.84
aac	N	0	2.16
aaa	K	3464	2744.00
aag	K	1051	1771.00
aga	R	0	1.38
agg	R	3	0.85
---	--	------	------
mPD		0.36	0.48
		nPD:	0.75
		N. weight:	0.12
		Sc. PD:	0.031
		Sc. rank:	162.8

,

HA

Pos.	439	obs:	exp:
gat	D	1	0.56
gac	D	0	0.44
gaa	E	3914	2681.00
gag	E	610	1843.00
---	--	------	------
mPD		0.23	0.48
		nPD:	0.48
		N. weight:	0.35
		Sc. PD:	0.0025
		Sc. rank:	-13.6

,

HA

Pos.	440	obs:	exp:
ttt	F	262	2028.00
ttc	F	4263	2497.00
---	--	------	------
mPD		0.11	0.49
		nPD:	0.22
		N. weight:	0.76
		Sc. PD:	-0.18
		Sc. rank:	-938.4

,

HA

Pos.	441	obs:	exp:
tct	S	0	32.80
tcc	S	0	30.82
tca	S	0	47.35
tcg	S	0	10.36
aat	N	479	2459.00
aac	N	3848	1868.00
agt	S	4	36.29
agc	S	191	37.37
gat	D	0	0.56
gac	D	1	0.44
ggt	G	0	0.31
ggc	G	2	0.25
gga	G	0	0.91
ggg	G	0	0.53
---	--	------	------
mPD		0.28	0.64
		nPD:	0.43
		N. weight:	1.
		Sc. PD:	-0.045
		Sc. rank:	-239.7

,

HA

Pos.	442	obs:	exp:
tct	S	0	25.90
tcc	S	0	24.34
tca	S	0	37.40
tcg	S	0	8.18
cat	H	425	441.30
cac	H	340	323.70
caa	Q	40	21.57
cag	Q	0	18.43
cgt	R	0	3.19
cgc	R	0	5.34
cga	R	0	10.15
cgg	R	0	7.30
att	I	0	0.71
atc	I	0	0.51
ata	I	2	0.78
aat	N	99	265.40
aac	N	368	201.60
aaa	K	2857	1787.00
aag	K	84	1154.00
agt	S	21	28.66
agc	S	133	29.51
aga	R	77	46.32
agg	R	24	28.71
gat	D	34	24.19
gac	D	9	18.81
gaa	E	11	6.52
gag	E	0	4.48
ggt	G	0	0.16
ggc	G	1	0.13
gga	G	0	0.45
ggg	G	0	0.26
---	--	------	------
mPD		0.93	1.2
		nPD:	0.8
		N. weight:	0.66
		Sc. PD:	0.2
		Sc. rank:	1024.8

,

HA

Pos.	443	obs:	exp:
ttt	F	3	1.34
ttc	F	0	1.66
tta	L	1073	491.40
ttg	L	2980	848.30
tgg	W	1	1.00
ctt	L	25	912.10
ctc	L	0	657.10
cta	L	128	788.50
ctg	L	310	818.60
atg	M	4	4.00
gtt	V	0	0.22
gtc	V	0	0.19
gta	V	1	0.25
gtg	V	0	0.34
---	--	------	------
mPD		0.59	1.1
		nPD:	0.51
		N. weight:	1.7
		Sc. PD:	0.06
		Sc. rank:	201.9

,

HA

Pos.	444	obs:	exp:
gtt	V	0	0.22
gtc	V	0	0.19
gta	V	1	0.25
gtg	V	0	0.34
gaa	E	4152	2681.00
gag	E	372	1843.00
---	--	------	------
mPD		0.15	0.48
		nPD:	0.31
		N. weight:	0.53
		Sc. PD:	-0.082
		Sc. rank:	-452.6

,

HA

Pos.	445	obs:	exp:
caa	Q	1	0.54
cag	Q	0	0.46
cgt	R	0	109.20
cgc	R	0	182.80
cga	R	168	347.70
cgg	R	11	249.90
att	I	0	6.74
atc	I	0	4.87
ata	I	19	7.39
act	T	0	0.25
acc	T	0	0.22
aca	T	1	0.45
acg	T	0	0.08
aaa	K	959	635.10
aag	K	86	409.90
aga	R	3005	1586.00
agg	R	275	983.10
---	--	------	------
mPD		0.59	1.2
		nPD:	0.48
		N. weight:	0.74
		Sc. PD:	0.0051
		Sc. rank:	-30.3

,

HA

Pos.	446	obs:	exp:
cgt	R	0	142.80
cgc	R	0	239.20
cga	R	0	454.80
cgg	R	18	326.90
aga	R	2968	2075.00
agg	R	1539	1286.00
---	--	------	------
mPD		0.46	0.94
		nPD:	0.49
		N. weight:	0.56
		Sc. PD:	0.007
		Sc. rank:	17.0

,

HA

Pos.	447	obs:	exp:
ttt	F	7	3.14
ttc	F	0	3.86
tta	L	0	68.78
ttg	L	54	118.70
ctt	L	1	127.60
ctc	L	0	91.95
cta	L	3	110.40
ctg	L	574	114.60
att	I	224	503.70
atc	I	5	364.20
ata	I	1191	552.10
atg	M	2454	2454.00
gtt	V	2	2.66
gtc	V	0	2.26
gta	V	3	3.03
gtg	V	7	4.04
---	--	------	------
mPD		0.71	0.85
		nPD:	0.84
		N. weight:	0.7
		Sc. PD:	0.24
		Sc. rank:	1153.4

,

HA

Pos.	448	obs:	exp:
aaa	K	6	3.65
aag	K	0	2.35
gtt	V	0	0.44
gtc	V	0	0.38
gta	V	2	0.50
gtg	V	0	0.67
gct	A	1	3.43
gcc	A	0	3.21
gca	A	3	6.31
gcg	A	10	1.05
gat	D	3	2.25
gac	D	1	1.75
gaa	E	3406	2647.00
gag	E	1061	1820.00
ggt	G	0	5.02
ggc	G	0	4.05
gga	G	13	14.49
ggg	G	19	8.43
---	--	------	------
mPD		0.39	0.51
		nPD:	0.77
		N. weight:	0.15
		Sc. PD:	0.04
		Sc. rank:	204.4

HA

Pos.	449	obs:	exp:
tct	S	0	7.06
tcc	S	0	6.64
tca	S	0	10.20
tcg	S	0	2.23
aat	N	1835	2548.00
aac	N	2648	1935.00
agt	S	40	7.82
agc	S	2	8.05
---	--	------	------
mPD		0.50	0.52
		nPD:	0.96
		N. weight:	0.13
		Sc. PD:	0.059
		Sc. rank:	251.5

HA

Pos.	450	obs:	exp:
ttt	F	2	0.90
ttc	F	0	1.10
tta	L	2661	492.20
ttg	L	940	849.60
ctt	L	11	913.50
ctc	L	0	658.10
cta	L	797	789.80
ctg	L	114	819.90
---	--	------	------
mPD		0.69	1.1
		nPD:	0.6
		N. weight:	1.9
		Sc. PD:	0.22
		Sc. rank:	1227.7

HA

Pos.	451	obs:	exp:
tat	Y	1	0.56
tac	Y	0	0.44
aat	N	3040	2571.00
aac	N	1484	1953.00
---	--	------	------
mPD		0.44	0.49
		nPD:	0.9
		N. weight:	0.046
		Sc. PD:	0.018
		Sc. rank:	85.9

HA

Pos.	452	obs:	exp:
cgt	R	0	14.08
cgc	R	0	23.57
cga	R	0	44.83
cgg	R	0	32.22
aat	N	43	24.44
aac	N	0	18.56
aaa	K	3495	2453.00
aag	K	541	1583.00
aga	R	445	204.50
agg	R	1	126.80
---	--	------	------
mPD		0.41	0.72
		nPD:	0.56
		N. weight:	0.45
		Sc. PD:	0.036
		Sc. rank:	191.6

HA

Pos.	453	obs:	exp:
cgt	R	0	1.52
cgc	R	0	2.54
cga	R	0	4.82
cgg	R	0	3.47
aaa	K	809	2721.00
aag	K	3668	1756.00
aga	R	0	22.01
agg	R	48	13.64
---	--	------	------
mPD		0.31	0.50
		nPD:	0.62
		N. weight:	0.78
		Sc. PD:	0.11
		Sc. rank:	570.8

HA

Pos.	454	obs:	exp:
att	I	49	19.16
atc	I	5	13.85
ata	I	0	20.99
gtt	V	4101	992.70
gtc	V	324	843.70
gta	V	45	1129.00
gtg	V	1	1506.00
---	--	------	------
mPD		0.18	0.76
		nPD:	0.23
		N. weight:	2.3
		Sc. PD:	-0.54
		Sc. rank:	-2709.7

HA

Pos.	455	obs:	exp:
aat	N	3	1.71
aac	N	0	1.29
gat	D	4019	2543.00
gac	D	503	1979.00
---	--	------	------
mPD		0.20	0.49
		nPD:	0.4
		N. weight:	0.5
		Sc. PD:	-0.035
		Sc. rank:	-242.4

HA

Pos.	456	obs:	exp:
gat	D	4380	2545.00
gac	D	145	1980.00
---	--	------	------
mPD		0.062	0.49
		nPD:	0.13
		N. weight:	0.87
		Sc. PD:	-0.29
		Sc. rank:	-1335.1

HA

Pos.	457	obs:	exp:
cgt	R	0	0.03
cgc	R	0	0.05
cga	R	0	0.10
cgg	R	0	0.07
aga	R	0	0.46
agg	R	1	0.28
gaa	E	0	0.59
gag	E	1	0.41
ggt	G	918	709.40
ggc	G	443	573.10
gga	G	301	2049.00
ggg	G	2861	1192.00
---	--	------	------
mPD		0.55	0.69
		nPD:	0.8
		N. weight:	0.9
		Sc. PD:	0.27
		Sc. rank:	1364.0

HA

Pos.	458	obs:	exp:
ttt	F	4044	1998.00
ttc	F	415	2461.00
tta	L	0	7.18
ttg	L	5	12.40
ctt	L	58	13.33
ctc	L	3	9.60
cta	L	0	11.52
ctg	L	0	11.96
---	--	------	------
mPD		0.20	0.52
		nPD:	0.37
		N. weight:	0.95
		Sc. PD:	-0.092
		Sc. rank:	-533.1

HA

Pos.	459	obs:	exp:
tta	L	105	474.80
ttg	L	635	819.50
ctt	L	79	881.20
ctc	L	8	634.80
cta	L	2594	761.80
ctg	L	942	790.90
caa	Q	1	0.54
cag	Q	0	0.46
att	I	3	45.05
atc	I	0	32.57
ata	I	124	49.37
gtt	V	1	7.55
gtc	V	0	6.42
gta	V	7	8.58
gtg	V	26	11.45
---	--	------	------
mPD		0.81	1.2
		nPD:	0.69
		N. weight:	1.3
		Sc. PD:	0.26
		Sc. rank:	1394.4

HA

Pos.	460	obs:	exp:
gat	D	1232	2545.00
gac	D	3292	1979.00
ggt	G	0	0.16
ggc	G	0	0.13
gga	G	1	0.45
ggg	G	0	0.26
---	--	------	------
mPD		0.40	0.49
		nPD:	0.81
		N. weight:	0.34
		Sc. PD:	0.11
		Sc. rank:	531.0

HA

Pos.	461	obs:	exp:
tta	L	1	0.33
ttg	L	0	0.56
ctt	L	2	0.61
ctc	L	0	0.44
cta	L	0	0.52
ctg	L	0	0.54
caa	Q	1	0.54
cag	Q	0	0.46
att	I	3396	1219.00
atc	I	16	881.30
ata	I	24	1336.00
atg	M	1	1.00
gtt	V	841	240.70
gtc	V	14	204.50
gta	V	212	273.60
gtg	V	17	365.10
---	--	------	------
mPD		0.49	1.1
		nPD:	0.45
		N. weight:	1.8
		Sc. PD:	-0.039
		Sc. rank:	-253.5

HA

Pos.	462	obs:	exp:
tta	L	0	0.11
ttg	L	1	0.19
tgg	W	4524	4524.00
ctt	L	0	0.20
ctc	L	0	0.15
cta	L	0	0.17
ctg	L	0	0.18
---	--	------	------
mPD		0.00044	0.0010
		nPD:	0.43
		N. weight:	0.00073
		Sc. PD:	-0.000033
		Sc. rank:	-0.2

HA

Pos.	463	obs:	exp:
att	I	1	0.35
atc	I	0	0.26
ata	I	0	0.39
act	T	515	1113.00
acc	T	399	978.30
aca	T	3381	2056.00
acg	T	229	377.20
---	--	------	------
mPD		0.42	0.68
		nPD:	0.62
		N. weight:	0.36
		Sc. PD:	0.047
		Sc. rank:	245.8

HA

Pos.	464	obs:	exp:
tat	Y	1624	2535.00
tac	Y	2901	1990.00
---	--	------	------
mPD		0.46	0.49
		nPD:	0.93
		N. weight:	0.16
		Sc. PD:	0.07
		Sc. rank:	311.1

HA

Pos.	465	obs:	exp:
aat	N	3825	2572.00
aac	N	700	1953.00
---	--	------	------
mPD		0.26	0.49
		nPD:	0.53
		N. weight:	0.35
		Sc. PD:	0.019
		Sc. rank:	88.1

HA			
Pos.	466	obs:	exp:
act	T	0	3.44
acc	T	0	3.03
aca	T	14	6.36
acg	T	0	1.17
gct	A	454	1106.00
gcc	A	1181	1033.00
gca	A	2832	2033.00
gcg	A	43	338.20
gat	D	1	0.56
gac	D	0	0.44
---	--	------	------
mPD		0.53	0.69
		nPD:	0.78
		N. weight:	0.27
		Sc. PD:	0.077
		Sc. rank:	395.9

,

HA			
Pos.	467	obs:	exp:
cgt	R	0	0.03
cgc	R	0	0.05
cga	R	0	0.10
cgg	R	0	0.07
aaa	K	1	0.61
aag	K	0	0.39
aga	R	1	0.46
agg	R	0	0.28
gtt	V	0	0.22
gtc	V	0	0.19
gta	V	1	0.25
gtg	V	0	0.34
gct	A	0	0.25
gcc	A	1	0.23
gca	A	0	0.45
gcg	A	0	0.07
gaa	E	2629	2679.00
gag	E	1892	1842.00
---	--	------	------
mPD		0.49	0.49
		nPD:	1.01
		N. weight:	0.0042
		Sc. PD:	0.0021
		Sc. rank:	8.3

,

HA			
Pos.	468	obs:	exp:
ttt	F	2	0.90
ttc	F	0	1.10
tta	L	180	489.40
ttg	L	3054	844.70
ctt	L	23	908.20
ctc	L	48	654.30
cta	L	241	785.20
ctg	L	951	815.20
atg	M	26	26.00
---	--	------	------
mPD		0.61	1.1
		nPD:	0.53
		N. weight:	1.5
		Sc. PD:	0.077
		Sc. rank:	352.4

,

HA			
Pos.	469	obs:	exp:
ttt	F	2	1.34
ttc	F	1	1.66
tta	L	1075	491.30
ttg	L	2869	848.10
tct	S	0	0.17
tcc	S	0	0.16
tca	S	0	0.24
tcg	S	1	0.05
ctt	L	93	911.90
ctc	L	220	656.90
cta	L	4	788.40
ctg	L	254	818.40
att	I	0	2.13
atc	I	6	1.54
ata	I	0	2.33
agt	S	0	0.19
agc	S	0	0.19
---	--	------	------
mPD		0.69	1.1
		nPD:	0.6
		N. weight:	1.6
		Sc. PD:	0.18
		Sc. rank:	998.6

,

HA			
Pos.	470	obs:	exp:
att	I	391	251.50
atc	I	60	181.90
ata	I	258	275.60
gtt	V	3553	847.30
gtc	V	127	720.10
gta	V	134	963.30
gtg	V	2	1285.00
---	--	------	------
mPD		0.50	1.0
		nPD:	0.49
		N. weight:	2.
		Sc. PD:	0.028
		Sc. rank:	89.6

,

HA			
Pos.	471	obs:	exp:
tta	L	149	492.20
ttg	L	164	849.60
ctt	L	50	913.50
ctc	L	400	658.10
cta	L	3461	789.80
ctg	L	299	819.90
att	I	0	0.35
atc	I	0	0.26
ata	I	1	0.39
gtt	V	0	0.22
gtc	V	0	0.19
gta	V	1	0.25
gtg	V	0	0.34
---	--	------	------
mPD		0.47	1.1
		nPD:	0.42
		N. weight:	1.8
		Sc. PD:	-0.099
		Sc. rank:	-663.0

,

HA			
Pos.	472	obs:	exp:
tta	L	106	488.70
ttg	L	1339	843.60
ctt	L	32	907.00
ctc	L	217	653.40
cta	L	30	784.20
ctg	L	2767	814.10
atg	M	33	33.00
gtt	V	0	0.22
gtc	V	0	0.19
gta	V	0	0.25
gtg	V	1	0.34
---	--	------	------
mPD		0.60	1.1
		nPD:	0.53
		N. weight:	1.5
		Sc. PD:	0.072
		Sc. rank:	248.5

,

HA			
Pos.	473	obs:	exp:
gaa	E	4170	2681.00
gag	E	355	1844.00
---	--	------	------
mPD		0.14	0.48
		nPD:	0.3
		N. weight:	0.55
		Sc. PD:	-0.091
		Sc. rank:	-480.6

,

HA			
Pos.	474	obs:	exp:
tct	S	0	0.17
tcc	S	0	0.16
tca	S	0	0.24
tcg	S	0	0.05
aat	N	4236	2571.00
aac	N	288	1953.00
agt	S	1	0.19
agc	S	0	0.19
---	--	------	------
mPD		0.12	0.49
		nPD:	0.24
		N. weight:	0.68
		Sc. PD:	-0.15
		Sc. rank:	-735.3

,

HA			
Pos.	475	obs:	exp:
aaa	K	1	0.61
aag	K	0	0.39
gat	D	1	0.56
gac	D	0	0.44
gaa	E	4256	2680.00
gag	E	267	1843.00
---	--	------	------
mPD		0.11	0.48
		nPD:	0.23
		N. weight:	0.63
		Sc. PD:	-0.15
		Sc. rank:	-743.8

,

HA			
Pos.	476	obs:	exp:
cgt	R	0	142.40
cgc	R	0	238.50
cga	R	20	453.50
cgg	R	9	326.00
aaa	K	10	7.90
aag	K	3	5.10
aga	R	2282	2069.00
agg	R	2201	1282.00
---	--	------	------
mPD		0.52	0.94
		nPD:	0.55
		N. weight:	0.58
		Sc. PD:	0.04
		Sc. rank:	191.7

,

HA			
Pos.	477	obs:	exp:
act	T	3895	1113.00
acc	T	520	978.50
aca	T	106	2056.00
acg	T	4	377.30
---	--	------	------
mPD		0.25	0.68
		nPD:	0.36
		N. weight:	1.8
		Sc. PD:	-0.2
		Sc. rank:	-1130.5

,

HA			
Pos.	478	obs:	exp:
tta	L	111	492.30
ttg	L	3792	849.80
tat	Y	1	0.56
tac	Y	0	0.44
ctt	L	8	913.70
ctc	L	26	658.20
cta	L	471	789.90
ctg	L	116	820.10
---	--	------	------
mPD		0.47	1.1
		nPD:	0.42
		N. weight:	2.1
		Sc. PD:	-0.12
		Sc. rank:	-778.3

,

HA			
Pos.	479	obs:	exp:
aat	N	0	0.57
aac	N	1	0.43
gat	D	2687	2545.00
gac	D	1837	1979.00
---	--	------	------
mPD		0.48	0.49
		nPD:	0.98
		N. weight:	0.0063
		Sc. PD:	0.003
		Sc. rank:	12.3

,

HA			
Pos.	480	obs:	exp:
ttt	F	360	1666.00
ttc	F	3358	2052.00
tta	L	223	36.56
ttg	L	111	63.11
tat	Y	78	263.30
tac	Y	392	206.70
ctt	L	1	67.86
ctc	L	1	48.89
cta	L	0	58.67
ctg	L	0	60.91
cat	H	0	0.58
cac	H	1	0.42
---	--	------	------
mPD		0.49	0.83
		nPD:	0.59
		N. weight:	0.75
		Sc. PD:	0.079
		Sc. rank:	440.9

,

HA			
Pos.	481	obs:	exp:
cat	H	2226	2610.00
cac	H	2299	1915.00
---	--	------	------
mPD		0.50	0.49
		nPD:	1.02
		N. weight:	0.031
		Sc. PD:	0.016
		Sc. rank:	60.5

,

HA			
Pos.	482	obs:	exp:
gat	D	1920	2539.00
gac	D	2594	1975.00
gaa	E	7	6.52
gag	E	4	4.48
---	--	------	------
mPD		0.49	0.49
		nPD:	0.99
		N. weight:	0.077
		Sc. PD:	0.037
		Sc. rank:	151.3

,

HA			
Pos.	483	obs:	exp:
ttt	F	189	102.60
ttc	F	40	126.40
tta	L	0	1.20
ttg	L	0	2.07
tct	S	44	718.00
tcc	S	2615	674.70
tca	S	1311	1037.00
tcg	S	299	226.90
ctt	L	10	2.22
ctc	L	1	1.60
cta	L	0	1.92
ctg	L	0	1.99
act	T	0	0.25
acc	T	0	0.22
aca	T	1	0.45
acg	T	0	0.08
agt	S	0	794.60
agc	S	0	818.10
gct	A	0	3.68
gcc	A	13	3.44
gca	A	2	6.76
gcg	A	0	1.12
---	--	------	------
mPD		0.67	1.7
		nPD:	0.4
		N. weight:	1.7
		Sc. PD:	-0.12
		Sc. rank:	-829.1

,

HA			
Pos.	484	obs:	exp:
tat	Y	0	0.56
tac	Y	1	0.44
cat	H	1	0.58
cac	H	0	0.42
aat	N	3296	2569.00
aac	N	1224	1951.00
aaa	K	3	1.82
aag	K	0	1.18
---	--	------	------
mPD		0.40	0.49
		nPD:	0.81
		N. weight:	0.11
		Sc. PD:	0.035
		Sc. rank:	174.2

,

HA			
Pos.	485	obs:	exp:
tta	L	0	0.11
ttg	L	1	0.19
ctt	L	0	0.20
ctc	L	0	0.15
cta	L	0	0.17
ctg	L	0	0.18
att	I	0	4.97
atc	I	0	3.59
ata	I	14	5.44
atg	M	1	1.00
gtt	V	9	1001.00
gtc	V	2	850.50
gta	V	653	1138.00
gtg	V	3843	1518.00
gaa	E	0	0.59
gag	E	1	0.41
ggt	G	0	0.16
ggc	G	0	0.13
gga	G	0	0.45
ggg	G	1	0.26
---	--	------	------
mPD		0.26	0.75
		nPD:	0.35
		N. weight:	1.4
		Sc. PD:	-0.16
		Sc. rank:	-903.8

,

HA			
Pos.	486	obs:	exp:
tat	Y	0	0.56
tac	Y	1	0.44
caa	Q	1	0.54
cag	Q	0	0.46
cgt	R	0	2.05
cgc	R	0	3.44
cga	R	0	6.53
cgg	R	0	4.70
atg	M	5	5.00
aat	N	1	0.57
aac	N	0	0.43
aaa	K	423	2704.00
aag	K	4027	1746.00
aga	R	20	29.81
agg	R	45	18.47
gaa	E	0	1.19
gag	E	2	0.81
---	--	------	------
mPD		0.21	0.52
		nPD:	0.41
		N. weight:	1.1
		Sc. PD:	-0.076
		Sc. rank:	-526.8

,

HA			
Pos.	487	obs:	exp:
tct	S	0	313.40
tcc	S	0	294.40
tca	S	0	452.40
tcg	S	0	99.01
att	I	4	2.13
atc	I	2	1.54
ata	I	0	2.33
act	T	2	13.28
acc	T	51	11.68
aca	T	1	24.54
acg	T	0	4.50
aat	N	1815	1469.00
aac	N	769	1115.00
aaa	K	1	0.61
aag	K	0	0.39
agt	S	1603	346.70
agc	S	260	357.00
gtt	V	1	0.22
gtc	V	0	0.19
gta	V	0	0.25
gtg	V	0	0.34
gct	A	0	0.25
gcc	A	1	0.23
gca	A	0	0.45
gcg	A	0	0.07
gat	D	1	1.69
gac	D	2	1.31
ggt	G	3	1.88
ggc	G	8	1.52
gga	G	1	5.44
ggg	G	0	3.16
---	--	------	------
mPD		0.88	1.6
		nPD:	0.56
		N. weight:	1.1
		Sc. PD:	0.086
		Sc. rank:	462.2

,

HA			
Pos.	488	obs:	exp:
tta	L	795	491.90
ttg	L	267	849.00
ctt	L	4	912.90
ctc	L	1	657.60
cta	L	532	789.20
ctg	L	2921	819.40
att	I	0	1.06
atc	I	0	0.77
ata	I	3	1.17
atg	M	2	2.00
---	--	------	------
mPD		0.78	1.1
		nPD:	0.68
		N. weight:	1.5
		Sc. PD:	0.3
		Sc. rank:	1610.4

,

HA			
Pos.	489	obs:	exp:
tat	Y	3055	2535.00
tac	Y	1470	1990.00
---	--	------	------
mPD		0.44	0.49
		nPD:	0.89
		N. weight:	0.056
		Sc. PD:	0.022
		Sc. rank:	101.0

,

HA			
Pos.	490	obs:	exp:
aat	N	0	3.98
aac	N	7	3.02
gtt	V	0	0.22
gtc	V	0	0.19
gta	V	0	0.25
gtg	V	1	0.34
gat	D	6	10.12
gac	D	12	7.88
gaa	E	1014	2663.00
gag	E	3480	1831.00
ggt	G	0	0.78
ggc	G	0	0.63
gga	G	0	2.26
ggg	G	5	1.32
---	--	------	------
mPD		0.36	0.49
		nPD:	0.73
		N. weight:	0.55
		Sc. PD:	0.13
		Sc. rank:	726.0

,

HA			
Pos.	491	obs:	exp:
cgt	R	0	5.24
cgc	R	0	8.77
cga	R	4	16.69
cgg	R	5	11.99
aat	N	1	0.57
aac	N	0	0.43
aaa	K	3817	2645.00
aag	K	535	1707.00
aga	R	102	76.13
agg	R	55	47.18
ggt	G	0	0.94
ggc	G	0	0.76
gga	G	5	2.72
ggg	G	1	1.58
---	--	------	------
mPD		0.31	0.58
		nPD:	0.54
		N. weight:	0.36
		Sc. PD:	0.021
		Sc. rank:	94.9

,

HA			
Pos.	492	obs:	exp:
att	I	3	53.57
atc	I	3	38.73
ata	I	145	58.70
atg	M	1	1.00
act	T	0	0.49
acc	T	0	0.43
aca	T	2	0.91
acg	T	0	0.17
gtt	V	30	970.50
gtc	V	200	824.80
gta	V	3494	1103.00
gtg	V	647	1472.00
---	--	------	------
mPD		0.40	0.81
		nPD:	0.49
		N. weight:	1.4
		Sc. PD:	0.021
		Sc. rank:	75.0

,

HA			
Pos.	493	obs:	exp:
tct	S	0	0.17
tcc	S	0	0.16
tca	S	1	0.24
tcg	S	0	0.05
cgt	R	0	40.94
cgc	R	0	68.55
cga	R	2	130.40
cgg	R	0	93.70
aaa	K	2353	1961.00
aag	K	873	1265.00
agt	S	0	0.19
agc	S	0	0.19
aga	R	1196	594.80
agg	R	99	368.60
ggt	G	0	0.16
ggc	G	0	0.13
gga	G	1	0.45
ggg	G	0	0.26
---	--	------	------
mPD		0.75	1.0
		nPD:	0.71
		N. weight:	0.35
		Sc. PD:	0.079
		Sc. rank:	430.5

,

HA			
Pos.	494	obs:	exp:
ttt	F	0	0.45
ttc	F	1	0.55
tta	L	24	2.83
ttg	L	2	4.88
tct	S	0	557.20
tcc	S	0	523.60
tca	S	200	804.50
tcg	S	4	176.10
ctt	L	0	5.25
ctc	L	0	3.78
cta	L	0	4.54
ctg	L	0	4.71
cgt	R	0	0.54
cgc	R	4	0.90
cga	R	0	1.71
cgg	R	0	1.23
att	I	1	8.51
atc	I	23	6.16
ata	I	0	9.33
act	T	0	1.97
acc	T	6	1.73
aca	T	2	3.64
acg	T	0	0.67
aat	N	18	409.80
aac	N	703	311.20
agt	S	114	616.60
agc	S	2995	634.90
aga	R	5	7.80
agg	R	8	4.83
gtt	V	0	1.55
gtc	V	7	1.32
gta	V	0	1.77
gtg	V	0	2.36
gct	A	0	14.71
gcc	A	54	13.75
gca	A	6	27.04
gcg	A	0	4.50
gat	D	1	1.12
gac	D	1	0.88
ggt	G	107	54.27
ggc	G	238	43.84
gga	G	1	156.70
ggg	G	0	91.17
---	--	------	------
mPD		0.84	1.9
		nPD:	0.44
		N. weight:	2.3
		Sc. PD:	-0.077
		Sc. rank:	-416.5

,

HA			
Pos.	495	obs:	exp:
caa	Q	3377	2440.00
cag	Q	1147	2084.00
atg	M	1	1.00
---	--	------	------
mPD		0.38	0.50
		nPD:	0.76
		N. weight:	0.18
		Sc. PD:	0.049
		Sc. rank:	254.1

,

HA			
Pos.	496	obs:	exp:
ttt	F	1	0.45
ttc	F	0	0.55
tta	L	2318	492.10
ttg	L	164	849.40
ctt	L	1	913.30
ctc	L	4	657.90
cta	L	1823	789.60
ctg	L	212	819.70
att	I	0	0.71
atc	I	0	0.51
ata	I	2	0.78
---	--	------	------
mPD		0.65	1.1
		nPD:	0.57
		N. weight:	2.
		Sc. PD:	0.18
		Sc. rank:	988.0

,

HA			
Pos.	497	obs:	exp:
tta	L	1	0.11
ttg	L	0	0.19
ctt	L	0	0.20
ctc	L	0	0.15
cta	L	0	0.17
ctg	L	0	0.18
caa	Q	0	0.54
cag	Q	1	0.46
cgt	R	0	35.07
cgc	R	0	58.72
cga	R	1	111.70
cgg	R	1	80.26
aaa	K	1551	2074.00
aag	K	1861	1338.00
aga	R	176	509.50
agg	R	933	315.80
---	--	------	------
mPD		0.84	0.99
		nPD:	0.86
		N. weight:	0.43
		Sc. PD:	0.15
		Sc. rank:	743.2

,

HA			
Pos.	498	obs:	exp:
tct	S	0	1.01
tcc	S	0	0.95
tca	S	0	1.46
tcg	S	0	0.32
act	T	0	1.97
acc	T	8	1.73
aca	T	0	3.64
acg	T	0	0.67
aat	N	2796	2500.00
aac	N	1603	1899.00
agt	S	4	1.12
agc	S	2	1.15
gat	D	42	63.00
gac	D	70	49.00
---	--	------	------
mPD		0.52	0.55
		nPD:	0.95
		N. weight:	0.033
		Sc. PD:	0.015
		Sc. rank:	63.5

,

HA			
Pos.	499	obs:	exp:
aat	N	4438	2571.00
aac	N	85	1952.00
aaa	K	0	1.22
aag	K	2	0.78
---	--	------	------
mPD		0.038	0.49
		nPD:	0.08
		N. weight:	0.94
		Sc. PD:	-0.35
		Sc. rank:	-1650.4

,

HA			
Pos.	500	obs:	exp:
gtt	V	0	0.44
gtc	V	2	0.38
gta	V	0	0.50
gtg	V	0	0.67
gct	A	2095	1108.00
gcc	A	2240	1036.00
gca	A	125	2038.00
gcg	A	62	339.10
ggt	G	0	0.16
ggc	G	1	0.13
gga	G	0	0.45
ggg	G	0	0.26
---	--	------	------
mPD		0.54	0.68
		nPD:	0.79
		N. weight:	1.1
		Sc. PD:	0.34
		Sc. rank:	1725.6

,

HA

Pos.	501	obs:	exp:
cgt	R	0	1.36
cgc	R	0	2.27
cga	R	0	4.32
cgg	R	0	3.11
aaa	K	3045	2723.00
aag	K	1436	1758.00
aga	R	20	19.72
agg	R	23	12.22
gaa	E	1	0.59
gag	E	0	0.41
---	--	------	------
mPD		0.46	0.50
		nPD:	0.91
		N. weight:	0.031
		Sc. PD:	0.013
		Sc. rank:	58.5

HA

Pos.	502	obs:	exp:
gtt	V	0	0.22
gtc	V	0	0.19
gta	V	1	0.25
gtg	V	0	0.34
gaa	E	4434	2681.00
gag	E	90	1843.00
---	--	------	------
mPD		0.039	0.48
		nPD:	0.08
		N. weight:	0.85
		Sc. PD:	-0.32
		Sc. rank:	-1471.2

HA

Pos.	503	obs:	exp:
tta	L	3	0.33
ttg	L	0	0.56
ctt	L	0	0.61
ctc	L	0	0.44
cta	L	0	0.52
ctg	L	0	0.54
att	I	1275	1530.00
atc	I	166	1106.00
ata	I	2872	1677.00
atg	M	3	3.00
act	T	0	0.98
acc	T	1	0.86
aca	T	3	1.82
acg	T	0	0.33
aat	N	1	0.57
aac	N	0	0.43
gtt	V	14	44.63
gtc	V	4	37.93
gta	V	181	50.74
gtg	V	2	67.70
---	--	------	------
mPD		0.55	0.76
		nPD:	0.73
		N. weight:	0.53
		Sc. PD:	0.12
		Sc. rank:	673.6

HA

Pos.	504	obs:	exp:
cgt	R	0	0.03
cgc	R	0	0.05
cga	R	1	0.10
cgg	R	0	0.07
aga	R	0	0.46
agg	R	0	0.28
ggt	G	114	709.60
ggc	G	3	573.20
gga	G	2971	2049.00
ggg	G	1436	1192.00
---	--	------	------
mPD		0.47	0.69
		nPD:	0.68
		N. weight:	0.5
		Sc. PD:	0.098
		Sc. rank:	524.9

HA

Pos.	505	obs:	exp:
tat	Y	0	0.56
tac	Y	1	0.44
aat	N	2678	2495.00
aac	N	1711	1894.00
gat	D	2	75.93
gac	D	133	59.07
---	--	------	------
mPD		0.54	0.55
		nPD:	0.99
		N. weight:	0.053
		Sc. PD:	0.026
		Sc. rank:	105.0

HA

Pos.	506	obs:	exp:
gct	A	0	0.25
gcc	A	0	0.23
gca	A	0	0.45
gcg	A	1	0.07
ggt	G	106	709.60
ggc	G	891	573.20
gga	G	383	2049.00
ggg	G	3144	1192.00
---	--	------	------
mPD		0.47	0.69
		nPD:	0.69
		N. weight:	1.1
		Sc. PD:	0.23
		Sc. rank:	1198.7

HA

Pos.	507	obs:	exp:
tgt	C	1969	1948.00
tgc	C	2556	2577.00
---	--	------	------
mPD		0.49	0.49
		nPD:	1.
		N. weight:	0.002
		Sc. PD:	0.001
		Sc. rank:	4.0

HA

Pos.	508	obs:	exp:
ttt	F	4449	2028.00
ttc	F	76	2497.00
---	--	------	------
mPD		0.033	0.49
		nPD:	0.07
		N. weight:	1.4
		Sc. PD:	-0.54
		Sc. rank:	-2515.2

HA

Pos.	509	obs:	exp:
aaa	K	0	0.61
aag	K	1	0.39
gaa	E	4195	2681.00
gag	E	329	1843.00
---	--	------	------
mPD		0.14	0.48
		nPD:	0.28
		N. weight:	0.57
		Sc. PD:	-0.11
		Sc. rank:	-557.7

HA

Pos.	510	obs:	exp:
ttt	F	1001	2027.00
ttc	F	3523	2497.00
att	I	0	0.35
atc	I	1	0.26
ata	I	0	0.39
---	--	------	------
mPD		0.35	0.50
		nPD:	0.7
		N. weight:	0.22
		Sc. PD:	0.046
		Sc. rank:	256.9

HA

Pos.	511	obs:	exp:
tat	Y	3051	2535.00
tac	Y	1473	1989.00
cat	H	1	0.58
cac	H	0	0.42
---	--	------	------
mPD		0.44	0.49
		nPD:	0.89
		N. weight:	0.056
		Sc. PD:	0.022
		Sc. rank:	100.4

HA

Pos.	512	obs:	exp:
cat	H	1108	2610.00
cac	H	3417	1915.00
---	--	------	------
mPD		0.37	0.49
		nPD:	0.76
		N. weight:	0.45
		Sc. PD:	0.12
		Sc. rank:	626.8

HA

Pos.	513	obs:	exp:
cgt	R	0	0.47
cgc	R	0	0.79
cga	R	0	1.51
cgg	R	0	1.08
act	T	0	0.25
acc	T	0	0.22
aca	T	1	0.45
acg	T	0	0.08
aaa	K	1398	2740.00
aag	K	3110	1768.00
aga	R	11	6.88
agg	R	4	4.26
ggt	G	0	0.16
ggc	G	0	0.13
gga	G	0	0.45
ggg	G	1	0.26
---	--	------	------
mPD		0.44	0.49
		nPD:	0.9
		N. weight:	0.36
		Sc. PD:	0.14
		Sc. rank:	664.5

HA

Pos.	514	obs:	exp:
tgt	C	4269	1948.00
tgc	C	256	2577.00
---	--	------	------
mPD		0.11	0.49
		nPD:	0.22
		N. weight:	1.2
		Sc. PD:	-0.29
		Sc. rank:	-1495.6

HA

Pos.	515	obs:	exp:
tct	S	0	0.17
tcc	S	0	0.16
tca	S	0	0.24
tcg	S	0	0.05
aat	N	56	1204.00
aac	N	2062	914.20
agt	S	0	0.19
agc	S	1	0.19
gat	D	1392	1352.00
gac	D	1012	1052.00
ggt	G	2	0.31
ggc	G	0	0.25
gga	G	0	0.91
ggg	G	0	0.53
---	--	------	------
mPD		0.94	0.99
		nPD:	0.94
		N. weight:	0.66
		Sc. PD:	0.29
		Sc. rank:	1270.5

HA			
Pos.	516	obs:	exp:
tct	S	0	0.17
tcc	S	0	0.16
tca	S	0	0.24
tcg	S	0	0.05
aat	N	508	1611.00
aac	N	2326	1223.00
agt	S	0	0.19
agc	S	1	0.19
gat	D	1455	950.00
gac	D	234	739.00
ggt	G	1	0.16
ggc	G	0	0.13
gga	G	0	0.45
ggg	G	0	0.26
---	--	------	------
mPD		0.96	0.96
		nPD:	1.
		N. weight:	0.55
		Sc. PD:	0.27
		Sc. rank:	1091.0

,

HA			
Pos.	517	obs:	exp:
tta	L	1	0.11
ttg	L	0	0.19
tct	S	0	0.84
tcc	S	0	0.79
tca	S	2	1.21
tcg	S	3	0.27
ctt	L	0	0.20
ctc	L	0	0.15
cta	L	0	0.17
ctg	L	0	0.18
att	I	1	28.73
atc	I	68	20.78
ata	I	12	31.49
atg	M	130	130.00
act	T	0	235.90
acc	T	247	207.40
aca	T	461	435.80
acg	T	251	79.97
aat	N	0	0.57
aac	N	1	0.43
aaa	K	9	9.12
aag	K	6	5.88
agt	S	0	0.93
agc	S	0	0.96
gtt	V	0	3.77
gtc	V	0	3.21
gta	V	4	4.29
gtg	V	13	5.73
gct	A	0	8.82
gcc	A	15	8.25
gca	A	17	16.23
gcg	A	4	2.70
gat	D	15	12.37
gac	D	7	9.63
gaa	E	3167	1929.00
gag	E	88	1326.00
ggt	G	0	0.47
ggc	G	2	0.38
gga	G	1	1.36
ggg	G	0	0.79
---	--	------	------
mPD		1.1	1.4
		nPD:	0.81
		N. weight:	0.79
		Sc. PD:	0.25
		Sc. rank:	1243.0

,

HA			
Pos.	518	obs:	exp:
tgt	C	607	1948.00
tgc	C	3918	2577.00
---	--	------	------
mPD		0.23	0.49
		nPD:	0.47
		N. weight:	0.41
		Sc. PD:	-0.0009
		Sc. rank:	-31.5

,

HA			
Pos.	519	obs:	exp:
att	I	3	3.55
atc	I	0	2.56
ata	I	7	3.89
atg	M	4515	4515.00
---	--	------	------
mPD		0.0044	0.0044
		nPD:	1.
		N. weight:	0.0021
		Sc. PD:	0.0011
		Sc. rank:	4.2

,

HA			
Pos.	520	obs:	exp:
aat	N	6	4.55
aac	N	2	3.45
aaa	K	1	0.61
aag	K	0	0.39
gat	D	685	485.40
gac	D	178	377.60
gaa	E	1497	2164.00
gag	E	2155	1488.00
ggt	G	0	0.16
ggc	G	0	0.13
gga	G	1	0.45
ggg	G	0	0.26
---	--	------	------
mPD		0.64	0.65
		nPD:	0.99
		N. weight:	0.16
		Sc. PD:	0.076
		Sc. rank:	307.7

,

HA			
Pos.	521	obs:	exp:
tct	S	0	758.40
tcc	S	0	712.60
tca	S	0	1095.00
tcg	S	0	239.60
aat	N	16	9.09
aac	N	0	6.91
agt	S	3842	839.20
agc	S	667	864.10
---	--	------	------
mPD		0.26	1.6
		nPD:	0.16
		N. weight:	2.5
		Sc. PD:	-0.74
		Sc. rank:	-3616.0

,

HA			
Pos.	522	obs:	exp:
att	I	7	48.95
atc	I	7	35.40
ata	I	124	53.65
gtt	V	674	974.10
gtc	V	875	827.80
gta	V	882	1107.00
gtg	V	1956	1478.00
---	--	------	------
mPD		0.76	0.80
		nPD:	0.95
		N. weight:	0.11
		Sc. PD:	0.048
		Sc. rank:	204.3

,

HA			
Pos.	523	obs:	exp:
tct	S	0	0.17
tcc	S	0	0.16
tca	S	1	0.24
tcg	S	0	0.05
cgt	R	0	26.77
cgc	R	0	44.82
cga	R	0	85.23
cgg	R	0	61.26
aaa	K	3343	2233.00
aag	K	332	1442.00
agt	S	0	0.19
agc	S	0	0.19
aga	R	795	388.90
agg	R	53	241.00
gct	A	0	0.25
gcc	A	0	0.23
gca	A	1	0.45
gcg	A	0	0.07
---	--	------	------
mPD		0.46	0.89
		nPD:	0.52
		N. weight:	0.59
		Sc. PD:	0.024
		Sc. rank:	87.0

,

HA			
Pos.	524	obs:	exp:
tct	S	0	1.68
tcc	S	0	1.58
tca	S	0	2.43
tcg	S	0	0.53
aat	N	4387	2562.00
aac	N	120	1945.00
agt	S	10	1.86
agc	S	0	1.92
gat	D	7	3.94
gac	D	0	3.06
ggt	G	1	0.16
ggc	G	0	0.13
gga	G	0	0.45
ggg	G	0	0.26
---	--	------	------
mPD		0.060	0.50
		nPD:	0.12
		N. weight:	0.89
		Sc. PD:	-0.3
		Sc. rank:	-1397.8

,

HA			
Pos.	525	obs:	exp:
gaa	E	2	10.67
gag	E	16	7.33
ggt	G	81	706.90
ggc	G	225	571.10
gga	G	2478	2041.00
ggg	G	1723	1188.00
---	--	------	------
mPD		0.56	0.69
		nPD:	0.8
		N. weight:	0.33
		Sc. PD:	0.1
		Sc. rank:	511.2

,

HA

Pos.	526	obs:	exp:
tct	S	1	0.34
tcc	S	0	0.32
tca	S	1	0.49
tcg	S	0	0.11
att	I	1	0.35
atc	I	0	0.26
ata	I	0	0.39
act	T	3573	1111.00
acc	T	680	976.80
aca	T	242	2052.00
acg	T	22	376.70
aat	N	5	2.84
aac	N	0	2.16
agt	S	0	0.37
agc	S	0	0.38
---	--	------	------
mPD		0.35	0.68
		nPD:	0.52
		N. weight:	1.5
		Sc. PD:	0.054
		Sc. rank:	198.9

,

HA

Pos.	527	obs:	exp:
tat	Y	4461	2535.00
tac	Y	64	1990.00
---	--	------	------
mPD		0.028	0.49
		nPD:	0.06
		N. weight:	1.
		Sc. PD:	-0.4
		Sc. rank:	-1834.6

,

HA

Pos.	528	obs:	exp:
tct	S	0	0.17
tcc	S	0	0.16
tca	S	0	0.24
tcg	S	0	0.05
tat	Y	0	1.12
tac	Y	2	0.88
cat	H	0	1.73
cac	H	3	1.27
aat	N	546	366.00
aac	N	98	278.00
aaa	K	1	0.61
aag	K	0	0.39
agt	S	1	0.19
agc	S	0	0.19
gat	D	740	2168.00
gac	D	3114	1686.00
gaa	E	15	11.26
gag	E	4	7.74
ggt	G	0	0.16
ggc	G	1	0.13
gga	G	0	0.45
ggg	G	0	0.26
---	--	------	------
mPD		0.66	0.74
		nPD:	0.89
		N. weight:	0.54
		Sc. PD:	0.21
		Sc. rank:	974.4

,

HA

Pos.	529	obs:	exp:
tat	Y	3331	2535.00
tac	Y	1194	1990.00
---	--	------	------
mPD		0.39	0.49
		nPD:	0.79
		N. weight:	0.13
		Sc. PD:	0.039
		Sc. rank:	199.0

,

HA

Pos.	530	obs:	exp:
tta	L	11	45.49
ttg	L	0	78.51
tct	S	3	4.21
tcc	S	10	3.95
tca	S	11	6.07
tcg	S	1	1.33
ctt	L	0	84.42
ctc	L	1	60.82
cta	L	404	72.99
ctg	L	2	75.77
cct	P	79	1196.00
ccc	P	335	816.20
cca	P	3571	1605.00
ccg	P	69	436.10
cat	H	1	3.46
cac	H	5	2.54
caa	Q	1	0.54
cag	Q	0	0.46
att	I	0	2.13
atc	I	0	1.54
ata	I	6	2.33
act	T	0	0.98
acc	T	1	0.86
aca	T	3	1.82
acg	T	0	0.33
aaa	K	11	6.69
aag	K	0	4.31
agt	S	0	4.65
agc	S	0	4.79
---	--	------	------
mPD		0.41	0.97
		nPD:	0.42
		N. weight:	1.3
		Sc. PD:	-0.067
		Sc. rank:	-400.5

,

HA

Pos.	531	obs:	exp:
caa	Q	2	1.08
cag	Q	0	0.92
cgt	R	0	7.45
cgc	R	0	12.47
cga	R	18	23.72
cgg	R	0	17.05
act	T	0	31.49
acc	T	0	27.68
aca	T	127	58.16
acg	T	1	10.67
aat	N	0	1.71
aac	N	3	1.29
aaa	K	4011	2517.00
aag	K	130	1624.00
aga	R	200	108.20
agg	R	18	67.08
gaa	E	15	8.89
gag	E	0	6.11
---	--	------	------
mPD		0.23	0.68
		nPD:	0.34
		N. weight:	0.76
		Sc. PD:	-0.097
		Sc. rank:	-560.8

,

HA

Pos.	532	obs:	exp:
ttt	F	0	0.45
ttc	F	1	0.55
tat	Y	2850	2535.00
tac	Y	1674	1989.00
---	--	------	------
mPD		0.47	0.49
		nPD:	0.95
		N. weight:	0.022
		Sc. PD:	0.0097
		Sc. rank:	42.2

,

HA

Pos.	533	obs:	exp:
ttt	F	4	3.58
ttc	F	4	4.42
tta	L	4	0.44
ttg	L	0	0.75
tct	S	208	671.30
tcc	S	1987	630.70
tca	S	1784	969.20
tcg	S	12	212.10
tat	Y	370	207.30
tac	Y	0	162.70
ctt	L	0	0.81
ctc	L	0	0.58
cta	L	0	0.70
ctg	L	0	0.73
cat	H	1	0.58
cac	H	0	0.42
act	T	0	0.98
acc	T	2	0.86
aca	T	2	1.82
acg	T	0	0.33
aat	N	75	42.63
aac	N	0	32.37
aaa	K	1	43.15
aag	K	70	27.85
agt	S	0	742.80
agc	S	0	764.90
gaa	E	0	0.59
gag	E	1	0.41
---	--	------	------
mPD		0.90	1.7
		nPD:	0.52
		N. weight:	1.5
		Sc. PD:	0.064
		Sc. rank:	234.9

,

HA

Pos.	534	obs:	exp:
tat	Y	0	0.56
tac	Y	1	0.44
atg	M	1	1.00
aaa	K	14	12.76
aag	K	7	8.24
gct	A	0	0.49
gcc	A	0	0.46
gca	A	1	0.90
gcg	A	1	0.15
gat	D	16	152.40
gac	D	255	118.60
gaa	E	3513	2503.00
gag	E	711	1721.00
ggt	G	0	0.78
ggc	G	0	0.63
gga	G	4	2.26
ggg	G	1	1.32
---	--	------	------
mPD		0.38	0.56
		nPD:	0.68
		N. weight:	0.32
		Sc. PD:	0.06
		Sc. rank:	317.4

,

HA

Pos.	535	obs:	exp:
gtt	V	0	0.22
gtc	V	0	0.19
gta	V	1	0.25
gtg	V	0	0.34
gaa	E	4192	2681.00
gag	E	332	1843.00
---	--	------	------
mPD		0.14	0.48
		nPD:	0.28
		N. weight:	0.57
		Sc. PD:	-0.1
		Sc. rank:	-552.9

,

HA

Pos. 536		obs:	exp:
tct	S	86	681.70
tcc	S	223	640.50
tca	S	3718	984.20
tcg	S	26	215.40
act	T	0	0.25
acc	T	0	0.22
aca	T	1	0.45
acg	T	0	0.08
agt	S	0	754.40
agc	S	0	776.70
gct	A	0	115.50
gcc	A	4	107.90
gca	A	467	212.30
gcg	A	0	35.32
---	--	------	------
mPD		0.33	1.7
		nPD:	0.19
		N. weight:	2.1
		Sc. PD:	-0.56
		Sc. rank:	-2816.5

,

HA

Pos. 537		obs:	exp:
tct	S	0	6.73
tcc	S	0	6.32
tca	S	0	9.71
tcg	S	0	2.13
cgt	R	0	11.96
cgc	R	0	20.03
cga	R	2	38.09
cgg	R	0	27.38
atg	M	1	1.00
act	T	0	0.98
acc	T	0	0.86
aca	T	1	1.82
acg	T	3	0.33
aat	N	3	1.71
aac	N	0	1.29
aaa	K	2285	2490.00
aag	K	1812	1607.00
agt	S	33	7.44
agc	S	7	7.67
aga	R	334	173.80
agg	R	43	107.70
gaa	E	0	0.59
gag	E	1	0.41
---	--	------	------
mPD		0.67	0.72
		nPD:	0.93
		N. weight:	0.11
		Sc. PD:	0.049
		Sc. rank:	217.1

,

HA

Pos. 538		obs:	exp:
ttt	F	3	1.34
ttc	F	0	1.66
tta	L	3206	490.10
ttg	L	246	846.00
ctt	L	146	909.60
ctc	L	30	655.30
cta	L	851	786.50
ctg	L	25	816.50
att	I	0	3.55
atc	I	0	2.56
ata	I	10	3.89
gtt	V	0	1.55
gtc	V	0	1.32
gta	V	0	1.77
gtg	V	7	2.36
gct	A	0	0.25
gcc	A	0	0.23
gca	A	0	0.45
gcg	A	1	0.07
---	--	------	------
mPD		0.55	1.1
		nPD:	0.48
		N. weight:	2.3
		Sc. PD:	0.011
		Sc. rank:	-119.3

,

HA

Pos. 539		obs:	exp:
tct	S	0	10.26
tcc	S	0	9.64
tca	S	0	14.81
tcg	S	0	3.24
tat	Y	0	0.56
tac	Y	1	0.44
cat	H	0	0.58
cac	H	1	0.42
cgt	R	0	0.03
cgc	R	0	0.05
cga	R	0	0.10
cgg	R	0	0.07
act	T	1	3.20
acc	T	9	2.81
aca	T	0	5.91
acg	T	3	1.08
aat	N	253	2505.00
aac	N	4155	1903.00
aaa	K	15	24.31
aag	K	25	15.69
agt	S	3	11.35
agc	S	58	11.69
aga	R	0	0.46
agg	R	1	0.28
---	--	------	------
mPD		0.16	0.56
		nPD:	0.29
		N. weight:	1.2
		Sc. PD:	-0.22
		Sc. rank:	-1164.6

,

HA

Pos. 540		obs:	exp:
cgt	R	0	142.50
cgc	R	0	238.60
cga	R	0	453.80
cgg	R	0	326.20
aaa	K	0	6.08
aag	K	10	3.92
aga	R	1670	2071.00
agg	R	2845	1283.00
---	--	------	------
mPD		0.47	0.94
		nPD:	0.5
		N. weight:	0.83
		Sc. PD:	0.018
		Sc. rank:	59.6

,

HA

Pos. 541		obs:	exp:
cgt	R	0	0.25
cgc	R	0	0.42
cga	R	0	0.80
cgg	R	0	0.58
att	I	0	3.55
atc	I	0	2.56
ata	I	10	3.89
aat	N	3	1.71
aac	N	0	1.29
aaa	K	27	23.09
aag	K	11	14.91
aga	R	8	3.67
agg	R	0	2.27
gtt	V	0	0.67
gtc	V	0	0.57
gta	V	2	0.76
gtg	V	1	1.01
gaa	E	2182	2629.00
gag	E	2255	1808.00
ggt	G	0	4.08
ggc	G	0	3.29
gga	G	25	11.78
ggg	G	1	6.85
---	--	------	------
mPD		0.55	0.53
		nPD:	1.03
		N. weight:	0.059
		Sc. PD:	0.031
		Sc. rank:	117.6

,

HA

Pos. 542		obs:	exp:
caa	Q	49	26.42
cag	Q	0	22.58
cgt	R	1	0.60
cgc	R	0	1.00
cga	R	1	1.91
cgg	R	0	1.37
aaa	K	3036	1862.00
aag	K	28	1202.00
aga	R	17	8.71
agg	R	0	5.40
gtt	V	0	0.22
gtc	V	0	0.19
gta	V	1	0.25
gtg	V	0	0.34
gct	A	0	0.74
gcc	A	0	0.69
gca	A	3	1.35
gcg	A	0	0.22
gaa	E	1263	822.40
gag	E	125	565.60
ggt	G	0	0.16
ggc	G	0	0.13
gga	G	1	0.45
ggg	G	0	0.26
---	--	------	------
mPD		0.52	0.93
		nPD:	0.56
		N. weight:	0.78
		Sc. PD:	0.058
		Sc. rank:	298.1

,

HA

Pos. 543		obs:	exp:
tta	L	0	0.22
ttg	L	0	0.38
ctt	L	0	0.40
ctc	L	0	0.29
cta	L	2	0.35
ctg	L	0	0.36
att	I	2938	1567.00
atc	I	32	1133.00
ata	I	1446	1717.00
gtt	V	97	23.76
gtc	V	0	20.19
gta	V	10	27.01
gtg	V	0	36.04
---	--	------	------
mPD		0.49	0.71
		nPD:	0.7
		N. weight:	0.71
		Sc. PD:	0.15
		Sc. rank:	813.8

,

HA

Pos. 544		obs:	exp:
tta	L	0	0.11
ttg	L	0	0.19
tat	Y	1	0.56
tac	Y	0	0.44
ctt	L	1	0.20
ctc	L	0	0.15
cta	L	0	0.17
ctg	L	0	0.18
aat	N	43	31.26
aac	N	12	23.74
gat	D	4066	2451.00
gac	D	291	1906.00
gaa	E	100	63.99
gag	E	8	44.01
ggt	G	3	0.47
ggc	G	0	0.38
gga	G	0	1.36
ggg	G	0	0.79
---	--	------	------
mPD		0.20	0.54
		nPD:	0.36
		N. weight:	0.68
		Sc. PD:	-0.075
		Sc. rank:	-433.4

,

HA

Pos. 545		obs:	exp:
tct	S	0	0.67
tcc	S	0	0.63
tca	S	0	0.97
tcg	S	0	0.21
cgt	R	0	0.28
cgc	R	0	0.48
cga	R	0	0.90
cgg	R	0	0.65
agt	S	4	0.74
agc	S	0	0.77
aga	R	9	4.13
agg	R	0	2.56
gaa	E	1	0.59
gag	E	0	0.41
ggt	G	230	707.60
ggc	G	10	571.60
gga	G	3747	2043.00
ggg	G	524	1189.00
---	--	------	------
mPD		0.30	0.69
		nPD:	0.43
		N. weight:	0.68
		Sc. PD:	-0.027
		Sc. rank:	-137.8

,

HA

Pos.	546	obs:	exp:
tta	L	0	1.96
ttg	L	3	3.38
ctt	L	15	3.64
ctc	L	0	2.62
cta	L	0	3.14
ctg	L	0	3.26
att	I	10	7.09
atc	I	0	5.13
ata	I	10	7.78
gtt	V	932	996.30
gtc	V	22	846.70
gta	V	752	1133.00
gtg	V	2781	1511.00
---	--	------	------
mPD		0.57	0.75
		nPD:	0.75
		N. weight:	0.56
		Sc. PD:	0.14
		Sc. rank:	768.8

,

HA

Pos.	547	obs:	exp:
cgt	R	0	0.44
cgc	R	0	0.74
cga	R	0	1.41
cgg	R	0	1.01
aat	N	7	3.98
aac	N	0	3.02
aaa	K	3124	2564.00
aag	K	1095	1655.00
aga	R	7	6.42
agg	R	7	3.98
gtt	V	0	0.22
gtc	V	0	0.19
gta	V	1	0.25
gtg	V	0	0.34
gaa	E	279	168.30
gag	E	5	115.70
---	--	------	------
mPD		0.50	0.61
		nPD:	0.82
		N. weight:	0.13
		Sc. PD:	0.043
		Sc. rank:	215.4

,

HA

Pos.	548	obs:	exp:
ttt	F	1	0.45
ttc	F	0	0.55
tta	L	76	492.30
ttg	L	3692	849.80
ctt	L	2	913.70
ctc	L	0	658.20
cta	L	211	789.90
ctg	L	543	820.10
---	--	------	------
mPD		0.40	1.1
		nPD:	0.35
		N. weight:	2.1
		Sc. PD:	-0.25
		Sc. rank:	-1400.8

,

HA

Pos.	549	obs:	exp:
cgt	R	0	0.06
cgc	R	0	0.11
cga	R	0	0.20
cgg	R	0	0.14
aat	N	1	11.37
aac	N	19	8.63
aaa	K	284	172.60
aag	K	0	111.40
aga	R	2	0.92
agg	R	0	0.57
gat	D	456	1132.00
gac	D	1556	880.30
gaa	E	1897	1307.00
gag	E	308	898.50
ggt	G	0	0.31
ggc	G	0	0.25
gga	G	2	0.91
ggg	G	0	0.53
---	--	------	------
mPD		0.76	0.87
		nPD:	0.87
		N. weight:	0.44
		Sc. PD:	0.17
		Sc. rank:	783.3

,

HA

Pos.	550	obs:	exp:
tta	L	12	1.31
ttg	L	0	2.25
tct	S	6	752.90
tcc	S	0	707.40
tca	S	4317	1087.00
tcg	S	153	237.90
ctt	L	0	2.42
ctc	L	0	1.75
cta	L	0	2.10
ctg	L	0	2.18
cct	P	0	3.84
ccc	P	0	2.62
cca	P	13	5.15
ccg	P	0	1.40
caa	Q	1	0.54
cag	Q	0	0.46
act	T	0	4.92
acc	T	0	4.32
aca	T	20	9.09
acg	T	0	1.67
agt	S	0	833.10
agc	S	0	857.80
gtt	V	0	0.22
gtc	V	0	0.19
gta	V	1	0.25
gtg	V	0	0.34
gct	A	0	0.49
gcc	A	0	0.46
gca	A	2	0.90
gcg	A	0	0.15
---	--	------	------
mPD		0.090	1.6
		nPD:	0.06
		N. weight:	2.6
		Sc. PD:	-1.
		Sc. rank:	-4784.9

,

HA

Pos.	551	obs:	exp:
tta	L	3	0.87
ttg	L	5	1.50
ctt	L	0	1.62
ctc	L	0	1.16
cta	L	0	1.40
ctg	L	0	1.45
att	I	6	15.96
atc	I	0	11.54
ata	I	39	17.49
atg	M	3077	3077.00
act	T	0	295.20
acc	T	0	259.50
aca	T	1187	545.30
acg	T	13	100.10
aaa	K	0	6.08
aag	K	10	3.92
gtt	V	0	33.31
gtc	V	0	28.30
gta	V	8	37.87
gtg	V	142	50.52
gct	A	0	8.58
gcc	A	0	8.02
gca	A	35	15.78
gcg	A	0	2.62
---	--	------	------
mPD		0.89	0.94
		nPD:	0.94
		N. weight:	0.48
		Sc. PD:	0.21
		Sc. rank:	930.4

,

HA

Pos.	552	obs:	exp:
caa	Q	2	1.62
cag	Q	1	1.38
cgt	R	0	37.47
cgc	R	0	62.74
cga	R	65	119.30
cgg	R	0	85.75
act	T	0	0.25
acc	T	0	0.22
aca	T	0	0.45
acg	T	1	0.08
aaa	K	1	9.72
aag	K	15	6.28
aga	R	251	544.40
agg	R	871	337.40
gtt	V	0	0.22
gtc	V	0	0.19
gta	V	0	0.25
gtg	V	1	0.34
gat	D	6	3.37
gac	D	0	2.63
gaa	E	87	69.92
gag	E	31	48.08
ggt	G	9	500.80
ggc	G	2	404.60
gga	G	1584	1446.00
ggg	G	1598	841.30
---	--	------	------
mPD		0.96	1.1
		nPD:	0.84
		N. weight:	0.76
		Sc. PD:	0.26
		Sc. rank:	1278.8

,

HA

Pos.	553	obs:	exp:
ttt	F	0	1.79
ttc	F	4	2.21
tta	L	1	0.11
ttg	L	0	0.19
tct	S	0	1.01
tcc	S	0	0.95
tca	S	0	1.46
tcg	S	0	0.32
ctt	L	0	0.20
ctc	L	0	0.15
cta	L	0	0.17
ctg	L	0	0.18
att	I	780	292.00
atc	I	42	211.10
ata	I	1	320.00
atg	M	1	1.00
act	T	6	1.48
acc	T	0	1.30
aca	T	0	2.73
acg	T	0	0.50
agt	S	6	1.12
agc	S	0	1.15
gtt	V	724	815.50
gtc	V	2677	693.10
gta	V	202	927.20
gtg	V	70	1237.00
gct	A	6	2.70
gcc	A	4	2.52
gca	A	1	4.96
gcg	A	0	0.82
---	--	------	------
mPD		0.83	1.1
		nPD:	0.79
		N. weight:	1.6
		Sc. PD:	0.48
		Sc. rank:	2449.9

,

HA

Pos.	554	obs:	exp:
ttt	F	0	0.45
ttc	F	1	0.55
tct	S	1	0.17
tcc	S	0	0.16
tca	S	0	0.24
tcg	S	0	0.05
tat	Y	3427	2396.00
tac	Y	850	1881.00
cat	H	71	124.00
cac	H	144	90.98
caa	Q	26	14.02
cag	Q	0	11.98
aat	N	3	2.27
aac	N	1	1.73
agt	S	0	0.19
agc	S	0	0.19
gat	D	1	0.56
gac	D	0	0.44
---	--	------	------
mPD		0.46	0.60
		nPD:	0.76
		N. weight:	0.26
		Sc. PD:	0.07
		Sc. rank:	364.4

,

HA

Pos.	555	obs:	exp:
tct	S	0	0.17
tcc	S	0	0.16
tca	S	0	0.24
tcg	S	0	0.05
tgg	W	10	10.00
cct	P	0	0.30
ccc	P	0	0.20
cca	P	0	0.40
ccg	P	1	0.11
cat	H	15	9.23
cac	H	1	6.77
caa	Q	194	1103.00
cag	Q	1851	942.20
cgt	R	0	46.31
cgc	R	0	77.54
cga	R	38	147.50
cgg	R	1422	106.00
aat	N	975	558.20
aac	N	7	423.80
aaa	K	1	0.61
aag	K	0	0.39
agt	S	1	0.19
agc	S	0	0.19
aga	R	0	672.80
agg	R	7	417.00
gat	D	2	1.12
gac	D	0	0.88
---	--	------	------
mPD		1.2	1.6
		nPD:	0.74
		N. weight:	2.2
		Sc. PD:	0.56
		Sc. rank:	3017.3

,

HA			
Pos.	556	obs:	exp:
ttt	F	1	0.45
ttc	F	0	0.55
tct	S	0	0.17
tcc	S	0	0.16
tca	S	0	0.24
tcg	S	0	0.05
att	I	3302	1597.00
atc	I	1183	1155.00
ata	I	17	1750.00
atg	M	1	1.00
agt	S	1	0.19
agc	S	0	0.19
gtt	V	18	4.44
gtc	V	2	3.77
gta	V	0	5.05
gtg	V	0	6.74
---	--	------	------
mPD		0.40	0.67
		nPD:	0.6
		N. weight:	1.
		Sc. PD:	0.12
		Sc. rank:	650.9

HA			
Pos.	557	obs:	exp:
tta	L	16	490.30
ttg	L	1482	846.20
ctt	L	22	909.80
ctc	L	0	655.50
cta	L	65	786.60
ctg	L	2920	816.60
atg	M	20	20.00
---	--	------	------
mPD		0.49	1.1
		nPD:	0.43
		N. weight:	1.9
		Sc. PD:	-0.078
		Sc. rank:	-419.1

HA			
Pos.	558	obs:	exp:
tct	S	26	104.50
tcc	S	0	98.14
tca	S	88	150.80
tcg	S	507	33.00
act	T	0	3.44
acc	T	0	3.03
aca	T	0	6.36
acg	T	14	1.17
agt	S	0	115.60
agc	S	0	119.00
gtt	V	0	1.11
gtc	V	0	0.94
gta	V	0	1.26
gtg	V	5	1.68
gct	A	75	952.30
gcc	A	0	890.10
gca	A	253	1751.00
gcg	A	3557	291.40
---	--	------	------
mPD		0.42	1.0
		nPD:	0.41
		N. weight:	4.2
		Sc. PD:	-0.27
		Sc. rank:	-1806.3

HA			
Pos.	559	obs:	exp:
att	I	304	1584.00
atc	I	3461	1145.00
ata	I	700	1736.00
act	T	0	0.25
acc	T	1	0.22
aca	T	0	0.45
acg	T	0	0.08
gtt	V	32	13.10
gtc	V	7	11.13
gta	V	19	14.89
gtg	V	1	19.87
---	--	------	------
mPD		0.41	0.69
		nPD:	0.59
		N. weight:	1.2
		Sc. PD:	0.13
		Sc. rank:	737.9

HA			
Pos.	560	obs:	exp:
ttt	F	1	0.45
ttc	F	0	0.55
tat	Y	1558	2535.00
tac	Y	2966	1989.00
---	--	------	------
mPD		0.45	0.49
		nPD:	0.92
		N. weight:	0.19
		Sc. PD:	0.078
		Sc. rank:	354.7

HA			
Pos.	561	obs:	exp:
tct	S	30	760.80
tcc	S	207	714.80
tca	S	4200	1098.00
tcg	S	86	240.40
agt	S	0	841.80
agc	S	0	866.80
gct	A	1	0.49
gcc	A	0	0.46
gca	A	1	0.90
gcg	A	0	0.15
---	--	------	------
mPD		0.14	1.6
		nPD:	0.08
		N. weight:	2.3
		Sc. PD:	-0.84
		Sc. rank:	-3826.7

HA			
Pos.	562	obs:	exp:
tct	S	0	0.67
tcc	S	0	0.63
tca	S	0	0.97
tcg	S	0	0.21
act	T	2793	1105.00
acc	T	309	971.60
aca	T	1310	2042.00
acg	T	81	374.70
agt	S	4	0.74
agc	S	0	0.77
gct	A	28	6.86
gcc	A	0	6.42
gca	A	0	12.62
gcg	A	0	2.10
---	--	------	------
mPD		0.54	0.69
		nPD:	0.77
		N. weight:	0.69
		Sc. PD:	0.19
		Sc. rank:	980.3

HA			
Pos.	563	obs:	exp:
att	I	13	241.90
atc	I	668	174.90
ata	I	1	265.10
act	T	0	1.48
acc	T	6	1.30
aca	T	0	2.73
acg	T	0	0.50
gtt	V	959	755.10
gtc	V	2435	641.80
gta	V	7	858.50
gtg	V	0	1146.00
gct	A	158	106.60
gcc	A	277	99.67
gca	A	0	196.10
gcg	A	0	32.62
gat	D	0	0.56
gac	D	1	0.44
---	--	------	------
mPD		0.81	1.2
		nPD:	0.69
		N. weight:	2.
		Sc. PD:	0.4
		Sc. rank:	2166.8

HA			
Pos.	564	obs:	exp:
tct	S	0	0.84
tcc	S	5	0.79
tca	S	0	1.21
tcg	S	0	0.27
act	T	0	0.25
acc	T	1	0.22
aca	T	0	0.45
acg	T	0	0.08
agt	S	0	0.93
agc	S	0	0.96
gtt	V	0	0.44
gtc	V	2	0.38
gta	V	0	0.50
gtg	V	0	0.67
gct	A	31	1107.00
gcc	A	4481	1035.00
gca	A	5	2036.00
gcg	A	0	338.80
---	--	------	------
mPD		0.019	0.68
		nPD:	0.03
		N. weight:	2.8
		Sc. PD:	-1.2
		Sc. rank:	-5359.6

HA			
Pos.	565	obs:	exp:
tct	S	0	760.80
tcc	S	0	714.80
tca	S	0	1098.00
tcg	S	0	240.40
att	I	2	0.71
atc	I	0	0.51
ata	I	0	0.78
agt	S	4334	841.80
agc	S	189	866.80
---	--	------	------
mPD		0.081	1.6
		nPD:	0.05
		N. weight:	3.
		Sc. PD:	-1.2
		Sc. rank:	-5612.8

HA			
Pos.	566	obs:	exp:
tct	S	125	760.10
tcc	S	2746	714.20
tca	S	1560	1097.00
tcg	S	88	240.20
act	T	0	1.48
acc	T	1	1.30
aca	T	4	2.73
acg	T	1	0.50
agt	S	0	841.10
agc	S	0	866.10
---	--	------	------
mPD		0.51	1.6
		nPD:	0.31
		N. weight:	1.7
		Sc. PD:	-0.26
		Sc. rank:	-1436.7

HA			
Pos.	567	obs:	exp:
ttt	F	1	0.45
ttc	F	0	0.55
tta	L	334	490.70
ttg	L	969	846.90
tct	S	0	0.50
tcc	S	0	0.47
tca	S	0	0.73
tcg	S	3	0.16
ctt	L	10	910.60
ctc	L	2	656.00
cta	L	1488	787.30
ctg	L	1706	817.40
att	I	0	3.55
atc	I	0	2.56
ata	I	10	3.89
atg	M	2	2.00
agt	S	0	0.56
agc	S	0	0.57
---	--	------	------
mPD		0.90	1.1
		nPD:	0.79
		N. weight:	0.95
		Sc. PD:	0.28
		Sc. rank:	1428.0

HA			
Pos.	568	obs:	exp:
tta	L	0	0.11
ttg	L	0	0.19
ctt	L	1	0.20
ctc	L	0	0.15
cta	L	0	0.17
ctg	L	0	0.18
att	I	13	9.22
atc	I	7	6.67
ata	I	6	10.11
atg	M	7	7.00
act	T	0	1.72
acc	T	0	1.51
aca	T	0	3.18
acg	T	7	0.58
gtt	V	2651	995.60
gtc	V	193	846.10
gta	V	1199	1132.00
gtg	V	441	1510.00
---	--	------	------
mPD		0.59	0.76
		nPD:	0.78
		N. weight:	0.81
		Sc. PD:	0.23
		Sc. rank:	1199.8

HA			
Pos.	569	obs:	exp:
ttt	F	39	17.48
ttc	F	0	21.52
tta	L	30	483.30
ttg	L	225	834.20
ctt	L	2827	896.90
ctc	L	192	646.10
cta	L	398	775.50
ctg	L	769	805.00
att	I	14	7.09
atc	I	0	5.13
ata	I	6	7.78
atg	M	8	8.00
gtt	V	0	3.77
gtc	V	0	3.21
gta	V	17	4.29
gtg	V	0	5.73
---	--	------	------
mPD		0.67	1.2
		nPD:	0.58
		N. weight:	1.
		Sc. PD:	0.11
		Sc. rank:	598.0

HA			
Pos.	570	obs:	exp:
tta	L	2048	446.90
ttg	L	1808	771.40
tct	S	0	1.35
tcc	S	0	1.26
tca	S	6	1.94
tcg	S	2	0.43
ctt	L	0	829.50
ctc	L	0	597.60
cta	L	52	717.10
ctg	L	199	744.50
att	I	0	10.29
atc	I	0	7.44
ata	I	29	11.27
act	T	0	0.74
acc	T	0	0.65
aca	T	3	1.36
acg	T	0	0.25
agt	S	0	1.49
agc	S	0	1.53
gtt	V	0	83.71
gtc	V	0	71.14
gta	V	371	95.17
gtg	V	6	127.00
gct	A	0	0.25
gcc	A	0	0.23
gca	A	1	0.45
gcg	A	0	0.07
---	--	------	------
mPD		0.76	1.2
		nPD:	0.61
		N. weight:	2.1
		Sc. PD:	0.27
		Sc. rank:	1415.5

,

HA			
Pos.	571	obs:	exp:
att	I	0	90.81
atc	I	256	65.66
ata	I	0	99.52
act	T	0	0.25
acc	T	1	0.22
aca	T	0	0.45
acg	T	0	0.08
gtt	V	0	946.10
gtc	V	4261	804.00
gta	V	0	1076.00
gtg	V	0	1435.00
gct	A	0	1.72
gcc	A	7	1.60
gca	A	0	3.16
gcg	A	0	0.52
---	--	------	------
mPD		0.11	0.85
		nPD:	0.13
		N. weight:	3.2
		Sc. PD:	-1.1
		Sc. rank:	-4908.0

,

HA			
Pos.	572	obs:	exp:
ttt	F	0	1.34
ttc	F	3	1.66
tct	S	779	742.40
tcc	S	3614	697.60
tca	S	21	1072.00
tcg	S	0	234.60
act	T	0	20.42
acc	T	83	17.95
aca	T	0	37.71
acg	T	0	6.92
agt	S	0	821.60
agc	S	0	845.90
gct	A	0	6.13
gcc	A	25	5.73
gca	A	0	11.27
gcg	A	0	1.87
---	--	------	------
mPD		0.34	1.6
		nPD:	0.21
		N. weight:	2.6
		Sc. PD:	-0.67
		Sc. rank:	-3410.2

,

HA			
Pos.	573	obs:	exp:
tta	L	0	487.50
ttg	L	15	841.50
ctt	L	8	904.80
ctc	L	0	651.80
cta	L	79	782.30
ctg	L	4378	812.10
atg	M	45	45.00
---	--	------	------
mPD		0.064	1.1
		nPD:	0.06
		N. weight:	3.1
		Sc. PD:	-1.2
		Sc. rank:	-5676.9

,

HA			
Pos.	574	obs:	exp:
ggt	G	0	709.80
ggc	G	0	573.40
gga	G	5	2050.00
ggg	G	4520	1192.00
---	--	------	------
mPD		0.0022	0.68
		nPD:	0.
		N. weight:	2.6
		Sc. PD:	-1.2
		Sc. rank:	-5158.3

,

HA			
Pos.	575	obs:	exp:
act	T	0	0.25
acc	T	0	0.22
aca	T	1	0.45
acg	T	0	0.08
gtt	V	0	0.22
gtc	V	0	0.19
gta	V	1	0.25
gtg	V	0	0.34
gct	A	2	1109.00
gcc	A	0	1036.00
gca	A	4517	2039.00
gcg	A	4	339.20
---	--	------	------
mPD		0.0035	0.68
		nPD:	0.01
		N. weight:	1.5
		Sc. PD:	-0.69
		Sc. rank:	-3053.8

,

HA			
Pos.	576	obs:	exp:
tta	L	0	46.58
ttg	L	0	80.39
tct	S	0	0.17
tcc	S	1	0.16
tca	S	0	0.24
tcg	S	0	0.05
ctt	L	0	86.44
ctc	L	428	62.27
cta	L	0	74.73
ctg	L	0	77.58
att	I	20	1360.00
atc	I	3814	983.40
ata	I	0	1491.00
act	T	1	19.92
acc	T	80	17.52
aca	T	0	36.81
acg	T	0	6.75
agt	S	0	0.19
agc	S	0	0.19
gtt	V	0	39.97
gtc	V	180	33.97
gta	V	0	45.44
gtg	V	0	60.63
gct	A	0	0.25
gcc	A	1	0.23
gca	A	0	0.45
gcg	A	0	0.07
---	--	------	------
mPD		0.29	0.97
		nPD:	0.3
		N. weight:	2.8
		Sc. PD:	-0.47
		Sc. rank:	-2458.7

,

HA			
Pos.	577	obs:	exp:
ttt	F	0	1.79
ttc	F	4	2.21
tct	S	0	623.90
tcc	S	0	586.20
tca	S	0	900.70
tcg	S	0	197.10
tgt	C	18	334.00
tgc	C	758	442.00
att	I	0	0.35
atc	I	1	0.26
ata	I	0	0.39
act	T	0	0.49
acc	T	2	0.43
aca	T	0	0.91
acg	T	0	0.17
agt	S	1378	690.30
agc	S	2331	710.80
ggt	G	9	5.18
ggc	G	24	4.18
gga	G	0	14.95
ggg	G	0	8.70
---	--	------	------
mPD		0.73	1.6
		nPD:	0.45
		N. weight:	1.8
		Sc. PD:	-0.041
		Sc. rank:	-278.9

,

HA			
Pos.	578	obs:	exp:
ttt	F	557	2015.00
ttc	F	3939	2481.00
tta	L	28	3.16
ttg	L	0	5.45
ctt	L	0	5.86
ctc	L	1	4.22
cta	L	0	5.06
ctg	L	0	5.26
---	--	------	------
mPD		0.23	0.51
		nPD:	0.45
		N. weight:	0.51
		Sc. PD:	-0.014
		Sc. rank:	-86.5

,

HA			
Pos.	579	obs:	exp:
tgg	W	4525	4525.00
---	--	------	------
mPD		0	0
		nPD:	1.
		N. weight:	0.
		Sc. PD:	0
		Sc. rank:	0

,

HA			
Pos.	580	obs:	exp:
tta	L	0	0.11
ttg	L	1	0.19
ctt	L	0	0.20
ctc	L	0	0.15
cta	L	0	0.17
ctg	L	0	0.18
atg	M	4524	4524.00
---	--	------	------
mPD		0.00044	0.00072
		nPD:	0.61
		N. weight:	0.00073
		Sc. PD:	0.000094
		Sc. rank:	0.5

,

HA			
Pos.	581	obs:	exp:
tgt	C	3635	1948.00
tgc	C	890	2577.00
---	--	------	------
mPD		0.32	0.49
		nPD:	0.64
		N. weight:	0.58
		Sc. PD:	0.091
		Sc. rank:	470.0

,

HA			
Pos.	582	obs:	exp:
ttt	F	0	0.45
ttc	F	1	0.55
tct	S	1700	759.80
tcc	S	2802	713.90
tca	S	13	1097.00
tcg	S	2	240.10
tat	Y	3	2.24
tac	Y	1	1.76
act	T	0	0.49
acc	T	2	0.43
aca	T	0	0.91
acg	T	0	0.17
agt	S	0	840.70
agc	S	0	865.70
gct	A	1	0.25
gcc	A	0	0.23
gca	A	0	0.45
gcg	A	0	0.07
---	--	------	------
mPD		0.48	1.6
		nPD:	0.29
		N. weight:	2.2
		Sc. PD:	-0.39
		Sc. rank:	-2038.9

,

HA			
Pos.	583	obs:	exp:
aat	N	4495	2572.00
aac	N	30	1953.00
---	--	------	------
mPD		0.013	0.49
		nPD:	0.03
		N. weight:	1.
		Sc. PD:	-0.44
		Sc. rank:	-2004.6

,

HA			
Pos.	584	obs:	exp:
ggt	G	39	709.80
ggc	G	1	573.40
gga	G	90	2050.00
ggg	G	4395	1192.00
---	--	------	------
mPD		0.056	0.68
		nPD:	0.08
		N. weight:	2.3
		Sc. PD:	-0.86
		Sc. rank:	-3963.2

,

HA			
Pos.	585	obs:	exp:
ttt	F	22	9.86
ttc	F	0	12.14
tct	S	4155	757.10
tcc	S	22	711.30
tca	S	243	1093.00
tcg	S	81	239.20
act	T	0	0.25
acc	T	0	0.22
aca	T	1	0.45
acg	T	0	0.08
agt	S	0	837.70
agc	S	0	862.60
gct	A	0	0.25
gcc	A	0	0.23
gca	A	1	0.45
gcg	A	0	0.07
---	--	------	------
mPD		0.16	1.6
		nPD:	0.09
		N. weight:	2.9
		Sc. PD:	-1.
		Sc. rank:	-4710.1

,

HA			
Pos.	586	obs:	exp:
tta	L	1757	492.40
ttg	L	2338	849.90
ctt	L	0	913.90
ctc	L	0	658.40
cta	L	424	790.10
ctg	L	6	820.30
---	--	------	------
mPD		0.67	1.1
		nPD:	0.59
		N. weight:	1.9
		Sc. PD:	0.2
		Sc. rank:	1132.8

,

HA			
Pos.	587	obs:	exp:
tta	L	0	0.11
ttg	L	0	0.19
ctt	L	0	0.20
ctc	L	0	0.15
cta	L	1	0.17
ctg	L	0	0.18
cat	H	1	0.58
cac	H	0	0.42
caa	Q	2369	2439.00
cag	Q	2153	2083.00
cgt	R	0	0.03
cgc	R	0	0.05
cga	R	1	0.10
cgg	R	0	0.07
aga	R	0	0.46
agg	R	0	0.28
---	--	------	------
mPD		0.50	0.50
		nPD:	1.
		N. weight:	0.0049
		Sc. PD:	0.0024
		Sc. rank:	9.6

,

HA			
Pos.	588	obs:	exp:
tgt	C	2782	1948.00
tgc	C	1743	2577.00
---	--	------	------
mPD		0.47	0.49
		nPD:	0.97
		N. weight:	0.14
		Sc. PD:	0.063
		Sc. rank:	267.8

,

HA			
Pos.	589	obs:	exp:
tct	S	0	0.34
tcc	S	0	0.32
tca	S	0	0.49
tcg	S	0	0.11
cgt	R	0	140.10
cgc	R	0	234.70
cga	R	20	446.30
cgg	R	0	320.80
act	T	0	0.49
acc	T	0	0.43
aca	T	2	0.91
acg	T	0	0.17
aaa	K	79	48.62
aag	K	1	31.38
agt	S	2	0.37
agc	S	0	0.38
aga	R	4355	2036.00
agg	R	65	1262.00
gtt	V	0	0.22
gtc	V	0	0.19
gta	V	1	0.25
gtg	V	0	0.34
---	--	------	------
mPD		0.075	0.97
		nPD:	0.08
		N. weight:	1.3
		Sc. PD:	-0.51
		Sc. rank:	-2332.2

,

HA			
Pos.	590	obs:	exp:
tta	L	1	0.11
ttg	L	0	0.19
ctt	L	0	0.20
ctc	L	0	0.15
cta	L	0	0.17
ctg	L	0	0.18
att	I	3	1538.00
atc	I	1	1112.00
ata	I	4332	1686.00
gtt	V	13	41.74
gtc	V	0	35.48
gta	V	166	47.46
gtg	V	9	63.32
---	--	------	------
mPD		0.092	0.75
		nPD:	0.12
		N. weight:	1.8
		Sc. PD:	-0.62
		Sc. rank:	-2859.5

,

HA			
Pos.	591	obs:	exp:
ttt	F	0	0.45
ttc	F	1	0.55
tgt	C	1084	1947.00
tgc	C	3440	2577.00
---	--	------	------
mPD		0.36	0.49
		nPD:	0.74
		N. weight:	0.16
		Sc. PD:	0.04
		Sc. rank:	212.8

,

HA			
Pos.	592	obs:	exp:
tta	L	0	0.22
ttg	L	0	0.38
ctt	L	0	0.40
ctc	L	2	0.29
cta	L	0	0.35
ctg	L	0	0.36
att	I	1385	1605.00
atc	I	3071	1160.00
ata	I	67	1758.00
---	--	------	------
mPD		0.45	0.66
		nPD:	0.68
		N. weight:	1.1
		Sc. PD:	0.21
		Sc. rank:	1127.7

,

HA			
Pos.	593	obs:	exp:
taa	.	3060	1925.00
tag	.	41	1192.00
tga	.	1424	1408.00
---	--	------	------
mPD		0.45	0.82
		nPD:	0.55
		N. weight:	0.57
		Sc. PD:	0.04
		Sc. rank:	190.2

}

Amino acid level conservation per codon

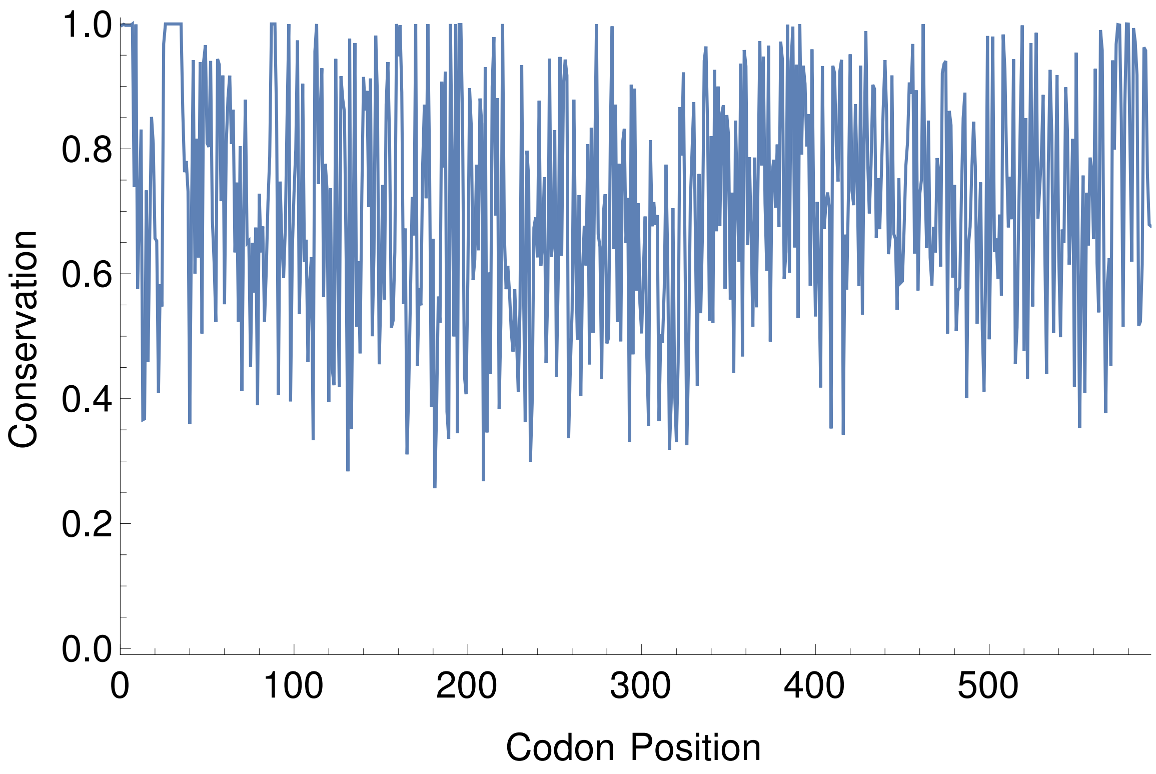

Conservation analysis plots

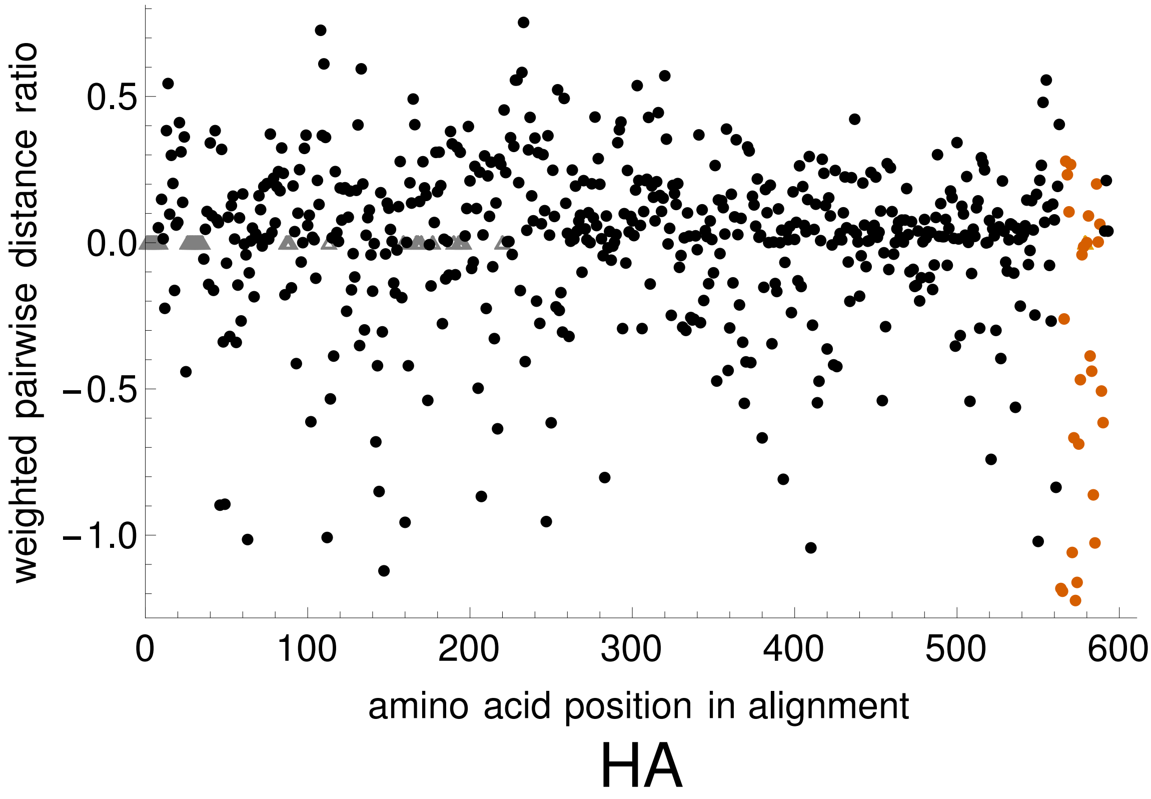

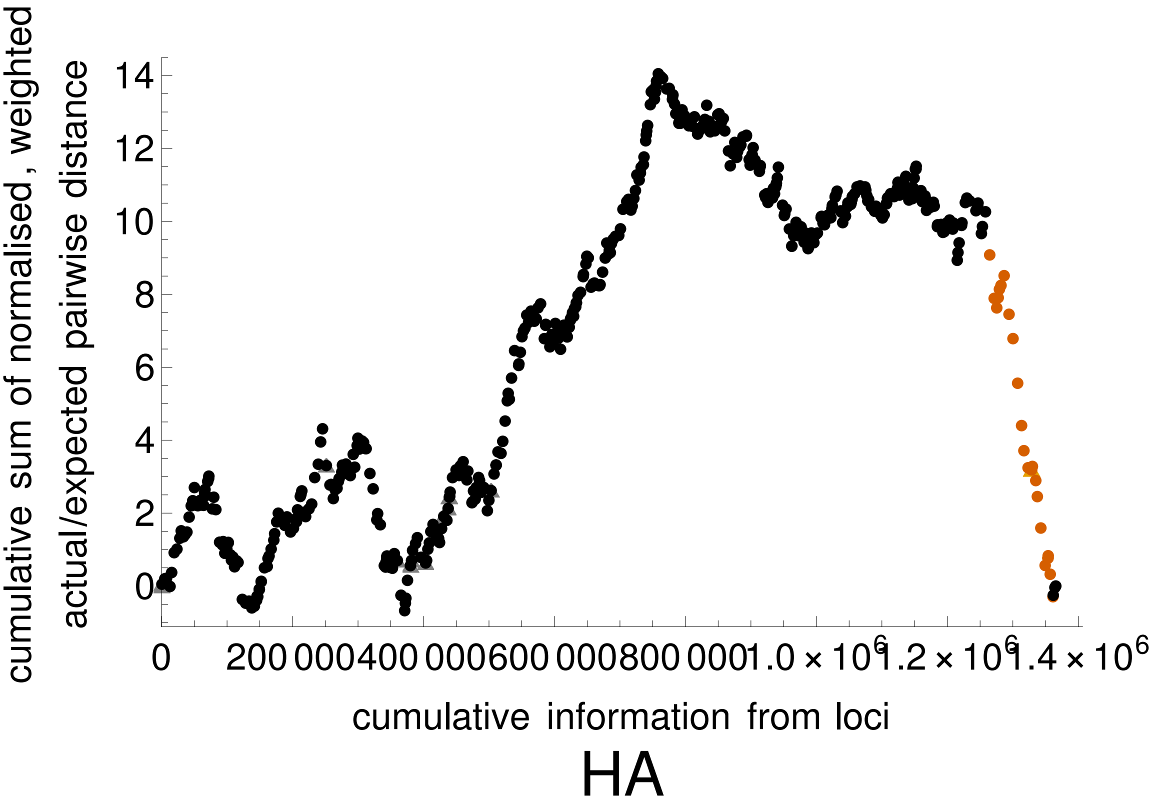

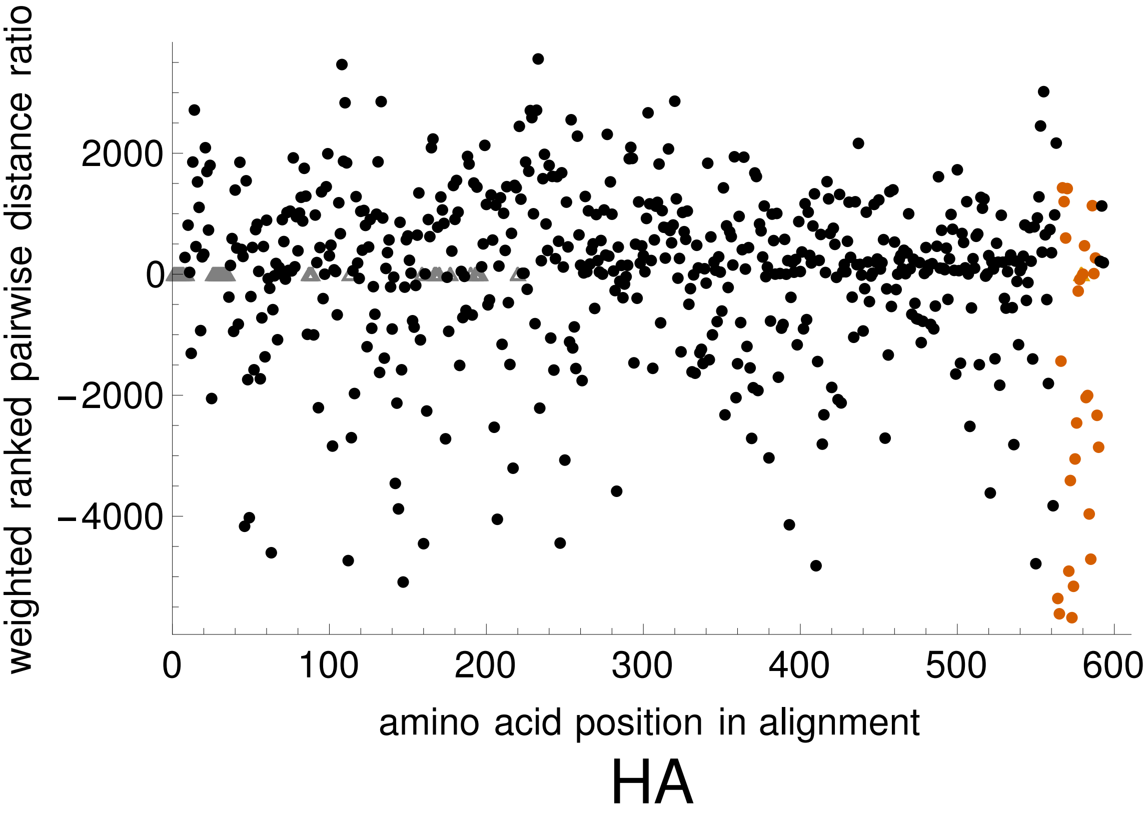
weighted ranked pairwise distance ratio
2000
0
−2000
−4000
0
100
200
300
400
500
600
amino acid position in alignment
HA

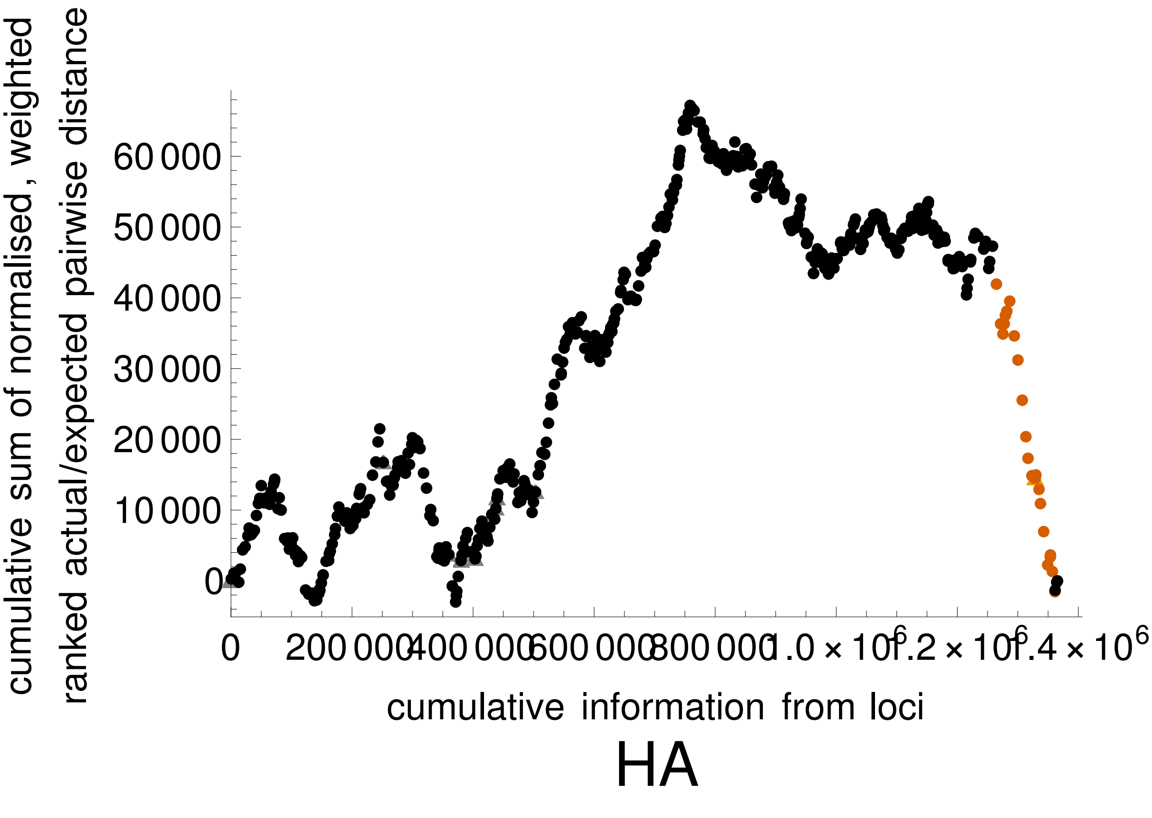
cumulative sum of normalised, weighted ranked actual/expected pairwise distance
60 000
50 000
40 000
30 000
20 000
10 000
0
0
200 000
400 000
600 000
800 000
1.0 × 10^6
1.2 × 10^6
1.4 × 10^6
cumulative information from loci
HA

NP

Gene length histogram

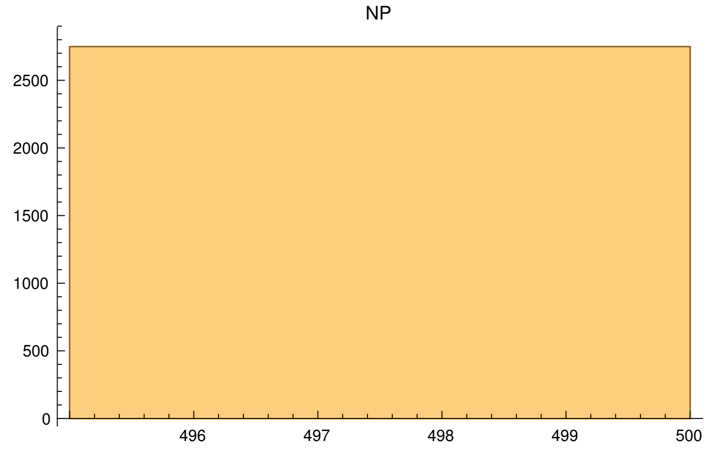

Information vs. nPD

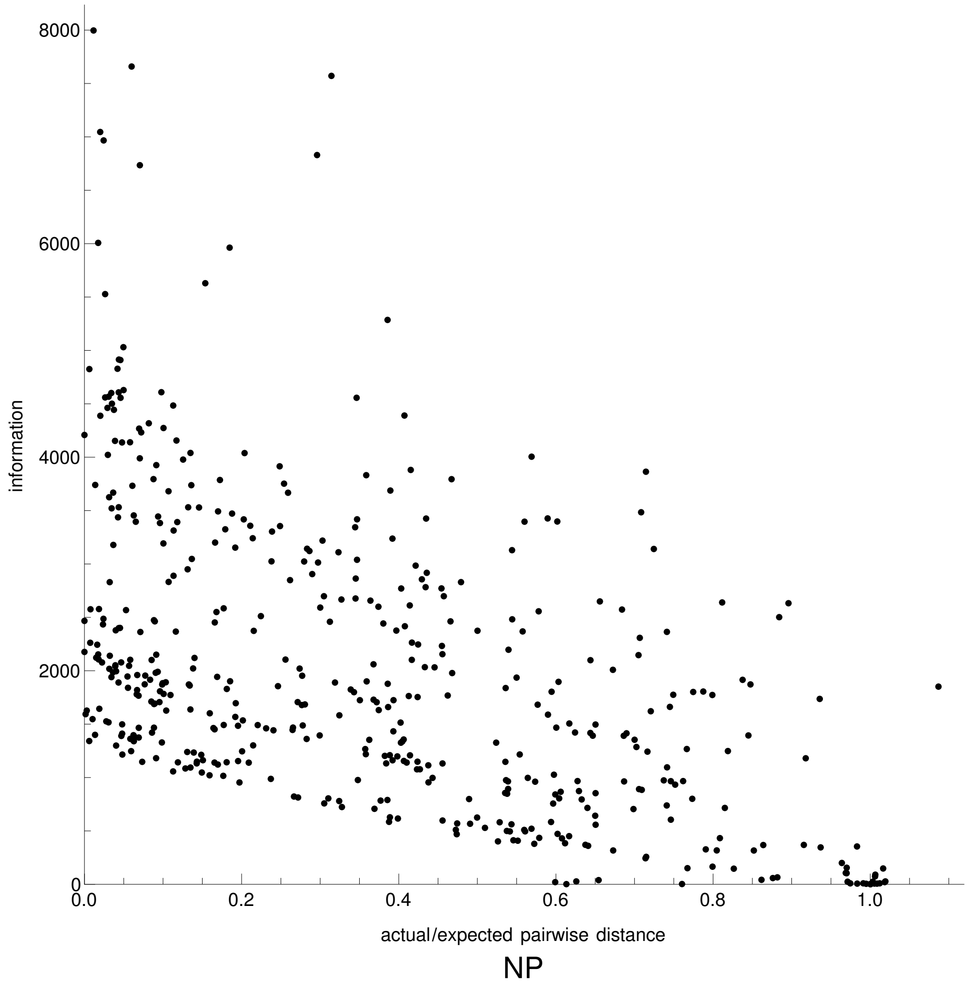

Example sequences highlighted by regions found in analyses to be conserved

Interesting points (by weighted raw PD) highlighted for gene NP:

------------------ATG

GCGTCTCAAGGCACCAAACGATCTTATGAGCAGATGGAAACTGGTGGAGAACGCC⋱
AGAATGCTACTGAAATCAGAGCATCTGTTGGGAGAATGGTTGGAGGAATTGGAAGGTTCTACATACAGAT⋱
GTGCACTGAACTCCAACTCAGTGACTATGAAGGGAGGCTGATCCAGAACAGCATAACAATAGAGAGAATG⋱
GTTCTCTCTGCGTTTGACGAGAGGAGAAACAAATACCTAGAAGAACATCCCAGTGCGGGGAAGGATCCAA⋱
AGAAAACTGGAGGTCCAATCTACAAAAAGAGAGACGGAAAATGGGTGAGAGAGCTGATTCTGTATGACAA⋱
AGATGAGATCAGGAGAATTTGGCGCCAAGCAAACAATGGTGAAGATGCTACTGCTGGTCTCACTCACCTG⋱
ATGATTTGGCACTCCAACCTGAATGATGCCACATATCAAAGAACAAGAGCTCTAGTGCGTACTGGGATGG⋱
ACCCCAGAATGTGCTCTCTGATGCAAGGATCAACTCTCCCGAGGAGATCTGGAGCTGCAGGTGCAGCAGT⋱
AAAGGGAGTTGGAACGATGGTAATGGAACTGATTCGGATGATAAAGAGGGGGATCAATGATAGGAACTTC⋱
TGGAGAGGCGAGAATGGACGAAGAACAAGAATTGCATATGAGAGAATGTGCAACATCCTCAAAGGGAAAT⋱
TTCAAACAGCATCGCAACGAGCAATGATGGACCAGGTGCGAGAAAGCAGAAATCCTGGGAATGCTGAGAT⋱
TGAAGACCTTATCTTCCTGGCAGGATCTGCACTCATTCTGAGAGGATCAGTGGCTCATAAATCCTGCCTG⋱
CCTGCCTGTGTATATGGACTTGTTGTGGCAAGTGGATATGACTTTGAAAGAGAAGGGTATTCTCTAGTTG⋱
GAATAGATCCTTTCCGTCTACTCCAGAACAGCCAGGTGTTCAGCCTCATTAGACCAAATGAGAATCCAGT⋱
GCATAAGAGTCAGTTGATATGGATGGCATGCCACTCTGCAGCATTTGAAGATCTGAGAGTGTCCAGTTTC⋱
ATCAGAGGGACAAAAGTAGTCCCAAGAGGACAACTGTCCACCAGAGGAGTTCAAATAGCTTCAAATGAAA⋱
ACATGGAAACAATGAACTCCATTACTCTTGAACTGAGAAGCAAATACTGGGCTATAAGAACCAGGAGCGG⋱
AGGAAACACCAATCAACAGAGAGCATCTGCAGGGCAAATCAGTGTACAACCTACTTTCTCAGTACAGAGA⋱
AACCTTCCTTTCGAGAGAGCGACCATCATGGCAGCATTTATAGGGAACACTGAAGGCAGAACATCTGACA⋱
TGAGAACTGAAATTATAAGAATGATGGAAAGTGCCAGA
CCAGAAGATGTGTCCTTCCAAGGGCGGGGAGTCTTCGAGTTCTCGGACGAAAAGG
CAACGAACCCGATCGTGCCTTCCTTTGACATGAGTAATGAAGGATCTTATTT
CTTCGGAGACAATGCAGAGGAGTATGACAATTAA

Interesting points (by weighted ranked PD) highlighted for gene NP:

------------------ATG

GCGTCTCAAGGCACCAAACGATCTTATGAGCAGATGGAAACTGGTGGAGAACGCCAGAATGCTACTGAA⋱
ATCAGAGCATCTGTTGGGAGAATGGTTGGAGGAATTGGAAGGTTCTACATACAGATGTGCACTGAACTCC⋱
AACTCAGTGACTATGAAGGGAGGCTGATCCAGAACAGCATAACAATAGAGAGAATGGTTCTCTCTGCGTT⋱
TGACGAGAGGAGAAACAAATACCTAGAAGAACATCCCAGTGCGGGGAAGGATCCAAAGAAAACTGGAGGT⋱
CCAATCTACAAAAAGAGAGACGGAAAATGGGTGAGAGAGCTGATTCTGTATGACAAAGATGAGATCAGGA⋱
GAATTTGGCGCCAAGCAAACAATGGTGAAGATGCTACTGCTGGTCTCACTCACCTGATGATTTGGCACTC⋱
CAACCTGAATGATGCCACATATCAAAGAACAAGAGCTCTAGTGCGTACTGGGATGGACCCCAGAATGTGC⋱
TCTCTGATGCAAGGATCAACTCTCCCGAGGAGATCTGGAGCTGCAGGTGCAGCAGTAAAGGGAGTTGGAA⋱
CGATGGTAATGGAACTGATTCGGATGATAAAGAGGGGGATCAATGATAGGAACTTCTGGAGAGGCGAGAA⋱
TGGACGAAGAACAAGAATTGCATATGAGAGAATGTGCAACATCCTCAAAGGGAAATTTCAAACAGCATCG⋱
CAACGAGCAATGATGGACCAGGTGCGAGAAAGCAGAAATCCTGGGAATGCTGAGATTGAAGACCTTATCT⋱
TCCTGGCAGGATCTGCACTCATTCTGAGAGGATCAGTGGCTCATAAATCCTGCCTGCCTGCCTGTGTATA⋱
TGGACTTGTTGTGGCAAGTGGATATGACTTTGAAAGAGAAGGGTATTCTCTAGTTGGAATAGATCCTTTC⋱
CGTCTACTCCAGAACAGCCAGGTGTTCAGCCTCATTAGACCAAATGAGAATCCAGTGCATAAGAGTCAGT⋱
TGATATGGATGGCATGCCACTCTGCAGCATTTGAAGATCTGAGAGTGTCCAGTTTCATCAGAGGGACAAA⋱
AGTAGTCCCAAGAGGACAACTGTCCACCAGAGGAGTTCAAATAGCTTCAAATGAAAACATGGAAACAATG⋱

AACTCCATTACTCTTGAACTGAGAAGCAAATACTGGGCTATAAGAACCAGGAGCGGAGGAAACACCAATC∵.
AACAGAGAGCATCTGCAGGGCAAATCAGTGTACAACCTACTTTCTCAGTACAGAGAAACCTTCCTTTCGA∵.
GAGAGCGACCATCATGGCAGCATTTATAGGGAACACTGAAGGCAGAACATCTGACATGAGAACTGAAATT∵.
ATAAGAATGATGGAAAGTGCCAGACCAGAAGATGTGTCCTTCCAAGGG

CGGGGAGTCTTCGAGTTCTCGGACGAAAAGGCAACGAACCCGATCGTGCCTTCCT
TTGACATGAGTAATGAAGGATCTTATTTCTTCGGAGACAATGCA

GAGGAGTATGACAATTAA

Per codon conservation report

NP			
Pos.	1	obs:	exp:
atg	M	1375	1375.00
---	-	1373	1373.00
---	--	------	------
mPD		0	0
		nPD:	1.
		N. weight:	0.
		Sc. PD:	0
		Sc. rank:	0

NP			
Pos.	2	obs:	exp:
tct	S	0	231.30
tcc	S	0	217.30
tca	S	0	333.90
tcg	S	0	73.08
agt	S	1375	255.90
agc	S	0	263.50
---	-	1373	1373.00
---	--	------	------
mPD		0	0.41
		nPD:	0.
		N. weight:	0.
		Sc. PD:	0
		Sc. rank:	0

NP			
Pos.	3	obs:	exp:
gat	D	0	773.40
gac	D	1375	601.60
---	-	1373	1373.00
---	--	------	------
mPD		0	0.12
		nPD:	0.
		N. weight:	0.
		Sc. PD:	0
		Sc. rank:	0

NP			
Pos.	4	obs:	exp:
att	I	79	487.80
atc	I	1295	352.70
ata	I	1	534.60
---	-	1373	1373.00
---	--	------	------
mPD		0.027	0.16
		nPD:	0.17
		N. weight:	0.
		Sc. PD:	0
		Sc. rank:	0

NP			
Pos.	5	obs:	exp:
aaa	K	1	0.61
aag	K	0	0.39
gat	D	1	0.56
gac	D	0	0.44
gaa	E	1371	812.30
gag	E	0	558.70
ggt	G	0	0.31
ggc	G	0	0.25
gga	G	2	0.91
ggg	G	0	0.53
---	-	1373	1373.00
---	--	------	------
mPD		0.0015	0.12
		nPD:	0.01
		N. weight:	0.
		Sc. PD:	0
		Sc. rank:	0

NP			
Pos.	6	obs:	exp:
tct	S	0	2.52
tcc	S	15	2.37
tca	S	0	3.64
tcg	S	0	0.80
att	I	0	0.35
atc	I	1	0.26
ata	I	0	0.39
act	T	0	8.61
acc	T	35	7.57
aca	T	0	15.90
acg	T	0	2.92
aat	N	0	1.14
aac	N	2	0.86
agt	S	0	2.79
agc	S	0	2.87
gtt	V	0	12.66
gtc	V	57	10.76
gta	V	0	14.39
gtg	V	0	19.20
gct	A	25	310.10
gcc	A	1240	289.80
gca	A	0	570.20
gcg	A	0	94.87
---	-	1373	1373.00
---	--	------	------
mPD		0.049	0.21
		nPD:	0.23
		N. weight:	0.
		Sc. PD:	0
		Sc. rank:	0

NP			
Pos.	7	obs:	exp:
atg	M	2748	2748.00
---	--	------	------
mPD		0	0
		nPD:	1.
		N. weight:	0.
		Sc. PD:	0
		Sc. rank:	0

NP			
Pos.	8	obs:	exp:
gtt	V	0	1.11
gtc	V	0	0.94
gta	V	0	1.26
gtg	V	5	1.68
gct	A	4	672.40
gcc	A	0	628.50
gca	A	14	1236.00
gcg	A	2725	205.70
---	--	------	------
mPD		0.017	0.68
		nPD:	0.02
		N. weight:	3.7
		Sc. PD:	-0.81
		Sc. rank:	-5021.5

NP			
Pos.	9	obs:	exp:
tct	S	2670	459.20
tcc	S	58	431.50
tca	S	2	662.90
tcg	S	0	145.10
cct	P	2	0.59
ccc	P	0	0.40
cca	P	0	0.79
ccg	P	0	0.22
act	T	15	3.69
acc	T	0	3.24
aca	T	0	6.82
acg	T	0	1.25
agt	S	0	508.10
agc	S	0	523.20
gct	A	1	0.25
gcc	A	0	0.23
gca	A	0	0.45
gcg	A	0	0.07
---	--	------	------
mPD		0.056	1.6
		nPD:	0.03
		N. weight:	2.4
		Sc. PD:	-0.52
		Sc. rank:	-2974.0

NP			
Pos.	10	obs:	exp:
cat	H	4	2.88
cac	H	1	2.12
caa	Q	2735	1479.00
cag	Q	8	1264.00
---	--	------	------
mPD		0.0094	0.50
		nPD:	0.02
		N. weight:	0.86
		Sc. PD:	-0.2
		Sc. rank:	-1260.0

NP			
Pos.	11	obs:	exp:
ggt	G	24	431.00
ggc	G	2723	348.20
gga	G	1	1245.00
ggg	G	0	724.10
---	--	------	------
mPD		0.018	0.68
		nPD:	0.03
		N. weight:	2.9
		Sc. PD:	-0.64
		Sc. rank:	-3883.8

NP			
Pos.	12	obs:	exp:
act	T	0	675.90
acc	T	2748	594.20
aca	T	0	1249.00
acg	T	0	229.10
---	--	------	------
mPD		0	0.68
		nPD:	0.
		N. weight:	2.2
		Sc. PD:	-0.54
		Sc. rank:	-3631.4

NP			
Pos.	13	obs:	exp:
aaa	K	2744	1670.00
aag	K	4	1078.00
---	--	------	------
mPD		0.0029	0.48
		nPD:	0.01
		N. weight:	0.7
		Sc. PD:	-0.17
		Sc. rank:	-1132.7

NP			
Pos.	14	obs:	exp:
cgt	R	1	86.74
cgc	R	0	145.20
cga	R	2533	276.20
cgg	R	214	198.50
aga	R	0	1260.00
agg	R	0	781.10
---	--	------	------
mPD		0.14	0.94
		nPD:	0.15
		N. weight:	3.
		Sc. PD:	-0.29
		Sc. rank:	-356.7

NP			
Pos.	15	obs:	exp:
tct	S	403	404.00
tcc	S	11	379.60
tca	S	1966	583.30
tcg	S	22	127.70
cct	P	0	0.30
ccc	P	0	0.20
cca	P	1	0.40
ccg	P	0	0.11
act	T	0	84.86
acc	T	0	74.60
aca	T	345	156.80
acg	T	0	28.77
agt	S	0	447.10
agc	S	0	460.30
---	--	------	------
mPD		0.49	1.6
		nPD:	0.3
		N. weight:	1.4
		Sc. PD:	0.054
		Sc. rank:	571.2

NP			
Pos.	16	obs:	exp:
tat	Y	2741	1540.00
tac	Y	7	1208.00
---	--	------	------
mPD		0.0051	0.49
		nPD:	0.01
		N. weight:	0.81
		Sc. PD:	-0.19
		Sc. rank:	-1274.7

NP			
Pos.	17	obs:	exp:
gaa	E	2528	1628.00
gag	E	220	1120.00
---	--	------	------
mPD		0.15	0.48
		nPD:	0.31
		N. weight:	0.4
		Sc. PD:	0.018
		Sc. rank:	176.1

NP			
Pos.	18	obs:	exp:
caa	Q	2321	1482.00
cag	Q	427	1266.00
---	--	------	------
mPD		0.26	0.50
		nPD:	0.53
		N. weight:	0.3
		Sc. PD:	0.078
		Sc. rank:	341.2

NP			
Pos.	19	obs:	exp:
atg	M	2748	2748.00
---	--	------	------
mPD		0	0
		nPD:	1.
		N. weight:	0.
		Sc. PD:	0
		Sc. rank:	0

NP			
Pos.	20	obs:	exp:
gat	D	105	59.62
gac	D	1	46.38
gaa	E	500	1565.00
gag	E	2142	1077.00
---	--	------	------
mPD		0.36	0.52
		nPD:	0.69
		N. weight:	0.51
		Sc. PD:	0.21
		Sc. rank:	712.7

NP			
Pos.	21	obs:	exp:
att	I	21	7.45
atc	I	0	5.39
ata	I	0	8.16
act	T	2584	670.80
acc	T	75	589.70
aca	T	21	1239.00
acg	T	47	227.40
---	--	------	------
mPD		0.12	0.69
		nPD:	0.17
		N. weight:	1.7
		Sc. PD:	-0.15
		Sc. rank:	-131.8

,

NP			
Pos.	22	obs:	exp:
tct	S	0	7.23
tcc	S	0	6.80
tca	S	0	10.44
tcg	S	0	2.29
agt	S	43	8.00
agc	S	0	8.24
gat	D	2	1.12
gac	D	0	0.88
ggt	G	2696	423.80
ggc	G	1	342.40
gga	G	2	1224.00
ggg	G	3	711.90
---	-	1	1.00
---	--	------	------
mPD		0.037	0.74
		nPD:	0.05
		N. weight:	2.6
		Sc. PD:	-0.52
		Sc. rank:	-2424.6

,

NP			
Pos.	23	obs:	exp:
gaa	E	1	0.59
gag	E	0	0.41
ggt	G	22	430.90
ggc	G	0	348.10
gga	G	444	1244.00
ggg	G	2281	723.80
---	--	------	------
mPD		0.29	0.69
		nPD:	0.42
		N. weight:	1.1
		Sc. PD:	0.17
		Sc. rank:	989.3

,

NP			
Pos.	24	obs:	exp:
gat	D	1	0.56
gac	D	0	0.44
gaa	E	949	1627.00
gag	E	1797	1119.00
ggt	G	0	0.16
ggc	G	0	0.13
gga	G	0	0.45
ggg	G	1	0.26
---	--	------	------
mPD		0.45	0.48
		nPD:	0.94
		N. weight:	0.18
		Sc. PD:	0.12
		Sc. rank:	296.3

,

NP			
Pos.	25	obs:	exp:
cgt	R	15	86.74
cgc	R	2733	145.20
cga	R	0	276.20
cgg	R	0	198.50
aga	R	0	1260.00
agg	R	0	781.10
---	--	------	------
mPD		0.011	0.94
		nPD:	0.01
		N. weight:	4.2
		Sc. PD:	-0.99
		Sc. rank:	-6474.0

,

NP			
Pos.	26	obs:	exp:
caa	Q	328	1482.00
cag	Q	2420	1266.00
---	--	------	------
mPD		0.21	0.50
		nPD:	0.42
		N. weight:	0.56
		Sc. PD:	0.089
		Sc. rank:	519.0

,

NP			
Pos.	27	obs:	exp:
aat	N	383	233.60
aac	N	28	177.40
gat	D	2299	1311.00
gac	D	32	1020.00
gaa	E	6	3.56
gag	E	0	2.44
---	--	------	------
mPD		0.30	0.75
		nPD:	0.4
		N. weight:	0.7
		Sc. PD:	0.096
		Sc. rank:	561.9

,

NP			
Pos.	28	obs:	exp:
act	T	265	71.58
acc	T	24	62.93
aca	T	2	132.20
acg	T	0	24.27
gtt	V	9	2.44
gtc	V	2	2.08
gta	V	0	2.78
gtg	V	0	3.71
gct	A	437	599.60
gcc	A	1976	560.40
gca	A	24	1103.00
gcg	A	9	183.40
---	--	------	------
mPD		0.60	0.88
		nPD:	0.68
		N. weight:	1.3
		Sc. PD:	0.55
		Sc. rank:	1892.5

,

NP			
Pos.	29	obs:	exp:
tct	S	0	0.34
tcc	S	1	0.32
tca	S	1	0.49
tcg	S	0	0.11
act	T	366	675.20
acc	T	48	593.60
aca	T	2274	1247.00
acg	T	57	228.90
aat	N	0	0.57
aac	N	1	0.43
agt	S	0	0.37
agc	S	0	0.38
---	--	------	------
mPD		0.30	0.68
		nPD:	0.44
		N. weight:	0.5
		Sc. PD:	0.086
		Sc. rank:	498.4

,

NP			
Pos.	30	obs:	exp:
gaa	E	2558	1628.00
gag	E	190	1120.00
---	--	------	------
mPD		0.13	0.48
		nPD:	0.27
		N. weight:	0.43
		Sc. PD:	0.0035
		Sc. rank:	123.9

,

NP			
Pos.	31	obs:	exp:
att	I	18	974.80
atc	I	2688	704.80
ata	I	42	1068.00
---	--	------	------
mPD		0.043	0.66
		nPD:	0.07
		N. weight:	1.8
		Sc. PD:	-0.33
		Sc. rank:	-1358.2

,

NP			
Pos.	32	obs:	exp:
cgt	R	0	86.43
cgc	R	0	144.70
cga	R	1	275.20
cgg	R	0	197.80
aaa	K	10	6.08
aag	K	0	3.92
aga	R	2619	1256.00
agg	R	118	778.20
---	--	------	------
mPD		0.090	0.94
		nPD:	0.1
		N. weight:	0.9
		Sc. PD:	-0.14
		Sc. rank:	-442.7

,

NP			
Pos.	33	obs:	exp:
tct	S	0	0.50
tcc	S	0	0.47
tca	S	3	0.73
tcg	S	0	0.16
act	T	0	0.49
acc	T	0	0.43
aca	T	2	0.91
acg	T	0	0.17
agt	S	0	0.56
agc	S	0	0.57
gct	A	7	672.40
gcc	A	1	628.50
gca	A	2618	1236.00
gcg	A	117	205.70
---	--	------	------
mPD		0.091	0.68
		nPD:	0.13
		N. weight:	0.98
		Sc. PD:	-0.12
		Sc. rank:	-238.6

,

NP			
Pos.	34	obs:	exp:
tct	S	2688	462.20
tcc	S	15	434.30
tca	S	41	667.30
tcg	S	4	146.10
agt	S	0	511.50
agc	S	0	526.60
---	--	------	------
mPD		0.043	1.6
		nPD:	0.03
		N. weight:	2.4
		Sc. PD:	-0.53
		Sc. rank:	-3249.1

,

NP			
Pos.	35	obs:	exp:
gtt	V	551	610.20
gtc	V	2139	518.50
gta	V	56	693.70
gtg	V	2	925.60
---	--	------	------
mPD		0.35	0.74
		nPD:	0.48
		N. weight:	1.5
		Sc. PD:	0.31
		Sc. rank:	1625.1

,

NP			
Pos.	36	obs:	exp:
ggt	G	0	431.00
ggc	G	6	348.20
gga	G	2324	1245.00
ggg	G	418	724.10
---	--	------	------
mPD		0.26	0.68
		nPD:	0.38
		N. weight:	0.63
		Sc. PD:	0.075
		Sc. rank:	441.5

,

NP			
Pos.	37	obs:	exp:
cgt	R	0	78.63
cgc	R	0	131.70
cga	R	40	250.40
cgg	R	0	180.00
aaa	K	92	156.20
aag	K	165	100.80
aga	R	2390	1142.00
agg	R	61	708.00
---	--	------	------
mPD		0.35	1.1
		nPD:	0.32
		N. weight:	0.83
		Sc. PD:	0.052
		Sc. rank:	419.6

,

NP			
Pos.	38	obs:	exp:
atg	M	2748	2748.00
---	--	------	------
mPD		0	0
		nPD:	1.
		N. weight:	0.
		Sc. PD:	0
		Sc. rank:	0

,

NP			
Pos.	39	obs:	exp:
att	I	2230	817.30
atc	I	70	591.00
ata	I	4	895.70
gtt	V	432	98.58
gtc	V	5	83.78
gta	V	5	112.10
gtg	V	2	149.60
---	--	------	------
mPD		0.33	0.96
		nPD:	0.34
		N. weight:	1.4
		Sc. PD:	0.12
		Sc. rank:	772.4

,

NP			
Pos.	40	obs:	exp:
tct	S	0	10.60
tcc	S	0	9.96
tca	S	0	15.30
tcg	S	0	3.35
act	T	3	0.74
acc	T	0	0.65
aca	T	0	1.36
acg	T	0	0.25
aat	N	15	8.53
aac	N	0	6.47
agt	S	57	11.73
agc	S	6	12.07
gtt	V	0	0.22
gtc	V	0	0.19
gta	V	1	0.25
gtg	V	0	0.34
gct	A	418	103.20
gcc	A	1	96.46
gca	A	0	189.80
gcg	A	2	31.57
gat	D	43	24.19
gac	D	0	18.81
gaa	E	275	163.50
gag	E	1	112.50
ggt	G	1586	302.10
ggc	G	190	244.00
gga	G	141	872.40
ggg	G	9	507.50
---	--	------	------
mPD		0.87	1.2
		nPD:	0.72
		N. weight:	1.6
		Sc. PD:	0.73
		Sc. rank:	2463.5

,

NP			
Pos.	41	obs:	exp:
ggt	G	0	431.00
ggc	G	4	348.20
gga	G	2641	1245.00
ggg	G	103	724.10
---	--	------	------
mPD		0.075	0.68
		nPD:	0.11
		N. weight:	0.93
		Sc. PD:	-0.13
		Sc. rank:	-345.4

,

NP			
Pos.	42	obs:	exp:
att	I	675	973.80
atc	I	2035	704.10
ata	I	35	1067.00
gtt	V	1	0.67
gtc	V	2	0.57
gta	V	0	0.76
gtg	V	0	1.01
---	--	------	------
mPD		0.39	0.66
		nPD:	0.59
		N. weight:	0.95
		Sc. PD:	0.3
		Sc. rank:	1210.0

,

NP			
Pos.	43	obs:	exp:
ggt	G	3	431.00
ggc	G	1	348.20
gga	G	545	1245.00
ggg	G	2199	724.10
---	--	------	------
mPD		0.32	0.68
		nPD:	0.47
		N. weight:	1.
		Sc. PD:	0.21
		Sc. rank:	1120.5

,

NP			
Pos.	44	obs:	exp:
cgt	R	0	57.67
cgc	R	0	96.57
cga	R	290	183.60
cgg	R	11	132.00
aaa	K	902	559.70
aag	K	19	361.30
aga	R	1283	837.90
agg	R	243	519.30
---	--	------	------
mPD		0.82	1.3
		nPD:	0.65
		N. weight:	0.45
		Sc. PD:	0.17
		Sc. rank:	615.3

,

NP			
Pos.	45	obs:	exp:
ttt	F	450	1227.00
ttc	F	2289	1512.00
tat	Y	0	5.04
tac	Y	9	3.96
---	--	------	------
mPD		0.28	0.50
		nPD:	0.56
		N. weight:	0.27
		Sc. PD:	0.077
		Sc. rank:	320.9

,

NP			
Pos.	46	obs:	exp:
tat	Y	39	1540.00
tac	Y	2709	1208.00
---	--	------	------
mPD		0.028	0.49
		nPD:	0.06
		N. weight:	1.1
		Sc. PD:	-0.21
		Sc. rank:	-938.3

,

NP			
Pos.	47	obs:	exp:
att	I	38	968.40
atc	I	2258	700.20
ata	I	434	1061.00
atg	M	1	1.00
gtt	V	0	3.77
gtc	V	9	3.21
gta	V	8	4.29
gtg	V	0	5.73
---	--	------	------
mPD		0.31	0.67
		nPD:	0.46
		N. weight:	1.1
		Sc. PD:	0.21
		Sc. rank:	1164.7

,

NP			
Pos.	48	obs:	exp:
caa	Q	2280	1482.00
cag	Q	468	1266.00
---	--	------	------
mPD		0.28	0.50
		nPD:	0.57
		N. weight:	0.27
		Sc. PD:	0.081
		Sc. rank:	332.9

,

NP			
Pos.	49	obs:	exp:
atg	M	2748	2748.00
---	--	------	------
mPD		0	0
		nPD:	1.
		N. weight:	0.
		Sc. PD:	0
		Sc. rank:	0

,

NP			
Pos.	50	obs:	exp:
tgt	C	352	1183.00
tgc	C	2396	1565.00
---	--	------	------
mPD		0.22	0.49
		nPD:	0.46
		N. weight:	0.31
		Sc. PD:	0.059
		Sc. rank:	324.7

,

NP			
Pos.	51	obs:	exp:
act	T	2656	675.90
acc	T	61	594.20
aca	T	25	1249.00
acg	T	6	229.10
---	--	------	------
mPD		0.065	0.68
		nPD:	0.1
		N. weight:	1.8
		Sc. PD:	-0.27
		Sc. rank:	-857.3

,

NP			
Pos.	52	obs:	exp:
gaa	E	2460	1628.00
gag	E	288	1120.00
---	--	------	------
mPD		0.19	0.48
		nPD:	0.39
		N. weight:	0.33
		Sc. PD:	0.041
		Sc. rank:	245.3

,

NP			
Pos.	53	obs:	exp:
ttt	F	0	0.45
ttc	F	1	0.55
tta	L	0	298.60
ttg	L	0	515.40
ctt	L	28	554.20
ctc	L	2675	399.20
cta	L	40	479.10
ctg	L	1	497.40
att	I	0	1.06
atc	I	3	0.77
ata	I	0	1.17
---	--	------	------
mPD		0.052	1.1
		nPD:	0.05
		N. weight:	2.6
		Sc. PD:	-0.52
		Sc. rank:	-2554.8

,

NP			
Pos.	54	obs:	exp:
caa	Q	395	220.00
cag	Q	13	188.00
cgt	R	0	0.03
cgc	R	0	0.05
cga	R	0	0.10
cgg	R	0	0.07
aaa	K	2267	1421.00
aag	K	72	917.50
aga	R	1	0.46
agg	R	0	0.28
---	--	------	------
mPD		0.31	0.73
		nPD:	0.43
		N. weight:	0.57
		Sc. PD:	0.091
		Sc. rank:	531.3

,

NP			
Pos.	55	obs:	exp:
tta	L	0	299.00
ttg	L	0	516.20
ctt	L	50	555.00
ctc	L	2678	399.80
cta	L	20	479.80
ctg	L	0	498.10
---	--	------	------
mPD		0.050	1.1
		nPD:	0.04
		N. weight:	2.6
		Sc. PD:	-0.53
		Sc. rank:	-2621.7

,

NP			
Pos.	56	obs:	exp:
tct	S	0	455.30
tcc	S	0	427.80
tca	S	0	657.40
tcg	S	0	143.90
att	I	1	0.35
atc	I	0	0.26
ata	I	0	0.39
aat	N	16	9.66
aac	N	1	7.34
agt	S	2682	503.80
agc	S	25	518.80
ggt	G	23	3.61
ggc	G	0	2.91
gga	G	0	10.42
ggg	G	0	6.06
---	--	------	------
mPD		0.048	1.7
		nPD:	0.03
		N. weight:	2.3
		Sc. PD:	-0.51
		Sc. rank:	-3087.0

,

NP			
Pos.	57	obs:	exp:
aat	N	0	0.57
aac	N	1	0.43
gat	D	1967	1543.00
gac	D	776	1200.00
gaa	E	4	2.37
gag	E	0	1.63
---	--	------	------
mPD		0.41	0.49
		nPD:	0.83
		N. weight:	0.077
		Sc. PD:	0.042
		Sc. rank:	121.6

,

NP			
Pos.	58	obs:	exp:
tat	Y	2680	1534.00
tac	Y	58	1204.00
tgt	C	1	0.43
tgc	C	0	0.57
cat	H	6	3.46
cac	H	0	2.54
caa	Q	0	0.54
cag	Q	1	0.46
aat	N	2	1.14
aac	N	0	0.86
---	--	------	------
mPD		0.049	0.50
		nPD:	0.1
		N. weight:	0.7
		Sc. PD:	-0.11
		Sc. rank:	-318.1

,

NP			
Pos.	59	obs:	exp:
gat	D	295	168.20
gac	D	4	130.80
gaa	E	963	1451.00
gag	E	1486	997.90
---	--	------	------
mPD		0.57	0.58
		nPD:	0.98
		N. weight:	0.19
		Sc. PD:	0.13
		Sc. rank:	304.4

,

NP			
Pos.	60	obs:	exp:
ggt	G	2	431.00
ggc	G	1	348.20
gga	G	2265	1245.00
ggg	G	480	724.10
---	--	------	------
mPD		0.29	0.68
		nPD:	0.42
		N. weight:	0.6
		Sc. PD:	0.095
		Sc. rank:	555.8

,

NP			
Pos.	61	obs:	exp:
cgt	R	4	86.62
cgc	R	3	145.00
cga	R	2218	275.80
cgg	R	79	198.20
act	T	0	0.98
acc	T	0	0.86
aca	T	0	1.82
acg	T	4	0.33
aga	R	106	1258.00
agg	R	334	779.90
---	--	------	------
mPD		0.54	0.94
		nPD:	0.57
		N. weight:	2.1
		Sc. PD:	0.62
		Sc. rank:	2590.4

,

NP			
Pos.	62	obs:	exp:
tta	L	213	298.60
ttg	L	59	515.40
ctt	L	1	554.20
ctc	L	0	399.20
cta	L	2080	479.10
ctg	L	391	497.40
act	T	4	0.98
acc	T	0	0.86
aca	T	0	1.82
acg	T	0	0.33
---	--	------	------
mPD		0.46	1.1
		nPD:	0.4
		N. weight:	1.5
		Sc. PD:	0.2
		Sc. rank:	1187.4

,

NP			
Pos.	63	obs:	exp:
tta	L	0	0.11
ttg	L	0	0.19
ctt	L	0	0.20
ctc	L	1	0.15
cta	L	0	0.17
ctg	L	0	0.18
att	I	382	973.10
atc	I	2351	703.50
ata	I	10	1066.00
aat	N	4	2.27
aac	N	0	1.73
---	--	------	------
mPD		0.25	0.66
		nPD:	0.38
		N. weight:	1.3
		Sc. PD:	0.15
		Sc. rank:	891.4

,

NP			
Pos.	64	obs:	exp:
tct	S	0	0.67
tcc	S	0	0.63
tca	S	4	0.97
tcg	S	0	0.21
cct	P	0	0.89
ccc	P	0	0.60
cca	P	3	1.19
ccg	P	0	0.32
caa	Q	1351	1478.00
cag	Q	1390	1263.00
agt	S	0	0.74
agc	S	0	0.77
---	--	------	------
mPD		0.51	0.51
		nPD:	1.
		N. weight:	0.013
		Sc. PD:	0.0091
		Sc. rank:	21.0

,

NP			
Pos.	65	obs:	exp:
aat	N	2125	1562.00
aac	N	623	1186.00
---	--	------	------
mPD		0.35	0.49
		nPD:	0.71
		N. weight:	0.13
		Sc. PD:	0.059
		Sc. rank:	198.9

,

NP			
Pos.	66	obs:	exp:
tct	S	0	462.20
tcc	S	0	434.30
tca	S	0	667.30
tcg	S	0	146.10
agt	S	337	511.50
agc	S	2411	526.60
---	--	------	------
mPD		0.22	1.6
		nPD:	0.13
		N. weight:	1.9
		Sc. PD:	-0.22
		Sc. rank:	-462.0

,

NP			
Pos.	67	obs:	exp:
tta	L	2	0.44
ttg	L	1	0.75
ctt	L	0	0.81
ctc	L	0	0.58
cta	L	1	0.70
ctg	L	0	0.73
att	I	4	896.10
atc	I	0	647.90
ata	I	2522	982.00
atg	M	211	211.00
gtt	V	0	1.55
gtc	V	0	1.32
gta	V	7	1.77
gtg	V	0	2.36
---	--	------	------
mPD		0.15	0.71
		nPD:	0.22
		N. weight:	1.2
		Sc. PD:	-0.051
		Sc. rank:	189.4

,

NP			
Pos.	68	obs:	exp:
act	T	2	675.90
acc	T	9	594.20
aca	T	2461	1249.00
acg	T	276	229.10
---	--	------	------
mPD		0.19	0.68
		nPD:	0.28
		N. weight:	0.88
		Sc. PD:	0.015
		Sc. rank:	271.8

,

NP			
Pos.	69	obs:	exp:
tta	L	1	0.11
ttg	L	0	0.19
ctt	L	0	0.20
ctc	L	0	0.15
cta	L	0	0.17
ctg	L	0	0.18
att	I	2	969.90
atc	I	1	701.20
ata	I	2731	1063.00
atg	M	2	2.00
gtt	V	0	2.44
gtc	V	0	2.08
gta	V	11	2.78
gtg	V	0	3.71
---	--	------	------
mPD		0.012	0.67
		nPD:	0.02
		N. weight:	1.4
		Sc. PD:	-0.31
		Sc. rank:	-1983.5

,

NP			
Pos.	70	obs:	exp:
gaa	E	95	1628.00
gag	E	2653	1120.00
---	--	------	------
mPD		0.067	0.48
		nPD:	0.14
		N. weight:	1.1
		Sc. PD:	-0.12
		Sc. rank:	-194.3

,

NP			
Pos.	71	obs:	exp:
cgt	R	0	86.39
cgc	R	0	144.70
cga	R	1	275.10
cgg	R	0	197.70
aaa	K	11	6.69
aag	K	0	4.31
aga	R	1465	1255.00
agg	R	1271	777.90
---	--	------	------
mPD		0.51	0.95
		nPD:	0.54
		N. weight:	0.45
		Sc. PD:	0.12
		Sc. rank:	503.7

,

NP			
Pos.	72	obs:	exp:
atg	M	2748	2748.00
---	--	------	------
mPD		0	0
		nPD:	1.
		N. weight:	0.
		Sc. PD:	0
		Sc. rank:	0

,

NP			
Pos.	73	obs:	exp:
tta	L	0	0.11
ttg	L	0	0.19
ctt	L	0	0.20
ctc	L	0	0.15
cta	L	0	0.17
ctg	L	1	0.18
gtt	V	97	609.30
gtc	V	312	517.80
gta	V	236	692.70
gtg	V	2099	924.30
gct	A	1	0.74
gcc	A	1	0.69
gca	A	0	1.35
gcg	A	1	0.22
---	--	------	------
mPD		0.40	0.74
		nPD:	0.54
		N. weight:	0.6
		Sc. PD:	0.16
		Sc. rank:	677.8

,

NP			
Pos.	74	obs:	exp:
tta	L	3	299.00
ttg	L	0	516.20
ctt	L	1270	555.00
ctc	L	1422	399.80
cta	L	28	479.80
ctg	L	25	498.10
---	--	------	------
mPD		0.52	1.1
		nPD:	0.46
		N. weight:	1.4
		Sc. PD:	0.27
		Sc. rank:	1479.7

,

NP			
Pos.	75	obs:	exp:
tct	S	2668	462.20
tcc	S	20	434.30
tca	S	20	667.30
tcg	S	40	146.10
agt	S	0	511.50
agc	S	0	526.60
---	--	------	------
mPD		0.057	1.6
		nPD:	0.04
		N. weight:	2.4
		Sc. PD:	-0.5
		Sc. rank:	-2828.7

,

NP			
Pos.	76	obs:	exp:
tct	S	5	3.36
tcc	S	0	3.16
tca	S	2	4.86
tcg	S	13	1.06
agt	S	0	3.72
agc	S	0	3.83
gct	A	2308	668.70
gcc	A	86	625.10
gca	A	319	1230.00
gcg	A	15	204.60
---	--	------	------
mPD		0.29	0.70
		nPD:	0.42
		N. weight:	1.2
		Sc. PD:	0.18
		Sc. rank:	1074.5

,

NP			
Pos.	77	obs:	exp:
ttt	F	2090	1218.00
ttc	F	629	1501.00
tat	Y	1	16.25
tac	Y	28	12.75
---	--	------	------
mPD		0.38	0.52
		nPD:	0.75
		N. weight:	0.32
		Sc. PD:	0.15
		Sc. rank:	483.9

,

NP			
Pos.	78	obs:	exp:
gat	D	2362	1546.00
gac	D	386	1202.00
---	--	------	------
mPD		0.24	0.49
		nPD:	0.49
		N. weight:	0.3
		Sc. PD:	0.066
		Sc. rank:	326.9

,

NP			
Pos.	79	obs:	exp:
gaa	E	62	1628.00
gag	E	2686	1120.00
---	--	------	------
mPD		0.044	0.48
		nPD:	0.09
		N. weight:	1.1
		Sc. PD:	-0.18
		Sc. rank:	-600.7

,

NP			
Pos.	80	obs:	exp:
cgt	R	0	86.74
cgc	R	0	145.20
cga	R	5	276.20
cgg	R	3	198.50
aga	R	2441	1260.00
agg	R	299	781.10
---	--	------	------
mPD		0.20	0.94
		nPD:	0.21
		N. weight:	0.68
		Sc. PD:	-0.028
		Sc. rank:	98.3

,

NP			
Pos.	81	obs:	exp:
cgt	R	0	86.74
cgc	R	0	145.20
cga	R	5	276.20
cgg	R	1	198.50
aga	R	2635	1260.00
agg	R	107	781.10
---	--	------	------
mPD		0.080	0.94
		nPD:	0.09
		N. weight:	0.9
		Sc. PD:	-0.15
		Sc. rank:	-547.1

,

NP			
Pos.	82	obs:	exp:
aat	N	2243	1562.00
aac	N	505	1186.00
---	--	------	------
mPD		0.30	0.49
		nPD:	0.61
		N. weight:	0.2
		Sc. PD:	0.068
		Sc. rank:	266.5

,

NP			
Pos.	83	obs:	exp:
cgt	R	0	0.41
cgc	R	0	0.69
cga	R	0	1.31
cgg	R	0	0.94
aaa	K	2074	1662.00
aag	K	661	1073.00
aga	R	12	5.96
agg	R	1	3.69
---	--	------	------
mPD		0.38	0.49
		nPD:	0.77
		N. weight:	0.079
		Sc. PD:	0.038
		Sc. rank:	122.2

,

NP			
Pos.	84	obs:	exp:
tat	Y	32	1540.00
tac	Y	2716	1208.00
---	--	------	------
mPD		0.023	0.49
		nPD:	0.05
		N. weight:	1.1
		Sc. PD:	-0.22
		Sc. rank:	-1056.5

,

NP			
Pos.	85	obs:	exp:
tta	L	77	299.00
ttg	L	270	516.20
ctt	L	1	555.00
ctc	L	20	399.80
cta	L	2255	479.80
ctg	L	125	498.10
---	--	------	------
mPD		0.48	1.1
		nPD:	0.42
		N. weight:	1.6
		Sc. PD:	0.24
		Sc. rank:	1432.7

,

NP			
Pos.	86	obs:	exp:
gaa	E	2708	1628.00
gag	E	40	1120.00
---	--	------	------
mPD		0.029	0.48
		nPD:	0.06
		N. weight:	0.65
		Sc. PD:	-0.12
		Sc. rank:	-552.0

,

NP			
Pos.	87	obs:	exp:
gat	D	3	2.81
gac	D	2	2.19
gaa	E	506	1625.00
gag	E	2236	1117.00
ggt	G	0	0.16
ggc	G	0	0.13
gga	G	1	0.45
ggg	G	0	0.26
---	--	------	------
mPD		0.30	0.49
		nPD:	0.63
		N. weight:	0.51
		Sc. PD:	0.18
		Sc. rank:	677.8

,

NP			
Pos.	88	obs:	exp:
cat	H	2510	1585.00
cac	H	238	1163.00
---	--	------	------
mPD		0.16	0.49
		nPD:	0.32
		N. weight:	0.41
		Sc. PD:	0.026
		Sc. rank:	204.5

,

NP			
Pos.	89	obs:	exp:
tct	S	0	0.34
tcc	S	2	0.32
tca	S	0	0.49
tcg	S	0	0.11
cct	P	62	810.00
ccc	P	2657	552.70
cca	P	26	1087.00
ccg	P	0	295.30
cat	H	0	0.58
cac	H	1	0.42
agt	S	0	0.37
agc	S	0	0.38
---	--	------	------
mPD		0.065	0.71
		nPD:	0.09
		N. weight:	2.1
		Sc. PD:	-0.33
		Sc. rank:	-1069.0

,

NP			
Pos.	90	obs:	exp:
tct	S	0	415.10
tcc	S	0	390.00
tca	S	0	599.30
tcg	S	0	131.20
aat	N	280	159.10
aac	N	0	120.90
agt	S	2440	459.40
agc	S	28	473.00
---	--	------	------
mPD		0.20	1.7
		nPD:	0.12
		N. weight:	2.2
		Sc. PD:	-0.29
		Sc. rank:	-673.2

,

NP			
Pos.	91	obs:	exp:
tct	S	25	4.54
tcc	S	0	4.27
tca	S	0	6.56
tcg	S	2	1.43
act	T	16	5.90
acc	T	0	5.19
aca	T	0	10.91
acg	T	8	2.00
agt	S	0	5.03
agc	S	0	5.17
gtt	V	1	0.22
gtc	V	0	0.19
gta	V	0	0.25
gtg	V	0	0.34
gct	A	2272	660.60
gcc	A	25	617.50
gca	A	46	1215.00
gcg	A	352	202.10
ggt	G	0	0.16
ggc	G	0	0.13
gga	G	0	0.45
ggg	G	1	0.26
---	--	------	------
mPD		0.31	0.72
		nPD:	0.43
		N. weight:	1.5
		Sc. PD:	0.24
		Sc. rank:	1423.4

,

NP			
Pos.	92	obs:	exp:
ggt	G	3	431.00
ggc	G	0	348.20
gga	G	132	1245.00
ggg	G	2613	724.10
---	--	------	------
mPD		0.094	0.68
		nPD:	0.14
		N. weight:	1.6
		Sc. PD:	-0.19
		Sc. rank:	-304.0

,

NP			
Pos.	93	obs:	exp:
aaa	K	588	1670.00
aag	K	2160	1078.00
---	--	------	------
mPD		0.34	0.48
		nPD:	0.71
		N. weight:	0.47
		Sc. PD:	0.2
		Sc. rank:	672.0

,

NP			
Pos.	94	obs:	exp:
gat	D	1462	1546.00
gac	D	1286	1202.00
---	--	------	------
mPD		0.50	0.49
		nPD:	1.01
		N. weight:	0.0049
		Sc. PD:	0.0035
		Sc. rank:	8.0

,

NP			
Pos.	95	obs:	exp:
cct	P	2266	810.60
ccc	P	49	553.10
cca	P	402	1088.00
ccg	P	30	295.50
cgt	R	0	0.03
cgc	R	0	0.05
cga	R	0	0.10
cgg	R	1	0.07
aga	R	0	0.46
agg	R	0	0.28
---	--	------	------
mPD		0.30	0.71
		nPD:	0.42
		N. weight:	0.92
		Sc. PD:	0.15
		Sc. rank:	854.4

,

NP

Pos.	96	obs:	exp:
cgt	R	0	0.09
cgc	R	0	0.16
cga	R	0	0.30
cgg	R	0	0.22
aaa	K	13	1668.00
aag	K	2732	1077.00
aga	R	0	1.38
agg	R	3	0.85
---	--	------	------
mPD		0.012	0.48
		nPD:	0.02
		N. weight:	1.3
		Sc. PD:	-0.29
		Sc. rank:	-1823.4

,

NP

Pos.	97	obs:	exp:
aaa	K	2699	1670.00
aag	K	49	1078.00
---	--	------	------
mPD		0.035	0.48
		nPD:	0.07
		N. weight:	0.6
		Sc. PD:	-0.11
		Sc. rank:	-390.9

,

NP

Pos.	98	obs:	exp:
act	T	1435	675.90
acc	T	2	594.20
aca	T	1300	1249.00
acg	T	11	229.10
---	--	------	------
mPD		0.50	0.68
		nPD:	0.74
		N. weight:	0.57
		Sc. PD:	0.26
		Sc. rank:	869.5

,

NP

Pos.	99	obs:	exp:
gaa	E	0	0.59
gag	E	1	0.41
ggt	G	11	430.90
ggc	G	0	348.10
gga	G	2524	1244.00
ggg	G	212	723.80
---	--	------	------
mPD		0.15	0.69
		nPD:	0.22
		N. weight:	0.78
		Sc. PD:	-0.028
		Sc. rank:	123.0

,

NP

Pos.	100	obs:	exp:
ggt	G	398	431.00
ggc	G	35	348.20
gga	G	1979	1245.00
ggg	G	336	724.10
---	--	------	------
mPD		0.45	0.68
		nPD:	0.65
		N. weight:	0.29
		Sc. PD:	0.11
		Sc. rank:	402.7

,

NP

Pos.	101	obs:	exp:
tct	S	0	0.67
tcc	S	4	0.63
tca	S	0	0.97
tcg	S	0	0.21
cct	P	39	809.70
ccc	P	2140	552.40
cca	P	549	1087.00
ccg	P	16	295.20
agt	S	0	0.74
agc	S	0	0.77
---	--	------	------
mPD		0.35	0.71
		nPD:	0.5
		N. weight:	1.2
		Sc. PD:	0.29
		Sc. rank:	1382.3

,

NP

Pos.	102	obs:	exp:
tta	L	0	0.11
ttg	L	0	0.19
ctt	L	0	0.20
ctc	L	1	0.15
cta	L	0	0.17
ctg	L	0	0.18
att	I	60	974.50
atc	I	346	704.60
ata	I	2341	1068.00
---	--	------	------
mPD		0.26	0.66
		nPD:	0.39
		N. weight:	0.75
		Sc. PD:	0.097
		Sc. rank:	584.4

,

NP

Pos.	103	obs:	exp:
tat	Y	2212	1540.00
tac	Y	536	1208.00
---	--	------	------
mPD		0.31	0.49
		nPD:	0.64
		N. weight:	0.19
		Sc. PD:	0.07
		Sc. rank:	260.6

,

NP

Pos.	104	obs:	exp:
cgt	R	0	73.70
cgc	R	0	123.40
cga	R	0	234.70
cgg	R	0	168.70
aaa	K	407	251.00
aag	K	6	162.00
aga	R	2305	1071.00
agg	R	30	663.70
---	--	------	------
mPD		0.28	1.1
		nPD:	0.25
		N. weight:	0.97
		Sc. PD:	-0.011
		Sc. rank:	201.7

,

NP

Pos.	105	obs:	exp:
cgt	R	0	74.90
cgc	R	0	125.40
cga	R	2	238.50
cgg	R	0	171.40
aaa	K	9	227.90
aag	K	366	147.10
aga	R	2266	1088.00
agg	R	105	674.50
---	--	------	------
mPD		0.52	1.1
		nPD:	0.46
		N. weight:	0.93
		Sc. PD:	0.18
		Sc. rank:	975.3

,

NP

Pos.	106	obs:	exp:
tta	L	16	2.61
ttg	L	0	4.51
ctt	L	0	4.85
ctc	L	0	3.49
cta	L	2	4.19
ctg	L	6	4.35
cgt	R	0	12.88
cgc	R	0	21.57
cga	R	1	41.01
cgg	R	0	29.48
att	I	0	387.00
atc	I	0	279.80
ata	I	1091	424.10
atg	M	94	94.00
act	T	0	1.72
acc	T	0	1.51
aca	T	7	3.18
acg	T	0	0.58
aaa	K	7	4.25
aag	K	0	2.75
aga	R	391	187.10
agg	R	16	116.00
gtt	V	1	246.70
gtc	V	4	209.60
gta	V	1037	280.50
gtg	V	69	374.20
gct	A	0	1.47
gcc	A	0	1.37
gca	A	6	2.70
gcg	A	0	0.45
---	--	------	------
mPD		0.89	1.5
		nPD:	0.58
		N. weight:	1.3
		Sc. PD:	0.41
		Sc. rank:	1679.4

,

NP

Pos.	107	obs:	exp:
aat	N	0	1.14
aac	N	2	0.86
gat	D	382	1465.00
gac	D	2222	1139.00
gaa	E	133	83.55
gag	E	8	57.45
ggt	G	0	0.16
ggc	G	1	0.13
gga	G	0	0.45
ggg	G	0	0.26
---	--	------	------
mPD		0.33	0.54
		nPD:	0.6
		N. weight:	0.54
		Sc. PD:	0.17
		Sc. rank:	691.9

,

NP

Pos.	108	obs:	exp:
cgt	R	0	0.06
cgc	R	0	0.11
cga	R	0	0.20
cgg	R	0	0.14
aga	R	2	0.92
agg	R	0	0.57
ggt	G	4	430.70
ggc	G	1	347.90
gga	G	2554	1244.00
ggg	G	187	723.50
---	--	------	------
mPD		0.13	0.69
		nPD:	0.19
		N. weight:	0.82
		Sc. PD:	-0.052
		Sc. rank:	49.2

,

NP

Pos.	109	obs:	exp:
aaa	K	592	1670.00
aag	K	2156	1078.00
---	--	------	------
mPD		0.34	0.48
		nPD:	0.71
		N. weight:	0.46
		Sc. PD:	0.2
		Sc. rank:	677.4

,

NP

Pos.	110	obs:	exp:
tgt	C	1	0.43
tgc	C	0	0.57
tgg	W	2747	2747.00
---	--	------	------
mPD		0.00073	0.00073
		nPD:	1.
		N. weight:	0.00044
		Sc. PD:	0.00031
		Sc. rank:	0.7

,

NP			
Pos.	111	obs:	exp:
tta	L	0	10.66
ttg	L	1	18.41
ctt	L	0	19.79
ctc	L	0	14.26
cta	L	2	17.11
ctg	L	95	17.76
att	I	2	10.29
atc	I	0	7.44
ata	I	27	11.27
atg	M	2458	2458.00
act	T	5	4.67
acc	T	0	4.11
aca	T	0	8.63
acg	T	14	1.58
gtt	V	0	31.97
gtc	V	0	27.17
gta	V	1	36.35
gtg	V	143	48.50
---	--	------	------
mPD		0.20	0.32
		nPD:	0.64
		N. weight:	0.19
		Sc. PD:	0.069
		Sc. rank:	254.7

,

NP			
Pos.	112	obs:	exp:
cgt	R	0	86.71
cgc	R	0	145.20
cga	R	0	276.10
cgg	R	0	198.50
aaa	K	1	0.61
aag	K	0	0.39
aga	R	2703	1260.00
agg	R	44	780.80
---	--	------	------
mPD		0.032	0.94
		nPD:	0.03
		N. weight:	1.
		Sc. PD:	-0.22
		Sc. rank:	-1247.3

,

NP			
Pos.	113	obs:	exp:
gaa	E	2293	1628.00
gag	E	455	1120.00
---	--	------	------
mPD		0.28	0.48
		nPD:	0.57
		N. weight:	0.2
		Sc. PD:	0.06
		Sc. rank:	245.5

,

NP			
Pos.	114	obs:	exp:
tta	L	0	298.90
ttg	L	4	516.00
ctt	L	60	554.80
ctc	L	2278	399.70
cta	L	18	479.70
ctg	L	387	498.00
att	I	0	0.35
atc	I	1	0.26
ata	I	0	0.39
---	--	------	------
mPD		0.30	1.1
		nPD:	0.26
		N. weight:	1.9
		Sc. PD:	0.0016
		Sc. rank:	509.4

,

NP			
Pos.	115	obs:	exp:
tta	L	0	0.11
ttg	L	0	0.19
ctt	L	1	0.20
ctc	L	0	0.15
cta	L	0	0.17
ctg	L	0	0.18
att	I	1290	752.40
atc	I	829	544.00
ata	I	2	824.60
atg	M	1	1.00
act	T	15	141.20
acc	T	559	124.10
aca	T	0	260.80
acg	T	0	47.86
gtt	V	48	11.32
gtc	V	2	9.62
gta	V	0	12.87
gtg	V	1	17.18
---	--	------	------
mPD		0.87	1.0
		nPD:	0.84
		N. weight:	1.
		Sc. PD:	0.55
		Sc. rank:	1597.2

,

NP			
Pos.	116	obs:	exp:
tta	L	1	298.80
ttg	L	15	515.80
tgt	C	1	0.43
tgc	C	0	0.57
ctt	L	2298	554.60
ctc	L	90	399.50
cta	L	35	479.50
ctg	L	307	497.80
att	I	1	0.35
atc	I	0	0.26
ata	I	0	0.39
---	--	------	------
mPD		0.30	1.1
		nPD:	0.26
		N. weight:	1.5
		Sc. PD:	0.0049
		Sc. rank:	410.3

,

NP			
Pos.	117	obs:	exp:
tat	Y	2704	1540.00
tac	Y	44	1208.00
---	--	------	------
mPD		0.032	0.49
		nPD:	0.06
		N. weight:	0.72
		Sc. PD:	-0.13
		Sc. rank:	-560.4

,

NP			
Pos.	118	obs:	exp:
gat	D	88	1542.00
gac	D	2654	1200.00
gaa	E	6	3.56
gag	E	0	2.44
---	--	------	------
mPD		0.066	0.49
		nPD:	0.13
		N. weight:	0.98
		Sc. PD:	-0.12
		Sc. rank:	-231.1

,

NP			
Pos.	119	obs:	exp:
aaa	K	2672	1670.00
aag	K	76	1078.00
---	--	------	------
mPD		0.054	0.48
		nPD:	0.11
		N. weight:	0.55
		Sc. PD:	-0.077
		Sc. rank:	-204.0

,

NP			
Pos.	120	obs:	exp:
aat	N	1	0.57
aac	N	0	0.43
gat	D	353	218.20
gac	D	35	169.80
gaa	E	2309	1398.00
gag	E	50	961.20
---	--	------	------
mPD		0.28	0.61
		nPD:	0.46
		N. weight:	0.59
		Sc. PD:	0.11
		Sc. rank:	613.9

,

NP			
Pos.	121	obs:	exp:
gat	D	0	0.56
gac	D	1	0.44
gaa	E	2510	1628.00
gag	E	237	1119.00
---	--	------	------
mPD		0.16	0.48
		nPD:	0.33
		N. weight:	0.38
		Sc. PD:	0.025
		Sc. rank:	196.3

,

NP			
Pos.	122	obs:	exp:
att	I	1	974.10
atc	I	391	704.30
ata	I	2354	1068.00
gtt	V	0	0.44
gtc	V	1	0.38
gta	V	1	0.50
gtg	V	0	0.67
---	--	------	------
mPD		0.25	0.66
		nPD:	0.37
		N. weight:	0.86
		Sc. PD:	0.095
		Sc. rank:	585.9

,

NP			
Pos.	123	obs:	exp:
cgt	R	0	86.52
cgc	R	0	144.90
cga	R	0	275.50
cgg	R	4	198.00
aaa	K	0	4.25
aag	K	7	2.75
aga	R	225	1257.00
agg	R	2512	779.10
---	--	------	------
mPD		0.16	0.94
		nPD:	0.17
		N. weight:	1.3
		Sc. PD:	-0.12
		Sc. rank:	-93.3

,

NP			
Pos.	124	obs:	exp:
cgt	R	0	86.33
cgc	R	0	144.60
cga	R	5	274.90
cgg	R	0	197.60
aaa	K	13	7.90
aag	K	0	5.10
aga	R	2659	1254.00
agg	R	71	777.40
---	--	------	------
mPD		0.063	0.95
		nPD:	0.07
		N. weight:	0.95
		Sc. PD:	-0.17
		Sc. rank:	-715.9

,

NP			
Pos.	125	obs:	exp:
att	I	389	156.40
atc	I	49	113.10
ata	I	3	171.40
act	T	1	0.25
acc	T	0	0.22
aca	T	0	0.45
acg	T	0	0.08
gtt	V	2242	512.00
gtc	V	20	435.10
gta	V	30	582.10
gtg	V	14	776.70
---	--	------	------
mPD		0.35	1.0
		nPD:	0.35
		N. weight:	1.8
		Sc. PD:	0.15
		Sc. rank:	1071.7

,

NP			
Pos.	126	obs:	exp:
tgg	W	2748	2748.00
---	--	------	------
mPD		0	0
		nPD:	1.
		N. weight:	0.
		Sc. PD:	0
		Sc. rank:	0

,

NP			
Pos.	127	obs:	exp:
cgt	R	45	86.74
cgc	R	2669	145.20
cga	R	24	276.20
cgg	R	10	198.50
aga	R	0	1260.00
agg	R	0	781.10
---	--	------	------
mPD		0.056	0.94
		nPD:	0.06
		N. weight:	4.
		Sc. PD:	-0.76
		Sc. rank:	-3284.6

,

NP			
Pos.	128	obs:	exp:
caa	Q	2688	1482.00
cag	Q	60	1266.00
---	--	------	------
mPD		0.043	0.50
		nPD:	0.09
		N. weight:	0.75
		Sc. PD:	-0.12
		Sc. rank:	-445.1

,

NP			
Pos.	129	obs:	exp:
tct	S	0	0.34
tcc	S	0	0.32
tca	S	2	0.49
tcg	S	0	0.11
agt	S	0	0.37
agc	S	0	0.38
gct	A	5	673.10
gcc	A	7	629.20
gca	A	2684	1238.00
gcg	A	50	205.90
---	--	------	------
mPD		0.046	0.68
		nPD:	0.07
		N. weight:	1.
		Sc. PD:	-0.19
		Sc. rank:	-764.4

,

NP			
Pos.	130	obs:	exp:
aat	N	271	1562.00
aac	N	2477	1186.00
---	--	------	------
mPD		0.18	0.49
		nPD:	0.36
		N. weight:	0.71
		Sc. PD:	0.071
		Sc. rank:	454.1

,

NP			
Pos.	131	obs:	exp:
tct	S	0	0.84
tcc	S	0	0.79
tca	S	0	1.21
tcg	S	0	0.27
aat	N	2700	1556.00
aac	N	37	1181.00
aaa	K	5	3.04
aag	K	0	1.96
agt	S	5	0.93
agc	S	0	0.96
gat	D	1	0.56
gac	D	0	0.44
---	--	------	------
mPD		0.035	0.50
		nPD:	0.07
		N. weight:	0.72
		Sc. PD:	-0.13
		Sc. rank:	-528.4

,

NP			
Pos.	132	obs:	exp:
tct	S	0	0.34
tcc	S	0	0.32
tca	S	0	0.49
tcg	S	0	0.11
aat	N	4	2.27
aac	N	0	1.73
agt	S	2	0.37
agc	S	0	0.38
ggt	G	1458	430.10
ggc	G	1085	347.40
gga	G	192	1242.00
ggg	G	7	722.50
---	--	------	------
mPD		0.56	0.69
		nPD:	0.81
		N. weight:	1.4
		Sc. PD:	0.73
		Sc. rank:	2182.5

,

NP			
Pos.	133	obs:	exp:
gat	D	14	14.06
gac	D	11	10.94
gaa	E	2648	1610.00
gag	E	70	1108.00
ggt	G	0	0.78
ggc	G	0	0.63
gga	G	5	2.26
ggg	G	0	1.32
---	--	------	------
mPD		0.071	0.50
		nPD:	0.14
		N. weight:	0.59
		Sc. PD:	-0.065
		Sc. rank:	-99.7

,

NP			
Pos.	134	obs:	exp:
aat	N	5	2.84
aac	N	0	2.16
gat	D	2685	1521.00
gac	D	20	1184.00
gaa	E	33	20.74
gag	E	2	14.26
ggt	G	3	0.47
ggc	G	0	0.38
gga	G	0	1.36
ggg	G	0	0.79
---	--	------	------
mPD		0.045	0.51
		nPD:	0.09
		N. weight:	0.77
		Sc. PD:	-0.12
		Sc. rank:	-439.2

,

NP			
Pos.	135	obs:	exp:
tct	S	0	1.01
tcc	S	0	0.95
tca	S	6	1.46
tcg	S	0	0.32
act	T	0	0.74
acc	T	0	0.65
aca	T	3	1.36
acg	T	0	0.25
agt	S	0	1.12
agc	S	0	1.15
gct	A	340	671.20
gcc	A	17	627.30
gca	A	2363	1234.00
gcg	A	18	205.30
ggt	G	0	0.16
ggc	G	0	0.13
gga	G	1	0.45
ggg	G	0	0.26
---	--	------	------
mPD		0.25	0.69
		nPD:	0.36
		N. weight:	0.64
		Sc. PD:	0.061
		Sc. rank:	393.4

,

NP			
Pos.	136	obs:	exp:
tct	S	0	0.17
tcc	S	0	0.16
tca	S	1	0.24
tcg	S	0	0.05
act	T	428	675.20
acc	T	1	593.60
aca	T	2307	1247.00
acg	T	9	228.90
agt	S	0	0.19
agc	S	0	0.19
gct	A	0	0.49
gcc	A	0	0.46
gca	A	2	0.90
gcg	A	0	0.15
---	--	------	------
mPD		0.27	0.68
		nPD:	0.4
		N. weight:	0.63
		Sc. PD:	0.084
		Sc. rank:	500.0

,

NP			
Pos.	137	obs:	exp:
tct	S	0	0.50
tcc	S	1	0.47
tca	S	2	0.73
tcg	S	0	0.16
cct	P	1	0.30
ccc	P	0	0.20
cca	P	0	0.40
ccg	P	0	0.11
act	T	1	0.25
acc	T	0	0.22
aca	T	0	0.45
acg	T	0	0.08
agt	S	0	0.56
agc	S	0	0.57
gct	A	1402	672.40
gcc	A	39	628.50
gca	A	1293	1236.00
gcg	A	9	205.70
---	--	------	------
mPD		0.52	0.68
		nPD:	0.76
		N. weight:	0.51
		Sc. PD:	0.24
		Sc. rank:	779.2

,

NP			
Pos.	138	obs:	exp:
ggt	G	1977	431.00
ggc	G	729	348.20
gga	G	30	1245.00
ggg	G	12	724.10
---	--	------	------
mPD		0.41	0.68
		nPD:	0.6
		N. weight:	1.8
		Sc. PD:	0.58
		Sc. rank:	2325.6

,

NP			
Pos.	139	obs:	exp:
tta	L	1	299.00
ttg	L	0	516.20
ctt	L	2305	555.00
ctc	L	438	399.80
cta	L	4	479.80
ctg	L	0	498.10
---	--	------	------
mPD		0.27	1.1
		nPD:	0.24
		N. weight:	1.7
		Sc. PD:	-0.032
		Sc. rank:	339.6

,

NP			
Pos.	140	obs:	exp:
act	T	2556	675.90
acc	T	185	594.20
aca	T	5	1249.00
acg	T	2	229.10
---	--	------	------
mPD		0.13	0.68
		nPD:	0.19
		N. weight:	1.7
		Sc. PD:	-0.1
		Sc. rank:	90.0

,

NP			
Pos.	141	obs:	exp:
cat	H	2130	1585.00
cac	H	617	1162.00
aat	N	1	0.57
aac	N	0	0.43
---	--	------	------
mPD		0.35	0.49
		nPD:	0.71
		N. weight:	0.13
		Sc. PD:	0.056
		Sc. rank:	186.9

,

NP			
Pos.	142	obs:	exp:
tta	L	4	44.07
ttg	L	20	76.07
ctt	L	7	81.79
ctc	L	0	58.93
cta	L	17	70.72
ctg	L	357	73.42
att	I	101	817.00
atc	I	1715	590.70
ata	I	487	895.30
atg	M	4	4.00
gtt	V	0	7.99
gtc	V	0	6.79
gta	V	36	9.09
gtg	V	0	12.13
---	--	------	------
mPD		0.83	0.98
		nPD:	0.85
		N. weight:	0.98
		Sc. PD:	0.55
		Sc. rank:	1572.3

,

NP			
Pos.	143	obs:	exp:
atg	M	2748	2748.00
---	--	------	------
mPD		0	0
		nPD:	1.
		N. weight:	0.
		Sc. PD:	0
		Sc. rank:	0

,

NP			
Pos.	144	obs:	exp:
att	I	2665	974.50
atc	I	65	704.60
ata	I	17	1068.00
gtt	V	1	0.22
gtc	V	0	0.19
gta	V	0	0.25
gtg	V	0	0.34
---	--	------	------
mPD		0.059	0.66
		nPD:	0.09
		N. weight:	1.3
		Sc. PD:	-0.21
		Sc. rank:	-711.5

,

NP			
Pos.	145	obs:	exp:
tgg	W	2748	2748.00
---	--	------	------
mPD		0	0
		nPD:	1.
		N. weight:	0.
		Sc. PD:	0
		Sc. rank:	0

,

NP			
Pos.	146	obs:	exp:
cat	H	2473	1585.00
cac	H	275	1163.00
---	--	------	------
mPD		0.18	0.49
		nPD:	0.37
		N. weight:	0.37
		Sc. PD:	0.039
		Sc. rank:	246.4

,

NP			
Pos.	147	obs:	exp:
tct	S	189	462.20
tcc	S	2551	434.30
tca	S	8	667.30
tcg	S	0	146.10
agt	S	0	511.50
agc	S	0	526.60
---	--	------	------
mPD		0.13	1.6
		nPD:	0.08
		N. weight:	2.3
		Sc. PD:	-0.38
		Sc. rank:	-1408.3

,

NP			
Pos.	148	obs:	exp:
aat	N	1219	1562.00
aac	N	1529	1186.00
---	--	------	------
mPD		0.49	0.49
		nPD:	1.01
		N. weight:	0.047
		Sc. PD:	0.034
		Sc. rank:	77.6

,

NP			
Pos.	149	obs:	exp:
tta	L	9	299.00
ttg	L	285	516.20
ctt	L	88	555.00
ctc	L	1	399.80
cta	L	104	479.80
ctg	L	2261	498.10
---	--	------	------
mPD		0.33	1.1
		nPD:	0.29
		N. weight:	1.5
		Sc. PD:	0.046
		Sc. rank:	568.1

,

NP			
Pos.	150	obs:	exp:
tct	S	0	0.17
tcc	S	0	0.16
tca	S	0	0.24
tcg	S	0	0.05
aat	N	2706	1561.00
aac	N	41	1186.00
agt	S	1	0.19
agc	S	0	0.19
---	--	------	------
mPD		0.030	0.49
		nPD:	0.06
		N. weight:	0.71
		Sc. PD:	-0.13
		Sc. rank:	-568.5

,

NP			
Pos.	151	obs:	exp:
gat	D	2642	1546.00
gac	D	106	1202.00
---	--	------	------
mPD		0.074	0.49
		nPD:	0.15
		N. weight:	0.61
		Sc. PD:	-0.062
		Sc. rank:	-85.4

NP			
Pos.	152	obs:	exp:
tct	S	0	2.35
tcc	S	14	2.21
tca	S	0	3.40
tcg	S	0	0.74
act	T	0	7.87
acc	T	31	6.92
aca	T	1	14.54
acg	T	0	2.67
agt	S	0	2.61
agc	S	0	2.68
gtt	V	0	0.44
gtc	V	2	0.38
gta	V	0	0.50
gtg	V	0	0.67
gct	A	42	661.90
gcc	A	2602	618.60
gca	A	56	1217.00
gcg	A	0	202.50
---	--	------	------
mPD		0.10	0.72
		nPD:	0.15
		N. weight:	1.9
		Sc. PD:	-0.2
		Sc. rank:	-281.1

NP			
Pos.	153	obs:	exp:
act	T	37	675.90
acc	T	67	594.20
aca	T	1661	1249.00
acg	T	983	229.10
---	--	------	------
mPD		0.51	0.68
		nPD:	0.75
		N. weight:	0.87
		Sc. PD:	0.4
		Sc. rank:	1323.8

NP			
Pos.	154	obs:	exp:
tat	Y	1568	1540.00
tac	Y	1180	1208.00
---	--	------	------
mPD		0.49	0.49
		nPD:	0.99
		N. weight:	0.0025
		Sc. PD:	0.0018
		Sc. rank:	4.1

NP			
Pos.	155	obs:	exp:
caa	Q	25	1482.00
cag	Q	2723	1266.00
---	--	------	------
mPD		0.018	0.50
		nPD:	0.04
		N. weight:	1.
		Sc. PD:	-0.22
		Sc. rank:	-1240.8

NP			
Pos.	156	obs:	exp:
cgt	R	0	86.74
cgc	R	0	145.20
cga	R	2	276.20
cgg	R	0	198.50
aga	R	2691	1260.00
agg	R	55	781.10
---	--	------	------
mPD		0.041	0.94
		nPD:	0.04
		N. weight:	0.99
		Sc. PD:	-0.2
		Sc. rank:	-1060.7

NP			
Pos.	157	obs:	exp:
act	T	12	675.90
acc	T	1	594.20
aca	T	2710	1249.00
acg	T	25	229.10
---	--	------	------
mPD		0.027	0.68
		nPD:	0.04
		N. weight:	1.
		Sc. PD:	-0.22
		Sc. rank:	-1161.0

NP			
Pos.	158	obs:	exp:
cgt	R	0	86.74
cgc	R	0	145.20
cga	R	2	276.20
cgg	R	0	198.50
aga	R	2515	1260.00
agg	R	231	781.10
---	--	------	------
mPD		0.16	0.94
		nPD:	0.17
		N. weight:	0.76
		Sc. PD:	-0.067
		Sc. rank:	-74.5

NP			
Pos.	159	obs:	exp:
gct	A	947	673.60
gcc	A	89	629.60
gca	A	285	1239.00
gcg	A	1427	206.10
---	--	------	------
mPD		0.60	0.68
		nPD:	0.88
		N. weight:	1.3
		Sc. PD:	0.78
		Sc. rank:	2116.4

NP			
Pos.	160	obs:	exp:
tta	L	299	299.00
ttg	L	6	516.20
ctt	L	1992	555.00
ctc	L	380	399.80
cta	L	69	479.80
ctg	L	2	498.10
---	--	------	------
mPD		0.64	1.1
		nPD:	0.56
		N. weight:	1.2
		Sc. PD:	0.35
		Sc. rank:	1489.7

NP			
Pos.	161	obs:	exp:
att	I	1	0.35
atc	I	0	0.26
ata	I	0	0.39
gtt	V	2264	609.90
gtc	V	77	518.40
gta	V	23	693.40
gtg	V	383	925.30
---	--	------	------
mPD		0.30	0.74
		nPD:	0.41
		N. weight:	1.3
		Sc. PD:	0.18
		Sc. rank:	1082.2

NP			
Pos.	162	obs:	exp:
cgt	R	384	86.74
cgc	R	2303	145.20
cga	R	52	276.20
cgg	R	9	198.50
aga	R	0	1260.00
agg	R	0	781.10
---	--	------	------
mPD		0.28	0.94
		nPD:	0.3
		N. weight:	3.6
		Sc. PD:	0.13
		Sc. rank:	1419.3

NP			
Pos.	163	obs:	exp:
tct	S	0	6.90
tcc	S	41	6.48
tca	S	0	9.96
tcg	S	0	2.18
cct	P	1	0.30
ccc	P	0	0.20
cca	P	0	0.40
ccg	P	0	0.11
act	T	1375	664.90
acc	T	1244	584.50
aca	T	50	1228.00
acg	T	34	225.40
agt	S	0	7.63
agc	S	0	7.86
gct	A	0	0.74
gcc	A	3	0.69
gca	A	0	1.35
gcg	A	0	0.22
---	--	------	------
mPD		0.56	0.71
		nPD:	0.79
		N. weight:	0.95
		Sc. PD:	0.48
		Sc. rank:	1474.2

NP			
Pos.	164	obs:	exp:
ggt	G	7	431.00
ggc	G	1	348.20
gga	G	2291	1245.00
ggg	G	449	724.10
---	--	------	------
mPD		0.28	0.68
		nPD:	0.41
		N. weight:	0.61
		Sc. PD:	0.086
		Sc. rank:	503.1

NP			
Pos.	165	obs:	exp:
tta	L	0	0.11
ttg	L	1	0.19
ctt	L	0	0.20
ctc	L	0	0.15
cta	L	0	0.17
ctg	L	0	0.18
atg	M	2747	2747.00
---	--	------	------
mPD		0.00073	0.0012
		nPD:	0.61
		N. weight:	0.00088
		Sc. PD:	0.0003
		Sc. rank:	1.2

NP			
Pos.	166	obs:	exp:
gat	D	2628	1546.00
gac	D	120	1202.00
---	--	------	------
mPD		0.084	0.49
		nPD:	0.17
		N. weight:	0.59
		Sc. PD:	-0.05
		Sc. rank:	-34.9

NP			
Pos.	167	obs:	exp:
cct	P	43	810.90
ccc	P	2701	553.30
cca	P	4	1088.00
ccg	P	0	295.60
---	--	------	------
mPD		0.034	0.70
		nPD:	0.05
		N. weight:	2.2
		Sc. PD:	-0.44
		Sc. rank:	-2062.7

NP			
Pos.	168	obs:	exp:
cgt	R	0	86.74
cgc	R	0	145.20
cga	R	0	276.20
cgg	R	0	198.50
aga	R	2517	1260.00
agg	R	231	781.10
---	--	------	------
mPD		0.15	0.94
		nPD:	0.16
		N. weight:	0.77
		Sc. PD:	-0.069
		Sc. rank:	-82.0

NP			
Pos.	169	obs:	exp:
atg	M	2748	2748.00
---	--	------	------
mPD		0	0
		nPD:	1.
		N. weight:	0.
		Sc. PD:	0
		Sc. rank:	0

NP			
Pos.	170	obs:	exp:
tgt	C	369	1183.00
tgc	C	2379	1565.00
---	--	------	------
mPD		0.23	0.49
		nPD:	0.47
		N. weight:	0.3
		Sc. PD:	0.062
		Sc. rank:	324.4

NP			
Pos.	171	obs:	exp:
tct	S	2576	462.20
tcc	S	163	434.30
tca	S	9	667.30
tcg	S	0	146.10
agt	S	0	511.50
agc	S	0	526.60
---	--	------	------
mPD		0.12	1.6
		nPD:	0.07
		N. weight:	2.2
		Sc. PD:	-0.39
		Sc. rank:	-1451.3

NP			
Pos.	172	obs:	exp:
tta	L	29	299.00
ttg	L	23	516.20
ctt	L	51	555.00
ctc	L	1	399.80
cta	L	2257	479.80
ctg	L	387	498.10
---	--	------	------
mPD		0.32	1.1
		nPD:	0.28
		N. weight:	1.6
		Sc. PD:	0.039
		Sc. rank:	580.4

NP			
Pos.	173	obs:	exp:
atg	M	2748	2748.00
---	--	------	------
mPD		0	0
		nPD:	1.
		N. weight:	0.
		Sc. PD:	0
		Sc. rank:	0

NP			
Pos.	174	obs:	exp:
caa	Q	2700	1482.00
cag	Q	48	1266.00
---	--	------	------
mPD		0.034	0.50
		nPD:	0.07
		N. weight:	0.77
		Sc. PD:	-0.14
		Sc. rank:	-559.4

NP			
Pos.	175	obs:	exp:
ggt	G	2546	431.00
ggc	G	31	348.20
gga	G	101	1245.00
ggg	G	70	724.10
---	--	------	------
mPD		0.14	0.68
		nPD:	0.2
		N. weight:	2.1
		Sc. PD:	-0.11
		Sc. rank:	239.4

NP			
Pos.	176	obs:	exp:
tct	S	29	462.20
tcc	S	18	434.30
tca	S	2669	667.30
tcg	S	32	146.10
agt	S	0	511.50
agc	S	0	526.60
---	--	------	------
mPD		0.056	1.6
		nPD:	0.03
		N. weight:	1.8
		Sc. PD:	-0.39
		Sc. rank:	-2241.3

,

NP			
Pos.	177	obs:	exp:
act	T	404	675.90
acc	T	19	594.20
aca	T	2261	1249.00
acg	T	64	229.10
---	--	------	------
mPD		0.30	0.68
		nPD:	0.44
		N. weight:	0.52
		Sc. PD:	0.092
		Sc. rank:	522.1

,

NP			
Pos.	178	obs:	exp:
tta	L	0	299.00
ttg	L	0	516.20
ctt	L	2004	555.00
ctc	L	744	399.80
cta	L	0	479.80
ctg	L	0	498.10
---	--	------	------
mPD		0.40	1.1
		nPD:	0.35
		N. weight:	1.6
		Sc. PD:	0.13
		Sc. rank:	934.1

,

NP			
Pos.	179	obs:	exp:
cct	P	70	810.90
ccc	P	2282	553.30
cca	P	372	1088.00
ccg	P	24	295.60
---	--	------	------
mPD		0.29	0.70
		nPD:	0.41
		N. weight:	1.4
		Sc. PD:	0.2
		Sc. rank:	1195.4

,

NP			
Pos.	180	obs:	exp:
cgt	R	0	86.74
cgc	R	0	145.20
cga	R	16	276.20
cgg	R	0	198.50
aga	R	2194	1260.00
agg	R	538	781.10
---	--	------	------
mPD		0.33	0.94
		nPD:	0.35
		N. weight:	0.51
		Sc. PD:	0.044
		Sc. rank:	307.9

,

NP			
Pos.	181	obs:	exp:
cgt	R	0	86.74
cgc	R	0	145.20
cga	R	10	276.20
cgg	R	7	198.50
aga	R	516	1260.00
agg	R	2215	781.10
---	--	------	------
mPD		0.32	0.94
		nPD:	0.34
		N. weight:	0.94
		Sc. PD:	0.076
		Sc. rank:	499.8

,

NP			
Pos.	182	obs:	exp:
tct	S	2679	460.70
tcc	S	31	432.90
tca	S	12	665.10
tcg	S	17	145.60
agt	S	0	509.80
agc	S	0	524.90
gct	A	8	2.21
gcc	A	0	2.06
gca	A	1	4.06
gcg	A	0	0.67
---	--	------	------
mPD		0.050	1.6
		nPD:	0.03
		N. weight:	2.4
		Sc. PD:	-0.52
		Sc. rank:	-3076.6

,

NP			
Pos.	183	obs:	exp:
ggt	G	1322	431.00
ggc	G	4	348.20
gga	G	556	1245.00
ggg	G	866	724.10
---	--	------	------
mPD		0.63	0.68
		nPD:	0.92
		N. weight:	0.62
		Sc. PD:	0.39
		Sc. rank:	1007.3

,

NP			
Pos.	184	obs:	exp:
gct	A	523	673.60
gcc	A	2122	629.60
gca	A	103	1239.00
gcg	A	0	206.10
---	--	------	------
mPD		0.37	0.68
		nPD:	0.54
		N. weight:	1.2
		Sc. PD:	0.31
		Sc. rank:	1330.4

,

NP			
Pos.	185	obs:	exp:
gct	A	411	673.60
gcc	A	5	629.60
gca	A	2322	1239.00
gcg	A	10	206.10
---	--	------	------
mPD		0.26	0.68
		nPD:	0.39
		N. weight:	0.63
		Sc. PD:	0.079
		Sc. rank:	471.2

,

NP			
Pos.	186	obs:	exp:
ggt	G	2708	431.00
ggc	G	12	348.20
gga	G	25	1245.00
ggg	G	3	724.10
---	--	------	------
mPD		0.029	0.68
		nPD:	0.04
		N. weight:	2.5
		Sc. PD:	-0.52
		Sc. rank:	-2756.2

,

NP			
Pos.	187	obs:	exp:
tct	S	0	0.34
tcc	S	0	0.32
tca	S	2	0.49
tcg	S	0	0.11
agt	S	0	0.37
agc	S	0	0.38
gct	A	1705	672.90
gcc	A	114	628.90
gca	A	509	1237.00
gcg	A	417	205.90
ggt	G	0	0.16
ggc	G	0	0.13
gga	G	0	0.45
ggg	G	1	0.26
---	--	------	------
mPD		0.56	0.68
		nPD:	0.82
		N. weight:	0.65
		Sc. PD:	0.35
		Sc. rank:	1036.2

,

NP			
Pos.	188	obs:	exp:
gct	A	24	673.60
gcc	A	1	629.60
gca	A	1514	1239.00
gcg	A	1209	206.10
---	--	------	------
mPD		0.50	0.68
		nPD:	0.74
		N. weight:	1.2
		Sc. PD:	0.57
		Sc. rank:	1871.8

,

NP			
Pos.	189	obs:	exp:
att	I	0	21.64
atc	I	0	15.65
ata	I	61	23.71
atg	M	2	2.00
act	T	0	0.25
acc	T	0	0.22
aca	T	1	0.45
acg	T	0	0.08
gtt	V	2	595.90
gtc	V	1	506.50
gta	V	939	677.50
gtg	V	1742	904.00
---	--	------	------
mPD		0.51	0.79
		nPD:	0.65
		N. weight:	0.78
		Sc. PD:	0.29
		Sc. rank:	1076.3

,

NP			
Pos.	190	obs:	exp:
aaa	K	2344	1670.00
aag	K	404	1078.00
---	--	------	------
mPD		0.25	0.48
		nPD:	0.53
		N. weight:	0.21
		Sc. PD:	0.054
		Sc. rank:	235.6

,

NP			
Pos.	191	obs:	exp:
ggt	G	42	431.00
ggc	G	41	348.20
gga	G	2617	1245.00
ggg	G	48	724.10
---	--	------	------
mPD		0.092	0.68
		nPD:	0.13
		N. weight:	0.86
		Sc. PD:	-0.1
		Sc. rank:	-198.4

,

NP			
Pos.	192	obs:	exp:
att	I	79	28.38
atc	I	1	20.52
ata	I	0	31.10
gtt	V	2459	592.40
gtc	V	200	503.40
gta	V	5	673.50
gtg	V	4	898.70
---	--	------	------
mPD		0.20	0.80
		nPD:	0.25
		N. weight:	1.8
		Sc. PD:	-0.016
		Sc. rank:	408.6

,

NP			
Pos.	193	obs:	exp:
ggt	G	7	431.00
ggc	G	0	348.20
gga	G	2246	1245.00
ggg	G	495	724.10
---	--	------	------
mPD		0.30	0.68
		nPD:	0.44
		N. weight:	0.58
		Sc. PD:	0.1
		Sc. rank:	579.9

,

NP			
Pos.	194	obs:	exp:
act	T	3	675.90
acc	T	4	594.20
aca	T	2652	1249.00
acg	T	89	229.10
---	--	------	------
mPD		0.068	0.68
		nPD:	0.1
		N. weight:	0.99
		Sc. PD:	-0.15
		Sc. rank:	-438.3

,

NP			
Pos.	195	obs:	exp:
tta	L	12	31.34
ttg	L	0	54.10
ctt	L	0	58.16
ctc	L	0	41.90
cta	L	270	50.29
ctg	L	6	52.21
att	I	10	813.40
atc	I	0	588.10
ata	I	2283	891.40
atg	M	62	62.00
gtt	V	0	23.31
gtc	V	0	19.81
gta	V	32	26.51
gtg	V	73	35.37
---	--	------	------
mPD		0.36	0.96
		nPD:	0.37
		N. weight:	1.4
		Sc. PD:	0.15
		Sc. rank:	926.0

,

NP

Pos.	196	obs:	exp:
tct	S	0	0.17
tcc	S	0	0.16
tca	S	1	0.24
tcg	S	0	0.05
att	I	0	0.35
atc	I	0	0.26
ata	I	1	0.39
act	T	0	53.62
acc	T	0	47.14
aca	T	215	99.06
acg	T	3	18.18
agt	S	0	0.19
agc	S	0	0.19
gtt	V	1	91.03
gtc	V	1	77.37
gta	V	351	103.50
gtg	V	57	138.10
gct	A	23	519.20
gcc	A	0	485.30
gca	A	2072	954.70
gcg	A	23	158.80
---	--	------	------
mPD		0.48	1.1
		nPD:	0.43
		N. weight:	1.1
		Sc. PD:	0.18
		Sc. rank:	1020.9

,

NP

Pos.	197	obs:	exp:
atg	M	2748	2748.00
---	--	------	------
mPD		0	0
		nPD:	1.
		N. weight:	0.
		Sc. PD:	0
		Sc. rank:	0

,

NP

Pos.	198	obs:	exp:
gaa	E	1186	1628.00
gag	E	1562	1120.00
---	--	------	------
mPD		0.49	0.48
		nPD:	1.02
		N. weight:	0.078
		Sc. PD:	0.056
		Sc. rank:	127.4

,

NP

Pos.	199	obs:	exp:
tta	L	1198	298.80
ttg	L	484	515.80
ctt	L	0	554.60
ctc	L	1	399.50
cta	L	672	479.50
ctg	L	391	497.80
atg	M	1	1.00
gtt	V	0	0.22
gtc	V	0	0.19
gta	V	1	0.25
gtg	V	0	0.34
---	--	------	------
mPD		0.91	1.1
		nPD:	0.8
		N. weight:	0.93
		Sc. PD:	0.48
		Sc. rank:	1454.7

,

NP

Pos.	200	obs:	exp:
att	I	401	973.80
atc	I	2329	704.10
ata	I	15	1067.00
gtt	V	2	0.67
gtc	V	1	0.57
gta	V	0	0.76
gtg	V	0	1.01
---	--	------	------
mPD		0.26	0.66
		nPD:	0.4
		N. weight:	1.2
		Sc. PD:	0.16
		Sc. rank:	986.7

,

NP

Pos.	201	obs:	exp:
tta	L	0	0.22
ttg	L	0	0.38
ctt	L	0	0.40
ctc	L	0	0.29
cta	L	2	0.35
ctg	L	0	0.36
cgt	R	2	86.68
cgc	R	2	145.10
cga	R	303	276.00
cgg	R	87	198.40
aga	R	2278	1259.00
agg	R	74	780.50
---	--	------	------
mPD		0.36	0.94
		nPD:	0.38
		N. weight:	0.59
		Sc. PD:	0.071
		Sc. rank:	418.0

,

NP

Pos.	202	obs:	exp:
atg	M	2748	2748.00
---	--	------	------
mPD		0	0
		nPD:	1.
		N. weight:	0.
		Sc. PD:	0
		Sc. rank:	0

,

NP

Pos.	203	obs:	exp:
att	I	10	973.10
atc	I	2103	703.50
ata	I	630	1066.00
atg	M	1	1.00
gtt	V	0	0.89
gtc	V	2	0.75
gta	V	2	1.01
gtg	V	0	1.35
---	--	------	------
mPD		0.36	0.66
		nPD:	0.55
		N. weight:	1.
		Sc. PD:	0.28
		Sc. rank:	1203.7

,

NP

Pos.	204	obs:	exp:
cgt	R	0	0.03
cgc	R	0	0.05
cga	R	0	0.10
cgg	R	0	0.07
aaa	K	2391	1669.00
aag	K	356	1078.00
aga	R	1	0.46
agg	R	0	0.28
---	--	------	------
mPD		0.23	0.48
		nPD:	0.47
		N. weight:	0.25
		Sc. PD:	0.051
		Sc. rank:	266.6

,

NP

Pos.	205	obs:	exp:
cgt	R	2288	86.74
cgc	R	33	145.20
cga	R	35	276.20
cgg	R	380	198.50
aga	R	1	1260.00
agg	R	11	781.10
---	--	------	------
mPD		0.30	0.94
		nPD:	0.31
		N. weight:	4.
		Sc. PD:	0.21
		Sc. rank:	1907.0

,

NP

Pos.	206	obs:	exp:
ggt	G	301	431.00
ggc	G	22	348.20
gga	G	1378	1245.00
ggg	G	1047	724.10
---	--	------	------
mPD		0.59	0.68
		nPD:	0.86
		N. weight:	0.19
		Sc. PD:	0.11
		Sc. rank:	309.4

,

NP

Pos.	207	obs:	exp:
att	I	20	971.30
atc	I	2690	702.30
ata	I	28	1064.00
gtt	V	0	2.22
gtc	V	10	1.89
gta	V	0	2.52
gtg	V	0	3.37
---	--	------	------
mPD		0.042	0.67
		nPD:	0.06
		N. weight:	1.8
		Sc. PD:	-0.34
		Sc. rank:	-1419.9

,

NP

Pos.	208	obs:	exp:
aat	N	2716	1562.00
aac	N	32	1186.00
---	--	------	------
mPD		0.023	0.49
		nPD:	0.05
		N. weight:	0.73
		Sc. PD:	-0.15
		Sc. rank:	-703.0

,

NP

Pos.	209	obs:	exp:
gat	D	430	1546.00
gac	D	2318	1202.00
---	--	------	------
mPD		0.26	0.49
		nPD:	0.54
		N. weight:	0.51
		Sc. PD:	0.14
		Sc. rank:	581.2

,

NP

Pos.	210	obs:	exp:
cgt	R	59	86.74
cgc	R	3	145.20
cga	R	2199	276.20
cgg	R	356	198.50
aga	R	77	1260.00
agg	R	54	781.10
---	--	------	------
mPD		0.38	0.94
		nPD:	0.41
		N. weight:	2.3
		Sc. PD:	0.33
		Sc. rank:	1946.8

,

NP

Pos.	211	obs:	exp:
tat	Y	0	0.56
tac	Y	1	0.44
aat	N	1417	1561.00
aac	N	1330	1186.00
---	--	------	------
mPD		0.50	0.49
		nPD:	1.02
		N. weight:	0.011
		Sc. PD:	0.0077
		Sc. rank:	17.5

,

NP

Pos.	212	obs:	exp:
ttt	F	105	1231.00
ttc	F	2643	1517.00
---	--	------	------
mPD		0.074	0.49
		nPD:	0.15
		N. weight:	0.64
		Sc. PD:	-0.066
		Sc. rank:	-93.9

,

NP

Pos.	213	obs:	exp:
tgg	W	2748	2748.00
---	--	------	------
mPD		0	0
		nPD:	1.
		N. weight:	0.
		Sc. PD:	0
		Sc. rank:	0

,

NP

Pos.	214	obs:	exp:
cgt	R	0	86.74
cgc	R	0	145.20
cga	R	1	276.20
cgg	R	6	198.50
aga	R	1410	1260.00
agg	R	1331	781.10
---	--	------	------
mPD		0.50	0.94
		nPD:	0.54
		N. weight:	0.44
		Sc. PD:	0.12
		Sc. rank:	507.5

,

NP

Pos.	215	obs:	exp:
ggt	G	2328	431.00
ggc	G	336	348.20
gga	G	82	1245.00
ggg	G	2	724.10
---	--	------	------
mPD		0.27	0.68
		nPD:	0.39
		N. weight:	1.9
		Sc. PD:	0.24
		Sc. rank:	1465.5

,

NP

Pos.	216	obs:	exp:
gat	D	0	0.56
gac	D	1	0.44
gaa	E	2387	1628.00
gag	E	360	1119.00
---	--	------	------
mPD		0.23	0.48
		nPD:	0.47
		N. weight:	0.27
		Sc. PD:	0.055
		Sc. rank:	289.4

,

NP

Pos.	217	obs:	exp:
tct	S	0	0.17
tcc	S	0	0.16
tca	S	0	0.24
tcg	S	0	0.05
aat	N	2708	1561.00
aac	N	39	1186.00
agt	S	1	0.19
agc	S	0	0.19
---	--	------	------
mPD		0.029	0.49
		nPD:	0.06
		N. weight:	0.71
		Sc. PD:	-0.14
		Sc. rank:	-606.1

,

NP

Pos.	218	obs:	exp:
ggt	G	40	431.00
ggc	G	7	348.20
gga	G	2553	1245.00
ggg	G	148	724.10
---	--	------	------
mPD		0.13	0.68
		nPD:	0.2
		N. weight:	0.78
		Sc. PD:	-0.047
		Sc. rank:	55.0

,

NP

Pos.	219	obs:	exp:
cgt	R	1	86.74
cgc	R	31	145.20
cga	R	2251	276.20
cgg	R	335	198.50
aga	R	129	1260.00
agg	R	1	781.10
---	--	------	------
mPD		0.33	0.94
		nPD:	0.35
		N. weight:	2.4
		Sc. PD:	0.2
		Sc. rank:	1375.6

,

NP

Pos.	220	obs:	exp:
cgt	R	0	86.27
cgc	R	0	144.50
cga	R	10	274.70
cgg	R	147	197.40
aaa	K	12	9.12
aag	K	3	5.88
aga	R	1249	1253.00
agg	R	1327	776.80
---	--	------	------
mPD		0.62	0.95
		nPD:	0.65
		N. weight:	0.34
		Sc. PD:	0.13
		Sc. rank:	460.1

NP			
Pos.	221	obs:	exp:
tct	S	0	0.34
tcc	S	0	0.32
tca	S	2	0.49
tcg	S	0	0.11
act	T	0	674.70
acc	T	3	593.10
aca	T	2731	1246.00
acg	T	9	228.70
agt	S	0	0.37
agc	S	0	0.38
gct	A	0	0.74
gcc	A	0	0.69
gca	A	3	1.35
gcg	A	0	0.22
---	--	------	------
mPD		0.012	0.68
		nPD:	0.02
		N. weight:	1.1
		Sc. PD:	-0.25
		Sc. rank:	-1630.1

,

NP			
Pos.	222	obs:	exp:
cgt	R	0	86.43
cgc	R	0	144.70
cga	R	0	275.20
cgg	R	0	197.80
aaa	K	3	6.08
aag	K	7	3.92
aga	R	638	1256.00
agg	R	2100	778.20
---	--	------	------
mPD		0.37	0.94
		nPD:	0.39
		N. weight:	0.87
		Sc. PD:	0.11
		Sc. rank:	641.4

,

NP			
Pos.	223	obs:	exp:
tta	L	0	0.54
ttg	L	0	0.94
tct	S	0	0.50
tcc	S	0	0.47
tca	S	0	0.73
tcg	S	0	0.16
ctt	L	5	1.01
ctc	L	0	0.73
cta	L	0	0.87
ctg	L	0	0.91
att	I	1217	438.50
atc	I	12	317.00
ata	I	7	480.50
act	T	24	6.40
acc	T	1	5.62
aca	T	1	11.81
acg	T	0	2.17
aat	N	2	1.14
aac	N	0	0.86
agt	S	3	0.56
agc	S	0	0.57
gtt	V	1449	322.20
gtc	V	0	273.80
gta	V	1	366.30
gtg	V	1	488.70
gct	A	24	5.88
gcc	A	0	5.50
gca	A	0	10.82
gcg	A	0	1.80
ggt	G	1	0.16
ggc	G	0	0.13
gga	G	0	0.45
ggg	G	0	0.26
---	--	------	------
mPD		0.56	1.3
		nPD:	0.43
		N. weight:	1.8
		Sc. PD:	0.3
		Sc. rank:	1757.7

,

NP			
Pos.	224	obs:	exp:
gct	A	1274	673.60
gcc	A	29	629.60
gca	A	1441	1239.00
gcg	A	4	206.10
---	--	------	------
mPD		0.51	0.68
		nPD:	0.75
		N. weight:	0.49
		Sc. PD:	0.23
		Sc. rank:	750.7

,

NP			
Pos.	225	obs:	exp:
ttt	F	3	1.34
ttc	F	0	1.66
tat	Y	2705	1538.00
tac	Y	40	1207.00
---	--	------	------
mPD		0.031	0.49
		nPD:	0.06
		N. weight:	0.73
		Sc. PD:	-0.14
		Sc. rank:	-582.6

,

NP			
Pos.	226	obs:	exp:
gaa	E	1683	1628.00
gag	E	1065	1120.00
---	--	------	------
mPD		0.47	0.48
		nPD:	0.98
		N. weight:	0.0034
		Sc. PD:	0.0023
		Sc. rank:	5.5

,

NP			
Pos.	227	obs:	exp:
cgt	R	0	86.74
cgc	R	0	145.20
cga	R	15	276.20
cgg	R	0	198.50
aga	R	2657	1260.00
agg	R	76	781.10
---	--	------	------
mPD		0.065	0.94
		nPD:	0.07
		N. weight:	0.93
		Sc. PD:	-0.17
		Sc. rank:	-683.3

,

NP			
Pos.	228	obs:	exp:
tta	L	0	1.85
ttg	L	1	3.19
ctt	L	0	3.43
ctc	L	0	2.47
cta	L	0	2.97
ctg	L	16	3.08
att	I	0	0.35
atc	I	0	0.26
ata	I	1	0.39
atg	M	2730	2730.00
---	--	------	------
mPD		0.013	0.021
		nPD:	0.63
		N. weight:	0.014
		Sc. PD:	0.005
		Sc. rank:	19.0

,

NP			
Pos.	229	obs:	exp:
tgt	C	43	1183.00
tgc	C	2705	1565.00
---	--	------	------
mPD		0.031	0.49
		nPD:	0.06
		N. weight:	0.7
		Sc. PD:	-0.13
		Sc. rank:	-547.6

,

NP			
Pos.	230	obs:	exp:
tct	S	0	0.17
tcc	S	0	0.16
tca	S	0	0.24
tcg	S	0	0.05
cat	H	0	0.58
cac	H	1	0.42
aat	N	2011	1561.00
aac	N	735	1185.00
agt	S	1	0.19
agc	S	0	0.19
---	--	------	------
mPD		0.39	0.49
		nPD:	0.8
		N. weight:	0.087
		Sc. PD:	0.045
		Sc. rank:	135.5

,

NP			
Pos.	231	obs:	exp:
att	I	486	974.10
atc	I	2155	704.30
ata	I	105	1068.00
gtt	V	1	0.44
gtc	V	1	0.38
gta	V	0	0.50
gtg	V	0	0.67
---	--	------	------
mPD		0.35	0.66
		nPD:	0.54
		N. weight:	0.96
		Sc. PD:	0.26
		Sc. rank:	1092.1

,

NP			
Pos.	232	obs:	exp:
ttt	F	0	0.45
ttc	F	1	0.55
tta	L	0	298.90
ttg	L	0	516.00
ctt	L	685	554.80
ctc	L	2030	399.70
cta	L	19	479.70
ctg	L	13	498.00
---	--	------	------
mPD		0.39	1.1
		nPD:	0.34
		N. weight:	1.8
		Sc. PD:	0.14
		Sc. rank:	948.8

,

NP			
Pos.	233	obs:	exp:
cgt	R	0	0.03
cgc	R	0	0.05
cga	R	0	0.10
cgg	R	0	0.07
aaa	K	2716	1669.00
aag	K	31	1078.00
aga	R	1	0.46
agg	R	0	0.28
---	--	------	------
mPD		0.023	0.48
		nPD:	0.05
		N. weight:	0.64
		Sc. PD:	-0.13
		Sc. rank:	-596.7

,

NP			
Pos.	234	obs:	exp:
cgt	R	0	0.03
cgc	R	0	0.05
cga	R	0	0.10
cgg	R	0	0.07
aga	R	1	0.46
agg	R	0	0.28
ggt	G	2	430.90
ggc	G	2	348.10
gga	G	2013	1244.00
ggg	G	730	723.80
---	--	------	------
mPD		0.39	0.69
		nPD:	0.57
		N. weight:	0.5
		Sc. PD:	0.15
		Sc. rank:	624.0

,

NP			
Pos.	235	obs:	exp:
aaa	K	2465	1670.00
aag	K	283	1078.00
---	--	------	------
mPD		0.18	0.48
		nPD:	0.39
		N. weight:	0.31
		Sc. PD:	0.038
		Sc. rank:	226.6

,

NP

Pos.	236	obs:	exp:
ttt	F	1989	1231.00
ttc	F	759	1517.00
---	--	------	------
mPD		0.40	0.49
		nPD:	0.81
		N. weight:	0.23
		Sc. PD:	0.12
		Sc. rank:	355.5

NP

Pos.	237	obs:	exp:
cat	H	6	3.46
cac	H	0	2.54
caa	Q	1629	1479.00
cag	Q	1113	1263.00
---	--	------	------
mPD		0.48	0.50
		nPD:	0.97
		N. weight:	0.013
		Sc. PD:	0.0086
		Sc. rank:	20.8

NP

Pos.	238	obs:	exp:
tct	S	0	0.17
tcc	S	0	0.16
tca	S	1	0.24
tcg	S	0	0.05
att	I	0	1.06
atc	I	0	0.77
ata	I	3	1.17
act	T	169	674.50
acc	T	1	592.90
aca	T	2506	1246.00
acg	T	66	228.60
agt	S	0	0.19
agc	S	0	0.19
gct	A	0	0.49
gcc	A	0	0.46
gca	A	2	0.90
gcg	A	0	0.15
---	--	------	------
mPD		0.16	0.68
		nPD:	0.24
		N. weight:	0.76
		Sc. PD:	-0.013
		Sc. rank:	152.0

NP

Pos.	239	obs:	exp:
act	T	0	0.49
acc	T	0	0.43
aca	T	2	0.91
acg	T	0	0.17
gct	A	1677	673.10
gcc	A	23	629.20
gca	A	1033	1238.00
gcg	A	13	205.90
---	--	------	------
mPD		0.49	0.68
		nPD:	0.72
		N. weight:	0.65
		Sc. PD:	0.28
		Sc. rank:	963.9

NP

Pos.	240	obs:	exp:
tct	S	0	0.67
tcc	S	3	0.63
tca	S	0	0.97
tcg	S	1	0.21
act	T	0	0.25
acc	T	1	0.22
aca	T	0	0.45
acg	T	0	0.08
agt	S	0	0.74
agc	S	0	0.77
gtt	V	0	0.22
gtc	V	1	0.19
gta	V	0	0.25
gtg	V	0	0.34
gct	A	30	672.20
gcc	A	1857	628.30
gca	A	545	1236.00
gcg	A	310	205.60
---	--	------	------
mPD		0.49	0.68
		nPD:	0.72
		N. weight:	0.85
		Sc. PD:	0.37
		Sc. rank:	1262.2

NP

Pos.	241	obs:	exp:
cat	H	0	1.15
cac	H	2	0.85
caa	Q	834	1481.00
cag	Q	1912	1265.00
---	--	------	------
mPD		0.42	0.50
		nPD:	0.85
		N. weight:	0.17
		Sc. PD:	0.094
		Sc. rank:	266.0

NP

Pos.	242	obs:	exp:
cgt	R	0	86.27
cgc	R	0	144.50
cga	R	364	274.70
cgg	R	31	197.40
aaa	K	0	9.12
aag	K	15	5.88
aga	R	415	1253.00
agg	R	1923	776.80
---	--	------	------
mPD		0.66	0.95
		nPD:	0.7
		N. weight:	0.71
		Sc. PD:	0.3
		Sc. rank:	1009.9

NP

Pos.	243	obs:	exp:
act	T	0	0.98
acc	T	0	0.86
aca	T	4	1.82
acg	T	0	0.33
gct	A	168	672.60
gcc	A	8	628.70
gca	A	2547	1237.00
gcg	A	21	205.80
---	--	------	------
mPD		0.14	0.68
		nPD:	0.2
		N. weight:	0.81
		Sc. PD:	-0.043
		Sc. rank:	70.4

NP

Pos.	244	obs:	exp:
att	I	0	1.06
atc	I	1	0.77
ata	I	2	1.17
atg	M	2745	2745.00
---	--	------	------
mPD		0.0022	0.0022
		nPD:	1.
		N. weight:	0.0011
		Sc. PD:	0.0008
		Sc. rank:	1.8

NP

Pos.	245	obs:	exp:
att	I	0	0.35
atc	I	0	0.26
ata	I	1	0.39
atg	M	2733	2733.00
act	T	0	0.74
acc	T	0	0.65
aca	T	0	1.36
acg	T	3	0.25
gtt	V	0	2.44
gtc	V	0	2.08
gta	V	0	2.78
gtg	V	11	3.71
---	--	------	------
mPD		0.011	0.018
		nPD:	0.6
		N. weight:	0.011
		Sc. PD:	0.0035
		Sc. rank:	13.7

NP

Pos.	246	obs:	exp:
gat	D	2317	1546.00
gac	D	431	1202.00
---	--	------	------
mPD		0.26	0.49
		nPD:	0.54
		N. weight:	0.26
		Sc. PD:	0.07
		Sc. rank:	298.2

NP

Pos.	247	obs:	exp:
caa	Q	2349	1482.00
cag	Q	399	1266.00
---	--	------	------
mPD		0.25	0.50
		nPD:	0.5
		N. weight:	0.33
		Sc. PD:	0.075
		Sc. rank:	361.3

NP

Pos.	248	obs:	exp:
att	I	0	1.06
atc	I	0	0.77
ata	I	3	1.17
gtt	V	17	609.50
gtc	V	0	518.00
gta	V	1319	692.90
gtg	V	1409	924.60
---	--	------	------
mPD		0.51	0.74
		nPD:	0.69
		N. weight:	0.73
		Sc. PD:	0.3
		Sc. rank:	1026.6

NP

Pos.	249	obs:	exp:
cgt	R	0	86.74
cgc	R	0	145.20
cga	R	356	276.20
cgg	R	29	198.50
aga	R	2336	1260.00
agg	R	27	781.10
---	--	------	------
mPD		0.28	0.94
		nPD:	0.3
		N. weight:	0.73
		Sc. PD:	0.029
		Sc. rank:	300.8

NP

Pos.	250	obs:	exp:
gaa	E	2660	1628.00
gag	E	88	1120.00
---	--	------	------
mPD		0.062	0.48
		nPD:	0.13
		N. weight:	0.57
		Sc. PD:	-0.07
		Sc. rank:	-157.0

NP

Pos.	251	obs:	exp:
tct	S	0	461.90
tcc	S	0	434.00
tca	S	0	666.80
tcg	S	0	145.90
act	T	0	0.25
acc	T	1	0.22
aca	T	0	0.45
acg	T	0	0.08
agt	S	2027	511.10
agc	S	719	526.30
ggt	G	1	0.16
ggc	G	0	0.13
gga	G	0	0.45
ggg	G	0	0.26
---	--	------	------
mPD		0.39	1.6
		nPD:	0.24
		N. weight:	1.6
		Sc. PD:	-0.031
		Sc. rank:	292.9

NP

Pos.	252	obs:	exp:
cgt	R	2	86.71
cgc	R	0	145.20
cga	R	1850	276.10
cgg	R	476	198.50
aaa	K	1	0.61
aag	K	0	0.39
aga	R	406	1260.00
agg	R	13	780.80
---	--	------	------
mPD		0.55	0.94
		nPD:	0.59
		N. weight:	1.8
		Sc. PD:	0.57
		Sc. rank:	2274.6

NP

Pos.	253	obs:	exp:
tct	S	0	42.39
tcc	S	0	39.83
tca	S	0	61.20
tcg	S	0	13.39
act	T	2	2.46
acc	T	8	2.16
aca	T	0	4.54
acg	T	0	0.83
aat	N	440	1413.00
aac	N	2046	1073.00
agt	S	5	46.90
agc	S	247	48.30
---	--	------	------
mPD		0.45	0.80
		nPD:	0.55
		N. weight:	0.64
		Sc. PD:	0.18
		Sc. rank:	759.6

NP

Pos.	254	obs:	exp:
tta	L	0	0.22
ttg	L	0	0.38
ctt	L	0	0.40
ctc	L	0	0.29
cta	L	0	0.35
ctg	L	2	0.36
cct	P	126	810.30
ccc	P	42	552.90
cca	P	2565	1087.00
ccg	P	13	295.40
---	--	------	------
mPD		0.13	0.71
		nPD:	0.18
		N. weight:	0.96
		Sc. PD:	-0.07
		Sc. rank:	4.5

NP

Pos.	255	obs:	exp:
ggt	G	9	431.00
ggc	G	2	348.20
gga	G	2511	1245.00
ggg	G	226	724.10
---	--	------	------
mPD		0.16	0.68
		nPD:	0.23
		N. weight:	0.77
		Sc. PD:	-0.02
		Sc. rank:	130.9

NP

Pos.	256	obs:	exp:
aat	N	1387	1561.00
aac	N	1360	1186.00
gat	D	1	0.56
gac	D	0	0.44
mPD		0.50	0.49
		nPD:	1.02
		N. weight:	0.014
		Sc. PD:	0.01
		Sc. rank:	23.3

NP

Pos.	257	obs:	exp:
gtt	V	3	0.67
gtc	V	0	0.57
gta	V	0	0.76
gtg	V	0	1.01
gct	A	2639	672.90
gcc	A	51	628.90
gca	A	41	1237.00
gcg	A	14	205.90
mPD		0.077	0.68
		nPD:	0.11
		N. weight:	1.7
		Sc. PD:	-0.24
		Sc. rank:	-575.6

NP

Pos.	258	obs:	exp:
gaa	E	129	1628.00
gag	E	2619	1120.00
mPD		0.090	0.48
		nPD:	0.19
		N. weight:	1.
		Sc. PD:	-0.069
		Sc. rank:	31.6

NP

Pos.	259	obs:	exp:
att	I	2666	974.50
atc	I	68	704.60
ata	I	13	1068.00
gtt	V	1	0.22
gtc	V	0	0.19
gta	V	0	0.25
gtg	V	0	0.34
mPD		0.058	0.66
		nPD:	0.09
		N. weight:	1.3
		Sc. PD:	-0.21
		Sc. rank:	-747.2

NP

Pos.	260	obs:	exp:
gaa	E	2721	1628.00
gag	E	27	1120.00
mPD		0.019	0.48
		nPD:	0.04
		N. weight:	0.68
		Sc. PD:	-0.14
		Sc. rank:	-752.9

NP

Pos.	261	obs:	exp:
gat	D	1493	1546.00
gac	D	1255	1202.00
mPD		0.50	0.49
		nPD:	1.01
		N. weight:	0.0033
		Sc. PD:	0.0023
		Sc. rank:	5.3

NP

Pos.	262	obs:	exp:
tta	L	0	299.00
ttg	L	0	516.20
ctt	L	162	555.00
ctc	L	2560	399.80
cta	L	26	479.80
ctg	L	0	498.10
mPD		0.13	1.1
		nPD:	0.11
		N. weight:	2.4
		Sc. PD:	-0.33
		Sc. rank:	-828.9

NP

Pos.	263	obs:	exp:
att	I	2010	973.40
atc	I	729	703.80
ata	I	5	1067.00
act	T	0	0.25
acc	T	1	0.22
aca	T	0	0.45
acg	T	0	0.08
gtt	V	1	0.67
gtc	V	2	0.57
gta	V	0	0.76
gtg	V	0	1.01
mPD		0.40	0.66
		nPD:	0.6
		N. weight:	0.77
		Sc. PD:	0.25
		Sc. rank:	995.3

NP

Pos.	264	obs:	exp:
ttt	F	506	1231.00
ttc	F	2242	1517.00
mPD		0.30	0.49
		nPD:	0.61
		N. weight:	0.23
		Sc. PD:	0.075
		Sc. rank:	297.9

NP

Pos.	265	obs:	exp:
tta	L	3	298.10
ttg	L	139	514.50
ctt	L	5	553.20
ctc	L	3	398.50
cta	L	176	478.30
ctg	L	2413	496.50
atg	M	8	8.00
gtt	V	0	0.22
gtc	V	0	0.19
gta	V	0	0.25
gtg	V	1	0.34
mPD		0.23	1.1
		nPD:	0.2
		N. weight:	1.8
		Sc. PD:	-0.095
		Sc. rank:	177.8

NP

Pos.	266	obs:	exp:
tct	S	0	0.50
tcc	S	0	0.47
tca	S	2	0.73
tcg	S	1	0.16
act	T	2	70.10
acc	T	0	61.63
aca	T	276	129.50
acg	T	7	23.77
agt	S	0	0.56
agc	S	0	0.57
gtt	V	0	0.22
gtc	V	0	0.19
gta	V	1	0.25
gtg	V	0	0.34
gct	A	8	602.80
gcc	A	0	563.40
gca	A	2396	1108.00
gcg	A	55	184.40
mPD		0.24	0.87
		nPD:	0.28
		N. weight:	1.
		Sc. PD:	0.018
		Sc. rank:	323.2

NP

Pos.	267	obs:	exp:
cgt	R	4	86.71
cgc	R	1	145.20
cga	R	526	276.10
cgg	R	2156	198.50
aga	R	13	1260.00
agg	R	47	780.80
ggt	G	0	0.16
ggc	G	0	0.13
gga	G	1	0.45
ggg	G	0	0.26
mPD		0.36	0.94
		nPD:	0.39
		N. weight:	2.8
		Sc. PD:	0.34
		Sc. rank:	2008.8

NP

Pos.	268	obs:	exp:
tct	S	321	462.20
tcc	S	8	434.30
tca	S	2415	667.30
tcg	S	4	146.10
agt	S	0	511.50
agc	S	0	526.60
mPD		0.21	1.6
		nPD:	0.13
		N. weight:	1.5
		Sc. PD:	-0.19
		Sc. rank:	-404.7

NP

Pos.	269	obs:	exp:
tct	S	0	0.17
tcc	S	0	0.16
tca	S	1	0.24
tcg	S	0	0.05
agt	S	0	0.19
agc	S	0	0.19
gct	A	3	673.40
gcc	A	4	629.40
gca	A	2674	1238.00
gcg	A	66	206.00
mPD		0.053	0.68
		nPD:	0.08
		N. weight:	1.
		Sc. PD:	-0.18
		Sc. rank:	-652.8

NP

Pos.	270	obs:	exp:
tta	L	16	298.90
ttg	L	1	516.00
ctt	L	1049	554.80
ctc	L	1623	399.70
cta	L	56	479.70
ctg	L	2	498.00
cct	P	1	0.30
ccc	P	0	0.20
cca	P	0	0.40
ccg	P	0	0.11
mPD		0.52	1.1
		nPD:	0.45
		N. weight:	1.5
		Sc. PD:	0.27
		Sc. rank:	1475.8

NP

Pos.	271	obs:	exp:
att	I	2583	953.20
atc	I	100	689.20
ata	I	4	1045.00
gtt	V	60	13.54
gtc	V	1	11.51
gta	V	0	15.40
gtg	V	0	20.55
mPD		0.12	0.71
		nPD:	0.17
		N. weight:	1.3
		Sc. PD:	-0.11
		Sc. rank:	-115.1

NP

Pos.	272	obs:	exp:
tta	L	5	299.00
ttg	L	56	516.20
ctt	L	31	555.00
ctc	L	3	399.80
cta	L	1043	479.80
ctg	L	1610	498.10
mPD		0.53	1.1
		nPD:	0.47
		N. weight:	1.3
		Sc. PD:	0.26
		Sc. rank:	1369.2

NP

Pos.	273	obs:	exp:
cgt	R	0	86.74
cgc	R	0	145.20
cga	R	1	276.20
cgg	R	2	198.50
aga	R	539	1260.00
agg	R	2206	781.10
mPD		0.32	0.94
		nPD:	0.34
		N. weight:	0.96
		Sc. PD:	0.074
		Sc. rank:	500.7

NP

Pos.	274	obs:	exp:
ggt	G	9	431.00
ggc	G	1	348.20
gga	G	2451	1245.00
ggg	G	287	724.10
mPD		0.19	0.68
		nPD:	0.28
		N. weight:	0.71
		Sc. PD:	0.017
		Sc. rank:	243.7

NP

Pos.	275	obs:	exp:
tct	S	43	461.90
tcc	S	39	434.00
tca	S	2650	666.80
tcg	S	14	145.90
agt	S	0	511.10
agc	S	0	526.30
gct	A	0	0.49
gcc	A	0	0.46
gca	A	2	0.90
gcg	A	0	0.15
mPD		0.070	1.6
		nPD:	0.04
		N. weight:	1.8
		Sc. PD:	-0.37
		Sc. rank:	-1940.6

NP			
Pos.	276	obs:	exp:
att	I	1	0.71
atc	I	0	0.51
ata	I	1	0.78
atg	M	1	1.00
gtt	V	2318	609.50
gtc	V	5	518.00
gta	V	28	692.90
gtg	V	394	924.60
---	--	------	------
mPD		0.27	0.74
		nPD:	0.36
		N. weight:	1.4
		Sc. PD:	0.14
		Sc. rank:	904.3

,

NP			
Pos.	277	obs:	exp:
gct	A	400	673.60
gcc	A	18	629.60
gca	A	2320	1239.00
gcg	A	10	206.10
---	--	------	------
mPD		0.27	0.68
		nPD:	0.39
		N. weight:	0.61
		Sc. PD:	0.078
		Sc. rank:	470.7

,

NP			
Pos.	278	obs:	exp:
tat	Y	1	0.56
tac	Y	0	0.44
cat	H	2521	1585.00
cac	H	226	1162.00
---	--	------	------
mPD		0.15	0.49
		nPD:	0.31
		N. weight:	0.42
		Sc. PD:	0.021
		Sc. rank:	188.2

,

NP			
Pos.	279	obs:	exp:
aaa	K	1932	1670.00
aag	K	816	1078.00
---	--	------	------
mPD		0.42	0.48
		nPD:	0.88
		N. weight:	0.031
		Sc. PD:	0.018
		Sc. rank:	49.1

,

NP			
Pos.	280	obs:	exp:
tct	S	1037	462.20
tcc	S	1678	434.30
tca	S	33	667.30
tcg	S	0	146.10
agt	S	0	511.50
agc	S	0	526.60
---	--	------	------
mPD		0.48	1.6
		nPD:	0.3
		N. weight:	1.6
		Sc. PD:	0.059
		Sc. rank:	642.6

,

NP			
Pos.	281	obs:	exp:
tct	S	0	0.50
tcc	S	0	0.47
tca	S	0	0.73
tcg	S	0	0.16
tgt	C	400	1181.00
tgc	C	2344	1563.00
cgt	R	0	0.03
cgc	R	1	0.05
cga	R	0	0.10
cgg	R	0	0.07
agt	S	0	0.56
agc	S	3	0.57
aga	R	0	0.46
agg	R	0	0.28
---	--	------	------
mPD		0.25	0.49
		nPD:	0.51
		N. weight:	0.28
		Sc. PD:	0.066
		Sc. rank:	308.3

,

NP			
Pos.	282	obs:	exp:
tta	L	12	298.60
ttg	L	168	515.40
ctt	L	14	554.20
ctc	L	0	399.20
cta	L	142	479.10
ctg	L	2408	497.40
atg	M	4	4.00
---	--	------	------
mPD		0.24	1.1
		nPD:	0.21
		N. weight:	1.8
		Sc. PD:	-0.079
		Sc. rank:	226.2

,

NP			
Pos.	283	obs:	exp:
cct	P	2641	810.90
ccc	P	24	553.30
cca	P	82	1088.00
ccg	P	1	295.60
---	--	------	------
mPD		0.075	0.70
		nPD:	0.11
		N. weight:	1.5
		Sc. PD:	-0.21
		Sc. rank:	-560.8

,

NP			
Pos.	284	obs:	exp:
tct	S	2	0.34
tcc	S	0	0.32
tca	S	0	0.49
tcg	S	0	0.11
act	T	2	0.49
acc	T	0	0.43
aca	T	0	0.91
acg	T	0	0.17
agt	S	0	0.37
agc	S	0	0.38
gct	A	2633	672.60
gcc	A	107	628.70
gca	A	3	1237.00
gcg	A	1	205.80
---	--	------	------
mPD		0.081	0.68
		nPD:	0.12
		N. weight:	1.8
		Sc. PD:	-0.24
		Sc. rank:	-528.8

,

NP			
Pos.	285	obs:	exp:
tgt	C	2737	1183.00
tgc	C	11	1565.00
---	--	------	------
mPD		0.0080	0.49
		nPD:	0.02
		N. weight:	1.2
		Sc. PD:	-0.27
		Sc. rank:	-1778.2

,

NP			
Pos.	286	obs:	exp:
tta	L	0	0.11
ttg	L	0	0.19
ctt	L	0	0.20
ctc	L	0	0.15
cta	L	0	0.17
ctg	L	1	0.18
att	I	0	3.19
atc	I	0	2.31
ata	I	9	3.50
gtt	V	13	607.90
gtc	V	0	516.70
gta	V	398	691.20
gtg	V	2327	922.20
---	--	------	------
mPD		0.27	0.75
		nPD:	0.36
		N. weight:	1.
		Sc. PD:	0.096
		Sc. rank:	633.1

,

NP			
Pos.	287	obs:	exp:
tat	Y	2631	1540.00
tac	Y	117	1208.00
---	--	------	------
mPD		0.082	0.49
		nPD:	0.17
		N. weight:	0.6
		Sc. PD:	-0.053
		Sc. rank:	-61.5

,

NP			
Pos.	288	obs:	exp:
ggt	G	5	431.00
ggc	G	43	348.20
gga	G	768	1245.00
ggg	G	1932	724.10
---	--	------	------
mPD		0.43	0.68
		nPD:	0.62
		N. weight:	0.75
		Sc. PD:	0.26
		Sc. rank:	994.1

,

NP			
Pos.	289	obs:	exp:
ttt	F	1	0.45
ttc	F	0	0.55
tta	L	0	298.50
ttg	L	0	515.20
ctt	L	2605	554.00
ctc	L	83	399.10
cta	L	55	479.00
ctg	L	0	497.20
cct	P	2	0.59
ccc	P	0	0.40
cca	P	0	0.79
ccg	P	0	0.22
att	I	2	0.71
atc	I	0	0.51
ata	I	0	0.78
---	--	------	------
mPD		0.10	1.1
		nPD:	0.09
		N. weight:	2.
		Sc. PD:	-0.32
		Sc. rank:	-1163.1

,

NP			
Pos.	290	obs:	exp:
att	I	296	106.40
atc	I	4	76.95
ata	I	0	116.60
gtt	V	107	23.98
gtc	V	0	20.38
gta	V	1	27.26
gtg	V	0	36.38
gct	A	33	573.60
gcc	A	2	536.20
gca	A	2006	1055.00
gcg	A	299	175.50
---	--	------	------
mPD		0.88	1.1
		nPD:	0.77
		N. weight:	0.94
		Sc. PD:	0.47
		Sc. rank:	1465.2

,

NP			
Pos.	291	obs:	exp:
att	I	0	3.19
atc	I	0	2.31
ata	I	9	3.50
atg	M	2	2.00
gtt	V	19	607.30
gtc	V	0	516.10
gta	V	1409	690.40
gtg	V	1307	921.20
gct	A	0	0.49
gcc	A	0	0.46
gca	A	1	0.90
gcg	A	1	0.15
---	--	------	------
mPD		0.52	0.75
		nPD:	0.69
		N. weight:	0.74
		Sc. PD:	0.31
		Sc. rank:	1049.7

,

NP			
Pos.	292	obs:	exp:
tct	S	0	0.17
tcc	S	1	0.16
tca	S	0	0.24
tcg	S	0	0.05
agt	S	0	0.19
agc	S	0	0.19
gtt	V	0	0.22
gtc	V	0	0.19
gta	V	1	0.25
gtg	V	0	0.34
gct	A	7	673.10
gcc	A	15	629.20
gca	A	2654	1238.00
gcg	A	70	205.90
---	--	------	------
mPD		0.067	0.68
		nPD:	0.1
		N. weight:	0.98
		Sc. PD:	-0.15
		Sc. rank:	-441.8

,

NP			
Pos.	293	obs:	exp:
tct	S	0	401.50
tcc	S	0	377.20
tca	S	0	579.70
tcg	S	0	126.90
cgt	R	0	0.03
cgc	R	0	0.05
cga	R	0	0.10
cgg	R	0	0.07
act	T	1	0.25
acc	T	0	0.22
aca	T	0	0.45
acg	T	0	0.08
aat	N	358	203.50
aac	N	0	154.50
agt	S	2281	444.30
agc	S	106	457.50
aga	R	0	0.46
agg	R	1	0.28
ggt	G	1	0.16
ggc	G	0	0.13
gga	G	0	0.45
ggg	G	0	0.26
---	--	------	------
mPD		0.30	1.8
		nPD:	0.17
		N. weight:	2.
		Sc. PD:	-0.16
		Sc. rank:	-68.2

,

NP			
Pos.	294	obs:	exp:
ggt	G	6	431.00
ggc	G	82	348.20
gga	G	1264	1245.00
ggg	G	1396	724.10
---	--	------	------
mPD		0.53	0.68
		nPD:	0.77
		N. weight:	0.42
		Sc. PD:	0.21
		Sc. rank:	648.7

,

NP			
Pos.	295	obs:	exp:
tat	Y	476	273.40
tac	Y	12	214.60
cat	H	2237	1304.00
cac	H	23	956.40
---	--	------	------
mPD		0.32	0.78
		nPD:	0.41
		N. weight:	0.71
		Sc. PD:	0.1
		Sc. rank:	587.9

,

NP			
Pos.	296	obs:	exp:
aat	N	0	3.41
aac	N	6	2.59
gat	D	55	1540.00
gac	D	2683	1198.00
gaa	E	4	2.37
gag	E	0	1.63
---	--	------	------
mPD		0.046	0.50
		nPD:	0.09
		N. weight:	1.
		Sc. PD:	-0.16
		Sc. rank:	-534.8

NP			
Pos.	297	obs:	exp:
ttt	F	2690	1231.00
ttc	F	58	1517.00
---	--	------	------
mPD		0.041	0.49
		nPD:	0.08
		N. weight:	1.
		Sc. PD:	-0.17
		Sc. rank:	-618.3

NP			
Pos.	298	obs:	exp:
gat	D	1	0.56
gac	D	0	0.44
gaa	E	2624	1622.00
gag	E	113	1115.00
ggt	G	0	1.57
ggc	G	0	1.27
gga	G	10	4.53
ggg	G	0	2.63
---	--	------	------
mPD		0.087	0.49
		nPD:	0.18
		N. weight:	0.53
		Sc. PD:	-0.041
		Sc. rank:	-16.4

NP			
Pos.	299	obs:	exp:
cgt	R	0	86.33
cgc	R	0	144.60
cga	R	2	274.90
cgg	R	7	197.60
aaa	K	8	7.90
aag	K	5	5.10
aga	R	568	1254.00
agg	R	2158	777.40
---	--	------	------
mPD		0.35	0.95
		nPD:	0.37
		N. weight:	0.91
		Sc. PD:	0.095
		Sc. rank:	600.2

NP			
Pos.	300	obs:	exp:
aaa	K	0	0.61
aag	K	1	0.39
gaa	E	2646	1628.00
gag	E	101	1119.00
---	--	------	------
mPD		0.072	0.48
		nPD:	0.15
		N. weight:	0.55
		Sc. PD:	-0.057
		Sc. rank:	-79.1

NP			
Pos.	301	obs:	exp:
ggt	G	11	431.00
ggc	G	2	348.20
gga	G	884	1245.00
ggg	G	1851	724.10
---	--	------	------
mPD		0.44	0.68
		nPD:	0.65
		N. weight:	0.73
		Sc. PD:	0.27
		Sc. rank:	995.8

NP			
Pos.	302	obs:	exp:
tat	Y	431	1538.00
tac	Y	2314	1207.00
cat	H	0	1.73
cac	H	3	1.27
---	--	------	------
mPD		0.27	0.49
		nPD:	0.54
		N. weight:	0.51
		Sc. PD:	0.14
		Sc. rank:	578.4

NP			
Pos.	303	obs:	exp:
tct	S	74	462.00
tcc	S	361	434.10
tca	S	2288	667.10
tcg	S	24	146.00
act	T	0	0.25
acc	T	0	0.22
aca	T	1	0.45
acg	T	0	0.08
agt	S	0	511.30
agc	S	0	526.50
---	--	------	------
mPD		0.29	1.6
		nPD:	0.18
		N. weight:	1.4
		Sc. PD:	-0.1
		Sc. rank:	-29.8

NP			
Pos.	304	obs:	exp:
tta	L	16	299.00
ttg	L	29	516.20
ctt	L	25	555.00
ctc	L	0	399.80
cta	L	444	479.80
ctg	L	2234	498.10
---	--	------	------
mPD		0.33	1.1
		nPD:	0.29
		N. weight:	1.6
		Sc. PD:	0.044
		Sc. rank:	594.0

NP			
Pos.	305	obs:	exp:
gtt	V	200	610.20
gtc	V	2410	518.50
gta	V	125	693.70
gtg	V	13	925.60
---	--	------	------
mPD		0.22	0.74
		nPD:	0.3
		N. weight:	1.7
		Sc. PD:	0.072
		Sc. rank:	728.1

NP			
Pos.	306	obs:	exp:
ggt	G	3	431.00
ggc	G	0	348.20
gga	G	331	1245.00
ggg	G	2414	724.10
---	--	------	------
mPD		0.21	0.68
		nPD:	0.31
		N. weight:	1.3
		Sc. PD:	0.067
		Sc. rank:	585.8

NP			
Pos.	307	obs:	exp:
tta	L	0	0.33
ttg	L	0	0.56
ctt	L	0	0.61
ctc	L	0	0.44
cta	L	3	0.52
ctg	L	0	0.54
att	I	3	959.20
atc	I	4	693.50
ata	I	2697	1051.00
gtt	V	0	9.10
gtc	V	0	7.74
gta	V	41	10.35
gtg	V	0	13.81
---	--	------	------
mPD		0.037	0.69
		nPD:	0.05
		N. weight:	1.3
		Sc. PD:	-0.26
		Sc. rank:	-1204.5

NP			
Pos.	308	obs:	exp:
gat	D	530	1546.00
gac	D	2218	1202.00
---	--	------	------
mPD		0.31	0.49
		nPD:	0.63
		N. weight:	0.42
		Sc. PD:	0.15
		Sc. rank:	559.6

NP			
Pos.	309	obs:	exp:
tct	S	0	0.17
tcc	S	0	0.16
tca	S	1	0.24
tcg	S	0	0.05
cct	P	456	810.60
ccc	P	27	553.10
cca	P	2014	1088.00
ccg	P	250	295.50
agt	S	0	0.19
agc	S	0	0.19
---	--	------	------
mPD		0.43	0.71
		nPD:	0.61
		N. weight:	0.45
		Sc. PD:	0.15
		Sc. rank:	599.3

NP			
Pos.	310	obs:	exp:
ttt	F	1488	1212.00
ttc	F	1217	1493.00
tta	L	42	4.57
ttg	L	0	7.89
tct	S	0	0.17
tcc	S	0	0.16
tca	S	1	0.24
tcg	S	0	0.05
ctt	L	0	8.48
ctc	L	0	6.11
cta	L	0	7.33
ctg	L	0	7.61
agt	S	0	0.19
agc	S	0	0.19
---	--	------	------
mPD		0.51	0.53
		nPD:	0.97
		N. weight:	0.081
		Sc. PD:	0.055
		Sc. rank:	133.2

NP			
Pos.	311	obs:	exp:
cgt	R	393	13.04
cgc	R	14	21.83
cga	R	1	41.51
cgg	R	1	29.84
aat	N	44	25.01
aac	N	0	18.99
aaa	K	2068	1392.00
aag	K	223	898.70
aga	R	4	189.40
agg	R	0	117.40
---	--	------	------
mPD		0.91	0.84
		nPD:	1.09
		N. weight:	0.97
		Sc. PD:	0.77
		Sc. rank:	1597.8

NP			
Pos.	312	obs:	exp:
tta	L	2166	299.00
ttg	L	193	516.20
ctt	L	0	555.00
ctc	L	0	399.80
cta	L	101	479.80
ctg	L	288	498.10
---	--	------	------
mPD		0.53	1.1
		nPD:	0.47
		N. weight:	2.
		Sc. PD:	0.4
		Sc. rank:	2135.4

NP			
Pos.	313	obs:	exp:
tta	L	1	299.00
ttg	L	0	516.20
ctt	L	894	555.00
ctc	L	1736	399.80
cta	L	108	479.80
ctg	L	9	498.10
---	--	------	------
mPD		0.49	1.1
		nPD:	0.43
		N. weight:	1.5
		Sc. PD:	0.24
		Sc. rank:	1410.8

NP			
Pos.	314	obs:	exp:
caa	Q	2245	1482.00
cag	Q	503	1266.00
---	--	------	------
mPD		0.30	0.50
		nPD:	0.6
		N. weight:	0.25
		Sc. PD:	0.081
		Sc. rank:	323.6

NP			
Pos.	315	obs:	exp:
tct	S	0	0.34
tcc	S	0	0.32
tca	S	0	0.49
tcg	S	0	0.11
aat	N	38	1561.00
aac	N	2708	1185.00
agt	S	0	0.37
agc	S	2	0.38
---	--	------	------
mPD		0.029	0.49
		nPD:	0.06
		N. weight:	1.1
		Sc. PD:	-0.21
		Sc. rank:	-940.5

NP			
Pos.	316	obs:	exp:
tct	S	0	460.90
tcc	S	0	433.00
tca	S	0	665.40
tcg	S	0	145.60
tgt	C	0	0.43
tgc	C	1	0.57
act	T	3	1.72
acc	T	4	1.51
aca	T	0	3.18
acg	T	0	0.58
agt	S	1188	510.00
agc	S	1552	525.10
---	--	------	------
mPD		0.50	1.6
		nPD:	0.3
		N. weight:	1.4
		Sc. PD:	0.063
		Sc. rank:	623.3

NP			
Pos.	317	obs:	exp:
caa	Q	2597	1482.00
cag	Q	151	1266.00
---	--	------	------
mPD		0.10	0.50
		nPD:	0.21
		N. weight:	0.6
		Sc. PD:	-0.028
		Sc. rank:	69.9

NP			
Pos.	318	obs:	exp:
att	I	22	9.22
atc	I	2	6.67
ata	I	2	10.11
gtt	V	78	604.40
gtc	V	16	513.60
gta	V	160	687.10
gtg	V	2468	916.80
---	--	------	------
mPD		0.21	0.76
		nPD:	0.27
		N. weight:	1.1
		Sc. PD:	0.016
		Sc. rank:	321.1

NP			
Pos.	319	obs:	exp:
ttt	F	8	648.80
ttc	F	1440	799.20
tat	Y	0	4.48
tac	Y	8	3.52
att	I	1	15.96
atc	I	44	11.54
ata	I	0	17.49
gtt	V	3	276.90
gtc	V	1241	235.30
gta	V	3	314.80
gtg	V	0	420.00
---	--	------	------
mPD		0.53	1.2
		nPD:	0.44
		N. weight:	1.5
		Sc. PD:	0.26
		Sc. rank:	1512.3

NP			
Pos.	320	obs:	exp:
tct	S	0	462.00
tcc	S	0	434.10
tca	S	0	667.10
tcg	S	0	146.00
tgt	C	0	0.43
tgc	C	1	0.57
agt	S	264	511.30
agc	S	2483	526.50
---	--	------	------
mPD		0.17	1.6
		nPD:	0.11
		N. weight:	1.9
		Sc. PD:	-0.28
		Sc. rank:	-747.7

NP			
Pos.	321	obs:	exp:
tta	L	4	299.00
ttg	L	35	516.20
ctt	L	122	555.00
ctc	L	329	399.80
cta	L	98	479.80
ctg	L	2160	498.10
---	--	------	------
mPD		0.37	1.1
		nPD:	0.33
		N. weight:	1.4
		Sc. PD:	0.092
		Sc. rank:	719.9

,

NP			
Pos.	322	obs:	exp:
att	I	374	516.90
atc	I	83	373.70
ata	I	1000	566.40
atg	M	1245	1245.00
act	T	1	0.25
acc	T	0	0.22
aca	T	0	0.45
acg	T	0	0.08
gtt	V	0	9.99
gtc	V	0	8.49
gta	V	4	11.36
gtg	V	41	15.16
---	--	------	------
mPD		0.66	0.72
		nPD:	0.92
		N. weight:	0.19
		Sc. PD:	0.12
		Sc. rank:	314.1

,

NP			
Pos.	323	obs:	exp:
cgt	R	0	86.74
cgc	R	0	145.20
cga	R	46	276.20
cgg	R	0	198.50
aga	R	2610	1260.00
agg	R	92	781.10
---	--	------	------
mPD		0.098	0.94
		nPD:	0.1
		N. weight:	0.85
		Sc. PD:	-0.13
		Sc. rank:	-341.2

,

NP			
Pos.	324	obs:	exp:
tta	L	0	0.11
ttg	L	0	0.19
tct	S	1	1.18
tcc	S	0	1.11
tca	S	3	1.70
tcg	S	3	0.37
ctt	L	0	0.20
ctc	L	0	0.15
cta	L	1	0.17
ctg	L	0	0.18
cct	P	46	807.40
ccc	P	1	550.80
cca	P	2305	1083.00
ccg	P	384	294.30
caa	Q	2	2.16
cag	Q	2	1.84
agt	S	0	1.30
agc	S	0	1.34
---	--	------	------
mPD		0.28	0.72
		nPD:	0.39
		N. weight:	0.9
		Sc. PD:	0.12
		Sc. rank:	708.7

,

NP			
Pos.	325	obs:	exp:
tct	S	0	0.67
tcc	S	0	0.63
tca	S	0	0.97
tcg	S	0	0.21
aat	N	2715	1559.00
aac	N	28	1184.00
aaa	K	1	0.61
aag	K	0	0.39
agt	S	4	0.74
agc	S	0	0.77
---	--	------	------
mPD		0.024	0.50
		nPD:	0.05
		N. weight:	0.74
		Sc. PD:	-0.15
		Sc. rank:	-699.7

,

NP			
Pos.	326	obs:	exp:
gaa	E	2319	1628.00
gag	E	429	1120.00
---	--	------	------
mPD		0.26	0.48
		nPD:	0.55
		N. weight:	0.22
		Sc. PD:	0.059
		Sc. rank:	255.0

,

NP			
Pos.	327	obs:	exp:
tct	S	0	0.17
tcc	S	0	0.16
tca	S	0	0.24
tcg	S	0	0.05
act	T	0	0.25
acc	T	1	0.22
aca	T	0	0.45
acg	T	0	0.08
aat	N	602	1560.00
aac	N	2143	1185.00
agt	S	1	0.19
agc	S	0	0.19
gat	D	0	0.56
gac	D	1	0.44
---	--	------	------
mPD		0.34	0.49
		nPD:	0.7
		N. weight:	0.37
		Sc. PD:	0.16
		Sc. rank:	524.0

,

NP			
Pos.	328	obs:	exp:
cct	P	2	810.90
ccc	P	1	553.30
cca	P	2725	1088.00
ccg	P	20	295.60
---	--	------	------
mPD		0.017	0.70
		nPD:	0.02
		N. weight:	1.3
		Sc. PD:	-0.29
		Sc. rank:	-1795.7

,

NP			
Pos.	329	obs:	exp:
tct	S	2	0.34
tcc	S	0	0.32
tca	S	0	0.49
tcg	S	0	0.11
act	T	6	1.48
acc	T	0	1.30
aca	T	0	2.73
acg	T	0	0.50
agt	S	0	0.37
agc	S	0	0.38
gtt	V	36	81.71
gtc	V	248	69.44
gta	V	78	92.90
gtg	V	6	124.00
gct	A	2278	581.50
gcc	A	51	543.50
gca	A	42	1069.00
gcg	A	1	177.90
---	--	------	------
mPD		0.51	0.94
		nPD:	0.54
		N. weight:	1.6
		Sc. PD:	0.45
		Sc. rank:	1918.6

,

NP			
Pos.	330	obs:	exp:
cat	H	453	1585.00
cac	H	2295	1163.00
---	--	------	------
mPD		0.28	0.49
		nPD:	0.56
		N. weight:	0.52
		Sc. PD:	0.15
		Sc. rank:	636.6

,

NP			
Pos.	331	obs:	exp:
aaa	K	95	1670.00
aag	K	2653	1078.00
---	--	------	------
mPD		0.067	0.48
		nPD:	0.14
		N. weight:	1.1
		Sc. PD:	-0.13
		Sc. rank:	-191.0

,

NP			
Pos.	332	obs:	exp:
tct	S	0	462.00
tcc	S	0	434.10
tca	S	0	667.10
tcg	S	0	146.00
agt	S	2580	511.30
agc	S	167	526.50
ggt	G	1	0.16
ggc	G	0	0.13
gga	G	0	0.45
ggg	G	0	0.26
---	--	------	------
mPD		0.11	1.6
		nPD:	0.07
		N. weight:	2.1
		Sc. PD:	-0.37
		Sc. rank:	-1463.6

,

NP			
Pos.	333	obs:	exp:
caa	Q	2229	1482.00
cag	Q	519	1266.00
---	--	------	------
mPD		0.31	0.50
		nPD:	0.62
		N. weight:	0.24
		Sc. PD:	0.081
		Sc. rank:	313.0

,

NP			
Pos.	334	obs:	exp:
ttt	F	2	0.90
ttc	F	0	1.10
tta	L	969	298.70
ttg	L	1338	515.60
ctt	L	40	554.40
ctc	L	31	399.40
cta	L	42	479.30
ctg	L	325	497.60
atg	M	1	1.00
---	--	------	------
mPD		0.77	1.1
		nPD:	0.67
		N. weight:	1.1
		Sc. PD:	0.42
		Sc. rank:	1467.0

,

NP			
Pos.	335	obs:	exp:
att	I	1	59.60
atc	I	0	43.09
ata	I	167	65.31
atg	M	2	2.00
gtt	V	22	572.40
gtc	V	50	486.50
gta	V	518	650.80
gtg	V	1988	868.30
---	--	------	------
mPD		0.53	0.86
		nPD:	0.62
		N. weight:	0.79
		Sc. PD:	0.27
		Sc. rank:	1049.2

,

NP			
Pos.	336	obs:	exp:
tgg	W	2748	2748.00
---	--	------	------
mPD		0	0
		nPD:	1.
		N. weight:	0.
		Sc. PD:	0
		Sc. rank:	0

,

NP			
Pos.	337	obs:	exp:
tta	L	0	3.37
ttg	L	23	5.82
ctt	L	0	6.26
ctc	L	0	4.51
cta	L	0	5.41
ctg	L	8	5.62
att	I	3	2.13
atc	I	0	1.54
ata	I	3	2.33
atg	M	2711	2711.00
---	--	------	------
mPD		0.027	0.041
		nPD:	0.65
		N. weight:	0.021
		Sc. PD:	0.0078
		Sc. rank:	28.3

,

NP			
Pos.	338	obs:	exp:
tct	S	0	0.17
tcc	S	0	0.16
tca	S	1	0.24
tcg	S	0	0.05
agt	S	0	0.19
agc	S	0	0.19
gct	A	47	673.40
gcc	A	5	629.40
gca	A	2675	1238.00
gcg	A	20	206.00
---	--	------	------
mPD		0.052	0.68
		nPD:	0.08
		N. weight:	0.98
		Sc. PD:	-0.17
		Sc. rank:	-632.1

,

NP			
Pos.	339	obs:	exp:
tgt	C	100	1183.00
tgc	C	2648	1565.00
---	--	------	------
mPD		0.070	0.49
		nPD:	0.14
		N. weight:	0.6
		Sc. PD:	-0.066
		Sc. rank:	-98.7

,

NP			
Pos.	340	obs:	exp:
cat	H	520	1585.00
cac	H	2227	1162.00
aat	N	0	0.57
aac	N	1	0.43
---	--	------	------
mPD		0.31	0.49
		nPD:	0.63
		N. weight:	0.46
		Sc. PD:	0.16
		Sc. rank:	613.5

,

NP

Pos.	341	obs :	exp :
tct	S	2662	462.00
tcc	S	29	434.10
tca	S	51	667.10
tcg	S	5	146.00
agt	S	0	511.30
agc	S	0	526.50
gct	A	1	0.25
gcc	A	0	0.23
gca	A	0	0.45
gcg	A	0	0.07
---	--	------	------
mPD		0.061	1.6
		nPD :	0.04
		N. weight :	2.3
		Sc. PD :	-0.49
		Sc. rank :	-2686.5

NP

Pos.	342	obs :	exp :
tct	S	3	0.50
tcc	S	0	0.47
tca	S	0	0.73
tcg	S	0	0.16
agt	S	0	0.56
agc	S	0	0.57
gct	A	2273	672.90
gcc	A	14	628.90
gca	A	450	1237.00
gcg	A	8	205.90
---	--	------	------
mPD		0.29	0.68
		nPD :	0.42
		N. weight :	1.2
		Sc. PD :	0.19
		Sc. rank :	1103.3

NP

Pos.	343	obs :	exp :
gct	A	65	673.60
gcc	A	5	629.60
gca	A	2656	1239.00
gcg	A	22	206.10
---	--	------	------
mPD		0.065	0.68
		nPD :	0.1
		N. weight :	0.95
		Sc. PD :	-0.15
		Sc. rank :	-452.2

NP

Pos.	344	obs :	exp :
ttt	F	2721	1231.00
ttc	F	27	1517.00
---	--	------	------
mPD		0.019	0.49
		nPD :	0.04
		N. weight :	1.1
		Sc. PD :	-0.22
		Sc. rank :	-1209.7

NP

Pos.	345	obs :	exp :
gaa	E	1708	1628.00
gag	E	1040	1120.00
---	--	------	------
mPD		0.47	0.48
		nPD :	0.97
		N. weight :	0.0047
		Sc. PD :	0.0032
		Sc. rank :	7.7

NP

Pos.	346	obs :	exp :
gat	D	2577	1546.00
gac	D	171	1202.00
---	--	------	------
mPD		0.12	0.49
		nPD :	0.24
		N. weight :	0.52
		Sc. PD :	-0.01
		Sc. rank :	90.2

NP

Pos.	347	obs :	exp :
tta	L	1228	298.70
ttg	L	203	515.60
ctt	L	1	554.40
ctc	L	1	399.40
cta	L	1005	479.30
ctg	L	307	497.60
att	I	0	1.06
atc	I	0	0.77
ata	I	3	1.17
---	--	------	------
mPD		0.80	1.1
		nPD :	0.71
		N. weight :	1.1
		Sc. PD :	0.48
		Sc. rank :	1614.4

NP

Pos.	348	obs :	exp :
cgt	R	0	86.74
cgc	R	0	145.20
cga	R	41	276.20
cgg	R	49	198.50
aga	R	1486	1260.00
agg	R	1172	781.10
---	--	------	------
mPD		0.56	0.94
		nPD :	0.59
		N. weight :	0.31
		Sc. PD :	0.098
		Sc. rank :	389.6

NP

Pos.	349	obs :	exp :
tta	L	19	2.18
ttg	L	1	3.76
ctt	L	0	4.04
ctc	L	0	2.91
cta	L	0	3.49
ctg	L	0	3.63
att	I	0	13.13
atc	I	0	9.49
ata	I	37	14.38
gtt	V	8	597.50
gtc	V	6	507.80
gta	V	2161	679.30
gtg	V	516	906.40
---	--	------	------
mPD		0.35	0.78
		nPD :	0.45
		N. weight :	1.2
		Sc. PD :	0.22
		Sc. rank :	1197.8

NP

Pos.	350	obs :	exp :
tta	L	22	2.39
ttg	L	0	4.13
tct	S	6	458.30
tcc	S	4	430.70
tca	S	2664	661.70
tcg	S	51	144.80
ctt	L	0	4.44
ctc	L	0	3.20
cta	L	0	3.84
ctg	L	0	3.99
act	T	0	0.25
acc	T	0	0.22
aca	T	1	0.45
acg	T	0	0.08
agt	S	0	507.20
agc	S	0	522.20
---	--	------	------
mPD		0.060	1.6
		nPD :	0.04
		N. weight :	1.9
		Sc. PD :	-0.41
		Sc. rank :	-2267.8

NP

Pos.	351	obs :	exp :
tct	S	0	459.90
tcc	S	0	432.10
tca	S	0	663.90
tcg	S	0	145.30
act	T	0	0.25
acc	T	1	0.22
aca	T	0	0.45
acg	T	0	0.08
agt	S	2620	508.90
agc	S	114	524.00
ggt	G	6	2.04
ggc	G	6	1.65
gga	G	0	5.89
ggg	G	1	3.43
---	--	------	------
mPD		0.095	1.6
		nPD :	0.06
		N. weight :	2.2
		Sc. PD :	-0.41
		Sc. rank :	-1867.5

NP

Pos.	352	obs :	exp :
ttt	F	284	1231.00
ttc	F	2463	1516.00
tta	L	0	0.11
ttg	L	0	0.19
ctt	L	1	0.20
ctc	L	0	0.15
cta	L	0	0.17
ctg	L	0	0.18
---	--	------	------
mPD		0.19	0.50
		nPD :	0.38
		N. weight :	0.41
		Sc. PD :	0.047
		Sc. rank :	282.6

NP

Pos.	353	obs :	exp :
att	I	18	974.50
atc	I	408	704.60
ata	I	2321	1068.00
gtt	V	0	0.22
gtc	V	0	0.19
gta	V	1	0.25
gtg	V	0	0.34
---	--	------	------
mPD		0.26	0.66
		nPD :	0.4
		N. weight :	0.79
		Sc. PD :	0.11
		Sc. rank :	638.0

NP

Pos.	354	obs :	exp :
cgt	R	0	86.62
cgc	R	0	145.00
cga	R	8	275.80
cgg	R	0	198.20
aaa	K	4	2.43
aag	K	0	1.57
aga	R	2675	1258.00
agg	R	61	779.90
---	--	------	------
mPD		0.052	0.94
		nPD :	0.06
		N. weight :	0.97
		Sc. PD :	-0.19
		Sc. rank :	-850.7

NP

Pos.	355	obs :	exp :
ggt	G	12	431.00
ggc	G	2	348.20
gga	G	1328	1245.00
ggg	G	1406	724.10
---	--	------	------
mPD		0.50	0.68
		nPD :	0.74
		N. weight :	0.51
		Sc. PD :	0.23
		Sc. rank :	765.3

NP

Pos.	356	obs :	exp :
cgt	R	0	0.47
cgc	R	0	0.79
cga	R	0	1.51
cgg	R	0	1.08
att	I	0	0.35
atc	I	0	0.26
ata	I	1	0.39
act	T	2	97.90
acc	T	11	86.06
aca	T	373	180.80
acg	T	12	33.19
aaa	K	158	1406.00
aag	K	2155	907.30
aga	R	11	6.88
agg	R	4	4.26
gct	A	0	2.94
gcc	A	1	2.75
gca	A	11	5.41
gcg	A	0	0.90
gaa	E	9	5.33
gag	E	0	3.67
---	--	------	------
mPD		0.61	0.82
		nPD :	0.75
		N. weight :	0.93
		Sc. PD :	0.44
		Sc. rank :	1424.9

NP

Pos.	357	obs :	exp :
cgt	R	0	0.98
cgc	R	0	1.64
cga	R	0	3.12
cgg	R	0	2.24
aaa	K	2664	1651.00
aag	K	53	1066.00
aga	R	31	14.22
agg	R	0	8.81
---	--	------	------
mPD		0.060	0.51
		nPD :	0.12
		N. weight :	0.6
		Sc. PD :	-0.08
		Sc. rank :	-175.7

NP

Pos.	358	obs :	exp :
tta	L	0	0.33
ttg	L	3	0.56
ctt	L	0	0.61
ctc	L	0	0.44
cta	L	0	0.52
ctg	L	0	0.54
att	I	0	0.35
atc	I	0	0.26
ata	I	1	0.39
atg	M	11	11.00
gtt	V	24	606.60
gtc	V	0	515.50
gta	V	246	689.70
gtg	V	2462	920.20
gct	A	0	0.25
gcc	A	0	0.23
gca	A	1	0.45
gcg	A	0	0.07
---	--	------	------
mPD		0.19	0.75
		nPD :	0.26
		N. weight :	1.1
		Sc. PD :	-0.0025
		Sc. rank :	278.5

NP

Pos.	359	obs :	exp :
ttt	F	2	0.90
ttc	F	0	1.10
tta	L	0	62.68
ttg	L	0	108.20
tct	S	1	0.17
tcc	S	0	0.16
tca	S	0	0.24
tcg	S	0	0.05
ctt	L	564	116.30
ctc	L	11	83.81
cta	L	1	100.60
ctg	L	0	104.40
cat	H	0	0.58
cac	H	1	0.42
att	I	1626	710.20
atc	I	338	513.50
ata	I	38	778.30
agt	S	0	0.19
agc	S	0	0.19
gtt	V	27	36.86
gtc	V	114	31.32
gta	V	25	41.90
gtg	V	0	55.91
---	--	------	------
mPD		0.74	1.2
		nPD :	0.64
		N. weight :	1.1
		Sc. PD :	0.4
		Sc. rank :	1496.4

NP

Pos.	360	obs :	exp :
tct	S	0	0.17
tcc	S	0	0.16
tca	S	1	0.24
tcg	S	0	0.05
cct	P	4	809.70
ccc	P	4	552.40
cca	P	2709	1087.00
ccg	P	27	295.20
act	T	0	0.74
acc	T	0	0.65
aca	T	3	1.36
acg	T	0	0.25
agt	S	0	0.19
agc	S	0	0.19
---	--	------	------
mPD		0.028	0.71
		nPD :	0.04
		N. weight :	1.2
		Sc. PD :	-0.26
		Sc. rank :	-1392.6

NP			
Pos.	361	obs:	exp:
cgt	R	0	86.74
cgc	R	0	145.20
cga	R	15	276.20
cgg	R	3	198.50
aga	R	2633	1260.00
agg	R	97	781.10
---	--	------	------
mPD		0.083	0.94
		nPD:	0.09
		N. weight:	0.89
		Sc. PD:	-0.14
		Sc. rank:	-500.4

,

NP			
Pos.	362	obs:	exp:
ggt	G	0	431.00
ggc	G	0	348.20
gga	G	2356	1245.00
ggg	G	392	724.10
---	--	------	------
mPD		0.24	0.68
		nPD:	0.36
		N. weight:	0.66
		Sc. PD:	0.063
		Sc. rank:	406.1

,

NP			
Pos.	363	obs:	exp:
caa	Q	372	204.90
cag	Q	8	175.10
cgt	R	0	0.54
cgc	R	0	0.90
cga	R	0	1.71
cgg	R	0	1.23
aaa	K	298	1429.00
aag	K	2053	922.20
aga	R	2	7.80
agg	R	15	4.83
---	--	------	------
mPD		0.62	0.73
		nPD:	0.84
		N. weight:	0.73
		Sc. PD:	0.41
		Sc. rank:	1166.3

,

NP			
Pos.	364	obs:	exp:
tta	L	24	299.00
ttg	L	297	516.20
ctt	L	1894	555.00
ctc	L	397	399.80
cta	L	12	479.80
ctg	L	124	498.10
---	--	------	------
mPD		0.69	1.1
		nPD:	0.6
		N. weight:	0.99
		Sc. PD:	0.33
		Sc. rank:	1305.9

,

NP			
Pos.	365	obs:	exp:
tta	L	14	1.52
ttg	L	0	2.63
tct	S	49	459.40
tcc	S	2647	431.60
tca	S	35	663.20
tcg	S	0	145.10
tat	Y	0	0.56
tac	Y	1	0.44
ctt	L	0	2.83
ctc	L	0	2.04
cta	L	0	2.44
ctg	L	0	2.54
act	T	0	0.49
acc	T	2	0.43
aca	T	0	0.91
acg	T	0	0.17
agt	S	0	508.30
agc	S	0	523.40
---	--	------	------
mPD		0.082	1.6
		nPD:	0.05
		N. weight:	2.4
		Sc. PD:	-0.48
		Sc. rank:	-2192.4

,

NP			
Pos.	366	obs:	exp:
act	T	27	675.90
acc	T	382	594.20
aca	T	2063	1249.00
acg	T	276	229.10
---	--	------	------
mPD		0.41	0.68
		nPD:	0.6
		N. weight:	0.44
		Sc. PD:	0.14
		Sc. rank:	568.6

,

NP			
Pos.	367	obs:	exp:
cgt	R	0	86.74
cgc	R	0	145.20
cga	R	7	276.20
cgg	R	0	198.50
aga	R	2628	1260.00
agg	R	113	781.10
---	--	------	------
mPD		0.084	0.94
		nPD:	0.09
		N. weight:	0.89
		Sc. PD:	-0.14
		Sc. rank:	-496.4

,

NP			
Pos.	368	obs:	exp:
ggt	G	10	431.00
ggc	G	0	348.20
gga	G	755	1245.00
ggg	G	1983	724.10
---	--	------	------
mPD		0.40	0.68
		nPD:	0.59
		N. weight:	0.83
		Sc. PD:	0.26
		Sc. rank:	1059.3

,

NP			
Pos.	369	obs:	exp:
att	I	108	77.33
atc	I	110	55.91
ata	I	0	84.75
gtt	V	669	548.70
gtc	V	1790	466.30
gta	V	9	623.80
gtg	V	3	832.30
gct	A	34	14.46
gcc	A	24	13.52
gca	A	1	26.59
gcg	A	0	4.42
---	--	------	------
mPD		0.61	0.93
		nPD:	0.66
		N. weight:	1.4
		Sc. PD:	0.53
		Sc. rank:	1925.2

,

NP			
Pos.	370	obs:	exp:
caa	Q	497	1482.00
cag	Q	2251	1266.00
---	--	------	------
mPD		0.30	0.50
		nPD:	0.6
		N. weight:	0.4
		Sc. PD:	0.13
		Sc. rank:	508.4

,

NP			
Pos.	371	obs:	exp:
att	I	2639	972.30
atc	I	23	703.00
ata	I	79	1066.00
gtt	V	7	1.55
gtc	V	0	1.32
gta	V	0	1.77
gtg	V	0	2.36
---	--	------	------
mPD		0.077	0.66
		nPD:	0.12
		N. weight:	1.2
		Sc. PD:	-0.17
		Sc. rank:	-401.1

,

NP			
Pos.	372	obs:	exp:
gct	A	2658	673.60
gcc	A	79	629.60
gca	A	4	1239.00
gcg	A	7	206.10
---	--	------	------
mPD		0.064	0.68
		nPD:	0.09
		N. weight:	1.8
		Sc. PD:	-0.28
		Sc. rank:	-904.3

,

NP			
Pos.	373	obs:	exp:
tct	S	4	462.20
tcc	S	6	434.30
tca	S	2676	667.30
tcg	S	62	146.10
agt	S	0	511.50
agc	S	0	526.60
---	--	------	------
mPD		0.051	1.6
		nPD:	0.03
		N. weight:	1.9
		Sc. PD:	-0.41
		Sc. rank:	-2418.6

,

NP			
Pos.	374	obs:	exp:
tct	S	0	14.80
tcc	S	0	13.91
tca	S	0	21.37
tcg	S	0	4.68
act	T	30	7.63
acc	T	1	6.70
aca	T	0	14.09
acg	T	0	2.59
aat	N	2591	1494.00
aac	N	37	1134.00
aaa	K	0	0.61
aag	K	1	0.39
agt	S	88	16.38
agc	S	0	16.86
---	--	------	------
mPD		0.11	0.63
		nPD:	0.18
		N. weight:	0.78
		Sc. PD:	-0.06
		Sc. rank:	-13.2

,

NP			
Pos.	375	obs:	exp:
gaa	E	641	1628.00
gag	E	2107	1120.00
---	--	------	------
mPD		0.36	0.48
		nPD:	0.74
		N. weight:	0.39
		Sc. PD:	0.18
		Sc. rank:	581.5

,

NP

Pos.	376	obs:	exp:
tct	S	0	10.60
tcc	S	0	9.96
tca	S	0	15.30
tcg	S	0	3.35
act	T	1	0.98
acc	T	3	0.86
aca	T	0	1.82
acg	T	0	0.33
aat	N	2257	1524.00
aac	N	424	1157.00
agt	S	59	11.73
agc	S	4	12.07
---	--	------	------
mPD		0.31	0.57
		nPD:	0.54
		N. weight:	0.29
		Sc. PD:	0.08
		Sc. rank:	341.3

,

NP

Pos.	377	obs:	exp:
att	I	1	1.77
atc	I	0	1.28
ata	I	4	1.94
atg	M	432	432.00
gtt	V	5	513.10
gtc	V	0	436.10
gta	V	117	583.40
gtg	V	2189	778.40
---	--	------	------
mPD		0.36	0.97
		nPD:	0.37
		N. weight:	1.1
		Sc. PD:	0.11
		Sc. rank:	708.9

,

NP

Pos.	378	obs:	exp:
aaa	K	1	0.61
aag	K	0	0.39
gat	D	2	1.69
gac	D	1	1.31
gaa	E	2686	1626.00
gag	E	58	1118.00
---	--	------	------
mPD		0.044	0.48
		nPD:	0.09
		N. weight:	0.62
		Sc. PD:	-0.099
		Sc. rank:	-334.5

,

NP

Pos.	379	obs:	exp:
tct	S	0	1.35
tcc	S	3	1.26
tca	S	1	1.94
tcg	S	0	0.43
att	I	7	16.67
atc	I	40	12.05
ata	I	0	18.27
act	T	28	454.60
acc	T	1343	399.60
aca	T	475	839.70
acg	T	2	154.10
aat	N	5	19.33
aac	N	29	14.67
agt	S	0	1.49
agc	S	4	1.53
gtt	V	0	1.11
gtc	V	5	0.94
gta	V	0	1.26
gtg	V	0	1.68
gct	A	40	197.60
gcc	A	753	184.70
gca	A	12	363.30
gcg	A	1	60.45
---	--	------	------
mPD		0.82	1.2
		nPD:	0.71
		N. weight:	1.2
		Sc. PD:	0.52
		Sc. rank:	1749.4

,

NP

Pos.	380	obs:	exp:
tta	L	0	0.33
ttg	L	0	0.56
ctt	L	0	0.61
ctc	L	0	0.44
cta	L	0	0.52
ctg	L	3	0.54
att	I	0	11.00
atc	I	0	7.95
ata	I	31	12.05
atg	M	2707	2707.00
gtt	V	0	1.55
gtc	V	0	1.32
gta	V	0	1.77
gtg	V	7	2.36
---	--	------	------
mPD		0.030	0.034
		nPD:	0.86
		N. weight:	0.022
		Sc. PD:	0.013
		Sc. rank:	35.3

,

NP

Pos.	381	obs:	exp:
aat	N	3	24.44
aac	N	40	18.56
gct	A	0	0.25
gcc	A	1	0.23
gca	A	0	0.45
gcg	A	0	0.07
gat	D	1045	1507.00
gac	D	1635	1173.00
gaa	E	10	11.85
gag	E	10	8.15
ggt	G	0	0.63
ggc	G	3	0.51
gga	G	1	1.81
ggg	G	0	1.05
---	--	------	------
mPD		0.52	0.54
		nPD:	0.96
		N. weight:	0.1
		Sc. PD:	0.07
		Sc. rank:	171.4

,

NP

Pos.	382	obs:	exp:
ttt	F	0	0.45
ttc	F	1	0.55
tct	S	72	461.50
tcc	S	2642	433.70
tca	S	29	666.30
tcg	S	1	145.80
act	T	0	0.49
acc	T	2	0.43
aca	T	0	0.91
acg	T	0	0.17
agt	S	0	510.70
agc	S	0	525.90
gct	A	0	0.25
gcc	A	1	0.23
gca	A	0	0.45
gcg	A	0	0.07
---	--	------	------
mPD		0.075	1.6
		nPD:	0.05
		N. weight:	2.4
		Sc. PD:	-0.48
		Sc. rank:	-2333.4

,

NP

Pos.	383	obs:	exp:
ttt	F	2	0.90
ttc	F	0	1.10
tct	S	0	21.70
tcc	S	0	20.39
tca	S	0	31.33
tcg	S	0	6.86
att	I	236	119.20
atc	I	99	86.18
ata	I	1	130.60
atg	M	10	10.00
act	T	0	0.25
acc	T	1	0.22
aca	T	0	0.45
acg	T	0	0.08
aat	N	2109	1257.00
aac	N	102	954.30
agt	S	125	24.01
agc	S	4	24.72
gtt	V	57	13.10
gtc	V	2	11.13
gta	V	0	14.89
gtg	V	0	19.87
---	--	------	------
mPD		0.52	0.99
		nPD:	0.52
		N. weight:	0.7
		Sc. PD:	0.18
		Sc. rank:	776.6

,

NP

Pos.	384	obs:	exp:
act	T	632	675.90
acc	T	2088	594.20
aca	T	28	1249.00
acg	T	0	229.10
---	--	------	------
mPD		0.37	0.68
		nPD:	0.54
		N. weight:	1.3
		Sc. PD:	0.36
		Sc. rank:	1532.7

,

NP

Pos.	385	obs:	exp:
tta	L	898	299.00
ttg	L	80	516.20
ctt	L	402	555.00
ctc	L	1	399.80
cta	L	103	479.80
ctg	L	1264	498.10
---	--	------	------
mPD		1.1	1.1
		nPD:	0.94
		N. weight:	0.91
		Sc. PD:	0.59
		Sc. rank:	1487.3

,

NP

Pos.	386	obs:	exp:
gat	D	0	1.12
gac	D	2	0.88
gaa	E	1903	1627.00
gag	E	843	1119.00
---	--	------	------
mPD		0.43	0.48
		nPD:	0.88
		N. weight:	0.034
		Sc. PD:	0.02
		Sc. rank:	54.8

,

NP

Pos.	387	obs:	exp:
tta	L	46	299.00
ttg	L	46	516.20
ctt	L	303	555.00
ctc	L	4	399.80
cta	L	1249	479.80
ctg	L	1100	498.10
---	--	------	------
mPD		0.66	1.1
		nPD:	0.58
		N. weight:	0.88
		Sc. PD:	0.27
		Sc. rank:	1097.3

,

NP

Pos.	388	obs:	exp:
cgt	R	0	86.74
cgc	R	0	145.20
cga	R	10	276.20
cgg	R	0	198.50
aga	R	2660	1260.00
agg	R	78	781.10
---	--	------	------
mPD		0.062	0.94
		nPD:	0.07
		N. weight:	0.93
		Sc. PD:	-0.17
		Sc. rank:	-706.0

,

NP

Pos.	389	obs:	exp:
tct	S	0	462.00
tcc	S	0	434.10
tca	S	0	667.10
tcg	S	0	146.00
act	T	0	0.25
acc	T	1	0.22
aca	T	0	0.45
acg	T	0	0.08
agt	S	89	511.30
agc	S	2658	526.50
---	--	------	------
mPD		0.063	1.6
		nPD:	0.04
		N. weight:	2.2
		Sc. PD:	-0.46
		Sc. rank:	-2479.6

,

NP

Pos.	390	obs:	exp:
cgt	R	0	73.64
cgc	R	0	123.30
cga	R	0	234.50
cgg	R	0	168.50
aaa	K	411	251.60
aag	K	3	162.40
aga	R	2246	1070.00
agg	R	87	663.10
ggt	G	0	0.16
ggc	G	0	0.13
gga	G	0	0.45
ggg	G	1	0.26
---	--	------	------
mPD		0.32	1.1
		nPD:	0.28
		N. weight:	0.88
		Sc. PD:	0.019
		Sc. rank:	297.5

,

NP			
Pos.	391	obs:	exp:
tat	Y	203	1540.00
tac	Y	2545	1208.00
---	--	------	------
mPD		0.14	0.49
		nPD:	0.28
		N. weight:	0.78
		Sc. PD:	0.015
		Sc. rank:	250.1

,

NP			
Pos.	392	obs:	exp:
tgg	W	2748	2748.00
---	--	------	------
mPD		0	0
		nPD:	1.
		N. weight:	0.
		Sc. PD:	0
		Sc. rank:	0

,

NP			
Pos.	393	obs:	exp:
gct	A	351	673.60
gcc	A	2379	629.60
gca	A	13	1239.00
gcg	A	5	206.10
---	--	------	------
mPD		0.23	0.68
		nPD:	0.35
		N. weight:	1.5
		Sc. PD:	0.12
		Sc. rank:	841.0

,

NP			
Pos.	394	obs:	exp:
att	I	2	974.80
atc	I	38	704.80
ata	I	2708	1068.00
---	--	------	------
mPD		0.029	0.66
		nPD:	0.04
		N. weight:	1.3
		Sc. PD:	-0.26
		Sc. rank:	-1301.2

,

NP			
Pos.	395	obs:	exp:
cgt	R	0	86.65
cgc	R	0	145.10
cga	R	0	275.90
cgg	R	0	198.30
aaa	K	0	1.82
aag	K	3	1.18
aga	R	615	1259.00
agg	R	2130	780.20
---	--	------	------
mPD		0.35	0.94
		nPD:	0.37
		N. weight:	0.89
		Sc. PD:	0.097
		Sc. rank:	599.5

,

NP			
Pos.	396	obs:	exp:
act	T	10	675.90
acc	T	2720	594.20
aca	T	15	1249.00
acg	T	3	229.10
---	--	------	------
mPD		0.020	0.68
		nPD:	0.03
		N. weight:	2.1
		Sc. PD:	-0.46
		Sc. rank:	-2753.5

,

NP			
Pos.	397	obs:	exp:
cgt	R	0	86.33
cgc	R	0	144.60
cga	R	0	274.90
cgg	R	1	197.60
aaa	K	8	7.90
aag	K	5	5.10
aga	R	1362	1254.00
agg	R	1372	777.40
---	--	------	------
mPD		0.51	0.95
		nPD:	0.54
		N. weight:	0.47
		Sc. PD:	0.13
		Sc. rank:	537.3

,

NP			
Pos.	398	obs:	exp:
tct	S	0	462.20
tcc	S	0	434.30
tca	S	0	667.30
tcg	S	0	146.10
agt	S	2260	511.50
agc	S	488	526.60
---	--	------	------
mPD		0.29	1.6
		nPD:	0.18
		N. weight:	1.7
		Sc. PD:	-0.13
		Sc. rank:	-9.5

,

NP			
Pos.	399	obs:	exp:
ggt	G	7	431.00
ggc	G	2	348.20
gga	G	2650	1245.00
ggg	G	89	724.10
---	--	------	------
mPD		0.069	0.68
		nPD:	0.1
		N. weight:	0.94
		Sc. PD:	-0.14
		Sc. rank:	-385.3

,

NP			
Pos.	400	obs:	exp:
ggt	G	10	431.00
ggc	G	11	348.20
gga	G	2590	1245.00
ggg	G	137	724.10
---	--	------	------
mPD		0.11	0.68
		nPD:	0.16
		N. weight:	0.84
		Sc. PD:	-0.079
		Sc. rank:	-93.9

,

NP			
Pos.	401	obs:	exp:
tct	S	0	1.18
tcc	S	0	1.11
tca	S	0	1.70
tcg	S	0	0.37
act	T	0	0.25
acc	T	1	0.22
aca	T	0	0.45
acg	T	0	0.08
aat	N	2166	1557.00
aac	N	574	1183.00
agt	S	7	1.30
agc	S	0	1.34
---	--	------	------
mPD		0.34	0.50
		nPD:	0.67
		N. weight:	0.17
		Sc. PD:	0.066
		Sc. rank:	231.6

,

NP			
Pos.	402	obs:	exp:
act	T	39	672.80
acc	T	2459	591.40
aca	T	237	1243.00
acg	T	0	228.10
aat	N	0	2.27
aac	N	4	1.73
gct	A	0	2.21
gcc	A	9	2.06
gca	A	0	4.06
gcg	A	0	0.67
---	--	------	------
mPD		0.19	0.69
		nPD:	0.28
		N. weight:	1.6
		Sc. PD:	0.032
		Sc. rank:	524.7

,

NP			
Pos.	403	obs:	exp:
tct	S	0	1.68
tcc	S	0	1.58
tca	S	0	2.43
tcg	S	0	0.53
cat	H	1	0.58
cac	H	0	0.42
act	T	2	0.49
acc	T	0	0.43
aca	T	0	0.91
acg	T	0	0.17
aat	N	2306	1555.00
aac	N	429	1180.00
agt	S	8	1.86
agc	S	2	1.92
---	--	------	------
mPD		0.27	0.51
		nPD:	0.54
		N. weight:	0.26
		Sc. PD:	0.07
		Sc. rank:	300.4

,

NP			
Pos.	404	obs:	exp:
tta	L	0	0.22
ttg	L	0	0.38
ctt	L	0	0.40
ctc	L	0	0.29
cta	L	1	0.35
ctg	L	1	0.36
cat	H	0	1.15
cac	H	2	0.85
caa	Q	2600	1464.00
cag	Q	114	1250.00
cgt	R	0	0.03
cgc	R	0	0.05
cga	R	1	0.10
cgg	R	0	0.07
aaa	K	29	17.62
aag	K	0	11.38
aga	R	0	0.46
agg	R	0	0.28
---	--	------	------
mPD		0.10	0.52
		nPD:	0.2
		N. weight:	0.65
		Sc. PD:	-0.036
		Sc. rank:	53.7

,

NP			
Pos.	405	obs:	exp:
cat	H	4	2.31
cac	H	0	1.69
caa	Q	1542	1480.00
cag	Q	1202	1264.00
---	--	------	------
mPD		0.49	0.50
		nPD:	0.99
		N. weight:	0.0048
		Sc. PD:	0.0034
		Sc. rank:	7.9

,

NP			
Pos.	406	obs:	exp:
cgt	R	0	28.03
cgc	R	0	46.94
cga	R	0	89.26
cgg	R	0	64.15
aaa	K	1213	1130.00
aag	K	647	729.60
aga	R	685	407.20
agg	R	203	252.40
---	--	------	------
mPD		0.87	1.1
		nPD:	0.79
		N. weight:	0.17
		Sc. PD:	0.087
		Sc. rank:	267.4

,

NP			
Pos.	407	obs:	exp:
act	T	0	2.21
acc	T	0	1.95
aca	T	9	4.09
acg	T	0	0.75
gct	A	8	671.40
gcc	A	7	627.60
gca	A	2648	1235.00
gcg	A	76	205.40
---	--	------	------
mPD		0.071	0.69
		nPD:	0.1
		N. weight:	0.99
		Sc. PD:	-0.15
		Sc. rank:	-402.4

,

NP			
Pos.	408	obs:	exp:
tct	S	415	461.50
tcc	S	2173	433.70
tca	S	155	666.30
tcg	S	1	145.80
act	T	0	0.25
acc	T	1	0.22
aca	T	0	0.45
acg	T	0	0.08
agt	S	0	510.70
agc	S	0	525.90
gct	A	0	0.74
gcc	A	3	0.69
gca	A	0	1.35
gcg	A	0	0.22
---	--	------	------
mPD		0.35	1.6
		nPD:	0.21
		N. weight:	1.7
		Sc. PD:	-0.071
		Sc. rank:	237.2

,

NP			
Pos.	409	obs:	exp:
tct	S	1	0.17
tcc	S	0	0.16
tca	S	0	0.24
tcg	S	0	0.05
act	T	3	5.17
acc	T	0	4.54
aca	T	18	9.54
acg	T	0	1.75
agt	S	0	0.19
agc	S	0	0.19
gct	A	265	668.20
gcc	A	5	624.60
gca	A	1728	1229.00
gcg	A	728	204.40
---	--	------	------
mPD		0.53	0.69
		nPD:	0.77
		N. weight:	0.66
		Sc. PD:	0.32
		Sc. rank:	1024.3

,

NP			
Pos.	410	obs:	exp:
tct	S	0	0.50
tcc	S	0	0.47
tca	S	0	0.73
tcg	S	0	0.16
agt	S	0	0.56
agc	S	3	0.57
ggt	G	47	430.60
ggc	G	2291	347.80
gga	G	27	1243.00
ggg	G	380	723.30
---	--	------	------
mPD		0.29	0.69
		nPD:	0.42
		N. weight:	2.
		Sc. PD:	0.3
		Sc. rank:	1811.7

,

NP			
Pos.	411	obs:	exp:
caa	Q	1108	1482.00
cag	Q	1640	1266.00
---	--	------	------
mPD		0.48	0.50
		nPD:	0.97
		N. weight:	0.056
		Sc. PD:	0.038
		Sc. rank:	91.2

,

NP			
Pos.	412	obs:	exp:
att	I	30	973.10
atc	I	2708	703.50
ata	I	5	1066.00
gtt	V	0	1.11
gtc	V	5	0.94
gta	V	0	1.26
gtg	V	0	1.68
---	--	------	------
mPD		0.029	0.66
		nPD:	0.04
		N. weight:	1.9
		Sc. PD:	-0.38
		Sc. rank:	-1930.3

,

NP			
Pos.	413	obs:	exp:
tct	S	0	462.20
tcc	S	0	434.30
tca	S	0	667.30
tcg	S	0	146.10
agt	S	2702	511.50
agc	S	46	526.60
---	--	------	------
mPD		0.033	1.6
		nPD:	0.02
		N. weight:	2.3
		Sc. PD:	-0.52
		Sc. rank:	-3273.0

,

NP			
Pos.	414	obs:	exp:
att	I	0	1.06
atc	I	0	0.77
ata	I	3	1.17
atg	M	1	1.00
gtt	V	10	609.30
gtc	V	4	517.80
gta	V	490	692.70
gtg	V	2240	924.30
---	--	------	------
mPD		0.31	0.74
		nPD:	0.41
		N. weight:	0.92
		Sc. PD:	0.14
		Sc. rank:	798.7

,

NP			
Pos.	415	obs:	exp:
caa	Q	1423	1482.00
cag	Q	1325	1266.00
---	--	------	------
mPD		0.50	0.50
		nPD:	1.01
		N. weight:	0.0035
		Sc. PD:	0.0025
		Sc. rank:	5.8

,

NP			
Pos.	416	obs:	exp:
cct	P	2634	810.60
ccc	P	103	553.10
cca	P	8	1088.00
ccg	P	2	295.50
gct	A	1	0.25
gcc	A	0	0.23
gca	A	0	0.45
gcg	A	0	0.07
---	--	------	------
mPD		0.080	0.70
		nPD:	0.11
		N. weight:	1.5
		Sc. PD:	-0.21
		Sc. rank:	-519.2

,

NP			
Pos.	417	obs:	exp:
act	T	377	675.70
acc	T	100	594.00
aca	T	2211	1248.00
acg	T	59	229.10
gct	A	0	0.25
gcc	A	0	0.23
gca	A	0	0.45
gcg	A	1	0.07
---	--	------	------
mPD		0.33	0.68
		nPD:	0.49
		N. weight:	0.42
		Sc. PD:	0.092
		Sc. rank:	459.7

,

NP			
Pos.	418	obs:	exp:
ttt	F	129	1231.00
ttc	F	2619	1517.00
---	--	------	------
mPD		0.090	0.49
		nPD:	0.18
		N. weight:	0.6
		Sc. PD:	-0.044
		Sc. rank:	-0.9

,

NP			
Pos.	419	obs:	exp:
tct	S	4	462.20
tcc	S	4	434.30
tca	S	2717	667.30
tcg	S	23	146.10
agt	S	0	511.50
agc	S	0	526.60
---	--	------	------
mPD		0.022	1.6
		nPD:	0.01
		N. weight:	2.
		Sc. PD:	-0.46
		Sc. rank:	-2993.5

,

NP			
Pos.	420	obs:	exp:
att	I	0	0.35
atc	I	0	0.26
ata	I	1	0.39
gtt	V	5	609.90
gtc	V	4	518.40
gta	V	1040	693.40
gtg	V	1698	925.30
---	--	------	------
mPD		0.48	0.74
		nPD:	0.64
		N. weight:	0.74
		Sc. PD:	0.27
		Sc. rank:	1005.2

,

NP			
Pos.	421	obs:	exp:
caa	Q	1112	1482.00
cag	Q	1636	1266.00
---	--	------	------
mPD		0.48	0.50
		nPD:	0.97
		N. weight:	0.055
		Sc. PD:	0.037
		Sc. rank:	89.4

,

NP			
Pos.	422	obs:	exp:
cgt	R	27	86.74
cgc	R	0	145.20
cga	R	994	276.20
cgg	R	1131	198.50
aga	R	548	1260.00
agg	R	48	781.10
---	--	------	------
mPD		0.84	0.94
		nPD:	0.9
		N. weight:	1.4
		Sc. PD:	0.84
		Sc. rank:	2239.3

,

NP			
Pos.	423	obs:	exp:
tct	S	0	4.04
tcc	S	0	3.79
tca	S	0	5.83
tcg	S	0	1.28
act	T	14	3.44
acc	T	0	3.03
aca	T	0	6.36
acg	T	0	1.17
aat	N	2438	1540.00
aac	N	271	1169.00
agt	S	24	4.47
agc	S	0	4.60
ggt	G	1	0.16
ggc	G	0	0.13
gga	G	0	0.45
ggg	G	0	0.26
---	--	------	------
mPD		0.21	0.54
		nPD:	0.39
		N. weight:	0.41
		Sc. PD:	0.05
		Sc. rank:	292.6

,

NP			
Pos.	424	obs:	exp:
tta	L	0	298.80
ttg	L	0	515.80
ctt	L	426	554.60
ctc	L	2277	399.50
cta	L	42	479.50
ctg	L	1	497.80
att	I	0	0.71
atc	I	2	0.51
ata	I	0	0.78
---	--	------	------
mPD		0.29	1.1
		nPD:	0.25
		N. weight:	2.
		Sc. PD:	-0.0079
		Sc. rank:	482.3

,

NP			
Pos.	425	obs:	exp:
cct	P	2514	810.90
ccc	P	193	553.30
cca	P	22	1088.00
ccg	P	19	295.60
---	--	------	------
mPD		0.16	0.70
		nPD:	0.22
		N. weight:	1.3
		Sc. PD:	-0.042
		Sc. rank:	218.5

,

NP			
Pos.	426	obs:	exp:
ttt	F	2554	1231.00
ttc	F	194	1517.00
---	--	------	------
mPD		0.13	0.49
		nPD:	0.27
		N. weight:	0.77
		Sc. PD:	0.0053
		Sc. rank:	219.1

,

NP			
Pos.	427	obs:	exp:
gat	D	2	1.69
gac	D	1	1.31
gaa	E	2313	1626.00
gag	E	432	1119.00
---	--	------	------
mPD		0.27	0.48
		nPD:	0.55
		N. weight:	0.21
		Sc. PD:	0.06
		Sc. rank:	254.2

,

NP			
Pos.	428	obs:	exp:
cgt	R	0	86.65
cgc	R	0	145.10
cga	R	555	275.90
cgg	R	21	198.30
aaa	K	1	1.22
aag	K	1	0.78
aga	R	2125	1259.00
agg	R	44	780.20
ggt	G	0	0.16
ggc	G	0	0.13
gga	G	1	0.45
ggg	G	0	0.26
---	--	------	------
mPD		0.38	0.94
		nPD:	0.4
		N. weight:	0.7
		Sc. PD:	0.098
		Sc. rank:	576.5

,

NP			
Pos.	429	obs:	exp:
tct	S	0	3.53
tcc	S	0	3.32
tca	S	20	5.10
tcg	S	1	1.12
act	T	0	7.38
acc	T	0	6.49
aca	T	23	13.63
acg	T	7	2.50
agt	S	0	3.91
agc	S	0	4.02
gct	A	10	661.10
gcc	A	2	617.90
gca	A	2368	1216.00
gcg	A	317	202.30
---	--	------	------
mPD		0.25	0.72
		nPD:	0.35
		N. weight:	0.9
		Sc. PD:	0.08
		Sc. rank:	549.0

,

NP			
Pos.	430	obs:	exp:
att	I	0	1.06
atc	I	3	0.77
ata	I	0	1.17
act	T	142	675.00
acc	T	2580	593.40
aca	T	22	1247.00
acg	T	0	228.80
aat	N	0	0.57
aac	N	1	0.43
---	--	------	------
mPD		0.12	0.68
		nPD:	0.17
		N. weight:	1.8
		Sc. PD:	-0.15
		Sc. rank:	-86.6

,

NP			
Pos.	431	obs:	exp:
att	I	119	185.50
atc	I	386	134.10
ata	I	18	203.30
gtt	V	2167	494.00
gtc	V	55	419.90
gta	V	1	561.70
gtg	V	2	749.40
---	--	------	------
mPD		0.59	1.1
		nPD:	0.56
		N. weight:	1.8
		Sc. PD:	0.51
		Sc. rank:	2160.0

NP			
Pos.	432	obs:	exp:
tta	L	268	76.94
ttg	L	395	132.80
ctt	L	0	142.80
ctc	L	0	102.90
cta	L	5	123.50
ctg	L	39	128.20
att	I	2	1.77
atc	I	0	1.28
ata	I	3	1.94
atg	M	2034	2034.00
gtt	V	0	0.44
gtc	V	0	0.38
gta	V	0	0.50
gtg	V	2	0.67
---	--	------	------
mPD		0.57	0.70
		nPD:	0.81
		N. weight:	0.38
		Sc. PD:	0.2
		Sc. rank:	592.3

NP			
Pos.	433	obs:	exp:
tct	S	0	0.17
tcc	S	0	0.16
tca	S	1	0.24
tcg	S	0	0.05
agt	S	0	0.19
agc	S	0	0.19
gtt	V	1	0.22
gtc	V	0	0.19
gta	V	0	0.25
gtg	V	0	0.34
gct	A	2	673.10
gcc	A	6	629.20
gca	A	2712	1238.00
gcg	A	26	205.90
---	--	------	------
mPD		0.027	0.68
		nPD:	0.04
		N. weight:	1.1
		Sc. PD:	-0.22
		Sc. rank:	-1212.0

NP			
Pos.	434	obs:	exp:
act	T	1	5.90
acc	T	1	5.19
aca	T	22	10.91
acg	T	0	2.00
gct	A	761	667.70
gcc	A	263	624.10
gca	A	1652	1228.00
gcg	A	48	204.30
---	--	------	------
mPD		0.56	0.70
		nPD:	0.8
		N. weight:	0.17
		Sc. PD:	0.087
		Sc. rank:	260.7

NP			
Pos.	435	obs:	exp:
ttt	F	457	1231.00
ttc	F	2291	1517.00
---	--	------	------
mPD		0.28	0.49
		nPD:	0.56
		N. weight:	0.26
		Sc. PD:	0.075
		Sc. rank:	315.8

NP			
Pos.	436	obs:	exp:
tct	S	0	385.50
tcc	S	0	362.20
tca	S	1	556.60
tcg	S	0	121.80
cgt	R	0	0.03
cgc	R	0	0.05
cga	R	0	0.10
cgg	R	0	0.07
att	I	23	9.22
atc	I	2	6.67
ata	I	1	10.11
act	T	315	99.62
acc	T	2	87.58
aca	T	87	184.00
acg	T	1	33.77
aat	N	1	9.66
aac	N	16	7.34
agt	S	61	426.60
agc	S	2230	439.30
aga	R	1	0.46
agg	R	0	0.28
gtt	V	1	0.22
gtc	V	0	0.19
gta	V	0	0.25
gtg	V	0	0.34
gct	A	2	0.98
gcc	A	0	0.92
gca	A	2	1.80
gcg	A	0	0.30
ggt	G	1	0.31
ggc	G	1	0.25
gga	G	0	0.91
ggg	G	0	0.53
---	--	------	------
mPD		0.59	1.7
		nPD:	0.36
		N. weight:	2.
		Sc. PD:	0.19
		Sc. rank:	1263.4

NP			
Pos.	437	obs:	exp:
gtt	V	0	0.22
gtc	V	0	0.19
gta	V	1	0.25
gtg	V	0	0.34
gaa	E	0	1.19
gag	E	2	0.81
ggt	G	6	430.60
ggc	G	5	347.80
gga	G	498	1243.00
ggg	G	2236	723.30
---	--	------	------
mPD		0.31	0.69
		nPD:	0.45
		N. weight:	1.1
		Sc. PD:	0.19
		Sc. rank:	1071.9

NP			
Pos.	438	obs:	exp:
tct	S	0	2.35
tcc	S	0	2.21
tca	S	0	3.40
tcg	S	0	0.74
act	T	0	0.25
acc	T	1	0.22
aca	T	0	0.45
acg	T	0	0.08
aat	N	41	1551.00
aac	N	2688	1178.00
agt	S	0	2.61
agc	S	14	2.68
gat	D	0	1.69
gac	D	3	1.31
ggt	G	0	0.16
ggc	G	1	0.13
gga	G	0	0.45
ggg	G	0	0.26
---	--	------	------
mPD		0.044	0.51
		nPD:	0.09
		N. weight:	1.1
		Sc. PD:	-0.18
		Sc. rank:	-663.6

NP			
Pos.	439	obs:	exp:
tct	S	0	18.84
tcc	S	0	17.70
tca	S	1	27.20
tcg	S	0	5.95
att	I	1	0.35
atc	I	0	0.26
ata	I	0	0.39
act	T	426	107.00
acc	T	3	94.06
aca	T	3	197.70
acg	T	3	36.27
aat	N	2162	1241.00
aac	N	21	942.20
aaa	K	4	4.25
aag	K	3	2.75
agt	S	110	20.85
agc	S	1	21.46
gtt	V	1	0.22
gtc	V	0	0.19
gta	V	0	0.25
gtg	V	0	0.34
gct	A	8	2.21
gcc	A	0	2.06
gca	A	1	4.06
gcg	A	0	0.67
---	--	------	------
mPD		0.37	0.97
		nPD:	0.39
		N. weight:	0.99
		Sc. PD:	0.12
		Sc. rank:	720.1

NP			
Pos.	440	obs:	exp:
gat	D	1	1.12
gac	D	1	0.88
gaa	E	2610	1626.00
gag	E	135	1119.00
ggt	G	0	0.16
ggc	G	0	0.13
gga	G	1	0.45
ggg	G	0	0.26
---	--	------	------
mPD		0.096	0.48
		nPD:	0.2
		N. weight:	0.5
		Sc. PD:	-0.029
		Sc. rank:	39.0

NP			
Pos.	441	obs:	exp:
ggt	G	9	431.00
ggc	G	407	348.20
gga	G	1990	1245.00
ggg	G	342	724.10
---	--	------	------
mPD		0.44	0.68
		nPD:	0.64
		N. weight:	0.38
		Sc. PD:	0.14
		Sc. rank:	505.2

NP			
Pos.	442	obs:	exp:
cgt	R	30	86.74
cgc	R	1	145.20
cga	R	250	276.20
cgg	R	1981	198.50
aga	R	403	1260.00
agg	R	83	781.10
---	--	------	------
mPD		0.67	0.94
		nPD:	0.71
		N. weight:	2.
		Sc. PD:	0.88
		Sc. rank:	2987.5

NP			
Pos.	443	obs:	exp:
att	I	0	1.06
atc	I	0	0.77
ata	I	3	1.17
act	T	25	674.50
acc	T	4	592.90
aca	T	2696	1246.00
acg	T	17	228.60
gct	A	0	0.74
gcc	A	0	0.69
gca	A	3	1.35
gcg	A	0	0.22
---	--	------	------
mPD		0.037	0.68
		nPD:	0.05
		N. weight:	1.
		Sc. PD:	-0.2
		Sc. rank:	-906.0

NP			
Pos.	444	obs:	exp:
ttt	F	1	0.45
ttc	F	0	0.55
tct	S	399	461.90
tcc	S	2253	434.00
tca	S	92	666.80
tcg	S	2	145.90
agt	S	0	511.10
agc	S	0	526.30
gct	A	0	0.25
gcc	A	1	0.23
gca	A	0	0.45
gcg	A	0	0.07
---	--	------	------
mPD		0.31	1.6
		nPD:	0.19
		N. weight:	1.8
		Sc. PD:	-0.12
		Sc. rank:	79.4

NP			
Pos.	445	obs:	exp:
gat	D	1237	1546.00
gac	D	1511	1202.00
---	--	------	------
mPD		0.50	0.49
		nPD:	1.01
		N. weight:	0.039
		Sc. PD:	0.028
		Sc. rank:	63.5

NP

Pos.	446	obs:	exp:
atg	M	2748	2748.00
---	--	------	------
mPD		0	0
		nPD:	1.
		N. weight:	0.
		Sc. PD:	0
		Sc. rank:	0

NP

Pos.	447	obs:	exp:
cgt	R	1	86.74
cgc	R	2	145.20
cga	R	1929	276.20
cgg	R	372	198.50
aga	R	80	1260.00
agg	R	364	781.10
---	--	------	------
mPD		0.66	0.94
		nPD:	0.71
		N. weight:	1.8
		Sc. PD:	0.78
		Sc. rank:	2662.6

NP

Pos.	448	obs:	exp:
act	T	428	675.50
acc	T	5	593.80
aca	T	2294	1248.00
acg	T	19	229.00
gct	A	0	0.49
gcc	A	0	0.46
gca	A	2	0.90
gcg	A	0	0.15
---	--	------	------
mPD		0.28	0.68
		nPD:	0.41
		N. weight:	0.6
		Sc. PD:	0.087
		Sc. rank:	512.8

NP

Pos.	449	obs:	exp:
gaa	E	2638	1628.00
gag	E	110	1120.00
---	--	------	------
mPD		0.077	0.48
		nPD:	0.16
		N. weight:	0.54
		Sc. PD:	-0.051
		Sc. rank:	-62.8

NP

Pos.	450	obs:	exp:
att	I	475	183.00
atc	I	37	132.30
ata	I	4	200.60
gtt	V	2119	495.60
gtc	V	85	421.20
gta	V	15	563.40
gtg	V	13	751.80
---	--	------	------
mPD		0.41	1.1
		nPD:	0.39
		N. weight:	1.7
		Sc. PD:	0.22
		Sc. rank:	1305.5

NP

Pos.	451	obs:	exp:
att	I	1	973.40
atc	I	2	703.80
ata	I	2741	1067.00
gtt	V	0	0.89
gtc	V	0	0.75
gta	V	4	1.01
gtg	V	0	1.35
---	--	------	------
mPD		0.0051	0.66
		nPD:	0.01
		N. weight:	1.4
		Sc. PD:	-0.32
		Sc. rank:	-2129.6

NP

Pos.	452	obs:	exp:
cgt	R	0	79.48
cgc	R	0	133.10
cga	R	0	253.10
cgg	R	4	181.90
aaa	K	53	139.20
aag	K	176	89.83
aga	R	1548	1155.00
agg	R	966	715.70
ggt	G	0	0.16
ggc	G	0	0.13
gga	G	1	0.45
ggg	G	0	0.26
---	--	------	------
mPD		0.64	1.1
		nPD:	0.6
		N. weight:	0.42
		Sc. PD:	0.14
		Sc. rank:	555.4

NP

Pos.	453	obs:	exp:
att	I	2	1.06
atc	I	0	0.77
ata	I	1	1.17
atg	M	2745	2745.00
---	--	------	------
mPD		0.0022	0.0022
		nPD:	1.
		N. weight:	0.001
		Sc. PD:	0.00071
		Sc. rank:	1.6

NP

Pos.	454	obs:	exp:
atg	M	2748	2748.00
---	--	------	------
mPD		0	0
		nPD:	1.
		N. weight:	0.
		Sc. PD:	0
		Sc. rank:	0

NP

Pos.	455	obs:	exp:
gaa	E	2554	1628.00
gag	E	194	1120.00
---	--	------	------
mPD		0.13	0.48
		nPD:	0.27
		N. weight:	0.43
		Sc. PD:	0.0055
		Sc. rank:	126.3

NP

Pos.	456	obs:	exp:
tct	S	0	414.30
tcc	S	0	389.30
tca	S	0	598.10
tcg	S	0	130.90
att	I	6	2.13
atc	I	0	1.54
ata	I	0	2.33
act	T	6	1.72
acc	T	1	1.51
aca	T	0	3.18
acg	T	0	0.58
aat	N	242	148.90
aac	N	20	113.10
agt	S	2419	458.40
agc	S	44	472.00
ggt	G	10	1.57
ggc	G	0	1.27
gga	G	0	4.53
ggg	G	0	2.63
---	--	------	------
mPD		0.23	1.7
		nPD:	0.13
		N. weight:	2.1
		Sc. PD:	-0.25
		Sc. rank:	-460.2

NP

Pos.	457	obs:	exp:
tct	S	0	0.67
tcc	S	2	0.63
tca	S	2	0.97
tcg	S	0	0.21
act	T	0	2.21
acc	T	6	1.95
aca	T	3	4.09
acg	T	0	0.75
aaa	K	2	1.22
aag	K	0	0.78
agt	S	0	0.74
agc	S	0	0.77
gtt	V	0	0.67
gtc	V	0	0.57
gta	V	3	0.76
gtg	V	0	1.01
gct	A	4	669.20
gcc	A	405	625.50
gca	A	2296	1231.00
gcg	A	25	204.70
---	--	------	------
mPD		0.29	0.69
		nPD:	0.41
		N. weight:	0.63
		Sc. PD:	0.094
		Sc. rank:	555.5

NP

Pos.	458	obs:	exp:
cgt	R	0	12.88
cgc	R	0	21.57
cga	R	0	41.01
cgg	R	0	29.48
aaa	K	302	1421.00
aag	K	2037	917.50
aga	R	246	187.10
agg	R	162	116.00
ggt	G	0	0.16
ggc	G	0	0.13
gga	G	1	0.45
ggg	G	0	0.26
---	--	------	------
mPD		0.57	0.82
		nPD:	0.7
		N. weight:	0.67
		Sc. PD:	0.29
		Sc. rank:	962.3

NP

Pos.	459	obs:	exp:
tct	S	0	5.21
tcc	S	0	4.90
tca	S	30	7.53
tcg	S	1	1.65
cct	P	18	800.00
ccc	P	9	545.80
cca	P	2676	1074.00
ccg	P	8	291.60
act	T	0	1.48
acc	T	0	1.30
aca	T	6	2.73
acg	T	0	0.50
agt	S	0	5.77
agc	S	0	5.94
---	--	------	------
mPD		0.053	0.74
		nPD:	0.07
		N. weight:	1.2
		Sc. PD:	-0.22
		Sc. rank:	-828.3

NP

Pos.	460	obs:	exp:
gat	D	4	2.81
gac	D	1	2.19
gaa	E	2656	1624.00
gag	E	85	1117.00
ggt	G	0	0.31
ggc	G	0	0.25
gga	G	1	0.91
ggg	G	1	0.53
---	--	------	------
mPD		0.066	0.49
		nPD:	0.13
		N. weight:	0.57
		Sc. PD:	-0.067
		Sc. rank:	-122.4

NP

Pos.	461	obs:	exp:
aat	N	3	1.71
aac	N	0	1.29
gat	D	2656	1539.00
gac	D	81	1198.00
gaa	E	8	4.74
gag	E	0	3.26
---	--	------	------
mPD		0.065	0.50
		nPD:	0.13
		N. weight:	0.65
		Sc. PD:	-0.079
		Sc. rank:	-176.6

NP

Pos.	462	obs:	exp:
tta	L	9	253.60
ttg	L	2237	437.70
tct	S	0	0.17
tcc	S	0	0.16
tca	S	0	0.24
tcg	S	1	0.05
ctt	L	0	470.60
ctc	L	0	339.00
cta	L	0	406.80
ctg	L	84	422.40
att	I	0	0.35
atc	I	0	0.26
ata	I	1	0.39
atg	M	18	18.00
agt	S	0	0.19
agc	S	0	0.19
gtt	V	1	88.37
gtc	V	0	75.10
gta	V	1	100.50
gtg	V	396	134.10
---	--	------	------
mPD		0.32	1.3
		nPD:	0.25
		N. weight:	2.1
		Sc. PD:	-0.019
		Sc. rank:	453.1

NP

Pos.	463	obs:	exp:
tct	S	192	462.20
tcc	S	2413	434.30
tca	S	134	667.30
tcg	S	9	146.10
agt	S	0	511.50
agc	S	0	526.60
---	--	------	------
mPD		0.22	1.6
		nPD:	0.14
		N. weight:	2.
		Sc. PD:	-0.23
		Sc. rank:	-393.3

NP

Pos.	464	obs:	exp:
ttt	F	98	1231.00
ttc	F	2650	1517.00
---	--	------	------
mPD		0.069	0.49
		nPD:	0.14
		N. weight:	0.65
		Sc. PD:	-0.073
		Sc. rank:	-115.9

NP

Pos.	465	obs:	exp:
cat	H	10	5.77
cac	H	0	4.23
caa	Q	180	1474.00
cag	Q	2553	1259.00
cgt	R	1	0.13
cgc	R	0	0.21
cga	R	0	0.40
cgg	R	3	0.29
aat	N	0	0.57
aac	N	1	0.43
aga	R	0	1.83
agg	R	0	1.14
---	--	------	------
mPD		0.13	0.51
		nPD:	0.26
		N. weight:	0.76
		Sc. PD:	0.0048
		Sc. rank:	212.0

NP			
Pos.	466	obs:	exp:
ggt	G	0	431.00
ggc	G	0	348.20
gga	G	98	1245.00
ggg	G	2650	724.10
---	--	------	------
mPD		0.069	0.68
		nPD:	0.1
		N. weight:	1.7
		Sc. PD:	-0.25
		Sc. rank:	-724.5

NP			
Pos.	467	obs:	exp:
cgt	R	3	86.74
cgc	R	0	145.20
cga	R	17	276.20
cgg	R	2722	198.50
aga	R	0	1260.00
agg	R	6	781.10
---	--	------	------
mPD		0.019	0.94
		nPD:	0.02
		N. weight:	3.7
		Sc. PD:	-0.84
		Sc. rank:	-5310.9

NP			
Pos.	468	obs:	exp:
ggt	G	13	431.00
ggc	G	0	348.20
gga	G	2718	1245.00
ggg	G	17	724.10
---	--	------	------
mPD		0.022	0.68
		nPD:	0.03
		N. weight:	1.1
		Sc. PD:	-0.23
		Sc. rank:	-1339.0

NP			
Pos.	469	obs:	exp:
gtt	V	117	610.20
gtc	V	2615	518.50
gta	V	14	693.70
gtg	V	2	925.60
---	--	------	------
mPD		0.093	0.74
		nPD:	0.13
		N. weight:	2.1
		Sc. PD:	-0.26
		Sc. rank:	-583.4

NP			
Pos.	470	obs:	exp:
ttt	F	21	1231.00
ttc	F	2727	1517.00
---	--	------	------
mPD		0.015	0.49
		nPD:	0.03
		N. weight:	0.8
		Sc. PD:	-0.17
		Sc. rank:	-1034.3

NP			
Pos.	471	obs:	exp:
gaa	E	0	1628.00
gag	E	2748	1120.00
---	--	------	------
mPD		0	0.48
		nPD:	0.
		N. weight:	1.3
		Sc. PD:	-0.32
		Sc. rank:	-2128.9

NP			
Pos.	472	obs:	exp:
ttt	F	0	3.14
ttc	F	7	3.86
tta	L	7	298.10
ttg	L	1	514.50
ctt	L	20	553.20
ctc	L	2596	398.50
cta	L	113	478.30
ctg	L	2	496.50
att	I	0	0.71
atc	I	2	0.51
ata	I	0	0.78
---	--	------	------
mPD		0.11	1.1
		nPD:	0.1
		N. weight:	2.4
		Sc. PD:	-0.37
		Sc. rank:	-1114.5

NP			
Pos.	473	obs:	exp:
tta	L	0	0.11
ttg	L	1	0.19
tct	S	12	461.20
tcc	S	1	433.40
tca	S	482	665.90
tcg	S	2247	145.70
ctt	L	0	0.20
ctc	L	0	0.15
cta	L	0	0.17
ctg	L	0	0.18
act	T	0	0.98
acc	T	0	0.86
aca	T	0	1.82
acg	T	4	0.33
agt	S	0	510.40
agc	S	0	525.50
gct	A	0	0.25
gcc	A	0	0.23
gca	A	0	0.45
gcg	A	1	0.07
---	--	------	------
mPD		0.30	1.6
		nPD:	0.18
		N. weight:	3.1
		Sc. PD:	-0.22
		Sc. rank:	78.7

NP			
Pos.	474	obs:	exp:
gat	D	22	1546.00
gac	D	2726	1202.00
---	--	------	------
mPD		0.016	0.49
		nPD:	0.03
		N. weight:	1.1
		Sc. PD:	-0.24
		Sc. rank:	-1401.0

NP			
Pos.	475	obs:	exp:
gat	D	3	1.69
gac	D	0	1.31
gaa	E	2739	1626.00
gag	E	6	1119.00
---	--	------	------
mPD		0.0065	0.48
		nPD:	0.01
		N. weight:	0.73
		Sc. PD:	-0.17
		Sc. rank:	-1129.7

NP			
Pos.	476	obs:	exp:
cgt	R	0	0.03
cgc	R	0	0.05
cga	R	0	0.10
cgg	R	0	0.07
aaa	K	29	1669.00
aag	K	2718	1078.00
aga	R	0	0.46
agg	R	1	0.28
---	--	------	------
mPD		0.022	0.48
		nPD:	0.05
		N. weight:	1.3
		Sc. PD:	-0.26
		Sc. rank:	-1270.5

NP			
Pos.	477	obs:	exp:
gct	A	2	673.60
gcc	A	0	629.60
gca	A	2734	1239.00
gcg	A	12	206.10
---	--	------	------
mPD		0.010	0.68
		nPD:	0.01
		N. weight:	1.1
		Sc. PD:	-0.26
		Sc. rank:	-1690.5

NP			
Pos.	478	obs:	exp:
tct	S	0	1.51
tcc	S	0	1.42
tca	S	0	2.19
tcg	S	9	0.48
cgt	R	0	0.03
cgc	R	0	0.05
cga	R	0	0.10
cgg	R	0	0.07
atg	M	2	2.00
act	T	9	661.40
acc	T	0	581.50
aca	T	3	1222.00
acg	T	2677	224.20
agt	S	0	1.68
agc	S	0	1.72
aga	R	0	0.46
agg	R	1	0.28
gct	A	0	11.52
gcc	A	0	10.77
gca	A	0	21.19
gcg	A	47	3.52
---	--	------	------
mPD		0.051	0.72
		nPD:	0.07
		N. weight:	3.5
		Sc. PD:	-0.63
		Sc. rank:	-2389.1

NP			
Pos.	479	obs:	exp:
tct	S	0	162.50
tcc	S	0	152.70
tca	S	0	234.60
tcg	S	0	51.34
cat	H	0	2.31
cac	H	4	1.69
att	I	0	4.61
atc	I	13	3.33
ata	I	0	5.05
act	T	0	3.44
acc	T	14	3.03
aca	T	0	6.36
acg	T	0	1.17
aat	N	0	995.20
aac	N	1751	755.80
agt	S	0	179.80
agc	S	966	185.10
---	--	------	------
mPD		0.47	1.5
		nPD:	0.32
		N. weight:	1.6
		Sc. PD:	0.1
		Sc. rank:	811.2

NP			
Pos.	480	obs:	exp:
tta	L	0	0.11
ttg	L	0	0.19
ctt	L	0	0.20
ctc	L	0	0.15
cta	L	0	0.17
ctg	L	1	0.18
cct	P	1	810.60
ccc	P	0	553.10
cca	P	15	1088.00
ccg	P	2731	295.50
---	--	------	------
mPD		0.012	0.70
		nPD:	0.02
		N. weight:	3.2
		Sc. PD:	-0.72
		Sc. rank:	-4695.8

NP			
Pos.	481	obs:	exp:
att	I	1	954.60
atc	I	2688	690.20
ata	I	2	1046.00
act	T	0	0.25
acc	T	1	0.22
aca	T	0	0.45
acg	T	0	0.08
gtt	V	0	12.43
gtc	V	56	10.57
gta	V	0	14.14
gtg	V	0	18.86
---	--	------	------
mPD		0.043	0.70
		nPD:	0.06
		N. weight:	2.
		Sc. PD:	-0.37
		Sc. rank:	-1575.6

NP			
Pos.	482	obs:	exp:
atg	M	2	2.00
gtt	V	0	609.70
gtc	V	0	518.20
gta	V	31	693.20
gtg	V	2715	924.90
---	--	------	------
mPD		0.024	0.74
		nPD:	0.03
		N. weight:	1.5
		Sc. PD:	-0.32
		Sc. rank:	-1863.4

NP			
Pos.	483	obs:	exp:
cct	P	2712	810.90
ccc	P	36	553.30
cca	P	0	1088.00
ccg	P	0	295.60
---	--	------	------
mPD		0.026	0.70
		nPD:	0.04
		N. weight:	1.7
		Sc. PD:	-0.35
		Sc. rank:	-1946.9

NP			
Pos.	484	obs:	exp:
tct	S	92	462.00
tcc	S	2647	434.10
tca	S	8	667.10
tcg	S	0	146.00
tat	Y	0	0.56
tac	Y	1	0.44
agt	S	0	511.30
agc	S	0	526.50
---	--	------	------
mPD		0.071	1.6
		nPD:	0.04
		N. weight:	2.4
		Sc. PD:	-0.49
		Sc. rank:	-2547.7

NP			
Pos.	485	obs:	exp:
ttt	F	2736	1230.00
ttc	F	8	1514.00
tta	L	1	0.44
ttg	L	1	0.75
ctt	L	2	0.81
ctc	L	0	0.58
cta	L	0	0.70
ctg	L	0	0.73
---	--	------	------
mPD		0.0087	0.50
		nPD:	0.02
		N. weight:	1.1
		Sc. PD:	-0.26
		Sc. rank:	-1674.7

NP

Pos.	486	obs:	exp:
gat	D	1	1543.00
gac	D	2743	1201.00
gaa	E	4	2.37
gag	E	0	1.63
---	--	------	------
mPD		0.0036	0.49
		nPD:	0.01
		N. weight:	1.2
		Sc. PD:	-0.28
		Sc. rank:	-1880.7

NP

Pos.	487	obs:	exp:
tta	L	0	0.11
ttg	L	1	0.19
ctt	L	0	0.20
ctc	L	0	0.15
cta	L	0	0.17
ctg	L	0	0.18
att	I	0	0.35
atc	I	0	0.26
ata	I	1	0.39
atg	M	2746	2746.00
---	--	------	------
mPD		0.0015	0.0019
		nPD:	0.76
		N. weight:	0.0014
		Sc. PD:	0.00066
		Sc. rank:	2.1

NP

Pos.	488	obs:	exp:
tct	S	0	441.40
tcc	S	0	414.70
tca	S	0	637.20
tcg	S	0	139.50
cgt	R	1	0.03
cgc	R	0	0.05
cga	R	0	0.10
cgg	R	0	0.07
aat	N	121	69.34
aac	N	1	52.66
agt	S	2582	488.40
agc	S	42	502.90
aga	R	0	0.46
agg	R	0	0.28
ggt	G	1	0.16
ggc	G	0	0.13
gga	G	0	0.45
ggg	G	0	0.26
---	--	------	------
mPD		0.12	1.7
		nPD:	0.07
		N. weight:	2.2
		Sc. PD:	-0.4
		Sc. rank:	-1596.3

NP

Pos.	489	obs:	exp:
att	I	3	1.06
atc	I	0	0.77
ata	I	0	1.17
aat	N	2275	1559.00
aac	N	467	1183.00
aaa	K	2	1.22
aag	K	0	0.78
gat	D	1	0.56
gac	D	0	0.44
---	--	------	------
mPD		0.29	0.49
		nPD:	0.58
		N. weight:	0.23
		Sc. PD:	0.07
		Sc. rank:	286.0

NP

Pos.	490	obs:	exp:
gat	D	1	0.56
gac	D	0	0.44
gaa	E	2451	1627.00
gag	E	295	1119.00
ggt	G	0	0.16
ggc	G	0	0.13
gga	G	1	0.45
ggg	G	0	0.26
---	--	------	------
mPD		0.19	0.48
		nPD:	0.4
		N. weight:	0.32
		Sc. PD:	0.043
		Sc. rank:	257.8

NP

Pos.	491	obs:	exp:
tct	S	0	0.84
tcc	S	0	0.79
tca	S	0	1.21
tcg	S	0	0.27
agt	S	5	0.93
agc	S	0	0.96
gtt	V	0	0.22
gtc	V	0	0.19
gta	V	0	0.25
gtg	V	1	0.34
ggt	G	45	430.10
ggc	G	0	347.40
gga	G	1198	1242.00
ggg	G	1499	722.50
---	--	------	------
mPD		0.52	0.69
		nPD:	0.75
		N. weight:	0.51
		Sc. PD:	0.24
		Sc. rank:	771.1

NP

Pos.	492	obs:	exp:
ttt	F	2	0.90
ttc	F	0	1.10
tct	S	2734	461.70
tcc	S	10	433.80
tca	S	0	666.60
tcg	S	1	145.90
tat	Y	1	0.56
tac	Y	0	0.44
agt	S	0	510.90
agc	S	0	526.10
---	--	------	------
mPD		0.010	1.6
		nPD:	0.01
		N. weight:	2.5
		Sc. PD:	-0.61
		Sc. rank:	-4031.3

NP

Pos.	493	obs:	exp:
tct	S	1	0.17
tcc	S	0	0.16
tca	S	0	0.24
tcg	S	0	0.05
tat	Y	2747	1539.00
tac	Y	0	1208.00
agt	S	0	0.19
agc	S	0	0.19
---	--	------	------
mPD		0.00073	0.49
		nPD:	0.
		N. weight:	0.84
		Sc. PD:	-0.2
		Sc. rank:	-1353.5

NP

Pos.	494	obs:	exp:
ttt	F	1	1231.00
ttc	F	2746	1516.00
tct	S	0	0.17
tcc	S	1	0.16
tca	S	0	0.24
tcg	S	0	0.05
agt	S	0	0.19
agc	S	0	0.19
---	--	------	------
mPD		0.0015	0.50
		nPD:	0.
		N. weight:	0.85
		Sc. PD:	-0.21
		Sc. rank:	-1377.2

NP

Pos.	495	obs:	exp:
ttt	F	19	1231.00
ttc	F	2729	1517.00
---	--	------	------
mPD		0.014	0.49
		nPD:	0.03
		N. weight:	0.8
		Sc. PD:	-0.18
		Sc. rank:	-1068.2

NP

Pos.	496	obs:	exp:
ggt	G	0	431.00
ggc	G	0	348.20
gga	G	2748	1245.00
ggg	G	0	724.10
---	--	------	------
mPD		0	0.68
		nPD:	0.
		N. weight:	1.1
		Sc. PD:	-0.28
		Sc. rank:	-1878.1

NP

Pos.	497	obs:	exp:
gat	D	137	1546.00
gac	D	2611	1202.00
---	--	------	------
mPD		0.095	0.49
		nPD:	0.19
		N. weight:	0.89
		Sc. PD:	-0.056
		Sc. rank:	58.4

NP

Pos.	498	obs:	exp:
tct	S	0	2.52
tcc	S	0	2.37
tca	S	0	3.64
tcg	S	0	0.80
act	T	1	0.25
acc	T	0	0.22
aca	T	0	0.45
acg	T	0	0.08
aat	N	2714	1549.00
aac	N	12	1177.00
aaa	K	6	3.65
aag	K	0	2.35
agt	S	15	2.79
agc	S	0	2.87
---	--	------	------
mPD		0.025	0.51
		nPD:	0.05
		N. weight:	0.79
		Sc. PD:	-0.16
		Sc. rank:	-738.9

NP

Pos.	499	obs:	exp:
gct	A	17	673.60
gcc	A	0	629.60
gca	A	2727	1239.00
gcg	A	4	206.10
---	--	------	------
mPD		0.015	0.68
		nPD:	0.02
		N. weight:	1.1
		Sc. PD:	-0.24
		Sc. rank:	-1542.0

NP

Pos.	500	obs:	exp:
gct	A	0	0.25
gcc	A	0	0.23
gca	A	0	0.45
gcg	A	1	0.07
gat	D	1	1.12
gac	D	1	0.88
gaa	E	191	1626.00
gag	E	2554	1119.00
---	--	------	------
mPD		0.13	0.48
		nPD:	0.27
		N. weight:	0.9
		Sc. PD:	0.011
		Sc. rank:	262.5

NP

Pos.	501	obs:	exp:
gaa	E	117	1628.00
gag	E	2631	1120.00
---	--	------	------
mPD		0.082	0.48
		nPD:	0.17
		N. weight:	1.
		Sc. PD:	-0.087
		Sc. rank:	-64.3

NP

Pos.	502	obs:	exp:
ttt	F	27	12.10
ttc	F	0	14.90
tct	S	1	0.17
tcc	S	0	0.16
tca	S	0	0.24
tcg	S	0	0.05
tat	Y	2605	1524.00
tac	Y	115	1196.00
agt	S	0	0.19
agc	S	0	0.19
---	--	------	------
mPD		0.10	0.51
		nPD:	0.2
		N. weight:	0.61
		Sc. PD:	-0.036
		Sc. rank:	45.2

NP

Pos.	503	obs:	exp:
aat	N	0	0.57
aac	N	1	0.43
gct	A	0	0.49
gcc	A	2	0.46
gca	A	0	0.90
gcg	A	0	0.15
gat	D	58	1543.00
gac	D	2685	1200.00
gaa	E	1	0.59
gag	E	0	0.41
ggt	G	0	0.16
ggc	G	1	0.13
gga	G	0	0.45
ggg	G	0	0.26
---	--	------	------
mPD		0.045	0.50
		nPD:	0.09
		N. weight:	1.
		Sc. PD:	-0.17
		Sc. rank:	-565.3

NP

Pos.	504	obs:	exp:
tct	S	0	423.70
tcc	S	0	398.10
tca	S	0	611.70
tcg	S	0	133.90
cat	H	1	0.58
cac	H	0	0.42
att	I	1	0.71
atc	I	1	0.51
ata	I	0	0.78
aat	N	204	128.50
aac	N	22	97.54
agt	S	2515	468.80
agc	S	4	482.80
---	--	------	------
mPD		0.17	1.7
		nPD:	0.1
		N. weight:	2.2
		Sc. PD:	-0.34
		Sc. rank:	-937.0

NP

Pos.	505	obs:	exp:
taa	.	384	1169.00
tag	.	16	723.90
tga	.	2348	855.00
---	--	------	------
mPD		0.26	0.82
		nPD:	0.32
		N. weight:	0.99
		Sc. PD:	0.057
		Sc. rank:	482.4

Amino acid level conservation per codon

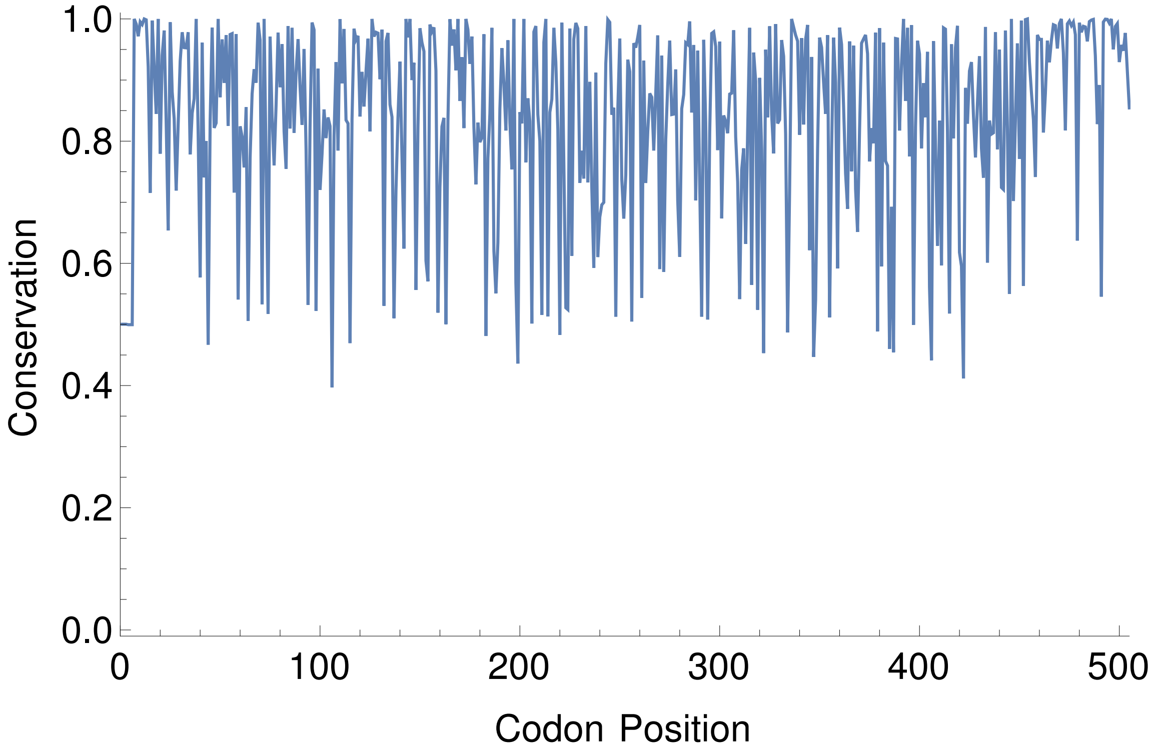

Conservation analysis plots

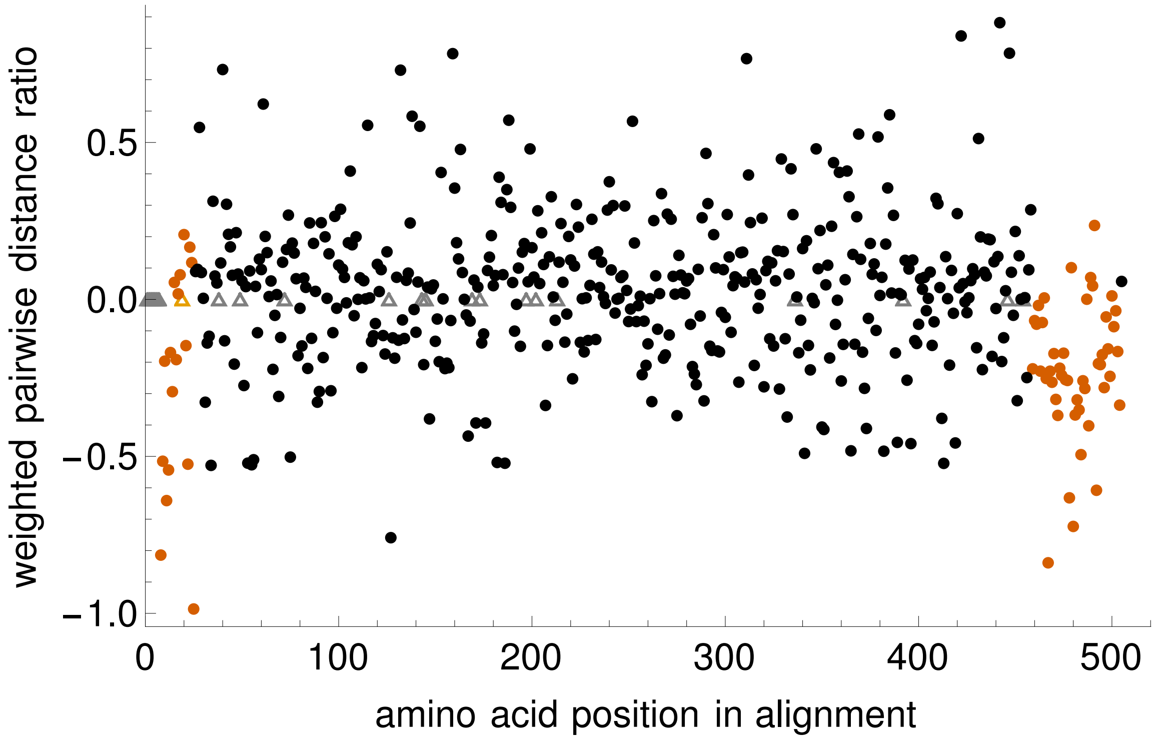

NP

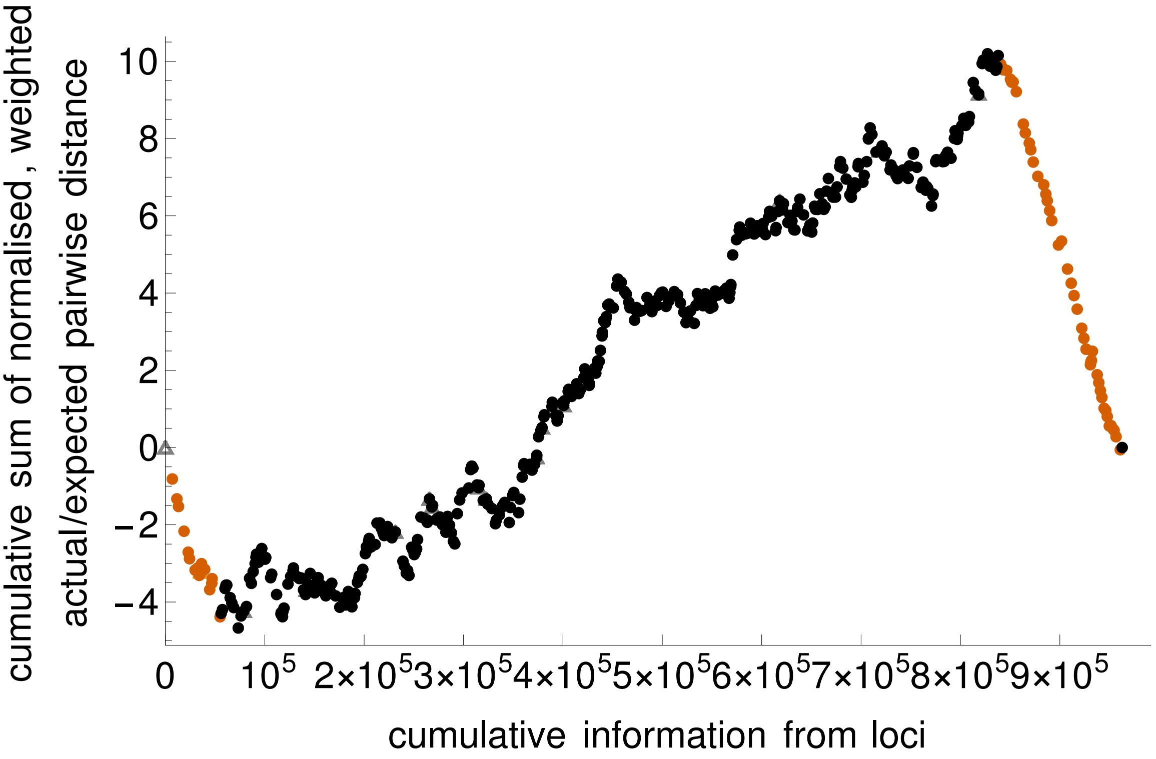

NP

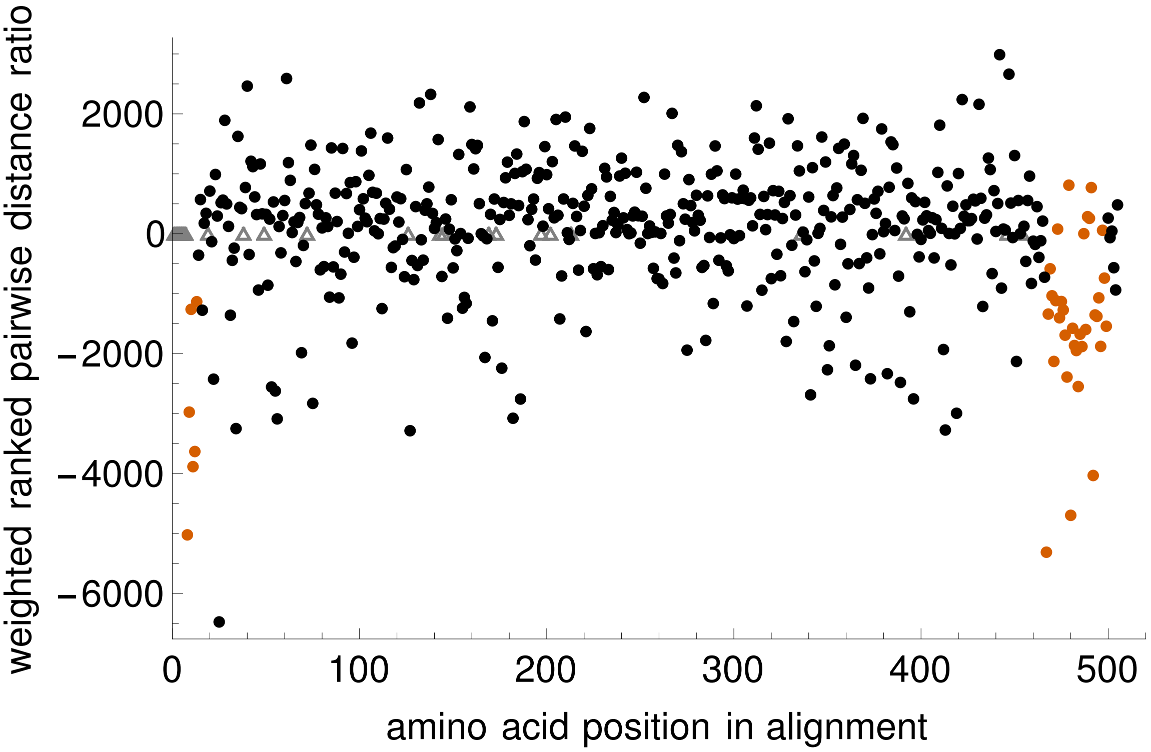
weighted ranked pairwise distance ratio
2000
0
−2000
−4000
−6000
0
100
200
300
400
500
amino acid position in alignment

NP

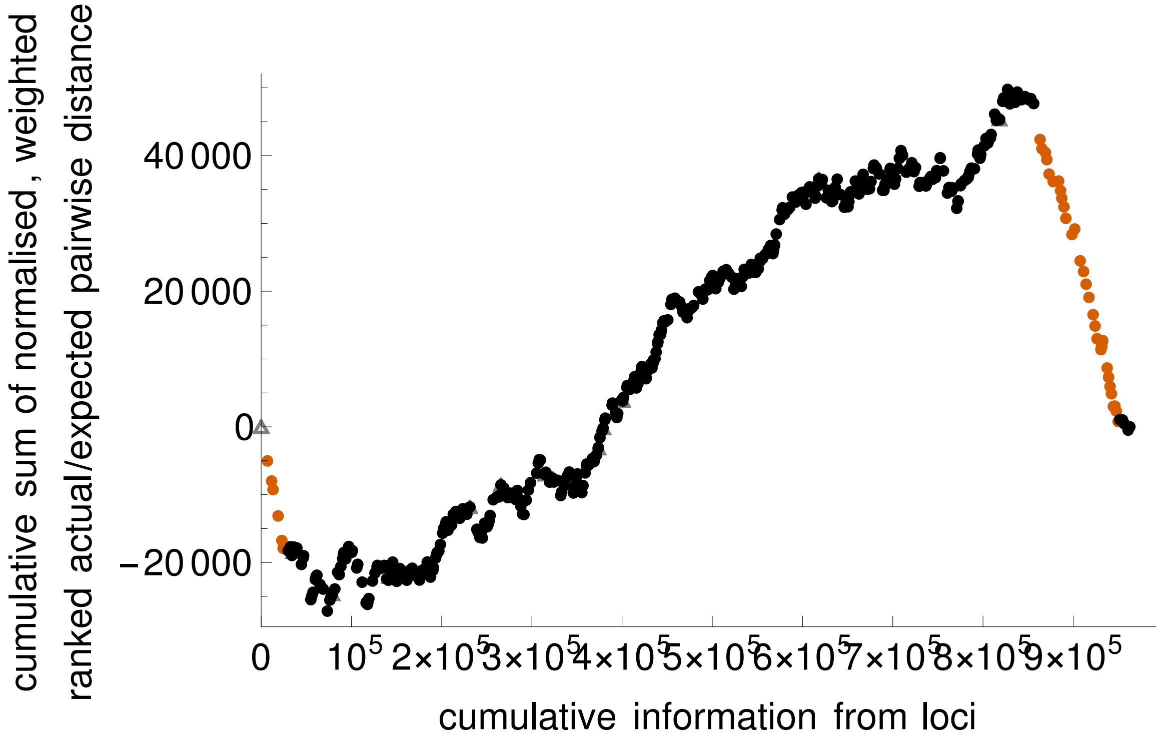
cumulative sum of normalised, weighted ranked actual/expected pairwise distance
40 000
20 000
0
−20 000
0
10^5
2×10^5
3×10^5
4×10^5
5×10^5
6×10^5
7×10^5
8×10^5
9×10^5
cumulative information from loci

NP

NA

Gene length histogram

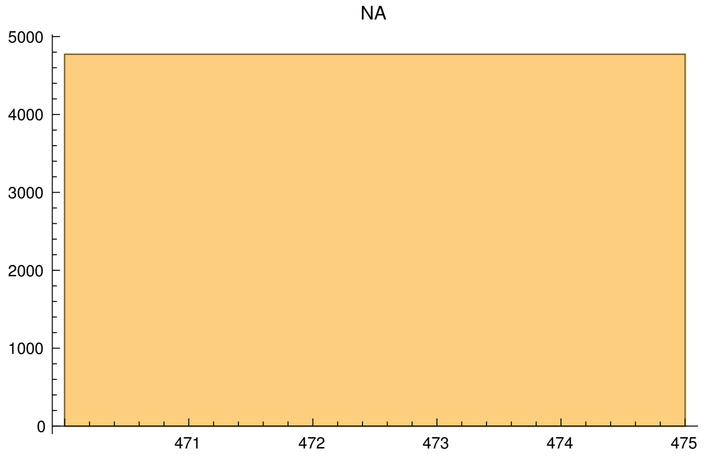

Information vs. nPD

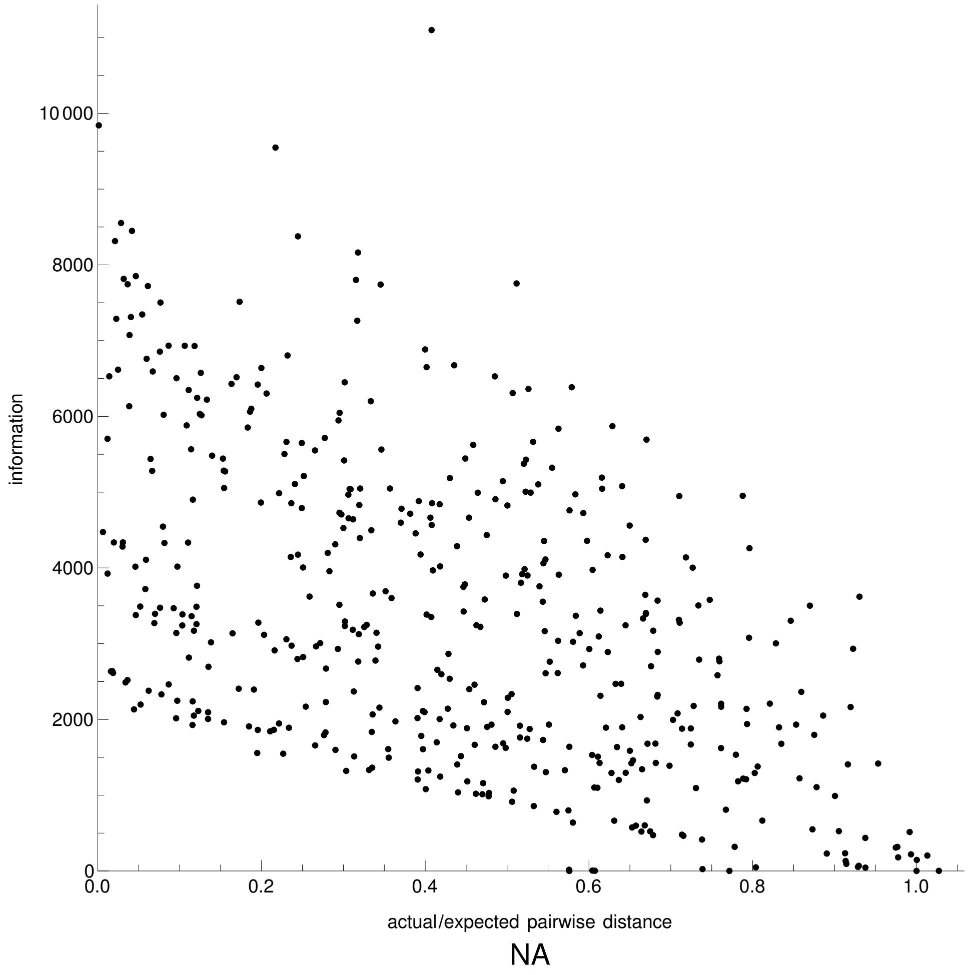

Example sequences highlighted by regions found in analyses to be conserved

Interesting points (by weighted raw PD) highlighted for gene NA:
------ATGAATCCAAATCAGAAGATAATAACAATTGGCTCTGTTTCTCTCTCCATTGCAGCAGCATGCTTCCTCA⋱
TGCAAATTGCCATCCTGATAACTACTGTAACATTACATTTCAAGCAGTATGAGTGCGGC ---TCC---CCTGCG----
--AACAAC---CAAGTAATGACATGTGAA ---TCAATAATAATAGAAAGAAACACAACTGAGATA ------------
GTGTACTTAACTAACACCACCATT ---------GAGAAA-----------------------------GAAAAATACCC⋱
CAAAACAGTGGAGTATAGAAATTGGTCAATGCCCCAATGCAAAATCACAGGGTTTGCACCCTTTTCCAAAGACAA⋱
TTCAATTCGGCTTTCTGCTGGTGGGGACATTTGGGTAACGAGAGAGCCTTATGTATCATGCGAACCTGGCAAATG⋱
TTACCAATTTGCACTCGGGCAAGGAACCACACTAGACAACAAACATTCAAATGACACAATACATGACAGAACCCC⋱
TTATCGAACTCTATTGATGAATGAGTTGGGT ---GTTCCATTTCATTTAGGAACAAGGCAAGTGTGTATAGCGTGG⋱
TCCAGCTCAAGTTGTTACGATGGAAAAGCATGGCTGCATGTTTGTATCACTGGGCATGATAAAAATGCAACTGCT⋱
AGCTTTATTTACGATGGTAGGCTTGTAGACAGTATTGGTTCGTGGTCTATGAATATCCTCAGAACCCAGGAATCG⋱
GAATGCGTTTGCATCAATGGGACTTGTACAGTAGTAATGACTGATGGAAGTGCTTCAGGAAAAGCTGATACCAGA⋱
ATATTGTTCATTGAAGAGGGGAAAATTGTTCATATTAGCCCGTTAGCAGGGAGTGCCCAACATGTAGAGGAGTGT⋱
TCCTGTTACCCTCGATATCCTGGGGTCAGATGTATTTGCAGAGACAACTGGAAAGGCTCTAACAGGCCCATTGTA⋱
GATATAAATGTAGAA ---GATTATAGCATTGACTCCAGTTATGTGTGTTCAGGACTTGTTGGCGACACACCCAGGA⋱
ACAACGACAGATCTAGTAGTAGCTATTGTCGTAATCCTAACAATGAGAAGGGGGGCCATGGGGTGAAAGGCTGGG⋱
CTTTTGACGATGGAAATGACCTGTGGATGGGAAGAACAATCAGCGAGGATTCACGCTCAGGTTATGAAACATTTA⋱
AAGTCATTGGTGGTTGGTCCACACCTAATTCCAAATTGCAGATAAACAGGCAAGTAATAGTTGATAGCAACAATA⋱
GGTCAGGTTATTCTGGTATTTTC ------------TCTGTTGAAAGCAAAAGCTGCATCAATAGATGTTTTTATGTG⋱
GAGTTGATAAGAGGAAGAAGAACAGAAGCTAGAGTCTGGTGGACC
TCAAACAGTATTGTTGTGTTTTGTGGCACTTCAGGTACCTATGGAACAGGCTCAT
GGCCTGATGGGGCAGACATCAATCTT ---ATGCCTATATAA

Interesting points (by weighted ranked PD) highlighted for gene NA:
------ATGAATCCAAATCAGAAGATAATAACAATTGGCTCTGTTTCTCTCTCCATTGCAGCAGCATGCTTCCTCATG⋱
CAAATTGCCATCCTGATAACTACTGTAACATTACATTTCAAGCAGTATGAGTGCGGC ---TCC---CCTGCG------
AACAAC---CAAGTAATGACATGTGAA ---TCAATAATAATAGAAAGAAACACAACTGAGATA ------------
GTGTACTTAACTAACACCACCATT ---------GAGAAA-----------------------------GAAAAATACCC⋱
CAAAACAGTGGAGTATAGAAATTGGTCAATGCCCCAATGCAAAATCACAGGGTTTGCACCCTTTTCCAAAGACAA⋱
TTCAATTCGGCTTTCTGCTGGTGGGGACATTTGGGTAACGAGAGAGCCTTATGTATCATGCGAACCTGGCAAATG⋱
TTACCAATTTGCACTCGGGCAAGGAACCACACTAGACAACAAACATTCAAATGACACAATACATGACAGAACCCC⋱
TTATCGAACTCTATTGATGAATGAGTTGGGT ---GTTCCATTTCATTTAGGAACAAGGCAAGTGTGTATAGCGTGG⋱
TCCAGCTCAAGTTGTTACGATGGAAAAGCATGGCTGCATGTTTGTATCACTGGGCATGATAAAAATGCAACTGCT⋱
AGCTTTATTTACGATGGTAGGCTTGTAGACAGTATTGGTTCGTGGTCTATGAATATCCTCAGAACCCAGGAATCG⋱
GAATGCGTTTGCATCAATGGGACTTGTACAGTAGTAATGACTGATGGAAGTGCTTCAGGAAAAGCTGATACCAGA⋱
ATATTGTTCATTGAAGAGGGGAAAATTGTTCATATTAGCCCGTTAGCAGGGAGTGCCCAACATGTAGAGGAGTGT⋱
TCCTGTTACCCTCGATATCCTGGGGTCAGATGTATTTGCAGAGACAACTGGAAAGGCTCTAACAGGCCCATTGTA⋱
GATATAAATGTAGAA ---GATTATAGCATTGACTCCAGTTATGTGTGTTCAGGACTTGTTGGCGACACACCCAGGA⋱
ACAACGACAGATCTAGTAGTAGCTATTGTCGTAATCCTAACAATGAGAAGGGGGGCCATGGGGTGAAAGGCTGGG⋱
CTTTTGACGATGGAAATGACCTGTGGATGGGAAGAACAATCAGCGAGGATTCACGCTCAGGTTATGAAACATTTA⋱
AAGTCATTGGTGGTTGGTCCACACCTAATTCCAAATTGCAGATAAACAGGCAAGTAATAGTTGATAGCAACAATA⋱
GGTCAGGTTATTCTGGTATTTTC ------------TCTGTTGAAAGCAAAAGCTGCATCAATAGATGTTTTTATGTG⋱
GAGTTGATAAGAGGAAGAAGAACAGAAGCTAGAGTCTGGTGGACC
TCAAACAGTATTGTTGTGTTTTGTGGCACTTCAGGTACCTATGGAACAGGCTCAT
GGCCTGATGGGGCAGACATCAATCTT ---ATGCCTATATAA

Per codon conservation report

NA			
Pos.	1	obs:	exp:
atg	M	7	7.00
---	-	4765	4765.00
---	--	------	------
mPD		0	0
		nPD:	1.
		N. weight:	0.
		Sc. PD:	0
		Sc. rank:	0

NA			
Pos.	2	obs:	exp:
aaa	K	7	4.25
aag	K	0	2.75
---	-	4765	4765.00
---	--	------	------
mPD		0	1.0×10^{-6}
		nPD:	0.
		N. weight:	0.
		Sc. PD:	0
		Sc. rank:	0

NA			
Pos.	3	obs:	exp:
atg	M	4772	4772.00
---	--	------	------
mPD		0	0
		nPD:	1.
		N. weight:	0.
		Sc. PD:	0
		Sc. rank:	0

NA			
Pos.	4	obs:	exp:
tct	S	0	0.17
tcc	S	0	0.16
tca	S	0	0.24
tcg	S	0	0.05
tat	Y	1	0.56
tac	Y	0	0.44
cat	H	2	1.15
cac	H	0	0.85
act	T	0	0.49
acc	T	2	0.43
aca	T	0	0.91
acg	T	0	0.17
aat	N	4625	2708.00
aac	N	139	2056.00
agt	S	1	0.19
agc	S	0	0.19
gat	D	2	1.12
gac	D	0	0.88
---	--	------	------
mPD		0.061	0.49
		nPD:	0.12
		N. weight:	0.69
		Sc. PD:	-0.15
		Sc. rank:	-982.3

NA			
Pos.	5	obs:	exp:
tct	S	0	4.21
tcc	S	0	3.95
tca	S	25	6.07
tcg	S	0	1.33
cct	P	8	1401.00
ccc	P	1	955.50
cca	P	4720	1879.00
ccg	P	17	510.50
agt	S	0	4.65
agc	S	0	4.79
gct	A	0	0.25
gcc	A	0	0.23
gca	A	1	0.45
gcg	A	0	0.07
---	--	------	------
mPD		0.022	0.72
		nPD:	0.03
		N. weight:	1.4
		Sc. PD:	-0.41
		Sc. rank:	-3343.7

NA			
Pos.	6	obs:	exp:
tct	S	0	1.35
tcc	S	0	1.26
tca	S	0	1.94
tcg	S	0	0.43
att	I	3	1.06
atc	I	0	0.77
ata	I	0	1.17
act	T	2	0.49
acc	T	0	0.43
aca	T	0	0.91
acg	T	0	0.17
aat	N	4678	2704.00
aac	N	79	2053.00
aaa	K	1	0.61
aag	K	0	0.39
agt	S	8	1.49
agc	S	0	1.53
gat	D	1	0.56
gac	D	0	0.44
---	--	------	------
mPD		0.039	0.50
		nPD:	0.08
		N. weight:	0.76
		Sc. PD:	-0.19
		Sc. rank:	-1418.5

NA			
Pos.	7	obs:	exp:
tct	S	0	0.17
tcc	S	0	0.16
tca	S	1	0.24
tcg	S	0	0.05
tat	Y	0	0.56
tac	Y	1	0.44
cct	P	0	0.59
ccc	P	0	0.40
cca	P	2	0.79
ccg	P	0	0.22
cat	H	0	1.15
cac	H	2	0.85
caa	Q	4295	2539.00
cag	Q	414	2170.00
cgt	R	0	0.09
cgc	R	0	0.16
cga	R	3	0.30
cgg	R	0	0.22
aaa	K	52	32.82
aag	K	2	21.18
agt	S	0	0.19
agc	S	0	0.19
aga	R	0	1.38
agg	R	0	0.85
---	--	------	------
mPD		0.19	0.52
		nPD:	0.35
		N. weight:	0.52
		Sc. PD:	0.0017
		Sc. rank:	119.5

NA			
Pos.	8	obs:	exp:
caa	Q	0	0.54
cag	Q	1	0.46
cgt	R	0	13.23
cgc	R	0	22.15
cga	R	0	42.11
cgg	R	0	30.27
atg	M	1	1.00
act	T	0	0.25
acc	T	0	0.22
aca	T	1	0.45
acg	T	0	0.08
aaa	K	251	2644.00
aag	K	4099	1706.00
aga	R	53	192.20
agg	R	366	119.10
---	--	------	------
mPD		0.28	0.69
		nPD:	0.41
		N. weight:	1.1
		Sc. PD:	0.056
		Sc. rank:	556.8

NA			
Pos.	9	obs:	exp:
tta	L	3	0.33
ttg	L	0	0.56
ctt	L	0	0.61
ctc	L	0	0.44
cta	L	0	0.52
ctg	L	0	0.54
att	I	3	1691.00
atc	I	2	1223.00
ata	I	4762	1853.00
atg	M	1	1.00
gtt	V	0	0.22
gtc	V	0	0.19
gta	V	1	0.25
gtg	V	0	0.34
---	--	------	------
mPD		0.0042	0.66
		nPD:	0.01
		N. weight:	1.5
		Sc. PD:	-0.46
		Sc. rank:	-3812.3

NA			
Pos.	10	obs:	exp:
att	I	83	1667.00
atc	I	4	1205.00
ata	I	4613	1827.00
atg	M	62	62.00
act	T	0	0.25
acc	T	0	0.22
aca	T	1	0.45
acg	T	0	0.08
aaa	K	1	0.61
aag	K	0	0.39
gtt	V	0	1.78
gtc	V	0	1.51
gta	V	8	2.02
gtg	V	0	2.69
---	--	------	------
mPD		0.065	0.67
		nPD:	0.1
		N. weight:	1.3
		Sc. PD:	-0.31
		Sc. rank:	-2238.5

NA			
Pos.	11	obs:	exp:
tct	S	0	0.34
tcc	S	0	0.32
tca	S	1	0.49
tcg	S	1	0.11
cct	P	0	0.30
ccc	P	0	0.20
cca	P	1	0.40
ccg	P	0	0.11
att	I	0	0.71
atc	I	0	0.51
ata	I	2	0.78
atg	M	2	2.00
act	T	13	1170.00
acc	T	4	1029.00
aca	T	3245	2162.00
acg	T	1496	396.80
agt	S	0	0.37
agc	S	0	0.38
gct	A	0	1.72
gcc	A	0	1.60
gca	A	6	3.16
gcg	A	1	0.52
---	--	------	------
mPD		0.44	0.69
		nPD:	0.64
		N. weight:	1.1
		Sc. PD:	0.29
		Sc. rank:	2100.0

NA			
Pos.	12	obs:	exp:
tta	L	0	0.33
ttg	L	0	0.56
ctt	L	3	0.61
ctc	L	0	0.44
cta	L	0	0.52
ctg	L	0	0.54
att	I	4591	1680.00
atc	I	142	1214.00
ata	I	2	1841.00
atg	M	16	16.00
gtt	V	18	4.00
gtc	V	0	3.40
gta	V	0	4.54
gtg	V	0	6.06
---	--	------	------
mPD		0.074	0.67
		nPD:	0.11
		N. weight:	1.4
		Sc. PD:	-0.31
		Sc. rank:	-2299.2

NA			
Pos.	13	obs:	exp:
tct	S	0	1.35
tcc	S	0	1.26
tca	S	0	1.94
tcg	S	0	0.43
agt	S	4	1.49
agc	S	4	1.53
ggt	G	536	747.20
ggc	G	4191	603.70
gga	G	34	2158.00
ggg	G	3	1255.00
---	--	------	------
mPD		0.22	0.69
		nPD:	0.32
		N. weight:	2.5
		Sc. PD:	-0.084
		Sc. rank:	-234.0

NA			
Pos.	14	obs:	exp:
ttt	F	49	22.40
ttc	F	1	27.60
tct	S	4335	791.00
tcc	S	277	743.30
tca	S	57	1142.00
tcg	S	34	250.00
tat	Y	1	1.12
tac	Y	1	0.88
act	T	17	4.18
acc	T	0	3.68
aca	T	0	7.72
acg	T	0	1.42
agt	S	0	875.30
agc	S	0	901.30
---	--	------	------
mPD		0.17	1.6
		nPD:	0.11
		N. weight:	2.3
		Sc. PD:	-0.51
		Sc. rank:	-3757.0

NA			
Pos.	15	obs:	exp:
ttt	F	24	10.75
ttc	F	0	13.25
tta	L	0	0.11
ttg	L	0	0.19
ctt	L	0	0.20
ctc	L	1	0.15
cta	L	0	0.17
ctg	L	0	0.18
att	I	996	359.00
atc	I	13	259.60
ata	I	3	393.40
atg	M	1	1.00
gtt	V	2818	791.80
gtc	V	581	672.90
gta	V	152	900.20
gtg	V	15	1201.00
gct	A	154	39.47
gcc	A	3	36.89
gca	A	4	72.57
gcg	A	0	12.07
gat	D	2	1.12
gac	D	0	0.88
ggt	G	5	0.78
ggc	G	0	0.63
gga	G	0	2.26
ggg	G	0	1.32
---	--	------	------
mPD		0.69	1.2
		nPD:	0.6
		N. weight:	1.4
		Sc. PD:	0.32
		Sc. rank:	2471.0

NA			
Pos.	16	obs:	exp:
tct	S	4020	802.50
tcc	S	742	754.00
tca	S	6	1159.00
tcg	S	3	253.60
tgt	C	1	0.43
tgc	C	0	0.57
agt	S	0	888.00
agc	S	0	914.30
---	--	------	------
mPD		0.27	1.6
		nPD:	0.16
		N. weight:	2.1
		Sc. PD:	-0.36
		Sc. rank:	-2486.1

,

NA			
Pos.	17	obs:	exp:
ttt	F	0	0.90
ttc	F	2	1.10
tta	L	31	476.30
ttg	L	3	822.10
ctt	L	1032	884.00
ctc	L	2998	636.80
cta	L	312	764.30
ctg	L	1	793.40
att	I	8	137.30
atc	I	355	99.26
ata	I	24	150.50
atg	M	4	4.00
gtt	V	0	0.44
gtc	V	2	0.38
gta	V	0	0.50
gtg	V	0	0.67
---	--	------	------
mPD		0.62	1.2
		nPD:	0.5
		N. weight:	1.6
		Sc. PD:	0.22
		Sc. rank:	1726.3

,

NA			
Pos.	18	obs:	exp:
ttt	F	0	0.45
ttc	F	1	0.55
tta	L	0	0.22
ttg	L	0	0.38
tct	S	4	14.30
tcc	S	80	13.43
tca	S	0	20.64
tcg	S	0	4.52
tgt	C	0	0.43
tgc	C	1	0.57
ctt	L	0	0.40
ctc	L	2	0.29
cta	L	0	0.35
ctg	L	0	0.36
att	I	508	1018.00
atc	I	2336	735.90
ata	I	25	1115.00
atg	M	34	34.00
act	T	334	415.70
acc	T	1344	365.40
aca	T	4	767.90
acg	T	8	140.90
aat	N	0	0.57
aac	N	1	0.43
agt	S	0	15.82
agc	S	1	16.29
gtt	V	2	6.88
gtc	V	29	5.85
gta	V	0	7.83
gtg	V	0	10.44
gct	A	44	14.22
gcc	A	14	13.29
gca	A	0	26.14
gcg	A	0	4.35
---	--	------	------
mPD		0.87	1.2
		nPD:	0.72
		N. weight:	1.4
		Sc. PD:	0.46
		Sc. rank:	3087.4

,

NA			
Pos.	19	obs:	exp:
ttt	F	15	6.72
ttc	F	0	8.28
tta	L	0	96.96
ttg	L	0	167.40
tct	S	0	0.17
tcc	S	0	0.16
tca	S	0	0.24
tcg	S	0	0.05
ctt	L	847	179.90
ctc	L	0	129.60
cta	L	3	155.60
ctg	L	41	161.50
att	I	3338	1322.00
atc	I	26	956.20
ata	I	364	1449.00
atg	M	11	11.00
act	T	68	18.20
acc	T	0	16.00
aca	T	6	33.63
acg	T	0	6.17
agt	S	1	0.19
agc	S	0	0.19
gtt	V	43	11.55
gtc	V	8	9.81
gta	V	1	13.13
gtg	V	0	17.52
---	--	------	------
mPD		0.53	1.1
		nPD:	0.5
		N. weight:	1.3
		Sc. PD:	0.17
		Sc. rank:	1373.9

,

NA			
Pos.	20	obs:	exp:
tct	S	0	2.35
tcc	S	12	2.21
tca	S	2	3.40
tcg	S	0	0.74
act	T	0	21.89
acc	T	11	19.25
aca	T	77	40.44
acg	T	1	7.42
agt	S	0	2.61
agc	S	0	2.68
gtt	V	0	0.22
gtc	V	1	0.19
gta	V	0	0.25
gtg	V	0	0.34
gct	A	83	1143.00
gcc	A	3593	1069.00
gca	A	941	2102.00
gcg	A	47	349.80
ggt	G	1	0.63
ggc	G	0	0.51
gga	G	3	1.81
ggg	G	0	1.05
---	--	------	------
mPD		0.42	0.73
		nPD:	0.58
		N. weight:	1.1
		Sc. PD:	0.24
		Sc. rank:	1853.3

,

NA			
Pos.	21	obs:	exp:
tta	L	1	0.65
ttg	L	0	1.13
tct	S	0	2.19
tcc	S	0	2.05
tca	S	13	3.16
tcg	S	0	0.69
ctt	L	0	1.21
ctc	L	0	0.87
cta	L	5	1.05
ctg	L	0	1.09
att	I	0	83.72
atc	I	7	60.53
ata	I	229	91.75
atg	M	63	63.00
act	T	8	863.10
acc	T	74	758.80
aca	T	3384	1594.00
acg	T	43	292.60
agt	S	0	2.42
agc	S	0	2.49
gtt	V	0	2.44
gtc	V	0	2.08
gta	V	11	2.78
gtg	V	0	3.71
gct	A	1	227.70
gcc	A	0	212.90
gca	A	924	418.70
gcg	A	4	69.67
ggt	G	0	0.78
ggc	G	0	0.63
gga	G	5	2.26
ggg	G	0	1.32
---	--	------	------
mPD		0.53	1.1
		nPD:	0.47
		N. weight:	1.1
		Sc. PD:	0.11
		Sc. rank:	977.1

,

NA			
Pos.	22	obs:	exp:
tta	L	64	14.26
ttg	L	11	24.61
ctt	L	0	26.46
ctc	L	0	19.06
cta	L	51	22.87
ctg	L	5	23.75
att	I	8	1006.00
atc	I	0	727.70
ata	I	2829	1103.00
atg	M	1601	1601.00
act	T	0	35.67
acc	T	0	31.35
aca	T	115	65.89
acg	T	30	12.09
gtt	V	1	11.77
gtc	V	0	10.00
gta	V	40	13.38
gtg	V	12	17.85
gct	A	3	1.23
gcc	A	0	1.15
gca	A	2	2.25
gcg	A	0	0.37
---	--	------	------
mPD		0.59	0.87
		nPD:	0.68
		N. weight:	0.94
		Sc. PD:	0.29
		Sc. rank:	2079.3

,

NA			
Pos.	23	obs:	exp:
tct	S	0	0.34
tcc	S	0	0.32
tca	S	0	0.49
tcg	S	0	0.11
tgt	C	734	2051.00
tgc	C	4032	2715.00
agt	S	0	0.37
agc	S	2	0.38
ggt	G	0	0.63
ggc	G	4	0.51
gga	G	0	1.81
ggg	G	0	1.05
---	--	------	------
mPD		0.26	0.49
		nPD:	0.53
		N. weight:	0.28
		Sc. PD:	0.047
		Sc. rank:	386.2

,

NA			
Pos.	24	obs:	exp:
ttt	F	607	1815.00
ttc	F	3443	2235.00
tta	L	371	76.83
ttg	L	16	132.60
tct	S	7	1.68
tcc	S	3	1.58
tca	S	0	2.43
tcg	S	0	0.53
ctt	L	29	142.60
ctc	L	285	102.70
cta	L	2	123.30
ctg	L	3	128.00
cct	P	1	0.30
ccc	P	0	0.20
cca	P	0	0.40
ccg	P	0	0.11
att	I	0	1.06
atc	I	3	0.77
ata	I	0	1.17
agt	S	0	1.86
agc	S	0	1.92
gtt	V	0	0.44
gtc	V	2	0.38
gta	V	0	0.50
gtg	V	0	0.67
---	--	------	------
mPD		0.49	0.78
		nPD:	0.63
		N. weight:	0.53
		Sc. PD:	0.14
		Sc. rank:	1025.7

,

NA			
Pos.	25	obs:	exp:
ttt	F	126	215.10
ttc	F	354	264.90
tta	L	19	461.20
ttg	L	0	796.00
tgt	C	0	1.29
tgc	C	3	1.71
ctt	L	3517	855.90
ctc	L	686	616.60
cta	L	12	740.00
ctg	L	4	768.20
att	I	35	17.74
atc	I	15	12.82
ata	I	0	19.44
gtt	V	0	0.22
gtc	V	1	0.19
gta	V	0	0.25
gtg	V	0	0.34
---	--	------	------
mPD		0.57	1.2
		nPD:	0.46
		N. weight:	1.6
		Sc. PD:	0.17
		Sc. rank:	1495.9

,

NA

Pos.	26	obs:	exp:
tta	L	0	0.87
ttg	L	8	1.50
ctt	L	0	1.62
ctc	L	0	1.16
cta	L	0	1.40
ctg	L	0	1.45
att	I	5	7.09
atc	I	0	5.13
ata	I	15	7.78
atg	M	4719	4719.00
act	T	0	4.92
acc	T	0	4.32
aca	T	11	9.09
acg	T	9	1.67
gtt	V	0	1.11
gtc	V	0	0.94
gta	V	0	1.26
gtg	V	5	1.68
---	--	------	------
mPD		0.027	0.033
		nPD:	0.8
		N. weight:	0.016
		Sc. PD:	0.0065
		Sc. rank:	38.6

,

NA

Pos.	27	obs:	exp:
caa	Q	4065	2573.00
cag	Q	707	2199.00
---	--	------	------
mPD		0.25	0.50
		nPD:	0.51
		N. weight:	0.35
		Sc. PD:	0.05
		Sc. rank:	397.4

,

NA

Pos.	28	obs:	exp:
tta	L	0	0.44
ttg	L	0	0.75
ctt	L	4	0.81
ctc	L	0	0.58
cta	L	0	0.70
ctg	L	0	0.73
att	I	3079	1102.00
atc	I	19	796.90
ata	I	9	1208.00
atg	M	153	153.00
act	T	716	177.60
acc	T	4	156.10
aca	T	1	328.10
acg	T	1	60.21
gtt	V	766	173.00
gtc	V	4	147.00
gta	V	0	196.60
gtg	V	9	262.40
gct	A	5	1.47
gcc	A	0	1.37
gca	A	0	2.70
gcg	A	1	0.45
ggt	G	1	0.16
ggc	G	0	0.13
gga	G	0	0.45
ggg	G	0	0.26
---	--	------	------
mPD		0.62	1.2
		nPD:	0.49
		N. weight:	1.7
		Sc. PD:	0.22
		Sc. rank:	1771.3

,

NA

Pos.	29	obs:	exp:
act	T	0	13.77
acc	T	56	12.11
aca	T	0	25.45
acg	T	0	4.67
gtt	V	1	39.74
gtc	V	176	33.78
gta	V	2	45.19
gtg	V	0	60.29
gct	A	725	1112.00
gcc	A	3556	1039.00
gca	A	253	2044.00
gcg	A	1	340.10
ggt	G	0	0.31
ggc	G	1	0.25
gga	G	0	0.91
ggg	G	1	0.53
---	--	------	------
mPD		0.44	0.78
		nPD:	0.56
		N. weight:	1.3
		Sc. PD:	0.25
		Sc. rank:	2033.4

,

NA

Pos.	30	obs:	exp:
tta	L	0	2.07
ttg	L	0	3.57
ctt	L	4	3.84
ctc	L	9	2.76
cta	L	6	3.32
ctg	L	0	3.44
att	I	1160	1673.00
atc	I	3195	1209.00
ata	I	360	1833.00
act	T	0	7.13
acc	T	29	6.27
aca	T	0	13.18
acg	T	0	2.42
gtt	V	0	2.00
gtc	V	9	1.70
gta	V	0	2.27
gtg	V	0	3.03
---	--	------	------
mPD		0.50	0.68
		nPD:	0.73
		N. weight:	0.71
		Sc. PD:	0.25
		Sc. rank:	1649.0

,

NA

Pos.	31	obs:	exp:
ttt	F	26	11.65
ttc	F	0	14.35
tta	L	22	512.80
ttg	L	127	885.10
ctt	L	69	951.60
ctc	L	2	685.60
cta	L	941	822.80
ctg	L	3551	854.20
att	I	1	1.06
atc	I	0	0.77
ata	I	2	1.17
atg	M	30	30.00
gtt	V	0	0.22
gtc	V	0	0.19
gta	V	0	0.25
gtg	V	1	0.34
---	--	------	------
mPD		0.44	1.2
		nPD:	0.38
		N. weight:	1.5
		Sc. PD:	0.043
		Sc. rank:	489.3

,

NA

Pos.	32	obs:	exp:
tta	L	7	17.85
ttg	L	62	30.80
ctt	L	0	33.12
ctc	L	0	23.86
cta	L	94	28.64
ctg	L	1	29.73
att	I	2	157.20
atc	I	2	113.60
ata	I	439	172.20
atg	M	17	17.00
act	T	0	6.89
acc	T	0	6.05
aca	T	28	12.72
acg	T	0	2.33
gtt	V	5	910.30
gtc	V	2	773.70
gta	V	3368	1035.00
gtg	V	725	1381.00
gct	A	0	4.90
gcc	A	0	4.58
gca	A	20	9.01
gcg	A	0	1.50
---	--	------	------
mPD		0.55	1.0
		nPD:	0.54
		N. weight:	1.3
		Sc. PD:	0.24
		Sc. rank:	1920.9

,

NA

Pos.	33	obs:	exp:
tct	S	3	0.67
tcc	S	0	0.63
tca	S	0	0.97
tcg	S	0	0.21
att	I	150	53.57
atc	I	1	38.73
ata	I	0	58.70
atg	M	4	4.00
act	T	4090	1127.00
acc	T	115	991.00
aca	T	29	2082.00
acg	T	349	382.20
aat	N	25	15.35
aac	N	2	11.65
agt	S	1	0.74
agc	S	0	0.77
gtt	V	0	0.44
gtc	V	0	0.38
gta	V	0	0.50
gtg	V	2	0.67
gct	A	1	0.25
gcc	A	0	0.23
gca	A	0	0.45
gcg	A	0	0.07
---	--	------	------
mPD		0.27	0.76
		nPD:	0.36
		N. weight:	1.6
		Sc. PD:	0.0085
		Sc. rank:	412.1

,

NA

Pos.	34	obs:	exp:
tct	S	1	0.34
tcc	S	0	0.32
tca	S	0	0.49
tcg	S	0	0.11
att	I	0	1.77
atc	I	5	1.28
ata	I	0	1.94
act	T	3547	919.20
acc	T	113	808.10
aca	T	71	1698.00
acg	T	6	311.60
aat	N	1014	583.70
aac	N	13	443.30
agt	S	1	0.37
agc	S	0	0.38
gct	A	1	0.25
gcc	A	0	0.23
gca	A	0	0.45
gcg	A	0	0.07
---	--	------	------
mPD		0.42	1.0
		nPD:	0.41
		N. weight:	1.6
		Sc. PD:	0.083
		Sc. rank:	918.3

,

NA

Pos.	35	obs:	exp:
tta	L	6	0.65
ttg	L	0	1.13
ctt	L	0	1.21
ctc	L	0	0.87
cta	L	0	1.05
ctg	L	0	1.09
att	I	5	154.70
atc	I	1	111.80
ata	I	430	169.50
atg	M	2	2.00
act	T	0	1.23
acc	T	0	1.08
aca	T	5	2.27
acg	T	0	0.42
gtt	V	87	958.10
gtc	V	5	814.20
gta	V	4026	1089.00
gtg	V	197	1453.00
gct	A	0	1.96
gcc	A	0	1.83
gca	A	8	3.61
gcg	A	0	0.60
---	--	------	------
mPD		0.29	0.92
		nPD:	0.32
		N. weight:	1.6
		Sc. PD:	-0.047
		Sc. rank:	50.7

,

NA

Pos.	36	obs:	exp:
tct	S	0	0.84
tcc	S	0	0.79
tca	S	5	1.21
tcg	S	0	0.27
cgt	R	0	0.03
cgc	R	0	0.05
cga	R	0	0.10
cgg	R	0	0.07
att	I	0	2.84
atc	I	0	2.05
ata	I	8	3.11
atg	M	41	41.00
act	T	3	1129.00
acc	T	25	992.80
aca	T	4259	2086.00
acg	T	304	382.80
aat	N	1	0.57
aac	N	0	0.43
aaa	K	98	63.81
aag	K	7	41.19
agt	S	0	0.93
agc	S	0	0.96
aga	R	1	0.46
agg	R	0	0.28
gct	A	0	4.66
gcc	A	0	4.35
gca	A	19	8.56
gcg	A	0	1.42
gaa	E	1	0.59
gag	E	0	0.41
---	--	------	------
mPD		0.22	0.76
		nPD:	0.29
		N. weight:	0.96
		Sc. PD:	-0.051
		Sc. rank:	-379.4

,

NA

Pos.	37	obs:	exp:
ttt	F	76	35.40
ttc	F	3	43.60
tta	L	1009	505.30
ttg	L	2920	872.10
tct	S	2	1.68
tcc	S	0	1.58
tca	S	1	2.43
tcg	S	7	0.53
tat	Y	0	1.12
tac	Y	2	0.88
tgg	W	2	2.00
ctt	L	0	937.70
ctc	L	1	675.50
cta	L	17	810.70
ctg	L	696	841.70
atg	M	30	30.00
agt	S	0	1.86
agc	S	0	1.92
gtt	V	0	1.33
gtc	V	0	1.13
gta	V	1	1.51
gtg	V	5	2.02
---	--	------	------
mPD		0.64	1.2
		nPD:	0.55
		N. weight:	1.3
		Sc. PD:	0.24
		Sc. rank:	1998.5

,

NA

Pos.	38	obs:	exp:
tta	L	0	0.11
ttg	L	0	0.19
tat	Y	1	1.68
tac	Y	2	1.32
ctt	L	1	0.20
ctc	L	0	0.15
cta	L	0	0.17
ctg	L	0	0.18
cat	H	3664	2709.00
cac	H	1032	1987.00
caa	Q	37	26.42
cag	Q	12	22.58
cgt	R	5	0.35
cgc	R	6	0.58
cga	R	0	1.11
cgg	R	0	0.79
aat	N	9	5.12
aac	N	0	3.88
aga	R	0	5.04
agg	R	0	3.13
gat	D	1	0.56
gac	D	0	0.44
---	-	2	2.00
---	--	------	------
mPD		0.37	0.51
		nPD:	0.71
		N. weight:	0.16
		Sc. PD:	0.052
		Sc. rank:	353.8

,

NA

Pos.	39	obs:	exp:
ttt	F	299	2044.00
ttc	F	4263	2518.00
tta	L	7	2.94
ttg	L	2	5.07
tct	S	0	9.08
tcc	S	54	8.53
tca	S	0	13.11
tcg	S	0	2.87
tat	Y	1	13.45
tac	Y	23	10.55
tgt	C	0	0.43
tgc	C	1	0.57
ctt	L	2	5.45
ctc	L	13	3.93
cta	L	3	4.71
ctg	L	0	4.89
cct	P	96	29.80
ccc	P	5	20.33
cca	P	0	40.00
ccg	P	0	10.86
agt	S	0	10.05
agc	S	0	10.35
gtt	V	0	0.22
gtc	V	1	0.19
gta	V	0	0.25
gtg	V	0	0.34
---	-	2	2.00
---	--	------	------
mPD		0.28	0.64
		nPD:	0.43
		N. weight:	0.63
		Sc. PD:	0.048
		Sc. rank:	444.9

,

NA

Pos.	40	obs:	exp:
tct	S	0	0.17
tcc	S	0	0.16
tca	S	0	0.24
tcg	S	0	0.05
cct	P	0	0.30
ccc	P	0	0.20
cca	P	0	0.40
ccg	P	1	0.11
caa	Q	1	0.54
cag	Q	0	0.46
cgt	R	0	17.87
cgc	R	0	29.92
cga	R	0	56.89
cgg	R	0	40.89
att	I	1	0.35
atc	I	0	0.26
ata	I	0	0.39
atg	M	3	3.00
act	T	1	0.25
acc	T	0	0.22
aca	T	0	0.45
acg	T	0	0.08
aat	N	976	559.30
aac	N	8	424.70
aaa	K	232	1948.00
aag	K	2974	1258.00
agt	S	1	0.19
agc	S	0	0.19
aga	R	340	259.60
agg	R	226	160.90
gat	D	2	1.12
gac	D	0	0.88
gaa	E	0	2.37
gag	E	4	1.63
---	-	2	2.00
---	--	------	------
mPD		0.71	0.93
		nPD:	0.76
		N. weight:	0.9
		Sc. PD:	0.34
		Sc. rank:	2155.4

,

NA			
Pos.	41	obs:	exp:
tta	L	0	3.26
ttg	L	0	5.63
ctt	L	0	6.06
ctc	L	0	4.36
cta	L	30	5.24
ctg	L	0	5.44
cat	H	10	25.38
cac	H	34	18.62
caa	Q	2880	2497.00
cag	Q	1750	2133.00
cgt	R	0	1.52
cgc	R	0	2.54
cga	R	41	4.82
cgg	R	7	3.47
aaa	K	6	9.72
aag	K	10	6.28
aga	R	0	22.01
agg	R	0	13.64
gaa	E	2	1.19
gag	E	0	0.81
---	-	2	2.00
---	--	------	------
mPD		0.52	0.57
		nPD:	0.91
		N. weight:	0.076
		Sc. PD:	0.039
		Sc. rank:	197.8

,

NA			
Pos.	42	obs:	exp:
ttt	F	7	3.14
ttc	F	0	3.86
tta	L	0	23.94
ttg	L	0	41.32
tct	S	0	0.50
tcc	S	0	0.47
tca	S	0	0.73
tcg	S	0	0.16
tat	Y	923	526.10
tac	Y	16	412.90
tgt	C	1068	462.30
tgc	C	6	611.70
ctt	L	219	44.43
ctc	L	1	32.01
cta	L	0	38.41
ctg	L	0	39.88
cct	P	63	35.12
ccc	P	56	23.96
cca	P	0	47.12
ccg	P	0	12.80
cat	H	1952	1358.00
cac	H	403	996.60
caa	Q	15	10.25
cag	Q	4	8.75
cgt	R	7	0.28
cgc	R	2	0.48
cga	R	0	0.90
cgg	R	0	0.65
aat	N	17	10.80
aac	N	2	8.20
aaa	K	1	0.61
aag	K	0	0.39
agt	S	3	0.56
agc	S	0	0.57
aga	R	0	4.13
agg	R	0	2.56
gat	D	4	2.81
gac	D	1	2.19
---	-	2	2.00
---	--	------	------
mPD		1.1	1.5
		nPD:	0.76
		N. weight:	0.71
		Sc. PD:	0.27
		Sc. rank:	1697.7

,

NA			
Pos.	43	obs:	exp:
tct	S	0	0.34
tcc	S	0	0.32
tca	S	0	0.49
tcg	S	0	0.11
tat	Y	4	2.24
tac	Y	0	1.76
cat	H	8	4.61
cac	H	0	3.39
aat	N	594	358.10
aac	N	36	271.90
aaa	K	15	11.55
aag	K	4	7.45
agt	S	1	0.37
agc	S	1	0.38
gtt	V	0	8.88
gtc	V	0	7.55
gta	V	3	10.10
gtg	V	37	13.47
gat	D	583	976.40
gac	D	1153	759.60
gaa	E	1570	1366.00
gag	E	736	939.70
ggt	G	2	3.92
ggc	G	9	3.17
gga	G	10	11.32
ggg	G	4	6.59
---	-	2	2.00
---	--	------	------
mPD		1.0	1.0
		nPD:	0.99
		N. weight:	0.17
		Sc. PD:	0.099
		Sc. rank:	443.5

,

NA			
Pos.	44	obs:	exp:
ttt	F	0	41.22
ttc	F	92	50.78
tct	S	0	34.48
tcc	S	5	32.40
tca	S	0	49.78
tcg	S	0	10.90
tat	Y	26	717.10
tac	Y	1254	562.90
tgt	C	56	1294.00
tgc	C	2951	1713.00
tgg	W	2	2.00
cat	H	0	5.19
cac	H	9	3.81
cgt	R	0	0.66
cgc	R	12	1.11
cga	R	6	2.11
cgg	R	0	1.52
aat	N	2	3.41
aac	N	4	2.59
agt	S	9	38.16
agc	S	191	39.29
aga	R	3	9.63
agg	R	0	5.97
gat	D	0	0.56
gac	D	1	0.44
ggt	G	0	22.90
ggc	G	146	18.50
gga	G	0	66.13
ggg	G	0	38.47
---	-	3	3.00
---	--	------	------
mPD		0.62	1.1
		nPD:	0.56
		N. weight:	0.99
		Sc. PD:	0.19
		Sc. rank:	1546.2

,

NA			
Pos.	45	obs:	exp:
tta	L	0	1.31
ttg	L	0	2.25
tct	S	0	11.77
tcc	S	0	11.06
tca	S	0	17.00
tcg	S	0	3.72
tat	Y	0	1.12
tac	Y	2	0.88
ctt	L	9	2.42
ctc	L	1	1.75
cta	L	2	2.10
ctg	L	0	2.18
cct	P	1	0.30
ccc	P	0	0.20
cca	P	0	0.40
ccg	P	0	0.11
cat	H	485	504.70
cac	H	390	370.30
caa	Q	4	2.16
cag	Q	0	1.84
cgt	R	1	0.06
cgc	R	1	0.11
cga	R	0	0.20
cgg	R	0	0.14
att	I	0	0.35
atc	I	1	0.26
ata	I	0	0.39
act	T	0	1.72
acc	T	7	1.51
aca	T	0	3.18
acg	T	0	0.58
aat	N	474	1356.00
aac	N	1912	1030.00
aaa	K	88	55.30
aag	K	3	35.70
agt	S	16	13.03
agc	S	54	13.42
aga	R	0	0.92
agg	R	0	0.57
gtt	V	0	0.44
gtc	V	1	0.38
gta	V	1	0.50
gtg	V	0	0.67
gct	A	0	0.25
gcc	A	1	0.23
gca	A	0	0.45
gcg	A	0	0.07
gat	D	135	444.30
gac	D	655	345.70
gaa	E	290	171.80
gag	E	0	118.20
ggt	G	18	37.02
ggc	G	116	29.90
gga	G	102	106.90
ggg	G	0	62.18
---	-	2	2.00
---	--	------	------
mPD		1.2	1.3
		nPD:	0.92
		N. weight:	0.46
		Sc. PD:	0.24
		Sc. rank:	1194.3

,

NA			
Pos.	46	obs:	exp:
tct	S	0	0.17
tcc	S	1	0.16
tca	S	0	0.24
tcg	S	0	0.05
agt	S	0	0.19
agc	S	0	0.19
---	-	4771	4771.00
---	--	------	------
mPD		0	7.2×10^{-8}
		nPD:	0.
		N. weight:	0.
		Sc. PD:	0
		Sc. rank:	0

,

NA			
Pos.	47	obs:	exp:
ttt	F	10	69.45
ttc	F	145	85.55
tta	L	0	0.22
ttg	L	0	0.38
tct	S	224	537.70
tcc	S	2878	505.30
tca	S	95	776.40
tcg	S	0	169.90
tat	Y	800	547.40
tac	Y	177	429.60
tgt	C	29	13.77
tgc	C	3	18.23
ctt	L	1	0.40
ctc	L	1	0.29
cta	L	0	0.35
ctg	L	0	0.36
cct	P	149	104.80
ccc	P	191	71.47
cca	P	15	140.60
ccg	P	0	38.19
cat	H	9	10.38
cac	H	9	7.62
cgt	R	3	0.09
cgc	R	0	0.16
cga	R	0	0.30
cgg	R	0	0.22
act	T	0	2.71
acc	T	11	2.38
aca	T	0	5.00
acg	T	0	0.92
aat	N	9	7.39
aac	N	4	5.61
agt	S	0	595.00
agc	S	0	612.70
aga	R	0	1.38
agg	R	0	0.85
gct	A	0	1.96
gcc	A	8	1.83
gca	A	0	3.61
gcg	A	0	0.60
---	-	1	1.00
---	--	------	------
mPD		0.97	1.8
		nPD:	0.54
		N. weight:	1.7
		Sc. PD:	0.29
		Sc. rank:	2338.2

,

NA			
Pos.	48	obs:	exp:
tta	L	0	0.11
ttg	L	0	0.19
ctt	L	0	0.20
ctc	L	1	0.15
cta	L	0	0.17
ctg	L	0	0.18
cct	P	0	2.66
ccc	P	9	1.81
cca	P	0	3.56
ccg	P	0	0.97
---	-	4762	4762.00
---	--	------	------
mPD		7.9×10^{-7}	4.2×10^{-6}
		nPD:	0.19
		N. weight:	0.
		Sc. PD:	0
		Sc. rank:	0

,

NA			
Pos.	49	obs:	exp:
ttt	F	0	6.72
ttc	F	15	8.28
tta	L	0	16.32
ttg	L	0	28.17
tct	S	251	284.10
tcc	S	1425	266.90
tca	S	13	410.20
tcg	S	0	89.77
tat	Y	4	2.24
tac	Y	0	1.76
ctt	L	38	30.29
ctc	L	101	21.82
cta	L	11	26.19
ctg	L	0	27.19
cct	P	547	841.90
ccc	P	2271	574.40
cca	P	34	1130.00
ccg	P	1	306.90
cat	H	2	10.38
cac	H	16	7.62
att	I	1	0.71
atc	I	1	0.51
ata	I	0	0.78
act	T	2	10.09
acc	T	39	8.87
aca	T	0	18.63
acg	T	0	3.42
agt	S	0	314.40
agc	S	0	323.70
---	--	------	------
mPD		0.86	1.5
		nPD:	0.55
		N. weight:	1.7
		Sc. PD:	0.33
		Sc. rank:	2670.5

,

NA			
Pos.	50	obs:	exp:
tta	L	77	16.21
ttg	L	0	27.99
tct	S	47	88.47
tcc	S	2	83.13
tca	S	471	127.70
tcg	S	6	27.96
ctt	L	0	30.09
ctc	L	0	21.68
cta	L	72	26.02
ctg	L	0	27.01
cct	P	4	948.10
ccc	P	1	646.90
cca	P	3174	1272.00
ccg	P	34	345.60
caa	Q	63	80.89
cag	Q	87	69.11
cgt	R	0	0.03
cgc	R	0	0.05
cga	R	0	0.10
cgg	R	0	0.07
act	T	1	19.19
acc	T	0	16.87
aca	T	74	35.44
acg	T	3	6.50
aat	N	1	0.57
aac	N	0	0.43
aaa	K	14	8.51
aag	K	0	5.49
agt	S	0	97.90
agc	S	0	100.80
aga	R	1	0.46
agg	R	0	0.28
gtt	V	0	3.77
gtc	V	0	3.21
gta	V	2	4.29
gtg	V	15	5.73
gct	A	63	150.80
gcc	A	0	140.90
gca	A	478	277.20
gcg	A	74	46.12
gaa	E	6	3.56
gag	E	0	2.44
---	-	2	2.00
---	--	------	------
mPD		0.71	1.4
		nPD:	0.52
		N. weight:	1.3
		Sc. PD:	0.2
		Sc. rank:	1672.1

,

NA			
Pos.	51	obs:	exp:
act	T	0	0.25
acc	T	0	0.22
aca	T	1	0.45
acg	T	0	0.08
aaa	K	30	18.23
aag	K	0	11.77
---	-	4741	4741.00
---	--	------	------
mPD		2.6×10^{-6}	0.000023
		nPD:	0.11
		N. weight:	0.
		Sc. PD:	0
		Sc. rank:	0

,

NA			
Pos.	52	obs:	exp:
aat	N	0	1.14
aac	N	2	0.86
---	-	4770	4770.00
---	--	------	------
mPD		0	8.6×10^{-8}
		nPD:	0.
		N. weight:	0.
		Sc. PD:	0
		Sc. rank:	0

,

NA			
Pos.	53	obs:	exp:
ttt	F	0	0.45
ttc	F	1	0.55
tct	S	0	41.38
tcc	S	0	38.88
tca	S	0	59.74
tcg	S	0	13.07
tat	Y	0	3.36
tac	Y	6	2.64
cat	H	0	1.15
cac	H	2	0.85
att	I	10	97.91
atc	I	263	70.79
ata	I	3	107.30
act	T	1	22.63
acc	T	83	19.89
aca	T	8	41.80
acg	T	0	7.67
aat	N	73	2266.00
aac	N	3914	1721.00
aaa	K	34	21.27
aag	K	1	13.73
agt	S	186	45.79
agc	S	60	47.15
gtt	V	1	5.33
gtc	V	23	4.53
gta	V	0	6.06
gtg	V	0	8.08
gat	D	0	3.94
gac	D	7	3.06
ggt	G	0	15.06
ggc	G	1	12.16
gga	G	95	43.48
ggg	G	0	25.29
---	--	------	------
mPD		0.49	0.95
		nPD:	0.52
		N. weight:	1.2
		Sc. PD:	0.19
		Sc. rank:	1505.1

,

NA			
Pos.	54	obs:	exp:
tct	S	0	16.65
tcc	S	0	15.65
tca	S	0	24.04
tcg	S	0	5.26
act	T	0	0.49
acc	T	2	0.43
aca	T	0	0.91
acg	T	0	0.17
aat	N	245	2650.00
aac	N	4417	2012.00
aaa	K	4	2.43
aag	K	0	1.57
agt	S	30	18.43
agc	S	69	18.97
gat	D	1	2.81
gac	D	4	2.19
---	--	------	------
mPD		0.15	0.57
		nPD:	0.27
		N. weight:	0.98
		Sc. PD:	-0.072
		Sc. rank:	-494.9

,

NA			
Pos.	55	obs:	exp:
tct	S	0	0.67
tcc	S	0	0.63
tca	S	0	0.97
tcg	S	0	0.21
agt	S	0	0.74
agc	S	4	0.77
---	-	4768	4768.00
---	--	------	------
mPD		0	1.1×10^{-6}
		nPD:	0.
		N. weight:	0.
		Sc. PD:	0
		Sc. rank:	0

,

NA

Pos.	56	obs:	exp:
tta	L	0	1.20
ttg	L	0	2.07
ctt	L	0	2.22
ctc	L	0	1.60
cta	L	0	1.92
ctg	L	11	1.99
cct	P	0	0.59
ccc	P	0	0.40
cca	P	2	0.79
ccg	P	0	0.22
cat	H	559	323.00
cac	H	1	237.00
caa	Q	3975	2243.00
cag	Q	185	1917.00
cgt	R	0	0.69
cgc	R	0	1.16
cga	R	22	2.21
cgg	R	0	1.59
aaa	K	17	10.33
aag	K	0	6.67
aga	R	0	10.09
agg	R	0	6.25
---	--	------	------
mPD		0.30	0.63
		nPD:	0.47
		N. weight:	0.73
		Sc. PD:	0.081
		Sc. rank:	686.3

,

NA

Pos.	57	obs:	exp:
tta	L	6	2.29
ttg	L	15	3.94
tct	S	0	9.25
tcc	S	0	8.69
tca	S	54	13.36
tcg	S	1	2.92
ctt	L	0	4.24
ctc	L	0	3.06
cta	L	0	3.67
ctg	L	0	3.81
cct	P	0	0.30
ccc	P	0	0.20
cca	P	1	0.40
ccg	P	0	0.11
att	I	0	114.90
atc	I	6	83.10
ata	I	318	126.00
atg	M	54	54.00
act	T	0	52.39
acc	T	0	46.06
aca	T	211	96.79
acg	T	2	17.76
aaa	K	1	0.61
aag	K	0	0.39
agt	S	0	10.24
agc	S	0	10.54
gtt	V	2	681.20
gtc	V	5	578.90
gta	V	2962	774.50
gtg	V	99	1033.00
gct	A	8	247.30
gcc	A	1	231.20
gca	A	992	454.80
gcg	A	8	75.67
gaa	E	1	0.59
gag	E	0	0.41
ggt	G	0	3.92
ggc	G	0	3.17
gga	G	25	11.32
ggg	G	0	6.59
---	--	------	------
mPD		0.73	1.4
		nPD:	0.52
		N. weight:	1.6
		Sc. PD:	0.26
		Sc. rank:	2083.8

,

NA

Pos.	58	obs:	exp:
tta	L	0	1.20
ttg	L	6	2.07
tct	S	0	0.17
tcc	S	0	0.16
tca	S	1	0.24
tcg	S	0	0.05
ctt	L	0	2.22
ctc	L	0	1.60
cta	L	0	1.92
ctg	L	5	1.99
cct	P	0	0.30
ccc	P	0	0.20
cca	P	0	0.40
ccg	P	1	0.11
cgt	R	0	0.09
cgc	R	0	0.16
cga	R	0	0.30
cgg	R	0	0.22
att	I	27	524.30
atc	I	1	379.10
ata	I	1450	574.60
atg	M	2668	2668.00
act	T	0	46.98
acc	T	0	41.30
aca	T	113	86.79
acg	T	78	15.93
aaa	K	0	3.65
aag	K	6	2.35
agt	S	0	0.19
agc	S	0	0.19
aga	R	0	1.38
agg	R	3	0.85
gtt	V	0	82.82
gtc	V	0	70.38
gta	V	310	94.16
gtg	V	63	125.60
gct	A	2	9.56
gcc	A	0	8.94
gca	A	26	17.58
gcg	A	11	2.92
gaa	E	0	0.59
gag	E	1	0.41
---	--	------	------
mPD		0.75	0.85
		nPD:	0.88
		N. weight:	0.59
		Sc. PD:	0.28
		Sc. rank:	1511.4

,

NA

Pos.	59	obs:	exp:
ttt	F	678	316.30
ttc	F	28	389.70
tta	L	0	291.10
ttg	L	30	502.50
tct	S	2	5.05
tcc	S	0	4.74
tca	S	7	7.29
tcg	S	21	1.59
tgg	W	2	2.00
ctt	L	434	540.20
ctc	L	3	389.20
cta	L	117	467.10
ctg	L	2091	484.90
cct	P	12	247.30
ccc	P	0	168.70
cca	P	330	331.90
ccg	P	496	90.14
caa	Q	7	28.04
cag	Q	45	23.96
cgt	R	0	1.07
cgc	R	0	1.80
cga	R	3	3.42
cgg	R	31	2.46
att	I	1	0.35
atc	I	0	0.26
ata	I	0	0.39
atg	M	190	190.00
act	T	9	58.79
acc	T	0	51.68
aca	T	215	108.60
acg	T	15	19.93
aaa	K	3	1.82
aag	K	0	1.18
agt	S	0	5.58
agc	S	0	5.75
aga	R	0	15.59
agg	R	0	9.66
gtt	V	1	0.22
gtc	V	0	0.19
gta	V	0	0.25
gtg	V	0	0.34
gct	A	0	0.25
gcc	A	0	0.23
gca	A	1	0.45
gcg	A	0	0.07
---	--	------	------
mPD		1.3	1.7
		nPD:	0.8
		N. weight:	1.4
		Sc. PD:	0.57
		Sc. rank:	3455.7

,

NA

Pos.	60	obs:	exp:
ttt	F	5	2.24
ttc	F	0	2.76
tct	S	0	0.50
tcc	S	0	0.47
tca	S	0	0.73
tcg	S	0	0.16
tat	Y	30	16.81
tac	Y	0	13.19
tgt	C	4624	2036.00
tgc	C	106	2694.00
cat	H	1	0.58
cac	H	0	0.42
cgt	R	1	0.03
cgc	R	0	0.05
cga	R	0	0.10
cgg	R	0	0.07
agt	S	3	0.56
agc	S	0	0.57
aga	R	0	0.46
agg	R	0	0.28
ggt	G	2	0.31
ggc	G	0	0.25
gga	G	0	0.91
ggg	G	0	0.53
---	--	------	------
mPD		0.061	0.51
		nPD:	0.12
		N. weight:	1.1
		Sc. PD:	-0.24
		Sc. rank:	-1682.1

,

NA

Pos.	61	obs:	exp:
aat	N	0	0.57
aac	N	1	0.43
aaa	K	324	215.70
aag	K	31	139.30
gtt	V	0	0.89
gtc	V	2	0.75
gta	V	0	1.01
gtg	V	2	1.35
gct	A	0	0.25
gcc	A	0	0.23
gca	A	1	0.45
gcg	A	0	0.07
gat	D	1	1.69
gac	D	2	1.31
gaa	E	4172	2604.00
gag	E	223	1791.00
ggt	G	0	2.04
ggc	G	0	1.65
gga	G	11	5.89
ggg	G	2	3.43
---	--	------	------
mPD		0.25	0.63
		nPD:	0.4
		N. weight:	0.52
		Sc. PD:	0.022
		Sc. rank:	213.7

,

NA

Pos.	62	obs:	exp:
cct	P	0	0.30
ccc	P	0	0.20
cca	P	1	0.40
ccg	P	0	0.11
---	-	4771	4771.00
---	--	------	------
mPD		0	3.1×10^{-8}
		nPD:	0.
		N. weight:	0.
		Sc. PD:	0
		Sc. rank:	0

,

NA

Pos.	63	obs:	exp:
tct	S	0	3.03
tcc	S	0	2.84
tca	S	17	4.37
tcg	S	1	0.96
cct	P	8	1401.00
ccc	P	1	955.50
cca	P	4672	1879.00
ccg	P	65	510.50
caa	Q	1	1.08
cag	Q	1	0.92
act	T	0	1.23
acc	T	0	1.08
aca	T	5	2.27
acg	T	0	0.42
agt	S	0	3.35
agc	S	0	3.45
gct	A	0	0.25
gcc	A	0	0.23
gca	A	1	0.45
gcg	A	0	0.07
---	--	------	------
mPD		0.042	0.72
		nPD:	0.06
		N. weight:	1.3
		Sc. PD:	-0.36
		Sc. rank:	-2782.0

,

NA

Pos.	64	obs:	exp:
tta	L	8	3.48
ttg	L	0	6.01
ctt	L	0	6.46
ctc	L	0	4.66
cta	L	24	5.59
ctg	L	0	5.80
cgt	R	0	0.09
cgc	R	0	0.16
cga	R	0	0.30
cgg	R	0	0.22
att	I	351	324.20
atc	I	2	234.40
ata	I	561	355.30
atg	M	36	36.00
act	T	89	913.80
acc	T	1	803.30
aca	T	3512	1688.00
acg	T	113	309.80
aaa	K	64	40.11
aag	K	2	25.89
aga	R	3	1.38
agg	R	0	0.85
gtt	V	0	1.33
gtc	V	0	1.13
gta	V	6	1.51
gtg	V	0	2.02
---	--	------	------
mPD		0.59	1.1
		nPD:	0.56
		N. weight:	0.85
		Sc. PD:	0.17
		Sc. rank:	1320.5

,

NA

Pos.	65	obs:	exp:
tta	L	15	1.85
ttg	L	0	3.19
ctt	L	0	3.43
ctc	L	0	2.47
cta	L	2	2.97
ctg	L	0	3.08
cgt	R	0	0.06
cgc	R	0	0.11
cga	R	0	0.20
cgg	R	0	0.14
att	I	24	1493.00
atc	I	34	1079.00
ata	I	4150	1636.00
atg	M	25	25.00
act	T	1	39.85
acc	T	0	35.03
aca	T	161	73.61
acg	T	0	13.51
aaa	K	100	60.77
aag	K	0	39.23
aga	R	2	0.92
agg	R	0	0.57
gtt	V	0	57.29
gtc	V	0	48.68
gta	V	255	65.13
gtg	V	3	86.90
---	--	------	------
mPD		0.25	0.89
		nPD:	0.28
		N. weight:	1.4
		Sc. PD:	-0.089
		Sc. rank:	-603.9

,

NA			
Pos.	66	obs:	exp:
tta	L	3	0.33
ttg	L	0	0.56
ctt	L	0	0.61
ctc	L	0	0.44
cta	L	0	0.52
ctg	L	0	0.54
cgt	R	0	0.03
cgc	R	0	0.05
cga	R	0	0.10
cgg	R	0	0.07
att	I	429	1635.00
atc	I	3	1182.00
ata	I	4178	1792.00
atg	M	22	22.00
act	T	0	5.41
acc	T	0	4.76
aca	T	22	10.00
acg	T	0	1.83
aga	R	1	0.46
agg	R	0	0.28
gtt	V	1	25.09
gtc	V	0	21.32
gta	V	112	28.53
gtg	V	0	38.06
---	-	1	1.00
---	--	------	------
mPD		0.23	0.72
		nPD:	0.32
		N. weight:	1.
		Sc. PD:	-0.03
		Sc. rank:	-18.8

,

NA			
Pos.	67	obs:	exp:
caa	Q	1	0.54
cag	Q	0	0.46
aat	N	1	0.57
aac	N	0	0.43
aaa	K	74	46.80
aag	K	3	30.20
gtt	V	0	0.22
gtc	V	0	0.19
gta	V	0	0.25
gtg	V	1	0.34
gat	D	1	9.56
gac	D	16	7.44
gaa	E	4126	2768.00
gag	E	546	1904.00
ggt	G	0	0.47
ggc	G	0	0.38
gga	G	3	1.36
ggg	G	0	0.79
---	--	------	------
mPD		0.24	0.52
		nPD:	0.47
		N. weight:	0.33
		Sc. PD:	0.036
		Sc. rank:	308.3

,

NA			
Pos.	68	obs:	exp:
tct	S	0	0.17
tcc	S	0	0.16
tca	S	0	0.24
tcg	S	0	0.05
cgt	R	0	128.20
cgc	R	0	214.60
cga	R	4	408.20
cgg	R	9	293.40
att	I	0	1.42
atc	I	0	1.03
ata	I	4	1.56
aaa	K	426	426.60
aag	K	276	275.40
agt	S	1	0.19
agc	S	0	0.19
aga	R	3435	1862.00
agg	R	613	1154.00
gaa	E	0	0.59
gag	E	1	0.41
ggt	G	0	0.31
ggc	G	0	0.25
gga	G	2	0.91
ggg	G	0	0.53
---	-	1	1.00
---	--	------	------
mPD		0.57	1.1
		nPD:	0.5
		N. weight:	0.55
		Sc. PD:	0.073
		Sc. rank:	583.0

,

NA			
Pos.	69	obs:	exp:
tct	S	0	0.34
tcc	S	0	0.32
tca	S	0	0.49
tcg	S	0	0.11
act	T	0	0.98
acc	T	4	0.86
aca	T	0	1.82
acg	T	0	0.33
aat	N	244	2680.00
aac	N	4471	2035.00
aaa	K	47	28.56
aag	K	0	18.44
agt	S	0	0.37
agc	S	2	0.38
gat	D	0	2.25
gac	D	4	1.75
---	--	------	------
mPD		0.12	0.51
		nPD:	0.24
		N. weight:	0.97
		Sc. PD:	-0.1
		Sc. rank:	-696.7

,

NA			
Pos.	70	obs:	exp:
tta	L	1	0.33
ttg	L	1	0.56
tct	S	0	4.71
tcc	S	0	4.43
tca	S	28	6.80
tcg	S	0	1.49
ctt	L	0	0.61
ctc	L	0	0.44
cta	L	1	0.52
ctg	L	0	0.54
cgt	R	0	2.40
cgc	R	0	4.02
cga	R	0	7.64
cgg	R	0	5.49
att	I	3	504.80
atc	I	1	365.00
ata	I	1419	553.20
atg	M	51	51.00
act	T	48	522.20
acc	T	4	459.10
aca	T	2070	964.70
acg	T	1	177.00
aaa	K	888	628.40
aag	K	146	405.60
agt	S	0	5.21
agc	S	0	5.37
aga	R	40	34.85
agg	R	36	21.60
gtt	V	0	7.33
gtc	V	0	6.23
gta	V	26	8.33
gtg	V	7	11.12
gct	A	0	0.25
gcc	A	0	0.23
gca	A	1	0.45
gcg	A	0	0.07
---	--	------	------
mPD		0.79	1.4
		nPD:	0.58
		N. weight:	0.99
		Sc. PD:	0.21
		Sc. rank:	1636.1

,

NA			
Pos.	71	obs:	exp:
cct	P	2	2.36
ccc	P	0	1.61
cca	P	1	3.17
ccg	P	5	0.86
cgt	R	0	0.13
cgc	R	0	0.21
cga	R	0	0.40
cgg	R	0	0.29
atg	M	1	1.00
act	T	112	1166.00
acc	T	48	1025.00
aca	T	3391	2154.00
acg	T	1190	395.30
aaa	K	14	9.72
aag	K	2	6.28
aga	R	4	1.83
agg	R	0	1.14
gct	A	0	0.49
gcc	A	0	0.46
gca	A	1	0.90
gcg	A	1	0.15
---	--	------	------
mPD		0.44	0.69
		nPD:	0.63
		N. weight:	0.81
		Sc. PD:	0.21
		Sc. rank:	1544.9

,

NA			
Pos.	72	obs:	exp:
tct	S	0	0.17
tcc	S	0	0.16
tca	S	0	0.24
tcg	S	0	0.05
cgt	R	0	16.38
cgc	R	0	27.43
cga	R	0	52.17
cgg	R	0	37.49
aat	N	4	2.27
aac	N	0	1.73
aaa	K	224	562.80
aag	K	702	363.20
agt	S	1	0.19
agc	S	0	0.19
aga	R	8	238.00
agg	R	511	147.50
gat	D	2	12.94
gac	D	21	10.06
gaa	E	694	1954.00
gag	E	2604	1344.00
ggt	G	0	0.16
ggc	G	0	0.13
gga	G	0	0.45
ggg	G	1	0.26
---	--	------	------
mPD		0.94	1.1
		nPD:	0.83
		N. weight:	0.62
		Sc. PD:	0.27
		Sc. rank:	1560.3

,

NA			
Pos.	73	obs:	exp:
ttt	F	4	1.79
ttc	F	0	2.21
tta	L	1	0.22
ttg	L	0	0.38
ctt	L	1	0.40
ctc	L	0	0.29
cta	L	0	0.35
ctg	L	0	0.36
cgt	R	0	0.03
cgc	R	0	0.05
cga	R	0	0.10
cgg	R	0	0.07
att	I	2627	1611.00
atc	I	16	1164.00
ata	I	1897	1765.00
atg	M	5	5.00
act	T	2	4.92
acc	T	0	4.32
aca	T	18	9.09
acg	T	0	1.67
aga	R	1	0.46
agg	R	0	0.28
gtt	V	22	44.41
gtc	V	0	37.74
gta	V	162	50.49
gtg	V	16	67.37
---	--	------	------
mPD		0.59	0.76
		nPD:	0.78
		N. weight:	0.5
		Sc. PD:	0.2
		Sc. rank:	1210.1

,

NA			
Pos.	74	obs:	exp:
---	-	4772	4772.00
---	--	------	------
mPD		0	0
		nPD:	1.
		N. weight:	0.
		Sc. PD:	0
		Sc. rank:	0

,

NA			
Pos.	75	obs:	exp:
---	-	4772	4772.00
---	--	------	------
mPD		0	0
		nPD:	1.
		N. weight:	0.
		Sc. PD:	0
		Sc. rank:	0

,

NA	Pos. 76	obs:	exp:
---	-	4772	4772.00
---	--	------	------
mPD		0	0
		nPD:	1.
		N. weight:	0.
		Sc. PD:	0
		Sc. rank:	0

,

NA	Pos. 77	obs:	exp:
---	-	4772	4772.00
---	--	------	------
mPD		0	0
		nPD:	1.
		N. weight:	0.
		Sc. PD:	0
		Sc. rank:	0

,

NA	Pos. 78	obs:	exp:
ttt	F	1	0.45
ttc	F	0	0.55
tta	L	2	0.33
ttg	L	1	0.56
ctt	L	0	0.61
ctc	L	0	0.44
cta	L	0	0.52
ctg	L	0	0.54
att	I	166	62.43
atc	I	0	45.14
ata	I	10	68.42
atg	M	52	52.00
gtt	V	175	1008.00
gtc	V	29	856.30
gta	V	253	1146.00
gtg	V	4081	1529.00
gct	A	0	0.49
gcc	A	0	0.46
gca	A	1	0.90
gcg	A	1	0.15
---	--	------	------
mPD		0.33	0.83
		nPD:	0.4
		N. weight:	1.1
		Sc. PD:	0.051
		Sc. rank:	526.6

,

NA	Pos. 79	obs:	exp:
ttt	F	3	1.34
ttc	F	0	1.66
tat	Y	4155	2648.00
tac	Y	572	2079.00
cat	H	6	20.77
cac	H	30	15.23
caa	Q	5	2.70
cag	Q	0	2.30
gtt	V	1	0.22
gtc	V	0	0.19
gta	V	0	0.25
gtg	V	0	0.34
---	--	------	------
mPD		0.24	0.51
		nPD:	0.47
		N. weight:	0.38
		Sc. PD:	0.042
		Sc. rank:	354.3

,

NA	Pos. 80	obs:	exp:
ttt	F	5	2.24
ttc	F	0	2.76
tta	L	387	510.70
ttg	L	1778	881.50
tct	S	0	0.50
tcc	S	0	0.47
tca	S	3	0.73
tcg	S	0	0.16
ctt	L	11	947.80
ctc	L	1	682.80
cta	L	106	819.50
ctg	L	2410	850.70
caa	Q	0	4.31
cag	Q	8	3.69
cgt	R	0	0.03
cgc	R	0	0.05
cga	R	0	0.10
cgg	R	1	0.07
att	I	6	2.48
atc	I	1	1.80
ata	I	0	2.72
atg	M	27	27.00
agt	S	0	0.56
agc	S	0	0.57
aga	R	0	0.46
agg	R	0	0.28
gtt	V	0	6.22
gtc	V	0	5.28
gta	V	4	7.07
gtg	V	24	9.43
---	--	------	------
mPD		0.71	1.2
		nPD:	0.61
		N. weight:	1.1
		Sc. PD:	0.27
		Sc. rank:	2026.4

,

NA	Pos. 81	obs:	exp:
ttt	F	3	3.14
ttc	F	4	3.86
tct	S	1	15.81
tcc	S	20	14.86
tca	S	72	22.83
tcg	S	0	5.00
cct	P	1	0.89
ccc	P	2	0.60
cca	P	0	1.19
ccg	P	0	0.32
att	I	5	62.43
atc	I	171	45.14
ata	I	0	68.42
act	T	241	964.00
acc	T	3575	847.40
aca	T	103	1781.00
acg	T	0	326.80
aat	N	330	225.60
aac	N	67	171.40
agt	S	1	17.50
agc	S	0	18.01
gtt	V	19	13.10
gtc	V	40	11.13
gta	V	0	14.89
gtg	V	0	19.87
gct	A	27	28.68
gcc	A	84	26.81
gca	A	6	52.74
gcg	A	0	8.77
---	--	------	------
mPD		0.64	1.0
		nPD:	0.62
		N. weight:	1.6
		Sc. PD:	0.4
		Sc. rank:	3046.5

,

NA	Pos. 82	obs:	exp:
tct	S	0	2.02
tcc	S	0	1.90
tca	S	0	2.91
tcg	S	0	0.64
cat	H	0	0.58
cac	H	1	0.42
aat	N	798	2697.00
aac	N	3947	2048.00
aaa	K	8	8.51
aag	K	6	5.49
agt	S	3	2.23
agc	S	9	2.30
---	--	------	------
mPD		0.29	0.50
		nPD:	0.58
		N. weight:	0.53
		Sc. PD:	0.11
		Sc. rank:	859.1

,

NA	Pos. 83	obs:	exp:
tct	S	0	11.27
tcc	S	64	10.59
tca	S	3	16.27
tcg	S	0	3.56
cct	P	0	0.59
ccc	P	2	0.40
cca	P	0	0.79
ccg	P	0	0.22
att	I	14	111.00
atc	I	298	80.28
ata	I	1	121.70
act	T	62	1056.00
acc	T	3718	928.80
aca	T	514	1952.00
acg	T	1	358.10
aat	N	0	9.66
aac	N	17	7.34
aaa	K	2	1.22
aag	K	0	0.78
agt	S	0	12.47
agc	S	0	12.84
gtt	V	0	13.54
gtc	V	61	11.51
gta	V	0	15.40
gtg	V	0	20.55
gct	A	1	2.21
gcc	A	8	2.06
gca	A	0	4.06
gcg	A	0	0.67
ggt	G	0	0.47
ggc	G	3	0.38
gga	G	0	1.36
ggg	G	0	0.79
---	-	3	3.00
---	--	------	------
mPD		0.43	0.89
		nPD:	0.49
		N. weight:	1.6
		Sc. PD:	0.2
		Sc. rank:	1661.1

,

NA	Pos. 84	obs:	exp:
tct	S	0	0.34
tcc	S	0	0.32
tca	S	0	0.49
tcg	S	0	0.11
att	I	97	65.98
atc	I	89	47.71
ata	I	0	72.31
act	T	1225	1127.00
acc	T	3334	991.00
aca	T	21	2082.00
acg	T	3	382.20
agt	S	0	0.37
agc	S	2	0.38
gct	A	1	0.25
gcc	A	0	0.23
gca	A	0	0.45
gcg	A	0	0.07
---	--	------	------
mPD		0.48	0.76
		nPD:	0.64
		N. weight:	1.4
		Sc. PD:	0.36
		Sc. rank:	2668.6

,

NA	Pos. 85	obs:	exp:
tta	L	4	0.65
ttg	L	1	1.13
ctt	L	0	1.21
ctc	L	0	0.87
cta	L	1	1.05
ctg	L	0	1.09
att	I	18	1302.00
atc	I	3	941.60
ata	I	3650	1427.00
atg	M	36	36.00
act	T	2	2.95
acc	T	0	2.59
aca	T	10	5.45
acg	T	0	1.00
gtt	V	11	232.50
gtc	V	2	197.60
gta	V	1008	264.30
gtg	V	26	352.70
---	--	------	------
mPD		0.39	1.1
		nPD:	0.37
		N. weight:	1.5
		Sc. PD:	0.026
		Sc. rank:	427.9

,

NA			
Pos.	86	obs:	exp:
gaa	E	1	1.78
gag	E	2	1.22
---	-	4769	4769.00
---	--	------	------
mPD		1.8×10^{-7}	1.9×10^{-7}
		nPD:	0.92
		N. weight:	0.
		Sc. PD:	0
		Sc. rank:	0

,

NA			
Pos.	87	obs:	exp:
---	-	4772	4772.00
---	--	------	------
mPD		0	0
		nPD:	1.
		N. weight:	0.
		Sc. PD:	0
		Sc. rank:	0

,

NA			
Pos.	88	obs:	exp:
aaa	K	0	1.82
aag	K	3	1.18
---	-	4769	4769.00
---	--	------	------
mPD		0	1.9×10^{-7}
		nPD:	0.
		N. weight:	0.
		Sc. PD:	0
		Sc. rank:	0

,

NA			
Pos.	89	obs:	exp:
tta	L	0	0.11
ttg	L	1	0.19
tat	Y	6	3.36
tac	Y	0	2.64
ctt	L	0	0.20
ctc	L	0	0.15
cta	L	0	0.17
ctg	L	0	0.18
att	I	2	0.71
atc	I	0	0.51
ata	I	0	0.78
aaa	K	12	69.28
aag	K	102	44.72
gtt	V	0	12.66
gtc	V	0	10.76
gta	V	15	14.39
gtg	V	42	19.20
gat	D	4	2.81
gac	D	1	2.19
gaa	E	234	2716.00
gag	E	4350	1868.00
ggt	G	0	0.31
ggc	G	0	0.25
gga	G	0	0.91
ggg	G	2	0.53
---	-	1	1.00
---	--	------	------
mPD		0.18	0.57
		nPD:	0.33
		N. weight:	1.1
		Sc. PD:	-0.025
		Sc. rank:	44.0

,

NA			
Pos.	90	obs:	exp:
cgt	R	0	15.15
cgc	R	0	25.37
cga	R	0	48.25
cgg	R	0	34.68
act	T	0	25.34
acc	T	0	22.27
aca	T	102	46.80
acg	T	1	8.59
aaa	K	1315	2543.00
aag	K	2869	1641.00
aga	R	371	220.10
agg	R	109	136.40
---	-	5	5.00
---	--	------	------
mPD		0.69	0.76
		nPD:	0.9
		N. weight:	0.32
		Sc. PD:	0.16
		Sc. rank:	838.0

,

NA			
Pos.	91	obs:	exp:
gaa	E	2	1.19
gag	E	0	0.81
---	-	4770	4770.00
---	--	------	------
mPD		0	8.5×10^{-8}
		nPD:	0.
		N. weight:	0.
		Sc. PD:	0
		Sc. rank:	0

,

NA			
Pos.	92	obs:	exp:
---	-	4772	4772.00
---	--	------	------
mPD		0	0
		nPD:	1.
		N. weight:	0.
		Sc. PD:	0
		Sc. rank:	0

,

NA			
Pos.	93	obs:	exp:
---	-	4772	4772.00
---	--	------	------
mPD		0	0
		nPD:	1.
		N. weight:	0.
		Sc. PD:	0
		Sc. rank:	0

,

NA			
Pos.	94	obs:	exp:
---	-	4772	4772.00
---	--	------	------
mPD		0	0
		nPD:	1.
		N. weight:	0.
		Sc. PD:	0
		Sc. rank:	0

,

NA			
Pos.	95	obs:	exp:
---	-	4772	4772.00
---	--	------	------
mPD		0	0
		nPD:	1.
		N. weight:	0.
		Sc. PD:	0
		Sc. rank:	0

,

NA			
Pos.	96	obs:	exp:
---	-	4772	4772.00
---	--	------	------
mPD		0	0
		nPD:	1.
		N. weight:	0.
		Sc. PD:	0
		Sc. rank:	0

,

NA			
Pos.	97	obs:	exp:
---	-	4772	4772.00
---	--	------	------
mPD		0	0
		nPD:	1.
		N. weight:	0.
		Sc. PD:	0
		Sc. rank:	0

,

NA			
Pos.	98	obs:	exp:
aaa	K	6	3.65
aag	K	0	2.35
---	-	4766	4766.00
---	--	------	------
mPD		0	7.5×10^{-7}
		nPD:	0.
		N. weight:	0.
		Sc. PD:	0
		Sc. rank:	0

,

NA			
Pos.	99	obs:	exp:
gaa	E	7	4.15
gag	E	0	2.85
---	-	4765	4765.00
---	--	------	------
mPD		0	1.0×10^{-6}
		nPD:	0.
		N. weight:	0.
		Sc. PD:	0
		Sc. rank:	0

,

NA			
Pos.	100	obs:	exp:
att	I	0	2.48
atc	I	0	1.80
ata	I	7	2.72
---	-	4765	4765.00
---	--	------	------
mPD		0	1.4×10^{-6}
		nPD:	0.
		N. weight:	0.
		Sc. PD:	0
		Sc. rank:	0

,

NA			
Pos.	101	obs:	exp:
aaa	K	14	10.33
aag	K	3	6.67
gct	A	0	0.74
gcc	A	0	0.69
gca	A	1	1.35
gcg	A	2	0.22
gaa	E	3774	2804.00
gag	E	959	1929.00
ggt	G	0	2.98
ggc	G	0	2.41
gga	G	16	8.61
ggg	G	3	5.01
---	--	------	------
mPD		0.34	0.50
		nPD:	0.68
		N. weight:	0.15
		Sc. PD:	0.046
		Sc. rank:	330.8

,

NA			
Pos.	102	obs:	exp:
tta	L	2	0.44
ttg	L	0	0.75
ctt	L	0	0.81
ctc	L	0	0.58
cta	L	2	0.70
ctg	L	0	0.73
cgt	R	0	0.03
cgc	R	0	0.05
cga	R	0	0.10
cgg	R	0	0.07
att	I	17	977.70
atc	I	0	706.90
ata	I	2739	1071.00
atg	M	62	62.00
act	T	0	111.20
acc	T	2	97.74
aca	T	449	205.40
acg	T	1	37.69
aat	N	10	5.68
aac	N	0	4.32
aaa	K	905	554.90
aag	K	8	358.10
aga	R	1	0.46
agg	R	0	0.28
gtt	V	2	114.30
gtc	V	0	97.18
gta	V	507	130.00
gtg	V	6	173.50
gct	A	0	13.73
gcc	A	0	12.83
gca	A	56	25.24
gcg	A	0	4.20
gaa	E	3	1.78
gag	E	0	1.22
---	--	------	------
mPD		0.72	1.4
		nPD:	0.52
		N. weight:	1.3
		Sc. PD:	0.2
		Sc. rank:	1636.0

,

NA			
Pos.	103	obs:	exp:
ttt	F	0	0.45
ttc	F	1	0.55
tct	S	0	0.34
tcc	S	0	0.32
tca	S	0	0.49
tcg	S	0	0.11
tat	Y	24	55.46
tac	Y	75	43.54
tgt	C	235	2008.00
tgc	C	4429	2656.00
tgg	W	3	3.00
cat	H	0	0.58
cac	H	1	0.42
aat	N	0	0.57
aac	N	1	0.43
agt	S	0	0.37
agc	S	2	0.38
gtt	V	0	0.22
gtc	V	1	0.19
gta	V	0	0.25
gtg	V	0	0.34
---	--	------	------
mPD		0.15	0.54
		nPD:	0.28
		N. weight:	0.59
		Sc. PD:	-0.04
		Sc. rank:	-291.7

,

NA			
Pos.	104	obs:	exp:
tta	L	0	1.52
ttg	L	0	2.63
tct	S	0	7.23
tcc	S	42	6.80
tca	S	1	10.44
tcg	S	0	2.29
ctt	L	1	2.83
ctc	L	13	2.04
cta	L	0	2.44
ctg	L	0	2.54
cct	P	74	1376.00
ccc	P	4430	938.80
cca	P	159	1847.00
ccg	P	0	501.60
cat	H	0	16.15
cac	H	28	11.85
act	T	0	2.95
acc	T	12	2.59
aca	T	0	5.45
acg	T	0	1.00
agt	S	0	8.00
agc	S	0	8.24
gat	D	11	6.75
gac	D	1	5.25
---	--	------	------
mPD		0.15	0.76
		nPD:	0.2
		N. weight:	2.1
		Sc. PD:	-0.3
		Sc. rank:	-2122.4

,

NA			
Pos.	105	obs:	exp:
caa	Q	55	29.66
cag	Q	0	25.34
cgt	R	0	0.63
cgc	R	0	1.06
cga	R	0	2.01
cgg	R	0	1.44
att	I	0	1.77
atc	I	0	1.28
ata	I	5	1.94
atg	M	1	1.00
act	T	1	7.63
acc	T	0	6.70
aca	T	30	14.09
acg	T	0	2.59
aat	N	12	32.40
aac	N	45	24.60
aaa	K	4483	2785.00
aag	K	99	1797.00
aga	R	20	9.17
agg	R	0	5.68
gaa	E	12	10.67
gag	E	6	7.33
ggt	G	0	0.16
ggc	G	0	0.13
gga	G	1	0.45
ggg	G	0	0.26
---	-	2	2.00
---	--	------	------
mPD		0.12	0.55
		nPD:	0.22
		N. weight:	0.64
		Sc. PD:	-0.076
		Sc. rank:	-546.8

,

NA			
Pos.	106	obs:	exp:
ttt	F	3	1.34
ttc	F	0	1.66
tta	L	518	319.20
ttg	L	51	550.90
tct	S	1	33.81
tcc	S	0	31.77
tca	S	196	48.81
tcg	S	4	10.68
ctt	L	5	592.40
ctc	L	7	426.70
cta	L	2308	512.10
ctg	L	44	531.70
cct	P	0	278.00
ccc	P	7	189.70
cca	P	922	373.00
ccg	P	13	101.30
caa	Q	18	9.71
cag	Q	0	8.29
att	I	31	22.70
atc	I	0	16.42
ata	I	33	24.88
atg	M	1	1.00
act	T	25	62.72
acc	T	0	55.14
aca	T	230	115.90
acg	T	0	21.26
aat	N	2	1.14
aac	N	0	0.86
aaa	K	2	1.82
aag	K	1	1.18
agt	S	0	37.41
agc	S	0	38.52
gtt	V	0	4.00
gtc	V	0	3.40
gta	V	17	4.54
gtg	V	1	6.06
gct	A	0	81.14
gcc	A	0	75.84
gca	A	331	149.20
gcg	A	0	24.82
gaa	E	1	0.59
gag	E	0	0.41
---	--	------	------
mPD		1.0	1.8
		nPD:	0.58
		N. weight:	1.6
		Sc. PD:	0.35
		Sc. rank:	2716.7

,

NA			
Pos.	107	obs:	exp:
tta	L	27	26.99
ttg	L	4	46.58
tct	S	0	14.13
tcc	S	0	13.28
tca	S	84	20.40
tcg	S	0	4.46
ctt	L	0	50.09
ctc	L	0	36.08
cta	L	204	43.30
ctg	L	13	44.96
cct	P	0	20.66
ccc	P	0	14.09
cca	P	70	27.72
ccg	P	0	7.53
caa	Q	35	18.87
cag	Q	0	16.13
cgt	R	0	0.03
cgc	R	0	0.05
cga	R	0	0.10
cgg	R	0	0.07
att	I	3	5.68
atc	I	0	4.10
ata	I	13	6.22
atg	M	15	15.00
act	T	2	136.30
acc	T	1	119.80
aca	T	540	251.70
acg	T	11	46.20
aaa	K	2	1.22
aag	K	0	0.78
agt	S	0	15.63
agc	S	0	16.10
aga	R	0	0.46
agg	R	1	0.28
gtt	V	16	137.00
gtc	V	0	116.40
gta	V	134	155.80
gtg	V	467	207.80
gct	A	7	740.10
gcc	A	2	691.70
gca	A	2785	1361.00
gcg	A	225	226.40
gaa	E	1	8.30
gag	E	13	5.70
ggt	G	0	15.21
ggc	G	0	12.29
gga	G	2	43.94
ggg	G	95	25.56
---	--	------	------
mPD		1.0	1.4
		nPD:	0.71
		N. weight:	1.1
		Sc. PD:	0.36
		Sc. rank:	2416.3

,

NA			
Pos.	108	obs:	exp:
cgt	R	0	0.06
cgc	R	0	0.11
cga	R	0	0.20
cgg	R	0	0.14
atg	M	1	1.00
aat	N	2	1.14
aac	N	0	0.86
aaa	K	278	173.80
aag	K	8	112.20
aga	R	0	0.92
agg	R	2	0.57
gtt	V	0	0.44
gtc	V	0	0.38
gta	V	0	0.50
gtg	V	2	0.67
gat	D	5	3.94
gac	D	2	3.06
gaa	E	4132	2558.00
gag	E	185	1759.00
ggt	G	0	24.31
ggc	G	0	19.64
gga	G	146	70.21
ggg	G	9	40.84
---	--	------	------
mPD		0.27	0.67
		nPD:	0.4
		N. weight:	0.58
		Sc. PD:	0.023
		Sc. rank:	229.6

,

NA			
Pos.	109	obs:	exp:
ttt	F	14	9.86
ttc	F	8	12.14
tat	Y	1089	2650.00
tac	Y	3642	2081.00
cat	H	0	10.96
cac	H	19	8.04
---	--	------	------
mPD		0.37	0.51
		nPD:	0.73
		N. weight:	0.36
		Sc. PD:	0.12
		Sc. rank:	829.9

,

NA			
Pos.	110	obs:	exp:
cgt	R	0	150.30
cgc	R	0	251.60
cga	R	0	478.50
cgg	R	0	344.00
att	I	0	0.35
atc	I	0	0.26
ata	I	1	0.39
aaa	K	1	6.08
aag	K	9	3.92
aga	R	3377	2183.00
agg	R	1384	1353.00
---	--	------	------
mPD		0.42	0.94
		nPD:	0.44
		N. weight:	0.49
		Sc. PD:	0.042
		Sc. rank:	376.5

,

NA

Pos.	111	obs:	exp:
tta	L	0	0.22
ttg	L	0	0.38
tct	S	0	78.38
tcc	S	0	73.65
tca	S	0	113.20
tcg	S	0	24.77
ctt	L	0	0.40
ctc	L	2	0.29
cta	L	0	0.35
ctg	L	0	0.36
cat	H	0	1.73
cac	H	3	1.27
cgt	R	0	0.41
cgc	R	8	0.69
cga	R	0	1.31
cgg	R	0	0.94
att	I	5	2.13
atc	I	1	1.54
ata	I	0	2.33
act	T	7	1.72
acc	T	0	1.51
aca	T	0	3.18
acg	T	0	0.58
aat	N	3386	2308.00
aac	N	675	1753.00
aaa	K	16	50.44
aag	K	67	32.56
agt	S	323	86.73
agc	S	143	89.31
aga	R	0	5.96
agg	R	5	3.69
gat	D	126	72.56
gac	D	3	56.44
gaa	E	0	0.59
gag	E	1	0.41
ggt	G	1	0.16
ggc	G	0	0.13
gga	G	0	0.45
ggg	G	0	0.26
---	--	------	------
mPD		0.56	0.90
		nPD:	0.63
		N. weight:	0.42
		Sc. PD:	0.11
		Sc. rank:	795.9

,

NA

Pos.	112	obs:	exp:
tgg	W	4772	4772.00
---	--	------	------
mPD		0	0
		nPD:	1.
		N. weight:	0.
		Sc. PD:	0
		Sc. rank:	0

,

NA

Pos.	113	obs:	exp:
tta	L	19	2.50
ttg	L	4	4.32
tct	S	3	739.10
tcc	S	17	694.40
tca	S	4243	1067.00
tcg	S	131	233.50
ctt	L	0	4.65
ctc	L	0	3.35
cta	L	0	4.02
ctg	L	0	4.17
cct	P	0	0.30
ccc	P	0	0.20
cca	P	1	0.40
ccg	P	0	0.11
act	T	0	0.49
acc	T	0	0.43
aca	T	2	0.91
acg	T	0	0.17
agt	S	0	817.80
agc	S	0	842.10
gtt	V	0	0.22
gtc	V	0	0.19
gta	V	1	0.25
gtg	V	0	0.34
gct	A	0	85.55
gcc	A	0	79.96
gca	A	349	157.30
gcg	A	0	26.17
gaa	E	2	1.19
gag	E	0	0.81
---	--	------	------
mPD		0.21	1.7
		nPD:	0.12
		N. weight:	2.
		Sc. PD:	-0.41
		Sc. rank:	-2763.8

,

NA

Pos.	114	obs:	exp:
tct	S	0	0.17
tcc	S	0	0.16
tca	S	0	0.24
tcg	S	0	0.05
cgt	R	0	0.22
cgc	R	0	0.37
cga	R	0	0.70
cgg	R	0	0.51
atg	M	86	86.00
aat	N	0	2.84
aac	N	5	2.16
aaa	K	914	2840.00
aag	K	3759	1833.00
agt	S	0	0.19
agc	S	1	0.19
aga	R	1	3.21
agg	R	6	1.99
---	--	------	------
mPD		0.35	0.52
		nPD:	0.67
		N. weight:	0.55
		Sc. PD:	0.16
		Sc. rank:	1168.1

,

NA

Pos.	115	obs:	exp:
tct	S	0	0.17
tcc	S	0	0.16
tca	S	0	0.24
tcg	S	1	0.05
cct	P	1233	1404.00
ccc	P	147	957.70
cca	P	715	1884.00
ccg	P	2662	511.70
caa	Q	0	1.62
cag	Q	3	1.38
cgt	R	0	0.03
cgc	R	0	0.05
cga	R	0	0.10
cgg	R	1	0.07
agt	S	0	0.19
agc	S	0	0.19
aga	R	0	0.46
agg	R	0	0.28
gtt	V	0	0.67
gtc	V	0	0.57
gta	V	0	0.76
gtg	V	3	1.01
gct	A	0	1.72
gcc	A	0	1.60
gca	A	0	3.16
gcg	A	7	0.52
---	--	------	------
mPD		0.60	0.71
		nPD:	0.85
		N. weight:	1.1
		Sc. PD:	0.49
		Sc. rank:	2736.8

,

NA

Pos.	116	obs:	exp:
caa	Q	4667	2573.00
cag	Q	105	2199.00
---	--	------	------
mPD		0.043	0.50
		nPD:	0.09
		N. weight:	0.8
		Sc. PD:	-0.2
		Sc. rank:	-1431.2

,

NA

Pos.	117	obs:	exp:
tgt	C	3910	2054.00
tgc	C	862	2718.00
---	--	------	------
mPD		0.30	0.49
		nPD:	0.6
		N. weight:	0.5
		Sc. PD:	0.12
		Sc. rank:	876.0

,

NA

Pos.	118	obs:	exp:
tct	S	0	2.35
tcc	S	0	2.21
tca	S	0	3.40
tcg	S	0	0.74
cat	H	2	4.61
cac	H	6	3.39
cgt	R	0	21.21
cgc	R	0	35.52
cga	R	0	67.54
cgg	R	0	48.55
att	I	0	1.06
atc	I	2	0.77
ata	I	1	1.17
act	T	0	6.40
acc	T	26	5.62
aca	T	0	11.81
acg	T	0	2.17
aat	N	83	1265.00
aac	N	2143	960.80
aaa	K	1447	1069.00
aag	K	312	690.00
agt	S	0	2.61
agc	S	14	2.68
aga	R	605	308.20
agg	R	67	191.00
gat	D	0	11.25
gac	D	20	8.75
gaa	E	43	25.48
gag	E	0	17.52
ggt	G	0	0.16
ggc	G	0	0.13
gga	G	1	0.45
ggg	G	0	0.26
---	--	------	------
mPD		0.87	1.1
		nPD:	0.79
		N. weight:	0.7
		Sc. PD:	0.28
		Sc. rank:	1713.2

,

NA

Pos.	119	obs:	exp:
att	I	3661	1689.00
atc	I	1075	1221.00
ata	I	24	1851.00
atg	M	2	2.00
gtt	V	8	2.22
gtc	V	1	1.89
gta	V	1	2.52
gtg	V	0	3.37
---	--	------	------
mPD		0.36	0.66
		nPD:	0.55
		N. weight:	0.85
		Sc. PD:	0.15
		Sc. rank:	1276.0

,

NA

Pos.	120	obs:	exp:
cgt	R	0	0.16
cgc	R	0	0.26
cga	R	0	0.50
cgg	R	0	0.36
atg	M	1	1.00
act	T	80	1109.00
acc	T	0	975.00
aca	T	4404	2049.00
acg	T	25	376.00
aat	N	2	1.14
aac	N	0	0.86
aaa	K	47	29.17
aag	K	1	18.83
aga	R	5	2.29
agg	R	0	1.42
gtt	V	0	0.89
gtc	V	0	0.75
gta	V	4	1.01
gtg	V	0	1.35
gct	A	3	49.27
gcc	A	0	46.05
gca	A	198	90.60
gcg	A	0	15.07
gaa	E	2	1.19
gag	E	0	0.81
---	--	------	------
mPD		0.15	0.79
		nPD:	0.2
		N. weight:	1.1
		Sc. PD:	-0.15
		Sc. rank:	-1064.6

,

NA			
Pos.	121	obs:	exp:
ggt	G	24	748.50
ggc	G	178	604.70
gga	G	3697	2161.00
ggg	G	873	1257.00
---	--	------	------
mPD		0.36	0.68
		nPD:	0.53
		N. weight:	0.45
		Sc. PD:	0.075
		Sc. rank:	622.5

NA			
Pos.	122	obs:	exp:
ttt	F	4710	2138.00
ttc	F	62	2634.00
---	--	------	------
mPD		0.026	0.49
		nPD:	0.05
		N. weight:	1.1
		Sc. PD:	-0.31
		Sc. rank:	-2431.9

NA			
Pos.	123	obs:	exp:
gtt	V	0	6.44
gtc	V	0	5.47
gta	V	29	7.32
gtg	V	0	9.77
gct	A	10	1163.00
gcc	A	0	1087.00
gca	A	4597	2138.00
gcg	A	136	355.70
---	--	------	------
mPD		0.072	0.69
		nPD:	0.1
		N. weight:	1.1
		Sc. PD:	-0.25
		Sc. rank:	-1861.7

NA			
Pos.	124	obs:	exp:
tct	S	2	0.34
tcc	S	0	0.32
tca	S	0	0.49
tcg	S	0	0.11
cct	P	4091	1407.00
ccc	P	495	960.10
cca	P	175	1889.00
ccg	P	8	513.00
act	T	1	0.25
acc	T	0	0.22
aca	T	0	0.45
acg	T	0	0.08
agt	S	0	0.37
agc	S	0	0.38
---	--	------	------
mPD		0.25	0.71
		nPD:	0.36
		N. weight:	1.2
		Sc. PD:	0.0081
		Sc. rank:	308.7

NA			
Pos.	125	obs:	exp:
ttt	F	4369	2138.00
ttc	F	403	2634.00
---	--	------	------
mPD		0.15	0.49
		nPD:	0.31
		N. weight:	0.77
		Sc. PD:	-0.027
		Sc. rank:	-95.9

NA			
Pos.	126	obs:	exp:
tct	S	2716	802.30
tcc	S	1983	753.90
tca	S	70	1158.00
tcg	S	1	253.50
agt	S	0	887.80
agc	S	0	914.20
gct	A	1	0.49
gcc	A	1	0.46
gca	A	0	0.90
gcg	A	0	0.15
---	--	------	------
mPD		0.50	1.6
		nPD:	0.31
		N. weight:	1.6
		Sc. PD:	-0.064
		Sc. rank:	-261.7

NA			
Pos.	127	obs:	exp:
cgt	R	0	5.11
cgc	R	0	8.56
cga	R	0	16.28
cgg	R	0	11.70
aaa	K	1108	2802.00
aag	K	3502	1808.00
aga	R	148	74.29
agg	R	14	46.04
---	--	------	------
mPD		0.45	0.56
		nPD:	0.81
		N. weight:	0.45
		Sc. PD:	0.19
		Sc. rank:	1122.5

NA			
Pos.	128	obs:	exp:
gat	D	91	2684.00
gac	D	4681	2088.00
---	--	------	------
mPD		0.037	0.49
		nPD:	0.08
		N. weight:	1.1
		Sc. PD:	-0.29
		Sc. rank:	-2153.0

NA			
Pos.	129	obs:	exp:
aat	N	3817	2712.00
aac	N	955	2060.00
---	--	------	------
mPD		0.32	0.49
		nPD:	0.65
		N. weight:	0.19
		Sc. PD:	0.052
		Sc. rank:	378.0

NA			
Pos.	130	obs:	exp:
ttt	F	1	0.45
ttc	F	0	0.55
tct	S	723	801.10
tcc	S	180	752.80
tca	S	2239	1157.00
tcg	S	1621	253.10
agt	S	0	886.50
agc	S	0	912.80
gct	A	4	1.96
gcc	A	4	1.83
gca	A	0	3.61
gcg	A	0	0.60
---	--	------	------
mPD		0.64	1.6
		nPD:	0.39
		N. weight:	1.4
		Sc. PD:	0.054
		Sc. rank:	529.6

NA			
Pos.	131	obs:	exp:
att	I	4190	1678.00
atc	I	484	1213.00
ata	I	56	1839.00
gtt	V	37	9.33
gtc	V	3	7.93
gta	V	2	10.60
gtg	V	0	14.15
---	--	------	------
mPD		0.22	0.68
		nPD:	0.33
		N. weight:	1.1
		Sc. PD:	-0.022
		Sc. rank:	56.3

NA			
Pos.	132	obs:	exp:
cgt	R	22	150.60
cgc	R	2	252.20
cga	R	128	479.60
cgg	R	3022	344.70
aga	R	220	2188.00
agg	R	1378	1356.00
---	--	------	------
mPD		0.59	0.94
		nPD:	0.63
		N. weight:	1.9
		Sc. PD:	0.49
		Sc. rank:	3651.3

NA			
Pos.	133	obs:	exp:
tta	L	0	519.30
ttg	L	0	896.30
ctt	L	4710	963.80
ctc	L	49	694.30
cta	L	3	833.30
ctg	L	10	865.00
---	--	------	------
mPD		0.026	1.1
		nPD:	0.02
		N. weight:	2.4
		Sc. PD:	-0.72
		Sc. rank:	-5850.8

NA			
Pos.	134	obs:	exp:
tct	S	3903	801.60
tcc	S	803	753.20
tca	S	37	1157.00
tcg	S	23	253.30
agt	S	0	887.10
agc	S	0	913.40
gct	A	5	1.47
gcc	A	0	1.37
gca	A	1	2.70
gcg	A	0	0.45
---	--	------	------
mPD		0.30	1.6
		nPD:	0.19
		N. weight:	2.
		Sc. PD:	-0.3
		Sc. rank:	-2116.4

NA			
Pos.	135	obs:	exp:
gtt	V	1	0.22
gtc	V	0	0.19
gta	V	0	0.25
gtg	V	0	0.34
gct	A	4242	1170.00
gcc	A	406	1093.00
gca	A	114	2151.00
gcg	A	9	357.80
---	--	------	------
mPD		0.20	0.68
		nPD:	0.3
		N. weight:	1.5
		Sc. PD:	-0.076
		Sc. rank:	-477.2

NA			
Pos.	136	obs:	exp:
tct	S	0	1.18
tcc	S	0	1.11
tca	S	0	1.70
tcg	S	0	0.37
agt	S	6	1.30
agc	S	1	1.34
ggt	G	4726	747.40
ggc	G	8	603.80
gga	G	17	2158.00
ggg	G	14	1256.00
---	--	------	------
mPD		0.020	0.69
		nPD:	0.03
		N. weight:	2.8
		Sc. PD:	-0.83
		Sc. rank:	-6719.8

NA			
Pos.	137	obs:	exp:
ggt	G	30	748.50
ggc	G	0	604.70
gga	G	319	2161.00
ggg	G	4423	1257.00
---	--	------	------
mPD		0.14	0.68
		nPD:	0.2
		N. weight:	1.6
		Sc. PD:	-0.22
		Sc. rank:	-1552.9

NA			
Pos.	138	obs:	exp:
tct	S	0	0.17
tcc	S	1	0.16
tca	S	0	0.24
tcg	S	0	0.05
cct	P	0	5.31
ccc	P	18	3.62
cca	P	0	7.13
ccg	P	0	1.94
agt	S	0	0.19
agc	S	0	0.19
gct	A	7	108.80
gcc	A	435	101.70
gca	A	2	200.10
gcg	A	0	33.30
gat	D	42	2423.00
gac	D	4265	1884.00
gaa	E	1	0.59
gag	E	0	0.41
ggt	G	0	0.16
ggc	G	1	0.13
gga	G	0	0.45
ggg	G	0	0.26
---	--	------	------
mPD		0.21	0.72
		nPD:	0.28
		N. weight:	1.3
		Sc. PD:	-0.081
		Sc. rank:	-551.5

NA			
Pos.	139	obs:	exp:
tta	L	0	0.11
ttg	L	0	0.19
ctt	L	0	0.20
ctc	L	1	0.15
cta	L	0	0.17
ctg	L	0	0.18
att	I	2414	1690.00
atc	I	2245	1222.00
ata	I	106	1852.00
gtt	V	5	1.33
gtc	V	1	1.13
gta	V	0	1.51
gtg	V	0	2.02
---	--	------	------
mPD		0.52	0.66
		nPD:	0.79
		N. weight:	0.63
		Sc. PD:	0.26
		Sc. rank:	1557.0

NA			
Pos.	140	obs:	exp:
tgg	W	4772	4772.00
---	--	------	------
mPD		0	0
		nPD:	1.
		N. weight:	0.
		Sc. PD:	0
		Sc. rank:	0

NA			
Pos.	141	obs:	exp:
att	I	5	6.03
atc	I	1	4.36
ata	I	11	6.61
atg	M	1	1.00
gtt	V	232	1055.00
gtc	V	235	896.90
gta	V	1133	1200.00
gtg	V	3153	1601.00
gct	A	0	0.25
gcc	A	0	0.23
gca	A	0	0.45
gcg	A	1	0.07
---	--	------	------
mPD		0.51	0.75
		nPD:	0.68
		N. weight:	0.47
		Sc. PD:	0.14
		Sc. rank:	1007.4

,

NA			
Pos.	142	obs:	exp:
act	T	69	1174.00
acc	T	3	1032.00
aca	T	2783	2168.00
acg	T	1917	397.90
---	--	------	------
mPD		0.50	0.68
		nPD:	0.73
		N. weight:	1.1
		Sc. PD:	0.4
		Sc. rank:	2670.9

,

NA			
Pos.	143	obs:	exp:
cgt	R	0	150.60
cgc	R	0	252.20
cga	R	4	479.50
cgg	R	0	344.70
aga	R	4300	2188.00
agg	R	467	1356.00
gaa	E	1	0.59
gag	E	0	0.41
---	--	------	------
mPD		0.18	0.94
		nPD:	0.19
		N. weight:	0.78
		Sc. PD:	-0.12
		Sc. rank:	-813.1

,

NA			
Pos.	144	obs:	exp:
gtt	V	0	0.22
gtc	V	0	0.19
gta	V	1	0.25
gtg	V	0	0.34
gaa	E	4392	2827.00
gag	E	379	1944.00
---	--	------	------
mPD		0.15	0.48
		nPD:	0.3
		N. weight:	0.43
		Sc. PD:	-0.019
		Sc. rank:	-97.9

,

NA			
Pos.	145	obs:	exp:
tta	L	0	0.11
ttg	L	0	0.19
ctt	L	1	0.20
ctc	L	0	0.15
cta	L	0	0.17
ctg	L	0	0.18
cct	P	4658	1408.00
ccc	P	96	960.50
cca	P	8	1889.00
ccg	P	9	513.20
---	--	------	------
mPD		0.047	0.70
		nPD:	0.07
		N. weight:	1.7
		Sc. PD:	-0.45
		Sc. rank:	-3412.6

,

NA			
Pos.	146	obs:	exp:
tta	L	0	0.11
ttg	L	0	0.19
tat	Y	4122	2673.00
tac	Y	649	2098.00
ctt	L	1	0.20
ctc	L	0	0.15
cta	L	0	0.17
ctg	L	0	0.18
---	--	------	------
mPD		0.24	0.49
		nPD:	0.48
		N. weight:	0.34
		Sc. PD:	0.039
		Sc. rank:	331.2

,

NA			
Pos.	147	obs:	exp:
att	I	0	12.06
atc	I	0	8.72
ata	I	34	13.22
gtt	V	258	1052.00
gtc	V	157	894.00
gta	V	451	1196.00
gtg	V	3872	1596.00
---	--	------	------
mPD		0.34	0.75
		nPD:	0.45
		N. weight:	0.78
		Sc. PD:	0.074
		Sc. rank:	667.6

,

NA			
Pos.	148	obs:	exp:
tct	S	40	802.50
tcc	S	13	754.00
tca	S	4620	1159.00
tcg	S	98	253.60
agt	S	0	888.00
agc	S	0	914.30
gct	A	0	0.25
gcc	A	1	0.23
gca	A	0	0.45
gcg	A	0	0.07
---	--	------	------
mPD		0.063	1.6
		nPD:	0.04
		N. weight:	2.
		Sc. PD:	-0.58
		Sc. rank:	-4576.0

,

NA			
Pos.	149	obs:	exp:
tgt	C	621	2054.00
tgc	C	4151	2718.00
---	--	------	------
mPD		0.23	0.49
		nPD:	0.46
		N. weight:	0.33
		Sc. PD:	0.034
		Sc. rank:	295.9

,

NA			
Pos.	150	obs:	exp:
tct	S	0	5.05
tcc	S	0	4.74
tca	S	0	7.29
tcg	S	0	1.59
act	T	1	0.25
acc	T	0	0.22
aca	T	0	0.45
acg	T	0	0.08
aat	N	2	1.71
aac	N	1	1.29
agt	S	29	5.58
agc	S	1	5.75
gtt	V	2	0.44
gtc	V	0	0.38
gta	V	0	0.50
gtg	V	0	0.67
gct	A	5	1.23
gcc	A	0	1.15
gca	A	0	2.25
gcg	A	0	0.37
gat	D	4319	2512.00
gac	D	147	1954.00
gaa	E	172	141.00
gag	E	66	96.98
ggt	G	19	4.24
ggc	G	0	3.42
gga	G	0	12.23
ggg	G	8	7.11
---	--	------	------
mPD		0.20	0.59
		nPD:	0.34
		N. weight:	0.67
		Sc. PD:	-0.0097
		Sc. rank:	76.0

,

NA

Pos.	151	obs:	exp:
ttt	F	2	4.93
ttc	F	9	6.07
tta	L	0	59.85
ttg	L	0	103.30
tct	S	116	22.03
tcc	S	14	20.70
tca	S	0	31.81
tcg	S	1	6.96
ctt	L	547	111.10
ctc	L	3	80.02
cta	L	0	96.04
ctg	L	0	99.70
cct	P	3257	1036.00
ccc	P	166	706.70
cca	P	77	1390.00
ccg	P	10	377.60
cat	H	13	295.30
cac	H	499	216.70
caa	Q	14	8.09
cag	Q	1	6.91
cgt	R	2	0.16
cgc	R	3	0.26
cga	R	0	0.50
cgg	R	0	0.36
act	T	18	4.67
acc	T	0	4.11
aca	T	0	8.63
acg	T	1	1.58
aat	N	5	10.80
aac	N	14	8.20
agt	S	0	24.38
agc	S	0	25.11
aga	R	0	2.29
agg	R	0	1.42
---	--	------	------
mPD		0.75	1.3
		nPD:	0.59
		N. weight:	1.5
		Sc. PD:	0.34
		Sc. rank:	2655.4

,

NA

Pos.	152	obs:	exp:
tct	S	0	63.75
tcc	S	0	59.90
tca	S	0	92.04
tcg	S	0	20.14
tat	Y	1	5.60
tac	Y	9	4.40
cgt	R	0	0.35
cgc	R	0	0.58
cga	R	0	1.11
cgg	R	0	0.79
att	I	0	0.71
atc	I	2	0.51
ata	I	0	0.78
act	T	1	0.25
acc	T	0	0.22
aca	T	0	0.45
acg	T	0	0.08
aat	N	8	162.00
aac	N	277	123.00
aaa	K	21	13.37
aag	K	1	8.63
agt	S	24	70.54
agc	S	355	72.63
aga	R	11	5.04
agg	R	0	3.13
gtt	V	6	3.77
gtc	V	10	3.21
gta	V	1	4.29
gtg	V	0	5.73
gat	D	1055	2046.00
gac	D	2583	1592.00
gaa	E	81	63.99
gag	E	27	44.01
ggt	G	3	46.90
ggc	G	226	37.89
gga	G	58	135.40
ggg	G	12	78.78
---	--	------	------
mPD		0.93	1.1
		nPD:	0.84
		N. weight:	0.55
		Sc. PD:	0.24
		Sc. rank:	1384.8

,

NA

Pos.	153	obs:	exp:
caa	Q	0	2.16
cag	Q	4	1.84
cgt	R	0	4.39
cgc	R	0	7.35
cga	R	0	13.97
cgg	R	0	10.04
act	T	0	0.25
acc	T	0	0.22
aca	T	0	0.45
acg	T	1	0.08
aat	N	4	2.27
aac	N	0	1.73
aaa	K	827	2810.00
aag	K	3797	1814.00
aga	R	30	63.74
agg	R	109	39.51
---	--	------	------
mPD		0.35	0.55
		nPD:	0.64
		N. weight:	0.62
		Sc. PD:	0.16
		Sc. rank:	1212.1

,

NA

Pos.	154	obs:	exp:
tgt	C	4634	2054.00
tgc	C	138	2718.00
---	--	------	------
mPD		0.056	0.49
		nPD:	0.11
		N. weight:	1.1
		Sc. PD:	-0.24
		Sc. rank:	-1715.2

,

NA

Pos.	155	obs:	exp:
ttt	F	8	4.03
ttc	F	1	4.97
tat	Y	3851	2668.00
tac	Y	912	2095.00
---	--	------	------
mPD		0.31	0.50
		nPD:	0.63
		N. weight:	0.22
		Sc. PD:	0.056
		Sc. rank:	413.1

,

NA

Pos.	156	obs:	exp:
cat	H	5	2.88
cac	H	0	2.12
caa	Q	3109	2571.00
cag	Q	1658	2196.00
---	--	------	------
mPD		0.45	0.50
		nPD:	0.91
		N. weight:	0.043
		Sc. PD:	0.022
		Sc. rank:	111.9

,

NA

Pos.	157	obs:	exp:
ttt	F	4688	2138.00
ttc	F	84	2634.00
---	--	------	------
mPD		0.035	0.49
		nPD:	0.07
		N. weight:	1.1
		Sc. PD:	-0.29
		Sc. rank:	-2142.6

,

NA

Pos.	158	obs:	exp:
tct	S	0	0.17
tcc	S	1	0.16
tca	S	0	0.24
tcg	S	0	0.05
agt	S	0	0.19
agc	S	0	0.19
gct	A	106	1170.00
gcc	A	3591	1093.00
gca	A	844	2151.00
gcg	A	230	357.80
---	--	------	------
mPD		0.40	0.68
		nPD:	0.59
		N. weight:	1.
		Sc. PD:	0.22
		Sc. rank:	1738.0

,

NA

Pos.	159	obs:	exp:
tta	L	160	519.10
ttg	L	0	896.00
ctt	L	3734	963.40
ctc	L	802	694.00
cta	L	56	832.90
ctg	L	18	864.70
cct	P	0	0.30
ccc	P	1	0.20
cca	P	0	0.40
ccg	P	0	0.11
att	I	1	0.35
atc	I	0	0.26
ata	I	0	0.39
---	--	------	------
mPD		0.42	1.1
		nPD:	0.37
		N. weight:	1.6
		Sc. PD:	0.028
		Sc. rank:	470.8

,

NA

Pos.	160	obs:	exp:
tct	S	0	0.17
tcc	S	0	0.16
tca	S	0	0.24
tcg	S	0	0.05
agt	S	0	0.19
agc	S	1	0.19
ggt	G	60	748.30
ggc	G	28	604.50
gga	G	1761	2161.00
ggg	G	2922	1257.00
---	--	------	------
mPD		0.49	0.69
		nPD:	0.71
		N. weight:	0.61
		Sc. PD:	0.2
		Sc. rank:	1391.5

,

NA

Pos.	161	obs:	exp:
cat	H	1	0.58
cac	H	0	0.42
caa	Q	1336	2572.00
cag	Q	3434	2198.00
cgt	R	0	0.03
cgc	R	0	0.05
cga	R	1	0.10
cgg	R	0	0.07
aga	R	0	0.46
agg	R	0	0.28
---	--	------	------
mPD		0.40	0.50
		nPD:	0.81
		N. weight:	0.22
		Sc. PD:	0.092
		Sc. rank:	541.6

,

NA

Pos.	162	obs:	exp:
ggt	G	6	748.50
ggc	G	0	604.70
gga	G	3949	2161.00
ggg	G	817	1257.00
---	--	------	------
mPD		0.29	0.68
		nPD:	0.42
		N. weight:	0.65
		Sc. PD:	0.04
		Sc. rank:	402.6

,

NA

Pos.	163	obs:	exp:
act	T	280	1174.00
acc	T	787	1032.00
aca	T	3689	2168.00
acg	T	16	397.90
---	--	------	------
mPD		0.37	0.68
		nPD:	0.55
		N. weight:	0.43
		Sc. PD:	0.077
		Sc. rank:	639.8

,

NA

Pos.	164	obs:	exp:
act	T	4	1174.00
acc	T	13	1032.00
aca	T	4599	2168.00
acg	T	155	397.80
aaa	K	1	0.61
aag	K	0	0.39
---	--	------	------
mPD		0.070	0.68
		nPD:	0.1
		N. weight:	1.1
		Sc. PD:	-0.24
		Sc. rank:	-1794.1

,

NA

Pos.	165	obs:	exp:
tta	L	2203	518.70
ttg	L	359	895.20
ctt	L	0	962.50
ctc	L	2	693.40
cta	L	2150	832.20
ctg	L	52	863.90
gtt	V	0	1.33
gtc	V	0	1.13
gta	V	5	1.51
gtg	V	1	2.02
---	--	------	------
mPD		0.66	1.1
		nPD:	0.58
		N. weight:	1.6
		Sc. PD:	0.32
		Sc. rank:	2520.6

,

NA			
Pos.	166	obs:	exp:
tct	S	0	4.04
tcc	S	0	3.79
tca	S	0	5.83
tcg	S	0	1.28
tat	Y	0	2.80
tac	Y	5	2.20
cat	H	0	2.31
cac	H	4	1.69
act	T	0	0.25
acc	T	1	0.22
aca	T	0	0.45
acg	T	0	0.08
aat	N	72	1962.00
aac	N	3380	1490.00
aaa	K	7	6.69
aag	K	4	4.31
agt	S	0	4.47
agc	S	24	4.60
gtt	V	0	0.22
gtc	V	1	0.19
gta	V	0	0.25
gtg	V	0	0.34
gct	A	0	0.49
gcc	A	2	0.46
gca	A	0	0.90
gcg	A	0	0.15
gat	D	58	714.90
gac	D	1213	556.10
gaa	E	1	0.59
gag	E	0	0.41
---	--	------	------
mPD		0.46	0.91
		nPD:	0.51
		N. weight:	1.1
		Sc. PD:	0.16
		Sc. rank:	1313.7

,

NA			
Pos.	167	obs:	exp:
act	T	0	0.25
acc	T	1	0.22
aca	T	0	0.45
acg	T	0	0.08
aat	N	267	2712.00
aac	N	4504	2059.00
---	--	------	------
mPD		0.11	0.49
		nPD:	0.22
		N. weight:	0.95
		Sc. PD:	-0.12
		Sc. rank:	-855.4

,

NA			
Pos.	168	obs:	exp:
cgt	R	0	64.11
cgc	R	0	107.30
cga	R	1	204.10
cgg	R	1	146.70
aaa	K	620	396.20
aag	K	32	255.80
aga	R	680	931.40
agg	R	1349	577.30
gtt	V	0	6.88
gtc	V	0	5.85
gta	V	2	7.83
gtg	V	29	10.44
gaa	E	3	1.78
gag	E	0	1.22
ggt	G	0	322.30
ggc	G	3	260.40
gga	G	1002	930.80
ggg	G	1050	541.50
---	--	------	------
mPD		1.2	1.5
		nPD:	0.85
		N. weight:	0.63
		Sc. PD:	0.29
		Sc. rank:	1604.8

,

NA			
Pos.	169	obs:	exp:
tta	L	0	0.11
ttg	L	0	0.19
ctt	L	1	0.20
ctc	L	0	0.15
cta	L	0	0.17
ctg	L	0	0.18
cat	H	4348	2752.00
cac	H	423	2019.00
---	--	------	------
mPD		0.16	0.49
		nPD:	0.33
		N. weight:	0.43
		Sc. PD:	-0.008
		Sc. rank:	25.1

,

NA			
Pos.	170	obs:	exp:
tct	S	3	802.50
tcc	S	2	754.00
tca	S	4436	1159.00
tcg	S	330	253.60
agt	S	0	888.00
agc	S	0	914.30
gct	A	0	0.25
gcc	A	0	0.23
gca	A	1	0.45
gcg	A	0	0.07
---	--	------	------
mPD		0.13	1.6
		nPD:	0.08
		N. weight:	2.
		Sc. PD:	-0.49
		Sc. rank:	-3593.1

,

NA			
Pos.	171	obs:	exp:
act	T	1	0.25
acc	T	0	0.22
aca	T	0	0.45
acg	T	0	0.08
aat	N	4732	2711.00
aac	N	38	2059.00
aaa	K	1	0.61
aag	K	0	0.39
---	--	------	------
mPD		0.017	0.49
		nPD:	0.03
		N. weight:	0.81
		Sc. PD:	-0.24
		Sc. rank:	-1902.3

,

NA			
Pos.	172	obs:	exp:
aat	N	122	970.20
aac	N	1585	736.80
gat	D	365	1689.00
gac	D	2637	1313.00
gaa	E	8	4.74
gag	E	0	3.26
ggt	G	7	8.63
ggc	G	43	6.97
gga	G	5	24.91
ggg	G	0	14.49
---	--	------	------
mPD		0.67	0.98
		nPD:	0.68
		N. weight:	0.76
		Sc. PD:	0.23
		Sc. rank:	1663.2

,

NA			
Pos.	173	obs:	exp:
att	I	5	1.77
atc	I	0	1.28
ata	I	0	1.94
act	T	2257	1172.00
acc	T	65	1031.00
aca	T	2285	2166.00
acg	T	159	397.40
gct	A	1	0.25
gcc	A	0	0.23
gca	A	0	0.45
gcg	A	0	0.07
---	--	------	------
mPD		0.55	0.68
		nPD:	0.8
		N. weight:	0.42
		Sc. PD:	0.18
		Sc. rank:	1050.9

,

NA			
Pos.	174	obs:	exp:
ttt	F	921	415.40
ttc	F	6	511.60
tta	L	6	0.65
ttg	L	0	1.13
ctt	L	0	1.21
ctc	L	0	0.87
cta	L	0	1.05
ctg	L	0	1.09
att	I	0	458.00
atc	I	3	331.10
ata	I	1288	501.90
atg	M	11	11.00
act	T	0	1.97
acc	T	0	1.73
aca	T	6	3.64
acg	T	2	0.67
gtt	V	4	558.20
gtc	V	10	474.40
gta	V	2422	634.60
gtg	V	78	846.80
gct	A	0	3.68
gcc	A	0	3.44
gca	A	15	6.76
gcg	A	0	1.12
---	--	------	------
mPD		0.96	1.4
		nPD:	0.71
		N. weight:	1.6
		Sc. PD:	0.53
		Sc. rank:	3634.1

,

NA			
Pos.	175	obs:	exp:
tat	Y	3	1.68
tac	Y	0	1.32
cct	P	2	0.59
ccc	P	0	0.40
cca	P	0	0.79
ccg	P	0	0.22
cat	H	4697	2740.00
cac	H	53	2010.00
caa	Q	1	0.54
cag	Q	0	0.46
cgt	R	8	0.25
cgc	R	0	0.42
cga	R	0	0.80
cgg	R	0	0.58
aat	N	8	4.55
aac	N	0	3.45
aga	R	0	3.67
agg	R	0	2.27
---	--	------	------
mPD		0.031	0.50
		nPD:	0.06
		N. weight:	0.78
		Sc. PD:	-0.21
		Sc. rank:	-1566.0

,

NA			
Pos.	176	obs:	exp:
aat	N	2	1.14
aac	N	0	0.86
gat	D	4530	2682.00
gac	D	239	2087.00
gaa	E	0	0.59
gag	E	1	0.41
---	--	------	------
mPD		0.096	0.49
		nPD:	0.2
		N. weight:	0.61
		Sc. PD:	-0.087
		Sc. rank:	-611.5

,

NA			
Pos.	177	obs:	exp:
cgt	R	0	150.60
cgc	R	0	252.20
cga	R	1	479.60
cgg	R	4	344.70
aga	R	1275	2188.00
agg	R	3492	1356.00
---	--	------	------
mPD		0.39	0.94
		nPD:	0.42
		N. weight:	0.85
		Sc. PD:	0.053
		Sc. rank:	558.5

,

NA			
Pos.	178	obs:	exp:
tct	S	0	0.17
tcc	S	0	0.16
tca	S	0	0.24
tcg	S	0	0.05
act	T	513	1172.00
acc	T	4224	1031.00
aca	T	26	2166.00
acg	T	3	397.40
aat	N	0	2.84
aac	N	5	2.16
agt	S	0	0.19
agc	S	1	0.19
---	--	------	------
mPD		0.21	0.68
		nPD:	0.3
		N. weight:	1.8
		Sc. PD:	-0.082
		Sc. rank:	-488.8

,

NA			
Pos.	179	obs:	exp:
tct	S	5	1.18
tcc	S	0	1.11
tca	S	2	1.70
tcg	S	0	0.37
cct	P	4315	1406.00
ccc	P	65	959.30
cca	P	357	1887.00
ccg	P	28	512.60
agt	S	0	1.30
agc	S	0	1.34
---	--	------	------
mPD		0.18	0.71
		nPD:	0.25
		N. weight:	1.3
		Sc. PD:	-0.12
		Sc. rank:	-781.1

,

NA			
Pos.	180	obs:	exp:
ttt	F	5	2.24
ttc	F	0	2.76
tat	Y	3083	2562.00
tac	Y	1490	2011.00
cat	H	192	111.90
cac	H	2	82.10
---	--	------	------
mPD		0.51	0.57
		nPD:	0.89
		N. weight:	0.075
		Sc. PD:	0.037
		Sc. rank:	194.3

,

NA

Pos.	181	obs:	exp:
cgt	R	28	150.60
cgc	R	4	252.20
cga	R	4194	479.60
cgg	R	262	344.70
aga	R	281	2188.00
agg	R	3	1356.00
---	--	------	------
mPD		0.23	0.94
		nPD:	0.24
		N. weight:	2.7
		Sc. PD:	-0.27
		Sc. rank:	-1769.5

,

NA

Pos.	182	obs:	exp:
act	T	345	1174.00
acc	T	4226	1032.00
aca	T	189	2168.00
acg	T	12	397.90
---	--	------	------
mPD		0.21	0.68
		nPD:	0.31
		N. weight:	1.6
		Sc. PD:	-0.066
		Sc. rank:	-290.5

,

NA

Pos.	183	obs:	exp:
tta	L	216	519.30
ttg	L	62	896.30
ctt	L	21	963.80
ctc	L	3	694.30
cta	L	3474	833.30
ctg	L	996	865.00
---	--	------	------
mPD		0.46	1.1
		nPD:	0.41
		N. weight:	1.5
		Sc. PD:	0.077
		Sc. rank:	757.0

,

NA

Pos.	184	obs:	exp:
tta	L	174	519.30
ttg	L	4419	896.30
ctt	L	0	963.80
ctc	L	0	694.30
cta	L	2	833.30
ctg	L	177	865.00
---	--	------	------
mPD		0.14	1.1
		nPD:	0.13
		N. weight:	2.1
		Sc. PD:	-0.45
		Sc. rank:	-2964.1

,

NA

Pos.	185	obs:	exp:
atg	M	4772	4772.00
---	--	------	------
mPD		0	0
		nPD:	1.
		N. weight:	0.
		Sc. PD:	0
		Sc. rank:	0

,

NA

Pos.	186	obs:	exp:
tct	S	0	33.14
tcc	S	0	31.13
tca	S	0	47.84
tcg	S	0	10.47
act	T	2	0.49
acc	T	0	0.43
aca	T	0	0.91
acg	T	0	0.17
aat	N	4377	2596.00
aac	N	191	1972.00
agt	S	193	36.67
agc	S	4	37.75
ggt	G	5	0.78
ggc	G	0	0.63
gga	G	0	2.26
ggg	G	0	1.32
---	--	------	------
mPD		0.16	0.64
		nPD:	0.25
		N. weight:	0.71
		Sc. PD:	-0.063
		Sc. rank:	-398.0

,

NA

Pos.	187	obs:	exp:
gat	D	2	3.37
gac	D	4	2.63
gaa	E	3117	2824.00
gag	E	1649	1942.00
---	--	------	------
mPD		0.45	0.48
		nPD:	0.94
		N. weight:	0.014
		Sc. PD:	0.0078
		Sc. rank:	38.0

,

NA

Pos.	188	obs:	exp:
tta	L	110	519.10
ttg	L	4534	896.00
ctt	L	0	963.40
ctc	L	0	694.00
cta	L	3	832.90
ctg	L	123	864.70
atg	M	1	1.00
gtt	V	0	0.22
gtc	V	0	0.19
gta	V	0	0.25
gtg	V	1	0.34
---	--	------	------
mPD		0.098	1.1
		nPD:	0.09
		N. weight:	2.3
		Sc. PD:	-0.55
		Sc. rank:	-4049.6

,

NA

Pos.	189	obs:	exp:
ggt	G	4367	748.50
ggc	G	26	604.70
gga	G	276	2161.00
ggg	G	103	1257.00
---	--	------	------
mPD		0.16	0.68
		nPD:	0.23
		N. weight:	2.2
		Sc. PD:	-0.24
		Sc. rank:	-1700.1

,

NA

Pos.	190	obs:	exp:
---	-	4772	4772.00
---	--	------	------
mPD		0	0
		nPD:	1.
		N. weight:	0.
		Sc. PD:	0
		Sc. rank:	0

,

NA

Pos.	191	obs:	exp:
att	I	1001	362.20
atc	I	16	261.90
ata	I	4	396.90
act	T	1	0.25
acc	T	0	0.22
aca	T	0	0.45
acg	T	0	0.08
gtt	V	3383	832.60
gtc	V	363	707.60
gta	V	4	946.60
gtg	V	0	1263.00
---	--	------	------
mPD		0.49	1.1
		nPD:	0.45
		N. weight:	1.8
		Sc. PD:	0.16
		Sc. rank:	1452.6

,

NA

Pos.	192	obs:	exp:
cct	P	30	1408.00
ccc	P	11	960.70
cca	P	4694	1890.00
ccg	P	37	513.30
---	--	------	------
mPD		0.032	0.70
		nPD:	0.05
		N. weight:	1.3
		Sc. PD:	-0.37
		Sc. rank:	-2865.2

,

NA

Pos.	193	obs:	exp:
ttt	F	3268	2138.00
ttc	F	1503	2633.00
tat	Y	0	0.56
tac	Y	1	0.44
---	--	------	------
mPD		0.43	0.50
		nPD:	0.87
		N. weight:	0.18
		Sc. PD:	0.086
		Sc. rank:	461.1

,

NA

Pos.	194	obs:	exp:
tat	Y	54	34.17
tac	Y	7	26.83
cat	H	4437	2716.00
cac	H	272	1993.00
aat	N	1	1.14
aac	N	1	0.86
---	--	------	------
mPD		0.14	0.51
		nPD:	0.27
		N. weight:	0.54
		Sc. PD:	-0.043
		Sc. rank:	-283.9

,

NA

Pos.	195	obs:	exp:
ttt	F	0	0.45
ttc	F	1	0.55
tta	L	1450	518.20
ttg	L	3172	894.50
ctt	L	0	961.70
ctc	L	0	692.90
cta	L	40	831.50
ctg	L	100	863.20
atg	M	7	7.00
act	T	0	0.25
acc	T	0	0.22
aca	T	0	0.45
acg	T	1	0.08
gtt	V	0	0.22
gtc	V	0	0.19
gta	V	0	0.25
gtg	V	1	0.34
---	--	------	------
mPD		0.49	1.1
		nPD:	0.43
		N. weight:	1.7
		Sc. PD:	0.12
		Sc. rank:	1189.6

,

NA

Pos.	196	obs:	exp:
ggt	G	3	748.50
ggc	G	96	604.70
gga	G	3798	2161.00
ggg	G	875	1257.00
---	--	------	------
mPD		0.33	0.68
		nPD:	0.49
		N. weight:	0.53
		Sc. PD:	0.066
		Sc. rank:	545.9

,

NA

Pos.	197	obs:	exp:
act	T	231	1173.00
acc	T	4232	1031.00
aca	T	281	2167.00
acg	T	26	397.80
gct	A	0	0.49
gcc	A	1	0.46
gca	A	0	0.90
gcg	A	1	0.15
---	--	------	------
mPD		0.21	0.68
		nPD:	0.31
		N. weight:	1.6
		Sc. PD:	-0.067
		Sc. rank:	-340.4

,

NA

Pos.	198	obs:	exp:
cgt	R	0	90.31
cgc	R	0	151.20
cga	R	7	287.60
cgg	R	38	206.70
act	T	0	0.25
acc	T	0	0.22
aca	T	0	0.45
acg	T	1	0.08
aaa	K	1345	1161.00
aag	K	565	749.20
aga	R	154	1312.00
agg	R	2662	813.20
---	--	------	------
mPD		0.93	1.3
		nPD:	0.73
		N. weight:	0.91
		Sc. PD:	0.32
		Sc. rank:	2134.9

,

NA

Pos.	199	obs:	exp:
caa	Q	4477	2573.00
cag	Q	295	2199.00
---	--	------	------
mPD		0.12	0.50
		nPD:	0.23
		N. weight:	0.62
		Sc. PD:	-0.067
		Sc. rank:	-468.3

,

NA

Pos.	200	obs:	exp:
att	I	0	1.42
atc	I	0	1.03
ata	I	4	1.56
gtt	V	160	1059.00
gtc	V	11	899.70
gta	V	410	1204.00
gtg	V	4187	1606.00
---	--	------	------
mPD		0.22	0.74
		nPD:	0.3
		N. weight:	1.1
		Sc. PD:	-0.048
		Sc. rank:	-256.4

,

NA			
Pos.	201	obs:	exp:
tgt	C	784	2054.00
tgc	C	3987	2717.00
cgt	R	0	0.03
cgc	R	1	0.05
cga	R	0	0.10
cgg	R	0	0.07
aga	R	0	0.46
agg	R	0	0.28
---	--	------	------
mPD		0.28	0.49
		nPD:	0.56
		N. weight:	0.25
		Sc. PD:	0.049
		Sc. rank:	392.4

NA			
Pos.	202	obs:	exp:
tta	L	0	0.11
ttg	L	0	0.19
ctt	L	0	0.20
ctc	L	0	0.15
cta	L	0	0.17
ctg	L	1	0.18
att	I	25	1454.00
atc	I	9	1052.00
ata	I	4066	1594.00
atg	M	646	646.00
gtt	V	0	5.55
gtc	V	0	4.72
gta	V	20	6.31
gtg	V	5	8.42
---	--	------	------
mPD		0.26	0.74
		nPD:	0.35
		N. weight:	1.2
		Sc. PD:	0.00026
		Sc. rank:	267.5

NA			
Pos.	203	obs:	exp:
tct	S	0	0.17
tcc	S	0	0.16
tca	S	0	0.24
tcg	S	1	0.05
agt	S	0	0.19
agc	S	0	0.19
gct	A	59	1170.00
gcc	A	6	1093.00
gca	A	4150	2151.00
gcg	A	556	357.80
---	--	------	------
mPD		0.23	0.68
		nPD:	0.34
		N. weight:	0.91
		Sc. PD:	-0.01
		Sc. rank:	122.3

NA			
Pos.	204	obs:	exp:
tgg	W	4760	4760.00
ggt	G	0	1.88
ggc	G	0	1.52
gga	G	0	5.44
ggg	G	12	3.16
---	--	------	------
mPD		0.0050	0.0087
		nPD:	0.58
		N. weight:	0.0052
		Sc. PD:	0.0011
		Sc. rank:	8.4

NA			
Pos.	205	obs:	exp:
tct	S	102	802.70
tcc	S	4586	754.20
tca	S	83	1159.00
tcg	S	1	253.60
agt	S	0	888.20
agc	S	0	914.50
---	--	------	------
mPD		0.076	1.6
		nPD:	0.05
		N. weight:	2.6
		Sc. PD:	-0.72
		Sc. rank:	-5501.5

NA			
Pos.	206	obs:	exp:
tct	S	0	802.50
tcc	S	0	754.00
tca	S	0	1159.00
tcg	S	0	253.60
aat	N	1	0.57
aac	N	0	0.43
agt	S	1919	888.00
agc	S	2852	914.30
---	--	------	------
mPD		0.48	1.6
		nPD:	0.3
		N. weight:	1.5
		Sc. PD:	-0.08
		Sc. rank:	-536.6

NA			
Pos.	207	obs:	exp:
tct	S	2	801.60
tcc	S	4	753.20
tca	S	4717	1157.00
tcg	S	43	253.30
act	T	0	1.48
acc	T	0	1.30
aca	T	6	2.73
acg	T	0	0.50
agt	S	0	887.10
agc	S	0	913.40
---	--	------	------
mPD		0.023	1.6
		nPD:	0.01
		N. weight:	2.1
		Sc. PD:	-0.66
		Sc. rank:	-5445.7

NA			
Pos.	208	obs:	exp:
tct	S	0	802.70
tcc	S	0	754.20
tca	S	0	1159.00
tcg	S	0	253.60
agt	S	4456	888.20
agc	S	316	914.50
---	--	------	------
mPD		0.12	1.6
		nPD:	0.08
		N. weight:	2.2
		Sc. PD:	-0.57
		Sc. rank:	-4274.6

NA			
Pos.	209	obs:	exp:
tgt	C	4420	2054.00
tgc	C	352	2718.00
---	--	------	------
mPD		0.14	0.49
		nPD:	0.28
		N. weight:	0.87
		Sc. PD:	-0.058
		Sc. rank:	-396.9

NA			
Pos.	210	obs:	exp:
tat	Y	157	202.80
tac	Y	205	159.20
cat	H	956	2544.00
cac	H	3454	1866.00
---	--	------	------
mPD		0.50	0.63
		nPD:	0.79
		N. weight:	0.39
		Sc. PD:	0.16
		Sc. rank:	965.9

NA			
Pos.	211	obs:	exp:
gtt	V	1	0.22
gtc	V	0	0.19
gta	V	0	0.25
gtg	V	0	0.34
gat	D	4753	2683.00
gac	D	18	2088.00
---	--	------	------
mPD		0.0079	0.49
		nPD:	0.02
		N. weight:	0.86
		Sc. PD:	-0.27
		Sc. rank:	-2190.4

NA			
Pos.	212	obs:	exp:
ggt	G	50	748.50
ggc	G	0	604.70
gga	G	3215	2161.00
ggg	G	1507	1257.00
---	--	------	------
mPD		0.45	0.68
		nPD:	0.65
		N. weight:	0.46
		Sc. PD:	0.13
		Sc. rank:	934.6

NA			
Pos.	213	obs:	exp:
caa	Q	1	1.62
cag	Q	2	1.38
cgt	R	0	2.43
cgc	R	0	4.07
cga	R	0	7.74
cgg	R	0	5.56
att	I	0	0.35
atc	I	0	0.26
ata	I	1	0.39
aaa	K	4604	2851.00
aag	K	87	1840.00
aga	R	77	35.31
agg	R	0	21.89
---	--	------	------
mPD		0.070	0.52
		nPD:	0.13
		N. weight:	0.65
		Sc. PD:	-0.13
		Sc. rank:	-872.3

NA			
Pos.	214	obs:	exp:
tct	S	0	3.53
tcc	S	0	3.32
tca	S	21	5.10
tcg	S	0	1.12
agt	S	0	3.91
agc	S	0	4.02
gct	A	89	1165.00
gcc	A	1	1089.00
gca	A	4327	2142.00
gcg	A	334	356.30
---	--	------	------
mPD		0.17	0.69
		nPD:	0.25
		N. weight:	0.92
		Sc. PD:	-0.085
		Sc. rank:	-541.6

NA			
Pos.	215	obs:	exp:
tgt	C	0	0.43
tgc	C	1	0.57
tgg	W	4771	4771.00
---	--	------	------
mPD		0.00042	0.00042
		nPD:	1.
		N. weight:	0.00018
		Sc. PD:	0.00011
		Sc. rank:	0.5

NA			
Pos.	216	obs:	exp:
tta	L	85	519.30
ttg	L	137	896.30
ctt	L	994	963.80
ctc	L	29	694.30
cta	L	185	833.30
ctg	L	3342	865.00
---	--	------	------
mPD		0.51	1.1
		nPD:	0.45
		N. weight:	1.2
		Sc. PD:	0.11
		Sc. rank:	986.2

NA			
Pos.	217	obs:	exp:
cat	H	3801	2753.00
cac	H	971	2019.00
---	--	------	------
mPD		0.32	0.49
		nPD:	0.66
		N. weight:	0.17
		Sc. PD:	0.049
		Sc. rank:	344.9

NA			
Pos.	218	obs:	exp:
att	I	60	22.70
atc	I	4	16.42
ata	I	0	24.88
gtt	V	4428	1045.00
gtc	V	174	888.40
gta	V	11	1188.00
gtg	V	95	1586.00
---	--	------	------
mPD		0.14	0.77
		nPD:	0.18
		N. weight:	1.9
		Sc. PD:	-0.3
		Sc. rank:	-2095.8

NA			
Pos.	219	obs:	exp:
tgt	C	4661	2054.00
tgc	C	111	2718.00
---	--	------	------
mPD		0.045	0.49
		nPD:	0.09
		N. weight:	1.1
		Sc. PD:	-0.27
		Sc. rank:	-2002.2

NA			
Pos.	220	obs:	exp:
att	I	975	1354.00
atc	I	469	979.30
ata	I	2374	1484.00
gtt	V	28	211.80
gtc	V	503	180.00
gta	V	411	240.80
gtg	V	12	321.30
---	--	------	------
mPD		0.89	1.0
		nPD:	0.88
		N. weight:	0.36
		Sc. PD:	0.18
		Sc. rank:	932.0

NA

Pos.	221	obs:	exp:
tct	S	1	0.17
tcc	S	0	0.16
tca	S	0	0.24
tcg	S	0	0.05
act	T	4514	1174.00
acc	T	131	1032.00
aca	T	113	2168.00
acg	T	13	397.80
agt	S	0	0.19
agc	S	0	0.19
---	--	------	------
mPD		0.10	0.68
		nPD:	0.15
		N. weight:	1.8
		Sc. PD:	-0.32
		Sc. rank:	-2251.8

,

NA

Pos.	222	obs:	exp:
gaa	E	0	1.78
gag	E	3	1.22
ggt	G	14	748.00
ggc	G	59	604.30
gga	G	804	2160.00
ggg	G	3892	1257.00
---	--	------	------
mPD		0.31	0.69
		nPD:	0.45
		N. weight:	1.1
		Sc. PD:	0.098
		Sc. rank:	878.3

,

NA

Pos.	223	obs:	exp:
ttt	F	6	2.69
ttc	F	0	3.31
tct	S	3	1.01
tcc	S	0	0.95
tca	S	0	1.46
tcg	S	0	0.32
tat	Y	233	131.10
tac	Y	1	102.90
cat	H	1789	1043.00
cac	H	20	765.50
aat	N	1080	632.00
aac	N	32	480.00
agt	S	3	1.12
agc	S	0	1.15
gat	D	1574	902.70
gac	D	31	702.30
---	--	------	------
mPD		0.73	1.2
		nPD:	0.61
		N. weight:	0.75
		Sc. PD:	0.18
		Sc. rank:	1369.4

,

NA

Pos.	224	obs:	exp:
gat	D	3684	2683.00
gac	D	1087	2088.00
ggt	G	0	0.16
ggc	G	1	0.13
gga	G	0	0.45
ggg	G	0	0.26
---	--	------	------
mPD		0.35	0.49
		nPD:	0.72
		N. weight:	0.15
		Sc. PD:	0.051
		Sc. rank:	344.3

,

NA

Pos.	225	obs:	exp:
tct	S	0	3.53
tcc	S	0	3.32
tca	S	0	5.10
tcg	S	0	1.12
tat	Y	0	1.68
tac	Y	3	1.32
cat	H	0	6.35
cac	H	11	4.65
caa	Q	18	10.25
cag	Q	1	8.75
cgt	R	0	2.08
cgc	R	0	3.49
cga	R	0	6.63
cgg	R	0	4.77
att	I	0	0.35
atc	I	0	0.26
ata	I	1	0.39
act	T	2	6.64
acc	T	1	5.84
aca	T	23	12.27
acg	T	1	2.25
aat	N	216	939.00
aac	N	1436	713.00
aaa	K	1290	801.60
aag	K	29	517.40
agt	S	1	3.91
agc	S	20	4.02
aga	R	63	30.27
agg	R	3	18.76
gtt	V	0	1.78
gtc	V	0	1.51
gta	V	8	2.02
gtg	V	0	2.69
gct	A	0	2.21
gcc	A	0	2.06
gca	A	9	4.06
gcg	A	0	0.67
gat	D	0	32.62
gac	D	58	25.38
gaa	E	1506	894.70
gag	E	4	615.30
ggt	G	0	10.67
ggc	G	1	8.62
gga	G	65	30.80
ggg	G	2	17.92
---	--	------	------
mPD		1.0	1.3
		nPD:	0.82
		N. weight:	0.72
		Sc. PD:	0.31
		Sc. rank:	1805.6

,

NA

Pos.	226	obs:	exp:
aat	N	4584	2712.00
aac	N	187	2059.00
gat	D	1	0.56
gac	D	0	0.44
---	--	------	------
mPD		0.076	0.49
		nPD:	0.15
		N. weight:	0.64
		Sc. PD:	-0.12
		Sc. rank:	-795.7

,

NA

Pos.	227	obs:	exp:
gct	A	59	1170.00
gcc	A	31	1093.00
gca	A	3621	2151.00
gcg	A	1061	357.90
---	--	------	------
mPD		0.37	0.68
		nPD:	0.55
		N. weight:	0.9
		Sc. PD:	0.17
		Sc. rank:	1369.4

,

NA

Pos.	228	obs:	exp:
att	I	1	0.35
atc	I	0	0.26
ata	I	0	0.39
act	T	3983	1173.00
acc	T	29	1031.00
aca	T	756	2167.00
acg	T	2	397.80
aat	N	1	0.57
aac	N	0	0.43
---	--	------	------
mPD		0.28	0.68
		nPD:	0.41
		N. weight:	1.3
		Sc. PD:	0.069
		Sc. rank:	768.3

,

NA

Pos.	229	obs:	exp:
gct	A	3207	1170.00
gcc	A	1462	1093.00
gca	A	38	2151.00
gcg	A	65	357.90
---	--	------	------
mPD		0.45	0.68
		nPD:	0.67
		N. weight:	1.1
		Sc. PD:	0.33
		Sc. rank:	2322.9

,

NA

Pos.	230	obs:	exp:
tct	S	0	802.30
tcc	S	0	753.90
tca	S	0	1158.00
tcg	S	0	253.50
cgt	R	0	0.03
cgc	R	0	0.05
cga	R	0	0.10
cgg	R	0	0.07
aat	N	0	0.57
aac	N	1	0.43
agt	S	478	887.80
agc	S	4292	914.20
aga	R	0	0.46
agg	R	1	0.28
---	--	------	------
mPD		0.18	1.6
		nPD:	0.11
		N. weight:	2.1
		Sc. PD:	-0.46
		Sc. rank:	-3321.9

,

NA			
Pos.	231	obs:	exp:
ttt	F	112	1599.00
ttc	F	3457	1970.00
tta	L	20	18.72
ttg	L	0	32.31
ctt	L	0	34.74
ctc	L	151	25.03
cta	L	0	30.03
ctg	L	1	31.18
att	I	14	360.40
atc	I	1001	260.60
ata	I	1	395.00
gtt	V	1	3.33
gtc	V	14	2.83
gta	V	0	3.79
gtg	V	0	5.05
---	--	------	------
mPD		0.45	0.97
		nPD:	0.46
		N. weight:	1.1
		Sc. PD:	0.11
		Sc. rank:	953.7

,

NA			
Pos.	232	obs:	exp:
att	I	4245	1693.00
atc	I	482	1224.00
ata	I	45	1855.00
---	--	------	------
mPD		0.20	0.66
		nPD:	0.3
		N. weight:	1.1
		Sc. PD:	-0.049
		Sc. rank:	-248.9

,

NA			
Pos.	233	obs:	exp:
tct	S	0	0.17
tcc	S	0	0.16
tca	S	1	0.24
tcg	S	0	0.05
tat	Y	883	2673.00
tac	Y	3888	2098.00
agt	S	0	0.19
agc	S	0	0.19
---	--	------	------
mPD		0.30	0.49
		nPD:	0.61
		N. weight:	0.47
		Sc. PD:	0.11
		Sc. rank:	835.4

,

NA			
Pos.	234	obs:	exp:
tct	S	0	2.02
tcc	S	0	1.90
tca	S	0	2.91
tcg	S	0	0.64
aat	N	3574	2146.00
aac	N	201	1629.00
aaa	K	1	1.22
aag	K	1	0.78
agt	S	12	2.23
agc	S	0	2.30
gat	D	619	545.60
gac	D	351	424.40
gaa	E	1	0.59
gag	E	0	0.41
ggt	G	5	1.88
ggc	G	7	1.52
gga	G	0	5.44
ggg	G	0	3.16
---	--	------	------
mPD		0.55	0.83
		nPD:	0.65
		N. weight:	0.48
		Sc. PD:	0.13
		Sc. rank:	963.5

,

NA			
Pos.	235	obs:	exp:
cgt	R	0	0.03
cgc	R	0	0.05
cga	R	0	0.10
cgg	R	0	0.07
aga	R	0	0.46
agg	R	1	0.28
gaa	E	1	16.00
gag	E	26	11.00
ggt	G	315	744.10
ggc	G	16	601.10
gga	G	1506	2149.00
ggg	G	2907	1250.00
---	--	------	------
mPD		0.53	0.70
		nPD:	0.76
		N. weight:	0.53
		Sc. PD:	0.2
		Sc. rank:	1266.2

,

NA			
Pos.	236	obs:	exp:
cgt	R	0	143.00
cgc	R	0	239.50
cga	R	0	455.40
cgg	R	81	327.30
att	I	0	0.35
atc	I	0	0.26
ata	I	1	0.39
atg	M	5	5.00
aaa	K	64	142.80
aag	K	171	92.18
aga	R	1178	2078.00
agg	R	3272	1288.00
---	--	------	------
mPD		0.51	1.0
		nPD:	0.51
		N. weight:	0.76
		Sc. PD:	0.11
		Sc. rank:	853.1

,

NA			
Pos.	237	obs:	exp:
ttt	F	1	0.45
ttc	F	0	0.55
tta	L	4	519.20
ttg	L	0	896.20
ctt	L	4057	963.60
ctc	L	40	694.20
cta	L	658	833.10
ctg	L	12	864.90
---	--	------	------
mPD		0.26	1.1
		nPD:	0.23
		N. weight:	1.8
		Sc. PD:	-0.2
		Sc. rank:	-1471.3

,

NA			
Pos.	238	obs:	exp:
tta	L	0	0.11
ttg	L	1	0.19
ctt	L	0	0.20
ctc	L	0	0.15
cta	L	0	0.17
ctg	L	0	0.18
att	I	0	22.70
atc	I	0	16.42
ata	I	64	24.88
atg	M	4	4.00
act	T	0	3.94
acc	T	0	3.46
aca	T	16	7.27
acg	T	0	1.33
gtt	V	177	1030.00
gtc	V	2	875.20
gta	V	3823	1171.00
gtg	V	636	1562.00
gct	A	0	11.28
gcc	A	0	10.54
gca	A	45	20.73
gcg	A	1	3.45
gaa	E	0	1.78
gag	E	3	1.22
---	--	------	------
mPD		0.36	0.80
		nPD:	0.45
		N. weight:	1.2
		Sc. PD:	0.11
		Sc. rank:	947.1

,

NA			
Pos.	239	obs:	exp:
gat	D	3634	2684.00
gac	D	1138	2088.00
---	--	------	------
mPD		0.36	0.49
		nPD:	0.74
		N. weight:	0.14
		Sc. PD:	0.048
		Sc. rank:	317.6

,

NA			
Pos.	240	obs:	exp:
tct	S	0	802.70
tcc	S	0	754.20
tca	S	0	1159.00
tcg	S	0	253.60
agt	S	4180	888.20
agc	S	592	914.50
---	--	------	------
mPD		0.22	1.6
		nPD:	0.13
		N. weight:	2.
		Sc. PD:	-0.41
		Sc. rank:	-2718.8

,

NA			
Pos.	241	obs:	exp:
tta	L	0	0.44
ttg	L	0	0.75
ctt	L	3	0.81
ctc	L	1	0.58
cta	L	0	0.70
ctg	L	0	0.73
att	I	4236	1680.00
atc	I	376	1215.00
ata	I	124	1841.00
gtt	V	24	7.11
gtc	V	1	6.04
gta	V	7	8.08
gtg	V	0	10.78
---	--	------	------
mPD		0.21	0.67
		nPD:	0.31
		N. weight:	1.
		Sc. PD:	-0.038
		Sc. rank:	-154.0

,

NA			
Pos.	242	obs:	exp:
tct	S	0	25.73
tcc	S	0	24.18
tca	S	0	37.15
tcg	S	0	8.13
cgt	R	66	2.90
cgc	R	0	4.86
cga	R	0	9.25
cgg	R	0	6.65
aat	N	3	1.71
aac	N	0	1.29
agt	S	148	28.48
agc	S	5	29.32
aga	R	24	42.19
agg	R	2	26.15
gtt	V	44	9.77
gtc	V	0	8.30
gta	V	0	11.11
gtg	V	0	14.82
gct	A	1	0.25
gcc	A	0	0.23
gca	A	0	0.45
gcg	A	0	0.07
ggt	G	4131	702.50
ggc	G	244	567.50
gga	G	61	2029.00
ggg	G	43	1180.00
---	--	------	------
mPD		0.27	0.85
		nPD:	0.32
		N. weight:	2.4
		Sc. PD:	-0.075
		Sc. rank:	-158.5

,

NA			
Pos.	243	obs:	exp:
tta	L	1	0.11
ttg	L	0	0.19
tct	S	104	802.50
tcc	S	30	754.00
tca	S	4093	1159.00
tcg	S	544	253.60
ctt	L	0	0.20
ctc	L	0	0.15
cta	L	0	0.17
ctg	L	0	0.18
agt	S	0	888.00
agc	S	0	914.30
---	--	------	------
mPD		0.25	1.6
		nPD:	0.15
		N. weight:	1.7
		Sc. PD:	-0.31
		Sc. rank:	-2154.5

,

NA			
Pos.	244	obs:	exp:
tgg	W	4771	4771.00
ggt	G	0	0.16
ggc	G	0	0.13
gga	G	0	0.45
ggg	G	1	0.26
---	--	------	------
mPD		0.00042	0.00073
		nPD:	0.58
		N. weight:	0.00044
		Sc. PD:	0.00009
		Sc. rank:	0.7

,

NA			
Pos.	245	obs:	exp:
tct	S	755	802.10
tcc	S	3989	753.70
tca	S	15	1158.00
tcg	S	10	253.50
agt	S	0	887.60
agc	S	0	914.00
gct	A	0	0.74
gcc	A	3	0.69
gca	A	0	1.35
gcg	A	0	0.22
---	--	------	------
mPD		0.28	1.6
		nPD:	0.17
		N. weight:	2.1
		Sc. PD:	-0.36
		Sc. rank:	-2449.2

,

NA			
Pos.	246	obs:	exp:
cat	H	10	13.84
cac	H	14	10.16
caa	Q	779	422.20
cag	Q	4	360.80
cgt	R	0	0.79
cgc	R	0	1.32
cga	R	13	2.51
cgg	R	0	1.81
atg	M	84	84.00
aat	N	5	5.12
aac	N	4	3.88
aaa	K	3709	2338.00
aag	K	138	1509.00
aga	R	12	11.46
agg	R	0	7.11
---	--	------	------
mPD		0.43	0.82
		nPD:	0.53
		N. weight:	0.61
		Sc. PD:	0.099
		Sc. rank:	820.7

,

NA			
Pos.	247	obs:	exp:
tat	Y	1	0.56
tac	Y	0	0.44
caa	Q	3	1.62
cag	Q	0	1.38
cgt	R	0	0.66
cgc	R	0	1.11
cga	R	0	2.11
cgg	R	0	1.52
aat	N	2884	1926.00
aac	N	504	1462.00
aaa	K	1276	794.30
aag	K	31	512.70
aga	R	21	9.63
agg	R	0	5.97
gat	D	41	27.56
gac	D	8	21.44
gaa	E	3	1.78
gag	E	0	1.22
---	--	------	------
mPD		0.57	0.73
		nPD:	0.78
		N. weight:	0.39
		Sc. PD:	0.15
		Sc. rank:	934.7

,

NA			
Pos.	248	obs:	exp:
att	I	468	1692.00
atc	I	800	1223.00
ata	I	3501	1854.00
gtt	V	1	0.67
gtc	V	0	0.57
gta	V	2	0.76
gtg	V	0	1.01
---	--	------	------
mPD		0.42	0.66
		nPD:	0.64
		N. weight:	0.42
		Sc. PD:	0.11
		Sc. rank:	841.0

,

NA			
Pos.	249	obs:	exp:
tta	L	20	519.30
ttg	L	0	896.30
ctt	L	221	963.80
ctc	L	3841	694.30
cta	L	688	833.30
ctg	L	2	865.00
---	--	------	------
mPD		0.34	1.1
		nPD:	0.3
		N. weight:	2.
		Sc. PD:	-0.1
		Sc. rank:	-645.2

,

NA			
Pos.	250	obs:	exp:
cgt	R	7	150.60
cgc	R	1	252.10
cga	R	33	479.40
cgg	R	428	344.60
act	T	0	0.25
acc	T	0	0.22
aca	T	0	0.45
acg	T	1	0.08
aaa	K	0	0.61
aag	K	1	0.39
aga	R	3102	2188.00
agg	R	1199	1356.00
---	--	------	------
mPD		0.63	0.94
		nPD:	0.67
		N. weight:	0.3
		Sc. PD:	0.089
		Sc. rank:	645.5

,

NA			
Pos.	251	obs:	exp:
act	T	443	1174.00
acc	T	3340	1032.00
aca	T	984	2168.00
acg	T	5	397.90
---	--	------	------
mPD		0.46	0.68
		nPD:	0.68
		N. weight:	0.88
		Sc. PD:	0.26
		Sc. rank:	1888.6

,

NA			
Pos.	252	obs:	exp:
cat	H	23	13.27
cac	H	0	9.73
caa	Q	147	2560.00
cag	Q	4601	2188.00
aaa	K	0	0.61
aag	K	1	0.39
---	--	------	------
mPD		0.069	0.50
		nPD:	0.14
		N. weight:	0.98
		Sc. PD:	-0.19
		Sc. rank:	-1285.5

,

NA			
Pos.	253	obs:	exp:
gaa	E	930	2828.00
gag	E	3842	1944.00
---	--	------	------
mPD		0.31	0.48
		nPD:	0.65
		N. weight:	0.52
		Sc. PD:	0.14
		Sc. rank:	1040.7

,

NA			
Pos.	254	obs:	exp:
tct	S	126	802.70
tcc	S	2	754.20
tca	S	923	1159.00
tcg	S	3721	253.60
agt	S	0	888.20
agc	S	0	914.50
---	--	------	------
mPD		0.35	1.6
		nPD:	0.22
		N. weight:	3.1
		Sc. PD:	-0.39
		Sc. rank:	-2702.7

,

NA			
Pos.	255	obs:	exp:
gaa	E	4495	2828.00
gag	E	277	1944.00
---	--	------	------
mPD		0.11	0.48
		nPD:	0.23
		N. weight:	0.5
		Sc. PD:	-0.058
		Sc. rank:	-423.3

,

NA			
Pos.	256	obs:	exp:
tgt	C	512	2054.00
tgc	C	4260	2718.00
---	--	------	------
mPD		0.19	0.49
		nPD:	0.39
		N. weight:	0.39
		Sc. PD:	0.014
		Sc. rank:	139.0

,

NA			
Pos.	257	obs:	exp:
att	I	31	13.13
atc	I	1	9.49
ata	I	5	14.38
gtt	V	3780	1051.00
gtc	V	901	893.50
gta	V	21	1195.00
gtg	V	33	1595.00
---	--	------	------
mPD		0.34	0.75
		nPD:	0.45
		N. weight:	1.5
		Sc. PD:	0.14
		Sc. rank:	1284.8

,

NA			
Pos.	258	obs:	exp:
tgt	C	4016	2054.00
tgc	C	756	2718.00
---	--	------	------
mPD		0.27	0.49
		nPD:	0.54
		N. weight:	0.56
		Sc. PD:	0.1
		Sc. rank:	810.1

,

NA			
Pos.	259	obs:	exp:
att	I	1056	1690.00
atc	I	3552	1222.00
ata	I	155	1852.00
atg	M	2	2.00
gtt	V	0	1.55
gtc	V	7	1.32
gta	V	0	1.77
gtg	V	0	2.36
---	--	------	------
mPD		0.40	0.66
		nPD:	0.6
		N. weight:	0.96
		Sc. PD:	0.22
		Sc. rank:	1670.9

,

NA			
Pos.	260	obs:	exp:
tct	S	0	2.02
tcc	S	0	1.90
tca	S	0	2.91
tcg	S	0	0.64
cat	H	1	0.58
cac	H	0	0.42
att	I	1	0.35
atc	I	0	0.26
ata	I	0	0.39
act	T	1	0.25
acc	T	0	0.22
aca	T	0	0.45
acg	T	0	0.08
aat	N	4395	2687.00
aac	N	333	2041.00
aaa	K	0	0.61
aag	K	1	0.39
agt	S	10	2.23
agc	S	2	2.30
gat	D	28	15.75
gac	D	0	12.25
---	--	------	------
mPD		0.15	0.51
		nPD:	0.29
		N. weight:	0.52
		Sc. PD:	-0.029
		Sc. rank:	-212.2

,

NA			
Pos.	261	obs:	exp:
tct	S	0	3.20
tcc	S	0	3.00
tca	S	0	4.61
tcg	S	0	1.01
cgt	R	1	3.16
cgc	R	0	5.29
cga	R	0	10.05
cgg	R	0	7.22
aaa	K	0	0.61
aag	K	1	0.39
agt	S	14	3.54
agc	S	5	3.64
aga	R	50	45.86
agg	R	49	28.42
gaa	E	24	14.22
gag	E	0	9.78
ggt	G	895	725.90
ggc	G	27	586.40
gga	G	3365	2096.00
ggg	G	341	1219.00
---	--	------	------
mPD		0.50	0.75
		nPD:	0.66
		N. weight:	0.44
		Sc. PD:	0.13
		Sc. rank:	892.5

,

NA			
Pos.	262	obs:	exp:
att	I	174	74.50
atc	I	29	53.86
ata	I	7	81.64
atg	M	1	1.00
act	T	3332	1118.00
acc	T	864	982.80
aca	T	79	2065.00
acg	T	270	379.00
gtt	V	0	3.55
gtc	V	16	3.02
gta	V	0	4.04
gtg	V	0	5.39
---	--	------	------
mPD		0.52	0.78
		nPD:	0.67
		N. weight:	1.1
		Sc. PD:	0.32
		Sc. rank:	2228.4

,

NA			
Pos.	263	obs:	exp:
tgt	C	3897	2054.00
tgc	C	875	2718.00
---	--	------	------
mPD		0.30	0.49
		nPD:	0.61
		N. weight:	0.49
		Sc. PD:	0.12
		Sc. rank:	875.8

,

NA			
Pos.	264	obs:	exp:
tct	S	0	0.17
tcc	S	0	0.16
tca	S	0	0.24
tcg	S	1	0.05
cgt	R	0	0.03
cgc	R	0	0.05
cga	R	0	0.10
cgg	R	0	0.07
att	I	0	40.80
atc	I	0	29.50
ata	I	115	44.71
atg	M	1	1.00
act	T	78	1116.00
acc	T	6	981.50
aca	T	4346	2062.00
acg	T	109	378.50
agt	S	0	0.19
agc	S	0	0.19
aga	R	1	0.46
agg	R	0	0.28
gtt	V	0	0.89
gtc	V	0	0.75
gta	V	4	1.01
gtg	V	0	1.35
gct	A	6	27.21
gcc	A	1	25.43
gca	A	104	50.03
gcg	A	0	8.32
---	--	------	------
mPD		0.18	0.78
		nPD:	0.23
		N. weight:	1.
		Sc. PD:	-0.11
		Sc. rank:	-807.0

,

NA			
Pos.	265	obs:	exp:
att	I	0	4.26
atc	I	0	3.08
ata	I	12	4.67
gtt	V	24	1057.00
gtc	V	0	898.20
gta	V	4170	1202.00
gtg	V	566	1603.00
---	--	------	------
mPD		0.22	0.74
		nPD:	0.3
		N. weight:	1.5
		Sc. PD:	-0.07
		Sc. rank:	-435.9

,

NA			
Pos.	266	obs:	exp:
tta	L	1	0.22
ttg	L	0	0.38
ctt	L	0	0.40
ctc	L	0	0.29
cta	L	1	0.35
ctg	L	0	0.36
att	I	1	11.35
atc	I	4	8.21
ata	I	27	12.44
aaa	K	1	0.61
aag	K	0	0.39
gtt	V	21	1052.00
gtc	V	641	893.90
gta	V	3778	1196.00
gtg	V	297	1596.00
---	--	------	------
mPD		0.36	0.75
		nPD:	0.47
		N. weight:	1.2
		Sc. PD:	0.13
		Sc. rank:	1118.9

,

NA			
Pos.	267	obs:	exp:
tta	L	0	0.54
ttg	L	0	0.94
ctt	L	0	1.01
ctc	L	0	0.73
cta	L	0	0.87
ctg	L	5	0.91
att	I	0	2.84
atc	I	0	2.05
ata	I	8	3.11
atg	M	4751	4751.00
aat	N	0	0.57
aac	N	1	0.43
gtt	V	0	1.55
gtc	V	0	1.32
gta	V	0	1.77
gtg	V	7	2.36
---	--	------	------
mPD		0.0092	0.012
		nPD:	0.74
		N. weight:	0.008
		Sc. PD:	0.0029
		Sc. rank:	18.8

,

NA			
Pos.	268	obs:	exp:
cgt	R	0	0.03
cgc	R	0	0.05
cga	R	0	0.10
cgg	R	0	0.07
act	T	4338	1174.00
acc	T	63	1032.00
aca	T	314	2168.00
acg	T	56	397.80
aga	R	0	0.46
agg	R	1	0.28
---	--	------	------
mPD		0.17	0.68
		nPD:	0.25
		N. weight:	1.6
		Sc. PD:	-0.15
		Sc. rank:	-955.5

,

NA			
Pos.	269	obs:	exp:
gtt	V	1	0.22
gtc	V	0	0.19
gta	V	0	0.25
gtg	V	0	0.34
gat	D	4729	2683.00
gac	D	42	2088.00
---	--	------	------
mPD		0.018	0.49
		nPD:	0.04
		N. weight:	0.82
		Sc. PD:	-0.24
		Sc. rank:	-1919.0

,

NA			
Pos.	270	obs:	exp:
gtt	V	0	0.22
gtc	V	0	0.19
gta	V	0	0.25
gtg	V	1	0.34
ggt	G	9	748.30
ggc	G	3	604.50
gga	G	2105	2161.00
ggg	G	2654	1257.00
---	--	------	------
mPD		0.50	0.69
		nPD:	0.72
		N. weight:	0.61
		Sc. PD:	0.21
		Sc. rank:	1411.0

,

NA			
Pos.	271	obs:	exp:
tct	S	0	798.30
tcc	S	0	750.10
tca	S	0	1153.00
tcg	S	0	252.20
aat	N	3	14.78
aac	N	23	11.22
agt	S	2430	883.30
agc	S	2316	909.60
---	--	------	------
mPD		0.51	1.6
		nPD:	0.31
		N. weight:	1.5
		Sc. PD:	-0.055
		Sc. rank:	-200.2

NA			
Pos.	272	obs:	exp:
tct	S	1	0.34
tcc	S	0	0.32
tca	S	1	0.49
tcg	S	0	0.11
cct	P	1	0.30
ccc	P	0	0.20
cca	P	0	0.40
ccg	P	0	0.11
agt	S	0	0.37
agc	S	0	0.38
gtt	V	2	0.44
gtc	V	0	0.38
gta	V	0	0.50
gtg	V	0	0.67
gct	A	4390	1169.00
gcc	A	102	1092.00
gca	A	258	2149.00
gcg	A	17	357.50
---	--	------	------
mPD		0.15	0.68
		nPD:	0.22
		N. weight:	1.6
		Sc. PD:	-0.19
		Sc. rank:	-1372.5

NA			
Pos.	273	obs:	exp:
ttt	F	1	0.45
ttc	F	0	0.55
tct	S	47	802.30
tcc	S	945	753.90
tca	S	3621	1158.00
tcg	S	157	253.50
agt	S	0	887.80
agc	S	0	914.20
gct	A	0	0.25
gcc	A	0	0.23
gca	A	1	0.45
gcg	A	0	0.07
---	--	------	------
mPD		0.38	1.6
		nPD:	0.24
		N. weight:	1.4
		Sc. PD:	-0.14
		Sc. rank:	-1007.3

NA			
Pos.	274	obs:	exp:
gaa	E	5	2.96
gag	E	0	2.04
ggt	G	86	747.70
ggc	G	4	604.00
gga	G	4092	2159.00
ggg	G	585	1256.00
---	--	------	------
mPD		0.25	0.69
		nPD:	0.36
		N. weight:	0.64
		Sc. PD:	0.0073
		Sc. rank:	173.6

NA			
Pos.	275	obs:	exp:
tct	S	0	0.34
tcc	S	0	0.32
tca	S	0	0.49
tcg	S	0	0.11
tgg	W	1	1.00
caa	Q	1	0.54
cag	Q	0	0.46
cgt	R	0	57.45
cgc	R	0	96.20
cga	R	4	182.90
cgg	R	0	131.50
att	I	0	25.90
atc	I	0	18.72
ata	I	73	28.38
act	T	0	0.49
acc	T	0	0.43
aca	T	2	0.91
acg	T	0	0.17
aat	N	3	1.71
aac	N	0	1.29
aaa	K	2779	1727.00
aag	K	62	1114.00
agt	S	2	0.37
agc	S	0	0.38
aga	R	1774	834.60
agg	R	42	517.30
gtt	V	0	0.67
gtc	V	0	0.57
gta	V	3	0.76
gtg	V	0	1.01
gaa	E	25	15.41
gag	E	1	10.59
---	--	------	------
mPD		0.55	1.2
		nPD:	0.46
		N. weight:	0.8
		Sc. PD:	0.08
		Sc. rank:	706.5

NA			
Pos.	276	obs:	exp:
tct	S	1	0.17
tcc	S	0	0.16
tca	S	0	0.24
tcg	S	0	0.05
agt	S	0	0.19
agc	S	0	0.19
gct	A	4323	1170.00
gcc	A	439	1093.00
gca	A	5	2151.00
gcg	A	4	357.80
---	--	------	------
mPD		0.17	0.68
		nPD:	0.25
		N. weight:	1.7
		Sc. PD:	-0.16
		Sc. rank:	-969.2

NA			
Pos.	277	obs:	exp:
tat	Y	2	1.12
tac	Y	0	0.88
aat	N	17	10.23
aac	N	1	7.77
gtt	V	1	0.22
gtc	V	0	0.19
gta	V	0	0.25
gtg	V	0	0.34
gat	D	4502	2666.00
gac	D	238	2074.00
gaa	E	11	6.52
gag	E	0	4.48
---	--	------	------
mPD		0.11	0.50
		nPD:	0.22
		N. weight:	0.61
		Sc. PD:	-0.076
		Sc. rank:	-553.5

NA			
Pos.	278	obs:	exp:
att	I	1	0.35
atc	I	0	0.26
ata	I	0	0.39
act	T	4042	1174.00
acc	T	651	1032.00
aca	T	43	2168.00
acg	T	35	397.80
---	--	------	------
mPD		0.26	0.68
		nPD:	0.39
		N. weight:	1.5
		Sc. PD:	0.049
		Sc. rank:	484.7

NA			
Pos.	279	obs:	exp:
cgt	R	0	26.99
cgc	R	0	45.19
cga	R	0	85.94
cgg	R	0	61.77
aaa	K	3845	2380.00
aag	K	72	1537.00
aga	R	797	392.10
agg	R	58	243.00
---	--	------	------
mPD		0.35	0.87
		nPD:	0.4
		N. weight:	0.69
		Sc. PD:	0.029
		Sc. rank:	277.0

NA			
Pos.	280	obs:	exp:
att	I	35	1657.00
atc	I	3	1198.00
ata	I	4633	1816.00
act	T	0	0.25
acc	T	0	0.22
aca	T	1	0.45
acg	T	0	0.08
gtt	V	0	22.20
gtc	V	0	18.87
gta	V	100	25.24
gtg	V	0	33.68
---	--	------	------
mPD		0.057	0.70
		nPD:	0.08
		N. weight:	1.4
		Sc. PD:	-0.35
		Sc. rank:	-2562.2

NA			
Pos.	281	obs:	exp:
tta	L	2450	491.10
ttg	L	41	847.70
ctt	L	11	911.40
ctc	L	0	656.60
cta	L	1867	788.00
ctg	L	144	818.10
att	I	0	89.04
atc	I	1	64.38
ata	I	250	97.58
atg	M	4	4.00
gtt	V	0	0.89
gtc	V	0	0.75
gta	V	4	1.01
gtg	V	0	1.35
---	--	------	------
mPD		0.63	1.2
		nPD:	0.52
		N. weight:	1.8
		Sc. PD:	0.27
		Sc. rank:	2183.1

NA			
Pos.	282	obs:	exp:
ttt	F	517	2138.00
ttc	F	4254	2633.00
tta	L	1	0.11
ttg	L	0	0.19
ctt	L	0	0.20
ctc	L	0	0.15
cta	L	0	0.17
ctg	L	0	0.18
---	--	------	------
mPD		0.19	0.50
		nPD:	0.39
		N. weight:	0.43
		Sc. PD:	0.016
		Sc. rank:	153.1

NA			
Pos.	283	obs:	exp:
tta	L	0	0.22
ttg	L	0	0.38
ctt	L	2	0.40
ctc	L	0	0.29
cta	L	0	0.35
ctg	L	0	0.36
att	I	3429	1267.00
atc	I	132	915.90
ata	I	10	1388.00
act	T	95	23.86
acc	T	0	20.98
aca	T	2	44.08
acg	T	0	8.09
gtt	V	1089	244.00
gtc	V	6	207.40
gta	V	2	277.40
gtg	V	2	370.20
gct	A	3	0.74
gcc	A	0	0.69
gca	A	0	1.35
gcg	A	0	0.22
---	--	------	------
mPD		0.46	1.1
		nPD:	0.42
		N. weight:	1.6
		Sc. PD:	0.096
		Sc. rank:	998.4

NA			
Pos.	284	obs:	exp:
caa	Q	0	1.62
cag	Q	3	1.38
cgt	R	0	0.44
cgc	R	0	0.74
cga	R	0	1.41
cgg	R	0	1.01
atg	M	14	14.00
aat	N	2	2.27
aac	N	2	1.73
aaa	K	120	123.40
aag	K	83	79.63
aga	R	14	6.42
agg	R	0	3.98
gtt	V	1	79.93
gtc	V	0	67.93
gta	V	102	90.88
gtg	V	257	121.30
gaa	E	2041	2473.00
gag	E	2132	1700.00
ggt	G	0	0.16
ggc	G	0	0.13
gga	G	1	0.45
ggg	G	0	0.26
---	--	------	------
mPD		0.75	0.76
		nPD:	0.98
		N. weight:	0.1
		Sc. PD:	0.06
		Sc. rank:	273.8

NA			
Pos.	285	obs:	exp:
caa	Q	0	0.54
cag	Q	1	0.46
aaa	K	0	0.61
aag	K	1	0.39
gaa	E	751	2825.00
gag	E	4016	1942.00
ggt	G	0	0.47
ggc	G	0	0.38
gga	G	0	1.36
ggg	G	3	0.79
---	--	------	------
mPD		0.27	0.49
		nPD:	0.55
		N. weight:	0.63
		Sc. PD:	0.12
		Sc. rank:	951.8

NA

Pos.	286	obs:	exp:
ggt	G	22	748.50
ggc	G	194	604.70
gga	G	299	2161.00
ggg	G	4257	1257.00
---	--	------	------
mPD		0.20	0.68
		nPD:	0.29
		N. weight:	1.4
		Sc. PD:	-0.079
		Sc. rank:	-580.2

,

NA

Pos.	287	obs:	exp:
cgt	R	0	1.26
cgc	R	0	2.11
cga	R	0	4.02
cgg	R	0	2.89
aat	N	1	1.71
aac	N	2	1.29
aaa	K	3137	2870.00
aag	K	1586	1853.00
aga	R	27	18.34
agg	R	13	11.37
gaa	E	5	3.56
gag	E	1	2.44
---	--	------	------
mPD		0.47	0.50
		nPD:	0.93
		N. weight:	0.018
		Sc. PD:	0.0093
		Sc. rank:	45.9

,

NA

Pos.	288	obs:	exp:
att	I	745	1623.00
atc	I	3708	1173.00
ata	I	122	1779.00
gtt	V	77	43.74
gtc	V	120	37.17
gta	V	0	49.73
gtg	V	0	66.36
---	--	------	------
mPD		0.41	0.75
		nPD:	0.54
		N. weight:	1.2
		Sc. PD:	0.21
		Sc. rank:	1657.7

,

NA

Pos.	289	obs:	exp:
tta	L	0	0.11
ttg	L	0	0.19
ctt	L	1	0.20
ctc	L	0	0.15
cta	L	0	0.17
ctg	L	0	0.18
att	I	2662	1021.00
atc	I	194	737.90
ata	I	21	1118.00
act	T	2	4.18
acc	T	14	3.68
aca	T	1	7.72
acg	T	0	1.42
aat	N	3	1.71
aac	N	0	1.29
aaa	K	1	0.61
aag	K	0	0.39
gtt	V	1422	412.50
gtc	V	405	350.60
gta	V	20	469.00
gtg	V	11	625.80
gct	A	14	3.68
gcc	A	1	3.44
gca	A	0	6.76
gcg	A	0	1.12
---	--	------	------
mPD		0.74	1.2
		nPD:	0.6
		N. weight:	1.3
		Sc. PD:	0.3
		Sc. rank:	2291.2

,

NA

Pos.	290	obs:	exp:
tat	Y	5	2.80
tac	Y	0	2.20
cat	H	3927	2644.00
cac	H	656	1939.00
cgt	R	37	1.17
cgc	R	0	1.96
cga	R	0	3.72
cgg	R	0	2.67
aat	N	3	1.71
aac	N	0	1.29
aga	R	0	16.97
agg	R	0	10.52
gat	D	0	80.99
gac	D	144	63.01
---	--	------	------
mPD		0.36	0.58
		nPD:	0.61
		N. weight:	0.36
		Sc. PD:	0.085
		Sc. rank:	636.1

,

NA

Pos.	291	obs:	exp:
att	I	3871	1495.00
atc	I	336	1081.00
ata	I	8	1639.00
atg	M	3	3.00
act	T	11	17.96
acc	T	62	15.79
aca	T	0	33.17
acg	T	0	6.09
gtt	V	467	105.50
gtc	V	8	89.63
gta	V	0	119.90
gtg	V	0	160.00
gct	A	6	1.47
gcc	A	0	1.37
gca	A	0	2.70
gcg	A	0	0.45
---	--	------	------
mPD		0.37	0.89
		nPD:	0.42
		N. weight:	1.3
		Sc. PD:	0.081
		Sc. rank:	847.4

,

NA

Pos.	292	obs:	exp:
tct	S	0	801.60
tcc	S	0	753.20
tca	S	0	1157.00
tcg	S	0	253.30
cgt	R	0	0.16
cgc	R	0	0.26
cga	R	0	0.50
cgg	R	0	0.36
agt	S	525	887.10
agc	S	4241	913.40
aga	R	4	2.29
agg	R	1	1.42
ggt	G	0	0.16
ggc	G	1	0.13
gga	G	0	0.45
ggg	G	0	0.26
---	--	------	------
mPD		0.20	1.6
		nPD:	0.12
		N. weight:	2.
		Sc. PD:	-0.43
		Sc. rank:	-2918.8

,

NA

Pos.	293	obs:	exp:
tct	S	0	1.35
tcc	S	0	1.26
tca	S	7	1.94
tcg	S	1	0.43
cct	P	15	682.90
ccc	P	21	465.90
cca	P	2003	916.40
ccg	P	275	248.90
caa	Q	118	63.63
cag	Q	0	54.37
cgt	R	1	0.25
cgc	R	0	0.42
cga	R	3	0.80
cgg	R	0	0.58
atg	M	3	3.00
act	T	5	446.00
acc	T	2	392.00
aca	T	1209	823.80
acg	T	597	151.20
aat	N	17	10.23
aac	N	1	7.77
aaa	K	62	251.00
aag	K	351	162.00
agt	S	0	1.49
agc	S	0	1.53
aga	R	2	3.67
agg	R	2	2.27
gct	A	0	17.89
gcc	A	0	16.73
gca	A	56	32.90
gcg	A	17	5.47
gaa	E	0	2.37
gag	E	4	1.63
---	--	------	------
mPD		1.1	1.4
		nPD:	0.8
		N. weight:	1.
		Sc. PD:	0.41
		Sc. rank:	2482.2

,

NA

Pos.	294	obs:	exp:
tta	L	421	519.20
ttg	L	4058	896.20
tgt	C	1	0.43
tgc	C	0	0.57
ctt	L	11	963.60
ctc	L	0	694.20
cta	L	9	833.10
ctg	L	272	864.90
---	--	------	------
mPD		0.28	1.1
		nPD:	0.25
		N. weight:	1.8
		Sc. PD:	-0.17
		Sc. rank:	-1157.4

,

NA

Pos.	295	obs:	exp:
ttt	F	7	3.14
ttc	F	0	3.86
tta	L	272	47.45
ttg	L	163	81.90
tct	S	9	519.20
tcc	S	4	487.90
tca	S	2723	749.60
tcg	S	351	164.10
ctt	L	0	88.05
ctc	L	0	63.44
cta	L	1	76.13
ctg	L	0	79.04
cgt	R	0	0.06
cgc	R	0	0.11
cga	R	0	0.20
cgg	R	0	0.14
att	I	0	217.50
atc	I	0	157.20
ata	I	613	238.30
atg	M	42	42.00
act	T	8	31.49
acc	T	0	27.68
aca	T	120	58.16
acg	T	0	10.67
aaa	K	11	7.29
aag	K	1	4.71
agt	S	0	574.60
agc	S	0	591.60
aga	R	2	0.92
agg	R	0	0.57
gtt	V	0	45.96
gtc	V	0	39.06
gta	V	206	52.25
gtg	V	1	69.72
gct	A	1	58.10
gcc	A	0	54.30
gca	A	226	106.80
gcg	A	10	17.77
gaa	E	1	0.59
gag	E	0	0.41
---	--	------	------
mPD		1.0	2.0
		nPD:	0.52
		N. weight:	1.8
		Sc. PD:	0.28
		Sc. rank:	2292.9

,

NA			
Pos.	296	obs:	exp:
gaa	E	1	0.59
gag	E	0	0.41
ggt	G	25	748.30
ggc	G	0	604.50
gga	G	4019	2161.00
ggg	G	727	1257.00
---	--	------	------
mPD		0.27	0.69
		nPD:	0.39
		N. weight:	0.66
		Sc. PD:	0.024
		Sc. rank:	224.1

,

NA			
Pos.	297	obs:	exp:
tct	S	0	785.70
tcc	S	0	738.20
tca	S	0	1134.00
tcg	S	0	248.30
cgt	R	8	0.60
cgc	R	0	1.00
cga	R	0	1.91
cgg	R	0	1.37
act	T	6	1.48
acc	T	0	1.30
aca	T	0	2.73
acg	T	0	0.50
aat	N	46	26.71
aac	N	1	20.29
agt	S	4647	869.40
agc	S	24	895.20
aga	R	11	8.71
agg	R	0	5.40
ggt	G	29	4.55
ggc	G	0	3.67
gga	G	0	13.14
ggg	G	0	7.64
---	--	------	------
mPD		0.052	1.7
		nPD:	0.03
		N. weight:	2.5
		Sc. PD:	-0.75
		Sc. rank:	-5996.0

,

NA			
Pos.	298	obs:	exp:
tct	S	0	0.17
tcc	S	0	0.16
tca	S	0	0.24
tcg	S	1	0.05
agt	S	0	0.19
agc	S	0	0.19
gct	A	2772	1169.00
gcc	A	646	1093.00
gca	A	512	2150.00
gcg	A	839	357.60
gat	D	2	1.12
gac	D	0	0.88
---	--	------	------
mPD		0.60	0.68
		nPD:	0.89
		N. weight:	0.67
		Sc. PD:	0.33
		Sc. rank:	1732.9

,

NA			
Pos.	299	obs:	exp:
cat	H	4	2.31
cac	H	0	1.69
caa	Q	929	2567.00
cag	Q	3832	2194.00
aaa	K	3	4.25
aag	K	4	2.75
---	--	------	------
mPD		0.32	0.50
		nPD:	0.64
		N. weight:	0.39
		Sc. PD:	0.1
		Sc. rank:	755.3

,

NA			
Pos.	300	obs:	exp:
cat	H	4188	2753.00
cac	H	584	2019.00
---	--	------	------
mPD		0.21	0.49
		nPD:	0.44
		N. weight:	0.34
		Sc. PD:	0.028
		Sc. rank:	255.8

,

NA			
Pos.	301	obs:	exp:
att	I	0	1.42
atc	I	2	1.03
ata	I	2	1.56
gtt	V	1412	1059.00
gtc	V	728	899.70
gta	V	1533	1204.00
gtg	V	1095	1606.00
---	--	------	------
mPD		0.73	0.74
		nPD:	0.99
		N. weight:	0.072
		Sc. PD:	0.042
		Sc. rank:	189.3

,

NA			
Pos.	302	obs:	exp:
gaa	E	400	2828.00
gag	E	4372	1944.00
---	--	------	------
mPD		0.15	0.48
		nPD:	0.32
		N. weight:	0.9
		Sc. PD:	-0.028
		Sc. rank:	-26.3

,

NA			
Pos.	303	obs:	exp:
gaa	E	671	2828.00
gag	E	4101	1944.00
---	--	------	------
mPD		0.24	0.48
		nPD:	0.5
		N. weight:	0.68
		Sc. PD:	0.094
		Sc. rank:	756.1

,

NA			
Pos.	304	obs:	exp:
tgt	C	998	2054.00
tgc	C	3774	2718.00
---	--	------	------
mPD		0.33	0.49
		nPD:	0.67
		N. weight:	0.17
		Sc. PD:	0.051
		Sc. rank:	363.8

,

NA			
Pos.	305	obs:	exp:
tct	S	1396	802.10
tcc	S	3315	753.70
tca	S	17	1158.00
tcg	S	41	253.50
cct	P	1	0.30
ccc	P	0	0.20
cca	P	0	0.40
ccg	P	0	0.11
act	T	2	0.49
acc	T	0	0.43
aca	T	0	0.91
acg	T	0	0.17
agt	S	0	887.60
agc	S	0	914.00
---	--	------	------
mPD		0.43	1.6
		nPD:	0.27
		N. weight:	1.8
		Sc. PD:	-0.14
		Sc. rank:	-961.3

,

NA			
Pos.	306	obs:	exp:
tgt	C	4571	2054.00
tgc	C	201	2718.00
---	--	------	------
mPD		0.081	0.49
		nPD:	0.16
		N. weight:	1.
		Sc. PD:	-0.18
		Sc. rank:	-1201.8

,

NA			
Pos.	307	obs:	exp:
ttt	F	3	1.34
ttc	F	0	1.66
tat	Y	3801	2672.00
tac	Y	968	2097.00
---	--	------	------
mPD		0.32	0.49
		nPD:	0.66
		N. weight:	0.2
		Sc. PD:	0.055
		Sc. rank:	395.1

,

NA			
Pos.	308	obs:	exp:
cct	P	3332	1408.00
ccc	P	1362	960.70
cca	P	74	1890.00
ccg	P	4	513.30
---	--	------	------
mPD		0.43	0.70
		nPD:	0.61
		N. weight:	1.
		Sc. PD:	0.24
		Sc. rank:	1808.1

,

NA			
Pos.	309	obs:	exp:
caa	Q	12	6.47
cag	Q	0	5.53
cgt	R	7	149.60
cgc	R	10	250.40
cga	R	3711	476.20
cgg	R	131	342.30
att	I	0	0.35
atc	I	0	0.26
ata	I	1	0.39
aaa	K	20	12.76
aag	K	1	8.24
aga	R	875	2173.00
agg	R	4	1347.00
---	--	------	------
mPD		0.38	0.95
		nPD:	0.4
		N. weight:	2.2
		Sc. PD:	0.1
		Sc. rank:	1009.4

,

NA			
Pos.	310	obs:	exp:
ttt	F	875	409.10
ttc	F	38	503.90
tct	S	204	34.82
tcc	S	3	32.71
tca	S	0	50.27
tcg	S	0	11.00
tat	Y	3100	1957.00
tac	Y	393	1536.00
cct	P	9	2.95
ccc	P	1	2.01
cca	P	0	3.96
ccg	P	0	1.08
cat	H	136	81.91
cac	H	6	60.09
caa	Q	0	2.16
cag	Q	4	1.84
act	T	1	0.25
acc	T	0	0.22
aca	T	0	0.45
acg	T	0	0.08
agt	S	0	38.53
agc	S	0	39.67
gat	D	2	1.12
gac	D	0	0.88
---	--	------	------
mPD		0.61	0.99
		nPD:	0.62
		N. weight:	0.62
		Sc. PD:	0.15
		Sc. rank:	1146.3

,

NA			
Pos.	311	obs:	exp:
tta	L	0	0.44
ttg	L	0	0.75
tct	S	88	14.80
tcc	S	0	13.91
tca	S	0	21.37
tcg	S	0	4.68
ctt	L	4	0.81
ctc	L	0	0.58
cta	L	0	0.70
ctg	L	0	0.73
cct	P	4488	1378.00
ccc	P	109	939.80
cca	P	13	1849.00
ccg	P	58	502.10
cat	H	8	4.61
cac	H	0	3.39
aat	N	4	2.27
aac	N	0	1.73
agt	S	0	16.38
agc	S	0	16.86
---	--	------	------
mPD		0.12	0.76
		nPD:	0.15
		N. weight:	1.6
		Sc. PD:	-0.3
		Sc. rank:	-2021.3

,

NA			
Pos.	312	obs:	exp:
tct	S	0	35.49
tcc	S	0	33.35
tca	S	0	51.24
tcg	S	0	11.21
tat	Y	1	0.56
tac	Y	0	0.44
cat	H	2	1.15
cac	H	0	0.85
cgt	R	1	0.06
cgc	R	0	0.11
cga	R	0	0.20
cgg	R	0	0.14
att	I	0	1.77
atc	I	5	1.28
ata	I	0	1.94
aat	N	14	30.69
aac	N	40	23.31
agt	S	64	39.27
agc	S	147	40.44
aga	R	1	0.92
agg	R	0	0.57
gat	D	396	307.70
gac	D	151	239.30
gaa	E	2	6.52
gag	E	9	4.48
ggt	G	3699	617.80
ggc	G	51	499.10
gga	G	158	1784.00
ggg	G	31	1038.00
---	--	------	------
mPD		0.56	1.1
		nPD:	0.51
		N. weight:	2.1
		Sc. PD:	0.29
		Sc. rank:	2356.1

,

NA			
Pos.	313	obs:	exp:
att	I	2	5.32
atc	I	11	3.85
ata	I	2	5.83
gtt	V	182	1056.00
gtc	V	4388	897.60
gta	V	185	1201.00
gtg	V	2	1602.00
---	--	------	------
mPD		0.15	0.74
		nPD:	0.21
		N. weight:	2.1
		Sc. PD:	-0.27
		Sc. rank:	-1898.5

,

NA			
Pos.	314	obs:	exp:
cgt	R	0	150.40
cgc	R	0	251.80
cga	R	4	478.80
cgg	R	0	344.20
att	I	0	0.35
atc	I	0	0.26
ata	I	1	0.39
aaa	K	7	4.25
aag	K	0	2.75
aga	R	4448	2185.00
agg	R	312	1354.00
---	--	------	------
mPD		0.13	0.94
		nPD:	0.14
		N. weight:	0.88
		Sc. PD:	-0.18
		Sc. rank:	-1157.2

,

NA			
Pos.	315	obs:	exp:
tgt	C	3064	2054.00
tgc	C	1708	2718.00
---	--	------	------
mPD		0.46	0.49
		nPD:	0.94
		N. weight:	0.14
		Sc. PD:	0.077
		Sc. rank:	375.0

,

NA			
Pos.	316	obs:	exp:
att	I	519	683.90
atc	I	1062	494.50
ata	I	347	749.50
gtt	V	339	631.50
gtc	V	2397	536.70
gta	V	106	717.90
gtg	V	2	957.90
---	--	------	------
mPD		0.92	1.2
		nPD:	0.75
		N. weight:	1.2
		Sc. PD:	0.43
		Sc. rank:	2754.8

,

NA			
Pos.	317	obs:	exp:
tgt	C	1223	2054.00
tgc	C	3548	2717.00
ggt	G	1	0.16
ggc	G	0	0.13
gga	G	0	0.45
ggg	G	0	0.26
---	--	------	------
mPD		0.38	0.49
		nPD:	0.78
		N. weight:	0.1
		Sc. PD:	0.041
		Sc. rank:	250.7

,

NA			
Pos.	318	obs:	exp:
cgt	R	0	150.60
cgc	R	0	252.20
cga	R	75	479.60
cgg	R	1	344.70
aga	R	4363	2188.00
agg	R	333	1356.00
---	--	------	------
mPD		0.16	0.94
		nPD:	0.17
		N. weight:	0.78
		Sc. PD:	-0.13
		Sc. rank:	-897.6

,

NA			
Pos.	319	obs:	exp:
gat	D	146	2684.00
gac	D	4626	2088.00
---	--	------	------
mPD		0.059	0.49
		nPD:	0.12
		N. weight:	1.1
		Sc. PD:	-0.23
		Sc. rank:	-1559.6

,

NA			
Pos.	320	obs:	exp:
aat	N	652	2712.00
aac	N	4120	2060.00
---	--	------	------
mPD		0.24	0.49
		nPD:	0.48
		N. weight:	0.63
		Sc. PD:	0.075
		Sc. rank:	625.3

,

NA			
Pos.	321	obs:	exp:
tgg	W	4771	4771.00
ggt	G	0	0.16
ggc	G	0	0.13
gga	G	0	0.45
ggg	G	1	0.26
---	--	------	------
mPD		0.00042	0.00073
		nPD:	0.58
		N. weight:	0.00044
		Sc. PD:	0.00009
		Sc. rank:	0.7

,

NA			
Pos.	322	obs:	exp:
caa	Q	4	2.16
cag	Q	0	1.84
cgt	R	0	6.34
cgc	R	0	10.62
cga	R	1	20.20
cgg	R	0	14.52
att	I	0	0.35
atc	I	0	0.26
ata	I	1	0.39
aaa	K	4248	2775.00
aag	K	318	1791.00
aga	R	120	92.18
agg	R	80	57.13
---	--	------	------
mPD		0.24	0.58
		nPD:	0.4
		N. weight:	0.43
		Sc. PD:	0.021
		Sc. rank:	208.3

,

NA			
Pos.	323	obs:	exp:
ggt	G	1156	748.50
ggc	G	3522	604.70
gga	G	72	2161.00
ggg	G	22	1257.00
---	--	------	------
mPD		0.40	0.68
		nPD:	0.58
		N. weight:	2.1
		Sc. PD:	0.44
		Sc. rank:	3426.9

,

NA			
Pos.	324	obs:	exp:
tct	S	751	802.70
tcc	S	3903	754.20
tca	S	117	1159.00
tcg	S	1	253.60
agt	S	0	888.20
agc	S	0	914.50
---	--	------	------
mPD		0.31	1.6
		nPD:	0.19
		N. weight:	2.
		Sc. PD:	-0.3
		Sc. rank:	-2088.0

,

NA			
Pos.	325	obs:	exp:
aat	N	4079	2712.00
aac	N	693	2060.00
---	--	------	------
mPD		0.25	0.49
		nPD:	0.51
		N. weight:	0.3
		Sc. PD:	0.042
		Sc. rank:	334.8

,

NA

Pos.	326	obs:	exp:
cgt	R	0	150.60
cgc	R	0	252.20
cga	R	1	479.50
cgg	R	10	344.70
aaa	K	0	0.61
aag	K	1	0.39
aga	R	932	2188.00
agg	R	3828	1356.00
---	--	------	------
mPD		0.32	0.94
		nPD:	0.34
		N. weight:	1.
		Sc. PD:	-0.01
		Sc. rank:	149.6

,

NA

Pos.	327	obs:	exp:
cct	P	219	1408.00
ccc	P	3641	960.70
cca	P	905	1890.00
ccg	P	7	513.30
---	--	------	------
mPD		0.38	0.70
		nPD:	0.54
		N. weight:	1.2
		Sc. PD:	0.21
		Sc. rank:	1737.3

,

NA

Pos.	328	obs:	exp:
ttt	F	0	1.34
ttc	F	3	1.66
att	I	737	1180.00
atc	I	1301	853.10
ata	I	1288	1293.00
act	T	0	0.49
acc	T	1	0.43
aca	T	1	0.91
acg	T	0	0.17
gtt	V	892	320.00
gtc	V	529	271.90
gta	V	18	363.80
gtg	V	2	485.40
---	--	------	------
mPD		1.1	1.1
		nPD:	0.95
		N. weight:	0.46
		Sc. PD:	0.26
		Sc. rank:	1221.0

,

NA

Pos.	329	obs:	exp:
tta	L	0	0.76
ttg	L	4	1.31
ctt	L	0	1.41
ctc	L	0	1.02
cta	L	0	1.22
ctg	L	3	1.27
att	I	2	13.13
atc	I	0	9.49
ata	I	35	14.38
atg	M	3	3.00
gtt	V	732	1049.00
gtc	V	0	891.20
gta	V	3581	1192.00
gtg	V	410	1591.00
gaa	E	2	1.19
gag	E	0	0.81
---	--	------	------
mPD		0.41	0.76
		nPD:	0.55
		N. weight:	1.
		Sc. PD:	0.19
		Sc. rank:	1523.9

,

NA

Pos.	330	obs:	exp:
tat	Y	2	81.80
tac	Y	144	64.20
aat	N	21	13.07
aac	N	2	9.93
aaa	K	1	0.61
aag	K	0	0.39
gat	D	3976	2587.00
gac	D	624	2013.00
gaa	E	2	1.19
gag	E	0	0.81
---	--	------	------
mPD		0.34	0.56
		nPD:	0.61
		N. weight:	0.36
		Sc. PD:	0.084
		Sc. rank:	636.4

,

NA

Pos.	331	obs:	exp:
att	I	29	1686.00
atc	I	1	1219.00
ata	I	4723	1848.00
gtt	V	0	4.22
gtc	V	0	3.59
gta	V	19	4.80
gtg	V	0	6.40
---	--	------	------
mPD		0.020	0.67
		nPD:	0.03
		N. weight:	1.4
		Sc. PD:	-0.42
		Sc. rank:	-3365.7

,

NA

Pos.	332	obs:	exp:
tct	S	0	1.85
tcc	S	0	1.74
tca	S	0	2.67
tcg	S	0	0.58
cgt	R	0	3.35
cgc	R	0	5.60
cga	R	0	10.65
cgg	R	0	7.66
att	I	1	0.35
atc	I	0	0.26
ata	I	0	0.39
aat	N	4389	2632.00
aac	N	241	1998.00
aaa	K	2	1.82
aag	K	1	1.18
agt	S	11	2.05
agc	S	0	2.11
aga	R	100	48.61
agg	R	6	30.13
gat	D	21	11.81
gac	D	0	9.19
---	--	------	------
mPD		0.20	0.58
		nPD:	0.33
		N. weight:	0.6
		Sc. PD:	-0.0092
		Sc. rank:	60.4

,

NA

Pos.	333	obs:	exp:
tta	L	14	4.14
ttg	L	7	7.14
ctt	L	0	7.67
ctc	L	1	5.53
cta	L	16	6.64
ctg	L	0	6.89
att	I	1	56.40
atc	I	0	40.78
ata	I	158	61.81
atg	M	543	543.00
gtt	V	2	895.20
gtc	V	1	760.80
gta	V	3870	1018.00
gtg	V	159	1358.00
---	--	------	------
mPD		0.52	0.98
		nPD:	0.53
		N. weight:	1.6
		Sc. PD:	0.27
		Sc. rank:	2215.3

,

NA

Pos.	334	obs:	exp:
caa	Q	1	4.31
cag	Q	7	3.69
cgt	R	0	1.14
cgc	R	0	1.90
cga	R	0	3.62
cgg	R	0	2.60
att	I	0	1.06
atc	I	0	0.77
ata	I	3	1.17
atg	M	7	7.00
act	T	0	3.20
acc	T	0	2.81
aca	T	11	5.91
acg	T	2	1.08
aat	N	11	7.39
aac	N	2	5.61
aaa	K	671	2706.00
aag	K	3781	1746.00
aga	R	4	16.51
agg	R	32	10.23
gat	D	1	0.56
gac	D	0	0.44
gaa	E	192	137.50
gag	E	40	94.54
ggt	G	0	1.10
ggc	G	0	0.89
gga	G	7	3.17
ggg	G	0	1.84
---	--	------	------
mPD		0.43	0.61
		nPD:	0.71
		N. weight:	0.68
		Sc. PD:	0.22
		Sc. rank:	1508.2

,

NA

Pos.	335	obs:	exp:
---	-	4772	4772.00
---	--	------	------
mPD		0	0
		nPD:	1.
		N. weight:	0.
		Sc. PD:	0
		Sc. rank:	0

,

NA

Pos.	336	obs:	exp:
tct	S	0	0.34
tcc	S	0	0.32
tca	S	0	0.49
tcg	S	0	0.11
act	T	2	0.49
acc	T	0	0.43
aca	T	0	0.91
acg	T	0	0.17
aat	N	729	419.50
aac	N	9	318.50
agt	S	2	0.37
agc	S	0	0.38
gat	D	3960	2260.00
gac	D	58	1758.00
gaa	E	2	5.33
gag	E	7	3.67
ggt	G	3	0.47
ggc	G	0	0.38
gga	G	0	1.36
ggg	G	0	0.79
---	--	------	------
mPD		0.30	0.76
		nPD:	0.39
		N. weight:	0.79
		Sc. PD:	0.029
		Sc. rank:	274.7

,

NA

Pos.	337	obs:	exp:
ttt	F	127	57.35
ttc	F	1	70.65
tat	Y	3968	2258.00
tac	Y	62	1772.00
cat	H	368	328.80
cac	H	202	241.20
cgt	R	42	1.33
cgc	R	0	2.22
cga	R	0	4.22
cgg	R	0	3.03
aaa	K	1	0.61
aag	K	0	0.39
aga	R	0	19.26
agg	R	0	11.94
gat	D	1	0.56
gac	D	0	0.44
---	--	------	------
mPD		0.40	0.80
		nPD:	0.5
		N. weight:	0.75
		Sc. PD:	0.1
		Sc. rank:	829.1

,

NA

Pos.	338	obs:	exp:
tct	S	0	766.80
tcc	S	0	720.50
tca	S	0	1107.00
tcg	S	0	242.30
cgt	R	0	2.27
cgc	R	3	3.81
cga	R	0	7.24
cgg	R	0	5.20
act	T	0	1.72
acc	T	7	1.51
aca	T	0	3.18
acg	T	0	0.58
aat	N	2	8.53
aac	N	13	6.47
aaa	K	102	62.60
aag	K	1	40.40
agt	S	181	848.50
agc	S	4378	873.70
aga	R	69	33.02
agg	R	0	20.46
ggt	G	0	2.51
ggc	G	16	2.03
gga	G	0	7.25
ggg	G	0	4.22
---	--	------	------
mPD		0.20	1.7
		nPD:	0.12
		N. weight:	2.3
		Sc. PD:	-0.49
		Sc. rank:	-3369.2

,

NA

Pos.	339	obs:	exp:
tct	S	0	0.67
tcc	S	0	0.63
tca	S	0	0.97
tcg	S	0	0.21
att	I	3709	1383.00
atc	I	188	1000.00
ata	I	2	1516.00
act	T	592	146.10
acc	T	2	128.40
aca	T	0	269.90
acg	T	0	49.53
agt	S	4	0.74
agc	S	0	0.77
gtt	V	256	57.29
gtc	V	2	48.68
gta	V	0	65.13
gtg	V	0	86.90
gct	A	17	4.17
gcc	A	0	3.90
gca	A	0	7.66
gcg	A	0	1.27
---	--	------	------
mPD		0.41	1.0
		nPD:	0.41
		N. weight:	1.5
		Sc. PD:	0.078
		Sc. rank:	838.9

,

NA

Pos.	340	obs:	exp:
ttt	F	7	3.14
ttc	F	0	3.86
tct	S	0	2.69
tcc	S	0	2.53
tca	S	0	3.89
tcg	S	0	0.85
tat	Y	1	0.56
tac	Y	0	0.44
cgt	R	0	0.03
cgc	R	0	0.05
cga	R	0	0.10
cgg	R	0	0.07
att	I	125	44.34
atc	I	0	32.06
ata	I	0	48.60
atg	M	12	12.00
act	T	23	5.66
acc	T	0	4.97
aca	T	0	10.45
acg	T	0	1.92
aat	N	93	143.20
aac	N	159	108.80
agt	S	16	2.98
agc	S	0	3.07
aga	R	0	0.46
agg	R	1	0.28
gtt	V	3173	713.00
gtc	V	38	605.90
gta	V	0	810.60
gtg	V	0	1082.00
gct	A	244	63.24
gcc	A	1	59.11
gca	A	2	116.30
gcg	A	11	19.35
gat	D	720	424.10
gac	D	34	329.90
gaa	E	2	1.78
gag	E	1	1.22
ggt	G	106	17.10
ggc	G	1	13.81
gga	G	0	49.37
ggg	G	2	28.72
---	--	------	------
mPD		0.73	1.4
		nPD:	0.53
		N. weight:	1.8
		Sc. PD:	0.31
		Sc. rank:	2549.0

,

NA

Pos.	341	obs:	exp:
tct	S	47	802.70
tcc	S	4689	754.20
tca	S	35	1159.00
tcg	S	1	253.60
agt	S	0	888.20
agc	S	0	914.50
---	--	------	------
mPD		0.034	1.6
		nPD:	0.02
		N. weight:	2.7
		Sc. PD:	-0.82
		Sc. rank:	-6742.3

,

NA

Pos.	342	obs:	exp:
tct	S	0	790.00
tcc	S	0	742.30
tca	S	0	1141.00
tcg	S	0	249.60
cat	H	3	1.73
cac	H	0	1.27
cgt	R	4	0.88
cgc	R	0	1.48
cga	R	0	2.81
cgg	R	0	2.02
att	I	2	0.71
atc	I	0	0.51
ata	I	0	0.78
act	T	2	0.49
acc	T	0	0.43
aca	T	0	0.91
acg	T	0	0.17
aat	N	15	9.09
aac	N	1	6.91
agt	S	4557	874.20
agc	S	140	900.20
aga	R	5	12.84
agg	R	19	7.96
ggt	G	24	3.76
ggc	G	0	3.04
gga	G	0	10.87
ggg	G	0	6.32
---	--	------	------
mPD		0.090	1.7
		nPD:	0.05
		N. weight:	2.4
		Sc. PD:	-0.66
		Sc. rank:	-5039.5

,

NA

Pos.	343	obs:	exp:
tat	Y	4544	2673.00
tac	Y	228	2099.00
---	--	------	------
mPD		0.091	0.49
		nPD:	0.18
		N. weight:	0.62
		Sc. PD:	-0.095
		Sc. rank:	-679.1

,

NA

Pos.	344	obs:	exp:
tta	L	36	4.46
ttg	L	2	7.70
ctt	L	0	8.28
ctc	L	0	5.97
cta	L	3	7.16
ctg	L	0	7.43
att	I	42	31.57
atc	I	0	22.83
ata	I	47	34.60
atg	M	208	208.00
gtt	V	81	982.30
gtc	V	4	834.80
gta	V	2326	1117.00
gtg	V	2013	1490.00
gct	A	0	2.45
gcc	A	0	2.29
gca	A	10	4.51
gcg	A	0	0.75
---	--	------	------
mPD		0.66	0.87
		nPD:	0.76
		N. weight:	0.72
		Sc. PD:	0.27
		Sc. rank:	1734.2

,

NA

Pos.	345	obs:	exp:
tgt	C	431	2054.00
tgc	C	4341	2718.00
---	--	------	------
mPD		0.16	0.49
		nPD:	0.34
		N. weight:	0.44
		Sc. PD:	-0.0066
		Sc. rank:	47.0

,

NA			
Pos.	346	obs:	exp:
tct	S	1158	802.70
tcc	S	17	754.20
tca	S	3514	1159.00
tcg	S	83	253.60
agt	S	0	888.20
agc	S	0	914.50
---	--	------	------
mPD		0.40	1.6
		nPD:	0.24
		N. weight:	1.4
		Sc. PD:	-0.13
		Sc. rank:	-921.4

,

NA			
Pos.	347	obs:	exp:
ggt	G	38	748.50
ggc	G	349	604.70
gga	G	2925	2161.00
ggg	G	1460	1257.00
---	--	------	------
mPD		0.53	0.68
		nPD:	0.77
		N. weight:	0.26
		Sc. PD:	0.1
		Sc. rank:	636.2

,

NA			
Pos.	348	obs:	exp:
ttt	F	3	1.34
ttc	F	0	1.66
tta	L	0	518.90
ttg	L	0	895.60
ctt	L	4665	962.90
ctc	L	62	693.70
cta	L	29	832.60
ctg	L	12	864.30
att	I	1	0.35
atc	I	0	0.26
ata	I	0	0.39
---	--	------	------
mPD		0.044	1.1
		nPD:	0.04
		N. weight:	2.3
		Sc. PD:	-0.66
		Sc. rank:	-5220.4

,

NA			
Pos.	349	obs:	exp:
att	I	1	0.35
atc	I	0	0.26
ata	I	0	0.39
act	T	1	0.25
acc	T	0	0.22
aca	T	0	0.45
acg	T	0	0.08
gtt	V	4652	1059.00
gtc	V	101	899.90
gta	V	8	1204.00
gtg	V	8	1606.00
gct	A	1	0.25
gcc	A	0	0.23
gca	A	0	0.45
gcg	A	0	0.07
---	--	------	------
mPD		0.050	0.74
		nPD:	0.07
		N. weight:	2.2
		Sc. PD:	-0.56
		Sc. rank:	-4211.6

,

NA			
Pos.	350	obs:	exp:
ggt	G	398	748.50
ggc	G	568	604.70
gga	G	3657	2161.00
ggg	G	149	1257.00
---	--	------	------
mPD		0.39	0.68
		nPD:	0.57
		N. weight:	0.43
		Sc. PD:	0.088
		Sc. rank:	693.3

,

NA			
Pos.	351	obs:	exp:
aat	N	0	0.57
aac	N	1	0.43
gat	D	711	2683.00
gac	D	4060	2088.00
---	--	------	------
mPD		0.25	0.49
		nPD:	0.52
		N. weight:	0.57
		Sc. PD:	0.087
		Sc. rank:	689.4

,

NA			
Pos.	352	obs:	exp:
act	T	33	1174.00
acc	T	17	1032.00
aca	T	4696	2168.00
acg	T	26	397.90
---	--	------	------
mPD		0.032	0.68
		nPD:	0.05
		N. weight:	1.1
		Sc. PD:	-0.31
		Sc. rank:	-2396.8

,

NA			
Pos.	353	obs:	exp:
cct	P	498	1408.00
ccc	P	4252	960.70
cca	P	22	1890.00
ccg	P	0	513.30
---	--	------	------
mPD		0.20	0.70
		nPD:	0.28
		N. weight:	1.9
		Sc. PD:	-0.13
		Sc. rank:	-892.5

,

NA			
Pos.	354	obs:	exp:
cgt	R	0	150.60
cgc	R	0	252.20
cga	R	5	479.50
cgg	R	0	344.70
aga	R	4509	2188.00
agg	R	257	1356.00
ggt	G	0	0.16
ggc	G	0	0.13
gga	G	1	0.45
ggg	G	0	0.26
---	--	------	------
mPD		0.10	0.94
		nPD:	0.11
		N. weight:	0.92
		Sc. PD:	-0.2
		Sc. rank:	-1465.0

,

NA			
Pos.	355	obs:	exp:
ttt	F	0	0.45
ttc	F	1	0.55
tta	L	0	0.11
ttg	L	0	0.19
tct	S	0	0.50
tcc	S	0	0.47
tca	S	0	0.73
tcg	S	0	0.16
tat	Y	0	5.04
tac	Y	9	3.96
ctt	L	0	0.20
ctc	L	0	0.15
cta	L	1	0.17
ctg	L	0	0.18
cat	H	0	1.73
cac	H	3	1.27
caa	Q	13	7.01
cag	Q	0	5.99
cgt	R	0	8.14
cgc	R	0	13.64
cga	R	23	25.93
cgg	R	0	18.64
att	I	0	32.28
atc	I	14	23.34
ata	I	77	35.38
atg	M	11	11.00
aat	N	122	438.80
aac	N	650	333.20
aaa	K	3218	2134.00
aag	K	293	1377.00
agt	S	2	0.56
agc	S	1	0.57
aga	R	223	118.30
agg	R	12	73.33
gtt	V	0	0.67
gtc	V	1	0.57
gta	V	0	0.76
gtg	V	2	1.01
gat	D	0	1.12
gac	D	2	0.88
gaa	E	85	54.51
gag	E	7	37.49
ggt	G	0	0.31
ggc	G	1	0.25
gga	G	1	0.91
ggg	G	0	0.53
---	--	------	------
mPD		0.60	0.86
		nPD:	0.7
		N. weight:	0.45
		Sc. PD:	0.14
		Sc. rank:	1000.9

,

NA

Pos.	356	obs:	exp:
tct	S	0	142.80
tcc	S	0	134.20
tca	S	0	206.20
tcg	S	0	45.12
tat	Y	1	1.12
tac	Y	1	0.88
cct	P	0	2.36
ccc	P	8	1.61
cca	P	0	3.17
ccg	P	0	0.86
att	I	9	6.39
atc	I	9	4.62
ata	I	0	7.00
act	T	1	25.34
acc	T	101	22.27
aca	T	1	46.80
acg	T	0	8.59
aat	N	425	1273.00
aac	N	1815	966.80
aaa	K	13	10.33
aag	K	4	6.67
agt	S	61	158.00
agc	S	788	162.70
gct	A	1	0.25
gcc	A	0	0.23
gca	A	0	0.45
gcg	A	0	0.07
gat	D	93	750.30
gac	D	1241	583.70
gaa	E	17	10.07
gag	E	0	6.93
ggt	G	1	28.70
ggc	G	182	23.19
gga	G	0	82.89
ggg	G	0	48.22
---	--	------	------
mPD		1.0	1.5
		nPD:	0.68
		N. weight:	1.
		Sc. PD:	0.31
		Sc. rank:	2229.5

,

NA

Pos.	357	obs:	exp:
aat	N	0	2.27
aac	N	4	1.73
gat	D	643	2682.00
gac	D	4125	2086.00
---	--	------	------
mPD		0.23	0.49
		nPD:	0.48
		N. weight:	0.62
		Sc. PD:	0.071
		Sc. rank:	607.2

,

NA

Pos.	358	obs:	exp:
tta	L	0	0.22
ttg	L	0	0.38
tct	S	0	399.30
tcc	S	0	375.20
tca	S	0	576.50
tcg	S	0	126.20
ctt	L	0	0.40
ctc	L	2	0.29
cta	L	0	0.35
ctg	L	0	0.36
cat	H	0	0.58
cac	H	1	0.42
caa	Q	1	0.54
cag	Q	0	0.46
cgt	R	0	35.67
cgc	R	91	59.73
cga	R	3	113.60
cgg	R	0	81.64
att	I	1	5.32
atc	I	11	3.85
ata	I	3	5.83
act	T	0	0.49
acc	T	0	0.43
aca	T	2	0.91
acg	T	0	0.17
aat	N	1	7.96
aac	N	13	6.04
aaa	K	990	615.60
aag	K	23	397.40
agt	S	132	441.90
agc	S	2242	455.00
aga	R	908	518.20
agg	R	128	321.20
gaa	E	3	3.56
gag	E	3	2.44
ggt	G	1	33.57
ggc	G	57	27.12
gga	G	54	96.93
ggg	G	102	56.39
---	--	------	------
mPD		1.0	1.9
		nPD:	0.55
		N. weight:	1.4
		Sc. PD:	0.25
		Sc. rank:	2081.2

,

NA

Pos.	359	obs:	exp:
ttt	F	50	585.20
ttc	F	1256	720.80
tta	L	26	47.99
ttg	L	1	82.83
tct	S	613	382.00
tcc	S	1645	358.90
tca	S	13	551.50
tcg	S	0	120.70
tat	Y	25	15.69
tac	Y	3	12.31
ctt	L	3	89.06
ctc	L	410	64.16
cta	L	1	77.00
ctg	L	0	79.94
caa	Q	1	0.54
cag	Q	0	0.46
att	I	0	0.35
atc	I	1	0.26
ata	I	0	0.39
act	T	5	19.19
acc	T	73	16.87
aca	T	0	35.44
acg	T	0	6.50
agt	S	0	422.70
agc	S	0	435.20
gtt	V	6	141.40
gtc	V	627	120.20
gta	V	4	160.80
gtg	V	0	214.60
gct	A	0	2.21
gcc	A	9	2.06
gca	A	0	4.06
gcg	A	0	0.67
---	--	------	------
mPD		1.2	1.9
		nPD:	0.62
		N. weight:	1.7
		Sc. PD:	0.41
		Sc. rank:	3105.6

,

NA

Pos.	360	obs:	exp:
tct	S	0	802.70
tcc	S	0	754.20
tca	S	0	1159.00
tcg	S	0	253.60
agt	S	409	888.20
agc	S	4363	914.50
---	--	------	------
mPD		0.16	1.6
		nPD:	0.1
		N. weight:	2.1
		Sc. PD:	-0.5
		Sc. rank:	-3669.9

,

NA

Pos.	361	obs:	exp:
tct	S	0	629.60
tcc	S	0	591.60
tca	S	0	908.90
tcg	S	0	198.90
cat	H	1	0.58
cac	H	0	0.42
caa	Q	0	22.65
cag	Q	42	19.35
cgt	R	0	0.98
cgc	R	0	1.64
cga	R	0	3.12
cgg	R	0	2.24
act	T	3	1.48
acc	T	3	1.30
aca	T	0	2.73
acg	T	0	0.50
aat	N	535	358.70
aac	N	96	272.30
aaa	K	14	187.20
aag	K	294	120.80
agt	S	3693	696.70
agc	S	50	717.30
aga	R	29	14.22
agg	R	2	8.81
ggt	G	10	1.57
ggc	G	0	1.27
gga	G	0	4.53
ggg	G	0	2.63
---	--	------	------
mPD		0.55	1.8
		nPD:	0.3
		N. weight:	2.1
		Sc. PD:	-0.096
		Sc. rank:	-535.0

,

NA

Pos.	362	obs:	exp:
tct	S	0	802.30
tcc	S	0	753.90
tca	S	0	1158.00
tcg	S	0	253.50
agt	S	2269	887.80
agc	S	2501	914.20
ggt	G	0	0.31
ggc	G	2	0.25
gga	G	0	0.91
ggg	G	0	0.53
---	--	------	------
mPD		0.50	1.6
		nPD:	0.31
		N. weight:	1.5
		Sc. PD:	-0.063
		Sc. rank:	-294.6

,

NA

Pos.	363	obs:	exp:
ttt	F	3	1.34
ttc	F	0	1.66
tct	S	0	0.17
tcc	S	0	0.16
tca	S	0	0.24
tcg	S	0	0.05
tat	Y	1284	769.20
tac	Y	89	603.80
cat	H	1085	651.80
cac	H	45	478.20
cgt	R	17	0.60
cgc	R	2	1.00
cga	R	0	1.91
cgg	R	0	1.37
aat	N	2017	1252.00
aac	N	185	950.40
agt	S	1	0.19
agc	S	0	0.19
aga	R	0	8.71
agg	R	0	5.40
gat	D	40	24.75
gac	D	4	19.25
---	--	------	------
mPD		0.78	1.1
		nPD:	0.68
		N. weight:	0.55
		Sc. PD:	0.17
		Sc. rank:	1185.3

,

NA

Pos.	364	obs:	exp:
tgt	C	1676	2054.00
tgc	C	3095	2717.00
ggt	G	1	0.16
ggc	G	0	0.13
gga	G	0	0.45
ggg	G	0	0.26
---	--	------	------
mPD		0.46	0.49
		nPD:	0.93
		N. weight:	0.022
		Sc. PD:	0.012
		Sc. rank:	58.5

,

NA

Pos.	365	obs:	exp:
ttt	F	1	0.45
ttc	F	0	0.55
tta	L	66	365.90
ttg	L	74	631.50
tct	S	0	0.34
tcc	S	0	0.32
tca	S	0	0.49
tcg	S	0	0.11
ctt	L	3	679.00
ctc	L	0	489.20
cta	L	2067	587.00
ctg	L	1152	609.40
cat	H	10	5.77
cac	H	0	4.23
caa	Q	260	143.40
cag	Q	6	122.60
cgt	R	165	22.82
cgc	R	58	38.21
cga	R	119	72.67
cgg	R	68	52.23
att	I	2	85.85
atc	I	0	62.07
ata	I	240	94.08
atg	M	21	21.00
act	T	0	0.25
acc	T	0	0.22
aca	T	1	0.45
acg	T	0	0.08
aaa	K	90	58.95
aag	K	7	38.05
agt	S	2	0.37
agc	S	0	0.38
aga	R	12	331.60
agg	R	301	205.50
gtt	V	0	10.44
gtc	V	0	8.87
gta	V	10	11.86
gtg	V	37	15.83
---	--	------	------
mPD		1.2	1.7
		nPD:	0.73
		N. weight:	1.3
		Sc. PD:	0.45
		Sc. rank:	3020.4

,

NA

Pos.	366	obs:	exp:
tct	S	0	3.03
tcc	S	0	2.84
tca	S	0	4.37
tcg	S	0	0.96
tat	Y	4	18.49
tac	Y	29	14.51
cat	H	1	0.58
cac	H	0	0.42
act	T	16	4.43
acc	T	2	3.89
aca	T	0	8.18
acg	T	0	1.50
aat	N	1281	1307.00
aac	N	1018	992.30
aaa	K	1	0.61
aag	K	0	0.39
agt	S	15	3.35
agc	S	3	3.45
gtt	V	0	4.44
gtc	V	20	3.77
gta	V	0	5.05
gtg	V	0	6.74
gct	A	1	0.49
gcc	A	1	0.46
gca	A	0	0.90
gcg	A	0	0.15
gat	D	1553	1326.00
gac	D	804	1031.00
gaa	E	0	7.11
gag	E	12	4.89
ggt	G	10	1.73
ggc	G	1	1.39
gga	G	0	4.98
ggg	G	0	2.90
---	--	------	------
mPD		1.0	1.0
		nPD:	0.98
		N. weight:	0.058
		Sc. PD:	0.034
		Sc. rank:	154.6

NA

Pos.	367	obs:	exp:
tct	S	3	0.50
tcc	S	0	0.47
tca	S	0	0.73
tcg	S	0	0.16
cct	P	4570	1407.00
ccc	P	121	959.70
cca	P	75	1888.00
ccg	P	1	512.80
agt	S	0	0.56
agc	S	0	0.57
gct	A	2	0.49
gcc	A	0	0.46
gca	A	0	0.90
gcg	A	0	0.15
---	--	------	------
mPD		0.082	0.71
		nPD:	0.12
		N. weight:	1.6
		Sc. PD:	-0.35
		Sc. rank:	-2450.2

NA

Pos.	368	obs:	exp:
aat	N	304	2712.00
aac	N	4467	2059.00
aaa	K	1	0.61
aag	K	0	0.39
---	--	------	------
mPD		0.12	0.49
		nPD:	0.24
		N. weight:	0.91
		Sc. PD:	-0.09
		Sc. rank:	-630.6

NA

Pos.	369	obs:	exp:
tct	S	0	1.01
tcc	S	0	0.95
tca	S	0	1.46
tcg	S	0	0.32
tat	Y	6	3.36
tac	Y	0	2.64
act	T	4	1.23
acc	T	1	1.08
aca	T	0	2.27
acg	T	0	0.42
aat	N	3765	2677.00
aac	N	945	2033.00
aaa	K	6	13.98
aag	K	17	9.02
agt	S	6	1.12
agc	S	0	1.15
gat	D	12	7.87
gac	D	2	6.13
gaa	E	1	3.56
gag	E	5	2.44
ggt	G	2	0.31
ggc	G	0	0.25
gga	G	0	0.91
ggg	G	0	0.53
---	--	------	------
mPD		0.35	0.52
		nPD:	0.67
		N. weight:	0.2
		Sc. PD:	0.057
		Sc. rank:	402.7

NA

Pos.	370	obs:	exp:
caa	Q	6	59.86
cag	Q	105	51.14
aaa	K	0	0.61
aag	K	1	0.39
gaa	E	476	2761.00
gag	E	4184	1899.00
---	--	------	------
mPD		0.23	0.53
		nPD:	0.43
		N. weight:	0.83
		Sc. PD:	0.06
		Sc. rank:	567.3

NA

Pos.	371	obs:	exp:
tct	S	0	22.71
tcc	S	0	21.34
tca	S	0	32.78
tcg	S	0	7.17
cgt	R	0	18.84
cgc	R	0	31.55
cga	R	0	60.01
cgg	R	0	43.13
att	I	0	0.71
atc	I	0	0.51
ata	I	2	0.78
act	T	0	0.25
acc	T	0	0.22
aca	T	1	0.45
acg	T	0	0.08
aat	N	71	59.68
aac	N	34	45.32
aaa	K	2268	1471.00
aag	K	153	949.70
agt	S	108	25.13
agc	S	27	25.87
aga	R	591	273.80
agg	R	6	169.70
gtt	V	0	0.22
gtc	V	0	0.19
gta	V	1	0.25
gtg	V	0	0.34
gct	A	0	0.49
gcc	A	0	0.46
gca	A	2	0.90
gcg	A	0	0.15
gat	D	0	1.12
gac	D	2	0.88
gaa	E	1492	892.30
gag	E	14	613.70
---	--	------	------
mPD		0.86	1.3
		nPD:	0.66
		N. weight:	0.66
		Sc. PD:	0.19
		Sc. rank:	1346.7

NA

Pos.	372	obs:	exp:
ggt	G	2290	748.50
ggc	G	6	604.70
gga	G	358	2161.00
ggg	G	2118	1257.00
---	--	------	------
mPD		0.57	0.68
		nPD:	0.83
		N. weight:	0.98
		Sc. PD:	0.43
		Sc. rank:	2465.9

NA

Pos.	373	obs:	exp:
tct	S	0	34.14
tcc	S	0	32.08
tca	S	0	49.30
tcg	S	0	10.79
cgt	R	0	0.09
cgc	R	0	0.16
cga	R	0	0.30
cgg	R	0	0.22
att	I	1	0.71
atc	I	0	0.51
ata	I	1	0.78
act	T	3	0.74
acc	T	0	0.65
aca	T	0	1.36
acg	T	0	0.25
aat	N	655	507.00
aac	N	237	385.00
aaa	K	0	3.04
aag	K	5	1.96
agt	S	154	37.78
agc	S	49	38.90
aga	R	0	1.38
agg	R	3	0.85
gtt	V	3	0.67
gtc	V	0	0.57
gta	V	0	0.76
gtg	V	0	1.01
gct	A	7	1.72
gcc	A	0	1.60
gca	A	0	3.16
gcg	A	0	0.52
gat	D	71	40.50
gac	D	1	31.50
gaa	E	16	435.50
gag	E	719	299.50
ggt	G	2550	446.60
ggc	G	50	360.70
gga	G	13	1290.00
ggg	G	234	750.10
---	--	------	------
mPD		1.3	1.6
		nPD:	0.79
		N. weight:	1.6
		Sc. PD:	0.65
		Sc. rank:	3939.8

NA

Pos.	374	obs:	exp:
tat	Y	1	0.56
tac	Y	0	0.44
cat	H	4631	2750.00
cac	H	137	2018.00
caa	Q	0	1.08
cag	Q	2	0.92
aat	N	1	0.57
aac	N	0	0.43
---	--	------	------
mPD		0.057	0.49
		nPD:	0.12
		N. weight:	0.67
		Sc. PD:	-0.14
		Sc. rank:	-1020.4

NA

Pos.	375	obs:	exp:
ggt	G	83	748.50
ggc	G	1	604.70
gga	G	1703	2161.00
ggg	G	2985	1257.00
---	--	------	------
mPD		0.48	0.68
		nPD:	0.7
		N. weight:	0.65
		Sc. PD:	0.21
		Sc. rank:	1441.7

NA

Pos.	376	obs:	exp:
ttt	F	1	0.45
ttc	F	0	0.55
att	I	117	41.86
atc	I	0	30.27
ata	I	1	45.87
atg	M	2	2.00
gtt	V	66	1033.00
gtc	V	0	877.60
gta	V	382	1174.00
gtg	V	4203	1567.00
---	--	------	------
mPD		0.27	0.79
		nPD:	0.34
		N. weight:	1.2
		Sc. PD:	-0.017
		Sc. rank:	149.8

,

NA

Pos.	377	obs:	exp:
att	I	1	0.35
atc	I	0	0.26
ata	I	0	0.39
aaa	K	4540	2900.00
aag	K	231	1871.00
---	--	------	------
mPD		0.093	0.48
		nPD:	0.19
		N. weight:	0.51
		Sc. PD:	-0.073
		Sc. rank:	-526.0

,

NA

Pos.	378	obs:	exp:
tct	S	0	0.34
tcc	S	0	0.32
tca	S	0	0.49
tcg	S	0	0.11
tgt	C	0	0.43
tgc	C	1	0.57
agt	S	0	0.37
agc	S	2	0.38
ggt	G	752	748.00
ggc	G	3821	604.30
gga	G	167	2160.00
ggg	G	29	1257.00
---	--	------	------
mPD		0.33	0.69
		nPD:	0.49
		N. weight:	2.1
		Sc. PD:	0.26
		Sc. rank:	2161.7

,

NA

Pos.	379	obs:	exp:
tgg	W	4771	4771.00
gaa	E	0	0.59
gag	E	1	0.41
---	--	------	------
mPD		0.00084	0.0011
		nPD:	0.77
		N. weight:	0.00029
		Sc. PD:	0.00011
		Sc. rank:	0.7

,

NA

Pos.	380	obs:	exp:
tct	S	0	0.17
tcc	S	1	0.16
tca	S	0	0.24
tcg	S	0	0.05
cct	P	1	0.30
ccc	P	0	0.20
cca	P	0	0.40
ccg	P	0	0.11
agt	S	0	0.19
agc	S	0	0.19
gct	A	160	1169.00
gcc	A	4560	1093.00
gca	A	49	2150.00
gcg	A	1	357.70
---	--	------	------
mPD		0.086	0.68
		nPD:	0.13
		N. weight:	2.
		Sc. PD:	-0.41
		Sc. rank:	-2670.8

,

NA

Pos.	381	obs:	exp:
ttt	F	4700	2118.00
ttc	F	27	2609.00
tta	L	44	4.90
ttg	L	0	8.45
ctt	L	1	9.09
ctc	L	0	6.55
cta	L	0	7.86
ctg	L	0	8.16
---	--	------	------
mPD		0.030	0.51
		nPD:	0.06
		N. weight:	1.2
		Sc. PD:	-0.33
		Sc. rank:	-2537.1

,

NA

Pos.	382	obs:	exp:
aat	N	7	3.98
aac	N	0	3.02
gat	D	3953	2672.00
gac	D	798	2079.00
gaa	E	14	8.30
gag	E	0	5.70
---	--	------	------
mPD		0.29	0.50
		nPD:	0.57
		N. weight:	0.26
		Sc. PD:	0.054
		Sc. rank:	417.5

,

NA

Pos.	383	obs:	exp:
tct	S	0	0.17
tcc	S	0	0.16
tca	S	0	0.24
tcg	S	0	0.05
tat	Y	1	0.56
tac	Y	0	0.44
att	I	3	1.06
atc	I	0	0.77
ata	I	0	1.17
aat	N	256	145.50
aac	N	0	110.50
agt	S	1	0.19
agc	S	0	0.19
gtt	V	1	0.22
gtc	V	0	0.19
gta	V	0	0.25
gtg	V	0	0.34
gct	A	3	0.74
gcc	A	0	0.69
gca	A	0	1.35
gcg	A	0	0.22
gat	D	4354	2515.00
gac	D	117	1956.00
gaa	E	11	20.74
gag	E	24	14.26
ggt	G	1	0.16
ggc	G	0	0.13
gga	G	0	0.45
ggg	G	0	0.26
---	--	------	------
mPD		0.17	0.61
		nPD:	0.28
		N. weight:	0.73
		Sc. PD:	-0.049
		Sc. rank:	-337.9

,

NA

Pos.	384	obs:	exp:
aaa	K	1	0.61
aag	K	0	0.39
gaa	E	82	48.59
gag	E	0	33.41
ggt	G	16	735.50
ggc	G	0	594.10
gga	G	3988	2124.00
ggg	G	685	1235.00
---	--	------	------
mPD		0.29	0.72
		nPD:	0.4
		N. weight:	0.68
		Sc. PD:	0.03
		Sc. rank:	283.8

,

NA

Pos.	385	obs:	exp:
tct	S	0	2.86
tcc	S	0	2.69
tca	S	0	4.13
tcg	S	0	0.90
cat	H	4	2.31
cac	H	0	1.69
cgt	R	0	0.09
cgc	R	0	0.16
cga	R	0	0.30
cgg	R	0	0.22
att	I	0	0.35
atc	I	0	0.26
ata	I	1	0.39
act	T	6	1.72
acc	T	0	1.51
aca	T	1	3.18
acg	T	0	0.58
aat	N	2927	1772.00
aac	N	191	1346.00
aaa	K	710	431.50
aag	K	0	278.50
agt	S	17	3.16
agc	S	0	3.26
aga	R	3	1.38
agg	R	0	0.85
gat	D	765	510.20
gac	D	142	396.80
gaa	E	1	0.59
gag	E	0	0.41
ggt	G	4	0.63
ggc	G	0	0.51
gga	G	0	1.81
ggg	G	0	1.05
---	--	------	------
mPD		0.69	0.95
		nPD:	0.72
		N. weight:	0.54
		Sc. PD:	0.19
		Sc. rank:	1248.5

,

NA

Pos.	386	obs:	exp:
cat	H	2	5.19
cac	H	7	3.81
aat	N	5	5.68
aac	N	5	4.32
gct	A	1	0.25
gcc	A	0	0.23
gca	A	0	0.45
gcg	A	0	0.07
gat	D	1581	2665.00
gac	D	3158	2074.00
gaa	E	10	5.93
gag	E	0	4.07
ggt	G	0	0.47
ggc	G	3	0.38
gga	G	0	1.36
ggg	G	0	0.79
---	--	------	------
mPD		0.46	0.50
		nPD:	0.91
		N. weight:	0.17
		Sc. PD:	0.087
		Sc. rank:	444.7

,

NA

Pos.	387	obs:	exp:
tta	L	4	77.92
ttg	L	621	134.50
ctt	L	1	144.60
ctc	L	0	104.20
cta	L	0	125.00
ctg	L	90	129.80
att	I	2	153.60
atc	I	2	111.10
ata	I	429	168.30
atg	M	50	50.00
act	T	0	21.65
acc	T	0	19.03
aca	T	87	39.99
acg	T	1	7.34
gtt	V	23	772.00
gtc	V	2	656.10
gta	V	260	877.70
gtg	V	3192	1171.00
gct	A	0	1.96
gcc	A	0	1.83
gca	A	5	3.61
gcg	A	3	0.60
---	--	------	------
mPD		0.76	1.2
		nPD:	0.62
		N. weight:	1.4
		Sc. PD:	0.34
		Sc. rank:	2544.3

,

NA

Pos.	388	obs:	exp:
tct	S	0	0.17
tcc	S	0	0.16
tca	S	0	0.24
tcg	S	0	0.05
tgg	W	4771	4771.00
agt	S	1	0.19
agc	S	0	0.19
---	--	------	------
mPD		0.00084	0.00082
		nPD:	1.03
		N. weight:	0.00055
		Sc. PD:	0.00034
		Sc. rank:	1.5

,

NA

Pos.	389	obs:	exp:
tta	L	0	0.11
ttg	L	1	0.19
ctt	L	0	0.20
ctc	L	0	0.15
cta	L	0	0.17
ctg	L	0	0.18
atg	M	4770	4770.00
gtt	V	0	0.22
gtc	V	0	0.19
gta	V	0	0.25
gtg	V	1	0.34
---	--	------	------
mPD		0.00084	0.0014
		nPD:	0.61
		N. weight:	0.0009
		Sc. PD:	0.00021
		Sc. rank:	1.6

,

NA

Pos.	390	obs:	exp:
ggt	G	5	748.50
ggc	G	1	604.70
gga	G	4610	2161.00
ggg	G	156	1257.00
---	--	------	------
mPD		0.066	0.68
		nPD:	0.1
		N. weight:	1.
		Sc. PD:	-0.24
		Sc. rank:	-1795.3

,

NA			
Pos.	391	obs:	exp:
cgt	R	0	150.60
cgc	R	0	252.20
cga	R	21	479.60
cgg	R	11	344.70
aga	R	3439	2188.00
agg	R	1301	1356.00
---	--	------	------
mPD		0.41	0.94
		nPD:	0.44
		N. weight:	0.46
		Sc. PD:	0.037
		Sc. rank:	345.1

,

NA			
Pos.	392	obs:	exp:
cct	P	0	0.30
ccc	P	0	0.20
cca	P	0	0.40
ccg	P	1	0.11
act	T	473	1173.00
acc	T	243	1031.00
aca	T	2172	2167.00
acg	T	1882	397.80
gct	A	0	0.25
gcc	A	0	0.23
gca	A	1	0.45
gcg	A	0	0.07
---	--	------	------
mPD		0.63	0.68
		nPD:	0.92
		N. weight:	0.71
		Sc. PD:	0.37
		Sc. rank:	1842.2

,

NA			
Pos.	393	obs:	exp:
tta	L	0	0.11
ttg	L	0	0.19
ctt	L	0	0.20
ctc	L	1	0.15
cta	L	0	0.17
ctg	L	0	0.18
att	I	32	1692.00
atc	I	4587	1223.00
ata	I	151	1854.00
gtt	V	0	0.22
gtc	V	1	0.19
gta	V	0	0.25
gtg	V	0	0.34
---	--	------	------
mPD		0.075	0.66
		nPD:	0.11
		N. weight:	1.8
		Sc. PD:	-0.4
		Sc. rank:	-2860.3

,

NA			
Pos.	394	obs:	exp:
tct	S	0	324.00
tcc	S	0	304.40
tca	S	0	467.70
tcg	S	0	102.40
cgt	R	0	0.47
cgc	R	1	0.79
cga	R	0	1.51
cgg	R	0	1.08
att	I	1	2.13
atc	I	5	1.54
ata	I	0	2.33
act	T	0	0.25
acc	T	1	0.22
aca	T	0	0.45
acg	T	0	0.08
aat	N	116	1540.00
aac	N	2594	1170.00
aaa	K	3	2.43
aag	K	1	1.57
agt	S	279	358.50
agc	S	1647	369.10
aga	R	14	6.88
agg	R	0	4.26
gat	D	3	46.12
gac	D	79	35.88
gaa	E	6	3.56
gag	E	0	2.44
ggt	G	0	3.45
ggc	G	21	2.79
gga	G	1	9.96
ggg	G	0	5.80
---	--	------	------
mPD		0.69	1.6
		nPD:	0.44
		N. weight:	1.4
		Sc. PD:	0.11
		Sc. rank:	1045.5

,

NA			
Pos.	395	obs:	exp:
tct	S	0	0.34
tcc	S	0	0.32
tca	S	0	0.49
tcg	S	0	0.11
cgt	R	0	0.03
cgc	R	0	0.05
cga	R	0	0.10
cgg	R	0	0.07
act	T	0	0.25
acc	T	0	0.22
aca	T	0	0.45
acg	T	1	0.08
aat	N	331	189.30
aac	N	2	143.70
aaa	K	334	291.10
aag	K	145	187.90
agt	S	2	0.37
agc	S	0	0.38
aga	R	1	0.46
agg	R	0	0.28
gat	D	33	18.56
gac	D	0	14.44
gaa	E	295	2324.00
gag	E	3627	1598.00
ggt	G	0	0.16
ggc	G	0	0.13
gga	G	1	0.45
ggg	G	0	0.26
---	--	------	------
mPD		0.64	0.84
		nPD:	0.76
		N. weight:	0.84
		Sc. PD:	0.31
		Sc. rank:	1996.0

,

NA			
Pos.	396	obs:	exp:
tct	S	0	11.10
tcc	S	0	10.43
tca	S	60	16.03
tcg	S	2	3.51
cgt	R	0	0.35
cgc	R	0	0.58
cga	R	0	1.11
cgg	R	0	0.79
atg	M	8	8.00
act	T	25	545.10
acc	T	1	479.20
aca	T	769	1007.00
acg	T	1421	184.80
aat	N	23	13.64
aac	N	1	10.36
aaa	K	76	715.90
aag	K	1102	462.10
agt	S	1	12.28
agc	S	3	12.65
aga	R	3	5.04
agg	R	8	3.13
gct	A	0	0.25
gcc	A	0	0.23
gca	A	0	0.45
gcg	A	1	0.07
gat	D	741	567.50
gac	D	268	441.50
gaa	E	81	148.10
gag	E	169	101.90
ggt	G	7	1.41
ggc	G	0	1.14
gga	G	0	4.08
ggg	G	2	2.37
---	--	------	------
mPD		1.5	1.6
		nPD:	0.93
		N. weight:	1.2
		Sc. PD:	0.63
		Sc. rank:	3110.1

,

NA			
Pos.	397	obs:	exp:
ttt	F	48	25.09
ttc	F	8	30.91
tta	L	2902	346.60
ttg	L	120	598.20
tct	S	5	235.00
tcc	S	1	220.80
tca	S	1372	339.20
tcg	S	19	74.25
tgg	W	1	1.00
ctt	L	7	643.20
ctc	L	0	463.40
cta	L	145	556.10
ctg	L	11	577.40
caa	Q	1	0.54
cag	Q	0	0.46
att	I	0	20.22
atc	I	0	14.62
ata	I	57	22.16
atg	M	4	4.00
act	T	0	0.98
acc	T	0	0.86
aca	T	4	1.82
acg	T	0	0.33
aaa	K	1	0.61
aag	K	0	0.39
agt	S	0	260.00
agc	S	0	267.70
gtt	V	1	13.99
gtc	V	0	11.89
gta	V	59	15.90
gtg	V	3	21.22
gct	A	0	0.74
gcc	A	0	0.69
gca	A	3	1.35
gcg	A	0	0.22
---	--	------	------
mPD		0.63	1.8
		nPD:	0.35
		N. weight:	2.5
		Sc. PD:	-0.013
		Sc. rank:	480.2

,

NA			
Pos.	398	obs:	exp:
cgt	R	930	150.60
cgc	R	3631	252.20
cga	R	137	479.50
cgg	R	72	344.70
aga	R	1	2188.00
agg	R	0	1356.00
ggt	G	1	0.16
ggc	G	0	0.13
gga	G	0	0.45
ggg	G	0	0.26
---	--	------	------
mPD		0.38	0.94
		nPD:	0.41
		N. weight:	3.6
		Sc. PD:	0.19
		Sc. rank:	1982.3

,

NA			
Pos.	399	obs:	exp:
ttt	F	2	6.27
ttc	F	12	7.73
tta	L	2720	308.30
ttg	L	69	532.10
tct	S	7	303.80
tcc	S	3	285.40
tca	S	1772	438.60
tcg	S	24	95.98
tat	Y	110	61.63
tac	Y	0	48.37
ctt	L	0	572.20
ctc	L	0	412.20
cta	L	44	494.70
ctg	L	0	513.50
act	T	0	1.23
acc	T	0	1.08
aca	T	5	2.27
acg	T	0	0.42
agt	S	0	336.10
agc	S	0	346.10
gct	A	0	0.98
gcc	A	0	0.92
gca	A	3	1.80
gcg	A	1	0.30
---	--	------	------
mPD		0.61	1.9
		nPD:	0.32
		N. weight:	2.7
		Sc. PD:	-0.082
		Sc. rank:	-103.1

,

NA			
Pos.	400	obs:	exp:
ggt	G	3656	748.50
ggc	G	1097	604.70
gga	G	15	2161.00
ggg	G	4	1257.00
---	--	------	------
mPD		0.36	0.68
		nPD:	0.53
		N. weight:	2.1
		Sc. PD:	0.33
		Sc. rank:	2773.4

,

NA			
Pos.	401	obs:	exp:
ttt	F	7	4.93
ttc	F	4	6.07
tat	Y	4372	2667.00
tac	Y	389	2094.00
---	--	------	------
mPD		0.16	0.50
		nPD:	0.31
		N. weight:	0.49
		Sc. PD:	-0.017
		Sc. rank:	-58.6

,

NA			
Pos.	402	obs:	exp:
gaa	E	4711	2828.00
gag	E	61	1944.00
---	--	------	------
mPD		0.025	0.48
		nPD:	0.05
		N. weight:	0.72
		Sc. PD:	-0.2
		Sc. rank:	-1525.3

,

NA			
Pos.	403	obs:	exp:
att	I	0	0.35
atc	I	1	0.26
ata	I	0	0.39
act	T	722	1174.00
acc	T	3762	1032.00
aca	T	275	2168.00
acg	T	12	397.80
---	--	------	------
mPD		0.35	0.68
		nPD:	0.52
		N. weight:	1.3
		Sc. PD:	0.2
		Sc. rank:	1567.5

,

NA			
Pos.	404	obs:	exp:
ttt	F	555	2136.00
ttc	F	4211	2630.00
tta	L	2	0.65
ttg	L	1	1.13
ctt	L	0	1.21
ctc	L	3	0.87
cta	L	0	1.05
ctg	L	0	1.09
---	--	------	------
mPD		0.21	0.50
		nPD:	0.42
		N. weight:	0.41
		Sc. PD:	0.025
		Sc. rank:	264.3

,

NA			
Pos.	405	obs:	exp:
cgt	R	0	2.30
cgc	R	0	3.86
cga	R	0	7.34
cgg	R	0	5.27
aaa	K	4242	2856.00
aag	K	457	1843.00
aga	R	63	33.48
agg	R	10	20.75
---	--	------	------
mPD		0.21	0.52
		nPD:	0.4
		N. weight:	0.35
		Sc. PD:	0.016
		Sc. rank:	155.8

,

NA			
Pos.	406	obs:	exp:
att	I	1	0.71
atc	I	1	0.51
ata	I	0	0.78
gtt	V	543	1059.00
gtc	V	4188	900.10
gta	V	38	1204.00
gtg	V	1	1607.00
---	--	------	------
mPD		0.22	0.74
		nPD:	0.29
		N. weight:	1.9
		Sc. PD:	-0.1
		Sc. rank:	-730.1

,

NA			
Pos.	407	obs:	exp:
tta	L	0	0.33
ttg	L	0	0.56
ctt	L	3	0.61
ctc	L	0	0.44
cta	L	0	0.52
ctg	L	0	0.54
att	I	3435	1454.00
atc	I	657	1051.00
ata	I	7	1594.00
act	T	0	0.74
acc	T	3	0.65
aca	T	0	1.36
acg	T	0	0.25
gtt	V	122	148.10
gtc	V	544	125.90
gta	V	1	168.40
gtg	V	0	224.70
---	--	------	------
mPD		0.62	0.93
		nPD:	0.67
		N. weight:	1.1
		Sc. PD:	0.32
		Sc. rank:	2327.7

,

NA			
Pos.	408	obs:	exp:
tct	S	0	1.51
tcc	S	0	1.42
tca	S	0	2.19
tcg	S	0	0.48
caa	Q	15	8.09
cag	Q	0	6.91
cgt	R	0	0.73
cgc	R	0	1.22
cga	R	0	2.31
cgg	R	0	1.66
act	T	0	0.25
acc	T	0	0.22
aca	T	1	0.45
acg	T	0	0.08
aat	N	18	12.50
aac	N	4	9.50
aaa	K	990	617.50
aag	K	26	398.50
agt	S	9	1.68
agc	S	0	1.72
aga	R	23	10.55
agg	R	0	6.54
gat	D	247	142.90
gac	D	7	111.10
gaa	E	2301	1398.00
gag	E	59	961.70
ggt	G	756	168.10
ggc	G	37	135.80
gga	G	183	485.60
ggg	G	96	282.50
---	--	------	------
mPD		1.1	1.3
		nPD:	0.86
		N. weight:	0.77
		Sc. PD:	0.36
		Sc. rank:	1973.7

,

NA			
Pos.	409	obs:	exp:
ggt	G	1216	748.50
ggc	G	2506	604.70
gga	G	722	2161.00
ggg	G	328	1257.00
---	--	------	------
mPD		0.63	0.68
		nPD:	0.92
		N. weight:	0.96
		Sc. PD:	0.5
		Sc. rank:	2506.8

,

NA			
Pos.	410	obs:	exp:
tgg	W	4772	4772.00
---	--	------	------
mPD		0	0
		nPD:	1.
		N. weight:	0.
		Sc. PD:	0
		Sc. rank:	0

,

NA			
Pos.	411	obs:	exp:
ttt	F	0	4.93
ttc	F	11	6.07
tct	S	142	795.40
tcc	S	4465	747.40
tca	S	119	1148.00
tcg	S	3	251.30
tat	Y	0	3.92
tac	Y	7	3.08
act	T	0	4.67
acc	T	16	4.11
aca	T	3	8.63
acg	T	0	1.58
agt	S	0	880.20
agc	S	0	906.30
gct	A	0	1.47
gcc	A	6	1.37
gca	A	0	2.70
gcg	A	0	0.45
---	--	------	------
mPD		0.13	1.6
		nPD:	0.08
		N. weight:	2.4
		Sc. PD:	-0.62
		Sc. rank:	-4585.9

,

NA			
Pos.	412	obs:	exp:
caa	Q	231	125.60
cag	Q	2	107.40
cgt	R	0	1.55
cgc	R	0	2.59
cga	R	0	4.93
cgg	R	0	3.54
att	I	0	36.89
atc	I	2	26.67
ata	I	102	40.43
act	T	20	116.80
acc	T	25	102.70
aca	T	429	215.80
acg	T	1	39.61
aat	N	7	21.60
aac	N	31	16.40
aaa	K	3591	2264.00
aag	K	134	1461.00
aga	R	48	22.47
agg	R	1	13.93
gtt	V	0	0.89
gtc	V	0	0.75
gta	V	4	1.01
gtg	V	0	1.35
gct	A	0	2.70
gcc	A	1	2.52
gca	A	10	4.96
gcg	A	0	0.82
gat	D	0	0.56
gac	D	1	0.44
gaa	E	131	78.21
gag	E	1	53.79
---	--	------	------
mPD		0.48	0.93
		nPD:	0.52
		N. weight:	0.63
		Sc. PD:	0.095
		Sc. rank:	746.6

,

NA			
Pos.	413	obs:	exp:
tct	S	1146	227.90
tcc	S	206	214.10
tca	S	3	329.00
tcg	S	0	72.02
tat	Y	0	0.56
tac	Y	1	0.44
tgt	C	1	0.43
tgc	C	0	0.57
cct	P	1435	716.50
ccc	P	930	488.80
cca	P	62	961.50
ccg	P	1	261.20
cat	H	1	0.58
cac	H	0	0.42
att	I	0	0.71
atc	I	2	0.51
ata	I	0	0.78
act	T	0	2.71
acc	T	11	2.38
aca	T	0	5.00
acg	T	0	0.92
agt	S	0	252.20
agc	S	0	259.70
gct	A	957	238.50
gcc	A	8	222.90
gca	A	5	438.60
gcg	A	3	72.97
---	--	------	------
mPD		1.0	1.6
		nPD:	0.65
		N. weight:	1.5
		Sc. PD:	0.41
		Sc. rank:	2982.6

,

NA			
Pos.	414	obs:	exp:
aat	N	1880	2692.00
aac	N	2856	2044.00
aaa	K	6	3.65
aag	K	0	2.35
gat	D	1	16.87
gac	D	29	13.13
---	--	------	------
mPD		0.49	0.50
		nPD:	0.97
		N. weight:	0.1
		Sc. PD:	0.058
		Sc. rank:	266.1

,

NA			
Pos.	415	obs:	exp:
ttt	F	0	0.90
ttc	F	2	1.10
tta	L	0	0.11
ttg	L	0	0.19
tct	S	191	800.80
tcc	S	4525	752.40
tca	S	45	1156.00
tcg	S	0	253.00
ctt	L	0	0.20
ctc	L	1	0.15
cta	L	0	0.17
ctg	L	0	0.18
act	T	0	1.72
acc	T	7	1.51
aca	T	0	3.18
acg	T	0	0.58
agt	S	0	886.10
agc	S	0	912.40
gct	A	0	0.25
gcc	A	1	0.23
gca	A	0	0.45
gcg	A	0	0.07
---	--	------	------
mPD		0.10	1.6
		nPD:	0.06
		N. weight:	2.5
		Sc. PD:	-0.67
		Sc. rank:	-5102.6

,

NA			
Pos.	416	obs:	exp:
cgt	R	0	0.09
cgc	R	0	0.16
cga	R	0	0.30
cgg	R	0	0.22
aaa	K	4721	2898.00
aag	K	48	1871.00
aga	R	3	1.38
agg	R	0	0.85
---	--	------	------
mPD		0.021	0.48
		nPD:	0.04
		N. weight:	0.7
		Sc. PD:	-0.2
		Sc. rank:	-1531.1

,

NA			
Pos.	417	obs:	exp:
ttt	F	211	137.60
ttc	F	96	169.40
tta	L	2148	407.10
ttg	L	1504	702.70
tct	S	27	34.14
tcc	S	3	32.08
tca	S	100	49.30
tcg	S	73	10.79
ctt	L	17	755.50
ctc	L	0	544.30
cta	L	50	653.20
ctg	L	22	678.10
att	I	5	56.05
atc	I	0	40.53
ata	I	153	61.43
atg	M	2	2.00
act	T	0	48.70
acc	T	0	42.82
aca	T	193	89.97
acg	T	5	16.51
aaa	K	1	0.61
aag	K	0	0.39
agt	S	0	37.78
agc	S	0	38.90
gtt	V	104	35.53
gtc	V	2	30.19
gta	V	44	40.39
gtg	V	10	53.89
gct	A	1	0.49
gcc	A	0	0.46
gca	A	0	0.90
gcg	A	1	0.15
---	--	------	------
mPD		0.95	1.5
		nPD:	0.64
		N. weight:	1.7
		Sc. PD:	0.44
		Sc. rank:	3235.7

,

NA			
Pos.	418	obs:	exp:
tgg	W	3	3.00
cat	H	0	0.58
cac	H	1	0.42
caa	Q	443	2570.00
cag	Q	4323	2196.00
aaa	K	0	1.22
aag	K	2	0.78
---	--	------	------
mPD		0.17	0.50
		nPD:	0.34
		N. weight:	0.7
		Sc. PD:	-0.0047
		Sc. rank:	115.0

,

NA			
Pos.	419	obs:	exp:
tta	L	1	0.11
ttg	L	0	0.19
tct	S	0	2.52
tcc	S	0	2.37
tca	S	15	3.64
tcg	S	0	0.80
ctt	L	0	0.20
ctc	L	0	0.15
cta	L	0	0.17
ctg	L	0	0.18
cgt	R	0	0.03
cgc	R	0	0.05
cga	R	0	0.10
cgg	R	0	0.07
att	I	1	573.60
atc	I	17	414.70
ata	I	1599	628.60
atg	M	36	36.00
act	T	87	743.10
acc	T	370	653.30
aca	T	2553	1373.00
acg	T	11	251.90
aaa	K	1	0.61
aag	K	0	0.39
agt	S	0	2.79
agc	S	0	2.87
aga	R	1	0.46
agg	R	0	0.28
gtt	V	0	14.43
gtc	V	1	12.27
gta	V	63	16.41
gtg	V	1	21.89
gct	A	0	3.68
gcc	A	1	3.44
gca	A	14	6.76
gcg	A	0	1.12
---	--	------	------
mPD		0.70	1.2
		nPD:	0.59
		N. weight:	0.89
		Sc. PD:	0.2
		Sc. rank:	1510.7

,

NA			
Pos.	420	obs:	exp:
tct	S	0	17.66
tcc	S	0	16.59
tca	S	0	25.50
tcg	S	0	5.58
tat	Y	8	4.48
tac	Y	0	3.52
cat	H	2	1.15
cac	H	0	0.85
att	I	1	0.35
atc	I	0	0.26
ata	I	0	0.39
aat	N	4374	2646.00
aac	N	281	2009.00
aaa	K	1	0.61
aag	K	0	0.39
agt	S	104	19.54
agc	S	1	20.12
---	--	------	------
mPD		0.16	0.57
		nPD:	0.28
		N. weight:	0.6
		Sc. PD:	-0.04
		Sc. rank:	-281.8

,

NA			
Pos.	421	obs:	exp:
cgt	R	0	150.00
cgc	R	0	251.20
cga	R	1	477.60
cgg	R	5	343.30
aaa	K	1	11.55
aag	K	18	7.45
aga	R	758	2179.00
agg	R	3988	1351.00
ggt	G	0	0.16
ggc	G	1	0.13
gga	G	0	0.45
ggg	G	0	0.26
---	--	------	------
mPD		0.28	0.95
		nPD:	0.3
		N. weight:	1.1
		Sc. PD:	-0.059
		Sc. rank:	-417.4

,

NA			
Pos.	422	obs:	exp:
caa	Q	4305	2573.00
cag	Q	467	2199.00
---	--	------	------
mPD		0.18	0.50
		nPD:	0.36
		N. weight:	0.49
		Sc. PD:	0.0018
		Sc. rank:	113.6

,

NA			
Pos.	423	obs:	exp:
ttt	F	0	0.45
ttc	F	1	0.55
tta	L	1	0.11
ttg	L	0	0.19
ctt	L	0	0.20
ctc	L	0	0.15
cta	L	0	0.17
ctg	L	0	0.18
att	I	12	69.17
atc	I	173	50.02
ata	I	10	75.81
act	T	0	0.25
acc	T	1	0.22
aca	T	0	0.45
acg	T	0	0.08
gtt	V	1150	1015.00
gtc	V	3093	862.70
gta	V	229	1154.00
gtg	V	100	1540.00
gct	A	2	0.49
gcc	A	0	0.46
gca	A	0	0.90
gcg	A	0	0.15
---	--	------	------
mPD		0.55	0.82
		nPD:	0.67
		N. weight:	1.2
		Sc. PD:	0.35
		Sc. rank:	2454.4

,

NA			
Pos.	424	obs:	exp:
tta	L	0	0.11
ttg	L	0	0.19
ctt	L	0	0.20
ctc	L	0	0.15
cta	L	1	0.17
ctg	L	0	0.18
att	I	106	1677.00
atc	I	47	1212.00
ata	I	4573	1837.00
atg	M	22	22.00
gtt	V	0	5.11
gtc	V	0	4.34
gta	V	23	5.81
gtg	V	0	7.75
---	--	------	------
mPD		0.081	0.67
		nPD:	0.12
		N. weight:	1.2
		Sc. PD:	-0.26
		Sc. rank:	-1785.7

,

NA			
Pos.	425	obs:	exp:
att	I	393	140.10
atc	I	2	101.30
ata	I	0	153.60
gtt	V	4314	971.90
gtc	V	55	825.90
gta	V	3	1105.00
gtg	V	5	1474.00
---	--	------	------
mPD		0.18	0.89
		nPD:	0.2
		N. weight:	2.2
		Sc. PD:	-0.3
		Sc. rank:	-2070.6

,

NA			
Pos.	426	obs:	exp:
tct	S	0	0.17
tcc	S	0	0.16
tca	S	0	0.24
tcg	S	0	0.05
aat	N	1	24.44
aac	N	42	18.56
aaa	K	25	15.19
aag	K	0	9.81
agt	S	1	0.19
agc	S	0	0.19
gat	D	907	1052.00
gac	D	964	818.60
gaa	E	2500	1538.00
gag	E	95	1057.00
ggt	G	105	37.17
ggc	G	87	30.03
gga	G	42	107.30
ggg	G	3	62.45
---	--	------	------
mPD		0.74	0.86
		nPD:	0.86
		N. weight:	0.4
		Sc. PD:	0.19
		Sc. rank:	1017.3

,

NA			
Pos.	427	obs:	exp:
tct	S	0	133.00
tcc	S	0	125.00
tca	S	0	192.10
tcg	S	0	42.04
caa	Q	7	3.77
cag	Q	0	3.23
cgt	R	0	34.50
cgc	R	2	57.77
cga	R	0	109.90
cgg	R	0	78.96
att	I	0	21.99
atc	I	4	15.90
ata	I	58	24.10
atg	M	1	1.00
aat	N	204	141.00
aac	N	44	107.00
aaa	K	1998	1370.00
aag	K	256	884.20
agt	S	151	147.20
agc	S	640	151.60
aga	R	1059	501.20
agg	R	32	310.70
gtt	V	0	0.44
gtc	V	0	0.38
gta	V	2	0.50
gtg	V	0	0.67
gat	D	2	2.81
gac	D	3	2.19
gaa	E	226	141.00
gag	E	12	96.98
ggt	G	2	11.14
ggc	G	23	9.00
gga	G	46	32.16
ggg	G	0	18.71
---	--	------	------
mPD		1.1	1.6
		nPD:	0.68
		N. weight:	0.75
		Sc. PD:	0.23
		Sc. rank:	1632.7

,

NA			
Pos.	428	obs:	exp:
tct	S	0	125.10
tcc	S	0	117.60
tca	S	0	180.70
tcg	S	0	39.54
tat	Y	39	21.85
tac	Y	0	17.15
cgt	R	1	0.09
cgc	R	0	0.16
cga	R	0	0.30
cgg	R	0	0.22
act	T	1	0.25
acc	T	0	0.22
aca	T	0	0.45
acg	T	0	0.08
aat	N	508	353.00
aac	N	113	268.00
aaa	K	1	0.61
aag	K	0	0.39
agt	S	699	138.50
agc	S	45	142.60
aga	R	2	1.38
agg	R	0	0.85
gtt	V	6	1.55
gtc	V	0	1.32
gta	V	0	1.77
gtg	V	1	2.36
gct	A	6	1.47
gcc	A	0	1.37
gca	A	0	2.70
gcg	A	0	0.45
gat	D	539	344.20
gac	D	73	267.80
gaa	E	4	2.96
gag	E	1	2.04
ggt	G	2597	428.70
ggc	G	116	346.30
gga	G	16	1238.00
ggg	G	4	720.10
---	--	------	------
mPD		0.96	1.7
		nPD:	0.56
		N. weight:	1.9
		Sc. PD:	0.37
		Sc. rank:	3009.5

,

NA			
Pos.	429	obs:	exp:
tct	S	0	5.72
tcc	S	0	5.37
tca	S	0	8.26
tcg	S	0	1.81
tat	Y	2	1.12
tac	Y	0	0.88
cat	H	2	1.73
cac	H	1	1.27
aat	N	1227	1470.00
aac	N	1360	1117.00
aaa	K	67	41.33
aag	K	1	26.67
agt	S	12	6.33
agc	S	22	6.52
gtt	V	1	0.22
gtc	V	0	0.19
gta	V	0	0.25
gtg	V	0	0.34
gat	D	1060	1161.00
gac	D	1005	903.50
gaa	E	8	4.74
gag	E	0	3.26
ggt	G	4	0.63
ggc	G	0	0.51
gga	G	0	1.81
ggg	G	0	1.05
---	--	------	------
mPD		1.0	1.0
		nPD:	1.
		N. weight:	0.048
		Sc. PD:	0.028
		Sc. rank:	126.0

,

NA			
Pos.	430	obs:	exp:
tta	L	0	0.22
ttg	L	2	0.38
tct	S	0	0.34
tcc	S	0	0.32
tca	S	0	0.49
tcg	S	0	0.11
tgg	W	309	309.00
ctt	L	0	0.40
ctc	L	0	0.29
cta	L	0	0.35
ctg	L	0	0.36
caa	Q	0	0.54
cag	Q	1	0.46
cgt	R	0	140.00
cgc	R	0	234.40
cga	R	0	445.70
cgg	R	45	320.30
atg	M	17	17.00
act	T	0	0.49
acc	T	0	0.43
aca	T	2	0.91
acg	T	0	0.17
aaa	K	1	3.04
aag	K	4	1.96
agt	S	1	0.37
agc	S	1	0.38
aga	R	838	2033.00
agg	R	3551	1260.00
---	--	------	------
mPD		0.44	1.0
		nPD:	0.43
		N. weight:	0.93
		Sc. PD:	0.066
		Sc. rank:	632.5

,

NA			
Pos.	431	obs:	exp:
tct	S	180	802.70
tcc	S	3581	754.20
tca	S	988	1159.00
tcg	S	23	253.60
agt	S	0	888.20
agc	S	0	914.50
---	--	------	------
mPD		0.39	1.6
		nPD:	0.24
		N. weight:	1.7
		Sc. PD:	-0.17
		Sc. rank:	-1167.0

,

NA			
Pos.	432	obs:	exp:
ggt	G	4703	748.50
ggc	G	52	604.70
gga	G	0	2161.00
ggg	G	17	1257.00
---	--	------	------
mPD		0.029	0.68
		nPD:	0.04
		N. weight:	2.8
		Sc. PD:	-0.79
		Sc. rank:	-6085.1

,

NA			
Pos.	433	obs:	exp:
tat	Y	4608	2673.00
tac	Y	164	2099.00
---	--	------	------
mPD		0.066	0.49
		nPD:	0.13
		N. weight:	0.68
		Sc. PD:	-0.14
		Sc. rank:	-905.5

,

NA			
Pos.	434	obs:	exp:
ttt	F	1	0.45
ttc	F	0	0.55
tta	L	0	0.11
ttg	L	1	0.19
tct	S	4628	802.10
tcc	S	60	753.70
tca	S	76	1158.00
tcg	S	5	253.50
ctt	L	0	0.20
ctc	L	0	0.15
cta	L	0	0.17
ctg	L	0	0.18
agt	S	0	887.60
agc	S	0	914.00
gct	A	1	0.25
gcc	A	0	0.23
gca	A	0	0.45
gcg	A	0	0.07
---	--	------	------
mPD		0.059	1.6
		nPD:	0.04
		N. weight:	2.5
		Sc. PD:	-0.73
		Sc. rank:	-5830.5

,

NA			
Pos.	435	obs:	exp:
ggt	G	3156	748.50
ggc	G	1535	604.70
gga	G	73	2161.00
ggg	G	8	1257.00
---	--	------	------
mPD		0.46	0.68
		nPD:	0.67
		N. weight:	1.9
		Sc. PD:	0.55
		Sc. rank:	3942.0

,

NA			
Pos.	436	obs:	exp:
tta	L	0	0.11
ttg	L	0	0.19
ctt	L	1	0.20
ctc	L	0	0.15
cta	L	0	0.17
ctg	L	0	0.18
att	I	4247	1525.00
atc	I	41	1102.00
ata	I	10	1671.00
atg	M	2	2.00
gtt	V	465	104.40
gtc	V	5	88.69
gta	V	0	118.60
gtg	V	0	158.30
gat	D	1	0.56
gac	D	0	0.44
---	--	------	------
mPD		0.20	0.86
		nPD:	0.24
		N. weight:	1.6
		Sc. PD:	-0.17
		Sc. rank:	-1153.3

,

NA			
Pos.	437	obs:	exp:
ttt	F	140	2138.00
ttc	F	4631	2633.00
tta	L	1	0.11
ttg	L	0	0.19
ctt	L	0	0.20
ctc	L	0	0.15
cta	L	0	0.17
ctg	L	0	0.18
---	--	------	------
mPD		0.057	0.50
		nPD:	0.12
		N. weight:	0.73
		Sc. PD:	-0.16
		Sc. rank:	-1131.4

,

NA			
Pos.	438	obs:	exp:
---	-	4772	4772.00
---	--	------	------
mPD		0	0
		nPD:	1.
		N. weight:	0.
		Sc. PD:	0
		Sc. rank:	0

,

NA			
Pos.	439	obs:	exp:
---	-	4772	4772.00
---	--	------	------
mPD		0	0
		nPD:	1.
		N. weight:	0.
		Sc. PD:	0
		Sc. rank:	0

,

NA			
Pos.	440	obs:	exp:
---	-	4772	4772.00
---	--	------	------
mPD		0	0
		nPD:	1.
		N. weight:	0.
		Sc. PD:	0
		Sc. rank:	0

,

NA			
Pos.	441	obs:	exp:
---	-	4772	4772.00
---	--	------	------
mPD		0	0
		nPD:	1.
		N. weight:	0.
		Sc. PD:	0
		Sc. rank:	0

,

NA			
Pos.	442	obs:	exp:
tct	S	2368	802.00
tcc	S	2175	753.50
tca	S	219	1158.00
tcg	S	6	253.40
act	T	1	0.98
acc	T	3	0.86
aca	T	0	1.82
acg	T	0	0.33
agt	S	0	887.40
agc	S	0	913.80
---	--	------	------
mPD		0.54	1.6
		nPD:	0.33
		N. weight:	1.5
		Sc. PD:	-0.023
		Sc. rank:	138.8

,

NA			
Pos.	443	obs:	exp:
att	I	881	313.60
atc	I	2	226.70
ata	I	1	343.70
act	T	1	0.25
acc	T	0	0.22
aca	T	0	0.45
acg	T	0	0.08
gtt	V	3775	863.10
gtc	V	94	733.50
gta	V	2	981.20
gtg	V	16	1309.00
---	--	------	------
mPD		0.35	1.0
		nPD:	0.33
		N. weight:	2.
		Sc. PD:	-0.033
		Sc. rank:	160.0

,

NA			
Pos.	444	obs:	exp:
gaa	E	3968	2828.00
gag	E	804	1944.00
---	--	------	------
mPD		0.28	0.48
		nPD:	0.58
		N. weight:	0.21
		Sc. PD:	0.044
		Sc. rank:	343.3

,

NA			
Pos.	445	obs:	exp:
tct	S	0	146.80
tcc	S	0	138.00
tca	S	0	212.00
tcg	S	0	46.40
cgt	R	0	0.03
cgc	R	0	0.05
cga	R	0	0.10
cgg	R	0	0.07
att	I	1	0.35
atc	I	0	0.26
ata	I	0	0.39
aat	N	5	56.27
aac	N	94	42.73
agt	S	67	162.50
agc	S	806	167.30
aga	R	1	0.46
agg	R	0	0.28
gtt	V	0	2.66
gtc	V	12	2.26
gta	V	0	3.03
gtg	V	0	4.04
gat	D	27	21.94
gac	D	12	17.06
ggt	G	870	587.70
ggc	G	2291	474.80
gga	G	335	1697.00
ggg	G	251	987.30
---	--	------	------
mPD		0.88	1.3
		nPD:	0.67
		N. weight:	1.4
		Sc. PD:	0.42
		Sc. rank:	2965.1

,

NA			
Pos.	446	obs:	exp:
tct	S	0	0.17
tcc	S	0	0.16
tca	S	0	0.24
tcg	S	0	0.05
caa	Q	3	1.62
cag	Q	0	1.38
cgt	R	0	0.32
cgc	R	0	0.53
cga	R	0	1.01
cgg	R	0	0.72
act	T	0	0.98
acc	T	0	0.86
aca	T	4	1.82
acg	T	0	0.33
aat	N	3	2.84
aac	N	2	2.16
aaa	K	4639	2882.00
aag	K	104	1861.00
agt	S	0	0.19
agc	S	1	0.19
aga	R	9	4.59
agg	R	1	2.84
gaa	E	3	2.37
gag	E	1	1.63
ggt	G	0	0.31
ggc	G	0	0.25
gga	G	2	0.91
ggg	G	0	0.53
---	--	------	------
mPD		0.057	0.49
		nPD:	0.12
		N. weight:	0.63
		Sc. PD:	-0.14
		Sc. rank:	-978.8

,

NA			
Pos.	447	obs:	exp:
tct	S	0	553.40
tcc	S	0	520.00
tca	S	0	798.90
tcg	S	0	174.90
cat	H	0	1.15
cac	H	2	0.85
cgt	R	1	0.25
cgc	R	0	0.42
cga	R	0	0.80
cgg	R	0	0.58
att	I	2	2.48
atc	I	5	1.80
ata	I	0	2.72
act	T	0	0.49
acc	T	2	0.43
aca	T	0	0.91
acg	T	0	0.17
aat	N	80	764.50
aac	N	1265	580.50
aaa	K	6	3.65
aag	K	0	2.35
agt	S	584	612.30
agc	S	2706	630.50
aga	R	7	3.67
agg	R	0	2.27
gtt	V	3	0.67
gtc	V	0	0.57
gta	V	0	0.76
gtg	V	0	1.01
gat	D	2	9.00
gac	D	14	7.00
ggt	G	41	14.59
ggc	G	52	11.78
gga	G	0	42.12
ggg	G	0	24.50
---	--	------	------
mPD		0.72	1.8
		nPD:	0.39
		N. weight:	1.6
		Sc. PD:	0.06
		Sc. rank:	595.9

,

NA			
Pos.	448	obs:	exp:
tct	S	0	0.34
tcc	S	0	0.32
tca	S	0	0.49
tcg	S	0	0.11
tgt	C	1615	2052.00
tgc	C	3153	2716.00
att	I	2	0.71
atc	I	0	0.51
ata	I	0	0.78
agt	S	2	0.37
agc	S	0	0.38
---	--	------	------
mPD		0.45	0.49
		nPD:	0.91
		N. weight:	0.031
		Sc. PD:	0.016
		Sc. rank:	79.3

,

NA			
Pos.	449	obs:	exp:
att	I	146	1684.00
atc	I	4546	1217.00
ata	I	54	1845.00
act	T	0	1.72
acc	T	7	1.51
aca	T	0	3.18
acg	T	0	0.58
gtt	V	4	4.22
gtc	V	15	3.59
gta	V	0	4.80
gtg	V	0	6.40
---	--	------	------
mPD		0.093	0.67
		nPD:	0.14
		N. weight:	1.8
		Sc. PD:	-0.35
		Sc. rank:	-2301.5

,

NA			
Pos.	450	obs:	exp:
tct	S	0	0.34
tcc	S	0	0.32
tca	S	0	0.49
tcg	S	0	0.11
cat	H	1	0.58
cac	H	0	0.42
act	T	24	5.90
acc	T	0	5.19
aca	T	0	10.91
acg	T	0	2.00
aat	N	4506	2696.00
aac	N	238	2048.00
agt	S	2	0.37
agc	S	0	0.38
gat	D	1	0.56
gac	D	0	0.44
---	--	------	------
mPD		0.11	0.51
		nPD:	0.21
		N. weight:	0.6
		Sc. PD:	-0.078
		Sc. rank:	-551.5

,

NA			
Pos.	451	obs:	exp:
cat	H	1	0.58
cac	H	0	0.42
cgt	R	86	150.50
cgc	R	4	252.10
cga	R	353	479.30
cgg	R	2202	344.50
att	I	0	0.35
atc	I	0	0.26
ata	I	1	0.39
act	T	0	0.25
acc	T	0	0.22
aca	T	1	0.45
acg	T	0	0.08
aga	R	491	2187.00
agg	R	1633	1355.00
---	--	------	------
mPD		0.82	0.94
		nPD:	0.87
		N. weight:	1.1
		Sc. PD:	0.55
		Sc. rank:	2937.1

,

NA			
Pos.	452	obs:	exp:
tct	S	0	0.50
tcc	S	0	0.47
tca	S	0	0.73
tcg	S	0	0.16
tgt	C	644	2053.00
tgc	C	4125	2716.00
agt	S	0	0.56
agc	S	3	0.57
---	--	------	------
mPD		0.23	0.49
		nPD:	0.48
		N. weight:	0.32
		Sc. PD:	0.037
		Sc. rank:	316.0

,

NA			
Pos.	453	obs:	exp:
ttt	F	4162	2138.00
ttc	F	610	2634.00
---	--	------	------
mPD		0.22	0.49
		nPD:	0.45
		N. weight:	0.61
		Sc. PD:	0.056
		Sc. rank:	506.6

,

NA			
Pos.	454	obs:	exp:
tat	Y	4655	2673.00
tac	Y	117	2099.00
---	--	------	------
mPD		0.048	0.49
		nPD:	0.1
		N. weight:	0.73
		Sc. PD:	-0.17
		Sc. rank:	-1262.3

,

NA			
Pos.	455	obs:	exp:
tta	L	0	0.11
ttg	L	1	0.19
ctt	L	0	0.20
ctc	L	0	0.15
cta	L	0	0.17
ctg	L	0	0.18
gtt	V	31	1059.00
gtc	V	0	900.30
gta	V	474	1204.00
gtg	V	4266	1607.00
---	--	------	------
mPD		0.19	0.74
		nPD:	0.26
		N. weight:	1.2
		Sc. PD:	-0.1
		Sc. rank:	-649.9

,

NA			
Pos.	456	obs:	exp:
gaa	E	330	2828.00
gag	E	4442	1944.00
---	--	------	------
mPD		0.13	0.48
		nPD:	0.27
		N. weight:	0.97
		Sc. PD:	-0.075
		Sc. rank:	-498.0

,

NA			
Pos.	457	obs:	exp:
tta	L	136	519.30
ttg	L	4118	896.30
ctt	L	4	963.80
ctc	L	0	694.30
cta	L	400	833.30
ctg	L	114	865.00
---	--	------	------
mPD		0.39	1.1
		nPD:	0.35
		N. weight:	1.8
		Sc. PD:	-0.0081
		Sc. rank:	379.9

,

NA			
Pos.	458	obs:	exp:
att	I	6	1692.00
atc	I	22	1223.00
ata	I	4741	1854.00
atg	M	1	1.00
gtt	V	0	0.44
gtc	V	0	0.38
gta	V	2	0.50
gtg	V	0	0.67
---	--	------	------
mPD		0.013	0.66
		nPD:	0.02
		N. weight:	1.4
		Sc. PD:	-0.43
		Sc. rank:	-3557.1

,

NA			
Pos.	459	obs:	exp:
cgt	R	0	150.60
cgc	R	0	252.20
cga	R	6	479.50
cgg	R	1	344.70
aga	R	2024	2188.00
agg	R	2740	1356.00
gaa	E	0	0.59
gag	E	1	0.41
---	--	------	------
mPD		0.49	0.94
		nPD:	0.52
		N. weight:	0.57
		Sc. PD:	0.091
		Sc. rank:	740.7

,

NA			
Pos.	460	obs:	exp:
cgt	R	0	0.03
cgc	R	0	0.05
cga	R	1	0.10
cgg	R	0	0.07
aga	R	0	0.46
agg	R	0	0.28
gaa	E	1	0.59
gag	E	0	0.41
ggt	G	0	748.20
ggc	G	49	604.40
gga	G	4657	2161.00
ggg	G	64	1257.00
---	--	------	------
mPD		0.047	0.69
		nPD:	0.07
		N. weight:	1.1
		Sc. PD:	-0.28
		Sc. rank:	-2077.9

,

NA			
Pos.	461	obs:	exp:
tgg	W	11	11.00
cgt	R	0	150.00
cgc	R	0	251.20
cga	R	0	477.60
cgg	R	12	343.30
atg	M	2	2.00
aaa	K	2	3.04
aag	K	3	1.96
aga	R	1206	2179.00
agg	R	3534	1351.00
ggt	G	0	0.31
ggc	G	0	0.25
gga	G	2	0.91
ggg	G	0	0.53
---	--	------	------
mPD		0.39	0.94
		nPD:	0.41
		N. weight:	0.87
		Sc. PD:	0.051
		Sc. rank:	527.2

,

NA			
Pos.	462	obs:	exp:
tct	S	0	0.34
tcc	S	0	0.32
tca	S	0	0.49
tcg	S	0	0.11
caa	Q	11	5.93
cag	Q	0	5.07
cgt	R	0	22.70
cgc	R	0	38.00
cga	R	32	72.27
cgg	R	0	51.94
att	I	0	0.35
atc	I	0	0.26
ata	I	1	0.39
aat	N	6	24.44
aac	N	37	18.56
aaa	K	3831	2423.00
aag	K	156	1564.00
agt	S	1	0.37
agc	S	1	0.38
aga	R	532	329.70
agg	R	155	204.40
gaa	E	7	4.15
gag	E	0	2.85
ggt	G	0	0.31
ggc	G	0	0.25
gga	G	2	0.91
ggg	G	0	0.53
---	--	------	------
mPD		0.42	0.84
		nPD:	0.5
		N. weight:	0.53
		Sc. PD:	0.072
		Sc. rank:	565.5

,

NA			
Pos.	463	obs:	exp:
tta	L	0	0.22
ttg	L	0	0.38
tct	S	0	22.71
tcc	S	0	21.34
tca	S	124	32.78
tcg	S	11	7.17
ctt	L	0	0.40
ctc	L	0	0.29
cta	L	0	0.35
ctg	L	2	0.36
cct	P	0	5.90
ccc	P	0	4.03
cca	P	19	7.92
ccg	P	1	2.15
cat	H	1	0.58
cac	H	0	0.42
caa	Q	330	1112.00
cag	Q	1733	950.50
cgt	R	0	1.52
cgc	R	0	2.54
cga	R	2	4.82
cgg	R	16	3.47
act	T	0	19.68
acc	T	0	17.30
aca	T	80	36.35
acg	T	0	6.67
aaa	K	1	13.98
aag	K	22	9.02
agt	S	0	25.13
agc	S	0	25.87
aga	R	0	22.01
agg	R	30	13.64
gct	A	0	0.98
gcc	A	0	0.92
gca	A	4	1.80
gcg	A	0	0.30
gat	D	38	21.37
gac	D	0	16.63
gaa	E	62	1397.00
gag	E	2295	960.40
ggt	G	0	0.16
ggc	G	0	0.13
gga	G	0	0.45
ggg	G	1	0.26
---	--	------	------
mPD		0.91	1.2
		nPD:	0.76
		N. weight:	0.91
		Sc. PD:	0.34
		Sc. rank:	2174.2

,

NA			
Pos.	464	obs:	exp:
aaa	K	2	1.22
aag	K	0	0.78
gaa	E	4659	2826.00
gag	E	111	1944.00
---	--	------	------
mPD		0.046	0.48
		nPD:	0.1
		N. weight:	0.66
		Sc. PD:	-0.15
		Sc. rank:	-1159.0

,

NA			
Pos.	465	obs:	exp:
tct	S	19	3.70
tcc	S	0	3.48
tca	S	0	5.34
tcg	S	1	1.17
cct	P	19	11.51
ccc	P	20	7.85
cca	P	0	15.44
ccg	P	0	4.20
cgt	R	0	0.06
cgc	R	0	0.11
cga	R	0	0.20
cgg	R	0	0.14
att	I	14	4.97
atc	I	0	3.59
ata	I	0	5.44
act	T	3602	901.80
acc	T	50	792.70
aca	T	5	1666.00
acg	T	9	305.70
aat	N	740	421.70
aac	N	2	320.30
aaa	K	3	1.82
aag	K	0	1.18
agt	S	2	4.09
agc	S	0	4.22
aga	R	0	0.92
agg	R	2	0.57
gtt	V	0	0.22
gtc	V	0	0.19
gta	V	0	0.25
gtg	V	1	0.34
gct	A	119	61.53
gcc	A	1	57.51
gca	A	2	113.10
gcg	A	129	18.82
gat	D	19	11.25
gac	D	1	8.75
gaa	E	0	7.11
gag	E	12	4.89
---	--	------	------
mPD		0.51	1.1
		nPD:	0.46
		N. weight:	1.8
		Sc. PD:	0.18
		Sc. rank:	1600.5

,

NA

Pos.	466	obs:	exp:
tct	S	0	0.34
tcc	S	0	0.32
tca	S	2	0.49
tcg	S	0	0.11
caa	Q	2	1.08
cag	Q	0	0.92
cgt	R	0	43.34
cgc	R	0	72.57
cga	R	18	138.00
cgg	R	0	99.19
att	I	0	0.71
atc	I	0	0.51
ata	I	2	0.78
act	T	0	2.95
acc	T	0	2.59
aca	T	12	5.45
acg	T	0	1.00
aat	N	0	1.14
aac	N	2	0.86
aaa	K	1843	1122.00
aag	K	4	724.50
agt	S	0	0.37
agc	S	0	0.38
aga	R	1343	629.70
agg	R	12	390.20
gtt	V	0	3.33
gtc	V	0	2.83
gta	V	15	3.79
gtg	V	0	5.05
gct	A	3	230.20
gcc	A	0	215.10
gca	A	934	423.30
gcg	A	2	70.42
gat	D	6	3.37
gac	D	0	2.63
gaa	E	567	337.10
gag	E	2	231.90
ggt	G	0	0.47
ggc	G	0	0.38
gga	G	3	1.36
ggg	G	0	0.79
---	--	-----	-----
mPD		1.1	1.7
		nPD:	0.62
		N. weight:	0.94
		Sc. PD:	0.24
		Sc. rank:	1774.2

,

NA

Pos.	467	obs:	exp:
att	I	1	0.71
atc	I	0	0.51
ata	I	1	0.78
gtt	V	103	1059.00
gtc	V	2738	900.10
gta	V	1820	1204.00
gtg	V	109	1607.00
---	--	-----	-----
mPD		0.53	0.74
		nPD:	0.71
		N. weight:	1.1
		Sc. PD:	0.35
		Sc. rank:	2418.8

,

NA

Pos.	468	obs:	exp:
ttt	F	12	5.38
ttc	F	0	6.62
tta	L	0	66.27
ttg	L	608	114.40
tat	Y	1	0.56
tac	Y	0	0.44
tgg	W	4030	4030.00
ctt	L	0	123.00
ctc	L	0	88.61
cta	L	0	106.30
ctg	L	1	110.40
ggt	G	0	18.82
ggc	G	0	15.21
gga	G	0	54.35
ggg	G	120	31.62
---	--	-----	-----
mPD		0.28	0.62
		nPD:	0.45
		N. weight:	0.39
		Sc. PD:	0.036
		Sc. rank:	319.6

,

NA

Pos.	469	obs:	exp:
tgg	W	4772	4772.00
---	--	-----	-----
mPD		0	0
		nPD:	1.
		N. weight:	0.
		Sc. PD:	0
		Sc. rank:	0

,

NA

Pos.	470	obs:	exp:
act	T	799	1174.00
acc	T	3844	1032.00
aca	T	54	2168.00
acg	T	75	397.90
---	--	-----	-----
mPD		0.32	0.68
		nPD:	0.48
		N. weight:	1.4
		Sc. PD:	0.16
		Sc. rank:	1406.4

,

NA

Pos.	471	obs:	exp:
tct	S	10	802.70
tcc	S	0	754.20
tca	S	4305	1159.00
tcg	S	457	253.60
agt	S	0	888.20
agc	S	0	914.50
---	--	-----	-----
mPD		0.18	1.6
		nPD:	0.11
		N. weight:	1.9
		Sc. PD:	-0.43
		Sc. rank:	-3148.4

,

NA

Pos.	472	obs:	exp:
tct	S	0	8.41
tcc	S	0	7.90
tca	S	0	12.14
tcg	S	0	2.66
aat	N	216	2684.00
aac	N	4506	2038.00
agt	S	0	9.31
agc	S	50	9.58
---	--	-----	-----
mPD		0.11	0.53
		nPD:	0.2
		N. weight:	1.
		Sc. PD:	-0.14
		Sc. rank:	-961.1

,

NA

Pos.	473	obs:	exp:
tct	S	0	802.70
tcc	S	0	754.20
tca	S	0	1159.00
tcg	S	0	253.60
agt	S	4609	888.20
agc	S	163	914.50
---	--	-----	-----
mPD		0.066	1.6
		nPD:	0.04
		N. weight:	2.4
		Sc. PD:	-0.68
		Sc. rank:	-5335.9

,

NA

Pos.	474	obs:	exp:
tta	L	0	1.63
ttg	L	0	2.82
ctt	L	15	3.03
ctc	L	0	2.18
cta	L	0	2.62
ctg	L	0	2.72
att	I	4642	1674.00
atc	I	45	1210.00
ata	I	31	1834.00
gtt	V	38	8.66
gtc	V	0	7.36
gta	V	1	9.85
gtg	V	0	13.14
---	--	-----	-----
mPD		0.054	0.68
		nPD:	0.08
		N. weight:	1.5
		Sc. PD:	-0.37
		Sc. rank:	-2743.0

,

NA

Pos.	475	obs:	exp:
tta	L	0	1.52
ttg	L	0	2.63
ctt	L	14	2.83
ctc	L	0	2.04
cta	L	0	2.44
ctg	L	0	2.54
att	I	7	2.48
atc	I	0	1.80
ata	I	0	2.72
gtt	V	4664	1054.00
gtc	V	75	895.40
gta	V	3	1198.00
gtg	V	3	1598.00
gct	A	6	1.47
gcc	A	0	1.37
gca	A	0	2.70
gcg	A	0	0.45
---	--	-----	-----
mPD		0.045	0.75
		nPD:	0.06
		N. weight:	2.2
		Sc. PD:	-0.59
		Sc. rank:	-4526.9

NA

Pos.	476	obs:	exp:
gtt	V	105	1056.00
gtc	V	332	897.40
gta	V	217	1201.00
gtg	V	4102	1602.00
gct	A	1	3.92
gcc	A	0	3.67
gca	A	0	7.21
gcg	A	15	1.20
---	--	-----	-----
mPD		0.26	0.74
		nPD:	0.34
		N. weight:	0.97
		Sc. PD:	-0.0079
		Sc. rank:	150.8

,

NA

Pos.	477	obs:	exp:
ttt	F	4629	2137.00
ttc	F	141	2633.00
tta	L	0	0.11
ttg	L	0	0.19
ctt	L	1	0.20
ctc	L	0	0.15
cta	L	0	0.17
ctg	L	0	0.18
gtt	V	1	0.22
gtc	V	0	0.19
gta	V	0	0.25
gtg	V	0	0.34
---	--	-----	-----
mPD		0.058	0.50
		nPD:	0.12
		N. weight:	1.
		Sc. PD:	-0.22
		Sc. rank:	-1566.5

,

NA

Pos.	478	obs:	exp:
tgt	C	4758	2054.00
tgc	C	14	2718.00
---	--	-----	-----
mPD		0.0059	0.49
		nPD:	0.01
		N. weight:	1.3
		Sc. PD:	-0.4
		Sc. rank:	-3303.0

,

NA

Pos.	479	obs:	exp:
tct	S	0	0.17
tcc	S	0	0.16
tca	S	0	0.24
tcg	S	0	0.05
agt	S	0	0.19
agc	S	1	0.19
ggt	G	595	748.30
ggc	G	3949	604.50
gga	G	162	2161.00
ggg	G	65	1257.00
---	--	-----	-----
mPD		0.30	0.69
		nPD:	0.44
		N. weight:	2.2
		Sc. PD:	0.17
		Sc. rank:	1596.0

,

NA

Pos.	480	obs:	exp:
cct	P	0	0.30
ccc	P	1	0.20
cca	P	0	0.40
ccg	P	0	0.11
act	T	1474	1174.00
acc	T	3164	1032.00
aca	T	53	2168.00
acg	T	80	397.80
---	--	-----	-----
mPD		0.46	0.68
		nPD:	0.68
		N. weight:	1.2
		Sc. PD:	0.36
		Sc. rank:	2545.1

,

NA

Pos.	481	obs:	exp:
tta	L	1	0.11
ttg	L	0	0.19
tct	S	25	800.30
tcc	S	10	752.00
tca	S	3613	1155.00
tcg	S	1110	252.90
ctt	L	0	0.20
ctc	L	0	0.15
cta	L	0	0.17
ctg	L	0	0.18
act	T	0	2.21
acc	T	0	1.95
aca	T	1	4.09
acg	T	8	0.75
agt	S	0	885.60
agc	S	0	911.90
gct	A	1	0.98
gcc	A	0	0.92
gca	A	3	1.80
gcg	A	0	0.30
---	--	------	------
mPD		0.38	1.6
		nPD:	0.23
		N. weight:	1.8
		Sc. PD:	-0.21
		Sc. rank:	-1458.4

,

NA

Pos.	482	obs:	exp:
tgt	C	2	0.86
tgc	C	0	1.14
cgt	R	0	0.09
cgc	R	3	0.16
cga	R	0	0.30
cgg	R	0	0.22
aga	R	0	1.38
agg	R	0	0.85
ggt	G	4477	747.70
ggc	G	210	604.00
gga	G	61	2159.00
ggg	G	19	1256.00
---	--	------	------
mPD		0.12	0.69
		nPD:	0.17
		N. weight:	2.5
		Sc. PD:	-0.4
		Sc. rank:	-2739.7

,

NA

Pos.	483	obs:	exp:
tct	S	0	0.84
tcc	S	0	0.79
tca	S	5	1.21
tcg	S	0	0.27
att	I	0	0.35
atc	I	0	0.26
ata	I	1	0.39
act	T	233	1172.00
acc	T	500	1031.00
aca	T	3045	2166.00
acg	T	988	397.40
agt	S	0	0.93
agc	S	0	0.96
---	--	------	------
mPD		0.54	0.68
		nPD:	0.79
		N. weight:	0.4
		Sc. PD:	0.16
		Sc. rank:	971.7

,

NA

Pos.	484	obs:	exp:
tat	Y	4751	2673.00
tac	Y	21	2099.00
---	--	------	------
mPD		0.0088	0.49
		nPD:	0.02
		N. weight:	0.86
		Sc. PD:	-0.26
		Sc. rank:	-2184.3

,

NA

Pos.	485	obs:	exp:
ggt	G	349	748.50
ggc	G	15	604.70
gga	G	4004	2161.00
ggg	G	404	1257.00
---	--	------	------
mPD		0.28	0.68
		nPD:	0.41
		N. weight:	0.55
		Sc. PD:	0.032
		Sc. rank:	332.1

,

NA

Pos.	486	obs:	exp:
tct	S	0	173.80
tcc	S	0	163.30
tca	S	1032	250.90
tcg	S	1	54.90
act	T	0	914.50
acc	T	0	804.00
aca	T	3716	1689.00
acg	T	2	310.00
agt	S	0	192.30
agc	S	0	198.00
gct	A	0	5.15
gcc	A	0	4.81
gca	A	21	9.47
gcg	A	0	1.57
---	--	------	------
mPD		0.35	1.1
		nPD:	0.32
		N. weight:	1.4
		Sc. PD:	-0.041
		Sc. rank:	19.2

,

NA

Pos.	487	obs:	exp:
ggt	G	2	748.50
ggc	G	4770	604.70
gga	G	0	2161.00
ggg	G	0	1257.00
---	--	------	------
mPD		0.00084	0.68
		nPD:	0.
		N. weight:	3.2
		Sc. PD:	-1.
		Sc. rank:	-8437.5

,

NA

Pos.	488	obs:	exp:
tct	S	0	788.40
tcc	S	10	740.70
tca	S	4674	1138.00
tcg	S	3	249.10
act	T	0	20.66
acc	T	0	18.16
aca	T	84	38.17
acg	T	0	7.00
agt	S	0	872.40
agc	S	0	898.30
gct	A	0	0.25
gcc	A	0	0.23
gca	A	1	0.45
gcg	A	0	0.07
---	--	------	------
mPD		0.040	1.6
		nPD:	0.02
		N. weight:	2.2
		Sc. PD:	-0.65
		Sc. rank:	-5262.7

,

NA

Pos.	489	obs:	exp:
tgg	W	4772	4772.00
---	--	------	------
mPD		0	0
		nPD:	1.
		N. weight:	0.
		Sc. PD:	0
		Sc. rank:	0

,

NA

Pos.	490	obs:	exp:
cct	P	4752	1408.00
ccc	P	20	960.70
cca	P	0	1890.00
ccg	P	0	513.30
---	--	------	------
mPD		0.0083	0.70
		nPD:	0.01
		N. weight:	1.9
		Sc. PD:	-0.58
		Sc. rank:	-4825.4

,

NA

Pos.	491	obs:	exp:
gat	D	4750	2684.00
gac	D	22	2088.00
---	--	------	------
mPD		0.0092	0.49
		nPD:	0.02
		N. weight:	0.85
		Sc. PD:	-0.26
		Sc. rank:	-2156.9

,

NA

Pos.	492	obs:	exp:
ggt	G	19	748.50
ggc	G	0	604.70
gga	G	1508	2161.00
ggg	G	3245	1257.00
---	--	------	------
mPD		0.44	0.68
		nPD:	0.64
		N. weight:	0.81
		Sc. PD:	0.21
		Sc. rank:	1560.1

,

NA

Pos.	493	obs:	exp:
tct	S	0	0.17
tcc	S	0	0.16
tca	S	0	0.24
tcg	S	1	0.05
agt	S	0	0.19
agc	S	0	0.19
gct	A	362	1169.00
gcc	A	0	1093.00
gca	A	620	2150.00
gcg	A	3787	357.60
ggt	G	0	0.31
ggc	G	0	0.25
gga	G	0	0.91
ggg	G	2	0.53
---	--	------	------
mPD		0.35	0.68
		nPD:	0.51
		N. weight:	2.5
		Sc. PD:	0.37
		Sc. rank:	2973.5

,

NA

Pos.	494	obs:	exp:
tat	Y	1	0.56
tac	Y	0	0.44
att	I	0	0.71
atc	I	0	0.51
ata	I	2	0.78
act	T	0	2.95
acc	T	0	2.59
aca	T	12	5.45
acg	T	0	1.00
aat	N	72	271.10
aac	N	405	205.90
aaa	K	7	4.25
aag	K	0	2.75
gat	D	2353	2402.00
gac	D	1918	1869.00
gaa	E	2	1.19
gag	E	0	0.81
---	--	------	------
mPD		0.70	0.69
		nPD:	1.01
		N. weight:	0.066
		Sc. PD:	0.04
		Sc. rank:	175.7

,

NA

Pos.	495	obs:	exp:
tta	L	0	0.33
ttg	L	0	0.56
ctt	L	0	0.61
ctc	L	3	0.44
cta	L	0	0.52
ctg	L	0	0.54
att	I	92	1690.00
atc	I	4517	1222.00
ata	I	155	1852.00
act	T	0	0.25
acc	T	1	0.22
aca	T	0	0.45
acg	T	0	0.08
gtt	V	0	0.89
gtc	V	4	0.75
gta	V	0	1.01
gtg	V	0	1.35
---	--	------	------
mPD		0.10	0.66
		nPD:	0.16
		N. weight:	1.7
		Sc. PD:	-0.31
		Sc. rank:	-2077.6

,

NA

Pos.	496	obs:	exp:
tct	S	0	56.01
tcc	S	0	52.63
tca	S	0	80.87
tcg	S	0	17.70
tat	Y	7	3.92
tac	Y	0	3.08
cat	H	2	1.73
cac	H	1	1.27
act	T	1	0.25
acc	T	0	0.22
aca	T	0	0.45
acg	T	0	0.08
aat	N	4181	2476.00
aac	N	176	1881.00
aaa	K	9	5.47
aag	K	0	3.53
agt	S	106	61.98
agc	S	227	63.82
gat	D	46	34.87
gac	D	16	27.13
---	--	------	------
mPD		0.32	0.76
		nPD:	0.43
		N. weight:	0.7
		Sc. PD:	0.049
		Sc. rank:	465.8

,

NA

Pos.	497	obs:	exp:
ttt	F	339	263.50
ttc	F	249	324.50
tta	L	3	435.20
ttg	L	0	751.10
ctt	L	242	807.60
ctc	L	3742	581.80
cta	L	12	698.30
ctg	L	0	724.90
att	I	0	64.21
atc	I	180	46.42
ata	I	1	70.37
gtt	V	1	0.89
gtc	V	2	0.75
gta	V	1	1.01
gtg	V	0	1.35
---	--	------	------
mPD		0.50	1.3
		nPD:	0.4
		N. weight:	2.2
		Sc. PD:	0.1
		Sc. rank:	985.0

,

NA			
Pos.	498	obs:	exp:
---	-	4772	4772.00
---	--	------	------
mPD		0	0
		nPD:	1.
		N. weight:	0.
		Sc. PD:	0
		Sc. rank:	0

,

NA			
Pos.	499	obs:	exp:
tta	L	0	0.11
ttg	L	1	0.19
ctt	L	0	0.20
ctc	L	0	0.15
cta	L	0	0.17
ctg	L	0	0.18
atg	M	4768	4768.00
gtt	V	0	0.67
gtc	V	0	0.57
gta	V	0	0.76
gtg	V	3	1.01
---	--	------	------
mPD		0.0017	0.0028
		nPD:	0.6
		N. weight:	0.0016
		Sc. PD:	0.00038
		Sc. rank:	2.8

,

NA			
Pos.	500	obs:	exp:
tta	L	0	0.22
ttg	L	0	0.38
tct	S	37	6.22
tcc	S	0	5.85
tca	S	0	8.99
tcg	S	0	1.97
ctt	L	2	0.40
ctc	L	0	0.29
cta	L	0	0.35
ctg	L	0	0.36
cct	P	4659	1396.00
ccc	P	0	952.70
cca	P	52	1874.00
ccg	P	21	509.00
cgt	R	1	0.03
cgc	R	0	0.05
cga	R	0	0.10
cgg	R	0	0.07
agt	S	0	6.89
agc	S	0	7.09
aga	R	0	0.46
agg	R	0	0.28
---	--	------	------
mPD		0.047	0.73
		nPD:	0.06
		N. weight:	1.8
		Sc. PD:	-0.47
		Sc. rank:	-3546.7

,

NA			
Pos.	501	obs:	exp:
tta	L	2	0.44
ttg	L	0	0.75
ctt	L	0	0.81
ctc	L	0	0.58
cta	L	2	0.70
ctg	L	0	0.73
att	I	1	1366.00
atc	I	1	988.00
ata	I	3850	1498.00
atg	M	41	41.00
act	T	0	1.23
acc	T	0	1.08
aca	T	5	2.27
acg	T	0	0.42
gtt	V	0	192.90
gtc	V	0	164.00
gta	V	869	219.40
gtg	V	0	292.70
ggt	G	0	0.16
ggc	G	0	0.13
gga	G	1	0.45
ggg	G	0	0.26
---	--	------	------
mPD		0.32	1.0
		nPD:	0.32
		N. weight:	1.6
		Sc. PD:	-0.046
		Sc. rank:	-2.8

,

NA			
Pos.	502	obs:	exp:
taa	.	3601	2030.00
tag	.	28	1257.00
tga	.	1143	1485.00
---	--	------	------
mPD		0.38	0.82
		nPD:	0.46
		N. weight:	0.54
		Sc. PD:	0.055
		Sc. rank:	481.5

}

Amino acid level conservation per codon

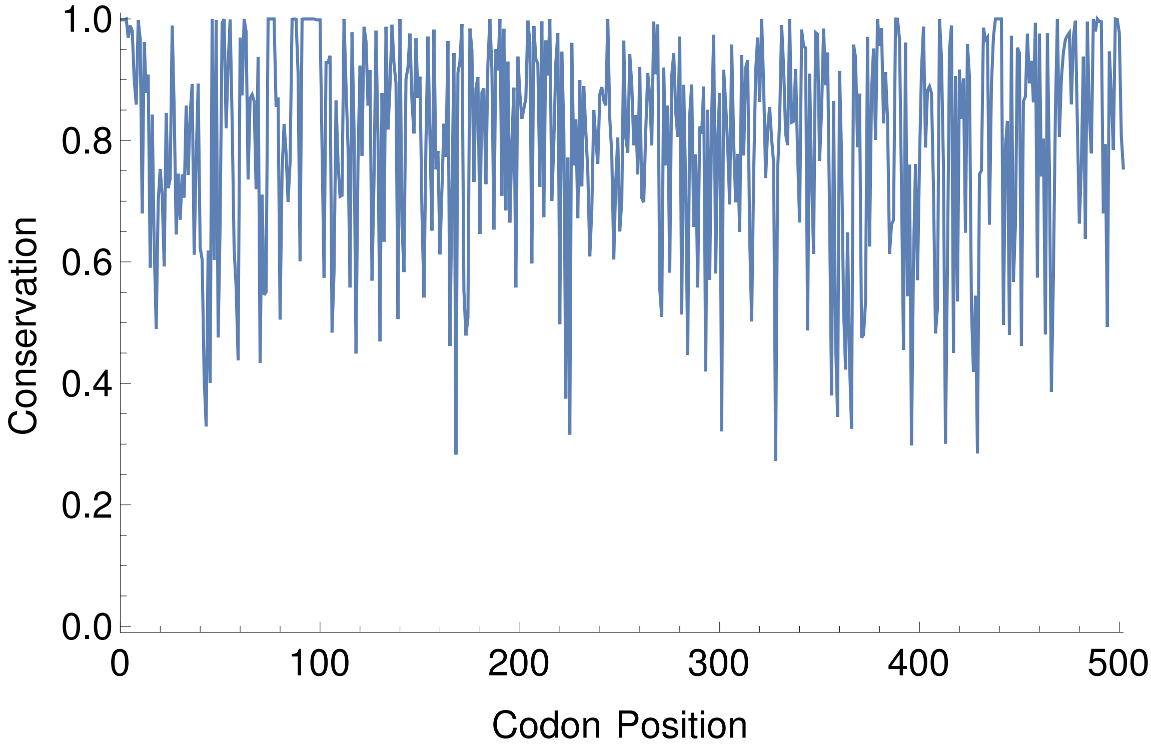

Conservation analysis plots

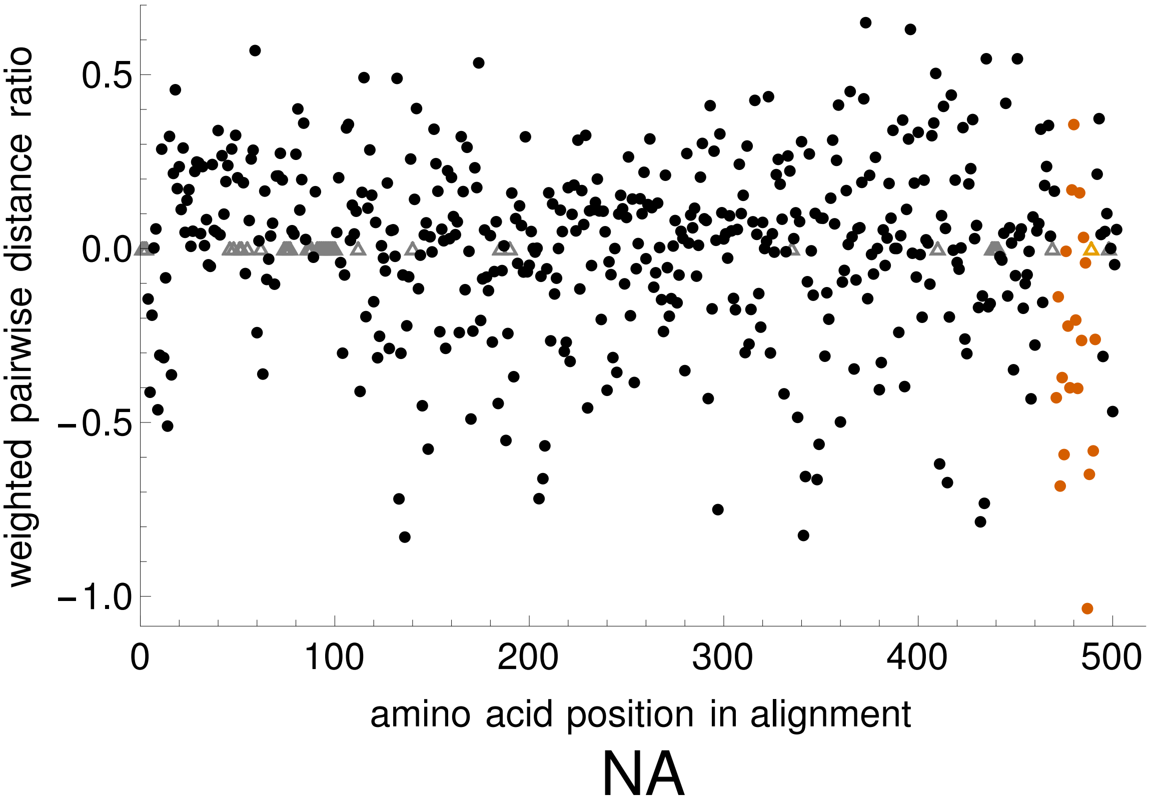

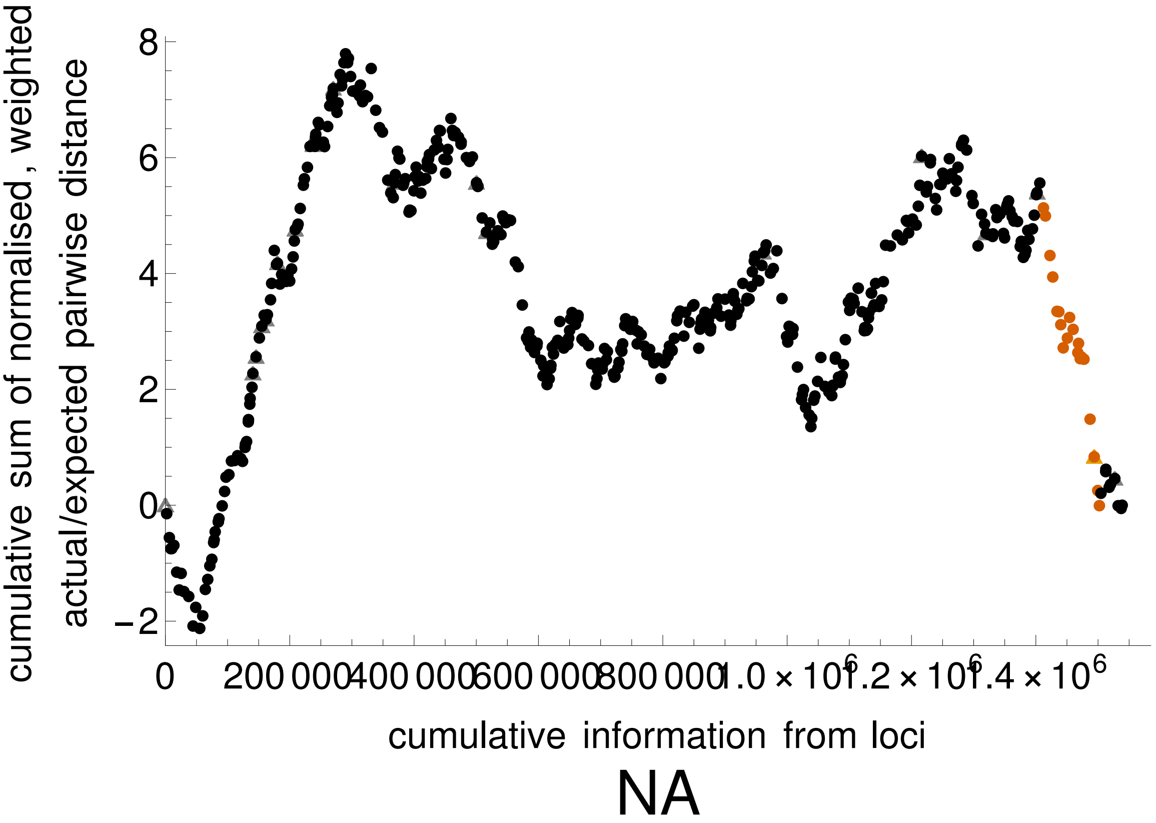

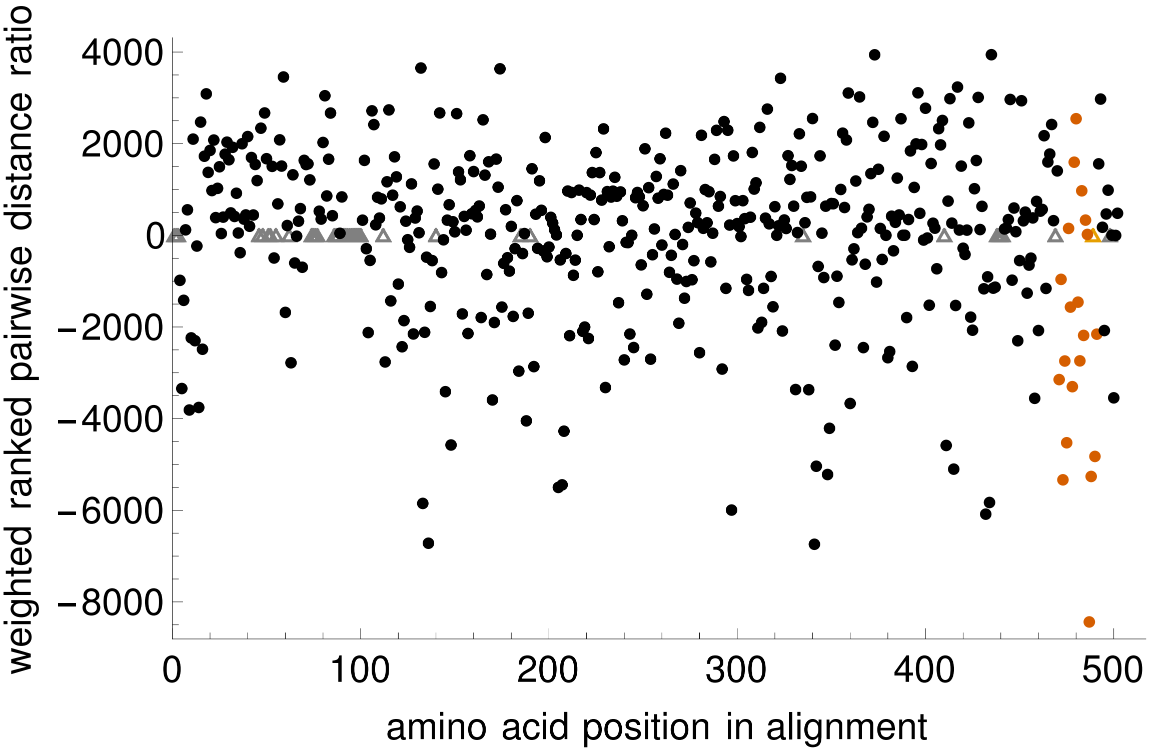
weighted ranked pairwise distance ratio
4000
2000
0
−2000
−4000
−6000
−8000
0
100
200
300
400
500
amino acid position in alignment

NA

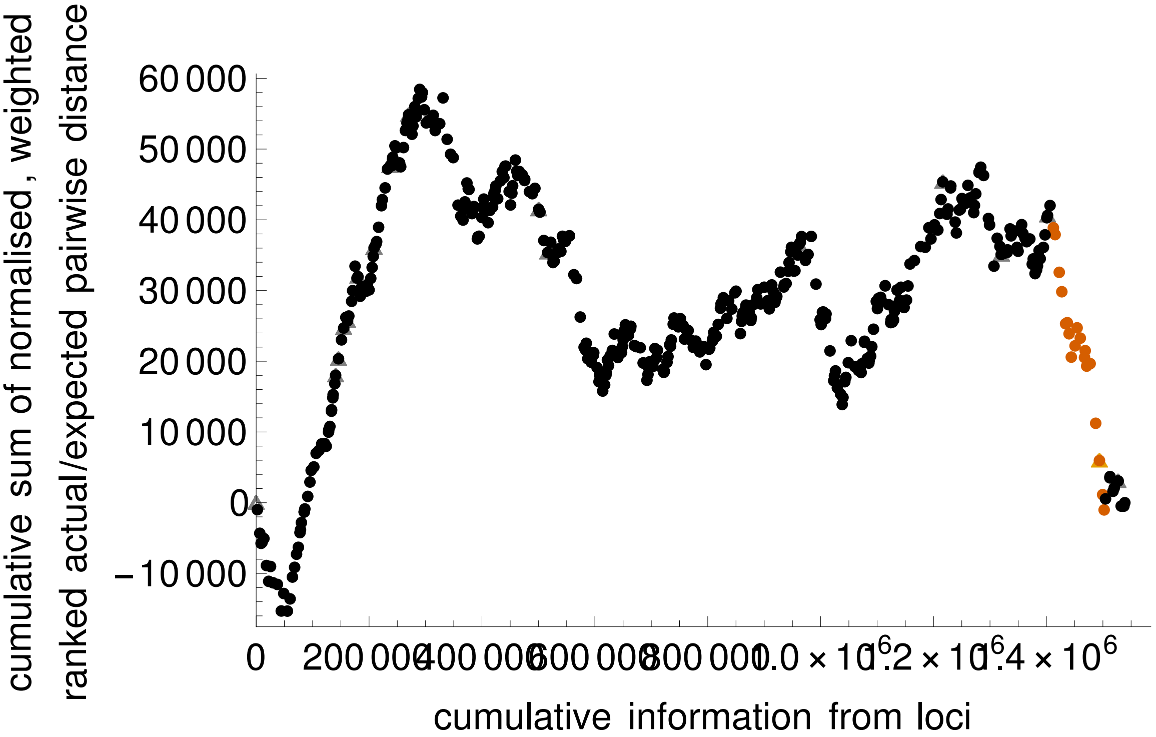
cumulative sum of normalised, weighted ranked actual/expected pairwise distance
60 000
50 000
40 000
30 000
20 000
10 000
0
−10 000
0
200 000
400 000
600 000
800 000
1.0 × 10^6
1.2 × 10^6
1.4 × 10^6
cumulative information from loci

NA

M1

Gene length histogram

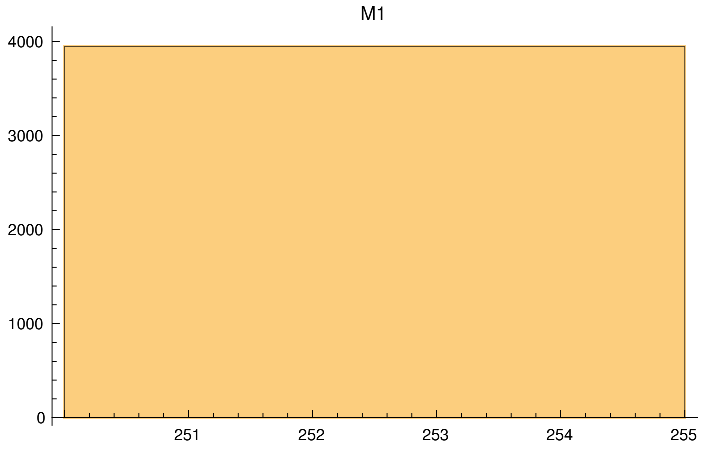

Information vs. nPD

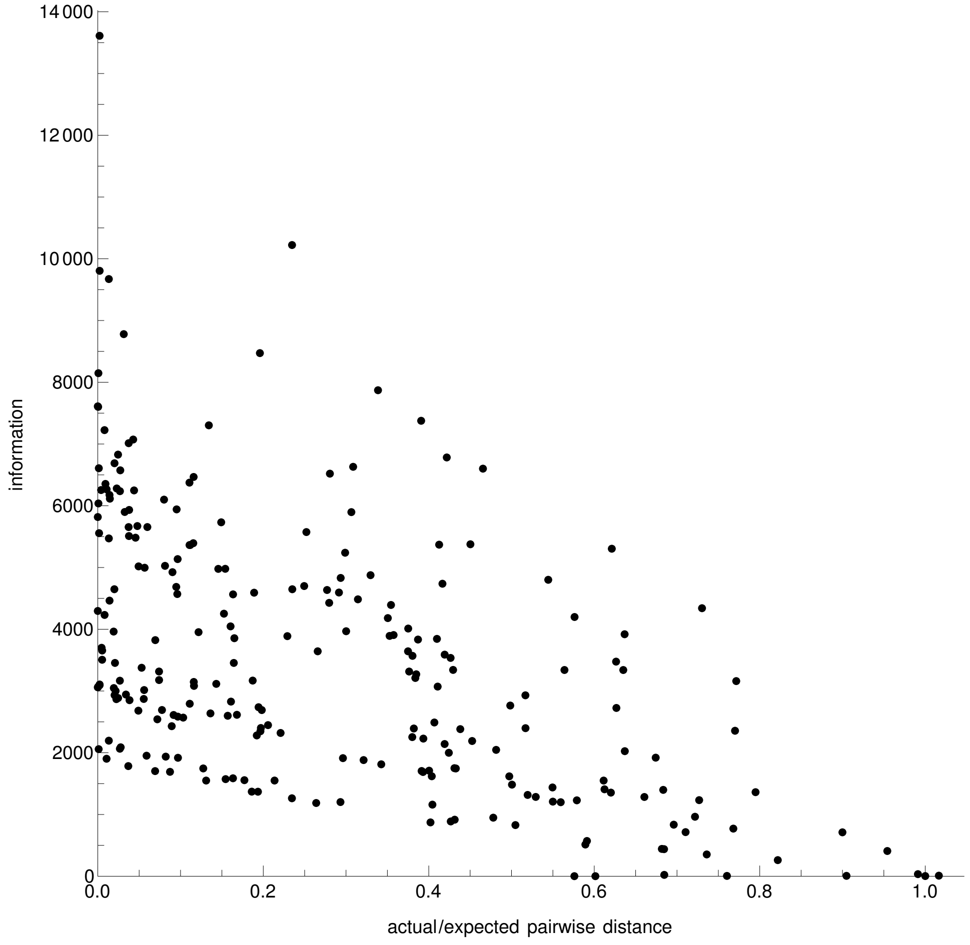

M1

Example sequences highlighted by regions found in analyses to be conserved

Interesting points (by weighted raw PD) highlighted for gene M1:

---ATG

AGTCTTCTAACCGAGGTCGAAACGTACGTTCTCTCTATCATCCCATCAGGCCCCC

TCAAAGCCGAGATCGCGCAGAGACTGGAAGATGTCTTTGCAGGGAAGAACACAGAT

CTAGAGGCTCTCATGGAGTGGCTAAAGACAAGACCAATCCTGTCACCTCTGACTA

AGGGGATTTTAGGATTTGTG

TTCACGCTCACCGTGCCCAGTGAGCGAGGACTGCAGCGTAGACGCTTTGTCCAAAATGC⋱

CCTAAATGGGAATGGAGACCCGAACAACATGGATAGAGCAGTCAAATTATACAAGAAGCTCAAAAGAGAA⋱

ATAACATTCCATGGAGCCAAGGAGGTGTCACTCAGCTACTCAACT

GGTGCACTTGCCAGCTGCATGGGCCTCATATACAACAGAATGGGAACAGTGACCACAGAAGTGG⋱

CTTTTGGTCTAGTGTGTGCCACTTGTGAGCAGATTGCTGATTCACAGCATCGGTCTCACAGACAAATGGC⋱

GACTACCACCAATCCATTAATCAGGCATGAAAACAGAATGGTGCTGGCTAGCACTACAGCTAAGGCTATG⋱

GAACAGATGGCTGGGTCGAGTGAACAGGCAGCGGAGGCCATGGAAGTTGCCAGTCAGGCCAGGCAGATGG⋱

TGCATGCGATGAGAACAATTGGGACTCATCCTAGCTCCAGTGCT

GGTCTGAAAGATGACCTTCTTGAAAATTTGCAGGCC

TACCAGAAACGGATGGGAGTGCAGATACAGCGATTCAAGTGA

Interesting points (by weighted ranked PD) highlighted for gene M1:

---ATG

AGTCTTCTAACCGAGGTCGAAACGTACGTTCTCTCTATCATCCCATCAGGCCCCC

TCAAAGCCGAGATCGCGCAGAGACTGGAAGATGTCTTTGCAGGGAAGAACACAGATCTAGAGGCTCT⋱

CATGGAGTGGCTAAAGACAAGACCAATCCTGTCACCTCTGACTAAGGGGATTTTAGGATTTGTGTTC

ACGCTCACCGTGCCCAGTGAGCGAGGACTGCAGCGTAGACGCTTTGTCCAAAATGCCCTAA⋱

ATGGGAATGGAGACCCGAACAACATGGATAGAGCAGTCAAATTATACAAGAAGCTCAAAAGAGAAATAAC⋱

ATTCCATGGAGCCAAGGAGGTGTCACTCAGCTACTCAACTGGTGCACTTGCCAGCTGCATGGGCCTCATA⋱

TACAACAGAATGGGAACAGTGACCACAGAAGTGGCTTTTGGTCTAGTGTGTGCCACTTGTGAGCAGATTG⋱

CTGATTCACAGCATCGGTCTCACAGACAAATGGCGACTACCACCAATCCATTAATCAGGCATGAAAACAG⋱

AATGGTGCTGGCTAGCACTACAGCTAAGGCTATGGAACAGATGGCTGGGTCGAGTGAACAGGCAGCGGAG⋱

GCCATGGAAGTTGCCAGTCAGGCCAGGCAGATGGTGCATGCGATGAGAACAATTGGGACTCATCCTAGCT⋱

CCAGTGCTGGTCTGAAAGATGACCTTCTTGAAAATTTGCAGGCCTACCAGAAACGGATGGGAGTGCAGAT⋱

ACAGCGATTCAAGTGA

Per codon conservation report

M1

Pos.	1	obs:	exp:
atg	M	1	1.00
---	-	3947	3947.00
mPD		0	0
		nPD:	1.
		N. weight:	0.
		Sc. PD:	0
		Sc. rank:	0

M1

Pos.	2	obs:	exp:
atg	M	3948	3948.00
mPD		0	0
		nPD:	1.
		N. weight:	0.
		Sc. PD:	0
		Sc. rank:	0

M1

Pos.	3	obs:	exp:
tct	S	0	663.00
tcc	S	0	623.00
tca	S	0	957.30
tcg	S	0	209.50
att	I	6	2.13
atc	I	0	1.54
ata	I	0	2.33
agt	S	3860	733.70
agc	S	82	755.50
mPD		0.044	1.6
		nPD:	0.03
		N. weight:	1.8
		Sc. PD:	-0.3
		Sc. rank:	-2621.2

M1

Pos.	4	obs:	exp:
tta	L	0	429.60
ttg	L	0	741.60
ctt	L	3939	797.30
ctc	L	8	574.40
cta	L	1	689.40
ctg	L	0	715.70
mPD		0.0046	1.1
		nPD:	0.
		N. weight:	1.8
		Sc. PD:	-0.34
		Sc. rank:	-4243.8

M1

Pos.	5	obs:	exp:
tta	L	1	429.10
ttg	L	0	740.60
ctt	L	6	796.30
ctc	L	0	573.70
cta	L	3886	688.50
ctg	L	50	714.80
cgt	R	0	0.16
cgc	R	0	0.26
cga	R	5	0.50
cgg	R	0	0.36
aga	R	0	2.29
agg	R	0	1.42
mPD		0.031	1.1
		nPD:	0.03
		N. weight:	1.9
		Sc. PD:	-0.31
		Sc. rank:	-2676.8

M1

Pos.	6	obs:	exp:
act	T	41	970.40
acc	T	3883	853.10
aca	T	21	1793.00
acg	T	0	329.00
gct	A	0	0.74
gcc	A	3	0.69
gca	A	0	1.35
gcg	A	0	0.22
mPD		0.033	0.68
		nPD:	0.05
		N. weight:	1.7
		Sc. PD:	-0.24
		Sc. rank:	-1481.4

M1

Pos.	7	obs:	exp:
tct	S	0	0.50
tcc	S	0	0.47
tca	S	0	0.73
tcg	S	3	0.16
agt	S	0	0.56
agc	S	0	0.57
gaa	E	14	2337.00
gag	E	3931	1608.00
mPD		0.010	0.49
		nPD:	0.02
		N. weight:	1.
		Sc. PD:	-0.17
		Sc. rank:	-1682.5

M1

Pos.	8	obs:	exp:
gtt	V	140	876.20
gtc	V	3804	744.60
gta	V	2	996.10
gtg	V	0	1329.00
gat	D	0	1.12
gac	D	2	0.88
mPD		0.070	0.74
		nPD:	0.1
		N. weight:	1.7
		Sc. PD:	-0.17
		Sc. rank:	-574.8

M1

Pos.	9	obs:	exp:
gaa	E	3947	2339.00
gag	E	1	1609.00
mPD		0.00051	0.48
		nPD:	0.
		N. weight:	0.6
		Sc. PD:	-0.11
		Sc. rank:	-1594.5

M1

Pos.	10	obs:	exp:
cct	P	0	0.30
ccc	P	0	0.20
cca	P	0	0.40
ccg	P	1	0.11
cgt	R	0	0.03
cgc	R	0	0.05
cga	R	0	0.10
cgg	R	0	0.07
act	T	0	970.40
acc	T	0	853.10
aca	T	0	1793.00
acg	T	3945	329.00
aaa	K	0	0.61
aag	K	1	0.39
aga	R	0	0.46
agg	R	1	0.28
mPD		0.0015	0.68
		nPD:	0.
		N. weight:	2.9
		Sc. PD:	-0.53
		Sc. rank:	-6836.3

M1

Pos.	11	obs:	exp:
tat	Y	568	2211.00
tac	Y	3379	1736.00
tgt	C	0	0.43
tgc	C	1	0.57
mPD		0.25	0.49
		nPD:	0.5
		N. weight:	0.44
		Sc. PD:	0.13
		Sc. rank:	1053.6

M1

Pos.	12	obs:	exp:
gtt	V	3893	876.60
gtc	V	53	745.00
gta	V	1	996.60
gtg	V	1	1330.00
mPD		0.027	0.74
		nPD:	0.04
		N. weight:	1.7
		Sc. PD:	-0.25
		Sc. rank:	-1995.2

M1

Pos.	13	obs:	exp:
tta	L	0	429.60
ttg	L	4	741.60
ctt	L	3333	797.30
ctc	L	581	574.40
cta	L	13	689.40
ctg	L	17	715.70
mPD		0.27	1.1
		nPD:	0.24
		N. weight:	1.4
		Sc. PD:	0.053
		Sc. rank:	1238.4

M1

Pos.	14	obs:	exp:
tct	S	3882	664.10
tcc	S	60	624.00
tca	S	6	958.70
tcg	S	0	209.80
agt	S	0	734.80
agc	S	0	756.60
mPD		0.033	1.6
		nPD:	0.02
		N. weight:	2.
		Sc. PD:	-0.33
		Sc. rank:	-3343.1

M1

Pos.	15	obs:	exp:
att	I	49	1397.00
atc	I	3873	1010.00
ata	I	15	1531.00
gtt	V	0	2.44
gtc	V	11	2.08
gta	V	0	2.78
gtg	V	0	3.71
mPD		0.038	0.66
		nPD:	0.06
		N. weight:	1.5
		Sc. PD:	-0.2
		Sc. rank:	-1085.5

M1

Pos.	16	obs:	exp:
att	I	32	1203.00
atc	I	2121	870.00
ata	I	1239	1319.00
atg	M	1	1.00
act	T	0	0.74
acc	T	0	0.65
aca	T	3	1.36
acg	T	0	0.25
gtt	V	94	122.60
gtc	V	457	104.20
gta	V	1	139.30
gtg	V	0	185.90
mPD		0.72	0.93
		nPD:	0.77
		N. weight:	0.69
		Sc. PD:	0.38
		Sc. rank:	2000.8

M1

Pos.	17	obs:	exp:
tct	S	0	0.17
tcc	S	0	0.16
tca	S	0	0.24
tcg	S	1	0.05
cct	P	15	1165.00
ccc	P	2	794.60
cca	P	469	1563.00
ccg	P	3461	424.60
agt	S	0	0.19
agc	S	0	0.19
mPD		0.22	0.70
		nPD:	0.31
		N. weight:	1.9
		Sc. PD:	0.21
		Sc. rank:	2628.8

M1

Pos.	18	obs:	exp:
tct	S	14	664.10
tcc	S	107	624.00
tca	S	3618	958.70
tcg	S	209	209.80
agt	S	0	734.80
agc	S	0	756.60
mPD		0.16	1.6
		nPD:	0.1
		N. weight:	1.3
		Sc. PD:	-0.13
		Sc. rank:	-400.4

M1

Pos.	19	obs:	exp:
ggt	G	0	619.30
ggc	G	3947	500.30
gga	G	0	1788.00
ggg	G	1	1040.00
mPD		0.00051	0.68
		nPD:	0.
		N. weight:	2.4
		Sc. PD:	-0.45
		Sc. rank:	-6443.2

M1

Pos.	20	obs:	exp:
tct	S	0	32.29
tcc	S	192	30.34
tca	S	0	46.62
tcg	S	0	10.20
cct	P	0	1108.00
ccc	P	3756	756.20
cca	P	0	1487.00
ccg	P	0	404.00
agt	S	0	35.74
agc	S	0	36.80
mPD		0.093	0.84
		nPD:	0.11
		N. weight:	1.9
		Sc. PD:	-0.15
		Sc. rank:	-321.3

M1

Pos . 21		obs :	exp :
tta	L	0	429.60
ttg	L	0	741.60
ctt	L	1	797.30
ctc	L	3947	574.40
cta	L	0	689.40
ctg	L	0	715.70
---	--	------	------
mPD		0.00051	1.1
		nPD :	0.
		N. weight :	2.2
		Sc. PD :	-0.42
		Sc. rank :	-6182.5

M1

Pos . 22		obs :	exp :
aaa	K	3938	2399.00
aag	K	10	1549.00
---	--	------	------
mPD		0.0051	0.48
		nPD :	0.01
		N. weight :	0.56
		Sc. PD :	-0.099
		Sc. rank :	-1174.7

M1

Pos . 23		obs :	exp :
gct	A	0	967.80
gcc	A	3948	904.60
gca	A	0	1780.00
gcg	A	0	296.10
[truncated: 327,844 more chars]
