# Supplementary material for: Locations and structures of influenza A virus packaging-associated signals and other functional elements via an in silico pipeline for predicting constrained features in RNA viruses
Source: PLoS Comput Biol. 2024 Apr 22;20(4):e1012009. doi: 10.1371/journal.pcbi.1012009 (PMC11034665; doi:10.1371/journal.pcbi.1012009)
Supplement: S4 Code — The content of the notebook follows the same pattern as that in S1 Code. (ZIP) [file pcbi.1012009.s117.zip › S4_Code.pdf]

H5N1 avian hosts

PB2

Gene length histogram

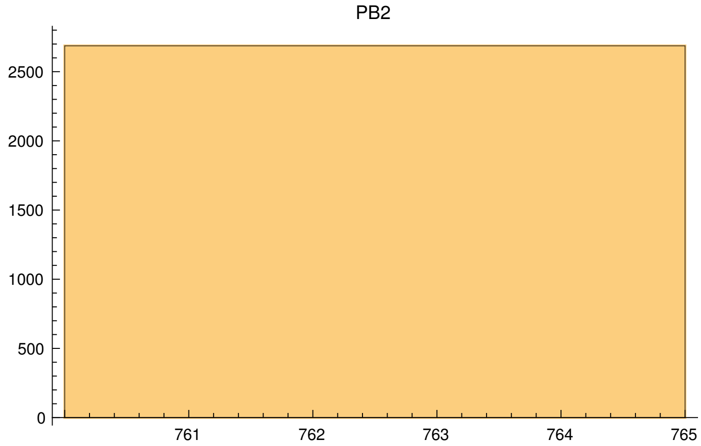

Information vs. nPD

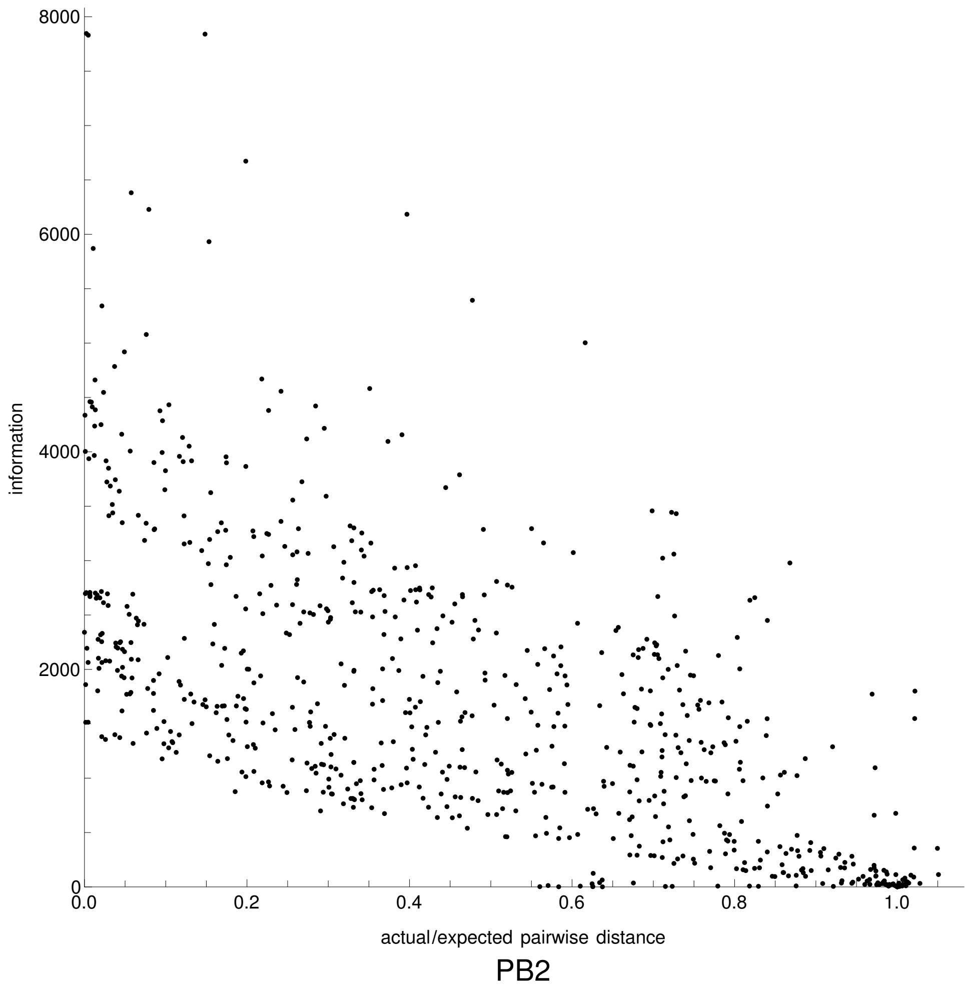

Example sequences highlighted by regions found in analyses to be conserved

Interesting points (by weighted raw PD) highlighted for gene PB2:

ATGGAGAGAATAAAAGAACTAAGAGATCTAATGTCACAGTCC
CGCACCCGCGAGATACTGACAAAAACCACTGTGGACCATATGGCCATAATCAAGAAA⋱
TACACATCAGGAAGACAAGAAAAGAACCCTGCCCTCAGAATGAAATGGATGATGGCAATGAAATATCCAA⋱
TCACAGCAGACAAGAGAATAATGGAGATGATCCCTGAACGGAATGAACAGGGACAGACGCTTTGGAGCAA⋱
GGCAAATGATGCTGGATCGGACAGGGTGATGGTGTCTCCCCTAGCTGTGACTTGGTGGAACAGGAATGGA⋱
CCGACAACAAGTACAGTCCATTATCCAAAGGTTTACAAAACATACTTTGAGAAGGTTGAAAGGTTAAAAC⋱
ATGGAACCTTTGGTCCCGTTCATTTCCGAAACCAAGTTAAGATACGCCGCCGAGTGGATATAAACCCGGG⋱
CCATGCAGATCTCAGTGCTAAAGAAGCACAAGATGTTATCATGGAGGTTGTTTACCCAAATGAAGTGGGA⋱
GCTAGAATATTGACATCAGAGTCGCAATTGACAATAACAAAAGAGAAGAAGGAAGAGCTCCAGGATTGTA⋱
AGATTGCTCCTTTAATGGTGGCATACATGCTGGAAAGAGAACTGGTCCGCAAAACCAGATTTCTACCGGT⋱
AGCAGGCGGAACAAGCAGTGTATACATTGAGGTATTGCATTTGACTCAAGGGACCTGTTGGGAACAGATG⋱
TACACTCCTGGTGGAGAAGTGAGAAATGATGATGTTGACCAAAGTTTGATCATCGCTGCCAGAAACATTG⋱
TTAGGAGAGCAACAGTATCGGCGGACCCACTGGCATCACTACTGGAGATGTGTCACAGCACACAAATTGG⋱
TGGAATAAGGATGGTA
GACATTCTTAGGCAAAATCCCACTGAGGAACAAGCTGTGGATATATGCAAAGCAG
CAATGGGCTTGAGGATCAGTTCATCTTTTAGCTTTGGAGGCTTCACTTTCAA
AAGAACAAGTGGGTCATCCGTAAAGAAAGAAGAAGTGCTTACAGGCAAC
CTCCAAACATTGAAAATAAGAGTGCATGAGGGGTATGAAGAATTCACAATGGT⋱
TGGGCGGAGAGCAACAGCTATCCTGAGGAAAGCAACTAGAAGGCTGATTCAATTGATAGTAAGTGGAAGA⋱
GATGAGCAATCAATCGCTGAAGCGATCATTGTAGCAATGGTGTTCTCACAGGAGGATTGCATGATAAAGG⋱
CAGTCCGAGGCGATCTGAATTTCGTAAACAGAGCAAACCAAAGATTGAACCCCATGCATCAACTATTGAG⋱
GCACTTCCAAAAAGATGCAAAAGTGCTGTTTCAGAACTGGGGAATTGAACCTATTGACAATGTCATGGGG⋱
ATGATCGGGATATTACCTGACATGACTCCAAGCACAGAGATGTCACTGAGAGGGGTGAGAGTTAGTAAGA⋱
TGGGAGTGGATGAATATTCCAGCACTGAGAGAGTAGTTGTGAGTATTGACCGTTTCTTGAGGGTCCGAGA⋱
TCAGCTAGGGAACGTACTCTTATCCCCTGAAGAGGTTAGTGAAACACAGGGAACAGAGAAGTTAACAATA⋱
ACATATTCATCCTCAATGATGTGGGAAATCAACGGCCCTGAGTCAGTGCTTGTTAATACTTATCAGTGGA⋱
TCATCAGGAATTGGGAGGCTGTAAAGATTCAATGGTCTCAAGATCCCGCAATGCTATACAATAAGATGGA⋱
ATTTGAACCATTCCAATCCTTGGTGCCCAAAGCCGCCAGAGGCCAATACAGTGGGTTTGTGAGAACACTG⋱
TTCCAACAGATGCGTGATGTTCTGGGGACGTTTGATACTGTTCAAATAATAAAGCTGCTACCATTTGCAG⋱
CAGCCCCACCGGAACAAAGCAGAATGCAGTTTTCTTCTCTAACTGTGAATGTGAGAGGTTCAGGAATGAG⋱
AATACTCGTGAGGGGTAACTCCCCCGTGTTCAACTACAACAAGGCAACTAAAAGGCTTACAGTCCTCGGA⋱
AAGGACGCAGGTGCATTAACAGAAGATCCAGACGAGGGAACAGCCGGGGTTGAATCTGCGGTACTGAGGG⋱
GATTCCTAATTCTAGGCAAGGAGGACAAAAGATATGGACCAGCATTG
AGCATCAATGAACTGAGCAATCTTGCAAAAGGGGAGAAGGCCAATGTGCTGATAG
GGCAAGGGGACGTGGTGTTGGTAATGAAACGGAAACGGGACTCTAGCATACT
TACTGACAGCCAGACAGCGACCAAAAGAATTCGGATGGCCATCAATTAG

Interesting points (by weighted ranked PD) highlighted for gene PB2:

ATGGAGAGAATAAAAGAACTAAGAGATCTAATGTCACAGTCC
CGCACCCGCGAGATACTGACAAAAACCACTGTGGACCATATGGCCATAATCAAGAAA⋱
TACACATCAGGAAGACAAGAAAAGAACCCTGCCCTCAGAATGAAATGGATGATGGCAATGAAATATCCAA⋱
TCACAGCAGACAAGAGAATAATGGAGATGATCCCTGAACGGAATGAACAGGGACAGACGCTTTGGAGCAA⋱
GGCAAATGATGCTGGATCGGACAGGGTGATGGTGTCTCCCCTAGCTGTGACTTGGTGGAACAGGAATGGA⋱

CCGACAACAAGTACAGTCCATTATCCAAAGGTTTACAAAACATACTTTGAGAAGGTTGAAAGGTTAAAAC⋱
ATGGAACCTTTGGTCCCGTTCATTTCCGAAACCAAGTTAAGATACGCCGCCGAGTGGATATAAACCCGGG⋱
CCATGCAGATCTCAGTGCTAAAGAAGCACAAGATGTTATCATGGAGGTTGTTTACCCAAATGAAGTGGGA⋱
GCTAGAATATTGACATCAGAGTCGCAATTGACAATAACAAAAGAGAAGAAGGAAGAGCTCCAGGATTGTA⋱
AGATTGCTCCTTTAATGGTGGCATACATGCTGGAAAGAGAACTGGTCCGCAAAACCAGATTTCTACCGGT⋱
AGCAGGCGGAACAAGCAGTGTATACATTGAGGTATTGCATTTGACTCAAGGGACCTGTTGGGAACAGATG⋱
TACACTCCTGGTGGAGAAGTGAGAAATGATGATGTTGACCAAAGTTTGATCATCGCTGCCAGAAACATTG⋱
TTAGGAGAGCAACAGTATCGGCGGACCCACTGGCATCACTACTGGAGATGTGTCACAGCACACAAATTGG⋱
TGGAATAAGGATGGTAGACATTCTTAGGCAAAATCCCACTGAGGAACAAGCTGTGGATATATGCAAAGCA⋱
GCAATGGGCTTGAGGATCAGTTCATCTTTTAGCTTTGGAGGCTTCACTTTCAAAAGAACAAGTGGGTCAT⋱
CCGTAAAGAAAGAAGAAGAAGTGCTTACAGGCAACCTCCAAACATTGAAAATAAGAGTGCATGAGGGGTA⋱
TGAAGAATTCACAATGGTTGGGCGGAGAGCAACAGCTATCCTGAGGAAAGCAACTAGAAGGCTGATTCAA⋱
TTGATAGTAAGTGGAAGAGATGAGCAATCAATCGCTGAAGCGATCATTGTAGCAATGGTGTTCTCACAGG⋱
AGGATTGCATGATAAAGGCAGTCCGAGGCGATCTGAATTTCGTAAACAGAGCAAACCAAAGATTGAACCC⋱
CATGCATCAACTATTGAGGCACTTCCAAAAAGATGCAAAAGTGCTGTTTCAGAACTGGGGAATTGAACCT⋱
ATTGACAATGTCATGGGGATGATCGGGATATTACCTGACATGACTCCAAGCACAGAGATGTCACTGAGAG⋱
GGGTGAGAGTTAGTAAGATGGGAGTGGATGAATATTCCAGCACTGAGAGAGTAGTTGTGAGTATTGACCG⋱
TTTCTTGAGGGTCCGAGATCAGCTAGGGAACGTACTCTTATCCCCTGAAGAGGTTAGTGAAACACAGGGA⋱
ACAGAGAAGTTAACAATAACATATTCATCCTCAATGATGTGGGAAATCAACGGCCCTGAGTCAGTGCTTG⋱
TTAATACTTATCAGTGGATCATCAGGAATTGGGAGGCTGTAAAGATTCAATGGTCTCAAGATCCCGCAAT⋱
GCTATACAATAAGATGGAATTTGAACCATTCCAATCCTTGGTGCCCAAAGCCGCCAGAGGCCAATACAGT⋱
GGGTTTGTGAGAACACTGTTCCAACAGATGCGTGATGTTCTGGGGACGTTTGATACTGTTCAAATAATAA⋱
AGCTGCTACCATTTGCAGCAGCCCCACCGGAACAAAGCAGAATGCAGTTTTCTTCTCTAACTGTGAATGT⋱
GAGAGGTTCAGGAATGAGAATACTCGTGAGGGGTAACTCCCCCGTGTTCAACTACAACAAGGCAACTAAA⋱
AGGCTTACAGTCCTCGGAAAGGACGCAGGTGCATTAACAGAAGATCCAGACGAGGGAACAGCCGGGGTTG⋱
AATCTGCGGTACTGAGGGGATTCCTAATTCTAGGCAAGGAGGACAAAAGATATGGACCAGCATTGAGCAT⋱
CAATGAACTGAGCAATCTTGCAAAAGGGGAGAAGGCCAATGTGCTGATAGGGCAA

GGGGACGTGGTGTTGGTAATGAAACGGAAACGGGACTCTAGCATACTTACTGACA
GCCAGACAGCGACCAAAAGAATTCGGATGGCCATCAATTAG

Per codon conservation report

PB2			
Pos.	1	obs:	exp:
atg	M	2687	2687.00
---	--	------	------
mPD		0	0
		nPD:	1.
		N. weight:	0.
		Sc. PD:	0
		Sc. rank:	0

PB2			
Pos.	2	obs:	exp:
cgt	R	0	0.04
cgc	R	0	0.05
cga	R	1	0.10
cgg	R	0	0.08
aat	N	1	0.55
aac	N	0	0.45
aga	R	0	0.47
agg	R	0	0.26
gat	D	0	1.08
gac	D	2	0.92
gaa	E	988	1503.00
gag	E	1693	1178.00
ggt	G	0	0.29
ggc	G	0	0.26
gga	G	2	0.88
ggg	G	0	0.57
---	--	------	------
mPD		0.47	0.50
		nPD:	0.95
		N. weight:	0.13
		Sc. PD:	0.078
		Sc. rank:	177.9

PB2			
Pos.	3	obs:	exp:
tgt	C	1	0.39
tgc	C	0	0.61
cgt	R	0	100.50
cgc	R	0	143.70
cga	R	741	270.60
cgg	R	4	219.20
aga	R	1926	1253.00
agg	R	14	697.50
ggt	G	0	0.14
ggc	G	0	0.13
gga	G	1	0.44
ggg	G	0	0.29
---	--	------	------
mPD		0.42	0.96
		nPD:	0.44
		N. weight:	0.91
		Sc. PD:	0.1
		Sc. rank:	526.9

PB2			
Pos.	4	obs:	exp:
tta	L	0	0.09
ttg	L	0	0.19
ctt	L	0	0.20
ctc	L	0	0.16
cta	L	1	0.15
ctg	L	0	0.21
cgt	R	0	0.11
cgc	R	0	0.16
cga	R	3	0.30
cgg	R	0	0.24
att	I	1	983.10
atc	I	0	716.90
ata	I	2678	979.00
atg	M	1	1.00
aaa	K	1	0.57
aag	K	0	0.43
aga	R	0	1.40
agg	R	0	0.78
gtt	V	0	0.43
gtc	V	0	0.42
gta	V	2	0.43
gtg	V	0	0.72
---	--	------	------
mPD		0.0089	0.67
		nPD:	0.01
		N. weight:	1.6
		Sc. PD:	-0.49
		Sc. rank:	-2017.7

PB2			
Pos.	5	obs:	exp:
cgt	R	0	0.52
cgc	R	0	0.75
cga	R	0	1.41
cgg	R	0	1.14
aaa	K	2443	1521.00
aag	K	229	1151.00
aga	R	14	6.54
agg	R	0	3.64
gaa	E	0	0.56
gag	E	1	0.44
---	--	------	------
mPD		0.17	0.50
		nPD:	0.33
		N. weight:	0.48
		Sc. PD:	0.0054
		Sc. rank:	111.2

PB2			
Pos.	6	obs:	exp:
aat	N	0	1.09
aac	N	2	0.91
aaa	K	7	3.99
aag	K	0	3.01
gaa	E	2217	1500.00
gag	E	459	1176.00
ggt	G	0	0.29
ggc	G	0	0.26
gga	G	2	0.88
ggg	G	0	0.57
---	--	------	------
mPD		0.29	0.50
		nPD:	0.58
		N. weight:	0.27
		Sc. PD:	0.069
		Sc. rank:	237.5

PB2			
Pos.	7	obs:	exp:
ttt	F	2	1.25
ttc	F	1	1.75
tta	L	1375	248.20
ttg	L	10	509.90
ctt	L	0	532.90
ctc	L	1	429.30
cta	L	1287	397.20
ctg	L	11	566.50
---	--	------	------
mPD		0.52	1.1
		nPD:	0.46
		N. weight:	2.3
		Sc. PD:	0.32
		Sc. rank:	1453.3

PB2			
Pos.	8	obs:	exp:
cgt	R	0	100.30
cgc	R	2	143.50
cga	R	140	270.20
cgg	R	3	218.90
aaa	K	6	3.42
aag	K	0	2.58
aga	R	2421	1252.00
agg	R	115	696.50
---	--	------	------
mPD		0.19	0.96
		nPD:	0.2
		N. weight:	0.78
		Sc. PD:	-0.092
		Sc. rank:	-210.1

PB2			
Pos.	9	obs:	exp:
tat	Y	2	0.96
tac	Y	0	1.04
aat	N	16	10.91
aac	N	4	9.09
gat	D	2609	1438.00
gac	D	54	1225.00
gaa	E	0	0.56
gag	E	1	0.44
ggt	G	1	0.14
ggc	G	0	0.13
gga	G	0	0.44
ggg	G	0	0.29
---	--	------	------
mPD		0.060	0.51
		nPD:	0.12
		N. weight:	0.84
		Sc. PD:	-0.17
		Sc. rank:	-532.0

PB2			
Pos.	10	obs:	exp:
tta	L	136	248.40
ttg	L	900	510.50
ctt	L	0	533.50
ctc	L	0	429.80
cta	L	1628	397.70
ctg	L	23	567.10
---	--	------	------
mPD		0.93	1.1
		nPD:	0.82
		N. weight:	1.6
		Sc. PD:	0.79
		Sc. rank:	2164.1

PB2			
Pos.	11	obs:	exp:
cgt	R	0	0.04
cgc	R	0	0.05
cga	R	0	0.10
cgg	R	0	0.08
atg	M	2685	2685.00
aga	R	0	0.47
agg	R	1	0.26
gtt	V	0	0.22
gtc	V	0	0.21
gta	V	0	0.21
gtg	V	1	0.36
---	--	------	------
mPD		0.0015	0.0027
		nPD:	0.56
		N. weight:	0.0014
		Sc. PD:	0.00034
		Sc. rank:	1.2

PB2			
Pos.	12	obs:	exp:
tta	L	1	0.09
ttg	L	0	0.19
tct	S	11	448.00
tcc	S	12	388.80
tca	S	1854	608.50
tcg	S	807	161.90
ctt	L	0	0.20
ctc	L	0	0.16
cta	L	0	0.15
ctg	L	0	0.21
cct	P	0	0.27
ccc	P	0	0.18
cca	P	1	0.41
ccg	P	0	0.14
act	T	0	0.26
acc	T	0	0.23
aca	T	0	0.42
acg	T	1	0.09
agt	S	0	582.20
agc	S	0	494.60
---	--	------	------
mPD		0.43	1.7
		nPD:	0.26
		N. weight:	2.
		Sc. PD:	-0.11
		Sc. rank:	-95.6

PB2			
Pos.	13	obs:	exp:
cat	H	0	4.05
cac	H	7	2.95
caa	Q	352	1338.00
cag	Q	2327	1341.00
gaa	E	0	0.56
gag	E	1	0.44
---	--	------	------
mPD		0.23	0.50
		nPD:	0.46
		N. weight:	0.5
		Sc. PD:	0.069
		Sc. rank:	319.4

PB2			
Pos.	14	obs:	exp:
ttt	F	0	0.42
ttc	F	1	0.58
tct	S	1315	447.20
tcc	S	1361	388.10
tca	S	3	607.30
tcg	S	0	161.60
cct	P	0	1.37
ccc	P	5	0.90
cca	P	0	2.03
ccg	P	0	0.70
agt	S	0	581.10
agc	S	0	493.70
gct	A	2	0.50
gcc	A	0	0.39
gca	A	0	0.89
gcg	A	0	0.22
---	--	------	------
mPD		0.51	1.7
		nPD:	0.31
		N. weight:	1.9
		Sc. PD:	-0.026
		Sc. rank:	254.1

PB2			
Pos.	15	obs:	exp:
cgt	R	3	100.60
cgc	R	2684	143.80
cga	R	0	270.80
cgg	R	0	219.40
aga	R	0	1254.00
agg	R	0	698.10
---	--	------	------
mPD		0.0022	0.95
		nPD:	0.
		N. weight:	4.7
		Sc. PD:	-1.5
		Sc. rank:	-6511.9

PB2			
Pos.	16	obs:	exp:
cct	P	7	1.91
ccc	P	0	1.26
cca	P	0	2.84
ccg	P	0	0.98
att	I	2	0.73
atc	I	0	0.54
ata	I	0	0.73
act	T	2331	701.50
acc	T	343	603.00
aca	T	1	1120.00
acg	T	1	251.80
gct	A	2	0.50
gcc	A	0	0.39
gca	A	0	0.89
gcg	A	0	0.22
---	--	------	------
mPD		0.23	0.70
		nPD:	0.33
		N. weight:	1.6
		Sc. PD:	0.013
		Sc. rank:	306.4

,

PB2			
Pos.	17	obs:	exp:
tgt	C	0	0.77
tgc	C	2	1.23
cgt	R	1	100.50
cgc	R	2681	143.70
cga	R	3	270.60
cgg	R	0	219.20
aga	R	0	1253.00
agg	R	0	697.50
---	--	------	------
mPD		0.0045	0.96
		nPD:	0.
		N. weight:	4.7
		Sc. PD:	-1.5
		Sc. rank:	-6352.6

,

PB2			
Pos.	18	obs:	exp:
gaa	E	2	1506.00
gag	E	2685	1181.00
---	--	------	------
mPD		0.0015	0.49
		nPD:	0.
		N. weight:	1.3
		Sc. PD:	-0.41
		Sc. rank:	-1814.2

,

PB2			
Pos.	19	obs:	exp:
att	I	0	985.30
atc	I	0	718.50
ata	I	2685	981.20
gtt	V	0	0.43
gtc	V	0	0.42
gta	V	2	0.43
gtg	V	0	0.72
---	--	------	------
mPD		0.0015	0.66
		nPD:	0.
		N. weight:	1.6
		Sc. PD:	-0.51
		Sc. rank:	-2275.0

,

PB2			
Pos.	20	obs:	exp:
tta	L	2	248.30
ttg	L	2	510.30
ctt	L	0	533.30
ctc	L	139	429.60
cta	L	1482	397.50
ctg	L	1061	566.90
cgt	R	0	0.04
cgc	R	0	0.05
cga	R	0	0.10
cgg	R	1	0.08
aga	R	0	0.47
agg	R	0	0.26
---	--	------	------
mPD		0.54	1.1
		nPD:	0.48
		N. weight:	1.5
		Sc. PD:	0.23
		Sc. rank:	1032.1

,

PB2			
Pos.	21	obs:	exp:
act	T	2	703.90
acc	T	185	605.00
aca	T	2490	1123.00
acg	T	8	252.60
gct	A	0	0.50
gcc	A	0	0.39
gca	A	2	0.89
gcg	A	0	0.22
---	--	------	------
mPD		0.14	0.70
		nPD:	0.2
		N. weight:	1.
		Sc. PD:	-0.13
		Sc. rank:	-335.0

,

PB2			
Pos.	22	obs:	exp:
cgt	R	0	0.97
cgc	R	0	1.39
cga	R	0	2.62
cgg	R	0	2.12
aat	N	1	0.55
aac	N	0	0.45
aaa	K	2572	1515.00
aag	K	88	1145.00
aga	R	24	12.14
agg	R	2	6.75
---	--	------	------
mPD		0.085	0.52
		nPD:	0.16
		N. weight:	0.7
		Sc. PD:	-0.11
		Sc. rank:	-299.0

,

PB2			
Pos.	23	obs:	exp:
ttt	F	1	0.42
ttc	F	0	0.58
tct	S	0	0.17
tcc	S	0	0.14
tca	S	0	0.23
tcg	S	0	0.06
act	T	17	703.90
acc	T	2661	605.00
aca	T	7	1123.00
acg	T	0	252.60
agt	S	0	0.22
agc	S	1	0.18
---	--	------	------
mPD		0.021	0.70
		nPD:	0.03
		N. weight:	2.3
		Sc. PD:	-0.66
		Sc. rank:	-2501.5

,

PB2			
Pos.	24	obs:	exp:
act	T	2230	704.40
acc	T	432	605.50
aca	T	3	1124.00
acg	T	22	252.80
---	--	------	------
mPD		0.29	0.70
		nPD:	0.41
		N. weight:	1.4
		Sc. PD:	0.12
		Sc. rank:	713.2

,

PB2			
Pos.	25	obs:	exp:
gtt	V	66	577.90
gtc	V	0	565.30
gta	V	8	572.90
gtg	V	2613	970.80
---	--	------	------
mPD		0.054	0.73
		nPD:	0.07
		N. weight:	1.5
		Sc. PD:	-0.35
		Sc. rank:	-1187.8

,

PB2			
Pos.	26	obs:	exp:
gat	D	12	1451.00
gac	D	2675	1236.00
---	--	------	------
mPD		0.0089	0.50
		nPD:	0.02
		N. weight:	1.2
		Sc. PD:	-0.36
		Sc. rank:	-1451.4

,

PB2			
Pos.	27	obs:	exp:
cct	P	0	0.27
ccc	P	1	0.18
cca	P	0	0.41
ccg	P	0	0.14
cat	H	2332	1555.00
cac	H	354	1131.00
---	--	------	------
mPD		0.23	0.49
		nPD:	0.47
		N. weight:	0.33
		Sc. PD:	0.048
		Sc. rank:	218.0

,

PB2			
Pos.	28	obs:	exp:
tta	L	0	0.09
ttg	L	0	0.19
ctt	L	1	0.20
ctc	L	0	0.16
cta	L	0	0.15
ctg	L	0	0.21
att	I	1	1.10
atc	I	0	0.80
ata	I	2	1.10
atg	M	2680	2680.00
act	T	0	0.26
acc	T	0	0.23
aca	T	0	0.42
acg	T	1	0.09
gtt	V	0	0.43
gtc	V	0	0.42
gta	V	0	0.43
gtg	V	2	0.72
---	--	------	------
mPD		0.0060	0.0073
		nPD:	0.82
		N. weight:	0.0048
		Sc. PD:	0.0023
		Sc. rank:	6.4

,

PB2			
Pos.	29	obs:	exp:
tta	L	1	0.09
ttg	L	0	0.19
ctt	L	0	0.20
ctc	L	0	0.16
cta	L	0	0.15
ctg	L	0	0.21
gct	A	14	676.40
gcc	A	2646	519.60
gca	A	22	1195.00
gcg	A	3	293.90
ggt	G	0	0.14
ggc	G	1	0.13
gga	G	0	0.44
ggg	G	0	0.29
---	--	------	------
mPD		0.032	0.69
		nPD:	0.05
		N. weight:	2.5
		Sc. PD:	-0.68
		Sc. rank:	-2416.5

,

PB2			
Pos.	30	obs:	exp:
tta	L	0	0.09
ttg	L	0	0.19
ctt	L	0	0.20
ctc	L	0	0.16
cta	L	1	0.15
ctg	L	0	0.21
att	I	0	983.80
atc	I	52	717.40
ata	I	2629	979.70
gtt	V	0	1.08
gtc	V	0	1.05
gta	V	5	1.07
gtg	V	0	1.81
---	--	------	------
mPD		0.042	0.67
		nPD:	0.06
		N. weight:	1.5
		Sc. PD:	-0.38
		Sc. rank:	-1259.0

,

PB2			
Pos.	31	obs:	exp:
att	I	237	985.70
atc	I	2311	718.80
ata	I	138	981.60
ggt	G	0	0.14
ggc	G	1	0.13
gga	G	0	0.44
ggg	G	0	0.29
---	--	------	------
mPD		0.25	0.66
		nPD:	0.38
		N. weight:	1.3
		Sc. PD:	0.072
		Sc. rank:	479.0

,

PB2			
Pos.	32	obs:	exp:
cgt	R	0	0.04
cgc	R	0	0.05
cga	R	0	0.10
cgg	R	0	0.08
aaa	K	196	1529.00
aag	K	2490	1157.00
aga	R	0	0.47
agg	R	1	0.26
---	--	------	------
mPD		0.14	0.49
		nPD:	0.28
		N. weight:	0.91
		Sc. PD:	-0.039
		Sc. rank:	0.4

,

PB2			
Pos.	33	obs:	exp:
aaa	K	2515	1530.00
aag	K	172	1157.00
---	--	------	------
mPD		0.12	0.49
		nPD:	0.24
		N. weight:	0.56
		Sc. PD:	-0.042
		Sc. rank:	-72.7

,

PB2			
Pos.	34	obs:	exp:
tat	Y	883	1288.00
tac	Y	1803	1398.00
tgt	C	0	0.39
tgc	C	1	0.61
---	--	------	------
mPD		0.44	0.50
		nPD:	0.88
		N. weight:	0.079
		Sc. PD:	0.043
		Sc. rank:	109.2

,

PB2			
Pos.	35	obs:	exp:
act	T	1	704.40
acc	T	216	605.50
aca	T	2180	1124.00
acg	T	290	252.80
---	--	------	------
mPD		0.32	0.70
		nPD:	0.46
		N. weight:	0.76
		Sc. PD:	0.11
		Sc. rank:	495.1

,

PB2			
Pos.	36	obs:	exp:
tct	S	3	448.50
tcc	S	1	389.20
tca	S	2240	609.10
tcg	S	443	162.10
agt	S	0	582.80
agc	S	0	495.20
---	--	------	------
mPD		0.28	1.7
		nPD:	0.17
		N. weight:	2.
		Sc. PD:	-0.3
		Sc. rank:	-851.2

,

PB2			
Pos.	37	obs:	exp:
ggt	G	7	388.60
ggc	G	0	352.90
gga	G	2527	1177.00
ggg	G	153	768.10
---	--	------	------
mPD		0.11	0.69
		nPD:	0.16
		N. weight:	1.
		Sc. PD:	-0.16
		Sc. rank:	-439.9

,

PB2			
Pos.	38	obs:	exp:
cgt	R	0	100.60
cgc	R	0	143.80
cga	R	12	270.80
cgg	R	0	219.40
aga	R	2149	1254.00
agg	R	526	698.10
---	--	------	------
mPD		0.32	0.95
		nPD:	0.34
		N. weight:	0.59
		Sc. PD:	0.011
		Sc. rank:	141.4

,

PB2			
Pos.	39	obs:	exp:
tta	L	0	0.09
ttg	L	0	0.19
ctt	L	0	0.20
ctc	L	0	0.16
cta	L	1	0.15
ctg	L	0	0.21
caa	Q	1633	1340.00
cag	Q	1051	1344.00
aaa	K	2	1.14
aag	K	0	0.86
---	--	------	------
mPD		0.48	0.50
		nPD:	0.95
		N. weight:	0.043
		Sc. PD:	0.027
		Sc. rank:	60.8

,

PB2			
Pos.	40	obs:	exp:
gat	D	1	0.54
gac	D	0	0.46
gaa	E	129	1506.00
gag	E	2557	1180.00
---	--	------	------
mPD		0.092	0.49
		nPD:	0.19
		N. weight:	1.
		Sc. PD:	-0.13
		Sc. rank:	-342.1

,

PB2			
Pos.	41	obs:	exp:
cgt	R	0	0.04
cgc	R	0	0.05
cga	R	0	0.10
cgg	R	0	0.08
aaa	K	61	1529.00
aag	K	2625	1157.00
aga	R	0	0.47
agg	R	1	0.26
---	--	------	------
mPD		0.045	0.49
		nPD:	0.09
		N. weight:	1.2
		Sc. PD:	-0.27
		Sc. rank:	-855.3

,

PB2			
Pos.	42	obs:	exp:
aat	N	1444	1465.00
aac	N	1243	1222.00
---	--	------	------
mPD		0.50	0.50
		nPD:	1.
		N. weight:	0.0027
		Sc. PD:	0.0018
		Sc. rank:	3.9

,

PB2			
Pos.	43	obs:	exp:
tta	L	0	0.09
ttg	L	0	0.19
ctt	L	1	0.20
ctc	L	0	0.16
cta	L	0	0.15
ctg	L	0	0.21
cct	P	2466	734.10
ccc	P	185	485.10
cca	P	34	1090.00
ccg	P	1	376.60
---	--	------	------
mPD		0.15	0.71
		nPD:	0.22
		N. weight:	1.6
		Sc. PD:	-0.17
		Sc. rank:	-377.2

,

PB2			
Pos.	44	obs:	exp:
tct	S	0	0.17
tcc	S	0	0.14
tca	S	1	0.23
tcg	S	0	0.06
agt	S	0	0.22
agc	S	0	0.18
gct	A	931	676.70
gcc	A	916	519.80
gca	A	621	1196.00
gcg	A	218	294.00
---	--	------	------
mPD		0.70	0.69
		nPD:	1.02
		N. weight:	0.22
		Sc. PD:	0.15
		Sc. rank:	306.6

,

PB2			
Pos.	45	obs:	exp:
ttt	F	0	0.42
ttc	F	1	0.58
tta	L	0	248.10
ttg	L	0	509.70
ctt	L	703	532.70
ctc	L	1968	429.20
cta	L	12	397.10
ctg	L	0	566.30
cct	P	0	0.27
ccc	P	1	0.18
cca	P	0	0.41
ccg	P	0	0.14
att	I	0	0.73
atc	I	2	0.54
ata	I	0	0.73
---	--	------	------
mPD		0.40	1.1
		nPD:	0.35
		N. weight:	1.9
		Sc. PD:	0.059
		Sc. rank:	550.3

,

PB2			
Pos.	46	obs:	exp:
cgt	R	0	100.60
cgc	R	0	143.80
cga	R	3	270.80
cgg	R	3	219.40
aga	R	1465	1254.00
agg	R	1216	698.10
---	--	------	------
mPD		0.50	0.95
		nPD:	0.52
		N. weight:	0.53
		Sc. PD:	0.11
		Sc. rank:	418.1

,

PB2			
Pos.	47	obs:	exp:
att	I	0	0.73
atc	I	0	0.54
ata	I	2	0.73
atg	M	2685	2685.00
---	--	------	------
mPD		0.0015	0.0015
		nPD:	1.
		N. weight:	0.0012
		Sc. PD:	0.00081
		Sc. rank:	1.7

,

PB2			
Pos.	48	obs:	exp:
aat	N	0	0.55
aac	N	1	0.45
aaa	K	2119	1529.00
aag	K	567	1157.00
---	--	------	------
mPD		0.33	0.49
		nPD:	0.68
		N. weight:	0.18
		Sc. PD:	0.062
		Sc. rank:	178.3

,

PB2			
Pos.	49	obs:	exp:
tgg	W	2687	2687.00
---	--	------	------
mPD		0	0
		nPD:	1.
		N. weight:	0.
		Sc. PD:	0
		Sc. rank:	0

,

PB2			
Pos.	50	obs:	exp:
att	I	0	0.73
atc	I	0	0.54
ata	I	2	0.73
atg	M	2682	2682.00
gtt	V	0	0.65
gtc	V	0	0.63
gta	V	0	0.64
gtg	V	3	1.08
---	--	------	------
mPD		0.0037	0.0051
		nPD:	0.72
		N. weight:	0.0031
		Sc. PD:	0.0012
		Sc. rank:	3.6

,

PB2			
Pos.	51	obs:	exp:
atg	M	2687	2687.00
---	--	------	------
mPD		0	0
		nPD:	1.
		N. weight:	0.
		Sc. PD:	0
		Sc. rank:	0

,

PB2			
Pos.	52	obs:	exp:
gtt	V	0	0.22
gtc	V	0	0.21
gta	V	1	0.21
gtg	V	0	0.36
gct	A	1	676.70
gcc	A	0	519.80
gca	A	2671	1196.00
gcg	A	14	294.00
---	--	------	------
mPD		0.012	0.69
		nPD:	0.02
		N. weight:	1.3
		Sc. PD:	-0.38
		Sc. rank:	-1524.5

,

PB2			
Pos.	53	obs:	exp:
atg	M	2686	2686.00
aaa	K	0	0.57
aag	K	1	0.43
---	--	------	------
mPD		0.00074	0.0012
		nPD:	0.64
		N. weight:	0.00051
		Sc. PD:	0.00016
		Sc. rank:	0.5

,

PB2			
Pos.	54	obs:	exp:
cgt	R	0	0.60
cgc	R	0	0.86
cga	R	0	1.61
cgg	R	0	1.31
aaa	K	2093	1521.00
aag	K	578	1150.00
aga	R	4	7.47
agg	R	12	4.16
---	--	------	------
mPD		0.35	0.51
		nPD:	0.7
		N. weight:	0.17
		Sc. PD:	0.065
		Sc. rank:	185.1

,

PB2			
Pos.	55	obs:	exp:
tat	Y	2442	1287.00
tac	Y	242	1397.00
cat	H	3	1.74
cac	H	0	1.26
---	--	------	------
mPD		0.17	0.50
		nPD:	0.33
		N. weight:	0.69
		Sc. PD:	0.0072
		Sc. rank:	141.6

,

PB2

Pos.	56	obs:	exp:
tct	S	0	0.17
tcc	S	0	0.14
tca	S	0	0.23
tcg	S	1	0.06
cct	P	99	734.10
ccc	P	0	485.10
cca	P	942	1090.00
ccg	P	1645	376.60
agt	S	0	0.22
agc	S	0	0.18
---	--	------	------
mPD		0.50	0.71
		nPD:	0.71
		N. weight:	1.3
		Sc. PD:	0.48
		Sc. rank:	1407.0

,

PB2

Pos.	57	obs:	exp:
att	I	1761	985.70
atc	I	922	718.80
ata	I	3	981.60
act	T	0	0.26
acc	T	1	0.23
aca	T	0	0.42
acg	T	0	0.09
---	--	------	------
mPD		0.45	0.66
		nPD:	0.69
		N. weight:	0.75
		Sc. PD:	0.27
		Sc. rank:	778.4

,

PB2

Pos.	58	obs:	exp:
tct	S	0	0.33
tcc	S	0	0.29
tca	S	2	0.45
tcg	S	0	0.12
cct	P	0	0.27
ccc	P	0	0.18
cca	P	1	0.41
ccg	P	0	0.14
act	T	6	701.80
acc	T	7	603.20
aca	T	2640	1120.00
acg	T	24	251.90
agt	S	0	0.43
agc	S	0	0.37
gct	A	0	1.76
gcc	A	0	1.35
gca	A	7	3.12
gcg	A	0	0.77
---	--	------	------
mPD		0.035	0.70
		nPD:	0.05
		N. weight:	1.3
		Sc. PD:	-0.35
		Sc. rank:	-1201.8

,

PB2

Pos.	59	obs:	exp:
gct	A	46	676.90
gcc	A	0	520.00
gca	A	1361	1196.00
gcg	A	1280	294.10
---	--	------	------
mPD		0.52	0.69
		nPD:	0.75
		N. weight:	1.2
		Sc. PD:	0.49
		Sc. rank:	1453.4

,

PB2

Pos.	60	obs:	exp:
aat	N	0	1.09
aac	N	2	0.91
gct	A	0	0.25
gcc	A	1	0.19
gca	A	0	0.45
gcg	A	0	0.11
gat	D	78	1447.00
gac	D	2602	1233.00
gaa	E	4	2.24
gag	E	0	1.76
---	--	------	------
mPD		0.062	0.50
		nPD:	0.12
		N. weight:	1.
		Sc. PD:	-0.2
		Sc. rank:	-603.9

,

PB2

Pos.	61	obs:	exp:
caa	Q	0	0.50
cag	Q	1	0.50
cgt	R	0	0.60
cgc	R	0	0.86
cga	R	0	1.61
cgg	R	0	1.31
aaa	K	870	1520.00
aag	K	1800	1150.00
aga	R	6	7.47
agg	R	10	4.16
---	--	------	------
mPD		0.45	0.51
		nPD:	0.89
		N. weight:	0.2
		Sc. PD:	0.11
		Sc. rank:	282.1

,

PB2

Pos.	62	obs:	exp:
cgt	R	0	99.58
cgc	R	0	142.40
cga	R	47	268.20
cgg	R	0	217.30
att	I	0	0.73
atc	I	0	0.54
ata	I	2	0.73
aaa	K	17	13.67
aag	K	7	10.33
aga	R	1720	1242.00
agg	R	894	691.30
---	--	------	------
mPD		0.50	0.97
		nPD:	0.51
		N. weight:	0.43
		Sc. PD:	0.083
		Sc. rank:	328.8

,

PB2

Pos.	63	obs:	exp:
att	I	0	983.80
atc	I	0	717.40
ata	I	2681	979.70
act	T	0	0.26
acc	T	0	0.23
aca	T	1	0.42
acg	T	0	0.09
gtt	V	0	1.08
gtc	V	0	1.05
gta	V	5	1.07
gtg	V	0	1.81
---	--	------	------
mPD		0.0045	0.67
		nPD:	0.01
		N. weight:	1.6
		Sc. PD:	-0.5
		Sc. rank:	-2171.6

,

PB2

Pos.	64	obs:	exp:
tta	L	3	0.28
ttg	L	0	0.57
ctt	L	0	0.60
ctc	L	0	0.48
cta	L	0	0.44
ctg	L	0	0.63
att	I	1	488.80
atc	I	4	356.40
ata	I	1327	486.80
atg	M	1339	1339.00
act	T	0	2.62
acc	T	0	2.25
aca	T	9	4.18
acg	T	1	0.94
gtt	V	0	0.65
gtc	V	0	0.63
gta	V	3	0.64
gtg	V	0	1.08
---	--	------	------
mPD		0.51	0.68
		nPD:	0.76
		N. weight:	0.8
		Sc. PD:	0.35
		Sc. rank:	1006.8

,

PB2

Pos.	65	obs:	exp:
aaa	K	0	3.42
aag	K	6	2.58
gat	D	762	421.70
gac	D	19	359.30
gaa	E	351	1063.00
gag	E	1545	833.10
ggt	G	0	0.58
ggc	G	0	0.53
gga	G	0	1.75
ggg	G	4	1.14
---	--	------	------
mPD		0.58	0.71
		nPD:	0.81
		N. weight:	0.59
		Sc. PD:	0.28
		Sc. rank:	784.5

,

PB2

Pos.	66	obs:	exp:
tta	L	0	0.18
ttg	L	2	0.38
ctt	L	0	0.40
ctc	L	0	0.32
cta	L	0	0.30
ctg	L	0	0.42
att	I	2	10.64
atc	I	0	7.76
ata	I	27	10.60
atg	M	2653	2653.00
act	T	0	0.26
acc	T	0	0.23
aca	T	0	0.42
acg	T	1	0.09
gtt	V	0	0.43
gtc	V	0	0.42
gta	V	0	0.43
gtg	V	2	0.72
---	--	------	------
mPD		0.025	0.028
		nPD:	0.91
		N. weight:	0.019
		Sc. PD:	0.011
		Sc. rank:	26.1

,

PB2

Pos.	67	obs:	exp:
tta	L	0	0.09
ttg	L	0	0.19
tct	S	0	0.17
tcc	S	1	0.14
tca	S	0	0.23
tcg	S	0	0.06
ctt	L	0	0.20
ctc	L	1	0.16
cta	L	0	0.15
ctg	L	0	0.21
att	I	1276	958.10
atc	I	1328	698.70
ata	I	7	954.20
act	T	2	0.52
acc	T	0	0.45
aca	T	0	0.84
acg	T	0	0.19
agt	S	0	0.22
agc	S	0	0.18
gtt	V	66	15.49
gtc	V	6	15.15
gta	V	0	15.35
gtg	V	0	26.01
---	--	------	------
mPD		0.56	0.72
		nPD:	0.77
		N. weight:	0.78
		Sc. PD:	0.35
		Sc. rank:	990.2

,

PB2

Pos.	68	obs:	exp:
tta	L	0	0.09
ttg	L	1	0.19
ctt	L	0	0.20
ctc	L	0	0.16
cta	L	0	0.15
ctg	L	0	0.21
cct	P	2451	734.10
ccc	P	4	485.10
cca	P	12	1090.00
ccg	P	219	376.60
---	--	------	------
mPD		0.16	0.71
		nPD:	0.23
		N. weight:	1.7
		Sc. PD:	-0.15
		Sc. rank:	-273.0

,

PB2

Pos.	69	obs:	exp:
aaa	K	2	1.14
aag	K	0	0.86
gtt	V	0	0.65
gtc	V	0	0.63
gta	V	3	0.64
gtg	V	0	1.08
gaa	E	2616	1502.00
gag	E	63	1177.00
ggt	G	0	0.43
ggc	G	0	0.39
gga	G	3	1.31
ggg	G	0	0.86
---	--	------	------
mPD		0.052	0.50
		nPD:	0.1
		N. weight:	0.77
		Sc. PD:	-0.16
		Sc. rank:	-509.7

,

PB2

Pos.	70	obs:	exp:
cgt	R	0	100.40
cgc	R	0	143.60
cga	R	5	270.50
cgg	R	11	219.20
aaa	K	1	1.71
aag	K	2	1.29
aga	R	1134	1253.00
agg	R	1534	697.30
---	--	------	------
mPD		0.50	0.96
		nPD:	0.53
		N. weight:	0.64
		Sc. PD:	0.13
		Sc. rank:	499.9

,

PB2

Pos. 71		obs:	exp:
aat	N	2469	1465.00
aac	N	218	1222.00
---	--	------	------
mPD		0.15	0.50
		nPD:	0.3
		N. weight:	0.55
		Sc. PD:	-0.011
		Sc. rank:	57.5

PB2

Pos. 72		obs:	exp:
gaa	E	1656	1506.00
gag	E	1030	1180.00
ggt	G	0	0.14
ggc	G	0	0.13
gga	G	0	0.44
ggg	G	1	0.29
---	--	------	------
mPD		0.47	0.49
		nPD:	0.96
		N. weight:	0.014
		Sc. PD:	0.0086
		Sc. rank:	19.2

PB2

Pos. 73		obs:	exp:
cat	H	6	4.63
cac	H	2	3.37
caa	Q	2561	1335.00
cag	Q	113	1339.00
aaa	K	4	2.28
aag	K	0	1.72
gaa	E	1	0.56
gag	E	0	0.44
---	--	------	------
mPD		0.090	0.51
		nPD:	0.18
		N. weight:	0.84
		Sc. PD:	-0.12
		Sc. rank:	-305.7

PB2

Pos. 74		obs:	exp:
gat	D	2	1.08
gac	D	0	0.92
ggt	G	880	388.30
ggc	G	154	352.70
gga	G	488	1177.00
ggg	G	1163	767.50
---	--	------	------
mPD		0.67	0.69
		nPD:	0.97
		N. weight:	0.4
		Sc. PD:	0.25
		Sc. rank:	561.9

PB2

Pos. 75		obs:	exp:
caa	Q	292	1341.00
cag	Q	2394	1345.00
aaa	K	1	0.57
aag	K	0	0.43
---	--	------	------
mPD		0.20	0.50
		nPD:	0.39
		N. weight:	0.57
		Sc. PD:	0.038
		Sc. rank:	229.8

PB2

Pos. 76		obs:	exp:
att	I	2	1.10
atc	I	0	0.80
ata	I	1	1.10
atg	M	3	3.00
act	T	814	702.00
acc	T	374	603.40
aca	T	824	1121.00
acg	T	666	252.00
aat	N	0	0.55
aac	N	1	0.45
gct	A	0	0.50
gcc	A	0	0.39
gca	A	0	0.89
gcg	A	2	0.22
---	--	------	------
mPD		0.74	0.70
		nPD:	1.05
		N. weight:	0.21
		Sc. PD:	0.15
		Sc. rank:	306.1

PB2

Pos. 77		obs:	exp:
ttt	F	0	0.42
ttc	F	1	0.58
tta	L	0	248.30
ttg	L	0	510.30
ctt	L	957	533.30
ctc	L	1685	429.60
cta	L	35	397.50
ctg	L	9	566.90
---	--	------	------
mPD		0.48	1.1
		nPD:	0.43
		N. weight:	1.7
		Sc. PD:	0.17
		Sc. rank:	920.2

PB2

Pos. 78		obs:	exp:
tgg	W	2687	2687.00
---	--	------	------
mPD		0	0
		nPD:	1.
		N. weight:	0.
		Sc. PD:	0
		Sc. rank:	0

PB2

Pos. 79		obs:	exp:
tct	S	0	410.10
tcc	S	0	355.90
tca	S	0	557.00
tcg	S	0	148.20
att	I	0	0.37
atc	I	1	0.27
ata	I	0	0.37
aat	N	0	1.09
aac	N	2	0.91
agt	S	35	532.90
agc	S	2422	452.80
ggt	G	0	32.83
ggc	G	227	29.82
gga	G	0	99.47
ggg	G	0	64.89
---	--	------	------
mPD		0.18	1.8
		nPD:	0.1
		N. weight:	2.7
		Sc. PD:	-0.57
		Sc. rank:	-1740.0

PB2

Pos. 80		obs:	exp:
caa	Q	0	0.50
cag	Q	1	0.50
cgt	R	0	3.14
cgc	R	0	4.50
cga	R	0	8.46
cgg	R	0	6.86
aat	N	0	0.55
aac	N	1	0.45
aaa	K	978	1481.00
aag	K	1623	1120.00
aga	R	17	39.21
agg	R	67	21.82
---	--	------	------
mPD		0.53	0.57
		nPD:	0.92
		N. weight:	0.16
		Sc. PD:	0.095
		Sc. rank:	224.3

PB2

Pos. 81		obs:	exp:
tct	S	0	0.17
tcc	S	0	0.14
tca	S	1	0.23
tcg	S	0	0.06
att	I	0	2.94
atc	I	0	2.14
ata	I	8	2.92
atg	M	1	1.00
act	T	0	692.90
acc	T	0	595.50
aca	T	2343	1106.00
acg	T	300	248.70
aaa	K	0	1.14
aag	K	2	0.86
agt	S	0	0.22
agc	S	0	0.18
gct	A	1	8.06
gcc	A	0	6.19
gca	A	31	14.24
gcg	A	0	3.50
---	--	------	------
mPD		0.23	0.73
		nPD:	0.32
		N. weight:	1.1
		Sc. PD:	-0.001
		Sc. rank:	186.3

PB2

Pos. 82		obs:	exp:
tct	S	0	6.51
tcc	S	0	5.65
tca	S	0	8.84
tcg	S	0	2.35
cat	H	7	4.05
cac	H	0	2.95
cgt	R	0	0.04
cgc	R	0	0.05
cga	R	0	0.10
cgg	R	0	0.08
act	T	2	0.52
acc	T	0	0.45
aca	T	0	0.84
acg	T	0	0.19
aat	N	2268	1438.00
aac	N	368	1198.00
aaa	K	2	1.14
aag	K	0	0.86
agt	S	37	8.46
agc	S	2	7.19
aga	R	1	0.47
agg	R	0	0.26
---	--	------	------
mPD		0.28	0.56
		nPD:	0.5
		N. weight:	0.4
		Sc. PD:	0.069
		Sc. rank:	293.7

PB2

Pos. 83		obs:	exp:
gat	D	2564	1451.00
gac	D	123	1236.00
---	--	------	------
mPD		0.087	0.50
		nPD:	0.18
		N. weight:	0.71
		Sc. PD:	-0.1
		Sc. rank:	-260.4

PB2

Pos. 84		obs:	exp:
act	T	1	0.26
acc	T	0	0.23
aca	T	0	0.42
acg	T	0	0.09
gtt	V	1	0.22
gtc	V	0	0.21
gta	V	0	0.21
gtg	V	0	0.36
gct	A	2562	676.40
gcc	A	101	519.60
gca	A	22	1195.00
gcg	A	0	293.90
---	--	------	------
mPD		0.089	0.69
		nPD:	0.13
		N. weight:	1.9
		Sc. PD:	-0.36
		Sc. rank:	-1067.9

PB2

Pos. 85		obs:	exp:
ggt	G	5	388.60
ggc	G	20	352.90
gga	G	1593	1177.00
ggg	G	1069	768.10
---	--	------	------
mPD		0.49	0.69
		nPD:	0.71
		N. weight:	0.46
		Sc. PD:	0.18
		Sc. rank:	526.8

PB2

Pos. 86		obs:	exp:
tct	S	19	448.50
tcc	S	0	389.20
tca	S	1064	609.10
tcg	S	1604	162.10
agt	S	0	582.80
agc	S	0	495.20
---	--	------	------
mPD		0.49	1.7
		nPD:	0.3
		N. weight:	2.5
		Sc. PD:	-0.064
		Sc. rank:	180.3

PB2

Pos. 87		obs:	exp:
aat	N	1	0.55
aac	N	0	0.45
gat	D	505	1449.00
gac	D	2178	1234.00
gaa	E	1	0.56
gag	E	0	0.44
ggt	G	2	0.29
ggc	G	0	0.26
gga	G	0	0.88
ggg	G	0	0.57
---	--	------	------
mPD		0.31	0.50
		nPD:	0.62
		N. weight:	0.43
		Sc. PD:	0.13
		Sc. rank:	406.0

PB2

Pos. 88		obs:	exp:
cgt	R	0	100.30
cgc	R	0	143.40
cga	R	178	270.10
cgg	R	3	218.80
aaa	K	0	3.42
aag	K	6	2.58
aga	R	1218	1251.00
agg	R	1281	696.20
ggt	G	0	0.14
ggc	G	0	0.13
gga	G	1	0.44
ggg	G	0	0.29
---	--	------	------
mPD		0.63	0.96
		nPD:	0.66
		N. weight:	0.41
		Sc. PD:	0.13
		Sc. rank:	399.1

PB2

Pos. 89		obs:	exp:
tta	L	0	0.83
ttg	L	8	1.71
ctt	L	0	1.79
ctc	L	0	1.44
cta	L	0	1.33
ctg	L	1	1.90
att	I	1	6.24
atc	I	0	4.55
ata	I	16	6.21
atg	M	3	3.00
gtt	V	93	571.50
gtc	V	0	559.00
gta	V	620	566.50
gtg	V	1944	960.00
gct	A	0	0.25
gcc	A	0	0.19
gca	A	1	0.45
gcg	A	0	0.11
---	--	------	------
mPD		0.43	0.76
		nPD:	0.58
		N. weight:	0.78
		Sc. PD:	0.2
		Sc. rank:	674.7

PB2

Pos. 90		obs:	exp:
att	I	1	1.83
atc	I	0	1.34
ata	I	4	1.83
atg	M	2678	2678.00
gtt	V	0	0.86
gtc	V	0	0.84
gta	V	0	0.85
gtg	V	4	1.45
---	--	------	------
mPD		0.0067	0.0086
		nPD:	0.78
		N. weight:	0.0045
		Sc. PD:	0.002
		Sc. rank:	5.8

PB2			
Pos.	91	obs:	exp:
gtt	V	3	577.90
gtc	V	7	565.30
gta	V	593	572.90
gtg	V	2084	970.80
---	--	------	------
mPD		0.35	0.73
		nPD:	0.48
		N. weight:	0.95
		Sc. PD:	0.15
		Sc. rank:	639.0

,

PB2			
Pos.	92	obs:	exp:
tct	S	580	448.20
tcc	S	900	388.90
tca	S	1012	608.70
tcg	S	193	162.00
act	T	0	0.26
acc	T	0	0.23
aca	T	1	0.42
acg	T	0	0.09
agt	S	0	582.40
agc	S	0	494.80
gct	A	1	0.25
gcc	A	0	0.19
gca	A	0	0.45
gcg	A	0	0.11
---	--	------	------
mPD		0.70	1.7
		nPD:	0.42
		N. weight:	0.89
		Sc. PD:	0.087
		Sc. rank:	473.1

,

PB2			
Pos.	93	obs:	exp:
tta	L	0	0.09
ttg	L	0	0.19
tct	S	2	1.00
tcc	S	4	0.87
tca	S	0	1.36
tcg	S	0	0.36
ctt	L	0	0.20
ctc	L	1	0.16
cta	L	0	0.15
ctg	L	0	0.21
cct	P	1229	732.10
ccc	P	1450	483.90
cca	P	0	1087.00
ccg	P	0	375.60
act	T	1	0.26
acc	T	0	0.23
aca	T	0	0.42
acg	T	0	0.09
agt	S	0	1.30
agc	S	0	1.11
---	--	------	------
mPD		0.50	0.72
		nPD:	0.7
		N. weight:	1.4
		Sc. PD:	0.51
		Sc. rank:	1466.8

,

PB2			
Pos.	94	obs:	exp:
tta	L	17	248.40
ttg	L	29	510.50
ctt	L	1	533.50
ctc	L	0	429.80
cta	L	1539	397.70
ctg	L	1101	567.10
---	--	------	------
mPD		0.52	1.1
		nPD:	0.47
		N. weight:	1.6
		Sc. PD:	0.23
		Sc. rank:	1064.2

,

PB2			
Pos.	95	obs:	exp:
gct	A	2606	676.90
gcc	A	36	520.00
gca	A	18	1196.00
gcg	A	27	294.10
---	--	------	------
mPD		0.059	0.69
		nPD:	0.09
		N. weight:	2.
		Sc. PD:	-0.46
		Sc. rank:	-1464.1

,

PB2			
Pos.	96	obs:	exp:
att	I	0	1.83
atc	I	0	1.34
ata	I	5	1.83
gtt	V	0	576.80
gtc	V	1	564.30
gta	V	1439	571.90
gtg	V	1242	969.00
---	--	------	------
mPD		0.50	0.74
		nPD:	0.68
		N. weight:	0.99
		Sc. PD:	0.35
		Sc. rank:	1009.3

,

PB2			
Pos.	97	obs:	exp:
act	T	1168	704.40
acc	T	316	605.50
aca	T	519	1124.00
acg	T	684	252.80
---	--	------	------
mPD		0.70	0.70
		nPD:	1.
		N. weight:	0.41
		Sc. PD:	0.27
		Sc. rank:	579.6

,

PB2			
Pos.	98	obs:	exp:
tgg	W	2687	2687.00
---	--	------	------
mPD		0	0
		nPD:	1.
		N. weight:	0.
		Sc. PD:	0
		Sc. rank:	0

,

PB2			
Pos.	99	obs:	exp:
tgg	W	2687	2687.00
---	--	------	------
mPD		0	0
		nPD:	1.
		N. weight:	0.
		Sc. PD:	0
		Sc. rank:	0

,

PB2			
Pos.	100	obs:	exp:
aat	N	2202	1465.00
aac	N	485	1222.00
---	--	------	------
mPD		0.30	0.50
		nPD:	0.6
		N. weight:	0.27
		Sc. PD:	0.074
		Sc. rank:	250.3

,

PB2			
Pos.	101	obs:	exp:
cgt	R	0	100.40
cgc	R	0	143.60
cga	R	0	270.50
cgg	R	0	219.20
aaa	K	0	1.71
aag	K	3	1.29
aga	R	1096	1253.00
agg	R	1588	697.30
---	--	------	------
mPD		0.49	0.96
		nPD:	0.51
		N. weight:	0.7
		Sc. PD:	0.13
		Sc. rank:	529.0

,

PB2			
Pos.	102	obs:	exp:
tct	S	0	22.20
tcc	S	0	19.27
tca	S	0	30.15
tcg	S	0	8.02
aat	N	2402	1390.00
aac	N	146	1158.00
aaa	K	6	3.42
aag	K	0	2.58
agt	S	132	28.85
agc	S	1	24.51
---	--	------	------
mPD		0.20	0.66
		nPD:	0.3
		N. weight:	0.74
		Sc. PD:	-0.013
		Sc. rank:	90.8

,

PB2			
Pos.	103	obs:	exp:
ggt	G	0	388.60
ggc	G	1	352.90
gga	G	884	1177.00
ggg	G	1802	768.10
---	--	------	------
mPD		0.44	0.69
		nPD:	0.64
		N. weight:	0.77
		Sc. PD:	0.24
		Sc. rank:	744.9

,

PB2			
Pos.	104	obs:	exp:
tct	S	0	0.17
tcc	S	0	0.14
tca	S	0	0.23
tcg	S	1	0.06
cct	P	203	734.10
ccc	P	3	485.10
cca	P	1273	1090.00
ccg	P	1207	376.60
agt	S	0	0.22
agc	S	0	0.18
---	--	------	------
mPD		0.57	0.71
		nPD:	0.8
		N. weight:	0.81
		Sc. PD:	0.38
		Sc. rank:	1057.8

,

PB2			
Pos.	105	obs:	exp:
cct	P	0	1.37
ccc	P	0	0.90
cca	P	0	2.03
ccg	P	5	0.70
cgt	R	0	0.04
cgc	R	0	0.05
cga	R	0	0.10
cgg	R	0	0.08
atg	M	7	7.00
act	T	9	656.40
acc	T	1	564.20
aca	T	864	1048.00
acg	T	1630	235.60
aaa	K	1	1.14
aag	K	1	0.86
aga	R	0	0.47
agg	R	1	0.26
gtt	V	0	0.22
gtc	V	0	0.21
gta	V	0	0.21
gtg	V	1	0.36
gct	A	0	42.07
gcc	A	1	32.32
gca	A	124	74.34
gcg	A	42	18.28
---	--	------	------
mPD		0.60	0.83
		nPD:	0.73
		N. weight:	1.8
		Sc. PD:	0.73
		Sc. rank:	2159.1

,

PB2

Pos.	106	obs:	exp:
tct	S	0	5.34
tcc	S	0	4.64
tca	S	32	7.25
tcg	S	0	1.93
att	I	0	0.37
atc	I	0	0.27
ata	I	1	0.37
act	T	18	653.50
acc	T	1	561.70
aca	T	2456	1043.00
acg	T	18	234.60
aaa	K	1	0.57
aag	K	0	0.43
agt	S	0	6.94
agc	S	0	5.90
gct	A	0	40.31
gcc	A	0	30.96
gca	A	160	71.22
gcg	A	0	17.51
---	--	------	------
mPD		0.16	0.83
		nPD:	0.2
		N. weight:	1.3
		Sc. PD:	-0.16
		Sc. rank:	-425.0

,

PB2

Pos.	107	obs:	exp:
tct	S	0	347.50
tcc	S	0	301.60
tca	S	0	472.00
tcg	S	0	125.60
tgt	C	1	0.39
tgc	C	0	0.61
aat	N	556	315.20
aac	N	22	262.80
agt	S	1997	451.60
agc	S	85	383.70
gat	D	17	9.18
gac	D	0	7.82
gaa	E	4	2.24
gag	E	0	1.76
ggt	G	5	0.72
ggc	G	0	0.66
gga	G	0	2.19
ggg	G	0	1.43
---	--	------	------
mPD		0.45	1.8
		nPD:	0.25
		N. weight:	1.9
		Sc. PD:	-0.14
		Sc. rank:	-232.5

,

PB2

Pos.	108	obs:	exp:
tct	S	0	0.17
tcc	S	0	0.14
tca	S	1	0.23
tcg	S	0	0.06
att	I	0	0.37
atc	I	0	0.27
ata	I	1	0.37
act	T	18	637.80
acc	T	103	548.20
aca	T	2202	1018.00
acg	T	110	228.90
agt	S	0	0.22
agc	S	0	0.18
gtt	V	0	4.30
gtc	V	0	4.21
gta	V	20	4.26
gtg	V	0	7.23
gct	A	1	58.45
gcc	A	1	44.89
gca	A	230	103.30
gcg	A	0	25.39
---	--	------	------
mPD		0.35	0.88
		nPD:	0.4
		N. weight:	0.97
		Sc. PD:	0.07
		Sc. rank:	410.4

,

PB2

Pos.	109	obs:	exp:
att	I	4	6.61
atc	I	8	4.82
ata	I	6	6.58
gtt	V	1345	573.20
gtc	V	1318	560.70
gta	V	2	568.20
gtg	V	0	962.90
gct	A	3	1.01
gcc	A	1	0.77
gca	A	0	1.78
gcg	A	0	0.44
---	--	------	------
mPD		0.52	0.75
		nPD:	0.69
		N. weight:	1.4
		Sc. PD:	0.5
		Sc. rank:	1441.9

,

PB2

Pos.	110	obs:	exp:
tat	Y	1	0.48
tac	Y	0	0.52
cat	H	2428	1551.00
cac	H	251	1128.00
cgt	R	3	0.11
cgc	R	0	0.16
cga	R	0	0.30
cgg	R	0	0.24
aat	N	4	2.18
aac	N	0	1.82
aga	R	0	1.40
agg	R	0	0.78
---	--	------	------
mPD		0.18	0.50
		nPD:	0.35
		N. weight:	0.44
		Sc. PD:	0.014
		Sc. rank:	130.1

,

PB2

Pos.	111	obs:	exp:
tat	Y	1947	1288.00
tac	Y	739	1398.00
tgt	C	1	0.39
tgc	C	0	0.61
---	--	------	------
mPD		0.40	0.50
		nPD:	0.8
		N. weight:	0.2
		Sc. PD:	0.096
		Sc. rank:	266.3

,

PB2

Pos.	112	obs:	exp:
tct	S	0	0.33
tcc	S	0	0.29
tca	S	2	0.45
tcg	S	0	0.12
cct	P	1	733.00
ccc	P	8	484.40
cca	P	2666	1089.00
ccg	P	7	376.00
caa	Q	1	0.50
cag	Q	0	0.50
act	T	0	0.52
acc	T	0	0.45
aca	T	2	0.84
acg	T	0	0.19
agt	S	0	0.43
agc	S	0	0.37
---	--	------	------
mPD		0.016	0.71
		nPD:	0.02
		N. weight:	1.4
		Sc. PD:	-0.41
		Sc. rank:	-1602.4

,

PB2

Pos.	113	obs:	exp:
cgt	R	0	0.37
cgc	R	0	0.54
cga	R	0	1.01
cgg	R	0	0.82
act	T	0	0.26
acc	T	0	0.23
aca	T	0	0.42
acg	T	1	0.09
aaa	K	1064	1524.00
aag	K	1612	1152.00
aga	R	1	4.67
agg	R	9	2.60
---	--	------	------
mPD		0.49	0.50
		nPD:	0.97
		N. weight:	0.11
		Sc. PD:	0.068
		Sc. rank:	150.1

,

PB2

Pos.	114	obs:	exp:
att	I	1	0.37
atc	I	0	0.27
ata	I	0	0.37
gtt	V	1413	577.70
gtc	V	1086	565.10
gta	V	172	572.70
gtg	V	15	970.50
---	--	------	------
mPD		0.56	0.73
		nPD:	0.76
		N. weight:	1.
		Sc. PD:	0.44
		Sc. rank:	1295.9

,

PB2

Pos.	115	obs:	exp:
tat	Y	758	1288.00
tac	Y	1927	1397.00
cat	H	0	0.58
cac	H	1	0.42
aat	N	0	0.55
aac	N	1	0.45
---	--	------	------
mPD		0.41	0.50
		nPD:	0.81
		N. weight:	0.13
		Sc. PD:	0.065
		Sc. rank:	179.6

,

PB2

Pos.	116	obs:	exp:
cgt	R	0	0.15
cgc	R	0	0.21
cga	R	0	0.40
cgg	R	0	0.33
aaa	K	2662	1528.00
aag	K	21	1155.00
aga	R	4	1.87
agg	R	0	1.04
---	--	------	------
mPD		0.018	0.49
		nPD:	0.04
		N. weight:	0.85
		Sc. PD:	-0.24
		Sc. rank:	-866.5

,

PB2

Pos.	117	obs:	exp:
tct	S	0	0.67
tcc	S	4	0.58
tca	S	0	0.91
tcg	S	0	0.24
cct	P	2	8.47
ccc	P	11	5.60
cca	P	18	12.58
ccg	P	0	4.35
atg	M	1	1.00
act	T	189	694.70
acc	T	980	597.10
aca	T	1452	1109.00
acg	T	29	249.30
aat	N	0	0.55
aac	N	1	0.45
agt	S	0	0.87
agc	S	0	0.74
---	--	------	------
mPD		0.59	0.72
		nPD:	0.81
		N. weight:	0.36
		Sc. PD:	0.17
		Sc. rank:	482.4

,

PB2

Pos.	118	obs:	exp:
tat	Y	207	1289.00
tac	Y	2480	1398.00
---	--	------	------
mPD		0.14	0.50
		nPD:	0.28
		N. weight:	0.63
		Sc. PD:	-0.022
		Sc. rank:	25.1

,

PB2

Pos.	119	obs:	exp:
ttt	F	1313	1121.00
ttc	F	1374	1566.00
---	--	------	------
mPD		0.50	0.49
		nPD:	1.03
		N. weight:	0.019
		Sc. PD:	0.013
		Sc. rank:	27.7

,

PB2

Pos.	120	obs:	exp:
gat	D	5	2.70
gac	D	0	2.30
gaa	E	1237	1473.00
gag	E	1391	1155.00
ggt	G	0	7.81
ggc	G	0	7.09
gga	G	1	23.66
ggg	G	53	15.44
---	--	------	------
mPD		0.54	0.54
		nPD:	1.
		N. weight:	0.068
		Sc. PD:	0.045
		Sc. rank:	97.0

,

PB2			
Pos.	121	obs:	exp:
cgt	R	0	0.11
cgc	R	0	0.16
cga	R	0	0.30
cgg	R	0	0.24
aaa	K	443	1528.00
aag	K	2241	1156.00
aga	R	0	1.40
agg	R	3	0.78
---	--	------	------
mPD		0.28	0.49
		nPD:	0.56
		N. weight:	0.57
		Sc. PD:	0.14
		Sc. rank:	478.4

,

PB2			
Pos.	122	obs:	exp:
ttt	F	1	0.42
ttc	F	0	0.58
att	I	4	4.04
atc	I	7	2.94
ata	I	0	4.02
atg	M	2	2.00
gtt	V	2076	574.10
gtc	V	484	561.50
gta	V	32	569.10
gtg	V	77	964.30
gct	A	0	1.01
gcc	A	4	0.77
gca	A	0	1.78
gcg	A	0	0.44
---	--	------	------
mPD		0.38	0.75
		nPD:	0.51
		N. weight:	1.4
		Sc. PD:	0.26
		Sc. rank:	1043.5

,

PB2			
Pos.	123	obs:	exp:
aaa	K	4	2.28
aag	K	0	1.72
gct	A	0	0.25
gcc	A	0	0.19
gca	A	1	0.45
gcg	A	0	0.11
gat	D	0	10.80
gac	D	20	9.20
gaa	E	2612	1492.00
gag	E	50	1170.00
---	--	------	------
mPD		0.055	0.50
		nPD:	0.11
		N. weight:	0.8
		Sc. PD:	-0.17
		Sc. rank:	-513.1

,

PB2			
Pos.	124	obs:	exp:
cgt	R	0	100.60
cgc	R	0	143.80
cga	R	0	270.80
cgg	R	0	219.40
aga	R	369	1254.00
agg	R	2318	698.10
---	--	------	------
mPD		0.24	0.95
		nPD:	0.25
		N. weight:	1.4
		Sc. PD:	-0.1
		Sc. rank:	-165.9

,

PB2			
Pos.	125	obs:	exp:
tta	L	2044	248.40
ttg	L	390	510.50
ctt	L	0	533.50
ctc	L	0	429.80
cta	L	253	397.70
ctg	L	0	567.10
---	--	------	------
mPD		0.42	1.1
		nPD:	0.37
		N. weight:	2.5
		Sc. PD:	0.13
		Sc. rank:	917.5

,

PB2			
Pos.	126	obs:	exp:
cgt	R	0	1.65
cgc	R	0	2.35
cga	R	0	4.43
cgg	R	0	3.59
aat	N	0	0.55
aac	N	1	0.45
aaa	K	2019	1504.00
aag	K	623	1138.00
aga	R	44	20.54
agg	R	0	11.43
---	--	------	------
mPD		0.39	0.53
		nPD:	0.73
		N. weight:	0.16
		Sc. PD:	0.062
		Sc. rank:	185.3

,

PB2			
Pos.	127	obs:	exp:
tat	Y	28	13.43
tac	Y	0	14.57
cat	H	2356	1537.00
cac	H	299	1118.00
caa	Q	1	0.50
cag	Q	0	0.50
aat	N	2	1.64
aac	N	1	1.36
---	--	------	------
mPD		0.22	0.51
		nPD:	0.43
		N. weight:	0.39
		Sc. PD:	0.043
		Sc. rank:	219.2

,

PB2			
Pos.	128	obs:	exp:
ggt	G	2	388.60
ggc	G	0	352.90
gga	G	2155	1177.00
ggg	G	530	768.10
---	--	------	------
mPD		0.32	0.69
		nPD:	0.46
		N. weight:	0.66
		Sc. PD:	0.092
		Sc. rank:	423.9

,

PB2			
Pos.	129	obs:	exp:
tct	S	0	0.17
tcc	S	0	0.14
tca	S	1	0.23
tcg	S	0	0.06
act	T	17	689.70
acc	T	2009	592.80
aca	T	605	1101.00
acg	T	0	247.50
aat	N	22	27.27
aac	N	28	22.73
agt	S	0	0.22
agc	S	0	0.18
gct	A	0	1.26
gcc	A	5	0.97
gca	A	0	2.23
gcg	A	0	0.55
---	--	------	------
mPD		0.41	0.74
		nPD:	0.56
		N. weight:	1.2
		Sc. PD:	0.29
		Sc. rank:	1030.7

,

PB2			
Pos.	130	obs:	exp:
ttt	F	398	1121.00
ttc	F	2288	1565.00
tat	Y	0	0.48
tac	Y	1	0.52
---	--	------	------
mPD		0.25	0.49
		nPD:	0.52
		N. weight:	0.28
		Sc. PD:	0.054
		Sc. rank:	213.0

,

PB2			
Pos.	131	obs:	exp:
ggt	G	1197	388.60
ggc	G	1483	352.90
gga	G	3	1177.00
ggg	G	4	768.10
---	--	------	------
mPD		0.50	0.69
		nPD:	0.72
		N. weight:	2.1
		Sc. PD:	0.82
		Sc. rank:	2415.6

,

PB2			
Pos.	132	obs:	exp:
cct	P	1016	734.30
ccc	P	1635	485.30
cca	P	22	1091.00
ccg	P	14	376.70
---	--	------	------
mPD		0.49	0.71
		nPD:	0.69
		N. weight:	1.3
		Sc. PD:	0.48
		Sc. rank:	1381.7

,

PB2			
Pos.	133	obs:	exp:
att	I	0	0.37
atc	I	1	0.27
ata	I	0	0.37
gtt	V	1177	577.50
gtc	V	1225	564.90
gta	V	247	572.50
gtg	V	36	970.10
gct	A	0	0.25
gcc	A	1	0.19
gca	A	0	0.45
gcg	A	0	0.11
---	--	------	------
mPD		0.59	0.74
		nPD:	0.81
		N. weight:	0.89
		Sc. PD:	0.42
		Sc. rank:	1178.3

,

PB2			
Pos.	134	obs:	exp:
cat	H	2014	1549.00
cac	H	661	1126.00
caa	Q	0	0.50
cag	Q	1	0.50
cgt	R	11	0.41
cgc	R	0	0.59
cga	R	0	1.11
cgg	R	0	0.90
aga	R	0	5.14
agg	R	0	2.86
---	--	------	------
mPD		0.38	0.51
		nPD:	0.75
		N. weight:	0.13
		Sc. PD:	0.056
		Sc. rank:	163.1

,

PB2			
Pos.	135	obs:	exp:
ttt	F	397	1121.00
ttc	F	2290	1566.00
---	--	------	------
mPD		0.25	0.49
		nPD:	0.52
		N. weight:	0.28
		Sc. PD:	0.054
		Sc. rank:	213.2

,

PB2			
Pos.	136	obs:	exp:
caa	Q	47	23.47
cag	Q	0	23.53
cgt	R	2	98.80
cgc	R	8	141.30
cga	R	1502	266.00
cgg	R	547	215.60
aga	R	578	1232.00
agg	R	3	685.90
---	--	------	------
mPD		0.71	1.0
		nPD:	0.71
		N. weight:	1.6
		Sc. PD:	0.61
		Sc. rank:	1781.5

,

PB2			
Pos.	137	obs:	exp:
tct	S	0	1.17
tcc	S	0	1.01
tca	S	0	1.59
tcg	S	0	0.42
act	T	0	0.26
acc	T	1	0.23
aca	T	0	0.42
acg	T	0	0.09
aat	N	1120	1460.00
aac	N	1558	1218.00
aaa	K	0	0.57
aag	K	1	0.43
agt	S	3	1.52
agc	S	4	1.29
---	--	------	------
mPD		0.49	0.51
		nPD:	0.97
		N. weight:	0.061
		Sc. PD:	0.039
		Sc. rank:	86.5

,

PB2			
Pos.	138	obs:	exp:
caa	Q	1781	1342.00
cag	Q	906	1345.00
---	--	------	------
mPD		0.45	0.50
		nPD:	0.89
		N. weight:	0.091
		Sc. PD:	0.051
		Sc. rank:	126.8

,

PB2			
Pos.	139	obs:	exp:
att	I	40	14.68
atc	I	0	10.70
ata	I	0	14.62
gtt	V	2285	569.30
gtc	V	358	556.90
gta	V	3	564.40
gtg	V	1	956.40
---	--	------	------
mPD		0.26	0.76
		nPD:	0.34
		N. weight:	1.8
		Sc. PD:	0.042
		Sc. rank:	497.0

,

PB2			
Pos.	140	obs:	exp:
cgt	R	0	0.15
cgc	R	0	0.21
cga	R	0	0.40
cgg	R	0	0.33
aaa	K	1947	1528.00
aag	K	736	1155.00
aga	R	0	1.87
agg	R	4	1.04
---	--	------	------
mPD		0.40	0.49
		nPD:	0.81
		N. weight:	0.09
		Sc. PD:	0.044
		Sc. rank:	120.4

,

PB2			
Pos.	141	obs:	exp:
att	I	3	984.90
atc	I	0	718.20
ata	I	2681	980.80
atg	M	1	1.00
act	T	0	0.26
acc	T	0	0.23
aca	T	1	0.42
acg	T	0	0.09
gtt	V	0	0.22
gtc	V	0	0.21
gta	V	1	0.21
gtg	V	0	0.36
---	--	------	------
mPD		0.0045	0.66
		nPD:	0.01
		N. weight:	1.6
		Sc. PD:	-0.5
		Sc. rank:	-2125.2

,

PB2			
Pos.	142	obs:	exp:
cgt	R	198	100.60
cgc	R	2307	143.80
cga	R	1	270.80
cgg	R	0	219.40
aga	R	180	1254.00
agg	R	1	698.10
---	--	------	------
mPD		0.38	0.95
		nPD:	0.4
		N. weight:	3.7
		Sc. PD:	0.28
		Sc. rank:	1646.0

,

PB2			
Pos.	143	obs:	exp:
cgt	R	61	100.60
cgc	R	2416	143.80
cga	R	28	270.80
cgg	R	178	219.40
aga	R	2	1254.00
agg	R	2	698.10
---	--	------	------
mPD		0.19	0.95
		nPD:	0.2
		N. weight:	4.
		Sc. PD:	-0.48
		Sc. rank:	-1191.2

,

PB2			
Pos.	144	obs:	exp:
cct	P	0	0.27
ccc	P	0	0.18
cca	P	1	0.41
ccg	P	0	0.14
caa	Q	2	1.00
cag	Q	0	1.00
cgt	R	0	100.40
cgc	R	0	143.60
cga	R	1380	270.50
cgg	R	96	219.20
aga	R	462	1253.00
agg	R	746	697.30
---	--	------	------
mPD		0.93	0.96
		nPD:	0.97
		N. weight:	1.1
		Sc. PD:	0.68
		Sc. rank:	1509.1

,

PB2			
Pos.	145	obs:	exp:
gtt	V	2467	577.90
gtc	V	189	565.30
gta	V	0	572.90
gtg	V	31	970.80
---	--	------	------
mPD		0.15	0.73
		nPD:	0.21
		N. weight:	2.
		Sc. PD:	-0.22
		Sc. rank:	-509.5

,

PB2			
Pos.	146	obs:	exp:
aat	N	0	1.64
aac	N	3	1.36
gat	D	1441	1447.00
gac	D	1239	1233.00
gaa	E	0	2.24
gag	E	4	1.76
---	--	------	------
mPD		0.50	0.50
		nPD:	1.
		N. weight:	0.0059
		Sc. PD:	0.004
		Sc. rank:	8.4

,

PB2			
Pos.	147	obs:	exp:
att	I	0	449.90
atc	I	1	328.10
ata	I	1225	448.00
atg	M	2	2.00
act	T	1	370.70
acc	T	2	318.60
aca	T	1408	591.70
acg	T	3	133.00
gtt	V	0	9.03
gtc	V	0	8.84
gta	V	42	8.96
gtg	V	0	15.17
gct	A	0	0.76
gcc	A	0	0.58
gca	A	3	1.34
gcg	A	0	0.33
---	--	------	------
mPD		0.54	1.2
		nPD:	0.44
		N. weight:	1.5
		Sc. PD:	0.18
		Sc. rank:	889.8

,

PB2			
Pos.	148	obs:	exp:
tct	S	0	0.33
tcc	S	0	0.29
tca	S	0	0.45
tcg	S	0	0.12
act	T	0	0.52
acc	T	2	0.45
aca	T	0	0.84
acg	T	0	0.19
aat	N	733	1463.00
aac	N	1949	1219.00
agt	S	0	0.43
agc	S	2	0.37
gat	D	0	0.54
gac	D	1	0.46
---	--	------	------
mPD		0.40	0.50
		nPD:	0.8
		N. weight:	0.25
		Sc. PD:	0.12
		Sc. rank:	330.1

,

PB2			
Pos.	149	obs:	exp:
cct	P	1401	734.30
ccc	P	51	485.30
cca	P	406	1091.00
ccg	P	829	376.70
---	--	------	------
mPD		0.61	0.71
		nPD:	0.86
		N. weight:	0.64
		Sc. PD:	0.34
		Sc. rank:	875.1

,

PB2			
Pos.	150	obs:	exp:
tct	S	0	0.33
tcc	S	0	0.29
tca	S	0	0.45
tcg	S	0	0.12
agt	S	0	0.43
agc	S	2	0.37
gtt	V	0	0.22
gtc	V	1	0.21
gta	V	0	0.21
gtg	V	0	0.36
ggt	G	215	388.10
ggc	G	2467	352.60
gga	G	2	1176.00
ggg	G	0	767.20
---	--	------	------
mPD		0.15	0.69
		nPD:	0.22
		N. weight:	2.8
		Sc. PD:	-0.28
		Sc. rank:	-611.1

,

PB2			
Pos.	151	obs:	exp:
cct	P	2	0.55
ccc	P	0	0.36
cca	P	0	0.81
ccg	P	0	0.28
cat	H	2528	1554.00
cac	H	156	1130.00
caa	Q	1	0.50
cag	Q	0	0.50
---	--	------	------
mPD		0.11	0.49
		nPD:	0.23
		N. weight:	0.56
		Sc. PD:	-0.051
		Sc. rank:	-95.0

,

PB2			
Pos.	152	obs:	exp:
tct	S	0	4.17
tcc	S	0	3.62
tca	S	25	5.67
tcg	S	0	1.51
act	T	0	0.26
acc	T	0	0.23
aca	T	1	0.42
acg	T	0	0.09
agt	S	0	5.42
agc	S	0	4.61
gtt	V	0	0.65
gtc	V	0	0.63
gta	V	3	0.64
gtg	V	0	1.08
gct	A	150	669.60
gcc	A	1	514.30
gca	A	2372	1183.00
gcg	A	135	290.90
---	--	------	------
mPD		0.22	0.72
		nPD:	0.3
		N. weight:	0.82
		Sc. PD:	-0.015
		Sc. rank:	88.2

,

PB2			
Pos.	153	obs:	exp:
cat	H	1	0.58
cac	H	0	0.42
aat	N	1	1.09
aac	N	1	0.91
gct	A	0	0.25
gcc	A	1	0.19
gca	A	0	0.45
gcg	A	0	0.11
gat	D	1899	1446.00
gac	D	779	1232.00
ggt	G	4	0.72
ggc	G	1	0.66
gga	G	0	2.19
ggg	G	0	1.43
---	--	------	------
mPD		0.42	0.51
		nPD:	0.83
		N. weight:	0.11
		Sc. PD:	0.053
		Sc. rank:	143.1

,

PB2			
Pos.	154	obs:	exp:
ttt	F	0	12.10
ttc	F	29	16.90
tta	L	0	245.80
ttg	L	0	504.90
ctt	L	307	527.70
ctc	L	2350	425.20
cta	L	1	393.40
ctg	L	0	561.00
---	--	------	------
mPD		0.22	1.1
		nPD:	0.2
		N. weight:	2.3
		Sc. PD:	-0.28
		Sc. rank:	-669.6

,

PB2			
Pos.	155	obs:	exp:
tct	S	0	443.80
tcc	S	0	385.20
tca	S	0	602.80
tcg	S	0	160.40
tgt	C	1	0.39
tgc	C	0	0.61
att	I	2	0.73
atc	I	0	0.54
ata	I	0	0.73
act	T	1	0.26
acc	T	0	0.23
aca	T	0	0.42
acg	T	0	0.09
aat	N	24	13.09
aac	N	0	10.91
agt	S	2627	576.70
agc	S	32	490.00
---	--	------	------
mPD		0.044	1.7
		nPD:	0.03
		N. weight:	2.4
		Sc. PD:	-0.68
		Sc. rank:	-2614.7

,

PB2			
Pos.	156	obs:	exp:
ttt	F	1	0.42
ttc	F	0	0.58
tct	S	2	0.33
tcc	S	0	0.29
tca	S	0	0.45
tcg	S	0	0.12
act	T	5	1.31
acc	T	0	1.13
aca	T	0	2.09
acg	T	0	0.47
agt	S	0	0.43
agc	S	0	0.37
gtt	V	12	2.80
gtc	V	1	2.74
gta	V	0	2.77
gtg	V	0	4.70
gct	A	2253	671.60
gcc	A	406	515.90
gca	A	0	1187.00
gcg	A	7	291.80
---	--	------	------
mPD		0.28	0.71
		nPD:	0.39
		N. weight:	1.6
		Sc. PD:	0.11
		Sc. rank:	669.9

PB2			
Pos.	157	obs:	exp:
cgt	R	0	0.07
cgc	R	0	0.11
cga	R	0	0.20
cgg	R	0	0.16
aaa	K	2658	1529.00
aag	K	27	1156.00
aga	R	2	0.93
agg	R	0	0.52
---	--	------	------
mPD		0.021	0.49
		nPD:	0.04
		N. weight:	0.83
		Sc. PD:	-0.23
		Sc. rank:	-817.0

PB2			
Pos.	158	obs:	exp:
gat	D	39	21.06
gac	D	0	17.94
gaa	E	2448	1484.00
gag	E	200	1164.00
---	--	------	------
mPD		0.16	0.51
		nPD:	0.32
		N. weight:	0.54
		Sc. PD:	0.0016
		Sc. rank:	93.3

PB2			
Pos.	159	obs:	exp:
gct	A	2	676.90
gcc	A	10	520.00
gca	A	2400	1196.00
gcg	A	275	294.10
---	--	------	------
mPD		0.19	0.69
		nPD:	0.28
		N. weight:	0.97
		Sc. PD:	-0.041
		Sc. rank:	16.2

PB2			
Pos.	160	obs:	exp:
caa	Q	2350	1342.00
cag	Q	337	1345.00
---	--	------	------
mPD		0.22	0.50
		nPD:	0.44
		N. weight:	0.52
		Sc. PD:	0.06
		Sc. rank:	301.0

PB2			
Pos.	161	obs:	exp:
gat	D	2185	1437.00
gac	D	476	1224.00
gaa	E	25	14.57
gag	E	1	11.43
---	--	------	------
mPD		0.31	0.51
		nPD:	0.61
		N. weight:	0.29
		Sc. PD:	0.082
		Sc. rank:	270.6

PB2			
Pos.	162	obs:	exp:
att	I	0	1.83
atc	I	5	1.34
ata	I	0	1.83
gtt	V	43	576.80
gtc	V	2448	564.30
gta	V	183	571.90
gtg	V	8	969.00
---	--	------	------
mPD		0.17	0.74
		nPD:	0.22
		N. weight:	2.
		Sc. PD:	-0.19
		Sc. rank:	-374.8

PB2			
Pos.	163	obs:	exp:
att	I	28	984.60
atc	I	2424	718.00
ata	I	231	980.50
gtt	V	0	0.86
gtc	V	4	0.84
gta	V	0	0.85
gtg	V	0	1.45
---	--	------	------
mPD		0.18	0.66
		nPD:	0.27
		N. weight:	1.5
		Sc. PD:	-0.077
		Sc. rank:	-36.6

PB2			
Pos.	164	obs:	exp:
att	I	0	0.37
atc	I	0	0.27
ata	I	1	0.37
atg	M	2686	2686.00
---	--	------	------
mPD		0.00074	0.00074
		nPD:	1.
		N. weight:	0.00061
		Sc. PD:	0.00041
		Sc. rank:	0.9

PB2			
Pos.	165	obs:	exp:
gaa	E	1301	1506.00
gag	E	1386	1181.00
---	--	------	------
mPD		0.50	0.49
		nPD:	1.01
		N. weight:	0.022
		Sc. PD:	0.015
		Sc. rank:	30.6

PB2			
Pos.	166	obs:	exp:
att	I	0	1.10
atc	I	3	0.80
ata	I	0	1.10
gtt	V	1056	577.10
gtc	V	1578	564.50
gta	V	26	572.10
gtg	V	23	969.40
gct	A	0	0.25
gcc	A	1	0.19
gca	A	0	0.45
gcg	A	0	0.11
---	--	------	------
mPD		0.50	0.74
		nPD:	0.68
		N. weight:	1.3
		Sc. PD:	0.45
		Sc. rank:	1303.9

PB2			
Pos.	167	obs:	exp:
att	I	3	1.10
atc	I	0	0.80
ata	I	0	1.10
gtt	V	2283	577.30
gtc	V	182	564.70
gta	V	219	572.30
gtg	V	0	969.70
---	--	------	------
mPD		0.27	0.74
		nPD:	0.36
		N. weight:	1.6
		Sc. PD:	0.069
		Sc. rank:	539.4

PB2			
Pos.	168	obs:	exp:
ttt	F	267	1121.00
ttc	F	2419	1565.00
tat	Y	0	0.48
tac	Y	1	0.52
---	--	------	------
mPD		0.18	0.49
		nPD:	0.37
		N. weight:	0.41
		Sc. PD:	0.019
		Sc. rank:	144.9

PB2			
Pos.	169	obs:	exp:
cct	P	1	734.30
ccc	P	1	485.30
cca	P	2645	1091.00
ccg	P	40	376.70
---	--	------	------
mPD		0.031	0.71
		nPD:	0.04
		N. weight:	1.4
		Sc. PD:	-0.37
		Sc. rank:	-1329.6

PB2			
Pos.	170	obs:	exp:
aat	N	2231	1465.00
aac	N	456	1222.00
---	--	------	------
mPD		0.28	0.50
		nPD:	0.57
		N. weight:	0.3
		Sc. PD:	0.072
		Sc. rank:	254.1

PB2			
Pos.	171	obs:	exp:
gaa	E	2079	1506.00
gag	E	608	1181.00
---	--	------	------
mPD		0.35	0.49
		nPD:	0.71
		N. weight:	0.16
		Sc. PD:	0.063
		Sc. rank:	184.2

PB2			
Pos.	172	obs:	exp:
atg	M	1	1.00
gtt	V	467	577.70
gtc	V	461	565.10
gta	V	154	572.70
gtg	V	1604	970.50
---	--	------	------
mPD		0.58	0.73
		nPD:	0.79
		N. weight:	0.25
		Sc. PD:	0.12
		Sc. rank:	330.2

PB2			
Pos.	173	obs:	exp:
cgt	R	0	0.15
cgc	R	0	0.21
cga	R	0	0.40
cgg	R	0	0.33
aga	R	4	1.87
agg	R	0	1.04
ggt	G	0	388.00
ggc	G	0	352.40
gga	G	2560	1176.00
ggg	G	123	766.90
---	--	------	------
mPD		0.090	0.69
		nPD:	0.13
		N. weight:	1.1
		Sc. PD:	-0.2
		Sc. rank:	-593.6

PB2			
Pos.	174	obs:	exp:
gct	A	1532	676.90
gcc	A	954	520.00
gca	A	61	1196.00
gcg	A	140	294.10
---	--	------	------
mPD		0.55	0.69
		nPD:	0.79
		N. weight:	0.94
		Sc. PD:	0.43
		Sc. rank:	1223.2

PB2			
Pos.	175	obs:	exp:
cgt	R	0	96.78
cgc	R	0	138.40
cga	R	19	260.60
cgg	R	0	211.20
att	I	0	1.47
atc	I	0	1.07
ata	I	4	1.46
aaa	K	46	54.09
aag	K	49	40.91
aga	R	1879	1207.00
agg	R	688	671.80
ggt	G	0	0.29
ggc	G	0	0.26
gga	G	2	0.88
ggg	G	0	0.57
---	--	------	------
mPD		0.48	1.0
		nPD:	0.48
		N. weight:	0.49
		Sc. PD:	0.076
		Sc. rank:	337.5

PB2			
Pos.	176	obs:	exp:
att	I	0	982.40
atc	I	7	716.40
ata	I	2670	978.30
atg	M	4	4.00
act	T	0	0.79
acc	T	0	0.68
aca	T	3	1.26
acg	T	0	0.28
gtt	V	0	0.65
gtc	V	0	0.63
gta	V	3	0.64
gtg	V	0	1.08
---	--	------	------
mPD		0.013	0.67
		nPD:	0.02
		N. weight:	1.6
		Sc. PD:	-0.48
		Sc. rank:	-1909.8

PB2			
Pos.	177	obs:	exp:
tta	L	28	248.30
ttg	L	2272	510.30
ctt	L	1	533.30
ctc	L	1	429.60
cta	L	127	397.50
ctg	L	257	566.90
cct	P	0	0.27
ccc	P	1	0.18
cca	P	0	0.41
ccg	P	0	0.14
---	--	------	------
mPD		0.36	1.1
		nPD:	0.32
		N. weight:	1.8
		Sc. PD:	-0.0029
		Sc. rank:	292.4

PB2			
Pos.	178	obs:	exp:
cct	P	1	0.27
ccc	P	0	0.18
cca	P	0	0.41
ccg	P	0	0.14
act	T	1136	702.60
acc	T	18	603.90
aca	T	1484	1121.00
acg	T	42	252.20
gct	A	4	1.51
gcc	A	0	1.16
gca	A	2	2.67
gcg	A	0	0.66
---	--	------	------
mPD		0.52	0.70
		nPD:	0.74
		N. weight:	0.51
		Sc. PD:	0.21
		Sc. rank:	613.1

PB2			
Pos.	179	obs:	exp:
tct	S	3	448.50
tcc	S	1	389.20
tca	S	1950	609.10
tcg	S	733	162.10
agt	S	0	582.80
agc	S	0	495.20
---	--	------	------
mPD		0.40	1.7
		nPD:	0.24
		N. weight:	2.
		Sc. PD:	-0.16
		Sc. rank:	-289.4

PB2			
Pos.	180	obs:	exp:
gaa	E	232	1506.00
gag	E	2455	1181.00
---	--	------	------
mPD		0.16	0.49
		nPD:	0.32
		N. weight:	0.83
		Sc. PD:	-0.0004
		Sc. rank:	139.9

PB2			
Pos.	181	obs:	exp:
ttt	F	0	0.42
ttc	F	1	0.58
tct	S	7	448.00
tcc	S	109	388.80
tca	S	1402	608.50
tcg	S	1166	161.90
act	T	0	0.52
acc	T	0	0.45
aca	T	0	0.84
acg	T	2	0.19
agt	S	0	582.20
agc	S	0	494.60
---	--	------	------
mPD		0.54	1.7
		nPD:	0.33
		N. weight:	2.
		Sc. PD:	0.012
		Sc. rank:	359.1

PB2			
Pos.	182	obs:	exp:
caa	Q	1546	1341.00
cag	Q	1140	1345.00
cgt	R	0	0.04
cgc	R	0	0.05
cga	R	1	0.10
cgg	R	0	0.08
aga	R	0	0.47
agg	R	0	0.26
---	--	------	------
mPD		0.49	0.50
		nPD:	0.98
		N. weight:	0.023
		Sc. PD:	0.015
		Sc. rank:	32.4

PB2			
Pos.	183	obs:	exp:
ttt	F	1	0.42
ttc	F	0	0.58
tta	L	937	248.30
ttg	L	1565	510.10
ctt	L	0	533.10
ctc	L	0	429.50
cta	L	7	397.40
ctg	L	176	566.70
atg	M	1	1.00
---	--	------	------
mPD		0.58	1.1
		nPD:	0.52
		N. weight:	1.7
		Sc. PD:	0.33
		Sc. rank:	1299.4

PB2			
Pos.	184	obs:	exp:
att	I	0	1.10
atc	I	0	0.80
ata	I	3	1.10
atg	M	1	1.00
act	T	1	658.50
acc	T	5	566.00
aca	T	2116	1051.00
acg	T	390	236.30
aaa	K	3	1.71
aag	K	0	1.29
gct	A	0	42.32
gcc	A	0	32.51
gca	A	168	74.78
gcg	A	0	18.39
---	--	------	------
mPD		0.38	0.82
		nPD:	0.46
		N. weight:	1.1
		Sc. PD:	0.15
		Sc. rank:	676.8

PB2			
Pos.	185	obs:	exp:
att	I	390	984.60
atc	I	43	718.00
ata	I	2250	980.50
act	T	0	0.26
acc	T	0	0.23
aca	T	1	0.42
acg	T	0	0.09
gtt	V	1	0.65
gtc	V	0	0.63
gta	V	2	0.64
gtg	V	0	1.08
---	--	------	------
mPD		0.28	0.66
		nPD:	0.42
		N. weight:	0.84
		Sc. PD:	0.082
		Sc. rank:	448.0

PB2			
Pos.	186	obs:	exp:
atg	M	15	15.00
act	T	3	700.20
acc	T	3	601.80
aca	T	1739	1118.00
acg	T	926	251.30
gct	A	0	0.25
gcc	A	0	0.19
gca	A	1	0.45
gcg	A	0	0.11
---	--	------	------
mPD		0.47	0.71
		nPD:	0.66
		N. weight:	1.2
		Sc. PD:	0.39
		Sc. rank:	1160.7

PB2			
Pos.	187	obs:	exp:
caa	Q	3	1.50
cag	Q	0	1.50
cgt	R	0	0.90
cgc	R	0	1.28
cga	R	0	2.42
cgg	R	0	1.96
aaa	K	2088	1515.00
aag	K	572	1145.00
aga	R	14	11.20
agg	R	10	6.24
---	--	------	------
mPD		0.36	0.52
		nPD:	0.7
		N. weight:	0.17
		Sc. PD:	0.064
		Sc. rank:	184.7

PB2			
Pos.	188	obs:	exp:
gaa	E	898	1501.00
gag	E	1779	1176.00
ggt	G	0	1.45
ggc	G	0	1.31
gga	G	6	4.38
ggg	G	4	2.86
---	--	------	------
mPD		0.45	0.50
		nPD:	0.91
		N. weight:	0.17
		Sc. PD:	0.098
		Sc. rank:	239.1

PB2			
Pos.	189	obs:	exp:
aaa	K	267	1530.00
aag	K	2420	1157.00
---	--	------	------
mPD		0.18	0.49
		nPD:	0.37
		N. weight:	0.8
		Sc. PD:	0.035
		Sc. rank:	263.6

PB2			
Pos.	190	obs:	exp:
cgt	R	0	0.26
cgc	R	0	0.37
cga	R	0	0.71
cgg	R	0	0.57
aat	N	1	0.55
aac	N	0	0.45
aaa	K	1560	1525.00
aag	K	1119	1154.00
aga	R	4	3.27
agg	R	3	1.82
---	--	------	------
mPD		0.49	0.50
		nPD:	0.99
		N. weight:	0.0056
		Sc. PD:	0.0037
		Sc. rank:	7.9

PB2			
Pos.	191	obs:	exp:
aaa	K	4	2.28
aag	K	0	1.72
gat	D	7	5.94
gac	D	4	5.06
gaa	E	1834	1493.00
gag	E	830	1171.00
ggt	G	0	1.16
ggc	G	0	1.05
gga	G	1	3.51
ggg	G	7	2.29
---	--	------	------
mPD		0.44	0.51
		nPD:	0.88
		N. weight:	0.065
		Sc. PD:	0.035
		Sc. rank:	89.3

PB2			
Pos.	192	obs:	exp:
aaa	K	0	1.71
aag	K	3	1.29
gat	D	1	0.54
gac	D	0	0.46
gaa	E	966	1504.00
gag	E	1717	1179.00
---	--	------	------
mPD		0.46	0.50
		nPD:	0.94
		N. weight:	0.14
		Sc. PD:	0.082
		Sc. rank:	190.7

PB2			
Pos.	193	obs:	exp:
ttt	F	0	0.42
ttc	F	1	0.58
tta	L	0	248.20
ttg	L	0	509.90
ctt	L	281	532.90
ctc	L	2393	429.30
cta	L	8	397.20
ctg	L	2	566.50
att	I	0	0.73
atc	I	2	0.54
ata	I	0	0.73
---	--	------	------
mPD		0.20	1.1
		nPD:	0.17
		N. weight:	2.4
		Sc. PD:	-0.34
		Sc. rank:	-875.4

PB2			
Pos.	194	obs:	exp:
cat	H	0	9.26
cac	H	16	6.74
caa	Q	1419	1330.00
cag	Q	1245	1334.00
cgt	R	0	0.04
cgc	R	0	0.05
cga	R	1	0.10
cgg	R	0	0.08
aaa	K	2	3.42
aag	K	4	2.58
aga	R	0	0.47
agg	R	0	0.26
---	--	------	------
mPD		0.51	0.51
		nPD:	1.
		N. weight:	0.017
		Sc. PD:	0.011
		Sc. rank:	24.0

PB2			
Pos.	195	obs:	exp:
tat	Y	0	0.48
tac	Y	1	0.52
aat	N	174	98.16
aac	N	6	81.84
aaa	K	4	2.28
aag	K	0	1.72
gat	D	1515	1226.00
gac	D	756	1045.00
gaa	E	169	94.74
gag	E	0	74.26
ggt	G	37	8.97
ggc	G	1	8.14
gga	G	24	27.17
ggg	G	0	17.72
---	--	------	------
mPD		0.67	0.75
		nPD:	0.91
		N. weight:	0.19
		Sc. PD:	0.11
		Sc. rank:	267.2

PB2			
Pos.	196	obs:	exp:
tct	S	0	0.33
tcc	S	0	0.29
tca	S	0	0.45
tcg	S	0	0.12
tat	Y	1	0.48
tac	Y	0	0.52
tgt	C	2233	1039.00
tgc	C	451	1645.00
agt	S	2	0.43
agc	S	0	0.37
---	--	------	------
mPD		0.28	0.48
		nPD:	0.59
		N. weight:	0.68
		Sc. PD:	0.18
		Sc. rank:	613.4

,

PB2			
Pos.	197	obs:	exp:
cgt	R	0	2.54
cgc	R	0	3.64
cga	R	0	6.85
cgg	R	0	5.55
atg	M	1	1.00
act	T	0	0.26
acc	T	0	0.23
aca	T	1	0.42
acg	T	0	0.09
aat	N	10	5.45
aac	N	0	4.55
aaa	K	545	1484.00
aag	K	2062	1123.00
aga	R	5	31.75
agg	R	63	17.67
---	--	------	------
mPD		0.38	0.56
		nPD:	0.68
		N. weight:	0.48
		Sc. PD:	0.17
		Sc. rank:	492.5

,

PB2			
Pos.	198	obs:	exp:
att	I	2481	984.90
atc	I	167	718.20
ata	I	36	980.80
act	T	1	0.26
acc	T	0	0.23
aca	T	0	0.42
acg	T	0	0.09
gtt	V	2	0.43
gtc	V	0	0.42
gta	V	0	0.43
gtg	V	0	0.72
---	--	------	------
mPD		0.14	0.66
		nPD:	0.22
		N. weight:	1.2
		Sc. PD:	-0.12
		Sc. rank:	-266.4

,

PB2			
Pos.	199	obs:	exp:
tct	S	25	4.17
tcc	S	0	3.62
tca	S	0	5.67
tcg	S	0	1.51
act	T	12	3.15
acc	T	0	2.70
aca	T	0	5.02
acg	T	0	1.13
agt	S	0	5.42
agc	S	0	4.61
gct	A	1816	667.60
gcc	A	809	512.80
gca	A	23	1180.00
gcg	A	2	290.00
---	--	------	------
mPD		0.46	0.72
		nPD:	0.64
		N. weight:	1.3
		Sc. PD:	0.4
		Sc. rank:	1241.6

,

PB2			
Pos.	200	obs:	exp:
cct	P	1245	734.30
ccc	P	1440	485.30
cca	P	2	1091.00
ccg	P	0	376.70
---	--	------	------
mPD		0.50	0.71
		nPD:	0.7
		N. weight:	1.3
		Sc. PD:	0.5
		Sc. rank:	1457.5

,

PB2			
Pos.	201	obs:	exp:
tta	L	1747	248.10
ttg	L	176	509.70
ctt	L	1	532.70
ctc	L	3	429.20
cta	L	616	397.10
ctg	L	140	566.30
att	I	0	1.47
atc	I	0	1.07
ata	I	4	1.46
---	--	------	------
mPD		0.62	1.1
		nPD:	0.55
		N. weight:	2.
		Sc. PD:	0.45
		Sc. rank:	1634.0

,

PB2			
Pos.	202	obs:	exp:
tta	L	0	0.09
ttg	L	0	0.19
ctt	L	0	0.20
ctc	L	0	0.16
cta	L	0	0.15
ctg	L	1	0.21
att	I	0	23.49
atc	I	0	17.13
ata	I	64	23.39
atg	M	2620	2620.00
gtt	V	0	0.43
gtc	V	0	0.42
gta	V	0	0.43
gtg	V	2	0.72
---	--	------	------
mPD		0.049	0.050
		nPD:	0.97
		N. weight:	0.041
		Sc. PD:	0.026
		Sc. rank:	57.7

,

PB2			
Pos.	203	obs:	exp:
tta	L	0	0.09
ttg	L	0	0.19
ctt	L	1	0.20
ctc	L	0	0.16
cta	L	0	0.15
ctg	L	0	0.21
att	I	3	1.10
atc	I	0	0.80
ata	I	0	1.10
gtt	V	1489	576.80
gtc	V	127	564.30
gta	V	12	571.90
gtg	V	1054	969.00
gct	A	1	0.25
gcc	A	0	0.19
gca	A	0	0.45
gcg	A	0	0.11
---	--	------	------
mPD		0.54	0.74
		nPD:	0.73
		N. weight:	0.77
		Sc. PD:	0.31
		Sc. rank:	924.8

,

PB2			
Pos.	204	obs:	exp:
act	T	0	0.26
acc	T	0	0.23
aca	T	1	0.42
acg	T	0	0.09
gct	A	3	676.70
gcc	A	24	519.80
gca	A	2649	1196.00
gcg	A	10	294.00
---	--	------	------
mPD		0.028	0.69
		nPD:	0.04
		N. weight:	1.2
		Sc. PD:	-0.33
		Sc. rank:	-1204.5

,

PB2			
Pos.	205	obs:	exp:
tat	Y	73	1289.00
tac	Y	2614	1398.00
---	--	------	------
mPD		0.053	0.50
		nPD:	0.11
		N. weight:	0.86
		Sc. PD:	-0.18
		Sc. rank:	-558.1

,

PB2			
Pos.	206	obs:	exp:
att	I	0	0.73
atc	I	0	0.54
ata	I	2	0.73
atg	M	2685	2685.00
---	--	------	------
mPD		0.0015	0.0015
		nPD:	1.
		N. weight:	0.0012
		Sc. PD:	0.00081
		Sc. rank:	1.7

,

PB2			
Pos.	207	obs:	exp:
tta	L	11	248.40
ttg	L	2449	510.50
ctt	L	0	533.50
ctc	L	0	429.80
cta	L	181	397.70
ctg	L	46	567.10
---	--	------	------
mPD		0.29	1.1
		nPD:	0.26
		N. weight:	2.1
		Sc. PD:	-0.14
		Sc. rank:	-178.3

,

PB2			
Pos.	208	obs:	exp:
gaa	E	1259	1506.00
gag	E	1428	1181.00
---	--	------	------
mPD		0.50	0.49
		nPD:	1.01
		N. weight:	0.03
		Sc. PD:	0.02
		Sc. rank:	42.8

,

PB2			
Pos.	209	obs:	exp:
cgt	R	0	100.60
cgc	R	0	143.80
cga	R	0	270.80
cgg	R	0	219.40
aga	R	1249	1254.00
agg	R	1438	698.10
---	--	------	------
mPD		0.50	0.95
		nPD:	0.52
		N. weight:	0.63
		Sc. PD:	0.12
		Sc. rank:	490.3

,

PB2			
Pos.	210	obs:	exp:
gaa	E	2446	1506.00
gag	E	241	1181.00
---	--	------	------
mPD		0.16	0.49
		nPD:	0.33
		N. weight:	0.49
		Sc. PD:	0.0051
		Sc. rank:	105.6

,

PB2			
Pos.	211	obs:	exp:
tta	L	110	248.30
ttg	L	112	510.10
ctt	L	6	533.10
ctc	L	0	429.50
cta	L	327	397.40
ctg	L	2130	566.70
atg	M	2	2.00
---	--	------	------
mPD		0.43	1.1
		nPD:	0.38
		N. weight:	1.5
		Sc. PD:	0.091
		Sc. rank:	589.4

,

PB2			
Pos.	212	obs:	exp:
att	I	0	2.57
atc	I	7	1.87
ata	I	0	2.56
gtt	V	1055	576.40
gtc	V	1623	563.80
gta	V	2	571.40
gtg	V	0	968.30
---	--	------	------
mPD		0.48	0.74
		nPD:	0.65
		N. weight:	1.4
		Sc. PD:	0.47
		Sc. rank:	1381.1

,

PB2			
Pos.	213	obs:	exp:
cgt	R	22	100.60
cgc	R	1914	143.80
cga	R	692	270.80
cgg	R	4	219.40
aga	R	55	1254.00
agg	R	0	698.10
---	--	------	------
mPD		0.46	0.95
		nPD:	0.48
		N. weight:	3.3
		Sc. PD:	0.5
		Sc. rank:	2230.7

,

PB2			
Pos.	214	obs:	exp:
caa	Q	1	0.50
cag	Q	0	0.50
cgt	R	0	0.15
cgc	R	0	0.21
cga	R	0	0.40
cgg	R	0	0.33
aaa	K	2492	1527.00
aag	K	190	1155.00
aga	R	4	1.87
agg	R	0	1.04
---	--	------	------
mPD		0.14	0.50
		nPD:	0.27
		N. weight:	0.53
		Sc. PD:	-0.025
		Sc. rank:	-11.7

,

PB2			
Pos.	215	obs:	exp:
cct	P	0	0.27
ccc	P	1	0.18
cca	P	0	0.41
ccg	P	0	0.14
att	I	0	0.37
atc	I	1	0.27
ata	I	0	0.37
act	T	27	703.10
acc	T	2428	604.30
aca	T	62	1122.00
acg	T	165	252.30
aat	N	0	1.64
aac	N	3	1.36
---	--	------	------
mPD		0.18	0.70
		nPD:	0.26
		N. weight:	1.8
		Sc. PD:	-0.12
		Sc. rank:	-168.0

,

PB2

Pos.	216	obs:	exp:
cgt	R	0	100.50
cgc	R	0	143.70
cga	R	0	270.70
cgg	R	0	219.30
aaa	K	1	0.57
aag	K	0	0.43
aga	R	2272	1254.00
agg	R	414	697.80
---	--	------	------
mPD		0.26	0.95
		nPD:	0.27
		N. weight:	0.69
		Sc. PD:	-0.032
		Sc. rank:	-6.9

PB2

Pos.	217	obs:	exp:
ttt	F	174	1121.00
ttc	F	2513	1566.00
---	--	------	------
mPD		0.12	0.49
		nPD:	0.25
		N. weight:	0.52
		Sc. PD:	-0.037
		Sc. rank:	-60.6

PB2

Pos.	218	obs:	exp:
tta	L	103	248.40
ttg	L	1	510.50
ctt	L	0	533.50
ctc	L	196	429.80
cta	L	1941	397.70
ctg	L	446	567.10
---	--	------	------
mPD		0.46	1.1
		nPD:	0.41
		N. weight:	1.6
		Sc. PD:	0.15
		Sc. rank:	849.1

PB2

Pos.	219	obs:	exp:
cct	P	60	734.30
ccc	P	4	485.30
cca	P	1083	1091.00
ccg	P	1540	376.70
---	--	------	------
mPD		0.51	0.71
		nPD:	0.72
		N. weight:	1.2
		Sc. PD:	0.47
		Sc. rank:	1390.6

PB2

Pos.	220	obs:	exp:
att	I	0	3.67
atc	I	0	2.68
ata	I	10	3.65
gtt	V	34	575.80
gtc	V	1	563.20
gta	V	2330	570.80
gtg	V	312	967.20
---	--	------	------
mPD		0.24	0.74
		nPD:	0.32
		N. weight:	1.7
		Sc. PD:	-0.0054
		Sc. rank:	263.5

PB2

Pos.	221	obs:	exp:
tct	S	0	3.34
tcc	S	0	2.90
tca	S	20	4.53
tcg	S	0	1.21
act	T	5	30.93
acc	T	0	26.59
aca	T	113	49.38
acg	T	0	11.10
agt	S	0	4.34
agc	S	0	3.69
gtt	V	9	3.01
gtc	V	0	2.95
gta	V	5	2.99
gtg	V	0	5.06
gct	A	1168	638.40
gcc	A	20	490.40
gca	A	1343	1128.00
gcg	A	3	277.40
gat	D	1	0.54
gac	D	0	0.46
---	--	------	------
mPD		0.61	0.81
		nPD:	0.76
		N. weight:	0.6
		Sc. PD:	0.26
		Sc. rank:	759.7

PB2

Pos.	222	obs:	exp:
cgt	R	0	0.19
cgc	R	5	0.27
cga	R	0	0.50
cgg	R	0	0.41
aga	R	0	2.33
agg	R	0	1.30
ggt	G	267	387.90
ggc	G	2394	352.30
gga	G	20	1175.00
ggg	G	1	766.70
---	--	------	------
mPD		0.20	0.69
		nPD:	0.28
		N. weight:	2.7
		Sc. PD:	-0.095
		Sc. rank:	99.9

PB2

Pos.	223	obs:	exp:
ggt	G	77	388.60
ggc	G	484	352.90
gga	G	1586	1177.00
ggg	G	540	768.10
---	--	------	------
mPD		0.58	0.69
		nPD:	0.84
		N. weight:	0.19
		Sc. PD:	0.099
		Sc. rank:	263.7

PB2

Pos.	224	obs:	exp:
att	I	0	1.83
atc	I	0	1.34
ata	I	5	1.83
act	T	7	703.10
acc	T	1	604.30
aca	T	2667	1122.00
acg	T	7	252.30
---	--	------	------
mPD		0.015	0.70
		nPD:	0.02
		N. weight:	1.4
		Sc. PD:	-0.4
		Sc. rank:	-1584.5

PB2

Pos.	225	obs:	exp:
tct	S	0	445.80
tcc	S	0	386.90
tca	S	0	605.50
tcg	S	0	161.10
cgt	R	0	0.04
cgc	R	1	0.05
cga	R	0	0.10
cgg	R	0	0.08
agt	S	445	579.40
agc	S	2226	492.20
aga	R	0	0.47
agg	R	0	0.26
ggt	G	0	2.17
ggc	G	15	1.97
gga	G	0	6.57
ggg	G	0	4.29
---	--	------	------
mPD		0.29	1.7
		nPD:	0.17
		N. weight:	2.
		Sc. PD:	-0.29
		Sc. rank:	-784.9

PB2

Pos.	226	obs:	exp:
tct	S	0	448.50
tcc	S	0	389.20
tca	S	0	609.10
tcg	S	0	162.10
agt	S	1873	582.80
agc	S	814	495.20
---	--	------	------
mPD		0.42	1.7
		nPD:	0.26
		N. weight:	1.6
		Sc. PD:	-0.1
		Sc. rank:	-153.7

PB2

Pos.	227	obs:	exp:
att	I	0	0.73
atc	I	0	0.54
ata	I	2	0.73
gtt	V	18	576.20
gtc	V	165	563.60
gta	V	763	571.20
gtg	V	1733	967.90
gct	A	0	1.51
gcc	A	0	1.16
gca	A	2	2.67
gcg	A	4	0.66
---	--	------	------
mPD		0.50	0.74
		nPD:	0.68
		N. weight:	0.59
		Sc. PD:	0.21
		Sc. rank:	603.8

PB2

Pos.	228	obs:	exp:
tat	Y	1124	1289.00
tac	Y	1563	1398.00
---	--	------	------
mPD		0.49	0.50
		nPD:	0.98
		N. weight:	0.015
		Sc. PD:	0.0095
		Sc. rank:	20.9

PB2

Pos.	229	obs:	exp:
tct	S	0	0.33
tcc	S	0	0.29
tca	S	0	0.45
tcg	S	0	0.12
att	I	1691	983.80
atc	I	981	717.40
ata	I	9	979.70
agt	S	0	0.43
agc	S	2	0.37
gtt	V	2	0.86
gtc	V	2	0.84
gta	V	0	0.85
gtg	V	0	1.45
---	--	------	------
mPD		0.47	0.67
		nPD:	0.71
		N. weight:	0.72
		Sc. PD:	0.28
		Sc. rank:	811.0

PB2

Pos.	230	obs:	exp:
gaa	E	811	1506.00
gag	E	1876	1181.00
---	--	------	------
mPD		0.42	0.49
		nPD:	0.86
		N. weight:	0.22
		Sc. PD:	0.12
		Sc. rank:	306.5

PB2

Pos.	231	obs:	exp:
att	I	0	23.49
atc	I	0	17.13
ata	I	64	23.39
gtt	V	13	563.90
gtc	V	2	551.60
gta	V	1619	559.10
gtg	V	988	947.30
gct	A	0	0.25
gcc	A	0	0.19
gca	A	1	0.45
gcg	A	0	0.11
---	--	------	------
mPD		0.52	0.78
		nPD:	0.66
		N. weight:	1.1
		Sc. PD:	0.36
		Sc. rank:	1060.0

PB2

Pos.	232	obs:	exp:
tta	L	238	248.40
ttg	L	1283	510.50
ctt	L	0	533.50
ctc	L	0	429.80
cta	L	17	397.70
ctg	L	1149	567.10
---	--	------	------
mPD		0.66	1.1
		nPD:	0.59
		N. weight:	1.2
		Sc. PD:	0.31
		Sc. rank:	1061.8

PB2

Pos.	233	obs:	exp:
tta	L	0	0.09
ttg	L	0	0.19
ctt	L	1	0.20
ctc	L	0	0.16
cta	L	0	0.15
ctg	L	0	0.21
cat	H	2012	1554.00
cac	H	673	1131.00
caa	Q	1	0.50
cag	Q	0	0.50
---	--	------	------
mPD		0.38	0.49
		nPD:	0.77
		N. weight:	0.11
		Sc. PD:	0.047
		Sc. rank:	135.0

PB2

Pos.	234	obs:	exp:
tta	L	159	248.20
ttg	L	2322	509.90
tgg	W	1	1.00
ctt	L	0	532.90
ctc	L	0	429.30
cta	L	2	397.20
ctg	L	201	566.50
gtt	V	0	0.43
gtc	V	0	0.42
gta	V	0	0.43
gtg	V	2	0.72
---	--	------	------
mPD		0.25	1.1
		nPD:	0.23
		N. weight:	2.
		Sc. PD:	-0.18
		Sc. rank:	-335.6

PB2

Pos.	235	obs:	exp:
act	T	2121	704.40
acc	T	562	605.50
aca	T	4	1124.00
acg	T	0	252.80
---	--	------	------
mPD		0.33	0.70
		nPD:	0.48
		N. weight:	1.4
		Sc. PD:	0.21
		Sc. rank:	952.3

PB2			
Pos.	236	obs:	exp:
cat	H	3	2.89
cac	H	2	2.11
caa	Q	2539	1339.00
cag	Q	143	1343.00
---	--	------	------
mPD		0.10	0.50
		nPD:	0.21
		N. weight:	0.79
		Sc. PD:	-0.088
		Sc. rank:	-201.4

PB2			
Pos.	237	obs:	exp:
cgt	R	0	0.07
cgc	R	0	0.11
cga	R	0	0.20
cgg	R	0	0.16
aga	R	1	0.93
agg	R	1	0.52
ggt	G	0	388.30
ggc	G	0	352.70
gga	G	1531	1177.00
ggg	G	1154	767.50
---	--	------	------
mPD		0.49	0.69
		nPD:	0.71
		N. weight:	0.53
		Sc. PD:	0.2
		Sc. rank:	607.4

PB2			
Pos.	238	obs:	exp:
att	I	0	0.37
atc	I	1	0.27
ata	I	0	0.37
act	T	25	704.10
acc	T	2466	605.20
aca	T	194	1124.00
acg	T	1	252.70
---	--	------	------
mPD		0.15	0.70
		nPD:	0.22
		N. weight:	1.8
		Sc. PD:	-0.18
		Sc. rank:	-381.5

PB2			
Pos.	239	obs:	exp:
tgt	C	200	1040.00
tgc	C	2487	1647.00
---	--	------	------
mPD		0.14	0.47
		nPD:	0.29
		N. weight:	0.42
		Sc. PD:	-0.013
		Sc. rank:	21.6

PB2			
Pos.	240	obs:	exp:
tgg	W	2687	2687.00
---	--	------	------
mPD		0	0
		nPD:	1.
		N. weight:	0.
		Sc. PD:	0
		Sc. rank:	0

PB2			
Pos.	241	obs:	exp:
aaa	K	1	0.57
aag	K	0	0.43
gaa	E	1698	1506.00
gag	E	988	1180.00
---	--	------	------
mPD		0.47	0.49
		nPD:	0.94
		N. weight:	0.02
		Sc. PD:	0.012
		Sc. rank:	28.0

PB2			
Pos.	242	obs:	exp:
caa	Q	1084	1342.00
cag	Q	1603	1345.00
---	--	------	------
mPD		0.48	0.50
		nPD:	0.96
		N. weight:	0.033
		Sc. PD:	0.02
		Sc. rank:	45.7

PB2			
Pos.	243	obs:	exp:
tta	L	0	1.76
ttg	L	18	3.61
ctt	L	0	3.77
ctc	L	0	3.04
cta	L	0	2.81
ctg	L	1	4.01
att	I	2	2.20
atc	I	0	1.61
ata	I	4	2.19
atg	M	2658	2658.00
gtt	V	0	0.86
gtc	V	0	0.84
gta	V	0	0.85
gtg	V	4	1.45
---	--	------	------
mPD		0.021	0.032
		nPD:	0.68
		N. weight:	0.022
		Sc. PD:	0.0075
		Sc. rank:	21.8

PB2			
Pos.	244	obs:	exp:
ttt	F	0	0.42
ttc	F	1	0.58
tat	Y	476	1288.00
tac	Y	2210	1398.00
---	--	------	------
mPD		0.29	0.50
		nPD:	0.58
		N. weight:	0.33
		Sc. PD:	0.085
		Sc. rank:	290.0

PB2			
Pos.	245	obs:	exp:
tct	S	1	0.17
tcc	S	0	0.14
tca	S	0	0.23
tcg	S	0	0.06
cct	P	1	0.27
ccc	P	0	0.18
cca	P	0	0.41
ccg	P	0	0.14
atg	M	1	1.00
act	T	1577	703.60
acc	T	89	604.80
aca	T	558	1123.00
acg	T	460	252.50
agt	S	0	0.22
agc	S	0	0.18
---	--	------	------
mPD		0.58	0.70
		nPD:	0.83
		N. weight:	0.6
		Sc. PD:	0.31
		Sc. rank:	817.8

PB2			
Pos.	246	obs:	exp:
cct	P	115	733.80
ccc	P	6	485.00
cca	P	2037	1090.00
ccg	P	527	376.40
caa	Q	2	1.00
cag	Q	0	1.00
---	--	------	------
mPD		0.39	0.71
		nPD:	0.54
		N. weight:	0.74
		Sc. PD:	0.16
		Sc. rank:	594.4

PB2			
Pos.	247	obs:	exp:
tct	S	0	0.50
tcc	S	0	0.43
tca	S	0	0.68
tcg	S	0	0.18
agt	S	0	0.65
agc	S	3	0.55
ggt	G	113	388.10
ggc	G	1355	352.60
gga	G	1209	1176.00
ggg	G	7	767.20
---	--	------	------
mPD		0.54	0.69
		nPD:	0.78
		N. weight:	1.
		Sc. PD:	0.47
		Sc. rank:	1321.2

PB2			
Pos.	248	obs:	exp:
cgt	R	0	0.04
cgc	R	0	0.05
cga	R	0	0.10
cgg	R	0	0.08
aga	R	1	0.47
agg	R	0	0.26
gaa	E	0	0.56
gag	E	1	0.44
ggt	G	10	388.30
ggc	G	0	352.70
gga	G	2010	1177.00
ggg	G	665	767.50
---	--	------	------
mPD		0.38	0.69
		nPD:	0.55
		N. weight:	0.57
		Sc. PD:	0.13
		Sc. rank:	473.6

PB2			
Pos.	249	obs:	exp:
aaa	K	69	48.40
aag	K	16	36.60
gat	D	1	1.08
gac	D	1	0.92
gaa	E	2281	1447.00
gag	E	300	1134.00
ggt	G	0	2.75
ggc	G	0	2.50
gga	G	10	8.33
ggg	G	9	5.43
---	--	------	------
mPD		0.29	0.57
		nPD:	0.51
		N. weight:	0.4
		Sc. PD:	0.073
		Sc. rank:	300.6

PB2			
Pos.	250	obs:	exp:
gtt	V	2	577.70
gtc	V	0	565.10
gta	V	1207	572.70
gtg	V	1477	970.50
gct	A	0	0.25
gcc	A	0	0.19
gca	A	1	0.45
gcg	A	0	0.11
---	--	------	------
mPD		0.50	0.73
		nPD:	0.68
		N. weight:	0.91
		Sc. PD:	0.32
		Sc. rank:	922.9

PB2			
Pos.	251	obs:	exp:
cgt	R	0	98.61
cgc	R	0	141.00
cga	R	0	265.50
cgg	R	0	215.20
aaa	K	50	29.61
aag	K	2	22.39
aga	R	2574	1230.00
agg	R	61	684.60
---	--	------	------
mPD		0.084	0.99
		nPD:	0.08
		N. weight:	1.1
		Sc. PD:	-0.25
		Sc. rank:	-815.1

PB2			
Pos.	252	obs:	exp:
tct	S	0	0.33
tcc	S	0	0.29
tca	S	0	0.45
tcg	S	0	0.12
cat	H	1	0.58
cac	H	0	0.42
aat	N	2438	1464.00
aac	N	246	1220.00
agt	S	0	0.43
agc	S	2	0.37
---	--	------	------
mPD		0.17	0.50
		nPD:	0.34
		N. weight:	0.52
		Sc. PD:	0.0098
		Sc. rank:	128.1

PB2			
Pos.	253	obs:	exp:
aat	N	2	1.64
aac	N	1	1.36
gat	D	1250	1449.00
gac	D	1434	1235.00
---	--	------	------
mPD		0.50	0.50
		nPD:	1.
		N. weight:	0.021
		Sc. PD:	0.014
		Sc. rank:	29.7

PB2			
Pos.	254	obs:	exp:
tat	Y	1	0.48
tac	Y	0	0.52
gtt	V	0	0.22
gtc	V	1	0.21
gta	V	0	0.21
gtg	V	0	0.36
gat	D	2039	1449.00
gac	D	645	1235.00
gaa	E	1	0.56
gag	E	0	0.44
---	--	------	------
mPD		0.37	0.50
		nPD:	0.74
		N. weight:	0.17
		Sc. PD:	0.07
		Sc. rank:	207.3

PB2			
Pos.	255	obs:	exp:
tta	L	0	17.57
ttg	L	0	36.09
ctt	L	190	37.72
ctc	L	0	30.39
cta	L	0	28.12
ctg	L	0	40.10
att	I	21	7.71
atc	I	0	5.62
ata	I	0	7.67
gtt	V	2252	531.90
gtc	V	40	520.30
gta	V	172	527.30
gtg	V	9	893.50
gct	A	3	0.76
gcc	A	0	0.58
gca	A	0	1.34
gcg	A	0	0.33
---	--	------	------
mPD		0.30	0.88
		nPD:	0.34
		N. weight:	2.
		Sc. PD:	0.039
		Sc. rank:	514.5

PB2			
Pos.	256	obs:	exp:
aat	N	0	0.55
aac	N	1	0.45
gat	D	663	1450.00
gac	D	2023	1236.00
---	--	------	------
mPD		0.37	0.50
		nPD:	0.75
		N. weight:	0.29
		Sc. PD:	0.12
		Sc. rank:	360.1

,

PB2			
Pos.	257	obs:	exp:
caa	Q	575	1341.00
cag	Q	2111	1345.00
cgt	R	0	0.04
cgc	R	0	0.05
cga	R	0	0.10
cgg	R	1	0.08
aga	R	0	0.47
agg	R	0	0.26
---	--	------	------
mPD		0.34	0.50
		nPD:	0.67
		N. weight:	0.28
		Sc. PD:	0.098
		Sc. rank:	283.5

,

PB2			
Pos.	258	obs:	exp:
tct	S	0	448.40
tcc	S	0	389.10
tca	S	0	608.90
tcg	S	0	162.00
agt	S	2600	582.60
agc	S	86	495.00
ggt	G	1	0.14
ggc	G	0	0.13
gga	G	0	0.44
ggg	G	0	0.29
---	--	------	------
mPD		0.063	1.7
		nPD:	0.04
		N. weight:	2.3
		Sc. PD:	-0.63
		Sc. rank:	-2298.4

,

PB2			
Pos.	259	obs:	exp:
tta	L	912	248.30
ttg	L	1604	510.30
ctt	L	0	533.30
ctc	L	0	429.60
cta	L	5	397.50
ctg	L	165	566.90
gtt	V	0	0.22
gtc	V	0	0.21
gta	V	0	0.21
gtg	V	1	0.36
---	--	------	------
mPD		0.57	1.1
		nPD:	0.51
		N. weight:	1.7
		Sc. PD:	0.31
		Sc. rank:	1266.0

,

PB2			
Pos.	260	obs:	exp:
att	I	1044	979.40
atc	I	1619	714.20
ata	I	6	975.40
gtt	V	0	3.87
gtc	V	18	3.79
gta	V	0	3.84
gtg	V	0	6.50
---	--	------	------
mPD		0.49	0.68
		nPD:	0.73
		N. weight:	0.84
		Sc. PD:	0.34
		Sc. rank:	992.3

,

PB2			
Pos.	261	obs:	exp:
att	I	1936	962.50
atc	I	497	701.90
ata	I	190	958.60
act	T	23	6.29
acc	T	0	5.41
aca	T	1	10.04
acg	T	0	2.26
gtt	V	40	8.60
gtc	V	0	8.42
gta	V	0	8.53
gtg	V	0	14.45
---	--	------	------
mPD		0.45	0.71
		nPD:	0.64
		N. weight:	0.59
		Sc. PD:	0.18
		Sc. rank:	561.1

,

PB2			
Pos.	262	obs:	exp:
gct	A	2655	676.90
gcc	A	20	520.00
gca	A	5	1196.00
gcg	A	7	294.10
---	--	------	------
mPD		0.024	0.69
		nPD:	0.03
		N. weight:	2.1
		Sc. PD:	-0.6
		Sc. rank:	-2223.3

,

PB2			
Pos.	263	obs:	exp:
tct	S	3	0.50
tcc	S	0	0.43
tca	S	0	0.68
tcg	S	0	0.18
agt	S	0	0.65
agc	S	0	0.55
gct	A	627	676.10
gcc	A	2052	519.40
gca	A	5	1195.00
gcg	A	0	293.80
---	--	------	------
mPD		0.36	0.69
		nPD:	0.53
		N. weight:	1.7
		Sc. PD:	0.34
		Sc. rank:	1319.3

,

PB2			
Pos.	264	obs:	exp:
cgt	R	0	100.60
cgc	R	0	143.80
cga	R	42	270.80
cgg	R	8	219.40
aga	R	2251	1254.00
agg	R	386	698.10
---	--	------	------
mPD		0.29	0.95
		nPD:	0.3
		N. weight:	0.6
		Sc. PD:	-0.012
		Sc. rank:	60.9

,

PB2			
Pos.	265	obs:	exp:
tct	S	0	0.83
tcc	S	0	0.72
tca	S	0	1.13
tcg	S	0	0.30
act	T	0	0.26
acc	T	1	0.23
aca	T	0	0.42
acg	T	0	0.09
aat	N	867	1460.00
aac	N	1811	1218.00
aaa	K	0	1.71
aag	K	3	1.29
agt	S	1	1.08
agc	S	4	0.92
---	--	------	------
mPD		0.44	0.50
		nPD:	0.88
		N. weight:	0.17
		Sc. PD:	0.093
		Sc. rank:	236.3

,

PB2			
Pos.	266	obs:	exp:
att	I	1865	986.00
atc	I	627	719.00
ata	I	195	981.90
---	--	------	------
mPD		0.46	0.66
		nPD:	0.69
		N. weight:	0.48
		Sc. PD:	0.18
		Sc. rank:	504.3

,

PB2			
Pos.	267	obs:	exp:
att	I	0	0.37
atc	I	0	0.27
ata	I	1	0.37
gtt	V	2298	577.70
gtc	V	202	565.10
gta	V	174	572.70
gtg	V	12	970.50
---	--	------	------
mPD		0.26	0.73
		nPD:	0.35
		N. weight:	1.6
		Sc. PD:	0.052
		Sc. rank:	482.8

,

PB2			
Pos.	268	obs:	exp:
cgt	R	0	100.50
cgc	R	0	143.70
cga	R	0	270.70
cgg	R	1	219.30
aaa	K	0	0.57
aag	K	1	0.43
aga	R	526	1254.00
agg	R	2159	697.80
---	--	------	------
mPD		0.32	0.95
		nPD:	0.33
		N. weight:	1.2
		Sc. PD:	0.012
		Sc. rank:	242.0

,

PB2			
Pos.	269	obs:	exp:
cgt	R	0	100.60
cgc	R	0	143.80
cga	R	1	270.80
cgg	R	0	219.40
aga	R	2428	1254.00
agg	R	258	698.10
---	--	------	------
mPD		0.17	0.95
		nPD:	0.18
		N. weight:	0.81
		Sc. PD:	-0.11
		Sc. rank:	-286.4

,

PB2			
Pos.	270	obs:	exp:
tct	S	0	0.17
tcc	S	0	0.14
tca	S	1	0.23
tcg	S	0	0.06
agt	S	0	0.22
agc	S	0	0.18
gct	A	210	676.70
gcc	A	6	519.80
gca	A	2451	1196.00
gcg	A	19	294.00
---	--	------	------
mPD		0.16	0.69
		nPD:	0.23
		N. weight:	0.87
		Sc. PD:	-0.074
		Sc. rank:	-136.2

,

PB2			
Pos.	271	obs:	exp:
att	I	0	0.37
atc	I	0	0.27
ata	I	1	0.37
atg	M	45	45.00
act	T	0	690.80
acc	T	3	593.70
aca	T	1273	1103.00
acg	T	1359	247.90
gct	A	0	1.51
gcc	A	0	1.16
gca	A	6	2.67
gcg	A	0	0.66
---	--	------	------
mPD		0.54	0.74
		nPD:	0.73
		N. weight:	1.5
		Sc. PD:	0.6
		Sc. rank:	1766.9

,

PB2			
Pos.	272	obs:	exp:
gtt	V	2	577.90
gtc	V	4	565.30
gta	V	2586	572.90
gtg	V	95	970.80
---	--	------	------
mPD		0.073	0.73
		nPD:	0.1
		N. weight:	2.2
		Sc. PD:	-0.48
		Sc. rank:	-1491.9

,

PB2			
Pos.	273	obs:	exp:
tct	S	4	448.50
tcc	S	17	389.20
tca	S	2615	609.10
tcg	S	51	162.10
agt	S	0	582.80
agc	S	0	495.20
---	--	------	------
mPD		0.052	1.7
		nPD:	0.03
		N. weight:	2.2
		Sc. PD:	-0.63
		Sc. rank:	-2348.5

,

PB2			
Pos.	274	obs:	exp:
tct	S	0	0.33
tcc	S	0	0.29
tca	S	0	0.45
tcg	S	2	0.12
act	T	0	3.15
acc	T	0	2.70
aca	T	10	5.02
acg	T	2	1.13
agt	S	0	0.43
agc	S	0	0.37
gct	A	4	673.40
gcc	A	3	517.30
gca	A	1426	1190.00
gcg	A	1240	292.60
---	--	------	------
mPD		0.51	0.70
		nPD:	0.73
		N. weight:	1.2
		Sc. PD:	0.49
		Sc. rank:	1465.7

,

PB2			
Pos.	275	obs:	exp:
gat	D	1459	1450.00
gac	D	1227	1236.00
gaa	E	1	0.56
gag	E	0	0.44
---	--	------	------
mPD		0.50	0.50
		nPD:	1.
		N. weight:	0.0029
		Sc. PD:	0.0019
		Sc. rank:	4.1

,

PB2			
Pos.	276	obs:	exp:
cct	P	5	734.30
ccc	P	0	485.30
cca	P	2219	1091.00
ccg	P	463	376.70
---	--	------	------
mPD		0.29	0.71
		nPD:	0.41
		N. weight:	1.
		Sc. PD:	0.085
		Sc. rank:	475.0

PB2			
Pos.	277	obs:	exp:
tta	L	36	248.30
ttg	L	1742	510.30
ctt	L	0	533.30
ctc	L	2	429.60
cta	L	281	397.50
ctg	L	625	566.90
atg	M	1	1.00
---	--	------	------
mPD		0.66	1.1
		nPD:	0.59
		N. weight:	1.2
		Sc. PD:	0.32
		Sc. rank:	1091.8

PB2			
Pos.	278	obs:	exp:
act	T	2	0.52
acc	T	0	0.45
aca	T	0	0.84
acg	T	0	0.19
gtt	V	0	1.08
gtc	V	0	1.05
gta	V	5	1.07
gtg	V	0	1.81
gct	A	841	675.10
gcc	A	1	518.60
gca	A	1823	1193.00
gcg	A	15	293.30
---	--	------	------
mPD		0.44	0.69
		nPD:	0.64
		N. weight:	0.56
		Sc. PD:	0.17
		Sc. rank:	535.5

PB2			
Pos.	279	obs:	exp:
tct	S	180	448.50
tcc	S	3	389.20
tca	S	1939	609.10
tcg	S	565	162.10
agt	S	0	582.80
agc	S	0	495.20
---	--	------	------
mPD		0.43	1.7
		nPD:	0.26
		N. weight:	1.7
		Sc. PD:	-0.099
		Sc. rank:	-123.2

PB2			
Pos.	280	obs:	exp:
tta	L	107	248.30
ttg	L	45	510.30
ctt	L	40	533.30
ctc	L	519	429.60
cta	L	709	397.50
ctg	L	1266	566.90
cat	H	1	0.58
cac	H	0	0.42
---	--	------	------
mPD		0.74	1.1
		nPD:	0.66
		N. weight:	0.75
		Sc. PD:	0.25
		Sc. rank:	733.8

PB2			
Pos.	281	obs:	exp:
tta	L	22	248.40
ttg	L	678	510.50
ctt	L	5	533.50
ctc	L	0	429.80
cta	L	13	397.70
ctg	L	1969	567.10
---	--	------	------
mPD		0.41	1.1
		nPD:	0.37
		N. weight:	1.5
		Sc. PD:	0.074
		Sc. rank:	553.0

PB2			
Pos.	282	obs:	exp:
gaa	E	340	1506.00
gag	E	2347	1181.00
---	--	------	------
mPD		0.22	0.49
		nPD:	0.45
		N. weight:	0.67
		Sc. PD:	0.084
		Sc. rank:	406.3

PB2			
Pos.	283	obs:	exp:
att	I	0	0.37
atc	I	0	0.27
ata	I	1	0.37
atg	M	2686	2686.00
---	--	------	------
mPD		0.00074	0.00074
		nPD:	1.
		N. weight:	0.00061
		Sc. PD:	0.00041
		Sc. rank:	0.9

PB2			
Pos.	284	obs:	exp:
tgt	C	1419	1040.00
tgc	C	1268	1647.00
---	--	------	------
mPD		0.50	0.47
		nPD:	1.05
		N. weight:	0.069
		Sc. PD:	0.049
		Sc. rank:	98.1

PB2			
Pos.	285	obs:	exp:
tat	Y	1	0.48
tac	Y	0	0.52
cat	H	928	1555.00
cac	H	1758	1131.00
---	--	------	------
mPD		0.45	0.49
		nPD:	0.93
		N. weight:	0.18
		Sc. PD:	0.11
		Sc. rank:	254.9

PB2			
Pos.	286	obs:	exp:
tct	S	0	446.50
tcc	S	0	387.50
tca	S	0	606.40
tcg	S	0	161.40
act	T	3	0.79
acc	T	0	0.68
aca	T	0	1.26
acg	T	0	0.28
agt	S	1095	580.20
agc	S	1580	493.00
ggt	G	4	1.30
ggc	G	5	1.18
gga	G	0	3.94
ggg	G	0	2.57
---	--	------	------
mPD		0.49	1.7
		nPD:	0.3
		N. weight:	1.5
		Sc. PD:	-0.035
		Sc. rank:	136.2

PB2			
Pos.	287	obs:	exp:
act	T	11	704.40
acc	T	3	605.50
aca	T	2624	1124.00
acg	T	49	252.80
---	--	------	------
mPD		0.046	0.70
		nPD:	0.07
		N. weight:	1.3
		Sc. PD:	-0.32
		Sc. rank:	-1051.0

PB2			
Pos.	288	obs:	exp:
tta	L	0	0.37
ttg	L	0	0.76
ctt	L	0	0.79
ctc	L	0	0.64
cta	L	1	0.59
ctg	L	3	0.84
caa	Q	2030	1328.00
cag	Q	629	1331.00
cgt	R	0	0.52
cgc	R	0	0.75
cga	R	11	1.41
cgg	R	3	1.14
aaa	K	10	5.69
aag	K	0	4.31
aga	R	0	6.54
agg	R	0	3.64
---	--	------	------
mPD		0.38	0.53
		nPD:	0.72
		N. weight:	0.26
		Sc. PD:	0.1
		Sc. rank:	301.1

PB2			
Pos.	289	obs:	exp:
att	I	2628	986.00
atc	I	52	719.00
ata	I	7	981.90
---	--	------	------
mPD		0.043	0.66
		nPD:	0.07
		N. weight:	1.5
		Sc. PD:	-0.37
		Sc. rank:	-1219.8

PB2			
Pos.	290	obs:	exp:
tct	S	0	0.83
tcc	S	0	0.72
tca	S	0	1.13
tcg	S	0	0.30
agt	S	5	1.08
agc	S	0	0.92
gct	A	1	0.25
gcc	A	0	0.19
gca	A	0	0.45
gcg	A	0	0.11
ggt	G	1348	387.70
ggc	G	1133	352.20
gga	G	41	1175.00
ggg	G	159	766.40
---	--	------	------
mPD		0.57	0.70
		nPD:	0.82
		N. weight:	1.6
		Sc. PD:	0.78
		Sc. rank:	2134.4

PB2			
Pos.	291	obs:	exp:
ggt	G	19	388.60
ggc	G	0	352.90
gga	G	667	1177.00
ggg	G	2001	768.10
---	--	------	------
mPD		0.38	0.69
		nPD:	0.56
		N. weight:	0.9
		Sc. PD:	0.21
		Sc. rank:	751.6

PB2			
Pos.	292	obs:	exp:
tta	L	1	0.28
ttg	L	0	0.57
ctt	L	0	0.60
ctc	L	0	0.48
cta	L	2	0.44
ctg	L	0	0.63
att	I	94	833.70
atc	I	1	608.00
ata	I	2177	830.30
atg	M	65	65.00
act	T	0	43.52
acc	T	0	37.40
aca	T	166	69.46
acg	T	0	15.62
gtt	V	6	38.28
gtc	V	3	37.45
gta	V	169	37.95
gtg	V	0	64.31
gct	A	0	0.76
gcc	A	0	0.58
gca	A	3	1.34
gcg	A	0	0.33
---	--	------	------
mPD		0.37	0.94
		nPD:	0.39
		N. weight:	1.4
		Sc. PD:	0.093
		Sc. rank:	554.8

PB2			
Pos.	293	obs:	exp:
cgt	R	0	100.20
cgc	R	0	143.30
cga	R	0	269.90
cgg	R	3	218.70
atg	M	1	1.00
aaa	K	1	4.56
aag	K	7	3.44
aga	R	513	1250.00
agg	R	2162	695.70
---	--	------	------
mPD		0.32	0.96
		nPD:	0.33
		N. weight:	1.2
		Sc. PD:	0.013
		Sc. rank:	273.8

PB2			
Pos.	294	obs:	exp:
att	I	0	0.37
atc	I	0	0.27
ata	I	1	0.37
atg	M	2686	2686.00
---	--	------	------
mPD		0.00074	0.00074
		nPD:	1.
		N. weight:	0.00061
		Sc. PD:	0.00041
		Sc. rank:	0.9

PB2			
Pos.	295	obs:	exp:
att	I	1	6.97
atc	I	0	5.08
ata	I	18	6.94
atg	M	165	165.00
act	T	1	0.26
acc	T	0	0.23
aca	T	0	0.42
acg	T	0	0.09
aaa	K	0	0.57
aag	K	1	0.43
gtt	V	12	537.90
gtc	V	114	526.20
gta	V	814	533.30
gtg	V	1561	903.60
---	--	------	------
mPD		0.62	0.85
		nPD:	0.73
		N. weight:	0.61
		Sc. PD:	0.24
		Sc. rank:	716.5

PB2			
Pos.	296	obs:	exp:
aat	N	0	2.18
aac	N	4	1.82
gat	D	26	1448.00
gac	D	2656	1234.00
gaa	E	1	0.56
gag	E	0	0.44
---	--	------	------
mPD		0.023	0.50
		nPD:	0.05
		N. weight:	1.2
		Sc. PD:	-0.32
		Sc. rank:	-1136.3

PB2			
Pos.	297	obs:	exp:
tta	L	0	0.09
ttg	L	0	0.19
ctt	L	0	0.20
ctc	L	0	0.16
cta	L	1	0.15
ctg	L	0	0.21
att	I	678	984.60
atc	I	1980	718.00
ata	I	25	980.50
gtt	V	0	0.65
gtc	V	3	0.63
gta	V	0	0.64
gtg	V	0	1.08
---	--	------	------
mPD		0.39	0.66
		nPD:	0.59
		N. weight:	1.
		Sc. PD:	0.27
		Sc. rank:	926.1

PB2			
Pos.	298	obs:	exp:
tta	L	0	248.30
ttg	L	0	510.30
ctt	L	2668	533.30
ctc	L	16	429.60
cta	L	2	397.50
ctg	L	0	566.90
att	I	1	0.37
atc	I	0	0.27
ata	I	0	0.37
---	--	------	------
mPD		0.014	1.1
		nPD:	0.01
		N. weight:	2.6
		Sc. PD:	-0.77
		Sc. rank:	-3207.5

PB2			
Pos.	299	obs:	exp:
cgt	R	0	98.16
cgc	R	0	140.40
cga	R	17	264.30
cgg	R	162	214.20
att	I	0	0.37
atc	I	0	0.27
ata	I	1	0.37
atg	M	2	2.00
act	T	0	0.52
acc	T	0	0.45
aca	T	0	0.84
acg	T	2	0.19
aaa	K	44	33.60
aag	K	15	25.40
aga	R	1217	1225.00
agg	R	1227	681.40
---	--	------	------
mPD		0.67	0.99
		nPD:	0.67
		N. weight:	0.39
		Sc. PD:	0.13
		Sc. rank:	388.0

PB2			
Pos.	300	obs:	exp:
cat	H	3	1.74
cac	H	0	1.26
caa	Q	1960	1340.00
cag	Q	723	1343.00
aaa	K	1	0.57
aag	K	0	0.43
---	--	------	------
mPD		0.40	0.50
		nPD:	0.79
		N. weight:	0.18
		Sc. PD:	0.084
		Sc. rank:	238.0

PB2			
Pos.	301	obs:	exp:
tat	Y	1	0.48
tac	Y	0	0.52
att	I	0	0.37
atc	I	1	0.27
ata	I	0	0.37
aat	N	1524	1464.00
aac	N	1160	1220.00
aaa	K	1	0.57
aag	K	0	0.43
---	--	------	------
mPD		0.49	0.50
		nPD:	0.99
		N. weight:	0.0057
		Sc. PD:	0.0038
		Sc. rank:	8.1

PB2			
Pos.	302	obs:	exp:
tta	L	0	0.09
ttg	L	0	0.19
tct	S	0	0.17
tcc	S	1	0.14
tca	S	0	0.23
tcg	S	0	0.06
ctt	L	0	0.20
ctc	L	0	0.16
cta	L	1	0.15
ctg	L	0	0.21
cct	P	6	733.80
ccc	P	15	485.00
cca	P	2651	1090.00
ccg	P	13	376.40
agt	S	0	0.22
agc	S	0	0.18
---	--	------	------
mPD		0.027	0.71
		nPD:	0.04
		N. weight:	1.4
		Sc. PD:	-0.38
		Sc. rank:	-1369.6

PB2			
Pos.	303	obs:	exp:
cct	P	1	0.27
ccc	P	0	0.18
cca	P	0	0.41
ccg	P	0	0.14
att	I	18	7.34
atc	I	0	5.35
ata	I	2	7.31
act	T	1445	698.90
acc	T	20	600.70
aca	T	1160	1116.00
acg	T	41	250.80
---	--	------	------
mPD		0.53	0.71
		nPD:	0.75
		N. weight:	0.59
		Sc. PD:	0.25
		Sc. rank:	728.5

PB2			
Pos.	304	obs:	exp:
gat	D	4	2.16
gac	D	0	1.84
gaa	E	1120	1504.00
gag	E	1563	1179.00
---	--	------	------
mPD		0.49	0.49
		nPD:	0.99
		N. weight:	0.071
		Sc. PD:	0.046
		Sc. rank:	100.2

PB2			
Pos.	305	obs:	exp:
gct	A	0	0.25
gcc	A	0	0.19
gca	A	1	0.45
gcg	A	0	0.11
gaa	E	1857	1506.00
gag	E	829	1180.00
---	--	------	------
mPD		0.43	0.49
		nPD:	0.87
		N. weight:	0.061
		Sc. PD:	0.033
		Sc. rank:	84.3

PB2			
Pos.	306	obs:	exp:
cct	P	0	0.27
ccc	P	0	0.18
cca	P	1	0.41
ccg	P	0	0.14
caa	Q	2676	1341.00
cag	Q	10	1345.00
---	--	------	------
mPD		0.0082	0.50
		nPD:	0.02
		N. weight:	1.1
		Sc. PD:	-0.33
		Sc. rank:	-1318.0

PB2			
Pos.	307	obs:	exp:
gct	A	1946	676.90
gcc	A	719	520.00
gca	A	22	1196.00
gcg	A	0	294.10
---	--	------	------
mPD		0.40	0.69
		nPD:	0.59
		N. weight:	1.3
		Sc. PD:	0.35
		Sc. rank:	1191.3

PB2			
Pos.	308	obs:	exp:
gtt	V	5	577.90
gtc	V	0	565.30
gta	V	62	572.90
gtg	V	2620	970.80
---	--	------	------
mPD		0.049	0.73
		nPD:	0.07
		N. weight:	1.5
		Sc. PD:	-0.37
		Sc. rank:	-1210.4

PB2			
Pos.	309	obs:	exp:
aat	N	14	8.18
aac	N	1	6.82
gat	D	2434	1434.00
gac	D	222	1222.00
gaa	E	1	0.56
gag	E	0	0.44
ggt	G	15	2.17
ggc	G	0	1.97
gga	G	0	6.57
ggg	G	0	4.29
---	--	------	------
mPD		0.18	0.52
		nPD:	0.33
		N. weight:	0.57
		Sc. PD:	0.0077
		Sc. rank:	136.1

PB2			
Pos.	310	obs:	exp:
att	I	1	986.00
atc	I	0	719.00
ata	I	2686	981.90
---	--	------	------
mPD		0.00074	0.66
		nPD:	0.
		N. weight:	1.6
		Sc. PD:	-0.51
		Sc. rank:	-2290.0

PB2			
Pos.	311	obs:	exp:
tat	Y	0	0.48
tac	Y	1	0.52
tgt	C	123	1040.00
tgc	C	2563	1646.00
---	--	------	------
mPD		0.088	0.48
		nPD:	0.19
		N. weight:	0.53
		Sc. PD:	-0.07
		Sc. rank:	-185.3

PB2			
Pos.	312	obs:	exp:
cgt	R	0	21.97
cgc	R	0	31.41
cga	R	2	59.15
cgg	R	0	47.93
aaa	K	1691	1196.00
aag	K	409	904.20
aga	R	578	274.00
agg	R	7	152.50
---	--	------	------
mPD		0.60	0.96
		nPD:	0.63
		N. weight:	0.41
		Sc. PD:	0.12
		Sc. rank:	383.5

PB2			
Pos.	313	obs:	exp:
gct	A	32	676.90
gcc	A	1	520.00
gca	A	2632	1196.00
gcg	A	22	294.10
---	--	------	------
mPD		0.040	0.69
		nPD:	0.06
		N. weight:	1.2
		Sc. PD:	-0.3
		Sc. rank:	-995.9

PB2			
Pos.	314	obs:	exp:
gct	A	11	676.90
gcc	A	1	520.00
gca	A	2667	1196.00
gcg	A	8	294.10
---	--	------	------
mPD		0.015	0.69
		nPD:	0.02
		N. weight:	1.2
		Sc. PD:	-0.37
		Sc. rank:	-1425.2

PB2			
Pos.	315	obs:	exp:
att	I	0	21.28
atc	I	1	15.52
ata	I	57	21.20
atg	M	2628	2628.00
aaa	K	0	0.57
aag	K	1	0.43
---	--	------	------
mPD		0.043	0.044
		nPD:	0.98
		N. weight:	0.033
		Sc. PD:	0.022
		Sc. rank:	47.5

PB2			
Pos.	316	obs:	exp:
ggt	G	2411	388.60
ggc	G	215	352.90
gga	G	9	1177.00
ggg	G	52	768.10
---	--	------	------
mPD		0.19	0.69
		nPD:	0.27
		N. weight:	2.5
		Sc. PD:	-0.12
		Sc. rank:	-31.3

,

PB2			
Pos.	317	obs:	exp:
tta	L	603	226.70
ttg	L	617	465.80
ctt	L	3	486.80
ctc	L	0	392.20
cta	L	452	362.90
ctg	L	777	517.50
caa	Q	0	0.50
cag	Q	1	0.50
att	I	0	0.37
atc	I	0	0.27
ata	I	1	0.37
atg	M	232	232.00
gtt	V	0	0.22
gtc	V	0	0.21
gta	V	1	0.21
gtg	V	0	0.36
---	--	------	------
mPD		1.1	1.2
		nPD:	0.89
		N. weight:	0.71
		Sc. PD:	0.4
		Sc. rank:	992.0

,

PB2			
Pos.	318	obs:	exp:
cgt	R	0	99.85
cgc	R	0	142.80
cga	R	0	268.90
cgg	R	1	217.90
aaa	K	3	10.82
aag	K	16	8.18
aga	R	659	1246.00
agg	R	2008	693.10
---	--	------	------
mPD		0.39	0.97
		nPD:	0.4
		N. weight:	1.
		Sc. PD:	0.081
		Sc. rank:	472.3

,

PB2			
Pos.	319	obs:	exp:
att	I	364	982.70
atc	I	2307	716.60
ata	I	7	978.70
act	T	1	0.26
acc	T	0	0.23
aca	T	0	0.42
acg	T	0	0.09
gtt	V	0	1.72
gtc	V	8	1.68
gta	V	0	1.71
gtg	V	0	2.89
---	--	------	------
mPD		0.25	0.67
		nPD:	0.37
		N. weight:	1.4
		Sc. PD:	0.066
		Sc. rank:	488.0

,

PB2			
Pos.	320	obs:	exp:
tct	S	0	448.40
tcc	S	0	389.10
tca	S	0	608.90
tcg	S	0	162.00
cgt	R	0	0.04
cgc	R	0	0.05
cga	R	0	0.10
cgg	R	0	0.08
agt	S	1735	582.60
agc	S	951	495.00
aga	R	1	0.47
agg	R	0	0.26
---	--	------	------
mPD		0.46	1.7
		nPD:	0.28
		N. weight:	1.5
		Sc. PD:	-0.065
		Sc. rank:	14.6

,

PB2			
Pos.	321	obs:	exp:
tct	S	141	448.20
tcc	S	2	388.90
tca	S	2532	608.70
tcg	S	10	162.00
cct	P	0	0.55
ccc	P	0	0.36
cca	P	2	0.81
ccg	P	0	0.28
agt	S	0	582.40
agc	S	0	494.80
---	--	------	------
mPD		0.11	1.7
		nPD:	0.07
		N. weight:	2.1
		Sc. PD:	-0.52
		Sc. rank:	-1703.4

,

PB2			
Pos.	322	obs:	exp:
tct	S	218	448.50
tcc	S	2365	389.20
tca	S	96	609.10
tcg	S	8	162.10
agt	S	0	582.80
agc	S	0	495.20
---	--	------	------
mPD		0.22	1.7
		nPD:	0.13
		N. weight:	2.4
		Sc. PD:	-0.44
		Sc. rank:	-1290.2

,

PB2			
Pos.	323	obs:	exp:
ttt	F	1485	1120.00
ttc	F	1200	1565.00
tta	L	0	0.18
ttg	L	0	0.38
ctt	L	0	0.40
ctc	L	2	0.32
cta	L	0	0.30
ctg	L	0	0.42
---	--	------	------
mPD		0.50	0.49
		nPD:	1.02
		N. weight:	0.065
		Sc. PD:	0.045
		Sc. rank:	92.6

,

PB2			
Pos.	324	obs:	exp:
tct	S	0	448.40
tcc	S	0	389.10
tca	S	0	608.90
tcg	S	0	162.00
cgt	R	0	0.04
cgc	R	0	0.05
cga	R	0	0.10
cgg	R	0	0.08
agt	S	1296	582.60
agc	S	1390	495.00
aga	R	1	0.47
agg	R	0	0.26
---	--	------	------
mPD		0.50	1.7
		nPD:	0.3
		N. weight:	1.5
		Sc. PD:	-0.026
		Sc. rank:	176.8

,

PB2			
Pos.	325	obs:	exp:
ttt	F	2648	1112.00
ttc	F	18	1554.00
tta	L	0	1.94
ttg	L	18	3.99
ctt	L	3	4.17
ctc	L	0	3.36
cta	L	0	3.11
ctg	L	0	4.43
---	--	------	------
mPD		0.029	0.50
		nPD:	0.06
		N. weight:	1.4
		Sc. PD:	-0.35
		Sc. rank:	-1196.8

,

PB2			
Pos.	326	obs:	exp:
ggt	G	182	388.60
ggc	G	19	352.90
gga	G	2394	1177.00
ggg	G	92	768.10
---	--	------	------
mPD		0.20	0.69
		nPD:	0.29
		N. weight:	0.8
		Sc. PD:	-0.023
		Sc. rank:	43.1

,

PB2			
Pos.	327	obs:	exp:
tct	S	0	1.84
tcc	S	0	1.59
tca	S	0	2.49
tcg	S	0	0.66
aat	N	0	8.18
aac	N	15	6.82
agt	S	0	2.39
agc	S	11	2.03
ggt	G	2005	384.80
ggc	G	455	349.50
gga	G	188	1166.00
ggg	G	13	760.60
---	--	------	------
mPD		0.44	0.73
		nPD:	0.6
		N. weight:	1.9
		Sc. PD:	0.51
		Sc. rank:	1712.1

,

PB2			
Pos.	328	obs:	exp:
ttt	F	17	1121.00
ttc	F	2670	1566.00
---	--	------	------
mPD		0.013	0.49
		nPD:	0.03
		N. weight:	0.82
		Sc. PD:	-0.24
		Sc. rank:	-916.1

,

PB2			
Pos.	329	obs:	exp:
att	I	41	15.41
atc	I	1	11.24
ata	I	0	15.35
act	T	2505	693.40
acc	T	134	596.00
aca	T	5	1107.00
acg	T	1	248.90
---	--	------	------
mPD		0.13	0.73
		nPD:	0.18
		N. weight:	1.8
		Sc. PD:	-0.25
		Sc. rank:	-650.4

,

PB2			
Pos.	330	obs:	exp:
ttt	F	237	1121.00
ttc	F	2450	1566.00
---	--	------	------
mPD		0.16	0.49
		nPD:	0.33
		N. weight:	0.44
		Sc. PD:	0.0044
		Sc. rank:	87.4

,

PB2			
Pos.	331	obs:	exp:
aaa	K	2549	1529.00
aag	K	137	1157.00
gaa	E	1	0.56
gag	E	0	0.44
---	--	------	------
mPD		0.098	0.49
		nPD:	0.2
		N. weight:	0.61
		Sc. PD:	-0.073
		Sc. rank:	-174.2

,

PB2			
Pos.	332	obs:	exp:
cgt	R	0	100.40
cgc	R	0	143.60
cga	R	12	270.40
cgg	R	4	219.10
aaa	K	2	2.28
aag	K	2	1.72
aga	R	1761	1253.00
agg	R	906	697.00
---	--	------	------
mPD		0.46	0.96
		nPD:	0.48
		N. weight:	0.48
		Sc. PD:	0.077
		Sc. rank:	336.1

,

PB2			
Pos.	333	obs:	exp:
att	I	0	0.37
atc	I	0	0.27
ata	I	1	0.37
act	T	0	704.10
acc	T	0	605.20
aca	T	2650	1124.00
acg	T	36	252.70
---	--	------	------
mPD		0.027	0.70
		nPD:	0.04
		N. weight:	1.3
		Sc. PD:	-0.37
		Sc. rank:	-1341.3

,

PB2			
Pos.	334	obs:	exp:
tct	S	0	447.00
tcc	S	0	387.90
tca	S	0	607.10
tcg	S	0	161.60
aat	N	4	2.18
aac	N	0	1.82
aaa	K	0	1.14
aag	K	2	0.86
agt	S	2224	580.90
agc	S	454	493.50
ggt	G	3	0.43
ggc	G	0	0.39
gga	G	0	1.31
ggg	G	0	0.86
---	--	------	------
mPD		0.29	1.7
		nPD:	0.17
		N. weight:	1.8
		Sc. PD:	-0.26
		Sc. rank:	-680.8

,

PB2			
Pos.	335	obs:	exp:
ggt	G	20	388.60
ggc	G	1	352.90
gga	G	1369	1177.00
ggg	G	1297	768.10
---	--	------	------
mPD		0.51	0.69
		nPD:	0.74
		N. weight:	0.5
		Sc. PD:	0.2
		Sc. rank:	604.9

,

PB2			
Pos.	336	obs:	exp:
ttt	F	2	0.83
ttc	F	0	1.17
tta	L	0	0.09
ttg	L	1	0.19
tct	S	8	445.80
tcc	S	0	386.90
tca	S	2388	605.50
tcg	S	275	161.10
ctt	L	0	0.20
ctc	L	0	0.16
cta	L	0	0.15
ctg	L	0	0.21
cct	P	0	0.27
ccc	P	0	0.18
cca	P	1	0.41
ccg	P	0	0.14
act	T	0	0.26
acc	T	0	0.23
aca	T	1	0.42
acg	T	0	0.09
agt	S	0	579.40
agc	S	0	492.20
gct	A	0	2.77
gcc	A	0	2.13
gca	A	11	4.90
gcg	A	0	1.20
---	--	------	------
mPD		0.20	1.7
		nPD:	0.12
		N. weight:	2.1
		Sc. PD:	-0.4
		Sc. rank:	-1217.6

PB2			
Pos.	337	obs:	exp:
tct	S	1327	448.20
tcc	S	1175	388.90
tca	S	182	608.70
tcg	S	1	162.00
cct	P	0	0.27
ccc	P	1	0.18
cca	P	0	0.41
ccg	P	0	0.14
agt	S	0	582.40
agc	S	0	494.80
gct	A	0	0.25
gcc	A	1	0.19
gca	A	0	0.45
gcg	A	0	0.11
---	--	------	------
mPD		0.56	1.7
		nPD:	0.34
		N. weight:	1.5
		Sc. PD:	0.029
		Sc. rank:	374.5

PB2			
Pos.	338	obs:	exp:
ttt	F	0	6.67
ttc	F	16	9.33
att	I	5	66.05
atc	I	167	48.17
ata	I	8	65.78
act	T	0	1.05
acc	T	4	0.90
aca	T	0	1.67
acg	T	0	0.38
gtt	V	157	532.10
gtc	V	2175	520.50
gta	V	142	527.50
gtg	V	0	893.90
gct	A	0	2.27
gcc	A	9	1.74
gca	A	0	4.01
gcg	A	0	0.99
gat	D	0	2.16
gac	D	4	1.84
---	--	------	------
mPD		0.36	0.89
		nPD:	0.41
		N. weight:	1.8
		Sc. PD:	0.15
		Sc. rank:	861.0

PB2			
Pos.	339	obs:	exp:
cgt	R	0	0.30
cgc	R	0	0.43
cga	R	0	0.81
cgg	R	0	0.65
att	I	0	1.10
atc	I	0	0.80
ata	I	3	1.10
atg	M	40	40.00
act	T	4	321.40
acc	T	0	276.30
aca	T	38	513.00
acg	T	1184	115.40
aat	N	1	1.09
aac	N	1	0.91
aaa	K	1168	782.90
aag	K	207	592.10
aga	R	7	3.73
agg	R	1	2.08
gtt	V	0	0.22
gtc	V	0	0.21
gta	V	0	0.21
gtg	V	1	0.36
gct	A	0	8.06
gcc	A	0	6.19
gca	A	0	14.24
gcg	A	32	3.50
---	--	------	------
mPD		1.0	1.2
		nPD:	0.87
		N. weight:	1.8
		Sc. PD:	0.97
		Sc. rank:	2487.8

PB2			
Pos.	340	obs:	exp:
cgt	R	0	43.52
cgc	R	0	62.24
cga	R	1	117.20
cgg	R	0	94.97
aat	N	0	0.55
aac	N	1	0.45
aaa	K	104	867.20
aag	K	1419	655.80
aga	R	916	542.90
agg	R	246	302.10
---	--	------	------
mPD		0.96	1.2
		nPD:	0.79
		N. weight:	0.79
		Sc. PD:	0.36
		Sc. rank:	1021.5

PB2			
Pos.	341	obs:	exp:
caa	Q	1	0.50
cag	Q	0	0.50
aaa	K	1	0.57
aag	K	0	0.43
gat	D	4	2.16
gac	D	0	1.84
gaa	E	2437	1503.00
gag	E	244	1178.00
---	--	------	------
mPD		0.17	0.50
		nPD:	0.34
		N. weight:	0.48
		Sc. PD:	0.0099
		Sc. rank:	127.6

PB2			
Pos.	342	obs:	exp:
gat	D	1	0.54
gac	D	0	0.46
gaa	E	848	1505.00
gag	E	1837	1180.00
ggt	G	0	0.14
ggc	G	0	0.13
gga	G	0	0.44
ggg	G	1	0.29
---	--	------	------
mPD		0.43	0.49
		nPD:	0.88
		N. weight:	0.2
		Sc. PD:	0.11
		Sc. rank:	279.0

PB2			
Pos.	343	obs:	exp:
gat	D	1	1.62
gac	D	2	1.38
gaa	E	2610	1505.00
gag	E	74	1179.00
---	--	------	------
mPD		0.056	0.49
		nPD:	0.11
		N. weight:	0.75
		Sc. PD:	-0.15
		Sc. rank:	-476.5

PB2			
Pos.	344	obs:	exp:
tta	L	0	5.73
ttg	L	61	11.78
ctt	L	0	12.31
ctc	L	0	9.92
cta	L	0	9.18
ctg	L	1	13.09
att	I	0	2.57
atc	I	0	1.87
ata	I	7	2.56
atg	M	31	31.00
gtt	V	44	556.40
gtc	V	0	544.30
gta	V	26	551.60
gtg	V	2517	934.70
---	--	------	------
mPD		0.13	0.80
		nPD:	0.16
		N. weight:	1.5
		Sc. PD:	-0.23
		Sc. rank:	-650.3

PB2			
Pos.	345	obs:	exp:
ttt	F	2	1.67
ttc	F	2	2.33
tta	L	0	247.40
ttg	L	0	508.40
ctt	L	1704	531.30
ctc	L	951	428.00
cta	L	19	396.00
ctg	L	2	564.80
cat	H	1	0.58
cac	H	0	0.42
att	I	3	2.20
atc	I	1	1.61
ata	I	2	2.19
---	--	------	------
mPD		0.48	1.1
		nPD:	0.42
		N. weight:	1.6
		Sc. PD:	0.16
		Sc. rank:	879.6

PB2			
Pos.	346	obs:	exp:
act	T	226	704.40
acc	T	424	605.50
aca	T	910	1124.00
acg	T	1127	252.80
---	--	------	------
mPD		0.68	0.70
		nPD:	0.97
		N. weight:	0.66
		Sc. PD:	0.42
		Sc. rank:	935.9

PB2			
Pos.	347	obs:	exp:
gct	A	0	0.25
gcc	A	1	0.19
gca	A	0	0.45
gcg	A	0	0.11
ggt	G	19	388.40
ggc	G	2667	352.80
gga	G	0	1177.00
ggg	G	0	767.80
---	--	------	------
mPD		0.015	0.69
		nPD:	0.02
		N. weight:	3.2
		Sc. PD:	-0.95
		Sc. rank:	-3704.3

PB2			
Pos.	348	obs:	exp:
tct	S	0	0.33
tcc	S	0	0.29
tca	S	0	0.45
tcg	S	0	0.12
act	T	0	0.26
acc	T	1	0.23
aca	T	0	0.42
acg	T	0	0.09
aat	N	334	1464.00
aac	N	2350	1220.00
agt	S	0	0.43
agc	S	2	0.37
---	--	------	------
mPD		0.22	0.50
		nPD:	0.44
		N. weight:	0.64
		Sc. PD:	0.075
		Sc. rank:	373.2

PB2			
Pos.	349	obs:	exp:
ttt	F	0	0.42
ttc	F	1	0.58
tta	L	0	248.30
ttg	L	0	510.30
ctt	L	146	533.30
ctc	L	2534	429.60
cta	L	5	397.50
ctg	L	1	566.90
---	--	------	------
mPD		0.11	1.1
		nPD:	0.1
		N. weight:	2.6
		Sc. PD:	-0.57
		Sc. rank:	-1789.4

PB2			
Pos.	350	obs:	exp:
cct	P	0	0.27
ccc	P	0	0.18
cca	P	1	0.41
ccg	P	0	0.14
caa	Q	2474	1341.00
cag	Q	212	1345.00
---	--	------	------
mPD		0.15	0.50
		nPD:	0.29
		N. weight:	0.68
		Sc. PD:	-0.019
		Sc. rank:	38.6

PB2

Pos.	351	obs:	exp:
cct	P	0	0.27
ccc	P	0	0.18
cca	P	1	0.41
ccg	P	0	0.14
act	T	2	697.80
acc	T	0	599.80
aca	T	2644	1114.00
acg	T	16	250.50
gct	A	0	6.05
gcc	A	0	4.64
gca	A	24	10.68
gcg	A	0	2.63
---	--	------	------
mPD		0.032	0.71
		nPD:	0.04
		N. weight:	1.4
		Sc. PD:	-0.37
		Sc. rank:	-1327.5

PB2

Pos.	352	obs:	exp:
tta	L	226	248.30
ttg	L	2243	510.30
tgg	W	1	1.00
ctt	L	0	533.30
ctc	L	0	429.60
cta	L	9	397.50
ctg	L	208	566.90
---	--	------	------
mPD		0.31	1.1
		nPD:	0.28
		N. weight:	1.9
		Sc. PD:	-0.083
		Sc. rank:	-5.6

PB2

Pos.	353	obs:	exp:
cgt	R	0	2.02
cgc	R	0	2.89
cga	R	0	5.44
cgg	R	0	4.41
aaa	K	2623	1499.00
aag	K	10	1134.00
aga	R	52	25.21
agg	R	2	14.03
---	--	------	------
mPD		0.048	0.54
		nPD:	0.09
		N. weight:	0.88
		Sc. PD:	-0.2
		Sc. rank:	-639.9

PB2

Pos.	354	obs:	exp:
tta	L	1	0.28
ttg	L	0	0.57
ctt	L	0	0.60
ctc	L	0	0.48
cta	L	2	0.44
ctg	L	0	0.63
att	I	1	968.00
atc	I	0	705.90
ata	I	2637	964.00
atg	M	12	12.00
gtt	V	6	7.31
gtc	V	0	7.15
gta	V	28	7.25
gtg	V	0	12.28
---	--	------	------
mPD		0.041	0.69
		nPD:	0.06
		N. weight:	1.6
		Sc. PD:	-0.42
		Sc. rank:	-1385.1

PB2

Pos.	355	obs:	exp:
tct	S	0	0.17
tcc	S	0	0.14
tca	S	0	0.23
tcg	S	0	0.06
cgt	R	0	95.58
cgc	R	0	136.70
cga	R	2	257.40
cgg	R	0	208.50
att	I	0	0.37
atc	I	0	0.27
ata	I	1	0.37
act	T	1	0.52
acc	T	0	0.45
aca	T	1	0.84
acg	T	0	0.19
aaa	K	115	68.33
aag	K	5	51.67
agt	S	1	0.22
agc	S	0	0.18
aga	R	2523	1192.00
agg	R	29	663.50
ggt	G	0	1.30
ggc	G	0	1.18
gga	G	9	3.94
ggg	G	0	2.57
---	--	------	------
mPD		0.12	1.0
		nPD:	0.12
		N. weight:	1.1
		Sc. PD:	-0.22
		Sc. rank:	-691.7

PB2

Pos.	356	obs:	exp:
att	I	0	57.25
atc	I	0	41.75
ata	I	156	57.01
atg	M	1	1.00
gtt	V	11	544.20
gtc	V	22	532.30
gta	V	2140	539.50
gtg	V	357	914.10
---	--	------	------
mPD		0.36	0.85
		nPD:	0.43
		N. weight:	1.6
		Sc. PD:	0.17
		Sc. rank:	882.2

PB2

Pos.	357	obs:	exp:
tat	Y	99	58.51
tac	Y	23	63.49
cat	H	2336	1485.00
cac	H	229	1080.00
---	--	------	------
mPD		0.26	0.58
		nPD:	0.45
		N. weight:	0.45
		Sc. PD:	0.055
		Sc. rank:	268.0

PB2

Pos.	358	obs:	exp:
aaa	K	0	0.57
aag	K	1	0.43
gat	D	1	1.08
gac	D	1	0.92
gaa	E	1071	1505.00
gag	E	1613	1179.00
---	--	------	------
mPD		0.48	0.49
		nPD:	0.97
		N. weight:	0.089
		Sc. PD:	0.057
		Sc. rank:	125.3

PB2

Pos.	359	obs:	exp:
ggt	G	5	388.60
ggc	G	2	352.90
gga	G	416	1177.00
ggg	G	2264	768.10
---	--	------	------
mPD		0.27	0.69
		nPD:	0.39
		N. weight:	1.2
		Sc. PD:	0.078
		Sc. rank:	477.8

PB2

Pos.	360	obs:	exp:
ttt	F	1	0.42
ttc	F	0	0.58
tat	Y	2561	1276.00
tac	Y	100	1385.00
tgt	C	1	0.39
tgc	C	0	0.61
cat	H	24	13.89
cac	H	0	10.11
---	--	------	------
mPD		0.091	0.52
		nPD:	0.18
		N. weight:	0.93
		Sc. PD:	-0.13
		Sc. rank:	-342.3

PB2

Pos.	361	obs:	exp:
gaa	E	1273	1506.00
gag	E	1414	1181.00
---	--	------	------
mPD		0.50	0.49
		nPD:	1.01
		N. weight:	0.027
		Sc. PD:	0.018
		Sc. rank:	38.5

PB2

Pos.	362	obs:	exp:
gaa	E	2476	1506.00
gag	E	211	1181.00
---	--	------	------
mPD		0.14	0.49
		nPD:	0.29
		N. weight:	0.53
		Sc. PD:	-0.014
		Sc. rank:	30.8

PB2

Pos.	363	obs:	exp:
ttt	F	63	1121.00
ttc	F	2623	1565.00
tta	L	0	0.09
ttg	L	0	0.19
ctt	L	0	0.20
ctc	L	1	0.16
cta	L	0	0.15
ctg	L	0	0.21
---	--	------	------
mPD		0.047	0.49
		nPD:	0.1
		N. weight:	0.71
		Sc. PD:	-0.16
		Sc. rank:	-504.9

PB2

Pos.	364	obs:	exp:
att	I	0	0.73
atc	I	0	0.54
ata	I	2	0.73
atg	M	4	4.00
act	T	13	702.60
acc	T	0	603.90
aca	T	2652	1121.00
acg	T	15	252.20
gct	A	0	0.25
gcc	A	0	0.19
gca	A	1	0.45
gcg	A	0	0.11
---	--	------	------
mPD		0.029	0.70
		nPD:	0.04
		N. weight:	1.3
		Sc. PD:	-0.36
		Sc. rank:	-1322.1

PB2

Pos.	365	obs:	exp:
att	I	0	2.57
atc	I	0	1.87
ata	I	7	2.56
atg	M	2678	2678.00
gtt	V	0	0.43
gtc	V	0	0.42
gta	V	0	0.43
gtg	V	2	0.72
---	--	------	------
mPD		0.0067	0.0076
		nPD:	0.88
		N. weight:	0.0055
		Sc. PD:	0.003
		Sc. rank:	7.6

PB2

Pos.	366	obs:	exp:
att	I	2	0.73
atc	I	0	0.54
ata	I	0	0.73
gtt	V	2106	577.10
gtc	V	562	564.50
gta	V	7	572.10
gtg	V	8	969.40
gct	A	2	0.50
gcc	A	0	0.39
gca	A	0	0.89
gcg	A	0	0.22
---	--	------	------
mPD		0.34	0.74
		nPD:	0.47
		N. weight:	1.6
		Sc. PD:	0.23
		Sc. rank:	1065.8

PB2

Pos.	367	obs:	exp:
gtt	V	0	0.65
gtc	V	0	0.63
gta	V	0	0.64
gtg	V	3	1.08
ggt	G	0	388.10
ggc	G	0	352.60
gga	G	463	1176.00
ggg	G	2221	767.20
---	--	------	------
mPD		0.29	0.69
		nPD:	0.42
		N. weight:	1.2
		Sc. PD:	0.11
		Sc. rank:	611.7

PB2

Pos.	368	obs:	exp:
cct	P	0	0.82
ccc	P	0	0.54
cca	P	0	1.22
ccg	P	3	0.42
cat	H	3	1.74
cac	H	0	1.26
caa	Q	182	599.10
cag	Q	1018	600.90
cgt	R	0	55.39
cgc	R	0	79.20
cga	R	292	149.10
cgg	R	544	120.90
aaa	K	1	0.57
aag	K	0	0.43
aga	R	391	690.90
agg	R	253	384.50
---	--	------	------
mPD		1.3	1.5
		nPD:	0.86
		N. weight:	0.62
		Sc. PD:	0.33
		Sc. rank:	852.0

PB2

Pos.	369	obs:	exp:
tct	S	0	0.17
tcc	S	0	0.14
tca	S	0	0.23
tcg	S	0	0.06
cgt	R	0	95.62
cgc	R	0	136.70
cga	R	3	257.50
cgg	R	0	208.60
act	T	0	0.26
acc	T	0	0.23
aca	T	1	0.42
acg	T	0	0.09
aaa	K	52	74.02
aag	K	78	55.98
agt	S	0	0.22
agc	S	1	0.18
aga	R	1287	1193.00
agg	R	1265	663.80
---	--	------	------
mPD		0.60	1.0
		nPD:	0.58
		N. weight:	0.55
		Sc. PD:	0.14
		Sc. rank:	480.3

PB2

Pos.	370	obs:	exp:
tct	S	0	0.17
tcc	S	0	0.14
tca	S	1	0.23
tcg	S	0	0.06
agt	S	0	0.22
agc	S	0	0.18
gct	A	130	676.70
gcc	A	3	519.80
gca	A	2541	1196.00
gcg	A	12	294.00
---	--	------	------
mPD		0.10	0.69
		nPD:	0.15
		N. weight:	1.
		Sc. PD:	-0.17
		Sc. rank:	-501.7

PB2			
Pos.	371	obs:	exp:
act	T	5	704.40
acc	T	3	605.50
aca	T	2476	1124.00
acg	T	203	252.80
---	--	------	------
mPD		0.15	0.70
		nPD:	0.21
		N. weight:	1.1
		Sc. PD:	-0.13
		Sc. rank:	-275.6

,

PB2			
Pos.	372	obs:	exp:
tct	S	0	0.17
tcc	S	0	0.14
tca	S	1	0.23
tcg	S	0	0.06
agt	S	0	0.22
agc	S	0	0.18
gct	A	1608	676.70
gcc	A	1071	519.80
gca	A	7	1196.00
gcg	A	0	294.00
---	--	------	------
mPD		0.48	0.69
		nPD:	0.7
		N. weight:	1.3
		Sc. PD:	0.48
		Sc. rank:	1388.3

,

PB2			
Pos.	373	obs:	exp:
tta	L	0	1.66
ttg	L	0	3.42
ctt	L	18	3.57
ctc	L	0	2.88
cta	L	0	2.66
ctg	L	0	3.80
att	I	1227	979.10
atc	I	1431	713.90
ata	I	10	975.00
atg	M	1	1.00
---	--	------	------
mPD		0.52	0.68
		nPD:	0.76
		N. weight:	0.76
		Sc. PD:	0.33
		Sc. rank:	959.6

,

PB2			
Pos.	374	obs:	exp:
tta	L	134	248.20
ttg	L	462	509.90
ctt	L	1	532.90
ctc	L	181	429.30
cta	L	1011	397.20
ctg	L	895	566.50
att	I	0	1.10
atc	I	0	0.80
ata	I	3	1.10
---	--	------	------
mPD		0.91	1.1
		nPD:	0.81
		N. weight:	0.65
		Sc. PD:	0.31
		Sc. rank:	859.9

,

PB2			
Pos.	375	obs:	exp:
tgg	W	1	1.00
cgt	R	0	100.40
cgc	R	0	143.60
cga	R	0	270.40
cgg	R	1	219.10
aaa	K	0	1.71
aag	K	3	1.29
aga	R	34	1253.00
agg	R	2648	697.00
---	--	------	------
mPD		0.029	0.96
		nPD:	0.03
		N. weight:	2.1
		Sc. PD:	-0.59
		Sc. rank:	-2201.5

,

PB2			
Pos.	376	obs:	exp:
cgt	R	0	0.07
cgc	R	0	0.11
cga	R	0	0.20
cgg	R	0	0.16
aaa	K	2528	1528.00
aag	K	156	1156.00
aga	R	2	0.93
agg	R	0	0.52
gaa	E	1	0.56
gag	E	0	0.44
---	--	------	------
mPD		0.11	0.49
		nPD:	0.23
		N. weight:	0.58
		Sc. PD:	-0.054
		Sc. rank:	-110.1

,

PB2			
Pos.	377	obs:	exp:
tct	S	0	0.50
tcc	S	0	0.43
tca	S	3	0.68
tcg	S	0	0.18
agt	S	0	0.65
agc	S	0	0.55
gct	A	126	676.10
gcc	A	2	519.40
gca	A	2545	1195.00
gcg	A	11	293.80
---	--	------	------
mPD		0.10	0.69
		nPD:	0.15
		N. weight:	1.
		Sc. PD:	-0.17
		Sc. rank:	-533.9

,

PB2			
Pos.	378	obs:	exp:
act	T	1483	704.10
acc	T	1201	605.20
aca	T	2	1124.00
acg	T	0	252.70
gct	A	0	0.25
gcc	A	1	0.19
gca	A	0	0.45
gcg	A	0	0.11
---	--	------	------
mPD		0.50	0.70
		nPD:	0.71
		N. weight:	1.2
		Sc. PD:	0.45
		Sc. rank:	1322.0

,

PB2			
Pos.	379	obs:	exp:
tct	S	0	0.17
tcc	S	0	0.14
tca	S	0	0.23
tcg	S	0	0.06
cgt	R	0	100.30
cgc	R	0	143.50
cga	R	2	270.20
cgg	R	0	218.90
aaa	K	5	2.85
aag	K	0	2.15
agt	S	1	0.22
agc	S	0	0.18
aga	R	2106	1252.00
agg	R	573	696.50
---	--	------	------
mPD		0.34	0.96
		nPD:	0.36
		N. weight:	0.59
		Sc. PD:	0.021
		Sc. rank:	189.0

,

PB2			
Pos.	380	obs:	exp:
cgt	R	0	100.30
cgc	R	0	143.40
cga	R	0	270.10
cgg	R	8	218.80
aaa	K	2	3.99
aag	K	5	3.01
aga	R	265	1251.00
agg	R	2407	696.20
---	--	------	------
mPD		0.19	0.96
		nPD:	0.2
		N. weight:	1.5
		Sc. PD:	-0.19
		Sc. rank:	-479.7

,

PB2			
Pos.	381	obs:	exp:
tta	L	0	247.90
ttg	L	366	509.30
ctt	L	4	532.30
ctc	L	0	428.80
cta	L	72	396.80
ctg	L	2239	565.90
atg	M	6	6.00
---	--	------	------
mPD		0.29	1.1
		nPD:	0.26
		N. weight:	1.7
		Sc. PD:	-0.099
		Sc. rank:	-102.2

,

PB2			
Pos.	382	obs:	exp:
att	I	1591	972.80
atc	I	1057	709.40
ata	I	3	968.80
act	T	0	0.26
acc	T	1	0.23
aca	T	0	0.42
acg	T	0	0.09
gtt	V	29	7.53
gtc	V	6	7.36
gta	V	0	7.46
gtg	V	0	12.65
---	--	------	------
mPD		0.51	0.69
		nPD:	0.73
		N. weight:	0.74
		Sc. PD:	0.3
		Sc. rank:	896.1

,

PB2			
Pos.	383	obs:	exp:
caa	Q	1191	1342.00
cag	Q	1496	1345.00
---	--	------	------
mPD		0.49	0.50
		nPD:	0.99
		N. weight:	0.013
		Sc. PD:	0.0083
		Sc. rank:	18.0

,

PB2			
Pos.	384	obs:	exp:
tta	L	159	248.40
ttg	L	2170	510.50
ctt	L	0	533.50
ctc	L	1	429.80
cta	L	176	397.70
ctg	L	181	567.10
---	--	------	------
mPD		0.45	1.1
		nPD:	0.4
		N. weight:	1.6
		Sc. PD:	0.13
		Sc. rank:	762.4

,

PB2			
Pos.	385	obs:	exp:
att	I	2	985.70
atc	I	3	718.80
ata	I	2681	981.60
gtt	V	0	0.22
gtc	V	0	0.21
gta	V	1	0.21
gtg	V	0	0.36
---	--	------	------
mPD		0.0045	0.66
		nPD:	0.01
		N. weight:	1.6
		Sc. PD:	-0.5
		Sc. rank:	-2105.1

,

PB2			
Pos.	386	obs:	exp:
gtt	V	0	577.90
gtc	V	3	565.30
gta	V	2067	572.90
gtg	V	617	970.80
---	--	------	------
mPD		0.36	0.73
		nPD:	0.48
		N. weight:	1.4
		Sc. PD:	0.23
		Sc. rank:	1008.7

,

PB2			
Pos.	387	obs:	exp:
tct	S	0	448.50
tcc	S	0	389.20
tca	S	0	609.10
tcg	S	0	162.10
agt	S	1700	582.80
agc	S	987	495.20
---	--	------	------
mPD		0.46	1.7
		nPD:	0.28
		N. weight:	1.5
		Sc. PD:	-0.058
		Sc. rank:	37.5

,

PB2			
Pos.	388	obs:	exp:
ggt	G	1	388.60
ggc	G	0	352.90
gga	G	2146	1177.00
ggg	G	540	768.10
---	--	------	------
mPD		0.32	0.69
		nPD:	0.47
		N. weight:	0.66
		Sc. PD:	0.096
		Sc. rank:	440.0

,

PB2			
Pos.	389	obs:	exp:
cgt	R	0	88.17
cgc	R	0	126.10
cga	R	83	237.40
cgg	R	2	192.40
att	I	0	0.37
atc	I	0	0.27
ata	I	1	0.37
aaa	K	327	187.90
aag	K	3	142.10
aga	R	2023	1100.00
agg	R	248	612.10
---	--	------	------
mPD		0.45	1.1
		nPD:	0.4
		N. weight:	0.66
		Sc. PD:	0.049
		Sc. rank:	281.5

,

PB2			
Pos.	390	obs:	exp:
act	T	2	0.52
acc	T	0	0.45
aca	T	0	0.84
acg	T	0	0.19
aat	N	290	650.00
aac	N	902	542.00
gat	D	27	805.60
gac	D	1465	686.40
gaa	E	1	0.56
gag	E	0	0.44
---	--	------	------
mPD		0.71	0.99
		nPD:	0.71
		N. weight:	0.76
		Sc. PD:	0.29
		Sc. rank:	853.4

,

PB2			
Pos.	391	obs :	exp :
caa	Q	1	1.00
cag	Q	1	1.00
gtt	V	0	0.22
gtc	V	0	0.21
gta	V	1	0.21
gtg	V	0	0.36
gat	D	16	9.18
gac	D	1	7.82
gaa	E	1488	1494.00
gag	E	1178	1172.00
ggt	G	0	0.14
ggc	G	0	0.13
gga	G	1	0.44
ggg	G	0	0.29
---	--	------	------
mPD		0.50	0.50
		nPD :	1.
		N. weight :	0.0091
		Sc. PD :	0.0061
		Sc. rank :	12.9

,

PB2			
Pos.	392	obs :	exp :
cat	H	3	1.74
cac	H	0	1.26
caa	Q	1507	1340.00
cag	Q	1177	1344.00
---	--	------	------
mPD		0.49	0.50
		nPD :	0.99
		N. weight :	0.016
		Sc. PD :	0.01
		Sc. rank :	22.8

,

PB2			
Pos.	393	obs :	exp :
tct	S	0	447.90
tcc	S	3	388.60
tca	S	2675	608.20
tcg	S	5	161.90
act	T	0	0.79
acc	T	0	0.68
aca	T	3	1.26
acg	T	0	0.28
agt	S	0	582.00
agc	S	0	494.40
gct	A	0	0.25
gcc	A	0	0.19
gca	A	1	0.45
gcg	A	0	0.11
---	--	------	------
mPD		0.0089	1.7
		nPD :	0.01
		N. weight :	2.4
		Sc. PD :	-0.74
		Sc. rank :	-3172.3

,

PB2			
Pos.	394	obs :	exp :
att	I	229	915.20
atc	I	2259	667.40
ata	I	6	911.40
atg	M	1	1.00
act	T	0	1.05
acc	T	4	0.90
aca	T	0	1.67
acg	T	0	0.38
gtt	V	7	40.44
gtc	V	180	39.55
gta	V	1	40.09
gtg	V	0	67.93
---	--	------	------
mPD		0.30	0.81
		nPD :	0.37
		N. weight :	1.6
		Sc. PD :	0.076
		Sc. rank :	573.0

,

PB2			
Pos.	395	obs :	exp :
tct	S	2	0.33
tcc	S	0	0.29
tca	S	0	0.45
tcg	S	0	0.12
act	T	12	3.41
acc	T	1	2.93
aca	T	0	5.44
acg	T	0	1.22
agt	S	0	0.43
agc	S	0	0.37
gtt	V	2	0.43
gtc	V	0	0.42
gta	V	0	0.43
gtg	V	0	0.72
gct	A	1597	672.60
gcc	A	1064	516.70
gca	A	5	1188.00
gcg	A	4	292.20
---	--	------	------
mPD		0.50	0.70
		nPD :	0.71
		N. weight :	1.3
		Sc. PD :	0.49
		Sc. rank :	1416.3

,

PB2			
Pos.	396	obs :	exp :
gaa	E	1051	1506.00
gag	E	1635	1180.00
ggt	G	0	0.14
ggc	G	0	0.13
gga	G	1	0.44
ggg	G	0	0.29
---	--	------	------
mPD		0.48	0.49
		nPD :	0.97
		N. weight :	0.097
		Sc. PD :	0.061
		Sc. rank :	135.8

,

PB2			
Pos.	397	obs :	exp :
act	T	0	0.52
acc	T	0	0.45
aca	T	0	0.84
acg	T	2	0.19
gtt	V	0	0.22
gtc	V	0	0.21
gta	V	1	0.21
gtg	V	0	0.36
gct	A	0	676.10
gcc	A	5	519.40
gca	A	2153	1195.00
gcg	A	526	293.80
---	--	------	------
mPD		0.32	0.69
		nPD :	0.46
		N. weight :	0.94
		Sc. PD :	0.13
		Sc. rank :	610.4

,

PB2			
Pos.	398	obs :	exp :
tta	L	0	0.09
ttg	L	0	0.19
ctt	L	0	0.20
ctc	L	1	0.16
cta	L	0	0.15
ctg	L	0	0.21
att	I	235	972.80
atc	I	2234	709.40
ata	I	182	968.80
atg	M	1	1.00
act	T	0	6.29
acc	T	24	5.41
aca	T	0	10.04
acg	T	0	2.26
gtt	V	0	2.15
gtc	V	9	2.10
gta	V	1	2.13
gtg	V	0	3.61
---	--	------	------
mPD		0.30	0.69
		nPD :	0.44
		N. weight :	1.2
		Sc. PD :	0.14
		Sc. rank :	695.9

,

PB2			
Pos.	399	obs :	exp :
att	I	1567	986.00
atc	I	106	719.00
ata	I	1014	981.90
---	--	------	------
mPD		0.52	0.66
		nPD :	0.78
		N. weight :	0.34
		Sc. PD :	0.15
		Sc. rank :	436.2

,

PB2			
Pos.	400	obs :	exp :
gtt	V	93	576.80
gtc	V	0	564.30
gta	V	1481	571.90
gtg	V	1108	969.00
gct	A	0	1.26
gcc	A	0	0.97
gca	A	5	2.23
gcg	A	0	0.55
---	--	------	------
mPD		0.53	0.74
		nPD :	0.71
		N. weight :	0.84
		Sc. PD :	0.33
		Sc. rank :	969.2

,

PB2			
Pos.	401	obs :	exp :
act	T	0	0.26
acc	T	1	0.23
aca	T	0	0.42
acg	T	0	0.09
gct	A	9	676.70
gcc	A	170	519.80
gca	A	1850	1196.00
gcg	A	657	294.00
---	--	------	------
mPD		0.46	0.69
		nPD :	0.67
		N. weight :	0.68
		Sc. PD :	0.23
		Sc. rank :	671.9

,

PB2			
Pos.	402	obs :	exp :
tta	L	0	2.13
ttg	L	23	4.37
ctt	L	0	4.57
ctc	L	0	3.68
cta	L	0	3.40
ctg	L	0	4.85
att	I	0	0.37
atc	I	0	0.27
ata	I	1	0.37
atg	M	2663	2663.00
---	--	------	------
mPD		0.018	0.028
		nPD :	0.63
		N. weight :	0.024
		Sc. PD :	0.0073
		Sc. rank :	22.4

,

PB2			
Pos.	403	obs :	exp :
gtt	V	143	577.90
gtc	V	0	565.30
gta	V	315	572.90
gtg	V	2229	970.80
---	--	------	------
mPD		0.30	0.73
		nPD :	0.4
		N. weight :	0.89
		Sc. PD :	0.071
		Sc. rank :	414.8

,

PB2			
Pos.	404	obs :	exp :
ttt	F	36	1108.00
ttc	F	2619	1547.00
tta	L	0	2.87
ttg	L	0	5.89
tct	S	0	0.17
tcc	S	1	0.14
tca	S	0	0.23
tcg	S	0	0.06
ctt	L	0	6.16
ctc	L	31	4.96
cta	L	0	4.59
ctg	L	0	6.54
agt	S	0	0.22
agc	S	0	0.18
---	--	------	------
mPD		0.050	0.51
		nPD :	0.1
		N. weight :	0.79
		Sc. PD :	-0.17
		Sc. rank :	-547.2

,

PB2			
Pos.	405	obs :	exp :
tct	S	424	448.00
tcc	S	39	388.80
tca	S	2196	608.50
tcg	S	25	161.90
cct	P	0	0.27
ccc	P	1	0.18
cca	P	0	0.41
ccg	P	0	0.14
act	T	0	0.52
acc	T	0	0.45
aca	T	2	0.84
acg	T	0	0.19
agt	S	0	582.20
agc	S	0	494.60
---	--	------	------
mPD		0.31	1.7
		nPD :	0.19
		N. weight :	1.6
		Sc. PD :	-0.21
		Sc. rank :	-555.5

,

PB2			
Pos.	406	obs:	exp:
caa	Q	1305	1342.00
cag	Q	1382	1345.00
---	--	------	------
mPD		0.50	0.50
		nPD:	1.
		N. weight:	0.0031
		Sc. PD:	0.0021
		Sc. rank:	4.4

,

PB2			
Pos.	407	obs:	exp:
gaa	E	139	1506.00
gag	E	2548	1181.00
---	--	------	------
mPD		0.098	0.49
		nPD:	0.2
		N. weight:	0.99
		Sc. PD:	-0.12
		Sc. rank:	-273.3

,

PB2			
Pos.	408	obs:	exp:
gat	D	2366	1450.00
gac	D	319	1235.00
gaa	E	0	1.12
gag	E	2	0.88
---	--	------	------
mPD		0.21	0.50
		nPD:	0.42
		N. weight:	0.44
		Sc. PD:	0.045
		Sc. rank:	237.0

,

PB2			
Pos.	409	obs:	exp:
tct	S	0	0.17
tcc	S	0	0.14
tca	S	0	0.23
tcg	S	0	0.06
tgt	C	533	1030.00
tgc	C	2128	1631.00
cgt	R	0	0.94
cgc	R	25	1.34
cga	R	0	2.52
cgg	R	0	2.04
agt	S	0	0.22
agc	S	1	0.18
aga	R	0	11.67
agg	R	0	6.49
---	--	------	------
mPD		0.34	0.50
		nPD:	0.67
		N. weight:	0.18
		Sc. PD:	0.061
		Sc. rank:	176.0

,

PB2			
Pos.	410	obs:	exp:
atg	M	2680	2680.00
gtt	V	0	1.51
gtc	V	0	1.47
gta	V	0	1.49
gtg	V	7	2.53
---	--	------	------
mPD		0.0052	0.0085
		nPD:	0.61
		N. weight:	0.0043
		Sc. PD:	0.0012
		Sc. rank:	4.0

,

PB2			
Pos.	411	obs:	exp:
tta	L	0	1.39
ttg	L	0	2.85
ctt	L	0	2.98
ctc	L	0	2.40
cta	L	15	2.22
ctg	L	0	3.17
att	I	43	906.40
atc	I	136	661.00
ata	I	2291	902.60
atg	M	6	6.00
gtt	V	1	42.16
gtc	V	0	41.24
gta	V	156	41.79
gtg	V	39	70.82
---	--	------	------
mPD		0.30	0.83
		nPD:	0.37
		N. weight:	1.2
		Sc. PD:	0.055
		Sc. rank:	408.0

,

PB2			
Pos.	412	obs:	exp:
cgt	R	0	0.07
cgc	R	0	0.11
cga	R	0	0.20
cgg	R	0	0.16
aaa	K	645	1529.00
aag	K	2040	1156.00
aga	R	0	0.93
agg	R	2	0.52
---	--	------	------
mPD		0.37	0.49
		nPD:	0.74
		N. weight:	0.37
		Sc. PD:	0.15
		Sc. rank:	450.8

,

PB2			
Pos.	413	obs:	exp:
gct	A	432	676.90
gcc	A	1	520.00
gca	A	2241	1196.00
gcg	A	13	294.10
---	--	------	------
mPD		0.28	0.69
		nPD:	0.4
		N. weight:	0.71
		Sc. PD:	0.058
		Sc. rank:	334.7

,

PB2			
Pos.	414	obs:	exp:
gtt	V	205	577.90
gtc	V	1456	565.30
gta	V	602	572.90
gtg	V	424	970.80
---	--	------	------
mPD		0.63	0.73
		nPD:	0.85
		N. weight:	0.52
		Sc. PD:	0.27
		Sc. rank:	706.7

,

PB2			
Pos.	415	obs:	exp:
cgt	R	0	100.60
cgc	R	3	143.80
cga	R	2351	270.80
cgg	R	19	219.40
aga	R	303	1254.00
agg	R	11	698.10
---	--	------	------
mPD		0.23	0.95
		nPD:	0.24
		N. weight:	2.8
		Sc. PD:	-0.21
		Sc. rank:	-363.8

,

PB2			
Pos.	416	obs:	exp:
tct	S	0	0.50
tcc	S	0	0.43
tca	S	0	0.68
tcg	S	0	0.18
agt	S	0	0.65
agc	S	3	0.55
gat	D	0	0.54
gac	D	1	0.46
ggt	G	1283	388.00
ggc	G	1394	352.40
gga	G	6	1176.00
ggg	G	0	766.90
---	--	------	------
mPD		0.50	0.69
		nPD:	0.73
		N. weight:	2.1
		Sc. PD:	0.83
		Sc. rank:	2461.6

,

PB2			
Pos.	417	obs:	exp:
aat	N	1	0.55
aac	N	0	0.45
gat	D	2635	1450.00
gac	D	51	1236.00
---	--	------	------
mPD		0.038	0.50
		nPD:	0.08
		N. weight:	0.85
		Sc. PD:	-0.21
		Sc. rank:	-670.4

,

PB2			
Pos.	418	obs:	exp:
tta	L	437	248.30
ttg	L	450	510.30
ctt	L	0	533.30
ctc	L	0	429.60
cta	L	985	397.50
ctg	L	814	566.90
gtt	V	0	0.22
gtc	V	0	0.21
gta	V	0	0.21
gtg	V	1	0.36
---	--	------	------
mPD		0.94	1.1
		nPD:	0.84
		N. weight:	0.84
		Sc. PD:	0.43
		Sc. rank:	1138.5

,

PB2			
Pos.	419	obs:	exp:
tct	S	0	0.17
tcc	S	0	0.14
tca	S	0	0.23
tcg	S	0	0.06
att	I	1	0.37
atc	I	0	0.27
ata	I	0	0.37
aat	N	2332	1464.00
aac	N	353	1221.00
agt	S	1	0.22
agc	S	0	0.18
---	--	------	------
mPD		0.23	0.50
		nPD:	0.46
		N. weight:	0.39
		Sc. PD:	0.054
		Sc. rank:	247.3

,

PB2			
Pos.	420	obs:	exp:
ttt	F	1084	1120.00
ttc	F	1600	1564.00
tat	Y	0	0.48
tac	Y	1	0.52
gtt	V	0	0.43
gtc	V	2	0.42
gta	V	0	0.43
gtg	V	0	0.72
---	--	------	------
mPD		0.48	0.49
		nPD:	0.99
		N. weight:	0.0054
		Sc. PD:	0.0035
		Sc. rank:	7.6

,

PB2			
Pos.	421	obs:	exp:
tta	L	1	0.09
ttg	L	0	0.19
ctt	L	0	0.20
ctc	L	0	0.16
cta	L	0	0.15
ctg	L	0	0.21
att	I	0	0.37
atc	I	0	0.27
ata	I	1	0.37
gtt	V	137	577.50
gtc	V	1059	564.90
gta	V	1442	572.50
gtg	V	47	970.10
---	--	------	------
mPD		0.55	0.73
		nPD:	0.75
		N. weight:	1.
		Sc. PD:	0.43
		Sc. rank:	1255.9

,

PB2			
Pos.	422	obs:	exp:
aat	N	718	1465.00
aac	N	1969	1222.00
---	--	------	------
mPD		0.39	0.50
		nPD:	0.79
		N. weight:	0.26
		Sc. PD:	0.12
		Sc. rank:	337.8

,

PB2			
Pos.	423	obs:	exp:
cgt	R	0	100.60
cgc	R	0	143.80
cga	R	1	270.80
cgg	R	1	219.40
aga	R	2255	1254.00
agg	R	430	698.10
---	--	------	------
mPD		0.27	0.95
		nPD:	0.28
		N. weight:	0.67
		Sc. PD:	-0.024
		Sc. rank:	18.3

,

PB2			
Pos.	424	obs:	exp:
gct	A	5	676.90
gcc	A	1	520.00
gca	A	1944	1196.00
gcg	A	737	294.10
---	--	------	------
mPD		0.40	0.69
		nPD:	0.58
		N. weight:	0.96
		Sc. PD:	0.25
		Sc. rank:	850.9

,

PB2			
Pos.	425	obs:	exp:
cat	H	0	0.58
cac	H	1	0.42
aat	N	833	1465.00
aac	N	1853	1221.00
---	--	------	------
mPD		0.43	0.50
		nPD:	0.86
		N. weight:	0.19
		Sc. PD:	0.099
		Sc. rank:	255.5

,

PB2

Pos.	426	obs:	exp:
cct	P	0	0.27
ccc	P	0	0.18
cca	P	1	0.41
ccg	P	0	0.14
cat	H	0	0.58
cac	H	1	0.42
caa	Q	1508	1341.00
cag	Q	1177	1344.00
---	--	------	------
mPD		0.49	0.50
		nPD:	0.98
		N. weight:	0.016
		Sc. PD:	0.011
		Sc. rank:	23.0

PB2

Pos.	427	obs:	exp:
tta	L	1	0.09
ttg	L	0	0.19
ctt	L	0	0.20
ctc	L	0	0.16
cta	L	0	0.15
ctg	L	0	0.21
cgt	R	0	100.40
cgc	R	0	143.60
cga	R	191	270.50
cgg	R	1017	219.20
att	I	0	0.37
atc	I	0	0.27
ata	I	1	0.37
aaa	K	1	0.57
aag	K	0	0.43
aga	R	1421	1253.00
agg	R	55	697.30
---	--	------	------
mPD		0.98	0.96
		nPD:	1.02
		N. weight:	0.93
		Sc. PD:	0.64
		Sc. rank:	1332.3

PB2

Pos.	428	obs:	exp:
tta	L	1350	248.30
ttg	L	84	510.30
tct	S	0	0.17
tcc	S	0	0.14
tca	S	1	0.23
tcg	S	0	0.06
ctt	L	1	533.30
ctc	L	436	429.60
cta	L	665	397.50
ctg	L	150	566.90
agt	S	0	0.22
agc	S	0	0.18
---	--	------	------
mPD		0.90	1.1
		nPD:	0.8
		N. weight:	1.4
		Sc. PD:	0.66
		Sc. rank:	1819.8

PB2

Pos.	429	obs:	exp:
tct	S	0	0.67
tcc	S	0	0.58
tca	S	0	0.91
tcg	S	0	0.24
tat	Y	1	0.48
tac	Y	0	0.52
aat	N	1569	1463.00
aac	N	1113	1219.00
agt	S	4	0.87
agc	S	0	0.74
---	--	------	------
mPD		0.49	0.50
		nPD:	0.97
		N. weight:	0.012
		Sc. PD:	0.0076
		Sc. rank:	16.7

PB2

Pos.	430	obs:	exp:
tct	S	0	0.67
tcc	S	4	0.58
tca	S	0	0.91
tcg	S	0	0.24
cct	P	616	702.10
ccc	P	1769	464.00
cca	P	184	1043.00
ccg	P	0	360.20
act	T	5	29.89
acc	T	109	25.69
aca	T	0	47.70
acg	T	0	10.73
agt	S	0	0.87
agc	S	0	0.74
---	--	------	------
mPD		0.54	0.79
		nPD:	0.68
		N. weight:	1.3
		Sc. PD:	0.45
		Sc. rank:	1291.6

PB2

Pos.	431	obs:	exp:
atg	M	2687	2687.00
---	--	------	------
mPD		0	0
		nPD:	1.
		N. weight:	0.
		Sc. PD:	0
		Sc. rank:	0

PB2

Pos.	432	obs:	exp:
tat	Y	3	1.44
tac	Y	0	1.56
cct	P	2	0.55
ccc	P	0	0.36
cca	P	0	0.81
ccg	P	0	0.28
cat	H	2534	1551.00
cac	H	146	1129.00
caa	Q	1	0.50
cag	Q	0	0.50
cgt	R	1	0.04
cgc	R	0	0.05
cga	R	0	0.10
cgg	R	0	0.08
aga	R	0	0.47
agg	R	0	0.26
---	--	------	------
mPD		0.11	0.49
		nPD:	0.22
		N. weight:	0.58
		Sc. PD:	-0.058
		Sc. rank:	-124.0

PB2

Pos.	433	obs:	exp:
caa	Q	2227	1342.00
cag	Q	460	1345.00
---	--	------	------
mPD		0.28	0.50
		nPD:	0.57
		N. weight:	0.39
		Sc. PD:	0.094
		Sc. rank:	329.3

PB2

Pos.	434	obs:	exp:
ttt	F	0	0.42
ttc	F	1	0.58
tta	L	0	248.30
ttg	L	0	510.30
ctt	L	157	533.30
ctc	L	2480	429.60
cta	L	47	397.50
ctg	L	2	566.90
---	--	------	------
mPD		0.14	1.1
		nPD:	0.13
		N. weight:	2.4
		Sc. PD:	-0.46
		Sc. rank:	-1383.9

PB2

Pos.	435	obs:	exp:
tta	L	8	248.20
ttg	L	231	509.90
ctt	L	1	532.90
ctc	L	0	429.30
cta	L	18	397.20
ctg	L	2426	566.50
atg	M	2	2.00
gtt	V	0	0.22
gtc	V	0	0.21
gta	V	0	0.21
gtg	V	1	0.36
---	--	------	------
mPD		0.18	1.1
		nPD:	0.16
		N. weight:	2.
		Sc. PD:	-0.3
		Sc. rank:	-850.8

PB2

Pos.	436	obs:	exp:
cgt	R	0	100.60
cgc	R	0	143.80
cga	R	0	270.80
cgg	R	0	219.40
aga	R	1567	1254.00
agg	R	1120	698.10
---	--	------	------
mPD		0.49	0.95
		nPD:	0.51
		N. weight:	0.53
		Sc. PD:	0.099
		Sc. rank:	401.1

PB2

Pos.	437	obs:	exp:
cat	H	2459	1555.00
cac	H	228	1132.00
---	--	------	------
mPD		0.16	0.49
		nPD:	0.32
		N. weight:	0.46
		Sc. PD:	-0.001
		Sc. rank:	71.7

PB2

Pos.	438	obs:	exp:
ttt	F	1447	1120.00
ttc	F	1237	1564.00
tta	L	2	0.18
ttg	L	0	0.38
tgt	C	1	0.39
tgc	C	0	0.61
ctt	L	0	0.40
ctc	L	0	0.32
cta	L	0	0.30
ctg	L	0	0.42
---	--	------	------
mPD		0.50	0.49
		nPD:	1.02
		N. weight:	0.055
		Sc. PD:	0.038
		Sc. rank:	77.7

PB2

Pos.	439	obs:	exp:
caa	Q	2465	1342.00
cag	Q	222	1345.00
---	--	------	------
mPD		0.15	0.50
		nPD:	0.3
		N. weight:	0.67
		Sc. PD:	-0.011
		Sc. rank:	80.4

PB2

Pos.	440	obs:	exp:
aaa	K	79	1530.00
aag	K	2608	1157.00
---	--	------	------
mPD		0.057	0.49
		nPD:	0.12
		N. weight:	1.1
		Sc. PD:	-0.23
		Sc. rank:	-722.6

PB2

Pos.	441	obs:	exp:
aat	N	2	7.09
aac	N	11	5.91
gat	D	1400	1443.00
gac	D	1272	1229.00
ggt	G	1	0.29
ggc	G	1	0.26
gga	G	0	0.88
ggg	G	0	0.57
---	--	------	------
mPD		0.51	0.51
		nPD:	1.
		N. weight:	0.0087
		Sc. PD:	0.0058
		Sc. rank:	12.3

PB2

Pos.	442	obs:	exp:
tct	S	0	0.67
tcc	S	0	0.58
tca	S	4	0.91
tcg	S	0	0.24
act	T	0	0.26
acc	T	0	0.23
aca	T	1	0.42
acg	T	0	0.09
agt	S	0	0.87
agc	S	0	0.74
gct	A	113	675.60
gcc	A	11	519.00
gca	A	2529	1194.00
gcg	A	29	293.60
---	--	------	------
mPD		0.11	0.69
		nPD:	0.16
		N. weight:	0.97
		Sc. PD:	-0.15
		Sc. rank:	-428.0

PB2

Pos.	443	obs:	exp:
cgt	R	0	0.30
cgc	R	0	0.43
cga	R	0	0.81
cgg	R	0	0.65
aaa	K	1112	1525.00
aag	K	1567	1154.00
aga	R	2	3.73
agg	R	6	2.08
---	--	------	------
mPD		0.49	0.50
		nPD:	0.99
		N. weight:	0.084
		Sc. PD:	0.055
		Sc. rank:	118.8

PB2

Pos.	444	obs:	exp:
att	I	0	1.83
atc	I	0	1.34
ata	I	5	1.83
atg	M	14	14.00
gtt	V	4	573.80
gtc	V	0	561.30
gta	V	472	568.90
gtg	V	2192	964.00
---	--	------	------
mPD		0.31	0.75
		nPD:	0.41
		N. weight:	1.
		Sc. PD:	0.093
		Sc. rank:	533.6

PB2

Pos.	445	obs:	exp:
tta	L	197	248.30
ttg	L	57	510.10
ctt	L	183	533.10
ctc	L	0	429.50
cta	L	1595	397.40
ctg	L	653	566.70
cct	P	0	0.27
ccc	P	0	0.18
cca	P	1	0.41
ccg	P	0	0.14
gtt	V	0	0.22
gtc	V	0	0.21
gta	V	0	0.21
gtg	V	1	0.36
---	--	------	------
mPD		0.65	1.1
		nPD:	0.58
		N. weight:	1.2
		Sc. PD:	0.3
		Sc. rank:	1039.2

PB2			
Pos.	446	obs:	exp:
ttt	F	1815	1113.00
ttc	F	853	1555.00
tta	L	0	1.76
ttg	L	0	3.61
ctt	L	18	3.77
ctc	L	1	3.04
cta	L	0	2.81
ctg	L	0	4.01
---	--	------	------
mPD		0.45	0.50
		nPD:	0.89
		N. weight:	0.25
		Sc. PD:	0.14
		Sc. rank:	343.2

,

PB2			
Pos.	447	obs:	exp:
tta	L	1	0.18
ttg	L	0	0.38
ctt	L	0	0.40
ctc	L	0	0.32
cta	L	1	0.30
ctg	L	0	0.42
cat	H	13	8.10
cac	H	1	5.90
caa	Q	1445	1333.00
cag	Q	1225	1337.00
aaa	K	1	0.57
aag	K	0	0.43
---	--	------	------
mPD		0.50	0.51
		nPD:	0.99
		N. weight:	0.014
		Sc. PD:	0.0092
		Sc. rank:	19.7

,

PB2			
Pos.	448	obs:	exp:
tct	S	0	0.67
tcc	S	0	0.58
tca	S	0	0.91
tcg	S	0	0.24
cat	H	1	0.58
cac	H	0	0.42
act	T	0	0.79
acc	T	3	0.68
aca	T	0	1.26
acg	T	0	0.28
aat	N	1507	1461.00
aac	N	1172	1218.00
agt	S	4	0.87
agc	S	0	0.74
---	--	------	------
mPD		0.50	0.50
		nPD:	0.99
		N. weight:	0.01
		Sc. PD:	0.0067
		Sc. rank:	14.5

,

PB2			
Pos.	449	obs:	exp:
tgg	W	2686	2686.00
ggt	G	0	0.14
ggc	G	0	0.13
gga	G	0	0.44
ggg	G	1	0.29
---	--	------	------
mPD		0.00074	0.0013
		nPD:	0.58
		N. weight:	0.00076
		Sc. PD:	0.0002
		Sc. rank:	0.7

,

PB2			
Pos.	450	obs:	exp:
gaa	E	1	0.56
gag	E	0	0.44
ggt	G	0	388.40
ggc	G	0	352.80
gga	G	2542	1177.00
ggg	G	144	767.80
---	--	------	------
mPD		0.10	0.69
		nPD:	0.15
		N. weight:	1.
		Sc. PD:	-0.18
		Sc. rank:	-525.1

,

PB2			
Pos.	451	obs:	exp:
tta	L	0	0.09
ttg	L	0	0.19
ctt	L	1	0.20
ctc	L	0	0.16
cta	L	0	0.15
ctg	L	0	0.21
att	I	2041	750.80
atc	I	4	547.50
ata	I	1	747.70
act	T	320	83.89
acc	T	0	72.10
aca	T	0	133.90
acg	T	0	30.11
aat	N	3	1.64
aac	N	0	1.36
gtt	V	316	68.18
gtc	V	1	66.69
gta	V	0	67.59
gtg	V	0	114.50
---	--	------	------
mPD		0.43	1.1
		nPD:	0.38
		N. weight:	1.8
		Sc. PD:	0.11
		Sc. rank:	685.8

,

PB2			
Pos.	452	obs:	exp:
gaa	E	2552	1506.00
gag	E	135	1181.00
---	--	------	------
mPD		0.095	0.49
		nPD:	0.19
		N. weight:	0.64
		Sc. PD:	-0.08
		Sc. rank:	-210.0

,

PB2			
Pos.	453	obs:	exp:
tta	L	0	0.18
ttg	L	0	0.38
tct	S	0	4.17
tcc	S	19	3.62
tca	S	6	5.67
tcg	S	0	1.51
ctt	L	0	0.40
ctc	L	2	0.32
cta	L	0	0.30
ctg	L	0	0.42
cct	P	51	699.60
ccc	P	2390	462.40
cca	P	119	1039.00
ccg	P	0	358.90
cat	H	1	1.16
cac	H	1	0.84
act	T	0	25.17
acc	T	96	21.63
aca	T	0	40.17
acg	T	0	9.03
agt	S	0	5.42
agc	S	0	4.61
gct	A	0	0.50
gcc	A	2	0.39
gca	A	0	0.89
gcg	A	0	0.22
---	--	------	------
mPD		0.22	0.81
		nPD:	0.27
		N. weight:	2.2
		Sc. PD:	-0.12
		Sc. rank:	-76.6

,

PB2			
Pos.	454	obs:	exp:
tta	L	0	0.09
ttg	L	0	0.19
ctt	L	1	0.20
ctc	L	0	0.16
cta	L	0	0.15
ctg	L	0	0.21
att	I	2637	982.40
atc	I	40	716.40
ata	I	0	978.30
act	T	3	0.79
acc	T	0	0.68
aca	T	0	1.26
acg	T	0	0.28
gtt	V	6	1.29
gtc	V	0	1.26
gta	V	0	1.28
gtg	V	0	2.17
---	--	------	------
mPD		0.037	0.67
		nPD:	0.06
		N. weight:	1.5
		Sc. PD:	-0.4
		Sc. rank:	-1368.8

,

PB2			
Pos.	455	obs:	exp:
gct	A	0	0.76
gcc	A	3	0.58
gca	A	0	1.34
gcg	A	0	0.33
gat	D	1379	1448.00
gac	D	1302	1233.00
gaa	E	1	1.68
gag	E	2	1.32
---	--	------	------
mPD		0.50	0.50
		nPD:	1.
		N. weight:	0.0083
		Sc. PD:	0.0056
		Sc. rank:	11.8

,

PB2			
Pos.	456	obs:	exp:
tct	S	0	3.67
tcc	S	0	3.19
tca	S	0	4.99
tcg	S	0	1.33
tat	Y	1	0.48
tac	Y	0	0.52
aat	N	2615	1439.00
aac	N	24	1200.00
aaa	K	1	0.57
aag	K	0	0.43
agt	S	22	4.77
agc	S	0	4.05
gat	D	24	12.96
gac	D	0	11.04
---	--	------	------
mPD		0.053	0.54
		nPD:	0.1
		N. weight:	0.92
		Sc. PD:	-0.2
		Sc. rank:	-628.7

,

PB2			
Pos.	457	obs:	exp:
att	I	0	5.50
atc	I	14	4.01
ata	I	1	5.48
gtt	V	11	574.70
gtc	V	2159	562.20
gta	V	348	569.70
gtg	V	154	965.40
---	--	------	------
mPD		0.34	0.75
		nPD:	0.45
		N. weight:	1.5
		Sc. PD:	0.19
		Sc. rank:	898.9

,

PB2			
Pos.	458	obs:	exp:
tta	L	0	0.18
ttg	L	0	0.38
ctt	L	0	0.40
ctc	L	0	0.32
cta	L	0	0.30
ctg	L	2	0.42
atg	M	2685	2685.00
---	--	------	------
mPD		0.0015	0.0024
		nPD:	0.63
		N. weight:	0.0019
		Sc. PD:	0.00056
		Sc. rank:	1.8

,

PB2			
Pos.	459	obs:	exp:
ggt	G	4	388.60
ggc	G	0	352.90
gga	G	594	1177.00
ggg	G	2089	768.10
---	--	------	------
mPD		0.35	0.69
		nPD:	0.5
		N. weight:	1.
		Sc. PD:	0.18
		Sc. rank:	741.5

,

PB2			
Pos.	460	obs:	exp:
atg	M	2687	2687.00
---	--	------	------
mPD		0	0
		nPD:	1.
		N. weight:	0.
		Sc. PD:	0
		Sc. rank:	0

,

PB2			
Pos.	461	obs:	exp:
att	I	185	961.10
atc	I	1744	700.80
ata	I	690	957.10
atg	M	1	1.00
act	T	0	0.26
acc	T	1	0.23
aca	T	0	0.42
acg	T	0	0.09
gtt	V	10	14.20
gtc	V	52	13.89
gta	V	4	14.07
gtg	V	0	23.85
---	--	------	------
mPD		0.53	0.72
		nPD:	0.74
		N. weight:	0.68
		Sc. PD:	0.28
		Sc. rank:	833.2

PB2			
Pos.	462	obs:	exp:
cgt	R	0	0.07
cgc	R	0	0.11
cga	R	0	0.20
cgg	R	0	0.16
aga	R	2	0.93
agg	R	0	0.52
ggt	G	1	388.30
ggc	G	0	352.70
gga	G	1718	1177.00
ggg	G	966	767.50
---	--	------	------
mPD		0.46	0.69
		nPD:	0.67
		N. weight:	0.53
		Sc. PD:	0.18
		Sc. rank:	523.2

PB2			
Pos.	463	obs:	exp:
tta	L	10	1.39
ttg	L	0	2.85
ctt	L	0	2.98
ctc	L	0	2.40
cta	L	5	2.22
ctg	L	0	3.17
att	I	0	922.20
atc	I	0	672.50
ata	I	2513	918.40
atg	M	14	14.00
gtt	V	0	31.19
gtc	V	0	30.51
gta	V	145	30.92
gtg	V	0	52.39
---	--	------	------
mPD		0.12	0.79
		nPD:	0.16
		N. weight:	1.7
		Sc. PD:	-0.27
		Sc. rank:	-766.6

PB2			
Pos.	464	obs:	exp:
tta	L	1134	240.60
ttg	L	759	494.30
tct	S	0	0.17
tcc	S	0	0.14
tca	S	1	0.23
tcg	S	0	0.06
ctt	L	0	516.60
ctc	L	0	416.20
cta	L	569	385.10
ctg	L	140	549.20
atg	M	84	84.00
agt	S	0	0.22
agc	S	0	0.18
---	--	------	------
mPD		0.90	1.2
		nPD:	0.78
		N. weight:	1.3
		Sc. PD:	0.58
		Sc. rank:	1646.8

PB2			
Pos.	465	obs:	exp:
tct	S	6	1.34
tcc	S	2	1.16
tca	S	0	1.81
tcg	S	0	0.48
cct	P	1924	730.00
ccc	P	731	482.40
cca	P	16	1084.00
ccg	P	0	374.50
cat	H	8	4.63
cac	H	0	3.37
agt	S	0	1.74
agc	S	0	1.47
---	--	------	------
mPD		0.42	0.72
		nPD:	0.58
		N. weight:	1.3
		Sc. PD:	0.32
		Sc. rank:	1116.2

PB2			
Pos.	466	obs:	exp:
gat	D	566	1450.00
gac	D	2119	1235.00
ggt	G	1	0.29
ggc	G	1	0.26
gga	G	0	0.88
ggg	G	0	0.57
---	--	------	------
mPD		0.33	0.50
		nPD:	0.67
		N. weight:	0.37
		Sc. PD:	0.13
		Sc. rank:	369.7

PB2			
Pos.	467	obs:	exp:
tta	L	0	2.31
ttg	L	19	4.75
ctt	L	0	4.96
ctc	L	0	4.00
cta	L	0	3.70
ctg	L	6	5.28
atg	M	2662	2662.00
---	--	------	------
mPD		0.018	0.030
		nPD:	0.62
		N. weight:	0.017
		Sc. PD:	0.0052
		Sc. rank:	16.4

PB2			
Pos.	468	obs:	exp:
act	T	1493	703.90
acc	T	927	605.00
aca	T	265	1123.00
acg	T	0	252.60
gct	A	2	0.50
gcc	A	0	0.39
gca	A	0	0.89
gcg	A	0	0.22
---	--	------	------
mPD		0.56	0.70
		nPD:	0.81
		N. weight:	0.69
		Sc. PD:	0.33
		Sc. rank:	918.2

PB2			
Pos.	469	obs:	exp:
tct	S	0	0.17
tcc	S	1	0.14
tca	S	0	0.23
tcg	S	0	0.06
cct	P	30	734.10
ccc	P	2239	485.10
cca	P	417	1090.00
ccg	P	0	376.60
agt	S	0	0.22
agc	S	0	0.18
---	--	------	------
mPD		0.28	0.71
		nPD:	0.4
		N. weight:	1.8
		Sc. PD:	0.13
		Sc. rank:	797.1

PB2			
Pos.	470	obs:	exp:
tct	S	0	415.80
tcc	S	0	360.80
tca	S	0	564.70
tcg	S	0	150.30
aat	N	2	106.90
aac	N	194	89.11
agt	S	90	540.30
agc	S	2401	459.10
---	--	------	------
mPD		0.20	1.7
		nPD:	0.12
		N. weight:	2.4
		Sc. PD:	-0.48
		Sc. rank:	-1485.5

PB2			
Pos.	471	obs:	exp:
tct	S	0	0.67
tcc	S	0	0.58
tca	S	0	0.91
tcg	S	4	0.24
cct	P	0	0.55
ccc	P	0	0.36
cca	P	2	0.81
ccg	P	0	0.28
att	I	2	1.83
atc	I	0	1.34
ata	I	3	1.83
act	T	191	699.70
acc	T	18	601.40
aca	T	2227	1117.00
acg	T	233	251.10
agt	S	0	0.87
agc	S	0	0.74
gct	A	1	1.76
gcc	A	0	1.35
gca	A	6	3.12
gcg	A	0	0.77
---	--	------	------
mPD		0.31	0.71
		nPD:	0.43
		N. weight:	0.75
		Sc. PD:	0.082
		Sc. rank:	420.3

PB2			
Pos.	472	obs:	exp:
aaa	K	2	1.14
aag	K	0	0.86
gat	D	4	2.70
gac	D	1	2.30
gaa	E	1625	1498.00
gag	E	1048	1175.00
ggt	G	0	1.01
ggc	G	0	0.92
gga	G	6	3.07
ggg	G	1	2.00
---	--	------	------
mPD		0.49	0.50
		nPD:	0.97
		N. weight:	0.014
		Sc. PD:	0.009
		Sc. rank:	19.9

PB2			
Pos.	473	obs:	exp:
tta	L	5	72.30
ttg	L	119	148.60
ctt	L	0	155.30
ctc	L	0	125.10
cta	L	21	115.70
ctg	L	637	165.10
caa	Q	2	1.50
cag	Q	1	1.50
att	I	0	4.77
atc	I	0	3.48
ata	I	13	4.75
atg	M	1729	1729.00
act	T	0	16.25
acc	T	0	13.97
aca	T	54	25.94
acg	T	8	5.83
gtt	V	0	21.08
gtc	V	0	20.62
gta	V	0	20.90
gtg	V	98	35.41
---	--	------	------
mPD		0.60	0.92
		nPD:	0.65
		N. weight:	0.57
		Sc. PD:	0.19
		Sc. rank:	553.0

PB2			
Pos.	474	obs:	exp:
tct	S	14	448.50
tcc	S	2	389.20
tca	S	2589	609.10
tcg	S	82	162.10
agt	S	0	582.80
agc	S	0	495.20
---	--	------	------
mPD		0.071	1.7
		nPD:	0.04
		N. weight:	2.2
		Sc. PD:	-0.6
		Sc. rank:	-2172.2

PB2			
Pos.	475	obs:	exp:
tta	L	13	248.00
ttg	L	379	509.50
ctt	L	2	532.50
ctc	L	0	429.00
cta	L	771	396.90
ctg	L	1517	566.10
atg	M	5	5.00
---	--	------	------
mPD		0.67	1.1
		nPD:	0.59
		N. weight:	1.1
		Sc. PD:	0.3
		Sc. rank:	1021.1

PB2			
Pos.	476	obs:	exp:
cgt	R	0	100.60
cgc	R	0	143.80
cga	R	2	270.80
cgg	R	0	219.40
aga	R	2306	1254.00
agg	R	379	698.10
---	--	------	------
mPD		0.24	0.95
		nPD:	0.26
		N. weight:	0.71
		Sc. PD:	-0.045
		Sc. rank:	-76.0

PB2			
Pos.	477	obs:	exp:
cgt	R	0	0.45
cgc	R	0	0.64
cga	R	0	1.21
cgg	R	0	0.98
aga	R	12	5.60
agg	R	0	3.12
ggt	G	3	386.80
ggc	G	3	351.40
gga	G	2517	1172.00
ggg	G	152	764.70
---	--	------	------
mPD		0.12	0.70
		nPD:	0.17
		N. weight:	1.
		Sc. PD:	-0.15
		Sc. rank:	-411.0

PB2			
Pos.	478	obs:	exp:
tta	L	0	0.18
ttg	L	2	0.38
ctt	L	0	0.40
ctc	L	0	0.32
cta	L	0	0.30
ctg	L	0	0.42
att	I	0	76.70
atc	I	0	55.93
ata	I	209	76.38
gtt	V	0	532.50
gtc	V	0	520.90
gta	V	893	527.90
gtg	V	1583	894.60
---	--	------	------
mPD		0.63	0.89
		nPD:	0.71
		N. weight:	0.96
		Sc. PD:	0.37
		Sc. rank:	1077.2

PB2			
Pos.	479	obs:	exp:
cgt	R	0	100.50
cgc	R	0	143.70
cga	R	5	270.70
cgg	R	4	219.30
aaa	K	1	0.57
aag	K	0	0.43
aga	R	2200	1254.00
agg	R	477	697.80
---	--	------	------
mPD		0.30	0.95
		nPD:	0.32
		N. weight:	0.62
		Sc. PD:	-0.0031
		Sc. rank:	88.4

PB2			
Pos.	480	obs:	exp:
ttt	F	1	0.42
ttc	F	0	0.58
att	I	80	32.29
atc	I	8	23.55
ata	I	0	32.16
gtt	V	1383	558.80
gtc	V	1204	546.60
gta	V	8	554.00
gtg	V	3	938.70
---	--	------	------
mPD		0.56	0.80
		nPD:	0.7
		N. weight:	1.3
		Sc. PD:	0.51
		Sc. rank:	1471.6

PB2			
Pos.	481	obs:	exp:
tct	S	0	448.50
tcc	S	0	389.20
tca	S	0	609.10
tcg	S	0	162.10
agt	S	2316	582.80
agc	S	371	495.20
---	--	------	------
mPD		0.24	1.7
		nPD:	0.14
		N. weight:	1.9
		Sc. PD:	-0.32
		Sc. rank:	-991.6

,

PB2			
Pos.	482	obs:	exp:
cgt	R	0	1.01
cgc	R	0	1.44
cga	R	0	2.72
cgg	R	0	2.20
att	I	0	0.37
atc	I	0	0.27
ata	I	1	0.37
act	T	0	0.26
acc	T	0	0.23
aca	T	1	0.42
acg	T	0	0.09
aaa	K	2536	1513.00
aag	K	122	1145.00
aga	R	27	12.60
agg	R	0	7.01
---	--	------	------
mPD		0.11	0.52
		nPD:	0.21
		N. weight:	0.64
		Sc. PD:	-0.071
		Sc. rank:	-154.4

,

PB2			
Pos.	483	obs:	exp:
att	I	0	3.30
atc	I	0	2.41
ata	I	9	3.29
atg	M	2636	2636.00
act	T	0	2.36
acc	T	0	2.03
aca	T	0	3.77
acg	T	9	0.85
aaa	K	0	0.57
aag	K	1	0.43
gtt	V	0	6.88
gtc	V	0	6.73
gta	V	0	6.82
gtg	V	32	11.56
---	--	------	------
mPD		0.038	0.059
		nPD:	0.64
		N. weight:	0.038
		Sc. PD:	0.012
		Sc. rank:	36.7

,

PB2			
Pos.	484	obs:	exp:
cgt	R	0	0.07
cgc	R	0	0.11
cga	R	2	0.20
cgg	R	0	0.16
aga	R	0	0.93
agg	R	0	0.52
ggt	G	3	388.30
ggc	G	0	352.70
gga	G	2520	1177.00
ggg	G	162	767.50
---	--	------	------
mPD		0.12	0.69
		nPD:	0.17
		N. weight:	1.
		Sc. PD:	-0.15
		Sc. rank:	-414.3

,

PB2			
Pos.	485	obs:	exp:
att	I	0	0.37
atc	I	0	0.27
ata	I	1	0.37
gtt	V	6	577.70
gtc	V	0	565.10
gta	V	840	572.70
gtg	V	1840	970.50
---	--	------	------
mPD		0.43	0.73
		nPD:	0.59
		N. weight:	0.89
		Sc. PD:	0.24
		Sc. rank:	803.4

,

PB2			
Pos.	486	obs:	exp:
tat	Y	1	0.48
tac	Y	0	0.52
gat	D	2613	1450.00
gac	D	73	1236.00
---	--	------	------
mPD		0.054	0.50
		nPD:	0.11
		N. weight:	0.81
		Sc. PD:	-0.17
		Sc. rank:	-519.3

,

PB2			
Pos.	487	obs:	exp:
gaa	E	2449	1506.00
gag	E	238	1181.00
---	--	------	------
mPD		0.16	0.49
		nPD:	0.33
		N. weight:	0.49
		Sc. PD:	0.0034
		Sc. rank:	88.8

,

PB2			
Pos.	488	obs:	exp:
tat	Y	2442	1287.00
tac	Y	242	1397.00
cat	H	3	1.74
cac	H	0	1.26
---	--	------	------
mPD		0.17	0.50
		nPD:	0.33
		N. weight:	0.69
		Sc. PD:	0.0072
		Sc. rank:	141.6

,

PB2			
Pos.	489	obs:	exp:
tct	S	48	447.00
tcc	S	2603	387.90
tca	S	27	607.10
tcg	S	0	161.60
cct	P	0	2.46
ccc	P	9	1.63
cca	P	0	3.65
ccg	P	0	1.26
agt	S	0	580.90
agc	S	0	493.50
---	--	------	------
mPD		0.061	1.7
		nPD:	0.04
		N. weight:	2.9
		Sc. PD:	-0.81
		Sc. rank:	-2971.6

,

PB2			
Pos.	490	obs:	exp:
tct	S	0	445.70
tcc	S	0	386.80
tca	S	0	605.30
tcg	S	0	161.10
tgt	C	0	0.77
tgc	C	2	1.23
cgt	R	0	0.04
cgc	R	0	0.05
cga	R	0	0.10
cgg	R	0	0.08
aat	N	2	7.63
aac	N	12	6.37
agt	S	1100	579.10
agc	S	1570	492.00
aga	R	1	0.47
agg	R	0	0.26
---	--	------	------
mPD		0.50	1.7
		nPD:	0.3
		N. weight:	1.5
		Sc. PD:	-0.032
		Sc. rank:	144.1

,

PB2			
Pos.	491	obs:	exp:
att	I	5	1.83
atc	I	0	1.34
ata	I	0	1.83
act	T	2448	703.10
acc	T	35	604.30
aca	T	182	1122.00
acg	T	17	252.30
---	--	------	------
mPD		0.17	0.70
		nPD:	0.24
		N. weight:	1.6
		Sc. PD:	-0.13
		Sc. rank:	-235.1

,

PB2			
Pos.	492	obs:	exp:
gat	D	2	1.08
gac	D	0	0.92
gaa	E	834	1505.00
gag	E	1851	1180.00
---	--	------	------
mPD		0.43	0.49
		nPD:	0.87
		N. weight:	0.21
		Sc. PD:	0.11
		Sc. rank:	289.6

,

PB2			
Pos.	493	obs:	exp:
cgt	R	0	99.77
cgc	R	0	142.70
cga	R	23	268.70
cgg	R	23	217.70
atg	M	1	1.00
aaa	K	4	11.39
aag	K	16	8.61
aga	R	2043	1245.00
agg	R	577	692.60
---	--	------	------
mPD		0.40	0.97
		nPD:	0.42
		N. weight:	0.49
		Sc. PD:	0.046
		Sc. rank:	258.5

,

PB2			
Pos.	494	obs:	exp:
att	I	0	75.23
atc	I	0	54.86
ata	I	205	74.92
gtt	V	4	533.80
gtc	V	0	522.20
gta	V	1459	529.20
gtg	V	1019	896.80
---	--	------	------
mPD		0.61	0.88
		nPD:	0.7
		N. weight:	1.1
		Sc. PD:	0.4
		Sc. rank:	1151.5

,

PB2			
Pos.	495	obs:	exp:
tta	L	0	0.09
ttg	L	0	0.19
ctt	L	0	0.20
ctc	L	1	0.16
cta	L	0	0.15
ctg	L	0	0.21
att	I	22	8.44
atc	I	1	6.15
ata	I	0	8.41
act	T	0	0.52
acc	T	2	0.45
aca	T	0	0.84
acg	T	0	0.19
gtt	V	1454	538.10
gtc	V	739	526.40
gta	V	186	533.50
gtg	V	123	904.00
gct	A	7	40.05
gcc	A	152	30.77
gca	A	0	70.77
gcg	A	0	17.40
---	--	------	------
mPD		0.71	0.87
		nPD:	0.82
		N. weight:	0.92
		Sc. PD:	0.45
		Sc. rank:	1227.9

,

PB2			
Pos.	496	obs:	exp:
gtt	V	3	576.40
gtc	V	4	563.80
gta	V	1503	571.40
gtg	V	1170	968.30
gct	A	0	1.76
gcc	A	1	1.35
gca	A	5	3.12
gcg	A	1	0.77
---	--	------	------
mPD		0.50	0.74
		nPD:	0.68
		N. weight:	1.
		Sc. PD:	0.35
		Sc. rank:	1009.3

PB2			
Pos.	497	obs:	exp:
tct	S	0	367.60
tcc	S	0	319.00
tca	S	0	499.20
tcg	S	0	132.80
cat	H	4	2.32
cac	H	0	1.68
cgt	R	2	0.15
cgc	R	0	0.21
cga	R	0	0.40
cgg	R	0	0.33
act	T	0	0.26
acc	T	1	0.23
aca	T	0	0.42
acg	T	0	0.09
aat	N	84	257.90
aac	N	389	215.10
agt	S	906	477.60
agc	S	1296	405.80
aga	R	2	1.87
agg	R	0	1.04
ggt	G	0	0.43
ggc	G	3	0.39
gga	G	0	1.31
ggg	G	0	0.86
---	--	------	------
mPD		0.77	1.8
		nPD:	0.43
		N. weight:	1.4
		Sc. PD:	0.14
		Sc. rank:	758.2

PB2			
Pos.	498	obs:	exp:
att	I	2411	985.30
atc	I	166	718.50
ata	I	108	981.20
atg	M	1	1.00
gtt	V	1	0.22
gtc	V	0	0.21
gta	V	0	0.21
gtg	V	0	0.36
---	--	------	------
mPD		0.19	0.66
		nPD:	0.29
		N. weight:	1.
		Sc. PD:	-0.034
		Sc. rank:	44.4

PB2			
Pos.	499	obs:	exp:
aat	N	0	1.09
aac	N	2	0.91
gtt	V	1	0.22
gtc	V	0	0.21
gta	V	0	0.21
gtg	V	0	0.36
gat	D	1100	1449.00
gac	D	1584	1235.00
---	--	------	------
mPD		0.49	0.50
		nPD:	0.97
		N. weight:	0.059
		Sc. PD:	0.038
		Sc. rank:	84.1

PB2			
Pos.	500	obs:	exp:
tgt	C	0	0.39
tgc	C	1	0.61
cgt	R	684	100.50
cgc	R	1536	143.70
cga	R	374	270.70
cgg	R	92	219.30
aga	R	0	1254.00
agg	R	0	697.80
---	--	------	------
mPD		0.59	0.95
		nPD:	0.62
		N. weight:	3.
		Sc. PD:	0.88
		Sc. rank:	2841.2

PB2			
Pos.	501	obs:	exp:
ttt	F	778	1121.00
ttc	F	1909	1566.00
---	--	------	------
mPD		0.41	0.49
		nPD:	0.85
		N. weight:	0.059
		Sc. PD:	0.031
		Sc. rank:	80.9

PB2			
Pos.	502	obs:	exp:
tta	L	1537	248.40
ttg	L	1138	510.50
ctt	L	0	533.50
ctc	L	0	429.80
cta	L	1	397.70
ctg	L	11	567.10
---	--	------	------
mPD		0.50	1.1
		nPD:	0.44
		N. weight:	2.2
		Sc. PD:	0.27
		Sc. rank:	1329.5

PB2			
Pos.	503	obs:	exp:
tgg	W	1	1.00
cgt	R	0	100.20
cgc	R	0	143.30
cga	R	0	269.90
cgg	R	9	218.70
aaa	K	0	4.56
aag	K	8	3.44
aga	R	472	1250.00
agg	R	2197	695.70
---	--	------	------
mPD		0.30	0.96
		nPD:	0.32
		N. weight:	1.2
		Sc. PD:	-0.0062
		Sc. rank:	182.3

PB2			
Pos.	504	obs:	exp:
ttt	F	0	0.42
ttc	F	1	0.58
att	I	1	0.37
atc	I	0	0.27
ata	I	0	0.37
gtt	V	1621	577.50
gtc	V	635	564.90
gta	V	429	572.50
gtg	V	0	970.10
---	--	------	------
mPD		0.56	0.74
		nPD:	0.76
		N. weight:	0.99
		Sc. PD:	0.42
		Sc. rank:	1230.2

PB2			
Pos.	505	obs:	exp:
cat	H	8	4.63
cac	H	0	3.37
cgt	R	4	100.30
cgc	R	0	143.40
cga	R	2279	270.00
cgg	R	261	218.80
aga	R	41	1251.00
agg	R	94	696.00
---	--	------	------
mPD		0.34	0.96
		nPD:	0.35
		N. weight:	2.8
		Sc. PD:	0.082
		Sc. rank:	777.7

PB2			
Pos.	506	obs:	exp:
gat	D	1372	1451.00
gac	D	1315	1236.00
---	--	------	------
mPD		0.50	0.50
		nPD:	1.01
		N. weight:	0.0053
		Sc. PD:	0.0036
		Sc. rank:	7.6

PB2			
Pos.	507	obs:	exp:
cat	H	4	2.89
cac	H	1	2.11
caa	Q	348	1339.00
cag	Q	2333	1342.00
cgt	R	0	0.04
cgc	R	0	0.05
cga	R	0	0.10
cgg	R	1	0.08
aga	R	0	0.47
agg	R	0	0.26
---	--	------	------
mPD		0.23	0.50
		nPD:	0.46
		N. weight:	0.5
		Sc. PD:	0.066
		Sc. rank:	310.6

PB2			
Pos.	508	obs:	exp:
tta	L	0	0.18
ttg	L	0	0.38
ctt	L	0	0.40
ctc	L	0	0.32
cta	L	1	0.30
ctg	L	1	0.42
caa	Q	2	13.98
cag	Q	26	14.02
cgt	R	0	98.39
cgc	R	4	140.70
cga	R	197	264.90
cgg	R	1295	214.70
att	I	0	0.73
atc	I	0	0.54
ata	I	2	0.73
aaa	K	2	14.24
aag	K	23	10.76
aga	R	933	1227.00
agg	R	200	683.00
ggt	G	0	0.14
ggc	G	0	0.13
gga	G	0	0.44
ggg	G	1	0.29
---	--	------	------
mPD		1.0	1.0
		nPD:	1.02
		N. weight:	1.1
		Sc. PD:	0.75
		Sc. rank:	1553.8

PB2			
Pos.	509	obs:	exp:
ggt	G	0	388.60
ggc	G	0	352.90
gga	G	1136	1177.00
ggg	G	1551	768.10
---	--	------	------
mPD		0.49	0.69
		nPD:	0.71
		N. weight:	0.64
		Sc. PD:	0.24
		Sc. rank:	711.2

PB2			
Pos.	510	obs:	exp:
tct	S	0	0.83
tcc	S	0	0.72
tca	S	0	1.13
tcg	S	0	0.30
aat	N	1852	1461.00
aac	N	828	1219.00
agt	S	0	1.08
agc	S	5	0.92
gat	D	1	1.08
gac	D	1	0.92
---	--	------	------
mPD		0.43	0.50
		nPD:	0.86
		N. weight:	0.08
		Sc. PD:	0.042
		Sc. rank:	109.4

PB2			
Pos. 511		obs:	exp:
att	I	0	29.36
atc	I	0	21.41
ata	I	80	29.24
gtt	V	0	560.70
gtc	V	0	548.50
gta	V	2183	555.90
gtg	V	424	941.90
---	--	------	------
mPD		0.32	0.79
		nPD:	0.41
		N. weight:	1.6
		Sc. PD:	0.14
		Sc. rank:	806.8

PB2			
Pos. 512		obs:	exp:
tta	L	3	248.40
ttg	L	0	510.50
ctt	L	13	533.50
ctc	L	2398	429.80
cta	L	271	397.70
ctg	L	2	567.10
---	--	------	------
mPD		0.20	1.1
		nPD:	0.17
		N. weight:	2.4
		Sc. PD:	-0.34
		Sc. rank:	-925.0

PB2			
Pos. 513		obs:	exp:
tta	L	1223	248.20
ttg	L	895	509.90
ctt	L	0	532.90
ctc	L	0	429.30
cta	L	234	397.20
ctg	L	332	566.50
att	I	0	1.10
atc	I	0	0.80
ata	I	3	1.10
---	--	------	------
mPD		0.83	1.1
		nPD:	0.74
		N. weight:	1.3
		Sc. PD:	0.54
		Sc. rank:	1591.8

PB2			
Pos. 514		obs:	exp:
tct	S	2449	448.50
tcc	S	58	389.20
tca	S	178	609.10
tcg	S	2	162.10
agt	S	0	582.80
agc	S	0	495.20
---	--	------	------
mPD		0.16	1.7
		nPD:	0.1
		N. weight:	2.3
		Sc. PD:	-0.5
		Sc. rank:	-1543.3

PB2			
Pos. 515		obs:	exp:
cct	P	757	734.10
ccc	P	1882	485.10
cca	P	39	1090.00
ccg	P	8	376.60
cat	H	0	0.58
cac	H	1	0.42
---	--	------	------
mPD		0.43	0.71
		nPD:	0.61
		N. weight:	1.5
		Sc. PD:	0.41
		Sc. rank:	1358.1

PB2			
Pos. 516		obs:	exp:
gat	D	1	0.54
gac	D	0	0.46
gaa	E	2468	1506.00
gag	E	218	1180.00
---	--	------	------
mPD		0.15	0.49
		nPD:	0.3
		N. weight:	0.52
		Sc. PD:	-0.0086
		Sc. rank:	64.8

PB2			
Pos. 517		obs:	exp:
caa	Q	0	0.50
cag	Q	1	0.50
gaa	E	768	1506.00
gag	E	1918	1180.00
---	--	------	------
mPD		0.41	0.49
		nPD:	0.83
		N. weight:	0.25
		Sc. PD:	0.13
		Sc. rank:	341.2

PB2			
Pos. 518		obs:	exp:
att	I	0	1.10
atc	I	3	0.80
ata	I	0	1.10
gtt	V	623	577.30
gtc	V	2055	564.70
gta	V	6	572.30
gtg	V	0	969.70
---	--	------	------
mPD		0.36	0.74
		nPD:	0.49
		N. weight:	1.6
		Sc. PD:	0.27
		Sc. rank:	1168.4

PB2			
Pos. 519		obs:	exp:
tct	S	0	448.50
tcc	S	0	389.20
tca	S	0	609.10
tcg	S	0	162.10
agt	S	1395	582.80
agc	S	1292	495.20
---	--	------	------
mPD		0.50	1.7
		nPD:	0.3
		N. weight:	1.5
		Sc. PD:	-0.027
		Sc. rank:	167.3

PB2			
Pos. 520		obs:	exp:
aaa	K	0	0.57
aag	K	1	0.43
gaa	E	2448	1506.00
gag	E	238	1180.00
---	--	------	------
mPD		0.16	0.49
		nPD:	0.33
		N. weight:	0.49
		Sc. PD:	0.0045
		Sc. rank:	96.3

PB2			
Pos. 521		obs:	exp:
tct	S	0	0.50
tcc	S	0	0.43
tca	S	0	0.68
tcg	S	0	0.18
act	T	98	700.20
acc	T	1328	601.80
aca	T	1236	1118.00
acg	T	9	251.30
aat	N	1	4.91
aac	N	8	4.09
aaa	K	1	0.57
aag	K	0	0.43
agt	S	0	0.65
agc	S	3	0.55
gct	A	0	0.76
gcc	A	2	0.58
gca	A	1	1.34
gcg	A	0	0.33
---	--	------	------
mPD		0.55	0.71
		nPD:	0.77
		N. weight:	0.59
		Sc. PD:	0.26
		Sc. rank:	751.0

PB2			
Pos. 522		obs:	exp:
tta	L	0	0.55
ttg	L	2	1.14
ctt	L	0	1.19
ctc	L	0	0.96
cta	L	0	0.89
ctg	L	4	1.27
cat	H	9	5.79
cac	H	1	4.21
caa	Q	284	1333.00
cag	Q	2386	1337.00
atg	M	1	1.00
---	--	------	------
mPD		0.20	0.51
		nPD:	0.4
		N. weight:	0.58
		Sc. PD:	0.043
		Sc. rank:	255.9

PB2			
Pos. 523		obs:	exp:
ggt	G	6	388.60
ggc	G	0	352.90
gga	G	2126	1177.00
ggg	G	555	768.10
---	--	------	------
mPD		0.33	0.69
		nPD:	0.48
		N. weight:	0.64
		Sc. PD:	0.1
		Sc. rank:	446.7

PB2			
Pos. 524		obs:	exp:
att	I	0	32.29
atc	I	0	23.55
ata	I	88	32.16
atg	M	2	2.00
act	T	3	676.90
acc	T	13	581.80
aca	T	2473	1080.00
acg	T	93	242.90
gtt	V	0	0.22
gtc	V	0	0.21
gta	V	1	0.21
gtg	V	0	0.36
gct	A	0	3.53
gcc	A	0	2.71
gca	A	14	6.23
gcg	A	0	1.53
---	--	------	------
mPD		0.16	0.77
		nPD:	0.2
		N. weight:	1.2
		Sc. PD:	-0.14
		Sc. rank:	-320.6

PB2			
Pos. 525		obs:	exp:
gct	A	0	0.25
gcc	A	0	0.19
gca	A	0	0.45
gcg	A	1	0.11
gat	D	0	0.54
gac	D	1	0.46
gaa	E	597	1505.00
gag	E	2088	1180.00
---	--	------	------
mPD		0.35	0.49
		nPD:	0.7
		N. weight:	0.39
		Sc. PD:	0.15
		Sc. rank:	423.4

PB2			
Pos. 526		obs:	exp:
cgt	R	0	8.31
cgc	R	0	11.88
cga	R	0	22.37
cgg	R	0	18.13
aat	N	0	0.55
aac	N	1	0.45
aaa	K	543	1403.00
aag	K	1921	1061.00
aga	R	26	103.60
agg	R	196	57.67
---	--	------	------
mPD		0.49	0.69
		nPD:	0.7
		N. weight:	0.5
		Sc. PD:	0.19
		Sc. rank:	543.9

PB2			
Pos. 527		obs:	exp:
tta	L	40	248.30
ttg	L	1574	510.30
ctt	L	1	533.30
ctc	L	0	429.60
cta	L	429	397.50
ctg	L	642	566.90
gtt	V	0	0.22
gtc	V	0	0.21
gta	V	0	0.21
gtg	V	1	0.36
---	--	------	------
mPD		0.77	1.1
		nPD:	0.69
		N. weight:	1.1
		Sc. PD:	0.39
		Sc. rank:	1137.6

PB2			
Pos. 528		obs:	exp:
cct	P	0	0.27
ccc	P	0	0.18
cca	P	1	0.41
ccg	P	0	0.14
att	I	0	0.37
atc	I	0	0.27
ata	I	1	0.37
act	T	202	703.10
acc	T	4	604.30
aca	T	1666	1122.00
acg	T	810	252.30
gct	A	0	0.76
gcc	A	0	0.58
gca	A	1	1.34
gcg	A	2	0.33
---	--	------	------
mPD		0.52	0.70
		nPD:	0.74
		N. weight:	0.81
		Sc. PD:	0.34
		Sc. rank:	996.2

PB2			
Pos. 529		obs:	exp:
att	I	2	977.20
atc	I	160	712.60
ata	I	2501	973.20
atg	M	1	1.00
gtt	V	0	4.95
gtc	V	0	4.84
gta	V	23	4.90
gtg	V	0	8.31
---	--	------	------
mPD		0.13	0.68
		nPD:	0.19
		N. weight:	1.3
		Sc. PD:	-0.16
		Sc. rank:	-430.5

PB2			
Pos. 530		obs:	exp:
att	I	0	1.83
atc	I	0	1.34
ata	I	5	1.83
act	T	399	703.10
acc	T	1	604.30
aca	T	2268	1122.00
acg	T	14	252.30
---	--	------	------
mPD		0.27	0.70
		nPD:	0.38
		N. weight:	0.81
		Sc. PD:	0.047
		Sc. rank:	307.0

PB2			
Pos.	531	obs:	exp:
tat	Y	2649	1289.00
tac	Y	38	1398.00
---	--	------	------
mPD		0.028	0.50
		nPD:	0.06
		N. weight:	1.1
		Sc. PD:	-0.28
		Sc. rank:	-964.9

,

PB2			
Pos.	532	obs:	exp:
tta	L	3	0.83
ttg	L	6	1.71
tct	S	2	446.50
tcc	S	2	387.50
tca	S	1591	606.40
tcg	S	1080	161.40
ctt	L	0	1.79
ctc	L	0	1.44
cta	L	0	1.33
ctg	L	0	1.90
cct	P	0	0.55
ccc	P	0	0.36
cca	P	0	0.81
ccg	P	2	0.28
act	T	0	0.26
acc	T	0	0.23
aca	T	0	0.42
acg	T	1	0.09
agt	S	0	580.20
agc	S	0	493.00
---	--	------	------
mPD		0.49	1.7
		nPD:	0.3
		N. weight:	2.2
		Sc. PD:	-0.05
		Sc. rank:	178.7

,

PB2			
Pos.	533	obs:	exp:
tta	L	1	0.09
ttg	L	0	0.19
tct	S	3	448.40
tcc	S	24	389.10
tca	S	2105	608.90
tcg	S	554	162.00
ctt	L	0	0.20
ctc	L	0	0.16
cta	L	0	0.15
ctg	L	0	0.21
agt	S	0	582.60
agc	S	0	495.00
---	--	------	------
mPD		0.34	1.7
		nPD:	0.21
		N. weight:	1.9
		Sc. PD:	-0.21
		Sc. rank:	-481.4

,

PB2			
Pos.	534	obs:	exp:
tct	S	50	447.20
tcc	S	986	388.10
tca	S	1638	607.30
tcg	S	5	161.60
cct	P	0	1.91
ccc	P	0	1.26
cca	P	7	2.84
ccg	P	0	0.98
agt	S	0	581.10
agc	S	0	493.70
gct	A	0	0.25
gcc	A	1	0.19
gca	A	0	0.45
gcg	A	0	0.11
---	--	------	------
mPD		0.50	1.7
		nPD:	0.3
		N. weight:	1.5
		Sc. PD:	-0.03
		Sc. rank:	146.3

,

PB2			
Pos.	535	obs:	exp:
tta	L	0	0.18
ttg	L	2	0.38
ctt	L	0	0.40
ctc	L	0	0.32
cta	L	0	0.30
ctg	L	0	0.42
att	I	0	0.37
atc	I	0	0.27
ata	I	1	0.37
atg	M	2684	2684.00
---	--	------	------
mPD		0.0022	0.0031
		nPD:	0.71
		N. weight:	0.0026
		Sc. PD:	0.001
		Sc. rank:	3.0

,

PB2			
Pos.	536	obs:	exp:
tta	L	0	0.18
ttg	L	1	0.38
ctt	L	0	0.40
ctc	L	0	0.32
cta	L	0	0.30
ctg	L	1	0.42
atg	M	2685	2685.00
---	--	------	------
mPD		0.0015	0.0024
		nPD:	0.63
		N. weight:	0.0015
		Sc. PD:	0.00046
		Sc. rank:	1.4

,

PB2			
Pos.	537	obs:	exp:
tgg	W	2687	2687.00
---	--	------	------
mPD		0	0
		nPD:	1.
		N. weight:	0.
		Sc. PD:	0
		Sc. rank:	0

,

PB2			
Pos.	538	obs:	exp:
gaa	E	2248	1506.00
gag	E	439	1181.00
---	--	------	------
mPD		0.27	0.49
		nPD:	0.56
		N. weight:	0.28
		Sc. PD:	0.065
		Sc. rank:	234.5

,

PB2			
Pos.	539	obs:	exp:
tta	L	1	0.28
ttg	L	0	0.57
ctt	L	0	0.60
ctc	L	2	0.48
cta	L	0	0.44
ctg	L	0	0.63
att	I	572	982.70
atc	I	2101	716.60
ata	I	5	978.70
act	T	0	0.26
acc	T	1	0.23
aca	T	0	0.42
acg	T	0	0.09
gtt	V	0	1.08
gtc	V	5	1.05
gta	V	0	1.07
gtg	V	0	1.81
---	--	------	------
mPD		0.35	0.67
		nPD:	0.52
		N. weight:	1.2
		Sc. PD:	0.23
		Sc. rank:	896.1

,

PB2			
Pos.	540	obs:	exp:
tct	S	0	0.17
tcc	S	0	0.14
tca	S	0	0.23
tcg	S	0	0.06
tat	Y	0	0.48
tac	Y	1	0.52
aat	N	1314	1464.00
aac	N	1371	1221.00
agt	S	0	0.22
agc	S	1	0.18
---	--	------	------
mPD		0.50	0.50
		nPD:	1.01
		N. weight:	0.014
		Sc. PD:	0.0095
		Sc. rank:	20.1

,

PB2			
Pos.	541	obs:	exp:
cgt	R	0	0.04
cgc	R	0	0.05
cga	R	0	0.10
cgg	R	1	0.08
aga	R	0	0.47
agg	R	0	0.26
gtt	V	1	0.22
gtc	V	0	0.21
gta	V	0	0.21
gtg	V	0	0.36
ggt	G	1718	388.30
ggc	G	850	352.70
gga	G	110	1177.00
ggg	G	7	767.50
---	--	------	------
mPD		0.49	0.69
		nPD:	0.71
		N. weight:	1.8
		Sc. PD:	0.7
		Sc. rank:	2070.8

,

PB2			
Pos.	542	obs:	exp:
cct	P	2058	734.10
ccc	P	55	485.10
cca	P	243	1090.00
ccg	P	330	376.60
cgt	R	1	0.04
cgc	R	0	0.05
cga	R	0	0.10
cgg	R	0	0.08
aga	R	0	0.47
agg	R	0	0.26
---	--	------	------
mPD		0.39	0.71
		nPD:	0.55
		N. weight:	0.97
		Sc. PD:	0.22
		Sc. rank:	789.9

,

PB2			
Pos.	543	obs:	exp:
gat	D	1	0.54
gac	D	0	0.46
gaa	E	1221	1506.00
gag	E	1465	1180.00
---	--	------	------
mPD		0.50	0.49
		nPD:	1.01
		N. weight:	0.04
		Sc. PD:	0.027
		Sc. rank:	56.1

,

PB2			
Pos.	544	obs:	exp:
tta	L	1	0.09
ttg	L	0	0.19
tct	S	4	448.40
tcc	S	102	389.10
tca	S	2390	608.90
tcg	S	190	162.00
ctt	L	0	0.20
ctc	L	0	0.16
cta	L	0	0.15
ctg	L	0	0.21
agt	S	0	582.60
agc	S	0	495.00
---	--	------	------
mPD		0.20	1.7
		nPD:	0.12
		N. weight:	1.9
		Sc. PD:	-0.37
		Sc. rank:	-1112.0

,

PB2			
Pos.	545	obs:	exp:
tta	L	0	0.09
ttg	L	1	0.19
ctt	L	0	0.20
ctc	L	0	0.16
cta	L	0	0.15
ctg	L	0	0.21
att	I	0	0.37
atc	I	0	0.27
ata	I	1	0.37
atg	M	3	3.00
gtt	V	21	576.80
gtc	V	0	564.30
gta	V	399	571.90
gtg	V	2262	969.00
---	--	------	------
mPD		0.27	0.74
		nPD:	0.37
		N. weight:	1.
		Sc. PD:	0.047
		Sc. rank:	352.7

,

PB2			
Pos.	546	obs:	exp:
tta	L	239	248.30
ttg	L	184	510.30
ctt	L	1448	533.30
ctc	L	27	429.60
cta	L	753	397.50
ctg	L	35	566.90
cct	P	1	0.27
ccc	P	0	0.18
cca	P	0	0.41
ccg	P	0	0.14
---	--	------	------
mPD		0.83	1.1
		nPD:	0.74
		N. weight:	0.95
		Sc. PD:	0.39
		Sc. rank:	1163.4

,

PB2			
Pos.	547	obs:	exp:
att	I	5	2.57
atc	I	2	1.87
ata	I	0	2.56
gtt	V	1111	576.40
gtc	V	1539	563.80
gta	V	0	571.40
gtg	V	30	968.30
---	--	------	------
mPD		0.50	0.74
		nPD:	0.68
		N. weight:	1.3
		Sc. PD:	0.47
		Sc. rank:	1355.4

,

PB2			
Pos.	548	obs:	exp:
aat	N	93	1465.00
aac	N	2594	1222.00
---	--	------	------
mPD		0.067	0.50
		nPD:	0.13
		N. weight:	1.
		Sc. PD:	-0.19
		Sc. rank:	-552.4

,

PB2			
Pos.	549	obs:	exp:
act	T	293	704.40
acc	T	1361	605.50
aca	T	1028	1124.00
acg	T	5	252.80
---	--	------	------
mPD		0.59	0.70
		nPD:	0.84
		N. weight:	0.45
		Sc. PD:	0.23
		Sc. rank:	613.3

,

PB2			
Pos.	550	obs:	exp:
tat	Y	1739	1289.00
tac	Y	948	1398.00
---	--	------	------
mPD		0.46	0.50
		nPD:	0.92
		N. weight:	0.095
		Sc. PD:	0.055
		Sc. rank:	132.4

,

PB2			
Pos.	551	obs:	exp:
caa	Q	2264	1342.00
cag	Q	423	1345.00
---	--	------	------
mPD		0.27	0.50
		nPD:	0.53
		N. weight:	0.42
		Sc. PD:	0.087
		Sc. rank:	335.4

,

PB2			
Pos.	552	obs:	exp:
tgg	W	2687	2687.00
---	--	------	------
mPD		0	0
		nPD:	1.
		N. weight:	0.
		Sc. PD:	0
		Sc. rank:	0

,

PB2			
Pos.	553	obs:	exp:
att	I	8	955.20
atc	I	2532	696.60
ata	I	63	951.20
gtt	V	0	18.07
gtc	V	84	17.67
gta	V	0	17.91
gtg	V	0	30.35
---	--	------	------
mPD		0.11	0.73
		nPD:	0.15
		N. weight:	1.9
		Sc. PD:	-0.32
		Sc. rank:	-914.6

,

PB2			
Pos.	554	obs:	exp:
tct	S	0	0.17
tcc	S	0	0.14
tca	S	0	0.23
tcg	S	0	0.06
att	I	532	985.30
atc	I	2141	718.50
ata	I	12	981.20
act	T	0	0.26
acc	T	1	0.23
aca	T	0	0.42
acg	T	0	0.09
agt	S	0	0.22
agc	S	1	0.18
---	--	------	------
mPD		0.33	0.66
		nPD:	0.49
		N. weight:	1.2
		Sc. PD:	0.2
		Sc. rank:	861.1

,

PB2			
Pos.	555	obs:	exp:
cgt	R	0	100.50
cgc	R	0	143.70
cga	R	23	270.60
cgg	R	0	219.20
aaa	K	2	1.14
aag	K	0	0.86
aga	R	2575	1253.00
agg	R	87	697.50
---	--	------	------
mPD		0.081	0.96
		nPD:	0.08
		N. weight:	0.98
		Sc. PD:	-0.23
		Sc. rank:	-747.6

,

PB2			
Pos.	556	obs:	exp:
tct	S	0	2.00
tcc	S	0	1.74
tca	S	0	2.72
tcg	S	0	0.72
aat	N	1002	1457.00
aac	N	1670	1215.00
aaa	K	2	1.14
aag	K	0	0.86
agt	S	7	2.60
agc	S	5	2.21
gat	D	0	0.54
gac	D	1	0.46
---	--	------	------
mPD		0.48	0.51
		nPD:	0.94
		N. weight:	0.11
		Sc. PD:	0.064
		Sc. rank:	148.0

,

PB2			
Pos.	557	obs:	exp:
tgg	W	2687	2687.00
---	--	------	------
mPD		0	0
		nPD:	1.
		N. weight:	0.
		Sc. PD:	0
		Sc. rank:	0

,

PB2			
Pos.	558	obs:	exp:
gaa	E	1958	1506.00
gag	E	728	1180.00
ggt	G	0	0.14
ggc	G	0	0.13
gga	G	1	0.44
ggg	G	0	0.29
---	--	------	------
mPD		0.40	0.49
		nPD:	0.8
		N. weight:	0.1
		Sc. PD:	0.048
		Sc. rank:	132.3

,

PB2			
Pos.	559	obs:	exp:
cct	P	2	0.55
ccc	P	0	0.36
cca	P	0	0.81
ccg	P	0	0.28
att	I	10	3.67
atc	I	0	2.68
ata	I	0	3.65
act	T	2025	678.70
acc	T	542	583.40
aca	T	19	1083.00
acg	T	3	243.60
aat	N	4	3.82
aac	N	3	3.18
gct	A	54	19.90
gcc	A	25	15.29
gca	A	0	35.16
gcg	A	0	8.65
---	--	------	------
mPD		0.42	0.77
		nPD:	0.54
		N. weight:	1.3
		Sc. PD:	0.29
		Sc. rank:	1063.7

,

PB2			
Pos.	560	obs:	exp:
tta	L	0	2.40
ttg	L	26	4.94
ctt	L	0	5.16
ctc	L	0	4.16
cta	L	0	3.85
ctg	L	0	5.49
att	I	0	1.83
atc	I	0	1.34
ata	I	5	1.83
atg	M	1	1.00
gtt	V	9	571.00
gtc	V	1	558.60
gta	V	367	566.10
gtg	V	2278	959.30
---	--	------	------
mPD		0.27	0.76
		nPD:	0.35
		N. weight:	1.1
		Sc. PD:	0.037
		Sc. rank:	340.9

,

PB2			
Pos.	561	obs:	exp:
aaa	K	456	1530.00
aag	K	2231	1157.00
---	--	------	------
mPD		0.28	0.49
		nPD:	0.57
		N. weight:	0.55
		Sc. PD:	0.14
		Sc. rank:	476.6

,

PB2			
Pos.	562	obs:	exp:
att	I	2530	984.60
atc	I	85	718.00
ata	I	68	980.50
act	T	1	0.26
acc	T	0	0.23
aca	T	0	0.42
acg	T	0	0.09
gtt	V	3	0.65
gtc	V	0	0.63
gta	V	0	0.64
gtg	V	0	1.08
---	--	------	------
mPD		0.11	0.66
		nPD:	0.17
		N. weight:	1.2
		Sc. PD:	-0.18
		Sc. rank:	-512.3

,

PB2			
Pos.	563	obs:	exp:
cat	H	0	0.58
cac	H	1	0.42
caa	Q	1799	1340.00
cag	Q	885	1344.00
cgt	R	0	0.04
cgc	R	0	0.05
cga	R	0	0.10
cgg	R	1	0.08
aaa	K	1	0.57
aag	K	0	0.43
aga	R	0	0.47
agg	R	0	0.26
---	--	------	------
mPD		0.44	0.50
		nPD:	0.88
		N. weight:	0.1
		Sc. PD:	0.056
		Sc. rank:	141.1

,

PB2			
Pos.	564	obs:	exp:
tgg	W	2687	2687.00
---	--	------	------
mPD		0	0
		nPD:	1.
		N. weight:	0.
		Sc. PD:	0
		Sc. rank:	0

,

PB2			
Pos.	565	obs:	exp:
ttt	F	1	0.42
ttc	F	0	0.58
tct	S	1474	448.20
tcc	S	1023	388.90
tca	S	187	608.70
tcg	S	1	162.00
tgt	C	1	0.39
tgc	C	0	0.61
agt	S	0	582.40
agc	S	0	494.80
---	--	------	------
mPD		0.55	1.7
		nPD:	0.33
		N. weight:	1.5
		Sc. PD:	0.019
		Sc. rank:	359.3

,

PB2			
Pos.	566	obs:	exp:
tta	L	0	0.09
ttg	L	0	0.19
ctt	L	0	0.20
ctc	L	0	0.16
cta	L	0	0.15
ctg	L	1	0.21
cct	P	0	0.27
ccc	P	0	0.18
cca	P	0	0.41
ccg	P	1	0.14
cat	H	3	1.74
cac	H	0	1.26
caa	Q	1470	1339.00
cag	Q	1212	1343.00
---	--	------	------
mPD		0.50	0.50
		nPD:	0.99
		N. weight:	0.013
		Sc. PD:	0.0088
		Sc. rank:	18.9

,

PB2			
Pos.	567	obs:	exp:
aat	N	1	1.09
aac	N	1	0.91
gat	D	971	1448.00
gac	D	1710	1233.00
gaa	E	2	1.68
gag	E	1	1.32
ggt	G	0	0.14
ggc	G	1	0.13
gga	G	0	0.44
ggg	G	0	0.29
---	--	------	------
mPD		0.47	0.50
		nPD:	0.93
		N. weight:	0.11
		Sc. PD:	0.065
		Sc. rank:	151.3

,

PB2			
Pos.	568	obs:	exp:
cct	P	967	734.10
ccc	P	1710	485.10
cca	P	8	1090.00
ccg	P	1	376.60
cgt	R	1	0.04
cgc	R	0	0.05
cga	R	0	0.10
cgg	R	0	0.08
aga	R	0	0.47
agg	R	0	0.26
---	--	------	------
mPD		0.47	0.71
		nPD:	0.66
		N. weight:	1.4
		Sc. PD:	0.48
		Sc. rank:	1405.9

,

PB2			
Pos.	569	obs:	exp:
tct	S	0	0.83
tcc	S	0	0.72
tca	S	0	1.13
tcg	S	5	0.30
act	T	172	702.60
acc	T	16	603.90
aca	T	1146	1121.00
acg	T	1346	252.20
agt	S	0	1.08
agc	S	0	0.92
gct	A	0	0.50
gcc	A	0	0.39
gca	A	2	0.89
gcg	A	0	0.22
---	--	------	------
mPD		0.57	0.70
		nPD:	0.81
		N. weight:	1.2
		Sc. PD:	0.58
		Sc. rank:	1599.0

,

PB2			
Pos.	570	obs:	exp:
att	I	3	31.93
atc	I	0	23.28
ata	I	84	31.79
atg	M	2580	2580.00
act	T	0	0.26
acc	T	0	0.23
aca	T	0	0.42
acg	T	1	0.09
gtt	V	0	4.09
gtc	V	0	4.00
gta	V	0	4.05
gtg	V	19	6.86
---	--	------	------
mPD		0.078	0.087
		nPD:	0.89
		N. weight:	0.059
		Sc. PD:	0.033
		Sc. rank:	82.2

,

PB2			
Pos.	571	obs:	exp:
tta	L	143	248.30
ttg	L	399	510.30
ctt	L	216	533.30
ctc	L	2	429.60
cta	L	731	397.50
ctg	L	1195	566.90
att	I	0	0.37
atc	I	0	0.27
ata	I	1	0.37
---	--	------	------
mPD		0.86	1.1
		nPD:	0.77
		N. weight:	0.59
		Sc. PD:	0.26
		Sc. rank:	739.5

,

PB2			
Pos.	572	obs:	exp:
tat	Y	274	1288.00
tac	Y	2412	1398.00
gat	D	0	0.54
gac	D	1	0.46
---	--	------	------
mPD		0.18	0.50
		nPD:	0.37
		N. weight:	0.54
		Sc. PD:	0.025
		Sc. rank:	185.6

,

PB2			
Pos.	573	obs:	exp:
tct	S	0	0.50
tcc	S	0	0.43
tca	S	0	0.68
tcg	S	0	0.18
aat	N	2020	1464.00
aac	N	664	1220.00
agt	S	3	0.65
agc	S	0	0.55
---	--	------	------
mPD		0.37	0.50
		nPD:	0.75
		N. weight:	0.15
		Sc. PD:	0.065
		Sc. rank:	190.4

,

PB2			
Pos.	574	obs:	exp:
cgt	R	0	0.30
cgc	R	0	0.43
cga	R	0	0.81
cgg	R	0	0.65
aaa	K	31	1525.00
aag	K	2648	1154.00
aga	R	0	3.73
agg	R	8	2.08
---	--	------	------
mPD		0.029	0.50
		nPD:	0.06
		N. weight:	1.3
		Sc. PD:	-0.33
		Sc. rank:	-1090.6

,

PB2			
Pos.	575	obs:	exp:
att	I	1	34.86
atc	I	0	25.42
ata	I	94	34.72
atg	M	2585	2585.00
gtt	V	0	1.51
gtc	V	0	1.47
gta	V	1	1.49
gtg	V	6	2.53
---	--	------	------
mPD		0.074	0.077
		nPD:	0.96
		N. weight:	0.058
		Sc. PD:	0.037
		Sc. rank:	82.1

,

PB2			
Pos.	576	obs:	exp:
gaa	E	716	1506.00
gag	E	1971	1181.00
---	--	------	------
mPD		0.39	0.49
		nPD:	0.79
		N. weight:	0.29
		Sc. PD:	0.14
		Sc. rank:	379.0

,

PB2			
Pos.	577	obs:	exp:
ttt	F	1728	1121.00
ttc	F	959	1566.00
---	--	------	------
mPD		0.46	0.49
		nPD:	0.94
		N. weight:	0.17
		Sc. PD:	0.1
		Sc. rank:	238.8

,

PB2			
Pos.	578	obs:	exp:
gaa	E	1275	1506.00
gag	E	1412	1181.00
---	--	------	------
mPD		0.50	0.49
		nPD:	1.01
		N. weight:	0.027
		Sc. PD:	0.018
		Sc. rank:	37.9

,

PB2			
Pos.	579	obs:	exp:
tct	S	0	0.83
tcc	S	0	0.72
tca	S	0	1.13
tcg	S	5	0.30
cct	P	464	733.00
ccc	P	562	484.40
cca	P	285	1089.00
ccg	P	1371	376.00
agt	S	0	1.08
agc	S	0	0.92
---	--	------	------
mPD		0.66	0.71
		nPD:	0.92
		N. weight:	0.78
		Sc. PD:	0.46
		Sc. rank:	1089.8

,

PB2			
Pos.	580	obs:	exp:
ttt	F	932	1120.00
ttc	F	1753	1565.00
tta	L	0	0.09
ttg	L	0	0.19
tct	S	0	0.17
tcc	S	1	0.14
tca	S	0	0.23
tcg	S	0	0.06
ctt	L	0	0.20
ctc	L	1	0.16
cta	L	0	0.15
ctg	L	0	0.21
agt	S	0	0.22
agc	S	0	0.18
---	--	------	------
mPD		0.45	0.49
		nPD:	0.93
		N. weight:	0.021
		Sc. PD:	0.013
		Sc. rank:	30.1

,

PB2			
Pos.	581	obs:	exp:
caa	Q	2065	1342.00
cag	Q	622	1345.00
---	--	------	------
mPD		0.36	0.50
		nPD:	0.71
		N. weight:	0.25
		Sc. PD:	0.096
		Sc. rank:	285.5

,

PB2			
Pos.	582	obs:	exp:
tct	S	218	448.20
tcc	S	2463	388.90
tca	S	4	608.70
tcg	S	0	162.00
cct	P	0	0.27
ccc	P	1	0.18
cca	P	0	0.41
ccg	P	0	0.14
agt	S	0	582.40
agc	S	0	494.80
gct	A	0	0.25
gcc	A	1	0.19
gca	A	0	0.45
gcg	A	0	0.11
---	--	------	------
mPD		0.15	1.7
		nPD:	0.09
		N. weight:	2.6
		Sc. PD:	-0.59
		Sc. rank:	-1884.5

,

PB2			
Pos.	583	obs:	exp:
tta	L	64	247.80
ttg	L	2112	509.10
ctt	L	1	532.10
ctc	L	0	428.70
cta	L	243	396.60
ctg	L	260	565.70
atg	M	7	7.00
---	--	------	------
mPD		0.51	1.1
		nPD:	0.46
		N. weight:	1.6
		Sc. PD:	0.21
		Sc. rank:	972.7

,

PB2			
Pos.	584	obs:	exp:
tta	L	1	0.09
ttg	L	0	0.19
ctt	L	0	0.20
ctc	L	0	0.16
cta	L	0	0.15
ctg	L	0	0.21
att	I	0	26.42
atc	I	0	19.27
ata	I	72	26.31
gtt	V	7	562.20
gtc	V	68	549.90
gta	V	1507	557.40
gtg	V	1032	944.50
---	--	------	------
mPD		0.56	0.79
		nPD:	0.71
		N. weight:	0.91
		Sc. PD:	0.35
		Sc. rank:	1009.3

,

PB2			
Pos.	585	obs:	exp:
tct	S	2	0.33
tcc	S	0	0.29
tca	S	0	0.45
tcg	S	0	0.12
cct	P	2293	733.80
ccc	P	382	485.00
cca	P	7	1090.00
ccg	P	3	376.40
agt	S	0	0.43
agc	S	0	0.37
---	--	------	------
mPD		0.25	0.71
		nPD:	0.35
		N. weight:	1.5
		Sc. PD:	0.05
		Sc. rank:	458.1

,

PB2			
Pos.	586	obs:	exp:
cgt	R	0	0.82
cgc	R	0	1.18
cga	R	0	2.22
cgg	R	0	1.80
aaa	K	1577	1517.00
aag	K	1088	1148.00
aga	R	7	10.27
agg	R	15	5.72
---	--	------	------
mPD		0.50	0.51
		nPD:	0.98
		N. weight:	0.012
		Sc. PD:	0.0079
		Sc. rank:	17.4

,

PB2			
Pos.	587	obs:	exp:
cct	P	1	0.27
ccc	P	0	0.18
cca	P	0	0.41
ccg	P	0	0.14
act	T	3	0.79
acc	T	0	0.68
aca	T	0	1.26
acg	T	0	0.28
gct	A	2037	675.90
gcc	A	34	519.20
gca	A	605	1194.00
gcg	A	7	293.70
---	--	------	------
mPD		0.38	0.69
		nPD:	0.54
		N. weight:	1.
		Sc. PD:	0.23
		Sc. rank:	838.8

,

PB2			
Pos.	588	obs:	exp:
tct	S	0	0.17
tcc	S	1	0.14
tca	S	0	0.23
tcg	S	0	0.06
att	I	4	16.51
atc	I	41	12.04
ata	I	0	16.44
act	T	0	159.60
acc	T	607	137.20
aca	T	2	254.80
acg	T	0	57.30
aat	N	0	1.09
aac	N	2	0.91
agt	S	0	0.22
agc	S	0	0.18
gtt	V	1	2.80
gtc	V	12	2.74
gta	V	0	2.77
gtg	V	0	4.70
gct	A	13	508.10
gcc	A	1999	390.30
gca	A	5	897.80
gcg	A	0	220.80
---	--	------	------
mPD		0.43	1.1
		nPD:	0.39
		N. weight:	2.5
		Sc. PD:	0.17
		Sc. rank:	1040.6

,

PB2			
Pos.	589	obs:	exp:
cgt	R	0	100.40
cgc	R	0	143.60
cga	R	5	270.50
cgg	R	0	219.20
aga	R	2619	1253.00
agg	R	60	697.30
ggt	G	0	0.43
ggc	G	0	0.39
gga	G	3	1.31
ggg	G	0	0.86
---	--	------	------
mPD		0.050	0.96
		nPD:	0.05
		N. weight:	1.1
		Sc. PD:	-0.28
		Sc. rank:	-979.7

PB2			
Pos.	590	obs:	exp:
tct	S	0	3.00
tcc	S	0	2.61
tca	S	0	4.08
tcg	S	0	1.09
tgt	C	42	16.64
tgc	C	1	26.36
agt	S	14	3.90
agc	S	4	3.32
ggt	G	1578	379.80
ggc	G	1046	344.90
gga	G	1	1151.00
ggg	G	1	750.60
---	--	------	------
mPD		0.52	0.75
		nPD:	0.7
		N. weight:	2.1
		Sc. PD:	0.77
		Sc. rank:	2233.7

PB2			
Pos.	591	obs:	exp:
caa	Q	1841	1340.00
cag	Q	842	1343.00
cgt	R	0	0.15
cgc	R	0	0.21
cga	R	3	0.40
cgg	R	1	0.33
aga	R	0	1.87
agg	R	0	1.04
---	--	------	------
mPD		0.43	0.51
		nPD:	0.86
		N. weight:	0.12
		Sc. PD:	0.065
		Sc. rank:	169.2

PB2			
Pos.	592	obs:	exp:
tat	Y	548	1289.00
tac	Y	2139	1398.00
---	--	------	------
mPD		0.32	0.50
		nPD:	0.65
		N. weight:	0.27
		Sc. PD:	0.087
		Sc. rank:	259.1

PB2			
Pos.	593	obs:	exp:
tct	S	0	448.40
tcc	S	0	389.10
tca	S	0	608.90
tcg	S	0	162.00
act	T	1	0.26
acc	T	0	0.23
aca	T	0	0.42
acg	T	0	0.09
agt	S	2660	582.60
agc	S	26	495.00
---	--	------	------
mPD		0.020	1.7
		nPD:	0.01
		N. weight:	2.4
		Sc. PD:	-0.73
		Sc. rank:	-3026.1

PB2			
Pos.	594	obs:	exp:
gct	A	0	0.25
gcc	A	0	0.19
gca	A	1	0.45
gcg	A	0	0.11
ggt	G	22	388.40
ggc	G	168	352.80
gga	G	2310	1177.00
ggg	G	186	767.80
---	--	------	------
mPD		0.25	0.69
		nPD:	0.37
		N. weight:	0.67
		Sc. PD:	0.03
		Sc. rank:	224.1

PB2			
Pos.	595	obs:	exp:
ttt	F	2507	1118.00
ttc	F	174	1563.00
tta	L	0	0.09
ttg	L	1	0.19
tgt	C	5	1.94
tgc	C	0	3.06
ctt	L	0	0.20
ctc	L	0	0.16
cta	L	0	0.15
ctg	L	0	0.21
---	--	------	------
mPD		0.13	0.49
		nPD:	0.26
		N. weight:	1.
		Sc. PD:	-0.064
		Sc. rank:	-103.7

PB2			
Pos.	596	obs:	exp:
att	I	0	0.37
atc	I	0	0.27
ata	I	1	0.37
act	T	0	0.26
acc	T	0	0.23
aca	T	1	0.42
acg	T	0	0.09
gtt	V	6	577.50
gtc	V	1	564.90
gta	V	577	572.50
gtg	V	2101	970.10
---	--	------	------
mPD		0.34	0.74
		nPD:	0.47
		N. weight:	0.97
		Sc. PD:	0.14
		Sc. rank:	646.5

PB2			
Pos.	597	obs:	exp:
cgt	R	0	100.50
cgc	R	0	143.70
cga	R	4	270.70
cgg	R	0	219.30
act	T	0	0.26
acc	T	0	0.23
aca	T	0	0.42
acg	T	1	0.09
aga	R	1541	1254.00
agg	R	1141	697.80
---	--	------	------
mPD		0.49	0.96
		nPD:	0.52
		N. weight:	0.53
		Sc. PD:	0.1
		Sc. rank:	398.7

PB2			
Pos.	598	obs:	exp:
att	I	0	2.20
atc	I	1	1.61
ata	I	5	2.19
atg	M	49	49.00
act	T	23	677.10
acc	T	447	582.00
aca	T	1633	1081.00
acg	T	480	243.00
gtt	V	0	0.65
gtc	V	1	0.63
gta	V	0	0.64
gtg	V	2	1.08
gct	A	0	11.59
gcc	A	0	8.90
gca	A	44	20.48
gcg	A	2	5.03
---	--	------	------
mPD		0.62	0.78
		nPD:	0.79
		N. weight:	0.52
		Sc. PD:	0.24
		Sc. rank:	670.8

PB2			
Pos.	599	obs:	exp:
tta	L	1447	248.30
ttg	L	158	510.30
ctt	L	11	533.30
ctc	L	1	429.60
cta	L	1017	397.50
ctg	L	52	566.90
gtt	V	0	0.22
gtc	V	0	0.21
gta	V	1	0.21
gtg	V	0	0.36
---	--	------	------
mPD		0.63	1.1
		nPD:	0.56
		N. weight:	1.9
		Sc. PD:	0.46
		Sc. rank:	1617.4

PB2			
Pos.	600	obs:	exp:
ttt	F	614	1121.00
ttc	F	2073	1566.00
---	--	------	------
mPD		0.35	0.49
		nPD:	0.73
		N. weight:	0.13
		Sc. PD:	0.052
		Sc. rank:	152.3

PB2			
Pos.	601	obs:	exp:
caa	Q	1373	1342.00
cag	Q	1314	1345.00
---	--	------	------
mPD		0.50	0.50
		nPD:	1.
		N. weight:	0.003
		Sc. PD:	0.002
		Sc. rank:	4.2

PB2			
Pos.	602	obs:	exp:
caa	Q	1529	1342.00
cag	Q	1158	1345.00
---	--	------	------
mPD		0.49	0.50
		nPD:	0.98
		N. weight:	0.018
		Sc. PD:	0.012
		Sc. rank:	26.0

PB2			
Pos.	603	obs:	exp:
tta	L	0	0.09
ttg	L	1	0.19
tgg	W	1	1.00
ctt	L	0	0.20
ctc	L	0	0.16
cta	L	0	0.15
ctg	L	0	0.21
att	I	0	0.73
atc	I	2	0.54
ata	I	0	0.73
atg	M	2682	2682.00
aat	N	1	0.55
aac	N	0	0.45
---	--	------	------
mPD		0.0052	0.0057
		nPD:	0.92
		N. weight:	0.003
		Sc. PD:	0.0017
		Sc. rank:	4.1

PB2			
Pos.	604	obs:	exp:
tta	L	0	0.09
ttg	L	0	0.19
tct	S	0	0.17
tcc	S	0	0.14
tca	S	0	0.23
tcg	S	0	0.06
ctt	L	1	0.20
ctc	L	0	0.16
cta	L	0	0.15
ctg	L	0	0.21
cgt	R	2489	100.30
cgc	R	2	143.40
cga	R	157	270.10
cgg	R	28	218.80
agt	S	1	0.22
agc	S	0	0.18
aga	R	4	1251.00
agg	R	0	696.20
gtt	V	1	0.22
gtc	V	0	0.21
gta	V	0	0.21
gtg	V	0	0.36
ggt	G	4	0.58
ggc	G	0	0.53
gga	G	0	1.75
ggg	G	0	1.14
---	--	------	------
mPD		0.14	0.96
		nPD:	0.15
		N. weight:	4.7
		Sc. PD:	-0.8
		Sc. rank:	-2411.7

,

PB2			
Pos.	605	obs:	exp:
gat	D	1857	1451.00
gac	D	830	1236.00
---	--	------	------
mPD		0.43	0.50
		nPD:	0.86
		N. weight:	0.08
		Sc. PD:	0.042
		Sc. rank:	109.3

,

PB2			
Pos.	606	obs:	exp:
att	I	0	5.14
atc	I	0	3.75
ata	I	14	5.12
gtt	V	19	574.90
gtc	V	2	562.40
gta	V	1340	570.00
gtg	V	1312	965.80
---	--	------	------
mPD		0.52	0.74
		nPD:	0.7
		N. weight:	0.9
		Sc. PD:	0.33
		Sc. rank:	950.4

,

PB2			
Pos.	607	obs:	exp:
tta	L	1	247.70
ttg	L	62	508.90
tgg	W	2	2.00
ctt	L	71	531.90
ctc	L	108	428.50
cta	L	783	396.50
ctg	L	1654	565.40
cgt	R	0	0.04
cgc	R	0	0.05
cga	R	0	0.10
cgg	R	1	0.08
att	I	0	1.47
atc	I	2	1.07
ata	I	2	1.46
aga	R	0	0.47
agg	R	0	0.26
gtt	V	0	0.22
gtc	V	0	0.21
gta	V	0	0.21
gtg	V	1	0.36
---	--	------	------
mPD		0.56	1.1
		nPD:	0.49
		N. weight:	1.1
		Sc. PD:	0.19
		Sc. rank:	837.5

,

PB2			
Pos.	608	obs:	exp:
ggt	G	1	388.60
ggc	G	0	352.90
gga	G	733	1177.00
ggg	G	1953	768.10
---	--	------	------
mPD		0.40	0.69
		nPD:	0.58
		N. weight:	0.89
		Sc. PD:	0.22
		Sc. rank:	778.1

,

PB2			
Pos.	609	obs:	exp:
act	T	63	704.40
acc	T	444	605.50
aca	T	2054	1124.00
acg	T	126	252.80
---	--	------	------
mPD		0.39	0.70
		nPD:	0.55
		N. weight:	0.53
		Sc. PD:	0.12
		Sc. rank:	433.9

,

PB2			
Pos.	610	obs:	exp:
ttt	F	2656	1120.00
ttc	F	29	1565.00
tta	L	2	0.18
ttg	L	0	0.38
ctt	L	0	0.40
ctc	L	0	0.32
cta	L	0	0.30
ctg	L	0	0.42
---	--	------	------
mPD		0.023	0.49
		nPD:	0.05
		N. weight:	1.3
		Sc. PD:	-0.36
		Sc. rank:	-1247.1

,

PB2			
Pos.	611	obs:	exp:
aat	N	3	2.73
aac	N	2	2.27
gat	D	1461	1445.00
gac	D	1215	1231.00
gaa	E	4	2.24
gag	E	0	1.76
ggt	G	0	0.29
ggc	G	2	0.26
gga	G	0	0.88
ggg	G	0	0.57
---	--	------	------
mPD		0.50	0.50
		nPD:	1.
		N. weight:	0.0071
		Sc. PD:	0.0047
		Sc. rank:	10.1

,

PB2			
Pos.	612	obs:	exp:
tct	S	1	0.50
tcc	S	0	0.43
tca	S	0	0.68
tcg	S	0	0.18
att	I	1	0.37
atc	I	0	0.27
ata	I	0	0.37
act	T	2537	703.40
acc	T	121	604.60
aca	T	21	1123.00
acg	T	4	252.40
agt	S	2	0.65
agc	S	0	0.55
---	--	------	------
mPD		0.11	0.70
		nPD:	0.15
		N. weight:	1.8
		Sc. PD:	-0.3
		Sc. rank:	-888.2

,

PB2			
Pos.	613	obs:	exp:
att	I	5	2.20
atc	I	1	1.61
ata	I	0	2.19
act	T	0	0.26
acc	T	1	0.23
aca	T	0	0.42
acg	T	0	0.09
gtt	V	314	565.70
gtc	V	2310	553.30
gta	V	6	560.80
gtg	V	0	950.20
gct	A	0	12.60
gcc	A	50	9.68
gca	A	0	22.26
gcg	A	0	5.47
---	--	------	------
mPD		0.26	0.78
		nPD:	0.33
		N. weight:	1.9
		Sc. PD:	0.015
		Sc. rank:	361.8

,

PB2			
Pos.	614	obs:	exp:
cat	H	1	0.58
cac	H	0	0.42
caa	Q	697	1341.00
cag	Q	1989	1345.00
---	--	------	------
mPD		0.38	0.50
		nPD:	0.77
		N. weight:	0.2
		Sc. PD:	0.087
		Sc. rank:	249.8

,

PB2			
Pos.	615	obs:	exp:
att	I	3	977.60
atc	I	0	712.90
ata	I	2661	973.50
act	T	0	0.26
acc	T	0	0.23
aca	T	1	0.42
acg	T	0	0.09
gtt	V	0	4.73
gtc	V	0	4.63
gta	V	22	4.69
gtg	V	0	7.95
---	--	------	------
mPD		0.019	0.68
		nPD:	0.03
		N. weight:	1.6
		Sc. PD:	-0.47
		Sc. rank:	-1774.9

,

PB2			
Pos.	616	obs:	exp:
att	I	4	983.80
atc	I	11	717.40
ata	I	2666	979.70
act	T	0	0.26
acc	T	0	0.23
aca	T	1	0.42
acg	T	0	0.09
gtt	V	0	1.08
gtc	V	0	1.05
gta	V	5	1.07
gtg	V	0	1.81
---	--	------	------
mPD		0.016	0.67
		nPD:	0.02
		N. weight:	1.6
		Sc. PD:	-0.46
		Sc. rank:	-1787.0

,

PB2			
Pos.	617	obs:	exp:
caa	Q	0	0.50
cag	Q	1	0.50
aaa	K	196	1529.00
aag	K	2490	1157.00
---	--	------	------
mPD		0.14	0.49
		nPD:	0.28
		N. weight:	0.91
		Sc. PD:	-0.039
		Sc. rank:	3.5

,

PB2			
Pos.	618	obs:	exp:
tta	L	2	237.40
ttg	L	3	487.90
ctt	L	28	509.90
ctc	L	296	410.80
cta	L	665	380.00
ctg	L	1574	542.00
atg	M	119	119.00
mPD		0.62	1.2
		nPD:	0.53
		N. weight:	1.1
		Sc. PD:	0.23
		Sc. rank:	897.0

PB2			
Pos.	619	obs:	exp:
tta	L	232	248.10
ttg	L	183	509.70
ctt	L	422	532.70
ctc	L	191	429.20
cta	L	1545	397.10
ctg	L	110	566.30
att	I	0	1.47
atc	I	0	1.07
ata	I	4	1.46
mPD		0.78	1.1
		nPD:	0.7
		N. weight:	0.9
		Sc. PD:	0.33
		Sc. rank:	952.2

PB2			
Pos.	620	obs:	exp:
cct	P	95	734.30
ccc	P	175	485.30
cca	P	2400	1091.00
ccg	P	17	376.70
mPD		0.20	0.71
		nPD:	0.28
		N. weight:	0.89
		Sc. PD:	-0.038
		Sc. rank:	11.6

PB2			
Pos.	621	obs:	exp:
ttt	F	2676	1121.00
ttc	F	11	1566.00
mPD		0.0082	0.49
		nPD:	0.02
		N. weight:	1.4
		Sc. PD:	-0.41
		Sc. rank:	-1656.8

PB2			
Pos.	622	obs:	exp:
tct	S	0	0.33
tcc	S	0	0.29
tca	S	2	0.45
tcg	S	0	0.12
act	T	0	0.79
acc	T	0	0.68
aca	T	3	1.26
acg	T	0	0.28
agt	S	0	0.43
agc	S	0	0.37
gct	A	204	675.60
gcc	A	6	519.00
gca	A	2466	1194.00
gcg	A	6	293.60
mPD		0.15	0.69
		nPD:	0.22
		N. weight:	0.91
		Sc. PD:	-0.09
		Sc. rank:	-180.6

PB2			
Pos.	623	obs:	exp:
tct	S	0	0.50
tcc	S	0	0.43
tca	S	3	0.68
tcg	S	0	0.18
act	T	0	0.26
acc	T	0	0.23
aca	T	1	0.42
acg	T	0	0.09
agt	S	0	0.65
agc	S	0	0.55
gct	A	160	675.60
gcc	A	23	519.00
gca	A	2491	1194.00
gcg	A	8	293.60
gaa	E	0	0.56
gag	E	1	0.44
mPD		0.14	0.69
		nPD:	0.2
		N. weight:	0.91
		Sc. PD:	-0.11
		Sc. rank:	-257.2

PB2			
Pos.	624	obs:	exp:
gct	A	106	676.90
gcc	A	1171	520.00
gca	A	1406	1196.00
gcg	A	4	294.10
mPD		0.53	0.69
		nPD:	0.78
		N. weight:	0.59
		Sc. PD:	0.26
		Sc. rank:	750.7

PB2			
Pos.	625	obs:	exp:
cct	P	2	734.10
ccc	P	8	485.10
cca	P	2668	1090.00
ccg	P	8	376.60
gct	A	0	0.25
gcc	A	0	0.19
gca	A	1	0.45
gcg	A	0	0.11
mPD		0.014	0.71
		nPD:	0.02
		N. weight:	1.4
		Sc. PD:	-0.41
		Sc. rank:	-1658.6

PB2			
Pos.	626	obs:	exp:
cct	P	792	734.30
ccc	P	66	485.30
cca	P	357	1091.00
ccg	P	1472	376.70
mPD		0.59	0.71
		nPD:	0.84
		N. weight:	0.93
		Sc. PD:	0.48
		Sc. rank:	1268.6

PB2			
Pos.	627	obs:	exp:
caa	Q	0	0.50
cag	Q	1	0.50
aaa	K	51	117.90
aag	K	156	89.13
gaa	E	1037	1390.00
gag	E	1442	1089.00
mPD		0.63	0.64
		nPD:	0.98
		N. weight:	0.092
		Sc. PD:	0.06
		Sc. rank:	130.9

PB2			
Pos.	628	obs:	exp:
tta	L	0	0.18
ttg	L	0	0.38
ctt	L	0	0.40
ctc	L	0	0.32
cta	L	0	0.30
ctg	L	2	0.42
cct	P	0	0.82
ccc	P	0	0.54
cca	P	1	1.22
ccg	P	2	0.42
cat	H	5	2.89
cac	H	0	2.11
caa	Q	304	1336.00
cag	Q	2372	1340.00
aaa	K	0	0.57
aag	K	1	0.43
mPD		0.21	0.51
		nPD:	0.41
		N. weight:	0.55
		Sc. PD:	0.05
		Sc. rank:	278.7

PB2			
Pos.	629	obs:	exp:
tct	S	0	447.20
tcc	S	0	388.10
tca	S	0	607.30
tcg	S	0	161.60
tgt	C	0	0.77
tgc	C	2	1.23
cgt	R	0	0.04
cgc	R	0	0.05
cga	R	0	0.10
cgg	R	0	0.08
aat	N	1	2.73
aac	N	4	2.27
agt	S	1032	581.10
agc	S	1647	493.70
aga	R	1	0.47
agg	R	0	0.26
mPD		0.48	1.7
		nPD:	0.29
		N. weight:	1.6
		Sc. PD:	-0.047
		Sc. rank:	77.3

PB2			
Pos.	630	obs:	exp:
cgt	R	0	99.17
cgc	R	0	141.80
cga	R	1	267.00
cgg	R	1	216.40
att	I	0	0.37
atc	I	0	0.27
ata	I	1	0.37
aaa	K	33	19.93
aag	K	2	15.07
aga	R	1577	1237.00
agg	R	1071	688.50
ggt	G	0	0.14
ggc	G	0	0.13
gga	G	1	0.44
ggg	G	0	0.29
mPD		0.51	0.98
		nPD:	0.52
		N. weight:	0.52
		Sc. PD:	0.1
		Sc. rank:	408.2

PB2			
Pos.	631	obs:	exp:
tta	L	0	7.58
ttg	L	1	15.58
ctt	L	1	16.28
ctc	L	0	13.12
cta	L	0	12.14
ctg	L	80	17.31
atg	M	2599	2599.00
aaa	K	0	0.57
aag	K	1	0.43
gtt	V	0	1.08
gtc	V	0	1.05
gta	V	0	1.07
gtg	V	5	1.81
mPD		0.064	0.10
		nPD:	0.63
		N. weight:	0.075
		Sc. PD:	0.023
		Sc. rank:	71.1

PB2			
Pos.	632	obs:	exp:
caa	Q	227	1342.00
cag	Q	2460	1345.00
mPD		0.15	0.50
		nPD:	0.31
		N. weight:	0.65
		Sc. PD:	-0.0073
		Sc. rank:	91.8

PB2			
Pos.	633	obs:	exp:
ttt	F	1800	1121.00
ttc	F	887	1566.00
mPD		0.44	0.49
		nPD:	0.91
		N. weight:	0.21
		Sc. PD:	0.12
		Sc. rank:	297.3

PB2			
Pos.	634	obs:	exp:
ttt	F	4	1.67
ttc	F	0	2.33
tct	S	2385	447.70
tcc	S	295	388.50
tca	S	0	608.00
tcg	S	2	161.80
act	T	1	0.26
acc	T	0	0.23
aca	T	0	0.42
acg	T	0	0.09
agt	S	0	581.70
agc	S	0	494.30
mPD		0.20	1.7
		nPD:	0.12
		N. weight:	2.4
		Sc. PD:	-0.46
		Sc. rank:	-1413.9

PB2			
Pos.	635	obs:	exp:
ttt	F	1	0.42
ttc	F	0	0.58
tct	S	2457	448.40
tcc	S	211	389.10
tca	S	18	608.90
tcg	S	0	162.00
agt	S	0	582.60
agc	S	0	495.00
mPD		0.16	1.7
		nPD:	0.1
		N. weight:	2.4
		Sc. PD:	-0.53
		Sc. rank:	-1691.0

PB2			
Pos.	636	obs:	exp:
tta	L	30	248.30
ttg	L	21	510.10
ctt	L	5	533.10
ctc	L	0	429.50
cta	L	2090	397.40
ctg	L	539	566.70
att	I	0	0.37
atc	I	0	0.27
ata	I	1	0.37
atg	M	1	1.00
mPD		0.37	1.1
		nPD:	0.33
		N. weight:	2.
		Sc. PD:	0.021
		Sc. rank:	428.5

PB2

Pos.	637	obs:	exp:
tct	S	1	0.17
tcc	S	0	0.14
tca	S	0	0.23
tcg	S	0	0.06
att	I	25	9.17
atc	I	0	6.69
ata	I	0	9.14
act	T	2613	697.10
acc	T	37	599.10
aca	T	4	1113.00
acg	T	5	250.20
agt	S	0	0.22
agc	S	0	0.18
gct	A	2	0.50
gcc	A	0	0.39
gca	A	0	0.89
gcg	A	0	0.22
mPD		0.054	0.72
		nPD:	0.08
		N. weight:	2.
		Sc. PD:	-0.49
		Sc. rank:	-1614.6

,

PB2

Pos.	638	obs:	exp:
tta	L	0	0.09
ttg	L	1	0.19
ctt	L	0	0.20
ctc	L	0	0.16
cta	L	0	0.15
ctg	L	0	0.21
gtt	V	5	577.70
gtc	V	0	565.10
gta	V	681	572.70
gtg	V	2000	970.50
mPD		0.38	0.73
		nPD:	0.52
		N. weight:	0.93
		Sc. PD:	0.18
		Sc. rank:	718.8

,

PB2

Pos.	639	obs:	exp:
tct	S	0	1.34
tcc	S	0	1.16
tca	S	0	1.81
tcg	S	0	0.48
aat	N	1694	1461.00
aac	N	985	1218.00
agt	S	1	1.74
agc	S	7	1.47
mPD		0.47	0.51
		nPD:	0.93
		N. weight:	0.034
		Sc. PD:	0.021
		Sc. rank:	48.2

,

PB2

Pos.	640	obs:	exp:
tta	L	0	0.18
ttg	L	2	0.38
ctt	L	0	0.40
ctc	L	0	0.32
cta	L	0	0.30
ctg	L	0	0.42
att	I	0	1.10
atc	I	0	0.80
ata	I	3	1.10
gtt	V	130	576.60
gtc	V	1	564.00
gta	V	843	571.70
gtg	V	1707	968.70
ggt	G	0	0.14
ggc	G	0	0.13
gga	G	1	0.44
ggg	G	0	0.29
mPD		0.50	0.74
		nPD:	0.68
		N. weight:	0.67
		Sc. PD:	0.23
		Sc. rank:	673.9

,

PB2

Pos.	641	obs:	exp:
cgt	R	0	100.40
cgc	R	0	143.60
cga	R	2	270.50
cgg	R	0	219.20
aaa	K	3	1.71
aag	K	0	1.29
aga	R	1188	1253.00
agg	R	1494	697.30
mPD		0.50	0.96
		nPD:	0.52
		N. weight:	0.65
		Sc. PD:	0.13
		Sc. rank:	503.7

,

PB2

Pos.	642	obs:	exp:
ggt	G	1058	388.60
ggc	G	1369	352.90
gga	G	228	1177.00
ggg	G	32	768.10
mPD		0.58	0.69
		nPD:	0.84
		N. weight:	1.5
		Sc. PD:	0.75
		Sc. rank:	2019.0

,

PB2

Pos.	643	obs:	exp:
tct	S	38	447.00
tcc	S	1	387.90
tca	S	2625	607.10
tcg	S	14	161.60
cct	P	0	0.27
ccc	P	0	0.18
cca	P	1	0.41
ccg	P	0	0.14
act	T	0	1.31
acc	T	0	1.13
aca	T	5	2.09
acg	T	0	0.47
agt	S	0	580.90
agc	S	0	493.50
gct	A	0	0.76
gcc	A	0	0.58
gca	A	3	1.34
gcg	A	0	0.33
mPD		0.046	1.7
		nPD:	0.03
		N. weight:	2.2
		Sc. PD:	-0.65
		Sc. rank:	-2466.1

,

PB2

Pos.	644	obs:	exp:
cgt	R	0	0.07
cgc	R	0	0.11
cga	R	2	0.20
cgg	R	0	0.16
aga	R	0	0.93
agg	R	0	0.52
ggt	G	1	388.30
ggc	G	1	352.70
gga	G	2643	1177.00
ggg	G	40	767.50
mPD		0.032	0.69
		nPD:	0.05
		N. weight:	1.2
		Sc. PD:	-0.33
		Sc. rank:	-1146.5

,

PB2

Pos.	645	obs:	exp:
ttt	F	1	0.42
ttc	F	0	0.58
tta	L	0	0.09
ttg	L	1	0.19
ctt	L	0	0.20
ctc	L	0	0.16
cta	L	0	0.15
ctg	L	0	0.21
att	I	0	0.73
atc	I	0	0.54
ata	I	2	0.73
atg	M	2682	2682.00
gtt	V	0	0.22
gtc	V	0	0.21
gta	V	0	0.21
gtg	V	1	0.36
mPD		0.0045	0.0054
		nPD:	0.83
		N. weight:	0.0034
		Sc. PD:	0.0017
		Sc. rank:	4.5

,

PB2

Pos.	646	obs:	exp:
tct	S	0	0.50
tcc	S	0	0.43
tca	S	0	0.68
tcg	S	0	0.18
cgt	R	0	100.30
cgc	R	0	143.50
cga	R	1	270.20
cgg	R	0	218.90
aaa	K	3	1.71
aag	K	0	1.29
agt	S	3	0.65
agc	S	0	0.55
aga	R	2505	1252.00
agg	R	175	696.50
mPD		0.13	0.96
		nPD:	0.13
		N. weight:	0.91
		Sc. PD:	-0.17
		Sc. rank:	-491.4

,

PB2

Pos.	647	obs:	exp:
tta	L	1	0.28
ttg	L	0	0.57
ctt	L	0	0.60
ctc	L	0	0.48
cta	L	2	0.44
ctg	L	0	0.63
att	I	0	982.00
atc	I	3	716.10
ata	I	2673	977.90
atg	M	4	4.00
act	T	0	0.26
acc	T	0	0.23
aca	T	1	0.42
acg	T	0	0.09
gtt	V	0	0.65
gtc	V	0	0.63
gta	V	3	0.64
gtg	V	0	1.08
mPD		0.010	0.67
		nPD:	0.02
		N. weight:	1.6
		Sc. PD:	-0.49
		Sc. rank:	-1968.4

,

PB2

Pos.	648	obs:	exp:
ttt	F	1	6.26
ttc	F	14	8.74
tta	L	0	228.40
ttg	L	0	469.20
ctt	L	1044	490.40
ctc	L	1246	395.10
cta	L	24	365.50
ctg	L	156	521.30
cct	P	47	51.65
ccc	P	142	34.14
cca	P	0	76.71
ccg	P	0	26.50
att	I	3	1.47
atc	I	1	1.07
ata	I	0	1.46
atg	M	6	6.00
gtt	V	3	0.65
gtc	V	0	0.63
gta	V	0	0.64
gtg	V	0	1.08
mPD		0.71	1.3
		nPD:	0.57
		N. weight:	1.3
		Sc. PD:	0.32
		Sc. rank:	1126.8

,

PB2

Pos.	649	obs:	exp:
att	I	0	116.70
atc	I	0	85.10
ata	I	318	116.20
atg	M	5	5.00
gtt	V	19	506.70
gtc	V	1	495.70
gta	V	1260	502.40
gtg	V	1076	851.20
gct	A	0	2.02
gcc	A	0	1.55
gca	A	7	3.56
gcg	A	1	0.88
mPD		0.71	0.96
		nPD:	0.74
		N. weight:	1.
		Sc. PD:	0.41
		Sc. rank:	1221.1

,

PB2

Pos.	650	obs:	exp:
cgt	R	0	100.50
cgc	R	0	143.70
cga	R	1	270.70
cgg	R	8	219.30
aga	R	1088	1254.00
agg	R	1589	697.80
ggt	G	0	0.14
ggc	G	0	0.13
gga	G	0	0.44
ggg	G	1	0.29
mPD		0.49	0.95
		nPD:	0.51
		N. weight:	0.68
		Sc. PD:	0.13
		Sc. rank:	515.2

,

PB2			
Pos.	651	obs:	exp:
ggt	G	62	388.60
ggc	G	2615	352.90
gga	G	9	1177.00
ggg	G	1	768.10
---	--	------	------
mPD		0.052	0.69
		nPD:	0.08
		N. weight:	3.1
		Sc. PD:	-0.74
		Sc. rank:	-2417.2

,

PB2			
Pos.	652	obs:	exp:
tct	S	0	1.17
tcc	S	0	1.01
tca	S	0	1.59
tcg	S	0	0.42
cat	H	2	1.16
cac	H	0	0.84
act	T	0	0.26
acc	T	1	0.23
aca	T	0	0.42
acg	T	0	0.09
aat	N	1902	1460.00
aac	N	775	1217.00
agt	S	7	1.52
agc	S	0	1.29
---	--	------	------
mPD		0.42	0.51
		nPD:	0.82
		N. weight:	0.1
		Sc. PD:	0.051
		Sc. rank:	138.3

,

PB2			
Pos.	653	obs:	exp:
ttt	F	0	0.42
ttc	F	1	0.58
tct	S	274	448.40
tcc	S	2389	389.10
tca	S	23	608.90
tcg	S	0	162.00
agt	S	0	582.60
agc	S	0	495.00
---	--	------	------
mPD		0.20	1.7
		nPD:	0.12
		N. weight:	2.5
		Sc. PD:	-0.49
		Sc. rank:	-1516.6

,

PB2			
Pos.	654	obs:	exp:
tct	S	8	1.34
tcc	S	0	1.16
tca	S	0	1.81
tcg	S	0	0.48
cct	P	2408	731.60
ccc	P	44	483.50
cca	P	223	1087.00
ccg	P	2	375.30
cat	H	1	0.58
cac	H	0	0.42
act	T	1	0.26
acc	T	0	0.23
aca	T	0	0.42
acg	T	0	0.09
agt	S	0	1.74
agc	S	0	1.47
---	--	------	------
mPD		0.19	0.72
		nPD:	0.27
		N. weight:	1.5
		Sc. PD:	-0.08
		Sc. rank:	-62.2

,

PB2			
Pos.	655	obs:	exp:
tta	L	0	0.09
ttg	L	1	0.19
ctt	L	0	0.20
ctc	L	0	0.16
cta	L	0	0.15
ctg	L	0	0.21
att	I	1	0.37
atc	I	0	0.27
ata	I	0	0.37
atg	M	1	1.00
gtt	V	215	572.50
gtc	V	1	560.00
gta	V	45	567.60
gtg	V	2401	961.80
gct	A	0	5.54
gcc	A	0	4.26
gca	A	0	9.79
gcg	A	22	2.41
---	--	------	------
mPD		0.20	0.75
		nPD:	0.26
		N. weight:	1.2
		Sc. PD:	-0.067
		Sc. rank:	-64.6

,

PB2			
Pos.	656	obs:	exp:
ttt	F	12	1120.00
ttc	F	2673	1565.00
tct	S	0	0.17
tcc	S	1	0.14
tca	S	0	0.23
tcg	S	0	0.06
tat	Y	0	0.48
tac	Y	1	0.52
agt	S	0	0.22
agc	S	0	0.18
---	--	------	------
mPD		0.010	0.49
		nPD:	0.02
		N. weight:	0.83
		Sc. PD:	-0.25
		Sc. rank:	-968.2

,

PB2			
Pos.	657	obs:	exp:
cat	H	0	1.16
cac	H	2	0.84
aat	N	513	1463.00
aac	N	2169	1219.00
aaa	K	2	1.14
aag	K	0	0.86
gat	D	0	0.54
gac	D	1	0.46
---	--	------	------
mPD		0.31	0.50
		nPD:	0.63
		N. weight:	0.43
		Sc. PD:	0.13
		Sc. rank:	410.0

,

PB2			
Pos.	658	obs:	exp:
tat	Y	848	1277.00
tac	Y	1814	1385.00
cat	H	22	13.89
cac	H	2	10.11
gat	D	1	0.54
gac	D	0	0.46
---	--	------	------
mPD		0.46	0.52
		nPD:	0.88
		N. weight:	0.094
		Sc. PD:	0.052
		Sc. rank:	130.0

,

PB2			
Pos.	659	obs:	exp:
tct	S	0	1.00
tcc	S	0	0.87
tca	S	0	1.36
tcg	S	0	0.36
att	I	0	0.73
atc	I	2	0.54
ata	I	0	0.73
aat	N	1658	1461.00
aac	N	1021	1218.00
agt	S	5	1.30
agc	S	1	1.11
---	--	------	------
mPD		0.48	0.51
		nPD:	0.95
		N. weight:	0.027
		Sc. PD:	0.016
		Sc. rank:	37.2

,

PB2			
Pos.	660	obs:	exp:
cgt	R	0	5.54
cgc	R	0	7.92
cga	R	0	14.91
cgg	R	0	12.09
aat	N	7	3.82
aac	N	0	3.18
aaa	K	160	1442.00
aag	K	2372	1090.00
aga	R	4	69.09
agg	R	144	38.45
---	--	------	------
mPD		0.22	0.63
		nPD:	0.35
		N. weight:	1.
		Sc. PD:	0.033
		Sc. rank:	304.3

,

PB2			
Pos.	661	obs:	exp:
tct	S	0	0.17
tcc	S	0	0.14
tca	S	1	0.23
tcg	S	0	0.06
act	T	0	19.40
acc	T	1	16.67
aca	T	69	30.96
acg	T	4	6.96
agt	S	0	0.22
agc	S	0	0.18
gtt	V	0	0.65
gtc	V	0	0.63
gta	V	3	0.64
gtg	V	0	1.08
gct	A	2	656.80
gcc	A	273	504.50
gca	A	2244	1160.00
gcg	A	88	285.30
gaa	E	2	1.12
gag	E	0	0.88
---	--	------	------
mPD		0.30	0.75
		nPD:	0.4
		N. weight:	0.76
		Sc. PD:	0.062
		Sc. rank:	358.9

,

PB2			
Pos.	662	obs:	exp:
att	I	0	1.10
atc	I	3	0.80
ata	I	0	1.10
act	T	45	679.20
acc	T	2317	583.80
aca	T	229	1084.00
acg	T	0	243.80
aat	N	0	50.72
aac	N	93	42.28
---	--	------	------
mPD		0.26	0.77
		nPD:	0.33
		N. weight:	1.7
		Sc. PD:	0.018
		Sc. rank:	377.3

,

PB2			
Pos.	663	obs:	exp:
caa	Q	5	3.00
cag	Q	1	3.00
cgt	R	0	1.80
cgc	R	0	2.57
cga	R	0	4.84
cgg	R	0	3.92
act	T	0	0.52
acc	T	0	0.45
aca	T	0	0.84
acg	T	2	0.19
aaa	K	1456	1498.00
aag	K	1175	1133.00
aga	R	47	22.41
agg	R	1	12.47
---	--	------	------
mPD		0.53	0.54
		nPD:	0.98
		N. weight:	0.028
		Sc. PD:	0.018
		Sc. rank:	39.3

,

PB2

Pos. 664		obs:	exp:
cgt	R	0	100.40
cgc	R	0	143.60
cga	R	1	270.50
cgg	R	1	219.20
aaa	K	0	1.71
aag	K	3	1.29
aga	R	369	1253.00
agg	R	2313	697.30
---	--	------	------
mPD		0.24	0.96
		nPD:	0.25
		N. weight:	1.4
		Sc. PD:	-0.094
		Sc. rank:	-154.7

,

PB2

Pos. 665		obs:	exp:
ttt	F	0	0.42
ttc	F	1	0.58
tta	L	0	248.10
ttg	L	0	509.70
ctt	L	1734	532.70
ctc	L	938	429.20
cta	L	10	397.10
ctg	L	1	566.30
cat	H	0	0.58
cac	H	1	0.42
att	I	1	0.37
atc	I	0	0.27
ata	I	0	0.37
gtt	V	1	0.22
gtc	V	0	0.21
gta	V	0	0.21
gtg	V	0	0.36
---	--	------	------
mPD		0.46	1.1
		nPD:	0.41
		N. weight:	1.7
		Sc. PD:	0.15
		Sc. rank:	844.1

,

PB2

Pos. 666		obs:	exp:
cct	P	0	0.27
ccc	P	0	0.18
cca	P	1	0.41
ccg	P	0	0.14
att	I	0	68.99
atc	I	4	50.31
ata	I	184	68.70
act	T	174	598.20
acc	T	1070	514.20
aca	T	1038	954.90
acg	T	0	214.70
aaa	K	1	0.57
aag	K	0	0.43
gtt	V	1	0.22
gtc	V	0	0.21
gta	V	0	0.21
gtg	V	0	0.36
gct	A	56	53.91
gcc	A	151	41.41
gca	A	7	95.26
gcg	A	0	23.42
---	--	------	------
mPD		0.85	0.97
		nPD:	0.88
		N. weight:	0.62
		Sc. PD:	0.34
		Sc. rank:	856.6

,

PB2

Pos. 667		obs:	exp:
att	I	48	20.92
atc	I	4	15.25
ata	I	5	20.83
gtt	V	1435	565.70
gtc	V	978	553.30
gta	V	210	560.80
gtg	V	7	950.20
---	--	------	------
mPD		0.60	0.78
		nPD:	0.77
		N. weight:	1.
		Sc. PD:	0.45
		Sc. rank:	1293.7

,

PB2

Pos. 668		obs:	exp:
tta	L	0	244.50
ttg	L	0	502.30
ctt	L	2434	525.00
ctc	L	210	422.90
cta	L	0	391.30
ctg	L	0	558.10
att	I	40	15.78
atc	I	3	11.51
ata	I	0	15.71
---	--	------	------
mPD		0.18	1.1
		nPD:	0.16
		N. weight:	2.2
		Sc. PD:	-0.36
		Sc. rank:	-1013.7

,

PB2

Pos. 669		obs:	exp:
ggt	G	3	388.60
ggc	G	2	352.90
gga	G	2422	1177.00
ggg	G	260	768.10
---	--	------	------
mPD		0.18	0.69
		nPD:	0.26
		N. weight:	0.87
		Sc. PD:	-0.053
		Sc. rank:	-69.7

,

PB2

Pos. 670		obs:	exp:
cgt	R	0	0.11
cgc	R	0	0.16
cga	R	0	0.30
cgg	R	0	0.24
aat	N	1	0.55
aac	N	0	0.45
aaa	K	209	1528.00
aag	K	2474	1155.00
aga	R	0	1.40
agg	R	3	0.78
---	--	------	------
mPD		0.15	0.49
		nPD:	0.3
		N. weight:	0.89
		Sc. PD:	-0.021
		Sc. rank:	66.1

,

PB2

Pos. 671		obs:	exp:
aat	N	3	1.64
aac	N	0	1.36
gat	D	1421	1449.00
gac	D	1262	1234.00
gaa	E	1	0.56
gag	E	0	0.44
---	--	------	------
mPD		0.50	0.50
		nPD:	1.
		N. weight:	0.0043
		Sc. PD:	0.0029
		Sc. rank:	6.1

,

PB2

Pos. 672		obs:	exp:
act	T	1	0.26
acc	T	0	0.23
aca	T	0	0.42
acg	T	0	0.09
gct	A	440	676.70
gcc	A	7	519.80
gca	A	2224	1196.00
gcg	A	15	294.00
---	--	------	------
mPD		0.29	0.69
		nPD:	0.42
		N. weight:	0.68
		Sc. PD:	0.065
		Sc. rank:	358.6

,

PB2

Pos. 673		obs:	exp:
cgt	R	1	0.04
cgc	R	0	0.05
cga	R	0	0.10
cgg	R	0	0.08
aga	R	0	0.47
agg	R	0	0.26
ggt	G	2641	388.40
ggc	G	21	352.80
gga	G	3	1177.00
ggg	G	21	767.80
---	--	------	------
mPD		0.034	0.69
		nPD:	0.05
		N. weight:	3.
		Sc. PD:	-0.79
		Sc. rank:	-2747.9

,

PB2

Pos. 674		obs:	exp:
tct	S	0	1.00
tcc	S	0	0.87
tca	S	6	1.36
tcg	S	0	0.36
act	T	1	0.26
acc	T	0	0.23
aca	T	0	0.42
acg	T	0	0.09
agt	S	0	1.30
agc	S	0	1.11
gtt	V	0	0.86
gtc	V	0	0.84
gta	V	4	0.85
gtg	V	0	1.45
gct	A	214	673.90
gcc	A	108	517.60
gca	A	2318	1191.00
gcg	A	35	292.80
gaa	E	1	0.56
gag	E	0	0.44
---	--	------	------
mPD		0.25	0.70
		nPD:	0.36
		N. weight:	0.65
		Sc. PD:	0.023
		Sc. rank:	209.4

,

PB2

Pos. 675		obs:	exp:
tta	L	1394	248.40
ttg	L	1223	510.50
ctt	L	4	533.50
ctc	L	1	429.80
cta	L	36	397.70
ctg	L	29	567.10
---	--	------	------
mPD		0.55	1.1
		nPD:	0.49
		N. weight:	2.
		Sc. PD:	0.33
		Sc. rank:	1418.3

,

PB2

Pos. 676		obs:	exp:
tta	L	0	0.18
ttg	L	0	0.38
tct	S	0	0.17
tcc	S	0	0.14
tca	S	1	0.23
tcg	S	0	0.06
ctt	L	2	0.40
ctc	L	0	0.32
cta	L	0	0.30
ctg	L	0	0.42
caa	Q	3	1.50
cag	Q	0	1.50
att	I	1	1.83
atc	I	0	1.34
ata	I	4	1.83
atg	M	216	216.00
act	T	65	633.60
acc	T	168	544.60
aca	T	2148	1011.00
acg	T	36	227.40
aaa	K	3	1.71
aag	K	0	1.29
agt	S	0	0.22
agc	S	0	0.18
gct	A	0	10.08
gcc	A	1	7.74
gca	A	39	17.80
gcg	A	0	4.38
---	--	------	------
mPD		0.51	0.91
		nPD:	0.56
		N. weight:	0.76
		Sc. PD:	0.18
		Sc. rank:	629.5

,

PB2

Pos. 677		obs:	exp:
aaa	K	3	2.28
aag	K	1	1.72
gtt	V	0	0.22
gtc	V	0	0.21
gta	V	1	0.21
gtg	V	0	0.36
gct	A	0	0.50
gcc	A	0	0.39
gca	A	1	0.89
gcg	A	1	0.22
gat	D	4	2.16
gac	D	0	1.84
gaa	E	1030	1391.00
gag	E	1452	1091.00
ggt	G	0	28.06
ggc	G	0	25.48
gga	G	154	85.01
ggg	G	40	55.46
---	--	------	------
mPD		0.63	0.65
		nPD:	0.97
		N. weight:	0.12
		Sc. PD:	0.077
		Sc. rank:	169.0

,

PB2			
Pos.	678	obs:	exp:
tct	S	0	0.17
tcc	S	0	0.14
tca	S	0	0.23
tcg	S	0	0.06
tat	Y	7	3.36
tac	Y	0	3.64
aat	N	95	51.81
aac	N	0	43.19
agt	S	1	0.22
agc	S	0	0.18
gat	D	1833	1384.00
gac	D	731	1180.00
gaa	E	3	7.29
gag	E	10	5.71
ggt	G	1	1.01
ggc	G	5	0.92
gga	G	1	3.07
ggg	G	0	2.00
---	--	------	------
mPD		0.49	0.58
		nPD:	0.83
		N. weight:	0.15
		Sc. PD:	0.075
		Sc. rank:	200.9

,

PB2			
Pos.	679	obs:	exp:
tta	L	0	0.46
ttg	L	0	0.95
tct	S	0	0.33
tcc	S	0	0.29
tca	S	2	0.45
tcg	S	0	0.12
ctt	L	0	0.99
ctc	L	0	0.80
cta	L	5	0.74
ctg	L	0	1.06
cct	P	1	728.30
ccc	P	17	481.30
cca	P	2585	1082.00
ccg	P	62	373.60
act	T	0	3.93
acc	T	0	3.38
aca	T	15	6.28
acg	T	0	1.41
agt	S	0	0.43
agc	S	0	0.37
---	--	------	------
mPD		0.074	0.73
		nPD:	0.1
		N. weight:	1.3
		Sc. PD:	-0.27
		Sc. rank:	-844.5

,

PB2			
Pos.	680	obs:	exp:
tat	Y	0	0.48
tac	Y	1	0.52
aat	N	6	21.81
aac	N	34	18.19
gat	D	1475	1302.00
gac	D	936	1109.00
gaa	E	4	3.92
gag	E	3	3.08
ggt	G	225	32.97
ggc	G	3	29.95
gga	G	0	99.91
ggg	G	0	65.17
---	--	------	------
mPD		0.65	0.74
		nPD:	0.88
		N. weight:	0.29
		Sc. PD:	0.16
		Sc. rank:	396.6

,

PB2			
Pos.	681	obs:	exp:
aaa	K	0	0.57
aag	K	1	0.43
gat	D	1	0.54
gac	D	0	0.46
gaa	E	219	1505.00
gag	E	2465	1179.00
ggt	G	0	0.14
ggc	G	0	0.13
gga	G	0	0.44
ggg	G	1	0.29
---	--	------	------
mPD		0.15	0.49
		nPD:	0.31
		N. weight:	0.85
		Sc. PD:	-0.011
		Sc. rank:	116.5

,

PB2			
Pos.	682	obs:	exp:
tct	S	0	0.17
tcc	S	0	0.14
tca	S	0	0.23
tcg	S	0	0.06
cgt	R	0	0.04
cgc	R	0	0.05
cga	R	0	0.10
cgg	R	0	0.08
agt	S	0	0.22
agc	S	1	0.18
aga	R	0	0.47
agg	R	1	0.26
ggt	G	0	388.30
ggc	G	180	352.70
gga	G	740	1177.00
ggg	G	1765	767.50
---	--	------	------
mPD		0.49	0.69
		nPD:	0.71
		N. weight:	0.61
		Sc. PD:	0.23
		Sc. rank:	684.5

,

PB2			
Pos.	683	obs:	exp:
tct	S	0	0.17
tcc	S	0	0.14
tca	S	1	0.23
tcg	S	0	0.06
act	T	0	661.90
acc	T	1	568.90
aca	T	2513	1057.00
acg	T	11	237.60
aaa	K	95	56.37
aag	K	4	42.63
agt	S	0	0.22
agc	S	0	0.18
gct	A	0	15.62
gcc	A	0	12.00
gca	A	62	27.60
gcg	A	0	6.79
---	--	------	------
mPD		0.13	0.81
		nPD:	0.16
		N. weight:	1.3
		Sc. PD:	-0.22
		Sc. rank:	-609.3

,

PB2			
Pos.	684	obs:	exp:
tct	S	1	4.67
tcc	S	25	4.06
tca	S	2	6.35
tcg	S	0	1.69
act	T	1	30.41
acc	T	103	26.14
aca	T	12	48.54
acg	T	0	10.91
aat	N	0	0.55
aac	N	1	0.45
agt	S	0	6.07
agc	S	0	5.16
gct	A	113	639.10
gcc	A	1384	490.90
gca	A	1039	1129.00
gcg	A	1	277.70
gat	D	3	2.16
gac	D	1	1.84
gaa	E	1	0.56
gag	E	0	0.44
---	--	------	------
mPD		0.63	0.81
		nPD:	0.79
		N. weight:	0.8
		Sc. PD:	0.37
		Sc. rank:	1032.6

,

PB2			
Pos.	685	obs:	exp:
cgt	R	0	0.07
cgc	R	0	0.11
cga	R	1	0.20
cgg	R	0	0.16
aga	R	1	0.93
agg	R	0	0.52
ggt	G	0	388.30
ggc	G	0	352.70
gga	G	2606	1177.00
ggg	G	79	767.50
---	--	------	------
mPD		0.059	0.69
		nPD:	0.08
		N. weight:	1.1
		Sc. PD:	-0.27
		Sc. rank:	-864.9

,

PB2			
Pos.	686	obs:	exp:
gtt	V	24	577.70
gtc	V	0	565.10
gta	V	271	572.70
gtg	V	2391	970.50
ggt	G	0	0.14
ggc	G	0	0.13
gga	G	0	0.44
ggg	G	1	0.29
---	--	------	------
mPD		0.20	0.73
		nPD:	0.27
		N. weight:	1.1
		Sc. PD:	-0.057
		Sc. rank:	-33.9

,

PB2			
Pos.	687	obs:	exp:
gaa	E	1947	1506.00
gag	E	740	1181.00
---	--	------	------
mPD		0.40	0.49
		nPD:	0.81
		N. weight:	0.095
		Sc. PD:	0.046
		Sc. rank:	127.0

,

PB2			
Pos.	688	obs:	exp:
tct	S	2634	448.50
tcc	S	45	389.20
tca	S	0	609.10
tcg	S	8	162.10
agt	S	0	582.80
agc	S	0	495.20
---	--	------	------
mPD		0.039	1.7
		nPD:	0.02
		N. weight:	2.7
		Sc. PD:	-0.8
		Sc. rank:	-3080.8

,

PB2			
Pos.	689	obs:	exp:
gct	A	185	676.90
gcc	A	3	520.00
gca	A	1589	1196.00
gcg	A	910	294.10
---	--	------	------
mPD		0.53	0.69
		nPD:	0.77
		N. weight:	0.74
		Sc. PD:	0.33
		Sc. rank:	945.4

,

PB2			
Pos.	690	obs:	exp:
att	I	0	0.37
atc	I	0	0.27
ata	I	1	0.37
gtt	V	231	577.70
gtc	V	6	565.10
gta	V	2404	572.70
gtg	V	45	970.50
---	--	------	------
mPD		0.19	0.73
		nPD:	0.26
		N. weight:	1.9
		Sc. PD:	-0.11
		Sc. rank:	-123.5

,

PB2			
Pos.	691	obs:	exp:
tta	L	503	248.40
ttg	L	736	510.50
ctt	L	1	533.50
ctc	L	0	429.80
cta	L	42	397.70
ctg	L	1405	567.10
---	--	------	------
mPD		0.82	1.1
		nPD:	0.73
		N. weight:	1.1
		Sc. PD:	0.44
		Sc. rank:	1311.7

,

PB2			
Pos.	692	obs:	exp:
cgt	R	0	100.60
cgc	R	0	143.80
cga	R	94	270.80
cgg	R	1	219.40
aga	R	1126	1254.00
agg	R	1466	698.10
---	--	------	------
mPD		0.56	0.95
		nPD:	0.59
		N. weight:	0.53
		Sc. PD:	0.14
		Sc. rank:	473.7

,

PB2			
Pos.	693	obs:	exp:
ggt	G	132	388.60
ggc	G	7	352.90
gga	G	1714	1177.00
ggg	G	834	768.10
---	--	------	------
mPD		0.49	0.69
		nPD:	0.72
		N. weight:	0.33
		Sc. PD:	0.13
		Sc. rank:	384.0

,

PB2			
Pos.	694	obs:	exp:
ttt	F	783	1121.00
ttc	F	1904	1566.00
---	--	------	------
mPD		0.41	0.49
		nPD:	0.85
		N. weight:	0.057
		Sc. PD:	0.03
		Sc. rank:	78.6

,

PB2			
Pos.	695	obs:	exp:
tta	L	153	248.40
ttg	L	6	510.50
ctt	L	1	533.50
ctc	L	182	429.80
cta	L	2123	397.70
ctg	L	222	567.10
---	--	------	------
mPD		0.38	1.1
		nPD:	0.34
		N. weight:	1.9
		Sc. PD:	0.037
		Sc. rank:	476.0

,

PB2			
Pos.	696	obs:	exp:
att	I	2183	983.50
atc	I	487	717.20
ata	I	10	979.40
gtt	V	7	1.51
gtc	V	0	1.47
gta	V	0	1.49
gtg	V	0	2.53
mPD		0.31	0.67
		nPD:	0.46
		N. weight:	0.92
		Sc. PD:	0.13
		Sc. rank:	589.7

PB2			
Pos.	697	obs:	exp:
tta	L	7	247.50
ttg	L	184	508.60
ctt	L	284	531.50
ctc	L	7	428.20
cta	L	1425	396.20
ctg	L	770	565.00
att	I	1	3.67
atc	I	0	2.68
ata	I	9	3.65
mPD		0.71	1.1
		nPD:	0.63
		N. weight:	1.
		Sc. PD:	0.31
		Sc. rank:	955.5

PB2			
Pos.	698	obs:	exp:
tct	S	0	0.33
tcc	S	0	0.29
tca	S	0	0.45
tcg	S	0	0.12
agt	S	0	0.43
agc	S	2	0.37
ggt	G	95	388.30
ggc	G	2463	352.70
gga	G	127	1177.00
ggg	G	0	767.50
mPD		0.16	0.69
		nPD:	0.23
		N. weight:	2.6
		Sc. PD:	-0.24
		Sc. rank:	-473.2

PB2			
Pos.	699	obs:	exp:
caa	Q	0	0.50
cag	Q	1	0.50
cgt	R	0	0.19
cgc	R	0	0.27
cga	R	0	0.50
cgg	R	0	0.41
act	T	0	0.26
acc	T	0	0.23
aca	T	1	0.42
acg	T	0	0.09
aat	N	7	3.82
aac	N	0	3.18
aaa	K	1221	1522.00
aag	K	1452	1151.00
aga	R	3	2.33
agg	R	2	1.30
mPD		0.50	0.50
		nPD:	1.01
		N. weight:	0.049
		Sc. PD:	0.033
		Sc. rank:	69.4

PB2			
Pos.	700	obs:	exp:
gaa	E	707	1506.00
gag	E	1979	1180.00
ggt	G	0	0.14
ggc	G	0	0.13
gga	G	0	0.44
ggg	G	1	0.29
mPD		0.39	0.49
		nPD:	0.79
		N. weight:	0.3
		Sc. PD:	0.14
		Sc. rank:	384.9

PB2			
Pos.	701	obs:	exp:
gat	D	29	1449.00
gac	D	2654	1234.00
gaa	E	4	2.24
gag	E	0	1.76
mPD		0.024	0.50
		nPD:	0.05
		N. weight:	1.2
		Sc. PD:	-0.31
		Sc. rank:	-1087.3

PB2			
Pos.	702	obs:	exp:
cgt	R	0	5.09
cgc	R	0	7.28
cga	R	0	13.71
cgg	R	0	11.11
att	I	0	1.83
atc	I	0	1.34
ata	I	5	1.83
act	T	0	0.26
acc	T	0	0.23
aca	T	1	0.42
acg	T	0	0.09
aaa	K	2349	1449.00
aag	K	196	1096.00
aga	R	136	63.49
agg	R	0	35.33
mPD		0.24	0.62
		nPD:	0.38
		N. weight:	0.55
		Sc. PD:	0.031
		Sc. rank:	205.1

PB2			
Pos.	703	obs:	exp:
cgt	R	0	100.50
cgc	R	0	143.70
cga	R	0	270.70
cgg	R	21	219.30
aga	R	2410	1254.00
agg	R	255	697.80
ggt	G	0	0.14
ggc	G	0	0.13
gga	G	1	0.44
ggg	G	0	0.29
mPD		0.20	0.95
		nPD:	0.21
		N. weight:	0.77
		Sc. PD:	-0.084
		Sc. rank:	-183.3

PB2			
Pos.	704	obs:	exp:
ttt	F	2	0.83
ttc	F	0	1.17
tat	Y	2648	1287.00
tac	Y	35	1396.00
tgt	C	1	0.39
tgc	C	0	0.61
gat	D	1	0.54
gac	D	0	0.46
mPD		0.029	0.50
		nPD:	0.06
		N. weight:	1.1
		Sc. PD:	-0.28
		Sc. rank:	-958.0

PB2			
Pos.	705	obs:	exp:
ggt	G	3	388.60
ggc	G	193	352.90
gga	G	2211	1177.00
ggg	G	280	768.10
mPD		0.31	0.69
		nPD:	0.45
		N. weight:	0.6
		Sc. PD:	0.073
		Sc. rank:	360.3

PB2			
Pos.	706	obs:	exp:
cct	P	171	734.30
ccc	P	3	485.30
cca	P	2495	1091.00
ccg	P	18	376.70
mPD		0.13	0.71
		nPD:	0.19
		N. weight:	1.1
		Sc. PD:	-0.14
		Sc. rank:	-356.2

PB2			
Pos.	707	obs:	exp:
tct	S	0	0.33
tcc	S	0	0.29
tca	S	2	0.45
tcg	S	0	0.12
agt	S	0	0.43
agc	S	0	0.37
gtt	V	0	0.22
gtc	V	0	0.21
gta	V	1	0.21
gtg	V	0	0.36
gct	A	0	675.90
gcc	A	0	519.20
gca	A	2242	1194.00
gcg	A	441	293.70
gaa	E	1	0.56
gag	E	0	0.44
mPD		0.28	0.69
		nPD:	0.4
		N. weight:	0.97
		Sc. PD:	0.076
		Sc. rank:	441.8

PB2			
Pos.	708	obs:	exp:
tta	L	31	248.40
ttg	L	1998	510.50
ctt	L	0	533.50
ctc	L	0	429.80
cta	L	8	397.70
ctg	L	650	567.10
mPD		0.40	1.1
		nPD:	0.36
		N. weight:	1.6
		Sc. PD:	0.056
		Sc. rank:	520.5

PB2			
Pos.	709	obs:	exp:
tct	S	0	448.20
tcc	S	0	388.90
tca	S	0	608.70
tcg	S	0	162.00
cgt	R	0	0.04
cgc	R	0	0.05
cga	R	0	0.10
cgg	R	0	0.08
agt	S	28	582.40
agc	S	2657	494.80
aga	R	1	0.47
agg	R	0	0.26
ggt	G	0	0.14
ggc	G	1	0.13
gga	G	0	0.44
ggg	G	0	0.29
mPD		0.022	1.7
		nPD:	0.01
		N. weight:	2.6
		Sc. PD:	-0.8
		Sc. rank:	-3249.7

PB2			
Pos.	710	obs:	exp:
att	I	44	984.90
atc	I	2620	718.20
ata	I	20	980.80
act	T	0	0.52
acc	T	2	0.45
aca	T	0	0.84
acg	T	0	0.19
gtt	V	0	0.22
gtc	V	1	0.21
gta	V	0	0.21
gtg	V	0	0.36
mPD		0.049	0.66
		nPD:	0.07
		N. weight:	1.9
		Sc. PD:	-0.47
		Sc. rank:	-1553.2

PB2			
Pos.	711	obs:	exp:
tct	S	0	6.84
tcc	S	0	5.94
tca	S	0	9.29
tcg	S	0	2.47
aat	N	2050	1440.00
aac	N	591	1201.00
agt	S	41	8.89
agc	S	0	7.56
gat	D	5	2.70
gac	D	0	2.30
mPD		0.38	0.55
		nPD:	0.68
		N. weight:	0.23
		Sc. PD:	0.08
		Sc. rank:	233.3

PB2			
Pos.	712	obs:	exp:
gaa	E	2467	1506.00
gag	E	219	1180.00
ggt	G	0	0.14
ggc	G	0	0.13
gga	G	1	0.44
ggg	G	0	0.29
mPD		0.15	0.49
		nPD:	0.31
		N. weight:	0.52
		Sc. PD:	-0.008
		Sc. rank:	65.7

PB2			
Pos.	713	obs:	exp:
tta	L	2	247.50
ttg	L	960	508.60
ctt	L	0	531.50
ctc	L	0	428.20
cta	L	30	396.20
ctg	L	1685	565.00
cgt	R	0	0.04
cgc	R	0	0.05
cga	R	0	0.10
cgg	R	1	0.08
atg	M	9	9.00
aga	R	0	0.47
agg	R	0	0.26
mPD		0.49	1.1
		nPD:	0.43
		N. weight:	1.4
		Sc. PD:	0.16
		Sc. rank:	813.9

PB2			
Pos.	714	obs:	exp:
tct	S	0	447.70
tcc	S	0	388.50
tca	S	0	608.00
tcg	S	0	161.80
aat	N	0	0.55
aac	N	1	0.45
agt	S	14	581.70
agc	S	2668	494.30
ggt	G	0	0.58
ggc	G	4	0.53
gga	G	0	1.75
ggg	G	0	1.14
mPD		0.014	1.7
		nPD:	0.01
		N. weight:	2.7
		Sc. PD:	-0.83
		Sc. rank:	-3487.7

PB2			
Pos.	715	obs:	exp:
tct	S	0	32.55
tcc	S	0	28.25
tca	S	0	44.21
tcg	S	0	11.76
cat	H	1	0.58
cac	H	0	0.42
att	I	1	0.37
atc	I	0	0.27
ata	I	0	0.37
act	T	2	0.52
acc	T	0	0.45
aca	T	0	0.84
acg	T	0	0.19
aat	N	2442	1357.00
aac	N	46	1131.00
agt	S	195	42.30
agc	S	0	35.94
---	--	------	------
mPD		0.17	0.74
		nPD:	0.23
		N. weight:	0.96
		Sc. PD:	-0.085
		Sc. rank:	-153.4

PB2			
Pos.	716	obs:	exp:
ttt	F	10	4.17
ttc	F	0	5.83
tta	L	0	247.40
ttg	L	7	508.40
ctt	L	2663	531.30
ctc	L	6	428.00
cta	L	0	396.00
ctg	L	0	564.80
att	I	1	0.37
atc	I	0	0.27
ata	I	0	0.37
---	--	------	------
mPD		0.023	1.1
		nPD:	0.02
		N. weight:	2.6
		Sc. PD:	-0.76
		Sc. rank:	-3015.9

PB2			
Pos.	717	obs:	exp:
tct	S	0	0.50
tcc	S	0	0.43
tca	S	3	0.68
tcg	S	0	0.18
act	T	6	10.22
acc	T	0	8.79
aca	T	17	16.32
acg	T	16	3.67
agt	S	0	0.65
agc	S	0	0.55
gtt	V	0	1.08
gtc	V	0	1.05
gta	V	3	1.07
gtg	V	2	1.81
gct	A	17	664.80
gcc	A	2	510.70
gca	A	1420	1175.00
gcg	A	1200	288.80
gaa	E	1	0.56
gag	E	0	0.44
---	--	------	------
mPD		0.54	0.73
		nPD:	0.75
		N. weight:	1.2
		Sc. PD:	0.49
		Sc. rank:	1447.6

PB2			
Pos.	718	obs:	exp:
cgt	R	0	1.16
cgc	R	0	1.66
cga	R	0	3.12
cgg	R	0	2.53
aaa	K	2343	1512.00
aag	K	313	1144.00
aga	R	19	14.47
agg	R	12	8.05
---	--	------	------
mPD		0.24	0.52
		nPD:	0.45
		N. weight:	0.38
		Sc. PD:	0.05
		Sc. rank:	235.4

PB2			
Pos.	719	obs:	exp:
cgt	R	0	0.07
cgc	R	0	0.11
cga	R	0	0.20
cgg	R	0	0.16
aga	R	0	0.93
agg	R	2	0.52
ggt	G	1	388.30
ggc	G	0	352.70
gga	G	218	1177.00
ggg	G	2466	767.50
---	--	------	------
mPD		0.15	0.69
		nPD:	0.22
		N. weight:	1.5
		Sc. PD:	-0.15
		Sc. rank:	-306.3

PB2			
Pos.	720	obs:	exp:
gaa	E	137	1506.00
gag	E	2549	1180.00
ggt	G	0	0.14
ggc	G	0	0.13
gga	G	0	0.44
ggg	G	1	0.29
---	--	------	------
mPD		0.098	0.49
		nPD:	0.2
		N. weight:	0.99
		Sc. PD:	-0.12
		Sc. rank:	-313.4

PB2			
Pos.	721	obs:	exp:
cgt	R	0	0.07
cgc	R	0	0.11
cga	R	0	0.20
cgg	R	0	0.16
aaa	K	1460	1528.00
aag	K	1223	1155.00
aga	R	2	0.93
agg	R	0	0.52
gaa	E	2	1.12
gag	E	0	0.88
---	--	------	------
mPD		0.50	0.49
		nPD:	1.01
		N. weight:	0.0062
		Sc. PD:	0.0042
		Sc. rank:	8.9

PB2			
Pos.	722	obs:	exp:
gtt	V	3	0.65
gtc	V	0	0.63
gta	V	0	0.64
gtg	V	0	1.08
gct	A	2605	676.10
gcc	A	44	519.40
gca	A	35	1195.00
gcg	A	0	293.80
---	--	------	------
mPD		0.060	0.69
		nPD:	0.09
		N. weight:	2.
		Sc. PD:	-0.46
		Sc. rank:	-1452.3

PB2			
Pos.	723	obs:	exp:
att	I	1	0.37
atc	I	0	0.27
ata	I	0	0.37
aat	N	2580	1465.00
aac	N	106	1221.00
---	--	------	------
mPD		0.077	0.50
		nPD:	0.15
		N. weight:	0.73
		Sc. PD:	-0.12
		Sc. rank:	-343.2

PB2			
Pos.	724	obs:	exp:
gtt	V	2	577.90
gtc	V	0	565.30
gta	V	125	572.90
gtg	V	2560	970.80
---	--	------	------
mPD		0.090	0.73
		nPD:	0.12
		N. weight:	1.4
		Sc. PD:	-0.27
		Sc. rank:	-793.2

PB2			
Pos.	725	obs:	exp:
tta	L	97	246.90
ttg	L	1024	507.20
ctt	L	1	530.10
ctc	L	0	427.10
cta	L	123	395.10
ctg	L	1425	563.50
atg	M	15	15.00
aaa	K	0	1.14
aag	K	2	0.86
---	--	------	------
mPD		0.65	1.1
		nPD:	0.57
		N. weight:	1.1
		Sc. PD:	0.27
		Sc. rank:	940.7

PB2			
Pos.	726	obs:	exp:
att	I	181	984.90
atc	I	7	718.20
ata	I	2496	980.80
gtt	V	0	0.65
gtc	V	0	0.63
gta	V	3	0.64
gtg	V	0	1.08
---	--	------	------
mPD		0.13	0.66
		nPD:	0.2
		N. weight:	1.2
		Sc. PD:	-0.14
		Sc. rank:	-329.8

PB2			
Pos.	727	obs:	exp:
cgt	R	0	0.49
cgc	R	0	0.70
cga	R	0	1.31
cgg	R	1	1.06
aga	R	2	6.07
agg	R	10	3.38
ggt	G	2	386.70
ggc	G	5	351.20
gga	G	469	1172.00
ggg	G	2198	764.40
---	--	------	------
mPD		0.30	0.70
		nPD:	0.43
		N. weight:	1.1
		Sc. PD:	0.13
		Sc. rank:	650.3

PB2			
Pos.	728	obs:	exp:
cct	P	0	0.27
ccc	P	0	0.18
cca	P	1	0.41
ccg	P	0	0.14
caa	Q	2472	1341.00
cag	Q	214	1345.00
---	--	------	------
mPD		0.15	0.50
		nPD:	0.29
		N. weight:	0.68
		Sc. PD:	-0.018
		Sc. rank:	41.5

PB2			
Pos.	729	obs:	exp:
cgt	R	0	0.04
cgc	R	0	0.05
cga	R	0	0.10
cgg	R	0	0.08
aga	R	1	0.47
agg	R	0	0.26
ggt	G	0	388.40
ggc	G	0	352.80
gga	G	2658	1177.00
ggg	G	28	767.80
---	--	------	------
mPD		0.021	0.69
		nPD:	0.03
		N. weight:	1.3
		Sc. PD:	-0.36
		Sc. rank:	-1333.3

PB2			
Pos.	730	obs:	exp:
aat	N	0	0.55
aac	N	1	0.45
gat	D	52	1450.00
gac	D	2634	1236.00
---	--	------	------
mPD		0.039	0.50
		nPD:	0.08
		N. weight:	1.1
		Sc. PD:	-0.26
		Sc. rank:	-860.0

PB2			
Pos.	731	obs:	exp:
tta	L	0	0.09
ttg	L	1	0.19
ctt	L	0	0.20
ctc	L	0	0.16
cta	L	0	0.15
ctg	L	0	0.21
att	I	0	1.10
atc	I	0	0.80
ata	I	3	1.10
atg	M	13	13.00
gtt	V	1	574.30
gtc	V	0	561.70
gta	V	162	569.30
gtg	V	2507	964.70
---	--	------	------
mPD		0.13	0.75
		nPD:	0.17
		N. weight:	1.3
		Sc. PD:	-0.19
		Sc. rank:	-534.9

PB2			
Pos.	732	obs:	exp:
gtt	V	2	577.90
gtc	V	0	565.30
gta	V	27	572.90
gtg	V	2658	970.80
---	--	------	------
mPD		0.021	0.73
		nPD:	0.03
		N. weight:	1.6
		Sc. PD:	-0.45
		Sc. rank:	-1694.9

PB2

Pos.	733	obs:	exp:
tta	L	6	248.30
ttg	L	2552	510.10
tgg	W	1	1.00
ctt	L	0	533.10
ctc	L	0	429.50
cta	L	0	397.40
ctg	L	127	566.70
atg	M	1	1.00
---	--	------	------
mPD		0.096	1.1
		nPD:	0.09
		N. weight:	2.4
		Sc. PD:	-0.54
		Sc. rank:	-1756.8

PB2

Pos.	734	obs:	exp:
att	I	0	0.37
atc	I	0	0.27
ata	I	1	0.37
gtt	V	0	577.50
gtc	V	0	564.90
gta	V	2194	572.50
gtg	V	491	970.10
gct	A	0	0.25
gcc	A	0	0.19
gca	A	1	0.45
gcg	A	0	0.11
---	--	------	------
mPD		0.30	0.74
		nPD:	0.41
		N. weight:	1.6
		Sc. PD:	0.14
		Sc. rank:	782.8

PB2

Pos.	735	obs:	exp:
att	I	1	0.37
atc	I	0	0.27
ata	I	0	0.37
atg	M	2686	2686.00
---	--	------	------
mPD		0.00074	0.00074
		nPD:	1.
		N. weight:	0.00061
		Sc. PD:	0.0004
		Sc. rank:	0.9

PB2

Pos.	736	obs:	exp:
cgt	R	0	0.04
cgc	R	0	0.05
cga	R	0	0.10
cgg	R	0	0.08
aaa	K	2685	1529.00
aag	K	0	1156.00
aga	R	0	0.47
agg	R	1	0.26
gaa	E	1	0.56
gag	E	0	0.44
---	--	------	------
mPD		0.0022	0.49
		nPD:	0.
		N. weight:	0.91
		Sc. PD:	-0.28
		Sc. rank:	-1244.5

PB2

Pos.	737	obs:	exp:
tta	L	0	0.18
ttg	L	0	0.38
ctt	L	0	0.40
ctc	L	0	0.32
cta	L	0	0.30
ctg	L	2	0.42
cct	P	0	0.55
ccc	P	0	0.36
cca	P	0	0.81
ccg	P	2	0.28
cgt	R	0	100.40
cgc	R	0	143.60
cga	R	205	270.40
cgg	R	2474	219.10
aga	R	0	1253.00
agg	R	4	697.00
---	--	------	------
mPD		0.15	0.96
		nPD:	0.15
		N. weight:	3.6
		Sc. PD:	-0.59
		Sc. rank:	-1724.5

PB2

Pos.	738	obs:	exp:
aaa	K	2647	1530.00
aag	K	40	1157.00
---	--	------	------
mPD		0.029	0.49
		nPD:	0.06
		N. weight:	0.8
		Sc. PD:	-0.2
		Sc. rank:	-676.6

PB2

Pos.	739	obs:	exp:
cct	P	0	0.82
ccc	P	0	0.54
cca	P	0	1.22
ccg	P	3	0.42
cgt	R	2	100.40
cgc	R	0	143.60
cga	R	89	270.50
cgg	R	2582	219.20
aga	R	0	1253.00
agg	R	11	697.30
---	--	------	------
mPD		0.076	0.96
		nPD:	0.08
		N. weight:	3.8
		Sc. PD:	-0.89
		Sc. rank:	-2883.4

PB2

Pos.	740	obs:	exp:
aat	N	0	8.73
aac	N	16	7.27
gat	D	1	1442.00
gac	D	2669	1228.00
ggt	G	0	0.14
ggc	G	1	0.13
gga	G	0	0.44
ggg	G	0	0.29
---	--	------	------
mPD		0.013	0.51
		nPD:	0.03
		N. weight:	1.3
		Sc. PD:	-0.36
		Sc. rank:	-1399.3

PB2

Pos.	741	obs:	exp:
ttt	F	4	1.67
ttc	F	0	2.33
tct	S	2658	447.90
tcc	S	17	388.60
tca	S	1	608.20
tcg	S	7	161.90
agt	S	0	582.00
agc	S	0	494.40
---	--	------	------
mPD		0.021	1.7
		nPD:	0.01
		N. weight:	2.8
		Sc. PD:	-0.85
		Sc. rank:	-3498.5

PB2

Pos.	742	obs:	exp:
tct	S	0	448.20
tcc	S	0	388.90
tca	S	0	608.70
tcg	S	0	162.00
act	T	0	0.26
acc	T	1	0.23
aca	T	0	0.42
acg	T	0	0.09
aat	N	0	0.55
aac	N	1	0.45
agt	S	13	582.40
agc	S	2672	494.80
---	--	------	------
mPD		0.011	1.7
		nPD:	0.01
		N. weight:	2.7
		Sc. PD:	-0.83
		Sc. rank:	-3550.9

PB2

Pos.	743	obs:	exp:
tta	L	2	1.57
ttg	L	0	3.23
ctt	L	0	3.38
ctc	L	0	2.72
cta	L	15	2.52
ctg	L	0	3.59
att	I	0	979.10
atc	I	0	713.90
ata	I	2668	975.00
atg	M	1	1.00
gtt	V	0	0.22
gtc	V	0	0.21
gta	V	1	0.21
gtg	V	0	0.36
---	--	------	------
mPD		0.014	0.68
		nPD:	0.02
		N. weight:	1.6
		Sc. PD:	-0.48
		Sc. rank:	-1917.0

PB2

Pos.	744	obs:	exp:
tta	L	0	248.40
ttg	L	0	510.50
ctt	L	2686	533.50
ctc	L	0	429.80
cta	L	1	397.70
ctg	L	0	567.10
---	--	------	------
mPD		0.00074	1.1
		nPD:	0.
		N. weight:	2.6
		Sc. PD:	-0.82
		Sc. rank:	-3722.1

PB2

Pos.	745	obs:	exp:
att	I	3	1.10
atc	I	0	0.80
ata	I	0	1.10
act	T	2654	703.60
acc	T	30	604.80
aca	T	0	1123.00
acg	T	0	252.50
---	--	------	------
mPD		0.024	0.70
		nPD:	0.03
		N. weight:	2.1
		Sc. PD:	-0.58
		Sc. rank:	-2158.4

PB2

Pos.	746	obs:	exp:
gat	D	3	1451.00
gac	D	2684	1236.00
---	--	------	------
mPD		0.0022	0.50
		nPD:	0.
		N. weight:	1.2
		Sc. PD:	-0.39
		Sc. rank:	-1700.7

PB2

Pos.	747	obs:	exp:
tct	S	0	448.20
tcc	S	0	388.90
tca	S	0	608.70
tcg	S	0	162.00
tgt	C	0	0.39
tgc	C	1	0.61
aat	N	0	0.55
aac	N	1	0.45
agt	S	129	582.40
agc	S	2556	494.80
---	--	------	------
mPD		0.093	1.7
		nPD:	0.06
		N. weight:	2.4
		Sc. PD:	-0.63
		Sc. rank:	-2156.8

PB2

Pos.	748	obs:	exp:
cct	P	0	0.27
ccc	P	0	0.18
cca	P	0	0.41
ccg	P	1	0.14
caa	Q	0	1341.00
cag	Q	2686	1345.00
---	--	------	------
mPD		0.00074	0.50
		nPD:	0.
		N. weight:	1.1
		Sc. PD:	-0.35
		Sc. rank:	-1574.4

PB2

Pos.	749	obs:	exp:
act	T	0	704.40
acc	T	0	605.50
aca	T	2687	1124.00
acg	T	0	252.80
---	--	------	------
mPD		0	0.70
		nPD:	0.
		N. weight:	1.4
		Sc. PD:	-0.45
		Sc. rank:	-2023.3

PB2

Pos.	750	obs:	exp:
gct	A	3	676.70
gcc	A	0	519.80
gca	A	6	1196.00
gcg	A	2677	294.00
ggt	G	0	0.14
ggc	G	0	0.13
gga	G	0	0.44
ggg	G	1	0.29
---	--	------	------
mPD		0.0074	0.69
		nPD:	0.01
		N. weight:	3.5
		Sc. PD:	-1.1
		Sc. rank:	-4509.9

PB2

Pos.	751	obs:	exp:
act	T	0	704.10
acc	T	2686	605.20
aca	T	0	1124.00
acg	T	0	252.70
aat	N	0	0.55
aac	N	1	0.45
---	--	------	------
mPD		0.00074	0.70
		nPD:	0.
		N. weight:	2.4
		Sc. PD:	-0.76
		Sc. rank:	-3414.2

PB2

Pos.	752	obs:	exp:
aat	N	1	0.55
aac	N	0	0.45
aaa	K	2686	1529.00
aag	K	0	1157.00
---	--	------	------
mPD		0.00074	0.49
		nPD:	0.
		N. weight:	0.91
		Sc. PD:	-0.29
		Sc. rank:	-1277.9

PB2

Pos.	753	obs:	exp:
tct	S	0	0.17
tcc	S	0	0.14
tca	S	0	0.23
tcg	S	0	0.06
cgt	R	0	100.50
cgc	R	0	143.70
cga	R	14	270.70
cgg	R	0	219.30
agt	S	1	0.22
agc	S	0	0.18
aga	R	2267	1254.00
agg	R	405	697.80
---	--	------	------
mPD		0.27	0.96
		nPD:	0.28
		N. weight:	0.66
		Sc. PD:	-0.027
		Sc. rank:	12.6

PB2

Pos.	754	obs:	exp:
ttt	F	1	0.42
ttc	F	0	0.58
tta	L	0	0.18
ttg	L	0	0.38
ctt	L	2	0.40
ctc	L	0	0.32
cta	L	0	0.30
ctg	L	0	0.42
att	I	2674	983.50
atc	I	2	717.20
ata	I	4	979.40
gtt	V	4	0.86
gtc	V	0	0.84
gta	V	0	0.85
gtg	V	0	1.45
---	--	------	------
mPD		0.0097	0.67
		nPD:	0.01
		N. weight:	1.6
		Sc. PD:	-0.48
		Sc. rank:	-1956.7

PB2

Pos.	755	obs:	exp:
cct	P	0	0.27
ccc	P	0	0.18
cca	P	0	0.41
ccg	P	1	0.14
caa	Q	0	1.50
cag	Q	3	1.50
cgt	R	0	100.40
cgc	R	0	143.60
cga	R	72	270.40
cgg	R	2611	219.10
aga	R	0	1253.00
agg	R	0	697.00
---	--	------	------
mPD		0.055	0.96
		nPD:	0.06
		N. weight:	3.9
		Sc. PD:	-1.
		Sc. rank:	-3343.7

PB2

Pos.	756	obs:	exp:
cgt	R	0	0.11
cgc	R	0	0.16
cga	R	0	0.30
cgg	R	0	0.24
atg	M	2677	2677.00
act	T	0	0.26
acc	T	0	0.23
aca	T	0	0.42
acg	T	1	0.09
aga	R	0	1.40
agg	R	3	0.78
gtt	V	0	1.29
gtc	V	0	1.26
gta	V	0	1.28
gtg	V	6	2.17
---	--	------	------
mPD		0.0074	0.013
		nPD:	0.57
		N. weight:	0.0076
		Sc. PD:	0.0019
		Sc. rank:	6.5

PB2

Pos.	757	obs:	exp:
cct	P	0	0.27
ccc	P	1	0.18
cca	P	0	0.41
ccg	P	0	0.14
gtt	V	0	1.51
gtc	V	7	1.47
gta	V	0	1.49
gtg	V	0	2.53
gct	A	0	674.60
gcc	A	2678	518.20
gca	A	0	1192.00
gcg	A	0	293.10
ggt	G	0	0.14
ggc	G	1	0.13
gga	G	0	0.44
ggg	G	0	0.29
---	--	------	------
mPD		0.0067	0.70
		nPD:	0.01
		N. weight:	2.7
		Sc. PD:	-0.81
		Sc. rank:	-3426.3

PB2

Pos.	758	obs:	exp:
ttt	F	0	0.42
ttc	F	1	0.58
att	I	34	983.50
atc	I	2646	717.20
ata	I	0	979.40
atg	M	1	1.00
act	T	0	0.52
acc	T	2	0.45
aca	T	0	0.84
acg	T	0	0.19
aat	N	0	0.55
aac	N	1	0.45
gtt	V	0	0.43
gtc	V	1	0.42
gta	V	0	0.43
gtg	V	1	0.72
---	--	------	------
mPD		0.031	0.67
		nPD:	0.05
		N. weight:	2.
		Sc. PD:	-0.54
		Sc. rank:	-1921.6

PB2

Pos.	759	obs:	exp:
ttt	F	1	0.42
ttc	F	0	0.58
tct	S	0	0.50
tcc	S	0	0.43
tca	S	0	0.68
tcg	S	0	0.18
tat	Y	1	0.48
tac	Y	0	0.52
att	I	2	4.04
atc	I	0	2.94
ata	I	9	4.02
aat	N	2665	1454.00
aac	N	2	1213.00
aaa	K	2	1.14
aag	K	0	0.86
agt	S	3	0.65
agc	S	0	0.55
gat	D	2	1.08
gac	D	0	0.92
---	--	------	------
mPD		0.024	0.51
		nPD:	0.05
		N. weight:	0.98
		Sc. PD:	-0.26
		Sc. rank:	-935.9

PB2

Pos.	760	obs:	exp:
taa	.	60	990.80
tag	.	2626	921.40
tga	.	1	774.80
---	--	------	------
mPD		0.045	0.86
		nPD:	0.05
		N. weight:	1.6
		Sc. PD:	-0.41
		Sc. rank:	-1417.7

Amino acid level conservation per codon

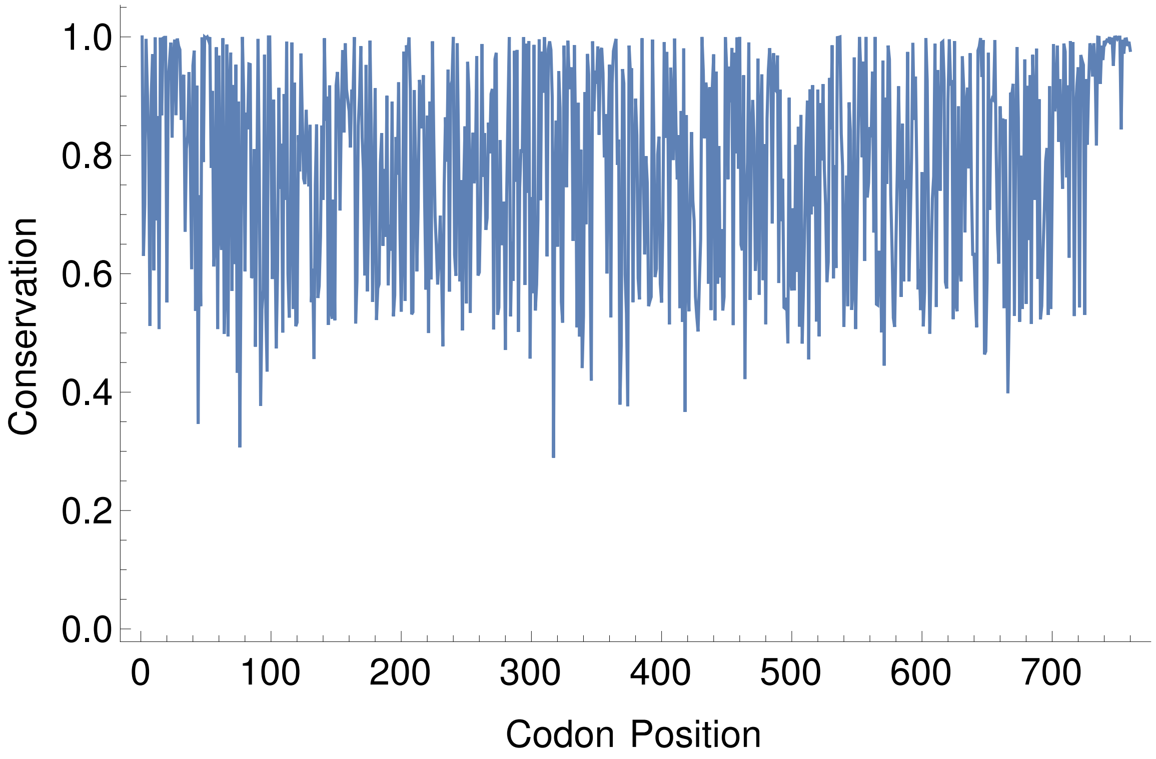

Conservation analysis plots

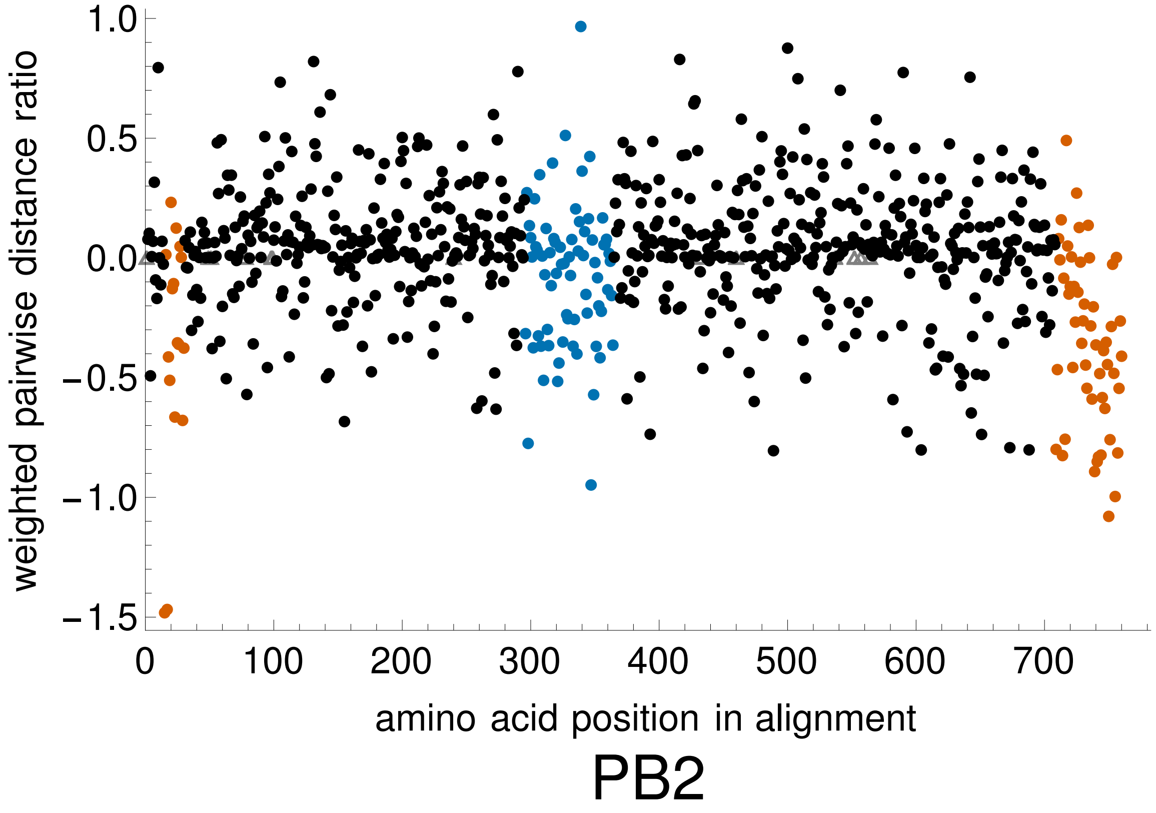

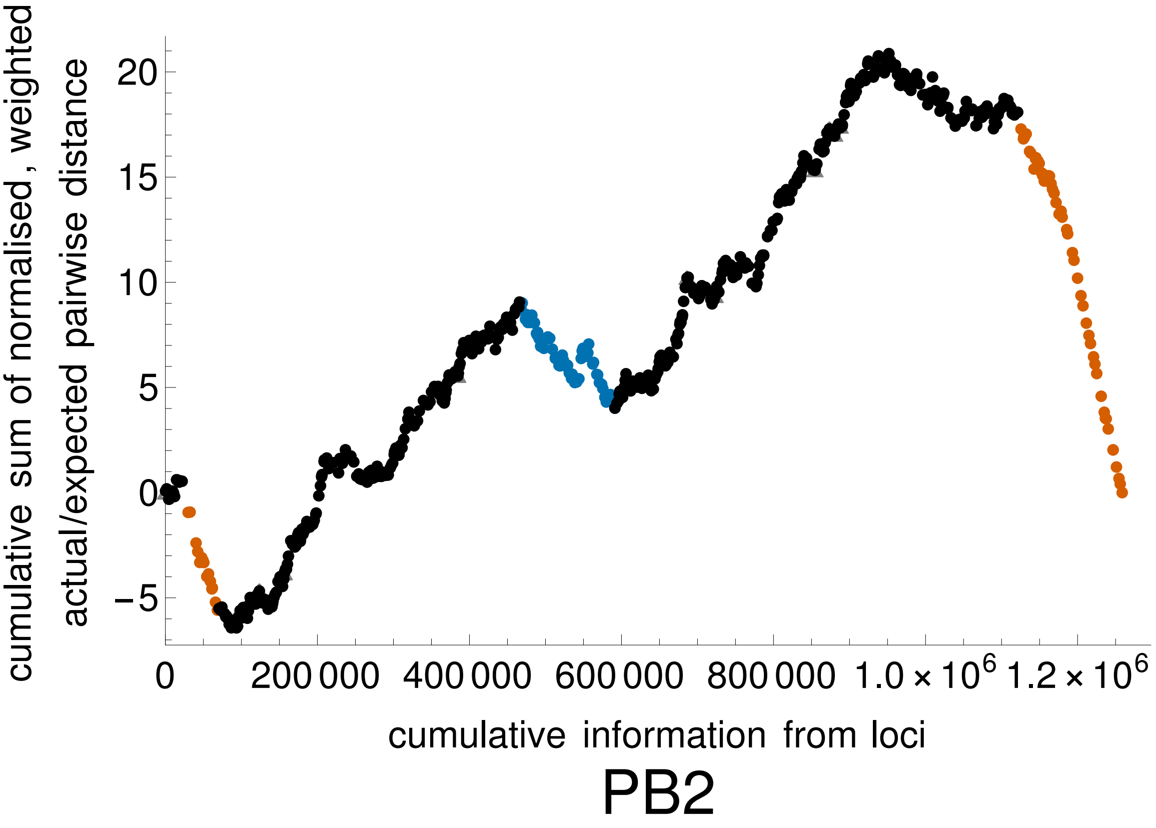

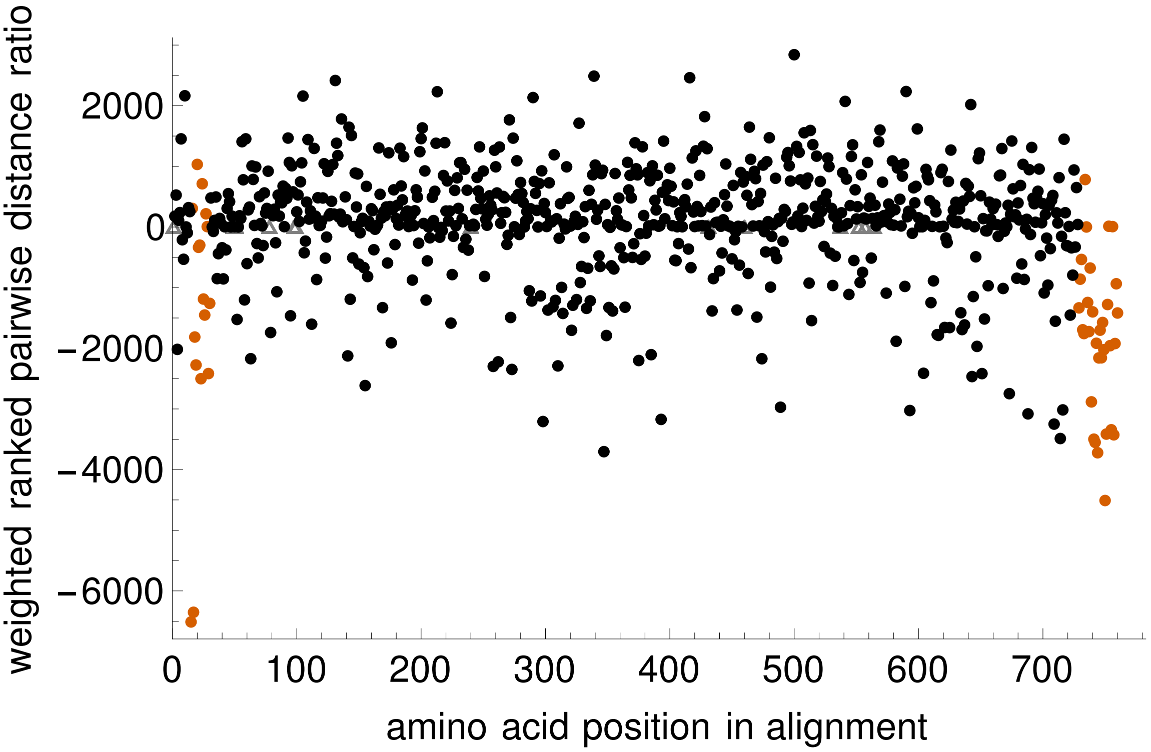
weighted ranked pairwise distance ratio
2000
0
−2000
−4000
−6000
0
100
200
300
400
500
600
700
amino acid position in alignment

PB2

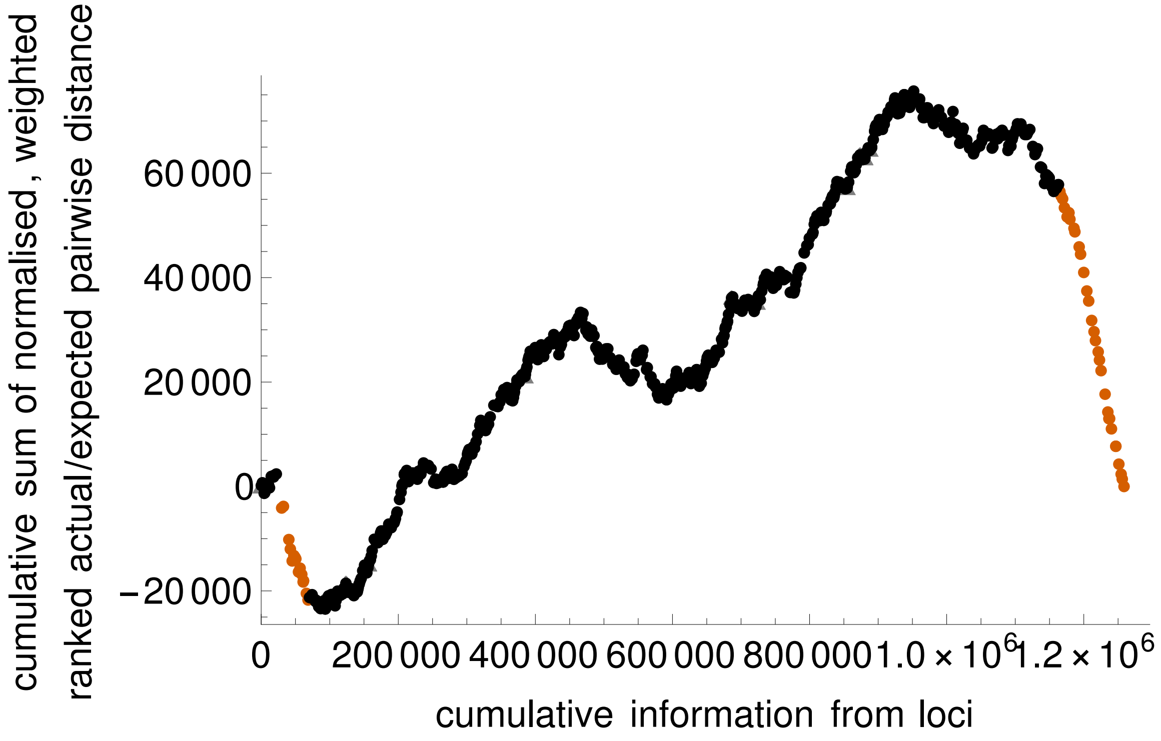
cumulative sum of normalised, weighted
ranked actual/expected pairwise distance
60 000
40 000
20 000
0
−20 000
0
200 000
400 000
600 000
800 000
1.0 × 10^6
1.2 × 10^6
cumulative information from loci

PB2

PB1

Gene length histogram

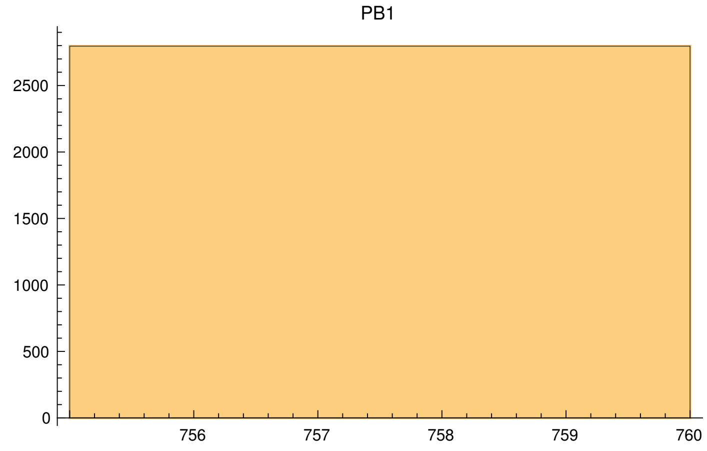

Information vs. nPD

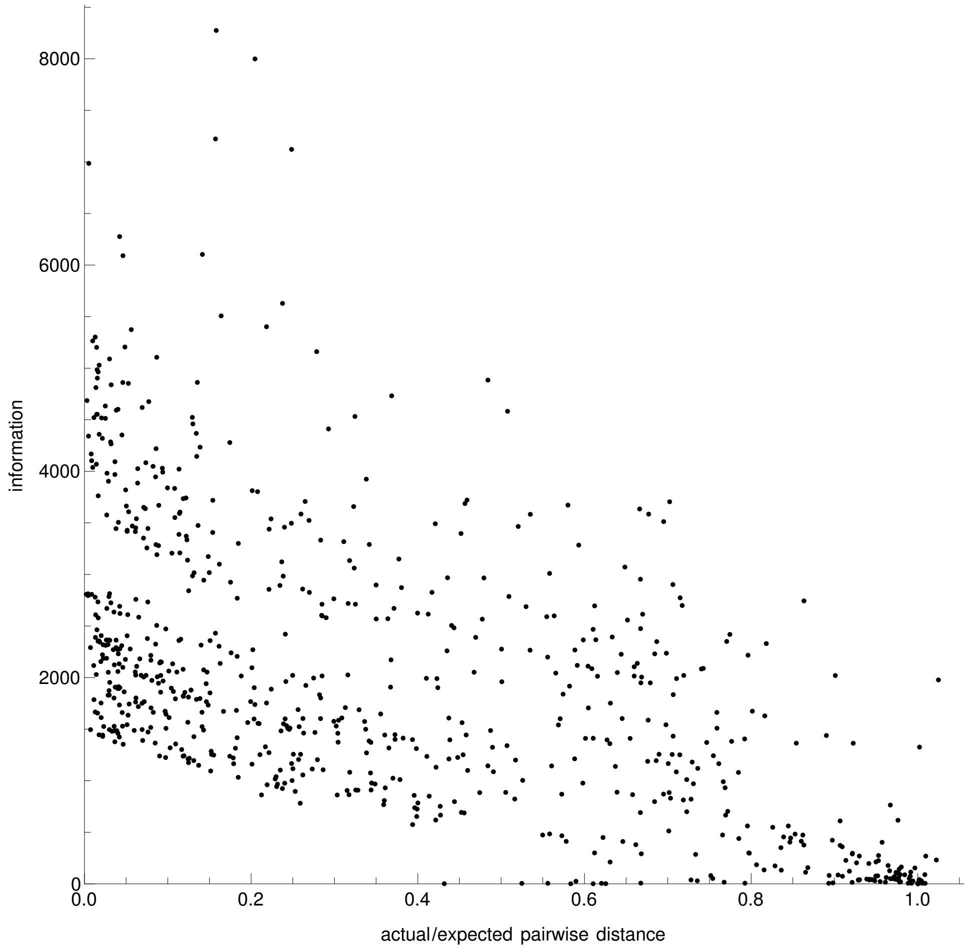

PB1

Example sequences highlighted by regions found in analyses to be conserved

```
Interesting points (by weighted raw PD) highlighted for gene PB1:
ATGGCTGTCAATCCGACTTTACTTTTCTTGAAAGTACCAGCACAAAATGCTATAAGTACCACATTCCCTTATACTG⋱
    GAGACCCTCCATACAGCCATGGAACAGGGACAGGATACACCATGGACACAGTCAACAGAACACACCAATATTCAG⋱
    AAAAGGGGAAGTGGACAACAAACACAGAGACTGGAGCACCCCAACTCAACCCGATTGATGGACCACTACCTGAGG⋱
    ATAATGAGCCCAGTGGGTATGCACAAACAGATTGTGTATTGGAAGCAATGGCTTTCCTTGAAGAATCCCACCCAG⋱
    GGATCTTTGAAAATTCGTGTCTTGAAACGATGGAAATTGTTCAACAAACAAGAGTGGATAAACTGACCCAAGGTC⋱
    GCCAGACCTATGACTGGACATTGAATAGAAACCAGCCGGCTGCAACTGCTTTGGCCAACACTATAGAAATCTTCA⋱
    GATCGAACGGTCTAACAGCCAATGAATCGGGACGACTAATAGATTTCCTCAAGGATGTGATGGAATCAATGGATA⋱
    AGGAAGAAATGGAAATAACAACACATTTCCAGAGAAAGAGAAGAGTGAGGGACAACATGACCAAGAAAATGGTCA⋱
    CACAAAGAACAATAGGGAAGAAAAAACAAAGGCTGAACAAAAAGAGCTACCTGATAAGAGCACTGACACTGAACA⋱
    CAATGACAAAAGATGCAGAAAGAGGCAAATTGAAGAGGCGAGCAATTGCAACACCCGGAATGCAAATCAGAGGAT⋱
    TCGTGTACTTTGTCGAAACCCTAGCGAGGAGTATCTGTGAGAAACTTGAGCAATCTGGACTCCCAGTCGGAGGGA⋱
    ATGAGAAGAAGGCTAAACTGGCAAACGTCGTGAGGAAGATGATGACTAGCTCACAGGATACTGAACTCTCCTTTA⋱
    CAATTACTGGAGACAACACCAAATGGAATGAGAATCAGAATCCTAGGATGTTTCTGGCAATGATAACGTACATCA⋱
    CAAGGAACCAACCAGAATGGTTTCGGAATGTCTTAAGCATTGCCCCTATAATGTTCTCAAACAAAATGGCGAGAT⋱
    TAGGAAAAGGATACATGTTCGAAAGTAAGAGCATGAAGTTACGGACACAAATACCAGCAGAAAGGCTTGCAAACA⋱
    TTGATCTGAAATACTTCAATGAATCAACGAGAAAGAAAATTGAAAAAATAAGACCTCTATTAATAGATGGTACAG⋱
    CCTCATTGAGCCCTGGAATGATGATGGGCATGTTCAACATGCTGAGTACAGTCCTAGGAGTCTCAATCCTGAATC⋱
    TTGGACAGAAAAGGTACACCAAAACCACATATTGGTGGGACGGACTCCAATCTTCTGATGATTTCGCTCTCATCG⋱
    TAAATGCACCGAATCATGAGGGAATACAAGCAGGAGTGGATAGGTTTTATAGGACTTGCAAACTAGTTGGAATCA⋱
    ATATGAGCAAGAAGAAGTCTTACATAAATCGGACAGGGACATTTGAATTCACGAGCTTTTTCTACCGCTATGGAT⋱
    TTGTAGCCAATTTCAGCATGGAGCTGCCCAGTTTTGGAGTGTCTGGAATTAATGAATCGGCCGACATGAGCATTG⋱
    GTGTTACAGTGATAAAGAACAATATGATAAACAACGACCTTGGACCAGCAACAGCTCAGATGGCTCTTCAGCTAT⋱
    TCATCAAGGACTACAGATACACATACCGATGCCACAGAGGGGATACGCAAATCCAAACGAGGAGATCATTCGAGC⋱
    TGAAGAAGCTGTGGGAGCAGACCCGTTCAAAGGCAGGACTGTTGGTTTCAGATGGAGGACCAAATCTATACAATA⋱
    TCCGAAATCTCCACATTCCTGAAGTCTGTTTGAAATGGGAATTGATGGATGAAGATTACCAGGGCAGACTGTGTA⋱
    ATCCTCTGAATCCGTTCGTCAGCCATAAGGAAATTGAATCGGTCAACAATGCTGTAGTAATGCCAGCTCATGGCC⋱
    CGGCCAAGAGTATGGAATATGATGCCGTTGCGACTACACATTCATGGATTCCTAAAAGGAATCGTTCCATTCTCA⋱
    ATACAAGTCAAAGGGGAATTCTTGAGGATGAACAGATGTACCAGAAGTGCTGCAATCTATTCGAGAAATTCTTCC⋱
    CCAGCAGTTCATATCGGAGG
   CCAGTTGGAATTTCCAGCATGGTGGAGGCCATGGTGTCTAGGGCCCGAATTGATG
     CACGAATTGATTTCGAGTCTGGAAGGATTAAGAAAGAAGAGTTTGCTGAGAT
     CATGAAGATCTGTTCCACCATTGAAGAGCTCAGACGGCAA---AAA---TAG

Interesting points (by weighted ranked PD) highlighted for gene PB1:
ATG
   GCTGTCAATCCGACTTTACTTTTCTTGAAAGTACCAGCACAAAATGCTATAAGTACCACATTCCCT⋱
    TATACTGGAGACCCTCCATACAGCCATGGAACAGGGACAGGATACACCATGGACACAGTCAACAGAACAC⋱
    ACCAATATTCAGAAAAGGGGAAGTGGACAACAAACACAGAGACTGGAGCACCCCAACTCAACCCGATTGA⋱
    TGGACCACTACCTGAGGATAATGAGCCCAGTGGGTATGCACAAACAGATTGTGTATTGGAAGCAATGGCT⋱
    TTCCTTGAAGAATCCCACCCAGGGATCTTTGAAAATTCGTGTCTTGAAACGATGGAAATTGTTCAACAAA⋱
    CAAGAGTGGATAAACTGACCCAAGGTCGCCAGACCTATGACTGGACATTGAATAGAAACCAGCCGGCTGC⋱
    AACTGCTTTGGCCAACACTATAGAAATCTTCAGATCGAACGGTCTAACAGCCAATGAATCGGGACGACTA⋱
    ATAGATTTCCTCAAGGATGTGATGGAATCAATGGATAAGGAAGAAATGGAAATAACAACACATTTCCAGA⋱
```

GAAAGAGAAGAGTGAGGGACAACATGACCAAGAAAATGGTCACACAAAGAACAATAGGGAAGAAAAAACA⋱
AAGGCTGAACAAAAAGAGCTACCTGATAAGAGCACTGACACTGAACACAATGACAAAAGATGCAGAAAGA⋱
GGCAAATTGAAGAGGCGAGCAATTGCAACACCCGGAATGCAAATCAGAGGATTCGTGTACTTTGTCGAAA⋱
CCCTAGCGAGGAGTATCTGTGAGAAACTTGAGCAATCTGGACTCCCAGTCGGAGGGAATGAGAAGAAGGC⋱
TAAACTGGCAAACGTCGTGAGGAAGATGATGACTAGCTCACAGGATACTGAACTCTCCTTTACAATTACT⋱
GGAGACAACACCAAATGGAATGAGAATCAGAATCCTAGGATGTTTCTGGCAATGATAACGTACATCACAA⋱
GGAACCAACCAGAATGGTTTCGGAATGTCTTAAGCATTGCCCCTATAATGTTCTCAAACAAAATGGCGAG⋱
ATTAGGAAAAGGATACATGTTCGAAAGTAAGAGCATGAAGTTACGGACACAAATACCAGCAGAAAGGCTT⋱
GCAAACATTGATCTGAAATACTTCAATGAATCAACGAGAAAGAAAATTGAAAAAATAAGACCTCTATTAA⋱
TAGATGGTACAGCCTCATTGAGCCCTGGAATGATGATGGGCATGTTCAACATGCTGAGTACAGTCCTAGG⋱
AGTCTCAATCCTGAATCTTGGACAGAAAAGGTACACCAAAACCACATATTGGTGGGACGGACTCCAATCT⋱
TCTGATGATTTCGCTCTCATCGTAAATGCACCGAATCATGAGGGAATACAAGCAGGAGTGGATAGGTTTT⋱
ATAGGACTTGCAAACTAGTTGGAATCAATATGAGCAAGAAGAAGTCTTACATAAATCGGACAGGGACATT⋱
TGAATTCACGAGCTTTTTCTACCGCTATGGATTTGTAGCCAATTTCAGCATGGAGCTGCCCAGTTTTGGA⋱
GTGTCTGGAATTAATGAATCGGCCGACATGAGCATTGGTGTTACAGTGATAAAGAACAATATGATAAACA⋱
ACGACCTTGGACCAGCAACAGCTCAGATGGCTCTTCAGCTATTCATCAAGGACTACAGATACACATACCG⋱
ATGCCACAGAGGGGATACGCAAATCCAAACGAGGAGATCATTCGAGCTGAAGAAGCTGTGGGAGCAGACC⋱
CGTTCAAAGGCAGGACTGTTGGTTTCAGATGGAGGACCAAATCTATACAATATCCGAAATCTCCACATTC⋱
CTGAAGTCTGTTTGAAATGGGAATTGATGGATGAAGATTACCAGGGCAGACTGTGTAATCCTCTGAATCC⋱
GTTCGTCAGCCATAAGGAAATTGAATCGGTCAACAATGCTGTAGTAATGCCAGCTCATGGCCCGGCCAAG⋱
AGTATGGAATATGATGCCGTTGCGACTACACATTCATGGATTCCTAAAAGGAATCGTTCCATTCTCAATA⋱
CAAGTCAAAGGGGAATTCTTGAGGATGAACAGATGTACCAGAAGTGCTGCAATCTATTCGAGAAATTCTT⋱
CCCCAGCAGTTCATATCGGAGGCCAGTTGGAATTTCCAGCATGGTGGAGGCCATGGTGTCTAGGGCCCGA⋱
ATTGATGCACGAATTGATTTCGAGTCTGGAAGGATTAAGAAAGAAGAGTTTGCT
GAGATCATGAAGATCTGTTCCACCATTGAAGAGCTCAGACGGCAA---AAA---TAG

Per codon conservation report

PB1

Pos.	1	obs:	exp:
atg	M	2796	2796.00
---	--	------	------
mPD		0	0
		nPD:	1.
		N. weight:	0.
		Sc. PD:	0
		Sc. rank:	0

PB1

Pos.	2	obs:	exp:
tat	Y	3	1.44
tac	Y	0	1.56
cat	H	2	1.16
cac	H	0	0.84
aat	N	2	1.09
aac	N	0	0.91
gtt	V	0	0.43
gtc	V	0	0.42
gta	V	2	0.43
gtg	V	0	0.72
gct	A	1	0.25
gcc	A	0	0.19
gca	A	0	0.45
gcg	A	0	0.11
gat	D	2741	1503.00
gac	D	43	1281.00
ggt	G	2	0.29
ggc	G	0	0.26
gga	G	0	0.88
ggg	G	0	0.57
---	--	------	------
mPD		0.040	0.51
		nPD:	0.08
		N. weight:	0.79
		Sc. PD:	-0.12
		Sc. rank:	-405.4

PB1

Pos.	3	obs:	exp:
att	I	0	0.37
atc	I	1	0.27
ata	I	0	0.37
gtt	V	2	600.70
gtc	V	2791	587.60
gta	V	0	595.50
gtg	V	0	1009.00
gat	D	0	1.08
gac	D	2	0.92
---	--	------	------
mPD		0.0036	0.74
		nPD:	0.
		N. weight:	2.3
		Sc. PD:	-0.51
		Sc. rank:	-3661.6

PB1

Pos.	4	obs:	exp:
tct	S	0	0.17
tcc	S	0	0.14
tca	S	0	0.23
tcg	S	0	0.06
cat	H	1	0.58
cac	H	0	0.42
att	I	0	0.37
atc	I	1	0.27
ata	I	0	0.37
aat	N	2236	1522.00
aac	N	555	1269.00
aaa	K	0	0.57
aag	K	1	0.43
agt	S	1	0.22
agc	S	0	0.18
gtt	V	0	0.22
gtc	V	0	0.21
gta	V	1	0.21
gtg	V	0	0.36
---	--	------	------
mPD		0.32	0.50
		nPD:	0.65
		N. weight:	0.21
		Sc. PD:	0.085
		Sc. rank:	282.2

PB1

Pos.	5	obs:	exp:
tct	S	0	0.17
tcc	S	0	0.14
tca	S	0	0.23
tcg	S	1	0.06
cct	P	1	763.60
ccc	P	0	504.60
cca	P	46	1134.00
ccg	P	2747	391.70
cgt	R	0	0.04
cgc	R	0	0.05
cga	R	0	0.10
cgg	R	1	0.08
agt	S	0	0.22
agc	S	0	0.18
aga	R	0	0.47
agg	R	0	0.26
---	--	------	------
mPD		0.035	0.71
		nPD:	0.05
		N. weight:	2.7
		Sc. PD:	-0.5
		Sc. rank:	-2234.1

PB1

Pos.	6	obs:	exp:
att	I	1	0.37
atc	I	0	0.27
ata	I	0	0.37
act	T	2739	732.40
acc	T	47	629.60
aca	T	0	1169.00
acg	T	8	262.90
gct	A	1	0.25
gcc	A	0	0.19
gca	A	0	0.45
gcg	A	0	0.11
---	--	------	------
mPD		0.040	0.70
		nPD:	0.06
		N. weight:	1.8
		Sc. PD:	-0.32
		Sc. rank:	-1299.4

PB1

Pos.	7	obs:	exp:
ttt	F	3	1.25
ttc	F	0	1.75
tta	L	2729	258.20
ttg	L	22	530.60
ctt	L	0	554.50
ctc	L	0	446.80
cta	L	42	413.30
ctg	L	0	589.50
---	--	------	------
mPD		0.047	1.1
		nPD:	0.04
		N. weight:	3.3
		Sc. PD:	-0.62
		Sc. rank:	-2998.2

PB1

Pos.	8	obs:	exp:
tta	L	0	258.30
ttg	L	0	530.80
ctt	L	2746	554.70
ctc	L	47	446.90
cta	L	0	413.50
ctg	L	1	589.70
att	I	2	0.73
atc	I	0	0.54
ata	I	0	0.73
---	--	------	------
mPD		0.035	1.1
		nPD:	0.03
		N. weight:	2.2
		Sc. PD:	-0.45
		Sc. rank:	-2496.8

PB1

Pos.	9	obs:	exp:
ttt	F	12	1165.00
ttc	F	2781	1628.00
tta	L	2	0.28
ttg	L	0	0.57
ctt	L	0	0.60
ctc	L	1	0.48
cta	L	0	0.44
ctg	L	0	0.63
---	--	------	------
mPD		0.011	0.49
		nPD:	0.02
		N. weight:	0.75
		Sc. PD:	-0.16
		Sc. rank:	-961.4

PB1

Pos.	10	obs:	exp:
tta	L	1019	258.10
ttg	L	1764	530.40
tct	S	0	0.33
tcc	S	0	0.29
tca	S	0	0.45
tcg	S	2	0.12
ctt	L	0	554.30
ctc	L	0	446.60
cta	L	3	413.20
ctg	L	6	589.30
agt	S	0	0.43
agc	S	0	0.37
gtt	V	0	0.43
gtc	V	0	0.42
gta	V	1	0.43
gtg	V	1	0.72
---	--	------	------
mPD		0.47	1.1
		nPD:	0.42
		N. weight:	1.8
		Sc. PD:	0.32
		Sc. rank:	1716.4

PB1

Pos.	11	obs:	exp:
cgt	R	0	0.30
cgc	R	0	0.43
cga	R	0	0.81
cgg	R	0	0.65
att	I	0	0.37
atc	I	0	0.27
ata	I	1	0.37
act	T	0	0.26
acc	T	0	0.23
aca	T	1	0.42
acg	T	0	0.09
aaa	K	2627	1586.00
aag	K	158	1199.00
aga	R	8	3.73
agg	R	0	2.08
gaa	E	1	0.56
gag	E	0	0.44
---	--	------	------
mPD		0.11	0.50
		nPD:	0.23
		N. weight:	0.53
		Sc. PD:	-0.0047
		Sc. rank:	198.5

PB1

Pos.	12	obs:	exp:
att	I	75	29.36
atc	I	1	21.41
ata	I	4	29.24
atg	M	2	2.00
gtt	V	72	583.10
gtc	V	0	570.40
gta	V	667	578.10
gtg	V	1972	979.50
gaa	E	0	1.12
gag	E	2	0.88
---	-	1	1.00
---	--	------	------
mPD		0.50	0.79
		nPD:	0.63
		N. weight:	0.73
		Sc. PD:	0.28
		Sc. rank:	935.2

PB1

Pos.	13	obs:	exp:
cct	P	13	763.60
ccc	P	118	504.60
cca	P	2490	1134.00
ccg	P	173	391.70
gct	A	0	0.25
gcc	A	0	0.19
gca	A	1	0.45
gcg	A	0	0.11
---	-	1	1.00
---	--	------	------
mPD		0.20	0.71
		nPD:	0.28
		N. weight:	0.84
		Sc. PD:	0.037
		Sc. rank:	504.1

PB1

Pos.	14	obs:	exp:
act	T	0	1.05
acc	T	0	0.90
aca	T	0	1.67
acg	T	4	0.38
gtt	V	0	352.70
gtc	V	0	345.00
gta	V	24	349.70
gtg	V	1616	592.50
gct	A	4	290.00
gcc	A	0	222.70
gca	A	80	512.30
gcg	A	1067	126.00
---	-	1	1.00
---	--	------	------
mPD		0.56	1.2
		nPD:	0.46
		N. weight:	1.9
		Sc. PD:	0.41
		Sc. rank:	1965.2

PB1

Pos.	15	obs:	exp:
cct	P	0	0.27
ccc	P	0	0.18
cca	P	1	0.41
ccg	P	0	0.14
cat	H	3	1.74
cac	H	0	1.26
caa	Q	2507	1394.00
cag	Q	285	1398.00
---	--	------	------
mPD		0.19	0.50
		nPD:	0.37
		N. weight:	0.53
		Sc. PD:	0.069
		Sc. rank:	459.0

PB1

Pos.	16	obs:	exp:
aat	N	2410	1326.00
aac	N	22	1106.00
gat	D	363	196.50
gac	D	1	167.50
mPD		0.24	0.72
		nPD:	0.34
		N. weight:	0.82
		Sc. PD:	0.078
		Sc. rank:	627.7

PB1

Pos.	17	obs:	exp:
cct	P	1	0.27
ccc	P	0	0.18
cca	P	0	0.41
ccg	P	0	0.14
gtt	V	1	0.22
gtc	V	0	0.21
gta	V	0	0.21
gtg	V	0	0.36
gct	A	1747	703.60
gcc	A	1037	540.50
gca	A	8	1243.00
gcg	A	1	305.70
ggt	G	1	0.14
ggc	G	0	0.13
gga	G	0	0.44
ggg	G	0	0.29
mPD		0.47	0.69
		nPD:	0.68
		N. weight:	1.2
		Sc. PD:	0.51
		Sc. rank:	1644.6

PB1

Pos.	18	obs:	exp:
tta	L	1	0.09
ttg	L	0	0.19
ctt	L	0	0.20
ctc	L	0	0.16
cta	L	0	0.15
ctg	L	0	0.21
cgt	R	0	0.04
cgc	R	0	0.05
cga	R	0	0.10
cgg	R	0	0.08
att	I	2	1016.00
atc	I	0	741.00
ata	I	2767	1012.00
aga	R	1	0.47
agg	R	0	0.26
gtt	V	0	5.38
gtc	V	0	5.26
gta	V	25	5.33
gtg	V	0	9.03
mPD		0.021	0.68
		nPD:	0.03
		N. weight:	1.5
		Sc. PD:	-0.3
		Sc. rank:	-1668.3

PB1

Pos.	19	obs:	exp:
tct	S	0	466.50
tcc	S	0	404.90
tca	S	0	633.60
tcg	S	0	168.60
tgt	C	1	0.39
tgc	C	0	0.61
agt	S	2476	606.20
agc	S	319	515.10
mPD		0.20	1.7
		nPD:	0.12
		N. weight:	1.7
		Sc. PD:	-0.19
		Sc. rank:	-253.5

PB1

Pos.	20	obs:	exp:
tct	S	0	4.17
tcc	S	25	3.62
tca	S	0	5.67
tcg	S	0	1.51
att	I	76	28.62
atc	I	1	20.87
ata	I	1	28.50
act	T	250	706.00
acc	T	2425	606.80
aca	T	18	1127.00
acg	T	0	253.40
agt	S	0	5.42
agc	S	0	4.61
mPD		0.29	0.77
		nPD:	0.38
		N. weight:	1.6
		Sc. PD:	0.22
		Sc. rank:	1443.5

PB1

Pos.	21	obs:	exp:
act	T	3	733.00
acc	T	0	630.00
aca	T	2756	1170.00
acg	T	37	263.10
mPD		0.028	0.70
		nPD:	0.04
		N. weight:	1.2
		Sc. PD:	-0.23
		Sc. rank:	-1144.7

PB1

Pos.	22	obs:	exp:
ttt	F	107	1166.00
ttc	F	2689	1630.00
mPD		0.074	0.49
		nPD:	0.15
		N. weight:	0.57
		Sc. PD:	-0.048
		Sc. rank:	43.4

PB1

Pos.	23	obs:	exp:
cct	P	2708	764.10
ccc	P	22	505.00
cca	P	17	1135.00
ccg	P	49	392.00
mPD		0.062	0.71
		nPD:	0.09
		N. weight:	1.7
		Sc. PD:	-0.24
		Sc. rank:	-702.7

PB1

Pos.	24	obs:	exp:
tat	Y	2164	1341.00
tac	Y	632	1455.00
mPD		0.35	0.50
		nPD:	0.7
		N. weight:	0.27
		Sc. PD:	0.12
		Sc. rank:	386.9

PB1

Pos.	25	obs:	exp:
att	I	1	0.37
atc	I	0	0.27
ata	I	0	0.37
act	T	2644	732.70
acc	T	60	629.80
aca	T	87	1170.00
acg	T	4	263.00
mPD		0.10	0.70
		nPD:	0.15
		N. weight:	1.6
		Sc. PD:	-0.14
		Sc. rank:	100.6

PB1

Pos.	26	obs:	exp:
ggt	G	0	404.30
ggc	G	2	367.30
gga	G	2774	1225.00
ggg	G	20	799.20
mPD		0.016	0.69
		nPD:	0.02
		N. weight:	1.1
		Sc. PD:	-0.24
		Sc. rank:	-1442.2

PB1

Pos.	27	obs:	exp:
gat	D	1145	1506.00
gac	D	1644	1283.00
gaa	E	6	3.36
gag	E	0	2.64
ggt	G	0	0.14
ggc	G	1	0.13
gga	G	0	0.44
ggg	G	0	0.29
mPD		0.49	0.50
		nPD:	0.97
		N. weight:	0.054
		Sc. PD:	0.039
		Sc. rank:	88.6

PB1

Pos.	28	obs:	exp:
cct	P	2758	764.10
ccc	P	38	505.00
cca	P	0	1135.00
ccg	P	0	392.00
mPD		0.027	0.71
		nPD:	0.04
		N. weight:	1.8
		Sc. PD:	-0.35
		Sc. rank:	-1843.2

PB1

Pos.	29	obs:	exp:
cct	P	0	764.10
ccc	P	76	505.00
cca	P	2702	1135.00
ccg	P	18	392.00
mPD		0.065	0.71
		nPD:	0.09
		N. weight:	1.1
		Sc. PD:	-0.16
		Sc. rank:	-415.9

PB1

Pos.	30	obs:	exp:
tct	S	0	0.17
tcc	S	1	0.14
tca	S	0	0.23
tcg	S	0	0.06
tat	Y	12	1340.00
tac	Y	2782	1454.00
cat	H	0	0.58
cac	H	1	0.42
agt	S	0	0.22
agc	S	0	0.18
mPD		0.010	0.50
		nPD:	0.02
		N. weight:	0.92
		Sc. PD:	-0.19
		Sc. rank:	-1203.4

PB1

Pos.	31	obs:	exp:
tct	S	0	466.50
tcc	S	0	404.90
tca	S	0	633.60
tcg	S	0	168.60
aat	N	0	0.55
aac	N	1	0.45
agt	S	6	606.20
agc	S	2789	515.10
mPD		0.0050	1.7
		nPD:	0.
		N. weight:	2.4
		Sc. PD:	-0.56
		Sc. rank:	-4023.1

PB1

Pos.	32	obs:	exp:
cat	H	2791	1619.00
cac	H	5	1177.00
mPD		0.0036	0.49
		nPD:	0.01
		N. weight:	0.78
		Sc. PD:	-0.18
		Sc. rank:	-1236.9

PB1

Pos.	33	obs:	exp:
ggt	G	0	404.30
ggc	G	0	367.30
gga	G	2384	1225.00
ggg	G	412	799.20
mPD		0.25	0.69
		nPD:	0.37
		N. weight:	0.69
		Sc. PD:	0.085
		Sc. rank:	575.0

PB1

Pos.	34	obs:	exp:
act	T	7	733.00
acc	T	0	630.00
aca	T	2771	1170.00
acg	T	18	263.10
mPD		0.018	0.70
		nPD:	0.03
		N. weight:	1.2
		Sc. PD:	-0.25
		Sc. rank:	-1489.7

PB1

Pos.	35	obs:	exp:
ggt	G	0	404.30
ggc	G	0	367.30
gga	G	494	1225.00
ggg	G	2302	799.20
mPD		0.29	0.69
		nPD:	0.42
		N. weight:	1.
		Sc. PD:	0.19
		Sc. rank:	987.3

PB1			
Pos.	36	obs:	exp:
cgt	R	0	0.04
cgc	R	0	0.05
cga	R	0	0.10
cgg	R	0	0.08
act	T	10	732.70
acc	T	2	629.80
aca	T	2730	1170.00
acg	T	53	263.00
aga	R	1	0.47
agg	R	0	0.26
---	--	------	------
mPD		0.046	0.70
		nPD:	0.07
		N. weight:	1.1
		Sc. PD:	-0.19
		Sc. rank:	-716.4

PB1			
Pos.	37	obs:	exp:
ggt	G	0	404.30
ggc	G	14	367.30
gga	G	1844	1225.00
ggg	G	938	799.20
---	--	------	------
mPD		0.45	0.69
		nPD:	0.66
		N. weight:	0.45
		Sc. PD:	0.18
		Sc. rank:	602.1

PB1			
Pos.	38	obs:	exp:
tat	Y	130	1340.00
tac	Y	2663	1453.00
cat	H	0	1.74
cac	H	3	1.26
---	--	------	------
mPD		0.091	0.50
		nPD:	0.18
		N. weight:	0.69
		Sc. PD:	-0.038
		Sc. rank:	142.7

PB1			
Pos.	39	obs:	exp:
cct	P	0	0.27
ccc	P	1	0.18
cca	P	0	0.41
ccg	P	0	0.14
act	T	116	732.40
acc	T	1937	629.60
aca	T	732	1169.00
acg	T	9	262.90
gct	A	0	0.25
gcc	A	1	0.19
gca	A	0	0.45
gcg	A	0	0.11
---	--	------	------
mPD		0.45	0.70
		nPD:	0.65
		N. weight:	0.84
		Sc. PD:	0.33
		Sc. rank:	1100.1

PB1			
Pos.	40	obs:	exp:
tta	L	0	1.85
ttg	L	1	3.80
ctt	L	0	3.97
ctc	L	0	3.20
cta	L	0	2.96
ctg	L	19	4.22
att	I	1	4.40
atc	I	0	3.21
ata	I	11	4.39
atg	M	2764	2764.00
---	--	------	------
mPD		0.023	0.031
		nPD:	0.73
		N. weight:	0.02
		Sc. PD:	0.0094
		Sc. rank:	29.8

PB1			
Pos.	41	obs:	exp:
gat	D	64	1509.00
gac	D	2731	1286.00
gaa	E	1	0.56
gag	E	0	0.44
---	--	------	------
mPD		0.045	0.50
		nPD:	0.09
		N. weight:	0.97
		Sc. PD:	-0.14
		Sc. rank:	-368.7

PB1			
Pos.	42	obs:	exp:
cct	P	0	0.27
ccc	P	0	0.18
cca	P	1	0.41
ccg	P	0	0.14
act	T	28	732.70
acc	T	38	629.80
aca	T	2569	1170.00
acg	T	160	263.00
---	--	------	------
mPD		0.15	0.70
		nPD:	0.22
		N. weight:	0.92
		Sc. PD:	-0.017
		Sc. rank:	299.6

PB1			
Pos.	43	obs:	exp:
ttt	F	0	0.42
ttc	F	1	0.58
att	I	0	0.37
atc	I	1	0.27
ata	I	0	0.37
gtt	V	56	600.90
gtc	V	2729	587.80
gta	V	9	595.80
gtg	V	0	1009.00
---	--	------	------
mPD		0.047	0.73
		nPD:	0.06
		N. weight:	2.1
		Sc. PD:	-0.36
		Sc. rank:	-1352.9

PB1			
Pos.	44	obs:	exp:
act	T	0	1.05
acc	T	4	0.90
aca	T	0	1.67
acg	T	0	0.38
aat	N	92	1522.00
aac	N	2699	1269.00
gat	D	0	0.54
gac	D	1	0.46
---	--	------	------
mPD		0.067	0.50
		nPD:	0.13
		N. weight:	0.93
		Sc. PD:	-0.094
		Sc. rank:	-49.8

PB1			
Pos.	45	obs:	exp:
cgt	R	0	104.60
cgc	R	0	149.60
cga	R	12	281.70
cgg	R	0	228.20
aga	R	2660	1305.00
agg	R	123	726.10
ggt	G	0	0.14
ggc	G	0	0.13
gga	G	1	0.44
ggg	G	0	0.29
---	--	------	------
mPD		0.093	0.95
		nPD:	0.1
		N. weight:	0.86
		Sc. PD:	-0.12
		Sc. rank:	-281.6

PB1			
Pos.	46	obs:	exp:
act	T	0	733.00
acc	T	1	630.00
aca	T	2754	1170.00
acg	T	41	263.10
---	--	------	------
mPD		0.030	0.70
		nPD:	0.04
		N. weight:	1.2
		Sc. PD:	-0.23
		Sc. rank:	-1070.0

PB1			
Pos.	47	obs:	exp:
tta	L	0	0.09
ttg	L	0	0.19
ctt	L	0	0.20
ctc	L	1	0.16
cta	L	0	0.15
ctg	L	0	0.21
cat	H	1165	1618.00
cac	H	1630	1177.00
---	--	------	------
mPD		0.49	0.49
		nPD:	1.
		N. weight:	0.08
		Sc. PD:	0.059
		Sc. rank:	132.4

PB1			
Pos.	48	obs:	exp:
tta	L	0	0.09
ttg	L	0	0.19
ctt	L	0	0.20
ctc	L	0	0.16
cta	L	1	0.15
ctg	L	0	0.21
cat	H	1	0.58
cac	H	0	0.42
caa	Q	2752	1391.00
cag	Q	34	1395.00
cgt	R	0	0.04
cgc	R	0	0.05
cga	R	1	0.10
cgg	R	0	0.08
aaa	K	7	3.99
aag	K	0	3.01
aga	R	0	0.47
agg	R	0	0.26
---	--	------	------
mPD		0.031	0.51
		nPD:	0.06
		N. weight:	0.92
		Sc. PD:	-0.16
		Sc. rank:	-641.9

PB1			
Pos.	49	obs:	exp:
tat	Y	1522	1341.00
tac	Y	1274	1455.00
---	--	------	------
mPD		0.50	0.50
		nPD:	0.99
		N. weight:	0.014
		Sc. PD:	0.011
		Sc. rank:	23.8

PB1			
Pos.	50	obs:	exp:
tct	S	0	466.50
tcc	S	0	404.90
tca	S	2677	633.60
tcg	S	118	168.60
act	T	0	0.26
acc	T	0	0.23
aca	T	1	0.42
acg	T	0	0.09
agt	S	0	606.20
agc	S	0	515.10
---	--	------	------
mPD		0.082	1.7
		nPD:	0.05
		N. weight:	2.
		Sc. PD:	-0.37
		Sc. rank:	-1612.1

PB1			
Pos.	51	obs:	exp:
gct	A	0	0.25
gcc	A	0	0.19
gca	A	0	0.45
gcg	A	1	0.11
gaa	E	1774	1567.00
gag	E	1021	1228.00
---	--	------	------
mPD		0.46	0.49
		nPD:	0.94
		N. weight:	0.02
		Sc. PD:	0.014
		Sc. rank:	32.5

PB1			
Pos.	52	obs:	exp:
caa	Q	0	0.50
cag	Q	1	0.50
cgt	R	0	0.94
cgc	R	0	1.34
cga	R	0	2.52
cgg	R	0	2.04
aat	N	1	0.55
aac	N	0	0.45
aaa	K	236	1577.00
aag	K	2533	1192.00
aga	R	0	11.67
agg	R	25	6.49
---	--	------	------
mPD		0.17	0.51
		nPD:	0.34
		N. weight:	0.78
		Sc. PD:	0.076
		Sc. rank:	600.4

PB1			
Pos.	53	obs:	exp:
ggt	G	15	404.30
ggc	G	0	367.30
gga	G	993	1225.00
ggg	G	1788	799.20
---	--	------	------
mPD		0.47	0.69
		nPD:	0.68
		N. weight:	0.62
		Sc. PD:	0.26
		Sc. rank:	861.7

PB1			
Pos.	54	obs:	exp:
cgt	R	0	3.18
cgc	R	0	4.55
cga	R	0	8.57
cgg	R	0	6.94
aat	N	0	0.55
aac	N	1	0.45
aaa	K	1059	1103.00
aag	K	878	834.10
aga	R	76	39.68
agg	R	9	22.08
gtt	V	0	1.29
gtc	V	0	1.26
gta	V	0	1.28
gtg	V	6	2.17
gaa	E	16	418.80
gag	E	731	328.20
ggt	G	0	2.89
ggc	G	0	2.63
gga	G	0	8.76
ggg	G	20	5.72
---	--	------	------
mPD		0.96	0.99
		nPD:	0.98
		N. weight:	0.32
		Sc. PD:	0.23
		Sc. rank:	528.9

PB1			
Pos.	55	obs:	exp:
tta	L	0	0.09
ttg	L	1	0.19
tgg	W	2795	2795.00
ctt	L	0	0.20
ctc	L	0	0.16
cta	L	0	0.15
ctg	L	0	0.21
---	--	------	------
mPD		0.00072	0.0017
		nPD:	0.43
		N. weight:	0.00087
		Sc. PD:	0.00016
		Sc. rank:	0.8

PB1			
Pos.	56	obs:	exp:
att	I	0	6.24
atc	I	0	4.55
ata	I	17	6.21
act	T	2	703.90
acc	T	1	605.00
aca	T	2672	1123.00
acg	T	10	252.60
gtt	V	0	0.22
gtc	V	0	0.21
gta	V	1	0.21
gtg	V	0	0.36
gct	A	0	23.43
gcc	A	0	18.00
gca	A	92	41.40
gcg	A	1	10.18
---	--	------	------
mPD		0.088	0.77
		nPD:	0.11
		N. weight:	1.2
		Sc. PD:	-0.15
		Sc. rank:	-307.6

,

PB1			
Pos.	57	obs:	exp:
cgt	R	0	0.07
cgc	R	0	0.11
cga	R	0	0.20
cgg	R	0	0.16
att	I	0	27.52
atc	I	0	20.07
ata	I	75	27.41
atg	M	1	1.00
act	T	0	676.90
acc	T	0	581.80
aca	T	2546	1080.00
acg	T	36	242.90
aaa	K	136	77.44
aag	K	0	58.56
aga	R	2	0.93
agg	R	0	0.52
---	--	------	------
mPD		0.17	0.84
		nPD:	0.2
		N. weight:	1.2
		Sc. PD:	-0.041
		Sc. rank:	319.5

,

PB1			
Pos.	58	obs:	exp:
tct	S	0	0.17
tcc	S	0	0.14
tca	S	0	0.23
tcg	S	0	0.06
cat	H	0	1.16
cac	H	2	0.84
aat	N	82	1523.00
aac	N	2711	1270.00
agt	S	0	0.22
agc	S	1	0.18
---	--	------	------
mPD		0.059	0.50
		nPD:	0.12
		N. weight:	0.95
		Sc. PD:	-0.11
		Sc. rank:	-179.9

,

PB1			
Pos.	59	obs:	exp:
tct	S	0	8.68
tcc	S	0	7.53
tca	S	52	11.79
tcg	S	0	3.14
att	I	0	0.73
atc	I	0	0.54
ata	I	2	0.73
act	T	9	716.20
acc	T	5	615.60
aca	T	2706	1143.00
acg	T	12	257.00
agt	S	0	11.28
agc	S	0	9.58
gct	A	0	2.52
gcc	A	0	1.94
gca	A	10	4.45
gcg	A	0	1.09
---	--	------	------
mPD		0.063	0.74
		nPD:	0.09
		N. weight:	1.2
		Sc. PD:	-0.18
		Sc. rank:	-562.4

,

PB1			
Pos.	60	obs:	exp:
aaa	K	0	0.57
aag	K	1	0.43
gat	D	1	0.54
gac	D	0	0.46
gaa	E	827	1566.00
gag	E	1967	1228.00
---	--	------	------
mPD		0.42	0.49
		nPD:	0.85
		N. weight:	0.21
		Sc. PD:	0.12
		Sc. rank:	337.9

,

PB1			
Pos.	61	obs:	exp:
tct	S	0	0.17
tcc	S	0	0.14
tca	S	0	0.23
tcg	S	0	0.06
act	T	950	732.70
acc	T	1823	629.80
aca	T	22	1170.00
acg	T	0	263.00
agt	S	1	0.22
agc	S	0	0.18
---	--	------	------
mPD		0.46	0.70
		nPD:	0.66
		N. weight:	1.1
		Sc. PD:	0.45
		Sc. rank:	1476.0

,

PB1			
Pos.	62	obs:	exp:
cgt	R	0	0.82
cgc	R	0	1.18
cga	R	0	2.22
cgg	R	0	1.80
aga	R	22	10.27
agg	R	0	5.72
gaa	E	1	1.12
gag	E	1	0.88
ggt	G	0	400.90
ggc	G	0	364.10
gga	G	2705	1215.00
ggg	G	67	792.40
---	--	------	------
mPD		0.065	0.70
		nPD:	0.09
		N. weight:	1.1
		Sc. PD:	-0.15
		Sc. rank:	-396.1

,

PB1			
Pos.	63	obs:	exp:
tct	S	0	0.50
tcc	S	0	0.43
tca	S	3	0.68
tcg	S	0	0.18
agt	S	0	0.65
agc	S	0	0.55
gct	A	1	703.60
gcc	A	3	540.50
gca	A	2761	1243.00
gcg	A	28	305.70
---	--	------	------
mPD		0.025	0.69
		nPD:	0.04
		N. weight:	1.1
		Sc. PD:	-0.22
		Sc. rank:	-1177.4

,

PB1			
Pos.	64	obs:	exp:
tta	L	0	4.53
ttg	L	0	9.31
tct	S	1	1.00
tcc	S	5	0.87
tca	S	0	1.36
tcg	S	0	0.36
ctt	L	2	9.73
ctc	L	47	7.84
cta	L	0	7.25
ctg	L	0	10.34
cct	P	849	749.10
ccc	P	1886	495.10
cca	P	6	1113.00
ccg	P	0	384.30
agt	S	0	1.30
agc	S	0	1.11
---	--	------	------
mPD		0.47	0.76
		nPD:	0.61
		N. weight:	1.4
		Sc. PD:	0.51
		Sc. rank:	1786.5

,

PB1			
Pos.	65	obs:	exp:
cct	P	0	0.82
ccc	P	0	0.54
cca	P	3	1.22
ccg	P	0	0.42
cat	H	1	0.58
cac	H	0	0.42
caa	Q	2661	1394.00
cag	Q	131	1398.00
---	--	------	------
mPD		0.092	0.50
		nPD:	0.18
		N. weight:	0.74
		Sc. PD:	-0.039
		Sc. rank:	162.7

,

PB1			
Pos.	66	obs:	exp:
tta	L	0	258.50
ttg	L	0	531.20
ctt	L	110	555.10
ctc	L	2683	447.20
cta	L	3	413.80
ctg	L	0	590.10
---	--	------	------
mPD		0.078	1.1
		nPD:	0.07
		N. weight:	2.4
		Sc. PD:	-0.4
		Sc. rank:	-1462.2

,

PB1			
Pos.	67	obs:	exp:
cat	H	0	0.58
cac	H	1	0.42
aat	N	331	1524.00
aac	N	2464	1271.00
---	--	------	------
mPD		0.21	0.50
		nPD:	0.42
		N. weight:	0.59
		Sc. PD:	0.11
		Sc. rank:	558.2

,

PB1			
Pos.	68	obs:	exp:
cct	P	181	763.80
ccc	P	9	504.80
cca	P	1354	1134.00
ccg	P	1251	391.90
caa	Q	0	0.50
cag	Q	1	0.50
---	--	------	------
mPD		0.56	0.71
		nPD:	0.79
		N. weight:	0.73
		Sc. PD:	0.4
		Sc. rank:	1153.7

,

PB1			
Pos.	69	obs:	exp:
att	I	2734	1021.00
atc	I	48	744.50
ata	I	0	1017.00
act	T	1	0.26
acc	T	0	0.23
aca	T	0	0.42
acg	T	0	0.09
gtt	V	13	2.80
gtc	V	0	2.74
gta	V	0	2.77
gtg	V	0	4.70
---	--	------	------
mPD		0.044	0.67
		nPD:	0.07
		N. weight:	1.3
		Sc. PD:	-0.23
		Sc. rank:	-855.7

,

PB1			
Pos.	70	obs:	exp:
gat	D	2686	1509.00
gac	D	108	1285.00
gaa	E	1	1.12
gag	E	1	0.88
---	--	------	------
mPD		0.076	0.50
		nPD:	0.15
		N. weight:	0.67
		Sc. PD:	-0.056
		Sc. rank:	52.9

,

PB1			
Pos.	71	obs:	exp:
ggt	G	0	404.30
ggc	G	6	367.30
gga	G	2197	1225.00
ggg	G	593	799.20
---	--	------	------
mPD		0.34	0.69
		nPD:	0.49
		N. weight:	0.57
		Sc. PD:	0.14
		Sc. rank:	614.4

,

PB1			
Pos.	72	obs:	exp:
cct	P	17	764.10
ccc	P	0	505.00
cca	P	2767	1135.00
ccg	P	12	392.00
---	--	------	------
mPD		0.021	0.71
		nPD:	0.03
		N. weight:	1.2
		Sc. PD:	-0.25
		Sc. rank:	-1446.2

,

PB1			
Pos.	73	obs:	exp:
tta	L	646	258.40
ttg	L	231	531.00
ctt	L	0	554.90
ctc	L	0	447.10
cta	L	1843	413.60
ctg	L	75	589.90
caa	Q	1	0.50
cag	Q	0	0.50
---	--	------	------
mPD		0.63	1.1
		nPD:	0.56
		N. weight:	1.6
		Sc. PD:	0.49
		Sc. rank:	1833.8

,

PB1			
Pos.	74	obs:	exp:
cct	P	2337	764.10
ccc	P	58	505.00
cca	P	224	1135.00
ccg	P	177	392.00
---	--	------	------
mPD		0.29	0.71
		nPD:	0.41
		N. weight:	1.
		Sc. PD:	0.17
		Sc. rank:	953.5

,

PB1			
Pos.	75	obs:	exp:
gtt	V	0	1.08
gtc	V	0	1.05
gta	V	0	1.07
gtg	V	5	1.81
gat	D	23	12.42
gac	D	0	10.58
gaa	E	201	1551.00
gag	E	2566	1216.00
ggt	G	0	0.14
ggc	G	0	0.13
gga	G	0	0.44
ggg	G	1	0.29
---	--	------	------
mPD		0.15	0.51
		nPD:	0.3
		N. weight:	0.8
		Sc. PD:	0.05
		Sc. rank:	534.1

,

PB1

Pos.	76	obs:	exp:
aat	N	19	10.91
aac	N	1	9.09
gat	D	1433	1498.00
gac	D	1341	1276.00
gaa	E	1	0.56
gag	E	0	0.44
ggt	G	0	0.14
ggc	G	1	0.13
gga	G	0	0.44
ggg	G	0	0.29
---	--	------	------
mPD		0.51	0.51
		nPD:	1.
		N. weight:	0.01
		Sc. PD:	0.0075
		Sc. rank:	16.5

PB1

Pos.	77	obs:	exp:
tct	S	0	1.34
tcc	S	0	1.16
tca	S	0	1.81
tcg	S	0	0.48
cat	H	0	1.16
cac	H	2	0.84
att	I	0	0.37
atc	I	1	0.27
ata	I	0	0.37
act	T	1	0.26
acc	T	0	0.23
aca	T	0	0.42
acg	T	0	0.09
aat	N	1137	1516.00
aac	N	1643	1264.00
agt	S	0	1.74
agc	S	8	1.47
gat	D	2	2.16
gac	D	2	1.84
---	--	------	------
mPD		0.49	0.51
		nPD:	0.97
		N. weight:	0.066
		Sc. PD:	0.047
		Sc. rank:	108.5

PB1

Pos.	78	obs:	exp:
gat	D	6	7.56
gac	D	8	6.44
gaa	E	848	1560.00
gag	E	1934	1222.00
---	--	------	------
mPD		0.43	0.50
		nPD:	0.86
		N. weight:	0.2
		Sc. PD:	0.12
		Sc. rank:	316.1

PB1

Pos.	79	obs:	exp:
cct	P	31	764.10
ccc	P	1392	505.00
cca	P	621	1135.00
ccg	P	752	392.00
---	--	------	------
mPD		0.63	0.71
		nPD:	0.89
		N. weight:	0.75
		Sc. PD:	0.48
		Sc. rank:	1215.0

PB1

Pos.	80	obs:	exp:
tct	S	0	465.70
tcc	S	0	404.10
tca	S	0	632.50
tcg	S	0	168.30
tgt	C	2	0.77
tgc	C	0	1.23
act	T	1	0.26
acc	T	0	0.23
aca	T	0	0.42
acg	T	0	0.09
aat	N	3	1.64
aac	N	0	1.36
agt	S	1759	605.20
agc	S	1031	514.20
---	--	------	------
mPD		0.47	1.7
		nPD:	0.28
		N. weight:	1.4
		Sc. PD:	0.061
		Sc. rank:	843.1

PB1

Pos.	81	obs:	exp:
ggt	G	277	404.30
ggc	G	4	367.30
gga	G	1123	1225.00
ggg	G	1392	799.20
---	--	------	------
mPD		0.58	0.69
		nPD:	0.84
		N. weight:	0.29
		Sc. PD:	0.17
		Sc. rank:	468.5

PB1

Pos.	82	obs:	exp:
tat	Y	2768	1340.00
tac	Y	27	1455.00
cgt	R	0	0.04
cgc	R	0	0.05
cga	R	0	0.10
cgg	R	0	0.08
aga	R	1	0.47
agg	R	0	0.26
---	--	------	------
mPD		0.021	0.50
		nPD:	0.04
		N. weight:	0.99
		Sc. PD:	-0.19
		Sc. rank:	-898.6

PB1

Pos.	83	obs:	exp:
gct	A	0	704.40
gcc	A	2	541.10
gca	A	2723	1245.00
gcg	A	71	306.00
---	--	------	------
mPD		0.051	0.69
		nPD:	0.07
		N. weight:	1.1
		Sc. PD:	-0.17
		Sc. rank:	-587.3

PB1

Pos.	84	obs:	exp:
caa	Q	2723	1395.00
cag	Q	72	1400.00
cgt	R	0	0.04
cgc	R	0	0.05
cga	R	1	0.10
cgg	R	0	0.08
aga	R	0	0.47
agg	R	0	0.26
---	--	------	------
mPD		0.051	0.50
		nPD:	0.1
		N. weight:	0.84
		Sc. PD:	-0.11
		Sc. rank:	-258.8

PB1

Pos.	85	obs:	exp:
act	T	3	733.00
acc	T	0	630.00
aca	T	2508	1170.00
acg	T	285	263.10
---	--	------	------
mPD		0.19	0.70
		nPD:	0.27
		N. weight:	1.
		Sc. PD:	0.027
		Sc. rank:	557.9

PB1

Pos.	86	obs:	exp:
gat	D	2670	1509.00
gac	D	125	1286.00
ggt	G	0	0.14
ggc	G	1	0.13
gga	G	0	0.44
ggg	G	0	0.29
---	--	------	------
mPD		0.087	0.50
		nPD:	0.17
		N. weight:	0.65
		Sc. PD:	-0.04
		Sc. rank:	114.9

PB1

Pos.	87	obs:	exp:
tgt	C	1822	1082.00
tgc	C	974	1714.00
---	--	------	------
mPD		0.45	0.47
		nPD:	0.96
		N. weight:	0.21
		Sc. PD:	0.15
		Sc. rank:	344.6

PB1

Pos.	88	obs:	exp:
gtt	V	0	601.40
gtc	V	85	588.20
gta	V	1616	596.20
gtg	V	1095	1010.00
---	--	------	------
mPD		0.51	0.73
		nPD:	0.7
		N. weight:	0.8
		Sc. PD:	0.36
		Sc. rank:	1157.1

PB1

Pos.	89	obs:	exp:
tta	L	475	258.50
ttg	L	2235	531.20
ctt	L	65	555.10
ctc	L	0	447.20
cta	L	9	413.80
ctg	L	12	590.10
---	--	------	------
mPD		0.38	1.1
		nPD:	0.34
		N. weight:	1.7
		Sc. PD:	0.17
		Sc. rank:	1356.6

PB1

Pos.	90	obs:	exp:
gaa	E	2771	1567.00
gag	E	25	1229.00
---	--	------	------
mPD		0.018	0.49
		nPD:	0.04
		N. weight:	0.77
		Sc. PD:	-0.15
		Sc. rank:	-820.3

PB1

Pos.	91	obs:	exp:
tct	S	0	0.17
tcc	S	0	0.14
tca	S	1	0.23
tcg	S	0	0.06
agt	S	0	0.22
agc	S	0	0.18
gct	A	0	703.90
gcc	A	0	540.70
gca	A	2710	1244.00
gcg	A	84	305.80
gat	D	0	0.54
gac	D	1	0.46
---	--	------	------
mPD		0.060	0.69
		nPD:	0.09
		N. weight:	1.
		Sc. PD:	-0.15
		Sc. rank:	-437.2

PB1

Pos.	92	obs:	exp:
tta	L	0	0.09
ttg	L	0	0.19
ctt	L	0	0.20
ctc	L	0	0.16
cta	L	0	0.15
ctg	L	1	0.21
att	I	4	1.47
atc	I	0	1.07
ata	I	0	1.46
atg	M	2791	2791.00
---	--	------	------
mPD		0.0036	0.0040
		nPD:	0.89
		N. weight:	0.0029
		Sc. PD:	0.0019
		Sc. rank:	4.7

PB1

Pos.	93	obs:	exp:
gct	A	2780	704.40
gcc	A	16	541.10
gca	A	0	1245.00
gcg	A	0	306.00
---	--	------	------
mPD		0.011	0.69
		nPD:	0.02
		N. weight:	2.
		Sc. PD:	-0.42
		Sc. rank:	-2680.7

PB1

Pos.	94	obs:	exp:
ttt	F	54	1154.00
ttc	F	2711	1611.00
tta	L	0	1.48
ttg	L	0	3.04
tct	S	0	2.00
tcc	S	12	1.74
tca	S	0	2.72
tcg	S	0	0.72
tat	Y	0	1.44
tac	Y	3	1.56
ctt	L	0	3.18
ctc	L	16	2.56
cta	L	0	2.37
ctg	L	0	3.38
agt	S	0	2.60
agc	S	0	2.21
---	--	------	------
mPD		0.060	0.51
		nPD:	0.12
		N. weight:	0.68
		Sc. PD:	-0.08
		Sc. rank:	-136.5

PB1

Pos.	95	obs:	exp:
tta	L	0	258.40
ttg	L	0	531.00
ctt	L	2745	554.90
ctc	L	45	447.10
cta	L	0	413.60
ctg	L	5	589.90
cat	H	1	0.58
cac	H	0	0.42
---	--	------	------
mPD		0.036	1.1
		nPD:	0.03
		N. weight:	2.2
		Sc. PD:	-0.45
		Sc. rank:	-2450.2

PB1			
Pos.	96	obs:	exp:
gat	D	0	1.62
gac	D	3	1.38
gaa	E	2613	1565.00
gag	E	179	1227.00
ggt	G	0	0.14
ggc	G	0	0.13
gga	G	0	0.44
ggg	G	1	0.29
---	--	------	------
mPD		0.12	0.49
		nPD:	0.25
		N. weight:	0.52
		Sc. PD:	0.0057
		Sc. rank:	257.2

,

PB1			
Pos.	97	obs:	exp:
cgt	R	0	0.04
cgc	R	0	0.05
cga	R	0	0.10
cgg	R	0	0.08
aat	N	3	1.64
aac	N	0	1.36
aaa	K	4	6.26
aag	K	7	4.74
aga	R	1	0.47
agg	R	0	0.26
gat	D	3	1.62
gac	D	0	1.38
gaa	E	1846	1557.00
gag	E	932	1221.00
---	--	------	------
mPD		0.46	0.51
		nPD:	0.91
		N. weight:	0.038
		Sc. PD:	0.025
		Sc. rank:	62.3

,

PB1			
Pos.	98	obs:	exp:
ttt	F	0	0.42
ttc	F	1	0.58
tct	S	61	465.40
tcc	S	2726	403.90
tca	S	1	632.00
tcg	S	0	168.20
tgt	C	0	0.39
tgc	C	1	0.61
cct	P	0	0.82
ccc	P	3	0.54
cca	P	0	1.22
ccg	P	0	0.42
act	T	0	0.52
acc	T	2	0.45
aca	T	0	0.84
acg	T	0	0.19
agt	S	0	604.70
agc	S	0	513.80
gct	A	1	0.25
gcc	A	0	0.19
gca	A	0	0.45
gcg	A	0	0.11
---	--	------	------
mPD		0.050	1.7
		nPD:	0.03
		N. weight:	2.7
		Sc. PD:	-0.54
		Sc. rank:	-3034.3

,

PB1			
Pos.	99	obs:	exp:
tat	Y	1	0.48
tac	Y	0	0.52
cat	H	352	1617.00
cac	H	2442	1177.00
caa	Q	0	0.50
cag	Q	1	0.50
---	--	------	------
mPD		0.22	0.49
		nPD:	0.45
		N. weight:	0.65
		Sc. PD:	0.14
		Sc. rank:	660.5

,

PB1			
Pos.	100	obs:	exp:
tct	S	0	0.83
tcc	S	0	0.72
tca	S	5	1.13
tcg	S	0	0.30
cct	P	0	762.70
ccc	P	5	504.10
cca	P	2781	1133.00
ccg	P	5	391.30
agt	S	0	1.08
agc	S	0	0.92
---	--	------	------
mPD		0.011	0.71
		nPD:	0.02
		N. weight:	1.3
		Sc. PD:	-0.28
		Sc. rank:	-1842.8

,

PB1			
Pos.	101	obs:	exp:
cgt	R	0	0.11
cgc	R	0	0.16
cga	R	0	0.30
cgg	R	1	0.24
aga	R	2	1.40
agg	R	0	0.78
gaa	E	3	1.68
gag	E	0	1.32
ggt	G	10	403.50
ggc	G	0	366.50
gga	G	859	1223.00
ggg	G	1921	797.50
---	--	------	------
mPD		0.44	0.69
		nPD:	0.63
		N. weight:	0.71
		Sc. PD:	0.27
		Sc. rank:	913.3

,

PB1			
Pos.	102	obs:	exp:
tta	L	0	0.09
ttg	L	0	0.19
ctt	L	0	0.20
ctc	L	1	0.16
cta	L	0	0.15
ctg	L	0	0.21
att	I	40	1026.00
atc	I	2748	747.90
ata	I	7	1021.00
---	--	------	------
mPD		0.034	0.66
		nPD:	0.05
		N. weight:	1.8
		Sc. PD:	-0.32
		Sc. rank:	-1407.5

,

PB1			
Pos.	103	obs:	exp:
ttt	F	2787	1166.00
ttc	F	8	1629.00
tta	L	0	0.09
ttg	L	0	0.19
ctt	L	1	0.20
ctc	L	0	0.16
cta	L	0	0.15
ctg	L	0	0.21
---	--	------	------
mPD		0.0064	0.49
		nPD:	0.01
		N. weight:	1.2
		Sc. PD:	-0.27
		Sc. rank:	-1860.5

,

PB1			
Pos.	104	obs:	exp:
gat	D	0	9.72
gac	D	18	8.28
gaa	E	2230	1279.00
gag	E	52	1003.00
ggt	G	0	71.73
ggc	G	0	65.15
gga	G	458	217.30
ggg	G	38	141.80
---	--	------	------
mPD		0.37	0.84
		nPD:	0.44
		N. weight:	0.73
		Sc. PD:	0.14
		Sc. rank:	713.8

,

PB1			
Pos.	105	obs:	exp:
tct	S	0	2.00
tcc	S	0	1.74
tca	S	0	2.72
tcg	S	0	0.72
cat	H	0	1.74
cac	H	3	1.26
act	T	68	18.35
acc	T	2	15.77
aca	T	0	29.29
acg	T	0	6.59
aat	N	976	1476.00
aac	N	1730	1230.00
agt	S	3	2.60
agc	S	9	2.21
gat	D	5	2.70
gac	D	0	2.30
---	--	------	------
mPD		0.53	0.58
		nPD:	0.92
		N. weight:	0.15
		Sc. PD:	0.1
		Sc. rank:	252.4

,

PB1			
Pos.	106	obs:	exp:
tct	S	978	466.70
tcc	S	18	405.00
tca	S	237	633.90
tcg	S	1563	168.70
agt	S	0	606.50
agc	S	0	515.30
---	--	------	------
mPD		0.56	1.7
		nPD:	0.34
		N. weight:	2.
		Sc. PD:	0.2
		Sc. rank:	1589.9

,

PB1			
Pos.	107	obs:	exp:
tgt	C	2745	1082.00
tgc	C	51	1714.00
---	--	------	------
mPD		0.036	0.47
		nPD:	0.08
		N. weight:	1.2
		Sc. PD:	-0.2
		Sc. rank:	-675.2

,

PB1			
Pos.	108	obs:	exp:
ttt	F	3	1.25
ttc	F	0	1.75
tta	L	0	256.80
ttg	L	0	527.60
ctt	L	2676	551.40
ctc	L	95	444.20
cta	L	6	411.00
ctg	L	0	586.10
cgt	R	2	0.07
cgc	R	0	0.11
cga	R	0	0.20
cgg	R	0	0.16
att	I	14	5.14
atc	I	0	3.75
ata	I	0	5.12
aga	R	0	0.93
agg	R	0	0.52
---	--	------	------
mPD		0.083	1.1
		nPD:	0.07
		N. weight:	2.1
		Sc. PD:	-0.34
		Sc. rank:	-1196.2

,

PB1			
Pos.	109	obs:	exp:
gtt	V	1	0.22
gtc	V	0	0.21
gta	V	0	0.21
gtg	V	0	0.36
gaa	E	2770	1567.00
gag	E	25	1228.00
---	--	------	------
mPD		0.019	0.49
		nPD:	0.04
		N. weight:	0.77
		Sc. PD:	-0.15
		Sc. rank:	-775.8

,

PB1			
Pos.	110	obs:	exp:
tct	S	1	0.17
tcc	S	0	0.14
tca	S	0	0.23
tcg	S	0	0.06
act	T	93	731.90
acc	T	0	629.10
aca	T	128	1168.00
acg	T	2571	262.70
agt	S	0	0.22
agc	S	0	0.18
gct	A	0	0.76
gcc	A	0	0.58
gca	A	1	1.34
gcg	A	2	0.33
---	--	------	------
mPD		0.15	0.70
		nPD:	0.22
		N. weight:	2.8
		Sc. PD:	-0.054
		Sc. rank:	957.7

,

PB1			
Pos.	111	obs:	exp:
tta	L	0	0.09
ttg	L	0	0.19
ctt	L	0	0.20
ctc	L	0	0.16
cta	L	0	0.15
ctg	L	1	0.21
att	I	4	6.97
atc	I	0	5.08
ata	I	15	6.94
atg	M	2774	2774.00
gtt	V	0	0.43
gtc	V	0	0.42
gta	V	0	0.43
gtg	V	2	0.72
---	--	------	------
mPD		0.016	0.017
		nPD:	0.92
		N. weight:	0.0075
		Sc. PD:	0.005
		Sc. rank:	12.3

PB1			
Pos.	112	obs:	exp:
caa	Q	3	1.50
cag	Q	0	1.50
gaa	E	2723	1565.00
gag	E	68	1226.00
ggt	G	0	0.29
ggc	G	0	0.26
gga	G	2	0.88
ggg	G	0	0.57
---	--	------	------
mPD		0.051	0.50
		nPD:	0.1
		N. weight:	0.69
		Sc. PD:	-0.091
		Sc. rank:	-209.7

PB1			
Pos.	113	obs:	exp:
att	I	1691	689.90
atc	I	185	503.10
ata	I	4	687.00
atg	M	1	1.00
act	T	1	0.26
acc	T	0	0.23
aca	T	0	0.42
acg	T	0	0.09
gtt	V	104	196.40
gtc	V	809	192.10
gta	V	0	194.70
gtg	V	0	329.90
gct	A	0	0.25
gcc	A	1	0.19
gca	A	0	0.45
gcg	A	0	0.11
---	--	------	------
mPD		0.90	1.2
		nPD:	0.77
		N. weight:	1.3
		Sc. PD:	0.66
		Sc. rank:	1973.6

PB1			
Pos.	114	obs:	exp:
att	I	25	11.38
atc	I	6	8.30
ata	I	0	11.33
gtt	V	2064	594.30
gtc	V	693	581.30
gta	V	4	589.10
gtg	V	2	998.30
gct	A	1	0.25
gcc	A	0	0.19
gca	A	0	0.45
gcg	A	0	0.11
gat	D	1	0.54
gac	D	0	0.46
---	--	------	------
mPD		0.40	0.76
		nPD:	0.53
		N. weight:	1.4
		Sc. PD:	0.4
		Sc. rank:	1592.3

PB1			
Pos.	115	obs:	exp:
cat	H	1	0.58
cac	H	0	0.42
caa	Q	1616	1395.00
cag	Q	1179	1400.00
---	--	------	------
mPD		0.49	0.50
		nPD:	0.98
		N. weight:	0.021
		Sc. PD:	0.015
		Sc. rank:	34.0

PB1			
Pos.	116	obs:	exp:
caa	Q	2771	1396.00
cag	Q	25	1400.00
---	--	------	------
mPD		0.018	0.50
		nPD:	0.04
		N. weight:	0.94
		Sc. PD:	-0.19
		Sc. rank:	-1006.9

PB1			
Pos.	117	obs:	exp:
act	T	0	729.30
acc	T	9	626.90
aca	T	2770	1164.00
acg	T	3	261.80
aaa	K	1	0.57
aag	K	0	0.43
gct	A	0	3.27
gcc	A	0	2.52
gca	A	13	5.79
gcg	A	0	1.42
---	--	------	------
mPD		0.019	0.71
		nPD:	0.03
		N. weight:	1.2
		Sc. PD:	-0.25
		Sc. rank:	-1516.3

PB1			
Pos.	118	obs:	exp:
cgt	R	0	104.50
cgc	R	0	149.50
cga	R	0	281.50
cgg	R	0	228.10
aaa	K	3	1.71
aag	K	0	1.29
aga	R	2755	1304.00
agg	R	38	725.60
---	--	------	------
mPD		0.029	0.96
		nPD:	0.03
		N. weight:	1.
		Sc. PD:	-0.21
		Sc. rank:	-1153.5

PB1			
Pos.	119	obs:	exp:
tta	L	0	0.46
ttg	L	5	0.95
ctt	L	0	0.99
ctc	L	0	0.80
cta	L	0	0.74
ctg	L	0	1.06
atg	M	4	4.00
gtt	V	243	598.60
gtc	V	0	585.50
gta	V	27	593.40
gtg	V	2513	1006.00
gct	A	0	0.76
gcc	A	0	0.58
gca	A	0	1.34
gcg	A	3	0.33
gaa	E	0	0.56
gag	E	1	0.44
---	--	------	------
mPD		0.19	0.74
		nPD:	0.25
		N. weight:	1.1
		Sc. PD:	0.012
		Sc. rank:	523.9

PB1			
Pos.	120	obs:	exp:
aat	N	0	1.09
aac	N	2	0.91
gat	D	713	1509.00
gac	D	2081	1285.00
---	--	------	------
mPD		0.38	0.50
		nPD:	0.77
		N. weight:	0.25
		Sc. PD:	0.13
		Sc. rank:	382.1

PB1			
Pos.	121	obs:	exp:
caa	Q	1	0.50
cag	Q	0	0.50
cgt	R	0	0.11
cgc	R	0	0.16
cga	R	0	0.30
cgg	R	0	0.24
aaa	K	2712	1590.00
aag	K	80	1202.00
aga	R	3	1.40
agg	R	0	0.78
---	--	------	------
mPD		0.058	0.49
		nPD:	0.12
		N. weight:	0.65
		Sc. PD:	-0.075
		Sc. rank:	-127.9

PB1			
Pos.	122	obs:	exp:
tta	L	162	258.50
ttg	L	80	531.20
ctt	L	14	555.10
ctc	L	0	447.20
cta	L	1228	413.80
ctg	L	1312	590.10
---	--	------	------
mPD		0.66	1.1
		nPD:	0.59
		N. weight:	1.1
		Sc. PD:	0.38
		Sc. rank:	1351.5

PB1			
Pos.	123	obs:	exp:
act	T	943	732.40
acc	T	1849	629.60
aca	T	2	1169.00
acg	T	0	262.90
gct	A	0	0.50
gcc	A	2	0.39
gca	A	0	0.89
gcg	A	0	0.22
---	--	------	------
mPD		0.45	0.70
		nPD:	0.64
		N. weight:	1.2
		Sc. PD:	0.46
		Sc. rank:	1523.9

PB1			
Pos.	124	obs:	exp:
caa	Q	2775	1395.00
cag	Q	19	1399.00
aaa	K	2	1.14
aag	K	0	0.86
---	--	------	------
mPD		0.015	0.50
		nPD:	0.03
		N. weight:	0.96
		Sc. PD:	-0.19
		Sc. rank:	-1105.6

PB1			
Pos.	125	obs:	exp:
tct	S	0	0.17
tcc	S	0	0.14
tca	S	0	0.23
tcg	S	0	0.06
agt	S	1	0.22
agc	S	0	0.18
ggt	G	2659	404.20
ggc	G	133	367.10
gga	G	3	1225.00
ggg	G	0	799.00
---	--	------	------
mPD		0.093	0.69
		nPD:	0.14
		N. weight:	2.5
		Sc. PD:	-0.25
		Sc. rank:	-83.4

PB1			
Pos.	126	obs:	exp:
cct	P	0	0.27
ccc	P	1	0.18
cca	P	0	0.41
ccg	P	0	0.14
cgt	R	361	104.60
cgc	R	2415	149.60
cga	R	19	281.70
cgg	R	0	228.20
aga	R	0	1305.00
agg	R	0	726.10
---	--	------	------
mPD		0.24	0.96
		nPD:	0.25
		N. weight:	3.7
		Sc. PD:	0.038
		Sc. rank:	1810.3

PB1			
Pos.	127	obs:	exp:
caa	Q	18	1395.00
cag	Q	2777	1400.00
cgt	R	0	0.04
cgc	R	0	0.05
cga	R	1	0.10
cgg	R	0	0.08
aga	R	0	0.47
agg	R	0	0.26
---	--	------	------
mPD		0.014	0.50
		nPD:	0.03
		N. weight:	0.95
		Sc. PD:	-0.19
		Sc. rank:	-1137.1

PB1			
Pos.	128	obs:	exp:
att	I	1	0.37
atc	I	0	0.27
ata	I	0	0.37
act	T	1756	732.70
acc	T	675	629.80
aca	T	364	1170.00
acg	T	0	263.00
---	--	------	------
mPD		0.53	0.70
		nPD:	0.76
		N. weight:	0.61
		Sc. PD:	0.31
		Sc. rank:	939.2

PB1			
Pos.	129	obs:	exp:
ttt	F	1	0.42
ttc	F	0	0.58
tat	Y	2767	1339.00
tac	Y	25	1453.00
aat	N	3	1.64
aac	N	0	1.36
---	--	------	------
mPD		0.021	0.50
		nPD:	0.04
		N. weight:	1.
		Sc. PD:	-0.19
		Sc. rank:	-944.0

PB1			
Pos.	130	obs:	exp:
gat	D	154	1510.00
gac	D	2642	1286.00
---	--	------	------
mPD		0.10	0.50
		nPD:	0.21
		N. weight:	0.81
		Sc. PD:	-0.022
		Sc. rank:	255.8

PB1			
Pos.	131	obs:	exp:
tgg	W	2796	2796.00
---	--	------	------
mPD		0	0
		nPD:	1.
		N. weight:	0.
		Sc. PD:	0
		Sc. rank:	0

PB1			
Pos.	132	obs:	exp:
act	T	10	733.00
acc	T	0	630.00
aca	T	2676	1170.00
acg	T	110	263.10
---	--	------	------
mPD		0.082	0.70
		nPD:	0.12
		N. weight:	1.1
		Sc. PD:	-0.13
		Sc. rank:	-209.8

PB1			
Pos.	133	obs:	exp:
tta	L	117	258.40
ttg	L	1609	531.00
ctt	L	0	554.90
ctc	L	0	447.10
cta	L	12	413.60
ctg	L	1057	589.90
gtt	V	0	0.22
gtc	V	0	0.21
gta	V	1	0.21
gtg	V	0	0.36
---	--	------	------
mPD		0.56	1.1
		nPD:	0.5
		N. weight:	1.2
		Sc. PD:	0.3
		Sc. rank:	1292.4

PB1			
Pos.	134	obs:	exp:
tct	S	0	0.17
tcc	S	0	0.14
tca	S	0	0.23
tcg	S	0	0.06
aat	N	2548	1524.00
aac	N	247	1271.00
agt	S	1	0.22
agc	S	0	0.18
---	--	------	------
mPD		0.16	0.50
		nPD:	0.33
		N. weight:	0.47
		Sc. PD:	0.04
		Sc. rank:	358.2

PB1			
Pos.	135	obs:	exp:
cgt	R	0	104.40
cgc	R	0	149.30
cga	R	4	281.10
cgg	R	1	227.70
aaa	K	7	3.99
aag	K	0	3.01
aga	R	2662	1302.00
agg	R	122	724.60
---	--	------	------
mPD		0.093	0.96
		nPD:	0.1
		N. weight:	0.87
		Sc. PD:	-0.12
		Sc. rank:	-300.6

PB1			
Pos.	136	obs:	exp:
aat	N	196	1525.00
aac	N	2600	1271.00
---	--	------	------
mPD		0.13	0.50
		nPD:	0.26
		N. weight:	0.76
		Sc. PD:	0.019
		Sc. rank:	414.1

PB1			
Pos.	137	obs:	exp:
caa	Q	2635	1396.00
cag	Q	161	1400.00
---	--	------	------
mPD		0.11	0.50
		nPD:	0.22
		N. weight:	0.69
		Sc. PD:	-0.014
		Sc. rank:	224.4

PB1			
Pos.	138	obs:	exp:
cct	P	7	763.80
ccc	P	5	504.80
cca	P	983	1134.00
ccg	P	1800	391.90
cgt	R	0	0.04
cgc	R	0	0.05
cga	R	0	0.10
cgg	R	1	0.08
aga	R	0	0.47
agg	R	0	0.26
---	--	------	------
mPD		0.46	0.71
		nPD:	0.65
		N. weight:	1.3
		Sc. PD:	0.54
		Sc. rank:	1774.0

PB1			
Pos.	139	obs:	exp:
gct	A	2735	704.40
gcc	A	20	541.10
gca	A	7	1245.00
gcg	A	34	306.00
---	--	------	------
mPD		0.043	0.69
		nPD:	0.06
		N. weight:	1.8
		Sc. PD:	-0.32
		Sc. rank:	-1231.9

PB1			
Pos.	140	obs:	exp:
act	T	0	0.26
acc	T	0	0.23
aca	T	1	0.42
acg	T	0	0.09
gct	A	46	704.10
gcc	A	1	540.90
gca	A	2649	1244.00
gcg	A	99	305.90
---	--	------	------
mPD		0.10	0.69
		nPD:	0.15
		N. weight:	0.92
		Sc. PD:	-0.082
		Sc. rank:	37.9

PB1			
Pos.	141	obs:	exp:
tct	S	1	0.17
tcc	S	0	0.14
tca	S	0	0.23
tcg	S	0	0.06
act	T	2503	732.40
acc	T	285	629.60
aca	T	6	1169.00
acg	T	0	262.90
aat	N	1	0.55
aac	N	0	0.45
agt	S	0	0.22
agc	S	0	0.18
---	--	------	------
mPD		0.19	0.70
		nPD:	0.27
		N. weight:	1.5
		Sc. PD:	0.046
		Sc. rank:	840.9

PB1			
Pos.	142	obs:	exp:
gct	A	1882	704.40
gcc	A	820	541.10
gca	A	92	1245.00
gcg	A	2	306.00
---	--	------	------
mPD		0.46	0.69
		nPD:	0.67
		N. weight:	1.
		Sc. PD:	0.43
		Sc. rank:	1399.8

PB1			
Pos.	143	obs:	exp:
tta	L	105	258.40
ttg	L	1948	531.00
tgg	W	1	1.00
ctt	L	0	554.90
ctc	L	0	447.10
cta	L	0	413.60
ctg	L	742	589.90
---	--	------	------
mPD		0.46	1.1
		nPD:	0.41
		N. weight:	1.4
		Sc. PD:	0.23
		Sc. rank:	1263.2

PB1			
Pos.	144	obs:	exp:
tct	S	0	0.83
tcc	S	5	0.72
tca	S	0	1.13
tcg	S	0	0.30
cct	P	0	0.27
ccc	P	1	0.18
cca	P	0	0.41
ccg	P	0	0.14
act	T	1	0.26
acc	T	0	0.23
aca	T	0	0.42
acg	T	0	0.09
agt	S	0	1.08
agc	S	0	0.92
gct	A	69	702.30
gcc	A	2565	539.50
gca	A	154	1241.00
gcg	A	0	305.20
ggt	G	0	0.14
ggc	G	1	0.13
gga	G	0	0.44
ggg	G	0	0.29
---	--	------	------
mPD		0.16	0.70
		nPD:	0.22
		N. weight:	1.8
		Sc. PD:	-0.025
		Sc. rank:	678.1

PB1			
Pos.	145	obs:	exp:
tct	S	0	0.67
tcc	S	0	0.58
tca	S	0	0.91
tcg	S	0	0.24
aat	N	86	1523.00
aac	N	2706	1269.00
agt	S	0	0.87
agc	S	4	0.74
---	--	------	------
mPD		0.063	0.50
		nPD:	0.12
		N. weight:	0.94
		Sc. PD:	-0.1
		Sc. rank:	-121.0

PB1			
Pos.	146	obs:	exp:
tct	S	0	0.17
tcc	S	0	0.14
tca	S	0	0.23
tcg	S	0	0.06
act	T	2643	732.70
acc	T	151	629.80
aca	T	1	1170.00
acg	T	0	263.00
agt	S	1	0.22
agc	S	0	0.18
---	--	------	------
mPD		0.10	0.70
		nPD:	0.15
		N. weight:	1.7
		Sc. PD:	-0.14
		Sc. rank:	94.4

PB1			
Pos.	147	obs:	exp:
att	I	11	1025.00
atc	I	1	747.40
ata	I	2781	1021.00
atg	M	3	3.00
---	--	------	------
mPD		0.011	0.66
		nPD:	0.02
		N. weight:	1.4
		Sc. PD:	-0.31
		Sc. rank:	-1983.9

PB1			
Pos.	148	obs:	exp:
gaa	E	1536	1567.00
gag	E	1259	1228.00
ggt	G	0	0.14
ggc	G	0	0.13
gga	G	0	0.44
ggg	G	1	0.29
---	--	------	------
mPD		0.50	0.49
		nPD:	1.01
		N. weight:	0.0032
		Sc. PD:	0.0024
		Sc. rank:	5.3

PB1			
Pos.	149	obs:	exp:
att	I	759	579.40
atc	I	815	422.50
ata	I	5	577.00
gtt	V	94	261.80
gtc	V	938	256.00
gta	V	28	259.50
gtg	V	157	439.70
---	--	------	------
mPD		1.0	1.2
		nPD:	0.82
		N. weight:	0.85
		Sc. PD:	0.48
		Sc. rank:	1351.0

PB1			
Pos.	150	obs:	exp:
ttt	F	74	1160.00
ttc	F	2706	1620.00
tta	L	13	1.39
ttg	L	0	2.85
tat	Y	0	0.48
tac	Y	1	0.52
ctt	L	0	2.98
ctc	L	2	2.40
cta	L	0	2.22
ctg	L	0	3.17
---	--	------	------
mPD		0.063	0.50
		nPD:	0.13
		N. weight:	0.64
		Sc. PD:	-0.069
		Sc. rank:	-73.8

PB1

Pos.	151	obs:	exp:
cgt	R	0	104.60
cgc	R	0	149.60
cga	R	0	281.80
cgg	R	0	228.30
aga	R	2777	1305.00
agg	R	19	726.40
---	--	------	------
mPD		0.014	0.95
		nPD:	0.01
		N. weight:	1.1
		Sc. PD:	-0.23
		Sc. rank:	-1557.5

,

PB1

Pos.	152	obs:	exp:
tta	L	5	6.66
ttg	L	66	13.68
tct	S	9	453.00
tcc	S	7	393.10
tca	S	1626	615.30
tcg	S	1072	163.70
ctt	L	0	14.30
ctc	L	0	11.52
cta	L	0	10.66
ctg	L	1	15.20
atg	M	1	1.00
act	T	0	1.57
acc	T	0	1.35
aca	T	0	2.51
acg	T	6	0.56
agt	S	0	588.70
agc	S	0	500.20
gct	A	0	0.76
gcc	A	0	0.58
gca	A	3	1.34
gcg	A	0	0.33
---	--	------	------
mPD		0.55	1.7
		nPD:	0.32
		N. weight:	1.9
		Sc. PD:	0.16
		Sc. rank:	1391.6

,

PB1

Pos.	153	obs:	exp:
aat	N	107	1525.00
aac	N	2689	1271.00
---	--	------	------
mPD		0.074	0.50
		nPD:	0.15
		N. weight:	0.9
		Sc. PD:	-0.079
		Sc. rank:	45.0

,

PB1

Pos.	154	obs:	exp:
tct	S	0	3.51
tcc	S	0	3.04
tca	S	0	4.76
tcg	S	0	1.27
tgt	C	3	1.16
tgc	C	0	1.84
aat	N	1	0.55
aac	N	0	0.45
agt	S	21	4.55
agc	S	0	3.87
gat	D	8	4.32
gac	D	0	3.68
ggt	G	2708	399.60
ggc	G	51	362.90
gga	G	0	1211.00
ggg	G	4	789.80
---	--	------	------
mPD		0.063	0.72
		nPD:	0.09
		N. weight:	2.7
		Sc. PD:	-0.39
		Sc. rank:	-1143.4

,

PB1

Pos.	155	obs:	exp:
tta	L	14	258.20
ttg	L	13	530.60
ctt	L	33	554.50
ctc	L	9	446.80
cta	L	1370	413.30
ctg	L	1354	589.50
att	I	0	1.10
atc	I	0	0.80
ata	I	3	1.10
---	--	------	------
mPD		0.54	1.1
		nPD:	0.48
		N. weight:	1.3
		Sc. PD:	0.31
		Sc. rank:	1411.0

,

PB1

Pos.	156	obs:	exp:
tct	S	0	0.17
tcc	S	0	0.14
tca	S	1	0.23
tcg	S	0	0.06
att	I	0	3.67
atc	I	0	2.68
ata	I	10	3.65
act	T	2	727.50
acc	T	0	625.30
aca	T	2760	1161.00
acg	T	13	261.10
aaa	K	2	1.14
aag	K	0	0.86
agt	S	0	0.22
agc	S	0	0.18
gtt	V	0	0.43
gtc	V	0	0.42
gta	V	2	0.43
gtg	V	0	0.72
gct	A	0	1.51
gcc	A	0	1.16
gca	A	6	2.67
gcg	A	0	0.66
---	--	------	------
mPD		0.027	0.71
		nPD:	0.04
		N. weight:	1.2
		Sc. PD:	-0.24
		Sc. rank:	-1251.4

,

PB1

Pos.	157	obs:	exp:
tct	S	2	1.50
tcc	S	6	1.30
tca	S	1	2.04
tcg	S	0	0.54
act	T	0	20.45
acc	T	78	17.58
aca	T	0	32.64
acg	T	0	7.34
agt	S	0	1.95
agc	S	0	1.66
gtt	V	0	1.08
gtc	V	4	1.05
gta	V	1	1.07
gtg	V	0	1.81
gct	A	75	681.20
gcc	A	1528	523.20
gca	A	1100	1204.00
gcg	A	1	296.00
---	--	------	------
mPD		0.57	0.76
		nPD:	0.76
		N. weight:	0.79
		Sc. PD:	0.4
		Sc. rank:	1213.9

,

PB1

Pos.	158	obs:	exp:
tct	S	0	59.09
tcc	S	0	51.28
tca	S	0	80.25
tcg	S	0	21.36
act	T	2	0.52
acc	T	0	0.45
aca	T	0	0.84
acg	T	0	0.19
aat	N	2393	1331.00
aac	N	47	1109.00
agt	S	354	76.78
agc	S	0	65.24
---	--	------	------
mPD		0.26	0.90
		nPD:	0.28
		N. weight:	0.94
		Sc. PD:	0.042
		Sc. rank:	578.1

,

PB1

Pos.	159	obs:	exp:
gat	D	1	2.70
gac	D	4	2.30
gaa	E	2489	1565.00
gag	E	302	1226.00
---	--	------	------
mPD		0.20	0.49
		nPD:	0.4
		N. weight:	0.39
		Sc. PD:	0.059
		Sc. rank:	346.1

,

PB1

Pos.	160	obs:	exp:
tct	S	105	466.70
tcc	S	1	405.00
tca	S	258	633.90
tcg	S	2432	168.70
agt	S	0	606.50
agc	S	0	515.30
---	--	------	------
mPD		0.23	1.7
		nPD:	0.14
		N. weight:	3.2
		Sc. PD:	-0.3
		Sc. rank:	56.8

,

PB1

Pos.	161	obs:	exp:
ggt	G	4	404.30
ggc	G	0	367.30
gga	G	1667	1225.00
ggg	G	1125	799.20
---	--	------	------
mPD		0.48	0.69
		nPD:	0.7
		N. weight:	0.46
		Sc. PD:	0.21
		Sc. rank:	669.2

,

PB1

Pos.	162	obs:	exp:
cct	P	0	0.27
ccc	P	0	0.18
cca	P	0	0.41
ccg	P	1	0.14
cgt	R	0	104.50
cgc	R	17	149.50
cga	R	19	281.50
cgg	R	1401	228.10
act	T	0	0.52
acc	T	0	0.45
aca	T	0	0.84
acg	T	2	0.19
aga	R	1042	1304.00
agg	R	314	725.60
---	--	------	------
mPD		0.98	0.96
		nPD:	1.02
		N. weight:	1.
		Sc. PD:	0.79
		Sc. rank:	1708.7

,

PB1

Pos.	163	obs:	exp:
tta	L	27	258.30
ttg	L	42	530.80
ctt	L	0	554.70
ctc	L	0	446.90
cta	L	1858	413.50
ctg	L	867	589.70
atg	M	2	2.00
---	--	------	------
mPD		0.49	1.1
		nPD:	0.44
		N. weight:	1.5
		Sc. PD:	0.3
		Sc. rank:	1502.3

,

PB1

Pos.	164	obs:	exp:
att	I	20	1025.00
atc	I	10	747.40
ata	I	2763	1021.00
atg	M	2	2.00
gtt	V	0	0.22
gtc	V	0	0.21
gta	V	1	0.21
gtg	V	0	0.36
---	--	------	------
mPD		0.023	0.66
		nPD:	0.04
		N. weight:	1.4
		Sc. PD:	-0.27
		Sc. rank:	-1478.5

,

PB1

Pos.	165	obs:	exp:
aat	N	1	0.55
aac	N	0	0.45
gat	D	2760	1509.00
gac	D	35	1286.00
---	--	------	------
mPD		0.025	0.50
		nPD:	0.05
		N. weight:	0.81
		Sc. PD:	-0.15
		Sc. rank:	-641.9

,

PB1			
Pos.	166	obs:	exp:
ttt	F	91	1164.00
ttc	F	2699	1626.00
tta	L	0	0.46
ttg	L	0	0.95
ctt	L	0	0.99
ctc	L	5	0.80
cta	L	0	0.74
ctg	L	0	1.06
att	I	0	0.37
atc	I	1	0.27
ata	I	0	0.37
---	--	------	------
mPD		0.067	0.49
		nPD:	0.14
		N. weight:	0.6
		Sc. PD:	-0.059
		Sc. rank:	-13.4

,

PB1			
Pos.	167	obs:	exp:
tta	L	0	258.40
ttg	L	0	531.00
ctt	L	22	554.90
ctc	L	2772	447.10
cta	L	1	413.60
ctg	L	0	589.90
att	I	0	0.37
atc	I	1	0.27
ata	I	0	0.37
---	--	------	------
mPD		0.017	1.1
		nPD:	0.02
		N. weight:	2.6
		Sc. PD:	-0.56
		Sc. rank:	-3699.6

,

PB1			
Pos.	168	obs:	exp:
cgt	R	0	1.16
cgc	R	0	1.66
cga	R	0	3.12
cgg	R	0	2.53
atg	M	1	1.00
aaa	K	205	1574.00
aag	K	2559	1190.00
aga	R	0	14.47
agg	R	31	8.05
---	--	------	------
mPD		0.16	0.52
		nPD:	0.3
		N. weight:	0.83
		Sc. PD:	0.054
		Sc. rank:	563.9

,

PB1			
Pos.	169	obs:	exp:
gat	D	2262	1510.00
gac	D	534	1286.00
---	--	------	------
mPD		0.31	0.50
		nPD:	0.62
		N. weight:	0.23
		Sc. PD:	0.088
		Sc. rank:	300.3

,

PB1			
Pos.	170	obs:	exp:
gtt	V	8	601.20
gtc	V	0	588.00
gta	V	308	596.00
gtg	V	2479	1010.00
ggt	G	0	0.14
ggc	G	0	0.13
gga	G	0	0.44
ggg	G	1	0.29
---	--	------	------
mPD		0.20	0.73
		nPD:	0.28
		N. weight:	1.
		Sc. PD:	0.037
		Sc. rank:	601.1

,

PB1			
Pos.	171	obs:	exp:
tta	L	0	0.18
ttg	L	1	0.38
ctt	L	0	0.40
ctc	L	0	0.32
cta	L	0	0.30
ctg	L	1	0.42
att	I	0	8.81
atc	I	0	6.42
ata	I	24	8.77
atg	M	2749	2749.00
act	T	0	0.79
acc	T	0	0.68
aca	T	0	1.26
acg	T	3	0.28
gtt	V	0	3.87
gtc	V	0	3.79
gta	V	0	3.84
gtg	V	18	6.50
---	--	------	------
mPD		0.033	0.044
		nPD:	0.75
		N. weight:	0.027
		Sc. PD:	0.014
		Sc. rank:	41.6

,

PB1			
Pos.	172	obs:	exp:
aaa	K	8	4.56
aag	K	0	3.44
gct	A	0	0.76
gcc	A	0	0.58
gca	A	3	1.34
gcg	A	0	0.33
gat	D	24	409.80
gac	D	735	349.20
gaa	E	1931	1116.00
gag	E	60	874.90
ggt	G	0	5.06
ggc	G	3	4.60
gga	G	6	15.34
ggg	G	26	10.00
---	--	------	------
mPD		0.48	0.73
		nPD:	0.65
		N. weight:	0.74
		Sc. PD:	0.3
		Sc. rank:	980.4

,

PB1			
Pos.	173	obs:	exp:
tct	S	2	466.70
tcc	S	1	405.00
tca	S	2773	633.90
tcg	S	20	168.70
agt	S	0	606.50
agc	S	0	515.30
---	--	------	------
mPD		0.016	1.7
		nPD:	0.01
		N. weight:	2.1
		Sc. PD:	-0.47
		Sc. rank:	-3242.5

,

PB1			
Pos.	174	obs:	exp:
tta	L	0	0.09
ttg	L	1	0.19
ctt	L	0	0.20
ctc	L	0	0.16
cta	L	0	0.15
ctg	L	0	0.21
att	I	0	0.37
atc	I	0	0.27
ata	I	1	0.37
atg	M	2780	2780.00
act	T	0	1.57
acc	T	0	1.35
aca	T	0	2.51
acg	T	6	0.56
gtt	V	0	1.72
gtc	V	0	1.68
gta	V	0	1.71
gtg	V	8	2.89
---	--	------	------
mPD		0.011	0.019
		nPD:	0.59
		N. weight:	0.013
		Sc. PD:	0.0045
		Sc. rank:	15.9

,

PB1			
Pos.	175	obs:	exp:
aat	N	23	13.09
aac	N	1	10.91
gtt	V	2	0.43
gtc	V	0	0.42
gta	V	0	0.43
gtg	V	0	0.72
gat	D	2481	1492.00
gac	D	283	1272.00
gaa	E	6	3.36
gag	E	0	2.64
---	--	------	------
mPD		0.20	0.52
		nPD:	0.4
		N. weight:	0.45
		Sc. PD:	0.069
		Sc. rank:	401.3

,

PB1			
Pos.	176	obs:	exp:
caa	Q	0	0.50
cag	Q	1	0.50
cgt	R	0	1.35
cgc	R	0	1.93
cga	R	0	3.63
cgg	R	1	2.94
act	T	0	74.45
acc	T	0	63.99
aca	T	284	118.80
acg	T	0	26.72
aat	N	1	0.55
aac	N	0	0.45
aaa	K	774	1409.00
aag	K	1700	1065.00
aga	R	7	16.81
agg	R	28	9.35
---	--	------	------
mPD		0.68	0.75
		nPD:	0.91
		N. weight:	0.32
		Sc. PD:	0.21
		Sc. rank:	517.8

,

PB1			
Pos.	177	obs:	exp:
aaa	K	30	17.08
aag	K	0	12.92
gat	D	6	3.24
gac	D	0	2.76
gaa	E	2527	1539.00
gag	E	219	1207.00
ggt	G	0	2.02
ggc	G	0	1.84
gga	G	11	6.13
ggg	G	3	4.00
---	--	------	------
mPD		0.18	0.53
		nPD:	0.34
		N. weight:	0.47
		Sc. PD:	0.049
		Sc. rank:	378.7

,

PB1			
Pos.	178	obs:	exp:
aaa	K	1	0.57
aag	K	0	0.43
gtt	V	0	0.22
gtc	V	0	0.21
gta	V	1	0.21
gtg	V	0	0.36
gat	D	0	1.62
gac	D	3	1.38
gaa	E	2627	1565.00
gag	E	164	1226.00
---	--	------	------
mPD		0.11	0.50
		nPD:	0.23
		N. weight:	0.54
		Sc. PD:	-0.0043
		Sc. rank:	204.0

,

PB1			
Pos.	179	obs:	exp:
tta	L	0	0.18
ttg	L	2	0.38
ctt	L	0	0.40
ctc	L	0	0.32
cta	L	0	0.30
ctg	L	0	0.42
att	I	2	68.99
atc	I	0	50.31
ata	I	186	68.70
atg	M	2594	2594.00
act	T	0	0.79
acc	T	0	0.68
aca	T	0	1.26
acg	T	3	0.28
gtt	V	0	1.94
gtc	V	0	1.89
gta	V	1	1.92
gtg	V	8	3.25
---	--	------	------
mPD		0.14	0.14
		nPD:	0.94
		N. weight:	0.1
		Sc. PD:	0.07
		Sc. rank:	168.4

,

PB1			
Pos.	180	obs:	exp:
gtt	V	0	0.22
gtc	V	0	0.21
gta	V	1	0.21
gtg	V	0	0.36
gct	A	0	0.25
gcc	A	0	0.19
gca	A	0	0.45
gcg	A	1	0.11
gat	D	5	5.94
gac	D	6	5.06
gaa	E	1182	1558.00
gag	E	1598	1222.00
ggt	G	0	0.43
ggc	G	0	0.39
gga	G	1	1.31
ggg	G	2	0.86
---	--	------	------
mPD		0.50	0.50
		nPD:	0.99
		N. weight:	0.06
		Sc. PD:	0.044
		Sc. rank:	98.1

,

PB1			
Pos.	181	obs:	exp:
att	I	19	1019.00
atc	I	2	743.10
ata	I	2756	1015.00
atg	M	5	5.00
gtt	V	0	3.01
gtc	V	0	2.95
gta	V	14	2.99
gtg	V	0	5.06
---	--	------	------
mPD		0.028	0.67
		nPD:	0.04
		N. weight:	1.4
		Sc. PD:	-0.27
		Sc. rank:	-1277.0

,

PB1			
Pos.	182	obs:	exp:
att	I	2	55.78
atc	I	0	40.67
ata	I	150	55.55
act	T	3	693.10
acc	T	0	595.80
aca	T	2609	1106.00
acg	T	32	248.80
---	--	------	------
mPD		0.13	0.80
		nPD:	0.16
		N. weight:	1.2
		Sc. PD:	-0.089
		Sc. rank:	176.6

,

PB1			
Pos.	183	obs:	exp:
att	I	0	1.10
atc	I	0	0.80
ata	I	3	1.10
act	T	1	729.80
acc	T	39	627.30
aca	T	2684	1165.00
acg	T	60	261.90
gct	A	0	2.27
gcc	A	0	1.74
gca	A	9	4.01
gcg	A	0	0.99
---	--	------	------
mPD		0.078	0.71
		nPD:	0.11
		N. weight:	1.1
		Sc. PD:	-0.13
		Sc. rank:	-273.5

,

PB1			
Pos.	184	obs:	exp:
tat	Y	2	0.96
tac	Y	0	1.04
cat	H	2708	1617.00
cac	H	86	1177.00
---	--	------	------
mPD		0.061	0.49
		nPD:	0.12
		N. weight:	0.61
		Sc. PD:	-0.068
		Sc. rank:	-76.9

,

PB1			
Pos.	185	obs:	exp:
ttt	F	25	1166.00
ttc	F	2771	1630.00
---	--	------	------
mPD		0.018	0.49
		nPD:	0.04
		N. weight:	0.72
		Sc. PD:	-0.14
		Sc. rank:	-759.2

,

PB1

Pos.	186	obs:	exp:
cat	H	3	1.74
cac	H	0	1.26
caa	Q	185	1394.00
cag	Q	2608	1399.00
---	--	------	------
mPD		0.13	0.50
		nPD:	0.25
		N. weight:	0.66
		Sc. PD:	0.0081
		Sc. rank:	329.2

PB1

Pos.	187	obs:	exp:
cgt	R	0	104.60
cgc	R	0	149.60
cga	R	3	281.80
cgg	R	1	228.30
aga	R	2621	1305.00
agg	R	171	726.40
---	--	------	------
mPD		0.12	0.95
		nPD:	0.12
		N. weight:	0.82
		Sc. PD:	-0.091
		Sc. rank:	-110.3

PB1

Pos.	188	obs:	exp:
cgt	R	0	0.19
cgc	R	0	0.27
cga	R	0	0.50
cgg	R	0	0.41
aaa	K	140	1589.00
aag	K	2651	1202.00
aga	R	0	2.33
agg	R	5	1.30
---	--	------	------
mPD		0.099	0.50
		nPD:	0.2
		N. weight:	0.92
		Sc. PD:	-0.035
		Sc. rank:	229.1

PB1

Pos.	189	obs:	exp:
cgt	R	0	104.60
cgc	R	0	149.60
cga	R	1	281.70
cgg	R	0	228.20
aaa	K	1	0.57
aag	K	0	0.43
aga	R	2731	1305.00
agg	R	63	726.10
---	--	------	------
mPD		0.045	0.95
		nPD:	0.05
		N. weight:	0.97
		Sc. PD:	-0.18
		Sc. rank:	-810.7

PB1

Pos.	190	obs:	exp:
cgt	R	0	104.50
cgc	R	0	149.40
cga	R	18	281.40
cgg	R	0	228.00
aaa	K	4	2.28
aag	K	0	1.72
aga	R	2593	1303.00
agg	R	181	725.30
---	--	------	------
mPD		0.14	0.96
		nPD:	0.14
		N. weight:	0.78
		Sc. PD:	-0.072
		Sc. rank:	16.5

PB1

Pos.	191	obs:	exp:
tta	L	0	2.13
ttg	L	0	4.37
ctt	L	0	4.57
ctc	L	0	3.68
cta	L	23	3.40
ctg	L	0	4.85
att	I	0	36.33
atc	I	0	26.49
ata	I	99	36.18
atg	M	2	2.00
gtt	V	1	574.70
gtc	V	0	562.20
gta	V	1907	569.70
gtg	V	764	965.40
---	--	------	------
mPD		0.48	0.82
		nPD:	0.59
		N. weight:	1.2
		Sc. PD:	0.4
		Sc. rank:	1440.9

PB1

Pos.	192	obs:	exp:
cgt	R	0	101.80
cgc	R	0	145.60
cga	R	0	274.10
cgg	R	4	222.10
aaa	K	0	43.28
aag	K	76	32.72
aga	R	878	1270.00
agg	R	1838	706.60
---	--	------	------
mPD		0.49	1.0
		nPD:	0.49
		N. weight:	0.78
		Sc. PD:	0.19
		Sc. rank:	835.9

PB1

Pos.	193	obs:	exp:
gat	D	153	1510.00
gac	D	2643	1286.00
---	--	------	------
mPD		0.10	0.50
		nPD:	0.21
		N. weight:	0.81
		Sc. PD:	-0.023
		Sc. rank:	253.4

PB1

Pos.	194	obs:	exp:
tct	S	0	1.34
tcc	S	0	1.16
tca	S	0	1.81
tcg	S	0	0.48
tat	Y	0	0.48
tac	Y	1	0.52
aat	N	34	1519.00
aac	N	2752	1267.00
agt	S	0	1.74
agc	S	8	1.47
gat	D	0	0.54
gac	D	1	0.46
---	--	------	------
mPD		0.031	0.51
		nPD:	0.06
		N. weight:	1.1
		Sc. PD:	-0.18
		Sc. rank:	-732.0

PB1

Pos.	195	obs:	exp:
tta	L	0	0.18
ttg	L	0	0.38
ctt	L	0	0.40
ctc	L	0	0.32
cta	L	0	0.30
ctg	L	2	0.42
att	I	26	16.88
atc	I	0	12.31
ata	I	20	16.81
atg	M	2718	2718.00
act	T	0	5.77
acc	T	0	4.96
aca	T	0	9.21
acg	T	22	2.07
gtt	V	0	1.72
gtc	V	0	1.68
gta	V	0	1.71
gtg	V	8	2.89
---	--	------	------
mPD		0.055	0.074
		nPD:	0.75
		N. weight:	0.042
		Sc. PD:	0.021
		Sc. rank:	64.0

PB1

Pos.	196	obs:	exp:
tct	S	0	0.17
tcc	S	1	0.14
tca	S	0	0.23
tcg	S	0	0.06
act	T	43	732.40
acc	T	2639	629.60
aca	T	112	1169.00
acg	T	0	262.90
agt	S	0	0.22
agc	S	0	0.18
gct	A	0	0.25
gcc	A	1	0.19
gca	A	0	0.45
gcg	A	0	0.11
---	--	------	------
mPD		0.11	0.70
		nPD:	0.15
		N. weight:	1.8
		Sc. PD:	-0.15
		Sc. rank:	159.1

PB1

Pos.	197	obs:	exp:
cgt	R	0	0.41
cgc	R	0	0.59
cga	R	0	1.11
cgg	R	0	0.90
aaa	K	345	1586.00
aag	K	2440	1199.00
aga	R	0	5.14
agg	R	11	2.86
---	--	------	------
mPD		0.22	0.50
		nPD:	0.45
		N. weight:	0.64
		Sc. PD:	0.13
		Sc. rank:	636.7

PB1

Pos.	198	obs:	exp:
cgt	R	0	0.79
cgc	R	0	1.12
cga	R	0	2.12
cgg	R	0	1.71
aaa	K	2320	1580.00
aag	K	454	1194.00
aga	R	11	9.80
agg	R	10	5.46
gaa	E	1	0.56
gag	E	0	0.44
---	--	------	------
mPD		0.29	0.51
		nPD:	0.57
		N. weight:	0.24
		Sc. PD:	0.079
		Sc. rank:	290.0

PB1

Pos.	199	obs:	exp:
tta	L	0	0.18
ttg	L	1	0.38
ctt	L	0	0.40
ctc	L	0	0.32
cta	L	0	0.30
ctg	L	1	0.42
att	I	1	1.47
atc	I	0	1.07
ata	I	3	1.46
atg	M	2789	2789.00
gtt	V	0	0.22
gtc	V	0	0.21
gta	V	0	0.21
gtg	V	1	0.36
---	--	------	------
mPD		0.0050	0.0063
		nPD:	0.79
		N. weight:	0.0032
		Sc. PD:	0.0017
		Sc. rank:	5.1

PB1

Pos.	200	obs:	exp:
att	I	0	23.49
atc	I	36	17.13
ata	I	28	23.39
gtt	V	5	587.60
gtc	V	1560	574.80
gta	V	1138	582.50
gtg	V	29	987.10
---	--	------	------
mPD		0.55	0.78
		nPD:	0.7
		N. weight:	1.2
		Sc. PD:	0.52
		Sc. rank:	1683.4

PB1

Pos.	201	obs:	exp:
atg	M	11	11.00
act	T	12	730.10
acc	T	2	627.50
aca	T	2039	1165.00
acg	T	732	262.00
---	--	------	------
mPD		0.41	0.71
		nPD:	0.57
		N. weight:	0.96
		Sc. PD:	0.31
		Sc. rank:	1148.2

PB1

Pos.	202	obs:	exp:
tta	L	0	0.28
ttg	L	0	0.57
ctt	L	0	0.60
ctc	L	0	0.48
cta	L	3	0.44
ctg	L	0	0.63
cct	P	0	0.27
ccc	P	0	0.18
cca	P	1	0.41
ccg	P	0	0.14
cat	H	1	1.16
cac	H	1	0.84
caa	Q	2746	1390.00
cag	Q	39	1395.00
cgt	R	0	0.19
cgc	R	0	0.27
cga	R	5	0.50
cgg	R	0	0.41
aga	R	0	2.33
agg	R	0	1.30
---	--	------	------
mPD		0.035	0.51
		nPD:	0.07
		N. weight:	0.91
		Sc. PD:	-0.15
		Sc. rank:	-567.0

PB1

Pos.	203	obs:	exp:
cgt	R	0	104.60
cgc	R	0	149.60
cga	R	34	281.70
cgg	R	0	228.20
aaa	K	1	0.57
aag	K	0	0.43
aga	R	2687	1305.00
agg	R	74	726.10
---	--	------	------
mPD		0.076	0.95
		nPD:	0.08
		N. weight:	0.89
		Sc. PD:	-0.14
		Sc. rank:	-443.9

PB1

Pos.	204	obs:	exp:
att	I	0	1.10
atc	I	0	0.80
ata	I	3	1.10
act	T	9	732.20
acc	T	0	629.30
aca	T	2757	1169.00
acg	T	27	262.80
---	--	------	------
mPD		0.028	0.70
		nPD:	0.04
		N. weight:	1.2
		Sc. PD:	-0.23
		Sc. rank:	-1181.1

PB1

Pos.	205	obs:	exp:
att	I	0	1023.00
atc	I	773	746.30
ata	I	2016	1019.00
atg	M	2	2.00
gtt	V	0	1.08
gtc	V	3	1.05
gta	V	2	1.07
gtg	V	0	1.81
---	--	------	------
mPD		0.41	0.66
		nPD:	0.61
		N. weight:	0.74
		Sc. PD:	0.27
		Sc. rank:	930.7

PB1			
Pos.	206	obs:	exp:
cgt	R	0	0.07
cgc	R	0	0.11
cga	R	0	0.20
cgg	R	0	0.16
aga	R	0	0.93
agg	R	2	0.52
ggt	G	0	404.10
ggc	G	0	367.00
gga	G	1064	1224.00
ggg	G	1730	798.70
---	--	------	------
mPD		0.47	0.69
		nPD:	0.69
		N. weight:	0.62
		Sc. PD:	0.27
		Sc. rank:	882.7

,

PB1			
Pos.	207	obs:	exp:
cgt	R	0	0.52
cgc	R	0	0.75
cga	R	0	1.41
cgg	R	0	1.14
aaa	K	6	1584.00
aag	K	2776	1198.00
aga	R	0	6.54
agg	R	14	3.64
---	--	------	------
mPD		0.014	0.50
		nPD:	0.03
		N. weight:	1.2
		Sc. PD:	-0.25
		Sc. rank:	-1447.4

,

PB1			
Pos.	208	obs:	exp:
cgt	R	0	0.60
cgc	R	0	0.86
cga	R	0	1.61
cgg	R	0	1.31
aaa	K	1642	1583.00
aag	K	1138	1197.00
aga	R	16	7.47
agg	R	0	4.16
---	--	------	------
mPD		0.49	0.51
		nPD:	0.98
		N. weight:	0.0099
		Sc. PD:	0.0071
		Sc. rank:	16.2

,

PB1			
Pos.	209	obs:	exp:
cgt	R	0	0.86
cgc	R	0	1.23
cga	R	0	2.32
cgg	R	0	1.88
aat	N	0	1.64
aac	N	3	1.36
aaa	K	2520	1577.00
aag	K	249	1192.00
aga	R	17	10.74
agg	R	6	5.98
gaa	E	1	0.56
gag	E	0	0.44
---	--	------	------
mPD		0.18	0.51
		nPD:	0.36
		N. weight:	0.42
		Sc. PD:	0.05
		Sc. rank:	346.4

,

PB1			
Pos.	210	obs:	exp:
tta	L	0	0.18
ttg	L	0	0.38
ctt	L	0	0.40
ctc	L	0	0.32
cta	L	1	0.30
ctg	L	1	0.42
cat	H	0	1.16
cac	H	2	0.84
caa	Q	2500	1394.00
cag	Q	292	1398.00
---	--	------	------
mPD		0.19	0.50
		nPD:	0.38
		N. weight:	0.53
		Sc. PD:	0.072
		Sc. rank:	464.7

,

PB1			
Pos.	211	obs:	exp:
cgt	R	0	104.30
cgc	R	0	149.20
cga	R	0	281.00
cgg	R	4	227.70
atg	M	1	1.00
aaa	K	1	3.99
aag	K	6	3.01
aga	R	57	1302.00
agg	R	2727	724.30
---	--	------	------
mPD		0.049	0.96
		nPD:	0.05
		N. weight:	1.8
		Sc. PD:	-0.33
		Sc. rank:	-1388.3

,

PB1			
Pos.	212	obs:	exp:
tta	L	50	258.40
ttg	L	93	531.00
ctt	L	2	554.90
ctc	L	0	447.10
cta	L	817	413.60
ctg	L	1833	589.90
atg	M	1	1.00
---	--	------	------
mPD		0.53	1.1
		nPD:	0.47
		N. weight:	1.2
		Sc. PD:	0.28
		Sc. rank:	1304.2

,

PB1			
Pos.	213	obs:	exp:
tct	S	0	2.50
tcc	S	0	2.17
tca	S	0	3.40
tcg	S	0	0.90
tat	Y	0	0.48
tac	Y	1	0.52
att	I	1	0.37
atc	I	0	0.27
ata	I	0	0.37
act	T	0	13.11
acc	T	49	11.27
aca	T	1	20.92
acg	T	0	4.70
aat	N	213	1473.00
aac	N	2488	1228.00
aaa	K	10	7.40
aag	K	3	5.60
agt	S	9	3.25
agc	S	6	2.76
gat	D	1	8.10
gac	D	14	6.90
---	--	------	------
mPD		0.21	0.58
		nPD:	0.37
		N. weight:	0.75
		Sc. PD:	0.099
		Sc. rank:	655.2

,

PB1			
Pos.	214	obs:	exp:
cgt	R	0	3.29
cgc	R	0	4.71
cga	R	0	8.87
cgg	R	0	7.19
aat	N	1	0.55
aac	N	0	0.45
aaa	K	1856	1541.00
aag	K	851	1166.00
aga	R	54	41.08
agg	R	34	22.86
---	--	------	------
mPD		0.49	0.57
		nPD:	0.87
		N. weight:	0.059
		Sc. PD:	0.036
		Sc. rank:	94.8

,

PB1			
Pos.	215	obs:	exp:
caa	Q	0	0.50
cag	Q	1	0.50
cgt	R	0	82.14
cgc	R	0	117.50
cga	R	0	221.20
cgg	R	0	179.20
aaa	K	17	341.10
aag	K	582	257.90
aga	R	64	1025.00
agg	R	2131	570.20
ggt	G	0	0.14
ggc	G	0	0.13
gga	G	0	0.44
ggg	G	1	0.29
---	--	------	------
mPD		0.39	1.2
		nPD:	0.32
		N. weight:	1.6
		Sc. PD:	0.13
		Sc. rank:	1175.9

,

PB1			
Pos.	216	obs:	exp:
tct	S	0	461.40
tcc	S	0	400.40
tca	S	0	626.60
tcg	S	0	166.80
tgt	C	1	0.77
tgc	C	1	1.23
cgt	R	0	0.11
cgc	R	0	0.16
cga	R	0	0.30
cgg	R	0	0.24
att	I	0	1.83
atc	I	5	1.34
ata	I	0	1.83
aat	N	1	9.27
aac	N	16	7.73
agt	S	167	599.50
agc	S	2597	509.40
aga	R	2	1.40
agg	R	1	0.78
ggt	G	1	0.72
ggc	G	4	0.66
gga	G	0	2.19
ggg	G	0	1.43
---	--	------	------
mPD		0.14	1.7
		nPD:	0.08
		N. weight:	2.1
		Sc. PD:	-0.32
		Sc. rank:	-1024.1

,

PB1			
Pos.	217	obs:	exp:
tat	Y	326	1340.00
tac	Y	2469	1455.00
cat	H	0	0.58
cac	H	1	0.42
---	--	------	------
mPD		0.21	0.50
		nPD:	0.41
		N. weight:	0.44
		Sc. PD:	0.076
		Sc. rank:	411.5

,

PB1			
Pos.	218	obs:	exp:
tta	L	1056	258.30
ttg	L	30	530.80
ctt	L	6	554.70
ctc	L	0	446.90
cta	L	147	413.50
ctg	L	1555	589.70
att	I	0	0.37
atc	I	0	0.27
ata	I	1	0.37
gtt	V	0	0.22
gtc	V	0	0.21
gta	V	1	0.21
gtg	V	0	0.36
---	--	------	------
mPD		0.97	1.1
		nPD:	0.86
		N. weight:	1.4
		Sc. PD:	0.87
		Sc. rank:	2312.3

,

PB1			
Pos.	219	obs:	exp:
att	I	1	1025.00
atc	I	0	747.70
ata	I	2793	1021.00
atg	M	2	2.00
---	--	------	------
mPD		0.0021	0.66
		nPD:	0.
		N. weight:	1.5
		Sc. PD:	-0.33
		Sc. rank:	-2398.6

,

PB1			
Pos.	220	obs:	exp:
cgt	R	0	104.60
cgc	R	4	149.60
cga	R	0	281.70
cgg	R	0	228.20
aaa	K	1	0.57
aag	K	0	0.43
aga	R	2598	1305.00
agg	R	193	726.10
---	--	------	------
mPD		0.13	0.95
		nPD:	0.14
		N. weight:	0.8
		Sc. PD:	-0.075
		Sc. rank:	3.1

,

PB1			
Pos.	221	obs:	exp:
tct	S	0	0.17
tcc	S	0	0.14
tca	S	1	0.23
tcg	S	0	0.06
act	T	0	0.26
acc	T	0	0.23
aca	T	0	0.42
acg	T	1	0.09
agt	S	0	0.22
agc	S	0	0.18
gtt	V	0	1.08
gtc	V	0	1.05
gta	V	2	1.07
gtg	V	3	1.81
gct	A	2	702.60
gcc	A	1	539.70
gca	A	1974	1241.00
gcg	A	812	305.30
---	--	------	------
mPD		0.42	0.69
		nPD:	0.6
		N. weight:	0.89
		Sc. PD:	0.32
		Sc. rank:	1112.5

,

PB1			
Pos.	222	obs:	exp:
tta	L	5	258.30
ttg	L	284	530.80
ctt	L	2	554.70
ctc	L	0	446.90
cta	L	22	413.50
ctg	L	2481	589.70
atg	M	2	2.00
---	--	------	------
mPD		0.21	1.1
		nPD:	0.18
		N. weight:	1.7
		Sc. PD:	-0.089
		Sc. rank:	406.9

,

PB1			
Pos.	223	obs:	exp:
act	T	4	732.70
acc	T	1	629.80
aca	T	2778	1170.00
acg	T	12	263.00
gct	A	0	0.25
gcc	A	0	0.19
gca	A	1	0.45
gcg	A	0	0.11
---	--	------	------
mPD		0.013	0.70
		nPD:	0.02
		N. weight:	1.2
		Sc. PD:	-0.26
		Sc. rank:	-1619.9

,

PB1			
Pos.	224	obs:	exp:
tta	L	3	258.50
ttg	L	459	531.20
ctt	L	2	555.10
ctc	L	0	447.20
cta	L	110	413.80
ctg	L	2222	590.10
---	--	------	------
mPD		0.35	1.1
		nPD:	0.32
		N. weight:	1.4
		Sc. PD:	0.11
		Sc. rank:	1005.9

,

PB1			
Pos.	225	obs:	exp:
aat	N	969	1525.00
aac	N	1827	1271.00
---	--	------	------
mPD		0.45	0.50
		nPD:	0.91
		N. weight:	0.12
		Sc. PD:	0.078
		Sc. rank:	193.3

,

PB1			
Pos.	226	obs:	exp:
tct	S	0	0.50
tcc	S	0	0.43
tca	S	3	0.68
tcg	S	0	0.18
act	T	6	732.20
acc	T	12	629.30
aca	T	2762	1169.00
acg	T	13	262.80
agt	S	0	0.65
agc	S	0	0.55
---	--	------	------
mPD		0.024	0.70
		nPD:	0.03
		N. weight:	1.2
		Sc. PD:	-0.23
		Sc. rank:	-1280.9

,

PB1			
Pos.	227	obs:	exp:
atg	M	2796	2796.00
---	--	------	------
mPD		0	0
		nPD:	1.
		N. weight:	0.
		Sc. PD:	0
		Sc. rank:	0

,

PB1			
Pos.	228	obs:	exp:
act	T	2	732.70
acc	T	1112	629.80
aca	T	1663	1170.00
acg	T	18	263.00
gct	A	0	0.25
gcc	A	0	0.19
gca	A	1	0.45
gcg	A	0	0.11
---	--	------	------
mPD		0.49	0.70
		nPD:	0.7
		N. weight:	0.61
		Sc. PD:	0.27
		Sc. rank:	879.5

,

PB1			
Pos.	229	obs:	exp:
aaa	K	1644	1592.00
aag	K	1152	1204.00
---	--	------	------
mPD		0.48	0.49
		nPD:	0.99
		N. weight:	0.0032
		Sc. PD:	0.0023
		Sc. rank:	5.3

,

PB1			
Pos.	230	obs:	exp:
gat	D	2622	1510.00
gac	D	174	1286.00
---	--	------	------
mPD		0.12	0.50
		nPD:	0.24
		N. weight:	0.58
		Sc. PD:	-0.0017
		Sc. rank:	224.3

,

PB1			
Pos.	231	obs:	exp:
gct	A	42	704.40
gcc	A	52	541.10
gca	A	2688	1245.00
gcg	A	14	306.00
---	--	------	------
mPD		0.075	0.69
		nPD:	0.11
		N. weight:	0.94
		Sc. PD:	-0.12
		Sc. rank:	-246.2

,

PB1			
Pos.	232	obs:	exp:
gaa	E	2780	1567.00
gag	E	16	1229.00
---	--	------	------
mPD		0.011	0.49
		nPD:	0.02
		N. weight:	0.8
		Sc. PD:	-0.17
		Sc. rank:	-1003.7

,

PB1			
Pos.	233	obs:	exp:
cgt	R	0	104.60
cgc	R	0	149.50
cga	R	3	281.60
cgg	R	1	228.10
aga	R	2280	1304.00
agg	R	510	725.90
gct	A	0	0.25
gcc	A	0	0.19
gca	A	1	0.45
gcg	A	0	0.11
gaa	E	1	0.56
gag	E	0	0.44
---	--	------	------
mPD		0.30	0.96
		nPD:	0.32
		N. weight:	0.56
		Sc. PD:	0.044
		Sc. rank:	406.9

,

PB1			
Pos.	234	obs:	exp:
tct	S	0	0.17
tcc	S	0	0.14
tca	S	0	0.23
tcg	S	0	0.06
agt	S	0	0.22
agc	S	1	0.18
ggt	G	348	404.20
ggc	G	2439	367.10
gga	G	7	1225.00
ggg	G	1	799.00
---	--	------	------
mPD		0.22	0.69
		nPD:	0.32
		N. weight:	2.4
		Sc. PD:	0.2
		Sc. rank:	1765.0

,

PB1			
Pos.	235	obs:	exp:
aaa	K	2708	1592.00
aag	K	88	1204.00
---	--	------	------
mPD		0.061	0.49
		nPD:	0.12
		N. weight:	0.63
		Sc. PD:	-0.07
		Sc. rank:	-83.5

,

PB1			
Pos.	236	obs:	exp:
tta	L	90	258.40
ttg	L	2650	531.00
tct	S	0	0.17
tcc	S	0	0.14
tca	S	0	0.23
tcg	S	1	0.06
ctt	L	0	554.90
ctc	L	0	447.10
cta	L	5	413.60
ctg	L	50	589.90
agt	S	0	0.22
agc	S	0	0.18
---	--	------	------
mPD		0.10	1.1
		nPD:	0.09
		N. weight:	2.1
		Sc. PD:	-0.3
		Sc. rank:	-760.3

,

PB1			
Pos.	237	obs:	exp:
cgt	R	0	0.79
cgc	R	0	1.12
cga	R	0	2.12
cgg	R	0	1.71
aat	N	0	0.55
aac	N	1	0.45
aaa	K	207	1580.00
aag	K	2567	1194.00
aga	R	0	9.80
agg	R	21	5.46
---	--	------	------
mPD		0.15	0.51
		nPD:	0.3
		N. weight:	0.82
		Sc. PD:	0.049
		Sc. rank:	547.3

,

PB1			
Pos.	238	obs:	exp:
cgt	R	0	104.40
cgc	R	0	149.40
cga	R	0	281.30
cgg	R	8	227.90
aaa	K	0	2.85
aag	K	5	2.15
aga	R	999	1303.00
agg	R	1784	725.10
---	--	------	------
mPD		0.47	0.96
		nPD:	0.49
		N. weight:	0.69
		Sc. PD:	0.17
		Sc. rank:	746.7

,

PB1			
Pos.	239	obs:	exp:
caa	Q	0	0.50
cag	Q	1	0.50
cgt	R	15	104.60
cgc	R	2	149.60
cga	R	1563	281.70
cgg	R	990	228.20
aga	R	96	1305.00
agg	R	129	726.10
---	--	------	------
mPD		0.64	0.96
		nPD:	0.67
		N. weight:	1.9
		Sc. PD:	0.79
		Sc. rank:	2575.2

,

PB1			
Pos.	240	obs:	exp:
act	T	0	0.26
acc	T	0	0.23
aca	T	1	0.42
acg	T	0	0.09
gct	A	1	704.10
gcc	A	1	540.90
gca	A	1905	1244.00
gcg	A	888	305.90
---	--	------	------
mPD		0.44	0.69
		nPD:	0.63
		N. weight:	0.91
		Sc. PD:	0.35
		Sc. rank:	1181.1

,

PB1			
Pos.	241	obs:	exp:
att	I	2771	1026.00
atc	I	25	748.20
ata	I	0	1022.00
---	--	------	------
mPD		0.018	0.66
		nPD:	0.03
		N. weight:	1.4
		Sc. PD:	-0.29
		Sc. rank:	-1685.9

,

PB1			
Pos.	242	obs:	exp:
gct	A	1	704.40
gcc	A	7	541.10
gca	A	2643	1245.00
gcg	A	145	306.00
---	--	------	------
mPD		0.10	0.69
		nPD:	0.15
		N. weight:	0.97
		Sc. PD:	-0.082
		Sc. rank:	65.8

,

PB1			
Pos.	243	obs:	exp:
act	T	0	733.00
acc	T	4	630.00
aca	T	2780	1170.00
acg	T	12	263.10
---	--	------	------
mPD		0.011	0.70
		nPD:	0.02
		N. weight:	1.2
		Sc. PD:	-0.26
		Sc. rank:	-1700.5

,

PB1			
Pos.	244	obs:	exp:
cct	P	101	764.10
ccc	P	1913	505.00
cca	P	780	1135.00
ccg	P	2	392.00
---	--	------	------
mPD		0.45	0.71
		nPD:	0.64
		N. weight:	1.1
		Sc. PD:	0.42
		Sc. rank:	1397.1

,

PB1			
Pos.	245	obs:	exp:
ggt	G	27	404.30
ggc	G	2	367.30
gga	G	1777	1225.00
ggg	G	990	799.20
---	--	------	------
mPD		0.47	0.69
		nPD:	0.68
		N. weight:	0.42
		Sc. PD:	0.18
		Sc. rank:	585.8

,

PB1			
Pos.	246	obs:	exp:
atg	M	2796	2796.00
---	--	------	------
mPD		0	0
		nPD:	1.
		N. weight:	0.
		Sc. PD:	0
		Sc. rank:	0

,

PB1			
Pos.	247	obs:	exp:
caa	Q	1798	1395.00
cag	Q	997	1400.00
act	T	0	0.26
acc	T	0	0.23
aca	T	1	0.42
acg	T	0	0.09
---	--	------	------
mPD		0.46	0.50
		nPD:	0.92
		N. weight:	0.064
		Sc. PD:	0.042
		Sc. rank:	104.2

,

PB1			
Pos.	248	obs:	exp:
tta	L	0	0.09
ttg	L	0	0.19
ctt	L	0	0.20
ctc	L	1	0.16
cta	L	0	0.15
ctg	L	0	0.21
att	I	966	1025.00
atc	I	1798	747.10
ata	I	28	1020.00
atg	M	1	1.00
act	T	0	0.26
acc	T	1	0.23
aca	T	0	0.42
acg	T	0	0.09
aat	N	1	0.55
aac	N	0	0.45
---	--	------	------
mPD		0.47	0.66
		nPD:	0.71
		N. weight:	0.75
		Sc. PD:	0.34
		Sc. rank:	1099.7

,

PB1			
Pos.	249	obs:	exp:
cgt	R	0	104.60
cgc	R	0	149.50
cga	R	4	281.60
cgg	R	0	228.10
act	T	0	0.26
acc	T	0	0.23
aca	T	1	0.42
acg	T	0	0.09
aaa	K	1	0.57
aag	K	0	0.43
aga	R	2742	1304.00
agg	R	48	725.90
---	--	------	------
mPD		0.038	0.96
		nPD:	0.04
		N. weight:	0.99
		Sc. PD:	-0.19
		Sc. rank:	-976.6

,

PB1			
Pos.	250	obs:	exp:
ggt	G	25	404.30
ggc	G	0	367.30
gga	G	2538	1225.00
ggg	G	233	799.20
---	--	------	------
mPD		0.17	0.69
		nPD:	0.25
		N. weight:	0.78
		Sc. PD:	0.0058
		Sc. rank:	359.1

,

PB1			
Pos.	251	obs:	exp:
ttt	F	1026	1166.00
ttc	F	1768	1628.00
tct	S	0	0.17
tcc	S	1	0.14
tca	S	0	0.23
tcg	S	0	0.06
att	I	0	0.37
atc	I	1	0.27
ata	I	0	0.37
agt	S	0	0.22
agc	S	0	0.18
---	--	------	------
mPD		0.47	0.49
		nPD:	0.95
		N. weight:	0.011
		Sc. PD:	0.008
		Sc. rank:	18.8

,

PB1			
Pos.	252	obs:	exp:
gtt	V	0	601.40
gtc	V	129	588.20
gta	V	283	596.20
gtg	V	2384	1010.00
---	--	------	------
mPD		0.26	0.73
		nPD:	0.36
		N. weight:	0.86
		Sc. PD:	0.098
		Sc. rank:	703.4

,

PB1			
Pos.	253	obs:	exp:
tat	Y	152	1339.00
tac	Y	2640	1453.00
cat	H	0	2.32
cac	H	4	1.68
---	--	------	------
mPD		0.11	0.50
		nPD:	0.21
		N. weight:	0.65
		Sc. PD:	-0.017
		Sc. rank:	208.2

,

PB1			
Pos.	254	obs:	exp:
ttt	F	2003	1160.00
ttc	F	777	1620.00
tgt	C	2	1.16
tgc	C	1	1.84
gtt	V	12	2.58
gtc	V	0	2.52
gta	V	0	2.56
gtg	V	0	4.34
gct	A	1	0.25
gcc	A	0	0.19
gca	A	0	0.45
gcg	A	0	0.11
---	--	------	------
mPD		0.41	0.50
		nPD:	0.83
		N. weight:	0.29
		Sc. PD:	0.16
		Sc. rank:	457.0

,

PB1			
Pos.	255	obs:	exp:
att	I	1	0.37
atc	I	0	0.27
ata	I	0	0.37
gtt	V	1803	600.30
gtc	V	963	587.20
gta	V	25	595.10
gtg	V	0	1008.00
gct	A	4	1.01
gcc	A	0	0.77
gca	A	0	1.78
gcg	A	0	0.44
---	--	------	------
mPD		0.47	0.74
		nPD:	0.63
		N. weight:	1.2
		Sc. PD:	0.48
		Sc. rank:	1620.2

,

PB1			
Pos.	256	obs:	exp:
caa	Q	1	0.50
cag	Q	0	0.50
gaa	E	2747	1567.00
gag	E	48	1228.00
---	--	------	------
mPD		0.034	0.49
		nPD:	0.07
		N. weight:	0.73
		Sc. PD:	-0.12
		Sc. rank:	-437.8

,

PB1			
Pos.	257	obs:	exp:
tct	S	0	0.50
tcc	S	0	0.43
tca	S	3	0.68
tcg	S	0	0.18
att	I	0	0.73
atc	I	0	0.54
ata	I	2	0.73
act	T	0	720.40
acc	T	1	619.20
aca	T	2743	1150.00
acg	T	4	258.60
agt	S	0	0.65
agc	S	0	0.55
gct	A	0	10.83
gcc	A	0	8.32
gca	A	43	19.14
gcg	A	0	4.71
---	--	------	------
mPD		0.037	0.73
		nPD:	0.05
		N. weight:	1.3
		Sc. PD:	-0.23
		Sc. rank:	-983.8

,

PB1			
Pos.	258	obs:	exp:
tta	L	444	258.20
ttg	L	8	530.60
ctt	L	4	554.50
ctc	L	0	446.80
cta	L	1539	413.30
ctg	L	798	589.50
att	I	0	0.37
atc	I	0	0.27
ata	I	1	0.37
gtt	V	0	0.43
gtc	V	0	0.42
gta	V	2	0.43
gtg	V	0	0.72
---	--	------	------
mPD		0.69	1.1
		nPD:	0.61
		N. weight:	1.3
		Sc. PD:	0.47
		Sc. rank:	1622.7

,

PB1			
Pos.	259	obs:	exp:
tct	S	0	0.17
tcc	S	0	0.14
tca	S	1	0.23
tcg	S	0	0.06
act	T	0	0.52
acc	T	0	0.45
aca	T	0	0.84
acg	T	2	0.19
agt	S	0	0.22
agc	S	0	0.18
gct	A	1	703.60
gcc	A	5	540.50
gca	A	926	1243.00
gcg	A	1861	305.70
---	--	------	------
mPD		0.45	0.69
		nPD:	0.65
		N. weight:	1.6
		Sc. PD:	0.64
		Sc. rank:	2121.1

,

PB1			
Pos.	260	obs:	exp:
cgt	R	0	104.20
cgc	R	0	149.00
cga	R	0	280.60
cgg	R	75	227.30
aaa	K	0	6.83
aag	K	12	5.17
aga	R	172	1300.00
agg	R	2537	723.30
---	--	------	------
mPD		0.18	0.96
		nPD:	0.18
		N. weight:	1.4
		Sc. PD:	-0.077
		Sc. rank:	327.1

,

PB1			
Pos.	261	obs:	exp:
tct	S	0	430.50
tcc	S	0	373.60
tca	S	0	584.70
tcg	S	0	155.60
tgt	C	126	49.15
tgc	C	1	77.85
cgt	R	2	0.19
cgc	R	0	0.27
cga	R	0	0.50
cgg	R	0	0.41
act	T	0	19.40
acc	T	0	16.67
aca	T	9	30.96
acg	T	65	6.96
aat	N	7	4.36
aac	N	1	3.64
agt	S	1430	559.40
agc	S	1149	475.30
aga	R	3	2.33
agg	R	0	1.30
ggt	G	0	0.43
ggc	G	2	0.39
gga	G	1	1.31
ggg	G	0	0.86
---	--	------	------
mPD		0.66	1.7
		nPD:	0.4
		N. weight:	1.4
		Sc. PD:	0.22
		Sc. rank:	1247.4

,

PB1			
Pos.	262	obs:	exp:
att	I	106	1025.00
atc	I	2677	747.40
ata	I	10	1021.00
gtt	V	0	0.65
gtc	V	3	0.63
gta	V	0	0.64
gtg	V	0	1.08
---	--	------	------
mPD		0.082	0.66
		nPD:	0.12
		N. weight:	1.6
		Sc. PD:	-0.18
		Sc. rank:	-226.8

,

PB1			
Pos.	263	obs:	exp:
tgt	C	2785	1082.00
tgc	C	11	1714.00
---	--	------	------
mPD		0.0078	0.47
		nPD:	0.02
		N. weight:	1.3
		Sc. PD:	-0.29
		Sc. rank:	-1829.7

,

PB1			
Pos.	264	obs:	exp:
caa	Q	0	0.50
cag	Q	1	0.50
gat	D	14	7.56
gac	D	0	6.44
gaa	E	1035	1559.00
gag	E	1746	1222.00
---	--	------	------
mPD		0.47	0.50
		nPD:	0.95
		N. weight:	0.11
		Sc. PD:	0.077
		Sc. rank:	181.5

,

PB1			
Pos.	265	obs:	exp:
cgt	R	0	0.11
cgc	R	0	0.16
cga	R	0	0.30
cgg	R	0	0.24
aaa	K	2569	1590.00
aag	K	224	1203.00
aga	R	3	1.40
agg	R	0	0.78
---	--	------	------
mPD		0.15	0.49
		nPD:	0.3
		N. weight:	0.45
		Sc. PD:	0.029
		Sc. rank:	301.6

,

PB1			
Pos.	266	obs:	exp:
tta	L	0	258.50
ttg	L	0	531.20
ctt	L	2616	555.10
ctc	L	159	447.20
cta	L	21	413.80
ctg	L	0	590.10
---	--	------	------
mPD		0.12	1.1
		nPD:	0.11
		N. weight:	2.
		Sc. PD:	-0.25
		Sc. rank:	-550.5

,

PB1			
Pos.	267	obs:	exp:
gaa	E	91	1567.00
gag	E	2705	1229.00
---	--	------	------
mPD		0.063	0.49
		nPD:	0.13
		N. weight:	0.98
		Sc. PD:	-0.11
		Sc. rank:	-109.6

,

PB1			
Pos.	268	obs:	exp:
cct	P	0	0.27
ccc	P	0	0.18
cca	P	1	0.41
ccg	P	0	0.14
caa	Q	2737	1395.00
cag	Q	58	1400.00
---	--	------	------
mPD		0.041	0.50
		nPD:	0.08
		N. weight:	0.87
		Sc. PD:	-0.13
		Sc. rank:	-417.4

,

PB1			
Pos.	269	obs:	exp:
tct	S	2705	466.70
tcc	S	83	405.00
tca	S	4	633.90
tcg	S	4	168.70
agt	S	0	606.50
agc	S	0	515.30
---	--	------	------
mPD		0.063	1.7
		nPD:	0.04
		N. weight:	2.4
		Sc. PD:	-0.47
		Sc. rank:	-2407.2

,

PB1			
Pos.	270	obs:	exp:
gaa	E	1	0.56
gag	E	0	0.44
ggt	G	1	404.20
ggc	G	8	367.10
gga	G	2540	1225.00
ggg	G	246	799.00
---	--	------	------
mPD		0.17	0.69
		nPD:	0.24
		N. weight:	0.8
		Sc. PD:	0.0036
		Sc. rank:	362.2

,

PB1			
Pos.	271	obs:	exp:
tta	L	1	258.50
ttg	L	0	531.20
ctt	L	261	555.10
ctc	L	2446	447.20
cta	L	85	413.80
ctg	L	3	590.10
---	--	------	------
mPD		0.23	1.1
		nPD:	0.2
		N. weight:	2.
		Sc. PD:	-0.071
		Sc. rank:	526.2

,

PB1			
Pos.	272	obs:	exp:
cct	P	313	763.80
ccc	P	790	504.80
cca	P	1680	1134.00
ccg	P	12	391.90
cat	H	0	0.58
cac	H	1	0.42
---	--	------	------
mPD		0.55	0.71
		nPD:	0.77
		N. weight:	0.37
		Sc. PD:	0.19
		Sc. rank:	571.7

,

PB1			
Pos.	273	obs:	exp:
att	I	7	2.57
atc	I	0	1.87
ata	I	0	2.56
gtt	V	1351	599.90
gtc	V	1431	586.80
gta	V	3	594.70
gtg	V	4	1008.00
---	--	------	------
mPD		0.51	0.74
		nPD:	0.69
		N. weight:	1.2
		Sc. PD:	0.54
		Sc. rank:	1742.0

,

PB1			
Pos.	274	obs:	exp:
cgt	R	0	0.04
cgc	R	0	0.05
cga	R	0	0.10
cgg	R	0	0.08
aga	R	1	0.47
agg	R	0	0.26
ggt	G	4	404.20
ggc	G	0	367.10
gga	G	2716	1225.00
ggg	G	75	799.00
---	--	------	------
mPD		0.056	0.69
		nPD:	0.08
		N. weight:	1.
		Sc. PD:	-0.16
		Sc. rank:	-508.6

,

PB1			
Pos.	275	obs:	exp:
ggt	G	1	404.30
ggc	G	68	367.30
gga	G	411	1225.00
ggg	G	2316	799.20
---	--	------	------
mPD		0.29	0.69
		nPD:	0.42
		N. weight:	0.99
		Sc. PD:	0.18
		Sc. rank:	947.9

,

PB1			
Pos.	276	obs:	exp:
aat	N	2754	1525.00
aac	N	42	1271.00
---	--	------	------
mPD		0.030	0.50
		nPD:	0.06
		N. weight:	0.78
		Sc. PD:	-0.13
		Sc. rank:	-554.5

,

PB1			
Pos.	277	obs:	exp:
gat	D	0	0.54
gac	D	1	0.46
gaa	E	227	1567.00
gag	E	2568	1228.00
---	--	------	------
mPD		0.15	0.49
		nPD:	0.3
		N. weight:	0.76
		Sc. PD:	0.049
		Sc. rank:	513.8

,

PB1			
Pos.	278	obs:	exp:
aaa	K	148	1592.00
aag	K	2648	1204.00
---	--	------	------
mPD		0.10	0.49
		nPD:	0.2
		N. weight:	0.91
		Sc. PD:	-0.03
		Sc. rank:	255.6

,

PB1			
Pos.	279	obs:	exp:
aaa	K	1122	1592.00
aag	K	1674	1204.00
---	--	------	------
mPD		0.48	0.49
		nPD:	0.98
		N. weight:	0.085
		Sc. PD:	0.062
		Sc. rank:	140.2

,

PB1			
Pos.	280	obs:	exp:
gct	A	2744	704.40
gcc	A	51	541.10
gca	A	1	1245.00
gcg	A	0	306.00
---	--	------	------
mPD		0.037	0.69
		nPD:	0.05
		N. weight:	1.9
		Sc. PD:	-0.34
		Sc. rank:	-1406.2

,

PB1			
Pos.	281	obs:	exp:
aaa	K	2703	1592.00
aag	K	93	1204.00
---	--	------	------
mPD		0.064	0.49
		nPD:	0.13
		N. weight:	0.62
		Sc. PD:	-0.065
		Sc. rank:	-48.4

,

PB1			
Pos.	282	obs:	exp:
tta	L	19	258.50
ttg	L	1793	531.20
ctt	L	0	555.10
ctc	L	0	447.20
cta	L	35	413.80
ctg	L	949	590.10
---	--	------	------
mPD		0.49	1.1
		nPD:	0.44
		N. weight:	1.3
		Sc. PD:	0.26
		Sc. rank:	1288.1

,

PB1			
Pos.	283	obs:	exp:
gct	A	8	704.40
gcc	A	2	541.10
gca	A	2777	1245.00
gcg	A	9	306.00
---	--	------	------
mPD		0.014	0.69
		nPD:	0.02
		N. weight:	1.1
		Sc. PD:	-0.24
		Sc. rank:	-1483.6

,

PB1			
Pos.	284	obs:	exp:
aat	N	1213	1525.00
aac	N	1583	1271.00
---	--	------	------
mPD		0.49	0.50
		nPD:	0.99
		N. weight:	0.039
		Sc. PD:	0.028
		Sc. rank:	63.5

,

PB1			
Pos.	285	obs:	exp:
gtt	V	222	601.40
gtc	V	2549	588.20
gta	V	24	596.20
gtg	V	1	1010.00
---	--	------	------
mPD		0.16	0.73
		nPD:	0.22
		N. weight:	1.8
		Sc. PD:	-0.029
		Sc. rank:	644.6

,

PB1			
Pos.	286	obs:	exp:
gtt	V	6	601.40
gtc	V	0	588.20
gta	V	196	596.20
gtg	V	2594	1010.00
---	--	------	------
mPD		0.13	0.73
		nPD:	0.18
		N. weight:	1.2
		Sc. PD:	-0.061
		Sc. rank:	249.6

PB1			
Pos.	287	obs:	exp:
cgt	R	0	104.60
cgc	R	0	149.60
cga	R	0	281.70
cgg	R	0	228.20
aaa	K	0	0.57
aag	K	1	0.43
aga	R	632	1305.00
agg	R	2163	726.10
---	--	------	------
mPD		0.35	0.95
		nPD:	0.37
		N. weight:	1.
		Sc. PD:	0.13
		Sc. rank:	836.7

PB1			
Pos.	288	obs:	exp:
aaa	K	1039	1592.00
aag	K	1757	1204.00
---	--	------	------
mPD		0.47	0.49
		nPD:	0.95
		N. weight:	0.12
		Sc. PD:	0.082
		Sc. rank:	192.1

PB1			
Pos.	289	obs:	exp:
atg	M	2796	2796.00
---	--	------	------
mPD		0	0
		nPD:	1.
		N. weight:	0.
		Sc. PD:	0
		Sc. rank:	0

PB1			
Pos.	290	obs:	exp:
atg	M	2796	2796.00
---	--	------	------
mPD		0	0
		nPD:	1.
		N. weight:	0.
		Sc. PD:	0
		Sc. rank:	0

PB1			
Pos.	291	obs:	exp:
att	I	2	2.20
atc	I	4	1.61
ata	I	0	2.19
act	T	1967	730.90
acc	T	809	628.20
aca	T	12	1167.00
acg	T	0	262.30
gct	A	2	0.50
gcc	A	0	0.39
gca	A	0	0.89
gcg	A	0	0.22
---	--	------	------
mPD		0.42	0.70
		nPD:	0.6
		N. weight:	1.1
		Sc. PD:	0.39
		Sc. rank:	1372.1

PB1			
Pos.	292	obs:	exp:
tct	S	0	3.84
tcc	S	0	3.33
tca	S	0	5.21
tcg	S	0	1.39
aat	N	349	1512.00
aac	N	2423	1260.00
agt	S	11	4.99
agc	S	12	4.24
gat	D	0	0.54
gac	D	1	0.46
---	--	------	------
mPD		0.24	0.53
		nPD:	0.46
		N. weight:	0.57
		Sc. PD:	0.12
		Sc. rank:	594.3

PB1			
Pos.	293	obs:	exp:
tta	L	1	0.09
ttg	L	0	0.19
tct	S	1	466.50
tcc	S	4	404.90
tca	S	2728	633.60
tcg	S	62	168.60
ctt	L	0	0.20
ctc	L	0	0.16
cta	L	0	0.15
ctg	L	0	0.21
agt	S	0	606.20
agc	S	0	515.10
---	--	------	------
mPD		0.048	1.7
		nPD:	0.03
		N. weight:	2.
		Sc. PD:	-0.41
		Sc. rank:	-2399.1

PB1			
Pos.	294	obs:	exp:
tta	L	0	0.09
ttg	L	0	0.19
ctt	L	0	0.20
ctc	L	0	0.16
cta	L	0	0.15
ctg	L	1	0.21
cat	H	3	1.74
cac	H	0	1.26
caa	Q	1698	1393.00
cag	Q	1092	1397.00
cgt	R	0	0.04
cgc	R	0	0.05
cga	R	1	0.10
cgg	R	0	0.08
aaa	K	1	0.57
aag	K	0	0.43
aga	R	0	0.47
agg	R	0	0.26
---	--	------	------
mPD		0.48	0.50
		nPD:	0.95
		N. weight:	0.04
		Sc. PD:	0.028
		Sc. rank:	66.2

PB1			
Pos.	295	obs:	exp:
gat	D	2459	1510.00
gac	D	337	1286.00
---	--	------	------
mPD		0.21	0.50
		nPD:	0.43
		N. weight:	0.39
		Sc. PD:	0.072
		Sc. rank:	375.4

PB1			
Pos.	296	obs:	exp:
att	I	2	0.73
atc	I	0	0.54
ata	I	0	0.73
act	T	1615	731.10
acc	T	7	628.40
aca	T	1166	1167.00
acg	T	1	262.40
aat	N	2	1.09
aac	N	0	0.91
gct	A	2	0.76
gcc	A	0	0.58
gca	A	1	1.34
gcg	A	0	0.33
---	--	------	------
mPD		0.49	0.70
		nPD:	0.71
		N. weight:	0.65
		Sc. PD:	0.3
		Sc. rank:	956.9

PB1			
Pos.	297	obs:	exp:
gat	D	1	0.54
gac	D	0	0.46
gaa	E	1607	1567.00
gag	E	1188	1228.00
---	--	------	------
mPD		0.49	0.49
		nPD:	0.99
		N. weight:	0.0031
		Sc. PD:	0.0023
		Sc. rank:	5.1

PB1			
Pos.	298	obs:	exp:
tta	L	0	254.10
ttg	L	0	522.00
ctt	L	166	545.60
ctc	L	2570	439.60
cta	L	12	406.70
ctg	L	0	580.00
att	I	0	16.51
atc	I	45	12.04
ata	I	0	16.44
gtt	V	0	0.65
gtc	V	3	0.63
gta	V	0	0.64
gtg	V	0	1.08
---	--	------	------
mPD		0.15	1.1
		nPD:	0.13
		N. weight:	2.3
		Sc. PD:	-0.23
		Sc. rank:	-131.0

PB1			
Pos.	299	obs:	exp:
tct	S	121	466.50
tcc	S	2669	404.90
tca	S	5	633.60
tcg	S	0	168.60
act	T	0	0.26
acc	T	1	0.23
aca	T	0	0.42
acg	T	0	0.09
agt	S	0	606.20
agc	S	0	515.10
---	--	------	------
mPD		0.087	1.7
		nPD:	0.05
		N. weight:	2.5
		Sc. PD:	-0.46
		Sc. rank:	-1912.1

PB1			
Pos.	300	obs:	exp:
ttt	F	1879	1166.00
ttc	F	917	1630.00
---	--	------	------
mPD		0.44	0.49
		nPD:	0.91
		N. weight:	0.19
		Sc. PD:	0.13
		Sc. rank:	316.5

PB1			
Pos.	301	obs:	exp:
act	T	1	733.00
acc	T	1	630.00
aca	T	2689	1170.00
acg	T	105	263.10
---	--	------	------
mPD		0.074	0.70
		nPD:	0.11
		N. weight:	1.1
		Sc. PD:	-0.14
		Sc. rank:	-320.8

PB1			
Pos.	302	obs:	exp:
att	I	2660	1024.00
atc	I	128	746.60
ata	I	2	1020.00
act	T	1	0.26
acc	T	0	0.23
aca	T	0	0.42
acg	T	0	0.09
gtt	V	5	1.08
gtc	V	0	1.05
gta	V	0	1.07
gtg	V	0	1.81
---	--	------	------
mPD		0.093	0.66
		nPD:	0.14
		N. weight:	1.2
		Sc. PD:	-0.12
		Sc. rank:	-4.0

PB1			
Pos.	303	obs:	exp:
act	T	2756	733.00
acc	T	17	630.00
aca	T	12	1170.00
acg	T	11	263.10
---	--	------	------
mPD		0.028	0.70
		nPD:	0.04
		N. weight:	1.8
		Sc. PD:	-0.35
		Sc. rank:	-1738.7

PB1			
Pos.	304	obs:	exp:
ggt	G	19	404.30
ggc	G	3	367.30
gga	G	1553	1225.00
ggg	G	1221	799.20
---	--	------	------
mPD		0.50	0.69
		nPD:	0.73
		N. weight:	0.43
		Sc. PD:	0.2
		Sc. rank:	647.2

PB1			
Pos.	305	obs:	exp:
aaa	K	1	0.57
aag	K	0	0.43
gat	D	741	1509.00
gac	D	2054	1286.00
---	--	------	------
mPD		0.39	0.50
		nPD:	0.79
		N. weight:	0.23
		Sc. PD:	0.12
		Sc. rank:	360.2

PB1			
Pos.	306	obs:	exp:
cat	H	1	0.58
cac	H	0	0.42
aat	N	1148	1524.00
aac	N	1647	1271.00
---	--	------	------
mPD		0.49	0.50
		nPD:	0.98
		N. weight:	0.056
		Sc. PD:	0.04
		Sc. rank:	91.3

PB1			
Pos.	307	obs:	exp:
act	T	360	733.00
acc	T	1052	630.00
aca	T	1222	1170.00
acg	T	162	263.10
---	--	------	------
mPD		0.65	0.70
		nPD:	0.93
		N. weight:	0.14
		Sc. PD:	0.095
		Sc. rank:	229.8

PB1			
Pos.	308	obs:	exp:
aaa	K	2767	1592.00
aag	K	29	1204.00
---	--	------	------
mPD		0.021	0.49
		nPD:	0.04
		N. weight:	0.74
		Sc. PD:	-0.14
		Sc. rank:	-693.8

PB1			
Pos.	309	obs:	exp:
tgg	W	2796	2796.00
---	--	------	------
mPD		0	0
		nPD:	1.
		N. weight:	0.
		Sc. PD:	0
		Sc. rank:	0

PB1			
Pos.	310	obs:	exp:
aat	N	2764	1525.00
aac	N	32	1271.00
---	--	------	------
mPD		0.023	0.50
		nPD:	0.05
		N. weight:	0.8
		Sc. PD:	-0.15
		Sc. rank:	-695.2

PB1			
Pos.	311	obs:	exp:
gaa	E	91	1567.00
gag	E	2705	1229.00
---	--	------	------
mPD		0.063	0.49
		nPD:	0.13
		N. weight:	0.98
		Sc. PD:	-0.11
		Sc. rank:	-109.6

PB1			
Pos.	312	obs:	exp:
aat	N	2684	1525.00
aac	N	112	1271.00
---	--	------	------
mPD		0.077	0.50
		nPD:	0.16
		N. weight:	0.65
		Sc. PD:	-0.053
		Sc. rank:	65.7

PB1			
Pos.	313	obs:	exp:
caa	Q	2028	1396.00
cag	Q	768	1400.00
---	--	------	------
mPD		0.40	0.50
		nPD:	0.8
		N. weight:	0.16
		Sc. PD:	0.085
		Sc. rank:	247.7

PB1			
Pos.	314	obs:	exp:
tct	S	0	8.35
tcc	S	0	7.24
tca	S	0	11.34
tcg	S	0	3.02
aat	N	2502	1497.00
aac	N	243	1248.00
aaa	K	1	0.57
aag	K	0	0.43
agt	S	50	10.85
agc	S	0	9.21
---	--	------	------
mPD		0.19	0.56
		nPD:	0.35
		N. weight:	0.51
		Sc. PD:	0.054
		Sc. rank:	405.2

PB1			
Pos.	315	obs:	exp:
tct	S	1	0.17
tcc	S	0	0.14
tca	S	0	0.23
tcg	S	0	0.06
cct	P	2688	763.80
ccc	P	104	504.80
cca	P	3	1134.00
ccg	P	0	391.90
agt	S	0	0.22
agc	S	0	0.18
---	--	------	------
mPD		0.074	0.71
		nPD:	0.1
		N. weight:	1.7
		Sc. PD:	-0.22
		Sc. rank:	-490.2

PB1			
Pos.	316	obs:	exp:
caa	Q	0	0.50
cag	Q	1	0.50
cgt	R	0	104.50
cgc	R	0	149.50
cga	R	66	281.50
cgg	R	1029	228.10
aaa	K	0	1.14
aag	K	2	0.86
aga	R	265	1304.00
agg	R	1433	725.60
---	--	------	------
mPD		0.69	0.96
		nPD:	0.72
		N. weight:	1.1
		Sc. PD:	0.49
		Sc. rank:	1586.5

PB1			
Pos.	317	obs:	exp:
att	I	2	5.87
atc	I	0	4.28
ata	I	14	5.85
atg	M	2569	2569.00
act	T	0	0.79
acc	T	0	0.68
aca	T	0	1.26
acg	T	3	0.28
gtt	V	2	44.74
gtc	V	1	43.76
gta	V	1	44.35
gtg	V	204	75.15
---	--	------	------
mPD		0.15	0.24
		nPD:	0.63
		N. weight:	0.11
		Sc. PD:	0.042
		Sc. rank:	142.2

PB1			
Pos.	318	obs:	exp:
ttt	F	2770	1166.00
ttc	F	26	1630.00
---	--	------	------
mPD		0.018	0.49
		nPD:	0.04
		N. weight:	1.2
		Sc. PD:	-0.23
		Sc. rank:	-1219.6

PB1			
Pos.	319	obs:	exp:
tta	L	8	258.50
ttg	L	211	531.20
ctt	L	78	555.10
ctc	L	0	447.20
cta	L	88	413.80
ctg	L	2411	590.10
---	--	------	------
mPD		0.26	1.1
		nPD:	0.23
		N. weight:	1.5
		Sc. PD:	-0.0051
		Sc. rank:	584.5

PB1			
Pos.	320	obs:	exp:
act	T	0	0.79
acc	T	0	0.68
aca	T	3	1.26
acg	T	0	0.28
gct	A	2	703.60
gcc	A	1	540.50
gca	A	2736	1243.00
gcg	A	54	305.70
---	--	------	------
mPD		0.042	0.69
		nPD:	0.06
		N. weight:	1.1
		Sc. PD:	-0.18
		Sc. rank:	-760.6

PB1			
Pos.	321	obs:	exp:
atg	M	2796	2796.00
---	--	------	------
mPD		0	0
		nPD:	1.
		N. weight:	0.
		Sc. PD:	0
		Sc. rank:	0

PB1			
Pos.	322	obs:	exp:
att	I	0	1025.00
atc	I	2	747.40
ata	I	2791	1021.00
gtt	V	0	0.65
gtc	V	0	0.63
gta	V	3	0.64
gtg	V	0	1.08
---	--	------	------
mPD		0.0036	0.66
		nPD:	0.01
		N. weight:	1.5
		Sc. PD:	-0.33
		Sc. rank:	-2330.3

PB1			
Pos.	323	obs:	exp:
atg	M	5	5.00
act	T	2	731.70
acc	T	1	628.90
aca	T	1306	1168.00
acg	T	1482	262.60
---	--	------	------
mPD		0.50	0.70
		nPD:	0.72
		N. weight:	1.4
		Sc. PD:	0.66
		Sc. rank:	2109.8

PB1			
Pos.	324	obs:	exp:
tat	Y	70	1341.00
tac	Y	2726	1455.00
---	--	------	------
mPD		0.049	0.50
		nPD:	0.1
		N. weight:	0.79
		Sc. PD:	-0.11
		Sc. rank:	-255.5

PB1			
Pos.	325	obs:	exp:
att	I	740	1025.00
atc	I	1851	747.70
ata	I	203	1021.00
gtt	V	0	0.43
gtc	V	2	0.42
gta	V	0	0.43
gtg	V	0	0.72
---	--	------	------
mPD		0.49	0.66
		nPD:	0.74
		N. weight:	0.58
		Sc. PD:	0.28
		Sc. rank:	887.1

PB1			
Pos.	326	obs:	exp:
act	T	1	733.00
acc	T	9	630.00
aca	T	2774	1170.00
acg	T	12	263.10
---	--	------	------
mPD		0.016	0.70
		nPD:	0.02
		N. weight:	1.2
		Sc. PD:	-0.25
		Sc. rank:	-1535.0

PB1			
Pos.	327	obs:	exp:
tgg	W	1	1.00
cgt	R	0	103.70
cgc	R	0	148.30
cga	R	0	279.30
cgg	R	3	226.30
aaa	K	8	12.53
aag	K	14	9.47
aga	R	1122	1294.00
agg	R	1647	720.10
ggt	G	0	0.14
ggc	G	0	0.13
gga	G	0	0.44
ggg	G	1	0.29
---	--	------	------
mPD		0.50	0.97
		nPD:	0.52
		N. weight:	0.63
		Sc. PD:	0.17
		Sc. rank:	700.7

PB1			
Pos.	328	obs:	exp:
tct	S	0	1.67
tcc	S	0	1.45
tca	S	0	2.27
tcg	S	0	0.60
cat	H	0	1.16
cac	H	2	0.84
cgt	R	0	0.04
cgc	R	0	0.05
cga	R	0	0.10
cgg	R	0	0.08
aat	N	62	1461.00
aac	N	2618	1219.00
aaa	K	97	55.23
aag	K	0	41.77
agt	S	0	2.17
agc	S	10	1.84
aga	R	1	0.47
agg	R	0	0.26
gat	D	0	3.24
gac	D	6	2.76
---	--	------	------
mPD		0.12	0.55
		nPD:	0.22
		N. weight:	0.99
		Sc. PD:	-0.013
		Sc. rank:	366.0

PB1			
Pos.	329	obs:	exp:
cct	P	0	0.27
ccc	P	0	0.18
cca	P	1	0.41
ccg	P	0	0.14
cat	H	1	0.58
cac	H	0	0.42
caa	Q	1172	1394.00
cag	Q	1620	1398.00
cgt	R	0	0.04
cgc	R	0	0.05
cga	R	0	0.10
cgg	R	1	0.08
aaa	K	0	0.57
aag	K	1	0.43
aga	R	0	0.47
agg	R	0	0.26
---	--	------	------
mPD		0.49	0.50
		nPD:	0.97
		N. weight:	0.023
		Sc. PD:	0.017
		Sc. rank:	38.1

PB1			
Pos.	330	obs:	exp:
cct	P	1224	764.10
ccc	P	58	505.00
cca	P	1432	1135.00
ccg	P	82	392.00
---	--	------	------
mPD		0.54	0.71
		nPD:	0.77
		N. weight:	0.35
		Sc. PD:	0.18
		Sc. rank:	539.2

PB1

Pos.	331	obs:	exp:
cgt	R	0	0.04
cgc	R	0	0.05
cga	R	0	0.10
cgg	R	0	0.08
aaa	K	163	96.80
aag	K	7	73.20
aga	R	1	0.47
agg	R	0	0.26
gtt	V	0	0.22
gtc	V	0	0.21
gta	V	1	0.21
gtg	V	0	0.36
gat	D	7	4.86
gac	D	2	4.14
gaa	E	2591	1463.00
gag	E	19	1147.00
ggt	G	0	0.72
ggc	G	0	0.66
gga	G	4	2.19
ggg	G	1	1.43
---	--	------	------
mPD		0.15	0.62
		nPD:	0.24
		N. weight:	0.77
		Sc. PD:	-0.0016
		Sc. rank:	304.2

PB1

Pos.	332	obs:	exp:
tgg	W	2796	2796.00
---	--	------	------
mPD		0	0
		nPD:	1.
		N. weight:	0.
		Sc. PD:	0
		Sc. rank:	0

PB1

Pos.	333	obs:	exp:
ttt	F	2567	1163.00
ttc	F	220	1624.00
tta	L	1	0.55
ttg	L	0	1.14
tct	S	3	0.50
tcc	S	0	0.43
tca	S	0	0.68
tcg	S	0	0.18
ctt	L	3	1.19
ctc	L	2	0.96
cta	L	0	0.89
ctg	L	0	1.27
agt	S	0	0.65
agc	S	0	0.55
---	--	------	------
mPD		0.15	0.49
		nPD:	0.31
		N. weight:	0.84
		Sc. PD:	0.058
		Sc. rank:	575.0

PB1

Pos.	334	obs:	exp:
caa	Q	1	0.50
cag	Q	0	0.50
cgt	R	0	104.60
cgc	R	0	149.50
cga	R	1103	281.60
cgg	R	513	228.10
aga	R	942	1304.00
agg	R	236	725.90
ggt	G	0	0.14
ggc	G	0	0.13
gga	G	0	0.44
ggg	G	1	0.29
---	--	------	------
mPD		0.88	0.96
		nPD:	0.92
		N. weight:	0.71
		Sc. PD:	0.47
		Sc. rank:	1162.2

PB1

Pos.	335	obs:	exp:
tct	S	0	0.50
tcc	S	0	0.43
tca	S	0	0.68
tcg	S	0	0.18
cat	H	1	0.58
cac	H	0	0.42
aat	N	2648	1523.00
aac	N	144	1269.00
agt	S	3	0.65
agc	S	0	0.55
---	--	------	------
mPD		0.10	0.50
		nPD:	0.2
		N. weight:	0.61
		Sc. PD:	-0.022
		Sc. rank:	154.9

PB1

Pos.	336	obs:	exp:
att	I	3	4.40
atc	I	9	3.21
ata	I	0	4.39
gtt	V	738	598.60
gtc	V	2020	585.50
gta	V	24	593.40
gtg	V	1	1006.00
gct	A	1	0.25
gcc	A	0	0.19
gca	A	0	0.45
gcg	A	0	0.11
---	--	------	------
mPD		0.41	0.74
		nPD:	0.55
		N. weight:	1.4
		Sc. PD:	0.42
		Sc. rank:	1562.2

PB1

Pos.	337	obs:	exp:
tta	L	1677	258.50
ttg	L	1061	531.20
ctt	L	0	555.10
ctc	L	0	447.20
cta	L	39	413.80
ctg	L	19	590.10
---	--	------	------
mPD		0.51	1.1
		nPD:	0.46
		N. weight:	1.9
		Sc. PD:	0.42
		Sc. rank:	2006.2

PB1

Pos.	338	obs:	exp:
tct	S	0	466.50
tcc	S	0	404.90
tca	S	0	633.60
tcg	S	0	168.60
att	I	0	0.37
atc	I	1	0.27
ata	I	0	0.37
agt	S	751	606.20
agc	S	2044	515.10
---	--	------	------
mPD		0.39	1.7
		nPD:	0.24
		N. weight:	1.6
		Sc. PD:	0.00075
		Sc. rank:	656.8

PB1

Pos.	339	obs:	exp:
att	I	2766	1020.00
atc	I	12	743.90
ata	I	2	1016.00
gtt	V	16	3.44
gtc	V	0	3.37
gta	V	0	3.41
gtg	V	0	5.78
---	--	------	------
mPD		0.021	0.67
		nPD:	0.03
		N. weight:	1.4
		Sc. PD:	-0.29
		Sc. rank:	-1578.8

PB1

Pos.	340	obs:	exp:
gct	A	210	704.10
gcc	A	2498	540.90
gca	A	80	1244.00
gcg	A	7	305.90
ggt	G	0	0.14
ggc	G	1	0.13
gga	G	0	0.44
ggg	G	0	0.29
---	--	------	------
mPD		0.20	0.69
		nPD:	0.28
		N. weight:	1.7
		Sc. PD:	0.077
		Sc. rank:	1061.8

PB1

Pos.	341	obs:	exp:
cct	P	2612	764.10
ccc	P	155	505.00
cca	P	28	1135.00
ccg	P	1	392.00
---	--	------	------
mPD		0.12	0.71
		nPD:	0.18
		N. weight:	1.5
		Sc. PD:	-0.093
		Sc. rank:	296.7

PB1

Pos.	342	obs:	exp:
att	I	1	1024.00
atc	I	0	746.90
ata	I	2790	1020.00
gtt	V	0	1.08
gtc	V	2	1.05
gta	V	3	1.07
gtg	V	0	1.81
---	--	------	------
mPD		0.0057	0.66
		nPD:	0.01
		N. weight:	1.5
		Sc. PD:	-0.33
		Sc. rank:	-2298.9

PB1

Pos.	343	obs:	exp:
atg	M	2796	2796.00
---	--	------	------
mPD		0	0
		nPD:	1.
		N. weight:	0.
		Sc. PD:	0
		Sc. rank:	0

PB1

Pos.	344	obs:	exp:
ttt	F	570	1166.00
ttc	F	2225	1629.00
tta	L	0	0.09
ttg	L	0	0.19
ctt	L	0	0.20
ctc	L	1	0.16
cta	L	0	0.15
ctg	L	0	0.21
---	--	------	------
mPD		0.33	0.49
		nPD:	0.67
		N. weight:	0.15
		Sc. PD:	0.064
		Sc. rank:	209.5

PB1

Pos.	345	obs:	exp:
tct	S	3	463.40
tcc	S	0	402.10
tca	S	2014	629.30
tcg	S	759	167.50
act	T	0	0.26
acc	T	0	0.23
aca	T	1	0.42
acg	T	0	0.09
agt	S	0	602.10
agc	S	0	511.60
gct	A	0	4.79
gcc	A	0	3.68
gca	A	19	8.46
gcg	A	0	2.08
---	--	------	------
mPD		0.41	1.7
		nPD:	0.25
		N. weight:	1.8
		Sc. PD:	0.018
		Sc. rank:	859.2

PB1

Pos.	346	obs:	exp:
aat	N	83	1524.00
aac	N	2711	1270.00
gat	D	0	1.08
gac	D	2	0.92
---	--	------	------
mPD		0.059	0.50
		nPD:	0.12
		N. weight:	0.95
		Sc. PD:	-0.11
		Sc. rank:	-167.4

PB1

Pos.	347	obs:	exp:
aat	N	1	0.55
aac	N	0	0.45
aaa	K	2347	1592.00
aag	K	448	1203.00
---	--	------	------
mPD		0.27	0.49
		nPD:	0.55
		N. weight:	0.25
		Sc. PD:	0.075
		Sc. rank:	284.1

PB1

Pos.	348	obs:	exp:
tta	L	0	16.37
ttg	L	177	33.63
ctt	L	0	35.14
ctc	L	0	28.31
cta	L	0	26.19
ctg	L	0	37.36
atg	M	2614	2614.00
act	T	0	0.26
acc	T	0	0.23
aca	T	0	0.42
acg	T	1	0.09
gtt	V	0	0.65
gtc	V	0	0.63
gta	V	0	0.64
gtg	V	3	1.08
gaa	E	0	0.56
gag	E	1	0.44
---	--	------	------
mPD		0.12	0.20
		nPD:	0.61
		N. weight:	0.16
		Sc. PD:	0.057
		Sc. rank:	198.0

PB1

Pos.	349	obs:	exp:
act	T	0	0.26
acc	T	0	0.23
aca	T	0	0.42
acg	T	1	0.09
gtt	V	0	0.43
gtc	V	0	0.42
gta	V	0	0.43
gtg	V	2	0.72
gct	A	5	703.60
gcc	A	0	540.50
gca	A	986	1243.00
gcg	A	1802	305.70
---	--	------	------
mPD		0.46	0.69
		nPD:	0.67
		N. weight:	1.5
		Sc. PD:	0.64
		Sc. rank:	2105.5

PB1

Pos.	350	obs:	exp:
cgt	R	0	104.40
cgc	R	0	149.40
cga	R	3	281.30
cgg	R	0	227.90
aaa	K	5	2.85
aag	K	0	2.15
aga	R	2697	1303.00
agg	R	91	725.10
---	--	------	------
mPD		0.069	0.96
		nPD:	0.07
		N. weight:	0.92
		Sc. PD:	-0.15
		Sc. rank:	-533.9

PB1			
Pos.	351	obs:	exp:
tta	L	986	258.50
ttg	L	932	531.20
ctt	L	0	555.10
ctc	L	0	447.20
cta	L	797	413.80
ctg	L	81	590.10
---	--	------	------
mPD		0.89	1.1
		nPD:	0.8
		N. weight:	1.2
		Sc. PD:	0.63
		Sc. rank:	1827.4

PB1			
Pos.	352	obs:	exp:
cgt	R	0	0.04
cgc	R	0	0.05
cga	R	0	0.10
cgg	R	0	0.08
aga	R	0	0.47
agg	R	1	0.26
gaa	E	0	6.17
gag	E	11	4.83
ggt	G	0	402.60
ggc	G	0	365.70
gga	G	1406	1220.00
ggg	G	1378	795.80
---	--	------	------
mPD		0.51	0.70
		nPD:	0.73
		N. weight:	0.51
		Sc. PD:	0.24
		Sc. rank:	767.6

PB1			
Pos.	353	obs:	exp:
cgt	R	0	9.73
cgc	R	0	13.91
cga	R	8	26.20
cgg	R	0	21.23
aaa	K	2522	1444.00
aag	K	14	1092.00
aga	R	252	121.40
agg	R	0	67.55
---	--	------	------
mPD		0.18	0.72
		nPD:	0.26
		N. weight:	0.8
		Sc. PD:	0.015
		Sc. rank:	408.9

PB1			
Pos.	354	obs:	exp:
ggt	G	97	404.30
ggc	G	1	367.30
gga	G	1888	1225.00
ggg	G	810	799.20
---	--	------	------
mPD		0.46	0.69
		nPD:	0.67
		N. weight:	0.36
		Sc. PD:	0.15
		Sc. rank:	490.3

PB1			
Pos.	355	obs:	exp:
tat	Y	184	1340.00
tac	Y	2610	1454.00
aat	N	0	1.09
aac	N	2	0.91
---	--	------	------
mPD		0.12	0.50
		nPD:	0.25
		N. weight:	0.61
		Sc. PD:	0.0062
		Sc. rank:	298.2

PB1			
Pos.	356	obs:	exp:
atg	M	2796	2796.00
---	--	------	------
mPD		0	0
		nPD:	1.
		N. weight:	0.
		Sc. PD:	0
		Sc. rank:	0

PB1			
Pos.	357	obs:	exp:
ttt	F	1040	1166.00
ttc	F	1756	1630.00
---	--	------	------
mPD		0.47	0.49
		nPD:	0.96
		N. weight:	0.0084
		Sc. PD:	0.0059
		Sc. rank:	13.7

PB1			
Pos.	358	obs:	exp:
aaa	K	2	1.14
aag	K	0	0.86
gtt	V	0	0.22
gtc	V	0	0.21
gta	V	1	0.21
gtg	V	0	0.36
gct	A	0	2.52
gcc	A	0	1.94
gca	A	10	4.45
gcg	A	0	1.09
gaa	E	2665	1560.00
gag	E	118	1223.00
---	--	------	------
mPD		0.090	0.50
		nPD:	0.18
		N. weight:	0.61
		Sc. PD:	-0.035
		Sc. rank:	124.6

PB1			
Pos.	359	obs:	exp:
tct	S	0	466.70
tcc	S	0	405.00
tca	S	0	633.90
tcg	S	0	168.70
agt	S	2616	606.50
agc	S	180	515.30
---	--	------	------
mPD		0.12	1.7
		nPD:	0.07
		N. weight:	1.9
		Sc. PD:	-0.3
		Sc. rank:	-1083.4

PB1			
Pos.	360	obs:	exp:
cgt	R	0	0.15
cgc	R	0	0.21
cga	R	0	0.40
cgg	R	0	0.33
act	T	0	0.26
acc	T	0	0.23
aca	T	0	0.42
acg	T	1	0.09
aat	N	5	2.73
aac	N	0	2.27
aaa	K	58	1584.00
aag	K	2724	1198.00
aga	R	0	1.87
agg	R	4	1.04
gaa	E	0	2.24
gag	E	4	1.76
---	--	------	------
mPD		0.051	0.50
		nPD:	0.1
		N. weight:	1.1
		Sc. PD:	-0.14
		Sc. rank:	-335.1

PB1			
Pos.	361	obs:	exp:
tct	S	0	462.90
tcc	S	0	401.70
tca	S	0	628.60
tcg	S	0	167.30
att	I	0	0.37
atc	I	1	0.27
ata	I	0	0.37
aat	N	0	12.00
aac	N	22	10.00
agt	S	36	601.50
agc	S	2737	511.00
---	--	------	------
mPD		0.042	1.7
		nPD:	0.03
		N. weight:	2.4
		Sc. PD:	-0.49
		Sc. rank:	-2919.5

PB1			
Pos.	362	obs:	exp:
tta	L	0	0.09
ttg	L	1	0.19
ctt	L	0	0.20
ctc	L	0	0.16
cta	L	0	0.15
ctg	L	0	0.21
atg	M	2795	2795.00
---	--	------	------
mPD		0.00072	0.0011
		nPD:	0.63
		N. weight:	0.00087
		Sc. PD:	0.00033
		Sc. rank:	1.1

PB1			
Pos.	363	obs:	exp:
cgt	R	0	5.69
cgc	R	0	8.13
cga	R	0	15.32
cgg	R	11	12.41
atg	M	2	2.00
aaa	K	263	1504.00
aag	K	2379	1138.00
aga	R	9	70.96
agg	R	132	39.49
---	--	------	------
mPD		0.29	0.63
		nPD:	0.46
		N. weight:	0.75
		Sc. PD:	0.16
		Sc. rank:	771.8

PB1			
Pos.	364	obs:	exp:
tta	L	1904	257.90
ttg	L	427	529.80
ctt	L	51	553.80
ctc	L	18	446.10
cta	L	374	412.80
ctg	L	15	588.70
cct	P	0	0.27
ccc	P	0	0.18
cca	P	1	0.41
ccg	P	0	0.14
att	I	0	2.20
atc	I	0	1.61
ata	I	6	2.19
---	--	------	------
mPD		0.59	1.1
		nPD:	0.52
		N. weight:	1.8
		Sc. PD:	0.5
		Sc. rank:	2037.8

PB1			
Pos.	365	obs:	exp:
cgt	R	2	104.60
cgc	R	0	149.60
cga	R	1660	281.70
cgg	R	838	228.20
aga	R	15	1305.00
agg	R	280	726.10
ggt	G	0	0.14
ggc	G	0	0.13
gga	G	1	0.44
ggg	G	0	0.29
---	--	------	------
mPD		0.67	0.95
		nPD:	0.7
		N. weight:	1.9
		Sc. PD:	0.87
		Sc. rank:	2816.8

PB1			
Pos.	366	obs:	exp:
att	I	0	0.37
atc	I	0	0.27
ata	I	1	0.37
act	T	0	732.40
acc	T	2	629.60
aca	T	2649	1169.00
acg	T	143	262.90
gct	A	0	0.25
gcc	A	0	0.19
gca	A	1	0.45
gcg	A	0	0.11
---	--	------	------
mPD		0.10	0.70
		nPD:	0.14
		N. weight:	1.1
		Sc. PD:	-0.1
		Sc. rank:	28.1

PB1			
Pos.	367	obs:	exp:
cct	P	0	0.27
ccc	P	0	0.18
cca	P	1	0.41
ccg	P	0	0.14
cat	H	1	0.58
cac	H	0	0.42
caa	Q	2584	1394.00
cag	Q	209	1399.00
cgt	R	0	0.04
cgc	R	0	0.05
cga	R	1	0.10
cgg	R	0	0.08
aga	R	0	0.47
agg	R	0	0.26
---	--	------	------
mPD		0.14	0.50
		nPD:	0.28
		N. weight:	0.63
		Sc. PD:	0.025
		Sc. rank:	373.9

PB1			
Pos.	368	obs:	exp:
att	I	9	1008.00
atc	I	3	735.10
ata	I	2735	1004.00
atg	M	1	1.00
act	T	0	0.52
acc	T	0	0.45
aca	T	2	0.84
acg	T	0	0.19
gtt	V	0	9.89
gtc	V	0	9.68
gta	V	46	9.81
gtg	V	0	16.62
---	--	------	------
mPD		0.043	0.70
		nPD:	0.06
		N. weight:	1.4
		Sc. PD:	-0.25
		Sc. rank:	-977.4

PB1			
Pos.	369	obs:	exp:
tct	S	0	0.50
tcc	S	0	0.43
tca	S	1	0.68
tcg	S	2	0.18
cct	P	757	763.00
ccc	P	1	504.30
cca	P	1782	1133.00
ccg	P	252	391.40
act	T	1	0.26
acc	T	0	0.23
aca	T	0	0.42
acg	T	0	0.09
agt	S	0	0.65
agc	S	0	0.55
---	--	------	------
mPD		0.51	0.71
		nPD:	0.72
		N. weight:	0.36
		Sc. PD:	0.17
		Sc. rank:	550.5

PB1			
Pos.	370	obs:	exp:
tct	S	0	3.51
tcc	S	0	3.04
tca	S	21	4.76
tcg	S	0	1.27
act	T	0	0.26
acc	T	0	0.23
aca	T	1	0.42
acg	T	0	0.09
agt	S	0	4.55
agc	S	0	3.87
gct	A	4	698.80
gcc	A	0	536.80
gca	A	2733	1235.00
gcg	A	37	303.60
---	--	------	------
mPD		0.045	0.71
		nPD:	0.06
		N. weight:	1.1
		Sc. PD:	-0.19
		Sc. rank:	-728.7

PB1			
Pos.	371	obs:	exp:
gaa	E	2557	1567.00
gag	E	239	1229.00
---	--	------	------
mPD		0.16	0.49
		nPD:	0.32
		N. weight:	0.45
		Sc. PD:	0.035
		Sc. rank:	320.5

PB1			
Pos.	372	obs:	exp:
tta	L	0	0.92
ttg	L	9	1.90
ctt	L	0	1.99
ctc	L	0	1.60
cta	L	0	1.48
ctg	L	1	2.11
cgt	R	0	0.04
cgc	R	0	0.05
cga	R	0	0.10
cgg	R	0	0.08
att	I	0	132.80
atc	I	0	96.87
ata	I	362	132.30
atg	M	2377	2377.00
act	T	0	0.79
acc	T	0	0.68
aca	T	3	1.26
acg	T	0	0.28
aga	R	0	0.47
agg	R	1	0.26
gtt	V	0	9.25
gtc	V	0	9.05
gta	V	1	9.17
gtg	V	42	15.54
---	--	------	------
mPD		0.27	0.30
		nPD:	0.9
		N. weight:	0.22
		Sc. PD:	0.14
		Sc. rank:	357.7

PB1			
Pos.	373	obs:	exp:
tta	L	0	257.50
ttg	L	0	529.10
ctt	L	2633	553.00
ctc	L	79	445.50
cta	L	66	412.20
ctg	L	7	587.80
att	I	11	4.04
atc	I	0	2.94
ata	I	0	4.02
---	--	------	------
mPD		0.11	1.1
		nPD:	0.1
		N. weight:	2.
		Sc. PD:	-0.27
		Sc. rank:	-633.1

PB1			
Pos.	374	obs:	exp:
tct	S	0	0.83
tcc	S	0	0.72
tca	S	5	1.13
tcg	S	0	0.30
act	T	0	3.41
acc	T	0	2.93
aca	T	13	5.44
acg	T	0	1.22
agt	S	0	1.08
agc	S	0	0.92
gtt	V	0	0.43
gtc	V	0	0.42
gta	V	2	0.43
gtg	V	0	0.72
gct	A	1	697.60
gcc	A	0	535.80
gca	A	2750	1233.00
gcg	A	18	303.10
gaa	E	4	3.92
gag	E	3	3.08
---	--	------	------
mPD		0.035	0.71
		nPD:	0.05
		N. weight:	1.1
		Sc. PD:	-0.21
		Sc. rank:	-928.3

PB1			
Pos.	375	obs:	exp:
tct	S	0	14.02
tcc	S	0	12.17
tca	S	0	19.04
tcg	S	0	5.07
cat	H	0	1.16
cac	H	2	0.84
act	T	0	10.49
acc	T	40	9.01
aca	T	0	16.74
acg	T	0	3.76
aat	N	58	1451.00
aac	N	2603	1210.00
aaa	K	4	2.28
aag	K	0	1.72
agt	S	29	18.22
agc	S	55	15.48
gat	D	1	2.70
gac	D	4	2.30
---	--	------	------
mPD		0.15	0.64
		nPD:	0.24
		N. weight:	1.
		Sc. PD:	0.0036
		Sc. rank:	459.8

PB1			
Pos.	376	obs:	exp:
att	I	2756	1025.00
atc	I	35	747.10
ata	I	1	1020.00
atg	M	1	1.00
gtt	V	3	0.65
gtc	V	0	0.63
gta	V	0	0.64
gtg	V	0	1.08
---	--	------	------
mPD		0.028	0.66
		nPD:	0.04
		N. weight:	1.4
		Sc. PD:	-0.26
		Sc. rank:	-1222.7

PB1			
Pos.	377	obs:	exp:
act	T	0	0.26
acc	T	1	0.23
aca	T	0	0.42
acg	T	0	0.09
aat	N	8	5.45
aac	N	2	4.55
gat	D	1542	1500.00
gac	D	1237	1279.00
gaa	E	1	0.56
gag	E	0	0.44
ggt	G	5	0.72
ggc	G	0	0.66
gga	G	0	2.19
ggg	G	0	1.43
---	--	------	------
mPD		0.51	0.51
		nPD:	0.99
		N. weight:	0.01
		Sc. PD:	0.0076
		Sc. rank:	17.0

PB1			
Pos.	378	obs:	exp:
ttt	F	1	0.42
ttc	F	0	0.58
tta	L	755	258.40
ttg	L	254	531.00
ctt	L	1313	554.90
ctc	L	283	447.10
cta	L	83	413.60
ctg	L	107	589.90
---	--	------	------
mPD		1.1	1.1
		nPD:	1.
		N. weight:	0.69
		Sc. PD:	0.51
		Sc. rank:	1141.5

PB1			
Pos.	379	obs:	exp:
cgt	R	0	0.11
cgc	R	0	0.16
cga	R	0	0.30
cgg	R	0	0.24
att	I	0	0.37
atc	I	0	0.27
ata	I	1	0.37
aaa	K	2522	1590.00
aag	K	270	1202.00
aga	R	3	1.40
agg	R	0	0.78
---	--	------	------
mPD		0.18	0.49
		nPD:	0.36
		N. weight:	0.4
		Sc. PD:	0.047
		Sc. rank:	328.4

PB1			
Pos.	380	obs:	exp:
tat	Y	354	1340.00
tac	Y	2441	1455.00
aat	N	0	0.55
aac	N	1	0.45
---	--	------	------
mPD		0.22	0.50
		nPD:	0.44
		N. weight:	0.42
		Sc. PD:	0.083
		Sc. rank:	412.9

PB1			
Pos.	381	obs:	exp:
ttt	F	14	1166.00
ttc	F	2781	1629.00
tct	S	0	0.17
tcc	S	1	0.14
tca	S	0	0.23
tcg	S	0	0.06
agt	S	0	0.22
agc	S	0	0.18
---	--	------	------
mPD		0.011	0.49
		nPD:	0.02
		N. weight:	0.75
		Sc. PD:	-0.16
		Sc. rank:	-950.6

PB1			
Pos.	382	obs:	exp:
tct	S	1	0.17
tcc	S	0	0.14
tca	S	0	0.23
tcg	S	0	0.06
aat	N	2547	1524.00
aac	N	248	1271.00
agt	S	0	0.22
agc	S	0	0.18
---	--	------	------
mPD		0.16	0.50
		nPD:	0.33
		N. weight:	0.47
		Sc. PD:	0.042
		Sc. rank:	358.5

PB1			
Pos.	383	obs:	exp:
aaa	K	18	10.25
aag	K	0	7.75
gat	D	4	56.69
gac	D	101	48.31
gaa	E	2517	1497.00
gag	E	154	1174.00
ggt	G	0	0.29
ggc	G	0	0.26
gga	G	2	0.88
ggg	G	0	0.57
---	--	------	------
mPD		0.19	0.54
		nPD:	0.34
		N. weight:	0.56
		Sc. PD:	0.057
		Sc. rank:	444.6

PB1			
Pos.	384	obs:	exp:
tta	L	1136	110.70
ttg	L	56	227.40
tct	S	0	215.30
tcc	S	0	186.90
tca	S	1287	292.40
tcg	S	3	77.83
ctt	L	0	237.70
ctc	L	0	191.50
cta	L	4	177.10
ctg	L	1	252.60
cct	P	0	2.19
ccc	P	0	1.44
cca	P	7	3.25
ccg	P	1	1.12
att	I	0	64.95
atc	I	0	47.36
ata	I	177	64.68
atg	M	1	1.00
act	T	1	30.67
acc	T	0	26.36
aca	T	114	48.96
acg	T	2	11.01
agt	S	0	279.80
agc	S	0	237.70
gtt	V	0	0.65
gtc	V	0	0.63
gta	V	2	0.64
gtg	V	1	1.08
gct	A	0	0.76
gcc	A	0	0.58
gca	A	3	1.34
gcg	A	0	0.33
---	--	------	------
mPD		0.75	2.0
		nPD:	0.37
		N. weight:	2.5
		Sc. PD:	0.31
		Sc. rank:	2114.3

PB1			
Pos.	385	obs:	exp:
cct	P	0	0.27
ccc	P	0	0.18
cca	P	0	0.41
ccg	P	1	0.14
act	T	44	732.70
acc	T	2	629.80
aca	T	1065	1170.00
acg	T	1684	263.00
---	--	------	------
mPD		0.49	0.70
		nPD:	0.71
		N. weight:	1.5
		Sc. PD:	0.69
		Sc. rank:	2223.7

PB1

Pos.	386	obs:	exp:
caa	Q	3	1.50
cag	Q	0	1.50
cgt	R	0	42.77
cgc	R	0	61.17
cga	R	0	115.20
cgg	R	0	93.33
aaa	K	1634	939.50
aag	K	16	710.50
aga	R	972	533.60
agg	R	171	296.90
---	--	------	------
mPD		0.61	1.2
		nPD:	0.51
		N. weight:	0.7
		Sc. PD:	0.18
		Sc. rank:	767.5

PB1

Pos.	387	obs:	exp:
cgt	R	0	20.40
cgc	R	0	29.17
cga	R	0	54.92
cgg	R	0	44.50
atg	M	5	5.00
aaa	K	4	1266.00
aag	K	2219	957.20
aga	R	3	254.40
agg	R	542	141.60
gaa	E	4	11.21
gag	E	16	8.79
ggt	G	0	0.43
ggc	G	0	0.39
gga	G	0	1.31
ggg	G	3	0.86
---	--	------	------
mPD		0.34	0.94
		nPD:	0.36
		N. weight:	1.3
		Sc. PD:	0.16
		Sc. rank:	1117.6

PB1

Pos.	388	obs:	exp:
cgt	R	0	0.19
cgc	R	0	0.27
cga	R	0	0.50
cgg	R	0	0.41
act	T	0	0.26
acc	T	0	0.23
aca	T	1	0.42
acg	T	0	0.09
aaa	K	2773	1589.00
aag	K	17	1201.00
aga	R	5	2.33
agg	R	0	1.30
---	--	------	------
mPD		0.016	0.50
		nPD:	0.03
		N. weight:	0.77
		Sc. PD:	-0.15
		Sc. rank:	-838.5

PB1

Pos.	389	obs:	exp:
att	I	1635	1024.00
atc	I	1084	746.90
ata	I	72	1020.00
gtt	V	2	1.08
gtc	V	3	1.05
gta	V	0	1.07
gtg	V	0	1.81
---	--	------	------
mPD		0.51	0.66
		nPD:	0.77
		N. weight:	0.52
		Sc. PD:	0.27
		Sc. rank:	799.8

PB1

Pos.	390	obs:	exp:
aaa	K	1	2.85
aag	K	4	2.15
gct	A	0	0.25
gcc	A	0	0.19
gca	A	0	0.45
gcg	A	1	0.11
gaa	E	1082	1561.00
gag	E	1703	1224.00
ggt	G	0	0.72
ggc	G	0	0.66
gga	G	4	2.19
ggg	G	1	1.43
---	--	------	------
mPD		0.48	0.50
		nPD:	0.96
		N. weight:	0.093
		Sc. PD:	0.066
		Sc. rank:	152.0

PB1

Pos.	391	obs:	exp:
cgt	R	0	9.99
cgc	R	0	14.29
cga	R	0	26.91
cgg	R	0	21.80
act	T	0	1.05
acc	T	0	0.90
aca	T	4	1.67
acg	T	0	0.38
aaa	K	1640	1437.00
aag	K	884	1087.00
aga	R	9	124.60
agg	R	258	69.36
gaa	E	1	0.56
gag	E	0	0.44
---	--	------	------
mPD		0.66	0.73
		nPD:	0.91
		N. weight:	0.19
		Sc. PD:	0.12
		Sc. rank:	305.3

PB1

Pos.	392	obs:	exp:
att	I	6	1018.00
atc	I	0	742.30
ata	I	2768	1014.00
gtt	V	0	4.73
gtc	V	0	4.63
gta	V	22	4.69
gtg	V	0	7.95
---	--	------	------
mPD		0.020	0.68
		nPD:	0.03
		N. weight:	1.5
		Sc. PD:	-0.3
		Sc. rank:	-1694.7

PB1

Pos.	393	obs:	exp:
cgt	R	0	104.40
cgc	R	0	149.30
cga	R	3	281.20
cgg	R	0	227.80
aaa	K	6	3.42
aag	K	0	2.58
aga	R	1841	1302.00
agg	R	946	724.80
---	--	------	------
mPD		0.45	0.96
		nPD:	0.47
		N. weight:	0.46
		Sc. PD:	0.11
		Sc. rank:	483.5

PB1

Pos.	394	obs:	exp:
tta	L	0	0.09
ttg	L	0	0.19
tct	S	6	1.00
tcc	S	0	0.87
tca	S	0	1.36
tcg	S	0	0.36
ctt	L	1	0.20
ctc	L	0	0.16
cta	L	0	0.15
ctg	L	0	0.21
cct	P	2247	759.70
ccc	P	532	502.10
cca	P	1	1128.00
ccg	P	0	389.80
cat	H	9	5.21
cac	H	0	3.79
agt	S	0	1.30
agc	S	0	1.11
---	--	------	------
mPD		0.32	0.72
		nPD:	0.44
		N. weight:	1.3
		Sc. PD:	0.26
		Sc. rank:	1283.8

PB1

Pos.	395	obs:	exp:
tta	L	28	258.40
ttg	L	4	531.00
ctt	L	253	554.90
ctc	L	85	447.10
cta	L	2230	413.60
ctg	L	195	589.90
caa	Q	0	0.50
cag	Q	1	0.50
---	--	------	------
mPD		0.36	1.1
		nPD:	0.32
		N. weight:	1.6
		Sc. PD:	0.13
		Sc. rank:	1174.8

PB1

Pos.	396	obs:	exp:
tta	L	1579	258.50
ttg	L	47	531.20
ctt	L	6	555.10
ctc	L	0	447.20
cta	L	941	413.80
ctg	L	223	590.10
---	--	------	------
mPD		0.67	1.1
		nPD:	0.59
		N. weight:	1.7
		Sc. PD:	0.59
		Sc. rank:	2108.5

PB1

Pos.	397	obs:	exp:
tta	L	67	6.19
ttg	L	0	12.73
ctt	L	0	13.30
ctc	L	0	10.72
cta	L	0	9.92
ctg	L	0	14.14
cgt	R	0	0.04
cgc	R	0	0.05
cga	R	0	0.10
cgg	R	0	0.08
att	I	245	992.60
atc	I	7	723.80
ata	I	2453	988.50
atg	M	18	18.00
aaa	K	0	0.57
aag	K	1	0.43
aga	R	1	0.47
agg	R	0	0.26
gtt	V	1	0.86
gtc	V	0	0.84
gta	V	3	0.85
gtg	V	0	1.45
---	--	------	------
mPD		0.23	0.72
		nPD:	0.32
		N. weight:	1.1
		Sc. PD:	0.08
		Sc. rank:	743.0

PB1

Pos.	398	obs:	exp:
tct	S	0	2.84
tcc	S	0	2.46
tca	S	0	3.85
tcg	S	0	1.03
aat	N	50	27.27
aac	N	0	22.73
agt	S	17	3.69
agc	S	0	3.13
gat	D	2652	1449.00
gac	D	31	1234.00
gaa	E	32	22.98
gag	E	9	18.02
ggt	G	5	0.72
ggc	G	0	0.66
gga	G	0	2.19
ggg	G	0	1.43
---	--	------	------
mPD		0.11	0.58
		nPD:	0.2
		N. weight:	0.82
		Sc. PD:	-0.033
		Sc. rank:	199.5

PB1

Pos.	399	obs:	exp:
tgt	C	1	0.39
tgc	C	0	0.61
gat	D	1	0.54
gac	D	0	0.46
ggt	G	1730	404.10
ggc	G	1040	367.00
gga	G	4	1224.00
ggg	G	20	798.70
---	--	------	------
mPD		0.48	0.69
		nPD:	0.7
		N. weight:	1.8
		Sc. PD:	0.81
		Sc. rank:	2628.4

PB1

Pos.	400	obs:	exp:
act	T	722	726.40
acc	T	23	624.40
aca	T	2000	1159.00
acg	T	26	260.70
aat	N	1	0.55
aac	N	0	0.45
gct	A	1	6.05
gcc	A	0	4.64
gca	A	23	10.68
gcg	A	0	2.63
---	--	------	------
mPD		0.43	0.71
		nPD:	0.6
		N. weight:	0.51
		Sc. PD:	0.18
		Sc. rank:	628.0

PB1			
Pos.	401	obs:	exp:
cct	P	0	0.27
ccc	P	1	0.18
cca	P	0	0.41
ccg	P	0	0.14
gtt	V	0	0.65
gtc	V	3	0.63
gta	V	0	0.64
gtg	V	0	1.08
gct	A	29	703.10
gcc	A	2752	540.10
gca	A	10	1242.00
gcg	A	0	305.50
gat	D	0	0.54
gac	D	1	0.46
---	--	------	------
mPD		0.031	0.69
		nPD:	0.04
		N. weight:	2.3
		Sc. PD:	-0.43
		Sc. rank:	-1995.6

,

PB1			
Pos.	402	obs:	exp:
tct	S	15	466.70
tcc	S	182	405.00
tca	S	2510	633.90
tcg	S	89	168.70
agt	S	0	606.50
agc	S	0	515.30
---	--	------	------
mPD		0.19	1.7
		nPD:	0.11
		N. weight:	1.7
		Sc. PD:	-0.2
		Sc. rank:	-364.1

,

PB1			
Pos.	403	obs:	exp:
tta	L	38	258.50
ttg	L	2652	531.20
ctt	L	0	555.10
ctc	L	0	447.20
cta	L	8	413.80
ctg	L	98	590.10
---	--	------	------
mPD		0.11	1.1
		nPD:	0.09
		N. weight:	2.1
		Sc. PD:	-0.29
		Sc. rank:	-734.2

,

PB1			
Pos.	404	obs:	exp:
tct	S	0	466.40
tcc	S	0	404.70
tca	S	0	633.40
tcg	S	0	168.60
cgt	R	0	0.04
cgc	R	0	0.05
cga	R	0	0.10
cgg	R	0	0.08
act	T	0	0.26
acc	T	1	0.23
aca	T	0	0.42
acg	T	0	0.09
agt	S	1051	606.00
agc	S	1743	514.90
aga	R	0	0.47
agg	R	1	0.26
---	--	------	------
mPD		0.47	1.7
		nPD:	0.29
		N. weight:	1.4
		Sc. PD:	0.065
		Sc. rank:	886.7

,

PB1			
Pos.	405	obs:	exp:
cct	P	2661	763.80
ccc	P	16	504.80
cca	P	39	1134.00
ccg	P	79	391.90
cat	H	1	0.58
cac	H	0	0.42
---	--	------	------
mPD		0.093	0.71
		nPD:	0.13
		N. weight:	1.6
		Sc. PD:	-0.16
		Sc. rank:	-106.1

,

PB1			
Pos.	406	obs:	exp:
gtt	V	0	0.22
gtc	V	0	0.21
gta	V	1	0.21
gtg	V	0	0.36
gaa	E	1	0.56
gag	E	0	0.44
ggt	G	4	404.10
ggc	G	1	367.00
gga	G	2726	1224.00
ggg	G	63	798.70
---	--	------	------
mPD		0.049	0.69
		nPD:	0.07
		N. weight:	1.
		Sc. PD:	-0.17
		Sc. rank:	-618.8

,

PB1			
Pos.	407	obs:	exp:
atg	M	2796	2796.00
---	--	------	------
mPD		0	0
		nPD:	1.
		N. weight:	0.
		Sc. PD:	0
		Sc. rank:	0

,

PB1			
Pos.	408	obs:	exp:
att	I	0	0.37
atc	I	1	0.27
ata	I	0	0.37
atg	M	2795	2795.00
---	--	------	------
mPD		0.00072	0.00072
		nPD:	1.
		N. weight:	0.00069
		Sc. PD:	0.00051
		Sc. rank:	1.1

,

PB1			
Pos.	409	obs:	exp:
cgt	R	0	0.04
cgc	R	0	0.05
cga	R	0	0.10
cgg	R	0	0.08
att	I	0	0.37
atc	I	0	0.27
ata	I	1	0.37
atg	M	2793	2793.00
act	T	0	0.26
acc	T	0	0.23
aca	T	0	0.42
acg	T	1	0.09
aga	R	0	0.47
agg	R	1	0.26
---	--	------	------
mPD		0.0021	0.0035
		nPD:	0.62
		N. weight:	0.0025
		Sc. PD:	0.00092
		Sc. rank:	3.2

,

PB1			
Pos.	410	obs:	exp:
ggt	G	48	404.30
ggc	G	2741	367.30
gga	G	6	1225.00
ggg	G	1	799.20
---	--	------	------
mPD		0.039	0.69
		nPD:	0.06
		N. weight:	2.8
		Sc. PD:	-0.5
		Sc. rank:	-2034.6

,

PB1			
Pos.	411	obs:	exp:
att	I	0	0.37
atc	I	0	0.27
ata	I	1	0.37
atg	M	2795	2795.00
---	--	------	------
mPD		0.00072	0.00072
		nPD:	1.
		N. weight:	0.00053
		Sc. PD:	0.00039
		Sc. rank:	0.9

,

PB1			
Pos.	412	obs:	exp:
ttt	F	11	1166.00
ttc	F	2784	1629.00
tta	L	1	0.09
ttg	L	0	0.19
ctt	L	0	0.20
ctc	L	0	0.16
cta	L	0	0.15
ctg	L	0	0.21
---	--	------	------
mPD		0.0086	0.49
		nPD:	0.02
		N. weight:	0.75
		Sc. PD:	-0.16
		Sc. rank:	-1018.7

,

PB1			
Pos.	413	obs:	exp:
aat	N	999	1525.00
aac	N	1797	1271.00
---	--	------	------
mPD		0.46	0.50
		nPD:	0.93
		N. weight:	0.11
		Sc. PD:	0.071
		Sc. rank:	173.7

,

PB1			
Pos.	414	obs:	exp:
att	I	0	0.37
atc	I	0	0.27
ata	I	1	0.37
atg	M	2795	2795.00
---	--	------	------
mPD		0.00072	0.00072
		nPD:	1.
		N. weight:	0.00053
		Sc. PD:	0.00039
		Sc. rank:	0.9

,

PB1			
Pos.	415	obs:	exp:
tta	L	7	258.40
ttg	L	27	531.00
ctt	L	3	554.90
ctc	L	0	447.10
cta	L	57	413.60
ctg	L	2701	589.90
cct	P	0	0.27
ccc	P	0	0.18
cca	P	0	0.41
ccg	P	1	0.14
---	--	------	------
mPD		0.072	1.1
		nPD:	0.06
		N. weight:	2.
		Sc. PD:	-0.34
		Sc. rank:	-1324.4

,

PB1			
Pos.	416	obs:	exp:
tct	S	0	466.70
tcc	S	0	405.00
tca	S	0	633.90
tcg	S	0	168.70
agt	S	2504	606.50
agc	S	292	515.30
---	--	------	------
mPD		0.19	1.7
		nPD:	0.11
		N. weight:	1.8
		Sc. PD:	-0.21
		Sc. rank:	-428.1

,

PB1			
Pos.	417	obs:	exp:
act	T	14	733.00
acc	T	8	630.00
aca	T	2583	1170.00
acg	T	191	263.10
---	--	------	------
mPD		0.14	0.70
		nPD:	0.2
		N. weight:	0.99
		Sc. PD:	-0.033
		Sc. rank:	275.6

,

PB1			
Pos.	418	obs:	exp:
att	I	0	0.73
atc	I	0	0.54
ata	I	2	0.73
gtt	V	481	600.90
gtc	V	1322	587.80
gta	V	969	595.80
gtg	V	22	1009.00
---	--	------	------
mPD		0.63	0.73
		nPD:	0.85
		N. weight:	0.71
		Sc. PD:	0.43
		Sc. rank:	1143.1

,

PB1			
Pos.	419	obs:	exp:
tta	L	1080	258.40
ttg	L	140	531.00
ctt	L	3	554.90
ctc	L	0	447.10
cta	L	1289	413.60
ctg	L	283	589.90
cgt	R	0	0.04
cgc	R	0	0.05
cga	R	0	0.10
cgg	R	1	0.08
aga	R	0	0.47
agg	R	0	0.26
---	--	------	------
mPD		0.75	1.1
		nPD:	0.67
		N. weight:	1.4
		Sc. PD:	0.57
		Sc. rank:	1892.8

,

PB1			
Pos.	420	obs:	exp:
ggt	G	5	404.30
ggc	G	0	367.30
gga	G	2544	1225.00
ggg	G	247	799.20
---	--	------	------
mPD		0.16	0.69
		nPD:	0.24
		N. weight:	0.81
		Sc. PD:	0.00067
		Sc. rank:	344.6

,

PB1			
Pos.	421	obs:	exp:
gtt	V	1768	601.40
gtc	V	940	588.20
gta	V	72	596.20
gtg	V	16	1010.00
---	--	------	------
mPD		0.49	0.73
		nPD:	0.66
		N. weight:	1.1
		Sc. PD:	0.46
		Sc. rank:	1505.6

PB1			
Pos.	422	obs:	exp:
tct	S	0	465.50
tcc	S	0	404.00
tca	S	2502	632.30
tcg	S	287	168.30
act	T	0	1.84
acc	T	0	1.58
aca	T	7	2.93
acg	T	0	0.66
agt	S	0	604.90
agc	S	0	514.00
---	--	------	------
mPD		0.19	1.7
		nPD:	0.11
		N. weight:	1.9
		Sc. PD:	-0.23
		Sc. rank:	-393.5

PB1			
Pos.	423	obs:	exp:
att	I	245	999.20
atc	I	2472	728.70
ata	I	6	995.10
gtt	V	0	15.70
gtc	V	73	15.36
gta	V	0	15.57
gtg	V	0	26.38
---	--	------	------
mPD		0.21	0.72
		nPD:	0.3
		N. weight:	1.4
		Sc. PD:	0.086
		Sc. rank:	954.8

PB1			
Pos.	424	obs:	exp:
tta	L	5	258.40
ttg	L	303	531.00
ctt	L	36	554.90
ctc	L	1	447.10
cta	L	904	413.60
ctg	L	1546	589.90
cct	P	0	0.27
ccc	P	0	0.18
cca	P	0	0.41
ccg	P	1	0.14
---	--	------	------
mPD		0.65	1.1
		nPD:	0.58
		N. weight:	1.
		Sc. PD:	0.33
		Sc. rank:	1210.4

PB1			
Pos.	425	obs:	exp:
aat	N	2433	1524.00
aac	N	362	1271.00
aaa	K	1	0.57
aag	K	0	0.43
---	--	------	------
mPD		0.23	0.50
		nPD:	0.46
		N. weight:	0.36
		Sc. PD:	0.076
		Sc. rank:	363.4

PB1			
Pos.	426	obs:	exp:
tta	L	1	257.90
ttg	L	0	529.80
ctt	L	2601	553.80
ctc	L	115	446.10
cta	L	68	412.80
ctg	L	4	588.70
att	I	7	2.57
atc	I	0	1.87
ata	I	0	2.56
---	--	------	------
mPD		0.13	1.1
		nPD:	0.12
		N. weight:	1.9
		Sc. PD:	-0.23
		Sc. rank:	-352.1

PB1			
Pos.	427	obs:	exp:
ggt	G	1	404.30
ggc	G	6	367.30
gga	G	1704	1225.00
ggg	G	1085	799.20
---	--	------	------
mPD		0.48	0.69
		nPD:	0.69
		N. weight:	0.45
		Sc. PD:	0.2
		Sc. rank:	647.5

PB1			
Pos.	428	obs:	exp:
caa	Q	1082	1395.00
cag	Q	1713	1400.00
aaa	K	1	0.57
aag	K	0	0.43
---	--	------	------
mPD		0.48	0.50
		nPD:	0.95
		N. weight:	0.039
		Sc. PD:	0.027
		Sc. rank:	64.6

PB1			
Pos.	429	obs:	exp:
cgt	R	0	3.22
cgc	R	0	4.60
cga	R	0	8.67
cgg	R	0	7.02
aaa	K	1544	1543.00
aag	K	1166	1167.00
aga	R	2	40.15
agg	R	84	22.34
---	--	------	------
mPD		0.55	0.57
		nPD:	0.97
		N. weight:	0.058
		Sc. PD:	0.041
		Sc. rank:	94.9

PB1			
Pos.	430	obs:	exp:
cgt	R	0	86.41
cgc	R	0	123.60
cga	R	0	232.70
cgg	R	1	188.50
atg	M	2	2.00
aat	N	27	14.72
aac	N	0	12.28
aaa	K	19	260.20
aag	K	438	196.80
aga	R	187	1078.00
agg	R	2121	599.90
ggt	G	0	0.14
ggc	G	0	0.13
gga	G	0	0.44
ggg	G	1	0.29
---	--	------	------
mPD		0.44	1.2
		nPD:	0.37
		N. weight:	1.4
		Sc. PD:	0.18
		Sc. rank:	1205.1

PB1			
Pos.	431	obs:	exp:
tat	Y	16	1334.00
tac	Y	2766	1448.00
cat	H	0	8.10
cac	H	14	5.90
---	--	------	------
mPD		0.021	0.51
		nPD:	0.04
		N. weight:	0.91
		Sc. PD:	-0.17
		Sc. rank:	-841.5

PB1			
Pos.	432	obs:	exp:
act	T	1	733.00
acc	T	2782	630.00
aca	T	12	1170.00
acg	T	1	263.10
---	--	------	------
mPD		0.010	0.70
		nPD:	0.01
		N. weight:	2.1
		Sc. PD:	-0.46
		Sc. rank:	-3102.6

PB1			
Pos.	433	obs:	exp:
caa	Q	1	0.50
cag	Q	0	0.50
cgt	R	0	0.19
cgc	R	0	0.27
cga	R	0	0.50
cgg	R	0	0.41
aaa	K	2769	1589.00
aag	K	21	1201.00
aga	R	4	2.33
agg	R	1	1.30
---	--	------	------
mPD		0.020	0.50
		nPD:	0.04
		N. weight:	0.76
		Sc. PD:	-0.15
		Sc. rank:	-745.7

PB1			
Pos.	434	obs:	exp:
cct	P	0	0.27
ccc	P	1	0.18
cca	P	0	0.41
ccg	P	0	0.14
act	T	49	732.70
acc	T	2658	629.80
aca	T	88	1170.00
acg	T	0	263.00
---	--	------	------
mPD		0.095	0.70
		nPD:	0.14
		N. weight:	1.8
		Sc. PD:	-0.18
		Sc. rank:	-45.2

PB1			
Pos.	435	obs:	exp:
cct	P	0	0.27
ccc	P	0	0.18
cca	P	1	0.41
ccg	P	0	0.14
att	I	0	6.97
atc	I	0	5.08
ata	I	19	6.94
act	T	1	727.70
acc	T	8	625.50
aca	T	2741	1162.00
acg	T	26	261.20
---	--	------	------
mPD		0.039	0.71
		nPD:	0.05
		N. weight:	1.2
		Sc. PD:	-0.21
		Sc. rank:	-876.7

PB1			
Pos.	436	obs:	exp:
tat	Y	1641	1341.00
tac	Y	1155	1455.00
---	--	------	------
mPD		0.49	0.50
		nPD:	0.97
		N. weight:	0.036
		Sc. PD:	0.026
		Sc. rank:	58.9

PB1			
Pos.	437	obs:	exp:
tgg	W	2795	2795.00
ggt	G	0	0.14
ggc	G	0	0.13
gga	G	0	0.44
ggg	G	1	0.29
---	--	------	------
mPD		0.00072	0.0012
		nPD:	0.58
		N. weight:	0.00065
		Sc. PD:	0.00022
		Sc. rank:	0.8

PB1			
Pos.	438	obs:	exp:
tct	S	0	0.17
tcc	S	0	0.14
tca	S	0	0.23
tcg	S	1	0.06
tgg	W	2794	2794.00
cgt	R	0	0.04
cgc	R	0	0.05
cga	R	0	0.10
cgg	R	1	0.08
agt	S	0	0.22
agc	S	0	0.18
aga	R	0	0.47
agg	R	0	0.26
---	--	------	------
mPD		0.0014	0.0026
		nPD:	0.56
		N. weight:	0.0028
		Sc. PD:	0.00086
		Sc. rank:	3.2

PB1			
Pos.	439	obs:	exp:
gat	D	1165	1510.00
gac	D	1631	1286.00
---	--	------	------
mPD		0.49	0.50
		nPD:	0.98
		N. weight:	0.047
		Sc. PD:	0.034
		Sc. rank:	76.8

PB1			
Pos.	440	obs:	exp:
gaa	E	1	0.56
gag	E	0	0.44
ggt	G	16	404.20
ggc	G	1	367.10
gga	G	1685	1225.00
ggg	G	1093	799.00
---	--	------	------
mPD		0.48	0.69
		nPD:	0.7
		N. weight:	0.43
		Sc. PD:	0.2
		Sc. rank:	632.3

PB1			
Pos.	441	obs:	exp:
tta	L	0	258.40
ttg	L	0	531.00
ctt	L	30	554.90
ctc	L	2746	447.10
cta	L	17	413.60
ctg	L	2	589.90
cct	P	1	0.27
ccc	P	0	0.18
cca	P	0	0.41
ccg	P	0	0.14
---	--	------	------
mPD		0.036	1.1
		nPD:	0.03
		N. weight:	2.5
		Sc. PD:	-0.51
		Sc. rank:	-2751.1

,

PB1			
Pos.	442	obs:	exp:
caa	Q	2758	1396.00
cag	Q	38	1400.00
---	--	------	------
mPD		0.027	0.50
		nPD:	0.05
		N. weight:	0.91
		Sc. PD:	-0.16
		Sc. rank:	-676.0

,

PB1			
Pos.	443	obs:	exp:
tct	S	190	466.70
tcc	S	2605	405.00
tca	S	1	633.90
tcg	S	0	168.70
agt	S	0	606.50
agc	S	0	515.30
---	--	------	------
mPD		0.13	1.7
		nPD:	0.08
		N. weight:	2.4
		Sc. PD:	-0.38
		Sc. rank:	-1258.7

,

PB1			
Pos.	444	obs:	exp:
tct	S	2764	466.70
tcc	S	20	405.00
tca	S	9	633.90
tcg	S	3	168.70
agt	S	0	606.50
agc	S	0	515.30
---	--	------	------
mPD		0.023	1.7
		nPD:	0.01
		N. weight:	2.5
		Sc. PD:	-0.55
		Sc. rank:	-3704.0

,

PB1			
Pos.	445	obs:	exp:
gat	D	2787	1510.00
gac	D	9	1286.00
---	--	------	------
mPD		0.0064	0.50
		nPD:	0.01
		N. weight:	0.87
		Sc. PD:	-0.19
		Sc. rank:	-1301.9

,

PB1			
Pos.	446	obs:	exp:
gat	D	2666	1510.00
gac	D	130	1286.00
---	--	------	------
mPD		0.089	0.50
		nPD:	0.18
		N. weight:	0.64
		Sc. PD:	-0.037
		Sc. rank:	129.0

,

PB1			
Pos.	447	obs:	exp:
ttt	F	184	1166.00
ttc	F	2612	1630.00
---	--	------	------
mPD		0.12	0.49
		nPD:	0.25
		N. weight:	0.47
		Sc. PD:	0.0068
		Sc. rank:	235.7

,

PB1			
Pos.	448	obs:	exp:
gtt	V	1	0.22
gtc	V	0	0.21
gta	V	0	0.21
gtg	V	0	0.36
gct	A	1986	703.90
gcc	A	804	540.70
gca	A	4	1244.00
gcg	A	0	305.80
ggt	G	1	0.14
ggc	G	0	0.13
gga	G	0	0.44
ggg	G	0	0.29
---	--	------	------
mPD		0.41	0.69
		nPD:	0.6
		N. weight:	1.2
		Sc. PD:	0.43
		Sc. rank:	1527.8

,

PB1			
Pos.	449	obs:	exp:
tta	L	1	258.40
ttg	L	0	531.00
ctt	L	521	554.90
ctc	L	2195	447.10
cta	L	74	413.60
ctg	L	4	589.90
cct	P	0	0.27
ccc	P	1	0.18
cca	P	0	0.41
ccg	P	0	0.14
---	--	------	------
mPD		0.35	1.1
		nPD:	0.31
		N. weight:	1.7
		Sc. PD:	0.12
		Sc. rank:	1198.8

,

PB1			
Pos.	450	obs:	exp:
att	I	834	1023.00
atc	I	768	745.80
ata	I	1185	1018.00
gtt	V	8	1.94
gtc	V	0	1.89
gta	V	1	1.92
gtg	V	0	3.25
---	--	------	------
mPD		0.66	0.67
		nPD:	0.99
		N. weight:	0.027
		Sc. PD:	0.02
		Sc. rank:	44.3

,

PB1			
Pos.	451	obs:	exp:
tta	L	51	4.72
ttg	L	0	9.69
ctt	L	0	10.13
ctc	L	0	8.16
cta	L	0	7.55
ctg	L	0	10.76
att	I	0	7.34
atc	I	0	5.35
ata	I	20	7.31
gtt	V	6	586.10
gtc	V	4	573.30
gta	V	2263	581.00
gtg	V	452	984.60
---	--	------	------
mPD		0.33	0.78
		nPD:	0.42
		N. weight:	1.5
		Sc. PD:	0.26
		Sc. rank:	1377.7

,

PB1			
Pos.	452	obs:	exp:
aat	N	2782	1525.00
aac	N	14	1271.00
---	--	------	------
mPD		0.010	0.50
		nPD:	0.02
		N. weight:	0.84
		Sc. PD:	-0.18
		Sc. rank:	-1098.6

,

PB1			
Pos.	453	obs:	exp:
act	T	0	0.26
acc	T	0	0.23
aca	T	1	0.42
acg	T	0	0.09
gct	A	80	704.10
gcc	A	567	540.90
gca	A	2098	1244.00
gcg	A	50	305.90
---	--	------	------
mPD		0.40	0.69
		nPD:	0.57
		N. weight:	0.45
		Sc. PD:	0.15
		Sc. rank:	540.3

,

PB1			
Pos.	454	obs:	exp:
tta	L	0	0.65
ttg	L	0	1.33
tct	S	2	2.17
tcc	S	0	1.88
tca	S	0	2.95
tcg	S	11	0.78
ctt	L	0	1.39
ctc	L	0	1.12
cta	L	0	1.04
ctg	L	7	1.48
cct	P	266	758.40
ccc	P	0	501.20
cca	P	241	1126.00
ccg	P	2268	389.10
caa	Q	0	0.50
cag	Q	1	0.50
agt	S	0	2.82
agc	S	0	2.40
---	--	------	------
mPD		0.33	0.73
		nPD:	0.45
		N. weight:	1.8
		Sc. PD:	0.37
		Sc. rank:	1778.5

,

PB1			
Pos.	455	obs:	exp:
tct	S	0	0.50
tcc	S	0	0.43
tca	S	0	0.68
tcg	S	0	0.18
aat	N	2623	1488.00
aac	N	106	1241.00
agt	S	2	0.65
agc	S	1	0.55
gat	D	64	34.56
gac	D	0	29.44
---	--	------	------
mPD		0.12	0.54
		nPD:	0.22
		N. weight:	0.66
		Sc. PD:	-0.011
		Sc. rank:	228.9

,

PB1			
Pos.	456	obs:	exp:
tat	Y	2	0.96
tac	Y	0	1.04
cat	H	2775	1611.00
cac	H	8	1172.00
caa	Q	4	2.00
cag	Q	0	2.00
cgt	R	6	0.22
cgc	R	0	0.32
cga	R	0	0.60
cgg	R	0	0.49
aat	N	1	0.55
aac	N	0	0.45
aga	R	0	2.80
agg	R	0	1.56
---	--	------	------
mPD		0.015	0.50
		nPD:	0.03
		N. weight:	0.78
		Sc. PD:	-0.16
		Sc. rank:	-900.7

,

PB1			
Pos.	457	obs:	exp:
aaa	K	26	15.37
aag	K	1	11.63
gtt	V	0	0.86
gtc	V	0	0.84
gta	V	0	0.85
gtg	V	4	1.45
gct	A	0	0.25
gcc	A	0	0.19
gca	A	1	0.45
gcg	A	0	0.11
gaa	E	793	1549.00
gag	E	1971	1215.00
---	--	------	------
mPD		0.44	0.52
		nPD:	0.85
		N. weight:	0.23
		Sc. PD:	0.14
		Sc. rank:	371.3

,

PB1			
Pos.	458	obs:	exp:
gaa	E	2	1.12
gag	E	0	0.88
ggt	G	4	404.10
ggc	G	2	367.00
gga	G	2499	1224.00
ggg	G	289	798.70
---	--	------	------
mPD		0.19	0.69
		nPD:	0.28
		N. weight:	0.77
		Sc. PD:	0.029
		Sc. rank:	444.9

,

PB1			
Pos.	459	obs:	exp:
att	I	5	1024.00
atc	I	0	746.90
ata	I	2786	1020.00
gtt	V	0	0.86
gtc	V	0	0.84
gta	V	3	0.85
gtg	V	1	1.45
gct	A	0	0.25
gcc	A	0	0.19
gca	A	1	0.45
gcg	A	0	0.11
---	--	------	------
mPD		0.0086	0.66
		nPD:	0.01
		N. weight:	1.5
		Sc. PD:	-0.32
		Sc. rank:	-2195.2

,

PB1			
Pos.	460	obs:	exp:
cct	P	0	0.27
ccc	P	0	0.18
cca	P	1	0.41
ccg	P	0	0.14
caa	Q	2750	1395.00
cag	Q	44	1399.00
aaa	K	1	0.57
aag	K	0	0.43
---	--	------	------
mPD		0.032	0.50
		nPD:	0.06
		N. weight:	0.9
		Sc. PD:	-0.15
		Sc. rank:	-574.0

,

PB1			
Pos.	461	obs:	exp:
gct	A	11	704.40
gcc	A	15	541.10
gca	A	2757	1245.00
gcg	A	13	306.00
---	--	------	------
mPD		0.028	0.69
		nPD:	0.04
		N. weight:	1.1
		Sc. PD:	-0.21
		Sc. rank:	-1055.6

PB1			
Pos.	462	obs:	exp:
cgt	R	0	0.07
cgc	R	0	0.11
cga	R	0	0.20
cgg	R	0	0.16
aga	R	1	0.93
agg	R	1	0.52
ggt	G	22	404.10
ggc	G	1	367.00
gga	G	1621	1224.00
ggg	G	1150	798.70
---	--	------	------
mPD		0.50	0.69
		nPD:	0.72
		N. weight:	0.42
		Sc. PD:	0.2
		Sc. rank:	636.6

PB1			
Pos.	463	obs:	exp:
att	I	0	1.10
atc	I	0	0.80
ata	I	3	1.10
gtt	V	160	600.50
gtc	V	0	587.40
gta	V	624	595.30
gtg	V	2008	1009.00
gct	A	0	0.25
gcc	A	0	0.19
gca	A	0	0.45
gcg	A	1	0.11
---	--	------	------
mPD		0.43	0.74
		nPD:	0.59
		N. weight:	0.63
		Sc. PD:	0.22
		Sc. rank:	767.4

PB1			
Pos.	464	obs:	exp:
aat	N	19	10.91
aac	N	1	9.09
gat	D	2608	1499.00
gac	D	168	1277.00
---	--	------	------
mPD		0.13	0.51
		nPD:	0.25
		N. weight:	0.58
		Sc. PD:	0.0069
		Sc. rank:	290.9

PB1			
Pos.	465	obs:	exp:
cgt	R	0	104.10
cgc	R	0	148.90
cga	R	2	280.40
cgg	R	2	227.20
aaa	K	1	7.40
aag	K	12	5.60
aga	R	795	1299.00
agg	R	1983	722.70
ggt	G	0	0.14
ggc	G	0	0.13
gga	G	0	0.44
ggg	G	1	0.29
---	--	------	------
mPD		0.42	0.96
		nPD:	0.44
		N. weight:	0.84
		Sc. PD:	0.16
		Sc. rank:	817.0

PB1			
Pos.	466	obs:	exp:
ttt	F	1774	1166.00
ttc	F	1020	1628.00
tta	L	0	0.09
ttg	L	1	0.19
tgt	C	1	0.39
tgc	C	0	0.61
ctt	L	0	0.20
ctc	L	0	0.16
cta	L	0	0.15
ctg	L	0	0.21
---	--	------	------
mPD		0.46	0.49
		nPD:	0.95
		N. weight:	0.14
		Sc. PD:	0.1
		Sc. rank:	234.8

PB1			
Pos.	467	obs:	exp:
tat	Y	2768	1341.00
tac	Y	28	1455.00
---	--	------	------
mPD		0.020	0.50
		nPD:	0.04
		N. weight:	0.99
		Sc. PD:	-0.19
		Sc. rank:	-982.1

PB1			
Pos.	468	obs:	exp:
cgt	R	0	104.50
cgc	R	0	149.50
cga	R	0	281.50
cgg	R	1	228.10
atg	M	2	2.00
aaa	K	0	0.57
aag	K	1	0.43
aga	R	181	1304.00
agg	R	2611	725.60
---	--	------	------
mPD		0.12	0.96
		nPD:	0.13
		N. weight:	1.6
		Sc. PD:	-0.16
		Sc. rank:	-140.9

PB1			
Pos.	469	obs:	exp:
tct	S	1	0.17
tcc	S	0	0.14
tca	S	0	0.23
tcg	S	0	0.06
att	I	1	0.37
atc	I	0	0.27
ata	I	0	0.37
act	T	1678	731.40
acc	T	1070	628.70
aca	T	41	1167.00
acg	T	1	262.50
agt	S	0	0.22
agc	S	0	0.18
gct	A	3	1.01
gcc	A	1	0.77
gca	A	0	1.78
gcg	A	0	0.44
---	--	------	------
mPD		0.50	0.70
		nPD:	0.71
		N. weight:	0.96
		Sc. PD:	0.44
		Sc. rank:	1412.8

PB1			
Pos.	470	obs:	exp:
tgt	C	1683	1081.00
tgc	C	1110	1712.00
tgg	W	2	2.00
gat	D	1	0.54
gac	D	0	0.46
---	--	------	------
mPD		0.48	0.48
		nPD:	1.01
		N. weight:	0.14
		Sc. PD:	0.11
		Sc. rank:	231.6

PB1			
Pos.	471	obs:	exp:
att	I	0	0.37
atc	I	0	0.27
ata	I	1	0.37
aat	N	0	0.55
aac	N	1	0.45
aaa	K	2620	1591.00
aag	K	174	1203.00
---	--	------	------
mPD		0.12	0.49
		nPD:	0.24
		N. weight:	0.51
		Sc. PD:	0.0011
		Sc. rank:	221.8

PB1			
Pos.	472	obs:	exp:
tta	L	308	258.50
ttg	L	160	531.20
ctt	L	1	555.10
ctc	L	2	447.20
cta	L	1357	413.80
ctg	L	968	590.10
---	--	------	------
mPD		0.76	1.1
		nPD:	0.68
		N. weight:	1.
		Sc. PD:	0.44
		Sc. rank:	1430.2

PB1			
Pos.	473	obs:	exp:
tta	L	0	0.09
ttg	L	0	0.19
ctt	L	1	0.20
ctc	L	0	0.16
cta	L	0	0.15
ctg	L	0	0.21
att	I	1	0.37
atc	I	0	0.27
ata	I	0	0.37
gtt	V	1985	600.90
gtc	V	802	587.80
gta	V	7	595.80
gtg	V	0	1009.00
---	--	------	------
mPD		0.41	0.73
		nPD:	0.56
		N. weight:	1.4
		Sc. PD:	0.43
		Sc. rank:	1596.2

PB1			
Pos.	474	obs:	exp:
ggt	G	7	404.30
ggc	G	0	367.30
gga	G	735	1225.00
ggg	G	2054	799.20
---	--	------	------
mPD		0.39	0.69
		nPD:	0.57
		N. weight:	0.8
		Sc. PD:	0.26
		Sc. rank:	953.0

PB1			
Pos.	475	obs:	exp:
att	I	38	1026.00
atc	I	2224	747.90
ata	I	533	1021.00
act	T	0	0.26
acc	T	1	0.23
aca	T	0	0.42
acg	T	0	0.09
---	--	------	------
mPD		0.33	0.66
		nPD:	0.5
		N. weight:	1.
		Sc. PD:	0.26
		Sc. rank:	1117.4

PB1			
Pos.	476	obs:	exp:
tct	S	0	0.17
tcc	S	0	0.14
tca	S	0	0.23
tcg	S	0	0.06
att	I	1	0.37
atc	I	0	0.27
ata	I	0	0.37
aat	N	2126	1524.00
aac	N	668	1270.00
agt	S	1	0.22
agc	S	0	0.18
---	--	------	------
mPD		0.37	0.50
		nPD:	0.73
		N. weight:	0.15
		Sc. PD:	0.072
		Sc. rank:	225.8

PB1			
Pos.	477	obs:	exp:
atg	M	2795	2795.00
gtt	V	0	0.22
gtc	V	0	0.21
gta	V	0	0.21
gtg	V	1	0.36
---	--	------	------
mPD		0.00072	0.0012
		nPD:	0.61
		N. weight:	0.00053
		Sc. PD:	0.00019
		Sc. rank:	0.7

PB1			
Pos.	478	obs:	exp:
tct	S	0	466.00
tcc	S	0	404.40
tca	S	0	632.90
tcg	S	0	168.40
cgt	R	0	0.04
cgc	R	0	0.05
cga	R	0	0.10
cgg	R	0	0.08
act	T	1	0.52
acc	T	1	0.45
aca	T	0	0.84
acg	T	0	0.19
aat	N	0	0.55
aac	N	1	0.45
agt	S	31	605.60
agc	S	2761	514.50
aga	R	1	0.47
agg	R	0	0.26
---	--	------	------
mPD		0.025	1.7
		nPD:	0.02
		N. weight:	2.4
		Sc. PD:	-0.51
		Sc. rank:	-3353.9

PB1			
Pos.	479	obs:	exp:
caa	Q	1	2.00
cag	Q	3	2.00
aaa	K	1249	1590.00
aag	K	1543	1202.00
---	--	------	------
mPD		0.50	0.49
		nPD:	1.01
		N. weight:	0.047
		Sc. PD:	0.035
		Sc. rank:	76.9

PB1			
Pos.	480	obs:	exp:
cgt	R	0	0.56
cgc	R	0	0.80
cga	R	0	1.51
cgg	R	0	1.22
aaa	K	90	1584.00
aag	K	2691	1197.00
aga	R	1	7.00
agg	R	14	3.90
---	--	------	------
mPD		0.074	0.50
		nPD:	0.15
		N. weight:	1.
		Sc. PD:	-0.091
		Sc. rank:	37.6

PB1

Pos.	481	obs:	exp:
aaa	K	193	1592.00
aag	K	2603	1204.00
---	--	------	------
mPD		0.13	0.49
		nPD:	0.26
		N. weight:	0.84
		Sc. PD:	0.02
		Sc. rank:	451.5

,

PB1

Pos.	482	obs:	exp:
tct	S	2584	466.50
tcc	S	192	404.90
tca	S	19	633.60
tcg	S	0	168.60
tat	Y	0	0.48
tac	Y	1	0.52
agt	S	0	606.20
agc	S	0	515.10
---	--	------	------
mPD		0.14	1.7
		nPD:	0.09
		N. weight:	2.2
		Sc. PD:	-0.33
		Sc. rank:	-983.2

,

PB1

Pos.	483	obs:	exp:
tat	Y	37	1341.00
tac	Y	2759	1455.00
---	--	------	------
mPD		0.026	0.50
		nPD:	0.05
		N. weight:	0.85
		Sc. PD:	-0.15
		Sc. rank:	-653.8

,

PB1

Pos.	484	obs:	exp:
att	I	0	1025.00
atc	I	0	747.10
ata	I	2792	1020.00
atg	M	3	3.00
gtt	V	0	0.22
gtc	V	0	0.21
gta	V	1	0.21
gtg	V	0	0.36
---	--	------	------
mPD		0.0029	0.66
		nPD:	0.
		N. weight:	1.5
		Sc. PD:	-0.33
		Sc. rank:	-2395.6

,

PB1

Pos.	485	obs:	exp:
aat	N	1766	1525.00
aac	N	1030	1271.00
---	--	------	------
mPD		0.47	0.50
		nPD:	0.94
		N. weight:	0.024
		Sc. PD:	0.017
		Sc. rank:	39.9

,

PB1

Pos.	486	obs:	exp:
cct	P	0	0.27
ccc	P	0	0.18
cca	P	0	0.41
ccg	P	1	0.14
cgt	R	0	104.50
cgc	R	0	149.50
cga	R	252	281.50
cgg	R	2459	228.10
aaa	K	0	0.57
aag	K	1	0.43
aga	R	6	1304.00
agg	R	76	725.60
ggt	G	0	0.14
ggc	G	0	0.13
gga	G	0	0.44
ggg	G	1	0.29
---	--	------	------
mPD		0.23	0.96
		nPD:	0.24
		N. weight:	2.9
		Sc. PD:	-0.00099
		Sc. rank:	1219.1

,

PB1

Pos.	487	obs:	exp:
act	T	734	733.00
acc	T	0	630.00
aca	T	2056	1170.00
acg	T	6	263.10
---	--	------	------
mPD		0.39	0.70
		nPD:	0.56
		N. weight:	0.6
		Sc. PD:	0.19
		Sc. rank:	698.8

,

PB1

Pos.	488	obs:	exp:
ggt	G	2	404.30
ggc	G	0	367.30
gga	G	1179	1225.00
ggg	G	1615	799.20
---	--	------	------
mPD		0.49	0.69
		nPD:	0.71
		N. weight:	0.57
		Sc. PD:	0.26
		Sc. rank:	837.4

,

PB1

Pos.	489	obs:	exp:
act	T	1	733.00
acc	T	0	630.00
aca	T	2779	1170.00
acg	T	16	263.10
---	--	------	------
mPD		0.012	0.70
		nPD:	0.02
		N. weight:	1.2
		Sc. PD:	-0.26
		Sc. rank:	-1664.8

,

PB1

Pos.	490	obs:	exp:
ttt	F	2733	1165.00
ttc	F	59	1627.00
tgt	C	4	1.55
tgc	C	0	2.45
---	--	------	------
mPD		0.044	0.49
		nPD:	0.09
		N. weight:	1.1
		Sc. PD:	-0.16
		Sc. rank:	-435.2

,

PB1

Pos.	491	obs:	exp:
gaa	E	1757	1567.00
gag	E	1039	1229.00
---	--	------	------
mPD		0.47	0.49
		nPD:	0.95
		N. weight:	0.016
		Sc. PD:	0.011
		Sc. rank:	26.2

,

PB1

Pos.	492	obs:	exp:
ttt	F	157	1166.00
ttc	F	2638	1629.00
tgt	C	0	0.39
tgc	C	1	0.61
---	--	------	------
mPD		0.11	0.49
		nPD:	0.22
		N. weight:	0.5
		Sc. PD:	-0.0092
		Sc. rank:	171.4

,

PB1

Pos.	493	obs:	exp:
cgt	R	0	0.04
cgc	R	0	0.05
cga	R	0	0.10
cgg	R	0	0.08
act	T	10	732.70
acc	T	3	629.80
aca	T	1226	1170.00
acg	T	1556	263.00
aga	R	1	0.47
agg	R	0	0.26
---	--	------	------
mPD		0.50	0.70
		nPD:	0.71
		N. weight:	1.4
		Sc. PD:	0.67
		Sc. rank:	2159.3

,

PB1

Pos.	494	obs:	exp:
tct	S	0	466.50
tcc	S	0	404.90
tca	S	0	633.60
tcg	S	0	168.60
aat	N	0	0.55
aac	N	1	0.45
agt	S	212	606.20
agc	S	2583	515.10
---	--	------	------
mPD		0.14	1.7
		nPD:	0.09
		N. weight:	2.1
		Sc. PD:	-0.31
		Sc. rank:	-965.6

,

PB1

Pos.	495	obs:	exp:
ttt	F	2496	1166.00
ttc	F	299	1629.00
tta	L	1	0.09
ttg	L	0	0.19
ctt	L	0	0.20
ctc	L	0	0.16
cta	L	0	0.15
ctg	L	0	0.21
---	--	------	------
mPD		0.19	0.49
		nPD:	0.39
		N. weight:	0.73
		Sc. PD:	0.11
		Sc. rank:	652.3

,

PB1

Pos.	496	obs:	exp:
ttt	F	172	1166.00
ttc	F	2624	1630.00
---	--	------	------
mPD		0.12	0.49
		nPD:	0.24
		N. weight:	0.48
		Sc. PD:	-0.00024
		Sc. rank:	194.0

,

PB1

Pos.	497	obs:	exp:
ttt	F	0	0.42
ttc	F	1	0.58
tat	Y	886	1340.00
tac	Y	1908	1454.00
tgt	C	0	0.39
tgc	C	1	0.61
---	--	------	------
mPD		0.43	0.50
		nPD:	0.87
		N. weight:	0.082
		Sc. PD:	0.05
		Sc. rank:	132.4

,

PB1

Pos.	498	obs:	exp:
cct	P	0	0.27
ccc	P	0	0.18
cca	P	1	0.41
ccg	P	0	0.14
cgt	R	16	104.60
cgc	R	2570	149.60
cga	R	209	281.70
cgg	R	0	228.20
aga	R	0	1305.00
agg	R	0	726.10
---	--	------	------
mPD		0.15	0.96
		nPD:	0.16
		N. weight:	3.8
		Sc. PD:	-0.3
		Sc. rank:	462.9

,

PB1

Pos.	499	obs:	exp:
tat	Y	2770	1341.00
tac	Y	26	1455.00
---	--	------	------
mPD		0.018	0.50
		nPD:	0.04
		N. weight:	1.
		Sc. PD:	-0.19
		Sc. rank:	-1028.5

PB1

Pos.	500	obs:	exp:
ggt	G	4	404.30
ggc	G	0	367.30
gga	G	2692	1225.00
ggg	G	100	799.20
---	--	------	------
mPD		0.072	0.69
		nPD:	0.1
		N. weight:	0.99
		Sc. PD:	-0.13
		Sc. rank:	-297.4

PB1

Pos.	501	obs:	exp:
ttt	F	1976	1166.00
ttc	F	820	1630.00
---	--	------	------
mPD		0.41	0.49
		nPD:	0.85
		N. weight:	0.25
		Sc. PD:	0.15
		Sc. rank:	403.6

PB1

Pos.	502	obs:	exp:
gtt	V	1	601.40
gtc	V	0	588.20
gta	V	1835	596.20
gtg	V	960	1010.00
---	--	------	------
mPD		0.45	0.73
		nPD:	0.62
		N. weight:	1.1
		Sc. PD:	0.39
		Sc. rank:	1344.5

PB1

Pos.	503	obs:	exp:
tct	S	0	0.17
tcc	S	1	0.14
tca	S	0	0.23
tcg	S	0	0.06
cct	P	0	0.27
ccc	P	1	0.18
cca	P	0	0.41
ccg	P	0	0.14
agt	S	0	0.22
agc	S	0	0.18
gct	A	984	703.90
gcc	A	1803	540.70
gca	A	7	1244.00
gcg	A	0	305.80
---	--	------	------
mPD		0.46	0.69
		nPD:	0.67
		N. weight:	1.3
		Sc. PD:	0.54
		Sc. rank:	1771.2

PB1

Pos.	504	obs:	exp:
cat	H	1	0.58
cac	H	0	0.42
aat	N	1756	1524.00
aac	N	1039	1271.00
---	--	------	------
mPD		0.47	0.50
		nPD:	0.94
		N. weight:	0.023
		Sc. PD:	0.016
		Sc. rank:	37.6

PB1

Pos.	505	obs:	exp:
ttt	F	130	1166.00
ttc	F	2664	1628.00
tta	L	0	0.09
ttg	L	1	0.19
tct	S	0	0.17
tcc	S	1	0.14
tca	S	0	0.23
tcg	S	0	0.06
ctt	L	0	0.20
ctc	L	0	0.16
cta	L	0	0.15
ctg	L	0	0.21
agt	S	0	0.22
agc	S	0	0.18
---	--	------	------
mPD		0.090	0.49
		nPD:	0.18
		N. weight:	0.54
		Sc. PD:	-0.028
		Sc. rank:	123.3

PB1

Pos.	506	obs:	exp:
tct	S	0	466.70
tcc	S	0	405.00
tca	S	0	633.90
tcg	S	0	168.70
agt	S	2621	606.50
agc	S	175	515.30
---	--	------	------
mPD		0.12	1.7
		nPD:	0.07
		N. weight:	1.9
		Sc. PD:	-0.31
		Sc. rank:	-1110.6

PB1

Pos.	507	obs:	exp:
atg	M	2794	2794.00
act	T	0	0.52
acc	T	0	0.45
aca	T	0	0.84
acg	T	2	0.19
---	--	------	------
mPD		0.0014	0.0027
		nPD:	0.52
		N. weight:	0.0025
		Sc. PD:	0.00069
		Sc. rank:	2.8

PB1

Pos.	508	obs:	exp:
gaa	E	177	1567.00
gag	E	2619	1229.00
---	--	------	------
mPD		0.12	0.49
		nPD:	0.24
		N. weight:	0.83
		Sc. PD:	0.0022
		Sc. rank:	366.6

PB1

Pos.	509	obs:	exp:
tta	L	223	258.50
ttg	L	79	531.20
ctt	L	6	555.10
ctc	L	0	447.20
cta	L	148	413.80
ctg	L	2340	590.10
---	--	------	------
mPD		0.43	1.1
		nPD:	0.38
		N. weight:	1.5
		Sc. PD:	0.21
		Sc. rank:	1329.7

PB1

Pos.	510	obs:	exp:
tct	S	0	0.17
tcc	S	1	0.14
tca	S	0	0.23
tcg	S	0	0.06
cct	P	22	763.80
ccc	P	2771	504.80
cca	P	2	1134.00
ccg	P	0	391.90
agt	S	0	0.22
agc	S	0	0.18
---	--	------	------
mPD		0.018	0.71
		nPD:	0.03
		N. weight:	2.4
		Sc. PD:	-0.5
		Sc. rank:	-3022.1

PB1

Pos.	511	obs:	exp:
tct	S	0	466.20
tcc	S	0	404.60
tca	S	0	633.20
tcg	S	0	168.50
aat	N	2	1.09
aac	N	0	0.91
agt	S	1743	605.80
agc	S	1050	514.70
ggt	G	1	0.14
ggc	G	0	0.13
gga	G	0	0.44
ggg	G	0	0.29
---	--	------	------
mPD		0.47	1.7
		nPD:	0.29
		N. weight:	1.4
		Sc. PD:	0.062
		Sc. rank:	863.9

PB1

Pos.	512	obs:	exp:
ttt	F	2775	1163.00
ttc	F	13	1625.00
tta	L	0	0.74
ttg	L	3	1.52
ctt	L	5	1.59
ctc	L	0	1.28
cta	L	0	1.18
ctg	L	0	1.69
---	--	------	------
mPD		0.015	0.49
		nPD:	0.03
		N. weight:	1.2
		Sc. PD:	-0.25
		Sc. rank:	-1387.9

PB1

Pos.	513	obs:	exp:
ggt	G	20	404.30
ggc	G	1	367.30
gga	G	2690	1225.00
ggg	G	85	799.20
---	--	------	------
mPD		0.073	0.69
		nPD:	0.11
		N. weight:	0.97
		Sc. PD:	-0.12
		Sc. rank:	-276.4

PB1

Pos.	514	obs:	exp:
gtt	V	735	601.40
gtc	V	0	588.20
gta	V	164	596.20
gtg	V	1897	1010.00
---	--	------	------
mPD		0.47	0.73
		nPD:	0.64
		N. weight:	0.59
		Sc. PD:	0.23
		Sc. rank:	772.4

PB1

Pos.	515	obs:	exp:
tct	S	2685	451.00
tcc	S	14	391.40
tca	S	1	612.50
tcg	S	2	163.00
tgt	C	1	0.39
tgc	C	0	0.61
cct	P	6	1.64
ccc	P	0	1.08
cca	P	0	2.44
ccg	P	0	0.84
agt	S	0	586.10
agc	S	0	497.90
gct	A	87	21.92
gcc	A	0	16.84
gca	A	0	38.73
gcg	A	0	9.52
---	--	------	------
mPD		0.077	1.7
		nPD:	0.05
		N. weight:	2.5
		Sc. PD:	-0.47
		Sc. rank:	-2182.3

PB1

Pos.	516	obs:	exp:
ggt	G	2	404.30
ggc	G	8	367.30
gga	G	1893	1225.00
ggg	G	893	799.20
---	--	------	------
mPD		0.44	0.69
		nPD:	0.64
		N. weight:	0.46
		Sc. PD:	0.18
		Sc. rank:	603.9

PB1

Pos.	517	obs:	exp:
att	I	1741	1026.00
atc	I	1048	747.90
ata	I	6	1021.00
gtt	V	1	0.22
gtc	V	0	0.21
gta	V	0	0.21
gtg	V	0	0.36
---	--	------	------
mPD		0.47	0.66
		nPD:	0.71
		N. weight:	0.65
		Sc. PD:	0.3
		Sc. rank:	970.1

PB1

Pos.	518	obs:	exp:
aat	N	2686	1525.00
aac	N	110	1271.00
---	--	------	------
mPD		0.076	0.50
		nPD:	0.15
		N. weight:	0.65
		Sc. PD:	-0.055
		Sc. rank:	53.5

PB1

Pos.	519	obs:	exp:
gaa	E	2487	1567.00
gag	E	309	1229.00
---	--	------	------
mPD		0.20	0.49
		nPD:	0.4
		N. weight:	0.38
		Sc. PD:	0.059
		Sc. rank:	342.4

PB1

Pos.	520	obs:	exp:
tct	S	67	466.70
tcc	S	1	405.00
tca	S	967	633.90
tcg	S	1761	168.70
agt	S	0	606.50
agc	S	0	515.30
---	--	------	------
mPD		0.48	1.7
		nPD:	0.29
		N. weight:	2.3
		Sc. PD:	0.12
		Sc. rank:	1509.4

PB1

Pos.	521	obs:	exp:
act	T	0	0.26
acc	T	1	0.23
aca	T	0	0.42
acg	T	0	0.09
gct	A	1166	704.10
gcc	A	1571	540.90
gca	A	44	1244.00
gcg	A	14	305.90
---	--	------	------
mPD		0.51	0.69
		nPD:	0.74
		N. weight:	1.1
		Sc. PD:	0.53
		Sc. rank:	1656.4

PB1

Pos.	522	obs:	exp:
gat	D	20	1510.00
gac	D	2776	1286.00
---	--	------	------
mPD		0.014	0.50
		nPD:	0.03
		N. weight:	1.1
		Sc. PD:	-0.22
		Sc. rank:	-1270.1

PB1

Pos.	523	obs:	exp:
att	I	0	2.57
atc	I	0	1.87
ata	I	7	2.56
atg	M	2788	2788.00
act	T	0	0.26
acc	T	0	0.23
aca	T	0	0.42
acg	T	1	0.09
---	--	------	------
mPD		0.0057	0.0064
		nPD:	0.9
		N. weight:	0.0049
		Sc. PD:	0.0032
		Sc. rank:	8.0

PB1

Pos.	524	obs:	exp:
tct	S	0	466.40
tcc	S	0	404.70
tca	S	0	633.40
tcg	S	0	168.60
agt	S	32	606.00
agc	S	2762	514.90
ggt	G	0	0.29
ggc	G	2	0.26
gga	G	0	0.88
ggg	G	0	0.57
---	--	------	------
mPD		0.024	1.7
		nPD:	0.01
		N. weight:	2.4
		Sc. PD:	-0.52
		Sc. rank:	-3446.4

PB1

Pos.	525	obs:	exp:
att	I	2656	976.50
atc	I	5	712.10
ata	I	0	972.40
gtt	V	135	29.04
gtc	V	0	28.40
gta	V	0	28.79
gtg	V	0	48.78
---	--	------	------
mPD		0.096	0.76
		nPD:	0.13
		N. weight:	1.5
		Sc. PD:	-0.16
		Sc. rank:	-176.3

PB1

Pos.	526	obs:	exp:
gtt	V	1	0.22
gtc	V	0	0.21
gta	V	0	0.21
gtg	V	0	0.36
ggt	G	1649	404.20
ggc	G	733	367.10
gga	G	398	1225.00
ggg	G	15	799.00
---	--	------	------
mPD		0.56	0.69
		nPD:	0.82
		N. weight:	1.2
		Sc. PD:	0.69
		Sc. rank:	1939.0

PB1

Pos.	527	obs:	exp:
att	I	141	141.30
atc	I	243	103.00
ata	I	1	140.70
atg	M	1	1.00
gtt	V	1606	518.30
gtc	V	714	507.00
gta	V	82	513.90
gtg	V	8	870.70
---	--	------	------
mPD		0.73	0.98
		nPD:	0.74
		N. weight:	1.1
		Sc. PD:	0.54
		Sc. rank:	1665.8

PB1

Pos.	528	obs:	exp:
tct	S	0	1.50
tcc	S	0	1.30
tca	S	9	2.04
tcg	S	0	0.54
act	T	15	729.80
acc	T	2	627.30
aca	T	2716	1165.00
acg	T	51	261.90
agt	S	0	1.95
agc	S	0	1.66
gct	A	0	0.76
gcc	A	0	0.58
gca	A	3	1.34
gcg	A	0	0.33
---	--	------	------
mPD		0.056	0.71
		nPD:	0.08
		N. weight:	1.1
		Sc. PD:	-0.17
		Sc. rank:	-568.4

PB1

Pos.	529	obs:	exp:
gtt	V	51	601.40
gtc	V	17	588.20
gta	V	107	596.20
gtg	V	2621	1010.00
---	--	------	------
mPD		0.12	0.73
		nPD:	0.16
		N. weight:	1.1
		Sc. PD:	-0.082
		Sc. rank:	177.2

PB1

Pos.	530	obs:	exp:
att	I	0	1026.00
atc	I	1	747.90
ata	I	2794	1021.00
gtt	V	0	0.22
gtc	V	0	0.21
gta	V	1	0.21
gtg	V	0	0.36
---	--	------	------
mPD		0.0014	0.66
		nPD:	0.
		N. weight:	1.5
		Sc. PD:	-0.34
		Sc. rank:	-2426.1

PB1

Pos.	531	obs:	exp:
cgt	R	0	2.13
cgc	R	0	3.05
cga	R	0	5.74
cgg	R	0	4.65
aaa	K	714	1560.00
aag	K	2025	1179.00
aga	R	34	26.61
agg	R	23	14.81
---	--	------	------
mPD		0.43	0.54
		nPD:	0.8
		N. weight:	0.29
		Sc. PD:	0.16
		Sc. rank:	461.5

PB1

Pos.	532	obs:	exp:
aat	N	149	1525.00
aac	N	2647	1271.00
---	--	------	------
mPD		0.10	0.50
		nPD:	0.2
		N. weight:	0.83
		Sc. PD:	-0.028
		Sc. rank:	228.0

PB1

Pos.	533	obs:	exp:
tct	S	0	1.00
tcc	S	0	0.87
tca	S	0	1.36
tcg	S	0	0.36
cat	H	1	0.58
cac	H	0	0.42
aat	N	1835	1521.00
aac	N	954	1268.00
agt	S	1	1.30
agc	S	5	1.11
---	--	------	------
mPD		0.46	0.50
		nPD:	0.9
		N. weight:	0.045
		Sc. PD:	0.029
		Sc. rank:	72.8

PB1

Pos.	534	obs:	exp:
atg	M	2796	2796.00
---	--	------	------
mPD		0	0
		nPD:	1.
		N. weight:	0.
		Sc. PD:	0
		Sc. rank:	0

PB1

Pos.	535	obs:	exp:
att	I	5	1023.00
atc	I	35	745.80
ata	I	2747	1018.00
gtt	V	0	1.94
gtc	V	0	1.89
gta	V	9	1.92
gtg	V	0	3.25
---	--	------	------
mPD		0.035	0.67
		nPD:	0.05
		N. weight:	1.4
		Sc. PD:	-0.25
		Sc. rank:	-1048.7

PB1

Pos.	536	obs:	exp:
aat	N	229	1524.00
aac	N	2566	1271.00
gat	D	0	0.54
gac	D	1	0.46
---	--	------	------
mPD		0.15	0.50
		nPD:	0.3
		N. weight:	0.72
		Sc. PD:	0.046
		Sc. rank:	485.6

PB1

Pos.	537	obs:	exp:
tct	S	0	0.67
tcc	S	0	0.58
tca	S	0	0.91
tcg	S	0	0.24
aat	N	1196	1521.00
aac	N	1594	1269.00
agt	S	4	0.87
agc	S	0	0.74
gat	D	0	1.08
gac	D	2	0.92
---	--	------	------
mPD		0.49	0.50
		nPD:	0.98
		N. weight:	0.046
		Sc. PD:	0.033
		Sc. rank:	75.6

PB1

Pos.	538	obs:	exp:
gat	D	259	1510.00
gac	D	2537	1286.00
---	--	------	------
mPD		0.17	0.50
		nPD:	0.34
		N. weight:	0.66
		Sc. PD:	0.065
		Sc. rank:	516.5

PB1

Pos.	539	obs:	exp:
ttt	F	1	0.42
ttc	F	0	0.58
tta	L	0	258.40
ttg	L	0	531.00
ctt	L	2762	554.90
ctc	L	4	447.10
cta	L	7	413.60
ctg	L	22	589.90
---	--	------	------
mPD		0.024	1.1
		nPD:	0.02
		N. weight:	2.3
		Sc. PD:	-0.47
		Sc. rank:	-2898.4

PB1

Pos.	540	obs:	exp:
ggt	G	287	404.30
ggc	G	125	367.30
gga	G	844	1225.00
ggg	G	1540	799.20
---	--	------	------
mPD		0.59	0.69
		nPD:	0.86
		N. weight:	0.25
		Sc. PD:	0.15
		Sc. rank:	397.3

PB1

Pos.	541	obs:	exp:
cct	P	7	763.80
ccc	P	4	504.80
cca	P	2776	1134.00
ccg	P	8	391.90
gct	A	0	0.25
gcc	A	0	0.19
gca	A	1	0.45
gcg	A	0	0.11
---	--	------	------
mPD		0.014	0.71
		nPD:	0.02
		N. weight:	1.3
		Sc. PD:	-0.27
		Sc. rank:	-1645.0

PB1

Pos.	542	obs:	exp:
gct	A	1	704.40
gcc	A	68	541.10
gca	A	2611	1245.00
gcg	A	116	306.00
---	--	------	------
mPD		0.13	0.69
		nPD:	0.18
		N. weight:	0.88
		Sc. PD:	-0.047
		Sc. rank:	185.9

PB1

Pos.	543	obs:	exp:
tct	S	0	0.17
tcc	S	0	0.14
tca	S	1	0.23
tcg	S	0	0.06
act	T	20	732.40
acc	T	10	629.60
aca	T	2756	1169.00
acg	T	8	262.90
aaa	K	1	0.57
aag	K	0	0.43
agt	S	0	0.22
agc	S	0	0.18
---	--	------	------
mPD		0.028	0.70
		nPD:	0.04
		N. weight:	1.2
		Sc. PD:	-0.22
		Sc. rank:	-1115.6

PB1			
Pos.	544	obs:	exp:
gct	A	2633	704.40
gcc	A	79	541.10
gca	A	84	1245.00
gcg	A	0	306.00
---	--	------	------
mPD		0.11	0.69
		nPD:	0.16
		N. weight:	1.6
		Sc. PD:	-0.12
		Sc. rank:	249.0

,

PB1			
Pos.	545	obs:	exp:
cat	H	1	0.58
cac	H	0	0.42
caa	Q	1143	1395.00
cag	Q	1652	1400.00
---	--	------	------
mPD		0.48	0.50
		nPD:	0.97
		N. weight:	0.026
		Sc. PD:	0.019
		Sc. rank:	43.3

,

PB1			
Pos.	546	obs:	exp:
atg	M	2796	2796.00
---	--	------	------
mPD		0	0
		nPD:	1.
		N. weight:	0.
		Sc. PD:	0
		Sc. rank:	0

,

PB1			
Pos.	547	obs:	exp:
gct	A	1948	704.40
gcc	A	848	541.10
gca	A	0	1245.00
gcg	A	0	306.00
---	--	------	------
mPD		0.42	0.69
		nPD:	0.61
		N. weight:	1.2
		Sc. PD:	0.45
		Sc. rank:	1575.3

,

PB1			
Pos.	548	obs:	exp:
tta	L	0	258.50
ttg	L	0	531.20
ctt	L	2592	555.10
ctc	L	174	447.20
cta	L	24	413.80
ctg	L	6	590.10
---	--	------	------
mPD		0.14	1.1
		nPD:	0.12
		N. weight:	2.
		Sc. PD:	-0.22
		Sc. rank:	-314.2

,

PB1			
Pos.	549	obs:	exp:
cct	P	0	0.27
ccc	P	0	0.18
cca	P	0	0.41
ccg	P	1	0.14
caa	Q	769	1395.00
cag	Q	2026	1400.00
---	--	------	------
mPD		0.40	0.50
		nPD:	0.8
		N. weight:	0.16
		Sc. PD:	0.084
		Sc. rank:	245.3

,

PB1			
Pos.	550	obs:	exp:
tta	L	157	258.40
ttg	L	1	531.00
ctt	L	2	554.90
ctc	L	0	447.10
cta	L	2584	413.60
ctg	L	51	589.90
cct	P	0	0.27
ccc	P	0	0.18
cca	P	1	0.41
ccg	P	0	0.14
---	--	------	------
mPD		0.15	1.1
		nPD:	0.13
		N. weight:	2.4
		Sc. PD:	-0.25
		Sc. rank:	-229.5

,

PB1			
Pos.	551	obs:	exp:
ttt	F	31	1166.00
ttc	F	2764	1629.00
tta	L	0	0.09
ttg	L	0	0.19
ctt	L	0	0.20
ctc	L	1	0.16
cta	L	0	0.15
ctg	L	0	0.21
---	--	------	------
mPD		0.023	0.49
		nPD:	0.05
		N. weight:	0.71
		Sc. PD:	-0.13
		Sc. rank:	-592.2

,

PB1			
Pos.	552	obs:	exp:
att	I	47	1023.00
atc	I	2729	746.30
ata	I	13	1019.00
act	T	0	0.52
acc	T	2	0.45
aca	T	0	0.84
acg	T	0	0.19
gtt	V	0	1.08
gtc	V	5	1.05
gta	V	0	1.07
gtg	V	0	1.81
---	--	------	------
mPD		0.047	0.67
		nPD:	0.07
		N. weight:	1.7
		Sc. PD:	-0.28
		Sc. rank:	-1042.5

,

PB1			
Pos.	553	obs:	exp:
cgt	R	0	0.04
cgc	R	0	0.05
cga	R	0	0.10
cgg	R	0	0.08
aaa	K	843	1591.00
aag	K	1951	1203.00
aga	R	1	0.47
agg	R	0	0.26
gat	D	1	0.54
gac	D	0	0.46
---	--	------	------
mPD		0.42	0.49
		nPD:	0.86
		N. weight:	0.22
		Sc. PD:	0.13
		Sc. rank:	346.6

,

PB1			
Pos.	554	obs:	exp:
gtt	V	0	0.22
gtc	V	1	0.21
gta	V	0	0.21
gtg	V	0	0.36
gat	D	822	1509.00
gac	D	1973	1286.00
---	--	------	------
mPD		0.42	0.50
		nPD:	0.84
		N. weight:	0.18
		Sc. PD:	0.11
		Sc. rank:	292.5

,

PB1			
Pos.	555	obs:	exp:
ttt	F	0	0.42
ttc	F	1	0.58
tat	Y	264	1340.00
tac	Y	2530	1454.00
cat	H	0	0.58
cac	H	1	0.42
---	--	------	------
mPD		0.17	0.50
		nPD:	0.34
		N. weight:	0.51
		Sc. PD:	0.053
		Sc. rank:	408.0

,

PB1			
Pos.	556	obs:	exp:
cgt	R	0	104.60
cgc	R	0	149.60
cga	R	4	281.80
cgg	R	16	228.30
aga	R	1685	1305.00
agg	R	1091	726.40
---	--	------	------
mPD		0.49	0.95
		nPD:	0.52
		N. weight:	0.43
		Sc. PD:	0.12
		Sc. rank:	479.2

,

PB1			
Pos.	557	obs:	exp:
tat	Y	6	1340.00
tac	Y	2788	1454.00
cat	H	0	1.16
cac	H	2	0.84
---	--	------	------
mPD		0.0057	0.50
		nPD:	0.01
		N. weight:	0.93
		Sc. PD:	-0.21
		Sc. rank:	-1416.5

,

PB1			
Pos.	558	obs:	exp:
act	T	7	733.00
acc	T	5	630.00
aca	T	1811	1170.00
acg	T	973	263.10
---	--	------	------
mPD		0.46	0.70
		nPD:	0.66
		N. weight:	1.1
		Sc. PD:	0.43
		Sc. rank:	1407.8

,

PB1			
Pos.	559	obs:	exp:
tct	S	0	0.17
tcc	S	1	0.14
tca	S	0	0.23
tcg	S	0	0.06
tat	Y	94	1340.00
tac	Y	2701	1455.00
agt	S	0	0.22
agc	S	0	0.18
---	--	------	------
mPD		0.066	0.50
		nPD:	0.13
		N. weight:	0.74
		Sc. PD:	-0.077
		Sc. rank:	-55.3

,

PB1			
Pos.	560	obs:	exp:
cgt	R	77	104.60
cgc	R	6	149.60
cga	R	2564	281.80
cgg	R	138	228.30
aga	R	11	1305.00
agg	R	0	726.40
---	--	------	------
mPD		0.16	0.95
		nPD:	0.16
		N. weight:	2.9
		Sc. PD:	-0.21
		Sc. rank:	492.5

,

PB1			
Pos.	561	obs:	exp:
ttt	F	0	0.42
ttc	F	1	0.58
tat	Y	0	0.48
tac	Y	1	0.52
tgt	C	290	1081.00
tgc	C	2504	1713.00
---	--	------	------
mPD		0.19	0.48
		nPD:	0.39
		N. weight:	0.3
		Sc. PD:	0.045
		Sc. rank:	268.2

,

PB1			
Pos.	562	obs:	exp:
cat	H	272	1619.00
cac	H	2524	1177.00
---	--	------	------
mPD		0.18	0.49
		nPD:	0.36
		N. weight:	0.75
		Sc. PD:	0.09
		Sc. rank:	621.4

,

PB1			
Pos.	563	obs:	exp:
cgt	R	0	104.60
cgc	R	0	149.60
cga	R	1	281.70
cgg	R	6	228.20
aga	R	2405	1305.00
agg	R	383	726.10
ggt	G	0	0.14
ggc	G	0	0.13
gga	G	1	0.44
ggg	G	0	0.29
---	--	------	------
mPD		0.25	0.95
		nPD:	0.26
		N. weight:	0.63
		Sc. PD:	0.012
		Sc. rank:	321.3

,

PB1			
Pos.	564	obs:	exp:
tgg	W	1	1.00
ggt	G	1083	404.20
ggc	G	14	367.10
gga	G	870	1225.00
ggg	G	828	799.00
---	--	------	------
mPD		0.67	0.69
		nPD:	0.97
		N. weight:	0.4
		Sc. PD:	0.28
		Sc. rank:	654.4

,

PB1			
Pos.	565	obs:	exp:
tat	Y	1	0.48
tac	Y	0	0.52
aat	N	0	0.55
aac	N	1	0.45
gat	D	1327	1509.00
gac	D	1467	1285.00
---	--	------	------
mPD		0.50	0.50
		nPD:	1.
		N. weight:	0.015
		Sc. PD:	0.011
		Sc. rank:	25.3

,

PB1			
Pos.	566	obs:	exp:
att	I	0	0.73
atc	I	0	0.54
ata	I	2	0.73
atg	M	134	134.00
act	T	162	695.00
acc	T	0	597.30
aca	T	998	1109.00
acg	T	1491	249.40
aaa	K	0	0.57
aag	K	1	0.43
gtt	V	0	0.43
gtc	V	0	0.42
gta	V	0	0.43
gtg	V	2	0.72
gct	A	0	1.51
gcc	A	0	1.16
gca	A	0	2.67
gcg	A	6	0.66
---	--	------	------
mPD		0.63	0.81
		nPD:	0.77
		N. weight:	1.2
		Sc. PD:	0.64
		Sc. rank:	1909.0

,

PB1

Pos.	567	obs:	exp:
cct	P	0	0.27
ccc	P	0	0.18
cca	P	1	0.41
ccg	P	0	0.14
cat	H	0	1.16
cac	H	2	0.84
caa	Q	2775	1391.00
cag	Q	11	1395.00
cgt	R	0	0.04
cgc	R	0	0.05
cga	R	1	0.10
cgg	R	0	0.08
aaa	K	5	2.85
aag	K	0	2.15
aga	R	0	0.47
agg	R	0	0.26
gaa	E	1	0.56
gag	E	0	0.44
---	--	------	------
mPD		0.015	0.51
		nPD:	0.03
		N. weight:	0.98
		Sc. PD:	-0.2
		Sc. rank:	-1135.9

,

PB1

Pos.	568	obs:	exp:
att	I	1199	1024.00
atc	I	1587	746.90
ata	I	5	1020.00
act	T	0	0.52
acc	T	2	0.45
aca	T	0	0.84
acg	T	0	0.19
gtt	V	2	0.65
gtc	V	1	0.63
gta	V	0	0.64
gtg	V	0	1.08
---	--	------	------
mPD		0.50	0.66
		nPD:	0.75
		N. weight:	0.72
		Sc. PD:	0.35
		Sc. rank:	1095.4

,

PB1

Pos.	569	obs:	exp:
cct	P	0	0.27
ccc	P	0	0.18
cca	P	1	0.41
ccg	P	0	0.14
cat	H	0	103.60
cac	H	179	75.38
caa	Q	2466	1305.00
cag	Q	147	1308.00
cgt	R	0	0.07
cgc	R	0	0.11
cga	R	2	0.20
cgg	R	0	0.16
aaa	K	1	0.57
aag	K	0	0.43
aga	R	0	0.93
agg	R	0	0.52
---	--	------	------
mPD		0.22	0.56
		nPD:	0.38
		N. weight:	0.74
		Sc. PD:	0.1
		Sc. rank:	656.5

,

PB1

Pos.	570	obs:	exp:
cgt	R	0	0.04
cgc	R	0	0.05
cga	R	0	0.10
cgg	R	0	0.08
act	T	12	732.70
acc	T	0	629.80
aca	T	290	1170.00
acg	T	2493	263.00
aga	R	0	0.47
agg	R	1	0.26
---	--	------	------
mPD		0.19	0.70
		nPD:	0.28
		N. weight:	2.7
		Sc. PD:	0.11
		Sc. rank:	1595.3

,

PB1

Pos.	571	obs:	exp:
cgt	R	0	103.70
cgc	R	0	148.20
cga	R	1	279.10
cgg	R	0	226.20
aaa	K	0	13.10
aag	K	23	9.90
aga	R	96	1293.00
agg	R	2673	719.60
ggt	G	0	0.43
ggc	G	0	0.39
gga	G	0	1.31
ggg	G	3	0.86
---	--	------	------
mPD		0.086	0.97
		nPD:	0.09
		N. weight:	1.7
		Sc. PD:	-0.25
		Sc. rank:	-693.8

,

PB1

Pos.	572	obs:	exp:
cgt	R	0	104.60
cgc	R	0	149.60
cga	R	0	281.80
cgg	R	0	228.30
aga	R	2711	1305.00
agg	R	85	726.40
---	--	------	------
mPD		0.059	0.95
		nPD:	0.06
		N. weight:	0.94
		Sc. PD:	-0.16
		Sc. rank:	-634.7

,

PB1

Pos.	573	obs:	exp:
tct	S	0	464.50
tcc	S	0	403.10
tca	S	2776	630.90
tcg	S	7	167.90
act	T	0	0.26
acc	T	0	0.23
aca	T	1	0.42
acg	T	0	0.09
agt	S	0	603.60
agc	S	0	512.90
gct	A	0	3.02
gcc	A	0	2.32
gca	A	12	5.34
gcg	A	0	1.31
---	--	------	------
mPD		0.014	1.7
		nPD:	0.01
		N. weight:	2.1
		Sc. PD:	-0.48
		Sc. rank:	-3340.4

,

PB1

Pos.	574	obs:	exp:
ttt	F	165	1166.00
ttc	F	2629	1628.00
tta	L	0	0.18
ttg	L	1	0.38
ctt	L	0	0.40
ctc	L	1	0.32
cta	L	0	0.30
ctg	L	0	0.42
---	--	------	------
mPD		0.11	0.49
		nPD:	0.23
		N. weight:	0.49
		Sc. PD:	-0.0036
		Sc. rank:	186.2

,

PB1

Pos.	575	obs:	exp:
gaa	E	29	1567.00
gag	E	2767	1229.00
---	--	------	------
mPD		0.021	0.49
		nPD:	0.04
		N. weight:	1.1
		Sc. PD:	-0.21
		Sc. rank:	-1043.0

,

PB1

Pos.	576	obs:	exp:
tta	L	2	258.30
ttg	L	55	530.80
ctt	L	59	554.70
ctc	L	0	446.90
cta	L	54	413.50
ctg	L	2624	589.70
att	I	0	0.37
atc	I	0	0.27
ata	I	1	0.37
atg	M	1	1.00
---	--	------	------
mPD		0.12	1.1
		nPD:	0.11
		N. weight:	1.9
		Sc. PD:	-0.23
		Sc. rank:	-494.9

,

PB1

Pos.	577	obs:	exp:
cgt	R	0	3.78
cgc	R	0	5.41
cga	R	0	10.18
cgg	R	0	8.25
aaa	K	179	1534.00
aag	K	2515	1160.00
aga	R	3	47.15
agg	R	98	26.24
gaa	E	0	0.56
gag	E	1	0.44
---	--	------	------
mPD		0.19	0.58
		nPD:	0.33
		N. weight:	0.88
		Sc. PD:	0.078
		Sc. rank:	670.0

,

PB1

Pos.	578	obs:	exp:
caa	Q	0	0.50
cag	Q	1	0.50
cgt	R	0	0.75
cgc	R	0	1.07
cga	R	0	2.02
cgg	R	0	1.63
act	T	0	0.26
acc	T	0	0.23
aca	T	0	0.42
acg	T	1	0.09
aat	N	0	0.55
aac	N	1	0.45
aaa	K	164	1578.00
aag	K	2607	1193.00
aga	R	5	9.34
agg	R	15	5.20
gaa	E	0	1.12
gag	E	2	0.88
---	--	------	------
mPD		0.13	0.51
		nPD:	0.26
		N. weight:	0.88
		Sc. PD:	0.016
		Sc. rank:	447.9

,

PB1

Pos.	579	obs:	exp:
tta	L	94	258.50
ttg	L	69	531.20
ctt	L	8	555.10
ctc	L	0	447.20
cta	L	1074	413.80
ctg	L	1551	590.10
---	--	------	------
mPD		0.60	1.1
		nPD:	0.53
		N. weight:	1.2
		Sc. PD:	0.34
		Sc. rank:	1348.3

,

PB1

Pos.	580	obs:	exp:
tgg	W	2796	2796.00
---	--	------	------
mPD		0	0
		nPD:	1.
		N. weight:	0.
		Sc. PD:	0
		Sc. rank:	0

,

PB1			
Pos.	581	obs:	exp:
aaa	K	0	1.14
aag	K	2	0.86
gat	D	22	14.04
gac	D	4	11.96
gaa	E	1586	1547.00
gag	E	1174	1213.00
ggt	G	0	1.16
ggc	G	0	1.05
gga	G	2	3.51
ggg	G	6	2.29
---	--	------	------
mPD		0.51	0.51
		nPD:	0.99
		N. weight:	0.01
		Sc. PD:	0.0076
		Sc. rank:	17.1

,

PB1			
Pos.	582	obs:	exp:
cat	H	0	1.74
cac	H	3	1.26
caa	Q	1670	1394.00
cag	Q	1123	1399.00
---	--	------	------
mPD		0.48	0.50
		nPD:	0.96
		N. weight:	0.032
		Sc. PD:	0.023
		Sc. rank:	52.6

,

PB1			
Pos.	583	obs:	exp:
tct	S	0	0.17
tcc	S	1	0.14
tca	S	0	0.23
tcg	S	0	0.06
act	T	33	732.70
acc	T	2760	629.80
aca	T	2	1170.00
acg	T	0	263.00
agt	S	0	0.22
agc	S	0	0.18
---	--	------	------
mPD		0.025	0.70
		nPD:	0.04
		N. weight:	2.1
		Sc. PD:	-0.41
		Sc. rank:	-2168.5

,

PB1			
Pos.	584	obs:	exp:
tta	L	0	0.37
ttg	L	0	0.76
tgt	C	1	0.39
tgc	C	0	0.61
ctt	L	4	0.79
ctc	L	0	0.64
cta	L	0	0.59
ctg	L	0	0.84
cat	H	146	85.10
cac	H	1	61.90
cgt	R	2453	98.95
cgc	R	187	141.50
cga	R	4	266.40
cgg	R	0	215.90
aga	R	0	1234.00
agg	R	0	686.90
---	--	------	------
mPD		0.23	1.1
		nPD:	0.2
		N. weight:	4.2
		Sc. PD:	-0.14
		Sc. rank:	1251.6

,

PB1			
Pos.	585	obs:	exp:
tct	S	28	466.70
tcc	S	72	405.00
tca	S	2612	633.90
tcg	S	84	168.70
agt	S	0	606.50
agc	S	0	515.30
---	--	------	------
mPD		0.13	1.7
		nPD:	0.08
		N. weight:	1.8
		Sc. PD:	-0.28
		Sc. rank:	-952.7

,

PB1			
Pos.	586	obs:	exp:
caa	Q	0	1.00
cag	Q	2	1.00
cgt	R	0	0.64
cgc	R	0	0.91
cga	R	0	1.71
cgg	R	0	1.39
aaa	K	271	1581.00
aag	K	2506	1196.00
aga	R	1	7.94
agg	R	16	4.42
---	--	------	------
mPD		0.19	0.51
		nPD:	0.37
		N. weight:	0.73
		Sc. PD:	0.096
		Sc. rank:	636.7

,

PB1			
Pos.	587	obs:	exp:
tct	S	0	0.17
tcc	S	0	0.14
tca	S	1	0.23
tcg	S	0	0.06
act	T	0	2.36
acc	T	0	2.03
aca	T	9	3.77
acg	T	0	0.85
agt	S	0	0.22
agc	S	0	0.18
gtt	V	0	0.43
gtc	V	0	0.42
gta	V	2	0.43
gtg	V	0	0.72
gct	A	1	701.30
gcc	A	4	538.70
gca	A	2770	1239.00
gcg	A	9	304.70
---	--	------	------
mPD		0.019	0.70
		nPD:	0.03
		N. weight:	1.1
		Sc. PD:	-0.23
		Sc. rank:	-1397.1

,

PB1			
Pos.	588	obs:	exp:
cgt	R	0	0.07
cgc	R	0	0.11
cga	R	0	0.20
cgg	R	0	0.16
aga	R	2	0.93
agg	R	0	0.52
gct	A	1	0.25
gcc	A	0	0.19
gca	A	0	0.45
gcg	A	0	0.11
gaa	E	1	0.56
gag	E	0	0.44
ggt	G	5	403.80
ggc	G	12	366.70
gga	G	2537	1223.00
ggg	G	238	798.10
---	--	------	------
mPD		0.17	0.69
		nPD:	0.25
		N. weight:	0.79
		Sc. PD:	0.0066
		Sc. rank:	364.9

,

PB1			
Pos.	589	obs:	exp:
tta	L	1	258.50
ttg	L	20	531.20
ctt	L	0	555.10
ctc	L	2	447.20
cta	L	702	413.80
ctg	L	2071	590.10
---	--	------	------
mPD		0.39	1.1
		nPD:	0.35
		N. weight:	1.5
		Sc. PD:	0.16
		Sc. rank:	1222.8

,

PB1			
Pos.	590	obs:	exp:
tta	L	293	258.50
ttg	L	2406	531.20
ctt	L	0	555.10
ctc	L	0	447.20
cta	L	26	413.80
ctg	L	71	590.10
---	--	------	------
mPD		0.27	1.1
		nPD:	0.24
		N. weight:	1.8
		Sc. PD:	0.0035
		Sc. rank:	782.0

,

PB1			
Pos.	591	obs:	exp:
att	I	40	14.68
atc	I	0	10.70
ata	I	0	14.62
gtt	V	1549	592.60
gtc	V	57	579.60
gta	V	283	587.40
gtg	V	866	995.40
gaa	E	1	0.56
gag	E	0	0.44
---	--	------	------
mPD		0.60	0.76
		nPD:	0.78
		N. weight:	0.56
		Sc. PD:	0.3
		Sc. rank:	884.7

,

PB1			
Pos.	592	obs:	exp:
tct	S	4	466.40
tcc	S	88	404.70
tca	S	2676	633.40
tcg	S	26	168.60
agt	S	0	606.00
agc	S	0	514.90
gct	A	0	0.50
gcc	A	0	0.39
gca	A	2	0.89
gcg	A	0	0.22
---	--	------	------
mPD		0.083	1.7
		nPD:	0.05
		N. weight:	1.9
		Sc. PD:	-0.35
		Sc. rank:	-1530.1

,

PB1			
Pos.	593	obs:	exp:
gtt	V	1	0.22
gtc	V	0	0.21
gta	V	0	0.21
gtg	V	0	0.36
gat	D	2747	1509.00
gac	D	47	1285.00
gaa	E	0	0.56
gag	E	1	0.44
---	--	------	------
mPD		0.034	0.50
		nPD:	0.07
		N. weight:	0.78
		Sc. PD:	-0.13
		Sc. rank:	-481.9

,

PB1			
Pos.	594	obs:	exp:
gct	A	0	0.25
gcc	A	0	0.19
gca	A	1	0.45
gcg	A	0	0.11
ggt	G	1	404.20
ggc	G	0	367.10
gga	G	2751	1225.00
ggg	G	43	799.00
---	--	------	------
mPD		0.032	0.69
		nPD:	0.05
		N. weight:	1.1
		Sc. PD:	-0.2
		Sc. rank:	-937.2

,

PB1			
Pos.	595	obs:	exp:
cgt	R	0	0.07
cgc	R	0	0.11
cga	R	1	0.20
cgg	R	0	0.16
aga	R	1	0.93
agg	R	0	0.52
ggt	G	0	404.10
ggc	G	8	367.00
gga	G	2708	1224.00
ggg	G	78	798.70
---	--	------	------
mPD		0.061	0.69
		nPD:	0.09
		N. weight:	1.
		Sc. PD:	-0.15
		Sc. rank:	-419.1

,

PB1			
Pos.	596	obs:	exp:
tta	L	0	0.09
ttg	L	0	0.19
ctt	L	0	0.20
ctc	L	0	0.16
cta	L	1	0.15
ctg	L	0	0.21
cct	P	6	763.80
ccc	P	2	504.80
cca	P	2715	1134.00
ccg	P	72	391.90
---	--	------	------
mPD		0.056	0.71
		nPD:	0.08
		N. weight:	1.2
		Sc. PD:	-0.18
		Sc. rank:	-586.5

,

PB1			
Pos.	597	obs:	exp:
cat	H	1	0.58
cac	H	0	0.42
att	I	1	0.37
atc	I	0	0.27
ata	I	0	0.37
aat	N	2483	1523.00
aac	N	309	1269.00
aaa	K	1	0.57
aag	K	0	0.43
gat	D	1	0.54
gac	D	0	0.46
---	--	------	------
mPD		0.20	0.50
		nPD:	0.4
		N. weight:	0.41
		Sc. PD:	0.065
		Sc. rank:	373.4

,

PB1			
Pos.	598	obs:	exp:
tta	L	180	258.10
ttg	L	5	530.20
ctt	L	0	554.20
ctc	L	1	446.40
cta	L	2511	413.00
ctg	L	94	589.10
cct	P	0	1.37
ccc	P	0	0.90
cca	P	5	2.03
ccg	P	0	0.70
---	--	------	------
mPD		0.20	1.1
		nPD:	0.17
		N. weight:	2.2
		Sc. PD:	-0.14
		Sc. rank:	419.3

,

PB1			
Pos.	599	obs:	exp:
tat	Y	31	1340.00
tac	Y	2764	1455.00
gat	D	0	0.54
gac	D	1	0.46
---	--	------	------
mPD		0.023	0.50
		nPD:	0.05
		N. weight:	0.87
		Sc. PD:	-0.16
		Sc. rank:	-758.4

,

PB1			
Pos.	600	obs:	exp:
tct	S	0	0.17
tcc	S	0	0.14
tca	S	0	0.23
tcg	S	0	0.06
cat	H	2	1.16
cac	H	0	0.84
att	I	1	0.37
atc	I	0	0.27
ata	I	0	0.37
aat	N	1631	1523.00
aac	N	1161	1269.00
agt	S	1	0.22
agc	S	0	0.18
---	--	------	------
mPD		0.49	0.50
		nPD:	0.98
		N. weight:	0.0085
		Sc. PD:	0.0061
		Sc. rank:	14.0

,

PB1			
Pos.	601	obs:	exp:
att	I	928	1025.00
atc	I	1860	747.40
ata	I	5	1021.00
act	T	0	0.26
acc	T	1	0.23
aca	T	0	0.42
acg	T	0	0.09
gtt	V	0	0.43
gtc	V	2	0.42
gta	V	0	0.43
gtg	V	0	0.72
---	--	------	------
mPD		0.45	0.66
		nPD:	0.68
		N. weight:	0.83
		Sc. PD:	0.35
		Sc. rank:	1154.5

,

PB1			
Pos.	602	obs:	exp:
cgt	R	2	104.60
cgc	R	0	149.60
cga	R	1760	281.80
cgg	R	1003	228.30
aga	R	24	1305.00
agg	R	7	726.40
---	--	------	------
mPD		0.48	0.95
		nPD:	0.51
		N. weight:	2.4
		Sc. PD:	0.63
		Sc. rank:	2649.6

,

PB1			
Pos.	603	obs:	exp:
tat	Y	1	0.48
tac	Y	0	0.52
aat	N	2436	1524.00
aac	N	359	1271.00
---	--	------	------
mPD		0.22	0.50
		nPD:	0.45
		N. weight:	0.36
		Sc. PD:	0.075
		Sc. rank:	363.4

,

PB1			
Pos.	604	obs:	exp:
ttt	F	0	0.83
ttc	F	2	1.17
tta	L	2	258.30
ttg	L	0	530.80
ctt	L	221	554.70
ctc	L	2562	446.90
cta	L	9	413.50
ctg	L	0	589.70
---	--	------	------
mPD		0.16	1.1
		nPD:	0.14
		N. weight:	2.2
		Sc. PD:	-0.21
		Sc. rank:	-19.0

,

PB1			
Pos.	605	obs:	exp:
cct	P	1	0.27
ccc	P	0	0.18
cca	P	0	0.41
ccg	P	0	0.14
cat	H	2321	1618.00
cac	H	474	1177.00
---	--	------	------
mPD		0.28	0.49
		nPD:	0.58
		N. weight:	0.21
		Sc. PD:	0.071
		Sc. rank:	257.1

,

PB1			
Pos.	606	obs:	exp:
tta	L	0	0.09
ttg	L	0	0.19
ctt	L	0	0.20
ctc	L	1	0.16
cta	L	0	0.15
ctg	L	0	0.21
att	I	1706	1025.00
atc	I	1068	747.70
ata	I	20	1021.00
gtt	V	1	0.22
gtc	V	0	0.21
gta	V	0	0.21
gtg	V	0	0.36
---	--	------	------
mPD		0.48	0.66
		nPD:	0.73
		N. weight:	0.62
		Sc. PD:	0.29
		Sc. rank:	932.3

,

PB1			
Pos.	607	obs:	exp:
tct	S	2	0.33
tcc	S	0	0.29
tca	S	0	0.45
tcg	S	0	0.12
cct	P	1603	763.60
ccc	P	15	504.60
cca	P	1112	1134.00
ccg	P	64	391.70
agt	S	0	0.43
agc	S	0	0.37
---	--	------	------
mPD		0.51	0.71
		nPD:	0.72
		N. weight:	0.53
		Sc. PD:	0.25
		Sc. rank:	795.2

,

PB1			
Pos.	608	obs:	exp:
cgt	R	0	0.04
cgc	R	0	0.05
cga	R	0	0.10
cgg	R	0	0.08
aaa	K	0	1.14
aag	K	2	0.86
aga	R	0	0.47
agg	R	1	0.26
gaa	E	256	1566.00
gag	E	2537	1227.00
---	--	------	------
mPD		0.17	0.50
		nPD:	0.34
		N. weight:	0.72
		Sc. PD:	0.073
		Sc. rank:	565.7

,

PB1			
Pos.	609	obs:	exp:
tct	S	3	0.50
tcc	S	0	0.43
tca	S	0	0.68
tcg	S	0	0.18
agt	S	0	0.65
agc	S	0	0.55
gtt	V	44	594.70
gtc	V	2704	581.70
gta	V	17	589.60
gtg	V	0	999.00
gct	A	25	7.05
gcc	A	3	5.42
gca	A	0	12.46
gcg	A	0	3.06
---	--	------	------
mPD		0.086	0.76
		nPD:	0.11
		N. weight:	2.1
		Sc. PD:	-0.25
		Sc. rank:	-488.8

,

PB1			
Pos.	610	obs:	exp:
tgt	C	36	1071.00
tgc	C	2732	1697.00
cgt	R	0	0.04
cgc	R	1	0.05
cga	R	0	0.10
cgg	R	0	0.08
aga	R	0	0.47
agg	R	0	0.26
ggt	G	0	3.90
ggc	G	27	3.55
gga	G	0	11.83
ggg	G	0	7.72
---	--	------	------
mPD		0.045	0.50
		nPD:	0.09
		N. weight:	0.65
		Sc. PD:	-0.093
		Sc. rank:	-255.0

,

PB1			
Pos.	611	obs:	exp:
tta	L	348	258.10
ttg	L	1674	530.40
ctt	L	7	554.30
ctc	L	0	446.60
cta	L	1	413.20
ctg	L	762	589.30
atg	M	3	3.00
gtt	V	0	0.22
gtc	V	0	0.21
gta	V	0	0.21
gtg	V	1	0.36
---	--	------	------
mPD		0.62	1.1
		nPD:	0.56
		N. weight:	1.1
		Sc. PD:	0.35
		Sc. rank:	1331.8

,

PB1			
Pos.	612	obs:	exp:
aaa	K	1813	1592.00
aag	K	983	1204.00
---	--	------	------
mPD		0.46	0.49
		nPD:	0.93
		N. weight:	0.021
		Sc. PD:	0.014
		Sc. rank:	34.5

,

PB1			
Pos.	613	obs:	exp:
tgg	W	2796	2796.00
---	--	------	------
mPD		0	0
		nPD:	1.
		N. weight:	0.
		Sc. PD:	0
		Sc. rank:	0

,

PB1			
Pos.	614	obs:	exp:
gat	D	1	1.08
gac	D	1	0.92
gaa	E	1878	1564.00
gag	E	912	1226.00
ggt	G	0	0.58
ggc	G	0	0.53
gga	G	3	1.75
ggg	G	1	1.14
---	--	------	------
mPD		0.44	0.50
		nPD:	0.89
		N. weight:	0.042
		Sc. PD:	0.027
		Sc. rank:	68.4

,

PB1			
Pos.	615	obs:	exp:
tta	L	27	258.40
ttg	L	1586	531.00
ctt	L	1	554.90
ctc	L	0	447.10
cta	L	193	413.60
ctg	L	988	589.90
cct	P	0	0.27
ccc	P	0	0.18
cca	P	0	0.41
ccg	P	1	0.14
---	--	------	------
mPD		0.63	1.1
		nPD:	0.57
		N. weight:	1.1
		Sc. PD:	0.34
		Sc. rank:	1260.4

,

PB1			
Pos.	616	obs:	exp:
atg	M	2796	2796.00
---	--	------	------
mPD		0	0
		nPD:	1.
		N. weight:	0.
		Sc. PD:	0
		Sc. rank:	0

,

PB1			
Pos.	617	obs:	exp:
gat	D	1986	1510.00
gac	D	810	1286.00
---	--	------	------
mPD		0.41	0.50
		nPD:	0.83
		N. weight:	0.091
		Sc. PD:	0.052
		Sc. rank:	145.0

,

PB1			
Pos.	618	obs:	exp:
att	I	0	0.37
atc	I	0	0.27
ata	I	1	0.37
aaa	K	17	9.68
aag	K	0	7.32
gtt	V	0	23.87
gtc	V	0	23.35
gta	V	106	23.67
gtg	V	5	40.11
gct	A	0	0.25
gcc	A	0	0.19
gca	A	1	0.45
gcg	A	0	0.11
gaa	E	2656	1492.00
gag	E	5	1169.00
ggt	G	0	0.72
ggc	G	0	0.66
gga	G	5	2.19
ggg	G	0	1.43
---	--	------	------
mPD		0.10	0.61
		nPD:	0.17
		N. weight:	0.87
		Sc. PD:	-0.06
		Sc. rank:	152.9

,

PB1			
Pos.	619	obs:	exp:
aat	N	3	7.09
aac	N	10	5.91
gat	D	1338	1494.00
gac	D	1429	1273.00
gaa	E	9	5.05
gag	E	0	3.95
ggt	G	4	1.01
ggc	G	3	0.92
gga	G	0	3.07
ggg	G	0	2.00
---	--	------	------
mPD		0.52	0.52
		nPD:	1.
		N. weight:	0.022
		Sc. PD:	0.016
		Sc. rank:	35.5

,

PB1			
Pos.	620	obs:	exp:
ttt	F	0	0.42
ttc	F	1	0.58
tat	Y	205	1340.00
tac	Y	2590	1455.00
---	--	------	------
mPD		0.14	0.50
		nPD:	0.27
		N. weight:	0.58
		Sc. PD:	0.02
		Sc. rank:	332.8

,

PB1			
Pos.	621	obs:	exp:
caa	Q	279	1336.00
cag	Q	2396	1339.00
cgt	R	0	3.74
cgc	R	0	5.35
cga	R	74	10.08
cgg	R	26	8.17
aaa	K	7	11.96
aag	K	14	9.04
aga	R	0	46.68
agg	R	0	25.98
---	--	------	------
mPD		0.31	0.64
		nPD:	0.48
		N. weight:	0.6
		Sc. PD:	0.14
		Sc. rank:	634.7

,

PB1			
Pos.	622	obs:	exp:
tct	S	0	1.00
tcc	S	0	0.87
tca	S	0	1.36
tcg	S	0	0.36
tgt	C	0	2.71
tgc	C	7	4.29
agt	S	0	1.30
agc	S	6	1.11
gat	D	0	0.54
gac	D	1	0.46
ggt	G	653	402.30
ggc	G	2055	365.40
gga	G	72	1219.00
ggg	G	2	795.20
---	--	------	------
mPD		0.41	0.70
		nPD:	0.58
		N. weight:	1.9
		Sc. PD:	0.64
		Sc. rank:	2310.1

,

PB1			
Pos.	623	obs:	exp:
cgt	R	0	104.60
cgc	R	0	149.60
cga	R	0	281.80
cgg	R	4	228.30
aga	R	1682	1305.00
agg	R	1110	726.40
---	--	------	------
mPD		0.48	0.95
		nPD:	0.51
		N. weight:	0.46
		Sc. PD:	0.12
		Sc. rank:	506.5

,

PB1			
Pos.	624	obs:	exp:
tta	L	4	258.50
ttg	L	778	531.20
ctt	L	87	555.10
ctc	L	0	447.20
cta	L	32	413.80
ctg	L	1895	590.10
---	--	------	------
mPD		0.49	1.1
		nPD:	0.44
		N. weight:	1.2
		Sc. PD:	0.23
		Sc. rank:	1135.6

,

PB1			
Pos.	625	obs:	exp:
tgt	C	1641	1082.00
tgc	C	1155	1714.00
---	--	------	------
mPD		0.49	0.47
		nPD:	1.02
		N. weight:	0.12
		Sc. PD:	0.092
		Sc. rank:	199.6

,

PB1			
Pos.	626	obs:	exp:
aat	N	2718	1525.00
aac	N	78	1271.00
---	--	------	------
mPD		0.054	0.50
		nPD:	0.11
		N. weight:	0.71
		Sc. PD:	-0.089
		Sc. rank:	-184.0

,

PB1			
Pos.	627	obs:	exp:
tct	S	4	0.67
tcc	S	0	0.58
tca	S	0	0.91
tcg	S	0	0.24
cct	P	2708	762.50
ccc	P	81	503.90
cca	P	1	1132.00
ccg	P	0	391.20
cat	H	2	1.16
cac	H	0	0.84
agt	S	0	0.87
agc	S	0	0.74
---	--	------	------
mPD		0.061	0.71
		nPD:	0.09
		N. weight:	1.7
		Sc. PD:	-0.25
		Sc. rank:	-782.9

,

PB1			
Pos.	628	obs:	exp:
tta	L	5	256.60
ttg	L	70	527.20
ctt	L	7	551.00
ctc	L	73	443.90
cta	L	4	410.70
ctg	L	2616	585.70
cct	P	0	0.27
ccc	P	0	0.18
cca	P	0	0.41
ccg	P	1	0.14
caa	Q	0	2.00
cag	Q	4	2.00
cgt	R	0	0.04
cgc	R	0	0.05
cga	R	0	0.10
cgg	R	1	0.08
atg	M	15	15.00
aga	R	0	0.47
agg	R	0	0.26
---	--	------	------
mPD		0.13	1.1
		nPD:	0.11
		N. weight:	1.9
		Sc. PD:	-0.23
		Sc. rank:	-421.2

,

PB1			
Pos.	629	obs:	exp:
aat	N	1750	1525.00
aac	N	1046	1271.00
---	--	------	------
mPD		0.47	0.50
		nPD:	0.94
		N. weight:	0.022
		Sc. PD:	0.015
		Sc. rank:	35.2

,

PB1			
Pos.	630	obs:	exp:
cct	P	4	764.10
ccc	P	14	505.00
cca	P	2021	1135.00
ccg	P	757	392.00
---	--	------	------
mPD		0.40	0.71
		nPD:	0.57
		N. weight:	0.84
		Sc. PD:	0.27
		Sc. rank:	993.4

,

PB1			
Pos.	631	obs:	exp:
ttt	F	1204	1166.00
ttc	F	1592	1630.00
---	--	------	------
mPD		0.49	0.49
		nPD:	1.01
		N. weight:	0.0027
		Sc. PD:	0.002
		Sc. rank:	4.5

,

PB1			
Pos.	632	obs:	exp:
att	I	0	4.04
atc	I	11	2.94
ata	I	0	4.02
gtt	V	123	596.90
gtc	V	2628	583.80
gta	V	24	591.70
gtg	V	0	1003.00
gct	A	0	2.52
gcc	A	10	1.94
gca	A	0	4.45
gcg	A	0	1.09
---	--	------	------
mPD		0.12	0.75
		nPD:	0.15
		N. weight:	1.9
		Sc. PD:	-0.16
		Sc. rank:	190.1

,

PB1			
Pos.	633	obs:	exp:
tct	S	0	452.50
tcc	S	0	392.70
tca	S	0	614.60
tcg	S	0	163.60
act	T	0	1.31
acc	T	5	1.13
aca	T	0	2.09
acg	T	0	0.47
aat	N	3	39.81
aac	N	70	33.19
agt	S	601	588.00
agc	S	2110	499.60
ggt	G	1	1.01
ggc	G	6	0.92
gga	G	0	3.07
ggg	G	0	2.00
---	--	------	------
mPD		0.40	1.7
		nPD:	0.24
		N. weight:	1.6
		Sc. PD:	-0.002
		Sc. rank:	652.0

,

PB1			
Pos.	634	obs:	exp:
tat	Y	1	0.48
tac	Y	0	0.52
cct	P	2	0.55
ccc	P	0	0.36
cca	P	0	0.81
ccg	P	0	0.28
cat	H	2627	1615.00
cac	H	162	1174.00
aat	N	4	2.18
aac	N	0	1.82
---	--	------	------
mPD		0.11	0.49
		nPD:	0.23
		N. weight:	0.5
		Sc. PD:	-0.0031
		Sc. rank:	192.1

,

PB1			
Pos.	635	obs:	exp:
cgt	R	0	32.86
cgc	R	0	46.99
cga	R	0	88.48
cgg	R	0	71.69
atg	M	5	5.00
aaa	K	703	1088.00
aag	K	1208	822.90
aga	R	119	409.90
agg	R	759	228.10
ggt	G	0	0.29
ggc	G	0	0.26
gga	G	0	0.88
ggg	G	2	0.57
---	--	------	------
mPD		0.85	1.1
		nPD:	0.77
		N. weight:	0.49
		Sc. PD:	0.25
		Sc. rank:	753.4

,

PB1			
Pos.	636	obs:	exp:
gaa	E	2736	1565.00
gag	E	55	1226.00
ggt	G	0	0.72
ggc	G	0	0.66
gga	G	5	2.19
ggg	G	0	1.43
---	--	------	------
mPD		0.042	0.50
		nPD:	0.08
		N. weight:	0.71
		Sc. PD:	-0.11
		Sc. rank:	-340.5

,

PB1			
Pos.	637	obs:	exp:
ttt	F	1	0.42
ttc	F	0	0.58
tta	L	0	0.37
ttg	L	0	0.76
ctt	L	4	0.79
ctc	L	0	0.64
cta	L	0	0.59
ctg	L	0	0.84
att	I	2510	1021.00
atc	I	204	744.70
ata	I	69	1017.00
gtt	V	8	1.72
gtc	V	0	1.68
gta	V	0	1.71
gtg	V	0	2.89
---	--	------	------
mPD		0.19	0.67
		nPD:	0.28
		N. weight:	0.96
		Sc. PD:	0.041
		Sc. rank:	573.4

,

PB1			
Pos.	638	obs:	exp:
gat	D	18	10.26
gac	D	1	8.74
gaa	E	1650	1556.00
gag	E	1126	1220.00
ggt	G	0	0.14
ggc	G	0	0.13
gga	G	1	0.44
ggg	G	0	0.29
---	--	------	------
mPD		0.49	0.50
		nPD:	0.98
		N. weight:	0.011
		Sc. PD:	0.0077
		Sc. rank:	17.5

,

PB1			
Pos.	639	obs:	exp:
tct	S	1565	466.70
tcc	S	1073	405.00
tca	S	152	633.90
tcg	S	6	168.70
agt	S	0	606.50
agc	S	0	515.30
---	--	------	------
mPD		0.54	1.7
		nPD:	0.33
		N. weight:	1.4
		Sc. PD:	0.12
		Sc. rank:	1064.6

,

PB1			
Pos.	640	obs:	exp:
tta	L	2	0.18
ttg	L	0	0.38
ctt	L	0	0.40
ctc	L	0	0.32
cta	L	0	0.30
ctg	L	0	0.42
att	I	3	3.30
atc	I	4	2.41
ata	I	2	3.29
gtt	V	14	598.80
gtc	V	1560	585.70
gta	V	988	593.60
gtg	V	222	1006.00
gct	A	0	0.25
gcc	A	0	0.19
gca	A	1	0.45
gcg	A	0	0.11
---	--	------	------
mPD		0.56	0.74
		nPD:	0.76
		N. weight:	0.87
		Sc. PD:	0.44
		Sc. rank:	1334.1

,

PB1			
Pos.	641	obs:	exp:
act	T	0	0.26
acc	T	1	0.23
aca	T	0	0.42
acg	T	0	0.09
aat	N	180	1524.00
aac	N	2615	1271.00
---	--	------	------
mPD		0.12	0.50
		nPD:	0.24
		N. weight:	0.79
		Sc. PD:	0.0046
		Sc. rank:	358.8

,

PB1			
Pos.	642	obs:	exp:
tct	S	0	16.69
tcc	S	0	14.49
tca	S	0	22.67
tcg	S	0	6.03
act	T	2	0.52
acc	T	0	0.45
aca	T	0	0.84
acg	T	0	0.19
aat	N	2670	1469.00
aac	N	23	1224.00
aaa	K	0	0.57
aag	K	1	0.43
agt	S	100	21.69
agc	S	0	18.43
---	--	------	------
mPD		0.087	0.62
		nPD:	0.14
		N. weight:	0.87
		Sc. PD:	-0.082
		Sc. rank:	0.4

,

PB1			
Pos.	643	obs:	exp:
tct	S	1	0.17
tcc	S	0	0.14
tca	S	0	0.23
tcg	S	0	0.06
act	T	6	1.57
acc	T	0	1.35
aca	T	0	2.51
acg	T	0	0.56
agt	S	0	0.22
agc	S	0	0.18
gct	A	2674	702.30
gcc	A	104	539.50
gca	A	6	1241.00
gcg	A	4	305.20
ggt	G	0	0.14
ggc	G	0	0.13
gga	G	1	0.44
ggg	G	0	0.29
---	--	------	------
mPD		0.085	0.70
		nPD:	0.12
		N. weight:	1.8
		Sc. PD:	-0.2
		Sc. rank:	-269.6

,

PB1			
Pos.	644	obs:	exp:
tta	L	0	0.28
ttg	L	3	0.57
ctt	L	0	0.60
ctc	L	0	0.48
cta	L	0	0.44
ctg	L	0	0.63
cct	P	0	0.82
ccc	P	0	0.54
cca	P	3	1.22
ccg	P	0	0.42
att	I	0	257.20
atc	I	0	187.60
ata	I	701	256.20
atg	M	422	422.00
act	T	0	5.51
acc	T	0	4.73
aca	T	19	8.79
acg	T	2	1.98
gtt	V	2	354.00
gtc	V	0	346.30
gta	V	437	351.00
gtg	V	1207	594.70
---	--	------	------
mPD		0.99	1.2
		nPD:	0.8
		N. weight:	0.87
		Sc. PD:	0.48
		Sc. rank:	1384.7

,

PB1			
Pos.	645	obs:	exp:
att	I	0	13.94
atc	I	0	10.17
ata	I	38	13.89
gtt	V	0	593.20
gtc	V	0	580.20
gta	V	1857	588.10
gtg	V	901	996.50
---	--	------	------
mPD		0.46	0.76
		nPD:	0.61
		N. weight:	1.1
		Sc. PD:	0.39
		Sc. rank:	1366.1

,

PB1			
Pos.	646	obs:	exp:
tta	L	0	0.28
ttg	L	1	0.57
ctt	L	0	0.60
ctc	L	0	0.48
cta	L	0	0.44
ctg	L	2	0.63
cgt	R	0	0.07
cgc	R	0	0.11
cga	R	0	0.20
cgg	R	0	0.16
att	I	2	2.20
atc	I	1	1.61
ata	I	3	2.19
atg	M	2778	2778.00
aga	R	2	0.93
agg	R	0	0.52
gtt	V	0	1.29
gtc	V	0	1.26
gta	V	0	1.28
gtg	V	6	2.17
gct	A	0	0.25
gcc	A	0	0.19
gca	A	0	0.45
gcg	A	1	0.11
---	--	------	------
mPD		0.015	0.020
		nPD:	0.77
		N. weight:	0.0082
		Sc. PD:	0.0042
		Sc. rank:	12.7

,

PB1			
Pos.	647	obs:	exp:
tct	S	0	0.67
tcc	S	0	0.58
tca	S	4	0.91
tcg	S	0	0.24
cct	P	8	763.00
ccc	P	3	504.30
cca	P	2642	1133.00
ccg	P	139	391.40
agt	S	0	0.87
agc	S	0	0.74
---	--	------	------
mPD		0.10	0.71
		nPD:	0.15
		N. weight:	1.1
		Sc. PD:	-0.095
		Sc. rank:	49.1

,

PB1			
Pos.	648	obs:	exp:
tct	S	4	0.67
tcc	S	0	0.58
tca	S	0	0.91
tcg	S	0	0.24
act	T	2	0.52
acc	T	0	0.45
aca	T	0	0.84
acg	T	0	0.19
agt	S	0	0.87
agc	S	0	0.74
gtt	V	6	1.72
gtc	V	2	1.68
gta	V	0	1.71
gtg	V	0	2.89
gct	A	1704	700.80
gcc	A	786	538.30
gca	A	278	1238.00
gcg	A	14	304.50
---	--	------	------
mPD		0.54	0.70
		nPD:	0.78
		N. weight:	0.72
		Sc. PD:	0.38
		Sc. rank:	1129.8

,

PB1			
Pos.	649	obs:	exp:
cat	H	2031	1619.00
cac	H	765	1177.00
---	--	------	------
mPD		0.40	0.49
		nPD:	0.82
		N. weight:	0.071
		Sc. PD:	0.04
		Sc. rank:	111.9

PB1			
Pos.	650	obs:	exp:
ggt	G	1784	404.30
ggc	G	997	367.30
gga	G	2	1225.00
ggg	G	13	799.20
---	--	------	------
mPD		0.47	0.69
		nPD:	0.68
		N. weight:	1.9
		Sc. PD:	0.8
		Sc. rank:	2622.4

PB1			
Pos.	651	obs:	exp:
cct	P	4	764.10
ccc	P	5	505.00
cca	P	1419	1135.00
ccg	P	1368	392.00
---	--	------	------
mPD		0.50	0.71
		nPD:	0.71
		N. weight:	1.
		Sc. PD:	0.48
		Sc. rank:	1539.9

PB1			
Pos.	652	obs:	exp:
act	T	0	0.52
acc	T	2	0.45
aca	T	0	0.84
acg	T	0	0.19
gtt	V	0	1.72
gtc	V	8	1.68
gta	V	0	1.71
gtg	V	0	2.89
gct	A	7	701.80
gcc	A	2776	539.10
gca	A	3	1240.00
gcg	A	0	304.90
---	--	------	------
mPD		0.014	0.70
		nPD:	0.02
		N. weight:	2.4
		Sc. PD:	-0.5
		Sc. rank:	-3054.6

PB1			
Pos.	653	obs:	exp:
caa	Q	0	1.00
cag	Q	2	1.00
cgt	R	0	0.30
cgc	R	0	0.43
cga	R	0	0.81
cgg	R	0	0.65
atg	M	1	1.00
aaa	K	807	1586.00
aag	K	1978	1199.00
aga	R	3	3.73
agg	R	5	2.08
---	--	------	------
mPD		0.42	0.50
		nPD:	0.84
		N. weight:	0.24
		Sc. PD:	0.14
		Sc. rank:	379.7

PB1			
Pos.	654	obs:	exp:
tct	S	0	449.40
tcc	S	0	389.90
tca	S	0	610.30
tcg	S	0	162.40
att	I	0	0.37
atc	I	1	0.27
ata	I	0	0.37
aat	N	34	22.36
aac	N	7	18.64
agt	S	1511	583.90
agc	S	1181	496.10
gct	A	42	10.83
gcc	A	0	8.32
gca	A	0	19.14
gcg	A	1	4.71
ggt	G	19	2.75
ggc	G	0	2.50
gga	G	0	8.33
ggg	G	0	5.43
---	--	------	------
mPD		0.59	1.7
		nPD:	0.35
		N. weight:	1.3
		Sc. PD:	0.15
		Sc. rank:	1091.8

PB1			
Pos.	655	obs:	exp:
cgt	R	0	0.04
cgc	R	0	0.05
cga	R	0	0.10
cgg	R	0	0.08
att	I	5	5.87
atc	I	0	4.28
ata	I	11	5.85
atg	M	2763	2763.00
act	T	0	0.79
acc	T	0	0.68
aca	T	0	1.26
acg	T	3	0.28
aga	R	0	0.47
agg	R	1	0.26
gtt	V	0	2.80
gtc	V	0	2.74
gta	V	0	2.77
gtg	V	13	4.70
---	--	------	------
mPD		0.024	0.032
		nPD:	0.74
		N. weight:	0.015
		Sc. PD:	0.0074
		Sc. rank:	23.2

PB1			
Pos.	656	obs:	exp:
gat	D	17	9.18
gac	D	0	7.82
gaa	E	2557	1557.00
gag	E	221	1221.00
ggt	G	0	0.14
ggc	G	0	0.13
gga	G	1	0.44
ggg	G	0	0.29
---	--	------	------
mPD		0.16	0.50
		nPD:	0.32
		N. weight:	0.47
		Sc. PD:	0.035
		Sc. rank:	329.8

PB1			
Pos.	657	obs:	exp:
tat	Y	2767	1341.00
tac	Y	29	1455.00
---	--	------	------
mPD		0.021	0.50
		nPD:	0.04
		N. weight:	0.99
		Sc. PD:	-0.19
		Sc. rank:	-930.5

PB1			
Pos.	658	obs:	exp:
gat	D	2785	1510.00
gac	D	11	1286.00
---	--	------	------
mPD		0.0078	0.50
		nPD:	0.02
		N. weight:	0.86
		Sc. PD:	-0.19
		Sc. rank:	-1206.4

PB1			
Pos.	659	obs:	exp:
gct	A	571	704.40
gcc	A	2216	541.10
gca	A	9	1245.00
gcg	A	0	306.00
---	--	------	------
mPD		0.33	0.69
		nPD:	0.48
		N. weight:	1.5
		Sc. PD:	0.36
		Sc. rank:	1641.2

PB1			
Pos.	660	obs:	exp:
att	I	300	110.50
atc	I	0	80.55
ata	I	1	110.00
gtt	V	2417	536.60
gtc	V	26	524.90
gta	V	40	532.00
gtg	V	12	901.50
---	--	------	------
mPD		0.25	0.94
		nPD:	0.26
		N. weight:	1.9
		Sc. PD:	0.05
		Sc. rank:	1066.7

PB1			
Pos.	661	obs:	exp:
act	T	7	2.88
acc	T	0	2.48
aca	T	3	4.60
acg	T	1	1.03
gtt	V	0	0.22
gtc	V	0	0.21
gta	V	0	0.21
gtg	V	1	0.36
gct	A	109	701.30
gcc	A	55	538.70
gca	A	1702	1239.00
gcg	A	918	304.70
---	--	------	------
mPD		0.53	0.70
		nPD:	0.75
		N. weight:	0.65
		Sc. PD:	0.33
		Sc. rank:	994.8

PB1			
Pos.	662	obs:	exp:
act	T	1783	732.20
acc	T	999	629.30
aca	T	10	1169.00
acg	T	1	262.80
aat	N	0	1.64
aac	N	3	1.36
---	--	------	------
mPD		0.47	0.70
		nPD:	0.67
		N. weight:	1.
		Sc. PD:	0.44
		Sc. rank:	1445.2

PB1			
Pos.	663	obs:	exp:
act	T	48	733.00
acc	T	3	630.00
aca	T	2455	1170.00
acg	T	290	263.10
---	--	------	------
mPD		0.22	0.70
		nPD:	0.31
		N. weight:	0.89
		Sc. PD:	0.065
		Sc. rank:	620.9

PB1			
Pos.	664	obs:	exp:
tta	L	0	0.09
ttg	L	0	0.19
ctt	L	1	0.20
ctc	L	0	0.16
cta	L	0	0.15
ctg	L	0	0.21
cat	H	1826	1618.00
cac	H	969	1177.00
---	--	------	------
mPD		0.45	0.49
		nPD:	0.93
		N. weight:	0.02
		Sc. PD:	0.013
		Sc. rank:	32.5

PB1			
Pos.	665	obs:	exp:
ttt	F	2	0.83
ttc	F	0	1.17
tta	L	1	0.09
ttg	L	0	0.19
tct	S	532	466.00
tcc	S	80	404.40
tca	S	2103	632.90
tcg	S	77	168.40
ctt	L	0	0.20
ctc	L	0	0.16
cta	L	0	0.15
ctg	L	0	0.21
cct	P	0	0.27
ccc	P	0	0.18
cca	P	1	0.41
ccg	P	0	0.14
agt	S	0	605.60
agc	S	0	514.50
---	--	------	------
mPD		0.40	1.7
		nPD:	0.24
		N. weight:	1.3
		Sc. PD:	0.0037
		Sc. rank:	561.7

PB1			
Pos.	666	obs:	exp:
tgg	W	2796	2796.00
---	--	------	------
mPD		0	0
		nPD:	1.
		N. weight:	0.
		Sc. PD:	0
		Sc. rank:	0

PB1			
Pos.	667	obs:	exp:
att	I	2352	939.40
atc	I	199	685.00
ata	I	9	935.50
act	T	136	36.70
acc	T	4	31.55
aca	T	0	58.58
acg	T	0	13.17
gtt	V	89	20.65
gtc	V	7	20.20
gta	V	0	20.47
gtg	V	0	34.69
---	--	------	------
mPD		0.31	0.83
		nPD:	0.37
		N. weight:	1.1
		Sc. PD:	0.14
		Sc. rank:	958.3

PB1

Pos.	668	obs:	exp:
tct	S	3	0.50
tcc	S	0	0.43
tca	S	0	0.68
tcg	S	0	0.18
cct	P	2648	763.00
ccc	P	57	504.30
cca	P	78	1133.00
ccg	P	9	391.40
cgt	R	1	0.04
cgc	R	0	0.05
cga	R	0	0.10
cgg	R	0	0.08
agt	S	0	0.65
agc	S	0	0.55
aga	R	0	0.47
agg	R	0	0.26
---	--	------	------
mPD		0.10	0.71
		nPD:	0.14
		N. weight:	1.5
		Sc. PD:	-0.14
		Sc. rank:	50.2

PB1

Pos.	669	obs:	exp:
cgt	R	0	0.15
cgc	R	0	0.21
cga	R	0	0.40
cgg	R	0	0.33
aaa	K	2466	1590.00
aag	K	326	1202.00
aga	R	0	1.87
agg	R	4	1.04
---	--	------	------
mPD		0.21	0.49
		nPD:	0.43
		N. weight:	0.35
		Sc. PD:	0.064
		Sc. rank:	333.1

PB1

Pos.	670	obs:	exp:
cgt	R	0	104.60
cgc	R	0	149.60
cga	R	0	281.80
cgg	R	10	228.30
aga	R	93	1305.00
agg	R	2693	726.40
---	--	------	------
mPD		0.071	0.95
		nPD:	0.07
		N. weight:	1.7
		Sc. PD:	-0.27
		Sc. rank:	-933.7

PB1

Pos.	671	obs:	exp:
aat	N	2218	1525.00
aac	N	578	1271.00
---	--	------	------
mPD		0.33	0.50
		nPD:	0.66
		N. weight:	0.2
		Sc. PD:	0.082
		Sc. rank:	266.4

PB1

Pos.	672	obs:	exp:
cgt	R	2567	104.60
cgc	R	221	149.60
cga	R	6	281.80
cgg	R	2	228.30
aga	R	0	1305.00
agg	R	0	726.40
---	--	------	------
mPD		0.15	0.95
		nPD:	0.16
		N. weight:	4.3
		Sc. PD:	-0.34
		Sc. rank:	611.7

PB1

Pos.	673	obs:	exp:
tct	S	29	466.70
tcc	S	2762	405.00
tca	S	5	633.90
tcg	S	0	168.70
agt	S	0	606.50
agc	S	0	515.30
---	--	------	------
mPD		0.024	1.7
		nPD:	0.01
		N. weight:	2.7
		Sc. PD:	-0.59
		Sc. rank:	-3906.3

PB1

Pos.	674	obs:	exp:
att	I	2663	1021.00
atc	I	117	744.20
ata	I	1	1016.00
gtt	V	0	3.23
gtc	V	15	3.16
gta	V	0	3.20
gtg	V	0	5.42
---	--	------	------
mPD		0.10	0.67
		nPD:	0.15
		N. weight:	1.2
		Sc. PD:	-0.1
		Sc. rank:	90.4

PB1

Pos.	675	obs:	exp:
ttt	F	0	0.83
ttc	F	2	1.17
tta	L	0	258.30
ttg	L	0	530.80
ctt	L	11	554.70
ctc	L	2770	446.90
cta	L	10	413.50
ctg	L	3	589.70
---	--	------	------
mPD		0.019	1.1
		nPD:	0.02
		N. weight:	2.6
		Sc. PD:	-0.56
		Sc. rank:	-3558.5

PB1

Pos.	676	obs:	exp:
aat	N	1747	1525.00
aac	N	1049	1271.00
---	--	------	------
mPD		0.47	0.50
		nPD:	0.95
		N. weight:	0.021
		Sc. PD:	0.014
		Sc. rank:	34.4

PB1

Pos.	677	obs:	exp:
act	T	65	732.70
acc	T	951	629.80
aca	T	431	1170.00
acg	T	1348	263.00
gct	A	0	0.25
gcc	A	1	0.19
gca	A	0	0.45
gcg	A	0	0.11
---	--	------	------
mPD		0.63	0.70
		nPD:	0.9
		N. weight:	1.1
		Sc. PD:	0.68
		Sc. rank:	1712.5

PB1

Pos.	678	obs:	exp:
tct	S	0	465.40
tcc	S	0	403.90
tca	S	0	632.00
tcg	S	0	168.20
cgt	R	0	0.07
cgc	R	0	0.11
cga	R	0	0.20
cgg	R	0	0.16
aat	N	4	2.18
aac	N	0	1.82
agt	S	2128	604.70
agc	S	660	513.80
aga	R	0	0.93
agg	R	2	0.52
ggt	G	2	0.29
ggc	G	0	0.26
gga	G	0	0.88
ggg	G	0	0.57
---	--	------	------
mPD		0.37	1.7
		nPD:	0.22
		N. weight:	1.5
		Sc. PD:	-0.024
		Sc. rank:	523.5

PB1

Pos.	679	obs:	exp:
tat	Y	1	0.48
tac	Y	0	0.52
cat	H	7	4.63
cac	H	1	3.37
caa	Q	2729	1390.00
cag	Q	56	1395.00
cgt	R	0	0.07
cgc	R	0	0.11
cga	R	0	0.20
cgg	R	2	0.16
aga	R	0	0.93
agg	R	0	0.52
---	--	------	------
mPD		0.049	0.51
		nPD:	0.1
		N. weight:	0.87
		Sc. PD:	-0.12
		Sc. rank:	-296.8

PB1

Pos.	680	obs:	exp:
cgt	R	0	103.90
cgc	R	0	148.60
cga	R	0	279.70
cgg	R	2	226.70
aaa	K	0	11.39
aag	K	20	8.61
aga	R	62	1296.00
agg	R	2712	721.20
---	--	------	------
mPD		0.059	0.97
		nPD:	0.06
		N. weight:	1.8
		Sc. PD:	-0.31
		Sc. rank:	-1250.7

PB1

Pos.	681	obs:	exp:
ggt	G	0	404.30
ggc	G	2	367.30
gga	G	2675	1225.00
ggg	G	119	799.20
---	--	------	------
mPD		0.083	0.69
		nPD:	0.12
		N. weight:	0.97
		Sc. PD:	-0.11
		Sc. rank:	-164.1

PB1

Pos.	682	obs:	exp:
att	I	2623	1022.00
atc	I	163	745.50
ata	I	0	1018.00
gtt	V	9	2.15
gtc	V	1	2.10
gta	V	0	2.13
gtg	V	0	3.61
---	--	------	------
mPD		0.12	0.67
		nPD:	0.18
		N. weight:	1.2
		Sc. PD:	-0.071
		Sc. rank:	233.2

PB1

Pos.	683	obs:	exp:
tta	L	0	258.50
ttg	L	0	531.20
ctt	L	2768	555.10
ctc	L	23	447.20
cta	L	4	413.80
ctg	L	1	590.10
---	--	------	------
mPD		0.020	1.1
		nPD:	0.02
		N. weight:	2.3
		Sc. PD:	-0.49
		Sc. rank:	-3023.9

PB1

Pos.	684	obs:	exp:
gaa	E	261	1567.00
gag	E	2535	1229.00
---	--	------	------
mPD		0.17	0.49
		nPD:	0.34
		N. weight:	0.72
		Sc. PD:	0.074
		Sc. rank:	569.5

PB1			
Pos.	685	obs:	exp:
gat	D	2742	1509.00
gac	D	53	1286.00
gaa	E	1	0.56
gag	E	0	0.44
---	--	------	------
mPD		0.038	0.50
		nPD:	0.08
		N. weight:	0.77
		Sc. PD:	-0.12
		Sc. rank:	-404.4

,

PB1			
Pos.	686	obs:	exp:
gaa	E	2336	1567.00
gag	E	460	1229.00
---	--	------	------
mPD		0.28	0.49
		nPD:	0.56
		N. weight:	0.25
		Sc. PD:	0.079
		Sc. rank:	295.2

,

PB1			
Pos.	687	obs:	exp:
cat	H	1	0.58
cac	H	0	0.42
caa	Q	189	1392.00
cag	Q	2599	1396.00
cgt	R	0	0.19
cgc	R	0	0.27
cga	R	0	0.50
cgg	R	5	0.41
aaa	K	0	1.14
aag	K	2	0.86
aga	R	0	2.33
agg	R	0	1.30
---	--	------	------
mPD		0.13	0.51
		nPD:	0.26
		N. weight:	0.66
		Sc. PD:	0.014
		Sc. rank:	340.1

,

PB1			
Pos.	688	obs:	exp:
att	I	0	1.83
atc	I	0	1.34
ata	I	5	1.83
atg	M	2791	2791.00
---	--	------	------
mPD		0.0036	0.0036
		nPD:	1.
		N. weight:	0.0026
		Sc. PD:	0.0019
		Sc. rank:	4.3

,

PB1			
Pos.	689	obs:	exp:
tct	S	0	1.17
tcc	S	7	1.01
tca	S	0	1.59
tcg	S	0	0.42
tat	Y	212	1338.00
tac	Y	2577	1451.00
agt	S	0	1.52
agc	S	0	1.29
---	--	------	------
mPD		0.15	0.51
		nPD:	0.29
		N. weight:	0.58
		Sc. PD:	0.027
		Sc. rank:	369.3

,

PB1			
Pos.	690	obs:	exp:
caa	Q	1177	1392.00
cag	Q	1612	1397.00
cgt	R	0	0.22
cgc	R	0	0.32
cga	R	2	0.60
cgg	R	4	0.49
aaa	K	0	0.57
aag	K	1	0.43
aga	R	0	2.80
agg	R	0	1.56
---	--	------	------
mPD		0.49	0.51
		nPD:	0.97
		N. weight:	0.026
		Sc. PD:	0.019
		Sc. rank:	43.1

,

PB1			
Pos.	691	obs:	exp:
cgt	R	0	0.26
cgc	R	0	0.37
cga	R	0	0.71
cgg	R	0	0.57
aaa	K	966	1588.00
aag	K	1823	1201.00
aga	R	2	3.27
agg	R	5	1.82
---	--	------	------
mPD		0.46	0.50
		nPD:	0.92
		N. weight:	0.15
		Sc. PD:	0.1
		Sc. rank:	246.7

,

PB1			
Pos.	692	obs:	exp:
tgt	C	148	1082.00
tgc	C	2647	1713.00
ggt	G	0	0.14
ggc	G	1	0.13
gga	G	0	0.44
ggg	G	0	0.29
---	--	------	------
mPD		0.10	0.48
		nPD:	0.21
		N. weight:	0.45
		Sc. PD:	-0.011
		Sc. rank:	144.2

,

PB1			
Pos.	693	obs:	exp:
tct	S	0	0.17
tcc	S	1	0.14
tca	S	0	0.23
tcg	S	0	0.06
tgt	C	183	1082.00
tgc	C	2612	1713.00
agt	S	0	0.22
agc	S	0	0.18
---	--	------	------
mPD		0.12	0.48
		nPD:	0.26
		N. weight:	0.41
		Sc. PD:	0.0083
		Sc. rank:	210.5

,

PB1			
Pos.	694	obs:	exp:
tct	S	0	137.20
tcc	S	0	119.10
tca	S	0	186.30
tcg	S	0	49.59
att	I	8	2.94
atc	I	0	2.14
ata	I	0	2.92
act	T	2	1.84
acc	T	5	1.58
aca	T	0	2.93
acg	T	0	0.66
aat	N	1621	976.70
aac	N	170	814.30
agt	S	801	178.30
agc	S	21	151.50
gat	D	1	0.54
gac	D	0	0.46
ggt	G	165	24.15
ggc	G	2	21.94
gga	G	0	73.18
ggg	G	0	47.74
---	--	------	------
mPD		0.71	1.5
		nPD:	0.47
		N. weight:	1.1
		Sc. PD:	0.24
		Sc. rank:	1113.9

,

PB1			
Pos.	695	obs:	exp:
tta	L	100	258.40
ttg	L	3	531.00
ctt	L	0	554.90
ctc	L	0	447.10
cta	L	2587	413.60
ctg	L	105	589.90
att	I	0	0.37
atc	I	0	0.27
ata	I	1	0.37
---	--	------	------
mPD		0.15	1.1
		nPD:	0.13
		N. weight:	2.3
		Sc. PD:	-0.24
		Sc. rank:	-186.8

,

PB1			
Pos.	696	obs:	exp:
ttt	F	304	1166.00
ttc	F	2492	1630.00
---	--	------	------
mPD		0.19	0.49
		nPD:	0.4
		N. weight:	0.34
		Sc. PD:	0.053
		Sc. rank:	307.9

,

PB1			
Pos.	697	obs:	exp:
gaa	E	318	1566.00
gag	E	2476	1228.00
ggt	G	0	0.29
ggc	G	0	0.26
gga	G	0	0.88
ggg	G	2	0.57
---	--	------	------
mPD		0.20	0.49
		nPD:	0.41
		N. weight:	0.64
		Sc. PD:	0.11
		Sc. rank:	593.2

,

PB1			
Pos.	698	obs:	exp:
cgt	R	0	0.07
cgc	R	0	0.11
cga	R	0	0.20
cgg	R	0	0.16
att	I	0	0.37
atc	I	0	0.27
ata	I	1	0.37
act	T	0	0.79
acc	T	0	0.68
aca	T	3	1.26
acg	T	0	0.28
aaa	K	2711	1589.00
aag	K	79	1201.00
aga	R	2	0.93
agg	R	0	0.52
---	--	------	------
mPD		0.059	0.50
		nPD:	0.12
		N. weight:	0.65
		Sc. PD:	-0.075
		Sc. rank:	-112.6

,

PB1			
Pos.	699	obs:	exp:
ttt	F	324	1166.00
ttc	F	2472	1630.00
---	--	------	------
mPD		0.20	0.49
		nPD:	0.42
		N. weight:	0.32
		Sc. PD:	0.058
		Sc. rank:	304.7

,

PB1			
Pos.	700	obs:	exp:
ttt	F	963	1166.00
ttc	F	1833	1630.00
---	--	------	------
mPD		0.45	0.49
		nPD:	0.93
		N. weight:	0.018
		Sc. PD:	0.012
		Sc. rank:	30.0

,

PB1			
Pos.	701	obs:	exp:
tta	L	0	0.09
ttg	L	0	0.19
ctt	L	0	0.20
ctc	L	1	0.16
cta	L	0	0.15
ctg	L	0	0.21
cct	P	124	763.00
ccc	P	2656	504.30
cca	P	12	1133.00
ccg	P	0	391.40
act	T	0	0.79
acc	T	3	0.68
aca	T	0	1.26
acg	T	0	0.28
---	--	------	------
mPD		0.096	0.71
		nPD:	0.13
		N. weight:	2.2
		Sc. PD:	-0.22
		Sc. rank:	-95.0

,

PB1

Pos.	702	obs:	exp:
tct	S	0	466.70
tcc	S	0	405.00
tca	S	0	633.90
tcg	S	0	168.70
agt	S	1688	606.50
agc	S	1108	515.30
mPD		0.48	1.7
		nPD:	0.29
		N. weight:	1.3
		Sc. PD:	0.068
		Sc. rank:	869.4

PB1

Pos.	703	obs:	exp:
tct	S	0	466.70
tcc	S	0	405.00
tca	S	0	633.90
tcg	S	0	168.70
agt	S	2732	606.50
agc	S	64	515.30
mPD		0.045	1.7
		nPD:	0.03
		N. weight:	2.1
		Sc. PD:	-0.43
		Sc. rank:	-2494.7

PB1

Pos.	704	obs:	exp:
tta	L	0	0.28
ttg	L	3	0.57
tct	S	5	465.00
tcc	S	5	403.60
tca	S	1899	631.60
tcg	S	877	168.10
ctt	L	0	0.60
ctc	L	0	0.48
cta	L	0	0.44
ctg	L	0	0.63
act	T	0	1.84
acc	T	0	1.58
aca	T	0	2.93
acg	T	7	0.66
agt	S	0	604.30
agc	S	0	513.40
mPD		0.45	1.7
		nPD:	0.27
		N. weight:	1.8
		Sc. PD:	0.056
		Sc. rank:	1036.7

PB1

Pos.	705	obs:	exp:
tat	Y	1619	1341.00
tac	Y	1177	1455.00
mPD		0.49	0.50
		nPD:	0.98
		N. weight:	0.031
		Sc. PD:	0.022
		Sc. rank:	51.2

PB1

Pos.	706	obs:	exp:
cgt	R	0	104.60
cgc	R	0	149.60
cga	R	16	281.70
cgg	R	1633	228.20
aaa	K	0	0.57
aag	K	1	0.43
aga	R	21	1305.00
agg	R	1125	726.10
mPD		0.51	0.95
		nPD:	0.54
		N. weight:	1.9
		Sc. PD:	0.54
		Sc. rank:	2147.8

PB1

Pos.	707	obs:	exp:
cgt	R	0	104.50
cgc	R	0	149.40
cga	R	0	281.40
cgg	R	0	228.00
aaa	K	0	0.57
aag	K	1	0.43
aga	R	1419	1303.00
agg	R	1373	725.30
ggt	G	0	0.43
ggc	G	0	0.39
gga	G	2	1.31
ggg	G	1	0.86
mPD		0.50	0.96
		nPD:	0.53
		N. weight:	0.52
		Sc. PD:	0.15
		Sc. rank:	591.3

PB1

Pos.	708	obs:	exp:
cct	P	20	763.80
ccc	P	2	504.80
cca	P	2704	1134.00
ccg	P	69	391.90
gct	A	0	0.25
gcc	A	0	0.19
gca	A	0	0.45
gcg	A	1	0.11
mPD		0.065	0.71
		nPD:	0.09
		N. weight:	1.1
		Sc. PD:	-0.16
		Sc. rank:	-432.1

PB1

Pos.	709	obs:	exp:
att	I	6	2.20
atc	I	0	1.61
ata	I	0	2.19
gtt	V	2758	600.10
gtc	V	16	587.00
gta	V	0	594.90
gtg	V	16	1008.00
mPD		0.027	0.74
		nPD:	0.04
		N. weight:	2.1
		Sc. PD:	-0.42
		Sc. rank:	-2216.5

PB1

Pos.	710	obs:	exp:
ggt	G	0	404.30
ggc	G	0	367.30
gga	G	2729	1225.00
ggg	G	67	799.20
mPD		0.047	0.69
		nPD:	0.07
		N. weight:	1.1
		Sc. PD:	-0.17
		Sc. rank:	-659.2

PB1

Pos.	711	obs:	exp:
ttt	F	1	0.42
ttc	F	0	0.58
att	I	2685	1026.00
atc	I	109	747.90
ata	I	1	1021.00
mPD		0.076	0.66
		nPD:	0.12
		N. weight:	1.2
		Sc. PD:	-0.15
		Sc. rank:	-252.3

PB1

Pos.	712	obs:	exp:
tct	S	22	466.70
tcc	S	2773	405.00
tca	S	1	633.90
tcg	S	0	168.70
agt	S	0	606.50
agc	S	0	515.30
mPD		0.016	1.7
		nPD:	0.01
		N. weight:	2.7
		Sc. PD:	-0.61
		Sc. rank:	-4252.8

PB1

Pos.	713	obs:	exp:
tct	S	0	465.90
tcc	S	0	404.30
tca	S	0	632.70
tcg	S	0	168.40
cgt	R	0	0.07
cgc	R	0	0.11
cga	R	0	0.20
cgg	R	0	0.16
act	T	3	0.79
acc	T	0	0.68
aca	T	0	1.26
acg	T	0	0.28
agt	S	868	605.40
agc	S	1923	514.30
aga	R	2	0.93
agg	R	0	0.52
mPD		0.43	1.7
		nPD:	0.26
		N. weight:	1.5
		Sc. PD:	0.034
		Sc. rank:	795.8

PB1

Pos.	714	obs:	exp:
att	I	1	0.73
atc	I	1	0.54
ata	I	0	0.73
atg	M	2794	2794.00
mPD		0.0014	0.0014
		nPD:	1.
		N. weight:	0.00085
		Sc. PD:	0.00063
		Sc. rank:	1.4

PB1

Pos.	715	obs:	exp:
tta	L	0	0.28
ttg	L	0	0.57
ctt	L	0	0.60
ctc	L	0	0.48
cta	L	0	0.44
ctg	L	3	0.63
atg	M	1	1.00
gtt	V	2	600.10
gtc	V	0	587.00
gta	V	95	594.90
gtg	V	2693	1008.00
gct	A	0	0.25
gcc	A	0	0.19
gca	A	1	0.45
gcg	A	0	0.11
ggt	G	0	0.14
ggc	G	0	0.13
gga	G	0	0.44
ggg	G	1	0.29
mPD		0.072	0.74
		nPD:	0.1
		N. weight:	1.3
		Sc. PD:	-0.18
		Sc. rank:	-428.1

PB1

Pos.	716	obs:	exp:
gat	D	0	1.08
gac	D	2	0.92
gaa	E	12	1566.00
gag	E	2781	1227.00
ggt	G	0	0.14
ggc	G	0	0.13
gga	G	0	0.44
ggg	G	1	0.29
mPD		0.011	0.49
		nPD:	0.02
		N. weight:	1.2
		Sc. PD:	-0.24
		Sc. rank:	-1484.6

PB1

Pos.	717	obs:	exp:
tct	S	0	0.17
tcc	S	1	0.14
tca	S	0	0.23
tcg	S	0	0.06
agt	S	0	0.22
agc	S	0	0.18
gct	A	8	704.10
gcc	A	2785	540.90
gca	A	2	1244.00
gcg	A	0	305.90
mPD		0.0078	0.69
		nPD:	0.01
		N. weight:	2.4
		Sc. PD:	-0.52
		Sc. rank:	-3594.6

PB1

Pos.	718	obs:	exp:
att	I	0	0.37
atc	I	1	0.27
ata	I	0	0.37
atg	M	2788	2788.00
aaa	K	0	3.99
aag	K	7	3.01
mPD		0.0057	0.0086
		nPD:	0.67
		N. weight:	0.0038
		Sc. PD:	0.0016
		Sc. rank:	5.2

PB1

Pos.	719	obs:	exp:
tta	L	0	0.09
ttg	L	1	0.19
ctt	L	0	0.20
ctc	L	0	0.16
cta	L	0	0.15
ctg	L	0	0.21
atg	M	17	17.00
gtt	V	89	596.20
gtc	V	1	583.20
gta	V	107	591.10
gtg	V	2575	1002.00
gct	A	0	1.51
gcc	A	0	1.16
gca	A	0	2.67
gcg	A	6	0.66
mPD		0.15	0.75
		nPD:	0.2
		N. weight:	1.1
		Sc. PD:	-0.039
		Sc. rank:	276.8

PB1			
Pos.	720	obs:	exp:
tct	S	2700	466.50
tcc	S	94	404.90
tca	S	1	633.60
tcg	S	0	168.60
tat	Y	1	0.48
tac	Y	0	0.52
agt	S	0	606.20
agc	S	0	515.10
---	--	------	------
mPD		0.066	1.7
		nPD:	0.04
		N. weight:	2.4
		Sc. PD:	-0.46
		Sc. rank:	-2326.1

PB1			
Pos.	721	obs:	exp:
tct	S	0	0.17
tcc	S	0	0.14
tca	S	0	0.23
tcg	S	0	0.06
cgt	R	0	104.50
cgc	R	0	149.40
cga	R	74	281.40
cgg	R	812	228.00
act	T	0	0.26
acc	T	0	0.23
aca	T	0	0.42
acg	T	1	0.09
aaa	K	0	1.14
aag	K	2	0.86
agt	S	1	0.22
agc	S	0	0.18
aga	R	0	1303.00
agg	R	1906	725.30
---	--	------	------
mPD		0.49	0.96
		nPD:	0.51
		N. weight:	1.5
		Sc. PD:	0.38
		Sc. rank:	1620.6

PB1			
Pos.	722	obs:	exp:
gct	A	265	703.60
gcc	A	2519	540.50
gca	A	9	1243.00
gcg	A	0	305.70
ggt	G	0	0.43
ggc	G	3	0.39
gga	G	0	1.31
ggg	G	0	0.86
---	--	------	------
mPD		0.18	0.69
		nPD:	0.26
		N. weight:	1.9
		Sc. PD:	0.04
		Sc. rank:	986.1

PB1			
Pos.	723	obs:	exp:
tta	L	0	0.09
ttg	L	0	0.19
ctt	L	0	0.20
ctc	L	0	0.16
cta	L	1	0.15
ctg	L	0	0.21
caa	Q	14	6.99
cag	Q	0	7.01
cgt	R	2	104.10
cgc	R	0	148.80
cga	R	2733	280.30
cgg	R	24	227.10
aga	R	22	1298.00
agg	R	0	722.50
---	--	------	------
mPD		0.045	0.97
		nPD:	0.05
		N. weight:	3.2
		Sc. PD:	-0.59
		Sc. rank:	-2675.0

PB1			
Pos.	724	obs:	exp:
att	I	2033	1026.00
atc	I	762	747.90
ata	I	0	1021.00
act	T	1	0.26
acc	T	0	0.23
aca	T	0	0.42
acg	T	0	0.09
---	--	------	------
mPD		0.40	0.66
		nPD:	0.6
		N. weight:	0.74
		Sc. PD:	0.26
		Sc. rank:	913.4

PB1			
Pos.	725	obs:	exp:
ttt	F	0	0.42
ttc	F	1	0.58
gtt	V	0	0.43
gtc	V	2	0.42
gta	V	0	0.43
gtg	V	0	0.72
gat	D	1305	1508.00
gac	D	1488	1285.00
---	--	------	------
mPD		0.50	0.50
		nPD:	1.
		N. weight:	0.02
		Sc. PD:	0.015
		Sc. rank:	32.2

PB1			
Pos.	726	obs:	exp:
tct	S	0	0.67
tcc	S	1	0.58
tca	S	3	0.91
tcg	S	0	0.24
cct	P	0	0.27
ccc	P	1	0.18
cca	P	0	0.41
ccg	P	0	0.14
agt	S	0	0.87
agc	S	0	0.74
gct	A	2	703.10
gcc	A	7	540.10
gca	A	2704	1242.00
gcg	A	78	305.50
---	--	------	------
mPD		0.065	0.69
		nPD:	0.09
		N. weight:	1.
		Sc. PD:	-0.14
		Sc. rank:	-357.2

PB1			
Pos.	727	obs:	exp:
cct	P	0	0.27
ccc	P	0	0.18
cca	P	1	0.41
ccg	P	0	0.14
cgt	R	10	104.60
cgc	R	855	149.60
cga	R	1869	281.70
cgg	R	49	228.20
aga	R	12	1305.00
agg	R	0	726.10
---	--	------	------
mPD		0.46	0.96
		nPD:	0.48
		N. weight:	2.5
		Sc. PD:	0.61
		Sc. rank:	2737.5

PB1			
Pos.	728	obs:	exp:
ttt	F	3	1.25
ttc	F	0	1.75
tta	L	0	0.09
ttg	L	0	0.19
ctt	L	1	0.20
ctc	L	0	0.16
cta	L	0	0.15
ctg	L	0	0.21
att	I	2713	1021.00
atc	I	70	744.70
ata	I	0	1017.00
act	T	2	0.52
acc	T	0	0.45
aca	T	0	0.84
acg	T	0	0.19
gtt	V	7	1.51
gtc	V	0	1.47
gta	V	0	1.49
gtg	V	0	2.53
---	--	------	------
mPD		0.058	0.67
		nPD:	0.09
		N. weight:	1.3
		Sc. PD:	-0.19
		Sc. rank:	-576.4

PB1			
Pos.	729	obs:	exp:
tat	Y	1	0.48
tac	Y	0	0.52
aat	N	1	0.55
aac	N	0	0.45
gat	D	2600	1508.00
gac	D	193	1285.00
gaa	E	1	0.56
gag	E	0	0.44
---	--	------	------
mPD		0.13	0.50
		nPD:	0.26
		N. weight:	0.55
		Sc. PD:	0.013
		Sc. rank:	295.3

PB1			
Pos.	730	obs:	exp:
ttt	F	66	1166.00
ttc	F	2728	1628.00
tta	L	0	0.09
ttg	L	0	0.19
tct	S	0	0.17
tcc	S	1	0.14
tca	S	0	0.23
tcg	S	0	0.06
ctt	L	0	0.20
ctc	L	1	0.16
cta	L	0	0.15
ctg	L	0	0.21
agt	S	0	0.22
agc	S	0	0.18
---	--	------	------
mPD		0.048	0.49
		nPD:	0.1
		N. weight:	0.64
		Sc. PD:	-0.087
		Sc. rank:	-215.7

PB1			
Pos.	731	obs:	exp:
aaa	K	0	0.57
aag	K	1	0.43
gaa	E	1129	1567.00
gag	E	1666	1228.00
---	--	------	------
mPD		0.48	0.49
		nPD:	0.98
		N. weight:	0.075
		Sc. PD:	0.054
		Sc. rank:	122.7

PB1

Pos.	732	obs:	exp:
ttt	F	2	0.83
ttc	F	0	1.17
tct	S	2760	463.00
tcc	S	11	401.80
tca	S	3	628.90
tcg	S	0	167.40
act	T	18	4.72
acc	T	0	4.06
aca	T	0	7.53
acg	T	0	1.69
agt	S	0	601.70
agc	S	0	511.20
gct	A	2	0.50
gcc	A	0	0.39
gca	A	0	0.89
gcg	A	0	0.22
---	--	------	------
mPD		0.026	1.7
		nPD:	0.02
		N. weight:	2.6
		Sc. PD:	-0.55
		Sc. rank:	-3583.7

,

PB1

Pos.	733	obs:	exp:
cgt	R	0	0.26
cgc	R	0	0.37
cga	R	0	0.71
cgg	R	0	0.57
aga	R	7	3.27
agg	R	0	1.82
gaa	E	1	0.56
gag	E	0	0.44
ggt	G	2	403.20
ggc	G	13	366.20
gga	G	2656	1222.00
ggg	G	117	797.00
---	--	------	------
mPD		0.096	0.69
		nPD:	0.14
		N. weight:	0.94
		Sc. PD:	-0.091
		Sc. rank:	-17.1

,

PB1

Pos.	734	obs:	exp:
cgt	R	0	104.50
cgc	R	0	149.40
cga	R	0	281.40
cgg	R	69	228.00
act	T	0	0.26
acc	T	0	0.23
aca	T	0	0.42
acg	T	1	0.09
aaa	K	0	1.14
aag	K	2	0.86
aga	R	11	1303.00
agg	R	2712	725.30
ggt	G	0	0.14
ggc	G	0	0.13
gga	G	0	0.44
ggg	G	1	0.29
---	--	------	------
mPD		0.059	0.96
		nPD:	0.06
		N. weight:	1.8
		Sc. PD:	-0.31
		Sc. rank:	-1234.4

,

PB1

Pos.	735	obs:	exp:
ttt	F	2	0.83
ttc	F	0	1.17
tct	S	0	0.33
tcc	S	0	0.29
tca	S	0	0.45
tcg	S	0	0.12
att	I	1833	1015.00
atc	I	920	739.90
ata	I	12	1010.00
atg	M	15	15.00
act	T	1	0.26
acc	T	0	0.23
aca	T	0	0.42
acg	T	0	0.09
agt	S	1	0.43
agc	S	1	0.37
gtt	V	8	2.15
gtc	V	2	2.10
gta	V	0	2.13
gtg	V	0	3.61
ggt	G	1	0.14
ggc	G	0	0.13
gga	G	0	0.44
ggg	G	0	0.29
---	--	------	------
mPD		0.47	0.68
		nPD:	0.69
		N. weight:	0.66
		Sc. PD:	0.29
		Sc. rank:	933.9

,

PB1

Pos.	736	obs:	exp:
tct	S	0	0.17
tcc	S	0	0.14
tca	S	0	0.23
tcg	S	0	0.06
caa	Q	0	1.00
cag	Q	2	1.00
cgt	R	0	0.34
cgc	R	0	0.48
cga	R	0	0.91
cgg	R	0	0.73
atg	M	3	3.00
aat	N	158	86.71
aac	N	1	72.29
aaa	K	60	1492.00
aag	K	2560	1128.00
agt	S	1	0.22
agc	S	0	0.18
aga	R	0	4.20
agg	R	9	2.34
gaa	E	0	1.12
gag	E	2	0.88
---	--	------	------
mPD		0.16	0.56
		nPD:	0.29
		N. weight:	1.1
		Sc. PD:	0.048
		Sc. rank:	664.2

,

PB1

Pos.	737	obs:	exp:
cgt	R	0	0.11
cgc	R	0	0.16
cga	R	0	0.30
cgg	R	0	0.24
act	T	0	0.52
acc	T	0	0.45
aca	T	1	0.84
acg	T	1	0.19
aat	N	0	1.09
aac	N	2	0.91
aaa	K	1988	1588.00
aag	K	801	1201.00
aga	R	1	1.40
agg	R	2	0.78
---	--	------	------
mPD		0.41	0.50
		nPD:	0.84
		N. weight:	0.069
		Sc. PD:	0.04
		Sc. rank:	110.6

,

PB1

Pos.	738	obs:	exp:
aaa	K	4	2.28
aag	K	0	1.72
gtt	V	0	0.43
gtc	V	0	0.42
gta	V	2	0.43
gtg	V	0	0.72
gat	D	0	2.16
gac	D	4	1.84
gaa	E	2326	1361.00
gag	E	101	1066.00
ggt	G	0	51.92
ggc	G	0	47.16
gga	G	358	157.30
ggg	G	1	102.60
---	--	------	------
mPD		0.30	0.76
		nPD:	0.4
		N. weight:	0.68
		Sc. PD:	0.11
		Sc. rank:	617.1

,

PB1

Pos.	739	obs:	exp:
tct	S	0	0.17
tcc	S	0	0.14
tca	S	0	0.23
tcg	S	0	0.06
aat	N	1	0.55
aac	N	0	0.45
aaa	K	0	2.28
aag	K	4	1.72
agt	S	1	0.22
agc	S	0	0.18
gtt	V	0	0.22
gtc	V	0	0.21
gta	V	0	0.21
gtg	V	1	0.36
gct	A	0	1.01
gcc	A	0	0.77
gca	A	0	1.78
gcg	A	4	0.44
gat	D	75	40.49
gac	D	0	34.51
gaa	E	54	1519.00
gag	E	2655	1190.00
ggt	G	0	0.14
ggc	G	0	0.13
gga	G	0	0.44
ggg	G	1	0.29
---	--	------	------
mPD		0.10	0.53
		nPD:	0.19
		N. weight:	1.1
		Sc. PD:	-0.051
		Sc. rank:	253.3

,

PB1

Pos.	740	obs:	exp:
ttt	F	2765	1163.00
ttc	F	23	1625.00
tta	L	1	0.65
ttg	L	0	1.33
tat	Y	1	0.48
tac	Y	0	0.52
ctt	L	6	1.39
ctc	L	0	1.12
cta	L	0	1.04
ctg	L	0	1.48
---	--	------	------
mPD		0.022	0.49
		nPD:	0.04
		N. weight:	1.2
		Sc. PD:	-0.23
		Sc. rank:	-1071.0

,

PB1

Pos.	741	obs:	exp:
tct	S	265	44.23
tcc	S	0	38.39
tca	S	0	60.08
tcg	S	0	15.99
act	T	7	1.84
acc	T	0	1.58
aca	T	0	2.93
acg	T	0	0.66
agt	S	0	57.48
agc	S	0	48.84
gtt	V	1	0.22
gtc	V	0	0.21
gta	V	0	0.21
gtg	V	0	0.36
gct	A	2494	635.60
gcc	A	25	488.20
gca	A	2	1123.00
gcg	A	2	276.10
---	--	------	------
mPD		0.20	0.95
		nPD:	0.21
		N. weight:	2.
		Sc. PD:	-0.059
		Sc. rank:	612.3

,

PB1

Pos.	742	obs:	exp:
aaa	K	0	1.71
aag	K	3	1.29
gat	D	1	0.54
gac	D	0	0.46
gaa	E	1	1565.00
gag	E	2791	1227.00
---	--	------	------
mPD		0.0036	0.50
		nPD:	0.01
		N. weight:	1.2
		Sc. PD:	-0.27
		Sc. rank:	-1899.2

,

PB1

Pos.	743	obs:	exp:
att	I	16	1023.00
atc	I	2771	746.10
ata	I	1	1019.00
atg	M	1	1.00
act	T	0	1.31
acc	T	5	1.13
aca	T	0	2.09
acg	T	0	0.47
gtt	V	0	0.43
gtc	V	2	0.42
gta	V	0	0.43
gtg	V	0	0.72
---	--	------	------
mPD		0.018	0.67
		nPD:	0.03
		N. weight:	1.9
		Sc. PD:	-0.38
		Sc. rank:	-2269.1

,

PB1

Pos.	744	obs:	exp:
tta	L	0	0.65
ttg	L	5	1.33
ctt	L	0	1.39
ctc	L	0	1.12
cta	L	0	1.04
ctg	L	2	1.48
att	I	1	38.16
atc	I	0	27.83
ata	I	103	38.01
atg	M	2649	2649.00
act	T	0	7.86
acc	T	0	6.76
aca	T	0	12.55
acg	T	30	2.82
aaa	K	0	0.57
aag	K	1	0.43
gtt	V	0	1.08
gtc	V	0	1.05
gta	V	0	1.07
gtg	V	5	1.81
---	--	------	------
mPD		0.10	0.13
		nPD:	0.81
		N. weight:	0.097
		Sc. PD:	0.053
		Sc. rank:	153.3

PB1

Pos.	745	obs:	exp:
cgt	R	0	5.20
cgc	R	0	7.44
cga	R	0	14.01
cgg	R	0	11.35
act	T	0	0.79
acc	T	0	0.68
aca	T	0	1.26
acg	T	3	0.28
aaa	K	0	1511.00
aag	K	2653	1142.00
aga	R	0	64.89
agg	R	139	36.11
gaa	E	0	0.56
gag	E	1	0.44
---	--	------	------
mPD		0.097	0.62
		nPD:	0.16
		N. weight:	1.3
		Sc. PD:	-0.1
		Sc. rank:	134.9

PB1

Pos.	746	obs:	exp:
att	I	3	993.40
atc	I	2703	724.40
ata	I	1	989.20
act	T	0	23.07
acc	T	88	19.83
aca	T	0	36.82
acg	T	0	8.28
gtt	V	0	0.22
gtc	V	1	0.21
gta	V	0	0.21
gtg	V	0	0.36
---	--	------	------
mPD		0.065	0.72
		nPD:	0.09
		N. weight:	1.9
		Sc. PD:	-0.28
		Sc. rank:	-760.4

PB1

Pos.	747	obs:	exp:
tgt	C	2787	1081.00
tgc	C	7	1713.00
cgt	R	2	0.07
cgc	R	0	0.11
cga	R	0	0.20
cgg	R	0	0.16
aga	R	0	0.93
agg	R	0	0.52
---	--	------	------
mPD		0.0064	0.48
		nPD:	0.01
		N. weight:	1.4
		Sc. PD:	-0.3
		Sc. rank:	-2022.9

PB1

Pos.	748	obs:	exp:
ttt	F	0	5.84
ttc	F	14	8.16
tct	S	5	463.50
tcc	S	2766	402.30
tca	S	6	629.50
tcg	S	0	167.50
cct	P	0	1.09
ccc	P	4	0.72
cca	P	0	1.62
ccg	P	0	0.56
agt	S	0	602.30
agc	S	0	511.80
gct	A	0	0.25
gcc	A	1	0.19
gca	A	0	0.45
gcg	A	0	0.11
---	--	------	------
mPD		0.021	1.7
		nPD:	0.01
		N. weight:	2.8
		Sc. PD:	-0.61
		Sc. rank:	-4153.7

PB1

Pos.	749	obs:	exp:
cct	P	0	1.91
ccc	P	7	1.26
cca	P	0	2.84
ccg	P	0	0.98
act	T	0	730.90
acc	T	2788	628.20
aca	T	0	1167.00
acg	T	0	262.30
gct	A	0	0.25
gcc	A	1	0.19
gca	A	0	0.45
gcg	A	0	0.11
---	--	------	------
mPD		0.0057	0.70
		nPD:	0.01
		N. weight:	2.2
		Sc. PD:	-0.49
		Sc. rank:	-3427.6

PB1

Pos.	750	obs:	exp:
tta	L	0	0.09
ttg	L	0	0.19
ctt	L	1	0.20
ctc	L	0	0.16
cta	L	0	0.15
ctg	L	0	0.21
att	I	2792	1025.00
atc	I	0	747.40
ata	I	1	1021.00
atg	M	1	1.00
act	T	1	0.26
acc	T	0	0.23
aca	T	0	0.42
acg	T	0	0.09
---	--	------	------
mPD		0.0029	0.66
		nPD:	0.
		N. weight:	1.5
		Sc. PD:	-0.33
		Sc. rank:	-2371.7

PB1

Pos.	751	obs:	exp:
aaa	K	2	1.14
aag	K	0	0.86
gaa	E	2771	1562.00
gag	E	16	1225.00
ggt	G	0	1.01
ggc	G	0	0.92
gga	G	7	3.07
ggg	G	0	2.00
---	--	------	------
mPD		0.018	0.50
		nPD:	0.04
		N. weight:	0.8
		Sc. PD:	-0.16
		Sc. rank:	-850.9

PB1

Pos.	752	obs:	exp:
aaa	K	2	1.14
aag	K	0	0.86
gtt	V	0	0.22
gtc	V	0	0.21
gta	V	1	0.21
gtg	V	0	0.36
gct	A	0	0.25
gcc	A	0	0.19
gca	A	1	0.45
gcg	A	0	0.11
gat	D	0	0.54
gac	D	1	0.46
gaa	E	329	1562.00
gag	E	2458	1225.00
ggt	G	0	0.58
ggc	G	0	0.53
gga	G	4	1.75
ggg	G	0	1.14
---	--	------	------
mPD		0.22	0.50
		nPD:	0.44
		N. weight:	0.63
		Sc. PD:	0.12
		Sc. rank:	617.1

PB1

Pos.	753	obs:	exp:
ttt	F	0	4.59
ttc	F	11	6.41
tta	L	0	257.00
ttg	L	0	528.10
ctt	L	11	552.00
ctc	L	2768	444.70
cta	L	1	411.40
ctg	L	0	586.80
cct	P	0	1.09
ccc	P	4	0.72
cca	P	0	1.62
ccg	P	0	0.56
att	I	0	0.37
atc	I	1	0.27
ata	I	0	0.37
---	--	------	------
mPD		0.020	1.1
		nPD:	0.02
		N. weight:	2.6
		Sc. PD:	-0.56
		Sc. rank:	-3515.0

PB1

Pos.	754	obs:	exp:
caa	Q	1	0.50
cag	Q	0	0.50
cgt	R	0	104.30
cgc	R	0	149.10
cga	R	2	280.80
cgg	R	0	227.50
aaa	K	3	1.71
aag	K	0	1.29
aga	R	2784	1301.00
agg	R	0	723.80
gaa	E	2	1.12
gag	E	0	0.88
ggt	G	0	0.58
ggc	G	0	0.53
gga	G	4	1.75
ggg	G	0	1.14
---	--	------	------
mPD		0.011	0.96
		nPD:	0.01
		N. weight:	1.1
		Sc. PD:	-0.24
		Sc. rank:	-1694.7

PB1

Pos.	755	obs:	exp:
tta	L	0	0.09
ttg	L	0	0.19
ctt	L	0	0.20
ctc	L	0	0.16
cta	L	0	0.15
ctg	L	1	0.21
cct	P	0	0.82
ccc	P	0	0.54
cca	P	0	1.22
ccg	P	3	0.42
caa	Q	0	0.50
cag	Q	1	0.50
cgt	R	0	104.40
cgc	R	2	149.40
cga	R	0	281.30
cgg	R	2789	227.90
aga	R	0	1303.00
agg	R	0	725.10
---	--	------	------
mPD		0.0050	0.96
		nPD:	0.01
		N. weight:	3.6
		Sc. PD:	-0.83
		Sc. rank:	-5834.0

PB1

Pos.	756	obs:	exp:
tta	L	0	0.09
ttg	L	0	0.19
ctt	L	0	0.20
ctc	L	0	0.16
cta	L	1	0.15
ctg	L	0	0.21
cct	P	2	75.43
ccc	P	137	49.85
cca	P	137	112.00
ccg	P	0	38.70
caa	Q	2436	1257.00
cag	Q	81	1260.00
cgt	R	0	0.07
cgc	R	0	0.11
cga	R	2	0.20
cgg	R	0	0.16
aga	R	0	0.93
agg	R	0	0.52
---	--	------	------
mPD		0.33	0.72
		nPD:	0.45
		N. weight:	0.82
		Sc. PD:	0.17
		Sc. rank:	822.4

PB1

Pos.	757	obs:	exp:
ggt	G	0	0.14
ggc	G	0	0.13
gga	G	0	0.44
ggg	G	1	0.29
---	-	2795	2795.00
---	--	------	------
mPD		0	8.8×10^{-8}
		nPD:	0.
		N. weight:	0.
		Sc. PD:	0
		Sc. rank:	0

,

PB1

Pos.	758	obs:	exp:
caa	Q	2	1.00
cag	Q	0	1.00
cgt	R	0	3.03
cgc	R	0	4.33
cga	R	0	8.16
cgg	R	0	6.61
act	T	0	2.10
acc	T	0	1.80
aca	T	8	3.35
acg	T	0	0.75
aat	N	7	3.82
aac	N	0	3.18
aaa	K	2496	1533.00
aag	K	197	1160.00
aga	R	74	37.81
agg	R	7	21.04
gaa	E	0	0.56
gag	E	1	0.44
ggt	G	0	0.58
ggc	G	0	0.53
gga	G	4	1.75
ggg	G	0	1.14
---	--	------	------
mPD		0.21	0.58
		nPD:	0.36
		N. weight:	0.49
		Sc. PD:	0.058
		Sc. rank:	401.9

,

PB1

Pos.	759	obs:	exp:
tgg	W	5	5.00
caa	Q	0	4.49
cag	Q	9	4.51
gtt	V	0	0.22
gtc	V	0	0.21
gta	V	0	0.21
gtg	V	1	0.36
gaa	E	0	15.14
gag	E	27	11.86
---	-	2754	2754.00
---	--	------	------
mPD		0.00017	0.00028
		nPD:	0.59
		N. weight:	0.
		Sc. PD:	0
		Sc. rank:	0

,

PB1

Pos.	760	obs:	exp:
taa	.	7	1031.00
tag	.	2746	958.80
tga	.	43	806.20
---	--	------	------
mPD		0.065	0.86
		nPD:	0.08
		N. weight:	1.4
		Sc. PD:	-0.22
		Sc. rank:	-766.9

}

Amino acid level conservation per codon

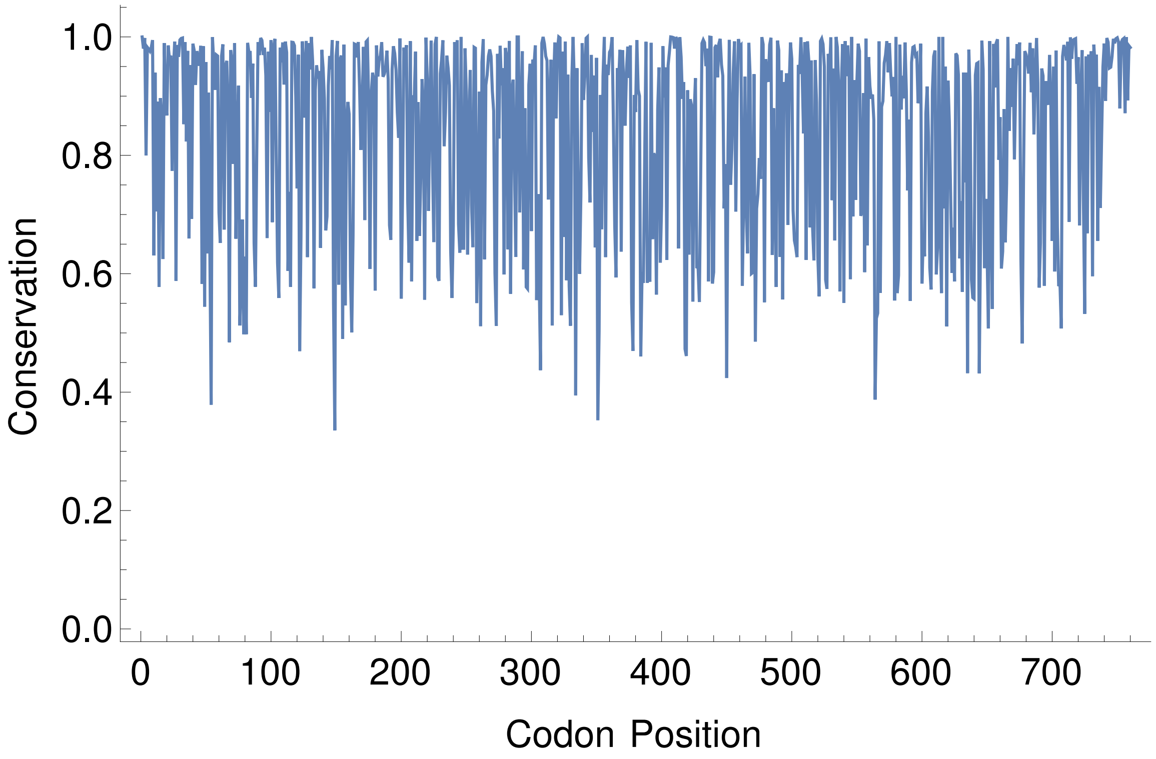

Conservation analysis plots

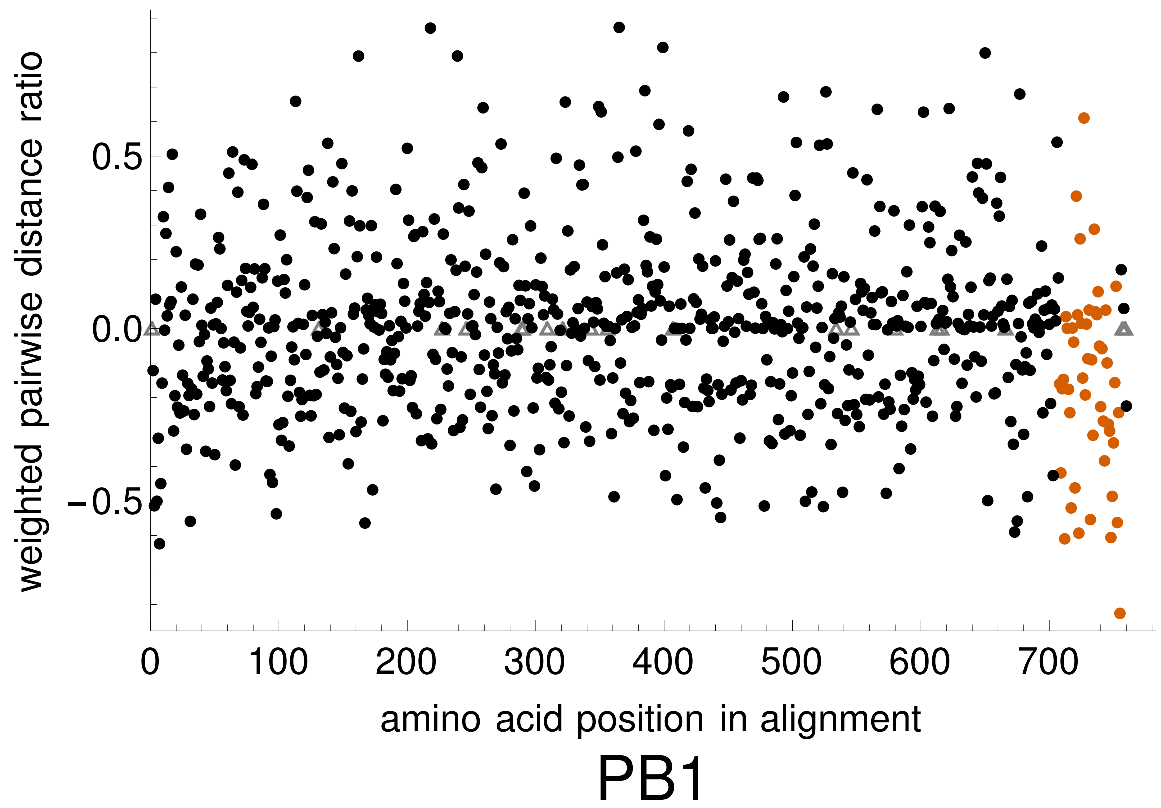

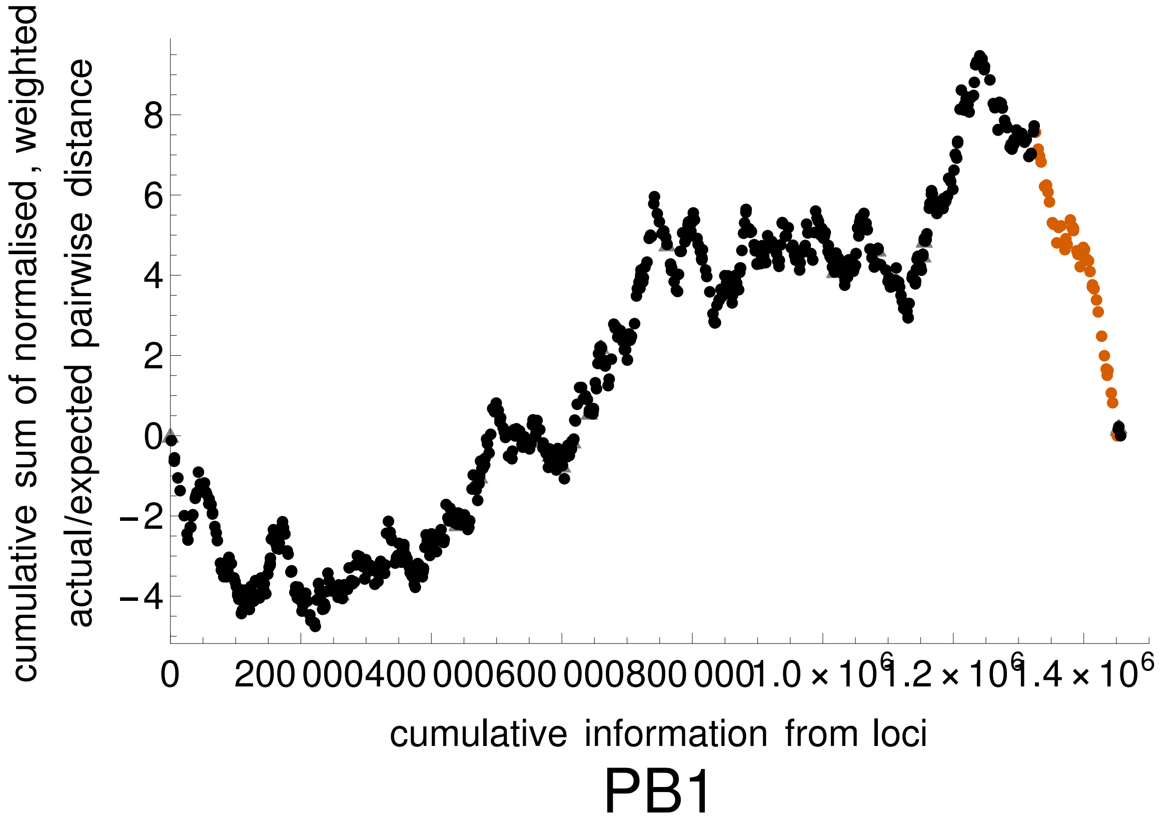

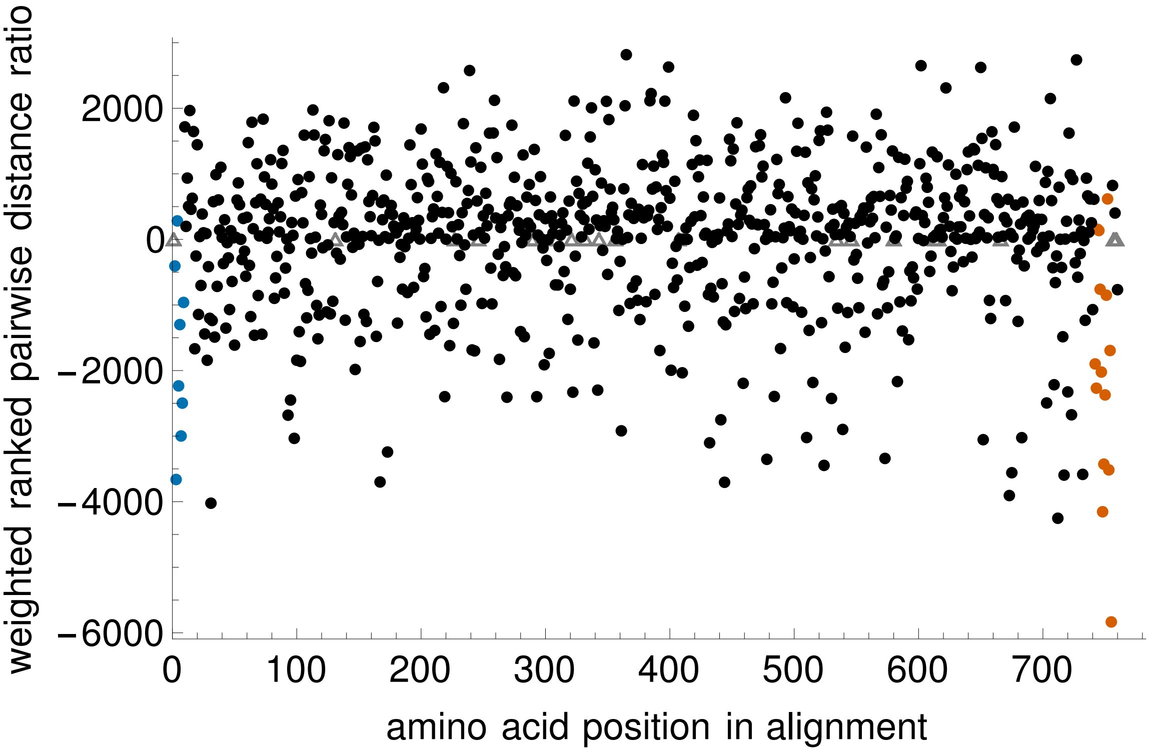
weighted ranked pairwise distance ratio
2000
0
−2000
−4000
−6000
0
100
200
300
400
500
600
700
amino acid position in alignment

PB1

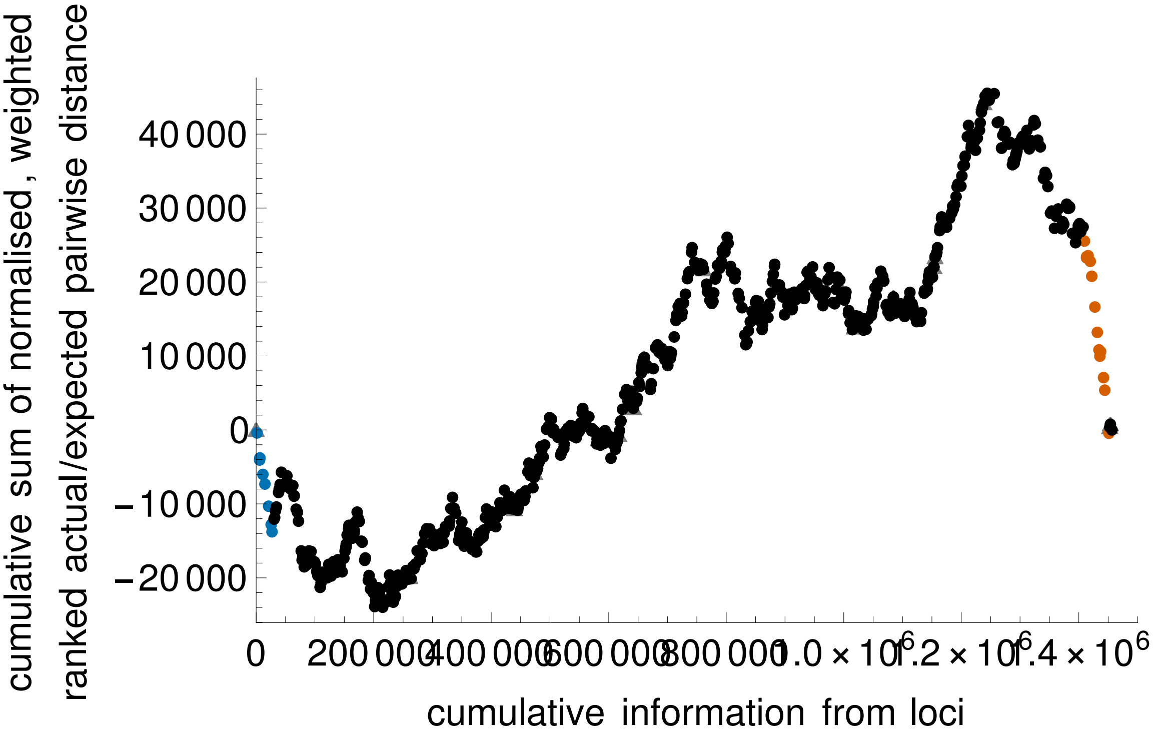
cumulative sum of normalised, weighted
ranked actual/expected pairwise distance
40 000
30 000
20 000
10 000
0
−10 000
−20 000
0
200 000
400 000
600 000
800 000
1.0 × 10^6
1.2 × 10^6
1.4 × 10^6
cumulative information from loci

PB1

PB1-F2

Gene length histogram

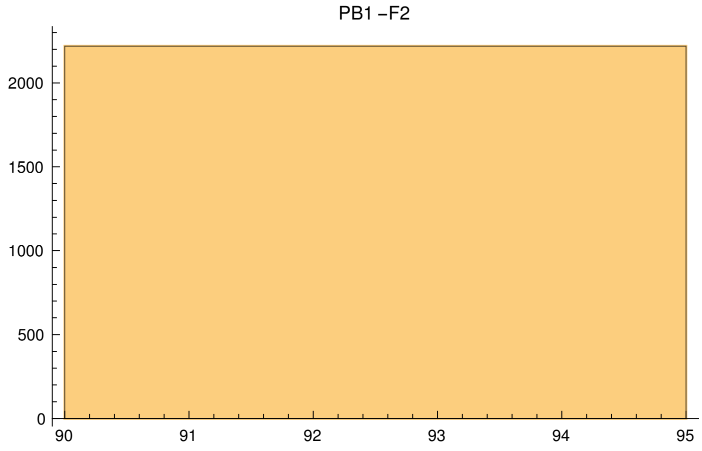

Information vs. nPD

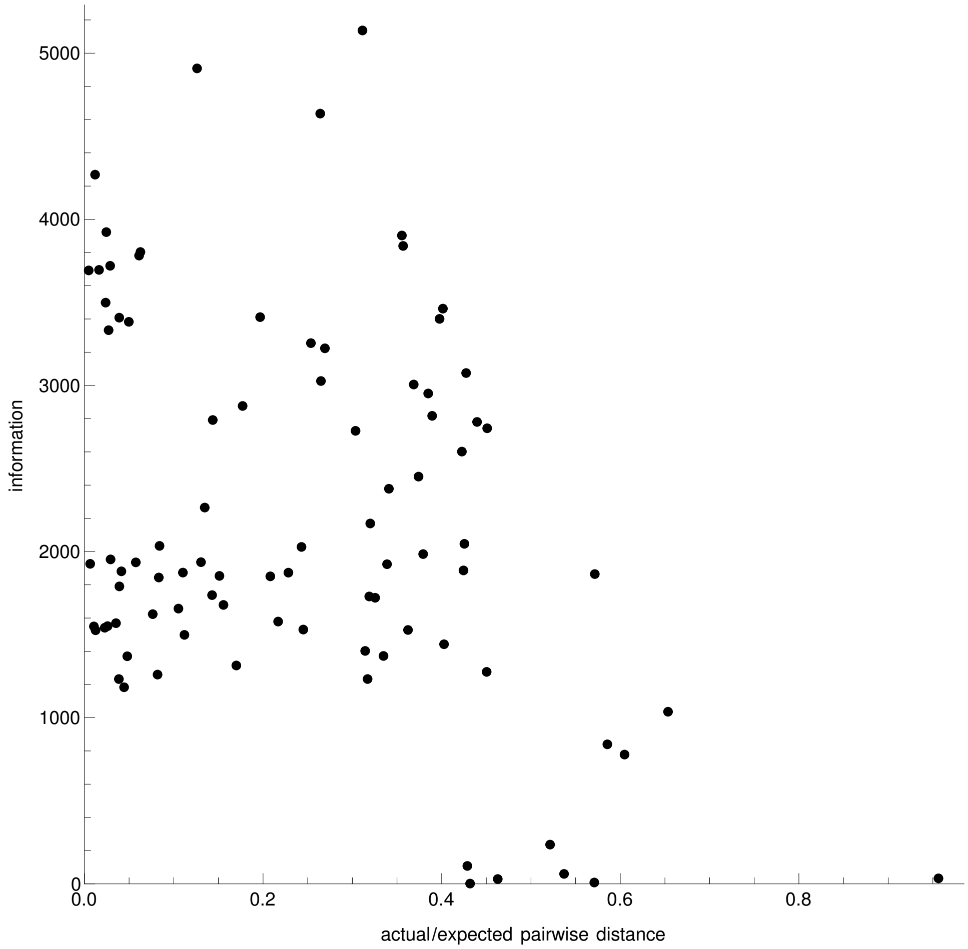

PB1-F2

Example sequences highlighted by regions found in analyses to be conserved

```
Interesting points (by weighted raw PD) highlighted for gene PB1-F2:
ATGGAACAGGGACAGGATACACCATGGACACAGTCAACAGAACACACCAATATTCAGAAAAGGGGAAGTGGACAACAA⋱
    ACACAGAGACTGGAGCACCCCAACTCAACCCGATTGATGGACCACTACCTGAGGATAATGAGCCCAGTGGGTATGCA⋱
    CAAACAGATTGTGTATTGGAAGCAATGGCTTTCCTTGAAGAATCCCACCCAGGGATCTTTGAAAATTCGTGTCTTGA⋱
    AACGATGGAAATTGTTCAACAAACAAGAGTGGATAAACTGA

Interesting points (by weighted ranked PD) highlighted for gene PB1-F2:
ATGGAACAGGGACAGGATACACCATGGACACAGTCAACAGAACACACCAATATTCAGAAAAGGGGAAGTGGACAACAA⋱
    ACACAGAGACTGGAGCACCCCAACTCAACCCGATTGATGGACCACTACCTGAGGATAATGAGCCCAGTGGGTATGCA⋱
    CAAACAGATTGTGTATTGG
```

AAGCAATGGCTTTCCTTGAAGAATCCCACCCAGGGATCTTTG

AAAATTCGTGTCTTGAAACGATGGAAATTGTTCAACAAACAAGAGTGGATAAACTGA

Per codon conservation report

PB1-F2			
Pos.	1	obs:	exp:
atg	M	2219	2219.00
---	--	------	------
mPD		0	0
		nPD:	1.
		N. weight:	0.
		Sc. PD:	0
		Sc. rank:	0

PB1-F2			
Pos.	2	obs:	exp:
gaa	E	1864	1045.00
gag	E	0	819.10
ggt	G	0	51.34
ggc	G	0	46.63
gga	G	355	155.60
ggg	G	0	101.50
---	--	------	------
mPD		0.27	0.80
		nPD:	0.33
		N. weight:	0.64
		Sc. PD:	0.077
		Sc. rank:	482.6

PB1-F2			
Pos.	3	obs:	exp:
tta	L	0	0.37
ttg	L	0	0.76
ctt	L	0	0.79
ctc	L	0	0.64
cta	L	0	0.59
ctg	L	4	0.84
caa	Q	0	1097.00
cag	Q	2198	1101.00
cgt	R	0	0.64
cgc	R	0	0.91
cga	R	0	1.71
cgg	R	17	1.39
aga	R	0	7.94
agg	R	0	4.42
---	--	------	------
mPD		0.019	0.53
		nPD:	0.04
		N. weight:	0.73
		Sc. PD:	-0.13
		Sc. rank:	-851.8

PB1-F2			
Pos.	4	obs:	exp:
gaa	E	442	247.80
gag	E	0	194.20
ggt	G	0	257.00
ggc	G	0	233.40
gga	G	1777	778.60
ggg	G	0	508.00
---	--	------	------
mPD		0.32	0.98
		nPD:	0.33
		N. weight:	0.8
		Sc. PD:	0.089
		Sc. rank:	584.8

PB1-F2			
Pos.	5	obs:	exp:
tta	L	0	0.09
ttg	L	0	0.19
ctt	L	0	0.20
ctc	L	0	0.16
cta	L	0	0.15
ctg	L	1	0.21
cct	P	0	0.55
ccc	P	0	0.36
cca	P	0	0.81
ccg	P	2	0.28
caa	Q	0	1083.00
cag	Q	2169	1086.00
cgt	R	0	1.76
cgc	R	0	2.52
cga	R	0	4.74
cgg	R	47	3.84
aga	R	0	21.94
agg	R	0	12.21
---	--	------	------
mPD		0.044	0.58
		nPD:	0.08
		N. weight:	0.76
		Sc. PD:	-0.099
		Sc. rank:	-515.5

PB1-F2			
Pos.	6	obs:	exp:
gat	D	1784	964.30
gac	D	2	821.70
ggt	G	433	62.62
ggc	G	0	56.88
gga	G	0	189.70
ggg	G	0	123.80
---	--	------	------
mPD		0.32	0.93
		nPD:	0.34
		N. weight:	0.9
		Sc. PD:	0.11
		Sc. rank:	709.7

PB1-F2			
Pos.	7	obs:	exp:
att	I	0	44.04
atc	I	0	32.11
ata	I	120	43.85
act	T	0	550.30
acc	T	1	473.00
aca	T	2097	878.30
acg	T	1	197.50
---	--	------	------
mPD		0.10	0.80
		nPD:	0.13
		N. weight:	0.9
		Sc. PD:	-0.071
		Sc. rank:	-322.6

PB1-F2			
Pos.	8	obs:	exp:
tta	L	0	10.08
ttg	L	0	20.71
ctt	L	0	21.64
ctc	L	0	17.44
cta	L	109	16.13
ctg	L	0	23.01
cct	P	1	375.00
ccc	P	18	247.80
cca	P	1353	556.90
ccg	P	0	192.40
caa	Q	729	364.00
cag	Q	0	365.00
cgt	R	0	0.34
cgc	R	0	0.48
cga	R	9	0.91
cgg	R	0	0.73
aga	R	0	4.20
agg	R	0	2.34
---	--	------	------
mPD		0.52	1.2
		nPD:	0.42
		N. weight:	0.88
		Sc. PD:	0.18
		Sc. rank:	1326.2

PB1-F2			
Pos.	9	obs:	exp:
tta	L	0	0.09
ttg	L	1	0.19
tgg	W	2218	2218.00
ctt	L	0	0.20
ctc	L	0	0.16
cta	L	0	0.15
ctg	L	0	0.21
---	--	------	------
mPD		0.00090	0.0021
		nPD:	0.43
		N. weight:	0.00077
		Sc. PD:	0.00017
		Sc. rank:	1.2

PB1-F2			
Pos.	10	obs:	exp:
att	I	0	1.10
atc	I	0	0.80
ata	I	3	1.10
act	T	0	580.70
acc	T	1	499.10
aca	T	2214	926.80
acg	T	0	208.40
aaa	K	1	0.57
aag	K	0	0.43
---	--	------	------
mPD		0.0045	0.70
		nPD:	0.01
		N. weight:	0.9
		Sc. PD:	-0.18
		Sc. rank:	-1594.8

PB1-F2			
Pos.	11	obs:	exp:
tta	L	0	2.59
ttg	L	0	5.32
ctt	L	0	5.56
ctc	L	0	4.48
cta	L	0	4.14
ctg	L	28	5.91
cct	P	0	2.73
ccc	P	0	1.81
cca	P	0	4.06
ccg	P	10	1.40
cat	H	1	0.58
cac	H	0	0.42
caa	Q	0	1012.00
cag	Q	2026	1014.00
cgt	R	0	5.76
cgc	R	0	8.24
cga	R	0	15.52
cgg	R	154	12.57
aga	R	0	71.89
agg	R	0	40.01
---	--	------	------
mPD		0.16	0.78
		nPD:	0.21
		N. weight:	0.86
		Sc. PD:	-0.0028
		Sc. rank:	16.5

PB1-F2			
Pos.	12	obs:	exp:
tta	L	51	4.72
ttg	L	0	9.69
tct	S	0	361.90
tcc	S	0	314.00
tca	S	2167	491.50
tcg	S	1	130.80
ctt	L	0	10.13
ctc	L	0	8.16
cta	L	0	7.55
ctg	L	0	10.76
agt	S	0	470.20
agc	S	0	399.50
---	--	------	------
mPD		0.046	1.7
		nPD:	0.03
		N. weight:	1.6
		Sc. PD:	-0.28
		Sc. rank:	-2023.2

PB1-F2			
Pos.	13	obs:	exp:
cct	P	0	1.09
ccc	P	0	0.72
cca	P	4	1.62
ccg	P	0	0.56
att	I	0	29.36
atc	I	0	21.41
ata	I	80	29.24
act	T	0	559.70
acc	T	12	481.10
aca	T	2122	893.40
acg	T	1	200.90
---	--	------	------
mPD		0.085	0.77
		nPD:	0.11
		N. weight:	0.87
		Sc. PD:	-0.086
		Sc. rank:	-450.8

PB1-F2			
Pos.	14	obs:	exp:
gaa	E	2097	1176.00
gag	E	0	921.50
ggt	G	0	17.64
ggc	G	0	16.03
gga	G	122	53.46
ggg	G	0	34.87
---	--	------	------
mPD		0.10	0.61
		nPD:	0.17
		N. weight:	0.61
		Sc. PD:	-0.025
		Sc. rank:	-83.2

PB1-F2

Pos.	15	obs:	exp:
cct	P	0	0.27
ccc	P	1	0.18
cca	P	0	0.41
ccg	P	0	0.14
cat	H	0	1275.00
cac	H	2202	927.30
cgt	R	0	0.60
cgc	R	16	0.86
cga	R	0	1.61
cgg	R	0	1.31
aga	R	0	7.47
agg	R	0	4.16
---	--	------	------
mPD		0.015	0.52
		nPD:	0.03
		N. weight:	0.91
		Sc. PD:	-0.16
		Sc. rank:	-1087.3

,

PB1-F2

Pos.	16	obs:	exp:
tct	S	0	0.17
tcc	S	1	0.14
tca	S	0	0.23
tcg	S	0	0.06
att	I	0	416.10
atc	I	1128	303.50
ata	I	6	414.40
act	T	0	284.20
acc	T	1084	244.30
aca	T	0	453.60
acg	T	0	102.00
agt	S	0	0.22
agc	S	0	0.18
---	--	------	------
mPD		0.51	1.2
		nPD:	0.43
		N. weight:	1.4
		Sc. PD:	0.3
		Sc. rank:	2301.1

,

PB1-F2

Pos.	17	obs:	exp:
tct	S	0	4.34
tcc	S	0	3.77
tca	S	0	5.89
tcg	S	0	1.57
tat	Y	1	0.48
tac	Y	0	0.52
att	I	1	0.37
atc	I	0	0.27
ata	I	0	0.37
aat	N	2190	1194.00
aac	N	0	995.70
agt	S	26	5.64
agc	S	0	4.79
gat	D	1	0.54
gac	D	0	0.46
---	--	------	------
mPD		0.026	0.54
		nPD:	0.05
		N. weight:	0.64
		Sc. PD:	-0.1
		Sc. rank:	-611.6

,

PB1-F2

Pos.	18	obs:	exp:
att	I	1075	394.50
atc	I	0	287.70
ata	I	0	392.80
act	T	1143	299.90
acc	T	0	257.80
aca	T	1	478.70
acg	T	0	107.60
---	--	------	------
mPD		0.50	1.2
		nPD:	0.42
		N. weight:	1.2
		Sc. PD:	0.25
		Sc. rank:	1785.4

,

PB1-F2

Pos.	19	obs:	exp:
caa	Q	0	1052.00
cag	Q	2107	1055.00
cgt	R	0	4.19
cgc	R	0	5.99
cga	R	0	11.29
cgg	R	112	9.15
aga	R	0	52.29
agg	R	0	29.10
---	--	------	------
mPD		0.096	0.67
		nPD:	0.14
		N. weight:	0.81
		Sc. PD:	-0.054
		Sc. rank:	-227.8

,

PB1-F2

Pos.	20	obs:	exp:
cgt	R	0	37.27
cgc	R	0	53.30
cga	R	1	100.40
cgg	R	0	81.33
aat	N	0	0.55
aac	N	1	0.45
aaa	K	1222	695.80
aag	K	0	526.20
aga	R	995	465.00
agg	R	0	258.80
---	--	------	------
mPD		0.50	1.2
		nPD:	0.4
		N. weight:	0.67
		Sc. PD:	0.12
		Sc. rank:	956.4

,

PB1-F2

Pos.	21	obs:	exp:
cgt	R	0	74.02
cgc	R	0	105.90
cga	R	0	199.30
cgg	R	0	161.50
atg	M	1	1.00
aaa	K	0	125.30
aag	K	220	94.73
aga	R	0	923.40
agg	R	1978	513.90
ggt	G	0	2.89
ggc	G	0	2.63
gga	G	0	8.76
ggg	G	20	5.72
---	--	------	------
mPD		0.20	1.1
		nPD:	0.18
		N. weight:	1.3
		Sc. PD:	-0.045
		Sc. rank:	-108.7

,

PB1-F2

Pos.	22	obs:	exp:
gtt	V	0	2.58
gtc	V	0	2.52
gta	V	5	2.56
gtg	V	7	4.34
gaa	E	968	543.20
gag	E	1	425.80
ggt	G	0	179.00
ggc	G	0	162.60
gga	G	946	542.50
ggg	G	292	353.90
---	--	------	------
mPD		0.73	1.1
		nPD:	0.65
		N. weight:	0.48
		Sc. PD:	0.21
		Sc. rank:	883.4

,

PB1-F2

Pos.	23	obs:	exp:
tct	S	0	180.40
tcc	S	0	156.60
tca	S	0	245.10
tcg	S	0	65.22
tgt	C	4	1.55
tgc	C	0	2.45
act	T	1	0.26
acc	T	0	0.23
aca	T	0	0.42
acg	T	0	0.09
aat	N	1041	567.70
aac	N	0	473.30
agt	S	1081	234.50
agc	S	0	199.20
gat	D	67	36.18
gac	D	0	30.82
ggt	G	25	3.62
ggc	G	0	3.28
gga	G	0	10.95
ggg	G	0	7.15
---	--	------	------
mPD		0.58	1.7
		nPD:	0.34
		N. weight:	1.1
		Sc. PD:	0.14
		Sc. rank:	927.4

,

PB1-F2

Pos.	24	obs:	exp:
ggt	G	0	320.90
ggc	G	0	291.50
gga	G	2130	972.30
ggg	G	89	634.30
---	--	------	------
mPD		0.077	0.69
		nPD:	0.11
		N. weight:	0.7
		Sc. PD:	-0.068
		Sc. rank:	-340.7

,

PB1-F2

Pos.	25	obs:	exp:
cct	P	0	0.27
ccc	P	0	0.18
cca	P	1	0.41
ccg	P	0	0.14
caa	Q	2213	1105.00
cag	Q	0	1108.00
cgt	R	0	0.19
cgc	R	0	0.27
cga	R	5	0.50
cgg	R	0	0.41
aga	R	0	2.33
agg	R	0	1.30
---	--	------	------
mPD		0.0054	0.51
		nPD:	0.01
		N. weight:	0.72
		Sc. PD:	-0.14
		Sc. rank:	-1261.6

,

PB1-F2

Pos.	26	obs:	exp:
cat	H	0	1.16
cac	H	2	0.84
caa	Q	2051	1024.00
cag	Q	0	1027.00
cgt	R	0	1.27
cgc	R	0	1.82
cga	R	34	3.43
cgg	R	0	2.78
aaa	K	132	75.16
aag	K	0	56.84
aga	R	0	15.87
agg	R	0	8.83
---	--	------	------
mPD		0.14	0.66
		nPD:	0.22
		N. weight:	0.74
		Sc. PD:	0.0038
		Sc. rank:	36.2

,

PB1-F2

Pos.	27	obs:	exp:
att	I	0	26.42
atc	I	0	19.27
ata	I	72	26.31
act	T	52	562.60
acc	T	0	483.60
aca	T	2084	898.00
acg	T	10	201.90
gct	A	0	0.25
gcc	A	0	0.19
gca	A	1	0.45
gcg	A	0	0.11
---	--	------	------
mPD		0.12	0.76
		nPD:	0.16
		N. weight:	0.78
		Sc. PD:	-0.042
		Sc. rank:	-125.8

,

PB1-F2

Pos.	28	obs:	exp:
tta	L	0	0.83
ttg	L	0	1.71
ctt	L	0	1.79
ctc	L	0	1.44
cta	L	0	1.33
ctg	L	9	1.90
cct	P	0	1.09
ccc	P	0	0.72
cca	P	0	1.62
ccg	P	4	0.56
caa	Q	0	1100.00
cag	Q	2204	1104.00
cgt	R	0	0.07
cgc	R	0	0.11
cga	R	0	0.20
cgg	R	2	0.16
aga	R	0	0.93
agg	R	0	0.52
---	--	------	------
mPD		0.013	0.52
		nPD:	0.03
		N. weight:	0.72
		Sc. PD:	-0.13
		Sc. rank:	-987.7

,

PB1-F2

Pos.	29	obs:	exp:
cgt	R	0	52.39
cgc	R	0	74.92
cga	R	0	141.10
cgg	R	0	114.30
att	I	0	0.37
atc	I	0	0.27
ata	I	1	0.37
aaa	K	818	465.80
aag	K	0	352.20
aga	R	1400	653.60
agg	R	0	363.70
---	--	------	------
mPD		0.47	1.3
		nPD:	0.36
		N. weight:	0.71
		Sc. PD:	0.1
		Sc. rank:	721.6

,

PB1-F2

Pos.	30	obs:	exp:
tta	L	0	82.75
ttg	L	0	170.00
ctt	L	0	177.70
ctc	L	0	143.20
cta	L	3	132.50
ctg	L	892	188.90
cct	P	0	357.70
ccc	P	0	236.40
cca	P	19	531.30
ccg	P	1290	183.50
caa	Q	0	7.49
cag	Q	15	7.51
---	--	------	------
mPD		0.51	1.4
		nPD:	0.36
		N. weight:	1.8
		Sc. PD:	0.25
		Sc. rank:	1761.1

,

PB1-F2

Pos.	31	obs:	exp:
cgt	R	0	0.04
cgc	R	0	0.05
cga	R	0	0.10
cgg	R	0	0.08
aaa	K	0	0.57
aag	K	1	0.43
aga	R	0	0.47
agg	R	1	0.26
gat	D	3	1.62
gac	D	0	1.38
gaa	E	0	1216.00
gag	E	2169	953.10
ggt	G	0	6.51
ggc	G	0	5.91
gga	G	0	19.72
ggg	G	45	12.86
---	--	------	------
mPD		0.045	0.54
		nPD:	0.08
		N. weight:	0.86
		Sc. PD:	-0.11
		Sc. rank:	-534.7

,

PB1-F2

Pos.	32	obs:	exp:
tta	L	0	0.09
ttg	L	0	0.19
ctt	L	0	0.20
ctc	L	1	0.16
cta	L	0	0.15
ctg	L	0	0.21
cct	P	0	0.27
ccc	P	1	0.18
cca	P	0	0.41
ccg	P	0	0.14
cat	H	6	1268.00
cac	H	2184	922.20
cgt	R	0	1.01
cgc	R	27	1.44
cga	R	0	2.72
cgg	R	0	2.20
aga	R	0	12.60
agg	R	0	7.01
---	--	------	------
mPD		0.031	0.54
		nPD:	0.06
		N. weight:	0.9
		Sc. PD:	-0.14
		Sc. rank:	-772.6

,

PB1-F2

Pos.	33	obs:	exp:
ttt	F	0	0.42
ttc	F	1	0.58
tta	L	0	77.48
ttg	L	0	159.20
tct	S	0	7.18
tcc	S	43	6.23
tca	S	0	9.75
tcg	S	0	2.59
ctt	L	0	166.40
ctc	L	838	134.00
cta	L	0	124.00
ctg	L	0	176.90
cct	P	0	363.70
ccc	P	1331	240.40
cca	P	0	540.20
ccg	P	0	186.60
cat	H	0	3.47
cac	H	6	2.53
agt	S	0	9.33
agc	S	0	7.92
---	--	------	------
mPD		0.51	1.4
		nPD:	0.36
		N. weight:	1.8
		Sc. PD:	0.25
		Sc. rank:	1657.0

,

PB1-F2

Pos.	34	obs:	exp:
tct	S	0	19.20
tcc	S	0	16.66
tca	S	0	26.07
tcg	S	0	6.94
cat	H	0	1.74
cac	H	3	1.26
att	I	0	0.37
atc	I	1	0.27
ata	I	0	0.37
aat	N	0	1145.00
aac	N	2100	954.80
agt	S	0	24.94
agc	S	115	21.19
---	--	------	------
mPD		0.10	0.67
		nPD:	0.15
		N. weight:	0.86
		Sc. PD:	-0.051
		Sc. rank:	-166.6

,

PB1-F2

Pos.	35	obs:	exp:
tta	L	100	9.25
ttg	L	0	19.00
tct	S	0	353.70
tcc	S	0	306.90
tca	S	2119	480.40
tcg	S	0	127.80
ctt	L	0	19.85
ctc	L	0	16.00
cta	L	0	14.80
ctg	L	0	21.11
agt	S	0	459.60
agc	S	0	390.50
---	--	------	------
mPD		0.086	1.7
		nPD:	0.05
		N. weight:	1.6
		Sc. PD:	-0.25
		Sc. rank:	-1408.7

,

PB1-F2

Pos.	36	obs:	exp:
att	I	0	111.20
atc	I	303	81.08
ata	I	0	110.70
act	T	0	502.30
acc	T	1916	431.70
aca	T	0	801.70
acg	T	0	180.30
---	--	------	------
mPD		0.24	0.93
		nPD:	0.25
		N. weight:	1.5
		Sc. PD:	0.062
		Sc. rank:	325.5

,

PB1-F2

Pos.	37	obs:	exp:
tta	L	0	14.70
ttg	L	0	30.21
ctt	L	0	31.57
ctc	L	0	25.43
cta	L	159	23.53
ctg	L	0	33.56
cct	P	0	2.46
ccc	P	0	1.63
cca	P	9	3.65
ccg	P	0	1.26
caa	Q	1196	603.10
cag	Q	12	604.90
cgt	R	0	31.55
cgc	R	0	45.11
cga	R	841	84.95
cgg	R	1	68.84
aga	R	1	393.50
agg	R	0	219.00
---	--	------	------
mPD		0.57	1.5
		nPD:	0.37
		N. weight:	1.4
		Sc. PD:	0.21
		Sc. rank:	1499.5

,

PB1-F2

Pos.	38	obs:	exp:
tta	L	0	201.70
ttg	L	2181	414.50
tct	S	0	6.18
tcc	S	0	5.36
tca	S	0	8.39
tcg	S	37	2.23
ctt	L	0	433.20
ctc	L	0	349.00
cta	L	0	322.90
ctg	L	1	460.50
agt	S	0	8.03
agc	S	0	6.82
---	--	------	------
mPD		0.034	1.2
		nPD:	0.03
		N. weight:	1.7
		Sc. PD:	-0.31
		Sc. rank:	-2135.6

,

PB1-F2

Pos.	39	obs:	exp:
cgt	R	0	0.04
cgc	R	0	0.05
cga	R	0	0.10
cgg	R	0	0.08
atg	M	2118	2118.00
act	T	0	25.95
acc	T	0	22.31
aca	T	0	41.43
acg	T	99	9.31
aaa	K	0	0.57
aag	K	1	0.43
aga	R	0	0.47
agg	R	1	0.26
---	--	------	------
mPD		0.087	0.17
		nPD:	0.52
		N. weight:	0.11
		Sc. PD:	0.033
		Sc. rank:	191.8

,

PB1-F2			
Pos.	40	obs:	exp:
gct	A	0	1.51
gcc	A	6	1.16
gca	A	0	2.67
gcg	A	0	0.66
gat	D	0	883.30
gac	D	1636	752.70
ggt	G	0	83.44
ggc	G	577	75.79
gga	G	0	252.80
ggg	G	0	164.90
---	--	------	------
mPD		0.39	1.0
		nPD:	0.37
		N. weight:	1.1
		Sc. PD:	0.18
		Sc. rank:	1276.2

,

PB1-F2			
Pos.	41	obs:	exp:
tta	L	0	1.48
ttg	L	0	3.04
ctt	L	14	3.18
ctc	L	2	2.56
cta	L	0	2.37
ctg	L	0	3.38
cat	H	368	1269.00
cac	H	1824	923.10
cgt	R	4	0.41
cgc	R	7	0.59
cga	R	0	1.11
cgg	R	0	0.90
aga	R	0	5.14
agg	R	0	2.86
---	--	------	------
mPD		0.31	0.53
		nPD:	0.59
		N. weight:	0.39
		Sc. PD:	0.14
		Sc. rank:	702.7

,

PB1-F2			
Pos.	42	obs:	exp:
tat	Y	0	924.70
tac	Y	1928	1003.00
tgt	C	0	112.20
tgc	C	290	177.80
aat	N	0	0.55
aac	N	1	0.45
---	--	------	------
mPD		0.23	0.73
		nPD:	0.31
		N. weight:	0.65
		Sc. PD:	0.065
		Sc. rank:	390.9

,

PB1-F2			
Pos.	43	obs:	exp:
tta	L	0	173.50
ttg	L	0	356.60
ctt	L	0	372.70
ctc	L	0	300.20
cta	L	0	277.80
ctg	L	1877	396.20
cct	P	0	14.21
ccc	P	0	9.39
cca	P	0	21.11
ccg	P	52	7.29
caa	Q	0	92.86
cag	Q	186	93.14
cgt	R	0	3.89
cgc	R	0	5.57
cga	R	0	10.48
cgg	R	104	8.49
aga	R	0	48.55
agg	R	0	27.02
---	--	------	------
mPD		0.27	1.4
		nPD:	0.2
		N. weight:	1.6
		Sc. PD:	-0.023
		Sc. rank:	-25.6

,

PB1-F2			
Pos.	44	obs:	exp:
tgg	W	5	5.00
cgt	R	0	76.90
cgc	R	0	110.00
cga	R	0	207.10
cgg	R	0	167.80
att	I	0	0.37
atc	I	0	0.27
ata	I	1	0.37
atg	M	14	14.00
aaa	K	0	81.43
aag	K	143	61.57
aga	R	16	959.40
agg	R	2039	533.90
ggt	G	0	0.14
ggc	G	0	0.13
gga	G	0	0.44
ggg	G	1	0.29
---	--	------	------
mPD		0.15	1.1
		nPD:	0.14
		N. weight:	1.3
		Sc. PD:	-0.086
		Sc. rank:	-296.8

,

PB1-F2			
Pos.	45	obs:	exp:
att	I	0	375.40
atc	I	2	273.80
ata	I	1021	373.80
atg	M	2	2.00
act	T	0	312.50
acc	T	0	268.60
aca	T	1190	498.80
acg	T	2	112.20
aaa	K	1	0.57
aag	K	0	0.43
gct	A	0	0.25
gcc	A	0	0.19
gca	A	1	0.45
gcg	A	0	0.11
---	--	------	------
mPD		0.50	1.2
		nPD:	0.43
		N. weight:	0.95
		Sc. PD:	0.2
		Sc. rank:	1475.9

,

PB1-F2			
Pos.	46	obs:	exp:
tta	L	0	0.09
ttg	L	0	0.19
tct	S	0	0.17
tcc	S	0	0.14
tca	S	0	0.23
tcg	S	1	0.06
ctt	L	0	0.20
ctc	L	0	0.16
cta	L	0	0.15
ctg	L	1	0.21
atg	M	1042	1042.00
act	T	0	305.90
acc	T	0	263.00
aca	T	0	488.30
acg	T	1167	109.80
agt	S	0	0.22
agc	S	0	0.18
gct	A	0	2.02
gcc	A	0	1.55
gca	A	0	3.56
gcg	A	8	0.88
---	--	------	------
mPD		0.51	1.2
		nPD:	0.44
		N. weight:	1.3
		Sc. PD:	0.29
		Sc. rank:	2152.0

,

PB1-F2			
Pos.	47	obs:	exp:
tct	S	0	234.50
tcc	S	0	203.50
tca	S	0	318.50
tcg	S	0	84.76
att	I	0	2.20
atc	I	6	1.61
ata	I	0	2.19
act	T	0	2.10
acc	T	8	1.80
aca	T	0	3.35
acg	T	0	0.75
aat	N	0	436.30
aac	N	800	363.70
agt	S	0	304.70
agc	S	1405	258.90
---	--	------	------
mPD		0.47	1.8
		nPD:	0.26
		N. weight:	1.4
		Sc. PD:	0.073
		Sc. rank:	508.4

,

PB1-F2			
Pos.	48	obs:	exp:
tta	L	0	1.48
ttg	L	0	3.04
ctt	L	0	3.18
ctc	L	0	2.56
cta	L	16	2.37
ctg	L	0	3.38
cct	P	2	270.00
ccc	P	0	178.40
cca	P	986	401.00
ccg	P	0	138.50
caa	Q	466	232.70
cag	Q	0	233.30
cgt	R	0	28.03
cgc	R	0	40.08
cga	R	749	75.48
cgg	R	0	61.16
aga	R	0	349.70
agg	R	0	194.60
---	--	------	------
mPD		0.65	1.7
		nPD:	0.38
		N. weight:	1.4
		Sc. PD:	0.23
		Sc. rank:	1666.0

,

PB1-F2			
Pos.	49	obs:	exp:
tta	L	0	0.09
ttg	L	0	0.19
ctt	L	0	0.20
ctc	L	0	0.16
cta	L	0	0.15
ctg	L	1	0.21
atg	M	1	1.00
gtt	V	0	260.00
gtc	V	0	254.40
gta	V	0	257.80
gtg	V	1209	436.80
gct	A	0	253.90
gcc	A	0	195.10
gca	A	0	448.70
gcg	A	1008	110.30
---	--	------	------
mPD		0.50	1.2
		nPD:	0.4
		N. weight:	1.6
		Sc. PD:	0.3
		Sc. rank:	2251.6

,

PB1-F2			
Pos.	50	obs:	exp:
gtt	V	269	57.86
gtc	V	0	56.59
gta	V	0	57.36
gtg	V	0	97.19
gct	A	1	0.25
gcc	A	0	0.19
gca	A	0	0.45
gcg	A	0	0.11
gat	D	1093	590.10
gac	D	0	502.90
gaa	E	1	0.56
gag	E	0	0.44
ggt	G	855	123.60
ggc	G	0	112.30
gga	G	0	374.60
ggg	G	0	244.40
---	--	------	------
mPD		0.59	1.3
		nPD:	0.45
		N. weight:	1.3
		Sc. PD:	0.3
		Sc. rank:	2220.4

,

PB1-F2			
Pos.	51	obs:	exp:
atg	M	2193	2193.00
act	T	0	6.55
acc	T	0	5.63
aca	T	0	10.46
acg	T	25	2.35
gaa	E	0	0.56
gag	E	1	0.44
---	--	------	------
mPD		0.024	0.045
		nPD:	0.54
		N. weight:	0.028
		Sc. PD:	0.0088
		Sc. rank:	48.7

,

PB1-F2			
Pos.	52	obs:	exp:
cct	P	0	0.55
ccc	P	2	0.36
cca	P	0	0.81
ccg	P	0	0.28
cat	H	0	1252.00
cac	H	2162	910.50
cgt	R	0	2.06
cgc	R	55	2.94
cga	R	0	5.54
cgg	R	0	4.49
aga	R	0	25.68
agg	R	0	14.29
---	--	------	------
mPD		0.050	0.60
		nPD:	0.08
		N. weight:	0.95
		Sc. PD:	-0.12
		Sc. rank:	-553.2

,

PB1-F2

Pos.	53	obs:	exp:
cgt	R	0	1.87
cgc	R	0	2.68
cga	R	0	5.04
cgg	R	0	4.08
aaa	K	2168	1234.00
aag	K	0	933.50
aga	R	50	23.34
agg	R	0	12.99
gaa	E	1	0.56
gag	E	0	0.44
---	--	------	------
mPD		0.045	0.55
		nPD:	0.08
		N. weight:	0.59
		Sc. PD:	-0.074
		Sc. rank:	-385.9

PB1-F2

Pos.	54	obs:	exp:
tta	L	0	0.18
ttg	L	0	0.38
ctt	L	0	0.40
ctc	L	0	0.32
cta	L	0	0.30
ctg	L	2	0.42
caa	Q	0	971.60
cag	Q	1946	974.40
cgt	R	0	10.14
cgc	R	0	14.50
cga	R	0	27.31
cgg	R	271	22.13
aga	R	0	126.50
agg	R	0	70.40
---	--	------	------
mPD		0.22	0.89
		nPD:	0.24
		N. weight:	0.95
		Sc. PD:	0.029
		Sc. rank:	116.7

PB1-F2

Pos.	55	obs:	exp:
att	I	2099	770.30
atc	I	0	561.70
ata	I	0	767.10
act	T	119	31.20
acc	T	0	26.81
aca	T	0	49.79
acg	T	0	11.20
gct	A	1	0.25
gcc	A	0	0.19
gca	A	0	0.45
gcg	A	0	0.11
---	--	------	------
mPD		0.10	0.77
		nPD:	0.13
		N. weight:	1.1
		Sc. PD:	-0.079
		Sc. rank:	-331.8

PB1-F2

Pos.	56	obs:	exp:
gtt	V	0	271.60
gtc	V	0	265.70
gta	V	0	269.30
gtg	V	1263	456.30
gct	A	0	240.80
gcc	A	0	185.00
gca	A	0	425.50
gcg	A	956	104.60
---	--	------	------
mPD		0.49	1.2
		nPD:	0.4
		N. weight:	1.6
		Sc. PD:	0.29
		Sc. rank:	2107.1

PB1-F2

Pos.	57	obs:	exp:
tct	S	16	14.19
tcc	S	69	12.31
tca	S	0	19.27
tcg	S	0	5.13
tat	Y	1082	521.30
tac	Y	5	565.70
tgt	C	1044	405.20
tgc	C	3	641.80
agt	S	0	18.44
agc	S	0	15.66
---	--	------	------
mPD		0.60	1.1
		nPD:	0.57
		N. weight:	0.87
		Sc. PD:	0.3
		Sc. rank:	1546.1

PB1-F2

Pos.	58	obs:	exp:
tta	L	0	6.01
ttg	L	65	12.35
tgg	W	2154	2154.00
ctt	L	0	12.91
ctc	L	0	10.40
cta	L	0	9.62
ctg	L	0	13.72
---	--	------	------
mPD		0.057	0.13
		nPD:	0.43
		N. weight:	0.05
		Sc. PD:	0.011
		Sc. rank:	80.9

PB1-F2

Pos.	59	obs:	exp:
cgt	R	0	0.86
cgc	R	0	1.23
cga	R	0	2.32
cgg	R	0	1.88
aat	N	1	0.55
aac	N	0	0.45
aaa	K	0	1250.00
aag	K	2195	945.10
aga	R	0	10.74
agg	R	23	5.98
---	--	------	------
mPD		0.021	0.52
		nPD:	0.04
		N. weight:	0.88
		Sc. PD:	-0.14
		Sc. rank:	-882.4

PB1-F2

Pos.	60	obs:	exp:
caa	Q	2146	1071.00
cag	Q	0	1075.00
cgt	R	0	2.69
cgc	R	0	3.85
cga	R	72	7.26
cgg	R	0	5.88
act	T	0	0.26
acc	T	0	0.23
aca	T	1	0.42
acg	T	0	0.09
aga	R	0	33.61
agg	R	0	18.71
---	--	------	------
mPD		0.065	0.61
		nPD:	0.11
		N. weight:	0.77
		Sc. PD:	-0.08
		Sc. rank:	-426.2

PB1-F2

Pos.	61	obs:	exp:
tgg	W	2219	2219.00
---	--	------	------
mPD		0	0
		nPD:	1.
		N. weight:	0.
		Sc. PD:	0
		Sc. rank:	0

PB1-F2

Pos.	62	obs:	exp:
tta	L	0	203.80
ttg	L	0	418.70
ctt	L	2189	437.60
ctc	L	15	352.50
cta	L	0	326.20
ctg	L	0	465.20
cct	P	15	4.10
ccc	P	0	2.71
cca	P	0	6.09
ccg	P	0	2.10
---	--	------	------
mPD		0.027	1.1
		nPD:	0.02
		N. weight:	1.6
		Sc. PD:	-0.3
		Sc. rank:	-2397.2

PB1-F2

Pos.	63	obs:	exp:
ttt	F	0	4.59
ttc	F	11	6.41
tct	S	0	366.70
tcc	S	2197	318.20
tca	S	0	498.10
tcg	S	0	132.50
cct	P	0	2.19
ccc	P	8	1.44
cca	P	0	3.25
ccg	P	0	1.12
act	T	0	0.79
acc	T	3	0.68
aca	T	0	1.26
acg	T	0	0.28
agt	S	0	476.50
agc	S	0	404.90
---	--	------	------
mPD		0.020	1.7
		nPD:	0.01
		N. weight:	2.
		Sc. PD:	-0.39
		Sc. rank:	-3313.8

PB1-F2

Pos.	64	obs:	exp:
tta	L	0	203.20
ttg	L	2198	417.60
tct	S	0	2.67
tcc	S	0	2.32
tca	S	0	3.63
tcg	S	16	0.97
tgg	W	5	5.00
ctt	L	0	436.40
ctc	L	0	351.60
cta	L	0	325.30
ctg	L	0	463.90
agt	S	0	3.47
agc	S	0	2.95
---	--	------	------
mPD		0.019	1.1
		nPD:	0.02
		N. weight:	1.7
		Sc. PD:	-0.33
		Sc. rank:	-2697.5

PB1-F2

Pos.	65	obs:	exp:
cgt	R	0	6.21
cgc	R	0	8.88
cga	R	0	16.73
cgg	R	0	13.55
act	T	0	0.79
acc	T	0	0.68
aca	T	0	1.26
acg	T	3	0.28
aaa	K	14	1167.00
aag	K	2035	882.30
aga	R	0	77.50
agg	R	166	43.13
ggt	G	0	0.14
ggc	G	0	0.13
gga	G	0	0.44
ggg	G	1	0.29
---	--	------	------
mPD		0.16	0.68
		nPD:	0.23
		N. weight:	0.87
		Sc. PD:	0.014
		Sc. rank:	74.1

PB1-F2

Pos.	66	obs:	exp:
tct	S	0	152.10
tcc	S	0	132.00
tca	S	0	206.50
tcg	S	0	54.96
att	I	6	2.20
atc	I	0	1.61
ata	I	0	2.19
aat	N	1299	708.40
aac	N	0	590.60
aaa	K	1	1.14
aag	K	1	0.86
agt	S	908	197.60
agc	S	3	167.90
gat	D	1	0.54
gac	D	0	0.46
---	--	------	------
mPD		0.49	1.5
		nPD:	0.32
		N. weight:	1.
		Sc. PD:	0.11
		Sc. rank:	703.5

PB1-F2

Pos.	67	obs:	exp:
tta	L	0	4.90
ttg	L	0	10.07
ctt	L	0	10.52
ctc	L	53	8.48
cta	L	0	7.84
ctg	L	0	11.19
cct	P	0	591.40
ccc	P	2164	390.90
cca	P	0	878.40
ccg	P	0	303.40
cat	H	0	0.58
cac	H	1	0.42
gct	A	0	0.25
gcc	A	1	0.19
gca	A	0	0.45
gcg	A	0	0.11
---	--	------	------
mPD		0.048	0.77
		nPD:	0.06
		N. weight:	1.8
		Sc. PD:	-0.26
		Sc. rank:	-1262.4

PB1-F2

Pos.	68	obs:	exp:
tct	S	0	0.17
tcc	S	0	0.14
tca	S	0	0.23
tcg	S	0	0.06
att	I	0	119.30
atc	I	325	86.97
ata	I	0	118.80
act	T	4	496.30
acc	T	1889	426.50
aca	T	0	792.10
acg	T	0	178.10
agt	S	0	0.22
agc	S	1	0.18
---	--	------	------
mPD		0.25	0.95
		nPD:	0.27
		N. weight:	1.5
		Sc. PD:	0.084
		Sc. rank:	633.7

PB1-F2

Pos.	69	obs:	exp:
cct	P	0	0.55
ccc	P	0	0.36
cca	P	0	0.81
ccg	P	2	0.28
cat	H	0	0.58
cac	H	1	0.42
caa	Q	2	1105.00
cag	Q	2212	1109.00
cgt	R	0	0.07
cgc	R	0	0.11
cga	R	0	0.20
cgg	R	2	0.16
aga	R	0	0.93
agg	R	0	0.52
---	--	------	------
mPD		0.0063	0.51
		nPD:	0.01
		N. weight:	0.71
		Sc. PD:	-0.14
		Sc. rank:	-1164.3

PB1-F2

Pos.	70	obs:	exp:
gtt	V	0	0.65
gtc	V	0	0.63
gta	V	3	0.64
gtg	V	0	1.08
gat	D	0	0.54
gac	D	1	0.46
gaa	E	419	234.90
gag	E	0	184.10
ggt	G	0	259.70
ggc	G	0	235.90
gga	G	1796	787.00
ggg	G	0	513.40
---	--	------	------
mPD		0.31	0.97
		nPD:	0.32
		N. weight:	0.81
		Sc. PD:	0.084
		Sc. rank:	527.8

PB1-F2

Pos.	71	obs:	exp:
ttt	F	39	16.27
ttc	F	0	22.73
tct	S	2173	362.90
tcc	S	1	314.90
tca	S	0	492.80
tcg	S	0	131.20
tat	Y	6	2.88
tac	Y	0	3.12
agt	S	0	471.50
agc	S	0	400.60
---	--	------	------
mPD		0.041	1.7
		nPD:	0.02
		N. weight:	1.8
		Sc. PD:	-0.33
		Sc. rank:	-2551.6

PB1-F2

Pos.	72	obs:	exp:
tta	L	0	204.60
ttg	L	2213	420.40
tct	S	0	1.00
tcc	S	0	0.87
tca	S	0	1.36
tcg	S	6	0.36
ctt	L	0	439.40
ctc	L	0	354.00
cta	L	0	327.50
ctg	L	0	467.10
agt	S	0	1.30
agc	S	0	1.11
---	--	------	------
mPD		0.0054	1.1
		nPD:	0.
		N. weight:	1.7
		Sc. PD:	-0.35
		Sc. rank:	-3120.0

PB1-F2

Pos.	73	obs:	exp:
cgt	R	0	0.71
cgc	R	0	1.02
cga	R	0	1.91
cgg	R	0	1.55
act	T	0	4.19
acc	T	0	3.61
aca	T	16	6.69
acg	T	0	1.51
aaa	K	1694	964.60
aag	K	0	729.40
aga	R	19	8.87
agg	R	0	4.94
gat	D	0	1.62
gac	D	3	1.38
gaa	E	449	251.70
gag	E	0	197.30
ggt	G	0	5.50
ggc	G	0	4.99
gga	G	38	16.65
ggg	G	0	10.86
---	--	------	------
mPD		0.41	0.91
		nPD:	0.45
		N. weight:	0.6
		Sc. PD:	0.14
		Sc. rank:	1002.1

PB1-F2

Pos.	74	obs:	exp:
tta	L	0	5.64
ttg	L	0	11.59
ctt	L	61	12.11
ctc	L	0	9.76
cta	L	0	9.03
ctg	L	0	12.88
cct	P	2	0.55
ccc	P	0	0.36
cca	P	0	0.81
ccg	P	0	0.28
att	I	509	186.80
atc	I	0	136.20
ata	I	0	186.00
act	T	1639	429.70
acc	T	0	369.30
aca	T	0	685.80
acg	T	0	154.20
gtt	V	1	0.22
gtc	V	0	0.21
gta	V	0	0.21
gtg	V	0	0.36
gct	A	7	1.76
gcc	A	0	1.35
gca	A	0	3.12
gcg	A	0	0.77
---	--	------	------
mPD		0.44	1.1
		nPD:	0.39
		N. weight:	1.3
		Sc. PD:	0.23
		Sc. rank:	1660.4

PB1-F2

Pos.	75	obs:	exp:
tta	L	0	88.67
ttg	L	0	182.20
ctt	L	959	190.40
ctc	L	0	153.40
cta	L	0	141.90
ctg	L	0	202.40
cct	P	18	4.92
ccc	P	0	3.25
cca	P	0	7.31
ccg	P	0	2.52
cat	H	189	109.40
cac	H	0	79.59
cgt	R	1053	39.41
cgc	R	0	56.35
cga	R	0	106.10
cgg	R	0	85.98
aga	R	0	491.60
agg	R	0	273.60
---	--	------	------
mPD		0.58	1.9
		nPD:	0.31
		N. weight:	2.4
		Sc. PD:	0.23
		Sc. rank:	1368.2

PB1-F2

Pos.	76	obs:	exp:
gtt	V	1	471.90
gtc	V	2186	461.60
gta	V	7	467.80
gtg	V	0	792.70
gct	A	0	6.30
gcc	A	25	4.84
gca	A	0	11.13
gcg	A	0	2.74
---	--	------	------
mPD		0.029	0.76
		nPD:	0.04
		N. weight:	1.6
		Sc. PD:	-0.27
		Sc. rank:	-1709.6

PB1-F2

Pos.	77	obs:	exp:
tta	L	0	197.10
ttg	L	2132	405.00
tct	S	0	14.19
tcc	S	0	12.31
tca	S	0	19.27
tcg	S	85	5.13
ctt	L	0	423.30
ctc	L	0	341.00
cta	L	0	315.50
ctg	L	0	450.00
agt	S	0	18.44
agc	S	0	15.66
gtt	V	0	0.43
gtc	V	0	0.42
gta	V	0	0.43
gtg	V	2	0.72
---	--	------	------
mPD		0.076	1.2
		nPD:	0.06
		N. weight:	1.8
		Sc. PD:	-0.26
		Sc. rank:	-1382.9

PB1-F2

Pos.	78	obs:	exp:
ttt	F	1	0.42
ttc	F	0	0.58
cgt	R	0	0.60
cgc	R	0	0.86
cga	R	0	1.61
cgg	R	0	1.31
aaa	K	2199	1254.00
aag	K	3	948.20
aga	R	16	7.47
agg	R	0	4.16
---	--	------	------
mPD		0.020	0.51
		nPD:	0.04
		N. weight:	0.58
		Sc. PD:	-0.097
		Sc. rank:	-655.6

PB1-F2

Pos.	79	obs:	exp:
tta	L	0	7.77
ttg	L	0	15.96
ctt	L	0	16.68
ctc	L	0	13.44
cta	L	84	12.43
ctg	L	0	17.73
caa	Q	86	42.94
cag	Q	0	43.06
cgt	R	0	76.68
cgc	R	0	109.70
cga	R	2047	206.50
cgg	R	2	167.30
aga	R	0	956.60
agg	R	0	532.30
---	--	------	------
mPD		0.15	1.2
		nPD:	0.13
		N. weight:	2.3
		Sc. PD:	-0.19
		Sc. rank:	-902.0

PB1-F2

Pos.	80	obs:	exp:
tta	L	0	0.37
ttg	L	4	0.76
tgt	C	0	1.16
tgc	C	3	1.84
tgg	W	2207	2207.00
ctt	L	0	0.79
ctc	L	0	0.64
cta	L	0	0.59
ctg	L	0	0.84
---	-	5	5.00
---	--	------	------
mPD		0.0063	0.011
		nPD:	0.57
		N. weight:	0.0038
		Sc. PD:	0.0013
		Sc. rank:	6.6

PB1-F2

Pos.	81	obs:	exp:
cgt	R	0	2.32
cgc	R	0	3.32
cga	R	0	6.25
cgg	R	0	5.06
aaa	K	1259	1224.00
aag	K	891	925.80
aga	R	51	28.94
agg	R	11	16.11
gaa	E	2	1.12
gag	E	0	0.88
---	-	5	5.00
---	--	------	------
mPD		0.54	0.56
		nPD:	0.96
		N. weight:	0.015
		Sc. PD:	0.011
		Sc. rank:	28.2

PB1-F2

Pos.	82	obs:	exp:
tta	L	15	114.30
ttg	L	1220	234.80
tct	S	0	162.20
tcc	S	0	140.80
tca	S	11	220.40
tcg	S	961	58.64
tgg	W	1	1.00
ctt	L	0	245.40
ctc	L	0	197.70
cta	L	0	182.90
ctg	L	1	260.90
cct	P	0	0.27
ccc	P	0	0.18
cca	P	0	0.41
ccg	P	1	0.14
agt	S	0	210.80
agc	S	0	179.10
---	-	9	9.00
---	--	------	------
mPD		0.51	1.9
		nPD:	0.26
		N. weight:	2.2
		Sc. PD:	0.11
		Sc. rank:	654.3

PB1-F2

Pos.	83	obs:	exp:
ttt	F	0	819.80
ttc	F	1965	1145.00
tta	L	0	0.09
ttg	L	0	0.19
tct	S	0	40.23
tcc	S	241	34.91
tca	S	0	54.63
tcg	S	0	14.54
tgt	C	0	0.77
tgc	C	2	1.23
ctt	L	0	0.20
ctc	L	1	0.16
cta	L	0	0.15
ctg	L	0	0.21
att	I	0	0.37
atc	I	1	0.27
ata	I	0	0.37
agt	S	0	52.27
agc	S	0	44.41
---	-	9	9.00
---	--	------	------
mPD		0.20	0.80
		nPD:	0.25
		N. weight:	0.71
		Sc. PD:	0.023
		Sc. rank:	108.9

PB1-F2

Pos.	84	obs:	exp:
tct	S	0	181.90
tcc	S	0	157.90
tca	S	0	247.10
tcg	S	0	65.76
att	I	0	0.37
atc	I	1	0.27
ata	I	0	0.37
aat	N	0	610.20
aac	N	1119	508.80
agt	S	0	236.40
agc	S	1090	200.90
---	-	9	9.00
---	--	------	------
mPD		0.50	1.6
		nPD:	0.3
		N. weight:	1.3
		Sc. PD:	0.11
		Sc. rank:	602.0

,

PB1-F2

Pos.	85	obs:	exp:
cgt	R	0	0.52
cgc	R	0	0.75
cga	R	0	1.41
cgg	R	0	1.14
aaa	K	2185	1250.00
aag	K	11	945.60
aga	R	14	6.54
agg	R	0	3.64
---	-	9	9.00
---	--	------	------
mPD		0.022	0.50
		nPD:	0.04
		N. weight:	0.55
		Sc. PD:	-0.09
		Sc. rank:	-542.6

,

PB1-F2

Pos.	86	obs:	exp:
cct	P	0	2.46
ccc	P	0	1.63
cca	P	9	3.65
ccg	P	0	1.26
caa	Q	2197	1097.00
cag	Q	0	1100.00
cgt	R	0	0.11
cgc	R	0	0.16
cga	R	3	0.30
cgg	R	0	0.24
aaa	K	1	0.57
aag	K	0	0.43
aga	R	0	1.40
agg	R	0	0.78
---	-	9	9.00
---	--	------	------
mPD		0.012	0.51
		nPD:	0.02
		N. weight:	0.72
		Sc. PD:	-0.13
		Sc. rank:	-1104.2

,

PB1-F2

Pos.	87	obs:	exp:
aaa	K	0	1.71
aag	K	3	1.29
gat	D	5	2.70
gac	D	0	2.30
gaa	E	4	1228.00
gag	E	2186	962.30
ggt	G	0	1.45
ggc	G	0	1.31
gga	G	0	4.38
ggg	G	10	2.86
---	-	11	11.00
---	--	------	------
mPD		0.020	0.50
		nPD:	0.04
		N. weight:	0.84
		Sc. PD:	-0.14
		Sc. rank:	-869.7

,

PB1-F2

Pos.	88	obs:	exp:
tta	L	0	1.20
ttg	L	13	2.47
tgg	W	2167	2167.00
ctt	L	0	2.58
ctc	L	0	2.08
cta	L	0	1.92
ctg	L	0	2.74
cgt	R	0	0.15
cgc	R	0	0.21
cga	R	0	0.40
cgg	R	3	0.33
aga	R	0	1.87
agg	R	1	1.04
---	-	35	35.00
---	--	------	------
mPD		0.015	0.032
		nPD:	0.46
		N. weight:	0.014
		Sc. PD:	0.0033
		Sc. rank:	23.5

,

PB1-F2

Pos.	89	obs:	exp:
att	I	0	240.70
atc	I	0	175.50
ata	I	656	239.70
act	T	0	400.60
acc	T	1	344.30
aca	T	1527	639.40
acg	T	0	143.80
---	-	35	35.00
---	--	------	------
mPD		0.41	1.1
		nPD:	0.38
		N. weight:	0.93
		Sc. PD:	0.15
		Sc. rank:	1068.4

,

PB1-F2

Pos.	90	obs:	exp:
tct	S	0	10.18
tcc	S	0	8.84
tca	S	0	13.83
tcg	S	0	3.68
aat	N	128	1156.00
aac	N	1992	963.90
agt	S	23	13.23
agc	S	38	11.24
gat	D	0	1.62
gac	D	3	1.38
gaa	E	0	1.12
gag	E	2	0.88
---	-	33	33.00
---	--	------	------
mPD		0.18	0.58
		nPD:	0.32
		N. weight:	0.58
		Sc. PD:	0.059
		Sc. rank:	357.2

,

PB1-F2

Pos.	91	obs:	exp:
taa	.	1241	818.20
tag	.	36	760.90
tga	.	942	639.90
---	--	------	------
mPD		0.52	0.86
		nPD:	0.6
		N. weight:	0.36
		Sc. PD:	0.14
		Sc. rank:	656.5

}

Amino acid level conservation per codon

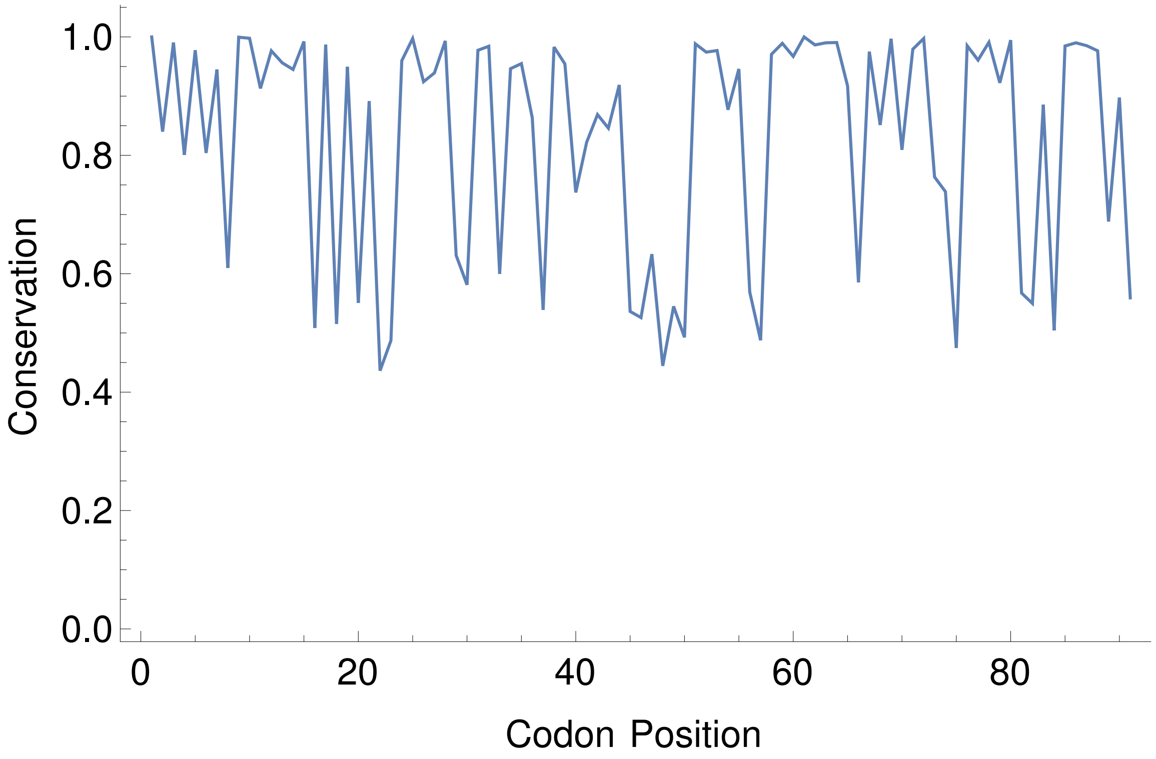

Conservation analysis plots

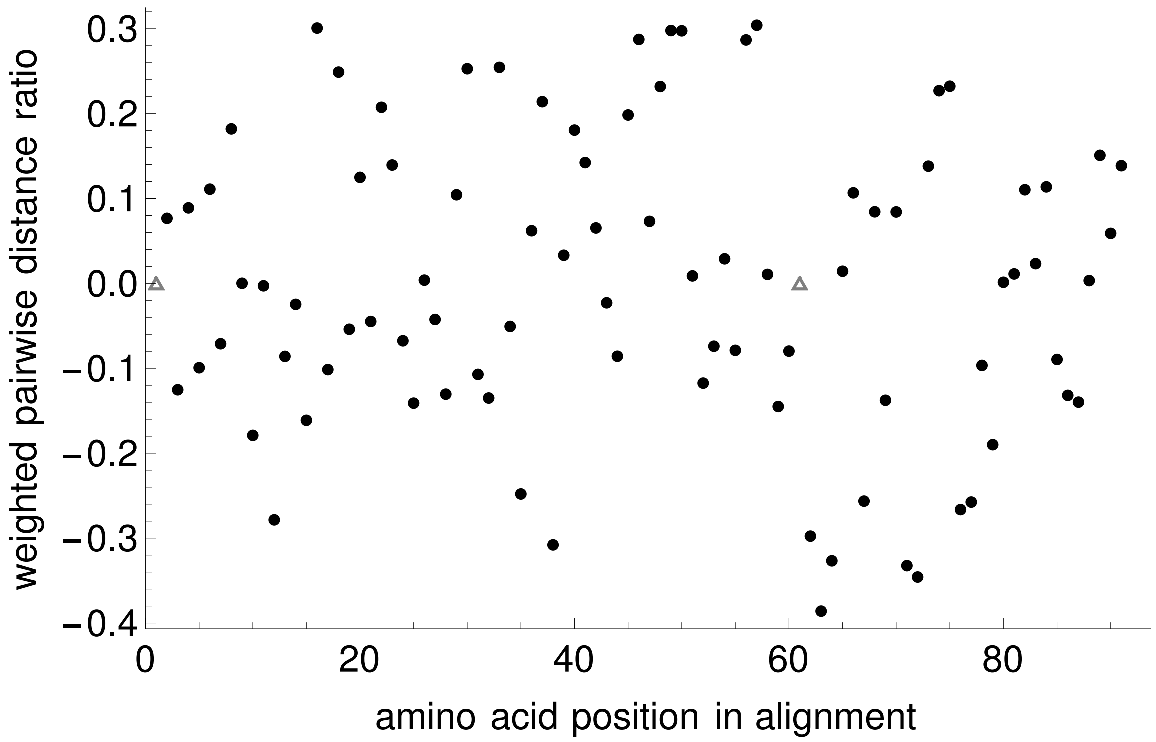

PB1−F2

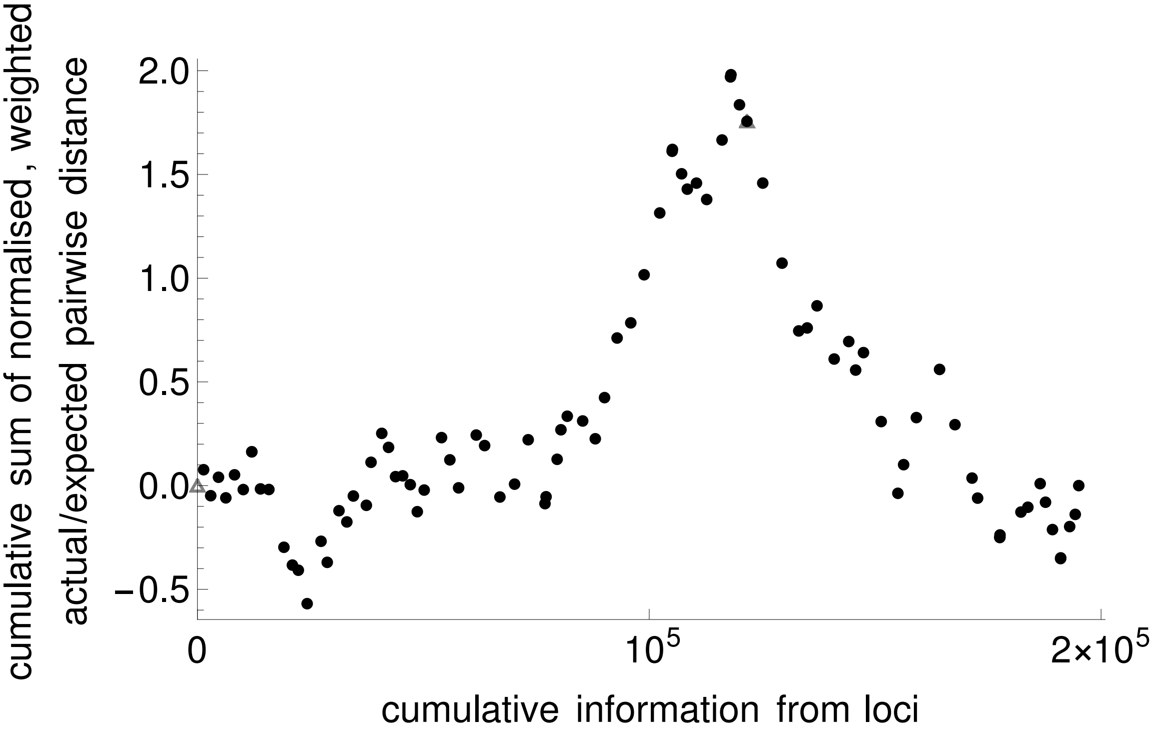

PB1−F2

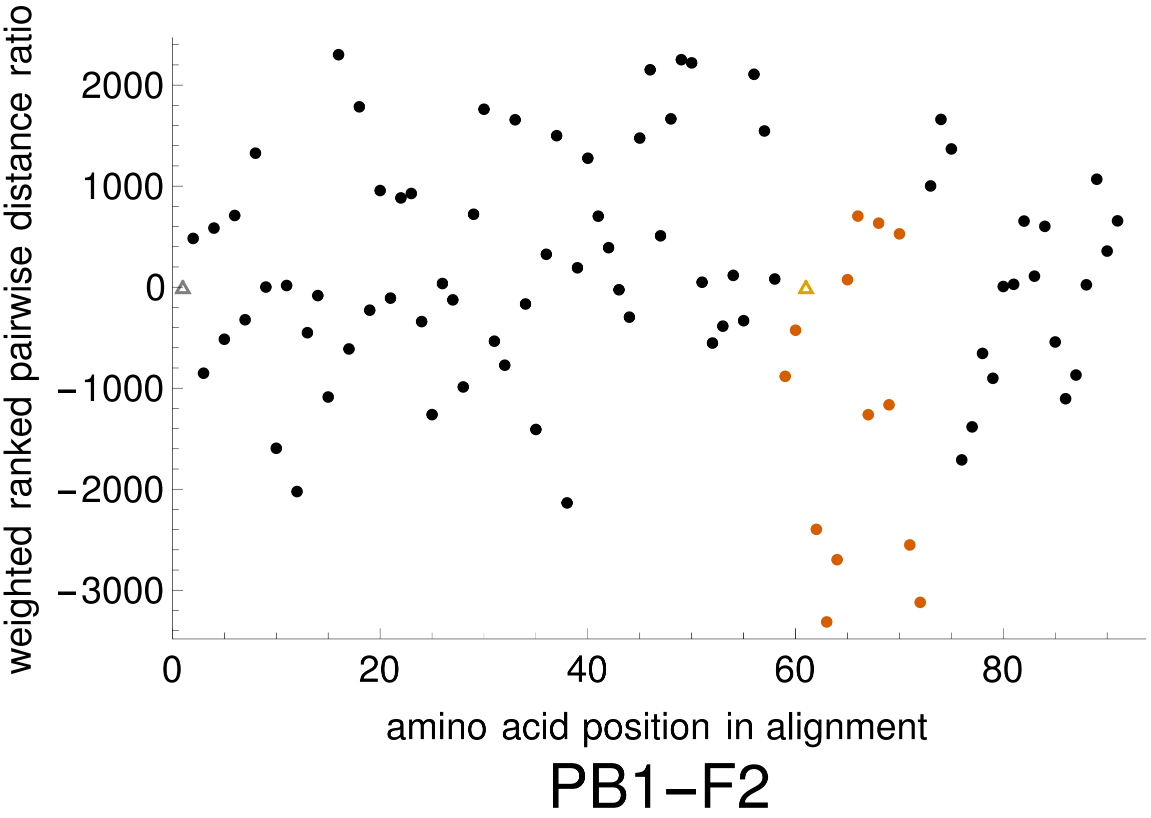
weighted ranked pairwise distance ratio
2000
1000
0
−1000
−2000
−3000
0
20
40
60
80
amino acid position in alignment
PB1−F2

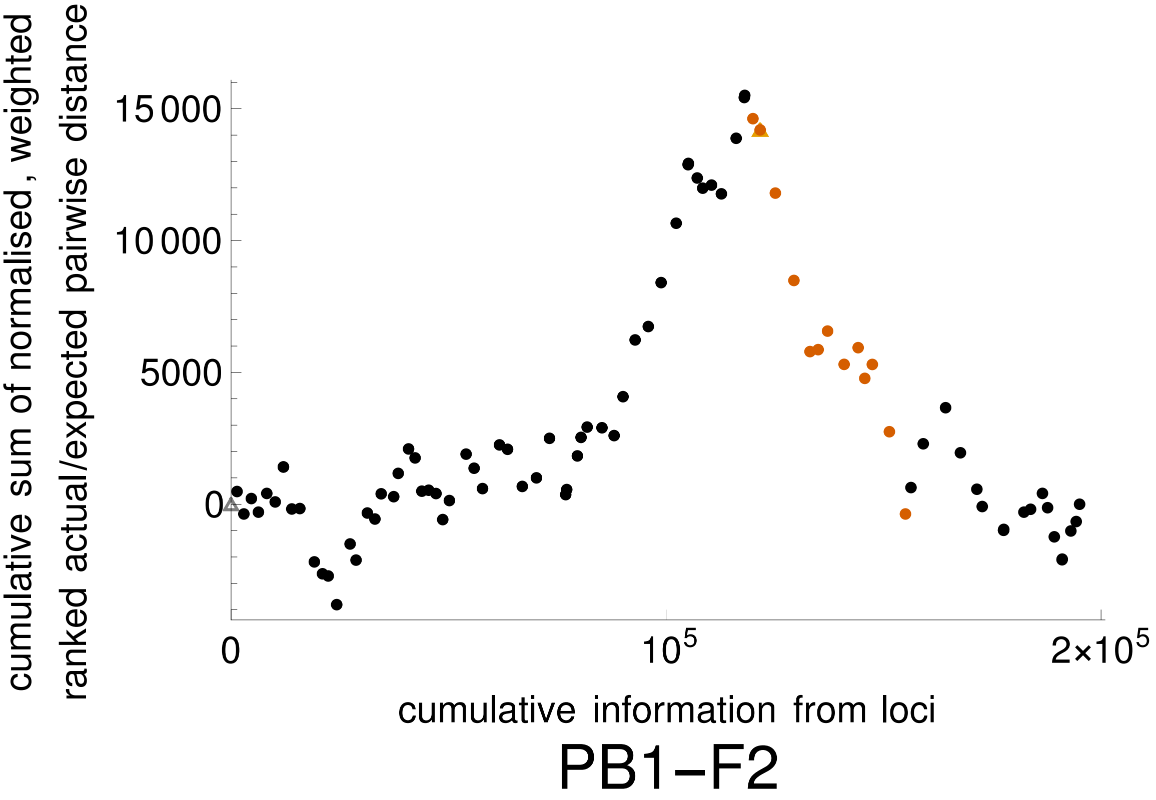
cumulative sum of normalised, weighted
ranked actual/expected pairwise distance
15 000
10 000
5000
0
0
10^5
2×10^5
cumulative information from loci
PB1−F2

PA

Gene length histogram

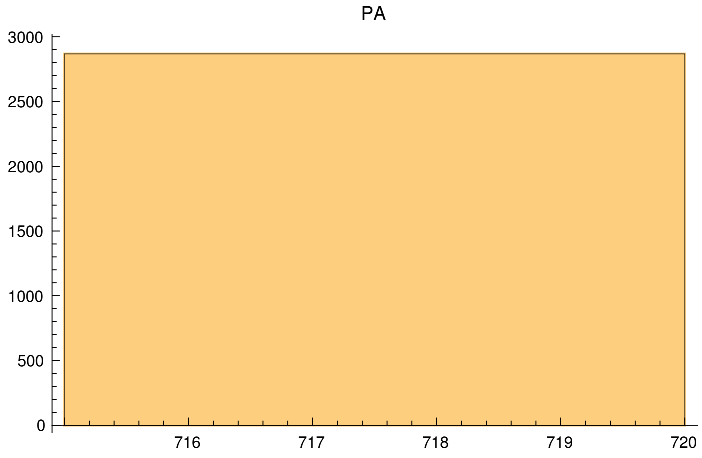

Information vs. nPD

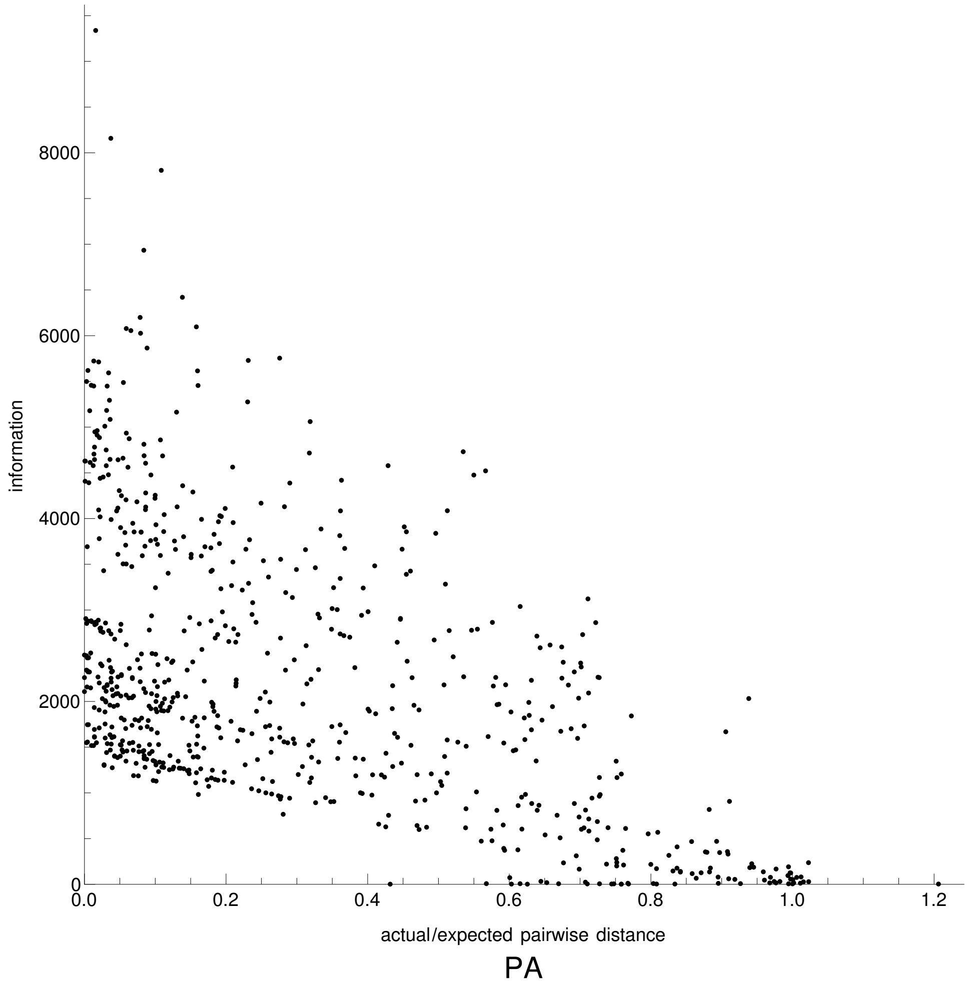

Example sequences highlighted by regions found in analyses to be conserved

Interesting points (by weighted raw PD) highlighted for gene PA:

ATGGAAGACTTTGTGCGACAATGCTTCAATCCAATGATCGTCGAGCTTGCGGAAAAGGCAATGAAAGAATATGGGG⋱
AAACTCCGAAAATCGAAACGAACAAATTCGCAGCAATATGCACACACTTAGAAGTCTGCTTCATGTATTCGGATT⋱
TCCACTTTATTGATGAACAAAGCGAATCAATAATTGTAGAATCTGGCGACCCGAATGCATTATTGAAACACCGAT⋱
TTGAGATAATTGAAGGGAGAGACCGAACAATGGCTTGGACAGTGGTAAATAGTATCTGCAACACCACAGGAGTCG⋱
ATAAGCCTAAATTCCTCCCAGATTTGTATGACTACAAAGAGAACCGATTCATTGAAATTGGAGTGACACGGAGGG⋱
AAGTTCACATATACTACCTAGAAAAAGCAAATAAGATAAAATCAGAGAAGACACACATTCACATATTCTCATTCA⋱
CTGGAGAGGAAATGGCCACCAAAGCAGATTATACCCTTGATGATGAGAGCAGAGCAAGAATCAAAACCAGGCTGT⋱
TCACTATAAGGCAAGAAATGGCCAGTAGGGGTCTATGG
GATTCCTTTCGTCAATCCGAGAGAGGCGAAGAGACAATTGAAGAAAGATTTGAAA
TCACAGGAACCATGCGCAGGCTTGCCGACCAAAGTCTCCCACCGAGCTTCTC
CAGCCTTGAAAATTTTAGAGCCTATGTGGATGGATTCGAACCGAACGGCTGC
ATTGAGGGCAAGCTTTCTCAAATGTCAAAAGAAGTGAACGCCAGAATTGAGCCATTT⋱
GTGAAGGAAACACCACGCCCTCTCAGACTACCTGATGGACCTCCCTGCTCTCAGCGGTCAAAGTTCTTAC⋱
TGATGGATGCCCTTAAATTAAGCATCGAAGATCCAAGCCATGAGGGGGAAGGTATACCGCTATATGATGC⋱
AATCAAATGCATGAAGACATTTTTCGGCTGGAAAGAGCCCAACATCGTAAAACCACATGAAAAAGGCATA⋱
AATCCCAATTACCTCCTGGCTTGGAAGCAGGTGCTGGCAGAACTCCAAGACATTGAAAATGAGGAGAAAA⋱
TCCCAAAAACAAAGAACATGAAGAAAACAAGCCAATTAAAGTGGGCACTTGGTGAGAACATGGCACCAGA⋱
AAAAGTAGACTTTGAGGACTGCAAAGATGTTAGCGATCTAAAACAGTATGACAGTGATGAACCAGAGCCT⋱
AGATCATTATCAAGCTGGATTCAGAGTGAATTCAACAAAGCATGCGAATTGACAGATTCGAGTTGGATTG⋱
AACTTGATGAAATAGGAGAAGATGTTGCTCCAATTGAGCACATTGCGAGTATGAGAAGAAACTATTTCAC⋱
AGCGGAAGTGTCTCATTGCAGGGCTACTGAATACATAATGAAGGGAGTGTATATAAATACAGCCCTGTTG⋱
AATGCATCCTGTGCAGCCATGGATGACTTTCAACTGATTCCAATGATAAGCAAGTGCAGAACCAAAGAAG⋱
GAAGACGGAAGACAAATCTGTATGGATTCATTATAAAAGGAAGATCCCATTTGAGGAATGACACCGATGT⋱
GGTAAACTTTGTGAGCATGGAATTCTCTCTTACTGACCCGAGGCTGGAACCACACAAGTGGGAAAAGTAC⋱
TGTGTTCTCGAGATAGGAGACATGCTCCTACGAACTGCAATAGGCCAAGTGTCAAGATCCATGTTCCTGT⋱
ATGTGAGAACCAATGGGACTTCCAAGATCAAGATGAAATGGGGCATGGAGATGAGGCGATGCCTTCTTCA⋱
ATCCCTTCAACAAATTGAGAGCATGATTGAGGCCGAGTCTTCTGTCAAAGAGAAAGACATGACCAAAGAA⋱
TTCTTTGTAACCAAATCAGAAACGTGGCCAATTGGGGAATCACCTAAAGGGGTGGAGGAAAGCTCCATTG⋱
GGAAGGTGTGCAGAACATTACTAGCAAAATCTGTATTCAACAGCCTATATGCATCTCCACAACTTGAGGG⋱
G
TTTTCAGCTGAATCAAGAAAATTGCTTCTCATTGTTCAGGCACTTAGGGACAACC
TGGAACCTGGAACCTTGGATCTTGGGGGGGTATATGAAGCAATTGAGGAGTG
CCTGATTAAGGAACCCTGGGTTTTGCTCAATGCGTCATGGTTCAACTCCTTC
CTCACACATGCACTGACATAG

Interesting points (by weighted ranked PD) highlighted for gene PA:

ATGGAAGACTTT
GTGCGACAATGCTTCAATCCAATGATCGTCGAGCTTGCGGAAAAGGCAATGAAAGAATATGG⋱
GGAAACTCCGAAAATCGAAACGAACAAATTCGCAGCAATATGCACACACTTAGAAGTCTGCTTCATGTAT⋱
TCGGATTTCCACTTTATTGATGAACAAAGCGAATCAATAATTGTAGAATCTGGCGACCCGAATGCATTAT⋱
TGAAACACCGATTTGAGATAATTGAAGGGAGAGACCGAACAATGGCTTGGACAGTGGTAAATAGTATCTG⋱
CAACACCACAGGAGTCGATAAGCCTAAATTCCTCCCAGATTTGTATGACTACAAAGAGAACCGATTCATT⋱
GAAATTGGAGTGACACGGAGGGAAGTTCACATATACTACCTAGAAAAAGCAAATAAGATAAAATCAGAGA⋱

AGACACACATTCACATATTCTCATTCACTGGAGAGGAAATGGCCACCAAAGCAGATTATACCCTTGATGA
TGAGAGCAGAGCAAGAATCAAAACCAGGCTGTTCACTATAAGGCAAGAAATGGCCAGTAGGGGTCTATGG
GATTCCTTTCGTCAATCCGAGAGAGGCGAAGAGACAATTGAAGAAAGATTTGAAA
TCACAGGAACCATGCGCAGGCTTGCCGACCAAAGTCTCCCACCGAGCTTCTC
CAGCCTTGAAAATTTTAGAGCCTATGTGGATGGATTCGAACCGAACGGCTGC
ATTGAGGGCAAGCTTTCTCAAATGTCAAAAGAAGTGAACGCCAGAATTGAGCCATTTGT
GAAGGAAACACCACGCCCTCTCAGACTACCTGATGGACCTCCCTGCTCTCAGCGGTCAAAGTTCTTACTG
ATGGATGCCCTTAAATTAAGCATCGAAGATCCAAGCCATGAGGGGGAAGGTATACCGCTATATGATGCAA
TCAAATGCATGAAGACATTTTTCGGCTGGAAAGAGCCCAACATCGTAAAACCACATGAAAAAGGCATAAA
TCCCAATTACCTCCTGGCTTGGAAGCAGGTGCTGGCAGAACTCCAAGACATTGAAAATGAGGAGAAAATC
CCAAAAACAAAGAACATGAAGAAAACAAGCCAATTAAAGTGGGCACTTGGTGAGAACATGGCACCAGAAA
AAGTAGACTTTGAGGACTGCAAAGATGTTAGCGATCTAAAACAGTATGACAGTGATGAACCAGAGCCTAG
ATCATTATCAAGCTGGATTCAGAGTGAATTCAACAAAGCATGCGAATTGACAGATTCGAGTTGGATTGAA
CTTGATGAAATAGGAGAAGATGTTGCTCCAATTGAGCACATTGCGAGTATGAGAAGAAACTATTTCACAG
CGGAAGTGTCTCATTGCAGGGCTACTGAATACATAATGAAGGGAGTGTATATAAATACAGCCCTGTTGAA
TGCATCCTGTGCAGCCATGGATGACTTTCAACTGATTCCAATGATAAGCAAGTGCAGAACCAAAGAAGGA
AGACGGAAGACAAATCTGTATGGATTCATTATAAAAGGAAGATCCCATTTGAGGAATGACACCGATGTGG
TAAACTTTGTGAGCATGGAATTCTCTCTTACTGACCCGAGGCTGGAACCACACAAGTGGGAAAAGTACTG
TGTTCTCGAGATAGGAGACATGCTCCTACGAACTGCAATAGGCCAAGTGTCAAGATCCATGTTCCTGTAT
GTGAGAACCAATGGGACTTCCAAGATCAAGATGAAATGGGGCATGGAGATGAGGCGATGC
CTTCTTCAATCCCTTCAACAAATTGAGAGCATGATTGAGGCCGAGTCTTCTGTCA
AAGAGAAAGACATGACCAAAGAATTCTTTGTAACCAAATCAGAAACGTGGCCAATTGGGGAATCACC
TAAAGGGGTGGAGGAAAGCTCCATTGGGAAGGTGTGCAGAACATTACTAGCAAAATCTGTATTCAACAGC
CTATATGCATCTCCACAACTTGAGGGGTTTTCAGCTGAATCAAGAAAATTG
CTTCTCATTGTTCAGGCACTTAGGGACAACCTGGAACCTGGAACCTTGGATCTTG
GGGGGGTATATGAAGCAATTGAGGAGTGCCTGATTAAGGAACCCTGGGTTTT
GCTCAATGCGTCATGGTTCAACTCCTTCCTCACACATGCACTGACATAG

Per codon conservation report

PA

Pos.	1	obs :	exp :
atg	M	2868	2868.00
---	--	------	------
mPD		0	0
		nPD :	1.
		N. weight :	0.
		Sc. PD :	0
		Sc. rank :	0

PA

Pos.	2	obs :	exp :
caa	Q	0	0.50
cag	Q	1	0.50
cgt	R	1	0.04
cgc	R	0	0.05
cga	R	0	0.10
cgg	R	0	0.08
aga	R	0	0.47
agg	R	0	0.26
gaa	E	2810	1606.00
gag	E	55	1259.00
ggt	G	0	0.14
ggc	G	0	0.13
gga	G	1	0.44
ggg	G	0	0.29
---	--	------	------
mPD		0.042	0.50
		nPD :	0.08
		N. weight :	0.65
		Sc. PD :	-0.075
		Sc. rank :	-261.7

PA

Pos.	3	obs :	exp :
cat	H	0	0.58
cac	H	1	0.42
aat	N	1	1.64
aac	N	2	1.36
gat	D	404	1542.00
gac	D	2452	1314.00
gaa	E	5	3.36
gag	E	1	2.64
ggt	G	0	0.29
ggc	G	2	0.26
gga	G	0	0.88
ggg	G	0	0.57
---	--	------	------
mPD		0.25	0.50
		nPD :	0.5
		N. weight :	0.46
		Sc. PD :	0.14
		Sc. rank :	659.5

PA

Pos.	4	obs :	exp :
ttt	F	2736	1192.00
ttc	F	122	1666.00
tta	L	0	0.46
ttg	L	0	0.95
tct	S	1	0.17
tcc	S	0	0.14
tca	S	0	0.23
tcg	S	0	0.06
tat	Y	0	0.48
tac	Y	1	0.52
tgt	C	2	0.77
tgc	C	0	1.23
ctt	L	5	0.99
ctc	L	0	0.80
cta	L	0	0.74
ctg	L	0	1.06
agt	S	0	0.22
agc	S	0	0.18
gtt	V	0	0.22
gtc	V	1	0.21
gta	V	0	0.21
gtg	V	0	0.36
---	--	------	------
mPD		0.090	0.49
		nPD :	0.18
		N. weight :	0.91
		Sc. PD :	-0.018
		Sc. rank :	387.2

PA

Pos.	5	obs :	exp :
tta	L	0	0.92
ttg	L	10	1.90
ctt	L	0	1.99
ctc	L	0	1.60
cta	L	0	1.48
ctg	L	0	2.11
atg	M	5	5.00
gtt	V	0	612.60
gtc	V	0	599.20
gta	V	12	607.30
gtg	V	2836	1029.00
gct	A	0	1.26
gcc	A	0	0.97
gca	A	0	2.23
gcg	A	5	0.55
---	--	------	------
mPD		0.022	0.75
		nPD :	0.03
		N. weight :	1.3
		Sc. PD :	-0.22
		Sc. rank :	-1647.1

PA

Pos.	6	obs :	exp :
cgt	R	10	107.30
cgc	R	0	153.50
cga	R	2786	289.00
cgg	R	20	234.20
aga	R	52	1339.00
agg	R	0	745.10
---	--	------	------
mPD		0.056	0.95
		nPD :	0.06
		N. weight :	2.8
		Sc. PD :	-0.39
		Sc. rank :	-1999.8

PA

Pos.	7	obs :	exp :
cct	P	0	0.82
ccc	P	0	0.54
cca	P	3	1.22
ccg	P	0	0.42
caa	Q	2830	1429.00
cag	Q	33	1434.00
cgt	R	0	0.07
cgc	R	0	0.11
cga	R	2	0.20
cgg	R	0	0.16
aga	R	0	0.93
agg	R	0	0.52
---	--	------	------
mPD		0.026	0.51
		nPD :	0.05
		N. weight :	0.84
		Sc. PD :	-0.12
		Sc. rank :	-716.1

PA

Pos.	8	obs :	exp :
tat	Y	0	0.96
tac	Y	2	1.04
tgt	C	25	1109.00
tgc	C	2841	1757.00
---	--	------	------
mPD		0.019	0.48
		nPD :	0.04
		N. weight :	0.59
		Sc. PD :	-0.094
		Sc. rank :	-593.2

PA

Pos.	9	obs :	exp :
ttt	F	29	1196.00
ttc	F	2838	1671.00
tct	S	0	0.17
tcc	S	1	0.14
tca	S	0	0.23
tcg	S	0	0.06
agt	S	0	0.22
agc	S	0	0.18
---	--	------	------
mPD		0.021	0.49
		nPD :	0.04
		N. weight :	0.65
		Sc. PD :	-0.1
		Sc. rank :	-634.7

PA

Pos.	10	obs :	exp :
tct	S	0	0.17
tcc	S	0	0.14
tca	S	0	0.23
tcg	S	0	0.06
cat	H	1	0.58
cac	H	0	0.42
att	I	0	0.37
atc	I	1	0.27
ata	I	0	0.37
act	T	0	0.26
acc	T	1	0.23
aca	T	0	0.42
acg	T	0	0.09
aat	N	2800	1561.00
aac	N	63	1302.00
agt	S	1	0.22
agc	S	0	0.18
gat	D	1	0.54
gac	D	0	0.46
---	--	------	------
mPD		0.048	0.50
		nPD :	0.1
		N. weight :	0.67
		Sc. PD :	-0.07
		Sc. rank :	-164.7

PA

Pos.	11	obs :	exp :
cct	P	1	783.80
ccc	P	2	518.00
cca	P	2859	1164.00
ccg	P	6	402.10
---	--	------	------
mPD		0.0063	0.71
		nPD :	0.01
		N. weight :	1.2
		Sc. PD :	-0.22
		Sc. rank :	-1936.4

PA

Pos.	12	obs :	exp :
tta	L	0	0.18
ttg	L	0	0.38
ctt	L	0	0.40
ctc	L	0	0.32
cta	L	2	0.30
ctg	L	0	0.42
att	I	1	10.27
atc	I	0	7.49
ata	I	27	10.23
atg	M	2838	2838.00
---	--	------	------
mPD		0.022	0.022
		nPD :	1.02
		N. weight :	0.013
		Sc. PD :	0.011
		Sc. rank :	24.8

PA

Pos.	13	obs :	exp :
att	I	1619	1027.00
atc	I	1177	748.70
ata	I	2	1023.00
act	T	51	13.63
acc	T	1	11.72
aca	T	0	21.76
acg	T	0	4.89
gtt	V	12	3.87
gtc	V	6	3.79
gta	V	0	3.84
gtg	V	0	6.50
---	--	------	------
mPD		0.53	0.71
		nPD :	0.75
		N. weight :	0.62
		Sc. PD :	0.34
		Sc. rank :	1132.9

PA

Pos.	14	obs :	exp :
att	I	0	0.73
atc	I	2	0.54
ata	I	0	0.73
gtt	V	48	611.50
gtc	V	2789	598.10
gta	V	6	606.20
gtg	V	0	1027.00
gct	A	2	5.79
gcc	A	21	4.45
gca	A	0	10.24
gcg	A	0	2.52
---	--	------	------
mPD		0.056	0.75
		nPD :	0.07
		N. weight :	1.9
		Sc. PD :	-0.24
		Sc. rank :	-1090.8

PA

Pos.	15	obs :	exp :
gat	D	4	2.16
gac	D	0	1.84
gaa	E	17	1602.00
gag	E	2841	1256.00
ggt	G	0	0.87
ggc	G	0	0.79
gga	G	0	2.63
ggg	G	6	1.72
---	--	------	------
mPD		0.019	0.50
		nPD :	0.04
		N. weight :	1.
		Sc. PD :	-0.17
		Sc. rank :	-1091.5

PA			
Pos.	16	obs:	exp:
tta	L	0	265.20
ttg	L	0	544.80
ctt	L	2867	569.40
ctc	L	0	458.80
cta	L	1	424.40
ctg	L	0	605.30
---	--	------	------
mPD		0.00070	1.1
		nPD:	0.
		N. weight:	2.1
		Sc. PD:	-0.42
		Sc. rank:	-3956.3

,

PA			
Pos.	17	obs:	exp:
gct	A	5	722.50
gcc	A	0	555.00
gca	A	163	1277.00
gcg	A	2700	313.90
---	--	------	------
mPD		0.11	0.69
		nPD:	0.16
		N. weight:	2.5
		Sc. PD:	-0.1
		Sc. rank:	767.5

,

PA			
Pos.	18	obs:	exp:
gaa	E	2759	1604.00
gag	E	102	1257.00
ggt	G	0	1.01
ggc	G	0	0.92
gga	G	7	3.07
ggg	G	0	2.00
---	--	------	------
mPD		0.073	0.50
		nPD:	0.15
		N. weight:	0.58
		Sc. PD:	-0.031
		Sc. rank:	103.3

,

PA			
Pos.	19	obs:	exp:
cgt	R	0	0.19
cgc	R	0	0.27
cga	R	0	0.50
cgg	R	0	0.41
aat	N	1	0.55
aac	N	0	0.45
aaa	K	644	1630.00
aag	K	2218	1232.00
aga	R	1	2.33
agg	R	4	1.30
---	--	------	------
mPD		0.35	0.50
		nPD:	0.71
		N. weight:	0.33
		Sc. PD:	0.17
		Sc. rank:	589.1

,

PA			
Pos.	20	obs:	exp:
cct	P	0	0.27
ccc	P	0	0.18
cca	P	1	0.41
ccg	P	0	0.14
att	I	0	0.37
atc	I	0	0.27
ata	I	1	0.37
act	T	0	229.90
acc	T	0	197.60
aca	T	877	367.00
acg	T	0	82.51
gct	A	0	501.10
gcc	A	0	384.90
gca	A	1988	885.30
gcg	A	1	217.70
---	--	------	------
mPD		0.43	1.1
		nPD:	0.38
		N. weight:	1.1
		Sc. PD:	0.2
		Sc. rank:	1304.3

,

PA			
Pos.	21	obs:	exp:
att	I	1	0.73
atc	I	0	0.54
ata	I	1	0.73
atg	M	2865	2865.00
act	T	0	0.26
acc	T	0	0.23
aca	T	0	0.42
acg	T	1	0.09
---	--	------	------
mPD		0.0021	0.0027
		nPD:	0.77
		N. weight:	0.0017
		Sc. PD:	0.00095
		Sc. rank:	3.1

,

PA			
Pos.	22	obs:	exp:
cgt	R	0	0.45
cgc	R	0	0.64
cga	R	0	1.21
cgg	R	0	0.98
act	T	0	0.26
acc	T	0	0.23
aca	T	1	0.42
acg	T	0	0.09
aaa	K	2797	1626.00
aag	K	58	1229.00
aga	R	12	5.60
agg	R	0	3.12
---	--	------	------
mPD		0.049	0.50
		nPD:	0.1
		N. weight:	0.63
		Sc. PD:	-0.065
		Sc. rank:	-151.4

,

PA			
Pos.	23	obs:	exp:
gtt	V	0	0.22
gtc	V	0	0.21
gta	V	1	0.21
gtg	V	0	0.36
gaa	E	2667	1607.00
gag	E	200	1260.00
---	--	------	------
mPD		0.13	0.49
		nPD:	0.26
		N. weight:	0.46
		Sc. PD:	0.028
		Sc. rank:	373.2

,

PA			
Pos.	24	obs:	exp:
ttt	F	2	0.83
ttc	F	0	1.17
tat	Y	2834	1372.00
tac	Y	26	1488.00
cat	H	5	2.89
cac	H	0	2.11
aat	N	1	0.55
aac	N	0	0.45
---	--	------	------
mPD		0.024	0.50
		nPD:	0.05
		N. weight:	0.91
		Sc. PD:	-0.14
		Sc. rank:	-859.7

,

PA			
Pos.	25	obs:	exp:
ggt	G	1	414.80
ggc	G	1	376.70
gga	G	153	1257.00
ggg	G	2713	819.80
---	--	------	------
mPD		0.10	0.69
		nPD:	0.15
		N. weight:	1.4
		Sc. PD:	-0.07
		Sc. rank:	259.8

,

PA			
Pos.	26	obs:	exp:
aaa	K	1	0.57
aag	K	0	0.43
gaa	E	2690	1607.00
gag	E	176	1259.00
ggt	G	0	0.14
ggc	G	0	0.13
gga	G	1	0.44
ggg	G	0	0.29
---	--	------	------
mPD		0.12	0.49
		nPD:	0.24
		N. weight:	0.48
		Sc. PD:	0.017
		Sc. rank:	345.0

,

PA			
Pos.	27	obs:	exp:
tct	S	0	97.82
tcc	S	0	84.88
tca	S	0	132.80
tcg	S	0	35.35
tat	Y	2	0.96
tac	Y	0	1.04
act	T	8	2.62
acc	T	2	2.25
aca	T	0	4.18
acg	T	0	0.94
aat	N	497	273.20
aac	N	4	227.80
agt	S	573	127.10
agc	S	13	108.00
gct	A	1	0.25
gcc	A	0	0.19
gca	A	0	0.45
gcg	A	0	0.11
gat	D	1651	953.50
gac	D	115	812.50
gaa	E	2	1.12
gag	E	0	0.88
---	--	------	------
mPD		0.90	1.4
		nPD:	0.62
		N. weight:	0.84
		Sc. PD:	0.35
		Sc. rank:	1364.5

,

PA			
Pos.	28	obs:	exp:
tta	L	0	0.65
ttg	L	0	1.33
ctt	L	0	1.39
ctc	L	0	1.12
cta	L	1	1.04
ctg	L	6	1.48
cct	P	10	780.20
ccc	P	2	515.70
cca	P	953	1159.00
ccg	P	1890	400.30
caa	Q	0	1.00
cag	Q	2	1.00
act	T	0	1.05
acc	T	0	0.90
aca	T	3	1.67
acg	T	1	0.38
---	--	------	------
mPD		0.46	0.72
		nPD:	0.64
		N. weight:	1.3
		Sc. PD:	0.54
		Sc. rank:	2082.6

,

PA			
Pos.	29	obs:	exp:
cgt	R	0	0.49
cgc	R	0	0.70
cga	R	0	1.31
cgg	R	0	1.06
att	I	0	0.37
atc	I	1	0.27
ata	I	0	0.37
aaa	K	2833	1622.00
aag	K	16	1227.00
aga	R	13	6.07
agg	R	0	3.38
gaa	E	4	2.24
gag	E	0	1.76
ggt	G	0	0.14
ggc	G	0	0.13
gga	G	1	0.44
ggg	G	0	0.29
---	--	------	------
mPD		0.026	0.51
		nPD:	0.05
		N. weight:	0.71
		Sc. PD:	-0.11
		Sc. rank:	-629.1

,

PA			
Pos.	30	obs:	exp:
tct	S	0	0.33
tcc	S	0	0.29
tca	S	0	0.45
tcg	S	0	0.12
att	I	193	1037.00
atc	I	2622	756.20
ata	I	11	1033.00
act	T	3	1.05
acc	T	1	0.90
aca	T	0	1.67
acg	T	0	0.38
agt	S	0	0.43
agc	S	2	0.37
gtt	V	1	7.74
gtc	V	35	7.57
gta	V	0	7.68
gtg	V	0	13.01
---	--	------	------
mPD		0.16	0.69
		nPD:	0.24
		N. weight:	1.4
		Sc. PD:	0.048
		Sc. rank:	989.6

,

PA			
Pos.	31	obs:	exp:
gaa	E	2163	1607.00
gag	E	703	1259.00
ggt	G	0	0.29
ggc	G	0	0.26
gga	G	2	0.88
ggg	G	0	0.57
---	--	------	------
mPD		0.37	0.49
		nPD:	0.75
		N. weight:	0.11
		Sc. PD:	0.06
		Sc. rank:	201.3

,

PA			
Pos.	32	obs:	exp:
tct	S	0	2.00
tcc	S	0	1.74
tca	S	11	2.72
tcg	S	0	0.72
atg	M	10	10.00
act	T	43	746.10
acc	T	3	641.30
aca	T	1141	1191.00
acg	T	1659	267.80
agt	S	1	2.60
agc	S	0	2.21
---	--	------	------
mPD		0.52	0.71
		nPD:	0.72
		N. weight:	1.3
		Sc. PD:	0.68
		Sc. rank:	2372.8

,

PA			
Pos.	33	obs:	exp:
tct	S	0	0.17
tcc	S	0	0.14
tca	S	0	0.23
tcg	S	0	0.06
aat	N	18	1563.00
aac	N	2848	1303.00
agt	S	0	0.22
agc	S	1	0.18
gat	D	0	0.54
gac	D	1	0.46
---	--	------	------
mPD		0.014	0.50
		nPD:	0.03
		N. weight:	1.
		Sc. PD:	-0.17
		Sc. rank:	-1276.3

,

PA			
Pos.	34	obs:	exp:
cgt	R	0	0.64
cgc	R	0	0.91
cga	R	0	1.71
cgg	R	0	1.39
aaa	K	2653	1623.00
aag	K	198	1228.00
aga	R	17	7.94
agg	R	0	4.42
---	--	------	------
mPD		0.14	0.51
		nPD:	0.28
		N. weight:	0.44
		Sc. PD:	0.033
		Sc. rank:	384.5

,

PA			
Pos.	35	obs:	exp:
ttt	F	1663	1188.00
ttc	F	1185	1660.00
tta	L	3	1.85
ttg	L	0	3.80
ctt	L	17	3.97
ctc	L	0	3.20
cta	L	0	2.96
ctg	L	0	4.22
---	--	------	------
mPD		0.50	0.50
		nPD:	0.99
		N. weight:	0.089
		Sc. PD:	0.069
		Sc. rank:	165.4

,

PA

Pos.	36	obs:	exp:
act	T	1	1.05
acc	T	3	0.90
aca	T	0	1.67
acg	T	0	0.38
gtt	V	1	0.43
gtc	V	1	0.42
gta	V	0	0.43
gtg	V	0	0.72
gct	A	598	721.00
gcc	A	2060	553.80
gca	A	203	1274.00
gcg	A	1	313.30
---	--	------	------
mPD		0.44	0.69
		nPD:	0.63
		N. weight:	1.
		Sc. PD:	0.44
		Sc. rank:	1695.1

PA

Pos.	37	obs:	exp:
tct	S	0	0.83
tcc	S	0	0.72
tca	S	5	1.13
tcg	S	0	0.30
agt	S	0	1.08
agc	S	0	0.92
gct	A	15	721.20
gcc	A	16	554.00
gca	A	2747	1274.00
gcg	A	85	313.40
---	--	------	------
mPD		0.082	0.69
		nPD:	0.12
		N. weight:	0.88
		Sc. PD:	-0.072
		Sc. rank:	0.3

PA

Pos.	38	obs:	exp:
att	I	0	1046.00
atc	I	1	762.70
ata	I	2849	1042.00
act	T	0	0.26
acc	T	0	0.23
aca	T	1	0.42
acg	T	0	0.09
gtt	V	0	3.66
gtc	V	0	3.58
gta	V	17	3.62
gtg	V	0	6.14
---	--	------	------
mPD		0.013	0.67
		nPD:	0.02
		N. weight:	1.3
		Sc. PD:	-0.24
		Sc. rank:	-1918.4

PA

Pos.	39	obs:	exp:
tgt	C	18	1110.00
tgc	C	2849	1757.00
cgt	R	0	0.04
cgc	R	1	0.05
cga	R	0	0.10
cgg	R	0	0.08
aga	R	0	0.47
agg	R	0	0.26
---	--	------	------
mPD		0.013	0.48
		nPD:	0.03
		N. weight:	0.61
		Sc. PD:	-0.1
		Sc. rank:	-780.8

PA

Pos.	40	obs:	exp:
act	T	27	751.10
acc	T	5	645.60
aca	T	2820	1199.00
acg	T	13	269.60
gct	A	0	0.76
gcc	A	0	0.58
gca	A	3	1.34
gcg	A	0	0.33
---	--	------	------
mPD		0.033	0.70
		nPD:	0.05
		N. weight:	1.1
		Sc. PD:	-0.16
		Sc. rank:	-961.0

PA

Pos.	41	obs:	exp:
cat	H	27	1660.00
cac	H	2841	1208.00
---	--	------	------
mPD		0.019	0.49
		nPD:	0.04
		N. weight:	1.1
		Sc. PD:	-0.17
		Sc. rank:	-1097.9

PA

Pos.	42	obs:	exp:
tta	L	2169	265.00
ttg	L	568	544.50
tct	S	0	0.17
tcc	S	0	0.14
tca	S	0	0.23
tcg	S	1	0.06
ctt	L	1	569.00
ctc	L	0	458.40
cta	L	124	424.10
ctg	L	4	604.90
att	I	0	0.37
atc	I	0	0.27
ata	I	1	0.37
agt	S	0	0.22
agc	S	0	0.18
---	--	------	------
mPD		0.41	1.1
		nPD:	0.36
		N. weight:	2.
		Sc. PD:	0.33
		Sc. rank:	2367.3

PA

Pos.	43	obs:	exp:
gat	D	0	0.54
gac	D	1	0.46
gaa	E	2378	1607.00
gag	E	488	1259.00
ggt	G	0	0.14
ggc	G	0	0.13
gga	G	0	0.44
ggg	G	1	0.29
---	--	------	------
mPD		0.28	0.49
		nPD:	0.58
		N. weight:	0.22
		Sc. PD:	0.081
		Sc. rank:	341.1

PA

Pos.	44	obs:	exp:
att	I	0	6.61
atc	I	18	4.82
ata	I	0	6.58
gtt	V	92	612.80
gtc	V	2713	599.40
gta	V	27	607.50
gtg	V	17	1029.00
gct	A	0	0.25
gcc	A	1	0.19
gca	A	0	0.45
gcg	A	0	0.11
---	--	------	------
mPD		0.10	0.75
		nPD:	0.14
		N. weight:	1.8
		Sc. PD:	-0.11
		Sc. rank:	267.9

PA

Pos.	45	obs:	exp:
tct	S	0	0.33
tcc	S	0	0.29
tca	S	0	0.45
tcg	S	0	0.12
tgt	C	1677	1109.00
tgc	C	1188	1756.00
tgg	W	1	1.00
agt	S	2	0.43
agc	S	0	0.37
---	--	------	------
mPD		0.49	0.48
		nPD:	1.02
		N. weight:	0.11
		Sc. PD:	0.089
		Sc. rank:	204.5

PA

Pos.	46	obs:	exp:
ttt	F	5	1196.00
ttc	F	2861	1670.00
tta	L	0	0.18
ttg	L	0	0.38
ctt	L	0	0.40
ctc	L	2	0.32
cta	L	0	0.30
ctg	L	0	0.42
---	--	------	------
mPD		0.0049	0.49
		nPD:	0.01
		N. weight:	0.7
		Sc. PD:	-0.13
		Sc. rank:	-1144.8

PA

Pos.	47	obs:	exp:
atg	M	2868	2868.00
---	--	------	------
mPD		0	0
		nPD:	1.
		N. weight:	0.
		Sc. PD:	0
		Sc. rank:	0

PA

Pos.	48	obs:	exp:
tat	Y	2809	1376.00
tac	Y	59	1492.00
---	--	------	------
mPD		0.040	0.50
		nPD:	0.08
		N. weight:	0.84
		Sc. PD:	-0.1
		Sc. rank:	-394.8

PA

Pos.	49	obs:	exp:
tct	S	10	477.60
tcc	S	0	414.40
tca	S	356	648.60
tcg	S	2495	172.60
tgg	W	6	6.00
cct	P	0	0.27
ccc	P	0	0.18
cca	P	0	0.41
ccg	P	1	0.14
agt	S	0	620.60
agc	S	0	527.20
---	--	------	------
mPD		0.23	1.7
		nPD:	0.14
		N. weight:	3.
		Sc. PD:	-0.18
		Sc. rank:	380.8

PA

Pos.	50	obs:	exp:
cat	H	1	0.58
cac	H	0	0.42
gat	D	2590	1548.00
gac	D	277	1319.00
---	--	------	------
mPD		0.18	0.50
		nPD:	0.35
		N. weight:	0.42
		Sc. PD:	0.062
		Sc. rank:	458.4

PA

Pos.	51	obs:	exp:
ttt	F	671	1196.00
ttc	F	2195	1670.00
tta	L	2	0.18
ttg	L	0	0.38
ctt	L	0	0.40
ctc	L	0	0.32
cta	L	0	0.30
ctg	L	0	0.42
---	--	------	------
mPD		0.36	0.49
		nPD:	0.74
		N. weight:	0.1
		Sc. PD:	0.054
		Sc. rank:	185.2

PA

Pos.	52	obs:	exp:
tta	L	0	0.09
ttg	L	0	0.19
ctt	L	0	0.20
ctc	L	1	0.16
cta	L	0	0.15
ctg	L	0	0.21
cat	H	854	1660.00
cac	H	2013	1207.00
---	--	------	------
mPD		0.42	0.49
		nPD:	0.86
		N. weight:	0.22
		Sc. PD:	0.14
		Sc. rank:	398.4

PA

Pos.	53	obs:	exp:
ttt	F	2764	1194.00
ttc	F	99	1669.00
tta	L	3	0.37
ttg	L	0	0.76
tct	S	1	0.17
tcc	S	0	0.14
tca	S	0	0.23
tcg	S	0	0.06
ctt	L	1	0.79
ctc	L	0	0.64
cta	L	0	0.59
ctg	L	0	0.84
agt	S	0	0.22
agc	S	0	0.18
---	--	------	------
mPD		0.070	0.49
		nPD:	0.14
		N. weight:	0.95
		Sc. PD:	-0.055
		Sc. rank:	158.6

PA

Pos.	54	obs:	exp:
tta	L	0	0.18
ttg	L	0	0.38
ctt	L	0	0.40
ctc	L	0	0.32
cta	L	0	0.30
ctg	L	2	0.42
att	I	1917	1046.00
atc	I	56	762.90
ata	I	878	1042.00
atg	M	7	7.00
act	T	2	0.52
acc	T	0	0.45
aca	T	0	0.84
acg	T	0	0.19
aaa	K	3	1.71
aag	K	0	1.29
gtt	V	3	0.65
gtc	V	0	0.63
gta	V	0	0.64
gtg	V	0	1.08
---	--	------	------
mPD		0.46	0.67
		nPD:	0.69
		N. weight:	0.41
		Sc. PD:	0.2
		Sc. rank:	706.3

PA

Pos.	55	obs:	exp:
aat	N	6	3.27
aac	N	0	2.73
gat	D	2719	1545.00
gac	D	143	1317.00
---	--	------	------
mPD		0.099	0.50
		nPD:	0.2
		N. weight:	0.57
		Sc. PD:	-0.0021
		Sc. rank:	302.1

PA			
Pos.	56	obs:	exp:
gat	D	0	2.70
gac	D	5	2.30
gaa	E	2765	1604.00
gag	E	97	1258.00
ggt	G	0	0.14
ggc	G	0	0.13
gga	G	1	0.44
ggg	G	0	0.29
---	--	------	------
mPD		0.069	0.50
		nPD:	0.14
		N. weight:	0.59
		Sc. PD:	-0.035
		Sc. rank:	90.9

,

PA			
Pos.	57	obs:	exp:
caa	Q	74	37.44
cag	Q	1	37.56
cgt	R	1	104.50
cgc	R	1	149.50
cga	R	2127	281.50
cgg	R	559	228.10
aga	R	103	1304.00
agg	R	2	725.60
---	--	------	------
mPD		0.44	1.0
		nPD:	0.43
		N. weight:	2.1
		Sc. PD:	0.48
		Sc. rank:	2705.0

,

PA			
Pos.	58	obs:	exp:
tct	S	0	85.96
tcc	S	0	74.60
tca	S	0	116.80
tcg	S	0	31.07
tgt	C	0	0.77
tgc	C	2	1.23
aat	N	1	0.55
aac	N	0	0.45
agt	S	475	111.70
agc	S	40	94.91
ggt	G	150	339.80
ggc	G	2138	308.70
gga	G	37	1030.00
ggg	G	25	671.70
---	--	------	------
mPD		0.67	1.2
		nPD:	0.55
		N. weight:	2.1
		Sc. PD:	0.71
		Sc. rank:	3150.8

,

PA			
Pos.	59	obs:	exp:
aat	N	3	1.64
aac	N	0	1.36
aaa	K	13	7.97
aag	K	1	6.03
gat	D	35	20.52
gac	D	3	17.48
gaa	E	2010	1571.00
gag	E	793	1232.00
ggt	G	1	1.45
ggc	G	0	1.31
gga	G	7	4.38
ggg	G	2	2.86
---	--	------	------
mPD		0.44	0.53
		nPD:	0.84
		N. weight:	0.082
		Sc. PD:	0.051
		Sc. rank:	150.0

,

PA			
Pos.	60	obs:	exp:
tct	S	4	476.60
tcc	S	0	413.60
tca	S	2820	647.20
tcg	S	31	172.20
cct	P	0	3.55
ccc	P	0	2.35
cca	P	13	5.28
ccg	P	0	1.82
agt	S	0	619.30
agc	S	0	526.10
---	--	------	------
mPD		0.033	1.7
		nPD:	0.02
		N. weight:	1.9
		Sc. PD:	-0.34
		Sc. rank:	-2700.5

,

PA			
Pos.	61	obs:	exp:
tta	L	0	0.09
ttg	L	0	0.19
tgg	W	1	1.00
ctt	L	0	0.20
ctc	L	0	0.16
cta	L	1	0.15
ctg	L	0	0.21
cgt	R	0	0.07
cgc	R	0	0.11
cga	R	0	0.20
cgg	R	0	0.16
att	I	0	783.10
atc	I	0	571.10
ata	I	2134	779.90
atg	M	596	596.00
act	T	3	31.98
acc	T	1	27.49
aca	T	59	51.05
acg	T	59	11.48
aga	R	1	0.93
agg	R	1	0.52
gtt	V	0	2.58
gtc	V	0	2.52
gta	V	12	2.56
gtg	V	0	4.34
---	--	------	------
mPD		0.45	0.84
		nPD:	0.54
		N. weight:	1.1
		Sc. PD:	0.35
		Sc. rank:	1572.0

,

PA			
Pos.	62	obs:	exp:
att	I	2648	1041.00
atc	I	186	759.20
ata	I	3	1037.00
atg	M	1	1.00
act	T	3	1.05
acc	T	1	0.90
aca	T	0	1.67
acg	T	0	0.38
gtt	V	24	5.59
gtc	V	2	5.47
gta	V	0	5.54
gtg	V	0	9.39
---	--	------	------
mPD		0.15	0.68
		nPD:	0.21
		N. weight:	1.
		Sc. PD:	0.013
		Sc. rank:	650.8

,

PA			
Pos.	63	obs:	exp:
tta	L	1	0.09
ttg	L	0	0.19
ctt	L	0	0.20
ctc	L	0	0.16
cta	L	0	0.15
ctg	L	0	0.21
att	I	0	5.50
atc	I	0	4.01
ata	I	15	5.48
act	T	0	4.19
acc	T	0	3.61
aca	T	16	6.69
acg	T	0	1.51
gtt	V	5	421.30
gtc	V	0	412.10
gta	V	1906	417.70
gtg	V	48	707.80
gct	A	0	215.40
gcc	A	0	165.50
gca	A	855	380.60
gcg	A	0	93.58
gaa	E	22	12.33
gag	E	0	9.67
---	--	------	------
mPD		0.49	1.2
		nPD:	0.41
		N. weight:	1.6
		Sc. PD:	0.33
		Sc. rank:	2012.7

,

PA			
Pos.	64	obs:	exp:
gct	A	0	0.76
gcc	A	0	0.58
gca	A	3	1.34
gcg	A	0	0.33
gat	D	5	8.10
gac	D	10	6.90
gaa	E	2808	1597.00
gag	E	41	1252.00
ggt	G	0	0.14
ggc	G	0	0.13
gga	G	1	0.44
ggg	G	0	0.29
---	--	------	------
mPD		0.041	0.50
		nPD:	0.08
		N. weight:	0.67
		Sc. PD:	-0.079
		Sc. rank:	-307.4

,

PA			
Pos.	65	obs:	exp:
ttt	F	18	7.51
ttc	F	0	10.49
tct	S	2736	470.70
tcc	S	51	408.50
tca	S	18	639.30
tcg	S	15	170.10
tat	Y	19	9.11
tac	Y	0	9.89
cct	P	9	2.46
ccc	P	0	1.63
cca	P	0	3.65
ccg	P	0	1.26
act	T	1	0.26
acc	T	0	0.23
aca	T	0	0.42
acg	T	0	0.09
agt	S	0	611.70
agc	S	0	519.70
gct	A	1	0.25
gcc	A	0	0.19
gca	A	0	0.45
gcg	A	0	0.11
---	--	------	------
mPD		0.091	1.7
		nPD:	0.05
		N. weight:	2.2
		Sc. PD:	-0.31
		Sc. rank:	-1748.8

,

PA			
Pos.	66	obs:	exp:
tct	S	0	1.00
tcc	S	0	0.87
tca	S	0	1.36
tcg	S	0	0.36
cgt	R	0	0.04
cgc	R	0	0.05
cga	R	0	0.10
cgg	R	0	0.08
agt	S	1	1.30
agc	S	5	1.11
aga	R	1	0.47
agg	R	0	0.26
gat	D	0	3.24
gac	D	6	2.76
gaa	E	2	1.12
gag	E	0	0.88
ggt	G	258	412.60
ggc	G	2097	374.70
gga	G	495	1250.00
ggg	G	3	815.50
---	--	------	------
mPD		0.43	0.70
		nPD:	0.62
		N. weight:	1.4
		Sc. PD:	0.57
		Sc. rank:	2267.7

,

PA			
Pos.	67	obs:	exp:
aat	N	11	6.00
aac	N	0	5.00
gat	D	2598	1542.00
gac	D	258	1314.00
ggt	G	1	0.14
ggc	G	0	0.13
gga	G	0	0.44
ggg	G	0	0.29
---	--	------	------
mPD		0.17	0.51
		nPD:	0.34
		N. weight:	0.44
		Sc. PD:	0.06
		Sc. rank:	466.6

,

PA			
Pos.	68	obs:	exp:
tta	L	0	0.55
ttg	L	0	1.14
tct	S	0	0.67
tcc	S	0	0.58
tca	S	3	0.91
tcg	S	1	0.24
ctt	L	0	1.19
ctc	L	0	0.96
cta	L	3	0.89
ctg	L	3	1.27
cct	P	0	780.80
ccc	P	12	516.00
cca	P	1017	1160.00
ccg	P	1828	400.60
cat	H	0	0.58
cac	H	1	0.42
agt	S	0	0.87
agc	S	0	0.74
---	--	------	------
mPD		0.47	0.72
		nPD:	0.66
		N. weight:	1.2
		Sc. PD:	0.55
		Sc. rank:	2035.0

,

PA			
Pos.	69	obs:	exp:
tct	S	0	0.33
tcc	S	0	0.29
tca	S	0	0.45
tcg	S	0	0.12
cat	H	3	1.74
cac	H	0	1.26
aat	N	2594	1561.00
aac	N	268	1301.00
aaa	K	1	0.57
aag	K	0	0.43
agt	S	1	0.43
agc	S	1	0.37
---	--	------	------
mPD		0.17	0.50
		nPD:	0.35
		N. weight:	0.42
		Sc. PD:	0.06
		Sc. rank:	445.5

,

PA			
Pos.	70	obs:	exp:
tct	S	0	0.17
tcc	S	0	0.14
tca	S	1	0.23
tcg	S	0	0.06
act	T	0	0.52
acc	T	0	0.45
aca	T	2	0.84
acg	T	0	0.19
agt	S	0	0.22
agc	S	0	0.18
gtt	V	1	6.45
gtc	V	0	6.31
gta	V	25	6.40
gtg	V	4	10.84
gct	A	93	713.40
gcc	A	14	548.00
gca	A	2709	1261.00
gcg	A	16	310.00
gaa	E	2	1.68
gag	E	1	1.32
---	--	------	------
mPD		0.11	0.72
		nPD:	0.16
		N. weight:	0.85
		Sc. PD:	-0.038
		Sc. rank:	204.3

,

PA

Pos.	71	obs:	exp:
tta	L	2762	265.20
ttg	L	54	544.80
ctt	L	2	569.40
ctc	L	20	458.80
cta	L	29	424.40
ctg	L	1	605.30
---	--	------	------
mPD		0.088	1.1
		nPD:	0.08
		N. weight:	2.9
		Sc. PD:	-0.35
		Sc. rank:	-1498.9

,

PA

Pos.	72	obs:	exp:
tta	L	180	265.10
ttg	L	2584	544.70
ctt	L	0	569.20
ctc	L	0	458.60
cta	L	43	424.30
ctg	L	60	605.10
aaa	K	0	0.57
aag	K	1	0.43
---	--	------	------
mPD		0.21	1.1
		nPD:	0.19
		N. weight:	1.7
		Sc. PD:	-0.018
		Sc. rank:	842.2

,

PA

Pos.	73	obs:	exp:
cgt	R	0	0.04
cgc	R	0	0.05
cga	R	0	0.10
cgg	R	0	0.08
act	T	0	0.26
acc	T	0	0.23
aca	T	1	0.42
acg	T	0	0.09
aaa	K	2795	1632.00
aag	K	71	1234.00
aga	R	1	0.47
agg	R	0	0.26
---	--	------	------
mPD		0.050	0.49
		nPD:	0.1
		N. weight:	0.61
		Sc. PD:	-0.06
		Sc. rank:	-102.4

,

PA

Pos.	74	obs:	exp:
ttt	F	0	0.42
ttc	F	1	0.58
cat	H	35	1660.00
cac	H	2832	1207.00
---	--	------	------
mPD		0.026	0.49
		nPD:	0.05
		N. weight:	1.1
		Sc. PD:	-0.16
		Sc. rank:	-887.1

,

PA

Pos.	75	obs:	exp:
tta	L	0	0.09
ttg	L	0	0.19
ctt	L	0	0.20
ctc	L	0	0.16
cta	L	1	0.15
ctg	L	0	0.21
cgt	R	4	107.30
cgc	R	4	153.40
cga	R	2744	288.90
cgg	R	31	234.10
aga	R	84	1338.00
agg	R	0	744.80
---	--	------	------
mPD		0.084	0.96
		nPD:	0.09
		N. weight:	2.7
		Sc. PD:	-0.3
		Sc. rank:	-875.3

,

PA

Pos.	76	obs:	exp:
ttt	F	2672	1196.00
ttc	F	194	1670.00
tat	Y	1	0.48
tac	Y	0	0.52
tgt	C	1	0.39
tgc	C	0	0.61
---	--	------	------
mPD		0.13	0.49
		nPD:	0.26
		N. weight:	0.8
		Sc. PD:	0.048
		Sc. rank:	647.4

,

PA

Pos.	77	obs:	exp:
gat	D	1	1.08
gac	D	1	0.92
gaa	E	560	1607.00
gag	E	2306	1259.00
---	--	------	------
mPD		0.32	0.49
		nPD:	0.64
		N. weight:	0.38
		Sc. PD:	0.16
		Sc. rank:	621.7

,

PA

Pos.	78	obs:	exp:
tta	L	17	2.13
ttg	L	0	4.37
ctt	L	0	4.57
ctc	L	0	3.68
cta	L	6	3.40
ctg	L	0	4.85
att	I	0	1015.00
atc	I	1	740.20
ata	I	2765	1011.00
atg	M	2	2.00
gtt	V	0	16.56
gtc	V	0	16.20
gta	V	77	16.42
gtg	V	0	27.82
---	--	------	------
mPD		0.070	0.74
		nPD:	0.09
		N. weight:	1.4
		Sc. PD:	-0.14
		Sc. rank:	-345.9

,

PA

Pos.	79	obs:	exp:
att	I	2225	1051.00
atc	I	638	766.40
ata	I	1	1047.00
gtt	V	3	0.86
gtc	V	1	0.84
gta	V	0	0.85
gtg	V	0	1.45
---	--	------	------
mPD		0.35	0.66
		nPD:	0.53
		N. weight:	0.72
		Sc. PD:	0.23
		Sc. rank:	1064.5

,

PA

Pos.	80	obs:	exp:
gaa	E	2830	1608.00
gag	E	38	1260.00
---	--	------	------
mPD		0.026	0.49
		nPD:	0.05
		N. weight:	0.68
		Sc. PD:	-0.099
		Sc. rank:	-561.9

,

PA

Pos.	81	obs:	exp:
cgt	R	0	0.07
cgc	R	0	0.11
cga	R	0	0.20
cgg	R	0	0.16
aga	R	0	0.93
agg	R	2	0.52
ggt	G	67	414.50
ggc	G	0	376.50
gga	G	440	1256.00
ggg	G	2359	819.20
---	--	------	------
mPD		0.30	0.69
		nPD:	0.43
		N. weight:	0.89
		Sc. PD:	0.2
		Sc. rank:	1140.7

,

PA

Pos.	82	obs:	exp:
tct	S	0	0.17
tcc	S	0	0.14
tca	S	0	0.23
tcg	S	0	0.06
cgt	R	0	107.30
cgc	R	0	153.40
cga	R	0	288.80
cgg	R	0	234.00
aaa	K	0	0.57
aag	K	1	0.43
agt	S	1	0.22
agc	S	0	0.18
aga	R	2828	1338.00
agg	R	38	744.60
---	--	------	------
mPD		0.028	0.96
		nPD:	0.03
		N. weight:	0.93
		Sc. PD:	-0.16
		Sc. rank:	-1164.0

,

PA

Pos.	83	obs:	exp:
tat	Y	0	0.48
tac	Y	1	0.52
aat	N	0	0.55
aac	N	1	0.45
gat	D	71	1546.00
gac	D	2793	1318.00
gaa	E	0	0.56
gag	E	1	0.44
ggt	G	0	0.14
ggc	G	1	0.13
gga	G	0	0.44
ggg	G	0	0.29
---	--	------	------
mPD		0.051	0.50
		nPD:	0.1
		N. weight:	0.87
		Sc. PD:	-0.085
		Sc. rank:	-137.4

,

PA

Pos.	84	obs:	exp:
cgt	R	5	107.30
cgc	R	19	153.50
cga	R	2777	289.00
cgg	R	37	234.20
aga	R	30	1339.00
agg	R	0	745.10
---	--	------	------
mPD		0.063	0.95
		nPD:	0.07
		N. weight:	2.8
		Sc. PD:	-0.38
		Sc. rank:	-1803.7

,

PA

Pos.	85	obs:	exp:
att	I	1	0.37
atc	I	0	0.27
ata	I	0	0.37
act	T	172	594.00
acc	T	103	510.60
aca	T	1546	948.20
acg	T	445	213.20
aat	N	1	0.55
aac	N	0	0.45
gct	A	0	151.20
gcc	A	1	116.10
gca	A	591	267.10
gcg	A	8	65.67
---	--	------	------
mPD		0.75	1.0
		nPD:	0.73
		N. weight:	0.54
		Sc. PD:	0.28
		Sc. rank:	978.7

,

PA			
Pos.	86	obs:	exp:
tta	L	0	0.09
ttg	L	1	0.19
tct	S	0	0.17
tcc	S	0	0.14
tca	S	0	0.23
tcg	S	0	0.06
ctt	L	0	0.20
ctc	L	0	0.16
cta	L	0	0.15
ctg	L	0	0.21
att	I	3	19.82
atc	I	0	14.45
ata	I	51	19.73
atg	M	2697	2697.00
act	T	0	0.26
acc	T	0	0.23
aca	T	0	0.42
acg	T	1	0.09
aaa	K	0	0.57
aag	K	1	0.43
agt	S	1	0.22
agc	S	0	0.18
gtt	V	0	24.30
gtc	V	0	23.77
gta	V	0	24.09
gtg	V	113	40.83
---	--	------	------
mPD		0.12	0.17
		nPD:	0.7
		N. weight:	0.077
		Sc. PD:	0.038
		Sc. rank:	133.3

PA			
Pos.	87	obs:	exp:
gtt	V	0	0.22
gtc	V	1	0.21
gta	V	0	0.21
gtg	V	0	0.36
gct	A	1131	722.00
gcc	A	1724	554.60
gca	A	10	1276.00
gcg	A	1	313.70
ggt	G	0	0.14
ggc	G	1	0.13
gga	G	0	0.44
ggg	G	0	0.29
---	--	------	------
mPD		0.48	0.69
		nPD:	0.7
		N. weight:	1.1
		Sc. PD:	0.55
		Sc. rank:	1953.9

PA			
Pos.	88	obs:	exp:
tgt	C	1	0.39
tgc	C	0	0.61
tgg	W	2864	2864.00
cgt	R	0	0.11
cgc	R	0	0.16
cga	R	0	0.30
cgg	R	2	0.24
aga	R	0	1.40
agg	R	1	0.78
---	--	------	------
mPD		0.0028	0.0042
		nPD:	0.67
		N. weight:	0.0029
		Sc. PD:	0.0013
		Sc. rank:	4.8

PA			
Pos.	89	obs:	exp:
atg	M	1	1.00
act	T	493	751.30
acc	T	7	645.80
aca	T	2186	1199.00
acg	T	180	269.70
gct	A	0	0.25
gcc	A	0	0.19
gca	A	1	0.45
gcg	A	0	0.11
---	--	------	------
mPD		0.39	0.70
		nPD:	0.55
		N. weight:	0.47
		Sc. PD:	0.16
		Sc. rank:	712.5

PA			
Pos.	90	obs:	exp:
tta	L	0	0.09
ttg	L	1	0.19
ctt	L	0	0.20
ctc	L	0	0.16
cta	L	0	0.15
ctg	L	0	0.21
att	I	0	2.20
atc	I	0	1.61
ata	I	6	2.19
atg	M	5	5.00
gtt	V	11	614.10
gtc	V	1	600.70
gta	V	215	608.80
gtg	V	2628	1032.00
ggt	G	0	0.14
ggc	G	0	0.13
gga	G	0	0.44
ggg	G	1	0.29
---	--	------	------
mPD		0.16	0.74
		nPD:	0.21
		N. weight:	1.
		Sc. PD:	0.013
		Sc. rank:	634.4

PA			
Pos.	91	obs:	exp:
att	I	0	16.88
atc	I	0	12.31
ata	I	46	16.81
atg	M	2	2.00
gtt	V	9	606.50
gtc	V	2	593.30
gta	V	1291	601.30
gtg	V	1518	1019.00
---	--	------	------
mPD		0.53	0.77
		nPD:	0.7
		N. weight:	0.74
		Sc. PD:	0.36
		Sc. rank:	1279.4

PA			
Pos.	92	obs:	exp:
tct	S	0	0.17
tcc	S	0	0.14
tca	S	0	0.23
tcg	S	0	0.06
aat	N	2807	1563.00
aac	N	60	1304.00
agt	S	1	0.22
agc	S	0	0.18
---	--	------	------
mPD		0.042	0.50
		nPD:	0.08
		N. weight:	0.68
		Sc. PD:	-0.078
		Sc. rank:	-292.1

PA			
Pos.	93	obs:	exp:
tct	S	0	478.10
tcc	S	0	414.90
tca	S	0	649.30
tcg	S	0	172.80
att	I	1	0.37
atc	I	0	0.27
ata	I	0	0.37
act	T	1	0.26
acc	T	0	0.23
aca	T	0	0.42
acg	T	0	0.09
aat	N	1	0.55
aac	N	0	0.45
agt	S	2742	621.20
agc	S	122	527.80
ggt	G	1	0.14
ggc	G	0	0.13
gga	G	0	0.44
ggg	G	0	0.29
---	--	------	------
mPD		0.084	1.7
		nPD:	0.05
		N. weight:	1.8
		Sc. PD:	-0.27
		Sc. rank:	-1572.0

PA			
Pos.	94	obs:	exp:
tta	L	0	1.20
ttg	L	0	2.47
ctt	L	11	2.58
ctc	L	2	2.08
cta	L	0	1.92
ctg	L	0	2.74
att	I	550	1024.00
atc	I	2188	746.90
ata	I	53	1020.00
atg	M	1	1.00
gtt	V	62	13.55
gtc	V	1	13.25
gta	V	0	13.43
gtg	V	0	22.76
---	--	------	------
mPD		0.42	0.72
		nPD:	0.59
		N. weight:	0.91
		Sc. PD:	0.35
		Sc. rank:	1438.6

PA			
Pos.	95	obs:	exp:
tct	S	0	4.34
tcc	S	0	3.77
tca	S	0	5.89
tcg	S	0	1.57
tgt	C	15	1100.00
tgc	C	2827	1742.00
agt	S	1	5.64
agc	S	25	4.79
---	--	------	------
mPD		0.029	0.50
		nPD:	0.06
		N. weight:	0.62
		Sc. PD:	-0.088
		Sc. rank:	-471.1

PA			
Pos.	96	obs:	exp:
cct	P	0	0.27
ccc	P	1	0.18
cca	P	0	0.41
ccg	P	0	0.14
att	I	0	0.73
atc	I	2	0.54
ata	I	0	0.73
act	T	0	0.26
acc	T	0	0.23
aca	T	1	0.42
acg	T	0	0.09
aat	N	9	1562.00
aac	N	2855	1302.00
---	--	------	------
mPD		0.010	0.50
		nPD:	0.02
		N. weight:	1.
		Sc. PD:	-0.18
		Sc. rank:	-1414.8

PA			
Pos.	97	obs:	exp:
tct	S	0	0.33
tcc	S	2	0.29
tca	S	0	0.45
tcg	S	0	0.12
att	I	0	1.10
atc	I	3	0.80
ata	I	0	1.10
act	T	38	748.20
acc	T	2798	643.10
aca	T	18	1194.00
acg	T	0	268.50
aat	N	0	3.27
aac	N	6	2.73
agt	S	0	0.43
agc	S	0	0.37
gct	A	0	0.76
gcc	A	3	0.58
gca	A	0	1.34
gcg	A	0	0.33
---	--	------	------
mPD		0.048	0.71
		nPD:	0.07
		N. weight:	1.8
		Sc. PD:	-0.24
		Sc. rank:	-1106.5

PA			
Pos.	98	obs:	exp:
cct	P	0	0.27
ccc	P	0	0.18
cca	P	1	0.41
ccg	P	0	0.14
act	T	3	750.50
acc	T	4	645.10
aca	T	2843	1198.00
acg	T	13	269.40
gct	A	0	1.01
gcc	A	0	0.77
gca	A	4	1.78
gcg	A	0	0.44
---	--	------	------
mPD		0.017	0.70
		nPD:	0.02
		N. weight:	1.1
		Sc. PD:	-0.19
		Sc. rank:	-1464.6

PA			
Pos.	99	obs:	exp:
cgt	R	0	0.49
cgc	R	0	0.70
cga	R	2	1.31
cgg	R	0	1.06
aga	R	8	6.07
agg	R	3	3.38
gtt	V	0	0.22
gtc	V	0	0.21
gta	V	0	0.21
gtg	V	1	0.36
gaa	E	20	11.21
gag	E	0	8.79
ggt	G	0	409.80
ggc	G	1	372.30
gga	G	2288	1242.00
ggg	G	545	810.10
---	--	------	------
mPD		0.33	0.71
		nPD:	0.47
		N. weight:	0.56
		Sc. PD:	0.15
		Sc. rank:	773.4

PA			
Pos.	100	obs:	exp:
tta	L	0	0.09
ttg	L	0	0.19
ctt	L	0	0.20
ctc	L	1	0.16
cta	L	0	0.15
ctg	L	0	0.21
att	I	4	9.54
atc	I	22	6.96
ata	I	0	9.50
gtt	V	192	605.50
gtc	V	2611	592.20
gta	V	12	600.20
gtg	V	0	1017.00
gct	A	7	6.55
gcc	A	19	5.03
gca	A	0	11.57
gcg	A	0	2.85
---	--	------	------
mPD		0.18	0.77
		nPD:	0.23
		N. weight:	1.7
		Sc. PD:	0.045
		Sc. rank:	1131.5

PA

Pos.	101	obs:	exp:
tct	S	0	1.34
tcc	S	0	1.16
tca	S	0	1.81
tcg	S	0	0.48
aat	N	484	271.00
aac	N	13	226.00
aaa	K	1	0.57
aag	K	0	0.43
agt	S	8	1.74
agc	S	0	1.47
gtt	V	0	1.72
gtc	V	0	1.68
gta	V	0	1.71
gtg	V	8	2.89
gat	D	939	518.90
gac	D	22	442.10
gaa	E	825	756.80
gag	E	525	593.20
ggt	G	33	6.22
ggc	G	3	5.65
gga	G	5	18.84
ggg	G	2	12.29
---	--	------	------
mPD		0.95	1.1
		nPD:	0.88
		N. weight:	0.38
		Sc. PD:	0.25
		Sc. rank:	698.8

,

PA

Pos.	102	obs:	exp:
cgt	R	0	0.04
cgc	R	0	0.05
cga	R	0	0.10
cgg	R	0	0.08
aaa	K	715	1633.00
aag	K	2152	1234.00
aga	R	1	0.47
agg	R	0	0.26
---	--	------	------
mPD		0.38	0.49
		nPD:	0.76
		N. weight:	0.28
		Sc. PD:	0.16
		Sc. rank:	516.6

,

PA

Pos.	103	obs:	exp:
cct	P	1887	783.80
ccc	P	899	518.00
cca	P	82	1164.00
ccg	P	0	402.10
---	--	------	------
mPD		0.47	0.71
		nPD:	0.66
		N. weight:	0.9
		Sc. PD:	0.41
		Sc. rank:	1515.3

,

PA

Pos.	104	obs:	exp:
cgt	R	0	0.34
cgc	R	0	0.48
cga	R	0	0.91
cgg	R	0	0.73
att	I	0	0.37
atc	I	0	0.27
ata	I	1	0.37
aaa	K	2804	1627.00
aag	K	54	1231.00
aga	R	9	4.20
agg	R	0	2.34
---	--	------	------
mPD		0.044	0.50
		nPD:	0.09
		N. weight:	0.63
		Sc. PD:	-0.071
		Sc. rank:	-213.1

,

PA

Pos.	105	obs:	exp:
ttt	F	543	1132.00
ttc	F	2170	1581.00
tta	L	5	1.85
ttg	L	0	3.80
tct	S	2	19.53
tcc	S	115	16.95
tca	S	0	26.52
tcg	S	0	7.06
tat	Y	0	8.63
tac	Y	18	9.37
ctt	L	7	3.97
ctc	L	8	3.20
cta	L	0	2.96
ctg	L	0	4.22
agt	S	0	25.38
agc	S	0	21.56
---	--	------	------
mPD		0.41	0.64
		nPD:	0.65
		N. weight:	0.25
		Sc. PD:	0.11
		Sc. rank:	418.5

,

PA

Pos.	106	obs:	exp:
tta	L	0	265.10
ttg	L	0	544.70
ctt	L	45	569.20
ctc	L	2821	458.60
cta	L	1	424.30
ctg	L	0	605.10
att	I	0	0.37
atc	I	1	0.27
ata	I	0	0.37
---	--	------	------
mPD		0.032	1.1
		nPD:	0.03
		N. weight:	2.3
		Sc. PD:	-0.39
		Sc. rank:	-2943.9

,

PA

Pos.	107	obs:	exp:
cct	P	808	783.80
ccc	P	23	518.00
cca	P	1972	1164.00
ccg	P	65	402.10
---	--	------	------
mPD		0.45	0.71
		nPD:	0.63
		N. weight:	0.41
		Sc. PD:	0.17
		Sc. rank:	673.8

,

PA

Pos.	108	obs:	exp:
aat	N	3	1.64
aac	N	0	1.36
gat	D	2155	1547.00
gac	D	710	1318.00
---	--	------	------
mPD		0.37	0.50
		nPD:	0.75
		N. weight:	0.13
		Sc. PD:	0.071
		Sc. rank:	236.6

,

PA

Pos.	109	obs:	exp:
tta	L	119	265.00
ttg	L	2685	544.50
tct	S	0	0.17
tcc	S	0	0.14
tca	S	0	0.23
tcg	S	1	0.06
ctt	L	0	569.00
ctc	L	0	458.40
cta	L	5	424.10
ctg	L	57	604.90
atg	M	1	1.00
agt	S	0	0.22
agc	S	0	0.18
---	--	------	------
mPD		0.13	1.1
		nPD:	0.11
		N. weight:	1.9
		Sc. PD:	-0.16
		Sc. rank:	-38.3

,

PA

Pos.	110	obs:	exp:
tat	Y	2802	1376.00
tac	Y	66	1492.00
---	--	------	------
mPD		0.045	0.50
		nPD:	0.09
		N. weight:	0.83
		Sc. PD:	-0.091
		Sc. rank:	-260.2

,

PA

Pos.	111	obs:	exp:
tat	Y	0	0.48
tac	Y	1	0.52
gtt	V	0	1.08
gtc	V	5	1.05
gta	V	0	1.07
gtg	V	0	1.81
gat	D	63	1545.00
gac	D	2799	1317.00
---	--	------	------
mPD		0.047	0.50
		nPD:	0.09
		N. weight:	0.89
		Sc. PD:	-0.094
		Sc. rank:	-240.7

,

PA

Pos.	112	obs:	exp:
tat	Y	28	1376.00
tac	Y	2840	1492.00
---	--	------	------
mPD		0.019	0.50
		nPD:	0.04
		N. weight:	0.8
		Sc. PD:	-0.13
		Sc. rank:	-810.4

,

PA

Pos.	113	obs:	exp:
cgt	R	0	0.67
cgc	R	0	0.96
cga	R	0	1.81
cgg	R	0	1.47
act	T	0	0.26
acc	T	0	0.23
aca	T	1	0.42
acg	T	0	0.09
aat	N	0	0.55
aac	N	1	0.45
aaa	K	2644	1622.00
aag	K	204	1226.00
aga	R	16	8.40
agg	R	2	4.68
---	--	------	------
mPD		0.15	0.51
		nPD:	0.29
		N. weight:	0.44
		Sc. PD:	0.038
		Sc. rank:	393.1

,

PA

Pos.	114	obs:	exp:
aaa	K	0	0.57
aag	K	1	0.43
gaa	E	79	1607.00
gag	E	2788	1260.00
---	--	------	------
mPD		0.054	0.49
		nPD:	0.11
		N. weight:	0.92
		Sc. PD:	-0.083
		Sc. rank:	-56.7

,

PA

Pos.	115	obs:	exp:
tct	S	0	4.34
tcc	S	0	3.77
tca	S	0	5.89
tcg	S	0	1.57
tat	Y	0	1.92
tac	Y	4	2.08
aat	N	179	1523.00
aac	N	2614	1270.00
agt	S	19	5.64
agc	S	7	4.79
gat	D	1	24.30
gac	D	44	20.70
---	--	------	------
mPD		0.18	0.56
		nPD:	0.32
		N. weight:	0.73
		Sc. PD:	0.087
		Sc. rank:	739.5

,

PA

Pos.	116	obs:	exp:
caa	Q	1	0.50
cag	Q	0	0.50
cgt	R	13	107.30
cgc	R	11	153.40
cga	R	2761	288.90
cgg	R	66	234.10
aga	R	12	1338.00
agg	R	4	744.80
---	--	------	------
mPD		0.076	0.96
		nPD:	0.08
		N. weight:	2.8
		Sc. PD:	-0.34
		Sc. rank:	-1401.5

,

PA

Pos.	117	obs:	exp:
ttt	F	128	1196.00
ttc	F	2740	1672.00
---	--	------	------
mPD		0.085	0.49
		nPD:	0.18
		N. weight:	0.5
		Sc. PD:	-0.013
		Sc. rank:	184.4

,

PA

Pos.	118	obs:	exp:
tta	L	0	0.09
ttg	L	0	0.19
ctt	L	1	0.20
ctc	L	0	0.16
cta	L	0	0.15
ctg	L	0	0.21
att	I	2580	998.50
atc	I	139	728.10
ata	I	2	994.40
act	T	14	24.38
acc	T	76	20.96
aca	T	3	38.91
acg	T	0	8.75
gtt	V	33	11.40
gtc	V	20	11.15
gta	V	0	11.30
gtg	V	0	19.15
---	--	------	------
mPD		0.25	0.77
		nPD:	0.33
		N. weight:	1.1
		Sc. PD:	0.14
		Sc. rank:	1132.3

,

PA

Pos.	119	obs:	exp:
gat	D	1	0.54
gac	D	0	0.46
gaa	E	2807	1607.00
gag	E	60	1260.00
---	--	------	------
mPD		0.042	0.49
		nPD:	0.08
		N. weight:	0.64
		Sc. PD:	-0.074
		Sc. rank:	-255.6

,

PA

Pos.	120	obs:	exp:
att	I	2849	1050.00
atc	I	10	765.60
ata	I	2	1046.00
gtt	V	5	1.51
gtc	V	0	1.47
gta	V	0	1.49
gtg	V	2	2.53
---	--	------	------
mPD		0.015	0.67
		nPD:	0.02
		N. weight:	1.3
		Sc. PD:	-0.23
		Sc. rank:	-1773.7

,

PA

Pos.	121	obs:	exp:
ggt	G	2	414.80
ggc	G	2	376.70
gga	G	2798	1257.00
ggg	G	66	819.80
---	--	------	------
mPD		0.048	0.69
		nPD:	0.07
		N. weight:	0.95
		Sc. PD:	-0.12
		Sc. rank:	-565.0

,

PA

Pos.	122	obs:	exp:
gtt	V	2	616.90
gtc	V	0	603.40
gta	V	850	611.50
gtg	V	2016	1036.00
---	--	------	------
mPD		0.42	0.73
		nPD:	0.57
		N. weight:	0.75
		Sc. PD:	0.27
		Sc. rank:	1156.2

,

PA

Pos.	123	obs:	exp:
act	T	2	751.80
acc	T	23	646.20
aca	T	2027	1200.00
acg	T	816	269.80
---	--	------	------
mPD		0.42	0.70
		nPD:	0.6
		N. weight:	0.87
		Sc. PD:	0.35
		Sc. rank:	1391.5

,

PA

Pos.	124	obs:	exp:
cgt	R	0	107.30
cgc	R	0	153.50
cga	R	1148	289.00
cgg	R	1639	234.20
aga	R	42	1339.00
agg	R	39	745.10
---	--	------	------
mPD		0.54	0.95
		nPD:	0.57
		N. weight:	2.1
		Sc. PD:	0.75
		Sc. rank:	3227.8

,

PA

Pos.	125	obs:	exp:
cgt	R	0	107.30
cgc	R	0	153.50
cga	R	5	289.00
cgg	R	670	234.20
aga	R	86	1339.00
agg	R	2107	745.10
---	--	------	------
mPD		0.42	0.95
		nPD:	0.44
		N. weight:	1.2
		Sc. PD:	0.29
		Sc. rank:	1594.9

,

PA

Pos.	126	obs:	exp:
gat	D	1	0.54
gac	D	0	0.46
gaa	E	2682	1607.00
gag	E	185	1260.00
---	--	------	------
mPD		0.12	0.49
		nPD:	0.25
		N. weight:	0.47
		Sc. PD:	0.021
		Sc. rank:	354.6

,

PA

Pos.	127	obs:	exp:
tta	L	0	0.28
ttg	L	0	0.57
ctt	L	3	0.60
ctc	L	0	0.48
cta	L	0	0.44
ctg	L	0	0.63
att	I	74	27.16
atc	I	0	19.80
ata	I	0	27.04
gtt	V	2653	599.20
gtc	V	131	586.10
gta	V	1	594.00
gtg	V	1	1007.00
gct	A	4	1.26
gcc	A	1	0.97
gca	A	0	2.23
gcg	A	0	0.55
---	--	------	------
mPD		0.14	0.79
		nPD:	0.18
		N. weight:	1.8
		Sc. PD:	-0.032
		Sc. rank:	776.4

,

PA

Pos.	128	obs:	exp:
tat	Y	0	0.48
tac	Y	1	0.52
cat	H	524	1657.00
cac	H	2339	1206.00
caa	Q	1	0.50
cag	Q	0	0.50
aat	N	2	1.64
aac	N	1	1.36
---	--	------	------
mPD		0.30	0.49
		nPD:	0.62
		N. weight:	0.44
		Sc. PD:	0.18
		Sc. rank:	712.2

,

PA

Pos.	129	obs:	exp:
tta	L	0	1.94
ttg	L	0	3.99
ctt	L	0	4.17
ctc	L	0	3.36
cta	L	21	3.11
ctg	L	0	4.43
att	I	5	827.90
atc	I	9	603.70
ata	I	2242	824.40
atg	M	28	28.00
act	T	0	135.50
acc	T	1	116.50
aca	T	511	216.30
acg	T	5	48.64
gtt	V	0	9.68
gtc	V	0	9.47
gta	V	45	9.60
gtg	V	0	16.26
gct	A	0	0.25
gcc	A	0	0.19
gca	A	1	0.45
gcg	A	0	0.11
---	--	------	------
mPD		0.38	1.0
		nPD:	0.37
		N. weight:	1.3
		Sc. PD:	0.21
		Sc. rank:	1465.3

,

PA

Pos.	130	obs:	exp:
tat	Y	119	1375.00
tac	Y	2748	1492.00
tgt	C	0	0.39
tgc	C	1	0.61
---	--	------	------
mPD		0.080	0.50
		nPD:	0.16
		N. weight:	0.64
		Sc. PD:	-0.026
		Sc. rank:	197.9

,

PA

Pos.	131	obs:	exp:
tat	Y	1694	1375.00
tac	Y	1173	1492.00
tgt	C	1	0.39
tgc	C	0	0.61
---	--	------	------
mPD		0.48	0.50
		nPD:	0.97
		N. weight:	0.035
		Sc. PD:	0.027
		Sc. rank:	65.9

,

PA

Pos.	132	obs:	exp:
tta	L	643	263.20
ttg	L	11	540.90
ctt	L	10	565.30
ctc	L	0	455.40
cta	L	1433	421.30
ctg	L	750	600.90
caa	Q	4	2.00
cag	Q	0	2.00
att	I	0	0.73
atc	I	0	0.54
ata	I	2	0.73
atg	M	15	15.00
---	--	------	------
mPD		0.76	1.1
		nPD:	0.68
		N. weight:	1.1
		Sc. PD:	0.53
		Sc. rank:	1921.5

,

PA

Pos.	133	obs:	exp:
gat	D	0	0.54
gac	D	1	0.46
gaa	E	2239	1607.00
gag	E	627	1259.00
ggt	G	0	0.14
ggc	G	0	0.13
gga	G	1	0.44
ggg	G	0	0.29
---	--	------	------
mPD		0.34	0.49
		nPD:	0.69
		N. weight:	0.14
		Sc. PD:	0.07
		Sc. rank:	249.1

,

PA

Pos.	134	obs:	exp:
cgt	R	0	0.04
cgc	R	0	0.05
cga	R	0	0.10
cgg	R	0	0.08
aaa	K	2484	1633.00
aag	K	383	1234.00
aga	R	1	0.47
agg	R	0	0.26
---	--	------	------
mPD		0.23	0.49
		nPD:	0.47
		N. weight:	0.28
		Sc. PD:	0.074
		Sc. rank:	388.0

,

PA

Pos.	135	obs:	exp:
tct	S	0	0.50
tcc	S	3	0.43
tca	S	0	0.68
tcg	S	0	0.18
agt	S	0	0.65
agc	S	0	0.55
gtt	V	0	0.22
gtc	V	1	0.21
gta	V	0	0.21
gtg	V	0	0.36
gct	A	14	721.20
gcc	A	1719	554.00
gca	A	1121	1274.00
gcg	A	9	313.40
gat	D	0	0.54
gac	D	1	0.46
---	--	------	------
mPD		0.49	0.69
		nPD:	0.71
		N. weight:	0.8
		Sc. PD:	0.4
		Sc. rank:	1414.4

,

PA			
Pos.	136	obs:	exp:
tct	S	0	1.67
tcc	S	0	1.45
tca	S	0	2.27
tcg	S	0	0.60
tgt	C	0	0.39
tgc	C	1	0.61
aat	N	325	1558.00
aac	N	2532	1299.00
agt	S	8	2.17
agc	S	2	1.84
---	--	------	------
mPD		0.21	0.51
		nPD:	0.42
		N. weight:	0.55
		Sc. PD:	0.12
		Sc. rank:	696.5

,

PA			
Pos.	137	obs:	exp:
cgt	R	0	0.04
cgc	R	0	0.05
cga	R	0	0.10
cgg	R	0	0.08
aaa	K	81	1633.00
aag	K	2786	1234.00
aga	R	0	0.47
agg	R	1	0.26
---	--	------	------
mPD		0.056	0.49
		nPD:	0.11
		N. weight:	0.94
		Sc. PD:	-0.082
		Sc. rank:	-14.6

,

PA			
Pos.	138	obs:	exp:
att	I	3	1052.00
atc	I	37	767.20
ata	I	2827	1048.00
aaa	K	1	0.57
aag	K	0	0.43
---	--	------	------
mPD		0.028	0.66
		nPD:	0.04
		N. weight:	1.2
		Sc. PD:	-0.19
		Sc. rank:	-1207.4

,

PA			
Pos.	139	obs:	exp:
cgt	R	0	0.04
cgc	R	0	0.05
cga	R	0	0.10
cgg	R	0	0.08
aaa	K	2659	1633.00
aag	K	208	1234.00
aga	R	0	0.47
agg	R	1	0.26
---	--	------	------
mPD		0.14	0.49
		nPD:	0.28
		N. weight:	0.43
		Sc. PD:	0.032
		Sc. rank:	366.3

,

PA			
Pos.	140	obs:	exp:
tct	S	43	478.60
tcc	S	525	415.30
tca	S	2237	650.00
tcg	S	62	173.00
act	T	0	0.26
acc	T	0	0.23
aca	T	1	0.42
acg	T	0	0.09
agt	S	0	621.90
agc	S	0	528.40
---	--	------	------
mPD		0.36	1.7
		nPD:	0.22
		N. weight:	1.3
		Sc. PD:	0.019
		Sc. rank:	808.0

,

PA			
Pos.	141	obs:	exp:
aaa	K	0	1.14
aag	K	2	0.86
gaa	E	51	1601.00
gag	E	2805	1255.00
ggt	G	0	1.45
ggc	G	0	1.31
gga	G	0	4.38
ggg	G	10	2.86
---	--	------	------
mPD		0.043	0.50
		nPD:	0.09
		N. weight:	0.97
		Sc. PD:	-0.11
		Sc. rank:	-360.5

,

PA			
Pos.	142	obs:	exp:
caa	Q	0	2.50
cag	Q	5	2.50
cgt	R	0	36.23
cgc	R	0	51.80
cga	R	0	97.55
cgg	R	0	79.04
atg	M	1	1.00
aat	N	9	4.91
aac	N	0	4.09
aaa	K	758	1070.00
aag	K	1121	809.10
aga	R	50	451.90
agg	R	918	251.50
ggt	G	0	0.87
ggc	G	0	0.79
gga	G	0	2.63
ggg	G	6	1.72
---	--	------	------
mPD		0.87	1.1
		nPD:	0.76
		N. weight:	0.56
		Sc. PD:	0.31
		Sc. rank:	1019.0

,

PA			
Pos.	143	obs:	exp:
atg	M	1	1.00
act	T	27	751.10
acc	T	25	645.60
aca	T	2772	1199.00
acg	T	41	269.60
gct	A	0	0.50
gcc	A	0	0.39
gca	A	2	0.89
gcg	A	0	0.22
---	--	------	------
mPD		0.066	0.70
		nPD:	0.09
		N. weight:	0.97
		Sc. PD:	-0.1
		Sc. rank:	-252.5

,

PA			
Pos.	144	obs:	exp:
tta	L	0	0.09
ttg	L	0	0.19
ctt	L	0	0.20
ctc	L	1	0.16
cta	L	0	0.15
ctg	L	0	0.21
cat	H	775	1658.00
cac	H	2089	1206.00
caa	Q	1	0.50
cag	Q	0	0.50
cgt	R	1	0.04
cgc	R	0	0.05
cga	R	0	0.10
cgg	R	0	0.08
aat	N	1	0.55
aac	N	0	0.45
aga	R	0	0.47
agg	R	0	0.26
---	--	------	------
mPD		0.40	0.49
		nPD:	0.81
		N. weight:	0.26
		Sc. PD:	0.16
		Sc. rank:	483.3

,

PA			
Pos.	145	obs:	exp:
att	I	2649	1052.00
atc	I	218	767.50
ata	I	1	1048.00
---	--	------	------
mPD		0.14	0.66
		nPD:	0.21
		N. weight:	1.
		Sc. PD:	0.012
		Sc. rank:	620.3

,

PA			
Pos.	146	obs:	exp:
cat	H	191	1660.00
cac	H	2676	1207.00
cgt	R	0	0.04
cgc	R	1	0.05
cga	R	0	0.10
cgg	R	0	0.08
aga	R	0	0.47
agg	R	0	0.26
---	--	------	------
mPD		0.13	0.49
		nPD:	0.26
		N. weight:	0.8
		Sc. PD:	0.043
		Sc. rank:	626.0

,

PA			
Pos.	147	obs:	exp:
tta	L	0	0.37
ttg	L	0	0.76
ctt	L	0	0.79
ctc	L	0	0.64
cta	L	4	0.59
ctg	L	0	0.84
att	I	26	1051.00
atc	I	48	766.40
ata	I	2790	1047.00
---	--	------	------
mPD		0.053	0.66
		nPD:	0.08
		N. weight:	1.2
		Sc. PD:	-0.14
		Sc. rank:	-552.2

,

PA			
Pos.	148	obs:	exp:
ttt	F	74	1196.00
ttc	F	2793	1671.00
tat	Y	0	0.48
tac	Y	1	0.52
---	--	------	------
mPD		0.051	0.49
		nPD:	0.1
		N. weight:	0.57
		Sc. PD:	-0.054
		Sc. rank:	-71.5

,

PA			
Pos.	149	obs:	exp:
tta	L	1	0.09
ttg	L	0	0.19
tct	S	6	478.10
tcc	S	12	414.90
tca	S	2815	649.30
tcg	S	31	172.80
ctt	L	0	0.20
ctc	L	0	0.16
cta	L	0	0.15
ctg	L	0	0.21
cct	P	0	0.27
ccc	P	0	0.18
cca	P	1	0.41
ccg	P	0	0.14
act	T	0	0.52
acc	T	0	0.45
aca	T	2	0.84
acg	T	0	0.19
agt	S	0	621.20
agc	S	0	527.80
---	--	------	------
mPD		0.037	1.7
		nPD:	0.02
		N. weight:	1.9
		Sc. PD:	-0.33
		Sc. rank:	-2529.2

,

PA			
Pos.	150	obs:	exp:
ttt	F	32	1196.00
ttc	F	2835	1671.00
tat	Y	0	0.48
tac	Y	1	0.52
---	--	------	------
mPD		0.023	0.49
		nPD:	0.05
		N. weight:	0.64
		Sc. PD:	-0.098
		Sc. rank:	-606.2

,

PA			
Pos.	151	obs:	exp:
att	I	7	3.67
atc	I	0	2.68
ata	I	3	3.65
act	T	2341	746.90
acc	T	17	642.00
aca	T	484	1192.00
acg	T	7	268.10
aat	N	6	3.27
aac	N	0	2.73
gct	A	3	0.76
gcc	A	0	0.58
gca	A	0	1.34
gcg	A	0	0.33
---	--	------	------
mPD		0.31	0.71
		nPD:	0.44
		N. weight:	1.
		Sc. PD:	0.23
		Sc. rank:	1295.1

,

PA			
Pos.	152	obs:	exp:
ggt	G	0	414.80
ggc	G	0	376.70
gga	G	2230	1257.00
ggg	G	638	819.80
---	--	------	------
mPD		0.35	0.69
		nPD:	0.5
		N. weight:	0.52
		Sc. PD:	0.15
		Sc. rank:	741.7

,

PA			
Pos.	153	obs:	exp:
gaa	E	165	1608.00
gag	E	2703	1260.00
---	--	------	------
mPD		0.11	0.49
		nPD:	0.22
		N. weight:	0.78
		Sc. PD:	0.015
		Sc. rank:	503.2

,

PA			
Pos.	154	obs:	exp:
aaa	K	1	0.57
aag	K	0	0.43
gaa	E	2776	1607.00
gag	E	91	1260.00
---	--	------	------
mPD		0.062	0.49
		nPD:	0.13
		N. weight:	0.59
		Sc. PD:	-0.044
		Sc. rank:	31.3

,

PA			
Pos.	155	obs:	exp:
tta	L	0	0.09
ttg	L	1	0.19
ctt	L	0	0.20
ctc	L	0	0.16
cta	L	0	0.15
ctg	L	0	0.21
att	I	1	0.73
atc	I	0	0.54
ata	I	1	0.73
atg	M	2865	2865.00
---	--	------	------
mPD		0.0021	0.0025
		nPD:	0.83
		N. weight:	0.0014
		Sc. PD:	0.00086
		Sc. rank:	2.5

,

PA

Pos.	156	obs:	exp:
act	T	0	0.26
acc	T	0	0.23
aca	T	1	0.42
acg	T	0	0.09
gtt	V	0	0.65
gtc	V	3	0.63
gta	V	0	0.64
gtg	V	0	1.08
gct	A	117	721.50
gcc	A	2427	554.20
gca	A	320	1275.00
gcg	A	0	313.50
---	--	------	------
mPD		0.27	0.69
		nPD:	0.39
		N. weight:	1.4
		Sc. PD:	0.26
		Sc. rank:	1640.6

PA

Pos.	157	obs:	exp:
tct	S	0	0.17
tcc	S	0	0.14
tca	S	0	0.23
tcg	S	0	0.06
act	T	721	751.30
acc	T	2091	645.80
aca	T	50	1199.00
acg	T	4	269.70
aat	N	0	0.55
aac	N	1	0.45
agt	S	0	0.22
agc	S	1	0.18
---	--	------	------
mPD		0.41	0.70
		nPD:	0.58
		N. weight:	1.
		Sc. PD:	0.39
		Sc. rank:	1640.3

PA

Pos.	158	obs:	exp:
cgt	R	0	1.68
cgc	R	0	2.41
cga	R	0	4.53
cgg	R	0	3.67
aaa	K	1977	1607.00
aag	K	846	1216.00
aga	R	45	21.01
agg	R	0	11.69
---	--	------	------
mPD		0.45	0.53
		nPD:	0.84
		N. weight:	0.065
		Sc. PD:	0.041
		Sc. rank:	120.0

PA

Pos.	159	obs:	exp:
tct	S	1	0.33
tcc	S	0	0.29
tca	S	0	0.45
tcg	S	1	0.12
act	T	0	0.52
acc	T	0	0.45
aca	T	0	0.84
acg	T	2	0.19
agt	S	0	0.43
agc	S	0	0.37
gct	A	881	721.50
gcc	A	8	554.20
gca	A	133	1275.00
gcg	A	1842	313.50
---	--	------	------
mPD		0.49	0.69
		nPD:	0.71
		N. weight:	1.4
		Sc. PD:	0.73
		Sc. rank:	2562.8

PA

Pos.	160	obs:	exp:
aat	N	0	1.64
aac	N	3	1.36
gat	D	107	1441.00
gac	D	2561	1227.00
gaa	E	197	110.40
gag	E	0	86.57
---	--	------	------
mPD		0.20	0.56
		nPD:	0.35
		N. weight:	0.8
		Sc. PD:	0.12
		Sc. rank:	859.9

PA

Pos.	161	obs:	exp:
ttt	F	2	0.83
ttc	F	0	1.17
tat	Y	1191	1374.00
tac	Y	1673	1490.00
tgt	C	0	0.39
tgc	C	1	0.61
cat	H	1	0.58
cac	H	0	0.42
---	--	------	------
mPD		0.49	0.50
		nPD:	0.97
		N. weight:	0.014
		Sc. PD:	0.011
		Sc. rank:	26.3

PA

Pos.	162	obs:	exp:
att	I	2	2.94
atc	I	6	2.14
ata	I	0	2.92
act	T	1081	747.90
acc	T	1766	642.90
aca	T	1	1194.00
acg	T	5	268.40
aat	N	0	1.09
aac	N	2	0.91
gct	A	0	1.26
gcc	A	5	0.97
gca	A	0	2.23
gcg	A	0	0.55
---	--	------	------
mPD		0.48	0.71
		nPD:	0.68
		N. weight:	1.
		Sc. PD:	0.48
		Sc. rank:	1730.0

PA

Pos.	163	obs:	exp:
tta	L	0	263.90
ttg	L	0	542.20
ctt	L	2788	566.70
ctc	L	59	456.50
cta	L	5	422.40
ctg	L	2	602.40
att	I	13	5.14
atc	I	1	3.75
ata	I	0	5.12
---	--	------	------
mPD		0.055	1.1
		nPD:	0.05
		N. weight:	2.
		Sc. PD:	-0.3
		Sc. rank:	-1781.4

PA

Pos.	164	obs:	exp:
aat	N	1	0.55
aac	N	0	0.45
gat	D	2812	1547.00
gac	D	54	1319.00
ggt	G	1	0.14
ggc	G	0	0.13
gga	G	0	0.44
ggg	G	0	0.29
---	--	------	------
mPD		0.038	0.50
		nPD:	0.08
		N. weight:	0.7
		Sc. PD:	-0.086
		Sc. rank:	-380.2

PA

Pos.	165	obs:	exp:
gat	D	12	8.64
gac	D	4	7.36
gaa	E	1706	1599.00
gag	E	1146	1253.00
---	--	------	------
mPD		0.49	0.50
		nPD:	0.98
		N. weight:	0.0071
		Sc. PD:	0.0055
		Sc. rank:	13.3

PA

Pos.	166	obs:	exp:
gaa	E	167	1607.00
gag	E	2700	1260.00
ggt	G	0	0.14
ggc	G	0	0.13
gga	G	0	0.44
ggg	G	1	0.29
---	--	------	------
mPD		0.11	0.49
		nPD:	0.22
		N. weight:	0.78
		Sc. PD:	0.017
		Sc. rank:	510.8

PA

Pos.	167	obs:	exp:
tct	S	0	478.70
tcc	S	0	415.40
tca	S	0	650.20
tcg	S	0	173.00
agt	S	263	622.10
agc	S	2605	528.50
---	--	------	------
mPD		0.17	1.7
		nPD:	0.1
		N. weight:	1.8
		Sc. PD:	-0.18
		Sc. rank:	-314.1

PA

Pos.	168	obs:	exp:
cgt	R	0	107.30
cgc	R	0	153.50
cga	R	1	289.00
cgg	R	1	234.20
aga	R	2335	1339.00
agg	R	531	745.10
---	--	------	------
mPD		0.30	0.95
		nPD:	0.32
		N. weight:	0.52
		Sc. PD:	0.059
		Sc. rank:	510.8

PA

Pos.	169	obs:	exp:
gct	A	5	722.20
gcc	A	7	554.80
gca	A	2744	1276.00
gcg	A	111	313.80
gaa	E	1	0.56
gag	E	0	0.44
---	--	------	------
mPD		0.083	0.69
		nPD:	0.12
		N. weight:	0.9
		Sc. PD:	-0.071
		Sc. rank:	19.4

PA

Pos.	170	obs:	exp:
cgt	R	0	107.30
cgc	R	0	153.40
cga	R	10	288.90
cgg	R	0	234.10
aga	R	2742	1338.00
agg	R	115	744.80
ggt	G	0	0.14
ggc	G	0	0.13
gga	G	1	0.44
ggg	G	0	0.29
---	--	------	------
mPD		0.085	0.95
		nPD:	0.09
		N. weight:	0.8
		Sc. PD:	-0.089
		Sc. rank:	-254.0

PA

Pos.	171	obs:	exp:
att	I	1110	1052.00
atc	I	808	767.50
ata	I	950	1048.00
---	--	------	------
mPD		0.66	0.66
		nPD:	1.
		N. weight:	0.0072
		Sc. PD:	0.0057
		Sc. rank:	13.4

PA

Pos.	172	obs:	exp:
cct	P	0	0.27
ccc	P	0	0.18
cca	P	1	0.41
ccg	P	0	0.14
cgt	R	0	0.04
cgc	R	0	0.05
cga	R	0	0.10
cgg	R	0	0.08
act	T	0	0.26
acc	T	0	0.23
aca	T	1	0.42
acg	T	0	0.09
aaa	K	2741	1631.00
aag	K	124	1234.00
aga	R	1	0.47
agg	R	0	0.26
---	--	------	------
mPD		0.086	0.49
		nPD:	0.17
		N. weight:	0.53
		Sc. PD:	-0.015
		Sc. rank:	195.4

PA

Pos.	173	obs:	exp:
act	T	16	751.80
acc	T	2772	646.20
aca	T	78	1200.00
acg	T	2	269.80
---	--	------	------
mPD		0.065	0.70
		nPD:	0.09
		N. weight:	1.7
		Sc. PD:	-0.19
		Sc. rank:	-498.0

PA

Pos.	174	obs:	exp:
cgt	R	0	107.20
cgc	R	0	153.30
cga	R	27	288.70
cgg	R	1	233.90
act	T	0	0.52
acc	T	0	0.45
aca	T	0	0.84
acg	T	2	0.19
aaa	K	0	0.57
aag	K	1	0.43
aga	R	757	1337.00
agg	R	2080	744.30
---	--	------	------
mPD		0.42	0.96
		nPD:	0.44
		N. weight:	0.77
		Sc. PD:	0.18
		Sc. rank:	989.5

PA

Pos.	175	obs:	exp:
tta	L	1	264.90
ttg	L	34	544.30
ctt	L	8	568.80
ctc	L	0	458.30
cta	L	120	424.00
ctg	L	2702	604.70
cgt	R	1	0.04
cgc	R	0	0.05
cga	R	0	0.10
cgg	R	0	0.08
atg	M	2	2.00
aga	R	0	0.47
agg	R	0	0.26
---	--	------	------
mPD		0.11	1.1
		nPD:	0.1
		N. weight:	1.7
		Sc. PD:	-0.17
		Sc. rank:	-326.0

PA

Pos.	176	obs:	exp:
ttt	F	29	1194.00
ttc	F	2832	1667.00
tct	S	0	0.17
tcc	S	1	0.14
tca	S	0	0.23
tcg	S	0	0.06
tat	Y	0	2.40
tac	Y	5	2.60
att	I	0	0.37
atc	I	1	0.27
ata	I	0	0.37
agt	S	0	0.22
agc	S	0	0.18
---	--	------	------
mPD		0.025	0.49
		nPD:	0.05
		N. weight:	0.65
		Sc. PD:	-0.096
		Sc. rank:	-575.2

,

PA

Pos.	177	obs:	exp:
cgt	R	0	0.04
cgc	R	0	0.05
cga	R	0	0.10
cgg	R	0	0.08
act	T	2310	751.60
acc	T	486	646.00
aca	T	70	1200.00
acg	T	1	269.70
aga	R	1	0.47
agg	R	0	0.26
---	--	------	------
mPD		0.32	0.70
		nPD:	0.46
		N. weight:	1.
		Sc. PD:	0.27
		Sc. rank:	1445.4

,

PA

Pos.	178	obs:	exp:
att	I	2	1049.00
atc	I	4	764.80
ata	I	2852	1044.00
gtt	V	0	2.15
gtc	V	0	2.10
gta	V	10	2.13
gtg	V	0	3.61
---	--	------	------
mPD		0.011	0.67
		nPD:	0.02
		N. weight:	1.3
		Sc. PD:	-0.24
		Sc. rank:	-1945.7

,

PA

Pos.	179	obs:	exp:
cgt	R	0	107.30
cgc	R	0	153.50
cga	R	0	289.00
cgg	R	0	234.20
aga	R	2160	1339.00
agg	R	708	745.10
---	--	------	------
mPD		0.37	0.95
		nPD:	0.39
		N. weight:	0.46
		Sc. PD:	0.086
		Sc. rank:	554.9

,

PA

Pos.	180	obs:	exp:
cct	P	0	0.27
ccc	P	0	0.18
cca	P	1	0.41
ccg	P	0	0.14
cat	H	2	3.47
cac	H	4	2.53
caa	Q	2319	1428.00
cag	Q	542	1433.00
---	--	------	------
mPD		0.31	0.50
		nPD:	0.62
		N. weight:	0.28
		Sc. PD:	0.11
		Sc. rank:	451.6

,

PA

Pos.	181	obs:	exp:
gaa	E	2027	1608.00
gag	E	841	1260.00
---	--	------	------
mPD		0.41	0.49
		nPD:	0.84
		N. weight:	0.062
		Sc. PD:	0.039
		Sc. rank:	113.8

,

PA

Pos.	182	obs:	exp:
atg	M	2868	2868.00
---	--	------	------
mPD		0	0
		nPD:	1.
		N. weight:	0.
		Sc. PD:	0
		Sc. rank:	0

,

PA

Pos.	183	obs:	exp:
tct	S	0	5.34
tcc	S	32	4.64
tca	S	0	7.25
tcg	S	0	1.93
agt	S	0	6.94
agc	S	0	5.90
gct	A	16	714.20
gcc	A	2690	548.60
gca	A	129	1262.00
gcg	A	0	310.30
ggt	G	0	0.14
ggc	G	1	0.13
gga	G	0	0.44
ggg	G	0	0.29
---	--	------	------
mPD		0.12	0.72
		nPD:	0.17
		N. weight:	1.9
		Sc. PD:	-0.066
		Sc. rank:	624.4

,

PA

Pos.	184	obs:	exp:
tct	S	0	472.20
tcc	S	0	409.80
tca	S	0	641.30
tcg	S	0	170.70
tgt	C	2	0.77
tgc	C	0	1.23
att	I	4	1.47
atc	I	0	1.07
ata	I	0	1.46
act	T	1	0.26
acc	T	0	0.23
aca	T	0	0.42
acg	T	0	0.09
aat	N	18	10.36
aac	N	1	8.64
agt	S	2596	613.60
agc	S	233	521.40
ggt	G	10	1.88
ggc	G	3	1.71
gga	G	0	5.70
ggg	G	0	3.72
---	--	------	------
mPD		0.18	1.7
		nPD:	0.11
		N. weight:	1.7
		Sc. PD:	-0.15
		Sc. rank:	-182.5

,

PA

Pos.	185	obs:	exp:
cgt	R	0	105.00
cgc	R	0	150.20
cga	R	0	282.80
cgg	R	0	229.10
aaa	K	4	34.73
aag	K	57	26.27
aga	R	684	1310.00
agg	R	2122	729.00
ggt	G	0	0.14
ggc	G	0	0.13
gga	G	0	0.44
ggg	G	1	0.29
---	--	------	------
mPD		0.41	0.99
		nPD:	0.41
		N. weight:	0.86
		Sc. PD:	0.18
		Sc. rank:	1081.8

,

PA

Pos.	186	obs:	exp:
tct	S	0	0.17
tcc	S	0	0.14
tca	S	0	0.23
tcg	S	0	0.06
agt	S	1	0.22
agc	S	0	0.18
gct	A	0	0.25
gcc	A	1	0.19
gca	A	0	0.45
gcg	A	0	0.11
ggt	G	2552	414.50
ggc	G	268	376.50
gga	G	18	1256.00
ggg	G	28	819.20
---	--	------	------
mPD		0.20	0.69
		nPD:	0.29
		N. weight:	2.
		Sc. PD:	0.18
		Sc. rank:	1853.5

,

PA

Pos.	187	obs:	exp:
tta	L	112	265.00
ttg	L	2	544.50
ctt	L	6	569.00
ctc	L	22	458.40
cta	L	2530	424.10
ctg	L	194	604.90
att	I	0	0.73
atc	I	0	0.54
ata	I	2	0.73
---	--	------	------
mPD		0.22	1.1
		nPD:	0.2
		N. weight:	1.9
		Sc. PD:	-0.0048
		Sc. rank:	1031.7

,

PA

Pos.	188	obs:	exp:
tgg	W	2868	2868.00
---	--	------	------
mPD		0	0
		nPD:	1.
		N. weight:	0.
		Sc. PD:	0
		Sc. rank:	0

,

PA

Pos.	189	obs:	exp:
gat	D	2838	1548.00
gac	D	29	1319.00
ggt	G	1	0.14
ggc	G	0	0.13
gga	G	0	0.44
ggg	G	0	0.29
---	--	------	------
mPD		0.021	0.50
		nPD:	0.04
		N. weight:	0.75
		Sc. PD:	-0.12
		Sc. rank:	-736.8

,

PA

Pos.	190	obs:	exp:
tct	S	0	476.90
tcc	S	2845	413.90
tca	S	12	647.70
tcg	S	0	172.40
cct	P	0	2.73
ccc	P	10	1.81
cca	P	0	4.06
ccg	P	0	1.40
act	T	0	0.26
acc	T	1	0.23
aca	T	0	0.42
acg	T	0	0.09
agt	S	0	619.70
agc	S	0	526.50
---	--	------	------
mPD		0.016	1.7
		nPD:	0.01
		N. weight:	2.5
		Sc. PD:	-0.48
		Sc. rank:	-4126.1

,

PA

Pos.	191	obs:	exp:
ttt	F	2868	1196.00
ttc	F	0	1672.00
---	--	------	------
mPD		0	0.49
		nPD:	0.
		N. weight:	1.2
		Sc. PD:	-0.23
		Sc. rank:	-2168.7

,

PA

Pos.	192	obs:	exp:
tgt	C	13	5.03
tgc	C	0	7.97
cct	P	1	0.27
ccc	P	0	0.18
cca	P	0	0.41
ccg	P	0	0.14
cat	H	1	0.58
cac	H	0	0.42
cgt	R	2846	106.80
cgc	R	7	152.70
cga	R	0	287.50
cgg	R	0	233.00
aga	R	0	1332.00
agg	R	0	741.20
---	--	------	------
mPD		0.015	0.97
		nPD:	0.02
		N. weight:	4.3
		Sc. PD:	-0.79
		Sc. rank:	-6405.1

,

PA

Pos.	193	obs:	exp:
caa	Q	1627	1432.00
cag	Q	1241	1436.00
---	--	------	------
mPD		0.49	0.50
		nPD:	0.98
		N. weight:	0.014
		Sc. PD:	0.011
		Sc. rank:	26.6

,

PA

Pos.	194	obs:	exp:
ttt	F	0	0.42
ttc	F	1	0.58
tct	S	556	478.40
tcc	S	2307	415.20
tca	S	2	649.70
tcg	S	1	172.90
tat	Y	0	0.48
tac	Y	1	0.52
agt	S	0	621.60
agc	S	0	528.20
---	--	------	------
mPD		0.32	1.7
		nPD:	0.19
		N. weight:	1.9
		Sc. PD:	-0.018
		Sc. rank:	929.2

,

PA

Pos.	195	obs:	exp:
gaa	E	280	1608.00
gag	E	2588	1260.00
---	--	------	------
mPD		0.18	0.49
		nPD:	0.36
		N. weight:	0.64
		Sc. PD:	0.098
		Sc. rank:	704.0

,

PA

Pos. 196		obs:	exp:
tct	S	0	0.17
tcc	S	0	0.14
tca	S	0	0.23
tcg	S	0	0.06
cgt	R	0	107.10
cgc	R	0	153.20
cga	R	7	288.50
cgg	R	0	233.80
aaa	K	4	2.28
aag	K	0	1.72
agt	S	1	0.22
agc	S	0	0.18
aga	R	2856	1337.00
agg	R	0	743.80
---	--	------	------
mPD		0.0084	0.96
		nPD:	0.01
		N. weight:	1.
		Sc. PD:	-0.19
		Sc. rank:	-1650.3

,

PA

Pos. 197		obs:	exp:
tct	S	0	0.33
tcc	S	0	0.29
tca	S	0	0.45
tcg	S	0	0.12
tgt	C	0	0.39
tgc	C	1	0.61
agt	S	0	0.43
agc	S	2	0.37
gat	D	0	0.54
gac	D	1	0.46
ggt	G	14	414.20
ggc	G	2848	376.20
gga	G	1	1255.00
ggg	G	1	818.70
---	--	------	------
mPD		0.014	0.69
		nPD:	0.02
		N. weight:	2.6
		Sc. PD:	-0.47
		Sc. rank:	-3731.5

,

PA

Pos. 198		obs:	exp:
caa	Q	1	0.50
cag	Q	0	0.50
aaa	K	2	1.14
aag	K	0	0.86
gaa	E	2858	1606.00
gag	E	7	1259.00
---	--	------	------
mPD		0.0070	0.49
		nPD:	0.01
		N. weight:	0.75
		Sc. PD:	-0.14
		Sc. rank:	-1163.5

,

PA

Pos. 199		obs:	exp:
aaa	K	0	0.57
aag	K	1	0.43
gat	D	1	0.54
gac	D	0	0.46
gaa	E	211	1605.00
gag	E	2652	1258.00
ggt	G	0	0.43
ggc	G	0	0.39
gga	G	0	1.31
ggg	G	3	0.86
---	--	------	------
mPD		0.14	0.50
		nPD:	0.28
		N. weight:	0.72
		Sc. PD:	0.057
		Sc. rank:	629.3

,

PA

Pos. 200		obs:	exp:
act	T	2	751.60
acc	T	1	646.00
aca	T	2864	1200.00
acg	T	0	269.70
aaa	K	1	0.57
aag	K	0	0.43
---	--	------	------
mPD		0.0028	0.70
		nPD:	0.
		N. weight:	1.1
		Sc. PD:	-0.22
		Sc. rank:	-2019.7

,

PA

Pos. 201		obs:	exp:
tta	L	0	0.18
ttg	L	0	0.38
ctt	L	2	0.40
ctc	L	0	0.32
cta	L	0	0.30
ctg	L	0	0.42
att	I	2649	975.80
atc	I	8	711.50
ata	I	2	971.70
act	T	195	51.12
acc	T	0	43.94
aca	T	0	81.59
acg	T	0	18.35
aat	N	2	1.09
aac	N	0	0.91
gtt	V	10	2.15
gtc	V	0	2.10
gta	V	0	2.13
gtg	V	0	3.61
---	--	------	------
mPD		0.14	0.80
		nPD:	0.18
		N. weight:	1.3
		Sc. PD:	-0.03
		Sc. rank:	528.2

,

PA

Pos. 202		obs:	exp:
gat	D	2	1.08
gac	D	0	0.92
gaa	E	2858	1607.00
gag	E	8	1259.00
---	--	------	------
mPD		0.0070	0.49
		nPD:	0.01
		N. weight:	0.75
		Sc. PD:	-0.14
		Sc. rank:	-1156.6

,

PA

Pos. 203		obs:	exp:
gaa	E	2731	1608.00
gag	E	137	1260.00
---	--	------	------
mPD		0.091	0.49
		nPD:	0.18
		N. weight:	0.53
		Sc. PD:	-0.0087
		Sc. rank:	237.5

,

PA

Pos. 204		obs:	exp:
tct	S	0	0.17
tcc	S	0	0.14
tca	S	0	0.23
tcg	S	0	0.06
cgt	R	0	87.72
cgc	R	0	125.40
cga	R	10	236.20
cgg	R	0	191.40
aaa	K	517	297.80
aag	K	6	225.20
agt	S	1	0.22
agc	S	0	0.18
aga	R	1415	1094.00
agg	R	919	609.00
---	--	------	------
mPD		0.74	1.2
		nPD:	0.62
		N. weight:	0.46
		Sc. PD:	0.19
		Sc. rank:	740.4

,

PA

Pos. 205		obs:	exp:
ttt	F	2866	1196.00
ttc	F	1	1671.00
tta	L	0	0.09
ttg	L	1	0.19
ctt	L	0	0.20
ctc	L	0	0.16
cta	L	0	0.15
ctg	L	0	0.21
---	--	------	------
mPD		0.0014	0.49
		nPD:	0.
		N. weight:	1.2
		Sc. PD:	-0.23
		Sc. rank:	-2085.1

,

PA

Pos. 206		obs:	exp:
aaa	K	3	1.71
aag	K	0	1.29
gct	A	0	0.50
gcc	A	0	0.39
gca	A	2	0.89
gcg	A	0	0.22
gat	D	0	2.16
gac	D	4	1.84
gaa	E	2847	1602.00
gag	E	10	1255.00
ggt	G	0	0.29
ggc	G	0	0.26
gga	G	2	0.88
ggg	G	0	0.57
---	--	------	------
mPD		0.015	0.50
		nPD:	0.03
		N. weight:	0.74
		Sc. PD:	-0.13
		Sc. rank:	-930.6

,

PA

Pos. 207		obs:	exp:
tta	L	0	0.46
ttg	L	0	0.95
ctt	L	0	0.99
ctc	L	5	0.80
cta	L	0	0.74
ctg	L	0	1.06
att	I	245	1050.00
atc	I	2616	765.90
ata	I	1	1046.00
gtt	V	0	0.22
gtc	V	1	0.21
gta	V	0	0.21
gtg	V	0	0.36
---	--	------	------
mPD		0.16	0.66
		nPD:	0.24
		N. weight:	1.3
		Sc. PD:	0.054
		Sc. rank:	979.9

,

PA

Pos. 208		obs:	exp:
cgt	R	0	0.11
cgc	R	0	0.16
cga	R	0	0.30
cgg	R	0	0.24
att	I	9	4.04
atc	I	0	2.94
ata	I	2	4.02
act	T	487	741.40
acc	T	10	637.20
aca	T	2323	1183.00
acg	T	8	266.10
aaa	K	18	10.25
aag	K	0	7.75
aga	R	3	1.40
agg	R	0	0.78
gct	A	1	2.02
gcc	A	0	1.55
gca	A	7	3.56
gcg	A	0	0.88
---	--	------	------
mPD		0.32	0.73
		nPD:	0.45
		N. weight:	0.61
		Sc. PD:	0.15
		Sc. rank:	811.3

,

PA

Pos. 209		obs:	exp:
ggt	G	1	414.80
ggc	G	0	376.70
gga	G	2758	1257.00
ggg	G	109	819.80
---	--	------	------
mPD		0.074	0.69
		nPD:	0.11
		N. weight:	0.9
		Sc. PD:	-0.084
		Sc. rank:	-94.5

,

PA

Pos. 210		obs:	exp:
att	I	0	1.10
atc	I	3	0.80
ata	I	0	1.10
act	T	31	749.80
acc	T	2786	644.40
aca	T	43	1197.00
acg	T	0	269.10
aat	N	0	1.09
aac	N	2	0.91
gct	A	0	0.76
gcc	A	3	0.58
gca	A	0	1.34
gcg	A	0	0.33
---	--	------	------
mPD		0.056	0.70
		nPD:	0.08
		N. weight:	1.8
		Sc. PD:	-0.21
		Sc. rank:	-855.4

,

PA

Pos.	211	obs:	exp:
tta	L	0	0.28
ttg	L	1	0.57
ctt	L	0	0.60
ctc	L	0	0.48
cta	L	0	0.44
ctg	L	2	0.63
att	I	1	2.20
atc	I	0	1.61
ata	I	5	2.19
atg	M	2857	2857.00
act	T	0	0.26
acc	T	0	0.23
aca	T	0	0.42
acg	T	1	0.09
gtt	V	0	0.22
gtc	V	0	0.21
gta	V	0	0.21
gtg	V	1	0.36
---	--	------	------
mPD		0.0077	0.010
		nPD :	0.77
		N. weight :	0.0052
		Sc. PD :	0.0029
		Sc. rank :	9.6

,

PA

Pos.	212	obs:	exp:
tgt	C	0	0.77
tgc	C	2	1.23
cat	H	0	2.32
cac	H	4	1.68
cgt	R	44	107.10
cgc	R	2816	153.20
cga	R	2	288.40
cgg	R	0	233.70
aga	R	0	1336.00
agg	R	0	743.50
---	--	------	------
mPD		0.036	0.96
		nPD :	0.04
		N. weight :	3.8
		Sc. PD :	-0.61
		Sc. rank :	-3995.8

,

PA

Pos.	213	obs:	exp:
tct	S	0	0.33
tcc	S	0	0.29
tca	S	0	0.45
tcg	S	0	0.12
tgg	W	1	1.00
cgt	R	0	106.90
cgc	R	0	152.90
cga	R	1	287.90
cgg	R	19	233.30
act	T	0	0.26
acc	T	0	0.23
aca	T	0	0.42
acg	T	1	0.09
aaa	K	0	3.99
aag	K	7	3.01
agt	S	0	0.43
agc	S	2	0.37
aga	R	483	1334.00
agg	R	2354	742.20
---	--	------	------
mPD		0.30	0.96
		nPD :	0.31
		N. weight :	1.
		Sc. PD :	0.11
		Sc. rank :	987.4

,

PA

Pos.	214	obs:	exp:
ttt	F	2	0.83
ttc	F	0	1.17
tta	L	0	265.00
ttg	L	0	544.50
ctt	L	2832	569.00
ctc	L	26	458.40
cta	L	1	424.10
ctg	L	7	604.90
---	--	------	------
mPD		0.025	1.1
		nPD :	0.02
		N. weight :	2.1
		Sc. PD :	-0.36
		Sc. rank :	-2772.3

,

PA

Pos.	215	obs:	exp:
act	T	0	0.52
acc	T	2	0.45
aca	T	0	0.84
acg	T	0	0.19
gtt	V	0	0.22
gtc	V	1	0.21
gta	V	0	0.21
gtg	V	0	0.36
gct	A	1026	721.70
gcc	A	1661	554.40
gca	A	177	1275.00
gcg	A	1	313.60
---	--	------	------
mPD		0.53	0.69
		nPD :	0.77
		N. weight :	0.85
		Sc. PD :	0.48
		Sc. rank :	1562.0

,

PA

Pos.	216	obs:	exp:
aat	N	2	26.18
aac	N	46	21.82
gat	D	153	1511.00
gac	D	2646	1288.00
gaa	E	13	7.85
gag	E	1	6.15
ggt	G	0	1.01
ggc	G	7	0.92
gga	G	0	3.07
ggg	G	0	2.00
---	--	------	------
mPD		0.15	0.54
		nPD :	0.28
		N. weight :	0.75
		Sc. PD :	0.055
		Sc. rank :	630.9

,

PA

Pos.	217	obs:	exp:
caa	Q	2848	1431.00
cag	Q	18	1435.00
aaa	K	1	0.57
aag	K	0	0.43
ggt	G	0	0.14
ggc	G	0	0.13
gga	G	1	0.44
ggg	G	0	0.29
---	--	------	------
mPD		0.015	0.50
		nPD :	0.03
		N. weight :	0.87
		Sc. PD :	-0.15
		Sc. rank :	-1103.9

,

PA

Pos.	218	obs:	exp:
tct	S	0	478.40
tcc	S	0	415.20
tca	S	0	649.70
tcg	S	0	172.90
cgt	R	0	0.04
cgc	R	0	0.05
cga	R	0	0.10
cgg	R	0	0.08
agt	S	1858	621.60
agc	S	1008	528.20
aga	R	1	0.47
agg	R	0	0.26
ggt	G	1	0.14
ggc	G	0	0.13
gga	G	0	0.44
ggg	G	0	0.29
---	--	------	------
mPD		0.46	1.7
		nPD :	0.28
		N. weight :	1.2
		Sc. PD :	0.093
		Sc. rank :	1066.8

,

PA

Pos.	219	obs:	exp:
ttt	F	0	0.42
ttc	F	1	0.58
tta	L	0	264.80
ttg	L	0	544.10
ctt	L	262	568.60
ctc	L	2597	458.10
cta	L	5	423.90
ctg	L	0	604.50
att	I	0	1.10
atc	I	3	0.80
ata	I	0	1.10
---	--	------	------
mPD		0.17	1.1
		nPD :	0.15
		N. weight :	2.
		Sc. PD :	-0.094
		Sc. rank :	464.3

,

PA

Pos.	220	obs:	exp:
cct	P	87	783.80
ccc	P	219	518.00
cca	P	2538	1164.00
ccg	P	24	402.10
---	--	------	------
mPD		0.21	0.71
		nPD :	0.3
		N. weight :	0.71
		Sc. PD :	0.067
		Sc. rank :	665.5

,

PA

Pos.	221	obs:	exp:
tta	L	0	0.09
ttg	L	0	0.19
tct	S	0	0.50
tcc	S	0	0.43
tca	S	0	0.68
tcg	S	3	0.18
ctt	L	0	0.20
ctc	L	0	0.16
cta	L	0	0.15
ctg	L	1	0.21
cct	P	22	782.40
ccc	P	0	517.10
cca	P	6	1162.00
ccg	P	2835	401.40
act	T	0	0.26
acc	T	0	0.23
aca	T	0	0.42
acg	T	1	0.09
agt	S	0	0.65
agc	S	0	0.55
---	--	------	------
mPD		0.023	0.71
		nPD :	0.03
		N. weight :	2.5
		Sc. PD :	-0.42
		Sc. rank :	-2971.2

,

PA

Pos.	222	obs:	exp:
tct	S	0	7.51
tcc	S	0	6.52
tca	S	0	10.20
tcg	S	0	2.71
cat	H	0	2.32
cac	H	4	1.68
aat	N	46	1531.00
aac	N	2762	1277.00
aaa	K	10	5.69
aag	K	0	4.31
agt	S	0	9.76
agc	S	45	8.29
ggt	G	0	0.14
ggc	G	1	0.13
gga	G	0	0.44
ggg	G	0	0.29
---	--	------	------
mPD		0.073	0.56
		nPD :	0.13
		N. weight :	0.96
		Sc. PD :	-0.065
		Sc. rank :	98.4

,

PA

Pos.	223	obs:	exp:
ttt	F	0	1196.00
ttc	F	2866	1670.00
tat	Y	0	0.96
tac	Y	2	1.04
---	--	------	------
mPD		0.0014	0.49
		nPD :	0.
		N. weight :	0.72
		Sc. PD :	-0.14
		Sc. rank :	-1296.1

,

PA

Pos.	224	obs:	exp:
ttt	F	0	1.25
ttc	F	3	1.75
tct	S	0	455.90
tcc	S	2729	395.60
tca	S	2	619.10
tcg	S	0	164.80
tat	Y	0	0.48
tac	Y	1	0.52
tgt	C	0	0.39
tgc	C	1	0.61
cct	P	0	33.34
ccc	P	122	22.04
cca	P	0	49.52
ccg	P	0	17.10
act	T	0	1.05
acc	T	4	0.90
aca	T	0	1.67
acg	T	0	0.38
agt	S	0	592.40
agc	S	0	503.30
gct	A	0	1.51
gcc	A	6	1.16
gca	A	0	2.67
gcg	A	0	0.66
---	--	------	------
mPD		0.093	1.7
		nPD :	0.06
		N. weight :	2.5
		Sc. PD :	-0.37
		Sc. rank :	-1995.2

,

PA

Pos.	225	obs:	exp:
tct	S	0	474.60
tcc	S	0	411.80
tca	S	0	644.50
tcg	S	0	171.50
aat	N	0	7.63
aac	N	14	6.37
agt	S	9	616.70
agc	S	2834	523.90
ggt	G	0	1.59
ggc	G	11	1.44
gga	G	0	4.82
ggg	G	0	3.14
---	--	------	------
mPD		0.024	1.7
		nPD :	0.01
		N. weight :	2.2
		Sc. PD :	-0.41
		Sc. rank :	-3411.5

,

PA			
Pos.	226	obs:	exp:
ttt	F	15	6.26
ttc	F	0	8.74
tta	L	0	260.80
ttg	L	0	535.90
ctt	L	2817	560.10
ctc	L	4	451.20
cta	L	0	417.50
ctg	L	0	595.40
att	I	32	11.74
atc	I	0	8.56
ata	I	0	11.69
---	--	------	------
mPD		0.035	1.1
		nPD:	0.03
		N. weight:	2.1
		Sc. PD:	-0.36
		Sc. rank:	-2569.7

PA			
Pos.	227	obs:	exp:
cgt	R	0	0.07
cgc	R	0	0.11
cga	R	0	0.20
cgg	R	0	0.16
aaa	K	19	10.82
aag	K	0	8.18
aga	R	2	0.93
agg	R	0	0.52
gaa	E	2617	1499.00
gag	E	57	1175.00
ggt	G	0	25.02
ggc	G	0	22.72
gga	G	173	75.81
ggg	G	0	49.45
---	--	------	------
mPD		0.17	0.64
		nPD:	0.26
		N. weight:	0.67
		Sc. PD:	0.041
		Sc. rank:	543.9

PA			
Pos.	228	obs:	exp:
tct	S	0	4.17
tcc	S	0	3.62
tca	S	0	5.67
tcg	S	0	1.51
act	T	2	0.52
acc	T	0	0.45
aca	T	0	0.84
acg	T	0	0.19
aat	N	1123	1528.00
aac	N	1679	1274.00
aaa	K	30	17.65
aag	K	1	13.35
agt	S	0	5.42
agc	S	25	4.61
gat	D	0	3.24
gac	D	6	2.76
gaa	E	2	1.12
gag	E	0	0.88
---	--	------	------
mPD		0.51	0.54
		nPD:	0.95
		N. weight:	0.087
		Sc. PD:	0.063
		Sc. rank:	161.1

PA			
Pos.	229	obs:	exp:
ttt	F	2842	1195.00
ttc	F	23	1670.00
tta	L	0	0.28
ttg	L	0	0.57
ctt	L	3	0.60
ctc	L	0	0.48
cta	L	0	0.44
ctg	L	0	0.63
---	--	------	------
mPD		0.018	0.49
		nPD:	0.04
		N. weight:	1.1
		Sc. PD:	-0.18
		Sc. rank:	-1182.6

PA			
Pos.	230	obs:	exp:
cgt	R	0	107.30
cgc	R	0	153.40
cga	R	0	288.90
cgg	R	0	234.10
aaa	K	1	0.57
aag	K	0	0.43
aga	R	2863	1338.00
agg	R	4	744.80
---	--	------	------
mPD		0.0035	0.95
		nPD:	0.
		N. weight:	1.
		Sc. PD:	-0.19
		Sc. rank:	-1774.9

PA			
Pos.	231	obs:	exp:
att	I	0	1.83
atc	I	5	1.34
ata	I	0	1.83
act	T	0	22.02
acc	T	84	18.93
aca	T	0	35.15
acg	T	0	7.90
gtt	V	0	6.67
gtc	V	31	6.52
gta	V	0	6.61
gtg	V	0	11.20
gct	A	1	692.30
gcc	A	2747	531.80
gca	A	0	1223.00
gcg	A	0	300.80
---	--	------	------
mPD		0.086	0.78
		nPD:	0.11
		N. weight:	2.2
		Sc. PD:	-0.19
		Sc. rank:	-109.5

PA			
Pos.	232	obs:	exp:
tat	Y	2868	1376.00
tac	Y	0	1492.00
---	--	------	------
mPD		0	0.50
		nPD:	0.
		N. weight:	0.98
		Sc. PD:	-0.19
		Sc. rank:	-1822.8

PA			
Pos.	233	obs:	exp:
gtt	V	0	616.90
gtc	V	0	603.40
gta	V	2	611.50
gtg	V	2866	1036.00
---	--	------	------
mPD		0.0014	0.73
		nPD:	0.
		N. weight:	1.3
		Sc. PD:	-0.26
		Sc. rank:	-2444.4

PA			
Pos.	234	obs:	exp:
gat	D	2864	1548.00
gac	D	3	1319.00
gaa	E	1	0.56
gag	E	0	0.44
---	--	------	------
mPD		0.0028	0.50
		nPD:	0.01
		N. weight:	0.81
		Sc. PD:	-0.16
		Sc. rank:	-1382.5

PA			
Pos.	235	obs:	exp:
cgt	R	0	0.04
cgc	R	0	0.05
cga	R	0	0.10
cgg	R	0	0.08
aga	R	1	0.47
agg	R	0	0.26
ggt	G	0	414.60
ggc	G	0	376.60
gga	G	2849	1256.00
ggg	G	18	819.50
---	--	------	------
mPD		0.013	0.69
		nPD:	0.02
		N. weight:	1.1
		Sc. PD:	-0.19
		Sc. rank:	-1513.2

PA			
Pos.	236	obs:	exp:
ttt	F	49	1195.00
ttc	F	2816	1670.00
tta	L	1	0.09
ttg	L	0	0.19
tct	S	0	0.17
tcc	S	1	0.14
tca	S	0	0.23
tcg	S	0	0.06
tgt	C	0	0.39
tgc	C	1	0.61
ctt	L	0	0.20
ctc	L	0	0.16
cta	L	0	0.15
ctg	L	0	0.21
agt	S	0	0.22
agc	S	0	0.18
---	--	------	------
mPD		0.036	0.49
		nPD:	0.07
		N. weight:	0.61
		Sc. PD:	-0.078
		Sc. rank:	-351.2

PA			
Pos.	237	obs:	exp:
cgt	R	0	0.15
cgc	R	0	0.21
cga	R	0	0.40
cgg	R	0	0.33
aaa	K	419	239.20
aag	K	1	180.80
aga	R	4	1.87
agg	R	0	1.04
gtt	V	0	0.22
gtc	V	0	0.21
gta	V	0	0.21
gtg	V	1	0.36
gct	A	0	0.50
gcc	A	0	0.39
gca	A	2	0.89
gcg	A	0	0.22
gaa	E	2424	1368.00
gag	E	17	1073.00
---	--	------	------
mPD		0.27	0.75
		nPD:	0.36
		N. weight:	0.72
		Sc. PD:	0.11
		Sc. rank:	797.7

PA			
Pos.	238	obs:	exp:
tct	S	0	0.33
tcc	S	0	0.29
tca	S	0	0.45
tcg	S	2	0.12
cct	P	0	783.00
ccc	P	0	517.50
cca	P	2	1163.00
ccg	P	2863	401.70
cgt	R	0	0.04
cgc	R	0	0.05
cga	R	0	0.10
cgg	R	1	0.08
agt	S	0	0.43
agc	S	0	0.37
aga	R	0	0.47
agg	R	0	0.26
---	--	------	------
mPD		0.0035	0.71
		nPD:	0.
		N. weight:	2.6
		Sc. PD:	-0.5
		Sc. rank:	-4493.6

PA			
Pos.	239	obs:	exp:
aat	N	32	1564.00
aac	N	2836	1304.00
---	--	------	------
mPD		0.022	0.50
		nPD:	0.04
		N. weight:	0.97
		Sc. PD:	-0.15
		Sc. rank:	-932.6

PA			
Pos.	240	obs:	exp:
ggt	G	3	414.80
ggc	G	2834	376.70
gga	G	31	1257.00
ggg	G	0	819.80
---	--	------	------
mPD		0.023	0.69
		nPD:	0.03
		N. weight:	2.6
		Sc. PD:	-0.43
		Sc. rank:	-2964.0

PA			
Pos.	241	obs:	exp:
ttt	F	0	2.09
ttc	F	5	2.91
tct	S	0	0.17
tcc	S	0	0.14
tca	S	0	0.23
tcg	S	0	0.06
tat	Y	0	56.11
tac	Y	117	60.89
tgt	C	5	1062.00
tgc	C	2740	1683.00
agt	S	0	0.22
agc	S	1	0.18
---	--	------	------
mPD		0.086	0.56
		nPD:	0.15
		N. weight:	0.65
		Sc. PD:	-0.03
		Sc. rank:	152.8

PA			
Pos.	242	obs:	exp:
att	I	2865	1052.00
atc	I	2	767.50
ata	I	1	1048.00
---	--	------	------
mPD		0.0021	0.66
		nPD:	0.
		N. weight:	1.3
		Sc. PD:	-0.26
		Sc. rank:	-2352.8

PA			
Pos.	243	obs:	exp:
gaa	E	5	1608.00
gag	E	2863	1260.00
---	--	------	------
mPD		0.0035	0.49
		nPD:	0.01
		N. weight:	1.1
		Sc. PD:	-0.21
		Sc. rank:	-1814.9

PA			
Pos.	244	obs:	exp:
ggt	G	4	414.80
ggc	G	2855	376.70
gga	G	9	1257.00
ggg	G	0	819.80
---	--	------	------
mPD		0.0090	0.69
		nPD:	0.01
		N. weight:	2.7
		Sc. PD:	-0.49
		Sc. rank:	-4222.9

PA			
Pos.	245	obs:	exp:
cgt	R	0	0.07
cgc	R	0	0.11
cga	R	0	0.20
cgg	R	0	0.16
aaa	K	298	1632.00
aag	K	2568	1234.00
aga	R	1	0.93
agg	R	1	0.52
---	--	------	------
mPD		0.19	0.49
		nPD:	0.38
		N. weight:	0.64
		Sc. PD:	0.11
		Sc. rank:	761.9

PA			
Pos.	246	obs:	exp:
tta	L	0	265.10
ttg	L	0	544.70
ctt	L	2866	569.20
ctc	L	1	458.60
cta	L	0	424.30
ctg	L	0	605.10
cct	P	1	0.27
ccc	P	0	0.18
cca	P	0	0.41
ccg	P	0	0.14
---	--	------	------
mPD		0.0014	1.1
		nPD:	0.
		N. weight:	2.1
		Sc. PD:	-0.42
		Sc. rank:	-3909.1

,

PA			
Pos.	247	obs:	exp:
tct	S	2825	476.90
tcc	S	32	413.90
tca	S	0	647.70
tcg	S	0	172.40
agt	S	0	619.70
agc	S	0	526.50
gct	A	11	2.77
gcc	A	0	2.13
gca	A	0	4.90
gcg	A	0	1.20
---	--	------	------
mPD		0.030	1.7
		nPD:	0.02
		N. weight:	2.3
		Sc. PD:	-0.42
		Sc. rank:	-3323.9

,

PA			
Pos.	248	obs:	exp:
cct	P	0	0.27
ccc	P	0	0.18
cca	P	1	0.41
ccg	P	0	0.14
caa	Q	2853	1431.00
cag	Q	14	1436.00
---	--	------	------
mPD		0.010	0.50
		nPD:	0.02
		N. weight:	0.88
		Sc. PD:	-0.16
		Sc. rank:	-1241.0

,

PA			
Pos.	249	obs:	exp:
atg	M	2868	2868.00
---	--	------	------
mPD		0	0
		nPD:	1.
		N. weight:	0.
		Sc. PD:	0
		Sc. rank:	0

,

PA			
Pos.	250	obs:	exp:
tta	L	1	0.09
ttg	L	0	0.19
tct	S	0	472.60
tcc	S	27	410.10
tca	S	2779	641.80
tcg	S	25	170.80
ctt	L	0	0.20
ctc	L	0	0.16
cta	L	0	0.15
ctg	L	0	0.21
cct	P	0	9.57
ccc	P	1	6.32
cca	P	34	14.21
ccg	P	0	4.91
act	T	0	0.26
acc	T	1	0.23
aca	T	0	0.42
acg	T	0	0.09
agt	S	0	614.10
agc	S	0	521.70
---	--	------	------
mPD		0.063	1.7
		nPD:	0.04
		N. weight:	1.8
		Sc. PD:	-0.3
		Sc. rank:	-1913.7

,

PA			
Pos.	251	obs:	exp:
cgt	R	0	0.75
cgc	R	0	1.07
cga	R	0	2.02
cgg	R	0	1.63
aaa	K	1860	1622.00
aag	K	988	1226.00
aga	R	13	9.34
agg	R	7	5.20
---	--	------	------
mPD		0.47	0.51
		nPD:	0.92
		N. weight:	0.025
		Sc. PD:	0.018
		Sc. rank:	46.2

,

PA			
Pos.	252	obs:	exp:
aaa	K	3	1.71
aag	K	0	1.29
gat	D	2	1.08
gac	D	0	0.92
gaa	E	2819	1602.00
gag	E	39	1256.00
ggt	G	0	0.72
ggc	G	0	0.66
gga	G	4	2.19
ggg	G	1	1.43
---	--	------	------
mPD		0.034	0.50
		nPD:	0.07
		N. weight:	0.68
		Sc. PD:	-0.089
		Sc. rank:	-405.9

,

PA			
Pos.	253	obs:	exp:
gtt	V	0	616.90
gtc	V	0	603.40
gta	V	138	611.50
gtg	V	2730	1036.00
---	--	------	------
mPD		0.092	0.73
		nPD:	0.12
		N. weight:	1.1
		Sc. PD:	-0.085
		Sc. rank:	50.6

,

PA			
Pos.	254	obs:	exp:
tct	S	0	0.50
tcc	S	0	0.43
tca	S	0	0.68
tcg	S	0	0.18
act	T	3	0.79
acc	T	0	0.68
aca	T	0	1.26
acg	T	0	0.28
aat	N	820	1542.00
aac	N	2008	1286.00
aaa	K	34	19.36
aag	K	0	14.64
agt	S	0	0.65
agc	S	3	0.55
---	--	------	------
mPD		0.43	0.51
		nPD:	0.84
		N. weight:	0.19
		Sc. PD:	0.12
		Sc. rank:	348.5

,

PA			
Pos.	255	obs:	exp:
gct	A	673	722.50
gcc	A	2162	555.00
gca	A	33	1277.00
gcg	A	0	313.90
---	--	------	------
mPD		0.38	0.69
		nPD:	0.55
		N. weight:	1.3
		Sc. PD:	0.44
		Sc. rank:	1942.0

,

PA			
Pos.	256	obs:	exp:
cgt	R	0	106.70
cgc	R	0	152.50
cga	R	4	287.20
cgg	R	0	232.70
att	I	0	0.37
atc	I	0	0.27
ata	I	1	0.37
aaa	K	12	9.11
aag	K	4	6.89
aga	R	2637	1330.00
agg	R	209	740.40
ggt	G	0	0.14
ggc	G	0	0.13
gga	G	1	0.44
ggg	G	0	0.29
---	--	------	------
mPD		0.15	0.96
		nPD:	0.16
		N. weight:	0.71
		Sc. PD:	-0.03
		Sc. rank:	188.4

,

PA			
Pos.	257	obs:	exp:
att	I	2846	1049.00
atc	I	10	764.80
ata	I	2	1044.00
gtt	V	10	2.15
gtc	V	0	2.10
gta	V	0	2.13
gtg	V	0	3.61
---	--	------	------
mPD		0.015	0.67
		nPD:	0.02
		N. weight:	1.3
		Sc. PD:	-0.23
		Sc. rank:	-1734.4

,

PA			
Pos.	258	obs:	exp:
aaa	K	0	0.57
aag	K	1	0.43
gaa	E	124	1607.00
gag	E	2743	1260.00
---	--	------	------
mPD		0.083	0.49
		nPD:	0.17
		N. weight:	0.84
		Sc. PD:	-0.027
		Sc. rank:	296.8

,

PA			
Pos.	259	obs:	exp:
tct	S	0	0.33
tcc	S	0	0.29
tca	S	2	0.45
tcg	S	0	0.12
cct	P	1	762.20
ccc	P	3	503.70
cca	P	2752	1132.00
ccg	P	33	391.00
caa	Q	77	38.44
cag	Q	0	38.56
agt	S	0	0.43
agc	S	0	0.37
---	--	------	------
mPD		0.079	0.76
		nPD:	0.1
		N. weight:	1.1
		Sc. PD:	-0.11
		Sc. rank:	-142.6

,

PA			
Pos.	260	obs:	exp:
ttt	F	2660	1195.00
ttc	F	205	1670.00
tta	L	1	0.09
ttg	L	0	0.19
tct	S	1	0.17
tcc	S	0	0.14
tca	S	0	0.23
tcg	S	0	0.06
ctt	L	0	0.20
ctc	L	0	0.16
cta	L	0	0.15
ctg	L	0	0.21
agt	S	0	0.22
agc	S	0	0.18
gtt	V	1	0.22
gtc	V	0	0.21
gta	V	0	0.21
gtg	V	0	0.36
---	--	------	------
mPD		0.13	0.49
		nPD:	0.28
		N. weight:	0.79
		Sc. PD:	0.058
		Sc. rank:	666.4

,

PA

Pos.	261	obs:	exp:
ttt	F	7	2.92
ttc	F	0	4.08
tta	L	110	152.70
ttg	L	400	313.60
ctt	L	1	327.80
ctc	L	0	264.10
cta	L	7	244.30
ctg	L	1133	348.50
caa	Q	0	27.96
cag	Q	56	28.04
cgt	R	0	0.07
cgc	R	0	0.11
cga	R	0	0.20
cgg	R	0	0.16
att	I	0	0.37
atc	I	0	0.27
ata	I	1	0.37
atg	M	999	999.00
act	T	0	1.31
acc	T	0	1.13
aca	T	1	2.09
acg	T	4	0.47
aaa	K	0	2.28
aag	K	4	1.72
aga	R	0	0.93
agg	R	2	0.52
gtt	V	0	30.76
gtc	V	0	30.09
gta	V	0	30.49
gtg	V	143	51.67
---	--	------	------
mPD		0.80	1.3
		nPD:	0.62
		N. weight:	0.73
		Sc. PD:	0.3
		Sc. rank:	1190.0

,

PA

Pos.	262	obs:	exp:
cgt	R	0	11.53
cgc	R	0	16.48
cga	R	0	31.04
cgg	R	0	25.15
aat	N	1	0.55
aac	N	0	0.45
aaa	K	158	1457.00
aag	K	2400	1101.00
aga	R	4	143.80
agg	R	304	80.02
gaa	E	0	0.56
gag	E	1	0.44
---	--	------	------
mPD		0.30	0.75
		nPD:	0.4
		N. weight:	0.89
		Sc. PD:	0.17
		Sc. rank:	1084.8

,

PA

Pos.	263	obs:	exp:
tct	S	0	0.50
tcc	S	0	0.43
tca	S	1	0.68
tcg	S	0	0.18
act	T	0	718.80
acc	T	46	617.80
aca	T	2684	1147.00
acg	T	12	258.00
aat	N	0	2.73
aac	N	5	2.27
aaa	K	38	21.64
aag	K	0	16.36
agt	S	0	0.65
agc	S	2	0.55
gct	A	0	19.15
gcc	A	0	14.71
gca	A	76	33.83
gcg	A	0	8.32
gaa	E	4	2.24
gag	E	0	1.76
---	--	------	------
mPD		0.13	0.79
		nPD:	0.17
		N. weight:	1.
		Sc. PD:	-0.033
		Sc. rank:	367.9

,

PA

Pos.	264	obs:	exp:
tct	S	0	0.17
tcc	S	0	0.14
tca	S	1	0.23
tcg	S	0	0.06
act	T	0	751.60
acc	T	5	646.00
aca	T	2386	1200.00
acg	T	476	269.70
agt	S	0	0.22
agc	S	0	0.18
---	--	------	------
mPD		0.28	0.70
		nPD:	0.4
		N. weight:	0.88
		Sc. PD:	0.17
		Sc. rank:	1082.4

,

PA

Pos.	265	obs:	exp:
tta	L	0	0.09
ttg	L	0	0.19
tct	S	0	1.34
tcc	S	0	1.16
tca	S	8	1.81
tcg	S	0	0.48
ctt	L	0	0.20
ctc	L	0	0.16
cta	L	1	0.15
ctg	L	0	0.21
cct	P	6	781.30
ccc	P	1	516.40
cca	P	2832	1160.00
ccg	P	20	400.80
agt	S	0	1.74
agc	S	0	1.47
---	--	------	------
mPD		0.025	0.72
		nPD:	0.03
		N. weight:	1.1
		Sc. PD:	-0.19
		Sc. rank:	-1284.1

,

PA

Pos.	266	obs:	exp:
tct	S	0	2.34
tcc	S	0	2.03
tca	S	0	3.17
tcg	S	0	0.84
tgt	C	0	1.16
tgc	C	3	1.84
cat	H	1	1.16
cac	H	1	0.84
cgt	R	122	106.60
cgc	R	2712	152.50
cga	R	2	287.10
cgg	R	13	232.60
agt	S	0	3.04
agc	S	14	2.58
aga	R	0	1330.00
agg	R	0	740.10
---	--	------	------
mPD		0.11	0.97
		nPD:	0.11
		N. weight:	3.6
		Sc. PD:	-0.33
		Sc. rank:	-268.5

,

PA

Pos.	267	obs:	exp:
tta	L	0	0.74
ttg	L	0	1.52
tct	S	7	1.17
tcc	S	0	1.01
tca	S	0	1.59
tcg	S	0	0.42
ctt	L	8	1.59
ctc	L	0	1.28
cta	L	0	1.18
ctg	L	0	1.69
cct	P	2806	778.60
ccc	P	38	514.60
cca	P	5	1156.00
ccg	P	0	399.40
cat	H	4	2.32
cac	H	0	1.68
agt	S	0	1.52
agc	S	0	1.29
---	--	------	------
mPD		0.043	0.73
		nPD:	0.06
		N. weight:	1.6
		Sc. PD:	-0.23
		Sc. rank:	-1176.4

,

PA

Pos.	268	obs:	exp:
ttt	F	0	2.92
ttc	F	7	4.08
tta	L	0	264.20
ttg	L	1	542.80
ctt	L	21	567.30
ctc	L	2727	457.00
cta	L	88	422.80
ctg	L	20	603.00
att	I	0	1.47
atc	I	2	1.07
ata	I	2	1.46
---	--	------	------
mPD		0.097	1.1
		nPD:	0.09
		N. weight:	2.1
		Sc. PD:	-0.24
		Sc. rank:	-767.9

,

PA

Pos.	269	obs:	exp:
cgt	R	0	97.26
cgc	R	0	139.10
cga	R	1	261.90
cgg	R	0	212.20
att	I	0	1.10
atc	I	0	0.80
ata	I	3	1.10
aaa	K	260	150.90
aag	K	5	114.10
aga	R	2563	1213.00
agg	R	35	675.20
ggt	G	0	0.14
ggc	G	0	0.13
gga	G	1	0.44
ggg	G	0	0.29
---	--	------	------
mPD		0.20	1.1
		nPD:	0.18
		N. weight:	0.9
		Sc. PD:	-0.018
		Sc. rank:	377.3

,

PA

Pos.	270	obs:	exp:
tta	L	1563	264.40
ttg	L	80	543.30
ctt	L	0	567.90
ctc	L	0	457.50
cta	L	1202	423.30
ctg	L	15	603.60
cct	P	0	0.82
ccc	P	0	0.54
cca	P	3	1.22
ccg	P	0	0.42
att	I	0	1.10
atc	I	0	0.80
ata	I	3	1.10
atg	M	2	2.00
---	--	------	------
mPD		0.56	1.1
		nPD:	0.5
		N. weight:	1.8
		Sc. PD:	0.52
		Sc. rank:	2526.1

,

PA

Pos.	271	obs:	exp:
cct	P	2763	783.00
ccc	P	74	517.50
cca	P	12	1163.00
ccg	P	16	401.70
cat	H	2	1.16
cac	H	0	0.84
gct	A	1	0.25
gcc	A	0	0.19
gca	A	0	0.45
gcg	A	0	0.11
---	--	------	------
mPD		0.071	0.71
		nPD:	0.1
		N. weight:	1.5
		Sc. PD:	-0.15
		Sc. rank:	-303.2

,

PA

Pos.	272	obs:	exp:
aat	N	13	7.09
aac	N	0	5.91
gat	D	1929	1045.00
gac	D	6	890.20
gaa	E	26	481.50
gag	E	833	377.50
ggt	G	56	8.82
ggc	G	0	8.01
gga	G	0	26.73
ggg	G	5	17.44
---	--	------	------
mPD		0.48	0.76
		nPD:	0.63
		N. weight:	0.86
		Sc. PD:	0.36
		Sc. rank:	1399.2

,

PA

Pos.	273	obs:	exp:
ggt	G	3	414.80
ggc	G	168	376.70
gga	G	51	1257.00
ggg	G	2646	819.80
---	--	------	------
mPD		0.15	0.69
		nPD:	0.21
		N. weight:	1.3
		Sc. PD:	0.012
		Sc. rank:	784.3

,

PA

Pos.	274	obs:	exp:
tta	L	0	0.09
ttg	L	0	0.19
tct	S	10	1.67
tcc	S	0	1.45
tca	S	0	2.27
tcg	S	0	0.60
ctt	L	1	0.20
ctc	L	0	0.16
cta	L	0	0.15
ctg	L	0	0.21
cct	P	2811	780.00
ccc	P	35	515.50
cca	P	0	1158.00
ccg	P	8	400.10
cat	H	3	1.74
cac	H	0	1.26
agt	S	0	2.17
agc	S	0	1.84
---	--	------	------
mPD		0.039	0.72
		nPD:	0.05
		N. weight:	1.6
		Sc. PD:	-0.23
		Sc. rank:	-1295.6

,

PA

Pos.	275	obs:	exp:
tta	L	0	3.14
ttg	L	0	6.46
tct	S	19	4.17
tcc	S	6	3.62
tca	S	0	5.67
tcg	S	0	1.51
ctt	L	21	6.75
ctc	L	13	5.44
cta	L	0	5.03
ctg	L	0	7.18
cct	P	719	767.40
ccc	P	2070	507.20
cca	P	19	1140.00
ccg	P	0	393.70
cat	H	1	0.58
cac	H	0	0.42
agt	S	0	5.42
agc	S	0	4.61
---	--	------	------
mPD		0.44	0.76
		nPD:	0.58
		N. weight:	1.3
		Sc. PD:	0.49
		Sc. rank:	2062.6

,

PA			
Pos.	276	obs:	exp:
tgt	C	825	1110.00
tgc	C	2043	1758.00
---	--	------	------
mPD		0.41	0.47
		nPD:	0.86
		N. weight:	0.031
		Sc. PD:	0.02
		Sc. rank:	56.5

PA			
Pos.	277	obs:	exp:
ttt	F	20	8.34
ttc	F	0	11.66
tta	L	0	0.28
ttg	L	0	0.57
tct	S	1919	425.30
tcc	S	625	369.10
tca	S	3	577.60
tcg	S	1	153.70
tat	Y	2	1.92
tac	Y	2	2.08
tgt	C	1	0.39
tgc	C	0	0.61
ctt	L	1	0.60
ctc	L	2	0.48
cta	L	0	0.44
ctg	L	0	0.63
cct	P	12	77.61
ccc	P	272	51.29
cca	P	0	115.30
ccg	P	0	39.82
cat	H	0	1.16
cac	H	2	0.84
agt	S	0	552.70
agc	S	0	469.60
gct	A	6	1.51
gcc	A	0	1.16
gca	A	0	2.67
gcg	A	0	0.66
---	--	------	------
mPD		0.64	1.7
		nPD:	0.37
		N. weight:	1.7
		Sc. PD:	0.28
		Sc. rank:	1994.2

PA			
Pos.	278	obs:	exp:
caa	Q	49	1432.00
cag	Q	2819	1436.00
---	--	------	------
mPD		0.034	0.50
		nPD:	0.07
		N. weight:	0.81
		Sc. PD:	-0.11
		Sc. rank:	-503.8

PA			
Pos.	279	obs:	exp:
caa	Q	0	1.00
cag	Q	2	1.00
cgt	R	2	107.30
cgc	R	9	153.40
cga	R	410	288.80
cgg	R	2432	234.00
aga	R	3	1338.00
agg	R	10	744.60
---	--	------	------
mPD		0.26	0.96
		nPD:	0.28
		N. weight:	2.7
		Sc. PD:	0.2
		Sc. rank:	2235.1

PA			
Pos.	280	obs:	exp:
tct	S	44	478.40
tcc	S	1	415.20
tca	S	164	649.70
tcg	S	2657	172.90
cct	P	0	0.27
ccc	P	0	0.18
cca	P	0	0.41
ccg	P	1	0.14
act	T	0	0.26
acc	T	0	0.23
aca	T	0	0.42
acg	T	1	0.09
agt	S	0	621.60
agc	S	0	528.20
---	--	------	------
mPD		0.14	1.7
		nPD:	0.08
		N. weight:	3.2
		Sc. PD:	-0.37
		Sc. rank:	-1396.2

PA			
Pos.	281	obs:	exp:
aaa	K	629	1633.00
aag	K	2239	1235.00
---	--	------	------
mPD		0.34	0.49
		nPD:	0.7
		N. weight:	0.34
		Sc. PD:	0.17
		Sc. rank:	592.3

PA			
Pos.	282	obs:	exp:
ttt	F	114	1196.00
ttc	F	2754	1672.00
---	--	------	------
mPD		0.076	0.49
		nPD:	0.16
		N. weight:	0.51
		Sc. PD:	-0.022
		Sc. rank:	125.6

PA			
Pos.	283	obs:	exp:
tta	L	1041	265.10
ttg	L	1292	544.70
tgg	W	1	1.00
ctt	L	0	569.20
ctc	L	0	458.60
cta	L	57	424.30
ctg	L	477	605.10
---	--	------	------
mPD		0.78	1.1
		nPD:	0.69
		N. weight:	1.1
		Sc. PD:	0.52
		Sc. rank:	1854.8

PA			
Pos.	284	obs:	exp:
tta	L	0	264.90
ttg	L	34	544.30
ctt	L	5	568.80
ctc	L	0	458.30
cta	L	32	424.00
ctg	L	2794	604.70
caa	Q	0	0.50
cag	Q	1	0.50
atg	M	2	2.00
---	--	------	------
mPD		0.051	1.1
		nPD:	0.05
		N. weight:	1.9
		Sc. PD:	-0.29
		Sc. rank:	-1810.7

PA			
Pos.	285	obs:	exp:
atg	M	2862	2862.00
act	T	0	0.26
acc	T	0	0.23
aca	T	0	0.42
acg	T	1	0.09
aaa	K	0	2.85
aag	K	5	2.15
---	--	------	------
mPD		0.0042	0.0068
		nPD:	0.62
		N. weight:	0.003
		Sc. PD:	0.0012
		Sc. rank:	4.9

PA			
Pos.	286	obs:	exp:
gat	D	2865	1549.00
gac	D	3	1319.00
---	--	------	------
mPD		0.0021	0.50
		nPD:	0.
		N. weight:	0.81
		Sc. PD:	-0.16
		Sc. rank:	-1420.4

PA			
Pos.	287	obs:	exp:
tct	S	2	23.04
tcc	S	136	19.99
tca	S	0	31.28
tcg	S	0	8.33
tgt	C	0	2.71
tgc	C	7	4.29
agt	S	0	29.93
agc	S	0	25.43
gct	A	202	659.30
gcc	A	2385	506.40
gca	A	30	1165.00
gcg	A	0	286.40
ggt	G	4	15.33
ggc	G	102	13.92
gga	G	0	46.45
ggg	G	0	30.30
---	--	------	------
mPD		0.33	0.90
		nPD:	0.36
		N. weight:	1.8
		Sc. PD:	0.28
		Sc. rank:	1973.1

PA			
Pos.	288	obs:	exp:
tta	L	0	265.00
ttg	L	13	544.50
ctt	L	2459	569.00
ctc	L	333	458.40
cta	L	12	424.10
ctg	L	49	604.90
cct	P	1	0.27
ccc	P	0	0.18
cca	P	0	0.41
ccg	P	0	0.14
gtt	V	1	0.22
gtc	V	0	0.21
gta	V	0	0.21
gtg	V	0	0.36
---	--	------	------
mPD		0.26	1.1
		nPD:	0.23
		N. weight:	1.5
		Sc. PD:	0.046
		Sc. rank:	1069.1

PA			
Pos.	289	obs:	exp:
aaa	K	1758	1633.00
aag	K	1110	1235.00
---	--	------	------
mPD		0.47	0.49
		nPD:	0.97
		N. weight:	0.0071
		Sc. PD:	0.0054
		Sc. rank:	13.3

PA			
Pos.	290	obs:	exp:
tta	L	1911	265.20
ttg	L	831	544.80
ctt	L	21	569.40
ctc	L	0	458.80
cta	L	103	424.40
ctg	L	2	605.30
---	--	------	------
mPD		0.51	1.1
		nPD:	0.45
		N. weight:	1.8
		Sc. PD:	0.45
		Sc. rank:	2426.9

PA			
Pos.	291	obs:	exp:
tct	S	0	476.40
tcc	S	0	413.40
tca	S	0	647.00
tcg	S	0	172.20
tgt	C	0	1.55
tgc	C	4	2.45
aat	N	2	4.36
aac	N	6	3.64
agt	S	499	619.00
agc	S	2355	526.00
ggt	G	1	0.29
ggc	G	1	0.26
gga	G	0	0.88
ggg	G	0	0.57
---	--	------	------
mPD		0.30	1.7
		nPD:	0.18
		N. weight:	1.6
		Sc. PD:	-0.033
		Sc. rank:	654.9

,

PA			
Pos.	292	obs:	exp:
att	I	788	1048.00
atc	I	2040	764.50
ata	I	29	1044.00
gtt	V	5	2.15
gtc	V	5	2.10
gta	V	0	2.13
gtg	V	0	3.61
---	-	1	1.00
---	--	------	------
mPD		0.42	0.67
		nPD:	0.63
		N. weight:	0.78
		Sc. PD:	0.33
		Sc. rank:	1287.2

,

PA			
Pos.	293	obs:	exp:
gaa	E	2673	1607.00
gag	E	194	1260.00
---	-	1	1.00
---	--	------	------
mPD		0.13	0.49
		nPD:	0.26
		N. weight:	0.46
		Sc. PD:	0.025
		Sc. rank:	364.5

,

PA			
Pos.	294	obs:	exp:
aat	N	1	1.09
aac	N	1	0.91
gtt	V	0	0.22
gtc	V	1	0.21
gta	V	0	0.21
gtg	V	0	0.36
gat	D	1120	1545.00
gac	D	1742	1317.00
gaa	E	2	1.12
gag	E	0	0.88
ggt	G	1	0.14
ggc	G	0	0.13
gga	G	0	0.44
ggg	G	0	0.29
---	--	------	------
mPD		0.48	0.50
		nPD:	0.96
		N. weight:	0.063
		Sc. PD:	0.047
		Sc. rank:	117.8

,

PA			
Pos.	295	obs:	exp:
cct	P	621	783.20
ccc	P	0	517.60
cca	P	1194	1163.00
ccg	P	1051	401.80
caa	Q	2	1.00
cag	Q	0	1.00
---	--	------	------
mPD		0.65	0.71
		nPD:	0.91
		N. weight:	0.42
		Sc. PD:	0.29
		Sc. rank:	779.2

,

PA			
Pos.	296	obs:	exp:
tct	S	0	476.20
tcc	S	0	413.30
tca	S	0	646.80
tcg	S	0	172.10
cgt	R	0	0.04
cgc	R	0	0.05
cga	R	0	0.10
cgg	R	0	0.08
aat	N	1	6.54
aac	N	11	5.46
aaa	K	1	0.57
aag	K	0	0.43
agt	S	605	618.80
agc	S	2248	525.80
aga	R	1	0.47
agg	R	0	0.26
ggt	G	0	0.14
ggc	G	1	0.13
gga	G	0	0.44
ggg	G	0	0.29
---	--	------	------
mPD		0.34	1.7
		nPD:	0.21
		N. weight:	1.5
		Sc. PD:	0.0097
		Sc. rank:	858.5

,

PA			
Pos.	297	obs:	exp:
tat	Y	8	3.84
tac	Y	0	4.16
cat	H	2842	1656.00
cac	H	18	1204.00
---	--	------	------
mPD		0.018	0.49
		nPD:	0.04
		N. weight:	0.68
		Sc. PD:	-0.11
		Sc. rank:	-736.4

,

PA			
Pos.	298	obs:	exp:
gat	D	6	3.24
gac	D	0	2.76
gaa	E	412	1604.00
gag	E	2450	1258.00
---	--	------	------
mPD		0.25	0.49
		nPD:	0.5
		N. weight:	0.5
		Sc. PD:	0.15
		Sc. rank:	715.5

,

PA			
Pos.	299	obs:	exp:
cgt	R	0	0.04
cgc	R	0	0.05
cga	R	0	0.10
cgg	R	0	0.08
aga	R	0	0.47
agg	R	1	0.26
ggt	G	0	414.60
ggc	G	20	376.60
gga	G	148	1256.00
ggg	G	2699	819.50
---	--	------	------
mPD		0.11	0.69
		nPD:	0.16
		N. weight:	1.3
		Sc. PD:	-0.051
		Sc. rank:	417.5

,

PA			
Pos.	300	obs:	exp:
aaa	K	0	0.57
aag	K	1	0.43
gaa	E	1519	1606.00
gag	E	1346	1259.00
ggt	G	0	0.29
ggc	G	0	0.26
gga	G	2	0.88
ggg	G	0	0.57
---	--	------	------
mPD		0.50	0.49
		nPD:	1.01
		N. weight:	0.0056
		Sc. PD:	0.0045
		Sc. rank:	10.4

,

PA			
Pos.	301	obs:	exp:
tgt	C	0	1.16
tgc	C	3	1.84
ggt	G	1197	414.30
ggc	G	1109	376.30
gga	G	68	1255.00
ggg	G	491	819.00
---	--	------	------
mPD		0.65	0.69
		nPD:	0.94
		N. weight:	0.94
		Sc. PD:	0.68
		Sc. rank:	1750.1

,

PA			
Pos.	302	obs:	exp:
att	I	1	1052.00
atc	I	20	767.20
ata	I	2846	1048.00
gtt	V	0	0.22
gtc	V	0	0.21
gta	V	1	0.21
gtg	V	0	0.36
---	--	------	------
mPD		0.015	0.66
		nPD:	0.02
		N. weight:	1.3
		Sc. PD:	-0.23
		Sc. rank:	-1704.2

,

PA			
Pos.	303	obs:	exp:
cct	P	8	783.80
ccc	P	5	518.00
cca	P	1387	1164.00
ccg	P	1468	402.10
---	--	------	------
mPD		0.50	0.71
		nPD:	0.71
		N. weight:	0.97
		Sc. PD:	0.49
		Sc. rank:	1722.5

,

PA			
Pos.	304	obs:	exp:
tta	L	8	264.90
ttg	L	5	544.30
ctt	L	16	568.80
ctc	L	0	458.30
cta	L	1969	424.00
ctg	L	867	604.70
att	I	0	1.10
atc	I	0	0.80
ata	I	3	1.10
---	--	------	------
mPD		0.44	1.1
		nPD:	0.39
		N. weight:	1.5
		Sc. PD:	0.28
		Sc. rank:	1822.6

,

PA			
Pos.	305	obs:	exp:
ttt	F	21	8.76
ttc	F	0	12.24
tat	Y	2359	1351.00
tac	Y	458	1466.00
tgt	C	4	2.71
tgc	C	3	4.29
cat	H	10	13.31
cac	H	13	9.69
---	--	------	------
mPD		0.31	0.53
		nPD:	0.58
		N. weight:	0.37
		Sc. PD:	0.14
		Sc. rank:	587.1

,

PA			
Pos.	306	obs:	exp:
gat	D	2733	1549.00
gac	D	135	1319.00
---	--	------	------
mPD		0.090	0.50
		nPD:	0.18
		N. weight:	0.58
		Sc. PD:	-0.012
		Sc. rank:	239.7

,

PA			
Pos.	307	obs:	exp:
tct	S	0	0.33
tcc	S	0	0.29
tca	S	2	0.45
tcg	S	0	0.12
agt	S	0	0.43
agc	S	0	0.37
gct	A	0	722.00
gcc	A	0	554.60
gca	A	2822	1276.00
gcg	A	44	313.70
---	--	------	------
mPD		0.032	0.69
		nPD:	0.05
		N. weight:	1.
		Sc. PD:	-0.15
		Sc. rank:	-952.6

,

PA			
Pos.	308	obs:	exp:
tta	L	0	0.55
ttg	L	0	1.14
ctt	L	0	1.19
ctc	L	2	0.96
cta	L	4	0.89
ctg	L	0	1.27
att	I	6	1022.00
atc	I	2615	745.50
ata	I	165	1018.00
act	T	0	10.75
acc	T	41	9.24
aca	T	0	17.16
acg	T	0	3.86
gtt	V	0	7.53
gtc	V	35	7.36
gta	V	0	7.46
gtg	V	0	12.65
---	--	------	------
mPD		0.17	0.72
		nPD:	0.24
		N. weight:	1.4
		Sc. PD:	0.051
		Sc. rank:	1043.4

,

PA			
Pos.	309	obs:	exp:
caa	Q	1	0.50
cag	Q	0	0.50
cgt	R	0	0.15
cgc	R	0	0.21
cga	R	0	0.40
cgg	R	0	0.33
aat	N	0	1.64
aac	N	3	1.36
aaa	K	2807	1628.00
aag	K	52	1231.00
aga	R	4	1.87
agg	R	0	1.04
gaa	E	1	0.56
gag	E	0	0.44
---	--	------	------
mPD		0.042	0.50
		nPD:	0.08
		N. weight:	0.64
		Sc. PD:	-0.073
		Sc. rank:	-257.7

,

PA			
Pos.	310	obs:	exp:
tgt	C	19	1110.00
tgc	C	2849	1758.00
---	--	------	------
mPD		0.013	0.47
		nPD:	0.03
		N. weight:	0.6
		Sc. PD:	-0.1
		Sc. rank:	-774.7

,

PA

Pos.	311	obs:	exp:
tta	L	0	0.09
ttg	L	0	0.19
ctt	L	0	0.20
ctc	L	0	0.16
cta	L	1	0.15
ctg	L	0	0.21
att	I	3	2.57
atc	I	0	1.87
ata	I	4	2.56
atg	M	2858	2858.00
gtt	V	0	0.43
gtc	V	0	0.42
gta	V	0	0.43
gtg	V	2	0.72
---	--	------	------
mPD		0.0077	0.0083
		nPD:	0.93
		N. weight:	0.0034
		Sc. PD:	0.0025
		Sc. rank:	6.4

,

PA

Pos.	312	obs:	exp:
cgt	R	0	0.04
cgc	R	0	0.05
cga	R	0	0.10
cgg	R	0	0.08
aat	N	1	0.55
aac	N	0	0.45
aaa	K	72	1632.00
aag	K	2794	1234.00
aga	R	0	0.47
agg	R	1	0.26
---	--	------	------
mPD		0.050	0.49
		nPD:	0.1
		N. weight:	0.96
		Sc. PD:	-0.093
		Sc. rank:	-145.5

,

PA

Pos.	313	obs:	exp:
act	T	217	751.80
acc	T	30	646.20
aca	T	2612	1200.00
acg	T	9	269.80
---	--	------	------
mPD		0.16	0.70
		nPD:	0.24
		N. weight:	0.76
		Sc. PD:	0.027
		Sc. rank:	546.9

,

PA

Pos.	314	obs:	exp:
ttt	F	2812	1196.00
ttc	F	56	1672.00
---	--	------	------
mPD		0.038	0.49
		nPD:	0.08
		N. weight:	1.
		Sc. PD:	-0.12
		Sc. rank:	-530.2

,

PA

Pos.	315	obs:	exp:
ttt	F	848	1187.00
ttc	F	1998	1659.00
tta	L	2	1.66
ttg	L	2	3.42
tat	Y	0	1.92
tac	Y	4	2.08
ctt	L	0	3.57
ctc	L	14	2.88
cta	L	0	2.66
ctg	L	0	3.80
---	--	------	------
mPD		0.43	0.50
		nPD:	0.86
		N. weight:	0.054
		Sc. PD:	0.035
		Sc. rank:	99.8

,

PA

Pos.	316	obs:	exp:
gat	D	0	0.54
gac	D	1	0.46
ggt	G	113	414.60
ggc	G	2360	376.60
gga	G	85	1256.00
ggg	G	309	819.50
---	--	------	------
mPD		0.31	0.69
		nPD:	0.45
		N. weight:	1.7
		Sc. PD:	0.41
		Sc. rank:	2259.0

,

PA

Pos.	317	obs:	exp:
tgg	W	2867	2867.00
cgt	R	0	0.04
cgc	R	0	0.05
cga	R	0	0.10
cgg	R	1	0.08
aga	R	0	0.47
agg	R	0	0.26
---	--	------	------
mPD		0.00070	0.0012
		nPD:	0.6
		N. weight:	0.0012
		Sc. PD:	0.00046
		Sc. rank:	1.8

,

PA

Pos.	318	obs:	exp:
cgt	R	0	0.07
cgc	R	0	0.11
cga	R	0	0.20
cgg	R	0	0.16
aaa	K	2395	1631.00
aag	K	470	1234.00
aga	R	2	0.93
agg	R	0	0.52
gaa	E	1	0.56
gag	E	0	0.44
---	--	------	------
mPD		0.28	0.49
		nPD:	0.56
		N. weight:	0.22
		Sc. PD:	0.077
		Sc. rank:	334.6

,

PA

Pos.	319	obs:	exp:
aaa	K	0	0.57
aag	K	1	0.43
gat	D	142	77.21
gac	D	1	65.79
gaa	E	167	1526.00
gag	E	2556	1197.00
ggt	G	0	0.14
ggc	G	0	0.13
gga	G	0	0.44
ggg	G	1	0.29
---	--	------	------
mPD		0.20	0.54
		nPD:	0.37
		N. weight:	0.77
		Sc. PD:	0.13
		Sc. rank:	903.9

,

PA

Pos.	320	obs:	exp:
cct	P	199	783.80
ccc	P	2657	518.00
cca	P	12	1164.00
ccg	P	0	402.10
---	--	------	------
mPD		0.14	0.71
		nPD:	0.19
		N. weight:	1.9
		Sc. PD:	-0.015
		Sc. rank:	967.0

,

PA

Pos.	321	obs:	exp:
tct	S	0	7.85
tcc	S	0	6.81
tca	S	0	10.65
tcg	S	0	2.84
tat	Y	0	0.48
tac	Y	1	0.52
cat	H	0	0.58
cac	H	1	0.42
att	I	0	0.37
atc	I	1	0.27
ata	I	0	0.37
act	T	1	1.84
acc	T	4	1.58
aca	T	2	2.93
acg	T	0	0.66
aat	N	94	1509.00
aac	N	2673	1258.00
aaa	K	25	14.24
aag	K	0	10.76
agt	S	0	10.19
agc	S	47	8.66
gat	D	0	1.62
gac	D	3	1.38
ggt	G	0	2.31
ggc	G	16	2.10
gga	G	0	7.01
ggg	G	0	4.57
---	--	------	------
mPD		0.15	0.60
		nPD:	0.24
		N. weight:	0.88
		Sc. PD:	0.036
		Sc. rank:	651.1

,

PA

Pos.	322	obs:	exp:
tta	L	0	17.94
ttg	L	0	36.85
ctt	L	1	38.52
ctc	L	193	31.03
cta	L	0	28.71
ctg	L	0	40.95
att	I	504	960.70
atc	I	2064	700.60
ata	I	50	956.70
gtt	V	1	12.04
gtc	V	41	11.78
gta	V	14	11.94
gtg	V	0	20.23
---	--	------	------
mPD		0.49	0.85
		nPD:	0.58
		N. weight:	1.
		Sc. PD:	0.37
		Sc. rank:	1566.6

,

PA

Pos.	323	obs:	exp:
att	I	0	27.89
atc	I	32	20.34
ata	I	44	27.77
gtt	V	1	587.20
gtc	V	44	574.40
gta	V	2166	582.10
gtg	V	519	986.40
gct	A	0	15.62
gcc	A	0	12.00
gca	A	62	27.60
gcg	A	0	6.79
---	--	------	------
mPD		0.43	0.83
		nPD:	0.52
		N. weight:	1.2
		Sc. PD:	0.36
		Sc. rank:	1699.6

,

PA

Pos.	324	obs:	exp:
aaa	K	2156	1633.00
aag	K	712	1235.00
---	--	------	------
mPD		0.37	0.49
		nPD:	0.76
		N. weight:	0.098
		Sc. PD:	0.054
		Sc. rank:	178.3

,

PA

Pos.	325	obs:	exp:
tta	L	0	0.37
ttg	L	0	0.76
tct	S	0	3.34
tcc	S	0	2.90
tca	S	19	4.53
tcg	S	1	1.21
ctt	L	0	0.79
ctc	L	0	0.64
cta	L	2	0.59
ctg	L	2	0.84
cct	P	4	776.40
ccc	P	0	513.10
cca	P	1845	1153.00
ccg	P	992	398.30
caa	Q	1	0.50
cag	Q	0	0.50
act	T	0	0.52
acc	T	0	0.45
aca	T	0	0.84
acg	T	2	0.19
agt	S	0	4.34
agc	S	0	3.69
---	--	------	------
mPD		0.47	0.73
		nPD:	0.65
		N. weight:	0.83
		Sc. PD:	0.36
		Sc. rank:	1389.9

,

PA			
Pos.	326	obs:	exp:
cat	H	2859	1660.00
cac	H	8	1207.00
caa	Q	1	0.50
cag	Q	0	0.50
---	--	------	------
mPD		0.0063	0.49
		nPD:	0.01
		N. weight:	0.7
		Sc. PD:	-0.13
		Sc. rank:	-1129.4

,

PA			
Pos.	327	obs:	exp:
aaa	K	11	6.83
aag	K	1	5.17
gtt	V	0	0.43
gtc	V	0	0.42
gta	V	2	0.43
gtg	V	0	0.72
gat	D	1	9.72
gac	D	17	8.28
gaa	E	2070	1576.00
gag	E	742	1236.00
ggt	G	0	3.47
ggc	G	0	3.15
gga	G	16	10.52
ggg	G	8	6.86
---	--	------	------
mPD		0.42	0.53
		nPD:	0.8
		N. weight:	0.1
		Sc. PD:	0.06
		Sc. rank:	185.0

,

PA			
Pos.	328	obs:	exp:
cgt	R	0	0.30
cgc	R	0	0.43
cga	R	0	0.81
cgg	R	0	0.65
aaa	K	1470	1629.00
aag	K	1390	1231.00
aga	R	3	3.73
agg	R	5	2.08
---	--	------	------
mPD		0.51	0.50
		nPD:	1.02
		N. weight:	0.013
		Sc. PD:	0.01
		Sc. rank:	23.3

,

PA			
Pos.	329	obs:	exp:
tct	S	0	0.17
tcc	S	0	0.14
tca	S	0	0.23
tcg	S	0	0.06
agt	S	0	0.22
agc	S	1	0.18
ggt	G	616	414.60
ggc	G	2223	376.60
gga	G	25	1256.00
ggg	G	3	819.50
---	--	------	------
mPD		0.35	0.69
		nPD:	0.51
		N. weight:	1.9
		Sc. PD:	0.58
		Sc. rank:	2766.4

,

PA			
Pos.	330	obs:	exp:
tta	L	1	0.09
ttg	L	0	0.19
ctt	L	0	0.20
ctc	L	0	0.16
cta	L	0	0.15
ctg	L	0	0.21
cgt	R	0	0.19
cgc	R	0	0.27
cga	R	0	0.50
cgg	R	0	0.41
att	I	17	1003.00
atc	I	0	731.60
ata	I	2717	999.10
act	T	0	27.00
acc	T	0	23.21
aca	T	102	43.10
acg	T	1	9.69
aga	R	5	2.33
agg	R	0	1.30
gtt	V	5	5.38
gtc	V	0	5.26
gta	V	20	5.33
gtg	V	0	9.03
---	--	------	------
mPD		0.11	0.76
		nPD:	0.14
		N. weight:	1.3
		Sc. PD:	-0.077
		Sc. rank:	207.9

,

PA			
Pos.	331	obs:	exp:
act	T	1	0.52
acc	T	1	0.45
aca	T	0	0.84
acg	T	0	0.19
aat	N	1310	1563.00
aac	N	1556	1303.00
---	--	------	------
mPD		0.50	0.50
		nPD:	1.
		N. weight:	0.024
		Sc. PD:	0.019
		Sc. rank:	44.1

,

PA			
Pos.	332	obs:	exp:
tta	L	0	2.31
ttg	L	0	4.75
tct	S	0	7.51
tcc	S	45	6.52
tca	S	0	10.20
tcg	S	0	2.71
ctt	L	1	4.96
ctc	L	24	4.00
cta	L	0	3.70
ctg	L	0	5.28
cct	P	904	762.50
ccc	P	1851	503.90
cca	P	35	1132.00
ccg	P	0	391.20
cat	H	2	1.16
cac	H	0	0.84
act	T	0	1.57
acc	T	6	1.35
aca	T	0	2.51
acg	T	0	0.56
agt	S	0	9.76
agc	S	0	8.29
---	--	------	------
mPD		0.50	0.78
		nPD:	0.64
		N. weight:	1.2
		Sc. PD:	0.52
		Sc. rank:	1996.9

,

PA			
Pos.	333	obs:	exp:
tct	S	0	0.33
tcc	S	0	0.29
tca	S	0	0.45
tcg	S	0	0.12
cat	H	1	0.58
cac	H	0	0.42
att	I	1	0.73
atc	I	0	0.54
ata	I	1	0.73
aat	N	2830	1561.00
aac	N	33	1302.00
agt	S	2	0.43
agc	S	0	0.37
---	--	------	------
mPD		0.027	0.50
		nPD:	0.05
		N. weight:	0.73
		Sc. PD:	-0.11
		Sc. rank:	-597.2

,

PA			
Pos.	334	obs:	exp:
ttt	F	0	0.83
ttc	F	2	1.17
tat	Y	117	1375.00
tac	Y	2749	1491.00
---	--	------	------
mPD		0.080	0.50
		nPD:	0.16
		N. weight:	0.65
		Sc. PD:	-0.027
		Sc. rank:	174.0

,

PA			
Pos.	335	obs:	exp:
ttt	F	0	1.25
ttc	F	3	1.75
tta	L	1	264.50
ttg	L	0	543.50
ctt	L	225	568.00
ctc	L	2627	457.60
cta	L	8	423.40
ctg	L	0	603.90
cct	P	0	0.27
ccc	P	1	0.18
cca	P	0	0.41
ccg	P	0	0.14
att	I	0	1.10
atc	I	3	0.80
ata	I	0	1.10
---	--	------	------
mPD		0.16	1.1
		nPD:	0.14
		N. weight:	2.
		Sc. PD:	-0.12
		Sc. rank:	288.7

,

PA			
Pos.	336	obs:	exp:
ttt	F	2	0.83
ttc	F	0	1.17
tta	L	0	264.20
ttg	L	25	542.80
ctt	L	1	567.30
ctc	L	0	457.00
cta	L	38	422.80
ctg	L	2793	603.00
atg	M	9	9.00
---	--	------	------
mPD		0.053	1.1
		nPD:	0.05
		N. weight:	1.9
		Sc. PD:	-0.29
		Sc. rank:	-1780.3

,

PA			
Pos.	337	obs:	exp:
act	T	301	96.73
acc	T	64	83.15
aca	T	4	154.40
acg	T	0	34.72
gtt	V	8	1.72
gtc	V	0	1.68
gta	V	0	1.71
gtg	V	0	2.89
gct	A	2474	627.30
gcc	A	12	481.80
gca	A	3	1108.00
gcg	A	1	272.50
gat	D	1	0.54
gac	D	0	0.46
---	--	------	------
mPD		0.29	0.92
		nPD:	0.31
		N. weight:	1.7
		Sc. PD:	0.19
		Sc. rank:	1628.8

,

PA			
Pos.	338	obs:	exp:
tta	L	0	0.09
ttg	L	1	0.19
tgg	W	2867	2867.00
ctt	L	0	0.20
ctc	L	0	0.16
cta	L	0	0.15
ctg	L	0	0.21
---	--	------	------
mPD		0.00070	0.0016
		nPD:	0.43
		N. weight:	0.00077
		Sc. PD:	0.00017
		Sc. rank:	1.0

,

PA			
Pos.	339	obs:	exp:
cgt	R	0	0.15
cgc	R	0	0.21
cga	R	0	0.40
cgg	R	0	0.33
act	T	0	0.79
acc	T	0	0.68
aca	T	0	1.26
acg	T	3	0.28
aaa	K	306	1629.00
aag	K	2555	1232.00
aga	R	0	1.87
agg	R	4	1.04
---	--	------	------
mPD		0.20	0.50
		nPD:	0.39
		N. weight:	0.63
		Sc. PD:	0.12
		Sc. rank:	771.7

,

PA			
Pos.	340	obs:	exp:
cct	P	0	0.55
ccc	P	0	0.36
cca	P	1	0.81
ccg	P	1	0.28
caa	Q	609	1430.00
cag	Q	2256	1435.00
gaa	E	1	0.56
gag	E	0	0.44
---	--	------	------
mPD		0.34	0.50
		nPD:	0.67
		N. weight:	0.24
		Sc. PD:	0.11
		Sc. rank:	396.9

,

PA

Pos.	341	obs:	exp:
tta	L	0	0.28
ttg	L	3	0.57
ctt	L	0	0.60
ctc	L	0	0.48
cta	L	0	0.44
ctg	L	0	0.63
gtt	V	3	616.00
gtc	V	0	602.50
gta	V	103	610.70
gtg	V	2758	1035.00
gct	A	0	0.25
gcc	A	0	0.19
gca	A	0	0.45
gcg	A	1	0.11
---	--	------	------
mPD		0.074	0.74
		nPD:	0.1
		N. weight:	1.2
		Sc. PD:	-0.12
		Sc. rank:	-228.1

,

PA

Pos.	342	obs:	exp:
tta	L	18	261.80
ttg	L	8	537.80
ctt	L	28	562.10
ctc	L	2	452.80
cta	L	547	419.00
ctg	L	2228	597.50
atg	M	37	37.00
---	--	------	------
mPD		0.38	1.1
		nPD:	0.33
		N. weight:	1.4
		Sc. PD:	0.17
		Sc. rank:	1419.0

,

PA

Pos.	343	obs:	exp:
tct	S	0	140.20
tcc	S	0	121.70
tca	S	836	190.40
tcg	S	4	50.68
act	T	0	37.23
acc	T	0	32.00
aca	T	142	59.42
acg	T	0	13.36
agt	S	0	182.20
agc	S	0	154.80
gtt	V	0	0.22
gtc	V	0	0.21
gta	V	1	0.21
gtg	V	0	0.36
gct	A	7	474.10
gcc	A	1	364.20
gca	A	1865	837.70
gcg	A	9	206.00
gat	D	0	0.54
gac	D	1	0.46
gaa	E	1	0.56
gag	E	0	0.44
ggt	G	0	0.14
ggc	G	0	0.13
gga	G	1	0.44
ggg	G	0	0.29
---	--	------	------
mPD		0.50	1.4
		nPD:	0.35
		N. weight:	1.3
		Sc. PD:	0.19
		Sc. rank:	1385.3

,

PA

Pos.	344	obs:	exp:
aaa	K	3	1.71
aag	K	0	1.29
gaa	E	2787	1605.00
gag	E	77	1259.00
ggt	G	0	0.14
ggc	G	0	0.13
gga	G	1	0.44
ggg	G	0	0.29
---	--	------	------
mPD		0.055	0.50
		nPD:	0.11
		N. weight:	0.62
		Sc. PD:	-0.055
		Sc. rank:	-29.0

,

PA

Pos.	345	obs:	exp:
ttt	F	1	1.25
ttc	F	2	1.75
tta	L	0	264.20
ttg	L	0	542.90
ctt	L	643	567.50
ctc	L	2193	457.20
cta	L	19	423.00
ctg	L	3	603.20
att	I	0	2.57
atc	I	7	1.87
ata	I	0	2.56
---	--	------	------
mPD		0.37	1.1
		nPD:	0.33
		N. weight:	1.6
		Sc. PD:	0.2
		Sc. rank:	1645.7

,

PA

Pos.	346	obs:	exp:
cct	P	0	0.27
ccc	P	0	0.18
cca	P	1	0.41
ccg	P	0	0.14
cat	H	0	0.58
cac	H	1	0.42
caa	Q	2798	1429.00
cag	Q	64	1433.00
cgt	R	0	0.15
cgc	R	0	0.21
cga	R	4	0.40
cgg	R	0	0.33
aga	R	0	1.87
agg	R	0	1.04
---	--	------	------
mPD		0.048	0.51
		nPD:	0.09
		N. weight:	0.79
		Sc. PD:	-0.083
		Sc. rank:	-209.3

,

PA

Pos.	347	obs:	exp:
aat	N	2	3.82
aac	N	5	3.18
gat	D	1292	1539.00
gac	D	1559	1312.00
gaa	E	9	5.05
gag	E	0	3.95
ggt	G	0	0.14
ggc	G	1	0.13
gga	G	0	0.44
ggg	G	0	0.29
---	--	------	------
mPD		0.50	0.51
		nPD:	1.
		N. weight:	0.026
		Sc. PD:	0.021
		Sc. rank:	48.8

,

PA

Pos.	348	obs:	exp:
ttt	F	2	0.83
ttc	F	0	1.17
tta	L	0	4.53
ttg	L	0	9.31
ctt	L	49	9.73
ctc	L	0	7.84
cta	L	0	7.25
ctg	L	0	10.34
att	I	2775	1026.00
atc	I	21	748.50
ata	I	1	1022.00
atg	M	5	5.00
gtt	V	13	3.23
gtc	V	2	3.16
gta	V	0	3.20
gtg	V	0	5.42
---	--	------	------
mPD		0.065	0.71
		nPD:	0.09
		N. weight:	1.3
		Sc. PD:	-0.14
		Sc. rank:	-389.4

,

PA

Pos.	349	obs:	exp:
gct	A	0	1.51
gcc	A	0	1.16
gca	A	5	2.67
gcg	A	1	0.66
gaa	E	2470	1602.00
gag	E	388	1256.00
ggt	G	0	0.58
ggc	G	0	0.53
gga	G	3	1.75
ggg	G	1	1.14
---	--	------	------
mPD		0.24	0.50
		nPD:	0.48
		N. weight:	0.29
		Sc. PD:	0.08
		Sc. rank:	405.4

,

PA

Pos.	350	obs:	exp:
tct	S	0	2.00
tcc	S	0	1.74
tca	S	0	2.72
tcg	S	0	0.72
cat	H	5	2.89
cac	H	0	2.11
att	I	0	0.37
atc	I	1	0.27
ata	I	0	0.37
act	T	2	0.52
acc	T	0	0.45
aca	T	0	0.84
acg	T	0	0.19
aat	N	2800	1552.00
aac	N	46	1294.00
aaa	K	2	1.14
aag	K	0	0.86
agt	S	12	2.60
agc	S	0	2.21
---	--	------	------
mPD		0.048	0.52
		nPD:	0.09
		N. weight:	0.71
		Sc. PD:	-0.076
		Sc. rank:	-208.8

,

PA

Pos.	351	obs:	exp:
aaa	K	0	0.57
aag	K	1	0.43
gct	A	0	0.25
gcc	A	0	0.19
gca	A	0	0.45
gcg	A	1	0.11
gat	D	41	22.14
gac	D	0	18.86
gaa	E	945	1578.00
gag	E	1870	1237.00
ggt	G	0	1.45
ggc	G	0	1.31
gga	G	1	4.38
ggg	G	9	2.86
---	--	------	------
mPD		0.47	0.52
		nPD:	0.91
		N. weight:	0.15
		Sc. PD:	0.11
		Sc. rank:	283.6

,

PA

Pos.	352	obs:	exp:
tat	Y	1	0.48
tac	Y	0	0.52
aat	N	5	2.73
aac	N	0	2.27
gat	D	881	484.30
gac	D	16	412.70
gaa	E	19	1100.00
gag	E	1943	862.10
ggt	G	2	0.43
ggc	G	0	0.39
gga	G	0	1.31
ggg	G	1	0.86
---	--	------	------
mPD		0.45	0.72
		nPD:	0.63
		N. weight:	0.92
		Sc. PD:	0.39
		Sc. rank:	1502.4

,

PA

Pos.	353	obs:	exp:
cgt	R	0	4.45
cgc	R	0	6.37
cga	R	0	11.99
cgg	R	0	9.72
att	I	0	0.73
atc	I	0	0.54
ata	I	2	0.73
aaa	K	2666	1563.00
aag	K	79	1182.00
aga	R	119	55.55
agg	R	0	30.92
gaa	E	2	1.12
gag	E	0	0.88
---	--	------	------
mPD		0.14	0.60
		nPD:	0.23
		N. weight:	0.61
		Sc. PD:	0.015
		Sc. rank:	398.1

,

PA

Pos.	354	obs:	exp:
ttt	F	225	97.62
ttc	F	9	136.40
tta	L	0	0.83
ttg	L	0	1.71
ctt	L	0	1.79
ctc	L	9	1.44
cta	L	0	1.33
ctg	L	0	1.90
att	I	596	950.80
atc	I	1991	693.30
ata	I	4	946.90
act	T	3	2.10
acc	T	5	1.80
aca	T	0	3.35
acg	T	0	0.75
gtt	V	0	5.59
gtc	V	26	5.47
gta	V	0	5.54
gtg	V	0	9.39
---	--	------	------
mPD		0.59	0.84
		nPD:	0.7
		N. weight:	0.94
		Sc. PD:	0.46
		Sc. rank:	1636.2

,

PA

Pos.	355	obs:	exp:
tta	L	0	0.09
ttg	L	0	0.19
tct	S	0	0.17
tcc	S	0	0.14
tca	S	1	0.23
tcg	S	0	0.06
ctt	L	0	0.20
ctc	L	0	0.16
cta	L	1	0.15
ctg	L	0	0.21
cct	P	0	783.20
ccc	P	3	517.60
cca	P	2748	1163.00
ccg	P	115	401.80
agt	S	0	0.22
agc	S	0	0.18
---	--	------	------
mPD		0.080	0.71
		nPD:	0.11
		N. weight:	1.
		Sc. PD:	-0.089
		Sc. rank:	-10.5

,

PA			
Pos.	356	obs:	exp:
cgt	R	0	0.26
cgc	R	0	0.37
cga	R	0	0.71
cgg	R	0	0.57
aaa	K	2760	1629.00
aag	K	101	1232.00
aga	R	6	3.27
agg	R	1	1.82
---	--	------	------
mPD		0.073	0.50
		nPD:	0.15
		N. weight:	0.56
		Sc. PD:	-0.029
		Sc. rank:	103.7

,

PA			
Pos.	357	obs:	exp:
att	I	0	6.61
atc	I	0	4.82
ata	I	18	6.58
act	T	0	742.70
acc	T	2	638.40
aca	T	2797	1185.00
acg	T	34	266.50
aaa	K	2	1.14
aag	K	0	0.86
gtt	V	0	2.58
gtc	V	0	2.52
gta	V	12	2.56
gtg	V	0	4.34
gct	A	0	0.76
gcc	A	0	0.58
gca	A	3	1.34
gcg	A	0	0.33
---	--	------	------
mPD		0.057	0.73
		nPD:	0.08
		N. weight:	1.1
		Sc. PD:	-0.13
		Sc. rank:	-587.9

,

PA			
Pos.	358	obs:	exp:
cgt	R	0	0.07
cgc	R	0	0.11
cga	R	0	0.20
cgg	R	0	0.16
aaa	K	242	1632.00
aag	K	2624	1234.00
aga	R	0	0.93
agg	R	2	0.52
---	--	------	------
mPD		0.16	0.49
		nPD:	0.32
		N. weight:	0.71
		Sc. PD:	0.08
		Sc. rank:	689.1

,

PA			
Pos.	359	obs:	exp:
tct	S	0	0.67
tcc	S	0	0.58
tca	S	0	0.91
tcg	S	0	0.24
cat	H	0	0.58
cac	H	1	0.42
act	T	0	0.26
acc	T	1	0.23
aca	T	0	0.42
acg	T	0	0.09
aat	N	42	1560.00
aac	N	2819	1301.00
aaa	K	1	0.57
aag	K	0	0.43
agt	S	0	0.87
agc	S	4	0.74
---	--	------	------
mPD		0.034	0.50
		nPD:	0.07
		N. weight:	0.95
		Sc. PD:	-0.13
		Sc. rank:	-595.3

,

PA			
Pos.	360	obs:	exp:
tta	L	0	0.09
ttg	L	0	0.19
ctt	L	0	0.20
ctc	L	0	0.16
cta	L	0	0.15
ctg	L	1	0.21
att	I	0	0.73
atc	I	0	0.54
ata	I	2	0.73
atg	M	2863	2863.00
gtt	V	0	0.43
gtc	V	0	0.42
gta	V	0	0.43
gtg	V	2	0.72
---	--	------	------
mPD		0.0035	0.0048
		nPD:	0.73
		N. weight:	0.0026
		Sc. PD:	0.0013
		Sc. rank:	4.7

,

PA			
Pos.	361	obs:	exp:
cgt	R	0	0.49
cgc	R	0	0.70
cga	R	0	1.31
cgg	R	0	1.06
aaa	K	556	1625.00
aag	K	2298	1229.00
aga	R	0	6.07
agg	R	13	3.38
gaa	E	1	0.56
gag	E	0	0.44
---	--	------	------
mPD		0.32	0.50
		nPD:	0.64
		N. weight:	0.4
		Sc. PD:	0.17
		Sc. rank:	664.6

,

PA			
Pos.	362	obs:	exp:
cgt	R	0	1.12
cgc	R	0	1.61
cga	R	0	3.02
cgg	R	0	2.45
aaa	K	2799	1616.00
aag	K	39	1222.00
aga	R	30	14.01
agg	R	0	7.79
---	--	------	------
mPD		0.048	0.52
		nPD:	0.09
		N. weight:	0.66
		Sc. PD:	-0.071
		Sc. rank:	-197.8

,

PA			
Pos.	363	obs:	exp:
tct	S	0	0.17
tcc	S	0	0.14
tca	S	1	0.23
tcg	S	0	0.06
act	T	3	751.30
acc	T	4	645.80
aca	T	2815	1199.00
acg	T	44	269.70
agt	S	0	0.22
agc	S	0	0.18
gct	A	0	0.25
gcc	A	0	0.19
gca	A	1	0.45
gcg	A	0	0.11
---	--	------	------
mPD		0.036	0.70
		nPD:	0.05
		N. weight:	1.1
		Sc. PD:	-0.16
		Sc. rank:	-897.2

,

PA			
Pos.	364	obs:	exp:
tct	S	0	449.40
tcc	S	0	389.90
tca	S	0	610.30
tcg	S	0	162.40
tgt	C	0	0.77
tgc	C	2	1.23
att	I	0	0.73
atc	I	2	0.54
ata	I	0	0.73
agt	S	39	583.90
agc	S	2653	496.10
gtt	V	0	0.43
gtc	V	2	0.42
gta	V	0	0.43
gtg	V	0	0.72
gct	A	0	0.25
gcc	A	1	0.19
gca	A	0	0.45
gcg	A	0	0.11
ggt	G	2	24.44
ggc	G	167	22.20
gga	G	0	74.05
ggg	G	0	48.31
---	--	------	------
mPD		0.15	1.7
		nPD:	0.08
		N. weight:	2.2
		Sc. PD:	-0.25
		Sc. rank:	-886.2

,

PA			
Pos.	365	obs:	exp:
cct	P	0	0.27
ccc	P	0	0.18
cca	P	1	0.41
ccg	P	0	0.14
cat	H	4	4.05
cac	H	3	2.95
caa	Q	2349	1428.00
cag	Q	511	1432.00
---	--	------	------
mPD		0.30	0.50
		nPD:	0.59
		N. weight:	0.3
		Sc. PD:	0.12
		Sc. rank:	474.3

,

PA			
Pos.	366	obs:	exp:
tta	L	1159	265.20
ttg	L	1660	544.80
ctt	L	0	569.40
ctc	L	0	458.80
cta	L	8	424.40
ctg	L	41	605.30
---	--	------	------
mPD		0.52	1.1
		nPD:	0.46
		N. weight:	1.6
		Sc. PD:	0.41
		Sc. rank:	2176.8

,

PA			
Pos.	367	obs:	exp:
cgt	R	0	11.56
cgc	R	0	16.54
cga	R	0	31.14
cgg	R	0	25.23
atg	M	2	2.00
aat	N	1	0.55
aac	N	0	0.45
aaa	K	48	1455.00
aag	K	2508	1101.00
aga	R	19	144.30
agg	R	290	80.28
---	--	------	------
mPD		0.24	0.75
		nPD:	0.32
		N. weight:	1.
		Sc. PD:	0.12
		Sc. rank:	1046.5

,

PA			
Pos.	368	obs:	exp:
tgg	W	2867	2867.00
cgt	R	0	0.04
cgc	R	1	0.05
cga	R	0	0.10
cgg	R	0	0.08
aga	R	0	0.47
agg	R	0	0.26
---	--	------	------
mPD		0.0014	0.0012
		nPD:	1.21
		N. weight:	0.0014
		Sc. PD:	0.0013
		Sc. rank:	2.5

,

PA			
Pos.	369	obs:	exp:
act	T	0	3.41
acc	T	0	2.93
aca	T	13	5.44
acg	T	0	1.22
gtt	V	0	30.97
gtc	V	0	30.30
gta	V	144	30.70
gtg	V	0	52.03
gct	A	0	683.00
gcc	A	3	524.60
gca	A	2694	1207.00
gcg	A	14	296.70
---	--	------	------
mPD		0.12	0.80
		nPD:	0.15
		N. weight:	1.1
		Sc. PD:	-0.06
		Sc. rank:	187.3

,

PA			
Pos.	370	obs:	exp:
tta	L	0	265.20
ttg	L	0	544.80
ctt	L	2363	569.40
ctc	L	494	458.80
cta	L	9	424.40
ctg	L	2	605.30
---	--	------	------
mPD		0.29	1.1
		nPD:	0.26
		N. weight:	1.6
		Sc. PD:	0.09
		Sc. rank:	1246.6

,

PA			
Pos.	371	obs:	exp:
ggt	G	2855	414.80
ggc	G	6	376.70
gga	G	2	1257.00
ggg	G	5	819.80
---	--	------	------
mPD		0.0090	0.69
		nPD:	0.01
		N. weight:	2.5
		Sc. PD:	-0.47
		Sc. rank:	-3987.8

,

PA			
Pos.	372	obs:	exp:
gat	D	6	3.24
gac	D	0	2.76
gaa	E	214	1604.00
gag	E	2648	1258.00
---	--	------	------
mPD		0.14	0.49
		nPD:	0.29
		N. weight:	0.72
		Sc. PD:	0.061
		Sc. rank:	644.5

,

PA			
Pos.	373	obs:	exp:
tct	S	0	0.67
tcc	S	0	0.58
tca	S	0	0.91
tcg	S	0	0.24
aat	N	1067	1561.00
aac	N	1795	1301.00
aaa	K	1	0.57
aag	K	0	0.43
agt	S	1	0.87
agc	S	3	0.74
gat	D	0	0.54
gac	D	1	0.46
---	--	------	------
mPD		0.47	0.50
		nPD:	0.94
		N. weight:	0.085
		Sc. PD:	0.061
		Sc. rank:	157.1

,

PA			
Pos.	374	obs:	exp:
tta	L	0	0.37
ttg	L	2	0.76
ctt	L	0	0.79
ctc	L	0	0.64
cta	L	0	0.59
ctg	L	2	0.84
cgt	R	0	0.90
cgc	R	0	1.28
cga	R	0	2.42
cgg	R	0	1.96
att	I	1	2.20
atc	I	0	1.61
ata	I	5	2.19
atg	M	2803	2803.00
act	T	0	0.52
acc	T	0	0.45
aca	T	0	0.84
acg	T	2	0.19
aga	R	0	11.20
agg	R	24	6.24
gtt	V	0	6.24
gtc	V	0	6.10
gta	V	1	6.18
gtg	V	28	10.48
---	--	------	------
mPD		0.046	0.076
		nPD:	0.6
		N. weight:	0.034
		Sc. PD:	0.013
		Sc. rank:	53.5

,

PA			
Pos.	375	obs:	exp:
gct	A	353	722.50
gcc	A	8	555.00
gca	A	2434	1277.00
gcg	A	73	313.90
---	--	------	------
mPD		0.26	0.69
		nPD:	0.38
		N. weight:	0.55
		Sc. PD:	0.099
		Sc. rank:	656.4

,

PA			
Pos.	376	obs:	exp:
cct	P	49	783.80
ccc	P	0	518.00
cca	P	2798	1164.00
ccg	P	21	402.10
---	--	------	------
mPD		0.048	0.71
		nPD:	0.07
		N. weight:	1.
		Sc. PD:	-0.14
		Sc. rank:	-649.4

,

PA			
Pos.	377	obs:	exp:
aaa	K	1	0.57
aag	K	0	0.43
gaa	E	1927	1607.00
gag	E	940	1260.00
---	--	------	------
mPD		0.44	0.49
		nPD:	0.89
		N. weight:	0.037
		Sc. PD:	0.025
		Sc. rank:	67.7

,

PA			
Pos.	378	obs:	exp:
aaa	K	2781	1633.00
aag	K	87	1235.00
---	--	------	------
mPD		0.059	0.49
		nPD:	0.12
		N. weight:	0.58
		Sc. PD:	-0.047
		Sc. rank:	9.9

,

PA			
Pos.	379	obs:	exp:
tta	L	4	0.37
ttg	L	0	0.76
ctt	L	0	0.79
ctc	L	0	0.64
cta	L	0	0.59
ctg	L	0	0.84
att	I	0	8.81
atc	I	0	6.42
ata	I	24	8.77
atg	M	3	3.00
gtt	V	1	609.80
gtc	V	2	596.40
gta	V	1954	604.50
gtg	V	878	1024.00
gct	A	0	0.50
gcc	A	0	0.39
gca	A	1	0.89
gcg	A	1	0.22
---	--	------	------
mPD		0.45	0.76
		nPD:	0.59
		N. weight:	1.
		Sc. PD:	0.39
		Sc. rank:	1606.0

,

PA			
Pos.	380	obs:	exp:
aat	N	0	0.55
aac	N	1	0.45
gat	D	115	1546.00
gac	D	2749	1318.00
ggt	G	0	0.43
ggc	G	3	0.39
gga	G	0	1.31
ggg	G	0	0.86
---	--	------	------
mPD		0.080	0.50
		nPD:	0.16
		N. weight:	0.8
		Sc. PD:	-0.033
		Sc. rank:	219.2

,

PA			
Pos.	381	obs:	exp:
ttt	F	2864	1196.00
ttc	F	4	1672.00
---	--	------	------
mPD		0.0028	0.49
		nPD:	0.01
		N. weight:	1.1
		Sc. PD:	-0.22
		Sc. rank:	-1954.8

,

PA			
Pos.	382	obs:	exp:
caa	Q	0	0.50
cag	Q	1	0.50
aaa	K	0	0.57
aag	K	1	0.43
gat	D	12	6.48
gac	D	0	5.52
gaa	E	75	1599.00
gag	E	2777	1253.00
ggt	G	0	0.29
ggc	G	0	0.26
gga	G	0	0.88
ggg	G	2	0.57
---	--	------	------
mPD		0.062	0.50
		nPD:	0.12
		N. weight:	0.92
		Sc. PD:	-0.071
		Sc. rank:	33.0

,

PA			
Pos.	383	obs:	exp:
aat	N	1	1.09
aac	N	1	0.91
gat	D	252	1545.00
gac	D	2609	1316.00
gaa	E	0	2.24
gag	E	4	1.76
ggt	G	0	0.14
ggc	G	1	0.13
gga	G	0	0.44
ggg	G	0	0.29
---	--	------	------
mPD		0.17	0.50
		nPD:	0.33
		N. weight:	0.62
		Sc. PD:	0.079
		Sc. rank:	647.2

,

PA			
Pos.	384	obs:	exp:
tat	Y	0	0.96
tac	Y	2	1.04
tgt	C	204	1109.00
tgc	C	2662	1757.00
---	--	------	------
mPD		0.13	0.48
		nPD:	0.28
		N. weight:	0.35
		Sc. PD:	0.028
		Sc. rank:	307.9

,

PA			
Pos.	385	obs:	exp:
caa	Q	2	1.00
cag	Q	0	1.00
cgt	R	0	0.52
cgc	R	0	0.75
cga	R	0	1.41
cgg	R	0	1.14
att	I	0	0.37
atc	I	0	0.27
ata	I	1	0.37
aaa	K	2656	1623.00
aag	K	194	1227.00
aga	R	14	6.54
agg	R	0	3.64
gaa	E	1	0.56
gag	E	0	0.44
---	--	------	------
mPD		0.14	0.51
		nPD:	0.27
		N. weight:	0.45
		Sc. PD:	0.032
		Sc. rank:	369.7

,

PA Pos. 386		obs:	exp:
aat	N	8	4.36
aac	N	0	3.64
gat	D	2661	1538.00
gac	D	187	1310.00
ggt	G	11	1.74
ggc	G	1	1.58
gga	G	0	5.26
ggg	G	0	3.43
---	--	------	------
mPD		0.14	0.51
		nPD:	0.27
		N. weight:	0.52
		Sc. PD:	0.033
		Sc. rank:	428.3

,

PA Pos. 387		obs:	exp:
att	I	157	59.81
atc	I	6	43.62
ata	I	0	59.57
gtt	V	2633	581.60
gtc	V	69	568.90
gta	V	0	576.60
gtg	V	2	977.00
gct	A	1	0.25
gcc	A	0	0.19
gca	A	0	0.45
gcg	A	0	0.11
---	--	------	------
mPD		0.16	0.85
		nPD:	0.19
		N. weight:	1.8
		Sc. PD:	-0.022
		Sc. rank:	872.1

,

PA Pos. 388		obs:	exp:
tct	S	0	455.00
tcc	S	0	394.90
tca	S	0	618.00
tcg	S	0	164.50
tgt	C	0	0.39
tgc	C	1	0.61
cgt	R	0	2.51
cgc	R	1	3.59
cga	R	0	6.75
cgg	R	0	5.47
att	I	0	1.47
atc	I	4	1.07
ata	I	0	1.46
aat	N	2	12.00
aac	N	20	10.00
agt	S	333	591.30
agc	S	2393	502.40
aga	R	66	31.28
agg	R	0	17.41
gat	D	0	1.08
gac	D	2	0.92
ggt	G	6	6.65
ggc	G	37	6.04
gga	G	3	20.16
ggg	G	0	13.15
---	--	------	------
mPD		0.30	1.7
		nPD:	0.18
		N. weight:	1.7
		Sc. PD:	-0.038
		Sc. rank:	648.5

,

PA Pos. 389		obs:	exp:
gat	D	2822	1549.00
gac	D	46	1319.00
---	--	------	------
mPD		0.032	0.50
		nPD:	0.06
		N. weight:	0.71
		Sc. PD:	-0.097
		Sc. rank:	-469.8

,

PA Pos. 390		obs:	exp:
tta	L	23	265.20
ttg	L	10	544.80
ctt	L	8	569.40
ctc	L	1	458.80
cta	L	2739	424.40
ctg	L	87	605.30
---	--	------	------
mPD		0.094	1.1
		nPD:	0.08
		N. weight:	2.2
		Sc. PD:	-0.26
		Sc. rank:	-935.2

,

PA Pos. 391		obs:	exp:
caa	Q	2	1.00
cag	Q	0	1.00
cgt	R	0	59.02
cgc	R	0	84.39
cga	R	170	158.90
cgg	R	0	128.80
att	I	0	0.37
atc	I	0	0.27
ata	I	1	0.37
act	T	0	4.72
acc	T	0	4.06
aca	T	18	7.53
acg	T	0	1.69
aaa	K	1242	723.20
aag	K	28	546.80
aga	R	1373	736.20
agg	R	34	409.70
---	--	------	------
mPD		0.66	1.3
		nPD:	0.51
		N. weight:	0.65
		Sc. PD:	0.2
		Sc. rank:	930.6

,

PA Pos. 392		obs:	exp:
caa	Q	316	1432.00
cag	Q	2552	1436.00
---	--	------	------
mPD		0.20	0.50
		nPD:	0.39
		N. weight:	0.46
		Sc. PD:	0.087
		Sc. rank:	555.2

,

PA Pos. 393		obs:	exp:
tat	Y	2036	1376.00
tac	Y	832	1492.00
---	--	------	------
mPD		0.41	0.50
		nPD:	0.83
		N. weight:	0.15
		Sc. PD:	0.09
		Sc. rank:	269.0

,

PA Pos. 394		obs:	exp:
cat	H	0	1.16
cac	H	2	0.84
aat	N	1	21.27
aac	N	38	17.73
gtt	V	0	0.22
gtc	V	1	0.21
gta	V	0	0.21
gtg	V	0	0.36
gct	A	0	0.25
gcc	A	1	0.19
gca	A	0	0.45
gcg	A	0	0.11
gat	D	34	1515.00
gac	D	2772	1291.00
gaa	E	8	5.61
gag	E	2	4.39
ggt	G	1	1.30
ggc	G	8	1.18
gga	G	0	3.94
ggg	G	0	2.57
---	--	------	------
mPD		0.068	0.54
		nPD:	0.13
		N. weight:	0.95
		Sc. PD:	-0.071
		Sc. rank:	47.0

,

PA Pos. 395		obs:	exp:
tct	S	0	470.70
tcc	S	0	408.50
tca	S	0	639.30
tcg	S	0	170.10
tgt	C	1	0.39
tgc	C	0	0.61
att	I	1	0.37
atc	I	0	0.27
ata	I	0	0.37
aat	N	42	22.90
aac	N	0	19.10
agt	S	2694	611.70
agc	S	126	519.70
ggt	G	4	0.58
ggc	G	0	0.53
gga	G	0	1.75
ggg	G	0	1.14
---	--	------	------
mPD		0.12	1.7
		nPD:	0.07
		N. weight:	1.8
		Sc. PD:	-0.23
		Sc. rank:	-1036.5

,

PA			
Pos.	396	obs:	exp:
aat	N	13	7.09
aac	N	0	5.91
gtt	V	0	0.22
gtc	V	0	0.21
gta	V	1	0.21
gtg	V	0	0.36
gat	D	1916	1530.00
gac	D	918	1304.00
gaa	E	12	8.41
gag	E	3	6.59
ggt	G	5	0.72
ggc	G	0	0.66
gga	G	0	2.19
ggg	G	0	1.43
---	--	------	------
mPD		0.46	0.52
		nPD:	0.88
		N. weight:	0.063
		Sc. PD:	0.042
		Sc. rank:	115.5

PA			
Pos.	397	obs:	exp:
aaa	K	2	1.14
aag	K	0	0.86
gat	D	4	2.16
gac	D	0	1.84
gaa	E	2711	1603.00
gag	E	149	1257.00
ggt	G	0	0.29
ggc	G	0	0.26
gga	G	2	0.88
ggg	G	0	0.57
---	--	------	------
mPD		0.10	0.50
		nPD:	0.21
		N. weight:	0.52
		Sc. PD:	0.004
		Sc. rank:	294.4

PA			
Pos.	398	obs:	exp:
tct	S	0	0.17
tcc	S	0	0.14
tca	S	1	0.23
tcg	S	0	0.06
cct	P	22	783.50
ccc	P	5	517.80
cca	P	2760	1164.00
ccg	P	80	402.00
agt	S	0	0.22
agc	S	0	0.18
---	--	------	------
mPD		0.073	0.71
		nPD:	0.1
		N. weight:	1.
		Sc. PD:	-0.097
		Sc. rank:	-143.1

PA			
Pos.	399	obs:	exp:
aaa	K	7	43.84
aag	K	70	33.16
gat	D	4	69.11
gac	D	124	58.89
gaa	E	829	1487.00
gag	E	1824	1166.00
ggt	G	0	1.45
ggc	G	0	1.31
gga	G	1	4.38
ggg	G	9	2.86
---	--	------	------
mPD		0.53	0.60
		nPD:	0.89
		N. weight:	0.22
		Sc. PD:	0.15
		Sc. rank:	401.6

PA			
Pos.	400	obs:	exp:
ttt	F	7	2.92
ttc	F	0	4.08
tta	L	0	0.46
ttg	L	0	0.95
tct	S	1401	239.90
tcc	S	32	208.20
tca	S	4	325.80
tcg	S	0	86.70
tat	Y	1	0.48
tac	Y	0	0.52
ctt	L	3	0.99
ctc	L	2	0.80
cta	L	0	0.74
ctg	L	0	1.06
cct	P	1279	358.60
ccc	P	27	237.00
cca	P	3	532.50
ccg	P	3	183.90
caa	Q	20	10.48
cag	Q	1	10.52
act	T	63	16.52
acc	T	0	14.20
aca	T	0	26.36
acg	T	0	5.93
agt	S	0	311.70
agc	S	0	264.80
gct	A	22	5.54
gcc	A	0	4.26
gca	A	0	9.79
gcg	A	0	2.41
---	--	------	------
mPD		0.61	1.7
		nPD:	0.36
		N. weight:	1.9
		Sc. PD:	0.3
		Sc. rank:	2167.8

PA			
Pos.	401	obs:	exp:
tct	S	0	0.17
tcc	S	0	0.14
tca	S	0	0.23
tcg	S	0	0.06
cgt	R	0	105.30
cgc	R	0	150.60
cga	R	1	283.60
cgg	R	0	229.80
aaa	K	50	30.18
aag	K	3	22.82
agt	S	1	0.22
agc	S	0	0.18
aga	R	2337	1314.00
agg	R	476	731.10
---	--	------	------
mPD		0.32	0.99
		nPD:	0.32
		N. weight:	0.54
		Sc. PD:	0.063
		Sc. rank:	544.6

PA			
Pos.	402	obs:	exp:
tct	S	1	478.60
tcc	S	1	415.30
tca	S	2404	650.00
tcg	S	461	173.00
aaa	K	1	0.57
aag	K	0	0.43
agt	S	0	621.90
agc	S	0	528.40
---	--	------	------
mPD		0.27	1.7
		nPD:	0.17
		N. weight:	1.7
		Sc. PD:	-0.059
		Sc. rank:	545.7

PA			
Pos.	403	obs:	exp:
tta	L	1129	264.60
ttg	L	7	543.70
ctt	L	0	568.20
ctc	L	0	457.80
cta	L	1689	423.60
ctg	L	37	604.10
att	I	0	2.20
atc	I	0	1.61
ata	I	6	2.19
---	--	------	------
mPD		0.51	1.1
		nPD:	0.45
		N. weight:	1.8
		Sc. PD:	0.45
		Sc. rank:	2424.5

PA			
Pos.	404	obs:	exp:
tct	S	0	188.50
tcc	S	0	163.50
tca	S	1123	255.90
tcg	S	6	68.11
cct	P	0	0.55
ccc	P	0	0.36
cca	P	2	0.81
ccg	P	0	0.28
act	T	0	1.57
acc	T	0	1.35
aca	T	6	2.51
acg	T	0	0.56
agt	S	0	244.90
agc	S	0	208.10
gtt	V	0	0.43
gtc	V	0	0.42
gta	V	2	0.43
gtg	V	0	0.72
gct	A	1	435.60
gcc	A	0	334.60
gca	A	1702	769.60
gcg	A	26	189.20
---	--	------	------
mPD		0.51	1.5
		nPD:	0.33
		N. weight:	1.4
		Sc. PD:	0.17
		Sc. rank:	1417.2

PA			
Pos.	405	obs:	exp:
tta	L	0	0.18
ttg	L	2	0.38
tct	S	0	470.20
tcc	S	0	408.10
tca	S	0	638.60
tcg	S	0	170.00
tgt	C	4	11.22
tgc	C	25	17.78
ctt	L	0	0.40
ctc	L	0	0.32
cta	L	0	0.30
ctg	L	0	0.42
aat	N	0	4.36
aac	N	8	3.64
agt	S	27	611.00
agc	S	2790	519.10
ggt	G	1	1.74
ggc	G	11	1.58
gga	G	0	5.26
ggg	G	0	3.43
---	--	------	------
mPD		0.060	1.7
		nPD:	0.04
		N. weight:	2.2
		Sc. PD:	-0.35
		Sc. rank:	-2365.1

PA			
Pos.	406	obs:	exp:
tgg	W	2864	2864.00
cgt	R	0	0.11
cgc	R	0	0.16
cga	R	0	0.30
cgg	R	1	0.24
aga	R	0	1.40
agg	R	2	0.78
ggt	G	0	0.14
ggc	G	0	0.13
gga	G	1	0.44
ggg	G	0	0.29
---	--	------	------
mPD		0.0035	0.0047
		nPD:	0.75
		N. weight:	0.0023
		Sc. PD:	0.0012
		Sc. rank:	4.1

PA			
Pos.	407	obs:	exp:
ttt	F	1	0.42
ttc	F	0	0.58
att	I	2064	1037.00
atc	I	762	756.50
ata	I	1	1033.00
act	T	2	0.79
acc	T	1	0.68
aca	T	0	1.26
acg	T	0	0.28
gtt	V	31	7.96
gtc	V	6	7.78
gta	V	0	7.89
gtg	V	0	13.37
---	--	------	------
mPD		0.42	0.69
		nPD:	0.61
		N. weight:	0.68
		Sc. PD:	0.27
		Sc. rank:	1090.0

PA			
Pos.	408	obs:	exp:
caa	Q	110	1432.00
cag	Q	2758	1436.00
---	--	------	------
mPD		0.074	0.50
		nPD:	0.15
		N. weight:	0.7
		Sc. PD:	-0.037
		Sc. rank:	128.4

PA			
Pos.	409	obs:	exp:
tct	S	0	472.40
tcc	S	0	409.90
tca	S	0	641.60
tcg	S	0	170.70
act	T	0	0.26
acc	T	1	0.23
aca	T	0	0.42
acg	T	0	0.09
aat	N	19	14.72
aac	N	8	12.28
agt	S	1547	613.80
agc	S	1283	521.50
ggt	G	8	1.45
ggc	G	2	1.31
gga	G	0	4.38
ggg	G	0	2.86
---	--	------	------
mPD		0.52	1.7
		nPD:	0.31
		N. weight:	1.2
		Sc. PD:	0.13
		Sc. rank:	1169.0

PA			
Pos.	410	obs:	exp:
gat	D	1	0.54
gac	D	0	0.46
gaa	E	2825	1607.00
gag	E	41	1259.00
ggt	G	0	0.14
ggc	G	0	0.13
gga	G	1	0.44
ggg	G	0	0.29
---	--	------	------
mPD		0.030	0.49
		nPD:	0.06
		N. weight:	0.68
		Sc. PD:	-0.094
		Sc. rank:	-469.3

PA			
Pos.	411	obs:	exp:
ttt	F	28	1196.00
ttc	F	2838	1670.00
tct	S	0	0.17
tcc	S	1	0.14
tca	S	0	0.23
tcg	S	0	0.06
tat	Y	0	0.48
tac	Y	1	0.52
agt	S	0	0.22
agc	S	0	0.18
---	--	------	------
mPD		0.021	0.49
		nPD:	0.04
		N. weight:	0.65
		Sc. PD:	-0.1
		Sc. rank:	-638.9

,

PA			
Pos.	412	obs:	exp:
aat	N	37	1564.00
aac	N	2831	1304.00
---	--	------	------
mPD		0.025	0.50
		nPD:	0.05
		N. weight:	0.95
		Sc. PD:	-0.14
		Sc. rank:	-824.9

,

PA			
Pos.	413	obs:	exp:
aat	N	0	1.64
aac	N	3	1.36
aaa	K	1307	1631.00
aag	K	1558	1234.00
---	--	------	------
mPD		0.50	0.49
		nPD:	1.01
		N. weight:	0.037
		Sc. PD:	0.03
		Sc. rank:	69.5

,

PA			
Pos.	414	obs:	exp:
cct	P	0	0.27
ccc	P	0	0.18
cca	P	1	0.41
ccg	P	0	0.14
gct	A	27	722.20
gcc	A	1	554.80
gca	A	2829	1276.00
gcg	A	10	313.80
---	--	------	------
mPD		0.027	0.69
		nPD:	0.04
		N. weight:	0.99
		Sc. PD:	-0.16
		Sc. rank:	-993.9

,

PA			
Pos.	415	obs:	exp:
tgt	C	575	1110.00
tgc	C	2292	1757.00
tgg	W	1	1.00
---	--	------	------
mPD		0.32	0.47
		nPD:	0.68
		N. weight:	0.11
		Sc. PD:	0.051
		Sc. rank:	185.8

,

PA			
Pos.	416	obs:	exp:
aaa	K	1	0.57
aag	K	0	0.43
gaa	E	2845	1607.00
gag	E	22	1260.00
---	--	------	------
mPD		0.016	0.49
		nPD:	0.03
		N. weight:	0.71
		Sc. PD:	-0.12
		Sc. rank:	-832.7

,

PA			
Pos.	417	obs:	exp:
tta	L	45	265.10
ttg	L	2338	544.70
ctt	L	0	569.20
ctc	L	0	458.60
cta	L	26	424.30
ctg	L	458	605.10
gtt	V	0	0.22
gtc	V	0	0.21
gta	V	0	0.21
gtg	V	1	0.36
---	--	------	------
mPD		0.33	1.1
		nPD:	0.29
		N. weight:	1.5
		Sc. PD:	0.13
		Sc. rank:	1335.8

,

PA			
Pos.	418	obs:	exp:
tct	S	0	1.50
tcc	S	0	1.30
tca	S	9	2.04
tcg	S	0	0.54
act	T	39	749.50
acc	T	36	644.20
aca	T	2782	1196.00
acg	T	2	269.00
agt	S	0	1.95
agc	S	0	1.66
---	--	------	------
mPD		0.059	0.70
		nPD:	0.08
		N. weight:	0.99
		Sc. PD:	-0.11
		Sc. rank:	-447.3

,

PA			
Pos.	419	obs:	exp:
aat	N	9	4.91
aac	N	0	4.09
gat	D	2406	1536.00
gac	D	439	1309.00
gaa	E	11	6.73
gag	E	1	5.27
ggt	G	2	0.29
ggc	G	0	0.26
gga	G	0	0.88
ggg	G	0	0.57
---	--	------	------
mPD		0.27	0.51
		nPD:	0.54
		N. weight:	0.29
		Sc. PD:	0.095
		Sc. rank:	428.4

,

PA			
Pos.	420	obs:	exp:
tct	S	89	478.70
tcc	S	0	415.40
tca	S	338	650.20
tcg	S	2441	173.00
agt	S	0	622.10
agc	S	0	528.50
---	--	------	------
mPD		0.26	1.7
		nPD:	0.16
		N. weight:	2.8
		Sc. PD:	-0.12
		Sc. rank:	731.0

,

PA			
Pos.	421	obs:	exp:
tct	S	0	450.20
tcc	S	0	390.70
tca	S	0	611.40
tcg	S	0	162.70
att	I	8	49.17
atc	I	126	35.86
ata	I	0	48.97
agt	S	2654	585.00
agc	S	43	497.00
ggt	G	37	5.35
ggc	G	0	4.86
gga	G	0	16.21
ggg	G	0	10.58
---	--	------	------
mPD		0.23	1.7
		nPD:	0.13
		N. weight:	1.9
		Sc. PD:	-0.13
		Sc. rank:	178.1

,

PA			
Pos.	422	obs:	exp:
tgg	W	2868	2868.00
---	--	------	------
mPD		0	0
		nPD:	1.
		N. weight:	0.
		Sc. PD:	0
		Sc. rank:	0

,

PA			
Pos.	423	obs:	exp:
tct	S	0	0.17
tcc	S	0	0.14
tca	S	0	0.23
tcg	S	0	0.06
att	I	2690	1013.00
atc	I	8	738.80
ata	I	63	1009.00
atg	M	2	2.00
act	T	9	2.36
acc	T	0	2.03
aca	T	0	3.77
acg	T	0	0.85
agt	S	1	0.22
agc	S	0	0.18
gtt	V	92	20.43
gtc	V	0	19.99
gta	V	3	20.26
gtg	V	0	34.32
---	--	------	------
mPD		0.12	0.74
		nPD:	0.17
		N. weight:	1.2
		Sc. PD:	-0.042
		Sc. rank:	409.2

,

PA			
Pos.	424	obs:	exp:
gat	D	0	1.62
gac	D	3	1.38
gaa	E	2770	1606.00
gag	E	95	1259.00
---	--	------	------
mPD		0.066	0.49
		nPD:	0.13
		N. weight:	0.59
		Sc. PD:	-0.039
		Sc. rank:	64.4

,

PA			
Pos.	425	obs:	exp:
ttt	F	89	37.96
ttc	F	2	53.04
tta	L	0	256.80
ttg	L	3	527.60
ctt	L	2727	551.40
ctc	L	35	444.20
cta	L	4	411.00
ctg	L	8	586.10
---	--	------	------
mPD		0.099	1.1
		nPD:	0.09
		N. weight:	2.
		Sc. PD:	-0.22
		Sc. rank:	-693.2

,

PA			
Pos.	426	obs:	exp:
gat	D	2779	1545.00
gac	D	83	1317.00
gaa	E	1	3.36
gag	E	5	2.64
---	--	------	------
mPD		0.060	0.50
		nPD:	0.12
		N. weight:	0.65
		Sc. PD:	-0.052
		Sc. rank:	16.3

,

PA			
Pos.	427	obs:	exp:
gaa	E	2082	1608.00
gag	E	786	1260.00
---	--	------	------
mPD		0.40	0.49
		nPD:	0.81
		N. weight:	0.079
		Sc. PD:	0.047
		Sc. rank:	145.4

,

PA			
Pos.	428	obs:	exp:
tta	L	0	0.09
ttg	L	0	0.19
ctt	L	0	0.20
ctc	L	0	0.16
cta	L	1	0.15
ctg	L	0	0.21
att	I	1	1052.00
atc	I	1	766.90
ata	I	2864	1047.00
atg	M	1	1.00
---	--	------	------
mPD		0.0028	0.66
		nPD:	0.
		N. weight:	1.3
		Sc. PD:	-0.26
		Sc. rank:	-2326.6

,

PA			
Pos.	429	obs:	exp:
cgt	R	0	0.07
cgc	R	0	0.11
cga	R	0	0.20
cgg	R	0	0.16
aga	R	1	0.93
agg	R	1	0.52
ggt	G	0	414.50
ggc	G	0	376.50
gga	G	1607	1256.00
ggg	G	1259	819.20
---	--	------	------
mPD		0.49	0.69
		nPD:	0.72
		N. weight:	0.44
		Sc. PD:	0.22
		Sc. rank:	778.7

,

PA			
Pos.	430	obs:	exp:
aaa	K	0	0.57
aag	K	1	0.43
gct	A	0	0.25
gcc	A	0	0.19
gca	A	1	0.45
gcg	A	0	0.11
gaa	E	2486	1604.00
gag	E	376	1258.00
ggt	G	0	0.58
ggc	G	0	0.53
gga	G	2	1.75
ggg	G	2	1.14
---	--	------	------
mPD		0.23	0.50
		nPD:	0.47
		N. weight:	0.3
		Sc. PD:	0.079
		Sc. rank:	413.6

,

PA			
Pos.	431	obs:	exp:
aat	N	1	0.55
aac	N	0	0.45
gat	D	1550	1548.00
gac	D	1317	1319.00
---	--	------	------
mPD		0.50	0.50
		nPD:	1.
		N. weight:	0.0022
		Sc. PD:	0.0018
		Sc. rank:	4.2

,

PA			
Pos.	432	obs:	exp:
tta	L	0	2.31
ttg	L	0	4.75
ctt	L	0	4.96
ctc	L	0	4.00
cta	L	24	3.70
ctg	L	1	5.28
att	I	219	82.93
atc	I	5	60.48
ata	I	2	82.59
gtt	V	1801	562.70
gtc	V	652	550.40
gta	V	163	557.80
gtg	V	0	945.20
gct	A	1	0.25
gcc	A	0	0.19
gca	A	0	0.45
gcg	A	0	0.11
---	--	------	------
mPD		0.61	0.90
		nPD:	0.67
		N. weight:	1.
		Sc. PD:	0.49
		Sc. rank:	1777.4

,

PA			
Pos.	433	obs:	exp:
gct	A	2785	722.50
gcc	A	80	555.00
gca	A	0	1277.00
gcg	A	3	313.90
---	--	------	------
mPD		0.056	0.69
		nPD:	0.08
		N. weight:	1.7
		Sc. PD:	-0.2
		Sc. rank:	-765.9

,

PA			
Pos.	434	obs:	exp:
cct	P	5	783.80
ccc	P	2	518.00
cca	P	2764	1164.00
ccg	P	97	402.10
---	--	------	------
mPD		0.070	0.71
		nPD:	0.1
		N. weight:	1.
		Sc. PD:	-0.1
		Sc. rank:	-240.2

,

PA			
Pos.	435	obs:	exp:
tta	L	0	0.09
ttg	L	0	0.19
ctt	L	1	0.20
ctc	L	0	0.16
cta	L	0	0.15
ctg	L	0	0.21
cct	P	0	0.27
ccc	P	1	0.18
cca	P	0	0.41
ccg	P	0	0.14
att	I	2208	1052.00
atc	I	652	766.90
ata	I	6	1047.00
---	--	------	------
mPD		0.36	0.66
		nPD:	0.54
		N. weight:	0.7
		Sc. PD:	0.23
		Sc. rank:	1050.7

,

PA			
Pos.	436	obs:	exp:
gtt	V	0	0.22
gtc	V	0	0.21
gta	V	0	0.21
gtg	V	1	0.36
gaa	E	905	1607.00
gag	E	1962	1260.00
---	--	------	------
mPD		0.43	0.49
		nPD:	0.88
		N. weight:	0.16
		Sc. PD:	0.11
		Sc. rank:	302.3

,

PA			
Pos.	437	obs:	exp:
tat	Y	0	0.96
tac	Y	2	1.04
cat	H	25	1658.00
cac	H	2840	1207.00
aat	N	0	0.55
aac	N	1	0.45
---	--	------	------
mPD		0.019	0.49
		nPD:	0.04
		N. weight:	1.1
		Sc. PD:	-0.17
		Sc. rank:	-1078.8

,

PA			
Pos.	438	obs:	exp:
att	I	2811	1052.00
atc	I	54	766.90
ata	I	1	1047.00
gtt	V	2	0.43
gtc	V	0	0.42
gta	V	0	0.43
gtg	V	0	0.72
---	--	------	------
mPD		0.039	0.66
		nPD:	0.06
		N. weight:	1.2
		Sc. PD:	-0.17
		Sc. rank:	-890.2

,

PA			
Pos.	439	obs:	exp:
gct	A	1	722.50
gcc	A	0	555.00
gca	A	1490	1277.00
gcg	A	1377	313.90
---	--	------	------
mPD		0.50	0.69
		nPD:	0.73
		N. weight:	1.
		Sc. PD:	0.54
		Sc. rank:	1886.5

,

PA			
Pos.	440	obs:	exp:
tct	S	0	478.60
tcc	S	0	415.30
tca	S	0	650.00
tcg	S	0	173.00
aat	N	1	0.55
aac	N	0	0.45
agt	S	2726	621.90
agc	S	141	528.40
---	--	------	------
mPD		0.094	1.7
		nPD:	0.06
		N. weight:	1.8
		Sc. PD:	-0.25
		Sc. rank:	-1374.2

,

PA			
Pos.	441	obs:	exp:
cgt	R	0	0.04
cgc	R	0	0.05
cga	R	0	0.10
cgg	R	0	0.08
att	I	2	1.10
atc	I	0	0.80
ata	I	1	1.10
atg	M	2536	2536.00
act	T	0	6.82
acc	T	0	5.86
aca	T	0	10.88
acg	T	26	2.45
aaa	K	0	1.14
aag	K	2	0.86
aga	R	0	0.47
agg	R	1	0.26
gtt	V	0	64.52
gtc	V	0	63.12
gta	V	0	63.97
gtg	V	300	108.40
---	--	------	------
mPD		0.21	0.35
		nPD:	0.59
		N. weight:	0.17
		Sc. PD:	0.067
		Sc. rank:	272.8

,

PA			
Pos.	442	obs:	exp:
cgt	R	0	107.10
cgc	R	0	153.10
cga	R	0	288.30
cgg	R	0	233.60
act	T	0	0.26
acc	T	0	0.23
aca	T	1	0.42
acg	T	0	0.09
aaa	K	5	3.42
aag	K	1	2.58
aga	R	1854	1336.00
agg	R	1007	743.30
---	--	------	------
mPD		0.46	0.96
		nPD:	0.48
		N. weight:	0.43
		Sc. PD:	0.12
		Sc. rank:	597.3

,

PA			
Pos.	443	obs:	exp:
cgt	R	0	107.30
cgc	R	0	153.40
cga	R	34	288.90
cgg	R	2	234.10
aaa	K	1	0.57
aag	K	0	0.43
aga	R	1613	1338.00
agg	R	1218	744.80
---	--	------	------
mPD		0.51	0.95
		nPD:	0.54
		N. weight:	0.38
		Sc. PD:	0.13
		Sc. rank:	573.7

,

PA			
Pos.	444	obs:	exp:
tct	S	0	0.17
tcc	S	0	0.14
tca	S	0	0.23
tcg	S	0	0.06
aat	N	323	1561.00
aac	N	2540	1302.00
agt	S	0	0.22
agc	S	1	0.18
gat	D	0	2.16
gac	D	4	1.84
---	--	------	------
mPD		0.20	0.50
		nPD:	0.41
		N. weight:	0.56
		Sc. PD:	0.11
		Sc. rank:	687.4

,

PA			
Pos.	445	obs:	exp:
ttt	F	3	1.25
ttc	F	0	1.75
tat	Y	2748	1373.00
tac	Y	114	1489.00
cat	H	3	1.74
cac	H	0	1.26
---	--	------	------
mPD		0.081	0.50
		nPD:	0.16
		N. weight:	0.75
		Sc. PD:	-0.031
		Sc. rank:	218.3

,

PA			
Pos.	446	obs:	exp:
ttt	F	500	1196.00
ttc	F	2368	1672.00
---	--	------	------
mPD		0.29	0.49
		nPD:	0.59
		N. weight:	0.18
		Sc. PD:	0.07
		Sc. rank:	287.1

,

PA			
Pos.	447	obs:	exp:
cct	P	0	0.55
ccc	P	0	0.36
cca	P	2	0.81
ccg	P	0	0.28
act	T	14	750.80
acc	T	8	645.30
aca	T	2677	1198.00
acg	T	165	269.50
gct	A	0	0.50
gcc	A	0	0.39
gca	A	2	0.89
gcg	A	0	0.22
---	--	------	------
mPD		0.13	0.70
		nPD:	0.18
		N. weight:	0.92
		Sc. PD:	-0.02
		Sc. rank:	371.9

,

PA			
Pos.	448	obs:	exp:
act	T	0	2.88
acc	T	0	2.48
aca	T	0	4.60
acg	T	11	1.03
gtt	V	0	0.43
gtc	V	0	0.42
gta	V	0	0.43
gtg	V	2	0.72
gct	A	30	703.60
gcc	A	1	540.50
gca	A	145	1243.00
gcg	A	2617	305.70
gaa	E	5	34.76
gag	E	57	27.24
---	--	------	------
mPD		0.17	0.74
		nPD:	0.23
		N. weight:	2.4
		Sc. PD:	0.07
		Sc. rank:	1660.2

,

PA			
Pos.	449	obs:	exp:
aaa	K	1	0.57
aag	K	0	0.43
gaa	E	2044	1607.00
gag	E	823	1260.00
---	--	------	------
mPD		0.41	0.49
		nPD:	0.83
		N. weight:	0.068
		Sc. PD:	0.042
		Sc. rank:	123.9

,

PA			
Pos.	450	obs:	exp:
att	I	0	4.04
atc	I	0	2.94
ata	I	11	4.02
gtt	V	6	613.40
gtc	V	0	600.00
gta	V	1488	608.10
gtg	V	1358	1030.00
gct	A	0	1.26
gcc	A	0	0.97
gca	A	2	2.23
gcg	A	3	0.55
---	--	------	------
mPD		0.51	0.75
		nPD:	0.69
		N. weight:	0.79
		Sc. PD:	0.38
		Sc. rank:	1354.2

,

PA			
Pos.	451	obs:	exp:
ttt	F	0	0.42
ttc	F	1	0.58
tct	S	1151	475.40
tcc	S	1696	412.50
tca	S	0	645.60
tcg	S	1	171.80
tat	Y	1	0.48
tac	Y	0	0.52
act	T	1	2.36
acc	T	8	2.03
aca	T	0	3.77
acg	T	0	0.85
agt	S	0	617.70
agc	S	0	524.90
gct	A	0	2.27
gcc	A	9	1.74
gca	A	0	4.01
gcg	A	0	0.99
---	--	------	------
mPD		0.50	1.7
		nPD:	0.3
		N. weight:	1.6
		Sc. PD:	0.15
		Sc. rank:	1500.9

,

PA			
Pos.	452	obs:	exp:
tat	Y	3	1.44
tac	Y	0	1.56
cat	H	2786	1654.00
cac	H	71	1203.00
caa	Q	1	0.50
cag	Q	0	0.50
cgt	R	6	0.22
cgc	R	0	0.32
cga	R	0	0.60
cgg	R	0	0.49
aat	N	1	0.55
aac	N	0	0.45
aga	R	0	2.80
agg	R	0	1.56
---	--	------	------
mPD		0.056	0.50
		nPD:	0.11
		N. weight:	0.59
		Sc. PD:	-0.052
		Sc. rank:	-23.4

,

PA			
Pos.	453	obs:	exp:
ttt	F	0	0.42
ttc	F	1	0.58
tgt	C	47	1110.00
tgc	C	2820	1757.00
---	--	------	------
mPD		0.033	0.48
		nPD:	0.07
		N. weight:	0.55
		Sc. PD:	-0.072
		Sc. rank:	-324.7

,

PA			
Pos.	454	obs:	exp:
cgt	R	0	107.20
cgc	R	0	153.30
cga	R	0	288.60
cgg	R	0	233.90
atg	M	1	1.00
act	T	0	0.52
acc	T	0	0.45
aca	T	0	0.84
acg	T	2	0.19
aaa	K	1	0.57
aag	K	0	0.43
aga	R	458	1337.00
agg	R	2406	744.00
---	--	------	------
mPD		0.27	0.96
		nPD:	0.28
		N. weight:	1.1
		Sc. PD:	0.088
		Sc. rank:	962.9

,

PA			
Pos.	455	obs:	exp:
gct	A	705	722.50
gcc	A	2141	555.00
gca	A	22	1277.00
gcg	A	0	313.90
---	--	------	------
mPD		0.38	0.69
		nPD:	0.55
		N. weight:	1.3
		Sc. PD:	0.45
		Sc. rank:	1976.3

,

PA			
Pos.	456	obs:	exp:
tct	S	0	0.17
tcc	S	0	0.14
tca	S	0	0.23
tcg	S	0	0.06
act	T	2800	751.60
acc	T	39	646.00
aca	T	28	1200.00
acg	T	0	269.70
agt	S	1	0.22
agc	S	0	0.18
---	--	------	------
mPD		0.047	0.70
		nPD:	0.07
		N. weight:	1.6
		Sc. PD:	-0.21
		Sc. rank:	-1021.5

,

PA			
Pos.	457	obs:	exp:
gaa	E	2728	1608.00
gag	E	140	1260.00
---	--	------	------
mPD		0.093	0.49
		nPD:	0.19
		N. weight:	0.53
		Sc. PD:	-0.0066
		Sc. rank:	245.2

,

PA			
Pos.	458	obs:	exp:
tat	Y	77	1376.00
tac	Y	2791	1492.00
---	--	------	------
mPD		0.052	0.50
		nPD:	0.1
		N. weight:	0.71
		Sc. PD:	-0.067
		Sc. rank:	-86.0

,

PA			
Pos.	459	obs:	exp:
tta	L	0	0.55
ttg	L	0	1.14
ctt	L	0	1.19
ctc	L	0	0.96
cta	L	6	0.89
ctg	L	0	1.27
att	I	3	1048.00
atc	I	0	764.30
ata	I	2853	1044.00
atg	M	5	5.00
gtt	V	0	0.22
gtc	V	0	0.21
gta	V	1	0.21
gtg	V	0	0.36
---	--	------	------
mPD		0.010	0.67
		nPD:	0.02
		N. weight:	1.3
		Sc. PD:	-0.24
		Sc. rank:	-1995.8

,

PA			
Pos.	460	obs:	exp:
att	I	0	0.37
atc	I	0	0.27
ata	I	1	0.37
atg	M	2866	2866.00
gtt	V	0	0.22
gtc	V	0	0.21
gta	V	0	0.21
gtg	V	1	0.36
---	--	------	------
mPD		0.0014	0.0018
		nPD:	0.76
		N. weight:	0.00094
		Sc. PD:	0.00051
		Sc. rank:	1.7

,

PA			
Pos.	461	obs:	exp:
cgt	R	0	0.26
cgc	R	0	0.37
cga	R	0	0.71
cgg	R	0	0.57
aat	N	1	0.55
aac	N	0	0.45
aaa	K	217	1629.00
aag	K	2643	1231.00
aga	R	2	3.27
agg	R	5	1.82
---	--	------	------
mPD		0.15	0.50
		nPD:	0.29
		N. weight:	0.74
		Sc. PD:	0.068
		Sc. rank:	680.2

,

PA			
Pos.	462	obs:	exp:
ggt	G	31	414.80
ggc	G	0	376.70
gga	G	2754	1257.00
ggg	G	83	819.80
---	--	------	------
mPD		0.077	0.69
		nPD:	0.11
		N. weight:	0.88
		Sc. PD:	-0.077
		Sc. rank:	-37.5

,

PA			
Pos.	463	obs:	exp:
att	I	1	0.37
atc	I	0	0.27
ata	I	0	0.37
gtt	V	30	616.20
gtc	V	26	602.80
gta	V	940	610.90
gtg	V	1869	1035.00
gaa	E	0	0.56
gag	E	1	0.44
ggt	G	0	0.14
ggc	G	0	0.13
gga	G	1	0.44
ggg	G	0	0.29
---	--	------	------
mPD		0.47	0.74
		nPD:	0.64
		N. weight:	0.63
		Sc. PD:	0.27
		Sc. rank:	1030.9

,

PA			
Pos.	464	obs:	exp:
tat	Y	1138	1374.00
tac	Y	1726	1490.00
cat	H	3	1.74
cac	H	0	1.26
aat	N	0	0.55
aac	N	1	0.45
---	--	------	------
mPD		0.48	0.50
		nPD:	0.96
		N. weight:	0.021
		Sc. PD:	0.016
		Sc. rank:	39.5

,

PA			
Pos.	465	obs:	exp:
tta	L	0	0.46
ttg	L	0	0.95
ctt	L	0	0.99
ctc	L	3	0.80
cta	L	2	0.74
ctg	L	0	1.06
att	I	4	1037.00
atc	I	1	756.50
ata	I	2822	1033.00
atg	M	10	10.00
act	T	0	4.46
acc	T	0	3.83
aca	T	17	7.11
acg	T	0	1.60
gtt	V	0	1.51
gtc	V	0	1.47
gta	V	7	1.49
gtg	V	0	2.53
gct	A	0	0.50
gcc	A	0	0.39
gca	A	2	0.89
gcg	A	0	0.22
---	--	------	------
mPD		0.035	0.69
		nPD:	0.05
		N. weight:	1.3
		Sc. PD:	-0.19
		Sc. rank:	-1130.4

,

PA

Pos.	466	obs:	exp:
tct	S	0	1.34
tcc	S	0	1.16
tca	S	0	1.81
tcg	S	0	0.48
cat	H	0	0.58
cac	H	1	0.42
act	T	0	0.26
acc	T	1	0.23
aca	T	0	0.42
acg	T	0	0.09
aat	N	338	1559.00
aac	N	2520	1299.00
agt	S	0	1.74
agc	S	8	1.47
---	--	------	------
mPD		0.21	0.51
		nPD:	0.42
		N. weight:	0.54
		Sc. PD:	0.12
		Sc. rank:	683.8

,

PA

Pos.	467	obs:	exp:
act	T	11	751.80
acc	T	4	646.20
aca	T	2731	1200.00
acg	T	122	269.80
---	--	------	------
mPD		0.091	0.70
		nPD:	0.13
		N. weight:	0.97
		Sc. PD:	-0.067
		Sc. rank:	95.0

,

PA

Pos.	468	obs:	exp:
gct	A	247	722.50
gcc	A	2616	555.00
gca	A	5	1277.00
gcg	A	0	313.90
---	--	------	------
mPD		0.16	0.69
		nPD:	0.23
		N. weight:	1.7
		Sc. PD:	0.055
		Sc. rank:	1239.9

,

PA

Pos.	469	obs:	exp:
tta	L	28	264.30
ttg	L	51	543.10
ctt	L	9	567.70
ctc	L	2	457.30
cta	L	1584	423.10
ctg	L	1185	603.40
atg	M	9	9.00
---	--	------	------
mPD		0.56	1.1
		nPD:	0.49
		N. weight:	1.2
		Sc. PD:	0.36
		Sc. rank:	1747.0

,

PA

Pos.	470	obs:	exp:
tta	L	17	265.20
ttg	L	2767	544.80
ctt	L	1	569.40
ctc	L	62	458.80
cta	L	1	424.40
ctg	L	20	605.30
---	--	------	------
mPD		0.11	1.1
		nPD:	0.1
		N. weight:	2.
		Sc. PD:	-0.2
		Sc. rank:	-413.1

,

PA

Pos.	471	obs:	exp:
tct	S	0	0.33
tcc	S	0	0.29
tca	S	0	0.45
tcg	S	0	0.12
act	T	1	0.26
acc	T	0	0.23
aca	T	0	0.42
acg	T	0	0.09
aat	N	2840	1562.00
aac	N	25	1303.00
agt	S	2	0.43
agc	S	0	0.37
---	--	------	------
mPD		0.019	0.50
		nPD:	0.04
		N. weight:	0.74
		Sc. PD:	-0.12
		Sc. rank:	-753.2

,

PA

Pos.	472	obs:	exp:
gtt	V	0	0.22
gtc	V	0	0.21
gta	V	1	0.21
gtg	V	0	0.36
gct	A	47	722.20
gcc	A	1	554.80
gca	A	2805	1276.00
gcg	A	14	313.80
---	--	------	------
mPD		0.043	0.69
		nPD:	0.06
		N. weight:	0.94
		Sc. PD:	-0.13
		Sc. rank:	-641.0

,

PA

Pos.	473	obs:	exp:
ttt	F	0	0.83
ttc	F	2	1.17
tct	S	140	478.10
tcc	S	2711	414.90
tca	S	10	649.30
tcg	S	3	172.80
tat	Y	0	0.48
tac	Y	1	0.52
act	T	0	0.26
acc	T	1	0.23
aca	T	0	0.42
acg	T	0	0.09
agt	S	0	621.20
agc	S	0	527.80
---	--	------	------
mPD		0.10	1.7
		nPD:	0.06
		N. weight:	2.3
		Sc. PD:	-0.31
		Sc. rank:	-1505.9

,

PA

Pos.	474	obs:	exp:
tct	S	0	24.37
tcc	S	0	21.15
tca	S	0	33.10
tcg	S	0	8.81
tgt	C	2705	1054.00
tgc	C	17	1668.00
agt	S	145	31.67
agc	S	1	26.91
---	--	------	------
mPD		0.11	0.59
		nPD:	0.18
		N. weight:	1.2
		Sc. PD:	-0.021
		Sc. rank:	554.5

,

PA

Pos.	475	obs:	exp:
tct	S	0	0.17
tcc	S	0	0.14
tca	S	1	0.23
tcg	S	0	0.06
cct	P	0	0.82
ccc	P	0	0.54
cca	P	3	1.22
ccg	P	0	0.42
act	T	0	0.52
acc	T	0	0.45
aca	T	2	0.84
acg	T	0	0.19
agt	S	0	0.22
agc	S	0	0.18
gtt	V	0	0.22
gtc	V	0	0.21
gta	V	1	0.21
gtg	V	0	0.36
gct	A	7	720.70
gcc	A	57	553.60
gca	A	2757	1273.00
gcg	A	40	313.10
---	--	------	------
mPD		0.076	0.69
		nPD:	0.11
		N. weight:	0.88
		Sc. PD:	-0.08
		Sc. rank:	-61.3

,

PA

Pos.	476	obs:	exp:
tct	S	0	1.00
tcc	S	6	0.87
tca	S	0	1.36
tcg	S	0	0.36
agt	S	0	1.30
agc	S	0	1.11
gtt	V	0	0.22
gtc	V	1	0.21
gta	V	0	0.21
gtg	V	0	0.36
gct	A	164	720.70
gcc	A	2482	553.60
gca	A	47	1273.00
gcg	A	168	313.10
---	--	------	------
mPD		0.24	0.70
		nPD:	0.35
		N. weight:	1.5
		Sc. PD:	0.22
		Sc. rank:	1640.0

,

PA

Pos.	477	obs:	exp:
tta	L	0	0.09
ttg	L	0	0.19
ctt	L	0	0.20
ctc	L	0	0.16
cta	L	0	0.15
ctg	L	1	0.21
atg	M	2867	2867.00
---	--	------	------
mPD		0.00070	0.0011
		nPD:	0.63
		N. weight:	0.00072
		Sc. PD:	0.0003
		Sc. rank:	1.2

,

PA

Pos.	478	obs:	exp:
aat	N	1	0.55
aac	N	0	0.45
gat	D	2755	1522.00
gac	D	63	1296.00
gaa	E	15	26.91
gag	E	33	21.09
ggt	G	1	0.14
ggc	G	0	0.13
gga	G	0	0.44
ggg	G	0	0.29
---	--	------	------
mPD		0.077	0.52
		nPD:	0.15
		N. weight:	0.68
		Sc. PD:	-0.035
		Sc. rank:	132.3

,

PA

Pos.	479	obs:	exp:
aat	N	0	1.64
aac	N	3	1.36
gat	D	55	1542.00
gac	D	2801	1314.00
gaa	E	8	4.48
gag	E	0	3.52
ggt	G	0	0.14
ggc	G	1	0.13
gga	G	0	0.44
ggg	G	0	0.29
---	--	------	------
mPD		0.046	0.50
		nPD:	0.09
		N. weight:	0.9
		Sc. PD:	-0.098
		Sc. rank:	-278.9

,

PA

Pos.	480	obs:	exp:
ttt	F	690	1196.00
ttc	F	2178	1672.00
---	--	------	------
mPD		0.37	0.49
		nPD:	0.75
		N. weight:	0.093
		Sc. PD:	0.05
		Sc. rank:	169.0

,

PA			
Pos.	481	obs:	exp:
cct	P	0	0.27
ccc	P	0	0.18
cca	P	1	0.41
ccg	P	0	0.14
caa	Q	2539	1431.00
cag	Q	328	1436.00
---	--	------	------
mPD		0.20	0.50
		nPD:	0.41
		N. weight:	0.45
		Sc. PD:	0.091
		Sc. rank:	558.7

PA			
Pos.	482	obs:	exp:
tta	L	3	263.80
ttg	L	772	542.00
ctt	L	3	566.50
ctc	L	0	456.40
cta	L	23	422.20
ctg	L	2052	602.20
atg	M	15	15.00
---	--	------	------
mPD		0.42	1.1
		nPD:	0.37
		N. weight:	1.3
		Sc. PD:	0.21
		Sc. rank:	1480.3

PA			
Pos.	483	obs:	exp:
tta	L	0	0.09
ttg	L	0	0.19
ctt	L	1	0.20
ctc	L	0	0.16
cta	L	0	0.15
ctg	L	0	0.21
att	I	2746	1050.00
atc	I	115	765.90
ata	I	1	1046.00
atg	M	1	1.00
gtt	V	4	0.86
gtc	V	0	0.84
gta	V	0	0.85
gtg	V	0	1.45
---	--	------	------
mPD		0.082	0.66
		nPD:	0.12
		N. weight:	1.1
		Sc. PD:	-0.087
		Sc. rank:	34.8

PA			
Pos.	484	obs:	exp:
tta	L	0	0.09
ttg	L	0	0.19
ctt	L	0	0.20
ctc	L	0	0.16
cta	L	1	0.15
ctg	L	0	0.21
cct	P	168	783.20
ccc	P	2	517.60
cca	P	2671	1163.00
ccg	P	25	401.80
act	T	0	0.26
acc	T	1	0.23
aca	T	0	0.42
acg	T	0	0.09
---	--	------	------
mPD		0.13	0.71
		nPD:	0.18
		N. weight:	0.88
		Sc. PD:	-0.016
		Sc. rank:	375.6

PA			
Pos.	485	obs:	exp:
tta	L	0	0.09
ttg	L	1	0.19
ctt	L	0	0.20
ctc	L	0	0.16
cta	L	0	0.15
ctg	L	0	0.21
att	I	0	2.94
atc	I	0	2.14
ata	I	8	2.92
atg	M	2858	2858.00
aaa	K	0	0.57
aag	K	1	0.43
---	--	------	------
mPD		0.0070	0.0078
		nPD:	0.9
		N. weight:	0.0049
		Sc. PD:	0.0033
		Sc. rank:	9.0

PA			
Pos.	486	obs:	exp:
tta	L	3	0.28
ttg	L	0	0.57
ctt	L	0	0.60
ctc	L	0	0.48
cta	L	0	0.44
ctg	L	0	0.63
att	I	1	1048.00
atc	I	19	764.00
ata	I	2835	1043.00
atg	M	6	6.00
gtt	V	0	0.86
gtc	V	0	0.84
gta	V	4	0.85
gtg	V	0	1.45
---	--	------	------
mPD		0.023	0.67
		nPD:	0.03
		N. weight:	1.3
		Sc. PD:	-0.21
		Sc. rank:	-1461.1

PA			
Pos.	487	obs:	exp:
tct	S	0	478.60
tcc	S	0	415.30
tca	S	0	650.00
tcg	S	0	173.00
aat	N	0	0.55
aac	N	1	0.45
agt	S	294	621.90
agc	S	2573	528.40
---	--	------	------
mPD		0.18	1.7
		nPD:	0.11
		N. weight:	1.8
		Sc. PD:	-0.16
		Sc. rank:	-54.0

PA			
Pos.	488	obs:	exp:
caa	Q	1	0.50
cag	Q	0	0.50
cgt	R	0	0.07
cgc	R	0	0.11
cga	R	0	0.20
cgg	R	0	0.16
aaa	K	759	1631.00
aag	K	2106	1234.00
aga	R	2	0.93
agg	R	0	0.52
---	--	------	------
mPD		0.39	0.49
		nPD:	0.8
		N. weight:	0.26
		Sc. PD:	0.15
		Sc. rank:	468.5

PA			
Pos.	489	obs:	exp:
tct	S	0	0.33
tcc	S	0	0.29
tca	S	0	0.45
tcg	S	0	0.12
tgt	C	64	1108.00
tgc	C	2800	1756.00
agt	S	0	0.43
agc	S	2	0.37
ggt	G	0	0.29
ggc	G	2	0.26
gga	G	0	0.88
ggg	G	0	0.57
---	--	------	------
mPD		0.046	0.48
		nPD:	0.1
		N. weight:	0.53
		Sc. PD:	-0.054
		Sc. rank:	-125.5

PA			
Pos.	490	obs:	exp:
cgt	R	0	107.30
cgc	R	0	153.40
cga	R	1	288.80
cgg	R	0	234.00
act	T	0	0.26
acc	T	0	0.23
aca	T	1	0.42
acg	T	0	0.09
aaa	K	1	0.57
aag	K	0	0.43
aga	R	2759	1338.00
agg	R	106	744.60
---	--	------	------
mPD		0.073	0.96
		nPD:	0.08
		N. weight:	0.83
		Sc. PD:	-0.1
		Sc. rank:	-455.9

PA			
Pos.	491	obs:	exp:
cct	P	0	0.27
ccc	P	1	0.18
cca	P	0	0.41
ccg	P	0	0.14
att	I	1	1.83
atc	I	4	1.34
ata	I	0	1.83
act	T	375	749.80
acc	T	2333	644.40
aca	T	151	1197.00
acg	T	1	269.10
aat	N	0	1.09
aac	N	2	0.91
---	--	------	------
mPD		0.32	0.70
		nPD:	0.46
		N. weight:	1.1
		Sc. PD:	0.28
		Sc. rank:	1540.9

PA			
Pos.	492	obs:	exp:
cgt	R	0	0.15
cgc	R	0	0.21
cga	R	0	0.40
cgg	R	0	0.33
aat	N	0	0.55
aac	N	1	0.45
aaa	K	2830	1630.00
aag	K	33	1233.00
aga	R	3	1.87
agg	R	1	1.04
---	--	------	------
mPD		0.027	0.49
		nPD:	0.05
		N. weight:	0.67
		Sc. PD:	-0.097
		Sc. rank:	-540.7

PA			
Pos.	493	obs:	exp:
aaa	K	2	1.14
aag	K	0	0.86
gaa	E	2735	1607.00
gag	E	131	1259.00
---	--	------	------
mPD		0.089	0.49
		nPD:	0.18
		N. weight:	0.54
		Sc. PD:	-0.012
		Sc. rank:	214.7

PA			
Pos.	494	obs:	exp:
gaa	E	1	0.56
gag	E	0	0.44
ggt	G	0	414.60
ggc	G	4	376.60
gga	G	2670	1256.00
ggg	G	193	819.50
---	--	------	------
mPD		0.13	0.69
		nPD:	0.19
		N. weight:	0.8
		Sc. PD:	-0.011
		Sc. rank:	359.9

PA			
Pos.	495	obs:	exp:
cgt	R	0	107.20
cgc	R	0	153.30
cga	R	0	288.70
cgg	R	21	233.90
aaa	K	2	1.71
aag	K	1	1.29
aga	R	2641	1337.00
agg	R	203	744.30
---	--	------	------
mPD		0.16	0.96
		nPD:	0.17
		N. weight:	0.69
		Sc. PD:	-0.022
		Sc. rank:	239.8

PA			
Pos.	496	obs:	exp:
cgt	R	3	107.30
cgc	R	0	153.40
cga	R	197	288.80
cgg	R	2533	234.00
act	T	0	0.26
acc	T	0	0.23
aca	T	0	0.42
acg	T	1	0.09
aaa	K	0	0.57
aag	K	1	0.43
aga	R	0	1338.00
agg	R	133	744.60
---	--	------	------
mPD		0.22	0.96
		nPD:	0.23
		N. weight:	2.7
		Sc. PD:	0.079
		Sc. rank:	1839.7

PA			
Pos.	497	obs:	exp:
cgt	R	0	1.35
cgc	R	0	1.93
cga	R	0	3.63
cgg	R	0	2.94
aaa	K	946	1613.00
aag	K	1886	1219.00
aga	R	9	16.81
agg	R	27	9.35
---	--	------	------
mPD		0.47	0.52
		nPD:	0.9
		N. weight:	0.16
		Sc. PD:	0.11
		Sc. rank:	296.8

PA			
Pos.	498	obs:	exp:
att	I	0	0.37
atc	I	0	0.27
ata	I	1	0.37
act	T	500	751.60
acc	T	7	646.00
aca	T	2341	1200.00
acg	T	19	269.70
---	--	------	------
mPD		0.30	0.70
		nPD:	0.44
		N. weight:	0.6
		Sc. PD:	0.14
		Sc. rank:	770.1

PA			
Pos.	499	obs:	exp:
tat	Y	1	0.48
tac	Y	0	0.52
aat	N	2746	1563.00
aac	N	121	1304.00
---	--	------	------
mPD		0.082	0.50
		nPD:	0.16
		N. weight:	0.59
		Sc. PD:	-0.021
		Sc. rank:	186.9

PA			
Pos.	500	obs:	exp:
tta	L	182	265.20
ttg	L	13	544.80
ctt	L	5	569.40
ctc	L	13	458.80
cta	L	248	424.40
ctg	L	2407	605.30
---	--	------	------
mPD		0.39	1.1
		nPD:	0.35
		N. weight:	1.4
		Sc. PD:	0.2
		Sc. rank:	1513.4

PA			
Pos.	501	obs:	exp:
tat	Y	2189	1376.00
tac	Y	679	1492.00
---	--	------	------
mPD		0.36	0.50
		nPD:	0.72
		N. weight:	0.23
		Sc. PD:	0.12
		Sc. rank:	403.5

PA			
Pos.	502	obs:	exp:
ggt	G	7	414.80
ggc	G	6	376.70
gga	G	2508	1257.00
ggg	G	347	819.80
---	--	------	------
mPD		0.22	0.69
		nPD:	0.32
		N. weight:	0.64
		Sc. PD:	0.076
		Sc. rank:	652.3

PA			
Pos.	503	obs:	exp:
ttt	F	60	1196.00
ttc	F	2807	1671.00
tgt	C	0	0.39
tgc	C	1	0.61
---	--	------	------
mPD		0.042	0.49
		nPD:	0.09
		N. weight:	0.59
		Sc. PD:	-0.068
		Sc. rank:	-228.9

PA			
Pos.	504	obs:	exp:
ttt	F	1	0.42
ttc	F	0	0.58
tta	L	0	0.09
ttg	L	0	0.19
ctt	L	1	0.20
ctc	L	0	0.16
cta	L	0	0.15
ctg	L	0	0.21
att	I	2596	1051.00
atc	I	248	766.70
ata	I	21	1047.00
atg	M	1	1.00
---	--	------	------
mPD		0.17	0.66
		nPD:	0.26
		N. weight:	0.92
		Sc. PD:	0.055
		Sc. rank:	747.7

PA			
Pos.	505	obs:	exp:
att	I	1	1050.00
atc	I	0	765.30
ata	I	2859	1045.00
atg	M	1	1.00
gtt	V	0	1.51
gtc	V	0	1.47
gta	V	7	1.49
gtg	V	0	2.53
---	--	------	------
mPD		0.0063	0.67
		nPD:	0.01
		N. weight:	1.3
		Sc. PD:	-0.25
		Sc. rank:	-2196.4

PA			
Pos.	506	obs:	exp:
aat	N	1	0.55
aac	N	0	0.45
aaa	K	2789	1633.00
aag	K	78	1234.00
---	--	------	------
mPD		0.054	0.49
		nPD:	0.11
		N. weight:	0.59
		Sc. PD:	-0.054
		Sc. rank:	-39.5

PA			
Pos.	507	obs:	exp:
cgt	R	0	0.04
cgc	R	0	0.05
cga	R	0	0.10
cgg	R	0	0.08
aga	R	1	0.47
agg	R	0	0.26
ggt	G	2	414.60
ggc	G	3	376.60
gga	G	2667	1256.00
ggg	G	195	819.50
---	--	------	------
mPD		0.13	0.69
		nPD:	0.19
		N. weight:	0.79
		Sc. PD:	-0.009
		Sc. rank:	379.3

PA			
Pos.	508	obs:	exp:
tct	S	0	0.17
tcc	S	0	0.14
tca	S	0	0.23
tcg	S	0	0.06
cgt	R	0	107.20
cgc	R	0	153.30
cga	R	3	288.60
cgg	R	0	233.90
aaa	K	2	1.14
aag	K	0	0.86
agt	S	1	0.22
agc	S	0	0.18
aga	R	1913	1337.00
agg	R	948	744.00
ggt	G	0	0.14
ggc	G	0	0.13
gga	G	1	0.44
ggg	G	0	0.29
---	--	------	------
mPD		0.45	0.96
		nPD:	0.47
		N. weight:	0.42
		Sc. PD:	0.11
		Sc. rank:	585.3

PA			
Pos.	509	obs:	exp:
tct	S	74	478.60
tcc	S	2792	415.30
tca	S	0	650.00
tcg	S	1	173.00
agt	S	0	621.90
agc	S	0	528.40
gct	A	0	0.25
gcc	A	1	0.19
gca	A	0	0.45
gcg	A	0	0.11
---	--	------	------
mPD		0.052	1.7
		nPD:	0.03
		N. weight:	2.4
		Sc. PD:	-0.4
		Sc. rank:	-2870.5

PA			
Pos.	510	obs:	exp:
cct	P	1	0.27
ccc	P	0	0.18
cca	P	0	0.41
ccg	P	0	0.14
cat	H	2344	1660.00
cac	H	523	1207.00
---	--	------	------
mPD		0.30	0.49
		nPD:	0.61
		N. weight:	0.17
		Sc. PD:	0.071
		Sc. rank:	279.7

PA			
Pos.	511	obs:	exp:
tta	L	28	265.10
ttg	L	2813	544.70
ctt	L	0	569.20
ctc	L	0	458.60
cta	L	0	424.30
ctg	L	26	605.10
atg	M	1	1.00
---	--	------	------
mPD		0.038	1.1
		nPD:	0.03
		N. weight:	2.1
		Sc. PD:	-0.34
		Sc. rank:	-2401.4

PA			
Pos.	512	obs:	exp:
cgt	R	0	107.30
cgc	R	0	153.50
cga	R	0	289.00
cgg	R	1	234.20
aga	R	329	1339.00
agg	R	2538	745.10
---	--	------	------
mPD		0.20	0.95
		nPD:	0.21
		N. weight:	1.2
		Sc. PD:	0.015
		Sc. rank:	751.1

PA			
Pos.	513	obs:	exp:
tct	S	0	1.00
tcc	S	0	0.87
tca	S	0	1.36
tcg	S	0	0.36
tat	Y	1	0.48
tac	Y	0	0.52
aat	N	1953	1560.00
aac	N	907	1300.00
agt	S	1	1.30
agc	S	5	1.11
gat	D	1	0.54
gac	D	0	0.46
---	--	------	------
mPD		0.44	0.50
		nPD:	0.87
		N. weight:	0.058
		Sc. PD:	0.039
		Sc. rank:	107.5

PA			
Pos.	514	obs:	exp:
gat	D	644	1547.00
gac	D	2222	1319.00
gaa	E	1	0.56
gag	E	0	0.44
ggt	G	0	0.14
ggc	G	1	0.13
gga	G	0	0.44
ggg	G	0	0.29
---	--	------	------
mPD		0.35	0.50
		nPD:	0.7
		N. weight:	0.28
		Sc. PD:	0.14
		Sc. rank:	487.9

PA			
Pos.	515	obs:	exp:
tct	S	0	0.33
tcc	S	1	0.29
tca	S	0	0.45
tcg	S	0	0.12
cct	P	0	0.27
ccc	P	1	0.18
cca	P	0	0.41
ccg	P	0	0.14
act	T	146	751.10
acc	T	2709	645.60
aca	T	10	1199.00
acg	T	0	269.60
agt	S	0	0.43
agc	S	1	0.37
---	--	------	------
mPD		0.11	0.70
		nPD:	0.15
		N. weight:	1.7
		Sc. PD:	-0.083
		Sc. rank:	356.2

PA			
Pos.	516	obs:	exp:
tat	Y	2	0.96
tac	Y	0	1.04
gat	D	2842	1547.00
gac	D	24	1319.00
---	--	------	------
mPD		0.018	0.50
		nPD:	0.04
		N. weight:	0.76
		Sc. PD:	-0.12
		Sc. rank:	-840.8

PA			
Pos.	517	obs:	exp:
atg	M	2	2.00
gtt	V	2	616.00
gtc	V	1	602.50
gta	V	33	610.70
gtg	V	2828	1035.00
gct	A	0	0.25
gcc	A	0	0.19
gca	A	0	0.45
gcg	A	1	0.11
ggt	G	0	0.14
ggc	G	0	0.13
gga	G	0	0.44
ggg	G	1	0.29
---	--	------	------
mPD		0.028	0.74
		nPD:	0.04
		N. weight:	1.3
		Sc. PD:	-0.2
		Sc. rank:	-1332.4

PA			
Pos.	518	obs:	exp:
tta	L	1	0.09
ttg	L	0	0.19
ctt	L	0	0.20
ctc	L	0	0.16
cta	L	0	0.15
ctg	L	0	0.21
gtt	V	11	614.10
gtc	V	3	600.70
gta	V	2530	608.80
gtg	V	311	1032.00
gct	A	0	2.77
gcc	A	0	2.13
gca	A	11	4.90
gcg	A	0	1.20
ggt	G	0	0.14
ggc	G	0	0.13
gga	G	1	0.44
ggg	G	0	0.29
---	--	------	------
mPD		0.21	0.74
		nPD:	0.28
		N. weight:	1.5
		Sc. PD:	0.12
		Sc. rank:	1323.0

PA			
Pos.	519	obs:	exp:
tct	S	0	0.17
tcc	S	0	0.14
tca	S	0	0.23
tcg	S	0	0.06
tat	Y	0	0.48
tac	Y	1	0.52
aat	N	647	1562.00
aac	N	2218	1303.00
aaa	K	0	0.57
aag	K	1	0.43
agt	S	0	0.22
agc	S	1	0.18
---	--	------	------
mPD		0.35	0.50
		nPD:	0.71
		N. weight:	0.29
		Sc. PD:	0.14
		Sc. rank:	502.9

PA			
Pos.	520	obs:	exp:
ttt	F	2822	1187.00
ttc	F	24	1659.00
tta	L	0	0.09
ttg	L	1	0.19
tat	Y	21	10.07
tac	Y	0	10.93
ctt	L	0	0.20
ctc	L	0	0.16
cta	L	0	0.15
ctg	L	0	0.21
---	--	------	------
mPD		0.032	0.50
		nPD:	0.06
		N. weight:	1.1
		Sc. PD:	-0.15
		Sc. rank:	-723.4

PA			
Pos.	521	obs:	exp:
tta	L	0	0.55
ttg	L	6	1.14
ctt	L	0	1.19
ctc	L	0	0.96
cta	L	0	0.89
ctg	L	0	1.27
att	I	0	0.73
atc	I	0	0.54
ata	I	2	0.73
atg	M	1	1.00
gtt	V	18	614.70
gtc	V	0	601.30
gta	V	74	609.40
gtg	V	2766	1033.00
gaa	E	0	0.56
gag	E	1	0.44
---	--	------	------
mPD		0.071	0.74
		nPD:	0.1
		N. weight:	1.2
		Sc. PD:	-0.12
		Sc. rank:	-290.2

,

PA			
Pos.	522	obs:	exp:
tct	S	0	478.40
tcc	S	0	415.20
tca	S	0	649.70
tcg	S	0	172.90
cgt	R	0	0.07
cgc	R	0	0.11
cga	R	0	0.20
cgg	R	0	0.16
agt	S	513	621.60
agc	S	2353	528.20
aga	R	2	0.93
agg	R	0	0.52
---	--	------	------
mPD		0.29	1.7
		nPD:	0.18
		N. weight:	1.6
		Sc. PD:	-0.035
		Sc. rank:	616.3

,

PA			
Pos.	523	obs:	exp:
atg	M	2868	2868.00
---	--	------	------
mPD		0	0
		nPD:	1.
		N. weight:	0.
		Sc. PD:	0
		Sc. rank:	0

,

PA			
Pos.	524	obs:	exp:
gaa	E	2796	1608.00
gag	E	72	1260.00
---	--	------	------
mPD		0.049	0.49
		nPD:	0.1
		N. weight:	0.62
		Sc. PD:	-0.063
		Sc. rank:	-143.3

,

PA			
Pos.	525	obs:	exp:
ttt	F	336	1196.00
ttc	F	2532	1672.00
---	--	------	------
mPD		0.21	0.49
		nPD:	0.43
		N. weight:	0.29
		Sc. PD:	0.064
		Sc. rank:	367.1

,

PA			
Pos.	526	obs:	exp:
tct	S	2817	478.70
tcc	S	50	415.40
tca	S	0	650.20
tcg	S	1	173.00
agt	S	0	622.10
agc	S	0	528.50
---	--	------	------
mPD		0.035	1.7
		nPD:	0.02
		N. weight:	2.3
		Sc. PD:	-0.4
		Sc. rank:	-3111.9

,

PA			
Pos.	527	obs:	exp:
ttt	F	2	0.83
ttc	F	0	1.17
tta	L	3	264.90
ttg	L	0	544.30
ctt	L	1696	568.80
ctc	L	555	458.30
cta	L	611	424.00
ctg	L	0	604.70
att	I	1	0.37
atc	I	0	0.27
ata	I	0	0.37
---	--	------	------
mPD		0.57	1.1
		nPD:	0.51
		N. weight:	1.
		Sc. PD:	0.31
		Sc. rank:	1448.5

,

PA			
Pos.	528	obs:	exp:
tct	S	6	1.00
tcc	S	0	0.87
tca	S	0	1.36
tcg	S	0	0.36
act	T	2820	750.30
acc	T	34	644.90
aca	T	6	1198.00
acg	T	2	269.30
agt	S	0	1.30
agc	S	0	1.11
---	--	------	------
mPD		0.033	0.70
		nPD:	0.05
		N. weight:	1.7
		Sc. PD:	-0.25
		Sc. rank:	-1538.4

,

PA			
Pos.	529	obs:	exp:
aat	N	0	0.55
aac	N	1	0.45
gct	A	0	0.25
gcc	A	1	0.19
gca	A	0	0.45
gcg	A	0	0.11
gat	D	658	1547.00
gac	D	2208	1319.00
---	--	------	------
mPD		0.36	0.50
		nPD:	0.71
		N. weight:	0.27
		Sc. PD:	0.14
		Sc. rank:	480.1

,

PA			
Pos.	530	obs:	exp:
cct	P	177	783.20
ccc	P	0	517.60
cca	P	49	1163.00
ccg	P	2640	401.80
cgt	R	0	0.07
cgc	R	0	0.11
cga	R	0	0.20
cgg	R	2	0.16
aga	R	0	0.93
agg	R	0	0.52
---	--	------	------
mPD		0.15	0.71
		nPD:	0.21
		N. weight:	2.1
		Sc. PD:	0.017
		Sc. rank:	1228.1

,

PA			
Pos.	531	obs:	exp:
tct	S	0	0.50
tcc	S	0	0.43
tca	S	0	0.68
tcg	S	0	0.18
cgt	R	0	105.60
cgc	R	0	151.10
cga	R	0	284.50
cgg	R	1	230.50
atg	M	5	5.00
aaa	K	8	20.50
aag	K	28	15.50
agt	S	3	0.65
agc	S	0	0.55
aga	R	250	1318.00
agg	R	2572	733.40
ggt	G	0	0.14
ggc	G	0	0.13
gga	G	0	0.44
ggg	G	1	0.29
---	--	------	------
mPD		0.20	0.98
		nPD:	0.2
		N. weight:	1.3
		Sc. PD:	-0.003
		Sc. rank:	718.7

,

PA			
Pos.	532	obs:	exp:
tta	L	10	260.20
ttg	L	431	534.60
ctt	L	4	558.70
ctc	L	0	450.10
cta	L	690	416.50
ctg	L	1679	593.90
att	I	0	1.47
atc	I	0	1.07
ata	I	4	1.46
atg	M	49	49.00
gtt	V	0	0.22
gtc	V	0	0.21
gta	V	0	0.21
gtg	V	1	0.36
---	--	------	------
mPD		0.66	1.1
		nPD:	0.58
		N. weight:	0.91
		Sc. PD:	0.34
		Sc. rank:	1430.5

,

PA			
Pos.	533	obs:	exp:
gct	A	0	0.25
gcc	A	0	0.19
gca	A	1	0.45
gcg	A	0	0.11
gat	D	8	4.86
gac	D	1	4.14
gaa	E	1294	1602.00
gag	E	1564	1256.00
---	--	------	------
mPD		0.50	0.50
		nPD:	1.01
		N. weight:	0.035
		Sc. PD:	0.028
		Sc. rank:	65.0

,

PA			
Pos.	534	obs:	exp:
tta	L	0	0.37
ttg	L	0	0.76
tct	S	0	6.18
tcc	S	0	5.36
tca	S	37	8.39
tcg	S	0	2.23
ctt	L	0	0.79
ctc	L	0	0.64
cta	L	4	0.59
ctg	L	0	0.84
cct	P	3	769.90
ccc	P	3	508.80
cca	P	2786	1143.00
ccg	P	25	395.00
caa	Q	4	2.00
cag	Q	0	2.00
act	T	0	1.31
acc	T	0	1.13
aca	T	5	2.09
acg	T	0	0.47
agt	S	0	8.03
agc	S	0	6.82
gct	A	0	0.25
gcc	A	0	0.19
gca	A	1	0.45
gcg	A	0	0.11
---	--	------	------
mPD		0.057	0.76
		nPD:	0.07
		N. weight:	1.1
		Sc. PD:	-0.14
		Sc. rank:	-633.7

,

PA			
Pos.	535	obs:	exp:
tta	L	0	0.09
ttg	L	0	0.19
tat	Y	1	15.83
tac	Y	32	17.17
ctt	L	0	0.20
ctc	L	1	0.16
cta	L	0	0.15
ctg	L	0	0.21
cct	P	0	0.82
ccc	P	3	0.54
cca	P	0	1.22
ccg	P	0	0.42
cat	H	45	1635.00
cac	H	2779	1189.00
caa	Q	3	1.50
cag	Q	0	1.50
cgt	R	0	0.07
cgc	R	2	0.11
cga	R	0	0.20
cgg	R	0	0.16
aat	N	0	1.09
aac	N	2	0.91
aga	R	0	0.93
agg	R	0	0.52
---	--	------	------
mPD		0.062	0.52
		nPD:	0.12
		N. weight:	1.
		Sc. PD:	-0.084
		Sc. rank:	14.4

,

PA			
Pos.	536	obs:	exp:
cgt	R	0	0.04
cgc	R	0	0.05
cga	R	0	0.10
cgg	R	0	0.08
aaa	K	1223	1632.00
aag	K	1643	1234.00
aga	R	0	0.47
agg	R	1	0.26
gaa	E	0	0.56
gag	E	1	0.44
---	--	------	------
mPD		0.49	0.49
		nPD:	1.
		N. weight:	0.057
		Sc. PD:	0.045
		Sc. rank:	106.9

,

PA			
Pos.	537	obs:	exp:
tgg	W	2868	2868.00
---	--	------	------
mPD		0	0
		nPD:	1.
		N. weight:	0.
		Sc. PD:	0
		Sc. rank:	0

,

PA			
Pos.	538	obs:	exp:
aaa	K	3	1.71
aag	K	0	1.29
gtt	V	0	10.32
gtc	V	0	10.10
gta	V	48	10.23
gtg	V	0	17.34
gat	D	0	1.08
gac	D	2	0.92
gaa	E	2602	1539.00
gag	E	143	1206.00
ggt	G	0	10.12
ggc	G	0	9.19
gga	G	70	30.67
ggg	G	0	20.01
---	--	------	------
mPD		0.18	0.59
		nPD:	0.3
		N. weight:	0.56
		Sc. PD:	0.055
		Sc. rank:	525.0

,

PA			
Pos.	539	obs:	exp:
cgt	R	0	0.22
cgc	R	0	0.32
cga	R	0	0.60
cgg	R	0	0.49
aaa	K	134	1630.00
aag	K	2728	1232.00
aga	R	0	2.80
agg	R	6	1.56
---	--	------	------
mPD		0.093	0.50
		nPD:	0.19
		N. weight:	0.86
		Sc. PD:	-0.011
		Sc. rank:	395.1

,

PA			
Pos.	540	obs:	exp:
tat	Y	21	1376.00
tac	Y	2847	1492.00
---	--	------	------
mPD		0.015	0.50
		nPD:	0.03
		N. weight:	0.81
		Sc. PD:	-0.14
		Sc. rank:	-1023.1

,

PA			
Pos.	541	obs:	exp:
tct	S	0	8.85
tcc	S	0	7.68
tca	S	0	12.02
tcg	S	0	3.20
tgt	C	2752	1089.00
tgc	C	62	1725.00
cgt	R	1	0.04
cgc	R	0	0.05
cga	R	0	0.10
cgg	R	0	0.08
agt	S	53	11.50
agc	S	0	9.77
aga	R	0	0.47
agg	R	0	0.26
---	--	------	------
mPD		0.079	0.52
		nPD:	0.15
		N. weight:	1.1
		Sc. PD:	-0.054
		Sc. rank:	251.5

,

PA			
Pos.	542	obs:	exp:
att	I	2	0.73
atc	I	0	0.54
ata	I	0	0.73
gtt	V	2146	616.40
gtc	V	714	603.00
gta	V	0	611.10
gtg	V	6	1036.00
---	--	------	------
mPD		0.38	0.73
		nPD:	0.52
		N. weight:	1.3
		Sc. PD:	0.4
		Sc. rank:	1887.3

,

PA			
Pos.	543	obs:	exp:
tta	L	0	263.60
ttg	L	0	541.60
tct	S	2	0.33
tcc	S	0	0.29
tca	S	0	0.45
tcg	S	0	0.12
ctt	L	1927	566.10
ctc	L	914	456.00
cta	L	8	421.90
ctg	L	2	601.80
cct	P	9	2.46
ccc	P	0	1.63
cca	P	0	3.65
ccg	P	0	1.26
att	I	1	2.20
atc	I	5	1.61
ata	I	0	2.19
agt	S	0	0.43
agc	S	0	0.37
---	--	------	------
mPD		0.45	1.1
		nPD:	0.4
		N. weight:	1.4
		Sc. PD:	0.27
		Sc. rank:	1697.8

,

PA			
Pos.	544	obs:	exp:
caa	Q	0	2.50
cag	Q	5	2.50
cgt	R	0	0.94
cgc	R	0	1.34
cga	R	0	2.52
cgg	R	25	2.04
aga	R	0	11.67
agg	R	0	6.49
gaa	E	956	1591.00
gag	E	1882	1247.00
---	--	------	------
mPD		0.48	0.53
		nPD:	0.91
		N. weight:	0.17
		Sc. PD:	0.12
		Sc. rank:	306.9

,

PA			
Pos.	545	obs:	exp:
att	I	20	987.10
atc	I	16	719.80
ata	I	2654	983.00
gtt	V	0	38.28
gtc	V	0	37.45
gta	V	167	37.95
gtg	V	11	64.31
---	--	------	------
mPD		0.15	0.79
		nPD:	0.19
		N. weight:	1.3
		Sc. PD:	-0.017
		Sc. rank:	579.5

,

PA			
Pos.	546	obs:	exp:
cgt	R	0	0.04
cgc	R	0	0.05
cga	R	0	0.10
cgg	R	0	0.08
aga	R	1	0.47
agg	R	0	0.26
ggt	G	5	414.60
ggc	G	14	376.60
gga	G	2725	1256.00
ggg	G	123	819.50
---	--	------	------
mPD		0.095	0.69
		nPD:	0.14
		N. weight:	0.84
		Sc. PD:	-0.052
		Sc. rank:	111.4

,

PA			
Pos.	547	obs:	exp:
aat	N	3	15.81
aac	N	26	13.19
gat	D	41	1531.00
gac	D	2795	1305.00
gaa	E	3	1.68
gag	E	0	1.32
---	--	------	------
mPD		0.052	0.52
		nPD:	0.1
		N. weight:	0.93
		Sc. PD:	-0.092
		Sc. rank:	-152.3

,

PA			
Pos.	548	obs:	exp:
att	I	0	0.37
atc	I	0	0.27
ata	I	1	0.37
atg	M	2866	2866.00
gtt	V	0	0.22
gtc	V	0	0.21
gta	V	0	0.21
gtg	V	1	0.36
---	--	------	------
mPD		0.0014	0.0018
		nPD:	0.76
		N. weight:	0.00094
		Sc. PD:	0.00051
		Sc. rank:	1.7

,

PA			
Pos.	549	obs:	exp:
ttt	F	0	0.83
ttc	F	2	1.17
tta	L	0	256.20
ttg	L	0	526.40
ctt	L	15	550.20
ctc	L	1835	443.20
cta	L	920	410.10
ctg	L	1	584.90
cat	H	0	0.58
cac	H	1	0.42
att	I	0	34.13
atc	I	1	24.89
ata	I	92	33.99
gtt	V	0	0.22
gtc	V	0	0.21
gta	V	1	0.21
gtg	V	0	0.36
---	--	------	------
mPD		0.53	1.2
		nPD:	0.45
		N. weight:	1.6
		Sc. PD:	0.39
		Sc. rank:	2117.4

,

PA			
Pos.	550	obs:	exp:
tta	L	867	263.40
ttg	L	17	541.20
ctt	L	6	565.70
ctc	L	487	455.70
cta	L	1416	421.60
ctg	L	56	601.30
att	I	0	6.97
atc	I	0	5.08
ata	I	19	6.94
---	--	------	------
mPD		0.76	1.1
		nPD:	0.67
		N. weight:	1.2
		Sc. PD:	0.56
		Sc. rank:	2040.2

,

PA			
Pos.	551	obs:	exp:
cgt	R	10	107.30
cgc	R	0	153.50
cga	R	1150	289.00
cgg	R	1672	234.20
aga	R	7	1339.00
agg	R	29	745.10
---	--	------	------
mPD		0.51	0.95
		nPD:	0.53
		N. weight:	2.2
		Sc. PD:	0.72
		Sc. rank:	3264.4

PA			
Pos.	552	obs:	exp:
att	I	2	0.73
atc	I	0	0.54
ata	I	0	0.73
act	T	2442	731.90
acc	T	300	629.10
aca	T	2	1168.00
acg	T	48	262.70
gct	A	73	18.39
gcc	A	0	14.13
gca	A	0	32.49
gcg	A	0	7.99
ggt	G	1	0.14
ggc	G	0	0.13
gga	G	0	0.44
ggg	G	0	0.29
---	--	------	------
mPD		0.27	0.75
		nPD:	0.36
		N. weight:	1.3
		Sc. PD:	0.2
		Sc. rank:	1430.7

PA			
Pos.	553	obs:	exp:
tct	S	3	1.50
tcc	S	0	1.30
tca	S	6	2.04
tcg	S	0	0.54
agt	S	0	1.95
agc	S	0	1.66
gct	A	124	720.00
gcc	A	3	553.10
gca	A	2712	1272.00
gcg	A	19	312.80
ggt	G	0	0.14
ggc	G	0	0.13
gga	G	1	0.44
ggg	G	0	0.29
---	--	------	------
mPD		0.11	0.70
		nPD:	0.15
		N. weight:	0.83
		Sc. PD:	-0.04
		Sc. rank:	179.6

PA			
Pos.	554	obs:	exp:
tta	L	2	0.18
ttg	L	0	0.38
ctt	L	0	0.40
ctc	L	0	0.32
cta	L	0	0.30
ctg	L	0	0.42
att	I	9	823.80
atc	I	0	600.80
ata	I	2236	820.40
atg	M	6	6.00
act	T	0	0.26
acc	T	0	0.23
aca	T	1	0.42
acg	T	0	0.09
gtt	V	1	131.80
gtc	V	0	129.00
gta	V	578	130.70
gtg	V	34	221.50
ggt	G	0	0.14
ggc	G	0	0.13
gga	G	1	0.44
ggg	G	0	0.29
---	--	------	------
mPD		0.37	1.0
		nPD:	0.36
		N. weight:	1.4
		Sc. PD:	0.21
		Sc. rank:	1531.7

PA			
Pos.	555	obs:	exp:
tct	S	0	2.17
tcc	S	0	1.88
tca	S	0	2.95
tcg	S	0	0.78
agt	S	0	2.82
agc	S	13	2.40
gct	A	1	0.50
gcc	A	1	0.39
gca	A	0	0.89
gcg	A	0	0.22
ggt	G	345	412.60
ggc	G	2506	374.70
gga	G	2	1250.00
ggg	G	0	815.50
---	--	------	------
mPD		0.22	0.70
		nPD:	0.32
		N. weight:	2.2
		Sc. PD:	0.25
		Sc. rank:	2157.5

PA			
Pos.	556	obs:	exp:
cct	P	0	0.27
ccc	P	0	0.18
cca	P	1	0.41
ccg	P	0	0.14
cat	H	1	0.58
cac	H	0	0.42
caa	Q	2134	1429.00
cag	Q	728	1433.00
cgt	R	0	0.04
cgc	R	0	0.05
cga	R	0	0.10
cgg	R	1	0.08
aaa	K	2	1.71
aag	K	1	1.29
aga	R	0	0.47
agg	R	0	0.26
---	--	------	------
mPD		0.38	0.50
		nPD:	0.76
		N. weight:	0.17
		Sc. PD:	0.095
		Sc. rank:	314.2

PA			
Pos.	557	obs:	exp:
tta	L	0	0.09
ttg	L	1	0.19
ctt	L	0	0.20
ctc	L	0	0.16
cta	L	0	0.15
ctg	L	0	0.21
att	I	1	0.37
atc	I	0	0.27
ata	I	0	0.37
atg	M	6	6.00
gtt	V	488	614.90
gtc	V	1	601.50
gta	V	92	609.60
gtg	V	2278	1033.00
gaa	E	0	0.56
gag	E	1	0.44
---	--	------	------
mPD		0.34	0.74
		nPD:	0.46
		N. weight:	0.7
		Sc. PD:	0.18
		Sc. rank:	968.4

PA			
Pos.	558	obs:	exp:
tta	L	1	0.18
ttg	L	0	0.38
tct	S	0	459.50
tcc	S	0	398.80
tca	S	2112	624.10
tcg	S	641	166.10
ctt	L	0	0.40
ctc	L	0	0.32
cta	L	1	0.30
ctg	L	0	0.42
cct	P	1	30.06
ccc	P	0	19.87
cca	P	107	44.65
ccg	P	2	15.42
agt	S	0	597.10
agc	S	0	507.30
gct	A	0	0.76
gcc	A	0	0.58
gca	A	3	1.34
gcg	A	0	0.33
---	--	------	------
mPD		0.43	1.7
		nPD:	0.25
		N. weight:	1.6
		Sc. PD:	0.083
		Sc. rank:	1270.3

PA			
Pos.	559	obs:	exp:
cgt	R	0	107.20
cgc	R	0	153.30
cga	R	4	288.60
cgg	R	5	233.90
aaa	K	1	1.71
aag	K	2	1.29
aga	R	1148	1337.00
agg	R	1707	744.00
ggt	G	0	0.14
ggc	G	0	0.13
gga	G	0	0.44
ggg	G	1	0.29
---	--	------	------
mPD		0.49	0.96
		nPD:	0.51
		N. weight:	0.56
		Sc. PD:	0.17
		Sc. rank:	815.7

PA			
Pos.	560	obs:	exp:
tct	S	0	2.34
tcc	S	14	2.03
tca	S	0	3.17
tcg	S	0	0.84
cct	P	19	776.70
ccc	P	2768	513.30
cca	P	55	1154.00
ccg	P	0	398.50
act	T	0	2.62
acc	T	10	2.25
aca	T	0	4.18
acg	T	0	0.94
agt	S	0	3.04
agc	S	0	2.58
gct	A	0	0.50
gcc	A	2	0.39
gca	A	0	0.89
gcg	A	0	0.22
---	--	------	------
mPD		0.069	0.73
		nPD:	0.09
		N. weight:	2.1
		Sc. PD:	-0.22
		Sc. rank:	-570.7

PA			
Pos.	561	obs:	exp:
tta	L	0	0.37
ttg	L	2	0.76
ctt	L	0	0.79
ctc	L	0	0.64
cta	L	0	0.59
ctg	L	2	0.84
att	I	1	1.10
atc	I	0	0.80
ata	I	2	1.10
atg	M	2851	2851.00
act	T	0	0.26
acc	T	0	0.23
aca	T	0	0.42
acg	T	1	0.09
gtt	V	0	1.94
gtc	V	0	1.89
gta	V	0	1.92
gtg	V	9	3.25
---	--	------	------
mPD		0.012	0.018
		nPD:	0.65
		N. weight:	0.0084
		Sc. PD:	0.0037
		Sc. rank:	14.0

PA			
Pos.	562	obs:	exp:
ttt	F	67	1186.00
ttc	F	2776	1657.00
tta	L	1	0.09
ttg	L	0	0.19
tat	Y	0	11.51
tac	Y	24	12.49
ctt	L	0	0.20
ctc	L	0	0.16
cta	L	0	0.15
ctg	L	0	0.21
---	--	------	------
mPD		0.063	0.50
		nPD:	0.12
		N. weight:	0.58
		Sc. PD:	-0.044
		Sc. rank:	22.7

PA			
Pos.	563	obs:	exp:
tta	L	1	265.20
ttg	L	127	544.80
ctt	L	14	569.40
ctc	L	0	458.80
cta	L	29	424.40
ctg	L	2697	605.30
---	--	------	------
mPD		0.12	1.1
		nPD:	0.1
		N. weight:	1.7
		Sc. PD:	-0.17
		Sc. rank:	-230.7

PA			
Pos.	564	obs:	exp:
ttt	F	4	1.67
ttc	F	0	2.33
tct	S	5	0.83
tcc	S	0	0.72
tca	S	0	1.13
tcg	S	0	0.30
tat	Y	2625	1371.00
tac	Y	234	1488.00
agt	S	0	1.08
agc	S	0	0.92
---	--	------	------
mPD		0.16	0.51
		nPD:	0.31
		N. weight:	0.6
		Sc. PD:	0.063
		Sc. rank:	565.1

PA			
Pos.	565	obs:	exp:
att	I	2	6.61
atc	I	0	4.82
ata	I	16	6.58
atg	M	28	28.00
gtt	V	39	606.70
gtc	V	0	593.50
gta	V	196	601.50
gtg	V	2586	1019.00
gct	A	0	0.25
gcc	A	0	0.19
gca	A	0	0.45
gcg	A	1	0.11
---	--	------	------
mPD		0.20	0.76
		nPD:	0.26
		N. weight:	0.97
		Sc. PD:	0.052
		Sc. rank:	759.7

PA			
Pos.	566	obs:	exp:
cgt	R	0	107.20
cgc	R	0	153.30
cga	R	0	288.70
cgg	R	0	233.90
act	T	0	0.26
acc	T	0	0.23
aca	T	1	0.42
acg	T	0	0.09
aaa	K	2	1.14
aag	K	0	0.86
aga	R	2833	1337.00
agg	R	32	744.30
---	--	------	------
mPD		0.024	0.96
		nPD:	0.03
		N. weight:	0.94
		Sc. PD:	-0.16
		Sc. rank:	-1239.4

,

PA			
Pos.	567	obs:	exp:
act	T	116	751.60
acc	T	2734	646.00
aca	T	17	1200.00
acg	T	0	269.70
gct	A	0	0.25
gcc	A	1	0.19
gca	A	0	0.45
gcg	A	0	0.11
---	--	------	------
mPD		0.090	0.70
		nPD:	0.13
		N. weight:	1.7
		Sc. PD:	-0.12
		Sc. rank:	119.9

,

PA			
Pos.	568	obs:	exp:
att	I	0	0.37
atc	I	1	0.27
ata	I	0	0.37
aat	N	2521	1562.00
aac	N	344	1303.00
aaa	K	2	1.14
aag	K	0	0.86
---	--	------	------
mPD		0.21	0.50
		nPD:	0.43
		N. weight:	0.35
		Sc. PD:	0.079
		Sc. rank:	446.3

,

PA			
Pos.	569	obs:	exp:
tgg	W	1	1.00
gtt	V	0	0.43
gtc	V	0	0.42
gta	V	0	0.43
gtg	V	2	0.72
gaa	E	1	1.12
gag	E	1	0.88
ggt	G	10	414.00
ggc	G	1	376.10
gga	G	824	1255.00
ggg	G	2028	818.40
---	--	------	------
mPD		0.42	0.69
		nPD:	0.61
		N. weight:	0.68
		Sc. PD:	0.27
		Sc. rank:	1080.8

,

PA			
Pos.	570	obs:	exp:
att	I	1	0.37
atc	I	0	0.27
ata	I	0	0.37
act	T	1411	750.30
acc	T	614	644.90
aca	T	27	1198.00
acg	T	810	269.30
aat	N	1	0.55
aac	N	0	0.45
gct	A	3	1.01
gcc	A	0	0.77
gca	A	0	1.78
gcg	A	1	0.44
---	--	------	------
mPD		0.63	0.70
		nPD:	0.91
		N. weight:	0.77
		Sc. PD:	0.54
		Sc. rank:	1429.8

,

PA			
Pos.	571	obs:	exp:
tct	S	66	478.70
tcc	S	2780	415.40
tca	S	22	650.20
tcg	S	0	173.00
agt	S	0	622.10
agc	S	0	528.50
---	--	------	------
mPD		0.060	1.7
		nPD:	0.04
		N. weight:	2.4
		Sc. PD:	-0.38
		Sc. rank:	-2558.6

,

PA			
Pos.	572	obs:	exp:
caa	Q	0	1.00
cag	Q	2	1.00
aaa	K	152	1632.00
aag	K	2714	1234.00
---	--	------	------
mPD		0.10	0.49
		nPD:	0.21
		N. weight:	0.83
		Sc. PD:	0.0046
		Sc. rank:	462.0

,

PA			
Pos.	573	obs:	exp:
att	I	922	1041.00
atc	I	1911	759.40
ata	I	5	1037.00
act	T	0	0.52
acc	T	2	0.45
aca	T	0	0.84
acg	T	0	0.19
gtt	V	1	6.02
gtc	V	27	5.89
gta	V	0	5.97
gtg	V	0	10.12
---	--	------	------
mPD		0.46	0.68
		nPD:	0.67
		N. weight:	0.78
		Sc. PD:	0.36
		Sc. rank:	1310.6

,

PA			
Pos.	574	obs:	exp:
caa	Q	1	0.50
cag	Q	0	0.50
aaa	K	1660	1633.00
aag	K	1207	1234.00
---	--	------	------
mPD		0.49	0.49
		nPD:	0.99
		N. weight:	0.0025
		Sc. PD:	0.002
		Sc. rank:	4.7

,

PA			
Pos.	575	obs:	exp:
atg	M	2868	2868.00
---	--	------	------
mPD		0	0
		nPD:	1.
		N. weight:	0.
		Sc. PD:	0
		Sc. rank:	0

,

PA			
Pos.	576	obs:	exp:
att	I	0	0.37
atc	I	0	0.27
ata	I	1	0.37
aaa	K	2856	1633.00
aag	K	11	1234.00
---	--	------	------
mPD		0.0083	0.49
		nPD:	0.02
		N. weight:	0.72
		Sc. PD:	-0.13
		Sc. rank:	-1054.6

,

PA			
Pos.	577	obs:	exp:
tgt	C	2	0.77
tgc	C	0	1.23
tgg	W	2865	2865.00
ggt	G	0	0.14
ggc	G	0	0.13
gga	G	0	0.44
ggg	G	1	0.29
---	--	------	------
mPD		0.0021	0.0026
		nPD:	0.81
		N. weight:	0.0015
		Sc. PD:	0.00087
		Sc. rank:	2.7

,

PA			
Pos.	578	obs:	exp:
tct	S	0	0.67
tcc	S	0	0.58
tca	S	0	0.91
tcg	S	0	0.24
agt	S	0	0.87
agc	S	4	0.74
gtt	V	0	0.22
gtc	V	1	0.21
gta	V	0	0.21
gtg	V	0	0.36
gat	D	0	0.54
gac	D	1	0.46
ggt	G	91	413.90
ggc	G	2734	375.90
gga	G	34	1254.00
ggg	G	3	818.10
---	--	------	------
mPD		0.090	0.69
		nPD:	0.13
		N. weight:	2.4
		Sc. PD:	-0.17
		Sc. rank:	198.9

,

PA			
Pos.	579	obs:	exp:
tta	L	0	0.09
ttg	L	0	0.19
ctt	L	0	0.20
ctc	L	0	0.16
cta	L	0	0.15
ctg	L	1	0.21
att	I	3	1.83
atc	I	0	1.34
ata	I	2	1.83
atg	M	2837	2837.00
act	T	0	0.52
acc	T	0	0.45
aca	T	0	0.84
acg	T	2	0.19
gtt	V	0	4.95
gtc	V	0	4.84
gta	V	0	4.90
gtg	V	23	8.31
---	--	------	------
mPD		0.021	0.033
		nPD:	0.64
		N. weight:	0.015
		Sc. PD:	0.0066
		Sc. rank:	25.0

,

PA			
Pos.	580	obs:	exp:
gat	D	0	0.54
gac	D	1	0.46
gaa	E	594	1607.00
gag	E	2273	1260.00
---	--	------	------
mPD		0.33	0.49
		nPD:	0.67
		N. weight:	0.35
		Sc. PD:	0.16
		Sc. rank:	589.3

,

PA			
Pos.	581	obs:	exp:
tta	L	0	0.09
ttg	L	0	0.19
ctt	L	0	0.20
ctc	L	0	0.16
cta	L	0	0.15
ctg	L	1	0.21
atg	M	2864	2864.00
act	T	0	0.52
acc	T	0	0.45
aca	T	0	0.84
acg	T	2	0.19
gtt	V	0	0.22
gtc	V	0	0.21
gta	V	0	0.21
gtg	V	1	0.36
---	--	------	------
mPD		0.0028	0.0049
		nPD:	0.57
		N. weight:	0.0034
		Sc. PD:	0.0012
		Sc. rank:	5.2

,

PA			
Pos.	582	obs:	exp:
tct	S	0	0.17
tcc	S	0	0.14
tca	S	0	0.23
tcg	S	0	0.06
cgt	R	0	107.30
cgc	R	0	153.40
cga	R	0	288.80
cgg	R	0	234.00
aaa	K	0	0.57
aag	K	1	0.43
agt	S	0	0.22
agc	S	1	0.18
aga	R	865	1338.00
agg	R	2001	744.60
---	--	------	------
mPD		0.42	0.96
		nPD:	0.44
		N. weight:	0.75
		Sc. PD:	0.18
		Sc. rank:	971.5

,

PA			
Pos.	583	obs:	exp:
cgt	R	27	107.30
cgc	R	36	153.50
cga	R	2637	289.00
cgg	R	152	234.20
aga	R	16	1339.00
agg	R	0	745.10
---	--	------	------
mPD		0.15	0.95
		nPD:	0.16
		N. weight:	2.6
		Sc. PD:	-0.11
		Sc. rank:	745.5

,

PA

Pos.	584	obs:	exp:
tct	S	0	0.67
tcc	S	3	0.58
tca	S	0	0.91
tcg	S	0	0.24
tgt	C	66	1108.00
tgc	C	2797	1755.00
agt	S	0	0.87
agc	S	1	0.74
ggt	G	0	0.14
ggc	G	1	0.13
gga	G	0	0.44
ggg	G	0	0.29
---	--	------	------
mPD		0.048	0.48
		nPD:	0.1
		N. weight:	0.52
		Sc. PD:	-0.052
		Sc. rank:	-84.6

PA

Pos.	585	obs:	exp:
ttt	F	4	2.50
ttc	F	2	3.50
tta	L	0	264.50
ttg	L	0	543.50
ctt	L	2827	568.00
ctc	L	34	457.60
cta	L	0	423.40
ctg	L	0	603.90
cct	P	1	0.27
ccc	P	0	0.18
cca	P	0	0.41
ccg	P	0	0.14
---	--	------	------
mPD		0.030	1.1
		nPD:	0.03
		N. weight:	2.1
		Sc. PD:	-0.36
		Sc. rank:	-2683.2

PA

Pos.	586	obs:	exp:
ttt	F	5	2.50
ttc	F	1	3.50
tta	L	0	264.60
ttg	L	0	543.70
ctt	L	2783	568.20
ctc	L	65	457.80
cta	L	12	423.60
ctg	L	2	604.10
---	--	------	------
mPD		0.059	1.1
		nPD:	0.05
		N. weight:	2.
		Sc. PD:	-0.29
		Sc. rank:	-1631.5

PA

Pos.	587	obs:	exp:
cct	P	0	0.27
ccc	P	0	0.18
cca	P	1	0.41
ccg	P	0	0.14
cat	H	1	0.58
cac	H	0	0.42
caa	Q	1920	1430.00
cag	Q	944	1434.00
aaa	K	2	1.14
aag	K	0	0.86
---	--	------	------
mPD		0.44	0.50
		nPD:	0.88
		N. weight:	0.082
		Sc. PD:	0.055
		Sc. rank:	151.9

PA

Pos.	588	obs:	exp:
tct	S	140	478.20
tcc	S	2721	415.00
tca	S	3	649.50
tcg	S	1	172.80
cct	P	0	0.82
ccc	P	3	0.54
cca	P	0	1.22
ccg	P	0	0.42
agt	S	0	621.40
agc	S	0	528.00
---	--	------	------
mPD		0.098	1.7
		nPD:	0.06
		N. weight:	2.3
		Sc. PD:	-0.32
		Sc. rank:	-1596.2

PA

Pos.	589	obs:	exp:
tta	L	0	264.70
ttg	L	0	543.90
ctt	L	2481	568.40
ctc	L	367	458.00
cta	L	10	423.70
ctg	L	5	604.30
cct	P	1	0.27
ccc	P	0	0.18
cca	P	0	0.41
ccg	P	0	0.14
cat	H	3	1.74
cac	H	0	1.26
att	I	1	0.37
atc	I	0	0.27
ata	I	0	0.37
---	--	------	------
mPD		0.24	1.1
		nPD:	0.21
		N. weight:	1.6
		Sc. PD:	0.013
		Sc. rank:	962.6

PA

Pos.	590	obs:	exp:
cct	P	0	0.82
ccc	P	0	0.54
cca	P	3	1.22
ccg	P	0	0.42
caa	Q	2827	1430.00
cag	Q	37	1434.00
aaa	K	1	0.57
aag	K	0	0.43
---	--	------	------
mPD		0.028	0.50
		nPD:	0.06
		N. weight:	0.83
		Sc. PD:	-0.12
		Sc. rank:	-650.0

PA

Pos.	591	obs:	exp:
tta	L	0	0.09
ttg	L	0	0.19
ctt	L	0	0.20
ctc	L	0	0.16
cta	L	1	0.15
ctg	L	0	0.21
cct	P	0	0.55
ccc	P	0	0.36
cca	P	2	0.81
ccg	P	0	0.28
caa	Q	2792	1430.00
cag	Q	73	1435.00
---	--	------	------
mPD		0.052	0.50
		nPD:	0.1
		N. weight:	0.77
		Sc. PD:	-0.074
		Sc. rank:	-113.7

PA

Pos.	592	obs:	exp:
att	I	2858	1049.00
atc	I	0	765.10
ata	I	1	1045.00
gtt	V	9	1.94
gtc	V	0	1.89
gta	V	0	1.92
gtg	V	0	3.25
---	--	------	------
mPD		0.0070	0.67
		nPD:	0.01
		N. weight:	1.3
		Sc. PD:	-0.25
		Sc. rank:	-2157.2

PA

Pos.	593	obs:	exp:
gat	D	1	0.54
gac	D	0	0.46
gaa	E	530	1607.00
gag	E	2337	1260.00
---	--	------	------
mPD		0.30	0.49
		nPD:	0.61
		N. weight:	0.4
		Sc. PD:	0.16
		Sc. rank:	639.4

PA

Pos.	594	obs:	exp:
tct	S	0	477.40
tcc	S	0	414.30
tca	S	0	648.40
tcg	S	0	172.50
cgt	R	0	0.07
cgc	R	0	0.11
cga	R	0	0.20
cgg	R	0	0.16
act	T	0	0.26
acc	T	1	0.23
aca	T	0	0.42
acg	T	0	0.09
aat	N	0	2.18
aac	N	4	1.82
agt	S	24	620.30
agc	S	2836	527.10
aga	R	1	0.93
agg	R	1	0.52
ggt	G	0	0.14
ggc	G	1	0.13
gga	G	0	0.44
ggg	G	0	0.29
---	--	------	------
mPD		0.022	1.7
		nPD:	0.01
		N. weight:	2.2
		Sc. PD:	-0.4
		Sc. rank:	-3418.3

PA

Pos.	595	obs:	exp:
tta	L	0	0.37
ttg	L	0	0.76
ctt	L	1	0.79
ctc	L	0	0.64
cta	L	0	0.59
ctg	L	3	0.84
att	I	4	2.20
atc	I	0	1.61
ata	I	2	2.19
atg	M	2851	2851.00
act	T	0	1.57
acc	T	0	1.35
aca	T	0	2.51
acg	T	6	0.56
gtt	V	0	0.22
gtc	V	0	0.21
gta	V	0	0.21
gtg	V	1	0.36
---	--	------	------
mPD		0.013	0.018
		nPD:	0.71
		N. weight:	0.011
		Sc. PD:	0.0054
		Sc. rank:	19.1

PA

Pos.	596	obs:	exp:
att	I	2820	1042.00
atc	I	18	759.70
ata	I	1	1037.00
aat	N	1	0.55
aac	N	0	0.45
gtt	V	26	6.02
gtc	V	2	5.89
gta	V	0	5.97
gtg	V	0	10.12
---	--	------	------
mPD		0.035	0.68
		nPD:	0.05
		N. weight:	1.3
		Sc. PD:	-0.19
		Sc. rank:	-1130.4

PA

Pos.	597	obs:	exp:
gtt	V	0	0.43
gtc	V	0	0.42
gta	V	0	0.43
gtg	V	2	0.72
gaa	E	686	1606.00
gag	E	2179	1259.00
ggt	G	0	0.14
ggc	G	0	0.13
gga	G	0	0.44
ggg	G	1	0.29
---	--	------	------
mPD		0.37	0.50
		nPD:	0.74
		N. weight:	0.29
		Sc. PD:	0.15
		Sc. rank:	520.0

PA

Pos.	598	obs:	exp:
tct	S	0	0.17
tcc	S	1	0.14
tca	S	0	0.23
tcg	S	0	0.06
act	T	0	0.79
acc	T	3	0.68
aca	T	0	1.26
acg	T	0	0.28
agt	S	0	0.22
agc	S	0	0.18
gct	A	266	721.50
gcc	A	2565	554.20
gca	A	32	1275.00
gcg	A	1	313.50
---	--	------	------
mPD		0.19	0.69
		nPD:	0.28
		N. weight:	1.6
		Sc. PD:	0.12
		Sc. rank:	1422.5

PA

Pos.	599	obs:	exp:
gat	D	1	0.54
gac	D	0	0.46
gaa	E	1050	1606.00
gag	E	1815	1259.00
ggt	G	0	0.29
ggc	G	0	0.26
gga	G	0	0.88
ggg	G	2	0.57
---	--	------	------
mPD		0.47	0.49
		nPD:	0.94
		N. weight:	0.1
		Sc. PD:	0.076
		Sc. rank:	193.9

PA

Pos.	600	obs:	exp:
tct	S	2825	478.70
tcc	S	41	415.40
tca	S	2	650.20
tcg	S	0	173.00
agt	S	0	622.10
agc	S	0	528.50
---	--	------	------
mPD		0.030	1.7
		nPD:	0.02
		N. weight:	2.3
		Sc. PD:	-0.41
		Sc. rank:	-3318.2

PA

Pos.	601	obs:	exp:
ttt	F	1	0.42
ttc	F	0	0.58
tct	S	2794	478.60
tcc	S	50	415.30
tca	S	16	650.00
tcg	S	7	173.00
agt	S	0	621.90
agc	S	0	528.40
---	--	------	------
mPD		0.051	1.7
		nPD:	0.03
		N. weight:	2.2
		Sc. PD:	-0.37
		Sc. rank:	-2690.6

PA

Pos.	602	obs:	exp:
att	I	0	8.44
atc	I	23	6.15
ata	I	0	8.41
gtt	V	71	611.90
gtc	V	2773	598.50
gta	V	1	606.60
gtg	V	0	1028.00
---	--	------	------
mPD		0.065	0.75
		nPD:	0.09
		N. weight:	1.9
		Sc. PD:	-0.22
		Sc. rank:	-649.3

PA

Pos.	603	obs:	exp:
cgt	R	0	0.52
cgc	R	0	0.75
cga	R	0	1.41
cgg	R	0	1.14
aaa	K	2792	1625.00
aag	K	62	1229.00
aga	R	12	6.54
agg	R	2	3.64
---	--	------	------
mPD		0.053	0.50
		nPD:	0.11
		N. weight:	0.62
		Sc. PD:	-0.058
		Sc. rank:	-73.2

PA

Pos.	604	obs:	exp:
aaa	K	1	0.57
aag	K	0	0.43
gaa	E	199	1607.00
gag	E	2668	1260.00
---	--	------	------
mPD		0.13	0.49
		nPD:	0.26
		N. weight:	0.74
		Sc. PD:	0.046
		Sc. rank:	603.8

,

PA

Pos.	605	obs:	exp:
cgt	R	0	0.15
cgc	R	0	0.21
cga	R	0	0.40
cgg	R	0	0.33
aaa	K	1225	1631.00
aag	K	1639	1233.00
aga	R	0	1.87
agg	R	4	1.04
---	--	------	------
mPD		0.49	0.49
		nPD:	1.
		N. weight:	0.058
		Sc. PD:	0.046
		Sc. rank:	108.2

,

PA

Pos.	606	obs:	exp:
aat	N	0	0.55
aac	N	1	0.45
gat	D	19	1547.00
gac	D	2846	1318.00
gaa	E	2	1.12
gag	E	0	0.88
---	--	------	------
mPD		0.015	0.50
		nPD:	0.03
		N. weight:	0.98
		Sc. PD:	-0.16
		Sc. rank:	-1207.1

,

PA

Pos.	607	obs:	exp:
tta	L	0	0.46
ttg	L	0	0.95
ctt	L	0	0.99
ctc	L	0	0.80
cta	L	2	0.74
ctg	L	3	1.06
atg	M	2861	2861.00
aaa	K	0	0.57
aag	K	1	0.43
gtt	V	0	0.22
gtc	V	0	0.21
gta	V	0	0.21
gtg	V	1	0.36
---	--	------	------
mPD		0.0063	0.0078
		nPD:	0.8
		N. weight:	0.0037
		Sc. PD:	0.0022
		Sc. rank:	6.8

,

PA

Pos.	608	obs:	exp:
tct	S	0	63.10
tcc	S	377	54.76
tca	S	1	85.69
tcg	S	0	22.80
act	T	41	652.50
acc	T	2434	560.80
aca	T	8	1041.00
acg	T	6	234.20
aat	N	0	0.55
aac	N	1	0.45
agt	S	0	81.99
agc	S	0	69.66
---	--	------	------
mPD		0.27	0.95
		nPD:	0.28
		N. weight:	1.9
		Sc. PD:	0.15
		Sc. rank:	1686.8

,

PA

Pos.	609	obs:	exp:
cgt	R	0	0.26
cgc	R	0	0.37
cga	R	0	0.71
cgg	R	0	0.57
aaa	K	1894	1629.00
aag	K	967	1232.00
aga	R	7	3.27
agg	R	0	1.82
---	--	------	------
mPD		0.45	0.50
		nPD:	0.91
		N. weight:	0.028
		Sc. PD:	0.02
		Sc. rank:	52.1

,

PA

Pos.	610	obs:	exp:
aaa	K	1	0.57
aag	K	0	0.43
gat	D	1	13.50
gac	D	24	11.50
gaa	E	2770	1593.00
gag	E	72	1249.00
---	--	------	------
mPD		0.067	0.50
		nPD:	0.13
		N. weight:	0.62
		Sc. PD:	-0.042
		Sc. rank:	66.4

,

PA

Pos.	611	obs:	exp:
ttt	F	324	1194.00
ttc	F	2539	1669.00
tta	L	4	0.37
ttg	L	0	0.76
ctt	L	0	0.79
ctc	L	0	0.64
cta	L	0	0.59
ctg	L	0	0.84
att	I	0	0.37
atc	I	1	0.27
ata	I	0	0.37
---	--	------	------
mPD		0.20	0.49
		nPD:	0.42
		N. weight:	0.3
		Sc. PD:	0.064
		Sc. rank:	382.0

,

PA

Pos.	612	obs:	exp:
ttt	F	2852	1196.00
ttc	F	15	1671.00
tat	Y	1	0.48
tac	Y	0	0.52
---	--	------	------
mPD		0.011	0.49
		nPD:	0.02
		N. weight:	1.1
		Sc. PD:	-0.2
		Sc. rank:	-1498.7

,

PA

Pos.	613	obs:	exp:
tta	L	6	0.55
ttg	L	0	1.14
ctt	L	0	1.19
ctc	L	0	0.96
cta	L	0	0.89
ctg	L	0	1.27
gtt	V	0	25.59
gtc	V	0	25.04
gta	V	119	25.37
gtg	V	0	43.00
gat	D	0	1.62
gac	D	3	1.38
gaa	E	2702	1535.00
gag	E	37	1204.00
ggt	G	0	0.14
ggc	G	0	0.13
gga	G	1	0.44
ggg	G	0	0.29
---	--	------	------
mPD		0.12	0.60
		nPD:	0.19
		N. weight:	0.74
		Sc. PD:	-0.0064
		Sc. rank:	372.2

,

PA

Pos.	614	obs:	exp:
ttt	F	0	0.42
ttc	F	1	0.58
tct	S	0	3.17
tcc	S	6	2.75
tca	S	0	4.31
tcg	S	0	1.15
att	I	0	0.73
atc	I	2	0.54
ata	I	0	0.73
act	T	3	276.60
acc	T	1050	237.70
aca	T	2	441.40
acg	T	0	99.26
aat	N	16	964.70
aac	N	1753	804.30
agt	S	0	4.12
agc	S	13	3.50
gct	A	0	4.03
gcc	A	16	3.10
gca	A	0	7.12
gcg	A	0	1.75
gat	D	0	3.24
gac	D	6	2.76
---	--	------	------
mPD		0.51	1.1
		nPD:	0.45
		N. weight:	1.3
		Sc. PD:	0.32
		Sc. rank:	1760.8

,

PA

Pos.	615	obs:	exp:
caa	Q	1	0.50
cag	Q	0	0.50
cgt	R	0	6.32
cgc	R	0	9.04
cga	R	0	17.03
cgg	R	0	13.80
aaa	K	2633	1536.00
aag	K	65	1162.00
aga	R	169	78.90
agg	R	0	43.90
---	--	------	------
mPD		0.16	0.64
		nPD:	0.24
		N. weight:	0.63
		Sc. PD:	0.027
		Sc. rank:	471.3

,

PA

Pos.	616	obs:	exp:
tta	L	0	0.65
ttg	L	7	1.33
tct	S	94	477.40
tcc	S	6	414.30
tca	S	2571	648.40
tcg	S	189	172.50
ctt	L	0	1.39
ctc	L	0	1.12
cta	L	0	1.04
ctg	L	0	1.48
agt	S	0	620.30
agc	S	0	527.10
gct	A	0	0.25
gcc	A	0	0.19
gca	A	1	0.45
gcg	A	0	0.11
---	--	------	------
mPD		0.20	1.7
		nPD:	0.12
		N. weight:	1.6
		Sc. PD:	-0.13
		Sc. rank:	13.5

,

PA			
Pos.	617	obs:	exp:
caa	Q	1	0.50
cag	Q	0	0.50
gtt	V	0	0.43
gtc	V	0	0.42
gta	V	2	0.43
gtg	V	0	0.72
gaa	E	2751	1605.00
gag	E	112	1258.00
ggt	G	0	0.29
ggc	G	0	0.26
gga	G	2	0.88
ggg	G	0	0.57
---	--	------	------
mPD		0.079	0.50
		nPD:	0.16
		N. weight:	0.57
		Sc. PD:	-0.024
		Sc. rank:	148.0

PA			
Pos.	618	obs:	exp:
tct	S	0	0.50
tcc	S	0	0.43
tca	S	0	0.68
tcg	S	3	0.18
caa	Q	0	7.99
cag	Q	16	8.01
atg	M	32	32.00
act	T	2	711.50
acc	T	0	611.50
aca	T	1731	1136.00
acg	T	981	255.40
aaa	K	4	3.42
aag	K	2	2.58
agt	S	0	0.65
agc	S	0	0.55
gtt	V	0	0.43
gtc	V	0	0.42
gta	V	0	0.43
gtg	V	2	0.72
gct	A	0	23.93
gcc	A	0	18.38
gca	A	3	42.29
gcg	A	92	10.40
---	--	------	------
mPD		0.59	0.82
		nPD:	0.73
		N. weight:	1.
		Sc. PD:	0.54
		Sc. rank:	1888.6

PA			
Pos.	619	obs:	exp:
tgg	W	2868	2868.00
---	--	------	------
mPD		0	0
		nPD:	1.
		N. weight:	0.
		Sc. PD:	0
		Sc. rank:	0

PA			
Pos.	620	obs:	exp:
cct	P	0	783.80
ccc	P	23	518.00
cca	P	2441	1164.00
ccg	P	404	402.10
---	--	------	------
mPD		0.26	0.71
		nPD:	0.36
		N. weight:	0.81
		Sc. PD:	0.13
		Sc. rank:	905.9

PA			
Pos.	621	obs:	exp:
tct	S	0	0.17
tcc	S	0	0.14
tca	S	0	0.23
tcg	S	0	0.06
att	I	2536	1047.00
atc	I	312	763.20
ata	I	4	1042.00
atg	M	1	1.00
agt	S	1	0.22
agc	S	0	0.18
gtt	V	14	3.01
gtc	V	0	2.95
gta	V	0	2.99
gtg	V	0	5.06
---	--	------	------
mPD		0.21	0.67
		nPD:	0.31
		N. weight:	0.91
		Sc. PD:	0.097
		Sc. rank:	869.4

PA			
Pos.	622	obs:	exp:
ggt	G	3	414.80
ggc	G	0	376.70
gga	G	1425	1257.00
ggg	G	1440	819.80
---	--	------	------
mPD		0.50	0.69
		nPD:	0.73
		N. weight:	0.45
		Sc. PD:	0.24
		Sc. rank:	823.1

PA			
Pos.	623	obs:	exp:
att	I	1	0.37
atc	I	0	0.27
ata	I	0	0.37
gtt	V	3	0.65
gtc	V	0	0.63
gta	V	0	0.64
gtg	V	0	1.08
gaa	E	2619	1605.00
gag	E	244	1258.00
ggt	G	0	0.14
ggc	G	0	0.13
gga	G	1	0.44
ggg	G	0	0.29
---	--	------	------
mPD		0.16	0.50
		nPD:	0.33
		N. weight:	0.41
		Sc. PD:	0.051
		Sc. rank:	425.1

PA			
Pos.	624	obs:	exp:
tct	S	3	478.70
tcc	S	531	415.40
tca	S	2302	650.20
tcg	S	32	173.00
agt	S	0	622.10
agc	S	0	528.50
---	--	------	------
mPD		0.32	1.7
		nPD:	0.19
		N. weight:	1.4
		Sc. PD:	-0.0089
		Sc. rank:	726.3

PA			
Pos.	625	obs:	exp:
cct	P	1294	783.80
ccc	P	1570	518.00
cca	P	0	1164.00
ccg	P	4	402.10
---	--	------	------
mPD		0.50	0.71
		nPD:	0.7
		N. weight:	1.1
		Sc. PD:	0.54
		Sc. rank:	1925.9

PA			
Pos.	626	obs:	exp:
cgt	R	0	2.92
cgc	R	0	4.17
cga	R	0	7.86
cgg	R	0	6.37
act	T	0	1.84
acc	T	0	1.58
aca	T	7	2.93
acg	T	0	0.66
aat	N	11	12.00
aac	N	11	10.00
aaa	K	2352	1556.00
aag	K	380	1176.00
aga	R	38	36.41
agg	R	40	20.26
gaa	E	29	16.26
gag	E	0	12.74
---	--	------	------
mPD		0.34	0.59
		nPD:	0.57
		N. weight:	0.28
		Sc. PD:	0.1
		Sc. rank:	431.7

PA			
Pos.	627	obs:	exp:
cgt	R	0	0.22
cgc	R	0	0.32
cga	R	0	0.60
cgg	R	0	0.49
aaa	K	0	1.14
aag	K	2	0.86
aga	R	0	2.80
agg	R	6	1.56
gaa	E	1	1.12
gag	E	1	0.88
ggt	G	2	413.30
ggc	G	0	375.40
gga	G	571	1252.00
ggg	G	2285	817.00
---	--	------	------
mPD		0.33	0.70
		nPD:	0.47
		N. weight:	0.88
		Sc. PD:	0.24
		Sc. rank:	1233.9

PA			
Pos.	628	obs:	exp:
tta	L	0	0.46
ttg	L	3	0.95
ctt	L	0	0.99
ctc	L	0	0.80
cta	L	0	0.74
ctg	L	2	1.06
att	I	0	2.57
atc	I	0	1.87
ata	I	7	2.56
atg	M	6	6.00
gtt	V	188	613.00
gtc	V	0	599.60
gta	V	72	607.70
gtg	V	2590	1030.00
---	--	------	------
mPD		0.19	0.75
		nPD:	0.25
		N. weight:	0.94
		Sc. PD:	0.043
		Sc. rank:	709.4

PA			
Pos.	629	obs:	exp:
gaa	E	909	1608.00
gag	E	1959	1260.00
---	--	------	------
mPD		0.43	0.49
		nPD:	0.88
		N. weight:	0.16
		Sc. PD:	0.11
		Sc. rank:	298.5

PA

Pos.	630	obs :	exp :
gtt	V	0	1.51
gtc	V	0	1.47
gta	V	7	1.49
gtg	V	0	2.53
gat	D	0	1.08
gac	D	2	0.92
gaa	E	2812	1602.00
gag	E	45	1255.00
ggt	G	0	0.29
ggc	G	0	0.26
gga	G	2	0.88
ggg	G	0	0.57
---	--	------	------
mPD		0.039	0.50
		nPD :	0.08
		N. weight :	0.67
		Sc. PD :	-0.082
		Sc. rank :	-366.9

,

PA

Pos.	631	obs :	exp :
tct	S	0	186.10
tcc	S	0	161.50
tca	S	0	252.80
tcg	S	0	67.27
att	I	0	0.37
atc	I	1	0.27
ata	I	0	0.37
agt	S	4	241.80
agc	S	1111	205.50
ggt	G	30	253.40
ggc	G	1688	230.10
gga	G	34	767.70
ggg	G	0	500.80
---	--	------	------
mPD		0.52	1.6
		nPD :	0.32
		N. weight :	2.3
		Sc. PD :	0.27
		Sc. rank :	2349.8

,

PA

Pos.	632	obs :	exp :
ttt	F	0	0.42
ttc	F	1	0.58
tct	S	602	475.40
tcc	S	2243	412.50
tca	S	3	645.60
tcg	S	0	171.80
tat	Y	0	0.48
tac	Y	1	0.52
tgt	C	0	0.77
tgc	C	2	1.23
cct	P	1	4.10
ccc	P	14	2.71
cca	P	0	6.09
ccg	P	0	2.10
act	T	0	0.26
acc	T	1	0.23
aca	T	0	0.42
acg	T	0	0.09
agt	S	0	617.70
agc	S	0	524.90
---	--	------	------
mPD		0.35	1.7
		nPD :	0.21
		N. weight :	1.8
		Sc. PD :	0.016
		Sc. rank :	1097.8

,

PA

Pos.	633	obs :	exp :
att	I	2261	1048.00
atc	I	589	764.30
ata	I	6	1044.00
gtt	V	0	2.58
gtc	V	12	2.52
gta	V	0	2.56
gtg	V	0	4.34
---	--	------	------
mPD		0.34	0.67
		nPD :	0.51
		N. weight :	0.73
		Sc. PD :	0.22
		Sc. rank :	1061.6

,

PA

Pos.	634	obs :	exp :
cgt	R	0	0.15
cgc	R	0	0.21
cga	R	0	0.40
cgg	R	4	0.33
aga	R	0	1.87
agg	R	0	1.04
ggt	G	2	414.20
ggc	G	0	376.20
gga	G	1507	1255.00
ggg	G	1355	818.70
---	--	------	------
mPD		0.50	0.69
		nPD :	0.73
		N. weight :	0.45
		Sc. PD :	0.23
		Sc. rank :	805.9

,

PA

Pos.	635	obs :	exp :
aaa	K	662	1633.00
aag	K	2206	1235.00
---	--	------	------
mPD		0.36	0.49
		nPD :	0.72
		N. weight :	0.32
		Sc. PD :	0.16
		Sc. rank :	569.7

,

PA

Pos.	636	obs :	exp :
gtt	V	15	615.60
gtc	V	0	602.10
gta	V	879	610.30
gtg	V	1968	1034.00
gct	A	0	0.25
gcc	A	0	0.19
gca	A	1	0.45
gcg	A	0	0.11
ggt	G	0	0.72
ggc	G	0	0.66
gga	G	0	2.19
ggg	G	5	1.43
---	--	------	------
mPD		0.44	0.74
		nPD :	0.59
		N. weight :	0.72
		Sc. PD :	0.28
		Sc. rank :	1132.8

,

PA

Pos.	637	obs :	exp :
tat	Y	0	0.48
tac	Y	1	0.52
tgt	C	49	1108.00
tgc	C	2815	1756.00
ggt	G	0	0.43
ggc	G	3	0.39
gga	G	0	1.31
ggg	G	0	0.86
---	--	------	------
mPD		0.036	0.48
		nPD :	0.08
		N. weight :	0.55
		Sc. PD :	-0.068
		Sc. rank :	-304.4

,

PA

Pos.	638	obs :	exp :
cgt	R	0	107.30
cgc	R	0	153.50
cga	R	3	289.00
cgg	R	0	234.20
aga	R	2767	1339.00
agg	R	98	745.10
---	--	------	------
mPD		0.068	0.95
		nPD :	0.07
		N. weight :	0.83
		Sc. PD :	-0.11
		Sc. rank :	-480.7

,

PA

Pos.	639	obs :	exp :
act	T	20	750.30
acc	T	540	644.90
aca	T	2262	1198.00
acg	T	40	269.30
gct	A	0	1.51
gcc	A	0	1.16
gca	A	6	2.67
gcg	A	0	0.66
---	--	------	------
mPD		0.34	0.70
		nPD :	0.49
		N. weight :	0.56
		Sc. PD :	0.16
		Sc. rank :	786.2

,

PA

Pos.	640	obs :	exp :
ttt	F	3	1.25
ttc	F	0	1.75
tta	L	1304	264.80
ttg	L	1453	544.10
tgg	W	1	1.00
ctt	L	0	568.60
ctc	L	0	458.10
cta	L	62	423.90
ctg	L	45	604.50
---	--	------	------
mPD		0.57	1.1
		nPD :	0.51
		N. weight :	1.5
		Sc. PD :	0.46
		Sc. rank :	2197.4

,

PA

Pos.	641	obs :	exp :
tta	L	24	264.80
ttg	L	19	544.10
ctt	L	3	568.60
ctc	L	16	458.10
cta	L	2136	423.90
ctg	L	666	604.50
cct	P	0	1.09
ccc	P	0	0.72
cca	P	4	1.62
ccg	P	0	0.56
---	--	------	------
mPD		0.41	1.1
		nPD :	0.36
		N. weight :	1.6
		Sc. PD :	0.24
		Sc. rank :	1760.0

,

PA

Pos.	642	obs :	exp :
tct	S	0	0.17
tcc	S	0	0.14
tca	S	1	0.23
tcg	S	0	0.06
act	T	0	0.26
acc	T	0	0.23
aca	T	1	0.42
acg	T	0	0.09
agt	S	0	0.22
agc	S	0	0.18
gct	A	72	722.00
gcc	A	1	554.60
gca	A	2372	1276.00
gcg	A	421	313.70
---	--	------	------
mPD		0.29	0.69
		nPD :	0.43
		N. weight :	0.66
		Sc. PD :	0.15
		Sc. rank :	839.3

,

PA

Pos.	643	obs :	exp :
cgt	R	0	3.70
cgc	R	0	5.30
cga	R	0	9.98
cgg	R	0	8.08
act	T	0	0.79
acc	T	0	0.68
aca	T	3	1.26
acg	T	0	0.28
aat	N	1	0.55
aac	N	0	0.45
aaa	K	1352	1574.00
aag	K	1413	1191.00
aga	R	2	46.22
agg	R	97	25.72
---	--	------	------
mPD		0.57	0.58
		nPD :	0.98
		N. weight :	0.078
		Sc. PD :	0.059
		Sc. rank :	144.3

,

PA

Pos.	644	obs :	exp :
tct	S	2833	478.60
tcc	S	8	415.30
tca	S	4	650.00
tcg	S	22	173.00
agt	S	0	621.90
agc	S	0	528.40
gct	A	1	0.25
gcc	A	0	0.19
gca	A	0	0.45
gcg	A	0	0.11
---	--	------	------
mPD		0.024	1.7
		nPD :	0.01
		N. weight :	2.3
		Sc. PD :	-0.42
		Sc. rank :	-3461.6

,

PA

Pos.	645	obs :	exp :
tta	L	0	0.09
ttg	L	0	0.19
ctt	L	0	0.20
ctc	L	0	0.16
cta	L	1	0.15
ctg	L	0	0.21
gtt	V	141	616.60
gtc	V	18	603.20
gta	V	1329	611.30
gtg	V	1379	1036.00
---	--	------	------
mPD		0.55	0.73
		nPD :	0.75
		N. weight :	0.54
		Sc. PD :	0.29
		Sc. rank :	984.5

,

PA

Pos.	646	obs :	exp :
ttt	F	20	1196.00
ttc	F	2846	1670.00
tct	S	0	0.33
tcc	S	2	0.29
tca	S	0	0.45
tcg	S	0	0.12
agt	S	0	0.43
agc	S	0	0.37
---	--	------	------
mPD		0.015	0.49
		nPD :	0.03
		N. weight :	0.67
		Sc. PD :	-0.11
		Sc. rank :	-806.5

,

PA

Pos.	647	obs :	exp :
act	T	0	0.52
acc	T	1	0.45
aca	T	1	0.84
acg	T	0	0.19
aat	N	31	1563.00
aac	N	2835	1303.00
---	--	------	------
mPD		0.023	0.50
		nPD :	0.05
		N. weight :	0.97
		Sc. PD :	-0.15
		Sc. rank :	-897.9

,

PA			
Pos.	648	obs:	exp:
tct	S	0	472.20
tcc	S	0	409.80
tca	S	0	641.30
tcg	S	0	170.70
tgt	C	0	6.19
tgc	C	16	9.81
cgt	R	0	0.04
cgc	R	1	0.05
cga	R	0	0.10
cgg	R	0	0.08
aat	N	5	10.91
aac	N	15	9.09
agt	S	621	613.60
agc	S	2208	521.40
aga	R	0	0.47
agg	R	0	0.26
gct	A	1	0.25
gcc	A	0	0.19
gca	A	0	0.45
gcg	A	0	0.11
ggt	G	1	0.14
ggc	G	0	0.13
gga	G	0	0.44
ggg	G	0	0.29
---	--	------	------
mPD		0.37	1.7
		nPD:	0.22
		N. weight:	1.5
		Sc. PD:	0.032
		Sc. rank:	969.6

,

PA			
Pos.	649	obs:	exp:
tta	L	485	264.90
ttg	L	53	544.30
ctt	L	0	568.80
ctc	L	1	458.30
cta	L	2278	424.00
ctg	L	48	604.70
att	I	0	1.10
atc	I	0	0.80
ata	I	3	1.10
---	--	------	------
mPD		0.38	1.1
		nPD:	0.33
		N. weight:	1.8
		Sc. PD:	0.24
		Sc. rank:	1909.5

,

PA			
Pos.	650	obs:	exp:
ttt	F	1	0.42
ttc	F	0	0.58
tat	Y	2838	1375.00
tac	Y	29	1492.00
---	--	------	------
mPD		0.021	0.50
		nPD:	0.04
		N. weight:	0.9
		Sc. PD:	-0.14
		Sc. rank:	-891.7

,

PA			
Pos.	651	obs:	exp:
tct	S	0	0.83
tcc	S	0	0.72
tca	S	5	1.13
tcg	S	0	0.30
act	T	0	0.26
acc	T	0	0.23
aca	T	1	0.42
acg	T	0	0.09
agt	S	0	1.08
agc	S	0	0.92
gct	A	73	721.00
gcc	A	4	553.80
gca	A	2765	1274.00
gcg	A	20	313.30
---	--	------	------
mPD		0.070	0.69
		nPD:	0.1
		N. weight:	0.89
		Sc. PD:	-0.088
		Sc. rank:	-161.5

,

PA			
Pos.	652	obs:	exp:
tct	S	2715	478.10
tcc	S	147	414.90
tca	S	2	649.30
tcg	S	0	172.80
act	T	4	1.05
acc	T	0	0.90
aca	T	0	1.67
acg	T	0	0.38
agt	S	0	621.20
agc	S	0	527.80
---	--	------	------
mPD		0.10	1.7
		nPD:	0.06
		N. weight:	2.1
		Sc. PD:	-0.29
		Sc. rank:	-1444.8

,

PA			
Pos.	653	obs:	exp:
tct	S	1	49.24
tcc	S	0	42.73
tca	S	294	66.88
tcg	S	0	17.80
cct	P	1	701.50
ccc	P	9	463.60
cca	P	2550	1042.00
ccg	P	7	359.90
act	T	0	1.05
acc	T	0	0.90
aca	T	4	1.67
acg	T	0	0.38
agt	S	0	63.99
agc	S	0	54.37
gct	A	0	0.50
gcc	A	0	0.39
gca	A	2	0.89
gcg	A	0	0.22
---	--	------	------
mPD		0.20	0.99
		nPD:	0.2
		N. weight:	1.2
		Sc. PD:	0.0031
		Sc. rank:	682.8

,

PA			
Pos.	654	obs:	exp:
caa	Q	2810	1432.00
cag	Q	58	1436.00
---	--	------	------
mPD		0.040	0.50
		nPD:	0.08
		N. weight:	0.79
		Sc. PD:	-0.095
		Sc. rank:	-394.7

,

PA			
Pos.	655	obs:	exp:
tta	L	0	264.80
ttg	L	0	544.10
ctt	L	1199	568.60
ctc	L	1635	458.10
cta	L	30	423.90
ctg	L	0	604.50
cct	P	1	0.27
ccc	P	0	0.18
cca	P	0	0.41
ccg	P	0	0.14
att	I	0	1.10
atc	I	3	0.80
ata	I	0	1.10
---	--	------	------
mPD		0.50	1.1
		nPD:	0.45
		N. weight:	1.3
		Sc. PD:	0.33
		Sc. rank:	1775.7

,

PA			
Pos.	656	obs:	exp:
gaa	E	25	1607.00
gag	E	2841	1259.00
ggt	G	0	0.29
ggc	G	0	0.26
gga	G	0	0.88
ggg	G	2	0.57
---	--	------	------
mPD		0.019	0.49
		nPD:	0.04
		N. weight:	1.
		Sc. PD:	-0.17
		Sc. rank:	-1052.0

,

PA			
Pos.	657	obs:	exp:
ggt	G	8	414.80
ggc	G	0	376.70
gga	G	274	1257.00
ggg	G	2586	819.80
---	--	------	------
mPD		0.18	0.69
		nPD:	0.26
		N. weight:	1.2
		Sc. PD:	0.066
		Sc. rank:	928.1

,

PA			
Pos.	658	obs:	exp:
ttt	F	2820	1196.00
ttc	F	48	1672.00
---	--	------	------
mPD		0.033	0.49
		nPD:	0.07
		N. weight:	1.
		Sc. PD:	-0.14
		Sc. rank:	-640.6

,

PA			
Pos.	659	obs:	exp:
tct	S	3	478.40
tcc	S	15	415.20
tca	S	2651	649.70
tcg	S	197	172.90
act	T	0	0.26
acc	T	0	0.23
aca	T	1	0.42
acg	T	0	0.09
agt	S	0	621.60
agc	S	0	528.20
gct	A	0	0.25
gcc	A	0	0.19
gca	A	1	0.45
gcg	A	0	0.11
---	--	------	------
mPD		0.14	1.7
		nPD:	0.09
		N. weight:	1.7
		Sc. PD:	-0.2
		Sc. rank:	-666.5

,

PA			
Pos.	660	obs:	exp:
tct	S	6	1.00
tcc	S	0	0.87
tca	S	0	1.36
tcg	S	0	0.36
cct	P	1	0.27
ccc	P	0	0.18
cca	P	0	0.41
ccg	P	0	0.14
agt	S	0	1.30
agc	S	0	1.11
gct	A	2663	720.70
gcc	A	184	553.60
gca	A	14	1273.00
gcg	A	0	313.10
---	--	------	------
mPD		0.13	0.70
		nPD:	0.19
		N. weight:	1.5
		Sc. PD:	-0.013
		Sc. rank:	762.6

,

PA			
Pos.	661	obs:	exp:
gaa	E	2761	1606.00
gag	E	104	1259.00
ggt	G	0	0.43
ggc	G	0	0.39
gga	G	3	1.31
ggg	G	0	0.86
---	--	------	------
mPD		0.072	0.50
		nPD:	0.15
		N. weight:	0.58
		Sc. PD:	-0.032
		Sc. rank:	101.0

,

PA			
Pos.	662	obs:	exp:
tct	S	4	478.70
tcc	S	66	415.40
tca	S	2725	650.20
tcg	S	73	173.00
agt	S	0	622.10
agc	S	0	528.50
---	--	------	------
mPD		0.096	1.7
		nPD:	0.06
		N. weight:	1.7
		Sc. PD:	-0.24
		Sc. rank:	-1309.7

,

PA

Pos.	663	obs:	exp:
cgt	R	0	107.30
cgc	R	0	153.40
cga	R	1	288.90
cgg	R	0	234.10
aaa	K	1	0.57
aag	K	0	0.43
aga	R	2826	1338.00
agg	R	40	744.80
---	--	------	------
mPD		0.029	0.95
		nPD:	0.03
		N. weight:	0.92
		Sc. PD:	-0.16
		Sc. rank:	-1144.6

PA

Pos.	664	obs:	exp:
cgt	R	0	0.19
cgc	R	0	0.27
cga	R	0	0.50
cgg	R	0	0.41
act	T	0	0.26
acc	T	0	0.23
aca	T	1	0.42
acg	T	0	0.09
aat	N	0	5.45
aac	N	10	4.55
aaa	K	2769	1624.00
aag	K	83	1228.00
aga	R	4	2.33
agg	R	1	1.30
---	--	------	------
mPD		0.068	0.50
		nPD:	0.14
		N. weight:	0.59
		Sc. PD:	-0.038
		Sc. rank:	66.1

PA

Pos.	665	obs:	exp:
ttt	F	0	0.42
ttc	F	1	0.58
tta	L	78	264.50
ttg	L	2582	543.50
ctt	L	0	568.00
ctc	L	2	457.60
cta	L	3	423.40
ctg	L	196	603.90
atg	M	6	6.00
---	--	------	------
mPD		0.19	1.1
		nPD:	0.17
		N. weight:	1.7
		Sc. PD:	-0.053
		Sc. rank:	626.3

PA

Pos.	666	obs:	exp:
tta	L	3	265.10
ttg	L	0	544.70
ctt	L	2774	569.20
ctc	L	77	458.60
cta	L	13	424.30
ctg	L	0	605.10
att	I	1	0.37
atc	I	0	0.27
ata	I	0	0.37
---	--	------	------
mPD		0.066	1.1
		nPD:	0.06
		N. weight:	1.9
		Sc. PD:	-0.27
		Sc. rank:	-1450.6

PA

Pos.	667	obs:	exp:
tta	L	0	265.10
ttg	L	0	544.70
ctt	L	8	569.20
ctc	L	2856	458.60
cta	L	3	424.30
ctg	L	0	605.10
att	I	0	0.37
atc	I	1	0.27
ata	I	0	0.37
---	--	------	------
mPD		0.0083	1.1
		nPD:	0.01
		N. weight:	2.4
		Sc. PD:	-0.46
		Sc. rank:	-4018.1

PA

Pos.	668	obs:	exp:
tta	L	0	0.09
ttg	L	1	0.19
ctt	L	0	0.20
ctc	L	0	0.16
cta	L	0	0.15
ctg	L	0	0.21
att	I	2855	1050.00
atc	I	6	765.60
ata	I	0	1046.00
act	T	1	0.26
acc	T	0	0.23
aca	T	0	0.42
acg	T	0	0.09
gtt	V	5	1.08
gtc	V	0	1.05
gta	V	0	1.07
gtg	V	0	1.81
---	--	------	------
mPD		0.0097	0.67
		nPD:	0.01
		N. weight:	1.3
		Sc. PD:	-0.24
		Sc. rank:	-2002.2

PA

Pos.	669	obs:	exp:
att	I	17	6.24
atc	I	0	4.55
ata	I	0	6.21
act	T	2	0.52
acc	T	0	0.45
aca	T	0	0.84
acg	T	0	0.19
gtt	V	2491	542.70
gtc	V	31	530.80
gta	V	1	538.00
gtg	V	0	911.60
gct	A	325	82.13
gcc	A	0	63.08
gca	A	1	145.10
gcg	A	0	35.68
---	--	------	------
mPD		0.24	0.96
		nPD:	0.25
		N. weight:	1.9
		Sc. PD:	0.092
		Sc. rank:	1474.4

PA

Pos.	670	obs:	exp:
caa	Q	8	1431.00
cag	Q	2859	1436.00
cgt	R	0	0.04
cgc	R	1	0.05
cga	R	0	0.10
cgg	R	0	0.08
aga	R	0	0.47
agg	R	0	0.26
---	--	------	------
mPD		0.0070	0.50
		nPD:	0.01
		N. weight:	0.9
		Sc. PD:	-0.17
		Sc. rank:	-1400.1

PA

Pos.	671	obs:	exp:
gct	A	2	722.50
gcc	A	3	555.00
gca	A	2836	1277.00
gcg	A	27	313.90
---	--	------	------
mPD		0.022	0.69
		nPD:	0.03
		N. weight:	1.
		Sc. PD:	-0.17
		Sc. rank:	-1181.0

PA

Pos.	672	obs:	exp:
ttt	F	11	4.59
ttc	F	0	6.41
tta	L	0	264.20
ttg	L	0	542.80
ctt	L	2855	567.30
ctc	L	2	457.00
cta	L	0	422.80
ctg	L	0	603.00
---	--	------	------
mPD		0.0090	1.1
		nPD:	0.01
		N. weight:	2.1
		Sc. PD:	-0.41
		Sc. rank:	-3555.4

PA

Pos.	673	obs:	exp:
cgt	R	0	106.70
cgc	R	0	152.50
cga	R	0	287.20
cgg	R	8	232.70
atg	M	11	11.00
aaa	K	0	3.42
aag	K	6	2.58
aga	R	3	1330.00
agg	R	2839	740.40
ggt	G	0	0.14
ggc	G	0	0.13
gga	G	0	0.44
ggg	G	1	0.29
---	--	------	------
mPD		0.020	0.97
		nPD:	0.02
		N. weight:	1.8
		Sc. PD:	-0.31
		Sc. rank:	-2437.9

PA

Pos.	674	obs:	exp:
gat	D	29	1549.00
gac	D	2839	1319.00
---	--	------	------
mPD		0.020	0.50
		nPD:	0.04
		N. weight:	0.96
		Sc. PD:	-0.15
		Sc. rank:	-949.7

PA

Pos.	675	obs:	exp:
tct	S	0	1.00
tcc	S	0	0.87
tca	S	0	1.36
tcg	S	0	0.36
act	T	0	0.26
acc	T	1	0.23
aca	T	0	0.42
acg	T	0	0.09
aat	N	36	1560.00
aac	N	2825	1301.00
agt	S	0	1.30
agc	S	6	1.11
---	--	------	------
mPD		0.030	0.50
		nPD:	0.06
		N. weight:	0.96
		Sc. PD:	-0.13
		Sc. rank:	-709.2

PA

Pos.	676	obs:	exp:
tta	L	0	265.20
ttg	L	1	544.80
ctt	L	6	569.40
ctc	L	0	458.80
cta	L	3	424.40
ctg	L	2858	605.30
---	--	------	------
mPD		0.0070	1.1
		nPD:	0.01
		N. weight:	2.
		Sc. PD:	-0.39
		Sc. rank:	-3443.9

PA

Pos.	677	obs:	exp:
aaa	K	1	0.57
aag	K	0	0.43
gat	D	2	1.62
gac	D	1	1.38
gaa	E	2841	1605.00
gag	E	23	1259.00
---	--	------	------
mPD		0.019	0.49
		nPD:	0.04
		N. weight:	0.71
		Sc. PD:	-0.11
		Sc. rank:	-732.9

PA

Pos.	678	obs:	exp:
cct	P	2864	783.80
ccc	P	3	518.00
cca	P	0	1164.00
ccg	P	1	402.10
---	--	------	------
mPD		0.0028	0.71
		nPD:	0.
		N. weight:	1.7
		Sc. PD:	-0.33
		Sc. rank:	-3021.2

PA

Pos.	679	obs:	exp:
tgg	W	1	1.00
cgt	R	0	0.07
cgc	R	0	0.11
cga	R	0	0.20
cgg	R	0	0.16
aga	R	2	0.93
agg	R	0	0.52
ggt	G	29	414.30
ggc	G	1	376.30
gga	G	1732	1255.00
ggg	G	1103	819.00
---	--	------	------
mPD		0.49	0.69
		nPD:	0.71
		N. weight:	0.38
		Sc. PD:	0.19
		Sc. rank:	663.9

PA

Pos.	680	obs:	exp:
tct	S	0	0.33
tcc	S	1	0.29
tca	S	0	0.45
tcg	S	0	0.12
act	T	23	750.00
acc	T	2786	644.70
aca	T	52	1197.00
acg	T	0	269.20
agt	S	0	0.43
agc	S	1	0.37
gct	A	0	1.26
gcc	A	5	0.97
gca	A	0	2.23
gcg	A	0	0.55
---	--	------	------
mPD		0.056	0.70
		nPD:	0.08
		N. weight:	1.8
		Sc. PD:	-0.21
		Sc. rank:	-871.6

PA			
Pos.	681	obs:	exp:
ttt	F	118	1183.00
ttc	F	2717	1652.00
tta	L	0	3.05
ttg	L	33	6.27
ctt	L	0	6.55
ctc	L	0	5.28
cta	L	0	4.88
ctg	L	0	6.97
mPD		0.10	0.51
		nPD:	0.2
		N. weight:	0.53
		Sc. PD:	-0.002
		Sc. rank:	278.7

PA			
Pos.	682	obs:	exp:
aat	N	5	2.73
aac	N	0	2.27
gat	D	2853	1545.00
gac	D	9	1317.00
gaa	E	0	0.56
gag	E	1	0.44
mPD		0.010	0.50
		nPD:	0.02
		N. weight:	0.79
		Sc. PD:	-0.14
		Sc. rank:	-1110.3

PA			
Pos.	683	obs:	exp:
tta	L	0	264.10
ttg	L	0	542.60
ctt	L	2849	567.10
ctc	L	4	456.80
cta	L	2	422.70
ctg	L	1	602.80
cct	P	0	0.27
ccc	P	0	0.18
cca	P	1	0.41
ccg	P	0	0.14
att	I	11	4.04
atc	I	0	2.94
ata	I	0	4.02
mPD		0.014	1.1
		nPD:	0.01
		N. weight:	2.1
		Sc. PD:	-0.4
		Sc. rank:	-3415.8

PA			
Pos.	684	obs:	exp:
cgt	R	0	0.15
cgc	R	0	0.21
cga	R	0	0.40
cgg	R	0	0.33
aga	R	1	1.87
agg	R	3	1.04
gct	A	0	0.50
gcc	A	0	0.39
gca	A	2	0.89
gcg	A	0	0.22
gaa	E	8	10.09
gag	E	10	7.91
ggt	G	0	411.30
ggc	G	0	373.60
gga	G	541	1246.00
ggg	G	2303	813.00
mPD		0.33	0.70
		nPD:	0.47
		N. weight:	0.91
		Sc. PD:	0.24
		Sc. rank:	1256.7

PA			
Pos.	685	obs:	exp:
ggt	G	0	414.80
ggc	G	0	376.70
gga	G	27	1257.00
ggg	G	2841	819.80
mPD		0.019	0.69
		nPD:	0.03
		N. weight:	1.6
		Sc. PD:	-0.27
		Sc. rank:	-2053.0

PA			
Pos.	686	obs:	exp:
tta	L	11	262.40
ttg	L	0	539.10
ctt	L	0	563.50
ctc	L	1	454.00
cta	L	2810	420.00
ctg	L	16	599.00
gtt	V	0	6.45
gtc	V	0	6.31
gta	V	30	6.40
gtg	V	0	10.84
mPD		0.040	1.1
		nPD:	0.04
		N. weight:	2.5
		Sc. PD:	-0.4
		Sc. rank:	-2742.8

PA			
Pos.	687	obs:	exp:
tat	Y	2842	1376.00
tac	Y	26	1492.00
mPD		0.018	0.50
		nPD:	0.04
		N. weight:	0.91
		Sc. PD:	-0.15
		Sc. rank:	-1011.0

PA			
Pos.	688	obs:	exp:
gat	D	2	21.60
gac	D	38	18.40
gaa	E	2670	1574.00
gag	E	137	1233.00
ggt	G	0	3.04
ggc	G	0	2.76
gga	G	21	9.20
ggg	G	0	6.00
mPD		0.13	0.52
		nPD:	0.25
		N. weight:	0.54
		Sc. PD:	0.027
		Sc. rank:	410.2

PA			
Pos.	689	obs:	exp:
tct	S	0	0.17
tcc	S	0	0.14
tca	S	1	0.23
tcg	S	0	0.06
cct	P	0	0.27
ccc	P	0	0.18
cca	P	1	0.41
ccg	P	0	0.14
agt	S	0	0.22
agc	S	0	0.18
gct	A	0	722.00
gcc	A	6	554.60
gca	A	2836	1276.00
gcg	A	24	313.70
mPD		0.022	0.69
		nPD:	0.03
		N. weight:	1.
		Sc. PD:	-0.17
		Sc. rank:	-1204.1

PA			
Pos.	690	obs:	exp:
att	I	2843	1051.00
atc	I	22	766.70
ata	I	0	1047.00
atg	M	1	1.00
gtt	V	2	0.43
gtc	V	0	0.42
gta	V	0	0.43
gtg	V	0	0.72
mPD		0.017	0.66
		nPD:	0.03
		N. weight:	1.3
		Sc. PD:	-0.22
		Sc. rank:	-1673.8

PA			
Pos.	691	obs:	exp:
gaa	E	3	1608.00
gag	E	2865	1260.00
mPD		0.0021	0.49
		nPD:	0.
		N. weight:	1.1
		Sc. PD:	-0.21
		Sc. rank:	-1886.3

PA			
Pos.	692	obs:	exp:
gaa	E	2	1608.00
gag	E	2866	1260.00
mPD		0.0014	0.49
		nPD:	0.
		N. weight:	1.1
		Sc. PD:	-0.21
		Sc. rank:	-1964.7

PA			
Pos.	693	obs:	exp:
tgt	C	113	1110.00
tgc	C	2754	1757.00
tgg	W	1	1.00
mPD		0.076	0.47
		nPD:	0.16
		N. weight:	0.46
		Sc. PD:	-0.018
		Sc. rank:	140.8

PA			
Pos.	694	obs:	exp:
tta	L	0	264.80
ttg	L	41	544.10
ctt	L	0	568.60
ctc	L	0	458.10
cta	L	214	423.90
ctg	L	2609	604.50
atg	M	1	1.00
gtt	V	0	0.65
gtc	V	0	0.63
gta	V	0	0.64
gtg	V	3	1.08
mPD		0.17	1.1
		nPD:	0.15
		N. weight:	1.7
		Sc. PD:	-0.083
		Sc. rank:	338.2

PA			
Pos.	695	obs:	exp:
att	I	2863	1051.00
atc	I	0	766.10
ata	I	0	1046.00
gtt	V	5	1.08
gtc	V	0	1.05
gta	V	0	1.07
gtg	V	0	1.81
mPD		0.0035	0.66
		nPD:	0.01
		N. weight:	1.3
		Sc. PD:	-0.26
		Sc. rank:	-2284.5

PA			
Pos.	696	obs:	exp:
aat	N	1244	1547.00
aac	N	1593	1290.00
aaa	K	0	17.65
aag	K	31	13.35
mPD		0.50	0.51
		nPD:	0.99
		N. weight:	0.044
		Sc. PD:	0.034
		Sc. rank:	82.2

PA			
Pos.	697	obs:	exp:
aat	N	2	1.09
aac	N	0	0.91
gat	D	2825	1531.00
gac	D	10	1304.00
gaa	E	31	17.38
gag	E	0	13.62
mPD		0.030	0.51
		nPD:	0.06
		N. weight:	0.79
		Sc. PD:	-0.11
		Sc. rank:	-598.3

PA

Pos.	698	obs:	exp:
cct	P	49	783.80
ccc	P	2819	518.00
cca	P	0	1164.00
ccg	P	0	402.10
---	--	------	------
mPD		0.034	0.71
		nPD:	0.05
		N. weight:	2.2
		Sc. PD:	-0.33
		Sc. rank:	-1943.3

,

PA

Pos.	699	obs:	exp:
tta	L	0	0.09
ttg	L	0	0.19
tgg	W	2865	2865.00
ctt	L	0	0.20
ctc	L	0	0.16
cta	L	0	0.15
ctg	L	1	0.21
cgt	R	0	0.07
cgc	R	0	0.11
cga	R	0	0.20
cgg	R	0	0.16
aga	R	0	0.93
agg	R	2	0.52
---	--	------	------
mPD		0.0028	0.0039
		nPD:	0.71
		N. weight:	0.002
		Sc. PD:	0.00099
		Sc. rank:	3.5

,

PA

Pos.	700	obs:	exp:
gtt	V	2867	616.60
gtc	V	0	603.20
gta	V	0	611.30
gtg	V	0	1036.00
ggt	G	1	0.14
ggc	G	0	0.13
gga	G	0	0.44
ggg	G	0	0.29
---	--	------	------
mPD		0.00070	0.73
		nPD:	0.
		N. weight:	2.
		Sc. PD:	-0.4
		Sc. rank:	-3745.4

,

PA

Pos.	701	obs:	exp:
tta	L	7	265.00
ttg	L	2847	544.50
tct	S	0	0.33
tcc	S	2	0.29
tca	S	0	0.45
tcg	S	0	0.12
ctt	L	0	569.00
ctc	L	0	458.40
cta	L	0	424.10
ctg	L	12	604.90
agt	S	0	0.43
agc	S	0	0.37
---	--	------	------
mPD		0.016	1.1
		nPD:	0.01
		N. weight:	2.2
		Sc. PD:	-0.4
		Sc. rank:	-3290.1

,

PA

Pos.	702	obs:	exp:
tta	L	0	265.10
ttg	L	0	544.70
ctt	L	2722	569.20
ctc	L	145	458.60
cta	L	0	424.30
ctg	L	0	605.10
cct	P	1	0.27
ccc	P	0	0.18
cca	P	0	0.41
ccg	P	0	0.14
---	--	------	------
mPD		0.097	1.1
		nPD:	0.09
		N. weight:	1.9
		Sc. PD:	-0.22
		Sc. rank:	-713.9

,

PA

Pos.	703	obs:	exp:
att	I	1	0.37
atc	I	0	0.27
ata	I	0	0.37
aat	N	2858	1562.00
aac	N	7	1303.00
aaa	K	2	1.14
aag	K	0	0.86
---	--	------	------
mPD		0.0070	0.50
		nPD:	0.01
		N. weight:	0.79
		Sc. PD:	-0.14
		Sc. rank:	-1223.4

,

PA

Pos.	704	obs:	exp:
gct	A	18	722.50
gcc	A	1	555.00
gca	A	1124	1277.00
gcg	A	1725	313.90
---	--	------	------
mPD		0.48	0.69
		nPD:	0.7
		N. weight:	1.3
		Sc. PD:	0.63
		Sc. rank:	2222.8

,

PA

Pos.	705	obs:	exp:
tct	S	2613	477.40
tcc	S	217	414.30
tca	S	30	648.40
tcg	S	0	172.50
tat	Y	8	3.84
tac	Y	0	4.16
agt	S	0	620.30
agc	S	0	527.10
---	--	------	------
mPD		0.16	1.7
		nPD:	0.1
		N. weight:	2.
		Sc. PD:	-0.2
		Sc. rank:	-430.0

,

PA

Pos.	706	obs:	exp:
tta	L	1	0.09
ttg	L	0	0.19
tgg	W	2866	2866.00
ctt	L	0	0.20
ctc	L	0	0.16
cta	L	0	0.15
ctg	L	0	0.21
ggt	G	0	0.14
ggc	G	0	0.13
gga	G	0	0.44
ggg	G	1	0.29
---	--	------	------
mPD		0.0021	0.0028
		nPD:	0.74
		N. weight:	0.0017
		Sc. PD:	0.0009
		Sc. rank:	3.1

,

PA

Pos.	707	obs:	exp:
ttt	F	9	1184.00
ttc	F	2830	1655.00
tta	L	4	2.59
ttg	L	0	5.32
tct	S	0	0.17
tcc	S	1	0.14
tca	S	0	0.23
tcg	S	0	0.06
ctt	L	0	5.56
ctc	L	24	4.48
cta	L	0	4.14
ctg	L	0	5.91
agt	S	0	0.22
agc	S	0	0.18
---	--	------	------
mPD		0.026	0.51
		nPD:	0.05
		N. weight:	0.71
		Sc. PD:	-0.1
		Sc. rank:	-602.6

,

PA

Pos.	708	obs:	exp:
aat	N	0	1564.00
aac	N	2868	1304.00
---	--	------	------
mPD		0	0.50
		nPD:	0.
		N. weight:	1.
		Sc. PD:	-0.21
		Sc. rank:	-1955.3

,

PA

Pos.	709	obs:	exp:
tct	S	6	478.60
tcc	S	2861	415.30
tca	S	0	650.00
tcg	S	0	173.00
cct	P	0	0.27
ccc	P	1	0.18
cca	P	0	0.41
ccg	P	0	0.14
agt	S	0	621.90
agc	S	0	528.40
---	--	------	------
mPD		0.0049	1.7
		nPD:	0.
		N. weight:	2.5
		Sc. PD:	-0.5
		Sc. rank:	-4552.8

,

PA

Pos.	710	obs:	exp:
ttt	F	0	1175.00
ttc	F	2816	1641.00
tta	L	0	0.28
ttg	L	0	0.57
tct	S	0	0.17
tcc	S	1	0.14
tca	S	0	0.23
tcg	S	0	0.06
tgt	C	0	18.58
tgc	C	48	29.42
ctt	L	0	0.60
ctc	L	3	0.48
cta	L	0	0.44
ctg	L	0	0.63
agt	S	0	0.22
agc	S	0	0.18
---	--	------	------
mPD		0.036	0.52
		nPD:	0.07
		N. weight:	0.72
		Sc. PD:	-0.094
		Sc. rank:	-432.1

,

PA

Pos.	711	obs:	exp:
ttt	F	0	0.83
ttc	F	2	1.17
tta	L	0	254.30
ttg	L	0	522.40
ctt	L	69	546.00
ctc	L	2681	439.90
cta	L	0	407.00
ctg	L	0	580.40
att	I	0	42.57
atc	I	116	31.04
ata	I	0	42.39
---	--	------	------
mPD		0.13	1.2
		nPD:	0.11
		N. weight:	2.3
		Sc. PD:	-0.21
		Sc. rank:	-209.6

,

PA

Pos.	712	obs:	exp:
cct	P	0	0.55
ccc	P	0	0.36
cca	P	2	0.81
ccg	P	0	0.28
cgt	R	0	0.07
cgc	R	0	0.11
cga	R	0	0.20
cgg	R	0	0.16
att	I	0	4.77
atc	I	0	3.48
ata	I	13	4.75
act	T	0	622.30
acc	T	0	534.90
aca	T	2373	993.40
acg	T	1	223.40
aaa	K	2	1.14
aag	K	0	0.86
aga	R	2	0.93
agg	R	0	0.52
gtt	V	0	0.22
gtc	V	0	0.21
gta	V	1	0.21
gtg	V	0	0.36
gct	A	0	119.40
gcc	A	2	91.72
gca	A	472	211.00
gcg	A	0	51.88
---	--	------	------
mPD		0.29	0.99
		nPD:	0.3
		N. weight:	1.1
		Sc. PD:	0.11
		Sc. rank:	1056.6

,

{

PA

Pos.	713	obs:	exp:
cat	H	2865	1660.00
cac	H	2	1207.00
cgt	R	1	0.04
cgc	R	0	0.05
cga	R	0	0.10
cgg	R	0	0.08
aga	R	0	0.47
agg	R	0	0.26
---	--	-----	-----
mPD		0.0021	0.49
		nPD:	0.
		N. weight:	0.72
		Sc. PD:	-0.14
		Sc. rank:	-1254.0

,

PA

Pos.	714	obs:	exp:
tct	S	0	0.17
tcc	S	0	0.14
tca	S	1	0.23
tcg	S	0	0.06
act	T	0	0.52
acc	T	0	0.45
aca	T	2	0.84
acg	T	0	0.19
agt	S	0	0.22
agc	S	0	0.18
gtt	V	0	0.43
gtc	V	0	0.42
gta	V	2	0.43
gtg	V	0	0.72
gct	A	0	721.20
gcc	A	0	554.00
gca	A	2863	1274.00
gcg	A	0	313.40
---	--	-----	-----
mPD		0.0035	0.69
		nPD:	0.01
		N. weight:	1.1
		Sc. PD:	-0.21
		Sc. rank:	-1852.1

,

PA

Pos.	715	obs:	exp:
tta	L	4	264.90
ttg	L	4	544.30
ctt	L	0	568.80
ctc	L	0	458.30
cta	L	201	424.00
ctg	L	2656	604.70
cgt	R	0	0.04
cgc	R	0	0.05
cga	R	0	0.10
cgg	R	1	0.08
aga	R	0	0.47
agg	R	0	0.26
gaa	E	2	1.12
gag	E	0	0.88
---	--	-----	-----
mPD		0.14	1.1
		nPD:	0.13
		N. weight:	1.7
		Sc. PD:	-0.13
		Sc. rank:	107.5

,

PA

Pos.	716	obs:	exp:
cgt	R	0	1.27
cgc	R	0	1.82
cga	R	0	3.43
cgg	R	0	2.78
att	I	0	0.37
atc	I	0	0.27
ata	I	1	0.37
act	T	0	7.86
acc	T	0	6.76
aca	T	30	12.55
acg	T	0	2.82
aat	N	63	34.36
aac	N	0	28.64
aaa	K	2678	1531.00
aag	K	11	1158.00
aga	R	34	15.87
agg	R	0	8.83
gat	D	1	0.54
gac	D	0	0.46
gaa	E	50	28.03
gag	E	0	21.97
---	--	-----	-----
mPD		0.13	0.60
		nPD:	0.22
		N. weight:	0.73
		Sc. PD:	0.011
		Sc. rank:	459.3

,

PA

Pos.	717	obs:	exp:
taa	.	152	1058.00
tag	.	2716	983.50
tga	.	0	827.00
---	--	-----	-----
mPD		0.10	0.86
		nPD:	0.12
		N. weight:	1.1
		Sc. PD:	-0.095
		Sc. rank:	-4.9

}

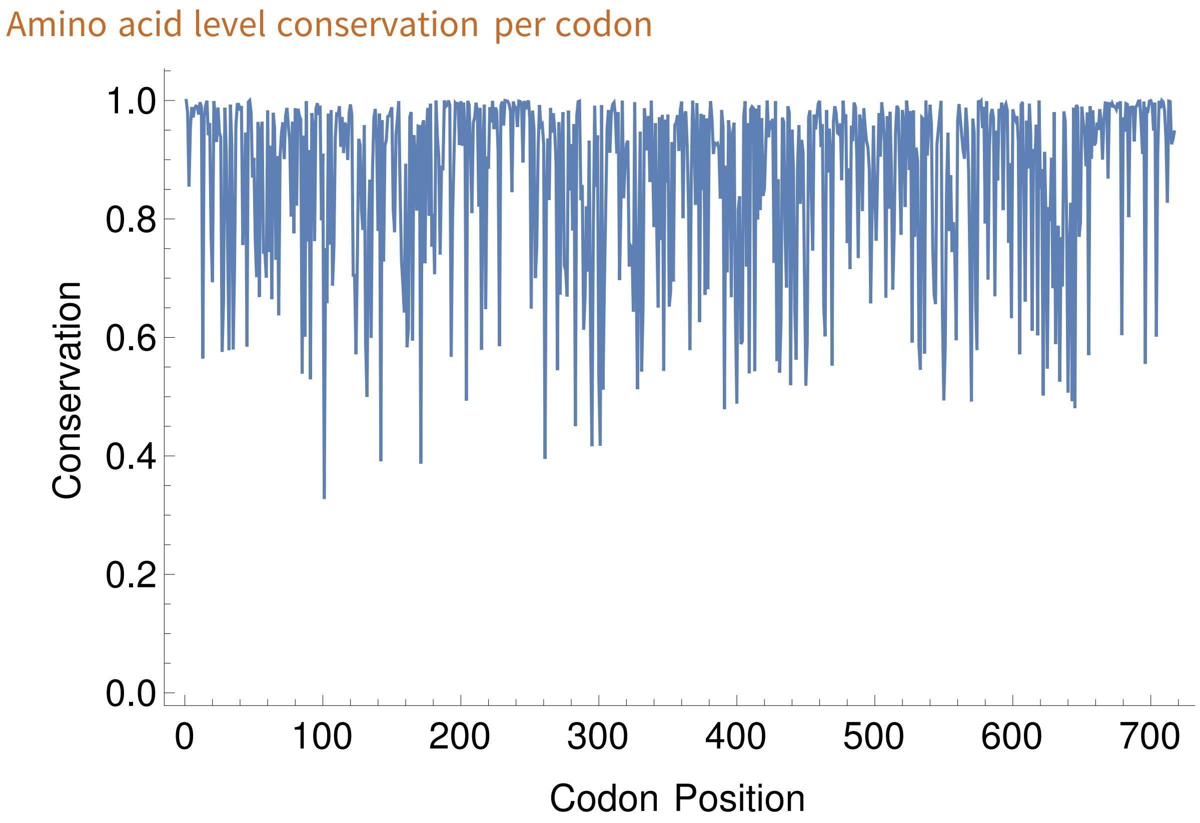

Amino acid level conservation per codon
Conservation
1.0
0.8
0.6
0.4
0.2
0.0
0
100
200
300
400
500
600
700
Codon Position

Conservation analysis plots

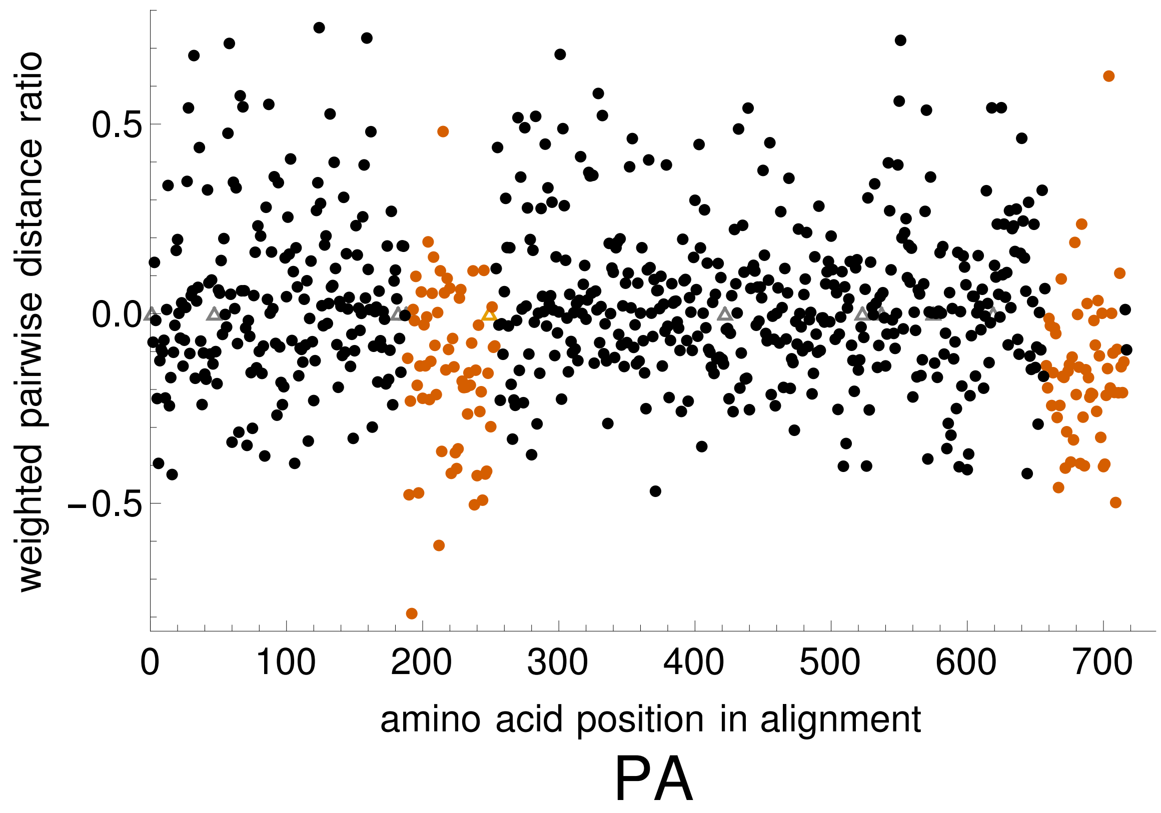

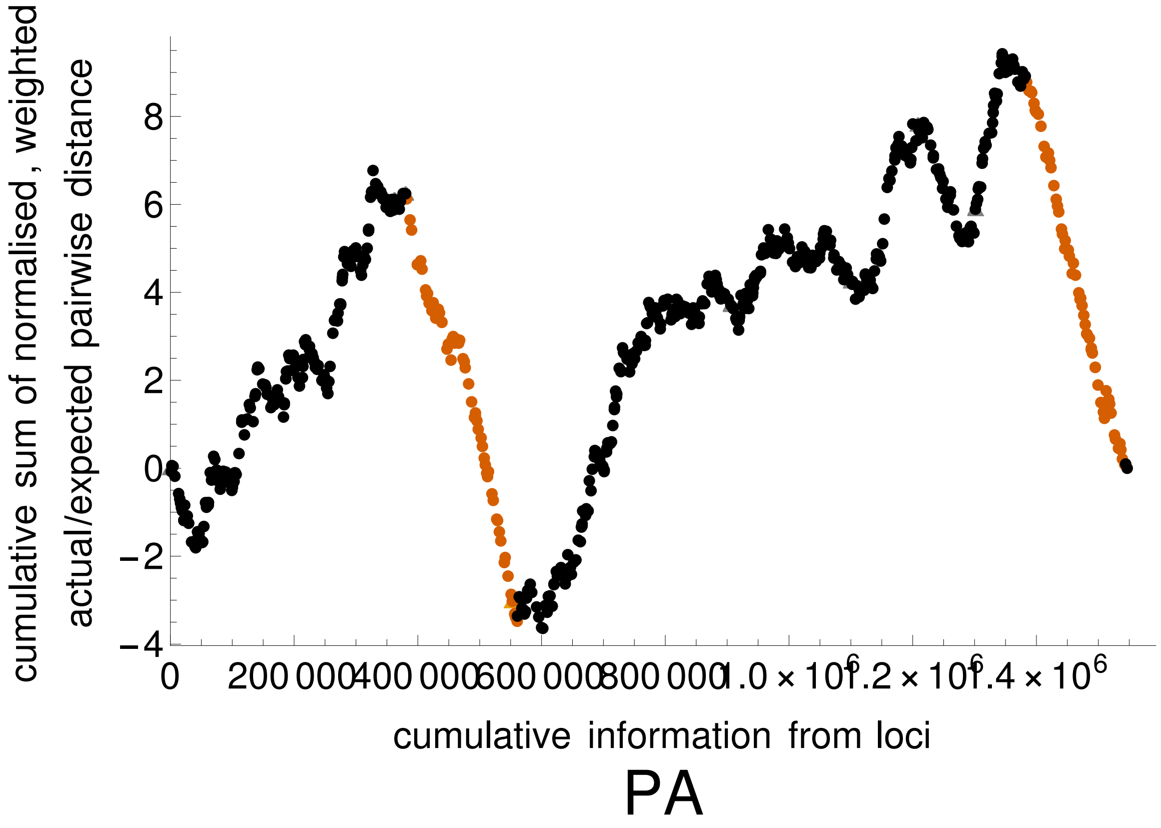

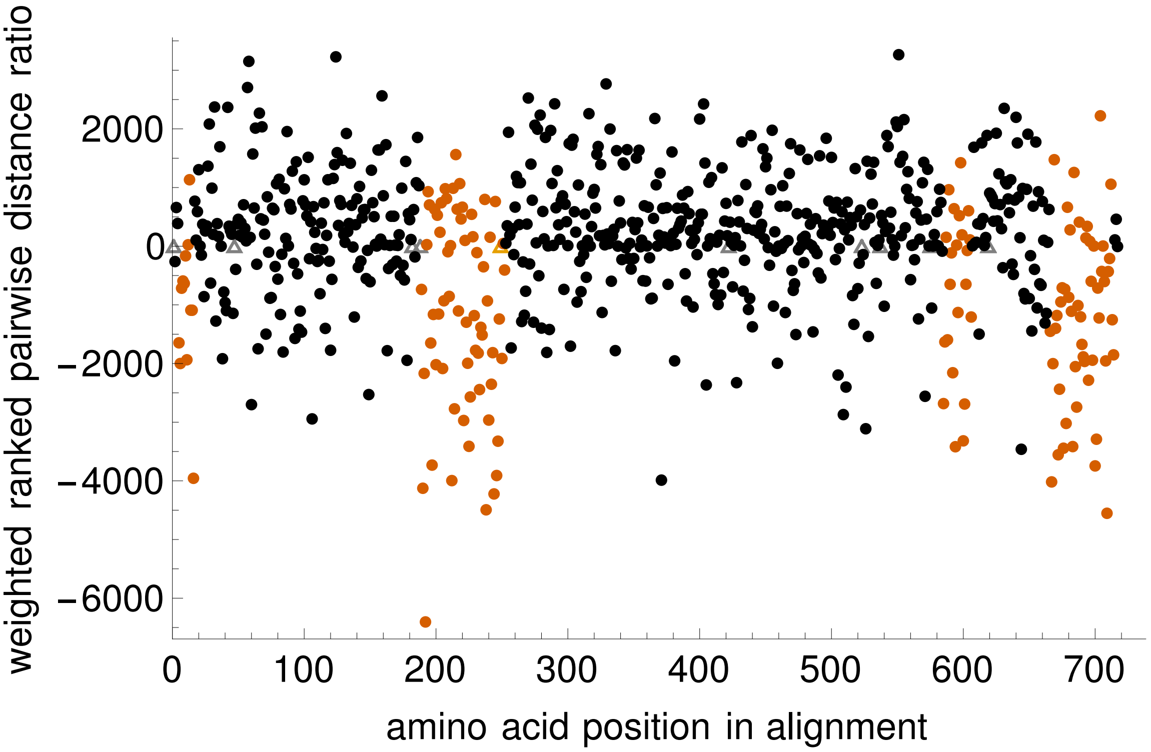
weighted ranked pairwise distance ratio
2000
0
−2000
−4000
−6000
0
100
200
300
400
500
600
700
amino acid position in alignment

PA

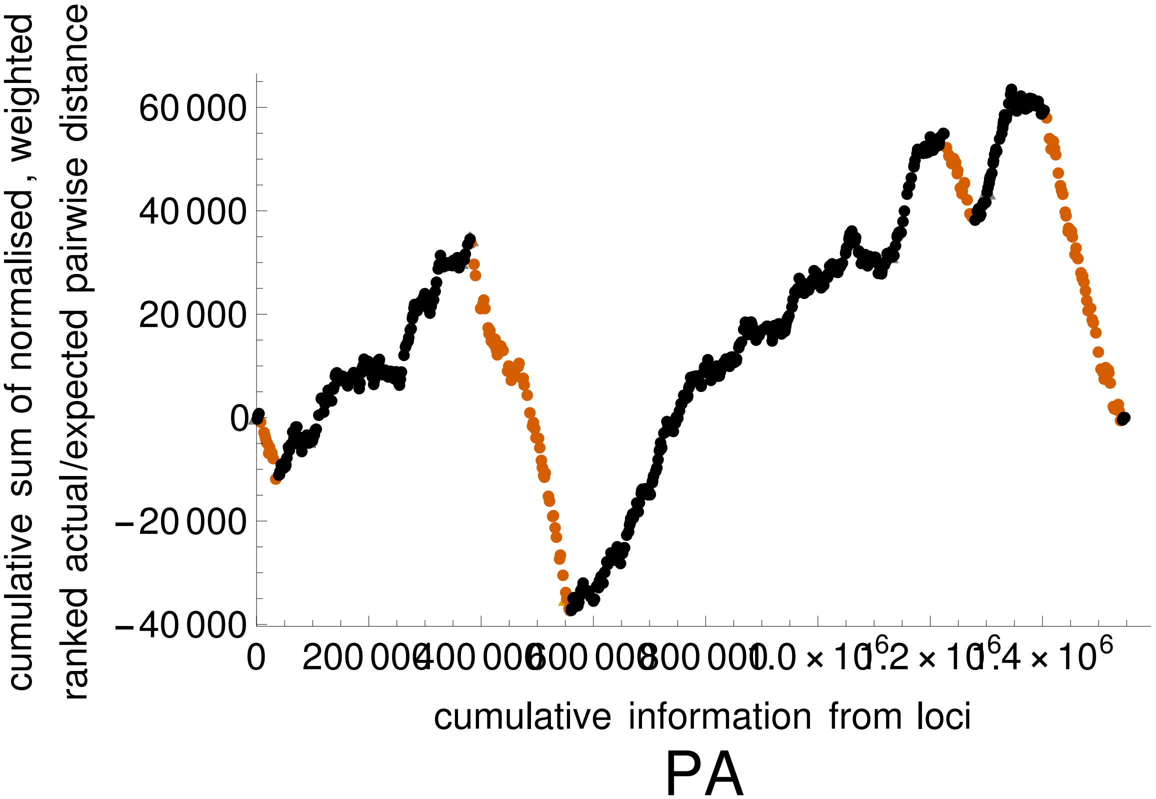
cumulative sum of normalised, weighted
ranked actual/expected pairwise distance
60 000
40 000
20 000
0
−20 000
−40 000
cumulative information from loci

PA

PA-X

Gene length histogram

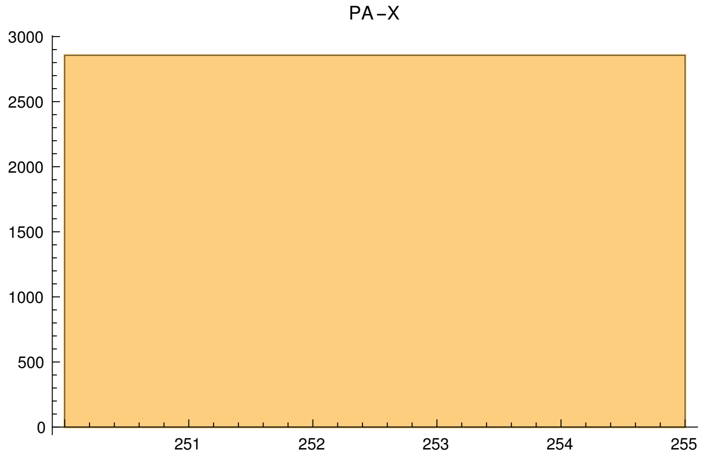

Information vs. nPD

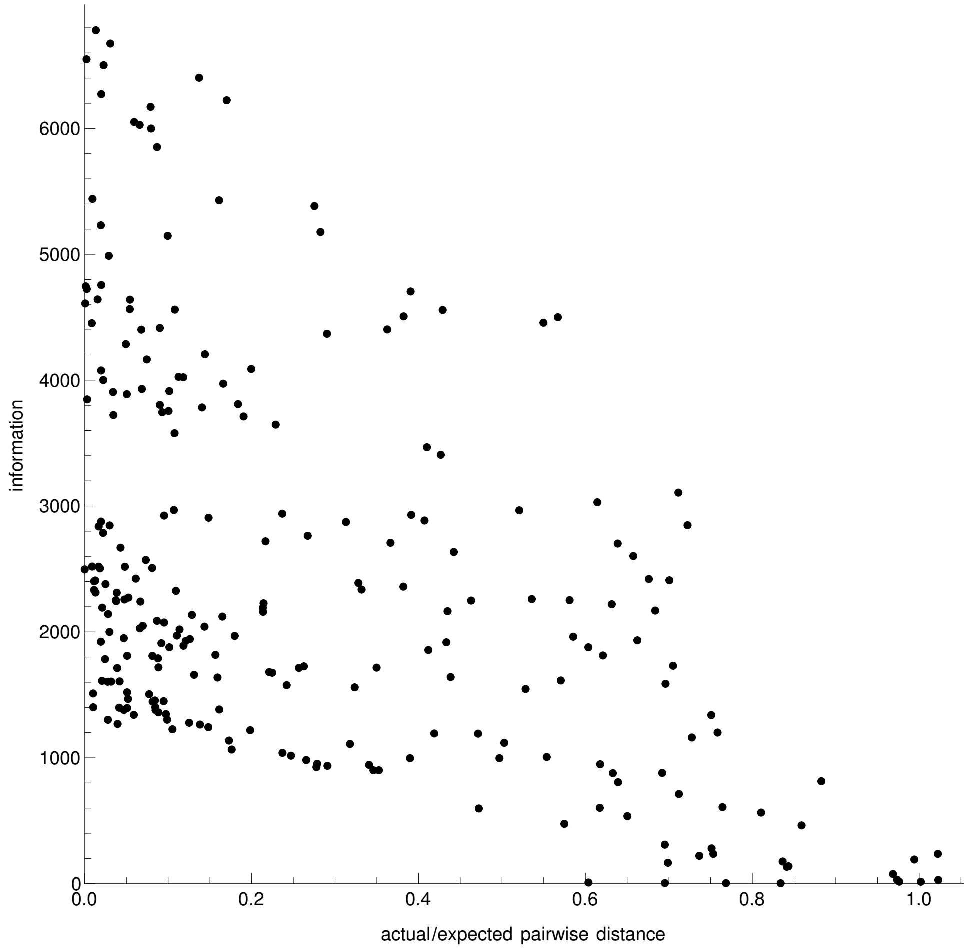

PA-X

Example sequences highlighted by regions found in analyses to be conserved

Interesting points (by weighted raw PD) highlighted for gene PA-X:

ATGGAAGACTTTGTGCGACAATGCTTCAATCCAATGATCGTCGAGCTTGCGGAAAAGGCAATGAAAGAATATGGGGAA
ACTCCGAAAATCGAAACGAACAAATTCGCAGCAATATGCACACACTTAGAAGTCTGCTTCATGTATTCGGATTTCCA
CTTTATTGATGAACAAAGCGAATCAATAATTGTAGAATCTGGCGACCCGAATGCATTATTGAAACACCGATTTGAGA
TAATTGAAGGGAGAGACCGAACAATGGCTTGGACAGTGGTAAATAGTATCTGCAACACCACAGGAGTCGATAAGCCT
AAATTCCTCCCAGATTTGTATGACTACAAAGAGAACCGATTCATTGAAATTGGAGTGACACGGAGGGAAGTTCACAT
ATACTACCTAGAAAAAGCAAATAAGATAAAATCAGAGAAGACACACATTCACATATTCTCATTCACTGGAGAGGAAA
TGGCCACCAAAGCAGATTATACCCTTGATGATGAGAGCAGAGCAAGAATCAAAACCAGGCTGTTCACTATAAGGCAA
GAAATGGCCAGTAGGGGTCTATGG
GATTCCTTTGTCAATCCGAGAGAGGCGAAGAGACAATTGAAGAAAGATTTGAAAT
CACAGGAACCATGCGCAGGCTTGCCGACCAAAGTCTCCCAC
CGAGCTTCTCCAGCCTTGAAAATTTTAGAGCCTATGTGGATGGATTCGAACCGAA
CGGCTGCATTGAGGGCAAGCTTTCTCAAATGTCAAAAGAAGTGA

Interesting points (by weighted ranked PD) highlighted for gene PA-X:

ATGGAAGACTTT **GTGCGACAATGCTTCAATCCAATGATCGTCGAGCTT**
GCGGAAAAGGCAATGAAAGAATATGGGGAAACTCCGAAAATCGAAACGAACAAATTCGCAGCA
ATATGCACACACTTAGAAGTCTGCTTCATGTATTCGGATTTCCACTTTATTGATGAACAAAGCGAATCAAT
AATTGTAGAATCTGGCGACCCGAATGCATTATTGAAACACCGATTTGAGATAATTGAAGGGAGAGACCGA
ACAATGGCTTGGACAGTGGTAAATAGTATCTGCAACACCACAGGAGTCGATAAGCCTAAATTCCTCCCAG
ATTTGTATGACTACAAAGAGAACCGATTCATTGAAATTGGAGTGACACGGAGGGAAGTTCACATATACTA
CCTAGAAAAAGCAAATAAGATAAAATCAGAGAAGACACACATTCACATATTCTCATTCACTGGAGAGGAA
ATGGCCACCAAAGCAGATTATACCCTTGATGATGAGAGCAGAGCAAGAATCAAAACCAGGCTGTTCACTA
TAAGGCAAGAAATGGCCAGTAGGGGTCTATGG
GATTCCTTTGTCAATCCGAGA **GAGGCGAAGAGACAATTGAAGAAAGATTTGAAA**
TCACAGGAACCATGCGCAGGCTTGCCGACCAAAGTCTCCCAC
CGAGCTTCTCCAGCCTTGAAAATTTTAGAGCCTATGTGGATGGATTCGAACCGAA
CGGCTGCATTGAGGGCAAGCTTTCTCAAATGTCAAAAGAAGTGA

Per codon conservation report

PA-X

Pos.	1	obs :	exp :
atg	M	2856	2856.00
---	--	------	------
mPD		0	0
		nPD :	1.
		N. weight :	0.
		Sc. PD :	0
		Sc. rank :	0

PA-X

Pos.	2	obs :	exp :
caa	Q	0	0.50
cag	Q	1	0.50
cgt	R	1	0.04
cgc	R	0	0.05
cga	R	0	0.10
cgg	R	0	0.08
aga	R	0	0.47
agg	R	0	0.26
gaa	E	2798	1599.00
gag	E	55	1254.00
ggt	G	0	0.14
ggc	G	0	0.13
gga	G	1	0.44
ggg	G	0	0.29
---	--	------	------
mPD		0.042	0.50
		nPD :	0.08
		N. weight :	0.6
		Sc. PD :	-0.061
		Sc. rank :	-170.1

PA-X

Pos.	3	obs :	exp :
cat	H	0	0.58
cac	H	1	0.42
aat	N	1	1.64
aac	N	2	1.36
gat	D	402	1536.00
gac	D	2442	1308.00
gaa	E	5	3.36
gag	E	1	2.64
ggt	G	0	0.29
ggc	G	2	0.26
gga	G	0	0.88
ggg	G	0	0.57
---	--	------	------
mPD		0.25	0.50
		nPD :	0.5
		N. weight :	0.42
		Sc. PD :	0.13
		Sc. rank :	653.3

PA-X

Pos.	4	obs :	exp :
ttt	F	2726	1187.00
ttc	F	120	1659.00
tta	L	0	0.46
ttg	L	0	0.95
tct	S	1	0.17
tcc	S	0	0.14
tca	S	0	0.23
tcg	S	0	0.06
tat	Y	0	0.48
tac	Y	1	0.52
tgt	C	2	0.77
tgc	C	0	1.23
ctt	L	5	0.99
ctc	L	0	0.80
cta	L	0	0.74
ctg	L	0	1.06
agt	S	0	0.22
agc	S	0	0.18
gtt	V	0	0.22
gtc	V	1	0.21
gta	V	0	0.21
gtg	V	0	0.36
---	--	------	------
mPD		0.089	0.49
		nPD :	0.18
		N. weight :	0.84
		Sc. PD :	-0.0074
		Sc. rank :	551.9

PA-X

Pos.	5	obs :	exp :
tta	L	0	0.92
ttg	L	10	1.90
ctt	L	0	1.99
ctc	L	0	1.60
cta	L	0	1.48
ctg	L	0	2.11
atg	M	5	5.00
gtt	V	0	610.00
gtc	V	0	596.70
gta	V	12	604.70
gtg	V	2824	1025.00
gct	A	0	1.26
gcc	A	0	0.97
gca	A	0	2.23
gcg	A	5	0.55
---	--	------	------
mPD		0.022	0.75
		nPD :	0.03
		N. weight :	1.2
		Sc. PD :	-0.19
		Sc. rank :	-1380.3

PA-X

Pos.	6	obs :	exp :
cgt	R	10	106.90
cgc	R	0	152.80
cga	R	2774	287.80
cgg	R	20	233.20
aga	R	52	1333.00
agg	R	0	742.00
---	--	------	------
mPD		0.057	0.95
		nPD :	0.06
		N. weight :	2.6
		Sc. PD :	-0.32
		Sc. rank :	-1653.5

PA-X

Pos.	7	obs :	exp :
cct	P	0	0.55
ccc	P	0	0.36
cca	P	2	0.81
ccg	P	0	0.28
caa	Q	2819	1424.00
cag	Q	33	1428.00
cgt	R	0	0.07
cgc	R	0	0.11
cga	R	2	0.20
cgg	R	0	0.16
aga	R	0	0.93
agg	R	0	0.52
---	--	------	------
mPD		0.026	0.50
		nPD :	0.05
		N. weight :	0.77
		Sc. PD :	-0.1
		Sc. rank :	-601.8

PA-X

Pos.	8	obs :	exp :
tat	Y	0	0.96
tac	Y	2	1.04
tgt	C	25	1105.00
tgc	C	2829	1749.00
---	--	------	------
mPD		0.019	0.48
		nPD :	0.04
		N. weight :	0.54
		Sc. PD :	-0.079
		Sc. rank :	-520.5

PA-X

Pos.	9	obs :	exp :
ttt	F	28	1191.00
ttc	F	2827	1664.00
tct	S	0	0.17
tcc	S	1	0.14
tca	S	0	0.23
tcg	S	0	0.06
agt	S	0	0.22
agc	S	0	0.18
---	--	------	------
mPD		0.020	0.49
		nPD :	0.04
		N. weight :	0.6
		Sc. PD :	-0.086
		Sc. rank :	-567.7

PA-X

Pos.	10	obs :	exp :
tct	S	0	0.17
tcc	S	0	0.14
tca	S	0	0.23
tcg	S	0	0.06
cat	H	1	0.58
cac	H	0	0.42
att	I	0	0.37
atc	I	1	0.27
ata	I	0	0.37
act	T	0	0.26
acc	T	1	0.23
aca	T	0	0.42
acg	T	0	0.09
aat	N	2789	1555.00
aac	N	62	1296.00
agt	S	1	0.22
agc	S	0	0.18
gat	D	1	0.54
gac	D	0	0.46
---	--	------	------
mPD		0.047	0.50
		nPD :	0.09
		N. weight :	0.62
		Sc. PD :	-0.057
		Sc. rank :	-51.4

PA-X

Pos.	11	obs :	exp :
cct	P	1	780.50
ccc	P	2	515.80
cca	P	2847	1159.00
ccg	P	6	400.40
---	--	------	------
mPD		0.0063	0.71
		nPD :	0.01
		N. weight :	1.1
		Sc. PD :	-0.19
		Sc. rank :	-1943.6

PA-X

Pos.	12	obs :	exp :
tta	L	0	0.18
ttg	L	0	0.38
ctt	L	0	0.40
ctc	L	0	0.32
cta	L	2	0.30
ctg	L	0	0.42
att	I	1	10.27
atc	I	0	7.49
ata	I	27	10.23
atg	M	2826	2826.00
---	--	------	------
mPD		0.022	0.022
		nPD :	1.02
		N. weight :	0.012
		Sc. PD :	0.0099
		Sc. rank :	24.7

PA-X

Pos.	13	obs :	exp :
att	I	1613	1023.00
atc	I	1173	746.10
ata	I	2	1019.00
act	T	49	13.11
acc	T	1	11.27
aca	T	0	20.92
acg	T	0	4.70
gtt	V	12	3.87
gtc	V	6	3.79
gta	V	0	3.84
gtg	V	0	6.50
---	--	------	------
mPD		0.53	0.71
		nPD :	0.75
		N. weight :	0.57
		Sc. PD :	0.31
		Sc. rank :	1132.0

PA-X

Pos.	14	obs :	exp :
att	I	0	0.73
atc	I	2	0.54
ata	I	0	0.73
gtt	V	48	608.90
gtc	V	2777	595.60
gta	V	6	603.60
gtg	V	0	1023.00
gct	A	2	5.79
gcc	A	21	4.45
gca	A	0	10.24
gcg	A	0	2.52
---	--	------	------
mPD		0.056	0.75
		nPD :	0.07
		N. weight :	1.8
		Sc. PD :	-0.2
		Sc. rank :	-776.1

PA-X

Pos.	15	obs :	exp :
gat	D	4	2.16
gac	D	0	1.84
gaa	E	17	1595.00
gag	E	2829	1251.00
ggt	G	0	0.87
ggc	G	0	0.79
gga	G	0	2.63
ggg	G	6	1.72
---	--	------	------
mPD		0.019	0.50
		nPD :	0.04
		N. weight :	0.96
		Sc. PD :	-0.14
		Sc. rank :	-955.2

PA-X

Pos.	16	obs:	exp:
tta	L	0	264.10
ttg	L	0	542.60
ctt	L	2855	567.10
ctc	L	0	456.80
cta	L	1	422.70
ctg	L	0	602.80
---	--	------	------
mPD		0.00070	1.1
		nPD:	0.
		N. weight:	2.
		Sc. PD:	-0.36
		Sc. rank:	-3916.7

,

PA-X

Pos.	17	obs:	exp:
gct	A	5	719.50
gcc	A	0	552.70
gca	A	163	1271.00
gcg	A	2688	312.60
---	--	------	------
mPD		0.11	0.69
		nPD:	0.16
		N. weight:	2.3
		Sc. PD:	-0.063
		Sc. rank:	1240.1

,

PA-X

Pos.	18	obs:	exp:
gaa	E	2747	1597.00
gag	E	102	1252.00
ggt	G	0	1.01
ggc	G	0	0.92
gga	G	7	3.07
ggg	G	0	2.00
---	--	------	------
mPD		0.074	0.50
		nPD:	0.15
		N. weight:	0.53
		Sc. PD:	-0.021
		Sc. rank:	241.2

,

PA-X

Pos.	19	obs:	exp:
cgt	R	0	0.19
cgc	R	0	0.27
cga	R	0	0.50
cgg	R	0	0.41
aat	N	1	0.55
aac	N	0	0.45
aaa	K	641	1623.00
aag	K	2209	1227.00
aga	R	1	2.33
agg	R	4	1.30
---	--	------	------
mPD		0.35	0.50
		nPD:	0.71
		N. weight:	0.3
		Sc. PD:	0.15
		Sc. rank:	590.5

,

PA-X

Pos.	20	obs:	exp:
cct	P	0	0.27
ccc	P	0	0.18
cca	P	1	0.41
ccg	P	0	0.14
att	I	0	0.37
atc	I	0	0.27
ata	I	1	0.37
act	T	0	228.90
acc	T	0	196.70
aca	T	873	365.30
acg	T	0	82.14
gct	A	0	499.00
gcc	A	0	383.30
gca	A	1980	881.80
gcg	A	1	216.80
---	--	------	------
mPD		0.43	1.1
		nPD:	0.38
		N. weight:	1.
		Sc. PD:	0.19
		Sc. rank:	1244.8

,

PA-X

Pos.	21	obs:	exp:
att	I	1	0.73
atc	I	0	0.54
ata	I	1	0.73
atg	M	2853	2853.00
act	T	0	0.26
acc	T	0	0.23
aca	T	0	0.42
acg	T	1	0.09
---	--	------	------
mPD		0.0021	0.0027
		nPD:	0.77
		N. weight:	0.0016
		Sc. PD:	0.00088
		Sc. rank:	3.1

,

PA-X

Pos.	22	obs:	exp:
cgt	R	0	0.45
cgc	R	0	0.64
cga	R	0	1.21
cgg	R	0	0.98
act	T	0	0.26
acc	T	0	0.23
aca	T	1	0.42
acg	T	0	0.09
aaa	K	2785	1619.00
aag	K	58	1224.00
aga	R	12	5.60
agg	R	0	3.12
---	--	------	------
mPD		0.049	0.50
		nPD:	0.1
		N. weight:	0.57
		Sc. PD:	-0.051
		Sc. rank:	-22.9

,

PA-X

Pos.	23	obs:	exp:
gtt	V	0	0.22
gtc	V	0	0.21
gta	V	1	0.21
gtg	V	0	0.36
gaa	E	2655	1600.00
gag	E	200	1255.00
---	--	------	------
mPD		0.13	0.49
		nPD:	0.27
		N. weight:	0.42
		Sc. PD:	0.031
		Sc. rank:	390.1

,

PA-X

Pos.	24	obs:	exp:
ttt	F	2	0.83
ttc	F	0	1.17
tat	Y	2822	1366.00
tac	Y	26	1482.00
cat	H	5	2.89
cac	H	0	2.11
aat	N	1	0.55
aac	N	0	0.45
---	--	------	------
mPD		0.024	0.50
		nPD:	0.05
		N. weight:	0.83
		Sc. PD:	-0.11
		Sc. rank:	-756.6

,

PA-X

Pos.	25	obs:	exp:
ggt	G	1	413.00
ggc	G	1	375.10
gga	G	152	1251.00
ggg	G	2702	816.40
---	--	------	------
mPD		0.10	0.69
		nPD:	0.15
		N. weight:	1.2
		Sc. PD:	-0.049
		Sc. rank:	588.8

,

PA-X

Pos.	26	obs:	exp:
aaa	K	1	0.57
aag	K	0	0.43
gaa	E	2678	1600.00
gag	E	176	1254.00
ggt	G	0	0.14
ggc	G	0	0.13
gga	G	1	0.44
ggg	G	0	0.29
---	--	------	------
mPD		0.12	0.49
		nPD:	0.24
		N. weight:	0.44
		Sc. PD:	0.021
		Sc. rank:	391.5

,

PA-X

Pos.	27	obs:	exp:
tct	S	0	97.48
tcc	S	0	84.60
tca	S	0	132.40
tcg	S	0	35.23
tat	Y	2	0.96
tac	Y	0	1.04
act	T	8	2.62
acc	T	2	2.25
aca	T	0	4.18
acg	T	0	0.94
aat	N	493	271.00
aac	N	4	226.00
agt	S	571	126.70
agc	S	13	107.60
gct	A	1	0.25
gcc	A	0	0.19
gca	A	0	0.45
gcg	A	0	0.11
gat	D	1646	950.30
gac	D	114	809.70
gaa	E	2	1.12
gag	E	0	0.88
---	--	------	------
mPD		0.89	1.4
		nPD:	0.62
		N. weight:	0.77
		Sc. PD:	0.32
		Sc. rank:	1359.4

,

PA-X

Pos.	28	obs:	exp:
tta	L	0	0.65
ttg	L	0	1.33
ctt	L	0	1.39
ctc	L	0	1.12
cta	L	1	1.04
ctg	L	6	1.48
cct	P	10	777.20
ccc	P	2	513.70
cca	P	950	1154.00
ccg	P	1882	398.70
caa	Q	0	1.00
cag	Q	2	1.00
act	T	0	0.79
acc	T	0	0.68
aca	T	2	1.26
acg	T	1	0.28
---	--	------	------
mPD		0.46	0.72
		nPD:	0.64
		N. weight:	1.2
		Sc. PD:	0.5
		Sc. rank:	2071.7

,

PA-X

Pos.	29	obs:	exp:
cgt	R	0	0.49
cgc	R	0	0.70
cga	R	0	1.31
cgg	R	0	1.06
att	I	0	0.37
atc	I	1	0.27
ata	I	0	0.37
aaa	K	2821	1615.00
aag	K	16	1222.00
aga	R	13	6.07
agg	R	0	3.38
gaa	E	4	2.24
gag	E	0	1.76
ggt	G	0	0.14
ggc	G	0	0.13
gga	G	1	0.44
ggg	G	0	0.29
---	--	------	------
mPD		0.026	0.51
		nPD:	0.05
		N. weight:	0.65
		Sc. PD:	-0.087
		Sc. rank:	-519.6

,

PA-X

Pos.	30	obs:	exp:
tct	S	0	0.33
tcc	S	0	0.29
tca	S	0	0.45
tcg	S	0	0.12
att	I	192	1033.00
atc	I	2611	753.00
ata	I	11	1028.00
act	T	3	1.05
acc	T	1	0.90
aca	T	0	1.67
acg	T	0	0.38
agt	S	0	0.43
agc	S	2	0.37
gtt	V	1	7.74
gtc	V	35	7.57
gta	V	0	7.68
gtg	V	0	13.01
---	--	------	------
mPD		0.16	0.69
		nPD:	0.24
		N. weight:	1.3
		Sc. PD:	0.058
		Sc. rank:	1098.9

,

PA-X

Pos.	31	obs:	exp:
gaa	E	2152	1600.00
gag	E	702	1254.00
ggt	G	0	0.29
ggc	G	0	0.26
gga	G	2	0.88
ggg	G	0	0.57
---	--	------	------
mPD		0.37	0.49
		nPD:	0.75
		N. weight:	0.1
		Sc. PD:	0.055
		Sc. rank:	200.1

,

PA-X

Pos.	32	obs:	exp:
tct	S	0	2.00
tcc	S	0	1.74
tca	S	11	2.72
tcg	S	0	0.72
atg	M	10	10.00
act	T	43	742.90
acc	T	3	638.60
aca	T	1137	1186.00
acg	T	1651	266.60
agt	S	1	2.60
agc	S	0	2.21
---	--	------	------
mPD		0.52	0.71
		nPD:	0.72
		N. weight:	1.2
		Sc. PD:	0.63
		Sc. rank:	2384.2

,

PA-X

Pos.	33	obs:	exp:
tct	S	0	0.17
tcc	S	0	0.14
tca	S	0	0.23
tcg	S	0	0.06
aat	N	18	1556.00
aac	N	2836	1298.00
agt	S	0	0.22
agc	S	1	0.18
gat	D	0	0.54
gac	D	1	0.46
---	--	------	------
mPD		0.014	0.50
		nPD:	0.03
		N. weight:	0.91
		Sc. PD:	-0.14
		Sc. rank:	-1100.2

,

PA-X

Pos.	34	obs:	exp:
cgt	R	0	0.64
cgc	R	0	0.91
cga	R	0	1.71
cgg	R	0	1.39
aaa	K	2641	1617.00
aag	K	198	1222.00
aga	R	17	7.94
agg	R	0	4.42
---	--	------	------
mPD		0.14	0.51
		nPD:	0.28
		N. weight:	0.41
		Sc. PD:	0.035
		Sc. rank:	406.2

,

PA-X

Pos.	35	obs:	exp:
ttt	F	1657	1183.00
ttc	F	1179	1653.00
tta	L	3	1.85
ttg	L	0	3.80
ctt	L	17	3.97
ctc	L	0	3.20
cta	L	0	2.96
ctg	L	0	4.22
---	--	------	------
mPD		0.50	0.50
		nPD:	0.99
		N. weight:	0.082
		Sc. PD:	0.064
		Sc. rank:	164.7

,

PA-X

Pos.	36	obs:	exp:
act	T	1	1.05
acc	T	3	0.90
aca	T	0	1.67
acg	T	0	0.38
gtt	V	1	0.43
gtc	V	1	0.42
gta	V	0	0.43
gtg	V	0	0.72
gct	A	595	718.00
gcc	A	2051	551.50
gca	A	203	1269.00
gcg	A	1	311.90
mPD		0.44	0.69
		nPD:	0.63
		N. weight:	0.94
		Sc. PD:	0.41
		Sc. rank:	1678.8

PA-X

Pos.	37	obs:	exp:
tct	S	0	0.83
tcc	S	0	0.72
tca	S	5	1.13
tcg	S	0	0.30
agt	S	0	1.08
agc	S	0	0.92
gct	A	15	718.20
gcc	A	16	551.70
gca	A	2735	1269.00
gcg	A	85	312.00
mPD		0.082	0.69
		nPD:	0.12
		N. weight:	0.8
		Sc. PD:	-0.055
		Sc. rank:	213.3

PA-X

Pos.	38	obs:	exp:
att	I	0	1041.00
atc	I	1	759.40
ata	I	2837	1037.00
act	T	0	0.26
acc	T	0	0.23
aca	T	1	0.42
acg	T	0	0.09
gtt	V	0	3.66
gtc	V	0	3.58
gta	V	17	3.62
gtg	V	0	6.14
mPD		0.013	0.67
		nPD:	0.02
		N. weight:	1.2
		Sc. PD:	-0.2
		Sc. rank:	-1824.4

PA-X

Pos.	39	obs:	exp:
tgt	C	18	1105.00
tgc	C	2837	1750.00
cgt	R	0	0.04
cgc	R	1	0.05
cga	R	0	0.10
cgg	R	0	0.08
aga	R	0	0.47
agg	R	0	0.26
mPD		0.013	0.48
		nPD:	0.03
		N. weight:	0.55
		Sc. PD:	-0.087
		Sc. rank:	-676.8

PA-X

Pos.	40	obs:	exp:
act	T	27	747.90
acc	T	5	642.90
aca	T	2808	1194.00
acg	T	13	268.40
gct	A	0	0.76
gcc	A	0	0.58
gca	A	3	1.34
gcg	A	0	0.33
mPD		0.033	0.70
		nPD:	0.05
		N. weight:	0.96
		Sc. PD:	-0.13
		Sc. rank:	-851.9

PA-X

Pos.	41	obs:	exp:
cat	H	27	1653.00
cac	H	2829	1203.00
mPD		0.019	0.49
		nPD:	0.04
		N. weight:	0.98
		Sc. PD:	-0.14
		Sc. rank:	-968.1

PA-X

Pos.	42	obs:	exp:
tta	L	2161	263.90
ttg	L	565	542.20
tct	S	0	0.17
tcc	S	0	0.14
tca	S	0	0.23
tcg	S	1	0.06
ctt	L	1	566.70
ctc	L	0	456.50
cta	L	123	422.40
ctg	L	4	602.40
att	I	0	0.37
atc	I	0	0.27
ata	I	1	0.37
agt	S	0	0.22
agc	S	0	0.18
mPD		0.41	1.1
		nPD:	0.36
		N. weight:	1.9
		Sc. PD:	0.32
		Sc. rank:	2257.8

PA-X

Pos.	43	obs:	exp:
gat	D	0	0.54
gac	D	1	0.46
gaa	E	2369	1600.00
gag	E	485	1254.00
ggt	G	0	0.14
ggc	G	0	0.13
gga	G	0	0.44
ggg	G	1	0.29
mPD		0.28	0.49
		nPD:	0.57
		N. weight:	0.2
		Sc. PD:	0.076
		Sc. rank:	339.0

PA-X

Pos.	44	obs:	exp:
att	I	0	6.61
atc	I	18	4.82
ata	I	0	6.58
gtt	V	92	610.20
gtc	V	2701	596.90
gta	V	27	604.90
gtg	V	17	1025.00
gct	A	0	0.25
gcc	A	1	0.19
gca	A	0	0.45
gcg	A	0	0.11
mPD		0.11	0.75
		nPD:	0.14
		N. weight:	1.6
		Sc. PD:	-0.076
		Sc. rank:	651.7

PA-X

Pos.	45	obs:	exp:
tct	S	0	0.33
tcc	S	0	0.29
tca	S	0	0.45
tcg	S	0	0.12
tgt	C	1671	1104.00
tgc	C	1182	1749.00
tgg	W	1	1.00
agt	S	2	0.43
agc	S	0	0.37
mPD		0.49	0.48
		nPD:	1.02
		N. weight:	0.1
		Sc. PD:	0.082
		Sc. rank:	203.5

PA-X

Pos.	46	obs:	exp:
ttt	F	5	1191.00
ttc	F	2849	1663.00
tta	L	0	0.18
ttg	L	0	0.38
ctt	L	0	0.40
ctc	L	2	0.32
cta	L	0	0.30
ctg	L	0	0.42
mPD		0.0049	0.49
		nPD:	0.01
		N. weight:	0.64
		Sc. PD:	-0.11
		Sc. rank:	-1135.3

PA-X

Pos.	47	obs:	exp:
atg	M	2856	2856.00
mPD		0	0
		nPD:	1.
		N. weight:	0.
		Sc. PD:	0
		Sc. rank:	0

PA-X

Pos.	48	obs:	exp:
tat	Y	2797	1370.00
tac	Y	59	1486.00
mPD		0.040	0.50
		nPD:	0.08
		N. weight:	0.77
		Sc. PD:	-0.081
		Sc. rank:	-242.4

PA-X

Pos.	49	obs:	exp:
tct	S	10	475.90
tcc	S	0	413.00
tca	S	353	646.30
tcg	S	2488	172.00
tgg	W	4	4.00
cct	P	0	0.27
ccc	P	0	0.18
cca	P	0	0.41
ccg	P	1	0.14
agt	S	0	618.40
agc	S	0	525.40
mPD		0.23	1.7
		nPD:	0.14
		N. weight:	2.7
		Sc. PD:	-0.14
		Sc. rank:	1008.8

PA-X

Pos.	50	obs:	exp:
cat	H	1	0.58
cac	H	0	0.42
gat	D	2579	1542.00
gac	D	276	1313.00
mPD		0.18	0.50
		nPD:	0.35
		N. weight:	0.38
		Sc. PD:	0.061
		Sc. rank:	450.4

PA-X

Pos.	51	obs:	exp:
ttt	F	667	1191.00
ttc	F	2187	1663.00
tta	L	2	0.18
ttg	L	0	0.38
ctt	L	0	0.40
ctc	L	0	0.32
cta	L	0	0.30
ctg	L	0	0.42
mPD		0.36	0.49
		nPD:	0.74
		N. weight:	0.094
		Sc. PD:	0.05
		Sc. rank:	185.8

PA-X

Pos.	52	obs:	exp:
tta	L	0	0.09
ttg	L	0	0.19
ctt	L	0	0.20
ctc	L	1	0.16
cta	L	0	0.15
ctg	L	0	0.21
cat	H	853	1653.00
cac	H	2002	1202.00
mPD		0.42	0.49
		nPD:	0.86
		N. weight:	0.2
		Sc. PD:	0.13
		Sc. rank:	396.1

PA-X

Pos.	53	obs:	exp:
ttt	F	2752	1189.00
ttc	F	99	1662.00
tta	L	3	0.37
ttg	L	0	0.76
tct	S	1	0.17
tcc	S	0	0.14
tca	S	0	0.23
tcg	S	0	0.06
ctt	L	1	0.79
ctc	L	0	0.64
cta	L	0	0.59
ctg	L	0	0.84
agt	S	0	0.22
agc	S	0	0.18
mPD		0.070	0.49
		nPD:	0.14
		N. weight:	0.87
		Sc. PD:	-0.038
		Sc. rank:	363.9

PA-X

Pos.	54	obs:	exp:
tta	L	0	0.18
ttg	L	0	0.38
ctt	L	0	0.40
ctc	L	0	0.32
cta	L	0	0.30
ctg	L	2	0.42
att	I	1909	1042.00
atc	I	56	759.70
ata	I	874	1037.00
atg	M	7	7.00
act	T	2	0.52
acc	T	0	0.45
aca	T	0	0.84
acg	T	0	0.19
aaa	K	3	1.71
aag	K	0	1.29
gtt	V	3	0.65
gtc	V	0	0.63
gta	V	0	0.64
gtg	V	0	1.08
mPD		0.46	0.67
		nPD:	0.69
		N. weight:	0.37
		Sc. PD:	0.18
		Sc. rank:	703.3

PA-X

Pos.	55	obs:	exp:
aat	N	6	3.27
aac	N	0	2.73
gat	D	2707	1539.00
gac	D	143	1311.00
mPD		0.099	0.50
		nPD:	0.2
		N. weight:	0.52
		Sc. PD:	0.0048
		Sc. rank:	373.0

PA-X

Pos.	56	obs:	exp:
gat	D	0	2.70
gac	D	5	2.30
gaa	E	2755	1598.00
gag	E	95	1252.00
ggt	G	0	0.14
ggc	G	0	0.13
gga	G	1	0.44
ggg	G	0	0.29
---	--	------	------
mPD		0.068	0.50
		nPD:	0.14
		N. weight:	0.54
		Sc. PD:	-0.027
		Sc. rank:	203.8

,

PA-X

Pos.	57	obs:	exp:
caa	Q	74	37.44
cag	Q	1	37.56
cgt	R	1	104.10
cgc	R	1	148.80
cga	R	2118	280.30
cgg	R	556	227.10
aga	R	103	1298.00
agg	R	2	722.50
---	--	------	------
mPD		0.44	1.0
		nPD:	0.43
		N. weight:	1.9
		Sc. PD:	0.45
		Sc. rank:	2809.1

,

PA-X

Pos.	58	obs:	exp:
tct	S	0	85.46
tcc	S	0	74.17
tca	S	0	116.10
tcg	S	0	30.89
tgt	C	0	0.77
tgc	C	2	1.23
aat	N	1	0.55
aac	N	0	0.45
agt	S	472	111.10
agc	S	40	94.36
ggt	G	149	338.50
ggc	G	2130	307.50
gga	G	37	1026.00
ggg	G	25	669.20
---	--	------	------
mPD		0.67	1.2
		nPD:	0.55
		N. weight:	1.9
		Sc. PD:	0.67
		Sc. rank:	3081.0

,

PA-X

Pos.	59	obs:	exp:
aat	N	3	1.64
aac	N	0	1.36
aaa	K	12	7.40
aag	K	1	5.60
gat	D	35	20.52
gac	D	3	17.48
gaa	E	2003	1565.00
gag	E	789	1227.00
ggt	G	1	1.45
ggc	G	0	1.31
gga	G	7	4.38
ggg	G	2	2.86
---	--	------	------
mPD		0.44	0.53
		nPD:	0.84
		N. weight:	0.075
		Sc. PD:	0.047
		Sc. rank:	150.3

,

PA-X

Pos.	60	obs:	exp:
tct	S	4	474.70
tcc	S	0	412.00
tca	S	2809	644.70
tcg	S	31	171.60
cct	P	0	3.28
ccc	P	0	2.17
cca	P	12	4.87
ccg	P	0	1.68
agt	S	0	616.90
agc	S	0	524.10
---	--	------	------
mPD		0.033	1.7
		nPD:	0.02
		N. weight:	1.7
		Sc. PD:	-0.29
		Sc. rank:	-2537.6

,

PA-X

Pos.	61	obs:	exp:
tta	L	0	0.09
ttg	L	0	0.19
tgg	W	1	1.00
ctt	L	0	0.20
ctc	L	0	0.16
cta	L	1	0.15
ctg	L	0	0.21
cgt	R	0	0.07
cgc	R	0	0.11
cga	R	0	0.20
cgg	R	0	0.16
att	I	0	779.80
atc	I	0	568.60
ata	I	2125	776.60
atg	M	593	593.00
act	T	3	31.98
acc	T	1	27.49
aca	T	59	51.05
acg	T	59	11.48
aga	R	1	0.93
agg	R	1	0.52
gtt	V	0	2.58
gtc	V	0	2.52
gta	V	12	2.56
gtg	V	0	4.34
---	--	------	------
mPD		0.45	0.84
		nPD:	0.54
		N. weight:	0.96
		Sc. PD:	0.33
		Sc. rank:	1533.6

,

PA-X

Pos.	62	obs:	exp:
att	I	2637	1037.00
atc	I	185	756.00
ata	I	3	1032.00
atg	M	1	1.00
act	T	3	1.05
acc	T	1	0.90
aca	T	0	1.67
acg	T	0	0.38
gtt	V	24	5.59
gtc	V	2	5.47
gta	V	0	5.54
gtg	V	0	9.39
---	--	------	------
mPD		0.15	0.68
		nPD:	0.21
		N. weight:	0.95
		Sc. PD:	0.023
		Sc. rank:	750.6

,

PA-X

Pos.	63	obs:	exp:
tta	L	1	0.09
ttg	L	0	0.19
ctt	L	0	0.20
ctc	L	0	0.16
cta	L	0	0.15
ctg	L	0	0.21
att	I	0	5.50
atc	I	0	4.01
ata	I	15	5.48
act	T	0	4.19
acc	T	0	3.61
aca	T	16	6.69
acg	T	0	1.51
gtt	V	5	419.60
gtc	V	0	410.50
gta	V	1898	416.00
gtg	V	48	704.90
gct	A	0	214.40
gcc	A	0	164.70
gca	A	851	378.80
gcg	A	0	93.14
gaa	E	22	12.33
gag	E	0	9.67
---	--	------	------
mPD		0.49	1.2
		nPD:	0.41
		N. weight:	1.5
		Sc. PD:	0.32
		Sc. rank:	2026.0

,

PA-X

Pos.	64	obs:	exp:
gct	A	0	0.50
gcc	A	0	0.39
gca	A	2	0.89
gcg	A	0	0.22
gat	D	5	8.10
gac	D	10	6.90
gaa	E	2797	1591.00
gag	E	41	1247.00
ggt	G	0	0.14
ggc	G	0	0.13
gga	G	1	0.44
ggg	G	0	0.29
---	--	------	------
mPD		0.041	0.50
		nPD:	0.08
		N. weight:	0.62
		Sc. PD:	-0.064
		Sc. rank:	-187.6

,

PA-X

Pos.	65	obs:	exp:
ttt	F	17	7.09
ttc	F	0	9.91
tct	S	2725	468.90
tcc	S	51	406.90
tca	S	18	636.80
tcg	S	15	169.50
tat	Y	19	9.11
tac	Y	0	9.89
cct	P	9	2.46
ccc	P	0	1.63
cca	P	0	3.65
ccg	P	0	1.26
act	T	1	0.26
acc	T	0	0.23
aca	T	0	0.42
acg	T	0	0.09
agt	S	0	609.30
agc	S	0	517.70
gct	A	1	0.25
gcc	A	0	0.19
gca	A	0	0.45
gcg	A	0	0.11
---	--	------	------
mPD		0.090	1.7
		nPD:	0.05
		N. weight:	2.
		Sc. PD:	-0.26
		Sc. rank:	-1367.8

,

PA-X

Pos.	66	obs:	exp:
tct	S	0	1.00
tcc	S	0	0.87
tca	S	0	1.36
tcg	S	0	0.36
cgt	R	0	0.04
cgc	R	0	0.05
cga	R	0	0.10
cgg	R	0	0.08
agt	S	1	1.30
agc	S	5	1.11
aga	R	1	0.47
agg	R	0	0.26
gat	D	0	3.24
gac	D	6	2.76
gaa	E	2	1.12
gag	E	0	0.88
ggt	G	256	410.90
ggc	G	2090	373.20
gga	G	492	1245.00
ggg	G	3	812.10
---	--	------	------
mPD		0.43	0.70
		nPD:	0.61
		N. weight:	1.3
		Sc. PD:	0.53
		Sc. rank:	2242.4

,

PA-X

Pos.	67	obs:	exp:
aat	N	11	6.00
aac	N	0	5.00
gat	D	2587	1536.00
gac	D	257	1308.00
ggt	G	1	0.14
ggc	G	0	0.13
gga	G	0	0.44
ggg	G	0	0.29
---	--	------	------
mPD		0.17	0.51
		nPD:	0.34
		N. weight:	0.4
		Sc. PD:	0.059
		Sc. rank:	462.0

,

PA-X

Pos.	68	obs:	exp:
tta	L	0	0.55
ttg	L	0	1.14
tct	S	0	0.67
tcc	S	0	0.58
tca	S	3	0.91
tcg	S	1	0.24
ctt	L	0	1.19
ctc	L	0	0.96
cta	L	3	0.89
ctg	L	3	1.27
cct	P	0	777.80
ccc	P	12	514.00
cca	P	1015	1155.00
ccg	P	1819	399.00
agt	S	0	0.87
agc	S	0	0.74
---	--	------	------
mPD		0.47	0.72
		nPD:	0.66
		N. weight:	1.1
		Sc. PD:	0.51
		Sc. rank:	2025.5

,

PA-X

Pos.	69	obs:	exp:
tct	S	0	0.33
tcc	S	0	0.29
tca	S	0	0.45
tcg	S	0	0.12
cat	H	2	1.16
cac	H	0	0.84
aat	N	2585	1555.00
aac	N	266	1296.00
aaa	K	1	0.57
aag	K	0	0.43
agt	S	1	0.43
agc	S	1	0.37
---	--	------	------
mPD		0.17	0.50
		nPD:	0.35
		N. weight:	0.38
		Sc. PD:	0.059
		Sc. rank:	443.5

,

PA-X

Pos.	70	obs:	exp:
tct	S	0	0.17
tcc	S	0	0.14
tca	S	1	0.23
tcg	S	0	0.06
act	T	0	0.52
acc	T	0	0.45
aca	T	2	0.84
acg	T	0	0.19
agt	S	0	0.22
agc	S	0	0.18
gtt	V	1	6.45
gtc	V	0	6.31
gta	V	25	6.40
gtg	V	4	10.84
gct	A	93	710.40
gcc	A	14	545.70
gca	A	2697	1255.00
gcg	A	16	308.70
gaa	E	2	1.68
gag	E	1	1.32
---	--	------	------
mPD		0.11	0.72
		nPD:	0.16
		N. weight:	0.77
		Sc. PD:	-0.024
		Sc. rank:	377.8

,

PA-X

Pos.	71	obs:	exp:
tta	L	2750	264.10
ttg	L	54	542.60
ctt	L	2	567.10
ctc	L	20	456.80
cta	L	29	422.70
ctg	L	1	602.80
---	--	------	------
mPD		0.089	1.1
		nPD:	0.08
		N. weight:	2.6
		Sc. PD:	-0.28
		Sc. rank:	-1012.1

,

PA-X

Pos.	72	obs:	exp:
tta	L	178	264.00
ttg	L	2574	542.40
ctt	L	0	566.90
ctc	L	0	456.70
cta	L	43	422.50
ctg	L	60	602.60
aaa	K	0	0.57
aag	K	1	0.43
---	--	------	------
mPD		0.21	1.1
		nPD:	0.19
		N. weight:	1.6
		Sc. PD:	0.0023
		Sc. rank:	1122.3

,

PA-X

Pos.	73	obs:	exp:
cgt	R	0	0.04
cgc	R	0	0.05
cga	R	0	0.10
cgg	R	0	0.08
aaa	K	2785	1626.00
aag	K	70	1229.00
aga	R	1	0.47
agg	R	0	0.26
---	--	------	------
mPD		0.049	0.49
		nPD:	0.1
		N. weight:	0.55
		Sc. PD:	-0.049
		Sc. rank:	-17.2

,

PA-X

Pos.	74	obs:	exp:
ttt	F	0	0.42
ttc	F	1	0.58
cat	H	35	1653.00
cac	H	2820	1202.00
---	--	------	------
mPD		0.026	0.49
		nPD:	0.05
		N. weight:	0.97
		Sc. PD:	-0.13
		Sc. rank:	-730.8

,

PA-X

Pos.	75	obs:	exp:
tta	L	0	0.09
ttg	L	0	0.19
ctt	L	0	0.20
ctc	L	0	0.16
cta	L	1	0.15
ctg	L	0	0.21
cgt	R	4	106.80
cgc	R	4	152.80
cga	R	2735	287.70
cgg	R	30	233.10
aga	R	82	1333.00
agg	R	0	741.70
---	--	------	------
mPD		0.083	0.96
		nPD:	0.09
		N. weight:	2.5
		Sc. PD:	-0.25
		Sc. rank:	-551.7

,

PA-X

Pos.	76	obs:	exp:
ttt	F	2660	1191.00
ttc	F	194	1663.00
tat	Y	1	0.48
tac	Y	0	0.52
tgt	C	1	0.39
tgc	C	0	0.61
---	--	------	------
mPD		0.13	0.49
		nPD:	0.26
		N. weight:	0.74
		Sc. PD:	0.053
		Sc. rank:	681.1

,

PA-X

Pos.	77	obs:	exp:
gat	D	1	0.54
gac	D	0	0.46
gaa	E	558	1600.00
gag	E	2297	1255.00
---	--	------	------
mPD		0.32	0.49
		nPD:	0.64
		N. weight:	0.34
		Sc. PD:	0.15
		Sc. rank:	619.8

,

PA-X

Pos.	78	obs:	exp:
tta	L	17	2.13
ttg	L	0	4.37
ctt	L	0	4.57
ctc	L	0	3.68
cta	L	6	3.40
ctg	L	0	4.85
att	I	0	1011.00
atc	I	1	737.00
ata	I	2753	1006.00
atg	M	2	2.00
gtt	V	0	16.56
gtc	V	0	16.20
gta	V	77	16.42
gtg	V	0	27.82
---	--	------	------
mPD		0.070	0.74
		nPD:	0.1
		N. weight:	1.2
		Sc. PD:	-0.11
		Sc. rank:	-78.8

,

PA-X

Pos.	79	obs:	exp:
att	I	2214	1047.00
atc	I	637	763.20
ata	I	1	1042.00
gtt	V	3	0.86
gtc	V	1	0.84
gta	V	0	0.85
gtg	V	0	1.45
---	--	------	------
mPD		0.35	0.66
		nPD:	0.53
		N. weight:	0.66
		Sc. PD:	0.22
		Sc. rank:	1039.0

,

PA-X

Pos.	80	obs:	exp:
gaa	E	2819	1601.00
gag	E	37	1255.00
---	--	------	------
mPD		0.026	0.49
		nPD:	0.05
		N. weight:	0.62
		Sc. PD:	-0.083
		Sc. rank:	-481.5

,

PA-X

Pos.	81	obs:	exp:
cgt	R	0	0.07
cgc	R	0	0.11
cga	R	0	0.20
cgg	R	0	0.16
aga	R	0	0.93
agg	R	2	0.52
ggt	G	66	412.70
ggc	G	0	374.90
gga	G	437	1251.00
ggg	G	2351	815.80
---	--	------	------
mPD		0.30	0.69
		nPD:	0.43
		N. weight:	0.82
		Sc. PD:	0.19
		Sc. rank:	1192.6

,

PA-X

Pos.	82	obs:	exp:
tct	S	0	0.17
tcc	S	0	0.14
tca	S	0	0.23
tcg	S	0	0.06
cgt	R	0	106.80
cgc	R	0	152.70
cga	R	0	287.60
cgg	R	0	233.00
aaa	K	0	0.57
aag	K	1	0.43
agt	S	1	0.22
agc	S	0	0.18
aga	R	2816	1332.00
agg	R	38	741.40
---	--	------	------
mPD		0.028	0.96
		nPD:	0.03
		N. weight:	0.85
		Sc. PD:	-0.13
		Sc. rank:	-986.4

,

PA-X

Pos.	83	obs:	exp:
tat	Y	0	0.48
tac	Y	1	0.52
gat	D	71	1540.00
gac	D	2782	1313.00
gaa	E	0	0.56
gag	E	1	0.44
ggt	G	0	0.14
ggc	G	1	0.13
gga	G	0	0.44
ggg	G	0	0.29
---	--	------	------
mPD		0.051	0.50
		nPD:	0.1
		N. weight:	0.8
		Sc. PD:	-0.068
		Sc. rank:	55.6

,

PA-X

Pos.	84	obs:	exp:
cgt	R	5	106.90
cgc	R	19	152.80
cga	R	2765	287.80
cgg	R	37	233.20
aga	R	30	1333.00
agg	R	0	742.00
---	--	------	------
mPD		0.063	0.95
		nPD:	0.07
		N. weight:	2.6
		Sc. PD:	-0.31
		Sc. rank:	-1498.9

,

PA-X

Pos.	85	obs:	exp:
att	I	1	0.37
atc	I	0	0.27
ata	I	0	0.37
act	T	172	591.10
acc	T	103	508.10
aca	T	1538	943.60
acg	T	442	212.20
aat	N	1	0.55
aac	N	0	0.45
gct	A	0	150.90
gcc	A	1	115.90
gca	A	590	266.60
gcg	A	8	65.56
---	--	------	------
mPD		0.75	1.0
		nPD:	0.73
		N. weight:	0.49
		Sc. PD:	0.26
		Sc. rank:	976.2

,

PA-X			
Pos.	86	obs:	exp:
tta	L	0	0.09
ttg	L	1	0.19
tct	S	0	0.17
tcc	S	0	0.14
tca	S	0	0.23
tcg	S	0	0.06
ctt	L	0	0.20
ctc	L	0	0.16
cta	L	0	0.15
ctg	L	0	0.21
att	I	3	19.82
atc	I	0	14.45
ata	I	51	19.73
atg	M	2685	2685.00
act	T	0	0.26
acc	T	0	0.23
aca	T	0	0.42
acg	T	1	0.09
aaa	K	0	0.57
aag	K	1	0.43
agt	S	1	0.22
agc	S	0	0.18
gtt	V	0	24.30
gtc	V	0	23.77
gta	V	0	24.09
gtg	V	113	40.83
---	--	------	------
mPD		0.12	0.17
		nPD:	0.7
		N. weight:	0.071
		Sc. PD:	0.035
		Sc. rank:	133.5

,

PA-X			
Pos.	87	obs:	exp:
gtt	V	0	0.22
gtc	V	1	0.21
gta	V	0	0.21
gtg	V	0	0.36
gct	A	1125	719.00
gcc	A	1718	552.30
gca	A	10	1270.00
gcg	A	1	312.40
ggt	G	0	0.14
ggc	G	1	0.13
gga	G	0	0.44
ggg	G	0	0.29
---	--	------	------
mPD		0.48	0.69
		nPD:	0.7
		N. weight:	1.
		Sc. PD:	0.51
		Sc. rank:	1959.0

,

PA-X			
Pos.	88	obs:	exp:
tgt	C	1	0.39
tgc	C	0	0.61
tgg	W	2853	2853.00
cgt	R	0	0.07
cgc	R	0	0.11
cga	R	0	0.20
cgg	R	1	0.16
aga	R	0	0.93
agg	R	1	0.52
---	--	------	------
mPD		0.0021	0.0030
		nPD:	0.7
		N. weight:	0.0017
		Sc. PD:	0.00086
		Sc. rank:	3.3

,

PA-X			
Pos.	89	obs:	exp:
atg	M	1	1.00
act	T	490	748.20
acc	T	7	643.10
aca	T	2177	1194.00
acg	T	180	268.50
gct	A	0	0.25
gcc	A	0	0.19
gca	A	1	0.45
gcg	A	0	0.11
---	--	------	------
mPD		0.39	0.70
		nPD:	0.55
		N. weight:	0.43
		Sc. PD:	0.15
		Sc. rank:	698.6

,

PA-X			
Pos.	90	obs:	exp:
tta	L	0	0.09
ttg	L	1	0.19
ctt	L	0	0.20
ctc	L	0	0.16
cta	L	0	0.15
ctg	L	0	0.21
att	I	0	2.20
atc	I	0	1.61
ata	I	6	2.19
atg	M	5	5.00
gtt	V	11	611.50
gtc	V	1	598.10
gta	V	213	606.20
gtg	V	2618	1027.00
ggt	G	0	0.14
ggc	G	0	0.13
gga	G	0	0.44
ggg	G	1	0.29
---	--	------	------
mPD		0.16	0.74
		nPD:	0.21
		N. weight:	0.93
		Sc. PD:	0.022
		Sc. rank:	710.5

,

PA-X			
Pos.	91	obs:	exp:
att	I	0	16.15
atc	I	0	11.77
ata	I	44	16.08
atg	M	2	2.00
gtt	V	9	604.40
gtc	V	2	591.20
gta	V	1285	599.20
gtg	V	1514	1015.00
---	--	------	------
mPD		0.53	0.77
		nPD:	0.7
		N. weight:	0.68
		Sc. PD:	0.33
		Sc. rank:	1278.9

,

PA-X			
Pos.	92	obs:	exp:
tct	S	0	0.17
tcc	S	0	0.14
tca	S	0	0.23
tcg	S	0	0.06
aat	N	2795	1557.00
aac	N	60	1298.00
agt	S	1	0.22
agc	S	0	0.18
---	--	------	------
mPD		0.042	0.50
		nPD:	0.08
		N. weight:	0.62
		Sc. PD:	-0.063
		Sc. rank:	-182.7

,

PA-X			
Pos.	93	obs:	exp:
tct	S	0	476.10
tcc	S	0	413.10
tca	S	0	646.50
tcg	S	0	172.10
att	I	1	0.37
atc	I	0	0.27
ata	I	0	0.37
act	T	1	0.26
acc	T	0	0.23
aca	T	0	0.42
acg	T	0	0.09
aat	N	1	0.55
aac	N	0	0.45
agt	S	2732	618.60
agc	S	120	525.60
ggt	G	1	0.14
ggc	G	0	0.13
gga	G	0	0.44
ggg	G	0	0.29
---	--	------	------
mPD		0.083	1.7
		nPD:	0.05
		N. weight:	1.7
		Sc. PD:	-0.22
		Sc. rank:	-1346.5

,

PA-X			
Pos.	94	obs:	exp:
tta	L	0	1.20
ttg	L	0	2.47
ctt	L	11	2.58
ctc	L	2	2.08
cta	L	0	1.92
ctg	L	0	2.74
att	I	547	1020.00
atc	I	2179	743.70
ata	I	53	1016.00
atg	M	1	1.00
gtt	V	62	13.55
gtc	V	1	13.25
gta	V	0	13.43
gtg	V	0	22.76
---	--	------	------
mPD		0.42	0.72
		nPD:	0.59
		N. weight:	0.84
		Sc. PD:	0.32
		Sc. rank:	1423.9

,

PA-X			
Pos.	95	obs:	exp:
tct	S	0	4.34
tcc	S	0	3.77
tca	S	0	5.89
tcg	S	0	1.57
tgt	C	15	1095.00
tgc	C	2815	1735.00
agt	S	1	5.64
agc	S	25	4.79
---	--	------	------
mPD		0.029	0.50
		nPD:	0.06
		N. weight:	0.57
		Sc. PD:	-0.072
		Sc. rank:	-390.2

,

PA-X			
Pos.	96	obs:	exp:
cct	P	0	0.27
ccc	P	1	0.18
cca	P	0	0.41
ccg	P	0	0.14
att	I	0	0.73
atc	I	2	0.54
ata	I	0	0.73
act	T	0	0.26
acc	T	0	0.23
aca	T	1	0.42
acg	T	0	0.09
aat	N	9	1555.00
aac	N	2843	1297.00
---	--	------	------
mPD		0.010	0.50
		nPD:	0.02
		N. weight:	0.93
		Sc. PD:	-0.15
		Sc. rank:	-1270.0

,

PA-X			
Pos.	97	obs:	exp:
tct	S	0	0.33
tcc	S	2	0.29
tca	S	0	0.45
tcg	S	0	0.12
att	I	0	1.10
atc	I	3	0.80
ata	I	0	1.10
act	T	38	745.00
acc	T	2786	640.40
aca	T	18	1189.00
acg	T	0	267.40
aat	N	0	3.27
aac	N	6	2.73
agt	S	0	0.43
agc	S	0	0.37
gct	A	0	0.76
gcc	A	3	0.58
gca	A	0	1.34
gcg	A	0	0.33
---	--	------	------
mPD		0.048	0.71
		nPD:	0.07
		N. weight:	1.7
		Sc. PD:	-0.2
		Sc. rank:	-833.0

,

PA-X			
Pos.	98	obs:	exp:
cct	P	0	0.27
ccc	P	0	0.18
cca	P	1	0.41
ccg	P	0	0.14
act	T	3	747.40
acc	T	4	642.40
aca	T	2831	1193.00
acg	T	13	268.20
gct	A	0	1.01
gcc	A	0	0.77
gca	A	4	1.78
gcg	A	0	0.44
---	--	------	------
mPD		0.017	0.70
		nPD:	0.02
		N. weight:	1.
		Sc. PD:	-0.16
		Sc. rank:	-1257.4

,

PA-X			
Pos.	99	obs:	exp:
cgt	R	0	0.49
cgc	R	0	0.70
cga	R	2	1.31
cgg	R	0	1.06
aga	R	8	6.07
agg	R	3	3.38
gtt	V	0	0.22
gtc	V	0	0.21
gta	V	0	0.21
gtg	V	1	0.36
gaa	E	20	11.21
gag	E	0	8.79
ggt	G	0	408.10
ggc	G	1	370.70
gga	G	2276	1237.00
ggg	G	545	806.70
---	--	------	------
mPD		0.33	0.71
		nPD:	0.47
		N. weight:	0.51
		Sc. PD:	0.14
		Sc. rank:	775.5

,

PA-X			
Pos.	100	obs:	exp:
tta	L	0	0.09
ttg	L	0	0.19
ctt	L	0	0.20
ctc	L	1	0.16
cta	L	0	0.15
ctg	L	0	0.21
att	I	4	9.54
atc	I	22	6.96
ata	I	0	9.50
gtt	V	192	602.90
gtc	V	2599	589.70
gta	V	12	597.70
gtg	V	0	1013.00
gct	A	7	6.55
gcc	A	19	5.03
gca	A	0	11.57
gcg	A	0	2.85
---	--	------	------
mPD		0.18	0.77
		nPD:	0.23
		N. weight:	1.6
		Sc. PD:	0.06
		Sc. rank:	1332.2

,

PA-X			
Pos.	101	obs:	exp:
tct	S	0	1.34
tcc	S	0	1.16
tca	S	0	1.81
tcg	S	0	0.48
aat	N	483	270.50
aac	N	13	225.50
aaa	K	1	0.57
aag	K	0	0.43
agt	S	8	1.74
agc	S	0	1.47
gtt	V	0	1.72
gtc	V	0	1.68
gta	V	0	1.71
gtg	V	8	2.89
gat	D	932	515.10
gac	D	22	438.90
gaa	E	824	754.50
gag	E	522	591.50
ggt	G	33	6.22
ggc	G	3	5.65
gga	G	5	18.84
ggg	G	2	12.29
---	--	------	------
mPD		0.95	1.1
		nPD:	0.88
		N. weight:	0.35
		Sc. PD:	0.23
		Sc. rank:	698.8

,

PA-X			
Pos.	102	obs:	exp:
cgt	R	0	0.04
cgc	R	0	0.05
cga	R	0	0.10
cgg	R	0	0.08
aaa	K	712	1626.00
aag	K	2143	1229.00
aga	R	1	0.47
agg	R	0	0.26
---	--	------	------
mPD		0.38	0.49
		nPD:	0.76
		N. weight:	0.26
		Sc. PD:	0.14
		Sc. rank:	518.0

,

PA-X			
Pos.	103	obs:	exp:
cct	P	1876	780.50
ccc	P	898	515.80
cca	P	82	1159.00
ccg	P	0	400.40
---	--	------	------
mPD		0.47	0.71
		nPD:	0.66
		N. weight:	0.82
		Sc. PD:	0.38
		Sc. rank:	1515.2

,

PA-X			
Pos.	104	obs:	exp:
cgt	R	0	0.34
cgc	R	0	0.48
cga	R	0	0.91
cgg	R	0	0.73
att	I	0	0.37
atc	I	0	0.27
ata	I	1	0.37
aaa	K	2792	1621.00
aag	K	54	1225.00
aga	R	9	4.20
agg	R	0	2.34
---	--	------	------
mPD		0.044	0.50
		nPD:	0.09
		N. weight:	0.58
		Sc. PD:	-0.057
		Sc. rank:	-115.8

,

PA-X			
Pos.	105	obs:	exp:
ttt	F	540	1128.00
ttc	F	2163	1575.00
tta	L	5	1.85
ttg	L	0	3.80
tct	S	2	19.20
tcc	S	113	16.66
tca	S	0	26.07
tcg	S	0	6.94
tat	Y	0	8.63
tac	Y	18	9.37
ctt	L	7	3.97
ctc	L	8	3.20
cta	L	0	2.96
ctg	L	0	4.22
agt	S	0	24.94
agc	S	0	21.19
---	--	------	------
mPD		0.41	0.64
		nPD:	0.65
		N. weight:	0.23
		Sc. PD:	0.1
		Sc. rank:	413.4

,

PA-X			
Pos.	106	obs:	exp:
tta	L	0	264.00
ttg	L	0	542.40
ctt	L	45	566.90
ctc	L	2809	456.70
cta	L	1	422.50
ctg	L	0	602.60
att	I	0	0.37
atc	I	1	0.27
ata	I	0	0.37
---	--	------	------
mPD		0.032	1.1
		nPD:	0.03
		N. weight:	2.1
		Sc. PD:	-0.33
		Sc. rank:	-2489.7

,

PA-X			
Pos.	107	obs:	exp:
cct	P	807	780.50
ccc	P	23	515.80
cca	P	1961	1159.00
ccg	P	65	400.40
---	--	------	------
mPD		0.45	0.71
		nPD:	0.63
		N. weight:	0.37
		Sc. PD:	0.16
		Sc. rank:	666.1

,

PA-X			
Pos.	108	obs:	exp:
aat	N	3	1.64
aac	N	0	1.36
gat	D	2146	1540.00
gac	D	707	1313.00
---	--	------	------
mPD		0.37	0.50
		nPD:	0.75
		N. weight:	0.12
		Sc. PD:	0.065
		Sc. rank:	236.8

,

PA-X			
Pos.	109	obs:	exp:
tta	L	118	263.90
ttg	L	2674	542.20
tct	S	0	0.17
tcc	S	0	0.14
tca	S	0	0.23
tcg	S	1	0.06
ctt	L	0	566.70
ctc	L	0	456.50
cta	L	5	422.40
ctg	L	57	602.40
atg	M	1	1.00
agt	S	0	0.22
agc	S	0	0.18
---	--	------	------
mPD		0.13	1.1
		nPD:	0.11
		N. weight:	1.7
		Sc. PD:	-0.13
		Sc. rank:	361.4

,

PA-X			
Pos.	110	obs:	exp:
tat	Y	2792	1370.00
tac	Y	64	1486.00
---	--	------	------
mPD		0.044	0.50
		nPD:	0.09
		N. weight:	0.76
		Sc. PD:	-0.075
		Sc. rank:	-159.4

,

PA-X			
Pos.	111	obs:	exp:
tat	Y	0	0.48
tac	Y	1	0.52
gtt	V	0	0.65
gtc	V	3	0.63
gta	V	0	0.64
gtg	V	0	1.08
gat	D	63	1540.00
gac	D	2789	1312.00
---	--	------	------
mPD		0.046	0.50
		nPD:	0.09
		N. weight:	0.81
		Sc. PD:	-0.077
		Sc. rank:	-96.6

,

PA-X			
Pos.	112	obs:	exp:
tat	Y	28	1370.00
tac	Y	2828	1486.00
---	--	------	------
mPD		0.019	0.50
		nPD:	0.04
		N. weight:	0.73
		Sc. PD:	-0.11
		Sc. rank:	-709.0

,

PA-X			
Pos.	113	obs:	exp:
cgt	R	0	0.67
cgc	R	0	0.96
cga	R	0	1.81
cgg	R	0	1.47
act	T	0	0.26
acc	T	0	0.23
aca	T	1	0.42
acg	T	0	0.09
aat	N	0	0.55
aac	N	1	0.45
aaa	K	2632	1615.00
aag	K	204	1221.00
aga	R	16	8.40
agg	R	2	4.68
---	--	------	------
mPD		0.15	0.51
		nPD:	0.29
		N. weight:	0.4
		Sc. PD:	0.04
		Sc. rank:	427.7

,

PA-X			
Pos.	114	obs:	exp:
aaa	K	0	0.57
aag	K	1	0.43
gaa	E	79	1600.00
gag	E	2776	1255.00
---	--	------	------
mPD		0.055	0.49
		nPD:	0.11
		N. weight:	0.84
		Sc. PD:	-0.064
		Sc. rank:	153.8

,

PA-X			
Pos.	115	obs:	exp:
tct	S	0	4.34
tcc	S	0	3.77
tca	S	0	5.89
tcg	S	0	1.57
tat	Y	0	1.92
tac	Y	4	2.08
aat	N	179	1517.00
aac	N	2602	1264.00
agt	S	19	5.64
agc	S	7	4.79
gat	D	1	24.30
gac	D	44	20.70
---	--	------	------
mPD		0.18	0.56
		nPD:	0.32
		N. weight:	0.66
		Sc. PD:	0.087
		Sc. rank:	738.1

,

PA-X

Pos.	116	obs:	exp:
caa	Q	1	0.50
cag	Q	0	0.50
cgt	R	13	106.80
cgc	R	11	152.80
cga	R	2749	287.70
cgg	R	66	233.10
aga	R	12	1333.00
agg	R	4	741.70
---	--	------	------
mPD		0.076	0.96
		nPD:	0.08
		N. weight:	2.6
		Sc. PD:	-0.27
		Sc. rank:	-879.1

PA-X

Pos.	117	obs:	exp:
ttt	F	128	1191.00
ttc	F	2728	1665.00
---	--	------	------
mPD		0.086	0.49
		nPD:	0.18
		N. weight:	0.45
		Sc. PD:	-0.0056
		Sc. rank:	292.7

PA-X

Pos.	118	obs:	exp:
tta	L	0	0.09
ttg	L	0	0.19
ctt	L	1	0.20
ctc	L	0	0.16
cta	L	0	0.15
ctg	L	0	0.21
att	I	2568	994.10
atc	I	139	724.90
ata	I	2	990.00
act	T	14	24.38
acc	T	76	20.96
aca	T	3	38.91
acg	T	0	8.75
gtt	V	33	11.40
gtc	V	20	11.15
gta	V	0	11.30
gtg	V	0	19.15
---	--	------	------
mPD		0.25	0.77
		nPD:	0.33
		N. weight:	0.99
		Sc. PD:	0.14
		Sc. rank:	1137.8

PA-X

Pos.	119	obs:	exp:
gat	D	1	0.54
gac	D	0	0.46
gaa	E	2795	1600.00
gag	E	60	1255.00
---	--	------	------
mPD		0.042	0.49
		nPD:	0.08
		N. weight:	0.59
		Sc. PD:	-0.059
		Sc. rank:	-162.0

PA-X

Pos.	120	obs:	exp:
att	I	2837	1045.00
atc	I	10	762.40
ata	I	2	1041.00
gtt	V	5	1.51
gtc	V	0	1.47
gta	V	0	1.49
gtg	V	2	2.53
---	--	------	------
mPD		0.015	0.67
		nPD:	0.02
		N. weight:	1.2
		Sc. PD:	-0.19
		Sc. rank:	-1591.2

PA-X

Pos.	121	obs:	exp:
ggt	G	2	413.00
ggc	G	2	375.10
gga	G	2786	1251.00
ggg	G	66	816.40
---	--	------	------
mPD		0.048	0.69
		nPD:	0.07
		N. weight:	0.87
		Sc. PD:	-0.1
		Sc. rank:	-421.9

PA-X

Pos.	122	obs:	exp:
gtt	V	1	614.30
gtc	V	0	600.90
gta	V	849	609.00
gtg	V	2006	1032.00
---	--	------	------
mPD		0.42	0.73
		nPD:	0.57
		N. weight:	0.69
		Sc. PD:	0.26
		Sc. rank:	1149.5

PA-X

Pos.	123	obs:	exp:
act	T	2	748.70
acc	T	23	643.50
aca	T	2016	1195.00
acg	T	815	268.70
---	--	------	------
mPD		0.42	0.70
		nPD:	0.6
		N. weight:	0.8
		Sc. PD:	0.32
		Sc. rank:	1373.1

PA-X

Pos.	124	obs:	exp:
cgt	R	0	106.90
cgc	R	0	152.80
cga	R	1145	287.80
cgg	R	1630	233.20
aga	R	42	1333.00
agg	R	39	742.00
---	--	------	------
mPD		0.54	0.95
		nPD:	0.57
		N. weight:	1.9
		Sc. PD:	0.71
		Sc. rank:	3183.2

PA-X

Pos.	125	obs:	exp:
cgt	R	0	106.90
cgc	R	0	152.80
cga	R	5	287.80
cgg	R	668	233.20
aga	R	86	1333.00
agg	R	2097	742.00
---	--	------	------
mPD		0.42	0.95
		nPD:	0.44
		N. weight:	1.1
		Sc. PD:	0.28
		Sc. rank:	1688.0

PA-X

Pos.	126	obs:	exp:
gat	D	1	0.54
gac	D	0	0.46
gaa	E	2670	1600.00
gag	E	185	1255.00
---	--	------	------
mPD		0.12	0.49
		nPD:	0.25
		N. weight:	0.43
		Sc. PD:	0.025
		Sc. rank:	390.8

PA-X

Pos.	127	obs:	exp:
tta	L	0	0.28
ttg	L	0	0.57
ctt	L	3	0.60
ctc	L	0	0.48
cta	L	0	0.44
ctg	L	0	0.63
att	I	74	27.16
atc	I	0	19.80
ata	I	0	27.04
gtt	V	2641	596.60
gtc	V	131	583.60
gta	V	1	591.50
gtg	V	1	1002.00
gct	A	4	1.26
gcc	A	1	0.97
gca	A	0	2.23
gcg	A	0	0.55
---	--	------	------
mPD		0.15	0.79
		nPD:	0.18
		N. weight:	1.6
		Sc. PD:	-0.0081
		Sc. rank:	1110.7

PA-X

Pos.	128	obs:	exp:
tat	Y	0	0.48
tac	Y	1	0.52
cat	H	522	1650.00
cac	H	2329	1201.00
caa	Q	1	0.50
cag	Q	0	0.50
aat	N	2	1.64
aac	N	1	1.36
---	--	------	------
mPD		0.30	0.49
		nPD:	0.62
		N. weight:	0.4
		Sc. PD:	0.17
		Sc. rank:	706.3

PA-X

Pos.	129	obs:	exp:
tta	L	0	1.94
ttg	L	0	3.99
ctt	L	0	4.17
ctc	L	0	3.36
cta	L	21	3.11
ctg	L	0	4.43
att	I	5	824.60
atc	I	9	601.30
ata	I	2233	821.10
atg	M	28	28.00
act	T	0	134.70
acc	T	1	115.80
aca	T	508	215.10
acg	T	5	48.36
gtt	V	0	9.68
gtc	V	0	9.47
gta	V	45	9.60
gtg	V	0	16.26
gct	A	0	0.25
gcc	A	0	0.19
gca	A	1	0.45
gcg	A	0	0.11
---	--	------	------
mPD		0.37	1.0
		nPD:	0.37
		N. weight:	1.2
		Sc. PD:	0.2
		Sc. rank:	1410.0

PA-X

Pos.	130	obs:	exp:
tat	Y	119	1369.00
tac	Y	2736	1486.00
tgt	C	0	0.39
tgc	C	1	0.61
---	--	------	------
mPD		0.081	0.50
		nPD:	0.16
		N. weight:	0.59
		Sc. PD:	-0.016
		Sc. rank:	321.6

PA-X

Pos.	131	obs:	exp:
tat	Y	1688	1369.00
tac	Y	1167	1486.00
tgt	C	1	0.39
tgc	C	0	0.61
---	--	------	------
mPD		0.48	0.50
		nPD:	0.97
		N. weight:	0.033
		Sc. PD:	0.025
		Sc. rank:	65.8

PA-X

Pos.	132	obs:	exp:
tta	L	642	262.10
ttg	L	11	538.60
ctt	L	10	562.90
ctc	L	0	453.50
cta	L	1428	419.60
ctg	L	744	598.40
caa	Q	4	2.00
cag	Q	0	2.00
att	I	0	0.73
atc	I	0	0.54
ata	I	2	0.73
atg	M	15	15.00
---	--	------	------
mPD		0.76	1.1
		nPD:	0.68
		N. weight:	1.
		Sc. PD:	0.49
		Sc. rank:	1914.1

PA-X

Pos.	133	obs:	exp:
gat	D	0	0.54
gac	D	1	0.46
gaa	E	2229	1600.00
gag	E	625	1254.00
ggt	G	0	0.14
ggc	G	0	0.13
gga	G	1	0.44
ggg	G	0	0.29
---	--	------	------
mPD		0.34	0.49
		nPD:	0.7
		N. weight:	0.13
		Sc. PD:	0.065
		Sc. rank:	247.8

PA-X

Pos.	134	obs:	exp:
cgt	R	0	0.04
cgc	R	0	0.05
cga	R	0	0.10
cgg	R	0	0.08
aaa	K	2474	1626.00
aag	K	381	1229.00
aga	R	1	0.47
agg	R	0	0.26
---	--	------	------
mPD		0.23	0.49
		nPD:	0.47
		N. weight:	0.25
		Sc. PD:	0.07
		Sc. rank:	389.6

PA-X

Pos.	135	obs:	exp:
tct	S	0	0.50
tcc	S	3	0.43
tca	S	0	0.68
tcg	S	0	0.18
agt	S	0	0.65
agc	S	0	0.55
gtt	V	0	0.22
gtc	V	1	0.21
gta	V	0	0.21
gtg	V	0	0.36
gct	A	13	718.20
gcc	A	1714	551.70
gca	A	1115	1269.00
gcg	A	9	312.00
gat	D	0	0.54
gac	D	1	0.46
---	--	------	------
mPD		0.49	0.69
		nPD:	0.71
		N. weight:	0.74
		Sc. PD:	0.37
		Sc. rank:	1415.9

PA-X

Pos.	136	obs:	exp:
tct	S	0	1.67
tcc	S	0	1.45
tca	S	0	2.27
tcg	S	0	0.60
tgt	C	0	0.39
tgc	C	1	0.61
aat	N	323	1551.00
aac	N	2522	1294.00
agt	S	8	2.17
agc	S	2	1.84
---	--	------	------
mPD		0.21	0.51
		nPD:	0.42
		N. weight:	0.51
		Sc. PD:	0.11
		Sc. rank:	707.7

,

PA-X

Pos.	137	obs:	exp:
cgt	R	0	0.04
cgc	R	0	0.05
cga	R	0	0.10
cgg	R	0	0.08
aaa	K	81	1626.00
aag	K	2774	1229.00
aga	R	0	0.47
agg	R	1	0.26
---	--	------	------
mPD		0.056	0.49
		nPD:	0.11
		N. weight:	0.86
		Sc. PD:	-0.063
		Sc. rank:	193.1

,

PA-X

Pos.	138	obs:	exp:
att	I	3	1048.00
atc	I	37	764.00
ata	I	2815	1043.00
aaa	K	1	0.57
aag	K	0	0.43
---	--	------	------
mPD		0.028	0.66
		nPD:	0.04
		N. weight:	1.1
		Sc. PD:	-0.16
		Sc. rank:	-1050.8

,

PA-X

Pos.	139	obs:	exp:
cgt	R	0	0.04
cgc	R	0	0.05
cga	R	0	0.10
cgg	R	0	0.08
aaa	K	2647	1626.00
aag	K	208	1229.00
aga	R	0	0.47
agg	R	1	0.26
---	--	------	------
mPD		0.14	0.49
		nPD:	0.28
		N. weight:	0.39
		Sc. PD:	0.034
		Sc. rank:	392.3

,

PA-X

Pos.	140	obs:	exp:
tct	S	43	476.60
tcc	S	522	413.60
tca	S	2228	647.20
tcg	S	62	172.20
act	T	0	0.26
acc	T	0	0.23
aca	T	1	0.42
acg	T	0	0.09
agt	S	0	619.30
agc	S	0	526.10
---	--	------	------
mPD		0.36	1.7
		nPD:	0.22
		N. weight:	1.2
		Sc. PD:	0.031
		Sc. rank:	938.0

,

PA-X

Pos.	141	obs:	exp:
aaa	K	0	1.14
aag	K	2	0.86
gaa	E	51	1594.00
gag	E	2793	1250.00
ggt	G	0	1.45
ggc	G	0	1.31
gga	G	0	4.38
ggg	G	10	2.86
---	--	------	------
mPD		0.043	0.50
		nPD:	0.09
		N. weight:	0.89
		Sc. PD:	-0.088
		Sc. rank:	-232.4

,

PA-X

Pos.	142	obs:	exp:
caa	Q	0	2.50
cag	Q	5	2.50
cgt	R	0	36.08
cgc	R	0	51.59
cga	R	0	97.15
cgg	R	0	78.72
atg	M	1	1.00
aat	N	9	4.91
aac	N	0	4.09
aaa	K	755	1065.00
aag	K	1116	805.60
aga	R	50	450.00
agg	R	914	250.40
ggt	G	0	0.87
ggc	G	0	0.79
gga	G	0	2.63
ggg	G	6	1.72
---	--	------	------
mPD		0.87	1.1
		nPD:	0.76
		N. weight:	0.51
		Sc. PD:	0.28
		Sc. rank:	1020.6

,

PA-X

Pos.	143	obs:	exp:
atg	M	1	1.00
act	T	27	747.90
acc	T	25	642.90
aca	T	2760	1194.00
acg	T	41	268.40
gct	A	0	0.50
gcc	A	0	0.39
gca	A	2	0.89
gcg	A	0	0.22
---	--	------	------
mPD		0.066	0.70
		nPD:	0.1
		N. weight:	0.88
		Sc. PD:	-0.081
		Sc. rank:	-43.4

,

PA-X

Pos.	144	obs:	exp:
tta	L	0	0.09
ttg	L	0	0.19
ctt	L	0	0.20
ctc	L	1	0.16
cta	L	0	0.15
ctg	L	0	0.21
cat	H	773	1651.00
cac	H	2079	1201.00
caa	Q	1	0.50
cag	Q	0	0.50
cgt	R	1	0.04
cgc	R	0	0.05
cga	R	0	0.10
cgg	R	0	0.08
aat	N	1	0.55
aac	N	0	0.45
aga	R	0	0.47
agg	R	0	0.26
---	--	------	------
mPD		0.40	0.49
		nPD:	0.81
		N. weight:	0.24
		Sc. PD:	0.15
		Sc. rank:	482.0

,

PA-X

Pos.	145	obs:	exp:
att	I	2638	1048.00
atc	I	217	764.30
ata	I	1	1044.00
---	--	------	------
mPD		0.14	0.66
		nPD:	0.21
		N. weight:	0.92
		Sc. PD:	0.022
		Sc. rank:	713.7

,

PA-X

Pos.	146	obs:	exp:
cat	H	191	1653.00
cac	H	2664	1202.00
cgt	R	0	0.04
cgc	R	1	0.05
cga	R	0	0.10
cgg	R	0	0.08
aga	R	0	0.47
agg	R	0	0.26
---	--	------	------
mPD		0.13	0.49
		nPD:	0.26
		N. weight:	0.73
		Sc. PD:	0.048
		Sc. rank:	667.4

,

PA-X

Pos.	147	obs:	exp:
tta	L	0	0.37
ttg	L	0	0.76
ctt	L	0	0.79
ctc	L	0	0.64
cta	L	4	0.59
ctg	L	0	0.84
att	I	26	1047.00
atc	I	48	763.20
ata	I	2778	1042.00
---	--	------	------
mPD		0.054	0.66
		nPD:	0.08
		N. weight:	1.1
		Sc. PD:	-0.11
		Sc. rank:	-349.1

,

PA-X

Pos.	148	obs:	exp:
ttt	F	74	1191.00
ttc	F	2781	1664.00
tat	Y	0	0.48
tac	Y	1	0.52
---	--	------	------
mPD		0.051	0.49
		nPD:	0.11
		N. weight:	0.52
		Sc. PD:	-0.043
		Sc. rank:	40.7

,

PA-X

Pos.	149	obs:	exp:
tta	L	1	0.09
ttg	L	0	0.19
tct	S	6	476.10
tcc	S	12	413.10
tca	S	2803	646.50
tcg	S	31	172.10
ctt	L	0	0.20
ctc	L	0	0.16
cta	L	0	0.15
ctg	L	0	0.21
cct	P	0	0.27
ccc	P	0	0.18
cca	P	1	0.41
ccg	P	0	0.14
act	T	0	0.52
acc	T	0	0.45
aca	T	2	0.84
acg	T	0	0.19
agt	S	0	618.60
agc	S	0	525.60
---	--	------	------
mPD		0.037	1.7
		nPD:	0.02
		N. weight:	1.7
		Sc. PD:	-0.28
		Sc. rank:	-2238.4

,

PA-X

Pos.	150	obs:	exp:
ttt	F	32	1191.00
ttc	F	2823	1664.00
tat	Y	0	0.48
tac	Y	1	0.52
---	--	------	------
mPD		0.023	0.49
		nPD:	0.05
		N. weight:	0.59
		Sc. PD:	-0.081
		Sc. rank:	-529.8

,

PA-X

Pos.	151	obs:	exp:
att	I	7	3.67
atc	I	0	2.68
ata	I	3	3.65
act	T	2332	743.70
acc	T	17	639.30
aca	T	481	1187.00
acg	T	7	266.90
aat	N	6	3.27
aac	N	0	2.73
gct	A	3	0.76
gcc	A	0	0.58
gca	A	0	1.34
gcg	A	0	0.33
---	--	------	------
mPD		0.31	0.71
		nPD:	0.43
		N. weight:	0.92
		Sc. PD:	0.22
		Sc. rank:	1359.9

,

PA-X

Pos.	152	obs:	exp:
ggt	G	0	413.00
ggc	G	0	375.10
gga	G	2221	1251.00
ggg	G	635	816.40
---	--	------	------
mPD		0.35	0.69
		nPD:	0.5
		N. weight:	0.48
		Sc. PD:	0.15
		Sc. rank:	736.7

,

PA-X

Pos.	153	obs:	exp:
gaa	E	165	1601.00
gag	E	2691	1255.00
---	--	------	------
mPD		0.11	0.49
		nPD:	0.22
		N. weight:	0.72
		Sc. PD:	0.022
		Sc. rank:	588.1

,

PA-X

Pos.	154	obs:	exp:
aaa	K	1	0.57
aag	K	0	0.43
gaa	E	2765	1600.00
gag	E	90	1255.00
---	--	------	------
mPD		0.062	0.49
		nPD:	0.13
		N. weight:	0.54
		Sc. PD:	-0.034
		Sc. rank:	156.2

,

PA-X

Pos.	155	obs:	exp:
tta	L	0	0.09
ttg	L	1	0.19
ctt	L	0	0.20
ctc	L	0	0.16
cta	L	0	0.15
ctg	L	0	0.21
att	I	1	0.73
atc	I	0	0.54
ata	I	1	0.73
atg	M	2853	2853.00
---	--	------	------
mPD		0.0021	0.0025
		nPD:	0.83
		N. weight:	0.0013
		Sc. PD:	0.0008
		Sc. rank:	2.5

,

PA-X

Pos. 156		obs:	exp:
act	T	0	0.26
acc	T	0	0.23
aca	T	1	0.42
acg	T	0	0.09
gtt	V	0	0.65
gtc	V	3	0.63
gta	V	0	0.64
gtg	V	0	1.08
gct	A	117	718.50
gcc	A	2417	551.90
gca	A	318	1269.00
gcg	A	0	312.20
---	--	------	------
mPD		0.27	0.69
		nPD:	0.39
		N. weight:	1.2
		Sc. PD:	0.25
		Sc. rank:	1657.6

,

PA-X

Pos. 157		obs:	exp:
tct	S	0	0.17
tcc	S	0	0.14
tca	S	0	0.23
tcg	S	0	0.06
act	T	718	748.20
acc	T	2082	643.10
aca	T	50	1194.00
acg	T	4	268.50
aat	N	0	0.55
aac	N	1	0.45
agt	S	0	0.22
agc	S	1	0.18
---	--	------	------
mPD		0.41	0.70
		nPD:	0.58
		N. weight:	0.96
		Sc. PD:	0.37
		Sc. rank:	1621.5

,

PA-X

Pos. 158		obs:	exp:
cgt	R	0	1.61
cgc	R	0	2.30
cga	R	0	4.33
cgg	R	0	3.51
aaa	K	1968	1602.00
aag	K	845	1211.00
aga	R	43	20.07
agg	R	0	11.17
---	--	------	------
mPD		0.45	0.53
		nPD:	0.84
		N. weight:	0.059
		Sc. PD:	0.037
		Sc. rank:	117.9

,

PA-X

Pos. 159		obs:	exp:
tct	S	1	0.33
tcc	S	0	0.29
tca	S	0	0.45
tcg	S	1	0.12
act	T	0	0.52
acc	T	0	0.45
aca	T	0	0.84
acg	T	2	0.19
agt	S	0	0.43
agc	S	0	0.37
gct	A	877	718.50
gcc	A	8	551.90
gca	A	133	1269.00
gcg	A	1834	312.20
---	--	------	------
mPD		0.49	0.69
		nPD:	0.71
		N. weight:	1.3
		Sc. PD:	0.67
		Sc. rank:	2568.9

,

PA-X

Pos. 160		obs:	exp:
aat	N	0	1.64
aac	N	3	1.36
gat	D	107	1435.00
gac	D	2550	1222.00
gaa	E	196	109.90
gag	E	0	86.13
---	--	------	------
mPD		0.20	0.56
		nPD:	0.35
		N. weight:	0.73
		Sc. PD:	0.11
		Sc. rank:	853.5

,

PA-X

Pos. 161		obs:	exp:
ttt	F	2	0.83
ttc	F	0	1.17
tat	Y	1183	1368.00
tac	Y	1669	1484.00
tgt	C	0	0.39
tgc	C	1	0.61
cat	H	1	0.58
cac	H	0	0.42
---	--	------	------
mPD		0.49	0.50
		nPD:	0.97
		N. weight:	0.013
		Sc. PD:	0.01
		Sc. rank:	26.7

,

PA-X

Pos. 162		obs:	exp:
att	I	2	2.94
atc	I	6	2.14
ata	I	0	2.92
act	T	1077	744.80
acc	T	1758	640.20
aca	T	1	1189.00
acg	T	5	267.30
aat	N	0	1.09
aac	N	2	0.91
gct	A	0	1.26
gcc	A	5	0.97
gca	A	0	2.23
gcg	A	0	0.55
---	--	------	------
mPD		0.48	0.71
		nPD:	0.68
		N. weight:	0.92
		Sc. PD:	0.44
		Sc. rank:	1729.6

,

PA-X

Pos. 163		obs:	exp:
tta	L	0	262.80
ttg	L	0	539.90
ctt	L	2776	564.30
ctc	L	59	454.60
cta	L	5	420.60
ctg	L	2	599.90
att	I	13	5.14
atc	I	1	3.75
ata	I	0	5.12
---	--	------	------
mPD		0.056	1.1
		nPD:	0.05
		N. weight:	1.8
		Sc. PD:	-0.25
		Sc. rank:	-1532.7

,

PA-X

Pos. 164		obs:	exp:
aat	N	1	0.55
aac	N	0	0.45
gat	D	2800	1541.00
gac	D	54	1313.00
ggt	G	1	0.14
ggc	G	0	0.13
gga	G	0	0.44
ggg	G	0	0.29
---	--	------	------
mPD		0.039	0.50
		nPD:	0.08
		N. weight:	0.64
		Sc. PD:	-0.07
		Sc. rank:	-273.9

,

PA-X

Pos. 165		obs:	exp:
gat	D	12	8.64
gac	D	4	7.36
gaa	E	1701	1592.00
gag	E	1139	1248.00
---	--	------	------
mPD		0.49	0.50
		nPD:	0.98
		N. weight:	0.0067
		Sc. PD:	0.0051
		Sc. rank:	13.5

,

PA-X

Pos. 166		obs:	exp:
gaa	E	167	1600.00
gag	E	2688	1255.00
ggt	G	0	0.14
ggc	G	0	0.13
gga	G	0	0.44
ggg	G	1	0.29
---	--	------	------
mPD		0.11	0.49
		nPD:	0.22
		N. weight:	0.71
		Sc. PD:	0.025
		Sc. rank:	594.3

,

PA-X

Pos. 167		obs:	exp:
tct	S	0	476.70
tcc	S	0	413.70
tca	S	0	647.50
tcg	S	0	172.30
agt	S	263	619.50
agc	S	2593	526.30
---	--	------	------
mPD		0.17	1.7
		nPD:	0.1
		N. weight:	1.7
		Sc. PD:	-0.14
		Sc. rank:	94.4

,

PA-X

Pos. 168		obs:	exp:
cgt	R	0	106.90
cgc	R	0	152.80
cga	R	1	287.80
cgg	R	1	233.20
aga	R	2326	1333.00
agg	R	528	742.00
---	--	------	------
mPD		0.30	0.95
		nPD:	0.32
		N. weight:	0.47
		Sc. PD:	0.059
		Sc. rank:	520.0

,

PA-X

Pos. 169		obs:	exp:
gct	A	5	719.20
gcc	A	7	552.50
gca	A	2732	1271.00
gcg	A	111	312.50
gaa	E	1	0.56
gag	E	0	0.44
---	--	------	------
mPD		0.083	0.69
		nPD:	0.12
		N. weight:	0.82
		Sc. PD:	-0.054
		Sc. rank:	228.4

,

PA-X

Pos. 170		obs:	exp:
cgt	R	0	106.80
cgc	R	0	152.80
cga	R	10	287.70
cgg	R	0	233.10
aga	R	2731	1333.00
agg	R	114	741.70
ggt	G	0	0.14
ggc	G	0	0.13
gga	G	1	0.44
ggg	G	0	0.29
---	--	------	------
mPD		0.084	0.95
		nPD:	0.09
		N. weight:	0.73
		Sc. PD:	-0.072
		Sc. rank:	-137.6

,

PA-X

Pos. 171		obs:	exp:
att	I	1103	1048.00
atc	I	804	764.30
ata	I	949	1044.00
---	--	------	------
mPD		0.66	0.66
		nPD:	1.
		N. weight:	0.0064
		Sc. PD:	0.0051
		Sc. rank:	12.9

,

PA-X

Pos. 172		obs:	exp:
cct	P	0	0.27
ccc	P	0	0.18
cca	P	1	0.41
ccg	P	0	0.14
cgt	R	0	0.04
cgc	R	0	0.05
cga	R	0	0.10
cgg	R	0	0.08
aaa	K	2730	1625.00
aag	K	124	1229.00
aga	R	1	0.47
agg	R	0	0.26
---	--	------	------
mPD		0.085	0.49
		nPD:	0.17
		N. weight:	0.48
		Sc. PD:	-0.0075
		Sc. rank:	308.8

,

PA-X

Pos. 173		obs:	exp:
act	T	16	748.70
acc	T	2761	643.50
aca	T	77	1195.00
acg	T	2	268.70
---	--	------	------
mPD		0.065	0.70
		nPD:	0.09
		N. weight:	1.6
		Sc. PD:	-0.15
		Sc. rank:	-148.7

,

PA-X

Pos. 174		obs:	exp:
cgt	R	0	106.80
cgc	R	0	152.70
cga	R	27	287.50
cgg	R	1	233.00
act	T	0	0.52
acc	T	0	0.45
aca	T	0	0.84
acg	T	2	0.19
aaa	K	0	0.57
aag	K	1	0.43
aga	R	755	1332.00
agg	R	2070	741.20
---	--	------	------
mPD		0.42	0.96
		nPD:	0.44
		N. weight:	0.7
		Sc. PD:	0.17
		Sc. rank:	1039.0

,

PA-X

Pos. 175		obs:	exp:
tta	L	1	263.90
ttg	L	34	542.20
ctt	L	8	566.70
ctc	L	0	456.50
cta	L	120	422.40
ctg	L	2691	602.40
cgt	R	1	0.04
cgc	R	0	0.05
cga	R	0	0.10
cgg	R	0	0.08
atg	M	1	1.00
aga	R	0	0.47
agg	R	0	0.26
---	--	------	------
mPD		0.11	1.1
		nPD:	0.1
		N. weight:	1.6
		Sc. PD:	-0.14
		Sc. rank:	47.8

,

PA-X

Pos.	176	obs:	exp:
ttt	F	29	1189.00
ttc	F	2820	1660.00
tct	S	0	0.17
tcc	S	1	0.14
tca	S	0	0.23
tcg	S	0	0.06
tat	Y	0	2.40
tac	Y	5	2.60
att	I	0	0.37
atc	I	1	0.27
ata	I	0	0.37
agt	S	0	0.22
agc	S	0	0.18
---	--	------	------
mPD		0.025	0.49
		nPD:	0.05
		N. weight:	0.59
		Sc. PD:	-0.08
		Sc. rank:	-471.2

,

PA-X

Pos.	177	obs:	exp:
cgt	R	0	0.04
cgc	R	0	0.05
cga	R	0	0.10
cgg	R	0	0.08
act	T	2300	748.40
acc	T	484	643.30
aca	T	70	1195.00
acg	T	1	268.60
aga	R	1	0.47
agg	R	0	0.26
---	--	------	------
mPD		0.32	0.70
		nPD:	0.46
		N. weight:	0.96
		Sc. PD:	0.26
		Sc. rank:	1455.7

,

PA-X

Pos.	178	obs:	exp:
att	I	2	1044.00
atc	I	4	761.60
ata	I	2840	1040.00
gtt	V	0	2.15
gtc	V	0	2.10
gta	V	10	2.13
gtg	V	0	3.61
---	--	------	------
mPD		0.011	0.67
		nPD:	0.02
		N. weight:	1.2
		Sc. PD:	-0.2
		Sc. rank:	-1903.5

,

PA-X

Pos.	179	obs:	exp:
cgt	R	0	106.90
cgc	R	0	152.80
cga	R	0	287.80
cgg	R	0	233.20
aga	R	2151	1333.00
agg	R	705	742.00
---	--	------	------
mPD		0.37	0.95
		nPD:	0.39
		N. weight:	0.42
		Sc. PD:	0.083
		Sc. rank:	541.8

,

PA-X

Pos.	180	obs:	exp:
cct	P	0	0.27
ccc	P	0	0.18
cca	P	1	0.41
ccg	P	0	0.14
cat	H	2	3.47
cac	H	4	2.53
caa	Q	2310	1422.00
cag	Q	539	1427.00
---	--	------	------
mPD		0.31	0.50
		nPD:	0.62
		N. weight:	0.26
		Sc. PD:	0.11
		Sc. rank:	446.7

,

PA-X

Pos.	181	obs:	exp:
gaa	E	2019	1601.00
gag	E	837	1255.00
---	--	------	------
mPD		0.41	0.49
		nPD:	0.84
		N. weight:	0.057
		Sc. PD:	0.036
		Sc. rank:	114.0

,

PA-X

Pos.	182	obs:	exp:
atg	M	2856	2856.00
---	--	------	------
mPD		0	0
		nPD:	1.
		N. weight:	0.
		Sc. PD:	0
		Sc. rank:	0

,

PA-X

Pos.	183	obs:	exp:
tct	S	0	5.34
tcc	S	32	4.64
tca	S	0	7.25
tcg	S	0	1.93
agt	S	0	6.94
agc	S	0	5.90
gct	A	16	711.20
gcc	A	2678	546.30
gca	A	129	1257.00
gcg	A	0	309.00
ggt	G	0	0.14
ggc	G	1	0.13
gga	G	0	0.44
ggg	G	0	0.29
---	--	------	------
mPD		0.12	0.72
		nPD:	0.17
		N. weight:	1.7
		Sc. PD:	-0.038
		Sc. rank:	993.9

,

PA-X

Pos.	184	obs:	exp:
tct	S	0	470.20
tcc	S	0	408.10
tca	S	0	638.60
tcg	S	0	170.00
tgt	C	2	0.77
tgc	C	0	1.23
att	I	4	1.47
atc	I	0	1.07
ata	I	0	1.46
act	T	1	0.26
acc	T	0	0.23
aca	T	0	0.42
acg	T	0	0.09
aat	N	18	10.36
aac	N	1	8.64
agt	S	2584	611.00
agc	S	233	519.10
ggt	G	10	1.88
ggc	G	3	1.71
gga	G	0	5.70
ggg	G	0	3.72
---	--	------	------
mPD		0.18	1.7
		nPD:	0.11
		N. weight:	1.5
		Sc. PD:	-0.12
		Sc. rank:	186.9

,

PA-X

Pos.	185	obs:	exp:
cgt	R	0	104.60
cgc	R	0	149.50
cga	R	0	281.60
cgg	R	0	228.10
aaa	K	4	34.73
aag	K	57	26.27
aga	R	682	1304.00
agg	R	2112	725.90
ggt	G	0	0.14
ggc	G	0	0.13
gga	G	0	0.44
ggg	G	1	0.29
---	--	------	------
mPD		0.41	0.99
		nPD:	0.41
		N. weight:	0.79
		Sc. PD:	0.17
		Sc. rank:	1094.4

,

PA-X

Pos.	186	obs:	exp:
tct	S	0	0.17
tcc	S	0	0.14
tca	S	0	0.23
tcg	S	0	0.06
agt	S	1	0.22
agc	S	0	0.18
gct	A	0	0.25
gcc	A	1	0.19
gca	A	0	0.45
gcg	A	0	0.11
ggt	G	2541	412.70
ggc	G	267	374.90
gga	G	18	1251.00
ggg	G	28	815.80
---	--	------	------
mPD		0.20	0.69
		nPD:	0.29
		N. weight:	1.9
		Sc. PD:	0.18
		Sc. rank:	1984.8

,

PA-X

Pos.	187	obs:	exp:
tta	L	112	263.90
ttg	L	2	542.20
ctt	L	6	566.70
ctc	L	22	456.50
cta	L	2518	422.40
ctg	L	194	602.40
att	I	0	0.73
atc	I	0	0.54
ata	I	2	0.73
---	--	------	------
mPD		0.22	1.1
		nPD:	0.2
		N. weight:	1.7
		Sc. PD:	0.018
		Sc. rank:	1299.6

,

PA-X

Pos.	188	obs:	exp:
tgg	W	2856	2856.00
---	--	------	------
mPD		0	0
		nPD:	1.
		N. weight:	0.
		Sc. PD:	0
		Sc. rank:	0

,

PA-X

Pos.	189	obs:	exp:
gat	D	2826	1542.00
gac	D	29	1313.00
ggt	G	1	0.14
ggc	G	0	0.13
gga	G	0	0.44
ggg	G	0	0.29
---	--	------	------
mPD		0.021	0.50
		nPD:	0.04
		N. weight:	0.68
		Sc. PD:	-0.098
		Sc. rank:	-645.0

,

PA-X

Pos.	190	obs:	exp:
tct	S	0	474.90
tcc	S	2834	412.10
tca	S	11	645.00
tcg	S	0	171.60
cct	P	0	2.73
ccc	P	10	1.81
cca	P	0	4.06
ccg	P	0	1.40
act	T	0	0.26
acc	T	1	0.23
aca	T	0	0.42
acg	T	0	0.09
agt	S	0	617.10
agc	S	0	524.30
---	--	------	------
mPD		0.015	1.7
		nPD:	0.01
		N. weight:	2.3
		Sc. PD:	-0.4
		Sc. rank:	-4111.1

,

PA-X

Pos.	191	obs:	exp:
ttt	F	2856	1191.00
ttc	F	0	1665.00
mPD		0	0.49
		nPD:	0.
		N. weight:	1.1
		Sc. PD:	-0.2
		Sc. rank:	-2155.0

,

PA-X

Pos.	192	obs:	exp:
tta	L	0	0.09
ttg	L	0	0.19
ctt	L	0	0.20
ctc	L	1	0.16
cta	L	0	0.15
ctg	L	0	0.21
att	I	0	0.37
atc	I	1	0.27
ata	I	0	0.37
gtt	V	0	612.30
gtc	V	2847	599.00
gta	V	0	607.10
gtg	V	0	1029.00
gct	A	0	1.76
gcc	A	7	1.35
gca	A	0	3.12
gcg	A	0	0.77
mPD		0.0063	0.74
		nPD:	0.01
		N. weight:	1.9
		Sc. PD:	-0.33
		Sc. rank:	-3467.9

,

PA-X

Pos.	193	obs:	exp:
tct	S	0	206.60
tcc	S	0	179.30
tca	S	0	280.70
tcg	S	0	74.69
aat	N	1618	882.40
aac	N	0	735.60
agt	S	1238	268.50
agc	S	0	228.10
mPD		0.49	1.6
		nPD:	0.31
		N. weight:	1.2
		Sc. PD:	0.15
		Sc. rank:	1337.2

,

PA-X

Pos.	194	obs:	exp:
tta	L	0	51.22
ttg	L	0	105.20
tct	S	0	0.17
tcc	S	0	0.14
tca	S	0	0.23
tcg	S	1	0.06
ctt	L	0	110.00
ctc	L	0	88.62
cta	L	0	81.99
ctg	L	554	116.90
cct	P	0	628.00
ccc	P	0	415.10
cca	P	0	932.70
ccg	P	2298	322.20
caa	Q	0	1.00
cag	Q	2	1.00
cgt	R	0	0.04
cgc	R	0	0.05
cga	R	0	0.10
cgg	R	1	0.08
agt	S	0	0.22
agc	S	0	0.18
aga	R	0	0.47
agg	R	0	0.26
mPD		0.32	1.1
		nPD:	0.28
		N. weight:	2.3
		Sc. PD:	0.19
		Sc. rank:	2266.6

,

PA-X

Pos.	195	obs:	exp:
cgt	R	0	96.40
cgc	R	0	137.90
cga	R	0	259.60
cgg	R	0	210.30
aat	N	0	3.82
aac	N	7	3.18
aaa	K	273	155.40
aag	K	0	117.60
aga	R	2576	1203.00
agg	R	0	669.20
mPD		0.18	1.1
		nPD:	0.16
		N. weight:	0.9
		Sc. PD:	-0.021
		Sc. rank:	506.3

,

PA-X

Pos.	196	obs:	exp:
aaa	K	0	2.28
aag	K	4	1.72
gtt	V	0	0.22
gtc	V	0	0.21
gta	V	0	0.21
gtg	V	1	0.36
gat	D	1	0.54
gac	D	0	0.46
gaa	E	2	1598.00
gag	E	2848	1252.00
mPD		0.0056	0.50
		nPD:	0.01
		N. weight:	0.99
		Sc. PD:	-0.17
		Sc. rank:	-1710.9

,

PA-X

Pos.	197	obs:	exp:
act	T	0	0.26
acc	T	0	0.23
aca	T	0	0.42
acg	T	1	0.09
gtt	V	0	3.01
gtc	V	0	2.95
gta	V	0	2.99
gtg	V	14	5.06
gct	A	0	715.20
gcc	A	1	549.40
gca	A	2	1264.00
gcg	A	2836	310.70
gaa	E	0	0.56
gag	E	1	0.44
ggt	G	0	0.14
ggc	G	0	0.13
gga	G	0	0.44
ggg	G	1	0.29
mPD		0.014	0.70
		nPD:	0.02
		N. weight:	2.7
		Sc. PD:	-0.44
		Sc. rank:	-3702.7

,

PA-X

Pos.	198	obs:	exp:
cgt	R	0	0.26
cgc	R	0	0.37
cga	R	0	0.71
cgg	R	0	0.57
aaa	K	1	1622.00
aag	K	2848	1227.00
aga	R	0	3.27
agg	R	7	1.82
mPD		0.0056	0.50
		nPD:	0.01
		N. weight:	1.
		Sc. PD:	-0.18
		Sc. rank:	-1777.8

,

PA-X

Pos.	199	obs:	exp:
cgt	R	0	98.98
cgc	R	0	141.50
cga	R	0	266.50
cgg	R	0	216.00
att	I	0	0.37
atc	I	0	0.27
ata	I	1	0.37
aaa	K	207	117.90
aag	K	0	89.13
aga	R	2645	1235.00
agg	R	0	687.20
ggt	G	0	0.43
ggc	G	0	0.39
gga	G	3	1.31
ggg	G	0	0.86
mPD		0.14	1.1
		nPD:	0.13
		N. weight:	0.91
		Sc. PD:	-0.053
		Sc. rank:	286.2

,

PA-X

Pos.	200	obs:	exp:
tta	L	0	0.18
ttg	L	0	0.38
ctt	L	0	0.40
ctc	L	0	0.32
cta	L	2	0.30
ctg	L	0	0.42
cct	P	0	0.27
ccc	P	0	0.18
cca	P	1	0.41
ccg	P	0	0.14
cat	H	0	0.58
cac	H	1	0.42
caa	Q	2842	1424.00
cag	Q	10	1428.00
mPD		0.0098	0.50
		nPD:	0.02
		N. weight:	0.82
		Sc. PD:	-0.13
		Sc. rank:	-1235.3

,

PA-X

Pos.	201	obs:	exp:
tta	L	0	263.20
ttg	L	2652	540.90
tct	S	0	1.34
tcc	S	0	1.16
tca	S	0	1.81
tcg	S	8	0.48
ctt	L	0	565.30
ctc	L	0	455.40
cta	L	0	421.30
ctg	L	195	600.90
atg	M	1	1.00
agt	S	0	1.74
agc	S	0	1.47
mPD		0.13	1.1
		nPD:	0.12
		N. weight:	1.7
		Sc. PD:	-0.12
		Sc. rank:	431.9

,

PA-X

Pos.	202	obs:	exp:
cgt	R	0	0.26
cgc	R	0	0.37
cga	R	0	0.71
cgg	R	0	0.57
atg	M	2	2.00
aaa	K	0	1621.00
aag	K	2847	1226.00
aga	R	0	3.27
agg	R	7	1.82
mPD		0.0063	0.50
		nPD:	0.01
		N. weight:	1.
		Sc. PD:	-0.18
		Sc. rank:	-1749.4

,

PA-X

Pos.	203	obs:	exp:
cgt	R	0	5.09
cgc	R	0	7.28
cga	R	0	13.71
cgg	R	0	11.11
aat	N	0	5.45
aac	N	10	4.55
aaa	K	2710	1543.00
aag	K	0	1167.00
aga	R	136	63.49
agg	R	0	35.33
mPD		0.098	0.61
		nPD:	0.16
		N. weight:	0.7
		Sc. PD:	-0.02
		Sc. rank:	348.2

,

PA-X

Pos.	204	obs:	exp:
tct	S	0	0.83
tcc	S	0	0.72
tca	S	0	1.13
tcg	S	0	0.30
aat	N	514	280.30
aac	N	0	233.70
agt	S	5	1.08
agc	S	0	0.92
gtt	V	1	0.22
gtc	V	0	0.21
gta	V	0	0.21
gtg	V	0	0.36
gat	D	1421	767.20
gac	D	0	653.80
ggt	G	915	132.30
ggc	G	0	120.20
gga	G	0	400.90
ggg	G	0	261.60
mPD		0.73	1.4
		nPD:	0.52
		N. weight:	1.3
		Sc. PD:	0.41
		Sc. rank:	1979.1

,

PA-X

Pos.	205	obs:	exp:
tta	L	3	264.00
ttg	L	2852	542.40
tct	S	0	0.17
tcc	S	0	0.14
tca	S	0	0.23
tcg	S	1	0.06
ctt	L	0	566.90
ctc	L	0	456.70
cta	L	0	422.50
ctg	L	0	602.60
agt	S	0	0.22
agc	S	0	0.18
mPD		0.0028	1.1
		nPD:	0.
		N. weight:	2.
		Sc. PD:	-0.36
		Sc. rank:	-3794.0

,

PA-X

Pos.	206	obs:	exp:
caa	Q	1	0.50
cag	Q	0	0.50
cgt	R	0	0.37
cgc	R	0	0.54
cga	R	0	1.01
cgg	R	0	0.82
act	T	0	1.05
acc	T	0	0.90
aca	T	4	1.67
acg	T	0	0.38
aat	N	0	2.73
aac	N	5	2.27
aaa	K	2833	1614.00
aag	K	1	1220.00
aga	R	10	4.67
agg	R	0	2.60
gaa	E	2	1.12
gag	E	0	0.88
---	--	------	------
mPD		0.016	0.51
		nPD:	0.03
		N. weight:	0.68
		Sc. PD:	-0.1
		Sc. rank:	-739.9

,

PA-X

Pos.	207	obs:	exp:
tta	L	238	22.56
ttg	L	6	46.35
tct	S	0	436.00
tcc	S	0	378.40
tca	S	2610	592.10
tcg	S	2	157.60
ctt	L	0	48.45
ctc	L	0	39.03
cta	L	0	36.11
ctg	L	0	51.50
agt	S	0	566.60
agc	S	0	481.40
---	--	------	------
mPD		0.16	1.8
		nPD:	0.09
		N. weight:	1.9
		Sc. PD:	-0.18
		Sc. rank:	-296.8

,

PA-X

Pos.	208	obs:	exp:
tta	L	0	45.77
ttg	L	9	94.04
ctt	L	0	98.28
ctc	L	0	79.18
cta	L	0	73.26
ctg	L	486	104.50
cct	P	0	2.19
ccc	P	0	1.44
cca	P	0	3.25
ccg	P	8	1.12
caa	Q	0	1160.00
cag	Q	2324	1164.00
cgt	R	0	0.30
cgc	R	0	0.43
cga	R	0	0.81
cgg	R	8	0.65
aaa	K	0	10.25
aag	K	18	7.75
aga	R	0	3.73
agg	R	0	2.08
gaa	E	0	1.68
gag	E	3	1.32
---	--	------	------
mPD		0.32	0.97
		nPD:	0.33
		N. weight:	1.
		Sc. PD:	0.14
		Sc. rank:	1147.0

,

PA-X

Pos.	209	obs:	exp:
gtt	V	0	0.22
gtc	V	0	0.21
gta	V	1	0.21
gtg	V	0	0.36
gaa	E	2744	1540.00
gag	E	3	1207.00
ggt	G	0	15.62
ggc	G	0	14.19
gga	G	108	47.32
ggg	G	0	30.87
---	--	------	------
mPD		0.076	0.58
		nPD:	0.13
		N. weight:	0.71
		Sc. PD:	-0.04
		Sc. rank:	230.5

,

PA-X

Pos.	210	obs:	exp:
tta	L	0	2.87
ttg	L	0	5.89
tct	S	0	0.50
tcc	S	0	0.43
tca	S	3	0.68
tcg	S	0	0.18
ctt	L	0	6.16
ctc	L	0	4.96
cta	L	31	4.59
ctg	L	0	6.54
cct	P	1	759.20
ccc	P	2	501.80
cca	P	2774	1128.00
ccg	P	1	389.50
caa	Q	43	21.47
cag	Q	0	21.53
act	T	0	0.26
acc	T	0	0.23
aca	T	1	0.42
acg	T	0	0.09
agt	S	0	0.65
agc	S	0	0.55
---	--	------	------
mPD		0.056	0.77
		nPD:	0.07
		N. weight:	1.1
		Sc. PD:	-0.12
		Sc. rank:	-510.4

,

PA-X

Pos.	211	obs:	exp:
ttt	F	0	0.42
ttc	F	1	0.58
tat	Y	0	2.40
tac	Y	5	2.60
tgt	C	0	1103.00
tgc	C	2849	1746.00
cgt	R	0	0.04
cgc	R	1	0.05
cga	R	0	0.10
cgg	R	0	0.08
aga	R	0	0.47
agg	R	0	0.26
---	--	------	------
mPD		0.0049	0.48
		nPD:	0.01
		N. weight:	0.6
		Sc. PD:	-0.1
		Sc. rank:	-1046.9

,

PA-X

Pos.	212	obs:	exp:
act	T	0	1.05
acc	T	0	0.90
aca	T	4	1.67
acg	T	0	0.38
gtt	V	0	9.46
gtc	V	0	9.26
gta	V	44	9.38
gtg	V	0	15.90
gct	A	1	706.90
gcc	A	19	543.00
gca	A	2786	1249.00
gcg	A	0	307.10
gaa	E	2	1.12
gag	E	0	0.88
---	--	------	------
mPD		0.048	0.73
		nPD:	0.07
		N. weight:	0.95
		Sc. PD:	-0.11
		Sc. rank:	-529.3

,

PA-X

Pos.	213	obs:	exp:
tct	S	0	1.17
tcc	S	0	1.01
tca	S	0	1.59
tcg	S	0	0.42
cgt	R	0	0.04
cgc	R	1	0.05
cga	R	0	0.10
cgg	R	0	0.08
agt	S	0	1.52
agc	S	7	1.29
aga	R	0	0.47
agg	R	0	0.26
gct	A	0	0.50
gcc	A	2	0.39
gca	A	0	0.89
gcg	A	0	0.22
gat	D	0	259.70
gac	D	481	221.30
ggt	G	2	342.00
ggc	G	2363	310.60
gga	G	0	1036.00
ggg	G	0	676.00
---	--	------	------
mPD		0.29	1.0
		nPD:	0.28
		N. weight:	2.2
		Sc. PD:	0.2
		Sc. rank:	2286.2

,

PA-X

Pos.	214	obs:	exp:
tta	L	1	261.10
ttg	L	2823	536.50
tct	S	0	4.34
tcc	S	0	3.77
tca	S	0	5.89
tcg	S	26	1.57
tgg	W	6	6.00
ctt	L	0	560.70
ctc	L	0	451.70
cta	L	0	417.90
ctg	L	0	596.10
agt	S	0	5.64
agc	S	0	4.79
---	--	------	------
mPD		0.023	1.2
		nPD:	0.02
		N. weight:	2.
		Sc. PD:	-0.33
		Sc. rank:	-2894.3

,

PA-X

Pos.	215	obs:	exp:
tta	L	0	94.40
ttg	L	0	194.00
ctt	L	0	202.70
ctc	L	0	163.30
cta	L	11	151.10
ctg	L	1010	215.50
cct	P	0	452.80
ccc	P	0	299.30
cca	P	34	672.60
ccg	P	1623	232.30
caa	Q	2	88.37
cag	Q	175	88.63
cgt	R	0	0.04
cgc	R	0	0.05
cga	R	0	0.10
cgg	R	1	0.08
aga	R	0	0.47
agg	R	0	0.26
---	--	------	------
mPD		0.56	1.4
		nPD:	0.39
		N. weight:	2.
		Sc. PD:	0.39
		Sc. rank:	2621.9

,

PA-X

Pos.	216	obs:	exp:
att	I	0	56.51
atc	I	154	41.21
ata	I	0	56.28
act	T	0	703.10
acc	T	2680	604.30
aca	T	1	1122.00
acg	T	1	252.30
aat	N	0	7.09
aac	N	13	5.91
gct	A	0	1.76
gcc	A	7	1.35
gca	A	0	3.12
gcg	A	0	0.77
---	--	------	------
mPD		0.12	0.81
		nPD:	0.14
		N. weight:	1.8
		Sc. PD:	-0.078
		Sc. rank:	801.0

,

PA-X

Pos.	217	obs:	exp:
cgt	R	0	0.67
cgc	R	0	0.96
cga	R	0	1.81
cgg	R	0	1.47
aaa	K	2836	1615.00
aag	K	1	1222.00
aga	R	18	8.40
agg	R	0	4.68
gaa	E	1	0.56
gag	E	0	0.44
---	--	------	------
mPD		0.014	0.51
		nPD:	0.03
		N. weight:	0.68
		Sc. PD:	-0.11
		Sc. rank:	-840.2

,

PA-X

Pos.	218	obs:	exp:
gtt	V	0	398.50
gtc	V	1850	389.80
gta	V	3	395.10
gtg	V	0	669.50
gct	A	1	252.40
gcc	A	1001	193.90
gca	A	0	446.00
gcg	A	0	109.70
gat	D	0	0.54
gac	D	1	0.46
---	--	------	------
mPD		0.46	1.2
		nPD:	0.38
		N. weight:	1.9
		Sc. PD:	0.36
		Sc. rank:	2436.7

,

PA-X

Pos.	219	obs:	exp:
ttt	F	0	109.30
ttc	F	262	152.70
tct	S	0	432.20
tcc	S	2589	375.00
tca	S	0	586.90
tcg	S	0	156.20
tat	Y	0	2.40
tac	Y	5	2.60
agt	S	0	561.60
agc	S	0	477.10
---	--	------	------
mPD		0.17	1.7
		nPD:	0.1
		N. weight:	2.2
		Sc. PD:	-0.19
		Sc. rank:	9.2

,

PA-X

Pos.	220	obs:	exp:
tta	L	0	7.95
ttg	L	0	16.34
ctt	L	0	17.08
ctc	L	86	13.76
cta	L	0	12.73
ctg	L	0	18.15
cct	P	0	59.30
ccc	P	217	39.19
cca	P	0	88.08
ccg	P	0	30.42
cat	H	3	1464.00
cac	H	2526	1065.00
cgt	R	0	0.90
cgc	R	24	1.28
cga	R	0	2.42
cgg	R	0	1.96
aga	R	0	11.20
agg	R	0	6.24
---	--	------	------
mPD		0.21	0.79
		nPD:	0.27
		N. weight:	1.2
		Sc. PD:	0.09
		Sc. rank:	1120.4

,

PA-X			
Pos.	221	obs:	exp:
tta	L	0	1.94
ttg	L	0	3.99
ctt	L	0	4.17
ctc	L	0	3.36
cta	L	21	3.11
ctg	L	0	4.43
caa	Q	6	3.00
cag	Q	0	3.00
cgt	R	0	105.90
cgc	R	4	151.40
cga	R	2824	285.10
cgg	R	1	231.00
aga	R	0	1321.00
agg	R	0	735.00
---	--	------	------
mPD		0.022	0.98
		nPD:	0.02
		N. weight:	2.8
		Sc. PD:	-0.45
		Sc. rank:	-3514.7

PA-X			
Pos.	222	obs:	exp:
att	I	44	16.15
atc	I	0	11.77
ata	I	0	16.08
act	T	2756	722.50
acc	T	0	621.00
aca	T	0	1153.00
acg	T	0	259.30
aat	N	10	5.45
aac	N	0	4.55
gct	A	46	11.59
gcc	A	0	8.90
gca	A	0	20.48
gcg	A	0	5.03
---	--	------	------
mPD		0.069	0.77
		nPD:	0.09
		N. weight:	1.6
		Sc. PD:	-0.16
		Sc. rank:	-213.6

PA-X			
Pos.	223	obs:	exp:
tct	S	2723	476.60
tcc	S	122	413.60
tca	S	4	647.20
tcg	S	6	172.20
act	T	1	0.26
acc	T	0	0.23
aca	T	0	0.42
acg	T	0	0.09
agt	S	0	619.30
agc	S	0	526.10
---	--	------	------
mPD		0.089	1.7
		nPD:	0.05
		N. weight:	1.9
		Sc. PD:	-0.26
		Sc. rank:	-1407.2

PA-X			
Pos.	224	obs:	exp:
tct	S	0	0.50
tcc	S	0	0.43
tca	S	3	0.68
tcg	S	0	0.18
cct	P	0	778.90
ccc	P	0	514.80
cca	P	2839	1157.00
ccg	P	11	399.60
caa	Q	2	1.00
cag	Q	0	1.00
act	T	0	0.26
acc	T	0	0.23
aca	T	1	0.42
acg	T	0	0.09
agt	S	0	0.65
agc	S	0	0.55
---	--	------	------
mPD		0.012	0.71
		nPD:	0.02
		N. weight:	1.1
		Sc. PD:	-0.18
		Sc. rank:	-1710.0

PA-X			
Pos.	225	obs:	exp:
act	T	1	3.67
acc	T	13	3.15
aca	T	0	5.86
acg	T	0	1.32
gtt	V	0	1.94
gtc	V	9	1.89
gta	V	0	1.92
gtg	V	0	3.25
gct	A	14	713.70
gcc	A	2788	548.20
gca	A	31	1261.00
gcg	A	0	310.10
---	--	------	------
mPD		0.048	0.71
		nPD:	0.07
		N. weight:	1.9
		Sc. PD:	-0.22
		Sc. rank:	-983.2

PA-X			
Pos.	226	obs:	exp:
tta	L	21	263.70
ttg	L	2831	541.80
tct	S	0	0.67
tcc	S	0	0.58
tca	S	0	0.91
tcg	S	4	0.24
ctt	L	0	566.30
ctc	L	0	456.20
cta	L	0	422.10
ctg	L	0	602.00
agt	S	0	0.87
agc	S	0	0.74
---	--	------	------
mPD		0.017	1.1
		nPD:	0.02
		N. weight:	2.
		Sc. PD:	-0.33
		Sc. rank:	-3185.9

PA-X			
Pos.	227	obs:	exp:
cgt	R	0	2.10
cgc	R	0	3.00
cga	R	0	5.64
cgg	R	0	4.57
aaa	K	2620	1495.00
aag	K	6	1131.00
aga	R	54	26.14
agg	R	2	14.55
gaa	E	174	97.54
gag	E	0	76.46
---	--	------	------
mPD		0.16	0.65
		nPD:	0.24
		N. weight:	0.67
		Sc. PD:	0.035
		Sc. rank:	601.6

PA-X			
Pos.	228	obs:	exp:
tta	L	0	0.18
ttg	L	0	0.38
tct	S	0	0.17
tcc	S	0	0.14
tca	S	0	0.23
tcg	S	0	0.06
ctt	L	2	0.40
ctc	L	0	0.32
cta	L	0	0.30
ctg	L	0	0.42
att	I	1113	409.20
atc	I	2	298.40
ata	I	0	407.50
act	T	1680	440.70
acc	T	1	378.80
aca	T	0	703.40
acg	T	0	158.20
aat	N	32	17.45
aac	N	0	14.55
agt	S	1	0.22
agc	S	0	0.18
gct	A	25	6.30
gcc	A	0	4.84
gca	A	0	11.13
gcg	A	0	2.74
---	--	------	------
mPD		0.51	1.2
		nPD:	0.43
		N. weight:	1.5
		Sc. PD:	0.34
		Sc. rank:	2054.8

PA-X			
Pos.	229	obs:	exp:
tta	L	2834	262.00
ttg	L	0	538.40
tct	S	0	3.67
tcc	S	0	3.19
tca	S	22	4.99
tcg	S	0	1.33
ctt	L	0	562.70
ctc	L	0	453.30
cta	L	0	419.40
ctg	L	0	598.20
agt	S	0	4.77
agc	S	0	4.05
---	--	------	------
mPD		0.015	1.1
		nPD:	0.01
		N. weight:	2.9
		Sc. PD:	-0.49
		Sc. rank:	-4745.6

PA-X			
Pos.	230	obs:	exp:
aaa	K	0	0.57
aag	K	1	0.43
gaa	E	87	1598.00
gag	E	2764	1253.00
ggt	G	0	0.58
ggc	G	0	0.53
gga	G	0	1.75
ggg	G	4	1.14
---	--	------	------
mPD		0.063	0.50
		nPD:	0.13
		N. weight:	0.83
		Sc. PD:	-0.051
		Sc. rank:	248.4

PA-X			
Pos.	231	obs:	exp:
tta	L	0	0.09
ttg	L	0	0.19
tct	S	36	6.01
tcc	S	0	5.21
tca	S	0	8.16
tcg	S	0	2.17
ctt	L	1	0.20
ctc	L	0	0.16
cta	L	0	0.15
ctg	L	0	0.21
cct	P	2819	770.40
ccc	P	0	509.20
cca	P	0	1144.00
ccg	P	0	395.20
agt	S	0	7.81
agc	S	0	6.63
---	--	------	------
mPD		0.026	0.74
		nPD:	0.03
		N. weight:	1.6
		Sc. PD:	-0.24
		Sc. rank:	-1633.2

PA-X			
Pos.	232	obs:	exp:
atg	M	2856	2856.00
---	--	------	------
mPD		0	0
		nPD:	1.
		N. weight:	0.
		Sc. PD:	0
		Sc. rank:	0

PA-X			
Pos.	233	obs:	exp:
tgg	W	2856	2856.00
---	--	------	------
mPD		0	0
		nPD:	1.
		N. weight:	0.
		Sc. PD:	0
		Sc. rank:	0

PA-X			
Pos.	234	obs:	exp:
att	I	0	0.37
atc	I	0	0.27
ata	I	1	0.37
atg	M	2851	2851.00
act	T	0	0.79
acc	T	0	0.68
aca	T	0	1.26
acg	T	3	0.28
aaa	K	0	0.57
aag	K	1	0.43
---	--	------	------
mPD		0.0035	0.0058
		nPD:	0.6
		N. weight:	0.0038
		Sc. PD:	0.0015
		Sc. rank:	6.5

PA-X

Pos.	235	obs:	exp:
gat	D	2838	1532.00
gac	D	0	1306.00
ggt	G	18	2.60
ggc	G	0	2.36
gga	G	0	7.89
ggg	G	0	5.15
---	--	------	------
mPD		0.013	0.51
		nPD:	0.02
		N. weight:	0.76
		Sc. PD:	-0.12
		Sc. rank:	-954.9

,

PA-X

Pos.	236	obs:	exp:
tta	L	5	4.53
ttg	L	44	9.31
tct	S	0	468.40
tcc	S	0	406.50
tca	S	417	636.10
tcg	S	2389	169.30
ctt	L	0	9.73
ctc	L	0	7.84
cta	L	0	7.25
ctg	L	0	10.34
cct	P	0	0.27
ccc	P	0	0.18
cca	P	0	0.41
ccg	P	1	0.14
agt	S	0	608.60
agc	S	0	517.10
---	--	------	------
mPD		0.29	1.7
		nPD:	0.17
		N. weight:	2.6
		Sc. PD:	-0.048
		Sc. rank:	1670.0

,

PA-X

Pos.	237	obs:	exp:
tct	S	0	3.00
tcc	S	0	2.61
tca	S	0	4.08
tcg	S	0	1.09
tgt	C	0	0.39
tgc	C	1	0.61
cat	H	0	1.16
cac	H	2	0.84
aat	N	2	1544.00
aac	N	2829	1287.00
agt	S	0	3.90
agc	S	18	3.32
gat	D	0	2.16
gac	D	4	1.84
---	--	------	------
mPD		0.020	0.52
		nPD:	0.04
		N. weight:	0.96
		Sc. PD:	-0.14
		Sc. rank:	-973.0

,

PA-X

Pos.	238	obs:	exp:
caa	Q	2	1.00
cag	Q	0	1.00
cgt	R	0	106.80
cgc	R	0	152.70
cga	R	2853	287.50
cgg	R	0	233.00
aga	R	0	1332.00
agg	R	0	741.20
ggt	G	0	0.14
ggc	G	0	0.13
gga	G	1	0.44
ggg	G	0	0.29
---	--	------	------
mPD		0.0021	0.96
		nPD:	0.
		N. weight:	2.8
		Sc. PD:	-0.51
		Sc. rank:	-5349.5

,

PA-X

Pos.	239	obs:	exp:
atg	M	32	32.00
act	T	0	740.30
acc	T	0	636.30
aca	T	0	1182.00
acg	T	2824	265.70
---	--	------	------
mPD		0.022	0.72
		nPD:	0.03
		N. weight:	2.8
		Sc. PD:	-0.44
		Sc. rank:	-3107.6

,

PA-X

Pos.	240	obs:	exp:
gtt	V	3	0.65
gtc	V	0	0.63
gta	V	0	0.64
gtg	V	0	1.08
gct	A	2821	710.90
gcc	A	0	546.10
gca	A	1	1256.00
gcg	A	0	308.90
gat	D	31	16.74
gac	D	0	14.26
---	--	------	------
mPD		0.024	0.71
		nPD:	0.03
		N. weight:	1.7
		Sc. PD:	-0.25
		Sc. rank:	-1755.8

,

PA-X

Pos.	241	obs:	exp:
tct	S	0	0.83
tcc	S	0	0.72
tca	S	5	1.13
tcg	S	0	0.30
act	T	0	30.41
acc	T	0	26.14
aca	T	116	48.54
acg	T	0	10.91
agt	S	0	1.08
agc	S	0	0.92
gtt	V	0	1.08
gtc	V	0	1.05
gta	V	5	1.07
gtg	V	0	1.81
gct	A	0	687.70
gcc	A	0	528.30
gca	A	2730	1215.00
gcg	A	0	298.80
---	--	------	------
mPD		0.085	0.78
		nPD:	0.11
		N. weight:	0.99
		Sc. PD:	-0.077
		Sc. rank:	168.1

,

PA-X

Pos.	242	obs:	exp:
tta	L	0	263.90
ttg	L	2854	542.20
tct	S	0	0.33
tcc	S	0	0.29
tca	S	0	0.45
tcg	S	2	0.12
ctt	L	0	566.70
ctc	L	0	456.50
cta	L	0	422.40
ctg	L	0	602.40
agt	S	0	0.43
agc	S	0	0.37
---	--	------	------
mPD		0.0014	1.1
		nPD:	0.
		N. weight:	2.
		Sc. PD:	-0.37
		Sc. rank:	-3966.8

,

PA-X

Pos.	243	obs:	exp:
cgt	R	0	106.70
cgc	R	0	152.60
cga	R	0	287.40
cgg	R	0	232.90
aaa	K	0	2.28
aag	K	4	1.72
aga	R	0	1331.00
agg	R	2852	740.90
---	--	------	------
mPD		0.0028	0.96
		nPD:	0.
		N. weight:	1.6
		Sc. PD:	-0.3
		Sc. rank:	-3046.5

,

PA-X

Pos.	244	obs:	exp:
gtt	V	0	0.86
gtc	V	0	0.84
gta	V	4	0.85
gtg	V	0	1.45
gct	A	0	716.20
gcc	A	0	550.10
gca	A	2843	1265.00
gcg	A	0	311.20
gaa	E	9	5.05
gag	E	0	3.95
---	--	------	------
mPD		0.0091	0.70
		nPD:	0.01
		N. weight:	0.98
		Sc. PD:	-0.17
		Sc. rank:	-1664.2

,

PA-X

Pos.	245	obs:	exp:
tct	S	0	426.70
tcc	S	0	370.20
tca	S	0	579.40
tcg	S	0	154.20
aat	N	0	162.50
aac	N	298	135.50
agt	S	0	554.40
agc	S	2556	471.00
gat	D	0	0.54
gac	D	1	0.46
ggt	G	0	0.14
ggc	G	1	0.13
gga	G	0	0.44
ggg	G	0	0.29
---	--	------	------
mPD		0.19	1.7
		nPD:	0.11
		N. weight:	1.9
		Sc. PD:	-0.15
		Sc. rank:	298.8

,

PA-X

Pos.	246	obs:	exp:
ttt	F	2843	1186.00
ttc	F	0	1657.00
tta	L	0	1.11
ttg	L	11	2.28
tct	S	1	0.17
tcc	S	0	0.14
tca	S	0	0.23
tcg	S	0	0.06
ctt	L	1	2.38
ctc	L	0	1.92
cta	L	0	1.78
ctg	L	0	2.53
agt	S	0	0.22
agc	S	0	0.18
---	--	------	------
mPD		0.0091	0.50
		nPD:	0.02
		N. weight:	1.1
		Sc. PD:	-0.18
		Sc. rank:	-1661.8

,

PA-X

Pos.	247	obs:	exp:
tta	L	0	261.10
ttg	L	0	536.50
ctt	L	0	560.70
ctc	L	2824	451.70
cta	L	0	417.90
ctg	L	0	596.10
cct	P	0	8.75
ccc	P	32	5.78
cca	P	0	12.99
ccg	P	0	4.49
---	--	------	------
mPD		0.022	1.1
		nPD:	0.02
		N. weight:	2.2
		Sc. PD:	-0.37
		Sc. rank:	-3390.8

,

PA-X

Pos.	248	obs:	exp:
caa	Q	1	0.50
cag	Q	0	0.50
cgt	R	0	0.52
cgc	R	0	0.75
cga	R	0	1.41
cgg	R	0	1.14
aaa	K	2841	1618.00
aag	K	0	1223.00
aga	R	14	6.54
agg	R	0	3.64
---	--	------	------
mPD		0.010	0.50
		nPD:	0.02
		N. weight:	0.69
		Sc. PD:	-0.11
		Sc. rank:	-943.5

,

PA-X

Pos.	249	obs:	exp:
tgt	C	2822	1105.00
tgc	C	33	1750.00
---	-	1	1.00
---	--	------	------
mPD		0.023	0.47
		nPD:	0.05
		N. weight:	1.1
		Sc. PD:	-0.15
		Sc. rank:	-931.6

,

PA-X			
Pos.	250	obs:	exp:
cct	P	0	7.65
ccc	P	0	5.06
cca	P	28	11.36
ccg	P	0	3.93
caa	Q	2801	1398.00
cag	Q	0	1403.00
cgt	R	0	0.94
cgc	R	0	1.34
cga	R	25	2.52
cgg	R	0	2.04
aga	R	0	11.67
agg	R	0	6.49
---	-	2	2.00
---	--	------	------
mPD		0.037	0.55
		nPD:	0.07
		N. weight:	0.86
		Sc. PD:	-0.1
		Sc. rank:	-492.3

,

PA-X			
Pos.	251	obs:	exp:
cgt	R	0	36.90
cgc	R	0	52.77
cga	R	0	99.36
cgg	R	0	80.51
aaa	K	0	1052.00
aag	K	1848	795.70
aga	R	3	460.30
agg	R	983	256.20
gaa	E	0	7.29
gag	E	13	5.71
ggt	G	0	1.01
ggc	G	0	0.92
gga	G	0	3.07
ggg	G	7	2.00
---	-	2	2.00
---	--	------	------
mPD		0.47	1.2
		nPD:	0.41
		N. weight:	1.2
		Sc. PD:	0.26
		Sc. rank:	1656.5

,

PA-X			
Pos.	252	obs:	exp:
cgt	R	0	1.46
cgc	R	0	2.09
cga	R	0	3.93
cgg	R	0	3.18
atg	M	2	2.00
aaa	K	0	1599.00
aag	K	2808	1209.00
aga	R	0	18.21
agg	R	39	10.13
gaa	E	0	2.24
gag	E	4	1.76
ggt	G	0	0.14
ggc	G	0	0.13
gga	G	0	0.44
ggg	G	1	0.29
---	-	2	2.00
---	--	------	------
mPD		0.032	0.53
		nPD:	0.06
		N. weight:	1.
		Sc. PD:	-0.13
		Sc. rank:	-645.0

,

PA-X			
Pos.	253	obs:	exp:
taa	.	138	1053.00
tag	.	0	979.40
tga	.	2718	823.50
---	--	------	------
mPD		0.092	0.86
		nPD:	0.11
		N. weight:	1.3
		Sc. PD:	-0.1
		Sc. rank:	124.1

}

Amino acid level conservation per codon

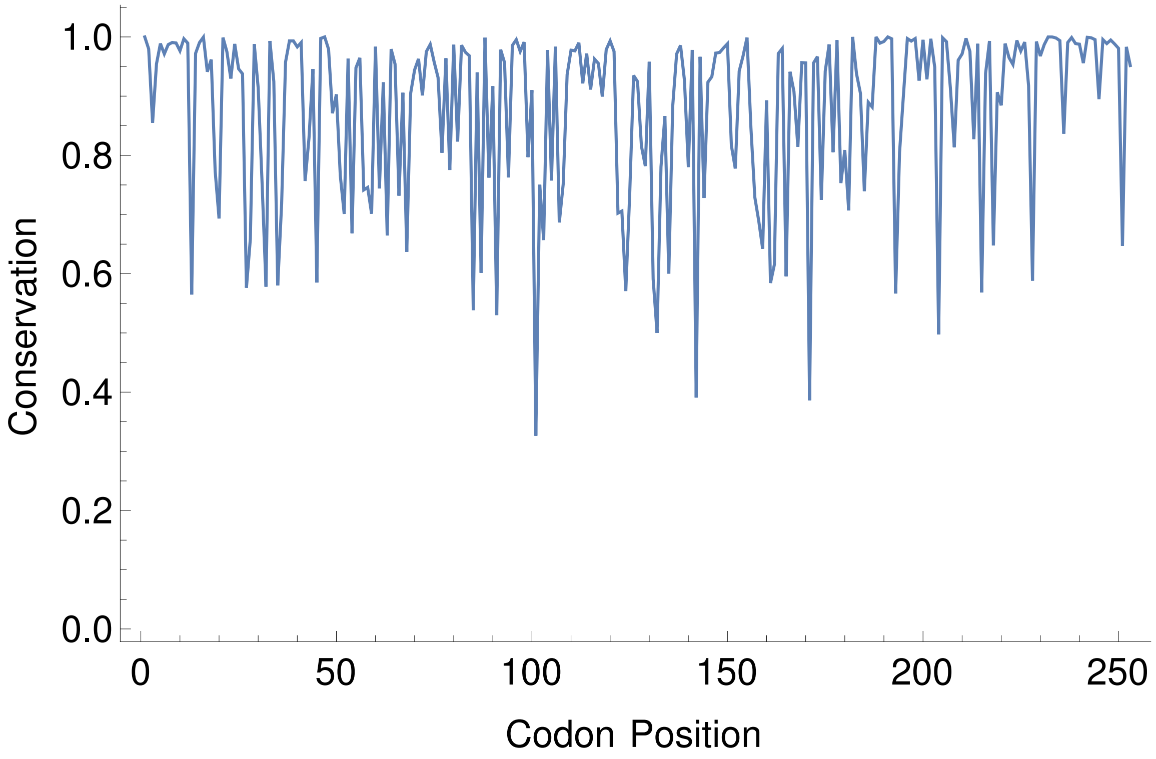

Conservation analysis plots

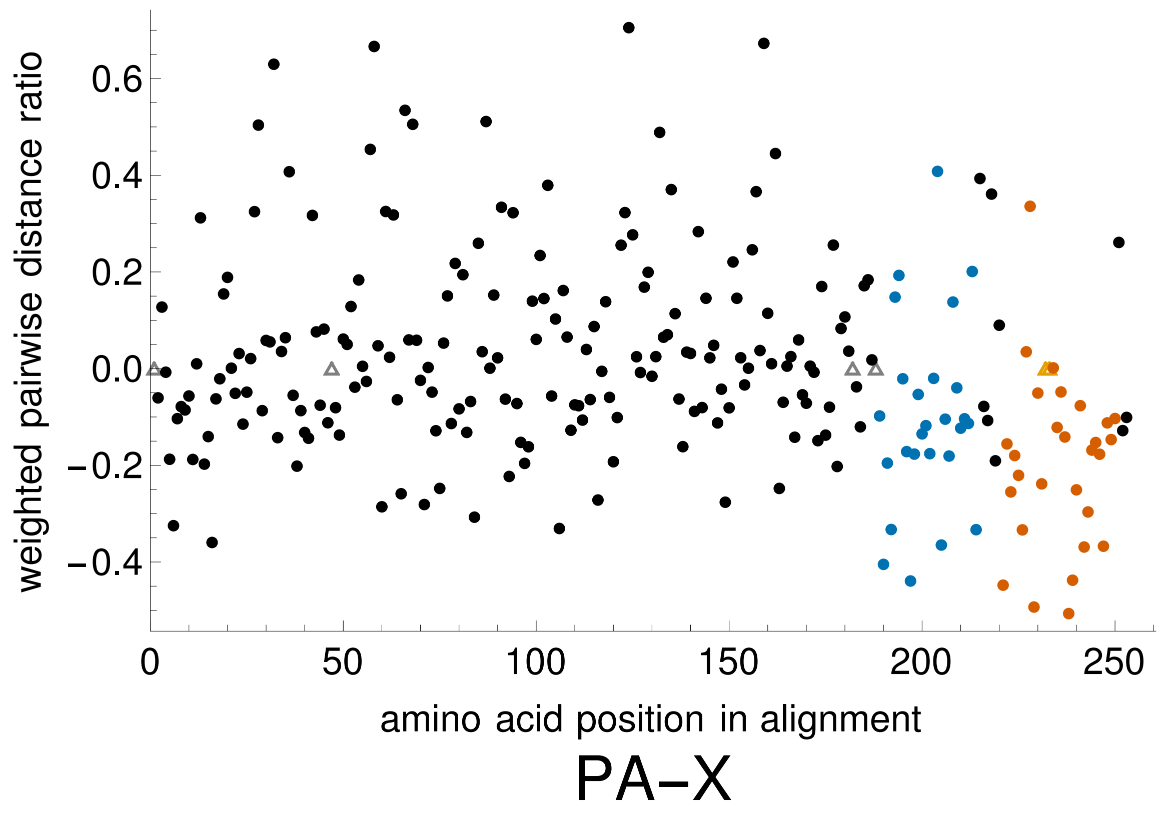

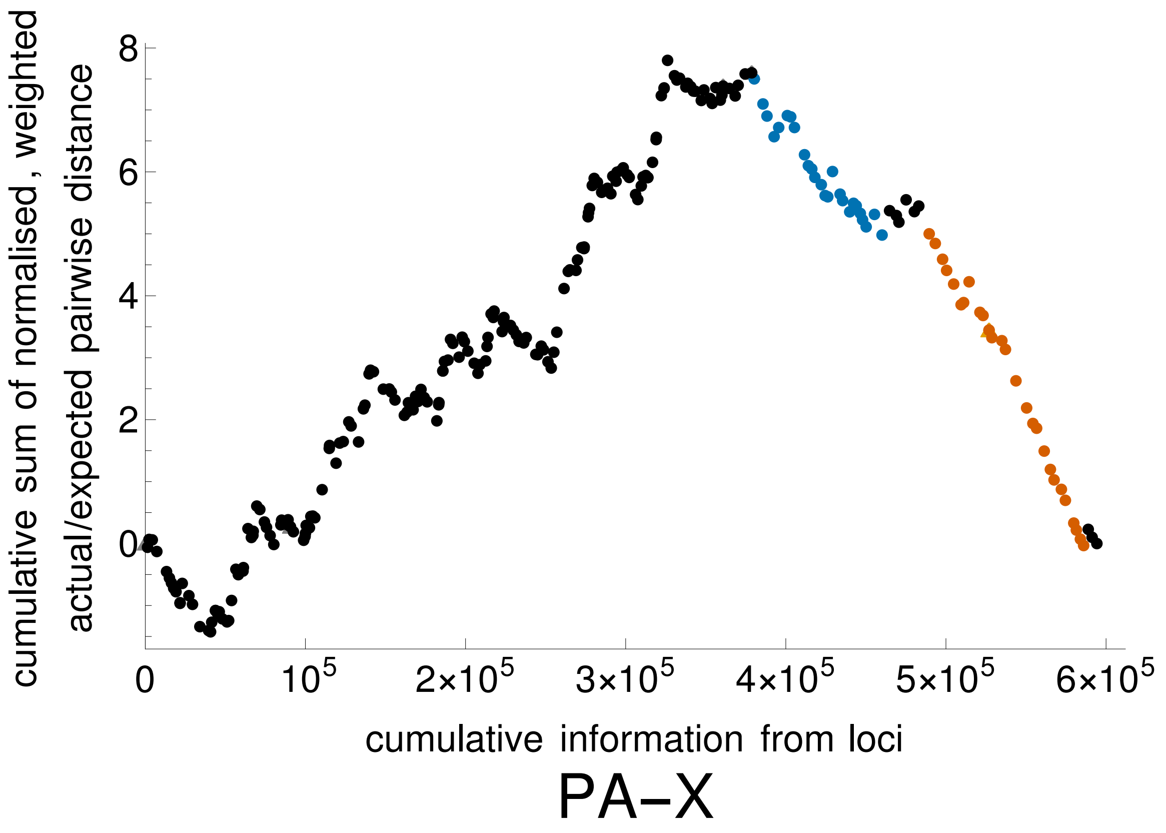

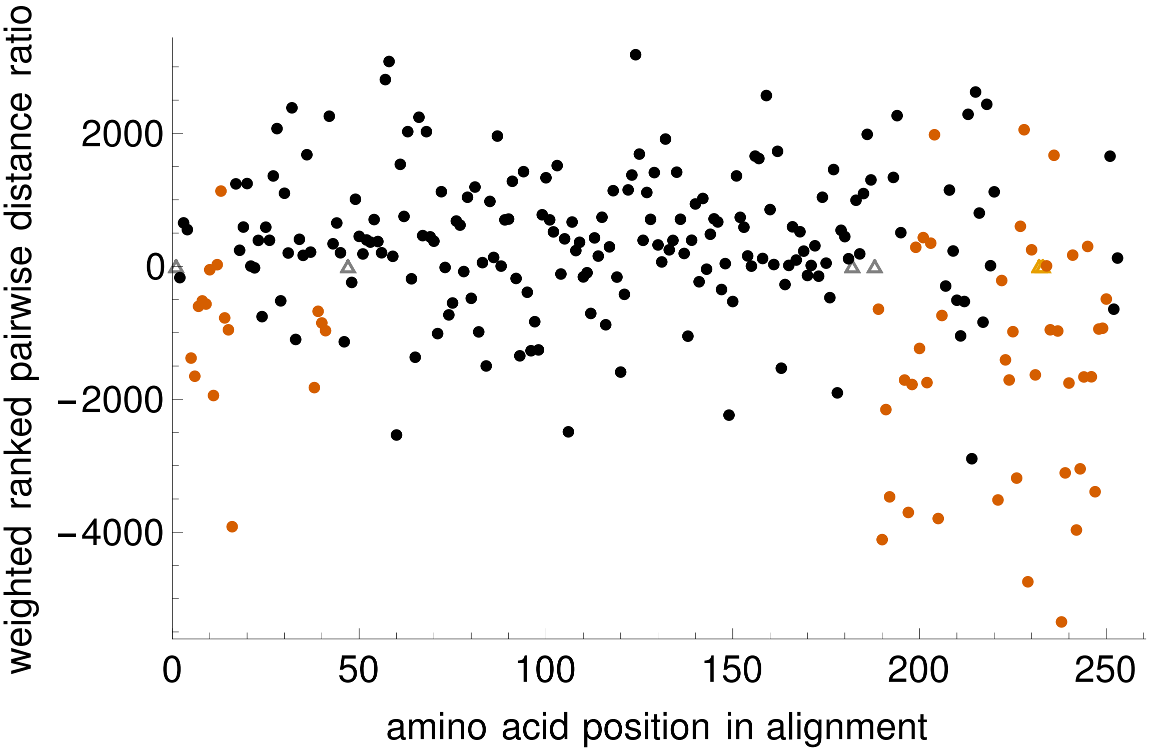
weighted ranked pairwise distance ratio
2000
0
−2000
−4000
0
50
100
150
200
250
amino acid position in alignment

PA–X

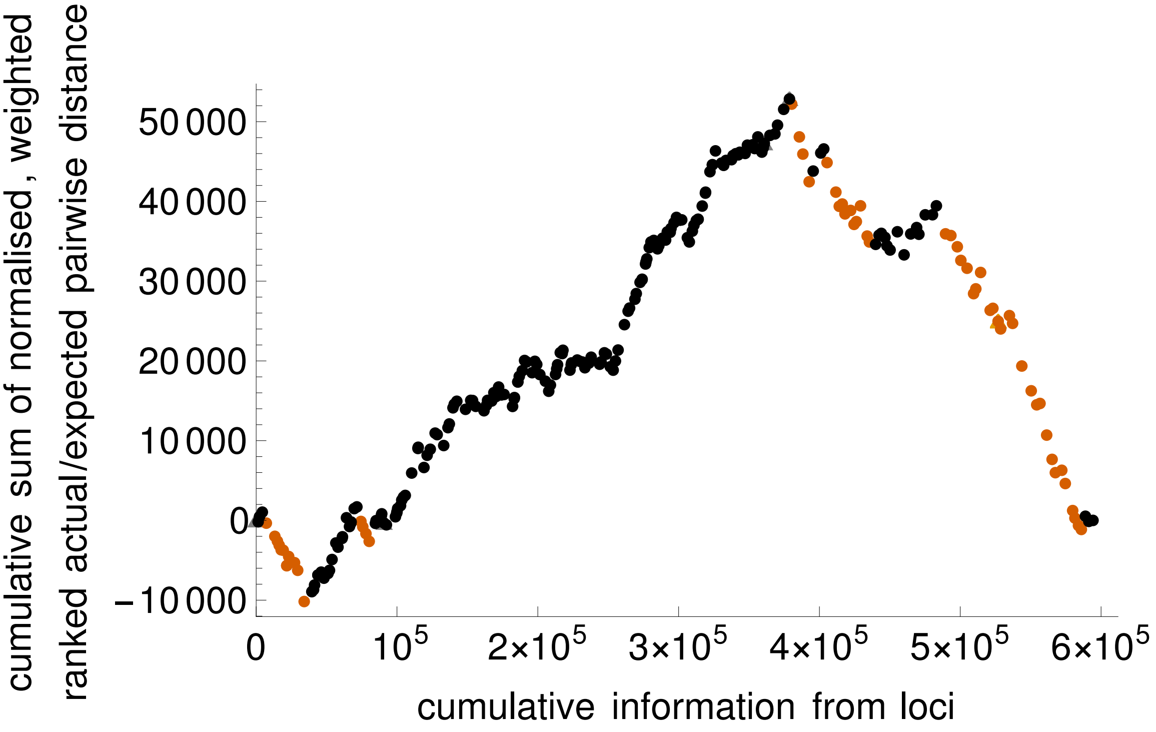
cumulative sum of normalised, weighted ranked actual/expected pairwise distance
50 000
40 000
30 000
20 000
10 000
0
−10 000
0
10^5
2×10^5
3×10^5
4×10^5
5×10^5
6×10^5
cumulative information from loci

PA–X

HA

Gene length histogram

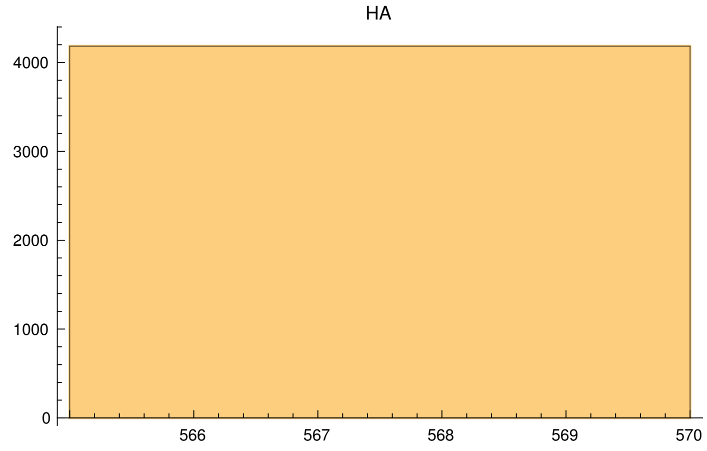

Information vs. nPD

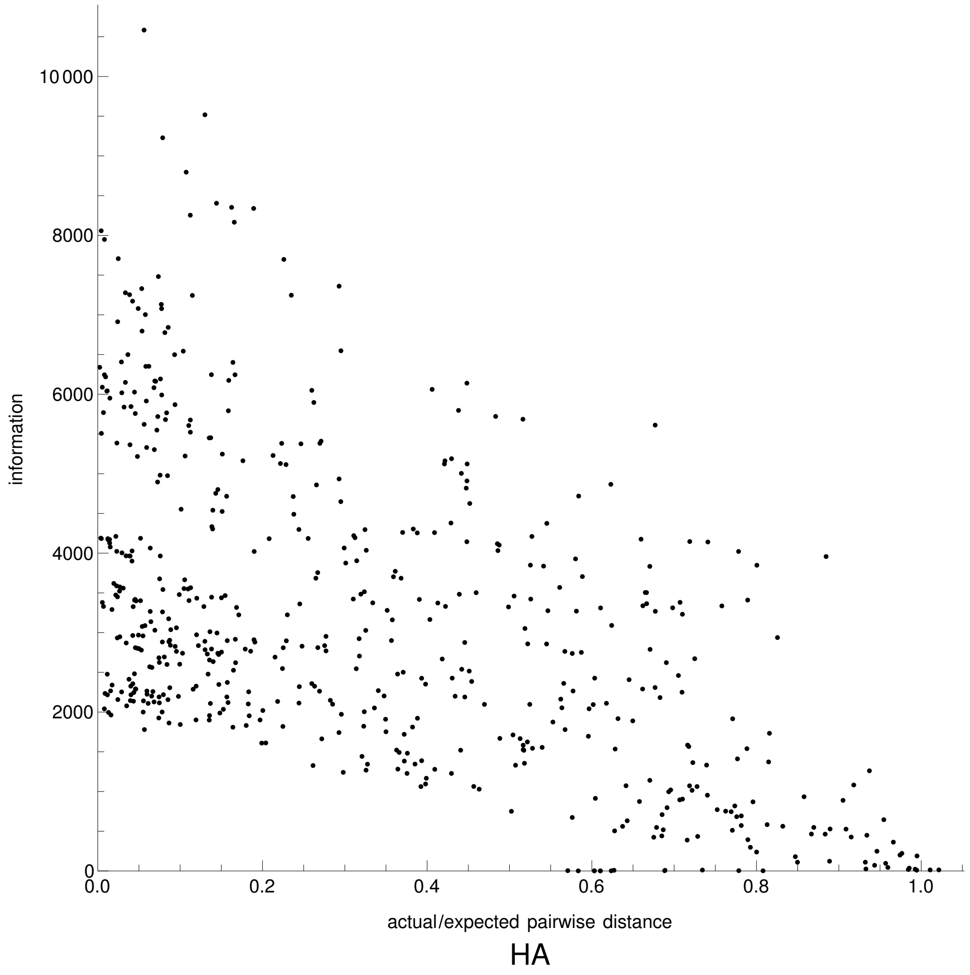

Example sequences highlighted by regions found in analyses to be conserved

Interesting points (by weighted raw PD) highlighted for gene HA:

------ATGGAGAAAATAGTGCTTCTTCTTGCAATAATCAGCCTTGTTAAAAGTGATCAGATTTGCATTGGTTACC⋱
ATGCAAACAACTCGACAGAGCAGGTTGACACAATAATGGAAAAGAACGTTACTGTTACACATGCTCAAGATATAC⋱
TGGAAAAGACACACAACGGGAAGCTCTGCGATCTAGATGGAGTGAAGCCTCTGATTTTAAGAGATTGTAGTGTAG⋱
CTGGATGGCTCCTCGGAAACCCAATGTGTGATGAATTCATCAATGTGCCGGAATGGTCTTACATAGTGGAGAAGG⋱
CCAACCCAGCCAATGACCTCTGTTACCCAGGGAATTTCAACGACTATGAAGAACTGAAACACCTATTGAGCAGGA⋱
TAAACCATTTTGAGAAAATTCAGATCATCCCCAAAAGTTCTTGGTCCGATCATGAAGCC ---
TCATCAGGGGTGAGCTCAGCATGTCCA ---TACCAGGGAGTGCCCTCCTTTTTCAGAAATGTGGTATGGCTTATC --
-AAAAAGAACAATACATACCCAACAATAAAGAGAAGCTACAATAATACCAACCAGGAAGATCTTTTGATACTGTGG⋱
GGGATTCATCATTCTAATGATGCAGCAGAGCAGACAAAGCTCTATCAAAACCCAACCACCTATATTTCCGTTGGG⋱
ACATCAACACTGAACCAGAGATTGGTACCAAAAATAGCTACTAGATCCAAAGTAAACGGGCAAAGTGGAAGGATG⋱
GATTTCTTCTGGACAATTTTAAAACCGAATGATGCAATCAACTTCGAGAGTAATGGAAATTTCATTGCTCCAGAA⋱
TATGCATACAAAATTGTCAAGAAAGGGGACTCGGCAATTATGAAAAGTGAGGTGGAGTATGGTAACTGCAACACC⋱
AAGTGTCAAACTCCAATAGGGGCGATAAACTCTAGTATGCCATTCCACAACATACACCCTCTCACCATCGGGGAA⋱
TGCCCCAAATATGTGAAATCAAACAAATTAGTCCTTGCGACTGGGCTCAGAAATAGTCCTCTA ------AGAGAA--
-------AGAAGAAGA---AAA---AGAGGACTATTTGGAGCTATAGCAGGTTTTATAGAGGGCGGATGGCAGGGAA⋱
TGGTAGATGGTTGGTATGGGTTTCACCATAGCAATGAGCAGGGGAGTGGGTACGCTGCAGACAAAGAATCCACTC⋱
AAAAGGCAATAGATGGAGTCACCAATAAGGTCAACTCAATCATTGACAAAATGAACACTCAGTTTGAGGCCGTTG⋱
GAAGGGAATTTAATAACTTAGAGAGGAGAATAGAGAATTTAAACAAGAAAATGGAAGACGGATTCCTAGATGTCT⋱
GGACTTATAATGCTGAACTTCTGGTTCTCATGGAAAATGAGAGAACTCTAGACTTCCATGATTCAAATGTCAAGA⋱
ACCTTTACGACAAGGTCCGACTACAGCTTAGGGATAATGCAAAGGAGCTGGGTAACGGTTGTTTCGAGTTCTATC⋱
ACAAATGTGATAATGAATGCATGGAAAGTGTAAGAAACGGAACGTATGACTACCCGCAGTATTCAGAAGAAGCAA⋱
GATTAAAAAGAGAGGAAATAAGTGGAGTAAAATTGGAATCAATAGGAACTTACCAA
ATACTGTCAATTTATTCAACAGTTGCGAGTTCTCTAGCACTGGCAATCATGGTGG
CTGGTCTATCTTTATGGATGTGCTCCAATGGGTCGTTACAATGCAGAATTTG
CATTTAA

Interesting points (by weighted ranked PD) highlighted for gene HA:

------ATGGAGAAAATAGTGCTTCTTCTTGCAATAATCAGCCTTGTTAAAAGTGATCAGATTTGCATTGGTTACCAT⋱
GCAAACAACTCGACAGAGCAGGTTGACACAATAATGGAAAAGAACGTTACTGTTACACATGCTCAAGATATACTG⋱
GAAAAGACACACAACGGGAAGCTCTGCGATCTAGATGGAGTGAAGCCTCTGATTTTAAGAGATTGTAGTGTAGCT⋱
GGATGGCTCCTCGGAAACCCAATGTGTGATGAATTCATCAATGTGCCGGAATGGTCTTACATAGTGGAGAAGGCC⋱
AACCCAGCCAATGACCTCTGTTACCCAGGGAATTTCAACGACTATGAAGAACTGAAACACCTATTGAGCAGGATA⋱
AACCATTTTGAGAAAATTCAGATCATCCCCAAAAGTTCTTGGTCCGATCATGAAGCC ---
TCATCAGGGGTGAGCTCAGCATGTCCA ---TACCAGGGAGTGCCCTCCTTTTTCAGAAATGTGGTATGGCTTATC --
-AAAAAGAACAATACATACCCAACAATAAAGAGAAGCTACAATAATACCAACCAGGAAGATCTTTTGATACTGTGG⋱
GGGATTCATCATTCTAATGATGCAGCAGAGCAGACAAAGCTCTATCAAAACCCAACCACCTATATTTCCGTTGGG⋱
ACATCAACACTGAACCAGAGATTGGTACCAAAAATAGCTACTAGATCCAAAGTAAACGGGCAAAGTGGAAGGATG⋱
GATTTCTTCTGGACAATTTTAAAACCGAATGATGCAATCAACTTCGAGAGTAATGGAAATTTCATTGCTCCAGAA⋱
TATGCATACAAAATTGTCAAGAAAGGGGACTCGGCAATTATGAAAAGTGAGGTGGAGTATGGTAACTGCAACACC⋱
AAGTGTCAAACTCCAATAGGGGCGATAAACTCTAGTATGCCATTCCACAACATACACCCTCTCACCATCGGGGAA⋱
TGCCCCAAATATGTGAAATCAAACAAATTAGTCCTTGCGACTGGGCTCAGAAATAGTCCTCTA ------AGAGAA--
-------AGAAGAAGA---AAA---AGAGGACTATTTGGAGCTATAGCAGGTTTTATAGAGGGCGGATGGCAGGGAA⋱
TGGTAGATGGTTGGTATGGGTTTCACCATAGCAATGAGCAGGGGAGTGGGTACGCTGCAGACAAAGAATCCACTC⋱

AAAAGGCAATAGATGGAGTCACCAATAAGGTCAACTCAATCATTGACAAAATGAACACTCAGTTTGAGGCCGTTG⋱

GAAGGGAATTTAATAACTTAGAGAGGAGAATAGAGAATTTAAACAAGAAAATGGAAGACGGATTCCTAGATGTCT⋱

GGACTTATAATGCTGAACTTCTGGTTCTCATGGAAAATGAGAGAACTCTAGACTTCCATGATTCAAATGTCAAGA⋱

ACCTTTACGACAAGGTCCGACTACAGCTTAGGGATAATGCAAAGGAGCTGGGTAACGGTTGTTTCGAGTTCTATC⋱

ACAAATGTGATAATGAATGCATGGAAAGTGTAAGAAACGGAACGTATGACTACCCGCAGTATTCAGAAGAAGCAA⋱

GATTAAAAAGAGAGGAAATAAGTGGAGTAAAATTGGAATCAATAGGAACTTACCAA

ATACTGTCAATTTATTCAACAGTTGCGAGTTCTCTAGCACTGGCAATCATGGTGG
CTGGTCTATCTTTATGGATGTGCTCCAATGGGTCGTTACAATGCAGAATTTG
CATTTAA

Per codon conservation report

HA

Pos.	1	obs:	exp:
atg	M	17	17.00
---	-	4167	4167.00
---	--	------	------
mPD		0	0
		nPD:	1.
		N. weight:	0.
		Sc. PD:	0
		Sc. rank:	0

HA

Pos.	2	obs:	exp:
aaa	K	17	9.68
aag	K	0	7.32
---	-	4167	4167.00
---	--	------	------
mPD		0	8.1×10^{-6}
		nPD:	0.
		N. weight:	0.
		Sc. PD:	0
		Sc. rank:	0

HA

Pos.	3	obs:	exp:
atg	M	4184	4184.00
---	--	------	------
mPD		0	0
		nPD:	1.
		N. weight:	0.
		Sc. PD:	0
		Sc. rank:	0

HA

Pos.	4	obs:	exp:
cct	P	0	0.27
ccc	P	0	0.18
cca	P	0	0.41
ccg	P	1	0.14
cat	H	6	3.47
cac	H	0	2.53
aaa	K	9	46.69
aag	K	73	35.31
gat	D	3	2.16
gac	D	1	1.84
gaa	E	110	2292.00
gag	E	3979	1797.00
ggt	G	0	0.14
ggc	G	0	0.13
gga	G	0	0.44
ggg	G	1	0.29
---	-	1	1.00
---	--	------	------
mPD		0.10	0.54
		nPD:	0.19
		N. weight:	0.95
		Sc. PD:	-0.041
		Sc. rank:	398.2

HA

Pos.	5	obs:	exp:
tct	S	0	1.67
tcc	S	0	1.45
tca	S	0	2.27
tcg	S	0	0.60
tat	Y	0	0.48
tac	Y	1	0.52
cat	H	1	0.58
cac	H	0	0.42
caa	Q	1	0.50
cag	Q	0	0.50
cgt	R	0	2.66
cgc	R	0	3.80
cga	R	0	7.15
cgg	R	0	5.80
act	T	0	1.05
acc	T	0	0.90
aca	T	4	1.67
acg	T	0	0.38
aat	N	3	468.40
aac	N	856	390.60
aaa	K	3079	1827.00
aag	K	130	1382.00
agt	S	1	2.17
agc	S	9	1.84
aga	R	68	33.15
agg	R	3	18.45
gaa	E	27	15.14
gag	E	0	11.86
---	-	1	1.00
---	--	------	------
mPD		0.43	0.73
		nPD:	0.6
		N. weight:	0.68
		Sc. PD:	0.24
		Sc. rank:	1427.8

HA

Pos.	6	obs:	exp:
tta	L	1	1.20
ttg	L	0	2.47
ctt	L	0	2.58
ctc	L	0	2.08
cta	L	12	1.92
ctg	L	0	2.74
cgt	R	0	0.04
cgc	R	0	0.05
cga	R	0	0.10
cgg	R	0	0.08
att	I	2	1509.00
atc	I	1	1100.00
ata	I	4108	1502.00
atg	M	5	5.00
act	T	0	12.32
acc	T	0	10.59
aca	T	47	19.67
acg	T	0	4.42
aaa	K	0	0.57
aag	K	1	0.43
aga	R	1	0.47
agg	R	0	0.26
gtt	V	0	1.08
gtc	V	0	1.05
gta	V	5	1.07
gtg	V	0	1.81
---	-	1	1.00
---	--	------	------
mPD		0.036	0.69
		nPD:	0.05
		N. weight:	1.4
		Sc. PD:	-0.25
		Sc. rank:	-2120.1

HA

Pos.	7	obs:	exp:
ttt	F	1	0.42
ttc	F	0	0.58
tta	L	0	0.37
ttg	L	3	0.76
ctt	L	0	0.79
ctc	L	0	0.64
cta	L	0	0.59
ctg	L	1	0.84
att	I	127	52.48
atc	I	2	38.27
ata	I	14	52.26
atg	M	67	67.00
act	T	0	2.10
acc	T	0	1.80
aca	T	0	3.35
acg	T	8	0.75
gtt	V	670	829.80
gtc	V	4	811.70
gta	V	911	822.60
gtg	V	2273	1394.00
gct	A	1	24.94
gcc	A	0	19.16
gca	A	2	44.07
gcg	A	96	10.84
gaa	E	0	1.12
gag	E	2	0.88
ggt	G	0	0.29
ggc	G	0	0.26
gga	G	0	0.88
ggg	G	2	0.57
---	--	------	------
mPD		0.72	0.89
		nPD:	0.81
		N. weight:	0.45
		Sc. PD:	0.25
		Sc. rank:	1159.0

HA

Pos.	8	obs:	exp:
ttt	F	27	11.68
ttc	F	1	16.32
tta	L	0	373.40
ttg	L	2	767.30
ctt	L	3640	801.90
ctc	L	46	646.10
cta	L	315	597.70
ctg	L	36	852.50
cct	P	10	2.73
ccc	P	0	1.81
cca	P	0	4.06
ccg	P	0	1.40
att	I	58	21.65
atc	I	1	15.79
ata	I	0	21.56
act	T	5	1.31
acc	T	0	1.13
aca	T	0	2.09
acg	T	0	0.47
gtt	V	42	9.03
gtc	V	0	8.84
gta	V	0	8.96
gtg	V	0	15.17
gct	A	1	0.25
gcc	A	0	0.19
gca	A	0	0.45
gcg	A	0	0.11
---	--	------	------
mPD		0.25	1.2
		nPD:	0.21
		N. weight:	1.7
		Sc. PD:	-0.038
		Sc. rank:	808.4

HA

Pos.	9	obs:	exp:
ttt	F	13	7.09
ttc	F	4	9.91
tta	L	0	378.50
ttg	L	0	777.80
tct	S	0	0.17
tcc	S	1	0.14
tca	S	0	0.23
tcg	S	0	0.06
ctt	L	3095	812.90
ctc	L	878	654.90
cta	L	120	605.90
ctg	L	1	864.10
cct	P	4	1.09
ccc	P	0	0.72
cca	P	0	1.62
ccg	P	0	0.56
att	I	30	11.38
atc	I	1	8.30
ata	I	0	11.33
agt	S	0	0.22
agc	S	0	0.18
gtt	V	1	0.22
gtc	V	0	0.21
gta	V	0	0.21
gtg	V	0	0.36
gct	A	0	9.07
gcc	A	36	6.97
gca	A	0	16.02
gcg	A	0	3.94
---	--	------	------
mPD		0.45	1.2
		nPD:	0.38
		N. weight:	1.4
		Sc. PD:	0.2
		Sc. rank:	1894.7

HA

Pos.	10	obs:	exp:
ttt	F	1913	810.60
ttc	F	30	1132.00
tta	L	8	207.00
ttg	L	2	425.40
tgt	C	1	0.39
tgc	C	0	0.61
ctt	L	2191	444.60
ctc	L	36	358.10
cta	L	1	331.40
ctg	L	1	472.60
att	I	1	0.37
atc	I	0	0.27
ata	I	0	0.37
---	--	------	------
mPD		0.54	1.2
		nPD:	0.45
		N. weight:	1.6
		Sc. PD:	0.33
		Sc. rank:	2712.6

HA

Pos.	11	obs :	exp :
tct	S	0	3.67
tcc	S	0	3.19
tca	S	22	4.99
tcg	S	0	1.33
cct	P	0	0.55
ccc	P	0	0.36
cca	P	2	0.81
ccg	P	0	0.28
atg	M	1	1.00
act	T	0	39.06
acc	T	0	33.57
aca	T	145	62.35
acg	T	4	14.02
agt	S	0	4.77
agc	S	0	4.05
gtt	V	0	4.30
gtc	V	0	4.21
gta	V	5	4.26
gtg	V	15	7.23
gct	A	7	1005.00
gcc	A	2	771.90
gca	A	3780	1776.00
gcg	A	200	436.60
ggt	G	0	0.14
ggc	G	1	0.13
gga	G	0	0.44
ggg	G	0	0.29
---	--	------	------
mPD		0.19	0.79
		nPD :	0.25
		N. weight :	0.94
		Sc. PD :	0.011
		Sc. rank :	655.9

,

HA

Pos.	12	obs :	exp :
tta	L	1	0.46
ttg	L	0	0.95
tct	S	0	0.50
tcc	S	0	0.43
tca	S	3	0.68
tcg	S	0	0.18
ctt	L	0	0.99
ctc	L	0	0.80
cta	L	3	0.74
ctg	L	1	1.06
att	I	3	848.80
atc	I	0	619.00
ata	I	2310	845.30
atg	M	58	58.00
act	T	0	367.30
acc	T	0	315.70
aca	T	1400	586.20
acg	T	1	131.80
agt	S	0	0.65
agc	S	0	0.55
gtt	V	0	9.68
gtc	V	0	9.47
gta	V	8	9.60
gtg	V	37	16.26
gct	A	1	90.44
gcc	A	0	69.47
gca	A	358	159.80
gcg	A	0	39.29
---	--	------	------
mPD		0.71	1.4
		nPD :	0.53
		N. weight :	1.3
		Sc. PD :	0.36
		Sc. rank :	2428.5

,

HA

Pos.	13	obs :	exp :
ttt	F	3	7.51
ttc	F	15	10.49
tta	L	0	0.18
ttg	L	0	0.38
ctt	L	0	0.40
ctc	L	2	0.32
cta	L	0	0.30
ctg	L	0	0.42
att	I	99	643.30
atc	I	1654	469.10
ata	I	0	640.60
act	T	4	51.38
acc	T	192	44.16
aca	T	0	82.01
acg	T	0	18.44
gtt	V	896	469.10
gtc	V	1285	458.90
gta	V	0	465.00
gtg	V	0	788.00
gct	A	0	8.57
gcc	A	34	6.58
gca	A	0	15.13
gcg	A	0	3.72
---	--	------	------
mPD		0.97	1.4
		nPD :	0.72
		N. weight :	1.4
		Sc. PD :	0.64
		Sc. rank :	3330.1

,

HA

Pos.	14	obs :	exp :
tct	S	0	663.30
tcc	S	0	575.70
tca	S	0	900.90
tcg	S	0	239.80
tgt	C	1	0.39
tgc	C	0	0.61
cgt	R	4	0.26
cgc	R	1	0.37
cga	R	0	0.71
cgg	R	0	0.57
act	T	1	0.52
acc	T	1	0.45
aca	T	0	0.84
acg	T	0	0.19
aat	N	104	82.35
aac	N	47	68.65
agt	S	1178	862.00
agc	S	2796	732.40
aga	R	1	3.27
agg	R	1	1.82
ggt	G	42	7.09
ggc	G	7	6.44
gga	G	0	21.47
ggg	G	0	14.01
---	--	------	------
mPD		0.53	1.7
		nPD :	0.31
		N. weight :	1.4
		Sc. PD :	0.1
		Sc. rank :	1432.1

,

HA

Pos.	15	obs :	exp :
ttt	F	13	5.42
ttc	F	0	7.58
tta	L	0	349.60
ttg	L	0	718.30
ctt	L	3737	750.70
ctc	L	13	604.80
cta	L	29	559.60
ctg	L	2	798.00
cct	P	2	0.55
ccc	P	0	0.36
cca	P	0	0.81
ccg	P	0	0.28
att	I	349	130.30
atc	I	6	95.00
ata	I	0	129.70
act	T	14	3.93
acc	T	0	3.38
aca	T	0	6.28
acg	T	1	1.41
gtt	V	18	3.87
gtc	V	0	3.79
gta	V	0	3.84
gtg	V	0	6.50
---	--	------	------
mPD		0.21	1.2
		nPD :	0.17
		N. weight :	2.1
		Sc. PD :	-0.14
		Sc. rank :	560.4

,

HA

Pos.	16	obs :	exp :
ttt	F	1	0.42
ttc	F	0	0.58
tta	L	0	0.09
ttg	L	0	0.19
ctt	L	1	0.20
ctc	L	0	0.16
cta	L	0	0.15
ctg	L	0	0.21
att	I	4	1.47
atc	I	0	1.07
ata	I	0	1.46
act	T	4	1.05
acc	T	0	0.90
aca	T	0	1.67
acg	T	0	0.38
gtt	V	3227	893.50
gtc	V	899	873.90
gta	V	25	885.70
gtg	V	3	1501.00
gct	A	14	4.79
gcc	A	5	3.68
gca	A	0	8.46
gcg	A	0	2.08
ggt	G	0	0.14
ggc	G	1	0.13
gga	G	0	0.44
ggg	G	0	0.29
---	--	------	------
mPD		0.37	0.75
		nPD :	0.49
		N. weight :	1.4
		Sc. PD :	0.33
		Sc. rank :	2429.9

,

HA

Pos.	17	obs :	exp :
caa	Q	1	0.50
cag	Q	0	0.50
cgt	R	0	2.99
cgc	R	0	4.28
cga	R	0	8.06
cgg	R	0	6.53
act	T	0	0.52
acc	T	0	0.45
aca	T	2	0.84
acg	T	0	0.19
aat	N	0	2.18
aac	N	4	1.82
aaa	K	4020	2326.00
aag	K	65	1759.00
aga	R	66	37.35
agg	R	14	20.78
gaa	E	12	6.73
gag	E	0	5.27
---	--	------	------
mPD		0.084	0.55
		nPD :	0.15
		N. weight :	0.67
		Sc. PD :	-0.054
		Sc. rank :	55.4

,

HA

Pos.	18	obs :	exp :
tct	S	0	681.20
tcc	S	0	591.20
tca	S	0	925.20
tcg	S	0	246.20
cgt	R	0	0.07
cgc	R	0	0.11
cga	R	0	0.20
cgg	R	0	0.16
agt	S	2412	885.20
agc	S	1669	752.10
aga	R	1	0.93
agg	R	1	0.52
gct	A	0	0.25
gcc	A	1	0.19
gca	A	0	0.45
gcg	A	0	0.11
ggt	G	81	14.46
ggc	G	19	13.14
gga	G	0	43.82
ggg	G	0	28.59
---	--	------	------
mPD		0.53	1.7
		nPD :	0.31
		N. weight :	1.3
		Sc. PD :	0.099
		Sc. rank :	1366.1

,

HA

Pos.	19	obs :	exp :
gtt	V	2	0.43
gtc	V	0	0.42
gta	V	0	0.43
gtg	V	0	0.72
gat	D	3987	2257.00
gac	D	194	1924.00
ggt	G	1	0.14
ggc	G	0	0.13
gga	G	0	0.44
ggg	G	0	0.29
---	--	------	------
mPD		0.090	0.50
		nPD :	0.18
		N. weight :	0.61
		Sc. PD :	-0.032
		Sc. rank :	203.9

,

HA

Pos.	20	obs :	exp :
tat	Y	1	0.48
tac	Y	0	0.52
cat	H	1650	960.90
cac	H	10	699.10
caa	Q	67	1254.00
cag	Q	2445	1258.00
cgt	R	3	0.41
cgc	R	0	0.59
cga	R	0	1.11
cgg	R	8	0.90
aga	R	0	5.14
agg	R	0	2.86
---	--	------	------
mPD		0.51	0.75
		nPD :	0.68
		N. weight :	0.76
		Sc. PD :	0.33
		Sc. rank :	1754.2

,

HA

Pos.	21	obs :	exp :
att	I	3937	1535.00
atc	I	240	1119.00
ata	I	5	1528.00
atg	M	1	1.00
aat	N	1	0.55
aac	N	0	0.45
---	--	------	------
mPD		0.11	0.66
		nPD :	0.17
		N. weight :	1.1
		Sc. PD :	-0.071
		Sc. rank :	326.6

,

HA

Pos.	22	obs :	exp :
tat	Y	0	0.48
tac	Y	1	0.52
tgt	C	2	1617.00
tgc	C	4176	2561.00
ggt	G	0	0.72
ggc	G	5	0.66
gga	G	0	2.19
ggg	G	0	1.43
---	--	------	------
mPD		0.0038	0.48
		nPD :	0.01
		N. weight :	0.68
		Sc. PD :	-0.15
		Sc. rank :	-1672.6

,

HA

Pos.	23	obs :	exp :
att	I	4024	1526.00
atc	I	16	1113.00
ata	I	119	1520.00
gtt	V	22	5.38
gtc	V	0	5.26
gta	V	0	5.33
gtg	V	3	9.03
---	--	------	------
mPD		0.076	0.67
		nPD :	0.11
		N. weight :	1.2
		Sc. PD :	-0.14
		Sc. rank :	-418.3

,

HA

Pos.	24	obs :	exp :
ttt	F	1	0.42
ttc	F	0	0.58
gat	D	1	0.54
gac	D	0	0.46
ggt	G	4014	604.80
ggc	G	120	549.30
gga	G	47	1832.00
ggg	G	1	1195.00
---	--	------	------
mPD		0.079	0.69
		nPD :	0.11
		N. weight :	2.4
		Sc. PD :	-0.28
		Sc. rank :	-798.1

,

HA

Pos.	25	obs :	exp :
tat	Y	1540	2007.00
tac	Y	2644	2177.00
---	--	------	------
mPD		0.47	0.50
		nPD :	0.93
		N. weight :	0.036
		Sc. PD :	0.025
		Sc. rank :	94.7

,

HA

Pos.	26	obs:	exp:
tat	Y	1	0.48
tac	Y	0	0.52
cat	H	4172	2421.00
cac	H	11	1762.00
---	--	------	------
mPD		0.0057	0.49
		nPD:	0.01
		N. weight:	0.73
		Sc. PD:	-0.16
		Sc. rank:	-1730.6

HA

Pos.	27	obs:	exp:
gct	A	1	1054.00
gcc	A	2	809.50
gca	A	4174	1862.00
gcg	A	6	457.80
ggt	G	0	0.14
ggc	G	0	0.13
gga	G	1	0.44
ggg	G	0	0.29
---	--	------	------
mPD		0.0048	0.69
		nPD:	0.01
		N. weight:	1.1
		Sc. PD:	-0.24
		Sc. rank:	-2735.7

HA

Pos.	28	obs:	exp:
tct	S	0	0.33
tcc	S	0	0.29
tca	S	0	0.45
tcg	S	0	0.12
aat	N	1575	2280.00
aac	N	2605	1900.00
agt	S	0	0.43
agc	S	2	0.37
gat	D	2	1.08
gac	D	0	0.92
---	--	------	------
mPD		0.47	0.50
		nPD:	0.95
		N. weight:	0.082
		Sc. PD:	0.056
		Sc. rank:	213.8

HA

Pos.	29	obs:	exp:
tat	Y	0	0.48
tac	Y	1	0.52
att	I	1	0.37
atc	I	0	0.27
ata	I	0	0.37
aat	N	1214	2280.00
aac	N	2966	1900.00
aaa	K	1	0.57
aag	K	0	0.43
gat	D	0	0.54
gac	D	1	0.46
---	--	------	------
mPD		0.41	0.50
		nPD:	0.83
		N. weight:	0.19
		Sc. PD:	0.11
		Sc. rank:	478.0

HA

Pos.	30	obs:	exp:
tta	L	0	0.09
ttg	L	1	0.19
tct	S	2	697.60
tcc	S	1	605.30
tca	S	502	947.40
tcg	S	3674	252.10
tgg	W	2	2.00
ctt	L	0	0.20
ctc	L	0	0.16
cta	L	0	0.15
ctg	L	0	0.21
cct	P	0	0.55
ccc	P	0	0.36
cca	P	0	0.81
ccg	P	2	0.28
agt	S	0	906.40
agc	S	0	770.10
---	--	------	------
mPD		0.21	1.7
		nPD:	0.13
		N. weight:	3.2
		Sc. PD:	-0.32
		Sc. rank:	-752.5

HA

Pos.	31	obs:	exp:
cct	P	0	0.55
ccc	P	0	0.36
cca	P	2	0.81
ccg	P	0	0.28
act	T	0	1096.00
acc	T	1	942.30
aca	T	4115	1750.00
acg	T	66	393.50
---	--	------	------
mPD		0.032	0.70
		nPD:	0.05
		N. weight:	1.1
		Sc. PD:	-0.21
		Sc. rank:	-1805.5

HA

Pos.	32	obs:	exp:
tct	S	0	0.17
tcc	S	0	0.14
tca	S	0	0.23
tcg	S	0	0.06
caa	Q	0	0.50
cag	Q	1	0.50
cgt	R	0	0.07
cgc	R	0	0.11
cga	R	0	0.20
cgg	R	0	0.16
aat	N	0	2.18
aac	N	4	1.82
aaa	K	5	225.50
aag	K	391	170.50
agt	S	0	0.22
agc	S	1	0.18
aga	R	0	0.93
agg	R	2	0.52
gtt	V	0	0.22
gtc	V	0	0.21
gta	V	0	0.21
gtg	V	1	0.36
gat	D	1	0.54
gac	D	0	0.46
gaa	E	78	2118.00
gag	E	3700	1660.00
---	--	------	------
mPD		0.22	0.67
		nPD:	0.33
		N. weight:	1.
		Sc. PD:	0.087
		Sc. rank:	1134.8

HA

Pos.	33	obs:	exp:
tta	L	0	0.18
ttg	L	0	0.38
ctt	L	0	0.40
ctc	L	0	0.32
cta	L	0	0.30
ctg	L	2	0.42
cat	H	3	1.74
cac	H	0	1.26
caa	Q	454	2033.00
cag	Q	3618	2039.00
cgt	R	0	0.71
cgc	R	0	1.02
cga	R	0	1.91
cgg	R	19	1.55
aaa	K	1	50.11
aag	K	87	37.89
aga	R	0	8.87
agg	R	0	4.94
---	--	------	------
mPD		0.25	0.56
		nPD:	0.44
		N. weight:	0.5
		Sc. PD:	0.1
		Sc. rank:	790.7

HA

Pos.	34	obs:	exp:
att	I	10	3.67
atc	I	0	2.68
ata	I	0	3.65
gtt	V	4053	897.50
gtc	V	28	877.90
gta	V	2	889.80
gtg	V	90	1508.00
gct	A	0	0.25
gcc	A	1	0.19
gca	A	0	0.45
gcg	A	0	0.11
---	--	------	------
mPD		0.062	0.74
		nPD:	0.08
		N. weight:	1.9
		Sc. PD:	-0.28
		Sc. rank:	-1459.3

HA

Pos.	35	obs:	exp:
gat	D	51	2259.00
gac	D	4132	1924.00
ggt	G	0	0.14
ggc	G	1	0.13
gga	G	0	0.44
ggg	G	0	0.29
---	--	------	------
mPD		0.025	0.50
		nPD:	0.05
		N. weight:	0.98
		Sc. PD:	-0.18
		Sc. rank:	-1523.7

HA

Pos.	36	obs:	exp:
cct	P	0	0.55
ccc	P	0	0.36
cca	P	2	0.81
ccg	P	0	0.28
act	T	0	1096.00
acc	T	1	942.10
aca	T	3119	1749.00
acg	T	1061	393.40
gct	A	0	0.25
gcc	A	0	0.19
gca	A	1	0.45
gcg	A	0	0.11
---	--	------	------
mPD		0.38	0.70
		nPD:	0.55
		N. weight:	0.95
		Sc. PD:	0.28
		Sc. rank:	1851.9

HA

Pos.	37	obs:	exp:
tta	L	1	0.09
ttg	L	0	0.19
ctt	L	0	0.20
ctc	L	0	0.16
cta	L	0	0.15
ctg	L	0	0.21
att	I	0	1534.00
atc	I	214	1119.00
ata	I	3966	1528.00
atg	M	1	1.00
act	T	0	0.26
acc	T	0	0.23
aca	T	1	0.42
acg	T	0	0.09
gtt	V	0	0.22
gtc	V	1	0.21
gta	V	0	0.21
gtg	V	0	0.36
---	--	------	------
mPD		0.099	0.66
		nPD:	0.15
		N. weight:	1.1
		Sc. PD:	-0.094
		Sc. rank:	44.2

HA

Pos.	38	obs:	exp:
tta	L	0	0.09
ttg	L	1	0.19
ctt	L	0	0.20
ctc	L	0	0.16
cta	L	0	0.15
ctg	L	0	0.21
att	I	0	0.73
atc	I	0	0.54
ata	I	2	0.73
atg	M	4178	4178.00
act	T	0	0.26
acc	T	0	0.23
aca	T	0	0.42
acg	T	1	0.09
aaa	K	0	0.57
aag	K	1	0.43
gtt	V	0	0.22
gtc	V	0	0.21
gta	V	0	0.21
gtg	V	1	0.36
---	--	------	------
mPD		0.0029	0.0042
		nPD:	0.69
		N. weight:	0.0026
		Sc. PD:	0.0011
		Sc. rank:	6.1

HA

Pos.	39	obs:	exp:
aaa	K	2	1.14
aag	K	0	0.86
gaa	E	4126	2344.00
gag	E	56	1838.00
---	--	------	------
mPD		0.027	0.49
		nPD:	0.06
		N. weight:	0.71
		Sc. PD:	-0.12
		Sc. rank:	-1019.5

HA

Pos.	40	obs:	exp:
cgt	R	0	0.30
cgc	R	0	0.43
cga	R	0	0.81
cgg	R	0	0.65
aaa	K	93	2378.00
aag	K	4083	1798.00
aga	R	0	3.73
agg	R	8	2.08
---	--	------	------
mPD		0.047	0.50
		nPD:	0.1
		N. weight:	1.
		Sc. PD:	-0.14
		Sc. rank:	-611.8

HA

Pos.	41	obs:	exp:
tat	Y	0	0.48
tac	Y	1	0.52
act	T	0	0.26
acc	T	1	0.23
aca	T	0	0.42
acg	T	0	0.09
aat	N	148	2280.00
aac	N	4033	1901.00
gat	D	0	0.54
gac	D	1	0.46
---	--	------	------
mPD		0.070	0.50
		nPD:	0.14
		N. weight:	0.87
		Sc. PD:	-0.08
		Sc. rank:	-55.1

,

HA

Pos.	42	obs:	exp:
att	I	7	2.57
atc	I	0	1.87
ata	I	0	2.56
gtt	V	2533	898.20
gtc	V	1585	878.60
gta	V	18	890.40
gtg	V	40	1509.00
gct	A	0	0.25
gcc	A	1	0.19
gca	A	0	0.45
gcg	A	0	0.11
---	--	------	------
mPD		0.49	0.74
		nPD:	0.67
		N. weight:	1.1
		Sc. PD:	0.46
		Sc. rank:	2520.9

,

HA

Pos.	43	obs:	exp:
att	I	1	0.37
atc	I	0	0.27
ata	I	0	0.37
act	T	4113	1097.00
acc	T	17	942.50
aca	T	15	1750.00
acg	T	38	393.60
---	--	------	------
mPD		0.034	0.70
		nPD:	0.05
		N. weight:	1.7
		Sc. PD:	-0.31
		Sc. rank:	-2743.7

,

HA

Pos.	44	obs:	exp:
gtt	V	4005	899.70
gtc	V	173	880.00
gta	V	1	891.90
gtg	V	4	1511.00
gct	A	0	0.25
gcc	A	1	0.19
gca	A	0	0.45
gcg	A	0	0.11
---	--	------	------
mPD		0.083	0.73
		nPD:	0.11
		N. weight:	1.9
		Sc. PD:	-0.22
		Sc. rank:	-717.0

,

HA

Pos.	45	obs:	exp:
att	I	0	0.37
atc	I	0	0.27
ata	I	1	0.37
act	T	9	1097.00
acc	T	10	942.50
aca	T	4149	1750.00
acg	T	15	393.60
---	--	------	------
mPD		0.017	0.70
		nPD:	0.02
		N. weight:	1.1
		Sc. PD:	-0.23
		Sc. rank:	-2443.7

,

HA

Pos.	46	obs:	exp:
tta	L	0	0.09
ttg	L	0	0.19
ctt	L	1	0.20
ctc	L	0	0.16
cta	L	0	0.15
ctg	L	0	0.21
cat	H	3463	2379.00
cac	H	647	1731.00
caa	Q	29	34.95
cag	Q	41	35.05
gat	D	1	1.62
gac	D	2	1.38
---	--	------	------
mPD		0.29	0.51
		nPD:	0.58
		N. weight:	0.22
		Sc. PD:	0.073
		Sc. rank:	453.0

,

HA

Pos.	47	obs:	exp:
tct	S	0	1.84
tcc	S	11	1.59
tca	S	0	2.49
tcg	S	0	0.66
agt	S	0	2.39
agc	S	0	2.03
gtt	V	1	0.43
gtc	V	1	0.42
gta	V	0	0.43
gtg	V	0	0.72
gct	A	419	1051.00
gcc	A	3749	807.10
gca	A	3	1857.00
gcg	A	0	456.50
---	--	------	------
mPD		0.19	0.70
		nPD:	0.27
		N. weight:	1.8
		Sc. PD:	0.059
		Sc. rank:	1486.6

,

HA

Pos.	48	obs:	exp:
tta	L	0	0.09
ttg	L	0	0.19
ctt	L	0	0.20
ctc	L	0	0.16
cta	L	1	0.15
ctg	L	0	0.21
cct	P	0	0.27
ccc	P	0	0.18
cca	P	1	0.41
ccg	P	0	0.14
cat	H	1	0.58
cac	H	0	0.42
caa	Q	4095	2066.00
cag	Q	43	2072.00
cgt	R	0	0.67
cgc	R	0	0.96
cga	R	18	1.81
cgg	R	0	1.47
aaa	K	24	14.24
aag	K	1	10.76
aga	R	0	8.40
agg	R	0	4.68
---	--	------	------
mPD		0.043	0.53
		nPD:	0.08
		N. weight:	0.89
		Sc. PD:	-0.13
		Sc. rank:	-730.2

,

HA

Pos.	49	obs:	exp:
cat	H	0	0.58
cac	H	1	0.42
aat	N	1	6.54
aac	N	11	5.46
gat	D	309	2252.00
gac	D	3862	1919.00
---	--	------	------
mPD		0.14	0.50
		nPD:	0.29
		N. weight:	0.69
		Sc. PD:	0.033
		Sc. rank:	620.9

,

HA

Pos.	50	obs:	exp:
tta	L	0	0.09
ttg	L	0	0.19
ctt	L	0	0.20
ctc	L	0	0.16
cta	L	1	0.15
ctg	L	0	0.21
att	I	0	1535.00
atc	I	4	1119.00
ata	I	4178	1528.00
act	T	0	0.26
acc	T	0	0.23
aca	T	1	0.42
acg	T	0	0.09
---	--	------	------
mPD		0.0029	0.66
		nPD:	0.
		N. weight:	1.4
		Sc. PD:	-0.31
		Sc. rank:	-3514.3

,

HA

Pos.	51	obs:	exp:
tta	L	6	386.50
ttg	L	36	794.10
ctt	L	3	829.90
ctc	L	0	668.60
cta	L	142	618.60
ctg	L	3993	882.30
cct	P	0	0.27
ccc	P	0	0.18
cca	P	0	0.41
ccg	P	1	0.14
cgt	R	0	0.04
cgc	R	0	0.05
cga	R	0	0.10
cgg	R	0	0.08
att	I	0	0.37
atc	I	0	0.27
ata	I	1	0.37
aga	R	0	0.47
agg	R	1	0.26
gtt	V	0	0.22
gtc	V	0	0.21
gta	V	0	0.21
gtg	V	1	0.36
---	--	------	------
mPD		0.092	1.1
		nPD:	0.08
		N. weight:	1.9
		Sc. PD:	-0.28
		Sc. rank:	-1470.1

,

HA

Pos.	52	obs:	exp:
gaa	E	3962	2345.00
gag	E	222	1839.00
---	--	------	------
mPD		0.10	0.49
		nPD:	0.2
		N. weight:	0.53
		Sc. PD:	-0.016
		Sc. rank:	234.1

,

HA

Pos.	53	obs:	exp:
cgt	R	0	2.32
cgc	R	0	3.32
cga	R	0	6.25
cgg	R	0	5.06
aaa	K	949	2347.00
aag	K	3173	1775.00
aga	R	0	28.94
agg	R	62	16.11
---	--	------	------
mPD		0.38	0.53
		nPD:	0.72
		N. weight:	0.35
		Sc. PD:	0.17
		Sc. rank:	856.4

,

HA

Pos.	54	obs:	exp:
act	T	5	1074.00
acc	T	0	922.70
aca	T	4056	1713.00
acg	T	34	385.30
aaa	K	3	6.26
aag	K	8	4.74
gct	A	0	10.58
gcc	A	0	8.13
gca	A	42	18.70
gcg	A	0	4.60
gaa	E	15	19.06
gag	E	19	14.94
ggt	G	0	0.29
ggc	G	0	0.26
gga	G	0	0.88
ggg	G	2	0.57
---	--	------	------
mPD		0.091	0.76
		nPD:	0.12
		N. weight:	1.1
		Sc. PD:	-0.13
		Sc. rank:	-334.4

,

HA

Pos.	55	obs:	exp:
cat	H	190	2421.00
cac	H	3992	1761.00
cgt	R	0	0.07
cgc	R	2	0.11
cga	R	0	0.20
cgg	R	0	0.16
aga	R	0	0.93
agg	R	0	0.52
---	--	------	------
mPD		0.088	0.49
		nPD:	0.18
		N. weight:	0.92
		Sc. PD:	-0.05
		Sc. rank:	305.9

,

HA			
Pos.	56	obs:	exp:
aat	N	400	2281.00
aac	N	3783	1902.00
aaa	K	1	0.57
aag	K	0	0.43
---	--	------	------
mPD		0.17	0.50
		nPD:	0.35
		N. weight:	0.63
		Sc. PD:	0.07
		Sc. rank:	750.5

,

HA			
Pos.	57	obs:	exp:
cgt	R	0	0.11
cgc	R	0	0.16
cga	R	0	0.30
cgg	R	0	0.24
aga	R	0	1.40
agg	R	3	0.78
gaa	E	0	0.56
gag	E	1	0.44
ggt	G	0	604.50
ggc	G	0	549.10
gga	G	206	1832.00
ggg	G	3974	1195.00
---	--	------	------
mPD		0.096	0.69
		nPD:	0.14
		N. weight:	1.4
		Sc. PD:	-0.13
		Sc. rank:	-139.9

,

HA			
Pos.	58	obs:	exp:
cgt	R	0	0.97
cgc	R	0	1.39
cga	R	0	2.62
cgg	R	0	2.12
act	T	0	0.26
acc	T	0	0.23
aca	T	0	0.42
acg	T	1	0.09
aat	N	1	0.55
aac	N	0	0.45
aaa	K	616	2366.00
aag	K	3540	1790.00
aga	R	1	12.14
agg	R	25	6.75
---	--	------	------
mPD		0.26	0.51
		nPD:	0.52
		N. weight:	0.54
		Sc. PD:	0.15
		Sc. rank:	1010.6

,

HA			
Pos.	59	obs:	exp:
tta	L	1	386.60
ttg	L	0	794.30
ctt	L	100	830.10
ctc	L	4067	668.80
cta	L	11	618.80
ctg	L	2	882.50
cct	P	0	0.27
ccc	P	1	0.18
cca	P	0	0.41
ccg	P	0	0.14
cat	H	0	0.58
cac	H	1	0.42
att	I	0	0.37
atc	I	1	0.27
ata	I	0	0.37
---	--	------	------
mPD		0.055	1.1
		nPD:	0.05
		N. weight:	2.3
		Sc. PD:	-0.42
		Sc. rank:	-3653.9

,

HA			
Pos.	60	obs:	exp:
tgt	C	1171	1619.00
tgc	C	3013	2565.00
---	--	------	------
mPD		0.40	0.47
		nPD:	0.85
		N. weight:	0.036
		Sc. PD:	0.022
		Sc. rank:	93.6

,

HA			
Pos.	61	obs:	exp:
tct	S	0	11.68
tcc	S	0	10.14
tca	S	0	15.87
tcg	S	0	4.22
aat	N	312	193.60
aac	N	43	161.40
agt	S	43	15.18
agc	S	27	12.90
gct	A	3	2.27
gcc	A	6	1.74
gca	A	0	4.01
gcg	A	0	0.99
gat	D	3524	2025.00
gac	D	226	1725.00
---	--	------	------
mPD		0.35	0.72
		nPD:	0.49
		N. weight:	0.55
		Sc. PD:	0.13
		Sc. rank:	991.1

,

HA			
Pos.	62	obs:	exp:
tta	L	549	386.60
ttg	L	9	794.30
ctt	L	41	830.10
ctc	L	10	668.80
cta	L	3502	618.80
ctg	L	70	882.50
att	I	3	1.10
atc	I	0	0.80
ata	I	0	1.10
---	--	------	------
mPD		0.29	1.1
		nPD:	0.26
		N. weight:	2.
		Sc. PD:	0.051
		Sc. rank:	1482.6

,

HA			
Pos.	63	obs:	exp:
tat	Y	2	0.96
tac	Y	0	1.04
aat	N	2596	1423.00
aac	N	14	1187.00
aaa	K	39	25.62
aag	K	6	19.38
gat	D	1406	824.50
gac	D	121	702.50
---	--	------	------
mPD		0.55	0.97
		nPD:	0.56
		N. weight:	0.68
		Sc. PD:	0.22
		Sc. rank:	1366.3

,

HA			
Pos.	64	obs:	exp:
ggt	G	5	605.10
ggc	G	0	549.60
gga	G	3219	1833.00
ggg	G	960	1196.00
---	--	------	------
mPD		0.36	0.69
		nPD:	0.52
		N. weight:	0.52
		Sc. PD:	0.14
		Sc. rank:	973.2

,

HA			
Pos.	65	obs:	exp:
tta	L	0	0.18
ttg	L	2	0.38
ctt	L	0	0.40
ctc	L	0	0.32
cta	L	0	0.30
ctg	L	0	0.42
att	I	26	9.54
atc	I	0	6.96
ata	I	0	9.50
atg	M	1	1.00
gtt	V	149	893.70
gtc	V	0	874.20
gta	V	48	886.00
gtg	V	3958	1501.00
---	--	------	------
mPD		0.12	0.75
		nPD:	0.15
		N. weight:	1.1
		Sc. PD:	-0.089
		Sc. rank:	106.3

,

HA			
Pos.	66	obs:	exp:
cgt	R	0	7.33
cgc	R	0	10.49
cga	R	0	19.75
cgg	R	0	16.00
aaa	K	401	2270.00
aag	K	3586	1717.00
aga	R	3	91.50
agg	R	193	50.92
gaa	E	0	0.56
gag	E	1	0.44
---	--	------	------
mPD		0.26	0.61
		nPD:	0.43
		N. weight:	0.73
		Sc. PD:	0.14
		Sc. rank:	1120.8

,

HA			
Pos.	67	obs:	exp:
tct	S	1	0.17
tcc	S	0	0.14
tca	S	0	0.23
tcg	S	0	0.06
cct	P	4074	1143.00
ccc	P	86	755.50
cca	P	21	1698.00
ccg	P	2	586.50
agt	S	0	0.22
agc	S	0	0.18
---	--	------	------
mPD		0.051	0.71
		nPD:	0.07
		N. weight:	1.6
		Sc. PD:	-0.25
		Sc. rank:	-1760.4

,

HA			
Pos.	68	obs:	exp:
tta	L	21	386.60
ttg	L	48	794.30
ctt	L	2	830.10
ctc	L	180	668.80
cta	L	1134	618.80
ctg	L	2796	882.50
caa	Q	0	1.00
cag	Q	2	1.00
atg	M	1	1.00
---	--	------	------
mPD		0.49	1.1
		nPD:	0.44
		N. weight:	1.2
		Sc. PD:	0.23
		Sc. rank:	1806.9

,

HA			
Pos.	69	obs:	exp:
att	I	4143	1534.00
atc	I	36	1118.00
ata	I	0	1527.00
gtt	V	5	1.08
gtc	V	0	1.05
gta	V	0	1.07
gtg	V	0	1.81
---	--	------	------
mPD		0.019	0.66
		nPD:	0.03
		N. weight:	1.3
		Sc. PD:	-0.26
		Sc. rank:	-2655.7

,

HA			
Pos.	70	obs:	exp:
tta	L	3653	386.90
ttg	L	440	794.80
ctt	L	0	830.70
ctc	L	0	669.30
cta	L	30	619.20
ctg	L	61	883.10
---	--	------	------
mPD		0.25	1.1
		nPD:	0.23
		N. weight:	2.5
		Sc. PD:	-0.023
		Sc. rank:	1419.7

,

HA			
Pos.	71	obs:	exp:
cgt	R	0	56.96
cgc	R	0	81.45
cga	R	2	153.40
cgg	R	0	124.30
att	I	0	0.37
atc	I	0	0.27
ata	I	1	0.37
aaa	K	1586	1504.00
aag	K	1056	1138.00
aga	R	1459	710.50
agg	R	61	395.40
gaa	E	16	8.97
gag	E	0	7.03
ggt	G	0	0.43
ggc	G	0	0.39
gga	G	3	1.31
ggg	G	0	0.86
---	--	------	------
mPD		0.87	1.2
		nPD:	0.74
		N. weight:	0.32
		Sc. PD:	0.15
		Sc. rank:	774.0

,

HA			
Pos.	72	obs:	exp:
tat	Y	1	0.48
tac	Y	0	0.52
aat	N	17	9.27
aac	N	0	7.73
gat	D	4022	2248.00
gac	D	142	1916.00
ggt	G	2	0.29
ggc	G	0	0.26
gga	G	0	0.88
ggg	G	0	0.57
---	--	------	------
mPD		0.075	0.51
		nPD:	0.15
		N. weight:	0.66
		Sc. PD:	-0.055
		Sc. rank:	18.8

,

HA			
Pos.	73	obs:	exp:
tgt	C	4104	1619.00
tgc	C	80	2565.00
---	--	------	------
mPD		0.038	0.47
		nPD:	0.08
		N. weight:	1.2
		Sc. PD:	-0.18
		Sc. rank:	-977.4

,

HA			
Pos.	74	obs:	exp:
tct	S	0	698.10
tcc	S	0	605.80
tca	S	0	948.10
tcg	S	0	252.30
cgt	R	0	0.04
cgc	R	0	0.05
cga	R	0	0.10
cgg	R	0	0.08
agt	S	4151	907.10
agc	S	31	770.70
aga	R	1	0.47
agg	R	0	0.26
ggt	G	1	0.14
ggc	G	0	0.13
gga	G	0	0.44
ggg	G	0	0.29
---	--	------	------
mPD		0.016	1.7
		nPD:	0.01
		N. weight:	2.1
		Sc. PD:	-0.45
		Sc. rank:	-4958.1

,

HA			
Pos.	75	obs:	exp:
att	I	0	0.37
atc	I	0	0.27
ata	I	1	0.37
gtt	V	7	899.50
gtc	V	2	879.80
gta	V	4132	891.70
gtg	V	41	1511.00
gaa	E	1	0.56
gag	E	0	0.44
---	--	------	------
mPD		0.025	0.73
		nPD:	0.03
		N. weight:	2.
		Sc. PD:	-0.4
		Sc. rank:	-3984.0

,

HA			
Pos.	76	obs:	exp:
cct	P	0	0.27
ccc	P	0	0.18
cca	P	1	0.41
ccg	P	0	0.14
act	T	2	0.52
acc	T	0	0.45
aca	T	0	0.84
acg	T	0	0.19
gtt	V	0	0.65
gtc	V	0	0.63
gta	V	3	0.64
gtg	V	0	1.08
gct	A	2454	1052.00
gcc	A	59	808.30
gca	A	1429	1859.00
gcg	A	235	457.20
gat	D	1	0.54
gac	D	0	0.46
---	--	------	------
mPD		0.54	0.69
		nPD:	0.78
		N. weight:	0.47
		Sc. PD:	0.24
		Sc. rank:	1166.4

,

HA			
Pos.	77	obs:	exp:
cgt	R	0	0.04
cgc	R	0	0.05
cga	R	0	0.10
cgg	R	0	0.08
aga	R	1	0.47
agg	R	0	0.26
ggt	G	3	604.90
ggc	G	6	549.40
gga	G	4105	1833.00
ggg	G	69	1196.00
---	--	------	------
mPD		0.037	0.69
		nPD:	0.05
		N. weight:	1.
		Sc. PD:	-0.18
		Sc. rank:	-1486.9

,

HA			
Pos.	78	obs:	exp:
tgg	W	4183	4183.00
cgt	R	0	0.04
cgc	R	0	0.05
cga	R	0	0.10
cgg	R	1	0.08
aga	R	0	0.47
agg	R	0	0.26
---	--	------	------
mPD		0.00048	0.00079
		nPD:	0.6
		N. weight:	0.00083
		Sc. PD:	0.00029
		Sc. rank:	1.8

,

HA			
Pos.	79	obs:	exp:
tta	L	8	386.90
ttg	L	1	794.80
ctt	L	259	830.70
ctc	L	3847	669.30
cta	L	69	619.20
ctg	L	0	883.10
---	--	------	------
mPD		0.15	1.1
		nPD:	0.14
		N. weight:	2.1
		Sc. PD:	-0.19
		Sc. rank:	-266.9

,

HA			
Pos.	80	obs:	exp:
tta	L	0	386.90
ttg	L	0	794.80
ctt	L	185	830.70
ctc	L	3984	669.30
cta	L	14	619.20
ctg	L	1	883.10
---	--	------	------
mPD		0.091	1.1
		nPD:	0.08
		N. weight:	2.2
		Sc. PD:	-0.33
		Sc. rank:	-1791.4

,

HA			
Pos.	81	obs:	exp:
ggt	G	1	605.10
ggc	G	0	549.60
gga	G	3154	1833.00
ggg	G	1029	1196.00
---	--	------	------
mPD		0.37	0.69
		nPD:	0.54
		N. weight:	0.51
		Sc. PD:	0.15
		Sc. rank:	997.8

,

HA			
Pos.	82	obs:	exp:
tct	S	0	0.17
tcc	S	0	0.14
tca	S	0	0.23
tcg	S	0	0.06
cat	H	0	0.58
cac	H	1	0.42
aat	N	946	2280.00
aac	N	3235	1901.00
agt	S	0	0.22
agc	S	1	0.18
gat	D	1	0.54
gac	D	0	0.46
---	--	------	------
mPD		0.35	0.50
		nPD:	0.71
		N. weight:	0.3
		Sc. PD:	0.13
		Sc. rank:	702.5

,

HA			
Pos.	83	obs:	exp:
cct	P	23	1143.00
ccc	P	39	755.70
cca	P	4116	1698.00
ccg	P	6	586.60
---	--	------	------
mPD		0.032	0.71
		nPD:	0.05
		N. weight:	1.1
		Sc. PD:	-0.21
		Sc. rank:	-1871.1

,

HA			
Pos.	84	obs:	exp:
ttt	F	3	1.25
ttc	F	0	1.75
tta	L	7	89.87
ttg	L	963	184.70
tct	S	0	6.34
tcc	S	0	5.50
tca	S	1	8.61
tcg	S	37	2.29
tgg	W	41	41.00
ctt	L	0	193.00
ctc	L	0	155.50
cta	L	0	143.80
ctg	L	2	205.20
cgt	R	0	0.19
cgc	R	0	0.27
cga	R	0	0.50
cgg	R	0	0.41
att	I	0	0.37
atc	I	0	0.27
ata	I	1	0.37
atg	M	3115	3115.00
act	T	0	1.57
acc	T	0	1.35
aca	T	0	2.51
acg	T	6	0.56
aaa	K	0	1.71
aag	K	3	1.29
agt	S	0	8.24
agc	S	0	7.00
aga	R	0	2.33
agg	R	5	1.30
---	--	------	------
mPD		0.43	0.72
		nPD:	0.6
		N. weight:	0.56
		Sc. PD:	0.2
		Sc. rank:	1182.6

,

HA			
Pos.	85	obs:	exp:
tgt	C	2992	1618.00
tgc	C	1188	2562.00
tgg	W	3	3.00
cgt	R	1	0.04
cgc	R	0	0.05
cga	R	0	0.10
cgg	R	0	0.08
aga	R	0	0.47
agg	R	0	0.26
---	--	------	------
mPD		0.41	0.48
		nPD:	0.86
		N. weight:	0.31
		Sc. PD:	0.19
		Sc. rank:	794.8

,

HA			
Pos.	86	obs:	exp:
tct	S	0	2.84
tcc	S	0	2.46
tca	S	0	3.85
tcg	S	0	1.03
agt	S	0	3.69
agc	S	17	3.13
gtt	V	0	0.22
gtc	V	1	0.21
gta	V	0	0.21
gtg	V	0	0.36
gct	A	0	0.25
gcc	A	1	0.19
gca	A	0	0.45
gcg	A	0	0.11
gat	D	254	2121.00
gac	D	3674	1807.00
ggt	G	0	34.27
ggc	G	219	31.13
gga	G	18	103.80
ggg	G	0	67.75
---	--	------	------
mPD		0.25	0.66
		nPD:	0.37
		N. weight:	0.83
		Sc. PD:	0.11
		Sc. rank:	1071.6

,

HA			
Pos.	87	obs:	exp:
aaa	K	44	25.05
aag	K	0	18.95
gct	A	0	2.27
gcc	A	0	1.74
gca	A	9	4.01
gcg	A	0	0.99
gat	D	1	5.94
gac	D	10	5.06
gaa	E	4076	2309.00
gag	E	43	1810.00
ggt	G	0	0.14
ggc	G	0	0.13
gga	G	1	0.44
ggg	G	0	0.29
---	--	------	------
mPD		0.051	0.52
		nPD:	0.1
		N. weight:	0.73
		Sc. PD:	-0.097
		Sc. rank:	-428.4

,

HA			
Pos.	88	obs:	exp:
ttt	F	89	1745.00
ttc	F	4094	2438.00
tct	S	0	0.17
tcc	S	1	0.14
tca	S	0	0.23
tcg	S	0	0.06
agt	S	0	0.22
agc	S	0	0.18
---	--	------	------
mPD		0.042	0.49
		nPD:	0.09
		N. weight:	0.62
		Sc. PD:	-0.089
		Sc. rank:	-426.3

,

HA			
Pos.	89	obs:	exp:
ttt	F	0	0.83
ttc	F	2	1.17
tta	L	0	65.74
ttg	L	0	135.10
tct	S	0	0.17
tcc	S	0	0.14
tca	S	0	0.23
tcg	S	0	0.06
ctt	L	5	141.20
ctc	L	669	113.70
cta	L	0	105.20
ctg	L	37	150.10
cct	P	0	15.30
ccc	P	56	10.11
cca	P	0	22.73
ccg	P	0	7.85
att	I	13	973.50
atc	I	2639	709.90
ata	I	1	969.50
act	T	0	188.20
acc	T	716	161.80
aca	T	2	300.40
acg	T	0	67.55
aat	N	0	19.09
aac	N	35	15.91
agt	S	0	0.22
agc	S	1	0.18
gtt	V	0	0.22
gtc	V	1	0.21
gta	V	0	0.21
gtg	V	0	0.36
gct	A	0	1.51
gcc	A	6	1.16
gca	A	0	2.67
gcg	A	0	0.66
gat	D	0	0.54
gac	D	1	0.46
---	--	------	------
mPD		0.65	1.3
		nPD:	0.48
		N. weight:	1.9
		Sc. PD:	0.45
		Sc. rank:	3319.7

,

HA			
Pos.	90	obs:	exp:
tct	S	0	0.17
tcc	S	0	0.14
tca	S	0	0.23
tcg	S	0	0.06
cat	H	3	1.74
cac	H	0	1.26
cgt	R	0	33.34
cgc	R	0	47.68
cga	R	0	89.79
cgg	R	0	72.76
aat	N	3134	1722.00
aac	N	23	1435.00
aaa	K	20	13.10
aag	K	3	9.90
agt	S	1	0.22
agc	S	0	0.18
aga	R	856	416.00
agg	R	35	231.50
gat	D	107	58.85
gac	D	2	50.15
---	--	------	------
mPD		0.74	1.2
		nPD:	0.65
		N. weight:	0.8
		Sc. PD:	0.32
		Sc. rank:	1749.5

,

HA			
Pos.	91	obs:	exp:
atg	M	1	1.00
gtt	V	8	899.30
gtc	V	9	879.60
gta	V	458	891.50
gtg	V	3706	1511.00
gct	A	0	0.25
gcc	A	0	0.19
gca	A	0	0.45
gcg	A	1	0.11
gaa	E	0	0.56
gag	E	1	0.44
---	--	------	------
mPD		0.20	0.73
		nPD:	0.28
		N. weight:	0.98
		Sc. PD:	0.039
		Sc. rank:	853.1

,

HA			
Pos.	92	obs:	exp:
tct	S	1	22.87
tcc	S	0	19.85
tca	S	27	31.06
tcg	S	109	8.27
cct	P	3	1104.00
ccc	P	15	729.70
cca	P	1956	1640.00
ccg	P	2066	566.40
caa	Q	1	3.49
cag	Q	6	3.51
agt	S	0	29.72
agc	S	0	25.25
---	--	------	------
mPD		0.57	0.80
		nPD:	0.71
		N. weight:	1.1
		Sc. PD:	0.49
		Sc. rank:	2564.1

,

HA			
Pos.	93	obs:	exp:
aaa	K	2	1.14
aag	K	0	0.86
gtt	V	0	0.22
gtc	V	0	0.21
gta	V	1	0.21
gtg	V	0	0.36
gaa	E	4057	2343.00
gag	E	123	1837.00
ggt	G	0	0.14
ggc	G	0	0.13
gga	G	1	0.44
ggg	G	0	0.29
---	--	------	------
mPD		0.059	0.49
		nPD:	0.12
		N. weight:	0.63
		Sc. PD:	-0.071
		Sc. rank:	-201.6

,

HA			
Pos.	94	obs:	exp:
tgg	W	4184	4184.00
---	--	------	------
mPD		0	0
		nPD:	1.
		N. weight:	0.
		Sc. PD:	0
		Sc. rank:	0

,

HA			
Pos.	95	obs:	exp:
tct	S	3148	698.40
tcc	S	996	606.10
tca	S	40	948.50
tcg	S	0	252.40
agt	S	0	907.50
agc	S	0	771.10
---	--	------	------
mPD		0.38	1.7
		nPD:	0.23
		N. weight:	1.7
		Sc. PD:	-0.011
		Sc. rank:	969.3

,

HA			
Pos.	96	obs:	exp:
tat	Y	148	2007.00
tac	Y	4036	2177.00
---	--	------	------
mPD		0.068	0.50
		nPD:	0.14
		N. weight:	0.7
		Sc. PD:	-0.067
		Sc. rank:	-114.4

,

HA			
Pos.	97	obs:	exp:
tta	L	2	0.18
ttg	L	0	0.38
ctt	L	0	0.40
ctc	L	0	0.32
cta	L	0	0.30
ctg	L	0	0.42
att	I	20	1534.00
atc	I	35	1119.00
ata	I	4126	1528.00
atg	M	1	1.00
---	--	------	------
mPD		0.027	0.66
		nPD:	0.04
		N. weight:	1.3
		Sc. PD:	-0.24
		Sc. rank:	-2287.8

,

HA			
Pos.	98	obs:	exp:
tta	L	0	0.28
ttg	L	0	0.57
ctt	L	0	0.60
ctc	L	0	0.48
cta	L	0	0.44
ctg	L	3	0.63
att	I	0	0.73
atc	I	0	0.54
ata	I	2	0.73
gtt	V	8	898.60
gtc	V	0	879.00
gta	V	1693	890.90
gtg	V	2477	1510.00
ggt	G	0	0.14
ggc	G	0	0.13
gga	G	0	0.44
ggg	G	1	0.29
---	--	------	------
mPD		0.49	0.74
		nPD:	0.66
		N. weight:	0.76
		Sc. PD:	0.31
		Sc. rank:	1685.2

,

HA			
Pos.	99	obs:	exp:
aaa	K	0	0.57
aag	K	1	0.43
gaa	E	129	2345.00
gag	E	4054	1838.00
---	--	------	------
mPD		0.060	0.49
		nPD:	0.12
		N. weight:	0.94
		Sc. PD:	-0.1
		Sc. rank:	-268.4

,

HA			
Pos.	100	obs:	exp:
cgt	R	0	33.53
cgc	R	0	47.95
cga	R	1	90.29
cgg	R	846	73.16
aaa	K	178	1872.00
aag	K	3110	1416.00
aga	R	1	418.30
agg	R	48	232.80
---	--	------	------
mPD		0.74	0.95
		nPD:	0.78
		N. weight:	1.3
		Sc. PD:	0.7
		Sc. rank:	3340.7

,

HA

Pos.	101	obs:	exp:
tct	S	0	0.33
tcc	S	2	0.29
tca	S	0	0.45
tcg	S	0	0.12
cct	P	0	0.27
ccc	P	1	0.18
cca	P	0	0.41
ccg	P	0	0.14
att	I	4	230.80
atc	I	584	168.30
ata	I	41	229.90
atg	M	1	1.00
act	T	9	25.43
acc	T	88	21.86
aca	T	0	40.59
acg	T	0	9.13
aat	N	0	2.73
aac	N	5	2.27
agt	S	0	0.43
agc	S	0	0.37
gtt	V	0	7.53
gtc	V	35	7.36
gta	V	0	7.46
gtg	V	0	12.65
gct	A	875	841.70
gcc	A	2463	646.50
gca	A	3	1487.00
gcg	A	0	365.70
gat	D	39	39.41
gac	D	34	33.59
---	--	------	------
mPD		0.95	1.3
		nPD:	0.74
		N. weight:	1.4
		Sc. PD:	0.67
		Sc. rank:	3378.9

,

HA

Pos.	102	obs:	exp:
tct	S	0	12.52
tcc	S	0	10.86
tca	S	0	17.00
tcg	S	0	4.52
cat	H	5	3.47
cac	H	1	2.53
att	I	1	0.37
atc	I	0	0.27
ata	I	0	0.37
aat	N	3313	2176.00
aac	N	677	1814.00
aaa	K	2	51.82
aag	K	89	39.18
agt	S	71	16.27
agc	S	4	13.82
gat	D	16	9.72
gac	D	2	8.28
ggt	G	3	0.43
ggc	G	0	0.39
gga	G	0	1.31
ggg	G	0	0.86
---	--	------	------
mPD		0.36	0.59
		nPD:	0.6
		N. weight:	0.3
		Sc. PD:	0.11
		Sc. rank:	644.1

,

HA

Pos.	103	obs:	exp:
cct	P	1	1143.00
ccc	P	4	755.70
cca	P	4144	1698.00
ccg	P	35	586.60
---	--	------	------
mPD		0.019	0.71
		nPD:	0.03
		N. weight:	1.2
		Sc. PD:	-0.24
		Sc. rank:	-2437.3

,

HA

Pos.	104	obs:	exp:
tct	S	0	6.84
tcc	S	41	5.94
tca	S	0	9.29
tcg	S	0	2.47
cct	P	0	0.27
ccc	P	1	0.18
cca	P	0	0.41
ccg	P	0	0.14
att	I	0	10.27
atc	I	28	7.49
ata	I	0	10.23
act	T	16	55.05
acc	T	194	47.32
aca	T	0	87.87
acg	T	0	19.76
aat	N	0	1.64
aac	N	3	1.36
agt	S	0	8.89
agc	S	0	7.56
gtt	V	5	77.21
gtc	V	353	75.53
gta	V	1	76.55
gtg	V	0	129.70
gct	A	709	891.50
gcc	A	2824	684.80
gca	A	6	1575.00
gcg	A	0	387.40
gat	D	0	1.62
gac	D	3	1.38
---	--	------	------
mPD		0.59	1.0
		nPD:	0.58
		N. weight:	1.6
		Sc. PD:	0.53
		Sc. rank:	3253.5

,

HA

Pos.	105	obs:	exp:
tct	S	0	0.17
tcc	S	0	0.14
tca	S	0	0.23
tcg	S	0	0.06
tat	Y	3	1.44
tac	Y	0	1.56
cat	H	1	0.58
cac	H	0	0.42
aat	N	4105	2278.00
aac	N	72	1899.00
agt	S	1	0.22
agc	S	0	0.18
gat	D	2	1.08
gac	D	0	0.92
---	--	------	------
mPD		0.037	0.50
		nPD:	0.07
		N. weight:	0.73
		Sc. PD:	-0.11
		Sc. rank:	-744.5

,

HA

Pos.	106	obs:	exp:
tct	S	0	0.50
tcc	S	0	0.43
tca	S	0	0.68
tcg	S	0	0.18
agt	S	0	0.65
agc	S	3	0.55
gat	D	68	1972.00
gac	D	3585	1681.00
ggt	G	11	76.36
ggc	G	517	69.35
gga	G	0	231.40
ggg	G	0	150.90
---	--	------	------
mPD		0.26	0.80
		nPD:	0.32
		N. weight:	1.2
		Sc. PD:	0.099
		Sc. rank:	1291.8

,

HA

Pos.	107	obs:	exp:
tta	L	0	385.40
ttg	L	1	791.80
ctt	L	27	827.60
ctc	L	4105	666.70
cta	L	10	616.80
ctg	L	25	879.70
caa	Q	0	5.99
cag	Q	12	6.01
att	I	0	1.47
atc	I	4	1.07
ata	I	0	1.46
---	--	------	------
mPD		0.044	1.1
		nPD:	0.04
		N. weight:	2.4
		Sc. PD:	-0.46
		Sc. rank:	-4439.1

,

HA

Pos.	108	obs:	exp:
tgt	C	4071	1619.00
tgc	C	113	2565.00
---	--	------	------
mPD		0.053	0.47
		nPD:	0.11
		N. weight:	1.1
		Sc. PD:	-0.14
		Sc. rank:	-476.7

,

HA

Pos.	109	obs:	exp:
tat	Y	510	2006.00
tac	Y	3673	2177.00
gat	D	0	0.54
gac	D	1	0.46
---	--	------	------
mPD		0.21	0.50
		nPD:	0.43
		N. weight:	0.41
		Sc. PD:	0.076
		Sc. rank:	613.8

,

HA

Pos.	110	obs:	exp:
tta	L	0	0.09
ttg	L	0	0.19
ctt	L	0	0.20
ctc	L	0	0.16
cta	L	1	0.15
ctg	L	0	0.21
cct	P	3	1143.00
ccc	P	10	755.50
cca	P	4150	1698.00
ccg	P	20	586.50
---	--	------	------
mPD		0.016	0.71
		nPD:	0.02
		N. weight:	1.2
		Sc. PD:	-0.24
		Sc. rank:	-2597.2

,

HA

Pos.	111	obs:	exp:
ggt	G	3	605.10
ggc	G	0	549.60
gga	G	585	1833.00
ggg	G	3596	1196.00
---	--	------	------
mPD		0.24	0.69
		nPD:	0.35
		N. weight:	1.1
		Sc. PD:	0.12
		Sc. rank:	1305.5

,

HA

Pos.	112	obs:	exp:
tct	S	0	181.80
tcc	S	0	157.70
tca	S	0	246.90
tcg	S	0	65.70
tat	Y	1	0.48
tac	Y	0	0.52
att	I	4	2.20
atc	I	2	1.61
ata	I	0	2.19
atg	M	3	3.00
act	T	45	11.80
acc	T	0	10.14
aca	T	0	18.83
acg	T	0	4.23
aat	N	2565	1455.00
aac	N	103	1213.00
aaa	K	3	4.56
aag	K	5	3.44
agt	S	232	236.20
agc	S	857	200.70
gtt	V	163	35.06
gtc	V	0	34.29
gta	V	0	34.76
gtg	V	0	58.89
gct	A	1	0.25
gcc	A	0	0.19
gca	A	0	0.45
gcg	A	0	0.11
gat	D	150	107.40
gac	D	49	91.55
gaa	E	0	0.56
gag	E	1	0.44
---	--	------	------
mPD		0.99	1.5
		nPD:	0.67
		N. weight:	0.92
		Sc. PD:	0.39
		Sc. rank:	2111.0

,

HA

Pos.	113	obs:	exp:
ttt	F	14	1335.00
ttc	F	3185	1864.00
tta	L	6	90.24
ttg	L	0	185.40
tct	S	0	0.33
tcc	S	2	0.29
tca	S	0	0.45
tcg	S	0	0.12
tat	Y	0	0.48
tac	Y	1	0.52
ctt	L	2	193.80
ctc	L	963	156.10
cta	L	5	144.40
ctg	L	0	206.00
cct	P	0	0.27
ccc	P	1	0.18
cca	P	0	0.41
ccg	P	0	0.14
att	I	0	0.37
atc	I	1	0.27
ata	I	0	0.37
agt	S	0	0.43
agc	S	0	0.37
gtt	V	0	0.86
gtc	V	4	0.84
gta	V	0	0.85
gtg	V	0	1.45
---	--	------	------
mPD		0.37	0.90
		nPD:	0.41
		N. weight:	1.1
		Sc. PD:	0.19
		Sc. rank:	1613.0

,

HA

Pos.	114	obs:	exp:
tct	S	0	4.34
tcc	S	0	3.77
tca	S	0	5.89
tcg	S	0	1.57
tat	Y	0	1.44
tac	Y	3	1.56
cgt	R	0	0.04
cgc	R	0	0.05
cga	R	0	0.10
cgg	R	0	0.08
aat	N	1339	2264.00
aac	N	2813	1888.00
aaa	K	1	0.57
aag	K	0	0.43
agt	S	2	5.64
agc	S	24	4.79
aga	R	1	0.47
agg	R	0	0.26
gat	D	0	0.54
gac	D	1	0.46
---	--	------	------
mPD		0.45	0.52
		nPD:	0.87
		N. weight:	0.15
		Sc. PD:	0.094
		Sc. rank:	395.7

,

HA

Pos.	115	obs:	exp:
tct	S	0	0.17
tcc	S	0	0.14
tca	S	0	0.23
tcg	S	0	0.06
aat	N	10	68.17
aac	N	115	56.83
agt	S	0	0.22
agc	S	1	0.18
gct	A	0	0.25
gcc	A	1	0.19
gca	A	0	0.45
gcg	A	0	0.11
gat	D	1692	2187.00
gac	D	2358	1863.00
gaa	E	4	2.24
gag	E	0	1.76
ggt	G	2	0.43
ggc	G	1	0.39
gga	G	0	1.31
ggg	G	0	0.86
---	--	------	------
mPD		0.54	0.56
		nPD:	0.97
		N. weight:	0.066
		Sc. PD:	0.047
		Sc. rank:	172.6

,

HA

Pos.	116	obs:	exp:
tat	Y	4128	2006.00
tac	Y	55	2177.00
aat	N	1	0.55
aac	N	0	0.45
---	--	------	------
mPD		0.026	0.50
		nPD:	0.05
		N. weight:	0.92
		Sc. PD:	-0.16
		Sc. rank:	-1390.5

,

HA

Pos.	117	obs:	exp:
gaa	E	3815	2345.00
gag	E	368	1838.00
---	-	1	1.00
---	--	------	------
mPD		0.16	0.49
		nPD:	0.33
		N. weight:	0.42
		Sc. PD:	0.037
		Sc. rank:	477.1

,

HA

Pos.	118	obs:	exp:
caa	Q	3	1.50
cag	Q	0	1.50
aaa	K	1	0.57
aag	K	0	0.43
gaa	E	4142	2343.00
gag	E	38	1837.00
---	--	------	------
mPD		0.020	0.49
		nPD:	0.04
		N. weight:	0.73
		Sc. PD:	-0.14
		Sc. rank:	-1322.0

,

HA

Pos.	119	obs:	exp:
tta	L	90	386.70
ttg	L	1558	794.50
ctt	L	13	830.30
ctc	L	0	668.90
cta	L	284	618.90
ctg	L	2237	882.70
atg	M	1	1.00
gtt	V	0	0.22
gtc	V	0	0.21
gta	V	0	0.21
gtg	V	1	0.36
---	--	------	------
mPD		0.65	1.1
		nPD:	0.58
		N. weight:	0.91
		Sc. PD:	0.3
		Sc. rank:	1849.1

,

HA

Pos.	120	obs:	exp:
cgt	R	0	0.11
cgc	R	0	0.16
cga	R	0	0.30
cgg	R	0	0.24
aaa	K	4103	2380.00
aag	K	76	1799.00
aga	R	2	1.40
agg	R	1	0.78
gaa	E	2	1.12
gag	E	0	0.88
---	--	------	------
mPD		0.039	0.49
		nPD:	0.08
		N. weight:	0.66
		Sc. PD:	-0.1
		Sc. rank:	-589.5

,

HA

Pos.	121	obs:	exp:
tat	Y	0	0.48
tac	Y	1	0.52
cct	P	0	0.27
ccc	P	1	0.18
cca	P	0	0.41
ccg	P	0	0.14
cat	H	197	2421.00
cac	H	3985	1761.00
---	--	------	------
mPD		0.091	0.49
		nPD:	0.19
		N. weight:	0.92
		Sc. PD:	-0.044
		Sc. rank:	332.7

,

HA

Pos.	122	obs:	exp:
tta	L	63	358.80
ttg	L	5	737.30
ctt	L	0	770.60
ctc	L	1	620.80
cta	L	3200	574.40
ctg	L	612	819.10
cct	P	0	0.27
ccc	P	0	0.18
cca	P	1	0.41
ccg	P	0	0.14
atg	M	301	301.00
gtt	V	0	0.22
gtc	V	0	0.21
gta	V	1	0.21
gtg	V	0	0.36
---	--	------	------
mPD		0.51	1.2
		nPD:	0.43
		N. weight:	1.7
		Sc. PD:	0.32
		Sc. rank:	2620.8

,

HA

Pos.	123	obs:	exp:
tta	L	62	383.20
ttg	L	4035	787.40
tct	S	0	0.33
tcc	S	0	0.29
tca	S	0	0.45
tcg	S	2	0.12
ctt	L	0	823.00
ctc	L	0	663.00
cta	L	0	613.40
ctg	L	48	874.90
atg	M	37	37.00
agt	S	0	0.43
agc	S	0	0.37
---	--	------	------
mPD		0.070	1.1
		nPD:	0.06
		N. weight:	2.1
		Sc. PD:	-0.35
		Sc. rank:	-2656.9

,

HA

Pos.	124	obs:	exp:
tct	S	0	697.20
tcc	S	0	605.10
tca	S	0	946.90
tcg	S	0	252.00
aat	N	0	2.18
aac	N	4	1.82
agt	S	123	906.00
agc	S	4054	769.80
ggt	G	1	0.29
ggc	G	1	0.26
gga	G	0	0.88
ggg	G	0	0.57
---	-	1	1.00
---	--	------	------
mPD		0.060	1.7
		nPD:	0.04
		N. weight:	2.2
		Sc. PD:	-0.41
		Sc. rank:	-4064.9

,

HA

Pos.	125	obs:	exp:
tct	S	0	10.68
tcc	S	0	9.27
tca	S	0	14.51
tcg	S	0	3.86
cgt	R	0	154.00
cgc	R	0	220.20
cga	R	19	414.60
cgg	R	2	335.90
att	I	0	0.37
atc	I	1	0.27
ata	I	0	0.37
act	T	0	0.52
acc	T	0	0.45
aca	T	2	0.84
acg	T	0	0.19
aat	N	0	0.55
aac	N	1	0.45
aaa	K	1	0.57
aag	K	0	0.43
agt	S	4	13.88
agc	S	60	11.79
aga	R	2384	1921.00
agg	R	1709	1069.00
---	-	1	1.00
---	--	------	------
mPD		0.51	0.99
		nPD:	0.52
		N. weight:	0.45
		Sc. PD:	0.12
		Sc. rank:	837.1

,

HA			
Pos.	126	obs:	exp:
att	I	8	1508.00
atc	I	13	1100.00
ata	I	4089	1502.00
act	T	0	18.87
acc	T	0	16.22
aca	T	72	30.13
acg	T	0	6.77
gtt	V	0	0.22
gtc	V	0	0.21
gta	V	1	0.21
gtg	V	0	0.36
---	-	1	1.00
---	--	------	------
mPD		0.044	0.70
		nPD:	0.06
		N. weight:	1.3
		Sc. PD:	-0.22
		Sc. rank:	-1660.1

,

HA			
Pos.	127	obs:	exp:
act	T	0	1.05
acc	T	0	0.90
aca	T	4	1.67
acg	T	0	0.38
aat	N	938	2272.00
aac	N	3229	1895.00
aaa	K	11	6.83
aag	K	1	5.17
gat	D	0	0.54
gac	D	1	0.46
---	--	------	------
mPD		0.36	0.50
		nPD:	0.71
		N. weight:	0.3
		Sc. PD:	0.14
		Sc. rank:	718.7

,

HA			
Pos.	128	obs:	exp:
cat	H	3977	2362.00
cac	H	103	1718.00
cgt	R	55	3.89
cgc	R	0	5.57
cga	R	0	10.48
cgg	R	49	8.49
aga	R	0	48.55
agg	R	0	27.02
---	--	------	------
mPD		0.12	0.59
		nPD:	0.2
		N. weight:	0.67
		Sc. PD:	-0.023
		Sc. rank:	290.2

,

HA			
Pos.	129	obs:	exp:
ttt	F	4154	1739.00
ttc	F	15	2430.00
tta	L	0	0.92
ttg	L	2	1.90
tat	Y	3	1.44
tac	Y	0	1.56
tgt	C	1	0.39
tgc	C	0	0.61
ctt	L	8	1.99
ctc	L	0	1.60
cta	L	0	1.48
ctg	L	0	2.11
cct	P	0	0.27
ccc	P	1	0.18
cca	P	0	0.41
ccg	P	0	0.14
---	--	------	------
mPD		0.015	0.49
		nPD:	0.03
		N. weight:	1.2
		Sc. PD:	-0.23
		Sc. rank:	-2348.5

,

HA			
Pos.	130	obs:	exp:
aaa	K	0	5.12
aag	K	9	3.88
gtt	V	0	0.22
gtc	V	0	0.21
gta	V	0	0.21
gtg	V	1	0.36
gat	D	2	1.08
gac	D	0	0.92
gaa	E	55	2338.00
gag	E	4116	1833.00
ggt	G	0	0.14
ggc	G	0	0.13
gga	G	0	0.44
ggg	G	1	0.29
---	--	------	------
mPD		0.032	0.50
		nPD:	0.06
		N. weight:	1.
		Sc. PD:	-0.17
		Sc. rank:	-1272.0

,

HA			
Pos.	131	obs:	exp:
cgt	R	0	0.04
cgc	R	0	0.05
cga	R	0	0.10
cgg	R	0	0.08
aaa	K	3290	2382.00
aag	K	893	1801.00
aga	R	1	0.47
agg	R	0	0.26
---	--	------	------
mPD		0.34	0.49
		nPD:	0.68
		N. weight:	0.15
		Sc. PD:	0.063
		Sc. rank:	340.4

,

HA			
Pos.	132	obs:	exp:
tta	L	2	0.18
ttg	L	0	0.38
ctt	L	0	0.40
ctc	L	0	0.32
cta	L	0	0.30
ctg	L	0	0.42
att	I	2472	1520.00
atc	I	15	1108.00
ata	I	1655	1514.00
act	T	25	7.86
acc	T	0	6.76
aca	T	5	12.55
acg	T	0	2.82
aaa	K	1	0.57
aag	K	0	0.43
gtt	V	5	1.94
gtc	V	0	1.89
gta	V	4	1.92
gtg	V	0	3.25
---	--	------	------
mPD		0.50	0.68
		nPD:	0.74
		N. weight:	0.44
		Sc. PD:	0.21
		Sc. rank:	1080.5

,

HA			
Pos.	133	obs:	exp:
tta	L	0	82.29
ttg	L	0	169.10
ctt	L	76	176.70
ctc	L	0	142.40
cta	L	4	131.70
ctg	L	810	187.80
cat	H	3	2.89
cac	H	2	2.11
caa	Q	204	1379.00
cag	Q	2559	1384.00
cgt	R	18	16.99
cgc	R	0	24.30
cga	R	24	45.75
cgg	R	412	37.07
atg	M	3	3.00
aaa	K	5	31.32
aag	K	50	23.68
aga	R	0	211.90
agg	R	0	117.90
gaa	E	0	7.29
gag	E	13	5.71
ggt	G	0	0.14
ggc	G	0	0.13
gga	G	0	0.44
ggg	G	1	0.29
---	--	------	------
mPD		0.67	1.3
		nPD:	0.5
		N. weight:	1.1
		Sc. PD:	0.28
		Sc. rank:	1984.8

,

HA			
Pos.	134	obs:	exp:
ttt	F	0	0.42
ttc	F	1	0.58
att	I	35	1527.00
atc	I	4064	1113.00
ata	I	61	1520.00
act	T	1	0.52
acc	T	1	0.45
aca	T	0	0.84
acg	T	0	0.19
gtt	V	0	4.52
gtc	V	21	4.42
gta	V	0	4.48
gtg	V	0	7.59
---	--	------	------
mPD		0.057	0.67
		nPD:	0.08
		N. weight:	1.6
		Sc. PD:	-0.24
		Sc. rank:	-1221.9

,

HA			
Pos.	135	obs:	exp:
ttt	F	0	19.61
ttc	F	47	27.39
tta	L	0	0.37
ttg	L	0	0.76
ctt	L	0	0.79
ctc	L	4	0.64
cta	L	0	0.59
ctg	L	0	0.84
att	I	539	1515.00
atc	I	3585	1105.00
ata	I	4	1509.00
act	T	0	0.79
acc	T	3	0.68
aca	T	0	1.26
acg	T	0	0.28
gtt	V	0	0.22
gtc	V	1	0.21
gta	V	0	0.21
gtg	V	0	0.36
gat	D	1	0.54
gac	D	0	0.46
---	--	------	------
mPD		0.25	0.69
		nPD:	0.37
		N. weight:	1.2
		Sc. PD:	0.16
		Sc. rank:	1553.9

,

HA			
Pos.	136	obs:	exp:
tta	L	0	0.09
ttg	L	0	0.19
tct	S	0	2.84
tcc	S	17	2.46
tca	S	0	3.85
tcg	S	0	1.03
ctt	L	0	0.20
ctc	L	1	0.16
cta	L	0	0.15
ctg	L	0	0.21
cct	P	47	1138.00
ccc	P	4103	751.90
cca	P	13	1690.00
ccg	P	0	583.70
cat	H	1	0.58
cac	H	0	0.42
cgt	R	0	0.07
cgc	R	2	0.11
cga	R	0	0.20
cgg	R	0	0.16
agt	S	0	3.69
agc	S	0	3.13
aga	R	0	0.93
agg	R	0	0.52
---	--	------	------
mPD		0.039	0.72
		nPD:	0.05
		N. weight:	2.2
		Sc. PD:	-0.39
		Sc. rank:	-3304.2

,

HA			
Pos.	137	obs:	exp:
caa	Q	2	1.00
cag	Q	0	1.00
cgt	R	0	4.57
cgc	R	0	6.53
cga	R	0	12.29
cgg	R	0	9.96
att	I	0	0.73
atc	I	0	0.54
ata	I	2	0.73
aat	N	2	1.64
aac	N	1	1.36
aaa	K	2991	2309.00
aag	K	1064	1746.00
aga	R	73	56.95
agg	R	49	31.69
---	--	------	------
mPD		0.45	0.57
		nPD:	0.79
		N. weight:	0.099
		Sc. PD:	0.053
		Sc. rank:	249.4

,

HA			
Pos.	138	obs:	exp:
tct	S	0	357.70
tcc	S	0	310.40
tca	S	0	485.80
tcg	S	0	129.30
tat	Y	0	0.48
tac	Y	1	0.52
att	I	2	0.73
atc	I	0	0.54
ata	I	0	0.73
act	T	1	0.26
acc	T	0	0.23
aca	T	0	0.42
acg	T	0	0.09
aat	N	210	135.80
aac	N	39	113.20
aaa	K	1	0.57
aag	K	0	0.43
agt	S	2116	464.80
agc	S	27	394.90
gat	D	1558	948.10
gac	D	198	807.90
gaa	E	10	12.89
gag	E	13	10.11
ggt	G	5	1.16
ggc	G	0	1.05
gga	G	0	3.51
ggg	G	3	2.29
---	--	------	------
mPD		1.1	1.9
		nPD:	0.59
		N. weight:	1.2
		Sc. PD:	0.42
		Sc. rank:	2578.4

,

HA			
Pos.	139	obs:	exp:
ttt	F	18	7.51
ttc	F	0	10.49
tct	S	3242	687.40
tcc	S	870	596.50
tca	S	5	933.60
tcg	S	1	248.40
tat	Y	24	11.51
tac	Y	0	12.49
cct	P	1	0.27
ccc	P	0	0.18
cca	P	0	0.41
ccg	P	0	0.14
cat	H	16	9.26
cac	H	0	6.74
act	T	2	0.52
acc	T	0	0.45
aca	T	0	0.84
acg	T	0	0.19
agt	S	0	893.20
agc	S	0	758.90
gct	A	1	0.25
gcc	A	0	0.19
gca	A	0	0.45
gcg	A	0	0.11
gat	D	4	2.16
gac	D	0	1.84
---	--	------	------
mPD		0.37	1.7
		nPD:	0.22
		N. weight:	1.8
		Sc. PD:	-0.021
		Sc. rank:	913.6

,

HA			
Pos.	140	obs:	exp:
tgg	W	4184	4184.00
---	--	------	------
mPD		0	0
		nPD:	1.
		N. weight:	0.
		Sc. PD:	0
		Sc. rank:	0

,

HA			
Pos.	141	obs:	exp:
ttt	F	1	0.42
ttc	F	0	0.58
tta	L	6	0.55
ttg	L	0	1.14
tct	S	15	478.70
tcc	S	713	415.40
tca	S	2111	650.20
tcg	S	29	173.00
ctt	L	0	1.19
ctc	L	0	0.96
cta	L	0	0.89
ctg	L	0	1.27
cct	P	9	348.20
ccc	P	230	230.10
cca	P	1031	517.10
ccg	P	4	178.60
act	T	0	4.46
acc	T	0	3.83
aca	T	17	7.11
acg	T	0	1.60
agt	S	0	622.10
agc	S	0	528.50
gtt	V	0	0.65
gtc	V	3	0.63
gta	V	0	0.64
gtg	V	0	1.08
gct	A	0	3.78
gcc	A	14	2.90
gca	A	1	6.68
gcg	A	0	1.64
---	--	------	------
mPD		0.81	1.8
		nPD:	0.46
		N. weight:	1.2
		Sc. PD:	0.25
		Sc. rank:	2001.7

,

HA			
Pos.	142	obs:	exp:
tct	S	0	37.39
tcc	S	0	32.45
tca	S	0	50.78
tcg	S	0	13.51
tat	Y	4	1.92
tac	Y	0	2.08
act	T	0	0.26
acc	T	1	0.23
aca	T	0	0.42
acg	T	0	0.09
aat	N	1054	592.80
aac	N	33	494.20
agt	S	212	48.59
agc	S	12	41.28
gat	D	2709	1503.00
gac	D	75	1281.00
gaa	E	70	40.36
gag	E	2	31.64
ggt	G	11	1.74
ggc	G	1	1.58
gga	G	0	5.26
ggg	G	0	3.43
---	--	------	------
mPD		0.63	1.1
		nPD:	0.58
		N. weight:	0.75
		Sc. PD:	0.25
		Sc. rank:	1535.0

,

HA			
Pos.	143	obs:	exp:
tat	Y	1	0.48
tac	Y	0	0.52
cat	H	4159	2421.00
cac	H	23	1761.00
cgt	R	1	0.04
cgc	R	0	0.05
cga	R	0	0.10
cgg	R	0	0.08
aga	R	0	0.47
agg	R	0	0.26
---	--	------	------
mPD		0.012	0.49
		nPD:	0.02
		N. weight:	0.71
		Sc. PD:	-0.15
		Sc. rank:	-1508.5

,

HA			
Pos.	144	obs:	exp:
aat	N	16	8.73
aac	N	0	7.27
gat	D	72	41.03
gac	D	4	34.97
gaa	E	4071	2294.00
gag	E	21	1798.00
---	--	------	------
mPD		0.060	0.52
		nPD:	0.12
		N. weight:	0.76
		Sc. PD:	-0.087
		Sc. rank:	-246.8

,

HA			
Pos.	145	obs:	exp:
tct	S	0	3.51
tcc	S	21	3.04
tca	S	0	4.76
tcg	S	0	1.27
act	T	1	248.00
acc	T	54	213.20
aca	T	891	395.80
acg	T	0	89.01
agt	S	0	4.55
agc	S	0	3.87
gtt	V	0	2.58
gtc	V	12	2.52
gta	V	0	2.56
gtg	V	0	4.34
gct	A	17	807.40
gcc	A	3147	620.20
gca	A	41	1427.00
gcg	A	0	350.80
---	--	------	------
mPD		0.72	1.1
		nPD:	0.68
		N. weight:	1.9
		Sc. PD:	0.79
		Sc. rank:	4294.4

,

HA			
Pos.	146	obs:	exp:
tct	S	0	0.17
tcc	S	1	0.14
tca	S	0	0.23
tcg	S	0	0.06
cct	P	0	0.27
ccc	P	1	0.18
cca	P	0	0.41
ccg	P	0	0.14
agt	S	0	0.22
agc	S	0	0.18
---	-	4182	4182.00
---	--	------	------
mPD		1.1×10^{-7}	3.8×10^{-7}
		nPD:	0.3
		N. weight:	0.
		Sc. PD:	0
		Sc. rank:	0

,

HA			
Pos.	147	obs:	exp:
ttt	F	0	13.77
ttc	F	33	19.23
tct	S	8	692.40
tcc	S	9	600.90
tca	S	4075	940.40
tcg	S	56	250.30
cct	P	0	0.27
ccc	P	0	0.18
cca	P	1	0.41
ccg	P	0	0.14
act	T	0	0.52
acc	T	0	0.45
aca	T	2	0.84
acg	T	0	0.19
agt	S	0	899.70
agc	S	0	764.40
---	--	------	------
mPD		0.067	1.7
		nPD:	0.04
		N. weight:	1.9
		Sc. PD:	-0.36
		Sc. rank:	-3451.0

,

HA			
Pos.	148	obs:	exp:
tta	L	260	272.90
ttg	L	1623	560.80
tct	S	0	142.60
tcc	S	0	123.70
tca	S	852	193.60
tcg	S	2	51.52
ctt	L	3	586.10
ctc	L	0	472.20
cta	L	861	436.90
ctg	L	205	623.10
cct	P	1	0.27
ccc	P	0	0.18
cca	P	0	0.41
ccg	P	0	0.14
cgt	R	0	0.11
cgc	R	0	0.16
cga	R	3	0.30
cgg	R	0	0.24
att	I	0	0.37
atc	I	0	0.27
ata	I	1	0.37
atg	M	59	59.00
aaa	K	0	1.14
aag	K	2	0.86
agt	S	0	185.20
agc	S	0	157.40
aga	R	0	1.40
agg	R	0	0.78
gct	A	0	0.25
gcc	A	0	0.19
gca	A	1	0.45
gcg	A	0	0.11
ggt	G	0	0.29
ggc	G	0	0.26
gga	G	2	0.88
ggg	G	0	0.57
---	-	309	309.00
---	--	------	------
mPD		1.1	1.4
		nPD:	0.76
		N. weight:	1.1
		Sc. PD:	0.56
		Sc. rank:	2735.5

,

HA			
Pos.	149	obs:	exp:
tgg	W	1	1.00
cgt	R	0	0.11
cgc	R	0	0.16
cga	R	3	0.30
cgg	R	0	0.24
aga	R	0	1.40
agg	R	0	0.78
ggt	G	4	604.50
ggc	G	0	549.10
gga	G	779	1832.00
ggg	G	3397	1195.00
---	--	------	------
mPD		0.31	0.69
		nPD:	0.45
		N. weight:	0.95
		Sc. PD:	0.19
		Sc. rank:	1526.0

,

HA			
Pos.	150	obs:	exp:
atg	M	2	2.00
gtt	V	9	899.30
gtc	V	0	879.60
gta	V	49	891.50
gtg	V	4123	1511.00
gct	A	0	0.25
gcc	A	0	0.19
gca	A	0	0.45
gcg	A	1	0.11
---	--	------	------
mPD		0.029	0.73
		nPD:	0.04
		N. weight:	1.3
		Sc. PD:	-0.25
		Sc. rank:	-2388.9

,

HA			
Pos.	151	obs:	exp:
tct	S	0	698.10
tcc	S	0	605.80
tca	S	0	948.10
tcg	S	0	252.30
cgt	R	0	0.04
cgc	R	0	0.05
cga	R	0	0.10
cgg	R	0	0.08
agt	S	608	907.10
agc	S	3574	770.70
aga	R	0	0.47
agg	R	1	0.26
ggt	G	1	0.14
ggc	G	0	0.13
gga	G	0	0.44
ggg	G	0	0.29
---	--	------	------
mPD		0.25	1.7
		nPD:	0.15
		N. weight:	1.7
		Sc. PD:	-0.14
		Sc. rank:	132.4

,

HA			
Pos.	152	obs:	exp:
tct	S	25	232.90
tcc	S	5	202.10
tca	S	1333	316.20
tcg	S	32	84.16
agt	S	0	302.60
agc	S	0	257.10
gtt	V	0	0.22
gtc	V	0	0.21
gta	V	1	0.21
gtg	V	0	0.36
gct	A	5	702.30
gcc	A	65	539.50
gca	A	2697	1241.00
gcg	A	21	305.20
---	--	------	------
mPD		0.52	1.4
		nPD:	0.36
		N. weight:	1.2
		Sc. PD:	0.15
		Sc. rank:	1509.6

,

HA			
Pos.	153	obs:	exp:
tct	S	1	0.50
tcc	S	0	0.43
tca	S	2	0.68
tcg	S	0	0.18
cct	P	0	0.27
ccc	P	0	0.18
cca	P	1	0.41
ccg	P	0	0.14
act	T	1	0.79
acc	T	0	0.68
aca	T	2	1.26
acg	T	0	0.28
agt	S	0	0.65
agc	S	0	0.55
gtt	V	0	0.43
gtc	V	0	0.42
gta	V	2	0.43
gtg	V	0	0.72
gct	A	881	1052.00
gcc	A	3	807.90
gca	A	3237	1858.00
gcg	A	54	457.00
---	--	------	------
mPD		0.36	0.69
		nPD:	0.52
		N. weight:	0.51
		Sc. PD:	0.14
		Sc. rank:	936.0

,

HA			
Pos.	154	obs:	exp:
tgt	C	3888	1619.00
tgc	C	296	2565.00
---	--	------	------
mPD		0.13	0.47
		nPD:	0.28
		N. weight:	0.92
		Sc. PD:	0.037
		Sc. rank:	807.9

,

HA			
Pos.	155	obs:	exp:
tta	L	3	1.76
ttg	L	0	3.61
tct	S	3	193.30
tcc	S	32	167.70
tca	S	1121	262.50
tcg	S	2	69.86
ctt	L	0	3.77
ctc	L	0	3.04
cta	L	15	2.81
ctg	L	1	4.01
cct	P	61	814.70
ccc	P	11	538.40
cca	P	2861	1210.00
ccg	P	48	417.90
cat	H	1	0.58
cac	H	0	0.42
caa	Q	21	10.48
cag	Q	0	10.52
act	T	0	1.05
acc	T	0	0.90
aca	T	4	1.67
acg	T	0	0.38
agt	S	0	251.20
agc	S	0	213.40
---	--	------	------
mPD		0.50	1.4
		nPD:	0.36
		N. weight:	1.2
		Sc. PD:	0.15
		Sc. rank:	1551.6

,

HA			
Pos.	156	obs:	exp:
tat	Y	3	1.44
tac	Y	0	1.56
---	-	4181	4181.00
---	--	------	------
mPD		0	2.6×10^{-7}
		nPD:	0.
		N. weight:	0.
		Sc. PD:	0
		Sc. rank:	0

,

HA			
Pos.	157	obs:	exp:
ttt	F	0	0.42
ttc	F	1	0.58
tat	Y	428	1989.00
tac	Y	3720	2159.00
tgt	C	1	0.39
tgc	C	0	0.61
cat	H	5	18.52
cac	H	27	13.48
gat	D	0	1.08
gac	D	2	0.92
---	--	------	------
mPD		0.20	0.52
		nPD:	0.39
		N. weight:	0.46
		Sc. PD:	0.07
		Sc. rank:	632.6

,

HA			
Pos.	158	obs:	exp:
tta	L	0	29.59
ttg	L	4	60.79
tct	S	0	0.17
tcc	S	0	0.14
tca	S	0	0.23
tcg	S	0	0.06
ctt	L	1	63.54
ctc	L	0	51.19
cta	L	3	47.36
ctg	L	312	67.54
cat	H	24	13.89
cac	H	0	10.11
caa	Q	174	1884.00
cag	Q	3600	1890.00
att	I	8	2.94
atc	I	0	2.14
ata	I	0	2.92
atg	M	1	1.00
act	T	2	0.52
acc	T	0	0.45
aca	T	0	0.84
acg	T	0	0.19
aat	N	54	29.45
aac	N	0	24.55
agt	S	1	0.22
agc	S	0	0.18
---	--	------	------
mPD		0.30	0.76
		nPD:	0.39
		N. weight:	0.8
		Sc. PD:	0.12
		Sc. rank:	1102.2

,

HA			
Pos.	159	obs:	exp:
cgt	R	0	0.30
cgc	R	0	0.43
cga	R	0	0.81
cgg	R	0	0.65
aga	R	8	3.73
agg	R	0	2.08
gat	D	1	0.54
gac	D	0	0.46
gaa	E	2	12.33
gag	E	20	9.67
ggt	G	28	600.60
ggc	G	12	545.50
gga	G	3982	1820.00
ggg	G	131	1187.00
---	--	------	------
mPD		0.10	0.70
		nPD:	0.15
		N. weight:	0.9
		Sc. PD:	-0.077
		Sc. rank:	14.1

,

HA			
Pos.	160	obs:	exp:
tct	S	0	35.39
tcc	S	0	30.71
tca	S	0	48.06
tcg	S	0	12.79
caa	Q	1	36.95
cag	Q	73	37.05
cgt	R	0	24.29
cgc	R	0	34.73
cga	R	0	65.40
cgg	R	0	52.99
att	I	0	1.10
atc	I	0	0.80
ata	I	3	1.10
atg	M	59	59.00
act	T	2	46.92
acc	T	0	40.33
aca	T	50	74.90
acg	T	127	16.84
aat	N	1633	893.80
aac	N	6	745.20
aaa	K	18	148.00
aag	K	242	112.00
agt	S	212	45.98
agc	S	0	39.07
aga	R	378	303.00
agg	R	271	168.60
gtt	V	0	30.33
gtc	V	0	29.66
gta	V	0	30.06
gtg	V	141	50.94
gct	A	5	217.90
gcc	A	0	167.40
gca	A	11	385.00
gcg	A	849	94.68
gaa	E	4	6.17
gag	E	7	4.83
ggt	G	0	13.30
ggc	G	0	12.08
gga	G	91	40.31
ggg	G	1	26.30
---	--	------	------
mPD		1.7	1.9
		nPD:	0.88
		N. weight:	1.3
		Sc. PD:	0.82
		Sc. rank:	3388.3

,

HA

Pos.	161	obs:	exp:
tta	L	0	0.37
ttg	L	0	0.76
tct	S	41	426.50
tcc	S	2509	370.10
tca	S	5	579.20
tcg	S	0	154.10
ctt	L	0	0.79
ctc	L	4	0.64
cta	L	0	0.59
ctg	L	0	0.84
cct	P	18	442.20
ccc	P	1599	292.20
cca	P	1	656.70
ccg	P	0	226.80
cat	H	1	0.58
cac	H	0	0.42
act	T	0	1.31
acc	T	5	1.13
aca	T	0	2.09
acg	T	0	0.47
agt	S	0	554.20
agc	S	0	470.90
gct	A	0	0.25
gcc	A	1	0.19
gca	A	0	0.45
gcg	A	0	0.11
---	--	------	------
mPD		0.51	1.7
		nPD:	0.29
		N. weight:	2.4
		Sc. PD:	0.14
		Sc. rank:	2279.7

,

HA

Pos.	162	obs:	exp:
tct	S	320	698.10
tcc	S	3795	605.80
tca	S	66	948.10
tcg	S	1	252.30
tat	Y	0	0.48
tac	Y	1	0.52
cct	P	0	0.27
ccc	P	1	0.18
cca	P	0	0.41
ccg	P	0	0.14
agt	S	0	907.10
agc	S	0	770.70
---	--	------	------
mPD		0.17	1.7
		nPD:	0.1
		N. weight:	2.2
		Sc. PD:	-0.28
		Sc. rank:	-1135.0

,

HA

Pos.	163	obs:	exp:
ttt	F	2601	1745.00
ttc	F	1581	2437.00
tta	L	1	0.09
ttg	L	0	0.19
tct	S	1	0.17
tcc	S	0	0.14
tca	S	0	0.23
tcg	S	0	0.06
ctt	L	0	0.20
ctc	L	0	0.16
cta	L	0	0.15
ctg	L	0	0.21
agt	S	0	0.22
agc	S	0	0.18
---	--	------	------
mPD		0.47	0.49
		nPD:	0.97
		N. weight:	0.12
		Sc. PD:	0.085
		Sc. rank:	313.1

,

HA

Pos.	164	obs:	exp:
ttt	F	940	1702.00
ttc	F	3139	2377.00
tta	L	1	0.18
ttg	L	0	0.38
tat	Y	101	48.92
tac	Y	1	53.08
tgt	C	1	0.39
tgc	C	0	0.61
ctt	L	0	0.40
ctc	L	1	0.32
cta	L	0	0.30
ctg	L	0	0.42
---	--	------	------
mPD		0.42	0.54
		nPD:	0.79
		N. weight:	0.13
		Sc. PD:	0.07
		Sc. rank:	329.4

,

HA

Pos.	165	obs:	exp:
cgt	R	0	153.30
cgc	R	0	219.30
cga	R	143	412.90
cgg	R	0	334.50
aaa	K	9	5.12
aag	K	0	3.88
aga	R	3849	1913.00
agg	R	105	1064.00
ggt	G	0	11.28
ggc	G	0	10.25
gga	G	78	34.18
ggg	G	0	22.30
---	--	------	------
mPD		0.15	0.98
		nPD:	0.16
		N. weight:	0.79
		Sc. PD:	-0.059
		Sc. rank:	101.3

,

HA

Pos.	166	obs:	exp:
tct	S	0	0.50
tcc	S	0	0.43
tca	S	0	0.68
tcg	S	0	0.18
att	I	1	0.37
atc	I	0	0.27
ata	I	0	0.37
act	T	2	0.52
acc	T	0	0.45
aca	T	0	0.84
acg	T	0	0.19
aat	N	4093	2277.00
aac	N	82	1898.00
agt	S	3	0.65
agc	S	0	0.55
gat	D	3	1.62
gac	D	0	1.38
---	--	------	------
mPD		0.043	0.50
		nPD:	0.09
		N. weight:	0.71
		Sc. PD:	-0.1
		Sc. rank:	-524.9

,

HA

Pos.	167	obs:	exp:
tta	L	0	0.46
ttg	L	5	0.95
ctt	L	0	0.99
ctc	L	0	0.80
cta	L	0	0.74
ctg	L	0	1.06
atg	M	1	1.00
gtt	V	111	898.60
gtc	V	0	879.00
gta	V	160	890.90
gtg	V	3907	1510.00
---	--	------	------
mPD		0.13	0.74
		nPD:	0.17
		N. weight:	1.1
		Sc. PD:	-0.066
		Sc. rank:	327.7

,

HA

Pos.	168	obs:	exp:
tta	L	0	4.53
ttg	L	49	9.31
ctt	L	0	9.73
ctc	L	0	7.84
cta	L	0	7.25
ctg	L	0	10.34
att	I	0	5.87
atc	I	0	4.28
ata	I	16	5.85
atg	M	1	1.00
gtt	V	9	885.50
gtc	V	0	866.20
gta	V	2288	877.80
gtg	V	1820	1487.00
gct	A	0	0.25
gcc	A	0	0.19
gca	A	1	0.45
gcg	A	0	0.11
---	--	------	------
mPD		0.53	0.77
		nPD:	0.69
		N. weight:	0.87
		Sc. PD:	0.38
		Sc. rank:	2033.5

,

HA

Pos.	169	obs:	exp:
tgg	W	4184	4184.00
---	--	------	------
mPD		0	0
		nPD:	1.
		N. weight:	0.
		Sc. PD:	0
		Sc. rank:	0

,

HA

Pos.	170	obs:	exp:
tta	L	0	385.20
ttg	L	37	791.40
ctt	L	4055	827.20
ctc	L	66	666.40
cta	L	6	616.50
ctg	L	2	879.30
att	I	18	6.61
atc	I	0	4.82
ata	I	0	6.58
---	--	------	------
mPD		0.078	1.1
		nPD:	0.07
		N. weight:	2.
		Sc. PD:	-0.33
		Sc. rank:	-2337.4

,

HA

Pos.	171	obs:	exp:
tta	L	0	5.36
ttg	L	0	11.02
ctt	L	0	11.52
ctc	L	58	9.28
cta	L	0	8.58
ctg	L	0	12.24
att	I	24	1374.00
atc	I	3709	1002.00
ata	I	12	1369.00
act	T	1	85.72
acc	T	326	73.68
aca	T	0	136.80
acg	T	0	30.77
gtt	V	0	11.61
gtc	V	54	11.36
gta	V	0	11.51
gtg	V	0	19.51
---	--	------	------
mPD		0.21	0.87
		nPD:	0.25
		N. weight:	1.8
		Sc. PD:	0.019
		Sc. rank:	1231.4

,

HA

Pos.	172	obs:	exp:
cgt	R	0	0.04
cgc	R	0	0.05
cga	R	0	0.10
cgg	R	0	0.08
aga	R	1	0.47
agg	R	0	0.26
---	-	4183	4183.00
---	--	------	------
mPD		0	5.5×10^{-8}
		nPD:	0.
		N. weight:	0.
		Sc. PD:	0
		Sc. rank:	0

,

HA

Pos.	173	obs:	exp:
caa	Q	2	1.00
cag	Q	0	1.00
att	I	0	0.37
atc	I	0	0.27
ata	I	1	0.37
act	T	0	1.31
acc	T	0	1.13
aca	T	5	2.09
acg	T	0	0.47
aat	N	0	1.09
aac	N	2	0.91
aaa	K	3968	2374.00
aag	K	202	1796.00
gaa	E	3	2.24
gag	E	1	1.76
---	--	------	------
mPD		0.099	0.50
		nPD:	0.2
		N. weight:	0.53
		Sc. PD:	-0.019
		Sc. rank:	228.0

,

HA

Pos.	174	obs:	exp:
cgt	R	0	0.41
cgc	R	0	0.59
cga	R	0	1.11
cgg	R	0	0.90
aaa	K	34	2376.00
aag	K	4139	1797.00
aga	R	0	5.14
agg	R	11	2.86
---	--	------	------
mPD		0.021	0.50
		nPD:	0.04
		N. weight:	1.1
		Sc. PD:	-0.2
		Sc. rank:	-1886.0

,

HA

Pos.	175	obs:	exp:
tct	S	0	5.34
tcc	S	0	4.64
tca	S	0	7.25
tcg	S	0	1.93
aat	N	67	1515.00
aac	N	2711	1263.00
agt	S	0	6.94
agc	S	32	5.90
gat	D	3	704.60
gac	D	1302	600.40
ggt	G	0	9.98
ggc	G	69	9.06
gga	G	0	30.23
ggg	G	0	19.72
---	--	------	------
mPD		0.52	1.0
		nPD:	0.52
		N. weight:	1.
		Sc. PD:	0.28
		Sc. rank:	1894.1

,

HA

Pos.	176	obs:	exp:
tct	S	0	76.78
tcc	S	0	66.63
tca	S	0	104.30
tcg	S	0	27.75
cat	H	1	0.58
cac	H	0	0.42
act	T	1	0.26
acc	T	0	0.23
aca	T	0	0.42
acg	T	0	0.09
aat	N	2337	1284.00
aac	N	17	1070.00
agt	S	450	99.78
agc	S	10	84.77
gct	A	13	3.27
gcc	A	0	2.52
gca	A	0	5.79
gcg	A	0	1.42
gat	D	1345	726.20
gac	D	0	618.80
ggt	G	10	1.45
ggc	G	0	1.31
gga	G	0	4.38
ggg	G	0	2.86
---	--	------	------
mPD		0.66	1.3
		nPD:	0.52
		N. weight:	0.95
		Sc. PD:	0.26
		Sc. rank:	1786.9

,

HA

Pos.	177	obs:	exp:
tct	S	0	3.17
tcc	S	0	2.75
tca	S	19	4.31
tcg	S	0	1.15
att	I	0	0.73
atc	I	0	0.54
ata	I	2	0.73
atg	M	1	1.00
act	T	2	295.20
acc	T	2	253.70
aca	T	1103	471.20
acg	T	19	105.90
aaa	K	5	2.85
aag	K	0	2.15
agt	S	0	4.12
agc	S	0	3.50
gtt	V	0	0.86
gtc	V	0	0.84
gta	V	4	0.85
gtg	V	0	1.45
gct	A	1	762.30
gcc	A	5	585.60
gca	A	2972	1347.00
gcg	A	48	331.20
gaa	E	1	0.56
gag	E	0	0.44
---	--	------	------
mPD		0.44	1.1
		nPD:	0.4
		N. weight:	1.
		Sc. PD:	0.17
		Sc. rank:	1467.0

,

HA

Pos.	178	obs:	exp:
tat	Y	217	2007.00
tac	Y	3967	2177.00
---	--	------	------
mPD		0.098	0.50
		nPD:	0.2
		N. weight:	0.63
		Sc. PD:	-0.023
		Sc. rank:	266.3

,

HA

Pos.	179	obs:	exp:
tta	L	0	0.09
ttg	L	0	0.19
tct	S	0	0.17
tcc	S	0	0.14
tca	S	1	0.23
tcg	S	0	0.06
ctt	L	0	0.20
ctc	L	0	0.16
cta	L	1	0.15
ctg	L	0	0.21
cct	P	7	1143.00
ccc	P	4	755.20
cca	P	4155	1697.00
ccg	P	15	586.20
act	T	0	0.26
acc	T	0	0.23
aca	T	1	0.42
acg	T	0	0.09
agt	S	0	0.22
agc	S	0	0.18
---	--	------	------
mPD		0.014	0.71
		nPD:	0.02
		N. weight:	1.2
		Sc. PD:	-0.25
		Sc. rank:	-2659.7

,

HA

Pos.	180	obs:	exp:
tta	L	0	0.28
ttg	L	0	0.57
tct	S	0	0.17
tcc	S	0	0.14
tca	S	1	0.23
tcg	S	0	0.06
ctt	L	0	0.60
ctc	L	0	0.48
cta	L	3	0.44
ctg	L	0	0.63
cct	P	0	7.93
ccc	P	0	5.24
cca	P	29	11.77
ccg	P	0	4.07
att	I	0	32.66
atc	I	0	23.82
ata	I	89	32.52
act	T	23	1064.00
acc	T	2	914.80
aca	T	4028	1699.00
acg	T	7	382.00
aaa	K	2	1.14
aag	K	0	0.86
agt	S	0	0.22
agc	S	0	0.18
---	--	------	------
mPD		0.075	0.76
		nPD:	0.1
		N. weight:	1.2
		Sc. PD:	-0.15
		Sc. rank:	-666.3

,

HA

Pos.	181	obs:	exp:
tta	L	0	0.28
ttg	L	0	0.57
ctt	L	0	0.60
ctc	L	0	0.48
cta	L	3	0.44
ctg	L	0	0.63
att	I	4	1533.00
atc	I	9	1118.00
ata	I	4164	1526.00
gtt	V	0	0.86
gtc	V	0	0.84
gta	V	4	0.85
gtg	V	0	1.45
---	--	------	------
mPD		0.0095	0.66
		nPD:	0.01
		N. weight:	1.4
		Sc. PD:	-0.29
		Sc. rank:	-3129.0

,

HA

Pos.	182	obs:	exp:
caa	Q	0	1.50
cag	Q	3	1.50
cgt	R	0	0.49
cgc	R	0	0.70
cga	R	0	1.31
cgg	R	0	1.06
act	T	0	1.84
acc	T	0	1.58
aca	T	0	2.93
acg	T	7	0.66
aat	N	3	1.64
aac	N	0	1.36
aaa	K	112	2356.00
aag	K	4026	1782.00
aga	R	0	6.07
agg	R	13	3.38
gaa	E	1	11.21
gag	E	19	8.79
---	--	------	------
mPD		0.074	0.51
		nPD:	0.14
		N. weight:	0.99
		Sc. PD:	-0.087
		Sc. rank:	-14.9

,

HA

Pos.	183	obs:	exp:
tct	S	0	5.17
tcc	S	0	4.49
tca	S	0	7.03
tcg	S	0	1.87
cgt	R	0	32.63
cgc	R	0	46.67
cga	R	0	87.87
cgg	R	0	71.20
att	I	1	354.90
atc	I	0	258.80
ata	I	966	353.40
atg	M	24	24.00
act	T	0	0.26
acc	T	0	0.23
aca	T	1	0.42
acg	T	0	0.09
aaa	K	2178	1261.00
aag	K	36	953.30
agt	S	31	6.72
agc	S	0	5.71
aga	R	543	407.10
agg	R	329	226.50
gtt	V	0	6.02
gtc	V	0	5.89
gta	V	1	5.97
gtg	V	27	10.12
gct	A	0	0.25
gcc	A	0	0.19
gca	A	1	0.45
gcg	A	0	0.11
gaa	E	44	24.67
gag	E	0	19.33
ggt	G	0	0.29
ggc	G	0	0.26
gga	G	2	0.88
ggg	G	0	0.57
---	--	------	------
mPD		0.83	1.4
		nPD:	0.6
		N. weight:	0.8
		Sc. PD:	0.29
		Sc. rank:	1708.5

,

HA

Pos.	184	obs:	exp:
tct	S	0	492.30
tcc	S	0	427.20
tca	S	0	668.50
tcg	S	0	177.90
tat	Y	3	1.44
tac	Y	0	1.56
cgt	R	0	0.11
cgc	R	2	0.16
cga	R	0	0.30
cgg	R	0	0.24
att	I	0	1.47
atc	I	4	1.07
ata	I	0	1.46
act	T	2	15.73
acc	T	58	13.52
aca	T	0	25.11
acg	T	0	5.65
aat	N	7	39.81
aac	N	66	33.19
agt	S	877	639.60
agc	S	2072	543.50
aga	R	1	1.40
agg	R	0	0.78
gtt	V	0	0.65
gtc	V	3	0.63
gta	V	0	0.64
gtg	V	0	1.08
gat	D	3	84.77
gac	D	154	72.23
gaa	E	2	1.12
gag	E	0	0.88
ggt	G	10	134.50
ggc	G	919	122.20
gga	G	1	407.50
ggg	G	0	265.80
---	--	------	------
mPD		0.86	1.9
		nPD:	0.45
		N. weight:	1.7
		Sc. PD:	0.35
		Sc. rank:	2856.5

,

HA

Pos.	185	obs:	exp:
tat	Y	47	2007.00
tac	Y	4137	2177.00
---	--	------	------
mPD		0.022	0.50
		nPD:	0.04
		N. weight:	0.82
		Sc. PD:	-0.15
		Sc. rank:	-1390.1

,

HA

Pos.	186	obs:	exp:
tct	S	0	1.17
tcc	S	0	1.01
tca	S	0	1.59
tcg	S	0	0.42
tat	Y	1	0.48
tac	Y	0	0.52
cat	H	100	57.89
cac	H	0	42.11
act	T	1	8.65
acc	T	32	7.44
aca	T	0	13.81
acg	T	0	3.10
aat	N	3807	2179.00
aac	N	188	1816.00
aaa	K	16	26.19
aag	K	30	19.81
agt	S	7	1.52
agc	S	0	1.29
gat	D	2	1.08
gac	D	0	0.92
---	--	------	------
mPD		0.19	0.58
		nPD:	0.32
		N. weight:	0.6
		Sc. PD:	0.051
		Sc. rank:	659.6

,

HA

Pos.	187	obs:	exp:
act	T	1	0.26
acc	T	0	0.23
aca	T	0	0.42
acg	T	0	0.09
aat	N	3718	2281.00
aac	N	465	1902.00
---	--	------	------
mPD		0.20	0.50
		nPD:	0.4
		N. weight:	0.39
		Sc. PD:	0.061
		Sc. rank:	537.1

,

HA

Pos.	188	obs:	exp:
att	I	0	4.40
atc	I	12	3.21
ata	I	0	4.39
act	T	187	1086.00
acc	T	3909	933.80
aca	T	48	1734.00
acg	T	0	389.90
aat	N	0	0.55
aac	N	1	0.45
gct	A	0	6.80
gcc	A	27	5.22
gca	A	0	12.02
gcg	A	0	2.96
---	--	------	------
mPD		0.13	0.72
		nPD:	0.18
		N. weight:	1.7
		Sc. PD:	-0.098
		Sc. rank:	551.2

,

HA

Pos.	189	obs:	exp:
tct	S	0	4.01
tcc	S	0	3.48
tca	S	0	5.44
tcg	S	0	1.45
cat	H	0	1.74
cac	H	3	1.26
aat	N	1383	2241.00
aac	N	2726	1868.00
agt	S	0	5.21
agc	S	24	4.42
gat	D	0	18.90
gac	D	35	16.10
ggt	G	0	1.88
ggc	G	13	1.71
gga	G	0	5.70
ggg	G	0	3.72
---	--	------	------
mPD		0.48	0.55
		nPD:	0.88
		N. weight:	0.15
		Sc. PD:	0.096
		Sc. rank:	395.2

,

HA

Pos.	190	obs:	exp:
tta	L	0	0.09
ttg	L	0	0.19
ctt	L	0	0.20
ctc	L	0	0.16
cta	L	0	0.15
ctg	L	1	0.21
cct	P	0	0.82
ccc	P	0	0.54
cca	P	1	1.22
ccg	P	2	0.42
cat	H	2	1.74
cac	H	1	1.26
caa	Q	1922	1121.00
cag	Q	323	1124.00
cgt	R	0	67.62
cgc	R	0	96.70
cga	R	891	182.10
cgg	R	858	147.60
att	I	0	0.37
atc	I	0	0.27
ata	I	1	0.37
act	T	0	0.79
acc	T	0	0.68
aca	T	3	1.26
acg	T	0	0.28
aaa	K	85	48.40
aag	K	0	36.60
aga	R	39	843.60
agg	R	19	469.40
gtt	V	0	7.74
gtc	V	0	7.57
gta	V	36	7.68
gtg	V	0	13.01
---	--	------	------
mPD		1.0	1.5
		nPD:	0.67
		N. weight:	1.2
		Sc. PD:	0.48
		Sc. rank:	2614.2

,

HA

Pos.	191	obs:	exp:
aaa	K	17	10.25
aag	K	1	7.75
gtt	V	0	0.86
gtc	V	0	0.84
gta	V	4	0.85
gtg	V	0	1.45
gat	D	0	0.54
gac	D	1	0.46
gaa	E	4136	2331.00
gag	E	23	1828.00
ggt	G	0	0.29
ggc	G	0	0.26
gga	G	2	0.88
ggg	G	0	0.57
---	--	------	------
mPD		0.023	0.50
		nPD:	0.05
		N. weight:	0.76
		Sc. PD:	-0.14
		Sc. rank:	-1223.3

,

HA

Pos.	192	obs:	exp:
aat	N	5	2.73
aac	N	0	2.27
gtt	V	1	0.22
gtc	V	0	0.21
gta	V	0	0.21
gtg	V	0	0.36
gat	D	4099	2254.00
gac	D	76	1921.00
ggt	G	3	0.43
ggc	G	0	0.39
gga	G	0	1.31
ggg	G	0	0.86
---	--	------	------
mPD		0.040	0.50
		nPD:	0.08
		N. weight:	0.73
		Sc. PD:	-0.11
		Sc. rank:	-607.4

,

HA

Pos.	193	obs:	exp:
tta	L	0	386.10
ttg	L	0	793.30
ctt	L	1700	829.10
ctc	L	2414	668.00
cta	L	37	618.00
ctg	L	25	881.40
att	I	3	2.94
atc	I	5	2.14
ata	I	0	2.92
---	--	------	------
mPD		0.50	1.1
		nPD:	0.45
		N. weight:	1.4
		Sc. PD:	0.28
		Sc. rank:	2244.4

,

HA

Pos.	194	obs:	exp:
tta	L	111	386.90
ttg	L	4046	794.80
ctt	L	1	830.70
ctc	L	0	669.30
cta	L	1	619.20
ctg	L	25	883.10
---	--	------	------
mPD		0.065	1.1
		nPD:	0.06
		N. weight:	2.1
		Sc. PD:	-0.36
		Sc. rank:	-2808.3

,

HA

Pos.	195	obs:	exp:
att	I	0	837.00
atc	I	18	610.40
ata	I	2263	833.60
gtt	V	6	409.30
gtc	V	2	400.40
gta	V	1789	405.80
gtg	V	106	687.60
---	--	------	------
mPD		0.56	1.2
		nPD:	0.45
		N. weight:	1.5
		Sc. PD:	0.32
		Sc. rank:	2611.7

,

HA

Pos.	196	obs:	exp:
tta	L	11	364.30
ttg	L	148	748.50
ctt	L	66	782.30
ctc	L	2	630.20
cta	L	313	583.10
ctg	L	3400	831.60
att	I	0	15.05
atc	I	0	10.97
ata	I	41	14.98
atg	M	203	203.00
---	--	------	------
mPD		0.37	1.2
		nPD:	0.31
		N. weight:	1.4
		Sc. PD:	0.1
		Sc. rank:	1440.9

,

HA

Pos.	197	obs:	exp:
tgg	W	4183	4183.00
cgt	R	0	0.04
cgc	R	0	0.05
cga	R	0	0.10
cgg	R	0	0.08
aga	R	0	0.47
agg	R	1	0.26
---	--	------	------
mPD		0.00048	0.00079
		nPD:	0.6
		N. weight:	0.00045
		Sc. PD:	0.00016
		Sc. rank:	0.9

,

HA

Pos.	198	obs:	exp:
ggt	G	1	605.10
ggc	G	0	549.60
gga	G	423	1833.00
ggg	G	3760	1196.00
---	--	------	------
mPD		0.18	0.69
		nPD:	0.26
		N. weight:	1.2
		Sc. PD:	0.035
		Sc. rank:	948.4

,

HA

Pos.	199	obs:	exp:
att	I	2902	1533.00
atc	I	1247	1118.00
ata	I	29	1527.00
gtt	V	6	1.29
gtc	V	0	1.26
gta	V	0	1.28
gtg	V	0	2.17
---	--	------	------
mPD		0.43	0.66
		nPD:	0.65
		N. weight:	0.63
		Sc. PD:	0.25
		Sc. rank:	1376.2

,

HA

Pos.	200	obs:	exp:
cat	H	1384	2421.00
cac	H	2798	1761.00
caa	Q	1	1.00
cag	Q	1	1.00
---	--	------	------
mPD		0.44	0.49
		nPD:	0.91
		N. weight:	0.17
		Sc. PD:	0.11
		Sc. rank:	451.9

,

HA			
Pos.	201	obs:	exp:
cat	H	3854	2422.00
cac	H	330	1762.00
---	--	------	------
mPD		0.15	0.49
		nPD:	0.3
		N. weight:	0.41
		Sc. PD:	0.025
		Sc. rank:	402.1

,

HA			
Pos.	202	obs:	exp:
tct	S	342	207.30
tcc	S	893	179.90
tca	S	7	281.60
tcg	S	0	74.93
cct	P	2203	795.80
ccc	P	45	526.00
cca	P	644	1182.00
ccg	P	20	408.30
act	T	29	7.60
acc	T	0	6.53
aca	T	0	12.13
acg	T	0	2.73
agt	S	0	269.40
agc	S	0	228.90
gct	A	1	0.25
gcc	A	0	0.19
gca	A	0	0.45
gcg	A	0	0.11
---	--	------	------
mPD		0.97	1.4
		nPD:	0.7
		N. weight:	1.1
		Sc. PD:	0.49
		Sc. rank:	2588.5

,

HA			
Pos.	203	obs:	exp:
tct	S	0	0.33
tcc	S	0	0.29
tca	S	0	0.45
tcg	S	0	0.12
aat	N	3191	2280.00
aac	N	990	1901.00
aaa	K	0	0.57
aag	K	1	0.43
agt	S	2	0.43
agc	S	0	0.37
---	--	------	------
mPD		0.36	0.50
		nPD:	0.73
		N. weight:	0.14
		Sc. PD:	0.069
		Sc. rank:	352.1

,

HA			
Pos.	204	obs:	exp:
aat	N	1024	572.10
aac	N	25	476.90
gat	D	3111	1687.00
gac	D	14	1438.00
ggt	G	10	1.45
ggc	G	0	1.31
gga	G	0	4.38
ggg	G	0	2.86
---	--	------	------
mPD		0.40	0.88
		nPD:	0.45
		N. weight:	0.79
		Sc. PD:	0.17
		Sc. rank:	1352.4

,

HA			
Pos.	205	obs:	exp:
atg	M	4	4.00
aat	N	3	1.64
aac	N	0	1.36
aaa	K	3	22.78
aag	K	37	17.22
gtt	V	0	0.86
gtc	V	0	0.84
gta	V	0	0.85
gtg	V	4	1.45
gct	A	265	567.80
gcc	A	1	436.20
gca	A	1227	1003.00
gcg	A	761	246.70
gat	D	1	0.54
gac	D	0	0.46
gaa	E	692	1050.00
gag	E	1181	823.00
ggt	G	1	0.72
ggc	G	0	0.66
gga	G	0	2.19
ggg	G	4	1.43
---	--	------	------
mPD		1.1	1.2
		nPD:	0.92
		N. weight:	0.36
		Sc. PD:	0.24
		Sc. rank:	930.4

,

HA			
Pos.	206	obs:	exp:
act	T	0	18.87
acc	T	0	16.22
aca	T	72	30.13
acg	T	0	6.77
aaa	K	5	2.85
aag	K	0	2.15
gtt	V	0	1.72
gtc	V	0	1.68
gta	V	8	1.71
gtg	V	0	2.89
gct	A	57	792.00
gcc	A	2	608.40
gca	A	3031	1399.00
gcg	A	54	344.10
gaa	E	944	534.80
gag	E	10	419.20
ggt	G	0	0.14
ggc	G	0	0.13
gga	G	1	0.44
ggg	G	0	0.29
---	--	------	------
mPD		0.45	1.1
		nPD:	0.42
		N. weight:	0.88
		Sc. PD:	0.16
		Sc. rank:	1283.1

,

HA			
Pos.	207	obs:	exp:
cat	H	1	0.58
cac	H	0	0.42
aaa	K	1	1.71
aag	K	2	1.29
gaa	E	443	2343.00
gag	E	3737	1837.00
---	--	------	------
mPD		0.19	0.49
		nPD:	0.39
		N. weight:	0.64
		Sc. PD:	0.094
		Sc. rank:	860.3

,

HA			
Pos.	208	obs:	exp:
cat	H	0	0.58
cac	H	1	0.42
caa	Q	169	2088.00
cag	Q	4013	2094.00
aaa	K	0	0.57
aag	K	1	0.43
---	--	------	------
mPD		0.078	0.50
		nPD:	0.16
		N. weight:	0.72
		Sc. PD:	-0.055
		Sc. rank:	82.1

,

HA			
Pos.	209	obs:	exp:
att	I	0	99.08
atc	I	0	72.25
ata	I	270	98.67
atg	M	15	15.00
act	T	1	1005.00
acc	T	5	863.50
aca	T	3746	1603.00
acg	T	80	360.50
aaa	K	29	16.51
aag	K	0	12.49
gtt	V	0	3.23
gtc	V	0	3.16
gta	V	15	3.20
gtg	V	0	5.42
gct	A	0	5.54
gcc	A	0	4.26
gca	A	22	9.79
gcg	A	0	2.41
gaa	E	1	0.56
gag	E	0	0.44
---	--	------	------
mPD		0.21	0.86
		nPD:	0.25
		N. weight:	1.1
		Sc. PD:	0.011
		Sc. rank:	752.0

,

HA			
Pos.	210	obs:	exp:
tct	S	0	0.33
tcc	S	0	0.29
tca	S	0	0.45
tcg	S	0	0.12
caa	Q	3	7.49
cag	Q	12	7.51
cgt	R	0	71.25
cgc	R	0	101.90
cga	R	0	191.90
cgg	R	1	155.50
atg	M	94	94.00
act	T	3	10.22
acc	T	0	8.79
aca	T	0	16.32
acg	T	36	3.67
aat	N	891	489.20
aac	N	6	407.80
aaa	K	136	673.00
aag	K	1046	509.00
agt	S	2	0.43
agc	S	0	0.37
aga	R	46	888.90
agg	R	1857	494.60
gct	A	0	1.01
gcc	A	0	0.77
gca	A	0	1.78
gcg	A	4	0.44
gat	D	28	15.12
gac	D	0	12.88
gaa	E	0	2.24
gag	E	4	1.76
ggt	G	0	2.17
ggc	G	0	1.97
gga	G	0	6.57
ggg	G	15	4.29
---	--	------	------
mPD		0.97	1.5
		nPD:	0.66
		N. weight:	1.2
		Sc. PD:	0.48
		Sc. rank:	2602.5

,

HA			
Pos.	211	obs:	exp:
tta	L	3	376.70
ttg	L	0	774.00
ctt	L	336	808.90
ctc	L	3462	651.70
cta	L	273	602.90
ctg	L	0	859.90
att	I	0	39.63
atc	I	108	28.90
ata	I	0	39.47
gtt	V	1	0.43
gtc	V	1	0.42
gta	V	0	0.43
gtg	V	0	0.72
---	--	------	------
mPD		0.31	1.2
		nPD:	0.27
		N. weight:	1.8
		Sc. PD:	0.062
		Sc. rank:	1523.1

HA			
Pos.	212	obs:	exp:
tat	Y	3318	2007.00
tac	Y	866	2177.00
---	--	------	------
mPD		0.33	0.50
		nPD:	0.66
		N. weight:	0.29
		Sc. PD:	0.12
		Sc. rank:	637.8

HA			
Pos.	213	obs:	exp:
cat	H	6	3.47
cac	H	0	2.53
caa	Q	3135	1591.00
cag	Q	52	1596.00
cgt	R	0	0.07
cgc	R	0	0.11
cga	R	2	0.20
cgg	R	0	0.16
aaa	K	989	563.10
aag	K	0	425.90
aga	R	0	0.93
agg	R	0	0.52
---	--	------	------
mPD		0.39	0.86
		nPD:	0.45
		N. weight:	0.83
		Sc. PD:	0.17
		Sc. rank:	1409.0

HA			
Pos.	214	obs:	exp:
tct	S	0	0.33
tcc	S	0	0.29
tca	S	0	0.45
tcg	S	0	0.12
cat	H	1	1.74
cac	H	2	1.26
act	T	0	0.52
acc	T	2	0.45
aca	T	0	0.84
acg	T	0	0.19
aat	N	308	2277.00
aac	N	3867	1898.00
agt	S	0	0.43
agc	S	2	0.37
gat	D	1	1.08
gac	D	1	0.92
---	--	------	------
mPD		0.14	0.50
		nPD:	0.28
		N. weight:	0.71
		Sc. PD:	0.032
		Sc. rank:	630.9

HA			
Pos.	215	obs:	exp:
tta	L	1	2.50
ttg	L	0	5.13
tct	S	0	12.19
tcc	S	0	10.57
tca	S	39	16.55
tcg	S	34	4.40
ctt	L	0	5.36
ctc	L	0	4.32
cta	L	26	4.00
ctg	L	0	5.70
cct	P	0	1115.00
ccc	P	3	737.10
cca	P	4046	1656.00
ccg	P	32	572.20
caa	Q	2	1.00
cag	Q	0	1.00
att	I	0	0.37
atc	I	0	0.27
ata	I	1	0.37
agt	S	0	15.83
agc	S	0	13.45
---	--	------	------
mPD		0.082	0.78
		nPD:	0.11
		N. weight:	1.2
		Sc. PD:	-0.15
		Sc. rank:	-609.6

HA			
Pos.	216	obs:	exp:
tct	S	5	1.00
tcc	S	0	0.87
tca	S	0	1.36
tcg	S	0	0.36
cct	P	0	0.55
ccc	P	1	0.36
cca	P	1	0.81
ccg	P	0	0.28
att	I	13	25.32
atc	I	56	18.46
ata	I	0	25.22
act	T	1217	1038.00
acc	T	2733	892.10
aca	T	5	1657.00
acg	T	4	372.50
aat	N	22	67.08
aac	N	101	55.92
aaa	K	0	1.71
aag	K	3	1.29
agt	S	1	1.30
agc	S	0	1.11
gct	A	10	4.03
gcc	A	6	3.10
gca	A	0	7.12
gcg	A	0	1.75
gat	D	1	3.24
gac	D	5	2.76
---	--	------	------
mPD		0.53	0.81
		nPD:	0.66
		N. weight:	1.1
		Sc. PD:	0.46
		Sc. rank:	2467.0

HA			
Pos.	217	obs:	exp:
cct	P	0	0.82
ccc	P	3	0.54
cca	P	0	1.22
ccg	P	0	0.42
act	T	162	1095.00
acc	T	4013	941.00
aca	T	0	1747.00
acg	T	1	392.90
gct	A	0	1.26
gcc	A	5	0.97
gca	A	0	2.23
gcg	A	0	0.55
---	--	------	------
mPD		0.079	0.70
		nPD:	0.11
		N. weight:	1.8
		Sc. PD:	-0.22
		Sc. rank:	-667.4

HA			
Pos.	218	obs:	exp:
ttt	F	1	0.42
ttc	F	0	0.58
tct	S	1	0.17
tcc	S	0	0.14
tca	S	0	0.23
tcg	S	0	0.06
tat	Y	3068	2003.00
tac	Y	1108	2173.00
cat	H	6	3.47
cac	H	0	2.53
agt	S	0	0.22
agc	S	0	0.18
---	--	------	------
mPD		0.39	0.50
		nPD:	0.78
		N. weight:	0.19
		Sc. PD:	0.1
		Sc. rank:	475.7

HA			
Pos.	219	obs:	exp:
att	I	3576	1430.00
atc	I	313	1043.00
ata	I	8	1424.00
act	T	2	0.52
acc	T	0	0.45
aca	T	0	0.84
acg	T	0	0.19
gtt	V	244	61.30
gtc	V	2	59.96
gta	V	1	60.77
gtg	V	38	103.00
---	--	------	------
mPD		0.29	0.80
		nPD:	0.36
		N. weight:	1.
		Sc. PD:	0.12
		Sc. rank:	1277.0

HA			
Pos.	220	obs:	exp:
ttt	F	0	0.42
ttc	F	1	0.58
tct	S	71	698.10
tcc	S	3208	605.80
tca	S	898	948.10
tcg	S	5	252.30
cct	P	0	0.27
ccc	P	1	0.18
cca	P	0	0.41
ccg	P	0	0.14
agt	S	0	907.10
agc	S	0	770.70
---	--	------	------
mPD		0.37	1.7
		nPD:	0.22
		N. weight:	1.7
		Sc. PD:	-0.022
		Sc. rank:	843.2

HA			
Pos.	221	obs:	exp:
att	I	1732	639.60
atc	I	9	466.40
ata	I	2	637.00
act	T	2	0.52
acc	T	0	0.45
aca	T	0	0.84
acg	T	0	0.19
gtt	V	2382	524.60
gtc	V	16	513.10
gta	V	38	520.10
gtg	V	3	881.20
---	--	------	------
mPD		0.52	1.2
		nPD:	0.42
		N. weight:	1.7
		Sc. PD:	0.3
		Sc. rank:	2489.7

HA			
Pos.	222	obs:	exp:
gaa	E	0	0.56
gag	E	1	0.44
ggt	G	2	604.90
ggc	G	0	549.40
gga	G	1277	1833.00
ggg	G	2904	1196.00
---	--	------	------
mPD		0.43	0.69
		nPD:	0.62
		N. weight:	0.7
		Sc. PD:	0.26
		Sc. rank:	1499.0

HA			
Pos.	223	obs:	exp:
act	T	805	1097.00
acc	T	9	942.80
aca	T	3225	1751.00
acg	T	145	393.70
---	--	------	------
mPD		0.37	0.70
		nPD:	0.53
		N. weight:	0.51
		Sc. PD:	0.14
		Sc. rank:	988.2

HA			
Pos.	224	obs:	exp:
tct	S	10	698.40
tcc	S	2	606.10
tca	S	4165	948.50
tcg	S	7	252.40
agt	S	0	907.50
agc	S	0	771.10
---	--	------	------
mPD		0.0091	1.7
		nPD:	0.01
		N. weight:	2.
		Sc. PD:	-0.45
		Sc. rank:	-5078.0

HA			
Pos.	225	obs:	exp:
att	I	0	0.37
atc	I	0	0.27
ata	I	1	0.37
atg	M	1	1.00
act	T	866	1096.00
acc	T	3	941.60
aca	T	3239	1749.00
acg	T	71	393.20
aaa	K	2	1.14
aag	K	0	0.86
gct	A	0	0.25
gcc	A	0	0.19
gca	A	1	0.45
gcg	A	0	0.11
---	--	------	------
mPD		0.36	0.70
		nPD:	0.51
		N. weight:	0.55
		Sc. PD:	0.15
		Sc. rank:	1009.7

HA

Pos.	226	obs:	exp:
tta	L	990	386.40
ttg	L	29	793.90
ctt	L	5	829.70
ctc	L	2	668.50
cta	L	2908	618.50
ctg	L	245	882.00
cct	P	0	0.27
ccc	P	0	0.18
cca	P	1	0.41
ccg	P	0	0.14
att	I	0	1.47
atc	I	0	1.07
ata	I	4	1.46
---	--	------	------
mPD		0.50	1.1
		nPD:	0.44
		N. weight:	1.7
		Sc. PD:	0.33
		Sc. rank:	2628.5

,

HA

Pos.	227	obs:	exp:
cat	H	1	0.58
cac	H	0	0.42
aat	N	142	2280.00
aac	N	4039	1901.00
gat	D	0	1.08
gac	D	2	0.92
---	--	------	------
mPD		0.067	0.50
		nPD:	0.14
		N. weight:	0.88
		Sc. PD:	-0.085
		Sc. rank:	-162.7

,

HA

Pos.	228	obs:	exp:
tta	L	0	6.93
ttg	L	0	14.25
ctt	L	0	14.89
ctc	L	0	12.00
cta	L	0	11.10
ctg	L	75	15.83
cat	H	1	1.74
cac	H	2	1.26
caa	Q	217	2050.00
cag	Q	3889	2056.00
---	--	------	------
mPD		0.13	0.55
		nPD:	0.24
		N. weight:	0.7
		Sc. PD:	0.006
		Sc. rank:	460.9

,

HA

Pos.	229	obs:	exp:
cgt	R	0	156.00
cgc	R	0	223.10
cga	R	8	420.00
cgg	R	0	340.30
att	I	0	0.37
atc	I	0	0.27
ata	I	1	0.37
aaa	K	13	7.97
aag	K	1	6.03
aga	R	3252	1946.00
agg	R	908	1083.00
ggt	G	0	0.14
ggc	G	0	0.13
gga	G	1	0.44
ggg	G	0	0.29
---	--	------	------
mPD		0.35	0.96
		nPD:	0.37
		N. weight:	0.49
		Sc. PD:	0.062
		Sc. rank:	623.9

,

HA

Pos.	230	obs:	exp:
tta	L	45	377.10
ttg	L	3998	774.90
tct	S	0	15.36
tcc	S	0	13.33
tca	S	47	20.86
tcg	S	45	5.55
ctt	L	0	809.90
ctc	L	0	652.50
cta	L	0	603.70
ctg	L	36	860.90
atg	M	5	5.00
act	T	0	0.52
acc	T	0	0.45
aca	T	2	0.84
acg	T	0	0.19
agt	S	0	19.96
agc	S	0	16.95
gtt	V	0	1.29
gtc	V	0	1.26
gta	V	0	1.28
gtg	V	6	2.17
---	--	------	------
mPD		0.11	1.2
		nPD:	0.09
		N. weight:	2.2
		Sc. PD:	-0.3
		Sc. rank:	-1374.3

,

HA

Pos.	231	obs:	exp:
tta	L	6	0.55
ttg	L	0	1.14
ctt	L	0	1.19
ctc	L	0	0.96
cta	L	0	0.89
ctg	L	0	1.27
att	I	2	32.29
atc	I	36	23.55
ata	I	50	32.16
atg	M	3	3.00
act	T	0	53.22
acc	T	0	45.74
aca	T	203	84.94
acg	T	0	19.10
gtt	V	19	760.10
gtc	V	34	743.50
gta	V	3287	753.50
gtg	V	194	1277.00
gct	A	0	87.16
gcc	A	0	66.95
gca	A	346	154.00
gcg	A	0	37.87
gat	D	0	0.54
gac	D	1	0.46
gaa	E	1	1.68
gag	E	2	1.32
---	--	------	------
mPD		0.49	1.1
		nPD:	0.45
		N. weight:	1.6
		Sc. PD:	0.33
		Sc. rank:	2589.9

,

HA

Pos.	232	obs:	exp:
cct	P	0	1143.00
ccc	P	360	755.70
cca	P	3819	1698.00
ccg	P	5	586.60
---	--	------	------
mPD		0.16	0.71
		nPD:	0.23
		N. weight:	0.93
		Sc. PD:	-0.0092
		Sc. rank:	496.8

,

HA

Pos.	233	obs:	exp:
caa	Q	1	0.50
cag	Q	0	0.50
cgt	R	0	12.16
cgc	R	0	17.39
cga	R	0	32.75
cgg	R	0	26.54
act	T	0	2.36
acc	T	0	2.03
aca	T	9	3.77
acg	T	0	0.85
aaa	K	3597	2151.00
aag	K	180	1626.00
aga	R	318	151.70
agg	R	7	84.43
gaa	E	70	40.36
gag	E	2	31.64
---	--	------	------
mPD		0.27	0.72
		nPD:	0.37
		N. weight:	0.57
		Sc. PD:	0.075
		Sc. rank:	739.7

,

HA

Pos.	234	obs:	exp:
tta	L	3	0.37
ttg	L	0	0.76
ctt	L	0	0.79
ctc	L	0	0.64
cta	L	1	0.59
ctg	L	0	0.84
att	I	5	1530.00
atc	I	1	1116.00
ata	I	4164	1524.00
atg	M	2	2.00
act	T	0	0.26
acc	T	0	0.23
aca	T	1	0.42
acg	T	0	0.09
gtt	V	0	1.51
gtc	V	0	1.47
gta	V	7	1.49
gtg	V	0	2.53
---	--	------	------
mPD		0.0095	0.67
		nPD:	0.01
		N. weight:	1.4
		Sc. PD:	-0.29
		Sc. rank:	-3180.2

,

HA

Pos.	235	obs:	exp:
tct	S	10	3.34
tcc	S	10	2.90
tca	S	0	4.53
tcg	S	0	1.21
act	T	10	4.72
acc	T	8	4.06
aca	T	0	7.53
acg	T	0	1.69
agt	S	0	4.34
agc	S	0	3.69
gtt	V	2	1.08
gtc	V	3	1.05
gta	V	0	1.07
gtg	V	0	1.81
gct	A	2448	1043.00
gcc	A	1690	801.30
gca	A	3	1843.00
gcg	A	0	453.20
---	--	------	------
mPD		0.50	0.71
		nPD:	0.71
		N. weight:	1.1
		Sc. PD:	0.51
		Sc. rank:	2664.8

,

HA

Pos.	236	obs:	exp:
att	I	9	3.30
atc	I	0	2.41
ata	I	0	3.29
act	T	4126	1092.00
acc	T	35	938.90
aca	T	0	1744.00
acg	T	6	392.10
aat	N	4	2.18
aac	N	0	1.82
gct	A	4	1.01
gcc	A	0	0.77
gca	A	0	1.78
gcg	A	0	0.44
---	--	------	------
mPD		0.028	0.70
		nPD:	0.04
		N. weight:	1.8
		Sc. PD:	-0.34
		Sc. rank:	-3254.5

,

HA

Pos.	237	obs:	exp:
cgt	R	0	156.20
cgc	R	0	223.30
cga	R	0	420.50
cgg	R	0	340.70
att	I	0	0.73
atc	I	0	0.54
ata	I	2	0.73
aat	N	1	0.55
aac	N	0	0.45
aaa	K	6	4.56
aag	K	2	3.44
aga	R	4012	1948.00
agg	R	161	1084.00
---	--	------	------
mPD		0.081	0.96
		nPD:	0.08
		N. weight:	0.86
		Sc. PD:	-0.13
		Sc. rank:	-651.1

,

HA

Pos.	238	obs:	exp:
ttt	F	2	1.67
ttc	F	2	2.33
tct	S	450	672.70
tcc	S	3560	583.80
tca	S	20	913.60
tcg	S	0	243.10
cct	P	9	39.90
ccc	P	136	26.37
cca	P	1	59.26
ccg	P	0	20.47
act	T	0	0.79
acc	T	3	0.68
aca	T	0	1.26
acg	T	0	0.28
agt	S	0	874.10
agc	S	0	742.70
gct	A	0	0.25
gcc	A	1	0.19
gca	A	0	0.45
gcg	A	0	0.11
---	--	------	------
mPD		0.28	1.7
		nPD:	0.16
		N. weight:	2.1
		Sc. PD:	-0.15
		Sc. rank:	467.2

,

HA

Pos.	239	obs:	exp:
caa	Q	916	457.30
cag	Q	0	458.70
cgt	R	0	2.54
cgc	R	0	3.64
cga	R	0	6.85
cgg	R	0	5.55
act	T	0	0.26
acc	T	0	0.23
aca	T	1	0.42
acg	T	0	0.09
aaa	K	2549	1821.00
aag	K	649	1377.00
aga	R	68	31.75
agg	R	0	17.67
gaa	E	1	0.56
gag	E	0	0.44
---	--	------	------
mPD		0.64	0.88
		nPD:	0.73
		N. weight:	0.35
		Sc. PD:	0.17
		Sc. rank:	859.9

,

HA

Pos.	240	obs:	exp:
tta	L	2	0.18
ttg	L	0	0.38
ctt	L	0	0.40
ctc	L	0	0.32
cta	L	0	0.30
ctg	L	0	0.42
att	I	0	582.00
atc	I	0	424.40
ata	I	1586	579.60
act	T	0	0.52
acc	T	0	0.45
aca	T	2	0.84
acg	T	0	0.19
gtt	V	2	557.70
gtc	V	3	545.50
gta	V	2504	552.90
gtg	V	84	936.90
gaa	E	1	0.56
gag	E	0	0.44
---	--	------	------
mPD		0.51	1.2
		nPD:	0.42
		N. weight:	1.7
		Sc. PD:	0.31
		Sc. rank:	2535.2

,

HA			
Pos.	241	obs:	exp:
tct	S	0	0.17
tcc	S	0	0.14
tca	S	0	0.23
tcg	S	0	0.06
cat	H	0	2.89
cac	H	5	2.11
aat	N	81	2276.00
aac	N	4092	1897.00
aaa	K	3	2.28
aag	K	1	1.72
agt	S	0	0.22
agc	S	1	0.18
gat	D	0	0.54
gac	D	1	0.46
---	--	------	------
mPD		0.043	0.50
		nPD:	0.09
		N. weight:	0.96
		Sc. PD:	-0.14
		Sc. rank:	-665.8

,

HA			
Pos.	242	obs:	exp:
cgt	R	0	0.11
cgc	R	0	0.16
cga	R	0	0.30
cgg	R	2	0.24
aga	R	0	1.40
agg	R	1	0.78
gaa	E	0	1.12
gag	E	2	0.88
ggt	G	1	604.30
ggc	G	10	548.90
gga	G	195	1831.00
ggg	G	3973	1195.00
---	--	------	------
mPD		0.096	0.69
		nPD:	0.14
		N. weight:	1.4
		Sc. PD:	-0.13
		Sc. rank:	-120.6

,

HA			
Pos.	243	obs:	exp:
cct	P	0	0.27
ccc	P	0	0.18
cca	P	1	0.41
ccg	P	0	0.14
caa	Q	4117	2088.00
cag	Q	66	2095.00
---	--	------	------
mPD		0.032	0.50
		nPD:	0.06
		N. weight:	0.85
		Sc. PD:	-0.14
		Sc. rank:	-1068.4

,

HA			
Pos.	244	obs:	exp:
tct	S	1	538.50
tcc	S	0	467.30
tca	S	0	731.30
tcg	S	0	194.60
cgt	R	893	35.63
cgc	R	1	50.95
cga	R	0	95.94
cgg	R	2	77.74
aat	N	1	0.55
aac	N	0	0.45
agt	S	3113	699.70
agc	S	112	594.50
aga	R	2	444.40
agg	R	54	247.30
ggt	G	5	0.72
ggc	G	0	0.66
gga	G	0	2.19
ggg	G	0	1.43
---	--	------	------
mPD		0.42	1.8
		nPD:	0.24
		N. weight:	2.4
		Sc. PD:	-0.00083
		Sc. rank:	1469.0

,

HA			
Pos.	245	obs:	exp:
gaa	E	3	1.68
gag	E	0	1.32
ggt	G	0	604.60
ggc	G	1570	549.20
gga	G	2387	1832.00
ggg	G	224	1195.00
---	--	------	------
mPD		0.53	0.69
		nPD:	0.77
		N. weight:	0.63
		Sc. PD:	0.33
		Sc. rank:	1578.1

,

HA			
Pos.	246	obs:	exp:
tgg	W	1	1.00
cgt	R	0	156.40
cgc	R	0	223.60
cga	R	11	421.00
cgg	R	1	341.20
act	T	0	0.26
acc	T	0	0.23
aca	T	0	0.42
acg	T	1	0.09
aaa	K	1	1.71
aag	K	2	1.29
aga	R	1159	1950.00
agg	R	3007	1085.00
ggt	G	0	0.14
ggc	G	0	0.13
gga	G	0	0.44
ggg	G	1	0.29
---	--	------	------
mPD		0.41	0.96
		nPD:	0.43
		N. weight:	0.8
		Sc. PD:	0.15
		Sc. rank:	1231.6

,

HA			
Pos.	247	obs:	exp:
tta	L	0	0.09
ttg	L	0	0.19
ctt	L	0	0.20
ctc	L	0	0.16
cta	L	0	0.15
ctg	L	1	0.21
att	I	16	629.30
atc	I	0	458.90
ata	I	1699	626.70
atg	M	2363	2363.00
gtt	V	0	22.37
gtc	V	0	21.88
gta	V	4	22.18
gtg	V	100	37.58
gat	D	1	0.54
gac	D	0	0.46
---	--	------	------
mPD		0.54	0.66
		nPD:	0.82
		N. weight:	0.57
		Sc. PD:	0.32
		Sc. rank:	1466.1

,

HA			
Pos.	248	obs:	exp:
tct	S	0	0.17
tcc	S	0	0.14
tca	S	0	0.23
tcg	S	0	0.06
act	T	0	0.79
acc	T	0	0.68
aca	T	3	1.26
acg	T	0	0.28
aat	N	0	0.55
aac	N	1	0.45
aaa	K	0	0.57
aag	K	1	0.43
agt	S	1	0.22
agc	S	0	0.18
gat	D	2008	1573.00
gac	D	905	1340.00
gaa	E	74	709.10
gag	E	1191	555.90
---	--	------	------
mPD		0.65	0.71
		nPD:	0.91
		N. weight:	0.29
		Sc. PD:	0.19
		Sc. rank:	762.0

,

HA			
Pos.	249	obs:	exp:
ttt	F	72	1744.00
ttc	F	4108	2436.00
tta	L	1	0.18
ttg	L	1	0.38
tct	S	1	0.17
tcc	S	0	0.14
tca	S	0	0.23
tcg	S	0	0.06
tgt	C	0	0.39
tgc	C	1	0.61
ctt	L	0	0.40
ctc	L	0	0.32
cta	L	0	0.30
ctg	L	0	0.42
agt	S	0	0.22
agc	S	0	0.18
---	--	------	------
mPD		0.036	0.49
		nPD:	0.07
		N. weight:	0.64
		Sc. PD:	-0.099
		Sc. rank:	-663.7

,

HA			
Pos.	250	obs:	exp:
ttt	F	864	1744.00
ttc	F	3317	2437.00
tta	L	0	0.09
ttg	L	0	0.19
tct	S	0	0.17
tcc	S	1	0.14
tca	S	0	0.23
tcg	S	0	0.06
tat	Y	0	0.48
tac	Y	1	0.52
ctt	L	1	0.20
ctc	L	0	0.16
cta	L	0	0.15
ctg	L	0	0.21
agt	S	0	0.22
agc	S	0	0.18
---	--	------	------
mPD		0.33	0.49
		nPD:	0.68
		N. weight:	0.14
		Sc. PD:	0.06
		Sc. rank:	321.4

,

HA			
Pos.	251	obs:	exp:
tgg	W	4183	4183.00
ggt	G	0	0.14
ggc	G	0	0.13
gga	G	0	0.44
ggg	G	1	0.29
---	--	------	------
mPD		0.00048	0.00082
		nPD:	0.58
		N. weight:	0.00041
		Sc. PD:	0.00014
		Sc. rank:	0.9

,

HA			
Pos.	252	obs:	exp:
att	I	0	3.30
atc	I	0	2.41
ata	I	9	3.29
act	T	3	1093.00
acc	T	0	939.60
aca	T	4145	1745.00
acg	T	22	392.30
gct	A	0	1.26
gcc	A	0	0.97
gca	A	5	2.23
gcg	A	0	0.55
---	--	------	------
mPD		0.019	0.70
		nPD:	0.03
		N. weight:	1.2
		Sc. PD:	-0.24
		Sc. rank:	-2423.8

,

HA			
Pos.	253	obs:	exp:
ttt	F	1	0.42
ttc	F	0	0.58
att	I	3693	1532.00
atc	I	442	1117.00
ata	I	40	1526.00
atg	M	4	4.00
gtt	V	2	0.86
gtc	V	1	0.84
gta	V	1	0.85
gtg	V	0	1.45
---	--	------	------
mPD		0.21	0.66
		nPD:	0.32
		N. weight:	0.9
		Sc. PD:	0.071
		Sc. rank:	961.4

,

HA			
Pos.	254	obs:	exp:
ttt	F	2	2.09
ttc	F	3	2.91
tta	L	3852	386.40
ttg	L	54	793.90
ctt	L	0	829.70
ctc	L	0	668.50
cta	L	247	618.50
ctg	L	26	882.00
---	--	------	------
mPD		0.16	1.1
		nPD:	0.14
		N. weight:	2.8
		Sc. PD:	-0.25
		Sc. rank:	-66.6

,

HA			
Pos.	255	obs:	exp:
cgt	R	0	0.60
cgc	R	0	0.86
cga	R	0	1.61
cgg	R	0	1.31
act	T	0	0.26
acc	T	0	0.23
aca	T	0	0.42
acg	T	1	0.09
aat	N	5	7.63
aac	N	9	6.37
aaa	K	4019	2364.00
aag	K	133	1788.00
aga	R	3	7.47
agg	R	13	4.16
gaa	E	1	0.56
gag	E	0	0.44
---	--	------	------
mPD		0.083	0.50
		nPD:	0.16
		N. weight:	0.6
		Sc. PD:	-0.041
		Sc. rank:	120.6

,

HA			
Pos.	256	obs:	exp:
tta	L	0	0.09
ttg	L	0	0.19
tct	S	0	86.13
tcc	S	0	74.75
tca	S	79	117.00
tcg	S	437	31.13
ctt	L	0	0.20
ctc	L	0	0.16
cta	L	0	0.15
ctg	L	1	0.21
cct	P	177	989.30
ccc	P	22	653.80
cca	P	1034	1469.00
ccg	P	2387	507.50
caa	Q	0	6.49
cag	Q	13	6.51
act	T	0	8.91
acc	T	0	7.66
aca	T	1	14.23
acg	T	33	3.20
agt	S	0	111.90
agc	S	0	95.09
---	--	------	------
mPD		0.69	1.1
		nPD:	0.66
		N. weight:	1.4
		Sc. PD:	0.57
		Sc. rank:	3063.0

,

HA			
Pos.	257	obs:	exp:
tat	Y	5	2.40
tac	Y	0	2.60
aat	N	3219	1783.00
aac	N	51	1487.00
aaa	K	3	2.28
aag	K	1	1.72
gat	D	846	487.60
gac	D	57	415.40
ggt	G	2	0.29
ggc	G	0	0.26
gga	G	0	0.88
ggg	G	0	0.57
---	--	------	------
mPD		0.39	0.84
		nPD:	0.47
		N. weight:	0.69
		Sc. PD:	0.16
		Sc. rank:	1204.8

,

HA			
Pos.	258	obs:	exp:
tat	Y	0	0.48
tac	Y	1	0.52
aat	N	1	1.09
aac	N	1	0.91
gat	D	3350	2257.00
gac	D	830	1923.00
gaa	E	1	0.56
gag	E	0	0.44
---	--	------	------
mPD		0.32	0.50
		nPD:	0.64
		N. weight:	0.21
		Sc. PD:	0.082
		Sc. rank:	457.6

,

HA			
Pos.	259	obs:	exp:
tct	S	6	1.84
tcc	S	0	1.59
tca	S	5	2.49
tcg	S	0	0.66
act	T	21	67.90
acc	T	1	58.36
aca	T	237	108.40
acg	T	0	24.37
agt	S	0	2.39
agc	S	0	2.03
gtt	V	0	3.66
gtc	V	3	3.58
gta	V	14	3.62
gtg	V	0	6.14
gct	A	40	981.70
gcc	A	50	754.10
gca	A	3802	1735.00
gcg	A	5	426.50
---	--	------	------
mPD		0.19	0.82
		nPD:	0.23
		N. weight:	0.96
		Sc. PD:	-0.006
		Sc. rank:	557.2

,

HA			
Pos.	260	obs:	exp:
tta	L	0	0.09
ttg	L	0	0.19
tct	S	0	0.17
tcc	S	1	0.14
tca	S	0	0.23
tcg	S	0	0.06
ctt	L	0	0.20
ctc	L	1	0.16
cta	L	0	0.15
ctg	L	0	0.21
att	I	25	1534.00
atc	I	3491	1119.00
ata	I	664	1528.00
agt	S	0	0.22
agc	S	0	0.18
gtt	V	0	0.43
gtc	V	1	0.42
gta	V	1	0.43
gtg	V	0	0.72
---	--	------	------
mPD		0.28	0.66
		nPD:	0.42
		N. weight:	1.1
		Sc. PD:	0.2
		Sc. rank:	1646.3

,

HA			
Pos.	261	obs:	exp:
tct	S	0	9.85
tcc	S	0	8.55
tca	S	0	13.38
tcg	S	0	3.56
tat	Y	1	2.88
tac	Y	5	3.12
cat	H	1184	1423.00
cac	H	1275	1036.00
caa	Q	3	1.50
cag	Q	0	1.50
act	T	2	0.52
acc	T	0	0.45
aca	T	0	0.84
acg	T	0	0.19
aat	N	277	902.50
aac	N	1378	752.50
agt	S	8	12.80
agc	S	51	10.87
---	--	------	------
mPD		0.97	1.0
		nPD:	0.95
		N. weight:	0.21
		Sc. PD:	0.15
		Sc. rank:	556.6

,

HA			
Pos.	262	obs:	exp:
ttt	F	786	1745.00
ttc	F	3398	2439.00
---	--	------	------
mPD		0.31	0.49
		nPD:	0.63
		N. weight:	0.17
		Sc. PD:	0.063
		Sc. rank:	362.6

,

HA			
Pos.	263	obs:	exp:
caa	Q	1	0.50
cag	Q	0	0.50
aaa	K	0	1.14
aag	K	2	0.86
gat	D	33	17.82
gac	D	0	15.18
gaa	E	92	2325.00
gag	E	4056	1823.00
---	--	------	------
mPD		0.060	0.50
		nPD:	0.12
		N. weight:	0.98
		Sc. PD:	-0.11
		Sc. rank:	-299.7

,

HA			
Pos.	264	obs:	exp:
tct	S	0	698.10
tcc	S	0	605.80
tca	S	0	948.10
tcg	S	0	252.30
act	T	2	0.52
acc	T	0	0.45
aca	T	0	0.84
acg	T	0	0.19
agt	S	3980	907.10
agc	S	202	770.70
---	--	------	------
mPD		0.093	1.7
		nPD:	0.06
		N. weight:	1.9
		Sc. PD:	-0.32
		Sc. rank:	-2586.8

,

HA			
Pos.	265	obs:	exp:
tct	S	0	0.17
tcc	S	0	0.14
tca	S	0	0.23
tcg	S	0	0.06
act	T	0	0.79
acc	T	3	0.68
aca	T	0	1.26
acg	T	0	0.28
aat	N	3764	2275.00
aac	N	408	1897.00
aaa	K	1	1.71
aag	K	2	1.29
agt	S	1	0.22
agc	S	0	0.18
gat	D	5	2.70
gac	D	0	2.30
---	--	------	------
mPD		0.18	0.50
		nPD:	0.36
		N. weight:	0.42
		Sc. PD:	0.053
		Sc. rank:	534.1

,

HA			
Pos.	266	obs:	exp:
gaa	E	3	1.68
gag	E	0	1.32
ggt	G	3	604.60
ggc	G	0	549.20
gga	G	3983	1832.00
ggg	G	195	1195.00
---	--	------	------
mPD		0.092	0.69
		nPD:	0.13
		N. weight:	0.9
		Sc. PD:	-0.089
		Sc. rank:	-201.0

,

HA			
Pos.	267	obs:	exp:
aat	N	3749	2281.00
aac	N	433	1901.00
aaa	K	1	0.57
aag	K	0	0.43
gat	D	1	0.54
gac	D	0	0.46
---	--	------	------
mPD		0.19	0.50
		nPD:	0.38
		N. weight:	0.41
		Sc. PD:	0.055
		Sc. rank:	531.3

,

HA			
Pos.	268	obs:	exp:
ttt	F	97	1742.00
ttc	F	4079	2434.00
tta	L	0	0.65
ttg	L	0	1.33
ctt	L	0	1.39
ctc	L	7	1.12
cta	L	0	1.04
ctg	L	0	1.48
att	I	0	0.37
atc	I	1	0.27
ata	I	0	0.37
---	--	------	------
mPD		0.049	0.49
		nPD:	0.1
		N. weight:	0.61
		Sc. PD:	-0.08
		Sc. rank:	-345.0

,

HA			
Pos.	269	obs:	exp:
ttt	F	1	0.42
ttc	F	0	0.58
tta	L	0	0.09
ttg	L	0	0.19
ctt	L	1	0.20
ctc	L	0	0.16
cta	L	0	0.15
ctg	L	0	0.21
att	I	4078	1533.00
atc	I	36	1118.00
ata	I	64	1527.00
gtt	V	4	0.86
gtc	V	0	0.84
gta	V	0	0.85
gtg	V	0	1.45
---	--	------	------
mPD		0.050	0.66
		nPD:	0.08
		N. weight:	1.2
		Sc. PD:	-0.19
		Sc. rank:	-1219.0

,

HA			
Pos.	270	obs:	exp:
cct	P	1	0.27
ccc	P	0	0.18
cca	P	0	0.41
ccg	P	0	0.14
act	T	2	0.52
acc	T	0	0.45
aca	T	0	0.84
acg	T	0	0.19
gtt	V	4	0.86
gtc	V	0	0.84
gta	V	0	0.85
gtg	V	0	1.45
gct	A	3968	1052.00
gcc	A	50	808.30
gca	A	159	1859.00
gcg	A	0	457.20
---	--	------	------
mPD		0.099	0.69
		nPD:	0.14
		N. weight:	1.6
		Sc. PD:	-0.14
		Sc. rank:	-77.2

,

HA			
Pos.	271	obs:	exp:
tct	S	0	0.17
tcc	S	1	0.14
tca	S	0	0.23
tcg	S	0	0.06
cct	P	38	1143.00
ccc	P	0	755.50
cca	P	4107	1698.00
ccg	P	38	586.50
agt	S	0	0.22
agc	S	0	0.18
---	--	------	------
mPD		0.037	0.71
		nPD:	0.05
		N. weight:	1.1
		Sc. PD:	-0.2
		Sc. rank:	-1711.0

,

HA			
Pos.	272	obs:	exp:
aaa	K	41	23.35
aag	K	0	17.65
gat	D	1	0.54
gac	D	0	0.46
gaa	E	3805	2321.00
gag	E	335	1819.00
ggt	G	0	0.29
ggc	G	0	0.26
gga	G	2	0.88
ggg	G	0	0.57
---	--	------	------
mPD		0.17	0.51
		nPD:	0.33
		N. weight:	0.44
		Sc. PD:	0.04
		Sc. rank:	512.4

,

HA			
Pos.	273	obs:	exp:
ttt	F	2	0.83
ttc	F	0	1.17
tct	S	1	0.17
tcc	S	0	0.14
tca	S	0	0.23
tcg	S	0	0.06
tat	Y	3511	1690.00
tac	Y	12	1833.00
cat	H	2	1.16
cac	H	0	0.84
aat	N	654	357.70
aac	N	2	298.30
agt	S	0	0.22
agc	S	0	0.18
---	--	------	------
mPD		0.27	0.77
		nPD:	0.36
		N. weight:	0.96
		Sc. PD:	0.11
		Sc. rank:	1162.6

,

HA			
Pos.	274	obs:	exp:
gct	A	0	1054.00
gcc	A	34	809.60
gca	A	4055	1862.00
gcg	A	95	457.90
---	--	------	------
mPD		0.060	0.69
		nPD:	0.09
		N. weight:	0.96
		Sc. PD:	-0.14
		Sc. rank:	-656.3

,

HA			
Pos.	275	obs:	exp:
ttt	F	0	0.42
ttc	F	1	0.58
tat	Y	450	2006.00
tac	Y	3733	2177.00
---	--	------	------
mPD		0.19	0.50
		nPD:	0.39
		N. weight:	0.45
		Sc. PD:	0.064
		Sc. rank:	593.9

,

HA			
Pos.	276	obs:	exp:
caa	Q	1	0.50
cag	Q	0	0.50
aaa	K	4164	2379.00
aag	K	14	1799.00
gaa	E	1	0.56
gag	E	0	0.44
---	-	4	4.00
---	--	------	------
mPD		0.0076	0.49
		nPD:	0.02
		N. weight:	0.75
		Sc. PD:	-0.16
		Sc. rank:	-1690.9

,

HA			
Pos.	277	obs:	exp:
att	I	4163	1534.00
atc	I	14	1119.00
ata	I	4	1528.00
aat	N	1	0.55
aac	N	0	0.45
gtt	V	2	0.43
gtc	V	0	0.42
gta	V	0	0.43
gtg	V	0	0.72
---	--	------	------
mPD		0.010	0.66
		nPD:	0.02
		N. weight:	1.3
		Sc. PD:	-0.29
		Sc. rank:	-3051.3

,

HA			
Pos.	278	obs:	exp:
att	I	2	25.32
atc	I	67	18.46
ata	I	0	25.22
gtt	V	39	884.80
gtc	V	4062	865.50
gta	V	13	877.20
gtg	V	0	1486.00
gct	A	0	0.25
gcc	A	1	0.19
gca	A	0	0.45
gcg	A	0	0.11
---	--	------	------
mPD		0.058	0.77
		nPD:	0.08
		N. weight:	2.
		Sc. PD:	-0.32
		Sc. rank:	-1960.4

,

HA			
Pos.	279	obs:	exp:
caa	Q	0	1.00
cag	Q	2	1.00
cgt	R	0	0.11
cgc	R	0	0.16
cga	R	0	0.30
cgg	R	1	0.24
aaa	K	239	2380.00
aag	K	3940	1799.00
aga	R	0	1.40
agg	R	2	0.78
---	--	------	------
mPD		0.11	0.49
		nPD:	0.22
		N. weight:	0.84
		Sc. PD:	-0.0091
		Sc. rank:	438.9

,

HA			
Pos.	280	obs:	exp:
caa	Q	0	2.50
cag	Q	5	2.50
cgt	R	0	0.37
cgc	R	0	0.54
cga	R	0	1.01
cgg	R	0	0.82
act	T	0	6.03
acc	T	0	5.18
aca	T	1	9.62
acg	T	22	2.16
aaa	K	3648	2356.00
aag	K	489	1781.00
aga	R	9	4.67
agg	R	1	2.60
gaa	E	8	5.05
gag	E	1	3.95
---	--	------	------
mPD		0.24	0.52
		nPD:	0.46
		N. weight:	0.34
		Sc. PD:	0.075
		Sc. rank:	589.5

,

HA			
Pos.	281	obs:	exp:
ggt	G	3	605.10
ggc	G	0	549.60
gga	G	1844	1833.00
ggg	G	2337	1196.00
---	--	------	------
mPD		0.49	0.69
		nPD:	0.72
		N. weight:	0.52
		Sc. PD:	0.24
		Sc. rank:	1249.7

,

HA			
Pos.	282	obs:	exp:
tct	S	0	0.67
tcc	S	0	0.58
tca	S	0	0.91
tcg	S	0	0.24
tat	Y	0	0.48
tac	Y	1	0.52
aat	N	1	1.09
aac	N	1	0.91
agt	S	0	0.87
agc	S	4	0.74
gat	D	167	2255.00
gac	D	4009	1921.00
gaa	E	1	0.56
gag	E	0	0.44
---	--	------	------
mPD		0.083	0.50
		nPD:	0.16
		N. weight:	0.84
		Sc. PD:	-0.057
		Sc. rank:	190.7

,

HA			
Pos.	283	obs:	exp:
ttt	F	0	0.83
ttc	F	2	1.17
tct	S	32	698.10
tcc	S	1054	605.80
tca	S	2944	948.10
tcg	S	152	252.30
agt	S	0	907.10
agc	S	0	770.70
---	--	------	------
mPD		0.44	1.7
		nPD:	0.27
		N. weight:	1.2
		Sc. PD:	0.038
		Sc. rank:	998.5

,

HA			
Pos.	284	obs:	exp:
tct	S	0	0.17
tcc	S	0	0.14
tca	S	1	0.23
tcg	S	0	0.06
act	T	2	910.40
acc	T	0	782.60
aca	T	3439	1453.00
acg	T	32	326.80
agt	S	0	0.22
agc	S	0	0.18
gtt	V	0	0.22
gtc	V	0	0.21
gta	V	0	0.21
gtg	V	1	0.36
gct	A	0	178.60
gcc	A	0	137.20
gca	A	700	315.60
gcg	A	9	77.60
---	--	------	------
mPD		0.30	0.98
		nPD:	0.31
		N. weight:	1.1
		Sc. PD:	0.082
		Sc. rank:	1147.2

,

HA			
Pos.	285	obs:	exp:
ttt	F	2	0.83
ttc	F	0	1.17
att	I	3157	1533.00
atc	I	1014	1118.00
ata	I	6	1526.00
act	T	0	0.26
acc	T	0	0.23
aca	T	1	0.42
acg	T	0	0.09
aat	N	1	0.55
aac	N	0	0.45
gtt	V	3	0.65
gtc	V	0	0.63
gta	V	0	0.64
gtg	V	0	1.08
---	--	------	------
mPD		0.37	0.66
		nPD:	0.56
		N. weight:	0.72
		Sc. PD:	0.23
		Sc. rank:	1434.5

,

HA			
Pos.	286	obs:	exp:
tta	L	0	0.28
ttg	L	3	0.57
ctt	L	0	0.60
ctc	L	0	0.48
cta	L	0	0.44
ctg	L	0	0.63
att	I	1	1.83
atc	I	2	1.34
ata	I	2	1.83
atg	M	4172	4172.00
gtt	V	0	0.86
gtc	V	0	0.84
gta	V	0	0.85
gtg	V	4	1.45
---	--	------	------
mPD		0.0057	0.0078
		nPD:	0.73
		N. weight:	0.0037
		Sc. PD:	0.0018
		Sc. rank:	9.2

,

HA			
Pos.	287	obs:	exp:
cgt	R	0	41.65
cgc	R	0	59.56
cga	R	0	112.20
cgg	R	0	90.88
aaa	K	3045	1749.00
aag	K	26	1322.00
aga	R	1093	519.60
agg	R	20	289.10
---	--	------	------
mPD		0.41	1.0
		nPD:	0.4
		N. weight:	0.78
		Sc. PD:	0.12
		Sc. rank:	1079.7

,

HA			
Pos.	288	obs:	exp:
tct	S	0	698.40
tcc	S	0	606.10
tca	S	0	948.50
tcg	S	0	252.40
agt	S	4176	907.50
agc	S	8	771.10
---	--	------	------
mPD		0.0038	1.7
		nPD:	0.
		N. weight:	2.1
		Sc. PD:	-0.47
		Sc. rank:	-5462.8

,

HA			
Pos.	289	obs:	exp:
cgt	R	0	0.04
cgc	R	0	0.05
cga	R	0	0.10
cgg	R	0	0.08
aaa	K	4	2.85
aag	K	1	2.15
aga	R	1	0.47
agg	R	0	0.26
gat	D	4	4.32
gac	D	4	3.68
gaa	E	2396	1839.00
gag	E	885	1442.00
ggt	G	7	128.60
ggc	G	19	116.80
gga	G	858	389.50
ggg	G	5	254.10
---	--	------	------
mPD		0.69	0.89
		nPD:	0.77
		N. weight:	0.27
		Sc. PD:	0.14
		Sc. rank:	675.0

,

HA

Pos.	290	obs:	exp:
tta	L	31	120.40
ttg	L	1236	247.30
tgg	W	1	1.00
ctt	L	0	258.50
ctc	L	0	208.30
cta	L	0	192.70
ctg	L	35	274.80
atg	M	36	36.00
gtt	V	1	609.10
gtc	V	0	595.80
gta	V	104	603.90
gtg	V	2727	1023.00
gct	A	0	2.77
gcc	A	0	2.13
gca	A	10	4.90
gcg	A	1	1.20
ggt	G	2	0.29
ggc	G	0	0.26
gga	G	0	0.88
ggg	G	0	0.57
---	--	------	------
mPD		0.52	1.2
		nPD:	0.43
		N. weight:	1.4
		Sc. PD:	0.27
		Sc. rank:	2184.1

HA

Pos.	291	obs:	exp:
caa	Q	29	14.48
cag	Q	0	14.52
aaa	K	67	38.15
aag	K	0	28.85
gct	A	0	0.25
gcc	A	0	0.19
gca	A	1	0.45
gcg	A	0	0.11
gat	D	2	1.08
gac	D	0	0.92
gaa	E	3862	2280.00
gag	E	206	1788.00
ggt	G	1	2.46
ggc	G	0	2.23
gga	G	15	7.45
ggg	G	1	4.86
---	--	------	------
mPD		0.15	0.55
		nPD:	0.27
		N. weight:	0.55
		Sc. PD:	0.019
		Sc. rank:	470.9

HA

Pos.	292	obs:	exp:
tat	Y	4133	2006.00
tac	Y	50	2177.00
cat	H	1	0.58
cac	H	0	0.42
---	--	------	------
mPD		0.024	0.50
		nPD:	0.05
		N. weight:	0.93
		Sc. PD:	-0.17
		Sc. rank:	-1465.8

HA

Pos.	293	obs:	exp:
tct	S	0	45.24
tcc	S	0	39.26
tca	S	0	61.44
tcg	S	0	16.35
tgt	C	2	0.77
tgc	C	0	1.23
agt	S	267	58.78
agc	S	4	49.94
gat	D	4	2.16
gac	D	0	1.84
gaa	E	1	0.56
gag	E	0	0.44
ggt	G	2686	564.90
ggc	G	1030	513.10
gga	G	181	1712.00
ggg	G	9	1117.00
---	--	------	------
mPD		0.56	0.90
		nPD:	0.62
		N. weight:	1.6
		Sc. PD:	0.6
		Sc. rank:	3479.8

HA

Pos.	294	obs:	exp:
tct	S	0	1.17
tcc	S	0	1.01
tca	S	0	1.59
tcg	S	0	0.42
tat	Y	0	0.96
tac	Y	2	1.04
cat	H	2	518.70
cac	H	894	377.30
act	T	0	0.52
acc	T	2	0.45
aca	T	0	0.84
acg	T	0	0.19
aat	N	278	1763.00
aac	N	2955	1470.00
aaa	K	2	1.14
aag	K	0	0.86
agt	S	0	1.52
agc	S	7	1.29
gat	D	1	22.68
gac	D	41	19.32
---	--	------	------
mPD		0.48	0.85
		nPD:	0.57
		N. weight:	0.78
		Sc. PD:	0.25
		Sc. rank:	1576.2

HA

Pos.	295	obs:	exp:
tat	Y	0	0.48
tac	Y	1	0.52
tgt	C	56	1619.00
tgc	C	4127	2564.00
---	--	------	------
mPD		0.027	0.47
		nPD:	0.06
		N. weight:	0.59
		Sc. PD:	-0.1
		Sc. rank:	-815.8

HA

Pos.	296	obs:	exp:
tct	S	0	29.38
tcc	S	0	25.49
tca	S	0	39.90
tcg	S	0	10.62
tat	Y	0	0.48
tac	Y	1	0.52
att	I	1	0.37
atc	I	0	0.27
ata	I	0	0.37
act	T	0	0.26
acc	T	1	0.23
aca	T	0	0.42
acg	T	0	0.09
aat	N	226	2158.00
aac	N	3732	1800.00
agt	S	0	38.18
agc	S	176	32.43
gat	D	2	25.38
gac	D	45	21.62
---	--	------	------
mPD		0.21	0.66
		nPD:	0.31
		N. weight:	0.84
		Sc. PD:	0.064
		Sc. rank:	880.9

HA

Pos.	297	obs:	exp:
tct	S	0	0.17
tcc	S	1	0.14
tca	S	0	0.23
tcg	S	0	0.06
att	I	0	0.37
atc	I	1	0.27
ata	I	0	0.37
act	T	23	1094.00
acc	T	4141	940.50
aca	T	10	1747.00
acg	T	0	392.70
agt	S	0	0.22
agc	S	0	0.18
gct	A	0	2.02
gcc	A	8	1.55
gca	A	0	3.56
gcg	A	0	0.88
---	--	------	------
mPD		0.020	0.70
		nPD:	0.03
		N. weight:	2.
		Sc. PD:	-0.4
		Sc. rank:	-4015.5

HA

Pos.	298	obs:	exp:
caa	Q	0	0.50
cag	Q	1	0.50
cgt	R	0	61.71
cgc	R	0	88.25
cga	R	0	166.20
cgg	R	1	134.70
aaa	K	981	1442.00
aag	K	1552	1091.00
aga	R	25	769.80
agg	R	1623	428.40
gaa	E	0	0.56
gag	E	1	0.44
---	--	------	------
mPD		0.84	1.2
		nPD:	0.71
		N. weight:	0.74
		Sc. PD:	0.34
		Sc. rank:	1778.1

HA

Pos.	299	obs:	exp:
tct	S	0	0.17
tcc	S	0	0.14
tca	S	0	0.23
tcg	S	0	0.06
tgt	C	3899	1619.00
tgc	C	283	2563.00
agt	S	1	0.22
agc	S	0	0.18
ggt	G	1	0.14
ggc	G	0	0.13
gga	G	0	0.44
ggg	G	0	0.29
---	--	------	------
mPD		0.13	0.48
		nPD:	0.27
		N. weight:	0.93
		Sc. PD:	0.029
		Sc. rank:	755.3

HA

Pos.	300	obs:	exp:
cat	H	0	0.58
cac	H	1	0.42
caa	Q	2273	2088.00
cag	Q	1910	2095.00
---	--	------	------
mPD		0.50	0.50
		nPD:	0.99
		N. weight:	0.0071
		Sc. PD:	0.0052
		Sc. rank:	18.6

HA

Pos.	301	obs:	exp:
tct	S	3	0.67
tcc	S	0	0.58
tca	S	0	0.91
tcg	S	0	0.24
act	T	3176	1096.00
acc	T	998	941.60
aca	T	3	1749.00
acg	T	2	393.20
aat	N	1	0.55
aac	N	0	0.45
agt	S	1	0.87
agc	S	0	0.74
---	--	------	------
mPD		0.37	0.70
		nPD:	0.53
		N. weight:	1.1
		Sc. PD:	0.32
		Sc. rank:	2170.7

HA

Pos.	302	obs:	exp:
cct	P	355	1143.00
ccc	P	2	755.70
cca	P	3293	1698.00
ccg	P	534	586.60
---	--	------	------
mPD		0.36	0.71
		nPD:	0.5
		N. weight:	0.57
		Sc. PD:	0.15
		Sc. rank:	1027.0

HA

Pos.	303	obs:	exp:
tta	L	13	2.77
ttg	L	16	5.70
ctt	L	0	5.96
ctc	L	0	4.80
cta	L	1	4.44
ctg	L	0	6.33
att	I	4	926.20
atc	I	1	675.40
ata	I	2519	922.40
atg	M	654	654.00
act	T	0	0.26
acc	T	0	0.23
aca	T	1	0.42
acg	T	0	0.09
gtt	V	0	209.70
gtc	V	0	205.10
gta	V	951	207.90
gtg	V	24	352.30
---	--	------	------
mPD		0.65	1.1
		nPD:	0.58
		N. weight:	1.3
		Sc. PD:	0.43
		Sc. rank:	2677.3

HA

Pos.	304	obs:	exp:
ggt	G	353	605.10
ggc	G	0	549.60
gga	G	733	1833.00
ggg	G	3098	1196.00
---	--	------	------
mPD		0.41	0.69
		nPD:	0.6
		N. weight:	0.69
		Sc. PD:	0.25
		Sc. rank:	1469.4

HA

Pos.	305	obs:	exp:
tct	S	0	0.17
tcc	S	0	0.14
tca	S	0	0.23
tcg	S	1	0.06
cct	P	0	0.27
ccc	P	0	0.18
cca	P	0	0.41
ccg	P	1	0.14
agt	S	0	0.22
agc	S	0	0.18
gct	A	69	1054.00
gcc	A	3	809.30
gca	A	93	1861.00
gcg	A	4017	457.70
---	--	------	------
mPD		0.078	0.69
		nPD:	0.11
		N. weight:	2.7
		Sc. PD:	-0.32
		Sc. rank:	-1088.9

,

HA

Pos.	306	obs:	exp:
tta	L	10	1.02
ttg	L	0	2.09
ctt	L	1	2.18
ctc	L	0	1.76
cta	L	0	1.63
ctg	L	0	2.32
att	I	1	1525.00
atc	I	1	1112.00
ata	I	4155	1519.00
atg	M	2	2.00
act	T	0	0.26
acc	T	0	0.23
aca	T	1	0.42
acg	T	0	0.09
aat	N	1	0.55
aac	N	0	0.45
gtt	V	0	2.58
gtc	V	0	2.52
gta	V	12	2.56
gtg	V	0	4.34
---	--	------	------
mPD		0.015	0.67
		nPD:	0.02
		N. weight:	1.4
		Sc. PD:	-0.29
		Sc. rank:	-3063.1

,

HA

Pos.	307	obs:	exp:
tct	S	0	1.17
tcc	S	0	1.01
tca	S	0	1.59
tcg	S	0	0.42
aat	N	1057	2275.00
aac	N	3115	1897.00
agt	S	0	1.52
agc	S	7	1.29
gat	D	4	2.70
gac	D	1	2.30
---	--	------	------
mPD		0.38	0.50
		nPD:	0.76
		N. weight:	0.25
		Sc. PD:	0.13
		Sc. rank:	617.7

,

HA

Pos.	308	obs:	exp:
ttt	F	1	0.42
ttc	F	0	0.58
tct	S	3547	696.70
tcc	S	624	604.60
tca	S	1	946.20
tcg	S	2	251.80
tat	Y	1	0.48
tac	Y	0	0.52
cct	P	0	0.27
ccc	P	1	0.18
cca	P	0	0.41
ccg	P	0	0.14
act	T	2	1.84
acc	T	5	1.58
aca	T	0	2.93
acg	T	0	0.66
agt	S	0	905.40
agc	S	0	769.20
---	--	------	------
mPD		0.26	1.7
		nPD:	0.16
		N. weight:	1.9
		Sc. PD:	-0.14
		Sc. rank:	292.6

,

HA

Pos.	309	obs:	exp:
tct	S	0	691.90
tcc	S	0	600.40
tca	S	0	939.70
tcg	S	0	250.10
cgt	R	0	0.07
cgc	R	0	0.11
cga	R	0	0.20
cgg	R	0	0.16
att	I	3	1.10
atc	I	0	0.80
ata	I	0	1.10
act	T	28	7.34
acc	T	0	6.31
aca	T	0	11.72
acg	T	0	2.63
aat	N	6	3.27
aac	N	0	2.73
agt	S	3786	899.10
agc	S	359	763.90
aga	R	0	0.93
agg	R	2	0.52
---	--	------	------
mPD		0.18	1.7
		nPD:	0.11
		N. weight:	1.7
		Sc. PD:	-0.22
		Sc. rank:	-841.8

,

HA

Pos.	310	obs:	exp:
tta	L	0	0.65
ttg	L	3	1.33
ctt	L	0	1.39
ctc	L	0	1.12
cta	L	0	1.04
ctg	L	4	1.48
atg	M	4176	4176.00
aaa	K	0	0.57
aag	K	1	0.43
---	--	------	------
mPD		0.0038	0.0061
		nPD:	0.63
		N. weight:	0.0028
		Sc. PD:	0.0011
		Sc. rank:	6.1

,

HA

Pos.	311	obs:	exp:
tta	L	0	0.09
ttg	L	0	0.19
ctt	L	0	0.20
ctc	L	0	0.16
cta	L	1	0.15
ctg	L	0	0.21
cct	P	5	1143.00
ccc	P	41	755.50
cca	P	4065	1698.00
ccg	P	72	586.50
---	--	------	------
mPD		0.056	0.71
		nPD:	0.08
		N. weight:	1.1
		Sc. PD:	-0.16
		Sc. rank:	-940.9

,

HA

Pos.	312	obs:	exp:
ttt	F	53	1744.00
ttc	F	4127	2436.00
tta	L	0	0.28
ttg	L	0	0.57
ctt	L	0	0.60
ctc	L	3	0.48
cta	L	0	0.44
ctg	L	0	0.63
att	I	0	0.37
atc	I	1	0.27
ata	I	0	0.37
---	--	------	------
mPD		0.027	0.49
		nPD:	0.06
		N. weight:	0.66
		Sc. PD:	-0.12
		Sc. rank:	-956.6

,

HA

Pos.	313	obs:	exp:
cct	P	0	1.09
ccc	P	2	0.72
cca	P	2	1.62
ccg	P	0	0.56
cat	H	159	2417.00
cac	H	4017	1759.00
caa	Q	1	0.50
cag	Q	0	0.50
cgt	R	0	0.07
cgc	R	2	0.11
cga	R	0	0.20
cgg	R	0	0.16
aga	R	0	0.93
agg	R	0	0.52
---	-	1	1.00
---	--	------	------
mPD		0.077	0.49
		nPD:	0.16
		N. weight:	0.96
		Sc. PD:	-0.072
		Sc. rank:	117.0

,

HA

Pos.	314	obs:	exp:
tat	Y	0	0.48
tac	Y	1	0.52
att	I	0	0.37
atc	I	1	0.27
ata	I	0	0.37
act	T	0	0.26
acc	T	0	0.23
aca	T	1	0.42
acg	T	0	0.09
aat	N	362	2279.00
aac	N	3817	1900.00
gat	D	1	1.08
gac	D	1	0.92
---	--	------	------
mPD		0.16	0.50
		nPD:	0.32
		N. weight:	0.66
		Sc. PD:	0.057
		Sc. rank:	729.6

,

HA

Pos.	315	obs:	exp:
tta	L	1	0.09
ttg	L	0	0.19
ctt	L	0	0.20
ctc	L	0	0.16
cta	L	0	0.15
ctg	L	0	0.21
att	I	17	1520.00
atc	I	643	1109.00
ata	I	3483	1514.00
act	T	0	0.26
acc	T	1	0.23
aca	T	0	0.42
acg	T	0	0.09
gtt	V	37	8.39
gtc	V	0	8.21
gta	V	2	8.32
gtg	V	0	14.09
---	--	------	------
mPD		0.30	0.68
		nPD:	0.44
		N. weight:	0.84
		Sc. PD:	0.17
		Sc. rank:	1339.8

,

HA

Pos.	316	obs:	exp:
tat	Y	0	0.48
tac	Y	1	0.52
cct	P	0	0.55
ccc	P	2	0.36
cca	P	0	0.81
ccg	P	0	0.28
cat	H	1466	2419.00
cac	H	2713	1760.00
aat	N	1	1.09
aac	N	1	0.91
---	--	------	------
mPD		0.46	0.49
		nPD:	0.93
		N. weight:	0.15
		Sc. PD:	0.1
		Sc. rank:	386.3

,

HA

Pos.	317	obs:	exp:
cct	P	3683	1143.00
ccc	P	490	755.20
cca	P	4	1697.00
ccg	P	4	586.20
cat	H	1	0.58
cac	H	0	0.42
act	T	0	0.52
acc	T	2	0.45
aca	T	0	0.84
acg	T	0	0.19
---	--	------	------
mPD		0.21	0.71
		nPD:	0.3
		N. weight:	1.3
		Sc. PD:	0.083
		Sc. rank:	1333.3

,

HA

Pos.	318	obs:	exp:
ttt	F	0	1.25
ttc	F	3	1.75
tta	L	0	385.80
ttg	L	0	792.80
ctt	L	62	828.50
ctc	L	4104	667.50
cta	L	7	617.60
ctg	L	0	880.80
cat	H	0	0.58
cac	H	1	0.42
att	I	0	2.57
atc	I	7	1.87
ata	I	0	2.56
---	--	------	------
mPD		0.038	1.1
		nPD:	0.03
		N. weight:	2.4
		Sc. PD:	-0.47
		Sc. rank:	-4662.8

,

HA

Pos.	319	obs:	exp:
tct	S	0	0.33
tcc	S	1	0.29
tca	S	1	0.45
tcg	S	0	0.12
act	T	657	1096.00
acc	T	3513	941.60
aca	T	7	1749.00
acg	T	2	393.20
agt	S	0	0.43
agc	S	0	0.37
gct	A	0	0.76
gcc	A	3	0.58
gca	A	0	1.34
gcg	A	0	0.33
---	--	------	------
mPD		0.27	0.70
		nPD:	0.39
		N. weight:	1.4
		Sc. PD:	0.21
		Sc. rank:	1896.5

,

HA			
Pos.	320	obs:	exp:
tct	S	0	0.17
tcc	S	0	0.14
tca	S	0	0.23
tcg	S	0	0.06
cct	P	0	0.27
ccc	P	0	0.18
cca	P	1	0.41
ccg	P	0	0.14
att	I	1293	1533.00
atc	I	2816	1118.00
ata	I	69	1527.00
aat	N	1	0.55
aac	N	0	0.45
agt	S	1	0.22
agc	S	0	0.18
gtt	V	0	0.65
gtc	V	3	0.63
gta	V	0	0.64
gtg	V	0	1.08
---	--	------	------
mPD		0.45	0.66
		nPD:	0.68
		N. weight:	0.72
		Sc. PD:	0.31
		Sc. rank:	1683.1

HA			
Pos.	321	obs:	exp:
tct	S	0	0.17
tcc	S	0	0.14
tca	S	0	0.23
tcg	S	1	0.06
cgt	R	0	0.04
cgc	R	0	0.05
cga	R	0	0.10
cgg	R	1	0.08
agt	S	0	0.22
agc	S	0	0.18
aga	R	0	0.47
agg	R	0	0.26
ggt	G	4	604.80
ggc	G	0	549.30
gga	G	1814	1832.00
ggg	G	2364	1195.00
---	--	------	------
mPD		0.49	0.69
		nPD:	0.72
		N. weight:	0.52
		Sc. PD:	0.24
		Sc. rank:	1262.1

HA			
Pos.	322	obs:	exp:
caa	Q	1	0.50
cag	Q	0	0.50
aaa	K	6	3.99
aag	K	1	3.01
gat	D	3	1.62
gac	D	0	1.38
gaa	E	4113	2339.00
gag	E	59	1833.00
ggt	G	0	0.14
ggc	G	0	0.13
gga	G	1	0.44
ggg	G	0	0.29
---	--	------	------
mPD		0.034	0.50
		nPD:	0.07
		N. weight:	0.7
		Sc. PD:	-0.11
		Sc. rank:	-830.6

HA			
Pos.	323	obs:	exp:
tgt	C	1674	1618.00
tgc	C	2507	2563.00
cgt	R	1	0.04
cgc	R	0	0.05
cga	R	0	0.10
cgg	R	0	0.08
aga	R	0	0.47
agg	R	0	0.26
ggt	G	0	0.29
ggc	G	2	0.26
gga	G	0	0.88
ggg	G	0	0.57
---	--	------	------
mPD		0.48	0.48
		nPD:	1.01
		N. weight:	0.0044
		Sc. PD:	0.0033
		Sc. rank:	11.5

HA			
Pos.	324	obs:	exp:
cct	P	21	1143.00
ccc	P	4148	755.70
cca	P	15	1698.00
ccg	P	0	586.60
---	--	------	------
mPD		0.017	0.71
		nPD:	0.02
		N. weight:	2.3
		Sc. PD:	-0.47
		Sc. rank:	-4848.4

HA			
Pos.	325	obs:	exp:
aat	N	1	0.55
aac	N	0	0.45
aaa	K	4143	2382.00
aag	K	40	1801.00
---	--	------	------
mPD		0.019	0.49
		nPD:	0.04
		N. weight:	0.71
		Sc. PD:	-0.13
		Sc. rank:	-1288.0

HA			
Pos.	326	obs:	exp:
tta	L	0	0.09
ttg	L	1	0.19
tat	Y	3257	2005.00
tac	Y	923	2175.00
tgt	C	0	0.77
tgc	C	2	1.23
ctt	L	0	0.20
ctc	L	0	0.16
cta	L	0	0.15
ctg	L	0	0.21
aat	N	1	0.55
aac	N	0	0.45
---	--	------	------
mPD		0.35	0.50
		nPD:	0.69
		N. weight:	0.26
		Sc. PD:	0.12
		Sc. rank:	618.5

HA			
Pos.	327	obs:	exp:
gtt	V	5	899.70
gtc	V	34	880.00
gta	V	126	891.90
gtg	V	4018	1511.00
ggt	G	0	0.14
ggc	G	0	0.13
gga	G	0	0.44
ggg	G	1	0.29
---	--	------	------
mPD		0.077	0.73
		nPD:	0.1
		N. weight:	1.2
		Sc. PD:	-0.15
		Sc. rank:	-604.1

HA			
Pos.	328	obs:	exp:
cgt	R	0	0.19
cgc	R	0	0.27
cga	R	0	0.50
cgg	R	0	0.41
att	I	0	0.37
atc	I	0	0.27
ata	I	1	0.37
aaa	K	3229	2379.00
aag	K	949	1799.00
aga	R	5	2.33
agg	R	0	1.30
---	--	------	------
mPD		0.35	0.49
		nPD:	0.72
		N. weight:	0.13
		Sc. PD:	0.06
		Sc. rank:	308.8

HA			
Pos.	329	obs:	exp:
tct	S	2	697.70
tcc	S	0	605.50
tca	S	4071	947.60
tcg	S	107	252.20
agt	S	0	906.70
agc	S	0	770.30
gct	A	0	1.01
gcc	A	0	0.77
gca	A	4	1.78
gcg	A	0	0.44
---	--	------	------
mPD		0.053	1.7
		nPD:	0.03
		N. weight:	1.9
		Sc. PD:	-0.38
		Sc. rank:	-3818.1

HA			
Pos.	330	obs:	exp:
tct	S	0	9.35
tcc	S	0	8.11
tca	S	0	12.70
tcg	S	0	3.38
act	T	0	32.77
acc	T	125	28.17
aca	T	0	52.30
acg	T	0	11.76
aat	N	23	2139.00
aac	N	3900	1784.00
aaa	K	2	1.71
aag	K	1	1.29
agt	S	4	12.15
agc	S	52	10.32
gat	D	33	41.03
gac	D	43	34.97
ggt	G	0	0.14
ggc	G	1	0.13
gga	G	0	0.44
ggg	G	0	0.29
---	--	------	------
mPD		0.15	0.65
		nPD:	0.23
		N. weight:	1.1
		Sc. PD:	-0.0054
		Sc. rank:	630.4

HA			
Pos.	331	obs:	exp:
cgt	R	0	46.26
cgc	R	0	66.15
cga	R	0	124.60
cgg	R	0	100.90
aaa	K	2089	1679.00
aag	K	859	1269.00
aga	R	1221	577.00
agg	R	15	321.10
---	--	------	------
mPD		0.75	1.1
		nPD:	0.69
		N. weight:	0.33
		Sc. PD:	0.15
		Sc. rank:	775.0

HA			
Pos.	332	obs:	exp:
tta	L	3040	386.90
ttg	L	916	794.80
ctt	L	0	830.70
ctc	L	0	669.30
cta	L	189	619.20
ctg	L	39	883.10
---	--	------	------
mPD		0.46	1.1
		nPD:	0.41
		N. weight:	2.
		Sc. PD:	0.33
		Sc. rank:	2845.8

HA			
Pos.	333	obs:	exp:
tct	S	0	0.17
tcc	S	1	0.14
tca	S	0	0.23
tcg	S	0	0.06
att	I	1	9.17
atc	I	24	6.69
ata	I	0	9.14
agt	S	0	0.22
agc	S	0	0.18
gtt	V	65	894.30
gtc	V	4078	874.80
gta	V	15	886.60
gtg	V	0	1502.00
---	--	------	------
mPD		0.051	0.75
		nPD:	0.07
		N. weight:	2.
		Sc. PD:	-0.33
		Sc. rank:	-2387.1

HA			
Pos.	334	obs:	exp:
ttt	F	1	0.42
ttc	F	0	0.58
tta	L	6	386.70
ttg	L	0	794.50
ctt	L	4122	830.30
ctc	L	35	668.90
cta	L	13	618.90
ctg	L	6	882.70
cct	P	1	0.27
ccc	P	0	0.18
cca	P	0	0.41
ccg	P	0	0.14
---	--	------	------
mPD		0.032	1.1
		nPD:	0.03
		N. weight:	2.1
		Sc. PD:	-0.42
		Sc. rank:	-4326.9

HA

Pos.	335	obs:	exp:
gtt	V	0	0.65
gtc	V	0	0.63
gta	V	0	0.64
gtg	V	3	1.08
gct	A	500	1053.00
gcc	A	3	808.70
gca	A	1141	1860.00
gcg	A	2535	457.40
gat	D	1	0.54
gac	D	0	0.46
ggt	G	1	0.14
ggc	G	0	0.13
gga	G	0	0.44
ggg	G	0	0.29
---	--	------	------
mPD		0.55	0.69
		nPD:	0.79
		N. weight:	1.1
		Sc. PD:	0.6
		Sc. rank:	2853.3

,

HA

Pos.	336	obs:	exp:
tct	S	1	0.17
tcc	S	0	0.14
tca	S	0	0.23
tcg	S	0	0.06
act	T	3860	1097.00
acc	T	66	942.50
aca	T	252	1750.00
acg	T	5	393.60
agt	S	0	0.22
agc	S	0	0.18
---	--	------	------
mPD		0.15	0.70
		nPD:	0.21
		N. weight:	1.4
		Sc. PD:	-0.036
		Sc. rank:	624.9

,

HA

Pos.	337	obs:	exp:
ggt	G	0	605.10
ggc	G	13	549.60
gga	G	280	1833.00
ggg	G	3891	1196.00
---	--	------	------
mPD		0.13	0.69
		nPD:	0.19
		N. weight:	1.3
		Sc. PD:	-0.058
		Sc. rank:	544.4

,

HA

Pos.	338	obs:	exp:
ttt	F	0	0.42
ttc	F	1	0.58
tta	L	0	386.10
ttg	L	0	793.30
ctt	L	70	829.10
ctc	L	4050	668.00
cta	L	56	618.00
ctg	L	0	881.40
cct	P	0	1.91
ccc	P	2	1.26
cca	P	5	2.84
ccg	P	0	0.98
---	--	------	------
mPD		0.065	1.1
		nPD:	0.06
		N. weight:	2.3
		Sc. PD:	-0.4
		Sc. rank:	-3140.4

,

HA

Pos.	339	obs:	exp:
tct	S	0	0.17
tcc	S	0	0.14
tca	S	0	0.23
tcg	S	0	0.06
cgt	R	0	156.50
cgc	R	0	223.80
cga	R	0	421.40
cgg	R	0	341.50
aaa	K	1	0.57
aag	K	0	0.43
agt	S	1	0.22
agc	S	0	0.18
aga	R	4029	1952.00
agg	R	153	1086.00
---	--	------	------
mPD		0.071	0.96
		nPD:	0.07
		N. weight:	0.87
		Sc. PD:	-0.13
		Sc. rank:	-879.6

,

HA

Pos.	340	obs:	exp:
aat	N	4121	2282.00
aac	N	63	1902.00
---	--	------	------
mPD		0.030	0.50
		nPD:	0.06
		N. weight:	0.74
		Sc. PD:	-0.12
		Sc. rank:	-949.1

,

HA

Pos.	341	obs:	exp:
tct	S	0	670.40
tcc	S	0	581.70
tca	S	0	910.40
tcg	S	0	242.30
tat	Y	0	0.96
tac	Y	2	1.04
att	I	3	5.87
atc	I	1	4.28
ata	I	12	5.85
act	T	0	4.46
acc	T	17	3.83
aca	T	0	7.11
acg	T	0	1.60
aat	N	36	21.81
aac	N	4	18.19
agt	S	2826	871.10
agc	S	1190	740.10
gtt	V	15	11.61
gtc	V	17	11.36
gta	V	22	11.51
gtg	V	0	19.51
gct	A	0	9.32
gcc	A	37	7.16
gca	A	0	16.47
gcg	A	0	4.05
ggt	G	1	0.29
ggc	G	1	0.26
gga	G	0	0.88
ggg	G	0	0.57
---	--	------	------
mPD		0.56	1.7
		nPD:	0.33
		N. weight:	1.3
		Sc. PD:	0.12
		Sc. rank:	1533.9

,

HA

Pos.	342	obs:	exp:
tta	L	0	0.55
ttg	L	0	1.14
tct	S	3	0.50
tcc	S	0	0.43
tca	S	0	0.68
tcg	S	0	0.18
ctt	L	6	1.19
ctc	L	0	0.96
cta	L	0	0.89
ctg	L	0	1.27
cct	P	4068	1138.00
ccc	P	89	752.10
cca	P	7	1690.00
ccg	P	0	583.80
cat	H	9	5.21
cac	H	0	3.79
agt	S	0	0.65
agc	S	0	0.55
gct	A	2	0.50
gcc	A	0	0.39
gca	A	0	0.89
gcg	A	0	0.22
---	--	------	------
mPD		0.054	0.72
		nPD:	0.08
		N. weight:	1.6
		Sc. PD:	-0.25
		Sc. rank:	-1627.0

,

HA

Pos.	343	obs:	exp:
tta	L	2	112.20
ttg	L	0	230.40
ctt	L	0	240.80
ctc	L	0	194.00
cta	L	1209	179.50
ctg	L	2	256.00
cat	H	1	0.58
cac	H	0	0.42
caa	Q	2936	1474.00
cag	Q	16	1478.00
cgt	R	0	0.15
cgc	R	0	0.21
cga	R	4	0.40
cgg	R	0	0.33
att	I	0	1.10
atc	I	0	0.80
ata	I	3	1.10
aaa	K	10	6.26
aag	K	1	4.74
aga	R	0	1.87
agg	R	0	1.04
---	--	------	------
mPD		0.43	1.2
		nPD:	0.37
		N. weight:	1.4
		Sc. PD:	0.18
		Sc. rank:	1814.8

,

HA

Pos.	344	obs:	exp:
cgt	R	0	0.04
cgc	R	0	0.05
cga	R	0	0.10
cgg	R	0	0.08
aga	R	1	0.47
agg	R	0	0.26
---	-	4183	4183.00
---	--	------	------
mPD		0	5.5×10^{-8}
		nPD:	0.
		N. weight:	0.
		Sc. PD:	0
		Sc. rank:	0

,

HA

Pos.	345	obs:	exp:
cgt	R	0	0.04
cgc	R	0	0.05
cga	R	0	0.10
cgg	R	0	0.08
aga	R	1	0.47
agg	R	0	0.26
---	-	4183	4183.00
---	--	------	------
mPD		0	5.5×10^{-8}
		nPD:	0.
		N. weight:	0.
		Sc. PD:	0
		Sc. rank:	0

,

HA

Pos.	346	obs:	exp:
tct	S	0	0.17
tcc	S	0	0.14
tca	S	0	0.23
tcg	S	0	0.06
cgt	R	0	125.30
cgc	R	0	179.10
cga	R	2	337.30
cgg	R	0	273.30
att	I	1	30.09
atc	I	0	21.94
ata	I	81	29.97
aaa	K	77	50.68
aag	K	12	38.32
agt	S	1	0.22
agc	S	0	0.18
aga	R	3307	1563.00
agg	R	38	869.50
gaa	E	5	4.48
gag	E	3	3.52
ggt	G	10	94.72
ggc	G	0	86.04
gga	G	638	287.00
ggg	G	7	187.20
---	-	2	2.00
---	--	------	------
mPD		0.38	1.2
		nPD:	0.32
		N. weight:	0.97
		Sc. PD:	0.077
		Sc. rank:	1031.4

,

HA

Pos.	347	obs:	exp:
cgt	R	0	0.26
cgc	R	0	0.37
cga	R	0	0.71
cgg	R	0	0.57
aaa	K	2	7.40
aag	K	11	5.60
aga	R	7	3.27
agg	R	0	1.82
gat	D	4	2.16
gac	D	0	1.84
gaa	E	1416	2330.00
gag	E	2740	1826.00
ggt	G	0	0.43
ggc	G	0	0.39
gga	G	1	1.31
ggg	G	2	0.86
---	-	1	1.00
---	--	------	------
mPD		0.46	0.51
		nPD:	0.91
		N. weight:	0.14
		Sc. PD:	0.093
		Sc. rank:	366.6

,

HA

Pos.	348	obs:	exp:
cgt	R	0	0.30
cgc	R	0	0.43
cga	R	0	0.81
cgg	R	0	0.65
act	T	0	15.73
acc	T	0	13.52
aca	T	57	25.11
acg	T	3	5.65
aaa	K	8	4.56
aag	K	0	3.44
aga	R	8	3.73
agg	R	0	2.08
gct	A	0	0.25
gcc	A	0	0.19
gca	A	0	0.45
gcg	A	1	0.11
ggt	G	0	0.29
ggc	G	0	0.26
gga	G	2	0.88
ggg	G	0	0.57
---	-	4105	4105.00
---	--	------	------
mPD		0.00019	0.00043
		nPD:	0.46
		N. weight:	0.
		Sc. PD:	0
		Sc. rank:	0

,

HA

Pos.	349	obs:	exp:
gaa	E	0	2.80
gag	E	5	2.20
---	-	4179	4179.00
---	--	------	------
mPD		0	7.0×10^{-7}
		nPD:	0.
		N. weight:	0.
		Sc. PD:	0
		Sc. rank:	0

,

HA

Pos.	350	obs:	exp:
ggt	G	0	0.72
ggc	G	0	0.66
gga	G	1	2.19
ggg	G	4	1.43
---	-	4179	4179.00
---	--	------	------
mPD		4.6×10^{-7}	9.8×10^{-7}
		nPD:	0.47
		N. weight:	0.
		Sc. PD:	0
		Sc. rank:	0

,

HA

Pos.	351	obs:	exp:
tct	S	0	31.38
tcc	S	0	27.23
tca	S	0	42.62
tcg	S	0	11.34
cgt	R	0	35.81
cgc	R	0	51.21
cga	R	0	96.44
cgg	R	0	78.14
aaa	K	261	151.50
aag	K	5	114.50
agt	S	14	40.78
agc	S	174	34.65
aga	R	933	446.80
agg	R	24	248.60
gaa	E	92	51.57
gag	E	0	40.43
ggt	G	0	26.32
ggc	G	0	23.91
gga	G	180	79.75
ggg	G	2	52.02
---	-	2499	2499.00
---	--	------	------
mPD		0.14	0.26
		nPD:	0.52
		N. weight:	0.
		Sc. PD:	0
		Sc. rank:	0

,

HA

Pos.	352	obs:	exp:
tct	S	0	0.17
tcc	S	0	0.14
tca	S	0	0.23
tcg	S	0	0.06
cgt	R	0	121.00
cgc	R	0	173.00
cga	R	0	325.80
cgg	R	0	264.00
aaa	K	17	496.50
aag	K	855	375.50
agt	S	0	0.22
agc	S	1	0.18
aga	R	3143	1509.00
agg	R	90	839.90
ggt	G	0	1.01
ggc	G	0	0.92
gga	G	7	3.07
ggg	G	0	2.00
---	-	71	71.00
---	--	------	------
mPD		0.67	1.2
		nPD:	0.57
		N. weight:	0.91
		Sc. PD:	0.29
		Sc. rank:	1852.6

,

HA

Pos.	353	obs:	exp:
cgt	R	0	153.60
cgc	R	0	219.60
cga	R	4	413.60
cgg	R	0	335.10
aaa	K	9	5.12
aag	K	0	3.88
aga	R	4066	1916.00
agg	R	34	1066.00
---	-	71	71.00
---	--	------	------
mPD		0.022	0.93
		nPD:	0.02
		N. weight:	0.97
		Sc. PD:	-0.2
		Sc. rank:	-2087.3

,

HA

Pos.	354	obs:	exp:
tct	S	0	0.83
tcc	S	0	0.72
tca	S	0	1.13
tcg	S	0	0.30
cgt	R	0	94.16
cgc	R	0	134.60
cga	R	4	253.50
cgg	R	0	205.40
aaa	K	1164	683.90
aag	K	37	517.10
agt	S	5	1.08
agc	S	0	0.92
aga	R	2490	1175.00
agg	R	22	653.60
gaa	E	1	0.56
gag	E	0	0.44
ggt	G	0	0.14
ggc	G	0	0.13
gga	G	1	0.44
ggg	G	0	0.29
---	-	460	460.00
---	--	------	------
mPD		0.38	1.0
		nPD:	0.37
		N. weight:	0.
		Sc. PD:	0
		Sc. rank:	0

,

HA

Pos.	355	obs:	exp:
cgt	R	0	0.67
cgc	R	0	0.96
cga	R	0	1.81
cgg	R	0	1.47
att	I	1	0.37
atc	I	0	0.27
ata	I	0	0.37
act	T	1	0.52
acc	T	0	0.45
aca	T	0	0.84
acg	T	1	0.19
aat	N	1	0.55
aac	N	0	0.45
aaa	K	2891	2331.00
aag	K	1202	1762.00
aga	R	9	8.40
agg	R	9	4.68
---	-	69	69.00
---	--	------	------
mPD		0.41	0.49
		nPD:	0.85
		N. weight:	0.06
		Sc. PD:	0.035
		Sc. rank:	153.0

,

HA

Pos.	356	obs:	exp:
cgt	R	0	0.15
cgc	R	0	0.21
cga	R	0	0.40
cgg	R	0	0.33
aga	R	4	1.87
agg	R	0	1.04
---	-	4180	4180.00
---	--	------	------
mPD		0	8.7×10^{-7}
		nPD:	0.
		N. weight:	0.
		Sc. PD:	0
		Sc. rank:	0

,

HA

Pos.	357	obs:	exp:
cgt	R	0	156.60
cgc	R	0	223.90
cga	R	2	421.60
cgg	R	0	341.60
aga	R	4114	1953.00
agg	R	68	1087.00
---	--	------	------
mPD		0.033	0.95
		nPD:	0.03
		N. weight:	0.95
		Sc. PD:	-0.18
		Sc. rank:	-1819.1

,

HA

Pos.	358	obs:	exp:
cgt	R	0	0.07
cgc	R	0	0.11
cga	R	1	0.20
cgg	R	0	0.16
aga	R	1	0.93
agg	R	0	0.52
gaa	E	1	0.56
gag	E	0	0.44
ggt	G	25	604.60
ggc	G	895	549.20
gga	G	3138	1832.00
ggg	G	123	1195.00
---	--	------	------
mPD		0.39	0.69
		nPD:	0.57
		N. weight:	0.59
		Sc. PD:	0.19
		Sc. rank:	1196.0

,

HA

Pos.	359	obs:	exp:
tta	L	268	386.60
ttg	L	518	794.30
ctt	L	1	830.10
ctc	L	0	668.80
cta	L	1457	618.80
ctg	L	1937	882.50
att	I	0	1.10
atc	I	0	0.80
ata	I	3	1.10
---	--	------	------
mPD		0.79	1.1
		nPD:	0.71
		N. weight:	0.81
		Sc. PD:	0.37
		Sc. rank:	1928.7

,

HA

Pos.	360	obs:	exp:
ttt	F	4042	1744.00
ttc	F	138	2436.00
tta	L	0	0.18
ttg	L	2	0.38
tct	S	1	0.17
tcc	S	0	0.14
tca	S	0	0.23
tcg	S	0	0.06
ctt	L	0	0.40
ctc	L	0	0.32
cta	L	0	0.30
ctg	L	0	0.42
agt	S	0	0.22
agc	S	0	0.18
ggt	G	0	0.14
ggc	G	0	0.13
gga	G	1	0.44
ggg	G	0	0.29
---	--	------	------
mPD		0.067	0.49
		nPD:	0.14
		N. weight:	1.
		Sc. PD:	-0.096
		Sc. rank:	-169.7

,

HA

Pos.	361	obs:	exp:
gct	A	0	0.25
gcc	A	0	0.19
gca	A	1	0.45
gcg	A	0	0.11
ggt	G	25	604.90
ggc	G	0	549.40
gga	G	3225	1833.00
ggg	G	933	1196.00
---	--	------	------
mPD		0.36	0.69
		nPD:	0.52
		N. weight:	0.5
		Sc. PD:	0.14
		Sc. rank:	935.9

,

HA

Pos.	362	obs:	exp:
gct	A	3060	1054.00
gcc	A	202	809.60
gca	A	65	1862.00
gcg	A	857	457.90
---	--	------	------
mPD		0.42	0.69
		nPD:	0.61
		N. weight:	1.1
		Sc. PD:	0.4
		Sc. rank:	2344.6

,

HA

Pos.	363	obs:	exp:
att	I	0	1531.00
atc	I	7	1116.00
ata	I	4165	1525.00
act	T	0	0.26
acc	T	0	0.23
aca	T	1	0.42
acg	T	0	0.09
aaa	K	11	6.26
aag	K	0	4.74
---	--	------	------
mPD		0.0091	0.67
		nPD:	0.01
		N. weight:	1.4
		Sc. PD:	-0.29
		Sc. rank:	-3187.4

,

HA

Pos.	364	obs:	exp:
tta	L	0	0.09
ttg	L	0	0.19
tct	S	0	0.33
tcc	S	0	0.29
tca	S	2	0.45
tcg	S	0	0.12
ctt	L	1	0.20
ctc	L	0	0.16
cta	L	0	0.15
ctg	L	0	0.21
agt	S	0	0.43
agc	S	0	0.37
gtt	V	2	0.43
gtc	V	0	0.42
gta	V	0	0.43
gtg	V	0	0.72
gct	A	9	1053.00
gcc	A	1	808.70
gca	A	4163	1860.00
gcg	A	6	457.40
---	--	------	------
mPD		0.012	0.69
		nPD:	0.02
		N. weight:	1.1
		Sc. PD:	-0.23
		Sc. rank:	-2437.7

,

HA

Pos.	365	obs:	exp:
tct	S	0	0.17
tcc	S	0	0.14
tca	S	0	0.23
tcg	S	0	0.06
cgt	R	0	0.07
cgc	R	0	0.11
cga	R	0	0.20
cgg	R	0	0.16
att	I	3	1.10
atc	I	0	0.80
ata	I	0	1.10
agt	S	1	0.22
agc	S	0	0.18
aga	R	0	0.93
agg	R	2	0.52
gtt	V	1	0.22
gtc	V	0	0.21
gta	V	0	0.21
gtg	V	0	0.36
ggt	G	2301	604.10
ggc	G	317	548.70
gga	G	96	1830.00
ggg	G	1463	1194.00
---	--	------	------
mPD		0.57	0.69
		nPD:	0.83
		N. weight:	0.97
		Sc. PD:	0.55
		Sc. rank:	2494.2

,

HA

Pos.	366	obs:	exp:
ttt	F	4008	1741.00
ttc	F	166	2433.00
tta	L	0	0.18
ttg	L	2	0.38
tct	S	2	0.33
tcc	S	0	0.29
tca	S	0	0.45
tcg	S	0	0.12
tat	Y	1	0.48
tac	Y	0	0.52
tgt	C	2	0.77
tgc	C	0	1.23
ctt	L	0	0.40
ctc	L	0	0.32
cta	L	0	0.30
ctg	L	0	0.42
cgt	R	2	0.07
cgc	R	0	0.11
cga	R	0	0.20
cgg	R	0	0.16
att	I	0	0.37
atc	I	0	0.27
ata	I	1	0.37
agt	S	0	0.43
agc	S	0	0.37
aga	R	0	0.93
agg	R	0	0.52
---	--	------	------
mPD		0.082	0.49
		nPD:	0.17
		N. weight:	0.97
		Sc. PD:	-0.064
		Sc. rank:	270.1

,

HA

Pos.	367	obs:	exp:
tta	L	0	0.28
ttg	L	0	0.57
ctt	L	1	0.60
ctc	L	2	0.48
cta	L	0	0.44
ctg	L	0	0.63
cgt	R	0	0.11
cgc	R	0	0.16
cga	R	0	0.30
cgg	R	0	0.24
att	I	2	1532.00
atc	I	3	1117.00
ata	I	4170	1526.00
act	T	0	0.26
acc	T	0	0.23
aca	T	1	0.42
acg	T	0	0.09
aaa	K	1	0.57
aag	K	0	0.43
aga	R	3	1.40
agg	R	0	0.78
gtt	V	0	0.22
gtc	V	0	0.21
gta	V	1	0.21
gtg	V	0	0.36
---	--	------	------
mPD		0.0081	0.67
		nPD:	0.01
		N. weight:	1.4
		Sc. PD:	-0.3
		Sc. rank:	-3224.1

,

HA

Pos.	368	obs:	exp:
caa	Q	0	0.50
cag	Q	1	0.50
cgt	R	0	0.07
cgc	R	0	0.11
cga	R	0	0.20
cgg	R	0	0.16
aaa	K	0	0.57
aag	K	1	0.43
aga	R	0	0.93
agg	R	2	0.52
gaa	E	277	2342.00
gag	E	3900	1835.00
ggt	G	0	0.43
ggc	G	0	0.39
gga	G	3	1.31
ggg	G	0	0.86
---	--	------	------
mPD		0.13	0.50
		nPD:	0.26
		N. weight:	0.78
		Sc. PD:	0.018
		Sc. rank:	562.1

,

HA

Pos.	369	obs:	exp:
cgt	R	0	0.04
cgc	R	0	0.05
cga	R	0	0.10
cgg	R	0	0.08
aga	R	1	0.47
agg	R	0	0.26
gaa	E	0	1.68
gag	E	3	1.32
ggt	G	31	604.50
ggc	G	146	549.10
gga	G	3992	1832.00
ggg	G	11	1195.00
---	--	------	------
mPD		0.090	0.69
		nPD:	0.13
		N. weight:	0.92
		Sc. PD:	-0.094
		Sc. rank:	-212.5

,

HA

Pos.	370	obs:	exp:
tct	S	0	0.17
tcc	S	0	0.14
tca	S	1	0.23
tcg	S	0	0.06
caa	Q	3	1.50
cag	Q	0	1.50
cgt	R	0	0.11
cgc	R	0	0.16
cga	R	0	0.30
cgg	R	0	0.24
agt	S	0	0.22
agc	S	0	0.18
aga	R	3	1.40
agg	R	0	0.78
ggt	G	39	604.10
ggc	G	6	548.70
gga	G	4104	1830.00
ggg	G	28	1194.00
---	--	------	------
mPD		0.040	0.69
		nPD:	0.06
		N. weight:	1.
		Sc. PD:	-0.18
		Sc. rank:	-1406.2

,

HA

Pos.	371	obs:	exp:
tgg	W	4179	4179.00
aaa	K	0	2.85
aag	K	5	2.15
---	--	------	------
mPD		0.0048	0.0061
		nPD:	0.78
		N. weight:	0.0014
		Sc. PD:	0.00073
		Sc. rank:	3.5

,

HA

Pos.	372	obs:	exp:
cct	P	0	0.27
ccc	P	0	0.18
cca	P	0	0.41
ccg	P	1	0.14
cat	H	2	1.16
cac	H	0	0.84
caa	Q	198	2087.00
cag	Q	3983	2094.00
---	--	------	------
mPD		0.092	0.50
		nPD:	0.18
		N. weight:	0.7
		Sc. PD:	-0.035
		Sc. rank:	238.4

,

HA

Pos.	373	obs:	exp:
gtt	V	0	0.22
gtc	V	0	0.21
gta	V	1	0.21
gtg	V	0	0.36
gaa	E	2	1.12
gag	E	0	0.88
ggt	G	59	604.60
ggc	G	33	549.20
gga	G	3714	1832.00
ggg	G	375	1195.00
---	--	------	------
mPD		0.20	0.69
		nPD:	0.3
		N. weight:	0.65
		Sc. PD:	0.038
		Sc. rank:	636.4

,

HA

Pos.	374	obs:	exp:
atg	M	4183	4183.00
gtt	V	0	0.22
gtc	V	0	0.21
gta	V	0	0.21
gtg	V	1	0.36
---	--	------	------
mPD		0.00048	0.00078
		nPD:	0.61
		N. weight:	0.00034
		Sc. PD:	0.00012
		Sc. rank:	0.7

,

HA

Pos.	375	obs:	exp:
tta	L	1	0.09
ttg	L	0	0.19
ctt	L	0	0.20
ctc	L	0	0.16
cta	L	0	0.15
ctg	L	0	0.21
att	I	0	12.48
atc	I	0	9.10
ata	I	34	12.42
gtt	V	905	892.20
gtc	V	6	872.70
gta	V	3221	884.50
gtg	V	16	1499.00
gat	D	1	0.54
gac	D	0	0.46
---	--	------	------
mPD		0.36	0.75
		nPD:	0.49
		N. weight:	1.4
		Sc. PD:	0.33
		Sc. rank:	2406.8

,

HA

Pos.	376	obs:	exp:
aat	N	4	2.18
aac	N	0	1.82
gat	D	4113	2256.00
gac	D	65	1922.00
gaa	E	0	0.56
gag	E	1	0.44
ggt	G	1	0.14
ggc	G	0	0.13
gga	G	0	0.44
ggg	G	0	0.29
---	--	------	------
mPD		0.033	0.50
		nPD:	0.07
		N. weight:	0.75
		Sc. PD:	-0.12
		Sc. rank:	-899.9

,

HA

Pos.	377	obs:	exp:
tgg	W	1	1.00
ggt	G	4077	604.90
ggc	G	68	549.40
gga	G	15	1833.00
ggg	G	23	1196.00
---	--	------	------
mPD		0.051	0.69
		nPD:	0.07
		N. weight:	2.5
		Sc. PD:	-0.39
		Sc. rank:	-2592.8

,

HA

Pos.	378	obs:	exp:
tgg	W	4184	4184.00
---	--	------	------
mPD		0	0
		nPD:	1.
		N. weight:	0.
		Sc. PD:	0
		Sc. rank:	0

,

HA

Pos.	379	obs:	exp:
ttt	F	8	3.34
ttc	F	0	4.66
tat	Y	4077	2003.00
tac	Y	99	2173.00
---	--	------	------
mPD		0.050	0.50
		nPD:	0.1
		N. weight:	0.86
		Sc. PD:	-0.11
		Sc. rank:	-491.7

,

HA

Pos.	380	obs:	exp:
att	I	2	0.73
atc	I	0	0.54
ata	I	0	0.73
gtt	V	2	0.43
gtc	V	0	0.42
gta	V	0	0.43
gtg	V	0	0.72
ggt	G	3	604.50
ggc	G	1	549.10
gga	G	137	1832.00
ggg	G	4039	1195.00
---	--	------	------
mPD		0.070	0.69
		nPD:	0.1
		N. weight:	1.5
		Sc. PD:	-0.2
		Sc. rank:	-831.6

,

HA

Pos.	381	obs:	exp:
ttt	F	155	68.00
ttc	F	8	95.00
tct	S	0	0.17
tcc	S	0	0.14
tca	S	0	0.23
tcg	S	1	0.06
tat	Y	104	1925.00
tac	Y	3910	2089.00
tgt	C	4	1.55
tgc	C	0	2.45
agt	S	0	0.22
agc	S	0	0.18
gtt	V	0	0.22
gtc	V	1	0.21
gta	V	0	0.21
gtg	V	0	0.36
gat	D	0	0.54
gac	D	1	0.46
---	--	------	------
mPD		0.20	0.58
		nPD:	0.34
		N. weight:	0.75
		Sc. PD:	0.076
		Sc. rank:	882.9

,

HA

Pos.	382	obs:	exp:
tta	L	0	0.46
ttg	L	0	0.95
tct	S	1	0.17
tcc	S	0	0.14
tca	S	0	0.23
tcg	S	0	0.06
ctt	L	2	0.99
ctc	L	3	0.80
cta	L	0	0.74
ctg	L	0	1.06
cat	H	1103	2416.00
cac	H	3070	1757.00
caa	Q	2	1.00
cag	Q	0	1.00
cgt	R	1	0.11
cgc	R	2	0.16
cga	R	0	0.30
cgg	R	0	0.24
agt	S	0	0.22
agc	S	0	0.18
aga	R	0	1.40
agg	R	0	0.78
---	--	------	------
mPD		0.39	0.50
		nPD:	0.8
		N. weight:	0.29
		Sc. PD:	0.16
		Sc. rank:	729.4

,

HA

Pos.	383	obs:	exp:
tct	S	0	0.17
tcc	S	0	0.14
tca	S	1	0.23
tcg	S	0	0.06
cat	H	2490	2418.00
cac	H	1687	1759.00
caa	Q	1	1.00
cag	Q	1	1.00
cgt	R	1	0.15
cgc	R	2	0.21
cga	R	0	0.40
cgg	R	1	0.33
agt	S	0	0.22
agc	S	0	0.18
aga	R	0	1.87
agg	R	0	1.04
---	--	------	------
mPD		0.49	0.49
		nPD:	0.98
		N. weight:	0.006
		Sc. PD:	0.0044
		Sc. rank:	15.8

,

HA

Pos.	384	obs:	exp:
tct	S	0	694.20
tcc	S	0	602.50
tca	S	0	942.80
tcg	S	0	250.90
caa	Q	0	1.00
cag	Q	2	1.00
cgt	R	0	0.07
cgc	R	2	0.11
cga	R	0	0.20
cgg	R	0	0.16
att	I	0	4.04
atc	I	11	2.94
ata	I	0	4.02
aat	N	0	2.73
aac	N	5	2.27
agt	S	223	902.10
agc	S	3936	766.50
aga	R	0	0.93
agg	R	0	0.52
gat	D	0	1.62
gac	D	3	1.38
ggt	G	1	0.29
ggc	G	1	0.26
gga	G	0	0.88
ggg	G	0	0.57
---	--	------	------
mPD		0.12	1.7
		nPD:	0.07
		N. weight:	2.
		Sc. PD:	-0.33
		Sc. rank:	-2279.8

,

HA

Pos.	385	obs:	exp:
cat	H	0	0.58
cac	H	1	0.42
caa	Q	5	2.50
cag	Q	0	2.50
att	I	1	0.37
atc	I	0	0.27
ata	I	0	0.37
aat	N	3289	2275.00
aac	N	883	1897.00
aaa	K	4	2.85
aag	K	1	2.15
---	--	------	------
mPD		0.34	0.50
		nPD:	0.68
		N. weight:	0.18
		Sc. PD:	0.078
		Sc. rank:	421.4

,

HA

Pos.	386	obs:	exp:
aat	N	0	0.55
aac	N	1	0.45
aaa	K	0	3.42
aag	K	6	2.58
gat	D	14	8.64
gac	D	2	7.36
gaa	E	846	2320.00
gag	E	3293	1819.00
ggt	G	0	3.18
ggc	G	0	2.89
gga	G	1	9.64
ggg	G	21	6.29
---	--	------	------
mPD		0.34	0.51
		nPD:	0.67
		N. weight:	0.38
		Sc. PD:	0.16
		Sc. rank:	860.4

,

HA

Pos.	387	obs:	exp:
cat	H	0	0.58
cac	H	1	0.42
caa	Q	331	2088.00
cag	Q	3851	2094.00
aaa	K	0	0.57
aag	K	1	0.43
---	--	------	------
mPD		0.15	0.50
		nPD:	0.29
		N. weight:	0.58
		Sc. PD:	0.032
		Sc. rank:	518.4

,

HA

Pos.	388	obs:	exp:
gct	A	0	0.25
gcc	A	0	0.19
gca	A	0	0.45
gcg	A	1	0.11
ggt	G	0	604.90
ggc	G	0	549.40
gga	G	553	1833.00
ggg	G	3630	1196.00
---	--	------	------
mPD		0.23	0.69
		nPD:	0.33
		N. weight:	1.1
		Sc. PD:	0.11
		Sc. rank:	1298.2

,

HA

Pos.	389	obs:	exp:
tct	S	0	697.10
tcc	S	0	604.90
tca	S	0	946.70
tcg	S	0	251.90
att	I	1	0.37
atc	I	0	0.27
ata	I	0	0.37
act	T	4	1.05
acc	T	0	0.90
aca	T	0	1.67
acg	T	0	0.38
aat	N	2	1.09
aac	N	0	0.91
agt	S	4021	905.80
agc	S	155	769.60
ggt	G	1	0.14
ggc	G	0	0.13
gga	G	0	0.44
ggg	G	0	0.29
---	--	------	------
mPD		0.075	1.7
		nPD:	0.05
		N. weight:	1.9
		Sc. PD:	-0.35
		Sc. rank:	-3119.2

,

HA

Pos.	390	obs:	exp:
ggt	G	839	605.10
ggc	G	8	549.60
gga	G	226	1833.00
ggg	G	3111	1196.00
---	--	------	------
mPD		0.40	0.69
		nPD:	0.59
		N. weight:	0.91
		Sc. PD:	0.31
		Sc. rank:	1904.3

,

HA

Pos.	391	obs:	exp:
ttt	F	0	0.42
ttc	F	1	0.58
tat	Y	247	2006.00
tac	Y	3935	2176.00
gtt	V	0	0.22
gtc	V	1	0.21
gta	V	0	0.21
gtg	V	0	0.36
---	--	------	------
mPD		0.11	0.50
		nPD:	0.22
		N. weight:	0.6
		Sc. PD:	-0.0062
		Sc. rank:	316.6

,

HA			
Pos.	392	obs:	exp:
tct	S	3	0.50
tcc	S	0	0.43
tca	S	0	0.68
tcg	S	0	0.18
cct	P	2	0.55
ccc	P	0	0.36
cca	P	0	0.81
ccg	P	0	0.28
act	T	1	0.26
acc	T	0	0.23
aca	T	0	0.42
acg	T	0	0.09
agt	S	0	0.65
agc	S	0	0.55
gct	A	4097	1053.00
gcc	A	13	808.50
gca	A	21	1860.00
gcg	A	47	457.30
---	--	------	------
mPD		0.041	0.69
		nPD:	0.06
		N. weight:	1.8
		Sc. PD:	-0.3
		Sc. rank:	-2297.6

HA			
Pos.	393	obs:	exp:
gct	A	115	1054.00
gcc	A	2	809.60
gca	A	3762	1862.00
gcg	A	305	457.90
---	--	------	------
mPD		0.19	0.69
		nPD:	0.27
		N. weight:	0.75
		Sc. PD:	0.024
		Sc. rank:	612.9

HA			
Pos.	394	obs:	exp:
tat	Y	0	0.96
tac	Y	2	1.04
aat	N	0	1.09
aac	N	2	0.91
gct	A	0	0.25
gcc	A	1	0.19
gca	A	0	0.45
gcg	A	0	0.11
gat	D	53	2256.00
gac	D	4126	1923.00
---	--	------	------
mPD		0.027	0.50
		nPD:	0.05
		N. weight:	0.98
		Sc. PD:	-0.17
		Sc. rank:	-1421.3

HA			
Pos.	395	obs:	exp:
caa	Q	1	0.50
cag	Q	0	0.50
cgt	R	0	1.50
cgc	R	0	2.14
cga	R	0	4.03
cgg	R	0	3.27
atg	M	6	6.00
act	T	0	0.26
acc	T	0	0.23
aca	T	1	0.42
acg	T	0	0.09
aaa	K	4039	2351.00
aag	K	90	1778.00
aga	R	40	18.67
agg	R	0	10.39
gaa	E	7	3.92
gag	E	0	3.08
---	--	------	------
mPD		0.071	0.52
		nPD:	0.14
		N. weight:	0.65
		Sc. PD:	-0.062
		Sc. rank:	-116.1

HA			
Pos.	396	obs:	exp:
aaa	K	2	4.56
aag	K	6	3.44
gaa	E	4111	2339.00
gag	E	62	1834.00
ggt	G	0	0.43
ggc	G	0	0.39
gga	G	3	1.31
ggg	G	0	0.86
---	--	------	------
mPD		0.037	0.50
		nPD:	0.07
		N. weight:	0.7
		Sc. PD:	-0.11
		Sc. rank:	-714.7

HA			
Pos.	397	obs:	exp:
tct	S	1600	697.10
tcc	S	2553	604.90
tca	S	23	946.70
tcg	S	0	251.90
tgt	C	1	0.39
tgc	C	0	0.61
act	T	5	1.84
acc	T	2	1.58
aca	T	0	2.93
acg	T	0	0.66
agt	S	0	905.80
agc	S	0	769.60
---	--	------	------
mPD		0.48	1.7
		nPD:	0.29
		N. weight:	1.6
		Sc. PD:	0.091
		Sc. rank:	1492.4

HA			
Pos.	398	obs:	exp:
tct	S	1	0.17
tcc	S	0	0.14
tca	S	0	0.23
tcg	S	0	0.06
act	T	3008	1096.00
acc	T	1169	942.30
aca	T	2	1750.00
acg	T	3	393.50
agt	S	0	0.22
agc	S	0	0.18
gct	A	1	0.25
gcc	A	0	0.19
gca	A	0	0.45
gcg	A	0	0.11
---	--	------	------
mPD		0.41	0.70
		nPD:	0.58
		N. weight:	1.1
		Sc. PD:	0.36
		Sc. rank:	2239.4

HA			
Pos.	399	obs:	exp:
caa	Q	4114	2088.00
cag	Q	68	2094.00
aaa	K	1	0.57
aag	K	0	0.43
gaa	E	1	0.56
gag	E	0	0.44
---	--	------	------
mPD		0.033	0.50
		nPD:	0.07
		N. weight:	0.85
		Sc. PD:	-0.14
		Sc. rank:	-1026.3

HA			
Pos.	400	obs:	exp:
cgt	R	0	6.85
cgc	R	0	9.79
cga	R	0	18.44
cgg	R	0	14.94
aaa	K	177	2278.00
aag	K	3824	1723.00
aga	R	2	85.43
agg	R	181	47.54
---	--	------	------
mPD		0.17	0.60
		nPD:	0.28
		N. weight:	0.94
		Sc. PD:	0.036
		Sc. rank:	811.1

HA			
Pos.	401	obs:	exp:
act	T	0	0.26
acc	T	0	0.23
aca	T	1	0.42
acg	T	0	0.09
gct	A	351	1054.00
gcc	A	2	809.50
gca	A	3026	1862.00
gcg	A	804	457.80
---	--	------	------
mPD		0.43	0.69
		nPD:	0.63
		N. weight:	0.51
		Sc. PD:	0.19
		Sc. rank:	1105.1

HA			
Pos.	402	obs:	exp:
tta	L	0	0.18
ttg	L	0	0.38
ctt	L	0	0.40
ctc	L	0	0.32
cta	L	2	0.30
ctg	L	0	0.42
att	I	12	1512.00
atc	I	36	1103.00
ata	I	4073	1506.00
atg	M	6	6.00
gtt	V	0	11.83
gtc	V	0	11.57
gta	V	55	11.73
gtg	V	0	19.87
---	--	------	------
mPD		0.052	0.69
		nPD:	0.08
		N. weight:	1.3
		Sc. PD:	-0.2
		Sc. rank:	-1279.4

HA			
Pos.	403	obs:	exp:
aat	N	13	13.63
aac	N	12	11.37
gat	D	2609	2246.00
gac	D	1550	1913.00
---	--	------	------
mPD		0.48	0.51
		nPD:	0.94
		N. weight:	0.024
		Sc. PD:	0.016
		Sc. rank:	61.3

HA			
Pos.	404	obs:	exp:
ggt	G	2	605.10
ggc	G	0	549.60
gga	G	3796	1833.00
ggg	G	386	1196.00
---	--	------	------
mPD		0.17	0.69
		nPD:	0.24
		N. weight:	0.77
		Sc. PD:	0.0069
		Sc. rank:	511.4

HA			
Pos.	405	obs:	exp:
att	I	1	63.48
atc	I	171	46.29
ata	I	1	63.22
act	T	0	0.26
acc	T	1	0.23
aca	T	0	0.42
acg	T	0	0.09
gtt	V	932	862.30
gtc	V	3067	843.40
gta	V	9	854.80
gtg	V	1	1448.00
gat	D	0	0.54
gac	D	1	0.46
---	--	------	------
mPD		0.43	0.82
		nPD:	0.53
		N. weight:	1.4
		Sc. PD:	0.39
		Sc. rank:	2688.2

HA			
Pos.	406	obs:	exp:
cct	P	0	0.82
ccc	P	3	0.54
cca	P	0	1.22
ccg	P	0	0.42
act	T	100	1096.00
acc	T	4075	941.60
aca	T	4	1749.00
acg	T	0	393.20
gct	A	0	0.50
gcc	A	2	0.39
gca	A	0	0.89
gcg	A	0	0.22
---	--	------	------
mPD		0.051	0.70
		nPD:	0.07
		N. weight:	1.9
		Sc. PD:	-0.3
		Sc. rank:	-2024.4

HA			
Pos.	407	obs:	exp:
act	T	11	2.88
acc	T	0	2.48
aca	T	0	4.60
acg	T	0	1.03
aat	N	4139	2276.00
aac	N	34	1897.00
---	--	------	------
mPD		0.021	0.50
		nPD:	0.04
		N. weight:	0.78
		Sc. PD:	-0.15
		Sc. rank:	-1343.7

HA			
Pos.	408	obs:	exp:
aat	N	1	1.09
aac	N	1	0.91
aaa	K	136	2381.00
aag	K	4046	1801.00
---	--	------	------
mPD		0.064	0.49
		nPD:	0.13
		N. weight:	0.96
		Sc. PD:	-0.097
		Sc. rank:	-255.7

HA			
Pos.	409	obs:	exp:
ttt	F	0	0.42
ttc	F	1	0.58
att	I	0	8.81
atc	I	24	6.42
ata	I	0	8.77
gtt	V	36	894.30
gtc	V	4062	874.80
gta	V	58	886.60
gtg	V	2	1502.00
ggt	G	1	0.14
ggc	G	0	0.13
gga	G	0	0.44
ggg	G	0	0.29
---	--	------	------
mPD		0.058	0.75
		nPD:	0.08
		N. weight:	2.
		Sc. PD:	-0.3
		Sc. rank:	-1776.9

HA			
Pos.	410	obs:	exp:
aat	N	1099	2281.00
aac	N	3084	1902.00
gat	D	0	0.54
gac	D	1	0.46
---	--	------	------
mPD		0.39	0.50
		nPD:	0.78
		N. weight:	0.23
		Sc. PD:	0.12
		Sc. rank:	576.8

HA			
Pos.	411	obs:	exp:
tct	S	44	698.40
tcc	S	1	606.10
tca	S	1617	948.50
tcg	S	2522	252.40
agt	S	0	907.50
agc	S	0	771.10
---	--	------	------
mPD		0.49	1.7
		nPD:	0.3
		N. weight:	2.2
		Sc. PD:	0.13
		Sc. rank:	2100.4

HA			
Pos.	412	obs:	exp:
att	I	199	1535.00
atc	I	3964	1119.00
ata	I	20	1529.00
---	-	1	1.00
---	--	------	------
mPD		0.10	0.66
		nPD:	0.15
		N. weight:	1.5
		Sc. PD:	-0.12
		Sc. rank:	90.7

HA			
Pos.	413	obs:	exp:
tta	L	0	0.09
ttg	L	0	0.19
ctt	L	1	0.20
ctc	L	0	0.16
cta	L	0	0.15
ctg	L	0	0.21
att	I	4152	1535.00
atc	I	28	1119.00
ata	I	2	1528.00
gtt	V	1	0.22
gtc	V	0	0.21
gta	V	0	0.21
gtg	V	0	0.36
---	--	------	------
mPD		0.015	0.66
		nPD:	0.02
		N. weight:	1.3
		Sc. PD:	-0.27
		Sc. rank:	-2895.8

HA			
Pos.	414	obs:	exp:
tat	Y	0	0.48
tac	Y	1	0.52
aat	N	2	9.82
aac	N	16	8.18
gat	D	39	2241.00
gac	D	4111	1909.00
gaa	E	5	2.80
gag	E	0	2.20
ggt	G	0	1.45
ggc	G	10	1.31
gga	G	0	4.38
ggg	G	0	2.86
---	--	------	------
mPD		0.036	0.51
		nPD:	0.07
		N. weight:	1.
		Sc. PD:	-0.16
		Sc. rank:	-1139.4

HA			
Pos.	415	obs:	exp:
cgt	R	0	0.04
cgc	R	0	0.05
cga	R	0	0.10
cgg	R	0	0.08
aaa	K	4140	2381.00
aag	K	42	1801.00
aga	R	0	0.47
agg	R	1	0.26
gaa	E	1	0.56
gag	E	0	0.44
---	--	------	------
mPD		0.021	0.49
		nPD:	0.04
		N. weight:	0.71
		Sc. PD:	-0.13
		Sc. rank:	-1206.7

HA			
Pos.	416	obs:	exp:
att	I	1	0.37
atc	I	0	0.27
ata	I	0	0.37
atg	M	4182	4182.00
act	T	0	0.26
acc	T	0	0.23
aca	T	0	0.42
acg	T	1	0.09
---	--	------	------
mPD		0.00096	0.0014
		nPD:	0.69
		N. weight:	0.0011
		Sc. PD:	0.00049
		Sc. rank:	2.6

HA			
Pos.	417	obs:	exp:
aat	N	82	2281.00
aac	N	4101	1902.00
gat	D	0	0.54
gac	D	1	0.46
---	--	------	------
mPD		0.039	0.50
		nPD:	0.08
		N. weight:	0.95
		Sc. PD:	-0.14
		Sc. rank:	-841.0

HA			
Pos.	418	obs:	exp:
att	I	2	1.10
atc	I	1	0.80
ata	I	0	1.10
act	T	3946	1095.00
acc	T	226	941.20
aca	T	5	1748.00
acg	T	0	393.00
aat	N	1	2.18
aac	N	3	1.82
---	--	------	------
mPD		0.11	0.70
		nPD:	0.16
		N. weight:	1.6
		Sc. PD:	-0.12
		Sc. rank:	166.6

HA			
Pos.	419	obs:	exp:
cat	H	0	0.58
cac	H	1	0.42
caa	Q	1088	2086.00
cag	Q	3091	2093.00
cgt	R	0	0.15
cgc	R	0	0.21
cga	R	4	0.40
cgg	R	0	0.33
aga	R	0	1.87
agg	R	0	1.04
---	--	------	------
mPD		0.39	0.50
		nPD:	0.77
		N. weight:	0.17
		Sc. PD:	0.088
		Sc. rank:	420.6

HA			
Pos.	420	obs:	exp:
ttt	F	4138	1744.00
ttc	F	43	2437.00
tta	L	0	0.09
ttg	L	1	0.19
ctt	L	0	0.20
ctc	L	0	0.16
cta	L	0	0.15
ctg	L	0	0.21
gtt	V	2	0.43
gtc	V	0	0.42
gta	V	0	0.43
gtg	V	0	0.72
---	--	------	------
mPD		0.022	0.49
		nPD:	0.04
		N. weight:	1.1
		Sc. PD:	-0.21
		Sc. rank:	-1877.5

HA			
Pos.	421	obs:	exp:
cgt	R	0	0.07
cgc	R	0	0.11
cga	R	0	0.20
cgg	R	0	0.16
aaa	K	0	1.14
aag	K	2	0.86
aga	R	0	0.93
agg	R	2	0.52
gat	D	0	0.54
gac	D	1	0.46
gaa	E	141	2343.00
gag	E	4038	1836.00
---	--	------	------
mPD		0.068	0.50
		nPD:	0.14
		N. weight:	0.92
		Sc. PD:	-0.087
		Sc. rank:	-111.7

HA			
Pos.	422	obs:	exp:
tct	S	0	0.17
tcc	S	1	0.14
tca	S	0	0.23
tcg	S	0	0.06
att	I	0	0.37
atc	I	1	0.27
ata	I	0	0.37
act	T	0	0.79
acc	T	3	0.68
aca	T	0	1.26
acg	T	0	0.28
agt	S	0	0.22
agc	S	0	0.18
gtt	V	0	0.22
gtc	V	1	0.21
gta	V	0	0.21
gtg	V	0	0.36
gct	A	1454	1053.00
gcc	A	1786	808.50
gca	A	938	1860.00
gcg	A	0	457.30
---	--	------	------
mPD		0.65	0.69
		nPD:	0.94
		N. weight:	0.42
		Sc. PD:	0.28
		Sc. rank:	1086.5

HA			
Pos.	423	obs:	exp:
att	I	10	7.71
atc	I	0	5.62
ata	I	11	7.67
gtt	V	2499	895.00
gtc	V	10	875.40
gta	V	1589	887.20
gtg	V	63	1503.00
gct	A	2	0.50
gcc	A	0	0.39
gca	A	0	0.89
gcg	A	0	0.22
---	--	------	------
mPD		0.50	0.74
		nPD:	0.68
		N. weight:	1.1
		Sc. PD:	0.46
		Sc. rank:	2511.5

HA			
Pos.	424	obs:	exp:
cgt	R	0	0.04
cgc	R	0	0.05
cga	R	0	0.10
cgg	R	0	0.08
aga	R	0	0.47
agg	R	1	0.26
gat	D	1	0.54
gac	D	0	0.46
gaa	E	1	14.57
gag	E	25	11.43
ggt	G	1	601.00
ggc	G	21	545.90
gga	G	3982	1821.00
ggg	G	152	1188.00
---	--	------	------
mPD		0.11	0.70
		nPD:	0.15
		N. weight:	0.91
		Sc. PD:	-0.075
		Sc. rank:	42.9

HA			
Pos.	425	obs:	exp:
cgt	R	0	144.20
cgc	R	0	206.20
cga	R	0	388.30
cgg	R	3	314.60
aaa	K	149	186.80
aag	K	179	141.20
aga	R	90	1799.00
agg	R	3760	1001.00
ggt	G	0	0.43
ggc	G	0	0.39
gga	G	0	1.31
ggg	G	3	0.86
---	--	------	------
mPD		0.26	1.1
		nPD:	0.24
		N. weight:	1.6
		Sc. PD:	0.0029
		Sc. rank:	977.3

HA			
Pos.	426	obs:	exp:
gat	D	1	0.54
gac	D	0	0.46
gaa	E	2830	2345.00
gag	E	1353	1838.00
---	--	------	------
mPD		0.44	0.49
		nPD:	0.89
		N. weight:	0.041
		Sc. PD:	0.026
		Sc. rank:	104.9

HA			
Pos.	427	obs:	exp:
ttt	F	3952	1745.00
ttc	F	231	2438.00
tct	S	1	0.17
tcc	S	0	0.14
tca	S	0	0.23
tcg	S	0	0.06
agt	S	0	0.22
agc	S	0	0.18
---	--	------	------
mPD		0.10	0.49
		nPD:	0.22
		N. weight:	0.89
		Sc. PD:	-0.017
		Sc. rank:	423.2

HA			
Pos.	428	obs:	exp:
tct	S	0	5.17
tcc	S	0	4.49
tca	S	0	7.03
tcg	S	0	1.87
act	T	2	0.52
acc	T	0	0.45
aca	T	0	0.84
acg	T	0	0.19
aat	N	3808	2263.00
aac	N	342	1887.00
agt	S	27	6.72
agc	S	4	5.71
gat	D	1	0.54
gac	D	0	0.46
---	--	------	------
mPD		0.17	0.52
		nPD:	0.32
		N. weight:	0.48
		Sc. PD:	0.039
		Sc. rank:	519.6

HA			
Pos.	429	obs:	exp:
tct	S	0	19.03
tcc	S	0	16.51
tca	S	0	25.84
tcg	S	0	6.88
tat	Y	0	0.48
tac	Y	1	0.52
cat	H	0	2.89
cac	H	5	2.11
cgt	R	0	0.04
cgc	R	0	0.05
cga	R	0	0.10
cgg	R	0	0.08
act	T	1	0.52
acc	T	1	0.45
aca	T	0	0.84
acg	T	0	0.19
aat	N	99	2206.00
aac	N	3946	1839.00
aaa	K	12	6.83
aag	K	0	5.17
agt	S	2	24.73
agc	S	112	21.01
aga	R	1	0.47
agg	R	0	0.26
gct	A	0	0.25
gcc	A	1	0.19
gca	A	0	0.45
gcg	A	0	0.11
gat	D	0	1.08
gac	D	2	0.92
gaa	E	1	0.56
gag	E	0	0.44
---	--	------	------
mPD		0.11	0.60
		nPD:	0.19
		N. weight:	0.96
		Sc. PD:	-0.043
		Sc. rank:	382.5

HA			
Pos.	430	obs:	exp:
ttt	F	1	1.25
ttc	F	2	1.75
tta	L	3968	386.60
ttg	L	146	794.30
ctt	L	0	830.10
ctc	L	0	668.80
cta	L	21	618.80
ctg	L	46	882.50
---	--	------	------
mPD		0.12	1.1
		nPD:	0.11
		N. weight:	2.9
		Sc. PD:	-0.36
		Sc. rank:	-1341.2

HA			
Pos.	431	obs:	exp:
caa	Q	0	1.00
cag	Q	2	1.00
aaa	K	4	2.28
aag	K	0	1.72
gaa	E	2384	2342.00
gag	E	1793	1835.00
ggt	G	0	0.14
ggc	G	0	0.13
gga	G	1	0.44
ggg	G	0	0.29
---	--	------	------
mPD		0.49	0.50
		nPD:	0.99
		N. weight:	0.0032
		Sc. PD:	0.0024
		Sc. rank:	8.4

HA			
Pos.	432	obs:	exp:
cgt	R	0	154.20
cgc	R	0	220.50
cga	R	0	415.30
cgg	R	32	336.50
aaa	K	1	34.73
aag	K	60	26.27
aga	R	201	1924.00
agg	R	3888	1071.00
ggt	G	0	0.29
ggc	G	0	0.26
gga	G	0	0.88
ggg	G	2	0.57
---	--	------	------
mPD		0.14	0.98
		nPD:	0.14
		N. weight:	1.5
		Sc. PD:	-0.14
		Sc. rank:	-106.9

HA			
Pos.	433	obs:	exp:
cgt	R	0	156.50
cgc	R	0	223.70
cga	R	0	421.30
cgg	R	0	341.40
aaa	K	3	1.71
aag	K	0	1.29
aga	R	3253	1952.00
agg	R	928	1086.00
---	--	------	------
mPD		0.35	0.96
		nPD:	0.36
		N. weight:	0.5
		Sc. PD:	0.062
		Sc. rank:	628.2

HA			
Pos.	434	obs:	exp:
tta	L	0	0.37
ttg	L	0	0.76
ctt	L	0	0.79
ctc	L	0	0.64
cta	L	4	0.59
ctg	L	0	0.84
att	I	4	1531.00
atc	I	0	1117.00
ata	I	4169	1525.00
atg	M	2	2.00
aaa	K	1	1.71
aag	K	2	1.29
gtt	V	0	0.43
gtc	V	0	0.42
gta	V	2	0.43
gtg	V	0	0.72
---	--	------	------
mPD		0.0081	0.67
		nPD:	0.01
		N. weight:	1.4
		Sc. PD:	-0.3
		Sc. rank:	-3248.0

HA			
Pos.	435	obs:	exp:
aaa	K	5	2.85
aag	K	0	2.15
gaa	E	1680	2341.00
gag	E	2496	1835.00
ggt	G	0	0.43
ggc	G	0	0.39
gga	G	0	1.31
ggg	G	3	0.86
---	--	------	------
mPD		0.49	0.50
		nPD:	0.98
		N. weight:	0.073
		Sc. PD:	0.052
		Sc. rank:	191.2

HA			
Pos.	436	obs:	exp:
act	T	1	0.26
acc	T	0	0.23
aca	T	0	0.42
acg	T	0	0.09
aat	N	4142	2280.00
aac	N	39	1901.00
gat	D	2	1.08
gac	D	0	0.92
---	--	------	------
mPD		0.020	0.50
		nPD:	0.04
		N. weight:	0.77
		Sc. PD:	-0.15
		Sc. rank:	-1392.0

HA			
Pos.	437	obs:	exp:
tta	L	2642	386.90
ttg	L	1471	794.80
ctt	L	0	830.70
ctc	L	0	669.30
cta	L	57	619.20
ctg	L	14	883.10
---	--	------	------
mPD		0.49	1.1
		nPD:	0.44
		N. weight:	1.9
		Sc. PD:	0.38
		Sc. rank:	2987.4

HA			
Pos.	438	obs:	exp:
att	I	1	0.37
atc	I	0	0.27
ata	I	0	0.37
aat	N	96	2280.00
aac	N	4085	1901.00
aaa	K	1	0.57
aag	K	0	0.43
gat	D	0	0.54
gac	D	1	0.46
---	--	------	------
mPD		0.047	0.50
		nPD:	0.09
		N. weight:	0.94
		Sc. PD:	-0.13
		Sc. rank:	-573.5

HA			
Pos.	439	obs:	exp:
cgt	R	0	0.75
cgc	R	0	1.07
cga	R	0	2.02
cgg	R	0	1.63
aat	N	4	2.73
aac	N	1	2.27
aaa	K	67	2368.00
aag	K	4092	1791.00
aga	R	0	9.34
agg	R	20	5.20
---	--	------	------
mPD		0.043	0.50
		nPD:	0.09
		N. weight:	1.1
		Sc. PD:	-0.15
		Sc. rank:	-741.4

HA			
Pos.	440	obs:	exp:
cgt	R	0	0.04
cgc	R	0	0.05
cga	R	0	0.10
cgg	R	0	0.08
aaa	K	1344	2382.00
aag	K	2839	1801.00
aga	R	0	0.47
agg	R	1	0.26
---	--	------	------
mPD		0.44	0.49
		nPD:	0.89
		N. weight:	0.17
		Sc. PD:	0.11
		Sc. rank:	452.7

HA			
Pos.	441	obs:	exp:
atg	M	4183	4183.00
gtt	V	0	0.22
gtc	V	0	0.21
gta	V	0	0.21
gtg	V	1	0.36
---	--	------	------
mPD		0.00048	0.00078
		nPD:	0.61
		N. weight:	0.00034
		Sc. PD:	0.00012
		Sc. rank:	0.7

HA			
Pos.	442	obs:	exp:
gaa	E	4120	2345.00
gag	E	64	1839.00
---	--	------	------
mPD		0.030	0.49
		nPD:	0.06
		N. weight:	0.7
		Sc. PD:	-0.12
		Sc. rank:	-895.4

HA			
Pos.	443	obs:	exp:
cat	H	0	0.58
cac	H	1	0.42
aat	N	0	1.09
aac	N	2	0.91
gat	D	106	2257.00
gac	D	4074	1923.00
gaa	E	1	0.56
gag	E	0	0.44
---	--	------	------
mPD		0.051	0.50
		nPD:	0.1
		N. weight:	0.91
		Sc. PD:	-0.12
		Sc. rank:	-493.1

HA			
Pos.	444	obs:	exp:
tta	L	1	0.09
ttg	L	0	0.19
ctt	L	0	0.20
ctc	L	0	0.16
cta	L	0	0.15
ctg	L	0	0.21
gaa	E	1	0.56
gag	E	0	0.44
ggt	G	1	604.80
ggc	G	0	549.30
gga	G	3627	1832.00
ggg	G	554	1195.00
---	--	------	------
mPD		0.23	0.69
		nPD:	0.34
		N. weight:	0.68
		Sc. PD:	0.066
		Sc. rank:	793.5

HA			
Pos.	445	obs:	exp:
ttt	F	595	1745.00
ttc	F	3588	2438.00
tgt	C	0	0.39
tgc	C	1	0.61
---	--	------	------
mPD		0.24	0.49
		nPD:	0.5
		N. weight:	0.25
		Sc. PD:	0.064
		Sc. rank:	449.1

HA			
Pos.	446	obs:	exp:
tta	L	65	386.60
ttg	L	2	794.30
ctt	L	5	830.10
ctc	L	10	668.80
cta	L	4059	618.80
ctg	L	40	882.50
caa	Q	1	0.50
cag	Q	0	0.50
att	I	0	0.37
atc	I	0	0.27
ata	I	1	0.37
gtt	V	0	0.22
gtc	V	0	0.21
gta	V	1	0.21
gtg	V	0	0.36
---	--	------	------
mPD		0.060	1.1
		nPD:	0.05
		N. weight:	2.4
		Sc. PD:	-0.43
		Sc. rank:	-3613.3

HA			
Pos.	447	obs:	exp:
cgt	R	0	0.04
cgc	R	0	0.05
cga	R	0	0.10
cgg	R	0	0.08
aat	N	8	4.36
aac	N	0	3.64
aga	R	1	0.47
agg	R	0	0.26
gat	D	4146	2254.00
gac	D	29	1921.00
---	--	------	------
mPD		0.019	0.50
		nPD:	0.04
		N. weight:	0.8
		Sc. PD:	-0.15
		Sc. rank:	-1503.4

HA			
Pos.	448	obs:	exp:
gtt	V	378	899.90
gtc	V	3735	880.30
gta	V	58	892.10
gtg	V	13	1512.00
---	--	------	------
mPD		0.19	0.73
		nPD:	0.27
		N. weight:	1.6
		Sc. PD:	0.047
		Sc. rank:	1273.9

HA			
Pos.	449	obs:	exp:
tgg	W	4184	4184.00
---	--	------	------
mPD		0	0
		nPD:	1.
		N. weight:	0.
		Sc. PD:	0
		Sc. rank:	0

HA			
Pos.	450	obs:	exp:
act	T	3234	1097.00
acc	T	940	942.80
aca	T	1	1751.00
acg	T	9	393.70
---	--	------	------
mPD		0.35	0.70
		nPD:	0.51
		N. weight:	1.1
		Sc. PD:	0.3
		Sc. rank:	2086.8

HA			
Pos.	451	obs:	exp:
tat	Y	3942	2007.00
tac	Y	242	2177.00
---	--	------	------
mPD		0.11	0.50
		nPD:	0.22
		N. weight:	0.71
		Sc. PD:	-0.012
		Sc. rank:	340.0

HA			
Pos.	452	obs:	exp:
tct	S	0	0.17
tcc	S	0	0.14
tca	S	0	0.23
tcg	S	0	0.06
aat	N	4136	2281.00
aac	N	47	1902.00
agt	S	1	0.22
agc	S	0	0.18
---	--	------	------
mPD		0.023	0.50
		nPD:	0.05
		N. weight:	0.76
		Sc. PD:	-0.14
		Sc. rank:	-1229.9

HA			
Pos.	453	obs:	exp:
gtt	V	4	1.08
gtc	V	0	1.05
gta	V	1	1.07
gtg	V	0	1.81
gct	A	3710	1053.00
gcc	A	209	808.50
gca	A	252	1860.00
gcg	A	7	457.30
ggt	G	1	0.14
ggc	G	0	0.13
gga	G	0	0.44
ggg	G	0	0.29
---	--	------	------
mPD		0.21	0.69
		nPD:	0.3
		N. weight:	1.3
		Sc. PD:	0.081
		Sc. rank:	1285.9

HA			
Pos.	454	obs:	exp:
gaa	E	4154	2345.00
gag	E	30	1839.00
---	--	------	------
mPD		0.014	0.49
		nPD:	0.03
		N. weight:	0.75
		Sc. PD:	-0.15
		Sc. rank:	-1516.9

HA			
Pos.	455	obs:	exp:
tta	L	0	386.70
ttg	L	0	794.50
ctt	L	3952	830.30
ctc	L	216	668.90
cta	L	11	618.90
ctg	L	3	882.70
att	I	2	0.73
atc	I	0	0.54
ata	I	0	0.73
---	--	------	------
mPD		0.11	1.1
		nPD:	0.09
		N. weight:	1.9
		Sc. PD:	-0.27
		Sc. rank:	-1207.3

HA			
Pos.	456	obs:	exp:
tta	L	6	386.90
ttg	L	23	794.80
ctt	L	18	830.70
ctc	L	14	669.30
cta	L	896	619.20
ctg	L	3227	883.10
---	--	------	------
mPD		0.36	1.1
		nPD:	0.32
		N. weight:	1.4
		Sc. PD:	0.12
		Sc. rank:	1597.4

HA			
Pos.	457	obs:	exp:
att	I	3	1.10
atc	I	0	0.80
ata	I	0	1.10
gtt	V	4092	899.30
gtc	V	50	879.60
gta	V	3	891.50
gtg	V	36	1511.00
---	--	------	------
mPD		0.043	0.73
		nPD:	0.06
		N. weight:	2.
		Sc. PD:	-0.33
		Sc. rank:	-2581.1

HA			
Pos.	458	obs:	exp:
ttt	F	0	2.09
ttc	F	5	2.91
tta	L	0	386.20
ttg	L	0	793.50
ctt	L	16	829.30
ctc	L	4084	668.10
cta	L	75	618.20
ctg	L	2	881.60
att	I	0	0.37
atc	I	0	0.27
ata	I	1	0.37
gtt	V	0	0.22
gtc	V	1	0.21
gta	V	0	0.21
gtg	V	0	0.36
---	--	------	------
mPD		0.047	1.1
		nPD:	0.04
		N. weight:	2.4
		Sc. PD:	-0.44
		Sc. rank:	-4127.0

HA			
Pos.	459	obs:	exp:
tta	L	0	0.09
ttg	L	0	0.19
ctt	L	0	0.20
ctc	L	0	0.16
cta	L	0	0.15
ctg	L	1	0.21
atg	M	4182	4182.00
act	T	0	0.26
acc	T	0	0.23
aca	T	0	0.42
acg	T	1	0.09
---	--	------	------
mPD		0.00096	0.0017
		nPD:	0.57
		N. weight:	0.0013
		Sc. PD:	0.00042
		Sc. rank:	2.6

HA			
Pos.	460	obs:	exp:
gat	D	1	0.54
gac	D	0	0.46
gaa	E	3368	2345.00
gag	E	815	1838.00
---	--	------	------
mPD		0.31	0.49
		nPD:	0.64
		N. weight:	0.19
		Sc. PD:	0.072
		Sc. rank:	405.7

HA			
Pos.	461	obs:	exp:
aat	N	3273	2280.00
aac	N	908	1901.00
aaa	K	3	1.71
aag	K	0	1.29
---	--	------	------
mPD		0.34	0.50
		nPD:	0.69
		N. weight:	0.17
		Sc. PD:	0.075
		Sc. rank:	400.5

HA			
Pos.	462	obs:	exp:
gaa	E	156	2345.00
gag	E	4028	1839.00
---	--	------	------
mPD		0.072	0.49
		nPD:	0.15
		N. weight:	0.91
		Sc. PD:	-0.079
		Sc. rank:	-6.2

,

HA			
Pos.	463	obs:	exp:
cgt	R	0	156.50
cgc	R	0	223.70
cga	R	2	421.30
cgg	R	0	341.40
aaa	K	1	0.57
aag	K	0	0.43
aga	R	3207	1952.00
agg	R	972	1086.00
ggt	G	0	0.29
ggc	G	0	0.26
gga	G	2	0.88
ggg	G	0	0.57
---	--	------	------
mPD		0.36	0.96
		nPD:	0.38
		N. weight:	0.49
		Sc. PD:	0.067
		Sc. rank:	643.2

,

HA			
Pos.	464	obs:	exp:
act	T	4150	1097.00
acc	T	23	942.80
aca	T	11	1751.00
acg	T	0	393.70
---	--	------	------
mPD		0.016	0.70
		nPD:	0.02
		N. weight:	1.8
		Sc. PD:	-0.37
		Sc. rank:	-3848.1

,

HA			
Pos.	465	obs:	exp:
tta	L	88	386.90
ttg	L	12	794.80
ctt	L	1	830.70
ctc	L	1	669.30
cta	L	4011	619.20
ctg	L	71	883.10
---	--	------	------
mPD		0.087	1.1
		nPD:	0.08
		N. weight:	2.4
		Sc. PD:	-0.36
		Sc. rank:	-2207.3

,

HA			
Pos.	466	obs:	exp:
tat	Y	0	0.48
tac	Y	1	0.52
cat	H	1	0.58
cac	H	0	0.42
aat	N	0	1.64
aac	N	3	1.36
gat	D	1087	2256.00
gac	D	3092	1923.00
---	--	------	------
mPD		0.39	0.50
		nPD:	0.78
		N. weight:	0.23
		Sc. PD:	0.12
		Sc. rank:	563.8

,

HA			
Pos.	467	obs:	exp:
ttt	F	1096	1734.00
ttc	F	3060	2422.00
tta	L	0	0.09
ttg	L	0	0.19
tat	Y	0	11.99
tac	Y	25	13.01
ctt	L	0	0.20
ctc	L	1	0.16
cta	L	0	0.15
ctg	L	0	0.21
gtt	V	0	0.43
gtc	V	2	0.42
gta	V	0	0.43
gtg	V	0	0.72
---	--	------	------
mPD		0.40	0.50
		nPD:	0.8
		N. weight:	0.079
		Sc. PD:	0.043
		Sc. rank:	199.7

,

HA			
Pos.	468	obs:	exp:
cct	P	1	0.27
ccc	P	0	0.18
cca	P	0	0.41
ccg	P	0	0.14
cat	H	4175	2421.00
cac	H	8	1762.00
---	--	------	------
mPD		0.0043	0.49
		nPD:	0.01
		N. weight:	0.74
		Sc. PD:	-0.16
		Sc. rank:	-1795.2

,

HA			
Pos.	469	obs:	exp:
aat	N	0	0.55
aac	N	1	0.45
gat	D	1174	2258.00
gac	D	3008	1924.00
ggt	G	0	0.14
ggc	G	1	0.13
gga	G	0	0.44
ggg	G	0	0.29
---	--	------	------
mPD		0.40	0.50
		nPD:	0.81
		N. weight:	0.19
		Sc. PD:	0.11
		Sc. rank:	491.8

,

HA			
Pos.	470	obs:	exp:
tct	S	6	698.40
tcc	S	10	606.10
tca	S	4133	948.50
tcg	S	35	252.40
agt	S	0	907.50
agc	S	0	771.10
---	--	------	------
mPD		0.024	1.7
		nPD:	0.01
		N. weight:	2.
		Sc. PD:	-0.42
		Sc. rank:	-4476.9

,

HA			
Pos.	471	obs:	exp:
cat	H	1	0.58
cac	H	0	0.42
att	I	1	0.37
atc	I	0	0.27
ata	I	0	0.37
aat	N	4172	2280.00
aac	N	9	1901.00
aaa	K	1	0.57
aag	K	0	0.43
---	--	------	------
mPD		0.0057	0.50
		nPD:	0.01
		N. weight:	0.82
		Sc. PD:	-0.18
		Sc. rank:	-1939.0

,

HA			
Pos.	472	obs:	exp:
ttt	F	0	0.42
ttc	F	1	0.58
gtt	V	301	899.50
gtc	V	3686	879.80
gta	V	192	891.70
gtg	V	3	1511.00
ggt	G	0	0.14
ggc	G	1	0.13
gga	G	0	0.44
ggg	G	0	0.29
---	--	------	------
mPD		0.22	0.73
		nPD:	0.3
		N. weight:	1.5
		Sc. PD:	0.089
		Sc. rank:	1461.2

,

HA			
Pos.	473	obs:	exp:
cgt	R	0	20.36
cgc	R	0	29.11
cga	R	0	54.82
cgg	R	6	44.42
aaa	K	45	2072.00
aag	K	3594	1567.00
aga	R	7	254.00
agg	R	531	141.30
gaa	E	0	0.56
gag	E	1	0.44
---	--	------	------
mPD		0.25	0.80
		nPD:	0.32
		N. weight:	1.2
		Sc. PD:	0.094
		Sc. rank:	1250.5

,

HA			
Pos.	474	obs:	exp:
tat	Y	0	0.48
tac	Y	1	0.52
act	T	0	0.26
acc	T	1	0.23
aca	T	0	0.42
acg	T	0	0.09
aat	N	442	2281.00
aac	N	3740	1901.00
---	--	------	------
mPD		0.19	0.50
		nPD:	0.38
		N. weight:	0.6
		Sc. PD:	0.085
		Sc. rank:	789.4

,

HA			
Pos.	475	obs:	exp:
tta	L	0	386.80
ttg	L	0	794.70
ctt	L	3838	830.50
ctc	L	284	669.10
cta	L	40	619.10
ctg	L	21	882.90
att	I	1	0.37
atc	I	0	0.27
ata	I	0	0.37
---	--	------	------
mPD		0.15	1.1
		nPD:	0.14
		N. weight:	1.8
		Sc. PD:	-0.17
		Sc. rank:	-266.6

,

HA			
Pos.	476	obs:	exp:
tat	Y	483	2007.00
tac	Y	3701	2177.00
---	--	------	------
mPD		0.20	0.50
		nPD:	0.41
		N. weight:	0.42
		Sc. PD:	0.071
		Sc. rank:	607.7

,

HA			
Pos.	477	obs:	exp:
aat	N	0	2.73
aac	N	5	2.27
aaa	K	1	0.57
aag	K	0	0.43
gat	D	1037	2229.00
gac	D	3092	1900.00
gaa	E	48	27.47
gag	E	1	21.53
---	--	------	------
mPD		0.39	0.51
		nPD:	0.77
		N. weight:	0.25
		Sc. PD:	0.13
		Sc. rank:	612.9

,

HA			
Pos.	478	obs:	exp:
cgt	R	0	0.34
cgc	R	0	0.48
cga	R	0	0.91
cgg	R	0	0.73
aaa	K	960	2377.00
aag	K	3215	1798.00
aga	R	0	4.20
agg	R	9	2.34
---	--	------	------
mPD		0.36	0.50
		nPD:	0.72
		N. weight:	0.34
		Sc. PD:	0.16
		Sc. rank:	816.0

,

HA			
Pos.	479	obs:	exp:
att	I	0	1.10
atc	I	3	0.80
ata	I	0	1.10
gtt	V	54	899.00
gtc	V	4009	879.40
gta	V	16	891.30
gtg	V	101	1510.00
gct	A	0	0.25
gcc	A	1	0.19
gca	A	0	0.45
gcg	A	0	0.11
---	--	------	------
mPD		0.081	0.74
		nPD:	0.11
		N. weight:	1.9
		Sc. PD:	-0.22
		Sc. rank:	-804.2

,

HA			
Pos.	480	obs:	exp:
tct	S	0	0.17
tcc	S	0	0.14
tca	S	0	0.23
tcg	S	0	0.06
caa	Q	1	0.50
cag	Q	0	0.50
cgt	R	0	156.20
cgc	R	0	223.40
cga	R	2395	420.70
cgg	R	59	340.90
aaa	K	6	3.42
aag	K	0	2.58
agt	S	1	0.22
agc	S	0	0.18
aga	R	1717	1949.00
agg	R	4	1085.00
ggt	G	0	0.14
ggc	G	0	0.13
gga	G	1	0.44
ggg	G	0	0.29
---	--	------	------
mPD		0.52	0.96
		nPD:	0.54
		N. weight:	1.3
		Sc. PD:	0.38
		Sc. rank:	2476.3

,

HA			
Pos.	481	obs:	exp:
tta	L	551	386.40
ttg	L	41	793.90
ctt	L	0	829.70
ctc	L	37	668.50
cta	L	3528	618.50
ctg	L	22	882.00
cgt	R	0	0.07
cgc	R	0	0.11
cga	R	2	0.20
cgg	R	0	0.16
att	I	0	0.37
atc	I	0	0.27
ata	I	1	0.37
aga	R	0	0.93
agg	R	0	0.52
gtt	V	0	0.43
gtc	V	0	0.42
gta	V	2	0.43
gtg	V	0	0.72
---	--	------	------
mPD		0.29	1.1
		nPD:	0.26
		N. weight:	2.
		Sc. PD:	0.047
		Sc. rank:	1477.9

,

HA			
Pos.	482	obs:	exp:
tta	L	0	0.09
ttg	L	0	0.19
ctt	L	0	0.20
ctc	L	0	0.16
cta	L	0	0.15
ctg	L	1	0.21
caa	Q	128	2088.00
cag	Q	4055	2095.00
---	--	------	------
mPD		0.060	0.50
		nPD:	0.12
		N. weight:	0.77
		Sc. PD:	-0.086
		Sc. rank:	-241.3

,

HA			
Pos.	483	obs:	exp:
ttt	F	1	0.42
ttc	F	0	0.58
tta	L	0	386.30
ttg	L	0	793.70
ctt	L	3847	829.50
ctc	L	231	668.30
cta	L	0	618.30
ctg	L	100	881.80
cct	P	1	0.27
ccc	P	0	0.18
cca	P	0	0.41
ccg	P	0	0.14
att	I	2	1.47
atc	I	2	1.07
ata	I	0	1.46
---	--	------	------
mPD		0.15	1.1
		nPD:	0.14
		N. weight:	1.8
		Sc. PD:	-0.17
		Sc. rank:	-358.5

,

HA			
Pos.	484	obs:	exp:
cgt	R	0	96.74
cgc	R	0	138.30
cga	R	0	260.50
cgg	R	1	211.10
aat	N	1	0.55
aac	N	0	0.45
aaa	K	20	909.30
aag	K	1577	687.70
aga	R	94	1207.00
agg	R	2490	671.60
ggt	G	0	0.14
ggc	G	0	0.13
gga	G	0	0.44
ggg	G	1	0.29
---	--	------	------
mPD		0.53	1.3
		nPD:	0.41
		N. weight:	1.4
		Sc. PD:	0.24
		Sc. rank:	2017.3

,

HA			
Pos.	485	obs:	exp:
aat	N	5	2.73
aac	N	0	2.27
gat	D	3636	2255.00
gac	D	540	1921.00
gaa	E	0	0.56
gag	E	1	0.44
ggt	G	2	0.29
ggc	G	0	0.26
gga	G	0	0.88
ggg	G	0	0.57
---	--	------	------
mPD		0.23	0.50
		nPD:	0.46
		N. weight:	0.35
		Sc. PD:	0.075
		Sc. rank:	604.3

,

HA			
Pos.	486	obs:	exp:
aat	N	4115	2282.00
aac	N	69	1902.00
---	--	------	------
mPD		0.032	0.50
		nPD:	0.07
		N. weight:	0.73
		Sc. PD:	-0.12
		Sc. rank:	-887.4

,

HA			
Pos.	487	obs:	exp:
gct	A	8	1054.00
gcc	A	70	809.50
gca	A	4054	1862.00
gcg	A	51	457.80
ggt	G	0	0.14
ggc	G	0	0.13
gga	G	1	0.44
ggg	G	0	0.29
---	--	------	------
mPD		0.061	0.69
		nPD:	0.09
		N. weight:	0.94
		Sc. PD:	-0.13
		Sc. rank:	-627.6

,

HA			
Pos.	488	obs:	exp:
caa	Q	0	1.00
cag	Q	2	1.00
cgt	R	0	0.26
cgc	R	0	0.37
cga	R	0	0.71
cgg	R	0	0.57
act	T	0	0.26
acc	T	0	0.23
aca	T	1	0.42
acg	T	0	0.09
aaa	K	1775	2375.00
aag	K	2396	1796.00
aga	R	1	3.27
agg	R	6	1.82
gaa	E	3	1.68
gag	E	0	1.32
---	--	------	------
mPD		0.50	0.50
		nPD:	0.99
		N. weight:	0.063
		Sc. PD:	0.046
		Sc. rank:	163.3

,

HA			
Pos.	489	obs:	exp:
aaa	K	0	0.57
aag	K	1	0.43
gat	D	3	1.62
gac	D	0	1.38
gaa	E	288	2343.00
gag	E	3892	1837.00
---	--	------	------
mPD		0.13	0.49
		nPD:	0.26
		N. weight:	0.77
		Sc. PD:	0.021
		Sc. rank:	590.0

,

HA			
Pos.	490	obs:	exp:
tta	L	2	386.50
ttg	L	333	794.10
ctt	L	684	829.90
ctc	L	3	668.60
cta	L	34	618.60
ctg	L	3124	882.30
atg	M	3	3.00
gtt	V	1	0.22
gtc	V	0	0.21
gta	V	0	0.21
gtg	V	0	0.36
---	--	------	------
mPD		0.44	1.1
		nPD:	0.39
		N. weight:	1.1
		Sc. PD:	0.17
		Sc. rank:	1541.4

,

HA			
Pos.	491	obs:	exp:
ggt	G	3210	605.10
ggc	G	157	549.60
gga	G	780	1833.00
ggg	G	37	1196.00
---	--	------	------
mPD		0.38	0.69
		nPD:	0.55
		N. weight:	1.4
		Sc. PD:	0.43
		Sc. rank:	2854.6

,

HA			
Pos.	492	obs:	exp:
aat	N	827	2279.00
aac	N	3352	1900.00
gat	D	0	2.70
gac	D	5	2.30
---	--	------	------
mPD		0.32	0.50
		nPD:	0.64
		N. weight:	0.35
		Sc. PD:	0.14
		Sc. rank:	776.2

,

HA			
Pos.	493	obs:	exp:
ggt	G	3238	605.10
ggc	G	895	549.60
gga	G	49	1833.00
ggg	G	2	1196.00
---	--	------	------
mPD		0.36	0.69
		nPD:	0.52
		N. weight:	1.9
		Sc. PD:	0.51
		Sc. rank:	3478.2

,

HA			
Pos.	494	obs:	exp:
tgt	C	4052	1619.00
tgc	C	132	2565.00
---	--	------	------
mPD		0.061	0.47
		nPD:	0.13
		N. weight:	1.1
		Sc. PD:	-0.11
		Sc. rank:	-303.9

,

HA			
Pos.	495	obs:	exp:
ttt	F	282	1744.00
ttc	F	3899	2437.00
tct	S	0	0.17
tcc	S	1	0.14
tca	S	0	0.23
tcg	S	0	0.06
att	I	0	0.37
atc	I	1	0.27
ata	I	0	0.37
agt	S	0	0.22
agc	S	0	0.18
gtt	V	0	0.22
gtc	V	0	0.21
gta	V	0	0.21
gtg	V	1	0.36
---	--	------	------
mPD		0.13	0.49
		nPD:	0.26
		N. weight:	0.44
		Sc. PD:	0.011
		Sc. rank:	325.9

,

HA			
Pos.	496	obs:	exp:
aaa	K	0	0.57
aag	K	1	0.43
gaa	E	918	2345.00
gag	E	3265	1838.00
---	--	------	------
mPD		0.34	0.49
		nPD:	0.7
		N. weight:	0.34
		Sc. PD:	0.15
		Sc. rank:	793.8

,

HA			
Pos.	497	obs:	exp:
ttt	F	34	1745.00
ttc	F	4148	2437.00
tta	L	0	0.09
ttg	L	0	0.19
tct	S	0	0.17
tcc	S	1	0.14
tca	S	0	0.23
tcg	S	0	0.06
ctt	L	0	0.20
ctc	L	1	0.16
cta	L	0	0.15
ctg	L	0	0.21
agt	S	0	0.22
agc	S	0	0.18
---	--	------	------
mPD		0.017	0.49
		nPD:	0.04
		N. weight:	0.69
		Sc. PD:	-0.13
		Sc. rank:	-1313.1

,

HA			
Pos.	498	obs:	exp:
tct	S	2	0.33
tcc	S	0	0.29
tca	S	0	0.45
tcg	S	0	0.12
tat	Y	4038	2000.00
tac	Y	133	2171.00
cat	H	11	6.37
cac	H	0	4.63
agt	S	0	0.43
agc	S	0	0.37
---	--	------	------
mPD		0.068	0.51
		nPD:	0.13
		N. weight:	0.82
		Sc. PD:	-0.08
		Sc. rank:	-176.3

,

HA			
Pos.	499	obs:	exp:
cct	P	0	0.27
ccc	P	1	0.18
cca	P	0	0.41
ccg	P	0	0.14
cat	H	65	2421.00
cac	H	4118	1762.00
---	--	------	------
mPD		0.031	0.49
		nPD:	0.06
		N. weight:	1.1
		Sc. PD:	-0.18
		Sc. rank:	-1347.4

,

HA			
Pos.	500	obs:	exp:
cgt	R	0	24.92
cgc	R	0	35.64
cga	R	0	67.11
cgg	R	0	54.38
att	I	0	0.37
atc	I	0	0.27
ata	I	1	0.37
act	T	0	0.26
acc	T	0	0.23
aca	T	1	0.42
acg	T	0	0.09
aat	N	1	1.09
aac	N	1	0.91
aaa	K	3463	2001.00
aag	K	51	1513.00
aga	R	658	310.90
agg	R	8	173.00
---	--	------	------
mPD		0.30	0.86
		nPD:	0.35
		N. weight:	0.73
		Sc. PD:	0.079
		Sc. rank:	861.6

,

HA			
Pos.	501	obs:	exp:
tgt	C	3534	1619.00
tgc	C	649	2564.00
cgt	R	1	0.04
cgc	R	0	0.05
cga	R	0	0.10
cgg	R	0	0.08
aga	R	0	0.47
agg	R	0	0.26
---	--	------	------
mPD		0.26	0.48
		nPD:	0.55
		N. weight:	0.62
		Sc. PD:	0.19
		Sc. rank:	1232.6

,

HA

Pos.	502	obs:	exp:
cat	H	1	0.58
cac	H	0	0.42
aat	N	1361	753.10
aac	N	20	627.90
gat	D	2714	1512.00
gac	D	87	1289.00
ggt	G	1	0.14
ggc	G	0	0.13
gga	G	0	0.44
ggg	G	0	0.29
---	--	------	------
mPD		0.49	0.94
		nPD:	0.52
		N. weight:	0.69
		Sc. PD:	0.19
		Sc. rank:	1315.2

HA

Pos.	503	obs:	exp:
tct	S	0	0.17
tcc	S	0	0.14
tca	S	0	0.23
tcg	S	0	0.06
aat	N	4006	2204.00
aac	N	35	1837.00
agt	S	1	0.22
agc	S	0	0.18
gat	D	142	76.67
gac	D	0	65.33
---	--	------	------
mPD		0.083	0.56
		nPD:	0.15
		N. weight:	0.78
		Sc. PD:	-0.066
		Sc. rank:	17.6

HA

Pos.	504	obs:	exp:
aaa	K	8	4.56
aag	K	0	3.44
gct	A	0	0.25
gcc	A	0	0.19
gca	A	1	0.45
gcg	A	0	0.11
gat	D	0	1.62
gac	D	3	1.38
gaa	E	4120	2326.00
gag	E	29	1823.00
ggt	G	0	3.33
ggc	G	0	3.02
gga	G	23	10.08
ggg	G	0	6.57
---	--	------	------
mPD		0.030	0.51
		nPD:	0.06
		N. weight:	0.75
		Sc. PD:	-0.13
		Sc. rank:	-971.2

HA

Pos.	505	obs:	exp:
tgt	C	3785	1619.00
tgc	C	398	2564.00
act	T	1	0.26
acc	T	0	0.23
aca	T	0	0.42
acg	T	0	0.09
---	--	------	------
mPD		0.17	0.48
		nPD:	0.36
		N. weight:	0.82
		Sc. PD:	0.1
		Sc. rank:	1029.3

HA

Pos.	506	obs:	exp:
ttt	F	0	0.42
ttc	F	1	0.58
att	I	5	17.61
atc	I	0	12.84
ata	I	43	17.54
atg	M	4134	4134.00
gtt	V	0	0.22
gtc	V	0	0.21
gta	V	0	0.21
gtg	V	1	0.36
---	--	------	------
mPD		0.024	0.024
		nPD:	0.99
		N. weight:	0.012
		Sc. PD:	0.0085
		Sc. rank:	30.7

HA

Pos.	507	obs:	exp:
caa	Q	1	0.50
cag	Q	0	0.50
gat	D	0	1.62
gac	D	3	1.38
gaa	E	4166	2343.00
gag	E	13	1836.00
ggt	G	0	0.14
ggc	G	0	0.13
gga	G	1	0.44
ggg	G	0	0.29
---	--	------	------
mPD		0.0086	0.49
		nPD:	0.02
		N. weight:	0.77
		Sc. PD:	-0.16
		Sc. rank:	-1728.1

HA

Pos.	508	obs:	exp:
tct	S	0	697.90
tcc	S	0	605.60
tca	S	0	947.80
tcg	S	0	252.20
aat	N	3	1.64
aac	N	0	1.36
agt	S	4156	906.90
agc	S	25	770.50
---	--	------	------
mPD		0.013	1.7
		nPD:	0.01
		N. weight:	2.1
		Sc. PD:	-0.45
		Sc. rank:	-5080.5

HA

Pos.	509	obs:	exp:
tta	L	1	0.09
ttg	L	0	0.19
ctt	L	0	0.20
ctc	L	0	0.16
cta	L	0	0.15
ctg	L	0	0.21
att	I	1	86.24
atc	I	0	62.89
ata	I	234	85.88
gtt	V	14	849.10
gtc	V	16	830.60
gta	V	2711	841.80
gtg	V	1207	1426.00
---	--	------	------
mPD		0.53	0.85
		nPD:	0.62
		N. weight:	1.
		Sc. PD:	0.38
		Sc. rank:	2219.5

HA

Pos.	510	obs:	exp:
cgt	R	0	152.50
cgc	R	3	218.00
cga	R	38	410.50
cgg	R	0	332.70
aaa	K	102	60.93
aag	K	5	46.07
aga	R	3989	1902.00
agg	R	44	1058.00
ggt	G	0	0.43
ggc	G	0	0.39
gga	G	3	1.31
ggg	G	0	0.86
---	--	------	------
mPD		0.095	1.0
		nPD:	0.1
		N. weight:	0.91
		Sc. PD:	-0.12
		Sc. rank:	-544.9

HA

Pos.	511	obs:	exp:
aat	N	1036	2281.00
aac	N	3146	1901.00
aaa	K	1	1.14
aag	K	1	0.86
---	--	------	------
mPD		0.37	0.50
		nPD:	0.75
		N. weight:	0.26
		Sc. PD:	0.13
		Sc. rank:	630.2

HA

Pos.	512	obs:	exp:
gct	A	0	1.01
gcc	A	0	0.77
gca	A	4	1.78
gcg	A	0	0.44
ggt	G	4	604.50
ggc	G	0	549.10
gga	G	2280	1832.00
ggg	G	1896	1195.00
---	--	------	------
mPD		0.50	0.69
		nPD:	0.72
		N. weight:	0.45
		Sc. PD:	0.21
		Sc. rank:	1098.6

HA

Pos.	513	obs:	exp:
cct	P	0	0.27
ccc	P	0	0.18
cca	P	0	0.41
ccg	P	1	0.14
atg	M	2	2.00
act	T	14	1096.00
acc	T	1	941.90
aca	T	277	1749.00
acg	T	3888	393.30
aaa	K	0	0.57
aag	K	1	0.43
---	--	------	------
mPD		0.13	0.70
		nPD:	0.19
		N. weight:	2.8
		Sc. PD:	-0.12
		Sc. rank:	1071.9

HA

Pos.	514	obs:	exp:
tat	Y	3983	2007.00
tac	Y	201	2177.00
---	--	------	------
mPD		0.091	0.50
		nPD:	0.18
		N. weight:	0.75
		Sc. PD:	-0.038
		Sc. rank:	260.8

HA

Pos.	515	obs:	exp:
tct	S	0	4.01
tcc	S	0	3.48
tca	S	0	5.44
tcg	S	0	1.45
tat	Y	0	0.48
tac	Y	1	0.52
aat	N	9	178.30
aac	N	318	148.70
agt	S	0	5.21
agc	S	24	4.42
gat	D	279	2065.00
gac	D	3546	1760.00
gaa	E	5	3.92
gag	E	2	3.08
---	--	------	------
mPD		0.30	0.67
		nPD:	0.45
		N. weight:	0.72
		Sc. PD:	0.15
		Sc. rank:	1166.3

HA

Pos.	516	obs:	exp:
tat	Y	432	2006.00
tac	Y	3750	2176.00
cat	H	0	1.16
cac	H	2	0.84
---	--	------	------
mPD		0.19	0.50
		nPD:	0.37
		N. weight:	0.46
		Sc. PD:	0.061
		Sc. rank:	596.7

HA

Pos.	517	obs:	exp:
tta	L	0	1.02
ttg	L	0	2.09
tct	S	7	1.50
tcc	S	0	1.30
tca	S	0	2.04
tcg	S	2	0.54
ctt	L	0	2.18
ctc	L	0	1.76
cta	L	0	1.63
ctg	L	11	2.32
cct	P	1045	1137.00
ccc	P	15	751.70
cca	P	282	1689.00
ccg	P	2820	583.50
caa	Q	2	1.00
cag	Q	0	1.00
agt	S	0	1.95
agc	S	0	1.66
---	--	------	------
mPD		0.48	0.72
		nPD:	0.67
		N. weight:	1.3
		Sc. PD:	0.53
		Sc. rank:	2887.4

HA

Pos.	518	obs:	exp:
cat	H	64	37.05
cac	H	0	26.95
caa	Q	333	2046.00
cag	Q	3766	2053.00
cgt	R	0	0.49
cgc	R	0	0.70
cga	R	2	1.31
cgg	R	11	1.06
aaa	K	1	4.56
aag	K	7	3.44
aga	R	0	6.07
agg	R	0	3.38
---	--	------	------
mPD		0.19	0.53
		nPD:	0.35
		N. weight:	0.58
		Sc. PD:	0.064
		Sc. rank:	691.7

HA

Pos.	519	obs:	exp:
ttt	F	1	0.42
ttc	F	0	0.58
tat	Y	4131	2006.00
tac	Y	51	2176.00
tgt	C	1	0.39
tgc	C	0	0.61
---	--	------	------
mPD		0.025	0.50
		nPD:	0.05
		N. weight:	0.93
		Sc. PD:	-0.17
		Sc. rank:	-1428.3

HA

Pos.	520	obs:	exp:
tct	S	14	697.20
tcc	S	0	605.10
tca	S	4148	946.90
tcg	S	15	252.00
caa	Q	0	0.50
cag	Q	1	0.50
act	T	0	0.26
acc	T	0	0.23
aca	T	1	0.42
acg	T	0	0.09
agt	S	0	906.00
agc	S	0	769.80
gct	A	0	1.26
gcc	A	0	0.97
gca	A	5	2.23
gcg	A	0	0.55
---	--	------	------
mPD		0.018	1.7
		nPD:	0.01
		N. weight:	2.
		Sc. PD:	-0.43
		Sc. rank:	-4779.8

HA

Pos.	521	obs:	exp:
aaa	K	32	18.22
aag	K	0	13.78
gat	D	3	21.60
gac	D	37	18.40
gaa	E	4088	2302.00
gag	E	19	1805.00
ggt	G	0	0.72
ggc	G	0	0.66
gga	G	5	2.19
ggg	G	0	1.43
---	--	------	------
mPD		0.045	0.52
		nPD:	0.09
		N. weight:	0.76
		Sc. PD:	-0.11
		Sc. rank:	-516.0

HA

Pos.	522	obs:	exp:
aaa	K	3	2.28
aag	K	1	1.72
gtt	V	0	1.72
gtc	V	0	1.68
gta	V	8	1.71
gtg	V	0	2.89
gaa	E	4139	2339.00
gag	E	33	1833.00
---	--	------	------
mPD		0.022	0.50
		nPD:	0.04
		N. weight:	0.74
		Sc. PD:	-0.14
		Sc. rank:	-1263.7

HA

Pos.	523	obs:	exp:
tct	S	0	38.73
tcc	S	0	33.61
tca	S	232	52.59
tcg	S	0	14.00
cct	P	0	0.55
ccc	P	0	0.36
cca	P	2	0.81
ccg	P	0	0.28
act	T	0	0.79
acc	T	0	0.68
aca	T	3	1.26
acg	T	0	0.28
agt	S	0	50.32
agc	S	0	42.75
gtt	V	0	0.65
gtc	V	0	0.63
gta	V	3	0.64
gtg	V	0	1.08
gct	A	3	993.60
gcc	A	1	763.20
gca	A	3926	1756.00
gcg	A	14	431.70
---	--	------	------
mPD		0.12	0.85
		nPD:	0.14
		N. weight:	1.1
		Sc. PD:	-0.11
		Sc. rank:	-126.0

HA

Pos.	524	obs:	exp:
cgt	R	0	155.20
cgc	R	0	221.90
cga	R	0	417.90
cgg	R	0	338.60
att	I	0	1.83
atc	I	0	1.34
ata	I	5	1.83
aaa	K	26	14.80
aag	K	0	11.20
aga	R	4102	1936.00
agg	R	45	1077.00
gat	D	1	0.54
gac	D	0	0.46
gaa	E	3	1.68
gag	E	0	1.32
ggt	G	0	0.29
ggc	G	0	0.26
gga	G	2	0.88
ggg	G	0	0.57
---	--	------	------
mPD		0.041	0.97
		nPD:	0.04
		N. weight:	0.98
		Sc. PD:	-0.18
		Sc. rank:	-1696.7

HA

Pos.	525	obs:	exp:
tta	L	3827	385.00
ttg	L	29	791.00
tct	S	0	2.34
tcc	S	0	2.03
tca	S	14	3.17
tcg	S	0	0.84
ctt	L	1	826.80
ctc	L	0	666.10
cta	L	255	616.20
ctg	L	52	878.90
att	I	0	1.47
atc	I	0	1.07
ata	I	4	1.46
agt	S	0	3.04
agc	S	0	2.58
gtt	V	0	0.43
gtc	V	0	0.42
gta	V	2	0.43
gtg	V	0	0.72
---	--	------	------
mPD		0.18	1.1
		nPD:	0.16
		N. weight:	2.8
		Sc. PD:	-0.19
		Sc. rank:	541.9

HA

Pos.	526	obs:	exp:
tct	S	0	0.83
tcc	S	0	0.72
tca	S	0	1.13
tcg	S	0	0.30
caa	Q	2	1.00
cag	Q	0	1.00
cgt	R	0	0.22
cgc	R	0	0.32
cga	R	0	0.60
cgg	R	0	0.49
act	T	0	0.52
acc	T	0	0.45
aca	T	2	0.84
acg	T	0	0.19
aat	N	32	63.80
aac	N	85	53.20
aaa	K	4011	2305.00
aag	K	37	1743.00
agt	S	0	1.08
agc	S	5	0.92
aga	R	5	2.80
agg	R	1	1.56
gat	D	0	0.54
gac	D	1	0.46
gaa	E	3	1.68
gag	E	0	1.32
---	--	------	------
mPD		0.084	0.53
		nPD:	0.16
		N. weight:	0.7
		Sc. PD:	-0.052
		Sc. rank:	95.0

HA

Pos.	527	obs:	exp:
cgt	R	0	156.40
cgc	R	0	223.60
cga	R	4	421.00
cgg	R	0	341.20
att	I	0	0.37
atc	I	0	0.27
ata	I	1	0.37
aaa	K	3	1.71
aag	K	0	1.29
aga	R	4130	1950.00
agg	R	44	1085.00
ggt	G	0	0.29
ggc	G	0	0.26
gga	G	2	0.88
ggg	G	0	0.57
---	--	------	------
mPD		0.026	0.96
		nPD:	0.03
		N. weight:	0.98
		Sc. PD:	-0.2
		Sc. rank:	-2019.7

HA

Pos.	528	obs:	exp:
aaa	K	3	2.85
aag	K	2	2.15
gtt	V	0	0.22
gtc	V	0	0.21
gta	V	1	0.21
gtg	V	0	0.36
gaa	E	1294	2338.00
gag	E	2876	1832.00
ggt	G	0	1.16
ggc	G	0	1.05
gga	G	0	3.51
ggg	G	8	2.29
---	--	------	------
mPD		0.43	0.50
		nPD:	0.87
		N. weight:	0.18
		Sc. PD:	0.11
		Sc. rank:	466.9

HA

Pos.	529	obs:	exp:
aaa	K	11	6.26
aag	K	0	4.74
gtt	V	0	0.22
gtc	V	0	0.21
gta	V	1	0.21
gtg	V	0	0.36
gat	D	7	3.78
gac	D	0	3.22
gaa	E	4038	2333.00
gag	E	124	1829.00
ggt	G	0	0.43
ggc	G	0	0.39
gga	G	3	1.31
ggg	G	0	0.86
---	--	------	------
mPD		0.068	0.50
		nPD:	0.14
		N. weight:	0.63
		Sc. PD:	-0.061
		Sc. rank:	-121.3

HA

Pos.	530	obs:	exp:
att	I	42	1534.00
atc	I	2	1119.00
ata	I	4136	1528.00
gtt	V	0	0.86
gtc	V	0	0.84
gta	V	4	0.85
gtg	V	0	1.45
---	--	------	------
mPD		0.023	0.66
		nPD:	0.03
		N. weight:	1.3
		Sc. PD:	-0.26
		Sc. rank:	-2525.5

HA

Pos.	531	obs:	exp:
tct	S	0	683.40
tcc	S	0	593.00
tca	S	0	928.10
tcg	S	0	247.00
cgt	R	1	0.04
cgc	R	0	0.05
cga	R	0	0.10
cgg	R	0	0.08
att	I	1	0.37
atc	I	0	0.27
ata	I	0	0.37
act	T	4	1.05
acc	T	0	0.90
aca	T	0	1.67
acg	T	0	0.38
aat	N	45	25.09
aac	N	1	20.91
agt	S	3181	888.00
agc	S	913	754.50
aga	R	0	0.47
agg	R	0	0.26
gat	D	1	18.36
gac	D	33	15.64
gaa	E	2	1.12
gag	E	0	0.88
ggt	G	1	0.29
ggc	G	1	0.26
gga	G	0	0.88
ggg	G	0	0.57
---	--	------	------
mPD		0.41	1.7
		nPD:	0.24
		N. weight:	1.4
		Sc. PD:	0.012
		Sc. rank:	928.6

HA

Pos.	532	obs:	exp:
cgt	R	0	0.04
cgc	R	0	0.05
cga	R	0	0.10
cgg	R	0	0.08
aga	R	1	0.47
agg	R	0	0.26
gaa	E	9	5.05
gag	E	0	3.95
ggt	G	0	603.60
ggc	G	0	548.30
gga	G	3927	1829.00
ggg	G	247	1193.00
---	--	------	------
mPD		0.12	0.69
		nPD:	0.17
		N. weight:	0.87
		Sc. PD:	-0.057
		Sc. rank:	249.5

HA

Pos.	533	obs:	exp:
att	I	0	3.67
atc	I	0	2.68
ata	I	10	3.65
atg	M	1	1.00
gtt	V	4	896.90
gtc	V	38	877.30
gta	V	3048	889.10
gtg	V	1080	1507.00
gct	A	0	0.76
gcc	A	0	0.58
gca	A	3	1.34
gcg	A	0	0.33
---	--	------	------
mPD		0.40	0.74
		nPD:	0.55
		N. weight:	1.1
		Sc. PD:	0.33
		Sc. rank:	2148.0

HA

Pos.	534	obs:	exp:
caa	Q	2	1.00
cag	Q	0	1.00
aaa	K	3732	2380.00
aag	K	448	1800.00
gaa	E	2	1.12
gag	E	0	0.88
---	--	------	------
mPD		0.19	0.49
		nPD:	0.39
		N. weight:	0.35
		Sc. PD:	0.053
		Sc. rank:	480.1

HA

Pos.	535	obs:	exp:
tta	L	881	386.10
ttg	L	2546	793.30
tct	S	0	0.50
tcc	S	0	0.43
tca	S	0	0.68
tcg	S	3	0.18
ctt	L	0	829.10
ctc	L	0	668.00
cta	L	4	618.00
ctg	L	745	881.40
atg	M	5	5.00
agt	S	0	0.65
agc	S	0	0.55
---	--	------	------
mPD		0.63	1.1
		nPD:	0.56
		N. weight:	1.2
		Sc. PD:	0.37
		Sc. rank:	2359.1

HA

Pos.	536	obs:	exp:
caa	Q	1	0.50
cag	Q	0	0.50
gtt	V	0	0.43
gtc	V	0	0.42
gta	V	2	0.43
gtg	V	0	0.72
gat	D	0	0.54
gac	D	1	0.46
gaa	E	3722	2343.00
gag	E	458	1837.00
---	--	------	------
mPD		0.20	0.49
		nPD:	0.4
		N. weight:	0.36
		Sc. PD:	0.057
		Sc. rank:	500.2

HA			
Pos. 537		obs:	exp:
tct	S	7	693.70
tcc	S	4	602.00
tca	S	3923	942.20
tcg	S	222	250.70
cct	P	0	6.56
ccc	P	0	4.33
cca	P	24	9.74
ccg	P	0	3.36
act	T	0	1.05
acc	T	0	0.90
aca	T	4	1.67
acg	T	0	0.38
agt	S	0	901.50
agc	S	0	765.90
---	--	------	------
mPD		0.12	1.7
		nPD:	0.07
		N. weight:	1.8
		Sc. PD:	-0.29
		Sc. rank:	-2022.2

HA			
Pos. 538		obs:	exp:
tta	L	0	0.18
ttg	L	1	0.38
ctt	L	0	0.40
ctc	L	0	0.32
cta	L	1	0.30
ctg	L	0	0.42
att	I	9	1370.00
atc	I	0	999.20
ata	I	3725	1365.00
atg	M	98	98.00
act	T	15	6.29
acc	T	0	5.41
aca	T	9	10.04
acg	T	0	2.26
gtt	V	0	69.69
gtc	V	0	68.17
gta	V	318	69.08
gtg	V	6	117.10
gct	A	0	0.50
gcc	A	0	0.39
gca	A	2	0.89
gcg	A	0	0.22
---	--	------	------
mPD		0.22	0.85
		nPD:	0.26
		N. weight:	1.4
		Sc. PD:	0.027
		Sc. rank:	987.7

HA			
Pos. 539		obs:	exp:
cgt	R	0	0.07
cgc	R	0	0.11
cga	R	0	0.20
cgg	R	0	0.16
aga	R	2	0.93
agg	R	0	0.52
gtt	V	0	6.88
gtc	V	0	6.73
gta	V	32	6.82
gtg	V	0	11.56
gct	A	0	0.50
gcc	A	1	0.39
gca	A	1	0.89
gcg	A	0	0.22
gaa	E	3	1.68
gag	E	0	1.32
ggt	G	1	599.40
ggc	G	35	544.50
gga	G	4052	1816.00
ggg	G	57	1185.00
---	--	------	------
mPD		0.063	0.71
		nPD:	0.09
		N. weight:	1.
		Sc. PD:	-0.14
		Sc. rank:	-661.8

HA			
Pos. 540		obs:	exp:
tta	L	0	3.79
ttg	L	0	7.79
ctt	L	0	8.14
ctc	L	41	6.56
cta	L	0	6.07
ctg	L	0	8.65
cct	P	0	0.27
ccc	P	1	0.18
cca	P	0	0.41
ccg	P	0	0.14
att	I	529	536.90
atc	I	934	391.50
ata	I	0	534.60
act	T	2184	601.60
acc	T	106	517.10
aca	T	0	960.30
acg	T	5	215.90
aat	N	1	0.55
aac	N	0	0.45
gtt	V	172	81.95
gtc	V	208	80.16
gta	V	1	81.24
gtg	V	0	137.70
gct	A	2	0.50
gcc	A	0	0.39
gca	A	0	0.89
gcg	A	0	0.22
---	--	------	------
mPD		1.1	1.4
		nPD:	0.8
		N. weight:	1.3
		Sc. PD:	0.7
		Sc. rank:	3241.7

HA			
Pos. 541		obs:	exp:
tat	Y	199	2005.00
tac	Y	3982	2176.00
cat	H	0	1.74
cac	H	3	1.26
---	--	------	------
mPD		0.092	0.50
		nPD:	0.18
		N. weight:	0.65
		Sc. PD:	-0.032
		Sc. rank:	229.6

HA			
Pos. 542		obs:	exp:
cct	P	0	0.55
ccc	P	0	0.36
cca	P	2	0.81
ccg	P	0	0.28
caa	Q	3263	2087.00
cag	Q	918	2094.00
cgt	R	0	0.04
cgc	R	0	0.05
cga	R	1	0.10
cgg	R	0	0.08
aga	R	0	0.47
agg	R	0	0.26
---	--	------	------
mPD		0.34	0.50
		nPD:	0.69
		N. weight:	0.23
		Sc. PD:	0.1
		Sc. rank:	547.7

HA			
Pos. 543		obs:	exp:
att	I	1	1535.00
atc	I	2	1119.00
ata	I	4179	1528.00
gtt	V	0	0.43
gtc	V	0	0.42
gta	V	2	0.43
gtg	V	0	0.72
---	--	------	------
mPD		0.0024	0.66
		nPD:	0.
		N. weight:	1.4
		Sc. PD:	-0.31
		Sc. rank:	-3592.5

HA			
Pos. 544		obs:	exp:
tta	L	29	386.60
ttg	L	418	794.30
ctt	L	8	830.10
ctc	L	1	668.80
cta	L	125	618.80
ctg	L	3600	882.50
cgt	R	0	0.04
cgc	R	0	0.05
cga	R	0	0.10
cgg	R	1	0.08
atg	M	2	2.00
aga	R	0	0.47
agg	R	0	0.26
---	--	------	------
mPD		0.27	1.1
		nPD:	0.24
		N. weight:	1.5
		Sc. PD:	0.0038
		Sc. rank:	950.9

HA			
Pos. 545		obs:	exp:
tct	S	1	697.90
tcc	S	0	605.60
tca	S	4144	947.80
tcg	S	36	252.20
cct	P	0	0.55
ccc	P	0	0.36
cca	P	2	0.81
ccg	P	0	0.28
agt	S	0	906.90
agc	S	0	770.50
gct	A	0	0.25
gcc	A	0	0.19
gca	A	1	0.45
gcg	A	0	0.11
---	--	------	------
mPD		0.019	1.7
		nPD:	0.01
		N. weight:	2.
		Sc. PD:	-0.43
		Sc. rank:	-4745.8

HA			
Pos. 546		obs:	exp:
ttt	F	1	0.42
ttc	F	0	0.58
tta	L	0	0.09
ttg	L	0	0.19
ctt	L	1	0.20
ctc	L	0	0.16
cta	L	0	0.15
ctg	L	0	0.21
att	I	4130	1532.00
atc	I	45	1117.00
ata	I	0	1526.00
act	T	7	1.84
acc	T	0	1.58
aca	T	0	2.93
acg	T	0	0.66
---	--	------	------
mPD		0.026	0.66
		nPD:	0.04
		N. weight:	1.3
		Sc. PD:	-0.25
		Sc. rank:	-2455.3

HA			
Pos. 547		obs:	exp:
tat	Y	4105	2006.00
tac	Y	78	2177.00
aat	N	1	0.55
aac	N	0	0.45
---	--	------	------
mPD		0.037	0.50
		nPD:	0.07
		N. weight:	0.89
		Sc. PD:	-0.14
		Sc. rank:	-917.6

HA			
Pos. 548		obs:	exp:
tct	S	234	697.60
tcc	S	7	605.30
tca	S	3933	947.40
tcg	S	5	252.10
agt	S	0	906.40
agc	S	0	770.10
gct	A	0	1.26
gcc	A	0	0.97
gca	A	5	2.23
gcg	A	0	0.55
---	--	------	------
mPD		0.11	1.7
		nPD:	0.07
		N. weight:	1.8
		Sc. PD:	-0.28
		Sc. rank:	-2042.3

HA			
Pos. 549		obs:	exp:
act	T	3	1097.00
acc	T	11	942.50
aca	T	4152	1750.00
acg	T	17	393.60
gct	A	0	0.25
gcc	A	0	0.19
gca	A	1	0.45
gcg	A	0	0.11
---	--	------	------
mPD		0.015	0.70
		nPD:	0.02
		N. weight:	1.1
		Sc. PD:	-0.24
		Sc. rank:	-2540.6

HA			
Pos. 550		obs:	exp:
att	I	11	4.40
atc	I	0	3.21
ata	I	1	4.39
gtt	V	301	702.70
gtc	V	0	687.30
gta	V	90	696.60
gtg	V	2876	1180.00
gct	A	13	227.50
gcc	A	0	174.70
gca	A	4	401.90
gcg	A	886	98.84
ggt	G	0	0.29
ggc	G	0	0.26
gga	G	0	0.88
ggg	G	2	0.57
---	--	------	------
mPD		0.53	1.1
		nPD:	0.49
		N. weight:	1.3
		Sc. PD:	0.32
		Sc. rank:	2372.2

HA			
Pos. 551		obs:	exp:
tct	S	0	0.33
tcc	S	0	0.29
tca	S	0	0.45
tcg	S	2	0.12
cct	P	0	0.27
ccc	P	0	0.18
cca	P	1	0.41
ccg	P	0	0.14
act	T	1	5.24
acc	T	0	4.51
aca	T	1	8.37
acg	T	18	1.88
agt	S	0	0.43
agc	S	0	0.37
gtt	V	0	1.08
gtc	V	0	1.05
gta	V	0	1.07
gtg	V	5	1.81
gct	A	7	1047.00
gcc	A	1	804.20
gca	A	761	1850.00
gcg	A	3387	454.90
---	--	------	------
mPD		0.32	0.70
		nPD:	0.45
		N. weight:	2.
		Sc. PD:	0.42
		Sc. rank:	3362.5

HA			
Pos.	552	obs:	exp:
tct	S	0	698.40
tcc	S	0	606.10
tca	S	0	948.50
tcg	S	0	252.40
agt	S	3599	907.50
agc	S	585	771.10
---	--	------	------
mPD		0.24	1.7
		nPD:	0.15
		N. weight:	1.6
		Sc. PD:	-0.14
		Sc. rank:	11.9

,

HA			
Pos.	553	obs:	exp:
tct	S	288	697.70
tcc	S	3868	605.50
tca	S	24	947.60
tcg	S	0	252.20
tat	Y	0	0.48
tac	Y	1	0.52
cct	P	0	0.55
ccc	P	2	0.36
cca	P	0	0.81
ccg	P	0	0.28
act	T	1	0.26
acc	T	0	0.23
aca	T	0	0.42
acg	T	0	0.09
agt	S	0	906.70
agc	S	0	770.30
---	--	------	------
mPD		0.14	1.7
		nPD:	0.09
		N. weight:	2.3
		Sc. PD:	-0.33
		Sc. rank:	-1619.4

,

HA			
Pos.	554	obs:	exp:
ttt	F	2	0.83
ttc	F	0	1.17
tta	L	50	386.70
ttg	L	0	794.50
ctt	L	0	830.30
ctc	L	1	668.90
cta	L	4127	618.90
ctg	L	4	882.70
---	--	------	------
mPD		0.028	1.1
		nPD:	0.02
		N. weight:	2.6
		Sc. PD:	-0.52
		Sc. rank:	-5330.4

,

HA			
Pos.	555	obs:	exp:
tta	L	1	0.18
ttg	L	0	0.38
tct	S	0	0.67
tcc	S	0	0.58
tca	S	4	0.91
tcg	S	0	0.24
ctt	L	0	0.40
ctc	L	0	0.32
cta	L	0	0.30
ctg	L	1	0.42
atg	M	1	1.00
act	T	0	6.55
acc	T	0	5.63
aca	T	25	10.46
acg	T	0	2.35
agt	S	0	0.87
agc	S	0	0.74
gtt	V	38	366.30
gtc	V	0	358.30
gta	V	627	363.10
gtg	V	1038	615.30
gct	A	3	615.70
gcc	A	2	472.90
gca	A	2410	1088.00
gcg	A	29	267.50
gaa	E	1	0.56
gag	E	0	0.44
ggt	G	0	0.58
ggc	G	0	0.53
gga	G	1	1.75
ggg	G	3	1.14
---	--	------	------
mPD		0.90	1.2
		nPD:	0.72
		N. weight:	0.88
		Sc. PD:	0.42
		Sc. rank:	2158.9

,

HA			
Pos.	556	obs:	exp:
tta	L	0	386.30
ttg	L	94	793.70
ctt	L	5	829.50
ctc	L	0	668.30
cta	L	1	618.30
ctg	L	4078	881.80
cct	P	0	0.27
ccc	P	0	0.18
cca	P	0	0.41
ccg	P	1	0.14
cgt	R	0	0.07
cgc	R	0	0.11
cga	R	0	0.20
cgg	R	2	0.16
atg	M	1	1.00
aga	R	0	0.93
agg	R	0	0.52
gtt	V	0	0.22
gtc	V	0	0.21
gta	V	0	0.21
gtg	V	1	0.36
ggt	G	0	0.14
ggc	G	0	0.13
gga	G	0	0.44
ggg	G	1	0.29
---	--	------	------
mPD		0.050	1.1
		nPD:	0.04
		N. weight:	2.
		Sc. PD:	-0.37
		Sc. rank:	-3340.2

,

HA			
Pos.	557	obs:	exp:
tct	S	0	0.17
tcc	S	0	0.14
tca	S	1	0.23
tcg	S	0	0.06
act	T	0	0.79
acc	T	0	0.68
aca	T	3	1.26
acg	T	0	0.28
agt	S	0	0.22
agc	S	0	0.18
gct	A	0	1053.00
gcc	A	1	808.50
gca	A	4177	1860.00
gcg	A	0	457.30
gat	D	0	0.54
gac	D	1	0.46
gaa	E	1	0.56
gag	E	0	0.44
---	--	------	------
mPD		0.0038	0.69
		nPD:	0.01
		N. weight:	1.1
		Sc. PD:	-0.25
		Sc. rank:	-2807.4

,

HA			
Pos.	558	obs:	exp:
tta	L	0	0.09
ttg	L	0	0.19
tct	S	0	0.33
tcc	S	0	0.29
tca	S	0	0.45
tcg	S	0	0.12
ctt	L	0	0.20
ctc	L	1	0.16
cta	L	0	0.15
ctg	L	0	0.21
att	I	1	1534.00
atc	I	4179	1119.00
ata	I	0	1528.00
act	T	0	0.26
acc	T	0	0.23
aca	T	1	0.42
acg	T	0	0.09
agt	S	0	0.43
agc	S	2	0.37
---	--	------	------
mPD		0.0029	0.66
		nPD:	0.
		N. weight:	1.8
		Sc. PD:	-0.41
		Sc. rank:	-4649.4

,

HA			
Pos.	559	obs:	exp:
tta	L	0	0.18
ttg	L	1	0.38
ctt	L	0	0.40
ctc	L	0	0.32
cta	L	0	0.30
ctg	L	1	0.42
att	I	19	17.25
atc	I	0	12.58
ata	I	28	17.18
atg	M	4131	4131.00
act	T	0	0.26
acc	T	0	0.23
aca	T	0	0.42
acg	T	1	0.09
aaa	K	0	1.71
aag	K	3	1.29
---	--	------	------
mPD		0.025	0.027
		nPD:	0.93
		N. weight:	0.0083
		Sc. PD:	0.0056
		Sc. rank:	21.6

,

HA			
Pos.	560	obs:	exp:
tta	L	0	0.28
ttg	L	3	0.57
ctt	L	0	0.60
ctc	L	0	0.48
cta	L	0	0.44
ctg	L	0	0.63
att	I	0	238.90
atc	I	0	174.20
ata	I	651	237.90
atg	M	2141	2141.00
act	T	0	0.26
acc	T	0	0.23
aca	T	0	0.42
acg	T	1	0.09
aaa	K	0	0.57
aag	K	1	0.43
gtt	V	0	298.10
gtc	V	0	291.60
gta	V	270	295.50
gtg	V	1116	500.80
gct	A	0	0.25
gcc	A	0	0.19
gca	A	0	0.45
gcg	A	1	0.11
---	--	------	------
mPD		0.79	1.0
		nPD:	0.79
		N. weight:	0.51
		Sc. PD:	0.27
		Sc. rank:	1283.0

,

HA

Pos.	561	obs:	exp:
tct	S	5	0.83
tcc	S	0	0.72
tca	S	0	1.13
tcg	S	0	0.30
cct	P	1	0.27
ccc	P	0	0.18
cca	P	0	0.41
ccg	P	0	0.14
act	T	0	0.26
acc	T	0	0.23
aca	T	1	0.42
acg	T	0	0.09
agt	S	0	1.08
agc	S	0	0.92
gct	A	4175	1052.00
gcc	A	0	807.90
gca	A	0	1858.00
gcg	A	0	457.00
gat	D	1	0.54
gac	D	0	0.46
ggt	G	1	0.14
ggc	G	0	0.13
gga	G	0	0.44
ggg	G	0	0.29
---	--	------	------
mPD		0.0048	0.69
		nPD:	0.01
		N. weight:	1.9
		Sc. PD:	-0.42
		Sc. rank:	-4759.6

,

HA

Pos.	562	obs:	exp:
tct	S	0	0.33
tcc	S	0	0.29
tca	S	0	0.45
tcg	S	0	0.12
agt	S	2	0.43
agc	S	0	0.37
ggt	G	4178	604.80
ggc	G	3	549.30
gga	G	0	1832.00
ggg	G	1	1195.00
---	--	------	------
mPD		0.0029	0.69
		nPD:	0.
		N. weight:	2.7
		Sc. PD:	-0.6
		Sc. rank:	-6845.8

,

HA

Pos.	563	obs:	exp:
tta	L	13	385.90
ttg	L	0	792.90
ctt	L	11	828.70
ctc	L	16	667.70
cta	L	3997	617.70
ctg	L	137	881.00
cct	P	0	0.27
ccc	P	0	0.18
cca	P	1	0.41
ccg	P	0	0.14
cat	H	1	0.58
cac	H	0	0.42
caa	Q	1	0.50
cag	Q	0	0.50
cgt	R	0	0.04
cgc	R	0	0.05
cga	R	1	0.10
cgg	R	0	0.08
att	I	0	2.20
atc	I	0	1.61
ata	I	6	2.19
aga	R	0	0.47
agg	R	0	0.26
---	--	------	------
mPD		0.087	1.1
		nPD:	0.08
		N. weight:	2.3
		Sc. PD:	-0.36
		Sc. rank:	-2141.1

,

HA

Pos.	564	obs:	exp:
ttt	F	316	132.20
ttc	F	1	184.80
tta	L	1	0.18
ttg	L	0	0.38
tct	S	3578	645.20
tcc	S	275	559.90
tca	S	1	876.20
tcg	S	11	233.20
ctt	L	1	0.40
ctc	L	0	0.32
cta	L	0	0.30
ctg	L	0	0.42
agt	S	0	838.30
agc	S	0	712.30
---	--	------	------
mPD		0.27	1.7
		nPD:	0.16
		N. weight:	2.
		Sc. PD:	-0.15
		Sc. rank:	349.6

,

HA

Pos.	565	obs:	exp:
ttt	F	70	41.30
ttc	F	29	57.70
tta	L	3992	377.50
ttg	L	87	775.70
tgg	W	1	1.00
ctt	L	0	810.70
ctc	L	1	653.10
cta	L	3	604.30
ctg	L	0	861.80
att	I	0	0.37
atc	I	0	0.27
ata	I	1	0.37
---	--	------	------
mPD		0.090	1.1
		nPD:	0.08
		N. weight:	3.1
		Sc. PD:	-0.46
		Sc. rank:	-2578.7

,

HA

Pos.	566	obs:	exp:
tgt	C	0	0.77
tgc	C	2	1.23
tgg	W	4181	4181.00
ggt	G	0	0.14
ggc	G	0	0.13
gga	G	0	0.44
ggg	G	1	0.29
---	--	------	------
mPD		0.0014	0.0018
		nPD:	0.81
		N. weight:	0.00074
		Sc. PD:	0.00041
		Sc. rank:	1.9

,

HA

Pos.	567	obs:	exp:
atg	M	4182	4182.00
aaa	K	0	0.57
aag	K	1	0.43
gtt	V	0	0.22
gtc	V	0	0.21
gta	V	0	0.21
gtg	V	1	0.36
---	--	------	------
mPD		0.00096	0.0015
		nPD:	0.62
		N. weight:	0.00062
		Sc. PD:	0.00023
		Sc. rank:	1.3

,

HA

Pos.	568	obs:	exp:
tgt	C	1726	1619.00
tgc	C	2457	2564.00
cgt	R	1	0.04
cgc	R	0	0.05
cga	R	0	0.10
cgg	R	0	0.08
aga	R	0	0.47
agg	R	0	0.26
---	--	------	------
mPD		0.49	0.48
		nPD:	1.02
		N. weight:	0.0044
		Sc. PD:	0.0034
		Sc. rank:	11.6

,

HA

Pos.	569	obs:	exp:
ttt	F	0	0.83
ttc	F	2	1.17
tta	L	0	0.09
ttg	L	0	0.19
tct	S	18	697.10
tcc	S	4157	604.90
tca	S	1	946.70
tcg	S	0	251.90
tat	Y	0	0.48
tac	Y	1	0.52
tgt	C	0	1.55
tgc	C	4	2.45
ctt	L	0	0.20
ctc	L	1	0.16
cta	L	0	0.15
ctg	L	0	0.21
agt	S	0	905.80
agc	S	0	769.60
---	--	------	------
mPD		0.013	1.7
		nPD:	0.01
		N. weight:	2.6
		Sc. PD:	-0.58
		Sc. rank:	-6403.7

,

HA

Pos.	570	obs:	exp:
tct	S	0	0.17
tcc	S	0	0.14
tca	S	0	0.23
tcg	S	0	0.06
cat	H	1	0.58
cac	H	0	0.42
aat	N	2549	2280.00
aac	N	1631	1900.00
aaa	K	0	0.57
aag	K	1	0.43
agt	S	0	0.22
agc	S	1	0.18
gat	D	0	0.54
gac	D	1	0.46
---	--	------	------
mPD		0.48	0.50
		nPD:	0.96
		N. weight:	0.014
		Sc. PD:	0.01
		Sc. rank:	37.6

,

HA

Pos.	571	obs:	exp:
cgt	R	0	0.04
cgc	R	0	0.05
cga	R	0	0.10
cgg	R	0	0.08
aga	R	0	0.47
agg	R	1	0.26
ggt	G	86	604.90
ggc	G	0	549.40
gga	G	1184	1833.00
ggg	G	2913	1196.00
---	--	------	------
mPD		0.44	0.69
		nPD:	0.63
		N. weight:	0.63
		Sc. PD:	0.24
		Sc. rank:	1384.5

,

HA

Pos.	572	obs:	exp:
tct	S	83	697.90
tcc	S	1	605.60
tca	S	113	947.80
tcg	S	3984	252.20
cct	P	0	0.27
ccc	P	0	0.18
cca	P	0	0.41
ccg	P	1	0.14
act	T	1	0.26
acc	T	0	0.23
aca	T	0	0.42
acg	T	0	0.09
agt	S	0	906.90
agc	S	0	770.50
gct	A	0	0.25
gcc	A	0	0.19
gca	A	0	0.45
gcg	A	1	0.11
---	--	------	------
mPD		0.093	1.7
		nPD:	0.06
		N. weight:	3.5
		Sc. PD:	-0.61
		Sc. rank:	-4929.3

,

HA

Pos.	573	obs:	exp:
ttt	F	1	0.42
ttc	F	0	0.58
tta	L	3777	386.30
ttg	L	114	793.70
ctt	L	0	829.50
ctc	L	1	668.30
cta	L	284	618.30
ctg	L	2	881.80
caa	Q	2	1.00
cag	Q	0	1.00
gtt	V	0	0.65
gtc	V	0	0.63
gta	V	2	0.64
gtg	V	1	1.08
---	--	------	------
mPD		0.19	1.1
		nPD:	0.17
		N. weight:	2.7
		Sc. PD:	-0.18
		Sc. rank:	682.3

,

HA

Pos.	574	obs:	exp:
cct	P	0	0.27
ccc	P	0	0.18
cca	P	0	0.41
ccg	P	1	0.14
caa	Q	1657	2088.00
cag	Q	2526	2095.00
---	--	------	------
mPD		0.48	0.50
		nPD:	0.96
		N. weight:	0.032
		Sc. PD:	0.022
		Sc. rank:	82.8

,

HA

Pos.	575	obs:	exp:
tat	Y	0	0.96
tac	Y	2	1.04
tgt	C	14	1619.00
tgc	C	4168	2563.00
---	--	------	------
mPD		0.0076	0.48
		nPD:	0.02
		N. weight:	0.65
		Sc. PD:	-0.14
		Sc. rank:	-1460.7

,

HA			
Pos.	576	obs:	exp:
tct	S	0	0.17
tcc	S	0	0.14
tca	S	0	0.23
tcg	S	0	0.06
cgt	R	0	156.40
cgc	R	0	223.60
cga	R	3	421.00
cgg	R	0	341.20
act	T	0	0.26
acc	T	0	0.23
aca	T	1	0.42
acg	T	0	0.09
aaa	K	3	1.71
aag	K	0	1.29
agt	S	1	0.22
agc	S	0	0.18
aga	R	4091	1950.00
agg	R	84	1085.00
ggt	G	0	0.14
ggc	G	0	0.13
gga	G	1	0.44
ggg	G	0	0.29
---	--	------	------
mPD		0.044	0.96
		nPD:	0.05
		N. weight:	0.93
		Sc. PD:	-0.17
		Sc. rank:	-1514.5

,

HA			
Pos.	577	obs:	exp:
tta	L	0	0.28
ttg	L	1	0.57
ctt	L	1	0.60
ctc	L	0	0.48
cta	L	0	0.44
ctg	L	1	0.63
att	I	4127	1526.00
atc	I	30	1113.00
ata	I	1	1520.00
atg	M	3	3.00
gtt	V	20	4.30
gtc	V	0	4.21
gta	V	0	4.26
gtg	V	0	7.23
---	--	------	------
mPD		0.028	0.67
		nPD:	0.04
		N. weight:	1.3
		Sc. PD:	-0.25
		Sc. rank:	-2346.5

,

HA			
Pos.	578	obs:	exp:
tta	L	0	0.09
ttg	L	0	0.19
tgt	C	9	1618.00
tgc	C	4172	2563.00
ctt	L	0	0.20
ctc	L	1	0.16
cta	L	0	0.15
ctg	L	0	0.21
cgt	R	0	0.04
cgc	R	1	0.05
cga	R	0	0.10
cgg	R	0	0.08
aga	R	0	0.47
agg	R	0	0.26
ggt	G	0	0.14
ggc	G	1	0.13
gga	G	0	0.44
ggg	G	0	0.29
---	--	------	------
mPD		0.0062	0.48
		nPD:	0.01
		N. weight:	0.66
		Sc. PD:	-0.14
		Sc. rank:	-1537.1

,

HA			
Pos.	579	obs:	exp:
att	I	4027	1535.00
atc	I	150	1120.00
ata	I	7	1529.00
---	--	------	------
mPD		0.072	0.66
		nPD:	0.11
		N. weight:	1.2
		Sc. PD:	-0.14
		Sc. rank:	-528.7

,

HA			
Pos.	580	obs:	exp:
taa	.	3054	1543.00
tag	.	930	1435.00
tga	.	200	1206.00
---	--	------	------
mPD		0.44	0.86
		nPD:	0.51
		N. weight:	0.44
		Sc. PD:	0.12
		Sc. rank:	804.3

}

Amino acid level conservation per codon

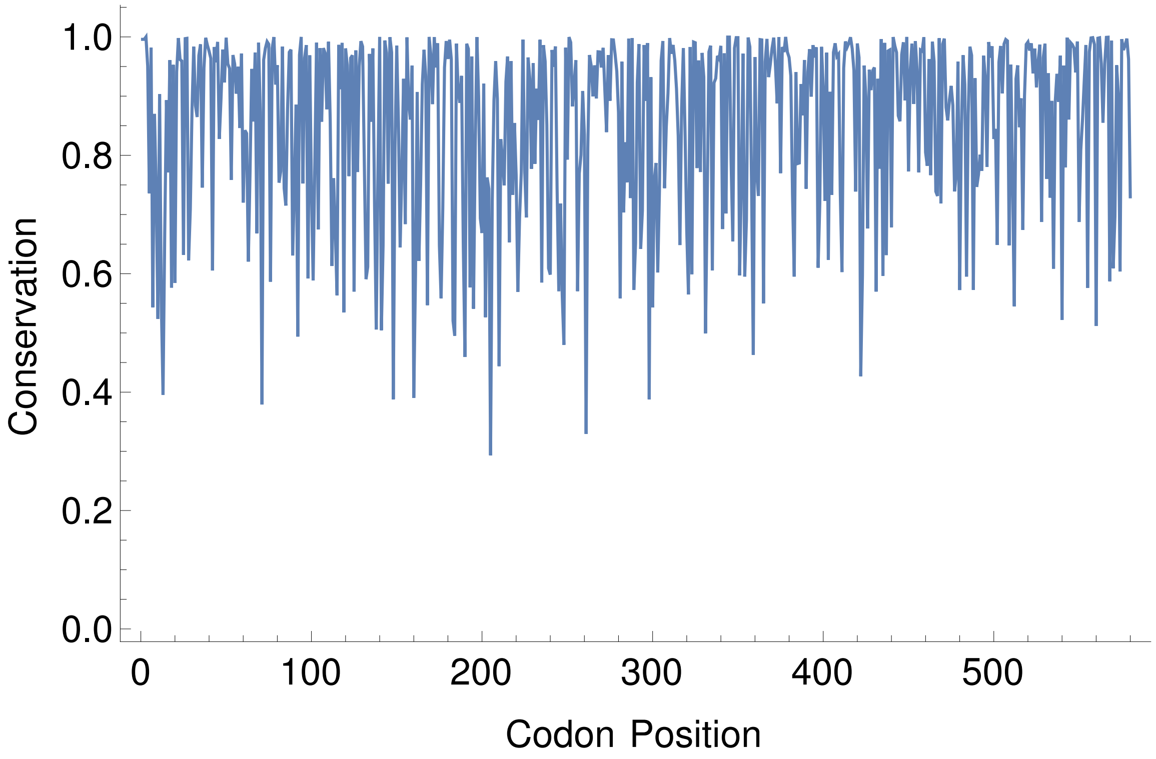

Conservation analysis plots

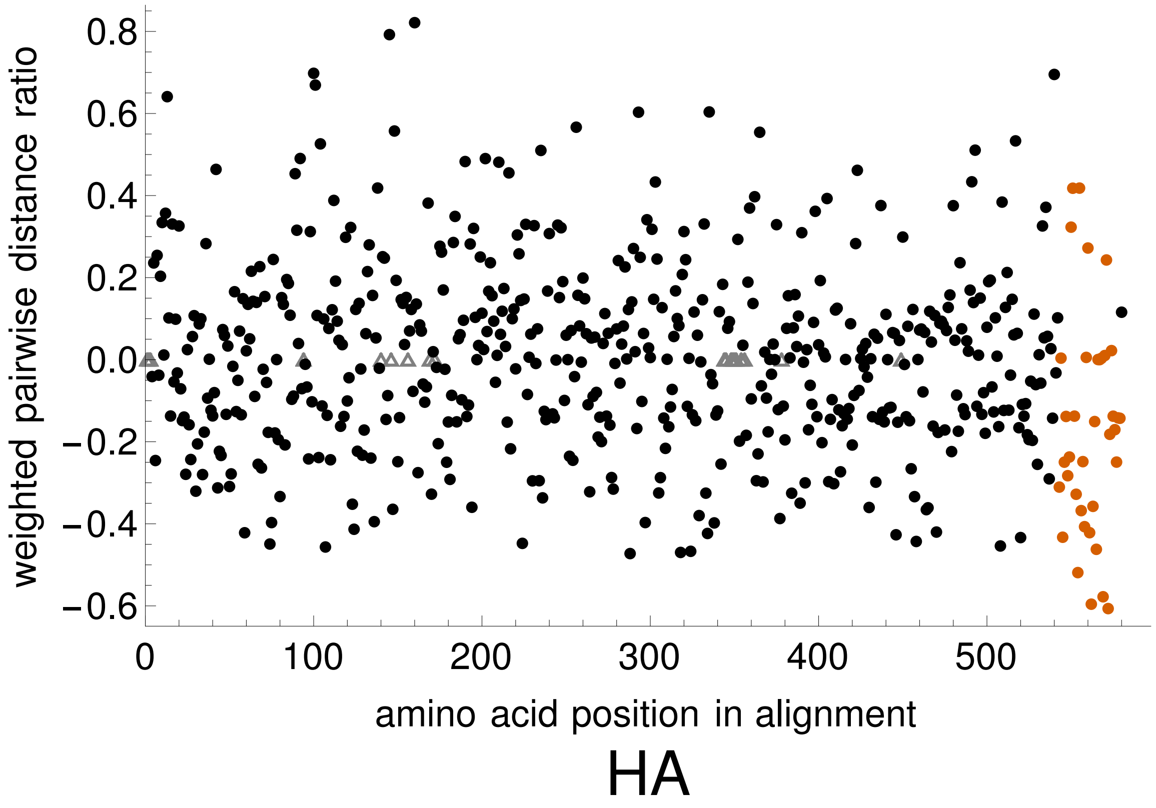

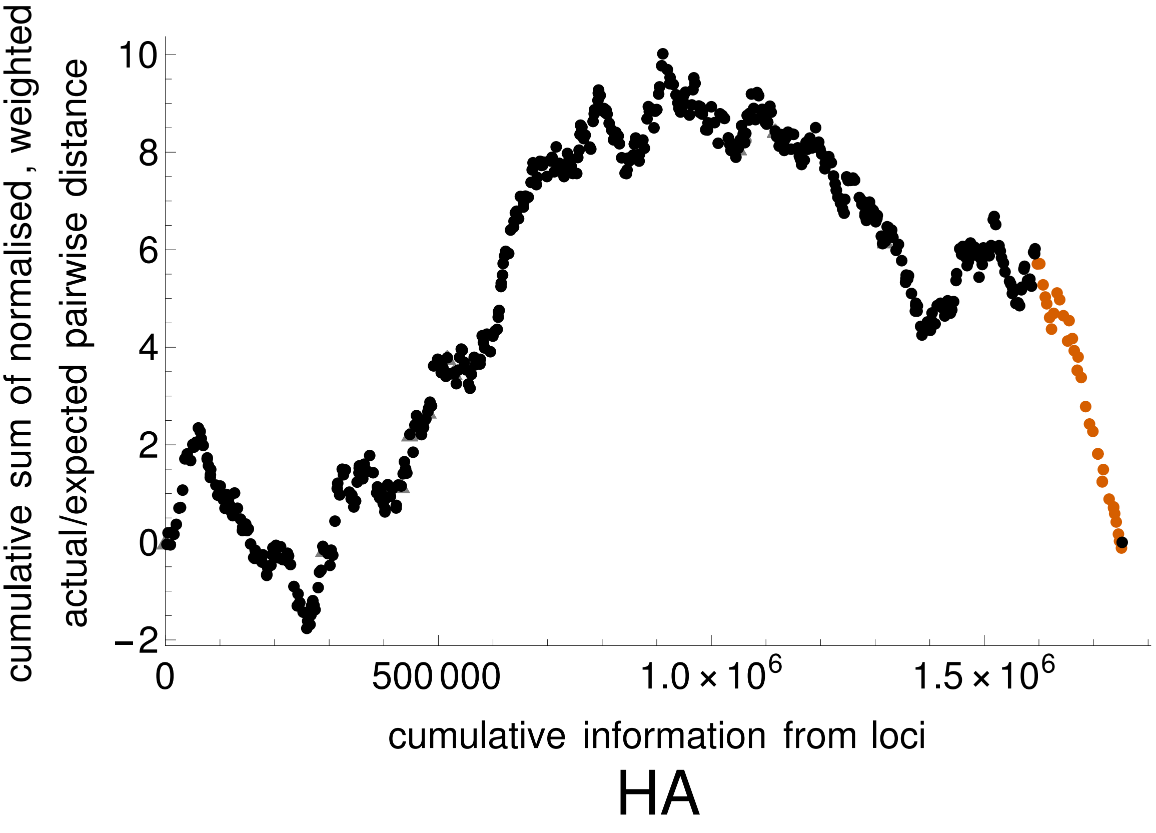

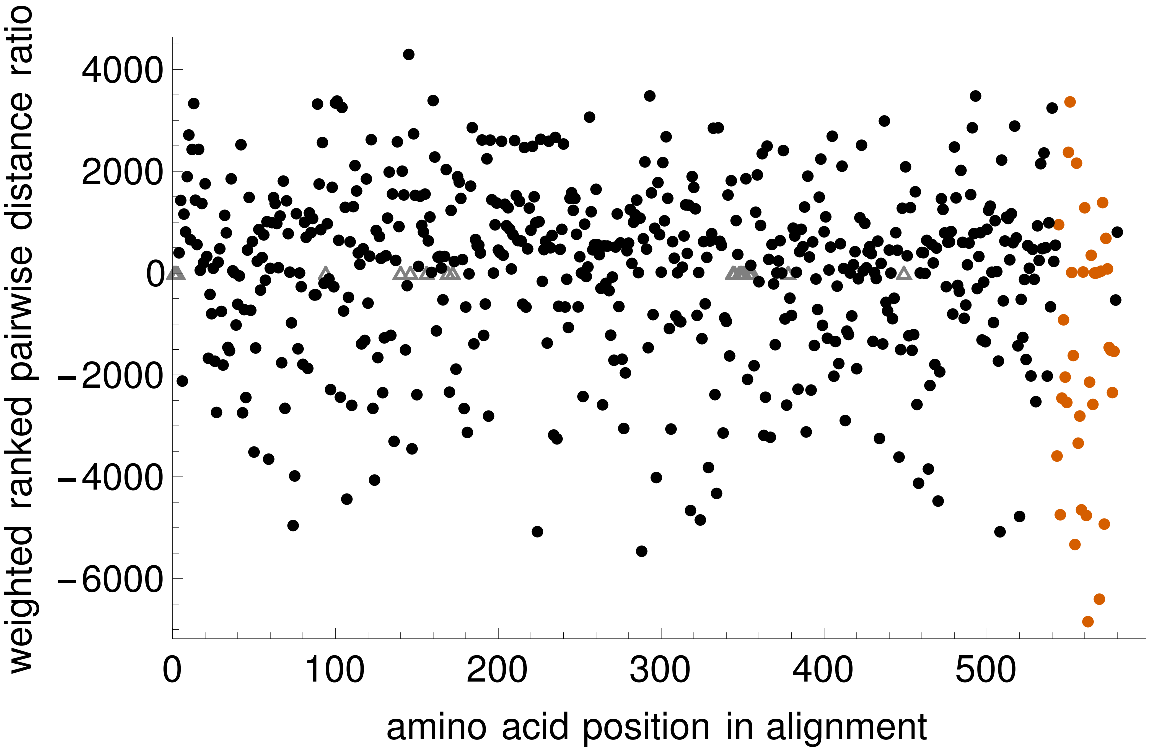
weighted ranked pairwise distance ratio
4000
2000
0
−2000
−4000
−6000
0
100
200
300
400
500
amino acid position in alignment

HA

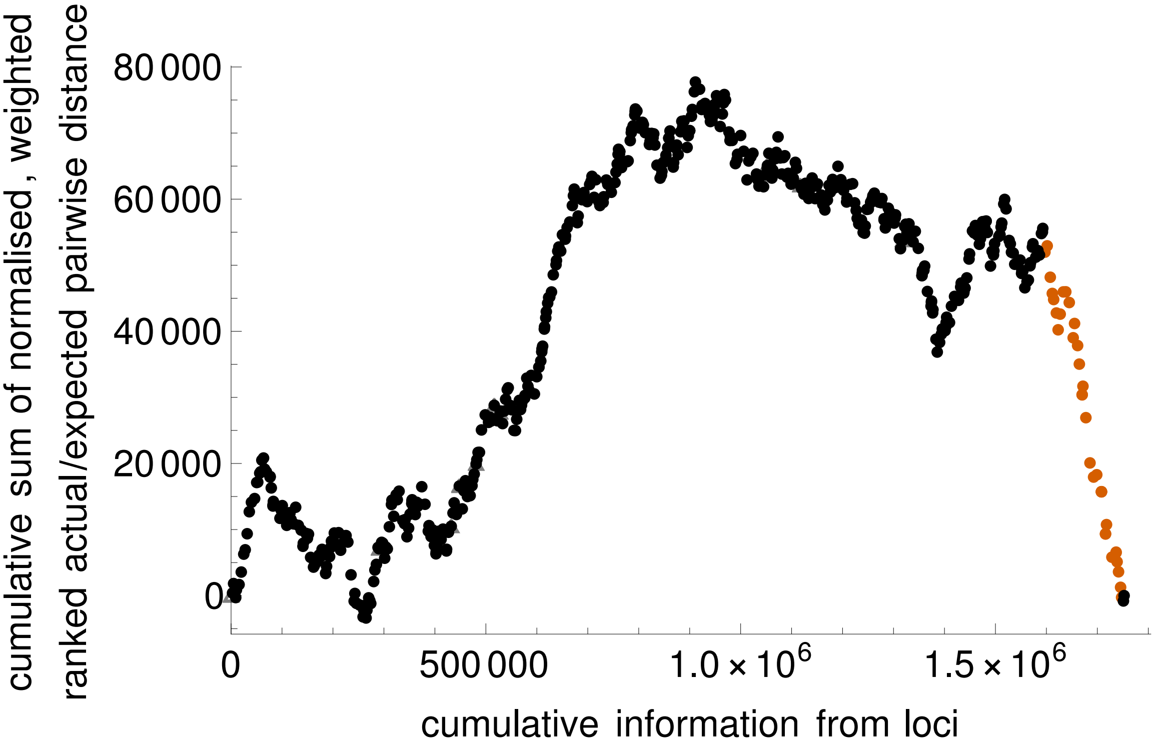
cumulative sum of normalised, weighted ranked actual/expected pairwise distance
80 000
60 000
40 000
20 000
0
0
500 000
1.0 × 10^6
1.5 × 10^6
cumulative information from loci

HA

NP

Gene length histogram

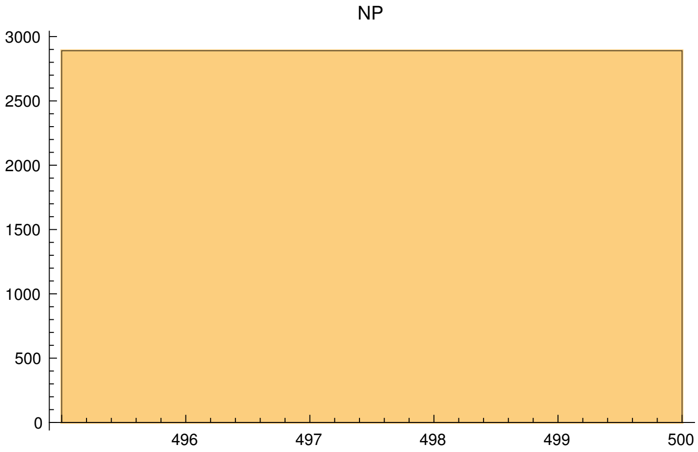

Information vs. nPD

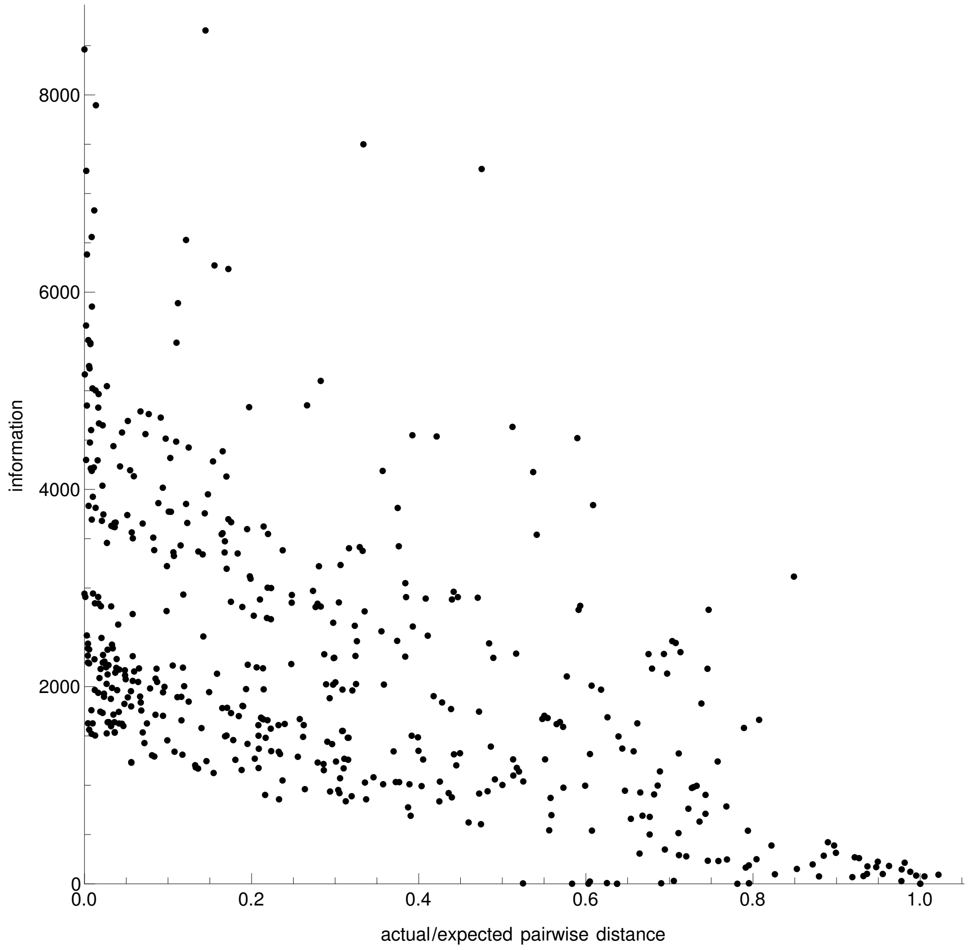

NP

Example sequences highlighted by regions found in analyses to be conserved

Interesting points (by weighted raw PD) highlighted for gene NP:

ATG

GCGTCTCAAGGCACCAAACGATCCTATGAACAGATGGAAACTGGTGGGGAACGCC AGAATGCCACAGAGATCAGAGCATCTGTTGGAAGAATGGTTGGTGGAATTGGGAGATTTTACATACAGA↵
TGTGCACTGAACTAAAACTCAGCGACTATGAAGGAAGGCTGATCCAGAACAGCATAACAATAGAGAGAAT↵
GGTTCTCTCTGCATTTGATGAAAGGAGAAACAAATACTTGGAAGAACATCCCAGTGCGGGGAAGGACCCG↵
AAGAAAACTGGAGGTCCAATTTATCGAAGGAGAGATGGGAAATGGATGAGAGAGCTGATTCTGTATGACA↵
AAGAGGAGATCAGGAGGATCTGGCGTCAAGCAAATAATGGGGAAGACGCAACTGCTGGTCTCACTCACCT↵
GATGATCTGGCATTCCAACCTAAATGATTCCACATACCAGAGAACAAGAGCTCTCGTGCGTACCGGGATG↵
GACCCCAGAATGTGCTCTCTGATGCAAGGATCAACTCTCCCGAGGAGATCTGGAGCTGCTGGTGCAGCAG↵
TAAAGGGAGTCGGGACGATGGTGATGGAACTAATTAGGATGATAAAGCGAGGGATTAATGATCGGAATTT↵
CTGGAGAGGAGAAAATGGACGAAGAACA

AGGATTGCATATGAGAGAATGTGCAACATCCTCAAAGGGAAATTCCAAACAGCAG CACAAAGGGCAATGATGGACCAGGTGCGAGAGAGCAGAAATCCTGGGAATGC TGAAATTGAAGATCTCATCTTCCTGGCACGGTCTGCACTCATCCTAAGAGGA TCAGTGGCTCATAAGTCCTGCTTGCCTGCTTGTGTGTACGGACTTGCTGTGG CCAGTGGATATGACTTTGAGAGAGAAGGGTATTCTCTAGTTGGAATAGATCC TTTCCGTCTGCTTCAAAACAGCCAGGTCTTCAGTCTCATTAGACCAAATGAG AACCCAGCACACAAGAGTCAACTGGTGTGGATGGCATGCCATTCTGCAGCAT TTGAGGACCTGAGAGTCTCAAGTTTCATCAGAGGGACAAGAATAGTCCCAAG AGGACAACTATCCACCAGAGGAGTTCAAATTGCTTCAAATGAGAACATGGAA ACAATGGACTCCAGCACTCTTGAACTGAGAAGCAGATATTGGGCTATAAGAA CCAGGAGTGGAGGAAACACCAACCAACAGAGAGCATCCGCAGGGCAAATCAGTGTA↵
CAGCCTACTTTCTCGGTACAGAGGAATCTTCCCTTCGAAAGAGCGACCATTATGGCAGCATTCACAGGGA↵
ATACTGAGGGCAGAACATCTGACATGAGA

ACTGAAATCATAAGGATGATGGAAAGTGCCAGACCAGAAGATGTGTCTTTCCAGG GGCGGGGAGTCTTCGAGCTCTCGGACGAAAAGGCAACGAACCCGATCGTGCC TTCCTTTGACATGAGTAATGAAGGATCTTATTTCTTCGGAGACAATGCAGAG GAGTATGAAAATTAA

Interesting points (by weighted ranked PD) highlighted for gene NP:

ATG

GCGTCTCAAGGCACCAAACGATCCTATGAACAGATGGAAACTGGTGGGGAACGCC AGAATGCCACAGAGATCAGAGCATCTGTTGGAAGAATGGTTGGTGGAATTGGGAGATTTTACATACAGA↵
TGTGCACTGAACTAAAACTCAGCGACTATGAAGGAAGGCTGATCCAGAACAGCATAACAATAGAGAGAAT↵
GGTTCTCTCTGCATTTGATGAAAGGAGAAACAAATACTTGGAAGAACATCCCAGTGCGGGGAAGGACCCG↵
AAGAAAACTGGAGGTCCAATTTATCGAAGGAGAGATGGGAAATGGATGAGAGAGCTGATTCTGTATGACA↵
AAGAGGAGATCAGGAGGATCTGGCGTCAAGCAAATAATGGGGAAGACGCAACTGCTGGTCTCACTCACCT↵
GATGATCTGGCATTCCAACCTAAATGATTCCACATACCAGAGAACAAGAGCTCTCGTGCGTACCGGGATG↵
GACCCCAGAATGTGCTCTCTGATGCAAGGATCAACTCTCCCGAGGAGATCTGGAGCTGCTGGTGCAGCAG↵
TAAAGGGAGTCGGGACGATGGTGATGGAACTAATTAGGATGATAAAGCGAGGGATTAATGATCGGAATTT↵
CTGGAGAGGAGAAAATGGACGAAGAACAAGGATTGCATATGAGAGAATGTGCAACATCCTCAAAGGGAAA↵
TTCCAAACAGCAGCACAAAGGGCAATGATGGACCAGGTGCGAGAGAGCAGAAATCCTGGGAATGCTGAAA↵
TTGAAGATCTCATCTTCCTGGCACGGTCTGCACTCATCCTAAGAGGATCAGTGGCTCATAAGTCCTGCTT↵

GCCTGCTTGTGTGTACGGACTTGCTGTGGCCAGTGGATATGACTTTGAGAGAGAAGGGTATTCTCTAGTT⋱
GGAATAGATCCTTTCCGTCTGCTTCAAAACAGCCAGGTCTTCAGTCTCATTAGACCAAATGAGAACCCAG⋱
CACACAAGAGTCAACTGGTGTGGATGGCATGCCATTCTGCAGCATTTGAGGACCTGAGAGTCTCAAGTTT⋱
CATCAGAGGGACAAGAATAGTCCCAAGAGGACAACTATCCACCAGAGGAGTTCAAATTGCTTCAAATGAG⋱
AACATGGAAACAATGGACTCCAGCACTCTTGAACTGAGAAGCAGATATTGGGCTATAAGAACCAGGAGTG⋱
GAGGAAACACCAACCAACAGAGAGCATCCGCAGGGCAAATCAGTGTACAGCCTACTTTCTCGGTACAGAG⋱
GAATCTTCCCTTCGAAAGAGCGACCATTATGGCAGCATTCACAGGGAATACTGAGGGCAGAACATCTGAC⋱
ATGAGAACTGAAATCATAAGGATGATGGAAAGTGCCAGACCA
GAAGATGTGTCTTTCCAGGGGCGGGGAGTCTTCGAGCTCTCGGACGAAAAGGCAA
CGAACCCGATCGTGCCTTCCTTTGACATGAGTAATGAAGGATCTTATTTCTT
CGGAGACAATGCAGAGGAGTATGAAAATTAA

Per codon conservation report

NP

Pos.	1	obs:	exp:
atg	M	2890	2890.00
mPD		0	0
		nPD:	1.
		N. weight:	0.
		Sc. PD:	0
		Sc. rank:	0

NP

Pos.	2	obs:	exp:
gtt	V	0	0.22
gtc	V	0	0.21
gta	V	0	0.21
gtg	V	1	0.36
gct	A	0	727.50
gcc	A	0	558.90
gca	A	1	1286.00
gcg	A	2887	316.10
ggt	G	0	0.14
ggc	G	0	0.13
gga	G	0	0.44
ggg	G	1	0.29
mPD		0.0021	0.69
		nPD:	0.
		N. weight:	2.9
		Sc. PD:	-0.55
		Sc. rank:	-5032.0

NP

Pos.	3	obs:	exp:
ttt	F	3	1.25
ttc	F	0	1.75
tct	S	2763	481.10
tcc	S	119	417.50
tca	S	0	653.40
tcg	S	0	173.90
tgt	C	1	0.39
tgc	C	0	0.61
cct	P	0	0.55
ccc	P	2	0.36
cca	P	0	0.81
ccg	P	0	0.28
agt	S	0	625.10
agc	S	0	531.10
gct	A	2	0.50
gcc	A	0	0.39
gca	A	0	0.89
gcg	A	0	0.22
mPD		0.086	1.7
		nPD:	0.05
		N. weight:	2.1
		Sc. PD:	-0.3
		Sc. rank:	-1348.8

NP

Pos.	4	obs:	exp:
cat	H	3	1.74
cac	H	0	1.26
caa	Q	2881	1441.00
cag	Q	6	1446.00
mPD		0.0062	0.50
		nPD:	0.01
		N. weight:	0.9
		Sc. PD:	-0.16
		Sc. rank:	-1196.7

NP

Pos.	5	obs:	exp:
tct	S	0	0.83
tcc	S	0	0.72
tca	S	0	1.13
tcg	S	0	0.30
tgt	C	0	0.39
tgc	C	1	0.61
agt	S	0	1.08
agc	S	5	0.92
gct	A	1	0.25
gcc	A	0	0.19
gca	A	0	0.45
gcg	A	0	0.11
ggt	G	0	416.90
ggc	G	2882	378.70
gga	G	0	1263.00
ggg	G	1	824.10
mPD		0.0062	0.70
		nPD:	0.01
		N. weight:	2.7
		Sc. PD:	-0.49
		Sc. rank:	-3830.2

NP

Pos.	6	obs:	exp:
cct	P	0	0.27
ccc	P	1	0.18
cca	P	0	0.41
ccg	P	0	0.14
act	T	1	757.40
acc	T	2888	651.00
aca	T	0	1209.00
acg	T	0	271.80
mPD		0.0014	0.70
		nPD:	0.
		N. weight:	2.
		Sc. PD:	-0.37
		Sc. rank:	-3532.2

NP

Pos.	7	obs:	exp:
cgt	R	0	0.07
cgc	R	0	0.11
cga	R	0	0.20
cgg	R	0	0.16
aat	N	1	0.55
aac	N	0	0.45
aaa	K	2887	1644.00
aag	K	0	1243.00
aga	R	2	0.93
agg	R	0	0.52
mPD		0.0021	0.49
		nPD:	0.
		N. weight:	0.74
		Sc. PD:	-0.14
		Sc. rank:	-1261.5

NP

Pos.	8	obs:	exp:
cgt	R	0	108.20
cgc	R	0	154.70
cga	R	2878	291.20
cgg	R	12	236.00
aga	R	0	1349.00
agg	R	0	750.80
mPD		0.0083	0.95
		nPD:	0.01
		N. weight:	3.
		Sc. PD:	-0.55
		Sc. rank:	-4434.3

NP

Pos.	9	obs:	exp:
ttt	F	1	0.42
ttc	F	0	0.58
tct	S	2253	479.60
tcc	S	591	416.20
tca	S	16	651.30
tcg	S	13	173.30
tat	Y	1	0.48
tac	Y	0	0.52
cct	P	10	4.10
ccc	P	5	2.71
cca	P	0	6.09
ccg	P	0	2.10
agt	S	0	623.20
agc	S	0	529.50
mPD		0.36	1.7
		nPD:	0.21
		N. weight:	1.7
		Sc. PD:	0.027
		Sc. rank:	950.2

NP

Pos.	10	obs:	exp:
tat	Y	2821	1358.00
tac	Y	10	1473.00
cat	H	59	34.15
cac	H	0	24.85
mPD		0.047	0.54
		nPD:	0.09
		N. weight:	0.93
		Sc. PD:	-0.1
		Sc. rank:	-321.4

NP

Pos.	11	obs:	exp:
gaa	E	2864	1620.00
gag	E	26	1270.00
mPD		0.018	0.49
		nPD:	0.04
		N. weight:	0.7
		Sc. PD:	-0.11
		Sc. rank:	-572.9

NP

Pos.	12	obs:	exp:
tta	L	0	0.09
ttg	L	0	0.19
ctt	L	0	0.20
ctc	L	0	0.16
cta	L	0	0.15
ctg	L	1	0.21
caa	Q	359	1442.00
cag	Q	2530	1447.00
mPD		0.22	0.50
		nPD:	0.44
		N. weight:	0.42
		Sc. PD:	0.097
		Sc. rank:	564.4

NP

Pos.	13	obs:	exp:
atg	M	2890	2890.00
mPD		0	0
		nPD:	1.
		N. weight:	0.
		Sc. PD:	0
		Sc. rank:	0

NP

Pos.	14	obs:	exp:
gaa	E	2118	1620.00
gag	E	772	1270.00
mPD		0.39	0.49
		nPD:	0.8
		N. weight:	0.085
		Sc. PD:	0.05
		Sc. rank:	158.9

NP

Pos.	15	obs:	exp:
act	T	2885	757.60
acc	T	4	651.20
aca	T	1	1209.00
acg	T	0	271.90
mPD		0.0035	0.70
		nPD:	0.
		N. weight:	1.7
		Sc. PD:	-0.33
		Sc. rank:	-2898.7

NP

Pos.	16	obs:	exp:
tct	S	0	21.20
tcc	S	0	18.40
tca	S	0	28.79
tcg	S	0	7.66
aat	N	3	1.64
aac	N	0	1.36
agt	S	126	27.55
agc	S	1	23.40
ggt	G	2755	399.10
ggc	G	1	362.50
gga	G	4	1209.00
ggg	G	0	788.90
mPD		0.092	0.84
		nPD:	0.11
		N. weight:	2.5
		Sc. PD:	-0.21
		Sc. rank:	-321.0

NP

Pos.	17	obs:	exp:
ggt	G	54	417.90
ggc	G	1	379.60
gga	G	2271	1266.00
ggg	G	564	826.10
mPD		0.34	0.69
		nPD:	0.5
		N. weight:	0.46
		Sc. PD:	0.13
		Sc. rank:	675.8

NP

Pos.	18	obs:	exp:
gat	D	3	2.16
gac	D	1	1.84
gaa	E	1004	1618.00
gag	E	1882	1268.00
mPD		0.46	0.49
		nPD:	0.92
		N. weight:	0.12
		Sc. PD:	0.086
		Sc. rank:	231.6

NP

Pos.	19	obs:	exp:
cgt	R	0	108.20
cgc	R	2890	154.70
cga	R	0	291.20
cgg	R	0	236.00
aga	R	0	1349.00
agg	R	0	750.80
mPD		0	0.95
		nPD:	0.
		N. weight:	3.9
		Sc. PD:	-0.74
		Sc. rank:	-7270.0

NP

Pos.	20	obs:	exp:
cct	P	0	0.55
ccc	P	0	0.36
cca	P	0	0.81
ccg	P	2	0.28
caa	Q	10	1442.00
cag	Q	2878	1446.00
mPD		0.0083	0.50
		nPD:	0.02
		N. weight:	0.88
		Sc. PD:	-0.16
		Sc. rank:	-1086.6

NP			
Pos.	21	obs:	exp:
tct	S	0	1.34
tcc	S	0	1.16
tca	S	0	1.81
tcg	S	0	0.48
aat	N	2857	1572.00
aac	N	25	1310.00
agt	S	8	1.74
agc	S	0	1.47
mPD		0.023	0.51
		nPD:	0.04
		N. weight:	0.74
		Sc. PD:	-0.11
		Sc. rank:	-529.9

NP			
Pos.	22	obs:	exp:
tct	S	5	0.83
tcc	S	0	0.72
tca	S	0	1.13
tcg	S	0	0.30
act	T	47	12.58
acc	T	1	10.82
aca	T	0	20.08
acg	T	0	4.52
agt	S	0	1.08
agc	S	0	0.92
gct	A	1673	714.70
gcc	A	1161	549.00
gca	A	3	1263.00
gcg	A	0	310.50
mPD		0.52	0.73
		nPD:	0.71
		N. weight:	1.1
		Sc. PD:	0.54
		Sc. rank:	1923.9

NP			
Pos.	23	obs:	exp:
tct	S	1	0.17
tcc	S	0	0.14
tca	S	0	0.23
tcg	S	0	0.06
act	T	2805	756.80
acc	T	16	650.50
aca	T	4	1208.00
acg	T	62	271.60
aat	N	2	1.09
aac	N	0	0.91
agt	S	0	0.22
agc	S	0	0.18
mPD		0.058	0.70
		nPD:	0.08
		N. weight:	1.6
		Sc. PD:	-0.18
		Sc. rank:	-621.7

NP			
Pos.	24	obs:	exp:
gaa	E	441	1620.00
gag	E	2449	1270.00
mPD		0.26	0.49
		nPD:	0.53
		N. weight:	0.47
		Sc. PD:	0.15
		Sc. rank:	720.7

NP			
Pos.	25	obs:	exp:
att	I	28	1060.00
atc	I	2859	773.10
ata	I	2	1056.00
gtt	V	0	0.22
gtc	V	1	0.21
gta	V	0	0.21
gtg	V	0	0.36
mPD		0.021	0.66
		nPD:	0.03
		N. weight:	1.7
		Sc. PD:	-0.27
		Sc. rank:	-1499.8

NP			
Pos.	26	obs:	exp:
cgt	R	0	108.10
cgc	R	0	154.60
cga	R	1	291.00
cgg	R	1	235.80
aga	R	1253	1348.00
agg	R	1633	750.30
ggt	G	0	0.29
ggc	G	0	0.26
gga	G	0	0.88
ggg	G	2	0.57
mPD		0.49	0.96
		nPD:	0.52
		N. weight:	0.54
		Sc. PD:	0.17
		Sc. rank:	812.0

NP			
Pos.	27	obs:	exp:
tct	S	0	0.17
tcc	S	0	0.14
tca	S	1	0.23
tcg	S	0	0.06
act	T	0	0.52
acc	T	0	0.45
aca	T	2	0.84
acg	T	0	0.19
agt	S	0	0.22
agc	S	0	0.18
gct	A	1	727.30
gcc	A	18	558.70
gca	A	2863	1285.00
gcg	A	5	316.00
mPD		0.019	0.69
		nPD:	0.03
		N. weight:	1.
		Sc. PD:	-0.17
		Sc. rank:	-995.9

NP			
Pos.	28	obs:	exp:
tct	S	2499	482.20
tcc	S	159	418.50
tca	S	17	654.90
tcg	S	214	174.30
cct	P	1	0.27
ccc	P	0	0.18
cca	P	0	0.41
ccg	P	0	0.14
agt	S	0	626.60
agc	S	0	532.40
mPD		0.24	1.7
		nPD:	0.15
		N. weight:	1.8
		Sc. PD:	-0.088
		Sc. rank:	240.5

NP			
Pos.	29	obs:	exp:
gtt	V	2826	620.90
gtc	V	56	607.40
gta	V	4	615.60
gtg	V	1	1043.00
ggt	G	3	0.43
ggc	G	0	0.39
gga	G	0	1.31
ggg	G	0	0.86
mPD		0.043	0.74
		nPD:	0.06
		N. weight:	1.9
		Sc. PD:	-0.25
		Sc. rank:	-1000.1

NP			
Pos.	30	obs:	exp:
ggt	G	1	417.90
ggc	G	2	379.60
gga	G	2659	1266.00
ggg	G	228	826.10
mPD		0.15	0.69
		nPD:	0.21
		N. weight:	0.76
		Sc. PD:	0.012
		Sc. rank:	427.9

NP			
Pos.	31	obs:	exp:
cgt	R	0	108.20
cgc	R	0	154.70
cga	R	179	291.20
cgg	R	1	236.00
aga	R	2431	1349.00
agg	R	279	750.80
mPD		0.29	0.95
		nPD:	0.31
		N. weight:	0.49
		Sc. PD:	0.051
		Sc. rank:	461.3

NP			
Pos.	32	obs:	exp:
atg	M	2890	2890.00
mPD		0	0
		nPD:	1.
		N. weight:	0.
		Sc. PD:	0
		Sc. rank:	0

NP			
Pos.	33	obs:	exp:
att	I	251	93.58
atc	I	4	68.24
ata	I	0	93.19
gtt	V	2428	566.70
gtc	V	15	554.40
gta	V	0	561.80
gtg	V	192	952.00
mPD		0.30	0.90
		nPD:	0.33
		N. weight:	1.6
		Sc. PD:	0.2
		Sc. rank:	1659.6

NP			
Pos.	34	obs:	exp:
tct	S	0	293.10
tcc	S	0	254.40
tca	S	0	398.10
tcg	S	0	105.90
cgt	R	0	0.07
cgc	R	0	0.11
cga	R	0	0.20
cgg	R	0	0.16
aat	N	55	29.99
aac	N	0	25.01
agt	S	1748	380.90
agc	S	8	323.60
aga	R	1	0.93
agg	R	1	0.52
gat	D	1	0.54
gac	D	0	0.46
ggt	G	824	155.60
ggc	G	251	141.30
gga	G	1	471.50
ggg	G	0	307.60
mPD		0.67	1.9
		nPD:	0.36
		N. weight:	1.9
		Sc. PD:	0.3
		Sc. rank:	2189.7

NP			
Pos.	35	obs:	exp:
ggt	G	15	417.90
ggc	G	1745	379.60
gga	G	1125	1266.00
ggg	G	5	826.10
mPD		0.48	0.69
		nPD:	0.7
		N. weight:	1.1
		Sc. PD:	0.55
		Sc. rank:	1988.5

NP			
Pos.	36	obs:	exp:
att	I	2545	1059.00
atc	I	341	772.30
ata	I	0	1055.00
atg	M	1	1.00
gtt	V	0	0.65
gtc	V	3	0.63
gta	V	0	0.64
gtg	V	0	1.08
mPD		0.21	0.66
		nPD:	0.32
		N. weight:	0.89
		Sc. PD:	0.11
		Sc. rank:	913.3

NP			
Pos.	37	obs:	exp:
ggt	G	0	417.90
ggc	G	1	379.60
gga	G	241	1266.00
ggg	G	2648	826.10
mPD		0.15	0.69
		nPD:	0.22
		N. weight:	1.2
		Sc. PD:	0.03
		Sc. rank:	786.4

NP			
Pos.	38	obs:	exp:
cgt	R	0	108.10
cgc	R	0	154.60
cga	R	0	291.00
cgg	R	4	235.80
aaa	K	0	1.14
aag	K	2	0.86
aga	R	306	1348.00
agg	R	2578	750.30
mPD		0.19	0.96
		nPD:	0.2
		N. weight:	1.2
		Sc. PD:	0.0057
		Sc. rank:	614.8

NP			
Pos.	39	obs:	exp:
ttt	F	306	1205.00
ttc	F	2583	1684.00
tat	Y	0	0.48
tac	Y	1	0.52
mPD		0.19	0.49
		nPD:	0.39
		N. weight:	0.31
		Sc. PD:	0.059
		Sc. rank:	390.3

NP			
Pos.	40	obs:	exp:
tat	Y	54	1386.00
tac	Y	2835	1503.00
cat	H	0	0.58
cac	H	1	0.42
mPD		0.037	0.50
		nPD:	0.07
		N. weight:	0.74
		Sc. PD:	-0.089
		Sc. rank:	-317.8

NP			
Pos.	41	obs:	exp:
att	I	0	1015.00
atc	I	1	740.20
ata	I	2765	1011.00
gtt	V	0	26.67
gtc	V	0	26.09
gta	V	115	26.44
gtg	V	9	44.80
---	--	------	------
mPD		0.089	0.75
		nPD:	0.12
		N. weight:	1.3
		Sc. PD:	-0.1
		Sc. rank:	-73.2

,

NP			
Pos.	42	obs:	exp:
cct	P	0	0.55
ccc	P	0	0.36
cca	P	0	0.81
ccg	P	2	0.28
caa	Q	60	1441.00
cag	Q	2827	1446.00
cgt	R	0	0.04
cgc	R	0	0.05
cga	R	0	0.10
cgg	R	1	0.08
aga	R	0	0.47
agg	R	0	0.26
---	--	------	------
mPD		0.043	0.50
		nPD:	0.09
		N. weight:	0.78
		Sc. PD:	-0.086
		Sc. rank:	-286.3

,

NP			
Pos.	43	obs:	exp:
atg	M	2890	2890.00
---	--	------	------
mPD		0	0
		nPD:	1.
		N. weight:	0.
		Sc. PD:	0
		Sc. rank:	0

,

NP			
Pos.	44	obs:	exp:
tgt	C	152	1116.00
tgc	C	2732	1768.00
ggt	G	0	0.87
ggc	G	6	0.79
gga	G	0	2.63
ggg	G	0	1.72
---	--	------	------
mPD		0.10	0.48
		nPD:	0.22
		N. weight:	0.41
		Sc. PD:	0.0073
		Sc. rank:	240.6

,

NP			
Pos.	45	obs:	exp:
act	T	1061	757.60
acc	T	5	651.20
aca	T	1799	1209.00
acg	T	25	271.90
---	--	------	------
mPD		0.48	0.70
		nPD:	0.69
		N. weight:	0.45
		Sc. PD:	0.22
		Sc. rank:	791.6

,

NP			
Pos.	46	obs:	exp:
aaa	K	1	0.57
aag	K	0	0.43
gaa	E	2171	1620.00
gag	E	718	1269.00
---	--	------	------
mPD		0.37	0.49
		nPD:	0.76
		N. weight:	0.11
		Sc. PD:	0.058
		Sc. rank:	195.1

,

NP			
Pos.	47	obs:	exp:
ttt	F	0	0.42
ttc	F	1	0.58
tta	L	0	266.10
ttg	L	0	546.70
ctt	L	115	571.40
ctc	L	2760	460.40
cta	L	3	425.90
ctg	L	0	607.40
att	I	0	4.04
atc	I	11	2.94
ata	I	0	4.02
---	--	------	------
mPD		0.087	1.1
		nPD:	0.08
		N. weight:	2.2
		Sc. PD:	-0.26
		Sc. rank:	-894.7

,

NP			
Pos.	48	obs:	exp:
caa	Q	113	56.92
cag	Q	1	57.08
aaa	K	2646	1581.00
aag	K	130	1195.00
---	--	------	------
mPD		0.16	0.57
		nPD:	0.29
		N. weight:	0.53
		Sc. PD:	0.045
		Sc. rank:	444.9

,

NP			
Pos.	49	obs:	exp:
ttt	F	0	0.83
ttc	F	2	1.17
tta	L	0	266.90
ttg	L	0	548.50
ctt	L	5	573.20
ctc	L	2881	461.80
cta	L	1	427.30
ctg	L	0	609.30
cct	P	0	0.27
ccc	P	1	0.18
cca	P	0	0.41
ccg	P	0	0.14
---	--	------	------
mPD		0.0062	1.1
		nPD:	0.01
		N. weight:	2.4
		Sc. PD:	-0.45
		Sc. rank:	-3927.1

,

NP			
Pos.	50	obs:	exp:
tct	S	0	460.70
tcc	S	0	399.80
tca	S	0	625.70
tcg	S	0	166.50
cgt	R	0	4.15
cgc	R	1	5.94
cga	R	0	11.19
cgg	R	0	9.06
aat	N	2	1.09
aac	N	0	0.91
agt	S	2125	598.70
agc	S	635	508.60
aga	R	110	51.82
agg	R	0	28.84
gat	D	0	0.54
gac	D	1	0.46
ggt	G	4	2.31
ggc	G	2	2.10
gga	G	10	7.01
ggg	G	0	4.57
---	--	------	------
mPD		0.42	1.7
		nPD:	0.25
		N. weight:	1.3
		Sc. PD:	0.065
		Sc. rank:	967.5

,

NP			
Pos.	51	obs:	exp:
aat	N	0	39.81
aac	N	73	33.19
gtt	V	0	0.22
gtc	V	1	0.21
gta	V	0	0.21
gtg	V	0	0.36
gat	D	191	1471.00
gac	D	2533	1253.00
gaa	E	79	49.89
gag	E	10	39.11
ggt	G	0	0.43
ggc	G	3	0.39
gga	G	0	1.31
ggg	G	0	0.86
---	--	------	------
mPD		0.23	0.58
		nPD:	0.4
		N. weight:	0.68
		Sc. PD:	0.13
		Sc. rank:	864.0

,

NP			
Pos.	52	obs:	exp:
ttt	F	1	0.42
ttc	F	0	0.58
tat	Y	1642	872.90
tac	Y	178	947.10
cat	H	1066	618.20
cac	H	2	449.80
aat	N	0	0.55
aac	N	1	0.45
---	--	------	------
mPD		0.58	0.97
		nPD:	0.6
		N. weight:	0.6
		Sc. PD:	0.24
		Sc. rank:	989.6

,

NP			
Pos.	53	obs:	exp:
gaa	E	2864	1620.00
gag	E	25	1269.00
ggt	G	0	0.14
ggc	G	0	0.13
gga	G	1	0.44
ggg	G	0	0.29
---	--	------	------
mPD		0.018	0.49
		nPD:	0.04
		N. weight:	0.7
		Sc. PD:	-0.11
		Sc. rank:	-578.4

,

NP			
Pos.	54	obs:	exp:
ggt	G	2	417.90
ggc	G	0	379.60
gga	G	725	1266.00
ggg	G	2163	826.10
---	--	------	------
mPD		0.38	0.69
		nPD:	0.55
		N. weight:	0.76
		Sc. PD:	0.26
		Sc. rank:	1185.5

,

NP			
Pos.	55	obs:	exp:
cgt	R	0	108.20
cgc	R	0	154.70
cga	R	1	291.20
cgg	R	0	236.00
aga	R	1305	1349.00
agg	R	1584	750.80
---	--	------	------
mPD		0.50	0.95
		nPD:	0.52
		N. weight:	0.52
		Sc. PD:	0.16
		Sc. rank:	788.4

,

NP			
Pos.	56	obs:	exp:
tta	L	4	267.10
ttg	L	35	548.80
ctt	L	240	573.60
ctc	L	29	462.10
cta	L	83	427.60
ctg	L	2498	609.80
cct	P	0	0.27
ccc	P	0	0.18
cca	P	0	0.41
ccg	P	1	0.14
---	--	------	------
mPD		0.25	1.1
		nPD:	0.22
		N. weight:	1.4
		Sc. PD:	0.034
		Sc. rank:	893.4

,

NP			
Pos.	57	obs:	exp:
tta	L	0	0.37
ttg	L	0	0.76
ctt	L	0	0.79
ctc	L	4	0.64
cta	L	0	0.59
ctg	L	0	0.84
att	I	326	1057.00
atc	I	2542	770.70
ata	I	12	1052.00
act	T	0	0.26
acc	T	1	0.23
aca	T	0	0.42
acg	T	0	0.09
gtt	V	3	1.08
gtc	V	2	1.05
gta	V	0	1.07
gtg	V	0	1.81
---	--	------	------
mPD		0.22	0.67
		nPD:	0.32
		N. weight:	1.2
		Sc. PD:	0.15
		Sc. rank:	1229.5

,

NP			
Pos.	58	obs:	exp:
caa	Q	304	1443.00
cag	Q	2586	1447.00
---	--	------	------
mPD		0.19	0.50
		nPD:	0.38
		N. weight:	0.47
		Sc. PD:	0.082
		Sc. rank:	565.8

,

NP			
Pos.	59	obs:	exp:
aat	N	41	1575.00
aac	N	2848	1314.00
aaa	K	1	0.57
aag	K	0	0.43
---	--	------	------
mPD		0.029	0.50
		nPD:	0.06
		N. weight:	0.94
		Sc. PD:	-0.13
		Sc. rank:	-522.8

,

NP			
Pos.	60	obs:	exp:
tct	S	0	482.10
tcc	S	0	418.30
tca	S	0	654.70
tcg	S	0	174.20
aat	N	0	1.09
aac	N	2	0.91
agt	S	5	626.40
agc	S	2883	532.20
---	--	------	------
mPD		0.0048	1.7
		nPD:	0.
		N. weight:	2.2
		Sc. PD:	-0.42
		Sc. rank:	-3873.1

,

NP			
Pos.	61	obs:	exp:
att	I	1	1055.00
atc	I	14	769.10
ata	I	2859	1050.00
atg	M	9	9.00
act	T	0	0.26
acc	T	0	0.23
aca	T	1	0.42
acg	T	0	0.09
gtt	V	0	1.29
gtc	V	0	1.26
gta	V	6	1.28
gtg	V	0	2.17
---	--	------	------
mPD		0.021	0.67
		nPD:	0.03
		N. weight:	1.3
		Sc. PD:	-0.21
		Sc. rank:	-1178.5

NP			
Pos.	62	obs:	exp:
act	T	24	757.60
acc	T	164	651.20
aca	T	2687	1209.00
acg	T	15	271.90
---	--	------	------
mPD		0.13	0.70
		nPD:	0.19
		N. weight:	0.82
		Sc. PD:	-0.0064
		Sc. rank:	341.8

NP			
Pos.	63	obs:	exp:
tta	L	0	0.37
ttg	L	0	0.76
ctt	L	0	0.79
ctc	L	0	0.64
cta	L	4	0.59
ctg	L	0	0.84
att	I	1	1055.00
atc	I	0	769.30
ata	I	2874	1051.00
atg	M	1	1.00
gtt	V	0	2.15
gtc	V	0	2.10
gta	V	10	2.13
gtg	V	0	3.61
---	--	------	------
mPD		0.011	0.67
		nPD:	0.02
		N. weight:	1.3
		Sc. PD:	-0.23
		Sc. rank:	-1641.0

NP			
Pos.	64	obs:	exp:
aaa	K	1	0.57
aag	K	0	0.43
gaa	E	198	1619.00
gag	E	2690	1269.00
ggt	G	0	0.14
ggc	G	0	0.13
gga	G	0	0.44
ggg	G	1	0.29
---	--	------	------
mPD		0.13	0.49
		nPD:	0.26
		N. weight:	0.73
		Sc. PD:	0.046
		Sc. rank:	547.3

NP			
Pos.	65	obs:	exp:
cgt	R	0	108.00
cgc	R	0	154.40
cga	R	0	290.80
cgg	R	1	235.70
aaa	K	4	2.28
aag	K	0	1.72
aga	R	2541	1347.00
agg	R	344	749.80
---	--	------	------
mPD		0.21	0.96
		nPD:	0.22
		N. weight:	0.61
		Sc. PD:	0.015
		Sc. rank:	403.7

NP			
Pos.	66	obs:	exp:
atg	M	2890	2890.00
---	--	------	------
mPD		0	0
		nPD:	1.
		N. weight:	0.
		Sc. PD:	0
		Sc. rank:	0

NP			
Pos.	67	obs:	exp:
tta	L	0	0.09
ttg	L	0	0.19
ctt	L	1	0.20
ctc	L	0	0.16
cta	L	0	0.15
ctg	L	0	0.21
att	I	5	2.20
atc	I	0	1.61
ata	I	1	2.19
gtt	V	1044	618.60
gtc	V	18	605.10
gta	V	1756	613.20
gtg	V	58	1039.00
gct	A	1	1.76
gcc	A	0	1.35
gca	A	6	3.12
gcg	A	0	0.77
---	--	------	------
mPD		0.51	0.74
		nPD:	0.68
		N. weight:	0.99
		Sc. PD:	0.47
		Sc. rank:	1727.8

NP			
Pos.	68	obs:	exp:
ttt	F	1	0.42
ttc	F	0	0.58
tta	L	0	267.10
ttg	L	0	548.80
ctt	L	1215	573.60
ctc	L	1641	462.10
cta	L	31	427.60
ctg	L	2	609.80
---	--	------	------
mPD		0.50	1.1
		nPD:	0.45
		N. weight:	1.3
		Sc. PD:	0.32
		Sc. rank:	1847.1

NP			
Pos.	69	obs:	exp:
tct	S	2706	482.10
tcc	S	4	418.30
tca	S	24	654.70
tcg	S	154	174.20
tat	Y	1	0.48
tac	Y	0	0.52
cct	P	1	0.27
ccc	P	0	0.18
cca	P	0	0.41
ccg	P	0	0.14
agt	S	0	626.40
agc	S	0	532.20
---	--	------	------
mPD		0.12	1.7
		nPD:	0.07
		N. weight:	2.1
		Sc. PD:	-0.25
		Sc. rank:	-902.7

NP			
Pos.	70	obs:	exp:
gct	A	14	728.00
gcc	A	11	559.20
gca	A	2853	1286.00
gcg	A	12	316.30
---	--	------	------
mPD		0.025	0.69
		nPD:	0.04
		N. weight:	0.98
		Sc. PD:	-0.15
		Sc. rank:	-780.6

NP			
Pos.	71	obs:	exp:
ttt	F	2866	1205.00
ttc	F	23	1684.00
tta	L	0	0.09
ttg	L	0	0.19
ctt	L	1	0.20
ctc	L	0	0.16
cta	L	0	0.15
ctg	L	0	0.21
---	--	------	------
mPD		0.016	0.49
		nPD:	0.03
		N. weight:	1.1
		Sc. PD:	-0.17
		Sc. rank:	-960.9

NP			
Pos.	72	obs:	exp:
gat	D	2864	1560.00
gac	D	26	1330.00
---	--	------	------
mPD		0.018	0.50
		nPD:	0.04
		N. weight:	0.75
		Sc. PD:	-0.12
		Sc. rank:	-630.1

NP			
Pos.	73	obs:	exp:
gaa	E	2663	1620.00
gag	E	227	1270.00
---	--	------	------
mPD		0.14	0.49
		nPD:	0.29
		N. weight:	0.43
		Sc. PD:	0.04
		Sc. rank:	374.1

NP			
Pos.	74	obs:	exp:
cgt	R	0	108.20
cgc	R	0	154.70
cga	R	232	291.20
cgg	R	2	236.00
aga	R	1335	1349.00
agg	R	1321	750.80
---	--	------	------
mPD		0.65	0.95
		nPD:	0.68
		N. weight:	0.31
		Sc. PD:	0.14
		Sc. rank:	535.2

NP			
Pos.	75	obs:	exp:
cgt	R	0	108.20
cgc	R	0	154.70
cga	R	0	291.20
cgg	R	2	236.00
aga	R	470	1349.00
agg	R	2418	750.80
---	--	------	------
mPD		0.27	0.95
		nPD:	0.29
		N. weight:	1.1
		Sc. PD:	0.092
		Sc. rank:	907.2

NP			
Pos.	76	obs:	exp:
aat	N	82	1576.00
aac	N	2808	1314.00
---	--	------	------
mPD		0.055	0.50
		nPD:	0.11
		N. weight:	0.86
		Sc. PD:	-0.073
		Sc. rank:	-105.1

NP			
Pos.	77	obs:	exp:
cgt	R	0	66.09
cgc	R	0	94.51
cga	R	0	178.00
cgg	R	0	144.20
aaa	K	942	640.00
aag	K	182	484.00
aga	R	492	824.40
agg	R	1274	458.80
---	--	------	------
mPD		0.98	1.3
		nPD:	0.76
		N. weight:	0.57
		Sc. PD:	0.31
		Sc. rank:	1044.3

NP			
Pos.	78	obs:	exp:
tat	Y	803	1386.00
tac	Y	2087	1504.00
---	--	------	------
mPD		0.40	0.50
		nPD:	0.8
		N. weight:	0.11
		Sc. PD:	0.067
		Sc. rank:	211.6

NP			
Pos.	79	obs:	exp:
tta	L	1	267.00
ttg	L	56	548.60
ctt	L	5	573.40
ctc	L	1	462.00
cta	L	226	427.40
ctg	L	2599	609.60
cct	P	0	0.27
ccc	P	0	0.18
cca	P	0	0.41
ccg	P	1	0.14
atg	M	1	1.00
---	--	------	------
mPD		0.19	1.1
		nPD:	0.17
		N. weight:	1.6
		Sc. PD:	-0.046
		Sc. rank:	408.5

NP			
Pos.	80	obs:	exp:
gaa	E	2641	1620.00
gag	E	249	1270.00
---	--	------	------
mPD		0.16	0.49
		nPD:	0.32
		N. weight:	0.41
		Sc. PD:	0.048
		Sc. rank:	411.1

NP			
Pos.	81	obs:	exp:
gaa	E	2224	1620.00
gag	E	666	1270.00
---	--	------	------
mPD		0.35	0.49
		nPD:	0.72
		N. weight:	0.13
		Sc. PD:	0.065
		Sc. rank:	228.4

NP			
Pos.	82	obs:	exp:
cct	P	3	1.37
ccc	P	2	0.90
cca	P	0	2.03
ccg	P	0	0.70
cat	H	681	1669.00
cac	H	2202	1214.00
aat	N	1	1.09
aac	N	1	0.91
---	--	------	------
mPD		0.37	0.49
		nPD:	0.74
		N. weight:	0.32
		Sc. PD:	0.17
		Sc. rank:	589.7

NP			
Pos.	83	obs:	exp:
cct	P	14	789.80
ccc	P	2873	522.00
cca	P	3	1173.00
ccg	P	0	405.20
---	--	------	------
mPD		0.012	0.71
		nPD:	0.02
		N. weight:	2.2
		Sc. PD:	-0.39
		Sc. rank:	-2672.3

NP			
Pos.	84	obs:	exp:
tct	S	0	479.20
tcc	S	0	415.90
tca	S	0	650.90
tcg	S	0	173.20
cgt	R	1	0.04
cgc	R	0	0.05
cga	R	0	0.10
cgg	R	0	0.08
act	T	2	0.52
acc	T	0	0.45
aca	T	0	0.84
acg	T	0	0.19
aat	N	16	8.73
aac	N	0	7.27
agt	S	2852	622.70
agc	S	19	529.10
aga	R	0	0.47
agg	R	0	0.26
---	--	------	------
mPD		0.026	1.7
		nPD:	0.02
		N. weight:	2.
		Sc. PD:	-0.35
		Sc. rank:	-2442.2

NP			
Pos.	85	obs:	exp:
cct	P	14	3.83
ccc	P	0	2.53
cca	P	0	5.68
ccg	P	0	1.96
act	T	1	2.36
acc	T	0	2.03
aca	T	2	3.77
acg	T	6	0.85
aaa	K	0	3.42
aag	K	6	2.58
gct	A	390	720.50
gcc	A	22	553.40
gca	A	35	1273.00
gcg	A	2413	313.00
gat	D	0	0.54
gac	D	1	0.46
---	--	------	------
mPD		0.30	0.71
		nPD:	0.42
		N. weight:	2.1
		Sc. PD:	0.45
		Sc. rank:	2748.9

NP			
Pos.	86	obs:	exp:
gaa	E	0	3.36
gag	E	6	2.64
ggt	G	8	417.10
ggc	G	0	378.80
gga	G	222	1264.00
ggg	G	2654	824.40
---	--	------	------
mPD		0.15	0.69
		nPD:	0.22
		N. weight:	1.2
		Sc. PD:	0.024
		Sc. rank:	736.9

NP			
Pos.	87	obs:	exp:
cgt	R	0	0.04
cgc	R	0	0.05
cga	R	0	0.10
cgg	R	0	0.08
aaa	K	195	1645.00
aag	K	2694	1244.00
aga	R	0	0.47
agg	R	1	0.26
---	--	------	------
mPD		0.13	0.49
		nPD:	0.26
		N. weight:	0.76
		Sc. PD:	0.044
		Sc. rank:	559.7

NP			
Pos.	88	obs:	exp:
gat	D	480	1560.00
gac	D	2410	1330.00
---	--	------	------
mPD		0.28	0.50
		nPD:	0.56
		N. weight:	0.4
		Sc. PD:	0.14
		Sc. rank:	626.1

NP			
Pos.	89	obs:	exp:
cct	P	629	789.80
ccc	P	220	522.00
cca	P	179	1173.00
ccg	P	1862	405.20
---	--	------	------
mPD		0.53	0.71
		nPD:	0.75
		N. weight:	0.99
		Sc. PD:	0.53
		Sc. rank:	1821.2

NP			
Pos.	90	obs:	exp:
cgt	R	0	0.04
cgc	R	0	0.05
cga	R	0	0.10
cgg	R	0	0.08
aaa	K	109	1645.00
aag	K	2780	1244.00
aga	R	0	0.47
agg	R	1	0.26
---	--	------	------
mPD		0.073	0.49
		nPD:	0.15
		N. weight:	0.89
		Sc. PD:	-0.042
		Sc. rank:	124.3

NP			
Pos.	91	obs:	exp:
aaa	K	2516	1646.00
aag	K	374	1244.00
---	--	------	------
mPD		0.23	0.49
		nPD:	0.46
		N. weight:	0.28
		Sc. PD:	0.072
		Sc. rank:	397.3

NP			
Pos.	92	obs:	exp:
act	T	2852	757.60
acc	T	33	651.20
aca	T	1	1209.00
acg	T	4	271.90
---	--	------	------
mPD		0.026	0.70
		nPD:	0.04
		N. weight:	1.7
		Sc. PD:	-0.26
		Sc. rank:	-1314.4

NP			
Pos.	93	obs:	exp:
cgt	R	0	0.07
cgc	R	0	0.11
cga	R	0	0.20
cgg	R	0	0.16
aga	R	2	0.93
agg	R	0	0.52
ggt	G	0	417.60
ggc	G	2	379.30
gga	G	2709	1265.00
ggg	G	177	825.50
---	--	------	------
mPD		0.12	0.69
		nPD:	0.17
		N. weight:	0.81
		Sc. PD:	-0.021
		Sc. rank:	244.1

NP			
Pos.	94	obs:	exp:
ggt	G	2679	417.90
ggc	G	207	379.60
gga	G	1	1266.00
ggg	G	3	826.10
---	--	------	------
mPD		0.14	0.69
		nPD:	0.2
		N. weight:	2.2
		Sc. PD:	-0.0018
		Sc. rank:	1024.6

NP			
Pos.	95	obs:	exp:
cct	P	3	789.80
ccc	P	1	522.00
cca	P	2869	1173.00
ccg	P	17	405.20
---	--	------	------
mPD		0.014	0.71
		nPD:	0.02
		N. weight:	1.1
		Sc. PD:	-0.2
		Sc. rank:	-1286.2

NP			
Pos.	96	obs:	exp:
ttt	F	2	0.83
ttc	F	0	1.17
att	I	2678	1059.00
atc	I	201	772.00
ata	I	6	1054.00
act	T	1	0.26
acc	T	0	0.23
aca	T	0	0.42
acg	T	0	0.09
gtt	V	2	0.43
gtc	V	0	0.42
gta	V	0	0.43
gtg	V	0	0.72
---	--	------	------
mPD		0.14	0.66
		nPD:	0.21
		N. weight:	1.
		Sc. PD:	0.0079
		Sc. rank:	508.4

NP			
Pos.	97	obs:	exp:
tat	Y	2645	1386.00
tac	Y	245	1504.00
---	--	------	------
mPD		0.16	0.50
		nPD:	0.31
		N. weight:	0.58
		Sc. PD:	0.064
		Sc. rank:	568.0

NP			
Pos.	98	obs:	exp:
caa	Q	1	0.50
cag	Q	0	0.50
cgt	R	0	105.90
cgc	R	0	151.40
cga	R	1053	285.10
cgg	R	1252	231.00
aaa	K	7	34.16
aag	K	53	25.84
aga	R	67	1321.00
agg	R	457	735.00
---	--	------	------
mPD		0.84	0.99
		nPD:	0.85
		N. weight:	1.4
		Sc. PD:	0.9
		Sc. rank:	2668.2

NP			
Pos.	99	obs:	exp:
cgt	R	0	108.10
cgc	R	0	154.60
cga	R	0	291.00
cgg	R	0	235.80
aaa	K	0	1.14
aag	K	2	0.86
aga	R	264	1348.00
agg	R	2624	750.30
---	--	------	------
mPD		0.17	0.96
		nPD:	0.18
		N. weight:	1.3
		Sc. PD:	-0.029
		Sc. rank:	448.9

NP			
Pos.	100	obs:	exp:
cgt	R	0	108.00
cgc	R	0	154.50
cga	R	0	290.90
cgg	R	1	235.70
aaa	K	3	1.71
aag	K	0	1.29
aga	R	2637	1348.00
agg	R	249	750.00
---	--	------	------
mPD		0.16	0.96
		nPD:	0.17
		N. weight:	0.68
		Sc. PD:	-0.02
		Sc. rank:	179.5

NP			
Pos.	101	obs:	exp:
aat	N	0	3.82
aac	N	7	3.18
gat	D	1024	1483.00
gac	D	1723	1264.00
gaa	E	131	76.24
gag	E	5	59.76
---	--	------	------
mPD		0.52	0.55
		nPD:	0.95
		N. weight:	0.1
		Sc. PD:	0.075
		Sc. rank:	193.4

NP			
Pos.	102	obs:	exp:
cgt	R	0	0.04
cgc	R	0	0.05
cga	R	0	0.10
cgg	R	0	0.08
aga	R	1	0.47
agg	R	0	0.26
gtt	V	0	0.43
gtc	V	0	0.42
gta	V	2	0.43
gtg	V	0	0.72
ggt	G	2	417.50
ggc	G	153	379.20
gga	G	800	1265.00
ggg	G	1932	825.30
---	--	------	------
mPD		0.48	0.69
		nPD:	0.69
		N. weight:	0.52
		Sc. PD:	0.25
		Sc. rank:	907.8

NP			
Pos.	103	obs:	exp:
cgt	R	0	1.38
cgc	R	0	1.98
cga	R	1	3.73
cgg	R	0	3.02
aaa	K	2646	1625.00
aag	K	207	1228.00
aga	R	36	17.27
agg	R	0	9.61
---	--	------	------
mPD		0.16	0.52
		nPD:	0.3
		N. weight:	0.43
		Sc. PD:	0.045
		Sc. rank:	402.8

NP			
Pos.	104	obs:	exp:
tgg	W	2890	2890.00
---	--	------	------
mPD		0	0
		nPD:	1.
		N. weight:	0.
		Sc. PD:	0
		Sc. rank:	0

NP			
Pos.	105	obs:	exp:
tta	L	0	0.37
ttg	L	0	0.76
ctt	L	0	0.79
ctc	L	0	0.64
cta	L	4	0.59
ctg	L	0	0.84
att	I	2	2.57
atc	I	0	1.87
ata	I	5	2.56
atg	M	612	612.00
act	T	0	0.52
acc	T	0	0.45
aca	T	0	0.84
acg	T	2	0.19
gtt	V	24	485.90
gtc	V	0	475.30
gta	V	81	481.70
gtg	V	2154	816.20
gct	A	0	1.51
gcc	A	0	1.16
gca	A	2	2.67
gcg	A	4	0.66
---	--	------	------
mPD		0.42	1.0
		nPD:	0.42
		N. weight:	0.87
		Sc. PD:	0.19
		Sc. rank:	1139.7

NP			
Pos.	106	obs:	exp:
cgt	R	0	108.20
cgc	R	0	154.70
cga	R	1	291.20
cgg	R	29	236.00
aga	R	2094	1349.00
agg	R	766	750.80
---	--	------	------
mPD		0.42	0.95
		nPD:	0.44
		N. weight:	0.4
		Sc. PD:	0.094
		Sc. rank:	541.5

NP			
Pos.	107	obs:	exp:
gat	D	3	1.62
gac	D	0	1.38
gaa	E	685	1618.00
gag	E	2202	1269.00
---	--	------	------
mPD		0.36	0.49
		nPD:	0.74
		N. weight:	0.29
		Sc. PD:	0.15
		Sc. rank:	521.5

NP			
Pos.	108	obs:	exp:
tta	L	2	267.10
ttg	L	83	548.80
ctt	L	15	573.60
ctc	L	0	462.10
cta	L	214	427.60
ctg	L	2575	609.80
atg	M	1	1.00
---	--	------	------
mPD		0.21	1.1
		nPD:	0.18
		N. weight:	1.5
		Sc. PD:	-0.022
		Sc. rank:	586.6

NP			
Pos.	109	obs:	exp:
att	I	1995	1033.00
atc	I	819	753.00
ata	I	0	1028.00
act	T	5	1.84
acc	T	2	1.58
aca	T	0	2.93
acg	T	0	0.66
gtt	V	68	14.84
gtc	V	1	14.52
gta	V	0	14.71
gtg	V	0	24.93
---	--	------	------
mPD		0.46	0.72
		nPD:	0.64
		N. weight:	0.68
		Sc. PD:	0.29
		Sc. rank:	1151.4

NP			
Pos.	110	obs:	exp:
tta	L	2	267.20
ttg	L	21	549.00
ctt	L	124	573.80
ctc	L	136	462.30
cta	L	62	427.70
ctg	L	2545	610.00
---	--	------	------
mPD		0.22	1.1
		nPD:	0.2
		N. weight:	1.4
		Sc. PD:	0.0012
		Sc. rank:	686.3

NP			
Pos.	111	obs:	exp:
tat	Y	1063	1385.00
tac	Y	1825	1503.00
cat	H	0	1.16
cac	H	2	0.84
---	--	------	------
mPD		0.47	0.50
		nPD:	0.93
		N. weight:	0.036
		Sc. PD:	0.026
		Sc. rank:	67.9

NP			
Pos.	112	obs:	exp:
cat	H	0	1.74
cac	H	3	1.26
gat	D	10	1558.00
gac	D	2876	1328.00
ggt	G	0	0.14
ggc	G	1	0.13
gga	G	0	0.44
ggg	G	0	0.29
---	--	------	------
mPD		0.0097	0.50
		nPD:	0.02
		N. weight:	0.99
		Sc. PD:	-0.17
		Sc. rank:	-1142.1

NP			
Pos.	113	obs:	exp:
caa	Q	1	0.50
cag	Q	0	0.50
aaa	K	2871	1645.00
aag	K	18	1244.00
---	--	------	------
mPD		0.013	0.49
		nPD:	0.03
		N. weight:	0.7
		Sc. PD:	-0.12
		Sc. rank:	-704.2

NP			
Pos.	114	obs:	exp:
gct	A	0	0.25
gcc	A	0	0.19
gca	A	0	0.45
gcg	A	1	0.11
gat	D	3	3.78
gac	D	4	3.22
gaa	E	1015	1616.00
gag	E	1867	1266.00
---	--	------	------
mPD		0.46	0.50
		nPD:	0.93
		N. weight:	0.12
		Sc. PD:	0.084
		Sc. rank:	224.2

NP			
Pos.	115	obs:	exp:
aaa	K	0	1.14
aag	K	2	0.86
gat	D	2	1.08
gac	D	0	0.92
gaa	E	58	1618.00
gag	E	2828	1268.00
---	--	------	------
mPD		0.042	0.49
		nPD:	0.09
		N. weight:	0.95
		Sc. PD:	-0.1
		Sc. rank:	-340.6

NP			
Pos.	116	obs:	exp:
att	I	24	1059.00
atc	I	2855	772.30
ata	I	7	1055.00
gtt	V	0	0.86
gtc	V	4	0.84
gta	V	0	0.85
gtg	V	0	1.45
---	--	------	------
mPD		0.024	0.66
		nPD:	0.04
		N. weight:	1.6
		Sc. PD:	-0.26
		Sc. rank:	-1331.1

NP			
Pos.	117	obs:	exp:
cgt	R	0	108.00
cgc	R	0	154.40
cga	R	1	290.80
cgg	R	191	235.70
aaa	K	0	1.71
aag	K	3	1.29
aga	R	294	1347.00
agg	R	2400	749.80
ggt	G	0	0.14
ggc	G	0	0.13
gga	G	0	0.44
ggg	G	1	0.29
---	--	------	------
mPD		0.31	0.96
		nPD:	0.32
		N. weight:	1.1
		Sc. PD:	0.13
		Sc. rank:	1094.3

NP			
Pos.	118	obs:	exp:
cgt	R	0	108.00
cgc	R	0	154.40
cga	R	0	290.80
cgg	R	0	235.70
aaa	K	1	2.28
aag	K	3	1.72
aga	R	1047	1347.00
agg	R	1839	749.80
---	--	------	------
mPD		0.47	0.96
		nPD:	0.49
		N. weight:	0.63
		Sc. PD:	0.18
		Sc. rank:	928.5

NP			
Pos.	119	obs:	exp:
att	I	1978	1058.00
atc	I	896	771.50
ata	I	9	1054.00
act	T	2	0.52
acc	T	0	0.45
aca	T	0	0.84
acg	T	0	0.19
gtt	V	4	1.08
gtc	V	1	1.05
gta	V	0	1.07
gtg	V	0	1.81
---	--	------	------
mPD		0.44	0.67
		nPD:	0.66
		N. weight:	0.61
		Sc. PD:	0.27
		Sc. rank:	1044.1

NP			
Pos.	120	obs:	exp:
tgg	W	2890	2890.00
---	--	------	------
mPD		0	0
		nPD:	1.
		N. weight:	0.
		Sc. PD:	0
		Sc. rank:	0

NP			
Pos.	121	obs:	exp:
tct	S	0	0.17
tcc	S	0	0.14
tca	S	0	0.23
tcg	S	0	0.06
tgt	C	1	0.39
tgc	C	0	0.61
cat	H	2	1.16
cac	H	0	0.84
cgt	R	1892	108.00
cgc	R	989	154.40
cga	R	4	290.70
cgg	R	0	235.60
agt	S	1	0.22
agc	S	0	0.18
aga	R	0	1347.00
agg	R	0	749.50
ggt	G	1	0.14
ggc	G	0	0.13
gga	G	0	0.44
ggg	G	0	0.29
---	--	------	------
mPD		0.46	0.96
		nPD:	0.48
		N. weight:	3.3
		Sc. PD:	0.89
		Sc. rank:	4781.0

,

NP			
Pos.	122	obs:	exp:
cat	H	2	1.16
cac	H	0	0.84
caa	Q	2820	1440.00
cag	Q	65	1445.00
cgt	R	0	0.11
cgc	R	0	0.16
cga	R	3	0.30
cgg	R	0	0.24
aga	R	0	1.40
agg	R	0	0.78
---	--	------	------
mPD		0.047	0.50
		nPD:	0.09
		N. weight:	0.78
		Sc. PD:	-0.078
		Sc. rank:	-228.9

,

NP			
Pos.	123	obs:	exp:
cct	P	0	0.55
ccc	P	0	0.36
cca	P	0	0.81
ccg	P	2	0.28
gct	A	394	727.50
gcc	A	8	558.90
gca	A	50	1286.00
gcg	A	2436	316.10
---	--	------	------
mPD		0.27	0.69
		nPD:	0.39
		N. weight:	2.1
		Sc. PD:	0.39
		Sc. rank:	2615.8

,

NP			
Pos.	124	obs:	exp:
tct	S	0	0.17
tcc	S	0	0.14
tca	S	0	0.23
tcg	S	0	0.06
act	T	0	0.26
acc	T	1	0.23
aca	T	0	0.42
acg	T	0	0.09
aat	N	605	1575.00
aac	N	2283	1313.00
agt	S	0	0.22
agc	S	1	0.18
---	--	------	------
mPD		0.33	0.50
		nPD:	0.67
		N. weight:	0.32
		Sc. PD:	0.14
		Sc. rank:	540.8

,

NP			
Pos.	125	obs:	exp:
tct	S	0	0.50
tcc	S	0	0.43
tca	S	0	0.68
tcg	S	0	0.18
cat	H	1	0.58
cac	H	0	0.42
aat	N	2856	1573.00
aac	N	29	1312.00
agt	S	3	0.65
agc	S	0	0.55
gat	D	1	0.54
gac	D	0	0.46
---	--	------	------
mPD		0.023	0.50
		nPD:	0.05
		N. weight:	0.73
		Sc. PD:	-0.11
		Sc. rank:	-506.9

,

NP			
Pos.	126	obs:	exp:
ggt	G	2	417.90
ggc	G	0	379.60
gga	G	2866	1266.00
ggg	G	22	826.10
---	--	------	------
mPD		0.016	0.69
		nPD:	0.02
		N. weight:	1.
		Sc. PD:	-0.17
		Sc. rank:	-1061.0

,

NP			
Pos.	127	obs:	exp:
gat	D	0	0.54
gac	D	1	0.46
gaa	E	956	1619.00
gag	E	1932	1269.00
ggt	G	0	0.14
ggc	G	0	0.13
gga	G	0	0.44
ggg	G	1	0.29
---	--	------	------
mPD		0.44	0.49
		nPD:	0.9
		N. weight:	0.14
		Sc. PD:	0.098
		Sc. rank:	270.3

,

NP			
Pos.	128	obs:	exp:
aat	N	0	1.09
aac	N	2	0.91
gat	D	128	1554.00
gac	D	2750	1324.00
gaa	E	10	5.61
gag	E	0	4.39
---	--	------	------
mPD		0.093	0.50
		nPD:	0.18
		N. weight:	0.78
		Sc. PD:	-0.0099
		Sc. rank:	302.4

,

NP			
Pos.	129	obs:	exp:
tct	S	0	12.52
tcc	S	0	10.86
tca	S	75	17.00
tcg	S	0	4.52
agt	S	0	16.27
agc	S	0	13.82
gct	A	0	708.90
gcc	A	5	544.50
gca	A	2771	1253.00
gcg	A	38	308.00
ggt	G	0	0.14
ggc	G	0	0.13
gga	G	1	0.44
ggg	G	0	0.29
---	--	------	------
mPD		0.081	0.76
		nPD:	0.11
		N. weight:	1.
		Sc. PD:	-0.091
		Sc. rank:	-192.5

,

NP			
Pos.	130	obs:	exp:
act	T	2867	757.10
acc	T	20	650.70
aca	T	1	1208.00
acg	T	0	271.70
aat	N	1	0.55
aac	N	0	0.45
gct	A	1	0.25
gcc	A	0	0.19
gca	A	0	0.45
gcg	A	0	0.11
---	--	------	------
mPD		0.016	0.70
		nPD:	0.02
		N. weight:	1.7
		Sc. PD:	-0.29
		Sc. rank:	-1799.2

,

NP			
Pos.	131	obs:	exp:
act	T	1	0.26
acc	T	0	0.23
aca	T	0	0.42
acg	T	0	0.09
gct	A	2772	727.80
gcc	A	22	559.00
gca	A	29	1286.00
gcg	A	66	316.20
---	--	------	------
mPD		0.079	0.69
		nPD:	0.12
		N. weight:	1.6
		Sc. PD:	-0.13
		Sc. rank:	-139.9

,

NP			
Pos.	132	obs:	exp:
ggt	G	2037	417.90
ggc	G	840	379.60
gga	G	2	1266.00
ggg	G	11	826.10
---	--	------	------
mPD		0.42	0.69
		nPD:	0.61
		N. weight:	1.8
		Sc. PD:	0.7
		Sc. rank:	2926.3

,

NP			
Pos.	133	obs:	exp:
tta	L	0	267.20
ttg	L	0	549.00
ctt	L	1588	573.80
ctc	L	1301	462.30
cta	L	1	427.70
ctg	L	0	610.00
---	--	------	------
mPD		0.50	1.1
		nPD:	0.44
		N. weight:	1.3
		Sc. PD:	0.32
		Sc. rank:	1856.1

,

NP			
Pos.	134	obs:	exp:
act	T	1176	757.60
acc	T	1707	651.20
aca	T	7	1209.00
acg	T	0	271.90
---	--	------	------
mPD		0.49	0.70
		nPD:	0.7
		N. weight:	0.97
		Sc. PD:	0.47
		Sc. rank:	1714.2

,

NP			
Pos.	135	obs:	exp:
cat	H	355	1673.00
cac	H	2535	1217.00
---	--	------	------
mPD		0.22	0.49
		nPD:	0.44
		N. weight:	0.6
		Sc. PD:	0.14
		Sc. rank:	825.9

,

NP			
Pos.	136	obs:	exp:
tta	L	0	267.00
ttg	L	270	548.60
ctt	L	78	573.40
ctc	L	0	462.00
cta	L	136	427.40
ctg	L	2404	609.60
atg	M	1	1.00
gtt	V	0	0.22
gtc	V	0	0.21
gta	V	0	0.21
gtg	V	1	0.36
---	--	------	------
mPD		0.31	1.1
		nPD:	0.28
		N. weight:	1.3
		Sc. PD:	0.098
		Sc. rank:	1005.5

,

NP			
Pos.	137	obs:	exp:
atg	M	2890	2890.00
---	--	------	------
mPD		0	0
		nPD:	1.
		N. weight:	0.
		Sc. PD:	0
		Sc. rank:	0

,

NP			
Pos.	138	obs:	exp:
att	I	385	1061.00
atc	I	665	773.40
ata	I	1840	1056.00
---	--	------	------
mPD		0.52	0.66
		nPD:	0.79
		N. weight:	0.25
		Sc. PD:	0.14
		Sc. rank:	456.6

,

NP			
Pos.	139	obs:	exp:
tgg	W	2889	2889.00
cgt	R	0	0.04
cgc	R	0	0.05
cga	R	0	0.10
cgg	R	0	0.08
aga	R	0	0.47
agg	R	1	0.26
---	--	------	------
mPD		0.00069	0.0011
		nPD:	0.6
		N. weight:	0.00061
		Sc. PD:	0.00024
		Sc. rank:	1.0

,

NP			
Pos.	140	obs:	exp:
cct	P	2	0.55
ccc	P	0	0.36
cca	P	0	0.81
ccg	P	0	0.28
cat	H	2793	1671.00
cac	H	94	1216.00
cgt	R	1	0.04
cgc	R	0	0.05
cga	R	0	0.10
cgg	R	0	0.08
aga	R	0	0.47
agg	R	0	0.26
---	--	------	------
mPD		0.065	0.49
		nPD:	0.13
		N. weight:	0.55
		Sc. PD:	-0.035
		Sc. rank:	14.7

,

NP

Pos.	141	obs:	exp:
tct	S	425	482.40
tcc	S	2460	418.60
tca	S	5	655.20
tcg	S	0	174.40
agt	S	0	626.90
agc	S	0	532.60
---	--	------	------
mPD		0.25	1.7
		nPD:	0.15
		N. weight:	2.
		Sc. PD:	-0.084
		Sc. rank:	303.0

,

NP

Pos.	142	obs:	exp:
aat	N	2732	1575.00
aac	N	157	1314.00
gat	D	1	0.54
gac	D	0	0.46
---	--	------	------
mPD		0.10	0.50
		nPD:	0.21
		N. weight:	0.54
		Sc. PD:	0.0055
		Sc. rank:	279.9

,

NP

Pos.	143	obs:	exp:
tta	L	160	267.10
ttg	L	39	548.80
ctt	L	1	573.60
ctc	L	0	462.10
cta	L	1934	427.60
ctg	L	755	609.80
gtt	V	0	0.22
gtc	V	0	0.21
gta	V	1	0.21
gtg	V	0	0.36
---	--	------	------
mPD		0.53	1.1
		nPD:	0.47
		N. weight:	1.3
		Sc. PD:	0.35
		Sc. rank:	1865.5

,

NP

Pos.	144	obs:	exp:
aat	N	2870	1576.00
aac	N	20	1314.00
---	--	------	------
mPD		0.014	0.50
		nPD:	0.03
		N. weight:	0.75
		Sc. PD:	-0.12
		Sc. rank:	-712.8

,

NP

Pos.	145	obs:	exp:
aat	N	1	0.55
aac	N	0	0.45
gat	D	2860	1560.00
gac	D	29	1329.00
---	--	------	------
mPD		0.021	0.50
		nPD:	0.04
		N. weight:	0.74
		Sc. PD:	-0.11
		Sc. rank:	-551.4

,

NP

Pos.	146	obs:	exp:
tct	S	0	0.17
tcc	S	1	0.14
tca	S	0	0.23
tcg	S	0	0.06
act	T	0	4.46
acc	T	17	3.83
aca	T	0	7.11
acg	T	0	1.60
agt	S	0	0.22
agc	S	0	0.18
gtt	V	0	0.65
gtc	V	3	0.63
gta	V	0	0.64
gtg	V	0	1.08
gct	A	16	722.80
gcc	A	2844	555.20
gca	A	9	1277.00
gcg	A	0	314.00
---	--	------	------
mPD		0.032	0.70
		nPD:	0.04
		N. weight:	2.1
		Sc. PD:	-0.31
		Sc. rank:	-1460.8

,

NP

Pos.	147	obs:	exp:
tct	S	0	0.33
tcc	S	0	0.29
tca	S	2	0.45
tcg	S	0	0.12
act	T	1	757.10
acc	T	27	650.70
aca	T	2801	1208.00
acg	T	59	271.70
agt	S	0	0.43
agc	S	0	0.37
---	--	------	------
mPD		0.060	0.70
		nPD:	0.09
		N. weight:	0.99
		Sc. PD:	-0.11
		Sc. rank:	-349.5

,

NP

Pos.	148	obs:	exp:
tat	Y	1757	1386.00
tac	Y	1133	1504.00
---	--	------	------
mPD		0.48	0.50
		nPD:	0.96
		N. weight:	0.046
		Sc. PD:	0.034
		Sc. rank:	86.1

,

NP

Pos.	149	obs:	exp:
caa	Q	17	1443.00
cag	Q	2873	1447.00
---	--	------	------
mPD		0.012	0.50
		nPD:	0.02
		N. weight:	0.86
		Sc. PD:	-0.15
		Sc. rank:	-899.6

,

NP

Pos.	150	obs:	exp:
tct	S	0	0.17
tcc	S	0	0.14
tca	S	0	0.23
tcg	S	0	0.06
cgt	R	0	108.10
cgc	R	0	154.60
cga	R	0	291.10
cgg	R	0	235.90
agt	S	1	0.22
agc	S	0	0.18
aga	R	2138	1349.00
agg	R	751	750.50
---	--	------	------
mPD		0.39	0.96
		nPD:	0.4
		N. weight:	0.45
		Sc. PD:	0.09
		Sc. rank:	580.0

,

NP

Pos.	151	obs:	exp:
act	T	12	757.60
acc	T	226	651.20
aca	T	1537	1209.00
acg	T	1115	271.90
---	--	------	------
mPD		0.56	0.70
		nPD:	0.81
		N. weight:	0.76
		Sc. PD:	0.45
		Sc. rank:	1414.8

,

NP

Pos.	152	obs:	exp:
cgt	R	0	108.10
cgc	R	0	154.60
cga	R	0	291.10
cgg	R	0	235.90
aga	R	2590	1349.00
agg	R	299	750.50
ggt	G	0	0.14
ggc	G	0	0.13
gga	G	1	0.44
ggg	G	0	0.29
---	--	------	------
mPD		0.19	0.95
		nPD:	0.2
		N. weight:	0.65
		Sc. PD:	-0.0018
		Sc. rank:	285.0

,

NP

Pos.	153	obs:	exp:
cct	P	0	0.27
ccc	P	1	0.18
cca	P	0	0.41
ccg	P	0	0.14
gct	A	2473	727.80
gcc	A	231	559.00
gca	A	183	1286.00
gcg	A	2	316.20
---	--	------	------
mPD		0.26	0.69
		nPD:	0.37
		N. weight:	1.1
		Sc. PD:	0.19
		Sc. rank:	1319.2

,

NP

Pos.	154	obs:	exp:
ttt	F	0	0.42
ttc	F	1	0.58
tta	L	0	266.90
ttg	L	0	548.50
ctt	L	216	573.20
ctc	L	2590	461.80
cta	L	81	427.30
ctg	L	0	609.30
att	I	0	0.37
atc	I	1	0.27
ata	I	0	0.37
gtt	V	0	0.22
gtc	V	1	0.21
gta	V	0	0.21
gtg	V	0	0.36
---	--	------	------
mPD		0.19	1.1
		nPD:	0.17
		N. weight:	1.9
		Sc. PD:	-0.051
		Sc. rank:	522.4

,

NP

Pos.	155	obs:	exp:
gtt	V	4	621.60
gtc	V	5	608.00
gta	V	338	616.20
gtg	V	2543	1044.00
---	--	------	------
mPD		0.21	0.73
		nPD:	0.29
		N. weight:	0.92
		Sc. PD:	0.082
		Sc. rank:	795.0

,

NP

Pos.	156	obs:	exp:
cgt	R	2353	108.20
cgc	R	363	154.70
cga	R	1	291.20
cgg	R	172	236.00
aga	R	0	1349.00
agg	R	1	750.80
---	--	------	------
mPD		0.32	0.95
		nPD:	0.33
		N. weight:	3.4
		Sc. PD:	0.45
		Sc. rank:	3773.7

,

NP

Pos.	157	obs:	exp:
act	T	2030	757.60
acc	T	795	651.20
aca	T	61	1209.00
acg	T	4	271.90
---	--	------	------
mPD		0.43	0.70
		nPD:	0.62
		N. weight:	0.9
		Sc. PD:	0.37
		Sc. rank:	1506.4

,

NP

Pos.	158	obs:	exp:
ggt	G	4	417.90
ggc	G	0	379.60
gga	G	1871	1266.00
ggg	G	1015	826.10
---	--	------	------
mPD		0.46	0.69
		nPD:	0.67
		N. weight:	0.42
		Sc. PD:	0.19
		Sc. rank:	724.0

,

NP

Pos.	159	obs:	exp:
atg	M	2890	2890.00
---	--	------	------
mPD		0	0
		nPD:	1.
		N. weight:	0.
		Sc. PD:	0
		Sc. rank:	0

,

NP

Pos.	160	obs:	exp:
gat	D	170	1560.00
gac	D	2720	1330.00
---	--	------	------
mPD		0.11	0.50
		nPD:	0.22
		N. weight:	0.72
		Sc. PD:	0.017
		Sc. rank:	454.5

,

NP			
Pos.	161	obs:	exp:
cct	P	169	789.80
ccc	P	2533	522.00
cca	P	188	1173.00
ccg	P	0	405.20
---	--	------	------
mPD		0.22	0.71
		nPD:	0.32
		N. weight:	1.6
		Sc. PD:	0.18
		Sc. rank:	1569.6

,

NP			
Pos.	162	obs:	exp:
cgt	R	0	108.00
cgc	R	0	154.40
cga	R	0	290.70
cgg	R	1	235.60
att	I	0	1.10
atc	I	0	0.80
ata	I	3	1.10
atg	M	1	1.00
aaa	K	0	0.57
aag	K	1	0.43
aga	R	488	1347.00
agg	R	2396	749.50
---	--	------	------
mPD		0.29	0.96
		nPD:	0.3
		N. weight:	1.
		Sc. PD:	0.1
		Sc. rank:	954.8

,

NP			
Pos.	163	obs:	exp:
atg	M	2888	2888.00
act	T	0	0.52
acc	T	0	0.45
aca	T	0	0.84
acg	T	2	0.19
---	--	------	------
mPD		0.0014	0.0026
		nPD:	0.52
		N. weight:	0.0022
		Sc. PD:	0.00069
		Sc. rank:	3.3

,

NP			
Pos.	164	obs:	exp:
tgt	C	38	1118.00
tgc	C	2851	1771.00
ggt	G	0	0.14
ggc	G	1	0.13
gga	G	0	0.44
ggg	G	0	0.29
---	--	------	------
mPD		0.027	0.48
		nPD:	0.06
		N. weight:	0.56
		Sc. PD:	-0.078
		Sc. rank:	-336.7

,

NP			
Pos.	165	obs:	exp:
tct	S	2849	482.20
tcc	S	39	418.50
tca	S	1	654.90
tcg	S	0	174.30
tgt	C	1	0.39
tgc	C	0	0.61
agt	S	0	626.60
agc	S	0	532.40
---	--	------	------
mPD		0.028	1.7
		nPD:	0.02
		N. weight:	2.3
		Sc. PD:	-0.4
		Sc. rank:	-2686.8

,

NP			
Pos.	166	obs:	exp:
tta	L	4	267.20
ttg	L	3	549.00
ctt	L	599	573.80
ctc	L	8	462.30
cta	L	113	427.70
ctg	L	2163	610.00
---	--	------	------
mPD		0.40	1.1
		nPD:	0.36
		N. weight:	1.2
		Sc. PD:	0.18
		Sc. rank:	1322.2

,

NP			
Pos.	167	obs:	exp:
atg	M	2889	2889.00
aaa	K	0	0.57
aag	K	1	0.43
---	--	------	------
mPD		0.00069	0.0011
		nPD:	0.64
		N. weight:	0.00038
		Sc. PD:	0.00016
		Sc. rank:	0.6

,

NP			
Pos.	168	obs:	exp:
cct	P	0	0.27
ccc	P	0	0.18
cca	P	1	0.41
ccg	P	0	0.14
caa	Q	2849	1442.00
cag	Q	40	1447.00
---	--	------	------
mPD		0.028	0.50
		nPD:	0.06
		N. weight:	0.82
		Sc. PD:	-0.11
		Sc. rank:	-494.5

,

NP			
Pos.	169	obs:	exp:
gaa	E	1	0.56
gag	E	0	0.44
ggt	G	10	417.80
ggc	G	166	379.50
gga	G	2022	1266.00
ggg	G	691	825.80
---	--	------	------
mPD		0.45	0.69
		nPD:	0.65
		N. weight:	0.3
		Sc. PD:	0.13
		Sc. rank:	510.8

,

NP			
Pos.	170	obs:	exp:
tct	S	29	482.40
tcc	S	0	418.60
tca	S	2838	655.20
tcg	S	23	174.40
agt	S	0	626.90
agc	S	0	532.60
---	--	------	------
mPD		0.036	1.7
		nPD:	0.02
		N. weight:	1.8
		Sc. PD:	-0.32
		Sc. rank:	-2032.9

,

NP			
Pos.	171	obs:	exp:
act	T	2542	757.60
acc	T	346	651.20
aca	T	0	1209.00
acg	T	2	271.90
---	--	------	------
mPD		0.21	0.70
		nPD:	0.3
		N. weight:	1.3
		Sc. PD:	0.13
		Sc. rank:	1220.5

,

NP			
Pos.	172	obs:	exp:
tta	L	0	267.20
ttg	L	0	549.00
ctt	L	197	573.80
ctc	L	2673	462.30
cta	L	20	427.70
ctg	L	0	610.00
---	--	------	------
mPD		0.14	1.1
		nPD:	0.12
		N. weight:	2.
		Sc. PD:	-0.14
		Sc. rank:	32.8

,

NP			
Pos.	173	obs:	exp:
cct	P	155	789.80
ccc	P	1	522.00
cca	P	872	1173.00
ccg	P	1862	405.20
---	--	------	------
mPD		0.49	0.71
		nPD:	0.69
		N. weight:	1.1
		Sc. PD:	0.51
		Sc. rank:	1864.9

,

NP			
Pos.	174	obs:	exp:
cgt	R	0	108.20
cgc	R	0	154.70
cga	R	0	291.20
cgg	R	12	236.00
aga	R	36	1349.00
agg	R	2842	750.80
---	--	------	------
mPD		0.033	0.95
		nPD:	0.03
		N. weight:	1.7
		Sc. PD:	-0.26
		Sc. rank:	-1436.6

,

NP			
Pos.	175	obs:	exp:
cgt	R	0	108.20
cgc	R	0	154.70
cga	R	0	291.20
cgg	R	0	236.00
aga	R	2179	1349.00
agg	R	711	750.80
---	--	------	------
mPD		0.37	0.95
		nPD:	0.39
		N. weight:	0.46
		Sc. PD:	0.086
		Sc. rank:	569.9

,

NP			
Pos.	176	obs:	exp:
tct	S	2514	482.40
tcc	S	153	418.60
tca	S	113	655.20
tcg	S	110	174.40
agt	S	0	626.90
agc	S	0	532.60
---	--	------	------
mPD		0.24	1.7
		nPD:	0.14
		N. weight:	1.7
		Sc. PD:	-0.09
		Sc. rank:	146.4

,

NP			
Pos.	177	obs:	exp:
gaa	E	1	0.56
gag	E	0	0.44
ggt	G	0	417.80
ggc	G	3	379.50
gga	G	2661	1266.00
ggg	G	225	825.80
---	--	------	------
mPD		0.15	0.69
		nPD:	0.21
		N. weight:	0.76
		Sc. PD:	0.011
		Sc. rank:	419.5

,

NP			
Pos.	178	obs:	exp:
tct	S	1	0.17
tcc	S	0	0.14
tca	S	0	0.23
tcg	S	0	0.06
agt	S	0	0.22
agc	S	0	0.18
gtt	V	1	0.22
gtc	V	0	0.21
gta	V	0	0.21
gtg	V	0	0.36
gct	A	2838	727.30
gcc	A	46	558.70
gca	A	2	1285.00
gcg	A	1	316.00
gat	D	1	0.54
gac	D	0	0.46
---	--	------	------
mPD		0.035	0.69
		nPD:	0.05
		N. weight:	1.7
		Sc. PD:	-0.24
		Sc. rank:	-1113.6

,

NP			
Pos.	179	obs:	exp:
gct	A	1836	728.00
gcc	A	1048	559.20
gca	A	5	1286.00
gcg	A	1	316.30
---	--	------	------
mPD		0.47	0.69
		nPD:	0.67
		N. weight:	1.1
		Sc. PD:	0.49
		Sc. rank:	1831.8

,

NP			
Pos.	180	obs:	exp:
cgt	R	1	0.04
cgc	R	0	0.05
cga	R	0	0.10
cgg	R	0	0.08
aga	R	0	0.47
agg	R	0	0.26
ggt	G	2456	417.80
ggc	G	22	379.50
gga	G	406	1266.00
ggg	G	5	825.80
---	--	------	------
mPD		0.26	0.69
		nPD:	0.37
		N. weight:	1.7
		Sc. PD:	0.3
		Sc. rank:	2063.8

,

NP			
Pos.	181	obs:	exp:
tct	S	0	0.17
tcc	S	0	0.14
tca	S	1	0.23
tcg	S	0	0.06
agt	S	0	0.22
agc	S	0	0.18
gct	A	1	727.80
gcc	A	2	559.00
gca	A	2848	1286.00
gcg	A	38	316.20
---	--	------	------
mPD		0.029	0.69
		nPD:	0.04
		N. weight:	0.99
		Sc. PD:	-0.15
		Sc. rank:	-729.3

,

NP			
Pos.	182	obs:	exp:
tct	S	0	0.67
tcc	S	0	0.58
tca	S	4	0.91
tcg	S	0	0.24
act	T	0	0.52
acc	T	0	0.45
aca	T	2	0.84
acg	T	0	0.19
agt	S	0	0.87
agc	S	0	0.74
gct	A	6	726.50
gcc	A	3	558.10
gca	A	2861	1284.00
gcg	A	14	315.70
---	--	------	------
mPD		0.020	0.69
		nPD:	0.03
		N. weight:	1.
		Sc. PD:	-0.17
		Sc. rank:	-956.9

,

NP			
Pos.	183	obs:	exp:
tta	L	0	0.18
ttg	L	0	0.38
ctt	L	0	0.40
ctc	L	0	0.32
cta	L	0	0.30
ctg	L	2	0.42
att	I	0	16.51
atc	I	0	12.04
ata	I	45	16.44
atg	M	2	2.00
gtt	V	0	610.80
gtc	V	0	597.50
gta	V	1073	605.60
gtg	V	1767	1026.00
gct	A	0	0.25
gcc	A	0	0.19
gca	A	1	0.45
gcg	A	0	0.11
---	--	------	------
mPD		0.51	0.77
		nPD:	0.66
		N. weight:	0.74
		Sc. PD:	0.33
		Sc. rank:	1268.7

,

NP			
Pos.	184	obs:	exp:
cgt	R	0	0.04
cgc	R	0	0.05
cga	R	0	0.10
cgg	R	0	0.08
aaa	K	238	1645.00
aag	K	2651	1244.00
aga	R	0	0.47
agg	R	1	0.26
---	--	------	------
mPD		0.15	0.49
		nPD:	0.31
		N. weight:	0.71
		Sc. PD:	0.076
		Sc. rank:	688.5

,

NP			
Pos.	185	obs:	exp:
ggt	G	93	417.90
ggc	G	3	379.60
gga	G	1281	1266.00
ggg	G	1513	826.10
---	--	------	------
mPD		0.53	0.69
		nPD:	0.77
		N. weight:	0.36
		Sc. PD:	0.2
		Sc. rank:	662.0

,

NP			
Pos.	186	obs:	exp:
att	I	0	42.20
atc	I	18	30.77
ata	I	97	42.03
gtt	V	204	596.90
gtc	V	849	583.80
gta	V	1686	591.70
gtg	V	36	1003.00
---	--	------	------
mPD		0.60	0.81
		nPD:	0.74
		N. weight:	0.83
		Sc. PD:	0.44
		Sc. rank:	1516.1

,

NP			
Pos.	187	obs:	exp:
cgt	R	0	0.11
cgc	R	0	0.16
cga	R	0	0.30
cgg	R	0	0.24
aga	R	2	1.40
agg	R	1	0.78
ggt	G	1	417.50
ggc	G	0	379.20
gga	G	1507	1265.00
ggg	G	1379	825.30
---	--	------	------
mPD		0.50	0.69
		nPD:	0.73
		N. weight:	0.44
		Sc. PD:	0.23
		Sc. rank:	799.6

,

NP			
Pos.	188	obs:	exp:
cct	P	0	0.27
ccc	P	0	0.18
cca	P	1	0.41
ccg	P	0	0.14
caa	Q	1	0.50
cag	Q	0	0.50
act	T	4	757.10
acc	T	2	650.70
aca	T	2019	1208.00
acg	T	863	271.70
---	--	------	------
mPD		0.42	0.70
		nPD:	0.61
		N. weight:	0.92
		Sc. PD:	0.36
		Sc. rank:	1517.3

,

NP			
Pos.	189	obs:	exp:
att	I	0	9.91
atc	I	0	7.23
ata	I	27	9.87
atg	M	2862	2862.00
aaa	K	0	0.57
aag	K	1	0.43
---	--	------	------
mPD		0.019	0.020
		nPD:	0.98
		N. weight:	0.013
		Sc. PD:	0.0097
		Sc. rank:	24.2

,

NP			
Pos.	190	obs:	exp:
gtt	V	0	621.60
gtc	V	0	608.00
gta	V	364	616.20
gtg	V	2526	1044.00
---	--	------	------
mPD		0.22	0.73
		nPD:	0.3
		N. weight:	0.93
		Sc. PD:	0.093
		Sc. rank:	857.8

,

NP			
Pos.	191	obs:	exp:
atg	M	2889	2889.00
gaa	E	0	0.56
gag	E	1	0.44
---	--	------	------
mPD		0.0014	0.0018
		nPD:	0.78
		N. weight:	0.00037
		Sc. PD:	0.00021
		Sc. rank:	0.7

,

NP			
Pos.	192	obs:	exp:
gaa	E	1168	1620.00
gag	E	1722	1270.00
---	--	------	------
mPD		0.48	0.49
		nPD:	0.98
		N. weight:	0.067
		Sc. PD:	0.051
		Sc. rank:	126.3

,

NP			
Pos.	193	obs:	exp:
tta	L	6	267.10
ttg	L	165	548.80
ctt	L	1	573.60
ctc	L	0	462.10
cta	L	902	427.60
ctg	L	1815	609.80
cgt	R	0	0.04
cgc	R	0	0.05
cga	R	0	0.10
cgg	R	1	0.08
aga	R	0	0.47
agg	R	0	0.26
---	--	------	------
mPD		0.54	1.1
		nPD:	0.48
		N. weight:	1.1
		Sc. PD:	0.31
		Sc. rank:	1621.4

,

NP			
Pos.	194	obs:	exp:
ttt	F	1	0.42
ttc	F	0	0.58
att	I	2754	1043.00
atc	I	82	760.80
ata	I	7	1039.00
aat	N	1	0.55
aac	N	0	0.45
gtt	V	34	9.68
gtc	V	11	9.47
gta	V	0	9.60
gtg	V	0	16.26
---	--	------	------
mPD		0.099	0.70
		nPD:	0.14
		N. weight:	1.1
		Sc. PD:	-0.062
		Sc. rank:	82.9

,

NP			
Pos.	195	obs:	exp:
cgt	R	0	108.20
cgc	R	22	154.70
cga	R	760	291.20
cgg	R	1993	236.00
aga	R	43	1349.00
agg	R	72	750.80
---	--	------	------
mPD		0.49	0.95
		nPD:	0.51
		N. weight:	2.1
		Sc. PD:	0.65
		Sc. rank:	3157.1

,

NP			
Pos.	196	obs:	exp:
atg	M	2889	2889.00
aaa	K	0	0.57
aag	K	1	0.43
---	--	------	------
mPD		0.00069	0.0011
		nPD:	0.64
		N. weight:	0.00038
		Sc. PD:	0.00016
		Sc. rank:	0.6

,

NP			
Pos.	197	obs:	exp:
att	I	214	1059.00
atc	I	201	772.00
ata	I	2470	1054.00
atg	M	2	2.00
gtt	V	0	0.65
gtc	V	0	0.63
gta	V	3	0.64
gtg	V	0	1.08
---	--	------	------
mPD		0.26	0.66
		nPD:	0.39
		N. weight:	0.68
		Sc. PD:	0.13
		Sc. rank:	852.8

,

NP			
Pos.	198	obs:	exp:
cgt	R	0	0.07
cgc	R	0	0.11
cga	R	0	0.20
cgg	R	0	0.16
act	T	0	0.26
acc	T	0	0.23
aca	T	1	0.42
acg	T	0	0.09
aat	N	0	0.55
aac	N	1	0.45
aaa	K	2192	1643.00
aag	K	694	1243.00
aga	R	1	0.93
agg	R	1	0.52
---	--	------	------
mPD		0.37	0.49
		nPD:	0.75
		N. weight:	0.11
		Sc. PD:	0.057
		Sc. rank:	195.8

,

NP			
Pos.	199	obs:	exp:
cct	P	0	0.55
ccc	P	0	0.36
cca	P	2	0.81
ccg	P	0	0.28
caa	Q	2	1.00
cag	Q	0	1.00
cgt	R	4	108.00
cgc	R	1	154.40
cga	R	2731	290.80
cgg	R	112	235.70
aga	R	37	1347.00
agg	R	1	749.80
---	--	------	------
mPD		0.11	0.96
		nPD:	0.11
		N. weight:	2.7
		Sc. PD:	-0.22
		Sc. rank:	-271.9

,

NP			
Pos.	200	obs:	exp:
cgt	R	0	0.11
cgc	R	0	0.16
cga	R	0	0.30
cgg	R	2	0.24
aga	R	0	1.40
agg	R	1	0.78
ggt	G	4	417.50
ggc	G	0	379.20
gga	G	754	1265.00
ggg	G	2129	825.30
---	--	------	------
mPD		0.39	0.69
		nPD:	0.57
		N. weight:	0.74
		Sc. PD:	0.26
		Sc. rank:	1169.8

NP

Pos. 201		obs:	exp:
att	I	1445	949.30
atc	I	1142	692.30
ata	I	0	945.40
gtt	V	17	65.17
gtc	V	284	63.75
gta	V	2	64.61
gtg	V	0	109.50
mPD		0.69	0.87
		nPD:	0.79
		N. weight:	0.72
		Sc. PD:	0.41
		Sc. rank:	1337.9

NP

Pos. 202		obs:	exp:
tct	S	0	0.33
tcc	S	0	0.29
tca	S	0	0.45
tcg	S	0	0.12
cat	H	0	0.58
cac	H	1	0.42
act	T	0	0.26
acc	T	1	0.23
aca	T	0	0.42
acg	T	0	0.09
aat	N	1095	1574.00
aac	N	1791	1312.00
agt	S	0	0.43
agc	S	2	0.37
mPD		0.47	0.50
		nPD:	0.95
		N. weight:	0.077
		Sc. PD:	0.056
		Sc. rank:	146.3

NP

Pos. 203		obs:	exp:
aat	N	0	1.09
aac	N	2	0.91
gat	D	1064	1559.00
gac	D	1824	1329.00
mPD		0.47	0.50
		nPD:	0.94
		N. weight:	0.081
		Sc. PD:	0.058
		Sc. rank:	152.4

NP

Pos. 204		obs:	exp:
cct	P	0	0.82
ccc	P	0	0.54
cca	P	0	1.22
ccg	P	3	0.42
cgt	R	117	108.00
cgc	R	6	154.50
cga	R	27	290.90
cgg	R	2722	235.70
aga	R	7	1348.00
agg	R	8	750.00
mPD		0.12	0.96
		nPD:	0.12
		N. weight:	3.
		Sc. PD:	-0.22
		Sc. rank:	-74.8

NP

Pos. 205		obs:	exp:
tct	S	0	0.17
tcc	S	0	0.14
tca	S	0	0.23
tcg	S	0	0.06
aat	N	2251	1575.00
aac	N	638	1314.00
agt	S	0	0.22
agc	S	1	0.18
mPD		0.35	0.50
		nPD:	0.69
		N. weight:	0.16
		Sc. PD:	0.077
		Sc. rank:	278.8

NP

Pos. 206		obs:	exp:
ttt	F	57	1205.00
ttc	F	2832	1684.00
tta	L	1	0.09
ttg	L	0	0.19
ctt	L	0	0.20
ctc	L	0	0.16
cta	L	0	0.15
ctg	L	0	0.21
mPD		0.039	0.49
		nPD:	0.08
		N. weight:	0.59
		Sc. PD:	-0.068
		Sc. rank:	-238.1

NP

Pos. 207		obs:	exp:
tgg	W	2890	2890.00
mPD		0	0
		nPD:	1.
		N. weight:	0.
		Sc. PD:	0
		Sc. rank:	0

NP

Pos. 208		obs:	exp:
cgt	R	0	108.20
cgc	R	0	154.70
cga	R	0	291.20
cgg	R	0	236.00
aga	R	2853	1349.00
agg	R	37	750.80
mPD		0.025	0.95
		nPD:	0.03
		N. weight:	0.92
		Sc. PD:	-0.15
		Sc. rank:	-940.9

NP

Pos. 209		obs:	exp:
ggt	G	287	417.90
ggc	G	2596	379.60
gga	G	7	1266.00
ggg	G	0	826.10
mPD		0.18	0.69
		nPD:	0.27
		N. weight:	2.2
		Sc. PD:	0.15
		Sc. rank:	1693.8

NP

Pos. 210		obs:	exp:
caa	Q	1	0.50
cag	Q	0	0.50
gaa	E	2235	1620.00
gag	E	654	1269.00
mPD		0.35	0.49
		nPD:	0.71
		N. weight:	0.13
		Sc. PD:	0.066
		Sc. rank:	237.1

NP

Pos. 211		obs:	exp:
aat	N	2149	1575.00
aac	N	740	1314.00
gat	D	1	0.54
gac	D	0	0.46
mPD		0.38	0.50
		nPD:	0.77
		N. weight:	0.11
		Sc. PD:	0.063
		Sc. rank:	208.6

NP

Pos. 212		obs:	exp:
gaa	E	1	0.56
gag	E	0	0.44
ggt	G	39	417.80
ggc	G	0	379.50
gga	G	2562	1266.00
ggg	G	288	825.80
mPD		0.20	0.69
		nPD:	0.3
		N. weight:	0.65
		Sc. PD:	0.062
		Sc. rank:	569.6

NP

Pos. 213		obs:	exp:
cgt	R	0	108.10
cgc	R	0	154.60
cga	R	641	291.10
cgg	R	322	235.90
aaa	K	1	0.57
aag	K	0	0.43
aga	R	1795	1349.00
agg	R	131	750.50
mPD		0.71	0.95
		nPD:	0.74
		N. weight:	0.41
		Sc. PD:	0.22
		Sc. rank:	750.3

NP

Pos. 214		obs:	exp:
cgt	R	0	107.90
cgc	R	0	154.20
cga	R	2	290.40
cgg	R	0	235.30
aaa	K	8	4.56
aag	K	0	3.44
aga	R	2226	1345.00
agg	R	654	748.70
mPD		0.36	0.96
		nPD:	0.37
		N. weight:	0.47
		Sc. PD:	0.08
		Sc. rank:	548.6

NP

Pos. 215		obs:	exp:
act	T	154	757.10
acc	T	143	650.70
aca	T	2572	1208.00
acg	T	19	271.70
gct	A	0	0.50
gcc	A	0	0.39
gca	A	2	0.89
gcg	A	0	0.22
mPD		0.20	0.70
		nPD:	0.29
		N. weight:	0.66
		Sc. PD:	0.059
		Sc. rank:	569.5

NP

Pos. 216		obs:	exp:
tgg	W	1	1.00
cgt	R	0	107.90
cgc	R	0	154.20
cga	R	0	290.40
cgg	R	1	235.30
aaa	K	0	3.42
aag	K	6	2.58
aga	R	73	1345.00
agg	R	2808	748.70
ggt	G	0	0.14
ggc	G	0	0.13
gga	G	0	0.44
ggg	G	1	0.29
mPD		0.055	0.96
		nPD:	0.06
		N. weight:	1.6
		Sc. PD:	-0.22
		Sc. rank:	-870.8

NP

Pos. 217		obs:	exp:
att	I	2788	1034.00
atc	I	30	754.10
ata	I	0	1030.00
atg	M	5	5.00
act	T	40	10.49
acc	T	0	9.01
aca	T	0	16.74
acg	T	0	3.76
gtt	V	27	5.81
gtc	V	0	5.68
gta	V	0	5.76
gtg	V	0	9.76
mPD		0.070	0.71
		nPD:	0.1
		N. weight:	1.3
		Sc. PD:	-0.12
		Sc. rank:	-322.6

NP

Pos. 218		obs:	exp:
gct	A	35	728.00
gcc	A	233	559.20
gca	A	2606	1286.00
gcg	A	16	316.30
mPD		0.18	0.69
		nPD:	0.26
		N. weight:	0.68
		Sc. PD:	0.042
		Sc. rank:	502.8

NP

Pos. 219		obs:	exp:
tat	Y	2641	1386.00
tac	Y	249	1504.00
mPD		0.16	0.50
		nPD:	0.32
		N. weight:	0.57
		Sc. PD:	0.066
		Sc. rank:	570.7

NP

Pos. 220		obs:	exp:
gat	D	2	1.08
gac	D	0	0.92
gaa	E	41	1619.00
gag	E	2847	1269.00
mPD		0.029	0.49
		nPD:	0.06
		N. weight:	0.98
		Sc. PD:	-0.13
		Sc. rank:	-513.8

NP

Pos. 221		obs:	exp:
cgt	R	0	108.20
cgc	R	0	154.70
cga	R	0	291.20
cgg	R	0	236.00
aga	R	2720	1349.00
agg	R	170	750.80
mPD		0.11	0.95
		nPD:	0.12
		N. weight:	0.76
		Sc. PD:	-0.06
		Sc. rank:	-58.3

NP

Pos. 222		obs:	exp:
att	I	0	0.73
atc	I	0	0.54
ata	I	2	0.73
atg	M	2888	2888.00
mPD		0.0014	0.0014
		nPD:	1.
		N. weight:	0.00092
		Sc. PD:	0.00072
		Sc. rank:	1.7

NP

Pos. 223		obs:	exp:
tgt	C	39	1119.00
tgc	C	2851	1771.00
mPD		0.027	0.47
		nPD:	0.06
		N. weight:	0.56
		Sc. PD:	-0.077
		Sc. rank:	-332.8

NP

Pos. 224		obs:	exp:
aat	N	69	1576.00
aac	N	2821	1314.00
mPD		0.047	0.50
		nPD:	0.09
		N. weight:	0.89
		Sc. PD:	-0.089
		Sc. rank:	-255.0

NP

Pos. 225		obs:	exp:
att	I	106	1061.00
atc	I	2784	773.40
ata	I	0	1056.00
mPD		0.071	0.66
		nPD:	0.11
		N. weight:	1.5
		Sc. PD:	-0.13
		Sc. rank:	-254.0

NP

Pos.	226	obs:	exp:
tta	L	0	267.20
ttg	L	0	549.00
ctt	L	8	573.80
ctc	L	2880	462.30
cta	L	2	427.70
ctg	L	0	610.00
---	--	------	------
mPD		0.0069	1.1
		nPD:	0.01
		N. weight:	2.4
		Sc. PD:	-0.44
		Sc. rank:	-3815.5

,

NP

Pos.	227	obs:	exp:
cgt	R	0	0.04
cgc	R	0	0.05
cga	R	0	0.10
cgg	R	0	0.08
att	I	0	0.73
atc	I	0	0.54
ata	I	2	0.73
aaa	K	2500	1643.00
aag	K	386	1243.00
aga	R	1	0.47
agg	R	0	0.26
gaa	E	1	0.56
gag	E	0	0.44
---	--	------	------
mPD		0.23	0.49
		nPD:	0.47
		N. weight:	0.28
		Sc. PD:	0.074
		Sc. rank:	392.0

,

NP

Pos.	228	obs:	exp:
tgg	W	2	2.00
ggt	G	0	417.60
ggc	G	0	379.30
gga	G	200	1265.00
ggg	G	2688	825.50
---	--	------	------
mPD		0.13	0.69
		nPD:	0.19
		N. weight:	1.3
		Sc. PD:	-0.011
		Sc. rank:	516.8

,

NP

Pos.	229	obs:	exp:
act	T	0	0.26
acc	T	0	0.23
aca	T	1	0.42
acg	T	0	0.09
aaa	K	2689	1645.00
aag	K	200	1244.00
---	--	------	------
mPD		0.13	0.49
		nPD:	0.26
		N. weight:	0.44
		Sc. PD:	0.028
		Sc. rank:	327.8

,

NP

Pos.	230	obs:	exp:
ttt	F	98	1205.00
ttc	F	2791	1684.00
att	I	0	0.37
atc	I	1	0.27
ata	I	0	0.37
---	--	------	------
mPD		0.066	0.49
		nPD:	0.14
		N. weight:	0.53
		Sc. PD:	-0.032
		Sc. rank:	18.7

,

NP

Pos.	231	obs:	exp:
tta	L	0	0.18
ttg	L	0	0.38
ctt	L	0	0.40
ctc	L	2	0.32
cta	L	0	0.30
ctg	L	0	0.42
caa	Q	2875	1442.00
cag	Q	13	1446.00
---	--	------	------
mPD		0.012	0.50
		nPD:	0.02
		N. weight:	0.88
		Sc. PD:	-0.15
		Sc. rank:	-920.4

,

NP

Pos.	232	obs:	exp:
att	I	0	0.37
atc	I	0	0.27
ata	I	1	0.37
act	T	4	757.10
acc	T	11	650.70
aca	T	2862	1208.00
acg	T	11	271.70
gct	A	0	0.25
gcc	A	0	0.19
gca	A	1	0.45
gcg	A	0	0.11
---	--	------	------
mPD		0.019	0.70
		nPD:	0.03
		N. weight:	1.1
		Sc. PD:	-0.18
		Sc. rank:	-1046.7

,

NP

Pos.	233	obs:	exp:
act	T	1	0.26
acc	T	0	0.23
aca	T	0	0.42
acg	T	0	0.09
gct	A	908	727.80
gcc	A	2	559.00
gca	A	1951	1286.00
gcg	A	28	316.20
---	--	------	------
mPD		0.45	0.69
		nPD:	0.65
		N. weight:	0.43
		Sc. PD:	0.19
		Sc. rank:	730.7

,

NP

Pos.	234	obs:	exp:
tct	S	0	29.21
tcc	S	0	25.35
tca	S	174	39.67
tcg	S	1	10.56
agt	S	0	37.96
agc	S	0	32.25
gtt	V	0	0.22
gtc	V	0	0.21
gta	V	0	0.21
gtg	V	1	0.36
gct	A	2	683.70
gcc	A	4	525.20
gca	A	2289	1208.00
gcg	A	419	297.10
---	--	------	------
mPD		0.37	0.86
		nPD:	0.43
		N. weight:	0.84
		Sc. PD:	0.19
		Sc. rank:	1125.4

,

NP

Pos.	235	obs:	exp:
caa	Q	2867	1443.00
cag	Q	23	1447.00
---	--	------	------
mPD		0.016	0.50
		nPD:	0.03
		N. weight:	0.85
		Sc. PD:	-0.14
		Sc. rank:	-798.8

,

NP

Pos.	236	obs:	exp:
cgt	R	0	108.10
cgc	R	0	154.60
cga	R	224	291.10
cgg	R	25	235.90
aaa	K	1	0.57
aag	K	0	0.43
aga	R	2605	1349.00
agg	R	35	750.50
---	--	------	------
mPD		0.20	0.95
		nPD:	0.21
		N. weight:	0.68
		Sc. PD:	0.0069
		Sc. rank:	355.2

,

NP

Pos.	237	obs:	exp:
gct	A	1	728.00
gcc	A	0	559.20
gca	A	2886	1286.00
gcg	A	3	316.30
---	--	------	------
mPD		0.0028	0.69
		nPD:	0.
		N. weight:	1.1
		Sc. PD:	-0.2
		Sc. rank:	-1807.3

,

NP

Pos.	238	obs:	exp:
atg	M	2890	2890.00
---	--	------	------
mPD		0	0
		nPD:	1.
		N. weight:	0.
		Sc. PD:	0
		Sc. rank:	0

,

NP

Pos.	239	obs:	exp:
cgt	R	0	0.04
cgc	R	0	0.05
cga	R	0	0.10
cgg	R	0	0.08
atg	M	2867	2867.00
aga	R	0	0.47
agg	R	1	0.26
gtt	V	0	4.73
gtc	V	0	4.63
gta	V	0	4.69
gtg	V	22	7.95
---	--	------	------
mPD		0.016	0.026
		nPD:	0.6
		N. weight:	0.011
		Sc. PD:	0.0043
		Sc. rank:	17.8

,

NP

Pos.	240	obs:	exp:
att	I	1	0.37
atc	I	0	0.27
ata	I	0	0.37
gat	D	2010	1560.00
gac	D	879	1329.00
---	--	------	------
mPD		0.42	0.50
		nPD:	0.85
		N. weight:	0.069
		Sc. PD:	0.044
		Sc. rank:	129.6

,

NP

Pos.	241	obs:	exp:
tta	L	0	0.09
ttg	L	0	0.19
ctt	L	0	0.20
ctc	L	0	0.16
cta	L	1	0.15
ctg	L	0	0.21
caa	Q	1962	1442.00
cag	Q	927	1447.00
---	--	------	------
mPD		0.44	0.50
		nPD:	0.87
		N. weight:	0.09
		Sc. PD:	0.059
		Sc. rank:	169.0

,

NP

Pos.	242	obs:	exp:
gtt	V	0	621.60
gtc	V	1	608.00
gta	V	17	616.20
gtg	V	2872	1044.00
---	--	------	------
mPD		0.012	0.73
		nPD:	0.02
		N. weight:	1.3
		Sc. PD:	-0.23
		Sc. rank:	-1559.7

,

NP

Pos.	243	obs:	exp:
cgt	R	621	108.20
cgc	R	1	154.70
cga	R	2015	291.20
cgg	R	65	236.00
aga	R	44	1349.00
agg	R	144	750.80
---	--	------	------
mPD		0.56	0.95
		nPD:	0.59
		N. weight:	2.1
		Sc. PD:	0.79
		Sc. rank:	3341.4

,

NP

Pos.	244	obs:	exp:
gaa	E	1210	1620.00
gag	E	1679	1269.00
ggt	G	0	0.14
ggc	G	0	0.13
gga	G	0	0.44
ggg	G	1	0.29
---	--	------	------
mPD		0.49	0.49
		nPD:	0.99
		N. weight:	0.056
		Sc. PD:	0.043
		Sc. rank:	105.4

,

NP

Pos.	245	obs:	exp:
tct	S	0	482.20
tcc	S	0	418.50
tca	S	0	654.90
tcg	S	0	174.30
agt	S	41	626.60
agc	S	2848	532.40
ggt	G	0	0.14
ggc	G	1	0.13
gga	G	0	0.44
ggg	G	0	0.29
---	--	------	------
mPD		0.029	1.7
		nPD:	0.02
		N. weight:	2.1
		Sc. PD:	-0.37
		Sc. rank:	-2491.5

,

NP			
Pos.	246	obs:	exp:
cgt	R	0	108.10
cgc	R	0	154.60
cga	R	99	291.00
cgg	R	87	235.80
act	T	0	0.26
acc	T	0	0.23
aca	T	1	0.42
acg	T	0	0.09
aaa	K	1	0.57
aag	K	0	0.43
aga	R	1959	1348.00
agg	R	743	750.30
---	--	------	------
mPD		0.53	0.96
		nPD:	0.56
		N. weight:	0.25
		Sc. PD:	0.086
		Sc. rank:	389.4

,

NP			
Pos.	247	obs:	exp:
tct	S	0	2.34
tcc	S	0	2.03
tca	S	0	3.17
tcg	S	0	0.84
act	T	15	3.93
acc	T	0	3.38
aca	T	0	6.28
acg	T	0	1.41
aat	N	2730	1560.00
aac	N	131	1301.00
agt	S	13	3.04
agc	S	1	2.58
---	--	------	------
mPD		0.11	0.53
		nPD:	0.2
		N. weight:	0.58
		Sc. PD:	0.0033
		Sc. rank:	289.5

,

NP			
Pos.	248	obs:	exp:
cct	P	2802	789.80
ccc	P	85	522.00
cca	P	0	1173.00
ccg	P	3	405.20
---	--	------	------
mPD		0.059	0.71
		nPD:	0.08
		N. weight:	1.5
		Sc. PD:	-0.17
		Sc. rank:	-581.1

,

NP			
Pos.	249	obs:	exp:
ggt	G	3	417.90
ggc	G	627	379.60
gga	G	108	1266.00
ggg	G	2152	826.10
---	--	------	------
mPD		0.40	0.69
		nPD:	0.58
		N. weight:	0.96
		Sc. PD:	0.35
		Sc. rank:	1539.7

,

NP			
Pos.	250	obs:	exp:
tct	S	0	0.17
tcc	S	0	0.14
tca	S	0	0.23
tcg	S	0	0.06
aat	N	2869	1575.00
aac	N	20	1314.00
agt	S	1	0.22
agc	S	0	0.18
---	--	------	------
mPD		0.014	0.50
		nPD:	0.03
		N. weight:	0.75
		Sc. PD:	-0.12
		Sc. rank:	-703.2

,

NP			
Pos.	251	obs:	exp:
gtt	V	1	0.22
gtc	V	0	0.21
gta	V	0	0.21
gtg	V	0	0.36
gct	A	2880	727.80
gcc	A	8	559.00
gca	A	1	1286.00
gcg	A	0	316.20
---	--	------	------
mPD		0.0069	0.69
		nPD:	0.01
		N. weight:	1.8
		Sc. PD:	-0.33
		Sc. rank:	-2502.5

,

NP			
Pos.	252	obs:	exp:
gtt	V	0	0.43
gtc	V	0	0.42
gta	V	2	0.43
gtg	V	0	0.72
gaa	E	2749	1619.00
gag	E	139	1269.00
---	--	------	------
mPD		0.093	0.49
		nPD:	0.19
		N. weight:	0.53
		Sc. PD:	-0.005
		Sc. rank:	207.4

,

NP			
Pos.	253	obs:	exp:
att	I	2879	1059.00
atc	I	7	772.60
ata	I	1	1055.00
gtt	V	2	0.65
gtc	V	1	0.63
gta	V	0	0.64
gtg	V	0	1.08
---	--	------	------
mPD		0.0083	0.66
		nPD:	0.01
		N. weight:	1.3
		Sc. PD:	-0.23
		Sc. rank:	-1719.0

,

NP			
Pos.	254	obs:	exp:
gaa	E	2838	1620.00
gag	E	52	1270.00
---	--	------	------
mPD		0.035	0.49
		nPD:	0.07
		N. weight:	0.65
		Sc. PD:	-0.08
		Sc. rank:	-292.9

,

NP			
Pos.	255	obs:	exp:
gat	D	2754	1560.00
gac	D	136	1330.00
---	--	------	------
mPD		0.090	0.50
		nPD:	0.18
		N. weight:	0.57
		Sc. PD:	-0.0096
		Sc. rank:	213.6

,

NP			
Pos.	256	obs:	exp:
tta	L	0	267.00
ttg	L	0	548.60
ctt	L	73	573.40
ctc	L	2703	462.00
cta	L	112	427.40
ctg	L	0	609.60
cct	P	0	0.27
ccc	P	1	0.18
cca	P	0	0.41
ccg	P	0	0.14
att	I	0	0.37
atc	I	1	0.27
ata	I	0	0.37
---	--	------	------
mPD		0.12	1.1
		nPD:	0.11
		N. weight:	2.
		Sc. PD:	-0.18
		Sc. rank:	-301.2

,

NP			
Pos.	257	obs:	exp:
tta	L	0	0.46
ttg	L	0	0.95
ctt	L	5	0.99
ctc	L	0	0.80
cta	L	0	0.74
ctg	L	0	1.06
att	I	1809	1058.00
atc	I	1070	771.20
ata	I	3	1053.00
act	T	0	0.52
acc	T	2	0.45
aca	T	0	0.84
acg	T	0	0.19
gtt	V	0	0.22
gtc	V	1	0.21
gta	V	0	0.21
gtg	V	0	0.36
---	--	------	------
mPD		0.47	0.67
		nPD:	0.71
		N. weight:	0.6
		Sc. PD:	0.3
		Sc. rank:	1077.1

,

NP			
Pos.	258	obs:	exp:
ttt	F	2529	1205.00
ttc	F	360	1684.00
tta	L	0	0.09
ttg	L	0	0.19
ctt	L	1	0.20
ctc	L	0	0.16
cta	L	0	0.15
ctg	L	0	0.21
---	--	------	------
mPD		0.22	0.49
		nPD:	0.45
		N. weight:	0.6
		Sc. PD:	0.15
		Sc. rank:	844.1

,

NP			
Pos.	259	obs:	exp:
tta	L	0	267.20
ttg	L	9	549.00
ctt	L	17	573.80
ctc	L	157	462.30
cta	L	109	427.70
ctg	L	2598	610.00
---	--	------	------
mPD		0.19	1.1
		nPD:	0.17
		N. weight:	1.5
		Sc. PD:	-0.045
		Sc. rank:	376.7

,

NP			
Pos.	260	obs:	exp:
cct	P	0	0.27
ccc	P	0	0.18
cca	P	0	0.41
ccg	P	1	0.14
act	T	0	0.52
acc	T	0	0.45
aca	T	2	0.84
acg	T	0	0.19
gct	A	2	727.30
gcc	A	0	558.70
gca	A	2842	1285.00
gcg	A	43	316.00
---	--	------	------
mPD		0.033	0.69
		nPD:	0.05
		N. weight:	0.99
		Sc. PD:	-0.14
		Sc. rank:	-672.2

,

NP			
Pos.	261	obs:	exp:
cgt	R	1	108.20
cgc	R	0	154.70
cga	R	237	291.20
cgg	R	2633	236.00
aga	R	0	1349.00
agg	R	19	750.80
---	--	------	------
mPD		0.16	0.95
		nPD:	0.17
		N. weight:	2.8
		Sc. PD:	-0.071
		Sc. rank:	950.4

,

NP			
Pos.	262	obs:	exp:
tct	S	2867	482.40
tcc	S	9	418.60
tca	S	13	655.20
tcg	S	1	174.40
agt	S	0	626.90
agc	S	0	532.60
---	--	------	------
mPD		0.016	1.7
		nPD:	0.01
		N. weight:	2.3
		Sc. PD:	-0.42
		Sc. rank:	-3247.6

,

NP			
Pos.	263	obs:	exp:
tct	S	0	0.17
tcc	S	0	0.14
tca	S	1	0.23
tcg	S	0	0.06
agt	S	0	0.22
agc	S	0	0.18
gct	A	187	727.80
gcc	A	0	559.00
gca	A	2431	1286.00
gcg	A	271	316.20
---	--	------	------
mPD		0.28	0.69
		nPD:	0.41
		N. weight:	0.57
		Sc. PD:	0.12
		Sc. rank:	740.3

,

NP			
Pos.	264	obs:	exp:
tta	L	0	267.20
ttg	L	0	549.00
ctt	L	111	573.80
ctc	L	2777	462.30
cta	L	2	427.70
ctg	L	0	610.00
---	--	------	------
mPD		0.075	1.1
		nPD:	0.07
		N. weight:	2.2
		Sc. PD:	-0.28
		Sc. rank:	-1060.1

,

NP			
Pos.	265	obs:	exp:
att	I	87	1055.00
atc	I	2784	769.60
ata	I	5	1051.00
gtt	V	0	3.01
gtc	V	14	2.95
gta	V	0	2.99
gtg	V	0	5.06
---	--	------	------
mPD		0.071	0.67
		nPD:	0.11
		N. weight:	1.5
		Sc. PD:	-0.14
		Sc. rank:	-274.5

,

NP			
Pos.	266	obs:	exp:
tta	L	1	266.80
ttg	L	79	548.30
ctt	L	5	573.00
ctc	L	0	461.60
cta	L	117	427.10
ctg	L	2684	609.10
cct	P	0	0.27
ccc	P	0	0.18
cca	P	0	0.41
ccg	P	1	0.14
atg	M	1	1.00
gtt	V	0	0.43
gtc	V	0	0.42
gta	V	0	0.43
gtg	V	2	0.72
---	--	------	------
mPD		0.14	1.1
		nPD:	0.12
		N. weight:	1.7
		Sc. PD:	-0.12
		Sc. rank:	1.5

,

NP			
Pos.	267	obs:	exp:
cgt	R	0	108.20
cgc	R	0	154.70
cga	R	0	291.20
cgg	R	0	236.00
aga	R	2682	1349.00
agg	R	208	750.80
---	--	------	------
mPD		0.13	0.95
		nPD:	0.14
		N. weight:	0.72
		Sc. PD:	-0.041
		Sc. rank:	37.6

,

NP			
Pos.	268	obs:	exp:
cgt	R	0	0.04
cgc	R	0	0.05
cga	R	0	0.10
cgg	R	0	0.08
aga	R	1	0.47
agg	R	0	0.26
ggt	G	0	417.80
ggc	G	1	379.50
gga	G	2662	1266.00
ggg	G	226	825.80
---	--	------	------
mPD		0.15	0.69
		nPD:	0.21
		N. weight:	0.77
		Sc. PD:	0.01
		Sc. rank:	417.5

,

NP			
Pos.	269	obs:	exp:
tct	S	2	482.40
tcc	S	0	418.60
tca	S	2869	655.20
tcg	S	19	174.40
agt	S	0	626.90
agc	S	0	532.60
---	--	------	------
mPD		0.014	1.7
		nPD:	0.01
		N. weight:	1.9
		Sc. PD:	-0.35
		Sc. rank:	-2803.3

,

NP			
Pos.	270	obs:	exp:
att	I	0	13.58
atc	I	0	9.90
ata	I	37	13.52
atg	M	1	1.00
gtt	V	1	613.40
gtc	V	0	600.00
gta	V	378	608.10
gtg	V	2473	1030.00
---	--	------	------
mPD		0.27	0.76
		nPD:	0.36
		N. weight:	0.92
		Sc. PD:	0.14
		Sc. rank:	1066.5

,

NP			
Pos.	271	obs:	exp:
gct	A	811	727.50
gcc	A	2071	558.90
gca	A	6	1286.00
gcg	A	0	316.10
ggt	G	0	0.29
ggc	G	2	0.26
gga	G	0	0.88
ggg	G	0	0.57
---	--	------	------
mPD		0.41	0.69
		nPD:	0.59
		N. weight:	1.3
		Sc. PD:	0.48
		Sc. rank:	2066.4

,

NP			
Pos.	272	obs:	exp:
tta	L	0	0.18
ttg	L	0	0.38
ctt	L	2	0.40
ctc	L	0	0.32
cta	L	0	0.30
ctg	L	0	0.42
cat	H	2649	1672.00
cac	H	239	1216.00
---	--	------	------
mPD		0.15	0.49
		nPD:	0.31
		N. weight:	0.38
		Sc. PD:	0.043
		Sc. rank:	376.1

,

NP			
Pos.	273	obs:	exp:
aaa	K	47	1646.00
aag	K	2843	1244.00
---	--	------	------
mPD		0.032	0.49
		nPD:	0.07
		N. weight:	1.
		Sc. PD:	-0.13
		Sc. rank:	-506.6

,

NP			
Pos.	274	obs:	exp:
tct	S	236	482.40
tcc	S	2653	418.60
tca	S	1	655.20
tcg	S	0	174.40
agt	S	0	626.90
agc	S	0	532.60
---	--	------	------
mPD		0.15	1.7
		nPD:	0.09
		N. weight:	2.2
		Sc. PD:	-0.22
		Sc. rank:	-678.0

,

NP			
Pos.	275	obs:	exp:
tgt	C	168	1118.00
tgc	C	2720	1770.00
ggt	G	0	0.29
ggc	G	2	0.26
gga	G	0	0.88
ggg	G	0	0.57
---	--	------	------
mPD		0.11	0.48
		nPD:	0.23
		N. weight:	0.39
		Sc. PD:	0.013
		Sc. rank:	262.3

,

NP			
Pos.	276	obs:	exp:
ttt	F	2	0.83
ttc	F	0	1.17
tta	L	19	266.80
ttg	L	2405	548.30
ctt	L	223	573.00
ctc	L	8	461.60
cta	L	1	427.10
ctg	L	230	609.10
gtt	V	0	0.43
gtc	V	0	0.42
gta	V	0	0.43
gtg	V	2	0.72
---	--	------	------
mPD		0.43	1.1
		nPD:	0.38
		N. weight:	1.4
		Sc. PD:	0.25
		Sc. rank:	1698.1

,

NP			
Pos.	277	obs:	exp:
tta	L	0	0.09
ttg	L	0	0.19
ctt	L	1	0.20
ctc	L	0	0.16
cta	L	0	0.15
ctg	L	0	0.21
cct	P	2786	789.30
ccc	P	25	521.60
cca	P	67	1172.00
ccg	P	10	404.90
cgt	R	1	0.04
cgc	R	0	0.05
cga	R	0	0.10
cgg	R	0	0.08
aga	R	0	0.47
agg	R	0	0.26
---	--	------	------
mPD		0.070	0.71
		nPD:	0.1
		N. weight:	1.5
		Sc. PD:	-0.14
		Sc. rank:	-359.3

,

NP			
Pos.	278	obs:	exp:
gct	A	2681	728.00
gcc	A	152	559.20
gca	A	53	1286.00
gcg	A	4	316.30
---	--	------	------
mPD		0.14	0.69
		nPD:	0.2
		N. weight:	1.4
		Sc. PD:	0.000015
		Sc. rank:	676.4

,

NP			
Pos.	279	obs:	exp:
ttt	F	3	1.25
ttc	F	0	1.75
tgt	C	2672	1117.00
tgc	C	215	1770.00
---	--	------	------
mPD		0.14	0.48
		nPD:	0.29
		N. weight:	0.86
		Sc. PD:	0.08
		Sc. rank:	749.5

,

NP			
Pos.	280	obs:	exp:
att	I	0	0.37
atc	I	0	0.27
ata	I	1	0.37
gtt	V	7	620.90
gtc	V	0	607.40
gta	V	569	615.60
gtg	V	2311	1043.00
gct	A	0	0.25
gcc	A	0	0.19
gca	A	0	0.45
gcg	A	1	0.11
ggt	G	0	0.14
ggc	G	0	0.13
gga	G	0	0.44
ggg	G	1	0.29
---	--	------	------
mPD		0.32	0.74
		nPD:	0.44
		N. weight:	0.81
		Sc. PD:	0.19
		Sc. rank:	1092.0

,

NP			
Pos.	281	obs:	exp:
ttt	F	0	0.42
ttc	F	1	0.58
tat	Y	265	1385.00
tac	Y	2623	1503.00
tgt	C	0	0.39
tgc	C	1	0.61
---	--	------	------
mPD		0.17	0.50
		nPD:	0.34
		N. weight:	0.47
		Sc. PD:	0.063
		Sc. rank:	523.1

,

NP			
Pos.	282	obs:	exp:
ggt	G	0	417.90
ggc	G	0	379.60
gga	G	2628	1266.00
ggg	G	262	826.10
---	--	------	------
mPD		0.16	0.69
		nPD:	0.24
		N. weight:	0.74
		Sc. PD:	0.03
		Sc. rank:	515.0

,

NP			
Pos.	283	obs:	exp:
tta	L	0	267.10
ttg	L	0	548.80
ctt	L	2258	573.60
ctc	L	619	462.10
cta	L	9	427.60
ctg	L	3	609.80
cct	P	1	0.27
ccc	P	0	0.18
cca	P	0	0.41
ccg	P	0	0.14
---	--	------	------
mPD		0.34	1.1
		nPD:	0.31
		N. weight:	1.5
		Sc. PD:	0.16
		Sc. rank:	1409.5

,

NP			
Pos.	284	obs:	exp:
gct	A	1316	728.00
gcc	A	15	559.20
gca	A	1551	1286.00
gcg	A	8	316.30
---	--	------	------
mPD		0.50	0.69
		nPD:	0.73
		N. weight:	0.45
		Sc. PD:	0.24
		Sc. rank:	820.2

,

NP			
Pos.	285	obs:	exp:
att	I	0	0.37
atc	I	0	0.27
ata	I	1	0.37
gtt	V	8	621.20
gtc	V	0	607.60
gta	V	348	615.80
gtg	V	2532	1043.00
gct	A	0	0.25
gcc	A	0	0.19
gca	A	0	0.45
gcg	A	1	0.11
---	--	------	------
mPD		0.22	0.73
		nPD:	0.3
		N. weight:	0.92
		Sc. PD:	0.089
		Sc. rank:	818.2

,

NP			
Pos.	286	obs:	exp:
gtt	V	0	15.06
gtc	V	69	14.73
gta	V	1	14.93
gtg	V	0	25.29
gct	A	3	710.40
gcc	A	2785	545.70
gca	A	32	1255.00
gcg	A	0	308.70
---	--	------	------
mPD		0.072	0.74
		nPD:	0.1
		N. weight:	2.1
		Sc. PD:	-0.2
		Sc. rank:	-546.2

,

NP			
Pos.	287	obs:	exp:
tct	S	0	468.00
tcc	S	0	406.20
tca	S	0	635.70
tcg	S	0	169.20
aat	N	4	2.18
aac	N	0	1.82
agt	S	2803	608.20
agc	S	1	516.70
ggt	G	82	11.86
ggc	G	0	10.77
gga	G	0	35.93
ggg	G	0	23.44
---	--	------	------
mPD		0.059	1.7
		nPD:	0.03
		N. weight:	2.
		Sc. PD:	-0.32
		Sc. rank:	-1717.3

,

NP			
Pos.	288	obs:	exp:
gtt	V	0	0.43
gtc	V	0	0.42
gta	V	2	0.43
gtg	V	0	0.72
ggt	G	33	417.60
ggc	G	0	379.30
gga	G	2279	1265.00
ggg	G	576	825.50
---	--	------	------
mPD		0.34	0.69
		nPD:	0.49
		N. weight:	0.48
		Sc. PD:	0.14
		Sc. rank:	712.3

,

NP			
Pos.	289	obs:	exp:
tat	Y	2223	1385.00
tac	Y	665	1503.00
cat	H	2	1.16
cac	H	0	0.84
---	--	------	------
mPD		0.36	0.50
		nPD:	0.71
		N. weight:	0.23
		Sc. PD:	0.12
		Sc. rank:	418.1

,

NP			
Pos.	290	obs:	exp:
aat	N	0	3.27
aac	N	6	2.73
gat	D	86	1557.00
gac	D	2797	1326.00
ggt	G	0	0.14
ggc	G	1	0.13
gga	G	0	0.44
ggg	G	0	0.29
---	--	------	------
mPD		0.063	0.50
		nPD:	0.12
		N. weight:	0.84
		Sc. PD:	-0.06
		Sc. rank:	19.1

,

NP			
Pos.	291	obs:	exp:
ttt	F	2233	1206.00
ttc	F	657	1684.00
---	--	------	------
mPD		0.35	0.49
		nPD:	0.72
		N. weight:	0.35
		Sc. PD:	0.18
		Sc. rank:	625.0

,

NP			
Pos.	292	obs:	exp:
aaa	K	0	0.57
aag	K	1	0.43
gaa	E	245	1620.00
gag	E	2644	1269.00
---	--	------	------
mPD		0.16	0.49
		nPD:	0.32
		N. weight:	0.68
		Sc. PD:	0.078
		Sc. rank:	675.4

,

NP			
Pos.	293	obs:	exp:
cgt	R	0	107.30
cgc	R	0	153.40
cga	R	1	288.90
cgg	R	0	234.10
aaa	K	23	13.10
aag	K	0	9.90
aga	R	2811	1338.00
agg	R	55	744.80
---	--	------	------
mPD		0.054	0.97
		nPD:	0.06
		N. weight:	0.89
		Sc. PD:	-0.12
		Sc. rank:	-542.5

,

NP			
Pos.	294	obs:	exp:
gaa	E	2811	1619.00
gag	E	77	1269.00
ggt	G	0	0.29
ggc	G	0	0.26
gga	G	2	0.88
ggg	G	0	0.57
---	--	------	------
mPD		0.053	0.49
		nPD:	0.11
		N. weight:	0.61
		Sc. PD:	-0.054
		Sc. rank:	-99.5

,

NP			
Pos.	295	obs:	exp:
tgg	W	2	2.00
ggt	G	2	417.60
ggc	G	1	379.30
gga	G	1449	1265.00
ggg	G	1436	825.50
---	--	------	------
mPD		0.50	0.69
		nPD:	0.73
		N. weight:	0.45
		Sc. PD:	0.23
		Sc. rank:	808.0

,

NP			
Pos.	296	obs:	exp:
tat	Y	26	1385.00
tac	Y	2862	1503.00
cat	H	2	1.16
cac	H	0	0.84
---	--	------	------
mPD		0.021	0.50
		nPD:	0.04
		N. weight:	0.79
		Sc. PD:	-0.12
		Sc. rank:	-595.8

,

NP			
Pos.	297	obs:	exp:
tct	S	2620	482.40
tcc	S	270	418.60
tca	S	0	655.20
tcg	S	0	174.40
agt	S	0	626.90
agc	S	0	532.60
---	--	------	------
mPD		0.17	1.7
		nPD:	0.1
		N. weight:	2.
		Sc. PD:	-0.18
		Sc. rank:	-416.4

,

NP			
Pos.	298	obs:	exp:
tta	L	11	267.00
ttg	L	6	548.60
ctt	L	3	573.40
ctc	L	0	462.00
cta	L	950	427.40
ctg	L	1918	609.60
cct	P	0	0.55
ccc	P	0	0.36
cca	P	0	0.81
ccg	P	2	0.28
---	--	------	------
mPD		0.46	1.1
		nPD:	0.41
		N. weight:	1.3
		Sc. PD:	0.27
		Sc. rank:	1711.9

,

NP			
Pos.	299	obs:	exp:
att	I	0	0.37
atc	I	1	0.27
ata	I	0	0.37
gtt	V	2669	621.40
gtc	V	201	607.80
gta	V	14	616.00
gtg	V	5	1044.00
---	--	------	------
mPD		0.14	0.73
		nPD:	0.19
		N. weight:	1.6
		Sc. PD:	-0.0051
		Sc. rank:	714.2

,

NP			
Pos.	300	obs:	exp:
ggt	G	1	417.90
ggc	G	1	379.60
gga	G	2458	1266.00
ggg	G	430	826.10
---	--	------	------
mPD		0.25	0.69
		nPD:	0.37
		N. weight:	0.61
		Sc. PD:	0.1
		Sc. rank:	711.8

,

NP			
Pos.	301	obs:	exp:
tta	L	0	0.09
ttg	L	0	0.19
ctt	L	0	0.20
ctc	L	0	0.16
cta	L	1	0.15
ctg	L	0	0.21
att	I	194	1051.00
atc	I	4	766.70
ata	I	2667	1047.00
atg	M	2	2.00
gtt	V	0	4.73
gtc	V	0	4.63
gta	V	22	4.69
gtg	V	0	7.95
---	--	------	------
mPD		0.14	0.68
		nPD:	0.21
		N. weight:	1.
		Sc. PD:	0.015
		Sc. rank:	554.9

,

NP			
Pos.	302	obs:	exp:
gat	D	2817	1560.00
gac	D	73	1330.00
---	--	------	------
mPD		0.049	0.50
		nPD:	0.1
		N. weight:	0.66
		Sc. PD:	-0.064
		Sc. rank:	-158.9

,

NP			
Pos.	303	obs:	exp:
tta	L	0	0.09
ttg	L	0	0.19
tct	S	1	0.17
tcc	S	0	0.14
tca	S	0	0.23
tcg	S	0	0.06
ctt	L	1	0.20
ctc	L	0	0.16
cta	L	0	0.15
ctg	L	0	0.21
cct	P	2645	789.30
ccc	P	240	521.60
cca	P	2	1172.00
ccg	P	1	404.90
agt	S	0	0.22
agc	S	0	0.18
---	--	------	------
mPD		0.16	0.71
		nPD:	0.22
		N. weight:	1.4
		Sc. PD:	0.028
		Sc. rank:	843.1

,

NP			
Pos.	304	obs:	exp:
ttt	F	113	1206.00
ttc	F	2777	1684.00
---	--	------	------
mPD		0.075	0.49
		nPD:	0.15
		N. weight:	0.51
		Sc. PD:	-0.022
		Sc. rank:	81.5

,

NP			
Pos.	305	obs:	exp:
cat	H	2	1.16
cac	H	0	0.84
cgt	R	2674	108.10
cgc	R	214	154.60
cga	R	0	291.00
cgg	R	0	235.80
aga	R	0	1348.00
agg	R	0	750.30
---	--	------	------
mPD		0.14	0.96
		nPD:	0.14
		N. weight:	3.9
		Sc. PD:	-0.2
		Sc. rank:	455.7

,

NP			
Pos.	306	obs:	exp:
tta	L	3	267.20
ttg	L	114	549.00
ctt	L	3	573.80
ctc	L	0	462.30
cta	L	46	427.70
ctg	L	2724	610.00
---	--	------	------
mPD		0.11	1.1
		nPD:	0.1
		N. weight:	1.7
		Sc. PD:	-0.16
		Sc. rank:	-389.7

NP			
Pos.	307	obs:	exp:
tta	L	0	267.20
ttg	L	0	549.00
ctt	L	2579	573.80
ctc	L	297	462.30
cta	L	12	427.70
ctg	L	2	610.00
---	--	------	------
mPD		0.19	1.1
		nPD:	0.17
		N. weight:	1.7
		Sc. PD:	-0.042
		Sc. rank:	527.2

NP			
Pos.	308	obs:	exp:
caa	Q	2840	1443.00
cag	Q	50	1447.00
---	--	------	------
mPD		0.034	0.50
		nPD:	0.07
		N. weight:	0.8
		Sc. PD:	-0.1
		Sc. rank:	-379.0

NP			
Pos.	309	obs:	exp:
tct	S	0	0.33
tcc	S	0	0.29
tca	S	0	0.45
tcg	S	0	0.12
act	T	0	0.26
acc	T	1	0.23
aca	T	0	0.42
acg	T	0	0.09
aat	N	44	1574.00
aac	N	2843	1313.00
agt	S	0	0.43
agc	S	2	0.37
---	--	------	------
mPD		0.032	0.50
		nPD:	0.06
		N. weight:	0.93
		Sc. PD:	-0.12
		Sc. rank:	-481.8

NP			
Pos.	310	obs:	exp:
tct	S	0	482.20
tcc	S	0	418.50
tca	S	0	654.90
tcg	S	0	174.30
aat	N	0	0.55
aac	N	1	0.45
agt	S	243	626.60
agc	S	2646	532.40
---	--	------	------
mPD		0.15	1.7
		nPD:	0.09
		N. weight:	1.8
		Sc. PD:	-0.19
		Sc. rank:	-550.5

NP			
Pos.	311	obs:	exp:
caa	Q	218	1443.00
cag	Q	2672	1447.00
---	--	------	------
mPD		0.14	0.50
		nPD:	0.28
		N. weight:	0.56
		Sc. PD:	0.044
		Sc. rank:	448.6

NP			
Pos.	312	obs:	exp:
gtt	V	52	621.60
gtc	V	2831	608.00
gta	V	4	616.20
gtg	V	3	1044.00
---	--	------	------
mPD		0.040	0.73
		nPD:	0.05
		N. weight:	1.9
		Sc. PD:	-0.27
		Sc. rank:	-1177.4

NP			
Pos.	313	obs:	exp:
ttt	F	1549	1204.00
ttc	F	1338	1683.00
tta	L	0	0.18
ttg	L	0	0.38
tct	S	0	0.17
tcc	S	1	0.14
tca	S	0	0.23
tcg	S	0	0.06
ctt	L	2	0.40
ctc	L	0	0.32
cta	L	0	0.30
ctg	L	0	0.42
agt	S	0	0.22
agc	S	0	0.18
---	--	------	------
mPD		0.50	0.49
		nPD:	1.02
		N. weight:	0.042
		Sc. PD:	0.034
		Sc. rank:	79.9

NP			
Pos.	314	obs:	exp:
tct	S	0	481.70
tcc	S	0	418.10
tca	S	0	654.30
tcg	S	0	174.10
tgt	C	4	1.55
tgc	C	0	2.45
agt	S	2404	626.00
agc	S	482	531.90
---	--	------	------
mPD		0.28	1.7
		nPD:	0.17
		N. weight:	1.5
		Sc. PD:	-0.039
		Sc. rank:	427.8

NP			
Pos.	315	obs:	exp:
tta	L	0	267.00
ttg	L	0	548.60
ctt	L	27	573.40
ctc	L	2846	462.00
cta	L	14	427.40
ctg	L	1	609.60
cct	P	0	0.27
ccc	P	1	0.18
cca	P	0	0.41
ccg	P	0	0.14
att	I	0	0.37
atc	I	1	0.27
ata	I	0	0.37
---	--	------	------
mPD		0.030	1.1
		nPD:	0.03
		N. weight:	2.3
		Sc. PD:	-0.38
		Sc. rank:	-2289.1

NP			
Pos.	316	obs:	exp:
att	I	2690	1061.00
atc	I	197	773.40
ata	I	3	1056.00
---	--	------	------
mPD		0.13	0.66
		nPD:	0.2
		N. weight:	1.
		Sc. PD:	-0.0024
		Sc. rank:	454.0

NP			
Pos.	317	obs:	exp:
tct	S	0	0.17
tcc	S	0	0.14
tca	S	0	0.23
tcg	S	0	0.06
cgt	R	0	108.10
cgc	R	0	154.60
cga	R	0	291.00
cgg	R	3	235.80
aaa	K	1	0.57
aag	K	0	0.43
agt	S	0	0.22
agc	S	1	0.18
aga	R	1909	1348.00
agg	R	976	750.30
---	--	------	------
mPD		0.45	0.96
		nPD:	0.47
		N. weight:	0.42
		Sc. PD:	0.11
		Sc. rank:	592.4

NP			
Pos.	318	obs:	exp:
tta	L	0	0.37
ttg	L	0	0.76
tct	S	0	1.50
tcc	S	0	1.30
tca	S	9	2.04
tcg	S	0	0.54
ctt	L	0	0.79
ctc	L	0	0.64
cta	L	1	0.59
ctg	L	3	0.84
cct	P	12	783.80
ccc	P	2	518.00
cca	P	2719	1164.00
ccg	P	135	402.10
caa	Q	2	1.00
cag	Q	0	1.00
act	T	0	1.84
acc	T	0	1.58
aca	T	7	2.93
acg	T	0	0.66
agt	S	0	1.95
agc	S	0	1.66
---	--	------	------
mPD		0.12	0.73
		nPD:	0.16
		N. weight:	0.97
		Sc. PD:	-0.037
		Sc. rank:	182.8

NP			
Pos.	319	obs:	exp:
tct	S	0	0.33
tcc	S	0	0.29
tca	S	0	0.45
tcg	S	0	0.12
act	T	3	0.79
acc	T	0	0.68
aca	T	0	1.26
acg	T	0	0.28
aat	N	2537	1553.00
aac	N	311	1295.00
aaa	K	2	21.07
aag	K	35	15.93
agt	S	2	0.43
agc	S	0	0.37
---	--	------	------
mPD		0.22	0.51
		nPD:	0.42
		N. weight:	0.38
		Sc. PD:	0.084
		Sc. rank:	507.8

NP			
Pos.	320	obs:	exp:
gaa	E	1117	1620.00
gag	E	1772	1269.00
ggt	G	0	0.14
ggc	G	0	0.13
gga	G	0	0.44
ggg	G	1	0.29
---	--	------	------
mPD		0.48	0.49
		nPD:	0.96
		N. weight:	0.083
		Sc. PD:	0.062
		Sc. rank:	156.4

NP			
Pos.	321	obs:	exp:
aat	N	1876	1576.00
aac	N	1014	1314.00
---	--	------	------
mPD		0.46	0.50
		nPD:	0.92
		N. weight:	0.031
		Sc. PD:	0.022
		Sc. rank:	58.7

NP			
Pos.	322	obs:	exp:
tct	S	0	0.17
tcc	S	0	0.14
tca	S	1	0.23
tcg	S	0	0.06
cct	P	23	789.50
ccc	P	11	521.80
cca	P	2830	1173.00
ccg	P	25	405.00
agt	S	0	0.22
agc	S	0	0.18
---	--	------	------
mPD		0.041	0.71
		nPD:	0.06
		N. weight:	1.1
		Sc. PD:	-0.14
		Sc. rank:	-594.0

NP			
Pos.	323	obs:	exp:
tct	S	0	0.83
tcc	S	0	0.72
tca	S	5	1.13
tcg	S	0	0.30
agt	S	0	1.08
agc	S	0	0.92
gtt	V	0	0.65
gtc	V	0	0.63
gta	V	3	0.64
gtg	V	0	1.08
gct	A	152	726.00
gcc	A	0	557.70
gca	A	2704	1283.00
gcg	A	26	315.40
---	--	------	------
mPD		0.12	0.70
		nPD:	0.18
		N. weight:	0.79
		Sc. PD:	-0.017
		Sc. rank:	276.6

NP			
Pos.	324	obs:	exp:
cat	H	2136	1673.00
cac	H	754	1217.00
---	--	------	------
mPD		0.39	0.49
		nPD:	0.79
		N. weight:	0.075
		Sc. PD:	0.043
		Sc. rank:	139.7

NP			
Pos.	325	obs:	exp:
aaa	K	238	1646.00
aag	K	2652	1244.00
---	--	------	------
mPD		0.15	0.49
		nPD:	0.31
		N. weight:	0.71
		Sc. PD:	0.076
		Sc. rank:	679.6

NP			
Pos.	326	obs:	exp:
tct	S	0	482.40
tcc	S	0	418.60
tca	S	0	655.20
tcg	S	0	174.40
agt	S	2517	626.90
agc	S	373	532.60
---	--	------	------
mPD		0.22	1.7
		nPD:	0.14
		N. weight:	1.5
		Sc. PD:	-0.092
		Sc. rank:	71.7

,

NP			
Pos.	327	obs:	exp:
caa	Q	2692	1443.00
cag	Q	198	1447.00
---	--	------	------
mPD		0.13	0.50
		nPD:	0.26
		N. weight:	0.59
		Sc. PD:	0.033
		Sc. rank:	428.2

,

NP			
Pos.	328	obs:	exp:
ttt	F	1	0.42
ttc	F	0	0.58
tta	L	1757	267.00
ttg	L	926	548.60
ctt	L	0	573.40
ctc	L	0	462.00
cta	L	13	427.40
ctg	L	192	609.60
gtt	V	0	0.22
gtc	V	0	0.21
gta	V	1	0.21
gtg	V	0	0.36
---	--	------	------
mPD		0.61	1.1
		nPD:	0.54
		N. weight:	1.6
		Sc. PD:	0.54
		Sc. rank:	2500.1

,

NP			
Pos.	329	obs:	exp:
att	I	0	2.20
atc	I	0	1.61
ata	I	6	2.19
gtt	V	3	620.30
gtc	V	0	606.80
gta	V	628	614.90
gtg	V	2253	1042.00
---	--	------	------
mPD		0.35	0.74
		nPD:	0.47
		N. weight:	0.8
		Sc. PD:	0.21
		Sc. rank:	1127.4

,

NP			
Pos.	330	obs:	exp:
tgg	W	2890	2890.00
---	--	------	------
mPD		0	0
		nPD:	1.
		N. weight:	0.
		Sc. PD:	0
		Sc. rank:	0

,

NP			
Pos.	331	obs:	exp:
tta	L	0	0.28
ttg	L	0	0.57
ctt	L	0	0.60
ctc	L	0	0.48
cta	L	0	0.44
ctg	L	3	0.63
att	I	1	0.37
atc	I	0	0.27
ata	I	0	0.37
atg	M	2886	2886.00
---	--	------	------
mPD		0.0028	0.0040
		nPD:	0.69
		N. weight:	0.0026
		Sc. PD:	0.0012
		Sc. rank:	4.5

,

NP			
Pos.	332	obs:	exp:
gct	A	63	728.00
gcc	A	13	559.20
gca	A	2810	1286.00
gcg	A	4	316.30
---	--	------	------
mPD		0.054	0.69
		nPD:	0.08
		N. weight:	0.9
		Sc. PD:	-0.1
		Sc. rank:	-366.0

,

NP			
Pos.	333	obs:	exp:
tct	S	0	0.17
tcc	S	0	0.14
tca	S	0	0.23
tcg	S	0	0.06
tgt	C	773	1118.00
tgc	C	2116	1771.00
agt	S	0	0.22
agc	S	1	0.18
---	--	------	------
mPD		0.39	0.48
		nPD:	0.83
		N. weight:	0.044
		Sc. PD:	0.027
		Sc. rank:	82.8

,

NP			
Pos.	334	obs:	exp:
cat	H	933	1673.00
cac	H	1957	1217.00
---	--	------	------
mPD		0.44	0.49
		nPD:	0.9
		N. weight:	0.18
		Sc. PD:	0.12
		Sc. rank:	334.0

,

NP			
Pos.	335	obs:	exp:
tct	S	2447	481.90
tcc	S	196	418.20
tca	S	222	654.50
tcg	S	22	174.20
cct	P	1	0.27
ccc	P	0	0.18
cca	P	0	0.41
ccg	P	0	0.14
agt	S	0	626.20
agc	S	0	532.00
gct	A	2	0.50
gcc	A	0	0.39
gca	A	0	0.89
gcg	A	0	0.22
---	--	------	------
mPD		0.27	1.7
		nPD:	0.17
		N. weight:	1.6
		Sc. PD:	-0.051
		Sc. rank:	355.0

,

NP			
Pos.	336	obs:	exp:
tct	S	0	0.33
tcc	S	0	0.29
tca	S	2	0.45
tcg	S	0	0.12
agt	S	0	0.43
agc	S	0	0.37
gct	A	23	727.50
gcc	A	1	558.90
gca	A	2689	1286.00
gcg	A	175	316.10
---	--	------	------
mPD		0.13	0.69
		nPD:	0.19
		N. weight:	0.82
		Sc. PD:	-0.007
		Sc. rank:	337.4

,

NP			
Pos.	337	obs:	exp:
gct	A	20	728.00
gcc	A	0	559.20
gca	A	2841	1286.00
gcg	A	29	316.30
---	--	------	------
mPD		0.033	0.69
		nPD:	0.05
		N. weight:	0.96
		Sc. PD:	-0.14
		Sc. rank:	-649.2

,

NP			
Pos.	338	obs:	exp:
ttt	F	1974	1205.00
ttc	F	915	1684.00
tct	S	1	0.17
tcc	S	0	0.14
tca	S	0	0.23
tcg	S	0	0.06
agt	S	0	0.22
agc	S	0	0.18
---	--	------	------
mPD		0.43	0.49
		nPD:	0.89
		N. weight:	0.19
		Sc. PD:	0.13
		Sc. rank:	362.0

,

NP			
Pos.	339	obs:	exp:
gaa	E	245	1620.00
gag	E	2645	1270.00
---	--	------	------
mPD		0.16	0.49
		nPD:	0.32
		N. weight:	0.68
		Sc. PD:	0.077
		Sc. rank:	668.9

,

NP			
Pos.	340	obs:	exp:
tat	Y	0	0.48
tac	Y	1	0.52
gtt	V	0	0.22
gtc	V	1	0.21
gta	V	0	0.21
gtg	V	0	0.36
gct	A	0	0.25
gcc	A	1	0.19
gca	A	0	0.45
gcg	A	0	0.11
gat	D	826	1559.00
gac	D	2061	1328.00
---	--	------	------
mPD		0.41	0.50
		nPD:	0.82
		N. weight:	0.18
		Sc. PD:	0.11
		Sc. rank:	331.1

,

NP			
Pos.	341	obs:	exp:
ttt	F	1	0.42
ttc	F	0	0.58
tta	L	1	267.10
ttg	L	11	548.80
ctt	L	1385	573.60
ctc	L	426	462.10
cta	L	13	427.60
ctg	L	1053	609.80
---	--	------	------
mPD		0.62	1.1
		nPD:	0.55
		N. weight:	0.77
		Sc. PD:	0.27
		Sc. rank:	1204.5

,

NP			
Pos.	342	obs:	exp:
cgt	R	0	108.10
cgc	R	0	154.60
cga	R	1	291.10
cgg	R	0	235.90
aaa	K	1	0.57
aag	K	0	0.43
aga	R	2794	1349.00
agg	R	94	750.50
---	--	------	------
mPD		0.064	0.95
		nPD:	0.07
		N. weight:	0.84
		Sc. PD:	-0.11
		Sc. rank:	-401.6

,

NP			
Pos.	343	obs:	exp:
att	I	1	9.54
atc	I	25	6.96
ata	I	0	9.50
gtt	V	52	616.00
gtc	V	2616	602.50
gta	V	14	610.70
gtg	V	182	1035.00
---	--	------	------
mPD		0.18	0.75
		nPD:	0.24
		N. weight:	1.5
		Sc. PD:	0.059
		Sc. rank:	1065.7

,

NP			
Pos.	344	obs:	exp:
tta	L	13	1.20
ttg	L	0	2.47
tct	S	4	480.20
tcc	S	0	416.70
tca	S	2863	652.20
tcg	S	10	173.60
ctt	L	0	2.58
ctc	L	0	2.08
cta	L	0	1.92
ctg	L	0	2.74
agt	S	0	624.00
agc	S	0	530.20
---	--	------	------
mPD		0.019	1.7
		nPD:	0.01
		N. weight:	1.9
		Sc. PD:	-0.35
		Sc. rank:	-2644.1

,

NP			
Pos.	345	obs:	exp:
tct	S	0	482.40
tcc	S	0	418.60
tca	S	0	655.20
tcg	S	0	174.40
agt	S	2500	626.90
agc	S	390	532.60
---	--	------	------
mPD		0.23	1.7
		nPD:	0.14
		N. weight:	1.5
		Sc. PD:	-0.083
		Sc. rank:	97.1

,

NP			
Pos.	346	obs:	exp:
ttt	F	95	1205.00
ttc	F	2793	1683.00
tta	L	2	0.18
ttg	L	0	0.38
ctt	L	0	0.40
ctc	L	0	0.32
cta	L	0	0.30
ctg	L	0	0.42
---	--	------	------
mPD		0.065	0.49
		nPD:	0.13
		N. weight:	0.54
		Sc. PD:	-0.034
		Sc. rank:	16.7

,

NP			
Pos.	347	obs:	exp:
tta	L	0	0.09
ttg	L	0	0.19
ctt	L	0	0.20
ctc	L	1	0.16
cta	L	0	0.15
ctg	L	0	0.21
att	I	11	1056.00
atc	I	2855	770.10
ata	I	12	1052.00
gtt	V	0	2.37
gtc	V	11	2.31
gta	V	0	2.35
gtg	V	0	3.97
---	--	------	------
mPD		0.024	0.67
		nPD:	0.04
		N. weight:	1.7
		Sc. PD:	-0.26
		Sc. rank:	-1385.5

,

NP			
Pos.	348	obs:	exp:
cgt	R	0	108.00
cgc	R	0	154.50
cga	R	0	290.90
cgg	R	0	235.70
aaa	K	3	1.71
aag	K	0	1.29
aga	R	2865	1348.00
agg	R	22	750.00
---	--	------	------
mPD		0.017	0.96
		nPD:	0.02
		N. weight:	0.95
		Sc. PD:	-0.17
		Sc. rank:	-1106.8

,

NP			
Pos.	349	obs:	exp:
ggt	G	2	417.90
ggc	G	0	379.60
gga	G	952	1266.00
ggg	G	1936	826.10
---	--	------	------
mPD		0.44	0.69
		nPD:	0.64
		N. weight:	0.63
		Sc. PD:	0.27
		Sc. rank:	1059.5

,

NP			
Pos.	350	obs:	exp:
tct	S	0	0.83
tcc	S	0	0.72
tca	S	5	1.13
tcg	S	0	0.30
att	I	0	2.20
atc	I	0	1.61
ata	I	6	2.19
atg	M	1	1.00
act	T	0	600.60
acc	T	1	516.20
aca	T	2287	958.60
acg	T	3	215.60
agt	S	0	1.08
agc	S	0	0.92
gct	A	0	147.90
gcc	A	0	113.60
gca	A	587	261.30
gcg	A	0	64.25
---	--	------	------
mPD		0.33	1.0
		nPD:	0.33
		N. weight:	1.1
		Sc. PD:	0.14
		Sc. rank:	1182.6

,

NP			
Pos.	351	obs:	exp:
cgt	R	0	107.50
cgc	R	0	153.80
cga	R	0	289.50
cgg	R	0	234.60
aaa	K	11	9.68
aag	K	6	7.32
aga	R	2801	1341.00
agg	R	72	746.40
---	--	------	------
mPD		0.064	0.96
		nPD:	0.07
		N. weight:	0.87
		Sc. PD:	-0.11
		Sc. rank:	-434.7

,

NP			
Pos.	352	obs:	exp:
tta	L	0	0.09
ttg	L	1	0.19
ctt	L	0	0.20
ctc	L	0	0.16
cta	L	0	0.15
ctg	L	0	0.21
att	I	0	1.10
atc	I	0	0.80
ata	I	3	1.10
atg	M	19	19.00
gtt	V	0	616.60
gtc	V	0	603.20
gta	V	828	611.30
gtg	V	2039	1036.00
---	--	------	------
mPD		0.43	0.75
		nPD:	0.57
		N. weight:	0.75
		Sc. PD:	0.27
		Sc. rank:	1189.2

,

NP			
Pos.	353	obs:	exp:
tta	L	0	0.74
ttg	L	0	1.52
ctt	L	0	1.59
ctc	L	8	1.28
cta	L	0	1.18
ctg	L	0	1.69
att	I	170	79.26
atc	I	46	57.80
ata	I	0	78.94
gtt	V	525	572.50
gtc	V	2110	560.00
gta	V	27	567.60
gtg	V	0	961.80
gct	A	1	1.01
gcc	A	3	0.77
gca	A	0	1.78
gcg	A	0	0.44
---	--	------	------
mPD		0.53	0.89
		nPD:	0.59
		N. weight:	1.3
		Sc. PD:	0.49
		Sc. rank:	2109.0

,

NP			
Pos.	354	obs:	exp:
cct	P	8	789.80
ccc	P	24	522.00
cca	P	2856	1173.00
ccg	P	2	405.20
---	--	------	------
mPD		0.023	0.71
		nPD:	0.03
		N. weight:	1.1
		Sc. PD:	-0.18
		Sc. rank:	-992.9

,

NP			
Pos.	355	obs:	exp:
cgt	R	0	108.20
cgc	R	0	154.70
cga	R	8	291.20
cgg	R	0	236.00
aga	R	2817	1349.00
agg	R	65	750.80
---	--	------	------
mPD		0.050	0.95
		nPD:	0.05
		N. weight:	0.86
		Sc. PD:	-0.12
		Sc. rank:	-557.5

,

NP			
Pos.	356	obs:	exp:
cgt	R	0	0.07
cgc	R	0	0.11
cga	R	0	0.20
cgg	R	0	0.16
aga	R	2	0.93
agg	R	0	0.52
ggt	G	3	417.60
ggc	G	1	379.30
gga	G	2230	1265.00
ggg	G	654	825.50
---	--	------	------
mPD		0.35	0.69
		nPD:	0.51
		N. weight:	0.5
		Sc. PD:	0.15
		Sc. rank:	752.5

,

NP			
Pos.	357	obs:	exp:
tta	L	0	0.09
ttg	L	0	0.19
ctt	L	0	0.20
ctc	L	0	0.16
cta	L	1	0.15
ctg	L	0	0.21
caa	Q	1085	1440.00
cag	Q	1800	1445.00
cgt	R	0	0.07
cgc	R	0	0.11
cga	R	0	0.20
cgg	R	2	0.16
aaa	K	0	1.14
aag	K	2	0.86
aga	R	0	0.93
agg	R	0	0.52
---	--	------	------
mPD		0.47	0.50
		nPD:	0.94
		N. weight:	0.046
		Sc. PD:	0.033
		Sc. rank:	87.1

,

NP			
Pos.	358	obs:	exp:
tta	L	22	267.20
ttg	L	11	549.00
ctt	L	0	573.80
ctc	L	5	462.30
cta	L	2610	427.70
ctg	L	242	610.00
---	--	------	------
mPD		0.19	1.1
		nPD:	0.17
		N. weight:	2.
		Sc. PD:	-0.063
		Sc. rank:	468.3

,

NP			
Pos.	359	obs:	exp:
tta	L	0	0.09
ttg	L	1	0.19
tct	S	9	482.10
tcc	S	2874	418.30
tca	S	5	654.70
tcg	S	0	174.20
ctt	L	0	0.20
ctc	L	0	0.16
cta	L	0	0.15
ctg	L	0	0.21
agt	S	0	626.40
agc	S	0	532.20
gct	A	0	0.25
gcc	A	1	0.19
gca	A	0	0.45
gcg	A	0	0.11
---	--	------	------
mPD		0.012	1.7
		nPD:	0.01
		N. weight:	2.5
		Sc. PD:	-0.46
		Sc. rank:	-3870.3

,

NP			
Pos.	360	obs:	exp:
act	T	62	757.60
acc	T	2430	651.20
aca	T	398	1209.00
acg	T	0	271.90
---	--	------	------
mPD		0.27	0.70
		nPD:	0.39
		N. weight:	1.2
		Sc. PD:	0.23
		Sc. rank:	1511.1

,

NP

Pos.	361	obs:	exp:
cgt	R	0	108.10
cgc	R	0	154.60
cga	R	9	291.10
cgg	R	0	235.90
att	I	0	0.37
atc	I	0	0.27
ata	I	1	0.37
aga	R	2844	1349.00
agg	R	36	750.50
---	--	------	------
mPD		0.032	0.95
		nPD:	0.03
		N. weight:	0.91
		Sc. PD:	-0.15
		Sc. rank:	-807.0

,

NP

Pos.	362	obs:	exp:
ggt	G	1	417.90
ggc	G	2	379.60
gga	G	775	1266.00
ggg	G	2112	826.10
---	--	------	------
mPD		0.39	0.69
		nPD:	0.57
		N. weight:	0.73
		Sc. PD:	0.26
		Sc. rank:	1157.6

,

NP

Pos.	363	obs:	exp:
att	I	9	3.30
atc	I	0	2.41
ata	I	0	3.29
gtt	V	2844	619.70
gtc	V	34	606.10
gta	V	3	614.30
gtg	V	0	1041.00
---	--	------	------
mPD		0.032	0.74
		nPD:	0.04
		N. weight:	1.9
		Sc. PD:	-0.29
		Sc. rank:	-1392.6

,

NP

Pos.	364	obs:	exp:
caa	Q	2711	1443.00
cag	Q	179	1447.00
---	--	------	------
mPD		0.12	0.50
		nPD:	0.23
		N. weight:	0.61
		Sc. PD:	0.021
		Sc. rank:	407.9

,

NP

Pos.	365	obs:	exp:
tta	L	0	0.37
ttg	L	0	0.76
tct	S	0	0.17
tcc	S	0	0.14
tca	S	0	0.23
tcg	S	0	0.06
ctt	L	4	0.79
ctc	L	0	0.64
cta	L	0	0.59
ctg	L	0	0.84
att	I	2871	1058.00
atc	I	3	771.70
ata	I	10	1054.00
agt	S	1	0.22
agc	S	0	0.18
gtt	V	1	0.22
gtc	V	0	0.21
gta	V	0	0.21
gtg	V	0	0.36
---	--	------	------
mPD		0.013	0.67
		nPD:	0.02
		N. weight:	1.3
		Sc. PD:	-0.22
		Sc. rank:	-1462.9

,

NP

Pos.	366	obs:	exp:
tct	S	2	0.50
tcc	S	0	0.43
tca	S	0	0.68
tcg	S	0	0.18
agt	S	1	0.65
agc	S	0	0.55
gct	A	2820	727.30
gcc	A	58	558.70
gca	A	7	1285.00
gcg	A	2	316.00
---	--	------	------
mPD		0.048	0.69
		nPD:	0.07
		N. weight:	1.7
		Sc. PD:	-0.21
		Sc. rank:	-766.7

,

NP

Pos.	367	obs:	exp:
ttt	F	0	0.42
ttc	F	1	0.58
tct	S	0	482.20
tcc	S	0	418.50
tca	S	2873	654.90
tcg	S	16	174.30
agt	S	0	626.60
agc	S	0	532.40
---	--	------	------
mPD		0.012	1.7
		nPD:	0.01
		N. weight:	1.9
		Sc. PD:	-0.36
		Sc. rank:	-2944.1

,

NP

Pos.	368	obs:	exp:
tct	S	0	2.84
tcc	S	0	2.46
tca	S	0	3.85
tcg	S	0	1.03
cat	H	1	0.58
cac	H	0	0.42
aat	N	2864	1566.00
aac	N	8	1306.00
agt	S	17	3.69
agc	S	0	3.13
---	--	------	------
mPD		0.018	0.52
		nPD:	0.03
		N. weight:	0.78
		Sc. PD:	-0.12
		Sc. rank:	-676.1

,

NP

Pos.	369	obs:	exp:
gaa	E	362	1620.00
gag	E	2528	1270.00
---	--	------	------
mPD		0.22	0.49
		nPD:	0.44
		N. weight:	0.55
		Sc. PD:	0.13
		Sc. rank:	757.9

,

NP

Pos.	370	obs:	exp:
tct	S	0	1.34
tcc	S	0	1.16
tca	S	0	1.81
tcg	S	0	0.48
act	T	0	0.26
acc	T	1	0.23
aca	T	0	0.42
acg	T	0	0.09
aat	N	171	1571.00
aac	N	2710	1310.00
agt	S	0	1.74
agc	S	8	1.47
---	--	------	------
mPD		0.12	0.51
		nPD:	0.23
		N. weight:	0.73
		Sc. PD:	0.024
		Sc. rank:	486.8

,

NP

Pos.	371	obs:	exp:
tta	L	0	0.09
ttg	L	0	0.19
ctt	L	0	0.20
ctc	L	0	0.16
cta	L	0	0.15
ctg	L	1	0.21
att	I	31	25.32
atc	I	16	18.46
ata	I	22	25.22
atg	M	2575	2575.00
act	T	0	63.18
acc	T	0	54.30
aca	T	7	100.80
acg	T	234	22.68
gtt	V	1	0.86
gtc	V	0	0.84
gta	V	0	0.85
gtg	V	3	1.45
---	--	------	------
mPD		0.21	0.34
		nPD:	0.61
		N. weight:	0.25
		Sc. PD:	0.098
		Sc. rank:	407.8

,

NP

Pos.	372	obs:	exp:
gat	D	3	2.16
gac	D	1	1.84
gaa	E	2581	1618.00
gag	E	305	1268.00
---	--	------	------
mPD		0.19	0.49
		nPD:	0.39
		N. weight:	0.35
		Sc. PD:	0.065
		Sc. rank:	436.6

,

NP

Pos.	373	obs:	exp:
cct	P	0	0.55
ccc	P	1	0.36
cca	P	1	0.81
ccg	P	0	0.28
act	T	3	452.20
acc	T	0	388.70
aca	T	1701	721.80
acg	T	21	162.30
gtt	V	0	31.83
gtc	V	0	31.14
gta	V	148	31.56
gtg	V	0	53.47
gct	A	42	255.70
gcc	A	1	196.40
gca	A	970	451.80
gcg	A	2	111.10
---	--	------	------
mPD		0.63	1.3
		nPD:	0.49
		N. weight:	1.
		Sc. PD:	0.3
		Sc. rank:	1537.1

,

NP

Pos.	374	obs:	exp:
tta	L	0	0.09
ttg	L	0	0.19
ctt	L	0	0.20
ctc	L	0	0.16
cta	L	0	0.15
ctg	L	1	0.21
att	I	3	1.47
atc	I	0	1.07
ata	I	1	1.46
atg	M	2883	2883.00
aaa	K	0	0.57
aag	K	1	0.43
gtt	V	0	0.22
gtc	V	0	0.21
gta	V	0	0.21
gtg	V	1	0.36
---	--	------	------
mPD		0.0048	0.0061
		nPD:	0.8
		N. weight:	0.0028
		Sc. PD:	0.0016
		Sc. rank:	5.1

,

NP

Pos.	375	obs:	exp:
aat	N	0	1.09
aac	N	2	0.91
gat	D	30	1557.00
gac	D	2854	1327.00
gaa	E	1	1.68
gag	E	2	1.32
ggt	G	0	0.14
ggc	G	1	0.13
gga	G	0	0.44
ggg	G	0	0.29
---	--	------	------
mPD		0.025	0.50
		nPD:	0.05
		N. weight:	0.95
		Sc. PD:	-0.14
		Sc. rank:	-630.1

,

NP

Pos.	376	obs:	exp:
tct	S	15	482.40
tcc	S	2873	418.60
tca	S	2	655.20
tcg	S	0	174.40
agt	S	0	626.90
agc	S	0	532.60
---	--	------	------
mPD		0.012	1.7
		nPD:	0.01
		N. weight:	2.5
		Sc. PD:	-0.46
		Sc. rank:	-3909.4

,

NP

Pos.	377	obs:	exp:
tct	S	0	169.90
tcc	S	0	147.50
tca	S	0	230.80
tcg	S	0	61.42
cgt	R	0	0.04
cgc	R	0	0.05
cga	R	0	0.10
cgg	R	0	0.08
aat	N	43	1020.00
aac	N	1828	850.70
agt	S	163	220.80
agc	S	855	187.60
aga	R	1	0.47
agg	R	0	0.26
---	--	------	------
mPD		0.59	1.4
		nPD:	0.41
		N. weight:	1.1
		Sc. PD:	0.24
		Sc. rank:	1499.1

,

NP

Pos.	378	obs:	exp:
act	T	2216	757.60
acc	T	661	651.20
aca	T	12	1209.00
acg	T	1	271.90
---	--	------	------
mPD		0.36	0.70
		nPD:	0.52
		N. weight:	1.1
		Sc. PD:	0.33
		Sc. rank:	1607.9

,

NP

Pos.	379	obs:	exp:
tta	L	0	267.00
ttg	L	0	548.60
ctt	L	2877	573.40
ctc	L	10	462.00
cta	L	1	427.40
ctg	L	0	609.60
cct	P	1	0.27
ccc	P	0	0.18
cca	P	0	0.41
ccg	P	0	0.14
att	I	1	0.37
atc	I	0	0.27
ata	I	0	0.37
---	--	------	------
mPD		0.0090	1.1
		nPD:	0.01
		N. weight:	2.1
		Sc. PD:	-0.39
		Sc. rank:	-3182.3

,

NP

Pos.	380	obs:	exp:
gaa	E	2804	1620.00
gag	E	86	1270.00
---	--	------	------
mPD		0.058	0.49
		nPD:	0.12
		N. weight:	0.6
		Sc. PD:	-0.047
		Sc. rank:	-43.3

,

NP

Pos.	381	obs:	exp:
tta	L	9	267.20
ttg	L	102	549.00
ctt	L	3	573.80
ctc	L	1	462.30
cta	L	24	427.70
ctg	L	2751	610.00
---	--	------	------
mPD		0.099	1.1
		nPD:	0.09
		N. weight:	1.8
		Sc. PD:	-0.19
		Sc. rank:	-582.5

NP

Pos.	382	obs:	exp:
cgt	R	0	108.10
cgc	R	0	154.60
cga	R	0	291.10
cgg	R	0	235.90
aga	R	2288	1349.00
agg	R	601	750.50
ggt	G	0	0.14
ggc	G	0	0.13
gga	G	1	0.44
ggg	G	0	0.29
---	--	------	------
mPD		0.33	0.95
		nPD:	0.35
		N. weight:	0.49
		Sc. PD:	0.071
		Sc. rank:	553.5

NP

Pos.	383	obs:	exp:
tct	S	0	482.40
tcc	S	0	418.60
tca	S	0	655.20
tcg	S	0	174.40
agt	S	1638	626.90
agc	S	1252	532.60
---	--	------	------
mPD		0.49	1.7
		nPD:	0.3
		N. weight:	1.2
		Sc. PD:	0.12
		Sc. rank:	1083.5

NP

Pos.	384	obs:	exp:
cgt	R	0	107.90
cgc	R	0	154.20
cga	R	0	290.40
cgg	R	0	235.30
aaa	K	5	4.56
aag	K	3	3.44
aga	R	2636	1345.00
agg	R	246	748.70
---	--	------	------
mPD		0.16	0.96
		nPD:	0.17
		N. weight:	0.69
		Sc. PD:	-0.019
		Sc. rank:	193.8

NP

Pos.	385	obs:	exp:
tat	Y	2541	1386.00
tac	Y	348	1503.00
tgt	C	1	0.39
tgc	C	0	0.61
---	--	------	------
mPD		0.21	0.50
		nPD:	0.43
		N. weight:	0.47
		Sc. PD:	0.1
		Sc. rank:	631.1

NP

Pos.	386	obs:	exp:
tgg	W	2889	2889.00
ggt	G	0	0.14
ggc	G	0	0.13
gga	G	0	0.44
ggg	G	1	0.29
---	--	------	------
mPD		0.00069	0.0012
		nPD:	0.58
		N. weight:	0.00057
		Sc. PD:	0.00021
		Sc. rank:	0.9

NP

Pos.	387	obs:	exp:
tct	S	1	0.17
tcc	S	0	0.14
tca	S	0	0.23
tcg	S	0	0.06
agt	S	0	0.22
agc	S	0	0.18
gtt	V	1	0.22
gtc	V	0	0.21
gta	V	0	0.21
gtg	V	0	0.36
gct	A	2593	727.50
gcc	A	216	558.90
gca	A	4	1286.00
gcg	A	75	316.10
---	--	------	------
mPD		0.19	0.69
		nPD:	0.27
		N. weight:	1.4
		Sc. PD:	0.1
		Sc. rank:	1051.0

NP

Pos.	388	obs:	exp:
att	I	0	1060.00
atc	I	0	773.10
ata	I	2889	1056.00
gtt	V	0	0.22
gtc	V	0	0.21
gta	V	1	0.21
gtg	V	0	0.36
---	--	------	------
mPD		0.00069	0.66
		nPD:	0.
		N. weight:	1.3
		Sc. PD:	-0.25
		Sc. rank:	-2436.7

NP

Pos.	389	obs:	exp:
cgt	R	0	108.10
cgc	R	0	154.60
cga	R	3	291.00
cgg	R	0	235.80
aaa	K	1	0.57
aag	K	0	0.43
aga	R	2620	1348.00
agg	R	265	750.30
ggt	G	0	0.14
ggc	G	0	0.13
gga	G	1	0.44
ggg	G	0	0.29
---	--	------	------
mPD		0.17	0.96
		nPD:	0.18
		N. weight:	0.66
		Sc. PD:	-0.013
		Sc. rank:	244.6

NP

Pos.	390	obs:	exp:
cct	P	0	0.27
ccc	P	1	0.18
cca	P	0	0.41
ccg	P	0	0.14
act	T	0	757.10
acc	T	2714	650.70
aca	T	174	1208.00
acg	T	0	271.70
aat	N	0	0.55
aac	N	1	0.45
---	--	------	------
mPD		0.11	0.70
		nPD:	0.16
		N. weight:	1.6
		Sc. PD:	-0.053
		Sc. rank:	324.0

NP

Pos.	391	obs:	exp:
cgt	R	0	107.50
cgc	R	0	153.70
cga	R	0	289.40
cgg	R	0	234.50
att	I	0	0.37
atc	I	0	0.27
ata	I	1	0.37
aaa	K	13	9.68
aag	K	4	7.32
aga	R	1160	1341.00
agg	R	1712	746.10
---	--	------	------
mPD		0.49	0.96
		nPD:	0.51
		N. weight:	0.58
		Sc. PD:	0.18
		Sc. rank:	862.3

NP

Pos.	392	obs:	exp:
tct	S	0	482.20
tcc	S	0	418.50
tca	S	0	654.90
tcg	S	0	174.30
cgt	R	0	0.04
cgc	R	1	0.05
cga	R	0	0.10
cgg	R	0	0.08
agt	S	1034	626.60
agc	S	1855	532.40
aga	R	0	0.47
agg	R	0	0.26
---	--	------	------
mPD		0.46	1.7
		nPD:	0.28
		N. weight:	1.3
		Sc. PD:	0.1
		Sc. rank:	1030.2

NP

Pos.	393	obs:	exp:
ggt	G	7	417.90
ggc	G	0	379.60
gga	G	2716	1266.00
ggg	G	167	826.10
---	--	------	------
mPD		0.11	0.69
		nPD:	0.16
		N. weight:	0.81
		Sc. PD:	-0.026
		Sc. rank:	167.9

NP

Pos.	394	obs:	exp:
ggt	G	2	417.90
ggc	G	0	379.60
gga	G	2052	1266.00
ggg	G	836	826.10
---	--	------	------
mPD		0.41	0.69
		nPD:	0.6
		N. weight:	0.45
		Sc. PD:	0.18
		Sc. rank:	746.1

NP

Pos.	395	obs:	exp:
act	T	0	18.61
acc	T	71	16.00
aca	T	0	29.71
acg	T	0	6.68
aat	N	23	1536.00
aac	N	2793	1280.00
gat	D	0	1.62
gac	D	3	1.38
---	--	------	------
mPD		0.066	0.56
		nPD:	0.12
		N. weight:	1.
		Sc. PD:	-0.078
		Sc. rank:	-64.9

NP

Pos.	396	obs:	exp:
cct	P	0	0.27
ccc	P	1	0.18
cca	P	0	0.41
ccg	P	0	0.14
act	T	277	752.40
acc	T	2574	646.70
aca	T	19	1201.00
acg	T	0	270.00
aat	N	1	9.82
aac	N	17	8.18
gct	A	0	0.25
gcc	A	1	0.19
gca	A	0	0.45
gcg	A	0	0.11
---	--	------	------
mPD		0.20	0.71
		nPD:	0.28
		N. weight:	1.5
		Sc. PD:	0.12
		Sc. rank:	1190.8

NP

Pos.	397	obs:	exp:
tct	S	0	11.85
tcc	S	0	10.28
tca	S	0	16.10
tcg	S	0	4.28
cat	H	0	0.58
cac	H	1	0.42
att	I	0	0.73
atc	I	2	0.54
ata	I	0	0.73
aat	N	293	1536.00
aac	N	2523	1280.00
agt	S	1	15.40
agc	S	70	13.08
---	--	------	------
mPD		0.23	0.58
		nPD:	0.4
		N. weight:	0.61
		Sc. PD:	0.12
		Sc. rank:	787.3

NP

Pos.	398	obs:	exp:
cat	H	7	65.99
cac	H	107	48.01
caa	Q	1318	1385.00
cag	Q	1456	1389.00
cgt	R	0	0.07
cgc	R	0	0.11
cga	R	2	0.20
cgg	R	0	0.16
aga	R	0	0.93
agg	R	0	0.52
---	--	------	------
mPD		0.54	0.54
		nPD:	1.
		N. weight:	0.038
		Sc. PD:	0.03
		Sc. rank:	72.4

NP

Pos.	399	obs:	exp:
cat	H	1	0.58
cac	H	0	0.42
caa	Q	32	1442.00
cag	Q	2856	1446.00
atg	M	1	1.00
---	--	------	------
mPD		0.024	0.50
		nPD:	0.05
		N. weight:	0.83
		Sc. PD:	-0.12
		Sc. rank:	-572.6

NP

Pos.	400	obs:	exp:
cgt	R	0	102.90
cgc	R	0	147.10
cga	R	4	277.00
cgg	R	2	224.50
aaa	K	19	80.29
aag	K	122	60.71
aga	R	1078	1283.00
agg	R	1665	714.20
---	--	------	------
mPD		0.57	1.0
		nPD:	0.55
		N. weight:	0.58
		Sc. PD:	0.2
		Sc. rank:	900.7

NP

Pos.	401	obs:	exp:
act	T	4	3.67
acc	T	0	3.15
aca	T	10	5.86
acg	T	0	1.32
gct	A	73	724.50
gcc	A	0	556.50
gca	A	2795	1280.00
gcg	A	8	314.80
mPD		0.067	0.70
		nPD:	0.1
		N. weight:	0.91
		Sc. PD:	-0.09
		Sc. rank:	-256.0

NP

Pos.	402	obs:	exp:
ttt	F	2	0.83
ttc	F	0	1.17
tct	S	2858	481.90
tcc	S	25	418.20
tca	S	4	654.50
tcg	S	0	174.20
tat	Y	1	0.48
tac	Y	0	0.52
agt	S	0	626.20
agc	S	0	532.00
mPD		0.022	1.7
		nPD:	0.01
		N. weight:	2.3
		Sc. PD:	-0.41
		Sc. rank:	-2971.9

NP

Pos.	403	obs:	exp:
tct	S	0	0.33
tcc	S	0	0.29
tca	S	2	0.45
tcg	S	0	0.12
act	T	0	0.52
acc	T	0	0.45
aca	T	2	0.84
acg	T	0	0.19
agt	S	0	0.43
agc	S	0	0.37
gtt	V	0	0.22
gtc	V	0	0.21
gta	V	1	0.21
gtg	V	0	0.36
gct	A	4	726.80
gcc	A	4	558.30
gca	A	2868	1284.00
gcg	A	9	315.80
mPD		0.015	0.69
		nPD:	0.02
		N. weight:	1.
		Sc. PD:	-0.18
		Sc. rank:	-1099.0

NP

Pos.	404	obs:	exp:
ggt	G	2	417.90
ggc	G	4	379.60
gga	G	1814	1266.00
ggg	G	1070	826.10
mPD		0.47	0.69
		nPD:	0.68
		N. weight:	0.41
		Sc. PD:	0.19
		Sc. rank:	719.2

NP

Pos.	405	obs:	exp:
caa	Q	485	1443.00
cag	Q	2405	1447.00
mPD		0.28	0.50
		nPD:	0.56
		N. weight:	0.32
		Sc. PD:	0.11
		Sc. rank:	501.4

NP

Pos.	406	obs:	exp:
att	I	10	1057.00
atc	I	2634	770.90
ata	I	237	1053.00
gtt	V	0	1.94
gtc	V	5	1.89
gta	V	4	1.92
gtg	V	0	3.25
mPD		0.17	0.67
		nPD:	0.25
		N. weight:	1.3
		Sc. PD:	0.063
		Sc. rank:	928.5

NP

Pos.	407	obs:	exp:
tct	S	0	482.40
tcc	S	0	418.60
tca	S	0	655.20
tcg	S	0	174.40
agt	S	1071	626.90
agc	S	1819	532.60
mPD		0.47	1.7
		nPD:	0.28
		N. weight:	1.3
		Sc. PD:	0.11
		Sc. rank:	1074.9

NP

Pos.	408	obs:	exp:
tta	L	0	0.09
ttg	L	0	0.19
ctt	L	0	0.20
ctc	L	0	0.16
cta	L	0	0.15
ctg	L	1	0.21
att	I	290	107.20
atc	I	0	78.14
ata	I	2	106.70
gtt	V	1521	558.60
gtc	V	8	546.40
gta	V	1062	553.70
gtg	V	6	938.30
mPD		0.65	0.92
		nPD:	0.71
		N. weight:	1.1
		Sc. PD:	0.55
		Sc. rank:	1982.9

NP

Pos.	409	obs:	exp:
caa	Q	623	1442.00
cag	Q	2266	1447.00
cgt	R	0	0.04
cgc	R	0	0.05
cga	R	0	0.10
cgg	R	1	0.08
aga	R	0	0.47
agg	R	0	0.26
mPD		0.34	0.50
		nPD:	0.68
		N. weight:	0.23
		Sc. PD:	0.11
		Sc. rank:	393.9

NP

Pos.	410	obs:	exp:
cct	P	434	789.80
ccc	P	2437	522.00
cca	P	19	1173.00
ccg	P	0	405.20
mPD		0.27	0.71
		nPD:	0.38
		N. weight:	1.6
		Sc. PD:	0.27
		Sc. rank:	1871.9

NP

Pos.	411	obs:	exp:
cct	P	1	0.27
ccc	P	0	0.18
cca	P	0	0.41
ccg	P	0	0.14
act	T	2664	756.80
acc	T	38	650.50
aca	T	173	1208.00
acg	T	12	271.60
gct	A	2	0.50
gcc	A	0	0.39
gca	A	0	0.89
gcg	A	0	0.22
mPD		0.15	0.70
		nPD:	0.21
		N. weight:	1.3
		Sc. PD:	0.015
		Sc. rank:	706.6

NP

Pos.	412	obs:	exp:
ttt	F	894	1206.00
ttc	F	1996	1684.00
mPD		0.43	0.49
		nPD:	0.88
		N. weight:	0.034
		Sc. PD:	0.023
		Sc. rank:	64.8

NP

Pos.	413	obs:	exp:
tct	S	357	482.40
tcc	S	1	418.60
tca	S	67	655.20
tcg	S	2465	174.40
agt	S	0	626.90
agc	S	0	532.60
mPD		0.26	1.7
		nPD:	0.16
		N. weight:	2.9
		Sc. PD:	-0.12
		Sc. rank:	516.9

NP

Pos.	414	obs:	exp:
att	I	0	1.83
atc	I	0	1.34
ata	I	5	1.83
gtt	V	0	620.10
gtc	V	0	606.50
gta	V	2399	614.70
gtg	V	484	1042.00
gct	A	0	0.25
gcc	A	0	0.19
gca	A	0	0.45
gcg	A	1	0.11
ggt	G	0	0.14
ggc	G	0	0.13
gga	G	1	0.44
ggg	G	0	0.29
mPD		0.28	0.74
		nPD:	0.38
		N. weight:	1.3
		Sc. PD:	0.24
		Sc. rank:	1632.7

NP

Pos.	415	obs:	exp:
caa	Q	224	1443.00
cag	Q	2666	1447.00
mPD		0.14	0.50
		nPD:	0.29
		N. weight:	0.55
		Sc. PD:	0.048
		Sc. rank:	466.9

NP

Pos.	416	obs:	exp:
cgt	R	0	108.20
cgc	R	0	154.70
cga	R	0	291.20
cgg	R	0	236.00
aga	R	2835	1349.00
agg	R	55	750.80
mPD		0.037	0.95
		nPD:	0.04
		N. weight:	0.9
		Sc. PD:	-0.14
		Sc. rank:	-684.8

NP

Pos.	417	obs:	exp:
tct	S	0	3.67
tcc	S	0	3.19
tca	S	0	4.99
tcg	S	0	1.33
act	T	1	1.31
acc	T	4	1.13
aca	T	0	2.09
acg	T	0	0.47
aat	N	970	1561.00
aac	N	1892	1301.00
agt	S	4	4.77
agc	S	18	4.05
gat	D	0	0.54
gac	D	1	0.46
mPD		0.47	0.53
		nPD:	0.88
		N. weight:	0.13
		Sc. PD:	0.087
		Sc. rank:	244.5

NP

Pos.	418	obs:	exp:
tta	L	0	267.00
ttg	L	0	548.60
ctt	L	2476	573.40
ctc	L	405	462.00
cta	L	7	427.40
ctg	L	0	609.60
cct	P	1	0.27
ccc	P	0	0.18
cca	P	0	0.41
ccg	P	0	0.14
att	I	1	0.37
atc	I	0	0.27
ata	I	0	0.37
mPD		0.25	1.1
		nPD:	0.22
		N. weight:	1.6
		Sc. PD:	0.034
		Sc. rank:	1015.9

NP

Pos.	419	obs:	exp:
cct	P	132	789.80
ccc	P	2363	522.00
cca	P	388	1173.00
ccg	P	7	405.20
mPD		0.31	0.71
		nPD:	0.44
		N. weight:	1.3
		Sc. PD:	0.31
		Sc. rank:	1793.5

NP

Pos.	420	obs:	exp:
ttt	F	584	1205.00
ttc	F	2304	1683.00
tta	L	0	0.09
ttg	L	0	0.19
tat	Y	0	0.48
tac	Y	1	0.52
ctt	L	0	0.20
ctc	L	1	0.16
cta	L	0	0.15
ctg	L	0	0.21
mPD		0.32	0.49
		nPD:	0.66
		N. weight:	0.14
		Sc. PD:	0.063
		Sc. rank:	239.4

NP

Pos.	421	obs:	exp:
gaa	E	2626	1620.00
gag	E	264	1270.00
---	--	------	------
mPD		0.17	0.49
		nPD:	0.34
		N. weight:	0.39
		Sc. PD:	0.053
		Sc. rank:	437.7

,

NP

Pos.	422	obs:	exp:
cgt	R	0	108.20
cgc	R	0	154.70
cga	R	3	291.20
cgg	R	0	236.00
aga	R	2567	1349.00
agg	R	320	750.80
---	--	------	------
mPD		0.20	0.95
		nPD:	0.21
		N. weight:	0.62
		Sc. PD:	0.0065
		Sc. rank:	329.5

,

NP

Pos.	423	obs:	exp:
tct	S	0	0.17
tcc	S	0	0.14
tca	S	0	0.23
tcg	S	1	0.06
act	T	0	12.06
acc	T	0	10.37
aca	T	8	19.25
acg	T	38	4.33
agt	S	0	0.22
agc	S	0	0.18
gct	A	4	716.20
gcc	A	0	550.10
gca	A	262	1265.00
gcg	A	2577	311.20
---	--	------	------
mPD		0.20	0.72
		nPD:	0.28
		N. weight:	2.3
		Sc. PD:	0.19
		Sc. rank:	1926.8

,

NP

Pos.	424	obs:	exp:
tct	S	0	0.17
tcc	S	0	0.14
tca	S	0	0.23
tcg	S	0	0.06
att	I	0	0.37
atc	I	1	0.27
ata	I	0	0.37
act	T	9	756.60
acc	T	2782	650.30
aca	T	95	1208.00
acg	T	0	271.50
aat	N	0	0.55
aac	N	1	0.45
agt	S	0	0.22
agc	S	1	0.18
gct	A	0	0.25
gcc	A	1	0.19
gca	A	0	0.45
gcg	A	0	0.11
---	--	------	------
mPD		0.072	0.70
		nPD:	0.1
		N. weight:	1.7
		Sc. PD:	-0.16
		Sc. rank:	-341.3

,

NP

Pos.	425	obs:	exp:
att	I	2623	1041.00
atc	I	213	759.20
ata	I	1	1037.00
atg	M	2	2.00
gtt	V	51	10.97
gtc	V	0	10.73
gta	V	0	10.87
gtg	V	0	18.43
---	--	------	------
mPD		0.17	0.70
		nPD:	0.25
		N. weight:	1.
		Sc. PD:	0.049
		Sc. rank:	715.9

,

NP

Pos.	426	obs:	exp:
tta	L	0	0.46
ttg	L	0	0.95
ctt	L	0	0.99
ctc	L	0	0.80
cta	L	0	0.74
ctg	L	5	1.06
atg	M	2885	2885.00
---	--	------	------
mPD		0.0035	0.0055
		nPD:	0.63
		N. weight:	0.0035
		Sc. PD:	0.0015
		Sc. rank:	6.0

,

NP

Pos.	427	obs:	exp:
gct	A	22	728.00
gcc	A	0	559.20
gca	A	1999	1286.00
gcg	A	869	316.30
---	--	------	------
mPD		0.43	0.69
		nPD:	0.63
		N. weight:	0.77
		Sc. PD:	0.32
		Sc. rank:	1296.7

,

NP

Pos.	428	obs:	exp:
tct	S	0	1.50
tcc	S	0	1.30
tca	S	9	2.04
tcg	S	0	0.54
act	T	0	1.05
acc	T	0	0.90
aca	T	4	1.67
acg	T	0	0.38
agt	S	0	1.95
agc	S	0	1.66
gct	A	6	724.80
gcc	A	0	556.70
gca	A	2153	1281.00
gcg	A	718	314.90
---	--	------	------
mPD		0.39	0.70
		nPD:	0.55
		N. weight:	0.78
		Sc. PD:	0.27
		Sc. rank:	1212.8

,

NP

Pos.	429	obs:	exp:
ttt	F	1754	1206.00
ttc	F	1136	1684.00
---	--	------	------
mPD		0.48	0.49
		nPD:	0.98
		N. weight:	0.098
		Sc. PD:	0.074
		Sc. rank:	184.8

,

NP

Pos.	430	obs:	exp:
tct	S	0	0.33
tcc	S	0	0.29
tca	S	2	0.45
tcg	S	0	0.12
att	I	0	1.47
atc	I	0	1.07
ata	I	4	1.46
act	T	29	742.90
acc	T	8	638.60
aca	T	2652	1186.00
acg	T	145	266.60
agt	S	0	0.43
agc	S	0	0.37
gct	A	0	12.60
gcc	A	0	9.68
gca	A	50	22.26
gcg	A	0	5.47
---	--	------	------
mPD		0.16	0.73
		nPD:	0.21
		N. weight:	0.9
		Sc. PD:	0.014
		Sc. rank:	523.4

,

NP

Pos.	431	obs:	exp:
ggt	G	13	417.90
ggc	G	2	379.60
gga	G	2118	1266.00
ggg	G	757	826.10
---	--	------	------
mPD		0.39	0.69
		nPD:	0.57
		N. weight:	0.44
		Sc. PD:	0.16
		Sc. rank:	710.7

,

NP

Pos.	432	obs:	exp:
tct	S	0	11.18
tcc	S	0	9.71
tca	S	0	15.19
tcg	S	0	4.04
aat	N	2569	1539.00
aac	N	254	1284.00
agt	S	67	14.53
agc	S	0	12.35
---	--	------	------
mPD		0.21	0.58
		nPD:	0.36
		N. weight:	0.46
		Sc. PD:	0.071
		Sc. rank:	529.8

,

NP

Pos.	433	obs:	exp:
tct	S	0	0.17
tcc	S	0	0.14
tca	S	0	0.23
tcg	S	0	0.06
att	I	2	0.73
atc	I	0	0.54
ata	I	0	0.73
act	T	2690	712.50
acc	T	8	612.40
aca	T	19	1137.00
acg	T	1	255.70
aat	N	6	9.82
aac	N	12	8.18
agt	S	1	0.22
agc	S	0	0.18
gct	A	149	38.04
gcc	A	2	29.22
gca	A	0	67.21
gcg	A	0	16.53
---	--	------	------
mPD		0.14	0.81
		nPD:	0.18
		N. weight:	1.7
		Sc. PD:	-0.036
		Sc. rank:	606.9

,

NP

Pos.	434	obs:	exp:
gat	D	0	0.54
gac	D	1	0.46
gaa	E	1301	1620.00
gag	E	1588	1269.00
---	--	------	------
mPD		0.50	0.49
		nPD:	1.01
		N. weight:	0.034
		Sc. PD:	0.027
		Sc. rank:	65.2

,

NP

Pos.	435	obs:	exp:
ggt	G	702	417.90
ggc	G	2185	379.60
gga	G	3	1266.00
ggg	G	0	826.10
---	--	------	------
mPD		0.37	0.69
		nPD:	0.54
		N. weight:	1.9
		Sc. PD:	0.63
		Sc. rank:	2925.7

,

NP			
Pos.	436	obs:	exp:
cgt	R	0	108.20
cgc	R	0	154.70
cga	R	0	291.20
cgg	R	0	236.00
aga	R	2520	1349.00
agg	R	370	750.80
---	--	-----	-----
mPD		0.22	0.95
		nPD:	0.23
		N. weight:	0.6
		Sc. PD:	0.021
		Sc. rank:	405.5

,

NP			
Pos.	437	obs:	exp:
tct	S	0	0.33
tcc	S	0	0.29
tca	S	0	0.45
tcg	S	2	0.12
att	I	2	0.73
atc	I	0	0.54
ata	I	0	0.73
act	T	183	756.60
acc	T	5	650.30
aca	T	941	1208.00
acg	T	1757	271.50
agt	S	0	0.43
agc	S	0	0.37
---	--	-----	-----
mPD		0.52	0.70
		nPD:	0.75
		N. weight:	1.3
		Sc. PD:	0.68
		Sc. rank:	2333.8

,

NP			
Pos.	438	obs:	exp:
ttt	F	2	0.83
ttc	F	0	1.17
tct	S	1480	482.10
tcc	S	1231	418.30
tca	S	176	654.70
tcg	S	1	174.20
agt	S	0	626.40
agc	S	0	532.20
---	--	-----	-----
mPD		0.55	1.7
		nPD:	0.34
		N. weight:	1.3
		Sc. PD:	0.17
		Sc. rank:	1402.3

,

NP			
Pos.	439	obs:	exp:
gat	D	158	1560.00
gac	D	2732	1330.00
---	--	-----	-----
mPD		0.10	0.50
		nPD:	0.21
		N. weight:	0.73
		Sc. PD:	0.0073
		Sc. rank:	376.7

,

NP			
Pos.	440	obs:	exp:
atg	M	2890	2890.00
---	--	-----	-----
mPD		0	0
		nPD:	1.
		N. weight:	0.
		Sc. PD:	0
		Sc. rank:	0

,

NP			
Pos.	441	obs:	exp:
cgt	R	0	108.10
cgc	R	0	154.60
cga	R	0	291.00
cgg	R	0	235.80
aaa	K	0	0.57
aag	K	1	0.43
aga	R	493	1348.00
agg	R	2395	750.30
ggt	G	0	0.14
ggc	G	0	0.13
gga	G	1	0.44
ggg	G	0	0.29
---	--	-----	-----
mPD		0.28	0.96
		nPD:	0.3
		N. weight:	1.
		Sc. PD:	0.1
		Sc. rank:	945.2

,

NP			
Pos.	442	obs:	exp:
att	I	2	0.73
atc	I	0	0.54
ata	I	0	0.73
act	T	2832	756.00
acc	T	14	649.80
aca	T	36	1207.00
acg	T	2	271.30
gct	A	4	1.01
gcc	A	0	0.77
gca	A	0	1.78
gcg	A	0	0.44
---	--	-----	-----
mPD		0.040	0.70
		nPD:	0.06
		N. weight:	1.6
		Sc. PD:	-0.22
		Sc. rank:	-945.4

,

NP			
Pos.	443	obs:	exp:
aaa	K	1	0.57
aag	K	0	0.43
gaa	E	2710	1620.00
gag	E	179	1269.00
---	--	-----	-----
mPD		0.12	0.49
		nPD:	0.24
		N. weight:	0.48
		Sc. PD:	0.018
		Sc. rank:	324.8

,

NP			
Pos.	444	obs:	exp:
ttt	F	0	0.42
ttc	F	1	0.58
att	I	6	1060.00
atc	I	2870	773.10
ata	I	13	1056.00
---	--	-----	-----
mPD		0.014	0.66
		nPD:	0.02
		N. weight:	1.7
		Sc. PD:	-0.29
		Sc. rank:	-1876.9

,

NP			
Pos.	445	obs:	exp:
att	I	3	1052.00
atc	I	32	767.50
ata	I	2833	1048.00
gtt	V	0	4.73
gtc	V	0	4.63
gta	V	22	4.69
gtg	V	0	7.95
---	--	-----	-----
mPD		0.039	0.68
		nPD:	0.06
		N. weight:	1.2
		Sc. PD:	-0.17
		Sc. rank:	-714.0

,

NP			
Pos.	446	obs:	exp:
cgt	R	0	105.30
cgc	R	0	150.60
cga	R	0	283.70
cgg	R	0	229.90
aaa	K	71	42.71
aag	K	4	32.29
aga	R	2397	1314.00
agg	R	418	731.30
---	--	-----	-----
mPD		0.30	1.0
		nPD:	0.3
		N. weight:	0.57
		Sc. PD:	0.057
		Sc. rank:	522.9

,

NP			
Pos.	447	obs:	exp:
tta	L	0	1.11
ttg	L	12	2.28
ctt	L	0	2.38
ctc	L	0	1.92
cta	L	0	1.78
ctg	L	0	2.53
att	I	3	2.94
atc	I	0	2.14
ata	I	5	2.92
atg	M	2867	2867.00
act	T	0	0.52
acc	T	0	0.45
aca	T	0	0.84
acg	T	2	0.19
gtt	V	0	0.22
gtc	V	0	0.21
gta	V	0	0.21
gtg	V	1	0.36
---	--	-----	-----
mPD		0.016	0.023
		nPD:	0.71
		N. weight:	0.014
		Sc. PD:	0.0067
		Sc. rank:	24.0

,

NP			
Pos.	448	obs:	exp:
att	I	0	0.37
atc	I	0	0.27
ata	I	1	0.37
atg	M	2889	2889.00
---	--	-----	-----
mPD		0.00069	0.00069
		nPD:	1.
		N. weight:	0.00046
		Sc. PD:	0.00036
		Sc. rank:	0.9

,

NP			
Pos.	449	obs:	exp:
aaa	K	1	0.57
aag	K	0	0.43
gaa	E	2782	1620.00
gag	E	107	1269.00
---	--	-----	-----
mPD		0.072	0.49
		nPD:	0.15
		N. weight:	0.57
		Sc. PD:	-0.029
		Sc. rank:	68.0

,

NP			
Pos.	450	obs:	exp:
tct	S	0	441.80
tcc	S	0	383.40
tca	S	0	600.10
tcg	S	0	159.70
cgt	R	0	0.04
cgc	R	0	0.05
cga	R	0	0.10
cgg	R	0	0.08
att	I	1	0.37
atc	I	0	0.27
ata	I	0	0.37
act	T	2	0.52
acc	T	0	0.45
aca	T	0	0.84
acg	T	0	0.19
aat	N	233	127.10
aac	N	0	105.90
agt	S	2565	574.10
agc	S	82	487.80
aga	R	0	0.47
agg	R	1	0.26
ggt	G	5	0.87
ggc	G	1	0.79
gga	G	0	2.63
ggg	G	0	1.72
---	--	-----	-----
mPD		0.21	1.7
		nPD:	0.12
		N. weight:	1.8
		Sc. PD:	-0.13
		Sc. rank:	-20.7

,

NP

Pos.	451	obs:	exp:
tct	S	0	0.17
tcc	S	1	0.14
tca	S	0	0.23
tcg	S	0	0.06
tgt	C	0	0.39
tgc	C	1	0.61
act	T	0	0.26
acc	T	1	0.23
aca	T	0	0.42
acg	T	0	0.09
agt	S	0	0.22
agc	S	0	0.18
gtt	V	0	0.86
gtc	V	4	0.84
gta	V	0	0.85
gtg	V	0	1.45
gct	A	12	726.30
gcc	A	2869	557.90
gca	A	2	1283.00
gcg	A	0	315.60
---	--	------	------
mPD		0.015	0.70
		nPD:	0.02
		N. weight:	2.1
		Sc. PD:	-0.36
		Sc. rank:	-2294.2

,

NP

Pos.	452	obs:	exp:
caa	Q	0	0.50
cag	Q	1	0.50
cgt	R	0	81.66
cgc	R	0	116.80
cga	R	0	219.90
cgg	R	0	178.20
aaa	K	704	402.60
aag	K	3	304.40
aga	R	2168	1019.00
agg	R	14	566.90
---	--	------	------
mPD		0.38	1.2
		nPD:	0.31
		N. weight:	0.9
		Sc. PD:	0.097
		Sc. rank:	869.7

,

NP

Pos.	453	obs:	exp:
tta	L	0	0.09
ttg	L	0	0.19
tct	S	0	3.34
tcc	S	0	2.90
tca	S	20	4.53
tcg	S	0	1.21
ctt	L	0	0.20
ctc	L	0	0.16
cta	L	1	0.15
ctg	L	0	0.21
cct	P	185	782.70
ccc	P	0	517.30
cca	P	2673	1162.00
ccg	P	6	401.50
act	T	0	0.52
acc	T	0	0.45
aca	T	2	0.84
acg	T	0	0.19
agt	S	0	4.34
agc	S	0	3.69
gct	A	0	0.76
gcc	A	0	0.58
gca	A	3	1.34
gcg	A	0	0.33
---	--	------	------
mPD		0.14	0.73
		nPD:	0.19
		N. weight:	0.9
		Sc. PD:	-0.0039
		Sc. rank:	380.9

,

NP

Pos.	454	obs:	exp:
caa	Q	1	0.50
cag	Q	0	0.50
gat	D	7	4.32
gac	D	1	3.68
gaa	E	2867	1614.00
gag	E	12	1265.00
ggt	G	0	0.29
ggc	G	0	0.26
gga	G	2	0.88
ggg	G	0	0.57
---	--	------	------
mPD		0.016	0.50
		nPD:	0.03
		N. weight:	0.73
		Sc. PD:	-0.12
		Sc. rank:	-676.9

,

NP

Pos.	455	obs:	exp:
gct	A	1	0.25
gcc	A	0	0.19
gca	A	0	0.45
gcg	A	0	0.11
gat	D	2884	1559.00
gac	D	3	1328.00
gaa	E	0	1.12
gag	E	2	0.88
---	--	------	------
mPD		0.0041	0.50
		nPD:	0.01
		N. weight:	0.8
		Sc. PD:	-0.15
		Sc. rank:	-1212.8

,

NP

Pos.	456	obs:	exp:
tta	L	0	0.65
ttg	L	5	1.33
ctt	L	0	1.39
ctc	L	0	1.12
cta	L	0	1.04
ctg	L	2	1.48
atg	M	2	2.00
gtt	V	0	619.20
gtc	V	0	605.70
gta	V	0	613.90
gtg	V	2879	1040.00
gct	A	0	0.50
gcc	A	0	0.39
gca	A	0	0.89
gcg	A	2	0.22
---	--	------	------
mPD		0.0076	0.74
		nPD:	0.01
		N. weight:	1.3
		Sc. PD:	-0.24
		Sc. rank:	-1863.2

,

NP

Pos.	457	obs:	exp:
tct	S	841	482.40
tcc	S	237	418.60
tca	S	1763	655.20
tcg	S	49	174.40
agt	S	0	626.90
agc	S	0	532.60
---	--	------	------
mPD		0.54	1.7
		nPD:	0.33
		N. weight:	0.92
		Sc. PD:	0.11
		Sc. rank:	965.9

,

NP

Pos.	458	obs:	exp:
ttt	F	6	1206.00
ttc	F	2884	1684.00
---	--	------	------
mPD		0.0041	0.49
		nPD:	0.01
		N. weight:	0.69
		Sc. PD:	-0.13
		Sc. rank:	-1044.1

,

NP

Pos.	459	obs:	exp:
cat	H	3	1.74
cac	H	0	1.26
caa	Q	242	1441.00
cag	Q	2645	1446.00
---	--	------	------
mPD		0.16	0.50
		nPD:	0.31
		N. weight:	0.53
		Sc. PD:	0.058
		Sc. rank:	522.2

,

NP

Pos.	460	obs:	exp:
ggt	G	0	417.90
ggc	G	0	379.60
gga	G	27	1266.00
ggg	G	2863	826.10
---	--	------	------
mPD		0.019	0.69
		nPD:	0.03
		N. weight:	1.6
		Sc. PD:	-0.26
		Sc. rank:	-1537.1

,

NP

Pos.	461	obs:	exp:
cct	P	0	0.55
ccc	P	0	0.36
cca	P	0	0.81
ccg	P	2	0.28
cgt	R	0	108.10
cgc	R	0	154.60
cga	R	0	291.00
cgg	R	2887	235.80
aga	R	0	1348.00
agg	R	1	750.30
---	--	------	------
mPD		0.0021	0.96
		nPD:	0.
		N. weight:	3.3
		Sc. PD:	-0.63
		Sc. rank:	-5857.9

,

NP

Pos.	462	obs:	exp:
ggt	G	41	417.90
ggc	G	0	379.60
gga	G	2771	1266.00
ggg	G	78	826.10
---	--	------	------
mPD		0.080	0.69
		nPD:	0.12
		N. weight:	0.86
		Sc. PD:	-0.069
		Sc. rank:	-71.6

,

NP

Pos.	463	obs:	exp:
gtt	V	1	620.90
gtc	V	2883	607.40
gta	V	3	615.60
gtg	V	0	1043.00
gct	A	0	0.76
gcc	A	3	0.58
gca	A	0	1.34
gcg	A	0	0.33
---	--	------	------
mPD		0.0048	0.74
		nPD:	0.01
		N. weight:	2.
		Sc. PD:	-0.38
		Sc. rank:	-3234.9

,

NP

Pos.	464	obs:	exp:
ttt	F	9	1206.00
ttc	F	2881	1684.00
---	--	------	------
mPD		0.0062	0.49
		nPD:	0.01
		N. weight:	0.69
		Sc. PD:	-0.12
		Sc. rank:	-905.4

,

NP

Pos.	465	obs:	exp:
tat	Y	0	0.48
tac	Y	1	0.52
gaa	E	0	1618.00
gag	E	2887	1269.00
ggt	G	0	0.29
ggc	G	0	0.26
gga	G	0	0.88
ggg	G	2	0.57
---	--	------	------
mPD		0.0028	0.50
		nPD:	0.01
		N. weight:	1.1
		Sc. PD:	-0.2
		Sc. rank:	-1760.7

,

NP

Pos.	466	obs:	exp:
ttt	F	0	15.85
ttc	F	38	22.15
tta	L	0	263.40
ttg	L	0	541.20
ctt	L	511	565.70
ctc	L	2231	455.70
cta	L	107	421.60
ctg	L	0	601.30
att	I	0	1.10
atc	I	3	0.80
ata	I	0	1.10
---	--	------	------
mPD		0.38	1.1
		nPD:	0.33
		N. weight:	1.5
		Sc. PD:	0.2
		Sc. rank:	1659.1

,

NP

Pos.	467	obs:	exp:
tct	S	1	482.20
tcc	S	0	418.50
tca	S	31	654.90
tcg	S	2857	174.30
cct	P	0	0.27
ccc	P	0	0.18
cca	P	0	0.41
ccg	P	1	0.14
agt	S	0	626.60
agc	S	0	532.40
---	--	------	------
mPD		0.023	1.7
		nPD:	0.01
		N. weight:	3.6
		Sc. PD:	-0.64
		Sc. rank:	-4543.3

,

NP

Pos.	468	obs:	exp:
gat	D	20	1560.00
gac	D	2870	1330.00
---	--	------	------
mPD		0.014	0.50
		nPD:	0.03
		N. weight:	0.97
		Sc. PD:	-0.16
		Sc. rank:	-929.1

,

NP

Pos.	469	obs:	exp:
gaa	E	2883	1620.00
gag	E	7	1270.00
---	--	------	------
mPD		0.0048	0.49
		nPD:	0.01
		N. weight:	0.74
		Sc. PD:	-0.14
		Sc. rank:	-1047.6

,

NP

Pos.	470	obs:	exp:
cgt	R	0	0.04
cgc	R	0	0.05
cga	R	0	0.10
cgg	R	0	0.08
aat	N	0	0.55
aac	N	1	0.45
aaa	K	0	1644.00
aag	K	2887	1243.00
aga	R	0	0.47
agg	R	1	0.26
gaa	E	0	0.56
gag	E	1	0.44
---	--	------	------
mPD		0.0021	0.49
		nPD:	0.
		N. weight:	1.1
		Sc. PD:	-0.21
		Sc. rank:	-1878.0

,

NP

Pos.	471	obs:	exp:
gct	A	0	728.00
gcc	A	1	559.20
gca	A	2878	1286.00
gcg	A	11	316.30
---	--	------	------
mPD		0.0083	0.69
		nPD:	0.01
		N. weight:	1.
		Sc. PD:	-0.19
		Sc. rank:	-1393.4

NP

Pos.	472	obs:	exp:
act	T	0	754.50
acc	T	0	648.50
aca	T	0	1204.00
acg	T	2878	270.80
gct	A	0	3.02
gcc	A	0	2.32
gca	A	0	5.34
gcg	A	12	1.31
---	--	------	------
mPD		0.0083	0.70
		nPD:	0.01
		N. weight:	3.1
		Sc. PD:	-0.56
		Sc. rank:	-4202.9

NP

Pos.	473	obs:	exp:
tct	S	0	0.83
tcc	S	0	0.72
tca	S	0	1.13
tcg	S	0	0.30
act	T	0	2.10
acc	T	8	1.80
aca	T	0	3.35
acg	T	0	0.75
aat	N	1	1561.00
aac	N	2861	1301.00
agt	S	0	1.08
agc	S	5	0.92
gat	D	0	8.10
gac	D	15	6.90
---	--	------	------
mPD		0.020	0.52
		nPD:	0.04
		N. weight:	1.
		Sc. PD:	-0.16
		Sc. rank:	-801.8

NP

Pos.	474	obs:	exp:
cct	P	2	789.80
ccc	P	0	522.00
cca	P	0	1173.00
ccg	P	2888	405.20
---	--	------	------
mPD		0.0014	0.71
		nPD:	0.
		N. weight:	2.6
		Sc. PD:	-0.49
		Sc. rank:	-4690.2

NP

Pos.	475	obs:	exp:
att	I	0	1056.00
atc	I	2877	769.90
ata	I	0	1051.00
gtt	V	0	2.80
gtc	V	13	2.74
gta	V	0	2.77
gtg	V	0	4.70
---	--	------	------
mPD		0.0090	0.67
		nPD:	0.01
		N. weight:	1.7
		Sc. PD:	-0.31
		Sc. rank:	-2241.8

NP

Pos.	476	obs:	exp:
gtt	V	0	621.60
gtc	V	0	608.00
gta	V	0	616.20
gtg	V	2890	1044.00
---	--	------	------
mPD		0	0.73
		nPD:	0.
		N. weight:	1.3
		Sc. PD:	-0.26
		Sc. rank:	-2527.9

NP

Pos.	477	obs:	exp:
cct	P	2881	789.80
ccc	P	9	522.00
cca	P	0	1173.00
ccg	P	0	405.20
---	--	------	------
mPD		0.0062	0.71
		nPD:	0.01
		N. weight:	1.7
		Sc. PD:	-0.31
		Sc. rank:	-2451.1

NP

Pos.	478	obs:	exp:
ttt	F	0	0.42
ttc	F	1	0.58
tct	S	9	482.20
tcc	S	2879	418.50
tca	S	1	654.90
tcg	S	0	174.30
agt	S	0	626.60
agc	S	0	532.40
---	--	------	------
mPD		0.0076	1.7
		nPD:	0.
		N. weight:	2.5
		Sc. PD:	-0.47
		Sc. rank:	-4203.3

NP

Pos.	479	obs:	exp:
ttt	F	2888	1205.00
ttc	F	1	1684.00
gtt	V	1	0.22
gtc	V	0	0.21
gta	V	0	0.21
gtg	V	0	0.36
---	--	------	------
mPD		0.0014	0.49
		nPD:	0.
		N. weight:	1.1
		Sc. PD:	-0.22
		Sc. rank:	-2030.9

NP

Pos.	480	obs:	exp:
aat	N	0	0.55
aac	N	1	0.45
gat	D	0	1559.00
gac	D	2888	1329.00
ggt	G	0	0.14
ggc	G	0	0.13
gga	G	1	0.44
ggg	G	0	0.29
---	--	------	------
mPD		0.0021	0.50
		nPD:	0.
		N. weight:	1.
		Sc. PD:	-0.19
		Sc. rank:	-1744.5

NP

Pos.	481	obs:	exp:
tta	L	0	0.09
ttg	L	1	0.19
ctt	L	0	0.20
ctc	L	0	0.16
cta	L	0	0.15
ctg	L	0	0.21
cat	H	1	0.58
cac	H	0	0.42
att	I	0	0.37
atc	I	0	0.27
ata	I	1	0.37
atg	M	2885	2885.00
aaa	K	0	0.57
aag	K	1	0.43
gtt	V	0	0.22
gtc	V	0	0.21
gta	V	0	0.21
gtg	V	1	0.36
---	--	------	------
mPD		0.0048	0.0061
		nPD:	0.8
		N. weight:	0.0023
		Sc. PD:	0.0013
		Sc. rank:	4.3

NP

Pos.	482	obs:	exp:
tct	S	0	179.60
tcc	S	0	155.90
tca	S	0	243.90
tcg	S	0	64.92
act	T	1	0.26
acc	T	0	0.23
aca	T	0	0.42
acg	T	0	0.09
aat	N	1800	987.60
aac	N	11	823.40
agt	S	942	233.40
agc	S	134	198.30
gaa	E	1	0.56
gag	E	0	0.44
ggt	G	1	0.14
ggc	G	0	0.13
gga	G	0	0.44
ggg	G	0	0.29
---	--	------	------
mPD		0.57	1.5
		nPD:	0.38
		N. weight:	1.1
		Sc. PD:	0.19
		Sc. rank:	1272.1

NP

Pos.	483	obs:	exp:
tat	Y	1	0.48
tac	Y	0	0.52
aat	N	2839	1572.00
aac	N	44	1311.00
aaa	K	6	3.42
aag	K	0	2.58
---	--	------	------
mPD		0.035	0.50
		nPD:	0.07
		N. weight:	0.7
		Sc. PD:	-0.087
		Sc. rank:	-318.4

NP

Pos.	484	obs:	exp:
gtt	V	0	0.22
gtc	V	0	0.21
gta	V	1	0.21
gtg	V	0	0.36
gaa	E	2653	1619.00
gag	E	235	1269.00
ggt	G	0	0.14
ggc	G	0	0.13
gga	G	1	0.44
ggg	G	0	0.29
---	--	------	------
mPD		0.15	0.49
		nPD:	0.31
		N. weight:	0.42
		Sc. PD:	0.044
		Sc. rank:	394.8

NP

Pos.	485	obs:	exp:
cgt	R	0	0.04
cgc	R	0	0.05
cga	R	0	0.10
cgg	R	0	0.08
aga	R	1	0.47
agg	R	0	0.26
ggt	G	3	417.80
ggc	G	0	379.50
gga	G	2653	1266.00
ggg	G	233	825.80
---	--	------	------
mPD		0.15	0.69
		nPD:	0.22
		N. weight:	0.76
		Sc. PD:	0.016
		Sc. rank:	457.9

NP

Pos.	486	obs:	exp:
tct	S	2889	482.40
tcc	S	1	418.60
tca	S	0	655.20
tcg	S	0	174.40
agt	S	0	626.90
agc	S	0	532.60
---	--	------	------
mPD		0.00069	1.7
		nPD:	0.
		N. weight:	2.4
		Sc. PD:	-0.45
		Sc. rank:	-4349.8

NP

Pos.	487	obs:	exp:
tat	Y	2724	1386.00
tac	Y	165	1503.00
cat	H	1	0.58
cac	H	0	0.42
---	--	------	------
mPD		0.11	0.50
		nPD:	0.22
		N. weight:	0.67
		Sc. PD:	0.012
		Sc. rank:	398.5

NP

Pos.	488	obs:	exp:
ttt	F	0	1204.00
ttc	F	2887	1683.00
tct	S	1	0.50
tcc	S	2	0.43
tca	S	0	0.68
tcg	S	0	0.18
agt	S	0	0.65
agc	S	0	0.55
---	--	------	------
mPD		0.0028	0.49
		nPD:	0.01
		N. weight:	0.71
		Sc. PD:	-0.13
		Sc. rank:	-1154.0

NP

Pos.	489	obs:	exp:
ttt	F	60	1206.00
ttc	F	2830	1684.00
---	--	------	------
mPD		0.041	0.49
		nPD:	0.08
		N. weight:	0.59
		Sc. PD:	-0.065
		Sc. rank:	-219.1

NP

Pos.	490	obs:	exp:
cgt	R	0	0.07
cgc	R	0	0.11
cga	R	2	0.20
cgg	R	0	0.16
aga	R	0	0.93
agg	R	0	0.52
gaa	E	0	0.56
gag	E	1	0.44
ggt	G	0	417.50
ggc	G	0	379.20
gga	G	2887	1265.00
ggg	G	0	825.30
---	--	------	------
mPD		0.0028	0.69
		nPD:	0.
		N. weight:	1.1
		Sc. PD:	-0.21
		Sc. rank:	-1873.5

NP			
Pos.	491	obs:	exp:
cgt	R	0	0.04
cgc	R	0	0.05
cga	R	0	0.10
cgg	R	0	0.08
aga	R	1	0.47
agg	R	0	0.26
gat	D	1	1560.00
gac	D	2888	1329.00
---	--	------	------
mPD		0.0028	0.50
		nPD:	0.01
		N. weight:	1.
		Sc. PD:	-0.19
		Sc. rank:	-1664.3

,

NP			
Pos.	492	obs:	exp:
tct	S	0	1.50
tcc	S	0	1.30
tca	S	0	2.04
tcg	S	0	0.54
cat	H	2	1.16
cac	H	0	0.84
aat	N	2876	1569.00
aac	N	2	1309.00
agt	S	9	1.95
agc	S	0	1.66
gat	D	1	0.54
gac	D	0	0.46
---	--	------	------
mPD		0.0097	0.51
		nPD:	0.02
		N. weight:	0.8
		Sc. PD:	-0.14
		Sc. rank:	-921.0

,

NP			
Pos.	493	obs:	exp:
gct	A	15	728.00
gcc	A	2	559.20
gca	A	2864	1286.00
gcg	A	9	316.30
---	--	------	------
mPD		0.018	0.69
		nPD:	0.03
		N. weight:	1.
		Sc. PD:	-0.17
		Sc. rank:	-1026.1

,

NP			
Pos.	494	obs:	exp:
aaa	K	0	1.71
aag	K	3	1.29
gat	D	4	2.16
gac	D	0	1.84
gaa	E	8	1616.00
gag	E	2874	1266.00
ggt	G	0	0.14
ggc	G	0	0.13
gga	G	0	0.44
ggg	G	1	0.29
---	--	------	------
mPD		0.011	0.50
		nPD:	0.02
		N. weight:	1.1
		Sc. PD:	-0.18
		Sc. rank:	-1128.5

,

NP			
Pos.	495	obs:	exp:
gat	D	10	5.40
gac	D	0	4.60
gaa	E	77	1614.00
gag	E	2802	1265.00
ggt	G	0	0.14
ggc	G	0	0.13
gga	G	0	0.44
ggg	G	1	0.29
---	--	------	------
mPD		0.059	0.50
		nPD:	0.12
		N. weight:	0.91
		Sc. PD:	-0.07
		Sc. rank:	-43.7

,

NP			
Pos.	496	obs:	exp:
ttt	F	8	3.34
ttc	F	0	4.66
tat	Y	2483	1382.00
tac	Y	398	1499.00
cat	H	1	0.58
cac	H	0	0.42
---	--	------	------
mPD		0.24	0.51
		nPD:	0.48
		N. weight:	0.43
		Sc. PD:	0.12
		Sc. rank:	620.6

,

NP			
Pos.	497	obs:	exp:
tat	Y	0	0.96
tac	Y	2	1.04
gat	D	6	1549.00
gac	D	2862	1319.00
gaa	E	16	11.21
gag	E	4	8.79
---	--	------	------
mPD		0.019	0.51
		nPD:	0.04
		N. weight:	1.
		Sc. PD:	-0.16
		Sc. rank:	-778.1

,

NP			
Pos.	498	obs:	exp:
tct	S	0	1.50
tcc	S	0	1.30
tca	S	0	2.04
tcg	S	0	0.54
cat	H	1	0.58
cac	H	0	0.42
act	T	1	0.26
acc	T	0	0.23
aca	T	0	0.42
acg	T	0	0.09
aat	N	2874	1569.00
aac	N	4	1309.00
aaa	K	1	0.57
aag	K	0	0.43
agt	S	9	1.95
agc	S	0	1.66
---	--	------	------
mPD		0.011	0.51
		nPD:	0.02
		N. weight:	0.79
		Sc. PD:	-0.14
		Sc. rank:	-868.0

,

NP			
Pos.	499	obs:	exp:
taa	.	2839	1066.00
tag	.	4	991.00
tga	.	47	833.40
---	--	------	------
mPD		0.035	0.86
		nPD:	0.04
		N. weight:	1.2
		Sc. PD:	-0.18
		Sc. rank:	-905.6

}

Amino acid level conservation per codon

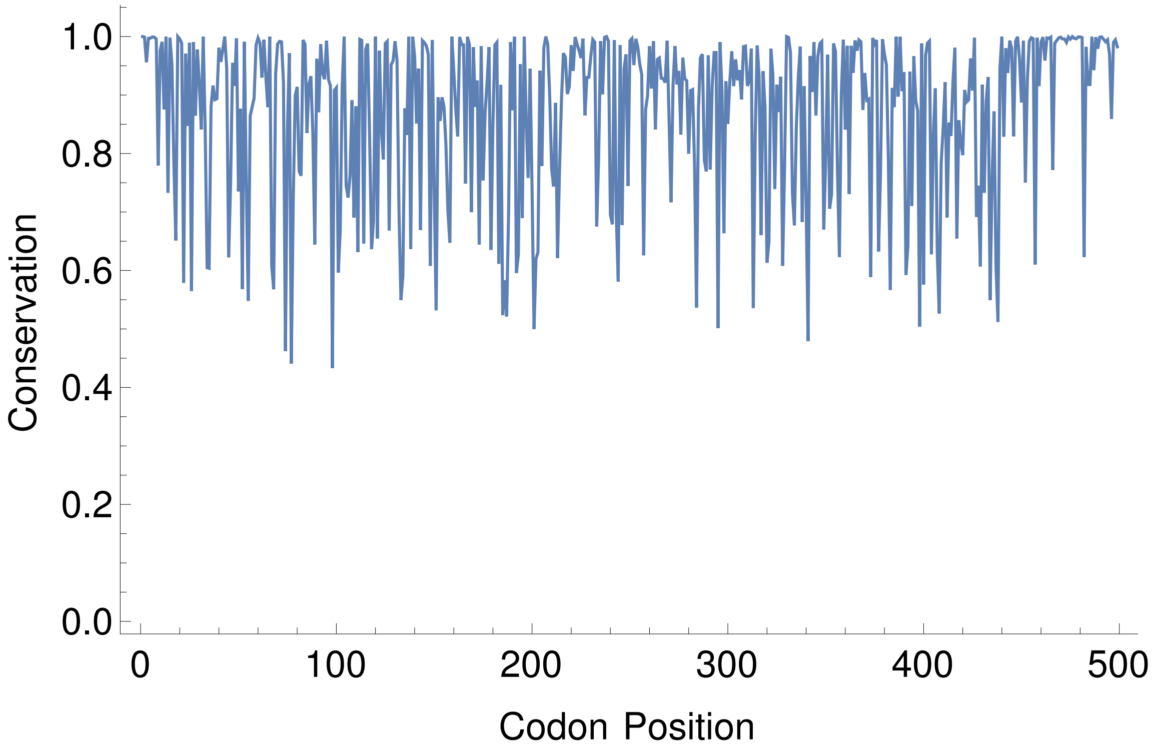

Conservation analysis plots

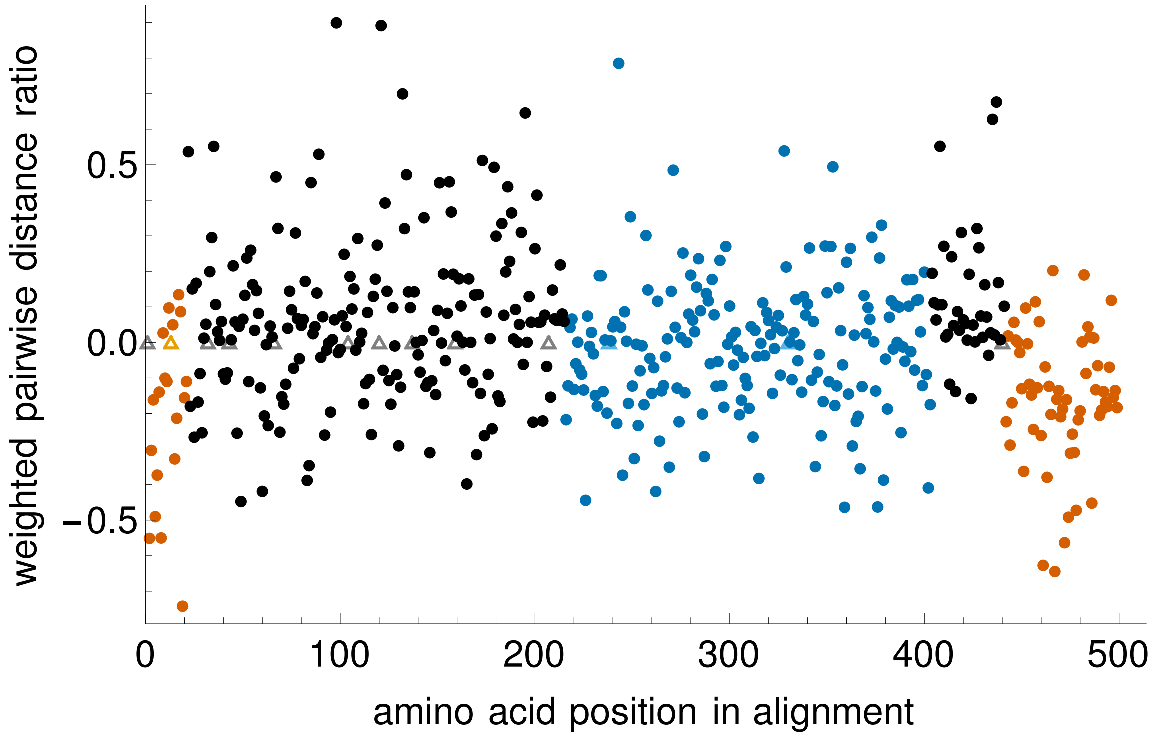

NP

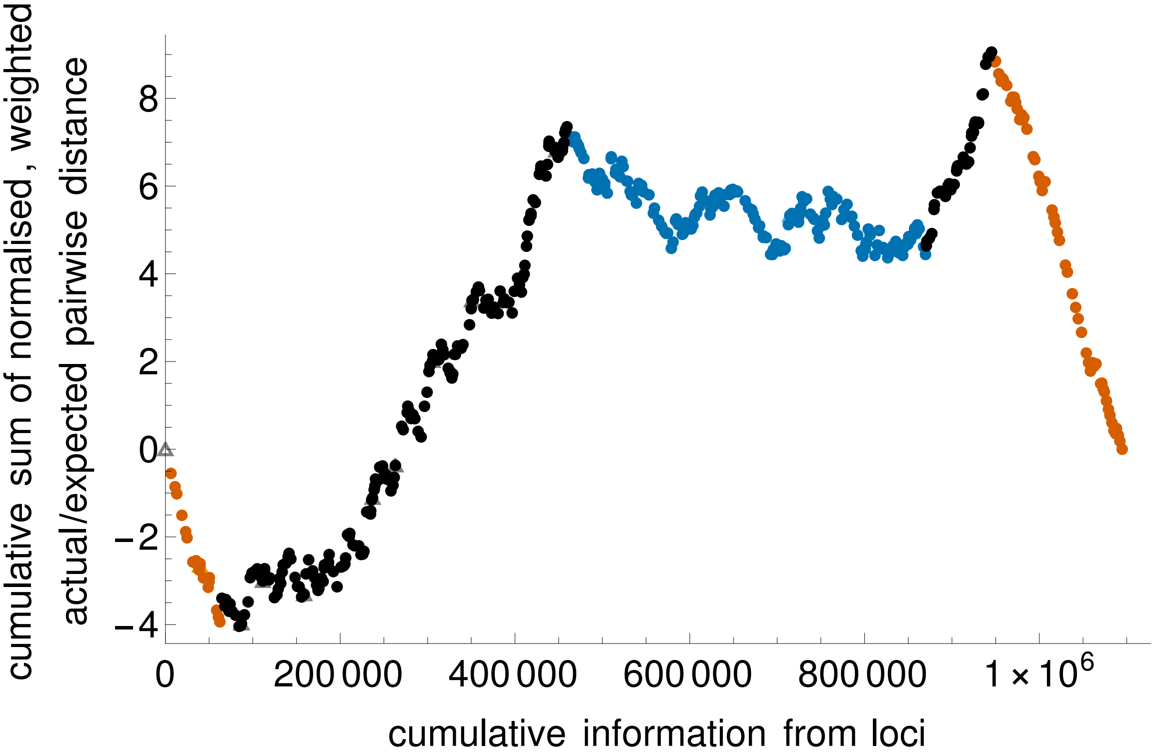

NP

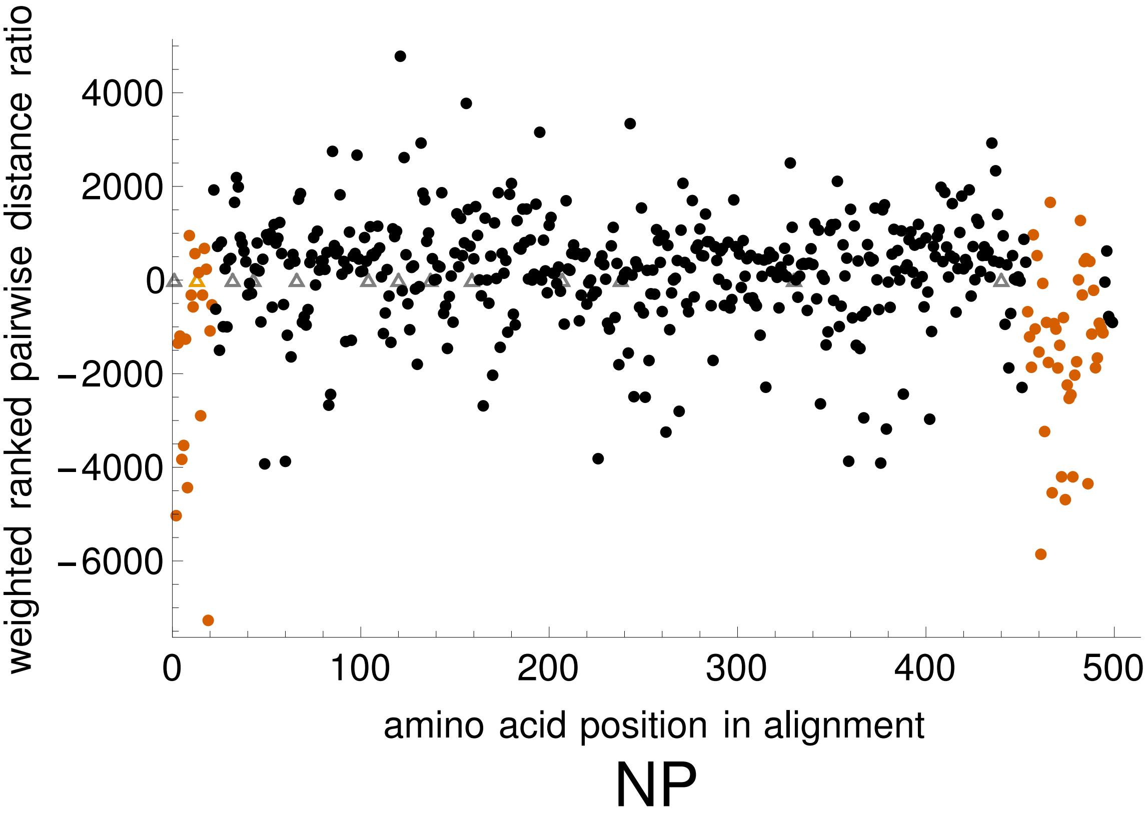
weighted ranked pairwise distance ratio
4000
2000
0
−2000
−4000
−6000
0
100
200
300
400
500
amino acid position in alignment
NP

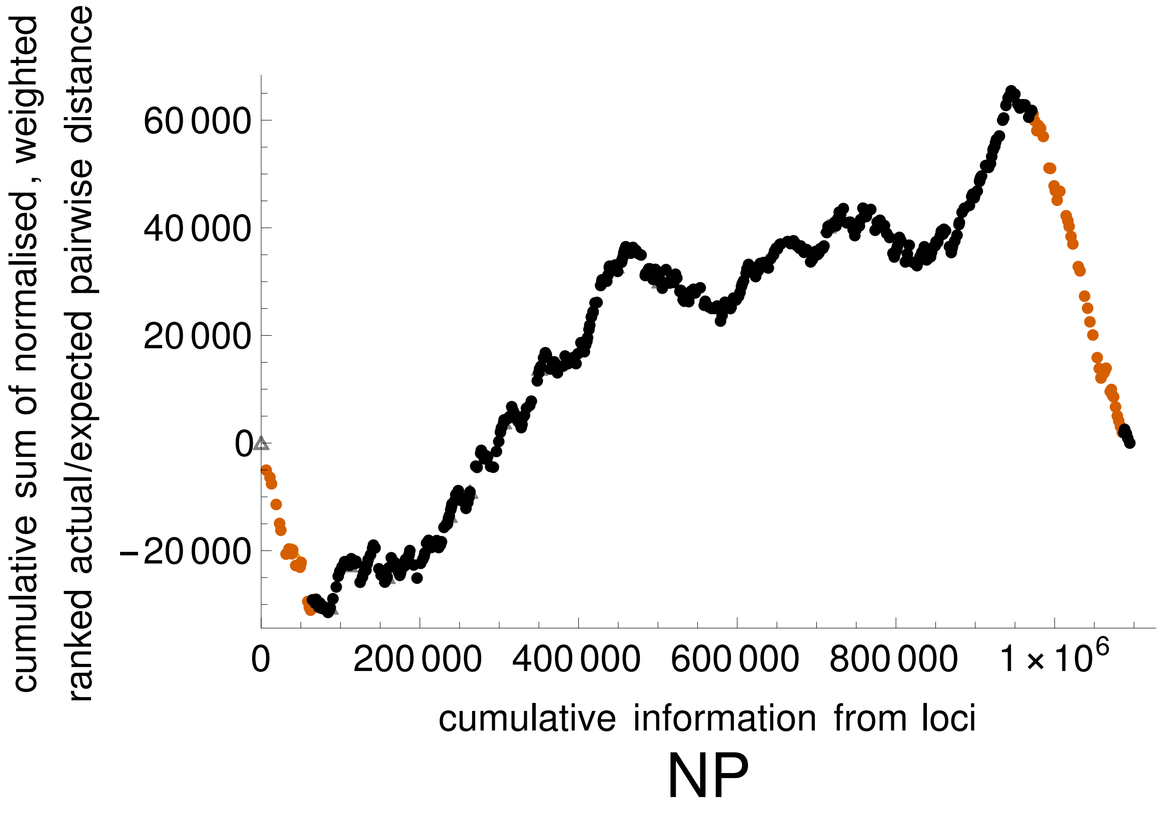
cumulative sum of normalised, weighted ranked actual/expected pairwise distance
60 000
40 000
20 000
0
−20 000
0
200 000
400 000
600 000
800 000
1 × 10^6
cumulative information from loci
NP

NA

Gene length histogram

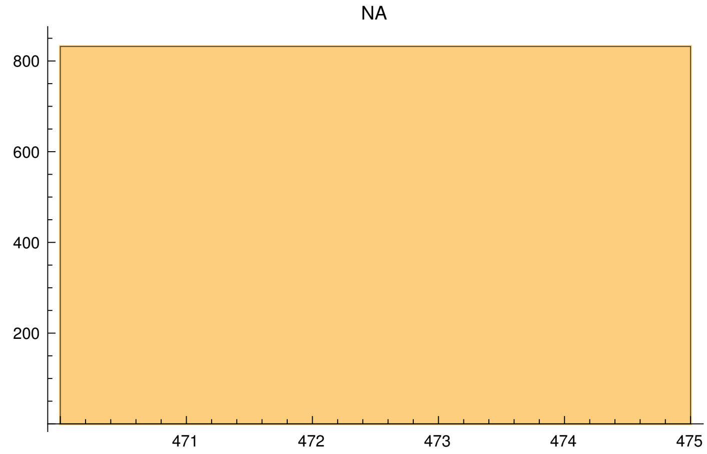

Information vs. nPD

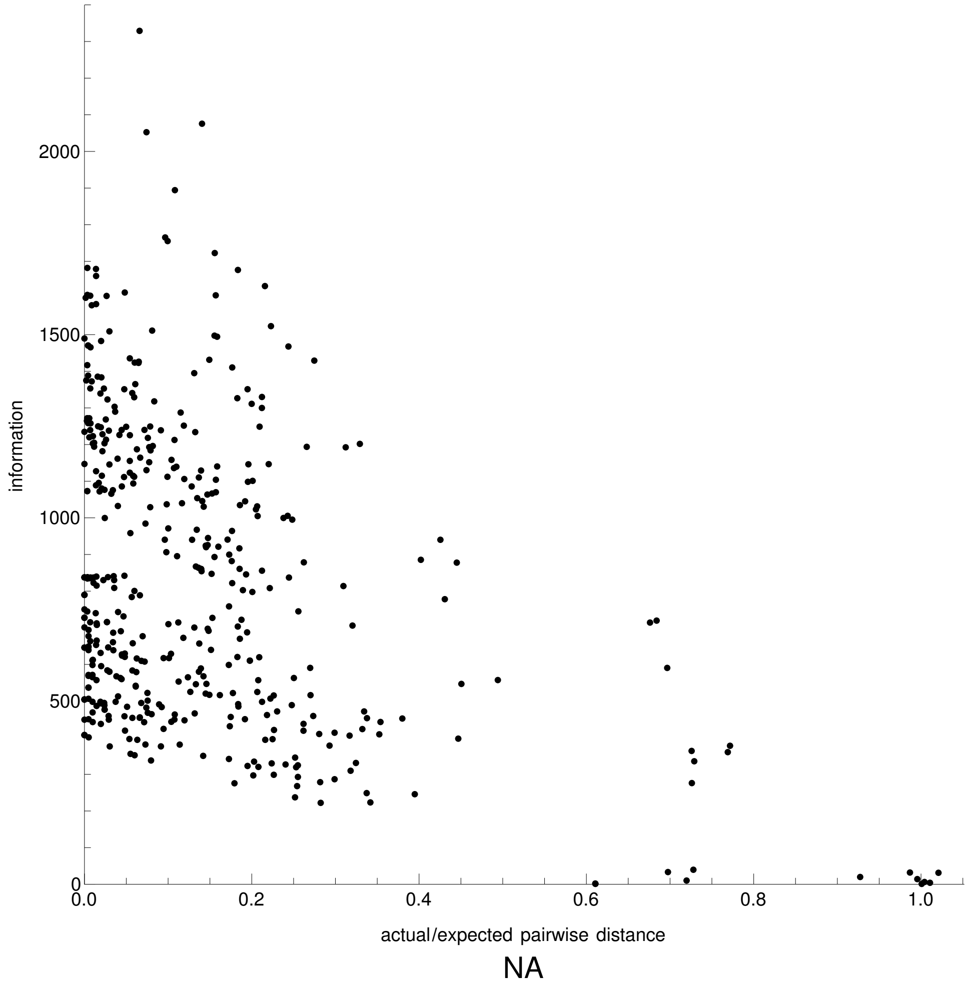

Example sequences highlighted by regions found in analyses to be conserved

Interesting points (by weighted raw PD) highlighted for gene NA:

ATGAATCCAAATCAAAAGATAATAACCATTGGATCAATCTGTATGGTAATTGGGATAGCCAGCTTGATACTACAAA⋱
TTGGGAACATAATCTCAATATGGGTTAGCCATTCAATTCAGACAGGAAATCAATACCAGCCTGAACCATGCAATC⋱
AAAGCGTCATTACCTATGAAAATAACACCTGGGTAAATCAGACGTATGTCAACATTAACAATACCAATTTTCTTG⋱
CTGAACAGACTGTTACTTCGGTGGCATTAGCGGGCAACTCATCTCTTTGCCCTATTAGTGGGTGGGCTATATACA⋱
GTAAGGATAACGGTATAAGAATTGGTTCCAAGGGGGATGTGTTTGTTATAAGAGAGCCATTCATCTCATGCTCCC⋱
ACTTGGAATGCAGGACCTTCTTTTTAACCCAGGGGGCTCTGCTGAATGACAAGCATTCTAATGGGACCGTCAAAG⋱
ATAGAAGTCCCTACAGAACTTTAATGAGTTGTCCTGTGGGTGAGGCTCCTTCCCCGTACAAT
TCAAGATTTGAGTCTGTTGCTTGGTCGGCAAGTGCTTGTCATGATGGCATCAGTT
GGTTGACAATCGGAATTTCTGGTCCAGACAATGGGGCTGTGGCTGTATTGAA
GTACAACGGCATAATAACGGACACTATCAAGAGTTGGAGGAATAACATTTTA
AGAACTCAAGAATCTGAGTGTGCATGTGTAAATGGCTCTTGCTTTACTGTAATGACTGATGGA⋱
CCAAGTAATGGGCAGGCCTCATACAAAATCTTCAAGATAGAAAAAGGGAAAGTAGTC
AAATCAGTTGAATTGAATGCCCCTAATTACCACTACGAGGAGTGCTCCTGTTATC
CTGATGCGGGCGAAATTATGTGTGTGTGCAGGGACAACTGGCATGGCTCAAATCGGCCGTGGGTATCTT⋱
TCAATCAAAATCTGGAGTATCAAATAGGATATATATGCAGTGGGGTTTTCGGGGACAATCCA
CGCCCCAATGATGGGACAGGCAGTTGCGGTCCAATGTCCTCTAGCGGGGCATATG
GAGTAAAAGGGTTTTCATTTAAATACGGCAATGGTGTTTGGATTGGAAGAACAAAAAGCACTAG⋱
TTCCAGAAGCGGCTTTGAGATGATTTGGGATCCGAACGGATGGACTGGGACGGACAATAGTTTCTCAGTG⋱
AAGCAAGACATCGTAGCAATAACTGACTGGTCAGGGTATAGTGGGAGTTTTGTCCAGCATCCAGAACTGA⋱
CAGGATTAGATTGCATGAGGCCTTGTTTCTGGGTTGAGCTAATCAGAGGGCGGCCCAAAGAA ---
AACACAATTTGGACTAGTGGG
AGCAGCATATCCTTTTGTGGTGTAAATAGTGACACTGTGGGTTGGTCTTGGCCAG
ACGGTGCTGAGTTGCCATTCACCATTGACAAGTAG

Interesting points (by weighted ranked PD) highlighted for gene NA:

ATGAATCCAAATCAAAAGATAATAACCATTGGATCAATCTGTATGGTAATTGGGATAGCCAGCTTGATACTACAAATT⋱
GGGAACATAATCTCAATATGGGTTAGCCATTCAATTCAGACAGGAAATCAATACCAGCCTGAACCATGCAATCAAAG⋱
CGTCATTACCTATGAAAATAACACCTGGGTAAATCAGACGTATGTCAACATTAACAATACCAATTTTCTTGCTGAAC⋱
AGACTGTTACTTCGGTGGCATTAGCGGGCAACTCATCTCTTTGCCCTATTAGTGGGTGGGCTATATACAGTAAGGAT⋱
AACGGTATAAGAATTGGTTCCAAGGGGGATGTGTTTGTTATAAGAGAGCCATTCATCTCATGCTCCCACTTGGAATG⋱
CAGGACCTTCTTTTTAACCCAGGGGGCTCTGCTGAATGACAAGCATTCTAATGGGACCGTCAAAGATAGAAGTCCCT⋱
ACAGAACTTTAATGAGTTGTCCTGTGGGTGAGGCTCCTTCCCCGTACAATTCAAGATTTGAGTCTGTTGCTTGGTCG⋱
GCAAGTGCTTGTCATGATGGCATCAGTTGGTTGACAATCGGAATTTCTGGTCCAGACAATGGGGCTGTGGCTGTATT⋱
GAAGTACAACGGCATAATAACGGACACTATCAAGAGTTGGAGGAATAACATTTTAAGAACTCAAGAATCTGAGTGTG⋱
CATGTGTAAATGGCTCTTGCTTTACTGTAATGACTGATGGACCAAGTAATGGGCAGGCCTCATACAAAATCTTCAAG⋱
ATAGAAAAAGGGAAAGTAGTCAAATCAGTTGAATTGAATGCCCCTAATTACCACTACGAGGAGTGCTCCTGTTATCC⋱
TGATGCGGGCGAAATTATGTGTGTGTGCAGGGACAACTGGCATGGCTCAAATCGGCCGTGGGTATCTTTCAATCAAA⋱
ATCTGGAGTATCAAATAGGATATATATGCAGTGGGGTTTTCGGGGACAATCCACGCCCCAATGATGGGACAGGCAGT⋱
TGCGGTCCAATGTCCTCTAGCGGGGCATATGGAGTAAAAGGGTTTTCATTTAAATACGGCAATGGTGTTTGGATTGG⋱
AAGAACAAAAAGCACTAGTTCCAGAAGCGGCTTTGAGATGATTTGGGATCCGAACGGATGGACTGGGACGGACAATA⋱
GTTTCTCAGTGAAGCAAGACATCGTAGCAATAACTGACTGGTCAGGGTATAGTGGGAGTTTTGTCCAGCATCCAGAA⋱
CTGACAGGATTAGATTGCATGAGGCCTTGTTTCTGGGTTGAGCTAATCAGAGGGCGGCCCAAAGAA ---
AACACAATTTGGACTAGTGGGAGCAGCATATCCTTTTGTGGTGTAAATAGT
GACACTGTGGGTTGGTCTTGGCCAGACGGTGCTGAGTTGCCATTCACCATTGACA
AGTAG

Per codon conservation report

NA			
Pos.	1	obs:	exp:
atg	M	832	832.00
---	--	------	------
mPD		0	0
		nPD:	1.
		N. weight:	0.
		Sc. PD:	0
		Sc. rank:	0

NA			
Pos.	2	obs:	exp:
aat	N	831	453.20
aac	N	0	377.80
---	-	1	1.00
---	--	------	------
mPD		0	0.49
		nPD:	0.
		N. weight:	0.64
		Sc. PD:	-0.063
		Sc. rank:	-434.9

NA			
Pos.	3	obs:	exp:
tct	S	0	0.33
tcc	S	0	0.29
tca	S	2	0.45
tcg	S	0	0.12
cct	P	0	226.60
ccc	P	0	149.70
cca	P	827	336.50
ccg	P	2	116.20
agt	S	0	0.43
agc	S	0	0.37
---	-	1	1.00
---	--	------	------
mPD		0.0096	0.71
		nPD:	0.01
		N. weight:	0.93
		Sc. PD:	-0.081
		Sc. rank:	-389.5

NA			
Pos.	4	obs:	exp:
aat	N	783	453.70
aac	N	49	378.30
---	--	------	------
mPD		0.11	0.50
		nPD:	0.22
		N. weight:	0.42
		Sc. PD:	0.048
		Sc. rank:	229.7

NA			
Pos.	5	obs:	exp:
caa	Q	816	415.40
cag	Q	16	416.60
---	--	------	------
mPD		0.038	0.50
		nPD:	0.08
		N. weight:	0.63
		Sc. PD:	-0.017
		Sc. rank:	25.8

NA			
Pos.	6	obs:	exp:
cgt	R	0	13.66
cgc	R	0	19.53
cga	R	0	36.78
cgg	R	0	29.80
aaa	K	0	265.90
aag	K	467	201.10
aga	R	0	170.40
agg	R	365	94.82
---	--	------	------
mPD		0.49	1.2
		nPD:	0.4
		N. weight:	1.1
		Sc. PD:	0.32
		Sc. rank:	733.5

NA			
Pos.	7	obs:	exp:
att	I	0	304.60
atc	I	0	222.10
ata	I	830	303.30
act	T	0	0.52
acc	T	0	0.45
aca	T	2	0.84
acg	T	0	0.19
---	--	------	------
mPD		0.0048	0.67
		nPD:	0.01
		N. weight:	1.1
		Sc. PD:	-0.098
		Sc. rank:	-512.7

NA			
Pos.	8	obs:	exp:
att	I	0	167.30
atc	I	1	122.00
ata	I	455	166.60
atg	M	4	4.00
act	T	0	97.26
acc	T	0	83.60
aca	T	371	155.20
acg	T	0	34.91
gtt	V	0	0.22
gtc	V	0	0.21
gta	V	1	0.21
gtg	V	0	0.36
---	--	------	------
mPD		0.51	1.2
		nPD:	0.43
		N. weight:	0.98
		Sc. PD:	0.31
		Sc. rank:	650.7

NA			
Pos.	9	obs:	exp:
att	I	0	0.37
atc	I	1	0.27
ata	I	0	0.37
act	T	37	217.60
acc	T	793	187.00
aca	T	0	347.30
acg	T	0	78.09
gct	A	0	0.25
gcc	A	1	0.19
gca	A	0	0.45
gcg	A	0	0.11
---	--	------	------
mPD		0.090	0.70
		nPD:	0.13
		N. weight:	1.4
		Sc. PD:	0.033
		Sc. rank:	282.0

NA			
Pos.	10	obs:	exp:
att	I	467	172.50
atc	I	3	125.80
ata	I	0	171.80
act	T	362	94.90
acc	T	0	81.57
aca	T	0	151.50
acg	T	0	34.06
---	--	------	------
mPD		0.50	1.2
		nPD:	0.43
		N. weight:	1.2
		Sc. PD:	0.37
		Sc. rank:	782.9

NA			
Pos.	11	obs:	exp:
ggt	G	3	120.30
ggc	G	0	109.30
gga	G	783	364.60
ggg	G	46	237.80
---	--	------	------
mPD		0.11	0.69
		nPD:	0.16
		N. weight:	0.65
		Sc. PD:	0.037
		Sc. rank:	242.5

NA			
Pos.	12	obs:	exp:
tta	L	2	0.18
ttg	L	0	0.38
tct	S	1	138.00
tcc	S	0	119.80
tca	S	825	187.50
tcg	S	1	49.89
ctt	L	0	0.40
ctc	L	0	0.32
cta	L	0	0.30
ctg	L	0	0.42
act	T	0	0.79
acc	T	0	0.68
aca	T	3	1.26
acg	T	0	0.28
agt	S	0	179.40
agc	S	0	152.40
---	--	------	------
mPD		0.017	1.7
		nPD:	0.01
		N. weight:	1.5
		Sc. PD:	-0.14
		Sc. rank:	-671.2

NA			
Pos.	13	obs:	exp:
att	I	4	304.90
atc	I	827	222.40
ata	I	0	303.70
gtt	V	0	0.22
gtc	V	1	0.21
gta	V	0	0.21
gtg	V	0	0.36
---	--	------	------
mPD		0.012	0.66
		nPD:	0.02
		N. weight:	1.4
		Sc. PD:	-0.11
		Sc. rank:	-474.4

NA			
Pos.	14	obs:	exp:
tgt	C	791	322.00
tgc	C	41	510.00
---	--	------	------
mPD		0.094	0.47
		nPD:	0.2
		N. weight:	0.77
		Sc. PD:	0.07
		Sc. rank:	363.7

NA			
Pos.	15	obs:	exp:
atg	M	832	832.00
---	--	------	------
mPD		0	0
		nPD:	1.
		N. weight:	0.
		Sc. PD:	0
		Sc. rank:	0

NA			
Pos.	16	obs:	exp:
tta	L	0	0.09
ttg	L	0	0.19
ctt	L	0	0.20
ctc	L	0	0.16
cta	L	1	0.15
ctg	L	0	0.21
att	I	0	2.20
atc	I	0	1.61
ata	I	6	2.19
gtt	V	0	171.60
gtc	V	0	167.90
gta	V	794	170.20
gtg	V	4	288.30
gct	A	0	6.80
gcc	A	0	5.22
gca	A	19	12.02
gcg	A	8	2.96
---	--	------	------
mPD		0.11	0.82
		nPD:	0.13
		N. weight:	1.6
		Sc. PD:	0.043
		Sc. rank:	347.7

NA			
Pos.	17	obs:	exp:
att	I	831	304.90
atc	I	0	222.40
ata	I	0	303.70
gtt	V	1	0.22
gtc	V	0	0.21
gta	V	0	0.21
gtg	V	0	0.36
---	--	------	------
mPD		0.0024	0.66
		nPD:	0.
		N. weight:	1.1
		Sc. PD:	-0.1
		Sc. rank:	-600.5

NA			
Pos.	18	obs:	exp:
ggt	G	0	120.30
ggc	G	0	109.30
gga	G	81	364.60
ggg	G	751	237.80
---	--	------	------
mPD		0.18	0.69
		nPD:	0.26
		N. weight:	0.94
		Sc. PD:	0.14
		Sc. rank:	557.7

NA			
Pos.	19	obs:	exp:
att	I	0	304.20
atc	I	0	221.80
ata	I	829	303.00
act	T	0	0.79
acc	T	0	0.68
aca	T	3	1.26
acg	T	0	0.28
---	--	------	------
mPD		0.0072	0.67
		nPD:	0.01
		N. weight:	1.1
		Sc. PD:	-0.094
		Sc. rank:	-456.2

NA			
Pos.	20	obs:	exp:
ttt	F	0	1.25
ttc	F	3	1.75
att	I	0	15.41
atc	I	4	11.24
ata	I	38	15.35
gtt	V	12	163.70
gtc	V	748	160.10
gta	V	1	162.30
gtg	V	0	275.00
gct	A	1	6.55
gcc	A	25	5.03
gca	A	0	11.57
gcg	A	0	2.85
---	--	------	------
mPD		0.28	0.90
		nPD:	0.31
		N. weight:	1.5
		Sc. PD:	0.3
		Sc. rank:	947.9

NA			
Pos.	21	obs:	exp:
tct	S	0	136.90
tcc	S	0	118.80
tca	S	0	185.90
tcg	S	0	49.47
aat	N	10	5.45
aac	N	0	4.55
agt	S	35	177.90
agc	S	785	151.10
ggt	G	0	0.29
ggc	G	2	0.26
gga	G	0	0.88
ggg	G	0	0.57
---	--	------	------
mPD		0.13	1.7
		nPD:	0.08
		N. weight:	1.6
		Sc. PD:	-0.038
		Sc. rank:	95.0

NA			
Pos.	22	obs:	exp:
tta	L	68	76.83
ttg	L	716	157.90
tct	S	0	0.17
tcc	S	0	0.14
tca	S	0	0.23
tcg	S	1	0.06
ctt	L	0	165.00
ctc	L	0	132.90
cta	L	7	123.00
ctg	L	40	175.40
agt	S	0	0.22
agc	S	0	0.18
---	--	------	------
mPD		0.27	1.1
		nPD:	0.24
		N. weight:	1.3
		Sc. PD:	0.17
		Sc. rank:	720.7

NA			
Pos.	23	obs:	exp:
att	I	0	1.83
atc	I	1	1.34
ata	I	4	1.83
atg	M	789	789.00
gtt	V	0	8.17
gtc	V	0	7.99
gta	V	4	8.10
gtg	V	34	13.73
---	--	------	------
mPD		0.11	0.16
		nPD:	0.7
		N. weight:	0.042
		Sc. PD:	0.024
		Sc. rank:	28.6

NA			
Pos.	24	obs:	exp:
tta	L	28	76.93
ttg	L	1	158.10
ctt	L	0	165.20
ctc	L	0	133.10
cta	L	47	123.10
ctg	L	756	175.60
---	--	------	------
mPD		0.23	1.1
		nPD:	0.21
		N. weight:	1.3
		Sc. PD:	0.13
		Sc. rank:	645.8

NA			
Pos.	25	obs:	exp:
caa	Q	830	415.40
cag	Q	2	416.60
---	--	------	------
mPD		0.0048	0.50
		nPD:	0.01
		N. weight:	0.71
		Sc. PD:	-0.064
		Sc. rank:	-326.6

NA			
Pos.	26	obs:	exp:
tta	L	0	0.09
ttg	L	0	0.19
ctt	L	1	0.20
ctc	L	0	0.16
cta	L	0	0.15
ctg	L	0	0.21
att	I	824	302.40
atc	I	0	220.50
ata	I	0	301.10
act	T	1	0.26
acc	T	0	0.23
aca	T	0	0.42
acg	T	0	0.09
gtt	V	6	1.29
gtc	V	0	1.26
gta	V	0	1.28
gtg	V	0	2.17
---	--	------	------
mPD		0.019	0.68
		nPD:	0.03
		N. weight:	1.1
		Sc. PD:	-0.077
		Sc. rank:	-260.3

NA			
Pos.	27	obs:	exp:
ggt	G	0	120.30
ggc	G	0	109.30
gga	G	41	364.60
ggg	G	791	237.80
---	--	------	------
mPD		0.094	0.69
		nPD:	0.14
		N. weight:	1.1
		Sc. PD:	0.034
		Sc. rank:	260.6

NA			
Pos.	28	obs:	exp:
aat	N	32	453.70
aac	N	800	378.30
---	--	------	------
mPD		0.074	0.50
		nPD:	0.15
		N. weight:	0.65
		Sc. PD:	0.029
		Sc. rank:	206.2

NA			
Pos.	29	obs:	exp:
att	I	0	301.60
atc	I	0	220.00
ata	I	822	300.40
atg	M	7	7.00
act	T	0	0.79
acc	T	0	0.68
aca	T	3	1.26
acg	T	0	0.28
---	--	------	------
mPD		0.024	0.67
		nPD:	0.04
		N. weight:	1.
		Sc. PD:	-0.068
		Sc. rank:	-208.5

NA			
Pos.	30	obs:	exp:
att	I	37	304.20
atc	I	790	221.80
ata	I	2	303.00
gtt	V	0	0.65
gtc	V	3	0.63
gta	V	0	0.64
gtg	V	0	1.08
---	--	------	------
mPD		0.097	0.67
		nPD:	0.14
		N. weight:	1.2
		Sc. PD:	0.047
		Sc. rank:	335.3

NA			
Pos.	31	obs:	exp:
tct	S	0	138.90
tcc	S	0	120.50
tca	S	832	188.60
tcg	S	0	50.20
agt	S	0	180.50
agc	S	0	153.30
---	--	------	------
mPD		0	1.7
		nPD:	0.
		N. weight:	1.6
		Sc. PD:	-0.16
		Sc. rank:	-1065.7

NA			
Pos.	32	obs:	exp:
att	I	0	305.30
atc	I	0	222.60
ata	I	832	304.00
---	--	------	------
mPD		0	0.66
		nPD:	0.
		N. weight:	1.1
		Sc. PD:	-0.11
		Sc. rank:	-722.9

NA			
Pos.	33	obs:	exp:
tgg	W	832	832.00
---	--	------	------
mPD		0	0
		nPD:	1.
		N. weight:	0.
		Sc. PD:	0
		Sc. rank:	0

NA			
Pos.	34	obs:	exp:
att	I	6	2.20
atc	I	0	1.61
ata	I	0	2.19
gtt	V	824	177.40
gtc	V	1	173.60
gta	V	0	175.90
gtg	V	0	298.10
gct	A	1	0.25
gcc	A	0	0.19
gca	A	0	0.45
gcg	A	0	0.11
---	--	------	------
mPD		0.019	0.75
		nPD:	0.03
		N. weight:	1.6
		Sc. PD:	-0.12
		Sc. rank:	-435.2

NA			
Pos.	35	obs:	exp:
tct	S	0	138.90
tcc	S	0	120.50
tca	S	0	188.60
tcg	S	0	50.20
agt	S	26	180.50
agc	S	806	153.30
---	--	------	------
mPD		0.061	1.7
		nPD:	0.04
		N. weight:	1.6
		Sc. PD:	-0.1
		Sc. rank:	-303.6

NA			
Pos.	36	obs:	exp:
cat	H	828	481.10
cac	H	3	349.90
caa	Q	0	0.50
cag	Q	1	0.50
---	--	------	------
mPD		0.0096	0.49
		nPD:	0.02
		N. weight:	0.55
		Sc. PD:	-0.045
		Sc. rank:	-184.1

NA			
Pos.	37	obs:	exp:
tct	S	0	138.70
tcc	S	6	120.40
tca	S	825	188.40
tcg	S	0	50.13
act	T	0	0.26
acc	T	0	0.23
aca	T	1	0.42
acg	T	0	0.09
agt	S	0	180.20
agc	S	0	153.10
---	--	------	------
mPD		0.017	1.7
		nPD:	0.01
		N. weight:	1.5
		Sc. PD:	-0.14
		Sc. rank:	-667.1

NA			
Pos.	38	obs:	exp:
att	I	42	305.30
atc	I	790	222.60
ata	I	0	304.00
---	--	------	------
mPD		0.096	0.66
		nPD:	0.15
		N. weight:	1.2
		Sc. PD:	0.047
		Sc. rank:	337.8

NA			
Pos.	39	obs:	exp:
caa	Q	782	414.90
cag	Q	49	416.10
cgt	R	0	0.04
cgc	R	0	0.05
cga	R	1	0.10
cgg	R	0	0.08
aga	R	0	0.47
agg	R	0	0.26
---	--	------	------
mPD		0.11	0.50
		nPD:	0.22
		N. weight:	0.5
		Sc. PD:	0.058
		Sc. rank:	276.4

NA			
Pos.	40	obs:	exp:
tct	S	1	0.17
tcc	S	0	0.14
tca	S	0	0.23
tcg	S	0	0.06
att	I	8	2.94
atc	I	0	2.14
ata	I	0	2.92
act	T	34	215.50
acc	T	0	185.20
aca	T	787	344.00
acg	T	1	77.34
agt	S	0	0.22
agc	S	0	0.18
gct	A	0	0.25
gcc	A	0	0.19
gca	A	1	0.45
gcg	A	0	0.11
---	--	------	------
mPD		0.12	0.72
		nPD:	0.17
		N. weight:	0.76
		Sc. PD:	0.05
		Sc. rank:	285.4

NA			
Pos.	41	obs:	exp:
cgt	R	0	0.04
cgc	R	0	0.05
cga	R	0	0.10
cgg	R	0	0.08
aga	R	0	0.47
agg	R	1	0.26
gaa	E	8	5.05
gag	E	1	3.95
ggt	G	0	118.70
ggc	G	0	107.80
gga	G	163	359.70
ggg	G	658	234.70
---	-	1	1.00
---	--	------	------
mPD		0.35	0.71
		nPD:	0.49
		N. weight:	0.7
		Sc. PD:	0.26
		Sc. rank:	472.3

,

NA			
Pos.	42	obs:	exp:
tct	S	0	6.84
tcc	S	0	5.94
tca	S	0	9.29
tcg	S	0	2.47
att	I	1	0.37
atc	I	0	0.27
ata	I	0	0.37
aat	N	787	430.30
aac	N	2	358.70
agt	S	21	8.89
agc	S	20	7.56
---	-	1	1.00
---	--	------	------
mPD		0.15	0.66
		nPD:	0.22
		N. weight:	0.64
		Sc. PD:	0.073
		Sc. rank:	348.4

,

NA			
Pos.	43	obs:	exp:
cat	H	1	0.58
cac	H	0	0.42
caa	Q	776	408.90
cag	Q	43	410.10
aaa	K	1	6.26
aag	K	10	4.74
---	-	1	1.00
---	--	------	------
mPD		0.15	0.53
		nPD:	0.28
		N. weight:	0.52
		Sc. PD:	0.088
		Sc. rank:	319.2

,

NA			
Pos.	44	obs:	exp:
tct	S	0	1.84
tcc	S	0	1.59
tca	S	0	2.49
tcg	S	0	0.66
tat	Y	0	370.30
tac	Y	772	401.70
tgt	C	0	0.39
tgc	C	1	0.61
cat	H	0	10.42
cac	H	18	7.58
aat	N	0	12.00
aac	N	22	10.00
agt	S	0	2.39
agc	S	11	2.03
gat	D	0	3.24
gac	D	6	2.76
ggt	G	0	0.14
ggc	G	1	0.13
gga	G	0	0.44
ggg	G	0	0.29
---	-	1	1.00
---	--	------	------
mPD		0.16	0.65
		nPD:	0.25
		N. weight:	0.71
		Sc. PD:	0.1
		Sc. rank:	414.9

,

NA			
Pos.	45	obs:	exp:
tat	Y	0	6.23
tac	Y	13	6.77
cct	P	0	0.27
ccc	P	1	0.18
cca	P	0	0.41
ccg	P	0	0.14
cat	H	5	15.05
cac	H	21	10.95
caa	Q	11	393.40
cag	Q	777	394.60
cgt	R	0	0.11
cgc	R	1	0.16
cga	R	0	0.30
cgg	R	2	0.24
aga	R	0	1.40
agg	R	0	0.78
---	-	1	1.00
---	--	------	------
mPD		0.16	0.59
		nPD:	0.27
		N. weight:	0.65
		Sc. PD:	0.1
		Sc. rank:	396.2

,

NA			
Pos.	46	obs:	exp:
tta	L	0	0.28
ttg	L	0	0.57
tct	S	6	1.00
tcc	S	0	0.87
tca	S	0	1.36
tcg	S	0	0.36
ctt	L	3	0.60
ctc	L	0	0.48
cta	L	0	0.44
ctg	L	0	0.63
cct	P	770	220.80
ccc	P	27	145.90
cca	P	11	328.00
ccg	P	0	113.30
cat	H	2	1.16
cac	H	0	0.84
agt	S	0	1.30
agc	S	0	1.11
gct	A	12	3.02
gcc	A	0	2.32
gca	A	0	5.34
gcg	A	0	1.31
---	-	1	1.00
---	--	------	------
mPD		0.14	0.77
		nPD:	0.19
		N. weight:	1.2
		Sc. PD:	0.091
		Sc. rank:	496.7

,

NA			
Pos.	47	obs:	exp:
aaa	K	3	1.71
aag	K	0	1.29
gaa	E	807	454.60
gag	E	4	356.40
ggt	G	0	2.60
ggc	G	0	2.36
gga	G	18	7.89
ggg	G	0	5.15
---	--	------	------
mPD		0.059	0.55
		nPD:	0.11
		N. weight:	0.59
		Sc. PD:	0.0025
		Sc. rank:	91.2

,

NA			
Pos.	48	obs:	exp:
tct	S	0	0.50
tcc	S	0	0.43
tca	S	3	0.68
tcg	S	0	0.18
cct	P	0	215.10
ccc	P	0	142.10
cca	P	777	319.40
ccg	P	10	110.30
att	I	0	0.37
atc	I	0	0.27
ata	I	1	0.37
act	T	0	10.49
acc	T	0	9.01
aca	T	32	16.74
acg	T	8	3.76
aaa	K	1	0.57
aag	K	0	0.43
agt	S	0	0.65
agc	S	0	0.55
---	--	------	------
mPD		0.15	0.82
		nPD:	0.18
		N. weight:	0.89
		Sc. PD:	0.068
		Sc. rank:	369.4

,

NA			
Pos.	49	obs:	exp:
tgt	C	0	322.00
tgc	C	832	510.00
---	--	------	------
mPD		0	0.47
		nPD:	0.
		N. weight:	0.51
		Sc. PD:	-0.051
		Sc. rank:	-351.5

,

NA			
Pos.	50	obs:	exp:
aat	N	827	453.70
aac	N	5	378.30
---	--	------	------
mPD		0.012	0.50
		nPD:	0.02
		N. weight:	0.6
		Sc. PD:	-0.046
		Sc. rank:	-169.6

,

NA			
Pos.	51	obs:	exp:
cat	H	0	0.58
cac	H	1	0.42
caa	Q	830	414.90
cag	Q	1	416.10
---	--	------	------
mPD		0.0048	0.50
		nPD:	0.01
		N. weight:	0.72
		Sc. PD:	-0.065
		Sc. rank:	-331.7

,

NA			
Pos.	52	obs:	exp:
tct	S	0	136.90
tcc	S	0	118.80
tca	S	0	185.90
tcg	S	0	49.47
cgt	R	0	0.22
cgc	R	0	0.32
cga	R	0	0.60
cgg	R	0	0.49
agt	S	40	177.90
agc	S	780	151.10
aga	R	6	2.80
agg	R	0	1.56
ggt	G	0	0.87
ggc	G	6	0.79
gga	G	0	2.63
ggg	G	0	1.72
---	--	------	------
mPD		0.12	1.7
		nPD:	0.07
		N. weight:	1.6
		Sc. PD:	-0.048
		Sc. rank:	25.5

,

NA			
Pos.	53	obs:	exp:
att	I	12	286.20
atc	I	768	208.70
ata	I	0	285.00
act	T	0	2.88
acc	T	11	2.48
aca	T	0	4.60
acg	T	0	1.03
gtt	V	0	8.39
gtc	V	39	8.21
gta	V	0	8.32
gtg	V	0	14.09
gct	A	0	0.50
gcc	A	2	0.39
gca	A	0	0.89
gcg	A	0	0.22
---	--	------	------
mPD		0.15	0.80
		nPD:	0.19
		N. weight:	1.3
		Sc. PD:	0.11
		Sc. rank:	593.4

,

NA			
Pos.	54	obs:	exp:
att	I	822	303.50
atc	I	5	221.30
ata	I	0	302.20
act	T	4	1.05
acc	T	0	0.90
aca	T	0	1.67
acg	T	0	0.38
gtt	V	1	0.22
gtc	V	0	0.21
gta	V	0	0.21
gtg	V	0	0.36
---	--	------	------
mPD		0.024	0.67
		nPD:	0.04
		N. weight:	1.
		Sc. PD:	-0.067
		Sc. rank:	-200.1

,

NA			
Pos.	55	obs:	exp:
act	T	51	217.10
acc	T	776	186.60
aca	T	1	346.50
acg	T	0	77.90
aat	N	2	1.09
aac	N	0	0.91
gct	A	0	0.50
gcc	A	2	0.39
gca	A	0	0.89
gcg	A	0	0.22
---	--	------	------
mPD		0.13	0.71
		nPD:	0.19
		N. weight:	1.3
		Sc. PD:	0.1
		Sc. rank:	573.0

,

NA			
Pos.	56	obs:	exp:
tat	Y	794	399.00
tac	Y	38	433.00
---	--	------	------
mPD		0.087	0.50
		nPD:	0.17
		N. weight:	0.58
		Sc. PD:	0.04
		Sc. rank:	223.8

,

NA			
Pos.	57	obs:	exp:
aaa	K	1	0.57
aag	K	0	0.43
gaa	E	78	465.80
gag	E	753	365.20
---	--	------	------
mPD		0.17	0.50
		nPD:	0.35
		N. weight:	0.52
		Sc. PD:	0.12
		Sc. rank:	335.1

,

NA			
Pos.	58	obs:	exp:
aat	N	4	453.70
aac	N	828	378.30
---	--	------	------
mPD		0.0096	0.50
		nPD:	0.02
		N. weight:	0.8
		Sc. PD:	-0.064
		Sc. rank:	-270.7

,

NA			
Pos.	59	obs:	exp:
aat	N	31	453.70
aac	N	801	378.30
---	--	------	------
mPD		0.072	0.50
		nPD:	0.14
		N. weight:	0.66
		Sc. PD:	0.026
		Sc. rank:	186.6

,

NA			
Pos.	60	obs:	exp:
att	I	0	1.47
atc	I	4	1.07
ata	I	0	1.46
act	T	43	217.10
acc	T	785	186.60
aca	T	0	346.50
acg	T	0	77.90
---	--	------	------
mPD		0.11	0.71
		nPD:	0.15
		N. weight:	1.3
		Sc. PD:	0.064
		Sc. rank:	438.0

,

NA			
Pos.	61	obs:	exp:
tgg	W	832	832.00
---	--	------	------
mPD		0	0
		nPD:	1.
		N. weight:	0.
		Sc. PD:	0
		Sc. rank:	0

,

NA			
Pos.	62	obs:	exp:
att	I	0	4.40
atc	I	0	3.21
ata	I	12	4.39
gtt	V	0	176.20
gtc	V	0	172.30
gta	V	780	174.60
gtg	V	39	295.90
gct	A	0	0.25
gcc	A	0	0.19
gca	A	1	0.45
gcg	A	0	0.11
---	--	------	------
mPD		0.12	0.77
		nPD:	0.16
		N. weight:	1.4
		Sc. PD:	0.072
		Sc. rank:	497.6

,

NA			
Pos.	63	obs:	exp:
aat	N	827	453.70
aac	N	5	378.30
---	--	------	------
mPD		0.012	0.50
		nPD:	0.02
		N. weight:	0.6
		Sc. PD:	-0.046
		Sc. rank:	-169.6

,

NA			
Pos.	64	obs:	exp:
cat	H	1	0.58
cac	H	0	0.42
caa	Q	18	414.90
cag	Q	813	416.10
---	--	------	------
mPD		0.045	0.50
		nPD:	0.09
		N. weight:	0.62
		Sc. PD:	-0.0085
		Sc. rank:	54.5

,

NA			
Pos.	65	obs:	exp:
act	T	0	218.10
acc	T	0	187.50
aca	T	68	348.10
acg	T	764	78.28
---	--	------	------
mPD		0.15	0.70
		nPD:	0.22
		N. weight:	2.1
		Sc. PD:	0.22
		Sc. rank:	1097.3

,

NA			
Pos.	66	obs:	exp:
tat	Y	791	398.60
tac	Y	40	432.40
aat	N	0	0.55
aac	N	1	0.45
---	--	------	------
mPD		0.096	0.50
		nPD:	0.19
		N. weight:	0.57
		Sc. PD:	0.048
		Sc. rank:	253.6

,

NA			
Pos.	67	obs:	exp:
tta	L	0	0.92
ttg	L	0	1.90
ctt	L	10	1.99
ctc	L	0	1.60
cta	L	0	1.48
ctg	L	0	2.11
att	I	9	17.98
atc	I	40	13.11
ata	I	0	17.91
gtt	V	2	166.30
gtc	V	771	162.60
gta	V	0	164.80
gtg	V	0	279.30
---	--	------	------
mPD		0.18	0.87
		nPD:	0.21
		N. weight:	1.6
		Sc. PD:	0.16
		Sc. rank:	806.8

,

NA			
Pos.	68	obs:	exp:
aat	N	1	453.70
aac	N	831	378.30
---	--	------	------
mPD		0.0024	0.50
		nPD:	0.
		N. weight:	0.82
		Sc. PD:	-0.078
		Sc. rank:	-433.4

,

NA			
Pos.	69	obs:	exp:
att	I	28	292.50
atc	I	730	213.30
ata	I	39	291.30
act	T	0	0.52
acc	T	2	0.45
aca	T	0	0.84
acg	T	0	0.19
gtt	V	0	7.10
gtc	V	33	6.94
gta	V	0	7.04
gtg	V	0	11.92
---	--	------	------
mPD		0.23	0.75
		nPD:	0.31
		N. weight:	1.
		Sc. PD:	0.2
		Sc. rank:	642.4

,

NA			
Pos.	70	obs:	exp:
tct	S	0	131.90
tcc	S	0	114.40
tca	S	0	179.10
tcg	S	0	47.66
cgt	R	0	0.04
cgc	R	0	0.05
cga	R	0	0.10
cgg	R	0	0.08
aat	N	1	22.36
aac	N	40	18.64
agt	S	37	171.40
agc	S	753	145.60
aga	R	1	0.47
agg	R	0	0.26
---	--	------	------
mPD		0.18	1.7
		nPD:	0.11
		N. weight:	1.5
		Sc. PD:	0.0061
		Sc. rank:	236.0

,

NA			
Pos.	71	obs:	exp:
aat	N	829	453.70
aac	N	3	378.30
---	--	------	------
mPD		0.0072	0.50
		nPD:	0.01
		N. weight:	0.61
		Sc. PD:	-0.053
		Sc. rank:	-234.4

,

NA			
Pos.	72	obs:	exp:
att	I	0	0.37
atc	I	1	0.27
ata	I	0	0.37
act	T	1	217.80
acc	T	816	187.20
aca	T	14	347.70
acg	T	0	78.19
---	--	------	------
mPD		0.038	0.70
		nPD:	0.05
		N. weight:	1.5
		Sc. PD:	-0.069
		Sc. rank:	-139.0

,

NA			
Pos.	73	obs:	exp:
att	I	7	2.57
atc	I	0	1.87
ata	I	0	2.56
aat	N	821	449.90
aac	N	4	375.10
---	--	------	------
mPD		0.026	0.52
		nPD:	0.05
		N. weight:	0.61
		Sc. PD:	-0.031
		Sc. rank:	-67.5

,

NA			
Pos.	74	obs:	exp:
ttt	F	788	330.40
ttc	F	4	461.60
tta	L	39	3.70
ttg	L	0	7.60
ctt	L	1	7.94
ctc	L	0	6.40
cta	L	0	5.92
ctg	L	0	8.44
---	--	------	------
mPD		0.10	0.58
		nPD:	0.17
		N. weight:	0.96
		Sc. PD:	0.064
		Sc. rank:	365.5

,

NA			
Pos.	75	obs:	exp:
tta	L	0	73.04
ttg	L	0	150.10
ctt	L	790	156.90
ctc	L	0	126.40
cta	L	0	116.90
ctg	L	0	166.70
cct	P	1	0.27
ccc	P	0	0.18
cca	P	0	0.41
ccg	P	0	0.14
att	I	37	13.94
atc	I	1	10.17
ata	I	0	13.89
gtt	V	3	0.65
gtc	V	0	0.63
gta	V	0	0.64
gtg	V	0	1.08
---	--	------	------
mPD		0.099	1.2
		nPD:	0.08
		N. weight:	1.7
		Sc. PD:	-0.032
		Sc. rank:	143.3

,

NA			
Pos.	76	obs:	exp:
tct	S	1	0.50
tcc	S	0	0.43
tca	S	2	0.68
tcg	S	0	0.18
act	T	27	7.08
acc	T	0	6.08
aca	T	0	11.30
acg	T	0	2.54
agt	S	0	0.65
agc	S	0	0.55
gtt	V	2	0.65
gtc	V	0	0.63
gta	V	1	0.64
gtg	V	0	1.08
gct	A	762	199.30
gcc	A	1	153.10
gca	A	28	352.10
gcg	A	0	86.58
ggt	G	0	1.16
ggc	G	0	1.05
gga	G	8	3.51
ggg	G	0	2.29
---	--	------	------
mPD		0.19	0.79
		nPD:	0.24
		N. weight:	1.3
		Sc. PD:	0.16
		Sc. rank:	710.4

,

NA			
Pos.	77	obs:	exp:
gtt	V	0	0.22
gtc	V	0	0.21
gta	V	0	0.21
gtg	V	1	0.36
gaa	E	54	458.60
gag	E	764	359.40
ggt	G	0	1.88
ggc	G	0	1.71
gga	G	11	5.70
ggg	G	2	3.72
---	--	------	------
mPD		0.18	0.53
		nPD:	0.33
		N. weight:	0.6
		Sc. PD:	0.13
		Sc. rank:	383.4

,

NA			
Pos.	78	obs:	exp:
caa	Q	2	408.40
cag	Q	816	409.60
cgt	R	0	0.07
cgc	R	0	0.11
cga	R	0	0.20
cgg	R	2	0.16
aaa	K	12	6.83
aag	K	0	5.17
aga	R	0	0.93
agg	R	0	0.52
---	--	------	------
mPD		0.066	0.54
		nPD:	0.12
		N. weight:	0.71
		Sc. PD:	0.014
		Sc. rank:	142.5

,

NA			
Pos.	79	obs:	exp:
act	T	13	3.41
acc	T	0	2.93
aca	T	0	5.44
acg	T	0	1.22
gtt	V	7	1.51
gtc	V	0	1.47
gta	V	0	1.49
gtg	V	0	2.53
gct	A	809	204.60
gcc	A	3	157.10
gca	A	0	361.40
gcg	A	0	88.88
---	--	------	------
mPD		0.055	0.74
		nPD:	0.07
		N. weight:	1.4
		Sc. PD:	-0.04
		Sc. rank:	42.0

,

NA			
Pos.	80	obs:	exp:
tta	L	1	0.18
ttg	L	0	0.38
ctt	L	0	0.40
ctc	L	0	0.32
cta	L	0	0.30
ctg	L	1	0.42
atg	M	1	1.00
gtt	V	769	177.90
gtc	V	5	174.00
gta	V	41	176.30
gtg	V	12	298.80
gct	A	0	0.25
gcc	A	1	0.19
gca	A	0	0.45
gcg	A	0	0.11
ggt	G	1	0.14
ggc	G	0	0.13
gga	G	0	0.44
ggg	G	0	0.29
---	--	------	------
mPD		0.15	0.75
		nPD:	0.2
		N. weight:	1.3
		Sc. PD:	0.13
		Sc. rank:	635.8

,

NA			
Pos.	81	obs:	exp:
cct	P	1	0.27
ccc	P	0	0.18
cca	P	0	0.41
ccg	P	0	0.14
att	I	1	0.37
atc	I	0	0.27
ata	I	0	0.37
act	T	770	202.10
acc	T	1	173.70
aca	T	0	322.60
acg	T	0	72.54
aat	N	3	1.64
aac	N	0	1.36
gtt	V	2	0.43
gtc	V	0	0.42
gta	V	0	0.43
gtg	V	0	0.72
gct	A	44	11.34
gcc	A	1	8.71
gca	A	0	20.03
gcg	A	0	4.93
gat	D	8	4.32
gac	D	0	3.68
ggt	G	1	0.14
ggc	G	0	0.13
gga	G	0	0.44
ggg	G	0	0.29
---	--	------	------
mPD		0.17	0.86
		nPD:	0.2
		N. weight:	1.4
		Sc. PD:	0.12
		Sc. rank:	645.6

,

NA			
Pos.	82	obs:	exp:
tct	S	0	132.40
tcc	S	0	114.90
tca	S	33	179.80
tcg	S	760	47.84
cct	P	0	10.66
ccc	P	0	7.04
cca	P	34	15.83
ccg	P	5	5.47
agt	S	0	172.00
agc	S	0	146.10
---	--	------	------
mPD		0.24	1.7
		nPD:	0.14
		N. weight:	2.6
		Sc. PD:	0.093
		Sc. rank:	710.4

,

NA			
Pos.	83	obs:	exp:
tta	L	1	0.09
ttg	L	0	0.19
ctt	L	0	0.20
ctc	L	0	0.16
cta	L	0	0.15
ctg	L	0	0.21
att	I	0	1.83
atc	I	0	1.34
ata	I	5	1.83
act	T	0	0.26
acc	T	0	0.23
aca	T	1	0.42
acg	T	0	0.09
aaa	K	2	1.14
aag	K	0	0.86
gtt	V	0	173.80
gtc	V	0	170.00
gta	V	781	172.30
gtg	V	27	291.90
gct	A	0	3.78
gcc	A	0	2.90
gca	A	15	6.68
gcg	A	0	1.64
---	--	------	------
mPD		0.13	0.80
		nPD:	0.16
		N. weight:	1.4
		Sc. PD:	0.076
		Sc. rank:	528.0

,

NA			
Pos.	84	obs:	exp:
tct	S	0	0.17
tcc	S	0	0.14
tca	S	1	0.23
tcg	S	0	0.06
act	T	0	210.20
acc	T	0	180.70
aca	T	799	335.60
acg	T	3	75.46
agt	S	0	0.22
agc	S	0	0.18
gct	A	0	7.31
gcc	A	0	5.61
gca	A	29	12.91
gcg	A	0	3.17
---	--	------	------
mPD		0.077	0.77
		nPD:	0.1
		N. weight:	0.9
		Sc. PD:	-0.0027
		Sc. rank:	119.5

,

NA			
Pos.	85	obs:	exp:
ttt	F	1	0.42
ttc	F	0	0.58
tta	L	785	76.83
ttg	L	6	157.90
ctt	L	0	165.00
ctc	L	0	132.90
cta	L	40	123.00
ctg	L	0	175.40
---	--	------	------
mPD		0.11	1.1
		nPD:	0.1
		N. weight:	2.2
		Sc. PD:	-0.015
		Sc. rank:	243.9

,

NA			
Pos.	86	obs:	exp:
gtt	V	0	0.43
gtc	V	0	0.42
gta	V	0	0.43
gtg	V	2	0.72
gct	A	1	208.10
gcc	A	0	159.80
gca	A	41	367.70
gcg	A	784	90.41
gaa	E	0	2.24
gag	E	4	1.76
---	--	------	------
mPD		0.11	0.70
		nPD:	0.16
		N. weight:	2.
		Sc. PD:	0.1
		Sc. rank:	708.3

,

NA			
Pos.	87	obs:	exp:
ggt	G	13	120.30
ggc	G	818	109.30
gga	G	1	364.60
ggg	G	0	237.80
---	--	------	------
mPD		0.033	0.69
		nPD:	0.05
		N. weight:	2.
		Sc. PD:	-0.11
		Sc. rank:	-250.7

,

NA			
Pos.	88	obs:	exp:
tct	S	0	0.17
tcc	S	0	0.14
tca	S	0	0.23
tcg	S	0	0.06
act	T	1	0.26
acc	T	0	0.23
aca	T	0	0.42
acg	T	0	0.09
aat	N	827	452.60
aac	N	3	377.40
agt	S	1	0.22
agc	S	0	0.18
---	--	------	------
mPD		0.012	0.50
		nPD:	0.02
		N. weight:	0.62
		Sc. PD:	-0.047
		Sc. rank:	-180.0

,

NA			
Pos.	89	obs:	exp:
tct	S	0	138.50
tcc	S	40	120.20
tca	S	790	188.20
tcg	S	0	50.07
agt	S	0	180.00
agc	S	0	153.00
gct	A	0	0.50
gcc	A	0	0.39
gca	A	2	0.89
gcg	A	0	0.22
---	--	------	------
mPD		0.096	1.7
		nPD:	0.06
		N. weight:	1.4
		Sc. PD:	-0.06
		Sc. rank:	-88.1

,

NA			
Pos.	90	obs:	exp:
ttt	F	1	0.42
ttc	F	0	0.58
tct	S	827	138.70
tcc	S	4	120.40
tca	S	0	188.40
tcg	S	0	50.13
agt	S	0	180.20
agc	S	0	153.10
---	--	------	------
mPD		0.012	1.7
		nPD:	0.01
		N. weight:	1.9
		Sc. PD:	-0.17
		Sc. rank:	-878.8

,

NA			
Pos.	91	obs:	exp:
tta	L	0	76.93
ttg	L	0	158.10
ctt	L	792	165.20
ctc	L	40	133.10
cta	L	0	123.10
ctg	L	0	175.60
---	--	------	------
mPD		0.092	1.1
		nPD:	0.08
		N. weight:	1.5
		Sc. PD:	-0.032
		Sc. rank:	122.7

,

NA			
Pos.	92	obs:	exp:
tgt	C	37	322.00
tgc	C	795	510.00
---	--	------	------
mPD		0.085	0.47
		nPD:	0.18
		N. weight:	0.35
		Sc. PD:	0.025
		Sc. rank:	141.4

,

NA			
Pos.	93	obs:	exp:
cct	P	774	227.40
ccc	P	47	150.30
cca	P	11	337.70
ccg	P	0	116.60
---	--	------	------
mPD		0.13	0.71
		nPD:	0.19
		N. weight:	1.1
		Sc. PD:	0.086
		Sc. rank:	469.8

,

NA			
Pos.	94	obs:	exp:
att	I	789	303.50
atc	I	38	221.30
ata	I	0	302.20
gtt	V	5	1.08
gtc	V	0	1.05
gta	V	0	1.07
gtg	V	0	1.81
---	--	------	------
mPD		0.099	0.67
		nPD:	0.15
		N. weight:	0.88
		Sc. PD:	0.037
		Sc. rank:	266.5

,

NA			
Pos.	95	obs:	exp:
tct	S	0	138.20
tcc	S	0	119.90
tca	S	0	187.70
tcg	S	0	49.95
aat	N	1	0.55
aac	N	0	0.45
agt	S	828	179.60
agc	S	0	152.60
ggt	G	3	0.43
ggc	G	0	0.39
gga	G	0	1.31
ggg	G	0	0.86
---	--	------	------
mPD		0.0096	1.7
		nPD:	0.01
		N. weight:	1.6
		Sc. PD:	-0.15
		Sc. rank:	-828.9

,

NA			
Pos.	96	obs:	exp:
ggt	G	0	120.30
ggc	G	0	109.30
gga	G	62	364.60
ggg	G	770	237.80
---	--	------	------
mPD		0.14	0.69
		nPD:	0.2
		N. weight:	1.
		Sc. PD:	0.094
		Sc. rank:	485.5

,

NA			
Pos.	97	obs:	exp:
tgg	W	832	832.00
---	--	------	------
mPD		0	0
		nPD:	1.
		N. weight:	0.
		Sc. PD:	0
		Sc. rank:	0

,

NA			
Pos.	98	obs:	exp:
gtt	V	1	0.22
gtc	V	0	0.21
gta	V	0	0.21
gtg	V	0	0.36
gct	A	420	209.30
gcc	A	1	160.80
gca	A	410	369.90
gcg	A	0	90.95
---	--	------	------
mPD		0.50	0.69
		nPD:	0.73
		N. weight:	0.42
		Sc. PD:	0.25
		Sc. rank:	289.3

,

NA			
Pos.	99	obs:	exp:
att	I	0	300.50
atc	I	0	219.20
ata	I	819	299.30
gtt	V	1	2.80
gtc	V	0	2.74
gta	V	12	2.77
gtg	V	0	4.70
---	--	------	------
mPD		0.033	0.69
		nPD:	0.05
		N. weight:	1.1
		Sc. PD:	-0.057
		Sc. rank:	-138.8

,

NA			
Pos.	100	obs:	exp:
tat	Y	1	399.00
tac	Y	831	433.00
---	--	------	------
mPD		0.0024	0.50
		nPD:	0.
		N. weight:	0.68
		Sc. PD:	-0.064
		Sc. rank:	-364.4

,

NA			
Pos.	101	obs:	exp:
tct	S	0	138.90
tcc	S	0	120.50
tca	S	0	188.60
tcg	S	0	50.20
agt	S	790	180.50
agc	S	42	153.30
---	--	------	------
mPD		0.096	1.7
		nPD:	0.06
		N. weight:	1.4
		Sc. PD:	-0.061
		Sc. rank:	-95.4

,

NA			
Pos.	102	obs:	exp:
aaa	K	0	473.80
aag	K	832	358.20
---	--	------	------
mPD		0	0.49
		nPD:	0.
		N. weight:	0.89
		Sc. PD:	-0.088
		Sc. rank:	-605.1

,

NA			
Pos.	103	obs:	exp:
aat	N	0	0.55
aac	N	1	0.45
gat	D	39	448.70
gac	D	792	382.30
---	--	------	------
mPD		0.092	0.50
		nPD:	0.18
		N. weight:	0.61
		Sc. PD:	0.047
		Sc. rank:	260.8

,

NA			
Pos.	104	obs:	exp:
aat	N	58	453.70
aac	N	774	378.30
---	--	------	------
mPD		0.13	0.50
		nPD:	0.26
		N. weight:	0.55
		Sc. PD:	0.084
		Sc. rank:	329.5

,

NA			
Pos.	105	obs:	exp:
tct	S	0	1.34
tcc	S	0	1.16
tca	S	0	1.81
tcg	S	0	0.48
agt	S	8	1.74
agc	S	0	1.47
ggt	G	824	119.20
ggc	G	0	108.20
gga	G	0	361.10
ggg	G	0	235.50
---	--	------	------
mPD		0.019	0.72
		nPD:	0.03
		N. weight:	2.
		Sc. PD:	-0.15
		Sc. rank:	-529.8

,

NA			
Pos.	106	obs:	exp:
att	I	0	303.80
atc	I	0	221.60
ata	I	828	302.60
gtt	V	0	0.86
gtc	V	0	0.84
gta	V	4	0.85
gtg	V	0	1.45
---	--	------	------
mPD		0.0096	0.67
		nPD:	0.01
		N. weight:	1.1
		Sc. PD:	-0.091
		Sc. rank:	-408.4

,

NA			
Pos.	107	obs:	exp:
cgt	R	0	31.14
cgc	R	0	44.52
cga	R	0	83.84
cgg	R	1	67.94
aga	R	808	388.40
agg	R	23	216.10
---	--	------	------
mPD		0.058	0.95
		nPD:	0.06
		N. weight:	0.68
		Sc. PD:	-0.028
		Sc. rank:	-24.7

,

NA			
Pos.	108	obs:	exp:
tta	L	0	0.09
ttg	L	0	0.19
ctt	L	1	0.20
ctc	L	0	0.16
cta	L	0	0.15
ctg	L	0	0.21
att	I	779	304.90
atc	I	13	222.40
ata	I	39	303.70
---	--	------	------
mPD		0.12	0.66
		nPD:	0.18
		N. weight:	0.78
		Sc. PD:	0.06
		Sc. rank:	323.7

,

NA			
Pos.	109	obs:	exp:
ggt	G	55	120.30
ggc	G	3	109.30
gga	G	10	364.60
ggg	G	764	237.80
---	--	------	------
mPD		0.15	0.69
		nPD:	0.22
		N. weight:	1.
		Sc. PD:	0.12
		Sc. rank:	553.8

,

NA			
Pos.	110	obs:	exp:
tct	S	2	138.50
tcc	S	826	120.20
tca	S	2	188.20
tcg	S	0	50.07
agt	S	0	180.00
agc	S	0	153.00
gct	A	0	0.50
gcc	A	2	0.39
gca	A	0	0.89
gcg	A	0	0.22
---	--	------	------
mPD		0.014	1.7
		nPD:	0.01
		N. weight:	2.
		Sc. PD:	-0.18
		Sc. rank:	-926.0

,

NA			
Pos.	111	obs:	exp:
aat	N	3	1.64
aac	N	0	1.36
aaa	K	26	472.00
aag	K	803	357.00
---	--	------	------
mPD		0.068	0.49
		nPD:	0.14
		N. weight:	0.73
		Sc. PD:	0.023
		Sc. rank:	179.6

,

NA			
Pos.	112	obs:	exp:
ggt	G	0	120.30
ggc	G	0	109.30
gga	G	42	364.60
ggg	G	790	237.80
---	--	------	------
mPD		0.096	0.69
		nPD:	0.14
		N. weight:	1.1
		Sc. PD:	0.038
		Sc. rank:	279.3

,

NA			
Pos.	113	obs:	exp:
gat	D	831	449.20
gac	D	1	382.80
---	--	------	------
mPD		0.0024	0.50
		nPD:	0.
		N. weight:	0.64
		Sc. PD:	-0.061
		Sc. rank:	-341.0

,

NA			
Pos.	114	obs:	exp:
gtt	V	0	178.90
gtc	V	0	175.00
gta	V	48	177.40
gtg	V	784	300.60
---	--	------	------
mPD		0.11	0.73
		nPD:	0.15
		N. weight:	0.87
		Sc. PD:	0.038
		Sc. rank:	269.6

,

NA			
Pos.	115	obs:	exp:
ttt	F	832	347.10
ttc	F	0	484.90
---	--	------	------
mPD		0	0.49
		nPD:	0.
		N. weight:	0.92
		Sc. PD:	-0.091
		Sc. rank:	-627.8

,

NA			
Pos.	116	obs:	exp:
gtt	V	788	178.90
gtc	V	44	175.00
gta	V	0	177.40
gtg	V	0	300.60
---	--	------	------
mPD		0.10	0.73
		nPD:	0.14
		N. weight:	1.4
		Sc. PD:	0.045
		Sc. rank:	340.7

,

NA			
Pos.	117	obs:	exp:
att	I	0	305.30
atc	I	32	222.60
ata	I	800	304.00
---	--	------	------
mPD		0.074	0.66
		nPD:	0.11
		N. weight:	0.9
		Sc. PD:	0.0076
		Sc. rank:	156.0

,

NA			
Pos.	118	obs:	exp:
cgt	R	0	31.14
cgc	R	0	44.52
cga	R	0	83.84
cgg	R	0	67.94
aga	R	824	388.40
agg	R	8	216.10
---	--	------	------
mPD		0.019	0.95
		nPD:	0.02
		N. weight:	0.75
		Sc. PD:	-0.06
		Sc. rank:	-244.8

,

NA			
Pos.	119	obs:	exp:
gaa	E	756	466.40
gag	E	76	365.60
---	--	------	------
mPD		0.17	0.49
		nPD:	0.34
		N. weight:	0.31
		Sc. PD:	0.071
		Sc. rank:	202.5

,

NA			
Pos.	120	obs:	exp:
cct	P	28	227.40
ccc	P	11	150.30
cca	P	23	337.70
ccg	P	770	116.60
---	--	------	------
mPD		0.14	0.71
		nPD:	0.2
		N. weight:	1.7
		Sc. PD:	0.15
		Sc. rank:	793.1

,

NA			
Pos.	121	obs:	exp:
ttt	F	55	347.10
ttc	F	777	484.90
---	--	------	------
mPD		0.12	0.49
		nPD:	0.25
		N. weight:	0.34
		Sc. PD:	0.049
		Sc. rank:	198.9

,

NA			
Pos.	122	obs:	exp:
att	I	40	303.10
atc	I	785	221.00
ata	I	1	301.90
gtt	V	0	1.29
gtc	V	6	1.26
gta	V	0	1.28
gtg	V	0	2.17
---	--	------	------
mPD		0.11	0.68
		nPD:	0.16
		N. weight:	1.2
		Sc. PD:	0.063
		Sc. rank:	430.5

,

NA			
Pos.	123	obs:	exp:
tct	S	0	138.70
tcc	S	0	120.40
tca	S	828	188.40
tcg	S	3	50.13
act	T	0	0.26
acc	T	0	0.23
aca	T	1	0.42
acg	T	0	0.09
agt	S	0	180.20
agc	S	0	153.10
---	--	------	------
mPD		0.0096	1.7
		nPD:	0.01
		N. weight:	1.5
		Sc. PD:	-0.14
		Sc. rank:	-788.0

,

NA			
Pos.	124	obs:	exp:
tgt	C	1	322.00
tgc	C	831	510.00
---	--	------	------
mPD		0.0024	0.47
		nPD:	0.01
		N. weight:	0.51
		Sc. PD:	-0.048
		Sc. rank:	-263.7

,

NA			
Pos.	125	obs:	exp:
tct	S	33	138.90
tcc	S	789	120.50
tca	S	0	188.60
tcg	S	10	50.20
agt	S	0	180.50
agc	S	0	153.30
---	--	------	------
mPD		0.099	1.7
		nPD:	0.06
		N. weight:	1.8
		Sc. PD:	-0.075
		Sc. rank:	-81.4

,

NA			
Pos.	126	obs:	exp:
cat	H	21	481.60
cac	H	811	350.40
---	--	------	------
mPD		0.049	0.49
		nPD:	0.1
		N. weight:	0.78
		Sc. PD:	-0.0018
		Sc. rank:	105.7

,

NA			
Pos.	127	obs:	exp:
tta	L	0	76.74
ttg	L	823	157.70
ctt	L	0	164.80
ctc	L	0	132.80
cta	L	0	122.80
ctg	L	7	175.20
atg	M	2	2.00
---	--	------	------
mPD		0.021	1.1
		nPD:	0.02
		N. weight:	1.7
		Sc. PD:	-0.14
		Sc. rank:	-578.1

,

NA			
Pos.	128	obs:	exp:
gaa	E	826	465.30
gag	E	4	364.70
ggt	G	0	0.29
ggc	G	0	0.26
gga	G	2	0.88
ggg	G	0	0.57
---	--	------	------
mPD		0.014	0.50
		nPD:	0.03
		N. weight:	0.58
		Sc. PD:	-0.042
		Sc. rank:	-140.3

,

NA			
Pos.	129	obs:	exp:
tgt	C	11	322.00
tgc	C	821	510.00
---	--	------	------
mPD		0.026	0.47
		nPD:	0.06
		N. weight:	0.45
		Sc. PD:	-0.021
		Sc. rank:	-38.6

,

NA			
Pos.	130	obs:	exp:
cgt	R	0	31.14
cgc	R	0	44.52
cga	R	0	83.84
cgg	R	0	67.94
aga	R	801	388.40
agg	R	31	216.10
---	--	------	------
mPD		0.072	0.95
		nPD:	0.08
		N. weight:	0.66
		Sc. PD:	-0.018
		Sc. rank:	25.7

,

NA			
Pos.	131	obs:	exp:
att	I	1	1.10
atc	I	2	0.80
ata	I	0	1.10
act	T	39	217.30
acc	T	788	186.80
aca	T	2	346.90
acg	T	0	78.00
---	--	------	------
mPD		0.10	0.70
		nPD:	0.15
		N. weight:	1.3
		Sc. PD:	0.056
		Sc. rank:	398.5

,

NA			
Pos.	132	obs:	exp:
ttt	F	753	347.10
ttc	F	79	484.90
---	--	------	------
mPD		0.17	0.49
		nPD:	0.35
		N. weight:	0.56
		Sc. PD:	0.13
		Sc. rank:	363.6

,

NA			
Pos.	133	obs:	exp:
ttt	F	454	347.10
ttc	F	378	484.90
---	--	------	------
mPD		0.50	0.49
		nPD:	1.02
		N. weight:	0.04
		Sc. PD:	0.035
		Sc. rank:	27.1

,

NA			
Pos.	134	obs:	exp:
tta	L	5	76.93
ttg	L	15	158.10
ctt	L	0	165.20
ctc	L	0	133.10
cta	L	44	123.10
ctg	L	768	175.60
---	--	------	------
mPD		0.16	1.1
		nPD:	0.14
		N. weight:	1.3
		Sc. PD:	0.047
		Sc. rank:	363.0

,

NA			
Pos.	135	obs:	exp:
act	T	65	218.10
acc	T	767	187.50
aca	T	0	348.10
acg	T	0	78.28
---	--	------	------
mPD		0.14	0.70
		nPD:	0.21
		N. weight:	1.3
		Sc. PD:	0.13
		Sc. rank:	636.4

,

NA			
Pos.	136	obs:	exp:
cct	P	0	0.27
ccc	P	0	0.18
cca	P	0	0.41
ccg	P	1	0.14
cat	H	1	0.58
cac	H	0	0.42
caa	Q	36	414.40
cag	Q	794	415.60
---	--	------	------
mPD		0.088	0.50
		nPD:	0.17
		N. weight:	0.54
		Sc. PD:	0.037
		Sc. rank:	210.5

,

NA			
Pos.	137	obs:	exp:
ggt	G	1	120.30
ggc	G	0	109.30
gga	G	760	364.60
ggg	G	71	237.80
---	--	------	------
mPD		0.16	0.69
		nPD:	0.23
		N. weight:	0.6
		Sc. PD:	0.073
		Sc. rank:	333.0

,

NA			
Pos.	138	obs:	exp:
gct	A	778	209.60
gcc	A	54	161.00
gca	A	0	370.30
gcg	A	0	91.06
---	--	------	------
mPD		0.12	0.69
		nPD:	0.18
		N. weight:	1.2
		Sc. PD:	0.085
		Sc. rank:	481.0

,

NA			
Pos.	139	obs:	exp:
tta	L	0	76.93
ttg	L	51	158.10
ctt	L	0	165.20
ctc	L	0	133.10
cta	L	19	123.10
ctg	L	762	175.60
---	--	------	------
mPD		0.16	1.1
		nPD:	0.14
		N. weight:	1.3
		Sc. PD:	0.049
		Sc. rank:	367.0

,

NA			
Pos.	140	obs:	exp:
tta	L	0	76.93
ttg	L	48	158.10
ctt	L	1	165.20
ctc	L	0	133.10
cta	L	0	123.10
ctg	L	783	175.60
---	--	------	------
mPD		0.11	1.1
		nPD:	0.1
		N. weight:	1.4
		Sc. PD:	-0.0058
		Sc. rank:	169.5

,

NA			
Pos.	141	obs:	exp:
tct	S	0	0.50
tcc	S	0	0.43
tca	S	0	0.68
tcg	S	0	0.18
aat	N	827	452.10
aac	N	2	376.90
agt	S	3	0.65
agc	S	0	0.55
---	--	------	------
mPD		0.012	0.51
		nPD:	0.02
		N. weight:	0.63
		Sc. PD:	-0.048
		Sc. rank:	-183.6

,

NA			
Pos.	142	obs:	exp:
gat	D	0	448.70
gac	D	831	382.30
ggt	G	0	0.14
ggc	G	1	0.13
gga	G	0	0.44
ggg	G	0	0.29
---	--	------	------
mPD		0.0024	0.50
		nPD:	0.
		N. weight:	0.82
		Sc. PD:	-0.078
		Sc. rank:	-444.4

,

NA			
Pos.	143	obs:	exp:
cgt	R	0	0.11
cgc	R	0	0.16
cga	R	0	0.30
cgg	R	0	0.24
aat	N	0	1.09
aac	N	2	0.91
aaa	K	769	470.90
aag	K	58	356.10
aga	R	3	1.40
agg	R	0	0.78
---	--	------	------
mPD		0.14	0.50
		nPD:	0.28
		N. weight:	0.35
		Sc. PD:	0.06
		Sc. rank:	217.2

,

NA			
Pos.	144	obs:	exp:
tat	Y	1	0.48
tac	Y	0	0.52
cat	H	813	481.10
cac	H	18	349.90
---	--	------	------
mPD		0.045	0.49
		nPD:	0.09
		N. weight:	0.48
		Sc. PD:	-0.0055
		Sc. rank:	44.6

,

NA			
Pos.	145	obs:	exp:
tct	S	798	138.90
tcc	S	33	120.50
tca	S	1	188.60
tcg	S	0	50.20
agt	S	0	180.50
agc	S	0	153.30
---	--	------	------
mPD		0.079	1.7
		nPD:	0.05
		N. weight:	1.7
		Sc. PD:	-0.092
		Sc. rank:	-228.1

,

NA			
Pos.	146	obs:	exp:
aat	N	832	453.70
aac	N	0	378.30
---	--	------	------
mPD		0	0.50
		nPD:	0.
		N. weight:	0.64
		Sc. PD:	-0.063
		Sc. rank:	-435.4

,

NA			
Pos.	147	obs:	exp:
ggt	G	0	120.30
ggc	G	0	109.30
gga	G	46	364.60
ggg	G	786	237.80
---	--	------	------
mPD		0.10	0.69
		nPD:	0.15
		N. weight:	1.1
		Sc. PD:	0.05
		Sc. rank:	343.7

,

NA			
Pos.	148	obs:	exp:
tct	S	0	0.17
tcc	S	1	0.14
tca	S	0	0.23
tcg	S	0	0.06
act	T	5	217.80
acc	T	825	187.20
aca	T	1	347.70
acg	T	0	78.19
agt	S	0	0.22
agc	S	0	0.18
---	--	------	------
mPD		0.017	0.70
		nPD:	0.02
		N. weight:	1.5
		Sc. PD:	-0.12
		Sc. rank:	-436.9

,

NA			
Pos.	149	obs:	exp:
att	I	1	0.37
atc	I	0	0.27
ata	I	0	0.37
gtt	V	797	178.70
gtc	V	34	174.80
gta	V	0	177.20
gtg	V	0	300.20
---	--	------	------
mPD		0.081	0.74
		nPD:	0.11
		N. weight:	1.4
		Sc. PD:	0.0089
		Sc. rank:	240.3

,

NA			
Pos.	150	obs:	exp:
aat	N	3	1.64
aac	N	0	1.36
aaa	K	87	466.90
aag	K	733	353.10
gaa	E	8	5.05
gag	E	1	3.95
---	--	------	------
mPD		0.23	0.52
		nPD:	0.45
		N. weight:	0.5
		Sc. PD:	0.17
		Sc. rank:	334.7

,

NA			
Pos.	151	obs:	exp:
gat	D	779	449.20
gac	D	53	382.80
---	--	------	------
mPD		0.12	0.50
		nPD:	0.24
		N. weight:	0.41
		Sc. PD:	0.054
		Sc. rank:	232.8

,

NA			
Pos.	152	obs:	exp:
cgt	R	0	31.14
cgc	R	0	44.52
cga	R	0	83.84
cgg	R	0	67.94
aga	R	820	388.40
agg	R	12	216.10
---	--	------	------
mPD		0.028	0.95
		nPD:	0.03
		N. weight:	0.73
		Sc. PD:	-0.052
		Sc. rank:	-171.1

,

NA			
Pos.	153	obs:	exp:
tct	S	0	138.90
tcc	S	0	120.50
tca	S	0	188.60
tcg	S	0	50.20
agt	S	3	180.50
agc	S	829	153.30
---	--	------	------
mPD		0.0072	1.7
		nPD:	0.
		N. weight:	1.8
		Sc. PD:	-0.17
		Sc. rank:	-984.2

,

NA			
Pos.	154	obs:	exp:
cct	P	338	227.40
ccc	P	493	150.30
cca	P	0	337.70
ccg	P	1	116.60
---	--	------	------
mPD		0.48	0.71
		nPD:	0.68
		N. weight:	0.91
		Sc. PD:	0.51
		Sc. rank:	614.6

,

NA			
Pos.	155	obs:	exp:
tat	Y	777	397.10
tac	Y	51	430.90
cat	H	0	2.32
cac	H	4	1.68
---	--	------	------
mPD		0.13	0.51
		nPD:	0.26
		N. weight:	0.53
		Sc. PD:	0.081
		Sc. rank:	314.6

,

NA			
Pos.	156	obs:	exp:
cgt	R	0	31.14
cgc	R	0	44.52
cga	R	0	83.84
cgg	R	0	67.94
aga	R	828	388.40
agg	R	4	216.10
---	--	------	------
mPD		0.0096	0.95
		nPD:	0.01
		N. weight:	0.77
		Sc. PD:	-0.069
		Sc. rank:	-346.4

,

NA			
Pos.	157	obs:	exp:
act	T	779	217.60
acc	T	39	187.00
aca	T	12	347.30
acg	T	0	78.09
gct	A	2	0.50
gcc	A	0	0.39
gca	A	0	0.89
gcg	A	0	0.22
---	--	------	------
mPD		0.12	0.70
		nPD:	0.17
		N. weight:	1.1
		Sc. PD:	0.076
		Sc. rank:	437.4

,

NA			
Pos.	158	obs:	exp:
tta	L	33	76.93
ttg	L	760	158.10
ctt	L	0	165.20
ctc	L	0	133.10
cta	L	28	123.10
ctg	L	11	175.60
---	--	------	------
mPD		0.23	1.1
		nPD:	0.2
		N. weight:	1.4
		Sc. PD:	0.13
		Sc. rank:	675.7

,

NA			
Pos.	159	obs:	exp:
atg	M	831	831.00
gtt	V	0	0.22
gtc	V	0	0.21
gta	V	0	0.21
gtg	V	1	0.36
---	--	------	------
mPD		0.0024	0.0039
		nPD:	0.61
		N. weight:	0.0013
		Sc. PD:	0.00063
		Sc. rank:	0.9

,

NA			
Pos.	160	obs:	exp:
tct	S	0	138.90
tcc	S	0	120.50
tca	S	0	188.60
tcg	S	0	50.20
agt	S	789	180.50
agc	S	43	153.30
---	--	------	------
mPD		0.098	1.7
		nPD:	0.06
		N. weight:	1.4
		Sc. PD:	-0.059
		Sc. rank:	-83.2

,

NA			
Pos.	161	obs:	exp:
tgt	C	808	322.00
tgc	C	24	510.00
---	--	------	------
mPD		0.056	0.47
		nPD:	0.12
		N. weight:	0.85
		Sc. PD:	0.012
		Sc. rank:	159.1

,

NA			
Pos.	162	obs:	exp:
tta	L	0	0.18
ttg	L	0	0.38
tct	S	0	0.33
tcc	S	2	0.29
tca	S	0	0.45
tcg	S	0	0.12
ctt	L	0	0.40
ctc	L	2	0.32
cta	L	0	0.30
ctg	L	0	0.42
cct	P	59	226.00
ccc	P	761	149.40
cca	P	7	335.70
ccg	P	0	115.90
act	T	0	0.26
acc	T	1	0.23
aca	T	0	0.42
acg	T	0	0.09
agt	S	0	0.43
agc	S	0	0.37
---	--	------	------
mPD		0.16	0.72
		nPD:	0.22
		N. weight:	1.4
		Sc. PD:	0.16
		Sc. rank:	781.4

,

NA			
Pos.	163	obs:	exp:
att	I	10	3.67
atc	I	0	2.68
ata	I	0	3.65
atg	M	5	5.00
gtt	V	32	175.70
gtc	V	0	171.90
gta	V	8	174.20
gtg	V	777	295.20
---	--	------	------
mPD		0.15	0.77
		nPD:	0.19
		N. weight:	0.87
		Sc. PD:	0.076
		Sc. rank:	395.2

,

NA			
Pos.	164	obs:	exp:
ggt	G	828	120.30
ggc	G	3	109.30
gga	G	0	364.60
ggg	G	1	237.80
---	--	------	------
mPD		0.0096	0.69
		nPD:	0.01
		N. weight:	2.
		Sc. PD:	-0.17
		Sc. rank:	-788.2

,

NA			
Pos.	165	obs:	exp:
gtt	V	0	1.51
gtc	V	0	1.47
gta	V	0	1.49
gtg	V	7	2.53
gat	D	1	0.54
gac	D	0	0.46
gaa	E	38	461.90
gag	E	786	362.10
---	--	------	------
mPD		0.11	0.51
		nPD:	0.21
		N. weight:	0.66
		Sc. PD:	0.066
		Sc. rank:	329.9

,

NA			
Pos.	166	obs:	exp:
tct	S	1	0.17
tcc	S	0	0.14
tca	S	0	0.23
tcg	S	0	0.06
act	T	2	0.52
acc	T	0	0.45
aca	T	0	0.84
acg	T	0	0.19
agt	S	0	0.22
agc	S	0	0.18
gtt	V	4	0.86
gtc	V	0	0.84
gta	V	0	0.85
gtg	V	0	1.45
gct	A	818	207.80
gcc	A	7	159.60
gca	A	0	367.20
gcg	A	0	90.30
---	--	------	------
mPD		0.033	0.71
		nPD:	0.05
		N. weight:	1.4
		Sc. PD:	-0.076
		Sc. rank:	-194.6

,

NA			
Pos.	167	obs:	exp:
cct	P	810	227.40
ccc	P	20	150.30
cca	P	0	337.70
ccg	P	2	116.60
---	--	------	------
mPD		0.052	0.71
		nPD:	0.07
		N. weight:	1.2
		Sc. PD:	-0.036
		Sc. rank:	29.2

,

NA			
Pos.	168	obs:	exp:
tct	S	47	138.90
tcc	S	785	120.50
tca	S	0	188.60
tcg	S	0	50.20
agt	S	0	180.50
agc	S	0	153.30
---	--	------	------
mPD		0.11	1.7
		nPD:	0.06
		N. weight:	1.8
		Sc. PD:	-0.067
		Sc. rank:	-37.5

,

NA			
Pos.	169	obs:	exp:
cct	P	0	227.40
ccc	P	0	150.30
cca	P	62	337.70
ccg	P	770	116.60
---	--	------	------
mPD		0.14	0.71
		nPD:	0.19
		N. weight:	1.7
		Sc. PD:	0.15
		Sc. rank:	787.2

,

NA			
Pos.	170	obs:	exp:
tat	Y	16	399.00
tac	Y	816	433.00
---	--	------	------
mPD		0.038	0.50
		nPD:	0.08
		N. weight:	0.59
		Sc. PD:	-0.016
		Sc. rank:	25.1

,

NA			
Pos.	171	obs:	exp:
tct	S	0	0.17
tcc	S	0	0.14
tca	S	0	0.23
tcg	S	0	0.06
att	I	2	0.73
atc	I	0	0.54
ata	I	0	0.73
act	T	1	0.26
acc	T	0	0.23
aca	T	0	0.42
acg	T	0	0.09
aat	N	740	451.50
aac	N	88	376.50
agt	S	0	0.22
agc	S	1	0.18
---	--	------	------
mPD		0.20	0.51
		nPD:	0.39
		N. weight:	0.31
		Sc. PD:	0.087
		Sc. rank:	202.6

,

NA			
Pos.	172	obs:	exp:
tct	S	0	138.90
tcc	S	0	120.50
tca	S	817	188.60
tcg	S	15	50.20
agt	S	0	180.50
agc	S	0	153.30
---	--	------	------
mPD		0.035	1.7
		nPD:	0.02
		N. weight:	1.5
		Sc. PD:	-0.12
		Sc. rank:	-459.8

,

NA			
Pos.	173	obs:	exp:
cgt	R	0	31.10
cgc	R	0	44.47
cga	R	0	83.74
cgg	R	0	67.86
aaa	K	1	0.57
aag	K	0	0.43
aga	R	776	387.90
agg	R	55	215.90
---	--	------	------
mPD		0.13	0.96
		nPD:	0.13
		N. weight:	0.59
		Sc. PD:	0.016
		Sc. rank:	128.7

,

NA			
Pos.	174	obs:	exp:
ttt	F	832	347.10
ttc	F	0	484.90
---	--	------	------
mPD		0	0.49
		nPD:	0.
		N. weight:	0.92
		Sc. PD:	-0.091
		Sc. rank:	-627.8

,

NA			
Pos.	175	obs:	exp:
gaa	E	1	466.40
gag	E	831	365.60
---	--	------	------
mPD		0.0024	0.49
		nPD:	0.
		N. weight:	0.86
		Sc. PD:	-0.081
		Sc. rank:	-450.2

,

NA			
Pos.	176	obs:	exp:
tct	S	829	138.90
tcc	S	0	120.50
tca	S	2	188.60
tcg	S	1	50.20
agt	S	0	180.50
agc	S	0	153.30
---	--	------	------
mPD		0.0072	1.7
		nPD:	0.
		N. weight:	1.9
		Sc. PD:	-0.18
		Sc. rank:	-1022.6

,

NA			
Pos.	177	obs:	exp:
gtt	V	831	178.90
gtc	V	1	175.00
gta	V	0	177.40
gtg	V	0	300.60
---	--	------	------
mPD		0.0024	0.73
		nPD:	0.
		N. weight:	1.6
		Sc. PD:	-0.15
		Sc. rank:	-969.8

,

NA			
Pos.	178	obs:	exp:
gct	A	819	209.60
gcc	A	13	161.00
gca	A	0	370.30
gcg	A	0	91.06
---	--	------	------
mPD		0.031	0.69
		nPD:	0.04
		N. weight:	1.4
		Sc. PD:	-0.077
		Sc. rank:	-205.7

,

NA			
Pos.	179	obs:	exp:
tgg	W	832	832.00
---	--	------	------
mPD		0	0
		nPD:	1.
		N. weight:	0.
		Sc. PD:	0
		Sc. rank:	0

,

NA			
Pos.	180	obs:	exp:
tct	S	6	138.90
tcc	S	0	120.50
tca	S	48	188.60
tcg	S	778	50.20
agt	S	0	180.50
agc	S	0	153.30
---	--	------	------
mPD		0.12	1.7
		nPD:	0.07
		N. weight:	2.6
		Sc. PD:	-0.073
		Sc. rank:	95.9

,

NA			
Pos.	181	obs:	exp:
gct	A	0	209.60
gcc	A	0	161.00
gca	A	830	370.30
gcg	A	2	91.06
---	--	------	------
mPD		0.0048	0.69
		nPD:	0.01
		N. weight:	0.84
		Sc. PD:	-0.078
		Sc. rank:	-412.0

,

NA			
Pos.	182	obs:	exp:
tct	S	0	138.70
tcc	S	0	120.40
tca	S	0	188.40
tcg	S	0	50.13
tgt	C	1	0.39
tgc	C	0	0.61
agt	S	829	180.20
agc	S	2	153.10
---	--	------	------
mPD		0.0072	1.7
		nPD:	0.
		N. weight:	1.6
		Sc. PD:	-0.15
		Sc. rank:	-867.9

,

NA			
Pos.	183	obs:	exp:
gct	A	826	209.60
gcc	A	6	161.00
gca	A	0	370.30
gcg	A	0	91.06
---	--	------	------
mPD		0.014	0.69
		nPD:	0.02
		N. weight:	1.4
		Sc. PD:	-0.11
		Sc. rank:	-439.9

,

NA			
Pos.	184	obs:	exp:
tgt	C	804	322.00
tgc	C	28	510.00
---	--	------	------
mPD		0.065	0.47
		nPD:	0.14
		N. weight:	0.83
		Sc. PD:	0.027
		Sc. rank:	205.4

,

NA			
Pos.	185	obs:	exp:
cat	H	819	481.60
cac	H	13	350.40
---	--	------	------
mPD		0.031	0.49
		nPD:	0.06
		N. weight:	0.5
		Sc. PD:	-0.019
		Sc. rank:	-13.0

,

NA			
Pos.	186	obs:	exp:
gat	D	820	449.20
gac	D	12	382.80
---	--	------	------
mPD		0.028	0.50
		nPD:	0.06
		N. weight:	0.57
		Sc. PD:	-0.025
		Sc. rank:	-43.7

NA			
Pos.	187	obs:	exp:
ggt	G	1	120.30
ggc	G	828	109.30
gga	G	3	364.60
ggg	G	0	237.80
---	--	------	------
mPD		0.0096	0.69
		nPD:	0.01
		N. weight:	2.1
		Sc. PD:	-0.18
		Sc. rank:	-826.4

NA			
Pos.	188	obs:	exp:
tct	S	0	0.33
tcc	S	0	0.29
tca	S	0	0.45
tcg	S	0	0.12
att	I	52	299.10
atc	I	761	218.10
ata	I	2	297.80
act	T	11	2.88
acc	T	0	2.48
aca	T	0	4.60
acg	T	0	1.03
agt	S	0	0.43
agc	S	2	0.37
gtt	V	0	0.86
gtc	V	4	0.84
gta	V	0	0.85
gtg	V	0	1.45
---	--	------	------
mPD		0.19	0.71
		nPD:	0.26
		N. weight:	1.1
		Sc. PD:	0.17
		Sc. rank:	665.0

NA			
Pos.	189	obs:	exp:
tct	S	0	137.70
tcc	S	0	119.50
tca	S	0	187.00
tcg	S	0	49.77
aat	N	5	2.73
aac	N	0	2.27
agt	S	814	178.90
agc	S	11	152.00
ggt	G	2	0.29
ggc	G	0	0.26
gga	G	0	0.88
ggg	G	0	0.57
---	--	------	------
mPD		0.043	1.7
		nPD:	0.03
		N. weight:	1.5
		Sc. PD:	-0.11
		Sc. rank:	-409.4

NA			
Pos.	190	obs:	exp:
tgg	W	832	832.00
---	--	------	------
mPD		0	0
		nPD:	1.
		N. weight:	0.
		Sc. PD:	0
		Sc. rank:	0

NA			
Pos.	191	obs:	exp:
tta	L	0	76.93
ttg	L	693	158.10
ctt	L	0	165.20
ctc	L	0	133.10
cta	L	0	123.10
ctg	L	139	175.60
---	--	------	------
mPD		0.28	1.1
		nPD:	0.25
		N. weight:	1.3
		Sc. PD:	0.18
		Sc. rank:	730.8

NA			
Pos.	192	obs:	exp:
act	T	0	218.10
acc	T	0	187.50
aca	T	830	348.10
acg	T	2	78.28
---	--	------	------
mPD		0.0048	0.70
		nPD:	0.01
		N. weight:	0.9
		Sc. PD:	-0.084
		Sc. rank:	-452.7

NA			
Pos.	193	obs:	exp:
att	I	61	304.90
atc	I	769	222.40
ata	I	1	303.70
gtt	V	0	0.22
gtc	V	1	0.21
gta	V	0	0.21
gtg	V	0	0.36
---	--	------	------
mPD		0.14	0.66
		nPD:	0.21
		N. weight:	1.1
		Sc. PD:	0.11
		Sc. rank:	566.7

NA			
Pos.	194	obs:	exp:
ggt	G	777	120.30
ggc	G	1	109.30
gga	G	20	364.60
ggg	G	34	237.80
---	--	------	------
mPD		0.13	0.69
		nPD:	0.18
		N. weight:	1.7
		Sc. PD:	0.13
		Sc. rank:	688.6

NA			
Pos.	195	obs:	exp:
att	I	829	304.90
atc	I	2	222.40
ata	I	0	303.70
gtt	V	1	0.22
gtc	V	0	0.21
gta	V	0	0.21
gtg	V	0	0.36
---	--	------	------
mPD		0.0072	0.66
		nPD:	0.01
		N. weight:	1.
		Sc. PD:	-0.092
		Sc. rank:	-445.1

NA			
Pos.	196	obs:	exp:
tct	S	789	138.90
tcc	S	43	120.50
tca	S	0	188.60
tcg	S	0	50.20
agt	S	0	180.50
agc	S	0	153.30
---	--	------	------
mPD		0.098	1.7
		nPD:	0.06
		N. weight:	1.7
		Sc. PD:	-0.071
		Sc. rank:	-99.5

NA			
Pos.	197	obs:	exp:
ggt	G	787	120.30
ggc	G	43	109.30
gga	G	1	364.60
ggg	G	1	237.80
---	--	------	------
mPD		0.10	0.69
		nPD:	0.15
		N. weight:	1.8
		Sc. PD:	0.08
		Sc. rank:	567.4

NA			
Pos.	198	obs:	exp:
cct	P	13	227.40
ccc	P	0	150.30
cca	P	819	337.70
ccg	P	0	116.60
---	--	------	------
mPD		0.031	0.71
		nPD:	0.04
		N. weight:	0.87
		Sc. PD:	-0.05
		Sc. rank:	-140.1

NA			
Pos.	199	obs:	exp:
gat	D	1	449.20
gac	D	831	382.80
---	--	------	------
mPD		0.0024	0.50
		nPD:	0.
		N. weight:	0.81
		Sc. PD:	-0.077
		Sc. rank:	-430.5

NA			
Pos.	200	obs:	exp:
tct	S	0	0.17
tcc	S	0	0.14
tca	S	0	0.23
tcg	S	0	0.06
act	T	1	0.26
acc	T	0	0.23
aca	T	0	0.42
acg	T	0	0.09
aat	N	828	452.60
aac	N	2	377.40
agt	S	1	0.22
agc	S	0	0.18
---	--	------	------
mPD		0.0096	0.50
		nPD:	0.02
		N. weight:	0.62
		Sc. PD:	-0.051
		Sc. rank:	-216.2

NA			
Pos.	201	obs:	exp:
gaa	E	8	4.48
gag	E	0	3.52
ggt	G	0	119.20
ggc	G	0	108.20
gga	G	744	361.10
ggg	G	80	235.50
---	--	------	------
mPD		0.19	0.71
		nPD:	0.27
		N. weight:	0.58
		Sc. PD:	0.095
		Sc. rank:	353.5

NA			
Pos.	202	obs:	exp:
gct	A	788	209.60
gcc	A	2	161.00
gca	A	25	370.30
gcg	A	17	91.06
---	--	------	------
mPD		0.10	0.69
		nPD:	0.15
		N. weight:	1.2
		Sc. PD:	0.051
		Sc. rank:	365.3

NA			
Pos.	203	obs:	exp:
gtt	V	0	178.90
gtc	V	0	175.00
gta	V	2	177.40
gtg	V	830	300.60
---	--	------	------
mPD		0.0048	0.73
		nPD:	0.01
		N. weight:	1.1
		Sc. PD:	-0.098
		Sc. rank:	-536.4

NA			
Pos.	204	obs:	exp:
gct	A	828	209.30
gcc	A	1	160.80
gca	A	2	369.90
gcg	A	0	90.95
ggt	G	1	0.14
ggc	G	0	0.13
gga	G	0	0.44
ggg	G	0	0.29
---	--	------	------
mPD		0.0096	0.69
		nPD:	0.01
		N. weight:	1.4
		Sc. PD:	-0.12
		Sc. rank:	-573.1

NA			
Pos.	205	obs:	exp:
att	I	0	0.37
atc	I	0	0.27
ata	I	1	0.37
gtt	V	0	178.70
gtc	V	0	174.80
gta	V	823	177.20
gtg	V	8	300.20
---	--	------	------
mPD		0.021	0.74
		nPD:	0.03
		N. weight:	1.6
		Sc. PD:	-0.11
		Sc. rank:	-368.4

NA			
Pos.	206	obs:	exp:
tta	L	5	76.37
ttg	L	821	156.90
ctt	L	0	164.00
ctc	L	0	132.10
cta	L	0	122.20
ctg	L	0	174.30
gtt	V	0	1.29
gtc	V	0	1.26
gta	V	0	1.28
gtg	V	6	2.17
---	--	------	------
mPD		0.026	1.1
		nPD:	0.02
		N. weight:	1.7
		Sc. PD:	-0.13
		Sc. rank:	-504.9

,

NA			
Pos.	207	obs:	exp:
aaa	K	54	473.80
aag	K	778	358.20
---	--	------	------
mPD		0.12	0.49
		nPD:	0.25
		N. weight:	0.62
		Sc. PD:	0.086
		Sc. rank:	356.8

,

NA			
Pos.	208	obs:	exp:
tat	Y	1	399.00
tac	Y	831	433.00
---	--	------	------
mPD		0.0024	0.50
		nPD:	0.
		N. weight:	0.68
		Sc. PD:	-0.064
		Sc. rank:	-364.4

,

NA			
Pos.	209	obs:	exp:
tct	S	0	0.17
tcc	S	0	0.14
tca	S	0	0.23
tcg	S	0	0.06
aat	N	816	447.20
aac	N	4	372.80
aaa	K	10	5.69
aag	K	0	4.31
agt	S	1	0.22
agc	S	0	0.18
gat	D	1	0.54
gac	D	0	0.46
---	--	------	------
mPD		0.038	0.51
		nPD:	0.07
		N. weight:	0.61
		Sc. PD:	-0.017
		Sc. rank:	15.4

,

NA			
Pos.	210	obs:	exp:
tct	S	0	0.17
tcc	S	0	0.14
tca	S	0	0.23
tcg	S	0	0.06
agt	S	0	0.22
agc	S	1	0.18
gat	D	0	1.08
gac	D	2	0.92
ggt	G	1	119.90
ggc	G	828	108.90
gga	G	0	363.30
ggg	G	0	237.00
---	--	------	------
mPD		0.0096	0.70
		nPD:	0.01
		N. weight:	2.1
		Sc. PD:	-0.18
		Sc. rank:	-862.5

,

NA			
Pos.	211	obs:	exp:
att	I	0	304.90
atc	I	0	222.40
ata	I	831	303.70
gtt	V	0	0.22
gtc	V	0	0.21
gta	V	1	0.21
gtg	V	0	0.36
---	--	------	------
mPD		0.0024	0.66
		nPD:	0.
		N. weight:	1.1
		Sc. PD:	-0.1
		Sc. rank:	-602.9

,

NA			
Pos.	212	obs:	exp:
att	I	0	305.30
atc	I	4	222.60
ata	I	828	304.00
---	--	------	------
mPD		0.0096	0.66
		nPD:	0.01
		N. weight:	1.
		Sc. PD:	-0.088
		Sc. rank:	-389.4

,

NA			
Pos.	213	obs:	exp:
act	T	0	218.10
acc	T	0	187.50
aca	T	57	348.10
acg	T	775	78.28
---	--	------	------
mPD		0.13	0.70
		nPD:	0.18
		N. weight:	2.1
		Sc. PD:	0.16
		Sc. rank:	893.6

,

NA			
Pos.	214	obs:	exp:
gat	D	775	448.10
gac	D	55	381.90
ggt	G	2	0.29
ggc	G	0	0.26
gga	G	0	0.88
ggg	G	0	0.57
---	--	------	------
mPD		0.13	0.50
		nPD:	0.26
		N. weight:	0.41
		Sc. PD:	0.06
		Sc. rank:	241.6

,

NA			
Pos.	215	obs:	exp:
act	T	826	218.10
acc	T	4	187.50
aca	T	0	348.10
acg	T	2	78.28
---	--	------	------
mPD		0.014	0.70
		nPD:	0.02
		N. weight:	1.4
		Sc. PD:	-0.11
		Sc. rank:	-431.8

,

NA			
Pos.	216	obs:	exp:
att	I	4	303.80
atc	I	823	221.60
ata	I	1	302.60
gtt	V	0	0.86
gtc	V	4	0.84
gta	V	0	0.85
gtg	V	0	1.45
---	--	------	------
mPD		0.022	0.67
		nPD:	0.03
		N. weight:	1.3
		Sc. PD:	-0.092
		Sc. rank:	-291.2

,

NA			
Pos.	217	obs:	exp:
aaa	K	7	473.80
aag	K	825	358.20
---	--	------	------
mPD		0.017	0.49
		nPD:	0.03
		N. weight:	0.83
		Sc. PD:	-0.056
		Sc. rank:	-175.1

,

NA			
Pos.	218	obs:	exp:
tct	S	0	138.70
tcc	S	0	120.40
tca	S	0	188.40
tcg	S	0	50.13
act	T	1	0.26
acc	T	0	0.23
aca	T	0	0.42
acg	T	0	0.09
agt	S	830	180.20
agc	S	1	153.10
---	--	------	------
mPD		0.0048	1.7
		nPD:	0.
		N. weight:	1.6
		Sc. PD:	-0.15
		Sc. rank:	-971.9

,

NA			
Pos.	219	obs:	exp:
tgg	W	832	832.00
---	--	------	------
mPD		0	0
		nPD:	1.
		N. weight:	0.
		Sc. PD:	0
		Sc. rank:	0

,

NA			
Pos.	220	obs:	exp:
cgt	R	0	31.10
cgc	R	0	44.47
cga	R	0	83.74
cgg	R	0	67.86
aga	R	807	387.90
agg	R	24	215.90
ggt	G	0	0.14
ggc	G	0	0.13
gga	G	1	0.44
ggg	G	0	0.29
---	--	------	------
mPD		0.058	0.96
		nPD:	0.06
		N. weight:	0.68
		Sc. PD:	-0.028
		Sc. rank:	-26.2

,

NA			
Pos.	221	obs:	exp:
tct	S	0	2.34
tcc	S	0	2.03
tca	S	0	3.17
tcg	S	0	0.84
aat	N	16	444.40
aac	N	799	370.60
agt	S	0	3.04
agc	S	14	2.58
gat	D	0	1.62
gac	D	3	1.38
---	--	------	------
mPD		0.078	0.56
		nPD:	0.14
		N. weight:	0.74
		Sc. PD:	0.026
		Sc. rank:	185.8

,

NA			
Pos.	222	obs:	exp:
act	T	0	0.26
acc	T	1	0.23
aca	T	0	0.42
acg	T	0	0.09
aat	N	22	452.60
aac	N	808	377.40
aaa	K	1	0.57
aag	K	0	0.43
---	--	------	------
mPD		0.056	0.50
		nPD:	0.11
		N. weight:	0.7
		Sc. PD:	0.0061
		Sc. rank:	122.2

,

NA			
Pos.	223	obs:	exp:
att	I	770	304.90
atc	I	0	222.40
ata	I	61	303.70
act	T	1	0.26
acc	T	0	0.23
aca	T	0	0.42
acg	T	0	0.09
---	--	------	------
mPD		0.14	0.66
		nPD:	0.21
		N. weight:	0.78
		Sc. PD:	0.079
		Sc. rank:	396.7

,

NA			
Pos.	224	obs:	exp:
tta	L	5	76.65
ttg	L	815	157.50
ctt	L	0	164.60
ctc	L	0	132.60
cta	L	0	122.70
ctg	L	9	175.00
atg	M	3	3.00
---	--	------	------
mPD		0.041	1.1
		nPD:	0.04
		N. weight:	1.6
		Sc. PD:	-0.11
		Sc. rank:	-314.5

,

NA			
Pos.	225	obs:	exp:
cgt	R	0	31.14
cgc	R	0	44.52
cga	R	0	83.84
cgg	R	0	67.94
aga	R	814	388.40
agg	R	18	216.10
---	--	------	------
mPD		0.042	0.95
		nPD:	0.04
		N. weight:	0.71
		Sc. PD:	-0.04
		Sc. rank:	-112.1

,

NA			
Pos.	226	obs:	exp:
act	T	789	217.60
acc	T	3	187.00
aca	T	38	347.30
acg	T	0	78.09
gct	A	0	0.50
gcc	A	0	0.39
gca	A	2	0.89
gcg	A	0	0.22
---	--	------	------
mPD		0.10	0.70
		nPD:	0.15
		N. weight:	1.2
		Sc. PD:	0.049
		Sc. rank:	350.8

,

NA			
Pos.	227	obs:	exp:
caa	Q	831	415.40
cag	Q	1	416.60
---	--	------	------
mPD		0.0024	0.50
		nPD:	0.
		N. weight:	0.72
		Sc. PD:	-0.068
		Sc. rank:	-390.7

,

NA			
Pos.	228	obs:	exp:
gaa	E	787	466.40
gag	E	45	365.60
---	--	------	------
mPD		0.10	0.49
		nPD:	0.21
		N. weight:	0.4
		Sc. PD:	0.041
		Sc. rank:	204.1

,

NA			
Pos.	229	obs:	exp:
tct	S	832	138.90
tcc	S	0	120.50
tca	S	0	188.60
tcg	S	0	50.20
agt	S	0	180.50
agc	S	0	153.30
---	--	------	------
mPD		0	1.7
		nPD:	0.
		N. weight:	1.9
		Sc. PD:	-0.19
		Sc. rank:	-1285.6

,

NA			
Pos.	230	obs:	exp:
gaa	E	795	466.40
gag	E	37	365.60
---	--	------	------
mPD		0.085	0.49
		nPD:	0.17
		N. weight:	0.43
		Sc. PD:	0.029
		Sc. rank:	163.6

,

NA			
Pos.	231	obs:	exp:
tgt	C	832	322.00
tgc	C	0	510.00
---	--	------	------
mPD		0	0.47
		nPD:	0.
		N. weight:	1.
		Sc. PD:	-0.099
		Sc. rank:	-681.7

NA			
Pos.	232	obs:	exp:
gtt	V	0	0.43
gtc	V	0	0.42
gta	V	0	0.43
gtg	V	2	0.72
gct	A	4	209.10
gcc	A	39	160.60
gca	A	30	369.40
gcg	A	757	90.85
---	--	------	------
mPD		0.17	0.69
		nPD:	0.24
		N. weight:	1.9
		Sc. PD:	0.25
		Sc. rank:	1062.0

NA			
Pos.	233	obs:	exp:
tgt	C	53	322.00
tgc	C	779	510.00
---	--	------	------
mPD		0.12	0.47
		nPD:	0.25
		N. weight:	0.3
		Sc. PD:	0.043
		Sc. rank:	175.1

NA			
Pos.	234	obs:	exp:
att	I	39	17.25
atc	I	0	12.58
ata	I	8	17.18
atg	M	1	1.00
gtt	V	0	167.50
gtc	V	0	163.90
gta	V	772	166.10
gtg	V	7	281.50
gct	A	0	1.26
gcc	A	0	0.97
gca	A	5	2.23
gcg	A	0	0.55
---	--	------	------
mPD		0.23	0.86
		nPD:	0.27
		N. weight:	1.5
		Sc. PD:	0.24
		Sc. rank:	910.1

NA			
Pos.	235	obs:	exp:
aat	N	830	453.20
aac	N	1	377.80
gat	D	1	0.54
gac	D	0	0.46
---	--	------	------
mPD		0.0048	0.50
		nPD:	0.01
		N. weight:	0.63
		Sc. PD:	-0.057
		Sc. rank:	-285.2

NA			
Pos.	236	obs:	exp:
ggt	G	45	120.30
ggc	G	785	109.30
gga	G	2	364.60
ggg	G	0	237.80
---	--	------	------
mPD		0.11	0.69
		nPD:	0.16
		N. weight:	1.9
		Sc. PD:	0.095
		Sc. rank:	636.6

NA			
Pos.	237	obs:	exp:
tct	S	829	138.90
tcc	S	0	120.50
tca	S	3	188.60
tcg	S	0	50.20
agt	S	0	180.50
agc	S	0	153.30
---	--	------	------
mPD		0.0072	1.7
		nPD:	0.
		N. weight:	1.9
		Sc. PD:	-0.18
		Sc. rank:	-1042.2

NA			
Pos.	238	obs:	exp:
tgt	C	12	322.00
tgc	C	820	510.00
---	--	------	------
mPD		0.028	0.47
		nPD:	0.06
		N. weight:	0.44
		Sc. PD:	-0.019
		Sc. rank:	-22.5

NA			
Pos.	239	obs:	exp:
ttt	F	76	347.10
ttc	F	756	484.90
---	--	------	------
mPD		0.17	0.49
		nPD:	0.34
		N. weight:	0.28
		Sc. PD:	0.065
		Sc. rank:	182.5

NA			
Pos.	240	obs:	exp:
act	T	496	217.80
acc	T	332	187.20
aca	T	3	347.70
acg	T	0	78.19
gct	A	1	0.25
gcc	A	0	0.19
gca	A	0	0.45
gcg	A	0	0.11
---	--	------	------
mPD		0.49	0.70
		nPD:	0.7
		N. weight:	0.75
		Sc. PD:	0.43
		Sc. rank:	505.9

NA			
Pos.	241	obs:	exp:
tta	L	0	0.18
ttg	L	0	0.38
ctt	L	0	0.40
ctc	L	0	0.32
cta	L	2	0.30
ctg	L	0	0.42
att	I	0	14.31
atc	I	0	10.44
ata	I	39	14.25
gtt	V	1	170.10
gtc	V	0	166.40
gta	V	789	168.70
gtg	V	1	285.80
---	--	------	------
mPD		0.099	0.83
		nPD:	0.12
		N. weight:	1.6
		Sc. PD:	0.023
		Sc. rank:	303.4

NA			
Pos.	242	obs:	exp:
att	I	0	0.37
atc	I	0	0.27
ata	I	1	0.37
atg	M	831	831.00
---	--	------	------
mPD		0.0024	0.0024
		nPD:	1.
		N. weight:	0.0013
		Sc. PD:	0.0011
		Sc. rank:	0.9

NA			
Pos.	243	obs:	exp:
act	T	828	218.10
acc	T	3	187.50
aca	T	1	348.10
acg	T	0	78.28
---	--	------	------
mPD		0.0096	0.70
		nPD:	0.01
		N. weight:	1.4
		Sc. PD:	-0.12
		Sc. rank:	-567.8

NA			
Pos.	244	obs:	exp:
gat	D	817	449.20
gac	D	15	382.80
---	--	------	------
mPD		0.035	0.50
		nPD:	0.07
		N. weight:	0.56
		Sc. PD:	-0.017
		Sc. rank:	6.5

NA			
Pos.	245	obs:	exp:
ggt	G	0	120.30
ggc	G	0	109.30
gga	G	822	364.60
ggg	G	10	237.80
---	--	------	------
mPD		0.024	0.69
		nPD:	0.03
		N. weight:	0.81
		Sc. PD:	-0.053
		Sc. rank:	-165.4

NA			
Pos.	246	obs:	exp:
cct	P	0	227.40
ccc	P	0	150.30
cca	P	824	337.70
ccg	P	8	116.60
---	--	------	------
mPD		0.019	0.71
		nPD:	0.03
		N. weight:	0.9
		Sc. PD:	-0.066
		Sc. rank:	-233.7

NA			
Pos.	247	obs:	exp:
tct	S	0	138.50
tcc	S	0	120.20
tca	S	0	188.20
tcg	S	0	50.07
aat	N	0	0.55
aac	N	1	0.45
agt	S	79	180.00
agc	S	751	153.00
ggt	G	0	0.14
ggc	G	1	0.13
gga	G	0	0.44
ggg	G	0	0.29
---	--	------	------
mPD		0.18	1.7
		nPD:	0.11
		N. weight:	1.4
		Sc. PD:	0.0048
		Sc. rank:	212.3

NA			
Pos.	248	obs:	exp:
aat	N	828	452.10
aac	N	1	376.90
gat	D	3	1.62
gac	D	0	1.38
---	--	------	------
mPD		0.0096	0.50
		nPD:	0.02
		N. weight:	0.63
		Sc. PD:	-0.051
		Sc. rank:	-219.2

NA			
Pos.	249	obs:	exp:
gct	A	0	0.25
gcc	A	1	0.19
gca	A	0	0.45
gcg	A	0	0.11
gat	D	1	2.16
gac	D	3	1.84
ggt	G	11	119.60
ggc	G	25	108.60
gga	G	1	362.40
ggg	G	790	236.40
---	--	------	------
mPD		0.11	0.70
		nPD:	0.16
		N. weight:	1.1
		Sc. PD:	0.056
		Sc. rank:	373.5

NA			
Pos.	250	obs:	exp:
caa	Q	1	415.40
cag	Q	831	416.60
---	--	------	------
mPD		0.0024	0.50
		nPD:	0.
		N. weight:	0.72
		Sc. PD:	-0.068
		Sc. rank:	-389.0

NA			
Pos.	251	obs:	exp:
gct	A	26	209.60
gcc	A	805	161.00
gca	A	1	370.30
gcg	A	0	91.06
mPD		0.063	0.69
		nPD:	0.09
		N. weight:	1.6
		Sc. PD:	-0.018
		Sc. rank:	144.7

,

NA			
Pos.	252	obs:	exp:
tct	S	0	138.70
tcc	S	0	120.40
tca	S	824	188.40
tcg	S	7	50.13
act	T	0	0.26
acc	T	0	0.23
aca	T	1	0.42
acg	T	0	0.09
agt	S	0	180.20
agc	S	0	153.10
mPD		0.019	1.7
		nPD:	0.01
		N. weight:	1.5
		Sc. PD:	-0.13
		Sc. rank:	-645.2

,

NA			
Pos.	253	obs:	exp:
tat	Y	766	399.00
tac	Y	66	433.00
mPD		0.15	0.50
		nPD:	0.29
		N. weight:	0.48
		Sc. PD:	0.087
		Sc. rank:	296.0

,

NA			
Pos.	254	obs:	exp:
cgt	R	0	0.04
cgc	R	0	0.05
cga	R	0	0.10
cgg	R	0	0.08
aaa	K	791	473.20
aag	K	40	357.80
aga	R	0	0.47
agg	R	1	0.26
mPD		0.096	0.49
		nPD:	0.19
		N. weight:	0.41
		Sc. PD:	0.036
		Sc. rank:	186.1

,

NA			
Pos.	255	obs:	exp:
att	I	36	304.90
atc	I	795	222.40
ata	I	0	303.70
gtt	V	0	0.22
gtc	V	1	0.21
gta	V	0	0.21
gtg	V	0	0.36
mPD		0.085	0.66
		nPD:	0.13
		N. weight:	1.2
		Sc. PD:	0.029
		Sc. rank:	248.3

,

NA			
Pos.	256	obs:	exp:
ttt	F	0	346.70
ttc	F	831	484.30
tta	L	0	0.09
ttg	L	0	0.19
ctt	L	0	0.20
ctc	L	1	0.16
cta	L	0	0.15
ctg	L	0	0.21
mPD		0.0024	0.49
		nPD:	0.
		N. weight:	0.57
		Sc. PD:	-0.054
		Sc. rank:	-297.1

,

NA			
Pos.	257	obs:	exp:
cgt	R	0	0.41
cgc	R	0	0.59
cga	R	0	1.11
cgg	R	0	0.90
aaa	K	32	467.50
aag	K	789	353.50
aga	R	4	5.14
agg	R	7	2.86
mPD		0.11	0.52
		nPD:	0.21
		N. weight:	0.7
		Sc. PD:	0.071
		Sc. rank:	354.2

,

NA			
Pos.	258	obs:	exp:
att	I	6	301.30
atc	I	0	219.70
ata	I	815	300.00
atg	M	6	6.00
gtt	V	0	1.08
gtc	V	0	1.05
gta	V	5	1.07
gtg	V	0	1.81
mPD		0.041	0.68
		nPD:	0.06
		N. weight:	1.
		Sc. PD:	-0.042
		Sc. rank:	-52.4

,

NA			
Pos.	259	obs:	exp:
gat	D	1	1.62
gac	D	2	1.38
gaa	E	72	464.70
gag	E	757	364.30
mPD		0.16	0.50
		nPD:	0.33
		N. weight:	0.54
		Sc. PD:	0.12
		Sc. rank:	343.3

,

NA			
Pos.	260	obs:	exp:
aaa	K	783	473.80
aag	K	49	358.20
mPD		0.11	0.49
		nPD:	0.23
		N. weight:	0.38
		Sc. PD:	0.045
		Sc. rank:	209.7

,

NA			
Pos.	261	obs:	exp:
cgt	R	0	0.04
cgc	R	0	0.05
cga	R	0	0.10
cgg	R	0	0.08
aga	R	0	0.47
agg	R	1	0.26
ggt	G	0	120.20
ggc	G	0	109.20
gga	G	32	364.10
ggg	G	799	237.50
mPD		0.076	0.69
		nPD:	0.11
		N. weight:	1.1
		Sc. PD:	0.0079
		Sc. rank:	192.5

,

NA			
Pos.	262	obs:	exp:
cgt	R	0	0.49
cgc	R	0	0.70
cga	R	0	1.31
cgg	R	0	1.06
aaa	K	770	466.30
aag	K	49	352.70
aga	R	0	6.07
agg	R	13	3.38
mPD		0.17	0.53
		nPD:	0.32
		N. weight:	0.39
		Sc. PD:	0.081
		Sc. rank:	247.3

,

NA			
Pos.	263	obs:	exp:
att	I	1	0.73
atc	I	0	0.54
ata	I	1	0.73
gtt	V	369	178.50
gtc	V	0	174.60
gta	V	461	177.00
gtg	V	0	299.90
mPD		0.50	0.74
		nPD:	0.68
		N. weight:	0.9
		Sc. PD:	0.5
		Sc. rank:	607.6

,

NA			
Pos.	264	obs:	exp:
att	I	0	1.83
atc	I	5	1.34
ata	I	0	1.83
gtt	V	16	177.90
gtc	V	799	174.00
gta	V	12	176.30
gtg	V	0	298.80
mPD		0.078	0.75
		nPD:	0.1
		N. weight:	1.5
		Sc. PD:	0.00084
		Sc. rank:	210.3

,

NA			
Pos.	265	obs:	exp:
aat	N	0	1.09
aac	N	2	0.91
aaa	K	830	472.60
aag	K	0	357.40
mPD		0.0048	0.49
		nPD:	0.01
		N. weight:	0.59
		Sc. PD:	-0.053
		Sc. rank:	-267.6

,

NA			
Pos.	266	obs:	exp:
tta	L	0	0.09
ttg	L	1	0.19
tct	S	0	138.50
tcc	S	0	120.20
tca	S	806	188.20
tcg	S	24	50.07
ctt	L	0	0.20
ctc	L	0	0.16
cta	L	0	0.15
ctg	L	0	0.21
act	T	0	0.26
acc	T	0	0.23
aca	T	0	0.42
acg	T	1	0.09
agt	S	0	180.00
agc	S	0	153.00
mPD		0.065	1.7
		nPD:	0.04
		N. weight:	1.5
		Sc. PD:	-0.09
		Sc. rank:	-261.4

,

NA			
Pos.	267	obs:	exp:
gtt	V	818	178.10
gtc	V	10	174.20
gta	V	0	176.60
gtg	V	0	299.20
gct	A	1	1.01
gcc	A	3	0.77
gca	A	0	1.78
gcg	A	0	0.44
mPD		0.040	0.74
		nPD:	0.05
		N. weight:	1.5
		Sc. PD:	-0.073
		Sc. rank:	-140.5

,

NA			
Pos.	268	obs:	exp:
gaa	E	808	466.40
gag	E	24	365.60
mPD		0.056	0.49
		nPD:	0.11
		N. weight:	0.48
		Sc. PD:	0.0048
		Sc. rank:	84.9

,

NA			
Pos.	269	obs:	exp:
tta	L	12	76.93
ttg	L	819	158.10
ctt	L	0	165.20
ctc	L	0	133.10
cta	L	0	123.10
ctg	L	1	175.60
mPD		0.031	1.1
		nPD:	0.03
		N. weight:	1.7
		Sc. PD:	-0.12
		Sc. rank:	-416.1

,

NA			
Pos.	270	obs:	exp:
tct	S	0	0.33
tcc	S	0	0.29
tca	S	0	0.45
tcg	S	0	0.12
aat	N	811	444.40
aac	N	4	370.60
agt	S	2	0.43
agc	S	0	0.37
gat	D	15	8.10
gac	D	0	6.90
mPD		0.050	0.54
		nPD:	0.09
		N. weight:	0.61
		Sc. PD:	-0.0065
		Sc. rank:	58.4

,

NA			
Pos.	271	obs:	exp:
gct	A	2	209.60
gcc	A	830	161.00
gca	A	0	370.30
gcg	A	0	91.06
mPD		0.0048	0.69
		nPD:	0.01
		N. weight:	1.7
		Sc. PD:	-0.16
		Sc. rank:	-840.3

,

NA			
Pos.	272	obs:	exp:
tct	S	2	0.33
tcc	S	0	0.29
tca	S	0	0.45
tcg	S	0	0.12
cct	P	820	226.80
ccc	P	10	149.90
cca	P	0	336.90
ccg	P	0	116.40
agt	S	0	0.43
agc	S	0	0.37
mPD		0.029	0.72
		nPD:	0.04
		N. weight:	1.3
		Sc. PD:	-0.08
		Sc. rank:	-227.4

,

NA			
Pos.	273	obs:	exp:
aat	N	832	453.70
aac	N	0	378.30
mPD		0	0.50
		nPD:	0.
		N. weight:	0.64
		Sc. PD:	-0.063
		Sc. rank:	-435.4

,

NA

Pos.	274	obs:	exp:
tat	Y	17	399.00
tac	Y	815	433.00
mPD		0.040	0.50
		nPD:	0.08
		N. weight:	0.59
		Sc. PD:	-0.013
		Sc. rank:	41.8

NA

Pos.	275	obs:	exp:
tat	Y	0	0.96
tac	Y	2	1.04
cat	H	1	480.50
cac	H	829	349.50
mPD		0.0072	0.49
		nPD:	0.01
		N. weight:	0.9
		Sc. PD:	-0.077
		Sc. rank:	-335.7

NA

Pos.	276	obs:	exp:
tat	Y	56	399.00
tac	Y	776	433.00
mPD		0.13	0.50
		nPD:	0.25
		N. weight:	0.44
		Sc. PD:	0.062
		Sc. rank:	255.7

NA

Pos.	277	obs:	exp:
gaa	E	10	466.40
gag	E	822	365.60
mPD		0.024	0.49
		nPD:	0.05
		N. weight:	0.8
		Sc. PD:	-0.042
		Sc. rank:	-94.1

NA

Pos.	278	obs:	exp:
gaa	E	776	466.40
gag	E	56	365.60
mPD		0.13	0.49
		nPD:	0.26
		N. weight:	0.37
		Sc. PD:	0.054
		Sc. rank:	218.3

NA

Pos.	279	obs:	exp:
tgt	C	16	322.00
tgc	C	816	510.00
mPD		0.038	0.47
		nPD:	0.08
		N. weight:	0.43
		Sc. PD:	-0.0098
		Sc. rank:	29.7

NA

Pos.	280	obs:	exp:
tct	S	1	138.90
tcc	S	831	120.50
tca	S	0	188.60
tcg	S	0	50.20
agt	S	0	180.50
agc	S	0	153.30
mPD		0.0024	1.7
		nPD:	0.
		N. weight:	2.
		Sc. PD:	-0.2
		Sc. rank:	-1249.6

NA

Pos.	281	obs:	exp:
tgt	C	832	322.00
tgc	C	0	510.00
mPD		0	0.47
		nPD:	0.
		N. weight:	1.
		Sc. PD:	-0.099
		Sc. rank:	-681.7

NA

Pos.	282	obs:	exp:
tat	Y	823	399.00
tac	Y	9	433.00
mPD		0.021	0.50
		nPD:	0.04
		N. weight:	0.71
		Sc. PD:	-0.041
		Sc. rank:	-116.0

NA

Pos.	283	obs:	exp:
cct	P	831	227.40
ccc	P	1	150.30
cca	P	0	337.70
ccg	P	0	116.60
mPD		0.0024	0.71
		nPD:	0.
		N. weight:	1.4
		Sc. PD:	-0.13
		Sc. rank:	-812.8

NA

Pos.	284	obs:	exp:
aat	N	12	6.54
aac	N	0	5.46
gat	D	817	442.70
gac	D	3	377.30
mPD		0.036	0.53
		nPD:	0.07
		N. weight:	0.63
		Sc. PD:	-0.021
		Sc. rank:	3.3

NA

Pos.	285	obs:	exp:
act	T	0	0.79
acc	T	0	0.68
aca	T	0	1.26
acg	T	3	0.28
gct	A	55	208.80
gcc	A	1	160.40
gca	A	10	369.00
gcg	A	763	90.74
mPD		0.16	0.70
		nPD:	0.22
		N. weight:	1.9
		Sc. PD:	0.22
		Sc. rank:	1058.1

NA

Pos.	286	obs:	exp:
tct	S	0	6.68
tcc	S	0	5.79
tca	S	0	9.07
tcg	S	0	2.41
agt	S	11	8.68
agc	S	29	7.37
ggt	G	758	114.50
ggc	G	31	104.00
gga	G	3	347.00
ggg	G	0	226.40
mPD		0.23	0.85
		nPD:	0.27
		N. weight:	1.8
		Sc. PD:	0.3
		Sc. rank:	1109.5

NA

Pos.	287	obs:	exp:
aaa	K	3	2.28
aag	K	1	1.72
gat	D	348	199.80
gac	D	22	170.20
gaa	E	43	256.70
gag	E	415	201.30
mPD		0.58	0.75
		nPD:	0.77
		N. weight:	0.48
		Sc. PD:	0.31
		Sc. rank:	327.0

NA

Pos.	288	obs:	exp:
att	I	763	289.90
atc	I	25	211.40
ata	I	2	288.70
gtt	V	3	9.03
gtc	V	0	8.84
gta	V	27	8.96
gtg	V	12	15.17
mPD		0.25	0.77
		nPD:	0.32
		N. weight:	0.89
		Sc. PD:	0.19
		Sc. rank:	565.9

NA

Pos.	289	obs:	exp:
tta	L	0	0.09
ttg	L	1	0.19
ctt	L	0	0.20
ctc	L	0	0.16
cta	L	0	0.15
ctg	L	0	0.21
att	I	1	1.47
atc	I	0	1.07
ata	I	3	1.46
atg	M	810	810.00
act	T	0	3.15
acc	T	0	2.70
aca	T	12	5.02
acg	T	0	1.13
gtt	V	0	1.08
gtc	V	0	1.05
gta	V	0	1.07
gtg	V	5	1.81
mPD		0.081	0.087
		nPD:	0.93
		N. weight:	0.025
		Sc. PD:	0.02
		Sc. rank:	17.2

NA

Pos.	290	obs:	exp:
tgt	C	824	322.00
tgc	C	8	510.00
mPD		0.019	0.47
		nPD:	0.04
		N. weight:	0.94
		Sc. PD:	-0.057
		Sc. rank:	-159.3

NA

Pos.	291	obs:	exp:
gtt	V	351	178.90
gtc	V	0	175.00
gta	V	34	177.40
gtg	V	447	300.60
mPD		0.53	0.73
		nPD:	0.73
		N. weight:	0.46
		Sc. PD:	0.27
		Sc. rank:	312.5

NA

Pos.	292	obs:	exp:
tgt	C	6	322.00
tgc	C	826	510.00
mPD		0.014	0.47
		nPD:	0.03
		N. weight:	0.48
		Sc. PD:	-0.033
		Sc. rank:	-104.6

NA

Pos.	293	obs:	exp:
cgt	R	0	31.14
cgc	R	0	44.52
cga	R	0	83.84
cgg	R	0	67.94
aga	R	40	388.40
agg	R	792	216.10
mPD		0.092	0.95
		nPD:	0.1
		N. weight:	1.2
		Sc. PD:	-0.0085
		Sc. rank:	122.2

NA

Pos.	294	obs:	exp:
gat	D	27	449.20
gac	D	805	382.80
mPD		0.063	0.50
		nPD:	0.13
		N. weight:	0.66
		Sc. PD:	0.015
		Sc. rank:	133.7

NA

Pos.	295	obs:	exp:
aat	N	765	453.70
aac	N	67	378.30
mPD		0.15	0.50
		nPD:	0.3
		N. weight:	0.36
		Sc. PD:	0.068
		Sc. rank:	224.7

NA

Pos.	296	obs:	exp:
tgg	W	832	832.00
mPD		0	0
		nPD:	1.
		N. weight:	0.
		Sc. PD:	0
		Sc. rank:	0

NA

Pos.	297	obs:	exp:
cat	H	830	481.60
cac	H	2	350.40
mPD		0.0048	0.49
		nPD:	0.01
		N. weight:	0.56
		Sc. PD:	-0.05
		Sc. rank:	-251.7

NA

Pos.	298	obs:	exp:
ggt	G	46	120.30
ggc	G	784	109.30
gga	G	2	364.60
ggg	G	0	237.80
---	--	------	------
mPD		0.11	0.69
		nPD:	0.16
		N. weight:	1.9
		Sc. PD:	0.1
		Sc. rank:	684.0

,

NA

Pos.	299	obs:	exp:
tct	S	0	138.90
tcc	S	1	120.50
tca	S	825	188.60
tcg	S	6	50.20
agt	S	0	180.50
agc	S	0	153.30
---	--	------	------
mPD		0.017	1.7
		nPD:	0.01
		N. weight:	1.5
		Sc. PD:	-0.14
		Sc. rank:	-673.6

,

NA

Pos.	300	obs:	exp:
aat	N	67	453.70
aac	N	765	378.30
---	--	------	------
mPD		0.15	0.50
		nPD:	0.3
		N. weight:	0.52
		Sc. PD:	0.098
		Sc. rank:	324.2

,

NA

Pos.	301	obs:	exp:
cgt	R	0	31.14
cgc	R	0	44.52
cga	R	40	83.84
cgg	R	789	67.94
aga	R	2	388.40
agg	R	1	216.10
---	--	------	------
mPD		0.10	0.95
		nPD:	0.11
		N. weight:	2.4
		Sc. PD:	0.011
		Sc. rank:	389.4

,

NA

Pos.	302	obs:	exp:
cct	P	0	227.40
ccc	P	0	150.30
cca	P	68	337.70
ccg	P	764	116.60
---	--	------	------
mPD		0.15	0.71
		nPD:	0.21
		N. weight:	1.7
		Sc. PD:	0.18
		Sc. rank:	875.4

,

NA

Pos.	303	obs:	exp:
tgg	W	832	832.00
---	--	------	------
mPD		0	0
		nPD:	1.
		N. weight:	0.
		Sc. PD:	0
		Sc. rank:	0

,

NA

Pos.	304	obs:	exp:
gtt	V	0	178.90
gtc	V	0	175.00
gta	V	781	177.40
gtg	V	51	300.60
---	--	------	------
mPD		0.12	0.73
		nPD:	0.16
		N. weight:	1.4
		Sc. PD:	0.07
		Sc. rank:	476.8

,

NA

Pos.	305	obs:	exp:
ttt	F	3	1.25
ttc	F	0	1.75
tct	S	791	138.40
tcc	S	38	120.10
tca	S	0	187.90
tcg	S	0	50.01
agt	S	0	179.80
agc	S	0	152.80
---	--	------	------
mPD		0.094	1.7
		nPD:	0.06
		N. weight:	1.7
		Sc. PD:	-0.075
		Sc. rank:	-132.3

,

NA

Pos.	306	obs:	exp:
ttt	F	757	347.10
ttc	F	75	484.90
---	--	------	------
mPD		0.16	0.49
		nPD:	0.34
		N. weight:	0.57
		Sc. PD:	0.13
		Sc. rank:	369.9

,

NA

Pos.	307	obs:	exp:
tct	S	0	0.17
tcc	S	0	0.14
tca	S	0	0.23
tcg	S	0	0.06
act	T	11	2.88
acc	T	0	2.48
aca	T	0	4.60
acg	T	0	1.03
aat	N	805	446.60
aac	N	14	372.40
agt	S	1	0.22
agc	S	0	0.18
gat	D	1	0.54
gac	D	0	0.46
---	--	------	------
mPD		0.064	0.54
		nPD:	0.12
		N. weight:	0.57
		Sc. PD:	0.0088
		Sc. rank:	111.7

NA

Pos.	308	obs:	exp:
cat	H	4	2.32
cac	H	0	1.68
caa	Q	752	397.40
cag	Q	44	398.60
aaa	K	32	18.22
aag	K	0	13.78
---	--	------	------
mPD		0.18	0.58
		nPD:	0.32
		N. weight:	0.51
		Sc. PD:	0.11
		Sc. rank:	323.2

,

NA

Pos.	309	obs:	exp:
aat	N	822	453.20
aac	N	9	377.80
gat	D	1	0.54
gac	D	0	0.46
---	--	------	------
mPD		0.024	0.50
		nPD:	0.05
		N. weight:	0.58
		Sc. PD:	-0.031
		Sc. rank:	-74.6

,

NA

Pos.	310	obs:	exp:
tta	L	4	76.93
ttg	L	14	158.10
ctt	L	0	165.20
ctc	L	0	133.10
cta	L	44	123.10
ctg	L	770	175.60
---	--	------	------
mPD		0.15	1.1
		nPD:	0.13
		N. weight:	1.3
		Sc. PD:	0.04
		Sc. rank:	313.7

,

NA

Pos.	311	obs:	exp:
gaa	E	31	466.40
gag	E	801	365.60
---	--	------	------
mPD		0.072	0.49
		nPD:	0.15
		N. weight:	0.69
		Sc. PD:	0.028
		Sc. rank:	202.2

,

NA

Pos.	312	obs:	exp:
tat	Y	830	399.00
tac	Y	2	433.00
---	--	------	------
mPD		0.0048	0.50
		nPD:	0.01
		N. weight:	0.76
		Sc. PD:	-0.068
		Sc. rank:	-344.1

,

NA

Pos.	313	obs:	exp:
caa	Q	795	401.90
cag	Q	10	403.10
cgt	R	0	0.97
cgc	R	0	1.39
cga	R	26	2.62
cgg	R	0	2.12
aaa	K	1	0.57
aag	K	0	0.43
aga	R	0	12.14
agg	R	0	6.75
---	--	------	------
mPD		0.087	0.61
		nPD:	0.14
		N. weight:	0.72
		Sc. PD:	0.027
		Sc. rank:	199.5

,

NA

Pos.	314	obs:	exp:
att	I	0	305.30
atc	I	0	222.60
ata	I	832	304.00
---	--	------	------
mPD		0	0.66
		nPD:	0.
		N. weight:	1.1
		Sc. PD:	-0.11
		Sc. rank:	-722.9

,

NA

Pos.	315	obs:	exp:
ggt	G	2	120.30
ggc	G	0	109.30
gga	G	818	364.60
ggg	G	12	237.80
---	--	------	------
mPD		0.033	0.69
		nPD:	0.05
		N. weight:	0.78
		Sc. PD:	-0.042
		Sc. rank:	-94.5

,

NA

Pos.	316	obs:	exp:
tat	Y	782	399.00
tac	Y	50	433.00
---	--	------	------
mPD		0.11	0.50
		nPD:	0.23
		N. weight:	0.53
		Sc. PD:	0.063
		Sc. rank:	296.3

,

NA

Pos.	317	obs:	exp:
att	I	0	305.30
atc	I	0	222.60
ata	I	832	304.00
---	--	------	------
mPD		0	0.66
		nPD:	0.
		N. weight:	1.1
		Sc. PD:	-0.11
		Sc. rank:	-722.9

,

NA

Pos.	318	obs:	exp:
tgt	C	0	322.00
tgc	C	832	510.00
---	--	------	------
mPD		0	0.47
		nPD:	0.
		N. weight:	0.51
		Sc. PD:	-0.051
		Sc. rank:	-351.5

,

NA

Pos.	319	obs:	exp:
tct	S	0	138.90
tcc	S	0	120.50
tca	S	0	188.60
tcg	S	0	50.20
agt	S	793	180.50
agc	S	39	153.30
---	--	------	------
mPD		0.089	1.7
		nPD:	0.05
		N. weight:	1.4
		Sc. PD:	-0.067
		Sc. rank:	-147.9

,

NA

Pos.	320	obs:	exp:
ggt	G	0	120.30
ggc	G	0	109.30
gga	G	54	364.60
ggg	G	778	237.80
---	--	------	------
mPD		0.12	0.69
		nPD:	0.18
		N. weight:	1.
		Sc. PD:	0.073
		Sc. rank:	413.1

,

NA

Pos.	321	obs:	exp:
ttt	F	1	0.42
ttc	F	0	0.58
att	I	11	4.04
atc	I	0	2.94
ata	I	0	4.02
gtt	V	816	176.40
gtc	V	4	172.50
gta	V	0	174.80
gtg	V	0	296.30
---	--	------	------
mPD		0.038	0.76
		nPD:	0.05
		N. weight:	1.6
		Sc. PD:	-0.081
		Sc. rank:	-176.9

,

NA

Pos.	322	obs:	exp:
ttt	F	43	347.10
ttc	F	789	484.90
---	--	------	------
mPD		0.098	0.49
		nPD:	0.2
		N. weight:	0.38
		Sc. PD:	0.035
		Sc. rank:	182.7

,

NA

Pos.	323	obs:	exp:
ggt	G	0	120.30
ggc	G	1	109.30
gga	G	443	364.60
ggg	G	388	237.80
---	--	------	------
mPD		0.50	0.69
		nPD:	0.73
		N. weight:	0.35
		Sc. PD:	0.21
		Sc. rank:	237.5

,

NA

Pos.	324	obs:	exp:
gat	D	12	449.20
gac	D	820	382.80
---	--	------	------
mPD		0.028	0.50
		nPD:	0.06
		N. weight:	0.74
		Sc. PD:	-0.033
		Sc. rank:	-56.2

,

NA

Pos.	325	obs:	exp:
tct	S	0	0.17
tcc	S	0	0.14
tca	S	0	0.23
tcg	S	0	0.06
aat	N	432	453.20
aac	N	399	377.80
agt	S	1	0.22
agc	S	0	0.18
---	--	------	------
mPD		0.50	0.50
		nPD:	1.
		N. weight:	0.0078
		Sc. PD:	0.0068
		Sc. rank:	5.4

,

NA

Pos.	326	obs:	exp:
cct	P	1	227.40
ccc	P	771	150.30
cca	P	32	337.70
ccg	P	28	116.60
---	--	------	------
mPD		0.14	0.71
		nPD:	0.2
		N. weight:	1.4
		Sc. PD:	0.13
		Sc. rank:	679.8

,

NA

Pos.	327	obs:	exp:
cgt	R	27	31.14
cgc	R	805	44.52
cga	R	0	83.84
cgg	R	0	67.94
aga	R	0	388.40
agg	R	0	216.10
---	--	------	------
mPD		0.063	0.95
		nPD:	0.07
		N. weight:	2.9
		Sc. PD:	-0.11
		Sc. rank:	-20.7

,

NA

Pos.	328	obs:	exp:
cct	P	1	227.40
ccc	P	831	150.30
cca	P	0	337.70
ccg	P	0	116.60
---	--	------	------
mPD		0.0024	0.71
		nPD:	0.
		N. weight:	1.8
		Sc. PD:	-0.17
		Sc. rank:	-1073.2

,

NA

Pos.	329	obs:	exp:
tct	S	0	1.50
tcc	S	0	1.30
tca	S	0	2.04
tcg	S	0	0.54
aat	N	823	448.80
aac	N	0	374.20
agt	S	9	1.95
agc	S	0	1.66
---	--	------	------
mPD		0.021	0.53
		nPD:	0.04
		N. weight:	0.65
		Sc. PD:	-0.039
		Sc. rank:	-111.7

,

NA

Pos.	330	obs:	exp:
gat	D	776	449.20
gac	D	56	382.80
---	--	------	------
mPD		0.13	0.50
		nPD:	0.25
		N. weight:	0.4
		Sc. PD:	0.058
		Sc. rank:	236.8

,

NA

Pos.	331	obs:	exp:
cgt	R	0	0.07
cgc	R	0	0.11
cga	R	0	0.20
cgg	R	0	0.16
aga	R	2	0.93
agg	R	0	0.52
ggt	G	0	120.00
ggc	G	0	109.00
gga	G	812	363.70
ggg	G	18	237.30
---	--	------	------
mPD		0.047	0.69
		nPD:	0.07
		N. weight:	0.77
		Sc. PD:	-0.026
		Sc. rank:	5.8

,

NA

Pos.	332	obs:	exp:
cct	P	0	17.49
ccc	P	0	11.56
cca	P	64	25.98
ccg	P	0	8.97
att	I	0	1.10
atc	I	0	0.80
ata	I	3	1.10
act	T	0	199.50
acc	T	0	171.50
aca	T	760	318.40
acg	T	1	71.60
gct	A	0	1.01
gcc	A	0	0.77
gca	A	4	1.78
gcg	A	0	0.44
---	--	------	------
mPD		0.16	0.86
		nPD:	0.19
		N. weight:	0.91
		Sc. PD:	0.074
		Sc. rank:	402.1

,

NA

Pos.	333	obs:	exp:
ggt	G	1	120.30
ggc	G	831	109.30
gga	G	0	364.60
ggg	G	0	237.80
---	--	------	------
mPD		0.0024	0.69
		nPD:	0.
		N. weight:	2.1
		Sc. PD:	-0.2
		Sc. rank:	-1223.2

,

NA

Pos.	334	obs:	exp:
tct	S	0	138.40
tcc	S	0	120.10
tca	S	0	187.90
tcg	S	0	50.01
cgt	R	0	0.07
cgc	R	0	0.11
cga	R	0	0.20
cgg	R	0	0.16
aat	N	1	0.55
aac	N	0	0.45
agt	S	827	179.80
agc	S	2	152.80
aga	R	0	0.93
agg	R	2	0.52
---	--	------	------
mPD		0.012	1.7
		nPD:	0.01
		N. weight:	1.6
		Sc. PD:	-0.15
		Sc. rank:	-762.9

,

NA

Pos.	335	obs:	exp:
tgt	C	60	322.00
tgc	C	772	510.00
---	--	------	------
mPD		0.13	0.47
		nPD:	0.28
		N. weight:	0.28
		Sc. PD:	0.048
		Sc. rank:	173.4

,

NA

Pos.	336	obs:	exp:
tct	S	0	123.50
tcc	S	0	107.20
tca	S	0	167.80
tcg	S	0	44.64
aat	N	13	7.09
aac	N	0	5.91
agt	S	740	160.50
agc	S	0	136.40
ggt	G	78	11.42
ggc	G	1	10.38
gga	G	0	34.62
ggg	G	0	22.58
---	--	------	------
mPD		0.21	1.8
		nPD:	0.11
		N. weight:	1.6
		Sc. PD:	0.018
		Sc. rank:	294.4

,

NA

Pos.	337	obs:	exp:
cct	P	0	227.40
ccc	P	0	150.30
cca	P	811	337.70
ccg	P	21	116.60
---	--	------	------
mPD		0.049	0.71
		nPD:	0.07
		N. weight:	0.86
		Sc. PD:	-0.028
		Sc. rank:	8.6

,

NA

Pos.	338	obs:	exp:
tta	L	0	0.09
ttg	L	0	0.19
ctt	L	0	0.20
ctc	L	0	0.16
cta	L	0	0.15
ctg	L	1	0.21
att	I	1	0.37
atc	I	0	0.27
ata	I	0	0.37
atg	M	774	774.00
gtt	V	0	12.04
gtc	V	0	11.78
gta	V	11	11.94
gtg	V	45	20.23
---	--	------	------
mPD		0.16	0.21
		nPD:	0.73
		N. weight:	0.05
		Sc. PD:	0.03
		Sc. rank:	34.1

,

NA

Pos.	339	obs:	exp:
ttt	F	0	0.83
ttc	F	2	1.17
tct	S	45	138.50
tcc	S	785	120.20
tca	S	0	188.20
tcg	S	0	50.07
agt	S	0	180.00
agc	S	0	153.00
---	--	------	------
mPD		0.11	1.7
		nPD:	0.06
		N. weight:	1.8
		Sc. PD:	-0.067
		Sc. rank:	-28.1

,

NA

Pos.	340	obs:	exp:
tct	S	818	136.50
tcc	S	0	118.50
tca	S	0	185.40
tcg	S	0	49.35
cct	P	13	3.55
ccc	P	0	2.35
cca	P	0	5.28
ccg	P	0	1.82
act	T	1	0.26
acc	T	0	0.23
aca	T	0	0.42
acg	T	0	0.09
agt	S	0	177.40
agc	S	0	150.70
---	--	------	------
mPD		0.033	1.7
		nPD:	0.02
		N. weight:	1.9
		Sc. PD:	-0.15
		Sc. rank:	-613.8

,

NA

Pos.	341	obs:	exp:
tct	S	0	0.17
tcc	S	0	0.14
tca	S	0	0.23
tcg	S	0	0.06
aat	N	354	448.30
aac	N	468	373.70
aaa	K	9	5.12
aag	K	0	3.88
agt	S	0	0.22
agc	S	1	0.18
---	--	------	------
mPD		0.50	0.51
		nPD:	0.99
		N. weight:	0.04
		Sc. PD:	0.034
		Sc. rank:	27.7

,

NA

Pos.	342	obs:	exp:
ggt	G	1	120.30
ggc	G	0	109.30
gga	G	6	364.60
ggg	G	825	237.80
---	--	------	------
mPD		0.017	0.69
		nPD:	0.02
		N. weight:	1.3
		Sc. PD:	-0.096
		Sc. rank:	-348.8

,

NA			
Pos.	343	obs:	exp:
gct	A	1	209.60
gcc	A	0	161.00
gca	A	828	370.30
gcg	A	3	91.06
---	--	------	------
mPD		0.0096	0.69
		nPD:	0.01
		N. weight:	0.83
		Sc. PD:	-0.071
		Sc. rank:	-330.1

NA			
Pos.	344	obs:	exp:
tat	Y	830	398.10
tac	Y	0	431.90
cat	H	2	1.16
cac	H	0	0.84
---	--	------	------
mPD		0.0048	0.50
		nPD:	0.01
		N. weight:	0.77
		Sc. PD:	-0.07
		Sc. rank:	-356.3

NA			
Pos.	345	obs:	exp:
ggt	G	0	120.30
ggc	G	0	109.30
gga	G	42	364.60
ggg	G	790	237.80
---	--	------	------
mPD		0.096	0.69
		nPD:	0.14
		N. weight:	1.1
		Sc. PD:	0.038
		Sc. rank:	279.3

NA			
Pos.	346	obs:	exp:
att	I	0	2.57
atc	I	0	1.87
ata	I	7	2.56
gtt	V	0	177.40
gtc	V	0	173.60
gta	V	818	175.90
gtg	V	7	298.10
---	--	------	------
mPD		0.033	0.75
		nPD:	0.04
		N. weight:	1.6
		Sc. PD:	-0.089
		Sc. rank:	-241.2

NA			
Pos.	347	obs:	exp:
aaa	K	802	473.80
aag	K	30	358.20
---	--	------	------
mPD		0.070	0.49
		nPD:	0.14
		N. weight:	0.44
		Sc. PD:	0.016
		Sc. rank:	122.2

NA			
Pos.	348	obs:	exp:
ggt	G	0	120.30
ggc	G	0	109.30
gga	G	16	364.60
ggg	G	816	237.80
---	--	------	------
mPD		0.038	0.69
		nPD:	0.05
		N. weight:	1.2
		Sc. PD:	-0.057
		Sc. rank:	-105.6

NA			
Pos.	349	obs:	exp:
ttt	F	829	347.10
ttc	F	3	484.90
---	--	------	------
mPD		0.0072	0.49
		nPD:	0.01
		N. weight:	0.89
		Sc. PD:	-0.076
		Sc. rank:	-331.4

NA			
Pos.	350	obs:	exp:
tct	S	2	138.90
tcc	S	1	120.50
tca	S	824	188.60
tcg	S	5	50.20
agt	S	0	180.50
agc	S	0	153.30
---	--	------	------
mPD		0.019	1.7
		nPD:	0.01
		N. weight:	1.5
		Sc. PD:	-0.13
		Sc. rank:	-632.9

NA			
Pos.	351	obs:	exp:
ttt	F	825	347.10
ttc	F	7	484.90
---	--	------	------
mPD		0.017	0.49
		nPD:	0.03
		N. weight:	0.87
		Sc. PD:	-0.058
		Sc. rank:	-179.8

NA			
Pos.	352	obs:	exp:
cgt	R	0	0.45
cgc	R	0	0.64
cga	R	0	1.21
cgg	R	0	0.98
aaa	K	67	466.90
aag	K	753	353.10
aga	R	12	5.60
agg	R	0	3.12
---	--	------	------
mPD		0.20	0.53
		nPD:	0.38
		N. weight:	0.57
		Sc. PD:	0.15
		Sc. rank:	372.3

NA			
Pos.	353	obs:	exp:
tat	Y	21	398.60
tac	Y	810	432.40
cat	H	1	0.58
cac	H	0	0.42
---	--	------	------
mPD		0.054	0.50
		nPD:	0.11
		N. weight:	0.57
		Sc. PD:	0.0022
		Sc. rank:	84.9

NA			
Pos.	354	obs:	exp:
tct	S	0	0.33
tcc	S	0	0.29
tca	S	0	0.45
tcg	S	0	0.12
agt	S	2	0.43
agc	S	0	0.37
ggt	G	808	120.00
ggc	G	22	109.00
gga	G	0	363.70
ggg	G	0	237.30
---	--	------	------
mPD		0.056	0.70
		nPD:	0.08
		N. weight:	1.9
		Sc. PD:	-0.041
		Sc. rank:	146.7

NA			
Pos.	355	obs:	exp:
tct	S	0	1.00
tcc	S	0	0.87
tca	S	0	1.36
tcg	S	0	0.36
aat	N	809	448.80
aac	N	14	374.20
aaa	K	1	1.14
aag	K	1	0.86
agt	S	6	1.30
agc	S	0	1.11
gat	D	1	0.54
gac	D	0	0.46
---	--	------	------
mPD		0.055	0.53
		nPD:	0.1
		N. weight:	0.56
		Sc. PD:	0.0002
		Sc. rank:	78.1

NA			
Pos.	356	obs:	exp:
tgg	W	1	1.00
ggt	G	75	120.20
ggc	G	1	109.20
gga	G	0	364.10
ggg	G	755	237.50
---	--	------	------
mPD		0.17	0.69
		nPD:	0.24
		N. weight:	1.1
		Sc. PD:	0.14
		Sc. rank:	609.0

NA			
Pos.	357	obs:	exp:
att	I	2	0.73
atc	I	0	0.54
ata	I	0	0.73
gtt	V	819	178.10
gtc	V	7	174.20
gta	V	0	176.60
gtg	V	2	299.20
ggt	G	2	0.29
ggc	G	0	0.26
gga	G	0	0.88
ggg	G	0	0.57
---	--	------	------
mPD		0.031	0.74
		nPD:	0.04
		N. weight:	1.5
		Sc. PD:	-0.092
		Sc. rank:	-255.9

NA			
Pos.	358	obs:	exp:
tgg	W	832	832.00
---	--	------	------
mPD		0	0
		nPD:	1.
		N. weight:	0.
		Sc. PD:	0
		Sc. rank:	0

NA			
Pos.	359	obs:	exp:
att	I	16	305.30
atc	I	776	222.60
ata	I	40	304.00
---	--	------	------
mPD		0.13	0.66
		nPD:	0.19
		N. weight:	1.1
		Sc. PD:	0.092
		Sc. rank:	483.5

NA			
Pos.	360	obs:	exp:
ggt	G	0	120.30
ggc	G	0	109.30
gga	G	819	364.60
ggg	G	13	237.80
---	--	------	------
mPD		0.031	0.69
		nPD:	0.04
		N. weight:	0.79
		Sc. PD:	-0.045
		Sc. rank:	-117.0

NA			
Pos.	361	obs:	exp:
cgt	R	0	31.14
cgc	R	0	44.52
cga	R	0	83.84
cgg	R	0	67.94
aga	R	820	388.40
agg	R	12	216.10
---	--	------	------
mPD		0.028	0.95
		nPD:	0.03
		N. weight:	0.73
		Sc. PD:	-0.052
		Sc. rank:	-171.1

NA			
Pos.	362	obs:	exp:
act	T	30	218.10
acc	T	24	187.50
aca	T	778	348.10
acg	T	0	78.28
---	--	------	------
mPD		0.12	0.70
		nPD:	0.18
		N. weight:	0.66
		Sc. PD:	0.047
		Sc. rank:	267.0

NA			
Pos.	363	obs:	exp:
aaa	K	822	473.80
aag	K	10	358.20
---	--	------	------
mPD		0.024	0.49
		nPD:	0.05
		N. weight:	0.53
		Sc. PD:	-0.028
		Sc. rank:	-61.8

NA			
Pos.	364	obs:	exp:
tct	S	0	138.90
tcc	S	0	120.50
tca	S	0	188.60
tcg	S	0	50.20
agt	S	57	180.50
agc	S	775	153.30
---	--	------	------
mPD		0.13	1.7
		nPD:	0.08
		N. weight:	1.5
		Sc. PD:	-0.038
		Sc. rank:	83.8

NA			
Pos.	365	obs:	exp:
att	I	3	1.10
atc	I	0	0.80
ata	I	0	1.10
act	T	822	217.30
acc	T	7	186.80
aca	T	0	346.90
acg	T	0	78.00
---	--	------	------
mPD		0.024	0.70
		nPD:	0.03
		N. weight:	1.4
		Sc. PD:	-0.091
		Sc. rank:	-288.4

NA			
Pos.	366	obs:	exp:
tct	S	0	135.20
tcc	S	0	117.30
tca	S	0	183.60
tcg	S	0	48.87
aat	N	21	11.45
aac	N	0	9.55
agt	S	745	175.70
agc	S	65	149.30
ggt	G	1	0.14
ggc	G	0	0.13
gga	G	0	0.44
ggg	G	0	0.29
---	--	------	------
mPD		0.20	1.7
		nPD:	0.12
		N. weight:	1.3
		Sc. PD:	0.017
		Sc. rank:	242.8

NA			
Pos.	367	obs:	exp:
tct	S	0	138.90
tcc	S	793	120.50
tca	S	39	188.60
tcg	S	0	50.20
agt	S	0	180.50
agc	S	0	153.30
---	--	------	------
mPD		0.089	1.7
		nPD:	0.05
		N. weight:	1.8
		Sc. PD:	-0.086
		Sc. rank:	-189.0

NA

Pos.	368	obs:	exp:
cgt	R	0	31.14
cgc	R	0	44.52
cga	R	0	83.84
cgg	R	0	67.94
aga	R	735	388.40
agg	R	97	216.10
mPD		0.21	0.95
		nPD:	0.22
		N. weight:	0.5
		Sc. PD:	0.054
		Sc. rank:	265.7

NA

Pos.	369	obs:	exp:
tct	S	0	137.40
tcc	S	0	119.20
tca	S	0	186.60
tcg	S	0	49.65
att	I	0	0.37
atc	I	1	0.27
ata	I	0	0.37
aat	N	1	4.36
aac	N	7	3.64
agt	S	45	178.50
agc	S	778	151.70
mPD		0.13	1.7
		nPD:	0.08
		N. weight:	1.5
		Sc. PD:	-0.041
		Sc. rank:	72.3

NA

Pos.	370	obs:	exp:
ggt	G	8	120.30
ggc	G	779	109.30
gga	G	11	364.60
ggg	G	34	237.80
mPD		0.12	0.69
		nPD:	0.18
		N. weight:	1.8
		Sc. PD:	0.13
		Sc. rank:	718.2

NA

Pos.	371	obs:	exp:
ttt	F	832	347.10
ttc	F	0	484.90
mPD		0	0.49
		nPD:	0.
		N. weight:	0.92
		Sc. PD:	-0.091
		Sc. rank:	-627.8

NA

Pos.	372	obs:	exp:
gaa	E	13	466.40
gag	E	819	365.60
mPD		0.031	0.49
		nPD:	0.06
		N. weight:	0.78
		Sc. PD:	-0.031
		Sc. rank:	-24.8

NA

Pos.	373	obs:	exp:
atg	M	830	830.00
gtt	V	0	0.43
gtc	V	0	0.42
gta	V	0	0.43
gtg	V	2	0.72
mPD		0.0048	0.0079
		nPD:	0.61
		N. weight:	0.0026
		Sc. PD:	0.0013
		Sc. rank:	1.7

NA

Pos.	374	obs:	exp:
att	I	816	303.50
atc	I	11	221.30
ata	I	0	302.20
gtt	V	5	1.08
gtc	V	0	1.05
gta	V	0	1.07
gtg	V	0	1.81
mPD		0.038	0.67
		nPD:	0.06
		N. weight:	0.99
		Sc. PD:	-0.045
		Sc. rank:	-82.2

NA

Pos.	375	obs:	exp:
tgg	W	832	832.00
mPD		0	0
		nPD:	1.
		N. weight:	0.
		Sc. PD:	0
		Sc. rank:	0

NA

Pos.	376	obs:	exp:
gat	D	812	449.20
gac	D	20	382.80
mPD		0.047	0.50
		nPD:	0.09
		N. weight:	0.54
		Sc. PD:	-0.0046
		Sc. rank:	52.1

NA

Pos.	377	obs:	exp:
cct	P	12	227.40
ccc	P	28	150.30
cca	P	26	337.70
ccg	P	766	116.60
mPD		0.15	0.71
		nPD:	0.21
		N. weight:	1.6
		Sc. PD:	0.17
		Sc. rank:	847.7

NA

Pos.	378	obs:	exp:
aat	N	818	451.50
aac	N	10	376.50
gat	D	4	2.16
gac	D	0	1.84
mPD		0.033	0.51
		nPD:	0.07
		N. weight:	0.57
		Sc. PD:	-0.021
		Sc. rank:	-3.1

NA

Pos.	379	obs:	exp:
ggt	G	3	120.30
ggc	G	0	109.30
gga	G	55	364.60
ggg	G	774	237.80
mPD		0.13	0.69
		nPD:	0.19
		N. weight:	1.
		Sc. PD:	0.084
		Sc. rank:	450.1

NA

Pos.	380	obs:	exp:
tgg	W	832	832.00
mPD		0	0
		nPD:	1.
		N. weight:	0.
		Sc. PD:	0
		Sc. rank:	0

NA

Pos.	381	obs:	exp:
act	T	787	215.50
acc	T	3	185.20
aca	T	21	344.00
acg	T	11	77.34
aat	N	2	1.09
aac	N	0	0.91
gct	A	0	2.02
gcc	A	0	1.55
gca	A	8	3.56
gcg	A	0	0.88
mPD		0.12	0.72
		nPD:	0.17
		N. weight:	1.2
		Sc. PD:	0.077
		Sc. rank:	445.9

NA

Pos.	382	obs:	exp:
aat	N	1	0.55
aac	N	0	0.45
gaa	E	7	458.60
gag	E	811	359.40
ggt	G	3	1.88
ggc	G	0	1.71
gga	G	9	5.70
ggg	G	1	3.72
mPD		0.080	0.53
		nPD:	0.15
		N. weight:	0.81
		Sc. PD:	0.037
		Sc. rank:	257.0

NA

Pos.	383	obs:	exp:
act	T	0	217.30
acc	T	0	186.80
aca	T	45	346.90
acg	T	784	78.00
aaa	K	0	1.71
aag	K	3	1.29
mPD		0.11	0.70
		nPD:	0.16
		N. weight:	2.2
		Sc. PD:	0.11
		Sc. rank:	746.3

NA

Pos.	384	obs:	exp:
gat	D	13	449.20
gac	D	819	382.80
mPD		0.031	0.50
		nPD:	0.06
		N. weight:	0.73
		Sc. PD:	-0.029
		Sc. rank:	-25.0

NA

Pos.	385	obs:	exp:
tct	S	0	136.00
tcc	S	0	118.10
tca	S	0	184.80
tcg	S	0	49.17
aat	N	2	9.27
aac	N	15	7.73
agt	S	791	176.80
agc	S	24	150.20
mPD		0.13	1.7
		nPD:	0.08
		N. weight:	1.5
		Sc. PD:	-0.036
		Sc. rank:	74.5

NA

Pos.	386	obs:	exp:
tct	S	0	137.50
tcc	S	0	119.40
tca	S	0	186.80
tcg	S	0	49.71
aat	N	7	3.82
aac	N	0	3.18
agt	S	817	178.70
agc	S	7	151.90
ggt	G	1	0.14
ggc	G	0	0.13
gga	G	0	0.44
ggg	G	0	0.29
mPD		0.036	1.7
		nPD:	0.02
		N. weight:	1.6
		Sc. PD:	-0.12
		Sc. rank:	-470.9

NA

Pos.	387	obs:	exp:
ttt	F	15	347.10
ttc	F	817	484.90
mPD		0.035	0.49
		nPD:	0.07
		N. weight:	0.48
		Sc. PD:	-0.014
		Sc. rank:	9.6

NA

Pos.	388	obs:	exp:
tta	L	2	0.18
ttg	L	0	0.38
tct	S	42	138.50
tcc	S	4	120.20
tca	S	775	188.20
tcg	S	9	50.07
ctt	L	0	0.40
ctc	L	0	0.32
cta	L	0	0.30
ctg	L	0	0.42
agt	S	0	180.00
agc	S	0	153.00
mPD		0.13	1.7
		nPD:	0.08
		N. weight:	1.3
		Sc. PD:	-0.031
		Sc. rank:	83.2

NA

Pos.	389	obs:	exp:
tta	L	0	0.28
ttg	L	3	0.57
ctt	L	0	0.60
ctc	L	0	0.48
cta	L	0	0.44
ctg	L	0	0.63
atg	M	6	6.00
gtt	V	0	177.00
gtc	V	0	173.10
gta	V	12	175.50
gtg	V	811	297.40
mPD		0.050	0.75
		nPD:	0.07
		N. weight:	1.
		Sc. PD:	-0.036
		Sc. rank:	-0.8

NA

Pos.	390	obs:	exp:
cgt	R	0	0.11
cgc	R	0	0.16
cga	R	0	0.30
cgg	R	0	0.24
aaa	K	17	472.00
aag	K	812	357.00
aga	R	0	1.40
agg	R	3	0.78
mPD		0.047	0.50
		nPD:	0.09
		N. weight:	0.78
		Sc. PD:	-0.0067
		Sc. rank:	77.5

NA

Pos.	391	obs:	exp:
caa	Q	758	415.40
cag	Q	74	416.60
mPD		0.16	0.50
		nPD:	0.32
		N. weight:	0.42
		Sc. PD:	0.089
		Sc. rank:	265.9

NA

Pos.	392	obs:	exp:
gat	D	37	447.60
gac	D	792	381.40
gaa	E	0	1.68
gag	E	3	1.32
mPD		0.092	0.50
		nPD:	0.18
		N. weight:	0.62
		Sc. PD:	0.048
		Sc. rank:	263.2

NA

Pos. 393		obs:	exp:
att	I	417	305.30
atc	I	409	222.60
ata	I	6	304.00
---	--	------	------
mPD		0.51	0.66
		nPD:	0.77
		N. weight:	0.46
		Sc. PD:	0.29
		Sc. rank:	311.4

NA

Pos. 394		obs:	exp:
tta	L	1	0.09
ttg	L	0	0.19
ctt	L	0	0.20
ctc	L	0	0.16
cta	L	0	0.15
ctg	L	0	0.21
att	I	0	2.20
atc	I	0	1.61
ata	I	6	2.19
gtt	V	10	177.40
gtc	V	0	173.60
gta	V	807	175.90
gtg	V	8	298.10
---	--	------	------
mPD		0.059	0.75
		nPD:	0.08
		N. weight:	1.5
		Sc. PD:	-0.035
		Sc. rank:	102.2

NA

Pos. 395		obs:	exp:
tct	S	0	0.17
tcc	S	0	0.14
tca	S	1	0.23
tcg	S	0	0.06
agt	S	0	0.22
agc	S	0	0.18
gct	A	10	119.70
gcc	A	0	91.92
gca	A	464	211.40
gcg	A	1	51.99
gaa	E	355	199.00
gag	E	0	156.00
ggt	G	0	0.14
ggc	G	0	0.13
gga	G	1	0.44
ggg	G	0	0.29
---	--	------	------
mPD		0.52	1.2
		nPD:	0.45
		N. weight:	0.69
		Sc. PD:	0.23
		Sc. rank:	462.0

NA

Pos. 396		obs:	exp:
tta	L	1	0.09
ttg	L	0	0.19
ctt	L	0	0.20
ctc	L	0	0.16
cta	L	0	0.15
ctg	L	0	0.21
att	I	0	301.60
atc	I	0	220.00
ata	I	822	300.40
atg	M	1	1.00
act	T	0	0.52
acc	T	0	0.45
aca	T	2	0.84
acg	T	0	0.19
gtt	V	0	1.29
gtc	V	0	1.26
gta	V	6	1.28
gtg	V	0	2.17
---	--	------	------
mPD		0.024	0.68
		nPD:	0.03
		N. weight:	1.1
		Sc. PD:	-0.07
		Sc. rank:	-214.5

NA

Pos. 397		obs:	exp:
act	T	825	218.10
acc	T	7	187.50
aca	T	0	348.10
acg	T	0	78.28
---	--	------	------
mPD		0.017	0.70
		nPD:	0.02
		N. weight:	1.4
		Sc. PD:	-0.1
		Sc. rank:	-385.4

NA

Pos. 398		obs:	exp:
gat	D	47	448.70
gac	D	784	382.30
ggt	G	0	0.14
ggc	G	1	0.13
gga	G	0	0.44
ggg	G	0	0.29
---	--	------	------
mPD		0.11	0.50
		nPD:	0.22
		N. weight:	0.58
		Sc. PD:	0.064
		Sc. rank:	312.3

NA

Pos. 399		obs:	exp:
tta	L	4	0.55
ttg	L	2	1.14
tgg	W	826	826.00
ctt	L	0	1.19
ctc	L	0	0.96
cta	L	0	0.89
ctg	L	0	1.27
---	--	------	------
mPD		0.024	0.033
		nPD:	0.72
		N. weight:	0.013
		Sc. PD:	0.0076
		Sc. rank:	8.7

NA

Pos. 400		obs:	exp:
tct	S	21	138.90
tcc	S	0	120.50
tca	S	811	188.60
tcg	S	0	50.20
agt	S	0	180.50
agc	S	0	153.30
---	--	------	------
mPD		0.049	1.7
		nPD:	0.03
		N. weight:	1.4
		Sc. PD:	-0.1
		Sc. rank:	-327.7

NA

Pos. 401		obs:	exp:
ggt	G	6	120.30
ggc	G	0	109.30
gga	G	819	364.60
ggg	G	7	237.80
---	--	------	------
mPD		0.031	0.69
		nPD:	0.04
		N. weight:	0.79
		Sc. PD:	-0.044
		Sc. rank:	-114.6

NA

Pos. 402		obs:	exp:
tat	Y	824	399.00
tac	Y	8	433.00
---	--	------	------
mPD		0.019	0.50
		nPD:	0.04
		N. weight:	0.72
		Sc. PD:	-0.045
		Sc. rank:	-130.8

NA

Pos. 403		obs:	exp:
tct	S	0	138.90
tcc	S	0	120.50
tca	S	0	188.60
tcg	S	0	50.20
agt	S	758	180.50
agc	S	74	153.30
---	--	------	------
mPD		0.16	1.7
		nPD:	0.1
		N. weight:	1.3
		Sc. PD:	-0.0065
		Sc. rank:	152.7

NA

Pos. 404		obs:	exp:
ggt	G	3	120.30
ggc	G	0	109.30
gga	G	39	364.60
ggg	G	790	237.80
---	--	------	------
mPD		0.096	0.69
		nPD:	0.14
		N. weight:	1.1
		Sc. PD:	0.038
		Sc. rank:	284.2

NA

Pos. 405		obs:	exp:
tct	S	0	77.45
tcc	S	0	67.21
tca	S	0	105.20
tcg	S	0	27.99
act	T	368	96.47
acc	T	0	82.92
aca	T	0	154.00
acg	T	0	34.62
agt	S	464	100.60
agc	S	0	85.51
---	--	------	------
mPD		0.49	1.5
		nPD:	0.33
		N. weight:	1.5
		Sc. PD:	0.33
		Sc. rank:	971.8

NA

Pos. 406		obs:	exp:
ttt	F	829	347.10
ttc	F	3	484.90
---	--	------	------
mPD		0.0072	0.49
		nPD:	0.01
		N. weight:	0.89
		Sc. PD:	-0.076
		Sc. rank:	-331.4

NA

Pos. 407		obs:	exp:
att	I	0	0.73
atc	I	2	0.54
ata	I	0	0.73
gtt	V	43	178.50
gtc	V	787	174.60
gta	V	0	177.00
gtg	V	0	299.90
---	--	------	------
mPD		0.10	0.74
		nPD:	0.14
		N. weight:	1.4
		Sc. PD:	0.049
		Sc. rank:	362.0

NA

Pos. 408		obs:	exp:
caa	Q	3	415.40
cag	Q	829	416.60
---	--	------	------
mPD		0.0072	0.50
		nPD:	0.01
		N. weight:	0.7
		Sc. PD:	-0.06
		Sc. rank:	-269.5

NA

Pos. 409		obs:	exp:
cat	H	468	481.60
cac	H	364	350.40
---	--	------	------
mPD		0.49	0.49
		nPD:	1.01
		N. weight:	0.0051
		Sc. PD:	0.0044
		Sc. rank:	3.5

NA

Pos. 410		obs:	exp:
cct	P	0	227.40
ccc	P	0	150.30
cca	P	824	337.70
ccg	P	8	116.60
---	--	------	------
mPD		0.019	0.71
		nPD:	0.03
		N. weight:	0.9
		Sc. PD:	-0.066
		Sc. rank:	-233.7

NA

Pos. 411		obs:	exp:
gaa	E	826	466.40
gag	E	6	365.60
---	--	------	------
mPD		0.014	0.49
		nPD:	0.03
		N. weight:	0.57
		Sc. PD:	-0.041
		Sc. rank:	-136.3

NA

Pos. 412		obs:	exp:
tta	L	3	75.91
ttg	L	6	156.00
ctt	L	2	163.00
ctc	L	1	131.30
cta	L	4	121.50
ctg	L	805	173.30
atg	M	11	11.00
---	--	------	------
mPD		0.071	1.1
		nPD:	0.06
		N. weight:	1.5
		Sc. PD:	-0.059
		Sc. rank:	-41.3

NA			
Pos.	413	obs:	exp:
act	T	2	218.10
acc	T	1	187.50
aca	T	801	348.10
acg	T	28	78.28
---	--	------	------
mPD		0.072	0.70
		nPD:	0.1
		N. weight:	0.79
		Sc. PD:	9.1×10^{-6}
		Sc. rank:	109.5

NA			
Pos.	414	obs:	exp:
ggt	G	0	120.30
ggc	G	0	109.30
gga	G	792	364.60
ggg	G	40	237.80
---	--	------	------
mPD		0.092	0.69
		nPD:	0.13
		N. weight:	0.69
		Sc. PD:	0.02
		Sc. rank:	155.6

NA			
Pos.	415	obs:	exp:
tta	L	788	76.74
ttg	L	27	157.70
tct	S	0	0.17
tcc	S	0	0.14
tca	S	1	0.23
tcg	S	0	0.06
ctt	L	0	164.80
ctc	L	0	132.80
cta	L	11	122.80
ctg	L	4	175.20
agt	S	0	0.22
agc	S	0	0.18
gtt	V	0	0.22
gtc	V	0	0.21
gta	V	1	0.21
gtg	V	0	0.36
---	--	------	------
mPD		0.11	1.1
		nPD:	0.1
		N. weight:	2.2
		Sc. PD:	-0.0086
		Sc. rank:	281.8

NA			
Pos.	416	obs:	exp:
tat	Y	1	0.48
tac	Y	0	0.52
gat	D	818	448.10
gac	D	12	381.90
ggt	G	1	0.14
ggc	G	0	0.13
gga	G	0	0.44
ggg	G	0	0.29
---	--	------	------
mPD		0.033	0.50
		nPD:	0.07
		N. weight:	0.58
		Sc. PD:	-0.021
		Sc. rank:	-2.1

NA			
Pos.	417	obs:	exp:
tgt	C	0	322.00
tgc	C	832	510.00
---	--	------	------
mPD		0	0.47
		nPD:	0.
		N. weight:	0.51
		Sc. PD:	-0.051
		Sc. rank:	-351.5

NA			
Pos.	418	obs:	exp:
att	I	0	5.14
atc	I	0	3.75
ata	I	14	5.12
atg	M	818	818.00
---	--	------	------
mPD		0.033	0.033
		nPD:	1.
		N. weight:	0.018
		Sc. PD:	0.015
		Sc. rank:	12.2

NA			
Pos.	419	obs:	exp:
cgt	R	0	31.14
cgc	R	0	44.52
cga	R	10	83.84
cgg	R	0	67.94
aga	R	21	388.40
agg	R	801	216.10
---	--	------	------
mPD		0.096	0.95
		nPD:	0.1
		N. weight:	1.2
		Sc. PD:	-0.0039
		Sc. rank:	160.3

NA			
Pos.	420	obs:	exp:
cct	P	831	227.40
ccc	P	1	150.30
cca	P	0	337.70
ccg	P	0	116.60
---	--	------	------
mPD		0.0024	0.71
		nPD:	0.
		N. weight:	1.4
		Sc. PD:	-0.13
		Sc. rank:	-812.8

NA			
Pos.	421	obs:	exp:
tgt	C	791	322.00
tgc	C	41	510.00
---	--	------	------
mPD		0.094	0.47
		nPD:	0.2
		N. weight:	0.77
		Sc. PD:	0.07
		Sc. rank:	363.7

NA			
Pos.	422	obs:	exp:
ttt	F	11	347.10
ttc	F	821	484.90
---	--	------	------
mPD		0.026	0.49
		nPD:	0.05
		N. weight:	0.5
		Sc. PD:	-0.024
		Sc. rank:	-54.6

NA			
Pos.	423	obs:	exp:
tgg	W	832	832.00
---	--	------	------
mPD		0	0
		nPD:	1.
		N. weight:	0.
		Sc. PD:	0
		Sc. rank:	0

NA			
Pos.	424	obs:	exp:
gtt	V	827	178.90
gtc	V	5	175.00
gta	V	0	177.40
gtg	V	0	300.60
---	--	------	------
mPD		0.012	0.73
		nPD:	0.02
		N. weight:	1.6
		Sc. PD:	-0.13
		Sc. rank:	-565.6

NA			
Pos.	425	obs:	exp:
gaa	E	3	466.40
gag	E	829	365.60
---	--	------	------
mPD		0.0072	0.49
		nPD:	0.01
		N. weight:	0.84
		Sc. PD:	-0.072
		Sc. rank:	-315.7

NA			
Pos.	426	obs:	exp:
tta	L	0	76.93
ttg	L	0	158.10
ctt	L	0	165.20
ctc	L	0	133.10
cta	L	438	123.10
ctg	L	394	175.60
---	--	------	------
mPD		0.50	1.1
		nPD:	0.45
		N. weight:	1.1
		Sc. PD:	0.36
		Sc. rank:	737.8

NA			
Pos.	427	obs:	exp:
att	I	750	305.30
atc	I	81	222.60
ata	I	1	304.00
---	--	------	------
mPD		0.18	0.66
		nPD:	0.27
		N. weight:	0.75
		Sc. PD:	0.12
		Sc. rank:	451.8

NA			
Pos.	428	obs:	exp:
cgt	R	0	31.14
cgc	R	0	44.52
cga	R	0	83.84
cgg	R	0	67.94
aga	R	821	388.40
agg	R	11	216.10
---	--	------	------
mPD		0.026	0.95
		nPD:	0.03
		N. weight:	0.74
		Sc. PD:	-0.054
		Sc. rank:	-187.2

NA			
Pos.	429	obs:	exp:
ggt	G	0	120.30
ggc	G	0	109.30
gga	G	40	364.60
ggg	G	792	237.80
---	--	------	------
mPD		0.092	0.69
		nPD:	0.13
		N. weight:	1.1
		Sc. PD:	0.031
		Sc. rank:	247.2

NA			
Pos.	430	obs:	exp:
cgt	R	1	30.76
cgc	R	0	43.99
cga	R	0	82.84
cgg	R	21	67.12
aaa	K	7	3.99
aag	K	0	3.01
aga	R	34	383.70
agg	R	766	213.50
ggt	G	0	0.43
ggc	G	0	0.39
gga	G	0	1.31
ggg	G	3	0.86
---	--	------	------
mPD		0.17	0.97
		nPD:	0.18
		N. weight:	1.1
		Sc. PD:	0.078
		Sc. rank:	436.0

NA			
Pos.	431	obs:	exp:
cct	P	2	227.40
ccc	P	826	150.30
cca	P	4	337.70
ccg	P	0	116.60
---	--	------	------
mPD		0.014	0.71
		nPD:	0.02
		N. weight:	1.7
		Sc. PD:	-0.14
		Sc. rank:	-560.0

NA			
Pos.	432	obs:	exp:
caa	Q	0	0.50
cag	Q	1	0.50
cgt	R	0	0.22
cgc	R	0	0.32
cga	R	0	0.60
cgg	R	0	0.49
aaa	K	788	469.20
aag	K	36	354.80
aga	R	6	2.80
agg	R	0	1.56
gaa	E	1	0.56
gag	E	0	0.44
---	--	------	------
mPD		0.10	0.51
		nPD:	0.2
		N. weight:	0.42
		Sc. PD:	0.04
		Sc. rank:	206.4

NA			
Pos.	433	obs:	exp:
gaa	E	15	466.40
gag	E	817	365.60
---	--	------	------
mPD		0.035	0.49
		nPD:	0.07
		N. weight:	0.77
		Sc. PD:	-0.023
		Sc. rank:	14.2

NA			
Pos.	434	obs:	exp:
aat	N	1	0.55
aac	N	0	0.45
---	-	831	831.00
---	--	------	------
mPD		0	7.2×10^{-7}
		nPD:	0.
		N. weight:	0.
		Sc. PD:	0
		Sc. rank:	0

NA			
Pos.	435	obs:	exp:
tct	S	0	2.34
tcc	S	0	2.03
tca	S	0	3.17
tcg	S	0	0.84
act	T	0	0.52
acc	T	2	0.45
aca	T	0	0.84
acg	T	0	0.19
aat	N	37	443.90
aac	N	777	370.10
agt	S	0	3.04
agc	S	14	2.58
gat	D	0	1.08
gac	D	2	0.92
---	--	------	------
mPD		0.13	0.56
		nPD:	0.23
		N. weight:	0.65
		Sc. PD:	0.077
		Sc. rank:	360.9

NA			
Pos.	436	obs:	exp:
act	T	0	217.80
acc	T	6	187.20
aca	T	815	347.70
acg	T	10	78.19
gct	A	0	0.25
gcc	A	0	0.19
gca	A	1	0.45
gcg	A	0	0.11
---	--	------	------
mPD		0.040	0.70
		nPD:	0.06
		N. weight:	0.83
		Sc. PD:	-0.037
		Sc. rank:	-59.7

NA			
Pos.	437	obs:	exp:
tta	L	0	1.20
ttg	L	0	2.47
ctt	L	13	2.58
ctc	L	0	2.08
cta	L	0	1.92
ctg	L	0	2.74
att	I	786	299.40
atc	I	30	218.40
ata	I	0	298.20
act	T	1	0.26
acc	T	0	0.23
aca	T	0	0.42
acg	T	0	0.09
gtt	V	2	0.43
gtc	V	0	0.42
gta	V	0	0.43
gtg	V	0	0.72
---	--	------	------
mPD		0.11	0.70
		nPD:	0.15
		N. weight:	0.92
		Sc. PD:	0.044
		Sc. rank:	301.0

,

NA			
Pos.	438	obs:	exp:
tgg	W	832	832.00
---	--	------	------
mPD		0	0
		nPD:	1.
		N. weight:	0.
		Sc. PD:	0
		Sc. rank:	0

,

NA			
Pos.	439	obs:	exp:
att	I	2	0.73
atc	I	0	0.54
ata	I	0	0.73
act	T	791	217.30
acc	T	38	186.80
aca	T	0	346.90
acg	T	0	78.00
gct	A	1	0.25
gcc	A	0	0.19
gca	A	0	0.45
gcg	A	0	0.11
---	--	------	------
mPD		0.094	0.70
		nPD:	0.13
		N. weight:	1.2
		Sc. PD:	0.036
		Sc. rank:	283.3

,

NA			
Pos.	440	obs:	exp:
tct	S	0	138.90
tcc	S	0	120.50
tca	S	0	188.60
tcg	S	0	50.20
agt	S	92	180.50
agc	S	740	153.30
---	--	------	------
mPD		0.20	1.7
		nPD:	0.12
		N. weight:	1.4
		Sc. PD:	0.021
		Sc. rank:	273.8

,

NA			
Pos.	441	obs:	exp:
ggt	G	0	120.30
ggc	G	0	109.30
gga	G	29	364.60
ggg	G	803	237.80
---	--	------	------
mPD		0.067	0.69
		nPD:	0.1
		N. weight:	1.1
		Sc. PD:	-0.0061
		Sc. rank:	129.1

,

NA			
Pos.	442	obs:	exp:
tct	S	0	138.90
tcc	S	0	120.50
tca	S	0	188.60
tcg	S	0	50.20
agt	S	6	180.50
agc	S	826	153.30
---	--	------	------
mPD		0.014	1.7
		nPD:	0.01
		N. weight:	1.7
		Sc. PD:	-0.16
		Sc. rank:	-814.3

,

NA			
Pos.	443	obs:	exp:
tct	S	0	137.50
tcc	S	0	119.40
tca	S	0	186.80
tcg	S	0	49.71
att	I	0	2.94
atc	I	8	2.14
ata	I	0	2.92
agt	S	3	178.70
agc	S	821	151.90
---	--	------	------
mPD		0.026	1.7
		nPD:	0.02
		N. weight:	1.8
		Sc. PD:	-0.15
		Sc. rank:	-634.9

,

NA			
Pos.	444	obs:	exp:
att	I	40	299.80
atc	I	0	218.60
ata	I	777	298.60
atg	M	12	12.00
gtt	V	0	0.65
gtc	V	0	0.63
gta	V	3	0.64
gtg	V	0	1.08
---	--	------	------
mPD		0.13	0.68
		nPD:	0.19
		N. weight:	0.85
		Sc. PD:	0.067
		Sc. rank:	367.8

,

NA			
Pos.	445	obs:	exp:
tct	S	21	138.90
tcc	S	811	120.50
tca	S	0	188.60
tcg	S	0	50.20
agt	S	0	180.50
agc	S	0	153.30
---	--	------	------
mPD		0.049	1.7
		nPD:	0.03
		N. weight:	1.9
		Sc. PD:	-0.13
		Sc. rank:	-431.6

,

NA			
Pos.	446	obs:	exp:
ttt	F	822	342.90
ttc	F	0	479.10
tta	L	0	0.92
ttg	L	2	1.90
ctt	L	8	1.99
ctc	L	0	1.60
cta	L	0	1.48
ctg	L	0	2.11
---	--	------	------
mPD		0.024	0.51
		nPD:	0.05
		N. weight:	0.92
		Sc. PD:	-0.05
		Sc. rank:	-131.8

,

NA			
Pos.	447	obs:	exp:
tgt	C	832	322.00
tgc	C	0	510.00
---	--	------	------
mPD		0	0.47
		nPD:	0.
		N. weight:	1.
		Sc. PD:	-0.099
		Sc. rank:	-681.7

,

NA			
Pos.	448	obs:	exp:
ggt	G	793	120.30
ggc	G	0	109.30
gga	G	29	364.60
ggg	G	10	237.80
---	--	------	------
mPD		0.090	0.69
		nPD:	0.13
		N. weight:	1.8
		Sc. PD:	0.047
		Sc. rank:	377.5

,

NA			
Pos.	449	obs:	exp:
gtt	V	2	178.90
gtc	V	0	175.00
gta	V	826	177.40
gtg	V	4	300.60
---	--	------	------
mPD		0.014	0.73
		nPD:	0.02
		N. weight:	1.6
		Sc. PD:	-0.13
		Sc. rank:	-527.4

,

NA			
Pos.	450	obs:	exp:
aat	N	824	449.90
aac	N	1	375.10
gat	D	7	3.78
gac	D	0	3.22
---	--	------	------
mPD		0.019	0.51
		nPD:	0.04
		N. weight:	0.63
		Sc. PD:	-0.04
		Sc. rank:	-116.1

,

NA			
Pos.	451	obs:	exp:
tct	S	0	135.70
tcc	S	0	117.80
tca	S	0	184.30
tcg	S	0	49.05
cgt	R	1	0.04
cgc	R	0	0.05
cga	R	0	0.10
cgg	R	0	0.08
agt	S	786	176.30
agc	S	27	149.80
aga	R	0	0.47
agg	R	0	0.26
ggt	G	16	2.60
ggc	G	2	2.36
gga	G	0	7.89
ggg	G	0	5.15
---	--	------	------
mPD		0.11	1.7
		nPD:	0.07
		N. weight:	1.5
		Sc. PD:	-0.052
		Sc. rank:	5.2

,

NA			
Pos.	452	obs:	exp:
aat	N	0	0.55
aac	N	1	0.45
gat	D	0	446.00
gac	D	826	380.00
gaa	E	4	2.24
gag	E	0	1.76
ggt	G	0	0.14
ggc	G	1	0.13
gga	G	0	0.44
ggg	G	0	0.29
---	--	------	------
mPD		0.014	0.51
		nPD:	0.03
		N. weight:	0.82
		Sc. PD:	-0.059
		Sc. rank:	-198.8

,

NA			
Pos.	453	obs:	exp:
att	I	2	0.73
atc	I	0	0.54
ata	I	0	0.73
act	T	827	217.60
acc	T	3	187.00
aca	T	0	347.30
acg	T	0	78.09
---	--	------	------
mPD		0.012	0.70
		nPD:	0.02
		N. weight:	1.4
		Sc. PD:	-0.11
		Sc. rank:	-490.1

,

NA			
Pos.	454	obs:	exp:
gtt	V	1	178.10
gtc	V	0	174.20
gta	V	2	176.60
gtg	V	825	299.20
ggt	G	0	0.58
ggc	G	0	0.53
gga	G	0	1.75
ggg	G	4	1.14
---	--	------	------
mPD		0.017	0.74
		nPD:	0.02
		N. weight:	1.
		Sc. PD:	-0.081
		Sc. rank:	-315.0

,

NA			
Pos.	455	obs:	exp:
gat	D	2	1.08
gac	D	0	0.92
ggt	G	830	120.00
ggc	G	0	109.00
gga	G	0	363.70
ggg	G	0	237.30
---	--	------	------
mPD		0.0048	0.69
		nPD:	0.01
		N. weight:	2.
		Sc. PD:	-0.19
		Sc. rank:	-1004.9

,

NA			
Pos.	456	obs:	exp:
tgg	W	832	832.00
---	--	------	------
mPD		0	0
		nPD:	1.
		N. weight:	0.
		Sc. PD:	0
		Sc. rank:	0

,

NA			
Pos.	457	obs:	exp:
tct	S	788	138.90
tcc	S	0	120.50
tca	S	0	188.60
tcg	S	44	50.20
agt	S	0	180.50
agc	S	0	153.30
mPD		0.10	1.7
		nPD:	0.06
		N. weight:	1.7
		Sc. PD:	-0.071
		Sc. rank:	-69.4

,

NA			
Pos.	458	obs:	exp:
tgg	W	832	832.00
mPD		0	0
		nPD:	1.
		N. weight:	0.
		Sc. PD:	0
		Sc. rank:	0

,

NA			
Pos.	459	obs:	exp:
cct	P	0	227.40
ccc	P	0	150.30
cca	P	832	337.70
ccg	P	0	116.60
mPD		0	0.71
		nPD:	0.
		N. weight:	0.95
		Sc. PD:	-0.094
		Sc. rank:	-647.5

,

NA			
Pos.	460	obs:	exp:
gat	D	0	449.20
gac	D	832	382.80
mPD		0	0.50
		nPD:	0.
		N. weight:	0.82
		Sc. PD:	-0.081
		Sc. rank:	-557.5

,

NA			
Pos.	461	obs:	exp:
tct	S	0	0.17
tcc	S	0	0.14
tca	S	0	0.23
tcg	S	0	0.06
agt	S	1	0.22
agc	S	0	0.18
ggt	G	831	120.20
ggc	G	0	109.20
gga	G	0	364.10
ggg	G	0	237.50
mPD		0.0024	0.69
		nPD:	0.
		N. weight:	2.
		Sc. PD:	-0.2
		Sc. rank:	-1182.2

,

NA			
Pos.	462	obs:	exp:
gct	A	832	209.60
gcc	A	0	161.00
gca	A	0	370.30
gcg	A	0	91.06
mPD		0	0.69
		nPD:	0.
		N. weight:	1.4
		Sc. PD:	-0.14
		Sc. rank:	-990.0

,

NA			
Pos.	463	obs:	exp:
gaa	E	46	466.40
gag	E	786	365.60
mPD		0.10	0.49
		nPD:	0.21
		N. weight:	0.63
		Sc. PD:	0.066
		Sc. rank:	330.9

,

NA			
Pos.	464	obs:	exp:
tta	L	0	76.93
ttg	L	831	158.10
ctt	L	0	165.20
ctc	L	0	133.10
cta	L	0	123.10
ctg	L	1	175.60
mPD		0.0024	1.1
		nPD:	0.
		N. weight:	1.7
		Sc. PD:	-0.17
		Sc. rank:	-1064.7

,

NA			
Pos.	465	obs:	exp:
cct	P	0	227.40
ccc	P	0	150.30
cca	P	831	337.70
ccg	P	1	116.60
mPD		0.0024	0.71
		nPD:	0.
		N. weight:	0.94
		Sc. PD:	-0.09
		Sc. rank:	-564.0

,

NA			
Pos.	466	obs:	exp:
ttt	F	0	347.10
ttc	F	832	484.90
mPD		0	0.49
		nPD:	0.
		N. weight:	0.57
		Sc. PD:	-0.056
		Sc. rank:	-387.7

,

NA			
Pos.	467	obs:	exp:
att	I	0	0.73
atc	I	2	0.54
ata	I	0	0.73
act	T	0	217.60
acc	T	830	187.00
aca	T	0	347.30
acg	T	0	78.09
mPD		0.0048	0.70
		nPD:	0.01
		N. weight:	1.6
		Sc. PD:	-0.15
		Sc. rank:	-788.8

,

NA			
Pos.	468	obs:	exp:
tta	L	0	0.09
ttg	L	0	0.19
ctt	L	1	0.20
ctc	L	0	0.16
cta	L	0	0.15
ctg	L	0	0.21
att	I	831	304.90
atc	I	0	222.40
ata	I	0	303.70
mPD		0.0024	0.66
		nPD:	0.
		N. weight:	1.1
		Sc. PD:	-0.1
		Sc. rank:	-603.8

,

NA			
Pos.	469	obs:	exp:
gat	D	0	449.20
gac	D	832	382.80
mPD		0	0.50
		nPD:	0.
		N. weight:	0.82
		Sc. PD:	-0.081
		Sc. rank:	-557.5

,

NA			
Pos.	470	obs:	exp:
aaa	K	1	473.80
aag	K	831	358.20
mPD		0.0024	0.49
		nPD:	0.
		N. weight:	0.88
		Sc. PD:	-0.083
		Sc. rank:	-459.2

,

NA			
Pos.	471	obs:	exp:
taa	.	50	306.80
tag	.	782	285.30
tga	.	0	239.90
mPD		0.11	0.86
		nPD:	0.13
		N. weight:	0.89
		Sc. PD:	0.024
		Sc. rank:	191.9

}

Amino acid level conservation per codon

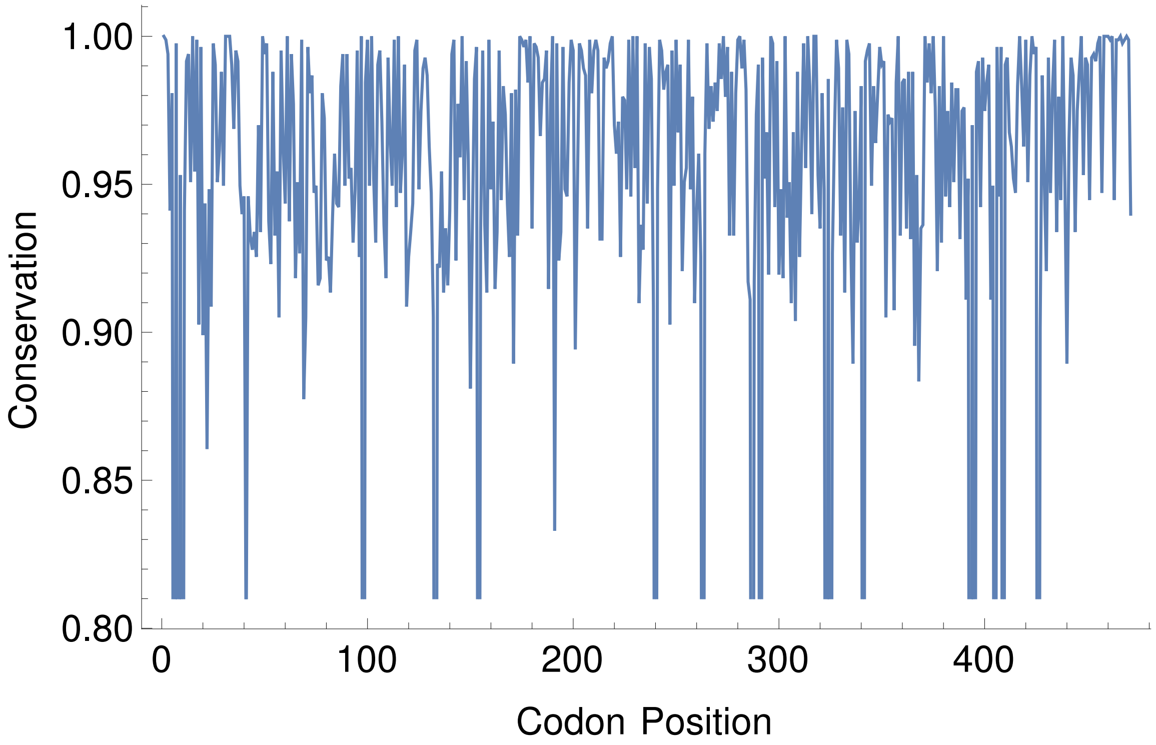

Conservation analysis plots

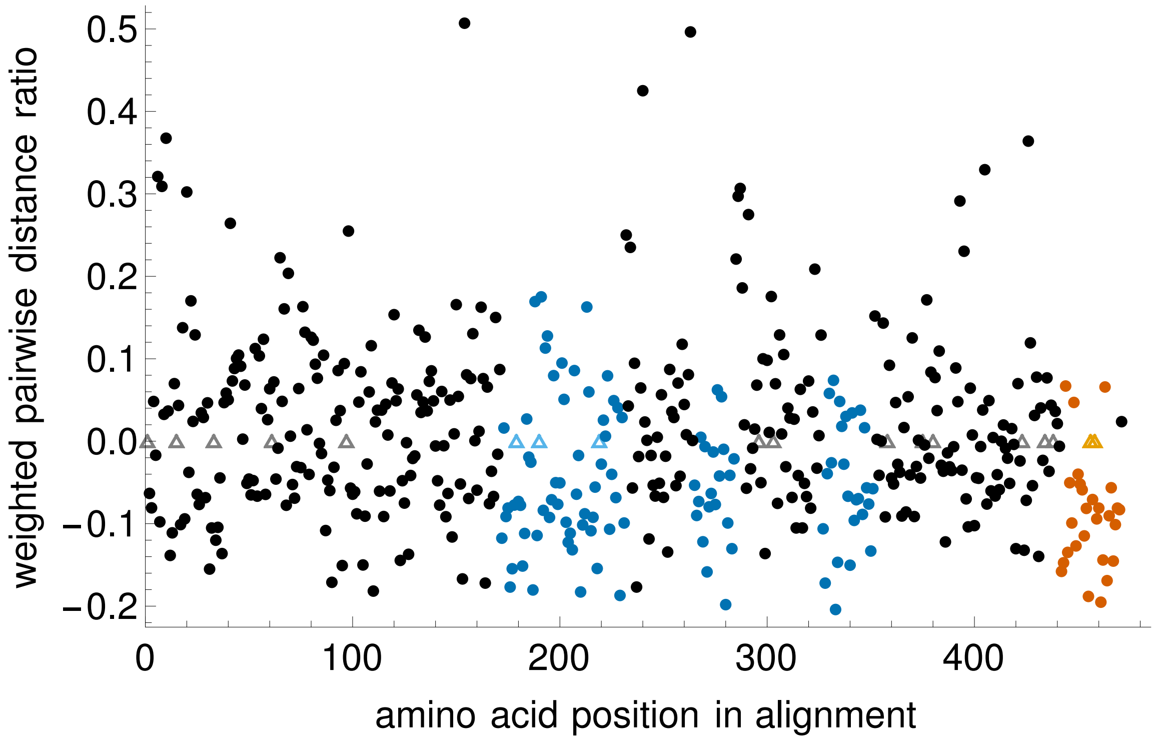

NA

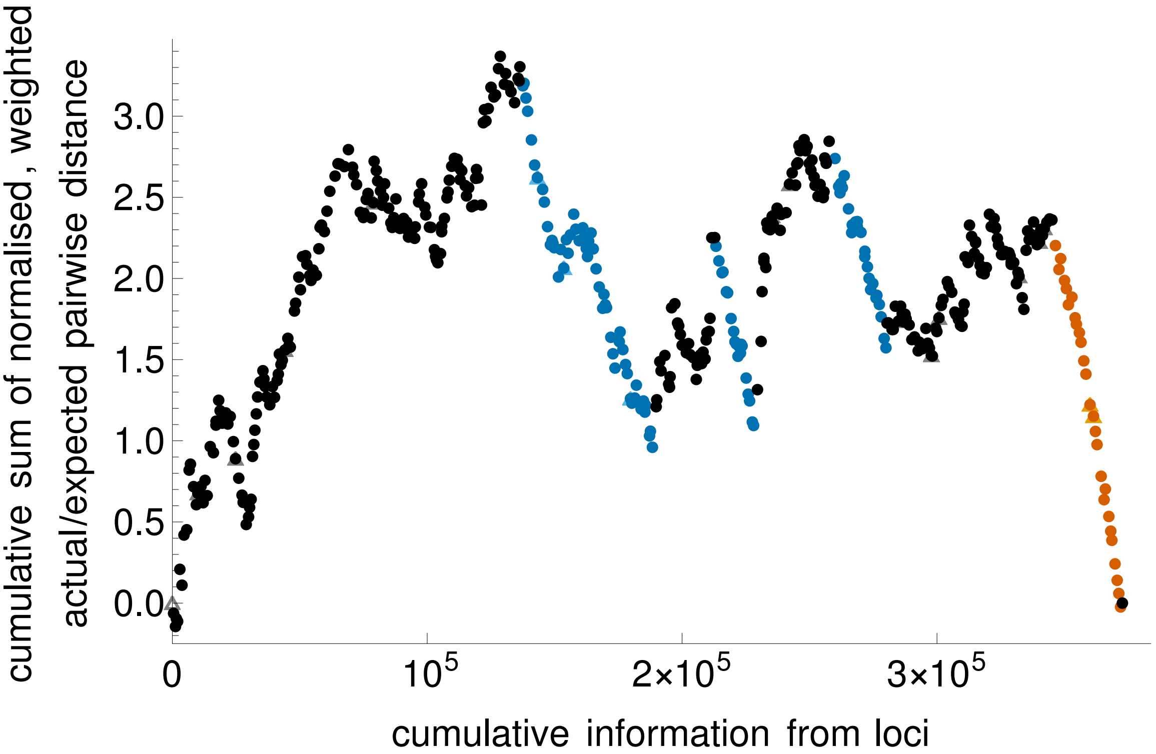

NA

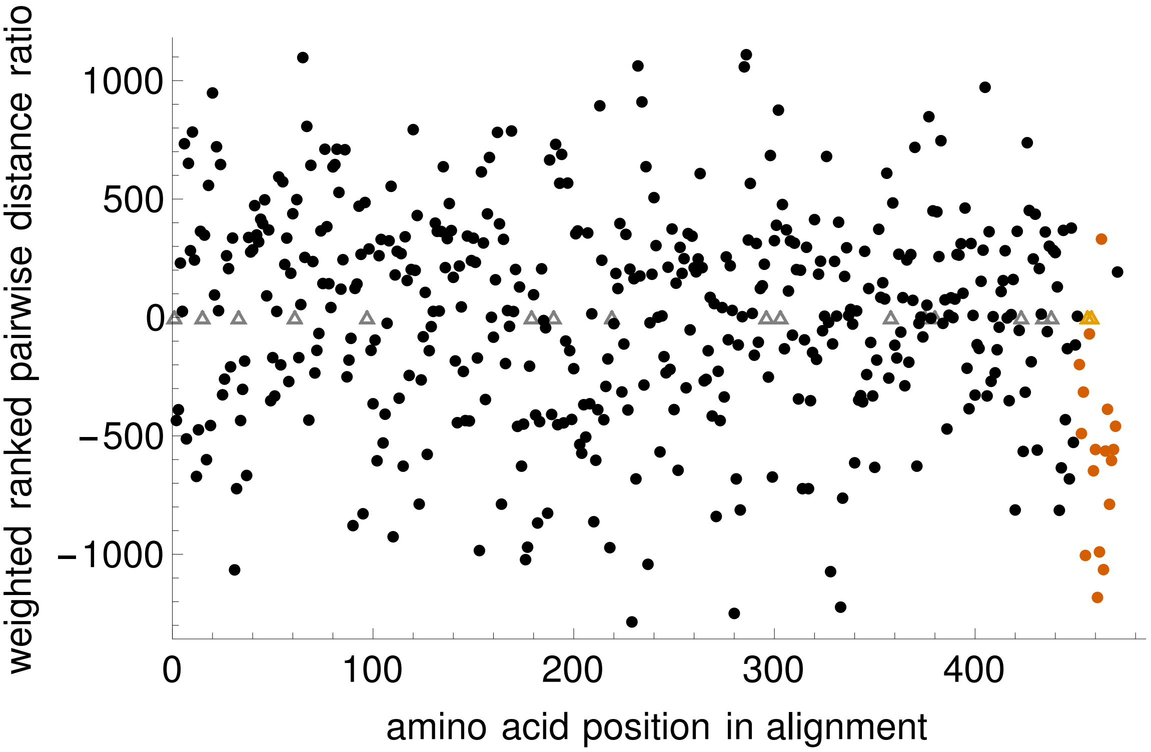
weighted ranked pairwise distance ratio
1000
500
0
−500
−1000
0
100
200
300
400
amino acid position in alignment

NA

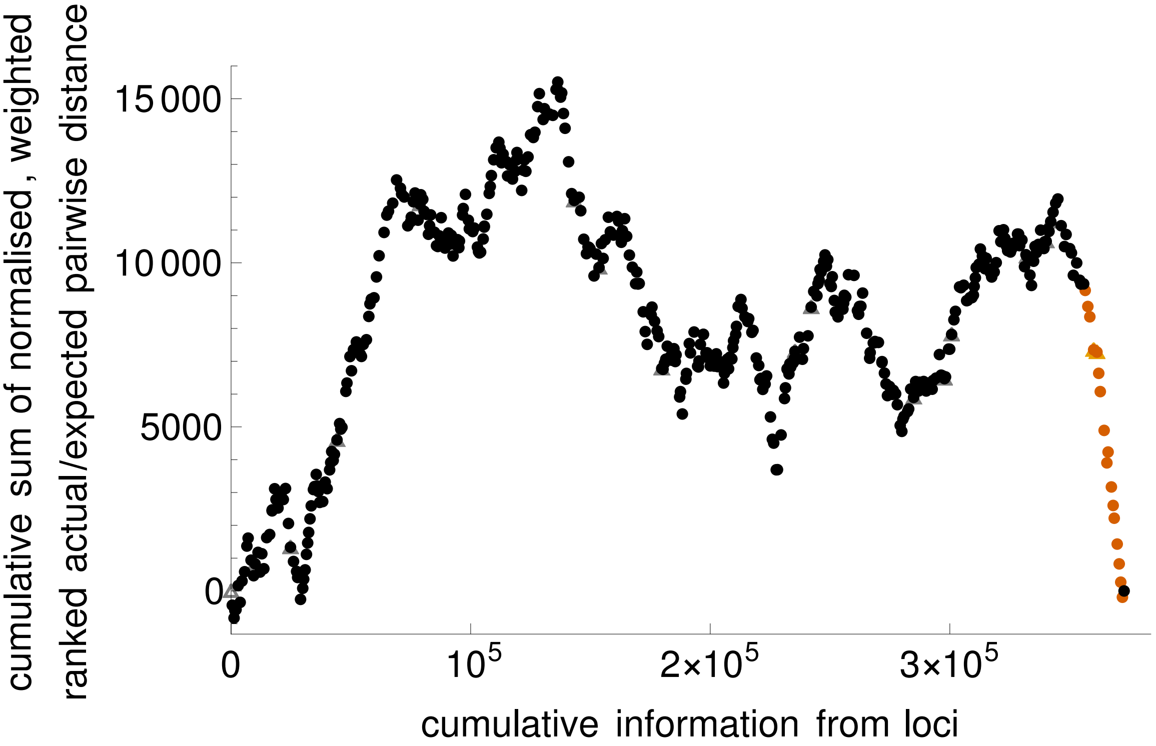
cumulative sum of normalised, weighted ranked actual/expected pairwise distance
15 000
10 000
5000
0
0
10^5
2×10^5
3×10^5
cumulative information from loci

NA

M1

Gene length histogram

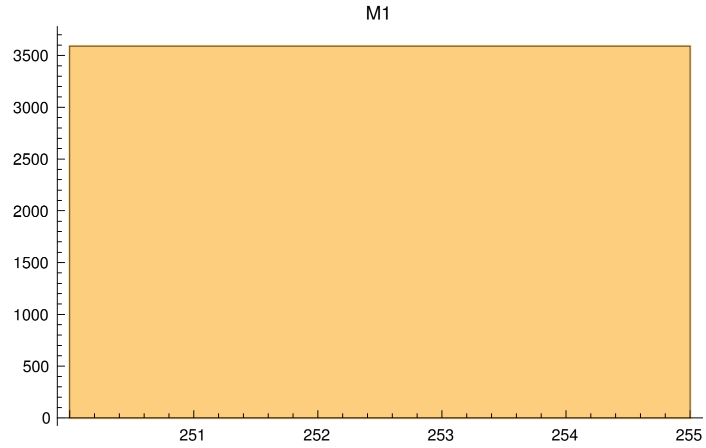

Information vs. nPD

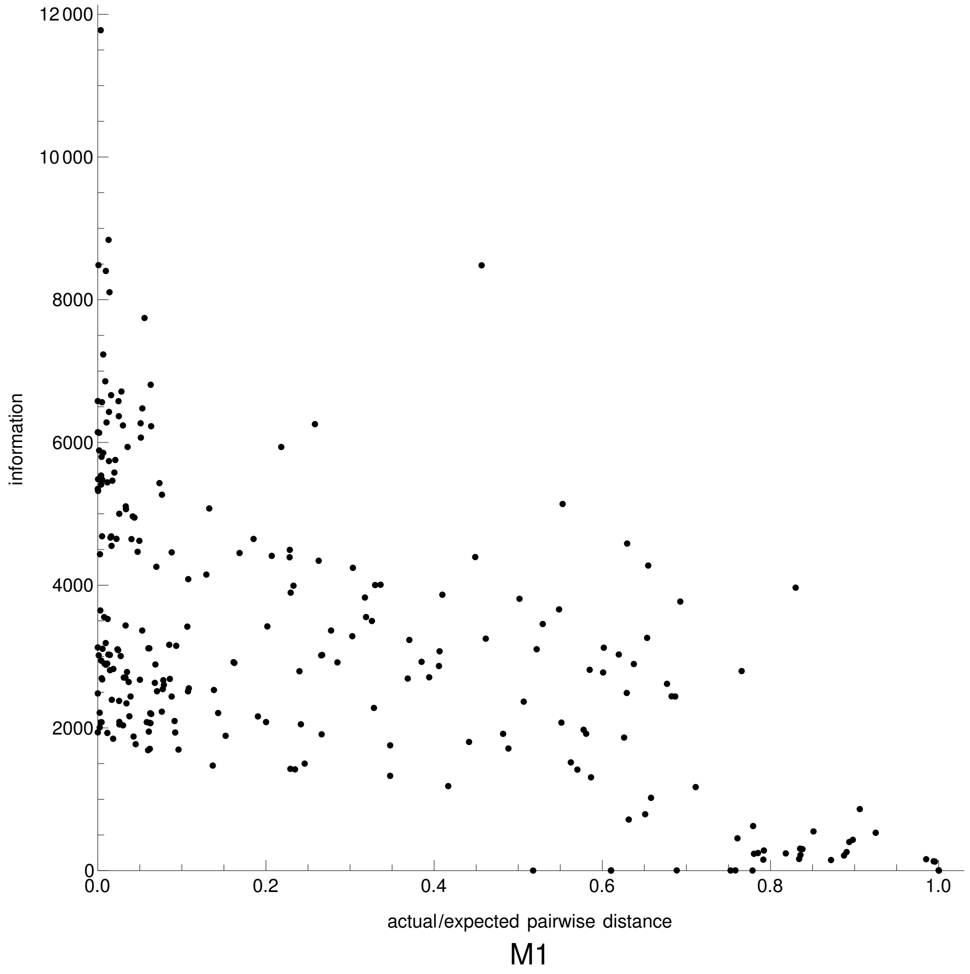

Example sequences highlighted by regions found in analyses to be conserved

Interesting points (by weighted raw PD) highlighted for gene M1:

ATG
AGTCTTCTAACCGAGGTCGAAACGTACGTTCTCTCTATCATCCCATCAGGCCCCCTCAAAG
CCGAGATCGCGCAGAAACTTGAGGATGTCTTTGCAGGAAAGAACACCGATCTC
GAGGCTCTCATGGAGTGGCTAAAGACAAGACCAATCCTGTCACCTCTGACTAAAG
GGATTTTGGGATTTGTATTCACGCTCACCGTGCCCAGTGAGCGAGGACTGCA
GCGTAGACGCTTTGTCCAGAATGCCCTAAATGGAAATGGAGATCCAAATAATATGGATAGGGCAGTTAA
GCTATATAAGAAGCTGAAACGAGAAATAACATTCCATGGGGCTAAGGAGGTCGCACTCAGCTACTCAACC
GGTGCACTTGCCAGTTGTATGGGTCTCATATACAACAGGATGGGAACGGTGACTACGGAAGTGGCTTTTG
GCCTAGTGTGTGCCACTTGTGAGCAGATTGCAGATTCACAGCATCGGTCTCACAGACAGATGGCGACCAT
CACCAACCCACTAATCAGGCACGAGAACAGAATGGTGCTGGCCAGCACTACAGCTAAGGCTATGGAGCAG
ATGGCGGGATCAAGCGAGCAGGCAGCGGAAGCCATGGAGGTCGCTAATCAGGCTAGGCAGATGGTGCAGG
CAATGAGGACAATTGGGACTCATCCTAACTCTAGTGCTGGTCTGAGAGATAATCTTCTTGAAAATTTGCA
GGCCTACCAGAAACGAATGGGAGTGCAGATGCAGCGATTCAAGTGA

Interesting points (by weighted ranked PD) highlighted for gene M1:

ATGAGT
CTTCTAACCGAGGTCGAAACGTACGTTCTCTCTATCATCCCATCAGGCCCCCTCA
AAGCC
GAGATCGCGCAGAAACTTGAGGATGTCTTTGCAGGAAAGAACACCGATCTCGAGGCTCTCATGGAGTGGCTA
AAGACAAGACCAATCCTGTCACCTCTGACTAAAGGGATTTTGGGATTTGTATTC
ACGCTCACCGTGCCCAGTGAGCGAGGACTGCAGCGTAGACGCTTTGTCCAGAATGCCCTAA
ATGGAAATGGAGATCCAAATAATATGGATAGGGCAGTTAAGCTATATAAGAAGCTGAAACGAGAAATAAC
ATTCCATGGGGCTAAGGAGGTCGCACTCAGCTACTCAACCGGTGCACTTGCCAGTTGTATGGGTCTCATA
TACAACAGGATGGGAACGGTGACTACGGAAGTGGCTTTTGGCCTAGTGTGT
GCCACTTGTGAGCAGATTGCAGATTCACAGCATCGGTCTCACAGACAGATGGCGACCAT
CACCAACCCACTAATCAGGCACGAGAACAGAATGGTGCTGGCCAGCACTACAGCTAAGGCTATGGAGCAG
ATGGCGGGATCAAGCGAGCAGGCAGCGGAAGCCATGGAGGTCGCTAATCAGGCTAGGCAGATGGTGCAGG
CAATGAGGACAATTGGGACTCATCCTAACTCTAGTGCTGGTCTGAGAGATAATCTTCTTGAAAATTTGCA
GGCCTACCAGAAACGAATGGGAGTGCAGATGCAGCGATTCAAGTGA

Per codon conservation report

M1

Pos.	1	obs:	exp:
atg	M	3590	3590.00
---	--	------	------
mPD		0	0
		nPD:	1.
		N. weight:	0.
		Sc. PD:	0
		Sc. rank:	0

M1

Pos.	2	obs:	exp:
tct	S	0	598.70
tcc	S	0	519.60
tca	S	0	813.20
tcg	S	0	216.40
att	I	3	1.10
atc	I	0	0.80
ata	I	0	1.10
agt	S	3456	778.00
agc	S	131	661.00
---	--	------	------
mPD		0.072	1.7
		nPD:	0.04
		N. weight:	1.6
		Sc. PD:	-0.17
		Sc. rank:	-170.6

M1

Pos.	3	obs:	exp:
ttt	F	6	2.50
ttc	F	0	3.50
tta	L	0	331.10
ttg	L	0	680.30
ctt	L	3581	711.00
ctc	L	0	572.80
cta	L	0	530.00
ctg	L	0	755.80
cct	P	2	0.55
ccc	P	0	0.36
cca	P	0	0.81
ccg	P	0	0.28
att	I	1	0.37
atc	I	0	0.27
ata	I	0	0.37
---	--	------	------
mPD		0.0050	1.1
		nPD:	0.
		N. weight:	1.8
		Sc. PD:	-0.27
		Sc. rank:	-3716.7

M1

Pos.	4	obs:	exp:
tta	L	13	331.70
ttg	L	0	681.40
ctt	L	3	712.20
ctc	L	2	573.80
cta	L	3558	530.90
ctg	L	11	757.10
cct	P	0	0.27
ccc	P	0	0.18
cca	P	1	0.41
ccg	P	0	0.14
att	I	0	0.73
atc	I	0	0.54
ata	I	2	0.73
---	--	------	------
mPD		0.018	1.1
		nPD:	0.02
		N. weight:	2.1
		Sc. PD:	-0.29
		Sc. rank:	-2248.2

M1

Pos.	5	obs:	exp:
tta	L	0	0.09
ttg	L	0	0.19
tct	S	0	0.17
tcc	S	1	0.14
tca	S	0	0.23
tcg	S	0	0.06
tat	Y	0	0.48
tac	Y	1	0.52
ctt	L	0	0.20
ctc	L	0	0.16
cta	L	0	0.15
ctg	L	1	0.21
cct	P	0	0.55
ccc	P	2	0.36
cca	P	0	0.81
ccg	P	0	0.28
att	I	0	0.73
atc	I	2	0.54
ata	I	0	0.73
act	T	407	938.50
acc	T	3142	806.70
aca	T	25	1498.00
acg	T	6	336.80
aat	N	0	1.09
aac	N	2	0.91
agt	S	0	0.22
agc	S	0	0.18
gct	A	0	0.25
gcc	A	1	0.19
gca	A	0	0.45
gcg	A	0	0.11
---	--	------	------
mPD		0.22	0.70
		nPD:	0.32
		N. weight:	1.2
		Sc. PD:	0.19
		Sc. rank:	2005.2

M1

Pos.	6	obs:	exp:
aaa	K	0	0.57
aag	K	1	0.43
gat	D	0	1.08
gac	D	2	0.92
gaa	E	7	2011.00
gag	E	3580	1576.00
---	--	------	------
mPD		0.0056	0.49
		nPD:	0.01
		N. weight:	0.92
		Sc. PD:	-0.13
		Sc. rank:	-1356.6

M1

Pos.	7	obs:	exp:
gtt	V	19	771.90
gtc	V	3568	755.10
gta	V	2	765.30
gtg	V	0	1297.00
ggt	G	1	0.14
ggc	G	0	0.13
gga	G	0	0.44
ggg	G	0	0.29
---	--	------	------
mPD		0.013	0.73
		nPD:	0.02
		N. weight:	1.7
		Sc. PD:	-0.24
		Sc. rank:	-1641.9

M1

Pos.	8	obs:	exp:
cgt	R	0	0.04
cgc	R	0	0.05
cga	R	1	0.10
cgg	R	0	0.08
aga	R	0	0.47
agg	R	0	0.26
gct	A	0	0.25
gcc	A	0	0.19
gca	A	1	0.45
gcg	A	0	0.11
gat	D	0	0.54
gac	D	1	0.46
gaa	E	3587	2011.00
gag	E	0	1576.00
---	--	------	------
mPD		0.0022	0.49
		nPD:	0.
		N. weight:	0.66
		Sc. PD:	-0.098
		Sc. rank:	-1323.9

M1

Pos.	9	obs:	exp:
act	T	0	940.90
acc	T	0	808.70
aca	T	0	1502.00
acg	T	3589	337.70
gct	A	0	0.25
gcc	A	0	0.19
gca	A	0	0.45
gcg	A	1	0.11
---	--	------	------
mPD		0.00056	0.70
		nPD:	0.
		N. weight:	2.7
		Sc. PD:	-0.41
		Sc. rank:	-6555.3

M1

Pos.	10	obs:	exp:
tat	Y	52	1721.00
tac	Y	3537	1868.00
cat	H	0	0.58
cac	H	1	0.42
---	--	------	------
mPD		0.029	0.50
		nPD:	0.06
		N. weight:	0.66
		Sc. PD:	-0.063
		Sc. rank:	133.0

M1

Pos.	11	obs:	exp:
gtt	V	3583	771.70
gtc	V	3	754.90
gta	V	1	765.10
gtg	V	1	1296.00
gct	A	1	0.25
gcc	A	0	0.19
gca	A	0	0.45
gcg	A	0	0.11
ggt	G	1	0.14
ggc	G	0	0.13
gga	G	0	0.44
ggg	G	0	0.29
---	--	------	------
mPD		0.0039	0.73
		nPD:	0.01
		N. weight:	1.7
		Sc. PD:	-0.26
		Sc. rank:	-3224.8

M1

Pos.	12	obs:	exp:
ttt	F	0	0.83
ttc	F	2	1.17
tta	L	0	331.70
ttg	L	0	681.60
ctt	L	98	712.40
ctc	L	3484	573.90
cta	L	6	531.00
ctg	L	0	757.30
---	--	------	------
mPD		0.057	1.1
		nPD:	0.05
		N. weight:	1.9
		Sc. PD:	-0.2
		Sc. rank:	130.1

M1

Pos.	13	obs:	exp:
ttt	F	2	0.83
ttc	F	0	1.17
tct	S	3559	598.20
tcc	S	25	519.20
tca	S	0	812.50
tcg	S	0	216.20
agt	S	0	777.40
agc	S	0	660.50
gct	A	4	1.01
gcc	A	0	0.77
gca	A	0	1.78
gcg	A	0	0.44
---	--	------	------
mPD		0.017	1.7
		nPD:	0.01
		N. weight:	2.
		Sc. PD:	-0.28
		Sc. rank:	-2975.8

M1

Pos.	14	obs:	exp:
att	I	5	1314.00
atc	I	3572	958.30
ata	I	4	1309.00
gtt	V	0	1.94
gtc	V	9	1.89
gta	V	0	1.92
gtg	V	0	3.25
---	--	------	------
mPD		0.010	0.67
		nPD:	0.02
		N. weight:	1.5
		Sc. PD:	-0.2
		Sc. rank:	-1641.7

M1

Pos.	15	obs:	exp:
att	I	270	952.60
atc	I	2296	694.70
ata	I	30	948.70
act	T	0	2.10
acc	T	8	1.80
aca	T	0	3.35
acg	T	0	0.75
gtt	V	7	212.10
gtc	V	978	207.40
gta	V	1	210.20
gtg	V	0	356.20
---	--	------	------
mPD		0.56	1.1
		nPD:	0.5
		N. weight:	1.2
		Sc. PD:	0.41
		Sc. rank:	2558.4

M1			
Pos.	16	obs:	exp:
tta	L	0	0.09
ttg	L	0	0.19
tct	S	0	0.33
tcc	S	0	0.29
tca	S	0	0.45
tcg	S	2	0.12
ctt	L	0	0.20
ctc	L	0	0.16
cta	L	0	0.15
ctg	L	1	0.21
cct	P	8	980.00
ccc	P	14	647.70
cca	P	1254	1456.00
ccg	P	2310	502.80
cgt	R	0	0.04
cgc	R	0	0.05
cga	R	0	0.10
cgg	R	1	0.08
agt	S	0	0.43
agc	S	0	0.37
aga	R	0	0.47
agg	R	0	0.26
---	--	------	------
mPD		0.46	0.71
		nPD:	0.65
		N. weight:	1.
		Sc. PD:	0.51
		Sc. rank:	2587.4

M1			
Pos.	17	obs:	exp:
tct	S	0	598.20
tcc	S	19	519.20
tca	S	2717	812.50
tcg	S	848	216.20
act	T	0	0.26
acc	T	0	0.23
aca	T	1	0.42
acg	T	0	0.09
agt	S	0	777.40
agc	S	0	660.50
gtt	V	0	1.08
gtc	V	5	1.05
gta	V	0	1.07
gtg	V	0	1.81
---	--	------	------
mPD		0.38	1.7
		nPD:	0.23
		N. weight:	1.4
		Sc. PD:	0.1
		Sc. rank:	1752.4

M1			
Pos.	18	obs:	exp:
tct	S	0	0.17
tcc	S	0	0.14
tca	S	0	0.23
tcg	S	0	0.06
agt	S	0	0.22
agc	S	1	0.18
ggt	G	0	519.00
ggc	G	3582	471.40
gga	G	0	1573.00
ggg	G	7	1026.00
---	--	------	------
mPD		0.0045	0.69
		nPD:	0.01
		N. weight:	2.3
		Sc. PD:	-0.34
		Sc. rank:	-4004.9

M1			
Pos.	19	obs:	exp:
cct	P	0	981.10
ccc	P	3590	648.40
cca	P	0	1457.00
ccg	P	0	503.30
---	--	------	------
mPD		0	0.71
		nPD:	0.
		N. weight:	1.9
		Sc. PD:	-0.3
		Sc. rank:	-5286.4

M1			
Pos.	20	obs:	exp:
ttt	F	0	2.09
ttc	F	5	2.91
tta	L	0	331.10
ttg	L	0	680.30
ctt	L	1	711.00
ctc	L	3580	572.80
cta	L	0	530.00
ctg	L	0	755.80
cct	P	0	0.27
ccc	P	1	0.18
cca	P	0	0.41
ccg	P	0	0.14
att	I	0	0.37
atc	I	1	0.27
ata	I	0	0.37
gtt	V	0	0.43
gtc	V	2	0.42
gta	V	0	0.43
gtg	V	0	0.72
---	--	------	------
mPD		0.0056	1.1
		nPD:	0.
		N. weight:	2.1
		Sc. PD:	-0.31
		Sc. rank:	-4046.5

M1			
Pos.	21	obs:	exp:
aaa	K	3588	2044.00
aag	K	2	1546.00
---	--	------	------
mPD		0.0011	0.49
		nPD:	0.
		N. weight:	0.63
		Sc. PD:	-0.096
		Sc. rank:	-1466.7

M1			
Pos.	22	obs:	exp:
act	T	0	0.26
acc	T	1	0.23
aca	T	0	0.42
acg	T	0	0.09
gct	A	1	904.10
gcc	A	3588	694.50
gca	A	0	1598.00
gcg	A	0	392.80
---	--	------	------
mPD		0.0011	0.69
		nPD:	0.
		N. weight:	1.9
		Sc. PD:	-0.28
		Sc. rank:	-4357.4

M1			
Pos.	23	obs:	exp:
att	I	0	0.37
atc	I	0	0.27
ata	I	1	0.37
gaa	E	68	2012.00
gag	E	3521	1577.00
---	--	------	------
mPD		0.039	0.49
		nPD:	0.08
		N. weight:	0.82
		Sc. PD:	-0.062
		Sc. rank:	538.5

M1			
Pos.	24	obs:	exp:
att	I	43	1317.00
atc	I	3062	960.70
ata	I	485	1312.00
---	--	------	------
mPD		0.25	0.66
		nPD:	0.39
		N. weight:	0.92
		Sc. PD:	0.21
		Sc. rank:	1718.4

M1			
Pos.	25	obs:	exp:
act	T	0	0.26
acc	T	0	0.23
aca	T	0	0.42
acg	T	1	0.09
gct	A	9	904.10
gcc	A	2	694.50
gca	A	341	1598.00
gcg	A	3237	392.80
---	--	------	------
mPD		0.18	0.69
		nPD:	0.26
		N. weight:	2.
		Sc. PD:	0.2
		Sc. rank:	2873.2

M1			
Pos.	26	obs:	exp:
caa	Q	55	1791.00
cag	Q	3533	1797.00
cgt	R	0	0.07
cgc	R	0	0.11
cga	R	0	0.20
cgg	R	2	0.16
aga	R	0	0.93
agg	R	0	0.52
---	--	------	------
mPD		0.031	0.50
		nPD:	0.06
		N. weight:	0.7
		Sc. PD:	-0.064
		Sc. rank:	216.6

M1			
Pos.	27	obs:	exp:
cgt	R	0	38.88
cgc	R	0	55.60
cga	R	0	104.70
cgg	R	0	84.84
att	I	0	0.37
atc	I	0	0.27
ata	I	1	0.37
aaa	K	2438	1452.00
aag	K	112	1098.00
aga	R	1039	485.00
agg	R	0	269.90
---	--	------	------
mPD		0.47	1.1
		nPD:	0.44
		N. weight:	0.57
		Sc. PD:	0.16
		Sc. rank:	1119.5

M1			
Pos.	28	obs:	exp:
tta	L	2	331.80
ttg	L	0	681.80
ctt	L	3552	712.60
ctc	L	16	574.10
cta	L	18	531.10
ctg	L	1	757.50
cat	H	1	0.58
cac	H	0	0.42
---	--	------	------
mPD		0.022	1.1
		nPD:	0.02
		N. weight:	1.8
		Sc. PD:	-0.24
		Sc. rank:	-1552.6

M1			
Pos.	29	obs:	exp:
gaa	E	1536	2012.00
gag	E	2054	1578.00
---	--	------	------
mPD		0.49	0.49
		nPD:	0.99
		N. weight:	0.042
		Sc. PD:	0.034
		Sc. rank:	113.2

M1			
Pos.	30	obs:	exp:
gat	D	3588	1937.00
gac	D	0	1651.00
gaa	E	2	1.12
gag	E	0	0.88
---	--	------	------
mPD		0.0011	0.50
		nPD:	0.
		N. weight:	0.7
		Sc. PD:	-0.11
		Sc. rank:	-1626.8

M1			
Pos.	31	obs:	exp:
att	I	0	1.10
atc	I	3	0.80
ata	I	0	1.10
gtt	V	128	771.30
gtc	V	2274	754.40
gta	V	1092	764.60
gtg	V	92	1296.00
gat	D	0	0.54
gac	D	1	0.46
---	--	------	------
mPD		0.51	0.74
		nPD:	0.69
		N. weight:	0.77
		Sc. PD:	0.41
		Sc. rank:	2003.4

M1			
Pos.	32	obs:	exp:
ttt	F	3528	1491.00
ttc	F	47	2084.00
tta	L	0	0.18
ttg	L	2	0.38
tct	S	13	2.17
tcc	S	0	1.88
tca	S	0	2.95
tcg	S	0	0.78
ctt	L	0	0.40
ctc	L	0	0.32
cta	L	0	0.30
ctg	L	0	0.42
agt	S	0	2.82
agc	S	0	2.40
---	--	------	------
mPD		0.034	0.50
		nPD:	0.07
		N. weight:	0.91
		Sc. PD:	-0.078
		Sc. rank:	426.2

M1			
Pos.	33	obs:	exp:
tct	S	0	0.17
tcc	S	0	0.14
tca	S	1	0.23
tcg	S	0	0.06
act	T	0	0.26
acc	T	0	0.23
aca	T	1	0.42
acg	T	0	0.09
agt	S	0	0.22
agc	S	0	0.18
gtt	V	0	1.29
gtc	V	0	1.26
gta	V	6	1.28
gtg	V	0	2.17
gct	A	1	902.40
gcc	A	0	693.20
gca	A	3578	1594.00
gcg	A	3	392.10
---	--	------	------
mPD		0.0067	0.69
		nPD:	0.01
		N. weight:	0.91
		Sc. PD:	-0.13
		Sc. rank:	-1406.0

M1			
Pos.	34	obs:	exp:
gaa	E	1	0.56
gag	E	0	0.44
ggt	G	0	519.00
ggc	G	0	471.40
gga	G	2061	1573.00
ggg	G	1528	1026.00
---	--	------	------
mPD		0.49	0.69
		nPD:	0.71
		N. weight:	0.37
		Sc. PD:	0.2
		Sc. rank:	973.8

M1			
Pos.	35	obs:	exp:
aaa	K	97	2044.00
aag	K	3492	1545.00
gaa	E	0	0.56
gag	E	1	0.44
---	--	------	------
mPD		0.053	0.49
		nPD:	0.11
		N. weight:	0.81
		Sc. PD:	-0.038
		Sc. rank:	717.8

M1

Pos.	36	obs:	exp:
tct	S	0	1.17
tcc	S	0	1.01
tca	S	0	1.59
tcg	S	0	0.42
aat	N	21	1954.00
aac	N	3562	1629.00
agt	S	0	1.52
agc	S	7	1.29
---	--	------	------
mPD		0.016	0.50
		nPD:	0.03
		N. weight:	0.85
		Sc. PD:	-0.11
		Sc. rank:	-391.9

,

M1

Pos.	37	obs:	exp:
att	I	1	0.73
atc	I	1	0.54
ata	I	0	0.73
act	T	571	892.40
acc	T	2658	767.00
aca	T	173	1424.00
acg	T	2	320.30
gtt	V	0	0.43
gtc	V	2	0.42
gta	V	0	0.43
gtg	V	0	0.72
gct	A	14	45.85
gcc	A	129	35.22
gca	A	39	81.01
gcg	A	0	19.92
---	--	------	------
mPD		0.47	0.80
		nPD:	0.58
		N. weight:	0.89
		Sc. PD:	0.38
		Sc. rank:	2069.3

,

M1

Pos.	38	obs:	exp:
gat	D	3567	1938.00
gac	D	22	1651.00
gaa	E	1	0.56
gag	E	0	0.44
---	--	------	------
mPD		0.013	0.50
		nPD:	0.03
		N. weight:	0.66
		Sc. PD:	-0.085
		Sc. rank:	-404.4

,

M1

Pos.	39	obs:	exp:
tta	L	0	331.80
ttg	L	0	681.80
tct	S	0	0.17
tcc	S	1	0.14
tca	S	0	0.23
tcg	S	0	0.06
ctt	L	1213	712.60
ctc	L	2345	574.10
cta	L	31	531.10
ctg	L	0	757.50
agt	S	0	0.22
agc	S	0	0.18
---	--	------	------
mPD		0.46	1.1
		nPD:	0.41
		N. weight:	1.2
		Sc. PD:	0.31
		Sc. rank:	2374.7

,

M1

Pos.	40	obs:	exp:
caa	Q	0	0.50
cag	Q	1	0.50
gat	D	2	1.08
gac	D	0	0.92
gaa	E	28	2011.00
gag	E	3559	1576.00
---	--	------	------
mPD		0.017	0.49
		nPD:	0.03
		N. weight:	0.88
		Sc. PD:	-0.1
		Sc. rank:	-274.2

,

M1

Pos.	41	obs:	exp:
gct	A	3540	904.40
gcc	A	8	694.70
gca	A	24	1598.00
gcg	A	18	392.90
---	--	------	------
mPD		0.028	0.69
		nPD:	0.04
		N. weight:	1.5
		Sc. PD:	-0.17
		Sc. rank:	-278.6

,

M1

Pos.	42	obs:	exp:
ttt	F	0	0.83
ttc	F	2	1.17
tta	L	0	330.20
ttg	L	0	678.40
ctt	L	24	709.00
ctc	L	3539	571.20
cta	L	8	528.50
ctg	L	0	753.70
att	I	0	6.24
atc	I	17	4.55
ata	I	0	6.21
---	--	------	------
mPD		0.028	1.1
		nPD:	0.03
		N. weight:	2.
		Sc. PD:	-0.26
		Sc. rank:	-1365.7

,

M1

Pos.	43	obs:	exp:
att	I	1	0.37
atc	I	0	0.27
ata	I	0	0.37
atg	M	3588	3588.00
act	T	0	0.26
acc	T	0	0.23
aca	T	0	0.42
acg	T	1	0.09
---	--	------	------
mPD		0.0011	0.0016
		nPD:	0.69
		N. weight:	0.0011
		Sc. PD:	0.00056
		Sc. rank:	2.8

,

M1

Pos.	44	obs:	exp:
aaa	K	0	0.57
aag	K	1	0.43
gaa	E	1486	2012.00
gag	E	2103	1577.00
---	--	------	------
mPD		0.49	0.49
		nPD:	0.99
		N. weight:	0.051
		Sc. PD:	0.042
		Sc. rank:	137.8

,

M1

Pos.	45	obs:	exp:
tgt	C	0	0.39
tgc	C	1	0.61
tgg	W	3588	3588.00
cgt	R	0	0.04
cgc	R	0	0.05
cga	R	0	0.10
cgg	R	0	0.08
aga	R	0	0.47
agg	R	1	0.26
---	--	------	------
mPD		0.0011	0.0015
		nPD:	0.75
		N. weight:	0.00058
		Sc. PD:	0.00034
		Sc. rank:	1.5

,

M1

Pos.	46	obs:	exp:
tta	L	31	327.30
ttg	L	0	672.50
ctt	L	0	702.90
ctc	L	0	566.30
cta	L	3480	523.90
ctg	L	29	747.20
att	I	0	18.35
atc	I	0	13.38
ata	I	50	18.27
---	--	------	------
mPD		0.060	1.1
		nPD:	0.05
		N. weight:	2.
		Sc. PD:	-0.21
		Sc. rank:	276.7

,

M1

Pos.	47	obs:	exp:
aaa	K	1	2044.00
aag	K	3589	1546.00
---	--	------	------
mPD		0.00056	0.49
		nPD:	0.
		N. weight:	0.95
		Sc. PD:	-0.15
		Sc. rank:	-2310.8

,

M1

Pos.	48	obs:	exp:
act	T	0	941.10
acc	T	0	808.90
aca	T	3590	1502.00
acg	T	0	337.80
---	--	------	------
mPD		0	0.70
		nPD:	0.
		N. weight:	0.99
		Sc. PD:	-0.15
		Sc. rank:	-2691.2

,

M1

Pos.	49	obs:	exp:
cgt	R	0	134.30
cgc	R	0	192.10
cga	R	0	361.80
cgg	R	0	293.10
aga	R	3581	1676.00
agg	R	9	932.70
---	--	------	------
mPD		0.0050	0.95
		nPD:	0.01
		N. weight:	0.85
		Sc. PD:	-0.13
		Sc. rank:	-1609.0

,

M1

Pos.	50	obs:	exp:
cct	P	1	981.10
ccc	P	0	648.40
cca	P	3559	1457.00
ccg	P	30	503.30
---	--	------	------
mPD		0.017	0.71
		nPD:	0.02
		N. weight:	0.98
		Sc. PD:	-0.13
		Sc. rank:	-749.3

,

M1

Pos.	51	obs:	exp:
att	I	4	1317.00
atc	I	3584	960.70
ata	I	2	1312.00
---	--	------	------
mPD		0.0033	0.66
		nPD:	0.01
		N. weight:	1.5
		Sc. PD:	-0.22
		Sc. rank:	-2841.0

,

M1

Pos.	52	obs:	exp:
tta	L	0	331.90
ttg	L	19	682.00
ctt	L	4	712.80
ctc	L	0	574.30
cta	L	0	531.30
ctg	L	3567	757.70
---	--	------	------
mPD		0.013	1.1
		nPD:	0.01
		N. weight:	1.7
		Sc. PD:	-0.24
		Sc. rank:	-2457.3

,

M1

Pos.	53	obs:	exp:
tct	S	0	599.30
tcc	S	0	520.00
tca	S	3589	813.90
tcg	S	1	216.60
agt	S	0	778.70
agc	S	0	661.60
---	--	------	------
mPD		0.00056	1.7
		nPD:	0.
		N. weight:	1.7
		Sc. PD:	-0.26
		Sc. rank:	-4208.6

,

M1

Pos.	54	obs:	exp:
tct	S	3	0.50
tcc	S	0	0.43
tca	S	0	0.68
tcg	S	0	0.18
cct	P	3569	979.70
ccc	P	0	647.50
cca	P	16	1455.00
ccg	P	0	502.60
cat	H	2	1.16
cac	H	0	0.84
agt	S	0	0.65
agc	S	0	0.55
---	--	------	------
mPD		0.012	0.71
		nPD:	0.02
		N. weight:	1.4
		Sc. PD:	-0.2
		Sc. rank:	-1444.6

,

M1

Pos.	55	obs:	exp:
tta	L	0	315.10
ttg	L	361	647.40
ctt	L	0	676.70
ctc	L	0	545.10
cta	L	215	504.40
ctg	L	2832	719.30
cct	P	0	0.55
ccc	P	0	0.36
cca	P	0	0.81
ccg	P	2	0.28
atg	M	180	180.00
---	--	------	------
mPD		0.38	1.2
		nPD:	0.33
		N. weight:	1.1
		Sc. PD:	0.19
		Sc. rank:	1886.1

,

M1			
Pos.	56	obs:	exp:
att	I	2	0.73
atc	I	0	0.54
ata	I	0	0.73
act	T	3570	940.60
acc	T	12	808.50
aca	T	6	1501.00
acg	T	0	337.60
---	--	------	------
mPD		0.011	0.70
		nPD:	0.02
		N. weight:	1.5
		Sc. PD:	-0.2
		Sc. rank:	-1533.1

,

M1			
Pos.	57	obs:	exp:
aat	N	1	0.55
aac	N	0	0.45
aaa	K	2561	2044.00
aag	K	1028	1545.00
---	--	------	------
mPD		0.41	0.49
		nPD:	0.83
		N. weight:	0.052
		Sc. PD:	0.035
		Sc. rank:	139.4

,

M1			
Pos.	58	obs:	exp:
gaa	E	0	1.12
gag	E	2	0.88
ggt	G	0	518.90
ggc	G	3	471.30
gga	G	264	1572.00
ggg	G	3321	1026.00
---	--	------	------
mPD		0.14	0.69
		nPD:	0.2
		N. weight:	1.1
		Sc. PD:	0.05
		Sc. rank:	1256.8

,

M1			
Pos.	59	obs:	exp:
att	I	1548	1163.00
atc	I	1259	848.00
ata	I	362	1158.00
atg	M	417	417.00
gtt	V	2	0.86
gtc	V	0	0.84
gta	V	0	0.85
gtg	V	2	1.45
---	--	------	------
mPD		0.67	0.72
		nPD:	0.93
		N. weight:	0.17
		Sc. PD:	0.13
		Sc. rank:	456.7

,

M1			
Pos.	60	obs:	exp:
tta	L	258	331.70
ttg	L	3305	681.60
tct	S	0	0.17
tcc	S	0	0.14
tca	S	1	0.23
tcg	S	0	0.06
tgg	W	1	1.00
ctt	L	0	712.40
ctc	L	0	573.90
cta	L	0	531.00
ctg	L	25	757.30
agt	S	0	0.22
agc	S	0	0.18
---	--	------	------
mPD		0.15	1.1
		nPD:	0.13
		N. weight:	1.6
		Sc. PD:	-0.036
		Sc. rank:	1526.9

,

M1			
Pos.	61	obs:	exp:
ggt	G	1	519.20
ggc	G	3	471.60
gga	G	3492	1573.00
ggg	G	94	1026.00
---	--	------	------
mPD		0.053	0.69
		nPD:	0.08
		N. weight:	0.8
		Sc. PD:	-0.062
		Sc. rank:	497.7

,

M1			
Pos.	62	obs:	exp:
ttt	F	3566	1496.00
ttc	F	21	2091.00
tta	L	0	0.09
ttg	L	0	0.19
ctt	L	1	0.20
ctc	L	0	0.16
cta	L	0	0.15
ctg	L	0	0.21
gtt	V	2	0.43
gtc	V	0	0.42
gta	V	0	0.43
gtg	V	0	0.72
---	--	------	------
mPD		0.013	0.49
		nPD:	0.03
		N. weight:	0.95
		Sc. PD:	-0.12
		Sc. rank:	-550.6

,

M1			
Pos.	63	obs:	exp:
att	I	0	0.37
atc	I	0	0.27
ata	I	1	0.37
gtt	V	1	771.90
gtc	V	7	755.10
gta	V	2305	765.30
gtg	V	1276	1297.00
---	--	------	------
mPD		0.46	0.73
		nPD:	0.63
		N. weight:	0.79
		Sc. PD:	0.37
		Sc. rank:	1909.2

,

M1			
Pos.	64	obs:	exp:
ttt	F	15	1497.00
ttc	F	3574	2092.00
tgt	C	0	0.39
tgc	C	1	0.61
---	--	------	------
mPD		0.0089	0.49
		nPD:	0.02
		N. weight:	0.58
		Sc. PD:	-0.079
		Sc. rank:	-548.0

,

M1			
Pos.	65	obs:	exp:
act	T	0	940.60
acc	T	0	808.50
aca	T	10	1501.00
acg	T	3578	337.60
gct	A	0	0.50
gcc	A	0	0.39
gca	A	0	0.89
gcg	A	2	0.22
---	--	------	------
mPD		0.0067	0.70
		nPD:	0.01
		N. weight:	2.7
		Sc. PD:	-0.38
		Sc. rank:	-4148.0

,

M1			
Pos.	66	obs:	exp:
tta	L	0	331.90
ttg	L	0	682.00
ctt	L	0	712.80
ctc	L	3590	574.30
cta	L	0	531.30
ctg	L	0	757.70
---	--	------	------
mPD		0	1.1
		nPD:	0.
		N. weight:	2.1
		Sc. PD:	-0.32
		Sc. rank:	-5661.6

,

M1			
Pos.	67	obs:	exp:
act	T	0	941.10
acc	T	3590	808.90
aca	T	0	1502.00
acg	T	0	337.80
---	--	------	------
mPD		0	0.70
		nPD:	0.
		N. weight:	1.7
		Sc. PD:	-0.26
		Sc. rank:	-4603.2

,

M1			
Pos.	68	obs:	exp:
atg	M	1	1.00
gtt	V	1	771.50
gtc	V	0	754.70
gta	V	0	764.80
gtg	V	3586	1296.00
gaa	E	0	1.12
gag	E	2	0.88
---	--	------	------
mPD		0.0022	0.74
		nPD:	0.
		N. weight:	1.2
		Sc. PD:	-0.17
		Sc. rank:	-2601.1

,

M1			
Pos.	69	obs:	exp:
tta	L	0	0.09
ttg	L	0	0.19
ctt	L	0	0.20
ctc	L	1	0.16
cta	L	0	0.15
ctg	L	0	0.21
cct	P	1	980.80
ccc	P	3588	648.20
cca	P	0	1457.00
ccg	P	0	503.20
---	--	------	------
mPD		0.0011	0.71
		nPD:	0.
		N. weight:	1.9
		Sc. PD:	-0.29
		Sc. rank:	-4618.7

,

M1			
Pos.	70	obs:	exp:
tct	S	0	599.10
tcc	S	0	519.90
tca	S	0	813.60
tcg	S	0	216.50
aat	N	1	0.55
aac	N	0	0.45
agt	S	3589	778.50
agc	S	0	661.40
---	--	------	------
mPD		0.00056	1.7
		nPD:	0.
		N. weight:	1.7
		Sc. PD:	-0.27
		Sc. rank:	-4401.9

,

M1			
Pos.	71	obs:	exp:
gaa	E	7	2012.00
gag	E	3583	1578.00
---	--	------	------
mPD		0.0039	0.49
		nPD:	0.01
		N. weight:	0.92
		Sc. PD:	-0.13
		Sc. rank:	-1566.2

,

M1			
Pos.	72	obs:	exp:
caa	Q	1	0.50
cag	Q	0	0.50
cgt	R	0	134.30
cgc	R	0	192.10
cga	R	3566	361.70
cgg	R	23	293.10
aga	R	0	1675.00
agg	R	0	932.40
---	--	------	------
mPD		0.013	0.95
		nPD:	0.01
		N. weight:	2.6
		Sc. PD:	-0.36
		Sc. rank:	-3035.6

,

M1			
Pos.	73	obs:	exp:
cgt	R	0	0.07
cgc	R	0	0.11
cga	R	0	0.20
cgg	R	0	0.16
aga	R	2	0.93
agg	R	0	0.52
ggt	G	0	518.90
ggc	G	3	471.30
gga	G	3585	1572.00
ggg	G	0	1026.00
---	--	------	------
mPD		0.0028	0.69
		nPD:	0.
		N. weight:	0.93
		Sc. PD:	-0.14
		Sc. rank:	-1923.3

,

M1			
Pos.	74	obs:	exp:
tta	L	0	331.80
ttg	L	0	681.80
ctt	L	0	712.60
ctc	L	0	574.10
cta	L	7	531.10
ctg	L	3582	757.50
atg	M	1	1.00
---	--	------	------
mPD		0.0045	1.1
		nPD:	0.
		N. weight:	1.7
		Sc. PD:	-0.26
		Sc. rank:	-3718.3

,

M1			
Pos.	75	obs:	exp:
cat	H	0	0.58
cac	H	1	0.42
caa	Q	14	1792.00
cag	Q	3575	1797.00
---	--	------	------
mPD		0.0083	0.50
		nPD:	0.02
		N. weight:	0.76
		Sc. PD:	-0.1
		Sc. rank:	-747.6

,

M1

Pos.	76	obs:	exp:
tct	S	0	0.67
tcc	S	0	0.58
tca	S	0	0.91
tcg	S	0	0.24
tgt	C	1	0.39
tgc	C	0	0.61
cgt	R	3584	134.20
cgc	R	1	191.90
cga	R	0	361.30
cgg	R	0	292.70
agt	S	4	0.87
agc	S	0	0.74
aga	R	0	1674.00
agg	R	0	931.40
mPD		0.0033	0.96
		nPD:	0.
		N. weight:	3.7
		Sc. PD:	-0.56
		Sc. rank:	-8050.1

M1

Pos.	77	obs:	exp:
cgt	R	0	134.30
cgc	R	0	192.00
cga	R	0	361.60
cgg	R	0	293.00
att	I	0	0.37
atc	I	0	0.27
ata	I	1	0.37
act	T	0	0.26
acc	T	0	0.23
aca	T	1	0.42
acg	T	0	0.09
aga	R	3582	1675.00
agg	R	6	932.10
mPD		0.0045	0.96
		nPD:	0.
		N. weight:	0.85
		Sc. PD:	-0.13
		Sc. rank:	-1700.3

M1

Pos.	78	obs:	exp:
cgt	R	1086	134.30
cgc	R	2469	192.10
cga	R	14	361.80
cgg	R	21	293.10
aga	R	0	1676.00
agg	R	0	932.70
mPD		0.44	0.95
		nPD:	0.46
		N. weight:	2.7
		Sc. PD:	0.8
		Sc. rank:	5500.9

M1

Pos.	79	obs:	exp:
ttt	F	3585	1497.00
ttc	F	4	2092.00
tta	L	0	0.09
ttg	L	0	0.19
ctt	L	1	0.20
ctc	L	0	0.16
cta	L	0	0.15
ctg	L	0	0.21
mPD		0.0028	0.49
		nPD:	0.01
		N. weight:	0.98
		Sc. PD:	-0.15
		Sc. rank:	-1809.1

M1

Pos.	80	obs:	exp:
gtt	V	262	772.10
gtc	V	3327	755.30
gta	V	1	765.50
gtg	V	0	1297.00
mPD		0.14	0.73
		nPD:	0.19
		N. weight:	1.5
		Sc. PD:	0.043
		Sc. rank:	1629.8

M1

Pos.	81	obs:	exp:
cat	H	0	0.58
cac	H	1	0.42
caa	Q	1065	1792.00
cag	Q	2524	1797.00
mPD		0.42	0.50
		nPD:	0.84
		N. weight:	0.097
		Sc. PD:	0.065
		Sc. rank:	262.7

M1

Pos.	82	obs:	exp:
tct	S	0	0.67
tcc	S	0	0.58
tca	S	0	0.91
tcg	S	0	0.24
tat	Y	1	0.48
tac	Y	0	0.52
cat	H	4	2.32
cac	H	0	1.68
act	T	1	0.26
acc	T	0	0.23
aca	T	0	0.42
acg	T	0	0.09
aat	N	3371	1952.00
aac	N	209	1628.00
agt	S	4	0.87
agc	S	0	0.74
mPD		0.12	0.50
		nPD:	0.23
		N. weight:	0.45
		Sc. PD:	0.033
		Sc. rank:	587.4

M1

Pos.	83	obs:	exp:
tct	S	0	4.34
tcc	S	26	3.77
tca	S	0	5.89
tcg	S	0	1.57
agt	S	0	5.64
agc	S	0	4.79
gct	A	897	897.80
gcc	A	2667	689.70
gca	A	0	1586.00
gcg	A	0	390.10
mPD		0.39	0.71
		nPD:	0.55
		N. weight:	1.2
		Sc. PD:	0.45
		Sc. rank:	2558.3

M1

Pos.	84	obs:	exp:
tta	L	23	331.80
ttg	L	0	681.80
tct	S	0	0.17
tcc	S	0	0.14
tca	S	1	0.23
tcg	S	0	0.06
ctt	L	40	712.60
ctc	L	4	574.10
cta	L	3487	531.10
ctg	L	35	757.50
agt	S	0	0.22
agc	S	0	0.18
mPD		0.057	1.1
		nPD:	0.05
		N. weight:	2.
		Sc. PD:	-0.2
		Sc. rank:	52.2

M1

Pos.	85	obs:	exp:
tct	S	0	5.51
tcc	S	0	4.78
tca	S	0	7.48
tcg	S	0	1.99
att	I	1	0.37
atc	I	0	0.27
ata	I	0	0.37
act	T	1	0.26
acc	T	0	0.23
aca	T	0	0.42
acg	T	0	0.09
aat	N	3530	1939.00
aac	N	25	1616.00
agt	S	33	7.16
agc	S	0	6.08
mPD		0.033	0.53
		nPD:	0.06
		N. weight:	0.65
		Sc. PD:	-0.06
		Sc. rank:	212.2

M1

Pos.	86	obs:	exp:
gaa	E	1	0.56
gag	E	0	0.44
ggt	G	0	519.00
ggc	G	0	471.40
gga	G	3337	1573.00
ggg	G	252	1026.00
mPD		0.13	0.69
		nPD:	0.19
		N. weight:	0.68
		Sc. PD:	0.024
		Sc. rank:	767.8

M1

Pos.	87	obs:	exp:
tct	S	0	0.17
tcc	S	0	0.14
tca	S	0	0.23
tcg	S	0	0.06
att	I	1	0.37
atc	I	0	0.27
ata	I	0	0.37
aat	N	3563	1957.00
aac	N	25	1631.00
agt	S	1	0.22
agc	S	0	0.18
mPD		0.015	0.50
		nPD:	0.03
		N. weight:	0.64
		Sc. PD:	-0.08
		Sc. rank:	-306.8

M1

Pos.	88	obs:	exp:
tct	S	0	0.17
tcc	S	0	0.14
tca	S	0	0.23
tcg	S	0	0.06
agt	S	1	0.22
agc	S	0	0.18
gct	A	0	0.25
gcc	A	0	0.19
gca	A	1	0.45
gcg	A	0	0.11
ggt	G	159	518.90
ggc	G	1	471.30
gga	G	3329	1572.00
ggg	G	99	1026.00
mPD		0.14	0.69
		nPD:	0.2
		N. weight:	0.66
		Sc. PD:	0.029
		Sc. rank:	749.0

M1

Pos.	89	obs:	exp:
cat	H	1	0.58
cac	H	0	0.42
gat	D	2622	1937.00
gac	D	966	1651.00
ggt	G	1	0.14
ggc	G	0	0.13
gga	G	0	0.44
ggg	G	0	0.29
mPD		0.39	0.50
		nPD:	0.79
		N. weight:	0.089
		Sc. PD:	0.056
		Sc. rank:	238.0

M1

Pos.	90	obs:	exp:
tct	S	0	0.17
tcc	S	0	0.14
tca	S	1	0.23
tcg	S	0	0.06
cct	P	0	980.30
ccc	P	1	647.90
cca	P	3579	1456.00
ccg	P	7	502.90
act	T	0	0.26
acc	T	1	0.23
aca	T	0	0.42
acg	T	0	0.09
agt	S	0	0.22
agc	S	0	0.18
gct	A	0	0.25
gcc	A	0	0.19
gca	A	1	0.45
gcg	A	0	0.11
mPD		0.0067	0.71
		nPD:	0.01
		N. weight:	1.
		Sc. PD:	-0.15
		Sc. rank:	-1632.3

M1			
Pos.	91	obs:	exp:
tct	S	0	0.33
tcc	S	0	0.29
tca	S	0	0.45
tcg	S	0	0.12
cat	H	1	0.58
cac	H	0	0.42
act	T	0	2.10
acc	T	8	1.80
aca	T	0	3.35
acg	T	0	0.75
aat	N	2533	1951.00
aac	N	1045	1627.00
agt	S	1	0.43
agc	S	1	0.37
gat	D	1	0.54
gac	D	0	0.46
---	--	------	------
mPD		0.42	0.50
		nPD:	0.84
		N. weight:	0.069
		Sc. PD:	0.046
		Sc. rank:	186.1

,

M1			
Pos.	92	obs:	exp:
cat	H	1	0.58
cac	H	0	0.42
aat	N	2455	1957.00
aac	N	1134	1632.00
---	--	------	------
mPD		0.43	0.50
		nPD:	0.87
		N. weight:	0.047
		Sc. PD:	0.033
		Sc. rank:	126.9

,

M1			
Pos.	93	obs:	exp:
att	I	0	0.37
atc	I	0	0.27
ata	I	1	0.37
atg	M	3589	3589.00
---	--	------	------
mPD		0.00056	0.00056
		nPD:	1.
		N. weight:	0.00032
		Sc. PD:	0.00027
		Sc. rank:	0.9

,

M1			
Pos.	94	obs:	exp:
aat	N	1	0.55
aac	N	0	0.45
gat	D	2571	1937.00
gac	D	1017	1651.00
ggt	G	0	0.14
ggc	G	1	0.13
gga	G	0	0.44
ggg	G	0	0.29
---	--	------	------
mPD		0.41	0.50
		nPD:	0.82
		N. weight:	0.076
		Sc. PD:	0.05
		Sc. rank:	203.8

,

M1			
Pos.	95	obs:	exp:
cgt	R	0	125.20
cgc	R	0	179.00
cga	R	0	337.10
cgg	R	0	273.10
aaa	K	6	139.50
aag	K	239	105.50
aga	R	627	1562.00
agg	R	2718	869.00
---	--	------	------
mPD		0.42	1.1
		nPD:	0.39
		N. weight:	0.86
		Sc. PD:	0.2
		Sc. rank:	1606.9

,

M1			
Pos.	96	obs:	exp:
gct	A	10	903.60
gcc	A	7	694.10
gca	A	3549	1597.00
gcg	A	21	392.60
ggt	G	0	0.43
ggc	G	0	0.39
gga	G	3	1.31
ggg	G	0	0.86
---	--	------	------
mPD		0.023	0.69
		nPD:	0.03
		N. weight:	0.85
		Sc. PD:	-0.1
		Sc. rank:	-376.0

,

M1			
Pos.	97	obs:	exp:
att	I	3	1.47
atc	I	1	1.07
ata	I	0	1.46
gtt	V	2440	771.30
gtc	V	1114	754.40
gta	V	8	764.60
gtg	V	24	1296.00
---	--	------	------
mPD		0.44	0.74
		nPD:	0.6
		N. weight:	0.99
		Sc. PD:	0.44
		Sc. rank:	2345.5

,

M1			
Pos.	98	obs:	exp:
aaa	K	725	2044.00
aag	K	2864	1545.00
gaa	E	0	0.56
gag	E	1	0.44
---	--	------	------
mPD		0.32	0.49
		nPD:	0.66
		N. weight:	0.32
		Sc. PD:	0.16
		Sc. rank:	821.3

,

M1			
Pos.	99	obs:	exp:
tta	L	73	331.30
ttg	L	306	680.70
ctt	L	12	711.40
ctc	L	1	573.10
cta	L	2521	530.30
ctg	L	670	756.30
att	I	0	1.10
atc	I	0	0.80
ata	I	3	1.10
atg	M	4	4.00
---	--	------	------
mPD		0.59	1.1
		nPD:	0.53
		N. weight:	1.1
		Sc. PD:	0.4
		Sc. rank:	2387.8

,

M1			
Pos.	100	obs:	exp:
tat	Y	2393	1721.00
tac	Y	1196	1868.00
tgt	C	1	0.39
tgc	C	0	0.61
---	--	------	------
mPD		0.45	0.50
		nPD:	0.89
		N. weight:	0.082
		Sc. PD:	0.06
		Sc. rank:	223.0

,

M1			
Pos.	101	obs:	exp:
cgt	R	0	28.52
cgc	R	0	40.78
cga	R	0	76.79
cgg	R	0	62.22
act	T	0	0.26
acc	T	0	0.23
aca	T	0	0.42
acg	T	1	0.09
aaa	K	272	1610.00
aag	K	2555	1217.00
aga	R	14	355.70
agg	R	748	198.00
---	--	------	------
mPD		0.48	0.95
		nPD:	0.51
		N. weight:	0.75
		Sc. PD:	0.26
		Sc. rank:	1602.6

,

M1			
Pos.	102	obs:	exp:
aaa	K	1174	2044.00
aag	K	2416	1546.00
---	--	------	------
mPD		0.44	0.49
		nPD:	0.9
		N. weight:	0.14
		Sc. PD:	0.1
		Sc. rank:	370.5

,

M1			
Pos.	103	obs:	exp:
tta	L	2	331.80
ttg	L	856	681.80
ctt	L	30	712.60
ctc	L	12	574.10
cta	L	50	531.10
ctg	L	2639	757.50
gtt	V	0	0.22
gtc	V	0	0.21
gta	V	0	0.21
gtg	V	1	0.36
---	--	------	------
mPD		0.42	1.1
		nPD:	0.37
		N. weight:	1.
		Sc. PD:	0.22
		Sc. rank:	1877.8

,

M1			
Pos.	104	obs:	exp:
aaa	K	2665	2044.00
aag	K	925	1546.00
---	--	------	------
mPD		0.38	0.49
		nPD:	0.78
		N. weight:	0.075
		Sc. PD:	0.046
		Sc. rank:	198.3

,

M1			
Pos.	105	obs:	exp:
cgt	R	0	134.30
cgc	R	0	192.10
cga	R	6	361.70
cgg	R	0	293.10
aaa	K	1	0.57
aag	K	0	0.43
aga	R	3455	1675.00
agg	R	128	932.40
---	--	------	------
mPD		0.073	0.95
		nPD:	0.08
		N. weight:	0.7
		Sc. PD:	-0.055
		Sc. rank:	398.1

,

M1			
Pos.	106	obs:	exp:
aaa	K	1	0.57
aag	K	0	0.43
gaa	E	2649	2012.00
gag	E	940	1577.00
---	--	------	------
mPD		0.39	0.49
		nPD:	0.79
		N. weight:	0.078
		Sc. PD:	0.049
		Sc. rank:	207.8

,

M1			
Pos.	107	obs:	exp:
att	I	381	1300.00
atc	I	13	947.80
ata	I	3148	1294.00
atg	M	48	48.00
---	--	------	------
mPD		0.22	0.67
		nPD:	0.33
		N. weight:	0.72
		Sc. PD:	0.12
		Sc. rank:	1240.8

,

M1			
Pos.	108	obs:	exp:
act	T	7	940.90
acc	T	3	808.70
aca	T	3572	1502.00
acg	T	7	337.70
aaa	K	1	0.57
aag	K	0	0.43
---	--	------	------
mPD		0.010	0.70
		nPD:	0.01
		N. weight:	0.95
		Sc. PD:	-0.13
		Sc. rank:	-1112.3

,

M1			
Pos.	109	obs:	exp:
ttt	F	52	1497.00
ttc	F	3537	2092.00
tta	L	0	0.09
ttg	L	1	0.19
ctt	L	0	0.20
ctc	L	0	0.16
cta	L	0	0.15
ctg	L	0	0.21
---	--	------	------
mPD		0.029	0.49
		nPD:	0.06
		N. weight:	0.53
		Sc. PD:	-0.05
		Sc. rank:	114.1

,

M1			
Pos.	110	obs:	exp:
tat	Y	4	1.92
tac	Y	0	2.08
cct	P	1	0.27
ccc	P	0	0.18
cca	P	0	0.41
ccg	P	0	0.14
cat	H	3580	2075.00
cac	H	5	1510.00
---	--	------	------
mPD		0.0056	0.49
		nPD:	0.01
		N. weight:	0.61
		Sc. PD:	-0.087
		Sc. rank:	-894.1

,

M1			
Pos.	111	obs:	exp:
ggt	G	0	519.20
ggc	G	0	471.60
gga	G	913	1573.00
ggg	G	2677	1026.00
---	--	------	------
mPD		0.38	0.69
		nPD:	0.55
		N. weight:	0.66
		Sc. PD:	0.26
		Sc. rank:	1458.9

,

M1			
Pos.	112	obs:	exp:
tct	S	4	0.67
tcc	S	0	0.58
tca	S	0	0.91
tcg	S	0	0.24
agt	S	0	0.87
agc	S	0	0.74
gct	A	3424	903.10
gcc	A	61	693.70
gca	A	99	1596.00
gcg	A	1	392.40
gat	D	1	0.54
gac	D	0	0.46
---	--	------	------
mPD		0.089	0.69
		nPD:	0.13
		N. weight:	1.3
		Sc. PD:	-0.034
		Sc. rank:	1202.7

,

M1			
Pos.	113	obs:	exp:
cgt	R	0	0.04
cgc	R	0	0.05
cga	R	0	0.10
cgg	R	0	0.08
aaa	K	1065	2044.00
aag	K	2524	1545.00
aga	R	0	0.47
agg	R	1	0.26
---	--	------	------
mPD		0.42	0.49
		nPD:	0.85
		N. weight:	0.17
		Sc. PD:	0.12
		Sc. rank:	469.7

,

M1			
Pos.	114	obs:	exp:
gaa	E	1174	2012.00
gag	E	2416	1578.00
---	--	------	------
mPD		0.44	0.49
		nPD:	0.89
		N. weight:	0.13
		Sc. PD:	0.093
		Sc. rank:	343.8

,

M1			
Pos.	115	obs:	exp:
att	I	1	0.37
atc	I	0	0.27
ata	I	0	0.37
gtt	V	1160	771.70
gtc	V	2349	754.90
gta	V	38	765.10
gtg	V	41	1296.00
ggt	G	0	0.14
ggc	G	0	0.13
gga	G	1	0.44
ggg	G	0	0.29
---	--	------	------
mPD		0.47	0.73
		nPD:	0.64
		N. weight:	0.91
		Sc. PD:	0.44
		Sc. rank:	2271.4

,

M1			
Pos.	116	obs:	exp:
tct	S	0	39.23
tcc	S	0	34.04
tca	S	234	53.27
tcg	S	1	14.18
cct	P	0	0.27
ccc	P	0	0.18
cca	P	1	0.41
ccg	P	0	0.14
agt	S	0	50.97
agc	S	0	43.31
gct	A	0	844.70
gcc	A	4	648.80
gca	A	3323	1492.00
gcg	A	26	367.00
ggt	G	0	0.14
ggc	G	0	0.13
gga	G	1	0.44
ggg	G	0	0.29
---	--	------	------
mPD		0.14	0.87
		nPD:	0.16
		N. weight:	0.92
		Sc. PD:	0.0056
		Sc. rank:	948.5

,

M1			
Pos.	117	obs:	exp:
tta	L	0	331.90
ttg	L	0	682.00
ctt	L	18	712.80
ctc	L	3529	574.30
cta	L	43	531.30
ctg	L	0	757.70
---	--	------	------
mPD		0.034	1.1
		nPD:	0.03
		N. weight:	2.
		Sc. PD:	-0.24
		Sc. rank:	-967.5

,

M1			
Pos.	118	obs:	exp:
tct	S	0	599.10
tcc	S	0	519.90
tca	S	0	813.60
tcg	S	0	216.50
aat	N	1	0.55
aac	N	0	0.45
agt	S	1879	778.50
agc	S	1710	661.40
---	--	------	------
mPD		0.50	1.7
		nPD:	0.3
		N. weight:	1.
		Sc. PD:	0.15
		Sc. rank:	1663.7

,

M1			
Pos.	119	obs:	exp:
ttt	F	0	0.42
ttc	F	1	0.58
tat	Y	235	1721.00
tac	Y	3354	1868.00
---	--	------	------
mPD		0.12	0.50
		nPD:	0.25
		N. weight:	0.47
		Sc. PD:	0.042
		Sc. rank:	668.5

,

M1			
Pos.	120	obs:	exp:
tct	S	15	599.30
tcc	S	2	520.00
tca	S	3513	813.90
tcg	S	60	216.60
agt	S	0	778.70
agc	S	0	661.60
---	--	------	------
mPD		0.042	1.7
		nPD:	0.03
		N. weight:	1.6
		Sc. PD:	-0.2
		Sc. rank:	-992.7

,

M1			
Pos.	121	obs:	exp:
cct	P	0	1.37
ccc	P	5	0.90
cca	P	0	2.03
ccg	P	0	0.70
act	T	165	939.30
acc	T	3278	807.30
aca	T	140	1499.00
acg	T	0	337.10
gct	A	0	0.50
gcc	A	2	0.39
gca	A	0	0.89
gcg	A	0	0.22
---	--	------	------
mPD		0.16	0.70
		nPD:	0.23
		N. weight:	1.3
		Sc. PD:	0.097
		Sc. rank:	1712.7

,

M1			
Pos.	122	obs:	exp:
ggt	G	3555	519.20
ggc	G	1	471.60
gga	G	2	1573.00
ggg	G	32	1026.00
---	--	------	------
mPD		0.019	0.69
		nPD:	0.03
		N. weight:	2.1
		Sc. PD:	-0.27
		Sc. rank:	-1131.8

,

M1			
Pos.	123	obs:	exp:
gtt	V	0	0.22
gtc	V	0	0.21
gta	V	1	0.21
gtg	V	0	0.36
gct	A	0	904.10
gcc	A	2	694.50
gca	A	3527	1598.00
gcg	A	60	392.80
---	--	------	------
mPD		0.035	0.69
		nPD:	0.05
		N. weight:	0.84
		Sc. PD:	-0.088
		Sc. rank:	-14.0

,

M1			
Pos.	124	obs:	exp:
tta	L	0	331.90
ttg	L	0	682.00
ctt	L	3113	712.80
ctc	L	455	574.30
cta	L	19	531.30
ctg	L	3	757.70
---	--	------	------
mPD		0.23	1.1
		nPD:	0.21
		N. weight:	1.4
		Sc. PD:	0.071
		Sc. rank:	1661.9

,

M1			
Pos.	125	obs:	exp:
tct	S	0	0.33
tcc	S	2	0.29
tca	S	0	0.45
tcg	S	0	0.12
act	T	0	1.05
acc	T	4	0.90
aca	T	0	1.67
acg	T	0	0.38
agt	S	0	0.43
agc	S	0	0.37
gct	A	13	902.90
gcc	A	3564	693.50
gca	A	7	1595.00
gcg	A	0	392.30
---	--	------	------
mPD		0.014	0.69
		nPD:	0.02
		N. weight:	1.8
		Sc. PD:	-0.24
		Sc. rank:	-1530.0

,

M1			
Pos.	126	obs:	exp:
tct	S	0	598.20
tcc	S	0	519.20
tca	S	0	812.50
tcg	S	0	216.20
agt	S	3488	777.40
agc	S	96	660.50
ggt	G	6	0.87
ggc	G	0	0.79
gga	G	0	2.63
ggg	G	0	1.72
---	--	------	------
mPD		0.055	1.7
		nPD:	0.03
		N. weight:	1.6
		Sc. PD:	-0.19
		Sc. rank:	-555.1

,

M1			
Pos.	127	obs:	exp:
tgt	C	899	1389.00
tgc	C	2691	2201.00
---	--	------	------
mPD		0.38	0.47
		nPD:	0.79
		N. weight:	0.049
		Sc. PD:	0.031
		Sc. rank:	129.8

,

M1			
Pos.	128	obs:	exp:
att	I	0	0.37
atc	I	0	0.27
ata	I	1	0.37
atg	M	3588	3588.00
aaa	K	0	0.57
aag	K	1	0.43
---	--	------	------
mPD		0.0011	0.0014
		nPD:	0.78
		N. weight:	0.00058
		Sc. PD:	0.00036
		Sc. rank:	1.6

,

M1			
Pos.	129	obs:	exp:
ggt	G	3297	519.20
ggc	G	289	471.60
gga	G	0	1573.00
ggg	G	4	1026.00
---	--	------	------
mPD		0.15	0.69
		nPD:	0.22
		N. weight:	1.9
		Sc. PD:	0.12
		Sc. rank:	2312.6

,

M1			
Pos.	130	obs:	exp:
tta	L	0	331.60
ttg	L	0	681.20
ctt	L	20	712.00
ctc	L	3563	573.60
cta	L	2	530.70
ctg	L	1	756.90
att	I	0	1.47
atc	I	4	1.07
ata	I	0	1.46
---	--	------	------
mPD		0.015	1.1
		nPD:	0.01
		N. weight:	2.
		Sc. PD:	-0.29
		Sc. rank:	-2520.1

,

M1

Pos.	131	obs:	exp:
att	I	9	1317.00
atc	I	0	960.70
ata	I	3581	1312.00
mPD		0.0050	0.66
		nPD:	0.01
		N. weight:	1.1
		Sc. PD:	-0.16
		Sc. rank:	-1940.1

M1

Pos.	132	obs:	exp:
tat	Y	33	1721.00
tac	Y	3556	1868.00
cat	H	0	0.58
cac	H	1	0.42
mPD		0.019	0.50
		nPD:	0.04
		N. weight:	0.68
		Sc. PD:	-0.08
		Sc. rank:	-162.8

M1

Pos.	133	obs:	exp:
cat	H	0	0.58
cac	H	1	0.42
aat	N	32	1957.00
aac	N	3557	1632.00
mPD		0.018	0.50
		nPD:	0.04
		N. weight:	0.84
		Sc. PD:	-0.098
		Sc. rank:	-211.4

M1

Pos.	134	obs:	exp:
cgt	R	0	134.30
cgc	R	0	192.00
cga	R	0	361.60
cgg	R	3	293.00
aaa	K	1	1.14
aag	K	1	0.86
aga	R	527	1675.00
agg	R	3058	932.10
mPD		0.25	0.96
		nPD:	0.27
		N. weight:	0.95
		Sc. PD:	0.1
		Sc. rank:	1433.0

M1

Pos.	135	obs:	exp:
atg	M	3587	3587.00
gtt	V	0	0.65
gtc	V	0	0.63
gta	V	0	0.64
gtg	V	3	1.08
mPD		0.0017	0.0027
		nPD:	0.61
		N. weight:	0.00096
		Sc. PD:	0.00043
		Sc. rank:	2.3

M1

Pos.	136	obs:	exp:
ggt	G	4	519.20
ggc	G	414	471.60
gga	G	2209	1573.00
ggg	G	963	1026.00
mPD		0.54	0.69
		nPD:	0.78
		N. weight:	0.2
		Sc. PD:	0.12
		Sc. rank:	525.1

M1

Pos.	137	obs:	exp:
act	T	47	916.20
acc	T	0	787.50
aca	T	766	1462.00
acg	T	2682	328.80
aaa	K	0	0.57
aag	K	1	0.43
gct	A	0	23.68
gcc	A	0	18.19
gca	A	19	41.84
gcg	A	75	10.29
mPD		0.41	0.75
		nPD:	0.55
		N. weight:	1.6
		Sc. PD:	0.64
		Sc. rank:	3670.8

M1

Pos.	138	obs:	exp:
tta	L	0	0.09
ttg	L	0	0.19
ctt	L	0	0.20
ctc	L	0	0.16
cta	L	0	0.15
ctg	L	1	0.21
atg	M	1	1.00
gtt	V	22	771.50
gtc	V	0	754.70
gta	V	90	764.80
gtg	V	3475	1296.00
ggt	G	0	0.14
ggc	G	0	0.13
gga	G	0	0.44
ggg	G	1	0.29
mPD		0.062	0.74
		nPD:	0.08
		N. weight:	1.
		Sc. PD:	-0.07
		Sc. rank:	676.8

M1

Pos.	139	obs:	exp:
act	T	2244	937.20
acc	T	1327	805.50
aca	T	4	1496.00
acg	T	0	336.40
aat	N	0	1.09
aac	N	2	0.91
gct	A	6	3.27
gcc	A	7	2.52
gca	A	0	5.79
gcg	A	0	1.42
mPD		0.48	0.70
		nPD:	0.68
		N. weight:	0.83
		Sc. PD:	0.43
		Sc. rank:	2121.5

M1

Pos.	140	obs:	exp:
act	T	45	704.10
acc	T	3	605.20
aca	T	802	1124.00
acg	T	1836	252.70
gct	A	0	227.70
gcc	A	0	174.90
gca	A	903	402.40
gcg	A	1	98.94
mPD		0.89	1.1
		nPD:	0.83
		N. weight:	1.3
		Sc. PD:	0.84
		Sc. rank:	3374.9

M1

Pos.	141	obs:	exp:
gaa	E	3552	2012.00
gag	E	38	1578.00
mPD		0.021	0.49
		nPD:	0.04
		N. weight:	0.59
		Sc. PD:	-0.066
		Sc. rank:	-84.9

M1

Pos.	142	obs:	exp:
gtt	V	0	761.40
gtc	V	0	744.80
gta	V	304	754.80
gtg	V	3236	1279.00
gct	A	0	0.25
gcc	A	0	0.19
gca	A	0	0.45
gcg	A	1	0.11
gaa	E	0	0.56
gag	E	1	0.44
ggt	G	0	6.94
ggc	G	0	6.30
gga	G	0	21.03
ggg	G	48	13.72
mPD		0.18	0.76
		nPD:	0.24
		N. weight:	0.88
		Sc. PD:	0.074
		Sc. rank:	1223.9

M1

Pos.	143	obs:	exp:
tct	S	3	0.50
tcc	S	0	0.43
tca	S	0	0.68
tcg	S	0	0.18
agt	S	0	0.65
agc	S	0	0.55
gtt	V	2	0.43
gtc	V	0	0.42
gta	V	0	0.43
gtg	V	0	0.72
gct	A	2547	903.10
gcc	A	94	693.70
gca	A	903	1596.00
gcg	A	41	392.40
mPD		0.43	0.69
		nPD:	0.63
		N. weight:	0.59
		Sc. PD:	0.27
		Sc. rank:	1419.8

M1

Pos.	144	obs:	exp:
ttt	F	2595	1116.00
ttc	F	80	1559.00
tta	L	2	84.60
ttg	L	856	173.80
ctt	L	57	181.70
ctc	L	0	146.40
cta	L	0	135.40
ctg	L	0	193.10
mPD		0.43	0.93
		nPD:	0.46
		N. weight:	1.
		Sc. PD:	0.31
		Sc. rank:	2131.0

M1

Pos.	145	obs:	exp:
ggt	G	41	519.20
ggc	G	3511	471.60
gga	G	38	1573.00
ggg	G	0	1026.00
mPD		0.043	0.69
		nPD:	0.06
		N. weight:	2.2
		Sc. PD:	-0.2
		Sc. rank:	799.3

M1

Pos.	146	obs:	exp:
tta	L	69	331.90
ttg	L	1	682.00
ctt	L	0	712.80
ctc	L	0	574.30
cta	L	3461	531.30
ctg	L	59	757.70
mPD		0.071	1.1
		nPD:	0.06
		N. weight:	2.
		Sc. PD:	-0.18
		Sc. rank:	814.9

M1

Pos.	147	obs:	exp:
atg	M	1	1.00
gtt	V	3	771.90
gtc	V	1	755.10
gta	V	223	765.30
gtg	V	3362	1297.00
mPD		0.12	0.73
		nPD:	0.16
		N. weight:	0.92
		Sc. PD:	0.0064
		Sc. rank:	963.4

M1

Pos.	148	obs:	exp:
tgt	C	3538	1389.00
tgc	C	52	2201.00
mPD		0.029	0.47
		nPD:	0.06
		N. weight:	0.98
		Sc. PD:	-0.093
		Sc. rank:	231.5

M1

Pos.	149	obs:	exp:
tct	S	0	0.17
tcc	S	1	0.14
tca	S	0	0.23
tcg	S	0	0.06
act	T	0	0.26
acc	T	1	0.23
aca	T	0	0.42
acg	T	0	0.09
agt	S	0	0.22
agc	S	0	0.18
gct	A	2	903.60
gcc	A	3582	694.10
gca	A	3	1597.00
gcg	A	0	392.60
gat	D	0	0.54
gac	D	1	0.46
mPD		0.0045	0.69
		nPD:	0.01
		N. weight:	1.8
		Sc. PD:	-0.27
		Sc. rank:	-3333.0

M1

Pos.	150	obs:	exp:
act	T	2262	941.10
acc	T	1283	808.90
aca	T	15	1502.00
acg	T	30	337.80
mPD		0.48	0.70
		nPD:	0.68
		N. weight:	0.77
		Sc. PD:	0.4
		Sc. rank:	1992.6

M1			
Pos.	151	obs:	exp:
tct	S	0	0.17
tcc	S	0	0.14
tca	S	0	0.23
tcg	S	0	0.06
tgt	C	3537	1389.00
tgc	C	52	2200.00
agt	S	1	0.22
agc	S	0	0.18
---	--	------	------
mPD		0.029	0.48
		nPD:	0.06
		N. weight:	0.98
		Sc. PD:	-0.092
		Sc. rank:	265.7

M1			
Pos.	152	obs:	exp:
gaa	E	8	2012.00
gag	E	3582	1578.00
---	--	------	------
mPD		0.0044	0.49
		nPD:	0.01
		N. weight:	0.91
		Sc. PD:	-0.13
		Sc. rank:	-1501.7

M1			
Pos.	153	obs:	exp:
caa	Q	424	1792.00
cag	Q	3166	1798.00
---	--	------	------
mPD		0.21	0.50
		nPD:	0.42
		N. weight:	0.37
		Sc. PD:	0.097
		Sc. rank:	730.7

M1			
Pos.	154	obs:	exp:
att	I	3576	1316.00
atc	I	9	959.90
ata	I	2	1311.00
act	T	2	0.52
acc	T	0	0.45
aca	T	0	0.84
acg	T	0	0.19
gtt	V	1	0.22
gtc	V	0	0.21
gta	V	0	0.21
gtg	V	0	0.36
---	--	------	------
mPD		0.0078	0.66
		nPD:	0.01
		N. weight:	1.1
		Sc. PD:	-0.16
		Sc. rank:	-1565.7

M1			
Pos.	155	obs:	exp:
gtt	V	0	0.22
gtc	V	0	0.21
gta	V	1	0.21
gtg	V	0	0.36
gct	A	993	904.10
gcc	A	3	694.50
gca	A	2587	1598.00
gcg	A	6	392.80
---	--	------	------
mPD		0.40	0.69
		nPD:	0.59
		N. weight:	0.41
		Sc. PD:	0.18
		Sc. rank:	965.3

M1			
Pos.	156	obs:	exp:
gat	D	3367	1938.00
gac	D	222	1651.00
gaa	E	0	0.56
gag	E	1	0.44
---	--	------	------
mPD		0.12	0.50
		nPD:	0.23
		N. weight:	0.45
		Sc. PD:	0.035
		Sc. rank:	613.0

M1			
Pos.	157	obs:	exp:
tct	S	4	590.90
tcc	S	45	512.80
tca	S	3480	802.50
tcg	S	11	213.60
agt	S	0	767.80
agc	S	0	652.40
gct	A	6	12.60
gcc	A	10	9.68
gca	A	34	22.26
gcg	A	0	5.47
---	--	------	------
mPD		0.069	1.7
		nPD:	0.04
		N. weight:	1.6
		Sc. PD:	-0.18
		Sc. rank:	-244.4

M1			
Pos.	158	obs:	exp:
cct	P	0	0.27
ccc	P	0	0.18
cca	P	0	0.41
ccg	P	1	0.14
cat	H	1	0.58
cac	H	0	0.42
caa	Q	82	1791.00
cag	Q	3506	1797.00
---	--	------	------
mPD		0.046	0.50
		nPD:	0.09
		N. weight:	0.66
		Sc. PD:	-0.042
		Sc. rank:	501.3

M1			
Pos.	159	obs:	exp:
cat	H	3535	2077.00
cac	H	53	1511.00
caa	Q	1	0.50
cag	Q	0	0.50
aat	N	1	0.55
aac	N	0	0.45
---	--	------	------
mPD		0.030	0.49
		nPD:	0.06
		N. weight:	0.54
		Sc. PD:	-0.05
		Sc. rank:	159.5

M1			
Pos.	160	obs:	exp:
cgt	R	4	134.30
cgc	R	0	192.10
cga	R	13	361.80
cgg	R	3568	293.10
aga	R	0	1676.00
agg	R	5	932.70
---	--	------	------
mPD		0.012	0.95
		nPD:	0.01
		N. weight:	2.8
		Sc. PD:	-0.39
		Sc. rank:	-3697.7

M1			
Pos.	161	obs:	exp:
tct	S	3482	599.30
tcc	S	102	520.00
tca	S	6	813.90
tcg	S	0	216.60
agt	S	0	778.70
agc	S	0	661.60
---	--	------	------
mPD		0.058	1.7
		nPD:	0.04
		N. weight:	1.9
		Sc. PD:	-0.22
		Sc. rank:	-508.6

M1			
Pos.	162	obs:	exp:
cat	H	11	2078.00
cac	H	3579	1512.00
---	--	------	------
mPD		0.0061	0.49
		nPD:	0.01
		N. weight:	0.96
		Sc. PD:	-0.14
		Sc. rank:	-1324.9

M1			
Pos.	163	obs:	exp:
cgt	R	0	134.30
cgc	R	0	192.10
cga	R	0	361.80
cgg	R	2	293.10
aga	R	2840	1676.00
agg	R	748	932.70
---	--	------	------
mPD		0.33	0.95
		nPD:	0.35
		N. weight:	0.42
		Sc. PD:	0.08
		Sc. rank:	749.5

M1			
Pos.	164	obs:	exp:
caa	Q	57	1792.00
cag	Q	3532	1797.00
cgt	R	0	0.04
cgc	R	0	0.05
cga	R	0	0.10
cgg	R	1	0.08
aga	R	0	0.47
agg	R	0	0.26
---	--	------	------
mPD		0.032	0.50
		nPD:	0.06
		N. weight:	0.69
		Sc. PD:	-0.063
		Sc. rank:	297.7

M1			
Pos.	165	obs:	exp:
att	I	0	314.10
atc	I	0	229.10
ata	I	856	312.80
atg	M	2733	2733.00
gtt	V	0	0.22
gtc	V	0	0.21
gta	V	0	0.21
gtg	V	1	0.36
---	--	------	------
mPD		0.36	0.40
		nPD:	0.91
		N. weight:	0.27
		Sc. PD:	0.2
		Sc. rank:	741.5

M1			
Pos.	166	obs:	exp:
act	T	0	0.52
acc	T	0	0.45
aca	T	2	0.84
acg	T	0	0.19
gtt	V	0	20.43
gtc	V	0	19.99
gta	V	36	20.26
gtg	V	59	34.32
gct	A	398	880.00
gcc	A	443	675.90
gca	A	2597	1555.00
gcg	A	55	382.30
---	--	------	------
mPD		0.49	0.75
		nPD:	0.65
		N. weight:	0.25
		Sc. PD:	0.12
		Sc. rank:	621.3

M1			
Pos.	167	obs:	exp:
att	I	1	0.73
atc	I	1	0.54
ata	I	0	0.73
act	T	763	936.90
acc	T	2764	805.30
aca	T	46	1495.00
acg	T	1	336.30
aat	N	5	3.27
aac	N	1	2.73
gct	A	5	2.02
gcc	A	1	1.55
gca	A	2	3.56
gcg	A	0	0.88
---	--	------	------
mPD		0.37	0.71
		nPD:	0.52
		N. weight:	0.98
		Sc. PD:	0.36
		Sc. rank:	2120.5

M1			
Pos.	168	obs:	exp:
att	I	16	893.20
atc	I	2418	651.30
ata	I	0	889.50
act	T	57	302.80
acc	T	1055	260.30
aca	T	43	483.30
acg	T	0	108.70
gtt	V	0	0.22
gtc	V	1	0.21
gta	V	0	0.21
gtg	V	0	0.36
---	--	------	------
mPD		0.50	1.1
		nPD:	0.45
		N. weight:	1.4
		Sc. PD:	0.4
		Sc. rank:	2769.3

M1			
Pos.	169	obs:	exp:
tct	S	0	0.33
tcc	S	1	0.29
tca	S	0	0.45
tcg	S	0	0.12
act	T	32	940.60
acc	T	3548	808.50
aca	T	8	1501.00
acg	T	0	337.60
agt	S	0	0.43
agc	S	1	0.37
---	--	------	------
mPD		0.023	0.70
		nPD:	0.03
		N. weight:	1.6
		Sc. PD:	-0.19
		Sc. rank:	-615.3

M1			
Pos.	170	obs:	exp:
act	T	0	0.26
acc	T	1	0.23
aca	T	0	0.42
acg	T	0	0.09
aat	N	79	1957.00
aac	N	3510	1632.00
---	--	------	------
mPD		0.044	0.50
		nPD:	0.09
		N. weight:	0.77
		Sc. PD:	-0.051
		Sc. rank:	548.7

M1			
Pos.	171	obs:	exp:
cct	P	0	980.80
ccc	P	0	648.20
cca	P	3560	1457.00
ccg	P	29	503.20
act	T	0	0.26
acc	T	0	0.23
aca	T	1	0.42
acg	T	0	0.09
---	--	------	------
mPD		0.017	0.71
		nPD:	0.02
		N. weight:	0.98
		Sc. PD:	-0.13
		Sc. rank:	-772.8

M1			
Pos.	172	obs:	exp:
tta	L	26	331.70
ttg	L	0	681.60
ctt	L	0	712.40
ctc	L	0	573.90
cta	L	3540	531.00
ctg	L	22	757.30
att	I	0	0.73
atc	I	0	0.54
ata	I	2	0.73
---	--	------	------
mPD		0.028	1.1
		nPD:	0.02
		N. weight:	2.1
		Sc. PD:	-0.27
		Sc. rank:	-1501.5

M1			
Pos.	173	obs:	exp:
att	I	53	1317.00
atc	I	3506	960.70
ata	I	31	1312.00
---	--	------	------
mPD		0.046	0.66
		nPD:	0.07
		N. weight:	1.3
		Sc. PD:	-0.11
		Sc. rank:	667.4

M1			
Pos.	174	obs:	exp:
cgt	R	0	134.20
cgc	R	0	191.90
cga	R	0	361.30
cgg	R	1	292.70
aaa	K	0	2.85
aag	K	5	2.15
aga	R	1311	1674.00
agg	R	2273	931.40
---	--	------	------
mPD		0.47	0.96
		nPD:	0.49
		N. weight:	0.54
		Sc. PD:	0.18
		Sc. rank:	1135.5

M1			
Pos.	175	obs:	exp:
cat	H	3550	2077.00
cac	H	38	1511.00
cgt	R	2	0.07
cgc	R	0	0.11
cga	R	0	0.20
cgg	R	0	0.16
aga	R	0	0.93
agg	R	0	0.52
---	--	------	------
mPD		0.022	0.49
		nPD:	0.05
		N. weight:	0.56
		Sc. PD:	-0.061
		Sc. rank:	-54.3

M1

Pos.	176	obs:	exp:
gaa	E	1545	2012.00
gag	E	2045	1578.00
---	--	------	------
mPD		0.49	0.49
		nPD:	1.
		N. weight:	0.04
		Sc. PD:	0.033
		Sc. rank:	109.1

,

M1

Pos.	177	obs:	exp:
tct	S	0	0.17
tcc	S	0	0.14
tca	S	0	0.23
tcg	S	0	0.06
aat	N	61	1957.00
aac	N	3527	1631.00
agt	S	0	0.22
agc	S	1	0.18
gtt	V	0	0.22
gtc	V	1	0.21
gta	V	0	0.21
gtg	V	0	0.36
---	--	------	------
mPD		0.035	0.50
		nPD:	0.07
		N. weight:	0.79
		Sc. PD:	-0.067
		Sc. rank:	407.7

,

M1

Pos.	178	obs:	exp:
cgt	R	0	134.30
cgc	R	0	192.10
cga	R	0	361.80
cgg	R	0	293.10
aga	R	3522	1676.00
agg	R	68	932.70
---	--	------	------
mPD		0.037	0.95
		nPD:	0.04
		N. weight:	0.77
		Sc. PD:	-0.089
		Sc. rank:	-170.9

,

M1

Pos.	179	obs:	exp:
atg	M	3589	3589.00
gtt	V	0	0.22
gtc	V	0	0.21
gta	V	0	0.21
gtg	V	1	0.36
---	--	------	------
mPD		0.00056	0.00091
		nPD:	0.61
		N. weight:	0.00032
		Sc. PD:	0.00014
		Sc. rank:	0.8

,

M1

Pos.	180	obs:	exp:
gtt	V	7	772.10
gtc	V	0	755.30
gta	V	120	765.50
gtg	V	3463	1297.00
---	--	------	------
mPD		0.068	0.73
		nPD:	0.09
		N. weight:	1.
		Sc. PD:	-0.061
		Sc. rank:	787.9

,

M1

Pos.	181	obs:	exp:
tta	L	0	331.70
ttg	L	859	681.60
ctt	L	13	712.40
ctc	L	1	573.90
cta	L	157	531.00
ctg	L	2558	757.30
atg	M	2	2.00
---	--	------	------
mPD		0.46	1.1
		nPD:	0.41
		N. weight:	0.97
		Sc. PD:	0.24
		Sc. rank:	1862.1

,

M1

Pos.	182	obs:	exp:
act	T	0	0.52
acc	T	2	0.45
aca	T	0	0.84
acg	T	0	0.19
gct	A	322	903.90
gcc	A	3175	694.30
gca	A	91	1597.00
gcg	A	0	392.70
---	--	------	------
mPD		0.21	0.69
		nPD:	0.3
		N. weight:	1.3
		Sc. PD:	0.2
		Sc. rank:	2188.0

,

M1

Pos.	183	obs:	exp:
tct	S	0	599.30
tcc	S	0	520.00
tca	S	0	813.90
tcg	S	0	216.60
agt	S	910	778.70
agc	S	2680	661.60
---	--	------	------
mPD		0.38	1.7
		nPD:	0.23
		N. weight:	1.2
		Sc. PD:	0.09
		Sc. rank:	1637.1

,

M1

Pos.	184	obs:	exp:
act	T	3530	941.10
acc	T	43	808.90
aca	T	16	1502.00
acg	T	1	337.80
---	--	------	------
mPD		0.033	0.70
		nPD:	0.05
		N. weight:	1.4
		Sc. PD:	-0.15
		Sc. rank:	-93.8

,

M1

Pos.	185	obs:	exp:
att	I	0	0.37
atc	I	0	0.27
ata	I	1	0.37
act	T	22	940.90
acc	T	18	808.70
aca	T	3410	1502.00
acg	T	139	337.70
---	--	------	------
mPD		0.096	0.70
		nPD:	0.14
		N. weight:	0.8
		Sc. PD:	-0.014
		Sc. rank:	783.4

,

M1

Pos.	186	obs:	exp:
cct	P	1	0.27
ccc	P	0	0.18
cca	P	0	0.41
ccg	P	0	0.14
act	T	1	0.26
acc	T	0	0.23
aca	T	0	0.42
acg	T	0	0.09
gct	A	3528	903.60
gcc	A	44	694.10
gca	A	12	1597.00
gcg	A	3	392.60
ggt	G	1	0.14
ggc	G	0	0.13
gga	G	0	0.44
ggg	G	0	0.29
---	--	------	------
mPD		0.034	0.69
		nPD:	0.05
		N. weight:	1.5
		Sc. PD:	-0.15
		Sc. rank:	-50.8

,

M1

Pos.	187	obs:	exp:
cgt	R	0	0.04
cgc	R	0	0.05
cga	R	0	0.10
cgg	R	0	0.08
aaa	K	69	2044.00
aag	K	3520	1545.00
aga	R	0	0.47
agg	R	1	0.26
---	--	------	------
mPD		0.038	0.49
		nPD:	0.08
		N. weight:	0.84
		Sc. PD:	-0.065
		Sc. rank:	537.3

,

M1

Pos.	188	obs:	exp:
gct	A	3145	904.40
gcc	A	405	694.70
gca	A	34	1598.00
gcg	A	6	392.90
---	--	------	------
mPD		0.22	0.69
		nPD:	0.32
		N. weight:	1.1
		Sc. PD:	0.18
		Sc. rank:	1889.0

,

M1

Pos.	189	obs:	exp:
att	I	0	0.37
atc	I	0	0.27
ata	I	1	0.37
atg	M	3589	3589.00
---	--	------	------
mPD		0.00056	0.00056
		nPD:	1.
		N. weight:	0.00032
		Sc. PD:	0.00027
		Sc. rank:	0.9

,

M1

Pos.	190	obs:	exp:
gaa	E	61	2012.00
gag	E	3529	1578.00
---	--	------	------
mPD		0.033	0.49
		nPD:	0.07
		N. weight:	0.83
		Sc. PD:	-0.072
		Sc. rank:	371.7

,

M1

Pos.	191	obs:	exp:
caa	Q	142	1792.00
cag	Q	3448	1798.00
---	--	------	------
mPD		0.076	0.50
		nPD:	0.15
		N. weight:	0.6
		Sc. PD:	-0.002
		Sc. rank:	601.4

,

M1

Pos.	192	obs:	exp:
atg	M	3588	3588.00
gtt	V	0	0.43
gtc	V	0	0.42
gta	V	0	0.43
gtg	V	2	0.72
---	--	------	------
mPD		0.0011	0.0018
		nPD:	0.61
		N. weight:	0.00064
		Sc. PD:	0.00029
		Sc. rank:	1.5

,

M1

Pos.	193	obs:	exp:
gct	A	971	904.10
gcc	A	0	694.50
gca	A	146	1598.00
gcg	A	2472	392.80
gaa	E	0	0.56
gag	E	1	0.44
---	--	------	------
mPD		0.45	0.69
		nPD:	0.65
		N. weight:	1.4
		Sc. PD:	0.67
		Sc. rank:	3431.2

,

M1

Pos.	194	obs:	exp:
gtt	V	0	0.22
gtc	V	0	0.21
gta	V	1	0.21
gtg	V	0	0.36
ggt	G	9	519.00
ggc	G	0	471.40
gga	G	3225	1573.00
ggg	G	355	1026.00
---	--	------	------
mPD		0.18	0.69
		nPD:	0.27
		N. weight:	0.6
		Sc. PD:	0.066
		Sc. rank:	915.5

,

M1

Pos.	195	obs:	exp:
tct	S	1	599.30
tcc	S	0	520.00
tca	S	2687	813.90
tcg	S	902	216.60
agt	S	0	778.70
agc	S	0	661.60
---	--	------	------
mPD		0.38	1.7
		nPD:	0.23
		N. weight:	1.4
		Sc. PD:	0.1
		Sc. rank:	1837.5

,

M1			
Pos.	196	obs:	exp:
tct	S	0	599.10
tcc	S	0	519.90
tca	S	0	813.60
tcg	S	0	216.50
aat	N	1	0.55
aac	N	0	0.45
agt	S	2319	778.50
agc	S	1270	661.40
---	--	------	------
mPD		0.46	1.7
		nPD:	0.28
		N. weight:	1.1
		Sc. PD:	0.13
		Sc. rank:	1659.0

M1			
Pos.	197	obs:	exp:
caa	Q	0	0.50
cag	Q	1	0.50
aaa	K	0	0.57
aag	K	1	0.43
gat	D	1	0.54
gac	D	0	0.46
gaa	E	337	2011.00
gag	E	3250	1576.00
---	--	------	------
mPD		0.17	0.49
		nPD:	0.35
		N. weight:	0.55
		Sc. PD:	0.11
		Sc. rank:	997.4

M1			
Pos.	198	obs:	exp:
cat	H	0	1.74
cac	H	3	1.26
caa	Q	1190	1791.00
cag	Q	2397	1796.00
---	--	------	------
mPD		0.44	0.50
		nPD:	0.89
		N. weight:	0.067
		Sc. PD:	0.049
		Sc. rank:	181.5

M1			
Pos.	199	obs:	exp:
gtt	V	0	0.22
gtc	V	0	0.21
gta	V	1	0.21
gtg	V	0	0.36
gct	A	5	904.10
gcc	A	1	694.50
gca	A	3572	1598.00
gcg	A	11	392.80
---	--	------	------
mPD		0.010	0.69
		nPD:	0.01
		N. weight:	0.89
		Sc. PD:	-0.12
		Sc. rank:	-1016.6

M1			
Pos.	200	obs:	exp:
tct	S	0	1.84
tcc	S	0	1.59
tca	S	8	2.49
tcg	S	3	0.66
atg	M	4	4.00
act	T	0	1.05
acc	T	0	0.90
aca	T	4	1.67
acg	T	0	0.38
agt	S	0	2.39
agc	S	0	2.03
gtt	V	0	81.73
gtc	V	0	79.95
gta	V	0	81.03
gtg	V	380	137.30
gct	A	51	803.90
gcc	A	0	617.50
gca	A	1014	1420.00
gcg	A	2126	349.30
---	--	------	------
mPD		0.63	0.91
		nPD:	0.69
		N. weight:	1.2
		Sc. PD:	0.63
		Sc. rank:	3125.9

M1			
Pos.	201	obs:	exp:
aaa	K	1	0.57
aag	K	0	0.43
gaa	E	3503	2012.00
gag	E	86	1577.00
---	--	------	------
mPD		0.047	0.49
		nPD:	0.1
		N. weight:	0.54
		Sc. PD:	-0.031
		Sc. rank:	430.4

M1			
Pos.	202	obs:	exp:
cct	P	0	0.27
ccc	P	1	0.18
cca	P	0	0.41
ccg	P	0	0.14
gct	A	88	904.10
gcc	A	3497	694.50
gca	A	4	1598.00
gcg	A	0	392.80
---	--	------	------
mPD		0.051	0.69
		nPD:	0.07
		N. weight:	1.7
		Sc. PD:	-0.14
		Sc. rank:	944.4

M1			
Pos.	203	obs:	exp:
att	I	7	4.40
atc	I	0	3.21
ata	I	5	4.39
atg	M	3578	3578.00
---	--	------	------
mPD		0.0067	0.0067
		nPD:	1.
		N. weight:	0.0017
		Sc. PD:	0.0014
		Sc. rank:	4.6

M1			
Pos.	204	obs:	exp:
gaa	E	228	2012.00
gag	E	3362	1578.00
---	--	------	------
mPD		0.12	0.49
		nPD:	0.24
		N. weight:	0.65
		Sc. PD:	0.055
		Sc. rank:	907.5

M1			
Pos.	205	obs:	exp:
att	I	382	337.20
atc	I	536	245.90
ata	I	1	335.80
gtt	V	1085	574.50
gtc	V	1585	561.90
gta	V	1	569.50
gtg	V	0	965.10
---	--	------	------
mPD		0.87	1.1
		nPD:	0.77
		N. weight:	0.88
		Sc. PD:	0.53
		Sc. rank:	2345.2

M1			
Pos.	206	obs:	exp:
cct	P	1	0.27
ccc	P	0	0.18
cca	P	0	0.41
ccg	P	0	0.14
gct	A	2583	904.10
gcc	A	935	694.50
gca	A	71	1598.00
gcg	A	0	392.80
---	--	------	------
mPD		0.41	0.69
		nPD:	0.6
		N. weight:	0.88
		Sc. PD:	0.39
		Sc. rank:	2066.3

M1			
Pos.	207	obs:	exp:
tct	S	0	183.60
tcc	S	0	159.30
tca	S	0	249.40
tcg	S	0	66.36
aat	N	2362	1357.00
aac	N	127	1132.00
aaa	K	1	0.57
aag	K	0	0.43
agt	S	1096	238.60
agc	S	4	202.70
---	--	------	------
mPD		0.50	1.3
		nPD:	0.37
		N. weight:	0.85
		Sc. PD:	0.18
		Sc. rank:	1544.6

M1			
Pos.	208	obs:	exp:
tct	S	0	0.17
tcc	S	0	0.14
tca	S	0	0.23
tcg	S	1	0.06
cat	H	0	0.58
cac	H	1	0.42
caa	Q	18	1790.00
cag	Q	3568	1796.00
cgt	R	0	0.07
cgc	R	0	0.11
cga	R	0	0.20
cgg	R	2	0.16
agt	S	0	0.22
agc	S	0	0.18
aga	R	0	0.93
agg	R	0	0.52
---	--	------	------
mPD		0.013	0.50
		nPD:	0.03
		N. weight:	0.75
		Sc. PD:	-0.097
		Sc. rank:	-497.8

M1			
Pos.	209	obs:	exp:
act	T	6	1.57
acc	T	0	1.35
aca	T	0	2.51
acg	T	0	0.56
gct	A	3478	902.90
gcc	A	95	693.50
gca	A	11	1595.00
gcg	A	0	392.30
---	--	------	------
mPD		0.061	0.69
		nPD:	0.09
		N. weight:	1.4
		Sc. PD:	-0.094
		Sc. rank:	1046.2

M1			
Pos.	210	obs:	exp:
cgt	R	0	134.20
cgc	R	0	191.90
cga	R	0	361.40
cgg	R	0	292.80
aaa	K	0	2.28
aag	K	4	1.72
aga	R	578	1674.00
agg	R	3008	931.60
---	--	------	------
mPD		0.27	0.96
		nPD:	0.28
		N. weight:	0.92
		Sc. PD:	0.12
		Sc. rank:	1457.4

M1			
Pos.	211	obs:	exp:
tta	L	0	0.09
ttg	L	0	0.19
ctt	L	0	0.20
ctc	L	0	0.16
cta	L	0	0.15
ctg	L	1	0.21
caa	Q	702	1790.00
cag	Q	2884	1796.00
aaa	K	1	1.71
aag	K	2	1.29
---	--	------	------
mPD		0.32	0.50
		nPD:	0.63
		N. weight:	0.23
		Sc. PD:	0.11
		Sc. rank:	557.3

M1			
Pos.	212	obs:	exp:
att	I	0	0.73
atc	I	1	0.54
ata	I	1	0.73
atg	M	3586	3586.00
gtt	V	0	0.43
gtc	V	0	0.42
gta	V	0	0.43
gtg	V	2	0.72
---	--	------	------
mPD		0.0022	0.0029
		nPD:	0.76
		N. weight:	0.0012
		Sc. PD:	0.00069
		Sc. rank:	3.1

M1			
Pos.	213	obs:	exp:
att	I	0	0.37
atc	I	0	0.27
ata	I	1	0.37
gtt	V	2	770.90
gtc	V	0	754.00
gta	V	62	764.20
gtg	V	3520	1295.00
gct	A	0	0.25
gcc	A	0	0.19
gca	A	0	0.45
gcg	A	1	0.11
gaa	E	0	0.56
gag	E	1	0.44
ggt	G	0	0.43
ggc	G	0	0.39
gga	G	0	1.31
ggg	G	3	0.86
---	--	------	------
mPD		0.039	0.74
		nPD:	0.05
		N. weight:	1.1
		Sc. PD:	-0.11
		Sc. rank:	96.6

M1			
Pos.	214	obs:	exp:
cat	H	1	2.89
cac	H	4	2.11
caa	Q	24	1789.00
cag	Q	3559	1794.00
cgt	R	0	0.07
cgc	R	0	0.11
cga	R	0	0.20
cgg	R	2	0.16
aga	R	0	0.93
agg	R	0	0.52
---	--	------	------
mPD		0.017	0.50
		nPD:	0.03
		N. weight:	0.74
		Sc. PD:	-0.089
		Sc. rank:	-245.0

M1			
Pos.	215	obs:	exp:
tct	S	0	0.17
tcc	S	0	0.14
tca	S	1	0.23
tcg	S	0	0.06
act	T	0	0.26
acc	T	1	0.23
aca	T	0	0.42
acg	T	0	0.09
agt	S	0	0.22
agc	S	0	0.18
gct	A	50	903.90
gcc	A	3	694.30
gca	A	2628	1597.00
gcg	A	907	392.70
---	--	------	------
mPD		0.40	0.69
		nPD:	0.58
		N. weight:	0.61
		Sc. PD:	0.26
		Sc. rank:	1399.0

M1

Pos.	216	obs:	exp:
atg	M	3590	3590.00
---	--	------	------
mPD		0	0
		nPD:	1.
		N. weight:	0.
		Sc. PD:	0
		Sc. rank:	0

M1

Pos.	217	obs:	exp:
cgt	R	0	134.20
cgc	R	0	192.00
cga	R	0	361.50
cgg	R	0	292.90
att	I	0	0.37
atc	I	0	0.27
ata	I	1	0.37
aaa	K	0	0.57
aag	K	1	0.43
aga	R	533	1675.00
agg	R	3054	931.90
ggt	G	0	0.14
ggc	G	0	0.13
gga	G	0	0.44
ggg	G	1	0.29
---	--	------	------
mPD		0.25	0.96
		nPD:	0.27
		N. weight:	0.95
		Sc. PD:	0.11
		Sc. rank:	1468.1

M1

Pos.	218	obs:	exp:
act	T	9	940.60
acc	T	910	808.50
aca	T	2647	1501.00
acg	T	22	337.60
gct	A	0	0.50
gcc	A	0	0.39
gca	A	2	0.89
gcg	A	0	0.22
---	--	------	------
mPD		0.39	0.70
		nPD:	0.56
		N. weight:	0.48
		Sc. PD:	0.19
		Sc. rank:	1088.8

M1

Pos.	219	obs:	exp:
att	I	3502	1297.00
atc	I	32	946.00
ata	I	1	1292.00
atg	M	2	2.00
gtt	V	6	11.40
gtc	V	43	11.15
gta	V	4	11.30
gtg	V	0	19.15
---	--	------	------
mPD		0.074	0.69
		nPD:	0.11
		N. weight:	1.1
		Sc. PD:	-0.052
		Sc. rank:	893.0

M1

Pos.	220	obs:	exp:
ggt	G	0	519.20
ggc	G	0	471.60
gga	G	982	1573.00
ggg	G	2608	1026.00
---	--	------	------
mPD		0.40	0.69
		nPD:	0.58
		N. weight:	0.62
		Sc. PD:	0.26
		Sc. rank:	1431.3

M1

Pos.	221	obs:	exp:
act	T	3562	941.10
acc	T	28	808.90
aca	T	0	1502.00
acg	T	0	337.80
---	--	------	------
mPD		0.015	0.70
		nPD:	0.02
		N. weight:	1.5
		Sc. PD:	-0.19
		Sc. rank:	-1189.5

M1

Pos.	222	obs:	exp:
cat	H	3466	2077.00
cac	H	122	1511.00
caa	Q	1	0.50
cag	Q	0	0.50
aat	N	1	0.55
aac	N	0	0.45
---	--	------	------
mPD		0.067	0.49
		nPD:	0.14
		N. weight:	0.47
		Sc. PD:	-0.0086
		Sc. rank:	447.7

M1

Pos.	223	obs:	exp:
cct	P	3448	981.10
ccc	P	133	648.40
cca	P	3	1457.00
ccg	P	6	503.30
---	--	------	------
mPD		0.076	0.71
		nPD:	0.11
		N. weight:	1.3
		Sc. PD:	-0.061
		Sc. rank:	1124.9

M1

Pos.	224	obs:	exp:
tct	S	0	160.90
tcc	S	0	139.60
tca	S	0	218.50
tcg	S	0	58.16
aat	N	226	1432.00
aac	N	2400	1194.00
agt	S	4	209.10
agc	S	960	177.70
---	--	------	------
mPD		0.51	1.3
		nPD:	0.41
		N. weight:	0.91
		Sc. PD:	0.22
		Sc. rank:	1718.3

M1

Pos.	225	obs:	exp:
tct	S	2459	598.70
tcc	S	1126	519.60
tca	S	1	813.20
tcg	S	1	216.40
tgt	C	1	0.39
tgc	C	0	0.61
act	T	0	0.52
acc	T	2	0.45
aca	T	0	0.84
acg	T	0	0.19
agt	S	0	778.00
agc	S	0	661.00
---	--	------	------
mPD		0.43	1.7
		nPD:	0.26
		N. weight:	1.4
		Sc. PD:	0.15
		Sc. rank:	2035.0

M1

Pos.	226	obs:	exp:
tct	S	0	599.30
tcc	S	0	520.00
tca	S	0	813.90
tcg	S	0	216.60
agt	S	3578	778.70
agc	S	12	661.60
---	--	------	------
mPD		0.0067	1.7
		nPD:	0.
		N. weight:	1.7
		Sc. PD:	-0.26
		Sc. rank:	-3570.2

M1

Pos.	227	obs:	exp:
cct	P	1	0.27
ccc	P	0	0.18
cca	P	0	0.41
ccg	P	0	0.14
act	T	23	18.61
acc	T	3	16.00
aca	T	45	29.71
acg	T	0	6.68
gct	A	2556	886.00
gcc	A	955	680.60
gca	A	4	1565.00
gcg	A	2	384.90
gat	D	1	0.54
gac	D	0	0.46
---	--	------	------
mPD		0.45	0.73
		nPD:	0.62
		N. weight:	0.96
		Sc. PD:	0.44
		Sc. rank:	2293.8

M1

Pos.	228	obs:	exp:
ggt	G	3579	519.20
ggc	G	6	471.60
gga	G	4	1573.00
ggg	G	1	1026.00
---	--	------	------
mPD		0.0061	0.69
		nPD:	0.01
		N. weight:	2.2
		Sc. PD:	-0.31
		Sc. rank:	-3599.6

M1

Pos.	229	obs:	exp:
tta	L	11	331.30
ttg	L	11	680.70
ctt	L	1	711.40
ctc	L	0	573.10
cta	L	332	530.30
ctg	L	3228	756.30
atg	M	5	5.00
gtt	V	0	0.43
gtc	V	0	0.42
gta	V	0	0.43
gtg	V	2	0.72
---	--	------	------
mPD		0.19	1.1
		nPD:	0.17
		N. weight:	1.4
		Sc. PD:	0.018
		Sc. rank:	1515.9

M1

Pos.	230	obs:	exp:
cgt	R	0	128.90
cgc	R	0	184.40
cga	R	0	347.20
cgg	R	0	281.30
aaa	K	111	82.56
aag	K	34	62.44
aga	R	3421	1608.00
agg	R	24	895.00
---	--	------	------
mPD		0.11	1.0
		nPD:	0.11
		N. weight:	0.79
		Sc. PD:	-0.038
		Sc. rank:	669.8

M1

Pos.	231	obs:	exp:
aat	N	1	0.55
aac	N	0	0.45
gat	D	3535	1937.00
gac	D	53	1651.00
gaa	E	1	0.56
gag	E	0	0.44
---	--	------	------
mPD		0.030	0.50
		nPD:	0.06
		N. weight:	0.62
		Sc. PD:	-0.058
		Sc. rank:	153.0

M1

Pos.	232	obs:	exp:
att	I	1	0.37
atc	I	0	0.27
ata	I	0	0.37
aat	N	2541	1412.00
aac	N	49	1178.00
gat	D	992	539.40
gac	D	7	459.60
---	--	------	------
mPD		0.43	0.90
		nPD:	0.48
		N. weight:	0.61
		Sc. PD:	0.2
		Sc. rank:	1264.9

M1

Pos.	233	obs:	exp:
ttt	F	1	0.42
ttc	F	0	0.58
tta	L	0	331.70
ttg	L	0	681.60
ctt	L	2712	712.40
ctc	L	876	573.90
cta	L	0	531.00
ctg	L	0	757.30
att	I	1	0.37
atc	I	0	0.27
ata	I	0	0.37
---	--	------	------
mPD		0.37	1.1
		nPD:	0.33
		N. weight:	1.3
		Sc. PD:	0.22
		Sc. rank:	2212.0

M1

Pos.	234	obs:	exp:
tta	L	0	330.20
ttg	L	0	678.40
ctt	L	3563	709.00
ctc	L	8	571.20
cta	L	0	528.50
ctg	L	0	753.70
att	I	19	6.97
atc	I	0	5.08
ata	I	0	6.94
---	--	------	------
mPD		0.015	1.1
		nPD:	0.01
		N. weight:	1.8
		Sc. PD:	-0.25
		Sc. rank:	-2329.8

M1

Pos.	235	obs:	exp:
aaa	K	1	0.57
aag	K	0	0.43
gaa	E	3587	2011.00
gag	E	0	1576.00
ggt	G	0	0.29
ggc	G	0	0.26
gga	G	2	0.88
ggg	G	0	0.57
---	--	------	------
mPD		0.0017	0.49
		nPD:	0.
		N. weight:	0.66
		Sc. PD:	-0.099
		Sc. rank:	-1473.5

M1			
Pos.	236	obs:	exp:
aat	N	3567	1957.00
aac	N	22	1632.00
gat	D	1	0.54
gac	D	0	0.46
---	--	------	------
mPD		0.013	0.50
		nPD:	0.03
		N. weight:	0.65
		Sc. PD:	-0.083
		Sc. rank:	-388.5

,

M1			
Pos.	237	obs:	exp:
tta	L	80	331.80
ttg	L	3433	681.80
ctt	L	0	712.60
ctc	L	0	574.10
cta	L	0	531.10
ctg	L	76	757.50
gtt	V	0	0.22
gtc	V	0	0.21
gta	V	0	0.21
gtg	V	1	0.36
---	--	------	------
mPD		0.086	1.1
		nPD:	0.08
		N. weight:	1.7
		Sc. PD:	-0.13
		Sc. rank:	1001.5

,

M1			
Pos.	238	obs:	exp:
caa	Q	0	1792.00
cag	Q	3590	1798.00
---	--	------	------
mPD		0	0.50
		nPD:	0.
		N. weight:	0.78
		Sc. PD:	-0.12
		Sc. rank:	-2136.6

,

M1			
Pos.	239	obs:	exp:
act	T	0	2.10
acc	T	8	1.80
aca	T	0	3.35
acg	T	0	0.75
gtt	V	0	1.72
gtc	V	8	1.68
gta	V	0	1.71
gtg	V	0	2.89
gct	A	60	899.90
gcc	A	3117	691.20
gca	A	393	1590.00
gcg	A	2	391.00
gat	D	0	1.08
gac	D	2	0.92
---	--	------	------
mPD		0.24	0.70
		nPD:	0.34
		N. weight:	1.3
		Sc. PD:	0.23
		Sc. rank:	2249.2

,

M1			
Pos.	240	obs:	exp:
ttt	F	0	0.83
ttc	F	2	1.17
tat	Y	54	1721.00
tac	Y	3534	1867.00
---	--	------	------
mPD		0.031	0.50
		nPD:	0.06
		N. weight:	0.65
		Sc. PD:	-0.061
		Sc. rank:	186.0

,

M1			
Pos.	241	obs:	exp:
cat	H	0	1.16
cac	H	2	0.84
caa	Q	915	1791.00
cag	Q	2673	1797.00
---	--	------	------
mPD		0.38	0.50
		nPD:	0.76
		N. weight:	0.14
		Sc. PD:	0.086
		Sc. rank:	377.3

,

M1			
Pos.	242	obs:	exp:
act	T	0	1.31
acc	T	1	1.13
aca	T	4	2.09
acg	T	0	0.47
aat	N	0	34.36
aac	N	63	28.64
aaa	K	3505	2005.00
aag	K	17	1517.00
---	--	------	------
mPD		0.047	0.51
		nPD:	0.09
		N. weight:	0.61
		Sc. PD:	-0.038
		Sc. rank:	471.0

,

M1			
Pos.	243	obs:	exp:
cgt	R	2	134.30
cgc	R	0	192.10
cga	R	2195	361.80
cgg	R	1015	293.10
aga	R	354	1676.00
agg	R	24	932.70
---	--	------	------
mPD		0.60	0.95
		nPD:	0.63
		N. weight:	1.4
		Sc. PD:	0.68
		Sc. rank:	3559.9

,

M1			
Pos.	244	obs:	exp:
cgt	R	0	0.04
cgc	R	0	0.05
cga	R	0	0.10
cgg	R	0	0.08
atg	M	3589	3589.00
aga	R	0	0.47
agg	R	1	0.26
---	--	------	------
mPD		0.00056	0.0011
		nPD:	0.52
		N. weight:	0.00043
		Sc. PD:	0.00015
		Sc. rank:	0.9

,

M1			
Pos.	245	obs:	exp:
ggt	G	5	519.20
ggc	G	1	471.60
gga	G	3567	1573.00
ggg	G	17	1026.00
---	--	------	------
mPD		0.013	0.69
		nPD:	0.02
		N. weight:	0.89
		Sc. PD:	-0.12
		Sc. rank:	-820.7

,

M1			
Pos.	246	obs:	exp:
tta	L	0	0.18
ttg	L	2	0.38
ctt	L	0	0.40
ctc	L	0	0.32
cta	L	0	0.30
ctg	L	0	0.42
gtt	V	10	771.50
gtc	V	0	754.70
gta	V	31	764.80
gtg	V	3546	1296.00
ggt	G	0	0.14
ggc	G	0	0.13
gga	G	0	0.44
ggg	G	1	0.29
---	--	------	------
mPD		0.024	0.74
		nPD:	0.03
		N. weight:	1.1
		Sc. PD:	-0.13
		Sc. rank:	-452.0

,

M1			
Pos.	247	obs:	exp:
caa	Q	1072	1792.00
cag	Q	2518	1798.00
---	--	------	------
mPD		0.42	0.50
		nPD:	0.84
		N. weight:	0.095
		Sc. PD:	0.064
		Sc. rank:	257.5

,

M1			
Pos.	248	obs:	exp:
tta	L	0	83.58
ttg	L	0	171.70
ctt	L	0	179.50
ctc	L	0	144.60
cta	L	0	133.80
ctg	L	904	190.80
att	I	8	8.81
atc	I	1	6.42
ata	I	15	8.77
atg	M	2661	2661.00
gtt	V	0	0.22
gtc	V	0	0.21
gta	V	0	0.21
gtg	V	1	0.36
---	--	------	------
mPD		0.39	0.68
		nPD:	0.57
		N. weight:	0.45
		Sc. PD:	0.18
		Sc. rank:	1020.2

,

M1			
Pos.	249	obs:	exp:
cat	H	84	48.63
cac	H	0	35.37
caa	Q	52	1749.00
cag	Q	3451	1754.00
cgt	R	0	0.11
cgc	R	0	0.16
cga	R	0	0.30
cgg	R	3	0.24
aga	R	0	1.40
agg	R	0	0.78
---	--	------	------
mPD		0.075	0.53
		nPD:	0.14
		N. weight:	0.7
		Sc. PD:	-0.0084
		Sc. rank:	694.2

,

M1			
Pos.	250	obs:	exp:
cgt	R	2	134.30
cgc	R	0	192.10
cga	R	3493	361.80
cgg	R	80	293.10
aga	R	15	1676.00
agg	R	0	932.70
---	--	------	------
mPD		0.053	0.95
		nPD:	0.06
		N. weight:	2.4
		Sc. PD:	-0.24
		Sc. rank:	460.4

,

M1			
Pos.	251	obs:	exp:
ttt	F	0	1498.00
ttc	F	3590	2092.00
---	--	------	------
mPD		0	0.49
		nPD:	0.
		N. weight:	0.61
		Sc. PD:	-0.094
		Sc. rank:	-1667.7

,

M1			
Pos.	252	obs:	exp:
cgt	R	0	0.45
cgc	R	0	0.64
cga	R	0	1.21
cgg	R	0	0.98
aaa	K	66	2037.00
aag	K	3512	1541.00
aga	R	0	5.60
agg	R	12	3.12
---	--	------	------
mPD		0.043	0.50
		nPD:	0.09
		N. weight:	0.85
		Sc. PD:	-0.058
		Sc. rank:	590.1

,

M1			
Pos.	253	obs:	exp:
taa	.	4	1324.00
tag	.	0	1231.00
tga	.	3586	1035.00
---	--	------	------
mPD		0.0022	0.86
		nPD:	0.
		N. weight:	1.4
		Sc. PD:	-0.21
		Sc. rank:	-3198.4

}

Amino acid level conservation per codon

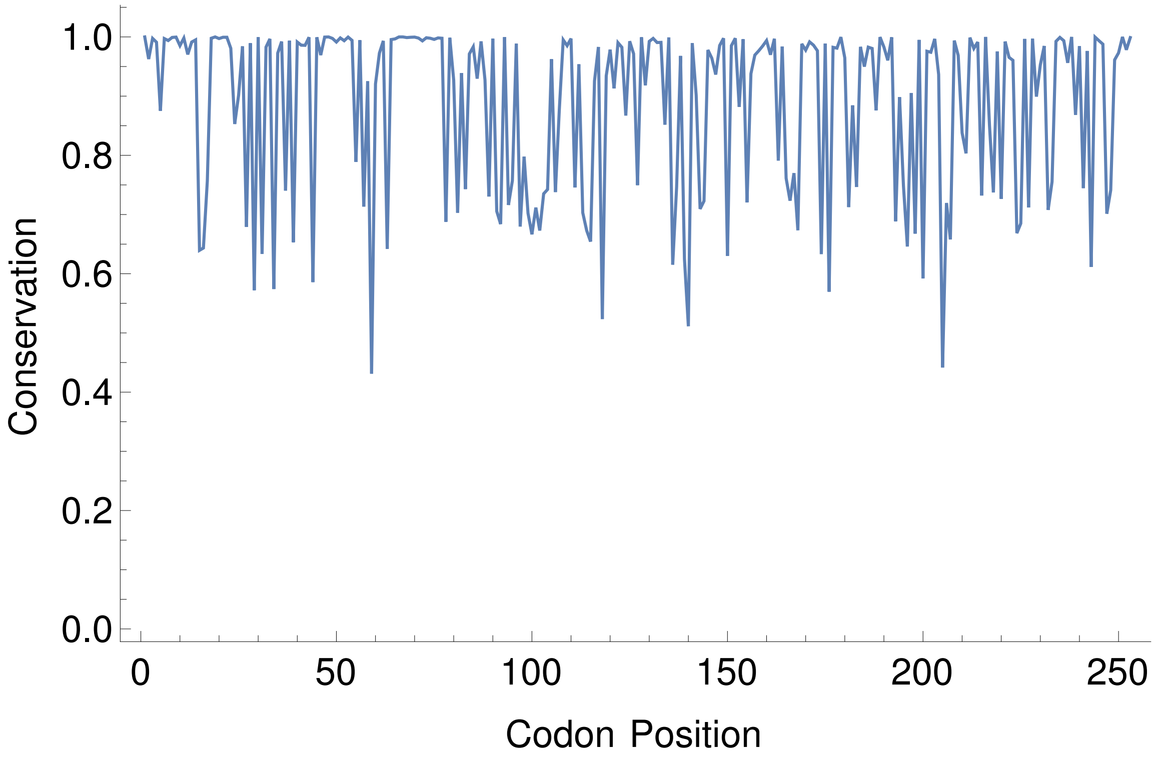

Conservation analysis plots

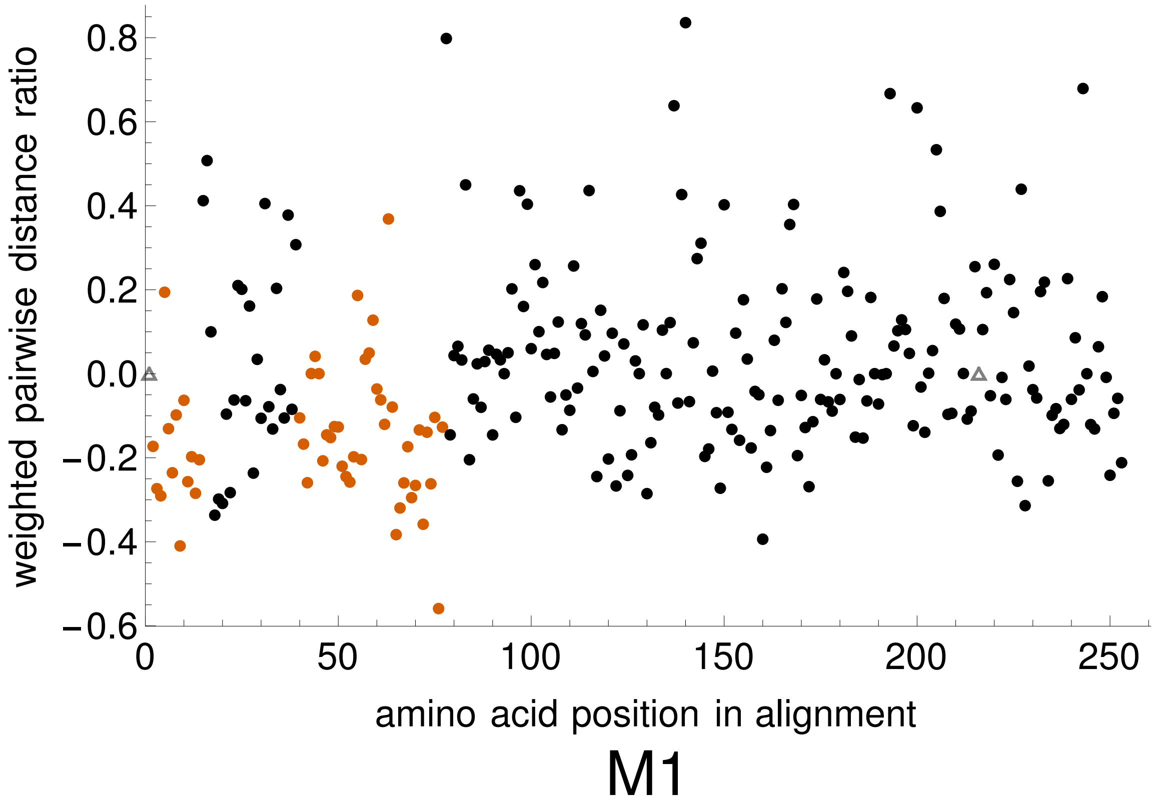

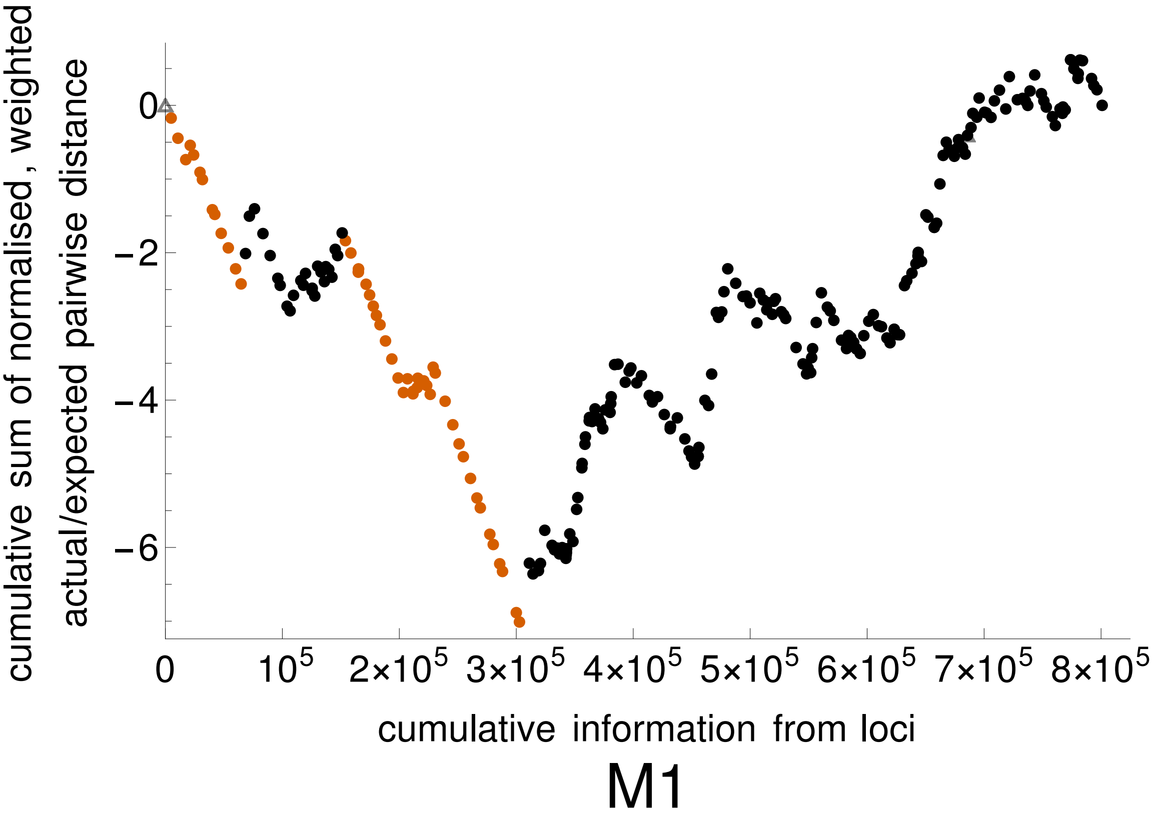

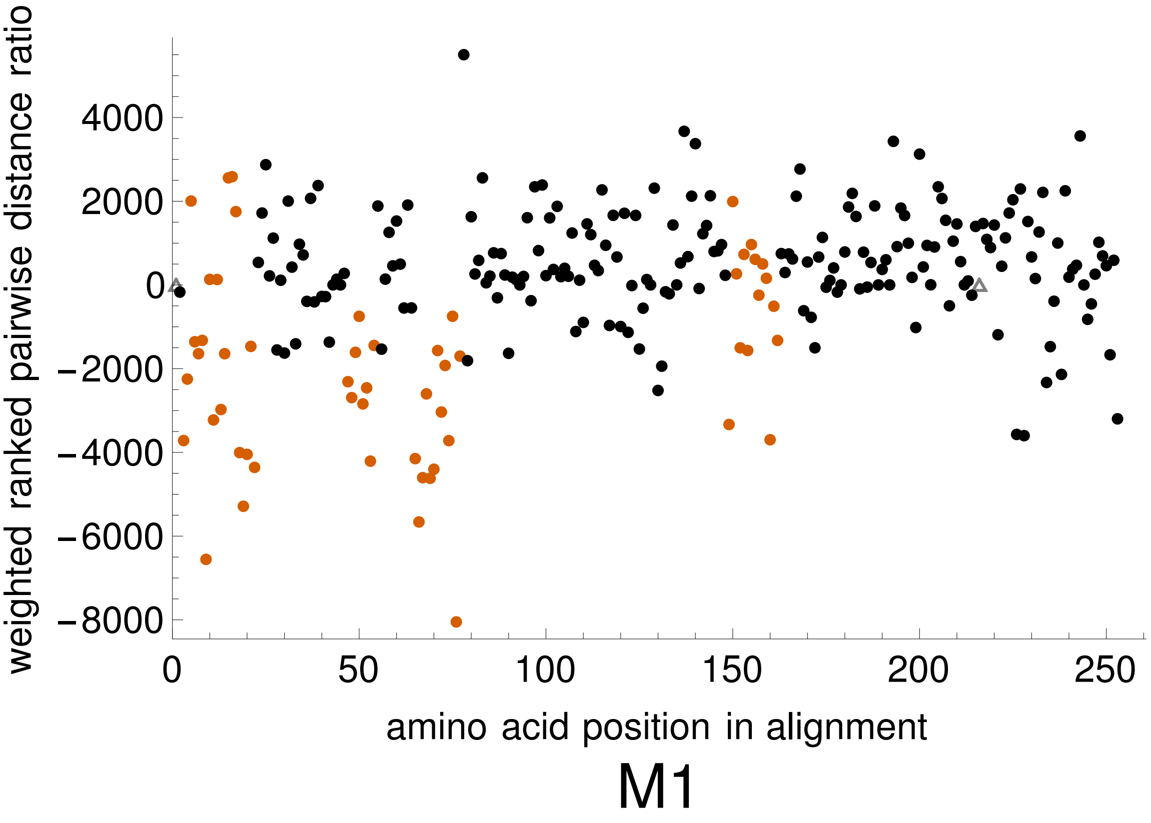
weighted ranked pairwise distance ratio
4000
2000
0
−2000
−4000
−6000
−8000
0
50
100
150
200
250
amino acid position in alignment
M1

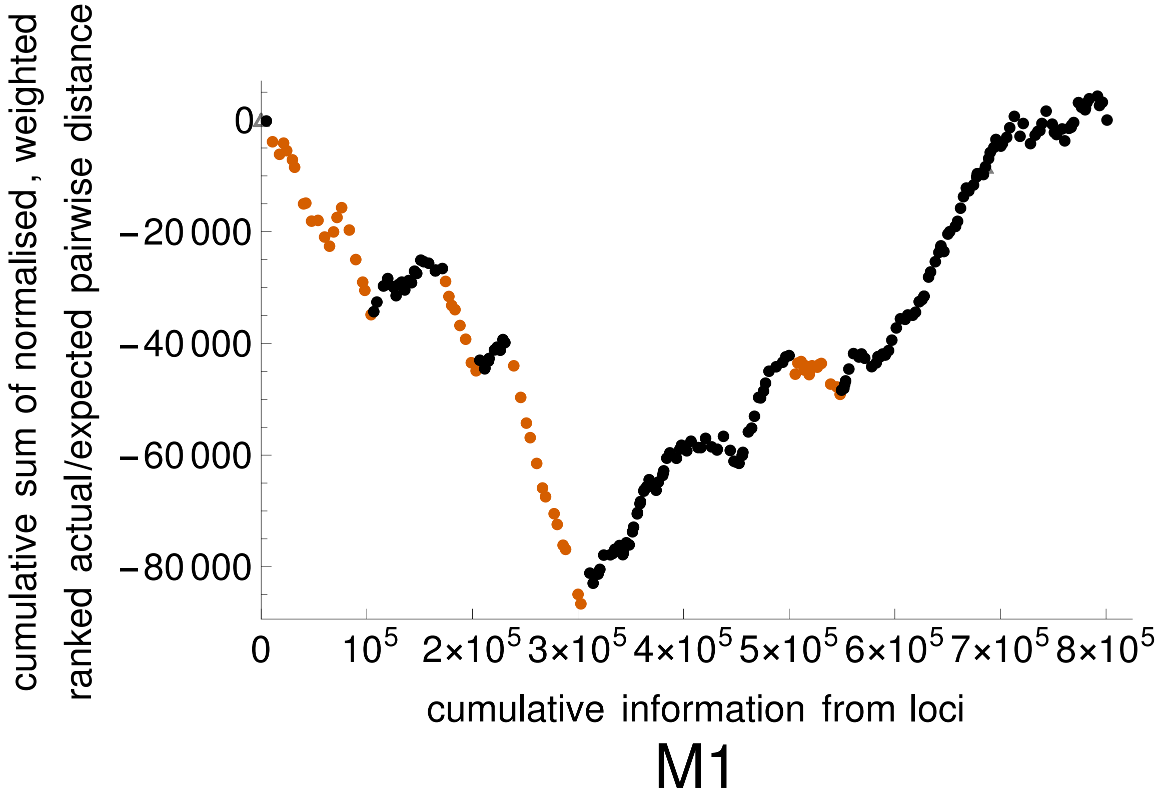
cumulative sum of normalised, weighted
ranked actual/expected pairwise distance
0
−20 000
−40 000
−60 000
−80 000
0
10^5
2×10^5
3×10^5
4×10^5
5×10^5
6×10^5
7×10^5
8×10^5
cumulative information from loci
M1

M2

Gene length histogram

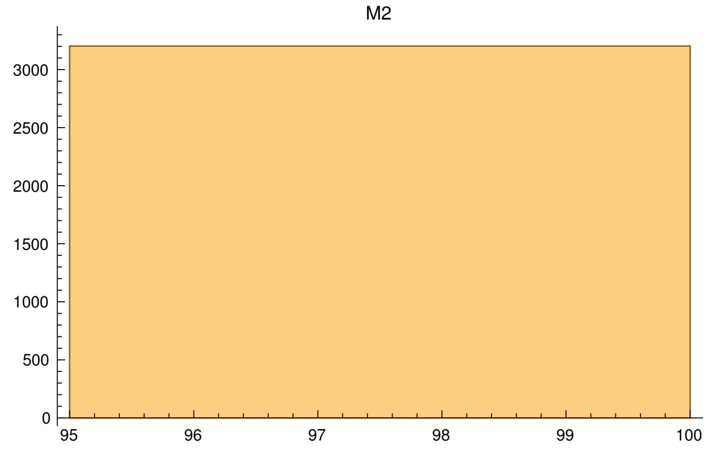

Information vs. nPD

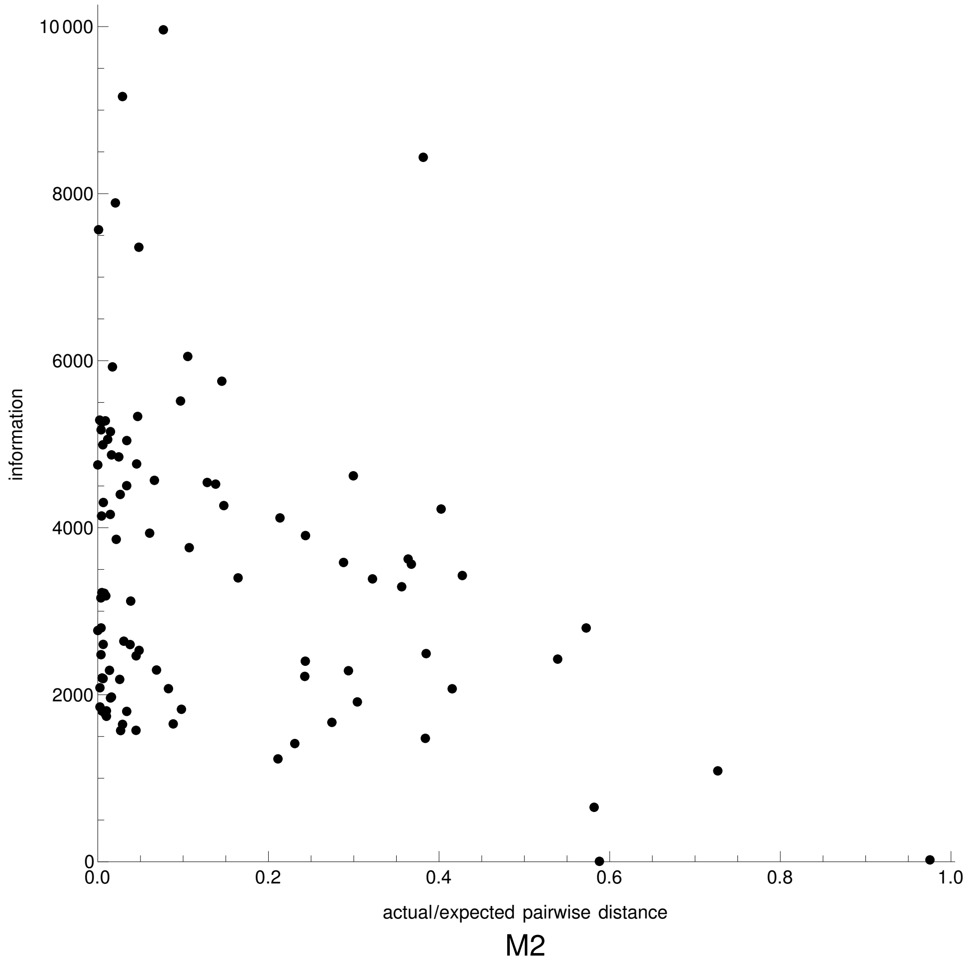

Example sequences highlighted by regions found in analyses to be conserved

Interesting points (by weighted raw PD) highlighted for gene M2:

ATGAGTCTTCTAACCGAGGTCGAAACGCCTACCAGAAACGAATGGGAGTGCAGATGCAGCGATTCAAGTGATCCTC⋱
TTGTTGTTGCCGCAAGT
ATCATTGGGATCTTGCACTTGATATTGTGGATTCTTGATCGTCTTTTCTTCAAATGCATTT⋱
ATCGTCGCCTTAAATACGGTTTGAAAAGAGGGCCTTCTACGGAAGGAGTACCTGAGTCTATGAGGGAAGA⋱
GTATCGGCAGGAACAGCAGAATGCTGTGGATGTTGACGATGGTCATTTTGTCAACATAGAATTGGAGTAA

Interesting points (by weighted ranked PD) highlighted for gene M2:

ATGAGTCTTCTAACCGAGGTCGAAACGCCTACCAGAAACGAATGGGAGTGCAGATGCAGCGATTCAAGTGATCCTCTT⋱
GTTGTTGCCGCAAGTATCATTGGGATCTTGCACTTGATATTGTGGATTCTTGATCGTCTTTTCTTCAAATGCATTTA⋱
TCGTCGCCTTAAATACGGTTTGAAAAGAGGGCCTTCTACGGAAGGAGTACCTGAGTCTATGAGGGAAGAGTATCGGC⋱
AGGAACAGCAGAATGCTGTGGATGTTGACGATGGTCATTTTGTCAACATAGAATTGGAGTAA

Per codon conservation report

M2			
Pos.	1	obs:	exp:
atg	M	3202	3202.00
---	--	------	------
mPD		0	0
		nPD:	1.
		N. weight:	0.
		Sc. PD:	0
		Sc. rank:	0

M2			
Pos.	2	obs:	exp:
tct	S	0	534.00
tcc	S	0	463.40
tca	S	0	725.20
tcg	S	0	193.00
att	I	3	1.10
atc	I	0	0.80
ata	I	0	1.10
agt	S	3110	693.90
agc	S	89	589.50
---	--	------	------
mPD		0.056	1.7
		nPD:	0.03
		N. weight:	1.3
		Sc. PD:	-0.094
		Sc. rank:	-207.9

M2			
Pos.	3	obs:	exp:
ttt	F	5	2.09
ttc	F	0	2.91
tta	L	0	295.40
ttg	L	0	607.00
ctt	L	3195	634.40
ctc	L	0	511.10
cta	L	0	472.80
ctg	L	0	674.40
cct	P	1	0.27
ccc	P	0	0.18
cca	P	0	0.41
ccg	P	0	0.14
att	I	1	0.37
atc	I	0	0.27
ata	I	0	0.37
---	--	------	------
mPD		0.0044	1.1
		nPD:	0.
		N. weight:	1.5
		Sc. PD:	-0.15
		Sc. rank:	-3514.0

M2			
Pos.	4	obs:	exp:
tta	L	13	295.90
ttg	L	0	607.90
ctt	L	3	635.40
ctc	L	2	511.90
cta	L	3171	473.60
ctg	L	11	675.40
cct	P	0	0.27
ccc	P	0	0.18
cca	P	1	0.41
ccg	P	0	0.14
att	I	0	0.37
atc	I	0	0.27
ata	I	1	0.37
---	--	------	------
mPD		0.019	1.1
		nPD:	0.02
		N. weight:	1.7
		Sc. PD:	-0.15
		Sc. rank:	-1589.0

M2			
Pos.	5	obs:	exp:
tct	S	0	0.17
tcc	S	1	0.14
tca	S	0	0.23
tcg	S	0	0.06
tat	Y	0	0.48
tac	Y	1	0.52
cct	P	0	0.27
ccc	P	1	0.18
cca	P	0	0.41
ccg	P	0	0.14
act	T	375	838.40
acc	T	2795	720.60
aca	T	22	1338.00
acg	T	6	300.90
agt	S	0	0.22
agc	S	0	0.18
gct	A	0	0.25
gcc	A	1	0.19
gca	A	0	0.45
gcg	A	0	0.11
---	--	------	------
mPD		0.23	0.70
		nPD:	0.32
		N. weight:	1.
		Sc. PD:	0.21
		Sc. rank:	2193.8

M2			
Pos.	6	obs:	exp:
gat	D	0	0.54
gac	D	1	0.46
gaa	E	4	1794.00
gag	E	3197	1407.00
---	--	------	------
mPD		0.0031	0.49
		nPD:	0.01
		N. weight:	0.77
		Sc. PD:	-0.075
		Sc. rank:	-1410.9

M2			
Pos.	7	obs:	exp:
gtt	V	17	688.70
gtc	V	3183	673.70
gta	V	2	682.70
gtg	V	0	1157.00
---	--	------	------
mPD		0.012	0.73
		nPD:	0.02
		N. weight:	1.4
		Sc. PD:	-0.13
		Sc. rank:	-1456.5

M2			
Pos.	8	obs:	exp:
gct	A	0	0.25
gcc	A	0	0.19
gca	A	1	0.45
gcg	A	0	0.11
gat	D	0	0.54
gac	D	1	0.46
gaa	E	3200	1794.00
gag	E	0	1406.00
---	--	------	------
mPD		0.0012	0.49
		nPD:	0.
		N. weight:	0.55
		Sc. PD:	-0.055
		Sc. rank:	-1390.6

M2			
Pos.	9	obs:	exp:
act	T	0	839.10
acc	T	0	721.30
aca	T	0	1339.00
acg	T	3201	301.20
gct	A	0	0.25
gcc	A	0	0.19
gca	A	0	0.45
gcg	A	1	0.11
---	--	------	------
mPD		0.00062	0.70
		nPD:	0.
		N. weight:	2.2
		Sc. PD:	-0.23
		Sc. rank:	-6040.3

M2			
Pos.	10	obs:	exp:
tta	L	0	4.99
ttg	L	0	10.26
tct	S	8	1.34
tcc	S	0	1.16
tca	S	0	1.81
tcg	S	0	0.48
ctt	L	54	10.72
ctc	L	0	8.64
cta	L	0	7.99
ctg	L	0	11.40
cct	P	2778	759.20
ccc	P	0	501.80
cca	P	0	1128.00
ccg	P	0	389.50
cat	H	361	209.00
cac	H	0	152.00
cgt	R	1	0.04
cgc	R	0	0.05
cga	R	0	0.10
cgg	R	0	0.08
agt	S	0	1.74
agc	S	0	1.47
aga	R	0	0.47
agg	R	0	0.26
---	--	------	------
mPD		0.24	0.97
		nPD:	0.24
		N. weight:	1.2
		Sc. PD:	0.15
		Sc. rank:	2175.2

M2			
Pos.	11	obs:	exp:
tct	S	0	0.33
tcc	S	2	0.29
tca	S	0	0.45
tcg	S	0	0.12
att	I	0	19.08
atc	I	52	13.92
ata	I	0	19.00
act	T	0	825.30
acc	T	3148	709.30
aca	T	0	1317.00
acg	T	0	296.20
agt	S	0	0.43
agc	S	0	0.37
---	--	------	------
mPD		0.033	0.73
		nPD:	0.05
		N. weight:	1.4
		Sc. PD:	-0.084
		Sc. rank:	309.3

,

M2			
Pos.	12	obs:	exp:
cgt	R	0	91.28
cgc	R	0	130.50
cga	R	0	245.80
cgg	R	0	199.20
aaa	K	763	434.50
aag	K	0	328.50
aga	R	2439	1139.00
agg	R	0	633.60
---	--	------	------
mPD		0.36	1.2
		nPD:	0.29
		N. weight:	0.67
		Sc. PD:	0.12
		Sc. rank:	1363.7

,

M2			
Pos.	13	obs:	exp:
tct	S	0	2.00
tcc	S	0	1.74
tca	S	0	2.72
tcg	S	0	0.72
cct	P	0	0.27
ccc	P	1	0.18
cca	P	0	0.41
ccg	P	0	0.14
cat	H	0	0.58
cac	H	1	0.42
cgt	R	0	0.15
cgc	R	0	0.21
cga	R	0	0.40
cgg	R	0	0.33
act	T	0	15.20
acc	T	58	13.07
aca	T	0	24.27
acg	T	0	5.46
aat	N	0	1502.00
aac	N	2755	1253.00
aaa	K	371	211.30
aag	K	0	159.70
agt	S	0	2.60
agc	S	12	2.21
aga	R	4	1.87
agg	R	0	1.04
---	--	------	------
mPD		0.25	0.66
		nPD:	0.38
		N. weight:	0.73
		Sc. PD:	0.2
		Sc. rank:	1911.1

,

M2			
Pos.	14	obs:	exp:
gtt	V	0	0.43
gtc	V	0	0.42
gta	V	2	0.43
gtg	V	0	0.72
gaa	E	2322	1302.00
gag	E	0	1020.00
ggt	G	0	127.00
ggc	G	0	115.30
gga	G	878	384.70
ggg	G	0	251.00
---	--	------	------
mPD		0.40	0.96
		nPD:	0.42
		N. weight:	0.61
		Sc. PD:	0.18
		Sc. rank:	1655.9

,

M2			
Pos.	15	obs:	exp:
tgg	W	3202	3202.00
---	--	------	------
mPD		0	0
		nPD:	1.
		N. weight:	0.
		Sc. PD:	0
		Sc. rank:	0

,

M2			
Pos.	16	obs:	exp:
gtt	V	2	1.08
gtc	V	0	1.05
gta	V	0	1.07
gtg	V	3	1.81
gct	A	0	0.25
gcc	A	0	0.19
gca	A	0	0.45
gcg	A	1	0.11
gaa	E	0	1782.00
gag	E	3179	1397.00
ggt	G	0	2.46
ggc	G	0	2.23
gga	G	0	7.45
ggg	G	17	4.86
---	--	------	------
mPD		0.016	0.51
		nPD:	0.03
		N. weight:	0.78
		Sc. PD:	-0.058
		Sc. rank:	-183.8

,

M2			
Pos.	17	obs:	exp:
ttt	F	0	2.50
ttc	F	6	3.50
tat	Y	0	13.91
tac	Y	29	15.09
tgt	C	0	1225.00
tgc	C	3166	1941.00
ggt	G	0	0.14
ggc	G	1	0.13
gga	G	0	0.44
ggg	G	0	0.29
---	--	------	------
mPD		0.022	0.50
		nPD:	0.04
		N. weight:	0.46
		Sc. PD:	-0.028
		Sc. rank:	43.0

,

M2			
Pos.	18	obs:	exp:
tct	S	0	0.17
tcc	S	0	0.14
tca	S	0	0.23
tcg	S	0	0.06
cgt	R	0	86.30
cgc	R	0	123.40
cga	R	0	232.40
cgg	R	0	188.30
aat	N	0	409.50
aac	N	751	341.50
aaa	K	143	82.00
aag	K	1	62.00
agt	S	0	0.22
agc	S	1	0.18
aga	R	2306	1077.00
agg	R	0	599.10
---	--	------	------
mPD		0.76	1.4
		nPD:	0.54
		N. weight:	0.71
		Sc. PD:	0.3
		Sc. rank:	2013.7

,

M2			
Pos.	19	obs:	exp:
ttt	F	0	2.92
ttc	F	7	4.08
tct	S	0	0.17
tcc	S	1	0.14
tca	S	0	0.23
tcg	S	0	0.06
tat	Y	0	6.23
tac	Y	13	6.77
tgt	C	0	1231.00
tgc	C	3181	1950.00
agt	S	0	0.22
agc	S	0	0.18
---	--	------	------
mPD		0.013	0.49
		nPD:	0.03
		N. weight:	0.46
		Sc. PD:	-0.036
		Sc. rank:	-219.1

,

M2			
Pos.	20	obs:	exp:
tct	S	0	512.80
tcc	S	0	445.00
tca	S	0	696.40
tcg	S	0	185.30
cgt	R	0	0.56
cgc	R	0	0.80
cga	R	0	1.51
cgg	R	0	1.22
att	I	0	23.12
atc	I	63	16.86
ata	I	0	23.02
aat	N	0	27.81
aac	N	51	23.19
agt	S	0	666.30
agc	S	3072	566.10
aga	R	15	7.00
agg	R	0	3.90
ggt	G	0	0.14
ggc	G	1	0.13
gga	G	0	0.44
ggg	G	0	0.29
---	--	------	------
mPD		0.079	1.7
		nPD:	0.05
		N. weight:	1.6
		Sc. PD:	-0.092
		Sc. rank:	494.1

,

M2			
Pos.	21	obs:	exp:
gtt	V	2	0.43
gtc	V	0	0.42
gta	V	0	0.43
gtg	V	0	0.72
gat	D	3126	1688.00
gac	D	0	1438.00
ggt	G	74	10.70
ggc	G	0	9.72
gga	G	0	32.43
ggg	G	0	21.15
---	--	------	------
mPD		0.046	0.56
		nPD:	0.08
		N. weight:	0.61
		Sc. PD:	-0.015
		Sc. rank:	544.6

,

M2			
Pos.	22	obs:	exp:
tct	S	0	534.50
tcc	S	0	463.80
tca	S	3202	725.90
tcg	S	0	193.20
agt	S	0	694.50
agc	S	0	590.10
---	--	------	------
mPD		0	1.7
		nPD:	0.
		N. weight:	1.4
		Sc. PD:	-0.14
		Sc. rank:	-4072.8

,

M2			
Pos.	23	obs:	exp:
tct	S	0	523.30
tcc	S	0	454.10
tca	S	0	710.70
tcg	S	0	189.10
aat	N	55	29.99
aac	N	0	25.01
agt	S	3135	680.00
agc	S	0	577.70
ggt	G	12	1.74
ggc	G	0	1.58
gga	G	0	5.26
ggg	G	0	3.43
---	--	------	------
mPD		0.041	1.7
		nPD:	0.02
		N. weight:	1.4
		Sc. PD:	-0.11
		Sc. rank:	-881.7

,

M2			
Pos.	24	obs:	exp:
aat	N	2	1.09
aac	N	0	0.91
gat	D	3130	1728.00
gac	D	70	1472.00
---	--	------	------
mPD		0.044	0.50
		nPD:	0.09
		N. weight:	0.49
		Sc. PD:	-0.0089
		Sc. rank:	448.0

,

M2			
Pos.	25	obs:	exp:
tta	L	0	0.92
ttg	L	0	1.90
tct	S	6	1.00
tcc	S	0	0.87
tca	S	0	1.36
tcg	S	0	0.36
ctt	L	10	1.99
ctc	L	0	1.60
cta	L	0	1.48
ctg	L	0	2.11
cct	P	3185	870.40
ccc	P	0	575.30
cca	P	0	1293.00
ccg	P	0	446.50
cat	H	1	0.58
cac	H	0	0.42
agt	S	0	1.30
agc	S	0	1.11
---	--	------	------
mPD		0.011	0.72
		nPD:	0.01
		N. weight:	1.2
		Sc. PD:	-0.11
		Sc. rank:	-1545.2

,

M2

Pos.	26	obs:	exp:
ttt	F	2	1.25
ttc	F	1	1.75
tta	L	0	277.80
ttg	L	0	570.90
ctt	L	2137	596.60
ctc	L	866	480.70
cta	L	2	444.70
ctg	L	0	634.30
att	I	192	70.46
atc	I	0	51.38
ata	I	0	70.16
gtt	V	2	0.43
gtc	V	0	0.42
gta	V	0	0.43
gtg	V	0	0.72
---	--	------	------
mPD		0.51	1.2
		nPD:	0.43
		N. weight:	1.
		Sc. PD:	0.31
		Sc. rank:	2801.1

,

M2

Pos.	27	obs:	exp:
att	I	434	159.30
atc	I	0	116.10
ata	I	0	158.60
act	T	1	0.26
acc	T	0	0.23
aca	T	0	0.42
acg	T	0	0.09
gtt	V	2636	568.70
gtc	V	6	556.30
gta	V	2	563.80
gtg	V	0	955.30
gct	A	90	23.18
gcc	A	1	17.80
gca	A	1	40.95
gcg	A	0	10.07
ggt	G	31	4.48
ggc	G	0	4.07
gga	G	0	13.58
ggg	G	0	8.86
---	--	------	------
mPD		0.32	1.1
		nPD:	0.3
		N. weight:	1.4
		Sc. PD:	0.25
		Sc. rank:	2865.8

,

M2

Pos.	28	obs:	exp:
ttt	F	129	53.82
ttc	F	0	75.18
tta	L	1	0.37
ttg	L	0	0.76
tct	S	0	0.17
tcc	S	0	0.14
tca	S	0	0.23
tcg	S	0	0.06
ctt	L	3	0.79
ctc	L	0	0.64
cta	L	0	0.59
ctg	L	0	0.84
att	I	1015	372.50
atc	I	0	271.60
ata	I	0	370.90
atg	M	1	1.00
agt	S	1	0.22
agc	S	0	0.18
gtt	V	2035	439.80
gtc	V	4	430.20
gta	V	6	436.00
gtg	V	0	738.90
gct	A	7	1.76
gcc	A	0	1.35
gca	A	0	3.12
gcg	A	0	0.77
---	--	------	------
mPD		0.50	1.2
		nPD:	0.4
		N. weight:	1.2
		Sc. PD:	0.35
		Sc. rank:	3330.9

,

M2

Pos.	29	obs:	exp:
tct	S	0	0.33
tcc	S	2	0.29
tca	S	0	0.45
tcg	S	0	0.12
act	T	1	2.36
acc	T	8	2.03
aca	T	0	3.77
acg	T	0	0.85
agt	S	0	0.43
agc	S	0	0.37
gtt	V	0	1.94
gtc	V	8	1.89
gta	V	1	1.92
gtg	V	0	3.25
gct	A	66	801.40
gcc	A	3044	615.60
gca	A	71	1416.00
gcg	A	0	348.20
gat	D	0	0.54
gac	D	1	0.46
---	--	------	------
mPD		0.097	0.70
		nPD:	0.14
		N. weight:	1.3
		Sc. PD:	0.039
		Sc. rank:	1843.3

,

M2

Pos.	30	obs:	exp:
tct	S	0	1.00
tcc	S	0	0.87
tca	S	6	1.36
tcg	S	0	0.36
act	T	0	1.84
acc	T	0	1.58
aca	T	7	2.93
acg	T	0	0.66
agt	S	0	1.30
agc	S	0	1.11
gct	A	37	803.40
gcc	A	1	617.10
gca	A	3124	1419.00
gcg	A	27	349.00
---	--	------	------
mPD		0.048	0.70
		nPD:	0.07
		N. weight:	0.68
		Sc. PD:	-0.025
		Sc. rank:	459.7

,

M2

Pos.	31	obs:	exp:
tct	S	0	466.50
tcc	S	0	404.90
tca	S	0	633.60
tcg	S	0	168.60
aat	N	370	220.30
aac	N	34	183.70
agt	S	2767	606.20
agc	S	28	515.10
ggt	G	3	0.43
ggc	G	0	0.39
gga	G	0	1.31
ggg	G	0	0.86
---	--	------	------
mPD		0.26	1.8
		nPD:	0.15
		N. weight:	1.3
		Sc. PD:	0.049
		Sc. rank:	1960.7

,

M2

Pos.	32	obs:	exp:
att	I	47	1170.00
atc	I	3137	853.10
ata	I	4	1165.00
act	T	0	0.26
acc	T	1	0.23
aca	T	0	0.42
acg	T	0	0.09
gtt	V	1	2.80
gtc	V	12	2.74
gta	V	0	2.77
gtg	V	0	4.70
---	--	------	------
mPD		0.041	0.67
		nPD:	0.06
		N. weight:	1.2
		Sc. PD:	-0.052
		Sc. rank:	647.3

,

M2

Pos.	33	obs:	exp:
ttt	F	2	0.83
ttc	F	0	1.17
tta	L	0	0.09
ttg	L	0	0.19
ctt	L	1	0.20
ctc	L	0	0.16
cta	L	0	0.15
ctg	L	0	0.21
att	I	3192	1173.00
atc	I	4	855.20
ata	I	0	1168.00
gtt	V	3	0.65
gtc	V	0	0.63
gta	V	0	0.64
gtg	V	0	1.08
---	--	------	------
mPD		0.0062	0.66
		nPD:	0.01
		N. weight:	0.94
		Sc. PD:	-0.089
		Sc. rank:	-1432.2

,

M2

Pos.	34	obs:	exp:
ggt	G	0	463.10
ggc	G	0	420.60
gga	G	24	1403.00
ggg	G	3178	915.30
---	--	------	------
mPD		0.015	0.69
		nPD:	0.02
		N. weight:	1.1
		Sc. PD:	-0.094
		Sc. rank:	-799.4

,

M2

Pos.	35	obs:	exp:
att	I	2	1167.00
atc	I	3083	850.70
ata	I	94	1162.00
gtt	V	0	4.95
gtc	V	23	4.84
gta	V	0	4.90
gtg	V	0	8.31
---	--	------	------
mPD		0.072	0.68
		nPD:	0.11
		N. weight:	1.1
		Sc. PD:	-0.00033
		Sc. rank:	1355.9

,

M2

Pos.	36	obs:	exp:
tta	L	0	296.10
ttg	L	3198	608.30
ctt	L	0	635.80
ctc	L	0	512.20
cta	L	0	473.90
ctg	L	4	675.80
---	--	------	------
mPD		0.0025	1.1
		nPD:	0.
		N. weight:	1.6
		Sc. PD:	-0.16
		Sc. rank:	-4075.7

,

M2

Pos.	37	obs:	exp:
cat	H	0	1854.00
cac	H	3202	1348.00
---	--	------	------
mPD		0	0.49
		nPD:	0.
		N. weight:	0.82
		Sc. PD:	-0.084
		Sc. rank:	-2373.3

,

M2

Pos.	38	obs:	exp:
ttt	F	10	4.17
ttc	F	0	5.83
tta	L	0	295.00
ttg	L	3187	606.20
ctt	L	0	633.60
ctc	L	0	510.40
cta	L	0	472.20
ctg	L	4	673.50
aaa	K	0	0.57
aag	K	1	0.43
---	--	------	------
mPD		0.010	1.1
		nPD:	0.01
		N. weight:	1.6
		Sc. PD:	-0.15
		Sc. rank:	-2510.8

,

M2

Pos.	39	obs:	exp:
cgt	R	0	0.04
cgc	R	0	0.05
cga	R	0	0.10
cgg	R	0	0.08
att	I	1	1172.00
atc	I	0	855.00
ata	I	3194	1168.00
atg	M	1	1.00
act	T	0	0.52
acc	T	0	0.45
aca	T	2	0.84
acg	T	0	0.19
aga	R	1	0.47
agg	R	0	0.26
gtt	V	0	0.65
gtc	V	0	0.63
gta	V	3	0.64
gtg	V	0	1.08
---	--	------	------
mPD		0.0050	0.66
		nPD:	0.01
		N. weight:	0.95
		Sc. PD:	-0.091
		Sc. rank:	-1617.3

,

M2

Pos.	40	obs:	exp:
tta	L	58	296.10
ttg	L	3140	608.30
ctt	L	0	635.80
ctc	L	0	512.20
cta	L	0	473.90
ctg	L	4	675.80
---	--	------	------
mPD		0.038	1.1
		nPD:	0.03
		N. weight:	1.5
		Sc. PD:	-0.11
		Sc. rank:	-53.4

,

M2

Pos.	41	obs:	exp:
tgg	W	3202	3202.00
mPD		0	0
		nPD:	1.
		N. weight:	0.
		Sc. PD:	0
		Sc. rank:	0

M2

Pos.	42	obs:	exp:
att	I	3160	1166.00
atc	I	17	850.20
ata	I	0	1161.00
act	T	19	4.98
acc	T	0	4.28
aca	T	0	7.95
acg	T	0	1.79
gtt	V	6	1.29
gtc	V	0	1.26
gta	V	0	1.28
gtg	V	0	2.17
mPD		0.026	0.68
		nPD:	0.04
		N. weight:	0.92
		Sc. PD:	-0.061
		Sc. rank:	59.8

M2

Pos.	43	obs:	exp:
ttt	F	21	8.76
ttc	F	0	12.24
tta	L	0	293.70
ttg	L	0	603.40
ctt	L	3175	630.60
ctc	L	1	508.00
cta	L	0	470.00
ctg	L	0	670.30
att	I	5	1.83
atc	I	0	1.34
ata	I	0	1.83
mPD		0.017	1.1
		nPD:	0.01
		N. weight:	1.5
		Sc. PD:	-0.14
		Sc. rank:	-1775.5

M2

Pos.	44	obs:	exp:
tat	Y	4	1.92
tac	Y	0	2.08
aat	N	3	1.64
aac	N	0	1.36
gtt	V	3	0.65
gtc	V	0	0.63
gta	V	0	0.64
gtg	V	0	1.08
gat	D	3190	1723.00
gac	D	2	1469.00
mPD		0.0075	0.50
		nPD:	0.01
		N. weight:	0.58
		Sc. PD:	-0.052
		Sc. rank:	-656.2

M2

Pos.	45	obs:	exp:
tta	L	0	0.09
ttg	L	0	0.19
tgt	C	1	0.39
tgc	C	0	0.61
ctt	L	1	0.20
ctc	L	0	0.16
cta	L	0	0.15
ctg	L	0	0.21
cat	H	32	19.68
cac	H	2	14.32
cgt	R	2426	118.50
cgc	R	740	169.40
cga	R	0	319.00
cgg	R	0	258.50
aga	R	0	1478.00
agg	R	0	822.50
mPD		0.38	0.99
		nPD:	0.38
		N. weight:	2.5
		Sc. PD:	0.66
		Sc. rank:	6292.8

M2

Pos.	46	obs:	exp:
tta	L	0	296.10
ttg	L	0	608.30
ctt	L	3181	635.80
ctc	L	21	512.20
cta	L	0	473.90
ctg	L	0	675.80
mPD		0.013	1.1
		nPD:	0.01
		N. weight:	1.5
		Sc. PD:	-0.14
		Sc. rank:	-2048.4

M2

Pos.	47	obs:	exp:
ttt	F	75	1247.00
ttc	F	2914	1742.00
tta	L	3	19.51
ttg	L	0	40.08
tct	S	0	0.33
tcc	S	2	0.29
tca	S	0	0.45
tcg	S	0	0.12
ctt	L	0	41.89
ctc	L	208	33.75
cta	L	0	31.23
ctg	L	0	44.54
agt	S	0	0.43
agc	S	0	0.37
mPD		0.17	0.62
		nPD:	0.27
		N. weight:	0.49
		Sc. PD:	0.079
		Sc. rank:	944.2

M2

Pos.	48	obs:	exp:
ttt	F	16	1333.00
ttc	F	3179	1862.00
tct	S	0	1.17
tcc	S	7	1.01
tca	S	0	1.59
tcg	S	0	0.42
agt	S	0	1.52
agc	S	0	1.29
mPD		0.014	0.49
		nPD:	0.03
		N. weight:	0.48
		Sc. PD:	-0.037
		Sc. rank:	-137.1

M2

Pos.	49	obs:	exp:
aaa	K	3195	1822.00
aag	K	5	1378.00
gaa	E	2	1.12
gag	E	0	0.88
mPD		0.0044	0.49
		nPD:	0.01
		N. weight:	0.52
		Sc. PD:	-0.049
		Sc. rank:	-825.0

M2

Pos.	50	obs:	exp:
ttt	F	0	73.84
ttc	F	177	103.20
tat	Y	0	9.59
tac	Y	20	10.41
tgt	C	33	1163.00
tgc	C	2972	1842.00
mPD		0.14	0.59
		nPD:	0.23
		N. weight:	0.42
		Sc. PD:	0.05
		Sc. rank:	725.6

M2

Pos.	51	obs:	exp:
tta	L	0	9.06
ttg	L	0	18.62
ctt	L	98	19.46
ctc	L	0	15.68
cta	L	0	14.50
ctg	L	0	20.68
att	I	2361	868.60
atc	I	6	633.40
ata	I	0	865.00
act	T	1	0.26
acc	T	0	0.23
aca	T	0	0.42
acg	T	0	0.09
gtt	V	732	157.40
gtc	V	0	154.00
gta	V	0	156.10
gtg	V	0	264.50
gct	A	4	1.01
gcc	A	0	0.77
gca	A	0	1.78
gcg	A	0	0.44
mPD		0.41	1.1
		nPD:	0.36
		N. weight:	1.1
		Sc. PD:	0.26
		Sc. rank:	2478.2

M2

Pos.	52	obs:	exp:
tat	Y	3191	1535.00
tac	Y	9	1665.00
tgt	C	1	0.39
tgc	C	0	0.61
cat	H	1	0.58
cac	H	0	0.42
mPD		0.0069	0.50
		nPD:	0.01
		N. weight:	0.67
		Sc. PD:	-0.061
		Sc. rank:	-901.2

M2

Pos.	53	obs:	exp:
cgt	R	3080	119.80
cgc	R	122	171.40
cga	R	0	322.70
cgg	R	0	261.50
aga	R	0	1495.00
agg	R	0	831.90
mPD		0.073	0.95
		nPD:	0.08
		N. weight:	2.9
		Sc. PD:	-0.087
		Sc. rank:	2510.1

M2

Pos.	54	obs:	exp:
tta	L	0	0.09
ttg	L	0	0.19
tgt	C	0	1.16
tgc	C	3	1.84
ctt	L	0	0.20
ctc	L	1	0.16
cta	L	0	0.15
ctg	L	0	0.21
cat	H	0	2.89
cac	H	5	2.11
cgt	R	33	119.50
cgc	R	3157	170.90
cga	R	3	321.80
cgg	R	0	260.70
aga	R	0	1491.00
agg	R	0	829.50
mPD		0.028	0.96
		nPD:	0.03
		N. weight:	2.7
		Sc. PD:	-0.2
		Sc. rank:	-841.6

M2

Pos.	55	obs:	exp:
ttt	F	95	40.47
ttc	F	2	56.53
tta	L	0	287.10
ttg	L	0	589.90
ctt	L	2959	616.50
ctc	L	146	496.70
cta	L	0	459.50
ctg	L	0	655.40
mPD		0.15	1.1
		nPD:	0.13
		N. weight:	1.3
		Sc. PD:	0.027
		Sc. rank:	1744.6

M2

Pos.	56	obs:	exp:
cgt	R	0	0.97
cgc	R	0	1.39
cga	R	0	2.62
cgg	R	0	2.12
aaa	K	3174	1808.00
aag	K	1	1367.00
aga	R	26	12.14
agg	R	0	6.75
gaa	E	1	0.56
gag	E	0	0.44
---	--	------	------
mPD		0.017	0.51
		nPD:	0.03
		N. weight:	0.53
		Sc. PD:	-0.038
		Sc. rank:	-66.3

,

M2

Pos.	57	obs:	exp:
tat	Y	1	1535.00
tac	Y	3200	1666.00
cat	H	0	0.58
cac	H	1	0.42
---	--	------	------
mPD		0.0012	0.50
		nPD:	0.
		N. weight:	0.61
		Sc. PD:	-0.062
		Sc. rank:	-1583.2

,

M2

Pos.	58	obs:	exp:
tct	S	0	1.00
tcc	S	0	0.87
tca	S	0	1.36
tcg	S	0	0.36
cgt	R	1	0.04
cgc	R	0	0.05
cga	R	0	0.10
cgg	R	0	0.08
agt	S	6	1.30
agc	S	0	1.11
aga	R	0	0.47
agg	R	0	0.26
gat	D	143	77.21
gac	D	0	65.79
gaa	E	0	0.56
gag	E	1	0.44
ggt	G	3011	441.20
ggc	G	2	400.80
gga	G	1	1337.00
ggg	G	37	872.10
---	--	------	------
mPD		0.12	0.80
		nPD:	0.15
		N. weight:	1.7
		Sc. PD:	0.062
		Sc. rank:	2517.8

,

M2

Pos.	59	obs:	exp:
tta	L	9	296.10
ttg	L	3193	608.30
ctt	L	0	635.80
ctc	L	0	512.20
cta	L	0	473.90
ctg	L	0	675.80
---	--	------	------
mPD		0.0056	1.1
		nPD:	0.
		N. weight:	1.6
		Sc. PD:	-0.15
		Sc. rank:	-3171.2

,

M2

Pos.	60	obs:	exp:
cgt	R	0	0.11
cgc	R	0	0.16
cga	R	0	0.30
cgg	R	0	0.24
aat	N	0	0.55
aac	N	1	0.45
aaa	K	3198	1821.00
aag	K	0	1377.00
aga	R	3	1.40
agg	R	0	0.78
---	--	------	------
mPD		0.0025	0.49
		nPD:	0.01
		N. weight:	0.53
		Sc. PD:	-0.052
		Sc. rank:	-1069.7

,

M2

Pos.	61	obs:	exp:
tct	S	0	2.50
tcc	S	0	2.17
tca	S	0	3.40
tcg	S	0	0.90
cgt	R	0	104.10
cgc	R	0	148.90
cga	R	0	280.50
cgg	R	0	227.20
att	I	0	2.57
atc	I	0	1.87
ata	I	7	2.56
aaa	K	11	6.83
aag	K	1	5.17
agt	S	0	3.25
agc	S	15	2.76
aga	R	2722	1299.00
agg	R	61	723.00
gaa	E	1	0.56
gag	E	0	0.44
ggt	G	0	55.53
ggc	G	0	50.44
gga	G	384	168.30
ggg	G	0	109.80
---	--	------	------
mPD		0.27	1.1
		nPD:	0.24
		N. weight:	0.65
		Sc. PD:	0.085
		Sc. rank:	1163.4

,

M2

Pos.	62	obs:	exp:
tct	S	0	0.83
tcc	S	0	0.72
tca	S	0	1.13
tcg	S	0	0.30
tgg	W	1	1.00
cgt	R	0	0.15
cgc	R	0	0.21
cga	R	0	0.40
cgg	R	0	0.33
agt	S	0	1.08
agc	S	5	0.92
aga	R	0	1.87
agg	R	4	1.04
gct	A	0	1.26
gcc	A	5	0.97
gca	A	0	2.23
gcg	A	0	0.55
ggt	G	3	460.90
ggc	G	147	418.60
gga	G	18	1396.00
ggg	G	3019	911.00
---	--	------	------
mPD		0.12	0.70
		nPD:	0.16
		N. weight:	1.
		Sc. PD:	0.055
		Sc. rank:	1622.8

,

M2

Pos.	63	obs:	exp:
tta	L	0	0.18
ttg	L	0	0.38
tct	S	1	0.17
tcc	S	0	0.14
tca	S	0	0.23
tcg	S	0	0.06
ctt	L	2	0.40
ctc	L	0	0.32
cta	L	0	0.30
ctg	L	0	0.42
cct	P	3197	874.30
ccc	P	2	577.80
cca	P	0	1298.00
ccg	P	0	448.50
agt	S	0	0.22
agc	S	0	0.18
---	--	------	------
mPD		0.0031	0.71
		nPD:	0.
		N. weight:	1.2
		Sc. PD:	-0.12
		Sc. rank:	-2723.9

,

M2

Pos.	64	obs:	exp:
ttt	F	29	12.10
ttc	F	0	16.90
tta	L	0	0.09
ttg	L	0	0.19
tct	S	2936	492.10
tcc	S	4	427.00
tca	S	8	668.30
tcg	S	0	177.90
tat	Y	7	3.36
tac	Y	0	3.64
ctt	L	1	0.20
ctc	L	0	0.16
cta	L	0	0.15
ctg	L	0	0.21
cct	P	3	0.82
ccc	P	0	0.54
cca	P	0	1.22
ccg	P	0	0.42
act	T	6	1.57
acc	T	0	1.35
aca	T	0	2.51
acg	T	0	0.56
agt	S	0	639.40
agc	S	0	543.30
gtt	V	13	2.80
gtc	V	0	2.74
gta	V	0	2.77
gtg	V	0	4.70
gct	A	193	49.12
gcc	A	2	37.73
gca	A	0	86.80
gcg	A	0	21.34
---	--	------	------
mPD		0.17	1.7
		nPD:	0.1
		N. weight:	1.6
		Sc. PD:	-0.016
		Sc. rank:	1655.3

,

M2

Pos.	65	obs:	exp:
tct	S	0	0.17
tcc	S	0	0.14
tca	S	0	0.23
tcg	S	1	0.06
cgt	R	0	0.15
cgc	R	0	0.21
cga	R	0	0.40
cgg	R	0	0.33
atg	M	15	15.00
act	T	3	829.70
acc	T	0	713.20
aca	T	16	1324.00
acg	T	3146	297.80
aaa	K	0	8.54
aag	K	15	6.46
agt	S	0	0.22
agc	S	0	0.18
aga	R	0	1.87
agg	R	4	1.04
gct	A	0	0.50
gcc	A	0	0.39
gca	A	0	0.89
gcg	A	2	0.22
---	--	------	------
mPD		0.035	0.72
		nPD:	0.05
		N. weight:	2.2
		Sc. PD:	-0.12
		Sc. rank:	963.3

,

M2

Pos.	66	obs:	exp:
act	T	0	4.98
acc	T	0	4.28
aca	T	19	7.95
acg	T	0	1.79
aaa	K	22	12.53
aag	K	0	9.47
gct	A	0	82.38
gcc	A	0	63.28
gca	A	326	145.60
gcg	A	1	35.79
gaa	E	2807	1575.00
gag	E	3	1235.00
ggt	G	0	3.47
ggc	G	0	3.15
gga	G	24	10.52
ggg	G	0	6.86
---	--	------	------
mPD		0.23	0.77
		nPD:	0.3
		N. weight:	0.56
		Sc. PD:	0.11
		Sc. rank:	1206.1

,

M2

Pos.	67	obs:	exp:
cgt	R	0	0.11
cgc	R	0	0.16
cga	R	0	0.30
cgg	R	0	0.24
aga	R	2	1.40
agg	R	1	0.78
ggt	G	0	462.60
ggc	G	0	420.20
gga	G	1658	1402.00
ggg	G	1541	914.40
---	--	------	------
mPD		0.50	0.69
		nPD:	0.73
		N. weight:	0.32
		Sc. PD:	0.19
		Sc. rank:	929.1

,

M2

Pos.	68	obs:	exp:
tta	L	3	0.37
ttg	L	0	0.76
ctt	L	0	0.79
ctc	L	0	0.64
cta	L	1	0.59
ctg	L	0	0.84
att	I	0	69.72
atc	I	0	50.84
ata	I	190	69.43
atg	M	1	1.00
gtt	V	0	646.80
gtc	V	0	632.60
gta	V	2649	641.20
gtg	V	358	1086.00
---	--	------	------
mPD		0.31	0.85
		nPD:	0.37
		N. weight:	1.
		Sc. PD:	0.26
		Sc. rank:	2502.0

,

M2

Pos.	69	obs:	exp:
tta	L	0	0.28
ttg	L	0	0.57
ctt	L	3	0.60
ctc	L	0	0.48
cta	L	0	0.44
ctg	L	0	0.63
cct	P	2302	874.30
ccc	P	3	577.80
cca	P	6	1298.00
ccg	P	888	448.50
---	--	------	------
mPD		0.41	0.71
		nPD:	0.57
		N. weight:	0.82
		Sc. PD:	0.37
		Sc. rank:	2363.9

,

M2

Pos.	70	obs:	exp:
caa	Q	1	0.50
cag	Q	0	0.50
aaa	K	0	1.71
aag	K	3	1.29
gct	A	0	0.50
gcc	A	0	0.39
gca	A	0	0.89
gcg	A	2	0.22
gaa	E	29	1792.00
gag	E	3167	1404.00
---	--	------	------
mPD		0.022	0.50
		nPD:	0.04
		N. weight:	0.73
		Sc. PD:	-0.044
		Sc. rank:	99.1

,

M2			
Pos.	71	obs:	exp:
ttt	F	2	0.83
ttc	F	0	1.17
tct	S	2473	534.20
tcc	S	726	463.50
tca	S	0	725.40
tcg	S	1	193.10
agt	S	0	694.10
agc	S	0	589.70
---	--	------	------
mPD		0.35	1.7
		nPD:	0.21
		N. weight:	1.2
		Sc. PD:	0.12
		Sc. rank:	2080.0

,

M2			
Pos.	72	obs:	exp:
tta	L	0	0.18
ttg	L	0	0.38
ctt	L	0	0.40
ctc	L	0	0.32
cta	L	0	0.30
ctg	L	2	0.42
atg	M	3199	3199.00
act	T	0	0.26
acc	T	0	0.23
aca	T	0	0.42
acg	T	1	0.09
---	--	------	------
mPD		0.0019	0.0032
		nPD:	0.59
		N. weight:	0.0016
		Sc. PD:	0.00075
		Sc. rank:	4.6

,

M2			
Pos.	73	obs:	exp:
cgt	R	0	119.50
cgc	R	0	170.90
cga	R	0	321.80
cgg	R	0	260.70
atg	M	1	1.00
aaa	K	0	4.56
aag	K	8	3.44
aga	R	1	1491.00
agg	R	3192	829.50
---	--	------	------
mPD		0.0062	0.96
		nPD:	0.01
		N. weight:	1.3
		Sc. PD:	-0.12
		Sc. rank:	-2235.7

,

M2			
Pos.	74	obs:	exp:
gaa	E	3194	1794.00
gag	E	7	1407.00
ggt	G	0	0.14
ggc	G	0	0.13
gga	G	1	0.44
ggg	G	0	0.29
---	--	------	------
mPD		0.0050	0.49
		nPD:	0.01
		N. weight:	0.53
		Sc. PD:	-0.05
		Sc. rank:	-795.9

,

M2			
Pos.	75	obs:	exp:
aaa	K	0	0.57
aag	K	1	0.43
gaa	E	337	1794.00
gag	E	2863	1406.00
ggt	G	0	0.14
ggc	G	0	0.13
gga	G	0	0.44
ggg	G	1	0.29
---	--	------	------
mPD		0.19	0.49
		nPD:	0.38
		N. weight:	0.44
		Sc. PD:	0.12
		Sc. rank:	1114.0

,

M2			
Pos.	76	obs:	exp:
tct	S	0	0.17
tcc	S	1	0.14
tca	S	0	0.23
tcg	S	0	0.06
tat	Y	564	1535.00
tac	Y	2637	1666.00
agt	S	0	0.22
agc	S	0	0.18
---	--	------	------
mPD		0.29	0.50
		nPD:	0.58
		N. weight:	0.19
		Sc. PD:	0.088
		Sc. rank:	553.1

,

M2			
Pos.	77	obs:	exp:
tta	L	0	0.46
ttg	L	0	0.95
ctt	L	0	0.99
ctc	L	0	0.80
cta	L	2	0.74
ctg	L	3	1.06
cct	P	0	0.55
ccc	P	0	0.36
cca	P	0	0.81
ccg	P	2	0.28
caa	Q	0	2.50
cag	Q	5	2.50
cgt	R	0	119.40
cgc	R	0	170.70
cga	R	17	321.50
cgg	R	3172	260.50
aga	R	0	1489.00
agg	R	1	828.70
---	--	------	------
mPD		0.020	0.97
		nPD:	0.02
		N. weight:	2.3
		Sc. PD:	-0.19
		Sc. rank:	-1791.9

,

M2			
Pos.	78	obs:	exp:
cct	P	0	0.27
ccc	P	0	0.18
cca	P	0	0.41
ccg	P	1	0.14
caa	Q	3	1598.00
cag	Q	3197	1602.00
gaa	E	0	0.56
gag	E	1	0.44
---	--	------	------
mPD		0.0031	0.50
		nPD:	0.01
		N. weight:	0.65
		Sc. PD:	-0.063
		Sc. rank:	-1218.8

,

M2			
Pos.	79	obs:	exp:
aaa	K	3	1.71
aag	K	0	1.29
gaa	E	3025	1792.00
gag	E	171	1404.00
ggt	G	0	0.43
ggc	G	0	0.39
gga	G	3	1.31
ggg	G	0	0.86
---	--	------	------
mPD		0.10	0.50
		nPD:	0.21
		N. weight:	0.36
		Sc. PD:	0.036
		Sc. rank:	596.1

,

M2			
Pos.	80	obs:	exp:
cct	P	0	0.27
ccc	P	0	0.18
cca	P	0	0.41
ccg	P	1	0.14
caa	Q	2	1598.00
cag	Q	3198	1602.00
aaa	K	0	0.57
aag	K	1	0.43
---	--	------	------
mPD		0.0025	0.50
		nPD:	0.
		N. weight:	0.65
		Sc. PD:	-0.064
		Sc. rank:	-1385.8

,

M2			
Pos.	81	obs:	exp:
tta	L	0	0.28
ttg	L	0	0.57
ctt	L	0	0.60
ctc	L	0	0.48
cta	L	0	0.44
ctg	L	3	0.63
cat	H	9	5.79
cac	H	1	4.21
caa	Q	5	1591.00
cag	Q	3181	1595.00
cgt	R	0	0.11
cgc	R	0	0.16
cga	R	0	0.30
cgg	R	3	0.24
aga	R	0	1.40
agg	R	0	0.78
---	--	------	------
mPD		0.013	0.51
		nPD:	0.03
		N. weight:	0.64
		Sc. PD:	-0.051
		Sc. rank:	-372.4

,

M2			
Pos.	82	obs:	exp:
tct	S	0	300.80
tcc	S	0	261.00
tca	S	0	408.50
tcg	S	0	108.70
att	I	1	0.37
atc	I	0	0.27
ata	I	0	0.37
aat	N	1394	761.80
aac	N	3	635.20
agt	S	1802	390.90
agc	S	0	332.10
gat	D	2	1.08
gac	D	0	0.92
---	--	------	------
mPD		0.50	1.7
		nPD:	0.29
		N. weight:	1.1
		Sc. PD:	0.18
		Sc. rank:	2093.8

,

M2			
Pos.	83	obs:	exp:
act	T	28	7.34
acc	T	0	6.31
aca	T	0	11.72
acg	T	0	2.63
gct	A	3172	799.60
gcc	A	1	614.20
gca	A	0	1413.00
gcg	A	1	347.40
---	--	------	------
mPD		0.019	0.71
		nPD:	0.03
		N. weight:	1.3
		Sc. PD:	-0.1
		Sc. rank:	-649.4

,

M2			
Pos.	84	obs:	exp:
gtt	V	0	688.30
gtc	V	0	673.20
gta	V	315	682.30
gtg	V	2885	1156.00
gct	A	0	0.25
gcc	A	0	0.19
gca	A	0	0.45
gcg	A	1	0.11
ggt	G	0	0.14
ggc	G	0	0.13
gga	G	0	0.44
ggg	G	1	0.29
---	--	------	------
mPD		0.18	0.73
		nPD:	0.24
		N. weight:	0.71
		Sc. PD:	0.092
		Sc. rank:	1288.8

,

M2			
Pos.	85	obs:	exp:
aat	N	6	3.27
aac	N	0	2.73
gct	A	2	0.50
gcc	A	0	0.39
gca	A	0	0.89
gcg	A	0	0.22
gat	D	3189	1722.00
gac	D	1	1468.00
ggt	G	4	0.58
ggc	G	0	0.53
gga	G	0	1.75
ggg	G	0	1.14
---	--	------	------
mPD		0.0081	0.51
		nPD:	0.02
		N. weight:	0.58
		Sc. PD:	-0.051
		Sc. rank:	-639.6

,

M2

Pos.	86	obs:	exp:
tta	L	0	0.18
ttg	L	0	0.38
ctt	L	2	0.40
ctc	L	0	0.32
cta	L	0	0.30
ctg	L	0	0.42
att	I	3	1.10
atc	I	0	0.80
ata	I	0	1.10
gtt	V	3122	687.40
gtc	V	73	672.40
gta	V	0	681.50
gtg	V	1	1155.00
gct	A	1	0.25
gcc	A	0	0.19
gca	A	0	0.45
gcg	A	0	0.11
mPD		0.049	0.74
		nPD:	0.07
		N. weight:	1.3
		Sc. PD:	-0.053
		Sc. rank:	859.8

M2

Pos.	87	obs:	exp:
gtt	V	0	0.22
gtc	V	1	0.21
gta	V	0	0.21
gtg	V	0	0.36
gat	D	1	1728.00
gac	D	3199	1472.00
ggt	G	0	0.14
ggc	G	1	0.13
gga	G	0	0.44
ggg	G	0	0.29
mPD		0.0019	0.50
		nPD:	0.
		N. weight:	0.73
		Sc. PD:	-0.073
		Sc. rank:	-1787.6

M2

Pos.	88	obs:	exp:
tat	Y	22	10.55
tac	Y	0	11.45
aat	N	13	9.27
aac	N	4	7.73
gat	D	3122	1701.00
gac	D	29	1450.00
gaa	E	0	0.56
gag	E	1	0.44
ggt	G	11	1.59
ggc	G	0	1.44
gga	G	0	4.82
ggg	G	0	3.14
mPD		0.052	0.53
		nPD:	0.1
		N. weight:	0.54
		Sc. PD:	-0.0049
		Sc. rank:	565.2

M2

Pos.	89	obs:	exp:
tct	S	0	17.19
tcc	S	0	14.92
tca	S	0	23.35
tcg	S	0	6.21
tgt	C	3	1.16
tgc	C	0	1.84
cgt	R	1	0.04
cgc	R	0	0.05
cga	R	0	0.10
cgg	R	0	0.08
agt	S	103	22.34
agc	S	0	18.98
aga	R	0	0.47
agg	R	0	0.26
gtt	V	3	0.65
gtc	V	0	0.63
gta	V	0	0.64
gtg	V	0	1.08
gct	A	1	0.25
gcc	A	0	0.19
gca	A	0	0.45
gcg	A	0	0.11
gat	D	20	10.80
gac	D	0	9.20
ggt	G	3061	444.10
ggc	G	8	403.40
gga	G	0	1346.00
ggg	G	2	877.80
mPD		0.086	0.81
		nPD:	0.11
		N. weight:	1.8
		Sc. PD:	-0.0035
		Sc. rank:	2063.1

M2

Pos.	90	obs:	exp:
cct	P	1	0.27
ccc	P	0	0.18
cca	P	0	0.41
ccg	P	0	0.14
cat	H	3194	1850.00
cac	H	2	1346.00
cgt	R	2	0.07
cgc	R	0	0.11
cga	R	0	0.20
cgg	R	0	0.16
aat	N	3	1.64
aac	N	0	1.36
aga	R	0	0.93
agg	R	0	0.52
mPD		0.0050	0.49
		nPD:	0.01
		N. weight:	0.51
		Sc. PD:	-0.048
		Sc. rank:	-751.4

M2

Pos.	91	obs:	exp:
ttt	F	3199	1335.00
ttc	F	0	1864.00
tta	L	0	0.18
ttg	L	1	0.38
tat	Y	1	0.48
tac	Y	0	0.52
ctt	L	1	0.40
ctc	L	0	0.32
cta	L	0	0.30
ctg	L	0	0.42
mPD		0.0019	0.49
		nPD:	0.
		N. weight:	0.82
		Sc. PD:	-0.082
		Sc. rank:	-1977.7

M2

Pos.	92	obs:	exp:
tta	L	1	0.09
ttg	L	0	0.19
tct	S	0	0.17
tcc	S	0	0.14
tca	S	1	0.23
tcg	S	0	0.06
ctt	L	0	0.20
ctc	L	0	0.16
cta	L	0	0.15
ctg	L	0	0.21
agt	S	0	0.22
agc	S	0	0.18
gtt	V	0	687.80
gtc	V	3198	672.80
gta	V	0	681.90
gtg	V	0	1155.00
gct	A	0	0.25
gcc	A	1	0.19
gca	A	0	0.45
gcg	A	0	0.11
ggt	G	0	0.14
ggc	G	1	0.13
gga	G	0	0.44
ggg	G	0	0.29
mPD		0.0044	0.74
		nPD:	0.01
		N. weight:	1.5
		Sc. PD:	-0.14
		Sc. rank:	-2830.8

M2

Pos.	93	obs:	exp:
tct	S	0	1.17
tcc	S	0	1.01
tca	S	0	1.59
tcg	S	0	0.42
tat	Y	0	0.96
tac	Y	2	1.04
cat	H	0	0.58
cac	H	1	0.42
act	T	0	0.52
acc	T	2	0.45
aca	T	0	0.84
acg	T	0	0.19
aat	N	0	1724.00
aac	N	3161	1437.00
agt	S	0	1.52
agc	S	7	1.29
gat	D	0	15.66
gac	D	29	13.34
mPD		0.025	0.52
		nPD:	0.05
		N. weight:	0.75
		Sc. PD:	-0.042
		Sc. rank:	364.6

M2

Pos.	94	obs:	exp:
tta	L	1	0.09
ttg	L	0	0.19
ctt	L	0	0.20
ctc	L	0	0.16
cta	L	0	0.15
ctg	L	0	0.21
cgt	R	0	0.04
cgc	R	0	0.05
cga	R	0	0.10
cgg	R	0	0.08
att	I	0	1173.00
atc	I	0	855.50
ata	I	3197	1168.00
atg	M	2	2.00
aga	R	1	0.47
agg	R	0	0.26
gtt	V	0	0.22
gtc	V	0	0.21
gta	V	1	0.21
gtg	V	0	0.36
mPD		0.0031	0.66
		nPD:	0.
		N. weight:	0.95
		Sc. PD:	-0.094
		Sc. rank:	-2066.4

M2

Pos.	95	obs:	exp:
caa	Q	1	0.50
cag	Q	0	0.50
cgt	R	0	0.04
cgc	R	0	0.05
cga	R	0	0.10
cgg	R	0	0.08
aaa	K	5	4.56
aag	K	3	3.44
aga	R	1	0.47
agg	R	0	0.26
gat	D	1	1.62
gac	D	2	1.38
gaa	E	1894	1780.00
gag	E	1282	1396.00
ggt	G	0	1.88
ggc	G	0	1.71
gga	G	10	5.70
ggg	G	3	3.72
mPD		0.50	0.51
		nPD:	0.98
		N. weight:	0.0067
		Sc. PD:	0.0056
		Sc. rank:	19.4

M2

Pos.	96	obs:	exp:
ttt	F	2	0.83
ttc	F	0	1.17
tta	L	15	295.40
ttg	L	2353	607.00
tct	S	0	0.17
tcc	S	0	0.14
tca	S	0	0.23
tcg	S	0	0.06
ctt	L	1	634.40
ctc	L	0	511.10
cta	L	4	472.80
ctg	L	822	674.40
atg	M	4	4.00
agt	S	1	0.22
agc	S	0	0.18
mPD		0.40	1.1
		nPD:	0.36
		N. weight:	0.97
		Sc. PD:	0.23
		Sc. rank:	2189.2

M2

Pos.	97	obs:	exp:
aaa	K	0	11.96
aag	K	21	9.04
gtt	V	0	0.43
gtc	V	0	0.42
gta	V	0	0.43
gtg	V	2	0.72
gat	D	0	0.54
gac	D	1	0.46
gaa	E	4	1777.00
gag	E	3166	1393.00
ggt	G	0	0.43
ggc	G	0	0.39
gga	G	0	1.31
ggg	G	3	0.86
---	-	5	5.00
mPD		0.019	0.51
		nPD:	0.04
		N. weight:	0.77
		Sc. PD:	-0.051
		Sc. rank:	7.7

M2

Pos.	98	obs:	exp:
taa	.	3197	1181.00
tag	.	5	1098.00
tga	.	0	923.30
mPD		0.0031	0.86
		nPD:	0.
		N. weight:	0.93
		Sc. PD:	-0.093
		Sc. rank:	-2318.3

Amino acid level conservation per codon

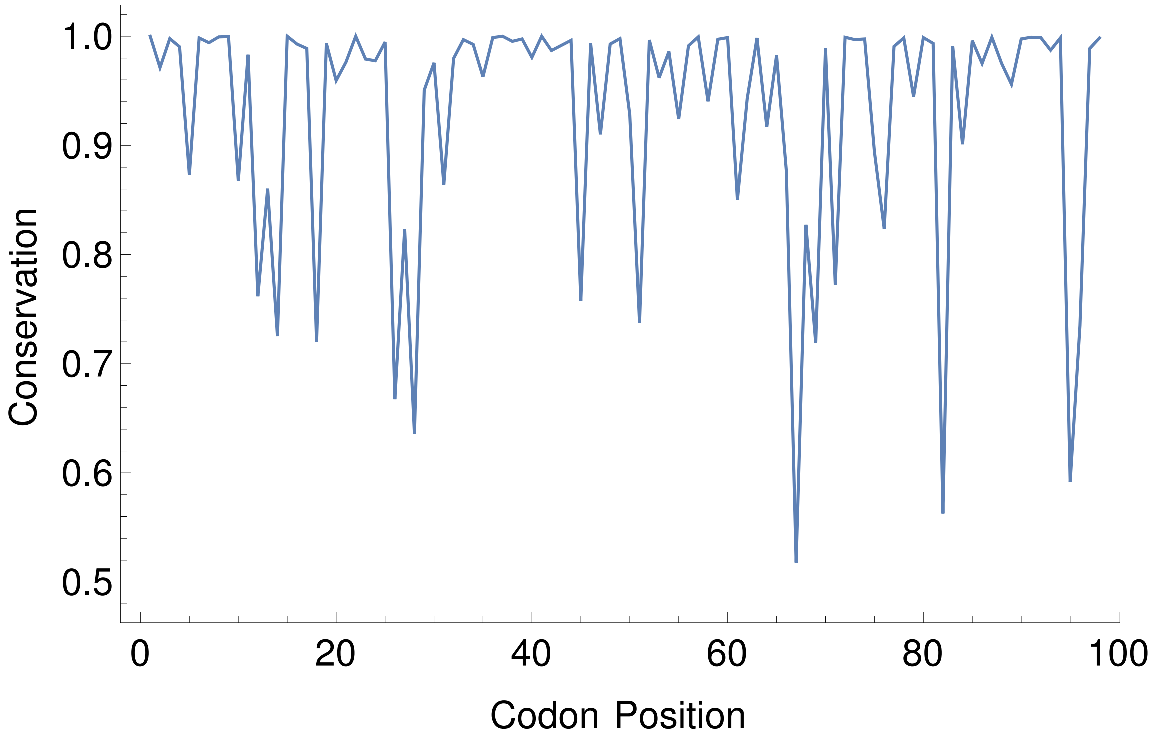

Conservation analysis plots

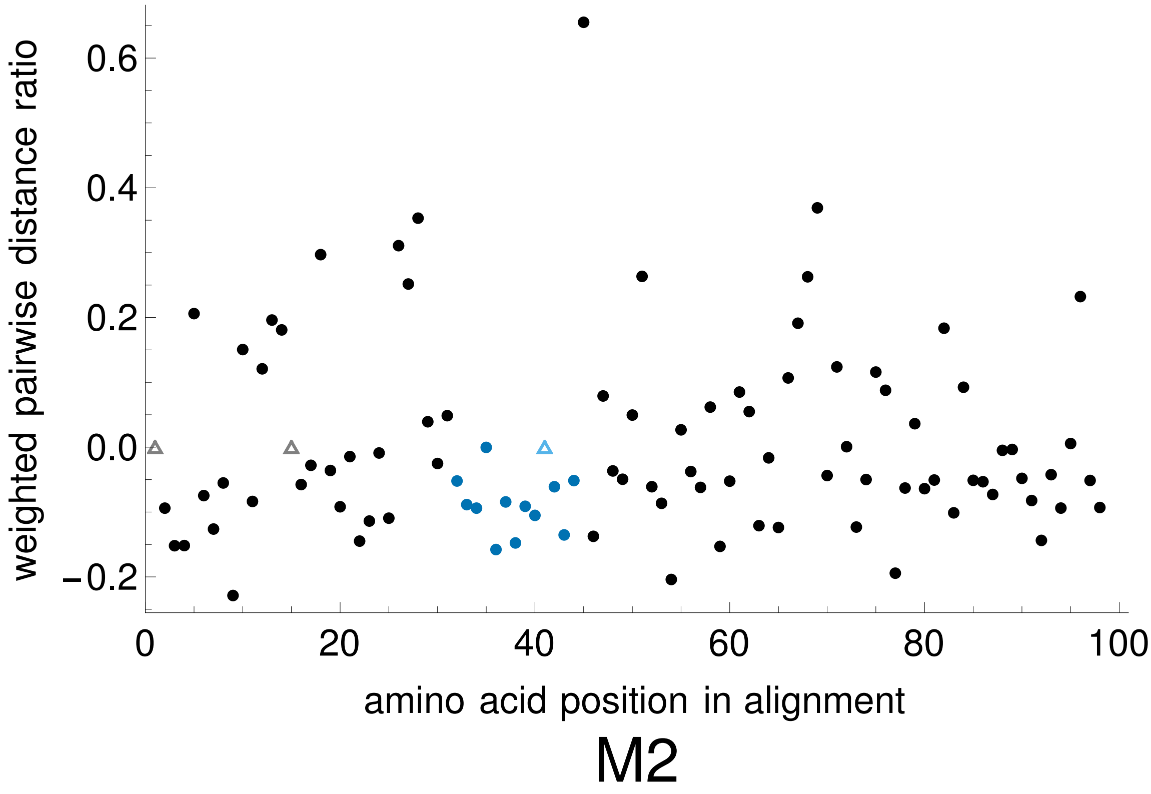

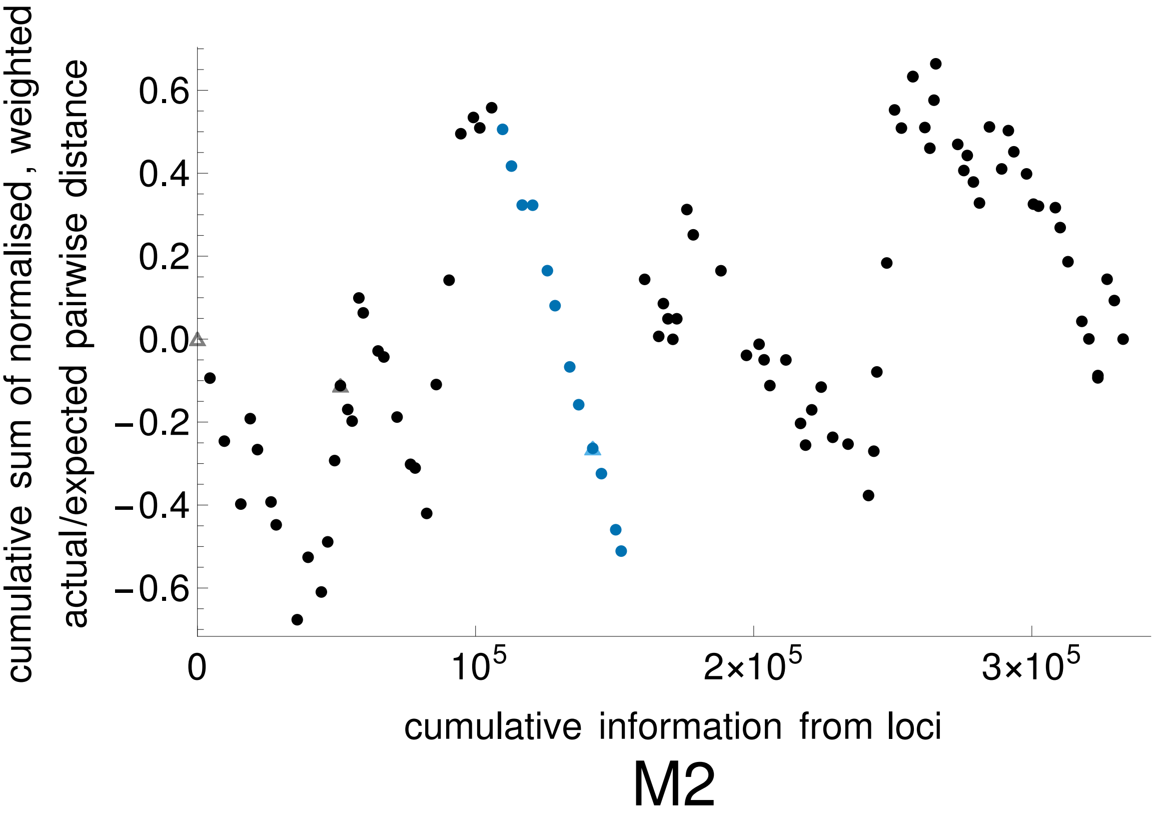

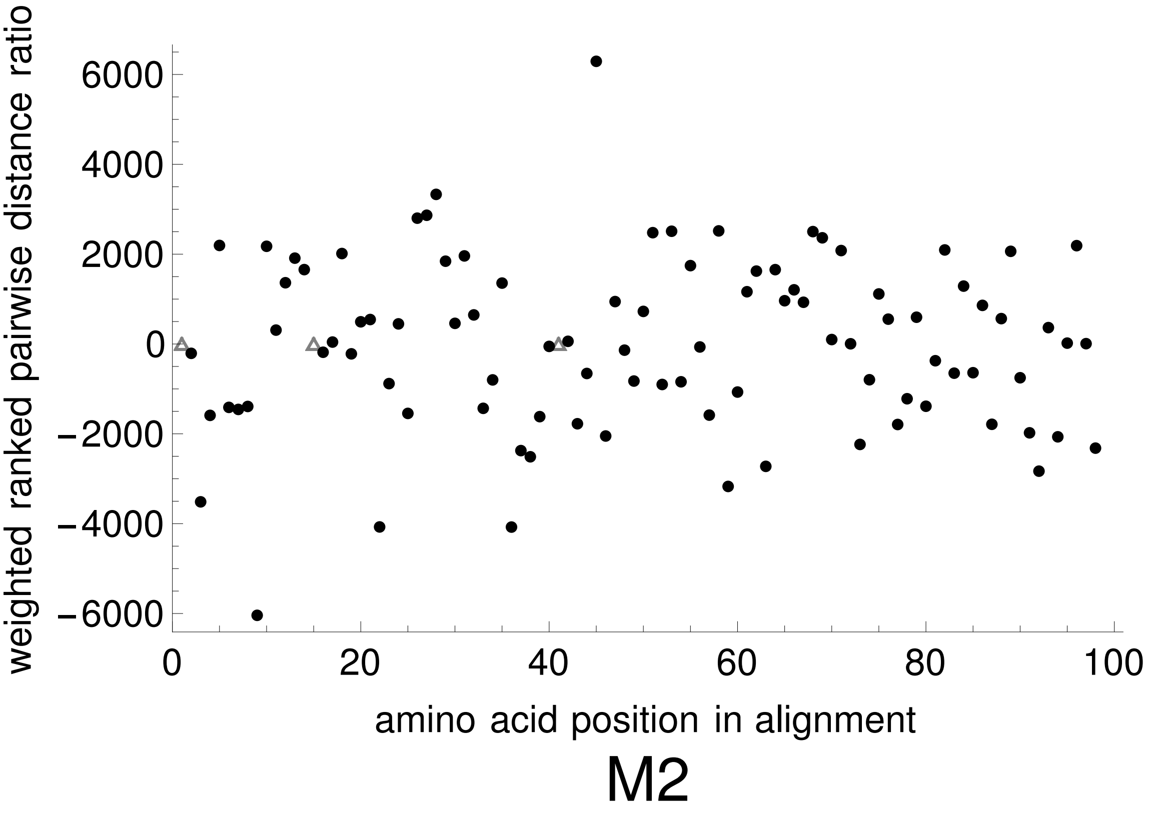
weighted ranked pairwise distance ratio
6000
4000
2000
0
−2000
−4000
−6000
0
20
40
60
80
100
amino acid position in alignment
M2

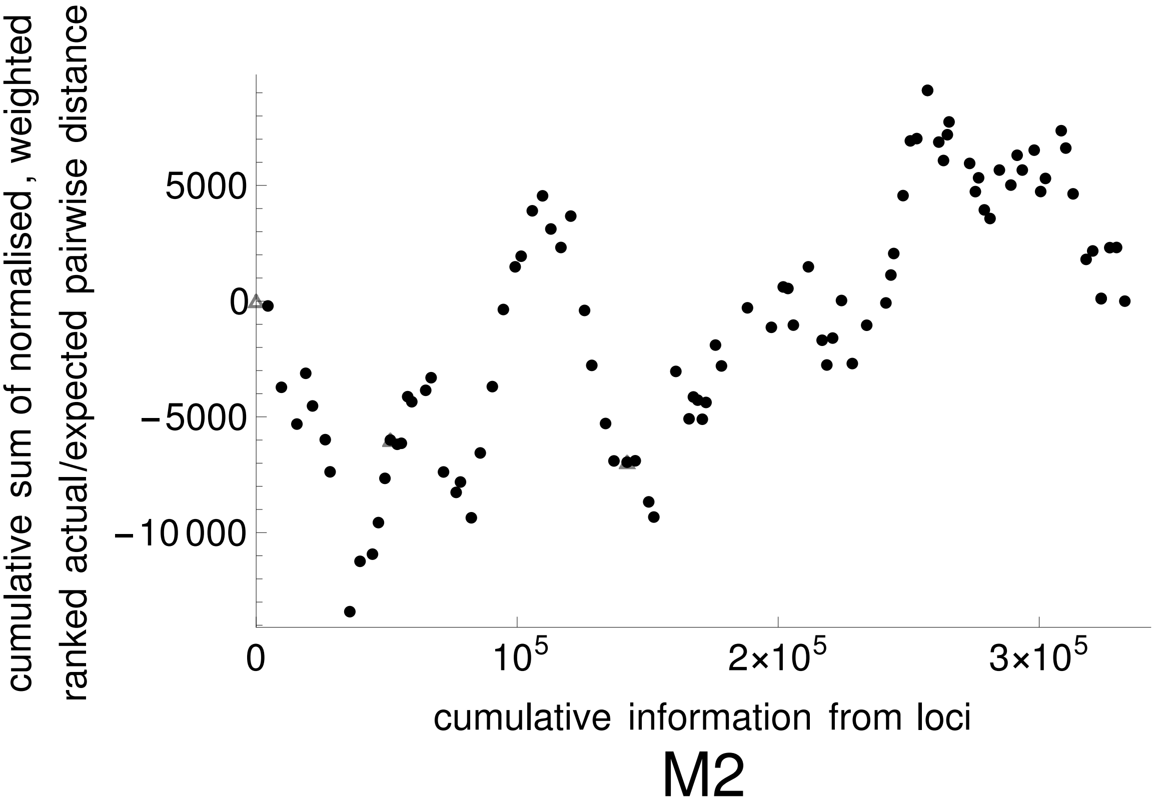
cumulative sum of normalised, weighted ranked actual/expected pairwise distance
5000
0
−5000
−10 000
0
10^5
2×10^5
3×10^5
cumulative information from loci
M2

NS1

Gene length histogram

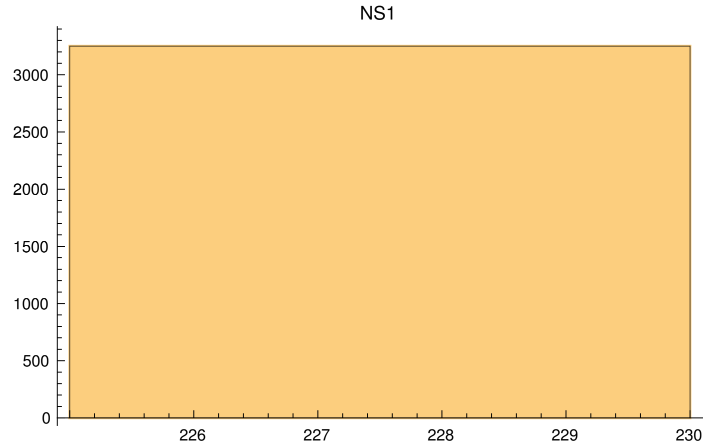

Information vs. nPD

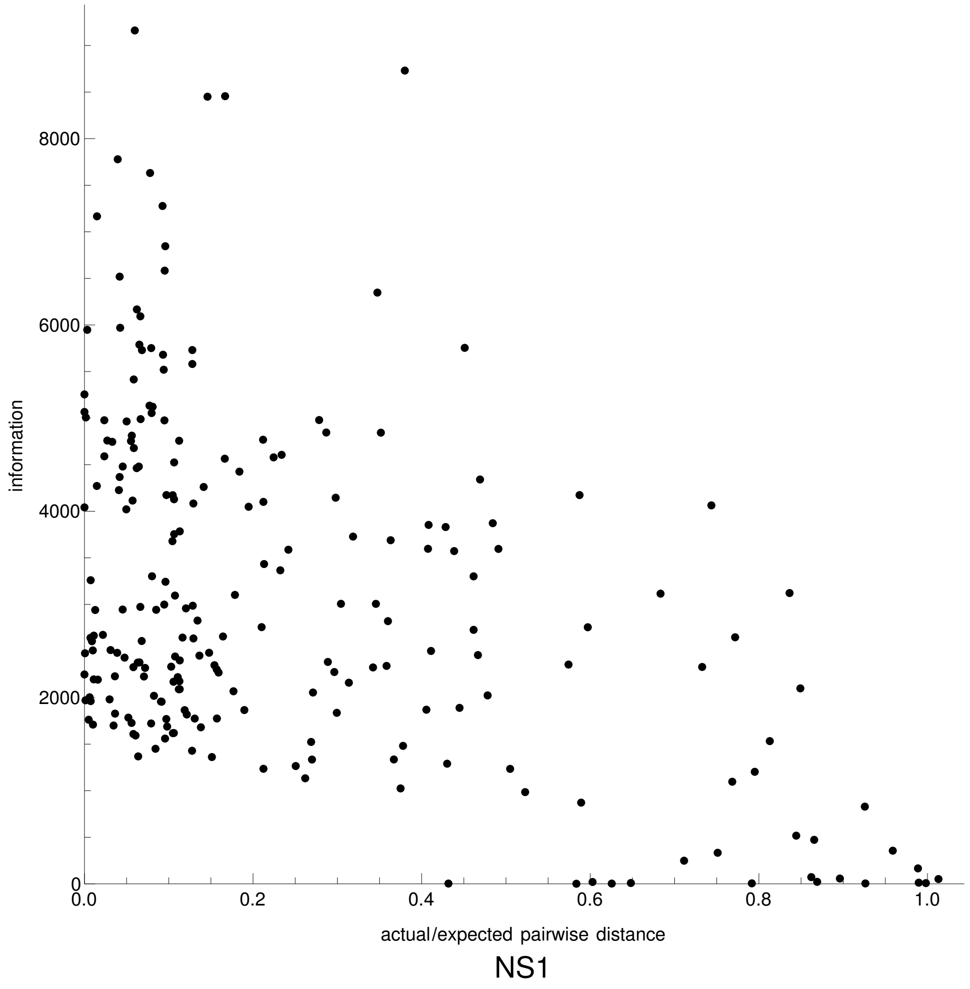

Example sequences highlighted by regions found in analyses to be conserved

```
Interesting points (by weighted raw PD) highlighted for gene NS1:
---ATG
```

GATTCCAACACTGTGTCAAGCTTTCAGGTAGACTGCTTTCTTTGGCATGTCCGCA AACGATTTGCAGACCAAGAACTGGGTGATGCCCCATTCCTTGACCGGCTTCG CCGAGATCAGAAGTCCCTAAGAGGAAGAGGCAACACTCTTGGTCTG

AACATCGAAACAGCTACTCGTGCGGGAAAGCAGATAGTGGAGCGGATTCTGGAGGAGGAGTCTGATGAGGCA
---------------CTTAAA---------------ATG---------------TCGGCTTCACGCTACCTAACTGACAT⋱
GACTCTCGAAGAAATGTCAAGGGACTGGTTCATGCTCATGCCCAAGCAGAAAGTGGCAGGTTCCCTTTGCATCAA⋱
AATGGACCAGGCAATAATGGATAAAAACATCATATTGAAAGCAAACTTCAGTGTGATTTTTGACCGGTTGGAAAC⋱
CCTAATACTACTT

AGAGCTTTCACAGAAGAAGGAGCAATCGTGGGAGAAATCTCACCGTTACCTTCTC TTCCAGGACATACTGATGAGGATGTCAAAAATGCA---ATTGGGGTCCTCAT CGGAGGACTTGAATGGAATGATAACACGGTTCGAGTCTCTGAAGCTCTACAGAGATTCG⋱
CTTGGAGAAGCAGTGATGAGAATGGGAGACCTCCACTCCCTCCAAATCAGAAACGGAAAATGGCGAGAAC⋱
AATTGAGTCAGAAGTT ---------------------TGA

```
Interesting points (by weighted ranked PD) highlighted for gene NS1:
```

---ATGGATTCCAACACTGTGTCAAGCTTTCAGGTAGACTGCTTTCTTTGGCATGTCCGCAAACGATTTGCAGACCAA⋱
GAACTGGGTGATGCCCCATTCCTTGACCGGCTTCGCCGAGATCAGAAGTCCCTAAGAGGAAGAGGCAACACTCTT⋱
GGTCTGAACATCGAAACAGCTACTCGTGCGGGAAAGCAGATAGTGGAGCGGATTCTGGAGGAGGAGTCTGATGAG⋱
GCA---------------CTTAAA---------------ATG---------------TCGGCTTCACGCTACCTAACTGA⋱
CATGACTCTCGAAGAAATGTCAAGGGACTGGTTCATGCTCATGCCCAAGCAGAAAGTGGCAGGTTCCCTTTGCAT⋱
CAAAATGGACCAGGCAATAATGGATAAAAACATCATATTGAAAGCAAACTTCAGTGTGATTTTTGACCGGTTGGA⋱
AACCCTAATACTACTTAGAGCTTTCACAGAAGAAGGAGCAATCGTGGGAGAAATCTCACCGTTACCTTCT

CTTCCAGGACATACTGATGAGGATGTCAAAAATGCA---ATTGGGGTCCTCATCG GAGGACTTGAATGGAATGATAACACGGTTCGAGTCTCTGAAGCTCTACAGAGATTCGCTTGG⋱
AGAAGCAGTGATGAGAATGGGAGACCTCCACTCCCTCCAAATCAGAAACGGAAAATGGCGAGAACAATTG⋱
AGTCAGAAGTT ---------------------TGA

Per codon conservation report

NS1			
Pos.	1	obs:	exp:
atg	M	4	4.00
---	-	3246	3246.00
---	--	------	------
mPD		0	0
		nPD:	1.
		N. weight:	0.
		Sc. PD:	0
		Sc. rank:	0

NS1			
Pos.	2	obs:	exp:
atg	M	3250	3250.00
---	--	------	------
mPD		0	0
		nPD:	1.
		N. weight:	0.
		Sc. PD:	0
		Sc. rank:	0

NS1			
Pos.	3	obs:	exp:
aat	N	8	4.36
aac	N	0	3.64
gtt	V	1	0.22
gtc	V	0	0.21
gta	V	0	0.21
gtg	V	0	0.36
gct	A	2	0.50
gcc	A	0	0.39
gca	A	0	0.89
gcg	A	0	0.22
gat	D	3168	1747.00
gac	D	68	1489.00
gaa	E	1	1.68
gag	E	2	1.32
---	--	------	------
mPD		0.050	0.51
		nPD:	0.1
		N. weight:	0.61
		Sc. PD:	-0.04
		Sc. rank:	-3.7

NS1			
Pos.	4	obs:	exp:
ttt	F	0	1.25
ttc	F	3	1.75
tct	S	25	492.40
tcc	S	2868	427.30
tca	S	57	668.80
tcg	S	0	178.00
tat	Y	0	0.48
tac	Y	1	0.52
cct	P	1	80.62
ccc	P	290	53.28
cca	P	4	119.70
ccg	P	0	41.36
act	T	0	0.26
acc	T	1	0.23
aca	T	0	0.42
acg	T	0	0.09
agt	S	0	639.90
agc	S	0	543.70
---	--	------	------
mPD		0.22	1.7
		nPD:	0.13
		N. weight:	2.1
		Sc. PD:	-0.081
		Sc. rank:	1065.6

NS1			
Pos.	5	obs:	exp:
ttt	F	0	0.42
ttc	F	1	0.58
tct	S	0	2.50
tcc	S	0	2.17
tca	S	0	3.40
tcg	S	0	0.90
aat	N	35	1762.00
aac	N	3196	1469.00
aaa	K	3	1.71
aag	K	0	1.29
agt	S	0	3.25
agc	S	15	2.76
---	--	------	------
mPD		0.034	0.51
		nPD:	0.07
		N. weight:	0.86
		Sc. PD:	-0.082
		Sc. rank:	-739.9

NS1			
Pos.	6	obs:	exp:
act	T	3126	851.70
acc	T	3	732.10
aca	T	74	1359.00
acg	T	46	305.70
gct	A	1	0.25
gcc	A	0	0.19
gca	A	0	0.45
gcg	A	0	0.11
---	--	------	------
mPD		0.074	0.70
		nPD:	0.11
		N. weight:	1.4
		Sc. PD:	-0.08
		Sc. rank:	255.2

NS1			
Pos.	7	obs:	exp:
att	I	0	16.15
atc	I	0	11.77
ata	I	44	16.08
atg	M	4	4.00
gtt	V	28	688.00
gtc	V	0	673.00
gta	V	11	682.10
gtg	V	3160	1156.00
gct	A	0	0.76
gcc	A	0	0.58
gca	A	2	1.34
gcg	A	1	0.33
---	--	------	------
mPD		0.082	0.77
		nPD:	0.11
		N. weight:	1.1
		Sc. PD:	-0.065
		Sc. rank:	234.4

NS1			
Pos.	8	obs:	exp:
ttt	F	0	0.42
ttc	F	1	0.58
tta	L	52	4.90
ttg	L	0	10.07
tct	S	1	525.60
tcc	S	1	456.10
tca	S	3141	713.90
tcg	S	6	190.00
ctt	L	0	10.52
ctc	L	0	8.48
cta	L	1	7.84
ctg	L	0	11.19
cct	P	0	0.27
ccc	P	0	0.18
cca	P	1	0.41
ccg	P	0	0.14
act	T	0	12.06
acc	T	45	10.37
aca	T	1	19.25
acg	T	0	4.33
agt	S	0	683.00
agc	S	0	580.30
---	--	------	------
mPD		0.095	1.7
		nPD:	0.06
		N. weight:	1.7
		Sc. PD:	-0.18
		Sc. rank:	-2171.8

NS1			
Pos.	9	obs:	exp:
tct	S	0	542.20
tcc	S	0	470.50
tca	S	0	736.30
tcg	S	45	196.00
cgt	R	0	0.04
cgc	R	0	0.05
cga	R	0	0.10
cgg	R	0	0.08
agt	S	655	704.50
agc	S	2548	598.60
aga	R	0	0.47
agg	R	1	0.26
ggt	G	0	0.14
ggc	G	1	0.13
gga	G	0	0.44
ggg	G	0	0.29
---	--	------	------
mPD		0.40	1.7
		nPD:	0.24
		N. weight:	1.3
		Sc. PD:	0.082
		Sc. rank:	1631.7

NS1			
Pos.	10	obs:	exp:
ttt	F	3158	1355.00
ttc	F	91	1894.00
tat	Y	1	0.48
tac	Y	0	0.52
---	--	------	------
mPD		0.055	0.49
		nPD:	0.11
		N. weight:	0.87
		Sc. PD:	-0.046
		Sc. rank:	313.6

NS1			
Pos.	11	obs:	exp:
caa	Q	0	1623.00
cag	Q	3250	1627.00
---	--	------	------
mPD		0	0.50
		nPD:	0.
		N. weight:	0.81
		Sc. PD:	-0.13
		Sc. rank:	-1932.9

NS1			
Pos.	12	obs:	exp:
gtt	V	2	699.00
gtc	V	0	683.80
gta	V	3248	693.00
gtg	V	0	1174.00
---	--	------	------
mPD		0.0012	0.73
		nPD:	0.
		N. weight:	1.8
		Sc. PD:	-0.28
		Sc. rank:	-4030.5

NS1			
Pos.	13	obs:	exp:
gct	A	0	0.25
gcc	A	1	0.19
gca	A	0	0.45
gcg	A	0	0.11
gat	D	93	1754.00
gac	D	3156	1495.00
---	--	------	------
mPD		0.056	0.50
		nPD:	0.11
		N. weight:	0.76
		Sc. PD:	-0.04
		Sc. rank:	240.9

NS1			
Pos.	14	obs:	exp:
tct	S	0	0.17
tcc	S	0	0.14
tca	S	0	0.23
tcg	S	0	0.06
tgt	C	48	1257.00
tgc	C	3200	1991.00
agt	S	0	0.22
agc	S	1	0.18
ggt	G	0	0.14
ggc	G	1	0.13
gga	G	0	0.44
ggg	G	0	0.29
---	--	------	------
mPD		0.030	0.48
		nPD:	0.06
		N. weight:	0.5
		Sc. PD:	-0.048
		Sc. rank:	-469.2

NS1			
Pos.	15	obs:	exp:
ttt	F	3013	1336.00
ttc	F	189	1866.00
tta	L	0	0.18
ttg	L	0	0.38
tat	Y	45	22.06
tac	Y	1	23.94
ctt	L	1	0.40
ctc	L	1	0.32
cta	L	0	0.30
ctg	L	0	0.42
---	--	------	------
mPD		0.14	0.52
		nPD:	0.27
		N. weight:	0.74
		Sc. PD:	0.066
		Sc. rank:	972.0

NS1			
Pos. 16		obs:	exp:
ttt	F	0	0.42
ttc	F	1	0.58
tta	L	1	299.90
ttg	L	14	616.30
ctt	L	2991	644.10
ctc	L	205	518.90
cta	L	30	480.10
ctg	L	3	684.70
att	I	5	1.83
atc	I	0	1.34
ata	I	0	1.83
---	--	------	------
mPD		0.16	1.1
		nPD:	0.14
		N. weight:	1.5
		Sc. PD:	-0.042
		Sc. rank:	1034.7

NS1			
Pos. 17		obs:	exp:
tta	L	0	0.09
ttg	L	1	0.19
tgt	C	4	1.55
tgc	C	0	2.45
tgg	W	3245	3245.00
ctt	L	0	0.20
ctc	L	0	0.16
cta	L	0	0.15
ctg	L	0	0.21
---	--	------	------
mPD		0.0031	0.0039
		nPD:	0.79
		N. weight:	0.002
		Sc. PD:	0.0011
		Sc. rank:	4.6

NS1			
[truncated: 187,508 more chars]
