# Supplementary material for: Locations and structures of influenza A virus packaging-associated signals and other functional elements via an in silico pipeline for predicting constrained features in RNA viruses
Source: PLoS Comput Biol. 2024 Apr 22;20(4):e1012009. doi: 10.1371/journal.pcbi.1012009 (PMC11034665; doi:10.1371/journal.pcbi.1012009)
Supplement: S5 Code — The content of the notebook follows the same pattern as that in S1 Code. (ZIP) [file pcbi.1012009.s118.zip › S5_Code.pdf]

H5N8 avian hosts

PB2

Gene length histogram

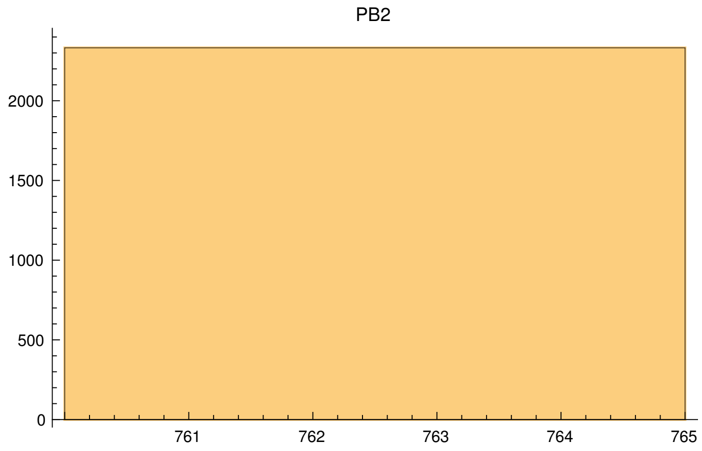

Information vs. nPD

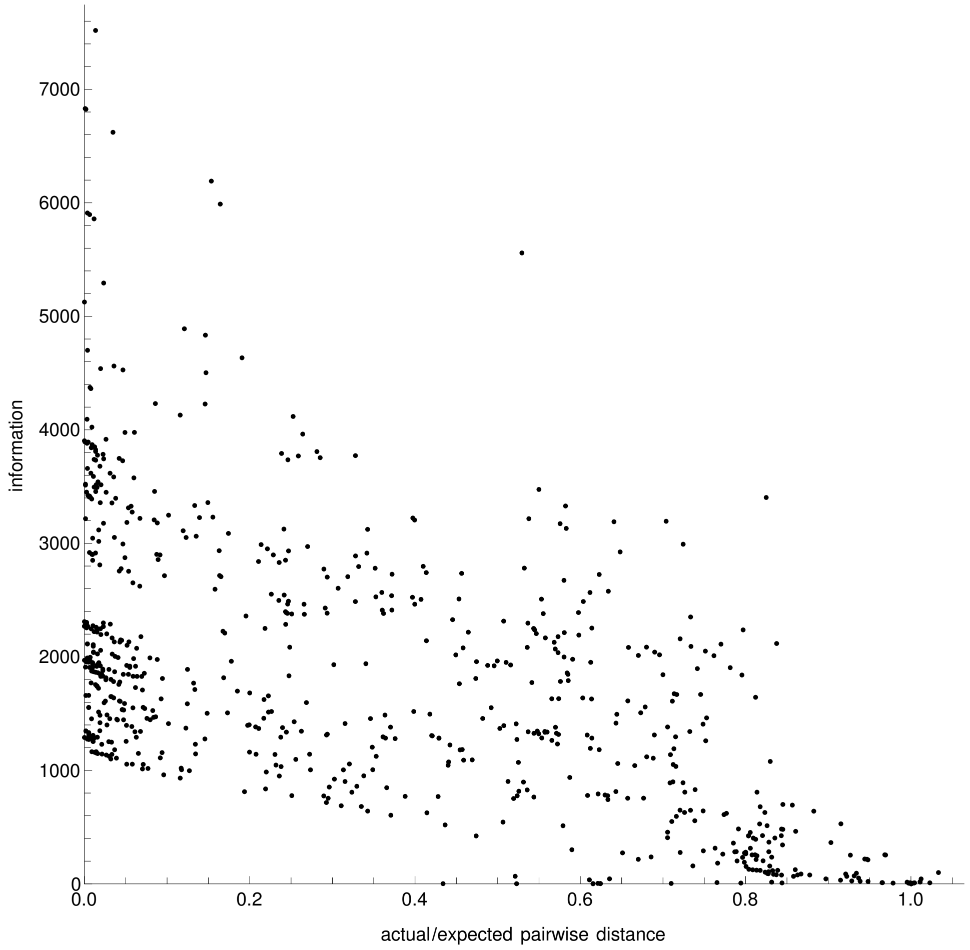

PB2

Example sequences highlighted by regions found in analyses to be conserved

Interesting points (by weighted raw PD) highlighted for gene PB2:

ATGGAGAGAATAAAAGAACTAAGAGATTTGATGTCGCAGTCT
CGCACTCGCGAGATACTGACAAAGACCACGGTGGACCATATGGCCATAATCAAGA
AATATACGTCAGGAAGACAGGAGAAGAATCCTGCACTTAGGATGAAATGGATGATGGCA
ATGAAATACCCGATTACAGCAGACAAAAGGATAATGGAGATGATTCCTGAAAGAAATGAGCAAGGTCAGA
CTCTTTGGAGCAAAACAAATGATGCTGGATCAGACAGAGTGATGGTGTCACCTCTGGCAGTGACGTGGTG
GAATAGAAATGGACCAACAACAAGTACAGTCCATTATCCAAAGGTTTACAAAACCTACTTTGAAAAGGTT
GAAAGGTTAAAGCATGGAACCTTCGGCCCTGTTCATTTTCGAAATCAGGTTAAGATACGCCGCAGGGTTG
ACATAAACCCGGGCCATGCAGATCTCAGTGCTAAAGAAGCACAAGACGTCATCATGGAGGTCGTTTTCCC
AAACGAAGTCGGAGCCAGGATTTTGACATCAGAGTCACAGTTGACAATAACAAAGGAAAAGAAGGAGGAG
CTTCAGGACTGTAAGATCGCCCCTTTAATGGTGGCATACATGTTGGAAAGAGAACTGGTTCGCAAAACCA
GATTCCTGCCAGTAGCTGGTGGGACAAGCAGCGTGTATATCGAGGTATTGCACTTGACTCAAGGGACCTG
CTGGGAACAAATGTACACACCGGGAGGAGAAGTGAGAAATGATGACATTGATCAGAGTTTAATTATTGCT
GCTAGAAATATTGTTAGGAGAGCAACAGTATCAGCAGACCCATTGGCTTCGCTCCTGGAGATGTGCCATA
GTACACACATTGGCGGGATAAGGATGGTAGACATCCTTAGACAGAACCCAACAGAAGAGCAAGCCGTGGA
TATATGCAAAGCAGCAATGGGTCTAAGAATCAGTTCATCCTTCAGCTTTGGAGGTTTCACTTTCAAAAGG
ACAAGTGGATCATCTGTCAAAAGAGAAGAGGAAGTGCTCACCGGCAACCTCCAAACATTGAAAATAAGAG
TACATGAAGGGTATGAGGAATTCACAATGGTTGGGCGAAGAGCAACAGCCATTCTAAGGAAAGCAACCAG
AAGGTTAATCCAATTGATAGTGAGTGGGAGAGACGAGCAGTCAATCGCCGAAGCGATCATAGTGGCAATG
GTGTTCTCCCAAGAGGATTGCATGATAAAAGCAGTACGGGGTGATTTGAATTTCGTCAATAGAGCGAATC
AGCGGCTCAATCCTATGCATCAACTTCTGAGGCATTTCCAGAAGGATGCAAAGGTACTGTTCCAAAACTG
GGGAGTTGAACCCATTGACAATGTAATGGGGATGATAGGAATATTGCCTGATATGACACCCAGTACAGAG
ATGTCAATAAGAGGGGTGAGAGTCAGCAAAATGGGAGTGGATGAATATTCCAGTACTGAGAGAGTGGTCG
TGAGTATTGATCGTTTTTTGAGGGTCCGAGACCAGAGAGGGAACGTGCTCCTGTCTCCTGAAGAGGTTAG
TGAAACACAGGGAACAGAGAAGCTGACGATAACATACTCATCATCTATGATGTGGGAAATTAACGGCCCG
GAATCAGTATTAGTTAACACATATCAATGGATCATTAGAAACTGGGAAACTGTGAAGATTCAATGGTCCC
AAGATCCGACAATGCTATACAACAAGATGGAGTTTGAGCCCTTTCAGTCCTTGGTGCCTAAGGCTGCCAG
AGGCCAGTATAGTGGATTTGTGAGGACGCTATTCCAGCAGATGCGTGATGTGCTGGGGACCTTTGACACT
GTCCAGATAATAAAGCTACTTCCATTTGCAGCAGCCCCACCGGAACAGAGTAGGATGCAGTTCTCTTCTC
TAACTGTAAACGTAAGAGGTTCAGGAATGAGAATACTTGTGAGAGGCAATTCCCCTGTGTTCAACTATAA
CAAGGCAACCAAGAGGCTTACAGTCCTTGGAAAGGATGCAGGTGCATTGACAGAAGACCCAGATGAGGGG
ACGGCAGGGGTGGAGTCTGCGGTATTAAGAGGGTTC
CTAATTCTGGGCAAAGAAGACAAAAGATATGGACCAGCATTGAGCATCAATGAAT
TGAGCAATCTTGCGAAGGGGGAGAAGGCTAATGTGTTGATAGGGCAAGGAGA
CGTGGTGTTGGTGATGAAACGGAAACGGGACTCTAGCATACTTACTGACAGC
CAGACAGCGACCAAAAGAATTCGGATGGCCATCAATTAG

Interesting points (by weighted ranked PD) highlighted for gene PB2:

ATGGAGAGAATAAAAGAACTAAGAGATTTGATGTCGCAGTCT
CGCACTCGCGAGATACTGACAAAGACCACGGTGGACCATATGGCCATAATCAAGAAA
TATACGTCAGGAAGACAGGAGAAGAATCCTGCACTTAGGATGAAATGGATGATGGCAATGAAATACCCGA
TTACAGCAGACAAAAGGATAATGGAGATGATTCCTGAAAGAAATGAGCAAGGTCAGACTCTTTGGAGCAA
AACAAATGATGCTGGATCAGACAGAGTGATGGTGTCACCTCTGGCAGTGACGTGGTGGAATAGAAATGGA
CCAACAACAAGTACAGTCCATTATCCAAAGGTTTACAAAACCTACTTTGAAAAGGTTGAAAGGTTAAAGC

ATGGAACCTTCGGCCCTGTTCATTTTCGAAATCAGGTTAAGATACGCCGCAGGGTTGACATAAACCCGGG⋱
CCATGCAGATCTCAGTGCTAAAGAAGCACAAGACGTCATCATGGAGGTCGTTTTCCCAAACGAAGTCGGA⋱
GCCAGGATTTTGACATCAGAGTCACAGTTGACAATAACAAAGGAAAAGAAGGAGGAGCTTCAGGACTGTA⋱
AGATCGCCCCTTTAATGGTGGCATACATGTTGGAAAGAGAACTGGTTCGCAAAACCAGATTCCTGCCAGT⋱
AGCTGGTGGGACAAGCAGCGTGTATATCGAGGTATTGCACTTGACTCAAGGGACCTGCTGGGAACAAATG⋱
TACACACCGGGAGGAGAAGTGAGAAATGATGACATTGATCAGAGTTTAATTATTGCTGCTAGAAATATTG⋱
TTAGGAGAGCAACAGTATCAGCAGACCCATTGGCTTCGCTCCTGGAGATGTGCCATAGTACACACATTGG⋱
CGGGATAAGGATGGTAGACATCCTTAGACAGAACCCAACAGAAGAGCAAGCCGTGGATATATGCAAAGCA⋱
GCAATGGGTCTAAGAATCAGTTCATCCTTCAGCTTTGGAGGTTTCACTTTCAAAAGGACAAGTGGATCAT⋱
CTGTCAAAAGAGAAGAGGAAGTGCTCACCGGCAACCTCCAAACATTGAAAATAAGAGTACATGAAGGGTA⋱
TGAGGAATTCACAATGGTTGGGCGAAGAGCAACAGCCATTCTAAGGAAAGCAACCAGAAGGTTAATCCAA⋱
TTGATAGTGAGTGGGAGAGACGAGCAGTCAATCGCCGAAGCGATCATAGTGGCAATGGTGTTCTCCCAAG⋱
AGGATTGCATGATAAAAGCAGTACGGGGTGATTTGAATTTCGTCAATAGAGCGAATCAGCGGCTCAATCC⋱
TATGCATCAACTTCTGAGGCATTTCCAGAAGGATGCAAAGGTACTGTTCCAAAACTGGGGAGTTGAACCC⋱
ATTGACAATGTAATGGGGATGATAGGAATATTGCCTGATATGACACCCAGTACAGAGATGTCAATAAGAG⋱
GGGTGAGAGTCAGCAAAATGGGAGTGGATGAATATTCCAGTACTGAGAGAGTGGTCGTGAGTATTGATCG⋱
TTTTTTGAGGGTCCGAGACCAGAGAGGGAACGTGCTCCTGTCTCCTGAAGAGGTTAGTGAAACACAGGGA⋱
ACAGAGAAGCTGACGATAACATACTCATCATCTATGATGTGGGAAATTAACGGCCCGGAATCAGTATTAG⋱
TTAACACATATCAATGGATCATTAGAAACTGGGAAACTGTGAAGATTCAATGGTCCCAAGATCCGACAAT⋱
GCTATACAACAAGATGGAGTTTGAGCCCTTTCAGTCCTTGGTGCCTAAGGCTGCCAGAGGCCAGTATAGT⋱
GGATTTGTGAGGACGCTATTCCAGCAGATGCGTGATGTGCTGGGGACCTTTGACACTGTCCAGATAATAA⋱
AGCTACTTCCATTTGCAGCAGCCCCACCGGAACAGAGTAGGATGCAGTTCTCTTCTCTAACTGTAAACGT⋱
AAGAGGTTCAGGAATGAGAATACTTGTGAGAGGCAATTCCCCTGTGTTCAACTATAACAAGGCAACCAAG⋱
AGGCTTACAGTCCTTGGAAAGGATGCAGGTGCATTGACAGAAGACCCAGATGAGGGGACGGCAGGGGTGG⋱
AGTCTGCGGTATTAAGAGGGTTCCTAATTCTGGGCAAAGAAGACAAAAGATATGGACCAGCATTGAGCAT⋱
CAATGAATTGAGCAATCTTGCGAAGGGGGAGAAGGCTAATGTGTTGATAGGGCAAGGAGACGTGGTGTTG⋱
GTGATG
AAACGGAAACGGGACTCTAGCATACTTACTGACAGCCAGACAGCGACCAAAAGAA
TTCGGATGGCCATCAATTAG

Per codon conservation report

PB2

Pos.	1	obs:	exp:
atg	M	2332	2332.00
---	--	------	------
mPD		0	0
		nPD:	1.
		N. weight:	0.
		Sc. PD:	0
		Sc. rank:	0

PB2

Pos.	2	obs:	exp:
gaa	E	23	1389.00
gag	E	2297	930.90
ggt	G	0	1.73
ggc	G	0	1.58
gga	G	11	5.24
ggg	G	1	3.45
---	--	------	------
mPD		0.039	0.49
		nPD:	0.08
		N. weight:	1.1
		Sc. PD:	-0.16
		Sc. rank:	-89.9

PB2

Pos.	3	obs:	exp:
tct	S	0	0.17
tcc	S	0	0.15
tca	S	0	0.23
tcg	S	0	0.06
cgt	R	0	87.98
cgc	R	0	123.60
cga	R	1	224.60
cgg	R	0	181.70
agt	S	1	0.20
agc	S	0	0.19
aga	R	2314	1064.00
agg	R	5	637.90
gaa	E	0	6.59
gag	E	11	4.41
---	--	------	------
mPD		0.034	0.97
		nPD:	0.04
		N. weight:	1.
		Sc. PD:	-0.19
		Sc. rank:	-463.8

PB2

Pos.	4	obs:	exp:
tta	L	1	2.07
ttg	L	0	3.57
ctt	L	0	3.56
ctc	L	0	3.28
cta	L	18	2.53
ctg	L	0	3.99
cgt	R	0	0.04
cgc	R	0	0.05
cga	R	0	0.10
cgg	R	0	0.08
att	I	0	856.70
atc	I	0	578.60
ata	I	2307	871.70
act	T	0	0.53
acc	T	0	0.44
aca	T	2	0.86
acg	T	0	0.17
aga	R	1	0.46
agg	R	0	0.27
gtt	V	0	0.65
gtc	V	0	0.63
gta	V	3	0.59
gtg	V	0	1.12
---	--	------	------
mPD		0.021	0.68
		nPD:	0.03
		N. weight:	1.3
		Sc. PD:	-0.24
		Sc. rank:	-668.9

PB2

Pos.	5	obs:	exp:
cgt	R	0	0.46
cgc	R	0	0.64
cga	R	0	1.16
cgg	R	0	0.94
att	I	0	0.74
atc	I	0	0.50
ata	I	2	0.76
act	T	0	5.03
acc	T	0	4.20
aca	T	19	8.16
acg	T	0	1.60
aat	N	2	1.59
aac	N	1	1.41
aaa	K	2294	1289.00
aag	K	2	1007.00
aga	R	12	5.50
agg	R	0	3.30
---	--	------	------
mPD		0.032	0.53
		nPD:	0.06
		N. weight:	0.77
		Sc. PD:	-0.12
		Sc. rank:	-120.7

PB2

Pos.	6	obs:	exp:
cgt	R	0	0.11
cgc	R	0	0.16
cga	R	0	0.29
cgg	R	0	0.23
aaa	K	1	0.56
aag	K	0	0.44
aga	R	3	1.38
agg	R	0	0.82
gat	D	0	1.15
gac	D	2	0.85
gaa	E	2294	1392.00
gag	E	30	932.50
ggt	G	0	0.29
ggc	G	0	0.26
gga	G	2	0.87
ggg	G	0	0.58
---	--	------	------
mPD		0.035	0.49
		nPD:	0.07
		N. weight:	0.6
		Sc. PD:	-0.088
		Sc. rank:	-58.3

PB2

Pos.	7	obs:	exp:
tta	L	111	254.40
ttg	L	0	437.40
ctt	L	1	437.10
ctc	L	0	402.20
cta	L	2213	310.10
ctg	L	6	489.80
att	I	0	0.37
atc	I	0	0.25
ata	I	1	0.38
---	--	------	------
mPD		0.098	1.1
		nPD:	0.09
		N. weight:	2.4
		Sc. PD:	-0.32
		Sc. rank:	-106.2

PB2

Pos.	8	obs:	exp:
cgt	R	0	88.36
cgc	R	0	124.10
cga	R	12	225.60
cgg	R	0	182.50
att	I	0	0.37
atc	I	0	0.25
ata	I	1	0.38
aga	R	2315	1069.00
agg	R	3	640.70
ggt	G	0	0.14
ggc	G	0	0.13
gga	G	1	0.44
ggg	G	0	0.29
---	--	------	------
mPD		0.015	0.95
		nPD:	0.02
		N. weight:	1.
		Sc. PD:	-0.2
		Sc. rank:	-805.6

PB2

Pos.	9	obs:	exp:
aat	N	2	1.06
aac	N	0	0.94
gat	D	2327	1339.00
gac	D	1	988.90
gaa	E	1	0.60
gag	E	0	0.40
ggt	G	1	0.14
ggc	G	0	0.13
gga	G	0	0.44
ggg	G	0	0.29
---	--	------	------
mPD		0.0043	0.49
		nPD:	0.01
		N. weight:	0.74
		Sc. PD:	-0.15
		Sc. rank:	-793.0

PB2

Pos.	10	obs:	exp:
tta	L	59	254.40
ttg	L	1594	437.40
ctt	L	0	437.10
ctc	L	0	402.20
cta	L	640	310.10
ctg	L	38	489.80
atg	M	1	1.00
---	--	------	------
mPD		0.83	1.1
		nPD:	0.73
		N. weight:	1.3
		Sc. PD:	0.67
		Sc. rank:	1861.7

PB2

Pos.	11	obs:	exp:
atg	M	2330	2330.00
gtt	V	0	0.43
gtc	V	0	0.42
gta	V	0	0.39
gtg	V	2	0.75
---	--	------	------
mPD		0.0017	0.0028
		nPD:	0.62
		N. weight:	0.0011
		Sc. PD:	0.00043
		Sc. rank:	1.4

PB2

Pos.	12	obs:	exp:
tta	L	0	0.33
ttg	L	3	0.56
tct	S	526	397.70
tcc	S	0	343.30
tca	S	102	525.00
tcg	S	1701	150.40
ctt	L	0	0.56
ctc	L	0	0.52
cta	L	0	0.40
ctg	L	0	0.63
agt	S	0	475.50
agc	S	0	437.00
---	--	------	------
mPD		0.42	1.6
		nPD:	0.25
		N. weight:	2.4
		Sc. PD:	0.07
		Sc. rank:	1139.2

PB2

Pos.	13	obs:	exp:
tat	Y	1	0.49
tac	Y	0	0.51
cat	H	4	2.38
cac	H	0	1.62
caa	Q	529	1180.00
cag	Q	1790	1139.00
cgt	R	0	0.19
cgc	R	0	0.27
cga	R	0	0.48
cgg	R	5	0.39
aaa	K	0	1.12
aag	K	2	0.88
aga	R	0	2.29
agg	R	0	1.37
gaa	E	0	0.60
gag	E	1	0.40
---	--	------	------
mPD		0.36	0.51
		nPD:	0.71
		N. weight:	0.23
		Sc. PD:	0.11
		Sc. rank:	307.0

PB2

Pos.	14	obs:	exp:
tct	S	1692	397.40
tcc	S	635	343.00
tca	S	0	524.50
tcg	S	0	150.30
tat	Y	2	0.97
tac	Y	0	1.03
cct	P	3	0.85
ccc	P	0	0.54
cca	P	0	1.22
ccg	P	0	0.39
agt	S	0	475.10
agc	S	0	436.60
---	--	------	------
mPD		0.40	1.6
		nPD:	0.24
		N. weight:	1.6
		Sc. PD:	0.034
		Sc. rank:	672.6

PB2

Pos.	15	obs:	exp:
cgt	R	0	88.44
cgc	R	2331	124.20
cga	R	1	225.80
cgg	R	0	182.60
aga	R	0	1070.00
agg	R	0	641.20
---	--	------	------
mPD		0.00086	0.95
		nPD:	0.
		N. weight:	3.9
		Sc. PD:	-0.84
		Sc. rank:	-5657.2

PB2			
Pos.	16	obs:	exp:
act	T	2324	617.70
acc	T	7	515.20
aca	T	1	1002.00
acg	T	0	197.00
---	--	------	------
mPD		0.0068	0.69
		nPD:	0.01
		N. weight:	1.7
		Sc. PD:	-0.36
		Sc. rank:	-1766.0

,

PB2			
Pos.	17	obs:	exp:
cgt	R	2	88.44
cgc	R	2330	124.20
cga	R	0	225.80
cgg	R	0	182.60
aga	R	0	1070.00
agg	R	0	641.20
---	--	------	------
mPD		0.0017	0.95
		nPD:	0.
		N. weight:	3.9
		Sc. PD:	-0.84
		Sc. rank:	-5386.1

,

PB2			
Pos.	18	obs:	exp:
gaa	E	2	1396.00
gag	E	2330	935.70
---	--	------	------
mPD		0.0017	0.48
		nPD:	0.
		N. weight:	1.2
		Sc. PD:	-0.26
		Sc. rank:	-1553.5

,

PB2			
Pos.	19	obs:	exp:
att	I	0	861.90
atc	I	0	582.10
ata	I	2321	877.00
atg	M	10	10.00
gtt	V	0	0.22
gtc	V	0	0.21
gta	V	1	0.20
gtg	V	0	0.37
---	--	------	------
mPD		0.0094	0.66
		nPD:	0.01
		N. weight:	1.3
		Sc. PD:	-0.26
		Sc. rank:	-1081.2

,

PB2			
Pos.	20	obs:	exp:
tta	L	0	254.50
ttg	L	1	437.60
ctt	L	0	437.30
ctc	L	4	402.30
cta	L	1464	310.30
ctg	L	863	490.00
---	--	------	------
mPD		0.47	1.1
		nPD:	0.41
		N. weight:	1.6
		Sc. PD:	0.29
		Sc. rank:	1256.4

,

PB2			
Pos.	21	obs:	exp:
act	T	3	617.20
acc	T	12	514.80
aca	T	2314	1001.00
acg	T	1	196.80
gct	A	0	0.51
gcc	A	0	0.39
gca	A	2	0.88
gcg	A	0	0.22
---	--	------	------
mPD		0.015	0.69
		nPD:	0.02
		N. weight:	1.1
		Sc. PD:	-0.21
		Sc. rank:	-682.8

,

PB2			
Pos.	22	obs:	exp:
cgt	R	0	0.27
cgc	R	0	0.37
cga	R	0	0.68
cgg	R	0	0.55
aaa	K	2151	1305.00
aag	K	174	1020.00
aga	R	6	3.21
agg	R	1	1.92
---	--	------	------
mPD		0.14	0.50
		nPD:	0.29
		N. weight:	0.44
		Sc. PD:	0.029
		Sc. rank:	246.2

,

PB2			
Pos.	23	obs:	exp:
act	T	1	617.70
acc	T	2331	515.20
aca	T	0	1002.00
acg	T	0	197.00
---	--	------	------
mPD		0.00086	0.69
		nPD:	0.
		N. weight:	2.
		Sc. PD:	-0.43
		Sc. rank:	-2849.1

,

PB2			
Pos.	24	obs:	exp:
act	T	2191	617.70
acc	T	1	515.20
aca	T	3	1002.00
acg	T	137	197.00
---	--	------	------
mPD		0.11	0.69
		nPD:	0.17
		N. weight:	1.6
		Sc. PD:	-0.086
		Sc. rank:	338.4

,

PB2			
Pos.	25	obs:	exp:
gtt	V	6	507.20
gtc	V	0	492.70
gta	V	1	458.80
gtg	V	2325	873.30
---	--	------	------
mPD		0.0060	0.73
		nPD:	0.01
		N. weight:	1.3
		Sc. PD:	-0.27
		Sc. rank:	-1418.5

,

PB2			
Pos.	26	obs:	exp:
gat	D	2	1341.00
gac	D	2330	990.60
---	--	------	------
mPD		0.0017	0.49
		nPD:	0.
		N. weight:	1.1
		Sc. PD:	-0.24
		Sc. rank:	-1476.3

,

PB2			
Pos.	27	obs:	exp:
cat	H	2299	1387.00
cac	H	33	945.40
---	--	------	------
mPD		0.028	0.48
		nPD:	0.06
		N. weight:	0.6
		Sc. PD:	-0.096
		Sc. rank:	-118.3

,

PB2			
Pos.	28	obs:	exp:
att	I	0	0.37
atc	I	0	0.25
ata	I	1	0.38
atg	M	2331	2331.00
---	--	------	------
mPD		0.00086	0.00086
		nPD:	1.
		N. weight:	0.00056
		Sc. PD:	0.00042
		Sc. rank:	0.8

,

PB2			
Pos.	29	obs:	exp:
gct	A	2	593.20
gcc	A	2321	454.80
gca	A	9	1025.00
gcg	A	0	258.90
---	--	------	------
mPD		0.0094	0.69
		nPD:	0.01
		N. weight:	2.1
		Sc. PD:	-0.43
		Sc. rank:	-1824.5

,

PB2			
Pos.	30	obs:	exp:
att	I	0	866.00
atc	I	0	584.90
ata	I	2332	881.20
---	--	------	------
mPD		0	0.66
		nPD:	0.
		N. weight:	1.3
		Sc. PD:	-0.28
		Sc. rank:	-1960.3

,

PB2			
Pos.	31	obs:	exp:
att	I	7	865.20
atc	I	849	584.40
ata	I	1474	880.40
gtt	V	0	0.43
gtc	V	1	0.42
gta	V	1	0.39
gtg	V	0	0.75
---	--	------	------
mPD		0.47	0.66
		nPD:	0.71
		N. weight:	0.6
		Sc. PD:	0.29
		Sc. rank:	804.8

,

PB2			
Pos.	32	obs:	exp:
aaa	K	36	1309.00
aag	K	2296	1023.00
---	--	------	------
mPD		0.030	0.49
		nPD:	0.06
		N. weight:	0.99
		Sc. PD:	-0.15
		Sc. rank:	-151.9

,

PB2			
Pos.	33	obs:	exp:
cgt	R	0	0.04
cgc	R	0	0.05
cga	R	0	0.10
cgg	R	0	0.08
aaa	K	2278	1308.00
aag	K	53	1023.00
aga	R	1	0.46
agg	R	0	0.27
---	--	------	------
mPD		0.045	0.49
		nPD:	0.09
		N. weight:	0.64
		Sc. PD:	-0.081
		Sc. rank:	-4.1

,

PB2			
Pos.	34	obs:	exp:
tat	Y	211	1134.00
tac	Y	2121	1198.00
---	--	------	------
mPD		0.16	0.50
		nPD:	0.33
		N. weight:	0.49
		Sc. PD:	0.052
		Sc. rank:	317.4

,

PB2			
Pos.	35	obs:	exp:
act	T	0	617.70
acc	T	0	515.20
aca	T	2142	1002.00
acg	T	190	197.00
---	--	------	------
mPD		0.15	0.69
		nPD:	0.22
		N. weight:	0.93
		Sc. PD:	-0.0043
		Sc. rank:	289.6

,

PB2			
Pos.	36	obs:	exp:
tct	S	0	398.30
tcc	S	0	343.80
tca	S	2320	525.70
tcg	S	12	150.60
agt	S	0	476.10
agc	S	0	437.50
---	--	------	------
mPD		0.010	1.6
		nPD:	0.01
		N. weight:	2.
		Sc. PD:	-0.41
		Sc. rank:	-2289.0

,

PB2			
Pos.	37	obs:	exp:
ggt	G	0	335.50
ggc	G	0	307.80
gga	G	2002	1018.00
ggg	G	330	670.70
---	--	------	------
mPD		0.24	0.69
		nPD:	0.35
		N. weight:	0.64
		Sc. PD:	0.082
		Sc. rank:	448.8

,

PB2			
Pos.	38	obs:	exp:
cgt	R	0	88.44
cgc	R	0	124.20
cga	R	0	225.80
cgg	R	0	182.60
aga	R	2295	1070.00
agg	R	37	641.20
---	--	------	------
mPD		0.031	0.95
		nPD:	0.03
		N. weight:	0.95
		Sc. PD:	-0.17
		Sc. rank:	-468.7

,

PB2			
Pos.	39	obs:	exp:
caa	Q	640	1185.00
cag	Q	1689	1144.00
cgt	R	0	0.04
cgc	R	0	0.05
cga	R	0	0.10
cgg	R	1	0.08
aaa	K	2	1.12
aag	K	0	0.88
aga	R	0	0.46
agg	R	0	0.27
---	--	------	------
mPD		0.40	0.50
		nPD:	0.8
		N. weight:	0.16
		Sc. PD:	0.087
		Sc. rank:	225.1

,

PB2			
Pos.	40	obs:	exp:
gat	D	0	0.58
gac	D	1	0.42
gaa	E	5	1396.00
gag	E	2326	935.30
---	--	------	------
mPD		0.0051	0.48
		nPD:	0.01
		N. weight:	1.2
		Sc. PD:	-0.25
		Sc. rank:	-1178.1

,

PB2

Pos.	41	obs:	exp:
cgt	R	0	0.08
cgc	R	0	0.11
cga	R	0	0.19
cgg	R	0	0.16
aaa	K	3	1308.00
aag	K	2327	1022.00
aga	R	0	0.92
agg	R	2	0.55
mPD		0.0043	0.49
		nPD:	0.01
		N. weight:	1.1
		Sc. PD:	-0.23
		Sc. rank:	-1181.7

PB2

Pos.	42	obs:	exp:
aat	N	530	1239.00
aac	N	1802	1093.00
mPD		0.35	0.50
		nPD:	0.71
		N. weight:	0.26
		Sc. PD:	0.12
		Sc. rank:	344.7

PB2

Pos.	43	obs:	exp:
cct	P	2324	657.60
ccc	P	7	422.00
cca	P	1	952.20
ccg	P	0	300.30
mPD		0.0068	0.70
		nPD:	0.01
		N. weight:	1.7
		Sc. PD:	-0.34
		Sc. rank:	-1723.2

PB2

Pos.	44	obs:	exp:
cct	P	0	0.28
ccc	P	1	0.18
cca	P	0	0.41
ccg	P	0	0.13
act	T	6	1.59
acc	T	0	1.33
aca	T	0	2.58
acg	T	0	0.51
gct	A	664	591.50
gcc	A	1457	453.40
gca	A	203	1022.00
gcg	A	1	258.10
mPD		0.53	0.70
		nPD:	0.75
		N. weight:	0.84
		Sc. PD:	0.43
		Sc. rank:	1185.2

PB2

Pos.	45	obs:	exp:
tta	L	0	254.50
ttg	L	0	437.60
ctt	L	218	437.30
ctc	L	2114	402.30
cta	L	0	310.30
ctg	L	0	490.00
mPD		0.17	1.1
		nPD:	0.15
		N. weight:	1.9
		Sc. PD:	-0.14
		Sc. rank:	304.2

PB2

Pos.	46	obs:	exp:
cgt	R	0	88.32
cgc	R	0	124.10
cga	R	0	225.50
cgg	R	0	182.40
aaa	K	0	1.68
aag	K	3	1.32
aga	R	663	1068.00
agg	R	1666	640.40
mPD		0.41	0.96
		nPD:	0.43
		N. weight:	0.74
		Sc. PD:	0.15
		Sc. rank:	602.9

PB2

Pos.	47	obs:	exp:
att	I	0	0.37
atc	I	0	0.25
ata	I	1	0.38
atg	M	2331	2331.00
mPD		0.00086	0.00086
		nPD:	1.
		N. weight:	0.00056
		Sc. PD:	0.00042
		Sc. rank:	0.8

PB2

Pos.	48	obs:	exp:
aaa	K	2323	1309.00
aag	K	9	1023.00
mPD		0.0077	0.49
		nPD:	0.02
		N. weight:	0.74
		Sc. PD:	-0.15
		Sc. rank:	-587.3

PB2

Pos.	49	obs:	exp:
tgg	W	2332	2332.00
mPD		0	0
		nPD:	1.
		N. weight:	0.
		Sc. PD:	0
		Sc. rank:	0

PB2

Pos.	50	obs:	exp:
atg	M	2332	2332.00
mPD		0	0
		nPD:	1.
		N. weight:	0.
		Sc. PD:	0
		Sc. rank:	0

PB2

Pos.	51	obs:	exp:
atg	M	2332	2332.00
mPD		0	0
		nPD:	1.
		N. weight:	0.
		Sc. PD:	0
		Sc. rank:	0

PB2

Pos.	52	obs:	exp:
gtt	V	0	0.22
gtc	V	0	0.21
gta	V	1	0.20
gtg	V	0	0.37
gct	A	3	593.00
gcc	A	0	454.60
gca	A	2297	1025.00
gcg	A	31	258.80
mPD		0.030	0.69
		nPD:	0.04
		N. weight:	1.
		Sc. PD:	-0.18
		Sc. rank:	-367.3

PB2

Pos.	53	obs:	exp:
atg	M	2332	2332.00
mPD		0	0
		nPD:	1.
		N. weight:	0.
		Sc. PD:	0
		Sc. rank:	0

PB2

Pos.	54	obs:	exp:
cgt	R	0	6.79
cgc	R	0	9.53
cga	R	0	17.33
cgg	R	0	14.02
aaa	K	2151	1208.00
aag	K	2	944.50
aga	R	179	82.11
agg	R	0	49.22
mPD		0.14	0.68
		nPD:	0.21
		N. weight:	0.79
		Sc. PD:	-0.0084
		Sc. rank:	233.2

PB2

Pos.	55	obs:	exp:
ttt	F	1	0.43
ttc	F	0	0.57
tat	Y	2172	1133.00
tac	Y	159	1198.00
mPD		0.13	0.50
		nPD:	0.26
		N. weight:	0.63
		Sc. PD:	0.021
		Sc. rank:	307.0

PB2

Pos.	56	obs:	exp:
cct	P	1467	657.60
ccc	P	8	422.00
cca	P	640	952.20
ccg	P	217	300.30
mPD		0.52	0.70
		nPD:	0.74
		N. weight:	0.48
		Sc. PD:	0.24
		Sc. rank:	661.3

PB2

Pos.	57	obs:	exp:
tta	L	0	13.53
ttg	L	0	23.27
ctt	L	0	23.25
ctc	L	124	21.39
cta	L	0	16.50
ctg	L	0	26.05
att	I	1698	819.90
atc	I	510	553.80
ata	I	0	834.30
mPD		0.50	0.77
		nPD:	0.64
		N. weight:	0.81
		Sc. PD:	0.33
		Sc. rank:	1008.6

PB2

Pos.	58	obs:	exp:
act	T	0	617.40
acc	T	0	515.00
aca	T	2328	1002.00
acg	T	3	196.90
gct	A	0	0.25
gcc	A	0	0.20
gca	A	1	0.44
gcg	A	0	0.11
mPD		0.0034	0.69
		nPD:	0.
		N. weight:	1.1
		Sc. PD:	-0.24
		Sc. rank:	-1364.0

PB2

Pos.	59	obs:	exp:
gct	A	1107	593.00
gcc	A	3	454.60
gca	A	1220	1025.00
gcg	A	1	258.80
ggt	G	0	0.14
ggc	G	0	0.13
gga	G	1	0.44
ggg	G	0	0.29
mPD		0.50	0.69
		nPD:	0.72
		N. weight:	0.51
		Sc. PD:	0.25
		Sc. rank:	695.1

PB2

Pos.	60	obs:	exp:
aat	N	0	1.06
aac	N	2	0.94
gat	D	30	1337.00
gac	D	2295	987.60
gaa	E	5	2.99
gag	E	0	2.01
mPD		0.031	0.49
		nPD:	0.06
		N. weight:	1.
		Sc. PD:	-0.16
		Sc. rank:	-143.5

PB2

Pos.	61	obs:	exp:
cgt	R	0	0.76
cgc	R	0	1.07
cga	R	0	1.94
cgg	R	0	1.57
aaa	K	1628	1298.00
aag	K	684	1014.00
aga	R	20	9.17
agg	R	0	5.50
mPD		0.43	0.51
		nPD:	0.84
		N. weight:	0.068
		Sc. PD:	0.041
		Sc. rank:	101.8

PB2

Pos.	62	obs:	exp:
cgt	R	0	88.02
cgc	R	0	123.60
cga	R	6	224.70
cgg	R	0	181.80
aaa	K	2	1.12
aag	K	0	0.88
aga	R	2086	1065.00
agg	R	229	638.20
ggt	G	0	1.29
ggc	G	0	1.19
gga	G	9	3.93
ggg	G	0	2.59
mPD		0.19	0.96
		nPD:	0.2
		N. weight:	0.67
		Sc. PD:	-0.014
		Sc. rank:	185.0

PB2

Pos.	63	obs:	exp:
att	I	0	863.00
atc	I	0	582.80
ata	I	2324	878.20
act	T	0	0.26
acc	T	0	0.22
aca	T	1	0.43
acg	T	0	0.08
gtt	V	0	1.52
gtc	V	0	1.48
gta	V	7	1.38
gtg	V	0	2.62
mPD		0.0068	0.66
		nPD:	0.01
		N. weight:	1.3
		Sc. PD:	-0.27
		Sc. rank:	-1303.5

PB2

Pos.	64	obs:	exp:
tta	L	0	0.11
ttg	L	0	0.19
ctt	L	0	0.19
ctc	L	0	0.17
cta	L	0	0.13
ctg	L	1	0.21
att	I	0	3.34
atc	I	0	2.26
ata	I	9	3.40
atg	M	2320	2320.00
act	T	0	0.53
acc	T	0	0.44
aca	T	0	0.86
acg	T	2	0.17
mPD		0.010	0.012
		nPD:	0.83
		N. weight:	0.0088
		Sc. PD:	0.0052
		Sc. rank:	13.0

PB2

Pos.	65	obs:	exp:
aaa	K	0	0.56
aag	K	1	0.44
gct	A	0	0.25
gcc	A	0	0.20
gca	A	0	0.44
gcg	A	1	0.11
gat	D	5	2.88
gac	D	0	2.12
gaa	E	570	1392.00
gag	E	1754	932.50
ggt	G	0	0.14
ggc	G	0	0.13
gga	G	1	0.44
ggg	G	0	0.29
mPD		0.38	0.49
		nPD:	0.77
		N. weight:	0.35
		Sc. PD:	0.19
		Sc. rank:	498.8

PB2			
Pos.	66	obs:	exp:
att	I	1	23.39
atc	I	0	15.80
ata	I	62	23.81
atg	M	2260	2260.00
gtt	V	0	1.96
gtc	V	0	1.90
gta	V	0	1.77
gtg	V	9	3.37
mPD		0.060	0.065
		nPD:	0.92
		N. weight:	0.038
		Sc. PD:	0.026
		Sc. rank:	56.9

PB2			
Pos.	67	obs:	exp:
att	I	834	861.50
atc	I	1486	581.80
ata	I	0	876.70
gtt	V	6	2.61
gtc	V	6	2.54
gta	V	0	2.36
gtg	V	0	4.49
mPD		0.47	0.67
		nPD:	0.71
		N. weight:	0.79
		Sc. PD:	0.37
		Sc. rank:	1045.9

PB2			
Pos.	68	obs:	exp:
cct	P	2295	657.60
ccc	P	36	422.00
cca	P	1	952.20
ccg	P	0	300.30
mPD		0.031	0.70
		nPD:	0.04
		N. weight:	1.6
		Sc. PD:	-0.27
		Sc. rank:	-542.1

PB2			
Pos.	69	obs:	exp:
aaa	K	3	1.68
aag	K	0	1.32
gaa	E	2265	1395.00
gag	E	64	934.50
mPD		0.056	0.48
		nPD:	0.12
		N. weight:	0.53
		Sc. PD:	-0.055
		Sc. rank:	18.5

PB2			
Pos.	70	obs:	exp:
cgt	R	0	88.25
cgc	R	0	123.90
cga	R	0	225.30
cgg	R	0	182.30
att	I	0	0.37
atc	I	0	0.25
ata	I	1	0.38
aaa	K	3	2.25
aag	K	1	1.75
aga	R	735	1067.00
agg	R	1592	639.80
mPD		0.44	0.96
		nPD:	0.46
		N. weight:	0.68
		Sc. PD:	0.16
		Sc. rank:	585.1

PB2			
Pos.	71	obs:	exp:
aat	N	2295	1239.00
aac	N	37	1093.00
mPD		0.031	0.50
		nPD:	0.06
		N. weight:	0.74
		Sc. PD:	-0.11
		Sc. rank:	-108.0

PB2			
Pos.	72	obs:	exp:
gaa	E	660	1396.00
gag	E	1672	935.70
mPD		0.41	0.48
		nPD:	0.84
		N. weight:	0.28
		Sc. PD:	0.17
		Sc. rank:	411.1

PB2			
Pos.	73	obs:	exp:
cat	H	0	0.59
cac	H	1	0.41
caa	Q	2304	1184.00
cag	Q	24	1144.00
aaa	K	3	1.68
aag	K	0	1.32
mPD		0.024	0.50
		nPD:	0.05
		N. weight:	0.83
		Sc. PD:	-0.14
		Sc. rank:	-244.9

PB2			
Pos.	74	obs:	exp:
tct	S	0	0.34
tcc	S	0	0.29
tca	S	0	0.45
tcg	S	0	0.13
agt	S	2	0.41
agc	S	0	0.38
ggt	G	1672	335.20
ggc	G	540	307.50
gga	G	111	1017.00
ggg	G	7	670.20
mPD		0.43	0.69
		nPD:	0.62
		N. weight:	1.6
		Sc. PD:	0.61
		Sc. rank:	1904.5

PB2			
Pos.	75	obs:	exp:
cat	H	5	2.97
cac	H	0	2.03
caa	Q	22	1184.00
cag	Q	2305	1143.00
mPD		0.023	0.50
		nPD:	0.05
		N. weight:	0.88
		Sc. PD:	-0.15
		Sc. rank:	-292.1

PB2			
Pos.	76	obs:	exp:
att	I	20	7.43
atc	I	0	5.02
ata	I	0	7.56
act	T	1661	609.00
acc	T	12	508.00
aca	T	1	987.90
acg	T	625	194.20
gct	A	13	3.31
gcc	A	0	2.54
gca	A	0	5.71
gcg	A	0	1.44
mPD		0.43	0.72
		nPD:	0.6
		N. weight:	1.4
		Sc. PD:	0.5
		Sc. rank:	1595.1

PB2			
Pos.	77	obs:	exp:
tta	L	0	254.40
ttg	L	0	437.40
ctt	L	842	437.10
ctc	L	1489	402.20
cta	L	0	310.10
ctg	L	0	489.80
att	I	1	0.37
atc	I	0	0.25
ata	I	0	0.38
mPD		0.46	1.1
		nPD:	0.41
		N. weight:	1.4
		Sc. PD:	0.26
		Sc. rank:	1130.2

PB2			
Pos.	78	obs:	exp:
tgg	W	2332	2332.00
mPD		0	0
		nPD:	1.
		N. weight:	0.
		Sc. PD:	0
		Sc. rank:	0

PB2			
Pos.	79	obs:	exp:
tct	S	0	393.60
tcc	S	0	339.80
tca	S	0	519.60
tcg	S	0	148.90
aat	N	0	3.19
aac	N	6	2.81
agt	S	185	470.60
agc	S	2120	432.50
ggt	G	0	3.02
ggc	G	21	2.77
gga	G	0	9.17
ggg	G	0	6.04
mPD		0.17	1.7
		nPD:	0.1
		N. weight:	1.9
		Sc. PD:	-0.22
		Sc. rank:	43.0

PB2			
Pos.	80	obs:	exp:
cgt	R	0	0.61
cgc	R	0	0.85
cga	R	0	1.55
cgg	R	0	1.25
aaa	K	1685	1300.00
aag	K	631	1016.00
aga	R	16	7.34
agg	R	0	4.40
mPD		0.41	0.51
		nPD:	0.8
		N. weight:	0.088
		Sc. PD:	0.049
		Sc. rank:	127.0

PB2			
Pos.	81	obs:	exp:
atg	M	1	1.00
act	T	0	616.40
acc	T	0	514.10
aca	T	2220	999.90
acg	T	107	196.60
gct	A	0	1.02
gcc	A	0	0.78
gca	A	4	1.76
gcg	A	0	0.44
mPD		0.093	0.69
		nPD:	0.13
		N. weight:	0.98
		Sc. PD:	-0.084
		Sc. rank:	91.6

PB2			
Pos.	82	obs:	exp:
tct	S	0	7.00
tcc	S	0	6.04
tca	S	0	9.24
tcg	S	0	2.65
aat	N	2282	1217.00
aac	N	9	1074.00
agt	S	41	8.37
agc	S	0	7.69
mPD		0.042	0.56
		nPD:	0.08
		N. weight:	0.84
		Sc. PD:	-0.12
		Sc. rank:	-71.5

PB2			
Pos.	83	obs:	exp:
aat	N	1	0.53
aac	N	0	0.47
gat	D	1869	1341.00
gac	D	462	990.20
mPD		0.32	0.49
		nPD:	0.65
		N. weight:	0.16
		Sc. PD:	0.065
		Sc. rank:	196.5

PB2			
Pos.	84	obs:	exp:
gct	A	2318	593.00
gcc	A	13	454.60
gca	A	0	1025.00
gcg	A	0	258.80
ggt	G	1	0.14
ggc	G	0	0.13
gga	G	0	0.44
ggg	G	0	0.29
mPD		0.012	0.69
		nPD:	0.02
		N. weight:	1.8
		Sc. PD:	-0.36
		Sc. rank:	-1333.2

PB2			
Pos.	85	obs:	exp:
ggt	G	7	335.50
ggc	G	5	307.80
gga	G	2314	1018.00
ggg	G	6	670.70
mPD		0.015	0.69
		nPD:	0.02
		N. weight:	1.
		Sc. PD:	-0.2
		Sc. rank:	-660.6

PB2

Pos.	86	obs:	exp:
tct	S	6	398.30
tcc	S	0	343.80
tca	S	2324	525.70
tcg	S	2	150.60
agt	S	0	476.10
agc	S	0	437.50
---	--	------	------
mPD		0.0068	1.6
		nPD:	0.
		N. weight:	2.
		Sc. PD:	-0.42
		Sc. rank:	-2417.1

,

PB2

Pos.	87	obs:	exp:
aat	N	0	12.22
aac	N	23	10.78
gat	D	12	1327.00
gac	D	2295	980.00
gaa	E	2	1.20
gag	E	0	0.80
---	--	------	------
mPD		0.031	0.51
		nPD:	0.06
		N. weight:	1.1
		Sc. PD:	-0.17
		Sc. rank:	-163.5

,

PB2

Pos.	88	obs:	exp:
cgt	R	0	87.68
cgc	R	0	123.20
cga	R	12	223.90
cgg	R	0	181.10
aaa	K	20	11.23
aag	K	0	8.77
aga	R	2188	1061.00
agg	R	112	635.70
---	--	------	------
mPD		0.12	0.97
		nPD:	0.12
		N. weight:	0.79
		Sc. PD:	-0.076
		Sc. rank:	47.7

,

PB2

Pos.	89	obs:	exp:
tta	L	0	0.11
ttg	L	0	0.19
ctt	L	0	0.19
ctc	L	0	0.17
cta	L	0	0.13
ctg	L	1	0.21
atg	M	46	46.00
gtt	V	0	496.50
gtc	V	0	482.40
gta	V	13	449.20
gtg	V	2270	854.90
gct	A	0	0.25
gcc	A	0	0.20
gca	A	0	0.44
gcg	A	1	0.11
ggt	G	0	0.14
ggc	G	0	0.13
gga	G	0	0.44
ggg	G	1	0.29
---	--	------	------
mPD		0.052	0.77
		nPD:	0.07
		N. weight:	1.2
		Sc. PD:	-0.19
		Sc. rank:	-141.1

,

PB2

Pos.	90	obs:	exp:
tta	L	0	1.42
ttg	L	13	2.44
ctt	L	0	2.44
ctc	L	0	2.24
cta	L	0	1.73
ctg	L	0	2.73
att	I	0	0.37
atc	I	0	0.25
ata	I	1	0.38
atg	M	2311	2311.00
act	T	0	1.06
acc	T	0	0.88
aca	T	0	1.72
acg	T	4	0.34
gtt	V	0	0.65
gtc	V	0	0.63
gta	V	0	0.59
gtg	V	3	1.12
---	--	------	------
mPD		0.018	0.029
		nPD:	0.61
		N. weight:	0.02
		Sc. PD:	0.0077
		Sc. rank:	24.1

,

PB2

Pos.	91	obs:	exp:
atg	M	1	1.00
gtt	V	2	506.70
gtc	V	832	492.30
gta	V	310	458.50
gtg	V	1186	872.50
gct	A	0	0.25
gcc	A	0	0.20
gca	A	0	0.44
gcg	A	1	0.11
---	--	------	------
mPD		0.60	0.73
		nPD:	0.82
		N. weight:	0.39
		Sc. PD:	0.23
		Sc. rank:	570.8

,

PB2

Pos.	92	obs:	exp:
tct	S	625	396.70
tcc	S	1	342.50
tca	S	1697	523.60
tcg	S	0	150.10
act	T	0	2.12
acc	T	0	1.77
aca	T	8	3.44
acg	T	0	0.68
agt	S	0	474.30
agc	S	0	435.80
gct	A	0	0.25
gcc	A	0	0.20
gca	A	1	0.44
gcg	A	0	0.11
---	--	------	------
mPD		0.40	1.6
		nPD:	0.24
		N. weight:	1.3
		Sc. PD:	0.027
		Sc. rank:	546.1

,

PB2

Pos.	93	obs:	exp:
tta	L	0	0.11
ttg	L	0	0.19
tct	S	1	0.51
tcc	S	2	0.44
tca	S	0	0.68
tcg	S	0	0.19
ctt	L	0	0.19
ctc	L	1	0.17
cta	L	0	0.13
ctg	L	0	0.21
cct	P	1658	656.20
ccc	P	669	421.10
cca	P	0	950.10
ccg	P	0	299.60
act	T	1	0.26
acc	T	0	0.22
aca	T	0	0.43
acg	T	0	0.08
agt	S	0	0.61
agc	S	0	0.56
---	--	------	------
mPD		0.41	0.71
		nPD:	0.58
		N. weight:	1.1
		Sc. PD:	0.37
		Sc. rank:	1217.6

,

PB2

Pos.	94	obs:	exp:
tta	L	0	253.30
ttg	L	2	435.50
ctt	L	26	435.20
ctc	L	0	400.40
cta	L	637	308.80
ctg	L	1656	487.70
cgt	R	0	0.04
cgc	R	0	0.05
cga	R	0	0.10
cgg	R	1	0.08
atg	M	10	10.00
aga	R	0	0.46
agg	R	0	0.27
---	--	------	------
mPD		0.42	1.1
		nPD:	0.37
		N. weight:	1.4
		Sc. PD:	0.2
		Sc. rank:	1024.0

,

PB2

Pos.	95	obs:	exp:
gct	A	2197	593.20
gcc	A	3	454.80
gca	A	132	1025.00
gcg	A	0	258.90
---	--	------	------
mPD		0.11	0.69
		nPD:	0.16
		N. weight:	1.5
		Sc. PD:	-0.093
		Sc. rank:	275.9

,

PB2

Pos.	96	obs:	exp:
gtt	V	2	506.90
gtc	V	0	492.50
gta	V	631	458.60
gtg	V	1698	872.90
gaa	E	0	0.60
gag	E	1	0.40
---	--	------	------
mPD		0.40	0.73
		nPD:	0.54
		N. weight:	0.76
		Sc. PD:	0.24
		Sc. rank:	761.9

,

PB2

Pos.	97	obs:	exp:
act	T	629	617.70
acc	T	1	515.20
aca	T	1512	1002.00
acg	T	190	197.00
---	--	------	------
mPD		0.50	0.69
		nPD:	0.73
		N. weight:	0.36
		Sc. PD:	0.18
		Sc. rank:	493.6

,

PB2

Pos.	98	obs:	exp:
tgg	W	2332	2332.00
---	--	------	------
mPD		0	0
		nPD:	1.
		N. weight:	0.
		Sc. PD:	0
		Sc. rank:	0

,

PB2

Pos.	99	obs:	exp:
tgt	C	1	0.43
tgc	C	0	0.57
tgg	W	2331	2331.00
---	--	------	------
mPD		0.00086	0.00086
		nPD:	1.
		N. weight:	0.00048
		Sc. PD:	0.00036
		Sc. rank:	0.7

,

PB2

Pos.	100	obs:	exp:
aat	N	2186	1239.00
aac	N	146	1093.00
---	--	------	------
mPD		0.12	0.50
		nPD:	0.24
		N. weight:	0.55
		Sc. PD:	0.0073
		Sc. rank:	201.2

,

PB2

Pos.	101	obs:	exp:
cgt	R	0	88.29
cgc	R	0	124.00
cga	R	1	225.40
cgg	R	0	182.30
aaa	K	4	2.25
aag	K	0	1.75
aga	R	2073	1068.00
agg	R	254	640.10
---	--	------	------
mPD		0.20	0.96
		nPD:	0.21
		N. weight:	0.66
		Sc. PD:	-0.0091
		Sc. rank:	188.3

,

PB2

Pos.	102	obs:	exp:
aat	N	2251	1238.00
aac	N	78	1091.00
aaa	K	1	1.12
aag	K	1	0.88
gat	D	1	0.58
gac	D	0	0.42
---	--	------	------
mPD		0.067	0.50
		nPD:	0.13
		N. weight:	0.66
		Sc. PD:	-0.056
		Sc. rank:	64.8

,

PB2

Pos.	103	obs:	exp:
ggt	G	3	335.50
ggc	G	11	307.80
gga	G	749	1018.00
ggg	G	1569	670.70
---	--	------	------
mPD		0.44	0.69
		nPD:	0.65
		N. weight:	0.61
		Sc. PD:	0.25
		Sc. rank:	758.8

,

PB2

Pos.	104	obs:	exp:
cct	P	0	657.60
ccc	P	0	422.00
cca	P	1827	952.20
ccg	P	505	300.30
---	--	------	------
mPD		0.34	0.70
		nPD:	0.48
		N. weight:	0.84
		Sc. PD:	0.21
		Sc. rank:	743.7

,

PB2

Pos.	105	obs:	exp:
cct	P	0	0.28
ccc	P	0	0.18
cca	P	1	0.41
ccg	P	0	0.13
att	I	0	1.86
atc	I	0	1.25
ata	I	5	1.89
atg	M	2	2.00
act	T	170	611.60
acc	T	0	510.20
aca	T	1830	992.20
acg	T	309	195.00
aat	N	8	4.25
aac	N	0	3.75
aaa	K	5	2.81
aag	K	0	2.19
gct	A	0	0.51
gcc	A	0	0.39
gca	A	0	0.88
gcg	A	2	0.22
---	--	------	------
mPD		0.37	0.71
		nPD:	0.53
		N. weight:	0.61
		Sc. PD:	0.18
		Sc. rank:	578.5

,

PB2

Pos.	106	obs:	exp:
cct	P	0	0.85
ccc	P	0	0.54
cca	P	3	1.22
ccg	P	0	0.39
act	T	3	477.60
acc	T	0	398.40
aca	T	1800	774.80
acg	T	0	152.30
gtt	V	0	0.22
gtc	V	0	0.21
gta	V	1	0.20
gtg	V	0	0.37
gct	A	0	133.60
gcc	A	0	102.40
gca	A	525	230.80
gcg	A	0	58.28
---	--	------	------
mPD		0.35	1.0
		nPD:	0.34
		N. weight:	1.1
		Sc. PD:	0.13
		Sc. rank:	732.2

,

PB2

Pos.	107	obs:	exp:
tct	S	0	396.90
tcc	S	0	342.60
tca	S	0	523.90
tcg	S	0	150.10
aat	N	6	3.19
aac	N	0	2.81
agt	S	2216	474.50
agc	S	108	436.00
ggt	G	2	0.29
ggc	G	0	0.26
gga	G	0	0.87
ggg	G	0	0.58
---	--	------	------
mPD		0.095	1.6
		nPD:	0.06
		N. weight:	1.9
		Sc. PD:	-0.3
		Sc. rank:	-371.9

,

PB2

Pos.	108	obs:	exp:
cct	P	0	0.85
ccc	P	0	0.54
cca	P	3	1.22
ccg	P	0	0.39
act	T	0	616.70
acc	T	0	514.40
aca	T	2324	1000.00
acg	T	4	196.60
gct	A	0	0.25
gcc	A	0	0.20
gca	A	1	0.44
gcg	A	0	0.11
---	--	------	------
mPD		0.0069	0.69
		nPD:	0.01
		N. weight:	1.1
		Sc. PD:	-0.23
		Sc. rank:	-1142.7

,

PB2

Pos.	109	obs:	exp:
att	I	0	2.97
atc	I	1	2.01
ata	I	7	3.02
gtt	V	11	504.80
gtc	V	1208	490.40
gta	V	1100	456.70
gtg	V	2	869.10
gct	A	0	0.76
gcc	A	0	0.59
gca	A	3	1.32
gcg	A	0	0.33
---	--	------	------
mPD		0.51	0.74
		nPD:	0.7
		N. weight:	1.2
		Sc. PD:	0.53
		Sc. rank:	1509.1

,

PB2

Pos.	110	obs:	exp:
tta	L	0	0.22
ttg	L	0	0.38
tat	Y	0	0.49
tac	Y	1	0.51
ctt	L	2	0.38
ctc	L	0	0.35
cta	L	0	0.27
ctg	L	0	0.42
cat	H	749	1384.00
cac	H	1578	943.40
caa	Q	1	0.51
cag	Q	0	0.49
cgt	R	0	0.04
cgc	R	1	0.05
cga	R	0	0.10
cgg	R	0	0.08
aga	R	0	0.46
agg	R	0	0.27
---	--	------	------
mPD		0.44	0.49
		nPD:	0.9
		N. weight:	0.21
		Sc. PD:	0.14
		Sc. rank:	313.1

,

PB2

Pos.	111	obs:	exp:
tat	Y	1168	1134.00
tac	Y	1164	1198.00
---	--	------	------
mPD		0.50	0.50
		nPD:	1.
		N. weight:	0.0029
		Sc. PD:	0.0022
		Sc. rank:	4.4

,

PB2

Pos.	112	obs:	exp:
cct	P	0	657.60
ccc	P	0	422.00
cca	P	2319	952.20
ccg	P	13	300.30
---	--	------	------
mPD		0.011	0.70
		nPD:	0.02
		N. weight:	1.2
		Sc. PD:	-0.23
		Sc. rank:	-910.3

,

PB2

Pos.	113	obs:	exp:
act	T	0	0.53
acc	T	0	0.44
aca	T	0	0.86
acg	T	2	0.17
aaa	K	1108	1308.00
aag	K	1222	1022.00
---	--	------	------
mPD		0.50	0.49
		nPD:	1.01
		N. weight:	0.025
		Sc. PD:	0.019
		Sc. rank:	37.6

,

PB2

Pos.	114	obs:	exp:
gtt	V	267	507.20
gtc	V	2050	492.70
gta	V	14	458.80
gtg	V	1	873.30
---	--	------	------
mPD		0.21	0.73
		nPD:	0.29
		N. weight:	1.6
		Sc. PD:	0.11
		Sc. rank:	902.5

,

PB2

Pos.	115	obs:	exp:
tct	S	0	0.17
tcc	S	1	0.15
tca	S	0	0.23
tcg	S	0	0.06
tat	Y	253	1133.00
tac	Y	2078	1198.00
agt	S	0	0.20
agc	S	0	0.19
---	--	------	------
mPD		0.19	0.50
		nPD:	0.39
		N. weight:	0.44
		Sc. PD:	0.071
		Sc. rank:	332.1

,

PB2

Pos.	116	obs:	exp:
cgt	R	0	0.42
cgc	R	0	0.59
cga	R	0	1.07
cgg	R	0	0.86
aaa	K	2321	1303.00
aag	K	0	1018.00
aga	R	11	5.05
agg	R	0	3.02
---	--	------	------
mPD		0.0094	0.50
		nPD:	0.02
		N. weight:	0.77
		Sc. PD:	-0.15
		Sc. rank:	-557.4

PB2

Pos.	117	obs:	exp:
tct	S	0	0.34
tcc	S	0	0.29
tca	S	2	0.45
tcg	S	0	0.13
cct	P	0	9.02
ccc	P	32	5.79
cca	P	0	13.07
ccg	P	0	4.12
att	I	0	1.11
atc	I	3	0.75
ata	I	0	1.13
act	T	123	607.40
acc	T	1544	506.60
aca	T	626	985.30
acg	T	0	193.70
aat	N	0	0.53
aac	N	1	0.47
agt	S	0	0.41
agc	S	0	0.38
gct	A	0	0.25
gcc	A	1	0.20
gca	A	0	0.44
gcg	A	0	0.11
---	--	------	------
mPD		0.50	0.72
		nPD:	0.69
		N. weight:	0.75
		Sc. PD:	0.34
		Sc. rank:	974.0

PB2

Pos.	118	obs:	exp:
ttt	F	0	0.43
ttc	F	1	0.57
tat	Y	17	1133.00
tac	Y	2314	1198.00
---	--	------	------
mPD		0.015	0.50
		nPD:	0.03
		N. weight:	0.83
		Sc. PD:	-0.16
		Sc. rank:	-436.6

PB2

Pos.	119	obs:	exp:
ttt	F	2321	992.30
ttc	F	11	1340.00
---	--	------	------
mPD		0.0094	0.49
		nPD:	0.02
		N. weight:	1.1
		Sc. PD:	-0.22
		Sc. rank:	-764.2

PB2

Pos.	120	obs:	exp:
gaa	E	1682	1396.00
gag	E	650	935.70
---	--	------	------
mPD		0.40	0.48
		nPD:	0.84
		N. weight:	0.046
		Sc. PD:	0.028
		Sc. rank:	68.3

PB2

Pos.	121	obs:	exp:
cgt	R	0	0.08
cgc	R	0	0.11
cga	R	0	0.19
cgg	R	0	0.16
aaa	K	72	1308.00
aag	K	2258	1022.00
aga	R	0	0.92
agg	R	2	0.55
---	--	------	------
mPD		0.062	0.49
		nPD:	0.12
		N. weight:	0.91
		Sc. PD:	-0.086
		Sc. rank:	64.8

PB2

Pos.	122	obs:	exp:
att	I	2	2.97
atc	I	5	2.01
ata	I	1	3.02
gtt	V	822	505.20
gtc	V	26	490.80
gta	V	1384	457.10
gtg	V	91	869.90
ggt	G	1	0.14
ggc	G	0	0.13
gga	G	0	0.44
ggg	G	0	0.29
---	--	------	------
mPD		0.53	0.74
		nPD:	0.72
		N. weight:	0.96
		Sc. PD:	0.46
		Sc. rank:	1293.0

PB2

Pos.	123	obs:	exp:
caa	Q	2	1.02
cag	Q	0	0.98
aaa	K	6	3.37
aag	K	0	2.63
gat	D	0	0.58
gac	D	1	0.42
gaa	E	2217	1391.00
gag	E	106	932.10
---	--	------	------
mPD		0.094	0.49
		nPD:	0.19
		N. weight:	0.47
		Sc. PD:	-0.013
		Sc. rank:	122.7

PB2

Pos.	124	obs:	exp:
cgt	R	0	88.44
cgc	R	0	124.20
cga	R	0	225.80
cgg	R	0	182.60
aga	R	207	1070.00
agg	R	2125	641.20
---	--	------	------
mPD		0.16	0.95
		nPD:	0.17
		N. weight:	1.3
		Sc. PD:	-0.064
		Sc. rank:	294.3

PB2

Pos.	125	obs:	exp:
ttt	F	1	0.43
ttc	F	0	0.57
tta	L	833	254.40
ttg	L	1492	437.40
ctt	L	0	437.10
ctc	L	0	402.20
cta	L	2	310.10
ctg	L	4	489.80
---	--	------	------
mPD		0.47	1.1
		nPD:	0.41
		N. weight:	1.6
		Sc. PD:	0.29
		Sc. rank:	1271.7

PB2

Pos.	126	obs:	exp:
aaa	K	1596	1309.00
aag	K	736	1023.00
---	--	------	------
mPD		0.43	0.49
		nPD:	0.88
		N. weight:	0.045
		Sc. PD:	0.029
		Sc. rank:	67.1

PB2

Pos.	127	obs:	exp:
tat	Y	1	0.49
tac	Y	0	0.51
cat	H	2313	1384.00
cac	H	14	943.40
cgt	R	2	0.08
cgc	R	0	0.11
cga	R	0	0.19
cgg	R	0	0.16
aat	N	2	1.06
aac	N	0	0.94
aga	R	0	0.92
agg	R	0	0.55
---	--	------	------
mPD		0.016	0.49
		nPD:	0.03
		N. weight:	0.65
		Sc. PD:	-0.12
		Sc. rank:	-320.6

PB2

Pos.	128	obs:	exp:
ggt	G	0	335.50
ggc	G	0	307.80
gga	G	2315	1018.00
ggg	G	17	670.70
---	--	------	------
mPD		0.014	0.69
		nPD:	0.02
		N. weight:	1.1
		Sc. PD:	-0.21
		Sc. rank:	-685.7

PB2

Pos.	129	obs:	exp:
att	I	0	0.74
atc	I	2	0.50
ata	I	0	0.76
act	T	1	617.20
acc	T	2321	514.80
aca	T	8	1001.00
acg	T	0	196.80
---	--	------	------
mPD		0.0094	0.69
		nPD:	0.01
		N. weight:	2.
		Sc. PD:	-0.4
		Sc. rank:	-1673.7

PB2

Pos.	130	obs:	exp:
ttt	F	1490	992.30
ttc	F	842	1340.00
---	--	------	------
mPD		0.46	0.49
		nPD:	0.94
		N. weight:	0.13
		Sc. PD:	0.088
		Sc. rank:	188.7

PB2

Pos.	131	obs:	exp:
ggt	G	2102	335.50
ggc	G	226	307.80
gga	G	3	1018.00
ggg	G	1	670.70
---	--	------	------
mPD		0.18	0.69
		nPD:	0.26
		N. weight:	2.2
		Sc. PD:	0.077
		Sc. rank:	1074.0

PB2

Pos.	132	obs:	exp:
cct	P	1656	657.60
ccc	P	675	422.00
cca	P	0	952.20
ccg	P	1	300.30
---	--	------	------
mPD		0.41	0.70
		nPD:	0.59
		N. weight:	1.1
		Sc. PD:	0.37
		Sc. rank:	1214.0

PB2

Pos.	133	obs:	exp:
gtt	V	2286	507.20
gtc	V	39	492.70
gta	V	1	458.80
gtg	V	6	873.30
---	--	------	------
mPD		0.039	0.73
		nPD:	0.05
		N. weight:	1.9
		Sc. PD:	-0.31
		Sc. rank:	-448.5

PB2

Pos.	134	obs:	exp:
cat	H	303	1387.00
cac	H	2029	945.40
---	--	------	------
mPD		0.23	0.48
		nPD:	0.47
		N. weight:	0.63
		Sc. PD:	0.15
		Sc. rank:	549.5

PB2

Pos.	135	obs:	exp:
ttt	F	1685	992.30
ttc	F	647	1340.00
---	--	------	------
mPD		0.40	0.49
		nPD:	0.82
		N. weight:	0.24
		Sc. PD:	0.14
		Sc. rank:	357.5

PB2

Pos.	136	obs:	exp:
caa	Q	7	3.56
cag	Q	0	3.44
cgt	R	0	88.17
cgc	R	0	123.80
cga	R	2190	225.10
cgg	R	123	182.10
aga	R	12	1066.00
agg	R	0	639.30
mPD		0.12	0.96
		nPD:	0.12
		N. weight:	2.8
		Sc. PD:	-0.28
		Sc. rank:	161.2

PB2

Pos.	137	obs:	exp:
tct	S	0	0.85
tcc	S	0	0.74
tca	S	0	1.13
tcg	S	0	0.32
aat	N	1697	1237.00
aac	N	630	1090.00
agt	S	4	1.02
agc	S	1	0.94
mPD		0.40	0.51
		nPD:	0.79
		N. weight:	0.12
		Sc. PD:	0.064
		Sc. rank:	166.0

PB2

Pos.	138	obs:	exp:
caa	Q	644	1187.00
cag	Q	1688	1145.00
mPD		0.40	0.50
		nPD:	0.8
		N. weight:	0.15
		Sc. PD:	0.085
		Sc. rank:	219.9

PB2

Pos.	139	obs:	exp:
att	I	526	195.30
atc	I	0	131.90
ata	I	0	198.80
gtt	V	1775	392.80
gtc	V	31	381.60
gta	V	0	355.30
gtg	V	0	676.30
mPD		0.38	1.1
		nPD:	0.34
		N. weight:	1.8
		Sc. PD:	0.21
		Sc. rank:	1203.7

PB2

Pos.	140	obs:	exp:
aaa	K	665	1309.00
aag	K	1667	1023.00
mPD		0.41	0.49
		nPD:	0.83
		N. weight:	0.21
		Sc. PD:	0.12
		Sc. rank:	311.8

PB2

Pos.	141	obs:	exp:
att	I	177	862.60
atc	I	0	582.60
ata	I	2146	877.80
gtt	V	0	1.96
gtc	V	0	1.90
gta	V	9	1.77
gtg	V	0	3.37
mPD		0.15	0.67
		nPD:	0.22
		N. weight:	0.95
		Sc. PD:	0.00056
		Sc. rank:	314.1

PB2

Pos.	142	obs:	exp:
cct	P	0	0.56
ccc	P	2	0.36
cca	P	0	0.82
ccg	P	0	0.26
cgt	R	150	88.36
cgc	R	2162	124.10
cga	R	5	225.60
cgg	R	1	182.50
aga	R	12	1069.00
agg	R	0	640.70
mPD		0.15	0.96
		nPD:	0.15
		N. weight:	3.6
		Sc. PD:	-0.24
		Sc. rank:	610.9

PB2

Pos.	143	obs:	exp:
cgt	R	5	88.44
cgc	R	2134	124.20
cga	R	181	225.80
cgg	R	12	182.60
aga	R	0	1070.00
agg	R	0	641.20
mPD		0.16	0.95
		nPD:	0.16
		N. weight:	3.4
		Sc. PD:	-0.19
		Sc. rank:	727.6

PB2

Pos.	144	obs:	exp:
cgt	R	0	88.44
cgc	R	0	124.20
cga	R	624	225.80
cgg	R	4	182.60
aga	R	32	1070.00
agg	R	1672	641.20
mPD		0.80	0.95
		nPD:	0.84
		N. weight:	1.2
		Sc. PD:	0.73
		Sc. rank:	1807.9

PB2

Pos.	145	obs:	exp:
gtt	V	2322	507.20
gtc	V	10	492.70
gta	V	0	458.80
gtg	V	0	873.30
mPD		0.0085	0.73
		nPD:	0.01
		N. weight:	2.
		Sc. PD:	-0.41
		Sc. rank:	-1853.6

PB2

Pos.	146	obs:	exp:
gat	D	130	1341.00
gac	D	2201	990.20
gaa	E	1	0.60
gag	E	0	0.40
mPD		0.11	0.49
		nPD:	0.22
		N. weight:	0.84
		Sc. PD:	-0.004
		Sc. rank:	257.0

PB2

Pos.	147	obs:	exp:
att	I	0	778.70
atc	I	0	525.90
ata	I	2097	792.40
act	T	0	57.22
acc	T	0	47.72
aca	T	215	92.82
acg	T	1	18.24
gtt	V	0	4.13
gtc	V	0	4.01
gta	V	19	3.74
gtg	V	0	7.11
mPD		0.19	0.85
		nPD:	0.22
		N. weight:	1.3
		Sc. PD:	-0.0044
		Sc. rank:	407.9

PB2

Pos.	148	obs:	exp:
tct	S	0	5.29
tcc	S	0	4.57
tca	S	0	6.99
tcg	S	0	2.00
aat	N	127	1223.00
aac	N	2174	1078.00
agt	S	0	6.33
agc	S	31	5.82
mPD		0.13	0.54
		nPD:	0.24
		N. weight:	0.74
		Sc. PD:	0.011
		Sc. rank:	275.9

PB2

Pos.	149	obs:	exp:
cct	P	5	657.60
ccc	P	0	422.00
cca	P	575	952.20
ccg	P	1752	300.30
mPD		0.37	0.70
		nPD:	0.53
		N. weight:	1.6
		Sc. PD:	0.48
		Sc. rank:	1540.2

PB2

Pos.	150	obs:	exp:
ggt	G	38	335.50
ggc	G	2294	307.80
gga	G	0	1018.00
ggg	G	0	670.70
mPD		0.032	0.69
		nPD:	0.05
		N. weight:	2.6
		Sc. PD:	-0.44
		Sc. rank:	-786.6

PB2

Pos.	151	obs:	exp:
cat	H	1932	1387.00
cac	H	400	945.40
mPD		0.28	0.48
		nPD:	0.59
		N. weight:	0.17
		Sc. PD:	0.062
		Sc. rank:	198.9

PB2

Pos.	152	obs:	exp:
tct	S	0	1.20
tcc	S	0	1.03
tca	S	7	1.58
tcg	S	0	0.45
att	I	0	0.37
atc	I	0	0.25
ata	I	1	0.38
act	T	0	0.53
acc	T	0	0.44
aca	T	2	0.86
acg	T	0	0.17
agt	S	0	1.43
agc	S	0	1.31
gtt	V	0	8.05
gtc	V	0	7.82
gta	V	37	7.28
gtg	V	0	13.86
gct	A	6	581.30
gcc	A	0	445.60
gca	A	2001	1004.00
gcg	A	278	253.70
mPD		0.26	0.74
		nPD:	0.35
		N. weight:	0.84
		Sc. PD:	0.1
		Sc. rank:	565.1

PB2

Pos.	153	obs:	exp:
aat	N	4	2.13
aac	N	0	1.87
gat	D	2257	1339.00
gac	D	71	988.90
mPD		0.062	0.49
		nPD:	0.13
		N. weight:	0.57
		Sc. PD:	-0.053
		Sc. rank:	44.4

PB2

Pos.	154	obs:	exp:
tta	L	0	254.40
ttg	L	0	437.40
ctt	L	33	437.10
ctc	L	2297	402.20
cta	L	1	310.10
ctg	L	0	489.80
att	I	0	0.37
atc	I	1	0.25
ata	I	0	0.38
mPD		0.030	1.1
		nPD:	0.03
		N. weight:	2.2
		Sc. PD:	-0.43
		Sc. rank:	-1253.9

PB2

Pos.	155	obs:	exp:
tct	S	0	377.30
tcc	S	0	325.60
tca	S	0	497.90
tcg	S	0	142.70
cgt	R	1	0.04
cgc	R	0	0.05
cga	R	0	0.10
cgg	R	0	0.08
aat	N	121	64.30
aac	N	0	56.70
agt	S	2207	451.00
agc	S	2	414.50
aga	R	0	0.46
agg	R	0	0.27
ggt	G	1	0.14
ggc	G	0	0.13
gga	G	0	0.44
ggg	G	0	0.29
mPD		0.10	1.7
		nPD:	0.06
		N. weight:	2.1
		Sc. PD:	-0.32
		Sc. rank:	-347.0

PB2			
Pos.	156	obs:	exp:
tct	S	0	0.17
tcc	S	1	0.15
tca	S	0	0.23
tcg	S	0	0.06
agt	S	0	0.20
agc	S	0	0.19
gtt	V	0	0.65
gtc	V	3	0.63
gta	V	0	0.59
gtg	V	0	1.12
gct	A	325	592.00
gcc	A	2001	453.80
gca	A	1	1023.00
gcg	A	0	258.30
ggt	G	0	0.14
ggc	G	1	0.13
gga	G	0	0.44
ggg	G	0	0.29
---	--	------	------
mPD		0.25	0.70
		nPD:	0.35
		N. weight:	1.6
		Sc. PD:	0.2
		Sc. rank:	1097.4

,

PB2			
Pos.	157	obs:	exp:
act	T	0	0.79
acc	T	0	0.66
aca	T	3	1.29
acg	T	0	0.25
aat	N	1	19.13
aac	N	35	16.87
aaa	K	2291	1287.00
aag	K	1	1005.00
gaa	E	1	0.60
gag	E	0	0.40
---	--	------	------
mPD		0.035	0.51
		nPD:	0.07
		N. weight:	0.77
		Sc. PD:	-0.12
		Sc. rank:	-90.8

,

PB2			
Pos.	158	obs:	exp:
aaa	K	4	2.25
aag	K	0	1.75
gaa	E	2315	1394.00
gag	E	13	934.10
---	--	------	------
mPD		0.015	0.48
		nPD:	0.03
		N. weight:	0.64
		Sc. PD:	-0.12
		Sc. rank:	-339.2

,

PB2			
Pos.	159	obs:	exp:
gct	A	0	593.20
gcc	A	0	454.80
gca	A	2321	1025.00
gcg	A	11	258.90
---	--	------	------
mPD		0.0094	0.69
		nPD:	0.01
		N. weight:	1.1
		Sc. PD:	-0.22
		Sc. rank:	-920.3

,

PB2			
Pos.	160	obs:	exp:
caa	Q	865	1187.00
cag	Q	1467	1145.00
---	--	------	------
mPD		0.47	0.50
		nPD:	0.93
		N. weight:	0.054
		Sc. PD:	0.037
		Sc. rank:	80.7

,

PB2			
Pos.	161	obs:	exp:
gat	D	2117	1341.00
gac	D	215	990.60
---	--	------	------
mPD		0.17	0.49
		nPD:	0.34
		N. weight:	0.37
		Sc. PD:	0.043
		Sc. rank:	247.6

,

PB2			
Pos.	162	obs:	exp:
gtt	V	1	506.90
gtc	V	2318	492.50
gta	V	11	458.60
gtg	V	1	872.90
gat	D	0	0.58
gac	D	1	0.42
---	--	------	------
mPD		0.012	0.73
		nPD:	0.02
		N. weight:	2.
		Sc. PD:	-0.41
		Sc. rank:	-1543.6

,

PB2			
Pos.	163	obs:	exp:
att	I	6	866.00
atc	I	2304	584.90
ata	I	22	881.20
---	--	------	------
mPD		0.024	0.66
		nPD:	0.04
		N. weight:	1.8
		Sc. PD:	-0.32
		Sc. rank:	-734.6

,

PB2			
Pos.	164	obs:	exp:
atg	M	2332	2332.00
---	--	------	------
mPD		0	0
		nPD:	1.
		N. weight:	0.
		Sc. PD:	0
		Sc. rank:	0

,

PB2			
Pos.	165	obs:	exp:
gaa	E	11	1396.00
gag	E	2321	935.70
---	--	------	------
mPD		0.0094	0.48
		nPD:	0.02
		N. weight:	1.2
		Sc. PD:	-0.23
		Sc. rank:	-794.5

,

PB2			
Pos.	166	obs:	exp:
att	I	0	0.37
atc	I	1	0.25
ata	I	0	0.38
gtt	V	987	506.90
gtc	V	1344	492.50
gta	V	0	458.60
gtg	V	0	872.90
---	--	------	------
mPD		0.49	0.73
		nPD:	0.67
		N. weight:	1.2
		Sc. PD:	0.5
		Sc. rank:	1468.8

,

PB2			
Pos.	167	obs:	exp:
att	I	0	0.37
atc	I	1	0.25
ata	I	0	0.38
gtt	V	2320	506.70
gtc	V	9	492.30
gta	V	1	458.50
gtg	V	0	872.50
gat	D	1	0.58
gac	D	0	0.42
---	--	------	------
mPD		0.011	0.73
		nPD:	0.02
		N. weight:	2.
		Sc. PD:	-0.4
		Sc. rank:	-1621.7

,

PB2			
Pos.	168	obs:	exp:
ttt	F	21	992.30
ttc	F	2311	1340.00
---	--	------	------
mPD		0.018	0.49
		nPD:	0.04
		N. weight:	0.68
		Sc. PD:	-0.12
		Sc. rank:	-282.0

,

PB2			
Pos.	169	obs:	exp:
tct	S	0	0.17
tcc	S	0	0.15
tca	S	1	0.23
tcg	S	0	0.06
cct	P	0	657.30
ccc	P	1	421.80
cca	P	2286	951.80
ccg	P	44	300.10
agt	S	0	0.20
agc	S	0	0.19
---	--	------	------
mPD		0.039	0.71
		nPD:	0.05
		N. weight:	1.1
		Sc. PD:	-0.18
		Sc. rank:	-237.8

,

PB2			
Pos.	170	obs:	exp:
aat	N	2145	1239.00
aac	N	186	1092.00
aaa	K	0	0.56
aag	K	1	0.44
---	--	------	------
mPD		0.15	0.50
		nPD:	0.3
		N. weight:	0.49
		Sc. PD:	0.035
		Sc. rank:	288.0

,

PB2			
Pos.	171	obs:	exp:
gaa	E	2327	1396.00
gag	E	5	935.70
---	--	------	------
mPD		0.0043	0.48
		nPD:	0.01
		N. weight:	0.67
		Sc. PD:	-0.14
		Sc. rank:	-712.2

,

PB2			
Pos.	172	obs:	exp:
gtt	V	1478	507.20
gtc	V	193	492.70
gta	V	24	458.80
gtg	V	637	873.30
---	--	------	------
mPD		0.52	0.73
		nPD:	0.71
		N. weight:	0.65
		Sc. PD:	0.31
		Sc. rank:	865.1

,

PB2			
Pos.	173	obs:	exp:
ggt	G	1	335.50
ggc	G	0	307.80
gga	G	2306	1018.00
ggg	G	25	670.70
---	--	------	------
mPD		0.022	0.69
		nPD:	0.03
		N. weight:	1.
		Sc. PD:	-0.19
		Sc. rank:	-515.8

,

PB2			
Pos.	174	obs:	exp:
gct	A	629	593.20
gcc	A	1691	454.80
gca	A	5	1025.00
gcg	A	7	258.90
---	--	------	------
mPD		0.40	0.69
		nPD:	0.58
		N. weight:	1.3
		Sc. PD:	0.44
		Sc. rank:	1426.3

,

PB2			
Pos.	175	obs:	exp:
tct	S	0	0.17
tcc	S	0	0.15
tca	S	0	0.23
tcg	S	0	0.06
cgt	R	0	88.40
cgc	R	0	124.20
cga	R	0	225.70
cgg	R	2	182.60
agt	S	1	0.20
agc	S	0	0.19
aga	R	698	1069.00
agg	R	1631	640.90
---	--	------	------
mPD		0.42	0.95
		nPD:	0.44
		N. weight:	0.7
		Sc. PD:	0.15
		Sc. rank:	583.2

,

PB2

Pos.	176	obs:	exp:
att	I	135	861.10
atc	I	1094	581.60
ata	I	1090	876.30
atg	M	1	1.00
gtt	V	3	2.61
gtc	V	9	2.54
gta	V	0	2.36
gtg	V	0	4.49
---	--	------	------
mPD		0.56	0.67
		nPD:	0.85
		N. weight:	0.4
		Sc. PD:	0.24
		Sc. rank:	599.7

,

PB2

Pos.	177	obs:	exp:
tta	L	11	254.50
ttg	L	2306	437.60
ctt	L	0	437.30
ctc	L	0	402.30
cta	L	5	310.30
ctg	L	10	490.00
---	--	------	------
mPD		0.026	1.1
		nPD:	0.02
		N. weight:	2.1
		Sc. PD:	-0.41
		Sc. rank:	-1251.4

,

PB2

Pos.	178	obs:	exp:
tct	S	0	0.34
tcc	S	0	0.29
tca	S	2	0.45
tcg	S	0	0.13
act	T	0	615.30
acc	T	121	513.30
aca	T	2184	998.20
acg	T	18	196.20
agt	S	0	0.41
agc	S	0	0.38
gct	A	0	1.78
gcc	A	0	1.37
gca	A	7	3.08
gcg	A	0	0.78
---	--	------	------
mPD		0.12	0.70
		nPD:	0.17
		N. weight:	0.86
		Sc. PD:	-0.041
		Sc. rank:	203.6

,

PB2

Pos.	179	obs:	exp:
tct	S	1	398.30
tcc	S	0	343.80
tca	S	2318	525.70
tcg	S	13	150.60
agt	S	0	476.10
agc	S	0	437.50
---	--	------	------
mPD		0.012	1.6
		nPD:	0.01
		N. weight:	2.
		Sc. PD:	-0.41
		Sc. rank:	-2202.9

,

PB2

Pos.	180	obs:	exp:
gaa	E	1481	1396.00
gag	E	851	935.70
---	--	------	------
mPD		0.46	0.48
		nPD:	0.97
		N. weight:	0.006
		Sc. PD:	0.0044
		Sc. rank:	9.1

,

PB2

Pos.	181	obs:	exp:
tct	S	0	398.30
tcc	S	0	343.80
tca	S	2229	525.70
tcg	S	103	150.60
agt	S	0	476.10
agc	S	0	437.50
---	--	------	------
mPD		0.084	1.6
		nPD:	0.05
		N. weight:	1.8
		Sc. PD:	-0.3
		Sc. rank:	-461.3

,

PB2

Pos.	182	obs:	exp:
caa	Q	1660	1187.00
cag	Q	672	1145.00
---	--	------	------
mPD		0.41	0.50
		nPD:	0.82
		N. weight:	0.12
		Sc. PD:	0.068
		Sc. rank:	170.7

,

PB2

Pos.	183	obs:	exp:
tta	L	1561	254.40
ttg	L	757	437.40
tct	S	0	0.17
tcc	S	0	0.15
tca	S	1	0.23
tcg	S	0	0.06
ctt	L	0	437.10
ctc	L	0	402.20
cta	L	1	310.10
ctg	L	12	489.80
agt	S	0	0.20
agc	S	0	0.19
---	--	------	------
mPD		0.45	1.1
		nPD:	0.4
		N. weight:	1.8
		Sc. PD:	0.32
		Sc. rank:	1424.7

,

PB2

Pos.	184	obs:	exp:
att	I	0	0.74
atc	I	0	0.50
ata	I	2	0.76
act	T	33	589.10
acc	T	0	491.40
aca	T	2159	955.70
acg	T	32	187.90
gct	A	1	26.97
gcc	A	0	20.67
gca	A	105	46.60
gcg	A	0	11.77
---	--	------	------
mPD		0.14	0.78
		nPD:	0.19
		N. weight:	0.97
		Sc. PD:	-0.035
		Sc. rank:	244.5

,

PB2

Pos.	185	obs:	exp:
tta	L	0	0.11
ttg	L	0	0.19
ctt	L	0	0.19
ctc	L	0	0.17
cta	L	1	0.13
ctg	L	0	0.21
att	I	0	865.60
atc	I	0	584.60
ata	I	2331	880.80
---	--	------	------
mPD		0.00086	0.66
		nPD:	0.
		N. weight:	1.3
		Sc. PD:	-0.28
		Sc. rank:	-1834.7

,

PB2

Pos.	186	obs:	exp:
act	T	0	617.40
acc	T	1	515.00
aca	T	1700	1002.00
acg	T	630	196.90
gct	A	0	0.25
gcc	A	0	0.20
gca	A	1	0.44
gcg	A	0	0.11
---	--	------	------
mPD		0.40	0.69
		nPD:	0.57
		N. weight:	0.94
		Sc. PD:	0.32
		Sc. rank:	1025.1

,

PB2

Pos.	187	obs:	exp:
cgt	R	0	37.32
cgc	R	0	52.41
cga	R	0	95.28
cgg	R	0	77.07
atg	M	100	100.00
aaa	K	687	700.50
aag	K	561	547.50
aga	R	2	451.40
agg	R	982	270.60
---	--	------	------
mPD		0.95	1.3
		nPD:	0.75
		N. weight:	0.72
		Sc. PD:	0.37
		Sc. rank:	1019.2

,

PB2

Pos.	188	obs:	exp:
gat	D	0	0.58
gac	D	1	0.42
gaa	E	1692	1395.00
gag	E	637	934.50
ggt	G	0	0.29
ggc	G	0	0.26
gga	G	2	0.87
ggg	G	0	0.58
---	--	------	------
mPD		0.40	0.48
		nPD:	0.83
		N. weight:	0.051
		Sc. PD:	0.03
		Sc. rank:	75.9

,

PB2

Pos.	189	obs:	exp:
aaa	K	527	1309.00
aag	K	1805	1023.00
---	--	------	------
mPD		0.35	0.49
		nPD:	0.71
		N. weight:	0.32
		Sc. PD:	0.15
		Sc. rank:	417.9

,

PB2

Pos.	190	obs:	exp:
cgt	R	0	0.99
cgc	R	0	1.38
cga	R	0	2.52
cgg	R	0	2.04
aaa	K	636	1294.00
aag	K	1670	1012.00
aga	R	0	11.93
agg	R	26	7.15
---	--	------	------
mPD		0.42	0.52
		nPD:	0.8
		N. weight:	0.24
		Sc. PD:	0.14
		Sc. rank:	351.5

,

PB2

Pos.	191	obs:	exp:
aaa	K	0	1.68
aag	K	3	1.32
gct	A	0	0.25
gcc	A	0	0.20
gca	A	1	0.44
gcg	A	0	0.11
gaa	E	1069	1394.00
gag	E	1259	934.10
---	--	------	------
mPD		0.50	0.48
		nPD:	1.03
		N. weight:	0.057
		Sc. PD:	0.045
		Sc. rank:	86.0

,

PB2

Pos.	192	obs:	exp:
gct	A	0	0.25
gcc	A	0	0.20
gca	A	0	0.44
gcg	A	1	0.11
gat	D	1	0.58
gac	D	0	0.42
gaa	E	2027	1395.00
gag	E	303	934.90
---	--	------	------
mPD		0.23	0.48
		nPD:	0.47
		N. weight:	0.24
		Sc. PD:	0.059
		Sc. rank:	213.7

,

PB2

Pos.	193	obs:	exp:
tta	L	0	254.50
ttg	L	0	437.60
ctt	L	1665	437.30
ctc	L	667	402.30
cta	L	0	310.30
ctg	L	0	490.00
---	--	------	------
mPD		0.41	1.1
		nPD:	0.36
		N. weight:	1.5
		Sc. PD:	0.2
		Sc. rank:	1034.1

,

PB2

Pos.	194	obs:	exp:
tta	L	0	0.11
ttg	L	0	0.19
ctt	L	0	0.19
ctc	L	0	0.17
cta	L	0	0.13
ctg	L	1	0.21
cat	H	3	1.78
cac	H	0	1.22
caa	Q	8	1183.00
cag	Q	2317	1142.00
cgt	R	0	0.08
cgc	R	0	0.11
cga	R	0	0.19
cgg	R	2	0.16
aaa	K	0	0.56
aag	K	1	0.44
aga	R	0	0.92
agg	R	0	0.55
---	--	------	------
mPD		0.013	0.51
		nPD:	0.03
		N. weight:	0.92
		Sc. PD:	-0.18
		Sc. rank:	-531.4

,

PB2

Pos.	195	obs:	exp:
aat	N	8	4.78
aac	N	1	4.22
gat	D	1709	1230.00
gac	D	429	908.20
gaa	E	1	0.60
gag	E	0	0.40
ggt	G	0	26.47
ggc	G	184	24.29
gga	G	0	80.32
ggg	G	0	52.92
---	--	------	------
mPD		0.54	0.70
		nPD:	0.78
		N. weight:	0.36
		Sc. PD:	0.19
		Sc. rank:	508.4

,

PB2

Pos.	196	obs:	exp:
tgt	C	717	1014.00
tgc	C	1614	1317.00
cgt	R	1	0.04
cgc	R	0	0.05
cga	R	0	0.10
cgg	R	0	0.08
aga	R	0	0.46
agg	R	0	0.27
---	--	------	------
mPD		0.43	0.49
		nPD:	0.87
		N. weight:	0.05
		Sc. PD:	0.031
		Sc. rank:	74.6

,

PB2

Pos.	197	obs:	exp:
caa	Q	0	0.51
cag	Q	1	0.49
cgt	R	0	0.49
cgc	R	0	0.69
cga	R	0	1.26
cgg	R	0	1.02
aat	N	1	0.53
aac	N	0	0.47
aaa	K	13	1301.00
aag	K	2304	1016.00
aga	R	0	5.96
agg	R	13	3.57
---	--	------	------
mPD		0.024	0.51
		nPD:	0.05
		N. weight:	1.1
		Sc. PD:	-0.18
		Sc. rank:	-316.2

,

PB2

Pos.	198	obs:	exp:
att	I	2298	865.60
atc	I	32	584.60
ata	I	1	880.80
gtt	V	1	0.22
gtc	V	0	0.21
gta	V	0	0.20
gtg	V	0	0.37
---	--	------	------
mPD		0.029	0.66
		nPD:	0.04
		N. weight:	1.2
		Sc. PD:	-0.21
		Sc. rank:	-427.4

,

PB2

Pos.	199	obs:	exp:
act	T	6	1.59
acc	T	0	1.33
aca	T	0	2.58
acg	T	0	0.51
gtt	V	1	0.22
gtc	V	0	0.21
gta	V	0	0.20
gtg	V	0	0.37
gct	A	2117	591.50
gcc	A	207	453.40
gca	A	1	1022.00
gcg	A	0	258.10
---	--	------	------
mPD		0.17	0.70
		nPD:	0.24
		N. weight:	1.5
		Sc. PD:	0.028
		Sc. rank:	582.7

,

PB2

Pos.	200	obs:	exp:
cct	P	2327	657.60
ccc	P	4	422.00
cca	P	0	952.20
ccg	P	1	300.30
---	--	------	------
mPD		0.0043	0.70
		nPD:	0.01
		N. weight:	1.7
		Sc. PD:	-0.35
		Sc. rank:	-1976.2

,

PB2

Pos.	201	obs:	exp:
tta	L	753	254.40
ttg	L	1502	437.40
ctt	L	0	437.10
ctc	L	0	402.20
cta	L	47	310.10
ctg	L	29	489.80
gtt	V	0	0.22
gtc	V	0	0.21
gta	V	0	0.20
gtg	V	1	0.37
---	--	------	------
mPD		0.51	1.1
		nPD:	0.45
		N. weight:	1.4
		Sc. PD:	0.32
		Sc. rank:	1218.7

,

PB2

Pos.	202	obs:	exp:
tta	L	0	0.11
ttg	L	0	0.19
ctt	L	0	0.19
ctc	L	0	0.17
cta	L	0	0.13
ctg	L	1	0.21
att	I	1	2.60
atc	I	0	1.76
ata	I	6	2.65
atg	M	2322	2322.00
gtt	V	0	0.43
gtc	V	0	0.42
gta	V	0	0.39
gtg	V	2	0.75
---	--	------	------
mPD		0.0086	0.010
		nPD:	0.84
		N. weight:	0.0048
		Sc. PD:	0.0029
		Sc. rank:	7.2

,

PB2

Pos.	203	obs:	exp:
atg	M	1	1.00
gtt	V	12	506.90
gtc	V	1	492.50
gta	V	3	458.60
gtg	V	2315	872.90
---	--	------	------
mPD		0.015	0.73
		nPD:	0.02
		N. weight:	1.3
		Sc. PD:	-0.25
		Sc. rank:	-836.6

,

PB2

Pos.	204	obs:	exp:
gct	A	3	593.20
gcc	A	0	454.80
gca	A	2325	1025.00
gcg	A	4	258.90
---	--	------	------
mPD		0.0060	0.69
		nPD:	0.01
		N. weight:	1.1
		Sc. PD:	-0.22
		Sc. rank:	-1161.2

,

PB2

Pos.	205	obs:	exp:
tat	Y	0	1132.00
tac	Y	2329	1197.00
cat	H	0	1.78
cac	H	3	1.22
---	--	------	------
mPD		0.0026	0.50
		nPD:	0.01
		N. weight:	0.89
		Sc. PD:	-0.19
		Sc. rank:	-1081.5

,

PB2

Pos.	206	obs:	exp:
atg	M	2332	2332.00
---	--	------	------
mPD		0	0
		nPD:	1.
		N. weight:	0.
		Sc. PD:	0
		Sc. rank:	0

,

PB2

Pos.	207	obs:	exp:
tta	L	1	254.50
ttg	L	2313	437.60
ctt	L	0	437.30
ctc	L	0	402.30
cta	L	11	310.30
ctg	L	7	490.00
---	--	------	------
mPD		0.026	1.1
		nPD:	0.02
		N. weight:	2.2
		Sc. PD:	-0.42
		Sc. rank:	-1347.7

,

PB2

Pos.	208	obs:	exp:
gaa	E	1655	1396.00
gag	E	677	935.70
---	--	------	------
mPD		0.41	0.48
		nPD:	0.86
		N. weight:	0.038
		Sc. PD:	0.023
		Sc. rank:	56.8

,

PB2

Pos.	209	obs:	exp:
cgt	R	0	88.40
cgc	R	0	124.20
cga	R	0	225.70
cgg	R	0	182.60
aaa	K	1	0.56
aag	K	0	0.44
aga	R	1973	1069.00
agg	R	358	640.90
---	--	------	------
mPD		0.26	0.95
		nPD:	0.27
		N. weight:	0.58
		Sc. PD:	0.029
		Sc. rank:	308.3

,

PB2

Pos.	210	obs:	exp:
gaa	E	2292	1396.00
gag	E	40	935.70
---	--	------	------
mPD		0.034	0.48
		nPD:	0.07
		N. weight:	0.58
		Sc. PD:	-0.086
		Sc. rank:	-59.6

,

PB2

Pos.	211	obs:	exp:
tta	L	0	254.50
ttg	L	13	437.60
ctt	L	0	437.30
ctc	L	0	402.30
cta	L	22	310.30
ctg	L	2297	490.00
---	--	------	------
mPD		0.030	1.1
		nPD:	0.03
		N. weight:	2.
		Sc. PD:	-0.38
		Sc. rank:	-1088.9

,

PB2

Pos.	212	obs:	exp:
gtt	V	1692	506.90
gtc	V	638	492.50
gta	V	1	458.60
gtg	V	0	872.90
gct	A	1	0.25
gcc	A	0	0.20
gca	A	0	0.44
gcg	A	0	0.11
---	--	------	------
mPD		0.40	0.73
		nPD:	0.55
		N. weight:	1.3
		Sc. PD:	0.4
		Sc. rank:	1279.4

,

PB2

Pos.	213	obs:	exp:
cct	P	0	0.56
ccc	P	2	0.36
cca	P	0	0.82
ccg	P	0	0.26
cat	H	0	0.59
cac	H	1	0.41
cgt	R	5	88.32
cgc	R	2293	124.10
cga	R	31	225.50
cgg	R	0	182.40
aga	R	0	1068.00
agg	R	0	640.40
---	--	------	------
mPD		0.033	0.96
		nPD:	0.03
		N. weight:	3.8
		Sc. PD:	-0.69
		Sc. rank:	-1738.6

,

PB2

Pos.	214	obs:	exp:
caa	Q	0	0.51
cag	Q	1	0.49
cgt	R	0	0.19
cgc	R	0	0.27
cga	R	0	0.48
cgg	R	0	0.39
atg	M	1	1.00
aaa	K	1213	1305.00
aag	K	1112	1020.00
aga	R	0	2.29
agg	R	5	1.37
---	--	------	------
mPD		0.51	0.50
		nPD:	1.01
		N. weight:	0.011
		Sc. PD:	0.0082
		Sc. rank:	16.1

,

PB2

Pos.	215	obs:	exp:
tct	S	0	0.17
tcc	S	1	0.15
tca	S	0	0.23
tcg	S	0	0.06
act	T	4	617.20
acc	T	2277	514.80
aca	T	37	1001.00
acg	T	12	196.80
agt	S	0	0.20
agc	S	0	0.19
gct	A	0	0.25
gcc	A	1	0.20
gca	A	0	0.44
gcg	A	0	0.11
---	--	------	------
mPD		0.046	0.69
		nPD:	0.07
		N. weight:	1.8
		Sc. PD:	-0.28
		Sc. rank:	-223.4

,

PB2

Pos.	216	obs:	exp:
cgt	R	0	88.44
cgc	R	0	124.20
cga	R	0	225.80
cgg	R	0	182.60
aga	R	2284	1070.00
agg	R	48	641.20
---	--	------	------
mPD		0.040	0.95
		nPD:	0.04
		N. weight:	0.92
		Sc. PD:	-0.16
		Sc. rank:	-336.5

,

PB2

Pos.	217	obs:	exp:
ttt	F	1482	992.30
ttc	F	850	1340.00
---	--	------	------
mPD		0.46	0.49
		nPD:	0.95
		N. weight:	0.12
		Sc. PD:	0.086
		Sc. rank:	182.8

,

PB2

Pos.	218	obs:	exp:
tta	L	1197	254.50
ttg	L	40	437.60
ctt	L	2	437.30
ctc	L	10	402.30
cta	L	893	310.30
ctg	L	190	490.00
---	--	------	------
mPD		0.69	1.1
		nPD:	0.6
		N. weight:	1.4
		Sc. PD:	0.53
		Sc. rank:	1680.5

,

PB2

Pos.	219	obs:	exp:
cct	P	0	657.60
ccc	P	0	422.00
cca	P	2218	952.20
ccg	P	114	300.30
---	--	------	------
mPD		0.093	0.70
		nPD:	0.13
		N. weight:	1.
		Sc. PD:	-0.089
		Sc. rank:	82.9

,

PB2

Pos.	220	obs:	exp:
gtt	V	4	507.20
gtc	V	2	492.70
gta	V	2316	458.80
gtg	V	10	873.30
---	--	------	------
mPD		0.014	0.73
		nPD:	0.02
		N. weight:	2.1
		Sc. PD:	-0.42
		Sc. rank:	-1502.7

,

PB2			
Pos.	221	obs:	exp:
tct	S	3	0.51
tcc	S	0	0.44
tca	S	0	0.68
tcg	S	0	0.19
act	T	3	0.79
acc	T	0	0.66
aca	T	0	1.29
acg	T	0	0.25
agt	S	0	0.61
agc	S	0	0.56
gtt	V	1	0.43
gtc	V	0	0.42
gta	V	1	0.39
gtg	V	0	0.75
gct	A	1689	591.20
gcc	A	10	453.20
gca	A	523	1022.00
gcg	A	102	258.00
---	--	------	------
mPD		0.43	0.70
		nPD:	0.61
		N. weight:	0.75
		Sc. PD:	0.28
		Sc. rank:	888.0

PB2			
Pos.	222	obs:	exp:
ggt	G	667	335.50
ggc	G	1601	307.80
gga	G	63	1018.00
ggg	G	1	670.70
---	--	------	------
mPD		0.45	0.69
		nPD:	0.65
		N. weight:	1.7
		Sc. PD:	0.69
		Sc. rank:	2104.1

PB2			
Pos.	223	obs:	exp:
cgt	R	0	0.04
cgc	R	0	0.05
cga	R	0	0.10
cgg	R	1	0.08
aga	R	0	0.46
agg	R	0	0.27
ggt	G	0	335.30
ggc	G	1	307.70
gga	G	1611	1018.00
ggg	G	719	670.40
---	--	------	------
mPD		0.43	0.69
		nPD:	0.62
		N. weight:	0.45
		Sc. PD:	0.18
		Sc. rank:	549.6

PB2			
Pos.	224	obs:	exp:
act	T	0	617.70
acc	T	0	515.20
aca	T	2332	1002.00
acg	T	0	197.00
---	--	------	------
mPD		0	0.69
		nPD:	0.
		N. weight:	1.1
		Sc. PD:	-0.24
		Sc. rank:	-1701.4

PB2			
Pos.	225	obs:	exp:
tct	S	0	397.70
tcc	S	0	343.30
tca	S	0	525.00
tcg	S	0	150.40
agt	S	3	475.50
agc	S	2326	437.00
ggt	G	0	0.43
ggc	G	3	0.40
gga	G	0	1.31
ggg	G	0	0.86
---	--	------	------
mPD		0.0051	1.6
		nPD:	0.
		N. weight:	2.2
		Sc. PD:	-0.47
		Sc. rank:	-2948.6

PB2			
Pos.	226	obs:	exp:
tct	S	0	398.30
tcc	S	0	343.80
tca	S	0	525.70
tcg	S	0	150.60
agt	S	656	476.10
agc	S	1676	437.50
---	--	------	------
mPD		0.40	1.6
		nPD:	0.25
		N. weight:	1.4
		Sc. PD:	0.033
		Sc. rank:	616.4

PB2			
Pos.	227	obs:	exp:
att	I	0	1.86
atc	I	0	1.25
ata	I	5	1.89
atg	M	1	1.00
gtt	V	0	505.40
gtc	V	11	491.00
gta	V	1428	457.30
gtg	V	885	870.30
gct	A	0	0.51
gcc	A	0	0.39
gca	A	2	0.88
gcg	A	0	0.22
---	--	------	------
mPD		0.48	0.74
		nPD:	0.66
		N. weight:	0.92
		Sc. PD:	0.39
		Sc. rank:	1165.3

PB2			
Pos.	228	obs:	exp:
tat	Y	858	1134.00
tac	Y	1474	1198.00
---	--	------	------
mPD		0.47	0.50
		nPD:	0.93
		N. weight:	0.04
		Sc. PD:	0.028
		Sc. rank:	60.5

PB2			
Pos.	229	obs:	exp:
att	I	848	866.00
atc	I	1472	584.90
ata	I	12	881.20
---	--	------	------
mPD		0.47	0.66
		nPD:	0.72
		N. weight:	0.74
		Sc. PD:	0.36
		Sc. rank:	998.7

PB2			
Pos.	230	obs:	exp:
gaa	E	2	1396.00
gag	E	2330	935.70
---	--	------	------
mPD		0.0017	0.48
		nPD:	0.
		N. weight:	1.2
		Sc. PD:	-0.26
		Sc. rank:	-1553.5

PB2			
Pos.	231	obs:	exp:
gtt	V	0	507.20
gtc	V	0	492.70
gta	V	2207	458.80
gtg	V	125	873.30
---	--	------	------
mPD		0.10	0.73
		nPD:	0.14
		N. weight:	1.9
		Sc. PD:	-0.15
		Sc. rank:	209.2

PB2			
Pos.	232	obs:	exp:
tta	L	15	254.50
ttg	L	1775	437.60
ctt	L	1	437.30
ctc	L	0	402.30
cta	L	2	310.30
ctg	L	539	490.00
---	--	------	------
mPD		0.37	1.1
		nPD:	0.33
		N. weight:	1.4
		Sc. PD:	0.15
		Sc. rank:	893.6

PB2			
Pos.	233	obs:	exp:
cat	H	1595	1387.00
cac	H	737	945.40
---	--	------	------
mPD		0.43	0.48
		nPD:	0.9
		N. weight:	0.025
		Sc. PD:	0.016
		Sc. rank:	37.7

PB2			
Pos.	234	obs:	exp:
tta	L	11	254.50
ttg	L	2311	437.60
ctt	L	0	437.30
ctc	L	0	402.30
cta	L	0	310.30
ctg	L	10	490.00
---	--	------	------
mPD		0.018	1.1
		nPD:	0.02
		N. weight:	2.2
		Sc. PD:	-0.43
		Sc. rank:	-1678.5

PB2			
Pos.	235	obs:	exp:
act	T	1817	617.70
acc	T	515	515.20
aca	T	0	1002.00
acg	T	0	197.00
---	--	------	------
mPD		0.34	0.69
		nPD:	0.5
		N. weight:	1.1
		Sc. PD:	0.3
		Sc. rank:	1021.3

PB2			
Pos.	236	obs:	exp:
cat	H	0	0.59
cac	H	1	0.41
caa	Q	2316	1186.00
cag	Q	15	1145.00
---	--	------	------
mPD		0.014	0.50
		nPD:	0.03
		N. weight:	0.85
		Sc. PD:	-0.16
		Sc. rank:	-457.6

PB2			
Pos.	237	obs:	exp:
ggt	G	0	335.50
ggc	G	1	307.80
gga	G	219	1018.00
ggg	G	2112	670.70
---	--	------	------
mPD		0.17	0.69
		nPD:	0.25
		N. weight:	1.2
		Sc. PD:	0.031
		Sc. rank:	557.0

PB2			
Pos.	238	obs:	exp:
act	T	4	617.40
acc	T	2305	515.00
aca	T	22	1002.00
acg	T	0	196.90
gct	A	0	0.25
gcc	A	1	0.20
gca	A	0	0.44
gcg	A	0	0.11
---	--	------	------
mPD		0.023	0.69
		nPD:	0.03
		N. weight:	1.9
		Sc. PD:	-0.35
		Sc. rank:	-928.6

PB2			
Pos.	239	obs:	exp:
tgt	C	7	1014.00
tgc	C	2325	1318.00
---	--	------	------
mPD		0.0060	0.49
		nPD:	0.01
		N. weight:	0.74
		Sc. PD:	-0.15
		Sc. rank:	-680.2

PB2			
Pos.	240	obs:	exp:
tgg	W	2332	2332.00
---	--	------	------
mPD		0	0
		nPD:	1.
		N. weight:	0.
		Sc. PD:	0
		Sc. rank:	0

PB2			
Pos.	241	obs:	exp:
gaa	E	2303	1396.00
gag	E	29	935.70
---	--	------	------
mPD		0.025	0.48
		nPD:	0.05
		N. weight:	0.6
		Sc. PD:	-0.1
		Sc. rank:	-157.1

PB2			
Pos.	242	obs:	exp:
caa	Q	1693	1187.00
cag	Q	639	1145.00
---	--	------	------
mPD		0.40	0.50
		nPD:	0.8
		N. weight:	0.13
		Sc. PD:	0.075
		Sc. rank:	192.3

PB2			
Pos.	243	obs:	exp:
tta	L	0	0.11
ttg	L	0	0.19
ctt	L	0	0.19
ctc	L	0	0.17
cta	L	0	0.13
ctg	L	1	0.21
atg	M	2331	2331.00
---	--	------	------
mPD		0.00086	0.0014
		nPD:	0.62
		N. weight:	0.0009
		Sc. PD:	0.00035
		Sc. rank:	1.1

PB2			
Pos.	244	obs:	exp:
tat	Y	30	1134.00
tac	Y	2302	1198.00
---	--	------	------
mPD		0.025	0.50
		nPD:	0.05
		N. weight:	0.8
		Sc. PD:	-0.13
		Sc. rank:	-213.5

PB2			
Pos.	245	obs:	exp:
act	T	633	617.70
acc	T	5	515.20
aca	T	1660	1002.00
acg	T	34	197.00
---	--	------	------
mPD		0.42	0.69
		nPD:	0.61
		N. weight:	0.45
		Sc. PD:	0.17
		Sc. rank:	528.3

PB2			
Pos. 246		obs:	exp:
cct	P	43	657.60
ccc	P	0	422.00
cca	P	1782	952.20
ccg	P	507	300.30
---	--	------	------
mPD		0.37	0.70
		nPD:	0.52
		N. weight:	0.73
		Sc. PD:	0.21
		Sc. rank:	684.5

,

PB2			
Pos. 247		obs:	exp:
ggt	G	0	335.50
ggc	G	628	307.80
gga	G	1702	1018.00
ggg	G	2	670.70
---	--	------	------
mPD		0.39	0.69
		nPD:	0.57
		N. weight:	0.76
		Sc. PD:	0.26
		Sc. rank:	824.1

,

PB2			
Pos. 248		obs:	exp:
ggt	G	0	335.50
ggc	G	0	307.80
gga	G	1002	1018.00
ggg	G	1330	670.70
---	--	------	------
mPD		0.49	0.69
		nPD:	0.71
		N. weight:	0.52
		Sc. PD:	0.25
		Sc. rank:	686.6

,

PB2			
Pos. 249		obs:	exp:
atg	M	1	1.00
aaa	K	81	46.03
aag	K	1	35.97
gtt	V	0	38.93
gtc	V	0	37.82
gta	V	0	35.22
gtg	V	179	67.03
gat	D	3	1.73
gac	D	0	1.27
gaa	E	751	1184.00
gag	E	1226	793.20
ggt	G	0	12.95
ggc	G	0	11.88
gga	G	0	39.29
ggg	G	90	25.89
---	--	------	------
mPD		0.74	0.81
		nPD:	0.92
		N. weight:	0.3
		Sc. PD:	0.2
		Sc. rank:	455.6

,

PB2			
Pos. 250		obs:	exp:
gtt	V	0	503.20
gtc	V	0	488.90
gta	V	2	455.30
gtg	V	2312	866.50
gct	A	0	4.58
gcc	A	0	3.51
gca	A	0	7.91
gcg	A	18	2.00
---	--	------	------
mPD		0.017	0.75
		nPD:	0.02
		N. weight:	1.3
		Sc. PD:	-0.26
		Sc. rank:	-805.0

,

PB2			
Pos. 251		obs:	exp:
cgt	R	0	88.25
cgc	R	0	123.90
cga	R	0	225.30
cgg	R	0	182.30
att	I	0	0.37
atc	I	0	0.25
ata	I	1	0.38
aaa	K	4	2.25
aag	K	0	1.75
aga	R	2232	1067.00
agg	R	95	639.80
---	--	------	------
mPD		0.082	0.96
		nPD:	0.09
		N. weight:	0.84
		Sc. PD:	-0.11
		Sc. rank:	-34.1

,

PB2			
Pos. 252		obs:	exp:
tct	S	0	0.34
tcc	S	0	0.29
tca	S	0	0.45
tcg	S	0	0.13
aat	N	2316	1238.00
aac	N	13	1091.00
aaa	K	0	0.56
aag	K	1	0.44
agt	S	2	0.41
agc	S	0	0.38
---	--	------	------
mPD		0.014	0.50
		nPD:	0.03
		N. weight:	0.8
		Sc. PD:	-0.15
		Sc. rank:	-433.2

,

PB2			
Pos. 253		obs:	exp:
aat	N	0	0.53
aac	N	1	0.47
gat	D	1695	1341.00
gac	D	636	990.20
---	--	------	------
mPD		0.40	0.49
		nPD:	0.81
		N. weight:	0.069
		Sc. PD:	0.04
		Sc. rank:	100.9

,

PB2			
Pos. 254		obs:	exp:
gat	D	2139	1341.00
gac	D	192	990.20
gaa	E	1	0.60
gag	E	0	0.40
---	--	------	------
mPD		0.15	0.49
		nPD:	0.31
		N. weight:	0.39
		Sc. PD:	0.034
		Sc. rank:	237.9

,

PB2			
Pos. 255		obs:	exp:
att	I	170	63.13
atc	I	0	42.64
ata	I	0	64.24
gtt	V	1966	469.80
gtc	V	187	456.40
gta	V	7	425.00
gtg	V	0	808.90
gct	A	2	0.51
gcc	A	0	0.39
gca	A	0	0.88
gcg	A	0	0.22
---	--	------	------
mPD		0.29	0.87
		nPD:	0.33
		N. weight:	1.6
		Sc. PD:	0.17
		Sc. rank:	1042.2

,

PB2			
Pos. 256		obs:	exp:
aat	N	5	2.66
aac	N	0	2.34
gat	D	1686	1339.00
gac	D	641	988.50
---	--	------	------
mPD		0.40	0.49
		nPD:	0.82
		N. weight:	0.068
		Sc. PD:	0.039
		Sc. rank:	99.7

,

PB2			
Pos. 257		obs:	exp:
caa	Q	10	1184.00
cag	Q	2318	1144.00
aaa	K	0	2.25
aag	K	4	1.75
---	--	------	------
mPD		0.012	0.50
		nPD:	0.02
		N. weight:	0.92
		Sc. PD:	-0.18
		Sc. rank:	-529.9

,

PB2			
Pos. 258		obs:	exp:
tct	S	0	398.30
tcc	S	0	343.80
tca	S	0	525.70
tcg	S	0	150.60
agt	S	2317	476.10
agc	S	15	437.50
---	--	------	------
mPD		0.013	1.6
		nPD:	0.01
		N. weight:	2.1
		Sc. PD:	-0.43
		Sc. rank:	-2323.7

,

PB2			
Pos. 259		obs:	exp:
tta	L	563	254.50
ttg	L	1764	437.60
ctt	L	0	437.30
ctc	L	0	402.30
cta	L	4	310.30
ctg	L	1	490.00
---	--	------	------
mPD		0.37	1.1
		nPD:	0.33
		N. weight:	1.7
		Sc. PD:	0.17
		Sc. rank:	1063.3

,

PB2			
Pos. 260		obs:	exp:
att	I	228	856.30
atc	I	2076	578.30
ata	I	2	871.40
gtt	V	0	5.65
gtc	V	26	5.49
gta	V	0	5.12
gtg	V	0	9.74
---	--	------	------
mPD		0.20	0.68
		nPD:	0.29
		N. weight:	1.4
		Sc. PD:	0.095
		Sc. rank:	787.9

,

PB2			
Pos. 261		obs:	exp:
att	I	1682	860.40
atc	I	633	581.10
ata	I	2	875.50
act	T	2	0.79
acc	T	1	0.66
aca	T	0	1.29
acg	T	0	0.25
gtt	V	11	2.61
gtc	V	1	2.54
gta	V	0	2.36
gtg	V	0	4.49
---	--	------	------
mPD		0.41	0.67
		nPD:	0.61
		N. weight:	0.68
		Sc. PD:	0.26
		Sc. rank:	815.7

,

PB2			
Pos. 262		obs:	exp:
gct	A	1808	593.20
gcc	A	6	454.80
gca	A	6	1025.00
gcg	A	512	258.90
---	--	------	------
mPD		0.35	0.69
		nPD:	0.51
		N. weight:	1.3
		Sc. PD:	0.37
		Sc. rank:	1216.4

,

PB2			
Pos. 263		obs:	exp:
act	T	1	0.26
acc	T	0	0.22
aca	T	0	0.43
acg	T	0	0.08
gct	A	1665	593.00
gcc	A	666	454.60
gca	A	0	1025.00
gcg	A	0	258.80
---	--	------	------
mPD		0.41	0.69
		nPD:	0.59
		N. weight:	1.1
		Sc. PD:	0.41
		Sc. rank:	1314.3

,

PB2			
Pos. 264		obs:	exp:
cgt	R	0	88.44
cgc	R	0	124.20
cga	R	1	225.80
cgg	R	3	182.60
aga	R	2280	1070.00
agg	R	48	641.20
---	--	------	------
mPD		0.046	0.95
		nPD:	0.05
		N. weight:	0.91
		Sc. PD:	-0.15
		Sc. rank:	-262.7

,

PB2			
Pos. 265		obs:	exp:
aat	N	1694	1239.00
aac	N	638	1093.00
---	--	------	------
mPD		0.40	0.50
		nPD:	0.8
		N. weight:	0.11
		Sc. PD:	0.061
		Sc. rank:	157.7

,

PB2			
Pos. 266		obs:	exp:
att	I	811	866.00
atc	I	672	584.90
ata	I	849	881.20
---	--	------	------
mPD		0.66	0.66
		nPD:	1.01
		N. weight:	0.0095
		Sc. PD:	0.0073
		Sc. rank:	14.3

,

PB2			
Pos. 267		obs:	exp:
att	I	10	3.71
atc	I	0	2.51
ata	I	0	3.78
gtt	V	2211	505.00
gtc	V	96	490.60
gta	V	11	456.90
gtg	V	4	869.50
---	--	------	------
mPD		0.10	0.74
		nPD:	0.14
		N. weight:	1.8
		Sc. PD:	-0.15
		Sc. rank:	185.6

,

PB2			
Pos. 268		obs:	exp:
cgt	R	0	88.44
cgc	R	0	124.20
cga	R	0	225.80
cgg	R	0	182.60
aga	R	15	1070.00
agg	R	2317	641.20
---	--	------	------
mPD		0.013	0.95
		nPD:	0.01
		N. weight:	1.7
		Sc. PD:	-0.34
		Sc. rank:	-1503.8

,

PB2			
Pos. 269		obs:	exp:
cgt	R	0	88.44
cgc	R	0	124.20
cga	R	0	225.80
cgg	R	0	182.60
aga	R	1222	1070.00
agg	R	1110	641.20
---	--	------	------
mPD		0.50	0.95
		nPD:	0.52
		N. weight:	0.45
		Sc. PD:	0.13
		Sc. rank:	418.4

,

PB2			
Pos. 270		obs:	exp:
gct	A	1	593.20
gcc	A	0	454.80
gca	A	2331	1025.00
gcg	A	0	258.90
---	--	------	------
mPD		0.00086	0.69
		nPD:	0.
		N. weight:	1.1
		Sc. PD:	-0.23
		Sc. rank:	-1571.1

,

PB2			
Pos.	271	obs:	exp:
act	T	0	617.70
acc	T	0	515.20
aca	T	1714	1002.00
acg	T	618	197.00
---	--	------	------
mPD		0.39	0.69
		nPD:	0.57
		N. weight:	0.94
		Sc. PD:	0.31
		Sc. rank:	989.2

,

PB2			
Pos.	272	obs:	exp:
att	I	0	1.11
atc	I	0	0.75
ata	I	3	1.13
gtt	V	0	506.50
gtc	V	0	492.10
gta	V	2322	458.30
gtg	V	7	872.10
---	--	------	------
mPD		0.0086	0.73
		nPD:	0.01
		N. weight:	2.1
		Sc. PD:	-0.44
		Sc. rank:	-2010.7

,

PB2			
Pos.	273	obs:	exp:
tct	S	0	398.30
tcc	S	9	343.80
tca	S	2321	525.70
tcg	S	2	150.60
agt	S	0	476.10
agc	S	0	437.50
---	--	------	------
mPD		0.0094	1.6
		nPD:	0.01
		N. weight:	2.
		Sc. PD:	-0.41
		Sc. rank:	-2320.8

,

PB2			
Pos.	274	obs:	exp:
act	T	0	0.26
acc	T	0	0.22
aca	T	1	0.43
acg	T	0	0.08
gtt	V	0	0.22
gtc	V	0	0.21
gta	V	1	0.20
gtg	V	0	0.37
gct	A	1	592.70
gcc	A	0	454.40
gca	A	1803	1024.00
gcg	A	526	258.70
---	--	------	------
mPD		0.35	0.69
		nPD:	0.51
		N. weight:	0.8
		Sc. PD:	0.22
		Sc. rank:	735.1

,

PB2			
Pos.	275	obs:	exp:
gat	D	666	1341.00
gac	D	1666	990.60
---	--	------	------
mPD		0.41	0.49
		nPD:	0.84
		N. weight:	0.23
		Sc. PD:	0.14
		Sc. rank:	343.6

,

PB2			
Pos.	276	obs:	exp:
cct	P	24	657.60
ccc	P	1	422.00
cca	P	2274	952.20
ccg	P	33	300.30
---	--	------	------
mPD		0.049	0.70
		nPD:	0.07
		N. weight:	1.
		Sc. PD:	-0.16
		Sc. rank:	-114.0

,

PB2			
Pos.	277	obs:	exp:
tta	L	9	254.50
ttg	L	1688	437.60
ctt	L	0	437.30
ctc	L	0	402.30
cta	L	8	310.30
ctg	L	627	490.00
---	--	------	------
mPD		0.41	1.1
		nPD:	0.36
		N. weight:	1.4
		Sc. PD:	0.19
		Sc. rank:	978.4

,

PB2			
Pos.	278	obs:	exp:
gtt	V	1	0.22
gtc	V	0	0.21
gta	V	0	0.20
gtg	V	0	0.37
gct	A	1649	593.00
gcc	A	42	454.60
gca	A	638	1025.00
gcg	A	2	258.80
---	--	------	------
mPD		0.43	0.69
		nPD:	0.61
		N. weight:	0.74
		Sc. PD:	0.28
		Sc. rank:	889.4

,

PB2			
Pos.	279	obs:	exp:
tct	S	15	398.30
tcc	S	0	343.80
tca	S	716	525.70
tcg	S	1601	150.60
agt	S	0	476.10
agc	S	0	437.50
---	--	------	------
mPD		0.43	1.6
		nPD:	0.26
		N. weight:	2.3
		Sc. PD:	0.093
		Sc. rank:	1156.4

,

PB2			
Pos.	280	obs:	exp:
tta	L	524	254.40
ttg	L	0	437.40
ctt	L	165	437.10
ctc	L	1486	402.20
cta	L	153	310.10
ctg	L	3	489.80
gtt	V	0	0.22
gtc	V	1	0.21
gta	V	0	0.20
gtg	V	0	0.37
---	--	------	------
mPD		0.85	1.1
		nPD:	0.75
		N. weight:	1.2
		Sc. PD:	0.6
		Sc. rank:	1654.6

,

PB2			
Pos.	281	obs:	exp:
tta	L	11	252.40
ttg	L	1999	433.80
ctt	L	0	433.50
ctc	L	0	398.90
cta	L	24	307.60
ctg	L	278	485.80
gtt	V	0	4.35
gtc	V	0	4.23
gta	V	0	3.94
gtg	V	20	7.49
---	--	------	------
mPD		0.27	1.1
		nPD:	0.24
		N. weight:	1.6
		Sc. PD:	0.022
		Sc. rank:	596.1

,

PB2			
Pos.	282	obs:	exp:
gat	D	4	2.30
gac	D	0	1.70
gaa	E	1530	1394.00
gag	E	798	934.10
---	--	------	------
mPD		0.45	0.48
		nPD:	0.94
		N. weight:	0.013
		Sc. PD:	0.0092
		Sc. rank:	19.9

,

PB2			
Pos.	283	obs:	exp:
att	I	0	2.23
atc	I	0	1.50
ata	I	6	2.27
atg	M	2326	2326.00
---	--	------	------
mPD		0.0051	0.0051
		nPD:	1.
		N. weight:	0.0033
		Sc. PD:	0.0025
		Sc. rank:	5.0

,

PB2			
Pos.	284	obs:	exp:
tgt	C	211	1014.00
tgc	C	2121	1318.00
---	--	------	------
mPD		0.16	0.49
		nPD:	0.34
		N. weight:	0.39
		Sc. PD:	0.043
		Sc. rank:	254.7

,

PB2			
Pos.	285	obs:	exp:
tat	Y	0	0.97
tac	Y	2	1.03
cat	H	221	1384.00
cac	H	2106	943.40
caa	Q	2	1.02
cag	Q	0	0.98
cgt	R	0	0.04
cgc	R	1	0.05
cga	R	0	0.10
cgg	R	0	0.08
aga	R	0	0.46
agg	R	0	0.27
---	--	------	------
mPD		0.18	0.49
		nPD:	0.36
		N. weight:	0.74
		Sc. PD:	0.1
		Sc. rank:	527.9

,

PB2			
Pos.	286	obs:	exp:
tct	S	0	397.90
tcc	S	0	343.50
tca	S	0	525.20
tcg	S	0	150.50
aat	N	1	0.53
aac	N	0	0.47
agt	S	1679	475.70
agc	S	651	437.20
ggt	G	1	0.14
ggc	G	0	0.13
gga	G	0	0.44
ggg	G	0	0.29
---	--	------	------
mPD		0.40	1.6
		nPD:	0.25
		N. weight:	1.4
		Sc. PD:	0.031
		Sc. rank:	588.3

,

PB2			
Pos.	287	obs:	exp:
act	T	0	617.70
acc	T	1	515.20
aca	T	2330	1002.00
acg	T	1	197.00
---	--	------	------
mPD		0.0017	0.69
		nPD:	0.
		N. weight:	1.1
		Sc. PD:	-0.24
		Sc. rank:	-1539.2

,

PB2			
Pos.	288	obs:	exp:
cat	H	0	12.49
cac	H	21	8.51
caa	Q	2284	1175.00
cag	Q	25	1134.00
aaa	K	2	1.12
aag	K	0	0.88
---	--	------	------
mPD		0.041	0.51
		nPD:	0.08
		N. weight:	0.83
		Sc. PD:	-0.11
		Sc. rank:	-62.5

,

PB2			
Pos.	289	obs:	exp:
att	I	2330	866.00
atc	I	2	584.90
ata	I	0	881.20
---	--	------	------
mPD		0.0017	0.66
		nPD:	0.
		N. weight:	1.3
		Sc. PD:	-0.28
		Sc. rank:	-1762.8

,

PB2			
Pos.	290	obs:	exp:
tct	S	0	0.17
tcc	S	0	0.15
tca	S	0	0.23
tcg	S	0	0.06
agt	S	1	0.20
agc	S	0	0.19
ggt	G	1713	335.30
ggc	G	421	307.70
gga	G	182	1018.00
ggg	G	15	670.40
---	--	------	------
mPD		0.42	0.69
		nPD:	0.61
		N. weight:	1.5
		Sc. PD:	0.56
		Sc. rank:	1749.9

,

PB2			
Pos.	291	obs:	exp:
ggt	G	0	335.50
ggc	G	1	307.80
gga	G	1473	1018.00
ggg	G	858	670.70
---	--	------	------
mPD		0.47	0.69
		nPD:	0.68
		N. weight:	0.43
		Sc. PD:	0.19
		Sc. rank:	553.2

,

PB2			
Pos.	292	obs:	exp:
att	I	0	63.87
atc	I	0	43.14
ata	I	172	64.99
atg	M	4	4.00
act	T	0	146.00
acc	T	0	121.70
aca	T	551	236.80
acg	T	0	46.54
gtt	V	0	348.20
gtc	V	0	338.30
gta	V	1079	315.00
gtg	V	522	599.50
gct	A	0	1.02
gcc	A	0	0.78
gca	A	4	1.76
gcg	A	0	0.44
---	--	------	------
mPD		1.1	1.5
		nPD:	0.74
		N. weight:	1.1
		Sc. PD:	0.55
		Sc. rank:	1515.1

,

PB2			
Pos.	293	obs:	exp:
cgt	R	0	87.91
cgc	R	0	123.50
cga	R	0	224.50
cgg	R	0	181.50
aaa	K	0	7.30
aag	K	13	5.70
aga	R	47	1063.00
agg	R	2271	637.40
ggt	G	0	0.14
ggc	G	0	0.13
gga	G	0	0.44
ggg	G	1	0.29
---	--	------	------
mPD		0.051	0.96
		nPD:	0.05
		N. weight:	1.6
		Sc. PD:	-0.26
		Sc. rank:	-362.9

,

PB2			
Pos.	294	obs:	exp:
tta	L	0	0.22
ttg	L	2	0.38
ctt	L	0	0.38
ctc	L	0	0.35
cta	L	0	0.27
ctg	L	0	0.42
atg	M	2330	2330.00
---	--	------	------
mPD		0.0017	0.0027
		nPD:	0.62
		N. weight:	0.0019
		Sc. PD:	0.00075
		Sc. rank:	2.3

,

PB2			
Pos.	295	obs:	exp:
att	I	0	11.14
atc	I	0	7.52
ata	I	30	11.34
atg	M	5	5.00
gtt	V	3	499.10
gtc	V	0	484.90
gta	V	369	451.60
gtg	V	1923	859.40
gct	A	0	0.51
gcc	A	0	0.39
gca	A	2	0.88
gcg	A	0	0.22
---	--	------	------
mPD		0.32	0.76
		nPD:	0.42
		N. weight:	0.86
		Sc. PD:	0.16
		Sc. rank:	693.4

,

PB2			
Pos.	296	obs:	exp:
gat	D	527	1341.00
gac	D	1805	990.60
---	--	------	------
mPD		0.35	0.49
		nPD:	0.72
		N. weight:	0.34
		Sc. PD:	0.16
		Sc. rank:	459.6

,

PB2			
Pos.	297	obs:	exp:
att	I	1551	863.70
atc	I	772	583.30
ata	I	3	878.90
gtt	V	4	1.30
gtc	V	2	1.27
gta	V	0	1.18
gtg	V	0	2.25
---	--	------	------
mPD		0.45	0.66
		nPD:	0.68
		N. weight:	0.64
		Sc. PD:	0.29
		Sc. rank:	827.2

,

PB2			
Pos.	298	obs:	exp:
ttt	F	1	0.43
ttc	F	0	0.57
tta	L	0	254.30
ttg	L	0	437.20
ctt	L	2321	436.90
ctc	L	7	402.00
cta	L	1	310.00
ctg	L	1	489.60
gtt	V	1	0.22
gtc	V	0	0.21
gta	V	0	0.20
gtg	V	0	0.37
---	--	------	------
mPD		0.0094	1.1
		nPD:	0.01
		N. weight:	2.2
		Sc. PD:	-0.46
		Sc. rank:	-2406.3

,

PB2			
Pos.	299	obs:	exp:
caa	Q	2	1.02
cag	Q	0	0.98
cgt	R	0	81.57
cgc	R	0	114.60
cga	R	0	208.30
cgg	R	12	168.50
aaa	K	170	100.50
aag	K	9	78.53
aga	R	405	986.70
agg	R	1734	591.50
---	--	------	------
mPD		0.53	1.1
		nPD:	0.49
		N. weight:	0.89
		Sc. PD:	0.23
		Sc. rank:	799.2

,

PB2			
Pos.	300	obs:	exp:
cat	H	4	2.38
cac	H	0	1.62
caa	Q	658	1183.00
cag	Q	1668	1143.00
cgt	R	0	0.08
cgc	R	0	0.11
cga	R	0	0.19
cgg	R	2	0.16
aga	R	0	0.92
agg	R	0	0.55
---	--	------	------
mPD		0.41	0.50
		nPD:	0.81
		N. weight:	0.15
		Sc. PD:	0.084
		Sc. rank:	213.5

,

PB2			
Pos.	301	obs:	exp:
tat	Y	1	0.49
tac	Y	0	0.51
aat	N	676	1239.00
aac	N	1655	1092.00
---	--	------	------
mPD		0.41	0.50
		nPD:	0.83
		N. weight:	0.16
		Sc. PD:	0.096
		Sc. rank:	240.0

,

PB2			
Pos.	302	obs:	exp:
cct	P	1	657.60
ccc	P	6	422.00
cca	P	2252	952.20
ccg	P	73	300.30
---	--	------	------
mPD		0.066	0.70
		nPD:	0.09
		N. weight:	1.
		Sc. PD:	-0.13
		Sc. rank:	4.2

,

PB2			
Pos.	303	obs:	exp:
att	I	1	0.37
atc	I	0	0.25
ata	I	0	0.38
act	T	624	617.40
acc	T	0	515.00
aca	T	1357	1002.00
acg	T	350	196.90
---	--	------	------
mPD		0.57	0.69
		nPD:	0.82
		N. weight:	0.36
		Sc. PD:	0.21
		Sc. rank:	529.2

,

PB2			
Pos.	304	obs:	exp:
gaa	E	860	1396.00
gag	E	1472	935.70
---	--	------	------
mPD		0.47	0.48
		nPD:	0.97
		N. weight:	0.15
		Sc. PD:	0.11
		Sc. rank:	219.6

,

PB2			
Pos.	305	obs:	exp:
gaa	E	636	1396.00
gag	E	1696	935.70
---	--	------	------
mPD		0.40	0.48
		nPD:	0.83
		N. weight:	0.29
		Sc. PD:	0.17
		Sc. rank:	434.1

,

PB2			
Pos.	306	obs:	exp:
caa	Q	2322	1187.00
cag	Q	10	1145.00
---	--	------	------
mPD		0.0085	0.50
		nPD:	0.02
		N. weight:	0.87
		Sc. PD:	-0.17
		Sc. rank:	-653.4

,

PB2			
Pos.	307	obs:	exp:
gct	A	2133	593.20
gcc	A	196	454.80
gca	A	3	1025.00
gcg	A	0	258.90
---	--	------	------
mPD		0.16	0.69
		nPD:	0.23
		N. weight:	1.5
		Sc. PD:	0.0059
		Sc. rank:	497.6

,

PB2			
Pos.	308	obs:	exp:
gtt	V	104	506.90
gtc	V	0	492.50
gta	V	6	458.60
gtg	V	2221	872.90
gaa	E	0	0.60
gag	E	1	0.40
---	--	------	------
mPD		0.091	0.73
		nPD:	0.12
		N. weight:	1.1
		Sc. PD:	-0.1
		Sc. rank:	81.8

,

PB2			
Pos.	309	obs:	exp:
aat	N	2	1.06
aac	N	0	0.94
gat	D	2199	1339.00
gac	D	129	988.90
ggt	G	2	0.29
ggc	G	0	0.26
gga	G	0	0.87
ggg	G	0	0.58
---	--	------	------
mPD		0.11	0.49
		nPD:	0.22
		N. weight:	0.48
		Sc. PD:	-0.0013
		Sc. rank:	152.5

,

PB2			
Pos.	310	obs:	exp:
att	I	0	866.00
atc	I	2	584.90
ata	I	2330	881.20
---	--	------	------
mPD		0.0017	0.66
		nPD:	0.
		N. weight:	1.3
		Sc. PD:	-0.28
		Sc. rank:	-1731.6

,

PB2

Pos.	311	obs:	exp:
tgt	C	38	1014.00
tgc	C	2294	1318.00
---	--	------	------
mPD		0.032	0.49
		nPD:	0.07
		N. weight:	0.66
		Sc. PD:	-0.1
		Sc. rank:	-88.5

PB2

Pos.	312	obs:	exp:
cgt	R	0	0.04
cgc	R	0	0.05
cga	R	0	0.10
cgg	R	0	0.08
aaa	K	2313	1308.00
aag	K	18	1023.00
aga	R	1	0.46
agg	R	0	0.27
---	--	------	------
mPD		0.016	0.49
		nPD:	0.03
		N. weight:	0.72
		Sc. PD:	-0.13
		Sc. rank:	-352.7

PB2

Pos.	313	obs:	exp:
tct	S	0	0.17
tcc	S	0	0.15
tca	S	1	0.23
tcg	S	0	0.06
agt	S	0	0.20
agc	S	0	0.19
gct	A	7	593.00
gcc	A	0	454.60
gca	A	2323	1025.00
gcg	A	1	258.80
---	--	------	------
mPD		0.0077	0.69
		nPD:	0.01
		N. weight:	1.1
		Sc. PD:	-0.22
		Sc. rank:	-1028.5

PB2

Pos.	314	obs:	exp:
gct	A	1	593.20
gcc	A	0	454.80
gca	A	2324	1025.00
gcg	A	7	258.90
---	--	------	------
mPD		0.0068	0.69
		nPD:	0.01
		N. weight:	1.1
		Sc. PD:	-0.22
		Sc. rank:	-1103.4

PB2

Pos.	315	obs:	exp:
tta	L	0	0.11
ttg	L	1	0.19
ctt	L	0	0.19
ctc	L	0	0.17
cta	L	0	0.13
ctg	L	0	0.21
att	I	0	0.37
atc	I	0	0.25
ata	I	1	0.38
atg	M	2329	2329.00
gtt	V	0	0.22
gtc	V	0	0.21
gta	V	0	0.20
gtg	V	1	0.37
---	--	------	------
mPD		0.0026	0.0036
		nPD:	0.71
		N. weight:	0.0021
		Sc. PD:	0.00099
		Sc. rank:	2.8

PB2

Pos.	316	obs:	exp:
ggt	G	2235	335.50
ggc	G	97	307.80
gga	G	0	1018.00
ggg	G	0	670.70
---	--	------	------
mPD		0.080	0.69
		nPD:	0.12
		N. weight:	2.4
		Sc. PD:	-0.24
		Sc. rank:	77.0

PB2

Pos.	317	obs:	exp:
tta	L	1498	254.40
ttg	L	508	437.40
ctt	L	0	437.10
ctc	L	0	402.20
cta	L	222	310.10
ctg	L	103	489.80
caa	Q	1	0.51
cag	Q	0	0.49
---	--	------	------
mPD		0.63	1.1
		nPD:	0.55
		N. weight:	1.4
		Sc. PD:	0.46
		Sc. rank:	1483.9

PB2

Pos.	318	obs:	exp:
cgt	R	0	88.36
cgc	R	0	124.10
cga	R	0	225.60
cgg	R	0	182.50
aaa	K	2	1.12
aag	K	0	0.88
aga	R	1668	1069.00
agg	R	662	640.70
---	--	------	------
mPD		0.41	0.95
		nPD:	0.43
		N. weight:	0.44
		Sc. PD:	0.088
		Sc. rank:	360.2

PB2

Pos.	319	obs:	exp:
att	I	530	864.10
atc	I	1797	583.60
ata	I	0	879.30
gtt	V	5	1.09
gtc	V	0	1.06
gta	V	0	0.98
gtg	V	0	1.87
---	--	------	------
mPD		0.36	0.66
		nPD:	0.54
		N. weight:	1.
		Sc. PD:	0.32
		Sc. rank:	1008.8

PB2

Pos.	320	obs:	exp:
tct	S	0	398.10
tcc	S	0	343.60
tca	S	0	525.40
tcg	S	0	150.60
aat	N	1	0.53
aac	N	0	0.47
agt	S	1959	475.90
agc	S	372	437.30
---	--	------	------
mPD		0.27	1.6
		nPD:	0.16
		N. weight:	1.6
		Sc. PD:	-0.088
		Sc. rank:	308.7

PB2

Pos.	321	obs:	exp:
tct	S	0	398.10
tcc	S	0	343.60
tca	S	2327	525.40
tcg	S	4	150.60
cct	P	0	0.28
ccc	P	0	0.18
cca	P	1	0.41
ccg	P	0	0.13
agt	S	0	475.90
agc	S	0	437.30
---	--	------	------
mPD		0.0043	1.6
		nPD:	0.
		N. weight:	2.
		Sc. PD:	-0.42
		Sc. rank:	-2690.3

PB2

Pos.	322	obs:	exp:
tct	S	51	398.10
tcc	S	2235	343.60
tca	S	45	525.40
tcg	S	0	150.60
agt	S	0	475.90
agc	S	0	437.30
gct	A	0	0.25
gcc	A	1	0.20
gca	A	0	0.44
gcg	A	0	0.11
---	--	------	------
mPD		0.081	1.6
		nPD:	0.05
		N. weight:	2.3
		Sc. PD:	-0.38
		Sc. rank:	-621.9

PB2

Pos.	323	obs:	exp:
ttt	F	632	992.30
ttc	F	1700	1340.00
---	--	------	------
mPD		0.40	0.49
		nPD:	0.81
		N. weight:	0.071
		Sc. PD:	0.04
		Sc. rank:	103.1

PB2

Pos.	324	obs:	exp:
tct	S	0	398.30
tcc	S	0	343.80
tca	S	0	525.70
tcg	S	0	150.60
agt	S	61	476.10
agc	S	2271	437.50
---	--	------	------
mPD		0.051	1.6
		nPD:	0.03
		N. weight:	2.1
		Sc. PD:	-0.39
		Sc. rank:	-1068.3

PB2

Pos.	325	obs:	exp:
ttt	F	2315	992.30
ttc	F	17	1340.00
---	--	------	------
mPD		0.014	0.49
		nPD:	0.03
		N. weight:	1.1
		Sc. PD:	-0.2
		Sc. rank:	-573.2

PB2

Pos.	326	obs:	exp:
ggt	G	10	335.50
ggc	G	3	307.80
gga	G	2311	1018.00
ggg	G	8	670.70
---	--	------	------
mPD		0.018	0.69
		nPD:	0.03
		N. weight:	1.
		Sc. PD:	-0.2
		Sc. rank:	-590.6

PB2

Pos.	327	obs:	exp:
ggt	G	1686	335.50
ggc	G	107	307.80
gga	G	535	1018.00
ggg	G	4	670.70
---	--	------	------
mPD		0.42	0.69
		nPD:	0.61
		N. weight:	1.3
		Sc. PD:	0.49
		Sc. rank:	1556.9

PB2

Pos.	328	obs:	exp:
ttt	F	180	991.90
ttc	F	2151	1339.00
tat	Y	0	0.49
tac	Y	1	0.51
---	--	------	------
mPD		0.14	0.49
		nPD:	0.29
		N. weight:	0.41
		Sc. PD:	0.028
		Sc. rank:	233.2

PB2

Pos.	329	obs:	exp:
att	I	2	1.11
atc	I	1	0.75
ata	I	0	1.13
act	T	1802	616.70
acc	T	526	514.40
aca	T	0	1000.00
acg	T	0	196.60
aat	N	1	0.53
aac	N	0	0.47
---	--	------	------
mPD		0.35	0.69
		nPD:	0.51
		N. weight:	1.1
		Sc. PD:	0.31
		Sc. rank:	1033.8

PB2

Pos.	330	obs:	exp:
ttt	F	11	991.90
ttc	F	2320	1339.00
tta	L	0	0.11
ttg	L	0	0.19
ctt	L	0	0.19
ctc	L	1	0.17
cta	L	0	0.13
ctg	L	0	0.21
---	--	------	------
mPD		0.010	0.49
		nPD:	0.02
		N. weight:	0.71
		Sc. PD:	-0.14
		Sc. rank:	-460.6

PB2

Pos.	331	obs:	exp:
aaa	K	2326	1309.00
aag	K	6	1023.00
---	--	------	------
mPD		0.0051	0.49
		nPD:	0.01
		N. weight:	0.75
		Sc. PD:	-0.15
		Sc. rank:	-747.7

PB2

Pos.	332	obs:	exp:
cgt	R	0	88.32
cgc	R	0	124.10
cga	R	0	225.50
cgg	R	0	182.40
aaa	K	0	0.56
aag	K	1	0.44
aga	R	663	1068.00
agg	R	1666	640.40
ggt	G	0	0.29
ggc	G	0	0.26
gga	G	0	0.87
ggg	G	2	0.58
---	--	------	------
mPD		0.41	0.96
		nPD:	0.43
		N. weight:	0.74
		Sc. PD:	0.15
		Sc. rank:	605.4

PB2

Pos.	333	obs:	exp:
act	T	1	617.70
acc	T	0	515.20
aca	T	2330	1002.00
acg	T	1	197.00
---	--	------	------
mPD		0.0017	0.69
		nPD:	0.
		N. weight:	1.1
		Sc. PD:	-0.24
		Sc. rank:	-1539.0

PB2

Pos.	334	obs:	exp:
tct	S	0	398.10
tcc	S	0	343.60
tca	S	0	525.40
tcg	S	0	150.60
aat	N	1	0.53
aac	N	0	0.47
agt	S	2293	475.90
agc	S	38	437.30
---	--	------	------
mPD		0.033	1.6
		nPD:	0.02
		N. weight:	2.
		Sc. PD:	-0.4
		Sc. rank:	-1323.1

PB2			
Pos.	335	obs:	exp:
ggt	G	512	335.50
ggc	G	0	307.80
gga	G	258	1018.00
ggg	G	1562	670.70
mPD		0.49	0.69
		nPD:	0.71
		N. weight:	0.68
		Sc. PD:	0.33
		Sc. rank:	912.9

PB2			
Pos.	336	obs:	exp:
tct	S	6	397.70
tcc	S	0	343.30
tca	S	820	525.00
tcg	S	1503	150.40
act	T	0	0.79
acc	T	0	0.66
aca	T	3	1.29
acg	T	0	0.25
agt	S	0	475.50
agc	S	0	437.00
mPD		0.46	1.6
		nPD:	0.28
		N. weight:	2.2
		Sc. PD:	0.13
		Sc. rank:	1187.9

PB2			
Pos.	337	obs:	exp:
tct	S	1733	398.30
tcc	S	455	343.80
tca	S	142	525.70
tcg	S	2	150.60
agt	S	0	476.10
agc	S	0	437.50
mPD		0.41	1.6
		nPD:	0.25
		N. weight:	1.4
		Sc. PD:	0.035
		Sc. rank:	642.9

PB2			
Pos.	338	obs:	exp:
att	I	28	20.42
atc	I	27	13.79
ata	I	0	20.78
gtt	V	503	474.30
gtc	V	1677	460.80
gta	V	1	429.10
gtg	V	0	816.70
gct	A	0	24.42
gcc	A	96	18.72
gca	A	0	42.20
gcg	A	0	10.66
mPD		0.48	0.86
		nPD:	0.56
		N. weight:	1.4
		Sc. PD:	0.44
		Sc. rank:	1419.9

PB2			
Pos.	339	obs:	exp:
tct	S	0	2.73
tcc	S	0	2.36
tca	S	0	3.61
tcg	S	0	1.03
caa	Q	1	0.51
cag	Q	0	0.49
cgt	R	0	19.30
cgc	R	0	27.11
cga	R	0	49.29
cgg	R	0	39.86
act	T	0	0.53
acc	T	0	0.44
aca	T	2	0.86
acg	T	0	0.17
aaa	K	1697	1013.00
aag	K	107	791.40
agt	S	16	3.27
agc	S	0	3.00
aga	R	509	233.50
agg	R	0	140.00
mPD		0.45	0.98
		nPD:	0.46
		N. weight:	0.63
		Sc. PD:	0.14
		Sc. rank:	543.5

PB2			
Pos.	340	obs:	exp:
cgt	R	0	55.29
cgc	R	0	77.66
cga	R	0	141.20
cgg	R	0	114.20
aaa	K	248	490.60
aag	K	626	383.40
aga	R	1317	668.80
agg	R	141	400.90
mPD		0.91	1.3
		nPD:	0.71
		N. weight:	0.51
		Sc. PD:	0.24
		Sc. rank:	674.9

PB2			
Pos.	341	obs:	exp:
gtt	V	0	0.22
gtc	V	0	0.21
gta	V	1	0.20
gtg	V	0	0.37
gat	D	0	1.15
gac	D	2	0.85
gaa	E	2317	1394.00
gag	E	11	934.10
ggt	G	0	0.14
ggc	G	0	0.13
gga	G	1	0.44
ggg	G	0	0.29
mPD		0.013	0.48
		nPD:	0.03
		N. weight:	0.65
		Sc. PD:	-0.12
		Sc. rank:	-356.5

PB2			
Pos.	342	obs:	exp:
gat	D	0	2.88
gac	D	5	2.12
gaa	E	1512	1393.00
gag	E	815	933.70
mPD		0.46	0.48
		nPD:	0.95
		N. weight:	0.012
		Sc. PD:	0.0086
		Sc. rank:	18.2

PB2			
Pos.	343	obs:	exp:
aaa	K	1	0.56
aag	K	0	0.44
gaa	E	2323	1396.00
gag	E	8	935.30
mPD		0.0077	0.48
		nPD:	0.02
		N. weight:	0.66
		Sc. PD:	-0.13
		Sc. rank:	-508.5

PB2			
Pos.	344	obs:	exp:
att	I	0	332.00
atc	I	0	224.20
ata	I	894	337.80
atg	M	4	4.00
gtt	V	0	311.60
gtc	V	0	302.80
gta	V	193	282.00
gtg	V	1240	536.60
gct	A	0	0.25
gcc	A	0	0.20
gca	A	1	0.44
gcg	A	0	0.11
mPD		0.97	1.2
		nPD:	0.8
		N. weight:	1.1
		Sc. PD:	0.59
		Sc. rank:	1518.8

PB2			
Pos.	345	obs:	exp:
ttt	F	0	0.43
ttc	F	1	0.57
tta	L	0	254.10
ttg	L	0	436.80
ctt	L	667	436.50
ctc	L	1460	401.70
cta	L	199	309.70
ctg	L	2	489.10
att	I	1	1.11
atc	I	1	0.75
ata	I	1	1.13
mPD		0.52	1.1
		nPD:	0.46
		N. weight:	1.2
		Sc. PD:	0.27
		Sc. rank:	1033.8

PB2			
Pos.	346	obs:	exp:
cct	P	0	0.28
ccc	P	0	0.18
cca	P	1	0.41
ccg	P	0	0.13
act	T	1108	617.40
acc	T	194	515.00
aca	T	1006	1002.00
acg	T	23	196.90
mPD		0.58	0.69
		nPD:	0.84
		N. weight:	0.24
		Sc. PD:	0.15
		Sc. rank:	362.7

PB2			
Pos.	347	obs:	exp:
ggt	G	28	335.50
ggc	G	2303	307.80
gga	G	1	1018.00
ggg	G	0	670.70
mPD		0.025	0.69
		nPD:	0.04
		N. weight:	2.6
		Sc. PD:	-0.47
		Sc. rank:	-1128.9

PB2			
Pos.	348	obs:	exp:
tct	S	0	0.17
tcc	S	0	0.15
tca	S	0	0.23
tcg	S	0	0.06
aat	N	170	1239.00
aac	N	2161	1092.00
agt	S	0	0.20
agc	S	1	0.19
mPD		0.14	0.50
		nPD:	0.27
		N. weight:	0.66
		Sc. PD:	0.032
		Sc. rank:	349.0

PB2			
Pos.	349	obs:	exp:
tta	L	0	254.50
ttg	L	0	437.60
ctt	L	12	437.30
ctc	L	2320	402.30
cta	L	0	310.30
ctg	L	0	490.00
mPD		0.010	1.1
		nPD:	0.01
		N. weight:	2.3
		Sc. PD:	-0.48
		Sc. rank:	-2425.4

PB2			
Pos.	350	obs:	exp:
caa	Q	2309	1187.00
cag	Q	23	1145.00
mPD		0.020	0.50
		nPD:	0.04
		N. weight:	0.83
		Sc. PD:	-0.15
		Sc. rank:	-328.1

PB2			
Pos.	351	obs:	exp:
att	I	0	0.37
atc	I	0	0.25
ata	I	1	0.38
atg	M	8	8.00
act	T	2	603.70
acc	T	0	503.50
aca	T	2161	979.30
acg	T	116	192.50
gct	A	0	11.19
gcc	A	0	8.58
gca	A	44	19.34
gcg	A	0	4.88
mPD		0.15	0.74
		nPD:	0.2
		N. weight:	0.96
		Sc. PD:	-0.021
		Sc. rank:	271.8

PB2			
Pos.	352	obs:	exp:
tta	L	5	254.50
ttg	L	822	437.60
ctt	L	1	437.30
ctc	L	0	402.30
cta	L	8	310.30
ctg	L	1496	490.00
mPD		0.47	1.1
		nPD:	0.41
		N. weight:	1.2
		Sc. PD:	0.23
		Sc. rank:	987.4

PB2			
Pos.	353	obs:	exp:
cgt	R	0	0.04
cgc	R	0	0.05
cga	R	0	0.10
cgg	R	0	0.08
aaa	K	2329	1308.00
aag	K	2	1023.00
aga	R	1	0.46
agg	R	0	0.27
mPD		0.0026	0.49
		nPD:	0.01
		N. weight:	0.76
		Sc. PD:	-0.16
		Sc. rank:	-920.0

PB2			
Pos.	354	obs:	exp:
tta	L	1	0.11
ttg	L	0	0.19
ctt	L	0	0.19
ctc	L	0	0.17
cta	L	0	0.13
ctg	L	0	0.21
att	I	1	552.90
atc	I	0	373.40
ata	I	1488	562.60
gtt	V	226	183.10
gtc	V	0	177.90
gta	V	615	165.70
gtg	V	1	315.30
mPD		0.64	1.2
		nPD:	0.54
		N. weight:	1.3
		Sc. PD:	0.4
		Sc. rank:	1287.5

PB2			
Pos.	355	obs:	exp:
cgt	R	0	87.83
cgc	R	0	123.40
cga	R	1	224.30
cgg	R	0	181.40
att	I	0	1.11
atc	I	0	0.75
ata	I	3	1.13
aaa	K	7	3.93
aag	K	0	3.07
aga	R	2248	1062.00
agg	R	67	636.80
ggt	G	0	0.86
ggc	G	0	0.79
gga	G	6	2.62
ggg	G	0	1.73
---	--	------	------
mPD		0.070	0.96
		nPD:	0.07
		N. weight:	0.89
		Sc. PD:	-0.13
		Sc. rank:	-78.7

,

PB2			
Pos.	356	obs:	exp:
att	I	0	394.00
atc	I	0	266.10
ata	I	1061	400.90
gtt	V	0	276.40
gtc	V	0	268.50
gta	V	1186	250.10
gtg	V	85	476.00
---	--	------	------
mPD		0.57	1.2
		nPD:	0.46
		N. weight:	1.6
		Sc. PD:	0.36
		Sc. rank:	1348.5

,

PB2			
Pos.	357	obs:	exp:
tat	Y	1	0.49
tac	Y	0	0.51
cat	H	2310	1385.00
cac	H	20	944.60
aat	N	1	0.53
aac	N	0	0.47
---	--	------	------
mPD		0.019	0.48
		nPD:	0.04
		N. weight:	0.64
		Sc. PD:	-0.11
		Sc. rank:	-252.8

,

PB2			
Pos.	358	obs:	exp:
aaa	K	1	1.12
aag	K	1	0.88
gaa	E	1686	1395.00
gag	E	644	934.90
---	--	------	------
mPD		0.40	0.48
		nPD:	0.83
		N. weight:	0.048
		Sc. PD:	0.029
		Sc. rank:	71.4

,

PB2			
Pos.	359	obs:	exp:
ggt	G	1	335.50
ggc	G	0	307.80
gga	G	1592	1018.00
ggg	G	739	670.70
---	--	------	------
mPD		0.43	0.69
		nPD:	0.63
		N. weight:	0.45
		Sc. PD:	0.18
		Sc. rank:	547.7

,

PB2			
Pos.	360	obs:	exp:
tat	Y	2329	1134.00
tac	Y	3	1198.00
---	--	------	------
mPD		0.0026	0.50
		nPD:	0.01
		N. weight:	0.95
		Sc. PD:	-0.2
		Sc. rank:	-1149.3

,

PB2			
Pos.	361	obs:	exp:
gaa	E	21	1396.00
gag	E	2311	935.70
---	--	------	------
mPD		0.018	0.48
		nPD:	0.04
		N. weight:	1.1
		Sc. PD:	-0.21
		Sc. rank:	-469.0

,

PB2			
Pos.	362	obs:	exp:
gaa	E	1797	1396.00
gag	E	535	935.70
---	--	------	------
mPD		0.35	0.48
		nPD:	0.74
		N. weight:	0.091
		Sc. PD:	0.045
		Sc. rank:	125.7

,

PB2			
Pos.	363	obs:	exp:
ttt	F	3	992.30
ttc	F	2329	1340.00
---	--	------	------
mPD		0.0026	0.49
		nPD:	0.01
		N. weight:	0.73
		Sc. PD:	-0.15
		Sc. rank:	-871.7

,

PB2			
Pos.	364	obs:	exp:
att	I	0	10.40
atc	I	0	7.02
ata	I	28	10.58
act	T	0	610.00
acc	T	1	508.80
aca	T	2271	989.60
acg	T	31	194.50
gct	A	0	0.25
gcc	A	0	0.20
gca	A	1	0.44
gcg	A	0	0.11
---	--	------	------
mPD		0.052	0.71
		nPD:	0.07
		N. weight:	1.1
		Sc. PD:	-0.15
		Sc. rank:	-98.3

,

PB2			
Pos.	365	obs:	exp:
att	I	0	3.71
atc	I	0	2.51
ata	I	10	3.78
atg	M	2322	2322.00
---	--	------	------
mPD		0.0085	0.0086
		nPD:	1.
		N. weight:	0.0056
		Sc. PD:	0.0042
		Sc. rank:	8.4

,

PB2			
Pos.	366	obs:	exp:
att	I	1	0.37
atc	I	0	0.25
ata	I	0	0.38
gtt	V	2283	506.70
gtc	V	46	492.30
gta	V	0	458.50
gtg	V	1	872.50
gct	A	1	0.25
gcc	A	0	0.20
gca	A	0	0.44
gcg	A	0	0.11
---	--	------	------
mPD		0.041	0.73
		nPD:	0.06
		N. weight:	1.9
		Sc. PD:	-0.31
		Sc. rank:	-398.0

,

PB2			
Pos.	367	obs:	exp:
ggt	G	0	335.50
ggc	G	1	307.80
gga	G	7	1018.00
ggg	G	2324	670.70
---	--	------	------
mPD		0.0068	0.69
		nPD:	0.01
		N. weight:	1.6
		Sc. PD:	-0.34
		Sc. rank:	-1642.3

,

PB2			
Pos.	368	obs:	exp:
tta	L	0	0.11
ttg	L	0	0.19
ctt	L	0	0.19
ctc	L	0	0.17
cta	L	1	0.13
ctg	L	0	0.21
caa	Q	4	4.07
cag	Q	4	3.93
cgt	R	0	88.06
cgc	R	0	123.70
cga	R	2106	224.80
cgg	R	191	181.90
aga	R	24	1065.00
agg	R	1	638.50
ggt	G	0	0.14
ggc	G	0	0.13
gga	G	1	0.44
ggg	G	0	0.29
---	--	------	------
mPD		0.18	0.96
		nPD:	0.19
		N. weight:	2.7
		Sc. PD:	-0.081
		Sc. rank:	695.4

,

PB2			
Pos.	369	obs:	exp:
tct	S	0	0.17
tcc	S	0	0.15
tca	S	0	0.23
tcg	S	0	0.06
cgt	R	0	88.32
cgc	R	0	124.10
cga	R	1	225.50
cgg	R	0	182.40
aaa	K	1	0.56
aag	K	0	0.44
agt	S	1	0.20
agc	S	0	0.19
aga	R	2315	1068.00
agg	R	13	640.40
ggt	G	0	0.14
ggc	G	0	0.13
gga	G	1	0.44
ggg	G	0	0.29
---	--	------	------
mPD		0.015	0.96
		nPD:	0.02
		N. weight:	1.
		Sc. PD:	-0.2
		Sc. rank:	-816.1

,

PB2			
Pos.	370	obs:	exp:
tct	S	1	0.17
tcc	S	0	0.15
tca	S	0	0.23
tcg	S	0	0.06
act	T	0	0.26
acc	T	0	0.22
aca	T	1	0.43
acg	T	0	0.08
agt	S	0	0.20
agc	S	0	0.19
gct	A	1444	592.70
gcc	A	12	454.40
gca	A	873	1024.00
gcg	A	1	258.70
---	--	------	------
mPD		0.48	0.69
		nPD:	0.69
		N. weight:	0.64
		Sc. PD:	0.29
		Sc. rank:	820.1

,

PB2			
Pos.	371	obs:	exp:
act	T	0	617.70
acc	T	0	515.20
aca	T	2320	1002.00
acg	T	12	197.00
---	--	------	------
mPD		0.010	0.69
		nPD:	0.01
		N. weight:	1.1
		Sc. PD:	-0.22
		Sc. rank:	-902.9

,

PB2			
Pos.	372	obs:	exp:
gct	A	113	593.20
gcc	A	2219	454.80
gca	A	0	1025.00
gcg	A	0	258.90
---	--	------	------
mPD		0.092	0.69
		nPD:	0.13
		N. weight:	1.9
		Sc. PD:	-0.16
		Sc. rank:	170.8

,

PB2			
Pos.	373	obs:	exp:
tta	L	0	0.11
ttg	L	0	0.19
ctt	L	1	0.19
ctc	L	0	0.17
cta	L	0	0.13
ctg	L	0	0.21
att	I	2320	865.60
atc	I	11	584.60
ata	I	0	880.80
---	--	------	------
mPD		0.010	0.66
		nPD:	0.02
		N. weight:	1.3
		Sc. PD:	-0.26
		Sc. rank:	-1025.4

,

PB2			
Pos.	374	obs:	exp:
tta	L	1	254.50
ttg	L	1476	437.60
ctt	L	0	437.30
ctc	L	12	402.30
cta	L	739	310.30
ctg	L	104	490.00
---	--	------	------
mPD		0.91	1.1
		nPD:	0.8
		N. weight:	1.3
		Sc. PD:	0.72
		Sc. rank:	1854.3

,

PB2			
Pos.	375	obs:	exp:
cgt	R	0	88.36
cgc	R	0	124.10
cga	R	0	225.60
cgg	R	0	182.50
att	I	0	0.37
atc	I	0	0.25
ata	I	1	0.38
atg	M	1	1.00
aga	R	201	1069.00
agg	R	2129	640.70
---	--	------	------
mPD		0.16	0.95
		nPD:	0.17
		N. weight:	1.3
		Sc. PD:	-0.067
		Sc. rank:	284.7

,

PB2			
Pos.	376	obs:	exp:
aaa	K	2315	1309.00
aag	K	17	1023.00
---	--	------	------
mPD		0.014	0.49
		nPD:	0.03
		N. weight:	0.72
		Sc. PD:	-0.13
		Sc. rank:	-383.2

,

PB2			
Pos.	377	obs:	exp:
tct	S	0	7.17
tcc	S	0	6.19
tca	S	42	9.47
tcg	S	0	2.71
agt	S	0	8.58
agc	S	0	7.88
gct	A	1	582.60
gcc	A	1	446.60
gca	A	2287	1007.00
gcg	A	1	254.20
---	--	------	------
mPD		0.038	0.74
		nPD:	0.05
		N. weight:	1.1
		Sc. PD:	-0.18
		Sc. rank:	-289.4

,

PB2			
Pos.	378	obs:	exp:
act	T	628	617.70
acc	T	1702	515.20
aca	T	2	1002.00
acg	T	0	197.00
---	--	------	------
mPD		0.39	0.69
		nPD:	0.57
		N. weight:	1.2
		Sc. PD:	0.4
		Sc. rank:	1271.9

,

PB2			
Pos.	379	obs:	exp:
cgt	R	0	86.77
cgc	R	0	121.90
cga	R	0	221.60
cgg	R	0	179.20
aaa	K	34	24.70
aag	K	10	19.30
aga	R	1206	1049.00
agg	R	1082	629.10
---	--	------	------
mPD		0.54	0.98
		nPD:	0.54
		N. weight:	0.44
		Sc. PD:	0.14
		Sc. rank:	438.0

,

PB2			
Pos.	380	obs:	exp:
cgt	R	0	88.40
cgc	R	0	124.20
cga	R	0	225.70
cgg	R	1	182.60
atg	M	1	1.00
aga	R	1128	1069.00
agg	R	1202	640.90
---	--	------	------
mPD		0.50	0.95
		nPD:	0.53
		N. weight:	0.47
		Sc. PD:	0.14
		Sc. rank:	442.0

,

PB2			
Pos.	381	obs:	exp:
tta	L	126	254.50
ttg	L	47	437.60
ctt	L	0	437.30
ctc	L	0	402.30
cta	L	4	310.30
ctg	L	2155	490.00
---	--	------	------
mPD		0.24	1.1
		nPD:	0.21
		N. weight:	1.7
		Sc. PD:	-0.014
		Sc. rank:	520.9

,

PB2			
Pos.	382	obs:	exp:
att	I	634	856.30
atc	I	1672	578.30
ata	I	0	871.40
gtt	V	0	5.65
gtc	V	26	5.49
gta	V	0	5.12
gtg	V	0	9.74
---	--	------	------
mPD		0.42	0.68
		nPD:	0.61
		N. weight:	0.93
		Sc. PD:	0.36
		Sc. rank:	1120.7

,

PB2			
Pos.	383	obs:	exp:
tta	L	0	0.11
ttg	L	0	0.19
ctt	L	0	0.19
ctc	L	0	0.17
cta	L	1	0.13
ctg	L	0	0.21
caa	Q	1670	1186.00
cag	Q	661	1145.00
---	--	------	------
mPD		0.41	0.50
		nPD:	0.81
		N. weight:	0.12
		Sc. PD:	0.071
		Sc. rank:	179.4

,

PB2			
Pos.	384	obs:	exp:
ttt	F	22	9.36
ttc	F	0	12.64
tta	L	1572	252.10
ttg	L	724	433.50
ctt	L	0	433.20
ctc	L	0	398.50
cta	L	11	307.30
ctg	L	3	485.40
---	--	------	------
mPD		0.45	1.1
		nPD:	0.4
		N. weight:	1.8
		Sc. PD:	0.32
		Sc. rank:	1412.6

,

PB2			
Pos.	385	obs:	exp:
att	I	0	865.20
atc	I	0	584.40
ata	I	2330	880.40
gtt	V	0	0.43
gtc	V	0	0.42
gta	V	2	0.39
gtg	V	0	0.75
---	--	------	------
mPD		0.0017	0.66
		nPD:	0.
		N. weight:	1.3
		Sc. PD:	-0.28
		Sc. rank:	-1763.6

,

PB2			
Pos.	386	obs:	exp:
tta	L	0	0.11
ttg	L	0	0.19
ctt	L	0	0.19
ctc	L	0	0.17
cta	L	0	0.13
ctg	L	1	0.21
atg	M	1	1.00
gtt	V	0	506.70
gtc	V	0	492.30
gta	V	561	458.50
gtg	V	1769	872.50
---	--	------	------
mPD		0.37	0.73
		nPD:	0.5
		N. weight:	0.79
		Sc. PD:	0.21
		Sc. rank:	714.4

,

PB2			
Pos.	387	obs:	exp:
tct	S	0	398.30
tcc	S	0	343.80
tca	S	0	525.70
tcg	S	0	150.60
agt	S	2325	476.10
agc	S	7	437.50
---	--	------	------
mPD		0.0060	1.6
		nPD:	0.
		N. weight:	2.1
		Sc. PD:	-0.45
		Sc. rank:	-2633.6

,

PB2			
Pos.	388	obs:	exp:
gtt	V	0	0.22
gtc	V	0	0.21
gta	V	1	0.20
gtg	V	0	0.37
ggt	G	1	335.30
ggc	G	0	307.70
gga	G	1799	1018.00
ggg	G	531	670.40
---	--	------	------
mPD		0.35	0.69
		nPD:	0.51
		N. weight:	0.52
		Sc. PD:	0.15
		Sc. rank:	479.1

,

PB2			
Pos.	389	obs:	exp:
cgt	R	0	88.02
cgc	R	0	123.60
cga	R	318	224.70
cgg	R	2	181.80
aaa	K	11	6.17
aag	K	0	4.83
aga	R	1996	1065.00
agg	R	5	638.20
---	--	------	------
mPD		0.25	0.96
		nPD:	0.26
		N. weight:	0.77
		Sc. PD:	0.03
		Sc. rank:	385.5

,

PB2			
Pos.	390	obs:	exp:
aat	N	0	0.53
aac	N	1	0.47
gat	D	180	1341.00
gac	D	2151	990.20
---	--	------	------
mPD		0.14	0.49
		nPD:	0.29
		N. weight:	0.75
		Sc. PD:	0.052
		Sc. rank:	429.2

,

PB2			
Pos.	391	obs:	exp:
gat	D	12	6.90
gac	D	0	5.10
gaa	E	673	1389.00
gag	E	1647	930.90
---	--	------	------
mPD		0.42	0.49
		nPD:	0.86
		N. weight:	0.27
		Sc. PD:	0.16
		Sc. rank:	397.5

,

PB2			
Pos.	392	obs:	exp:
caa	Q	629	1185.00
cag	Q	1701	1145.00
cgt	R	0	0.08
cgc	R	0	0.11
cga	R	2	0.19
cgg	R	0	0.16
aga	R	0	0.92
agg	R	0	0.55
---	--	------	------
mPD		0.40	0.50
		nPD:	0.79
		N. weight:	0.16
		Sc. PD:	0.09
		Sc. rank:	233.5

,

PB2			
Pos.	393	obs:	exp:
tct	S	177	398.30
tcc	S	1	343.80
tca	S	2147	525.70
tcg	S	7	150.60
agt	S	0	476.10
agc	S	0	437.50
---	--	------	------
mPD		0.15	1.6
		nPD:	0.09
		N. weight:	1.6
		Sc. PD:	-0.21
		Sc. rank:	-25.4

,

PB2			
Pos.	394	obs:	exp:
tta	L	0	0.11
ttg	L	0	0.19
ctt	L	0	0.19
ctc	L	1	0.17
cta	L	0	0.13
ctg	L	0	0.21
att	I	66	865.20
atc	I	2264	584.40
ata	I	0	880.40
gtt	V	1	0.22
gtc	V	0	0.21
gta	V	0	0.20
gtg	V	0	0.37
---	--	------	------
mPD		0.058	0.66
		nPD:	0.09
		N. weight:	1.7
		Sc. PD:	-0.22
		Sc. rank:	-56.2

,

PB2			
Pos.	395	obs:	exp:
act	T	2	0.53
acc	T	0	0.44
aca	T	0	0.86
acg	T	0	0.17
gct	A	2094	592.70
gcc	A	230	454.40
gca	A	6	1024.00
gcg	A	0	258.70
---	--	------	------
mPD		0.18	0.69
		nPD:	0.27
		N. weight:	1.4
		Sc. PD:	0.06
		Sc. rank:	726.8

PB2			
Pos.	396	obs:	exp:
gat	D	5	2.88
gac	D	0	2.12
gaa	E	2288	1393.00
gag	E	39	933.70
---	--	------	------
mPD		0.037	0.48
		nPD:	0.08
		N. weight:	0.58
		Sc. PD:	-0.082
		Sc. rank:	-48.5

PB2			
Pos.	397	obs:	exp:
tct	S	0	0.34
tcc	S	0	0.29
tca	S	0	0.45
tcg	S	2	0.13
act	T	0	0.79
acc	T	0	0.66
aca	T	3	1.29
acg	T	0	0.25
agt	S	0	0.41
agc	S	0	0.38
gct	A	1	592.00
gcc	A	0	453.80
gca	A	2134	1023.00
gcg	A	192	258.30
---	--	------	------
mPD		0.16	0.70
		nPD:	0.23
		N. weight:	0.87
		Sc. PD:	0.0037
		Sc. rank:	299.0

PB2			
Pos.	398	obs:	exp:
att	I	4	773.10
atc	I	2062	522.20
ata	I	16	786.70
gtt	V	1	54.37
gtc	V	247	52.82
gta	V	2	49.19
gtg	V	0	93.62
---	--	------	------
mPD		0.21	0.87
		nPD:	0.24
		N. weight:	1.8
		Sc. PD:	0.034
		Sc. rank:	705.4

PB2			
Pos.	399	obs:	exp:
att	I	119	866.00
atc	I	523	584.90
ata	I	1690	881.20
---	--	------	------
mPD		0.42	0.66
		nPD:	0.64
		N. weight:	0.47
		Sc. PD:	0.19
		Sc. rank:	577.7

PB2			
Pos.	400	obs:	exp:
gtt	V	2	501.10
gtc	V	0	486.80
gta	V	633	453.30
gtg	V	1669	862.80
gct	A	0	7.12
gcc	A	0	5.46
gca	A	26	12.31
gcg	A	2	3.11
---	--	------	------
mPD		0.43	0.75
		nPD:	0.57
		N. weight:	0.76
		Sc. PD:	0.26
		Sc. rank:	818.9

PB2			
Pos.	401	obs:	exp:
gct	A	103	593.20
gcc	A	716	454.80
gca	A	1387	1025.00
gcg	A	126	258.90
---	--	------	------
mPD		0.55	0.69
		nPD:	0.79
		N. weight:	0.28
		Sc. PD:	0.15
		Sc. rank:	398.2

PB2			
Pos.	402	obs:	exp:
tta	L	0	0.33
ttg	L	3	0.56
ctt	L	0	0.56
ctc	L	0	0.52
cta	L	0	0.40
ctg	L	0	0.63
att	I	21	7.80
atc	I	0	5.27
ata	I	0	7.94
atg	M	2308	2308.00
---	--	------	------
mPD		0.020	0.022
		nPD:	0.93
		N. weight:	0.015
		Sc. PD:	0.01
		Sc. rank:	22.3

PB2			
Pos.	403	obs:	exp:
att	I	1	0.37
atc	I	0	0.25
ata	I	0	0.38
gtt	V	1478	506.90
gtc	V	1	492.50
gta	V	16	458.60
gtg	V	836	872.90
---	--	------	------
mPD		0.47	0.73
		nPD:	0.64
		N. weight:	0.86
		Sc. PD:	0.35
		Sc. rank:	1067.0

PB2			
Pos.	404	obs:	exp:
ttt	F	6	992.30
ttc	F	2326	1340.00
---	--	------	------
mPD		0.0051	0.49
		nPD:	0.01
		N. weight:	0.72
		Sc. PD:	-0.15
		Sc. rank:	-714.9

PB2			
Pos.	405	obs:	exp:
tct	S	0	398.30
tcc	S	191	343.80
tca	S	2141	525.70
tcg	S	0	150.60
agt	S	0	476.10
agc	S	0	437.50
---	--	------	------
mPD		0.15	1.6
		nPD:	0.09
		N. weight:	1.7
		Sc. PD:	-0.21
		Sc. rank:	-14.8

PB2			
Pos.	406	obs:	exp:
cat	H	1	0.59
cac	H	0	0.41
caa	Q	597	1186.00
cag	Q	1734	1145.00
---	--	------	------
mPD		0.38	0.50
		nPD:	0.76
		N. weight:	0.18
		Sc. PD:	0.095
		Sc. rank:	255.7

PB2			
Pos.	407	obs:	exp:
gaa	E	23	1396.00
gag	E	2309	935.70
---	--	------	------
mPD		0.020	0.48
		nPD:	0.04
		N. weight:	1.1
		Sc. PD:	-0.2
		Sc. rank:	-442.2

PB2			
Pos.	408	obs:	exp:
gat	D	1836	1341.00
gac	D	496	990.60
---	--	------	------
mPD		0.34	0.49
		nPD:	0.69
		N. weight:	0.14
		Sc. PD:	0.061
		Sc. rank:	175.3

PB2			
Pos.	409	obs:	exp:
tgt	C	3	1014.00
tgc	C	2329	1318.00
---	--	------	------
mPD		0.0026	0.49
		nPD:	0.01
		N. weight:	0.75
		Sc. PD:	-0.16
		Sc. rank:	-899.9

PB2			
Pos.	410	obs:	exp:
atg	M	2332	2332.00
---	--	------	------
mPD		0	0
		nPD:	1.
		N. weight:	0.
		Sc. PD:	0
		Sc. rank:	0

PB2			
Pos.	411	obs:	exp:
att	I	12	862.60
atc	I	5	582.60
ata	I	2306	877.80
atg	M	2	2.00
aaa	K	1	0.56
aag	K	0	0.44
gtt	V	0	1.30
gtc	V	0	1.27
gta	V	6	1.18
gtg	V	0	2.25
---	--	------	------
mPD		0.022	0.66
		nPD:	0.03
		N. weight:	1.2
		Sc. PD:	-0.23
		Sc. rank:	-593.3

PB2			
Pos.	412	obs:	exp:
aaa	K	1661	1309.00
aag	K	671	1023.00
---	--	------	------
mPD		0.41	0.49
		nPD:	0.83
		N. weight:	0.067
		Sc. PD:	0.04
		Sc. rank:	99.2

PB2			
Pos.	413	obs:	exp:
gct	A	181	593.20
gcc	A	0	454.80
gca	A	2151	1025.00
gcg	A	0	258.90
---	--	------	------
mPD		0.14	0.69
		nPD:	0.21
		N. weight:	0.79
		Sc. PD:	-0.011
		Sc. rank:	225.9

PB2			
Pos.	414	obs:	exp:
gtt	V	11	506.90
gtc	V	632	492.50
gta	V	1663	458.60
gtg	V	25	872.90
gaa	E	1	0.60
gag	E	0	0.40
---	--	------	------
mPD		0.42	0.73
		nPD:	0.57
		N. weight:	1.2
		Sc. PD:	0.43
		Sc. rank:	1354.1

PB2			
Pos.	415	obs:	exp:
cgt	R	0	88.44
cgc	R	0	124.20
cga	R	2157	225.80
cgg	R	170	182.60
aga	R	5	1070.00
agg	R	0	641.20
---	--	------	------
mPD		0.14	0.95
		nPD:	0.15
		N. weight:	2.8
		Sc. PD:	-0.2
		Sc. rank:	378.7

PB2			
Pos.	416	obs:	exp:
tgt	C	5	2.17
tgc	C	0	2.83
ggt	G	1700	334.70
ggc	G	620	307.10
gga	G	7	1016.00
ggg	G	0	669.30
---	--	------	------
mPD		0.40	0.69
		nPD:	0.58
		N. weight:	1.8
		Sc. PD:	0.63
		Sc. rank:	2006.8

PB2			
Pos.	417	obs:	exp:
aat	N	5	2.66
aac	N	0	2.34
gat	D	2263	1339.00
gac	D	64	988.50
---	--	------	------
mPD		0.058	0.49
		nPD:	0.12
		N. weight:	0.58
		Sc. PD:	-0.06
		Sc. rank:	21.6

PB2			
Pos.	418	obs:	exp:
tta	L	30	254.50
ttg	L	1001	437.60
ctt	L	0	437.30
ctc	L	0	402.30
cta	L	511	310.30
ctg	L	790	490.00
---	--	------	------
mPD		0.85	1.1
		nPD:	0.75
		N. weight:	0.81
		Sc. PD:	0.41
		Sc. rank:	1129.9

PB2			
Pos.	419	obs:	exp:
tct	S	0	0.17
tcc	S	0	0.15
tca	S	0	0.23
tcg	S	0	0.06
att	I	1	0.37
atc	I	0	0.25
ata	I	0	0.38
aat	N	2321	1238.00
aac	N	9	1092.00
agt	S	1	0.20
agc	S	0	0.19
---	--	------	------
mPD		0.0094	0.50
		nPD:	0.02
		N. weight:	0.81
		Sc. PD:	-0.16
		Sc. rank:	-577.2

PB2

Pos.	420	obs:	exp:
ttt	F	2046	991.90
ttc	F	285	1339.00
tgt	C	1	0.43
tgc	C	0	0.57
mPD		0.22	0.49
		nPD:	0.44
		N. weight:	0.6
		Sc. PD:	0.13
		Sc. rank:	494.9

PB2

Pos.	421	obs:	exp:
gtt	V	363	507.20
gtc	V	1340	492.70
gta	V	105	458.80
gtg	V	524	873.30
mPD		0.59	0.73
		nPD:	0.81
		N. weight:	0.46
		Sc. PD:	0.27
		Sc. rank:	676.9

PB2

Pos.	422	obs:	exp:
aat	N	203	1239.00
aac	N	2128	1092.00
aaa	K	1	0.56
aag	K	0	0.44
mPD		0.16	0.50
		nPD:	0.32
		N. weight:	0.61
		Sc. PD:	0.058
		Sc. rank:	375.0

PB2

Pos.	423	obs:	exp:
cgt	R	0	88.44
cgc	R	0	124.20
cga	R	0	225.80
cgg	R	0	182.60
aga	R	2213	1070.00
agg	R	119	641.20
mPD		0.097	0.95
		nPD:	0.1
		N. weight:	0.81
		Sc. PD:	-0.095
		Sc. rank:	12.7

PB2

Pos.	424	obs:	exp:
gct	A	178	593.20
gcc	A	0	454.80
gca	A	643	1025.00
gcg	A	1511	258.90
mPD		0.50	0.69
		nPD:	0.72
		N. weight:	1.2
		Sc. PD:	0.6
		Sc. rank:	1681.4

PB2

Pos.	425	obs:	exp:
aat	N	1653	1239.00
aac	N	679	1093.00
mPD		0.41	0.50
		nPD:	0.83
		N. weight:	0.09
		Sc. PD:	0.053
		Sc. rank:	133.3

PB2

Pos.	426	obs:	exp:
caa	Q	627	1187.00
cag	Q	1705	1145.00
mPD		0.39	0.50
		nPD:	0.79
		N. weight:	0.16
		Sc. PD:	0.089
		Sc. rank:	231.9

PB2

Pos.	427	obs:	exp:
cgt	R	0	88.44
cgc	R	0	124.20
cga	R	12	225.80
cgg	R	1698	182.60
aga	R	619	1070.00
agg	R	3	641.20
mPD		0.79	0.95
		nPD:	0.82
		N. weight:	2.
		Sc. PD:	1.1
		Sc. rank:	2880.8

PB2

Pos.	428	obs:	exp:
tta	L	106	254.50
ttg	L	523	437.60
ctt	L	0	437.30
ctc	L	192	402.30
cta	L	1428	310.30
ctg	L	83	490.00
mPD		0.89	1.1
		nPD:	0.78
		N. weight:	1.1
		Sc. PD:	0.59
		Sc. rank:	1563.8

PB2

Pos.	429	obs:	exp:
aat	N	1608	1239.00
aac	N	724	1093.00
mPD		0.43	0.50
		nPD:	0.86
		N. weight:	0.072
		Sc. PD:	0.044
		Sc. rank:	107.2

PB2

Pos.	430	obs:	exp:
cct	P	1662	657.60
ccc	P	659	422.00
cca	P	10	952.20
ccg	P	1	300.30
mPD		0.41	0.70
		nPD:	0.59
		N. weight:	1.
		Sc. PD:	0.36
		Sc. rank:	1181.8

PB2

Pos.	431	obs:	exp:
atg	M	2332	2332.00
mPD		0	0
		nPD:	1.
		N. weight:	0.
		Sc. PD:	0
		Sc. rank:	0

PB2

Pos.	432	obs:	exp:
cat	H	2324	1387.00
cac	H	8	945.40
mPD		0.0068	0.48
		nPD:	0.01
		N. weight:	0.67
		Sc. PD:	-0.13
		Sc. rank:	-562.0

PB2

Pos.	433	obs:	exp:
caa	Q	2184	1187.00
cag	Q	148	1145.00
mPD		0.12	0.50
		nPD:	0.24
		N. weight:	0.59
		Sc. PD:	0.0092
		Sc. rank:	221.9

PB2

Pos.	434	obs:	exp:
ttt	F	0	0.43
ttc	F	1	0.57
tta	L	0	254.40
ttg	L	0	437.40
ctt	L	1629	437.10
ctc	L	702	402.20
cta	L	0	310.10
ctg	L	0	489.80
mPD		0.42	1.1
		nPD:	0.37
		N. weight:	1.5
		Sc. PD:	0.21
		Sc. rank:	1069.8

PB2

Pos.	435	obs:	exp:
tta	L	0	254.40
ttg	L	47	437.40
ctt	L	0	437.10
ctc	L	0	402.20
cta	L	3	310.10
ctg	L	2281	489.80
cct	P	0	0.28
ccc	P	0	0.18
cca	P	0	0.41
ccg	P	1	0.13
mPD		0.043	1.1
		nPD:	0.04
		N. weight:	1.9
		Sc. PD:	-0.35
		Sc. rank:	-780.0

PB2

Pos.	436	obs:	exp:
tct	S	0	0.34
tcc	S	0	0.29
tca	S	0	0.45
tcg	S	0	0.13
cgt	R	0	88.32
cgc	R	0	124.10
cga	R	0	225.50
cgg	R	3	182.40
atg	M	1	1.00
agt	S	0	0.41
agc	S	2	0.38
aga	R	638	1068.00
agg	R	1688	640.40
mPD		0.40	0.96
		nPD:	0.42
		N. weight:	0.75
		Sc. PD:	0.14
		Sc. rank:	609.1

PB2

Pos.	437	obs:	exp:
cat	H	1692	1387.00
cac	H	640	945.40
mPD		0.40	0.48
		nPD:	0.83
		N. weight:	0.052
		Sc. PD:	0.031
		Sc. rank:	77.2

PB2

Pos.	438	obs:	exp:
ttt	F	6	992.30
ttc	F	2326	1340.00
mPD		0.0051	0.49
		nPD:	0.01
		N. weight:	0.72
		Sc. PD:	-0.15
		Sc. rank:	-714.9

PB2

Pos.	439	obs:	exp:
caa	Q	2140	1187.00
cag	Q	192	1145.00
mPD		0.15	0.50
		nPD:	0.3
		N. weight:	0.53
		Sc. PD:	0.041
		Sc. rank:	315.1

PB2

Pos.	440	obs:	exp:
cgt	R	0	0.08
cgc	R	0	0.11
cga	R	0	0.19
cgg	R	0	0.16
aaa	K	688	1308.00
aag	K	1642	1022.00
aga	R	0	0.92
agg	R	2	0.55
mPD		0.42	0.49
		nPD:	0.84
		N. weight:	0.2
		Sc. PD:	0.12
		Sc. rank:	293.5

PB2

Pos.	441	obs:	exp:
gat	D	2332	1341.00
gac	D	0	990.60
mPD		0	0.49
		nPD:	0.
		N. weight:	0.74
		Sc. PD:	-0.16
		Sc. rank:	-1113.9

PB2

Pos.	442	obs:	exp:
tct	S	0	0.85
tcc	S	0	0.74
tca	S	5	1.13
tcg	S	0	0.32
act	T	0	0.26
acc	T	0	0.22
aca	T	1	0.43
acg	T	0	0.08
agt	S	0	1.02
agc	S	0	0.94
gct	A	1	591.70
gcc	A	0	453.60
gca	A	2324	1022.00
gcg	A	1	258.20
mPD		0.0069	0.70
		nPD:	0.01
		N. weight:	1.1
		Sc. PD:	-0.23
		Sc. rank:	-1127.6

PB2

Pos.	443	obs:	exp:
cgt	R	0	0.08
cgc	R	0	0.11
cga	R	0	0.19
cgg	R	0	0.16
aaa	K	1744	1308.00
aag	K	586	1022.00
aga	R	0	0.92
agg	R	2	0.55
mPD		0.38	0.49
		nPD:	0.77
		N. weight:	0.1
		Sc. PD:	0.055
		Sc. rank:	148.5

PB2

Pos.	444	obs:	exp:
tta	L	0	0.11
ttg	L	1	0.19
ctt	L	0	0.19
ctc	L	0	0.17
cta	L	0	0.13
ctg	L	0	0.21
att	I	0	0.37
atc	I	0	0.25
ata	I	1	0.38
atg	M	1	1.00
gtt	V	0	506.10
gtc	V	102	491.70
gta	V	190	457.90
gtg	V	2035	871.40
gct	A	0	0.51
gcc	A	0	0.39
gca	A	0	0.88
gcg	A	2	0.22
mPD		0.23	0.73
		nPD:	0.32
		N. weight:	0.81
		Sc. PD:	0.073
		Sc. rank:	494.5

PB2			
Pos.	445	obs:	exp:
tta	L	0	254.50
ttg	L	2	437.60
ctt	L	11	437.30
ctc	L	2	402.30
cta	L	4	310.30
ctg	L	2313	490.00
---	--	------	------
mPD		0.016	1.1
		nPD:	0.01
		N. weight:	2.
		Sc. PD:	-0.41
		Sc. rank:	-1665.9

PB2			
Pos.	446	obs:	exp:
ttt	F	1781	992.30
ttc	F	551	1340.00
---	--	------	------
mPD		0.36	0.49
		nPD:	0.74
		N. weight:	0.32
		Sc. PD:	0.16
		Sc. rank:	442.4

PB2			
Pos.	447	obs:	exp:
caa	Q	2188	1186.00
cag	Q	143	1145.00
aaa	K	1	0.56
aag	K	0	0.44
---	--	------	------
mPD		0.12	0.50
		nPD:	0.23
		N. weight:	0.6
		Sc. PD:	0.0057
		Sc. rank:	213.0

PB2			
Pos.	448	obs:	exp:
tct	S	0	5.81
tcc	S	0	5.01
tca	S	0	7.66
tcg	S	0	2.20
att	I	0	0.37
atc	I	1	0.25
ata	I	0	0.38
act	T	0	0.53
acc	T	2	0.44
aca	T	0	0.86
acg	T	0	0.17
aat	N	647	1218.00
aac	N	1645	1074.00
aaa	K	3	1.68
aag	K	0	1.32
agt	S	0	6.94
agc	S	34	6.38
---	--	------	------
mPD		0.43	0.55
		nPD:	0.79
		N. weight:	0.21
		Sc. PD:	0.11
		Sc. rank:	295.0

PB2			
Pos.	449	obs:	exp:
tta	L	0	0.11
ttg	L	1	0.19
tgg	W	2331	2331.00
ctt	L	0	0.19
ctc	L	0	0.17
cta	L	0	0.13
ctg	L	0	0.21
---	--	------	------
mPD		0.00086	0.0020
		nPD:	0.43
		N. weight:	0.00096
		Sc. PD:	0.0002
		Sc. rank:	0.8

PB2			
Pos.	450	obs:	exp:
ggt	G	0	335.50
ggc	G	0	307.80
gga	G	548	1018.00
ggg	G	1784	670.70
---	--	------	------
mPD		0.36	0.69
		nPD:	0.52
		N. weight:	0.81
		Sc. PD:	0.24
		Sc. rank:	756.3

PB2			
Pos.	451	obs:	exp:
att	I	2128	793.50
atc	I	9	535.90
ata	I	0	807.50
act	T	11	2.91
acc	T	0	2.43
aca	T	0	4.73
acg	T	0	0.93
gtt	V	184	40.02
gtc	V	0	38.88
gta	V	0	36.20
gtg	V	0	68.90
---	--	------	------
mPD		0.16	0.83
		nPD:	0.2
		N. weight:	1.4
		Sc. PD:	-0.035
		Sc. rank:	363.9

PB2			
Pos.	452	obs:	exp:
gat	D	0	0.58
gac	D	1	0.42
gaa	E	2325	1396.00
gag	E	6	935.30
---	--	------	------
mPD		0.0060	0.48
		nPD:	0.01
		N. weight:	0.66
		Sc. PD:	-0.14
		Sc. rank:	-605.0

PB2			
Pos.	453	obs:	exp:
tta	L	0	3.82
ttg	L	0	6.57
tct	S	5	22.20
tcc	S	0	19.16
tca	S	125	29.30
tcg	S	0	8.40
ctt	L	0	6.56
ctc	L	0	6.04
cta	L	35	4.66
ctg	L	0	7.35
cct	P	595	610.50
ccc	P	254	391.70
cca	P	1315	884.00
ccg	P	1	278.80
caa	Q	1	0.51
cag	Q	0	0.49
act	T	0	0.26
acc	T	1	0.22
aca	T	0	0.43
acg	T	0	0.08
agt	S	0	26.54
agc	S	0	24.39
---	--	------	------
mPD		0.66	0.90
		nPD:	0.73
		N. weight:	0.37
		Sc. PD:	0.18
		Sc. rank:	511.3

PB2			
Pos.	454	obs:	exp:
att	I	2332	866.00
atc	I	0	584.90
ata	I	0	881.20
---	--	------	------
mPD		0	0.66
		nPD:	0.
		N. weight:	1.3
		Sc. PD:	-0.29
		Sc. rank:	-1995.4

PB2			
Pos.	455	obs:	exp:
gct	A	0	0.25
gcc	A	1	0.20
gca	A	0	0.44
gcg	A	0	0.11
gat	D	628	1341.00
gac	D	1703	990.20
---	--	------	------
mPD		0.39	0.49
		nPD:	0.81
		N. weight:	0.26
		Sc. PD:	0.15
		Sc. rank:	376.8

PB2			
Pos.	456	obs:	exp:
tct	S	0	0.17
tcc	S	0	0.15
tca	S	0	0.23
tcg	S	0	0.06
aat	N	2327	1238.00
aac	N	3	1092.00
agt	S	1	0.20
agc	S	0	0.19
gat	D	1	0.58
gac	D	0	0.42
---	--	------	------
mPD		0.0043	0.50
		nPD:	0.01
		N. weight:	0.83
		Sc. PD:	-0.17
		Sc. rank:	-907.9

PB2			
Pos.	457	obs:	exp:
gtt	V	0	507.20
gtc	V	712	492.70
gta	V	1600	458.80
gtg	V	20	873.30
---	--	------	------
mPD		0.44	0.73
		nPD:	0.6
		N. weight:	1.3
		Sc. PD:	0.46
		Sc. rank:	1468.8

PB2			
Pos.	458	obs:	exp:
atg	M	2332	2332.00
---	--	------	------
mPD		0	0
		nPD:	1.
		N. weight:	0.
		Sc. PD:	0
		Sc. rank:	0

PB2			
Pos.	459	obs:	exp:
ggt	G	1	335.50
ggc	G	0	307.80
gga	G	47	1018.00
ggg	G	2284	670.70
---	--	------	------
mPD		0.040	0.69
		nPD:	0.06
		N. weight:	1.5
		Sc. PD:	-0.24
		Sc. rank:	-288.3

PB2			
Pos.	460	obs:	exp:
atg	M	2332	2332.00
---	--	------	------
mPD		0	0
		nPD:	1.
		N. weight:	0.
		Sc. PD:	0
		Sc. rank:	0

PB2			
Pos.	461	obs:	exp:
att	I	897	862.60
atc	I	1238	582.60
ata	I	188	877.80
gtt	V	2	1.96
gtc	V	2	1.90
gta	V	5	1.77
gtg	V	0	3.37
---	--	------	------
mPD		0.57	0.67
		nPD:	0.86
		N. weight:	0.4
		Sc. PD:	0.25
		Sc. rank:	595.4

PB2			
Pos.	462	obs:	exp:
ggt	G	0	335.50
ggc	G	0	307.80
gga	G	1164	1018.00
ggg	G	1168	670.70
---	--	------	------
mPD		0.50	0.69
		nPD:	0.73
		N. weight:	0.46
		Sc. PD:	0.23
		Sc. rank:	635.2

PB2			
Pos.	463	obs:	exp:
att	I	0	864.80
atc	I	0	584.10
ata	I	2329	880.10
atg	M	1	1.00
gtt	V	0	0.43
gtc	V	0	0.42
gta	V	2	0.39
gtg	V	0	0.75
---	--	------	------
mPD		0.0026	0.66
		nPD:	0.
		N. weight:	1.3
		Sc. PD:	-0.28
		Sc. rank:	-1612.7

PB2			
Pos.	464	obs:	exp:
tta	L	626	251.70
ttg	L	229	432.70
ctt	L	2	432.40
ctc	L	0	397.90
cta	L	0	306.80
ctg	L	1449	484.50
atg	M	26	26.00
---	--	------	------
mPD		0.87	1.1
		nPD:	0.76
		N. weight:	1.2
		Sc. PD:	0.6
		Sc. rank:	1634.5

PB2			
Pos.	465	obs:	exp:
cct	P	2055	657.00
ccc	P	37	421.60
cca	P	238	951.30
ccg	P	0	300.00
cat	H	2	1.19
cac	H	0	0.81
---	--	------	------
mPD		0.21	0.71
		nPD:	0.3
		N. weight:	1.1
		Sc. PD:	0.086
		Sc. rank:	657.0

PB2			
Pos.	466	obs:	exp:
gct	A	0	0.25
gcc	A	1	0.20
gca	A	0	0.44
gcg	A	0	0.11
gat	D	356	1340.00
gac	D	1974	989.80
gaa	E	1	0.60
gag	E	0	0.40
---	--	------	------
mPD		0.26	0.49
		nPD:	0.53
		N. weight:	0.51
		Sc. PD:	0.15
		Sc. rank:	493.3

PB2			
Pos.	467	obs:	exp:
att	I	0	0.37
atc	I	0	0.25
ata	I	1	0.38
atg	M	2331	2331.00
---	--	------	------
mPD		0.00086	0.00086
		nPD:	1.
		N. weight:	0.00056
		Sc. PD:	0.00042
		Sc. rank:	0.8

PB2			
Pos.	468	obs:	exp:
cct	P	0	0.28
ccc	P	1	0.18
cca	P	0	0.41
ccg	P	0	0.13
act	T	628	613.70
acc	T	1509	511.90
aca	T	174	995.60
acg	T	6	195.70
gct	A	0	3.56
gcc	A	1	2.73
gca	A	13	6.15
gcg	A	0	1.55
---	--	------	------
mPD		0.51	0.70
		nPD:	0.73
		N. weight:	0.77
		Sc. PD:	0.38
		Sc. rank:	1057.3

PB2			
Pos.	469	obs:	exp:
cct	P	2	657.60
ccc	P	1687	422.00
cca	P	641	952.20
ccg	P	2	300.30
---	--	------	------
mPD		0.40	0.70
		nPD:	0.57
		N. weight:	1.2
		Sc. PD:	0.4
		Sc. rank:	1269.9

PB2			
Pos.	470	obs:	exp:
tct	S	0	395.00
tcc	S	0	341.00
tca	S	0	521.40
tcg	S	0	149.40
act	T	0	0.79
acc	T	3	0.66
aca	T	0	1.29
acg	T	0	0.25
aat	N	0	8.50
aac	N	16	7.50
agt	S	235	472.30
agc	S	2078	434.00
---	--	------	------
mPD		0.20	1.7
		nPD:	0.12
		N. weight:	1.8
		Sc. PD:	-0.18
		Sc. rank:	82.7

PB2			
Pos.	471	obs:	exp:
cct	P	0	0.28
ccc	P	0	0.18
cca	P	1	0.41
ccg	P	0	0.13
act	T	13	616.70
acc	T	0	514.40
aca	T	2314	1000.00
acg	T	1	196.60
gct	A	0	0.76
gcc	A	0	0.59
gca	A	3	1.32
gcg	A	0	0.33
---	--	------	------
mPD		0.015	0.69
		nPD:	0.02
		N. weight:	1.1
		Sc. PD:	-0.21
		Sc. rank:	-692.2

PB2			
Pos.	472	obs:	exp:
aaa	K	1	0.56
aag	K	0	0.44
gaa	E	17	1396.00
gag	E	2314	935.30
---	--	------	------
mPD		0.016	0.48
		nPD:	0.03
		N. weight:	1.2
		Sc. PD:	-0.21
		Sc. rank:	-549.0

PB2			
Pos.	473	obs:	exp:
att	I	0	2.23
atc	I	0	1.50
ata	I	6	2.27
atg	M	2320	2320.00
aaa	K	0	0.56
aag	K	1	0.44
gtt	V	0	1.09
gtc	V	0	1.06
gta	V	0	0.98
gtg	V	5	1.87
---	--	------	------
mPD		0.010	0.013
		nPD:	0.77
		N. weight:	0.0066
		Sc. PD:	0.0035
		Sc. rank:	9.4

PB2			
Pos.	474	obs:	exp:
tct	S	0	396.20
tcc	S	1	342.00
tca	S	2295	523.00
tcg	S	24	149.90
act	T	0	3.18
acc	T	0	2.65
aca	T	12	5.16
acg	T	0	1.01
agt	S	0	473.70
agc	S	0	435.30
---	--	------	------
mPD		0.031	1.6
		nPD:	0.02
		N. weight:	1.9
		Sc. PD:	-0.38
		Sc. rank:	-1344.1

PB2			
Pos.	475	obs:	exp:
tta	L	0	252.80
ttg	L	8	434.60
ctt	L	1	434.30
ctc	L	0	399.60
cta	L	736	308.10
ctg	L	1571	486.60
att	I	0	4.83
atc	I	0	3.26
ata	I	13	4.91
atg	M	3	3.00
---	--	------	------
mPD		0.46	1.1
		nPD:	0.4
		N. weight:	1.4
		Sc. PD:	0.24
		Sc. rank:	1103.0

PB2			
Pos.	476	obs:	exp:
cgt	R	0	88.44
cgc	R	0	124.20
cga	R	1	225.80
cgg	R	0	182.60
aga	R	2296	1070.00
agg	R	35	641.20
---	--	------	------
mPD		0.030	0.95
		nPD:	0.03
		N. weight:	0.95
		Sc. PD:	-0.17
		Sc. rank:	-476.5

PB2			
Pos.	477	obs:	exp:
ggt	G	0	335.50
ggc	G	0	307.80
gga	G	2161	1018.00
ggg	G	171	670.70
---	--	------	------
mPD		0.14	0.69
		nPD:	0.2
		N. weight:	0.8
		Sc. PD:	-0.019
		Sc. rank:	217.9

PB2			
Pos.	478	obs:	exp:
att	I	0	4.83
atc	I	0	3.26
ata	I	13	4.91
gtt	V	1	504.30
gtc	V	0	490.00
gta	V	634	456.30
gtg	V	1684	868.40
---	--	------	------
mPD		0.41	0.74
		nPD:	0.56
		N. weight:	0.77
		Sc. PD:	0.25
		Sc. rank:	801.0

PB2			
Pos.	479	obs:	exp:
cgt	R	0	88.44
cgc	R	0	124.20
cga	R	0	225.80
cgg	R	0	182.60
aga	R	2292	1070.00
agg	R	40	641.20
---	--	------	------
mPD		0.034	0.95
		nPD:	0.04
		N. weight:	0.94
		Sc. PD:	-0.17
		Sc. rank:	-422.9

PB2			
Pos.	480	obs:	exp:
att	I	2	4.46
atc	I	10	3.01
ata	I	0	4.53
gtt	V	620	504.60
gtc	V	1699	490.20
gta	V	1	456.50
gtg	V	0	868.80
---	--	------	------
mPD		0.40	0.74
		nPD:	0.54
		N. weight:	1.3
		Sc. PD:	0.4
		Sc. rank:	1286.5

PB2			
Pos.	481	obs:	exp:
tct	S	0	398.30
tcc	S	0	343.80
tca	S	0	525.70
tcg	S	0	150.60
agt	S	754	476.10
agc	S	1578	437.50
---	--	------	------
mPD		0.44	1.6
		nPD:	0.27
		N. weight:	1.4
		Sc. PD:	0.058
		Sc. rank:	708.1

PB2			
Pos.	482	obs:	exp:
cgt	R	0	0.49
cgc	R	0	0.69
cga	R	0	1.26
cgg	R	0	1.02
aaa	K	2302	1302.00
aag	K	17	1017.00
aga	R	13	5.96
agg	R	0	3.57
---	--	------	------
mPD		0.026	0.51
		nPD:	0.05
		N. weight:	0.72
		Sc. PD:	-0.12
		Sc. rank:	-194.3

PB2			
Pos.	483	obs:	exp:
atg	M	2305	2305.00
act	T	0	7.15
acc	T	0	5.97
aca	T	0	11.60
acg	T	27	2.28
---	--	------	------
mPD		0.023	0.044
		nPD:	0.52
		N. weight:	0.038
		Sc. PD:	0.011
		Sc. rank:	35.7

PB2			
Pos.	484	obs:	exp:
ggt	G	0	335.50
ggc	G	0	307.80
gga	G	2317	1018.00
ggg	G	15	670.70
---	--	------	------
mPD		0.013	0.69
		nPD:	0.02
		N. weight:	1.1
		Sc. PD:	-0.21
		Sc. rank:	-774.9

PB2			
Pos.	485	obs:	exp:
gtt	V	2	507.20
gtc	V	0	492.70
gta	V	643	458.80
gtg	V	1687	873.30
---	--	------	------
mPD		0.40	0.73
		nPD:	0.55
		N. weight:	0.76
		Sc. PD:	0.24
		Sc. rank:	771.8

PB2			
Pos.	486	obs:	exp:
gat	D	2330	1341.00
gac	D	2	990.60
---	--	------	------
mPD		0.0017	0.49
		nPD:	0.
		N. weight:	0.73
		Sc. PD:	-0.16
		Sc. rank:	-950.4

PB2			
Pos.	487	obs:	exp:
gaa	E	2324	1396.00
gag	E	7	935.30
ggt	G	0	0.14
ggc	G	0	0.13
gga	G	1	0.44
ggg	G	0	0.29
---	--	------	------
mPD		0.0068	0.48
		nPD:	0.01
		N. weight:	0.66
		Sc. PD:	-0.13
		Sc. rank:	-555.1

PB2			
Pos.	488	obs:	exp:
tat	Y	2305	1134.00
tac	Y	27	1198.00
---	--	------	------
mPD		0.023	0.50
		nPD:	0.05
		N. weight:	0.88
		Sc. PD:	-0.15
		Sc. rank:	-289.4

PB2			
Pos.	489	obs:	exp:
tct	S	8	398.30
tcc	S	2317	343.80
tca	S	7	525.70
tcg	S	0	150.60
agt	S	0	476.10
agc	S	0	437.50
---	--	------	------
mPD		0.013	1.6
		nPD:	0.01
		N. weight:	2.5
		Sc. PD:	-0.52
		Sc. rank:	-2777.5

PB2

Pos.	490	obs:	exp:
tct	S	0	397.70
tcc	S	0	343.30
tca	S	0	525.00
tcg	S	0	150.40
cgt	R	0	0.04
cgc	R	0	0.05
cga	R	0	0.10
cgg	R	0	0.08
act	T	1	0.26
acc	T	0	0.22
aca	T	0	0.43
acg	T	0	0.08
aat	N	1	0.53
aac	N	0	0.47
agt	S	1686	475.50
agc	S	643	437.00
aga	R	0	0.46
agg	R	1	0.27
---	--	------	------
mPD		0.40	1.6
		nPD:	0.24
		N. weight:	1.4
		Sc. PD:	0.03
		Sc. rank:	578.2

PB2

Pos.	491	obs:	exp:
att	I	1	0.37
atc	I	0	0.25
ata	I	0	0.38
act	T	2252	617.20
acc	T	56	514.80
aca	T	14	1001.00
acg	T	8	196.80
gct	A	1	0.25
gcc	A	0	0.20
gca	A	0	0.44
gcg	A	0	0.11
---	--	------	------
mPD		0.067	0.69
		nPD:	0.1
		N. weight:	1.6
		Sc. PD:	-0.19
		Sc. rank:	19.4

PB2

Pos.	492	obs:	exp:
gat	D	1	0.58
gac	D	0	0.42
gaa	E	3	1395.00
gag	E	2326	934.50
ggt	G	0	0.29
ggc	G	0	0.26
gga	G	0	0.87
ggg	G	2	0.58
---	--	------	------
mPD		0.0051	0.48
		nPD:	0.01
		N. weight:	1.2
		Sc. PD:	-0.25
		Sc. rank:	-1191.6

PB2

Pos.	493	obs:	exp:
cgt	R	0	88.40
cgc	R	0	124.20
cga	R	4	225.70
cgg	R	2	182.60
aaa	K	1	0.56
aag	K	0	0.44
aga	R	2160	1069.00
agg	R	165	640.90
---	--	------	------
mPD		0.14	0.95
		nPD:	0.15
		N. weight:	0.73
		Sc. PD:	-0.054
		Sc. rank:	84.9

PB2

Pos.	494	obs:	exp:
gtt	V	8	507.20
gtc	V	0	492.70
gta	V	23	458.80
gtg	V	2301	873.30
---	--	------	------
mPD		0.026	0.73
		nPD:	0.04
		N. weight:	1.2
		Sc. PD:	-0.22
		Sc. rank:	-521.6

PB2

Pos.	495	obs:	exp:
tta	L	0	0.11
ttg	L	0	0.19
ctt	L	0	0.19
ctc	L	1	0.17
cta	L	0	0.13
ctg	L	0	0.21
att	I	1	0.74
atc	I	1	0.50
ata	I	0	0.76
atg	M	1	1.00
gtt	V	631	505.90
gtc	V	1682	491.50
gta	V	10	457.70
gtg	V	3	871.00
gct	A	1	0.51
gcc	A	1	0.39
gca	A	0	0.88
gcg	A	0	0.22
---	--	------	------
mPD		0.41	0.73
		nPD:	0.56
		N. weight:	1.2
		Sc. PD:	0.41
		Sc. rank:	1306.1

PB2

Pos.	496	obs:	exp:
atg	M	1	1.00
gtt	V	0	505.60
gtc	V	0	491.20
gta	V	636	457.50
gtg	V	1689	870.60
gct	A	0	1.53
gcc	A	0	1.17
gca	A	0	2.64
gcg	A	6	0.67
---	--	------	------
mPD		0.40	0.74
		nPD:	0.55
		N. weight:	0.77
		Sc. PD:	0.24
		Sc. rank:	783.4

PB2

Pos.	497	obs:	exp:
tct	S	0	382.70
tcc	S	0	330.40
tca	S	0	505.20
tcg	S	0	144.80
cat	H	0	0.59
cac	H	1	0.41
cgt	R	0	3.38
cgc	R	89	4.74
cga	R	0	8.62
cgg	R	0	6.97
aat	N	0	0.53
aac	N	1	0.47
agt	S	241	457.60
agc	S	2000	420.50
aga	R	0	40.82
agg	R	0	24.47
---	--	------	------
mPD		0.26	1.7
		nPD:	0.16
		N. weight:	1.9
		Sc. PD:	-0.12
		Sc. rank:	332.4

PB2

Pos.	498	obs:	exp:
att	I	2290	864.80
atc	I	39	584.10
ata	I	0	880.10
gtt	V	3	0.65
gtc	V	0	0.63
gta	V	0	0.59
gtg	V	0	1.12
---	--	------	------
mPD		0.035	0.66
		nPD:	0.05
		N. weight:	1.2
		Sc. PD:	-0.2
		Sc. rank:	-275.0

PB2

Pos.	499	obs:	exp:
gat	D	1676	1341.00
gac	D	656	990.60
---	--	------	------
mPD		0.40	0.49
		nPD:	0.83
		N. weight:	0.061
		Sc. PD:	0.036
		Sc. rank:	90.6

PB2

Pos.	500	obs:	exp:
cgt	R	1227	88.44
cgc	R	1091	124.20
cga	R	7	225.80
cgg	R	7	182.60
aga	R	0	1070.00
agg	R	0	641.20
---	--	------	------
mPD		0.50	0.95
		nPD:	0.53
		N. weight:	3.2
		Sc. PD:	0.95
		Sc. rank:	3051.1

PB2

Pos.	501	obs:	exp:
ttt	F	153	992.30
ttc	F	2179	1340.00
---	--	------	------
mPD		0.12	0.49
		nPD:	0.25
		N. weight:	0.45
		Sc. PD:	0.013
		Sc. rank:	210.9

PB2

Pos.	502	obs:	exp:
tta	L	104	254.50
ttg	L	2215	437.60
ctt	L	0	437.30
ctc	L	0	402.30
cta	L	0	310.30
ctg	L	13	490.00
---	--	------	------
mPD		0.096	1.1
		nPD:	0.08
		N. weight:	2.
		Sc. PD:	-0.26
		Sc. rank:	-105.9

PB2

Pos.	503	obs:	exp:
cgt	R	0	88.44
cgc	R	0	124.20
cga	R	12	225.80
cgg	R	513	182.60
aga	R	1303	1070.00
agg	R	504	641.20
---	--	------	------
mPD		0.84	0.95
		nPD:	0.88
		N. weight:	0.37
		Sc. PD:	0.24
		Sc. rank:	550.8

PB2

Pos.	504	obs:	exp:
gtt	V	635	507.20
gtc	V	1696	492.70
gta	V	1	458.80
gtg	V	0	873.30
---	--	------	------
mPD		0.40	0.73
		nPD:	0.54
		N. weight:	1.3
		Sc. PD:	0.4
		Sc. rank:	1292.2

PB2

Pos.	505	obs:	exp:
cgt	R	0	88.44
cgc	R	0	124.20
cga	R	2309	225.80
cgg	R	15	182.60
aga	R	5	1070.00
agg	R	3	641.20
---	--	------	------
mPD		0.022	0.95
		nPD:	0.02
		N. weight:	3.
		Sc. PD:	-0.59
		Sc. rank:	-1794.9

PB2

Pos.	506	obs:	exp:
aat	N	1	0.53
aac	N	0	0.47
gat	D	2097	1341.00
gac	D	234	990.20
---	--	------	------
mPD		0.18	0.49
		nPD:	0.37
		N. weight:	0.35
		Sc. PD:	0.05
		Sc. rank:	252.7

PB2

Pos.	507	obs:	exp:
cct	P	0	0.28
ccc	P	0	0.18
cca	P	0	0.41
ccg	P	1	0.13
cat	H	1	0.59
cac	H	0	0.41
caa	Q	190	1183.00
cag	Q	2135	1142.00
cgt	R	0	0.15
cgc	R	0	0.21
cga	R	4	0.39
cgg	R	0	0.31
aaa	K	1	0.56
aag	K	0	0.44
aga	R	0	1.83
agg	R	0	1.10
---	--	------	------
mPD		0.16	0.51
		nPD:	0.31
		N. weight:	0.58
		Sc. PD:	0.051
		Sc. rank:	348.6

PB2

Pos.	508	obs:	exp:
caa	Q	1	266.60
cag	Q	523	257.40
cgt	R	0	59.96
cgc	R	0	84.21
cga	R	3	153.10
cgg	R	110	123.80
aaa	K	179	127.40
aag	K	48	99.58
aga	R	415	725.20
agg	R	1053	434.70
---	--	------	------
mPD		1.2	1.5
		nPD:	0.83
		N. weight:	0.62
		Sc. PD:	0.37
		Sc. rank:	916.5

PB2

Pos.	509	obs:	exp:
ggt	G	3	335.50
ggc	G	0	307.80
gga	G	1529	1018.00
ggg	G	800	670.70
---	--	------	------
mPD		0.45	0.69
		nPD:	0.66
		N. weight:	0.43
		Sc. PD:	0.18
		Sc. rank:	543.7

PB2			
Pos.	510	obs:	exp:
aat	N	1163	1239.00
aac	N	1169	1093.00
---	--	------	------
mPD		0.50	0.50
		nPD:	1.
		N. weight:	0.0052
		Sc. PD:	0.004
		Sc. rank:	7.9

,

PB2			
Pos.	511	obs:	exp:
tta	L	3	0.33
ttg	L	0	0.56
ctt	L	0	0.56
ctc	L	0	0.52
cta	L	0	0.40
ctg	L	0	0.63
att	I	0	1.11
atc	I	0	0.75
ata	I	3	1.13
gtt	V	0	505.90
gtc	V	0	491.50
gta	V	654	457.70
gtg	V	1672	871.00
---	--	------	------
mPD		0.41	0.73
		nPD:	0.56
		N. weight:	0.77
		Sc. PD:	0.25
		Sc. rank:	808.4

,

PB2			
Pos.	512	obs:	exp:
tta	L	0	254.40
ttg	L	0	437.40
ctt	L	1123	437.10
ctc	L	1195	402.20
cta	L	13	310.10
ctg	L	0	489.80
att	I	0	0.37
atc	I	1	0.25
ata	I	0	0.38
---	--	------	------
mPD		0.51	1.1
		nPD:	0.45
		N. weight:	1.3
		Sc. PD:	0.29
		Sc. rank:	1116.4

,

PB2			
Pos.	513	obs:	exp:
tta	L	117	254.50
ttg	L	97	437.60
ctt	L	2	437.30
ctc	L	0	402.30
cta	L	514	310.30
ctg	L	1602	490.00
---	--	------	------
mPD		0.56	1.1
		nPD:	0.5
		N. weight:	1.1
		Sc. PD:	0.29
		Sc. rank:	994.0

,

PB2			
Pos.	514	obs:	exp:
tct	S	2249	397.70
tcc	S	68	343.30
tca	S	10	525.00
tcg	S	2	150.40
act	T	3	0.79
acc	T	0	0.66
aca	T	0	1.29
acg	T	0	0.25
agt	S	0	475.50
agc	S	0	437.00
---	--	------	------
mPD		0.069	1.6
		nPD:	0.04
		N. weight:	2.2
		Sc. PD:	-0.38
		Sc. rank:	-804.4

,

PB2			
Pos.	515	obs:	exp:
cct	P	1682	657.30
ccc	P	634	421.80
cca	P	15	951.80
ccg	P	0	300.10
cat	H	1	0.59
cac	H	0	0.41
---	--	------	------
mPD		0.41	0.71
		nPD:	0.58
		N. weight:	1.
		Sc. PD:	0.35
		Sc. rank:	1131.9

,

PB2			
Pos.	516	obs:	exp:
gaa	E	858	1396.00
gag	E	1474	935.70
---	--	------	------
mPD		0.47	0.48
		nPD:	0.97
		N. weight:	0.15
		Sc. PD:	0.11
		Sc. rank:	221.2

,

PB2			
Pos.	517	obs:	exp:
gaa	E	1452	1396.00
gag	E	880	935.70
---	--	------	------
mPD		0.47	0.48
		nPD:	0.98
		N. weight:	0.0039
		Sc. PD:	0.0029
		Sc. rank:	5.9

,

PB2			
Pos.	518	obs:	exp:
gtt	V	1653	507.20
gtc	V	636	492.70
gta	V	36	458.80
gtg	V	7	873.30
---	--	------	------
mPD		0.42	0.73
		nPD:	0.58
		N. weight:	1.1
		Sc. PD:	0.4
		Sc. rank:	1282.1

,

PB2			
Pos.	519	obs:	exp:
tct	S	0	395.40
tcc	S	0	341.30
tca	S	0	521.80
tcg	S	0	149.50
aat	N	17	9.03
aac	N	0	7.97
agt	S	1670	472.70
agc	S	645	434.30
---	--	------	------
mPD		0.41	1.7
		nPD:	0.25
		N. weight:	1.4
		Sc. PD:	0.038
		Sc. rank:	642.6

,

PB2			
Pos.	520	obs:	exp:
gaa	E	2277	1396.00
gag	E	55	935.70
---	--	------	------
mPD		0.046	0.48
		nPD:	0.1
		N. weight:	0.55
		Sc. PD:	-0.067
		Sc. rank:	3.5

,

PB2			
Pos.	521	obs:	exp:
att	I	0	0.74
atc	I	0	0.50
ata	I	2	0.76
act	T	0	568.20
acc	T	0	473.90
aca	T	2145	921.70
acg	T	0	181.20
aaa	K	1	0.56
aag	K	0	0.44
gct	A	1	46.81
gcc	A	0	35.88
gca	A	183	80.88
gcg	A	0	20.43
---	--	------	------
mPD		0.15	0.84
		nPD:	0.18
		N. weight:	1.1
		Sc. PD:	-0.048
		Sc. rank:	278.0

,

PB2			
Pos.	522	obs:	exp:
tta	L	1	57.19
ttg	L	1	98.33
tat	Y	17	8.26
tac	Y	0	8.74
ctt	L	0	98.26
ctc	L	0	90.41
cta	L	87	69.72
ctg	L	435	110.10
cat	H	820	487.60
cac	H	0	332.40
caa	Q	13	494.00
cag	Q	958	477.00
---	--	------	------
mPD		0.88	1.2
		nPD:	0.71
		N. weight:	0.96
		Sc. PD:	0.46
		Sc. rank:	1288.3

,

PB2			
Pos.	523	obs:	exp:
gaa	E	1	0.60
gag	E	0	0.40
ggt	G	0	335.30
ggc	G	0	307.70
gga	G	2327	1018.00
ggg	G	4	670.40
---	--	------	------
mPD		0.0043	0.69
		nPD:	0.01
		N. weight:	1.1
		Sc. PD:	-0.23
		Sc. rank:	-1286.0

,

PB2			
Pos.	524	obs:	exp:
act	T	2	617.20
acc	T	0	514.80
aca	T	2324	1001.00
acg	T	4	196.80
gct	A	0	0.51
gcc	A	0	0.39
gca	A	2	0.88
gcg	A	0	0.22
---	--	------	------
mPD		0.0069	0.69
		nPD:	0.01
		N. weight:	1.1
		Sc. PD:	-0.23
		Sc. rank:	-1129.6

,

PB2			
Pos.	525	obs:	exp:
gaa	E	548	1396.00
gag	E	1784	935.70
---	--	------	------
mPD		0.36	0.48
		nPD:	0.75
		N. weight:	0.37
		Sc. PD:	0.19
		Sc. rank:	516.6

,

PB2			
Pos.	526	obs:	exp:
cgt	R	0	0.57
cgc	R	0	0.80
cga	R	0	1.45
cgg	R	0	1.17
aaa	K	2069	1301.00
aag	K	248	1016.00
aga	R	3	6.88
agg	R	12	4.12
---	--	------	------
mPD		0.21	0.51
		nPD:	0.41
		N. weight:	0.36
		Sc. PD:	0.067
		Sc. rank:	289.1

,

PB2			
Pos.	527	obs:	exp:
tta	L	511	254.40
ttg	L	145	437.40
ctt	L	0	437.10
ctc	L	0	402.20
cta	L	256	310.10
ctg	L	1419	489.80
att	I	0	0.37
atc	I	0	0.25
ata	I	1	0.38
---	--	------	------
mPD		0.85	1.1
		nPD:	0.75
		N. weight:	0.96
		Sc. PD:	0.49
		Sc. rank:	1336.9

,

PB2			
Pos.	528	obs:	exp:
act	T	2	617.70
acc	T	0	515.20
aca	T	644	1002.00
acg	T	1686	197.00
---	--	------	------
mPD		0.40	0.69
		nPD:	0.58
		N. weight:	1.9
		Sc. PD:	0.67
		Sc. rank:	2159.7

,

PB2			
Pos.	529	obs:	exp:
att	I	0	865.60
atc	I	8	584.60
ata	I	2323	880.80
gtt	V	0	0.22
gtc	V	0	0.21
gta	V	1	0.20
gtg	V	0	0.37
---	--	------	------
mPD		0.0077	0.66
		nPD:	0.01
		N. weight:	1.3
		Sc. PD:	-0.26
		Sc. rank:	-1188.4

,

PB2			
Pos.	530	obs:	exp:
att	I	0	0.74
atc	I	0	0.50
ata	I	2	0.76
atg	M	2	2.00
act	T	14	616.70
acc	T	0	514.40
aca	T	1209	1000.00
acg	T	1105	196.60
---	--	------	------
mPD		0.51	0.69
		nPD:	0.73
		N. weight:	1.2
		Sc. PD:	0.6
		Sc. rank:	1662.1

,

PB2			
Pos.	531	obs:	exp:
tat	Y	2189	1134.00
tac	Y	143	1198.00
---	--	------	------
mPD		0.12	0.50
		nPD:	0.23
		N. weight:	0.65
		Sc. PD:	0.0054
		Sc. rank:	230.5

,

PB2			
Pos.	532	obs:	exp:
tct	S	0	398.30
tcc	S	2	343.80
tca	S	2315	525.70
tcg	S	15	150.60
agt	S	0	476.10
agc	S	0	437.50
---	--	------	------
mPD		0.014	1.6
		nPD:	0.01
		N. weight:	1.9
		Sc. PD:	-0.4
		Sc. rank:	-2079.7

,

PB2			
Pos.	533	obs:	exp:
tct	S	0	398.30
tcc	S	0	343.80
tca	S	876	525.70
tcg	S	1456	150.60
agt	S	0	476.10
agc	S	0	437.50
---	--	------	------
mPD		0.47	1.6
		nPD:	0.29
		N. weight:	2.2
		Sc. PD:	0.13
		Sc. rank:	1189.5

,

PB2			
Pos.	534	obs:	exp:
tct	S	985	398.30
tcc	S	708	343.80
tca	S	635	525.70
tcg	S	4	150.60
agt	S	0	476.10
agc	S	0	437.50
---	--	------	------
mPD		0.66	1.6
		nPD:	0.4
		N. weight:	0.87
		Sc. PD:	0.15
		Sc. rank:	668.6

,

PB2			
Pos.	535	obs:	exp:
atg	M	2332	2332.00
---	--	------	------
mPD		0	0
		nPD:	1.
		N. weight:	0.
		Sc. PD:	0
		Sc. rank:	0

,

PB2			
Pos.	536	obs:	exp:
atg	M	2332	2332.00
---	--	------	------
mPD		0	0
		nPD:	1.
		N. weight:	0.
		Sc. PD:	0
		Sc. rank:	0

,

PB2			
Pos.	537	obs:	exp:
tgg	W	2332	2332.00
---	--	------	------
mPD		0	0
		nPD:	1.
		N. weight:	0.
		Sc. PD:	0
		Sc. rank:	0

,

PB2			
Pos.	538	obs:	exp:
gaa	E	2314	1396.00
gag	E	18	935.70
---	--	------	------
mPD		0.015	0.48
		nPD:	0.03
		N. weight:	0.63
		Sc. PD:	-0.12
		Sc. rank:	-320.0

,

PB2			
Pos.	539	obs:	exp:
att	I	190	864.10
atc	I	2137	583.60
ata	I	0	879.30
act	T	0	0.79
acc	T	3	0.66
aca	T	0	1.29
acg	T	0	0.25
gtt	V	2	0.43
gtc	V	0	0.42
gta	V	0	0.39
gtg	V	0	0.75
---	--	------	------
mPD		0.16	0.66
		nPD:	0.24
		N. weight:	1.4
		Sc. PD:	0.018
		Sc. rank:	516.5

,

PB2			
Pos.	540	obs:	exp:
tct	S	0	0.17
tcc	S	0	0.15
tca	S	0	0.23
tcg	S	0	0.06
cat	H	1	0.59
cac	H	0	0.41
aat	N	2197	1238.00
aac	N	133	1092.00
agt	S	0	0.20
agc	S	1	0.19
---	--	------	------
mPD		0.11	0.50
		nPD:	0.22
		N. weight:	0.57
		Sc. PD:	-0.0011
		Sc. rank:	180.9

,

PB2			
Pos.	541	obs:	exp:
ggt	G	2116	335.50
ggc	G	198	307.80
gga	G	9	1018.00
ggg	G	9	670.70
---	--	------	------
mPD		0.17	0.69
		nPD:	0.25
		N. weight:	2.1
		Sc. PD:	0.05
		Sc. rank:	952.5

,

PB2			
Pos.	542	obs:	exp:
tct	S	0	0.17
tcc	S	0	0.15
tca	S	0	0.23
tcg	S	1	0.06
cct	P	638	657.30
ccc	P	0	421.80
cca	P	354	951.80
ccg	P	1339	300.10
agt	S	0	0.20
agc	S	0	0.19
---	--	------	------
mPD		0.57	0.71
		nPD:	0.81
		N. weight:	0.94
		Sc. PD:	0.54
		Sc. rank:	1374.2

,

PB2			
Pos.	543	obs:	exp:
aaa	K	1	0.56
aag	K	0	0.44
gat	D	0	0.58
gac	D	1	0.42
gaa	E	1673	1395.00
gag	E	657	934.90
---	--	------	------
mPD		0.41	0.48
		nPD:	0.84
		N. weight:	0.045
		Sc. PD:	0.027
		Sc. rank:	66.3

,

PB2			
Pos.	544	obs:	exp:
tct	S	14	398.10
tcc	S	1462	343.60
tca	S	836	525.40
tcg	S	19	150.60
agt	S	0	475.90
agc	S	0	437.30
gct	A	0	0.25
gcc	A	0	0.20
gca	A	0	0.44
gcg	A	1	0.11
---	--	------	------
mPD		0.48	1.6
		nPD:	0.29
		N. weight:	1.4
		Sc. PD:	0.094
		Sc. rank:	788.8

,

PB2			
Pos.	545	obs:	exp:
att	I	0	0.37
atc	I	0	0.25
ata	I	1	0.38
atg	M	1	1.00
gtt	V	4	506.70
gtc	V	0	492.30
gta	V	146	458.50
gtg	V	2180	872.50
---	--	------	------
mPD		0.12	0.73
		nPD:	0.17
		N. weight:	1.
		Sc. PD:	-0.054
		Sc. rank:	236.6

,

PB2			
Pos.	546	obs:	exp:
tta	L	229	254.50
ttg	L	16	437.60
ctt	L	102	437.30
ctc	L	0	402.30
cta	L	1459	310.30
ctg	L	526	490.00
---	--	------	------
mPD		0.61	1.1
		nPD:	0.54
		N. weight:	1.2
		Sc. PD:	0.36
		Sc. rank:	1161.5

,

PB2			
Pos.	547	obs:	exp:
att	I	3	1.86
atc	I	2	1.25
ata	I	0	1.89
gtt	V	304	506.10
gtc	V	2022	491.70
gta	V	1	457.90
gtg	V	0	871.40
---	--	------	------
mPD		0.23	0.73
		nPD:	0.32
		N. weight:	1.6
		Sc. PD:	0.15
		Sc. rank:	957.1

,

PB2			
Pos.	548	obs:	exp:
aat	N	42	1239.00
aac	N	2290	1093.00
---	--	------	------
mPD		0.035	0.50
		nPD:	0.07
		N. weight:	0.89
		Sc. PD:	-0.13
		Sc. rank:	-88.3

,

PB2			
Pos.	549	obs:	exp:
act	T	16	617.70
acc	T	626	515.20
aca	T	1687	1002.00
acg	T	3	197.00
---	--	------	------
mPD		0.40	0.69
		nPD:	0.59
		N. weight:	0.54
		Sc. PD:	0.19
		Sc. rank:	619.9

,

PB2			
Pos.	550	obs:	exp:
tat	Y	2115	1134.00
tac	Y	217	1198.00
---	--	------	------
mPD		0.17	0.50
		nPD:	0.34
		N. weight:	0.55
		Sc. PD:	0.062
		Sc. rank:	356.9

,

PB2			
Pos.	551	obs:	exp:
caa	Q	2251	1187.00
cag	Q	81	1145.00
---	--	------	------
mPD		0.067	0.50
		nPD:	0.13
		N. weight:	0.71
		Sc. PD:	-0.06
		Sc. rank:	67.8

,

PB2			
Pos.	552	obs:	exp:
tgg	W	2332	2332.00
---	--	------	------
mPD		0	0
		nPD:	1.
		N. weight:	0.
		Sc. PD:	0
		Sc. rank:	0

,

PB2			
Pos.	553	obs:	exp:
att	I	1	861.90
atc	I	2314	582.10
ata	I	6	877.00
gtt	V	0	2.39
gtc	V	11	2.32
gta	V	0	2.16
gtg	V	0	4.12
---	--	------	------
mPD		0.015	0.67
		nPD:	0.02
		N. weight:	1.8
		Sc. PD:	-0.35
		Sc. rank:	-1099.2

,

PB2			
Pos.	554	obs:	exp:
att	I	1664	865.20
atc	I	666	584.40
ata	I	0	880.40
act	T	2	0.53
acc	T	0	0.44
aca	T	0	0.86
acg	T	0	0.17
---	--	------	------
mPD		0.41	0.66
		nPD:	0.62
		N. weight:	0.68
		Sc. PD:	0.26
		Sc. rank:	821.8

,

PB2

Pos.	555	obs:	exp:
cgt	R	0	88.29
cgc	R	0	124.00
cga	R	0	225.40
cgg	R	0	182.30
aaa	K	2	1.12
aag	K	0	0.88
aga	R	2317	1068.00
agg	R	11	640.10
ggt	G	0	0.29
ggc	G	0	0.26
gga	G	2	0.87
ggg	G	0	0.58
mPD		0.013	0.96
		nPD:	0.01
		N. weight:	1.
		Sc. PD:	-0.2
		Sc. rank:	-912.2

PB2

Pos.	556	obs:	exp:
tct	S	0	54.65
tcc	S	0	47.17
tca	S	0	72.13
tcg	S	0	20.67
aat	N	1807	1069.00
aac	N	205	942.80
agt	S	299	65.34
agc	S	21	60.04
mPD		0.41	0.93
		nPD:	0.44
		N. weight:	0.62
		Sc. PD:	0.13
		Sc. rank:	510.2

PB2

Pos.	557	obs:	exp:
tgg	W	2332	2332.00
mPD		0	0
		nPD:	1.
		N. weight:	0.
		Sc. PD:	0
		Sc. rank:	0

PB2

Pos.	558	obs:	exp:
gaa	E	1862	1396.00
gag	E	470	935.70
mPD		0.32	0.48
		nPD:	0.67
		N. weight:	0.12
		Sc. PD:	0.054
		Sc. rank:	157.2

PB2

Pos.	559	obs:	exp:
tct	S	0	1.37
tcc	S	0	1.18
tca	S	0	1.80
tcg	S	0	0.52
att	I	514	191.60
atc	I	2	129.40
ata	I	0	195.00
act	T	1804	477.90
acc	T	0	398.60
aca	T	0	775.20
acg	T	0	152.40
aat	N	2	1.06
aac	N	0	0.94
agt	S	8	1.63
agc	S	0	1.50
gtt	V	2	0.43
gtc	V	0	0.42
gta	V	0	0.39
gtg	V	0	0.75
mPD		0.36	1.0
		nPD:	0.34
		N. weight:	1.7
		Sc. PD:	0.19
		Sc. rank:	1111.0

PB2

Pos.	560	obs:	exp:
att	I	0	0.37
atc	I	0	0.25
ata	I	1	0.38
gtt	V	1	506.90
gtc	V	0	492.50
gta	V	34	458.60
gtg	V	2296	872.90
mPD		0.031	0.73
		nPD:	0.04
		N. weight:	1.2
		Sc. PD:	-0.21
		Sc. rank:	-434.2

PB2

Pos.	561	obs:	exp:
aat	N	2	1.06
aac	N	0	0.94
aaa	K	148	1308.00
aag	K	2182	1022.00
mPD		0.12	0.49
		nPD:	0.24
		N. weight:	0.77
		Sc. PD:	0.017
		Sc. rank:	325.8

PB2

Pos.	562	obs:	exp:
att	I	2292	866.00
atc	I	30	584.90
ata	I	10	881.20
mPD		0.034	0.66
		nPD:	0.05
		N. weight:	1.2
		Sc. PD:	-0.2
		Sc. rank:	-298.7

PB2

Pos.	563	obs:	exp:
cat	H	1	1.19
cac	H	1	0.81
caa	Q	1091	1185.00
cag	Q	1238	1144.00
cgt	R	0	0.04
cgc	R	0	0.05
cga	R	0	0.10
cgg	R	1	0.08
aga	R	0	0.46
agg	R	0	0.27
mPD		0.50	0.50
		nPD:	1.
		N. weight:	0.0086
		Sc. PD:	0.0065
		Sc. rank:	12.9

PB2

Pos.	564	obs:	exp:
tgg	W	2332	2332.00
mPD		0	0
		nPD:	1.
		N. weight:	0.
		Sc. PD:	0
		Sc. rank:	0

PB2

Pos.	565	obs:	exp:
ttt	F	0	0.43
ttc	F	1	0.57
tct	S	631	397.60
tcc	S	1684	343.20
tca	S	13	524.80
tcg	S	0	150.40
act	T	0	0.79
acc	T	3	0.66
aca	T	0	1.29
acg	T	0	0.25
agt	S	0	475.30
agc	S	0	436.80
mPD		0.41	1.6
		nPD:	0.25
		N. weight:	1.7
		Sc. PD:	0.041
		Sc. rank:	768.6

PB2

Pos.	566	obs:	exp:
caa	Q	1234	1187.00
cag	Q	1098	1145.00
mPD		0.50	0.50
		nPD:	1.
		N. weight:	0.0035
		Sc. PD:	0.0026
		Sc. rank:	5.2

PB2

Pos.	567	obs:	exp:
aat	N	1	1.06
aac	N	1	0.94
gat	D	177	1339.00
gac	D	2151	988.90
gaa	E	2	1.20
gag	E	0	0.80
mPD		0.14	0.49
		nPD:	0.29
		N. weight:	0.76
		Sc. PD:	0.053
		Sc. rank:	442.6

PB2

Pos.	568	obs:	exp:
cct	P	1560	657.30
ccc	P	639	421.80
cca	P	1	951.80
ccg	P	131	300.10
cat	H	1	0.59
cac	H	0	0.41
mPD		0.47	0.71
		nPD:	0.67
		N. weight:	0.86
		Sc. PD:	0.38
		Sc. rank:	1103.0

PB2

Pos.	569	obs:	exp:
act	T	2	617.70
acc	T	0	515.20
aca	T	589	1002.00
acg	T	1741	197.00
mPD		0.38	0.69
		nPD:	0.55
		N. weight:	2.
		Sc. PD:	0.64
		Sc. rank:	2044.3

PB2

Pos.	570	obs:	exp:
cgt	R	0	0.04
cgc	R	0	0.05
cga	R	0	0.10
cgg	R	0	0.08
att	I	0	1.11
atc	I	1	0.75
ata	I	2	1.13
atg	M	2325	2325.00
act	T	0	0.26
acc	T	1	0.22
aca	T	0	0.43
acg	T	0	0.08
aga	R	0	0.46
agg	R	1	0.27
gtt	V	0	0.43
gtc	V	0	0.42
gta	V	0	0.39
gtg	V	2	0.75
mPD		0.0069	0.0086
		nPD:	0.79
		N. weight:	0.004
		Sc. PD:	0.0022
		Sc. rank:	5.8

PB2

Pos.	571	obs:	exp:
tta	L	81	254.50
ttg	L	1406	437.60
ctt	L	0	437.30
ctc	L	0	402.30
cta	L	216	310.30
ctg	L	629	490.00
mPD		0.68	1.1
		nPD:	0.6
		N. weight:	0.94
		Sc. PD:	0.35
		Sc. rank:	1101.3

PB2

Pos.	572	obs:	exp:
tat	Y	11	1134.00
tac	Y	2321	1198.00
mPD		0.0094	0.50
		nPD:	0.02
		N. weight:	0.85
		Sc. PD:	-0.17
		Sc. rank:	-601.0

PB2

Pos.	573	obs:	exp:
aat	N	2126	1239.00
aac	N	206	1093.00
mPD		0.16	0.50
		nPD:	0.32
		N. weight:	0.46
		Sc. PD:	0.046
		Sc. rank:	287.5

PB2

Pos.	574	obs:	exp:
cgt	R	0	1.14
cgc	R	0	1.60
cga	R	0	2.90
cgg	R	0	2.35
aaa	K	4	1292.00
aag	K	2298	1010.00
aga	R	0	13.76
agg	R	30	8.25
mPD		0.029	0.53
		nPD:	0.05
		N. weight:	1.1
		Sc. PD:	-0.18
		Sc. rank:	-241.2

PB2			
Pos.	575	obs:	exp:
atg	M	1805	1805.00
gtt	V	0	114.60
gtc	V	0	111.30
gta	V	1	103.70
gtg	V	526	197.30
---	--	------	------
mPD		0.35	0.61
		nPD:	0.58
		N. weight:	0.29
		Sc. PD:	0.1
		Sc. rank:	325.3

PB2			
Pos.	576	obs:	exp:
gaa	E	1572	1396.00
gag	E	760	935.70
---	--	------	------
mPD		0.44	0.48
		nPD:	0.91
		N. weight:	0.019
		Sc. PD:	0.012
		Sc. rank:	27.8

PB2			
Pos.	577	obs:	exp:
ttt	F	2311	992.30
ttc	F	21	1340.00
---	--	------	------
mPD		0.018	0.49
		nPD:	0.04
		N. weight:	1.1
		Sc. PD:	-0.19
		Sc. rank:	-446.1

PB2			
Pos.	578	obs:	exp:
gaa	E	625	1396.00
gag	E	1707	935.70
---	--	------	------
mPD		0.39	0.48
		nPD:	0.82
		N. weight:	0.3
		Sc. PD:	0.18
		Sc. rank:	442.9

PB2			
Pos.	579	obs:	exp:
cct	P	11	657.60
ccc	P	1682	422.00
cca	P	17	952.20
ccg	P	622	300.30
---	--	------	------
mPD		0.41	0.70
		nPD:	0.58
		N. weight:	1.5
		Sc. PD:	0.53
		Sc. rank:	1708.3

PB2			
Pos.	580	obs:	exp:
ttt	F	283	992.30
ttc	F	2049	1340.00
---	--	------	------
mPD		0.21	0.49
		nPD:	0.44
		N. weight:	0.3
		Sc. PD:	0.062
		Sc. rank:	245.2

PB2			
Pos.	581	obs:	exp:
caa	Q	2131	1187.00
cag	Q	201	1145.00
---	--	------	------
mPD		0.16	0.50
		nPD:	0.32
		N. weight:	0.52
		Sc. PD:	0.047
		Sc. rank:	314.0

PB2			
Pos.	582	obs:	exp:
tct	S	117	397.60
tcc	S	2210	343.20
tca	S	1	524.80
tcg	S	0	150.40
agt	S	0	475.30
agc	S	0	436.80
gct	A	0	1.02
gcc	A	4	0.78
gca	A	0	1.76
gcg	A	0	0.44
---	--	------	------
mPD		0.10	1.6
		nPD:	0.06
		N. weight:	2.3
		Sc. PD:	-0.36
		Sc. rank:	-365.2

PB2			
Pos.	583	obs:	exp:
tta	L	1	254.40
ttg	L	2317	437.40
ctt	L	0	437.10
ctc	L	0	402.20
cta	L	3	310.10
ctg	L	10	489.80
atg	M	1	1.00
---	--	------	------
mPD		0.015	1.1
		nPD:	0.01
		N. weight:	2.2
		Sc. PD:	-0.44
		Sc. rank:	-1889.8

PB2			
Pos.	584	obs:	exp:
att	I	0	1.49
atc	I	4	1.00
ata	I	0	1.51
gtt	V	3	506.30
gtc	V	7	491.90
gta	V	627	458.10
gtg	V	1691	871.80
---	--	------	------
mPD		0.41	0.73
		nPD:	0.55
		N. weight:	0.74
		Sc. PD:	0.24
		Sc. rank:	762.4

PB2			
Pos.	585	obs:	exp:
cct	P	851	657.60
ccc	P	1481	422.00
cca	P	0	952.20
ccg	P	0	300.30
---	--	------	------
mPD		0.46	0.70
		nPD:	0.66
		N. weight:	1.2
		Sc. PD:	0.51
		Sc. rank:	1511.4

PB2			
Pos.	586	obs:	exp:
cgt	R	0	0.49
cgc	R	0	0.69
cga	R	0	1.26
cgg	R	0	1.02
aaa	K	641	1302.00
aag	K	1678	1017.00
aga	R	1	5.96
agg	R	12	3.57
---	--	------	------
mPD		0.41	0.51
		nPD:	0.81
		N. weight:	0.23
		Sc. PD:	0.13
		Sc. rank:	336.2

PB2			
Pos.	587	obs:	exp:
gct	A	2294	593.20
gcc	A	0	454.80
gca	A	12	1025.00
gcg	A	26	258.90
---	--	------	------
mPD		0.032	0.69
		nPD:	0.05
		N. weight:	1.7
		Sc. PD:	-0.29
		Sc. rank:	-537.9

PB2			
Pos.	588	obs:	exp:
ttt	F	0	0.43
ttc	F	1	0.57
tct	S	0	1.54
tcc	S	9	1.33
tca	S	0	2.03
tcg	S	0	0.58
act	T	0	0.26
acc	T	1	0.22
aca	T	0	0.43
acg	T	0	0.08
agt	S	0	1.84
agc	S	0	1.69
gtt	V	0	7.39
gtc	V	34	7.18
gta	V	0	6.69
gtg	V	0	12.73
gct	A	843	581.80
gcc	A	1444	446.00
gca	A	0	1005.00
gcg	A	0	253.90
---	--	------	------
mPD		0.50	0.74
		nPD:	0.68
		N. weight:	1.2
		Sc. PD:	0.53
		Sc. rank:	1538.2

PB2			
Pos.	589	obs:	exp:
cgt	R	0	88.40
cgc	R	0	124.20
cga	R	10	225.70
cgg	R	0	182.60
aaa	K	1	0.56
aag	K	0	0.44
aga	R	2313	1069.00
agg	R	8	640.90
---	--	------	------
mPD		0.016	0.95
		nPD:	0.02
		N. weight:	0.99
		Sc. PD:	-0.2
		Sc. rank:	-747.2

PB2			
Pos.	590	obs:	exp:
tct	S	0	0.17
tcc	S	0	0.15
tca	S	0	0.23
tcg	S	0	0.06
tgt	C	0	0.43
tgc	C	1	0.57
agt	S	0	0.20
agc	S	1	0.19
gat	D	0	1.15
gac	D	2	0.85
ggt	G	118	334.90
ggc	G	2208	307.30
gga	G	2	1016.00
ggg	G	0	669.60
---	--	------	------
mPD		0.10	0.69
		nPD:	0.15
		N. weight:	2.4
		Sc. PD:	-0.18
		Sc. rank:	304.4

PB2			
Pos.	591	obs:	exp:
caa	Q	662	1187.00
cag	Q	1670	1145.00
---	--	------	------
mPD		0.41	0.50
		nPD:	0.81
		N. weight:	0.14
		Sc. PD:	0.082
		Sc. rank:	207.3

PB2			
Pos.	592	obs:	exp:
tat	Y	1646	1134.00
tac	Y	686	1198.00
---	--	------	------
mPD		0.42	0.50
		nPD:	0.83
		N. weight:	0.13
		Sc. PD:	0.08
		Sc. rank:	200.0

PB2			
Pos.	593	obs:	exp:
tct	S	0	398.30
tcc	S	0	343.80
tca	S	0	525.70
tcg	S	0	150.60
agt	S	2311	476.10
agc	S	21	437.50
---	--	------	------
mPD		0.018	1.6
		nPD:	0.01
		N. weight:	2.1
		Sc. PD:	-0.42
		Sc. rank:	-2003.1

,

PB2			
Pos.	594	obs:	exp:
ggt	G	6	335.50
ggc	G	20	307.80
gga	G	1355	1018.00
ggg	G	951	670.70
---	--	------	------
mPD		0.50	0.69
		nPD:	0.72
		N. weight:	0.37
		Sc. PD:	0.18
		Sc. rank:	503.6

,

PB2			
Pos.	595	obs:	exp:
ttt	F	2323	992.30
ttc	F	9	1340.00
---	--	------	------
mPD		0.0077	0.49
		nPD:	0.02
		N. weight:	1.1
		Sc. PD:	-0.22
		Sc. rank:	-873.7

,

PB2			
Pos.	596	obs:	exp:
att	I	0	0.74
atc	I	0	0.50
ata	I	2	0.76
gtt	V	21	506.50
gtc	V	0	492.10
gta	V	51	458.30
gtg	V	2257	872.10
gct	A	0	0.25
gcc	A	0	0.20
gca	A	0	0.44
gcg	A	1	0.11
---	--	------	------
mPD		0.064	0.73
		nPD:	0.09
		N. weight:	1.1
		Sc. PD:	-0.15
		Sc. rank:	-33.2

,

PB2			
Pos.	597	obs:	exp:
cgt	R	0	88.44
cgc	R	0	124.20
cga	R	0	225.80
cgg	R	1	182.60
aga	R	643	1070.00
agg	R	1688	641.20
---	--	------	------
mPD		0.40	0.95
		nPD:	0.42
		N. weight:	0.75
		Sc. PD:	0.14
		Sc. rank:	608.1

,

PB2			
Pos.	598	obs:	exp:
atg	M	1	1.00
act	T	28	617.40
acc	T	0	515.00
aca	T	2140	1002.00
acg	T	163	196.90
---	--	------	------
mPD		0.15	0.69
		nPD:	0.22
		N. weight:	0.87
		Sc. PD:	0.00062
		Sc. rank:	290.0

,

PB2			
Pos.	599	obs:	exp:
tta	L	1332	254.50
ttg	L	140	437.60
ctt	L	1	437.30
ctc	L	0	402.30
cta	L	333	310.30
ctg	L	526	490.00
---	--	------	------
mPD		0.87	1.1
		nPD:	0.77
		N. weight:	1.2
		Sc. PD:	0.65
		Sc. rank:	1724.3

,

PB2			
Pos.	600	obs:	exp:
ttt	F	69	992.30
ttc	F	2263	1340.00
---	--	------	------
mPD		0.057	0.49
		nPD:	0.12
		N. weight:	0.58
		Sc. PD:	-0.059
		Sc. rank:	22.6

,

PB2			
Pos.	601	obs:	exp:
caa	Q	1723	1187.00
cag	Q	609	1145.00
---	--	------	------
mPD		0.39	0.50
		nPD:	0.77
		N. weight:	0.15
		Sc. PD:	0.08
		Sc. rank:	214.0

,

PB2			
Pos.	602	obs:	exp:
caa	Q	654	1187.00
cag	Q	1678	1145.00
---	--	------	------
mPD		0.40	0.50
		nPD:	0.81
		N. weight:	0.15
		Sc. PD:	0.083
		Sc. rank:	212.4

,

PB2			
Pos.	603	obs:	exp:
atg	M	2331	2331.00
gtt	V	0	0.22
gtc	V	0	0.21
gta	V	0	0.20
gtg	V	1	0.37
---	--	------	------
mPD		0.00086	0.0014
		nPD:	0.62
		N. weight:	0.00056
		Sc. PD:	0.00022
		Sc. rank:	0.7

,

PB2			
Pos.	604	obs:	exp:
cgt	R	2317	88.44
cgc	R	3	124.20
cga	R	6	225.80
cgg	R	6	182.60
aga	R	0	1070.00
agg	R	0	641.20
---	--	------	------
mPD		0.013	0.95
		nPD:	0.01
		N. weight:	4.3
		Sc. PD:	-0.87
		Sc. rank:	-3805.1

,

PB2			
Pos.	605	obs:	exp:
gat	D	1628	1341.00
gac	D	704	990.60
---	--	------	------
mPD		0.42	0.49
		nPD:	0.86
		N. weight:	0.045
		Sc. PD:	0.028
		Sc. rank:	67.7

,

PB2			
Pos.	606	obs:	exp:
att	I	0	1.49
atc	I	0	1.00
ata	I	4	1.51
gtt	V	1	506.30
gtc	V	0	491.90
gta	V	613	458.10
gtg	V	1714	871.80
---	--	------	------
mPD		0.39	0.73
		nPD:	0.54
		N. weight:	0.77
		Sc. PD:	0.24
		Sc. rank:	753.5

,

PB2			
Pos.	607	obs:	exp:
tta	L	9	254.00
ttg	L	1336	436.60
ctt	L	12	436.30
ctc	L	0	401.50
cta	L	37	309.60
ctg	L	933	488.90
caa	Q	0	1.02
cag	Q	2	0.98
atg	M	3	3.00
---	--	------	------
mPD		0.54	1.1
		nPD:	0.47
		N. weight:	1.1
		Sc. PD:	0.28
		Sc. rank:	995.4

,

PB2			
Pos.	608	obs:	exp:
ggt	G	1	335.50
ggc	G	0	307.80
gga	G	54	1018.00
ggg	G	2277	670.70
---	--	------	------
mPD		0.046	0.69
		nPD:	0.07
		N. weight:	1.5
		Sc. PD:	-0.23
		Sc. rank:	-193.0

,

PB2			
Pos.	609	obs:	exp:
act	T	3	617.70
acc	T	204	515.20
aca	T	2121	1002.00
acg	T	4	197.00
---	--	------	------
mPD		0.17	0.69
		nPD:	0.24
		N. weight:	0.79
		Sc. PD:	0.014
		Sc. rank:	305.0

,

PB2			
Pos.	610	obs:	exp:
ttt	F	2328	992.30
ttc	F	4	1340.00
---	--	------	------
mPD		0.0034	0.49
		nPD:	0.01
		N. weight:	1.1
		Sc. PD:	-0.24
		Sc. rank:	-1279.1

,

PB2			
Pos.	611	obs:	exp:
aat	N	0	0.53
aac	N	1	0.47
gat	D	808	1340.00
gac	D	1522	989.80
ggt	G	0	0.14
ggc	G	1	0.13
gga	G	0	0.44
ggg	G	0	0.29
---	--	------	------
mPD		0.45	0.49
		nPD:	0.93
		N. weight:	0.15
		Sc. PD:	0.099
		Sc. rank:	218.0

,

PB2			
Pos.	612	obs:	exp:
act	T	2292	617.70
acc	T	2	515.20
aca	T	38	1002.00
acg	T	0	197.00
---	--	------	------
mPD		0.034	0.69
		nPD:	0.05
		N. weight:	1.6
		Sc. PD:	-0.28
		Sc. rank:	-464.4

,

PB2			
Pos.	613	obs:	exp:
tct	S	0	0.17
tcc	S	0	0.15
tca	S	0	0.23
tcg	S	0	0.06
att	I	511	191.20
atc	I	4	129.20
ata	I	0	194.60
agt	S	1	0.20
agc	S	0	0.19
gtt	V	22	394.50
gtc	V	1791	383.30
gta	V	1	356.90
gtg	V	0	679.30
gct	A	0	0.51
gcc	A	2	0.39
gca	A	0	0.88
gcg	A	0	0.22
---	--	------	------
mPD		0.70	1.1
		nPD:	0.64
		N. weight:	1.8
		Sc. PD:	0.74
		Sc. rank:	2263.1

,

PB2			
Pos.	614	obs:	exp:
caa	Q	1495	1187.00
cag	Q	837	1145.00
---	--	------	------
mPD		0.46	0.50
		nPD:	0.92
		N. weight:	0.05
		Sc. PD:	0.034
		Sc. rank:	74.9

,

PB2			
Pos.	615	obs:	exp:
tta	L	1	0.33
ttg	L	0	0.56
ctt	L	0	0.56
ctc	L	0	0.52
cta	L	2	0.40
ctg	L	0	0.63
att	I	0	860.00
atc	I	2	580.80
ata	I	2314	875.10
gtt	V	0	2.83
gtc	V	0	2.75
gta	V	13	2.56
gtg	V	0	4.87
---	--	------	------
mPD		0.015	0.67
		nPD:	0.02
		N. weight:	1.3
		Sc. PD:	-0.25
		Sc. rank:	-800.4

,

PB2			
Pos.	616	obs:	exp:
att	I	0	865.20
atc	I	0	584.40
ata	I	2330	880.40
atg	M	1	1.00
gtt	V	0	0.22
gtc	V	0	0.21
gta	V	1	0.20
gtg	V	0	0.37
---	--	------	------
mPD		0.0017	0.66
		nPD:	0.
		N. weight:	1.3
		Sc. PD:	-0.28
		Sc. rank:	-1755.6

,

PB2

Pos.	617	obs:	exp:
cgt	R	0	0.04
cgc	R	0	0.05
cga	R	0	0.10
cgg	R	0	0.08
aaa	K	363	1308.00
aag	K	1968	1023.00
aga	R	1	0.46
agg	R	0	0.27
mPD		0.26	0.49
		nPD:	0.54
		N. weight:	0.47
		Sc. PD:	0.14
		Sc. rank:	459.1

PB2

Pos.	618	obs:	exp:
tta	L	0	254.50
ttg	L	0	437.60
ctt	L	5	437.30
ctc	L	1486	402.30
cta	L	194	310.30
ctg	L	647	490.00
mPD		0.51	1.1
		nPD:	0.45
		N. weight:	1.2
		Sc. PD:	0.26
		Sc. rank:	972.8

PB2

Pos.	619	obs:	exp:
tta	L	530	253.90
ttg	L	2	436.50
ctt	L	131	436.20
ctc	L	35	401.30
cta	L	1415	309.50
ctg	L	213	488.70
att	I	1	2.23
atc	I	3	1.50
ata	I	2	2.27
mPD		0.65	1.1
		nPD:	0.57
		N. weight:	1.2
		Sc. PD:	0.41
		Sc. rank:	1300.3

PB2

Pos.	620	obs:	exp:
cct	P	3	657.60
ccc	P	12	422.00
cca	P	1693	952.20
ccg	P	624	300.30
mPD		0.40	0.70
		nPD:	0.57
		N. weight:	0.79
		Sc. PD:	0.27
		Sc. rank:	849.2

PB2

Pos.	621	obs:	exp:
ttt	F	2330	992.30
ttc	F	2	1340.00
mPD		0.0017	0.49
		nPD:	0.
		N. weight:	1.1
		Sc. PD:	-0.24
		Sc. rank:	-1482.0

PB2

Pos.	622	obs:	exp:
gct	A	12	593.20
gcc	A	5	454.80
gca	A	2311	1025.00
gcg	A	4	258.90
mPD		0.018	0.69
		nPD:	0.03
		N. weight:	1.
		Sc. PD:	-0.2
		Sc. rank:	-589.1

PB2

Pos.	623	obs:	exp:
act	T	0	0.26
acc	T	0	0.22
aca	T	1	0.43
acg	T	0	0.08
gct	A	8	593.00
gcc	A	4	454.60
gca	A	2316	1025.00
gcg	A	3	258.80
mPD		0.014	0.69
		nPD:	0.02
		N. weight:	1.
		Sc. PD:	-0.21
		Sc. rank:	-701.5

PB2

Pos.	624	obs:	exp:
gct	A	243	593.20
gcc	A	2089	454.80
gca	A	0	1025.00
gcg	A	0	258.90
mPD		0.19	0.69
		nPD:	0.27
		N. weight:	1.7
		Sc. PD:	0.08
		Sc. rank:	903.8

PB2

Pos.	625	obs:	exp:
cct	P	0	657.60
ccc	P	2	422.00
cca	P	2323	952.20
ccg	P	7	300.30
mPD		0.0077	0.70
		nPD:	0.01
		N. weight:	1.2
		Sc. PD:	-0.24
		Sc. rank:	-1126.5

PB2

Pos.	626	obs:	exp:
cct	P	7	657.60
ccc	P	0	422.00
cca	P	208	952.20
ccg	P	2117	300.30
mPD		0.17	0.70
		nPD:	0.24
		N. weight:	2.2
		Sc. PD:	0.035
		Sc. rank:	833.4

PB2

Pos.	627	obs:	exp:
gaa	E	1320	1396.00
gag	E	1012	935.70
mPD		0.49	0.48
		nPD:	1.02
		N. weight:	0.0053
		Sc. PD:	0.0041
		Sc. rank:	8.0

PB2

Pos.	628	obs:	exp:
tta	L	0	0.22
ttg	L	0	0.38
ctt	L	0	0.38
ctc	L	0	0.35
cta	L	0	0.27
ctg	L	2	0.42
cat	H	511	304.40
cac	H	1	207.60
caa	Q	46	925.00
cag	Q	1772	893.00
mPD		0.37	0.67
		nPD:	0.56
		N. weight:	0.77
		Sc. PD:	0.25
		Sc. rank:	803.0

PB2

Pos.	629	obs:	exp:
tct	S	0	396.70
tcc	S	0	342.50
tca	S	0	523.60
tcg	S	0	150.10
aat	N	9	4.78
aac	N	0	4.22
agt	S	1695	474.30
agc	S	628	435.80
mPD		0.40	1.6
		nPD:	0.24
		N. weight:	1.4
		Sc. PD:	0.029
		Sc. rank:	556.6

PB2

Pos.	630	obs:	exp:
cgt	R	0	75.28
cgc	R	0	105.70
cga	R	0	192.20
cgg	R	0	155.50
aaa	K	1	194.80
aag	K	346	152.20
aga	R	646	910.50
agg	R	1339	545.80
mPD		0.65	1.2
		nPD:	0.57
		N. weight:	0.72
		Sc. PD:	0.24
		Sc. rank:	768.7

PB2

Pos.	631	obs:	exp:
tta	L	0	0.22
ttg	L	0	0.38
ctt	L	0	0.38
ctc	L	0	0.35
cta	L	0	0.27
ctg	L	2	0.42
atg	M	2329	2329.00
gtt	V	0	0.22
gtc	V	0	0.21
gta	V	0	0.20
gtg	V	1	0.37
mPD		0.0026	0.0041
		nPD:	0.62
		N. weight:	0.0024
		Sc. PD:	0.00091
		Sc. rank:	2.8

PB2

Pos.	632	obs:	exp:
caa	Q	1751	1187.00
cag	Q	581	1145.00
mPD		0.37	0.50
		nPD:	0.75
		N. weight:	0.17
		Sc. PD:	0.085
		Sc. rank:	234.0

PB2

Pos.	633	obs:	exp:
ttt	F	2113	992.30
ttc	F	219	1340.00
mPD		0.17	0.49
		nPD:	0.35
		N. weight:	0.69
		Sc. PD:	0.085
		Sc. rank:	469.2

PB2

Pos.	634	obs:	exp:
tct	S	913	398.30
tcc	S	1419	343.80
tca	S	0	525.70
tcg	S	0	150.60
agt	S	0	476.10
agc	S	0	437.50
mPD		0.48	1.6
		nPD:	0.29
		N. weight:	1.6
		Sc. PD:	0.1
		Sc. rank:	891.5

PB2

Pos.	635	obs:	exp:
tct	S	1747	398.30
tcc	S	585	343.80
tca	S	0	525.70
tcg	S	0	150.60
agt	S	0	476.10
agc	S	0	437.50
mPD		0.38	1.6
		nPD:	0.23
		N. weight:	1.7
		Sc. PD:	0.011
		Sc. rank:	581.7

PB2

Pos.	636	obs:	exp:
tta	L	0	254.00
ttg	L	5	436.60
ctt	L	5	436.30
ctc	L	0	401.50
cta	L	223	309.60
ctg	L	2094	488.90
atg	M	5	5.00
mPD		0.19	1.1
		nPD:	0.16
		N. weight:	1.7
		Sc. PD:	-0.096
		Sc. rank:	323.2

PB2

Pos.	637	obs:	exp:
tct	S	0	0.51
tcc	S	0	0.44
tca	S	0	0.68
tcg	S	0	0.19
att	I	21	7.80
atc	I	0	5.27
ata	I	0	7.94
act	T	1793	611.10
acc	T	507	509.70
aca	T	3	991.30
acg	T	4	194.90
agt	S	3	0.61
agc	S	0	0.56
gct	A	1	0.25
gcc	A	0	0.20
gca	A	0	0.44
gcg	A	0	0.11
mPD		0.37	0.71
		nPD:	0.52
		N. weight:	1.1
		Sc. PD:	0.32
		Sc. rank:	1029.0

PB2

Pos.	638	obs:	exp:
att	I	0	0.37
atc	I	0	0.25
ata	I	1	0.38
gtt	V	177	506.70
gtc	V	0	492.30
gta	V	169	458.50
gtg	V	1984	872.50
ggt	G	0	0.14
ggc	G	0	0.13
gga	G	0	0.44
ggg	G	1	0.29
mPD		0.27	0.73
		nPD:	0.36
		N. weight:	0.74
		Sc. PD:	0.1
		Sc. rank:	532.1

PB2

Pos.	639	obs:	exp:
aat	N	647	1239.00
aac	N	1684	1092.00
gat	D	0	0.58
gac	D	1	0.42
mPD		0.40	0.50
		nPD:	0.81
		N. weight:	0.18
		Sc. PD:	0.1
		Sc. rank:	261.1

PB2

Pos.	640	obs:	exp:
att	I	1	3.34
atc	I	0	2.26
ata	I	8	3.40
gtt	V	20	505.20
gtc	V	2	490.80
gta	V	2175	457.10
gtg	V	126	869.90
mPD		0.13	0.74
		nPD:	0.17
		N. weight:	1.8
		Sc. PD:	-0.082
		Sc. rank:	429.8

PB2

Pos.	641	obs:	exp:
cgt	R	0	88.40
cgc	R	0	124.20
cga	R	1	225.70
cgg	R	0	182.60
aga	R	2265	1069.00
agg	R	65	640.90
ggt	G	0	0.14
ggc	G	0	0.13
gga	G	1	0.44
ggg	G	0	0.29
mPD		0.056	0.95
		nPD:	0.06
		N. weight:	0.89
		Sc. PD:	-0.14
		Sc. rank:	-165.4

PB2			
Pos.	642	obs:	exp:
ggt	G	1692	335.50
ggc	G	627	307.80
gga	G	12	1018.00
ggg	G	1	670.70
---	--	------	------
mPD		0.40	0.69
		nPD:	0.58
		N. weight:	1.8
		Sc. PD:	0.63
		Sc. rank:	2044.3

,

PB2			
Pos.	643	obs:	exp:
tct	S	3	398.10
tcc	S	5	343.60
tca	S	1816	525.40
tcg	S	507	150.60
agt	S	0	475.90
agc	S	0	437.30
gct	A	0	0.25
gcc	A	0	0.20
gca	A	1	0.44
gcg	A	0	0.11
---	--	------	------
mPD		0.35	1.6
		nPD:	0.21
		N. weight:	1.6
		Sc. PD:	-0.018
		Sc. rank:	478.7

,

PB2			
Pos.	644	obs:	exp:
tgg	W	1	1.00
ggt	G	0	335.30
ggc	G	0	307.70
gga	G	2207	1018.00
ggg	G	124	670.40
---	--	------	------
mPD		0.10	0.69
		nPD:	0.15
		N. weight:	0.86
		Sc. PD:	-0.062
		Sc. rank:	129.5

,

PB2			
Pos.	645	obs:	exp:
tta	L	0	3.93
ttg	L	6	6.76
ctt	L	0	6.75
ctc	L	0	6.21
cta	L	0	4.79
ctg	L	30	7.56
att	I	0	0.74
atc	I	0	0.50
ata	I	2	0.76
atg	M	2294	2294.00
---	--	------	------
mPD		0.032	0.051
		nPD:	0.64
		N. weight:	0.025
		Sc. PD:	0.01
		Sc. rank:	31.2

,

PB2			
Pos.	646	obs:	exp:
cgt	R	0	88.44
cgc	R	0	124.20
cga	R	0	225.80
cgg	R	0	182.60
aga	R	2283	1070.00
agg	R	49	641.20
---	--	------	------
mPD		0.041	0.95
		nPD:	0.04
		N. weight:	0.92
		Sc. PD:	-0.16
		Sc. rank:	-324.2

,

PB2			
Pos.	647	obs:	exp:
tta	L	2	0.22
ttg	L	0	0.38
ctt	L	0	0.38
ctc	L	0	0.35
cta	L	0	0.27
ctg	L	0	0.42
att	I	0	861.10
atc	I	2	581.60
ata	I	2317	876.30
atg	M	11	11.00
---	--	------	------
mPD		0.013	0.66
		nPD:	0.02
		N. weight:	1.3
		Sc. PD:	-0.25
		Sc. rank:	-887.2

,

PB2			
Pos.	648	obs:	exp:
tta	L	0	254.50
ttg	L	0	437.60
ctt	L	1694	437.30
ctc	L	626	402.30
cta	L	3	310.30
ctg	L	9	490.00
---	--	------	------
mPD		0.40	1.1
		nPD:	0.35
		N. weight:	1.5
		Sc. PD:	0.18
		Sc. rank:	1006.2

,

PB2			
Pos.	649	obs:	exp:
gtt	V	21	507.20
gtc	V	0	492.70
gta	V	656	458.80
gtg	V	1655	873.30
---	--	------	------
mPD		0.42	0.73
		nPD:	0.57
		N. weight:	0.71
		Sc. PD:	0.24
		Sc. rank:	762.6

,

PB2			
Pos.	650	obs:	exp:
cgt	R	0	88.44
cgc	R	0	124.20
cga	R	0	225.80
cgg	R	0	182.60
aga	R	739	1070.00
agg	R	1593	641.20
---	--	------	------
mPD		0.43	0.95
		nPD:	0.45
		N. weight:	0.68
		Sc. PD:	0.15
		Sc. rank:	577.7

,

PB2			
Pos.	651	obs:	exp:
ggt	G	516	335.50
ggc	G	1777	307.80
gga	G	39	1018.00
ggg	G	0	670.70
---	--	------	------
mPD		0.37	0.69
		nPD:	0.54
		N. weight:	1.8
		Sc. PD:	0.57
		Sc. rank:	1822.5

,

PB2			
Pos.	652	obs:	exp:
aat	N	224	1239.00
aac	N	2108	1093.00
---	--	------	------
mPD		0.17	0.50
		nPD:	0.35
		N. weight:	0.58
		Sc. PD:	0.071
		Sc. rank:	393.2

,

PB2			
Pos.	653	obs:	exp:
tct	S	7	398.10
tcc	S	2318	343.60
tca	S	6	525.40
tcg	S	0	150.60
agt	S	0	475.90
agc	S	0	437.30
---	-	1	1.00
---	--	------	------
mPD		0.011	1.6
		nPD:	0.01
		N. weight:	2.5
		Sc. PD:	-0.52
		Sc. rank:	-2871.7

,

PB2			
Pos.	654	obs:	exp:
cct	P	2297	657.60
ccc	P	21	422.00
cca	P	14	952.20
ccg	P	0	300.30
---	--	------	------
mPD		0.030	0.70
		nPD:	0.04
		N. weight:	1.6
		Sc. PD:	-0.28
		Sc. rank:	-581.4

,

PB2			
Pos.	655	obs:	exp:
atg	M	5	5.00
gtt	V	1	506.10
gtc	V	0	491.70
gta	V	49	457.90
gtg	V	2277	871.40
---	--	------	------
mPD		0.046	0.73
		nPD:	0.06
		N. weight:	1.2
		Sc. PD:	-0.18
		Sc. rank:	-167.9

,

PB2			
Pos.	656	obs:	exp:
ttt	F	2	992.30
ttc	F	2330	1340.00
---	--	------	------
mPD		0.0017	0.49
		nPD:	0.
		N. weight:	0.73
		Sc. PD:	-0.16
		Sc. rank:	-958.2

,

PB2			
Pos.	657	obs:	exp:
aat	N	49	1239.00
aac	N	2283	1093.00
---	--	------	------
mPD		0.041	0.50
		nPD:	0.08
		N. weight:	0.88
		Sc. PD:	-0.12
		Sc. rank:	-63.0

,

PB2			
Pos.	658	obs:	exp:
tat	Y	1688	1132.00
tac	Y	641	1197.00
cat	H	2	1.78
cac	H	1	1.22
---	--	------	------
mPD		0.40	0.50
		nPD:	0.8
		N. weight:	0.16
		Sc. PD:	0.09
		Sc. rank:	231.4

,

PB2			
Pos.	659	obs:	exp:
act	T	1	0.26
acc	T	0	0.22
aca	T	0	0.43
acg	T	0	0.08
aat	N	1467	1239.00
aac	N	864	1092.00
---	--	------	------
mPD		0.47	0.50
		nPD:	0.94
		N. weight:	0.029
		Sc. PD:	0.02
		Sc. rank:	43.9

,

PB2			
Pos.	660	obs:	exp:
cgt	R	0	6.75
cgc	R	0	9.48
cga	R	0	17.24
cgg	R	0	13.94
aaa	K	32	1209.00
aag	K	2122	944.90
aga	R	0	81.65
agg	R	178	48.94
---	--	------	------
mPD		0.17	0.68
		nPD:	0.25
		N. weight:	1.1
		Sc. PD:	0.026
		Sc. rank:	484.7

,

PB2			
Pos.	661	obs:	exp:
tct	S	0	0.17
tcc	S	0	0.15
tca	S	1	0.23
tcg	S	0	0.06
act	T	0	27.55
acc	T	101	22.98
aca	T	3	44.69
acg	T	0	8.78
agt	S	0	0.20
agc	S	0	0.19
gtt	V	0	1.30
gtc	V	6	1.27
gta	V	0	1.18
gtg	V	0	2.25
gct	A	0	565.00
gcc	A	1363	433.10
gca	A	858	976.30
gcg	A	0	246.60
---	--	------	------
mPD		0.56	0.78
		nPD:	0.71
		N. weight:	0.92
		Sc. PD:	0.44
		Sc. rank:	1227.9

,

PB2			
Pos.	662	obs:	exp:
act	T	92	617.40
acc	T	2229	515.00
aca	T	10	1002.00
acg	T	0	196.90
gct	A	0	0.25
gcc	A	1	0.20
gca	A	0	0.44
gcg	A	0	0.11
---	--	------	------
mPD		0.085	0.69
		nPD:	0.12
		N. weight:	1.8
		Sc. PD:	-0.17
		Sc. rank:	118.2

,

PB2			
Pos.	663	obs:	exp:
cgt	R	0	1.25
cgc	R	0	1.76
cga	R	0	3.20
cgg	R	0	2.58
aat	N	1	0.53
aac	N	0	0.47
aaa	K	634	1290.00
aag	K	1664	1008.00
aga	R	2	15.14
agg	R	31	9.07
---	--	------	------
mPD		0.43	0.53
		nPD:	0.8
		N. weight:	0.24
		Sc. PD:	0.14
		Sc. rank:	351.9

,

PB2			
Pos.	664	obs:	exp:
cgt	R	0	88.40
cgc	R	0	124.20
cga	R	0	225.70
cgg	R	0	182.60
aaa	K	1	0.56
aag	K	0	0.44
aga	R	1290	1069.00
agg	R	1041	640.90
---	--	------	------
mPD		0.50	0.95
		nPD:	0.52
		N. weight:	0.43
		Sc. PD:	0.12
		Sc. rank:	401.9

,

PB2			
Pos.	665	obs:	exp:
tta	L	0	254.50
ttg	L	0	437.60
ctt	L	795	437.30
ctc	L	1535	402.30
cta	L	1	310.30
ctg	L	1	490.00
---	--	------	------
mPD		0.45	1.1
		nPD:	0.4
		N. weight:	1.4
		Sc. PD:	0.25
		Sc. rank:	1095.9

,

PB2			
Pos.	666	obs:	exp:
tct	S	0	30.23
tcc	S	0	26.09
tca	S	177	39.90
tcg	S	0	11.43
act	T	0	432.80
acc	T	108	361.00
aca	T	1518	702.10
acg	T	8	138.00
agt	S	0	36.14
agc	S	0	33.21
gct	A	0	132.50
gcc	A	437	101.60
gca	A	84	229.00
gcg	A	0	57.84
---	--	------	------
mPD		0.82	1.2
		nPD:	0.7
		N. weight:	1.1
		Sc. PD:	0.49
		Sc. rank:	1382.4

,

PB2			
Pos.	667	obs:	exp:
att	I	346	132.90
atc	I	12	89.78
ata	I	0	135.30
gtt	V	1250	429.30
gtc	V	723	417.10
gta	V	1	388.40
gtg	V	0	739.20
---	--	------	------
mPD		0.69	1.0
		nPD:	0.69
		N. weight:	1.2
		Sc. PD:	0.53
		Sc. rank:	1521.5

,

PB2			
Pos.	668	obs:	exp:
ttt	F	1	0.43
ttc	F	0	0.57
tta	L	0	253.90
ttg	L	0	436.50
ctt	L	2314	436.20
ctc	L	11	401.30
cta	L	1	309.50
ctg	L	0	488.70
att	I	5	1.86
atc	I	0	1.25
ata	I	0	1.89
---	--	------	------
mPD		0.015	1.1
		nPD:	0.01
		N. weight:	2.2
		Sc. PD:	-0.44
		Sc. rank:	-1916.8

,

PB2			
Pos.	669	obs:	exp:
ggt	G	0	335.50
ggc	G	0	307.80
gga	G	2255	1018.00
ggg	G	77	670.70
---	--	------	------
mPD		0.064	0.69
		nPD:	0.09
		N. weight:	0.94
		Sc. PD:	-0.12
		Sc. rank:	-2.5

,

PB2			
Pos.	670	obs:	exp:
aaa	K	41	1309.00
aag	K	2291	1023.00
---	--	------	------
mPD		0.035	0.49
		nPD:	0.07
		N. weight:	0.98
		Sc. PD:	-0.14
		Sc. rank:	-102.7

,

PB2			
Pos.	671	obs:	exp:
aat	N	2	1.06
aac	N	0	0.94
gat	D	1705	1340.00
gac	D	625	989.80
---	--	------	------
mPD		0.39	0.49
		nPD:	0.8
		N. weight:	0.074
		Sc. PD:	0.042
		Sc. rank:	106.7

,

PB2			
Pos.	672	obs:	exp:
gct	A	33	593.20
gcc	A	1	454.80
gca	A	2298	1025.00
gcg	A	0	258.90
---	--	------	------
mPD		0.029	0.69
		nPD:	0.04
		N. weight:	1.
		Sc. PD:	-0.18
		Sc. rank:	-385.7

,

PB2			
Pos.	673	obs:	exp:
ggt	G	1243	335.50
ggc	G	1087	307.80
gga	G	1	1018.00
ggg	G	1	670.70
---	--	------	------
mPD		0.50	0.69
		nPD:	0.72
		N. weight:	1.7
		Sc. PD:	0.84
		Sc. rank:	2346.7

,

PB2			
Pos.	674	obs:	exp:
tct	S	0	1.71
tcc	S	10	1.47
tca	S	0	2.25
tcg	S	0	0.65
act	T	0	0.26
acc	T	1	0.22
aca	T	0	0.43
acg	T	0	0.08
agt	S	0	2.04
agc	S	0	1.88
gtt	V	0	0.65
gtc	V	1	0.63
gta	V	2	0.59
gtg	V	0	1.12
gct	A	5	589.70
gcc	A	1458	452.00
gca	A	854	1019.00
gcg	A	1	257.30
---	--	------	------
mPD		0.48	0.71
		nPD:	0.68
		N. weight:	0.89
		Sc. PD:	0.4
		Sc. rank:	1145.3

,

PB2			
Pos.	675	obs:	exp:
tta	L	635	254.50
ttg	L	1667	437.60
ctt	L	0	437.30
ctc	L	0	402.30
cta	L	2	310.30
ctg	L	28	490.00
---	--	------	------
mPD		0.42	1.1
		nPD:	0.37
		N. weight:	1.6
		Sc. PD:	0.23
		Sc. rank:	1167.8

,

PB2			
Pos.	676	obs:	exp:
att	I	0	4.08
atc	I	2	2.76
ata	I	9	4.16
act	T	8	612.70
acc	T	10	511.10
aca	T	2292	993.90
acg	T	3	195.40
gct	A	2	2.04
gcc	A	0	1.56
gca	A	6	3.52
gcg	A	0	0.89
---	--	------	------
mPD		0.038	0.71
		nPD:	0.05
		N. weight:	1.1
		Sc. PD:	-0.17
		Sc. rank:	-257.9

,

PB2			
Pos.	677	obs:	exp:
aaa	K	1	0.56
aag	K	0	0.44
gat	D	0	0.58
gac	D	1	0.42
gaa	E	1684	1386.00
gag	E	630	928.50
ggt	G	0	2.30
ggc	G	0	2.11
gga	G	16	6.98
ggg	G	0	4.60
---	--	------	------
mPD		0.41	0.50
		nPD:	0.82
		N. weight:	0.059
		Sc. PD:	0.034
		Sc. rank:	86.6

,

PB2			
Pos.	678	obs:	exp:
ttt	F	71	30.21
ttc	F	0	40.79
tat	Y	1013	492.90
tac	Y	1	521.10
tgt	C	2	0.87
tgc	C	0	1.13
cat	H	4	2.38
cac	H	0	1.62
aat	N	5	2.66
aac	N	0	2.34
gat	D	914	671.90
gac	D	254	496.10
ggt	G	67	9.78
ggc	G	1	8.98
gga	G	0	29.68
ggg	G	0	19.56
---	--	------	------
mPD		0.81	1.1
		nPD:	0.72
		N. weight:	0.59
		Sc. PD:	0.28
		Sc. rank:	798.9

,

PB2			
Pos.	679	obs:	exp:
tct	S	0	0.17
tcc	S	0	0.15
tca	S	1	0.23
tcg	S	0	0.06
cct	P	2	657.30
ccc	P	2	421.80
cca	P	1981	951.80
ccg	P	346	300.10
agt	S	0	0.20
agc	S	0	0.19
---	--	------	------
mPD		0.26	0.71
		nPD:	0.36
		N. weight:	0.85
		Sc. PD:	0.12
		Sc. rank:	613.6

,

PB2			
Pos.	680	obs:	exp:
gat	D	2276	1324.00
gac	D	26	977.90
gaa	E	27	16.77
gag	E	1	11.23
ggt	G	2	0.29
ggc	G	0	0.26
gga	G	0	0.87
ggg	G	0	0.58
---	--	------	------
mPD		0.047	0.50
		nPD:	0.09
		N. weight:	0.66
		Sc. PD:	-0.083
		Sc. rank:	-0.0

,

PB2	Pos. 681	obs:	exp:
gat	D	10	5.75
gac	D	0	4.25
gaa	E	24	1390.00
gag	E	2297	931.30
---	-	1	1.00
mPD		0.029	0.48
		nPD:	0.06
		N. weight:	1.1
		Sc. PD:	-0.18
		Sc. rank:	-201.8

PB2	Pos. 682	obs:	exp:
cgt	R	0	0.08
cgc	R	0	0.11
cga	R	0	0.19
cgg	R	0	0.16
aga	R	1	0.92
agg	R	1	0.55
gaa	E	3	1.80
gag	E	0	1.20
ggt	G	0	334.70
ggc	G	12	307.10
gga	G	1595	1016.00
ggg	G	720	669.30
mPD		0.44	0.69
		nPD:	0.63
		N. weight:	0.43
		Sc. PD:	0.17
		Sc. rank:	521.0

PB2	Pos. 683	obs:	exp:
att	I	0	0.37
atc	I	0	0.25
ata	I	1	0.38
atg	M	2	2.00
act	T	0	614.00
acc	T	0	512.20
aca	T	2092	996.00
acg	T	226	195.80
aaa	K	6	3.37
aag	K	0	2.63
gct	A	0	1.27
gcc	A	0	0.98
gca	A	5	2.20
gcg	A	0	0.56
mPD		0.19	0.70
		nPD:	0.27
		N. weight:	0.92
		Sc. PD:	0.042
		Sc. rank:	479.5

PB2	Pos. 684	obs:	exp:
tct	S	0	58.92
tcc	S	0	50.86
tca	S	345	77.77
tcg	S	0	22.28
act	T	0	139.10
acc	T	0	116.00
aca	T	525	225.60
acg	T	0	44.34
agt	S	0	70.44
agc	S	0	64.73
gct	A	12	371.70
gcc	A	102	284.90
gca	A	1346	642.20
gcg	A	1	162.20
gaa	E	1	0.60
gag	E	0	0.40
mPD		0.63	1.3
		nPD:	0.47
		N. weight:	1.
		Sc. PD:	0.25
		Sc. rank:	914.3

PB2	Pos. 685	obs:	exp:
cgt	R	0	0.04
cgc	R	0	0.05
cga	R	0	0.10
cgg	R	0	0.08
aga	R	1	0.46
agg	R	0	0.27
gtt	V	0	0.22
gtc	V	0	0.21
gta	V	1	0.20
gtg	V	0	0.37
gaa	E	2	1.20
gag	E	0	0.80
ggt	G	0	334.90
ggc	G	0	307.30
gga	G	2159	1016.00
ggg	G	169	669.60
mPD		0.14	0.69
		nPD:	0.2
		N. weight:	0.8
		Sc. PD:	-0.018
		Sc. rank:	221.2

PB2	Pos. 686	obs:	exp:
atg	M	1	1.00
gtt	V	3	506.90
gtc	V	0	492.50
gta	V	9	458.60
gtg	V	2319	872.90
mPD		0.011	0.73
		nPD:	0.02
		N. weight:	1.3
		Sc. PD:	-0.26
		Sc. rank:	-1018.8

PB2	Pos. 687	obs:	exp:
gaa	E	658	1396.00
gag	E	1674	935.70
mPD		0.41	0.48
		nPD:	0.84
		N. weight:	0.28
		Sc. PD:	0.17
		Sc. rank:	412.8

PB2	Pos. 688	obs:	exp:
tct	S	2240	398.10
tcc	S	84	343.60
tca	S	7	525.40
tcg	S	0	150.60
act	T	1	0.26
acc	T	0	0.22
aca	T	0	0.43
acg	T	0	0.08
agt	S	0	475.90
agc	S	0	437.30
mPD		0.076	1.6
		nPD:	0.05
		N. weight:	2.1
		Sc. PD:	-0.37
		Sc. rank:	-684.2

PB2	Pos. 689	obs:	exp:
gct	A	14	593.20
gcc	A	0	454.80
gca	A	2107	1025.00
gcg	A	211	258.90
mPD		0.18	0.69
		nPD:	0.25
		N. weight:	0.82
		Sc. PD:	0.025
		Sc. rank:	397.8

PB2	Pos. 690	obs:	exp:
tta	L	0	0.11
ttg	L	0	0.19
ctt	L	0	0.19
ctc	L	0	0.17
cta	L	0	0.13
ctg	L	1	0.21
gtt	V	11	506.90
gtc	V	1	492.50
gta	V	1834	458.60
gtg	V	485	872.90
mPD		0.34	0.73
		nPD:	0.46
		N. weight:	1.3
		Sc. PD:	0.3
		Sc. rank:	1112.0

PB2	Pos. 691	obs:	exp:
tta	L	1671	254.50
ttg	L	30	437.60
ctt	L	1	437.30
ctc	L	0	402.30
cta	L	6	310.30
ctg	L	624	490.00
mPD		0.80	1.1
		nPD:	0.7
		N. weight:	1.8
		Sc. PD:	0.86
		Sc. rank:	2410.1

PB2	Pos. 692	obs:	exp:
cgt	R	0	88.44
cgc	R	0	124.20
cga	R	0	225.80
cgg	R	0	182.60
aga	R	1808	1070.00
agg	R	524	641.20
mPD		0.35	0.95
		nPD:	0.37
		N. weight:	0.49
		Sc. PD:	0.068
		Sc. rank:	352.0

PB2	Pos. 693	obs:	exp:
gaa	E	0	0.60
gag	E	1	0.40
ggt	G	0	335.30
ggc	G	2	307.70
gga	G	1587	1018.00
ggg	G	742	670.40
mPD		0.44	0.69
		nPD:	0.63
		N. weight:	0.45
		Sc. PD:	0.18
		Sc. rank:	544.4

PB2	Pos. 694	obs:	exp:
ttt	F	1483	991.50
ttc	F	847	1339.00
tta	L	1	0.11
ttg	L	0	0.19
ctt	L	0	0.19
ctc	L	0	0.17
cta	L	0	0.13
ctg	L	0	0.21
att	I	0	0.37
atc	I	0	0.25
ata	I	1	0.38
mPD		0.46	0.49
		nPD:	0.95
		N. weight:	0.12
		Sc. PD:	0.088
		Sc. rank:	187.0

PB2	Pos. 695	obs:	exp:
tta	L	11	254.50
ttg	L	0	437.60
ctt	L	0	437.30
ctc	L	12	402.30
cta	L	2306	310.30
ctg	L	3	490.00
mPD		0.022	1.1
		nPD:	0.02
		N. weight:	2.6
		Sc. PD:	-0.51
		Sc. rank:	-1765.1

PB2	Pos. 696	obs:	exp:
att	I	2297	865.60
atc	I	33	584.60
ata	I	1	880.80
gtt	V	1	0.22
gtc	V	0	0.21
gta	V	0	0.20
gtg	V	0	0.37
mPD		0.030	0.66
		nPD:	0.05
		N. weight:	1.2
		Sc. PD:	-0.21
		Sc. rank:	-412.8

PB2	Pos. 697	obs:	exp:
tta	L	0	254.50
ttg	L	11	437.60
ctt	L	0	437.30
ctc	L	0	402.30
cta	L	111	310.30
ctg	L	2210	490.00
mPD		0.10	1.1
		nPD:	0.09
		N. weight:	1.8
		Sc. PD:	-0.24
		Sc. rank:	-40.2

PB2	Pos. 698	obs:	exp:
ggt	G	3	335.50
ggc	G	2329	307.80
gga	G	0	1018.00
ggg	G	0	670.70
mPD		0.0026	0.69
		nPD:	0.
		N. weight:	2.7
		Sc. PD:	-0.57
		Sc. rank:	-3353.7

PB2	Pos. 699	obs:	exp:
cgt	R	0	1.40
cgc	R	0	1.97
cga	R	0	3.58
cgg	R	0	2.90
aaa	K	1792	1288.00
aag	K	503	1007.00
aga	R	6	16.97
agg	R	31	10.17
mPD		0.38	0.53
		nPD:	0.72
		N. weight:	0.16
		Sc. PD:	0.077
		Sc. rank:	215.4

PB2	Pos. 700	obs:	exp:
gaa	E	234	1396.00
gag	E	2098	935.70
mPD		0.18	0.48
		nPD:	0.38
		N. weight:	0.73
		Sc. PD:	0.11
		Sc. rank:	550.0

PB2	Pos. 701	obs:	exp:
aat	N	0	1.59
aac	N	3	1.41
gat	D	7	1340.00
gac	D	2322	989.30
mPD		0.0086	0.49
		nPD:	0.02
		N. weight:	1.1
		Sc. PD:	-0.22
		Sc. rank:	-820.5

PB2	Pos. 702	obs:	exp:
cgt	R	0	19.87
cgc	R	0	27.91
cga	R	0	50.74
cgg	R	0	41.04
aaa	K	1795	1015.00
aag	K	13	793.20
aga	R	524	240.40
agg	R	0	144.10
mPD		0.36	0.97
		nPD:	0.37
		N. weight:	0.79
		Sc. PD:	0.11
		Sc. rank:	576.2

PB2	Pos. 703	obs:	exp:
cgt	R	0	88.44
cgc	R	0	124.20
cga	R	0	225.80
cgg	R	0	182.60
aga	R	2235	1070.00
agg	R	97	641.20
mPD		0.080	0.95
		nPD:	0.08
		N. weight:	0.84
		Sc. PD:	-0.11
		Sc. rank:	-57.7

PB2	Pos. 704	obs:	exp:
tat	Y	2304	1134.00
tac	Y	28	1198.00
mPD		0.024	0.50
		nPD:	0.05
		N. weight:	0.88
		Sc. PD:	-0.15
		Sc. rank:	-256.4

PB2

Pos.	705	obs:	exp:
ggt	G	0	335.50
ggc	G	12	307.80
gga	G	2315	1018.00
ggg	G	5	670.70
mPD		0.015	0.69
		nPD:	0.02
		N. weight:	1.1
		Sc. PD:	-0.21
		Sc. rank:	-681.5

PB2

Pos.	706	obs:	exp:
cct	P	0	657.60
ccc	P	0	422.00
cca	P	2323	952.20
ccg	P	9	300.30
mPD		0.0077	0.70
		nPD:	0.01
		N. weight:	1.2
		Sc. PD:	-0.24
		Sc. rank:	-1134.3

PB2

Pos.	707	obs:	exp:
tct	S	0	4.10
tcc	S	0	3.54
tca	S	24	5.41
tcg	S	0	1.55
agt	S	0	4.90
agc	S	0	4.50
gct	A	0	587.10
gcc	A	0	450.10
gca	A	2282	1015.00
gcg	A	26	256.20
mPD		0.042	0.72
		nPD:	0.06
		N. weight:	1.
		Sc. PD:	-0.17
		Sc. rank:	-190.7

PB2

Pos.	708	obs:	exp:
tta	L	26	254.50
ttg	L	1756	437.60
ctt	L	0	437.30
ctc	L	0	402.30
cta	L	521	310.30
ctg	L	29	490.00
mPD		0.72	1.1
		nPD:	0.63
		N. weight:	1.5
		Sc. PD:	0.59
		Sc. rank:	1817.7

PB2

Pos.	709	obs:	exp:
tct	S	0	395.70
tcc	S	0	341.60
tca	S	0	522.30
tcg	S	0	149.70
aat	N	0	6.91
aac	N	13	6.09
agt	S	3	473.10
agc	S	2314	434.70
ggt	G	0	0.29
ggc	G	2	0.26
gga	G	0	0.87
ggg	G	0	0.58
mPD		0.015	1.7
		nPD:	0.01
		N. weight:	2.2
		Sc. PD:	-0.46
		Sc. rank:	-2312.8

PB2

Pos.	710	obs:	exp:
att	I	1	840.30
atc	I	2259	567.50
ata	I	3	855.10
gtt	V	0	15.01
gtc	V	69	14.58
gta	V	0	13.58
gtg	V	0	25.84
mPD		0.061	0.72
		nPD:	0.08
		N. weight:	1.8
		Sc. PD:	-0.25
		Sc. rank:	-112.7

PB2

Pos.	711	obs:	exp:
act	T	0	0.26
acc	T	1	0.22
aca	T	0	0.43
acg	T	0	0.08
aat	N	1194	1239.00
aac	N	1137	1092.00
mPD		0.50	0.50
		nPD:	1.
		N. weight:	0.0042
		Sc. PD:	0.0032
		Sc. rank:	6.3

PB2

Pos.	712	obs:	exp:
aaa	K	1	0.56
aag	K	0	0.44
gaa	E	2320	1395.00
gag	E	9	934.50
ggt	G	0	0.29
ggc	G	0	0.26
gga	G	2	0.87
ggg	G	0	0.58
mPD		0.010	0.48
		nPD:	0.02
		N. weight:	0.66
		Sc. PD:	-0.13
		Sc. rank:	-421.2

PB2

Pos.	713	obs:	exp:
tta	L	2	254.30
ttg	L	1678	437.20
ctt	L	0	436.90
ctc	L	0	402.00
cta	L	5	310.00
ctg	L	645	489.60
atg	M	2	2.00
mPD		0.41	1.1
		nPD:	0.36
		N. weight:	1.4
		Sc. PD:	0.19
		Sc. rank:	978.9

PB2

Pos.	714	obs:	exp:
tct	S	0	395.70
tcc	S	0	341.60
tca	S	0	522.30
tcg	S	0	149.70
agt	S	9	473.10
agc	S	2308	434.70
ggt	G	0	2.16
ggc	G	15	1.98
gga	G	0	6.55
ggg	G	0	4.31
mPD		0.020	1.7
		nPD:	0.01
		N. weight:	2.2
		Sc. PD:	-0.45
		Sc. rank:	-2015.6

PB2

Pos.	715	obs:	exp:
tct	S	0	2.05
tcc	S	0	1.77
tca	S	0	2.70
tcg	S	0	0.78
tat	Y	1	0.49
tac	Y	0	0.51
cat	H	2	1.19
cac	H	0	0.81
aat	N	2298	1231.00
aac	N	18	1085.00
agt	S	12	2.45
agc	S	0	2.25
ggt	G	1	0.14
ggc	G	0	0.13
gga	G	0	0.44
ggg	G	0	0.29
mPD		0.030	0.52
		nPD:	0.06
		N. weight:	0.8
		Sc. PD:	-0.13
		Sc. rank:	-163.3

PB2

Pos.	716	obs:	exp:
ttt	F	2	0.85
ttc	F	0	1.15
tta	L	0	254.00
ttg	L	0	436.60
ctt	L	2326	436.30
ctc	L	1	401.50
cta	L	0	309.60
ctg	L	0	488.90
att	I	3	1.11
atc	I	0	0.75
ata	I	0	1.13
mPD		0.0051	1.1
		nPD:	0.
		N. weight:	2.2
		Sc. PD:	-0.47
		Sc. rank:	-2728.1

PB2

Pos.	717	obs:	exp:
tct	S	0	0.34
tcc	S	0	0.29
tca	S	0	0.45
tcg	S	2	0.13
act	T	0	1.06
acc	T	0	0.88
aca	T	1	1.72
acg	T	3	0.34
agt	S	0	0.41
agc	S	0	0.38
gtt	V	0	1.96
gtc	V	0	1.90
gta	V	0	1.77
gtg	V	9	3.37
gct	A	7	589.40
gcc	A	0	451.80
gca	A	103	1019.00
gcg	A	2207	257.20
mPD		0.10	0.71
		nPD:	0.15
		N. weight:	2.6
		Sc. PD:	-0.19
		Sc. rank:	379.3

PB2

Pos.	718	obs:	exp:
cgt	R	0	0.04
cgc	R	0	0.05
cga	R	0	0.10
cgg	R	0	0.08
aaa	K	2148	1308.00
aag	K	183	1023.00
aga	R	1	0.46
agg	R	0	0.27
mPD		0.15	0.49
		nPD:	0.29
		N. weight:	0.43
		Sc. PD:	0.031
		Sc. rank:	253.9

PB2

Pos.	719	obs:	exp:
ggt	G	0	335.30
ggc	G	0	307.70
gga	G	15	1018.00
ggg	G	2316	670.40
---	-	1	1.00
mPD		0.013	0.69
		nPD:	0.02
		N. weight:	1.6
		Sc. PD:	-0.32
		Sc. rank:	-1166.2

PB2

Pos.	720	obs:	exp:
gaa	E	1480	1396.00
gag	E	852	935.70
mPD		0.46	0.48
		nPD:	0.97
		N. weight:	0.006
		Sc. PD:	0.0043
		Sc. rank:	9.0

PB2

Pos.	721	obs:	exp:
aaa	K	644	1309.00
aag	K	1688	1023.00
mPD		0.40	0.49
		nPD:	0.81
		N. weight:	0.23
		Sc. PD:	0.13
		Sc. rank:	328.4

PB2

Pos.	722	obs:	exp:
gct	A	1622	593.20
gcc	A	710	454.80
gca	A	0	1025.00
gcg	A	0	258.90
mPD		0.42	0.69
		nPD:	0.61
		N. weight:	1.1
		Sc. PD:	0.42
		Sc. rank:	1338.6

PB2

Pos.	723	obs:	exp:
aat	N	1987	1239.00
aac	N	345	1093.00
mPD		0.25	0.50
		nPD:	0.51
		N. weight:	0.31
		Sc. PD:	0.086
		Sc. rank:	284.3

PB2

Pos.	724	obs:	exp:
gtt	V	0	507.20
gtc	V	0	492.70
gta	V	1	458.80
gtg	V	2331	873.30
mPD		0.00086	0.73
		nPD:	0.
		N. weight:	1.3
		Sc. PD:	-0.28
		Sc. rank:	-1884.5

PB2

Pos.	725	obs:	exp:
tta	L	181	254.50
ttg	L	1505	437.60
ctt	L	0	437.30
ctc	L	0	402.30
cta	L	14	310.30
ctg	L	632	490.00
mPD		0.55	1.1
		nPD:	0.49
		N. weight:	1.1
		Sc. PD:	0.29
		Sc. rank:	987.1

PB2

Pos.	726	obs:	exp:
att	I	12	862.60
atc	I	0	582.60
ata	I	2311	877.80
gtt	V	0	1.96
gtc	V	0	1.90
gta	V	9	1.77
gtg	V	0	3.37
mPD		0.018	0.67
		nPD:	0.03
		N. weight:	1.3
		Sc. PD:	-0.24
		Sc. rank:	-685.7

PB2

Pos.	727	obs:	exp:
ggt	G	1	335.50
ggc	G	0	307.80
gga	G	827	1018.00
ggg	G	1504	670.70
mPD		0.46	0.69
		nPD:	0.67
		N. weight:	0.6
		Sc. PD:	0.26
		Sc. rank:	757.5

PB2

Pos.	728	obs:	exp:
caa	Q	2329	1187.00
cag	Q	3	1145.00
mPD		0.0026	0.50
		nPD:	0.01
		N. weight:	0.89
		Sc. PD:	-0.19
		Sc. rank:	-1079.2

PB2			
Pos.	729	obs:	exp:
ggt	G	0	335.50
ggc	G	0	307.80
gga	G	2319	1018.00
ggg	G	13	670.70
---	--	------	------
mPD		0.011	0.69
		nPD:	0.02
		N. weight:	1.1
		Sc. PD:	-0.21
		Sc. rank:	-819.4

,

PB2			
Pos.	730	obs:	exp:
gat	D	2	1341.00
gac	D	2330	990.60
---	--	------	------
mPD		0.0017	0.49
		nPD:	0.
		N. weight:	1.1
		Sc. PD:	-0.24
		Sc. rank:	-1476.3

,

PB2			
Pos.	731	obs:	exp:
att	I	1	62.76
atc	I	0	42.38
ata	I	168	63.86
atg	M	7	7.00
gtt	V	1	468.90
gtc	V	0	455.50
gta	V	184	424.20
gtg	V	1971	807.40
---	--	------	------
mPD		0.40	0.88
		nPD:	0.45
		N. weight:	1.
		Sc. PD:	0.23
		Sc. rank:	860.2

,

PB2			
Pos.	732	obs:	exp:
gtt	V	0	507.20
gtc	V	1	492.70
gta	V	2	458.80
gtg	V	2329	873.30
---	--	------	------
mPD		0.0026	0.73
		nPD:	0.
		N. weight:	1.3
		Sc. PD:	-0.28
		Sc. rank:	-1674.3

,

PB2			
Pos.	733	obs:	exp:
tta	L	0	254.50
ttg	L	1808	437.60
ctt	L	0	437.30
ctc	L	0	402.30
cta	L	0	310.30
ctg	L	524	490.00
---	--	------	------
mPD		0.35	1.1
		nPD:	0.31
		N. weight:	1.5
		Sc. PD:	0.12
		Sc. rank:	898.1

,

PB2			
Pos.	734	obs:	exp:
att	I	0	0.74
atc	I	0	0.50
ata	I	2	0.76
gtt	V	5	506.70
gtc	V	0	492.30
gta	V	2126	458.50
gtg	V	199	872.50
---	--	------	------
mPD		0.16	0.73
		nPD:	0.22
		N. weight:	1.7
		Sc. PD:	-0.00097
		Sc. rank:	553.4

,

PB2			
Pos.	735	obs:	exp:
atg	M	2332	2332.00
---	--	------	------
mPD		0	0
		nPD:	1.
		N. weight:	0.
		Sc. PD:	0
		Sc. rank:	0

,

PB2			
Pos.	736	obs:	exp:
cgt	R	0	0.04
cgc	R	0	0.05
cga	R	0	0.10
cgg	R	0	0.08
aaa	K	2331	1308.00
aag	K	0	1023.00
aga	R	1	0.46
agg	R	0	0.27
---	--	------	------
mPD		0.00086	0.49
		nPD:	0.
		N. weight:	0.77
		Sc. PD:	-0.17
		Sc. rank:	-1077.4

,

PB2			
Pos.	737	obs:	exp:
cgt	R	1	88.44
cgc	R	0	124.20
cga	R	3	225.80
cgg	R	2328	182.60
aga	R	0	1070.00
agg	R	0	641.20
---	--	------	------
mPD		0.0034	0.95
		nPD:	0.
		N. weight:	3.4
		Sc. PD:	-0.72
		Sc. rank:	-4281.3

,

PB2			
Pos.	738	obs:	exp:
aaa	K	2329	1309.00
aag	K	3	1023.00
---	--	------	------
mPD		0.0026	0.49
		nPD:	0.01
		N. weight:	0.76
		Sc. PD:	-0.16
		Sc. rank:	-913.1

,

PB2			
Pos.	739	obs:	exp:
caa	Q	0	0.51
cag	Q	1	0.49
cgt	R	0	88.40
cgc	R	0	124.20
cga	R	6	225.70
cgg	R	2325	182.60
aga	R	0	1069.00
agg	R	0	640.90
---	--	------	------
mPD		0.0060	0.95
		nPD:	0.01
		N. weight:	3.4
		Sc. PD:	-0.71
		Sc. rank:	-3905.5

,

PB2			
Pos.	740	obs:	exp:
aat	N	0	2.13
aac	N	4	1.87
gat	D	0	1339.00
gac	D	2328	988.90
---	--	------	------
mPD		0.0034	0.49
		nPD:	0.01
		N. weight:	1.1
		Sc. PD:	-0.24
		Sc. rank:	-1305.6

,

PB2			
Pos.	741	obs:	exp:
tct	S	2327	398.10
tcc	S	4	343.60
tca	S	0	525.40
tcg	S	0	150.60
tgt	C	0	0.43
tgc	C	1	0.57
agt	S	0	475.90
agc	S	0	437.30
---	--	------	------
mPD		0.0051	1.6
		nPD:	0.
		N. weight:	2.3
		Sc. PD:	-0.5
		Sc. rank:	-3087.4

,

PB2			
Pos.	742	obs:	exp:
tct	S	0	398.30
tcc	S	0	343.80
tca	S	0	525.70
tcg	S	0	150.60
agt	S	1	476.10
agc	S	2331	437.50
---	--	------	------
mPD		0.00086	1.6
		nPD:	0.
		N. weight:	2.2
		Sc. PD:	-0.48
		Sc. rank:	-3260.4

,

PB2			
Pos.	743	obs:	exp:
att	I	0	866.00
atc	I	0	584.90
ata	I	2332	881.20
---	--	------	------
mPD		0	0.66
		nPD:	0.
		N. weight:	1.3
		Sc. PD:	-0.28
		Sc. rank:	-1960.3

,

PB2			
Pos.	744	obs:	exp:
tta	L	0	254.50
ttg	L	0	437.60
ctt	L	2332	437.30
ctc	L	0	402.30
cta	L	0	310.30
ctg	L	0	490.00
---	--	------	------
mPD		0	1.1
		nPD:	0.
		N. weight:	2.2
		Sc. PD:	-0.48
		Sc. rank:	-3371.6

,

PB2			
Pos.	745	obs:	exp:
act	T	2318	617.70
acc	T	14	515.20
aca	T	0	1002.00
acg	T	0	197.00
---	--	------	------
mPD		0.012	0.69
		nPD:	0.02
		N. weight:	1.7
		Sc. PD:	-0.34
		Sc. rank:	-1278.2

,

PB2			
Pos.	746	obs:	exp:
gat	D	1	1341.00
gac	D	2330	990.20
ggt	G	0	0.14
ggc	G	1	0.13
gga	G	0	0.44
ggg	G	0	0.29
---	--	------	------
mPD		0.0017	0.49
		nPD:	0.
		N. weight:	1.1
		Sc. PD:	-0.24
		Sc. rank:	-1495.7

,

PB2			
Pos.	747	obs:	exp:
tct	S	0	398.30
tcc	S	0	343.80
tca	S	0	525.70
tcg	S	0	150.60
agt	S	70	476.10
agc	S	2262	437.50
---	--	------	------
mPD		0.058	1.6
		nPD:	0.04
		N. weight:	2.1
		Sc. PD:	-0.37
		Sc. rank:	-908.5

,

PB2			
Pos.	748	obs:	exp:
caa	Q	0	1186.00
cag	Q	2331	1145.00
cgt	R	0	0.04
cgc	R	0	0.05
cga	R	0	0.10
cgg	R	1	0.08
aga	R	0	0.46
agg	R	0	0.27
---	--	------	------
mPD		0.00086	0.50
		nPD:	0.
		N. weight:	0.95
		Sc. PD:	-0.2
		Sc. rank:	-1330.5

,

PB2			
Pos.	749	obs:	exp:
act	T	0	617.40
acc	T	0	515.00
aca	T	2331	1002.00
acg	T	0	196.90
gct	A	0	0.25
gcc	A	0	0.20
gca	A	1	0.44
gcg	A	0	0.11
---	--	------	------
mPD		0.00086	0.69
		nPD:	0.
		N. weight:	1.1
		Sc. PD:	-0.24
		Sc. rank:	-1606.6

,

PB2			
Pos.	750	obs:	exp:
gct	A	0	593.20
gcc	A	0	454.80
gca	A	0	1025.00
gcg	A	2332	258.90
---	--	------	------
mPD		0	0.69
		nPD:	0.
		N. weight:	2.9
		Sc. PD:	-0.63
		Sc. rank:	-4427.4

,

PB2			
Pos.	751	obs:	exp:
cct	P	0	0.28
ccc	P	1	0.18
cca	P	0	0.41
ccg	P	0	0.13
act	T	0	617.40
acc	T	2331	515.00
aca	T	0	1002.00
acg	T	0	196.90
---	--	------	------
mPD		0.00086	0.69
		nPD:	0.
		N. weight:	2.
		Sc. PD:	-0.43
		Sc. rank:	-2881.0

,

PB2

Pos.	752	obs:	exp:
caa	Q	1	0.51
cag	Q	0	0.49
aaa	K	2331	1308.00
aag	K	0	1023.00
---	--	------	------
mPD		0.00086	0.49
		nPD:	0.
		N. weight:	0.77
		Sc. PD:	-0.17
		Sc. rank:	-1075.0

,

PB2

Pos.	753	obs:	exp:
cgt	R	0	88.40
cgc	R	0	124.20
cga	R	0	225.70
cgg	R	0	182.60
att	I	0	0.37
atc	I	0	0.25
ata	I	1	0.38
aga	R	2323	1069.00
agg	R	8	640.90
---	--	------	------
mPD		0.0077	0.95
		nPD:	0.01
		N. weight:	1.
		Sc. PD:	-0.21
		Sc. rank:	-1122.9

,

PB2

Pos.	754	obs:	exp:
tta	L	0	0.11
ttg	L	0	0.19
ctt	L	1	0.19
ctc	L	0	0.17
cta	L	0	0.13
ctg	L	0	0.21
att	I	2330	865.60
atc	I	1	584.60
ata	I	0	880.80
---	--	------	------
mPD		0.0017	0.66
		nPD:	0.
		N. weight:	1.3
		Sc. PD:	-0.28
		Sc. rank:	-1775.3

,

PB2

Pos.	755	obs:	exp:
cgt	R	0	88.44
cgc	R	0	124.20
cga	R	13	225.80
cgg	R	2319	182.60
aga	R	0	1070.00
agg	R	0	641.20
---	--	------	------
mPD		0.011	0.95
		nPD:	0.01
		N. weight:	3.4
		Sc. PD:	-0.69
		Sc. rank:	-3179.0

,

PB2

Pos.	756	obs:	exp:
cgt	R	0	0.04
cgc	R	0	0.05
cga	R	0	0.10
cgg	R	0	0.08
atg	M	2331	2331.00
aga	R	0	0.46
agg	R	1	0.27
---	--	------	------
mPD		0.00086	0.0016
		nPD:	0.52
		N. weight:	0.00074
		Sc. PD:	0.00022
		Sc. rank:	0.7

,

PB2

Pos.	757	obs:	exp:
tct	S	0	0.17
tcc	S	1	0.15
tca	S	0	0.23
tcg	S	0	0.06
cct	P	0	0.28
ccc	P	1	0.18
cca	P	0	0.41
ccg	P	0	0.13
cgt	R	0	0.04
cgc	R	1	0.05
cga	R	0	0.10
cgg	R	0	0.08
agt	S	0	0.20
agc	S	0	0.19
aga	R	0	0.46
agg	R	0	0.27
gtt	V	0	113.70
gtc	V	523	110.50
gta	V	0	102.90
gtg	V	0	195.80
gct	A	0	458.90
gcc	A	1804	351.80
gca	A	0	793.00
gcg	A	0	200.30
ggt	G	0	0.29
ggc	G	2	0.26
gga	G	0	0.87
ggg	G	0	0.58
---	--	------	------
mPD		0.35	1.1
		nPD:	0.33
		N. weight:	2.2
		Sc. PD:	0.22
		Sc. rank:	1374.3

,

PB2

Pos.	758	obs:	exp:
att	I	1	866.00
atc	I	2331	584.90
ata	I	0	881.20
---	--	------	------
mPD		0.00086	0.66
		nPD:	0.
		N. weight:	1.8
		Sc. PD:	-0.4
		Sc. rank:	-2586.2

,

PB2

Pos.	759	obs:	exp:
tct	S	0	1.02
tcc	S	0	0.88
tca	S	0	1.35
tcg	S	0	0.39
act	T	2	0.53
acc	T	0	0.44
aca	T	0	0.86
acg	T	0	0.17
aat	N	2308	1230.00
aac	N	6	1084.00
aaa	K	0	5.61
aag	K	10	4.39
agt	S	6	1.23
agc	S	0	1.13
---	--	------	------
mPD		0.021	0.51
		nPD:	0.04
		N. weight:	0.83
		Sc. PD:	-0.15
		Sc. rank:	-324.1

,

PB2

Pos.	760	obs:	exp:
taa	.	9	855.20
tag	.	2323	654.50
tga	.	0	822.40
---	--	------	------
mPD		0.0077	0.86
		nPD:	0.01
		N. weight:	1.7
		Sc. PD:	-0.34
		Sc. rank:	-1765.6

}

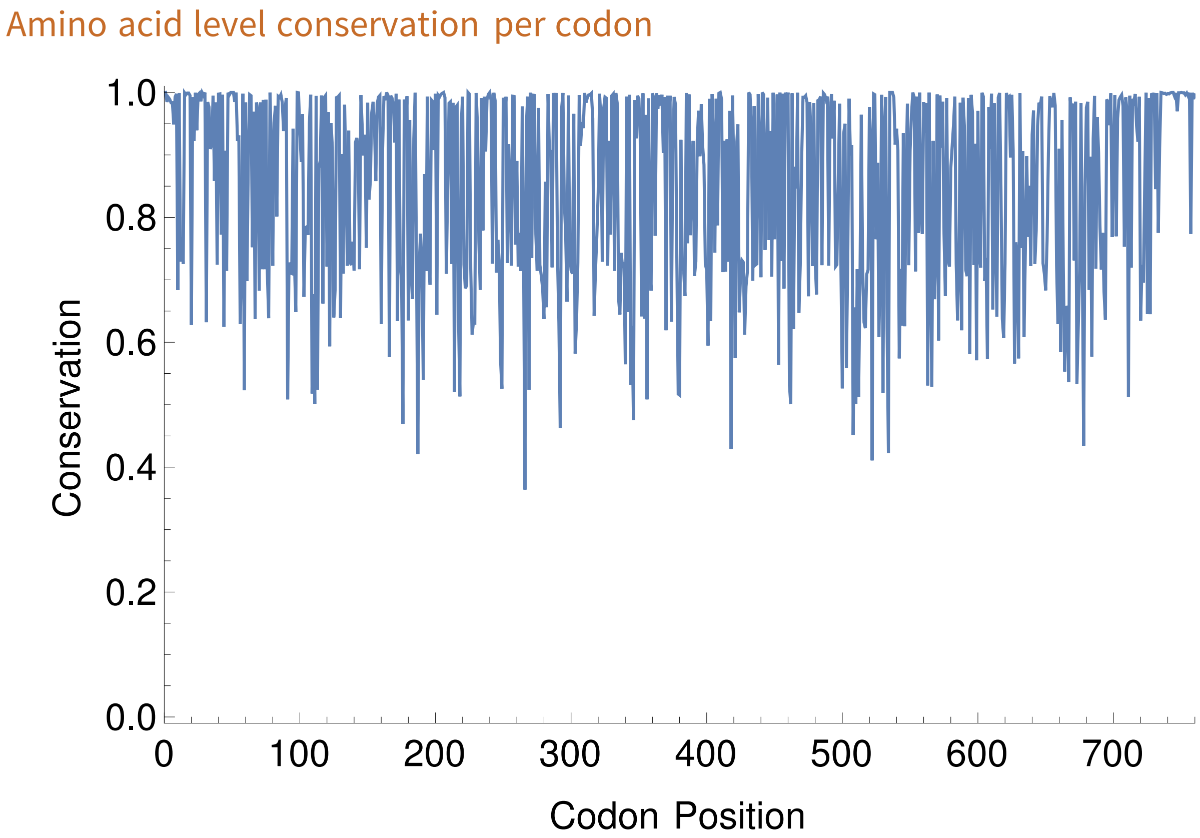
Amino acid level conservation per codon
Conservation
1.0
0.8
0.6
0.4
0.2
0.0
0
100
200
300
400
500
600
700
Codon Position

Conservation analysis plots

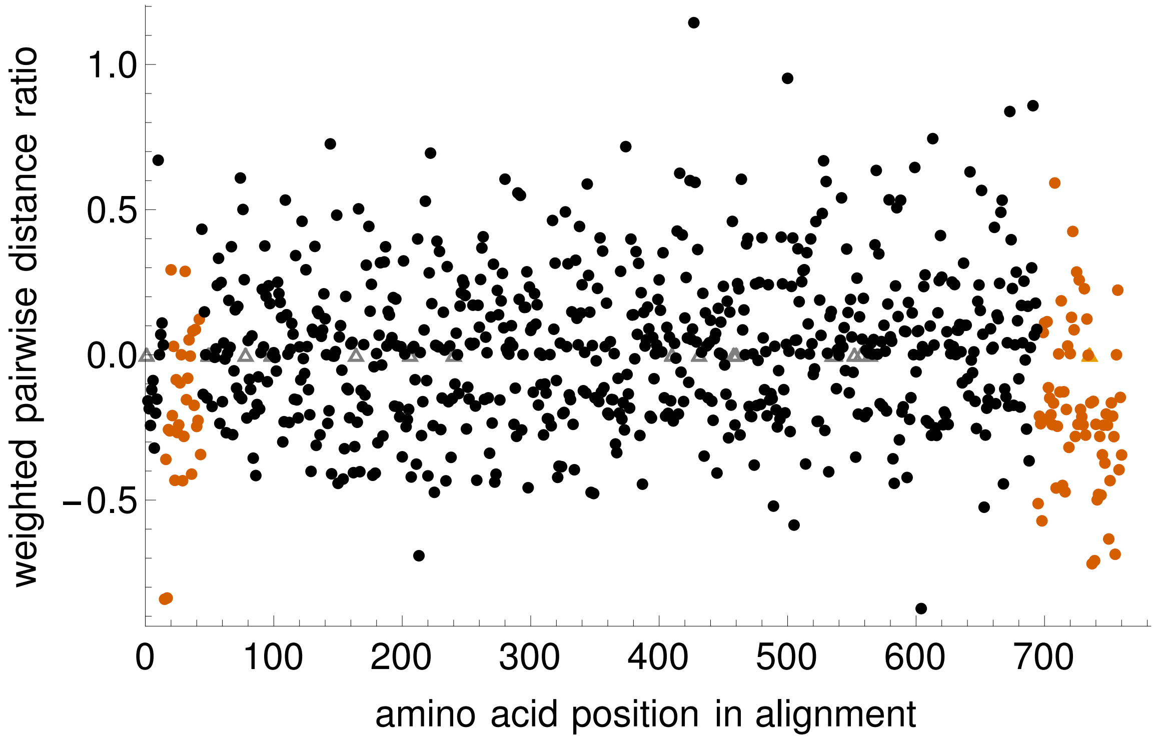

PB2

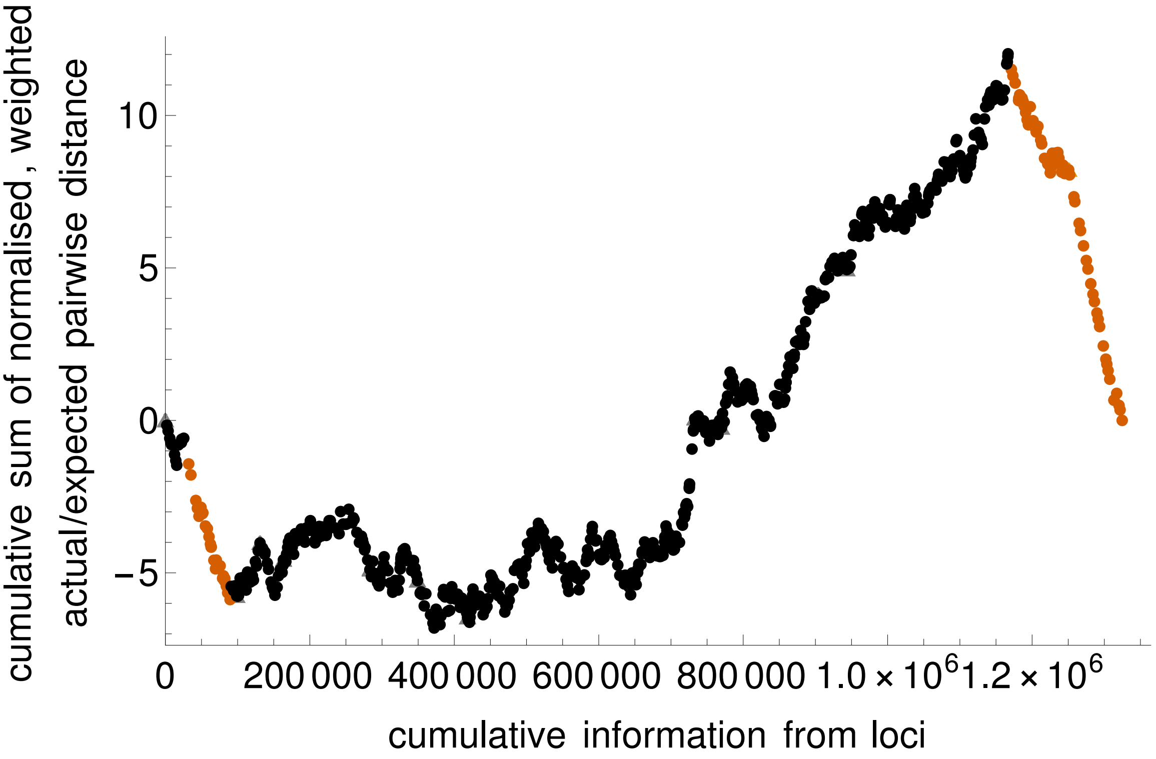

PB2

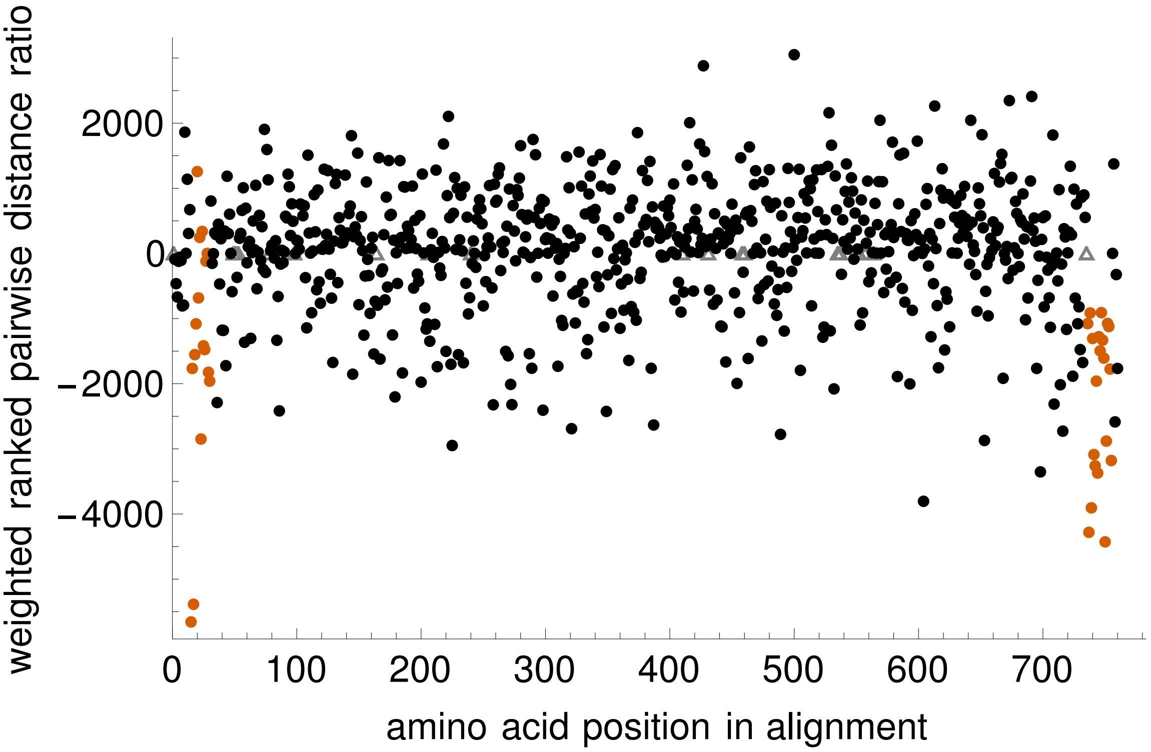
weighted ranked pairwise distance ratio
2000
0
−2000
−4000
0
100
200
300
400
500
600
700
amino acid position in alignment

PB2

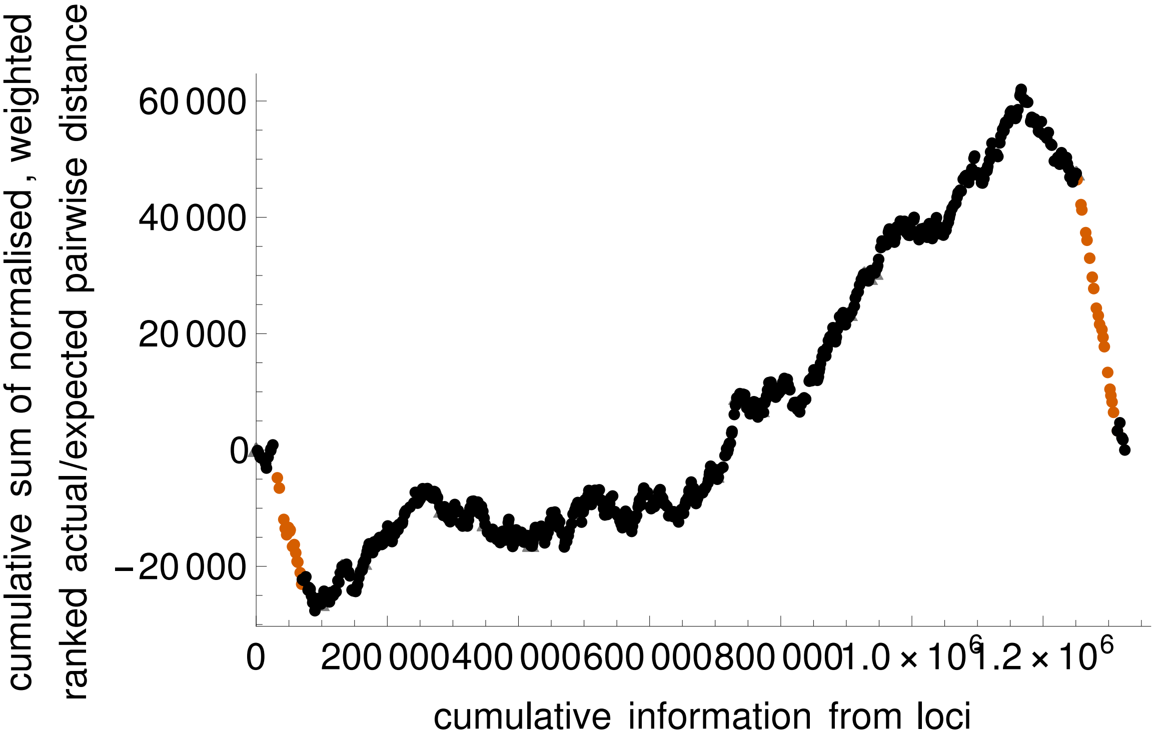
cumulative sum of normalised, weighted ranked actual/expected pairwise distance
60 000
40 000
20 000
0
−20 000
0
200 000
400 000
600 000
800 000
1.0 × 10^6
1.2 × 10^6
cumulative information from loci

PB2

PB1

Gene length histogram

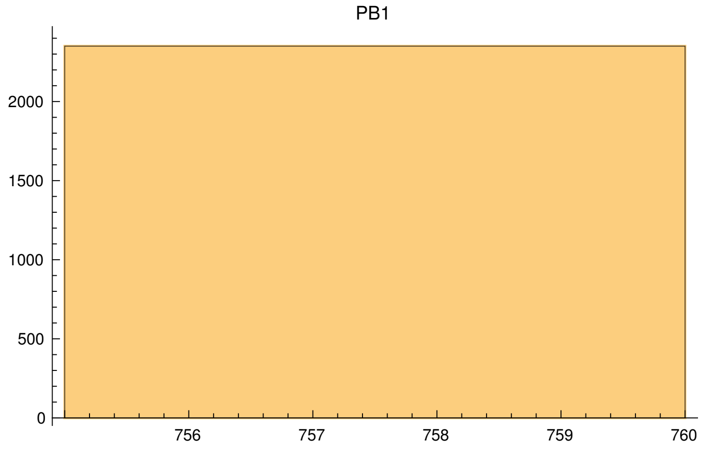

Information vs. nPD

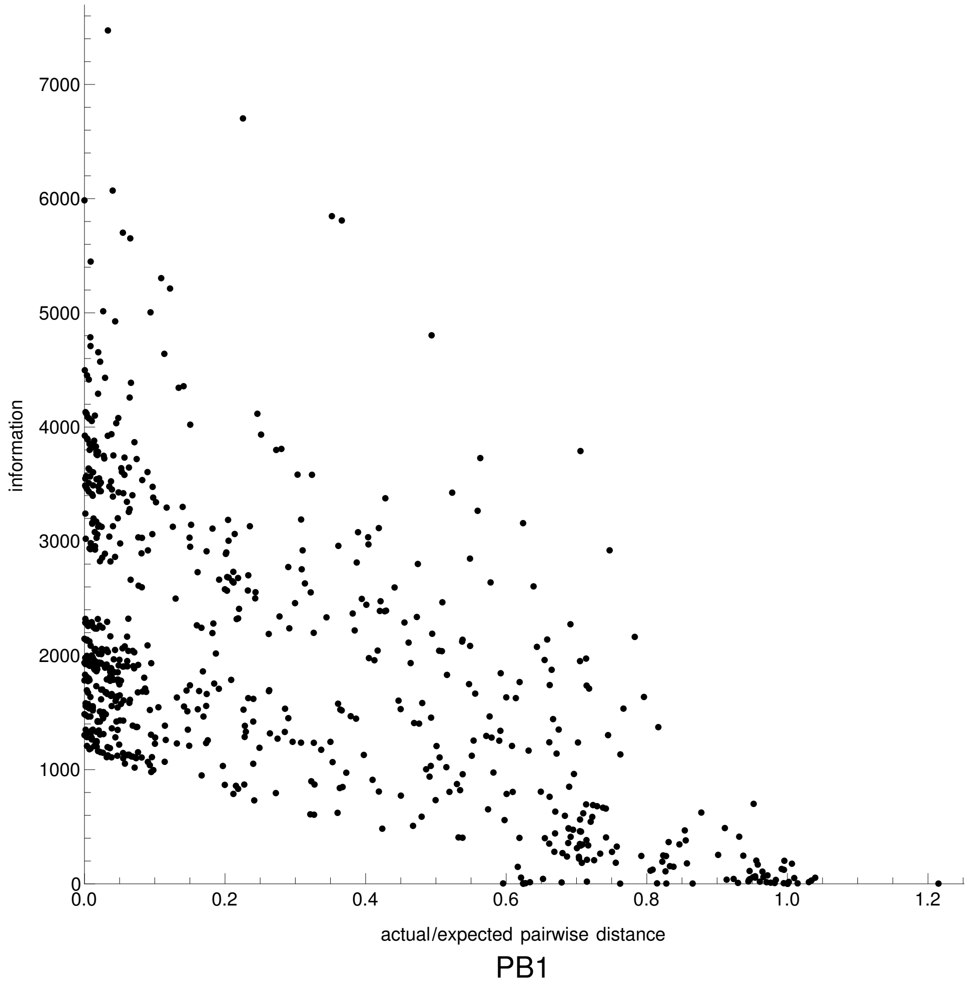

Example sequences highlighted by regions found in analyses to be conserved

Interesting points (by weighted raw PD) highlighted for gene PB1:

ATGGATGTCAATCCGACTTTACTTTTCTTAAAAGTGCCAGCGCAAAATGCTATAAGTACTACATTCCCTTACACTG⋱
GAGATCCTCCATACAGCCATGGAACAGGAACAGGATATACCATGGACACAGTAAACAGAACACATCAATACTCAG⋱
AAAAGGGAAAGTGGACAACAAACACAGAAACCGGAGCACCCCAACTCAACCCAATTGATGGACCATTACCAGAGG⋱
ACAACGAGCCAAGCGGATATGCACAAACTGATTGCGTGTTGGAAGCTATGGCTTTCCTTGAAGAATCCCACCCAG⋱
GGATATTTGAAAACTCTTGTCTTGAAGCGATGGAAGTCGTTCAGCAAACAAGAGTGGACAAACTAACCCAAGGTC⋱
GCCAGACTTATGACTGGACACTGAACAGAAACCAACCAGCTGCAACTGCTTTGGCCAACACTATAGAGGTGTTCA⋱
GATCGAATGGTCTGACAGCCAATGAATCAGGGAGACTGATAGATTTTCTCAGGGATGTGATGGAATCAATGGATA⋱
AAGAAGAAATGGAAATAACAACACATTTCCAGAGAAAAAGAAGAGTGAGGGACAACATGACCAAGAAGATGGTCA⋱
CACAAAGAACAATAGGAAAGAAGAAGCAGAGGCTGAACAAGAGGTGTTACTTAATAAGAGCACTGACATTGAACA⋱
CAATGACCAAAGATGCAGAAAGGGGCAAATTGAAAAGACGGGCAATTGCAACACCCGGGATGCAGATTAGAGGAT⋱
TCGTGTACTTTGTCGAAACACTAGCGAGGAGCATCTGTGAGAAACTCGAGCAATCTGGGCTCCCTGTTGGAGGGA⋱
ATGAGAAGAAGGCTAAATTGGCAAATGTCGTGAGAAAAATGATGACTAACTCACAAGATACAGAGCTCTCCTTTA⋱
CAATTACTGGAGACAACACCAAATGGAATGAGAATCAAAACCCTCGGATGTTTTTGGCAATGATAACATACATCA⋱
CAAGAAACCAACCTGAATGGTTTAGAAATGTCTTAAGCATTGCCCCTATAATGTTCTCAAACAAAATGGCGAGAT⋱
TAGGGAAAGGATACATGTTTGAAAGTAAGAGCATGAAGCTAAGGACACAAATACCAGCAGAGATGCTTACAAACA⋱
TTGATCTGAAATATTTCAACGAATCAACGAGAAAGAAAATCGAGAAAATAAGACCTCTGCTGATTGATGGCACGG⋱
CCTCATTGAGTCCTGGGATGATGATGGGCATGTTCAATATGCTGAGCACAGTATTAGGGGTCTCAATCCTGAATC⋱
TCGGGCAAAAGAGGTACACCAAAACCACATACTGGTGGGATGGACTTCAATCCTCTGATGATTTCGCTCTCATAG⋱
TGAATGCACCGAATCATGAGGGGATACAAGCAGGAGTGGATAGGTTCTATAGGACCTGCAAACTGGTTGGGATCA⋱
ACATGAGCAAAAAGAAGTCTTACATAAACCGAACAGGAACATTTGAGTTCACAAGTTTTTTCTACCGCTATGGAT⋱
TTGTAGCTAACTTCAGTATGGAATTACCCAGCTTTGGAGTGTCTGGAATCAATGAATCGGCTGACATGAGCATTG⋱
GAGTTACAGTGATAAAAAACAATATGATAAACAATGATCTTGGACCAGCAACAGCTCAAATGGCTCTTCAGTTAT⋱
TCATCAAAGACTATAGGTACACGTACCGATGCCACAGGGGTGATACACAAATTCAAACGAGGAGATCATTCGAGC⋱
TGAAGAAACTGTGGGAGCAGACCCGTTCAAAGGCAGGGCTGTTGGTATCAGACGGAGGACCAAACCTATACAACA⋱
TTCGGAATCTCCACATCCCAGAGGTCTGCTTGAAGTGGGAATTGATGGATGAAGACTACCAAGGCAGGCTGTGCA⋱
ATCCTCTGAATCCATTTGTCAGTCATAAAGAGATTGAGTCCGTAAACAATGCTGTAGTAATGCCAGCCCATGGCC⋱
CGGCCAAGAGCATGGAATATGATGCTGTTGCGACTACACACTCGTGGATTCCTAAGAGGAACCGTTCCATTCTCA⋱
ATACCAGCCAAAGGGGAATTCTTGAGGATGAGCAAATGTACCAAAAGTGCTGTAGTCTATTCGAGAGATTCTTCC⋱
CCAGCAGT
TCATACAGGAGGCCAGTTGGAATTTCCAGCATGGTGGAGGCCATGGTGTCTAGGG
CCCGAATTGATGCACGCATTGATTTCGAATCTGGAAGGATTAAGAAAGAAGA
GTTTGCTGAGATCATGAAGATCTGTTCCACCATTGAAGAGCTCAGACGGCAA
AAA---TAG

Interesting points (by weighted ranked PD) highlighted for gene PB1:

ATGGATGTCAATCCGACTTTACTTTTCTTAAAAGTGCCAGCGCAAAATGCTATAAGTACTACATTCCCTTACACTGGA⋱
GATCCTCCATACAGCCATGGAACAGGAACAGGATATACCATGGACACAGTAAACAGAACACATCAATACTCAGAAAA⋱
GGGAAAGTGGACAACAAACACAGAAACCGGAGCACCCCAACTCAACCCAATTGATGGACCATTACCAGAGGACAACG⋱
AGCCAAGCGGATATGCACAAACTGATTGCGTGTTGGAAGCTATGGCTTTCCTTGAAGAATCCCACCCAGGGATATTT⋱
GAAAACTCTTGTCTTGAAGCGATGGAAGTCGTTCAGCAAACAAGAGTGGACAAACTAACCCAAGGTCGCCAGACTTA⋱
TGACTGGACACTGAACAGAAACCAACCAGCTGCAACTGCTTTGGCCAACACTATAGAGGTGTTCAGATCGAATGGTC⋱
TGACAGCCAATGAATCAGGGAGACTGATAGATTTTCTCAGGGATGTGATGGAATCAATGGATAAAGAAGAAATGGAA⋱
ATAACAACACATTTCCAGAGAAAAAGAAGAGTGAGGGACAACATGACCAAGAAGATGGTCACACAAAGAACAATAGG⋱

AAAGAAGAAGCAGAGGCTGAACAAGAGGTGTTACTTAATAAGAGCACTGACATTGAACACAATGACCAAAGATGCAG⋱.
AAAGGGGCAAATTGAAAAGACGGGCAATTGCAACACCCGGGATGCAGATTAGAGGATTCGTGTACTTTGTCGAAACA⋱.
CTAGCGAGGAGCATCTGTGAGAAACTCGAGCAATCTGGGCTCCCTGTTGGAGGGAATGAGAAGAAGGCTAAATTGGC⋱.
AAATGTCGTGAGAAAAATGATGACTAACTCACAAGATACAGAGCTCTCCTTTACAATTACTGGAGACAACACCAAAT⋱.
GGAATGAGAATCAAAACCCTCGGATGTTTTTGGCAATGATAACATACATCACAAGAAACCAACCTGAATGGTTTAGA⋱.
AATGTCTTAAGCATTGCCCCTATAATGTTCTCAAACAAAATGGCGAGATTAGGGAAAGGATACATGTTTGAAAGTAA⋱.
GAGCATGAAGCTAAGGACACAAATACCAGCAGAGATGCTTACAAACATTGATCTGAAATATTTCAACGAATCAACGA⋱.
GAAAGAAAATCGAGAAAATAAGACCTCTGCTGATTGATGGCACGGCCTCATTGAGTCCTGGGATGATGATGGGCATG⋱.
TTCAATATGCTGAGCACAGTATTAGGGGTCTCAATCCTGAATCTCGGGCAAAAGAGGTACACCAAAACCACATACTG⋱.
GTGGGATGGACTTCAATCCTCTGATGATTTCGCTCTCATAGTGAATGCACCGAATCATGAGGGGATACAAGCAGGAG⋱.
TGGATAGGTTCTATAGGACCTGCAAACTGGTTGGGATCAACATGAGCAAAAAGAAGTCTTACATAAACCGAACAGGA⋱.
ACATTTGAGTTCACAAGTTTTTTCTACCGCTATGGATTTGTAGCTAACTTCAGTATGGAATTACCCAGCTTTGGAGT⋱.
GTCTGGAATCAATGAATCGGCTGACATGAGCATTGGAGTTACAGTGATAAAAAACAATATGATAAACAATGATCTTG⋱.
GACCAGCAACAGCTCAAATGGCTCTTCAGTTATTCATCAAAGACTATAGGTACACGTACCGATGCCACAGGGGTGAT⋱.
ACACAAATTCAAACGAGGAGATCATTCGAGCTGAAGAAACTGTGGGAGCAGACCCGTTCAAAGGCAGGGCTGTTGGT⋱.
ATCAGACGGAGGACCAAACCTATACAACATTCGGAATCTCCACATCCCAGAGGTCTGCTTGAAGTGGGAATTGATGG⋱.
ATGAAGACTACCAAGGCAGGCTGTGCAATCCTCTGAATCCATTTGTCAGTCATAAAGAGATTGAGTCCGTAAACAAT⋱.
GCTGTAGTAATGCCAGCCCATGGCCCGGCCAAGAGCATGGAATATGATGCTGTTGCGACTACACACTCGTGGATTCC⋱.
TAAGAGGAACCGTTCCATTCTCAATACCAGCCAAAGGGGAATTCTTGAGGATGAGCAAATGTACCAAAAGTGCTGTA⋱.
GTCTATTCGAGAGATTCTTCCCCAGCAGTTCATACAGGAGGCCAGTTGGAATTTCCAGCATGGTGGAGGCCATGGTG⋱.
TCTAGGGCCCGAATTGATGCACGCATTGATTTCGAATCTGGAAGGATTAAGAAAGAAGAGTTTGCT
GAGATCATGAAGATCTGTTCCACCATTGAAGAGCTCAGACGGCAAAAA---TAG

Per codon conservation report

PB1

Pos.	1	obs:	exp:
atg	M	2350	2350.00
mPD		0	0
		nPD:	1.
		N. weight:	0.
		Sc. PD:	0
		Sc. rank:	0

PB1

Pos.	2	obs:	exp:
cat	H	2	1.19
cac	H	0	0.81
gat	D	2343	1349.00
gac	D	3	996.50
gaa	E	1	0.60
gag	E	0	0.40
---	-	1	1.00
mPD		0.0051	0.49
		nPD:	0.01
		N. weight:	0.69
		Sc. PD:	-0.097
		Sc. rank:	-665.4

PB1

Pos.	3	obs:	exp:
att	I	0	0.37
atc	I	1	0.25
ata	I	0	0.38
gtt	V	2	510.40
gtc	V	2344	495.90
gta	V	1	461.80
gtg	V	0	878.90
gct	A	0	0.25
gcc	A	0	0.20
gca	A	1	0.44
gcg	A	0	0.11
---	-	1	1.00
mPD		0.0051	0.73
		nPD:	0.01
		N. weight:	2.
		Sc. PD:	-0.28
		Sc. rank:	-2265.9

PB1

Pos.	4	obs:	exp:
tat	Y	1	0.49
tac	Y	0	0.51
cat	H	3	2.97
cac	H	2	2.03
caa	Q	1	0.51
cag	Q	0	0.49
act	T	0	0.26
acc	T	1	0.22
aca	T	0	0.43
acg	T	0	0.08
aat	N	1821	1245.00
aac	N	521	1097.00
mPD		0.35	0.51
		nPD:	0.7
		N. weight:	0.17
		Sc. PD:	0.089
		Sc. rank:	253.5

PB1

Pos.	5	obs:	exp:
tct	S	0	0.17
tcc	S	0	0.15
tca	S	0	0.23
tcg	S	1	0.06
cct	P	0	661.80
ccc	P	0	424.70
cca	P	4	958.30
ccg	P	2343	302.20
cgt	R	0	0.08
cgc	R	0	0.11
cga	R	0	0.19
cgg	R	2	0.16
agt	S	0	0.20
agc	S	0	0.19
aga	R	0	0.92
agg	R	0	0.55
mPD		0.0060	0.71
		nPD:	0.01
		N. weight:	2.6
		Sc. PD:	-0.37
		Sc. rank:	-2764.9

PB1

Pos.	6	obs:	exp:
tct	S	0	0.17
tcc	S	0	0.15
tca	S	0	0.23
tcg	S	0	0.06
att	I	1	0.37
atc	I	0	0.25
ata	I	0	0.38
act	T	2335	621.20
acc	T	9	518.10
aca	T	0	1008.00
acg	T	1	198.10
agt	S	1	0.20
agc	S	0	0.19
gct	A	3	0.76
gcc	A	0	0.59
gca	A	0	1.32
gcg	A	0	0.33
mPD		0.013	0.69
		nPD:	0.02
		N. weight:	1.6
		Sc. PD:	-0.22
		Sc. rank:	-1062.4

PB1

Pos.	7	obs:	exp:
ttt	F	6	2.55
ttc	F	0	3.45
tta	L	2314	255.80
ttg	L	26	439.80
ctt	L	0	439.50
ctc	L	0	404.40
cta	L	4	311.90
ctg	L	0	492.50
mPD		0.030	1.1
		nPD:	0.03
		N. weight:	2.7
		Sc. PD:	-0.34
		Sc. rank:	-1123.7

PB1

Pos.	8	obs:	exp:
tta	L	0	256.30
ttg	L	0	440.60
ctt	L	1863	440.30
ctc	L	484	405.10
cta	L	0	312.40
ctg	L	1	493.30
att	I	2	0.74
atc	I	0	0.50
ata	I	0	0.76
mPD		0.33	1.1
		nPD:	0.29
		N. weight:	1.5
		Sc. PD:	0.2
		Sc. rank:	1407.0

PB1

Pos.	9	obs:	exp:
ttt	F	127	997.80
ttc	F	2218	1347.00
tta	L	5	0.55
ttg	L	0	0.94
ctt	L	0	0.94
ctc	L	0	0.86
cta	L	0	0.67
ctg	L	0	1.05
mPD		0.11	0.49
		nPD:	0.22
		N. weight:	0.46
		Sc. PD:	0.027
		Sc. rank:	353.7

PB1

Pos.	10	obs:	exp:
ttt	F	1	0.43
ttc	F	0	0.57
tta	L	1793	256.10
ttg	L	545	440.20
ctt	L	2	439.90
ctc	L	0	404.80
cta	L	5	312.10
ctg	L	1	492.90
gtt	V	0	0.65
gtc	V	0	0.63
gta	V	3	0.59
gtg	V	0	1.12
mPD		0.37	1.1
		nPD:	0.32
		N. weight:	1.9
		Sc. PD:	0.32
		Sc. rank:	1954.7

PB1

Pos.	11	obs:	exp:
cgt	R	0	0.11
cgc	R	0	0.16
cga	R	0	0.29
cgg	R	0	0.23
aaa	K	2338	1317.00
aag	K	8	1029.00
aga	R	2	1.38
agg	R	1	0.82
gaa	E	1	0.60
gag	E	0	0.40
mPD		0.011	0.50
		nPD:	0.02
		N. weight:	0.7
		Sc. PD:	-0.091
		Sc. rank:	-364.6

PB1

Pos.	12	obs:	exp:
atg	M	6	6.00
gtt	V	16	509.60
gtc	V	0	495.10
gta	V	9	461.00
gtg	V	2318	877.40
gct	A	0	0.25
gcc	A	0	0.20
gca	A	0	0.44
gcg	A	1	0.11
mPD		0.027	0.73
		nPD:	0.04
		N. weight:	1.2
		Sc. PD:	-0.13
		Sc. rank:	-284.0

PB1

Pos.	13	obs:	exp:
cct	P	1	662.70
ccc	P	4	425.20
cca	P	2333	959.50
ccg	P	12	302.60
mPD		0.014	0.70
		nPD:	0.02
		N. weight:	1.1
		Sc. PD:	-0.14
		Sc. rank:	-617.8

PB1

Pos.	14	obs:	exp:
tct	S	0	0.17
tcc	S	1	0.15
tca	S	0	0.23
tcg	S	0	0.06
act	T	0	0.53
acc	T	0	0.44
aca	T	0	0.86
acg	T	2	0.17
agt	S	0	0.20
agc	S	0	0.19
gtt	V	0	0.43
gtc	V	0	0.42
gta	V	0	0.39
gtg	V	2	0.75
gct	A	14	596.30
gcc	A	0	457.10
gca	A	74	1030.00
gcg	A	2256	260.20
ggt	G	0	0.14
ggc	G	1	0.13
gga	G	0	0.44
ggg	G	0	0.29
mPD		0.079	0.70
		nPD:	0.11
		N. weight:	2.5
		Sc. PD:	-0.1
		Sc. rank:	1153.7

PB1

Pos.	15	obs:	exp:
tta	L	0	0.11
ttg	L	0	0.19
ctt	L	0	0.19
ctc	L	0	0.17
cta	L	0	0.13
ctg	L	1	0.21
caa	Q	2344	1195.00
cag	Q	5	1154.00
mPD		0.0059	0.50
		nPD:	0.01
		N. weight:	0.84
		Sc. PD:	-0.12
		Sc. rank:	-754.5

PB1			
Pos.	16	obs:	exp:
tct	S	0	12.13
tcc	S	0	10.47
tca	S	0	16.00
tcg	S	0	4.59
tgt	C	1	0.43
tgc	C	0	0.57
act	T	0	0.26
acc	T	0	0.22
aca	T	1	0.43
acg	T	0	0.08
aat	N	2275	1210.00
aac	N	1	1066.00
aaa	K	1	0.56
aag	K	0	0.44
agt	S	71	14.50
agc	S	0	13.32
---	--	------	------
mPD		0.064	0.60
		nPD:	0.11
		N. weight:	0.83
		Sc. PD:	-0.04
		Sc. rank:	365.5

PB1			
Pos.	17	obs:	exp:
gct	A	2132	597.60
gcc	A	217	458.10
gca	A	0	1033.00
gcg	A	0	260.80
gat	D	1	0.58
gac	D	0	0.42
---	--	------	------
mPD		0.17	0.69
		nPD:	0.24
		N. weight:	1.4
		Sc. PD:	0.12
		Sc. rank:	1189.0

PB1			
Pos.	18	obs:	exp:
att	I	0	871.50
atc	I	0	588.60
ata	I	2347	886.90
gtt	V	0	0.65
gtc	V	0	0.63
gta	V	3	0.59
gtg	V	0	1.12
---	--	------	------
mPD		0.0026	0.66
		nPD:	0.
		N. weight:	1.2
		Sc. PD:	-0.18
		Sc. rank:	-1604.3

PB1			
Pos.	19	obs:	exp:
tct	S	0	401.30
tcc	S	0	346.40
tca	S	0	529.70
tcg	S	0	151.80
agt	S	2304	479.80
agc	S	46	440.90
---	--	------	------
mPD		0.038	1.6
		nPD:	0.02
		N. weight:	1.9
		Sc. PD:	-0.24
		Sc. rank:	-910.0

PB1			
Pos.	20	obs:	exp:
act	T	1679	622.00
acc	T	669	518.80
aca	T	0	1009.00
acg	T	0	198.30
gct	A	2	0.51
gcc	A	0	0.39
gca	A	0	0.88
gcg	A	0	0.22
---	--	------	------
mPD		0.41	0.69
		nPD:	0.59
		N. weight:	0.99
		Sc. PD:	0.42
		Sc. rank:	1398.3

PB1			
Pos.	21	obs:	exp:
act	T	0	622.50
acc	T	0	519.20
aca	T	2136	1010.00
acg	T	214	198.50
---	--	------	------
mPD		0.17	0.69
		nPD:	0.24
		N. weight:	0.87
		Sc. PD:	0.072
		Sc. rank:	742.7

PB1			
Pos.	22	obs:	exp:
ttt	F	32	1000.00
ttc	F	2318	1350.00
---	--	------	------
mPD		0.027	0.49
		nPD:	0.05
		N. weight:	0.62
		Sc. PD:	-0.06
		Sc. rank:	35.3

PB1			
Pos.	23	obs:	exp:
cct	P	2330	662.40
ccc	P	6	425.00
cca	P	5	959.10
ccg	P	8	302.50
gat	D	1	0.58
gac	D	0	0.42
---	--	------	------
mPD		0.018	0.71
		nPD:	0.03
		N. weight:	1.5
		Sc. PD:	-0.19
		Sc. rank:	-685.8

PB1			
Pos.	24	obs:	exp:
tat	Y	678	1142.00
tac	Y	1672	1208.00
---	--	------	------
mPD		0.41	0.50
		nPD:	0.82
		N. weight:	0.1
		Sc. PD:	0.068
		Sc. rank:	166.1

PB1			
Pos.	25	obs:	exp:
act	T	2336	622.50
acc	T	9	519.20
aca	T	5	1010.00
acg	T	0	198.50
---	--	------	------
mPD		0.012	0.69
		nPD:	0.02
		N. weight:	1.6
		Sc. PD:	-0.22
		Sc. rank:	-1106.5

PB1			
Pos.	26	obs:	exp:
ggt	G	0	338.10
ggc	G	0	310.20
gga	G	2349	1026.00
ggg	G	1	675.90
---	--	------	------
mPD		0.00085	0.69
		nPD:	0.
		N. weight:	1.
		Sc. PD:	-0.16
		Sc. rank:	-1588.1

PB1			
Pos.	27	obs:	exp:
gat	D	2229	1351.00
gac	D	120	997.80
ggt	G	1	0.14
ggc	G	0	0.13
gga	G	0	0.44
ggg	G	0	0.29
---	--	------	------
mPD		0.098	0.49
		nPD:	0.2
		N. weight:	0.47
		Sc. PD:	0.02
		Sc. rank:	317.7

PB1			
Pos.	28	obs:	exp:
cct	P	2039	662.70
ccc	P	311	425.20
cca	P	0	959.50
ccg	P	0	302.60
---	--	------	------
mPD		0.23	0.70
		nPD:	0.33
		N. weight:	1.2
		Sc. PD:	0.2
		Sc. rank:	1205.5

PB1			
Pos.	29	obs:	exp:
cct	P	0	662.70
ccc	P	0	425.20
cca	P	2315	959.50
ccg	P	35	302.60
---	--	------	------
mPD		0.029	0.70
		nPD:	0.04
		N. weight:	1.1
		Sc. PD:	-0.12
		Sc. rank:	-140.1

PB1			
Pos.	30	obs:	exp:
tat	Y	1	1142.00
tac	Y	2349	1208.00
---	--	------	------
mPD		0.00085	0.50
		nPD:	0.
		N. weight:	0.84
		Sc. PD:	-0.12
		Sc. rank:	-1231.0

PB1			
Pos.	31	obs:	exp:
tct	S	0	401.30
tcc	S	0	346.40
tca	S	0	529.70
tcg	S	0	151.80
agt	S	1	479.80
agc	S	2349	440.90
---	--	------	------
mPD		0.00085	1.6
		nPD:	0.
		N. weight:	2.1
		Sc. PD:	-0.32
		Sc. rank:	-3292.3

PB1			
Pos.	32	obs:	exp:
cat	H	2348	1397.00
cac	H	2	952.70
---	--	------	------
mPD		0.0017	0.48
		nPD:	0.
		N. weight:	0.65
		Sc. PD:	-0.096
		Sc. rank:	-888.6

PB1			
Pos.	33	obs:	exp:
ggt	G	0	338.10
ggc	G	0	310.20
gga	G	2204	1026.00
ggg	G	146	675.90
---	--	------	------
mPD		0.12	0.69
		nPD:	0.17
		N. weight:	0.79
		Sc. PD:	0.011
		Sc. rank:	489.7

PB1			
Pos.	34	obs:	exp:
act	T	0	622.50
acc	T	0	519.20
aca	T	2311	1010.00
acg	T	39	198.50
---	--	------	------
mPD		0.033	0.69
		nPD:	0.05
		N. weight:	1.
		Sc. PD:	-0.1
		Sc. rank:	-57.7

PB1			
Pos.	35	obs:	exp:
ggt	G	0	338.10
ggc	G	0	310.20
gga	G	2320	1026.00
ggg	G	30	675.90
---	--	------	------
mPD		0.025	0.69
		nPD:	0.04
		N. weight:	0.97
		Sc. PD:	-0.11
		Sc. rank:	-249.1

PB1			
Pos.	36	obs:	exp:
act	T	0	622.50
acc	T	0	519.20
aca	T	2349	1010.00
acg	T	1	198.50
---	--	------	------
mPD		0.00085	0.69
		nPD:	0.
		N. weight:	1.1
		Sc. PD:	-0.16
		Sc. rank:	-1629.0

PB1			
Pos.	37	obs:	exp:
ggt	G	16	338.10
ggc	G	0	310.20
gga	G	1374	1026.00
ggg	G	960	675.90
---	--	------	------
mPD		0.49	0.69
		nPD:	0.71
		N. weight:	0.38
		Sc. PD:	0.2
		Sc. rank:	575.8

PB1			
Pos.	38	obs:	exp:
tat	Y	1395	1142.00
tac	Y	954	1207.00
cat	H	1	0.59
cac	H	0	0.41
---	--	------	------
mPD		0.48	0.50
		nPD:	0.97
		N. weight:	0.032
		Sc. PD:	0.025
		Sc. rank:	51.3

PB1			
Pos.	39	obs:	exp:
cct	P	0	0.28
ccc	P	1	0.18
cca	P	0	0.41
ccg	P	0	0.13
act	T	2	622.00
acc	T	2340	518.80
aca	T	6	1009.00
acg	T	0	198.30
gct	A	0	0.25
gcc	A	1	0.20
gca	A	0	0.44
gcg	A	0	0.11
---	--	------	------
mPD		0.0085	0.69
		nPD:	0.01
		N. weight:	1.9
		Sc. PD:	-0.26
		Sc. rank:	-1635.1

PB1			
Pos.	40	obs:	exp:
tta	L	0	0.11
ttg	L	0	0.19
ctt	L	0	0.19
ctc	L	0	0.17
cta	L	0	0.13
ctg	L	1	0.21
att	I	0	2.60
atc	I	0	1.76
ata	I	7	2.65
atg	M	2342	2342.00
---	--	------	------
mPD		0.0068	0.0073
		nPD:	0.93
		N. weight:	0.0045
		Sc. PD:	0.0034
		Sc. rank:	7.2

PB1			
Pos.	41	obs:	exp:
gat	D	21	1351.00
gac	D	2328	997.80
ggt	G	0	0.14
ggc	G	1	0.13
gga	G	0	0.44
ggg	G	0	0.29
mPD		0.019	0.49
		nPD:	0.04
		N. weight:	1.
		Sc. PD:	-0.12
		Sc. rank:	-220.2

PB1			
Pos.	42	obs:	exp:
act	T	0	622.50
acc	T	1	519.20
aca	T	2340	1010.00
acg	T	9	198.50
mPD		0.0085	0.69
		nPD:	0.01
		N. weight:	1.
		Sc. PD:	-0.14
		Sc. rank:	-902.4

PB1			
Pos.	43	obs:	exp:
tta	L	1	0.11
ttg	L	0	0.19
ctt	L	0	0.19
ctc	L	0	0.17
cta	L	0	0.13
ctg	L	0	0.21
att	I	0	0.37
atc	I	1	0.25
ata	I	0	0.38
gtt	V	9	510.60
gtc	V	909	496.10
gta	V	1430	462.00
gtg	V	0	879.30
mPD		0.48	0.73
		nPD:	0.66
		N. weight:	1.2
		Sc. PD:	0.56
		Sc. rank:	1683.7

PB1			
Pos.	44	obs:	exp:
tct	S	0	0.34
tcc	S	0	0.29
tca	S	0	0.45
tcg	S	0	0.13
aat	N	1	1248.00
aac	N	2347	1100.00
agt	S	0	0.41
agc	S	2	0.38
mPD		0.0026	0.50
		nPD:	0.01
		N. weight:	0.96
		Sc. PD:	-0.14
		Sc. rank:	-1208.8

PB1			
Pos.	45	obs:	exp:
cgt	R	0	89.12
cgc	R	0	125.20
cga	R	0	227.60
cgg	R	0	184.10
aga	R	2342	1078.00
agg	R	8	646.20
mPD		0.0068	0.95
		nPD:	0.01
		N. weight:	0.96
		Sc. PD:	-0.14
		Sc. rank:	-1098.1

PB1			
Pos.	46	obs:	exp:
act	T	0	622.50
acc	T	0	519.20
aca	T	1858	1010.00
acg	T	492	198.50
mPD		0.33	0.69
		nPD:	0.48
		N. weight:	0.85
		Sc. PD:	0.27
		Sc. rank:	1071.5

PB1			
Pos.	47	obs:	exp:
cat	H	2328	1397.00
cac	H	22	952.70
mPD		0.019	0.48
		nPD:	0.04
		N. weight:	0.6
		Sc. PD:	-0.068
		Sc. rank:	-122.3

PB1			
Pos.	48	obs:	exp:
caa	Q	2343	1196.00
cag	Q	7	1154.00
mPD		0.0059	0.50
		nPD:	0.01
		N. weight:	0.83
		Sc. PD:	-0.12
		Sc. rank:	-745.4

PB1			
Pos.	49	obs:	exp:
tat	Y	27	1142.00
tac	Y	2323	1208.00
mPD		0.023	0.50
		nPD:	0.05
		N. weight:	0.77
		Sc. PD:	-0.081
		Sc. rank:	-60.5

PB1			
Pos.	50	obs:	exp:
tct	S	1	401.30
tcc	S	1	346.40
tca	S	2344	529.70
tcg	S	4	151.80
agt	S	0	479.80
agc	S	0	440.90
mPD		0.0051	1.6
		nPD:	0.
		N. weight:	1.9
		Sc. PD:	-0.28
		Sc. rank:	-2626.6

PB1			
Pos.	51	obs:	exp:
aaa	K	2	1.12
aag	K	0	0.88
gaa	E	2079	1405.00
gag	E	268	941.70
ggt	G	0	0.14
ggc	G	0	0.13
gga	G	1	0.44
ggg	G	0	0.29
mPD		0.20	0.48
		nPD:	0.42
		N. weight:	0.26
		Sc. PD:	0.068
		Sc. rank:	308.1

PB1			
Pos.	52	obs:	exp:
cgt	R	0	0.15
cgc	R	0	0.21
cga	R	0	0.39
cgg	R	0	0.31
act	T	0	0.26
acc	T	0	0.22
aca	T	0	0.43
acg	T	1	0.08
aaa	K	93	1316.00
aag	K	2252	1029.00
aga	R	0	1.83
agg	R	4	1.10
mPD		0.080	0.50
		nPD:	0.16
		N. weight:	0.82
		Sc. PD:	0.0051
		Sc. rank:	495.5

PB1			
Pos.	53	obs:	exp:
tct	S	0	0.17
tcc	S	0	0.15
tca	S	1	0.23
tcg	S	0	0.06
tgt	C	0	0.43
tgc	C	1	0.57
cgt	R	0	0.08
cgc	R	0	0.11
cga	R	0	0.19
cgg	R	0	0.16
agt	S	0	0.20
agc	S	0	0.19
aga	R	2	0.92
agg	R	0	0.55
ggt	G	0	337.50
ggc	G	1	309.70
gga	G	2283	1024.00
ggg	G	62	674.80
mPD		0.057	0.69
		nPD:	0.08
		N. weight:	0.91
		Sc. PD:	-0.064
		Sc. rank:	302.5

PB1			
Pos.	54	obs:	exp:
caa	Q	0	0.51
cag	Q	1	0.49
cgt	R	0	0.68
cgc	R	0	0.96
cga	R	0	1.74
cgg	R	0	1.41
aaa	K	193	1308.00
aag	K	2137	1022.00
aga	R	0	8.26
agg	R	18	4.95
gaa	E	0	0.60
gag	E	1	0.40
mPD		0.17	0.51
		nPD:	0.33
		N. weight:	0.67
		Sc. PD:	0.11
		Sc. rank:	679.2

PB1			
Pos.	55	obs:	exp:
tgg	W	2349	2349.00
cgt	R	0	0.04
cgc	R	0	0.05
cga	R	1	0.10
cgg	R	0	0.08
aga	R	0	0.46
agg	R	0	0.27
mPD		0.0017	0.0014
		nPD:	1.21
		N. weight:	0.0013
		Sc. PD:	0.0013
		Sc. rank:	2.0

PB1			
Pos.	56	obs:	exp:
att	I	0	0.74
atc	I	0	0.50
ata	I	2	0.76
act	T	0	620.40
acc	T	0	517.50
aca	T	2327	1006.00
acg	T	15	197.80
aaa	K	1	0.56
aag	K	0	0.44
gct	A	1	1.27
gcc	A	0	0.98
gca	A	2	2.20
gcg	A	2	0.56
mPD		0.022	0.70
		nPD:	0.03
		N. weight:	1.
		Sc. PD:	-0.12
		Sc. rank:	-354.2

PB1			
Pos.	57	obs:	exp:
att	I	0	1.11
atc	I	0	0.75
ata	I	3	1.13
act	T	0	611.60
acc	T	1	510.20
aca	T	2307	992.20
acg	T	1	195.00
aaa	K	37	20.77
aag	K	0	16.23
gct	A	0	0.25
gcc	A	0	0.20
gca	A	1	0.44
gcg	A	0	0.11
mPD		0.036	0.72
		nPD:	0.05
		N. weight:	1.1
		Sc. PD:	-0.11
		Sc. rank:	-5.9

PB1			
Pos.	58	obs:	exp:
tct	S	0	0.85
tcc	S	0	0.74
tca	S	0	1.13
tcg	S	0	0.32
att	I	0	0.37
atc	I	1	0.25
ata	I	0	0.38
aat	N	33	1246.00
aac	N	2311	1098.00
agt	S	0	1.02
agc	S	5	0.94
mPD		0.033	0.51
		nPD:	0.06
		N. weight:	0.87
		Sc. PD:	-0.076
		Sc. rank:	158.0

PB1			
Pos.	59	obs:	exp:
tct	S	0	2.05
tcc	S	0	1.77
tca	S	12	2.70
tcg	S	0	0.78
cct	P	0	0.28
ccc	P	0	0.18
cca	P	1	0.41
ccg	P	0	0.13
act	T	103	618.80
acc	T	421	516.10
aca	T	1811	1004.00
acg	T	1	197.30
aaa	K	1	0.56
aag	K	0	0.44
agt	S	0	2.45
agc	S	0	2.25
mPD		0.37	0.70
		nPD:	0.53
		N. weight:	0.44
		Sc. PD:	0.16
		Sc. rank:	585.6

PB1			
Pos.	60	obs:	exp:
gat	D	2	1.15
gac	D	0	0.85
gaa	E	1454	1406.00
gag	E	894	942.10
mPD		0.47	0.48
		nPD:	0.98
		N. weight:	0.0039
		Sc. PD:	0.0031
		Sc. rank:	6.3

PB1

Pos.	61	obs:	exp:
tct	S	0	0.17
tcc	S	0	0.15
tca	S	0	0.23
tcg	S	1	0.06
act	T	568	622.20
acc	T	1777	519.00
aca	T	4	1009.00
acg	T	0	198.40
agt	S	0	0.20
agc	S	0	0.19
---	--	------	------
mPD		0.37	0.69
		nPD:	0.54
		N. weight:	1.1
		Sc. PD:	0.42
		Sc. rank:	1519.9

,

PB1

Pos.	62	obs:	exp:
cgt	R	0	0.38
cgc	R	0	0.53
cga	R	1	0.97
cgg	R	0	0.78
aga	R	9	4.59
agg	R	0	2.75
gct	A	0	0.25
gcc	A	0	0.20
gca	A	1	0.44
gcg	A	0	0.11
ggt	G	2	336.50
ggc	G	0	308.70
gga	G	2101	1021.00
ggg	G	236	672.70
---	--	------	------
mPD		0.19	0.70
		nPD:	0.27
		N. weight:	0.69
		Sc. PD:	0.08
		Sc. rank:	626.3

,

PB1

Pos.	63	obs:	exp:
gct	A	0	597.80
gcc	A	0	458.30
gca	A	2149	1033.00
gcg	A	201	260.90
---	--	------	------
mPD		0.16	0.69
		nPD:	0.23
		N. weight:	0.82
		Sc. PD:	0.057
		Sc. rank:	663.7

,

PB1

Pos.	64	obs:	exp:
tta	L	0	1.09
ttg	L	0	1.88
tct	S	1	0.34
tcc	S	1	0.29
tca	S	0	0.45
tcg	S	0	0.13
ctt	L	0	1.88
ctc	L	10	1.73
cta	L	0	1.33
ctg	L	0	2.10
cct	P	10	656.80
ccc	P	2318	421.40
cca	P	1	950.90
ccg	P	0	299.90
cat	H	0	4.16
cac	H	7	2.84
cgt	R	0	0.04
cgc	R	1	0.05
cga	R	0	0.10
cgg	R	0	0.08
act	T	0	0.26
acc	T	1	0.22
aca	T	0	0.43
acg	T	0	0.08
agt	S	0	0.41
agc	S	0	0.38
aga	R	0	0.46
agg	R	0	0.27
---	--	------	------
mPD		0.028	0.73
		nPD:	0.04
		N. weight:	2.1
		Sc. PD:	-0.24
		Sc. rank:	-439.9

,

PB1

Pos.	65	obs:	exp:
tta	L	0	0.22
ttg	L	0	0.38
ctt	L	0	0.38
ctc	L	0	0.35
cta	L	2	0.27
ctg	L	0	0.42
caa	Q	2317	1195.00
cag	Q	31	1153.00
---	--	------	------
mPD		0.028	0.50
		nPD:	0.06
		N. weight:	0.77
		Sc. PD:	-0.075
		Sc. rank:	48.4

,

PB1

Pos.	66	obs:	exp:
tta	L	0	256.20
ttg	L	0	440.40
ctt	L	40	440.10
ctc	L	2307	404.90
cta	L	0	312.30
ctg	L	0	493.10
gtt	V	0	0.65
gtc	V	1	0.63
gta	V	0	0.59
gtg	V	2	1.12
---	--	------	------
mPD		0.038	1.1
		nPD:	0.03
		N. weight:	2.1
		Sc. PD:	-0.25
		Sc. rank:	-665.8

,

PB1

Pos.	67	obs:	exp:
tct	S	0	0.34
tcc	S	0	0.29
tca	S	0	0.45
tcg	S	0	0.13
aat	N	54	1248.00
aac	N	2294	1100.00
agt	S	0	0.41
agc	S	2	0.38
---	--	------	------
mPD		0.047	0.50
		nPD:	0.09
		N. weight:	0.82
		Sc. PD:	-0.049
		Sc. rank:	306.1

,

PB1

Pos.	68	obs:	exp:
cct	P	1	662.40
ccc	P	0	425.00
cca	P	2339	959.10
ccg	P	9	302.50
act	T	1	0.26
acc	T	0	0.22
aca	T	0	0.43
acg	T	0	0.08
---	--	------	------
mPD		0.010	0.71
		nPD:	0.01
		N. weight:	1.1
		Sc. PD:	-0.15
		Sc. rank:	-895.6

,

PB1

Pos.	69	obs:	exp:
att	I	2335	867.80
atc	I	0	586.10
ata	I	2	883.10
act	T	1	0.26
acc	T	0	0.22
aca	T	0	0.43
acg	T	0	0.08
aat	N	1	0.53
aac	N	0	0.47
gtt	V	11	2.39
gtc	V	0	2.32
gta	V	0	2.16
gtg	V	0	4.12
---	--	------	------
mPD		0.013	0.67
		nPD:	0.02
		N. weight:	1.3
		Sc. PD:	-0.17
		Sc. rank:	-785.0

,

PB1

Pos.	70	obs:	exp:
gat	D	2347	1351.00
gac	D	2	997.80
ggt	G	1	0.14
ggc	G	0	0.13
gga	G	0	0.44
ggg	G	0	0.29
---	--	------	------
mPD		0.0026	0.49
		nPD:	0.01
		N. weight:	0.69
		Sc. PD:	-0.1
		Sc. rank:	-863.1

,

PB1

Pos.	71	obs:	exp:
ggt	G	0	338.10
ggc	G	1	310.20
gga	G	2225	1026.00
ggg	G	124	675.90
---	--	------	------
mPD		0.10	0.69
		nPD:	0.15
		N. weight:	0.81
		Sc. PD:	-0.0067
		Sc. rank:	445.2

,

PB1

Pos.	72	obs:	exp:
tta	L	0	0.11
ttg	L	0	0.19
tct	S	0	5.81
tcc	S	0	5.01
tca	S	33	7.66
tcg	S	1	2.20
ctt	L	0	0.19
ctc	L	0	0.17
cta	L	1	0.13
ctg	L	0	0.21
cct	P	0	652.80
ccc	P	1	418.90
cca	P	2303	945.20
ccg	P	11	298.10
agt	S	0	6.94
agc	S	0	6.38
---	--	------	------
mPD		0.040	0.75
		nPD:	0.05
		N. weight:	1.1
		Sc. PD:	-0.11
		Sc. rank:	41.3

,

PB1

Pos.	73	obs:	exp:
ttt	F	1	0.43
ttc	F	0	0.57
tta	L	1946	256.30
ttg	L	222	440.60
ctt	L	0	440.30
ctc	L	0	405.10
cta	L	135	312.40
ctg	L	45	493.30
att	I	0	0.37
atc	I	0	0.25
ata	I	1	0.38
---	--	------	------
mPD		0.34	1.1
		nPD:	0.3
		N. weight:	1.9
		Sc. PD:	0.28
		Sc. rank:	1865.7

,

PB1

Pos.	74	obs:	exp:
cct	P	673	662.40
ccc	P	11	425.00
cca	P	1642	959.10
ccg	P	23	302.50
cgt	R	0	0.04
cgc	R	0	0.05
cga	R	1	0.10
cgg	R	0	0.08
aga	R	0	0.46
agg	R	0	0.27
---	--	------	------
mPD		0.43	0.71
		nPD:	0.61
		N. weight:	0.43
		Sc. PD:	0.19
		Sc. rank:	615.3

,

PB1

Pos.	75	obs:	exp:
aaa	K	0	1.68
aag	K	3	1.32
gat	D	100	57.52
gac	D	0	42.48
gaa	E	15	1344.00
gag	E	2229	900.40
ggt	G	0	0.43
ggc	G	0	0.40
gga	G	0	1.31
ggg	G	3	0.86
---	--	------	------
mPD		0.099	0.53
		nPD:	0.19
		N. weight:	1.1
		Sc. PD:	0.034
		Sc. rank:	723.6

,

PB1

Pos.	76	obs:	exp:
tat	Y	0	0.97
tac	Y	2	1.03
aat	N	0	1.06
aac	N	2	0.94
gat	D	522	1348.00
gac	D	1821	995.30
ggt	G	0	0.43
ggc	G	3	0.40
gga	G	0	1.31
ggg	G	0	0.86
mPD		0.35	0.50
		nPD:	0.71
		N. weight:	0.33
		Sc. PD:	0.18
		Sc. rank:	509.2

PB1

Pos.	77	obs:	exp:
tct	S	0	0.17
tcc	S	0	0.15
tca	S	0	0.23
tcg	S	0	0.06
aat	N	1136	1248.00
aac	N	1212	1100.00
aaa	K	1	0.56
aag	K	0	0.44
agt	S	0	0.20
agc	S	1	0.19
mPD		0.50	0.50
		nPD:	1.
		N. weight:	0.0092
		Sc. PD:	0.0075
		Sc. rank:	14.7

PB1

Pos.	78	obs:	exp:
gct	A	0	0.25
gcc	A	0	0.20
gca	A	0	0.44
gcg	A	1	0.11
gat	D	29	16.68
gac	D	0	12.32
gaa	E	147	1389.00
gag	E	2173	930.90
mPD		0.14	0.49
		nPD:	0.29
		N. weight:	0.83
		Sc. PD:	0.1
		Sc. rank:	772.5

PB1

Pos.	79	obs:	exp:
cct	P	0	662.70
ccc	P	0	425.20
cca	P	2337	959.50
ccg	P	13	302.60
mPD		0.011	0.70
		nPD:	0.02
		N. weight:	1.1
		Sc. PD:	-0.15
		Sc. rank:	-829.9

PB1

Pos.	80	obs:	exp:
tct	S	0	400.60
tcc	S	0	345.80
tca	S	0	528.80
tcg	S	0	151.50
cgt	R	0	0.08
cgc	R	0	0.11
cga	R	0	0.19
cgg	R	0	0.16
att	I	0	0.37
atc	I	1	0.25
ata	I	0	0.38
act	T	0	0.26
acc	T	1	0.22
aca	T	0	0.43
acg	T	0	0.08
agt	S	19	479.00
agc	S	2327	440.20
aga	R	0	0.92
agg	R	2	0.55
mPD		0.019	1.6
		nPD:	0.01
		N. weight:	2.1
		Sc. PD:	-0.29
		Sc. rank:	-1861.5

PB1

Pos.	81	obs:	exp:
gtt	V	0	0.22
gtc	V	0	0.21
gta	V	1	0.20
gtg	V	0	0.37
ggt	G	1	337.90
ggc	G	0	310.00
gga	G	2337	1025.00
ggg	G	11	675.60
mPD		0.011	0.69
		nPD:	0.02
		N. weight:	1.
		Sc. PD:	-0.14
		Sc. rank:	-759.3

PB1

Pos.	82	obs:	exp:
tat	Y	2336	1142.00
tac	Y	13	1207.00
gat	D	1	0.58
gac	D	0	0.42
mPD		0.012	0.50
		nPD:	0.02
		N. weight:	0.87
		Sc. PD:	-0.11
		Sc. rank:	-411.4

PB1

Pos.	83	obs:	exp:
gct	A	0	597.80
gcc	A	0	458.30
gca	A	2307	1033.00
gcg	A	43	260.90
mPD		0.036	0.69
		nPD:	0.05
		N. weight:	0.96
		Sc. PD:	-0.096
		Sc. rank:	11.8

PB1

Pos.	84	obs:	exp:
cat	H	1	0.59
cac	H	0	0.41
caa	Q	2347	1195.00
cag	Q	2	1154.00
mPD		0.0026	0.50
		nPD:	0.01
		N. weight:	0.85
		Sc. PD:	-0.12
		Sc. rank:	-1068.0

PB1

Pos.	85	obs:	exp:
act	T	1392	622.20
acc	T	2	519.00
aca	T	374	1009.00
acg	T	581	198.40
gat	D	1	0.58
gac	D	0	0.42
mPD		0.56	0.69
		nPD:	0.82
		N. weight:	0.74
		Sc. PD:	0.48
		Sc. rank:	1173.2

PB1

Pos.	86	obs:	exp:
gat	D	2311	1352.00
gac	D	39	998.20
mPD		0.033	0.49
		nPD:	0.07
		N. weight:	0.6
		Sc. PD:	-0.051
		Sc. rank:	128.3

PB1

Pos.	87	obs:	exp:
tgt	C	579	1022.00
tgc	C	1771	1328.00
mPD		0.37	0.49
		nPD:	0.76
		N. weight:	0.1
		Sc. PD:	0.058
		Sc. rank:	155.9

PB1

Pos.	88	obs:	exp:
gtt	V	1	510.90
gtc	V	15	496.30
gta	V	42	462.20
gtg	V	2291	879.60
gat	D	1	0.58
gac	D	0	0.42
mPD		0.050	0.73
		nPD:	0.07
		N. weight:	1.1
		Sc. PD:	-0.092
		Sc. rank:	251.1

PB1

Pos.	89	obs:	exp:
tta	L	42	256.50
ttg	L	2291	441.00
ctt	L	12	440.70
ctc	L	0	405.40
cta	L	0	312.70
ctg	L	5	493.80
mPD		0.059	1.1
		nPD:	0.05
		N. weight:	2.
		Sc. PD:	-0.2
		Sc. rank:	47.5

PB1

Pos.	90	obs:	exp:
gat	D	0	1.15
gac	D	2	0.85
gaa	E	2345	1406.00
gag	E	3	942.10
mPD		0.0043	0.48
		nPD:	0.01
		N. weight:	0.64
		Sc. PD:	-0.091
		Sc. rank:	-649.5

PB1

Pos.	91	obs:	exp:
tct	S	0	0.17
tcc	S	0	0.15
tca	S	1	0.23
tcg	S	0	0.06
agt	S	0	0.20
agc	S	0	0.19
gct	A	20	597.60
gcc	A	5	458.10
gca	A	2320	1033.00
gcg	A	4	260.80
mPD		0.025	0.69
		nPD:	0.04
		N. weight:	0.96
		Sc. PD:	-0.11
		Sc. rank:	-253.8

PB1

Pos.	92	obs:	exp:
att	I	0	1.49
atc	I	1	1.00
ata	I	3	1.51
atg	M	2345	2345.00
act	T	0	0.26
acc	T	1	0.22
aca	T	0	0.43
acg	T	0	0.08
mPD		0.0051	0.0050
		nPD:	1.01
		N. weight:	0.0024
		Sc. PD:	0.002
		Sc. rank:	3.8

PB1

Pos.	93	obs:	exp:
tct	S	1	0.17
tcc	S	0	0.15
tca	S	0	0.23
tcg	S	0	0.06
agt	S	0	0.20
agc	S	0	0.19
gct	A	2333	596.80
gcc	A	13	457.50
gca	A	0	1031.00
gcg	A	0	260.40
gat	D	3	1.73
gac	D	0	1.27
mPD		0.014	0.70
		nPD:	0.02
		N. weight:	1.7
		Sc. PD:	-0.22
		Sc. rank:	-951.2

PB1

Pos.	94	obs:	exp:
ttt	F	93	997.00
ttc	F	2250	1346.00
tta	L	1	0.33
ttg	L	0	0.56
tct	S	0	0.51
tcc	S	3	0.44
tca	S	0	0.68
tcg	S	0	0.19
ctt	L	0	0.56
ctc	L	1	0.52
cta	L	0	0.40
ctg	L	1	0.63
agt	S	0	0.61
agc	S	0	0.56
gtt	V	0	0.22
gtc	V	1	0.21
gta	V	0	0.20
gtg	V	0	0.37
mPD		0.083	0.50
		nPD:	0.17
		N. weight:	0.51
		Sc. PD:	0.0059
		Sc. rank:	313.5

PB1

Pos.	95	obs:	exp:
tta	L	0	256.20
ttg	L	0	440.40
tct	S	0	0.17
tcc	S	0	0.15
tca	S	0	0.23
tcg	S	0	0.06
ctt	L	2336	440.10
ctc	L	3	404.90
cta	L	0	312.30
ctg	L	8	493.10
cgt	R	1	0.08
cgc	R	0	0.11
cga	R	0	0.19
cgg	R	0	0.16
agt	S	1	0.20
agc	S	0	0.19
aga	R	0	0.92
agg	R	1	0.55
mPD		0.014	1.1
		nPD:	0.01
		N. weight:	2.1
		Sc. PD:	-0.29
		Sc. rank:	-1781.6

PB1			
Pos.	96	obs:	exp:
aat	N	1	0.53
aac	N	0	0.47
gat	D	0	0.58
gac	D	1	0.42
gaa	E	2336	1405.00
gag	E	10	941.30
ggt	G	0	0.29
ggc	G	0	0.26
gga	G	2	0.87
ggg	G	0	0.58
---	--	------	------
mPD		0.013	0.48
		nPD:	0.03
		N. weight:	0.62
		Sc. PD:	-0.077
		Sc. rank:	-266.4

,

PB1			
Pos.	97	obs:	exp:
aaa	K	3	3.37
aag	K	3	2.63
gat	D	0	0.58
gac	D	1	0.42
gaa	E	1583	1402.00
gag	E	759	939.70
ggt	G	0	0.14
ggc	G	0	0.13
gga	G	1	0.44
ggg	G	0	0.29
---	--	------	------
mPD		0.44	0.49
		nPD:	0.91
		N. weight:	0.02
		Sc. PD:	0.015
		Sc. rank:	31.6

,

PB1			
Pos.	98	obs:	exp:
ttt	F	0	0.43
ttc	F	1	0.57
tct	S	2	401.20
tcc	S	2343	346.30
tca	S	4	529.50
tcg	S	0	151.70
agt	S	0	479.60
agc	S	0	440.70
---	--	------	------
mPD		0.0059	1.6
		nPD:	0.
		N. weight:	2.4
		Sc. PD:	-0.35
		Sc. rank:	-3199.7

,

PB1			
Pos.	99	obs:	exp:
cat	H	5	1397.00
cac	H	2345	952.70
---	--	------	------
mPD		0.0042	0.48
		nPD:	0.01
		N. weight:	1.1
		Sc. PD:	-0.16
		Sc. rank:	-1145.6

,

PB1			
Pos.	100	obs:	exp:
tct	S	0	0.51
tcc	S	0	0.44
tca	S	3	0.68
tcg	S	0	0.19
cct	P	1	661.50
ccc	P	0	424.50
cca	P	2337	957.90
ccg	P	8	302.10
caa	Q	1	0.51
cag	Q	0	0.49
agt	S	0	0.61
agc	S	0	0.56
---	--	------	------
mPD		0.011	0.71
		nPD:	0.02
		N. weight:	1.1
		Sc. PD:	-0.15
		Sc. rank:	-849.4

,

PB1			
Pos.	101	obs:	exp:
gaa	E	0	1.20
gag	E	2	0.80
ggt	G	2	337.80
ggc	G	0	309.90
gga	G	14	1025.00
ggg	G	2332	675.30
---	--	------	------
mPD		0.015	0.69
		nPD:	0.02
		N. weight:	1.5
		Sc. PD:	-0.2
		Sc. rank:	-796.9

,

PB1			
Pos.	102	obs:	exp:
att	I	0	871.50
atc	I	1146	588.60
ata	I	1201	886.90
atg	M	2	2.00
gtt	V	0	0.22
gtc	V	0	0.21
gta	V	1	0.20
gtg	V	0	0.37
---	--	------	------
mPD		0.50	0.66
		nPD:	0.76
		N. weight:	0.61
		Sc. PD:	0.36
		Sc. rank:	959.0

,

PB1			
Pos.	103	obs:	exp:
ttt	F	2345	998.30
ttc	F	1	1348.00
tat	Y	1	0.49
tac	Y	0	0.51
tgt	C	1	0.43
tgc	C	0	0.57
gtt	V	1	0.43
gtc	V	0	0.42
gta	V	0	0.39
gtg	V	1	0.75
---	--	------	------
mPD		0.0051	0.49
		nPD:	0.01
		N. weight:	1.1
		Sc. PD:	-0.15
		Sc. rank:	-1044.0

,

PB1			
Pos.	104	obs:	exp:
tat	Y	1	0.49
tac	Y	0	0.51
gat	D	1	0.58
gac	D	0	0.42
gaa	E	2296	1406.00
gag	E	52	942.10
---	--	------	------
mPD		0.046	0.48
		nPD:	0.09
		N. weight:	0.53
		Sc. PD:	-0.031
		Sc. rank:	205.8

,

PB1			
Pos.	105	obs:	exp:
tct	S	0	0.17
tcc	S	0	0.15
tca	S	0	0.23
tcg	S	0	0.06
att	I	0	0.74
atc	I	2	0.50
ata	I	0	0.76
act	T	0	0.53
acc	T	2	0.44
aca	T	0	0.86
acg	T	0	0.17
aat	N	24	1246.00
aac	N	2320	1098.00
agt	S	0	0.20
agc	S	1	0.19
gat	D	0	0.58
gac	D	1	0.42
---	--	------	------
mPD		0.025	0.50
		nPD:	0.05
		N. weight:	0.89
		Sc. PD:	-0.091
		Sc. rank:	-1.6

,

PB1			
Pos.	106	obs:	exp:
tct	S	1804	401.30
tcc	S	3	346.40
tca	S	532	529.70
tcg	S	11	151.80
agt	S	0	479.80
agc	S	0	440.90
---	--	------	------
mPD		0.36	1.6
		nPD:	0.22
		N. weight:	1.4
		Sc. PD:	0.089
		Sc. rank:	1119.9

,

PB1			
Pos.	107	obs:	exp:
ttt	F	1	0.43
ttc	F	0	0.57
tgt	C	2345	1021.00
tgc	C	4	1328.00
---	--	------	------
mPD		0.0043	0.49
		nPD:	0.01
		N. weight:	1.
		Sc. PD:	-0.15
		Sc. rank:	-1071.7

,

PB1			
Pos.	108	obs:	exp:
ttt	F	4	1.70
ttc	F	0	2.30
tta	L	0	248.50
ttg	L	0	427.30
ctt	L	2246	427.00
ctc	L	30	392.90
cta	L	1	303.00
ctg	L	0	478.40
cgt	R	1	0.04
cgc	R	0	0.05
cga	R	0	0.10
cgg	R	0	0.08
att	I	67	25.25
atc	I	1	17.05
ata	I	0	25.70
aga	R	0	0.46
agg	R	0	0.27
---	--	------	------
mPD		0.087	1.2
		nPD:	0.07
		N. weight:	2.
		Sc. PD:	-0.16
		Sc. rank:	546.5

,

PB1			
Pos.	109	obs:	exp:
gaa	E	2346	1407.00
gag	E	4	942.90
---	--	------	------
mPD		0.0034	0.48
		nPD:	0.01
		N. weight:	0.64
		Sc. PD:	-0.091
		Sc. rank:	-728.3

,

PB1			
Pos.	110	obs:	exp:
act	T	1	284.00
acc	T	0	236.90
aca	T	492	460.60
acg	T	579	90.55
gct	A	1	325.10
gcc	A	0	249.20
gca	A	18	561.80
gcg	A	1259	141.90
---	--	------	------
mPD		0.84	1.2
		nPD:	0.71
		N. weight:	2.
		Sc. PD:	1.1
		Sc. rank:	3114.2

,

PB1			
Pos.	111	obs:	exp:
tta	L	0	0.11
ttg	L	1	0.19
ctt	L	0	0.19
ctc	L	0	0.17
cta	L	0	0.13
ctg	L	0	0.21
cgt	R	0	0.04
cgc	R	0	0.05
cga	R	0	0.10
cgg	R	0	0.08
att	I	0	0.37
atc	I	0	0.25
ata	I	1	0.38
atg	M	2346	2346.00
aga	R	1	0.46
agg	R	0	0.27
gtt	V	0	0.22
gtc	V	0	0.21
gta	V	0	0.20
gtg	V	1	0.37
---	--	------	------
mPD		0.0043	0.0052
		nPD:	0.81
		N. weight:	0.0024
		Sc. PD:	0.0015
		Sc. rank:	3.8

,

PB1			
Pos.	112	obs:	exp:
gat	D	1	0.58
gac	D	0	0.42
gaa	E	2148	1406.00
gag	E	200	942.10
ggt	G	0	0.14
ggc	G	0	0.13
gga	G	1	0.44
ggg	G	0	0.29
---	--	------	------
mPD		0.16	0.48
		nPD:	0.33
		N. weight:	0.33
		Sc. PD:	0.054
		Sc. rank:	333.4

,

PB1			
Pos.	113	obs:	exp:
att	I	533	541.40
atc	I	925	365.70
ata	I	0	550.90
act	T	0	1.32
acc	T	5	1.10
aca	T	0	2.15
acg	T	0	0.42
gtt	V	34	192.90
gtc	V	823	187.40
gta	V	30	174.50
gtg	V	0	332.20
---	--	------	------
mPD		0.86	1.2
		nPD:	0.71
		N. weight:	1.1
		Sc. PD:	0.58
		Sc. rank:	1630.5

,

PB1			
Pos.	114	obs:	exp:
att	I	14	5.20
atc	I	0	3.51
ata	I	0	5.29
gtt	V	2319	508.00
gtc	V	17	493.60
gta	V	0	459.60
gtg	V	0	874.80
---	--	------	------
mPD		0.026	0.74
		nPD:	0.04
		N. weight:	1.9
		Sc. PD:	-0.22
		Sc. rank:	-530.1

,

PB1			
Pos.	115	obs:	exp:
caa	Q	499	1196.00
cag	Q	1851	1154.00
---	--	------	------
mPD		0.33	0.50
		nPD:	0.67
		N. weight:	0.24
		Sc. PD:	0.12
		Sc. rank:	352.2

,

PB1			
Pos.	116	obs:	exp:
caa	Q	2332	1196.00
cag	Q	18	1154.00
---	--	------	------
mPD		0.015	0.50
		nPD:	0.03
		N. weight:	0.8
		Sc. PD:	-0.097
		Sc. rank:	-284.2

,

PB1			
Pos.	117	obs:	exp:
tct	S	0	0.34
tcc	S	0	0.29
tca	S	2	0.45
tcg	S	0	0.13
act	T	2	622.00
acc	T	0	518.80
aca	T	2341	1009.00
acg	T	5	198.30
agt	S	0	0.41
agc	S	0	0.38
---	--	------	------
mPD		0.0076	0.69
		nPD:	0.01
		N. weight:	1.
		Sc. PD:	-0.15
		Sc. rank:	-965.4

,

PB1			
Pos.	118	obs:	exp:
cgt	R	0	89.08
cgc	R	0	125.10
cga	R	0	227.50
cgg	R	0	184.00
aaa	K	1	0.56
aag	K	0	0.44
aga	R	2346	1077.00
agg	R	3	645.90
---	--	------	------
mPD		0.0034	0.95
		nPD:	0.
		N. weight:	0.98
		Sc. PD:	-0.14
		Sc. rank:	-1319.2

,

PB1			
Pos.	119	obs:	exp:
gtt	V	0	510.90
gtc	V	0	496.30
gta	V	14	462.20
gtg	V	2335	879.60
gct	A	0	0.25
gcc	A	0	0.20
gca	A	0	0.44
gcg	A	1	0.11
---	--	------	------
mPD		0.013	0.73
		nPD:	0.02
		N. weight:	1.2
		Sc. PD:	-0.16
		Sc. rank:	-803.2

,

PB1			
Pos.	120	obs:	exp:
cat	H	0	0.59
cac	H	1	0.41
gat	D	419	1351.00
gac	D	1930	997.80
---	--	------	------
mPD		0.29	0.49
		nPD:	0.6
		N. weight:	0.42
		Sc. PD:	0.18
		Sc. rank:	599.9

,

PB1			
Pos.	121	obs:	exp:
cgt	R	0	0.19
cgc	R	0	0.27
cga	R	0	0.48
cgg	R	0	0.39
aaa	K	2337	1316.00
aag	K	8	1029.00
aga	R	5	2.29
agg	R	0	1.37
---	--	------	------
mPD		0.011	0.50
		nPD:	0.02
		N. weight:	0.71
		Sc. PD:	-0.091
		Sc. rank:	-367.0

,

PB1			
Pos.	122	obs:	exp:
tta	L	49	255.70
ttg	L	14	439.60
ctt	L	0	439.30
ctc	L	0	404.20
cta	L	1763	311.70
ctg	L	517	492.30
atg	M	7	7.00
---	--	------	------
mPD		0.41	1.1
		nPD:	0.36
		N. weight:	1.6
		Sc. PD:	0.32
		Sc. rank:	1694.2

,

PB1			
Pos.	123	obs:	exp:
cat	H	0	0.59
cac	H	1	0.41
att	I	0	0.37
atc	I	1	0.25
ata	I	0	0.38
act	T	170	621.20
acc	T	2174	518.10
aca	T	1	1008.00
acg	T	0	198.10
gct	A	0	0.76
gcc	A	3	0.59
gca	A	0	1.32
gcg	A	0	0.33
---	--	------	------
mPD		0.14	0.69
		nPD:	0.2
		N. weight:	1.6
		Sc. PD:	0.071
		Sc. rank:	1093.5

,

PB1			
Pos.	124	obs:	exp:
caa	Q	2320	1196.00
cag	Q	30	1154.00
---	--	------	------
mPD		0.025	0.50
		nPD:	0.05
		N. weight:	0.77
		Sc. PD:	-0.078
		Sc. rank:	1.1

,

PB1			
Pos.	125	obs:	exp:
tct	S	0	0.17
tcc	S	0	0.15
tca	S	0	0.23
tcg	S	0	0.06
agt	S	1	0.20
agc	S	0	0.19
ggt	G	2326	337.90
ggc	G	23	310.00
gga	G	0	1025.00
ggg	G	0	675.60
---	--	------	------
mPD		0.020	0.69
		nPD:	0.03
		N. weight:	2.4
		Sc. PD:	-0.29
		Sc. rank:	-875.6

,

PB1			
Pos.	126	obs:	exp:
cat	H	0	0.59
cac	H	1	0.41
cgt	R	500	89.08
cgc	R	1849	125.10
cga	R	0	227.50
cgg	R	0	184.00
aga	R	0	1077.00
agg	R	0	645.90
---	--	------	------
mPD		0.34	0.95
		nPD:	0.35
		N. weight:	3.2
		Sc. PD:	0.6
		Sc. rank:	3302.2

,

PB1			
Pos.	127	obs:	exp:
caa	Q	5	1196.00
cag	Q	2345	1154.00
---	--	------	------
mPD		0.0042	0.50
		nPD:	0.01
		N. weight:	0.88
		Sc. PD:	-0.13
		Sc. rank:	-936.2

,

PB1			
Pos.	128	obs:	exp:
act	T	1631	622.50
acc	T	712	519.20
aca	T	7	1010.00
acg	T	0	198.50
---	--	------	------
mPD		0.43	0.69
		nPD:	0.62
		N. weight:	0.95
		Sc. PD:	0.43
		Sc. rank:	1359.2

,

PB1			
Pos.	129	obs:	exp:
tat	Y	2347	1142.00
tac	Y	3	1208.00
---	--	------	------
mPD		0.0026	0.50
		nPD:	0.01
		N. weight:	0.9
		Sc. PD:	-0.13
		Sc. rank:	-1132.8

,

PB1			
Pos.	130	obs:	exp:
gat	D	104	1352.00
gac	D	2246	998.20
---	--	------	------
mPD		0.085	0.49
		nPD:	0.17
		N. weight:	0.84
		Sc. PD:	0.015
		Sc. rank:	526.0

,

PB1

Pos.	131	obs:	exp:
tgg	W	2350	2350.00
---	--	------	------
mPD		0	0
		nPD:	1.
		N. weight:	0.
		Sc. PD:	0
		Sc. rank:	0

,

PB1

Pos.	132	obs:	exp:
act	T	0	622.50
acc	T	0	519.20
aca	T	2326	1010.00
acg	T	24	198.50
---	--	------	------
mPD		0.020	0.69
		nPD:	0.03
		N. weight:	1.
		Sc. PD:	-0.12
		Sc. rank:	-369.5

,

PB1

Pos.	133	obs:	exp:
tta	L	13	256.50
ttg	L	578	441.00
ctt	L	0	440.70
ctc	L	0	405.40
cta	L	4	312.70
ctg	L	1755	493.80
---	--	------	------
mPD		0.39	1.1
		nPD:	0.34
		N. weight:	1.3
		Sc. PD:	0.23
		Sc. rank:	1297.6

,

PB1

Pos.	134	obs:	exp:
aat	N	933	1230.00
aac	N	1381	1084.00
gat	D	1	20.71
gac	D	35	15.29
---	--	------	------
mPD		0.51	0.53
		nPD:	0.96
		N. weight:	0.058
		Sc. PD:	0.046
		Sc. rank:	92.7

,

PB1

Pos.	135	obs:	exp:
cgt	R	0	89.12
cgc	R	0	125.20
cga	R	0	227.60
cgg	R	0	184.10
aga	R	2214	1078.00
agg	R	136	646.20
---	--	------	------
mPD		0.11	0.95
		nPD:	0.11
		N. weight:	0.75
		Sc. PD:	-0.029
		Sc. rank:	348.4

,

PB1

Pos.	136	obs:	exp:
aat	N	26	1249.00
aac	N	2324	1101.00
---	--	------	------
mPD		0.022	0.50
		nPD:	0.04
		N. weight:	0.88
		Sc. PD:	-0.095
		Sc. rank:	-89.8

,

PB1

Pos.	137	obs:	exp:
caa	Q	1814	1196.00
cag	Q	536	1154.00
---	--	------	------
mPD		0.35	0.50
		nPD:	0.7
		N. weight:	0.19
		Sc. PD:	0.1
		Sc. rank:	283.6

,

PB1

Pos.	138	obs:	exp:
tct	S	0	0.34
tcc	S	0	0.29
tca	S	0	0.45
tcg	S	2	0.13
cct	P	1	662.10
ccc	P	1	424.90
cca	P	1687	958.70
ccg	P	659	302.30
agt	S	0	0.41
agc	S	0	0.38
---	--	------	------
mPD		0.41	0.71
		nPD:	0.58
		N. weight:	0.79
		Sc. PD:	0.32
		Sc. rank:	1094.6

,

PB1

Pos.	139	obs:	exp:
gct	A	2308	597.80
gcc	A	1	458.30
gca	A	40	1033.00
gcg	A	1	260.90
---	--	------	------
mPD		0.035	0.69
		nPD:	0.05
		N. weight:	1.6
		Sc. PD:	-0.16
		Sc. rank:	13.3

,

PB1

Pos.	140	obs:	exp:
gct	A	1	597.80
gcc	A	4	458.30
gca	A	2310	1033.00
gcg	A	35	260.90
---	--	------	------
mPD		0.034	0.69
		nPD:	0.05
		N. weight:	0.95
		Sc. PD:	-0.099
		Sc. rank:	-31.5

,

PB1

Pos.	141	obs:	exp:
cct	P	0	0.28
ccc	P	1	0.18
cca	P	0	0.41
ccg	P	0	0.13
act	T	1527	622.00
acc	T	819	518.80
aca	T	2	1009.00
acg	T	0	198.30
aat	N	1	0.53
aac	N	0	0.47
---	--	------	------
mPD		0.46	0.69
		nPD:	0.66
		N. weight:	0.94
		Sc. PD:	0.46
		Sc. rank:	1378.7

,

PB1

Pos.	142	obs:	exp:
tct	S	819	140.60
tcc	S	4	121.30
tca	S	0	185.50
tcg	S	0	53.16
cct	P	1	0.28
ccc	P	0	0.18
cca	P	0	0.41
ccg	P	0	0.13
agt	S	0	168.00
agc	S	0	154.40
gct	A	1361	388.20
gcc	A	146	297.60
gca	A	18	670.80
gcg	A	1	169.40
---	--	------	------
mPD		0.59	1.5
		nPD:	0.4
		N. weight:	1.6
		Sc. PD:	0.39
		Sc. rank:	1836.8

,

PB1

Pos.	143	obs:	exp:
tta	L	526	256.50
ttg	L	1668	441.00
ctt	L	0	440.70
ctc	L	0	405.40
cta	L	12	312.70
ctg	L	144	493.80
---	--	------	------
mPD		0.48	1.1
		nPD:	0.42
		N. weight:	1.3
		Sc. PD:	0.33
		Sc. rank:	1515.5

,

PB1

Pos.	144	obs:	exp:
tct	S	0	0.17
tcc	S	1	0.15
tca	S	0	0.23
tcg	S	0	0.06
agt	S	0	0.20
agc	S	0	0.19
gct	A	14	597.60
gcc	A	2335	458.10
gca	A	0	1033.00
gcg	A	0	260.80
---	--	------	------
mPD		0.013	0.69
		nPD:	0.02
		N. weight:	2.
		Sc. PD:	-0.27
		Sc. rank:	-1317.7

,

PB1

Pos.	145	obs:	exp:
aat	N	243	1249.00
aac	N	2107	1101.00
---	--	------	------
mPD		0.19	0.50
		nPD:	0.37
		N. weight:	0.52
		Sc. PD:	0.11
		Sc. rank:	570.8

,

PB1

Pos.	146	obs:	exp:
att	I	1	0.37
atc	I	0	0.25
ata	I	0	0.38
act	T	2338	620.60
acc	T	3	517.70
aca	T	2	1007.00
acg	T	0	197.90
aat	N	6	3.19
aac	N	0	2.81
---	--	------	------
mPD		0.010	0.70
		nPD:	0.01
		N. weight:	1.7
		Sc. PD:	-0.23
		Sc. rank:	-1333.3

,

PB1

Pos.	147	obs:	exp:
att	I	0	871.50
atc	I	0	588.60
ata	I	2347	886.90
gtt	V	0	0.65
gtc	V	0	0.63
gta	V	3	0.59
gtg	V	0	1.12
---	--	------	------
mPD		0.0026	0.66
		nPD:	0.
		N. weight:	1.2
		Sc. PD:	-0.18
		Sc. rank:	-1604.3

,

PB1

Pos.	148	obs:	exp:
gaa	E	677	1407.00
gag	E	1673	942.90
---	--	------	------
mPD		0.41	0.48
		nPD:	0.85
		N. weight:	0.25
		Sc. PD:	0.17
		Sc. rank:	401.5

,

PB1

Pos.	149	obs:	exp:
att	I	0	0.37
atc	I	1	0.25
ata	I	0	0.38
atg	M	1	1.00
gtt	V	0	510.60
gtc	V	932	496.10
gta	V	5	462.00
gtg	V	1411	879.30
---	--	------	------
mPD		0.48	0.73
		nPD:	0.66
		N. weight:	0.67
		Sc. PD:	0.33
		Sc. rank:	978.0

,

PB1

Pos.	150	obs:	exp:
ttt	F	2	994.00
ttc	F	2334	1342.00
tta	L	14	1.53
ttg	L	0	2.63
ctt	L	0	2.63
ctc	L	0	2.42
cta	L	0	1.86
ctg	L	0	2.94
---	--	------	------
mPD		0.014	0.50
		nPD:	0.03
		N. weight:	0.71
		Sc. PD:	-0.088
		Sc. rank:	-291.8

,

PB1

Pos.	151	obs:	exp:
cgt	R	0	89.12
cgc	R	0	125.20
cga	R	0	227.60
cgg	R	0	184.10
aga	R	2343	1078.00
agg	R	7	646.20
---	--	------	------
mPD		0.0059	0.95
		nPD:	0.01
		N. weight:	0.96
		Sc. PD:	-0.14
		Sc. rank:	-1146.1

,

PB1

Pos.	152	obs:	exp:
tta	L	0	2.51
ttg	L	23	4.32
tct	S	2	397.40
tcc	S	0	343.00
tca	S	55	524.50
tcg	S	2270	150.30
ctt	L	0	4.31
ctc	L	0	3.97
cta	L	0	3.06
ctg	L	0	4.83
agt	S	0	475.10
agc	S	0	436.60
---	--	------	------
mPD		0.067	1.7
		nPD:	0.04
		N. weight:	3.3
		Sc. PD:	-0.36
		Sc. rank:	-537.2

,

PB1

Pos.	153	obs:	exp:
aat	N	1457	1249.00
aac	N	893	1101.00
---	--	------	------
mPD		0.47	0.50
		nPD:	0.95
		N. weight:	0.022
		Sc. PD:	0.017
		Sc. rank:	35.8

,

PB1

Pos.	154	obs:	exp:
tct	S	0	1.54
tcc	S	0	1.33
tca	S	0	2.03
tcg	S	0	0.58
tgt	C	0	4.35
tgc	C	10	5.65
agt	S	9	1.84
agc	S	0	1.69
gat	D	1	0.58
gac	D	0	0.42
ggt	G	2304	335.20
ggc	G	22	307.50
gga	G	0	1017.00
ggg	G	4	670.20
---	--	------	------
mPD		0.047	0.71
		nPD:	0.07
		N. weight:	2.4
		Sc. PD:	-0.2
		Sc. rank:	498.6

,

PB1

Pos.	155	obs:	exp:
tta	L	1	255.80
ttg	L	2	439.80
ctt	L	2	439.50
ctc	L	0	404.40
cta	L	767	311.90
ctg	L	1572	492.50
atg	M	6	6.00
---	--	------	------
mPD		0.45	1.1
		nPD:	0.39
		N. weight:	1.3
		Sc. PD:	0.31
		Sc. rank:	1512.5

,

PB1			
Pos.	156	obs:	exp:
att	I	0	0.37
atc	I	0	0.25
ata	I	1	0.38
act	T	11	615.10
acc	T	0	513.00
aca	T	2310	997.80
acg	T	1	196.10
aaa	K	6	3.37
aag	K	0	2.63
gct	A	0	5.34
gcc	A	0	4.10
gca	A	20	9.23
gcg	A	1	2.33
---	--	------	------
mPD		0.035	0.71
		nPD:	0.05
		N. weight:	1.
		Sc. PD:	-0.11
		Sc. rank:	-21.5

,

PB1			
Pos.	157	obs:	exp:
act	T	0	1.06
acc	T	4	0.88
aca	T	0	1.72
acg	T	0	0.34
gct	A	63	596.30
gcc	A	2281	457.10
gca	A	0	1030.00
gcg	A	0	260.20
gat	D	0	1.15
gac	D	2	0.85
---	--	------	------
mPD		0.057	0.70
		nPD:	0.08
		N. weight:	1.9
		Sc. PD:	-0.13
		Sc. rank:	627.9

,

PB1			
Pos.	158	obs:	exp:
tct	S	0	0.34
tcc	S	0	0.29
tca	S	0	0.45
tcg	S	0	0.13
aat	N	2228	1248.00
aac	N	120	1100.00
agt	S	2	0.41
agc	S	0	0.38
---	--	------	------
mPD		0.099	0.50
		nPD:	0.2
		N. weight:	0.56
		Sc. PD:	0.023
		Sc. rank:	377.2

,

PB1			
Pos.	159	obs:	exp:
gat	D	35	21.28
gac	D	2	15.72
gaa	E	1718	1385.00
gag	E	595	928.10
---	--	------	------
mPD		0.40	0.50
		nPD:	0.81
		N. weight:	0.067
		Sc. PD:	0.043
		Sc. rank:	106.0

,

PB1			
Pos.	160	obs:	exp:
tct	S	3	401.30
tcc	S	0	346.40
tca	S	1945	529.70
tcg	S	402	151.80
agt	S	0	479.80
agc	S	0	440.90
---	--	------	------
mPD		0.29	1.6
		nPD:	0.17
		N. weight:	1.6
		Sc. PD:	0.029
		Sc. rank:	993.6

,

PB1			
Pos.	161	obs:	exp:
ggt	G	0	338.10
ggc	G	1	310.20
gga	G	158	1026.00
ggg	G	2191	675.90
---	--	------	------
mPD		0.13	0.69
		nPD:	0.18
		N. weight:	1.2
		Sc. PD:	0.034
		Sc. rank:	807.4

,

PB1			
Pos.	162	obs:	exp:
cgt	R	0	89.08
cgc	R	0	125.10
cga	R	1	227.50
cgg	R	0	184.00
aga	R	2308	1077.00
agg	R	40	645.90
gaa	E	1	0.60
gag	E	0	0.40
---	--	------	------
mPD		0.036	0.95
		nPD:	0.04
		N. weight:	0.89
		Sc. PD:	-0.1
		Sc. rank:	-206.6

,

PB1			
Pos.	163	obs:	exp:
tta	L	2	256.50
ttg	L	36	441.00
ctt	L	0	440.70
ctc	L	0	405.40
cta	L	314	312.70
ctg	L	1998	493.80
---	--	------	------
mPD		0.26	1.1
		nPD:	0.23
		N. weight:	1.5
		Sc. PD:	0.11
		Sc. rank:	1214.9

,

PB1			
Pos.	164	obs:	exp:
att	I	0	872.30
atc	I	0	589.10
ata	I	2349	887.60
gtt	V	0	0.22
gtc	V	0	0.21
gta	V	1	0.20
gtg	V	0	0.37
---	--	------	------
mPD		0.00085	0.66
		nPD:	0.
		N. weight:	1.2
		Sc. PD:	-0.18
		Sc. rank:	-1865.8

,

PB1			
Pos.	165	obs:	exp:
gat	D	2307	1352.00
gac	D	43	998.20
---	--	------	------
mPD		0.036	0.49
		nPD:	0.07
		N. weight:	0.59
		Sc. PD:	-0.047
		Sc. rank:	156.7

,

PB1			
Pos.	166	obs:	exp:
ttt	F	1516	997.40
ttc	F	828	1347.00
tta	L	0	0.65
ttg	L	0	1.13
ctt	L	0	1.13
ctc	L	6	1.04
cta	L	0	0.80
ctg	L	0	1.26
---	--	------	------
mPD		0.46	0.49
		nPD:	0.94
		N. weight:	0.13
		Sc. PD:	0.1
		Sc. rank:	212.4

,

PB1			
Pos.	167	obs:	exp:
tta	L	0	256.50
ttg	L	0	441.00
ctt	L	5	440.70
ctc	L	2344	405.40
cta	L	1	312.70
ctg	L	0	493.80
---	--	------	------
mPD		0.0051	1.1
		nPD:	0.
		N. weight:	2.2
		Sc. PD:	-0.32
		Sc. rank:	-2821.3

,

PB1			
Pos.	168	obs:	exp:
cgt	R	0	50.10
cgc	R	0	70.36
cga	R	0	127.90
cgg	R	0	103.50
act	T	0	0.26
acc	T	0	0.22
aca	T	0	0.43
acg	T	1	0.08
aaa	K	494	577.00
aag	K	534	451.00
aga	R	63	605.90
agg	R	1258	363.20
---	--	------	------
mPD		0.85	1.3
		nPD:	0.67
		N. weight:	0.78
		Sc. PD:	0.39
		Sc. rank:	1148.5

,

PB1			
Pos.	169	obs:	exp:
gtt	V	1	0.22
gtc	V	0	0.21
gta	V	0	0.20
gtg	V	0	0.37
gat	D	2349	1351.00
gac	D	0	997.80
---	--	------	------
mPD		0.00085	0.49
		nPD:	0.
		N. weight:	0.7
		Sc. PD:	-0.1
		Sc. rank:	-1019.7

,

PB1			
Pos.	170	obs:	exp:
gtt	V	3	510.90
gtc	V	0	496.30
gta	V	7	462.20
gtg	V	2339	879.60
gct	A	0	0.25
gcc	A	0	0.20
gca	A	0	0.44
gcg	A	1	0.11
---	--	------	------
mPD		0.0093	0.73
		nPD:	0.01
		N. weight:	1.2
		Sc. PD:	-0.17
		Sc. rank:	-1031.4

,

PB1			
Pos.	171	obs:	exp:
att	I	4	1.86
atc	I	0	1.25
ata	I	1	1.89
atg	M	2301	2301.00
gtt	V	0	9.57
gtc	V	0	9.30
gta	V	1	8.66
gtg	V	43	16.48
---	--	------	------
mPD		0.042	0.064
		nPD:	0.65
		N. weight:	0.023
		Sc. PD:	0.011
		Sc. rank:	33.9

,

PB1			
Pos.	172	obs:	exp:
gct	A	0	0.51
gcc	A	0	0.39
gca	A	2	0.88
gcg	A	0	0.22
gat	D	0	83.41
gac	D	145	61.59
gaa	E	1648	1312.00
gag	E	544	879.50
ggt	G	0	1.58
ggc	G	0	1.45
gga	G	8	4.80
ggg	G	3	3.16
---	--	------	------
mPD		0.46	0.55
		nPD:	0.82
		N. weight:	0.13
		Sc. PD:	0.087
		Sc. rank:	212.8

,

PB1			
Pos.	173	obs:	exp:
tct	S	0	401.30
tcc	S	0	346.40
tca	S	2348	529.70
tcg	S	2	151.80
agt	S	0	479.80
agc	S	0	440.90
---	--	------	------
mPD		0.0017	1.6
		nPD:	0.
		N. weight:	1.9
		Sc. PD:	-0.28
		Sc. rank:	-2903.9

,

PB1			
Pos.	174	obs:	exp:
tta	L	0	0.22
ttg	L	1	0.38
ctt	L	0	0.38
ctc	L	0	0.35
cta	L	0	0.27
ctg	L	1	0.42
att	I	1	0.37
atc	I	0	0.25
ata	I	0	0.38
atg	M	2336	2336.00
gtt	V	0	2.39
gtc	V	0	2.32
gta	V	0	2.16
gtg	V	11	4.12
---	--	------	------
mPD		0.012	0.019
		nPD:	0.63
		N. weight:	0.0077
		Sc. PD:	0.0036
		Sc. rank:	11.1

,

PB1			
Pos.	175	obs:	exp:
aat	N	144	76.52
aac	N	0	67.48
gat	D	1546	1264.00
gac	D	652	933.70
gaa	E	0	4.79
gag	E	8	3.21
---	--	------	------
mPD		0.52	0.61
		nPD:	0.86
		N. weight:	0.097
		Sc. PD:	0.066
		Sc. rank:	153.8

,

PB1			
Pos.	176	obs:	exp:
cgt	R	0	0.30
cgc	R	0	0.43
cga	R	0	0.77
cgg	R	0	0.63
aat	N	0	0.53
aac	N	1	0.47
aaa	K	2339	1314.00
aag	K	2	1027.00
aga	R	7	3.67
agg	R	1	2.20
---	--	------	------
mPD		0.010	0.50
		nPD:	0.02
		N. weight:	0.72
		Sc. PD:	-0.095
		Sc. rank:	-415.3

,

PB1			
Pos.	177	obs:	exp:
aaa	K	1	0.56
aag	K	0	0.44
gat	D	0	0.58
gac	D	1	0.42
gaa	E	2318	1401.00
gag	E	21	938.50
ggt	G	0	1.29
ggc	G	0	1.19
gga	G	9	3.93
ggg	G	0	2.59
---	--	------	------
mPD		0.027	0.49
		nPD:	0.06
		N. weight:	0.59
		Sc. PD:	-0.057
		Sc. rank:	35.3

,

PB1			
Pos.	178	obs:	exp:
gtt	V	0	0.22
gtc	V	0	0.21
gta	V	0	0.20
gtg	V	1	0.37
gat	D	1	0.58
gac	D	0	0.42
gaa	E	1249	1403.00
gag	E	1094	940.10
ggt	G	0	0.72
ggc	G	0	0.66
gga	G	4	2.18
ggg	G	1	1.44
---	--	------	------
mPD		0.50	0.49
		nPD:	1.03
		N. weight:	0.016
		Sc. PD:	0.014
		Sc. rank:	25.3

,

PB1			
Pos.	179	obs:	exp:
att	I	0	7.43
atc	I	0	5.02
ata	I	20	7.56
atg	M	2328	2328.00
gtt	V	0	0.43
gtc	V	0	0.42
gta	V	0	0.39
gtg	V	2	0.75
---	--	------	------
mPD		0.019	0.020
		nPD:	0.94
		N. weight:	0.012
		Sc. PD:	0.0089
		Sc. rank:	18.5

,

PB1			
Pos.	180	obs:	exp:
aaa	K	3	1.68
aag	K	0	1.32
gat	D	0	1.73
gac	D	3	1.27
gaa	E	2342	1402.00
gag	E	0	939.70
ggt	G	0	0.29
ggc	G	0	0.26
gga	G	2	0.87
ggg	G	0	0.58
---	--	------	------
mPD		0.0068	0.49
		nPD:	0.01
		N. weight:	0.65
		Sc. PD:	-0.089
		Sc. rank:	-530.1

,

PB1			
Pos.	181	obs:	exp:
tta	L	1	0.11
ttg	L	0	0.19
ctt	L	0	0.19
ctc	L	0	0.17
cta	L	0	0.13
ctg	L	0	0.21
att	I	0	565.20
atc	I	0	381.70
ata	I	1522	575.10
gtt	V	0	179.90
gtc	V	0	174.70
gta	V	825	162.70
gtg	V	2	309.70
---	--	------	------
mPD		0.46	1.2
		nPD:	0.39
		N. weight:	1.5
		Sc. PD:	0.34
		Sc. rank:	1686.3

,

PB1			
Pos.	182	obs:	exp:
att	I	0	1.11
atc	I	0	0.75
ata	I	3	1.13
act	T	1	621.70
acc	T	1	518.60
aca	T	2318	1009.00
acg	T	27	198.20
---	--	------	------
mPD		0.027	0.69
		nPD:	0.04
		N. weight:	1.
		Sc. PD:	-0.11
		Sc. rank:	-187.4

,

PB1			
Pos.	183	obs:	exp:
act	T	0	622.50
acc	T	0	519.20
aca	T	2336	1010.00
acg	T	14	198.50
---	--	------	------
mPD		0.012	0.69
		nPD:	0.02
		N. weight:	1.
		Sc. PD:	-0.14
		Sc. rank:	-714.3

,

PB1			
Pos.	184	obs:	exp:
tat	Y	1	0.49
tac	Y	0	0.51
cat	H	2337	1397.00
cac	H	12	952.30
---	--	------	------
mPD		0.011	0.48
		nPD:	0.02
		N. weight:	0.62
		Sc. PD:	-0.08
		Sc. rank:	-308.6

,

PB1			
Pos.	185	obs:	exp:
ttt	F	36	999.50
ttc	F	2313	1349.00
gtt	V	0	0.22
gtc	V	1	0.21
gta	V	0	0.20
gtg	V	0	0.37
---	--	------	------
mPD		0.031	0.49
		nPD:	0.06
		N. weight:	0.61
		Sc. PD:	-0.054
		Sc. rank:	95.9

,

PB1			
Pos.	186	obs:	exp:
cct	P	0	0.28
ccc	P	0	0.18
cca	P	0	0.41
ccg	P	1	0.13
caa	Q	546	1195.00
cag	Q	1803	1154.00
---	--	------	------
mPD		0.36	0.50
		nPD:	0.71
		N. weight:	0.21
		Sc. PD:	0.11
		Sc. rank:	317.1

,

PB1			
Pos.	187	obs:	exp:
tct	S	0	0.34
tcc	S	0	0.29
tca	S	0	0.45
tcg	S	0	0.13
cgt	R	0	89.01
cgc	R	0	125.00
cga	R	0	227.30
cgg	R	0	183.80
agt	S	2	0.41
agc	S	0	0.38
aga	R	2298	1077.00
agg	R	49	645.30
ggt	G	0	0.14
ggc	G	0	0.13
gga	G	1	0.44
ggg	G	0	0.29
---	--	------	------
mPD		0.043	0.96
		nPD:	0.05
		N. weight:	0.87
		Sc. PD:	-0.093
		Sc. rank:	-72.0

,

PB1			
Pos.	188	obs:	exp:
cgt	R	0	0.04
cgc	R	0	0.05
cga	R	0	0.10
cgg	R	0	0.08
aaa	K	1422	1319.00
aag	K	927	1030.00
aga	R	0	0.46
agg	R	1	0.27
---	--	------	------
mPD		0.48	0.49
		nPD:	0.97
		N. weight:	0.0079
		Sc. PD:	0.0063
		Sc. rank:	12.7

,

PB1			
Pos.	189	obs:	exp:
cgt	R	0	89.12
cgc	R	0	125.20
cga	R	0	227.60
cgg	R	0	184.10
aga	R	2323	1078.00
agg	R	27	646.20
---	--	------	------
mPD		0.023	0.95
		nPD:	0.02
		N. weight:	0.92
		Sc. PD:	-0.12
		Sc. rank:	-429.4

,

PB1			
Pos.	190	obs:	exp:
cgt	R	0	89.01
cgc	R	0	125.00
cga	R	1	227.30
cgg	R	0	183.80
aaa	K	3	1.68
aag	K	0	1.32
aga	R	2315	1077.00
agg	R	31	645.30
---	--	------	------
mPD		0.029	0.96
		nPD:	0.03
		N. weight:	0.9
		Sc. PD:	-0.11
		Sc. rank:	-317.7

,

PB1			
Pos.	191	obs:	exp:
att	I	0	23.02
atc	I	0	15.55
ata	I	62	23.43
gtt	V	0	497.40
gtc	V	0	483.20
gta	V	860	450.00
gtg	V	1427	856.40
gaa	E	0	0.60
gag	E	1	0.40
---	--	------	------
mPD		0.53	0.78
		nPD:	0.67
		N. weight:	0.73
		Sc. PD:	0.37
		Sc. rank:	1081.8

,

PB1			
Pos.	192	obs:	exp:
cgt	R	0	89.08
cgc	R	0	125.10
cga	R	0	227.50
cgg	R	0	184.00
att	I	0	0.37
atc	I	1	0.25
ata	I	0	0.38
aga	R	569	1077.00
agg	R	1780	645.90
---	--	------	------
mPD		0.37	0.95
		nPD:	0.39
		N. weight:	0.78
		Sc. PD:	0.18
		Sc. rank:	861.8

,

PB1			
Pos.	193	obs:	exp:
gtt	V	0	0.43
gtc	V	2	0.42
gta	V	0	0.39
gtg	V	0	0.75
gat	D	5	1351.00
gac	D	2343	997.40
---	--	------	------
mPD		0.0059	0.49
		nPD:	0.01
		N. weight:	1.1
		Sc. PD:	-0.15
		Sc. rank:	-944.1

,

PB1			
Pos.	194	obs:	exp:
tct	S	0	0.17
tcc	S	0	0.15
tca	S	0	0.23
tcg	S	0	0.06
aat	N	575	1248.00
aac	N	1774	1101.00
agt	S	0	0.20
agc	S	1	0.19
---	--	------	------
mPD		0.37	0.50
		nPD:	0.74
		N. weight:	0.22
		Sc. PD:	0.13
		Sc. rank:	341.0

,

PB1			
Pos.	195	obs:	exp:
att	I	2	2.60
atc	I	0	1.76
ata	I	5	2.65
atg	M	2206	2206.00
act	T	0	1.85
acc	T	0	1.55
aca	T	0	3.01
acg	T	7	0.59
gtt	V	0	28.27
gtc	V	0	27.47
gta	V	0	25.58
gtg	V	130	48.68
---	--	------	------
mPD		0.12	0.19
		nPD:	0.62
		N. weight:	0.08
		Sc. PD:	0.036
		Sc. rank:	114.1

,

PB1			
Pos.	196	obs:	exp:
tct	S	0	0.17
tcc	S	0	0.15
tca	S	0	0.23
tcg	S	0	0.06
act	T	832	622.20
acc	T	1517	519.00
aca	T	0	1009.00
acg	T	0	198.40
agt	S	1	0.20
agc	S	0	0.19
---	--	------	------
mPD		0.46	0.69
		nPD:	0.66
		N. weight:	1.
		Sc. PD:	0.5
		Sc. rank:	1488.9

,

PB1			
Pos.	197	obs:	exp:
cgt	R	0	1.63
cgc	R	0	2.29
cga	R	0	4.16
cgg	R	0	3.37
aat	N	1	0.53
aac	N	0	0.47
aaa	K	195	1294.00
aag	K	2111	1012.00
aga	R	0	19.72
agg	R	43	11.82
---	--	------	------
mPD		0.19	0.54
		nPD:	0.35
		N. weight:	0.67
		Sc. PD:	0.13
		Sc. rank:	693.3

,

PB1			
Pos.	198	obs:	exp:
cgt	R	0	0.49
cgc	R	0	0.69
cga	R	0	1.26
cgg	R	0	1.02
aat	N	1	0.53
aac	N	0	0.47
aaa	K	695	1311.00
aag	K	1641	1025.00
aga	R	3	5.96
agg	R	10	3.57
---	--	------	------
mPD		0.43	0.51
		nPD:	0.85
		N. weight:	0.19
		Sc. PD:	0.13
		Sc. rank:	296.3

,

PB1			
Pos.	199	obs:	exp:
att	I	2	1.11
atc	I	0	0.75
ata	I	1	1.13
atg	M	2346	2346.00
gtt	V	0	0.22
gtc	V	0	0.21
gta	V	0	0.20
gtg	V	1	0.37
---	--	------	------
mPD		0.0034	0.0039
		nPD:	0.87
		N. weight:	0.0015
		Sc. PD:	0.0011
		Sc. rank:	2.4

,

PB1			
Pos.	200	obs:	exp:
att	I	0	1.11
atc	I	3	0.75
ata	I	0	1.13
gtt	V	1	510.40
gtc	V	2345	495.90
gta	V	1	461.80
gtg	V	0	878.90
---	--	------	------
mPD		0.0043	0.73
		nPD:	0.01
		N. weight:	2.
		Sc. PD:	-0.28
		Sc. rank:	-2414.5

,

PB1			
Pos.	201	obs:	exp:
tct	S	0	0.17
tcc	S	0	0.15
tca	S	1	0.23
tcg	S	0	0.06
act	T	0	622.20
acc	T	1	519.00
aca	T	2344	1009.00
acg	T	4	198.40
agt	S	0	0.20
agc	S	0	0.19
---	--	------	------
mPD		0.0051	0.69
		nPD:	0.01
		N. weight:	1.1
		Sc. PD:	-0.15
		Sc. rank:	-1165.4

,

PB1			
Pos.	202	obs:	exp:
cat	H	1	1.19
cac	H	1	0.81
caa	Q	2334	1195.00
cag	Q	14	1153.00
---	--	------	------
mPD		0.014	0.50
		nPD:	0.03
		N. weight:	0.81
		Sc. PD:	-0.1
		Sc. rank:	-331.9

,

PB1			
Pos.	203	obs:	exp:
cgt	R	0	89.08
cgc	R	0	125.10
cga	R	1	227.50
cgg	R	0	184.00
aaa	K	1	0.56
aag	K	0	0.44
aga	R	2316	1077.00
agg	R	32	645.90
---	--	------	------
mPD		0.029	0.95
		nPD:	0.03
		N. weight:	0.9
		Sc. PD:	-0.11
		Sc. rank:	-323.6

,

PB1			
Pos.	204	obs:	exp:
att	I	0	0.37
atc	I	0	0.25
ata	I	1	0.38
atg	M	1	1.00
act	T	0	622.00
acc	T	0	518.80
aca	T	2144	1009.00
acg	T	204	198.30
---	--	------	------
mPD		0.16	0.69
		nPD:	0.23
		N. weight:	0.88
		Sc. PD:	0.066
		Sc. rank:	725.8

,

PB1			
Pos.	205	obs:	exp:
tta	L	3	0.33
ttg	L	0	0.56
ctt	L	0	0.56
ctc	L	0	0.52
cta	L	0	0.40
ctg	L	0	0.63
att	I	0	871.50
atc	I	0	588.60
ata	I	2347	886.90
---	--	------	------
mPD		0.0026	0.66
		nPD:	0.
		N. weight:	1.2
		Sc. PD:	-0.18
		Sc. rank:	-1612.0

,

PB1			
Pos.	206	obs:	exp:
ggt	G	0	338.10
ggc	G	0	310.20
gga	G	646	1026.00
ggg	G	1704	675.90
---	--	------	------
mPD		0.40	0.69
		nPD:	0.58
		N. weight:	0.69
		Sc. PD:	0.28
		Sc. rank:	962.6

,

PB1			
Pos.	207	obs:	exp:
cgt	R	0	0.04
cgc	R	0	0.05
cga	R	0	0.10
cgg	R	0	0.08
aaa	K	28	1319.00
aag	K	2321	1030.00
aga	R	0	0.46
agg	R	1	0.27
---	--	------	------
mPD		0.024	0.49
		nPD:	0.05
		N. weight:	0.96
		Sc. PD:	-0.098
		Sc. rank:	-13.5

,

PB1			
Pos.	208	obs:	exp:
cgt	R	0	0.08
cgc	R	0	0.11
cga	R	0	0.19
cgg	R	0	0.16
act	T	0	0.26
acc	T	0	0.22
aca	T	0	0.43
acg	T	1	0.08
aat	N	1	0.53
aac	N	0	0.47
aaa	K	540	1317.00
aag	K	1806	1029.00
aga	R	0	0.92
agg	R	2	0.55
---	--	------	------
mPD		0.36	0.50
		nPD:	0.72
		N. weight:	0.29
		Sc. PD:	0.16
		Sc. rank:	453.3

,

PB1			
Pos.	209	obs:	exp:
aaa	K	674	1319.00
aag	K	1676	1031.00
---	--	------	------
mPD		0.41	0.49
		nPD:	0.83
		N. weight:	0.2
		Sc. PD:	0.13
		Sc. rank:	313.4

,

PB1			
Pos.	210	obs:	exp:
tta	L	0	0.22
ttg	L	0	0.38
ctt	L	0	0.38
ctc	L	0	0.35
cta	L	0	0.27
ctg	L	2	0.42
caa	Q	301	1193.00
cag	Q	2043	1151.00
cgt	R	0	0.11
cgc	R	0	0.16
cga	R	0	0.29
cgg	R	3	0.23
aaa	K	0	0.56
aag	K	1	0.44
aga	R	0	1.38
agg	R	0	0.82
---	--	------	------
mPD		0.23	0.51
		nPD:	0.45
		N. weight:	0.42
		Sc. PD:	0.12
		Sc. rank:	506.6

,

PB1			
Pos.	211	obs:	exp:
cgt	R	0	88.29
cgc	R	0	124.00
cga	R	0	225.40
cgg	R	0	182.30
aaa	K	3	9.54
aag	K	14	7.46
aga	R	488	1068.00
agg	R	1840	640.10
ggt	G	0	0.72
ggc	G	0	0.66
gga	G	0	2.18
ggg	G	5	1.44
---	--	------	------
mPD		0.35	0.97
		nPD:	0.36
		N. weight:	0.85
		Sc. PD:	0.17
		Sc. rank:	897.3

,

PB1			
Pos.	212	obs:	exp:
tta	L	0	255.60
ttg	L	3	439.50
tct	S	0	0.34
tcc	S	0	0.29
tca	S	0	0.45
tcg	S	2	0.13
ctt	L	29	439.20
ctc	L	0	404.10
cta	L	175	311.60
ctg	L	2135	492.10
atg	M	6	6.00
agt	S	0	0.41
agc	S	0	0.38
---	--	------	------
mPD		0.17	1.1
		nPD:	0.15
		N. weight:	1.6
		Sc. PD:	-0.007
		Sc. rank:	907.0

,

PB1			
Pos.	213	obs:	exp:
tct	S	0	0.34
tcc	S	0	0.29
tca	S	0	0.45
tcg	S	0	0.13
tat	Y	0	0.49
tac	Y	1	0.51
att	I	0	5.94
atc	I	16	4.01
ata	I	0	6.05
act	T	0	0.26
acc	T	1	0.22
aca	T	0	0.43
acg	T	0	0.08
aat	N	210	1235.00
aac	N	2114	1089.00
aaa	K	2	1.12
aag	K	0	0.88
agt	S	0	0.41
agc	S	2	0.38
gat	D	0	2.30
gac	D	4	1.70
---	--	------	------
mPD		0.18	0.52
		nPD:	0.35
		N. weight:	0.57
		Sc. PD:	0.11
		Sc. rank:	603.5

,

PB1			
Pos.	214	obs:	exp:
cgt	R	0	3.03
cgc	R	0	4.26
cga	R	0	7.75
cgg	R	0	6.27
aaa	K	54	1274.00
aag	K	2216	995.80
aga	R	0	36.70
agg	R	80	22.00
---	--	------	------
mPD		0.11	0.58
		nPD:	0.19
		N. weight:	0.92
		Sc. PD:	0.032
		Sc. rank:	616.4

,

PB1			
Pos.	215	obs:	exp:
cgt	R	0	68.19
cgc	R	0	95.77
cga	R	0	174.10
cgg	R	0	140.80
aaa	K	5	309.30
aag	K	546	241.70
aga	R	192	824.70
agg	R	1606	494.40
gaa	E	0	0.60
gag	E	1	0.40
---	--	------	------
mPD		0.51	1.2
		nPD:	0.42
		N. weight:	1.1
		Sc. PD:	0.28
		Sc. rank:	1279.5

PB1

Pos.	216	obs:	exp:
tct	S	0	315.10
tcc	S	0	272.00
tca	S	0	415.90
tcg	S	0	119.20
tgt	C	3	1.30
tgc	C	0	1.70
att	I	0	1.11
atc	I	3	0.75
ata	I	0	1.13
aat	N	11	265.20
aac	N	488	233.80
agt	S	1402	376.70
agc	S	443	346.20
---	--	------	------
mPD		0.82	1.8
		nPD:	0.46
		N. weight:	1.2
		Sc. PD:	0.36
		Sc. rank:	1507.2

PB1

Pos.	217	obs:	exp:
ttt	F	0	0.85
ttc	F	2	1.15
tat	Y	78	1141.00
tac	Y	2270	1207.00
---	--	------	------
mPD		0.066	0.50
		nPD:	0.13
		N. weight:	0.66
		Sc. PD:	-0.015
		Sc. rank:	334.4

PB1

Pos.	218	obs:	exp:
tta	L	1497	256.50
ttg	L	832	441.00
ctt	L	0	440.70
ctc	L	0	405.40
cta	L	20	312.70
ctg	L	1	493.80
---	--	------	------
mPD		0.48	1.1
		nPD:	0.42
		N. weight:	1.7
		Sc. PD:	0.43
		Sc. rank:	1963.4

PB1

Pos.	219	obs:	exp:
att	I	0	728.60
atc	I	0	492.10
ata	I	1962	741.40
gtt	V	0	84.38
gtc	V	0	81.98
gta	V	370	76.34
gtg	V	18	145.30
---	--	------	------
mPD		0.29	0.97
		nPD:	0.3
		N. weight:	1.3
		Sc. PD:	0.19
		Sc. rank:	1269.0

PB1

Pos.	220	obs:	exp:
cgt	R	0	89.12
cgc	R	0	125.20
cga	R	5	227.60
cgg	R	0	184.10
aga	R	1831	1078.00
agg	R	514	646.20
---	--	------	------
mPD		0.35	0.95
		nPD:	0.36
		N. weight:	0.45
		Sc. PD:	0.092
		Sc. rank:	481.2

PB1

Pos.	221	obs:	exp:
act	T	0	0.79
acc	T	0	0.66
aca	T	3	1.29
acg	T	0	0.25
gtt	V	0	0.22
gtc	V	0	0.21
gta	V	1	0.20
gtg	V	0	0.37
gct	A	0	596.80
gcc	A	0	457.50
gca	A	2335	1031.00
gcg	A	11	260.40
---	--	------	------
mPD		0.013	0.70
		nPD:	0.02
		N. weight:	1.
		Sc. PD:	-0.13
		Sc. rank:	-668.4

PB1

Pos.	222	obs:	exp:
tta	L	1	256.40
ttg	L	20	440.80
ctt	L	0	440.50
ctc	L	0	405.30
cta	L	7	312.50
ctg	L	2321	493.50
atg	M	1	1.00
---	--	------	------
mPD		0.025	1.1
		nPD:	0.02
		N. weight:	1.9
		Sc. PD:	-0.24
		Sc. rank:	-962.2

PB1

Pos.	223	obs:	exp:
act	T	0	622.50
acc	T	71	519.20
aca	T	2278	1010.00
acg	T	1	198.50
---	--	------	------
mPD		0.059	0.69
		nPD:	0.09
		N. weight:	0.92
		Sc. PD:	-0.062
		Sc. rank:	314.7

PB1

Pos.	224	obs:	exp:
tta	L	1	256.50
ttg	L	1617	441.00
ctt	L	0	440.70
ctc	L	0	405.40
cta	L	4	312.70
ctg	L	728	493.80
---	--	------	------
mPD		0.43	1.1
		nPD:	0.38
		N. weight:	1.3
		Sc. PD:	0.28
		Sc. rank:	1399.9

PB1

Pos.	225	obs:	exp:
aat	N	963	1248.00
aac	N	1386	1101.00
gat	D	0	0.58
gac	D	1	0.42
---	--	------	------
mPD		0.48	0.50
		nPD:	0.97
		N. weight:	0.04
		Sc. PD:	0.032
		Sc. rank:	64.3

PB1

Pos.	226	obs:	exp:
act	T	1	622.50
acc	T	1	519.20
aca	T	2315	1010.00
acg	T	33	198.50
---	--	------	------
mPD		0.029	0.69
		nPD:	0.04
		N. weight:	1.
		Sc. PD:	-0.11
		Sc. rank:	-127.8

PB1

Pos.	227	obs:	exp:
atg	M	2350	2350.00
---	--	------	------
mPD		0	0
		nPD:	1.
		N. weight:	0.
		Sc. PD:	0
		Sc. rank:	0

PB1

Pos.	228	obs:	exp:
att	I	0	0.37
atc	I	1	0.25
ata	I	0	0.38
act	T	2	622.00
acc	T	1640	518.80
aca	T	705	1009.00
acg	T	1	198.30
gct	A	0	0.25
gcc	A	0	0.20
gca	A	1	0.44
gcg	A	0	0.11
---	--	------	------
mPD		0.42	0.69
		nPD:	0.61
		N. weight:	0.88
		Sc. PD:	0.39
		Sc. rank:	1246.6

PB1

Pos.	229	obs:	exp:
att	I	0	0.37
atc	I	0	0.25
ata	I	1	0.38
aaa	K	2293	1319.00
aag	K	56	1030.00
---	--	------	------
mPD		0.047	0.49
		nPD:	0.1
		N. weight:	0.6
		Sc. PD:	-0.034
		Sc. rank:	237.3

PB1

Pos.	230	obs:	exp:
aat	N	1	0.53
aac	N	0	0.47
gat	D	2308	1351.00
gac	D	41	997.80
---	--	------	------
mPD		0.035	0.49
		nPD:	0.07
		N. weight:	0.6
		Sc. PD:	-0.048
		Sc. rank:	154.4

PB1

Pos.	231	obs:	exp:
gct	A	7	597.80
gcc	A	13	458.30
gca	A	2324	1033.00
gcg	A	6	260.90
---	--	------	------
mPD		0.022	0.69
		nPD:	0.03
		N. weight:	0.97
		Sc. PD:	-0.12
		Sc. rank:	-323.8

PB1

Pos.	232	obs:	exp:
gtt	V	0	0.22
gtc	V	0	0.21
gta	V	1	0.20
gtg	V	0	0.37
gaa	E	2317	1406.00
gag	E	32	942.50
---	--	------	------
mPD		0.028	0.48
		nPD:	0.06
		N. weight:	0.57
		Sc. PD:	-0.054
		Sc. rank:	58.2

PB1

Pos.	233	obs:	exp:
cgt	R	0	89.12
cgc	R	0	125.20
cga	R	22	227.60
cgg	R	0	184.10
aga	R	2291	1078.00
agg	R	37	646.20
---	--	------	------
mPD		0.050	0.95
		nPD:	0.05
		N. weight:	0.85
		Sc. PD:	-0.085
		Sc. rank:	13.5

PB1

Pos.	234	obs:	exp:
tct	S	0	0.17
tcc	S	0	0.15
tca	S	0	0.23
tcg	S	0	0.06
agt	S	0	0.20
agc	S	1	0.19
gat	D	0	0.58
gac	D	1	0.42
ggt	G	5	337.80
ggc	G	2334	309.90
gga	G	9	1025.00
ggg	G	0	675.30
---	--	------	------
mPD		0.014	0.69
		nPD:	0.02
		N. weight:	2.5
		Sc. PD:	-0.33
		Sc. rank:	-1467.2

PB1

Pos.	235	obs:	exp:
aaa	K	1085	1319.00
aag	K	1265	1031.00
---	--	------	------
mPD		0.50	0.49
		nPD:	1.01
		N. weight:	0.027
		Sc. PD:	0.023
		Sc. rank:	44.1

PB1			
Pos.	236	obs:	exp:
tta	L	15	256.50
ttg	L	2324	441.00
ctt	L	0	440.70
ctc	L	0	405.40
cta	L	0	312.70
ctg	L	11	493.80
---	--	------	------
mPD		0.022	1.1
		nPD:	0.02
		N. weight:	2.
		Sc. PD:	-0.27
		Sc. rank:	-1237.4

PB1			
Pos.	237	obs:	exp:
cgt	R	0	0.23
cgc	R	0	0.32
cga	R	0	0.58
cgg	R	0	0.47
aaa	K	190	1316.00
aag	K	2154	1028.00
aga	R	0	2.75
agg	R	6	1.65
---	--	------	------
mPD		0.15	0.50
		nPD:	0.31
		N. weight:	0.67
		Sc. PD:	0.099
		Sc. rank:	645.6

PB1			
Pos.	238	obs:	exp:
cgt	R	0	89.12
cgc	R	0	125.20
cga	R	0	227.60
cgg	R	0	184.10
aga	R	1433	1078.00
agg	R	917	646.20
---	--	------	------
mPD		0.48	0.95
		nPD:	0.5
		N. weight:	0.4
		Sc. PD:	0.13
		Sc. rank:	507.0

PB1			
Pos.	239	obs:	exp:
cgt	R	30	89.12
cgc	R	0	125.20
cga	R	4	227.60
cgg	R	2301	184.10
aga	R	13	1078.00
agg	R	2	646.20
---	--	------	------
mPD		0.052	0.95
		nPD:	0.05
		N. weight:	3.1
		Sc. PD:	-0.3
		Sc. rank:	154.2

PB1			
Pos.	240	obs:	exp:
gct	A	0	597.80
gcc	A	0	458.30
gca	A	2338	1033.00
gcg	A	12	260.90
---	--	------	------
mPD		0.010	0.69
		nPD:	0.01
		N. weight:	1.
		Sc. PD:	-0.14
		Sc. rank:	-807.1

PB1			
Pos.	241	obs:	exp:
att	I	2342	872.60
atc	I	4	589.40
ata	I	4	888.00
---	--	------	------
mPD		0.0068	0.66
		nPD:	0.01
		N. weight:	1.2
		Sc. PD:	-0.17
		Sc. rank:	-1192.7

PB1			
Pos.	242	obs:	exp:
gct	A	0	597.80
gcc	A	0	458.30
gca	A	2349	1033.00
gcg	A	1	260.90
---	--	------	------
mPD		0.00085	0.69
		nPD:	0.
		N. weight:	1.
		Sc. PD:	-0.15
		Sc. rank:	-1597.9

PB1			
Pos.	243	obs:	exp:
act	T	0	622.50
acc	T	1	519.20
aca	T	2320	1010.00
acg	T	29	198.50
---	--	------	------
mPD		0.025	0.69
		nPD:	0.04
		N. weight:	1.
		Sc. PD:	-0.12
		Sc. rank:	-262.8

PB1			
Pos.	244	obs:	exp:
cct	P	1	662.70
ccc	P	2169	425.20
cca	P	150	959.50
ccg	P	30	302.60
---	--	------	------
mPD		0.14	0.70
		nPD:	0.2
		N. weight:	1.7
		Sc. PD:	0.082
		Sc. rank:	1233.9

PB1			
Pos.	245	obs:	exp:
ggt	G	0	338.10
ggc	G	0	310.20
gga	G	64	1026.00
ggg	G	2286	675.90
---	--	------	------
mPD		0.053	0.69
		nPD:	0.08
		N. weight:	1.4
		Sc. PD:	-0.11
		Sc. rank:	421.4

PB1			
Pos.	246	obs:	exp:
atg	M	2350	2350.00
---	--	------	------
mPD		0	0
		nPD:	1.
		N. weight:	0.
		Sc. PD:	0
		Sc. rank:	0

PB1			
Pos.	247	obs:	exp:
caa	Q	522	1196.00
cag	Q	1828	1154.00
---	--	------	------
mPD		0.35	0.50
		nPD:	0.69
		N. weight:	0.22
		Sc. PD:	0.12
		Sc. rank:	332.7

PB1			
Pos.	248	obs:	exp:
att	I	1663	871.50
atc	I	681	588.60
ata	I	3	886.90
act	T	0	0.26
acc	T	1	0.22
aca	T	0	0.43
acg	T	0	0.08
gtt	V	2	0.43
gtc	V	0	0.42
gta	V	0	0.39
gtg	V	0	0.75
---	--	------	------
mPD		0.42	0.66
		nPD:	0.63
		N. weight:	0.63
		Sc. PD:	0.29
		Sc. rank:	903.8

PB1			
Pos.	249	obs:	exp:
cgt	R	0	89.12
cgc	R	0	125.20
cga	R	1	227.60
cgg	R	0	184.10
aga	R	2342	1078.00
agg	R	7	646.20
---	--	------	------
mPD		0.0068	0.95
		nPD:	0.01
		N. weight:	0.96
		Sc. PD:	-0.14
		Sc. rank:	-1093.6

PB1			
Pos.	250	obs:	exp:
ggt	G	40	338.10
ggc	G	0	310.20
gga	G	1818	1026.00
ggg	G	492	675.90
---	--	------	------
mPD		0.36	0.69
		nPD:	0.52
		N. weight:	0.43
		Sc. PD:	0.15
		Sc. rank:	569.4

PB1			
Pos.	251	obs:	exp:
ttt	F	361	1000.00
ttc	F	1989	1350.00
---	--	------	------
mPD		0.26	0.49
		nPD:	0.53
		N. weight:	0.22
		Sc. PD:	0.08
		Sc. rank:	289.9

PB1			
Pos.	252	obs:	exp:
gtt	V	1	511.10
gtc	V	0	496.50
gta	V	12	462.40
gtg	V	2337	880.00
---	--	------	------
mPD		0.011	0.73
		nPD:	0.02
		N. weight:	1.2
		Sc. PD:	-0.16
		Sc. rank:	-928.8

PB1			
Pos.	253	obs:	exp:
tat	Y	69	1142.00
tac	Y	2280	1207.00
cat	H	0	0.59
cac	H	1	0.41
---	--	------	------
mPD		0.058	0.50
		nPD:	0.12
		N. weight:	0.68
		Sc. PD:	-0.026
		Sc. rank:	319.0

PB1			
Pos.	254	obs:	exp:
ttt	F	1467	997.00
ttc	F	876	1346.00
tta	L	2	0.44
ttg	L	0	0.75
tat	Y	0	0.49
tac	Y	1	0.51
ctt	L	1	0.75
ctc	L	1	0.69
cta	L	0	0.53
ctg	L	0	0.84
gtt	V	2	0.43
gtc	V	0	0.42
gta	V	0	0.39
gtg	V	0	0.75
---	--	------	------
mPD		0.47	0.50
		nPD:	0.96
		N. weight:	0.11
		Sc. PD:	0.085
		Sc. rank:	175.6

PB1			
Pos.	255	obs:	exp:
gtt	V	871	510.00
gtc	V	1473	495.50
gta	V	1	461.40
gtg	V	0	878.10
gct	A	3	1.27
gcc	A	2	0.98
gca	A	0	2.20
gcg	A	0	0.56
---	--	------	------
mPD		0.47	0.73
		nPD:	0.64
		N. weight:	1.1
		Sc. PD:	0.53
		Sc. rank:	1619.4

PB1			
Pos.	256	obs:	exp:
gaa	E	2332	1407.00
gag	E	18	942.90
---	--	------	------
mPD		0.015	0.48
		nPD:	0.03
		N. weight:	0.6
		Sc. PD:	-0.072
		Sc. rank:	-203.1

PB1			
Pos.	257	obs:	exp:
act	T	0	610.00
acc	T	1	508.80
aca	T	2297	989.60
acg	T	5	194.50
gct	A	0	11.96
gcc	A	0	9.17
gca	A	47	20.66
gcg	A	0	5.22
---	--	------	------
mPD		0.044	0.73
		nPD:	0.06
		N. weight:	1.1
		Sc. PD:	-0.096
		Sc. rank:	125.7

PB1			
Pos.	258	obs:	exp:
tta	L	515	256.50
ttg	L	0	441.00
ctt	L	0	440.70
ctc	L	1	405.40
cta	L	1690	312.70
ctg	L	144	493.80
---	--	------	------
mPD		0.46	1.1
		nPD:	0.4
		N. weight:	1.6
		Sc. PD:	0.4
		Sc. rank:	1864.5

PB1			
Pos.	259	obs:	exp:
gct	A	5	597.80
gcc	A	0	458.30
gca	A	215	1033.00
gcg	A	2130	260.90
---	--	------	------
mPD		0.17	0.69
		nPD:	0.25
		N. weight:	2.2
		Sc. PD:	0.2
		Sc. rank:	1937.5

PB1			
Pos.	260	obs:	exp:
cgt	R	0	89.01
cgc	R	0	125.00
cga	R	0	227.30
cgg	R	0	183.80
aaa	K	0	1.68
aag	K	3	1.32
aga	R	7	1077.00
agg	R	2340	645.30
---	--	------	------
mPD		0.0085	0.96
		nPD:	0.01
		N. weight:	1.6
		Sc. PD:	-0.23
		Sc. rank:	-1603.3

PB1			
Pos.	261	obs:	exp:
tct	S	0	397.40
tcc	S	0	343.00
tca	S	0	524.50
tcg	S	0	150.30
tgt	C	0	9.57
tgc	C	22	12.43
agt	S	4	475.10
agc	S	2323	436.60
ggt	G	0	0.14
ggc	G	1	0.13
gga	G	0	0.44
ggg	G	0	0.29
---	--	------	------
mPD		0.023	1.6
		nPD:	0.01
		N. weight:	2.1
		Sc. PD:	-0.29
		Sc. rank:	-1709.6

PB1			
Pos.	262	obs:	exp:
att	I	15	872.60
atc	I	2331	589.40
ata	I	4	888.00
---	--	------	------
mPD		0.016	0.66
		nPD:	0.02
		N. weight:	1.7
		Sc. PD:	-0.21
		Sc. rank:	-769.7

PB1			
Pos.	263	obs:	exp:
tgt	C	1862	1022.00
tgc	C	488	1328.00
---	--	------	------
mPD		0.33	0.49
		nPD:	0.67
		N. weight:	0.34
		Sc. PD:	0.17
		Sc. rank:	504.6

PB1			
Pos.	264	obs:	exp:
gaa	E	177	1407.00
gag	E	2173	942.90
---	--	------	------
mPD		0.14	0.48
		nPD:	0.29
		N. weight:	0.78
		Sc. PD:	0.1
		Sc. rank:	738.3

PB1			
Pos.	265	obs:	exp:
cgt	R	0	0.08
cgc	R	0	0.11
cga	R	0	0.19
cgg	R	0	0.16
aaa	K	2321	1318.00
aag	K	27	1030.00
aga	R	2	0.92
agg	R	0	0.55
---	--	------	------
mPD		0.024	0.49
		nPD:	0.05
		N. weight:	0.66
		Sc. PD:	-0.067
		Sc. rank:	-11.9

PB1			
Pos.	266	obs:	exp:
tta	L	13	255.60
ttg	L	0	439.50
ctt	L	795	439.20
ctc	L	711	404.10
cta	L	820	311.60
ctg	L	3	492.10
att	I	7	2.60
atc	I	0	1.76
ata	I	0	2.65
gtt	V	0	0.22
gtc	V	0	0.21
gta	V	1	0.20
gtg	V	0	0.37
---	--	------	------
mPD		0.68	1.1
		nPD:	0.6
		N. weight:	0.88
		Sc. PD:	0.38
		Sc. rank:	1242.7

PB1			
Pos.	267	obs:	exp:
gaa	E	19	1407.00
gag	E	2331	942.90
---	--	------	------
mPD		0.016	0.48
		nPD:	0.03
		N. weight:	1.1
		Sc. PD:	-0.13
		Sc. rank:	-318.0

PB1			
Pos.	268	obs:	exp:
caa	Q	2259	1196.00
cag	Q	91	1154.00
---	--	------	------
mPD		0.074	0.50
		nPD:	0.15
		N. weight:	0.65
		Sc. PD:	-0.0037
		Sc. rank:	360.1

PB1			
Pos.	269	obs:	exp:
tct	S	2344	401.20
tcc	S	4	346.30
tca	S	1	529.50
tcg	S	0	151.70
tgt	C	1	0.43
tgc	C	0	0.57
agt	S	0	479.60
agc	S	0	440.70
---	--	------	------
mPD		0.0051	1.6
		nPD:	0.
		N. weight:	2.2
		Sc. PD:	-0.33
		Sc. rank:	-3101.5

PB1			
Pos.	270	obs:	exp:
ggt	G	1	338.10
ggc	G	39	310.20
gga	G	399	1026.00
ggg	G	1911	675.90
---	--	------	------
mPD		0.31	0.69
		nPD:	0.45
		N. weight:	0.82
		Sc. PD:	0.24
		Sc. rank:	1002.1

PB1			
Pos.	271	obs:	exp:
tta	L	0	256.40
ttg	L	0	440.80
ctt	L	4	440.50
ctc	L	2341	405.30
cta	L	4	312.50
ctg	L	0	493.50
att	I	0	0.37
atc	I	1	0.25
ata	I	0	0.38
---	--	------	------
mPD		0.0076	1.1
		nPD:	0.01
		N. weight:	2.2
		Sc. PD:	-0.32
		Sc. rank:	-2580.2

PB1			
Pos.	272	obs:	exp:
cct	P	2154	662.70
ccc	P	180	425.20
cca	P	16	959.50
ccg	P	0	302.60
---	--	------	------
mPD		0.15	0.70
		nPD:	0.22
		N. weight:	1.3
		Sc. PD:	0.078
		Sc. rank:	981.1

PB1			
Pos.	273	obs:	exp:
att	I	5	1.86
atc	I	0	1.25
ata	I	0	1.89
gtt	V	2343	510.00
gtc	V	2	495.50
gta	V	0	461.40
gtg	V	0	878.10
---	--	------	------
mPD		0.0059	0.73
		nPD:	0.01
		N. weight:	1.9
		Sc. PD:	-0.27
		Sc. rank:	-2082.1

PB1			
Pos.	274	obs:	exp:
ggt	G	0	338.10
ggc	G	0	310.20
gga	G	2337	1026.00
ggg	G	13	675.90
---	--	------	------
mPD		0.011	0.69
		nPD:	0.02
		N. weight:	1.
		Sc. PD:	-0.14
		Sc. rank:	-753.5

PB1			
Pos.	275	obs:	exp:
ggt	G	0	338.10
ggc	G	14	310.20
gga	G	490	1026.00
ggg	G	1846	675.90
---	--	------	------
mPD		0.34	0.69
		nPD:	0.49
		N. weight:	0.78
		Sc. PD:	0.26
		Sc. rank:	992.8

PB1			
Pos.	276	obs:	exp:
act	T	1	0.26
acc	T	0	0.22
aca	T	0	0.43
acg	T	0	0.08
aat	N	2345	1248.00
aac	N	3	1100.00
aaa	K	1	0.56
aag	K	0	0.44
---	--	------	------
mPD		0.0043	0.50
		nPD:	0.01
		N. weight:	0.79
		Sc. PD:	-0.11
		Sc. rank:	-830.0

PB1			
Pos.	277	obs:	exp:
gaa	E	526	1407.00
gag	E	1824	942.90
---	--	------	------
mPD		0.35	0.48
		nPD:	0.72
		N. weight:	0.37
		Sc. PD:	0.21
		Sc. rank:	576.0

PB1			
Pos.	278	obs:	exp:
aaa	K	781	1319.00
aag	K	1569	1031.00
---	--	------	------
mPD		0.44	0.49
		nPD:	0.9
		N. weight:	0.14
		Sc. PD:	0.099
		Sc. rank:	218.0

PB1			
Pos.	279	obs:	exp:
aat	N	3	1.59
aac	N	0	1.41
aaa	K	5	1317.00
aag	K	2342	1030.00
---	--	------	------
mPD		0.0068	0.49
		nPD:	0.01
		N. weight:	1.
		Sc. PD:	-0.14
		Sc. rank:	-850.8

PB1			
Pos.	280	obs:	exp:
gct	A	2143	597.80
gcc	A	206	458.30
gca	A	1	1033.00
gcg	A	0	260.90
---	--	------	------
mPD		0.16	0.69
		nPD:	0.23
		N. weight:	1.4
		Sc. PD:	0.1
		Sc. rank:	1141.3

PB1			
Pos.	281	obs:	exp:
aat	N	1	0.53
aac	N	0	0.47
aaa	K	2210	1318.00
aag	K	138	1030.00
gaa	E	1	0.60
gag	E	0	0.40
---	--	------	------
mPD		0.11	0.49
		nPD:	0.23
		N. weight:	0.47
		Sc. PD:	0.033
		Sc. rank:	379.3

PB1			
Pos.	282	obs:	exp:
tta	L	3	256.10
ttg	L	2329	440.20
tct	S	0	0.68
tcc	S	0	0.59
tca	S	0	0.90
tcg	S	4	0.26
ctt	L	0	439.90
ctc	L	0	404.80
cta	L	1	312.10
ctg	L	13	492.90
agt	S	0	0.82
agc	S	0	0.75
---	--	------	------
mPD		0.019	1.1
		nPD:	0.02
		N. weight:	2.1
		Sc. PD:	-0.28
		Sc. rank:	-1520.8

PB1			
Pos.	283	obs:	exp:
gct	A	0	597.80
gcc	A	0	458.30
gca	A	2318	1033.00
gcg	A	32	260.90
---	--	------	------
mPD		0.027	0.69
		nPD:	0.04
		N. weight:	0.98
		Sc. PD:	-0.11
		Sc. rank:	-188.4

PB1			
Pos.	284	obs:	exp:
aat	N	2347	1249.00
aac	N	3	1101.00
---	--	------	------
mPD		0.0026	0.50
		nPD:	0.01
		N. weight:	0.79
		Sc. PD:	-0.11
		Sc. rank:	-988.7

PB1			
Pos.	285	obs:	exp:
gtt	V	866	510.90
gtc	V	1462	496.30
gta	V	11	462.20
gtg	V	10	879.60
gct	A	1	0.25
gcc	A	0	0.20
gca	A	0	0.44
gcg	A	0	0.11
---	--	------	------
mPD		0.48	0.73
		nPD:	0.65
		N. weight:	1.1
		Sc. PD:	0.51
		Sc. rank:	1536.5

PB1			
Pos.	286	obs:	exp:
gtt	V	0	511.10
gtc	V	1	496.50
gta	V	5	462.40
gtg	V	2344	880.00
---	--	------	------
mPD		0.0051	0.73
		nPD:	0.01
		N. weight:	1.2
		Sc. PD:	-0.18
		Sc. rank:	-1411.5

PB1			
Pos.	287	obs:	exp:
cgt	R	0	89.04
cgc	R	0	125.10
cga	R	0	227.40
cgg	R	0	183.90
aaa	K	2	1.12
aag	K	0	0.88
aga	R	2137	1077.00
agg	R	211	645.60
---	--	------	------
mPD		0.17	0.95
		nPD:	0.17
		N. weight:	0.66
		Sc. PD:	0.012
		Sc. rank:	414.0

PB1			
Pos.	288	obs:	exp:
caa	Q	1	0.51
cag	Q	0	0.49
aaa	K	1850	1319.00
aag	K	499	1030.00
---	--	------	------
mPD		0.34	0.49
		nPD:	0.68
		N. weight:	0.15
		Sc. PD:	0.074
		Sc. rank:	215.7

PB1			
Pos.	289	obs:	exp:
tta	L	0	0.33
ttg	L	3	0.56
ctt	L	0	0.56
ctc	L	0	0.52
cta	L	0	0.40
ctg	L	0	0.63
atg	M	2347	2347.00
---	--	------	------
mPD		0.0026	0.0041
		nPD:	0.62
		N. weight:	0.0027
		Sc. PD:	0.0012
		Sc. rank:	3.9

PB1			
Pos.	290	obs:	exp:
atg	M	2350	2350.00
---	--	------	------
mPD		0	0
		nPD:	1.
		N. weight:	0.
		Sc. PD:	0
		Sc. rank:	0

PB1			
Pos.	291	obs:	exp:
att	I	1	3.71
atc	I	9	2.51
ata	I	0	3.78
act	T	2291	619.60
acc	T	47	516.80
aca	T	0	1005.00
acg	T	1	197.60
gct	A	1	0.25
gcc	A	0	0.20
gca	A	0	0.44
gcg	A	0	0.11
---	--	------	------
mPD		0.057	0.70
		nPD:	0.08
		N. weight:	1.6
		Sc. PD:	-0.11
		Sc. rank:	477.4

PB1			
Pos.	292	obs:	exp:
tct	S	0	0.17
tcc	S	0	0.15
tca	S	0	0.23
tcg	S	0	0.06
tat	Y	0	0.49
tac	Y	1	0.51
aat	N	21	1248.00
aac	N	2327	1100.00
agt	S	0	0.20
agc	S	1	0.19
---	--	------	------
mPD		0.019	0.50
		nPD:	0.04
		N. weight:	0.9
		Sc. PD:	-0.1
		Sc. rank:	-176.8

PB1			
Pos.	293	obs:	exp:
tta	L	1	0.11
ttg	L	0	0.19
tct	S	0	401.00
tcc	S	1	346.10
tca	S	2146	529.30
tcg	S	201	151.70
ctt	L	0	0.19
ctc	L	0	0.17
cta	L	0	0.13
ctg	L	0	0.21
act	T	0	0.26
acc	T	0	0.22
aca	T	1	0.43
acg	T	0	0.08
agt	S	0	479.40
agc	S	0	440.50
---	--	------	------
mPD		0.16	1.6
		nPD:	0.1
		N. weight:	1.7
		Sc. PD:	-0.094
		Sc. rank:	666.3

PB1			
Pos.	294	obs:	exp:
caa	Q	2349	1196.00
cag	Q	1	1154.00
---	--	------	------
mPD		0.00085	0.50
		nPD:	0.
		N. weight:	0.85
		Sc. PD:	-0.13
		Sc. rank:	-1255.7

PB1			
Pos.	295	obs:	exp:
gat	D	1832	1352.00
gac	D	518	998.20
---	--	------	------
mPD		0.34	0.49
		nPD:	0.7
		N. weight:	0.12
		Sc. PD:	0.064
		Sc. rank:	179.6

PB1			
Pos.	296	obs:	exp:
cct	P	0	0.56
ccc	P	0	0.36
cca	P	2	0.82
ccg	P	0	0.26
att	I	0	0.74
atc	I	0	0.50
ata	I	2	0.76
act	T	2	621.40
acc	T	0	518.30
aca	T	2344	1008.00
acg	T	0	198.20
---	--	------	------
mPD		0.0051	0.69
		nPD:	0.01
		N. weight:	1.1
		Sc. PD:	-0.15
		Sc. rank:	-1178.6

PB1			
Pos.	297	obs:	exp:
gaa	E	14	1407.00
gag	E	2336	942.90
---	--	------	------
mPD		0.012	0.48
		nPD:	0.02
		N. weight:	1.1
		Sc. PD:	-0.14
		Sc. rank:	-501.2

PB1			
Pos.	298	obs:	exp:
tta	L	0	256.20
ttg	L	0	440.40
ctt	L	24	440.10
ctc	L	1824	404.90
cta	L	499	312.30
ctg	L	0	493.10
cct	P	0	0.28
ccc	P	1	0.18
cca	P	0	0.41
ccg	P	0	0.13
att	I	0	0.74
atc	I	2	0.50
ata	I	0	0.76
---	--	------	------
mPD		0.35	1.1
		nPD:	0.31
		N. weight:	1.6
		Sc. PD:	0.24
		Sc. rank:	1556.7

PB1			
Pos.	299	obs:	exp:
tct	S	39	401.30
tcc	S	2260	346.40
tca	S	51	529.70
tcg	S	0	151.80
agt	S	0	479.80
agc	S	0	440.90
---	--	------	------
mPD		0.074	1.6
		nPD:	0.05
		N. weight:	2.2
		Sc. PD:	-0.23
		Sc. rank:	-186.9

PB1			
Pos.	300	obs:	exp:
ttt	F	2173	1000.00
ttc	F	177	1350.00
---	--	------	------
mPD		0.14	0.49
		nPD:	0.29
		N. weight:	0.72
		Sc. PD:	0.091
		Sc. rank:	667.7

PB1			
Pos.	301	obs:	exp:
act	T	0	622.50
acc	T	1	519.20
aca	T	2347	1010.00
acg	T	2	198.50
---	--	------	------
mPD		0.0026	0.69
		nPD:	0.
		N. weight:	1.1
		Sc. PD:	-0.16
		Sc. rank:	-1408.1

PB1			
Pos.	302	obs:	exp:
att	I	2126	871.50
atc	I	221	588.60
ata	I	0	886.90
gtt	V	3	0.65
gtc	V	0	0.63
gta	V	0	0.59
gtg	V	0	1.12
---	--	------	------
mPD		0.17	0.66
		nPD:	0.26
		N. weight:	0.91
		Sc. PD:	0.095
		Sc. rank:	812.9

PB1			
Pos.	303	obs:	exp:
act	T	2158	622.50
acc	T	168	519.20
aca	T	23	1010.00
acg	T	1	198.50
---	--	------	------
mPD		0.15	0.69
		nPD:	0.22
		N. weight:	1.3
		Sc. PD:	0.082
		Sc. rank:	1023.4

PB1			
Pos.	304	obs:	exp:
ggt	G	0	338.10
ggc	G	0	310.20
gga	G	2320	1026.00
ggg	G	30	675.90
---	--	------	------
mPD		0.025	0.69
		nPD:	0.04
		N. weight:	0.97
		Sc. PD:	-0.11
		Sc. rank:	-249.1

PB1			
Pos.	305	obs:	exp:
gat	D	147	1352.00
gac	D	2203	998.20
---	--	------	------
mPD		0.12	0.49
		nPD:	0.24
		N. weight:	0.77
		Sc. PD:	0.063
		Sc. rank:	648.7

PB1			
Pos.	306	obs:	exp:
aat	N	523	1249.00
aac	N	1827	1101.00
---	--	------	------
mPD		0.35	0.50
		nPD:	0.7
		N. weight:	0.26
		Sc. PD:	0.13
		Sc. rank:	383.1

PB1			
Pos.	307	obs:	exp:
act	T	1	622.50
acc	T	2345	519.20
aca	T	4	1010.00
acg	T	0	198.50
---	--	------	------
mPD		0.0042	0.69
		nPD:	0.01
		N. weight:	1.9
		Sc. PD:	-0.27
		Sc. rank:	-2318.0

PB1			
Pos.	308	obs:	exp:
aaa	K	1862	1319.00
aag	K	488	1031.00
---	--	------	------
mPD		0.33	0.49
		nPD:	0.67
		N. weight:	0.15
		Sc. PD:	0.076
		Sc. rank:	223.6

PB1			
Pos.	309	obs:	exp:
tgg	W	2350	2350.00
---	--	------	------
mPD		0	0
		nPD:	1.
		N. weight:	0.
		Sc. PD:	0
		Sc. rank:	0

PB1			
Pos.	310	obs:	exp:
aat	N	1903	1249.00
aac	N	447	1101.00
---	--	------	------
mPD		0.31	0.50
		nPD:	0.62
		N. weight:	0.22
		Sc. PD:	0.098
		Sc. rank:	308.7

PB1

Pos.	311	obs:	exp:
gaa	E	88	1407.00
gag	E	2262	942.90
---	--	------	------
mPD		0.072	0.48
		nPD:	0.15
		N. weight:	0.94
		Sc. PD:	-0.0044
		Sc. rank:	527.9

,

PB1

Pos.	312	obs:	exp:
aat	N	2339	1249.00
aac	N	11	1101.00
---	--	------	------
mPD		0.0093	0.50
		nPD:	0.02
		N. weight:	0.77
		Sc. PD:	-0.1
		Sc. rank:	-484.4

,

PB1

Pos.	313	obs:	exp:
caa	Q	1810	1196.00
cag	Q	540	1154.00
---	--	------	------
mPD		0.35	0.50
		nPD:	0.71
		N. weight:	0.19
		Sc. PD:	0.1
		Sc. rank:	283.4

,

PB1

Pos.	314	obs:	exp:
tct	S	0	0.17
tcc	S	0	0.15
tca	S	0	0.23
tcg	S	0	0.06
aat	N	895	1248.00
aac	N	1454	1101.00
agt	S	0	0.20
agc	S	1	0.19
---	--	------	------
mPD		0.47	0.50
		nPD:	0.95
		N. weight:	0.061
		Sc. PD:	0.047
		Sc. rank:	97.1

,

PB1

Pos.	315	obs:	exp:
cct	P	2344	662.70
ccc	P	6	425.20
cca	P	0	959.50
ccg	P	0	302.60
---	--	------	------
mPD		0.0051	0.70
		nPD:	0.01
		N. weight:	1.6
		Sc. PD:	-0.23
		Sc. rank:	-1777.0

,

PB1

Pos.	316	obs:	exp:
cgt	R	4	89.12
cgc	R	0	125.20
cga	R	7	227.60
cgg	R	2223	184.10
aga	R	1	1078.00
agg	R	115	646.20
---	--	------	------
mPD		0.10	0.95
		nPD:	0.11
		N. weight:	2.9
		Sc. PD:	-0.13
		Sc. rank:	1288.1

,

PB1

Pos.	317	obs:	exp:
att	I	487	181.60
atc	I	0	122.60
ata	I	2	184.80
atg	M	1846	1846.00
act	T	1	0.53
acc	T	0	0.44
aca	T	0	0.86
acg	T	1	0.17
gtt	V	0	2.83
gtc	V	0	2.75
gta	V	0	2.56
gtg	V	13	4.87
---	--	------	------
mPD		0.34	0.38
		nPD:	0.91
		N. weight:	0.26
		Sc. PD:	0.19
		Sc. rank:	420.3

,

PB1

Pos.	318	obs:	exp:
ttt	F	2348	1000.00
ttc	F	2	1350.00
---	--	------	------
mPD		0.0017	0.49
		nPD:	0.
		N. weight:	1.1
		Sc. PD:	-0.16
		Sc. rank:	-1481.0

,

PB1

Pos.	319	obs:	exp:
tta	L	5	256.50
ttg	L	2184	441.00
ctt	L	0	440.70
ctc	L	0	405.40
cta	L	1	312.70
ctg	L	160	493.80
---	--	------	------
mPD		0.13	1.1
		nPD:	0.12
		N. weight:	1.8
		Sc. PD:	-0.066
		Sc. rank:	847.4

,

PB1

Pos.	320	obs:	exp:
gct	A	0	597.80
gcc	A	0	458.30
gca	A	2320	1033.00
gcg	A	30	260.90
---	--	------	------
mPD		0.025	0.69
		nPD:	0.04
		N. weight:	0.98
		Sc. PD:	-0.11
		Sc. rank:	-263.0

,

PB1

Pos.	321	obs:	exp:
atg	M	2350	2350.00
---	--	------	------
mPD		0	0
		nPD:	1.
		N. weight:	0.
		Sc. PD:	0
		Sc. rank:	0

,

PB1

Pos.	322	obs:	exp:
att	I	0	853.00
atc	I	0	576.10
ata	I	2297	868.00
gtt	V	0	11.53
gtc	V	0	11.20
gta	V	53	10.43
gtg	V	0	19.85
---	--	------	------
mPD		0.044	0.71
		nPD:	0.06
		N. weight:	1.3
		Sc. PD:	-0.11
		Sc. rank:	168.1

,

PB1

Pos.	323	obs:	exp:
act	T	1	622.50
acc	T	1	519.20
aca	T	2226	1010.00
acg	T	122	198.50
---	--	------	------
mPD		0.10	0.69
		nPD:	0.15
		N. weight:	0.91
		Sc. PD:	-0.0086
		Sc. rank:	495.6

,

PB1

Pos.	324	obs:	exp:
tat	Y	1331	1142.00
tac	Y	1019	1208.00
---	--	------	------
mPD		0.49	0.50
		nPD:	0.98
		N. weight:	0.019
		Sc. PD:	0.015
		Sc. rank:	29.7

,

PB1

Pos.	325	obs:	exp:
att	I	18	863.00
atc	I	2288	582.80
ata	I	18	878.20
gtt	V	0	5.65
gtc	V	26	5.49
gta	V	0	5.12
gtg	V	0	9.74
---	--	------	------
mPD		0.052	0.68
		nPD:	0.08
		N. weight:	1.6
		Sc. PD:	-0.12
		Sc. rank:	480.0

,

PB1

Pos.	326	obs:	exp:
act	T	0	622.50
acc	T	0	519.20
aca	T	2348	1010.00
acg	T	2	198.50
---	--	------	------
mPD		0.0017	0.69
		nPD:	0.
		N. weight:	1.1
		Sc. PD:	-0.16
		Sc. rank:	-1505.0

,

PB1

Pos.	327	obs:	exp:
cgt	R	0	88.06
cgc	R	0	123.70
cga	R	0	224.80
cgg	R	0	181.90
aaa	K	28	15.72
aag	K	0	12.28
aga	R	2172	1065.00
agg	R	150	638.50
---	--	------	------
mPD		0.14	0.97
		nPD:	0.15
		N. weight:	0.73
		Sc. PD:	-0.0055
		Sc. rank:	400.0

,

PB1

Pos.	328	obs:	exp:
cat	H	0	0.59
cac	H	1	0.41
aat	N	514	1247.00
aac	N	1833	1100.00
aaa	K	2	1.12
aag	K	0	0.88
---	--	------	------
mPD		0.34	0.50
		nPD:	0.69
		N. weight:	0.26
		Sc. PD:	0.14
		Sc. rank:	391.0

,

PB1

Pos.	329	obs:	exp:
caa	Q	2309	1196.00
cag	Q	41	1154.00
---	--	------	------
mPD		0.034	0.50
		nPD:	0.07
		N. weight:	0.75
		Sc. PD:	-0.063
		Sc. rank:	172.9

,

PB1

Pos.	330	obs:	exp:
cct	P	2112	662.70
ccc	P	203	425.20
cca	P	35	959.50
ccg	P	0	302.60
---	--	------	------
mPD		0.18	0.70
		nPD:	0.26
		N. weight:	1.2
		Sc. PD:	0.12
		Sc. rank:	1049.6

,

PB1

Pos.	331	obs:	exp:
aaa	K	6	3.37
aag	K	0	2.63
gct	A	0	0.25
gcc	A	0	0.20
gca	A	1	0.44
gcg	A	0	0.11
gat	D	0	1.73
gac	D	3	1.27
gaa	E	1729	1400.00
gag	E	609	938.10
ggt	G	0	0.29
ggc	G	0	0.26
gga	G	0	0.87
ggg	G	2	0.58
---	--	------	------
mPD		0.39	0.49
		nPD:	0.81
		N. weight:	0.062
		Sc. PD:	0.039
		Sc. rank:	98.3

,

PB1

Pos.	332	obs:	exp:
tgg	W	2350	2350.00
---	--	------	------
mPD		0	0
		nPD:	1.
		N. weight:	0.
		Sc. PD:	0
		Sc. rank:	0

,

PB1

Pos.	333	obs:	exp:
ttt	F	2347	1000.00
ttc	F	3	1350.00
---	--	------	------
mPD		0.0026	0.49
		nPD:	0.01
		N. weight:	1.1
		Sc. PD:	-0.16
		Sc. rank:	-1327.0

,

PB1

Pos.	334	obs:	exp:
cgt	R	0	89.12
cgc	R	0	125.20
cga	R	0	227.60
cgg	R	0	184.10
aga	R	2313	1078.00
agg	R	37	646.20
---	--	------	------
mPD		0.031	0.95
		nPD:	0.03
		N. weight:	0.9
		Sc. PD:	-0.11
		Sc. rank:	-292.6

,

PB1

Pos.	335	obs:	exp:
aat	N	2350	1249.00
aac	N	0	1101.00
---	--	------	------
mPD		0	0.50
		nPD:	0.
		N. weight:	0.8
		Sc. PD:	-0.12
		Sc. rank:	-1284.0

,

PB1			
Pos.	336	obs:	exp:
att	I	0	7.80
atc	I	1	5.27
ata	I	20	7.94
atg	M	1	1.00
gtt	V	4	505.40
gtc	V	1815	491.00
gta	V	19	457.30
gtg	V	486	870.30
gct	A	0	1.02
gcc	A	3	0.78
gca	A	0	1.76
gcg	A	1	0.44
---	--	------	------
mPD		0.38	0.75
		nPD:	0.5
		N. weight:	1.1
		Sc. PD:	0.37
		Sc. rank:	1420.0

,

PB1			
Pos.	337	obs:	exp:
tta	L	1904	256.50
ttg	L	441	441.00
ctt	L	0	440.70
ctc	L	0	405.40
cta	L	4	312.70
ctg	L	1	493.80
---	--	------	------
mPD		0.31	1.1
		nPD:	0.27
		N. weight:	2.
		Sc. PD:	0.23
		Sc. rank:	1866.0

,

PB1			
Pos.	338	obs:	exp:
tct	S	0	401.30
tcc	S	0	346.40
tca	S	0	529.70
tcg	S	0	151.80
agt	S	529	479.80
agc	S	1821	440.90
---	--	------	------
mPD		0.35	1.6
		nPD:	0.21
		N. weight:	1.4
		Sc. PD:	0.079
		Sc. rank:	1074.3

,

PB1			
Pos.	339	obs:	exp:
att	I	2308	870.00
atc	I	35	587.60
ata	I	0	885.30
gtt	V	1	1.52
gtc	V	6	1.48
gta	V	0	1.38
gtg	V	0	2.62
---	--	------	------
mPD		0.040	0.66
		nPD:	0.06
		N. weight:	1.2
		Sc. PD:	-0.11
		Sc. rank:	134.3

,

PB1			
Pos.	340	obs:	exp:
act	T	0	0.26
acc	T	1	0.22
aca	T	0	0.43
acg	T	0	0.08
gct	A	103	597.30
gcc	A	2231	457.90
gca	A	14	1032.00
gcg	A	0	260.70
ggt	G	0	0.14
ggc	G	1	0.13
gga	G	0	0.44
ggg	G	0	0.29
---	--	------	------
mPD		0.097	0.69
		nPD:	0.14
		N. weight:	1.8
		Sc. PD:	-0.026
		Sc. rank:	935.6

,

PB1			
Pos.	341	obs:	exp:
cct	P	2319	662.70
ccc	P	30	425.20
cca	P	0	959.50
ccg	P	1	302.60
---	--	------	------
mPD		0.026	0.70
		nPD:	0.04
		N. weight:	1.5
		Sc. PD:	-0.17
		Sc. rank:	-360.1

,

PB1			
Pos.	342	obs:	exp:
att	I	0	872.30
atc	I	0	589.10
ata	I	2349	887.60
atg	M	1	1.00
---	--	------	------
mPD		0.00085	0.66
		nPD:	0.
		N. weight:	1.2
		Sc. PD:	-0.18
		Sc. rank:	-1858.0

,

PB1			
Pos.	343	obs:	exp:
att	I	0	0.37
atc	I	0	0.25
ata	I	1	0.38
atg	M	2349	2349.00
---	--	------	------
mPD		0.00085	0.00085
		nPD:	1.
		N. weight:	0.00052
		Sc. PD:	0.00043
		Sc. rank:	0.8

,

PB1			
Pos.	344	obs:	exp:
ttt	F	0	1000.00
ttc	F	2350	1350.00
---	--	------	------
mPD		0	0.49
		nPD:	0.
		N. weight:	0.7
		Sc. PD:	-0.11
		Sc. rank:	-1125.8

,

PB1			
Pos.	345	obs:	exp:
tct	S	0	401.30
tcc	S	21	346.40
tca	S	2181	529.70
tcg	S	148	151.80
agt	S	0	479.80
agc	S	0	440.90
---	--	------	------
mPD		0.13	1.6
		nPD:	0.08
		N. weight:	1.6
		Sc. PD:	-0.12
		Sc. rank:	524.8

,

PB1			
Pos.	346	obs:	exp:
aat	N	38	1249.00
aac	N	2312	1101.00
---	--	------	------
mPD		0.032	0.50
		nPD:	0.06
		N. weight:	0.85
		Sc. PD:	-0.076
		Sc. rank:	137.5

,

PB1			
Pos.	347	obs:	exp:
aaa	K	2282	1319.00
aag	K	68	1031.00
---	--	------	------
mPD		0.056	0.49
		nPD:	0.11
		N. weight:	0.58
		Sc. PD:	-0.023
		Sc. rank:	267.1

,

PB1			
Pos.	348	obs:	exp:
tta	L	0	3.82
ttg	L	0	6.57
ctt	L	0	6.56
ctc	L	0	6.04
cta	L	0	4.66
ctg	L	35	7.35
atg	M	2315	2315.00
---	--	------	------
mPD		0.029	0.047
		nPD:	0.62
		N. weight:	0.029
		Sc. PD:	0.013
		Sc. rank:	42.0

,

PB1			
Pos.	349	obs:	exp:
gct	A	3	597.80
gcc	A	0	458.30
gca	A	768	1033.00
gcg	A	1579	260.90
---	--	------	------
mPD		0.44	0.69
		nPD:	0.64
		N. weight:	1.4
		Sc. PD:	0.66
		Sc. rank:	2026.2

,

PB1			
Pos.	350	obs:	exp:
cgt	R	0	89.08
cgc	R	0	125.10
cga	R	5	227.50
cgg	R	0	184.00
aaa	K	1	0.56
aag	K	0	0.44
aga	R	2343	1077.00
agg	R	1	645.90
---	--	------	------
mPD		0.0060	0.95
		nPD:	0.01
		N. weight:	0.97
		Sc. PD:	-0.14
		Sc. rank:	-1147.6

,

PB1			
Pos.	351	obs:	exp:
tta	L	2149	256.50
ttg	L	184	441.00
ctt	L	0	440.70
ctc	L	0	405.40
cta	L	15	312.70
ctg	L	2	493.80
---	--	------	------
mPD		0.16	1.1
		nPD:	0.14
		N. weight:	2.3
		Sc. PD:	-0.032
		Sc. rank:	1258.5

,

PB1			
Pos.	352	obs:	exp:
ggt	G	0	338.10
ggc	G	0	310.20
gga	G	446	1026.00
ggg	G	1904	675.90
---	--	------	------
mPD		0.31	0.69
		nPD:	0.45
		N. weight:	0.86
		Sc. PD:	0.25
		Sc. rank:	1047.5

,

PB1			
Pos.	353	obs:	exp:
caa	Q	6	3.05
cag	Q	0	2.95
aaa	K	2343	1316.00
aag	K	1	1028.00
---	--	------	------
mPD		0.0059	0.50
		nPD:	0.01
		N. weight:	0.73
		Sc. PD:	-0.1
		Sc. rank:	-647.8

,

PB1			
Pos.	354	obs:	exp:
ggt	G	0	338.10
ggc	G	0	310.20
gga	G	2346	1026.00
ggg	G	4	675.90
---	--	------	------
mPD		0.0034	0.69
		nPD:	0.
		N. weight:	1.
		Sc. PD:	-0.15
		Sc. rank:	-1312.7

,

PB1			
Pos.	355	obs:	exp:
tat	Y	2	1142.00
tac	Y	2348	1208.00
---	--	------	------
mPD		0.0017	0.50
		nPD:	0.
		N. weight:	0.84
		Sc. PD:	-0.12
		Sc. rank:	-1161.3

,

PB1			
Pos.	356	obs:	exp:
atg	M	2350	2350.00
---	--	------	------
mPD		0	0
		nPD:	1.
		N. weight:	0.
		Sc. PD:	0
		Sc. rank:	0

,

PB1			
Pos.	357	obs:	exp:
ttt	F	1812	1000.00
ttc	F	538	1350.00
---	--	------	------
mPD		0.35	0.49
		nPD:	0.72
		N. weight:	0.32
		Sc. PD:	0.17
		Sc. rank:	488.9

,

PB1			
Pos.	358	obs:	exp:
gat	D	0	4.60
gac	D	8	3.40
gaa	E	2293	1402.00
gag	E	48	939.30
ggt	G	0	0.14
ggc	G	0	0.13
gga	G	1	0.44
ggg	G	0	0.29
---	--	------	------
mPD		0.048	0.48
		nPD:	0.1
		N. weight:	0.54
		Sc. PD:	-0.03
		Sc. rank:	226.4

,

PB1			
Pos.	359	obs:	exp:
tct	S	0	401.30
tcc	S	0	346.40
tca	S	0	529.70
tcg	S	0	151.80
agt	S	2326	479.80
agc	S	24	440.90
---	--	------	------
mPD		0.020	1.6
		nPD:	0.01
		N. weight:	1.9
		Sc. PD:	-0.27
		Sc. rank:	-1704.5

,

PB1			
Pos.	360	obs:	exp:
aaa	K	8	1319.00
aag	K	2342	1031.00
---	--	------	------
mPD		0.0068	0.49
		nPD:	0.01
		N. weight:	1.
		Sc. PD:	-0.14
		Sc. rank:	-838.5

,

PB1

Pos.	361	obs:	exp:
tct	S	0	400.80
tcc	S	0	346.00
tca	S	0	529.00
tcg	S	0	151.60
cgt	R	0	0.04
cgc	R	0	0.05
cga	R	0	0.10
cgg	R	0	0.08
aat	N	0	1.06
aac	N	2	0.94
agt	S	6	479.20
agc	S	2341	440.30
aga	R	0	0.46
agg	R	1	0.27
---	--	------	------
mPD		0.0076	1.6
		nPD:	0.
		N. weight:	2.1
		Sc. PD:	-0.31
		Sc. rank:	-2666.2

,

PB1

Pos.	362	obs:	exp:
tta	L	0	0.11
ttg	L	0	0.19
ctt	L	0	0.19
ctc	L	0	0.17
cta	L	0	0.13
ctg	L	1	0.21
atg	M	2349	2349.00
---	--	------	------
mPD		0.00085	0.0014
		nPD:	0.62
		N. weight:	0.00084
		Sc. PD:	0.00038
		Sc. rank:	1.2

,

PB1

Pos.	363	obs:	exp:
cgt	R	0	0.04
cgc	R	0	0.05
cga	R	0	0.10
cgg	R	0	0.08
aaa	K	51	1319.00
aag	K	2298	1030.00
aga	R	0	0.46
agg	R	1	0.27
---	--	------	------
mPD		0.043	0.49
		nPD:	0.09
		N. weight:	0.91
		Sc. PD:	-0.059
		Sc. rank:	312.8

,

PB1

Pos.	364	obs:	exp:
tta	L	6	256.20
ttg	L	1	440.40
ctt	L	11	440.10
ctc	L	2	404.90
cta	L	2323	312.30
ctg	L	4	493.10
att	I	2	1.11
atc	I	0	0.75
ata	I	1	1.13
---	--	------	------
mPD		0.025	1.1
		nPD:	0.02
		N. weight:	2.5
		Sc. PD:	-0.32
		Sc. rank:	-1229.7

,

PB1

Pos.	365	obs:	exp:
cgt	R	0	89.12
cgc	R	0	125.20
cga	R	52	227.60
cgg	R	656	184.10
aga	R	926	1078.00
agg	R	716	646.20
---	--	------	------
mPD		0.91	0.95
		nPD:	0.95
		N. weight:	0.38
		Sc. PD:	0.29
		Sc. rank:	604.1

,

PB1

Pos.	366	obs:	exp:
act	T	1	622.00
acc	T	0	518.80
aca	T	2334	1009.00
acg	T	13	198.30
gct	A	0	0.51
gcc	A	0	0.39
gca	A	2	0.88
gcg	A	0	0.22
---	--	------	------
mPD		0.014	0.69
		nPD:	0.02
		N. weight:	1.
		Sc. PD:	-0.14
		Sc. rank:	-616.3

,

PB1

Pos.	367	obs:	exp:
caa	Q	2345	1196.00
cag	Q	5	1154.00
---	--	------	------
mPD		0.0042	0.50
		nPD:	0.01
		N. weight:	0.84
		Sc. PD:	-0.12
		Sc. rank:	-889.1

,

PB1

Pos.	368	obs:	exp:
att	I	1	870.00
atc	I	0	587.60
ata	I	2342	885.30
atg	M	1	1.00
gtt	V	0	1.30
gtc	V	0	1.27
gta	V	6	1.18
gtg	V	0	2.25
---	--	------	------
mPD		0.0068	0.66
		nPD:	0.01
		N. weight:	1.2
		Sc. PD:	-0.17
		Sc. rank:	-1213.7

,

PB1

Pos.	369	obs:	exp:
tct	S	0	1.71
tcc	S	0	1.47
tca	S	10	2.25
tcg	S	0	0.65
cct	P	200	659.90
ccc	P	1	423.40
cca	P	1870	955.40
ccg	P	269	301.30
agt	S	0	2.04
agc	S	0	1.88
---	--	------	------
mPD		0.35	0.72
		nPD:	0.49
		N. weight:	0.54
		Sc. PD:	0.17
		Sc. rank:	680.3

,

PB1

Pos.	370	obs:	exp:
gtt	V	0	0.22
gtc	V	0	0.21
gta	V	1	0.20
gtg	V	0	0.37
gct	A	0	597.60
gcc	A	5	458.10
gca	A	2336	1033.00
gcg	A	8	260.80
---	--	------	------
mPD		0.012	0.69
		nPD:	0.02
		N. weight:	1.
		Sc. PD:	-0.13
		Sc. rank:	-703.7

,

PB1

Pos.	371	obs:	exp:
gat	D	1	1.15
gac	D	1	0.85
gaa	E	960	1406.00
gag	E	1388	942.10
---	--	------	------
mPD		0.48	0.48
		nPD:	1.01
		N. weight:	0.095
		Sc. PD:	0.079
		Sc. rank:	152.4

,

PB1

Pos.	372	obs:	exp:
tta	L	0	0.44
ttg	L	1	0.75
ctt	L	0	0.75
ctc	L	0	0.69
cta	L	0	0.53
ctg	L	3	0.84
atg	M	2345	2345.00
aaa	K	0	0.56
aag	K	1	0.44
---	--	------	------
mPD		0.0043	0.0068
		nPD:	0.63
		N. weight:	0.0031
		Sc. PD:	0.0014
		Sc. rank:	4.5

,

PB1

Pos.	373	obs:	exp:
tta	L	0	256.20
ttg	L	0	440.40
ctt	L	2274	440.10
ctc	L	59	404.90
cta	L	13	312.30
ctg	L	1	493.10
att	I	1	1.11
atc	I	0	0.75
ata	I	2	1.13
---	--	------	------
mPD		0.065	1.1
		nPD:	0.06
		N. weight:	1.9
		Sc. PD:	-0.18
		Sc. rank:	167.7

,

PB1

Pos.	374	obs:	exp:
tct	S	0	0.17
tcc	S	0	0.15
tca	S	1	0.23
tcg	S	0	0.06
att	I	0	1.11
atc	I	0	0.75
ata	I	3	1.13
act	T	0	372.20
acc	T	1	310.40
aca	T	1404	603.70
acg	T	0	118.70
agt	S	0	0.20
agc	S	0	0.19
gct	A	0	239.40
gcc	A	0	183.50
gca	A	941	413.60
gcg	A	0	104.50
---	--	------	------
mPD		0.48	1.2
		nPD:	0.41
		N. weight:	1.1
		Sc. PD:	0.26
		Sc. rank:	1221.6

,

PB1

Pos.	375	obs:	exp:
tct	S	0	2.73
tcc	S	0	2.36
tca	S	0	3.61
tcg	S	0	1.03
act	T	55	130.10
acc	T	436	108.50
aca	T	0	211.00
acg	T	0	41.47
aat	N	871	958.20
aac	N	932	844.80
aaa	K	1	0.56
aag	K	0	0.44
agt	S	9	3.27
agc	S	7	3.00
gat	D	0	21.28
gac	D	37	15.72
gaa	E	2	1.20
gag	E	0	0.80
---	--	------	------
mPD		0.85	0.97
		nPD:	0.88
		N. weight:	0.34
		Sc. PD:	0.24
		Sc. rank:	536.5

,

PB1

Pos.	376	obs:	exp:
att	I	2176	871.50
atc	I	171	588.60
ata	I	0	886.90
atg	M	1	1.00
gtt	V	2	0.43
gtc	V	0	0.42
gta	V	0	0.39
gtg	V	0	0.75
---	--	------	------
mPD		0.14	0.66
		nPD:	0.21
		N. weight:	0.96
		Sc. PD:	0.05
		Sc. rank:	708.0

,

PB1

Pos.	377	obs:	exp:
tct	S	0	0.34
tcc	S	0	0.29
tca	S	0	0.45
tcg	S	0	0.13
aat	N	35	18.60
aac	N	0	16.40
agt	S	2	0.41
agc	S	0	0.38
gct	A	1	0.25
gcc	A	0	0.20
gca	A	0	0.44
gcg	A	0	0.11
gat	D	1424	1310.00
gac	D	853	967.20
gaa	E	31	18.56
gag	E	0	12.44
ggt	G	4	0.58
ggc	G	0	0.53
gga	G	0	1.75
ggg	G	0	1.15
---	--	------	------
mPD		0.52	0.54
		nPD:	0.95
		N. weight:	0.036
		Sc. PD:	0.028
		Sc. rank:	57.1

,

PB1

Pos.	378	obs:	exp:
tta	L	177	256.50
ttg	L	427	441.00
ctt	L	1	440.70
ctc	L	0	405.40
cta	L	2	312.70
ctg	L	1743	493.80
---	--	------	------
mPD		0.52	1.1
		nPD:	0.46
		N. weight:	1.1
		Sc. PD:	0.34
		Sc. rank:	1396.9

,

PB1

Pos.	379	obs:	exp:
cgt	R	0	0.15
cgc	R	0	0.21
cga	R	0	0.39
cgg	R	0	0.31
aat	N	2	1.59
aac	N	1	1.41
aaa	K	1479	1315.00
aag	K	863	1027.00
aga	R	1	1.83
agg	R	3	1.10
gaa	E	0	0.60
gag	E	1	0.40
---	--	------	------
mPD		0.47	0.50
		nPD:	0.95
		N. weight:	0.018
		Sc. PD:	0.014
		Sc. rank:	28.4

,

PB1

Pos.	380	obs:	exp:
tat	Y	1921	1142.00
tac	Y	429	1208.00
---	--	------	------
mPD		0.30	0.50
		nPD:	0.6
		N. weight:	0.3
		Sc. PD:	0.13
		Sc. rank:	423.7

,

PB1

Pos.	381	obs:	exp:
ttt	F	0	999.50
ttc	F	2349	1349.00
tta	L	0	0.11
ttg	L	0	0.19
ctt	L	0	0.19
ctc	L	1	0.17
cta	L	0	0.13
ctg	L	0	0.21
---	--	------	------
mPD		0.00085	0.49
		nPD:	0.
		N. weight:	0.7
		Sc. PD:	-0.1
		Sc. rank:	-1020.2

PB1

Pos.	382	obs:	exp:
aat	N	406	1249.00
aac	N	1944	1101.00
---	--	------	------
mPD		0.29	0.50
		nPD:	0.57
		N. weight:	0.35
		Sc. PD:	0.14
		Sc. rank:	486.1

PB1

Pos.	383	obs:	exp:
gaa	E	2204	1405.00
gag	E	142	941.30
ggt	G	0	0.58
ggc	G	0	0.53
gga	G	4	1.75
ggg	G	0	1.15
---	--	------	------
mPD		0.12	0.48
		nPD:	0.24
		N. weight:	0.39
		Sc. PD:	0.033
		Sc. rank:	335.8

PB1

Pos.	384	obs:	exp:
tta	L	1	0.11
ttg	L	0	0.19
tct	S	0	278.90
tcc	S	22	240.70
tca	S	1122	368.10
tcg	S	489	105.50
ctt	L	0	0.19
ctc	L	0	0.17
cta	L	0	0.13
ctg	L	0	0.21
cct	P	0	194.60
ccc	P	0	124.90
cca	P	690	281.70
ccg	P	0	88.85
caa	Q	6	3.05
cag	Q	0	2.95
act	T	0	5.30
acc	T	0	4.42
aca	T	20	8.59
acg	T	0	1.69
agt	S	0	333.40
agc	S	0	306.40
---	--	------	------
mPD		0.78	1.8
		nPD:	0.44
		N. weight:	1.4
		Sc. PD:	0.39
		Sc. rank:	1689.6

PB1

Pos.	385	obs:	exp:
cct	P	0	0.28
ccc	P	0	0.18
cca	P	0	0.41
ccg	P	1	0.13
act	T	0	622.20
acc	T	0	519.00
aca	T	734	1009.00
acg	T	1615	198.40
---	--	------	------
mPD		0.43	0.69
		nPD:	0.62
		N. weight:	1.7
		Sc. PD:	0.78
		Sc. rank:	2442.0

PB1

Pos.	386	obs:	exp:
cgt	R	0	88.66
cgc	R	0	124.50
cga	R	0	226.40
cgg	R	0	183.10
aaa	K	12	6.74
aag	K	0	5.26
aga	R	2296	1072.00
agg	R	42	642.90
---	--	------	------
mPD		0.045	0.96
		nPD:	0.05
		N. weight:	0.89
		Sc. PD:	-0.093
		Sc. rank:	-64.4

PB1

Pos.	387	obs:	exp:
cgt	R	0	0.11
cgc	R	0	0.16
cga	R	0	0.29
cgg	R	0	0.23
aat	N	25	13.29
aac	N	0	11.71
aaa	K	4	1302.00
aag	K	2316	1018.00
aga	R	0	1.38
agg	R	3	0.82
gaa	E	0	1.20
gag	E	2	0.80
---	--	------	------
mPD		0.029	0.51
		nPD:	0.06
		N. weight:	1.
		Sc. PD:	-0.098
		Sc. rank:	80.8

PB1

Pos.	388	obs:	exp:
aaa	K	2349	1319.00
aag	K	1	1031.00
---	--	------	------
mPD		0.00085	0.49
		nPD:	0.
		N. weight:	0.73
		Sc. PD:	-0.11
		Sc. rank:	-1062.5

PB1

Pos.	389	obs:	exp:
tta	L	0	0.22
ttg	L	0	0.38
ctt	L	0	0.38
ctc	L	2	0.35
cta	L	0	0.27
ctg	L	0	0.42
att	I	531	871.90
atc	I	1795	588.90
ata	I	22	887.20
---	--	------	------
mPD		0.37	0.66
		nPD:	0.56
		N. weight:	0.9
		Sc. PD:	0.35
		Sc. rank:	1222.6

PB1

Pos.	390	obs:	exp:
aaa	K	0	1.68
aag	K	3	1.32
gaa	E	15	1405.00
gag	E	2332	941.70
---	--	------	------
mPD		0.015	0.48
		nPD:	0.03
		N. weight:	1.1
		Sc. PD:	-0.13
		Sc. rank:	-383.1

PB1

Pos.	391	obs:	exp:
cgt	R	0	0.04
cgc	R	0	0.05
cga	R	0	0.10
cgg	R	0	0.08
act	T	0	0.26
acc	T	0	0.22
aca	T	1	0.43
acg	T	0	0.08
aat	N	11	5.85
aac	N	0	5.15
aaa	K	2324	1312.00
aag	K	13	1025.00
aga	R	1	0.46
agg	R	0	0.27
---	--	------	------
mPD		0.022	0.50
		nPD:	0.04
		N. weight:	0.69
		Sc. PD:	-0.075
		Sc. rank:	-68.3

PB1

Pos.	392	obs:	exp:
tta	L	1	0.11
ttg	L	0	0.19
ctt	L	0	0.19
ctc	L	0	0.17
cta	L	0	0.13
ctg	L	0	0.21
att	I	0	863.40
atc	I	1	583.10
ata	I	2324	878.50
atg	M	1	1.00
act	T	0	0.26
acc	T	0	0.22
aca	T	1	0.43
acg	T	0	0.08
gtt	V	0	4.78
gtc	V	0	4.65
gta	V	22	4.33
gtg	V	0	8.24
---	--	------	------
mPD		0.022	0.68
		nPD:	0.03
		N. weight:	1.2
		Sc. PD:	-0.15
		Sc. rank:	-408.4

PB1

Pos.	393	obs:	exp:
cgt	R	0	89.08
cgc	R	0	125.10
cga	R	1	227.50
cgg	R	0	184.00
aga	R	1820	1077.00
agg	R	528	645.90
ggt	G	0	0.14
ggc	G	0	0.13
gga	G	1	0.44
ggg	G	0	0.29
---	--	------	------
mPD		0.35	0.95
		nPD:	0.37
		N. weight:	0.46
		Sc. PD:	0.094
		Sc. rank:	496.4

PB1

Pos.	394	obs:	exp:
tta	L	0	0.22
ttg	L	0	0.38
ctt	L	2	0.38
ctc	L	0	0.35
cta	L	0	0.27
ctg	L	0	0.42
cct	P	2341	661.80
ccc	P	6	424.70
cca	P	0	958.30
ccg	P	0	302.20
cat	H	1	0.59
cac	H	0	0.41
---	--	------	------
mPD		0.0076	0.71
		nPD:	0.01
		N. weight:	1.6
		Sc. PD:	-0.22
		Sc. rank:	-1486.9

PB1

Pos.	395	obs:	exp:
tta	L	100	256.10
ttg	L	8	440.20
ctt	L	13	439.90
ctc	L	524	404.80
cta	L	34	312.10
ctg	L	1667	492.90
atg	M	4	4.00
---	--	------	------
mPD		0.53	1.1
		nPD:	0.46
		N. weight:	1.
		Sc. PD:	0.31
		Sc. rank:	1282.9

PB1			
Pos.	396	obs:	exp:
tta	L	4	256.40
ttg	L	3	440.80
ctt	L	0	440.50
ctc	L	0	405.30
cta	L	890	312.50
ctg	L	1452	493.50
att	I	0	0.37
atc	I	0	0.25
ata	I	1	0.38
---	--	------	------
mPD		0.48	1.1
		nPD:	0.42
		N. weight:	1.3
		Sc. PD:	0.35
		Sc. rank:	1576.9

PB1			
Pos.	397	obs:	exp:
tta	L	0	0.22
ttg	L	0	0.38
ctt	L	0	0.38
ctc	L	0	0.35
cta	L	2	0.27
ctg	L	0	0.42
att	I	1644	870.80
atc	I	21	588.10
ata	I	680	886.10
atg	M	2	2.00
gtt	V	1	0.22
gtc	V	0	0.21
gta	V	0	0.20
gtg	V	0	0.37
---	--	------	------
mPD		0.43	0.66
		nPD:	0.65
		N. weight:	0.43
		Sc. PD:	0.21
		Sc. rank:	630.4

PB1			
Pos.	398	obs:	exp:
aat	N	16	8.50
aac	N	0	7.50
gct	A	0	0.25
gcc	A	0	0.20
gca	A	1	0.44
gcg	A	0	0.11
gat	D	1802	1057.00
gac	D	35	780.30
gaa	E	486	292.20
gag	E	2	195.80
ggt	G	8	1.15
ggc	G	0	1.06
gga	G	0	3.49
ggg	G	0	2.30
---	--	------	------
mPD		0.37	0.68
		nPD:	0.55
		N. weight:	0.6
		Sc. PD:	0.23
		Sc. rank:	819.3

PB1			
Pos.	399	obs:	exp:
ggt	G	44	338.10
ggc	G	1780	310.20
gga	G	9	1026.00
ggg	G	517	675.90
---	--	------	------
mPD		0.38	0.69
		nPD:	0.55
		N. weight:	1.5
		Sc. PD:	0.59
		Sc. rank:	2068.7

PB1			
Pos.	400	obs:	exp:
act	T	66	621.40
acc	T	101	518.30
aca	T	813	1008.00
acg	T	1366	198.20
gct	A	0	1.02
gcc	A	0	0.78
gca	A	4	1.76
gcg	A	0	0.44
---	--	------	------
mPD		0.54	0.69
		nPD:	0.78
		N. weight:	1.2
		Sc. PD:	0.71
		Sc. rank:	1839.7

PB1			
Pos.	401	obs:	exp:
cct	P	0	0.28
ccc	P	1	0.18
cca	P	0	0.41
ccg	P	0	0.13
gct	A	40	597.60
gcc	A	2306	458.10
gca	A	3	1033.00
gcg	A	0	260.80
---	--	------	------
mPD		0.037	0.69
		nPD:	0.05
		N. weight:	1.9
		Sc. PD:	-0.19
		Sc. rank:	63.1

PB1			
Pos.	402	obs:	exp:
tct	S	0	401.30
tcc	S	4	346.40
tca	S	1860	529.70
tcg	S	486	151.80
agt	S	0	479.80
agc	S	0	440.90
---	--	------	------
mPD		0.33	1.6
		nPD:	0.2
		N. weight:	1.6
		Sc. PD:	0.07
		Sc. rank:	1078.4

PB1			
Pos.	403	obs:	exp:
tta	L	69	256.40
ttg	L	2210	440.80
tgg	W	1	1.00
ctt	L	0	440.50
ctc	L	0	405.30
cta	L	0	312.50
ctg	L	70	493.50
---	--	------	------
mPD		0.12	1.1
		nPD:	0.1
		N. weight:	1.8
		Sc. PD:	-0.093
		Sc. rank:	783.4

PB1			
Pos.	404	obs:	exp:
tct	S	0	401.30
tcc	S	0	346.40
tca	S	0	529.70
tcg	S	0	151.80
agt	S	2269	479.80
agc	S	81	440.90
---	--	------	------
mPD		0.067	1.6
		nPD:	0.04
		N. weight:	1.8
		Sc. PD:	-0.2
		Sc. rank:	-285.9

PB1			
Pos.	405	obs:	exp:
cct	P	2343	662.70
ccc	P	6	425.20
cca	P	0	959.50
ccg	P	1	302.60
---	--	------	------
mPD		0.0059	0.70
		nPD:	0.01
		N. weight:	1.6
		Sc. PD:	-0.22
		Sc. rank:	-1682.4

PB1			
Pos.	406	obs:	exp:
ggt	G	0	338.10
ggc	G	0	310.20
gga	G	936	1026.00
ggg	G	1414	675.90
---	--	------	------
mPD		0.48	0.69
		nPD:	0.7
		N. weight:	0.52
		Sc. PD:	0.27
		Sc. rank:	779.1

PB1			
Pos.	407	obs:	exp:
atg	M	2350	2350.00
---	--	------	------
mPD		0	0
		nPD:	1.
		N. weight:	0.
		Sc. PD:	0
		Sc. rank:	0

PB1			
Pos.	408	obs:	exp:
atg	M	2350	2350.00
---	--	------	------
mPD		0	0
		nPD:	1.
		N. weight:	0.
		Sc. PD:	0
		Sc. rank:	0

PB1			
Pos.	409	obs:	exp:
atg	M	2350	2350.00
---	--	------	------
mPD		0	0
		nPD:	1.
		N. weight:	0.
		Sc. PD:	0
		Sc. rank:	0

PB1			
Pos.	410	obs:	exp:
ggt	G	6	338.10
ggc	G	2343	310.20
gga	G	1	1026.00
ggg	G	0	675.90
---	--	------	------
mPD		0.0059	0.69
		nPD:	0.01
		N. weight:	2.5
		Sc. PD:	-0.36
		Sc. rank:	-2628.8

PB1			
Pos.	411	obs:	exp:
atg	M	2350	2350.00
---	--	------	------
mPD		0	0
		nPD:	1.
		N. weight:	0.
		Sc. PD:	0
		Sc. rank:	0

PB1			
Pos.	412	obs:	exp:
ttt	F	6	1000.00
ttc	F	2344	1350.00
---	--	------	------
mPD		0.0051	0.49
		nPD:	0.01
		N. weight:	0.68
		Sc. PD:	-0.096
		Sc. rank:	-656.1

PB1			
Pos.	413	obs:	exp:
aat	N	1785	1249.00
aac	N	565	1101.00
---	--	------	------
mPD		0.37	0.50
		nPD:	0.73
		N. weight:	0.14
		Sc. PD:	0.08
		Sc. rank:	221.3

PB1			
Pos.	414	obs:	exp:
tta	L	0	0.11
ttg	L	1	0.19
ctt	L	0	0.19
ctc	L	0	0.17
cta	L	0	0.13
ctg	L	0	0.21
atg	M	2349	2349.00
---	--	------	------
mPD		0.00085	0.0014
		nPD:	0.62
		N. weight:	0.0009
		Sc. PD:	0.00041
		Sc. rank:	1.3

PB1			
Pos.	415	obs:	exp:
tta	L	3	256.50
ttg	L	8	441.00
ctt	L	0	440.70
ctc	L	0	405.40
cta	L	9	312.70
ctg	L	2330	493.80
---	--	------	------
mPD		0.019	1.1
		nPD:	0.02
		N. weight:	1.9
		Sc. PD:	-0.26
		Sc. rank:	-1323.6

PB1			
Pos.	416	obs:	exp:
tct	S	0	401.20
tcc	S	0	346.30
tca	S	0	529.50
tcg	S	0	151.70
act	T	0	0.26
acc	T	1	0.22
aca	T	0	0.43
acg	T	0	0.08
agt	S	646	479.60
agc	S	1703	440.70
---	--	------	------
mPD		0.40	1.6
		nPD:	0.24
		N. weight:	1.3
		Sc. PD:	0.12
		Sc. rank:	1156.1

PB1			
Pos.	417	obs:	exp:
act	T	2	622.50
acc	T	0	519.20
aca	T	2347	1010.00
acg	T	1	198.50
---	--	------	------
mPD		0.0026	0.69
		nPD:	0.
		N. weight:	1.1
		Sc. PD:	-0.16
		Sc. rank:	-1407.2

PB1			
Pos.	418	obs:	exp:
att	I	0	0.37
atc	I	0	0.25
ata	I	1	0.38
gtt	V	21	510.60
gtc	V	498	496.10
gta	V	1749	462.00
gtg	V	80	879.30
gct	A	0	0.25
gcc	A	1	0.20
gca	A	0	0.44
gcg	A	0	0.11
---	--	------	------
mPD		0.40	0.73
		nPD:	0.55
		N. weight:	1.1
		Sc. PD:	0.43
		Sc. rank:	1518.8

PB1			
Pos.	419	obs:	exp:
tta	L	1290	256.50
ttg	L	1001	441.00
ctt	L	0	440.70
ctc	L	0	405.40
cta	L	56	312.70
ctg	L	3	493.80
---	--	------	------
mPD		0.54	1.1
		nPD:	0.47
		N. weight:	1.5
		Sc. PD:	0.47
		Sc. rank:	1883.2

PB1			
Pos.	420	obs:	exp:
ggt	G	0	338.10
ggc	G	0	310.20
gga	G	666	1026.00
ggg	G	1684	675.90
---	--	------	------
mPD		0.41	0.69
		nPD:	0.59
		N. weight:	0.68
		Sc. PD:	0.29
		Sc. rank:	945.5

PB1			
Pos.	421	obs:	exp:
gtt	V	17	511.10
gtc	V	1840	496.50
gta	V	483	462.40
gtg	V	10	880.00
---	--	------	------
mPD		0.34	0.73
		nPD:	0.47
		N. weight:	1.3
		Sc. PD:	0.39
		Sc. rank:	1563.0

PB1			
Pos.	422	obs:	exp:
tct	S	5	401.00
tcc	S	0	346.10
tca	S	2331	529.30
tcg	S	12	151.70
agt	S	0	479.40
agc	S	0	440.50
gct	A	0	0.51
gcc	A	0	0.39
gca	A	0	0.88
gcg	A	2	0.22
---	--	------	------
mPD		0.018	1.6
		nPD:	0.01
		N. weight:	1.8
		Sc. PD:	-0.26
		Sc. rank:	-1739.2

PB1			
Pos.	423	obs:	exp:
att	I	507	872.60
atc	I	1843	589.40
ata	I	0	888.00
---	--	------	------
mPD		0.34	0.66
		nPD:	0.52
		N. weight:	0.99
		Sc. PD:	0.35
		Sc. rank:	1291.9

PB1			
Pos.	424	obs:	exp:
tta	L	5	256.50
ttg	L	6	441.00
ctt	L	6	440.70
ctc	L	0	405.40
cta	L	531	312.70
ctg	L	1802	493.80
---	--	------	------
mPD		0.37	1.1
		nPD:	0.32
		N. weight:	1.4
		Sc. PD:	0.22
		Sc. rank:	1378.7

PB1			
Pos.	425	obs:	exp:
act	T	2	0.53
acc	T	0	0.44
aca	T	0	0.86
acg	T	0	0.17
aat	N	2337	1248.00
aac	N	11	1100.00
---	--	------	------
mPD		0.011	0.50
		nPD:	0.02
		N. weight:	0.77
		Sc. PD:	-0.099
		Sc. rank:	-405.7

PB1			
Pos.	426	obs:	exp:
ttt	F	1	0.43
ttc	F	0	0.57
tta	L	0	256.40
ttg	L	0	440.80
ctt	L	923	440.50
ctc	L	1408	405.30
cta	L	18	312.50
ctg	L	0	493.50
---	--	------	------
mPD		0.49	1.1
		nPD:	0.43
		N. weight:	1.3
		Sc. PD:	0.34
		Sc. rank:	1549.7

PB1			
Pos.	427	obs:	exp:
ggt	G	1	338.10
ggc	G	490	310.20
gga	G	264	1026.00
ggg	G	1595	675.90
---	--	------	------
mPD		0.48	0.69
		nPD:	0.7
		N. weight:	0.67
		Sc. PD:	0.35
		Sc. rank:	1004.9

PB1			
Pos.	428	obs:	exp:
caa	Q	2323	1183.00
cag	Q	3	1143.00
cgt	R	0	0.80
cgc	R	0	1.12
cga	R	21	2.03
cgg	R	0	1.64
aaa	K	2	1.12
aag	K	0	0.88
aga	R	0	9.63
agg	R	0	5.77
gct	A	0	0.25
gcc	A	0	0.20
gca	A	1	0.44
gcg	A	0	0.11
---	--	------	------
mPD		0.024	0.53
		nPD:	0.04
		N. weight:	0.86
		Sc. PD:	-0.093
		Sc. rank:	-82.1

PB1			
Pos.	429	obs:	exp:
cgt	R	0	0.08
cgc	R	0	0.11
cga	R	0	0.19
cgg	R	0	0.16
aaa	K	213	1318.00
aag	K	2135	1030.00
aga	R	0	0.92
agg	R	2	0.55
---	--	------	------
mPD		0.17	0.49
		nPD:	0.34
		N. weight:	0.63
		Sc. PD:	0.11
		Sc. rank:	649.5

PB1			
Pos.	430	obs:	exp:
cgt	R	0	88.36
cgc	R	0	124.10
cga	R	0	225.60
cgg	R	0	182.50
aaa	K	4	10.10
aag	K	14	7.90
aga	R	26	1069.00
agg	R	2304	640.70
ggt	G	0	0.29
ggc	G	0	0.26
gga	G	0	0.87
ggg	G	2	0.58
---	--	------	------
mPD		0.042	0.97
		nPD:	0.04
		N. weight:	1.5
		Sc. PD:	-0.17
		Sc. rank:	-180.1

PB1			
Pos.	431	obs:	exp:
tct	S	0	0.17
tcc	S	1	0.15
tca	S	0	0.23
tcg	S	0	0.06
tat	Y	9	1140.00
tac	Y	2337	1206.00
tgt	C	0	0.43
tgc	C	1	0.57
cat	H	0	1.19
cac	H	2	0.81
agt	S	0	0.20
agc	S	0	0.19
---	--	------	------
mPD		0.011	0.50
		nPD:	0.02
		N. weight:	0.81
		Sc. PD:	-0.11
		Sc. rank:	-433.7

PB1			
Pos.	432	obs:	exp:
cct	P	0	0.28
ccc	P	1	0.18
cca	P	0	0.41
ccg	P	0	0.13
act	T	17	622.20
acc	T	2331	519.00
aca	T	1	1009.00
acg	T	0	198.40
---	--	------	------
mPD		0.016	0.69
		nPD:	0.02
		N. weight:	1.9
		Sc. PD:	-0.24
		Sc. rank:	-905.3

PB1			
Pos.	433	obs:	exp:
caa	Q	1	0.51
cag	Q	0	0.49
cgt	R	0	0.08
cgc	R	0	0.11
cga	R	0	0.19
cgg	R	0	0.16
aat	N	1	0.53
aac	N	0	0.47
aaa	K	2344	1317.00
aag	K	2	1029.00
aga	R	2	0.92
agg	R	0	0.55
---	--	------	------
mPD		0.0051	0.50
		nPD:	0.01
		N. weight:	0.72
		Sc. PD:	-0.1
		Sc. rank:	-712.2

PB1			
Pos.	434	obs:	exp:
act	T	2	622.20
acc	T	2333	519.00
aca	T	14	1009.00
acg	T	0	198.40
aat	N	0	0.53
aac	N	1	0.47
---	--	------	------
mPD		0.014	0.69
		nPD:	0.02
		N. weight:	1.9
		Sc. PD:	-0.24
		Sc. rank:	-1019.1

PB1			
Pos.	435	obs:	exp:
att	I	0	2.60
atc	I	0	1.76
ata	I	7	2.65
act	T	3	620.60
acc	T	0	517.70
aca	T	2339	1007.00
acg	T	1	197.90
---	--	------	------
mPD		0.0093	0.70
		nPD:	0.01
		N. weight:	1.1
		Sc. PD:	-0.15
		Sc. rank:	-886.5

PB1			
Pos.	436	obs:	exp:
tat	Y	56	1142.00
tac	Y	2293	1207.00
aat	N	0	0.53
aac	N	1	0.47
---	--	------	------
mPD		0.047	0.50
		nPD:	0.09
		N. weight:	0.7
		Sc. PD:	-0.041
		Sc. rank:	272.8

PB1			
Pos.	437	obs:	exp:
tgt	C	1	0.43
tgc	C	0	0.57
tgg	W	2349	2349.00
---	--	------	------
mPD		0.00085	0.00085
		nPD:	1.
		N. weight:	0.00045
		Sc. PD:	0.00037
		Sc. rank:	0.7

PB1			
Pos.	438	obs:	exp:
tgg	W	2348	2348.00
cgt	R	0	0.04
cgc	R	0	0.05
cga	R	0	0.10
cgg	R	1	0.08
aga	R	0	0.46
agg	R	0	0.27
ggt	G	0	0.14
ggc	G	0	0.13
gga	G	0	0.44
ggg	G	1	0.29
---	--	------	------
mPD		0.0017	0.0029
		nPD:	0.6
		N. weight:	0.002
		Sc. PD:	0.00088
		Sc. rank:	2.9

PB1			
Pos.	439	obs:	exp:
gat	D	1820	1351.00
gac	D	529	997.80
gaa	E	1	0.60
gag	E	0	0.40
---	--	------	------
mPD		0.35	0.49
		nPD:	0.71
		N. weight:	0.11
		Sc. PD:	0.062
		Sc. rank:	175.1

PB1			
Pos.	440	obs:	exp:
ggt	G	0	338.10
ggc	G	4	310.20
gga	G	2287	1026.00
ggg	G	59	675.90
---	--	------	------
mPD		0.052	0.69
		nPD:	0.08
		N. weight:	0.9
		Sc. PD:	-0.069
		Sc. rank:	252.7

PB1			
Pos.	441	obs:	exp:
tta	L	0	256.50
ttg	L	0	441.00
ctt	L	1395	440.70
ctc	L	948	405.40
cta	L	2	312.70
ctg	L	5	493.80
---	--	------	------
mPD		0.49	1.1
		nPD:	0.43
		N. weight:	1.3
		Sc. PD:	0.34
		Sc. rank:	1529.3

PB1			
Pos.	442	obs:	exp:
caa	Q	2345	1196.00
cag	Q	5	1154.00
---	--	------	------
mPD		0.0042	0.50
		nPD:	0.01
		N. weight:	0.84
		Sc. PD:	-0.12
		Sc. rank:	-889.1

PB1			
Pos.	443	obs:	exp:
tct	S	96	401.30
tcc	S	2253	346.40
tca	S	1	529.70
tcg	S	0	151.80
agt	S	0	479.80
agc	S	0	440.90
---	--	------	------
mPD		0.079	1.6
		nPD:	0.05
		N. weight:	2.2
		Sc. PD:	-0.23
		Sc. rank:	-90.5

PB1			
Pos.	444	obs:	exp:
tct	S	2346	401.30
tcc	S	2	346.40
tca	S	2	529.70
tcg	S	0	151.80
agt	S	0	479.80
agc	S	0	440.90
---	--	------	------
mPD		0.0034	1.6
		nPD:	0.
		N. weight:	2.2
		Sc. PD:	-0.33
		Sc. rank:	-3172.8

PB1			
Pos.	445	obs:	exp:
gat	D	2314	1352.00
gac	D	36	998.20
---	--	------	------
mPD		0.030	0.49
		nPD:	0.06
		N. weight:	0.61
		Sc. PD:	-0.055
		Sc. rank:	78.4

PB1			
Pos.	446	obs:	exp:
gat	D	2331	1352.00
gac	D	19	998.20
mPD		0.016	0.49
		nPD:	0.03
		N. weight:	0.65
		Sc. PD:	-0.077
		Sc. rank:	-208.9

PB1			
Pos.	447	obs:	exp:
ttt	F	36	1000.00
ttc	F	2314	1350.00
mPD		0.030	0.49
		nPD:	0.06
		N. weight:	0.61
		Sc. PD:	-0.055
		Sc. rank:	77.0

PB1			
Pos.	448	obs:	exp:
act	T	7	1.85
acc	T	0	1.55
aca	T	0	3.01
acg	T	0	0.59
gtt	V	3	0.65
gtc	V	0	0.63
gta	V	0	0.59
gtg	V	0	1.12
gct	A	2182	595.30
gcc	A	154	456.30
gca	A	4	1029.00
gcg	A	0	259.80
mPD		0.13	0.70
		nPD:	0.19
		N. weight:	1.4
		Sc. PD:	0.051
		Sc. rank:	970.0

PB1			
Pos.	449	obs:	exp:
ttt	F	0	0.43
ttc	F	1	0.57
tta	L	0	256.30
ttg	L	0	440.60
ctt	L	12	440.30
ctc	L	2336	405.10
cta	L	0	312.40
ctg	L	0	493.30
cat	H	0	0.59
cac	H	1	0.41
mPD		0.012	1.1
		nPD:	0.01
		N. weight:	2.2
		Sc. PD:	-0.31
		Sc. rank:	-2083.2

PB1			
Pos.	450	obs:	exp:
att	I	3	872.60
atc	I	1	589.40
ata	I	2346	888.00
mPD		0.0034	0.66
		nPD:	0.01
		N. weight:	1.2
		Sc. PD:	-0.18
		Sc. rank:	-1518.3

PB1			
Pos.	451	obs:	exp:
att	I	0	0.37
atc	I	0	0.25
ata	I	1	0.38
atg	M	1	1.00
gtt	V	0	510.60
gtc	V	0	496.10
gta	V	522	462.00
gtg	V	1826	879.30
mPD		0.35	0.73
		nPD:	0.48
		N. weight:	0.76
		Sc. PD:	0.24
		Sc. rank:	945.7

PB1			
Pos.	452	obs:	exp:
aat	N	2349	1249.00
aac	N	1	1101.00
mPD		0.00085	0.50
		nPD:	0.
		N. weight:	0.8
		Sc. PD:	-0.12
		Sc. rank:	-1166.1

PB1			
Pos.	453	obs:	exp:
tct	S	0	0.17
tcc	S	0	0.15
tca	S	1	0.23
tcg	S	0	0.06
agt	S	0	0.20
agc	S	0	0.19
gct	A	13	597.60
gcc	A	2	458.10
gca	A	2309	1033.00
gcg	A	25	260.80
mPD		0.034	0.69
		nPD:	0.05
		N. weight:	0.94
		Sc. PD:	-0.096
		Sc. rank:	-9.5

PB1			
Pos.	454	obs:	exp:
tta	L	0	0.22
ttg	L	0	0.38
tct	S	0	4.44
tcc	S	0	3.83
tca	S	0	5.86
tcg	S	26	1.68
ctt	L	1	0.38
ctc	L	0	0.35
cta	L	0	0.27
ctg	L	1	0.42
cct	P	4	654.20
ccc	P	0	419.80
cca	P	85	947.30
ccg	P	2231	298.70
act	T	0	0.53
acc	T	0	0.44
aca	T	0	0.86
acg	T	2	0.17
agt	S	0	5.31
agc	S	0	4.88
mPD		0.099	0.74
		nPD:	0.13
		N. weight:	2.3
		Sc. PD:	-0.048
		Sc. rank:	1213.6

PB1			
Pos.	455	obs:	exp:
tct	S	0	1.02
tcc	S	0	0.88
tca	S	0	1.35
tcg	S	0	0.39
act	T	1	0.26
acc	T	0	0.22
aca	T	0	0.43
acg	T	0	0.08
aat	N	2288	1239.00
aac	N	44	1093.00
agt	S	6	1.23
agc	S	0	1.13
gat	D	11	6.33
gac	D	0	4.67
mPD		0.052	0.52
		nPD:	0.1
		N. weight:	0.69
		Sc. PD:	-0.036
		Sc. rank:	295.1

PB1			
Pos.	456	obs:	exp:
cat	H	2297	1391.00
cac	H	42	948.30
cgt	R	2	0.15
cgc	R	2	0.21
cga	R	0	0.39
cgg	R	0	0.31
aat	N	7	3.72
aac	N	0	3.28
aga	R	0	1.83
agg	R	0	1.10
mPD		0.046	0.50
		nPD:	0.09
		N. weight:	0.56
		Sc. PD:	-0.034
		Sc. rank:	207.1

PB1			
Pos.	457	obs:	exp:
gtt	V	0	0.22
gtc	V	0	0.21
gta	V	0	0.20
gtg	V	1	0.37
gaa	E	1	1406.00
gag	E	2348	942.50
mPD		0.0017	0.48
		nPD:	0.
		N. weight:	1.2
		Sc. PD:	-0.17
		Sc. rank:	-1567.4

PB1			
Pos.	458	obs:	exp:
ggt	G	0	338.10
ggc	G	1	310.20
gga	G	631	1026.00
ggg	G	1718	675.90
mPD		0.39	0.69
		nPD:	0.57
		N. weight:	0.7
		Sc. PD:	0.28
		Sc. rank:	963.8

PB1			
Pos.	459	obs:	exp:
att	I	1	870.80
atc	I	0	588.10
ata	I	2344	886.10
gtt	V	0	0.87
gtc	V	0	0.85
gta	V	4	0.79
gtg	V	0	1.50
gct	A	0	0.25
gcc	A	0	0.20
gca	A	1	0.44
gcg	A	0	0.11
mPD		0.0060	0.66
		nPD:	0.01
		N. weight:	1.2
		Sc. PD:	-0.17
		Sc. rank:	-1219.8

PB1			
Pos.	460	obs:	exp:
tta	L	0	0.11
ttg	L	0	0.19
ctt	L	0	0.19
ctc	L	0	0.17
cta	L	1	0.13
ctg	L	0	0.21
caa	Q	1799	1194.00
cag	Q	548	1153.00
aaa	K	2	1.12
aag	K	0	0.88
mPD		0.36	0.50
		nPD:	0.72
		N. weight:	0.18
		Sc. PD:	0.099
		Sc. rank:	279.8

PB1			
Pos.	461	obs:	exp:
cct	P	1	0.28
ccc	P	0	0.18
cca	P	0	0.41
ccg	P	0	0.13
gct	A	167	597.60
gcc	A	30	458.10
gca	A	2151	1033.00
gcg	A	1	260.80
mPD		0.16	0.69
		nPD:	0.23
		N. weight:	0.69
		Sc. PD:	0.049
		Sc. rank:	563.3

PB1			
Pos.	462	obs:	exp:
ggt	G	2	338.10
ggc	G	1	310.20
gga	G	2147	1026.00
ggg	G	200	675.90
mPD		0.16	0.69
		nPD:	0.23
		N. weight:	0.72
		Sc. PD:	0.052
		Sc. rank:	587.5

PB1			
Pos.	463	obs:	exp:
gtt	V	19	511.10
gtc	V	0	496.50
gta	V	150	462.40
gtg	V	2181	880.00
mPD		0.13	0.73
		nPD:	0.18
		N. weight:	0.95
		Sc. PD:	0.027
		Sc. rank:	624.9

PB1			
Pos.	464	obs:	exp:
aat	N	9	5.85
aac	N	2	5.15
gat	D	1806	1345.00
gac	D	533	993.60
mPD		0.36	0.50
		nPD:	0.73
		N. weight:	0.11
		Sc. PD:	0.062
		Sc. rank:	172.8

PB1			
Pos.	465	obs:	exp:
cgt	R	0	89.08
cgc	R	0	125.10
cga	R	0	227.50
cgg	R	0	184.00
aaa	K	1	0.56
aag	K	0	0.44
aga	R	881	1077.00
agg	R	1468	645.90
mPD		0.47	0.95
		nPD:	0.49
		N. weight:	0.56
		Sc. PD:	0.18
		Sc. rank:	702.5

PB1			
Pos.	466	obs:	exp:
ttt	F	2	1000.00
ttc	F	2348	1350.00
---	--	------	------
mPD		0.0017	0.49
		nPD:	0.
		N. weight:	0.69
		Sc. PD:	-0.1
		Sc. rank:	-957.6

,

PB1			
Pos.	467	obs:	exp:
tat	Y	2242	1142.00
tac	Y	107	1207.00
gat	D	1	0.58
gac	D	0	0.42
---	--	------	------
mPD		0.088	0.50
		nPD:	0.18
		N. weight:	0.68
		Sc. PD:	0.014
		Sc. rank:	433.5

,

PB1			
Pos.	468	obs:	exp:
cgt	R	0	89.12
cgc	R	0	125.20
cga	R	0	227.60
cgg	R	0	184.10
aga	R	76	1078.00
agg	R	2274	646.20
---	--	------	------
mPD		0.063	0.95
		nPD:	0.07
		N. weight:	1.4
		Sc. PD:	-0.12
		Sc. rank:	288.2

,

PB1			
Pos.	469	obs:	exp:
att	I	34	13.37
atc	I	2	9.03
ata	I	0	13.60
act	T	481	612.20
acc	T	1810	510.60
aca	T	20	993.00
acg	T	0	195.20
gct	A	0	0.76
gcc	A	3	0.59
gca	A	0	1.32
gcg	A	0	0.33
---	--	------	------
mPD		0.39	0.72
		nPD:	0.54
		N. weight:	1.2
		Sc. PD:	0.43
		Sc. rank:	1538.2

,

PB1			
Pos.	470	obs:	exp:
tgt	C	522	1022.00
tgc	C	1828	1328.00
---	--	------	------
mPD		0.35	0.49
		nPD:	0.7
		N. weight:	0.13
		Sc. PD:	0.068
		Sc. rank:	192.7

,

PB1			
Pos.	471	obs:	exp:
cgt	R	0	0.04
cgc	R	0	0.05
cga	R	0	0.10
cgg	R	0	0.08
aaa	K	2180	1319.00
aag	K	169	1030.00
aga	R	1	0.46
agg	R	0	0.27
---	--	------	------
mPD		0.13	0.49
		nPD:	0.27
		N. weight:	0.43
		Sc. PD:	0.049
		Sc. rank:	386.6

,

PB1			
Pos.	472	obs:	exp:
tta	L	1	256.50
ttg	L	705	441.00
ctt	L	0	440.70
ctc	L	0	405.40
cta	L	18	312.70
ctg	L	1626	493.80
---	--	------	------
mPD		0.44	1.1
		nPD:	0.38
		N. weight:	1.2
		Sc. PD:	0.27
		Sc. rank:	1318.3

,

PB1			
Pos.	473	obs:	exp:
gtt	V	2333	511.10
gtc	V	14	496.50
gta	V	1	462.40
gtg	V	2	880.00
---	--	------	------
mPD		0.014	0.73
		nPD:	0.02
		N. weight:	1.9
		Sc. PD:	-0.25
		Sc. rank:	-1081.6

,

PB1			
Pos.	474	obs:	exp:
gct	A	0	0.25
gcc	A	0	0.20
gca	A	0	0.44
gcg	A	1	0.11
ggt	G	1	337.90
ggc	G	0	310.00
gga	G	66	1025.00
ggg	G	2282	675.60
---	--	------	------
mPD		0.056	0.69
		nPD:	0.08
		N. weight:	1.4
		Sc. PD:	-0.1
		Sc. rank:	440.2

,

PB1			
Pos.	475	obs:	exp:
att	I	5	871.50
atc	I	2340	588.60
ata	I	2	886.90
gtt	V	0	0.65
gtc	V	3	0.63
gta	V	0	0.59
gtg	V	0	1.12
---	--	------	------
mPD		0.0085	0.66
		nPD:	0.01
		N. weight:	1.7
		Sc. PD:	-0.24
		Sc. rank:	-1455.0

,

PB1			
Pos.	476	obs:	exp:
att	I	0	0.37
atc	I	1	0.25
ata	I	0	0.38
aat	N	533	1248.00
aac	N	1816	1101.00
---	--	------	------
mPD		0.35	0.50
		nPD:	0.7
		N. weight:	0.25
		Sc. PD:	0.13
		Sc. rank:	375.0

,

PB1			
Pos.	477	obs:	exp:
tta	L	0	0.22
ttg	L	0	0.38
ctt	L	0	0.38
ctc	L	0	0.35
cta	L	0	0.27
ctg	L	2	0.42
atg	M	2348	2348.00
---	--	------	------
mPD		0.0017	0.0027
		nPD:	0.62
		N. weight:	0.0017
		Sc. PD:	0.00077
		Sc. rank:	2.4

,

PB1			
Pos.	478	obs:	exp:
tct	S	0	401.20
tcc	S	0	346.30
tca	S	0	529.50
tcg	S	0	151.70
att	I	0	0.37
atc	I	0	0.25
ata	I	1	0.38
agt	S	520	479.60
agc	S	1829	440.70
---	--	------	------
mPD		0.35	1.6
		nPD:	0.21
		N. weight:	1.4
		Sc. PD:	0.077
		Sc. rank:	1058.9

,

PB1			
Pos.	479	obs:	exp:
att	I	0	0.37
atc	I	0	0.25
ata	I	1	0.38
aaa	K	2305	1319.00
aag	K	44	1030.00
---	--	------	------
mPD		0.038	0.49
		nPD:	0.08
		N. weight:	0.62
		Sc. PD:	-0.048
		Sc. rank:	175.3

,

PB1			
Pos.	480	obs:	exp:
cgt	R	0	0.49
cgc	R	0	0.69
cga	R	0	1.26
cgg	R	0	1.02
aaa	K	1	1312.00
aag	K	2336	1025.00
aga	R	0	5.96
agg	R	13	3.57
---	--	------	------
mPD		0.012	0.51
		nPD:	0.02
		N. weight:	1.
		Sc. PD:	-0.13
		Sc. rank:	-496.3

,

PB1			
Pos.	481	obs:	exp:
aaa	K	503	1319.00
aag	K	1847	1031.00
---	--	------	------
mPD		0.34	0.49
		nPD:	0.68
		N. weight:	0.32
		Sc. PD:	0.16
		Sc. rank:	477.9

,

PB1			
Pos.	482	obs:	exp:
tct	S	1846	401.30
tcc	S	503	346.40
tca	S	0	529.70
tcg	S	1	151.80
agt	S	0	479.80
agc	S	0	440.90
---	--	------	------
mPD		0.34	1.6
		nPD:	0.2
		N. weight:	1.6
		Sc. PD:	0.079
		Sc. rank:	1174.3

,

PB1			
Pos.	483	obs:	exp:
tat	Y	587	1142.00
tac	Y	1763	1208.00
---	--	------	------
mPD		0.37	0.50
		nPD:	0.75
		N. weight:	0.15
		Sc. PD:	0.087
		Sc. rank:	236.4

,

PB1			
Pos.	484	obs:	exp:
tta	L	9	0.98
ttg	L	0	1.69
ctt	L	0	1.69
ctc	L	0	1.55
cta	L	0	1.20
ctg	L	0	1.89
att	I	2	868.20
atc	I	3	586.40
ata	I	2333	883.50
atg	M	2	2.00
gtt	V	0	0.22
gtc	V	0	0.21
gta	V	1	0.20
gtg	V	0	0.37
---	--	------	------
mPD		0.014	0.67
		nPD:	0.02
		N. weight:	1.2
		Sc. PD:	-0.16
		Sc. rank:	-654.1

,

PB1			
Pos.	485	obs:	exp:
aat	N	535	1249.00
aac	N	1815	1101.00
---	--	------	------
mPD		0.35	0.50
		nPD:	0.71
		N. weight:	0.25
		Sc. PD:	0.13
		Sc. rank:	376.3

,

PB1

Pos.	486	obs:	exp:
tta	L	0	0.11
ttg	L	0	0.19
ctt	L	0	0.19
ctc	L	0	0.17
cta	L	1	0.13
ctg	L	0	0.21
caa	Q	2	1.02
cag	Q	0	0.98
cgt	R	66	89.01
cgc	R	0	125.00
cga	R	1366	227.30
cgg	R	882	183.80
aga	R	0	1077.00
agg	R	33	645.30
---	--	------	------
mPD		0.54	0.96
		nPD:	0.56
		N. weight:	2.
		Sc. PD:	0.8
		Sc. rank:	2768.4

PB1

Pos.	487	obs:	exp:
act	T	6	622.50
acc	T	0	519.20
aca	T	2320	1010.00
acg	T	24	198.50
---	--	------	------
mPD		0.025	0.69
		nPD:	0.04
		N. weight:	1.
		Sc. PD:	-0.11
		Sc. rank:	-248.1

PB1

Pos.	488	obs:	exp:
ggt	G	0	338.10
ggc	G	0	310.20
gga	G	2316	1026.00
ggg	G	34	675.90
---	--	------	------
mPD		0.029	0.69
		nPD:	0.04
		N. weight:	0.96
		Sc. PD:	-0.11
		Sc. rank:	-131.6

PB1

Pos.	489	obs:	exp:
act	T	4	622.50
acc	T	0	519.20
aca	T	2345	1010.00
acg	T	1	198.50
---	--	------	------
mPD		0.0042	0.69
		nPD:	0.01
		N. weight:	1.1
		Sc. PD:	-0.15
		Sc. rank:	-1289.8

PB1

Pos.	490	obs:	exp:
ttt	F	2334	999.50
ttc	F	15	1349.00
tta	L	1	0.11
ttg	L	0	0.19
ctt	L	0	0.19
ctc	L	0	0.17
cta	L	0	0.13
ctg	L	0	0.21
---	--	------	------
mPD		0.014	0.49
		nPD:	0.03
		N. weight:	1.
		Sc. PD:	-0.13
		Sc. rank:	-409.5

PB1

Pos.	491	obs:	exp:
gaa	E	545	1407.00
gag	E	1805	942.90
---	--	------	------
mPD		0.36	0.48
		nPD:	0.74
		N. weight:	0.36
		Sc. PD:	0.2
		Sc. rank:	552.4

PB1

Pos.	492	obs:	exp:
ttt	F	0	999.50
ttc	F	2349	1349.00
att	I	0	0.37
atc	I	1	0.25
ata	I	0	0.38
---	--	------	------
mPD		0.00085	0.49
		nPD:	0.
		N. weight:	0.7
		Sc. PD:	-0.1
		Sc. rank:	-1024.1

PB1

Pos.	493	obs:	exp:
act	T	0	622.50
acc	T	2	519.20
aca	T	2347	1010.00
acg	T	1	198.50
---	--	------	------
mPD		0.0026	0.69
		nPD:	0.
		N. weight:	1.1
		Sc. PD:	-0.16
		Sc. rank:	-1407.4

PB1

Pos.	494	obs:	exp:
tct	S	0	401.00
tcc	S	0	346.10
tca	S	0	529.30
tcg	S	0	151.70
aaa	K	1	0.56
aag	K	0	0.44
agt	S	1419	479.40
agc	S	929	440.50
---	-	1	1.00
---	--	------	------
mPD		0.48	1.6
		nPD:	0.29
		N. weight:	1.2
		Sc. PD:	0.16
		Sc. rank:	1145.0

PB1

Pos.	495	obs:	exp:
ttt	F	2328	999.50
ttc	F	21	1349.00
gtt	V	1	0.22
gtc	V	0	0.21
gta	V	0	0.20
gtg	V	0	0.37
---	--	------	------
mPD		0.019	0.49
		nPD:	0.04
		N. weight:	1.
		Sc. PD:	-0.12
		Sc. rank:	-224.1

PB1

Pos.	496	obs:	exp:
ttt	F	9	1000.00
ttc	F	2341	1350.00
---	--	------	------
mPD		0.0076	0.49
		nPD:	0.02
		N. weight:	0.67
		Sc. PD:	-0.091
		Sc. rank:	-513.0

PB1

Pos.	497	obs:	exp:
tat	Y	211	1142.00
tac	Y	2139	1208.00
---	--	------	------
mPD		0.16	0.50
		nPD:	0.33
		N. weight:	0.47
		Sc. PD:	0.079
		Sc. rank:	480.0

PB1

Pos.	498	obs:	exp:
cgt	R	526	89.12
cgc	R	1822	125.20
cga	R	2	227.60
cgg	R	0	184.10
aga	R	0	1078.00
agg	R	0	646.20
---	--	------	------
mPD		0.35	0.95
		nPD:	0.37
		N. weight:	3.1
		Sc. PD:	0.64
		Sc. rank:	3394.4

PB1

Pos.	499	obs:	exp:
tat	Y	2322	1142.00
tac	Y	28	1208.00
---	--	------	------
mPD		0.024	0.50
		nPD:	0.05
		N. weight:	0.83
		Sc. PD:	-0.087
		Sc. rank:	-57.6

PB1

Pos.	500	obs:	exp:
ggt	G	0	338.10
ggc	G	0	310.20
gga	G	1844	1026.00
ggg	G	506	675.90
---	--	------	------
mPD		0.34	0.69
		nPD:	0.49
		N. weight:	0.51
		Sc. PD:	0.17
		Sc. rank:	637.5

PB1

Pos.	501	obs:	exp:
ttt	F	2346	1000.00
ttc	F	4	1350.00
---	--	------	------
mPD		0.0034	0.49
		nPD:	0.01
		N. weight:	1.1
		Sc. PD:	-0.15
		Sc. rank:	-1244.7

PB1

Pos.	502	obs:	exp:
gtt	V	0	511.10
gtc	V	0	496.50
gta	V	2182	462.40
gtg	V	168	880.00
---	--	------	------
mPD		0.13	0.73
		nPD:	0.18
		N. weight:	1.7
		Sc. PD:	0.044
		Sc. rank:	1093.0

PB1

Pos.	503	obs:	exp:
tct	S	6	1.02
tcc	S	0	0.88
tca	S	0	1.35
tcg	S	0	0.39
agt	S	0	1.23
agc	S	0	1.13
gct	A	1375	596.30
gcc	A	934	457.10
gca	A	31	1030.00
gcg	A	4	260.20
---	--	------	------
mPD		0.50	0.70
		nPD:	0.72
		N. weight:	0.92
		Sc. PD:	0.5
		Sc. rank:	1423.9

PB1

Pos.	504	obs:	exp:
aat	N	33	1249.00
aac	N	2317	1101.00
---	--	------	------
mPD		0.028	0.50
		nPD:	0.06
		N. weight:	0.87
		Sc. PD:	-0.084
		Sc. rank:	64.4

PB1

Pos.	505	obs:	exp:
ttt	F	53	1000.00
ttc	F	2297	1350.00
---	--	------	------
mPD		0.044	0.49
		nPD:	0.09
		N. weight:	0.58
		Sc. PD:	-0.036
		Sc. rank:	207.6

PB1

Pos.	506	obs:	exp:
tct	S	0	401.30
tcc	S	0	346.40
tca	S	0	529.70
tcg	S	0	151.80
agt	S	1863	479.80
agc	S	487	440.90
---	--	------	------
mPD		0.33	1.6
		nPD:	0.2
		N. weight:	1.4
		Sc. PD:	0.061
		Sc. rank:	953.6

PB1

Pos.	507	obs:	exp:
att	I	0	0.37
atc	I	0	0.25
ata	I	1	0.38
atg	M	2348	2348.00
gtt	V	0	0.22
gtc	V	0	0.21
gta	V	0	0.20
gtg	V	1	0.37
---	--	------	------
mPD		0.0017	0.0022
		nPD:	0.76
		N. weight:	0.0011
		Sc. PD:	0.00062
		Sc. rank:	1.7

PB1

Pos.	508	obs:	exp:
gaa	E	1293	1407.00
gag	E	1057	942.90
---	--	------	------
mPD		0.50	0.48
		nPD:	1.03
		N. weight:	0.0084
		Sc. PD:	0.0071
		Sc. rank:	13.4

PB1

Pos.	509	obs:	exp:
tta	L	1588	256.50
ttg	L	51	441.00
ctt	L	0	440.70
ctc	L	0	405.40
cta	L	39	312.70
ctg	L	672	493.80
---	--	------	------
mPD		0.85	1.1
		nPD:	0.75
		N. weight:	1.6
		Sc. PD:	0.91
		Sc. rank:	2464.3

PB1

Pos.	510	obs:	exp:
cct	P	6	662.70
ccc	P	2302	425.20
cca	P	42	959.50
ccg	P	0	302.60
---	--	------	------
mPD		0.040	0.70
		nPD:	0.06
		N. weight:	2.
		Sc. PD:	-0.19
		Sc. rank:	191.9

PB1			
Pos.	511	obs:	exp:
tct	S	0	401.20
tcc	S	0	346.30
tca	S	0	529.50
tcg	S	0	151.70
aat	N	0	0.53
aac	N	1	0.47
agt	S	13	479.60
agc	S	2336	440.70
---	--	------	------
mPD		0.012	1.6
		nPD:	0.01
		N. weight:	2.1
		Sc. PD:	-0.3
		Sc. rank:	-2344.2

,

PB1			
Pos.	512	obs:	exp:
ttt	F	2341	1000.00
ttc	F	9	1350.00
---	--	------	------
mPD		0.0076	0.49
		nPD:	0.02
		N. weight:	1.1
		Sc. PD:	-0.14
		Sc. rank:	-800.6

,

PB1			
Pos.	513	obs:	exp:
ggt	G	0	338.10
ggc	G	0	310.20
gga	G	2336	1026.00
ggg	G	14	675.90
---	--	------	------
mPD		0.012	0.69
		nPD:	0.02
		N. weight:	1.
		Sc. PD:	-0.13
		Sc. rank:	-689.9

,

PB1			
Pos.	514	obs:	exp:
gtt	V	519	511.10
gtc	V	3	496.50
gta	V	29	462.40
gtg	V	1799	880.00
---	--	------	------
mPD		0.37	0.73
		nPD:	0.5
		N. weight:	0.65
		Sc. PD:	0.22
		Sc. rank:	836.4

,

PB1			
Pos.	515	obs:	exp:
tct	S	2331	399.10
tcc	S	6	344.50
tca	S	0	526.80
tcg	S	0	151.00
cct	P	3	0.85
ccc	P	0	0.54
cca	P	0	1.22
ccg	P	0	0.39
cat	H	10	5.95
cac	H	0	4.05
agt	S	0	477.20
agc	S	0	438.50
---	--	------	------
mPD		0.025	1.7
		nPD:	0.01
		N. weight:	2.2
		Sc. PD:	-0.3
		Sc. rank:	-1744.4

,

PB1			
Pos.	516	obs:	exp:
ggt	G	4	338.10
ggc	G	0	310.20
gga	G	1788	1026.00
ggg	G	558	675.90
---	--	------	------
mPD		0.36	0.69
		nPD:	0.53
		N. weight:	0.47
		Sc. PD:	0.17
		Sc. rank:	622.7

,

PB1			
Pos.	517	obs:	exp:
att	I	531	868.20
atc	I	1801	586.40
ata	I	6	883.50
gtt	V	3	2.61
gtc	V	9	2.54
gta	V	0	2.36
gtg	V	0	4.49
---	--	------	------
mPD		0.37	0.67
		nPD:	0.55
		N. weight:	0.94
		Sc. PD:	0.36
		Sc. rank:	1264.3

,

PB1			
Pos.	518	obs:	exp:
aat	N	2327	1249.00
aac	N	23	1101.00
---	--	------	------
mPD		0.019	0.50
		nPD:	0.04
		N. weight:	0.73
		Sc. PD:	-0.083
		Sc. rank:	-139.6

,

PB1			
Pos.	519	obs:	exp:
gaa	E	2152	1407.00
gag	E	198	942.90
---	--	------	------
mPD		0.15	0.48
		nPD:	0.32
		N. weight:	0.33
		Sc. PD:	0.053
		Sc. rank:	327.0

,

PB1			
Pos.	520	obs:	exp:
tct	S	17	401.20
tcc	S	0	346.30
tca	S	1218	529.50
tcg	S	1114	151.70
cct	P	0	0.28
ccc	P	0	0.18
cca	P	0	0.41
ccg	P	1	0.13
agt	S	0	479.60
agc	S	0	440.70
---	--	------	------
mPD		0.51	1.6
		nPD:	0.31
		N. weight:	1.7
		Sc. PD:	0.26
		Sc. rank:	1678.4

,

PB1			
Pos.	521	obs:	exp:
gct	A	2321	597.80
gcc	A	16	458.30
gca	A	10	1033.00
gcg	A	3	260.90
---	--	------	------
mPD		0.024	0.69
		nPD:	0.04
		N. weight:	1.6
		Sc. PD:	-0.19
		Sc. rank:	-451.9

,

PB1			
Pos.	522	obs:	exp:
gat	D	2	1352.00
gac	D	2348	998.20
---	--	------	------
mPD		0.0017	0.49
		nPD:	0.
		N. weight:	1.1
		Sc. PD:	-0.16
		Sc. rank:	-1476.0

,

PB1			
Pos.	523	obs:	exp:
att	I	0	0.37
atc	I	0	0.25
ata	I	1	0.38
atg	M	2349	2349.00
---	--	------	------
mPD		0.00085	0.00085
		nPD:	1.
		N. weight:	0.00052
		Sc. PD:	0.00043
		Sc. rank:	0.8

,

PB1			
Pos.	524	obs:	exp:
tct	S	0	400.80
tcc	S	0	346.00
tca	S	0	529.00
tcg	S	0	151.60
agt	S	137	479.20
agc	S	2210	440.30
ggt	G	0	0.43
ggc	G	3	0.40
gga	G	0	1.31
ggg	G	0	0.86
---	--	------	------
mPD		0.11	1.6
		nPD:	0.07
		N. weight:	1.8
		Sc. PD:	-0.15
		Sc. rank:	405.6

,

PB1			
Pos.	525	obs:	exp:
att	I	2342	871.50
atc	I	3	588.60
ata	I	2	886.90
atg	M	2	2.00
gtt	V	1	0.22
gtc	V	0	0.21
gta	V	0	0.20
gtg	V	0	0.37
---	--	------	------
mPD		0.0068	0.66
		nPD:	0.01
		N. weight:	1.2
		Sc. PD:	-0.17
		Sc. rank:	-1208.3

,

PB1			
Pos.	526	obs:	exp:
ggt	G	1	338.10
ggc	G	0	310.20
gga	G	2344	1026.00
ggg	G	5	675.90
---	--	------	------
mPD		0.0051	0.69
		nPD:	0.01
		N. weight:	1.
		Sc. PD:	-0.15
		Sc. rank:	-1127.9

,

PB1			
Pos.	527	obs:	exp:
ttt	F	1	0.43
ttc	F	0	0.57
tta	L	0	0.33
ttg	L	0	0.56
ctt	L	3	0.56
ctc	L	0	0.52
cta	L	0	0.40
ctg	L	0	0.63
att	I	4	1.49
atc	I	0	1.00
ata	I	0	1.51
gtt	V	2297	508.70
gtc	V	26	494.20
gta	V	14	460.20
gtg	V	2	875.90
gct	A	3	0.76
gcc	A	0	0.59
gca	A	0	1.32
gcg	A	0	0.33
---	--	------	------
mPD		0.045	0.74
		nPD:	0.06
		N. weight:	1.8
		Sc. PD:	-0.17
		Sc. rank:	198.6

,

PB1			
Pos.	528	obs:	exp:
att	I	0	2.97
atc	I	0	2.01
ata	I	8	3.02
act	T	1	620.10
acc	T	3	517.20
aca	T	2333	1006.00
acg	T	4	197.70
gct	A	0	0.25
gcc	A	0	0.20
gca	A	1	0.44
gcg	A	0	0.11
---	--	------	------
mPD		0.014	0.70
		nPD:	0.02
		N. weight:	1.
		Sc. PD:	-0.14
		Sc. rank:	-582.4

,

PB1			
Pos.	529	obs:	exp:
atg	M	1	1.00
gtt	V	0	510.90
gtc	V	0	496.30
gta	V	513	462.20
gtg	V	1836	879.60
---	--	------	------
mPD		0.34	0.73
		nPD:	0.47
		N. weight:	0.76
		Sc. PD:	0.23
		Sc. rank:	938.0

,

PB1			
Pos.	530	obs:	exp:
att	I	1	872.30
atc	I	1	589.10
ata	I	2347	887.60
gtt	V	0	0.22
gtc	V	0	0.21
gta	V	1	0.20
gtg	V	0	0.37
---	--	------	------
mPD		0.0026	0.66
		nPD:	0.
		N. weight:	1.2
		Sc. PD:	-0.18
		Sc. rank:	-1580.0

,

PB1

Pos.	531	obs:	exp:
cgt	R	0	0.04
cgc	R	0	0.05
cga	R	0	0.10
cgg	R	0	0.08
aaa	K	1446	1319.00
aag	K	903	1030.00
aga	R	1	0.46
agg	R	0	0.27
---	--	------	------
mPD		0.47	0.49
		nPD:	0.96
		N. weight:	0.01
		Sc. PD:	0.008
		Sc. rank:	16.5

,

PB1

Pos.	532	obs:	exp:
aat	N	516	1248.00
aac	N	1833	1101.00
gat	D	0	0.58
gac	D	1	0.42
---	--	------	------
mPD		0.34	0.50
		nPD:	0.69
		N. weight:	0.26
		Sc. PD:	0.14
		Sc. rank:	388.9

,

PB1

Pos.	533	obs:	exp:
aat	N	1660	1248.00
aac	N	689	1101.00
gat	D	1	0.58
gac	D	0	0.42
---	--	------	------
mPD		0.42	0.50
		nPD:	0.83
		N. weight:	0.084
		Sc. PD:	0.055
		Sc. rank:	132.7

,

PB1

Pos.	534	obs:	exp:
atg	M	2350	2350.00
---	--	------	------
mPD		0	0
		nPD:	1.
		N. weight:	0.
		Sc. PD:	0
		Sc. rank:	0

,

PB1

Pos.	535	obs:	exp:
att	I	0	871.50
atc	I	3	588.60
ata	I	2344	886.90
atg	M	1	1.00
gtt	V	0	0.43
gtc	V	0	0.42
gta	V	2	0.39
gtg	V	0	0.75
---	--	------	------
mPD		0.0051	0.66
		nPD:	0.01
		N. weight:	1.2
		Sc. PD:	-0.17
		Sc. rank:	-1333.0

,

PB1

Pos.	536	obs:	exp:
att	I	1	0.37
atc	I	0	0.25
ata	I	0	0.38
aat	N	13	1248.00
aac	N	2336	1101.00
---	--	------	------
mPD		0.013	0.50
		nPD:	0.03
		N. weight:	0.92
		Sc. PD:	-0.12
		Sc. rank:	-402.0

,

PB1

Pos.	537	obs:	exp:
tgt	C	1	0.43
tgc	C	0	0.57
aat	N	1862	1248.00
aac	N	487	1101.00
---	--	------	------
mPD		0.33	0.50
		nPD:	0.66
		N. weight:	0.19
		Sc. PD:	0.093
		Sc. rank:	277.3

,

PB1

Pos.	538	obs:	exp:
gat	D	1983	1350.00
gac	D	364	997.00
gaa	E	1	0.60
gag	E	0	0.40
ggt	G	2	0.29
ggc	G	0	0.26
gga	G	0	0.87
ggg	G	0	0.58
---	--	------	------
mPD		0.26	0.49
		nPD:	0.54
		N. weight:	0.22
		Sc. PD:	0.081
		Sc. rank:	289.5

,

PB1

Pos.	539	obs:	exp:
tta	L	0	256.50
ttg	L	0	441.00
ctt	L	2345	440.70
ctc	L	5	405.40
cta	L	0	312.70
ctg	L	0	493.80
---	--	------	------
mPD		0.0042	1.1
		nPD:	0.
		N. weight:	2.1
		Sc. PD:	-0.31
		Sc. rank:	-2755.4

,

PB1

Pos.	540	obs:	exp:
ggt	G	0	338.10
ggc	G	0	310.20
gga	G	2344	1026.00
ggg	G	6	675.90
---	--	------	------
mPD		0.0051	0.69
		nPD:	0.01
		N. weight:	1.
		Sc. PD:	-0.15
		Sc. rank:	-1133.1

,

PB1

Pos.	541	obs:	exp:
cct	P	1	662.70
ccc	P	11	425.20
cca	P	2207	959.50
ccg	P	131	302.60
---	--	------	------
mPD		0.11	0.70
		nPD:	0.16
		N. weight:	0.91
		Sc. PD:	0.0073
		Sc. rank:	550.8

,

PB1

Pos.	542	obs:	exp:
gct	A	0	597.80
gcc	A	0	458.30
gca	A	2310	1033.00
gcg	A	40	260.90
---	--	------	------
mPD		0.033	0.69
		nPD:	0.05
		N. weight:	0.96
		Sc. PD:	-0.1
		Sc. rank:	-35.7

,

PB1

Pos.	543	obs:	exp:
act	T	0	622.50
acc	T	0	519.20
aca	T	2349	1010.00
acg	T	1	198.50
---	--	------	------
mPD		0.00085	0.69
		nPD:	0.
		N. weight:	1.1
		Sc. PD:	-0.16
		Sc. rank:	-1629.0

,

PB1

Pos.	544	obs:	exp:
gct	A	2334	597.80
gcc	A	16	458.30
gca	A	0	1033.00
gcg	A	0	260.90
---	--	------	------
mPD		0.014	0.69
		nPD:	0.02
		N. weight:	1.7
		Sc. PD:	-0.22
		Sc. rank:	-1011.1

,

PB1

Pos.	545	obs:	exp:
caa	Q	2306	1196.00
cag	Q	44	1154.00
---	--	------	------
mPD		0.037	0.50
		nPD:	0.07
		N. weight:	0.74
		Sc. PD:	-0.059
		Sc. rank:	193.7

,

PB1

Pos.	546	obs:	exp:
atg	M	2350	2350.00
---	--	------	------
mPD		0	0
		nPD:	1.
		N. weight:	0.
		Sc. PD:	0
		Sc. rank:	0

,

PB1

Pos.	547	obs:	exp:
gct	A	2211	597.80
gcc	A	139	458.30
gca	A	0	1033.00
gcg	A	0	260.90
---	--	------	------
mPD		0.11	0.69
		nPD:	0.16
		N. weight:	1.5
		Sc. PD:	0.0087
		Sc. rank:	879.5

,

PB1

Pos.	548	obs:	exp:
tta	L	0	256.20
ttg	L	0	440.40
ctt	L	2313	440.10
ctc	L	29	404.90
cta	L	5	312.30
ctg	L	0	493.10
att	I	3	1.11
atc	I	0	0.75
ata	I	0	1.13
---	--	------	------
mPD		0.031	1.1
		nPD:	0.03
		N. weight:	2.
		Sc. PD:	-0.25
		Sc. rank:	-809.8

,

PB1

Pos.	549	obs:	exp:
caa	Q	279	1196.00
cag	Q	2071	1154.00
---	--	------	------
mPD		0.21	0.50
		nPD:	0.42
		N. weight:	0.44
		Sc. PD:	0.11
		Sc. rank:	510.1

,

PB1

Pos.	550	obs:	exp:
tta	L	1444	256.50
ttg	L	3	441.00
ctt	L	0	440.70
ctc	L	0	405.40
cta	L	891	312.70
ctg	L	12	493.80
---	--	------	------
mPD		0.49	1.1
		nPD:	0.43
		N. weight:	1.8
		Sc. PD:	0.48
		Sc. rank:	2177.4

,

PB1

Pos.	551	obs:	exp:
ttt	F	501	1000.00
ttc	F	1849	1350.00
---	--	------	------
mPD		0.34	0.49
		nPD:	0.69
		N. weight:	0.13
		Sc. PD:	0.067
		Sc. rank:	191.9

,

PB1

Pos.	552	obs:	exp:
tta	L	0	0.11
ttg	L	0	0.19
ctt	L	0	0.19
ctc	L	1	0.17
cta	L	0	0.13
ctg	L	0	0.21
att	I	20	868.20
atc	I	2318	586.40
ata	I	0	883.50
act	T	0	1.32
acc	T	5	1.10
aca	T	0	2.15
acg	T	0	0.42
gtt	V	0	1.30
gtc	V	6	1.27
gta	V	0	1.18
gtg	V	0	2.25
---	--	------	------
mPD		0.027	0.67
		nPD:	0.04
		N. weight:	1.7
		Sc. PD:	-0.19
		Sc. rank:	-252.0

,

PB1

Pos.	553	obs:	exp:
aaa	K	1529	1319.00
aag	K	821	1031.00
---	--	------	------
mPD		0.45	0.49
		nPD:	0.92
		N. weight:	0.023
		Sc. PD:	0.017
		Sc. rank:	36.9

,

PB1

Pos.	554	obs:	exp:
gat	D	256	1352.00
gac	D	2094	998.20
---	--	------	------
mPD		0.19	0.49
		nPD:	0.4
		N. weight:	0.61
		Sc. PD:	0.14
		Sc. rank:	685.9

,

PB1

Pos.	555	obs:	exp:
ttt	F	1	0.43
ttc	F	0	0.57
tat	Y	1664	1142.00
tac	Y	685	1207.00
---	--	------	------
mPD		0.41	0.50
		nPD:	0.83
		N. weight:	0.13
		Sc. PD:	0.086
		Sc. rank:	208.3

,

PB1

Pos.	556	obs:	exp:
cgt	R	0	89.08
cgc	R	0	125.10
cga	R	0	227.50
cgg	R	1	184.00
aaa	K	0	0.56
aag	K	1	0.44
aga	R	552	1077.00
agg	R	1796	645.90
mPD		0.36	0.95
		nPD:	0.38
		N. weight:	0.79
		Sc. PD:	0.17
		Sc. rank:	863.8

PB1

Pos.	557	obs:	exp:
tat	Y	484	1142.00
tac	Y	1866	1208.00
mPD		0.33	0.50
		nPD:	0.65
		N. weight:	0.22
		Sc. PD:	0.1
		Sc. rank:	313.9

PB1

Pos.	558	obs:	exp:
act	T	3	622.50
acc	T	0	519.20
aca	T	1381	1010.00
acg	T	966	198.50
mPD		0.49	0.69
		nPD:	0.7
		N. weight:	1.1
		Sc. PD:	0.56
		Sc. rank:	1591.2

PB1

Pos.	559	obs:	exp:
tat	Y	537	1142.00
tac	Y	1813	1208.00
mPD		0.35	0.50
		nPD:	0.71
		N. weight:	0.18
		Sc. PD:	0.097
		Sc. rank:	275.6

PB1

Pos.	560	obs:	exp:
cgt	R	0	89.12
cgc	R	0	125.20
cga	R	2244	227.60
cgg	R	90	184.10
aga	R	13	1078.00
agg	R	3	646.20
mPD		0.090	0.95
		nPD:	0.09
		N. weight:	2.7
		Sc. PD:	-0.16
		Sc. rank:	1037.2

PB1

Pos.	561	obs:	exp:
tgt	C	311	1022.00
tgc	C	2039	1328.00
mPD		0.23	0.49
		nPD:	0.47
		N. weight:	0.27
		Sc. PD:	0.083
		Sc. rank:	337.3

PB1

Pos.	562	obs:	exp:
cat	H	40	1397.00
cac	H	2310	952.70
mPD		0.033	0.48
		nPD:	0.07
		N. weight:	1.
		Sc. PD:	-0.085
		Sc. rank:	242.4

PB1

Pos.	563	obs:	exp:
cgt	R	0	89.08
cgc	R	0	125.10
cga	R	0	227.50
cgg	R	0	184.00
aaa	K	0	0.56
aag	K	1	0.44
aga	R	203	1077.00
agg	R	2146	645.90
mPD		0.16	0.95
		nPD:	0.17
		N. weight:	1.2
		Sc. PD:	0.014
		Sc. rank:	737.6

PB1

Pos.	564	obs:	exp:
tct	S	0	0.17
tcc	S	0	0.15
tca	S	0	0.23
tcg	S	0	0.06
agt	S	1	0.20
agc	S	0	0.19
ggt	G	2297	337.90
ggc	G	36	310.00
gga	G	10	1025.00
ggg	G	6	675.60
mPD		0.044	0.69
		nPD:	0.06
		N. weight:	2.3
		Sc. PD:	-0.2
		Sc. rank:	391.7

PB1

Pos.	565	obs:	exp:
gat	D	2121	1352.00
gac	D	229	998.20
mPD		0.18	0.49
		nPD:	0.36
		N. weight:	0.34
		Sc. PD:	0.067
		Sc. rank:	352.5

PB1

Pos.	566	obs:	exp:
att	I	0	0.37
atc	I	0	0.25
ata	I	1	0.38
act	T	0	620.90
acc	T	0	517.90
aca	T	2343	1007.00
acg	T	1	198.00
gct	A	0	1.27
gcc	A	0	0.98
gca	A	5	2.20
gcg	A	0	0.56
mPD		0.0060	0.69
		nPD:	0.01
		N. weight:	1.1
		Sc. PD:	-0.15
		Sc. rank:	-1116.1

PB1

Pos.	567	obs:	exp:
caa	Q	2349	1196.00
cag	Q	1	1154.00
mPD		0.00085	0.50
		nPD:	0.
		N. weight:	0.85
		Sc. PD:	-0.13
		Sc. rank:	-1255.7

PB1

Pos.	568	obs:	exp:
tta	L	0	0.11
ttg	L	0	0.19
tct	S	0	0.17
tcc	S	0	0.15
tca	S	0	0.23
tcg	S	0	0.06
ctt	L	1	0.19
ctc	L	0	0.17
cta	L	0	0.13
ctg	L	0	0.21
att	I	1857	871.50
atc	I	3	588.60
ata	I	487	886.90
act	T	1	0.26
acc	T	0	0.22
aca	T	0	0.43
acg	T	0	0.08
agt	S	1	0.20
agc	S	0	0.19
mPD		0.33	0.66
		nPD:	0.51
		N. weight:	0.6
		Sc. PD:	0.2
		Sc. rank:	771.6

PB1

Pos.	569	obs:	exp:
cat	H	6	3.57
cac	H	0	2.43
caa	Q	2199	1193.00
cag	Q	145	1151.00
mPD		0.12	0.50
		nPD:	0.24
		N. weight:	0.57
		Sc. PD:	0.047
		Sc. rank:	478.2

PB1

Pos.	570	obs:	exp:
act	T	2	622.50
acc	T	0	519.20
aca	T	101	1010.00
acg	T	2247	198.50
mPD		0.084	0.69
		nPD:	0.12
		N. weight:	2.8
		Sc. PD:	-0.091
		Sc. rank:	1374.1

PB1

Pos.	571	obs:	exp:
cgt	R	0	87.49
cgc	R	0	122.90
cga	R	0	223.40
cgg	R	10	180.70
aaa	K	0	24.14
aag	K	43	18.86
aga	R	102	1058.00
agg	R	2195	634.30
mPD		0.13	0.98
		nPD:	0.13
		N. weight:	1.3
		Sc. PD:	-0.033
		Sc. rank:	675.7

PB1

Pos.	572	obs:	exp:
cgt	R	0	89.12
cgc	R	0	125.20
cga	R	1	227.60
cgg	R	0	184.10
aga	R	2331	1078.00
agg	R	18	646.20
mPD		0.016	0.95
		nPD:	0.02
		N. weight:	0.93
		Sc. PD:	-0.13
		Sc. rank:	-677.1

PB1

Pos.	573	obs:	exp:
tct	S	3	401.30
tcc	S	0	346.40
tca	S	2338	529.70
tcg	S	9	151.80
agt	S	0	479.80
agc	S	0	440.90
mPD		0.010	1.6
		nPD:	0.01
		N. weight:	1.9
		Sc. PD:	-0.27
		Sc. rank:	-2224.2

PB1

Pos.	574	obs:	exp:
ttt	F	44	998.30
ttc	F	2302	1348.00
tta	L	3	0.33
ttg	L	0	0.56
tct	S	0	0.17
tcc	S	1	0.15
tca	S	0	0.23
tcg	S	0	0.06
ctt	L	0	0.56
ctc	L	0	0.52
cta	L	0	0.40
ctg	L	0	0.63
agt	S	0	0.20
agc	S	0	0.19
mPD		0.040	0.49
		nPD:	0.08
		N. weight:	0.6
		Sc. PD:	-0.043
		Sc. rank:	184.0

PB1

Pos.	575	obs:	exp:
gaa	E	2	1407.00
gag	E	2348	942.90
mPD		0.0017	0.48
		nPD:	0.
		N. weight:	1.1
		Sc. PD:	-0.17
		Sc. rank:	-1556.3

PB1			
Pos.	576	obs:	exp:
tta	L	0	255.50
ttg	L	3	439.30
ctt	L	0	439.00
ctc	L	0	403.90
cta	L	525	311.50
ctg	L	1813	491.90
atg	M	8	8.00
gtt	V	0	0.22
gtc	V	0	0.21
gta	V	0	0.20
gtg	V	1	0.37
---	--	------	------
mPD		0.36	1.1
		nPD:	0.31
		N. weight:	1.4
		Sc. PD:	0.22
		Sc. rank:	1410.8

PB1			
Pos.	577	obs:	exp:
cgt	R	0	0.19
cgc	R	0	0.27
cga	R	0	0.48
cgg	R	0	0.39
aat	N	1	0.53
aac	N	0	0.47
aaa	K	2	1316.00
aag	K	2342	1028.00
aga	R	0	2.29
agg	R	5	1.37
---	--	------	------
mPD		0.0068	0.50
		nPD:	0.01
		N. weight:	1.
		Sc. PD:	-0.14
		Sc. rank:	-865.4

PB1			
Pos.	578	obs:	exp:
cgt	R	0	0.11
cgc	R	0	0.16
cga	R	0	0.29
cgg	R	0	0.23
aat	N	1	0.53
aac	N	0	0.47
aaa	K	160	1316.00
aag	K	2185	1029.00
aga	R	0	1.38
agg	R	3	0.82
gaa	E	0	0.60
gag	E	1	0.40
---	--	------	------
mPD		0.13	0.50
		nPD:	0.26
		N. weight:	0.71
		Sc. PD:	0.075
		Sc. rank:	639.6

PB1			
Pos.	579	obs:	exp:
tta	L	2	254.50
ttg	L	13	437.60
ctt	L	0	437.30
ctc	L	0	402.30
cta	L	18	310.30
ctg	L	2299	490.00
atg	M	18	18.00
---	--	------	------
mPD		0.045	1.1
		nPD:	0.04
		N. weight:	1.9
		Sc. PD:	-0.21
		Sc. rank:	-331.3

PB1			
Pos.	580	obs:	exp:
tgg	W	2350	2350.00
---	--	------	------
mPD		0	0
		nPD:	1.
		N. weight:	0.
		Sc. PD:	0
		Sc. rank:	0

PB1			
Pos.	581	obs:	exp:
gat	D	10	5.75
gac	D	0	4.25
gaa	E	15	1401.00
gag	E	2325	938.90
---	--	------	------
mPD		0.021	0.48
		nPD:	0.04
		N. weight:	1.1
		Sc. PD:	-0.12
		Sc. rank:	-136.1

PB1			
Pos.	582	obs:	exp:
cat	H	2	1.19
cac	H	0	0.81
caa	Q	20	1194.00
cag	Q	2327	1153.00
aaa	K	0	0.56
aag	K	1	0.44
---	--	------	------
mPD		0.019	0.50
		nPD:	0.04
		N. weight:	0.84
		Sc. PD:	-0.095
		Sc. rank:	-168.8

PB1			
Pos.	583	obs:	exp:
act	T	128	622.50
acc	T	2222	519.20
aca	T	0	1010.00
acg	T	0	198.50
---	--	------	------
mPD		0.10	0.69
		nPD:	0.15
		N. weight:	1.6
		Sc. PD:	-0.0086
		Sc. rank:	914.0

PB1			
Pos.	584	obs:	exp:
tta	L	0	0.11
ttg	L	0	0.19
ctt	L	1	0.19
ctc	L	0	0.17
cta	L	0	0.13
ctg	L	0	0.21
cat	H	3	2.38
cac	H	1	1.62
cgt	R	2313	88.93
cgc	R	23	124.90
cga	R	3	227.10
cgg	R	6	183.70
aga	R	0	1076.00
agg	R	0	644.80
---	--	------	------
mPD		0.032	0.96
		nPD:	0.03
		N. weight:	4.
		Sc. PD:	-0.48
		Sc. rank:	-1189.1

PB1			
Pos.	585	obs:	exp:
tct	S	0	401.30
tcc	S	2	346.40
tca	S	2343	529.70
tcg	S	5	151.80
agt	S	0	479.80
agc	S	0	440.90
---	--	------	------
mPD		0.0059	1.6
		nPD:	0.
		N. weight:	1.9
		Sc. PD:	-0.27
		Sc. rank:	-2504.6

PB1			
Pos.	586	obs:	exp:
cgt	R	0	0.38
cgc	R	0	0.53
cga	R	0	0.97
cgg	R	0	0.78
act	T	0	0.26
acc	T	0	0.22
aca	T	0	0.43
acg	T	1	0.08
aaa	K	63	1313.00
aag	K	2276	1026.00
aga	R	6	4.59
agg	R	4	2.75
---	--	------	------
mPD		0.066	0.50
		nPD:	0.13
		N. weight:	0.88
		Sc. PD:	-0.02
		Sc. rank:	447.2

PB1			
Pos.	587	obs:	exp:
act	T	0	11.65
acc	T	0	9.72
aca	T	44	18.91
acg	T	0	3.72
gtt	V	0	1.74
gtc	V	0	1.69
gta	V	8	1.57
gtg	V	0	3.00
gct	A	1	584.60
gcc	A	0	448.10
gca	A	2288	1010.00
gcg	A	9	255.10
---	--	------	------
mPD		0.052	0.74
		nPD:	0.07
		N. weight:	1.
		Sc. PD:	-0.083
		Sc. rank:	244.3

PB1			
Pos.	588	obs:	exp:
ggt	G	0	338.10
ggc	G	0	310.20
gga	G	701	1026.00
ggg	G	1649	675.90
---	--	------	------
mPD		0.42	0.69
		nPD:	0.61
		N. weight:	0.65
		Sc. PD:	0.29
		Sc. rank:	922.0

PB1			
Pos.	589	obs:	exp:
tta	L	2	256.50
ttg	L	17	441.00
ctt	L	1	440.70
ctc	L	0	405.40
cta	L	66	312.70
ctg	L	2264	493.80
---	--	------	------
mPD		0.073	1.1
		nPD:	0.06
		N. weight:	1.8
		Sc. PD:	-0.16
		Sc. rank:	315.1

PB1			
Pos.	590	obs:	exp:
tta	L	20	256.50
ttg	L	2312	441.00
ctt	L	0	440.70
ctc	L	0	405.40
cta	L	0	312.70
ctg	L	18	493.80
---	--	------	------
mPD		0.032	1.1
		nPD:	0.03
		N. weight:	2.
		Sc. PD:	-0.25
		Sc. rank:	-769.5

PB1			
Pos.	591	obs:	exp:
tta	L	0	0.22
ttg	L	0	0.38
ctt	L	0	0.38
ctc	L	0	0.35
cta	L	2	0.27
ctg	L	0	0.42
att	I	7	334.20
atc	I	0	225.70
ata	I	893	340.10
gtt	V	520	314.90
gtc	V	49	305.90
gta	V	738	284.90
gtg	V	141	542.20
---	--	------	------
mPD		0.94	1.2
		nPD:	0.77
		N. weight:	0.83
		Sc. PD:	0.49
		Sc. rank:	1301.3

PB1			
Pos.	592	obs:	exp:
tct	S	2	401.20
tcc	S	0	346.30
tca	S	2327	529.50
tcg	S	20	151.70
agt	S	0	479.60
agc	S	0	440.70
gct	A	0	0.25
gcc	A	0	0.20
gca	A	1	0.44
gcg	A	0	0.11
---	--	------	------
mPD		0.019	1.6
		nPD:	0.01
		N. weight:	1.8
		Sc. PD:	-0.25
		Sc. rank:	-1671.3

PB1			
Pos.	593	obs:	exp:
gat	D	968	1352.00
gac	D	1382	998.20
---	--	------	------
mPD		0.48	0.49
		nPD:	0.99
		N. weight:	0.07
		Sc. PD:	0.057
		Sc. rank:	112.6

PB1			
Pos.	594	obs:	exp:
ggt	G	2	338.10
ggc	G	0	310.20
gga	G	1528	1026.00
ggg	G	820	675.90
---	--	------	------
mPD		0.46	0.69
		nPD:	0.66
		N. weight:	0.41
		Sc. PD:	0.2
		Sc. rank:	602.2

PB1			
Pos.	595	obs:	exp:
gaa	E	1	0.60
gag	E	0	0.40
ggt	G	0	337.90
ggc	G	487	310.00
gga	G	1001	1025.00
ggg	G	861	675.60
---	--	------	------
mPD		0.64	0.69
		nPD:	0.93
		N. weight:	0.22
		Sc. PD:	0.17
		Sc. rank:	355.4

PB1

Pos.	596	obs:	exp:
cct	P	0	662.70
ccc	P	15	425.20
cca	P	2304	959.50
ccg	P	31	302.60
mPD		0.039	0.70
		nPD:	0.05
		N. weight:	1.
		Sc. PD:	-0.1
		Sc. rank:	55.9

PB1

Pos.	597	obs:	exp:
aat	N	271	1249.00
aac	N	2079	1101.00
mPD		0.20	0.50
		nPD:	0.41
		N. weight:	0.49
		Sc. PD:	0.12
		Sc. rank:	566.1

PB1

Pos.	598	obs:	exp:
tta	L	496	256.50
ttg	L	4	441.00
ctt	L	0	440.70
ctc	L	0	405.40
cta	L	1721	312.70
ctg	L	129	493.80
mPD		0.44	1.1
		nPD:	0.39
		N. weight:	1.7
		Sc. PD:	0.38
		Sc. rank:	1856.9

PB1

Pos.	599	obs:	exp:
tat	Y	4	1142.00
tac	Y	2346	1208.00
mPD		0.0034	0.50
		nPD:	0.01
		N. weight:	0.83
		Sc. PD:	-0.12
		Sc. rank:	-970.4

PB1

Pos.	600	obs:	exp:
aat	N	534	1249.00
aac	N	1816	1101.00
mPD		0.35	0.50
		nPD:	0.71
		N. weight:	0.25
		Sc. PD:	0.13
		Sc. rank:	374.8

PB1

Pos.	601	obs:	exp:
att	I	1826	872.60
atc	I	37	589.40
ata	I	487	888.00
mPD		0.35	0.66
		nPD:	0.54
		N. weight:	0.52
		Sc. PD:	0.19
		Sc. rank:	691.8

PB1

Pos.	602	obs:	exp:
caa	Q	1	0.51
cag	Q	0	0.49
cgt	R	0	89.08
cgc	R	62	125.10
cga	R	8	227.50
cgg	R	2277	184.00
aga	R	1	1077.00
agg	R	1	645.90
mPD		0.062	0.95
		nPD:	0.07
		N. weight:	3.
		Sc. PD:	-0.27
		Sc. rank:	593.3

PB1

Pos.	603	obs:	exp:
aat	N	2025	1248.00
aac	N	324	1101.00
gat	D	0	0.58
gac	D	1	0.42
mPD		0.24	0.50
		nPD:	0.48
		N. weight:	0.32
		Sc. PD:	0.1
		Sc. rank:	396.8

PB1

Pos.	604	obs:	exp:
tta	L	0	256.50
ttg	L	0	441.00
ctt	L	118	440.70
ctc	L	2224	405.40
cta	L	8	312.70
ctg	L	0	493.80
mPD		0.10	1.1
		nPD:	0.09
		N. weight:	1.9
		Sc. PD:	-0.12
		Sc. rank:	686.8

PB1

Pos.	605	obs:	exp:
cat	H	103	1397.00
cac	H	2247	952.70
mPD		0.084	0.48
		nPD:	0.17
		N. weight:	0.9
		Sc. PD:	0.017
		Sc. rank:	570.7

PB1

Pos.	606	obs:	exp:
tta	L	0	0.44
ttg	L	0	0.75
ctt	L	0	0.75
ctc	L	4	0.69
cta	L	0	0.53
ctg	L	0	0.84
att	I	66	870.80
atc	I	2278	588.10
ata	I	1	886.10
gtt	V	0	0.22
gtc	V	1	0.21
gta	V	0	0.20
gtg	V	0	0.37
mPD		0.060	0.66
		nPD:	0.09
		N. weight:	1.6
		Sc. PD:	-0.099
		Sc. rank:	578.1

PB1

Pos.	607	obs:	exp:
cct	P	1	662.70
ccc	P	36	425.20
cca	P	2278	959.50
ccg	P	35	302.60
mPD		0.060	0.70
		nPD:	0.09
		N. weight:	0.97
		Sc. PD:	-0.066
		Sc. rank:	328.3

PB1

Pos.	608	obs:	exp:
gaa	E	16	1407.00
gag	E	2334	942.90
mPD		0.014	0.48
		nPD:	0.03
		N. weight:	1.1
		Sc. PD:	-0.14
		Sc. rank:	-432.1

PB1

Pos.	609	obs:	exp:
att	I	0	0.37
atc	I	1	0.25
ata	I	0	0.38
gtt	V	10	510.60
gtc	V	2332	496.10
gta	V	6	462.00
gtg	V	0	879.30
gct	A	0	0.25
gcc	A	1	0.20
gca	A	0	0.44
gcg	A	0	0.11
mPD		0.015	0.73
		nPD:	0.02
		N. weight:	1.9
		Sc. PD:	-0.25
		Sc. rank:	-1036.6

PB1

Pos.	610	obs:	exp:
tgt	C	2	1022.00
tgc	C	2348	1328.00
mPD		0.0017	0.49
		nPD:	0.
		N. weight:	0.72
		Sc. PD:	-0.11
		Sc. rank:	-991.5

PB1

Pos.	611	obs:	exp:
tta	L	5	256.40
ttg	L	2212	440.80
tct	S	0	0.17
tcc	S	0	0.15
tca	S	0	0.23
tcg	S	1	0.06
ctt	L	0	440.50
ctc	L	0	405.30
cta	L	0	312.50
ctg	L	132	493.50
agt	S	0	0.20
agc	S	0	0.19
mPD		0.11	1.1
		nPD:	0.1
		N. weight:	1.8
		Sc. PD:	-0.1
		Sc. rank:	764.4

PB1

Pos.	612	obs:	exp:
aaa	K	85	1319.00
aag	K	2265	1031.00
mPD		0.070	0.49
		nPD:	0.14
		N. weight:	0.84
		Sc. PD:	-0.011
		Sc. rank:	451.4

PB1

Pos.	613	obs:	exp:
tgg	W	2350	2350.00
mPD		0	0
		nPD:	1.
		N. weight:	0.
		Sc. PD:	0
		Sc. rank:	0

PB1

Pos.	614	obs:	exp:
gat	D	6	3.45
gac	D	0	2.55
gaa	E	1178	1402.00
gag	E	1163	939.30
ggt	G	0	0.43
ggc	G	0	0.40
gga	G	3	1.31
ggg	G	0	0.86
mPD		0.51	0.49
		nPD:	1.04
		N. weight:	0.029
		Sc. PD:	0.025
		Sc. rank:	46.3

PB1

Pos.	615	obs:	exp:
tta	L	2	256.30
ttg	L	51	440.60
ctt	L	0	440.30
ctc	L	0	405.10
cta	L	255	312.40
ctg	L	2040	493.30
atg	M	2	2.00
mPD		0.24	1.1
		nPD:	0.21
		N. weight:	1.5
		Sc. PD:	0.081
		Sc. rank:	1101.0

PB1

Pos.	616	obs:	exp:
atg	M	2350	2350.00
mPD		0	0
		nPD:	1.
		N. weight:	0.
		Sc. PD:	0
		Sc. rank:	0

PB1

Pos.	617	obs:	exp:
gat	D	2217	1352.00
gac	D	133	998.20
mPD		0.11	0.49
		nPD:	0.22
		N. weight:	0.45
		Sc. PD:	0.028
		Sc. rank:	348.8

PB1

Pos.	618	obs:	exp:
aaa	K	2	1.12
aag	K	0	0.88
gat	D	1	0.58
gac	D	0	0.42
gaa	E	2339	1405.00
gag	E	7	941.30
ggt	G	0	0.14
ggc	G	0	0.13
gga	G	1	0.44
ggg	G	0	0.29
mPD		0.0093	0.48
		nPD:	0.02
		N. weight:	0.63
		Sc. PD:	-0.083
		Sc. rank:	-391.9

PB1

Pos.	619	obs:	exp:
att	I	0	0.37
atc	I	1	0.25
ata	I	0	0.38
act	T	0	0.26
acc	T	0	0.22
aca	T	1	0.43
acg	T	0	0.08
aat	N	0	0.53
aac	N	1	0.47
gat	D	697	1349.00
gac	D	1648	996.10
ggt	G	0	0.29
ggc	G	2	0.26
gga	G	0	0.87
ggg	G	0	0.58
mPD		0.42	0.50
		nPD:	0.86
		N. weight:	0.21
		Sc. PD:	0.14
		Sc. rank:	326.6

PB1

Pos.	620	obs:	exp:
tat	Y	15	1142.00
tac	Y	2335	1208.00
---	--	------	------
mPD		0.013	0.50
		nPD:	0.03
		N. weight:	0.8
		Sc. PD:	-0.1
		Sc. rank:	-352.0

,

PB1

Pos.	621	obs:	exp:
cct	P	0	0.28
ccc	P	0	0.18
cca	P	0	0.41
ccg	P	1	0.13
caa	Q	1428	1193.00
cag	Q	917	1152.00
cgt	R	0	0.08
cgc	R	0	0.11
cga	R	1	0.19
cgg	R	1	0.16
aaa	K	1	1.12
aag	K	1	0.88
aga	R	0	0.92
agg	R	0	0.55
---	--	------	------
mPD		0.48	0.51
		nPD:	0.95
		N. weight:	0.032
		Sc. PD:	0.024
		Sc. rank:	50.4

,

PB1

Pos.	622	obs:	exp:
gat	D	0	0.58
gac	D	1	0.42
ggt	G	582	337.90
ggc	G	1748	310.00
gga	G	17	1025.00
ggg	G	2	675.60
---	--	------	------
mPD		0.39	0.69
		nPD:	0.56
		N. weight:	1.8
		Sc. PD:	0.69
		Sc. rank:	2410.7

,

PB1

Pos.	623	obs:	exp:
cgt	R	0	89.04
cgc	R	0	125.10
cga	R	0	227.40
cgg	R	0	183.90
aaa	K	0	1.12
aag	K	2	0.88
aga	R	526	1077.00
agg	R	1822	645.60
---	--	------	------
mPD		0.35	0.95
		nPD:	0.37
		N. weight:	0.82
		Sc. PD:	0.17
		Sc. rank:	876.7

,

PB1

Pos.	624	obs:	exp:
tta	L	1	256.30
ttg	L	148	440.60
ctt	L	502	440.30
ctc	L	3	405.10
cta	L	1	312.40
ctg	L	1693	493.30
atg	M	2	2.00
---	--	------	------
mPD		0.46	1.1
		nPD:	0.4
		N. weight:	1.1
		Sc. PD:	0.26
		Sc. rank:	1226.3

,

PB1

Pos.	625	obs:	exp:
tgt	C	938	1022.00
tgc	C	1412	1328.00
---	--	------	------
mPD		0.48	0.49
		nPD:	0.98
		N. weight:	0.0055
		Sc. PD:	0.0044
		Sc. rank:	8.8

,

PB1

Pos.	626	obs:	exp:
atg	M	1	1.00
aat	N	2323	1248.00
aac	N	26	1101.00
---	--	------	------
mPD		0.024	0.50
		nPD:	0.05
		N. weight:	0.73
		Sc. PD:	-0.076
		Sc. rank:	-45.1

,

PB1

Pos.	627	obs:	exp:
tct	S	5	9.05
tcc	S	48	7.81
tca	S	0	11.95
tcg	S	0	3.42
cct	P	1822	647.70
ccc	P	475	415.60
cca	P	0	937.90
ccg	P	0	295.80
agt	S	0	10.82
agc	S	0	9.94
---	--	------	------
mPD		0.39	0.77
		nPD:	0.51
		N. weight:	1.1
		Sc. PD:	0.38
		Sc. rank:	1425.2

,

PB1

Pos.	628	obs:	exp:
tta	L	0	252.90
ttg	L	2	434.80
ctt	L	5	434.50
ctc	L	0	399.80
cta	L	36	308.30
ctg	L	2274	486.80
caa	Q	0	10.68
cag	Q	21	10.32
cgt	R	0	0.08
cgc	R	0	0.11
cga	R	0	0.19
cgg	R	2	0.16
atg	M	10	10.00
aga	R	0	0.92
agg	R	0	0.55
---	--	------	------
mPD		0.064	1.2
		nPD:	0.06
		N. weight:	1.8
		Sc. PD:	-0.18
		Sc. rank:	130.3

,

PB1

Pos.	629	obs:	exp:
tct	S	0	0.34
tcc	S	0	0.29
tca	S	0	0.45
tcg	S	0	0.13
aat	N	1649	1248.00
aac	N	699	1100.00
agt	S	2	0.41
agc	S	0	0.38
---	--	------	------
mPD		0.42	0.50
		nPD:	0.84
		N. weight:	0.081
		Sc. PD:	0.054
		Sc. rank:	128.4

,

PB1

Pos.	630	obs:	exp:
cct	P	1	662.70
ccc	P	523	425.20
cca	P	1584	959.50
ccg	P	242	302.60
---	--	------	------
mPD		0.49	0.70
		nPD:	0.69
		N. weight:	0.46
		Sc. PD:	0.24
		Sc. rank:	684.3

,

PB1

Pos.	631	obs:	exp:
ttt	F	2345	999.50
ttc	F	4	1349.00
tta	L	0	0.11
ttg	L	0	0.19
ctt	L	1	0.19
ctc	L	0	0.17
cta	L	0	0.13
ctg	L	0	0.21
---	--	------	------
mPD		0.0043	0.49
		nPD:	0.01
		N. weight:	1.1
		Sc. PD:	-0.15
		Sc. rank:	-1092.4

,

PB1

Pos.	632	obs:	exp:
att	I	0	2.60
atc	I	7	1.76
ata	I	0	2.65
gtt	V	23	508.90
gtc	V	2317	494.40
gta	V	0	460.40
gtg	V	0	876.30
gct	A	0	0.76
gcc	A	3	0.59
gca	A	0	1.32
gcg	A	0	0.33
---	--	------	------
mPD		0.028	0.74
		nPD:	0.04
		N. weight:	1.9
		Sc. PD:	-0.22
		Sc. rank:	-427.2

,

PB1

Pos.	633	obs:	exp:
tct	S	0	395.50
tcc	S	0	341.40
tca	S	0	522.10
tcg	S	0	149.60
tgt	C	21	9.13
tgc	C	0	11.87
aat	N	13	6.91
aac	N	0	6.09
agt	S	2254	472.90
agc	S	62	434.50
---	--	------	------
mPD		0.080	1.7
		nPD:	0.05
		N. weight:	1.8
		Sc. PD:	-0.19
		Sc. rank:	-46.7

,

PB1

Pos.	634	obs:	exp:
cat	H	2331	1397.00
cac	H	18	952.30
aat	N	1	0.53
aac	N	0	0.47
---	--	------	------
mPD		0.016	0.48
		nPD:	0.03
		N. weight:	0.61
		Sc. PD:	-0.072
		Sc. rank:	-189.4

,

PB1

Pos.	635	obs:	exp:
cgt	R	0	0.04
cgc	R	0	0.05
cga	R	0	0.10
cgg	R	0	0.08
aat	N	1	0.53
aac	N	0	0.47
aaa	K	1340	1318.00
aag	K	1008	1030.00
aga	R	1	0.46
agg	R	0	0.27
---	--	------	------
mPD		0.49	0.49
		nPD:	0.99
		N. weight:	0.0032
		Sc. PD:	0.0026
		Sc. rank:	5.1

,

PB1

Pos.	636	obs:	exp:
gaa	E	929	1406.00
gag	E	1419	942.10
ggt	G	0	0.29
ggc	G	0	0.26
gga	G	2	0.87
ggg	G	0	0.58
---	--	------	------
mPD		0.48	0.48
		nPD:	1.
		N. weight:	0.11
		Sc. PD:	0.089
		Sc. rank:	174.5

,

PB1

Pos.	637	obs:	exp:
ttt	F	4	1.70
ttc	F	0	2.30
att	I	2330	870.00
atc	I	1	587.60
ata	I	12	885.30
atg	M	3	3.00
---	--	------	------
mPD		0.017	0.66
		nPD:	0.03
		N. weight:	1.2
		Sc. PD:	-0.15
		Sc. rank:	-523.9

,

PB1

Pos.	638	obs:	exp:
gct	A	0	0.51
gcc	A	0	0.39
gca	A	0	0.88
gcg	A	2	0.22
gat	D	1	0.58
gac	D	0	0.42
gaa	E	41	1398.00
gag	E	2294	936.90
ggt	G	0	1.73
ggc	G	0	1.58
gga	G	0	5.24
ggg	G	12	3.45
---	--	------	------
mPD		0.047	0.49
		nPD:	0.1
		N. weight:	1.
		Sc. PD:	-0.061
		Sc. rank:	410.5

,

PB1

Pos.	639	obs:	exp:
ttt	F	0	0.43
ttc	F	1	0.57
tct	S	119	401.20
tcc	S	2205	346.30
tca	S	25	529.50
tcg	S	0	151.70
agt	S	0	479.60
agc	S	0	440.70
---	--	------	------
mPD		0.12	1.6
		nPD:	0.07
		N. weight:	2.1
		Sc. PD:	-0.17
		Sc. rank:	528.6

,

PB1

Pos.	640	obs:	exp:
att	I	0	11.88
atc	I	0	8.03
ata	I	32	12.09
gtt	V	0	504.10
gtc	V	1	489.80
gta	V	2293	456.10
gtg	V	24	868.00
---	--	------	------
mPD		0.048	0.76
		nPD :	0.06
		N. weight :	2.
		Sc. PD:	-0.17
		Sc. rank :	304.1

,

PB1

Pos.	641	obs:	exp:
aat	N	0	1249.00
aac	N	2350	1101.00
---	--	------	------
mPD		0	0.50
		nPD :	0.
		N. weight :	0.96
		Sc. PD:	-0.14
		Sc. rank :	-1539.6

,

PB1

Pos.	642	obs:	exp:
tct	S	0	1.54
tcc	S	0	1.33
tca	S	0	2.03
tcg	S	0	0.58
aat	N	2334	1244.00
aac	N	7	1097.00
agt	S	9	1.84
agc	S	0	1.69
---	--	------	------
mPD		0.014	0.51
		nPD :	0.03
		N. weight :	0.78
		Sc. PD:	-0.097
		Sc. rank :	-333.8

,

PB1

Pos.	643	obs:	exp:
gct	A	2341	597.80
gcc	A	3	458.30
gca	A	0	1033.00
gcg	A	6	260.90
---	--	------	------
mPD		0.0076	0.69
		nPD :	0.01
		N. weight :	1.7
		Sc. PD:	-0.24
		Sc. rank :	-1587.7

,

PB1

Pos.	644	obs:	exp:
att	I	0	0.74
atc	I	0	0.50
ata	I	2	0.76
gtt	V	3	510.20
gtc	V	1	495.70
gta	V	1933	461.60
gtg	V	409	878.50
gct	A	0	0.51
gcc	A	0	0.39
gca	A	2	0.88
gcg	A	0	0.22
---	--	------	------
mPD		0.29	0.73
		nPD :	0.4
		N. weight :	1.3
		Sc. PD:	0.32
		Sc. rank :	1492.1

,

PB1

Pos.	645	obs:	exp:
att	I	0	1.86
atc	I	0	1.25
ata	I	5	1.89
gtt	V	0	510.00
gtc	V	0	495.50
gta	V	1795	461.40
gtg	V	550	878.10
---	--	------	------
mPD		0.36	0.73
		nPD :	0.49
		N. weight :	1.2
		Sc. PD:	0.39
		Sc. rank :	1512.7

,

PB1

Pos.	646	obs:	exp:
tta	L	0	0.55
ttg	L	5	0.94
ctt	L	0	0.94
ctc	L	0	0.86
cta	L	0	0.67
ctg	L	0	1.05
att	I	2	0.74
atc	I	0	0.50
ata	I	0	0.76
atg	M	2341	2341.00
gtt	V	0	0.43
gtc	V	0	0.42
gta	V	0	0.39
gtg	V	2	0.75
---	--	------	------
mPD		0.0076	0.011
		nPD :	0.68
		N. weight :	0.0066
		Sc. PD:	0.0034
		Sc. rank :	9.9

,

PB1

Pos.	647	obs:	exp:
cct	P	1	662.10
ccc	P	824	424.90
cca	P	1493	958.70
ccg	P	30	302.30
cat	H	0	0.59
cac	H	1	0.41
caa	Q	1	0.51
cag	Q	0	0.49
---	--	------	------
mPD		0.47	0.71
		nPD :	0.67
		N. weight :	0.61
		Sc. PD:	0.31
		Sc. rank :	911.5

,

PB1

Pos.	648	obs:	exp:
tct	S	3	0.51
tcc	S	0	0.44
tca	S	0	0.68
tcg	S	0	0.19
act	T	0	5.03
acc	T	19	4.20
aca	T	0	8.16
acg	T	0	1.60
agt	S	0	0.61
agc	S	0	0.56
gct	A	185	592.20
gcc	A	2141	454.00
gca	A	2	1023.00
gcg	A	0	258.40
---	--	------	------
mPD		0.17	0.71
		nPD :	0.24
		N. weight :	1.7
		Sc. PD:	0.13
		Sc. rank :	1420.1

,

PB1

Pos.	649	obs:	exp:
cat	H	2223	1395.00
cac	H	124	951.50
caa	Q	3	1.53
cag	Q	0	1.47
---	--	------	------
mPD		0.10	0.48
		nPD :	0.21
		N. weight :	0.42
		Sc. PD:	0.023
		Sc. rank :	318.2

,

PB1

Pos.	650	obs:	exp:
ggt	G	552	338.10
ggc	G	1797	310.20
gga	G	1	1026.00
ggg	G	0	675.90
---	--	------	------
mPD		0.36	0.69
		nPD :	0.52
		N. weight :	1.8
		Sc. PD:	0.66
		Sc. rank :	2436.4

,

PB1

Pos.	651	obs:	exp:
tta	L	0	0.11
ttg	L	0	0.19
ctt	L	0	0.19
ctc	L	0	0.17
cta	L	0	0.13
ctg	L	1	0.21
cct	P	1	662.40
ccc	P	1	425.00
cca	P	664	959.10
ccg	P	1683	302.50
---	--	------	------
mPD		0.41	0.71
		nPD :	0.58
		N. weight :	1.4
		Sc. PD:	0.58
		Sc. rank :	1979.6

,

PB1

Pos.	652	obs:	exp:
act	T	0	0.53
acc	T	2	0.44
aca	T	0	0.86
acg	T	0	0.17
gtt	V	0	0.22
gtc	V	1	0.21
gta	V	0	0.20
gtg	V	0	0.37
gct	A	3	597.10
gcc	A	2336	457.70
gca	A	8	1032.00
gcg	A	0	260.60
---	--	------	------
mPD		0.012	0.69
		nPD :	0.02
		N. weight :	2.
		Sc. PD:	-0.27
		Sc. rank :	-1430.0

,

PB1

Pos.	653	obs:	exp:
cgt	R	0	0.04
cgc	R	0	0.05
cga	R	0	0.10
cgg	R	0	0.08
aaa	K	525	1319.00
aag	K	1824	1030.00
aga	R	0	0.46
agg	R	1	0.27
---	--	------	------
mPD		0.35	0.49
		nPD :	0.71
		N. weight :	0.3
		Sc. PD:	0.16
		Sc. rank :	459.9

,

PB1

Pos.	654	obs:	exp:
tct	S	0	392.10
tcc	S	0	338.50
tca	S	0	517.60
tcg	S	0	148.30
att	I	0	0.37
atc	I	1	0.25
ata	I	0	0.38
aat	N	0	20.19
aac	N	38	17.81
agt	S	28	468.80
agc	S	2268	430.80
ggt	G	0	2.16
ggc	G	15	1.98
gga	G	0	6.55
ggg	G	0	4.31
---	--	------	------
mPD		0.069	1.7
		nPD :	0.04
		N. weight :	2.
		Sc. PD:	-0.22
		Sc. rank :	-284.6

,

PB1

Pos.	655	obs:	exp:
att	I	0	2.23
atc	I	0	1.50
ata	I	6	2.27
atg	M	2344	2344.00
---	--	------	------
mPD		0.0051	0.0051
		nPD :	1.
		N. weight :	0.0031
		Sc. PD:	0.0026
		Sc. rank :	5.0

,

PB1

Pos.	656	obs:	exp:
gaa	E	2309	1407.00
gag	E	41	942.90
---	--	------	------
mPD		0.034	0.48
		nPD :	0.07
		N. weight :	0.55
		Sc. PD:	-0.045
		Sc. rank :	140.4

,

PB1

Pos.	657	obs:	exp:
tat	Y	2349	1142.00
tac	Y	0	1207.00
caa	Q	0	0.51
cag	Q	1	0.49
---	--	------	------
mPD		0.0017	0.50
		nPD :	0.
		N. weight :	0.91
		Sc. PD:	-0.13
		Sc. rank :	-1273.5

,

PB1

Pos.	658	obs:	exp:
gat	D	2348	1352.00
gac	D	2	998.20
---	--	------	------
mPD		0.0017	0.49
		nPD :	0.
		N. weight :	0.69
		Sc. PD:	-0.1
		Sc. rank :	-950.2

,

PB1

Pos.	659	obs:	exp:
gct	A	2341	597.80
gcc	A	4	458.30
gca	A	3	1033.00
gcg	A	2	260.90
---	--	------	------
mPD		0.0076	0.69
		nPD :	0.01
		N. weight :	1.7
		Sc. PD:	-0.24
		Sc. rank :	-1571.8

,

PB1

Pos.	660	obs:	exp:
att	I	1	0.37
atc	I	0	0.25
ata	I	0	0.38
gtt	V	2237	510.90
gtc	V	109	496.30
gta	V	0	462.20
gtg	V	3	879.60
---	--	------	------
mPD		0.092	0.73
		nPD :	0.13
		N. weight :	1.7
		Sc. PD:	-0.048
		Sc. rank :	836.3

,

PB1

Pos.	661	obs:	exp:
tct	S	0	0.34
tcc	S	0	0.29
tca	S	0	0.45
tcg	S	2	0.13
agt	S	0	0.41
agc	S	0	0.38
gct	A	10	597.30
gcc	A	4	457.90
gca	A	20	1032.00
gcg	A	2314	260.70
---	--	------	------
mPD		0.030	0.69
		nPD :	0.04
		N. weight :	2.7
		Sc. PD:	-0.29
		Sc. rank :	-280.0

,

PB1

Pos.	662	obs:	exp:
act	T	1004	622.20
acc	T	1340	519.00
aca	T	1	1009.00
acg	T	4	198.40
aat	N	1	0.53
aac	N	0	0.47
---	--	------	------
mPD		0.49	0.69
		nPD :	0.71
		N. weight :	0.94
		Sc. PD:	0.51
		Sc. rank :	1441.0

,

PB1

Pos.	663	obs:	exp:
act	T	3	622.50
acc	T	0	519.20
aca	T	2335	1010.00
acg	T	12	198.50
---	--	------	------
mPD		0.013	0.69
		nPD :	0.02
		N. weight :	1.
		Sc. PD:	-0.14
		Sc. rank :	-659.1

,

PB1			
Pos.	664	obs:	exp:
cat	H	341	1397.00
cac	H	2009	952.70
---	--	------	------
mPD		0.25	0.48
		nPD:	0.51
		N. weight:	0.55
		Sc. PD:	0.19
		Sc. rank:	719.0

PB1			
Pos.	665	obs:	exp:
tta	L	1	0.11
ttg	L	0	0.19
tct	S	4	401.20
tcc	S	15	346.30
tca	S	801	529.50
tcg	S	1529	151.70
ctt	L	0	0.19
ctc	L	0	0.17
cta	L	0	0.13
ctg	L	0	0.21
agt	S	0	479.60
agc	S	0	440.70
---	--	------	------
mPD		0.46	1.6
		nPD:	0.28
		N. weight:	2.1
		Sc. PD:	0.25
		Sc. rank:	1905.1

PB1			
Pos.	666	obs:	exp:
tgg	W	2350	2350.00
---	--	------	------
mPD		0	0
		nPD:	1.
		N. weight:	0.
		Sc. PD:	0
		Sc. rank:	0

PB1			
Pos.	667	obs:	exp:
att	I	1740	674.70
atc	I	76	455.70
ata	I	1	686.60
act	T	17	10.60
acc	T	23	8.84
aca	T	0	17.19
acg	T	0	3.38
gtt	V	7	107.20
gtc	V	486	104.20
gta	V	0	97.00
gtg	V	0	184.60
---	--	------	------
mPD		0.74	1.1
		nPD:	0.69
		N. weight:	1.2
		Sc. PD:	0.64
		Sc. rank:	1836.0

PB1			
Pos.	668	obs:	exp:
tta	L	0	0.11
ttg	L	0	0.19
tct	S	2	0.34
tcc	S	0	0.29
tca	S	0	0.45
tcg	S	0	0.13
ctt	L	1	0.19
ctc	L	0	0.17
cta	L	0	0.13
ctg	L	0	0.21
cct	P	2156	661.80
ccc	P	169	424.70
cca	P	22	958.30
ccg	P	0	302.20
agt	S	0	0.41
agc	S	0	0.38
---	--	------	------
mPD		0.15	0.71
		nPD:	0.22
		N. weight:	1.2
		Sc. PD:	0.075
		Sc. rank:	961.9

PB1			
Pos.	669	obs:	exp:
aaa	K	186	1319.00
aag	K	2164	1031.00
---	--	------	------
mPD		0.15	0.49
		nPD:	0.3
		N. weight:	0.67
		Sc. PD:	0.092
		Sc. rank:	638.3

PB1			
Pos.	670	obs:	exp:
cgt	R	0	89.12
cgc	R	0	125.20
cga	R	0	227.60
cgg	R	0	184.10
aga	R	17	1078.00
agg	R	2333	646.20
---	--	------	------
mPD		0.014	0.95
		nPD:	0.02
		N. weight:	1.6
		Sc. PD:	-0.21
		Sc. rank:	-1234.7

PB1			
Pos.	671	obs:	exp:
aat	N	156	1249.00
aac	N	2194	1101.00
---	--	------	------
mPD		0.12	0.50
		nPD:	0.25
		N. weight:	0.64
		Sc. PD:	0.059
		Sc. rank:	562.5

PB1			
Pos.	672	obs:	exp:
cgt	R	2063	89.12
cgc	R	283	125.20
cga	R	0	227.60
cgg	R	4	184.10
aga	R	0	1078.00
agg	R	0	646.20
---	--	------	------
mPD		0.21	0.95
		nPD:	0.23
		N. weight:	3.6
		Sc. PD:	0.25
		Sc. rank:	2904.0

PB1			
Pos.	673	obs:	exp:
tct	S	35	401.30
tcc	S	2312	346.40
tca	S	3	529.70
tcg	S	0	151.80
agt	S	0	479.80
agc	S	0	440.90
---	--	------	------
mPD		0.032	1.6
		nPD:	0.02
		N. weight:	2.3
		Sc. PD:	-0.3
		Sc. rank:	-1424.0

PB1			
Pos.	674	obs:	exp:
att	I	2125	870.00
atc	I	218	587.60
ata	I	0	885.30
gtt	V	7	1.52
gtc	V	0	1.48
gta	V	0	1.38
gtg	V	0	2.62
---	--	------	------
mPD		0.17	0.66
		nPD:	0.26
		N. weight:	0.91
		Sc. PD:	0.096
		Sc. rank:	820.4

PB1			
Pos.	675	obs:	exp:
tta	L	0	256.30
ttg	L	3	440.60
ctt	L	2	440.30
ctc	L	1825	405.10
cta	L	0	312.40
ctg	L	518	493.30
att	I	0	0.37
atc	I	1	0.25
ata	I	0	0.38
atg	M	1	1.00
---	--	------	------
mPD		0.35	1.1
		nPD:	0.31
		N. weight:	1.5
		Sc. PD:	0.22
		Sc. rank:	1458.5

PB1			
Pos.	676	obs:	exp:
act	T	1	0.26
acc	T	0	0.22
aca	T	0	0.43
acg	T	0	0.08
aat	N	2339	1248.00
aac	N	10	1101.00
---	--	------	------
mPD		0.0093	0.50
		nPD:	0.02
		N. weight:	0.77
		Sc. PD:	-0.1
		Sc. rank:	-488.9

PB1			
Pos.	677	obs:	exp:
act	T	38	622.50
acc	T	2298	519.20
aca	T	14	1010.00
acg	T	0	198.50
---	--	------	------
mPD		0.043	0.69
		nPD:	0.06
		N. weight:	1.8
		Sc. PD:	-0.16
		Sc. rank:	257.1

PB1			
Pos.	678	obs:	exp:
tct	S	0	400.00
tcc	S	0	345.30
tca	S	0	527.90
tcg	S	0	151.30
aat	N	0	4.25
aac	N	8	3.75
agt	S	493	478.20
agc	S	1849	439.40
---	--	------	------
mPD		0.34	1.6
		nPD:	0.21
		N. weight:	1.4
		Sc. PD:	0.071
		Sc. rank:	1057.2

PB1			
Pos.	679	obs:	exp:
caa	Q	2142	1195.00
cag	Q	207	1154.00
aaa	K	1	0.56
aag	K	0	0.44
---	--	------	------
mPD		0.16	0.50
		nPD:	0.32
		N. weight:	0.48
		Sc. PD:	0.079
		Sc. rank:	486.2

PB1			
Pos.	680	obs:	exp:
cgt	R	0	89.04
cgc	R	0	125.10
cga	R	0	227.40
cgg	R	0	183.90
aaa	K	0	1.12
aag	K	2	0.88
aga	R	522	1077.00
agg	R	1826	645.60
---	--	------	------
mPD		0.35	0.95
		nPD:	0.36
		N. weight:	0.82
		Sc. PD:	0.17
		Sc. rank:	877.9

PB1			
Pos.	681	obs:	exp:
ggt	G	0	338.10
ggc	G	0	310.20
gga	G	2345	1026.00
ggg	G	5	675.90
---	--	------	------
mPD		0.0042	0.69
		nPD:	0.01
		N. weight:	1.
		Sc. PD:	-0.15
		Sc. rank:	-1254.5

PB1			
Pos.	682	obs:	exp:
att	I	1695	837.00
atc	I	559	565.30
ata	I	0	851.70
gtt	V	96	20.88
gtc	V	0	20.28
gta	V	0	18.89
gtg	V	0	35.95
---	--	------	------
mPD		0.44	0.75
		nPD:	0.59
		N. weight:	0.72
		Sc. PD:	0.31
		Sc. rank:	1013.1

PB1			
Pos.	683	obs:	exp:
tta	L	0	256.40
ttg	L	0	440.80
ctt	L	2129	440.50
ctc	L	195	405.30
cta	L	0	312.50
ctg	L	25	493.50
att	I	1	0.37
atc	I	0	0.25
ata	I	0	0.38
---	--	------	------
mPD		0.17	1.1
		nPD:	0.15
		N. weight:	1.7
		Sc. PD:	-0.0052
		Sc. rank:	993.9

PB1			
Pos.	684	obs:	exp:
gaa	E	43	1406.00
gag	E	2306	942.50
ggt	G	0	0.14
ggc	G	0	0.13
gga	G	0	0.44
ggg	G	1	0.29
---	--	------	------
mPD		0.037	0.48
		nPD:	0.08
		N. weight:	1.
		Sc. PD:	-0.079
		Sc. rank:	296.1

PB1			
Pos.	685	obs:	exp:
gat	D	2340	1352.00
gac	D	10	998.20
---	--	------	------
mPD		0.0085	0.49
		nPD:	0.02
		N. weight:	0.67
		Sc. PD:	-0.089
		Sc. rank:	-449.1

PB1			
Pos.	686	obs:	exp:
gat	D	1	0.58
gac	D	0	0.42
gaa	E	540	1406.00
gag	E	1809	942.50
---	--	------	------
mPD		0.35	0.48
		nPD:	0.74
		N. weight:	0.36
		Sc. PD:	0.2
		Sc. rank:	558.4

PB1			
Pos.	687	obs:	exp:
caa	Q	594	1194.00
cag	Q	1753	1153.00
aaa	K	0	1.68
aag	K	3	1.32
---	--	------	------
mPD		0.38	0.50
		nPD:	0.76
		N. weight:	0.18
		Sc. PD:	0.1
		Sc. rank:	275.6

PB1

Pos. 688		obs:	exp:
att	I	0	0.74
atc	I	0	0.50
ata	I	2	0.76
atg	M	2347	2347.00
gtt	V	0	0.22
gtc	V	0	0.21
gta	V	0	0.20
gtg	V	1	0.37
---	--	------	------
mPD		0.0026	0.0031
		nPD:	0.83
		N. weight:	0.0016
		Sc. PD:	0.001
		Sc. rank:	2.5

PB1

Pos. 689		obs:	exp:
tat	Y	518	1142.00
tac	Y	1832	1208.00
---	--	------	------
mPD		0.34	0.50
		nPD:	0.69
		N. weight:	0.19
		Sc. PD:	0.1
		Sc. rank:	287.1

PB1

Pos. 690		obs:	exp:
caa	Q	1407	1193.00
cag	Q	937	1151.00
cgt	R	0	0.19
cgc	R	0	0.27
cga	R	4	0.48
cgg	R	1	0.39
aaa	K	1	0.56
aag	K	0	0.44
aga	R	0	2.29
agg	R	0	1.37
---	--	------	------
mPD		0.48	0.51
		nPD:	0.95
		N. weight:	0.029
		Sc. PD:	0.023
		Sc. rank:	47.0

PB1

Pos. 691		obs:	exp:
cgt	R	0	0.11
cgc	R	0	0.16
cga	R	0	0.29
cgg	R	0	0.23
aaa	K	137	1317.00
aag	K	2210	1030.00
aga	R	1	1.38
agg	R	2	0.82
---	--	------	------
mPD		0.11	0.50
		nPD:	0.23
		N. weight:	0.75
		Sc. PD:	0.053
		Sc. rank:	608.0

PB1

Pos. 692		obs:	exp:
tgt	C	5	1021.00
tgc	C	2344	1328.00
---	-	1	1.00
---	--	------	------
mPD		0.0042	0.49
		nPD:	0.01
		N. weight:	0.7
		Sc. PD:	-0.1
		Sc. rank:	-724.8

PB1

Pos. 693		obs:	exp:
tgt	C	1460	1022.00
tgc	C	890	1328.00
---	--	------	------
mPD		0.47	0.49
		nPD:	0.96
		N. weight:	0.091
		Sc. PD:	0.071
		Sc. rank:	145.6

PB1

Pos. 694		obs:	exp:
tct	S	0	307.60
tcc	S	0	265.50
tca	S	0	406.00
tcg	S	0	116.30
cgt	R	0	0.04
cgc	R	0	0.05
cga	R	0	0.10
cgg	R	0	0.08
att	I	1	0.37
atc	I	0	0.25
ata	I	0	0.38
aat	N	543	289.10
aac	N	1	254.90
agt	S	1772	367.70
agc	S	29	337.90
aga	R	0	0.46
agg	R	1	0.27
ggt	G	3	0.43
ggc	G	0	0.40
gga	G	0	1.31
ggg	G	0	0.86
---	--	------	------
mPD		0.39	1.8
		nPD:	0.21
		N. weight:	1.7
		Sc. PD:	0.094
		Sc. rank:	1259.1

PB1

Pos. 695		obs:	exp:
tta	L	193	255.80
ttg	L	0	439.80
tct	S	0	1.02
tcc	S	0	0.88
tca	S	6	1.35
tcg	S	0	0.39
ctt	L	0	439.50
ctc	L	1	404.40
cta	L	2137	311.90
ctg	L	13	492.50
agt	S	0	1.23
agc	S	0	1.13
---	--	------	------
mPD		0.17	1.1
		nPD:	0.15
		N. weight:	2.2
		Sc. PD:	-0.0093
		Sc. rank:	1255.4

PB1

Pos. 696		obs:	exp:
ttt	F	660	1000.00
ttc	F	1690	1350.00
---	--	------	------
mPD		0.40	0.49
		nPD:	0.83
		N. weight:	0.059
		Sc. PD:	0.038
		Sc. rank:	93.6

PB1

Pos. 697		obs:	exp:
gaa	E	532	1407.00
gag	E	1818	942.90
---	--	------	------
mPD		0.35	0.48
		nPD:	0.73
		N. weight:	0.37
		Sc. PD:	0.2
		Sc. rank:	568.6

PB1

Pos. 698		obs:	exp:
cgt	R	0	0.23
cgc	R	0	0.32
cga	R	0	0.58
cgg	R	0	0.47
aaa	K	2340	1316.00
aag	K	4	1028.00
aga	R	6	2.75
agg	R	0	1.65
---	--	------	------
mPD		0.0085	0.50
		nPD:	0.02
		N. weight:	0.72
		Sc. PD:	-0.096
		Sc. rank:	-518.6

PB1

Pos. 699		obs:	exp:
ttt	F	40	1000.00
ttc	F	2310	1350.00
---	--	------	------
mPD		0.033	0.49
		nPD:	0.07
		N. weight:	0.6
		Sc. PD:	-0.05
		Sc. rank:	134.4

PB1

Pos. 700		obs:	exp:
ttt	F	1	1000.00
ttc	F	2349	1350.00
---	--	------	------
mPD		0.00085	0.49
		nPD:	0.
		N. weight:	0.7
		Sc. PD:	-0.1
		Sc. rank:	-1011.3

PB1

Pos. 701		obs:	exp:
cct	P	540	662.70
ccc	P	1803	425.20
cca	P	7	959.50
ccg	P	0	302.60
---	--	------	------
mPD		0.36	0.70
		nPD:	0.51
		N. weight:	1.3
		Sc. PD:	0.46
		Sc. rank:	1732.0

PB1

Pos. 702		obs:	exp:
tct	S	0	401.30
tcc	S	0	346.40
tca	S	0	529.70
tcg	S	0	151.80
agt	S	828	479.80
agc	S	1522	440.90
---	--	------	------
mPD		0.46	1.6
		nPD:	0.28
		N. weight:	1.3
		Sc. PD:	0.15
		Sc. rank:	1160.3

PB1

Pos. 703		obs:	exp:
tct	S	0	401.30
tcc	S	0	346.40
tca	S	0	529.70
tcg	S	0	151.80
agt	S	1853	479.80
agc	S	497	440.90
---	--	------	------
mPD		0.33	1.6
		nPD:	0.2
		N. weight:	1.4
		Sc. PD:	0.064
		Sc. rank:	975.4

PB1

Pos. 704		obs:	exp:
tct	S	2	401.30
tcc	S	0	346.40
tca	S	2347	529.70
tcg	S	1	151.80
agt	S	0	479.80
agc	S	0	440.90
---	--	------	------
mPD		0.0026	1.6
		nPD:	0.
		N. weight:	1.9
		Sc. PD:	-0.28
		Sc. rank:	-2788.5

PB1

Pos. 705		obs:	exp:
tat	Y	7	1142.00
tac	Y	2343	1208.00
---	--	------	------
mPD		0.0059	0.50
		nPD:	0.01
		N. weight:	0.82
		Sc. PD:	-0.11
		Sc. rank:	-731.3

PB1

Pos. 706		obs:	exp:
cgt	R	0	89.12
cgc	R	0	125.20
cga	R	0	227.60
cgg	R	30	184.10
aga	R	3	1078.00
agg	R	2317	646.20
---	--	------	------
mPD		0.028	0.95
		nPD:	0.03
		N. weight:	1.6
		Sc. PD:	-0.19
		Sc. rank:	-586.9

PB1

Pos. 707		obs:	exp:
cgt	R	0	89.08
cgc	R	0	125.10
cga	R	0	227.50
cgg	R	1	184.00
atg	M	1	1.00
aga	R	193	1077.00
agg	R	2155	645.90
---	--	------	------
mPD		0.15	0.95
		nPD:	0.16
		N. weight:	1.2
		Sc. PD:	0.0059
		Sc. rank:	721.9

PB1

Pos. 708		obs:	exp:
cct	P	1	662.70
ccc	P	0	425.20
cca	P	2330	959.50
ccg	P	19	302.60
---	--	------	------
mPD		0.017	0.70
		nPD:	0.02
		N. weight:	1.1
		Sc. PD:	-0.14
		Sc. rank:	-502.9

PB1

Pos. 709		obs:	exp:
gtt	V	2348	511.10
gtc	V	1	496.50
gta	V	0	462.40
gtg	V	1	880.00
---	--	------	------
mPD		0.0017	0.73
		nPD:	0.
		N. weight:	1.9
		Sc. PD:	-0.29
		Sc. rank:	-2730.7

PB1

Pos. 710		obs:	exp:
ggt	G	0	338.10
ggc	G	0	310.20
gga	G	2345	1026.00
ggg	G	5	675.90
---	--	------	------
mPD		0.0042	0.69
		nPD:	0.01
		N. weight:	1.
		Sc. PD:	-0.15
		Sc. rank:	-1254.5

PB1

Pos.	711	obs:	exp:
att	I	1790	872.60
atc	I	559	589.40
ata	I	1	888.00
mPD		0.36	0.66
		nPD:	0.55
		N. weight:	0.68
		Sc. PD:	0.26
		Sc. rank:	918.2

PB1

Pos.	712	obs:	exp:
tct	S	3	401.30
tcc	S	2338	346.40
tca	S	9	529.70
tcg	S	0	151.80
agt	S	0	479.80
agc	S	0	440.90
mPD		0.010	1.6
		nPD:	0.01
		N. weight:	2.4
		Sc. PD:	-0.34
		Sc. rank:	-2859.8

PB1

Pos.	713	obs:	exp:
tct	S	0	400.60
tcc	S	0	345.80
tca	S	0	528.80
tcg	S	0	151.50
tgt	C	0	1.74
tgc	C	4	2.26
agt	S	491	479.00
agc	S	1855	440.20
mPD		0.33	1.6
		nPD:	0.2
		N. weight:	1.4
		Sc. PD:	0.068
		Sc. rank:	1029.7

PB1

Pos.	714	obs:	exp:
att	I	1	0.37
atc	I	0	0.25
ata	I	0	0.38
atg	M	2349	2349.00
mPD		0.00085	0.00085
		nPD:	1.
		N. weight:	0.00053
		Sc. PD:	0.00044
		Sc. rank:	0.9

PB1

Pos.	715	obs:	exp:
atg	M	1	1.00
gtt	V	33	510.60
gtc	V	0	496.10
gta	V	27	462.00
gtg	V	2288	879.30
ggt	G	0	0.14
ggc	G	0	0.13
gga	G	0	0.44
ggg	G	1	0.29
mPD		0.052	0.73
		nPD:	0.07
		N. weight:	1.1
		Sc. PD:	-0.089
		Sc. rank:	267.5

PB1

Pos.	716	obs:	exp:
gaa	E	1	1407.00
gag	E	2349	942.90
mPD		0.00085	0.48
		nPD:	0.
		N. weight:	1.2
		Sc. PD:	-0.17
		Sc. rank:	-1663.8

PB1

Pos.	717	obs:	exp:
gct	A	6	597.80
gcc	A	2344	458.30
gca	A	0	1033.00
gcg	A	0	260.90
mPD		0.0051	0.69
		nPD:	0.01
		N. weight:	2.
		Sc. PD:	-0.29
		Sc. rank:	-2281.0

PB1

Pos.	718	obs:	exp:
atg	M	2350	2350.00
mPD		0	0
		nPD:	1.
		N. weight:	0.
		Sc. PD:	0
		Sc. rank:	0

PB1

Pos.	719	obs:	exp:
tta	L	0	0.11
ttg	L	1	0.19
ctt	L	0	0.19
ctc	L	0	0.17
cta	L	0	0.13
ctg	L	0	0.21
atg	M	3	3.00
gtt	V	23	510.20
gtc	V	0	495.70
gta	V	23	461.60
gtg	V	2300	878.50
mPD		0.042	0.73
		nPD:	0.06
		N. weight:	1.1
		Sc. PD:	-0.11
		Sc. rank:	112.3

PB1

Pos.	720	obs:	exp:
tct	S	2145	401.30
tcc	S	201	346.40
tca	S	0	529.70
tcg	S	4	151.80
agt	S	0	479.80
agc	S	0	440.90
mPD		0.16	1.6
		nPD:	0.1
		N. weight:	1.9
		Sc. PD:	-0.11
		Sc. rank:	771.3

PB1

Pos.	721	obs:	exp:
cgt	R	0	89.12
cgc	R	0	125.20
cga	R	0	227.60
cgg	R	2	184.10
aga	R	0	1078.00
agg	R	2348	646.20
mPD		0.0017	0.95
		nPD:	0.
		N. weight:	1.6
		Sc. PD:	-0.24
		Sc. rank:	-2340.5

PB1

Pos.	722	obs:	exp:
tct	S	0	0.34
tcc	S	2	0.29
tca	S	0	0.45
tcg	S	0	0.13
act	T	0	0.26
acc	T	1	0.22
aca	T	0	0.43
acg	T	0	0.08
agt	S	0	0.41
agc	S	0	0.38
gtt	V	0	0.22
gtc	V	1	0.21
gta	V	0	0.20
gtg	V	0	0.37
gct	A	9	596.60
gcc	A	2336	457.30
gca	A	0	1031.00
gcg	A	0	260.30
ggt	G	0	0.14
ggc	G	1	0.13
gga	G	0	0.44
ggg	G	0	0.29
mPD		0.012	0.70
		nPD:	0.02
		N. weight:	2.
		Sc. PD:	-0.27
		Sc. rank:	-1455.8

PB1

Pos.	723	obs:	exp:
caa	Q	7	3.56
cag	Q	0	3.44
cgt	R	0	88.85
cgc	R	0	124.80
cga	R	2340	226.90
cgg	R	0	183.50
aga	R	3	1075.00
agg	R	0	644.20
mPD		0.0085	0.96
		nPD:	0.01
		N. weight:	2.9
		Sc. PD:	-0.42
		Sc. rank:	-2948.3

PB1

Pos.	724	obs:	exp:
att	I	2212	872.60
atc	I	138	589.40
ata	I	0	888.00
mPD		0.11	0.66
		nPD:	0.17
		N. weight:	1.
		Sc. PD:	0.013
		Sc. rank:	618.4

PB1

Pos.	725	obs:	exp:
gat	D	2329	1352.00
gac	D	21	998.20
mPD		0.018	0.49
		nPD:	0.04
		N. weight:	0.64
		Sc. PD:	-0.074
		Sc. rank:	-174.9

PB1

Pos.	726	obs:	exp:
gct	A	0	597.80
gcc	A	0	458.30
gca	A	2311	1033.00
gcg	A	39	260.90
mPD		0.033	0.69
		nPD:	0.05
		N. weight:	0.96
		Sc. PD:	-0.1
		Sc. rank:	-62.8

PB1

Pos.	727	obs:	exp:
cgt	R	195	89.12
cgc	R	1616	125.20
cga	R	15	227.60
cgg	R	524	184.10
aga	R	0	1078.00
agg	R	0	646.20
mPD		0.47	0.95
		nPD:	0.49
		N. weight:	2.6
		Sc. PD:	0.85
		Sc. rank:	3305.0

PB1

Pos.	728	obs:	exp:
att	I	959	865.20
atc	I	1371	584.40
ata	I	0	880.40
act	T	1	0.26
acc	T	0	0.22
aca	T	0	0.43
acg	T	0	0.08
gtt	V	19	4.13
gtc	V	0	4.01
gta	V	0	3.74
gtg	V	0	7.11
mPD		0.50	0.68
		nPD:	0.74
		N. weight:	0.7
		Sc. PD:	0.4
		Sc. rank:	1094.1

PB1

Pos.	729	obs:	exp:
gat	D	979	1352.00
gac	D	1371	998.20
mPD		0.49	0.49
		nPD:	1.
		N. weight:	0.066
		Sc. PD:	0.054
		Sc. rank:	106.4

PB1

Pos.	730	obs:	exp:
ttt	F	9	999.50
ttc	F	2340	1349.00
tta	L	0	0.11
ttg	L	0	0.19
ctt	L	0	0.19
ctc	L	1	0.17
cta	L	0	0.13
ctg	L	0	0.21
mPD		0.0085	0.49
		nPD:	0.02
		N. weight:	0.67
		Sc. PD:	-0.09
		Sc. rank:	-454.3

PB1

Pos.	731	obs:	exp:
gaa	E	1840	1407.00
gag	E	510	942.90
---	--	------	------
mPD		0.34	0.48
		nPD:	0.71
		N. weight:	0.099
		Sc. PD:	0.053
		Sc. rank:	151.5

PB1

Pos.	732	obs:	exp:
tct	S	2347	401.30
tcc	S	2	346.40
tca	S	1	529.70
tcg	S	0	151.80
agt	S	0	479.80
agc	S	0	440.90
---	--	------	------
mPD		0.0026	1.6
		nPD:	0.
		N. weight:	2.2
		Sc. PD:	-0.33
		Sc. rank:	-3309.7

PB1

Pos.	733	obs:	exp:
cgt	R	0	0.08
cgc	R	0	0.11
cga	R	0	0.19
cgg	R	0	0.16
aga	R	2	0.92
agg	R	0	0.55
gat	D	1	0.58
gac	D	0	0.42
ggt	G	485	337.60
ggc	G	104	309.80
gga	G	1750	1025.00
ggg	G	8	675.10
---	--	------	------
mPD		0.40	0.69
		nPD:	0.58
		N. weight:	0.53
		Sc. PD:	0.22
		Sc. rank:	733.7

PB1

Pos.	734	obs:	exp:
tct	S	0	0.17
tcc	S	0	0.15
tca	S	0	0.23
tcg	S	0	0.06
cgt	R	0	89.08
cgc	R	0	125.10
cga	R	0	227.50
cgg	R	15	184.00
agt	S	1	0.20
agc	S	0	0.19
aga	R	1	1077.00
agg	R	2333	645.90
---	--	------	------
mPD		0.014	0.95
		nPD:	0.02
		N. weight:	1.6
		Sc. PD:	-0.22
		Sc. rank:	-1236.4

PB1

Pos.	735	obs:	exp:
tct	S	0	0.34
tcc	S	0	0.29
tca	S	0	0.45
tcg	S	0	0.13
att	I	2176	822.50
atc	I	31	555.50
ata	I	8	837.00
agt	S	2	0.41
agc	S	0	0.38
gtt	V	132	28.92
gtc	V	1	28.10
gta	V	0	26.17
gtg	V	0	49.80
---	--	------	------
mPD		0.14	0.78
		nPD:	0.18
		N. weight:	1.2
		Sc. PD:	0.031
		Sc. rank:	763.2

PB1

Pos.	736	obs:	exp:
cgt	R	0	0.83
cgc	R	0	1.17
cga	R	0	2.13
cgg	R	0	1.72
act	T	0	1.85
acc	T	0	1.55
aca	T	0	3.01
acg	T	7	0.59
aat	N	1	0.53
aac	N	0	0.47
aaa	K	8	1302.00
aag	K	2312	1018.00
aga	R	0	10.09
agg	R	22	6.05
---	--	------	------
mPD		0.032	0.52
		nPD:	0.06
		N. weight:	1.
		Sc. PD:	-0.094
		Sc. rank:	127.2

PB1

Pos.	737	obs:	exp:
caa	Q	2	1.02
cag	Q	0	0.98
cgt	R	0	0.99
cgc	R	0	1.38
cga	R	0	2.52
cgg	R	0	2.04
act	T	0	0.53
acc	T	0	0.44
aca	T	2	0.86
acg	T	0	0.17
aat	N	0	31.89
aac	N	60	28.11
aaa	K	1663	1254.00
aag	K	571	980.00
aga	R	25	11.93
agg	R	1	7.15
gaa	E	26	15.57
gag	E	0	10.43
---	--	------	------
mPD		0.45	0.57
		nPD:	0.79
		N. weight:	0.13
		Sc. PD:	0.082
		Sc. rank:	207.9

PB1

Pos.	738	obs:	exp:
gat	D	0	3.45
gac	D	6	2.55
gaa	E	2323	1401.00
gag	E	16	938.50
ggt	G	0	0.72
ggc	G	0	0.66
gga	G	5	2.18
ggg	G	0	1.44
---	--	------	------
mPD		0.023	0.49
		nPD:	0.05
		N. weight:	0.6
		Sc. PD:	-0.064
		Sc. rank:	-46.2

PB1

Pos.	739	obs:	exp:
aaa	K	0	0.56
aag	K	1	0.44
gat	D	45	25.88
gac	D	0	19.12
gaa	E	6	1378.00
gag	E	2296	923.70
ggt	G	0	0.29
ggc	G	0	0.26
gga	G	0	0.87
ggg	G	2	0.58
---	--	------	------
mPD		0.045	0.50
		nPD:	0.09
		N. weight:	1.1
		Sc. PD:	-0.071
		Sc. rank:	403.1

PB1

Pos.	740	obs:	exp:
ttt	F	2311	983.40
ttc	F	0	1328.00
tta	L	0	4.04
ttg	L	0	6.94
tct	S	1	0.17
tcc	S	0	0.15
tca	S	0	0.23
tcg	S	0	0.06
ctt	L	37	6.94
ctc	L	0	6.38
cta	L	0	4.92
ctg	L	0	7.77
agt	S	0	0.20
agc	S	0	0.19
gtt	V	1	0.22
gtc	V	0	0.21
gta	V	0	0.20
gtg	V	0	0.37
---	--	------	------
mPD		0.033	0.52
		nPD:	0.06
		N. weight:	1.1
		Sc. PD:	-0.099
		Sc. rank:	152.8

PB1

Pos.	741	obs:	exp:
tct	S	1	0.17
tcc	S	0	0.15
tca	S	0	0.23
tcg	S	0	0.06
act	T	38	10.07
acc	T	0	8.40
aca	T	0	16.33
acg	T	0	3.21
agt	S	0	0.20
agc	S	0	0.19
gct	A	2309	587.90
gcc	A	2	450.70
gca	A	0	1016.00
gcg	A	0	256.60
---	--	------	------
mPD		0.034	0.72
		nPD:	0.05
		N. weight:	1.7
		Sc. PD:	-0.18
		Sc. rank:	-87.1

PB1

Pos.	742	obs:	exp:
gaa	E	0	1407.00
gag	E	2350	942.90
---	--	------	------
mPD		0	0.48
		nPD:	0.
		N. weight:	1.2
		Sc. PD:	-0.17
		Sc. rank:	-1854.8

PB1

Pos.	743	obs:	exp:
att	I	0	872.60
atc	I	2349	589.40
ata	I	1	888.00
---	--	------	------
mPD		0.00085	0.66
		nPD:	0.
		N. weight:	1.7
		Sc. PD:	-0.26
		Sc. rank:	-2624.0

PB1

Pos.	744	obs:	exp:
tta	L	0	0.65
ttg	L	6	1.13
ctt	L	0	1.13
ctc	L	0	1.04
cta	L	0	0.80
ctg	L	0	1.26
att	I	1	2.23
atc	I	0	1.50
ata	I	5	2.27
atg	M	2334	2334.00
act	T	0	0.26
acc	T	0	0.22
aca	T	0	0.43
acg	T	1	0.08
gtt	V	0	0.65
gtc	V	0	0.63
gta	V	0	0.59
gtg	V	3	1.12
---	--	------	------
mPD		0.014	0.019
		nPD:	0.71
		N. weight:	0.011
		Sc. PD:	0.0057
		Sc. rank:	16.2

PB1

Pos.	745	obs:	exp:
aaa	K	0	1319.00
aag	K	2350	1031.00
---	--	------	------
mPD		0	0.49
		nPD:	0.
		N. weight:	1.
		Sc. PD:	-0.16
		Sc. rank:	-1673.5

PB1

Pos.	746	obs:	exp:
att	I	0	872.30
atc	I	2337	589.10
ata	I	12	887.60
act	T	0	0.26
acc	T	1	0.22
aca	T	0	0.43
acg	T	0	0.08
---	--	------	------
mPD		0.011	0.66
		nPD:	0.02
		N. weight:	1.7
		Sc. PD:	-0.23
		Sc. rank:	-1247.6

PB1

Pos.	747	obs:	exp:
tgt	C	2348	1022.00
tgc	C	2	1328.00
---	--	------	------
mPD		0.0017	0.49
		nPD:	0.
		N. weight:	1.
		Sc. PD:	-0.15
		Sc. rank:	-1451.1

PB1

Pos.	748	obs:	exp:
ttt	F	0	0.43
ttc	F	1	0.57
tct	S	0	401.20
tcc	S	2349	346.30
tca	S	0	529.50
tcg	S	0	151.70
agt	S	0	479.60
agc	S	0	440.70
---	--	------	------
mPD		0.00085	1.6
		nPD:	0.
		N. weight:	2.4
		Sc. PD:	-0.36
		Sc. rank:	-3794.9

PB1

Pos.	749	obs:	exp:
tct	S	0	0.17
tcc	S	1	0.15
tca	S	0	0.23
tcg	S	0	0.06
act	T	0	622.20
acc	T	2349	519.00
aca	T	0	1009.00
acg	T	0	198.40
agt	S	0	0.20
agc	S	0	0.19
---	--	------	------
mPD		0.00085	0.69
		nPD:	0.
		N. weight:	1.9
		Sc. PD:	-0.29
		Sc. rank:	-2929.8

PB1

Pos.	750	obs:	exp:
att	I	2349	872.60
atc	I	0	589.40
ata	I	1	888.00
---	--	------	------
mPD		0.00085	0.66
		nPD:	0.
		N. weight:	1.3
		Sc. PD:	-0.19
		Sc. rank:	-1877.9

PB1

Pos.	751	obs:	exp:
gat	D	3	1.73
gac	D	0	1.27
gaa	E	2340	1402.00
gag	E	1	939.30
ggt	G	0	0.86
ggc	G	0	0.79
gga	G	6	2.62
ggg	G	0	1.73
---	--	------	------
mPD		0.0085	0.49
		nPD:	0.02
		N. weight:	0.65
		Sc. PD:	-0.086
		Sc. rank:	-429.4

PB1

Pos.	752	obs:	exp:
aaa	K	0	0.56
aag	K	1	0.44
gaa	E	3	1406.00
gag	E	2346	942.50
---	--	------	------
mPD		0.0034	0.48
		nPD:	0.01
		N. weight:	1.1
		Sc. PD:	-0.16
		Sc. rank:	-1314.6

PB1

Pos.	753	obs:	exp:
ttt	F	0	5.53
ttc	F	13	7.47
tta	L	0	202.60
ttg	L	0	348.30
ctt	L	0	348.00
ctc	L	1856	320.20
cta	L	0	246.90
ctg	L	0	390.00
att	I	0	178.20
atc	I	480	120.40
ata	I	0	181.40
act	T	0	0.26
acc	T	1	0.22
aca	T	0	0.43
acg	T	0	0.08
---	--	------	------
mPD		0.34	1.3
		nPD:	0.25
		N. weight:	2.1
		Sc. PD:	0.2
		Sc. rank:	1876.4

PB1

Pos.	754	obs:	exp:
cgt	R	0	89.08
cgc	R	0	125.10
cga	R	0	227.50
cgg	R	0	184.00
aaa	K	1	0.56
aag	K	0	0.44
aga	R	2349	1077.00
agg	R	0	645.90
---	--	------	------
mPD		0.00085	0.95
		nPD:	0.
		N. weight:	0.99
		Sc. PD:	-0.15
		Sc. rank:	-1532.2

PB1

Pos.	755	obs:	exp:
cgt	R	0	89.12
cgc	R	0	125.20
cga	R	0	227.60
cgg	R	2350	184.10
aga	R	0	1078.00
agg	R	0	646.20
---	--	------	------
mPD		0	0.95
		nPD:	0.
		N. weight:	3.2
		Sc. PD:	-0.49
		Sc. rank:	-5173.0

PB1

Pos.	756	obs:	exp:
cct	P	0	0.28
ccc	P	0	0.18
cca	P	1	0.41
ccg	P	0	0.13
caa	Q	2305	1195.00
cag	Q	44	1154.00
---	--	------	------
mPD		0.038	0.50
		nPD:	0.08
		N. weight:	0.74
		Sc. PD:	-0.057
		Sc. rank:	204.2

PB1

Pos.	757	obs:	exp:
cgt	R	0	0.53
cgc	R	0	0.75
cga	R	0	1.36
cgg	R	0	1.10
act	T	0	0.79
acc	T	0	0.66
aca	T	3	1.29
acg	T	0	0.25
aat	N	14	10.63
aac	N	6	9.37
aaa	K	2288	1298.00
aag	K	24	1014.00
aga	R	14	6.42
agg	R	0	3.85
gaa	E	1	0.60
gag	E	0	0.40
---	--	------	------
mPD		0.052	0.52
		nPD:	0.1
		N. weight:	0.66
		Sc. PD:	-0.035
		Sc. rank:	280.6

PB1

Pos.	758	obs:	exp:
tta	L	0	3.82
ttg	L	35	6.57
tgg	W	4	4.00
ctt	L	0	6.56
ctc	L	0	6.04
cta	L	0	4.66
ctg	L	0	7.35
caa	Q	0	253.90
cag	Q	499	245.10
---	-	1812	1812.00
---	--	------	------
mPD		0.014	0.037
		nPD:	0.38
		N. weight:	0.
		Sc. PD:	0
		Sc. rank:	0

PB1

Pos.	759	obs:	exp:
taa	.	0	861.80
tag	.	1835	659.50
tga	.	515	828.70
---	--	------	------
mPD		0.68	0.86
		nPD:	0.8
		N. weight:	0.88
		Sc. PD:	0.55
		Sc. rank:	1396.0

Amino acid level conservation per codon

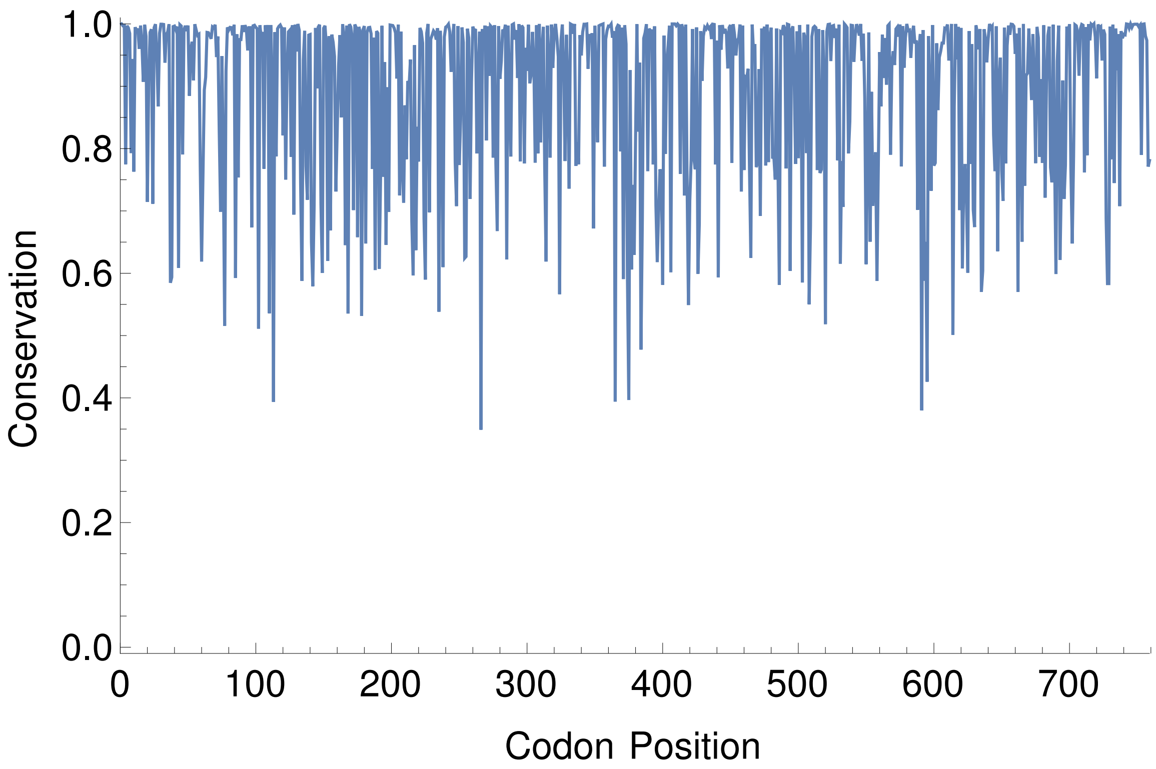

Conservation analysis plots

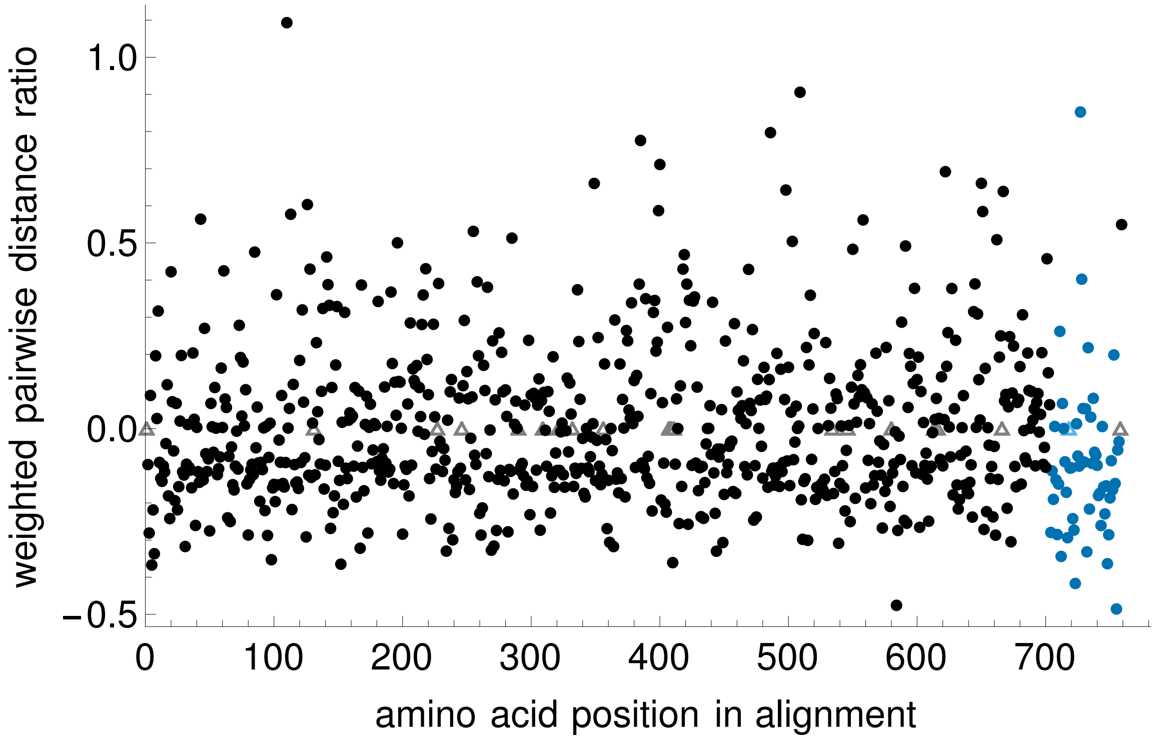

PB1

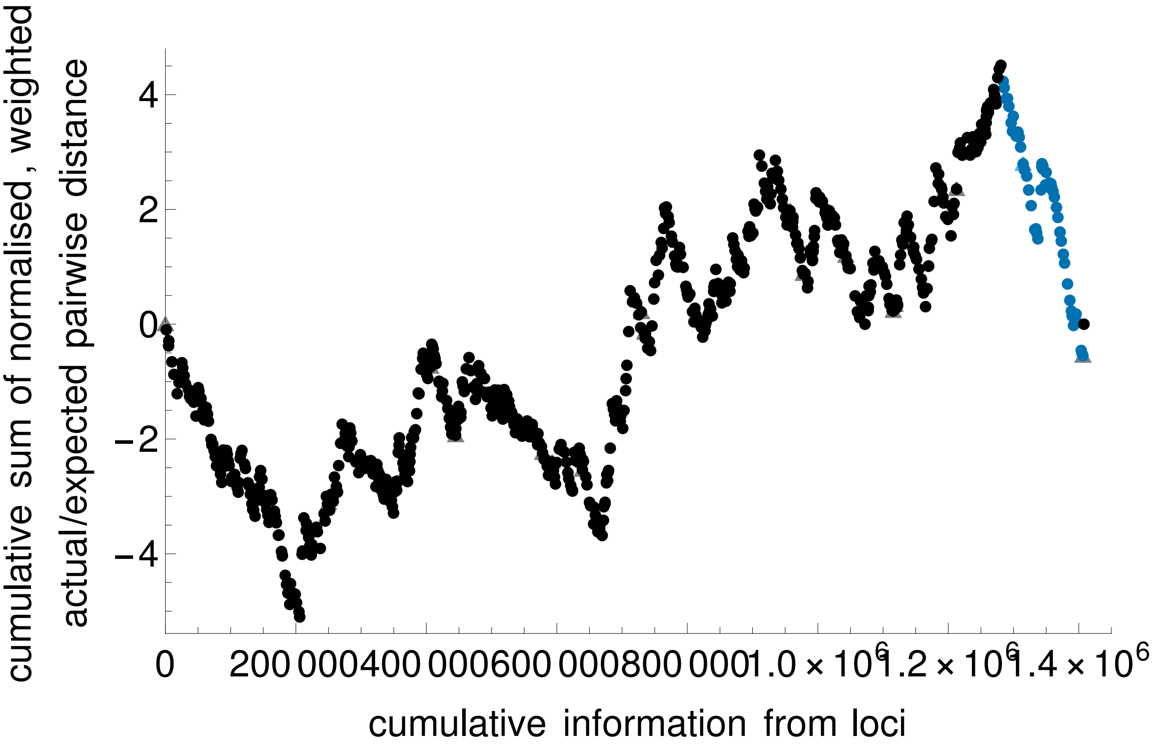

PB1

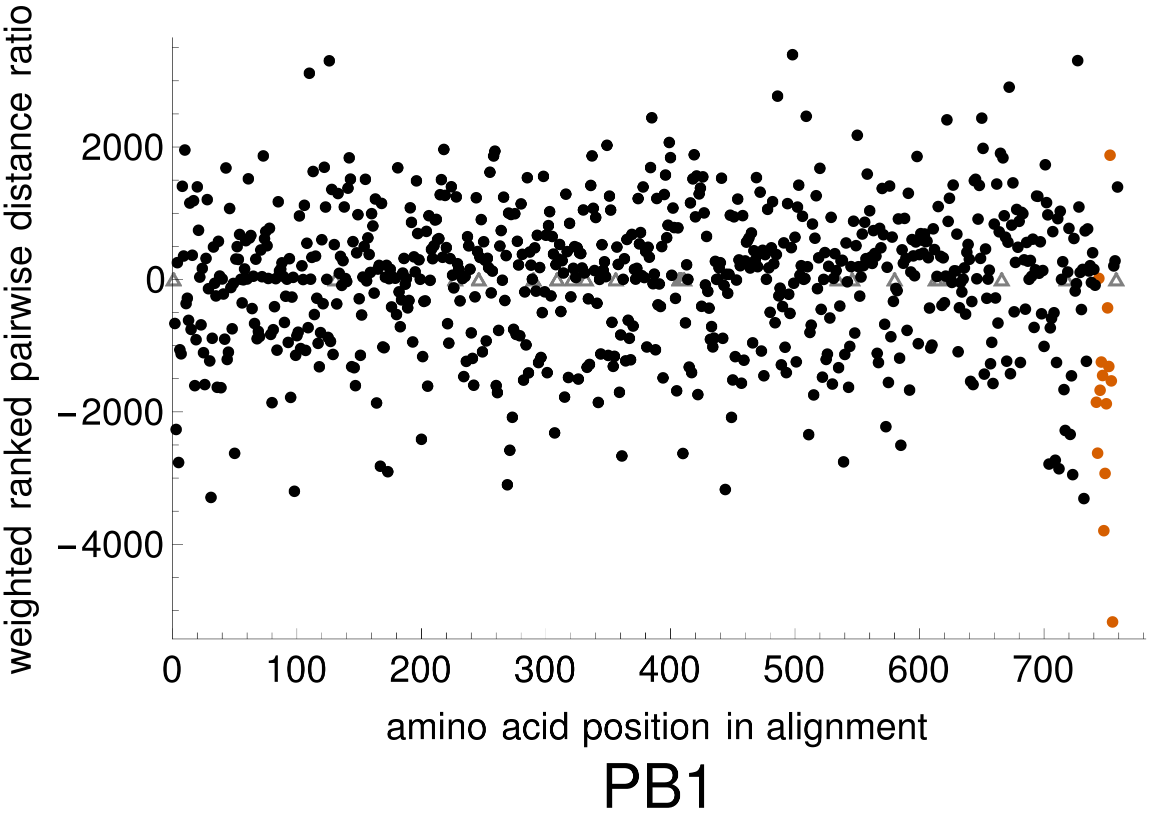
weighted ranked pairwise distance ratio
2000
0
−2000
−4000
0
100
200
300
400
500
600
700
amino acid position in alignment
PB1

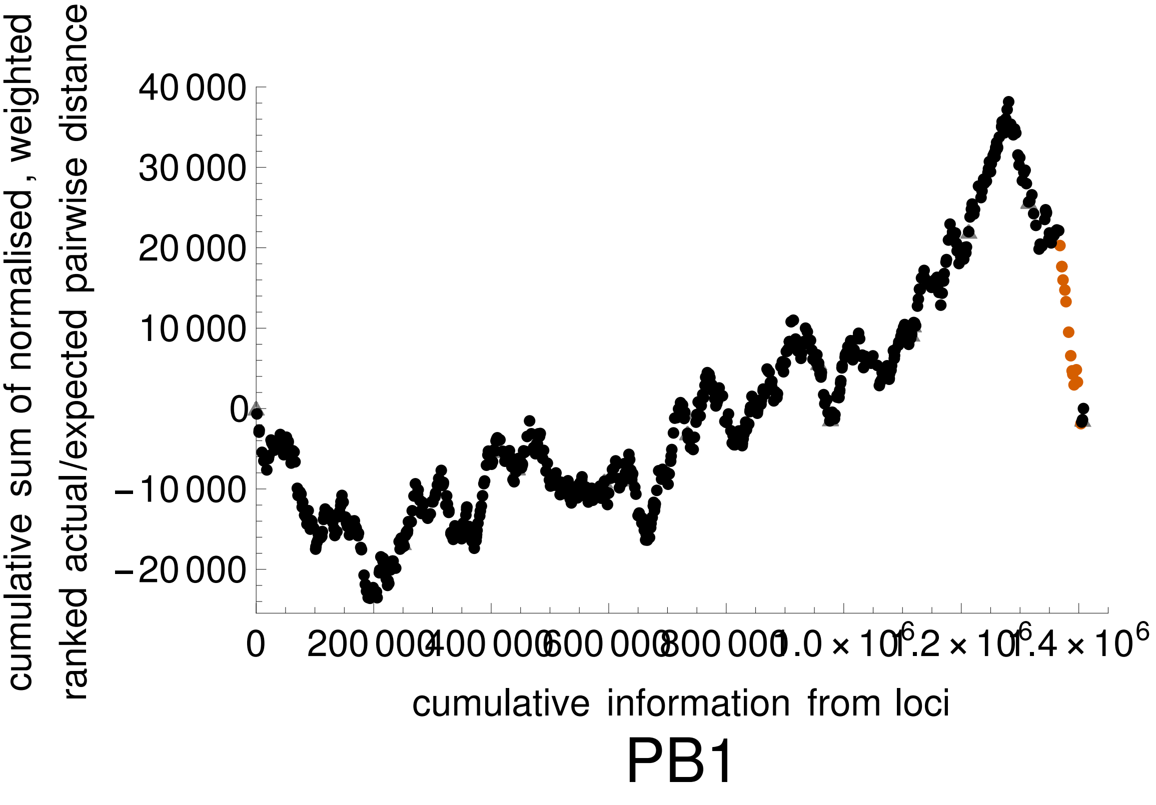
cumulative sum of normalised, weighted ranked actual/expected pairwise distance
40 000
30 000
20 000
10 000
0
−10 000
−20 000
0
200 000
400 000
600 000
800 000
1.0 × 10^6
1.2 × 10^6
1.4 × 10^6
cumulative information from loci
PB1

PB1-F2

Gene length histogram

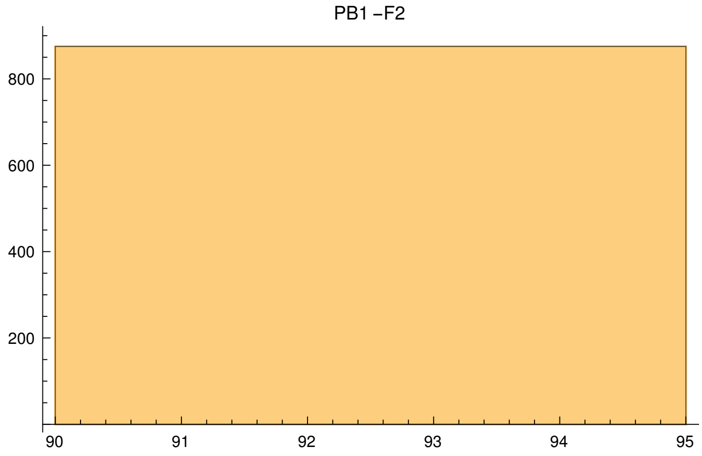

Information vs. nPD

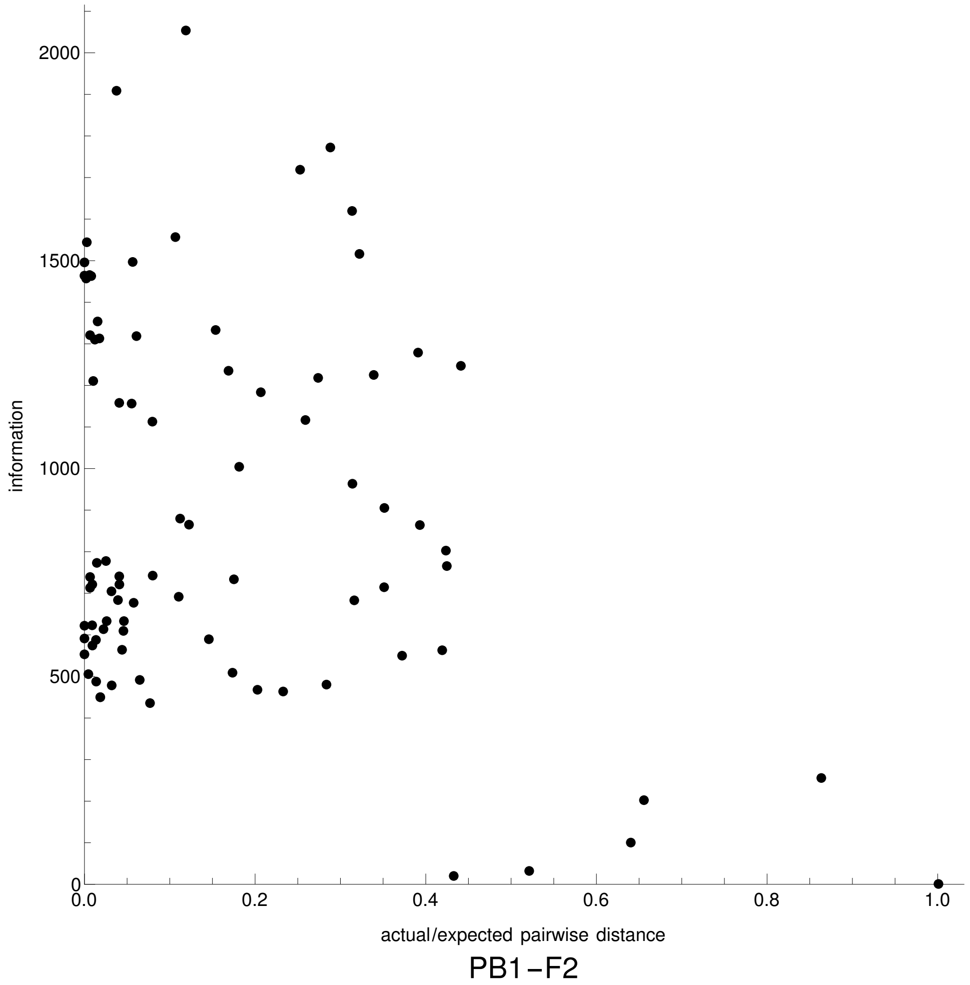

Example sequences highlighted by regions found in analyses to be conserved

Interesting points (by weighted raw PD) highlighted for gene PB1-F2:

ATGGAACAGGAACAGGATACACCATGGACACAGTCAACAGAACACATCAATACTCAGAAAAGGGGAAATGGACAACAA⋱
ACACAGAGACCGGAGCACCCCAACTCAACCCAATTGATGGACCACTGCCTGAGGACAACGAACCAAGCGGATATGCA⋱
CAAACAGATTGTGTGTTGGAAGCAATGGCTTTCCTTGAAGAGTCCCACCCAGGGATCTTTGAAAACTCTTGTCTTGA⋱
AGCGATGGAAGTTGTTCAGCAAACAAGAGTGGACAAACTAA

Interesting points (by weighted ranked PD) highlighted for gene PB1-F2:

ATGGAACAGGAACAGGATACACCATGGACACAGTCAACAGAACACATCAATACTCAGAAAAGGGGAAATGGACAACAA⋱
ACACAGAGACCGGAGCACCCCAACTCAACCCAATTGATGGACCACTGCCTGAGGACAACGAACCAAGCGGATATGCA⋱
CAAACAGATTGTGTGTTGG
AAGCAATGGCTTTCCTTGAAGAGTCCCACCCAGGGATCTTTG
AAAACTCTTGTCTTGAAGCGATGGAAGTTGTTCAGCAAACAAGAGTGGACAAACTAA

Per codon conservation report

{

PB1-F2

Pos.	1	obs:	exp:
atg	M	875	875.00
mPD		0	0
		nPD:	1.
		N. weight:	0.
		Sc. PD:	0
		Sc. rank:	0

,

PB1-F2

Pos.	2	obs:	exp:
gaa	E	775	464.00
gag	E	0	311.00
ggt	G	0	14.39
ggc	G	0	13.20
gga	G	100	43.65
ggg	G	0	28.76
mPD		0.20	0.71
		nPD:	0.28
		N. weight:	0.57
		Sc. PD:	0.08
		Sc. rank:	237.8

,

PB1-F2

Pos.	3	obs:	exp:
caa	Q	0	442.10
cag	Q	869	426.90
cgt	R	0	0.23
cgc	R	0	0.32
cga	R	0	0.58
cgg	R	6	0.47
aga	R	0	2.75
agg	R	0	1.65
mPD		0.014	0.52
		nPD:	0.03
		N. weight:	0.75
		Sc. PD:	-0.075
		Sc. rank:	-166.3

,

PB1-F2

Pos.	4	obs:	exp:
gaa	E	871	521.50
gag	E	0	349.50
ggt	G	0	0.58
ggc	G	0	0.53
gga	G	4	1.75
ggg	G	0	1.15
mPD		0.0091	0.49
		nPD:	0.02
		N. weight:	0.53
		Sc. PD:	-0.057
		Sc. rank:	-143.2

,

PB1-F2

Pos.	5	obs:	exp:
caa	Q	0	445.20
cag	Q	875	429.80
mPD		0	0.50
		nPD:	0.
		N. weight:	0.73
		Sc. PD:	-0.091
		Sc. rank:	-531.2

,

PB1-F2

Pos.	6	obs:	exp:
gtt	V	1	0.22
gtc	V	0	0.21
gta	V	0	0.20
gtg	V	0	0.37
gat	D	731	420.50
gac	D	0	310.50
ggt	G	143	20.57
ggc	G	0	18.87
gga	G	0	62.42
ggg	G	0	41.13
mPD		0.28	0.87
		nPD:	0.32
		N. weight:	0.81
		Sc. PD:	0.14
		Sc. rank:	415.4

,

PB1-F2

Pos.	7	obs:	exp:
att	I	0	10.03
atc	I	0	6.77
ata	I	27	10.20
act	T	0	224.60
acc	T	0	187.40
aca	T	848	364.40
acg	T	0	71.63
mPD		0.060	0.75
		nPD:	0.08
		N. weight:	0.88
		Sc. PD:	-0.043
		Sc. rank:	65.7

,

PB1-F2

Pos.	8	obs:	exp:
tta	L	0	0.11
ttg	L	0	0.19
ctt	L	0	0.19
ctc	L	0	0.17
cta	L	1	0.13
ctg	L	0	0.21
cct	P	0	244.80
ccc	P	1	157.10
cca	P	867	354.40
ccg	P	0	111.80
caa	Q	6	3.05
cag	Q	0	2.95
mPD		0.018	0.72
		nPD:	0.03
		N. weight:	0.92
		Sc. PD:	-0.092
		Sc. rank:	-215.3

,

PB1-F2

Pos.	9	obs:	exp:
tgg	W	875	875.00
mPD		0	0
		nPD:	1.
		N. weight:	0.
		Sc. PD:	0
		Sc. rank:	0

,

PB1-F2

Pos.	10	obs:	exp:
att	I	0	4.83
atc	I	0	3.26
ata	I	13	4.91
act	T	0	228.30
acc	T	0	190.50
aca	T	862	370.40
acg	T	0	72.81
mPD		0.029	0.72
		nPD:	0.04
		N. weight:	0.87
		Sc. PD:	-0.075
		Sc. rank:	-116.0

,

PB1-F2

Pos.	11	obs:	exp:
cct	P	0	0.28
ccc	P	0	0.18
cca	P	0	0.41
ccg	P	1	0.13
caa	Q	1	440.10
cag	Q	864	424.90
cgt	R	0	0.34
cgc	R	0	0.48
cga	R	0	0.87
cgg	R	9	0.70
aga	R	0	4.13
agg	R	0	2.47
mPD		0.025	0.54
		nPD:	0.05
		N. weight:	0.75
		Sc. PD:	-0.061
		Sc. rank:	-49.7

,

PB1-F2

Pos.	12	obs:	exp:
tta	L	9	0.98
ttg	L	0	1.69
tct	S	0	147.90
tcc	S	0	127.70
tca	S	866	195.20
tcg	S	0	55.94
ctt	L	0	1.69
ctc	L	0	1.55
cta	L	0	1.20
ctg	L	0	1.89
agt	S	0	176.80
agc	S	0	162.50
mPD		0.020	1.7
		nPD:	0.01
		N. weight:	1.5
		Sc. PD:	-0.17
		Sc. rank:	-563.0

,

PB1-F2

Pos.	13	obs:	exp:
att	I	0	0.37
atc	I	0	0.25
ata	I	1	0.38
act	T	0	231.20
acc	T	0	192.90
aca	T	873	375.10
acg	T	0	73.74
gct	A	0	0.25
gcc	A	0	0.20
gca	A	1	0.44
gcg	A	0	0.11
mPD		0.0046	0.69
		nPD:	0.01
		N. weight:	0.87
		Sc. PD:	-0.1
		Sc. rank:	-427.5

,

PB1-F2

Pos.	14	obs:	exp:
gaa	E	871	521.50
gag	E	0	349.50
ggt	G	0	0.58
ggc	G	0	0.53
gga	G	4	1.75
ggg	G	0	1.15
mPD		0.0091	0.49
		nPD:	0.02
		N. weight:	0.53
		Sc. PD:	-0.057
		Sc. rank:	-143.2

,

PB1-F2

Pos.	15	obs:	exp:
cat	H	0	232.50
cac	H	391	158.50
cgt	R	0	18.35
cgc	R	484	25.78
cga	R	0	46.87
cgg	R	0	37.91
aga	R	0	222.00
agg	R	0	133.10
mPD		0.49	1.7
		nPD:	0.29
		N. weight:	2.1
		Sc. PD:	0.31
		Sc. rank:	947.8

,

PB1-F2

Pos.	16	obs:	exp:
att	I	0	323.80
atc	I	872	218.70
ata	I	0	329.50
act	T	0	0.79
acc	T	3	0.66
aca	T	0	1.29
acg	T	0	0.25
mPD		0.0068	0.66
		nPD:	0.01
		N. weight:	1.4
		Sc. PD:	-0.16
		Sc. rank:	-555.9

,

PB1-F2

Pos.	17	obs:	exp:
aat	N	875	465.00
aac	N	0	410.00
mPD		0	0.50
		nPD:	0.
		N. weight:	0.65
		Sc. PD:	-0.081
		Sc. rank:	-472.4

,

PB1-F2

Pos.	18	obs:	exp:
att	I	13	4.83
atc	I	0	3.26
ata	I	0	4.91
act	T	862	228.30
acc	T	0	190.50
aca	T	0	370.40
acg	T	0	72.81
mPD		0.029	0.72
		nPD:	0.04
		N. weight:	1.4
		Sc. PD:	-0.12
		Sc. rank:	-181.3

,

PB1-F2

Pos.	19	obs:	exp:
tta	L	0	0.11
ttg	L	0	0.19
ctt	L	0	0.19
ctc	L	0	0.17
cta	L	0	0.13
ctg	L	1	0.21
caa	Q	2	443.70
cag	Q	870	428.30
cgt	R	0	0.08
cgc	R	0	0.11
cga	R	0	0.19
cgg	R	2	0.16
aga	R	0	0.92
agg	R	0	0.55
mPD		0.011	0.51
		nPD:	0.02
		N. weight:	0.72
		Sc. PD:	-0.075
		Sc. rank:	-180.6

,

PB1-F2

Pos.	20	obs:	exp:
cgt	R	0	8.38
cgc	R	0	11.77
cga	R	0	21.40
cgg	R	0	17.31
aaa	K	654	367.10
aag	K	0	286.90
aga	R	221	101.40
agg	R	0	60.77
mPD		0.38	1.0
		nPD:	0.37
		N. weight:	0.65
		Sc. PD:	0.15
		Sc. rank:	395.1

,

PB1-F2

Pos.	21	obs:	exp:
cgt	R	0	31.78
cgc	R	0	44.64
cga	R	0	81.15
cgg	R	0	65.63
aaa	K	0	20.21
aag	K	36	15.79
aga	R	0	384.40
agg	R	838	230.40
ggt	G	0	0.14
ggc	G	0	0.13
gga	G	0	0.44
ggg	G	1	0.29
---	--	------	------
mPD		0.081	1.0
		nPD:	0.08
		N. weight:	1.3
		Sc. PD:	-0.065
		Sc. rank:	79.9

,

PB1-F2

Pos.	22	obs:	exp:
gct	A	0	0.25
gcc	A	1	0.20
gca	A	0	0.44
gcg	A	0	0.11
gaa	E	817	489.20
gag	E	0	327.80
ggt	G	0	8.20
ggc	G	0	7.52
gga	G	57	24.88
ggg	G	0	16.39
---	--	------	------
mPD		0.13	0.62
		nPD:	0.2
		N. weight:	0.55
		Sc. PD:	0.036
		Sc. rank:	166.6

,

PB1-F2

Pos.	23	obs:	exp:
tct	S	0	121.80
tcc	S	0	105.10
tca	S	0	160.70
tcg	S	0	46.06
aat	N	162	86.09
aac	N	0	75.91
agt	S	713	145.60
agc	S	0	133.80
---	--	------	------
mPD		0.30	1.8
		nPD:	0.17
		N. weight:	1.5
		Sc. PD:	0.05
		Sc. rank:	364.6

,

PB1-F2

Pos.	24	obs:	exp:
ggt	G	0	125.90
ggc	G	0	115.50
gga	G	873	382.00
ggg	G	2	251.70
---	--	------	------
mPD		0.0046	0.69
		nPD:	0.01
		N. weight:	0.84
		Sc. PD:	-0.1
		Sc. rank:	-401.1

,

PB1-F2

Pos.	25	obs:	exp:
caa	Q	872	444.20
cag	Q	1	428.80
cgt	R	0	0.08
cgc	R	0	0.11
cga	R	2	0.19
cgg	R	0	0.16
aga	R	0	0.92
agg	R	0	0.55
---	--	------	------
mPD		0.0069	0.51
		nPD:	0.01
		N. weight:	0.69
		Sc. PD:	-0.078
		Sc. rank:	-244.8

,

PB1-F2

Pos.	26	obs:	exp:
cct	P	0	0.28
ccc	P	0	0.18
cca	P	1	0.41
ccg	P	0	0.13
caa	Q	836	425.40
cag	Q	0	410.60
cgt	R	0	0.04
cgc	R	0	0.05
cga	R	1	0.10
cgg	R	0	0.08
aaa	K	37	20.77
aag	K	0	16.23
aga	R	0	0.46
agg	R	0	0.27
---	--	------	------
mPD		0.086	0.59
		nPD:	0.15
		N. weight:	0.7
		Sc. PD:	0.0086
		Sc. rank:	140.0

,

PB1-F2

Pos.	27	obs:	exp:
act	T	12	231.80
acc	T	0	193.30
aca	T	863	376.00
acg	T	0	73.91
---	--	------	------
mPD		0.027	0.69
		nPD:	0.04
		N. weight:	0.81
		Sc. PD:	-0.071
		Sc. rank:	-121.6

,

PB1-F2

Pos.	28	obs:	exp:
tta	L	0	8.40
ttg	L	0	14.45
ctt	L	0	14.44
ctc	L	0	13.28
cta	L	0	10.24
ctg	L	77	16.18
cct	P	0	117.90
ccc	P	0	75.64
cca	P	0	170.70
ccg	P	418	53.82
caa	Q	0	193.30
cag	Q	380	186.70
---	--	------	------
mPD		0.58	1.3
		nPD:	0.44
		N. weight:	1.5
		Sc. PD:	0.43
		Sc. rank:	1051.1

,

PB1-F2

Pos.	29	obs:	exp:
cgt	R	0	32.20
cgc	R	0	45.22
cga	R	0	82.21
cgg	R	0	66.49
att	I	0	0.37
atc	I	0	0.25
ata	I	1	0.38
aaa	K	25	14.03
aag	K	0	10.97
aga	R	849	389.40
agg	R	0	233.40
---	--	------	------
mPD		0.058	1.0
		nPD:	0.06
		N. weight:	0.8
		Sc. PD:	-0.056
		Sc. rank:	-2.5

,

PB1-F2

Pos.	30	obs:	exp:
tta	L	0	57.52
ttg	L	0	98.89
ctt	L	0	98.82
ctc	L	0	90.92
cta	L	2	70.12
ctg	L	525	110.70
cct	P	0	97.85
ccc	P	0	62.79
cca	P	1	141.70
ccg	P	346	44.68
caa	Q	0	0.51
cag	Q	1	0.49
---	--	------	------
mPD		0.49	1.5
		nPD:	0.32
		N. weight:	1.8
		Sc. PD:	0.32
		Sc. rank:	973.7

,

PB1-F2

Pos.	31	obs:	exp:
gtt	V	0	0.43
gtc	V	0	0.42
gta	V	0	0.39
gtg	V	2	0.75
gaa	E	0	406.00
gag	E	678	272.00
ggt	G	0	28.05
ggc	G	0	25.74
gga	G	0	85.12
ggg	G	195	56.09
---	--	------	------
mPD		0.35	0.89
		nPD:	0.39
		N. weight:	1.
		Sc. PD:	0.25
		Sc. rank:	662.4

,

PB1-F2

Pos.	32	obs:	exp:
cat	H	0	424.50
cac	H	714	289.50
cgt	R	0	6.11
cgc	R	161	8.58
cga	R	0	15.59
cgg	R	0	12.61
aga	R	0	73.85
agg	R	0	44.27
---	--	------	------
mPD		0.30	1.2
		nPD:	0.26
		N. weight:	1.3
		Sc. PD:	0.16
		Sc. rank:	510.1

,

PB1-F2

Pos.	33	obs:	exp:
tta	L	0	0.76
ttg	L	0	1.31
tct	S	0	1.54
tcc	S	9	1.33
tca	S	0	2.03
tcg	S	0	0.58
ctt	L	0	1.31
ctc	L	7	1.21
cta	L	0	0.93
ctg	L	0	1.47
cct	P	0	241.40
ccc	P	856	154.90
cca	P	0	349.50
ccg	P	0	110.20
cat	H	0	0.59
cac	H	1	0.41
act	T	0	0.26
acc	T	1	0.22
aca	T	0	0.43
acg	T	0	0.08
agt	S	0	1.84
agc	S	0	1.69
gct	A	0	0.25
gcc	A	1	0.20
gca	A	0	0.44
gcg	A	0	0.11
---	--	------	------
mPD		0.043	0.76
		nPD:	0.06
		N. weight:	1.8
		Sc. PD:	-0.13
		Sc. rank:	-28.2

,

PB1-F2

Pos.	34	obs:	exp:
tct	S	0	5.29
tcc	S	0	4.57
tca	S	0	6.99
tcg	S	0	2.00
aat	N	0	448.50
aac	N	844	395.50
agt	S	0	6.33
agc	S	31	5.82
---	--	------	------
mPD		0.068	0.62
		nPD:	0.11
		N. weight:	0.82
		Sc. PD:	-0.017
		Sc. rank:	96.1

,

PB1-F2

Pos.	35	obs:	exp:
tta	L	13	1.42
ttg	L	0	2.44
tct	S	0	147.20
tcc	S	0	127.10
tca	S	862	194.30
tcg	S	0	55.68
ctt	L	0	2.44
ctc	L	0	2.24
cta	L	0	1.73
ctg	L	0	2.73
agt	S	0	176.00
agc	S	0	161.70
---	--	------	------
mPD		0.029	1.7
		nPD:	0.02
		N. weight:	1.5
		Sc. PD:	-0.17
		Sc. rank:	-431.1

,

PB1-F2

Pos.	36	obs:	exp:
att	I	0	7.43
atc	I	20	5.02
ata	I	0	7.56
act	T	0	226.50
acc	T	855	188.90
aca	T	0	367.40
acg	T	0	72.22
---	--	------	------
mPD		0.045	0.73
		nPD:	0.06
		N. weight:	1.6
		Sc. PD:	-0.1
		Sc. rank:	34.2

,

PB1-F2

Pos.	37	obs:	exp:
caa	Q	865	443.20
cag	Q	6	427.80
cgt	R	0	0.15
cgc	R	0	0.21
cga	R	4	0.39
cgg	R	0	0.31
aga	R	0	1.83
agg	R	0	1.10
---	--	------	------
mPD		0.023	0.52
		nPD:	0.04
		N. weight:	0.67
		Sc. PD:	-0.055
		Sc. rank:	-60.0

,

PB1-F2

Pos.	38	obs:	exp:
tta	L	0	95.51
ttg	L	874	164.20
ctt	L	0	164.10
ctc	L	0	151.00
cta	L	0	116.40
ctg	L	1	183.80
---	--	------	------
mPD		0.0023	1.1
		nPD:	0.
		N. weight:	1.7
		Sc. PD:	-0.21
		Sc. rank:	-1073.0

,

PB1-F2

Pos.	39	obs:	exp:
atg	M	875	875.00
---	--	------	------
mPD		0	0
		nPD:	1.
		N. weight:	0.
		Sc. PD:	0
		Sc. rank:	0

,

PB1-F2

Pos.	40	obs:	exp:
gct	A	0	0.25
gcc	A	1	0.20
gca	A	0	0.44
gcg	A	0	0.11
gat	D	33	449.20
gac	D	748	331.80
ggt	G	0	13.38
ggc	G	93	12.28
gga	G	0	40.60
ggg	G	0	26.75
---	--	------	------
mPD		0.26	0.75
		nPD:	0.35
		N. weight:	0.84
		Sc. PD:	0.17
		Sc. rank:	490.0

PB1-F2

Pos.	41	obs:	exp:
cct	P	1	0.28
ccc	P	0	0.18
cca	P	0	0.41
ccg	P	0	0.13
cat	H	707	519.10
cac	H	166	353.90
cgt	R	0	0.04
cgc	R	1	0.05
cga	R	0	0.10
cgg	R	0	0.08
aga	R	0	0.46
agg	R	0	0.27
---	--	------	------
mPD		0.31	0.49
		nPD:	0.64
		N. weight:	0.12
		Sc. PD:	0.056
		Sc. rank:	84.9

PB1-F2

Pos.	42	obs:	exp:
ttt	F	0	0.43
ttc	F	1	0.57
tat	Y	0	325.70
tac	Y	670	344.30
tgt	C	0	88.71
tgc	C	204	115.30
---	--	------	------
mPD		0.36	0.86
		nPD:	0.42
		N. weight:	0.66
		Sc. PD:	0.18
		Sc. rank:	438.7

PB1-F2

Pos.	43	obs:	exp:
tta	L	0	70.62
ttg	L	0	121.40
ctt	L	0	121.30
ctc	L	0	111.60
cta	L	0	86.08
ctg	L	647	135.90
cct	P	0	1.69
ccc	P	0	1.09
cca	P	0	2.45
ccg	P	6	0.77
caa	Q	0	102.30
cag	Q	201	98.73
cgt	R	0	0.80
cgc	R	0	1.12
cga	R	0	2.03
cgg	R	21	1.64
aga	R	0	9.63
agg	R	0	5.77
---	--	------	------
mPD		0.40	1.5
		nPD:	0.27
		N. weight:	1.4
		Sc. PD:	0.19
		Sc. rank:	589.8

PB1-F2

Pos.	44	obs:	exp:
cgt	R	0	29.35
cgc	R	0	41.23
cga	R	0	74.95
cgg	R	0	60.62
atg	M	96	96.00
aaa	K	0	1.68
aag	K	3	1.32
aga	R	0	355.00
agg	R	774	212.80
ggt	G	0	0.29
ggc	G	0	0.26
gga	G	0	0.87
ggg	G	2	0.58
---	--	------	------
mPD		0.21	1.1
		nPD:	0.18
		N. weight:	1.2
		Sc. PD:	0.054
		Sc. rank:	347.1

PB1-F2

Pos.	45	obs:	exp:
att	I	0	183.80
atc	I	0	124.10
ata	I	495	187.00
act	T	0	100.10
acc	T	0	83.52
aca	T	378	162.40
acg	T	0	31.93
gct	A	0	0.51
gcc	A	0	0.39
gca	A	2	0.88
gcg	A	0	0.22
---	--	------	------
mPD		0.50	1.2
		nPD:	0.42
		N. weight:	0.95
		Sc. PD:	0.26
		Sc. rank:	639.9

PB1-F2

Pos.	46	obs:	exp:
atg	M	44	44.00
act	T	0	220.10
acc	T	0	183.60
aca	T	0	357.10
acg	T	831	70.19
---	--	------	------
mPD		0.096	0.80
		nPD:	0.12
		N. weight:	2.4
		Sc. PD:	-0.031
		Sc. rank:	420.7

PB1-F2

Pos.	47	obs:	exp:
tct	S	0	125.00
tcc	S	0	107.90
tca	S	0	165.00
tcg	S	0	47.28
aat	N	0	75.99
aac	N	143	67.01
agt	S	0	149.50
agc	S	732	137.30
---	--	------	------
mPD		0.27	1.8
		nPD:	0.15
		N. weight:	1.6
		Sc. PD:	0.031
		Sc. rank:	356.6

PB1-F2

Pos.	48	obs:	exp:
caa	Q	864	439.60
cag	Q	0	424.40
cgt	R	0	0.42
cgc	R	0	0.59
cga	R	11	1.07
cgg	R	0	0.86
aga	R	0	5.05
agg	R	0	3.02
---	--	------	------
mPD		0.025	0.54
		nPD:	0.05
		N. weight:	0.72
		Sc. PD:	-0.059
		Sc. rank:	-56.5

PB1-F2

Pos.	49	obs:	exp:
gtt	V	0	2.61
gtc	V	0	2.54
gta	V	0	2.36
gtg	V	12	4.49
gct	A	0	219.50
gcc	A	0	168.30
gca	A	0	379.40
gcg	A	863	95.81
---	--	------	------
mPD		0.027	0.72
		nPD:	0.04
		N. weight:	2.3
		Sc. PD:	-0.2
		Sc. rank:	-368.8

PB1-F2

Pos.	50	obs:	exp:
gtt	V	1	0.22
gtc	V	0	0.21
gta	V	0	0.20
gtg	V	0	0.37
gat	D	872	501.60
gac	D	0	370.40
ggt	G	2	0.29
ggc	G	0	0.26
gga	G	0	0.87
ggg	G	0	0.58
---	--	------	------
mPD		0.0068	0.50
		nPD:	0.01
		N. weight:	0.58
		Sc. PD:	-0.064
		Sc. rank:	-197.8

PB1-F2

Pos.	51	obs:	exp:
atg	M	862	862.00
act	T	0	3.44
acc	T	0	2.87
aca	T	0	5.59
acg	T	13	1.10
---	--	------	------
mPD		0.029	0.056
		nPD:	0.52
		N. weight:	0.038
		Sc. PD:	0.014
		Sc. rank:	27.1

PB1-F2

Pos.	52	obs:	exp:
cat	H	0	498.30
cac	H	838	339.70
cgt	R	0	1.40
cgc	R	37	1.97
cga	R	0	3.58
cgg	R	0	2.90
aga	R	0	16.97
agg	R	0	10.17
---	--	------	------
mPD		0.081	0.66
		nPD:	0.12
		N. weight:	1.
		Sc. PD:	-0.0097
		Sc. rank:	194.0

PB1-F2

Pos.	53	obs:	exp:
cgt	R	0	0.04
cgc	R	0	0.05
cga	R	0	0.10
cgg	R	0	0.08
aaa	K	874	490.60
aag	K	0	383.40
aga	R	1	0.46
agg	R	0	0.27
---	--	------	------
mPD		0.0023	0.50
		nPD:	0.
		N. weight:	0.6
		Sc. PD:	-0.072
		Sc. rank:	-332.4

PB1-F2

Pos.	54	obs:	exp:
tta	L	0	0.55
ttg	L	0	0.94
ctt	L	0	0.94
ctc	L	0	0.86
cta	L	0	0.67
ctg	L	5	1.05
caa	Q	0	167.40
cag	Q	329	161.60
cgt	R	0	20.52
cgc	R	0	28.82
cga	R	0	52.39
cgg	R	541	42.37
aga	R	0	248.20
agg	R	0	148.80
---	--	------	------
mPD		0.48	1.5
		nPD:	0.31
		N. weight:	1.9
		Sc. PD:	0.32
		Sc. rank:	925.0

PB1-F2

Pos.	55	obs:	exp:
att	I	837	310.80
atc	I	0	209.90
ata	I	0	316.30
act	T	38	10.07
acc	T	0	8.40
aca	T	0	16.33
acg	T	0	3.21
---	--	------	------
mPD		0.083	0.74
		nPD:	0.11
		N. weight:	1.
		Sc. PD:	-0.02
		Sc. rank:	139.6

PB1-F2

Pos.	56	obs:	exp:
gtt	V	0	115.30
gtc	V	0	112.00
gta	V	0	104.30
gtg	V	530	198.50
gct	A	0	87.77
gcc	A	0	67.28
gca	A	0	151.70
gcg	A	345	38.30
---	--	------	------
mPD		0.48	1.2
		nPD:	0.39
		N. weight:	1.5
		Sc. PD:	0.37
		Sc. rank:	955.5

PB1-F2

Pos.	57	obs:	exp:
ttt	F	1	0.43
ttc	F	0	0.57
tct	S	1	2.56
tcc	S	14	2.21
tca	S	0	3.38
tcg	S	0	0.97
tat	Y	13	6.32
tac	Y	0	6.68
tgt	C	845	367.90
tgc	C	1	478.10
agt	S	0	3.06
agc	S	0	2.81
---	--	------	------
mPD		0.098	0.56
		nPD:	0.18
		N. weight:	0.87
		Sc. PD:	0.035
		Sc. rank:	237.0

PB1-F2

Pos.	58	obs:	exp:
tta	L	0	1.31
ttg	L	12	2.25
tgg	W	863	863.00
ctt	L	0	2.25
ctc	L	0	2.07
cta	L	0	1.60
ctg	L	0	2.52
---	--	------	------
mPD		0.027	0.063
		nPD:	0.43
		N. weight:	0.024
		Sc. PD:	0.0067
		Sc. rank:	16.4

PB1-F2

Pos.	59	obs:	exp:
cgt	R	0	0.04
cgc	R	0	0.05
cga	R	0	0.10
cgg	R	0	0.08
aat	N	1	0.53
aac	N	0	0.47
aaa	K	0	490.00
aag	K	873	383.00
aga	R	0	0.46
agg	R	1	0.27
---	--	------	------
mPD		0.0046	0.50
		nPD:	0.01
		N. weight:	0.85
		Sc. PD:	-0.099
		Sc. rank:	-360.1

PB1-F2

Pos.	60	obs:	exp:
caa	Q	875	445.20
cag	Q	0	429.80
---	--	------	------
mPD		0	0.50
		nPD:	0.
		N. weight:	0.7
		Sc. PD:	-0.087
		Sc. rank:	-504.9

PB1-F2

Pos.	61	obs:	exp:
tgt	C	1	0.43
tgc	C	0	0.57
tgg	W	874	874.00
---	--	------	------
mPD		0.0023	0.0023
		nPD:	1.
		N. weight:	0.00098
		Sc. PD:	0.0008
		Sc. rank:	0.7

PB1-F2

Pos.	62	obs:	exp:
tta	L	0	95.07
ttg	L	0	163.40
ctt	L	871	163.30
ctc	L	0	150.30
cta	L	0	115.90
ctg	L	0	183.00
cct	P	4	1.13
ccc	P	0	0.72
cca	P	0	1.63
ccg	P	0	0.52
---	--	------	------
mPD		0.0091	1.1
		nPD:	0.01
		N. weight:	1.7
		Sc. PD:	-0.2
		Sc. rank:	-774.6

PB1-F2

Pos.	63	obs:	exp:
ttt	F	0	36.59
ttc	F	86	49.41
tct	S	0	134.40
tcc	S	787	116.00
tca	S	0	177.40
tcg	S	0	50.84
tat	Y	0	0.49
tac	Y	1	0.51
cct	P	0	0.28
ccc	P	1	0.18
cca	P	0	0.41
ccg	P	0	0.13
agt	S	0	160.70
agc	S	0	147.70
---	--	------	------
mPD		0.18	1.7
		nPD:	0.11
		N. weight:	1.8
		Sc. PD:	-0.045
		Sc. rank:	192.1

PB1-F2

Pos.	64	obs:	exp:
tta	L	0	95.51
ttg	L	875	164.20
ctt	L	0	164.10
ctc	L	0	151.00
cta	L	0	116.40
ctg	L	0	183.80
---	--	------	------
mPD		0	1.1
		nPD:	0.
		N. weight:	1.7
		Sc. PD:	-0.22
		Sc. rank:	-1250.3

PB1-F2

Pos.	65	obs:	exp:
cgt	R	0	0.11
cgc	R	0	0.16
cga	R	0	0.29
cgg	R	0	0.23
act	T	0	0.26
acc	T	0	0.22
aca	T	0	0.43
acg	T	1	0.08
aaa	K	3	488.90
aag	K	868	382.10
aga	R	0	1.38
agg	R	3	0.82
---	--	------	------
mPD		0.016	0.50
		nPD:	0.03
		N. weight:	0.83
		Sc. PD:	-0.079
		Sc. rank:	-173.9

PB1-F2

Pos.	66	obs:	exp:
tct	S	0	112.50
tcc	S	0	97.15
tca	S	0	148.50
tcg	S	0	42.57
aat	N	216	114.80
aac	N	0	101.20
agt	S	659	134.60
agc	S	0	123.60
---	--	------	------
mPD		0.37	1.8
		nPD:	0.21
		N. weight:	1.4
		Sc. PD:	0.097
		Sc. rank:	453.0

PB1-F2

Pos.	67	obs:	exp:
cct	P	0	246.70
ccc	P	875	158.30
cca	P	0	357.30
ccg	P	0	112.70
---	--	------	------
mPD		0	0.70
		nPD:	0.
		N. weight:	1.8
		Sc. PD:	-0.22
		Sc. rank:	-1277.5

PB1-F2

Pos.	68	obs:	exp:
att	I	0	0.74
atc	I	2	0.50
ata	I	0	0.76
act	T	0	231.20
acc	T	873	192.90
aca	T	0	375.10
acg	T	0	73.74
---	--	------	------
mPD		0.0046	0.69
		nPD:	0.01
		N. weight:	1.6
		Sc. PD:	-0.18
		Sc. rank:	-785.8

PB1-F2

Pos.	69	obs:	exp:
tta	L	0	0.11
ttg	L	0	0.19
ctt	L	0	0.19
ctc	L	0	0.17
cta	L	0	0.13
ctg	L	1	0.21
caa	Q	0	444.20
cag	Q	873	428.80
aaa	K	0	0.56
aag	K	1	0.44
---	--	------	------
mPD		0.0046	0.51
		nPD:	0.01
		N. weight:	0.74
		Sc. PD:	-0.085
		Sc. rank:	-321.1

PB1-F2

Pos.	70	obs:	exp:
gaa	E	13	7.78
gag	E	0	5.22
ggt	G	0	124.00
ggc	G	0	113.80
gga	G	862	376.30
ggg	G	0	247.90
---	--	------	------
mPD		0.029	0.72
		nPD:	0.04
		N. weight:	0.85
		Sc. PD:	-0.073
		Sc. rank:	-85.8

PB1-F2

Pos.	71	obs:	exp:
tct	S	873	149.10
tcc	S	0	128.70
tca	S	0	196.80
tcg	S	0	56.39
tat	Y	2	0.97
tac	Y	0	1.03
agt	S	0	178.20
agc	S	0	163.80
---	--	------	------
mPD		0.0046	1.6
		nPD:	0.
		N. weight:	1.8
		Sc. PD:	-0.22
		Sc. rank:	-1083.7

PB1-F2

Pos.	72	obs:	exp:
tta	L	0	95.29
ttg	L	873	163.80
tct	S	0	0.17
tcc	S	0	0.15
tca	S	0	0.23
tcg	S	1	0.06
ctt	L	0	163.70
ctc	L	0	150.60
cta	L	0	116.20
ctg	L	0	183.40
atg	M	1	1.00
agt	S	0	0.20
agc	S	0	0.19
---	--	------	------
mPD		0.0046	1.1
		nPD:	0.
		N. weight:	1.7
		Sc. PD:	-0.21
		Sc. rank:	-978.8

PB1-F2

Pos.	73	obs:	exp:
cgt	R	0	1.90
cgc	R	0	2.66
cga	R	0	4.84
cgg	R	0	3.92
aaa	K	824	463.10
aag	K	1	361.90
aga	R	50	22.93
agg	R	0	13.75
---	--	------	------
mPD		0.11	0.63
		nPD:	0.17
		N. weight:	0.6
		Sc. PD:	0.023
		Sc. rank:	156.0

PB1-F2

Pos.	74	obs:	exp:
att	I	18	6.68
atc	I	0	4.51
ata	I	0	6.80
act	T	857	227.00
acc	T	0	189.40
aca	T	0	368.30
acg	T	0	72.39
---	--	------	------
mPD		0.040	0.73
		nPD:	0.06
		N. weight:	1.4
		Sc. PD:	-0.099
		Sc. rank:	-60.7

PB1-F2

Pos.	75	obs:	exp:
tta	L	0	39.73
ttg	L	0	68.30
ctt	L	364	68.26
ctc	L	0	62.80
cta	L	0	48.43
ctg	L	0	76.48
cat	H	500	297.30
cac	H	0	202.70
cgt	R	11	0.42
cgc	R	0	0.59
cga	R	0	1.07
cgg	R	0	0.86
aga	R	0	5.05
agg	R	0	3.02
---	--	------	------
mPD		0.50	1.4
		nPD:	0.35
		N. weight:	1.1
		Sc. PD:	0.22
		Sc. rank:	639.1

PB1-F2

Pos.	76	obs:	exp:
gtt	V	0	189.40
gtc	V	870	184.00
gta	V	1	171.40
gtg	V	0	326.20
gct	A	0	1.02
gcc	A	4	0.78
gca	A	0	1.76
gcg	A	0	0.44
---	--	------	------
mPD		0.011	0.74
		nPD:	0.02
		N. weight:	1.6
		Sc. PD:	-0.18
		Sc. rank:	-484.4

PB1-F2

Pos.	77	obs:	exp:
tta	L	0	95.18
ttg	L	872	163.60
tct	S	0	0.34
tcc	S	0	0.29
tca	S	0	0.45
tcg	S	2	0.13
ctt	L	0	163.50
ctc	L	0	150.40
cta	L	0	116.00
ctg	L	0	183.20
agt	S	0	0.41
agc	S	0	0.38
gtt	V	0	0.22
gtc	V	0	0.21
gta	V	0	0.20
gtg	V	1	0.37
---	--	------	------
mPD		0.0069	1.1
		nPD:	0.01
		N. weight:	1.7
		Sc. PD:	-0.21
		Sc. rank:	-915.3

PB1-F2

Pos.	78	obs:	exp:
cgt	R	0	0.08
cgc	R	0	0.11
cga	R	0	0.19
cgg	R	0	0.16
aaa	K	858	490.00
aag	K	15	383.00
aga	R	2	0.92
agg	R	0	0.55
---	--	------	------
mPD		0.038	0.50
		nPD:	0.08
		N. weight:	0.51
		Sc. PD:	-0.027
		Sc. rank:	20.4

PB1-F2

Pos.	79	obs:	exp:
tta	L	0	0.11
ttg	L	0	0.19
ctt	L	0	0.19
ctc	L	0	0.17
cta	L	1	0.13
ctg	L	0	0.21
caa	Q	488	248.80
cag	Q	1	240.20
cgt	R	0	14.60
cgc	R	0	20.51
cga	R	385	37.28
cgg	R	0	30.15
aga	R	0	176.60
agg	R	0	105.90
---	--	------	------
mPD		0.50	1.5
		nPD:	0.34
		N. weight:	1.4
		Sc. PD:	0.28
		Sc. rank:	820.5

PB1-F2

Pos.	80	obs:	exp:
tgg	W	875	875.00
---	--	------	------
mPD		0	0
		nPD:	1.
		N. weight:	0.
		Sc. PD:	0
		Sc. rank:	0

PB1-F2

Pos.	81	obs:	exp:
cgt	R	0	6.07
cgc	R	0	8.52
cga	R	0	15.49
cgg	R	0	12.53
aaa	K	532	401.30
aag	K	183	313.70
aga	R	1	73.39
agg	R	159	43.99
---	--	------	------
mPD		0.78	0.90
		nPD:	0.86
		N. weight:	0.3
		Sc. PD:	0.21
		Sc. rank:	218.9

PB1-F2

Pos.	82	obs:	exp:
tta	L	1	58.18
ttg	L	532	100.00
tct	S	0	53.45
tcc	S	0	46.14
tca	S	4	70.55
tcg	S	309	20.22
ctt	L	0	99.95
ctc	L	0	91.96
cta	L	0	70.92
ctg	L	0	112.00
agt	S	0	63.91
agc	S	0	58.72
---	-	29	29.00
---	--	------	------
mPD		0.45	1.8
		nPD:	0.25
		N. weight:	2.
		Sc. PD:	0.23
		Sc. rank:	742.1

PB1-F2

Pos.	83	obs:	exp:
ttt	F	0	357.00
ttc	F	839	482.00
tct	S	0	1.20
tcc	S	7	1.03
tca	S	0	1.58
tcg	S	0	0.45
agt	S	0	1.43
agc	S	0	1.31
---	-	29	29.00
---	--	------	------
mPD		0.015	0.48
		nPD:	0.03
		N. weight:	0.56
		Sc. PD:	-0.053
		Sc. rank:	-113.0

PB1-F2

Pos.	84	obs:	exp:
tct	S	0	60.11
tcc	S	0	51.89
tca	S	0	79.35
tcg	S	0	22.74
aat	N	0	262.50
aac	N	494	231.50
agt	S	0	71.87
agc	S	352	66.04
---	-	29	29.00
---	--	------	------
mPD		0.45	1.4
		nPD:	0.31
		N. weight:	1.1
		Sc. PD:	0.19
		Sc. rank:	571.1

PB1-F2

Pos.	85	obs:	exp:
cgt	R	0	0.57
cgc	R	0	0.80
cga	R	0	1.45
cgg	R	0	1.17
aaa	K	831	466.40
aag	K	0	364.60
aga	R	15	6.88
agg	R	0	4.12
---	-	29	29.00
---	--	------	------
mPD		0.033	0.50
		nPD:	0.06
		N. weight:	0.58
		Sc. PD:	-0.037
		Sc. rank:	18.2

PB1-F2

Pos.	86	obs:	exp:
tta	L	0	0.22
ttg	L	0	0.38
ctt	L	0	0.38
ctc	L	0	0.35
cta	L	2	0.27
ctg	L	0	0.42
caa	Q	844	429.40
cag	Q	0	414.60
---	-	29	29.00
---	--	------	------
mPD		0.0044	0.47
		nPD:	0.01
		N. weight:	0.68
		Sc. PD:	-0.078
		Sc. rank:	-279.3

PB1-F2

Pos.	87	obs:	exp:
aaa	K	0	0.56
aag	K	1	0.44
gaa	E	0	504.80
gag	E	843	338.20
ggt	G	0	0.29
ggc	G	0	0.26
gga	G	0	0.87
ggg	G	2	0.58
---	-	29	29.00
---	--	------	------
mPD		0.0066	0.46
		nPD:	0.01
		N. weight:	0.91
		Sc. PD:	-0.1
		Sc. rank:	-300.1

PB1-F2

Pos.	88	obs:	exp:
tgg	W	843	843.00
---	-	32	32.00
---	--	------	------
mPD		0	0
		nPD:	1.
		N. weight:	0.
		Sc. PD:	0
		Sc. rank:	0

PB1-F2

Pos.	89	obs:	exp:
att	I	0	154.50
atc	I	0	104.30
ata	I	416	157.20
act	T	0	113.10
acc	T	0	94.34
aca	T	427	183.50
acg	T	0	36.07
---	-	32	32.00
---	--	------	------
mPD		0.46	1.1
		nPD:	0.42
		N. weight:	0.9
		Sc. PD:	0.25
		Sc. rank:	623.4

PB1-F2

Pos.	90	obs:	exp:
tct	S	0	0.17
tcc	S	0	0.15
tca	S	0	0.23
tcg	S	0	0.06
aat	N	44	443.20
aac	N	790	390.80
aaa	K	7	3.93
aag	K	0	3.07
agt	S	0	0.20
agc	S	1	0.19
gat	D	0	0.58
gac	D	1	0.42
---	-	32	32.00
---	--	------	------
mPD		0.11	0.48
		nPD:	0.23
		N. weight:	0.55
		Sc. PD:	0.051
		Sc. rank:	182.3

PB1-F2

Pos.	91	obs:	exp:
taa	.	338	320.90
tag	.	34	245.60
tga	.	503	308.60
---	--	------	------
mPD		0.56	0.86
		nPD:	0.66
		N. weight:	0.24
		Sc. PD:	0.12
		Sc. rank:	172.1

Amino acid level conservation per codon

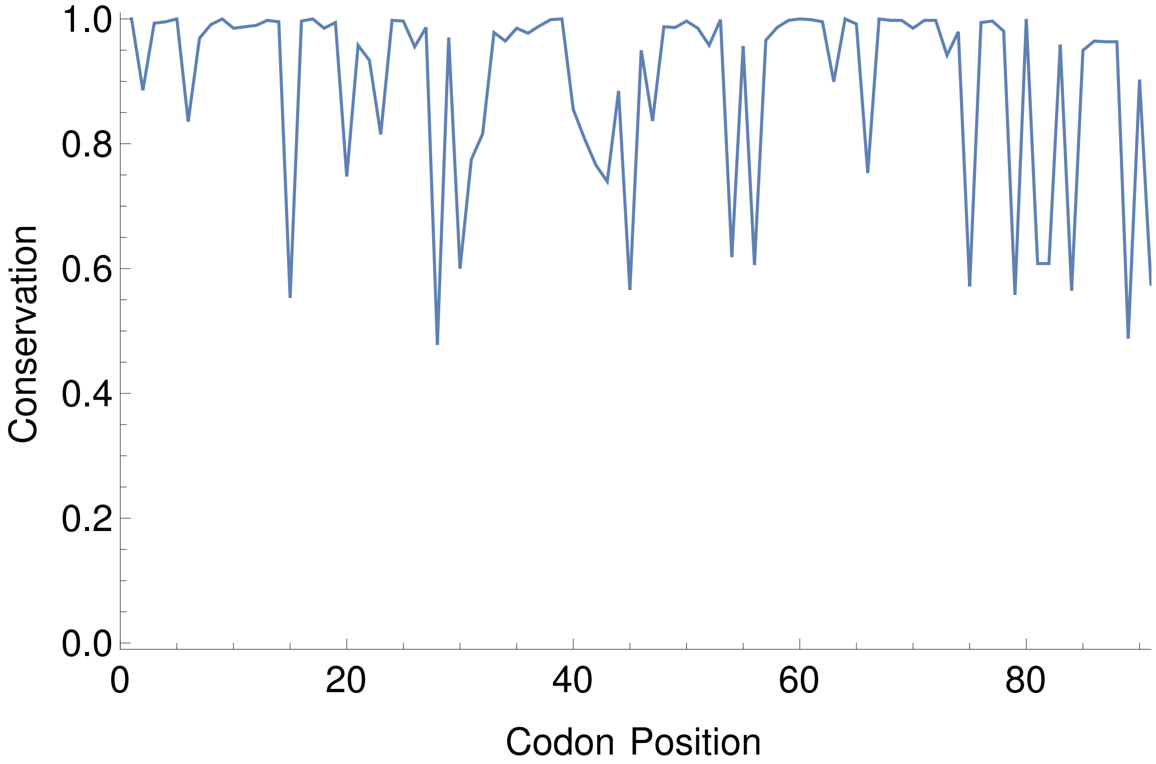

Conservation analysis plots

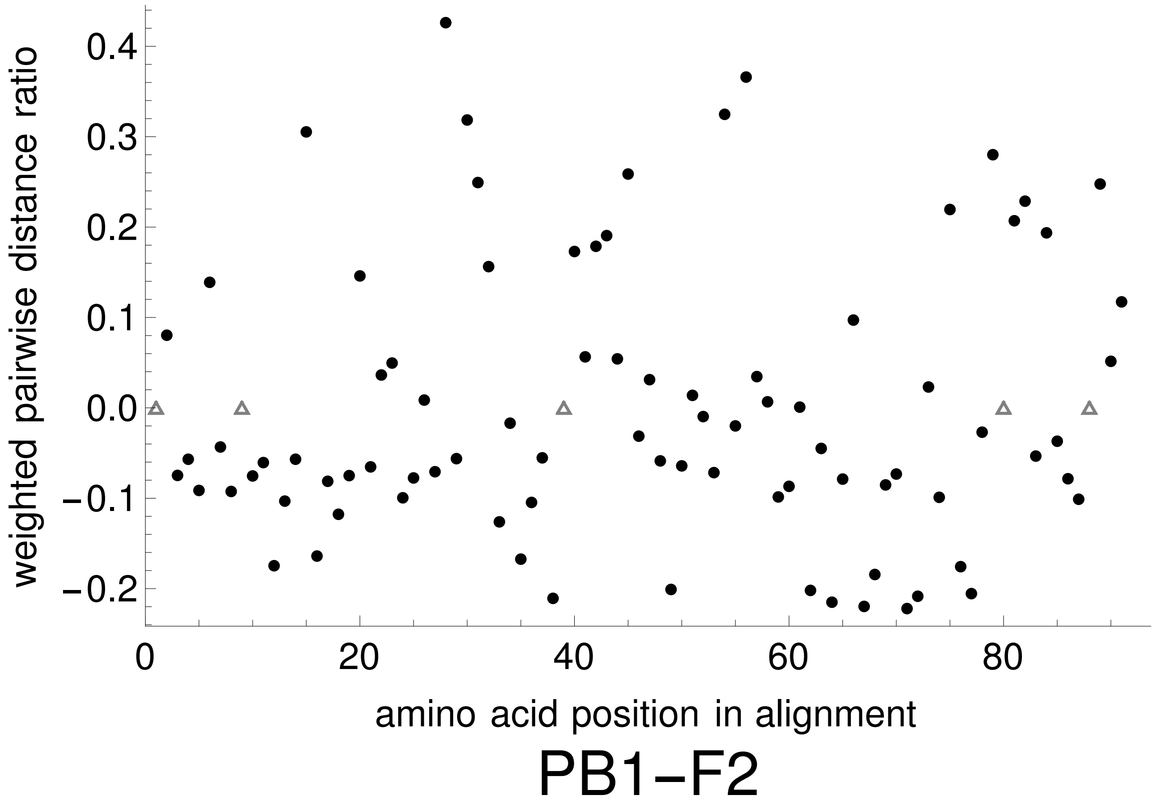

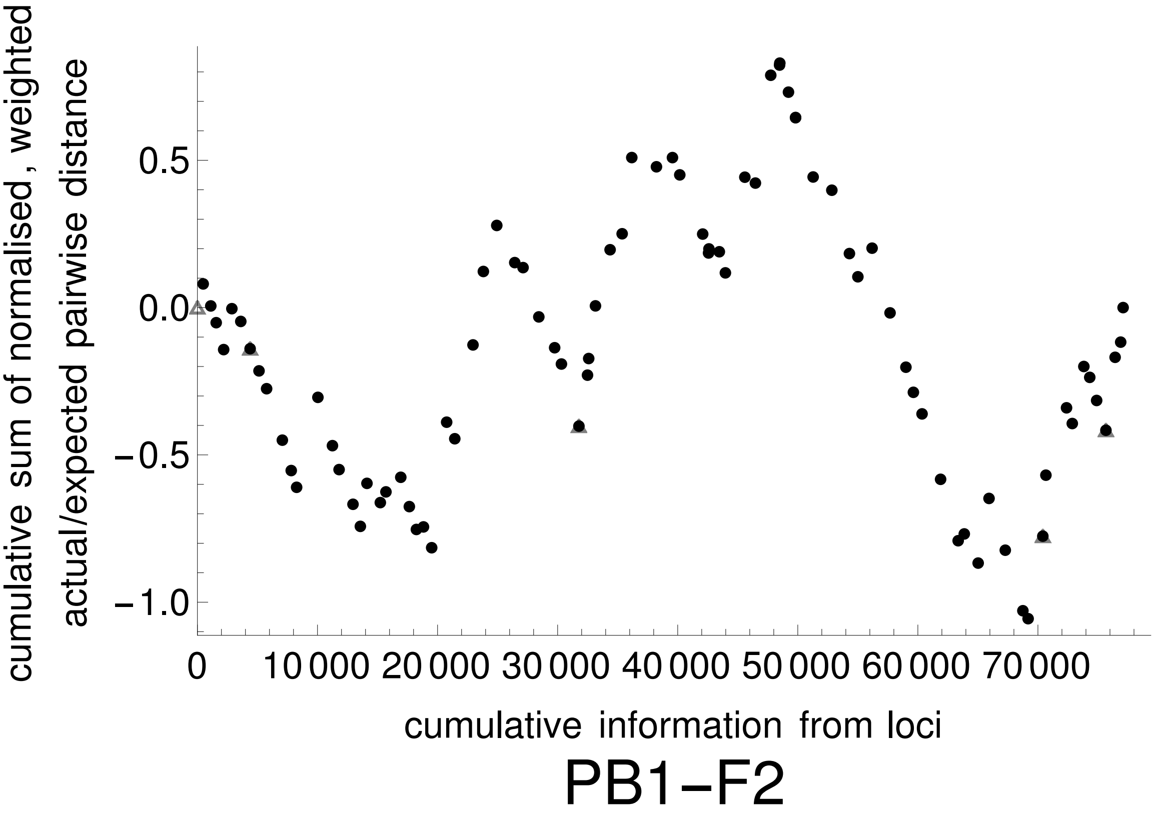

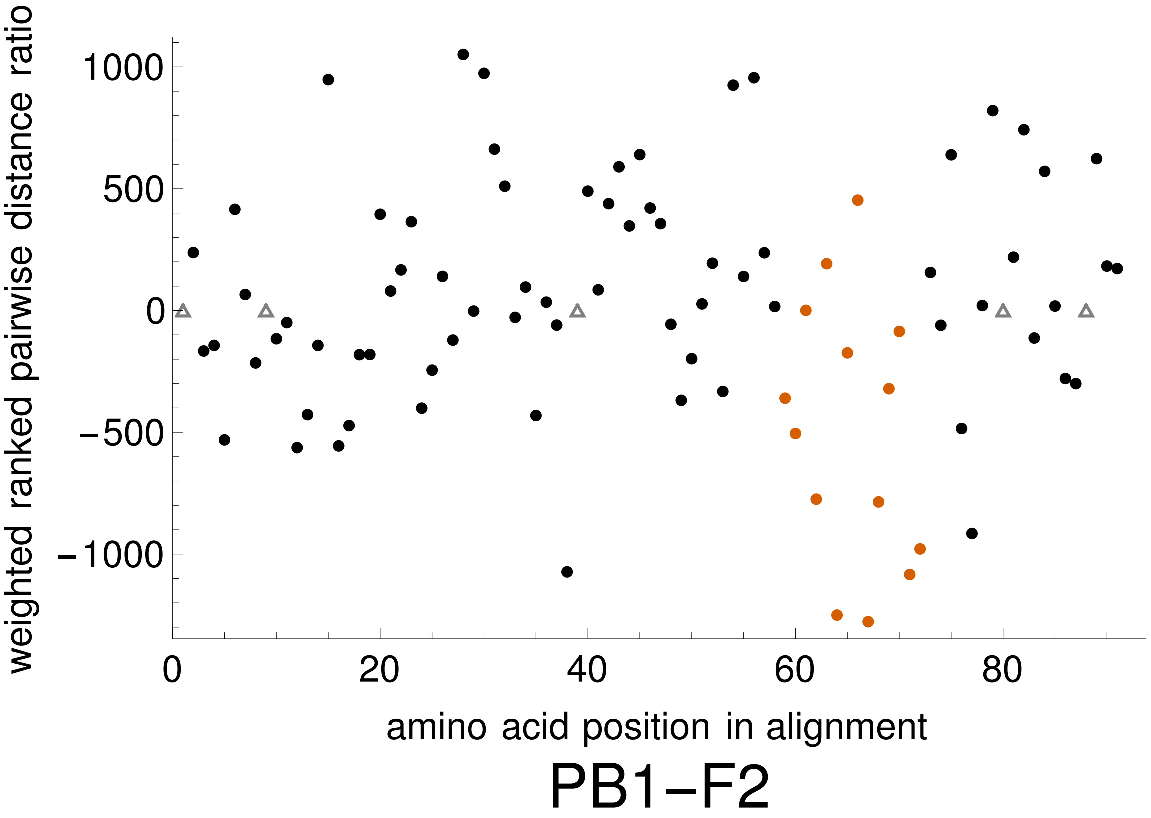

weighted ranked pairwise distance ratio
1000
500
0
−500
−1000
0
20
40
60
80
amino acid position in alignment
PB1−F2

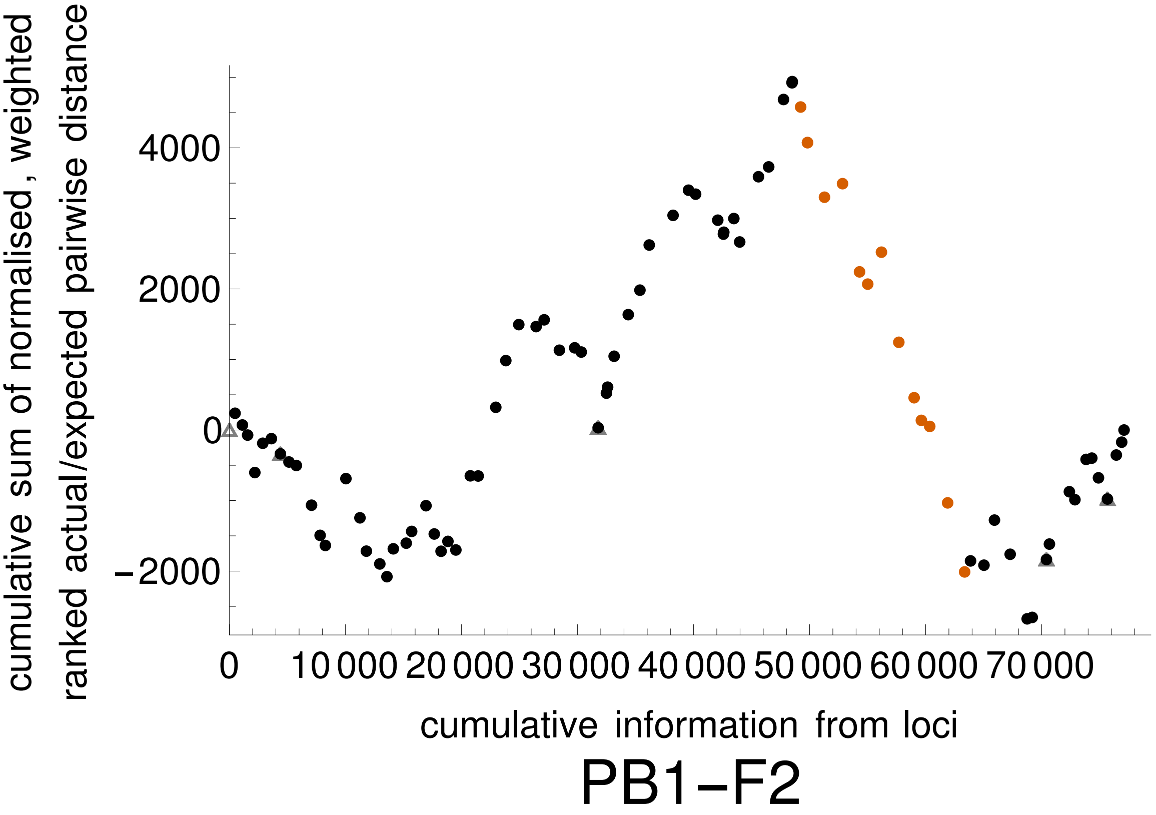

cumulative sum of normalised, weighted ranked actual/expected pairwise distance
4000
2000
0
−2000
0
10 000
20 000
30 000
40 000
50 000
60 000
70 000
cumulative information from loci
PB1−F2

PA

Gene length histogram

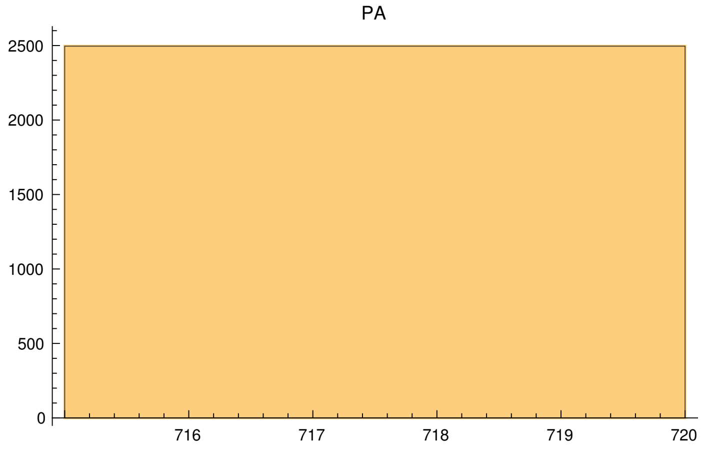

Information vs. nPD

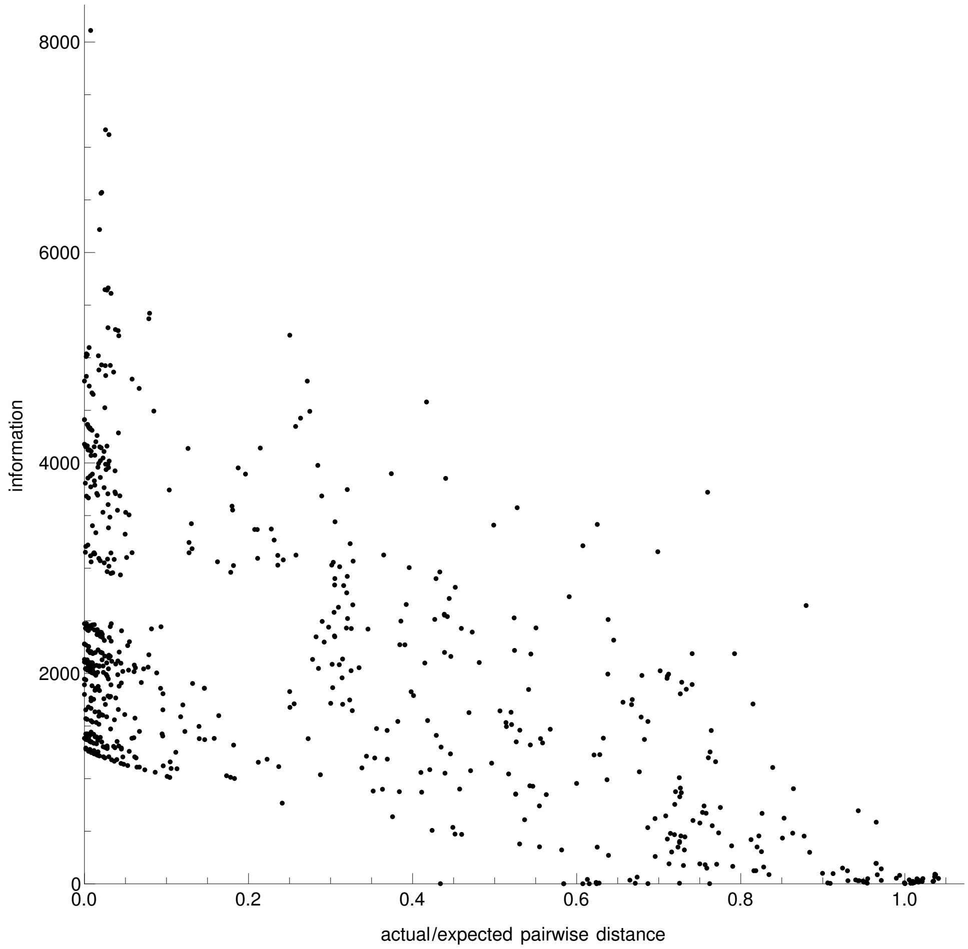

PA

Example sequences highlighted by regions found in analyses to be conserved

Interesting points (by weighted raw PD) highlighted for gene PA:

ATGGAAGACTTTGTGCGACAATGCTTCAATCCAATGATTGTCGAGCTTGCGGAAAAGGCAATGAAAGAATATGGGG⋱
AGGATCCGAAAATCGAAACGAACAAATTTGCAGCAATATGCACACACCTAGAAGTCTGTTTCATGTATTCGGATT⋱
TTCATTTTATTGATGAACGAGGTGGATCAATAATTGTAGAATCTGGCGACCCGAATGCATTATTGAAACACCGAT⋱
TTGAGATAATTGAAGGGAGAGACCGAACAATGGCCTGGACAGTGGTAAATAGTATTTGCCACACCACAGGGGTCG⋱
AGAAACCTAAATTCCTCCCAGATCTGTATGACTACAAAGAGAACCGATTCATCGAAATTGGAGTAACACGAAGGG⋱
AAGTTCACATATACTATCTAGAAAAAGCCAACAAGATAAAATCAGAGAAGACACACATTCACATATTCTCATTCA⋱
CTGGAGAGGAGATGGCCACCAAAGCGGACTACACCCTTGACGAGGAGAGCAGAGCAAGAATCAAAACCAGGCTGT⋱
TCACTATAAGGCAAGAAATGGCCAATAGGGGTCTATGG
GATTCCTTTCGTCAGTCCGAGAGAGGCGAAGAGACAATTGAAGAAAGATTTGAAA
TTACAGGAACCATGCGCAGGCTTGCCGACCAAAGTCTCCCACCGAACTTCTC
CAGCCTTGAAAACTTTAGAGCCTATGTGGATGGATTCGAACCGAACGGCTGC
ATTGAGGGCAAGCTTTCTCAAATGTCAAAAGAAGTGAACGCTAGGATTGAGCCATTT⋱
CTGAAGACAACACCACGCCCTCTCAGATTACCTGATGGGCCTCCCTGCTCTCAGCGATCGAAGTTCTTGC⋱
TGATGGATGCTCTTAAATTAAGCATCGAGGATCCGAGCCATGAAGGGGAAGGCATACCGCTATATGATGC⋱
AATAAAATGCATGAAGACATTTTTCGGCTGGAAAGAGCCCAACATCGTAAAACCGCATGAGAAAGGCATA⋱
AACCCAAATTACCTCCTGGCTTGGAAACAGGTGTTAGCAGAACTCCAAGATATTGAGAATGAGGAGAAAA⋱
TTCCAAAAACAAAGAACATGAAGAAAACAAGCCAATTGAAGTGGGCACTTGGTGAGAATATGGCACCAGA⋱
GAAAGTCGACTTTGAGGACTGCAAAGACGTTAGCGATCTACGACAATATGACAGTGATGAGCCAGAGTCT⋱
AGATCGCTAGCAAGCTGGATCCAGAGCGAATTCAACAAAGCATGCGAACTGACAGATTCAAGTTGGATTG⋱
AACTTGATGAAATAGGGGAAGACGTTGCCCCAATTGAGCACATTGCGAGTATGAGAAGAAACTATTTCAC⋱
AGCGGAAGTATCCCATAGCAGGGCCACTGAATACATAATGAAAGGAGTGTACATAAACACAGCTCTGTTG⋱
AATGCATCCTGTGCAGCCATGGATGACTTCCAACTGATTCCAATGATAAGCAAATGCAGAACCAAAGAAG⋱
GAAGGCGGAAGACAAATCTGTATGGGTACATCATAAAAGGAAGGTCCCATTTGAGGAATGACACCGATGT⋱
GGTAAACTTTGTGAGCATGGAATTCTCTCTTACTGACCCGAGGCTGGAGCCACACAAGTGGGAAAAGTAC⋱
TGTGTCCTCGAGATAGGAGACATGCTACTAAGGACCGCAATAGGCCAAGTGTCAAGGCCCATGTTCCTGT⋱
ATGTAAGAACCAATGGGACTTCCAAGATCAAGATGAAATGGGGCATGGAGATGAGGCGATGCCTTCTTCA⋱
ATCCCTCCAACAAATTGAGAGCATGATTGAGGCCGAGTCTTCTGTCAAAGAGAAGGACATGACCAAAGAA⋱
TTCTTTGAAAACAAATCGGAAACATGGCCAATTGGAGAATCACCTAAGGGGGTGGAGGAAGGCTCCATTG⋱
GAAAGGTGTGCAGAACATTGCTAGCAAAGTCTGTATTCAACAGCCTATATGCATCTCCACAACTCGAGGG⋱
GTTTTCGGCTGAATCAAGGAAATTG
CTTCTCATTGTTCAGGCACTTAGGGACAACCTGGAACCTGGGACCTTCGATCTTG
GGGGGCTATATGAAGCAATTGAGGAGTGCCTGATTAACGATCCATGGGTTTT
GCTTAATGCGTCTTGGTTCAACTCCTTCCTCACACATGCACTGAAATAG

Interesting points (by weighted ranked PD) highlighted for gene PA:

ATGGAAGACTTTGTGCGACAATGCTTCAATCCAATGATTGTCGAGCTTGCGGAAAAGGCAATGAAAGAATATGGGGAG⋱
GATCCGAAAATCGAAACGAACAAATTTGCAGCAATATGCACACACCTAGAAGTCTGTTTCATGTATTCGGATTTTCA⋱
TTTTATTGATGAACGAGGTGGATCAATAATTGTAGAATCTGGCGACCCGAATGCATTATTGAAACACCGATTTGAGA⋱
TAATTGAAGGGAGAGACCGAACAATGGCCTGGACAGTGGTAAATAGTATTTGCCACACCACAGGGGTCGAGAAACCT⋱
AAATTCCTCCCAGATCTGTATGACTACAAAGAGAACCGATTCATCGAAATTGGAGTAACACGAAGGGAAGTTCACAT⋱
ATACTATCTAGAAAAAGCCAACAAGATAAAATCAGAGAAGACACACATTCACATATTCTCATTCACTGGAGAGGAGA⋱
TGGCCACCAAAGCGGACTACACCCTTGACGAGGAGAGCAGAGCAAGAATCAAAACCAGGCTGTTCACTATAAGGCAA⋱
GAAATGGCCAATAGGGGTCTATGG

GATTCCTTTCGTCAGTCCGAGAGAGGCGAAGAGACAATTGAAGAAAGATTTGAAA TTACAGGAACCATGCGCAGGCTTGCCGACCAAAGTCTCCCACCGAACTTCTC CAGCCTTGAAAACTTTAGAGCCTATGTGGATGGATTCGAACCGAACGGCTGC ATTGAGGGCAAGCTTTCTCAAATGTCAAAAGAAGTGAACGCTAGGATTGAGCCATTTCTGAAG⋱.

ACAACACCACGCCCTCTCAGATTACCTGATGGGCCTCCCTGCTCTCAGCGATCGAAGTTCTTGCTGATGG⋱.

ATGCTCTTAAATTAAGCATCGAGGATCCGAGCCATGAAGGGGAAGGCATACCGCTATATGATGCAATAAA⋱.

ATGCATGAAGACATTTTTCGGCTGGAAAGAGCCCAACATCGTAAAACCGCATGAGAAAGGCATAAACCCA⋱.

AATTACCTCCTGGCTTGGAAACAGGTGTTAGCAGAACTCCAAGATATTGAGAATGAGGAGAAAATTCCAA⋱.

AAACAAAGAACATGAAGAAAACAAGCCAATTGAAGTGGGCACTTGGTGAGAATATGGCACCAGAGAAAGT⋱.

CGACTTTGAGGACTGCAAAGACGTTAGCGATCTACGACAATATGACAGTGATGAGCCAGAGTCTAGATCG⋱.

CTAGCAAGCTGGATCCAGAGCGAATTCAACAAAGCATGCGAACTGACAGATTCAAGTTGGATTGAACTTG⋱.

ATGAAATAGGGGAAGACGTTGCCCCAATTGAGCACATTGCGAGTATGAGAAGAAACTATTTCACAGCGGA⋱.

AGTATCCCATAGCAGGGCCACTGAATACATAATGAAAGGAGTGTACATAAACACAGCTCTGTTGAATGCA⋱.

TCCTGTGCAGCCATGGATGACTTCCAACTGATTCCAATGATAAGCAAATGCAGAACCAAAGAAGGAAGGC⋱.

GGAAGACAAATCTGTATGGGTACATCATAAAAGGAAGGTCCCATTTGAGGAATGACACCGATGTGGTAAA⋱.

CTTTGTGAGCATGGAATTCTCTCTTACTGACCCGAGGCTGGAGCCACACAAGTGGGAAAAGTACTGTGTC⋱.

CTCGAGATAGGAGACATGCTACTAAGGACCGCAATAGGCCAAGTGTCAAGGCCCATGTTCCTGTATGTAA⋱.

GAACCAATGGGACTTCCAAGATCAAGATGAAATGGGGCATGGAGATGAGGCGATGCCTTCTTCAATCCCT⋱.

CCAACAAATTGAGAGCATGATTGAGGCCGAGTCTTCTGTCAAAGAGAAGGACATGACCAAAGAATTCTTT⋱.

GAAAACAAATCGGAAACATGGCCAATTGGAGAATCACCTAAGGGGGTGGAGGAAGGCTCCATTGGAAAGG⋱.

TGTGCAGAACATTGCTAGCAAAGTCTGTATTCAACAGCCTATATGCATCTCCACAACTCGAGGGGTTTTC⋱.

GGCTGAATCAAGGAAATTGCTTCTCATTGTTCAGGCACTTAGGGACAACCTGGAACCTGGGACCTTCGAT⋱.

CTTGGG

GGGCTATATGAAGCAATTGAGGAGTGCCTGATTAACGATCCATGGGTTTTGCTTA ATGCGTCTTGGTTCAACTCCTTCCTCACACATGCACTGAAATAG

Per codon conservation report

PA

Pos.	1	obs:	exp:
atg	M	2496	2496.00
---	--	------	------
mPD		0	0
		nPD:	1.
		N. weight:	0.
		Sc. PD:	0
		Sc. rank:	0

PA

Pos.	2	obs:	exp:
caa	Q	0	5.60
cag	Q	11	5.40
gtt	V	0	0.22
gtc	V	0	0.21
gta	V	1	0.20
gtg	V	0	0.37
gaa	E	2473	1483.00
gag	E	4	993.90
ggt	G	0	1.01
ggc	G	0	0.92
gga	G	7	3.06
ggg	G	0	2.01
---	--	------	------
mPD		0.027	0.50
		nPD:	0.05
		N. weight:	0.63
		Sc. PD:	-0.058
		Sc. rank:	246.9

PA

Pos.	3	obs:	exp:
cgt	R	0	0.04
cgc	R	0	0.05
cga	R	0	0.10
cgg	R	0	0.08
aat	N	0	1.59
aac	N	3	1.41
aaa	K	9	5.05
aag	K	0	3.95
aga	R	1	0.46
agg	R	0	0.27
gct	A	0	0.51
gcc	A	0	0.39
gca	A	2	0.88
gcg	A	0	0.22
gat	D	12	1421.00
gac	D	2459	1050.00
gaa	E	6	3.59
gag	E	0	2.41
ggt	G	0	0.58
ggc	G	4	0.53
gga	G	0	1.75
ggg	G	0	1.15
---	--	------	------
mPD		0.040	0.51
		nPD:	0.08
		N. weight:	1.
		Sc. PD:	-0.072
		Sc. rank:	499.7

PA

Pos.	4	obs:	exp:
ttt	F	2477	1055.00
ttc	F	3	1425.00
tta	L	0	1.31
ttg	L	2	2.25
ctt	L	9	2.25
ctc	L	0	2.07
cta	L	1	1.60
ctg	L	0	2.52
att	I	2	0.74
atc	I	0	0.50
ata	I	0	0.76
act	T	1	0.26
acc	T	0	0.22
aca	T	0	0.43
acg	T	0	0.08
gtt	V	1	0.22
gtc	V	0	0.21
gta	V	0	0.20
gtg	V	0	0.37
---	--	------	------
mPD		0.017	0.50
		nPD:	0.03
		N. weight:	1.1
		Sc. PD:	-0.12
		Sc. rank:	130.3

PA

Pos.	5	obs:	exp:
tta	L	0	0.11
ttg	L	0	0.19
ctt	L	0	0.19
ctc	L	0	0.17
cta	L	0	0.13
ctg	L	1	0.21
atg	M	1	1.00
gtt	V	1	542.40
gtc	V	1	527.00
gta	V	0	490.70
gtg	V	2492	933.90
---	--	------	------
mPD		0.0032	0.73
		nPD:	0.
		N. weight:	1.2
		Sc. PD:	-0.17
		Sc. rank:	-1615.2

PA

Pos.	6	obs:	exp:
cgt	R	0	94.62
cgc	R	0	132.90
cga	R	2398	241.60
cgg	R	94	195.40
aga	R	3	1144.00
agg	R	0	686.00
ggt	G	0	0.14
ggc	G	0	0.13
gga	G	1	0.44
ggg	G	0	0.29
---	--	------	------
mPD		0.076	0.95
		nPD:	0.08
		N. weight:	2.7
		Sc. PD:	-0.18
		Sc. rank:	1400.7

PA

Pos.	7	obs:	exp:
caa	Q	2494	1269.00
cag	Q	1	1226.00
cgt	R	0	0.04
cgc	R	0	0.05
cga	R	0	0.10
cgg	R	1	0.08
aga	R	0	0.46
agg	R	0	0.27
---	--	------	------
mPD		0.0024	0.50
		nPD:	0.
		N. weight:	0.84
		Sc. PD:	-0.12
		Sc. rank:	-1097.7

PA

Pos.	8	obs:	exp:
tat	Y	0	0.49
tac	Y	1	0.51
tgt	C	0	1084.00
tgc	C	2494	1410.00
ggt	G	0	0.14
ggc	G	1	0.13
gga	G	0	0.44
ggg	G	0	0.29
---	--	------	------
mPD		0.0016	0.49
		nPD:	0.
		N. weight:	0.71
		Sc. PD:	-0.1
		Sc. rank:	-1038.7

PA

Pos.	9	obs:	exp:
ttt	F	1	1062.00
ttc	F	2494	1433.00
tgt	C	0	0.43
tgc	C	1	0.57
---	--	------	------
mPD		0.0016	0.49
		nPD:	0.
		N. weight:	0.69
		Sc. PD:	-0.098
		Sc. rank:	-996.6

PA

Pos.	10	obs:	exp:
att	I	1	0.37
atc	I	0	0.25
ata	I	0	0.38
aat	N	2491	1326.00
aac	N	4	1169.00
---	--	------	------
mPD		0.0040	0.50
		nPD:	0.01
		N. weight:	0.77
		Sc. PD:	-0.11
		Sc. rank:	-817.8

PA

Pos.	11	obs:	exp:
cct	P	0	703.60
ccc	P	1	451.50
cca	P	2490	1019.00
ccg	P	4	321.30
act	T	0	0.26
acc	T	1	0.22
aca	T	0	0.43
acg	T	0	0.08
---	--	------	------
mPD		0.0056	0.71
		nPD:	0.01
		N. weight:	1.1
		Sc. PD:	-0.15
		Sc. rank:	-1185.9

PA

Pos.	12	obs:	exp:
tta	L	0	0.11
ttg	L	0	0.19
tgg	W	1	1.00
ctt	L	0	0.19
ctc	L	0	0.17
cta	L	0	0.13
ctg	L	1	0.21
att	I	0	1.11
atc	I	0	0.75
ata	I	3	1.13
atg	M	2491	2491.00
---	--	------	------
mPD		0.0048	0.0053
		nPD:	0.91
		N. weight:	0.0022
		Sc. PD:	0.0017
		Sc. rank:	3.9

PA

Pos.	13	obs:	exp:
tta	L	0	0.11
ttg	L	0	0.19
ctt	L	1	0.19
ctc	L	0	0.17
cta	L	0	0.13
ctg	L	0	0.21
att	I	1893	925.40
atc	I	599	625.00
ata	I	0	941.70
act	T	0	0.26
acc	T	1	0.22
aca	T	0	0.43
acg	T	0	0.08
gtt	V	2	0.43
gtc	V	0	0.42
gta	V	0	0.39
gtg	V	0	0.75
---	--	------	------
mPD		0.37	0.66
		nPD:	0.56
		N. weight:	0.67
		Sc. PD:	0.27
		Sc. rank:	992.0

PA

Pos.	14	obs:	exp:
ttt	F	0	0.43
ttc	F	1	0.57
att	I	0	3.71
atc	I	10	2.51
ata	I	0	3.78
gtt	V	1	539.40
gtc	V	2478	524.00
gta	V	1	488.00
gtg	V	0	928.70
gct	A	0	1.27
gcc	A	5	0.98
gca	A	0	2.20
gcg	A	0	0.56
---	--	------	------
mPD		0.014	0.74
		nPD:	0.02
		N. weight:	1.9
		Sc. PD:	-0.24
		Sc. rank:	-926.5

PA

Pos.	15	obs:	exp:
aaa	K	0	0.56
aag	K	1	0.44
gaa	E	17	1494.00
gag	E	2478	1001.00
---	--	------	------
mPD		0.014	0.48
		nPD:	0.03
		N. weight:	1.1
		Sc. PD:	-0.13
		Sc. rank:	-13.4

PA

Pos.	16	obs:	exp:
ttt	F	1	0.43
ttc	F	0	0.57
tta	L	0	272.20
ttg	L	0	468.00
ctt	L	2492	467.70
ctc	L	1	430.30
cta	L	0	331.80
ctg	L	1	524.00
cgt	R	0	0.04
cgc	R	0	0.05
cga	R	0	0.10
cgg	R	1	0.08
aga	R	0	0.46
agg	R	0	0.27
mPD		0.0040	1.1
		nPD:	0.
		N. weight:	2.1
		Sc. PD:	-0.3
		Sc. rank:	-2885.8

PA

Pos.	17	obs:	exp:
gtt	V	0	0.22
gtc	V	0	0.21
gta	V	0	0.20
gtg	V	1	0.37
gct	A	1	634.50
gcc	A	0	486.30
gca	A	33	1096.00
gcg	A	2460	276.90
ggt	G	0	0.14
ggc	G	0	0.13
gga	G	0	0.44
ggg	G	1	0.29
mPD		0.028	0.69
		nPD:	0.04
		N. weight:	2.6
		Sc. PD:	-0.28
		Sc. rank:	654.5

PA

Pos.	18	obs:	exp:
aaa	K	2	1.12
aag	K	0	0.88
gtt	V	0	0.22
gtc	V	0	0.21
gta	V	0	0.20
gtg	V	1	0.37
gat	D	0	0.58
gac	D	1	0.42
gaa	E	2487	1492.00
gag	E	4	999.50
ggt	G	0	0.14
ggc	G	0	0.13
gga	G	1	0.44
ggg	G	0	0.29
mPD		0.0080	0.48
		nPD:	0.02
		N. weight:	0.63
		Sc. PD:	-0.081
		Sc. rank:	-383.9

PA

Pos.	19	obs:	exp:
cgt	R	0	11.00
cgc	R	0	15.45
cga	R	0	28.08
cgg	R	0	22.71
aat	N	1	0.53
aac	N	0	0.47
aaa	K	9	1238.00
aag	K	2196	967.30
aga	R	0	133.00
agg	R	290	79.74
mPD		0.21	0.77
		nPD:	0.28
		N. weight:	1.1
		Sc. PD:	0.13
		Sc. rank:	973.7

PA

Pos.	20	obs:	exp:
tct	S	0	0.17
tcc	S	0	0.15
tca	S	0	0.23
tcg	S	1	0.06
act	T	0	145.70
acc	T	0	121.50
aca	T	550	236.30
acg	T	0	46.46
agt	S	0	0.20
agc	S	0	0.19
gct	A	0	494.80
gcc	A	0	379.30
gca	A	1943	855.00
gcg	A	2	215.90
mPD		0.35	1.0
		nPD:	0.33
		N. weight:	1.
		Sc. PD:	0.19
		Sc. rank:	1180.7

PA

Pos.	21	obs:	exp:
atg	M	2495	2495.00
gtt	V	0	0.22
gtc	V	0	0.21
gta	V	0	0.20
gtg	V	1	0.37
mPD		0.00080	0.0013
		nPD:	0.62
		N. weight:	0.00049
		Sc. PD:	0.00022
		Sc. rank:	0.7

PA

Pos.	22	obs:	exp:
cgt	R	0	0.11
cgc	R	0	0.16
cga	R	0	0.29
cgg	R	0	0.23
aat	N	1	0.53
aac	N	0	0.47
aaa	K	2484	1399.00
aag	K	8	1093.00
aga	R	3	1.38
agg	R	0	0.82
mPD		0.0096	0.50
		nPD:	0.02
		N. weight:	0.69
		Sc. PD:	-0.088
		Sc. rank:	-340.4

PA

Pos.	23	obs:	exp:
gtt	V	0	0.22
gtc	V	0	0.21
gta	V	1	0.20
gtg	V	0	0.37
gaa	E	2482	1494.00
gag	E	13	1001.00
mPD		0.011	0.48
		nPD:	0.02
		N. weight:	0.6
		Sc. PD:	-0.074
		Sc. rank:	-190.8

PA

Pos.	24	obs:	exp:
ttt	F	2	0.85
ttc	F	0	1.15
tat	Y	2481	1209.00
tac	Y	6	1278.00
tgt	C	1	0.43
tgc	C	0	0.57
cat	H	6	3.57
cac	H	0	2.43
mPD		0.012	0.51
		nPD:	0.02
		N. weight:	0.88
		Sc. PD:	-0.11
		Sc. rank:	-274.2

PA

Pos.	25	obs:	exp:
gtt	V	0	0.22
gtc	V	0	0.21
gta	V	0	0.20
gtg	V	1	0.37
ggt	G	1	358.90
ggc	G	0	329.30
gga	G	26	1089.00
ggg	G	2468	717.60
mPD		0.022	0.69
		nPD:	0.03
		N. weight:	1.5
		Sc. PD:	-0.17
		Sc. rank:	115.2

PA

Pos.	26	obs:	exp:
gaa	E	2209	1495.00
gag	E	287	1001.00
mPD		0.20	0.48
		nPD:	0.42
		N. weight:	0.25
		Sc. PD:	0.068
		Sc. rank:	322.7

PA

Pos.	27	obs:	exp:
tct	S	0	0.17
tcc	S	0	0.15
tca	S	0	0.23
tcg	S	0	0.06
tat	Y	2	0.97
tac	Y	0	1.03
aat	N	587	311.90
aac	N	0	275.10
aaa	K	1	0.56
aag	K	0	0.44
agt	S	1	0.20
agc	S	0	0.19
gat	D	1900	1095.00
gac	D	3	808.40
ggt	G	2	0.29
ggc	G	0	0.26
gga	G	0	0.87
ggg	G	0	0.58
mPD		0.37	0.86
		nPD:	0.43
		N. weight:	0.7
		Sc. PD:	0.19
		Sc. rank:	907.6

PA

Pos.	28	obs:	exp:
tta	L	0	0.44
ttg	L	0	0.75
tct	S	2	0.34
tcc	S	0	0.29
tca	S	0	0.45
tcg	S	0	0.13
ctt	L	0	0.75
ctc	L	0	0.69
cta	L	0	0.53
ctg	L	4	0.84
cct	P	908	701.90
ccc	P	4	450.40
cca	P	834	1016.00
ccg	P	743	320.50
caa	Q	1	0.51
cag	Q	0	0.49
agt	S	0	0.41
agc	S	0	0.38
mPD		0.67	0.71
		nPD:	0.94
		N. weight:	0.35
		Sc. PD:	0.27
		Sc. rank:	598.7

PA

Pos.	29	obs:	exp:
cat	H	1	0.59
cac	H	0	0.41
cgt	R	0	0.27
cgc	R	0	0.37
cga	R	0	0.68
cgg	R	0	0.55
aat	N	1	0.53
aac	N	0	0.47
aaa	K	1575	1396.00
aag	K	912	1091.00
aga	R	7	3.21
agg	R	0	1.92
mPD		0.47	0.50
		nPD:	0.94
		N. weight:	0.019
		Sc. PD:	0.014
		Sc. rank:	32.1

PA

Pos.	30	obs:	exp:
tta	L	0	0.11
ttg	L	0	0.19
ctt	L	0	0.19
ctc	L	1	0.17
cta	L	0	0.13
ctg	L	0	0.21
att	I	22	919.40
atc	I	2454	621.00
ata	I	0	935.60
act	T	0	0.79
acc	T	3	0.66
aca	T	0	1.29
acg	T	0	0.25
aat	N	0	0.53
aac	N	1	0.47
gtt	V	0	3.26
gtc	V	15	3.17
gta	V	0	2.95
gtg	V	0	5.62
mPD		0.033	0.67
		nPD:	0.05
		N. weight:	1.7
		Sc. PD:	-0.16
		Sc. rank:	584.3

PA

Pos.	31	obs:	exp:
gaa	E	2489	1495.00
gag	E	7	1001.00
mPD		0.0056	0.48
		nPD:	0.01
		N. weight:	0.62
		Sc. PD:	-0.083
		Sc. rank:	-517.2

PA

Pos.	32	obs:	exp:
att	I	0	6.68
atc	I	0	4.51
ata	I	18	6.80
atg	M	2	2.00
act	T	8	579.30
acc	T	1	483.20
aca	T	1213	939.80
acg	T	965	184.70
gct	A	0	73.52
gcc	A	0	56.36
gca	A	108	127.00
gcg	A	181	32.08
mPD		0.72	0.91
		nPD:	0.79
		N. weight:	1.1
		Sc. PD:	0.68
		Sc. rank:	1851.7

PA

Pos.	33	obs:	exp:
aat	N	229	1326.00
aac	N	2266	1169.00
gct	A	0	0.25
gcc	A	0	0.20
gca	A	1	0.44
gcg	A	0	0.11
mPD		0.17	0.50
		nPD:	0.34
		N. weight:	0.55
		Sc. PD:	0.1
		Sc. rank:	634.8

PA

Pos.	34	obs:	exp:
cgt	R	0	0.04
cgc	R	0	0.05
cga	R	0	0.10
cgg	R	0	0.08
aat	N	1	0.53
aac	N	0	0.47
aaa	K	1333	1399.00
aag	K	1160	1094.00
aga	R	1	0.46
agg	R	0	0.27
gaa	E	0	0.60
gag	E	1	0.40
mPD		0.50	0.49
		nPD:	1.01
		N. weight:	0.005
		Sc. PD:	0.0042
		Sc. rank:	8.7

PA

Pos.	35	obs:	exp:
ttt	F	1896	1058.00
ttc	F	590	1428.00
tta	L	2	0.98
ttg	L	0	1.69
tgt	C	1	0.43
tgc	C	0	0.57
ctt	L	7	1.69
ctc	L	0	1.55
cta	L	0	1.20
ctg	L	0	1.89
mPD		0.37	0.50
		nPD:	0.74
		N. weight:	0.3
		Sc. PD:	0.17
		Sc. rank:	497.6

PA

Pos.	36	obs:	exp:
cct	P	0	0.28
ccc	P	1	0.18
cca	P	0	0.41
ccg	P	0	0.13
act	T	4	10.60
acc	T	36	8.84
aca	T	0	17.19
acg	T	0	3.38
gct	A	567	624.50
gcc	A	1561	478.70
gca	A	327	1079.00
gcg	A	0	272.50
mPD		0.55	0.72
		nPD:	0.76
		N. weight:	0.73
		Sc. PD:	0.44
		Sc. rank:	1223.3

PA

Pos.	37	obs:	exp:
gct	A	0	635.00
gcc	A	0	486.70
gca	A	2495	1097.00
gcg	A	1	277.10
mPD		0.00080	0.69
		nPD:	0.
		N. weight:	1.
		Sc. PD:	-0.15
		Sc. rank:	-1661.9

PA

Pos.	38	obs:	exp:
att	I	0	925.70
atc	I	0	625.20
ata	I	2493	942.00
gtt	V	0	0.65
gtc	V	0	0.63
gta	V	3	0.59
gtg	V	0	1.12
mPD		0.0024	0.66
		nPD:	0.
		N. weight:	1.2
		Sc. PD:	-0.17
		Sc. rank:	-1678.4

PA

Pos.	39	obs:	exp:
tgt	C	910	1085.00
tgc	C	1586	1411.00
mPD		0.46	0.49
		nPD:	0.94
		N. weight:	0.015
		Sc. PD:	0.011
		Sc. rank:	25.5

PA

Pos.	40	obs:	exp:
act	T	581	661.20
acc	T	14	551.50
aca	T	1864	1073.00
acg	T	37	210.80
mPD		0.39	0.69
		nPD:	0.56
		N. weight:	0.42
		Sc. PD:	0.17
		Sc. rank:	629.0

PA

Pos.	41	obs:	exp:
cat	H	27	1484.00
cac	H	2469	1012.00
mPD		0.021	0.48
		nPD:	0.04
		N. weight:	1.
		Sc. PD:	-0.11
		Sc. rank:	324.9

PA

Pos.	42	obs:	exp:
tta	L	2455	272.40
ttg	L	14	468.40
ctt	L	0	468.00
ctc	L	0	430.60
cta	L	27	332.10
ctg	L	0	524.40
mPD		0.033	1.1
		nPD:	0.03
		N. weight:	2.6
		Sc. PD:	-0.31
		Sc. rank:	-210.3

PA

Pos.	43	obs:	exp:
gaa	E	1949	1495.00
gag	E	547	1001.00
mPD		0.34	0.48
		nPD:	0.71
		N. weight:	0.095
		Sc. PD:	0.052
		Sc. rank:	153.1

PA

Pos.	44	obs:	exp:
att	I	0	2.60
atc	I	7	1.76
ata	I	0	2.65
gtt	V	8	540.70
gtc	V	2456	525.30
gta	V	14	489.10
gtg	V	8	930.90
gct	A	0	0.76
gcc	A	3	0.59
gca	A	0	1.32
gcg	A	0	0.33
mPD		0.032	0.74
		nPD:	0.04
		N. weight:	1.8
		Sc. PD:	-0.19
		Sc. rank:	549.0

PA

Pos.	45	obs:	exp:
tct	S	0	0.34
tcc	S	2	0.29
tca	S	0	0.45
tcg	S	0	0.13
tgt	C	1308	1084.00
tgc	C	1186	1410.00
agt	S	0	0.41
agc	S	0	0.38
mPD		0.50	0.49
		nPD:	1.01
		N. weight:	0.024
		Sc. PD:	0.02
		Sc. rank:	41.7

PA

Pos.	46	obs:	exp:
ttt	F	0	1062.00
ttc	F	2495	1433.00
gtt	V	0	0.22
gtc	V	1	0.21
gta	V	0	0.20
gtg	V	0	0.37
mPD		0.00080	0.49
		nPD:	0.
		N. weight:	0.69
		Sc. PD:	-0.1
		Sc. rank:	-1090.1

PA

Pos.	47	obs:	exp:
atg	M	2496	2496.00
mPD		0	0
		nPD:	1.
		N. weight:	0.
		Sc. PD:	0
		Sc. rank:	0

PA

Pos.	48	obs:	exp:
tat	Y	2491	1213.00
tac	Y	5	1283.00
mPD		0.0040	0.50
		nPD:	0.01
		N. weight:	0.88
		Sc. PD:	-0.12
		Sc. rank:	-938.3

PA

Pos.	49	obs:	exp:
tct	S	5	426.30
tcc	S	0	368.00
tca	S	37	562.60
tcg	S	2454	161.20
agt	S	0	509.60
agc	S	0	468.30
mPD		0.033	1.6
		nPD:	0.02
		N. weight:	3.3
		Sc. PD:	-0.41
		Sc. rank:	-1418.2

PA

Pos.	50	obs:	exp:
gat	D	2183	1436.00
gac	D	313	1060.00
mPD		0.22	0.49
		nPD:	0.45
		N. weight:	0.27
		Sc. PD:	0.078
		Sc. rank:	361.8

PA

Pos.	51	obs:	exp:
ttt	F	690	1062.00
ttc	F	1805	1433.00
tta	L	1	0.11
ttg	L	0	0.19
ctt	L	0	0.19
ctc	L	0	0.17
cta	L	0	0.13
ctg	L	0	0.21
mPD		0.40	0.49
		nPD:	0.82
		N. weight:	0.062
		Sc. PD:	0.041
		Sc. rank:	106.2

PA

Pos.	52	obs:	exp:
cat	H	1205	1484.00
cac	H	1291	1012.00
mPD		0.50	0.48
		nPD:	1.04
		N. weight:	0.034
		Sc. PD:	0.029
		Sc. rank:	58.4

PA

Pos.	53	obs:	exp:
ttt	F	1546	1062.00
ttc	F	949	1433.00
tat	Y	0	0.49
tac	Y	1	0.51
mPD		0.47	0.49
		nPD:	0.96
		N. weight:	0.097
		Sc. PD:	0.077
		Sc. rank:	167.7

PA

Pos.	54	obs:	exp:
tta	L	0	0.11
ttg	L	0	0.19
ctt	L	0	0.19
ctc	L	1	0.17
cta	L	0	0.13
ctg	L	0	0.21
att	I	2484	925.70
atc	I	7	625.20
ata	I	2	942.00
gtt	V	2	0.43
gtc	V	0	0.42
gta	V	0	0.39
gtg	V	0	0.75
mPD		0.010	0.66
		nPD:	0.02
		N. weight:	1.2
		Sc. PD:	-0.16
		Sc. rank:	-799.8

PA

Pos.	55	obs:	exp:
aat	N	5	2.66
aac	N	0	2.34
gat	D	2479	1433.00
gac	D	12	1058.00
mPD		0.014	0.49
		nPD:	0.03
		N. weight:	0.65
		Sc. PD:	-0.078
		Sc. rank:	-89.4

PA

Pos.	56	obs :	exp :
gat	D	0	2.30
gac	D	4	1.70
gaa	E	2478	1492.00
gag	E	13	999.50
ggt	G	0	0.14
ggc	G	0	0.13
gga	G	1	0.44
ggg	G	0	0.29
---	--	------	------
mPD		0.014	0.48
		nPD :	0.03
		N. weight :	0.6
		Sc. PD:	-0.07
		Sc. rank :	-10.6

PA

Pos.	57	obs :	exp :
caa	Q	10	5.09
cag	Q	0	4.91
cgt	R	1	94.28
cgc	R	0	132.40
cga	R	2461	240.70
cgg	R	21	194.70
aga	R	3	1140.00
agg	R	0	683.60
---	--	------	------
mPD		0.028	0.96
		nPD :	0.03
		N. weight :	2.8
		Sc. PD:	-0.33
		Sc. rank :	-149.9

PA

Pos.	58	obs :	exp :
tct	S	0	0.34
tcc	S	0	0.29
tca	S	0	0.45
tcg	S	0	0.13
agt	S	2	0.41
agc	S	0	0.38
ggt	G	773	358.80
ggc	G	1717	329.20
gga	G	4	1089.00
ggg	G	0	717.30
---	--	------	------
mPD		0.43	0.69
		nPD :	0.63
		N. weight :	1.7
		Sc. PD:	0.79
		Sc. rank :	2589.4

PA

Pos.	59	obs :	exp :
att	I	0	2.60
atc	I	0	1.76
ata	I	7	2.65
aat	N	0	1.59
aac	N	3	1.41
aaa	K	531	301.40
aag	K	6	235.60
gat	D	0	0.58
gac	D	1	0.42
gaa	E	1657	995.10
gag	E	5	666.90
ggt	G	0	41.14
ggc	G	0	37.75
gga	G	286	124.80
ggg	G	0	82.26
---	--	------	------
mPD		0.56	1.1
		nPD :	0.53
		N. weight :	0.67
		Sc. PD:	0.25
		Sc. rank :	967.7

PA

Pos.	60	obs :	exp :
tct	S	0	426.30
tcc	S	0	368.00
tca	S	2486	562.60
tcg	S	10	161.20
agt	S	0	509.60
agc	S	0	468.30
---	--	------	------
mPD		0.0080	1.6
		nPD :	0.
		N. weight :	1.8
		Sc. PD:	-0.26
		Sc. rank :	-2379.5

PA

Pos.	61	obs :	exp :
att	I	0	796.50
atc	I	0	538.00
ata	I	2145	810.50
atg	M	45	45.00
act	T	0	79.73
acc	T	0	66.51
aca	T	301	129.30
acg	T	0	25.42
aaa	K	2	1.12
aag	K	0	0.88
gtt	V	0	0.65
gtc	V	0	0.63
gta	V	3	0.59
gtg	V	0	1.12
---	--	------	------
mPD		0.25	0.89
		nPD :	0.28
		N. weight :	1.2
		Sc. PD:	0.15
		Sc. rank :	1078.3

PA

Pos.	62	obs :	exp :
att	I	1328	925.70
atc	I	1163	625.20
ata	I	2	942.00
act	T	2	0.53
acc	T	0	0.44
aca	T	0	0.86
acg	T	0	0.17
gtt	V	1	0.22
gtc	V	0	0.21
gta	V	0	0.20
gtg	V	0	0.37
---	--	------	------
mPD		0.50	0.66
		nPD :	0.76
		N. weight :	0.6
		Sc. PD:	0.36
		Sc. rank :	1001.9

PA

Pos.	63	obs :	exp :
tta	L	3	0.33
ttg	L	0	0.56
ctt	L	0	0.56
ctc	L	0	0.52
cta	L	0	0.40
ctg	L	0	0.63
att	I	1	7.43
atc	I	0	5.02
ata	I	19	7.56
atg	M	19	19.00
gtt	V	0	533.50
gtc	V	0	518.30
gta	V	2307	482.70
gtg	V	146	918.60
gct	A	0	0.25
gcc	A	0	0.20
gca	A	1	0.44
gcg	A	0	0.11
---	--	------	------
mPD		0.16	0.76
		nPD :	0.21
		N. weight :	1.7
		Sc. PD:	0.095
		Sc. rank :	1270.1

PA

Pos.	64	obs :	exp :
gaa	E	2481	1495.00
gag	E	15	1001.00
---	--	------	------
mPD		0.012	0.48
		nPD :	0.02
		N. weight :	0.6
		Sc. PD:	-0.073
		Sc. rank :	-154.2

PA

Pos.	65	obs :	exp :
ttt	F	2	0.85
ttc	F	0	1.15
tct	S	2477	425.60
tcc	S	9	367.40
tca	S	3	561.70
tcg	S	3	161.00
tat	Y	1	0.49
tac	Y	0	0.51
cct	P	1	0.28
ccc	P	0	0.18
cca	P	0	0.41
ccg	P	0	0.13
agt	S	0	508.80
agc	S	0	467.50
---	--	------	------
mPD		0.015	1.6
		nPD :	0.01
		N. weight :	2.2
		Sc. PD:	-0.29
		Sc. rank :	-2076.8

PA

Pos.	66	obs :	exp :
tct	S	0	0.68
tcc	S	0	0.59
tca	S	0	0.90
tcg	S	0	0.26
agt	S	0	0.82
agc	S	4	0.75
gat	D	0	167.40
gac	D	291	123.60
ggt	G	18	316.60
ggc	G	2155	290.50
gga	G	27	960.80
ggg	G	1	633.10
---	--	------	------
mPD		0.25	0.93
		nPD :	0.26
		N. weight :	2.2
		Sc. PD:	0.25
		Sc. rank :	1951.9

PA

Pos.	67	obs :	exp :
aat	N	1	0.53
aac	N	0	0.47
gat	D	1296	1434.00
gac	D	1197	1059.00
gaa	E	1	0.60
gag	E	0	0.40
ggt	G	0	0.14
ggc	G	1	0.13
gga	G	0	0.44
ggg	G	0	0.29
---	--	------	------
mPD		0.50	0.49
		nPD :	1.02
		N. weight :	0.011
		Sc. PD:	0.0097
		Sc. rank :	19.7

PA

Pos.	68	obs :	exp :
tta	L	0	0.33
ttg	L	0	0.56
ctt	L	0	0.56
ctc	L	0	0.52
cta	L	0	0.40
ctg	L	3	0.63
cct	P	23	703.00
ccc	P	0	451.10
cca	P	26	1018.00
ccg	P	2444	321.00
---	--	------	------
mPD		0.041	0.71
		nPD :	0.06
		N. weight :	2.4
		Sc. PD:	-0.21
		Sc. rank :	1008.0

PA

Pos.	69	obs :	exp :
aat	N	2458	1326.00
aac	N	38	1170.00
---	--	------	------
mPD		0.030	0.50
		nPD :	0.06
		N. weight :	0.69
		Sc. PD:	-0.06
		Sc. rank :	294.1

PA

Pos.	70	obs :	exp :
gtt	V	0	0.43
gtc	V	0	0.42
gta	V	2	0.39
gtg	V	0	0.75
gct	A	2	634.20
gcc	A	2	486.20
gca	A	2485	1096.00
gcg	A	4	276.80
gaa	E	1	0.60
gag	E	0	0.40
---	--	------	------
mPD		0.0088	0.69
		nPD :	0.01
		N. weight :	1.
		Sc. PD:	-0.13
		Sc. rank :	-764.6

PA

Pos.	71	obs :	exp :
tta	L	2443	272.40
ttg	L	18	468.40
ctt	L	0	468.00
ctc	L	7	430.60
cta	L	28	332.10
ctg	L	0	524.40
---	--	------	------
mPD		0.048	1.1
		nPD :	0.04
		N. weight :	2.6
		Sc. PD:	-0.27
		Sc. rank :	726.8

PA

Pos.	72	obs :	exp :
tta	L	0	272.40
ttg	L	2112	468.40
ctt	L	0	468.00
ctc	L	0	430.60
cta	L	9	332.10
ctg	L	375	524.40
---	--	------	------
mPD		0.27	1.1
		nPD :	0.24
		N. weight :	1.5
		Sc. PD:	0.13
		Sc. rank :	1236.1

PA

Pos.	73	obs :	exp :
aaa	K	1579	1401.00
aag	K	917	1095.00
---	--	------	------
mPD		0.47	0.49
		nPD :	0.94
		N. weight :	0.015
		Sc. PD:	0.012
		Sc. rank :	26.1

PA

Pos.	74	obs :	exp :
cat	H	8	1484.00
cac	H	2488	1012.00
---	--	------	------
mPD		0.0064	0.48
		nPD :	0.01
		N. weight :	1.1
		Sc. PD:	-0.15
		Sc. rank :	-815.1

PA

Pos.	75	obs :	exp :
cgt	R	17	94.66
cgc	R	1	133.00
cga	R	2463	241.70
cgg	R	13	195.50
aga	R	2	1145.00
agg	R	0	686.30
---	--	------	------
mPD		0.026	0.95
		nPD :	0.03
		N. weight :	2.8
		Sc. PD:	-0.33
		Sc. rank :	-393.9

PA

Pos.	76	obs:	exp:
ttt	F	2485	1062.00
ttc	F	10	1433.00
tgt	C	1	0.43
tgc	C	0	0.57
---	--	------	------
mPD		0.0088	0.49
		nPD:	0.02
		N. weight:	1.
		Sc. PD:	-0.13
		Sc. rank:	-549.9

PA

Pos.	77	obs:	exp:
gat	D	0	0.58
gac	D	1	0.42
gaa	E	36	1494.00
gag	E	2459	1001.00
---	--	------	------
mPD		0.029	0.48
		nPD:	0.06
		N. weight:	1.
		Sc. PD:	-0.09
		Sc. rank:	450.7

PA

Pos.	78	obs:	exp:
tta	L	0	0.98
ttg	L	0	1.69
ctt	L	0	1.69
ctc	L	0	1.55
cta	L	9	1.20
ctg	L	0	1.89
att	I	4	915.00
atc	I	3	618.00
ata	I	2457	931.10
gtt	V	0	5.00
gtc	V	0	4.86
gta	V	23	4.53
gtg	V	0	8.61
---	--	------	------
mPD		0.031	0.69
		nPD:	0.05
		N. weight:	1.2
		Sc. PD:	-0.12
		Sc. rank:	396.0

PA

Pos.	79	obs:	exp:
att	I	2488	924.60
atc	I	2	624.50
ata	I	0	940.90
aat	N	1	0.53
aac	N	0	0.47
gtt	V	5	1.09
gtc	V	0	1.06
gta	V	0	0.98
gtg	V	0	1.87
---	--	------	------
mPD		0.0064	0.66
		nPD:	0.01
		N. weight:	1.2
		Sc. PD:	-0.17
		Sc. rank:	-1161.6

PA

Pos.	80	obs:	exp:
gaa	E	2487	1495.00
gag	E	9	1001.00
---	--	------	------
mPD		0.0072	0.48
		nPD:	0.01
		N. weight:	0.61
		Sc. PD:	-0.08
		Sc. rank:	-424.8

PA

Pos.	81	obs:	exp:
ggt	G	0	359.10
ggc	G	0	329.40
gga	G	24	1090.00
ggg	G	2472	717.90
---	--	------	------
mPD		0.019	0.69
		nPD:	0.03
		N. weight:	1.5
		Sc. PD:	-0.18
		Sc. rank:	-187.2

PA

Pos.	82	obs:	exp:
cgt	R	0	94.66
cgc	R	0	133.00
cga	R	1	241.70
cgg	R	0	195.50
aga	R	2480	1145.00
agg	R	15	686.30
---	--	------	------
mPD		0.013	0.95
		nPD:	0.01
		N. weight:	0.93
		Sc. PD:	-0.12
		Sc. rank:	-684.5

PA

Pos.	83	obs:	exp:
gat	D	10	1435.00
gac	D	2485	1060.00
ggt	G	0	0.14
ggc	G	1	0.13
gga	G	0	0.44
ggg	G	0	0.29
---	--	------	------
mPD		0.0088	0.49
		nPD:	0.02
		N. weight:	1.
		Sc. PD:	-0.13
		Sc. rank:	-556.5

PA

Pos.	84	obs:	exp:
tta	L	0	0.22
ttg	L	0	0.38
ctt	L	0	0.38
ctc	L	0	0.35
cta	L	2	0.27
ctg	L	0	0.42
caa	Q	1	0.51
cag	Q	0	0.49
cgt	R	3	94.54
cgc	R	5	132.80
cga	R	2466	241.40
cgg	R	12	195.30
aga	R	7	1144.00
agg	R	0	685.50
---	--	------	------
mPD		0.024	0.96
		nPD:	0.03
		N. weight:	2.8
		Sc. PD:	-0.34
		Sc. rank:	-689.2

PA

Pos.	85	obs:	exp:
cct	P	0	0.28
ccc	P	0	0.18
cca	P	1	0.41
ccg	P	0	0.13
att	I	0	1.11
atc	I	0	0.75
ata	I	3	1.13
act	T	27	660.10
acc	T	4	550.60
aca	T	1934	1071.00
acg	T	527	210.50
---	--	------	------
mPD		0.36	0.69
		nPD:	0.51
		N. weight:	0.77
		Sc. PD:	0.27
		Sc. rank:	1074.2

PA

Pos.	86	obs:	exp:
tta	L	0	0.11
ttg	L	1	0.19
tct	S	0	0.17
tcc	S	0	0.15
tca	S	0	0.23
tcg	S	0	0.06
ctt	L	0	0.19
ctc	L	0	0.17
cta	L	0	0.13
ctg	L	0	0.21
att	I	1	2.23
atc	I	0	1.50
ata	I	5	2.27
atg	M	2460	2460.00
agt	S	1	0.20
agc	S	0	0.19
gtt	V	0	6.09
gtc	V	0	5.92
gta	V	0	5.51
gtg	V	28	10.49
---	--	------	------
mPD		0.029	0.044
		nPD:	0.66
		N. weight:	0.017
		Sc. PD:	0.0087
		Sc. rank:	26.9

PA

Pos.	87	obs:	exp:
gct	A	580	634.20
gcc	A	1906	486.20
gca	A	7	1096.00
gcg	A	0	276.80
gat	D	0	0.58
gac	D	1	0.42
ggt	G	0	0.29
ggc	G	2	0.26
gga	G	0	0.87
ggg	G	0	0.58
---	--	------	------
mPD		0.36	0.69
		nPD:	0.52
		N. weight:	1.3
		Sc. PD:	0.46
		Sc. rank:	1796.2

PA

Pos.	88	obs:	exp:
tgg	W	2496	2496.00
---	--	------	------
mPD		0	0
		nPD:	1.
		N. weight:	0.
		Sc. PD:	0
		Sc. rank:	0

PA

Pos.	89	obs:	exp:
atg	M	3	3.00
act	T	0	660.40
acc	T	0	550.80
aca	T	2255	1071.00
acg	T	238	210.60
---	--	------	------
mPD		0.18	0.69
		nPD:	0.26
		N. weight:	0.85
		Sc. PD:	0.088
		Sc. rank:	730.2

PA

Pos.	90	obs:	exp:
tta	L	0	0.11
ttg	L	1	0.19
ctt	L	0	0.19
ctc	L	0	0.17
cta	L	0	0.13
ctg	L	0	0.21
atg	M	8	8.00
gtt	V	1	540.90
gtc	V	0	525.50
gta	V	39	489.30
gtg	V	2447	931.30
---	--	------	------
mPD		0.039	0.74
		nPD:	0.05
		N. weight:	1.1
		Sc. PD:	-0.11
		Sc. rank:	422.5

PA

Pos.	91	obs:	exp:
att	I	0	0.37
atc	I	0	0.25
ata	I	1	0.38
gtt	V	0	542.20
gtc	V	0	526.70
gta	V	1263	490.50
gtg	V	1230	933.60
gct	A	0	0.51
gcc	A	0	0.39
gca	A	0	0.88
gcg	A	2	0.22
---	--	------	------
mPD		0.50	0.73
		nPD:	0.69
		N. weight:	0.77
		Sc. PD:	0.4
		Sc. rank:	1214.6

PA

Pos.	92	obs:	exp:
tct	S	0	0.17
tcc	S	0	0.15
tca	S	0	0.23
tcg	S	0	0.06
aat	N	2485	1326.00
aac	N	10	1169.00
agt	S	1	0.20
agc	S	0	0.19
---	--	------	------
mPD		0.0088	0.50
		nPD:	0.02
		N. weight:	0.76
		Sc. PD:	-0.097
		Sc. rank:	-411.3

PA

Pos.	93	obs:	exp:
tct	S	0	422.50
tcc	S	0	364.70
tca	S	0	557.70
tcg	S	0	159.80
agt	S	2418	505.10
agc	S	56	464.20
ggt	G	20	3.16
ggc	G	2	2.90
gga	G	0	9.60
ggg	G	0	6.33
---	--	------	------
mPD		0.063	1.7
		nPD:	0.04
		N. weight:	1.9
		Sc. PD:	-0.2
		Sc. rank:	375.4

PA

Pos.	94	obs:	exp:
att	I	292	926.50
atc	I	757	625.70
ata	I	1446	942.80
gtt	V	0	0.22
gtc	V	0	0.21
gta	V	1	0.20
gtg	V	0	0.37
---	--	------	------
mPD		0.56	0.66
		nPD:	0.85
		N. weight:	0.22
		Sc. PD:	0.15
		Sc. rank:	371.2

PA

Pos.	95	obs:	exp:
tgt	C	2	1085.00
tgc	C	2493	1410.00
cat	H	0	0.59
cac	H	1	0.41
---	--	------	------
mPD		0.0032	0.49
		nPD:	0.01
		N. weight:	0.7
		Sc. PD:	-0.098
		Sc. rank:	-837.7

PA

Pos. 96		obs:	exp:
cat	H	2	170.10
cac	H	284	115.90
aat	N	9	1174.00
aac	N	2201	1036.00
---	--	------	------
mPD		0.21	0.70
		nPD:	0.3
		N. weight:	0.93
		Sc. PD:	0.14
		Sc. rank:	916.3

PA

Pos. 97		obs:	exp:
tct	S	0	0.17
tcc	S	0	0.15
tca	S	0	0.23
tcg	S	0	0.06
act	T	1175	660.60
acc	T	1315	551.00
aca	T	4	1072.00
acg	T	0	210.70
aat	N	1	0.53
aac	N	0	0.47
agt	S	0	0.20
agc	S	1	0.19
---	--	------	------
mPD		0.50	0.69
		nPD:	0.73
		N. weight:	0.9
		Sc. PD:	0.51
		Sc. rank:	1467.1

PA

Pos. 98		obs:	exp:
act	T	3	661.20
acc	T	2	551.50
aca	T	2476	1073.00
acg	T	15	210.80
---	--	------	------
mPD		0.016	0.69
		nPD:	0.02
		N. weight:	1.
		Sc. PD:	-0.12
		Sc. rank:	-320.4

PA

Pos. 99		obs:	exp:
cgt	R	0	0.04
cgc	R	0	0.05
cga	R	0	0.10
cgg	R	0	0.08
aga	R	1	0.46
agg	R	0	0.27
gaa	E	8	4.79
gag	E	0	3.21
ggt	G	22	357.80
ggc	G	0	328.30
gga	G	2076	1086.00
ggg	G	389	715.30
---	--	------	------
mPD		0.29	0.70
		nPD:	0.41
		N. weight:	0.53
		Sc. PD:	0.13
		Sc. rank:	658.6

PA

Pos. 100		obs:	exp:
att	I	0	9.28
atc	I	25	6.27
ata	I	0	9.45
act	T	0	0.26
acc	T	1	0.22
aca	T	0	0.43
acg	T	0	0.08
gtt	V	57	514.10
gtc	V	2303	499.50
gta	V	4	465.10
gtg	V	0	885.20
gct	A	0	26.97
gcc	A	106	20.67
gca	A	0	46.60
gcg	A	0	11.77
---	--	------	------
mPD		0.15	0.84
		nPD:	0.18
		N. weight:	1.8
		Sc. PD:	0.053
		Sc. rank:	1266.6

PA

Pos. 101		obs:	exp:
gat	D	1071	643.10
gac	D	47	474.90
gaa	E	1219	823.90
gag	E	157	552.10
ggt	G	1	0.29
ggc	G	0	0.26
gga	G	1	0.87
ggg	G	0	0.58
---	--	------	------
mPD		0.57	0.74
		nPD:	0.78
		N. weight:	0.36
		Sc. PD:	0.22
		Sc. rank:	612.1

PA

Pos. 102		obs:	exp:
aaa	K	308	1401.00
aag	K	2188	1095.00
---	--	------	------
mPD		0.22	0.49
		nPD:	0.44
		N. weight:	0.53
		Sc. PD:	0.15
		Sc. rank:	692.6

PA

Pos. 103		obs:	exp:
cct	P	1295	703.80
ccc	P	1199	451.60
cca	P	2	1019.00
ccg	P	0	321.40
---	--	------	------
mPD		0.50	0.70
		nPD:	0.71
		N. weight:	0.98
		Sc. PD:	0.53
		Sc. rank:	1557.7

PA

Pos. 104		obs:	exp:
caa	Q	1	0.51
cag	Q	0	0.49
cgt	R	0	0.08
cgc	R	0	0.11
cga	R	0	0.19
cgg	R	0	0.16
aat	N	3	1.59
aac	N	0	1.41
aaa	K	1333	1398.00
aag	K	1157	1092.00
aga	R	2	0.92
agg	R	0	0.55
---	--	------	------
mPD		0.50	0.50
		nPD:	1.01
		N. weight:	0.0058
		Sc. PD:	0.0049
		Sc. rank:	10.1

PA

Pos. 105		obs:	exp:
ttt	F	20	1059.00
ttc	F	2469	1430.00
tta	L	2	0.65
ttg	L	0	1.13
tct	S	0	0.17
tcc	S	1	0.15
tca	S	0	0.23
tcg	S	0	0.06
ctt	L	1	1.13
ctc	L	3	1.04
cta	L	0	0.80
ctg	L	0	1.26
agt	S	0	0.20
agc	S	0	0.19
---	--	------	------
mPD		0.022	0.50
		nPD:	0.05
		N. weight:	0.64
		Sc. PD:	-0.065
		Sc. rank:	205.2

PA

Pos. 106		obs:	exp:
tta	L	0	272.40
ttg	L	0	468.40
ctt	L	1160	468.00
ctc	L	1335	430.60
cta	L	0	332.10
ctg	L	1	524.40
---	--	------	------
mPD		0.50	1.1
		nPD:	0.44
		N. weight:	1.3
		Sc. PD:	0.36
		Sc. rank:	1669.2

PA

Pos. 107		obs:	exp:
cct	P	1214	703.80
ccc	P	4	451.60
cca	P	1263	1019.00
ccg	P	15	321.40
---	--	------	------
mPD		0.51	0.70
		nPD:	0.72
		N. weight:	0.44
		Sc. PD:	0.24
		Sc. rank:	706.4

PA

Pos. 108		obs:	exp:
gat	D	2241	1436.00
gac	D	255	1060.00
---	--	------	------
mPD		0.18	0.49
		nPD:	0.38
		N. weight:	0.32
		Sc. PD:	0.07
		Sc. rank:	381.7

PA

Pos. 109		obs:	exp:
tta	L	16	272.40
ttg	L	2298	468.40
ctt	L	0	468.00
ctc	L	0	430.60
cta	L	0	332.10
ctg	L	182	524.40
---	--	------	------
mPD		0.15	1.1
		nPD:	0.13
		N. weight:	1.7
		Sc. PD:	-0.032
		Sc. rank:	1093.6

PA

Pos. 110		obs:	exp:
tat	Y	2480	1213.00
tac	Y	16	1283.00
---	--	------	------
mPD		0.013	0.50
		nPD:	0.03
		N. weight:	0.85
		Sc. PD:	-0.1
		Sc. rank:	-194.5

PA

Pos. 111		obs:	exp:
gat	D	603	1436.00
gac	D	1893	1060.00
---	--	------	------
mPD		0.37	0.49
		nPD:	0.75
		N. weight:	0.29
		Sc. PD:	0.17
		Sc. rank:	478.3

PA

Pos. 112		obs:	exp:
tat	Y	1	1213.00
tac	Y	2495	1283.00
---	--	------	------
mPD		0.00080	0.50
		nPD:	0.
		N. weight:	0.83
		Sc. PD:	-0.12
		Sc. rank:	-1311.0

PA

Pos. 113		obs:	exp:
cgt	R	0	41.60
cgc	R	0	58.43
cga	R	69	106.20
cgg	R	15	85.92
aaa	K	558	783.60
aag	K	838	612.40
aga	R	998	503.20
agg	R	15	301.60
ggt	G	0	0.43
ggc	G	0	0.40
gga	G	3	1.31
ggg	G	0	0.86
---	--	------	------
mPD		1.0	1.2
		nPD:	0.83
		N. weight:	0.33
		Sc. PD:	0.22
		Sc. rank:	570.2

PA

Pos. 114		obs:	exp:
gat	D	0	0.58
gac	D	1	0.42
gaa	E	1192	1490.00
gag	E	1296	998.30
ggt	G	0	1.01
ggc	G	0	0.92
gga	G	1	3.06
ggg	G	6	2.01
---	--	------	------
mPD		0.51	0.49
		nPD:	1.04
		N. weight:	0.042
		Sc. PD:	0.036
		Sc. rank:	72.5

PA

Pos. 115		obs:	exp:
tct	S	0	0.85
tcc	S	0	0.74
tca	S	0	1.13
tcg	S	0	0.32
act	T	0	0.26
acc	T	1	0.22
aca	T	0	0.43
acg	T	0	0.08
aat	N	49	1320.00
aac	N	2434	1163.00
agt	S	0	1.02
agc	S	5	0.94
gat	D	0	4.03
gac	D	7	2.97
---	--	------	------
mPD		0.049	0.51
		nPD:	0.1
		N. weight:	0.83
		Sc. PD:	-0.043
		Sc. rank:	464.6

PA

Pos.	116	obs:	exp:
caa	Q	2	1.02
cag	Q	0	0.98
cgt	R	3	94.58
cgc	R	5	132.80
cga	R	2457	241.50
cgg	R	26	195.30
aga	R	3	1144.00
agg	R	0	685.80
---	--	------	------
mPD		0.031	0.96
		nPD:	0.03
		N. weight:	2.8
		Sc. PD:	-0.32
		Sc. rank:	291.2

,

PA

Pos.	117	obs:	exp:
ttt	F	2	1062.00
ttc	F	2494	1434.00
---	--	------	------
mPD		0.0016	0.49
		nPD:	0.
		N. weight:	0.68
		Sc. PD:	-0.097
		Sc. rank:	-985.2

,

PA

Pos.	118	obs:	exp:
tta	L	0	0.11
ttg	L	0	0.19
ctt	L	1	0.19
ctc	L	0	0.17
cta	L	0	0.13
ctg	L	0	0.21
att	I	2203	923.50
atc	I	284	623.70
ata	I	0	939.80
atg	M	2	2.00
gtt	V	6	1.30
gtc	V	0	1.27
gta	V	0	1.18
gtg	V	0	2.25
---	--	------	------
mPD		0.21	0.66
		nPD:	0.31
		N. weight:	0.85
		Sc. PD:	0.14
		Sc. rank:	906.5

,

PA

Pos.	119	obs:	exp:
gaa	E	2486	1495.00
gag	E	10	1001.00
---	--	------	------
mPD		0.0080	0.48
		nPD:	0.02
		N. weight:	0.61
		Sc. PD:	-0.079
		Sc. rank:	-368.5

,

PA

Pos.	120	obs:	exp:
att	I	2468	917.20
atc	I	2	619.50
ata	I	0	933.30
gtt	V	26	5.65
gtc	V	0	5.49
gta	V	0	5.12
gtg	V	0	9.74
---	--	------	------
mPD		0.022	0.68
		nPD:	0.03
		N. weight:	1.2
		Sc. PD:	-0.14
		Sc. rank:	142.5

,

PA

Pos.	121	obs:	exp:
ggt	G	0	359.10
ggc	G	1	329.40
gga	G	2154	1090.00
ggg	G	341	717.90
---	--	------	------
mPD		0.24	0.69
		nPD:	0.34
		N. weight:	0.61
		Sc. PD:	0.11
		Sc. rank:	700.6

,

PA

Pos.	122	obs:	exp:
att	I	0	5.94
atc	I	0	4.01
ata	I	16	6.05
gtt	V	2	539.40
gtc	V	3	524.00
gta	V	1477	488.00
gtg	V	998	928.70
---	--	------	------
mPD		0.50	0.74
		nPD:	0.67
		N. weight:	0.85
		Sc. PD:	0.43
		Sc. rank:	1320.4

,

PA

Pos.	123	obs:	exp:
act	T	24	661.20
acc	T	4	551.50
aca	T	1281	1073.00
acg	T	1187	210.80
---	--	------	------
mPD		0.51	0.69
		nPD:	0.74
		N. weight:	1.1
		Sc. PD:	0.63
		Sc. rank:	1806.2

,

PA

Pos.	124	obs:	exp:
cgt	R	0	94.66
cgc	R	0	133.00
cga	R	1893	241.70
cgg	R	9	195.50
aga	R	13	1145.00
agg	R	581	686.30
---	--	------	------
mPD		0.72	0.95
		nPD:	0.76
		N. weight:	1.9
		Sc. PD:	1.1
		Sc. rank:	3106.4

,

PA

Pos.	125	obs:	exp:
cgt	R	0	94.66
cgc	R	0	133.00
cga	R	0	241.70
cgg	R	0	195.50
aga	R	13	1145.00
agg	R	2483	686.30
---	--	------	------
mPD		0.010	0.95
		nPD:	0.01
		N. weight:	1.6
		Sc. PD:	-0.21
		Sc. rank:	-1361.2

,

PA

Pos.	126	obs:	exp:
gaa	E	2467	1486.00
gag	E	14	995.50
ggt	G	0	2.16
ggc	G	0	1.98
gga	G	15	6.55
ggg	G	0	4.31
---	--	------	------
mPD		0.023	0.49
		nPD:	0.05
		N. weight:	0.6
		Sc. PD:	-0.06
		Sc. rank:	203.3

,

PA

Pos.	127	obs:	exp:
att	I	395	146.70
atc	I	0	99.06
ata	I	0	149.30
gtt	V	2037	448.70
gtc	V	23	435.90
gta	V	1	405.90
gtg	V	2	772.50
gct	A	37	9.67
gcc	A	1	7.41
gca	A	0	16.70
gcg	A	0	4.22
---	--	------	------
mPD		0.32	1.0
		nPD:	0.31
		N. weight:	1.7
		Sc. PD:	0.26
		Sc. rank:	1772.1

,

PA

Pos.	128	obs:	exp:
cat	H	17	1483.00
cac	H	2478	1012.00
caa	Q	1	0.51
cag	Q	0	0.49
---	--	------	------
mPD		0.014	0.48
		nPD:	0.03
		N. weight:	1.1
		Sc. PD:	-0.13
		Sc. rank:	-22.0

,

PA

Pos.	129	obs:	exp:
att	I	0	700.70
atc	I	0	473.20
ata	I	1887	713.00
atg	M	26	26.00
act	T	0	154.20
acc	T	0	128.60
aca	T	559	250.10
acg	T	23	49.16
gtt	V	0	0.22
gtc	V	0	0.21
gta	V	1	0.20
gtg	V	0	0.37
---	--	------	------
mPD		0.40	1.0
		nPD:	0.38
		N. weight:	1.1
		Sc. PD:	0.26
		Sc. rank:	1372.5

,

PA

Pos.	130	obs:	exp:
tat	Y	243	1213.00
tac	Y	2253	1283.00
---	--	------	------
mPD		0.18	0.50
		nPD:	0.35
		N. weight:	0.44
		Sc. PD:	0.087
		Sc. rank:	512.9

,

PA

Pos.	131	obs:	exp:
tat	Y	1903	1213.00
tac	Y	593	1283.00
---	--	------	------
mPD		0.36	0.50
		nPD:	0.73
		N. weight:	0.2
		Sc. PD:	0.11
		Sc. rank:	326.1

,

PA

Pos.	132	obs:	exp:
tta	L	16	272.30
ttg	L	0	468.20
ctt	L	915	467.90
ctc	L	0	430.50
cta	L	1547	332.00
ctg	L	17	524.20
att	I	1	0.37
atc	I	0	0.25
ata	I	0	0.38
---	--	------	------
mPD		0.49	1.1
		nPD:	0.43
		N. weight:	1.4
		Sc. PD:	0.39
		Sc. rank:	1861.0

,

PA

Pos.	133	obs:	exp:
aaa	K	2	1.12
aag	K	0	0.88
gct	A	0	0.25
gcc	A	0	0.20
gca	A	1	0.44
gcg	A	0	0.11
gat	D	1	0.58
gac	D	0	0.42
gaa	E	2455	1492.00
gag	E	36	999.50
ggt	G	0	0.14
ggc	G	0	0.13
gga	G	1	0.44
ggg	G	0	0.29
---	--	------	------
mPD		0.032	0.48
		nPD:	0.07
		N. weight:	0.55
		Sc. PD:	-0.045
		Sc. rank:	257.9

,

PA

Pos.	134	obs:	exp:
aaa	K	1861	1401.00
aag	K	635	1095.00
---	--	------	------
mPD		0.38	0.49
		nPD:	0.77
		N. weight:	0.093
		Sc. PD:	0.056
		Sc. rank:	156.9

,

PA

Pos.	135	obs:	exp:
act	T	0	0.26
acc	T	0	0.22
aca	T	1	0.43
acg	T	0	0.08
gtt	V	0	0.65
gtc	V	1	0.63
gta	V	2	0.59
gtg	V	0	1.12
gct	A	7	633.90
gcc	A	1908	486.00
gca	A	577	1095.00
gcg	A	0	276.70
---	--	------	------
mPD		0.36	0.70
		nPD:	0.52
		N. weight:	1.1
		Sc. PD:	0.4
		Sc. rank:	1582.5

,

PA			
Pos.	136	obs:	exp:
tct	S	0	1.54
tcc	S	0	1.33
tca	S	0	2.03
tcg	S	0	0.58
aat	N	581	1321.00
aac	N	1905	1165.00
agt	S	0	1.84
agc	S	9	1.69
gat	D	0	0.58
gac	D	1	0.42
---	--	------	------
mPD		0.37	0.51
		nPD:	0.71
		N. weight:	0.24
		Sc. PD:	0.13
		Sc. rank:	385.4

,

PA			
Pos.	137	obs:	exp:
aaa	K	105	1401.00
aag	K	2391	1095.00
---	--	------	------
mPD		0.081	0.49
		nPD:	0.16
		N. weight:	0.8
		Sc. PD:	0.011
		Sc. rank:	547.5

,

PA			
Pos.	138	obs:	exp:
att	I	0	926.50
atc	I	0	625.70
ata	I	2495	942.80
gtt	V	0	0.22
gtc	V	0	0.21
gta	V	1	0.20
gtg	V	0	0.37
---	--	------	------
mPD		0.00080	0.66
		nPD:	0.
		N. weight:	1.2
		Sc. PD:	-0.18
		Sc. rank:	-1967.4

,

PA			
Pos.	139	obs:	exp:
aat	N	1	0.53
aac	N	0	0.47
aaa	K	2378	1400.00
aag	K	117	1095.00
---	--	------	------
mPD		0.090	0.49
		nPD:	0.18
		N. weight:	0.5
		Sc. PD:	0.016
		Sc. rank:	364.1

,

PA			
Pos.	140	obs:	exp:
tta	L	23	2.51
ttg	L	0	4.32
tct	S	10	422.00
tcc	S	11	364.30
tca	S	2054	557.00
tcg	S	396	159.60
ctt	L	0	4.31
ctc	L	0	3.97
cta	L	0	3.06
ctg	L	0	4.83
cct	P	0	0.28
ccc	P	0	0.18
cca	P	1	0.41
ccg	P	0	0.13
caa	Q	0	0.51
cag	Q	1	0.49
agt	S	0	504.50
agc	S	0	463.60
---	--	------	------
mPD		0.30	1.7
		nPD:	0.18
		N. weight:	1.5
		Sc. PD:	0.047
		Sc. rank:	1091.4

,

PA			
Pos.	141	obs:	exp:
aaa	K	0	1.68
aag	K	3	1.32
gaa	E	78	1492.00
gag	E	2414	999.90
ggt	G	0	0.14
ggc	G	0	0.13
gga	G	0	0.44
ggg	G	1	0.29
---	--	------	------
mPD		0.064	0.48
		nPD:	0.13
		N. weight:	0.95
		Sc. PD:	-0.016
		Sc. rank:	620.1

,

PA			
Pos.	142	obs:	exp:
cgt	R	0	1.33
cgc	R	0	1.86
cga	R	0	3.39
cgg	R	0	2.74
act	T	0	0.53
acc	T	0	0.44
aca	T	2	0.86
acg	T	0	0.17
aaa	K	1160	1380.00
aag	K	1298	1078.00
aga	R	3	16.05
agg	R	32	9.62
gaa	E	1	0.60
gag	E	0	0.40
---	--	------	------
mPD		0.53	0.53
		nPD:	0.99
		N. weight:	0.04
		Sc. PD:	0.033
		Sc. rank:	69.7

,

PA			
Pos.	143	obs:	exp:
act	T	0	660.90
acc	T	0	551.30
aca	T	2494	1072.00
acg	T	1	210.70
gct	A	0	0.25
gcc	A	0	0.20
gca	A	1	0.44
gcg	A	0	0.11
---	--	------	------
mPD		0.0016	0.69
		nPD:	0.
		N. weight:	1.
		Sc. PD:	-0.15
		Sc. rank:	-1596.2

,

PA			
Pos.	144	obs:	exp:
tat	Y	0	0.49
tac	Y	1	0.51
cat	H	36	1483.00
cac	H	2458	1011.00
caa	Q	1	0.51
cag	Q	0	0.49
---	--	------	------
mPD		0.030	0.48
		nPD:	0.06
		N. weight:	1.
		Sc. PD:	-0.087
		Sc. rank:	457.1

,

PA			
Pos.	145	obs:	exp:
att	I	2471	926.50
atc	I	21	625.70
ata	I	3	942.80
gtt	V	1	0.22
gtc	V	0	0.21
gta	V	0	0.20
gtg	V	0	0.37
---	--	------	------
mPD		0.020	0.66
		nPD:	0.03
		N. weight:	1.2
		Sc. PD:	-0.14
		Sc. rank:	32.2

,

PA			
Pos.	146	obs:	exp:
tta	L	0	0.11
ttg	L	0	0.19
ctt	L	0	0.19
ctc	L	1	0.17
cta	L	0	0.13
ctg	L	0	0.21
cat	H	14	1483.00
cac	H	2481	1012.00
---	--	------	------
mPD		0.012	0.48
		nPD:	0.02
		N. weight:	1.1
		Sc. PD:	-0.13
		Sc. rank:	-293.9

,

PA			
Pos.	147	obs:	exp:
att	I	7	926.50
atc	I	12	625.70
ata	I	2476	942.80
atg	M	1	1.00
---	--	------	------
mPD		0.016	0.66
		nPD:	0.02
		N. weight:	1.2
		Sc. PD:	-0.14
		Sc. rank:	-323.7

,

PA			
Pos.	148	obs:	exp:
ttt	F	14	1062.00
ttc	F	2482	1434.00
---	--	------	------
mPD		0.011	0.49
		nPD:	0.02
		N. weight:	0.65
		Sc. PD:	-0.08
		Sc. rank:	-211.0

,

PA			
Pos.	149	obs:	exp:
tct	S	2	426.30
tcc	S	1	368.00
tca	S	2491	562.60
tcg	S	2	161.20
agt	S	0	509.60
agc	S	0	468.30
---	--	------	------
mPD		0.0040	1.6
		nPD:	0.
		N. weight:	1.8
		Sc. PD:	-0.26
		Sc. rank:	-2737.5

,

PA			
Pos.	150	obs:	exp:
ttt	F	4	1062.00
ttc	F	2492	1434.00
---	--	------	------
mPD		0.0032	0.49
		nPD:	0.01
		N. weight:	0.68
		Sc. PD:	-0.094
		Sc. rank:	-803.0

,

PA			
Pos.	151	obs:	exp:
att	I	3	1.11
atc	I	0	0.75
ata	I	0	1.13
act	T	2444	654.00
acc	T	18	545.50
aca	T	6	1061.00
acg	T	1	208.50
aat	N	24	12.75
aac	N	0	11.25
---	--	------	------
mPD		0.041	0.71
		nPD:	0.06
		N. weight:	1.6
		Sc. PD:	-0.14
		Sc. rank:	643.2

,

PA			
Pos.	152	obs:	exp:
ggt	G	0	359.10
ggc	G	0	329.40
gga	G	789	1090.00
ggg	G	1707	717.90
---	--	------	------
mPD		0.43	0.69
		nPD:	0.63
		N. weight:	0.61
		Sc. PD:	0.29
		Sc. rank:	932.7

,

PA			
Pos.	153	obs:	exp:
gaa	E	53	1494.00
gag	E	2442	1001.00
ggt	G	0	0.14
ggc	G	0	0.13
gga	G	0	0.44
ggg	G	1	0.29
---	--	------	------
mPD		0.042	0.48
		nPD:	0.09
		N. weight:	1.
		Sc. PD:	-0.06
		Sc. rank:	542.0

,

PA			
Pos.	154	obs:	exp:
gaa	E	1615	1495.00
gag	E	881	1001.00
---	--	------	------
mPD		0.46	0.48
		nPD:	0.95
		N. weight:	0.0082
		Sc. PD:	0.0064
		Sc. rank:	14.1

,

PA			
Pos.	155	obs:	exp:
atg	M	2496	2496.00
---	--	------	------
mPD		0	0
		nPD:	1.
		N. weight:	0.
		Sc. PD:	0
		Sc. rank:	0

,

PA			
Pos.	156	obs:	exp:
act	T	0	0.26
acc	T	0	0.22
aca	T	1	0.43
acg	T	0	0.08
gtt	V	0	0.22
gtc	V	1	0.21
gta	V	0	0.20
gtg	V	0	0.37
gct	A	9	634.50
gcc	A	2482	486.30
gca	A	3	1096.00
gcg	A	0	276.90
---	--	------	------
mPD		0.012	0.69
		nPD:	0.02
		N. weight:	2.
		Sc. PD:	-0.26
		Sc. rank:	-1153.2

,

PA			
Pos.	157	obs:	exp:
tct	S	0	0.17
tcc	S	1	0.15
tca	S	0	0.23
tcg	S	0	0.06
act	T	8	660.40
acc	T	2194	550.80
aca	T	291	1071.00
acg	T	0	210.60
agt	S	0	0.20
agc	S	0	0.19
gct	A	0	0.51
gcc	A	2	0.39
gca	A	0	0.88
gcg	A	0	0.22
---	--	------	------
mPD		0.21	0.69
		nPD:	0.31
		N. weight:	1.3
		Sc. PD:	0.2
		Sc. rank:	1362.0

,

PA			
Pos.	158	obs:	exp:
caa	Q	4	2.04
cag	Q	0	1.96
cgt	R	0	0.72
cgc	R	0	1.01
cga	R	0	1.84
cgg	R	0	1.49
aaa	K	1270	1386.00
aag	K	1200	1084.00
aga	R	2	8.72
agg	R	17	5.22
gaa	E	3	1.80
gag	E	0	1.20
---	--	------	------
mPD		0.52	0.52
		nPD:	1.01
		N. weight:	0.019
		Sc. PD:	0.016
		Sc. rank:	32.7

,

PA			
Pos.	159	obs:	exp:
tct	S	1	0.17
tcc	S	0	0.15
tca	S	0	0.23
tcg	S	0	0.06
agt	S	0	0.20
agc	S	0	0.19
gtt	V	0	0.22
gtc	V	0	0.21
gta	V	1	0.20
gtg	V	0	0.37
gct	A	594	634.20
gcc	A	78	486.20
gca	A	1492	1096.00
gcg	A	329	276.80
gat	D	1	0.58
gac	D	0	0.42
---	--	------	------
mPD		0.57	0.69
		nPD:	0.82
		N. weight:	0.17
		Sc. PD:	0.11
		Sc. rank:	297.7

,

PA			
Pos.	160	obs:	exp:
aat	N	1	1.59
aac	N	2	1.41
gat	D	1200	1433.00
gac	D	1292	1059.00
gaa	E	1	0.60
gag	E	0	0.40
---	--	------	------
mPD		0.50	0.49
		nPD:	1.02
		N. weight:	0.025
		Sc. PD:	0.021
		Sc. rank:	43.1

,

PA			
Pos.	161	obs:	exp:
tat	Y	978	1213.00
tac	Y	1517	1282.00
cat	H	1	0.59
cac	H	0	0.41
---	--	------	------
mPD		0.48	0.50
		nPD:	0.95
		N. weight:	0.025
		Sc. PD:	0.019
		Sc. rank:	42.5

,

PA			
Pos.	162	obs:	exp:
att	I	1	12.25
atc	I	32	8.28
ata	I	0	12.47
act	T	1168	652.10
acc	T	1294	544.00
aca	T	0	1058.00
acg	T	0	208.00
gtt	V	0	0.22
gtc	V	1	0.21
gta	V	0	0.20
gtg	V	0	0.37
---	--	------	------
mPD		0.53	0.72
		nPD:	0.73
		N. weight:	0.92
		Sc. PD:	0.53
		Sc. rank:	1517.8

,

PA			
Pos.	163	obs:	exp:
ttt	F	1	0.43
ttc	F	0	0.57
tta	L	0	269.60
ttg	L	0	463.50
ctt	L	2469	463.20
ctc	L	1	426.20
cta	L	0	328.60
ctg	L	0	519.00
att	I	25	9.28
atc	I	0	6.27
ata	I	0	9.45
---	--	------	------
mPD		0.021	1.1
		nPD:	0.02
		N. weight:	2.1
		Sc. PD:	-0.26
		Sc. rank:	-1037.8

,

PA			
Pos.	164	obs:	exp:
gat	D	1810	1436.00
gac	D	686	1060.00
---	--	------	------
mPD		0.40	0.49
		nPD:	0.82
		N. weight:	0.062
		Sc. PD:	0.04
		Sc. rank:	105.8

,

PA			
Pos.	165	obs:	exp:
gaa	E	1804	1495.00
gag	E	692	1001.00
---	--	------	------
mPD		0.40	0.48
		nPD:	0.83
		N. weight:	0.044
		Sc. PD:	0.029
		Sc. rank:	74.7

,

PA			
Pos.	166	obs:	exp:
aaa	K	0	0.56
aag	K	1	0.44
gaa	E	435	1494.00
gag	E	2060	1001.00
---	--	------	------
mPD		0.29	0.48
		nPD:	0.6
		N. weight:	0.48
		Sc. PD:	0.21
		Sc. rank:	714.1

,

PA			
Pos.	167	obs:	exp:
tct	S	0	426.10
tcc	S	0	367.80
tca	S	0	562.40
tcg	S	0	161.20
aat	N	0	0.53
aac	N	1	0.47
agt	S	8	509.40
agc	S	2487	468.10
---	--	------	------
mPD		0.0072	1.6
		nPD:	0.
		N. weight:	2.1
		Sc. PD:	-0.29
		Sc. rank:	-2745.0

,

PA			
Pos.	168	obs:	exp:
cgt	R	0	94.62
cgc	R	0	132.90
cga	R	0	241.60
cgg	R	0	195.40
aaa	K	1	0.56
aag	K	0	0.44
aga	R	2474	1144.00
agg	R	21	686.00
---	--	------	------
mPD		0.017	0.95
		nPD:	0.02
		N. weight:	0.92
		Sc. PD:	-0.12
		Sc. rank:	-482.1

,

PA			
Pos.	169	obs:	exp:
gct	A	4	635.00
gcc	A	1	486.70
gca	A	2484	1097.00
gcg	A	7	277.10
---	--	------	------
mPD		0.0096	0.69
		nPD:	0.01
		N. weight:	0.99
		Sc. PD:	-0.13
		Sc. rank:	-725.7

,

PA			
Pos.	170	obs:	exp:
cgt	R	0	94.62
cgc	R	0	132.90
cga	R	1	241.60
cgg	R	0	195.40
aaa	K	0	0.56
aag	K	1	0.44
aga	R	1897	1144.00
agg	R	597	686.00
---	--	------	------
mPD		0.37	0.95
		nPD:	0.38
		N. weight:	0.44
		Sc. PD:	0.1
		Sc. rank:	526.6

,

PA			
Pos.	171	obs:	exp:
att	I	11	926.50
atc	I	1263	625.70
ata	I	1221	942.80
aat	N	1	0.53
aac	N	0	0.47
---	--	------	------
mPD		0.51	0.66
		nPD:	0.77
		N. weight:	0.58
		Sc. PD:	0.35
		Sc. rank:	976.8

,

PA			
Pos.	172	obs:	exp:
aaa	K	2493	1401.00
aag	K	3	1095.00
---	--	------	------
mPD		0.0024	0.49
		nPD:	0.
		N. weight:	0.71
		Sc. PD:	-0.1
		Sc. rank:	-907.5

,

PA			
Pos.	173	obs:	exp:
act	T	9	661.20
acc	T	2461	551.50
aca	T	26	1073.00
acg	T	0	210.80
---	--	------	------
mPD		0.028	0.69
		nPD:	0.04
		N. weight:	1.8
		Sc. PD:	-0.19
		Sc. rank:	401.9

,

PA			
Pos.	174	obs:	exp:
cgt	R	0	94.66
cgc	R	0	133.00
cga	R	0	241.70
cgg	R	1	195.50
aga	R	28	1145.00
agg	R	2467	686.30
---	--	------	------
mPD		0.023	0.95
		nPD:	0.02
		N. weight:	1.5
		Sc. PD:	-0.19
		Sc. rank:	-435.4

,

PA			
Pos.	175	obs:	exp:
tta	L	1	272.40
ttg	L	542	468.40
ctt	L	2	468.00
ctc	L	0	430.60
cta	L	1211	332.10
ctg	L	740	524.40
---	--	------	------
mPD		0.84	1.1
		nPD:	0.74
		N. weight:	0.95
		Sc. PD:	0.55
		Sc. rank:	1559.0

,

PA			
Pos.	176	obs:	exp:
ttt	F	1	1061.00
ttc	F	2493	1433.00
tta	L	0	0.11
ttg	L	0	0.19
ctt	L	0	0.19
ctc	L	1	0.17
cta	L	0	0.13
ctg	L	0	0.21
gtt	V	0	0.22
gtc	V	1	0.21
gta	V	0	0.20
gtg	V	0	0.37
---	--	------	------
mPD		0.0024	0.49
		nPD:	0.
		N. weight:	0.69
		Sc. PD:	-0.097
		Sc. rank:	-875.9

,

PA			
Pos.	177	obs:	exp:
act	T	2468	661.20
acc	T	16	551.50
aca	T	12	1073.00
acg	T	0	210.80
---	--	------	------
mPD		0.022	0.69
		nPD:	0.03
		N. weight:	1.6
		Sc. PD:	-0.18
		Sc. rank:	134.7

,

PA			
Pos.	178	obs:	exp:
att	I	0	926.50
atc	I	578	625.70
ata	I	1917	942.80
gtt	V	0	0.22
gtc	V	1	0.21
gta	V	0	0.20
gtg	V	0	0.37
---	--	------	------
mPD		0.36	0.66
		nPD:	0.54
		N. weight:	0.66
		Sc. PD:	0.25
		Sc. rank:	962.6

,

PA			
Pos.	179	obs:	exp:
cgt	R	0	94.66
cgc	R	0	133.00
cga	R	1	241.70
cgg	R	0	195.50
aga	R	1249	1145.00
agg	R	1246	686.30
---	--	------	------
mPD		0.50	0.95
		nPD:	0.53
		N. weight:	0.43
		Sc. PD:	0.16
		Sc. rank:	609.1

,

PA			
Pos.	180	obs:	exp:
caa	Q	2488	1270.00
cag	Q	8	1226.00
---	--	------	------
mPD		0.0064	0.50
		nPD:	0.01
		N. weight:	0.82
		Sc. PD:	-0.11
		Sc. rank:	-616.9

,

PA

Pos.	181	obs:	exp:
gat	D	5	2.88
gac	D	0	2.12
gaa	E	1337	1492.00
gag	E	1154	999.50
---	--	------	------
mPD		0.50	0.48
		nPD:	1.04
		N. weight:	0.013
		Sc. PD:	0.011
		Sc. rank:	23.0

,

PA

Pos.	182	obs:	exp:
tta	L	0	0.44
ttg	L	4	0.75
ctt	L	0	0.75
ctc	L	0	0.69
cta	L	0	0.53
ctg	L	0	0.84
atg	M	2491	2491.00
aaa	K	0	0.56
aag	K	1	0.44
---	--	------	------
mPD		0.0040	0.0064
		nPD:	0.63
		N. weight:	0.0038
		Sc. PD:	0.0017
		Sc. rank:	5.7

,

PA

Pos.	183	obs:	exp:
gtt	V	0	1.96
gtc	V	9	1.90
gta	V	0	1.77
gtg	V	0	3.37
gct	A	0	632.70
gcc	A	2479	485.00
gca	A	8	1093.00
gcg	A	0	276.10
---	--	------	------
mPD		0.014	0.70
		nPD:	0.02
		N. weight:	2.
		Sc. PD:	-0.25
		Sc. rank:	-922.1

,

PA

Pos.	184	obs:	exp:
tct	S	0	110.00
tcc	S	0	94.94
tca	S	0	145.20
tcg	S	0	41.60
tat	Y	1	0.49
tac	Y	0	0.51
act	T	1	0.26
acc	T	0	0.22
aca	T	0	0.43
acg	T	0	0.08
aat	N	1846	983.10
aac	N	4	866.90
agt	S	638	131.50
agc	S	6	120.80
---	--	------	------
mPD		0.39	1.2
		nPD:	0.31
		N. weight:	1.1
		Sc. PD:	0.17
		Sc. rank:	1130.8

,

PA

Pos.	185	obs:	exp:
cgt	R	0	94.58
cgc	R	0	132.80
cga	R	0	241.50
cgg	R	0	195.30
aaa	K	1	1.12
aag	K	1	0.88
aga	R	11	1144.00
agg	R	2483	685.80
---	--	------	------
mPD		0.011	0.95
		nPD:	0.01
		N. weight:	1.6
		Sc. PD:	-0.21
		Sc. rank:	-1299.1

,

PA

Pos.	186	obs:	exp:
tct	S	0	7.34
tcc	S	0	6.34
tca	S	0	9.69
tcg	S	0	2.78
agt	S	39	8.78
agc	S	4	8.07
ggt	G	2437	352.90
ggc	G	11	323.80
gga	G	0	1071.00
ggg	G	5	705.50
---	--	------	------
mPD		0.050	0.75
		nPD:	0.07
		N. weight:	2.4
		Sc. PD:	-0.19
		Sc. rank:	1087.7

,

PA

Pos.	187	obs:	exp:
tta	L	6	272.10
ttg	L	0	467.80
ctt	L	35	467.50
ctc	L	7	430.10
cta	L	1914	331.70
ctg	L	531	523.80
att	I	0	1.11
atc	I	0	0.75
ata	I	3	1.13
---	--	------	------
mPD		0.37	1.1
		nPD:	0.32
		N. weight:	1.6
		Sc. PD:	0.27
		Sc. rank:	1804.1

,

PA

Pos.	188	obs:	exp:
tgg	W	2496	2496.00
---	--	------	------
mPD		0	0
		nPD:	1.
		N. weight:	0.
		Sc. PD:	0
		Sc. rank:	0

,

PA

Pos.	189	obs:	exp:
gat	D	2487	1435.00
gac	D	8	1060.00
gaa	E	0	0.60
gag	E	1	0.40
---	--	------	------
mPD		0.0072	0.49
		nPD:	0.01
		N. weight:	0.66
		Sc. PD:	-0.087
		Sc. rank:	-468.4

,

PA

Pos.	190	obs:	exp:
ttt	F	0	0.85
ttc	F	2	1.15
tct	S	1	425.20
tcc	S	2484	367.10
tca	S	5	561.30
tcg	S	0	160.80
cct	P	0	1.13
ccc	P	4	0.72
cca	P	0	1.63
ccg	P	0	0.52
agt	S	0	508.40
agc	S	0	467.20
---	--	------	------
mPD		0.0096	1.6
		nPD:	0.01
		N. weight:	2.4
		Sc. PD:	-0.33
		Sc. rank:	-2865.0

,

PA

Pos.	191	obs:	exp:
ttt	F	2494	1062.00
ttc	F	2	1434.00
---	--	------	------
mPD		0.0016	0.49
		nPD:	0.
		N. weight:	1.1
		Sc. PD:	-0.15
		Sc. rank:	-1523.5

,

PA

Pos.	192	obs:	exp:
tgt	C	1	0.43
tgc	C	0	0.57
cgt	R	2487	94.62
cgc	R	8	132.90
cga	R	0	241.60
cgg	R	0	195.40
aga	R	0	1144.00
agg	R	0	686.00
---	--	------	------
mPD		0.0072	0.95
		nPD:	0.01
		N. weight:	4.
		Sc. PD:	-0.56
		Sc. rank:	-4440.0

,

PA

Pos.	193	obs:	exp:
cat	H	1	0.59
cac	H	0	0.41
caa	Q	591	1268.00
cag	Q	1901	1224.00
aaa	K	0	1.68
aag	K	3	1.32
---	--	------	------
mPD		0.36	0.50
		nPD:	0.73
		N. weight:	0.2
		Sc. PD:	0.11
		Sc. rank:	317.5

,

PA

Pos.	194	obs:	exp:
tct	S	552	426.10
tcc	S	1941	367.80
tca	S	2	562.40
tcg	S	0	161.20
agt	S	0	509.40
agc	S	0	468.10
gct	A	0	0.25
gcc	A	1	0.20
gca	A	0	0.44
gcg	A	0	0.11
---	--	------	------
mPD		0.35	1.6
		nPD:	0.21
		N. weight:	1.7
		Sc. PD:	0.1
		Sc. rank:	1283.5

,

PA

Pos.	195	obs:	exp:
gaa	E	44	1495.00
gag	E	2452	1001.00
---	--	------	------
mPD		0.035	0.48
		nPD:	0.07
		N. weight:	1.
		Sc. PD:	-0.077
		Sc. rank:	488.1

,

PA

Pos.	196	obs:	exp:
cgt	R	0	94.66
cgc	R	0	133.00
cga	R	0	241.70
cgg	R	0	195.50
aga	R	2496	1145.00
agg	R	0	686.30
---	--	------	------
mPD		0	0.95
		nPD:	0.
		N. weight:	0.97
		Sc. PD:	-0.14
		Sc. rank:	-1680.1

,

PA

Pos.	197	obs:	exp:
ggt	G	0	359.10
ggc	G	2494	329.40
gga	G	2	1090.00
ggg	G	0	717.90
---	--	------	------
mPD		0.0016	0.69
		nPD:	0.
		N. weight:	2.5
		Sc. PD:	-0.36
		Sc. rank:	-3795.0

,

PA

Pos.	198	obs:	exp:
gaa	E	2491	1495.00
gag	E	5	1001.00
---	--	------	------
mPD		0.0040	0.48
		nPD:	0.01
		N. weight:	0.62
		Sc. PD:	-0.086
		Sc. rank:	-624.9

,

PA

Pos.	199	obs:	exp:
gat	D	2	1.15
gac	D	0	0.85
gaa	E	343	1493.00
gag	E	2150	1000.00
ggt	G	0	0.14
ggc	G	0	0.13
gga	G	0	0.44
ggg	G	1	0.29
---	--	------	------
mPD		0.24	0.48
		nPD:	0.5
		N. weight:	0.57
		Sc. PD:	0.19
		Sc. rank:	794.5

,

PA

Pos.	200	obs:	exp:
act	T	0	661.20
acc	T	0	551.50
aca	T	2496	1073.00
acg	T	0	210.80
---	--	------	------
mPD		0	0.69
		nPD:	0.
		N. weight:	1.1
		Sc. PD:	-0.15
		Sc. rank:	-1820.8

,

PA			
Pos.	201	obs:	exp:
ttt	F	1	0.43
ttc	F	0	0.57
att	I	2450	923.50
atc	I	37	623.70
ata	I	0	939.80
act	T	2	0.53
acc	T	0	0.44
aca	T	0	0.86
acg	T	0	0.17
gtt	V	6	1.30
gtc	V	0	1.27
gta	V	0	1.18
gtg	V	0	2.25
---	--	------	------
mPD		0.036	0.66
		nPD:	0.05
		N. weight:	1.1
		Sc. PD:	-0.11
		Sc. rank:	457.7

PA			
Pos.	202	obs:	exp:
gaa	E	2464	1495.00
gag	E	32	1001.00
---	--	------	------
mPD		0.025	0.48
		nPD:	0.05
		N. weight:	0.56
		Sc. PD:	-0.053
		Sc. rank:	211.3

PA			
Pos.	203	obs:	exp:
gaa	E	2489	1495.00
gag	E	7	1001.00
---	--	------	------
mPD		0.0056	0.48
		nPD:	0.01
		N. weight:	0.62
		Sc. PD:	-0.083
		Sc. rank:	-517.2

PA			
Pos.	204	obs:	exp:
cgt	R	0	94.62
cgc	R	0	132.90
cga	R	3	241.60
cgg	R	0	195.40
aaa	K	1	0.56
aag	K	0	0.44
aga	R	1943	1144.00
agg	R	549	686.00
---	--	------	------
mPD		0.35	0.95
		nPD:	0.36
		N. weight:	0.45
		Sc. PD:	0.094
		Sc. rank:	526.7

PA			
Pos.	205	obs:	exp:
ttt	F	2494	1062.00
ttc	F	1	1433.00
att	I	1	0.37
atc	I	0	0.25
ata	I	0	0.38
---	--	------	------
mPD		0.0016	0.49
		nPD:	0.
		N. weight:	1.1
		Sc. PD:	-0.15
		Sc. rank:	-1542.4

PA			
Pos.	206	obs:	exp:
aaa	K	56	31.43
aag	K	0	24.57
gat	D	0	7.48
gac	D	13	5.52
gaa	E	2421	1453.00
gag	E	6	973.80
---	--	------	------
mPD		0.059	0.53
		nPD:	0.11
		N. weight:	0.62
		Sc. PD:	-0.023
		Sc. rank:	369.7

PA			
Pos.	207	obs:	exp:
tta	L	0	0.55
ttg	L	0	0.94
ctt	L	0	0.94
ctc	L	5	0.86
cta	L	0	0.67
ctg	L	0	1.05
att	I	299	925.00
atc	I	2192	624.70
ata	I	0	941.30
---	--	------	------
mPD		0.21	0.66
		nPD:	0.33
		N. weight:	1.2
		Sc. PD:	0.21
		Sc. rank:	1367.0

PA			
Pos.	208	obs:	exp:
tct	S	0	0.17
tcc	S	0	0.15
tca	S	1	0.23
tcg	S	0	0.06
act	T	1	557.30
acc	T	8	464.90
aca	T	2095	904.10
acg	T	0	177.70
aaa	K	1	0.56
aag	K	0	0.44
agt	S	0	0.20
agc	S	0	0.19
gct	A	0	99.21
gcc	A	0	76.05
gca	A	390	171.40
gcg	A	0	43.30
---	--	------	------
mPD		0.27	0.96
		nPD:	0.29
		N. weight:	1.
		Sc. PD:	0.14
		Sc. rank:	955.2

PA			
Pos.	209	obs:	exp:
cgt	R	0	0.04
cgc	R	0	0.05
cga	R	0	0.10
cgg	R	0	0.08
aga	R	1	0.46
agg	R	0	0.27
ggt	G	0	358.90
ggc	G	0	329.30
gga	G	2492	1089.00
ggg	G	3	717.60
---	--	------	------
mPD		0.0032	0.69
		nPD:	0.
		N. weight:	1.
		Sc. PD:	-0.14
		Sc. rank:	-1342.1

PA			
Pos.	210	obs:	exp:
att	I	0	0.37
atc	I	1	0.25
ata	I	0	0.38
act	T	0	660.60
acc	T	2452	551.00
aca	T	42	1072.00
acg	T	0	210.70
gct	A	0	0.25
gcc	A	1	0.20
gca	A	0	0.44
gcg	A	0	0.11
---	--	------	------
mPD		0.035	0.69
		nPD:	0.05
		N. weight:	1.8
		Sc. PD:	-0.17
		Sc. rank:	635.8

PA			
Pos.	211	obs:	exp:
tta	L	0	3.93
ttg	L	36	6.76
ctt	L	0	6.75
ctc	L	0	6.21
cta	L	0	4.79
ctg	L	0	7.56
att	I	5	3.34
atc	I	0	2.26
ata	I	4	3.40
atg	M	2451	2451.00
---	--	------	------
mPD		0.036	0.053
		nPD:	0.67
		N. weight:	0.032
		Sc. PD:	0.016
		Sc. rank:	49.9

PA			
Pos.	212	obs:	exp:
cat	H	0	2.38
cac	H	4	1.62
cgt	R	25	94.50
cgc	R	2465	132.70
cga	R	2	241.30
cgg	R	0	195.20
aga	R	0	1143.00
agg	R	0	685.20
---	--	------	------
mPD		0.025	0.96
		nPD:	0.03
		N. weight:	3.6
		Sc. PD:	-0.43
		Sc. rank:	-755.4

PA			
Pos.	213	obs:	exp:
cgt	R	0	94.58
cgc	R	0	132.80
cga	R	0	241.50
cgg	R	0	195.30
aaa	K	0	1.12
aag	K	2	0.88
aga	R	13	1144.00
agg	R	2481	685.80
---	--	------	------
mPD		0.012	0.95
		nPD:	0.01
		N. weight:	1.6
		Sc. PD:	-0.21
		Sc. rank:	-1234.3

PA			
Pos.	214	obs:	exp:
tta	L	0	272.40
ttg	L	0	468.40
ctt	L	2487	468.00
ctc	L	9	430.60
cta	L	0	332.10
ctg	L	0	524.40
---	--	------	------
mPD		0.0072	1.1
		nPD:	0.01
		N. weight:	2.1
		Sc. PD:	-0.29
		Sc. rank:	-2455.0

PA			
Pos.	215	obs:	exp:
gct	A	961	635.00
gcc	A	1511	486.70
gca	A	24	1097.00
gcg	A	0	277.10
---	--	------	------
mPD		0.49	0.69
		nPD:	0.7
		N. weight:	1.
		Sc. PD:	0.54
		Sc. rank:	1608.5

PA			
Pos.	216	obs:	exp:
tat	Y	0	3.89
tac	Y	8	4.11
aat	N	0	8.50
aac	N	16	7.50
gat	D	2	1422.00
gac	D	2470	1050.00
---	--	------	------
mPD		0.021	0.51
		nPD:	0.04
		N. weight:	1.1
		Sc. PD:	-0.11
		Sc. rank:	250.3

PA			
Pos.	217	obs:	exp:
caa	Q	2356	1270.00
cag	Q	140	1226.00
---	--	------	------
mPD		0.11	0.50
		nPD:	0.21
		N. weight:	0.58
		Sc. PD:	0.035
		Sc. rank:	446.4

PA			
Pos.	218	obs:	exp:
tct	S	0	426.30
tcc	S	0	368.00
tca	S	0	562.60
tcg	S	0	161.20
agt	S	2379	509.60
agc	S	117	468.30
---	--	------	------
mPD		0.089	1.6
		nPD:	0.05
		N. weight:	1.8
		Sc. PD:	-0.16
		Sc. rank:	682.4

PA			
Pos.	219	obs:	exp:
tta	L	0	272.40
ttg	L	0	468.40
ctt	L	10	468.00
ctc	L	2486	430.60
cta	L	0	332.10
ctg	L	0	524.40
---	--	------	------
mPD		0.0080	1.1
		nPD:	0.01
		N. weight:	2.2
		Sc. PD:	-0.3
		Sc. rank:	-2457.6

PA			
Pos.	220	obs:	exp:
cct	P	0	703.60
ccc	P	0	451.50
cca	P	2492	1019.00
ccg	P	3	321.30
act	T	0	0.26
acc	T	0	0.22
aca	T	1	0.43
acg	T	0	0.08
---	--	------	------
mPD		0.0032	0.71
		nPD:	0.
		N. weight:	1.1
		Sc. PD:	-0.16
		Sc. rank:	-1463.9

PA

Pos.	221	obs:	exp:
tct	S	0	0.17
tcc	S	0	0.15
tca	S	0	0.23
tcg	S	1	0.06
cct	P	13	703.60
ccc	P	0	451.50
cca	P	1	1019.00
ccg	P	2481	321.30
agt	S	0	0.20
agc	S	0	0.19
---	--	------	------
mPD		0.012	0.71
		nPD:	0.02
		N. weight:	2.5
		Sc. PD:	-0.32
		Sc. rank:	-1472.8

,

PA

Pos.	222	obs:	exp:
tct	S	0	0.34
tcc	S	0	0.29
tca	S	0	0.45
tcg	S	0	0.13
aat	N	583	1325.00
aac	N	1911	1169.00
agt	S	1	0.41
agc	S	1	0.38
---	--	------	------
mPD		0.36	0.50
		nPD:	0.72
		N. weight:	0.23
		Sc. PD:	0.13
		Sc. rank:	376.2

,

PA

Pos.	223	obs:	exp:
ttt	F	1	1062.00
ttc	F	2495	1434.00
---	--	------	------
mPD		0.00080	0.49
		nPD:	0.
		N. weight:	0.69
		Sc. PD:	-0.099
		Sc. rank:	-1081.2

,

PA

Pos.	224	obs:	exp:
tct	S	0	267.60
tcc	S	1567	231.00
tca	S	0	353.20
tcg	S	0	101.20
cct	P	0	3.67
ccc	P	13	2.35
cca	P	0	5.31
ccg	P	0	1.67
act	T	0	3.71
acc	T	14	3.09
aca	T	0	6.02
acg	T	0	1.18
agt	S	0	319.90
agc	S	0	294.00
gct	A	0	229.50
gcc	A	898	175.90
gca	A	4	396.50
gcg	A	0	100.10
---	--	------	------
mPD		0.48	1.7
		nPD:	0.27
		N. weight:	2.2
		Sc. PD:	0.27
		Sc. rank:	2038.2

,

PA

Pos.	225	obs:	exp:
tct	S	0	421.50
tcc	S	0	363.80
tca	S	0	556.30
tcg	S	0	159.40
tgt	C	0	0.43
tgc	C	1	0.57
cgt	R	0	1.02
cgc	R	27	1.44
cga	R	0	2.61
cgg	R	0	2.11
agt	S	1	503.90
agc	S	2467	463.00
aga	R	0	12.38
agg	R	0	7.42
---	--	------	------
mPD		0.023	1.7
		nPD:	0.01
		N. weight:	2.1
		Sc. PD:	-0.28
		Sc. rank:	-1500.2

,

PA

Pos.	226	obs:	exp:
ttt	F	11	4.68
ttc	F	0	6.32
tta	L	0	270.80
ttg	L	0	465.50
ctt	L	2479	465.20
ctc	L	2	428.00
cta	L	0	330.10
ctg	L	0	521.30
cgt	R	1	0.04
cgc	R	0	0.05
cga	R	0	0.10
cgg	R	0	0.08
att	I	3	1.11
atc	I	0	0.75
ata	I	0	1.13
aga	R	0	0.46
agg	R	0	0.27
---	--	------	------
mPD		0.014	1.1
		nPD:	0.01
		N. weight:	2.1
		Sc. PD:	-0.28
		Sc. rank:	-1694.2

,

PA

Pos.	227	obs:	exp:
gaa	E	2493	1493.00
gag	E	1	1001.00
ggt	G	0	0.29
ggc	G	0	0.26
gga	G	2	0.87
ggg	G	0	0.58
---	--	------	------
mPD		0.0024	0.48
		nPD:	0.
		N. weight:	0.64
		Sc. PD:	-0.09
		Sc. rank:	-801.7

,

PA

Pos.	228	obs:	exp:
tct	S	0	0.17
tcc	S	0	0.15
tca	S	0	0.23
tcg	S	0	0.06
aat	N	670	1325.00
aac	N	1824	1169.00
aaa	K	1	0.56
aag	K	0	0.44
agt	S	0	0.20
agc	S	1	0.19
---	--	------	------
mPD		0.39	0.50
		nPD:	0.79
		N. weight:	0.18
		Sc. PD:	0.11
		Sc. rank:	304.7

,

PA

Pos.	229	obs:	exp:
ttt	F	2496	1062.00
ttc	F	0	1434.00
---	--	------	------
mPD		0	0.49
		nPD:	0.
		N. weight:	1.1
		Sc. PD:	-0.16
		Sc. rank:	-1841.9

,

PA

Pos.	230	obs:	exp:
cgt	R	0	94.62
cgc	R	0	132.90
cga	R	0	241.60
cgg	R	0	195.40
att	I	0	0.37
atc	I	0	0.25
ata	I	1	0.38
aga	R	2494	1144.00
agg	R	1	686.00
---	--	------	------
mPD		0.0016	0.95
		nPD:	0.
		N. weight:	0.97
		Sc. PD:	-0.14
		Sc. rank:	-1500.6

,

PA

Pos.	231	obs:	exp:
act	T	0	0.79
acc	T	3	0.66
aca	T	0	1.29
acg	T	0	0.25
gtt	V	0	0.65
gtc	V	3	0.63
gta	V	0	0.59
gtg	V	0	1.12
gct	A	0	633.40
gcc	A	2489	485.60
gca	A	1	1095.00
gcg	A	0	276.40
---	--	------	------
mPD		0.0056	0.70
		nPD:	0.01
		N. weight:	2.
		Sc. PD:	-0.28
		Sc. rank:	-2126.9

,

PA

Pos.	232	obs:	exp:
tat	Y	2496	1213.00
tac	Y	0	1283.00
---	--	------	------
mPD		0	0.50
		nPD:	0.
		N. weight:	0.9
		Sc. PD:	-0.13
		Sc. rank:	-1555.0

,

PA

Pos.	233	obs:	exp:
gtt	V	3	542.80
gtc	V	0	527.40
gta	V	0	491.10
gtg	V	2493	934.70
---	--	------	------
mPD		0.0024	0.73
		nPD:	0.
		N. weight:	1.2
		Sc. PD:	-0.17
		Sc. rank:	-1737.6

,

PA

Pos.	234	obs:	exp:
gat	D	2491	1436.00
gac	D	5	1060.00
---	--	------	------
mPD		0.0040	0.49
		nPD:	0.01
		N. weight:	0.67
		Sc. PD:	-0.093
		Sc. rank:	-685.7

,

PA

Pos.	235	obs:	exp:
ggt	G	2	359.10
ggc	G	0	329.40
gga	G	2485	1090.00
ggg	G	9	717.90
---	--	------	------
mPD		0.0088	0.69
		nPD:	0.01
		N. weight:	1.
		Sc. PD:	-0.13
		Sc. rank:	-759.5

,

PA

Pos.	236	obs:	exp:
ttt	F	914	1062.00
ttc	F	1582	1434.00
---	--	------	------
mPD		0.46	0.49
		nPD:	0.95
		N. weight:	0.011
		Sc. PD:	0.0087
		Sc. rank:	19.3

,

PA

Pos.	237	obs:	exp:
cgt	R	0	0.04
cgc	R	0	0.05
cga	R	0	0.10
cgg	R	0	0.08
aaa	K	603	338.50
aag	K	0	264.50
aga	R	1	0.46
agg	R	0	0.27
gaa	E	1889	1133.00
gag	E	3	759.10
---	--	------	------
mPD		0.37	0.85
		nPD:	0.43
		N. weight:	0.65
		Sc. PD:	0.18
		Sc. rank:	842.8

,

PA

Pos.	238	obs:	exp:
cct	P	0	703.00
ccc	P	0	451.10
cca	P	2	1018.00
ccg	P	2491	321.00
caa	Q	0	1.02
cag	Q	2	0.98
act	T	0	0.26
acc	T	0	0.22
aca	T	0	0.43
acg	T	1	0.08
---	--	------	------
mPD		0.0040	0.71
		nPD:	0.01
		N. weight:	2.5
		Sc. PD:	-0.36
		Sc. rank:	-3129.4

,

PA

Pos.	239	obs:	exp:
aat	N	18	1326.00
aac	N	2478	1170.00
---	--	------	------
mPD		0.014	0.50
		nPD:	0.03
		N. weight:	0.89
		Sc. PD:	-0.1
		Sc. rank:	-67.2

,

PA

Pos.	240	obs:	exp:
tct	S	0	0.34
tcc	S	0	0.29
tca	S	0	0.45
tcg	S	0	0.13
agt	S	0	0.41
agc	S	2	0.38
ggt	G	0	358.80
ggc	G	2465	329.20
gga	G	29	1089.00
ggg	G	0	717.30
---	--	------	------
mPD		0.025	0.69
		nPD:	0.04
		N. weight:	2.4
		Sc. PD:	-0.27
		Sc. rank:	345.6

,

PA			
Pos.	241	obs:	exp:
tat	Y	0	0.49
tac	Y	1	0.51
tgt	C	0	1085.00
tgc	C	2495	1410.00
---	--	------	------
mPD		0.00080	0.49
		nPD:	0.
		N. weight:	0.71
		Sc. PD:	-0.1
		Sc. rank:	-1124.0

,

PA			
Pos.	242	obs:	exp:
tta	L	0	57.85
ttg	L	0	99.45
ctt	L	530	99.38
ctc	L	0	91.44
cta	L	0	70.52
ctg	L	0	111.40
att	I	1966	730.00
atc	I	0	493.10
ata	I	0	742.90
---	--	------	------
mPD		0.33	1.1
		nPD:	0.32
		N. weight:	1.4
		Sc. PD:	0.23
		Sc. rank:	1515.7

,

PA			
Pos.	243	obs:	exp:
gaa	E	1	1495.00
gag	E	2495	1001.00
---	--	------	------
mPD		0.00080	0.48
		nPD:	0.
		N. weight:	1.1
		Sc. PD:	-0.16
		Sc. rank:	-1763.7

,

PA			
Pos.	244	obs:	exp:
ggt	G	0	359.10
ggc	G	2493	329.40
gga	G	3	1090.00
ggg	G	0	717.90
---	--	------	------
mPD		0.0024	0.69
		nPD:	0.
		N. weight:	2.5
		Sc. PD:	-0.36
		Sc. rank:	-3512.0

,

PA			
Pos.	245	obs:	exp:
aaa	K	5	1401.00
aag	K	2491	1095.00
---	--	------	------
mPD		0.0040	0.49
		nPD:	0.01
		N. weight:	1.
		Sc. PD:	-0.14
		Sc. rank:	-1047.4

,

PA			
Pos.	246	obs:	exp:
tta	L	0	272.40
ttg	L	0	468.40
ctt	L	2496	468.00
ctc	L	0	430.60
cta	L	0	332.10
ctg	L	0	524.40
---	--	------	------
mPD		0	1.1
		nPD:	0.
		N. weight:	2.1
		Sc. PD:	-0.3
		Sc. rank:	-3608.3

,

PA			
Pos.	247	obs:	exp:
tct	S	2496	426.30
tcc	S	0	368.00
tca	S	0	562.60
tcg	S	0	161.20
agt	S	0	509.60
agc	S	0	468.30
---	--	------	------
mPD		0	1.6
		nPD:	0.
		N. weight:	2.2
		Sc. PD:	-0.32
		Sc. rank:	-3809.8

,

PA			
Pos.	248	obs:	exp:
tta	L	0	0.22
ttg	L	0	0.38
ctt	L	0	0.38
ctc	L	0	0.35
cta	L	2	0.27
ctg	L	0	0.42
caa	Q	2491	1269.00
cag	Q	3	1225.00
---	--	------	------
mPD		0.0040	0.50
		nPD:	0.01
		N. weight:	0.83
		Sc. PD:	-0.11
		Sc. rank:	-893.2

,

PA			
Pos.	249	obs:	exp:
atg	M	2496	2496.00
---	--	------	------
mPD		0	0
		nPD:	1.
		N. weight:	0.
		Sc. PD:	0
		Sc. rank:	0

,

PA			
Pos.	250	obs:	exp:
tct	S	0	423.70
tcc	S	47	365.70
tca	S	2432	559.30
tcg	S	2	160.30
cct	P	0	4.23
ccc	P	1	2.71
cca	P	14	6.12
ccg	P	0	1.93
agt	S	0	506.60
agc	S	0	465.50
---	--	------	------
mPD		0.051	1.7
		nPD:	0.03
		N. weight:	1.7
		Sc. PD:	-0.2
		Sc. rank:	70.0

,

PA			
Pos.	251	obs:	exp:
cgt	R	0	0.08
cgc	R	0	0.11
cga	R	0	0.19
cgg	R	0	0.16
aaa	K	2487	1400.00
aag	K	7	1094.00
aga	R	2	0.92
agg	R	0	0.55
---	--	------	------
mPD		0.0072	0.49
		nPD:	0.01
		N. weight:	0.7
		Sc. PD:	-0.092
		Sc. rank:	-494.0

,

PA			
Pos.	252	obs:	exp:
gat	D	0	0.58
gac	D	1	0.42
gaa	E	2476	1494.00
gag	E	19	1001.00
---	--	------	------
mPD		0.016	0.48
		nPD:	0.03
		N. weight:	0.59
		Sc. PD:	-0.067
		Sc. rank:	69.6

,

PA			
Pos.	253	obs:	exp:
gtt	V	4	542.80
gtc	V	0	527.40
gta	V	10	491.10
gtg	V	2482	934.70
---	--	------	------
mPD		0.011	0.73
		nPD:	0.02
		N. weight:	1.2
		Sc. PD:	-0.15
		Sc. rank:	-803.3

,

PA			
Pos.	254	obs:	exp:
tct	S	0	2.22
tcc	S	0	1.92
tca	S	0	2.93
tcg	S	0	0.84
aat	N	1610	1313.00
aac	N	861	1158.00
aaa	K	8	5.05
aag	K	1	3.95
agt	S	12	2.65
agc	S	1	2.44
gat	D	3	1.73
gac	D	0	1.27
---	--	------	------
mPD		0.47	0.52
		nPD:	0.9
		N. weight:	0.05
		Sc. PD:	0.037
		Sc. rank:	86.2

,

PA			
Pos.	255	obs:	exp:
tct	S	0	0.17
tcc	S	1	0.15
tca	S	0	0.23
tcg	S	0	0.06
agt	S	0	0.20
agc	S	0	0.19
gct	A	286	634.70
gcc	A	2200	486.50
gca	A	9	1097.00
gcg	A	0	277.00
---	--	------	------
mPD		0.21	0.69
		nPD:	0.3
		N. weight:	1.5
		Sc. PD:	0.23
		Sc. rank:	1513.1

,

PA			
Pos.	256	obs:	exp:
cgt	R	0	94.66
cgc	R	0	133.00
cga	R	2	241.70
cgg	R	0	195.50
aga	R	2088	1145.00
agg	R	406	686.30
---	--	------	------
mPD		0.27	0.95
		nPD:	0.29
		N. weight:	0.52
		Sc. PD:	0.07
		Sc. rank:	485.1

,

PA			
Pos.	257	obs:	exp:
tta	L	0	0.11
ttg	L	0	0.19
ctt	L	1	0.19
ctc	L	0	0.17
cta	L	0	0.13
ctg	L	0	0.21
att	I	2489	926.50
atc	I	6	625.70
ata	I	0	942.80
---	--	------	------
mPD		0.0056	0.66
		nPD:	0.01
		N. weight:	1.2
		Sc. PD:	-0.17
		Sc. rank:	-1212.4

,

PA			
Pos.	258	obs:	exp:
aaa	K	0	0.56
aag	K	1	0.44
gaa	E	90	1494.00
gag	E	2405	1001.00
---	--	------	------
mPD		0.070	0.48
		nPD:	0.15
		N. weight:	0.93
		Sc. PD:	-0.0029
		Sc. rank:	616.0

,

PA			
Pos.	259	obs:	exp:
tct	S	0	0.51
tcc	S	0	0.44
tca	S	3	0.68
tcg	S	0	0.19
cct	P	1	703.00
ccc	P	28	451.10
cca	P	2462	1018.00
ccg	P	2	321.00
agt	S	0	0.61
agc	S	0	0.56
---	--	------	------
mPD		0.027	0.71
		nPD:	0.04
		N. weight:	1.
		Sc. PD:	-0.11
		Sc. rank:	216.8

,

PA			
Pos.	260	obs:	exp:
ttt	F	1940	1062.00
ttc	F	556	1434.00
---	--	------	------
mPD		0.35	0.49
		nPD:	0.71
		N. weight:	0.32
		Sc. PD:	0.18
		Sc. rank:	513.6

,

PA			
Pos.	261	obs:	exp:
tta	L	0	208.50
ttg	L	26	358.40
ctt	L	227	358.20
ctc	L	0	329.50
cta	L	4	254.10
ctg	L	1653	401.30
cgt	R	0	0.23
cgc	R	0	0.32
cga	R	0	0.58
cgg	R	6	0.47
atg	M	552	552.00
aga	R	0	2.75
agg	R	0	1.65
gtt	V	0	6.09
gtc	V	0	5.92
gta	V	1	5.51
gtg	V	27	10.49
---	--	------	------
mPD		0.55	1.3
		nPD:	0.44
		N. weight:	1.1
		Sc. PD:	0.31
		Sc. rank:	1445.5

,

PA			
Pos.	262	obs:	exp:
cgt	R	0	10.85
cgc	R	0	15.23
cga	R	0	27.69
cgg	R	0	22.40
aat	N	1	0.53
aac	N	0	0.47
aaa	K	46	1240.00
aag	K	2163	969.10
aga	R	0	131.20
agg	R	286	78.64
---	--	------	------
mPD		0.24	0.76
		nPD:	0.31
		N. weight:	0.98
		Sc. PD:	0.16
		Sc. rank:	1031.1

,

PA			
Pos.	263	obs:	exp:
att	I	0	0.37
atc	I	0	0.25
ata	I	1	0.38
act	T	56	645.30
acc	T	0	538.20
aca	T	2164	1047.00
acg	T	216	205.80
aat	N	20	10.63
aac	N	0	9.37
aaa	K	30	20.21
aag	K	6	15.79
gct	A	0	0.76
gcc	A	0	0.59
gca	A	3	1.32
gcg	A	0	0.33
---	--	------	------
mPD		0.26	0.74
		nPD:	0.36
		N. weight:	0.74
		Sc. PD:	0.15
		Sc. rank:	862.9

,

PA			
Pos.	264	obs:	exp:
act	T	1	661.20
acc	T	1	551.50
aca	T	2489	1073.00
acg	T	5	210.80
---	--	------	------
mPD		0.0056	0.69
		nPD:	0.01
		N. weight:	1.
		Sc. PD:	-0.14
		Sc. rank:	-1066.4

,

PA			
Pos.	265	obs:	exp:
cct	P	1	703.80
ccc	P	2	451.60
cca	P	2490	1019.00
ccg	P	3	321.40
---	--	------	------
mPD		0.0048	0.70
		nPD:	0.01
		N. weight:	1.1
		Sc. PD:	-0.15
		Sc. rank:	-1271.5

,

PA			
Pos.	266	obs:	exp:
tgt	C	0	0.87
tgc	C	2	1.13
cat	H	0	1.19
cac	H	2	0.81
cgt	R	20	94.50
cgc	R	2460	132.70
cga	R	11	241.30
cgg	R	1	195.20
aga	R	0	1143.00
agg	R	0	685.20
---	--	------	------
mPD		0.029	0.96
		nPD:	0.03
		N. weight:	3.6
		Sc. PD:	-0.41
		Sc. rank:	43.3

,

PA			
Pos.	267	obs:	exp:
tta	L	0	0.11
ttg	L	0	0.19
tct	S	1	0.17
tcc	S	0	0.15
tca	S	0	0.23
tcg	S	0	0.06
ctt	L	1	0.19
ctc	L	0	0.17
cta	L	0	0.13
ctg	L	0	0.21
cct	P	2479	702.70
ccc	P	4	450.90
cca	P	8	1017.00
ccg	P	1	320.90
cat	H	2	1.19
cac	H	0	0.81
agt	S	0	0.20
agc	S	0	0.19
---	--	------	------
mPD		0.014	0.71
		nPD:	0.02
		N. weight:	1.5
		Sc. PD:	-0.19
		Sc. rank:	-756.1

,

PA			
Pos.	268	obs:	exp:
tta	L	0	267.10
ttg	L	0	459.20
ctt	L	5	458.80
ctc	L	2435	422.20
cta	L	0	325.60
ctg	L	7	514.10
att	I	0	18.20
atc	I	49	12.29
ata	I	0	18.52
---	--	------	------
mPD		0.048	1.2
		nPD:	0.04
		N. weight:	2.1
		Sc. PD:	-0.23
		Sc. rank:	564.2

,

PA			
Pos.	269	obs:	exp:
caa	Q	1	0.51
cag	Q	0	0.49
cgt	R	0	77.89
cgc	R	0	109.40
cga	R	0	198.90
cgg	R	0	160.90
att	I	0	1.86
atc	I	0	1.25
ata	I	5	1.89
aaa	K	436	244.70
aag	K	0	191.30
aga	R	2049	942.20
agg	R	5	564.80
---	--	------	------
mPD		0.30	1.2
		nPD:	0.25
		N. weight:	0.91
		Sc. PD:	0.09
		Sc. rank:	760.8

,

PA			
Pos.	270	obs:	exp:
tta	L	2000	272.40
ttg	L	488	468.40
ctt	L	0	468.00
ctc	L	0	430.60
cta	L	4	332.10
ctg	L	4	524.40
---	--	------	------
mPD		0.32	1.1
		nPD:	0.28
		N. weight:	2.
		Sc. PD:	0.26
		Sc. rank:	1845.6

,

PA			
Pos.	271	obs:	exp:
cct	P	2457	703.60
ccc	P	16	451.50
cca	P	15	1019.00
ccg	P	7	321.30
cat	H	1	0.59
cac	H	0	0.41
---	--	------	------
mPD		0.031	0.71
		nPD:	0.04
		N. weight:	1.5
		Sc. PD:	-0.15
		Sc. rank:	447.5

,

PA			
Pos.	272	obs:	exp:
aat	N	1	0.53
aac	N	0	0.47
gtt	V	1	0.22
gtc	V	0	0.21
gta	V	0	0.20
gtg	V	0	0.37
gct	A	2	0.51
gcc	A	0	0.39
gca	A	0	0.88
gcg	A	0	0.22
gat	D	2430	1400.00
gac	D	4	1034.00
gaa	E	8	4.79
gag	E	0	3.21
ggt	G	50	7.19
ggc	G	0	6.60
gga	G	0	21.83
ggg	G	0	14.38
---	--	------	------
mPD		0.052	0.55
		nPD:	0.09
		N. weight:	0.71
		Sc. PD:	-0.038
		Sc. rank:	395.1

,

PA			
Pos.	273	obs:	exp:
ggt	G	873	359.10
ggc	G	6	329.40
gga	G	913	1090.00
ggg	G	704	717.90
---	--	------	------
mPD		0.66	0.69
		nPD:	0.97
		N. weight:	0.29
		Sc. PD:	0.23
		Sc. rank:	505.8

,

PA			
Pos.	274	obs:	exp:
tta	L	0	2.07
ttg	L	0	3.57
ctt	L	19	3.56
ctc	L	0	3.28
cta	L	0	2.53
ctg	L	0	3.99
cct	P	2463	698.50
ccc	P	12	448.20
cca	P	2	1011.00
ccg	P	0	318.90
---	--	------	------
mPD		0.026	0.72
		nPD:	0.04
		N. weight:	1.5
		Sc. PD:	-0.17
		Sc. rank:	235.4

,

PA			
Pos.	275	obs:	exp:
tta	L	0	0.33
ttg	L	0	0.56
tct	S	1	0.17
tcc	S	0	0.15
tca	S	0	0.23
tcg	S	0	0.06
ctt	L	0	0.56
ctc	L	3	0.52
cta	L	0	0.40
ctg	L	0	0.63
cct	P	597	702.20
ccc	P	1858	450.60
cca	P	35	1017.00
ccg	P	0	320.60
cat	H	2	1.19
cac	H	0	0.81
agt	S	0	0.20
agc	S	0	0.19
---	--	------	------
mPD		0.39	0.71
		nPD:	0.55
		N. weight:	1.2
		Sc. PD:	0.47
		Sc. rank:	1789.9

PA

Pos.	276	obs:	exp:
tgt	C	1252	1085.00
tgc	C	1244	1411.00
---	--	------	------
mPD		0.50	0.49
		nPD:	1.02
		N. weight:	0.013
		Sc. PD:	0.011
		Sc. rank:	23.0

PA

Pos.	277	obs:	exp:
ttt	F	8	4.68
ttc	F	3	6.32
tct	S	1304	421.10
tcc	S	1149	363.50
tca	S	13	555.90
tcg	S	0	159.30
tat	Y	5	4.86
tac	Y	5	5.14
cct	P	3	1.69
ccc	P	3	1.09
cca	P	0	2.45
ccg	P	0	0.77
cat	H	3	1.78
cac	H	0	1.22
agt	S	0	503.50
agc	S	0	462.70
---	--	------	------
mPD		0.53	1.7
		nPD:	0.32
		N. weight:	1.4
		Sc. PD:	0.23
		Sc. rank:	1496.2

PA

Pos.	278	obs:	exp:
caa	Q	31	1270.00
cag	Q	2465	1226.00
---	--	------	------
mPD		0.025	0.50
		nPD:	0.05
		N. weight:	0.8
		Sc. PD:	-0.079
		Sc. rank:	276.5

PA

Pos.	279	obs:	exp:
cgt	R	1	94.66
cgc	R	1	133.00
cga	R	320	241.70
cgg	R	2160	195.50
aga	R	4	1145.00
agg	R	10	686.30
---	--	------	------
mPD		0.24	0.95
		nPD:	0.25
		N. weight:	2.6
		Sc. PD:	0.26
		Sc. rank:	2203.5

PA

Pos.	280	obs:	exp:
tct	S	1	425.90
tcc	S	0	367.70
tca	S	41	562.20
tcg	S	2452	161.10
act	T	0	0.53
acc	T	0	0.44
aca	T	0	0.86
acg	T	2	0.17
agt	S	0	509.20
agc	S	0	467.90
---	--	------	------
mPD		0.035	1.6
		nPD:	0.02
		N. weight:	3.3
		Sc. PD:	-0.41
		Sc. rank:	-1234.8

PA

Pos.	281	obs:	exp:
aaa	K	638	1401.00
aag	K	1858	1095.00
---	--	------	------
mPD		0.38	0.49
		nPD:	0.77
		N. weight:	0.24
		Sc. PD:	0.15
		Sc. rank:	408.1

PA

Pos.	282	obs:	exp:
ttt	F	16	1062.00
ttc	F	2480	1434.00
---	--	------	------
mPD		0.013	0.49
		nPD:	0.03
		N. weight:	0.65
		Sc. PD:	-0.078
		Sc. rank:	-120.6

PA

Pos.	283	obs:	exp:
tta	L	1004	272.40
ttg	L	1479	468.40
ctt	L	0	468.00
ctc	L	0	430.60
cta	L	3	332.10
ctg	L	10	524.40
---	--	------	------
mPD		0.49	1.1
		nPD:	0.43
		N. weight:	1.5
		Sc. PD:	0.41
		Sc. rank:	1917.3

PA

Pos.	284	obs:	exp:
tta	L	1	272.30
ttg	L	15	468.20
ctt	L	61	467.90
ctc	L	0	430.50
cta	L	1186	332.00
ctg	L	1232	524.20
gtt	V	0	0.22
gtc	V	0	0.21
gta	V	0	0.20
gtg	V	1	0.37
---	--	------	------
mPD		0.54	1.1
		nPD:	0.47
		N. weight:	1.2
		Sc. PD:	0.38
		Sc. rank:	1648.3

PA

Pos.	285	obs:	exp:
atg	M	2496	2496.00
---	--	------	------
mPD		0	0
		nPD:	1.
		N. weight:	0.
		Sc. PD:	0
		Sc. rank:	0

PA

Pos.	286	obs:	exp:
gat	D	2495	1436.00
gac	D	1	1060.00
---	--	------	------
mPD		0.00080	0.49
		nPD:	0.
		N. weight:	0.69
		Sc. PD:	-0.099
		Sc. rank:	-1074.1

PA

Pos.	287	obs:	exp:
tct	S	0	18.44
tcc	S	108	15.92
tca	S	0	24.34
tcg	S	0	6.98
agt	S	0	22.05
agc	S	0	20.26
gct	A	303	607.50
gcc	A	2085	465.70
gca	A	0	1050.00
gcg	A	0	265.10
---	--	------	------
mPD		0.30	0.81
		nPD:	0.36
		N. weight:	1.6
		Sc. PD:	0.33
		Sc. rank:	1842.6

PA

Pos.	288	obs:	exp:
tta	L	0	272.30
ttg	L	4	468.20
ctt	L	2458	467.90
ctc	L	19	430.50
cta	L	0	332.00
ctg	L	14	524.20
att	I	1	0.37
atc	I	0	0.25
ata	I	0	0.38
---	--	------	------
mPD		0.033	1.1
		nPD:	0.03
		N. weight:	2.
		Sc. PD:	-0.23
		Sc. rank:	-50.7

PA

Pos.	289	obs:	exp:
cgt	R	0	0.04
cgc	R	0	0.05
cga	R	0	0.10
cgg	R	0	0.08
aaa	K	2470	1400.00
aag	K	25	1095.00
aga	R	1	0.46
agg	R	0	0.27
---	--	------	------
mPD		0.021	0.49
		nPD:	0.04
		N. weight:	0.65
		Sc. PD:	-0.069
		Sc. rank:	174.6

PA

Pos.	290	obs:	exp:
ttt	F	1	0.43
ttc	F	0	0.57
tta	L	472	272.30
ttg	L	1615	468.20
ctt	L	7	467.90
ctc	L	0	430.50
cta	L	401	332.00
ctg	L	0	524.20
---	--	------	------
mPD		0.73	1.1
		nPD:	0.65
		N. weight:	1.2
		Sc. PD:	0.56
		Sc. rank:	1785.7

PA

Pos.	291	obs:	exp:
tct	S	0	425.40
tcc	S	0	367.20
tca	S	0	561.50
tcg	S	0	160.90
tgt	C	0	0.87
tgc	C	2	1.13
aat	N	0	1.59
aac	N	3	1.41
agt	S	7	508.60
agc	S	2484	467.40
---	--	------	------
mPD		0.0096	1.6
		nPD:	0.01
		N. weight:	2.1
		Sc. PD:	-0.29
		Sc. rank:	-2477.7

PA

Pos.	292	obs:	exp:
att	I	1197	924.30
atc	I	1291	624.20
ata	I	1	940.50
gtt	V	2	1.52
gtc	V	5	1.48
gta	V	0	1.38
gtg	V	0	2.62
---	--	------	------
mPD		0.51	0.66
		nPD:	0.76
		N. weight:	0.63
		Sc. PD:	0.37
		Sc. rank:	1050.1

PA

Pos.	293	obs:	exp:
gaa	E	2187	1495.00
gag	E	309	1001.00
---	--	------	------
mPD		0.22	0.48
		nPD:	0.45
		N. weight:	0.24
		Sc. PD:	0.07
		Sc. rank:	319.2

PA

Pos.	294	obs:	exp:
tct	S	0	0.17
tcc	S	0	0.15
tca	S	0	0.23
tcg	S	0	0.06
aat	N	2	1.06
aac	N	0	0.94
agt	S	0	0.20
agc	S	1	0.19
gat	D	326	1434.00
gac	D	2167	1059.00
---	--	------	------
mPD		0.23	0.49
		nPD:	0.47
		N. weight:	0.54
		Sc. PD:	0.17
		Sc. rank:	737.7

PA

Pos.	295	obs:	exp:
cct	P	6	703.80
ccc	P	1	451.60
cca	P	837	1019.00
ccg	P	1652	321.40
---	--	------	------
mPD		0.45	0.70
		nPD:	0.64
		N. weight:	1.3
		Sc. PD:	0.6
		Sc. rank:	1928.8

PA			
Pos.	296	obs:	exp:
tct	S	0	422.00
tcc	S	0	364.30
tca	S	0	557.00
tcg	S	0	159.60
cgt	R	0	0.04
cgc	R	0	0.05
cga	R	0	0.10
cgg	R	0	0.08
act	T	0	4.77
acc	T	18	3.98
aca	T	0	7.73
acg	T	0	1.52
aat	N	0	1.06
aac	N	2	0.94
agt	S	22	504.50
agc	S	2449	463.60
aga	R	1	0.46
agg	R	0	0.27
ggt	G	0	0.58
ggc	G	4	0.53
gga	G	0	1.75
ggg	G	0	1.15
---	--	------	------
mPD		0.037	1.7
		nPD:	0.02
		N. weight:	2.
		Sc. PD:	-0.25
		Sc. rank:	-671.6

PA			
Pos.	297	obs:	exp:
cct	P	1	0.28
ccc	P	0	0.18
cca	P	0	0.41
ccg	P	0	0.13
cat	H	2021	1483.00
cac	H	474	1012.00
---	--	------	------
mPD		0.31	0.48
		nPD:	0.64
		N. weight:	0.14
		Sc. PD:	0.065
		Sc. rank:	208.0

PA			
Pos.	298	obs:	exp:
gaa	E	753	1495.00
gag	E	1743	1001.00
---	--	------	------
mPD		0.42	0.48
		nPD:	0.88
		N. weight:	0.23
		Sc. PD:	0.16
		Sc. rank:	388.6

PA			
Pos.	299	obs:	exp:
ggt	G	3	359.10
ggc	G	4	329.40
gga	G	590	1090.00
ggg	G	1899	717.90
---	--	------	------
mPD		0.37	0.69
		nPD:	0.53
		N. weight:	0.73
		Sc. PD:	0.27
		Sc. rank:	1056.7

PA			
Pos.	300	obs:	exp:
gaa	E	1254	1495.00
gag	E	1242	1001.00
---	--	------	------
mPD		0.50	0.48
		nPD:	1.04
		N. weight:	0.026
		Sc. PD:	0.022
		Sc. rank:	44.5

PA			
Pos.	301	obs:	exp:
gat	D	0	0.58
gac	D	1	0.42
ggt	G	1777	358.90
ggc	G	683	329.30
gga	G	17	1089.00
ggg	G	18	717.60
---	--	------	------
mPD		0.42	0.69
		nPD:	0.61
		N. weight:	1.6
		Sc. PD:	0.72
		Sc. rank:	2416.3

PA			
Pos.	302	obs:	exp:
att	I	0	926.10
atc	I	24	625.50
ata	I	2470	942.40
gtt	V	0	0.43
gtc	V	1	0.42
gta	V	1	0.39
gtg	V	0	0.75
---	--	------	------
mPD		0.021	0.66
		nPD:	0.03
		N. weight:	1.2
		Sc. PD:	-0.13
		Sc. rank:	126.1

PA			
Pos.	303	obs:	exp:
tta	L	0	0.11
ttg	L	0	0.19
ctt	L	0	0.19
ctc	L	0	0.17
cta	L	0	0.13
ctg	L	1	0.21
cct	P	7	703.60
ccc	P	0	451.50
cca	P	20	1019.00
ccg	P	2468	321.30
---	--	------	------
mPD		0.022	0.71
		nPD:	0.03
		N. weight:	2.5
		Sc. PD:	-0.28
		Sc. rank:	155.6

PA			
Pos.	304	obs:	exp:
tta	L	3	272.40
ttg	L	3	468.40
ctt	L	1	468.00
ctc	L	0	430.60
cta	L	1284	332.10
ctg	L	1205	524.40
---	--	------	------
mPD		0.50	1.1
		nPD:	0.44
		N. weight:	1.4
		Sc. PD:	0.39
		Sc. rank:	1815.7

PA			
Pos.	305	obs:	exp:
tat	Y	1581	1207.00
tac	Y	902	1276.00
tgt	C	0	3.91
tgc	C	9	5.09
cat	H	3	2.38
cac	H	1	1.62
---	--	------	------
mPD		0.47	0.51
		nPD:	0.93
		N. weight:	0.062
		Sc. PD:	0.047
		Sc. rank:	106.9

PA			
Pos.	306	obs:	exp:
gat	D	2479	1436.00
gac	D	17	1060.00
---	--	------	------
mPD		0.014	0.49
		nPD:	0.03
		N. weight:	0.64
		Sc. PD:	-0.076
		Sc. rank:	-79.1

PA			
Pos.	307	obs:	exp:
gct	A	0	635.00
gcc	A	0	486.70
gca	A	2487	1097.00
gcg	A	9	277.10
---	--	------	------
mPD		0.0072	0.69
		nPD:	0.01
		N. weight:	1.
		Sc. PD:	-0.14
		Sc. rank:	-904.3

PA			
Pos.	308	obs:	exp:
att	I	0	562.60
atc	I	1229	380.00
ata	I	286	572.50
act	T	0	259.60
acc	T	980	216.50
aca	T	0	421.10
acg	T	0	82.78
gtt	V	0	0.22
gtc	V	1	0.21
gta	V	0	0.20
gtg	V	0	0.37
---	--	------	------
mPD		0.68	1.2
		nPD:	0.59
		N. weight:	1.4
		Sc. PD:	0.59
		Sc. rank:	2038.5

PA			
Pos.	309	obs:	exp:
caa	Q	4	2.04
cag	Q	0	1.96
aat	N	0	0.53
aac	N	1	0.47
aaa	K	2491	1398.00
aag	K	0	1093.00
---	--	------	------
mPD		0.0040	0.50
		nPD:	0.01
		N. weight:	0.72
		Sc. PD:	-0.099
		Sc. rank:	-750.9

PA			
Pos.	310	obs:	exp:
tgt	C	10	1085.00
tgc	C	2486	1411.00
---	--	------	------
mPD		0.0080	0.49
		nPD:	0.02
		N. weight:	0.68
		Sc. PD:	-0.088
		Sc. rank:	-429.7

PA			
Pos.	311	obs:	exp:
tta	L	0	0.11
ttg	L	0	0.19
ctt	L	0	0.19
ctc	L	0	0.17
cta	L	0	0.13
ctg	L	1	0.21
att	I	0	0.74
atc	I	0	0.50
ata	I	2	0.76
atg	M	2492	2492.00
act	T	0	0.26
acc	T	0	0.22
aca	T	0	0.43
acg	T	1	0.08
---	--	------	------
mPD		0.0032	0.0044
		nPD:	0.73
		N. weight:	0.003
		Sc. PD:	0.0017
		Sc. rank:	4.8

PA			
Pos.	312	obs:	exp:
cgt	R	0	0.11
cgc	R	0	0.16
cga	R	0	0.29
cgg	R	0	0.23
aaa	K	3	1399.00
aag	K	2490	1094.00
aga	R	0	1.38
agg	R	3	0.82
---	--	------	------
mPD		0.0048	0.50
		nPD:	0.01
		N. weight:	1.
		Sc. PD:	-0.14
		Sc. rank:	-946.3

PA			
Pos.	313	obs:	exp:
act	T	1	660.90
acc	T	7	551.30
aca	T	1937	1072.00
acg	T	550	210.70
aaa	K	1	0.56
aag	K	0	0.44
---	--	------	------
mPD		0.35	0.69
		nPD:	0.51
		N. weight:	0.82
		Sc. PD:	0.29
		Sc. rank:	1148.8

PA			
Pos.	314	obs:	exp:
ttt	F	2103	1062.00
ttc	F	393	1434.00
---	--	------	------
mPD		0.27	0.49
		nPD:	0.54
		N. weight:	0.47
		Sc. PD:	0.18
		Sc. rank:	677.9

PA			
Pos.	315	obs:	exp:
ttt	F	1799	1061.00
ttc	F	694	1432.00
tta	L	0	0.22
ttg	L	2	0.38
tat	Y	0	0.49
tac	Y	1	0.51
ctt	L	0	0.38
ctc	L	0	0.35
cta	L	0	0.27
ctg	L	0	0.42
---	--	------	------
mPD		0.40	0.49
		nPD:	0.82
		N. weight:	0.23
		Sc. PD:	0.15
		Sc. rank:	387.2

PA

Pos.	316	obs :	exp :
ggt	G	579	359.10
ggc	G	1906	329.40
gga	G	9	1090.00
ggg	G	2	717.90
---	--	------	------
mPD		0.36	0.69
		nPD :	0.53
		N. weight :	1.8
		Sc. PD :	0.66
		Sc. rank :	2578.5

,

PA

Pos.	317	obs :	exp :
tgg	W	2495	2495.00
cgt	R	0	0.04
cgc	R	0	0.05
cga	R	0	0.10
cgg	R	0	0.08
aga	R	0	0.46
agg	R	1	0.27
---	--	------	------
mPD		0.00080	0.0013
		nPD :	0.61
		N. weight :	0.00064
		Sc. PD :	0.00029
		Sc. rank :	1.0

,

PA

Pos.	318	obs :	exp :
aaa	K	1568	1401.00
aag	K	928	1095.00
---	--	------	------
mPD		0.47	0.49
		nPD :	0.95
		N. weight :	0.014
		Sc. PD :	0.011
		Sc. rank :	23.4

,

PA

Pos.	319	obs :	exp :
gaa	E	3	1495.00
gag	E	2493	1001.00
---	--	------	------
mPD		0.0024	0.48
		nPD :	0.
		N. weight :	1.1
		Sc. PD :	-0.16
		Sc. rank :	-1415.9

,

PA

Pos.	320	obs :	exp :
cct	P	13	703.80
ccc	P	2478	451.60
cca	P	5	1019.00
ccg	P	0	321.40
---	--	------	------
mPD		0.014	0.70
		nPD :	0.02
		N. weight :	2.1
		Sc. PD :	-0.26
		Sc. rank :	-874.8

,

PA

Pos.	321	obs :	exp :
tct	S	0	0.68
tcc	S	0	0.59
tca	S	0	0.90
tcg	S	0	0.26
cat	H	0	0.59
cac	H	1	0.41
act	T	0	0.26
acc	T	1	0.22
aca	T	0	0.43
acg	T	0	0.08
aat	N	322	1322.00
aac	N	2165	1165.00
aaa	K	3	1.68
aag	K	0	1.32
agt	S	0	0.82
agc	S	4	0.75
---	--	------	------
mPD		0.23	0.51
		nPD :	0.46
		N. weight :	0.45
		Sc. PD :	0.14
		Sc. rank :	611.9

,

PA

Pos.	322	obs :	exp :
att	I	554	924.30
atc	I	1924	624.20
ata	I	11	940.50
gtt	V	2	1.52
gtc	V	5	1.48
gta	V	0	1.38
gtg	V	0	2.62
---	--	------	------
mPD		0.36	0.66
		nPD :	0.54
		N. weight :	0.92
		Sc. PD :	0.35
		Sc. rank :	1341.9

,

PA

Pos.	323	obs :	exp :
att	I	0	37.50
atc	I	11	25.33
ata	I	90	38.16
gtt	V	0	520.40
gtc	V	8	505.60
gta	V	831	470.80
gtg	V	1554	896.10
gct	A	0	0.51
gcc	A	0	0.39
gca	A	2	0.88
gcg	A	0	0.22
---	--	------	------
mPD		0.56	0.81
		nPD :	0.68
		N. weight :	0.69
		Sc. PD :	0.36
		Sc. rank :	1076.6

,

PA

Pos.	324	obs :	exp :
aaa	K	2486	1401.00
aag	K	10	1095.00
---	--	------	------
mPD		0.0080	0.49
		nPD :	0.02
		N. weight :	0.69
		Sc. PD :	-0.09
		Sc. rank :	-437.3

,

PA

Pos.	325	obs :	exp :
tta	L	0	5.78
ttg	L	0	9.95
tct	S	0	0.17
tcc	S	0	0.15
tca	S	1	0.23
tcg	S	0	0.06
ctt	L	0	9.94
ctc	L	0	9.14
cta	L	53	7.05
ctg	L	0	11.14
cct	P	0	688.10
ccc	P	0	441.50
cca	P	2154	996.30
ccg	P	286	314.20
caa	Q	1	0.51
cag	Q	0	0.49
act	T	0	0.26
acc	T	0	0.22
aca	T	1	0.43
acg	T	0	0.08
agt	S	0	0.20
agc	S	0	0.19
---	--	------	------
mPD		0.25	0.76
		nPD :	0.32
		N. weight :	0.87
		Sc. PD :	0.15
		Sc. rank :	968.4

,

PA

Pos.	326	obs :	exp :
cat	H	2492	1483.00
cac	H	3	1012.00
caa	Q	0	0.51
cag	Q	1	0.49
---	--	------	------
mPD		0.0032	0.48
		nPD :	0.01
		N. weight :	0.64
		Sc. PD :	-0.089
		Sc. rank :	-749.3

,

PA

Pos.	327	obs :	exp :
att	I	0	0.74
atc	I	0	0.50
ata	I	2	0.76
aaa	K	2	1.12
aag	K	0	0.88
gtt	V	0	124.60
gtc	V	0	121.10
gta	V	572	112.70
gtg	V	1	214.60
gat	D	0	0.58
gac	D	1	0.42
gaa	E	1896	1145.00
gag	E	17	767.60
ggt	G	0	0.72
ggc	G	0	0.66
gga	G	5	2.18
ggg	G	0	1.44
---	--	------	------
mPD		0.38	0.94
		nPD :	0.4
		N. weight :	0.91
		Sc. PD :	0.22
		Sc. rank :	1129.6

,

PA

Pos.	328	obs :	exp :
cgt	R	0	0.27
cgc	R	0	0.37
cga	R	0	0.68
cgg	R	0	0.55
act	T	0	0.26
acc	T	0	0.22
aca	T	0	0.43
acg	T	1	0.08
aaa	K	1300	1396.00
aag	K	1187	1091.00
aga	R	0	3.21
agg	R	7	1.92
gaa	E	1	0.60
gag	E	0	0.40
---	--	------	------
mPD		0.51	0.50
		nPD :	1.01
		N. weight :	0.012
		Sc. PD :	0.0099
		Sc. rank :	20.4

,

PA

Pos.	329	obs :	exp :
ggt	G	12	359.10
ggc	G	2478	329.40
gga	G	6	1090.00
ggg	G	0	717.90
---	--	------	------
mPD		0.014	0.69
		nPD :	0.02
		N. weight :	2.5
		Sc. PD :	-0.31
		Sc. rank :	-973.9

,

PA

Pos.	330	obs :	exp :
att	I	0	926.50
atc	I	2	625.70
ata	I	2493	942.80
atg	M	1	1.00
---	--	------	------
mPD		0.0024	0.66
		nPD :	0.
		N. weight :	1.2
		Sc. PD :	-0.17
		Sc. rank :	-1652.5

,

PA			
Pos.	331	obs:	exp:
tct	S	0	0.17
tcc	S	0	0.15
tca	S	0	0.23
tcg	S	0	0.06
aat	N	721	1326.00
aac	N	1774	1169.00
agt	S	1	0.20
agc	S	0	0.19
---	--	------	------
mPD		0.41	0.50
		nPD:	0.83
		N. weight:	0.15
		Sc. PD:	0.1
		Sc. rank:	260.4

,

PA			
Pos.	332	obs:	exp:
tta	L	0	0.11
ttg	L	0	0.19
tct	S	0	0.17
tcc	S	0	0.15
tca	S	1	0.23
tcg	S	0	0.06
ctt	L	0	0.19
ctc	L	1	0.17
cta	L	0	0.13
ctg	L	0	0.21
cct	P	1206	703.00
ccc	P	1005	451.10
cca	P	282	1018.00
ccg	P	0	321.00
cat	H	0	0.59
cac	H	1	0.41
agt	S	0	0.20
agc	S	0	0.19
---	--	------	------
mPD		0.59	0.71
		nPD:	0.84
		N. weight:	0.55
		Sc. PD:	0.37
		Sc. rank:	943.4

,

PA			
Pos.	333	obs:	exp:
aat	N	1917	1326.00
aac	N	579	1170.00
---	--	------	------
mPD		0.36	0.50
		nPD:	0.72
		N. weight:	0.15
		Sc. PD:	0.084
		Sc. rank:	243.5

,

PA			
Pos.	334	obs:	exp:
tat	Y	600	1213.00
tac	Y	1896	1283.00
---	--	------	------
mPD		0.37	0.50
		nPD:	0.73
		N. weight:	0.16
		Sc. PD:	0.091
		Sc. rank:	263.7

,

PA			
Pos.	335	obs:	exp:
tta	L	0	272.40
ttg	L	0	468.40
ctt	L	8	468.00
ctc	L	2488	430.60
cta	L	0	332.10
ctg	L	0	524.40
---	--	------	------
mPD		0.0064	1.1
		nPD:	0.01
		N. weight:	2.2
		Sc. PD:	-0.3
		Sc. rank:	-2687.3

,

PA			
Pos.	336	obs:	exp:
tta	L	6	272.00
ttg	L	546	467.60
ctt	L	5	467.30
ctc	L	0	429.90
cta	L	11	331.60
ctg	L	1924	523.60
caa	Q	0	1.02
cag	Q	2	0.98
cgt	R	0	0.08
cgc	R	0	0.11
cga	R	0	0.19
cgg	R	2	0.16
aga	R	0	0.92
agg	R	0	0.55
---	--	------	------
mPD		0.37	1.1
		nPD:	0.32
		N. weight:	1.3
		Sc. PD:	0.21
		Sc. rank:	1392.1

,

PA			
Pos.	337	obs:	exp:
tct	S	1	0.17
tcc	S	0	0.15
tca	S	0	0.23
tcg	S	0	0.06
act	T	15	3.97
acc	T	0	3.31
aca	T	0	6.45
acg	T	0	1.27
agt	S	0	0.20
agc	S	0	0.19
gtt	V	5	1.09
gtc	V	0	1.06
gta	V	0	0.98
gtg	V	0	1.87
gct	A	2470	629.60
gcc	A	2	482.60
gca	A	0	1088.00
gcg	A	3	274.80
---	--	------	------
mPD		0.021	0.71
		nPD:	0.03
		N. weight:	1.7
		Sc. PD:	-0.2
		Sc. rank:	-59.5

,

PA			
Pos.	338	obs:	exp:
tgg	W	2495	2495.00
ggt	G	0	0.14
ggc	G	0	0.13
gga	G	0	0.44
ggg	G	1	0.29
---	--	------	------
mPD		0.00080	0.0014
		nPD:	0.58
		N. weight:	0.00062
		Sc. PD:	0.00026
		Sc. rank:	0.9

,

PA			
Pos.	339	obs:	exp:
aaa	K	293	1401.00
aag	K	2203	1095.00
---	--	------	------
mPD		0.21	0.49
		nPD:	0.42
		N. weight:	0.54
		Sc. PD:	0.14
		Sc. rank:	688.5

,

PA			
Pos.	340	obs:	exp:
caa	Q	95	1270.00
cag	Q	2401	1226.00
---	--	------	------
mPD		0.073	0.50
		nPD:	0.15
		N. weight:	0.68
		Sc. PD:	-0.0019
		Sc. rank:	459.5

,

PA			
Pos.	341	obs:	exp:
atg	M	1	1.00
gtt	V	7	542.60
gtc	V	0	527.20
gta	V	12	490.90
gtg	V	2476	934.30
---	--	------	------
mPD		0.016	0.73
		nPD:	0.02
		N. weight:	1.2
		Sc. PD:	-0.15
		Sc. rank:	-414.6

,

PA			
Pos.	342	obs:	exp:
tta	L	289	271.60
ttg	L	26	466.90
ctt	L	0	466.50
ctc	L	0	429.30
cta	L	947	331.00
ctg	L	1226	522.80
atg	M	8	8.00
---	--	------	------
mPD		0.73	1.1
		nPD:	0.64
		N. weight:	1.
		Sc. PD:	0.47
		Sc. rank:	1524.8

,

PA			
Pos.	343	obs:	exp:
tct	S	0	155.40
tcc	S	0	134.20
tca	S	909	205.10
tcg	S	1	58.78
agt	S	0	185.80
agc	S	0	170.70
gtt	V	0	6.31
gtc	V	0	6.13
gta	V	29	5.71
gtg	V	0	10.86
gct	A	0	395.80
gcc	A	0	303.40
gca	A	1551	684.00
gcg	A	5	172.70
gaa	E	1	0.60
gag	E	0	0.40
---	--	------	------
mPD		0.49	1.5
		nPD:	0.33
		N. weight:	1.3
		Sc. PD:	0.23
		Sc. rank:	1507.7

,

PA			
Pos.	344	obs:	exp:
gaa	E	2432	1495.00
gag	E	64	1001.00
---	--	------	------
mPD		0.050	0.48
		nPD:	0.1
		N. weight:	0.5
		Sc. PD:	-0.022
		Sc. rank:	294.7

,

PA			
Pos.	345	obs:	exp:
tta	L	0	229.30
ttg	L	0	394.20
ctt	L	7	394.00
ctc	L	2086	362.50
cta	L	7	279.50
ctg	L	1	441.40
att	I	0	146.70
atc	I	395	99.06
ata	I	0	149.30
---	--	------	------
mPD		0.28	1.3
		nPD:	0.21
		N. weight:	2.1
		Sc. PD:	0.13
		Sc. rank:	1620.6

,

PA

Pos.	346	obs:	exp:
cat	H	62	38.05
cac	H	2	25.95
caa	Q	2417	1237.00
cag	Q	14	1194.00
cgt	R	0	0.04
cgc	R	0	0.05
cga	R	1	0.10
cgg	R	0	0.08
aga	R	0	0.46
agg	R	0	0.27
---	--	------	------
mPD		0.062	0.53
		nPD:	0.12
		N. weight:	0.79
		Sc. PD:	-0.025
		Sc. rank:	474.6

PA

Pos.	347	obs:	exp:
aat	N	4	2.13
aac	N	0	1.87
gat	D	1891	1433.00
gac	D	601	1059.00
---	--	------	------
mPD		0.37	0.49
		nPD:	0.75
		N. weight:	0.095
		Sc. PD:	0.056
		Sc. rank:	157.2

PA

Pos.	348	obs:	exp:
tta	L	0	0.55
ttg	L	0	0.94
ctt	L	5	0.94
ctc	L	0	0.86
cta	L	0	0.67
ctg	L	0	1.05
att	I	2486	923.90
atc	I	2	624.00
ata	I	0	940.10
act	T	1	0.26
acc	T	0	0.22
aca	T	0	0.43
acg	T	0	0.08
gtt	V	2	0.43
gtc	V	0	0.42
gta	V	0	0.39
gtg	V	0	0.75
---	--	------	------
mPD		0.0080	0.66
		nPD:	0.01
		N. weight:	1.2
		Sc. PD:	-0.16
		Sc. rank:	-986.0

PA

Pos.	349	obs:	exp:
gaa	E	2122	1495.00
gag	E	374	1001.00
---	--	------	------
mPD		0.25	0.48
		nPD:	0.53
		N. weight:	0.19
		Sc. PD:	0.07
		Sc. rank:	273.8

PA

Pos.	350	obs:	exp:
tct	S	0	11.78
tcc	S	0	10.17
tca	S	0	15.55
tcg	S	0	4.46
act	T	32	9.54
acc	T	4	7.95
aca	T	0	15.47
acg	T	0	3.04
aat	N	1223	1267.00
aac	N	1162	1118.00
aaa	K	4	3.37
aag	K	2	2.63
agt	S	68	14.09
agc	S	1	12.95
---	--	------	------
mPD		0.58	0.63
		nPD:	0.92
		N. weight:	0.075
		Sc. PD:	0.057
		Sc. rank:	129.1

PA

Pos.	351	obs:	exp:
gat	D	0	0.58
gac	D	1	0.42
gaa	E	545	1485.00
gag	E	1935	995.10
ggt	G	0	2.16
ggc	G	0	1.98
gga	G	4	6.55
ggg	G	11	4.31
---	--	------	------
mPD		0.36	0.49
		nPD:	0.72
		N. weight:	0.38
		Sc. PD:	0.21
		Sc. rank:	608.3

PA

Pos.	352	obs:	exp:
aaa	K	0	0.56
aag	K	1	0.44
gat	D	1	0.58
gac	D	0	0.42
gaa	E	8	1493.00
gag	E	2486	1001.00
---	--	------	------
mPD		0.0080	0.48
		nPD:	0.02
		N. weight:	1.1
		Sc. PD:	-0.14
		Sc. rank:	-674.3

PA

Pos.	353	obs:	exp:
cgt	R	0	0.08
cgc	R	0	0.11
cga	R	0	0.19
cgg	R	0	0.16
act	T	0	23.05
acc	T	0	19.22
aca	T	87	37.38
acg	T	0	7.35
aaa	K	2392	1351.00
aag	K	15	1056.00
aga	R	2	0.92
agg	R	0	0.55
---	--	------	------
mPD		0.081	0.58
		nPD:	0.14
		N. weight:	0.69
		Sc. PD:	-0.0064
		Sc. rank:	454.1

PA

Pos.	354	obs:	exp:
att	I	701	896.80
atc	I	1714	605.70
ata	I	0	912.60
act	T	26	7.68
acc	T	3	6.41
aca	T	0	12.46
acg	T	0	2.45
gtt	V	0	11.31
gtc	V	52	10.99
gta	V	0	10.23
gtg	V	0	19.47
---	--	------	------
mPD		0.48	0.73
		nPD:	0.66
		N. weight:	0.86
		Sc. PD:	0.43
		Sc. rank:	1334.3

PA

Pos.	355	obs:	exp:
tct	S	0	0.17
tcc	S	0	0.15
tca	S	1	0.23
tcg	S	0	0.06
cct	P	10	703.60
ccc	P	0	451.50
cca	P	2253	1019.00
ccg	P	232	321.30
agt	S	0	0.20
agc	S	0	0.19
---	--	------	------
mPD		0.18	0.71
		nPD:	0.25
		N. weight:	0.84
		Sc. PD:	0.083
		Sc. rank:	712.3

PA

Pos.	356	obs:	exp:
cgt	R	0	0.08
cgc	R	0	0.11
cga	R	0	0.19
cgg	R	0	0.16
act	T	0	1.85
acc	T	0	1.55
aca	T	7	3.01
acg	T	0	0.59
aaa	K	2471	1396.00
aag	K	16	1091.00
aga	R	2	0.92
agg	R	0	0.55
---	--	------	------
mPD		0.020	0.50
		nPD:	0.04
		N. weight:	0.68
		Sc. PD:	-0.072
		Sc. rank:	147.4

PA

Pos.	357	obs:	exp:
act	T	0	660.90
acc	T	0	551.30
aca	T	2487	1072.00
acg	T	8	210.70
gct	A	0	0.25
gcc	A	0	0.20
gca	A	1	0.44
gcg	A	0	0.11
---	--	------	------
mPD		0.0072	0.69
		nPD:	0.01
		N. weight:	1.
		Sc. PD:	-0.14
		Sc. rank:	-927.5

PA

Pos.	358	obs:	exp:
cgt	R	0	0.30
cgc	R	0	0.43
cga	R	0	0.77
cgg	R	0	0.63
aaa	K	546	1397.00
aag	K	1942	1091.00
aga	R	0	3.67
agg	R	8	2.20
---	--	------	------
mPD		0.35	0.50
		nPD:	0.7
		N. weight:	0.31
		Sc. PD:	0.16
		Sc. rank:	488.8

PA

Pos.	359	obs:	exp:
tct	S	0	0.34
tcc	S	0	0.29
tca	S	0	0.45
tcg	S	0	0.13
act	T	0	4.50
acc	T	17	3.76
aca	T	0	7.30
acg	T	0	1.44
aat	N	2	1316.00
aac	N	2475	1161.00
agt	S	0	0.41
agc	S	2	0.38
---	--	------	------
mPD		0.017	0.52
		nPD:	0.03
		N. weight:	0.94
		Sc. PD:	-0.11
		Sc. rank:	85.4

PA

Pos.	360	obs:	exp:
atg	M	2496	2496.00
---	--	------	------
mPD		0	0
		nPD:	1.
		N. weight:	0.
		Sc. PD:	0
		Sc. rank:	0

PA

Pos.	361	obs:	exp:
cgt	R	0	12.70
cgc	R	0	17.84
cga	R	0	32.44
cgg	R	0	26.24
aat	N	2	1.06
aac	N	0	0.94
aaa	K	34	1212.00
aag	K	2125	947.10
aga	R	1	153.70
agg	R	334	92.11
---	--	------	------
mPD		0.26	0.81
		nPD:	0.33
		N. weight:	1.
		Sc. PD:	0.17
		Sc. rank:	1135.4

PA

Pos.	362	obs:	exp:
cgt	R	0	0.08
cgc	R	0	0.11
cga	R	0	0.19
cgg	R	0	0.16
aaa	K	1316	1400.00
aag	K	1178	1094.00
aga	R	1	0.92
agg	R	1	0.55
---	--	------	------
mPD		0.50	0.49
		nPD:	1.01
		N. weight:	0.0056
		Sc. PD:	0.0047
		Sc. rank:	9.7

PA

Pos.	363	obs:	exp:
act	T	0	661.20
acc	T	0	551.50
aca	T	1563	1073.00
acg	T	933	210.80
---	--	------	------
mPD		0.47	0.69
		nPD:	0.68
		N. weight:	0.99
		Sc. PD:	0.51
		Sc. rank:	1550.5

PA

Pos.	364	obs:	exp:
tct	S	0	425.90
tcc	S	0	367.70
tca	S	0	562.20
tcg	S	0	161.10
tgt	C	1	0.43
tgc	C	0	0.57
cgt	R	0	0.04
cgc	R	0	0.05
cga	R	0	0.10
cgg	R	0	0.08
agt	S	392	509.20
agc	S	2102	467.90
aga	R	1	0.46
agg	R	0	0.27
---	--	------	------
mPD		0.27	1.6
		nPD:	0.16
		N. weight:	1.5
		Sc. PD:	0.019
		Sc. rank:	1042.5

PA

Pos.	365	obs:	exp:
cat	H	2	1.78
cac	H	1	1.22
caa	Q	2485	1268.00
cag	Q	8	1225.00
---	--	------	------
mPD		0.0088	0.50
		nPD:	0.02
		N. weight:	0.82
		Sc. PD:	-0.1
		Sc. rank:	-458.6

PA			
Pos. 366		obs:	exp:
tta	L	7	272.40
ttg	L	772	468.40
ctt	L	0	468.00
ctc	L	0	430.60
cta	L	537	332.10
ctg	L	1180	524.40
---	--	------	------
mPD		0.77	1.1
		nPD:	0.68
		N. weight:	0.79
		Sc. PD:	0.41
		Sc. rank:	1237.2

PA			
Pos. 367		obs:	exp:
caa	Q	0	0.51
cag	Q	1	0.49
cgt	R	0	1.14
cgc	R	0	1.60
cga	R	0	2.90
cgg	R	0	2.35
aat	N	1	0.53
aac	N	0	0.47
aaa	K	8	1383.00
aag	K	2456	1081.00
aga	R	0	13.76
agg	R	30	8.25
---	--	------	------
mPD		0.032	0.52
		nPD:	0.06
		N. weight:	1.
		Sc. PD:	-0.087
		Sc. rank:	432.0

PA			
Pos. 368		obs:	exp:
tgg	W	2496	2496.00
---	--	------	------
mPD		0	0
		nPD:	1.
		N. weight:	0.
		Sc. PD:	0
		Sc. rank:	0

PA			
Pos. 369		obs:	exp:
tct	S	0	0.51
tcc	S	0	0.44
tca	S	3	0.68
tcg	S	0	0.19
agt	S	0	0.61
agc	S	0	0.56
gtt	V	0	0.43
gtc	V	0	0.42
gta	V	2	0.39
gtg	V	0	0.75
gct	A	0	633.70
gcc	A	0	485.80
gca	A	2482	1095.00
gcg	A	9	276.50
---	--	------	------
mPD		0.011	0.70
		nPD:	0.02
		N. weight:	1.
		Sc. PD:	-0.13
		Sc. rank:	-648.9

PA			
Pos. 370		obs:	exp:
tta	L	0	272.30
ttg	L	0	468.20
ctt	L	1940	467.90
ctc	L	554	430.50
cta	L	1	332.00
ctg	L	0	524.20
cgt	R	1	0.04
cgc	R	0	0.05
cga	R	0	0.10
cgg	R	0	0.08
aga	R	0	0.46
agg	R	0	0.27
---	--	------	------
mPD		0.35	1.1
		nPD:	0.31
		N. weight:	1.4
		Sc. PD:	0.22
		Sc. rank:	1482.1

PA			
Pos. 371		obs:	exp:
ggt	G	2494	359.10
ggc	G	0	329.40
gga	G	0	1090.00
ggg	G	2	717.90
---	--	------	------
mPD		0.0016	0.69
		nPD:	0.
		N. weight:	2.4
		Sc. PD:	-0.35
		Sc. rank:	-3633.9

PA			
Pos. 372		obs:	exp:
gaa	E	1176	1495.00
gag	E	1320	1001.00
---	--	------	------
mPD		0.50	0.48
		nPD:	1.04
		N. weight:	0.043
		Sc. PD:	0.038
		Sc. rank:	75.0

PA			
Pos. 373		obs:	exp:
aat	N	1329	1326.00
aac	N	1167	1170.00
---	--	------	------
mPD		0.50	0.50
		nPD:	1.
		N. weight:	0.0021
		Sc. PD:	0.0017
		Sc. rank:	3.6

PA			
Pos. 374		obs:	exp:
cgt	R	0	0.08
cgc	R	0	0.11
cga	R	0	0.19
cgg	R	0	0.16
att	I	0	0.74
atc	I	0	0.50
ata	I	2	0.76
atg	M	2491	2491.00
aga	R	0	0.92
agg	R	2	0.55
gtt	V	0	0.22
gtc	V	0	0.21
gta	V	0	0.20
gtg	V	1	0.37
---	--	------	------
mPD		0.0040	0.0060
		nPD:	0.67
		N. weight:	0.0028
		Sc. PD:	0.0014
		Sc. rank:	4.3

PA			
Pos. 375		obs:	exp:
gct	A	2	635.00
gcc	A	0	486.70
gca	A	2488	1097.00
gcg	A	6	277.10
---	--	------	------
mPD		0.0064	0.69
		nPD:	0.01
		N. weight:	1.
		Sc. PD:	-0.14
		Sc. rank:	-956.4

PA			
Pos. 376		obs:	exp:
cct	P	580	703.80
ccc	P	0	451.60
cca	P	1904	1019.00
ccg	P	12	321.40
---	--	------	------
mPD		0.36	0.70
		nPD:	0.52
		N. weight:	0.52
		Sc. PD:	0.19
		Sc. rank:	735.1

PA			
Pos. 377		obs:	exp:
gaa	E	597	1495.00
gag	E	1899	1001.00
---	--	------	------
mPD		0.36	0.48
		nPD:	0.76
		N. weight:	0.34
		Sc. PD:	0.2
		Sc. rank:	557.1

PA			
Pos. 378		obs:	exp:
cgt	R	0	0.15
cgc	R	0	0.21
cga	R	0	0.39
cgg	R	0	0.31
aaa	K	2384	1399.00
aag	K	108	1093.00
aga	R	4	1.83
agg	R	0	1.10
---	--	------	------
mPD		0.086	0.50
		nPD:	0.17
		N. weight:	0.51
		Sc. PD:	0.012
		Sc. rank:	353.2

PA			
Pos. 379		obs:	exp:
tta	L	0	0.33
ttg	L	3	0.56
ctt	L	0	0.56
ctc	L	0	0.52
cta	L	0	0.40
ctg	L	0	0.63
att	I	0	0.37
atc	I	0	0.25
ata	I	1	0.38
atg	M	107	107.00
act	T	0	0.26
acc	T	0	0.22
aca	T	0	0.43
acg	T	1	0.08
gtt	V	2	518.50
gtc	V	145	503.70
gta	V	544	469.10
gtg	V	1693	892.70
---	--	------	------
mPD		0.51	0.81
		nPD:	0.64
		N. weight:	0.49
		Sc. PD:	0.23
		Sc. rank:	754.4

PA			
Pos. 380		obs:	exp:
aat	N	0	2.13
aac	N	4	1.87
gat	D	10	1433.00
gac	D	2482	1059.00
---	--	------	------
mPD		0.011	0.49
		nPD:	0.02
		N. weight:	1.
		Sc. PD:	-0.13
		Sc. rank:	-338.4

PA			
Pos. 381		obs:	exp:
ttt	F	2471	1062.00
ttc	F	25	1434.00
---	--	------	------
mPD		0.020	0.49
		nPD:	0.04
		N. weight:	0.99
		Sc. PD:	-0.11
		Sc. rank:	229.8

PA			
Pos. 382		obs:	exp:
gtt	V	0	0.87
gtc	V	0	0.85
gta	V	0	0.79
gtg	V	4	1.50
gat	D	11	6.33
gac	D	0	4.67
gaa	E	11	1486.00
gag	E	2470	995.50
---	--	------	------
mPD		0.021	0.49
		nPD:	0.04
		N. weight:	1.1
		Sc. PD:	-0.11
		Sc. rank:	318.2

PA			
Pos. 383		obs:	exp:
tat	Y	0	0.49
tac	Y	1	0.51
aat	N	0	0.53
aac	N	1	0.47
gat	D	19	1430.00
gac	D	2467	1056.00
gaa	E	8	4.79
gag	E	0	3.21
---	--	------	------
mPD		0.023	0.49
		nPD:	0.05
		N. weight:	1.
		Sc. PD:	-0.1
		Sc. rank:	337.3

PA			
Pos. 384		obs:	exp:
tgt	C	1208	1085.00
tgc	C	1288	1411.00
---	--	------	------
mPD		0.50	0.49
		nPD:	1.02
		N. weight:	0.0081
		Sc. PD:	0.0069
		Sc. rank:	14.1

PA			
Pos. 385		obs:	exp:
cgt	R	0	20.55
cgc	R	0	28.87
cga	R	0	52.48
cgg	R	0	42.45
aaa	K	1939	1097.00
aag	K	15	857.20
aga	R	540	248.60
agg	R	2	149.00
---	--	------	------
mPD		0.35	0.96
		nPD:	0.37
		N. weight:	0.73
		Sc. PD:	0.16
		Sc. rank:	862.2

PA			
Pos.	386	obs:	exp:
gat	D	1781	1414.00
gac	D	677	1044.00
gaa	E	0	20.96
gag	E	35	14.04
ggt	G	0	0.43
ggc	G	3	0.40
gga	G	0	1.31
ggg	G	0	0.86
---	--	------	------
mPD		0.42	0.51
		nPD:	0.83
		N. weight:	0.08
		Sc. PD:	0.053
		Sc. rank:	136.1

PA			
Pos.	387	obs:	exp:
att	I	24	8.91
atc	I	0	6.02
ata	I	0	9.07
gtt	V	2461	537.60
gtc	V	10	522.30
gta	V	0	486.40
gtg	V	1	925.70
---	--	------	------
mPD		0.028	0.75
		nPD:	0.04
		N. weight:	1.9
		Sc. PD:	-0.2
		Sc. rank:	313.3

PA			
Pos.	388	obs:	exp:
tct	S	0	419.90
tcc	S	0	362.50
tca	S	0	554.30
tcg	S	0	158.80
cgt	R	0	0.04
cgc	R	0	0.05
cga	R	0	0.10
cgg	R	0	0.08
att	I	1	1.49
atc	I	3	1.00
ata	I	0	1.51
aat	N	0	1.06
aac	N	2	0.94
agt	S	11	502.10
agc	S	2448	461.40
aga	R	1	0.46
agg	R	0	0.27
ggt	G	1	4.32
ggc	G	29	3.96
gga	G	0	13.10
ggg	G	0	8.63
---	--	------	------
mPD		0.040	1.7
		nPD:	0.02
		N. weight:	2.1
		Sc. PD:	-0.25
		Sc. rank:	-620.1

PA			
Pos.	389	obs:	exp:
gat	D	2489	1436.00
gac	D	7	1060.00
---	--	------	------
mPD		0.0056	0.49
		nPD:	0.01
		N. weight:	0.67
		Sc. PD:	-0.09
		Sc. rank:	-569.2

PA			
Pos.	390	obs:	exp:
tta	L	5	272.40
ttg	L	4	468.40
ctt	L	5	468.00
ctc	L	2	430.60
cta	L	2475	332.10
ctg	L	5	524.40
---	--	------	------
mPD		0.020	1.1
		nPD:	0.02
		N. weight:	2.4
		Sc. PD:	-0.31
		Sc. rank:	-1332.8

PA			
Pos.	391	obs:	exp:
caa	Q	1	0.51
cag	Q	0	0.49
cgt	R	0	79.79
cgc	R	0	112.10
cga	R	289	203.70
cgg	R	0	164.80
aaa	K	359	219.50
aag	K	32	171.50
aga	R	1783	965.10
agg	R	32	578.50
---	--	------	------
mPD		0.52	1.2
		nPD:	0.45
		N. weight:	0.62
		Sc. PD:	0.18
		Sc. rank:	828.9

PA			
Pos.	392	obs:	exp:
caa	Q	290	1270.00
cag	Q	2206	1226.00
---	--	------	------
mPD		0.21	0.50
		nPD:	0.41
		N. weight:	0.43
		Sc. PD:	0.11
		Sc. rank:	542.9

PA			
Pos.	393	obs:	exp:
tat	Y	1300	1213.00
tac	Y	1196	1283.00
---	--	------	------
mPD		0.50	0.50
		nPD:	1.
		N. weight:	0.0051
		Sc. PD:	0.0042
		Sc. rank:	8.8

PA			
Pos.	394	obs:	exp:
aat	N	0	5.31
aac	N	10	4.69
gat	D	11	1300.00
gac	D	2249	960.00
gaa	E	0	132.90
gag	E	222	89.07
ggt	G	0	0.58
ggc	G	4	0.53
gga	G	0	1.75
ggg	G	0	1.15
---	--	------	------
mPD		0.18	0.58
		nPD:	0.31
		N. weight:	1.
		Sc. PD:	0.16
		Sc. rank:	1084.3

PA			
Pos.	395	obs:	exp:
tct	S	0	426.30
tcc	S	0	368.00
tca	S	0	562.60
tcg	S	0	161.20
agt	S	2481	509.60
agc	S	15	468.30
---	--	------	------
mPD		0.012	1.6
		nPD:	0.01
		N. weight:	1.9
		Sc. PD:	-0.27
		Sc. rank:	-2186.7

PA			
Pos.	396	obs:	exp:
aat	N	1	1.59
aac	N	2	1.41
gat	D	1293	1427.00
gac	D	1188	1054.00
gaa	E	4	2.40
gag	E	0	1.60
ggt	G	1	1.15
ggc	G	7	1.06
gga	G	0	3.49
ggg	G	0	2.30
---	--	------	------
mPD		0.51	0.50
		nPD:	1.02
		N. weight:	0.018
		Sc. PD:	0.015
		Sc. rank:	31.1

PA			
Pos.	397	obs:	exp:
aaa	K	1	0.56
aag	K	0	0.44
gat	D	1	0.58
gac	D	0	0.42
gaa	E	2179	1492.00
gag	E	312	999.50
ggt	G	0	0.43
ggc	G	0	0.40
gga	G	3	1.31
ggg	G	0	0.86
---	--	------	------
mPD		0.22	0.48
		nPD:	0.46
		N. weight:	0.23
		Sc. PD:	0.071
		Sc. rank:	321.0

PA			
Pos.	398	obs:	exp:
cct	P	7	703.80
ccc	P	1	451.60
cca	P	2477	1019.00
ccg	P	11	321.40
---	--	------	------
mPD		0.015	0.70
		nPD:	0.02
		N. weight:	1.1
		Sc. PD:	-0.13
		Sc. rank:	-394.7

PA			
Pos.	399	obs:	exp:
aaa	K	0	0.56
aag	K	1	0.44
gaa	E	732	1493.00
gag	E	1761	1000.00
ggt	G	0	0.29
ggc	G	0	0.26
gga	G	0	0.87
ggg	G	2	0.58
---	--	------	------
mPD		0.42	0.48
		nPD:	0.86
		N. weight:	0.24
		Sc. PD:	0.17
		Sc. rank:	411.9

PA			
Pos.	400	obs:	exp:
ttt	F	1	0.43
ttc	F	0	0.57
tct	S	1776	321.10
tcc	S	103	277.10
tca	S	0	423.80
tcg	S	1	121.40
cct	P	29	171.40
ccc	P	579	110.00
cca	P	0	248.20
ccg	P	0	78.29
caa	Q	7	3.56
cag	Q	0	3.44
agt	S	0	383.80
agc	S	0	352.70
---	--	------	------
mPD		0.78	1.8
		nPD:	0.44
		N. weight:	1.9
		Sc. PD:	0.55
		Sc. rank:	2555.3

PA			
Pos.	401	obs:	exp:
tct	S	0	0.34
tcc	S	0	0.29
tca	S	0	0.45
tcg	S	0	0.13
cgt	R	0	94.35
cgc	R	0	132.50
cga	R	3	240.90
cgg	R	0	194.90
aaa	K	3	1.68
aag	K	0	1.32
agt	S	2	0.41
agc	S	0	0.38
aga	R	2476	1141.00
agg	R	9	684.10
ggt	G	0	0.43
ggc	G	0	0.40
gga	G	3	1.31
ggg	G	0	0.86
---	--	------	------
mPD		0.016	0.96
		nPD:	0.02
		N. weight:	0.94
		Sc. PD:	-0.12
		Sc. rank:	-562.0

PA			
Pos.	402	obs:	exp:
tct	S	0	426.30
tcc	S	2	368.00
tca	S	1736	562.60
tcg	S	758	161.20
agt	S	0	509.60
agc	S	0	468.30
---	--	------	------
mPD		0.42	1.6
		nPD:	0.26
		N. weight:	1.6
		Sc. PD:	0.16
		Sc. rank:	1361.2

PA			
Pos.	403	obs:	exp:
tta	L	581	272.20
ttg	L	0	468.00
ctt	L	0	467.70
ctc	L	3	430.30
cta	L	1906	331.80
ctg	L	4	524.00
att	I	0	0.74
atc	I	0	0.50
ata	I	2	0.76
---	--	------	------
mPD		0.36	1.1
		nPD:	0.32
		N. weight:	1.9
		Sc. PD:	0.31
		Sc. rank:	2057.2

PA			
Pos.	404	obs:	exp:
tct	S	0	108.30
tcc	S	0	93.46
tca	S	634	142.90
tcg	S	0	40.95
act	T	0	0.79
acc	T	0	0.66
aca	T	3	1.29
acg	T	0	0.25
agt	S	0	129.40
agc	S	0	119.00
gtt	V	0	0.22
gtc	V	0	0.21
gta	V	1	0.20
gtg	V	0	0.37
gct	A	4	472.70
gcc	A	0	362.30
gca	A	1852	816.70
gcg	A	2	206.30
---	--	------	------
mPD		0.39	1.3
		nPD:	0.3
		N. weight:	1.2
		Sc. PD:	0.18
		Sc. rank:	1173.3

PA			
Pos.	405	obs:	exp:
tct	S	0	425.40
tcc	S	0	367.20
tca	S	0	561.50
tcg	S	0	160.90
att	I	1	0.37
atc	I	0	0.25
ata	I	0	0.38
aat	N	1	1.59
aac	N	2	1.41
agt	S	1167	508.60
agc	S	1324	467.40
ggt	G	0	0.14
ggc	G	1	0.13
gga	G	0	0.44
ggg	G	0	0.29
---	--	------	------
mPD		0.50	1.6
		nPD:	0.3
		N. weight:	1.2
		Sc. PD:	0.18
		Sc. rank:	1180.6

PA			
Pos.	406	obs:	exp:
tgg	W	2495	2495.00
ggt	G	0	0.14
ggc	G	0	0.13
gga	G	0	0.44
ggg	G	1	0.29
mPD		0.00080	0.0014
		nPD:	0.58
		N. weight:	0.00062
		Sc. PD:	0.00026
		Sc. rank:	0.9

PA			
Pos.	407	obs:	exp:
att	I	418	915.00
atc	I	2045	618.00
ata	I	1	931.10
gtt	V	2	6.96
gtc	V	30	6.76
gta	V	0	6.30
gtg	V	0	11.98
mPD		0.31	0.69
		nPD:	0.45
		N. weight:	1.1
		Sc. PD:	0.31
		Sc. rank:	1455.8

PA			
Pos.	408	obs:	exp:
caa	Q	39	1270.00
cag	Q	2457	1226.00
mPD		0.031	0.50
		nPD:	0.06
		N. weight:	0.79
		Sc. PD:	-0.067
		Sc. rank:	346.6

PA			
Pos.	409	obs:	exp:
tct	S	0	425.80
tcc	S	0	367.50
tca	S	0	562.00
tcg	S	0	161.00
agt	S	1247	509.00
agc	S	1246	467.70
ggt	G	0	0.43
ggc	G	3	0.40
gga	G	0	1.31
ggg	G	0	0.86
mPD		0.50	1.6
		nPD:	0.31
		N. weight:	1.2
		Sc. PD:	0.18
		Sc. rank:	1191.4

PA			
Pos.	410	obs:	exp:
gaa	E	2467	1495.00
gag	E	29	1001.00
mPD		0.023	0.48
		nPD:	0.05
		N. weight:	0.57
		Sc. PD:	-0.056
		Sc. rank:	193.1

PA			
Pos.	411	obs:	exp:
ttt	F	1104	1062.00
ttc	F	1392	1434.00
mPD		0.49	0.49
		nPD:	1.01
		N. weight:	0.0028
		Sc. PD:	0.0023
		Sc. rank:	4.8

PA			
Pos.	412	obs:	exp:
aat	N	8	1326.00
aac	N	2488	1170.00
mPD		0.0064	0.50
		nPD:	0.01
		N. weight:	0.92
		Sc. PD:	-0.12
		Sc. rank:	-689.8

PA			
Pos.	413	obs:	exp:
aaa	K	1256	1401.00
aag	K	1240	1095.00
mPD		0.50	0.49
		nPD:	1.02
		N. weight:	0.011
		Sc. PD:	0.0089
		Sc. rank:	18.2

PA			
Pos.	414	obs:	exp:
gct	A	1161	635.00
gcc	A	6	486.70
gca	A	1324	1097.00
gcg	A	5	277.10
mPD		0.50	0.69
		nPD:	0.73
		N. weight:	0.45
		Sc. PD:	0.26
		Sc. rank:	740.7

PA			
Pos.	415	obs:	exp:
tgt	C	22	1085.00
tgc	C	2474	1411.00
mPD		0.017	0.49
		nPD:	0.04
		N. weight:	0.65
		Sc. PD:	-0.072
		Sc. rank:	94.9

PA			
Pos.	416	obs:	exp:
gat	D	3	1.73
gac	D	0	1.27
gaa	E	2457	1493.00
gag	E	36	1000.00
mPD		0.031	0.48
		nPD:	0.06
		N. weight:	0.55
		Sc. PD:	-0.046
		Sc. rank:	249.9

PA			
Pos.	417	obs:	exp:
tta	L	77	272.20
ttg	L	1389	468.00
ctt	L	0	467.70
ctc	L	0	430.30
cta	L	2	331.80
ctg	L	1026	524.00
gtt	V	0	0.43
gtc	V	0	0.42
gta	V	0	0.39
gtg	V	2	0.75
mPD		0.55	1.1
		nPD:	0.48
		N. weight:	1.1
		Sc. PD:	0.34
		Sc. rank:	1454.4

PA			
Pos.	418	obs:	exp:
tct	S	0	0.34
tcc	S	0	0.29
tca	S	2	0.45
tcg	S	0	0.13
act	T	6	660.60
acc	T	12	551.00
aca	T	2473	1072.00
acg	T	3	210.70
agt	S	0	0.41
agc	S	0	0.38
mPD		0.018	0.69
		nPD:	0.03
		N. weight:	0.99
		Sc. PD:	-0.12
		Sc. rank:	-171.3

PA			
Pos.	419	obs:	exp:
aat	N	4	3.19
aac	N	2	2.81
gat	D	2475	1432.00
gac	D	15	1058.00
mPD		0.018	0.49
		nPD:	0.04
		N. weight:	0.65
		Sc. PD:	-0.071
		Sc. rank:	110.8

PA			
Pos.	420	obs:	exp:
tct	S	3	426.30
tcc	S	0	368.00
tca	S	754	562.60
tcg	S	1739	161.20
agt	S	0	509.60
agc	S	0	468.30
mPD		0.42	1.6
		nPD:	0.26
		N. weight:	2.2
		Sc. PD:	0.23
		Sc. rank:	1877.7

PA			
Pos.	421	obs:	exp:
tct	S	0	424.70
tcc	S	0	366.60
tca	S	0	560.60
tcg	S	0	160.60
tgt	C	2	0.87
tgc	C	0	1.13
cgt	R	0	0.04
cgc	R	0	0.05
cga	R	0	0.10
cgg	R	0	0.08
att	I	3	1.11
atc	I	0	0.75
ata	I	0	1.13
aat	N	1	0.53
aac	N	0	0.47
agt	S	2476	507.80
agc	S	11	466.60
aga	R	0	0.46
agg	R	1	0.27
ggt	G	2	0.29
ggc	G	0	0.26
gga	G	0	0.87
ggg	G	0	0.58
mPD		0.016	1.6
		nPD:	0.01
		N. weight:	1.9
		Sc. PD:	-0.26
		Sc. rank:	-1819.9

PA			
Pos.	422	obs:	exp:
tta	L	0	0.11
ttg	L	1	0.19
tgg	W	2495	2495.00
ctt	L	0	0.19
ctc	L	0	0.17
cta	L	0	0.13
ctg	L	0	0.21
mPD		0.00080	0.0018
		nPD:	0.43
		N. weight:	0.00084
		Sc. PD:	0.00023
		Sc. rank:	1.1

PA			
Pos.	423	obs:	exp:
tta	L	0	0.11
ttg	L	0	0.19
ctt	L	1	0.19
ctc	L	0	0.17
cta	L	0	0.13
ctg	L	0	0.21
att	I	2053	780.20
atc	I	33	526.90
ata	I	15	793.90
atg	M	4	4.00
gtt	V	389	84.82
gtc	V	1	82.40
gta	V	0	76.74
gtg	V	0	146.00
mPD		0.31	0.96
		nPD:	0.32
		N. weight:	1.2
		Sc. PD:	0.2
		Sc. rank:	1307.0

PA			
Pos.	424	obs:	exp:
gat	D	0	0.58
gac	D	1	0.42
gaa	E	2434	1494.00
gag	E	61	1001.00
mPD		0.048	0.48
		nPD:	0.1
		N. weight:	0.51
		Sc. PD:	-0.024
		Sc. rank:	291.8

PA			
Pos.	425	obs:	exp:
ttt	F	6	2.55
ttc	F	0	3.45
tta	L	0	271.80
ttg	L	0	467.20
ctt	L	2455	466.90
ctc	L	33	429.60
cta	L	1	331.30
ctg	L	1	523.20
mPD		0.032	1.1
		nPD:	0.03
		N. weight:	2.
		Sc. PD:	-0.23
		Sc. rank:	-201.9

PA			
Pos.	426	obs:	exp:
gat	D	2493	1434.00
gac	D	0	1059.00
gaa	E	3	1.80
gag	E	0	1.20
mPD		0.0024	0.49
		nPD:	0.
		N. weight:	0.69
		Sc. PD:	-0.097
		Sc. rank:	-874.8

PA			
Pos.	427	obs:	exp:
gaa	E	2077	1495.00
gag	E	419	1001.00
mPD		0.28	0.48
		nPD:	0.58
		N. weight:	0.16
		Sc. PD:	0.068
		Sc. rank:	239.7

PA			
Pos.	428	obs:	exp:
att	I	2	926.50
atc	I	1	625.70
ata	I	2492	942.80
gtt	V	0	0.22
gtc	V	0	0.21
gta	V	1	0.20
gtg	V	0	0.37
mPD		0.0032	0.66
		nPD:	0.
		N. weight:	1.2
		Sc. PD:	-0.17
		Sc. rank:	-1542.2

PA			
Pos.	429	obs:	exp:
tgg	W	2	2.00
ggt	G	0	358.80
ggc	G	0	329.20
gga	G	582	1089.00
ggg	G	1912	717.30
mPD		0.36	0.69
		nPD:	0.52
		N. weight:	0.76
		Sc. PD:	0.27
		Sc. rank:	1071.3

PA			
Pos.	430	obs:	exp:
gaa	E	2469	1495.00
gag	E	27	1001.00
mPD		0.021	0.48
		nPD:	0.04
		N. weight:	0.57
		Sc. PD:	-0.058
		Sc. rank:	178.9

PA			
Pos.	431	obs:	exp:
gat	D	621	1436.00
gac	D	1875	1060.00
---	--	------	------
mPD		0.37	0.49
		nPD:	0.77
		N. weight:	0.28
		Sc. PD:	0.17
		Sc. rank:	464.1

PA			
Pos.	432	obs:	exp:
att	I	106	39.36
atc	I	0	26.58
ata	I	0	40.05
gtt	V	2386	519.10
gtc	V	1	504.30
gta	V	0	469.70
gtg	V	0	893.90
gct	A	3	0.76
gcc	A	0	0.59
gca	A	0	1.32
gcg	A	0	0.33
---	--	------	------
mPD		0.085	0.82
		nPD:	0.1
		N. weight:	1.9
		Sc. PD:	-0.084
		Sc. rank:	1086.3

PA			
Pos.	433	obs:	exp:
gct	A	2198	635.00
gcc	A	297	486.70
gca	A	1	1097.00
gcg	A	0	277.10
---	--	------	------
mPD		0.21	0.69
		nPD:	0.3
		N. weight:	1.3
		Sc. PD:	0.19
		Sc. rank:	1285.6

PA			
Pos.	434	obs:	exp:
tct	S	0	0.17
tcc	S	0	0.15
tca	S	1	0.23
tcg	S	0	0.06
cct	P	0	703.60
ccc	P	0	451.50
cca	P	2165	1019.00
ccg	P	330	321.30
agt	S	0	0.20
agc	S	0	0.19
---	--	------	------
mPD		0.23	0.71
		nPD:	0.33
		N. weight:	0.82
		Sc. PD:	0.14
		Sc. rank:	930.8

PA			
Pos.	435	obs:	exp:
att	I	2479	926.10
atc	I	15	625.50
ata	I	0	942.40
gtt	V	2	0.43
gtc	V	0	0.42
gta	V	0	0.39
gtg	V	0	0.75
---	--	------	------
mPD		0.014	0.66
		nPD:	0.02
		N. weight:	1.2
		Sc. PD:	-0.15
		Sc. rank:	-497.7

PA			
Pos.	436	obs:	exp:
gaa	E	1617	1495.00
gag	E	879	1001.00
---	--	------	------
mPD		0.46	0.48
		nPD:	0.95
		N. weight:	0.0084
		Sc. PD:	0.0065
		Sc. rank:	14.5

PA			
Pos.	437	obs:	exp:
tat	Y	0	1.46
tac	Y	3	1.54
cat	H	0	1482.00
cac	H	2493	1011.00
---	--	------	------
mPD		0.0024	0.48
		nPD:	0.
		N. weight:	1.1
		Sc. PD:	-0.16
		Sc. rank:	-1421.8

PA			
Pos.	438	obs:	exp:
att	I	2480	926.10
atc	I	13	625.50
ata	I	1	942.40
gtt	V	2	0.43
gtc	V	0	0.42
gta	V	0	0.39
gtg	V	0	0.75
---	--	------	------
mPD		0.013	0.66
		nPD:	0.02
		N. weight:	1.2
		Sc. PD:	-0.15
		Sc. rank:	-560.1

PA			
Pos.	439	obs:	exp:
gct	A	0	635.00
gcc	A	0	486.70
gca	A	33	1097.00
gcg	A	2463	277.10
---	--	------	------
mPD		0.026	0.69
		nPD:	0.04
		N. weight:	2.6
		Sc. PD:	-0.29
		Sc. rank:	509.7

PA			
Pos.	440	obs:	exp:
tct	S	0	426.30
tcc	S	0	368.00
tca	S	0	562.60
tcg	S	0	161.20
agt	S	2471	509.60
agc	S	25	468.30
---	--	------	------
mPD		0.020	1.6
		nPD:	0.01
		N. weight:	1.9
		Sc. PD:	-0.26
		Sc. rank:	-1544.5

PA			
Pos.	441	obs:	exp:
att	I	8	4.46
atc	I	0	3.01
ata	I	4	4.53
atg	M	2483	2483.00
gtt	V	0	0.22
gtc	V	0	0.21
gta	V	0	0.20
gtg	V	1	0.37
---	--	------	------
mPD		0.010	0.011
		nPD:	0.95
		N. weight:	0.0033
		Sc. PD:	0.0026
		Sc. rank:	5.7

PA			
Pos.	442	obs:	exp:
cgt	R	0	94.66
cgc	R	0	133.00
cga	R	0	241.70
cgg	R	1	195.50
aga	R	1244	1145.00
agg	R	1251	686.30
---	--	------	------
mPD		0.50	0.95
		nPD:	0.53
		N. weight:	0.43
		Sc. PD:	0.16
		Sc. rank:	610.2

PA			
Pos.	443	obs:	exp:
cgt	R	0	94.66
cgc	R	0	133.00
cga	R	35	241.70
cgg	R	1	195.50
aga	R	1230	1145.00
agg	R	1230	686.30
---	--	------	------
mPD		0.53	0.95
		nPD:	0.55
		N. weight:	0.37
		Sc. PD:	0.15
		Sc. rank:	545.8

PA			
Pos.	444	obs:	exp:
tct	S	0	0.17
tcc	S	0	0.15
tca	S	0	0.23
tcg	S	0	0.06
aat	N	19	1326.00
aac	N	2476	1169.00
agt	S	0	0.20
agc	S	1	0.19
---	--	------	------
mPD		0.016	0.50
		nPD:	0.03
		N. weight:	0.89
		Sc. PD:	-0.1
		Sc. rank:	59.7

PA			
Pos.	445	obs:	exp:
ttt	F	1	0.43
ttc	F	0	0.57
tat	Y	1920	1213.00
tac	Y	575	1282.00
---	--	------	------
mPD		0.36	0.50
		nPD:	0.71
		N. weight:	0.21
		Sc. PD:	0.12
		Sc. rank:	339.7

PA			
Pos.	446	obs:	exp:
ttt	F	15	1062.00
ttc	F	2481	1434.00
---	--	------	------
mPD		0.012	0.49
		nPD:	0.02
		N. weight:	0.65
		Sc. PD:	-0.079
		Sc. rank:	-179.7

PA			
Pos.	447	obs:	exp:
act	T	0	660.60
acc	T	4	551.00
aca	T	2490	1072.00
acg	T	0	210.70
aaa	K	2	1.12
aag	K	0	0.88
---	--	------	------
mPD		0.0048	0.69
		nPD:	0.01
		N. weight:	1.
		Sc. PD:	-0.14
		Sc. rank:	-1194.9

PA			
Pos.	448	obs:	exp:
tct	S	0	0.68
tcc	S	0	0.59
tca	S	4	0.90
tcg	S	0	0.26
act	T	0	0.53
acc	T	0	0.44
aca	T	0	0.86
acg	T	2	0.17
agt	S	0	0.82
agc	S	0	0.75
gct	A	6	632.90
gcc	A	2	485.20
gca	A	361	1094.00
gcg	A	2119	276.20
gaa	E	0	1.20
gag	E	2	0.80
---	--	------	------
mPD		0.26	0.70
		nPD:	0.37
		N. weight:	1.9
		Sc. PD:	0.43
		Sc. rank:	2329.1

PA			
Pos.	449	obs:	exp:
gaa	E	2474	1495.00
gag	E	22	1001.00
---	--	------	------
mPD		0.017	0.48
		nPD:	0.04
		N. weight:	0.58
		Sc. PD:	-0.064
		Sc. rank:	90.6

PA			
Pos.	450	obs:	exp:
att	I	0	14.11
atc	I	0	9.53
ata	I	38	14.36
gtt	V	5	534.60
gtc	V	0	519.40
gta	V	1814	483.60
gtg	V	639	920.40
---	--	------	------
mPD		0.41	0.76
		nPD:	0.54
		N. weight:	1.1
		Sc. PD:	0.42
		Sc. rank:	1598.5

PA			
Pos.	451	obs:	exp:
ttt	F	1	0.43
ttc	F	0	0.57
tct	S	633	426.10
tcc	S	1861	367.80
tca	S	1	562.40
tcg	S	0	161.20
agt	S	0	509.40
agc	S	0	468.10
---	--	------	------
mPD		0.38	1.6
		nPD:	0.23
		N. weight:	1.6
		Sc. PD:	0.13
		Sc. rank:	1309.3

PA			
Pos.	452	obs:	exp:
cat	H	2472	1483.00
cac	H	23	1012.00
cgt	R	1	0.04
cgc	R	0	0.05
cga	R	0	0.10
cgg	R	0	0.08
aga	R	0	0.46
agg	R	0	0.27
---	--	------	------
mPD		0.019	0.48
		nPD:	0.04
		N. weight:	0.59
		Sc. PD:	-0.063
		Sc. rank:	126.7

PA			
Pos.	453	obs:	exp:
ttt	F	0	0.43
ttc	F	1	0.57
tct	S	0	7.00
tcc	S	0	6.04
tca	S	0	9.24
tcg	S	0	2.65
tgt	C	3	1067.00
tgc	C	2451	1387.00
agt	S	0	8.37
agc	S	41	7.69
---	--	------	------
mPD		0.036	0.53
		nPD:	0.07
		N. weight:	0.72
		Sc. PD:	-0.058
		Sc. rank:	339.2

PA			
Pos.	454	obs:	exp:
cgt	R	0	94.66
cgc	R	0	133.00
cga	R	0	241.70
cgg	R	0	195.50
aga	R	21	1145.00
agg	R	2475	686.30
---	--	------	------
mPD		0.017	0.95
		nPD:	0.02
		N. weight:	1.5
		Sc. PD:	-0.2
		Sc. rank:	-881.3

PA			
Pos.	455	obs:	exp:
cct	P	0	0.28
ccc	P	1	0.18
cca	P	0	0.41
ccg	P	0	0.13
gct	A	2083	634.70
gcc	A	310	486.50
gca	A	101	1097.00
gcg	A	1	277.00
---	--	------	------
mPD		0.29	0.69
		nPD:	0.41
		N. weight:	1.
		Sc. PD:	0.27
		Sc. rank:	1313.3

PA			
Pos.	456	obs:	exp:
cct	P	1	0.28
ccc	P	0	0.18
cca	P	0	0.41
ccg	P	0	0.13
act	T	1329	660.90
acc	T	3	551.30
aca	T	1162	1072.00
acg	T	1	210.70
---	--	------	------
mPD		0.50	0.69
		nPD:	0.73
		N. weight:	0.5
		Sc. PD:	0.28
		Sc. rank:	815.4

PA			
Pos.	457	obs:	exp:
gaa	E	2342	1495.00
gag	E	154	1001.00
---	--	------	------
mPD		0.12	0.48
		nPD:	0.24
		N. weight:	0.38
		Sc. PD:	0.034
		Sc. rank:	314.8

PA			
Pos.	458	obs:	exp:
tct	S	0	0.17
tcc	S	1	0.15
tca	S	0	0.23
tcg	S	0	0.06
tat	Y	878	1213.00
tac	Y	1617	1282.00
agt	S	0	0.20
agc	S	0	0.19
---	--	------	------
mPD		0.46	0.50
		nPD:	0.91
		N. weight:	0.049
		Sc. PD:	0.036
		Sc. rank:	83.9

PA			
Pos.	459	obs:	exp:
tta	L	2	0.22
ttg	L	0	0.38
ctt	L	0	0.38
ctc	L	0	0.35
cta	L	0	0.27
ctg	L	0	0.42
att	I	0	925.70
atc	I	0	625.20
ata	I	2493	942.00
gtt	V	0	0.22
gtc	V	0	0.21
gta	V	1	0.20
gtg	V	0	0.37
---	--	------	------
mPD		0.0024	0.66
		nPD:	0.
		N. weight:	1.2
		Sc. PD:	-0.17
		Sc. rank:	-1672.0

PA			
Pos.	460	obs:	exp:
att	I	0	0.37
atc	I	0	0.25
ata	I	1	0.38
atg	M	2494	2494.00
gtt	V	0	0.22
gtc	V	0	0.21
gta	V	0	0.20
gtg	V	1	0.37
---	--	------	------
mPD		0.0016	0.0021
		nPD:	0.76
		N. weight:	0.00098
		Sc. PD:	0.00058
		Sc. rank:	1.6

PA			
Pos.	461	obs:	exp:
aaa	K	2022	1401.00
aag	K	474	1095.00
---	--	------	------
mPD		0.31	0.49
		nPD:	0.63
		N. weight:	0.17
		Sc. PD:	0.081
		Sc. rank:	263.0

PA			
Pos.	462	obs:	exp:
ggt	G	4	359.10
ggc	G	0	329.40
gga	G	2486	1090.00
ggg	G	6	717.90
---	--	------	------
mPD		0.0080	0.69
		nPD:	0.01
		N. weight:	1.
		Sc. PD:	-0.13
		Sc. rank:	-842.4

PA			
Pos.	463	obs:	exp:
tta	L	1	0.11
ttg	L	0	0.19
ctt	L	0	0.19
ctc	L	0	0.17
cta	L	0	0.13
ctg	L	0	0.21
gtt	V	541	542.60
gtc	V	6	527.20
gta	V	1213	490.90
gtg	V	735	934.30
---	--	------	------
mPD		0.63	0.73
		nPD:	0.86
		N. weight:	0.45
		Sc. PD:	0.31
		Sc. rank:	774.2

PA			
Pos.	464	obs:	exp:
tat	Y	583	1206.00
tac	Y	1898	1275.00
cat	H	0	8.92
cac	H	15	6.08
---	--	------	------
mPD		0.37	0.51
		nPD:	0.72
		N. weight:	0.17
		Sc. PD:	0.098
		Sc. rank:	281.5

PA			
Pos.	465	obs:	exp:
att	I	0	912.70
atc	I	0	616.50
ata	I	2458	928.80
atg	M	1	1.00
gtt	V	0	0.22
gtc	V	0	0.21
gta	V	1	0.20
gtg	V	0	0.37
gct	A	0	9.16
gcc	A	0	7.02
gca	A	36	15.83
gcg	A	0	4.00
---	--	------	------
mPD		0.058	0.72
		nPD:	0.08
		N. weight:	1.2
		Sc. PD:	-0.08
		Sc. rank:	633.1

PA			
Pos.	466	obs:	exp:
act	T	0	0.26
acc	T	1	0.22
aca	T	0	0.43
acg	T	0	0.08
aat	N	592	1326.00
aac	N	1903	1169.00
---	--	------	------
mPD		0.36	0.50
		nPD:	0.73
		N. weight:	0.23
		Sc. PD:	0.13
		Sc. rank:	370.6

PA			
Pos.	467	obs:	exp:
cct	P	0	0.28
ccc	P	0	0.18
cca	P	1	0.41
ccg	P	0	0.13
act	T	3	660.60
acc	T	2	551.00
aca	T	2487	1072.00
acg	T	2	210.70
gct	A	0	0.25
gcc	A	0	0.20
gca	A	1	0.44
gcg	A	0	0.11
---	--	------	------
mPD		0.0072	0.69
		nPD:	0.01
		N. weight:	1.
		Sc. PD:	-0.14
		Sc. rank:	-919.5

PA			
Pos.	468	obs:	exp:
gct	A	1330	635.00
gcc	A	1151	486.70
gca	A	15	1097.00
gcg	A	0	277.10
---	--	------	------
mPD		0.50	0.69
		nPD:	0.73
		N. weight:	0.96
		Sc. PD:	0.54
		Sc. rank:	1566.0

PA			
Pos.	469	obs:	exp:
tta	L	1159	272.10
ttg	L	15	467.80
ctt	L	0	467.50
ctc	L	1	430.10
cta	L	55	331.70
ctg	L	1263	523.80
atg	M	3	3.00
---	--	------	------
mPD		1.0	1.1
		nPD:	0.88
		N. weight:	1.3
		Sc. PD:	0.94
		Sc. rank:	2273.9

PA			
Pos.	470	obs:	exp:
tta	L	5	272.40
ttg	L	2470	468.40
ctt	L	0	468.00
ctc	L	17	430.60
cta	L	0	332.10
ctg	L	4	524.40
---	--	------	------
mPD		0.034	1.1
		nPD:	0.03
		N. weight:	2.
		Sc. PD:	-0.23
		Sc. rank:	43.9

PA			
Pos.	471	obs:	exp:
aat	N	2494	1326.00
aac	N	2	1170.00
---	--	------	------
mPD		0.0016	0.50
		nPD:	0.
		N. weight:	0.78
		Sc. PD:	-0.11
		Sc. rank:	-1141.5

PA			
Pos.	472	obs:	exp:
tct	S	0	92.56
tcc	S	0	79.90
tca	S	539	122.20
tcg	S	3	35.01
agt	S	0	110.70
agc	S	0	101.70
gct	A	6	497.10
gcc	A	4	381.00
gca	A	1935	858.90
gcg	A	9	216.90
---	--	------	------
mPD		0.36	1.2
		nPD:	0.29
		N. weight:	1.1
		Sc. PD:	0.16
		Sc. rank:	1098.4

PA			
Pos.	473	obs:	exp:
tct	S	16	426.10
tcc	S	2477	367.80
tca	S	2	562.40
tcg	S	0	161.20
agt	S	0	509.40
agc	S	0	468.10
gct	A	0	0.25
gcc	A	1	0.20
gca	A	0	0.44
gcg	A	0	0.11
---	--	------	------
mPD		0.015	1.6
		nPD:	0.01
		N. weight:	2.3
		Sc. PD:	-0.32
		Sc. rank:	-2282.7

PA			
Pos.	474	obs:	exp:
tct	S	0	0.85
tcc	S	0	0.74
tca	S	0	1.13
tcg	S	0	0.32
tgt	C	2489	1083.00
tgc	C	2	1408.00
agt	S	5	1.02
agc	S	0	0.94
---	--	------	------
mPD		0.0056	0.50
		nPD:	0.01
		N. weight:	1.
		Sc. PD:	-0.14
		Sc. rank:	-883.8

PA			
Pos.	475	obs:	exp:
act	T	0	8.74
acc	T	0	7.29
aca	T	33	14.18
acg	T	0	2.79
gct	A	29	626.60
gcc	A	1	480.30
gca	A	2409	1083.00
gcg	A	24	273.40
---	--	------	------
mPD		0.069	0.72
		nPD:	0.1
		N. weight:	0.9
		Sc. PD:	-0.047
		Sc. rank:	511.6

PA

Pos.	476	obs:	exp:
tct	S	0	0.17
tcc	S	1	0.15
tca	S	0	0.23
tcg	S	0	0.06
agt	S	0	0.20
agc	S	0	0.19
gtt	V	0	0.22
gtc	V	1	0.21
gta	V	0	0.20
gtg	V	0	0.37
gct	A	9	634.50
gcc	A	2473	486.30
gca	A	12	1096.00
gcg	A	0	276.90
---	--	------	------
mPD		0.018	0.69
		nPD:	0.03
		N. weight:	2.
		Sc. PD:	-0.24
		Sc. rank:	-348.3

,

PA

Pos.	477	obs:	exp:
tta	L	0	0.11
ttg	L	0	0.19
ctt	L	0	0.19
ctc	L	0	0.17
cta	L	0	0.13
ctg	L	1	0.21
atg	M	2495	2495.00
---	--	------	------
mPD		0.00080	0.0013
		nPD:	0.62
		N. weight:	0.00078
		Sc. PD:	0.00036
		Sc. rank:	1.2

,

PA

Pos.	478	obs:	exp:
tat	Y	1	0.49
tac	Y	0	0.51
gtt	V	1	0.22
gtc	V	0	0.21
gta	V	0	0.20
gtg	V	0	0.37
gat	D	2458	1435.00
gac	D	36	1059.00
---	--	------	------
mPD		0.030	0.49
		nPD:	0.06
		N. weight:	0.6
		Sc. PD:	-0.052
		Sc. rank:	263.3

,

PA

Pos.	479	obs:	exp:
aat	N	0	0.53
aac	N	1	0.47
gat	D	55	1434.00
gac	D	2438	1059.00
gaa	E	2	1.20
gag	E	0	0.80
---	--	------	------
mPD		0.045	0.49
		nPD:	0.09
		N. weight:	0.93
		Sc. PD:	-0.051
		Sc. rank:	506.5

,

PA

Pos.	480	obs:	exp:
ttt	F	15	1062.00
ttc	F	2480	1433.00
atg	M	1	1.00
---	--	------	------
mPD		0.014	0.49
		nPD:	0.03
		N. weight:	0.65
		Sc. PD:	-0.077
		Sc. rank:	-86.5

,

PA

Pos.	481	obs:	exp:
caa	Q	2479	1270.00
cag	Q	17	1226.00
---	--	------	------
mPD		0.014	0.50
		nPD:	0.03
		N. weight:	0.79
		Sc. PD:	-0.095
		Sc. rank:	-133.6

,

PA

Pos.	482	obs:	exp:
tta	L	0	272.20
ttg	L	588	468.00
tat	Y	1	0.49
tac	Y	0	0.51
ctt	L	4	467.70
ctc	L	0	430.30
cta	L	34	331.80
ctg	L	1868	524.00
atg	M	1	1.00
---	--	------	------
mPD		0.39	1.1
		nPD:	0.35
		N. weight:	1.2
		Sc. PD:	0.23
		Sc. rank:	1405.4

,

PA

Pos.	483	obs:	exp:
att	I	2483	926.90
atc	I	12	626.00
ata	I	1	943.20
---	--	------	------
mPD		0.010	0.66
		nPD:	0.02
		N. weight:	1.2
		Sc. PD:	-0.16
		Sc. rank:	-779.2

,

PA

Pos.	484	obs:	exp:
cct	P	0	703.60
ccc	P	0	451.50
cca	P	2491	1019.00
ccg	P	4	321.30
gct	A	0	0.25
gcc	A	0	0.20
gca	A	1	0.44
gcg	A	0	0.11
---	--	------	------
mPD		0.0040	0.71
		nPD:	0.01
		N. weight:	1.1
		Sc. PD:	-0.15
		Sc. rank:	-1352.4

,

PA

Pos.	485	obs:	exp:
tgg	W	1	1.00
cgt	R	0	0.04
cgc	R	0	0.05
cga	R	0	0.10
cgg	R	0	0.08
att	I	0	11.88
atc	I	0	8.03
ata	I	32	12.09
atg	M	2462	2462.00
aga	R	0	0.46
agg	R	1	0.27
---	--	------	------
mPD		0.028	0.029
		nPD:	0.97
		N. weight:	0.016
		Sc. PD:	0.013
		Sc. rank:	28.0

,

PA

Pos.	486	obs:	exp:
tta	L	1	0.11
ttg	L	0	0.19
ctt	L	0	0.19
ctc	L	0	0.17
cta	L	0	0.13
ctg	L	0	0.21
att	I	0	926.10
atc	I	0	625.50
ata	I	2494	942.40
gtt	V	0	0.22
gtc	V	0	0.21
gta	V	1	0.20
gtg	V	0	0.37
---	--	------	------
mPD		0.0016	0.66
		nPD:	0.
		N. weight:	1.2
		Sc. PD:	-0.17
		Sc. rank:	-1779.7

,

PA

Pos.	487	obs:	exp:
tct	S	0	425.90
tcc	S	0	367.70
tca	S	0	562.20
tcg	S	0	161.10
tgt	C	0	0.43
tgc	C	1	0.57
aat	N	0	0.53
aac	N	1	0.47
agt	S	3	509.20
agc	S	2491	467.90
---	--	------	------
mPD		0.0040	1.6
		nPD:	0.
		N. weight:	2.1
		Sc. PD:	-0.3
		Sc. rank:	-3064.7

,

PA

Pos.	488	obs:	exp:
aaa	K	1362	1401.00
aag	K	1134	1095.00
---	--	------	------
mPD		0.50	0.49
		nPD:	1.01
		N. weight:	0.0027
		Sc. PD:	0.0022
		Sc. rank:	4.6

,

PA

Pos.	489	obs:	exp:
tgt	C	13	1085.00
tgc	C	2483	1411.00
---	--	------	------
mPD		0.010	0.49
		nPD:	0.02
		N. weight:	0.67
		Sc. PD:	-0.084
		Sc. rank:	-264.1

,

PA

Pos.	490	obs:	exp:
cgt	R	0	94.58
cgc	R	0	132.80
cga	R	1	241.50
cgg	R	0	195.30
aaa	K	2	1.12
aag	K	0	0.88
aga	R	2475	1144.00
agg	R	18	685.80
---	--	------	------
mPD		0.017	0.95
		nPD:	0.02
		N. weight:	0.92
		Sc. PD:	-0.12
		Sc. rank:	-521.2

,

PA

Pos.	491	obs:	exp:
cct	P	0	0.28
ccc	P	1	0.18
cca	P	0	0.41
ccg	P	0	0.13
act	T	5	660.10
acc	T	2482	550.60
aca	T	5	1071.00
acg	T	0	210.50
aat	N	0	1.59
aac	N	3	1.41
---	--	------	------
mPD		0.011	0.69
		nPD:	0.02
		N. weight:	1.8
		Sc. PD:	-0.24
		Sc. rank:	-1176.0

,

PA

Pos.	492	obs:	exp:
cgt	R	0	0.08
cgc	R	0	0.11
cga	R	0	0.19
cgg	R	0	0.16
aaa	K	2381	1400.00
aag	K	113	1094.00
aga	R	2	0.92
agg	R	0	0.55
---	--	------	------
mPD		0.088	0.49
		nPD:	0.18
		N. weight:	0.51
		Sc. PD:	0.014
		Sc. rank:	349.4

,

PA

Pos.	493	obs:	exp:
gaa	E	2493	1495.00
gag	E	3	1001.00
---	--	------	------
mPD		0.0024	0.48
		nPD:	0.
		N. weight:	0.63
		Sc. PD:	-0.089
		Sc. rank:	-790.4

,

PA

Pos.	494	obs:	exp:
ggt	G	1	359.10
ggc	G	1	329.40
gga	G	1311	1090.00
ggg	G	1183	717.90
---	--	------	------
mPD		0.50	0.69
		nPD:	0.73
		N. weight:	0.41
		Sc. PD:	0.23
		Sc. rank:	670.7

,

PA

Pos.	495	obs:	exp:
cgt	R	0	94.66
cgc	R	0	133.00
cga	R	11	241.70
cgg	R	6	195.50
aga	R	2184	1145.00
agg	R	295	686.30
---	--	------	------
mPD		0.23	0.95
		nPD:	0.24
		N. weight:	0.56
		Sc. PD:	0.047
		Sc. rank:	456.2

,

PA			
Pos.	496	obs:	exp:
tgg	W	1	1.00
cgt	R	2	94.62
cgc	R	0	132.90
cga	R	16	241.60
cgg	R	2474	195.40
aga	R	0	1144.00
agg	R	3	686.00
---	--	------	------
mPD		0.018	0.95
		nPD:	0.02
		N. weight:	3.1
		Sc. PD:	-0.4
		Sc. rank:	-1585.1

PA			
Pos.	497	obs:	exp:
cgt	R	0	0.08
cgc	R	0	0.11
cga	R	0	0.19
cgg	R	0	0.16
aaa	K	26	1400.00
aag	K	2468	1094.00
aga	R	0	0.92
agg	R	2	0.55
---	--	------	------
mPD		0.022	0.49
		nPD:	0.04
		N. weight:	0.95
		Sc. PD:	-0.097
		Sc. rank:	302.9

PA			
Pos.	498	obs:	exp:
act	T	1	661.20
acc	T	0	551.50
aca	T	2492	1073.00
acg	T	3	210.80
---	--	------	------
mPD		0.0032	0.69
		nPD:	0.
		N. weight:	1.
		Sc. PD:	-0.15
		Sc. rank:	-1370.6

PA			
Pos.	499	obs:	exp:
tct	S	0	0.17
tcc	S	0	0.15
tca	S	0	0.23
tcg	S	0	0.06
aat	N	2487	1325.00
aac	N	7	1169.00
aaa	K	0	0.56
aag	K	1	0.44
agt	S	1	0.20
agc	S	0	0.19
---	--	------	------
mPD		0.0072	0.50
		nPD:	0.01
		N. weight:	0.77
		Sc. PD:	-0.1
		Sc. rank:	-544.8

PA			
Pos.	500	obs:	exp:
tta	L	79	272.40
ttg	L	10	468.40
ctt	L	0	468.00
ctc	L	3	430.60
cta	L	526	332.10
ctg	L	1878	524.40
---	--	------	------
mPD		0.44	1.1
		nPD:	0.39
		N. weight:	1.2
		Sc. PD:	0.29
		Sc. rank:	1515.5

PA			
Pos.	501	obs:	exp:
tat	Y	2491	1213.00
tac	Y	5	1283.00
---	--	------	------
mPD		0.0040	0.50
		nPD:	0.01
		N. weight:	0.88
		Sc. PD:	-0.12
		Sc. rank:	-938.3

PA			
Pos.	502	obs:	exp:
ggt	G	2	359.10
ggc	G	1156	329.40
gga	G	167	1090.00
ggg	G	1171	717.90
---	--	------	------
mPD		0.56	0.69
		nPD:	0.81
		N. weight:	0.85
		Sc. PD:	0.55
		Sc. rank:	1451.1

PA			
Pos.	503	obs:	exp:
ttt	F	2	1012.00
ttc	F	2376	1366.00
tat	Y	0	57.36
tac	Y	118	60.64
---	--	------	------
mPD		0.092	0.58
		nPD:	0.16
		N. weight:	0.69
		Sc. PD:	0.0059
		Sc. rank:	465.7

PA			
Pos.	504	obs:	exp:
ttt	F	0	0.85
ttc	F	2	1.15
att	I	1791	925.70
atc	I	694	625.20
ata	I	8	942.00
gtt	V	1	0.22
gtc	V	0	0.21
gta	V	0	0.20
gtg	V	0	0.37
---	--	------	------
mPD		0.41	0.66
		nPD:	0.62
		N. weight:	0.61
		Sc. PD:	0.28
		Sc. rank:	922.9

PA			
Pos.	505	obs:	exp:
att	I	0	916.80
atc	I	0	619.20
ata	I	2469	933.00
act	T	0	0.53
acc	T	0	0.44
aca	T	2	0.86
acg	T	0	0.17
gtt	V	0	5.44
gtc	V	0	5.28
gta	V	25	4.92
gtg	V	0	9.36
---	--	------	------
mPD		0.021	0.68
		nPD:	0.03
		N. weight:	1.2
		Sc. PD:	-0.14
		Sc. rank:	62.7

PA			
Pos.	506	obs:	exp:
aaa	K	2430	1400.00
aag	K	65	1095.00
gaa	E	1	0.60
gag	E	0	0.40
---	--	------	------
mPD		0.052	0.49
		nPD:	0.1
		N. weight:	0.58
		Sc. PD:	-0.025
		Sc. rank:	339.4

PA			
Pos.	507	obs:	exp:
ggt	G	0	359.10
ggc	G	0	329.40
gga	G	2487	1090.00
ggg	G	9	717.90
---	--	------	------
mPD		0.0072	0.69
		nPD:	0.01
		N. weight:	1.
		Sc. PD:	-0.14
		Sc. rank:	-893.3

PA			
Pos.	508	obs:	exp:
cgt	R	0	94.62
cgc	R	0	132.90
cga	R	1	241.60
cgg	R	0	195.40
aga	R	2342	1144.00
agg	R	152	686.00
ggt	G	0	0.14
ggc	G	0	0.13
gga	G	0	0.44
ggg	G	1	0.29
---	--	------	------
mPD		0.12	0.95
		nPD:	0.12
		N. weight:	0.72
		Sc. PD:	-0.019
		Sc. rank:	438.3

PA			
Pos.	509	obs:	exp:
tct	S	22	426.30
tcc	S	2474	368.00
tca	S	0	562.60
tcg	S	0	161.20
agt	S	0	509.60
agc	S	0	468.30
---	--	------	------
mPD		0.017	1.6
		nPD:	0.01
		N. weight:	2.3
		Sc. PD:	-0.31
		Sc. rank:	-2036.1

PA			
Pos.	510	obs:	exp:
cat	H	2487	1484.00
cac	H	9	1012.00
---	--	------	------
mPD		0.0072	0.48
		nPD:	0.01
		N. weight:	0.62
		Sc. PD:	-0.081
		Sc. rank:	-432.6

PA			
Pos.	511	obs:	exp:
tta	L	15	272.40
ttg	L	2478	468.40
ctt	L	0	468.00
ctc	L	0	430.60
cta	L	0	332.10
ctg	L	3	524.40
---	--	------	------
mPD		0.014	1.1
		nPD:	0.01
		N. weight:	2.
		Sc. PD:	-0.27
		Sc. rank:	-1564.5

PA			
Pos.	512	obs:	exp:
cgt	R	0	94.66
cgc	R	0	133.00
cga	R	0	241.70
cgg	R	0	195.50
aga	R	36	1145.00
agg	R	2460	686.30
---	--	------	------
mPD		0.028	0.95
		nPD:	0.03
		N. weight:	1.5
		Sc. PD:	-0.18
		Sc. rank:	-7.6

PA			
Pos.	513	obs:	exp:
tct	S	0	0.17
tcc	S	0	0.15
tca	S	0	0.23
tcg	S	0	0.06
aat	N	2476	1326.00
aac	N	19	1169.00
agt	S	1	0.20
agc	S	0	0.19
---	--	------	------
mPD		0.016	0.50
		nPD:	0.03
		N. weight:	0.73
		Sc. PD:	-0.084
		Sc. rank:	49.4

PA			
Pos.	514	obs:	exp:
gat	D	950	1436.00
gac	D	1546	1060.00
---	--	------	------
mPD		0.47	0.49
		nPD:	0.97
		N. weight:	0.097
		Sc. PD:	0.077
		Sc. rank:	168.2

PA			
Pos.	515	obs:	exp:
act	T	446	661.20
acc	T	2049	551.50
aca	T	0	1073.00
acg	T	1	210.80
---	--	------	------
mPD		0.29	0.69
		nPD:	0.43
		N. weight:	1.3
		Sc. PD:	0.34
		Sc. rank:	1603.6

PA			
Pos.	516	obs:	exp:
gat	D	2491	1436.00
gac	D	5	1060.00
---	--	------	------
mPD		0.0040	0.49
		nPD:	0.01
		N. weight:	0.67
		Sc. PD:	-0.093
		Sc. rank:	-685.7

PA			
Pos.	517	obs:	exp:
gtt	V	1	542.80
gtc	V	0	527.40
gta	V	14	491.10
gtg	V	2481	934.70
---	--	------	------
mPD		0.012	0.73
		nPD:	0.02
		N. weight:	1.2
		Sc. PD:	-0.15
		Sc. rank:	-728.4

PA			
Pos.	518	obs:	exp:
gtt	V	0	542.80
gtc	V	0	527.40
gta	V	2481	491.10
gtg	V	15	934.70
---	--	------	------
mPD		0.012	0.73
		nPD:	0.02
		N. weight:	2.
		Sc. PD:	-0.26
		Sc. rank:	-1228.4

PA			
Pos.	519	obs:	exp:
aat	N	598	1326.00
aac	N	1898	1170.00
---	--	------	------
mPD		0.36	0.50
		nPD:	0.73
		N. weight:	0.22
		Sc. PD:	0.13
		Sc. rank:	365.4

PA			
Pos.	520	obs:	exp:
ttt	F	2462	1054.00
ttc	F	16	1424.00
tat	Y	18	8.75
tac	Y	0	9.25
---	--	------	------
mPD		0.027	0.50
		nPD:	0.05
		N. weight:	1.
		Sc. PD:	-0.095
		Sc. rank:	386.7

PA			
Pos.	521	obs:	exp:
tta	L	0	9.93
ttg	L	73	17.08
ctt	L	0	17.06
ctc	L	0	15.70
cta	L	0	12.11
ctg	L	18	19.12
atg	M	1	1.00
gtt	V	0	522.80
gtc	V	0	507.90
gta	V	4	473.00
gtg	V	2400	900.20
---	--	------	------
mPD		0.075	0.80
		nPD:	0.09
		N. weight:	1.2
		Sc. PD:	-0.067
		Sc. rank:	673.2

PA			
Pos.	522	obs:	exp:
tct	S	0	426.30
tcc	S	0	368.00
tca	S	0	562.60
tcg	S	0	161.20
agt	S	297	509.60
agc	S	2199	468.30
---	--	------	------
mPD		0.21	1.6
		nPD:	0.13
		N. weight:	1.6
		Sc. PD:	-0.035
		Sc. rank:	1010.9

PA			
Pos.	523	obs:	exp:
atg	M	2496	2496.00
---	--	------	------
mPD		0	0
		nPD:	1.
		N. weight:	0.
		Sc. PD:	0
		Sc. rank:	0

PA			
Pos.	524	obs:	exp:
gaa	E	1898	1495.00
gag	E	598	1001.00
---	--	------	------
mPD		0.36	0.48
		nPD:	0.76
		N. weight:	0.075
		Sc. PD:	0.044
		Sc. rank:	123.9

PA			
Pos.	525	obs:	exp:
ttt	F	60	1062.00
ttc	F	2436	1434.00
---	--	------	------
mPD		0.047	0.49
		nPD:	0.1
		N. weight:	0.56
		Sc. PD:	-0.029
		Sc. rank:	319.0

PA			
Pos.	526	obs:	exp:
tct	S	2074	426.30
tcc	S	132	368.00
tca	S	290	562.60
tcg	S	0	161.20
agt	S	0	509.60
agc	S	0	468.30
---	--	------	------
mPD		0.29	1.6
		nPD:	0.18
		N. weight:	1.5
		Sc. PD:	0.042
		Sc. rank:	1032.0

PA			
Pos.	527	obs:	exp:
tta	L	0	272.40
ttg	L	0	468.40
ctt	L	1149	468.00
ctc	L	1344	430.60
cta	L	3	332.10
ctg	L	0	524.40
---	--	------	------
mPD		0.50	1.1
		nPD:	0.44
		N. weight:	1.3
		Sc. PD:	0.36
		Sc. rank:	1671.1

PA			
Pos.	528	obs:	exp:
act	T	2097	661.20
acc	T	395	551.50
aca	T	4	1073.00
acg	T	0	210.80
---	--	------	------
mPD		0.27	0.69
		nPD:	0.39
		N. weight:	1.1
		Sc. PD:	0.27
		Sc. rank:	1385.0

PA			
Pos.	529	obs:	exp:
gat	D	42	1435.00
gac	D	2453	1060.00
gaa	E	1	0.60
gag	E	0	0.40
---	--	------	------
mPD		0.034	0.49
		nPD:	0.07
		N. weight:	0.96
		Sc. PD:	-0.075
		Sc. rank:	452.4

PA			
Pos.	530	obs:	exp:
cct	P	8	703.80
ccc	P	0	451.60
cca	P	1169	1019.00
ccg	P	1319	321.40
---	--	------	------
mPD		0.50	0.70
		nPD:	0.71
		N. weight:	0.99
		Sc. PD:	0.55
		Sc. rank:	1600.1

PA			
Pos.	531	obs:	exp:
cgt	R	0	94.16
cgc	R	0	132.30
cga	R	0	240.40
cgg	R	1	194.50
aaa	K	0	6.74
aag	K	12	5.26
aga	R	20	1139.00
agg	R	2462	682.70
ggt	G	0	0.14
ggc	G	0	0.13
gga	G	0	0.44
ggg	G	1	0.29
---	--	------	------
mPD		0.027	0.96
		nPD:	0.03
		N. weight:	1.5
		Sc. PD:	-0.18
		Sc. rank:	-171.1

PA			
Pos.	532	obs:	exp:
tta	L	0	272.00
ttg	L	3	467.60
ctt	L	0	467.30
ctc	L	0	429.90
cta	L	34	331.60
ctg	L	2455	523.60
cct	P	0	0.28
ccc	P	0	0.18
cca	P	0	0.41
ccg	P	1	0.13
atg	M	3	3.00
---	--	------	------
mPD		0.032	1.1
		nPD:	0.03
		N. weight:	1.9
		Sc. PD:	-0.22
		Sc. rank:	-171.1

PA			
Pos.	533	obs:	exp:
gaa	E	592	1495.00
gag	E	1904	1001.00
---	--	------	------
mPD		0.36	0.48
		nPD:	0.75
		N. weight:	0.34
		Sc. PD:	0.2
		Sc. rank:	562.3

PA			
Pos.	534	obs:	exp:
cct	P	0	703.80
ccc	P	0	451.60
cca	P	2487	1019.00
ccg	P	9	321.40
---	--	------	------
mPD		0.0072	0.70
		nPD:	0.01
		N. weight:	1.1
		Sc. PD:	-0.15
		Sc. rank:	-991.8

PA			
Pos.	535	obs:	exp:
tat	Y	0	1.46
tac	Y	3	1.54
cat	H	11	1460.00
cac	H	2445	995.70
caa	Q	1	0.51
cag	Q	0	0.49
cgt	R	0	0.04
cgc	R	1	0.05
cga	R	0	0.10
cgg	R	0	0.08
aat	N	0	18.60
aac	N	35	16.40
aga	R	0	0.46
agg	R	0	0.27
---	--	------	------
mPD		0.040	0.51
		nPD:	0.08
		N. weight:	1.1
		Sc. PD:	-0.075
		Sc. rank:	548.1

PA			
Pos.	536	obs:	exp:
aaa	K	1237	1401.00
aag	K	1259	1095.00
---	--	------	------
mPD		0.50	0.49
		nPD:	1.02
		N. weight:	0.013
		Sc. PD:	0.011
		Sc. rank:	22.3

PA			
Pos.	537	obs:	exp:
tgg	W	2496	2496.00
---	--	------	------
mPD		0	0
		nPD:	1.
		N. weight:	0.
		Sc. PD:	0
		Sc. rank:	0

PA			
Pos.	538	obs:	exp:
gtt	V	0	0.43
gtc	V	0	0.42
gta	V	2	0.39
gtg	V	0	0.75
gaa	E	2443	1493.00
gag	E	50	1000.00
ggt	G	0	0.14
ggc	G	0	0.13
gga	G	1	0.44
ggg	G	0	0.29
---	--	------	------
mPD		0.042	0.48
		nPD:	0.09
		N. weight:	0.53
		Sc. PD:	-0.033
		Sc. rank:	284.2

PA			
Pos.	539	obs:	exp:
cgt	R	0	0.08
cgc	R	0	0.11
cga	R	0	0.19
cgg	R	0	0.16
aaa	K	251	1400.00
aag	K	2243	1094.00
aga	R	0	0.92
agg	R	2	0.55
---	--	------	------
mPD		0.18	0.49
		nPD:	0.37
		N. weight:	0.59
		Sc. PD:	0.13
		Sc. rank:	702.5

PA			
Pos.	540	obs:	exp:
tat	Y	11	1213.00
tac	Y	2485	1283.00
---	--	------	------
mPD		0.0088	0.50
		nPD:	0.02
		N. weight:	0.8
		Sc. PD:	-0.1
		Sc. rank:	-444.3

PA			
Pos.	541	obs:	exp:
tgt	C	2490	1085.00
tgc	C	6	1411.00
---	--	------	------
mPD		0.0048	0.49
		nPD:	0.01
		N. weight:	1.
		Sc. PD:	-0.14
		Sc. rank:	-933.5

PA			
Pos.	542	obs:	exp:
gtt	V	1274	542.80
gtc	V	298	527.40
gta	V	131	491.10
gtg	V	793	934.70
---	--	------	------
mPD		0.62	0.73
		nPD:	0.85
		N. weight:	0.31
		Sc. PD:	0.21
		Sc. rank:	533.4

PA			
Pos.	543	obs:	exp:
tta	L	0	272.20
ttg	L	0	468.00
ctt	L	1224	467.70
ctc	L	1267	430.30
cta	L	3	331.80
ctg	L	0	524.00
att	I	0	0.74
atc	I	2	0.50
ata	I	0	0.76
---	--	------	------
mPD		0.50	1.1
		nPD:	0.44
		N. weight:	1.3
		Sc. PD:	0.36
		Sc. rank:	1692.3

PA			
Pos.	544	obs:	exp:
gaa	E	578	1456.00
gag	E	1853	975.40
ggt	G	0	9.35
ggc	G	0	8.58
gga	G	0	28.37
ggg	G	65	18.70
---	--	------	------
mPD		0.41	0.54
		nPD:	0.76
		N. weight:	0.37
		Sc. PD:	0.22
		Sc. rank:	613.6

PA			
Pos.	545	obs:	exp:
att	I	0	576.30
atc	I	0	389.20
ata	I	1552	586.50
gtt	V	3	205.30
gtc	V	0	199.50
gta	V	938	185.70
gtg	V	3	353.50
mPD		0.48	1.2
		nPD:	0.4
		N. weight:	1.5
		Sc. PD:	0.36
		Sc. rank:	1852.8

PA			
Pos.	546	obs:	exp:
ggt	G	1	359.10
ggc	G	0	329.40
gga	G	2142	1090.00
ggg	G	353	717.90
mPD		0.24	0.69
		nPD:	0.35
		N. weight:	0.6
		Sc. PD:	0.12
		Sc. rank:	697.3

PA			
Pos.	547	obs:	exp:
aat	N	0	0.53
aac	N	1	0.47
gat	D	15	1434.00
gac	D	2478	1059.00
gaa	E	1	0.60
gag	E	0	0.40
ggt	G	0	0.14
ggc	G	1	0.13
gga	G	0	0.44
ggg	G	0	0.29
mPD		0.014	0.49
		nPD:	0.03
		N. weight:	1.
		Sc. PD:	-0.12
		Sc. rank:	-44.3

PA			
Pos.	548	obs:	exp:
att	I	0	0.74
atc	I	0	0.50
ata	I	2	0.76
atg	M	2494	2494.00
mPD		0.0016	0.0016
		nPD:	1.
		N. weight:	0.00097
		Sc. PD:	0.00081
		Sc. rank:	1.7

PA			
Pos.	549	obs:	exp:
tta	L	0	272.40
ttg	L	0	468.40
ctt	L	2	468.00
ctc	L	2207	430.60
cta	L	286	332.10
ctg	L	1	524.40
mPD		0.21	1.1
		nPD:	0.18
		N. weight:	1.8
		Sc. PD:	0.054
		Sc. rank:	1268.8

PA			
Pos.	550	obs:	exp:
tta	L	10	272.30
ttg	L	4	468.20
ctt	L	0	467.90
ctc	L	0	430.50
cta	L	2463	332.00
ctg	L	18	524.20
att	I	0	0.37
atc	I	0	0.25
ata	I	1	0.38
mPD		0.029	1.1
		nPD:	0.03
		N. weight:	2.4
		Sc. PD:	-0.29
		Sc. rank:	-457.8

PA			
Pos.	551	obs:	exp:
cgt	R	1	94.66
cgc	R	1	133.00
cga	R	580	241.70
cgg	R	1864	195.50
aga	R	0	1145.00
agg	R	50	686.30
mPD		0.40	0.95
		nPD:	0.42
		N. weight:	2.3
		Sc. PD:	0.6
		Sc. rank:	2890.7

PA			
Pos.	552	obs:	exp:
tct	S	0	0.17
tcc	S	0	0.15
tca	S	0	0.23
tcg	S	0	0.06
act	T	2215	659.30
acc	T	271	549.90
aca	T	0	1070.00
acg	T	3	210.20
aat	N	1	0.53
aac	N	0	0.47
agt	S	1	0.20
agc	S	0	0.19
gct	A	5	1.27
gcc	A	0	0.98
gca	A	0	2.20
gcg	A	0	0.56
mPD		0.20	0.69
		nPD:	0.29
		N. weight:	1.2
		Sc. PD:	0.17
		Sc. rank:	1185.2

PA			
Pos.	553	obs:	exp:
gct	A	0	635.00
gcc	A	2	486.70
gca	A	2490	1097.00
gcg	A	4	277.10
mPD		0.0048	0.69
		nPD:	0.01
		N. weight:	1.
		Sc. PD:	-0.14
		Sc. rank:	-1161.7

PA			
Pos.	554	obs:	exp:
att	I	13	925.70
atc	I	0	625.20
ata	I	2480	942.00
gtt	V	0	0.65
gtc	V	0	0.63
gta	V	3	0.59
gtg	V	0	1.12
mPD		0.013	0.66
		nPD:	0.02
		N. weight:	1.2
		Sc. PD:	-0.15
		Sc. rank:	-557.6

PA			
Pos.	555	obs:	exp:
ggt	G	21	359.10
ggc	G	2474	329.40
gga	G	1	1090.00
ggg	G	0	717.90
mPD		0.017	0.69
		nPD:	0.03
		N. weight:	2.5
		Sc. PD:	-0.3
		Sc. rank:	-571.8

PA			
Pos.	556	obs:	exp:
caa	Q	2098	1270.00
cag	Q	398	1226.00
mPD		0.27	0.50
		nPD:	0.54
		N. weight:	0.3
		Sc. PD:	0.11
		Sc. rank:	441.3

PA			
Pos.	557	obs:	exp:
att	I	0	13.37
atc	I	0	9.03
ata	I	36	13.60
atg	M	24	24.00
gtt	V	0	529.80
gtc	V	0	514.70
gta	V	533	479.30
gtg	V	1903	912.20
mPD		0.40	0.78
		nPD:	0.51
		N. weight:	0.75
		Sc. PD:	0.27
		Sc. rank:	1050.2

PA			
Pos.	558	obs:	exp:
tta	L	2	0.22
ttg	L	0	0.38
tct	S	1	425.90
tcc	S	5	367.70
tca	S	2449	562.20
tcg	S	39	161.10
ctt	L	0	0.38
ctc	L	0	0.35
cta	L	0	0.27
ctg	L	0	0.42
agt	S	0	509.20
agc	S	0	467.90
mPD		0.037	1.6
		nPD:	0.02
		N. weight:	1.8
		Sc. PD:	-0.22
		Sc. rank:	-603.2

PA			
Pos.	559	obs:	exp:
cgt	R	0	94.62
cgc	R	0	132.90
cga	R	2	241.60
cgg	R	2	195.40
aaa	K	1	0.56
aag	K	0	0.44
aga	R	585	1144.00
agg	R	1906	686.00
mPD		0.36	0.95
		nPD:	0.38
		N. weight:	0.77
		Sc. PD:	0.17
		Sc. rank:	926.3

PA			
Pos.	560	obs:	exp:
tct	S	0	18.44
tcc	S	108	15.92
tca	S	0	24.34
tcg	S	0	6.98
cct	P	24	672.80
ccc	P	2361	431.70
cca	P	1	974.20
ccg	P	0	307.20
act	T	0	0.26
acc	T	1	0.22
aca	T	0	0.43
acg	T	0	0.08
agt	S	0	22.05
agc	S	0	20.26
gct	A	0	0.25
gcc	A	1	0.20
gca	A	0	0.44
gcg	A	0	0.11
mPD		0.10	0.83
		nPD:	0.13
		N. weight:	2.1
		Sc. PD:	-0.047
		Sc. rank:	1273.1

PA			
Pos.	561	obs:	exp:
atg	M	2453	2453.00
gtt	V	0	9.35
gtc	V	0	9.09
gta	V	0	8.46
gtg	V	43	16.10
mPD		0.034	0.055
		nPD:	0.61
		N. weight:	0.021
		Sc. PD:	0.0095
		Sc. rank:	31.8

PA			
Pos.	562	obs:	exp:
ttt	F	611	1062.00
ttc	F	1885	1434.00
mPD		0.37	0.49
		nPD:	0.76
		N. weight:	0.091
		Sc. PD:	0.054
		Sc. rank:	150.7

PA			
Pos.	563	obs:	exp:
tta	L	0	272.40
ttg	L	309	468.40
ctt	L	551	468.00
ctc	L	2	430.60
cta	L	46	332.10
ctg	L	1588	524.40
mPD		0.59	1.1
		nPD:	0.52
		N. weight:	0.81
		Sc. PD:	0.29
		Sc. rank:	1151.0

PA			
Pos.	564	obs:	exp:
tat	Y	2377	1213.00
tac	Y	119	1283.00
mPD		0.091	0.50
		nPD:	0.18
		N. weight:	0.66
		Sc. PD:	0.021
		Sc. rank:	478.0

PA			
Pos.	565	obs:	exp:
att	I	0	0.74
atc	I	0	0.50
ata	I	2	0.76
gtt	V	9	542.40
gtc	V	0	527.00
gta	V	686	490.70
gtg	V	1799	933.90
---	--	------	------
mPD		0.41	0.73
		nPD:	0.56
		N. weight:	0.69
		Sc. PD:	0.27
		Sc. rank:	1019.9

,

PA			
Pos.	566	obs:	exp:
cgt	R	0	94.66
cgc	R	0	133.00
cga	R	0	241.70
cgg	R	0	195.50
aga	R	2459	1145.00
agg	R	37	686.30
---	--	------	------
mPD		0.029	0.95
		nPD:	0.03
		N. weight:	0.89
		Sc. PD:	-0.1
		Sc. rank:	28.2

,

PA			
Pos.	567	obs:	exp:
act	T	9	661.20
acc	T	2471	551.50
aca	T	16	1073.00
acg	T	0	210.80
---	--	------	------
mPD		0.020	0.69
		nPD:	0.03
		N. weight:	1.8
		Sc. PD:	-0.21
		Sc. rank:	-120.0

,

PA			
Pos.	568	obs:	exp:
aat	N	2490	1326.00
aac	N	6	1170.00
---	--	------	------
mPD		0.0048	0.50
		nPD:	0.01
		N. weight:	0.77
		Sc. PD:	-0.1
		Sc. rank:	-730.7

,

PA			
Pos.	569	obs:	exp:
ggt	G	12	359.10
ggc	G	0	329.40
gga	G	398	1090.00
ggg	G	2086	717.90
---	--	------	------
mPD		0.28	0.69
		nPD:	0.4
		N. weight:	0.89
		Sc. PD:	0.22
		Sc. rank:	1111.0

,

PA			
Pos.	570	obs:	exp:
act	T	2451	661.20
acc	T	45	551.50
aca	T	0	1073.00
acg	T	0	210.80
---	--	------	------
mPD		0.035	0.69
		nPD:	0.05
		N. weight:	1.5
		Sc. PD:	-0.15
		Sc. rank:	570.2

,

PA			
Pos.	571	obs:	exp:
tct	S	1194	426.30
tcc	S	1296	368.00
tca	S	6	562.60
tcg	S	0	161.20
agt	S	0	509.60
agc	S	0	468.30
---	--	------	------
mPD		0.50	1.6
		nPD:	0.3
		N. weight:	1.4
		Sc. PD:	0.22
		Sc. rank:	1432.9

,

PA			
Pos.	572	obs:	exp:
aaa	K	18	1401.00
aag	K	2478	1095.00
---	--	------	------
mPD		0.014	0.49
		nPD:	0.03
		N. weight:	0.97
		Sc. PD:	-0.11
		Sc. rank:	-47.0

,

PA			
Pos.	573	obs:	exp:
tta	L	0	0.11
ttg	L	0	0.19
ctt	L	0	0.19
ctc	L	1	0.17
cta	L	0	0.13
ctg	L	0	0.21
att	I	7	926.50
atc	I	2488	625.70
ata	I	0	942.80
---	--	------	------
mPD		0.0064	0.66
		nPD:	0.01
		N. weight:	1.7
		Sc. PD:	-0.23
		Sc. rank:	-1568.3

,

PA			
Pos.	574	obs:	exp:
aaa	K	1217	1401.00
aag	K	1279	1095.00
---	--	------	------
mPD		0.50	0.49
		nPD:	1.02
		N. weight:	0.016
		Sc. PD:	0.013
		Sc. rank:	27.2

,

PA			
Pos.	575	obs:	exp:
atg	M	2496	2496.00
---	--	------	------
mPD		0	0
		nPD:	1.
		N. weight:	0.
		Sc. PD:	0
		Sc. rank:	0

,

PA			
Pos.	576	obs:	exp:
cgt	R	0	0.04
cgc	R	0	0.05
cga	R	0	0.10
cgg	R	0	0.08
aaa	K	2488	1400.00
aag	K	7	1095.00
aga	R	1	0.46
agg	R	0	0.27
---	--	------	------
mPD		0.0064	0.49
		nPD:	0.01
		N. weight:	0.7
		Sc. PD:	-0.093
		Sc. rank:	-521.7

,

PA			
Pos.	577	obs:	exp:
tgg	W	2496	2496.00
---	--	------	------
mPD		0	0
		nPD:	1.
		N. weight:	0.
		Sc. PD:	0
		Sc. rank:	0

,

PA			
Pos.	578	obs:	exp:
ggt	G	926	359.10
ggc	G	1541	329.40
gga	G	29	1090.00
ggg	G	0	717.90
---	--	------	------
mPD		0.48	0.69
		nPD:	0.7
		N. weight:	1.6
		Sc. PD:	0.84
		Sc. rank:	2499.6

,

PA			
Pos.	579	obs:	exp:
att	I	8	5.57
atc	I	0	3.76
ata	I	7	5.67
atg	M	2478	2478.00
gtt	V	0	0.65
gtc	V	0	0.63
gta	V	0	0.59
gtg	V	3	1.12
---	--	------	------
mPD		0.014	0.016
		nPD:	0.91
		N. weight:	0.0045
		Sc. PD:	0.0033
		Sc. rank:	7.7

,

PA			
Pos.	580	obs:	exp:
gat	D	1	0.58
gac	D	0	0.42
gaa	E	90	1494.00
gag	E	2405	1001.00
---	--	------	------
mPD		0.070	0.48
		nPD:	0.15
		N. weight:	0.93
		Sc. PD:	-0.0029
		Sc. rank:	620.1

,

PA			
Pos.	581	obs:	exp:
atg	M	2496	2496.00
---	--	------	------
mPD		0	0
		nPD:	1.
		N. weight:	0.
		Sc. PD:	0
		Sc. rank:	0

,

PA			
Pos.	582	obs:	exp:
cgt	R	0	94.66
cgc	R	0	133.00
cga	R	0	241.70
cgg	R	0	195.50
aga	R	2	1145.00
agg	R	2494	686.30
---	--	------	------
mPD		0.0016	0.95
		nPD:	0.
		N. weight:	1.6
		Sc. PD:	-0.23
		Sc. rank:	-2470.3

,

PA			
Pos.	583	obs:	exp:
cgt	R	8	94.66
cgc	R	12	133.00
cga	R	2400	241.70
cgg	R	71	195.50
aga	R	5	1145.00
agg	R	0	686.30
---	--	------	------
mPD		0.075	0.95
		nPD:	0.08
		N. weight:	2.7
		Sc. PD:	-0.19
		Sc. rank:	1338.1

,

PA			
Pos.	584	obs:	exp:
tct	S	0	0.34
tcc	S	1	0.29
tca	S	0	0.45
tcg	S	0	0.13
tgt	C	26	1084.00
tgc	C	2468	1410.00
agt	S	0	0.41
agc	S	1	0.38
---	--	------	------
mPD		0.022	0.49
		nPD:	0.05
		N. weight:	0.64
		Sc. PD:	-0.066
		Sc. rank:	208.8

,

PA			
Pos.	585	obs:	exp:
tta	L	0	272.40
ttg	L	0	468.40
ctt	L	2099	468.00
ctc	L	397	430.60
cta	L	0	332.10
ctg	L	0	524.40
---	--	------	------
mPD		0.27	1.1
		nPD:	0.24
		N. weight:	1.6
		Sc. PD:	0.13
		Sc. rank:	1262.6

,

PA			
Pos.	586	obs:	exp:
tta	L	0	272.40
ttg	L	0	468.40
ctt	L	2442	468.00
ctc	L	54	430.60
cta	L	0	332.10
ctg	L	0	524.40
---	--	------	------
mPD		0.042	1.1
		nPD:	0.04
		N. weight:	2.
		Sc. PD:	-0.21
		Sc. rank:	354.9

,

PA			
Pos.	587	obs:	exp:
caa	Q	2435	1270.00
cag	Q	61	1226.00
---	--	------	------
mPD		0.048	0.50
		nPD:	0.1
		N. weight:	0.7
		Sc. PD:	-0.037
		Sc. rank:	391.9

,

PA			
Pos.	588	obs:	exp:
tct	S	51	426.10
tcc	S	2444	367.80
tca	S	0	562.40
tcg	S	0	161.20
cct	P	0	0.28
ccc	P	1	0.18
cca	P	0	0.41
ccg	P	0	0.13
agt	S	0	509.40
agc	S	0	468.10
---	--	------	------
mPD		0.041	1.6
		nPD:	0.02
		N. weight:	2.3
		Sc. PD:	-0.27
		Sc. rank:	-589.6

,

PA			
Pos.	589	obs:	exp:
tta	L	0	272.40
ttg	L	0	468.40
ctt	L	1662	468.00
ctc	L	833	430.60
cta	L	1	332.10
ctg	L	0	524.40
---	--	------	------
mPD		0.45	1.1
		nPD:	0.39
		N. weight:	1.3
		Sc. PD:	0.31
		Sc. rank:	1627.2

,

PA			
Pos.	590	obs:	exp:
caa	Q	2473	1270.00
cag	Q	23	1226.00
---	--	------	------
mPD		0.018	0.50
		nPD:	0.04
		N. weight:	0.78
		Sc. PD:	-0.086
		Sc. rank:	124.2

,

PA			
Pos.	591	obs:	exp:
caa	Q	2483	1270.00
cag	Q	13	1226.00
---	--	------	------
mPD		0.010	0.50
		nPD:	0.02
		N. weight:	0.8
		Sc. PD:	-0.1
		Sc. rank:	-327.1

,

PA			
Pos.	592	obs:	exp:
att	I	2490	925.70
atc	I	3	625.20
ata	I	0	942.00
gtt	V	3	0.65
gtc	V	0	0.63
gta	V	0	0.59
gtg	V	0	1.12
---	--	------	------
mPD		0.0048	0.66
		nPD:	0.01
		N. weight:	1.2
		Sc. PD:	-0.17
		Sc. rank:	-1376.5

,

PA			
Pos.	593	obs:	exp:
gaa	E	12	1495.00
gag	E	2484	1001.00
---	--	------	------
mPD		0.0096	0.48
		nPD:	0.02
		N. weight:	1.1
		Sc. PD:	-0.14
		Sc. rank:	-498.8

,

PA			
Pos.	594	obs:	exp:
tct	S	0	426.30
tcc	S	0	368.00
tca	S	0	562.60
tcg	S	0	161.20
agt	S	3	509.60
agc	S	2493	468.30
---	--	------	------
mPD		0.0024	1.6
		nPD:	0.
		N. weight:	2.1
		Sc. PD:	-0.3
		Sc. rank:	-3301.6

,

PA			
Pos.	595	obs:	exp:
tta	L	0	0.87
ttg	L	8	1.50
ctt	L	0	1.50
ctc	L	0	1.38
cta	L	0	1.06
ctg	L	0	1.68
atg	M	2488	2488.00
---	--	------	------
mPD		0.0064	0.010
		nPD:	0.62
		N. weight:	0.0067
		Sc. PD:	0.0031
		Sc. rank:	10.1

,

PA			
Pos.	596	obs:	exp:
att	I	2479	926.50
atc	I	16	625.70
ata	I	0	942.80
gtt	V	1	0.22
gtc	V	0	0.21
gta	V	0	0.20
gtg	V	0	0.37
---	--	------	------
mPD		0.014	0.66
		nPD:	0.02
		N. weight:	1.2
		Sc. PD:	-0.15
		Sc. rank:	-489.8

,

PA			
Pos.	597	obs:	exp:
gat	D	1	0.58
gac	D	0	0.42
gaa	E	1167	1494.00
gag	E	1328	1001.00
---	--	------	------
mPD		0.50	0.48
		nPD:	1.04
		N. weight:	0.046
		Sc. PD:	0.04
		Sc. rank:	79.3

,

PA			
Pos.	598	obs:	exp:
tct	S	0	0.17
tcc	S	1	0.15
tca	S	0	0.23
tcg	S	0	0.06
act	T	0	19.34
acc	T	73	16.13
aca	T	0	31.37
acg	T	0	6.17
agt	S	0	0.20
agc	S	0	0.19
gtt	V	0	5.87
gtc	V	27	5.70
gta	V	0	5.31
gtg	V	0	10.11
gct	A	124	609.30
gcc	A	1668	467.00
gca	A	603	1053.00
gcg	A	0	265.90
---	--	------	------
mPD		0.52	0.77
		nPD:	0.67
		N. weight:	0.87
		Sc. PD:	0.44
		Sc. rank:	1361.2

,

PA			
Pos.	599	obs:	exp:
gaa	E	12	1495.00
gag	E	2484	1001.00
---	--	------	------
mPD		0.0096	0.48
		nPD:	0.02
		N. weight:	1.1
		Sc. PD:	-0.14
		Sc. rank:	-498.8

,

PA			
Pos.	600	obs:	exp:
tct	S	2486	426.30
tcc	S	8	368.00
tca	S	2	562.60
tcg	S	0	161.20
agt	S	0	509.60
agc	S	0	468.30
---	--	------	------
mPD		0.0080	1.6
		nPD:	0.
		N. weight:	2.2
		Sc. PD:	-0.31
		Sc. rank:	-2796.0

,

PA			
Pos.	601	obs:	exp:
ttt	F	1	0.43
ttc	F	0	0.57
tct	S	2482	426.10
tcc	S	13	367.80
tca	S	0	562.40
tcg	S	0	161.20
agt	S	0	509.40
agc	S	0	468.10
---	--	------	------
mPD		0.011	1.6
		nPD:	0.01
		N. weight:	2.2
		Sc. PD:	-0.3
		Sc. rank:	-2519.5

,

PA			
Pos.	602	obs:	exp:
att	I	0	0.74
atc	I	2	0.50
ata	I	0	0.76
gtt	V	20	542.40
gtc	V	2474	527.00
gta	V	0	490.70
gtg	V	0	933.90
---	--	------	------
mPD		0.018	0.73
		nPD:	0.02
		N. weight:	1.9
		Sc. PD:	-0.23
		Sc. rank:	-551.2

,

PA			
Pos.	603	obs:	exp:
cgt	R	0	0.15
cgc	R	0	0.21
cga	R	0	0.39
cgg	R	0	0.31
aaa	K	2489	1399.00
aag	K	3	1093.00
aga	R	4	1.83
agg	R	0	1.10
---	--	------	------
mPD		0.0056	0.50
		nPD:	0.01
		N. weight:	0.71
		Sc. PD:	-0.096
		Sc. rank:	-611.3

,

PA			
Pos.	604	obs:	exp:
gaa	E	1346	1494.00
gag	E	1149	1001.00
ggt	G	0	0.14
ggc	G	0	0.13
gga	G	0	0.44
ggg	G	1	0.29
---	--	------	------
mPD		0.50	0.48
		nPD:	1.03
		N. weight:	0.012
		Sc. PD:	0.01
		Sc. rank:	20.2

,

PA			
Pos.	605	obs:	exp:
aaa	K	1836	1401.00
aag	K	660	1095.00
---	--	------	------
mPD		0.39	0.49
		nPD:	0.79
		N. weight:	0.083
		Sc. PD:	0.052
		Sc. rank:	140.3

,

PA			
Pos.	606	obs:	exp:
aat	N	0	0.53
aac	N	1	0.47
gat	D	1	1435.00
gac	D	2494	1060.00
---	--	------	------
mPD		0.0016	0.49
		nPD:	0.
		N. weight:	1.1
		Sc. PD:	-0.15
		Sc. rank:	-1536.4

,

PA			
Pos.	607	obs:	exp:
cgt	R	0	0.04
cgc	R	0	0.05
cga	R	0	0.10
cgg	R	0	0.08
att	I	1	0.37
atc	I	0	0.25
ata	I	0	0.38
atg	M	2494	2494.00
aga	R	0	0.46
agg	R	1	0.27
---	--	------	------
mPD		0.0016	0.0023
		nPD:	0.69
		N. weight:	0.0011
		Sc. PD:	0.0006
		Sc. rank:	1.8

,

PA			
Pos.	608	obs:	exp:
act	T	5	659.80
acc	T	2483	550.40
aca	T	3	1070.00
acg	T	0	210.40
gct	A	0	1.27
gcc	A	5	0.98
gca	A	0	2.20
gcg	A	0	0.56
---	--	------	------
mPD		0.010	0.69
		nPD:	0.01
		N. weight:	1.9
		Sc. PD:	-0.24
		Sc. rank:	-1268.7

,

PA			
Pos.	609	obs:	exp:
cgt	R	0	0.04
cgc	R	0	0.05
cga	R	0	0.10
cgg	R	0	0.08
aaa	K	689	1400.00
aag	K	1806	1095.00
aga	R	1	0.46
agg	R	0	0.27
---	--	------	------
mPD		0.40	0.49
		nPD:	0.81
		N. weight:	0.21
		Sc. PD:	0.14
		Sc. rank:	356.1

,

PA			
Pos.	610	obs:	exp:
aaa	K	23	12.91
aag	K	0	10.09
gct	A	0	0.51
gcc	A	0	0.39
gca	A	2	0.88
gcg	A	0	0.22
gat	D	2	1.15
gac	D	0	0.85
gaa	E	2429	1478.00
gag	E	40	990.70
---	--	------	------
mPD		0.053	0.50
		nPD:	0.11
		N. weight:	0.55
		Sc. PD:	-0.023
		Sc. rank:	322.5

,

PA			
Pos.	611	obs:	exp:
ttt	F	69	1061.00
ttc	F	2425	1433.00
tct	S	0	0.17
tcc	S	1	0.15
tca	S	0	0.23
tcg	S	0	0.06
agt	S	0	0.20
agc	S	0	0.19
gtt	V	0	0.22
gtc	V	1	0.21
gta	V	0	0.20
gtg	V	0	0.37
---	--	------	------
mPD		0.055	0.49
		nPD:	0.11
		N. weight:	0.55
		Sc. PD:	-0.02
		Sc. rank:	324.9

,

PA			
Pos.	612	obs:	exp:
ttt	F	2492	1062.00
ttc	F	4	1434.00
---	--	------	------
mPD		0.0032	0.49
		nPD:	0.01
		N. weight:	1.1
		Sc. PD:	-0.15
		Sc. rank:	-1245.0

,

PA			
Pos.	613	obs:	exp:
gaa	E	2451	1493.00
gag	E	43	1001.00
ggt	G	0	0.29
ggc	G	0	0.26
gga	G	2	0.87
ggg	G	0	0.58
---	--	------	------
mPD		0.035	0.48
		nPD:	0.07
		N. weight:	0.54
		Sc. PD:	-0.04
		Sc. rank:	260.3

,

PA			
Pos.	614	obs:	exp:
tct	S	0	0.34
tcc	S	0	0.29
tca	S	0	0.45
tcg	S	0	0.13
act	T	0	2.91
acc	T	11	2.43
aca	T	0	4.73
acg	T	0	0.93
aat	N	543	1318.00
aac	N	1938	1163.00
agt	S	0	0.41
agc	S	2	0.38
gat	D	0	1.15
gac	D	2	0.85
---	--	------	------
mPD		0.35	0.51
		nPD:	0.69
		N. weight:	0.27
		Sc. PD:	0.14
		Sc. rank:	419.6

,

PA			
Pos.	615	obs:	exp:
cgt	R	0	44.60
cgc	R	0	62.64
cga	R	0	113.90
cgg	R	0	92.10
aaa	K	1315	740.40
aag	K	4	578.60
aga	R	1152	539.40
agg	R	24	323.40
ggt	G	0	0.14
ggc	G	0	0.13
gga	G	1	0.44
ggg	G	0	0.29
---	--	------	------
mPD		0.52	1.2
		nPD:	0.42
		N. weight:	0.77
		Sc. PD:	0.2
		Sc. rank:	982.7

,

PA			
Pos.	616	obs:	exp:
tta	L	4	0.65
ttg	L	2	1.13
tct	S	0	424.60
tcc	S	0	366.50
tca	S	2193	560.40
tcg	S	293	160.60
ctt	L	0	1.13
ctc	L	0	1.04
cta	L	0	0.80
ctg	L	0	1.26
cct	P	0	0.28
ccc	P	0	0.18
cca	P	1	0.41
ccg	P	0	0.13
act	T	0	0.79
acc	T	0	0.66
aca	T	3	1.29
acg	T	0	0.25
agt	S	0	507.60
agc	S	0	466.40
---	--	------	------
mPD		0.22	1.7
		nPD:	0.13
		N. weight:	1.6
		Sc. PD:	-0.028
		Sc. rank:	1029.8

,

PA			
Pos.	617	obs:	exp:
gaa	E	2485	1495.00
gag	E	11	1001.00
---	--	------	------
mPD		0.0088	0.48
		nPD:	0.02
		N. weight:	0.61
		Sc. PD:	-0.078
		Sc. rank:	-321.8

,

PA			
Pos.	618	obs:	exp:
atg	M	1	1.00
act	T	38	641.80
acc	T	6	535.40
aca	T	1899	1041.00
acg	T	480	204.70
aaa	K	4	37.05
aag	K	62	28.95
gct	A	0	1.53
gcc	A	0	1.17
gca	A	6	2.64
gcg	A	0	0.67
---	--	------	------
mPD		0.42	0.75
		nPD:	0.57
		N. weight:	0.73
		Sc. PD:	0.3
		Sc. rank:	1092.1

,

PA			
Pos.	619	obs:	exp:
tgg	W	2496	2496.00
---	--	------	------
mPD		0	0
		nPD:	1.
		N. weight:	0.
		Sc. PD:	0
		Sc. rank:	0

,

PA			
Pos.	620	obs:	exp:
cct	P	7	703.80
ccc	P	7	451.60
cca	P	2472	1019.00
ccg	P	10	321.40
---	--	------	------
mPD		0.019	0.70
		nPD:	0.03
		N. weight:	1.
		Sc. PD:	-0.13
		Sc. rank:	-171.4

,

PA			
Pos.	621	obs:	exp:
att	I	2483	926.10
atc	I	9	625.50
ata	I	2	942.40
gtt	V	2	0.43
gtc	V	0	0.42
gta	V	0	0.39
gtg	V	0	0.75
---	--	------	------
mPD		0.010	0.66
		nPD:	0.02
		N. weight:	1.2
		Sc. PD:	-0.16
		Sc. rank:	-789.1

,

PA			
Pos.	622	obs:	exp:
ggt	G	0	359.10
ggc	G	0	329.40
gga	G	1869	1090.00
ggg	G	627	717.90
---	--	------	------
mPD		0.38	0.69
		nPD:	0.55
		N. weight:	0.46
		Sc. PD:	0.18
		Sc. rank:	679.9

,

PA			
Pos.	623	obs:	exp:
aaa	K	1	0.56
aag	K	0	0.44
gtt	V	0	0.22
gtc	V	0	0.21
gta	V	0	0.20
gtg	V	1	0.37
gat	D	2	1.15
gac	D	0	0.85
gaa	E	1652	1492.00
gag	E	839	999.50
ggt	G	0	0.14
ggc	G	0	0.13
gga	G	0	0.44
ggg	G	1	0.29
---	--	------	------
mPD		0.45	0.48
		nPD:	0.93
		N. weight:	0.015
		Sc. PD:	0.011
		Sc. rank:	25.8

,

PA			
Pos.	624	obs:	exp:
tct	S	0	426.30
tcc	S	7	368.00
tca	S	2200	562.60
tcg	S	289	161.20
agt	S	0	509.60
agc	S	0	468.30
---	--	------	------
mPD		0.21	1.6
		nPD:	0.13
		N. weight:	1.6
		Sc. PD:	-0.034
		Sc. rank:	992.3

,

PA			
Pos.	625	obs:	exp:
cct	P	1260	703.80
ccc	P	1235	451.60
cca	P	1	1019.00
ccg	P	0	321.40
---	--	------	------
mPD		0.50	0.70
		nPD:	0.71
		N. weight:	0.99
		Sc. PD:	0.54
		Sc. rank:	1580.2

,

PA			
Pos.	626	obs:	exp:
caa	Q	0	0.51
cag	Q	1	0.49
cgt	R	0	1.63
cgc	R	0	2.29
cga	R	0	4.16
cgg	R	0	3.37
act	T	0	0.53
acc	T	0	0.44
aca	T	2	0.86
acg	T	0	0.17
aat	N	2	2.13
aac	N	2	1.87
aaa	K	1328	1371.00
aag	K	1115	1072.00
aga	R	4	19.72
agg	R	39	11.82
gaa	E	0	1.80
gag	E	3	1.20
---	--	------	------
mPD		0.54	0.54
		nPD:	0.99
		N. weight:	0.027
		Sc. PD:	0.022
		Sc. rank:	46.2

,

PA

Pos.	627	obs:	exp:
cgt	R	0	0.04
cgc	R	0	0.05
cga	R	0	0.10
cgg	R	0	0.08
aga	R	1	0.46
agg	R	0	0.27
gaa	E	0	0.60
gag	E	1	0.40
ggt	G	1	358.80
ggc	G	0	329.20
gga	G	1228	1089.00
ggg	G	1265	717.30
---	--	------	------
mPD		0.50	0.69
		nPD:	0.73
		N. weight:	0.43
		Sc. PD:	0.24
		Sc. rank:	706.1

,

PA

Pos.	628	obs:	exp:
tta	L	0	0.44
ttg	L	2	0.75
ctt	L	0	0.75
ctc	L	0	0.69
cta	L	0	0.53
ctg	L	2	0.84
atg	M	1	1.00
gtt	V	0	541.70
gtc	V	0	526.30
gta	V	15	490.10
gtg	V	2476	932.80
---	--	------	------
mPD		0.016	0.73
		nPD:	0.02
		N. weight:	1.2
		Sc. PD:	-0.15
		Sc. rank:	-426.5

,

PA

Pos.	629	obs:	exp:
gaa	E	19	1495.00
gag	E	2477	1001.00
---	--	------	------
mPD		0.015	0.48
		nPD:	0.03
		N. weight:	1.1
		Sc. PD:	-0.12
		Sc. rank:	49.1

,

PA

Pos.	630	obs:	exp:
gaa	E	2495	1494.00
gag	E	0	1001.00
ggt	G	0	0.14
ggc	G	0	0.13
gga	G	1	0.44
ggg	G	0	0.29
---	--	------	------
mPD		0.00080	0.48
		nPD:	0.
		N. weight:	0.64
		Sc. PD:	-0.092
		Sc. rank:	-997.9

,

PA

Pos.	631	obs:	exp:
tct	S	0	99.05
tcc	S	0	85.50
tca	S	0	130.70
tcg	S	0	37.46
tgt	C	0	1.30
tgc	C	3	1.70
agt	S	1	118.40
agc	S	579	108.80
ggt	G	7	275.20
ggc	G	1904	252.50
gga	G	2	835.10
ggg	G	0	550.20
---	--	------	------
mPD		0.37	1.4
		nPD:	0.27
		N. weight:	2.4
		Sc. PD:	0.28
		Sc. rank:	2134.8

,

PA

Pos.	632	obs:	exp:
tct	S	555	419.40
tcc	S	1900	362.10
tca	S	1	553.60
tcg	S	0	158.60
cct	P	0	11.28
ccc	P	40	7.24
cca	P	0	16.33
ccg	P	0	5.15
agt	S	0	501.50
agc	S	0	460.80
---	--	------	------
mPD		0.38	1.7
		nPD:	0.23
		N. weight:	1.7
		Sc. PD:	0.13
		Sc. rank:	1338.1

,

PA

Pos.	633	obs:	exp:
att	I	2489	926.90
atc	I	7	626.00
ata	I	0	943.20
---	--	------	------
mPD		0.0056	0.66
		nPD:	0.01
		N. weight:	1.2
		Sc. PD:	-0.17
		Sc. rank:	-1202.2

,

PA

Pos.	634	obs:	exp:
ggt	G	0	359.10
ggc	G	0	329.40
gga	G	294	1090.00
ggg	G	2202	717.90
---	--	------	------
mPD		0.21	0.69
		nPD:	0.3
		N. weight:	1.
		Sc. PD:	0.15
		Sc. rank:	1020.7

,

PA

Pos.	635	obs:	exp:
aaa	K	76	1401.00
aag	K	2420	1095.00
---	--	------	------
mPD		0.059	0.49
		nPD:	0.12
		N. weight:	0.85
		Sc. PD:	-0.024
		Sc. rank:	511.8

,

PA

Pos.	636	obs:	exp:
gtt	V	0	542.80
gtc	V	0	527.40
gta	V	312	491.10
gtg	V	2184	934.70
---	--	------	------
mPD		0.22	0.73
		nPD:	0.3
		N. weight:	0.86
		Sc. PD:	0.13
		Sc. rank:	828.8

,

PA

Pos.	637	obs:	exp:
tgt	C	546	1085.00
tgc	C	1950	1411.00
---	--	------	------
mPD		0.34	0.49
		nPD:	0.7
		N. weight:	0.13
		Sc. PD:	0.069
		Sc. rank:	205.1

,

PA

Pos.	638	obs:	exp:
cgt	R	0	94.66
cgc	R	0	133.00
cga	R	0	241.70
cgg	R	0	195.50
aga	R	2485	1145.00
agg	R	11	686.30
---	--	------	------
mPD		0.0088	0.95
		nPD:	0.01
		N. weight:	0.94
		Sc. PD:	-0.13
		Sc. rank:	-916.7

,

PA

Pos.	639	obs:	exp:
act	T	8	661.20
acc	T	10	551.50
aca	T	2472	1073.00
acg	T	6	210.80
---	--	------	------
mPD		0.019	0.69
		nPD:	0.03
		N. weight:	0.99
		Sc. PD:	-0.12
		Sc. rank:	-116.7

,

PA

Pos.	640	obs:	exp:
tta	L	975	272.40
ttg	L	1477	468.40
ctt	L	0	468.00
ctc	L	0	430.60
cta	L	14	332.10
ctg	L	30	524.40
---	--	------	------
mPD		0.51	1.1
		nPD:	0.45
		N. weight:	1.4
		Sc. PD:	0.42
		Sc. rank:	1911.3

,

PA

Pos.	641	obs:	exp:
tta	L	98	272.40
ttg	L	1	468.40
ctt	L	0	468.00
ctc	L	0	430.60
cta	L	2373	332.10
ctg	L	24	524.40
---	--	------	------
mPD		0.096	1.1
		nPD:	0.08
		N. weight:	2.2
		Sc. PD:	-0.14
		Sc. rank:	1198.0

,

PA

Pos.	642	obs:	exp:
gct	A	0	635.00
gcc	A	0	486.70
gca	A	2494	1097.00
gcg	A	2	277.10
---	--	------	------
mPD		0.0016	0.69
		nPD:	0.
		N. weight:	1.
		Sc. PD:	-0.15
		Sc. rank:	-1553.7

,

PA

Pos.	643	obs:	exp:
cgt	R	0	0.11
cgc	R	0	0.16
cga	R	0	0.29
cgg	R	0	0.23
aaa	K	991	1399.00
aag	K	1502	1094.00
aga	R	0	1.38
agg	R	3	0.82
---	--	------	------
mPD		0.48	0.50
		nPD:	0.97
		N. weight:	0.071
		Sc. PD:	0.057
		Sc. rank:	123.1

,

PA

Pos.	644	obs:	exp:
tct	S	2489	426.30
tcc	S	5	368.00
tca	S	0	562.60
tcg	S	2	161.20
agt	S	0	509.60
agc	S	0	468.30
---	--	------	------
mPD		0.0056	1.6
		nPD:	0.
		N. weight:	2.2
		Sc. PD:	-0.31
		Sc. rank:	-3096.0

,

PA

Pos.	645	obs:	exp:
gtt	V	1	540.90
gtc	V	2	525.50
gta	V	873	489.30
gtg	V	1611	931.30
gct	A	0	2.29
gcc	A	0	1.76
gca	A	9	3.96
gcg	A	0	1.00
---	--	------	------
mPD		0.47	0.74
		nPD:	0.63
		N. weight:	0.69
		Sc. PD:	0.33
		Sc. rank:	1054.0

,

PA

Pos.	646	obs:	exp:
ttt	F	1	1062.00
ttc	F	2495	1434.00
---	--	------	------
mPD		0.00080	0.49
		nPD:	0.
		N. weight:	0.69
		Sc. PD:	-0.099
		Sc. rank:	-1081.2

,

PA

Pos.	647	obs:	exp:
aat	N	90	1326.00
aac	N	2406	1170.00
---	--	------	------
mPD		0.070	0.50
		nPD:	0.14
		N. weight:	0.75
		Sc. PD:	-0.0071
		Sc. rank:	490.0

,

PA

Pos.	648	obs:	exp:
tct	S	0	425.10
tcc	S	0	366.90
tca	S	0	561.10
tcg	S	0	160.80
aat	N	0	1.59
aac	N	3	1.41
agt	S	10	508.20
agc	S	2479	467.00
ggt	G	0	0.58
ggc	G	4	0.53
gga	G	0	1.75
ggg	G	0	1.15
---	--	------	------
mPD		0.014	1.6
		nPD:	0.01
		N. weight:	2.1
		Sc. PD:	-0.28
		Sc. rank:	-2065.0

,

PA

Pos.	649	obs:	exp:
tta	L	6	272.40
ttg	L	7	468.40
ctt	L	0	468.00
ctc	L	4	430.60
cta	L	2189	332.10
ctg	L	290	524.40
---	--	------	------
mPD		0.22	1.1
		nPD:	0.2
		N. weight:	1.9
		Sc. PD:	0.089
		Sc. rank:	1452.8

,

PA

Pos.	650	obs:	exp:
tat	Y	2470	1213.00
tac	Y	26	1283.00
---	--	------	------
mPD		0.021	0.50
		nPD:	0.04
		N. weight:	0.83
		Sc. PD:	-0.087
		Sc. rank:	209.6

,

PA

Pos.	651	obs:	exp:
gct	A	1	635.00
gcc	A	0	486.70
gca	A	2486	1097.00
gcg	A	9	277.10
---	--	------	------
mPD		0.0080	0.69
		nPD:	0.01
		N. weight:	1.
		Sc. PD:	-0.13
		Sc. rank:	-846.8

,

PA			
Pos.	652	obs:	exp:
tct	S	2488	426.10
tcc	S	7	367.80
tca	S	0	562.40
tcg	S	0	161.20
act	T	1	0.26
acc	T	0	0.22
aca	T	0	0.43
acg	T	0	0.08
agt	S	0	509.40
agc	S	0	468.10
mPD		0.0064	1.6
		nPD:	0.
		N. weight:	2.2
		Sc. PD:	-0.31
		Sc. rank:	-2965.5

PA			
Pos.	653	obs:	exp:
tct	S	0	0.34
tcc	S	0	0.29
tca	S	2	0.45
tcg	S	0	0.13
cct	P	0	703.30
ccc	P	1	451.30
cca	P	2489	1018.00
ccg	P	4	321.10
agt	S	0	0.41
agc	S	0	0.38
mPD		0.0056	0.71
		nPD:	0.01
		N. weight:	1.1
		Sc. PD:	-0.15
		Sc. rank:	-1192.0

PA			
Pos.	654	obs:	exp:
caa	Q	2482	1270.00
cag	Q	14	1226.00
mPD		0.011	0.50
		nPD:	0.02
		N. weight:	0.8
		Sc. PD:	-0.099
		Sc. rank:	-280.6

PA			
Pos.	655	obs:	exp:
tta	L	0	272.40
ttg	L	0	468.40
ctt	L	579	468.00
ctc	L	1905	430.60
cta	L	12	332.10
ctg	L	0	524.40
mPD		0.36	1.1
		nPD:	0.32
		N. weight:	1.5
		Sc. PD:	0.24
		Sc. rank:	1591.1

PA			
Pos.	656	obs:	exp:
gaa	E	13	1495.00
gag	E	2483	1001.00
mPD		0.010	0.48
		nPD:	0.02
		N. weight:	1.1
		Sc. PD:	-0.14
		Sc. rank:	-401.0

PA			
Pos.	657	obs:	exp:
ggt	G	3	359.10
ggc	G	2	329.40
gga	G	498	1090.00
ggg	G	1993	717.90
mPD		0.32	0.69
		nPD:	0.47
		N. weight:	0.81
		Sc. PD:	0.25
		Sc. rank:	1114.2

PA			
Pos.	658	obs:	exp:
ttt	F	2490	1062.00
ttc	F	5	1433.00
tct	S	1	0.17
tcc	S	0	0.15
tca	S	0	0.23
tcg	S	0	0.06
agt	S	0	0.20
agc	S	0	0.19
mPD		0.0048	0.49
		nPD:	0.01
		N. weight:	1.
		Sc. PD:	-0.14
		Sc. rank:	-956.1

PA			
Pos.	659	obs:	exp:
tct	S	5	426.30
tcc	S	0	368.00
tca	S	1813	562.60
tcg	S	678	161.20
agt	S	0	509.60
agc	S	0	468.30
mPD		0.40	1.6
		nPD:	0.24
		N. weight:	1.5
		Sc. PD:	0.14
		Sc. rank:	1275.0

PA			
Pos.	660	obs:	exp:
gct	A	2484	635.00
gcc	A	7	486.70
gca	A	2	1097.00
gcg	A	3	277.10
mPD		0.0096	0.69
		nPD:	0.01
		N. weight:	1.7
		Sc. PD:	-0.22
		Sc. rank:	-1208.4

PA			
Pos.	661	obs:	exp:
gaa	E	1930	1495.00
gag	E	566	1001.00
mPD		0.35	0.48
		nPD:	0.73
		N. weight:	0.087
		Sc. PD:	0.049
		Sc. rank:	142.7

PA			
Pos.	662	obs:	exp:
tct	S	0	426.30
tcc	S	0	368.00
tca	S	1938	562.60
tcg	S	558	161.20
agt	S	0	509.60
agc	S	0	468.30
mPD		0.35	1.6
		nPD:	0.21
		N. weight:	1.5
		Sc. PD:	0.093
		Sc. rank:	1190.8

PA			
Pos.	663	obs:	exp:
cgt	R	0	94.66
cgc	R	0	133.00
cga	R	0	241.70
cgg	R	0	195.50
aga	R	2195	1145.00
agg	R	301	686.30
mPD		0.21	0.95
		nPD:	0.22
		N. weight:	0.59
		Sc. PD:	0.042
		Sc. rank:	465.1

PA			
Pos.	664	obs:	exp:
cgt	R	0	0.04
cgc	R	0	0.05
cga	R	0	0.10
cgg	R	0	0.08
aaa	K	799	1400.00
aag	K	1696	1095.00
aga	R	0	0.46
agg	R	1	0.27
mPD		0.44	0.49
		nPD:	0.88
		N. weight:	0.15
		Sc. PD:	0.11
		Sc. rank:	257.8

PA			
Pos.	665	obs:	exp:
tta	L	587	271.80
ttg	L	1893	467.20
ctt	L	0	466.90
ctc	L	0	429.60
cta	L	0	331.30
ctg	L	10	523.20
atg	M	6	6.00
mPD		0.37	1.1
		nPD:	0.33
		N. weight:	1.5
		Sc. PD:	0.27
		Sc. rank:	1755.2

PA			
Pos.	666	obs:	exp:
tta	L	0	272.40
ttg	L	0	468.40
ctt	L	2459	468.00
ctc	L	36	430.60
cta	L	1	332.10
ctg	L	0	524.40
mPD		0.029	1.1
		nPD:	0.03
		N. weight:	2.
		Sc. PD:	-0.24
		Sc. rank:	-401.2

PA			
Pos.	667	obs:	exp:
tta	L	0	272.40
ttg	L	0	468.40
ctt	L	22	468.00
ctc	L	2474	430.60
cta	L	0	332.10
ctg	L	0	524.40
mPD		0.017	1.1
		nPD:	0.02
		N. weight:	2.1
		Sc. PD:	-0.28
		Sc. rank:	-1422.8

PA			
Pos.	668	obs:	exp:
att	I	2496	926.90
atc	I	0	626.00
ata	I	0	943.20
mPD		0	0.66
		nPD:	0.
		N. weight:	1.2
		Sc. PD:	-0.18
		Sc. rank:	-2135.5

PA			
Pos.	669	obs:	exp:
att	I	2	0.74
atc	I	0	0.50
ata	I	0	0.76
gtt	V	2489	542.40
gtc	V	5	527.00
gta	V	0	490.70
gtg	V	0	933.90
mPD		0.0056	0.73
		nPD:	0.01
		N. weight:	1.9
		Sc. PD:	-0.26
		Sc. rank:	-2048.3

PA			
Pos.	670	obs:	exp:
caa	Q	5	1270.00
cag	Q	2491	1226.00
mPD		0.0040	0.50
		nPD:	0.01
		N. weight:	0.87
		Sc. PD:	-0.12
		Sc. rank:	-928.2

PA			
Pos.	671	obs:	exp:
gct	A	2	635.00
gcc	A	0	486.70
gca	A	2493	1097.00
gcg	A	1	277.10
mPD		0.0024	0.69
		nPD:	0.
		N. weight:	1.
		Sc. PD:	-0.14
		Sc. rank:	-1430.8

PA			
Pos.	672	obs:	exp:
ttt	F	575	246.00
ttc	F	3	332.00
tta	L	0	209.30
ttg	L	0	359.90
ctt	L	1918	359.70
ctc	L	0	330.90
cta	L	0	255.20
ctg	L	0	403.00
mPD		0.36	1.2
		nPD:	0.29
		N. weight:	1.8
		Sc. PD:	0.25
		Sc. rank:	1740.8

PA			
Pos.	673	obs:	exp:
cgt	R	0	94.47
cgc	R	0	132.70
cga	R	0	241.20
cgg	R	0	195.10
aaa	K	0	2.81
aag	K	5	2.19
aga	R	0	1143.00
agg	R	2491	684.90
mPD		0.0040	0.96
		nPD:	0.
		N. weight:	1.6
		Sc. PD:	-0.23
		Sc. rank:	-2174.9

PA			
Pos.	674	obs:	exp:
gat	D	9	1436.00
gac	D	2487	1060.00
mPD		0.0072	0.49
		nPD:	0.01
		N. weight:	1.
		Sc. PD:	-0.14
		Sc. rank:	-725.6

PA			
Pos.	675	obs:	exp:
tct	S	0	0.17
tcc	S	0	0.15
tca	S	0	0.23
tcg	S	0	0.06
aat	N	22	1325.00
aac	N	2472	1169.00
agt	S	0	0.20
agc	S	1	0.19
gat	D	0	0.58
gac	D	1	0.42
mPD		0.019	0.50
		nPD:	0.04
		N. weight:	0.88
		Sc. PD:	-0.096
		Sc. rank:	187.1

PA			
Pos.	676	obs:	exp:
tta	L	0	272.40
ttg	L	2	468.40
ctt	L	1	468.00
ctc	L	0	430.60
cta	L	15	332.10
ctg	L	2478	524.40
mPD		0.014	1.1
		nPD:	0.01
		N. weight:	1.9
		Sc. PD:	-0.25
		Sc. rank:	-1474.0

PA			
Pos.	677	obs:	exp:
gaa	E	2101	1495.00
gag	E	395	1001.00
---	--	------	------
mPD		0.27	0.48
		nPD:	0.55
		N. weight:	0.18
		Sc. PD:	0.069
		Sc. rank:	259.5

PA			
Pos.	678	obs:	exp:
cct	P	2495	703.80
ccc	P	0	451.60
cca	P	1	1019.00
ccg	P	0	321.40
---	--	------	------
mPD		0.00080	0.70
		nPD:	0.
		N. weight:	1.6
		Sc. PD:	-0.23
		Sc. rank:	-2568.8

PA			
Pos.	679	obs:	exp:
ggt	G	7	359.10
ggc	G	0	329.40
gga	G	904	1090.00
ggg	G	1585	717.90
---	--	------	------
mPD		0.47	0.69
		nPD:	0.68
		N. weight:	0.53
		Sc. PD:	0.27
		Sc. rank:	829.2

PA			
Pos.	680	obs:	exp:
act	T	488	661.20
acc	T	2005	551.50
aca	T	3	1073.00
acg	T	0	210.80
---	--	------	------
mPD		0.32	0.69
		nPD:	0.46
		N. weight:	1.2
		Sc. PD:	0.37
		Sc. rank:	1656.1

PA			
Pos.	681	obs:	exp:
ttt	F	39	1062.00
ttc	F	2457	1434.00
---	--	------	------
mPD		0.031	0.49
		nPD:	0.06
		N. weight:	0.6
		Sc. PD:	-0.05
		Sc. rank:	268.2

PA			
Pos.	682	obs:	exp:
aat	N	38	20.19
aac	N	0	17.81
gat	D	2458	1414.00
gac	D	0	1044.00
---	--	------	------
mPD		0.030	0.52
		nPD:	0.06
		N. weight:	0.69
		Sc. PD:	-0.062
		Sc. rank:	277.4

PA			
Pos.	683	obs:	exp:
tta	L	0	268.20
ttg	L	0	461.00
ctt	L	2456	460.70
ctc	L	1	423.90
cta	L	0	326.90
ctg	L	0	516.20
att	I	8	2.97
atc	I	0	2.01
ata	I	0	3.02
gtt	V	31	6.74
gtc	V	0	6.55
gta	V	0	6.10
gtg	V	0	11.61
---	--	------	------
mPD		0.032	1.2
		nPD:	0.03
		N. weight:	2.1
		Sc. PD:	-0.25
		Sc. rank:	-318.8

PA			
Pos.	684	obs:	exp:
cgt	R	0	0.04
cgc	R	0	0.05
cga	R	0	0.10
cgg	R	0	0.08
aga	R	0	0.46
agg	R	1	0.27
gaa	E	0	1.80
gag	E	3	1.20
ggt	G	0	358.50
ggc	G	0	328.90
gga	G	26	1088.00
ggg	G	2466	716.80
---	--	------	------
mPD		0.024	0.69
		nPD:	0.03
		N. weight:	1.5
		Sc. PD:	-0.17
		Sc. rank:	192.7

PA			
Pos.	685	obs:	exp:
ggt	G	1	359.10
ggc	G	0	329.40
gga	G	6	1090.00
ggg	G	2489	717.90
---	--	------	------
mPD		0.0056	0.69
		nPD:	0.01
		N. weight:	1.5
		Sc. PD:	-0.21
		Sc. rank:	-1567.1

PA			
Pos.	686	obs:	exp:
tta	L	2	272.40
ttg	L	0	468.40
ctt	L	0	468.00
ctc	L	0	430.60
cta	L	2493	332.10
ctg	L	1	524.40
---	--	------	------
mPD		0.0024	1.1
		nPD:	0.
		N. weight:	2.5
		Sc. PD:	-0.36
		Sc. rank:	-3830.6

PA			
Pos.	687	obs:	exp:
tat	Y	2489	1213.00
tac	Y	7	1283.00
---	--	------	------
mPD		0.0056	0.50
		nPD:	0.01
		N. weight:	0.88
		Sc. PD:	-0.12
		Sc. rank:	-757.4

PA			
Pos.	688	obs:	exp:
gct	A	0	0.25
gcc	A	0	0.20
gca	A	1	0.44
gcg	A	0	0.11
gat	D	1	1.15
gac	D	1	0.85
gaa	E	2489	1492.00
gag	E	2	999.50
ggt	G	0	0.29
ggc	G	0	0.26
gga	G	2	0.87
ggg	G	0	0.58
---	--	------	------
mPD		0.0056	0.48
		nPD:	0.01
		N. weight:	0.63
		Sc. PD:	-0.085
		Sc. rank:	-534.6

PA			
Pos.	689	obs:	exp:
gct	A	36	635.00
gcc	A	1	486.70
gca	A	2459	1097.00
gcg	A	0	277.10
---	--	------	------
mPD		0.029	0.69
		nPD:	0.04
		N. weight:	0.94
		Sc. PD:	-0.098
		Sc. rank:	266.4

PA			
Pos.	690	obs:	exp:
att	I	2486	926.90
atc	I	10	626.00
ata	I	0	943.20
---	--	------	------
mPD		0.0080	0.66
		nPD:	0.01
		N. weight:	1.2
		Sc. PD:	-0.16
		Sc. rank:	-959.0

PA			
Pos.	691	obs:	exp:
gaa	E	0	1495.00
gag	E	2496	1001.00
---	--	------	------
mPD		0	0.48
		nPD:	0.
		N. weight:	1.1
		Sc. PD:	-0.17
		Sc. rank:	-1968.5

PA			
Pos.	692	obs:	exp:
gaa	E	0	1495.00
gag	E	2496	1001.00
---	--	------	------
mPD		0	0.48
		nPD:	0.
		N. weight:	1.1
		Sc. PD:	-0.17
		Sc. rank:	-1968.5

PA			
Pos.	693	obs:	exp:
tgt	C	3	1085.00
tgc	C	2493	1411.00
---	--	------	------
mPD		0.0024	0.49
		nPD:	0.
		N. weight:	0.7
		Sc. PD:	-0.099
		Sc. rank:	-894.2

PA			
Pos.	694	obs:	exp:
tta	L	0	272.40
ttg	L	0	468.40
ctt	L	0	468.00
ctc	L	0	430.60
cta	L	6	332.10
ctg	L	2490	524.40
---	--	------	------
mPD		0.0048	1.1
		nPD:	0.
		N. weight:	1.9
		Sc. PD:	-0.27
		Sc. rank:	-2586.7

PA			
Pos.	695	obs:	exp:
att	I	2494	926.10
atc	I	0	625.50
ata	I	0	942.40
gtt	V	2	0.43
gtc	V	0	0.42
gta	V	0	0.39
gtg	V	0	0.75
---	--	------	------
mPD		0.0016	0.66
		nPD:	0.
		N. weight:	1.2
		Sc. PD:	-0.18
		Sc. rank:	-1818.3

PA			
Pos.	696	obs:	exp:
tct	S	0	0.17
tcc	S	0	0.15
tca	S	0	0.23
tcg	S	0	0.06
aat	N	1009	1326.00
aac	N	1486	1169.00
agt	S	0	0.20
agc	S	1	0.19
---	--	------	------
mPD		0.48	0.50
		nPD:	0.97
		N. weight:	0.043
		Sc. PD:	0.034
		Sc. rank:	74.8

PA			
Pos.	697	obs:	exp:
gat	D	2492	1436.00
gac	D	4	1060.00
---	--	------	------
mPD		0.0032	0.49
		nPD:	0.01
		N. weight:	0.68
		Sc. PD:	-0.094
		Sc. rank:	-796.1

PA			
Pos.	698	obs:	exp:
cct	P	23	703.80
ccc	P	2187	451.60
cca	P	286	1019.00
ccg	P	0	321.40
---	--	------	------
mPD		0.22	0.70
		nPD:	0.31
		N. weight:	1.5
		Sc. PD:	0.24
		Sc. rank:	1580.1

PA

Pos.	699	obs:	exp:
tgg	W	2496	2496.00
---	--	------	------
mPD		0	0
		nPD:	1.
		N. weight:	0.
		Sc. PD:	0
		Sc. rank:	0

,

PA

Pos.	700	obs:	exp:
gtt	V	2495	542.60
gtc	V	0	527.20
gta	V	0	490.90
gtg	V	0	934.30
gct	A	1	0.25
gcc	A	0	0.20
gca	A	0	0.44
gcg	A	0	0.11
---	--	------	------
mPD		0.00080	0.73
		nPD:	0.
		N. weight:	1.9
		Sc. PD:	-0.27
		Sc. rank:	-3118.3

,

PA

Pos.	701	obs:	exp:
tta	L	2	272.40
ttg	L	2494	468.40
ctt	L	0	468.00
ctc	L	0	430.60
cta	L	0	332.10
ctg	L	0	524.40
---	--	------	------
mPD		0.0016	1.1
		nPD:	0.
		N. weight:	2.1
		Sc. PD:	-0.3
		Sc. rank:	-3328.6

,

PA

Pos.	702	obs:	exp:
tta	L	0	272.30
ttg	L	0	468.20
ctt	L	2494	467.90
ctc	L	0	430.50
cta	L	0	332.00
ctg	L	1	524.20
att	I	1	0.37
atc	I	0	0.25
ata	I	0	0.38
---	--	------	------
mPD		0.0016	1.1
		nPD:	0.
		N. weight:	2.1
		Sc. PD:	-0.3
		Sc. rank:	-3355.1

,

PA

Pos.	703	obs:	exp:
aat	N	2495	1326.00
aac	N	1	1170.00
---	--	------	------
mPD		0.00080	0.50
		nPD:	0.
		N. weight:	0.78
		Sc. PD:	-0.11
		Sc. rank:	-1241.7

,

PA

Pos.	704	obs:	exp:
gct	A	0	635.00
gcc	A	0	486.70
gca	A	553	1097.00
gcg	A	1943	277.10
---	--	------	------
mPD		0.35	0.69
		nPD:	0.5
		N. weight:	1.7
		Sc. PD:	0.58
		Sc. rank:	2376.0

,

PA

Pos.	705	obs:	exp:
tct	S	2496	426.30
tcc	S	0	368.00
tca	S	0	562.60
tcg	S	0	161.20
agt	S	0	509.60
agc	S	0	468.30
---	--	------	------
mPD		0	1.6
		nPD:	0.
		N. weight:	2.2
		Sc. PD:	-0.32
		Sc. rank:	-3809.8

,

PA

Pos.	706	obs:	exp:
tgg	W	2496	2496.00
---	--	------	------
mPD		0	0
		nPD:	1.
		N. weight:	0.
		Sc. PD:	0
		Sc. rank:	0

,

PA

Pos.	707	obs:	exp:
ttt	F	5	1062.00
ttc	F	2491	1434.00
---	--	------	------
mPD		0.0040	0.49
		nPD:	0.01
		N. weight:	0.67
		Sc. PD:	-0.093
		Sc. rank:	-689.5

,

PA

Pos.	708	obs:	exp:
aat	N	0	1326.00
aac	N	2496	1170.00
---	--	------	------
mPD		0	0.50
		nPD:	0.
		N. weight:	0.94
		Sc. PD:	-0.14
		Sc. rank:	-1634.1

,

PA

Pos.	709	obs:	exp:
tct	S	0	426.30
tcc	S	2496	368.00
tca	S	0	562.60
tcg	S	0	161.20
agt	S	0	509.60
agc	S	0	468.30
---	--	------	------
mPD		0	1.6
		nPD:	0.
		N. weight:	2.4
		Sc. PD:	-0.35
		Sc. rank:	-4126.9

,

PA

Pos.	710	obs:	exp:
ttt	F	0	1062.00
ttc	F	2496	1434.00
---	--	------	------
mPD		0	0.49
		nPD:	0.
		N. weight:	0.69
		Sc. PD:	-0.1
		Sc. rank:	-1194.9

,

PA

Pos.	711	obs:	exp:
tta	L	0	272.30
ttg	L	0	468.20
ctt	L	543	467.90
ctc	L	1951	430.50
cta	L	1	332.00
ctg	L	0	524.20
att	I	1	0.37
atc	I	0	0.25
ata	I	0	0.38
---	--	------	------
mPD		0.34	1.1
		nPD:	0.3
		N. weight:	1.5
		Sc. PD:	0.22
		Sc. rank:	1474.3

,

PA

Pos.	712	obs:	exp:
att	I	0	1.49
atc	I	0	1.00
ata	I	4	1.51
act	T	0	659.30
acc	T	0	549.90
aca	T	2488	1070.00
acg	T	1	210.20
aaa	K	1	0.56
aag	K	0	0.44
gct	A	0	0.51
gcc	A	0	0.39
gca	A	2	0.88
gcg	A	0	0.22
---	--	------	------
mPD		0.0064	0.69
		nPD:	0.01
		N. weight:	1.
		Sc. PD:	-0.14
		Sc. rank:	-1007.6

,

PA

Pos.	713	obs:	exp:
cat	H	2495	1484.00
cac	H	1	1012.00
---	--	------	------
mPD		0.00080	0.48
		nPD:	0.
		N. weight:	0.64
		Sc. PD:	-0.093
		Sc. rank:	-1007.5

,

PA

Pos.	714	obs:	exp:
gtt	V	0	0.22
gtc	V	0	0.21
gta	V	1	0.20
gtg	V	0	0.37
gct	A	0	634.20
gcc	A	0	486.20
gca	A	2493	1096.00
gcg	A	0	276.80
ggt	G	0	0.29
ggc	G	0	0.26
gga	G	2	0.87
ggg	G	0	0.58
---	--	------	------
mPD		0.0024	0.69
		nPD:	0.
		N. weight:	1.
		Sc. PD:	-0.15
		Sc. rank:	-1450.7

,

PA

Pos.	715	obs:	exp:
tta	L	0	236.20
ttg	L	13	406.10
ctt	L	3	405.80
ctc	L	0	373.40
cta	L	24	287.90
ctg	L	2124	454.70
caa	Q	0	0.51
cag	Q	1	0.49
cgt	R	0	12.55
cgc	R	0	17.63
cga	R	12	32.05
cgg	R	319	25.92
aga	R	0	151.80
agg	R	0	91.01
---	--	------	------
mPD		0.27	1.5
		nPD:	0.19
		N. weight:	2.
		Sc. PD:	0.073
		Sc. rank:	1456.2

,

PA

Pos.	716	obs:	exp:
caa	Q	222	113.00
cag	Q	0	109.00
cgt	R	0	0.04
cgc	R	0	0.05
cga	R	0	0.10
cgg	R	0	0.08
act	T	0	0.26
acc	T	0	0.22
aca	T	1	0.43
acg	T	0	0.08
aat	N	0	1.59
aac	N	3	1.41
aaa	K	2251	1272.00
aag	K	15	994.10
aga	R	1	0.46
agg	R	0	0.27
gaa	E	3	1.80
gag	E	0	1.20
---	--	------	------
mPD		0.18	0.66
		nPD:	0.27
		N. weight:	0.69
		Sc. PD:	0.083
		Sc. rank:	618.8

,

PA

Pos.	717	obs:	exp:
taa	.	8	915.30
tag	.	2488	700.50
tga	.	0	880.20
---	--	------	------
mPD		0.0064	0.86
		nPD:	0.01
		N. weight:	1.6
		Sc. PD:	-0.22
		Sc. rank:	-1737.1

}

Amino acid level conservation per codon

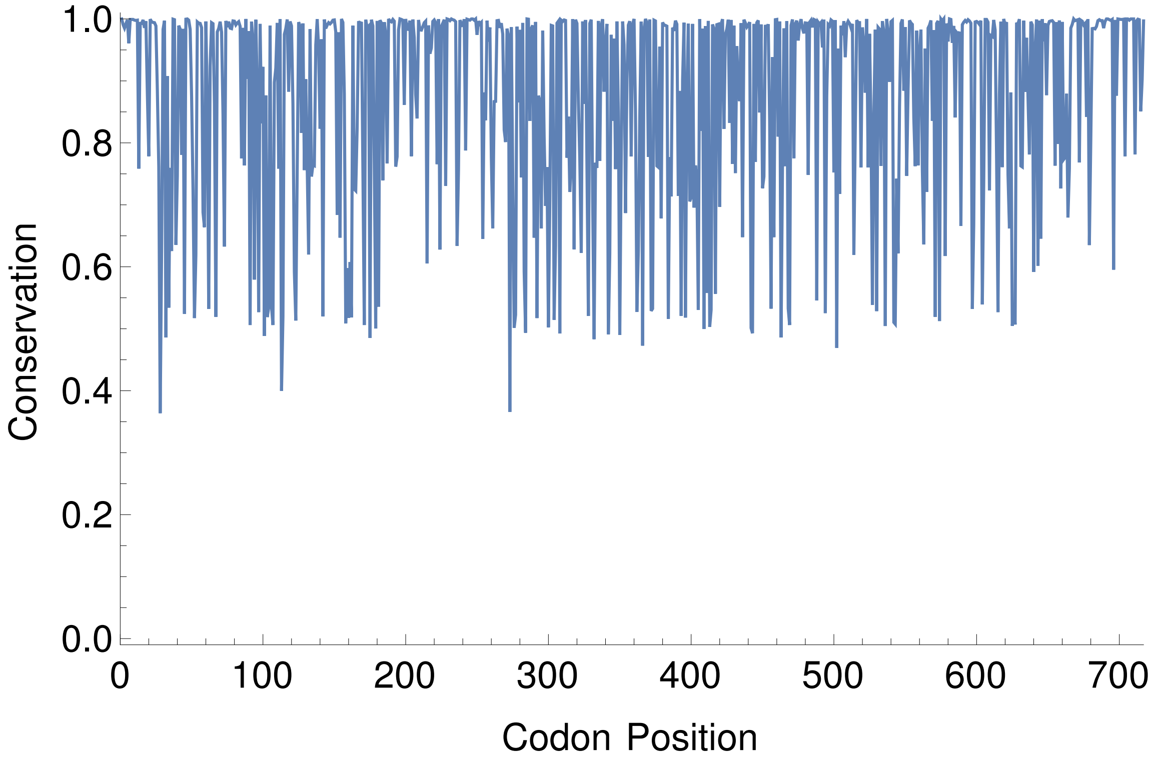

Conservation analysis plots

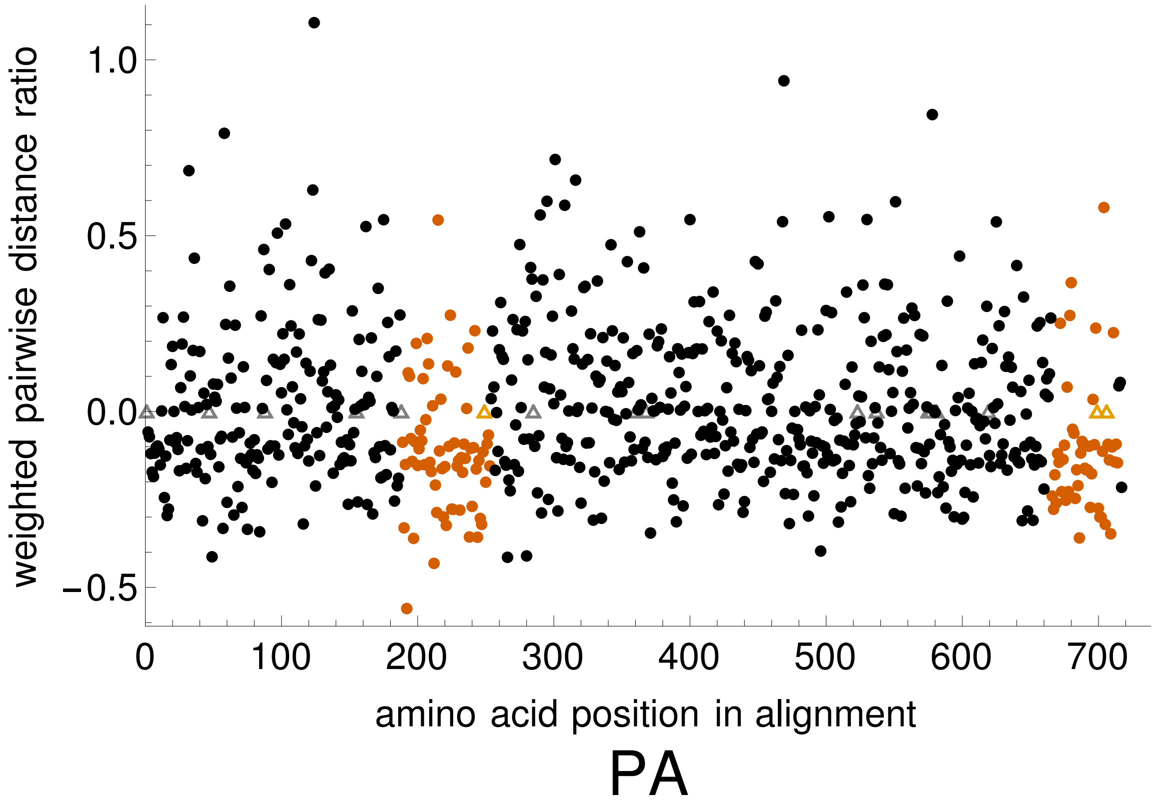

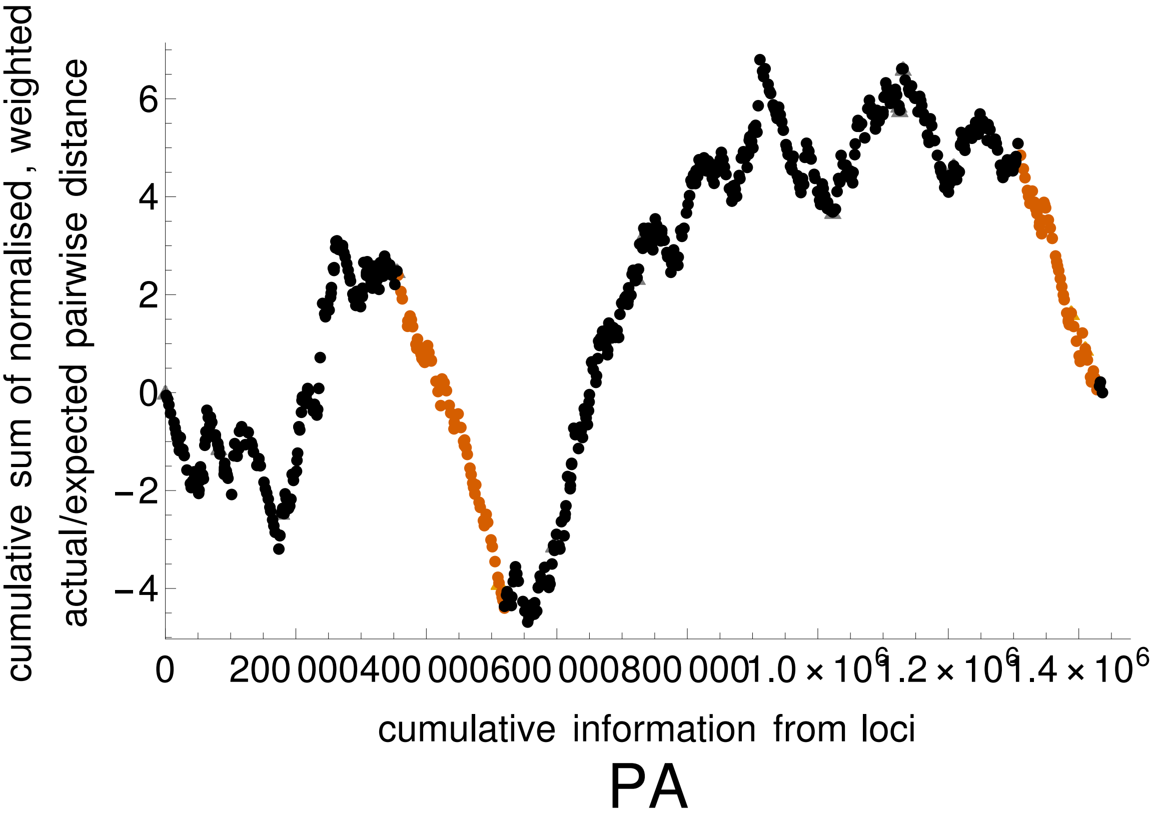

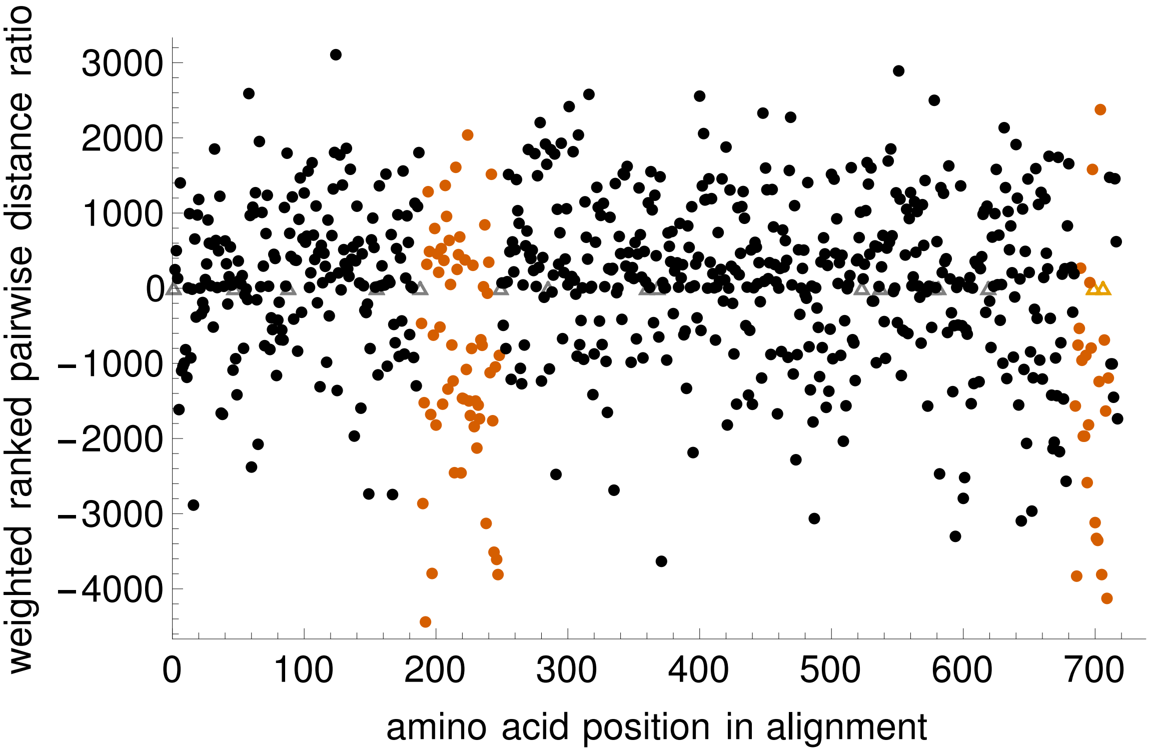
weighted ranked pairwise distance ratio
3000
2000
1000
0
−1000
−2000
−3000
−4000
0
100
200
300
400
500
600
700
amino acid position in alignment

PA

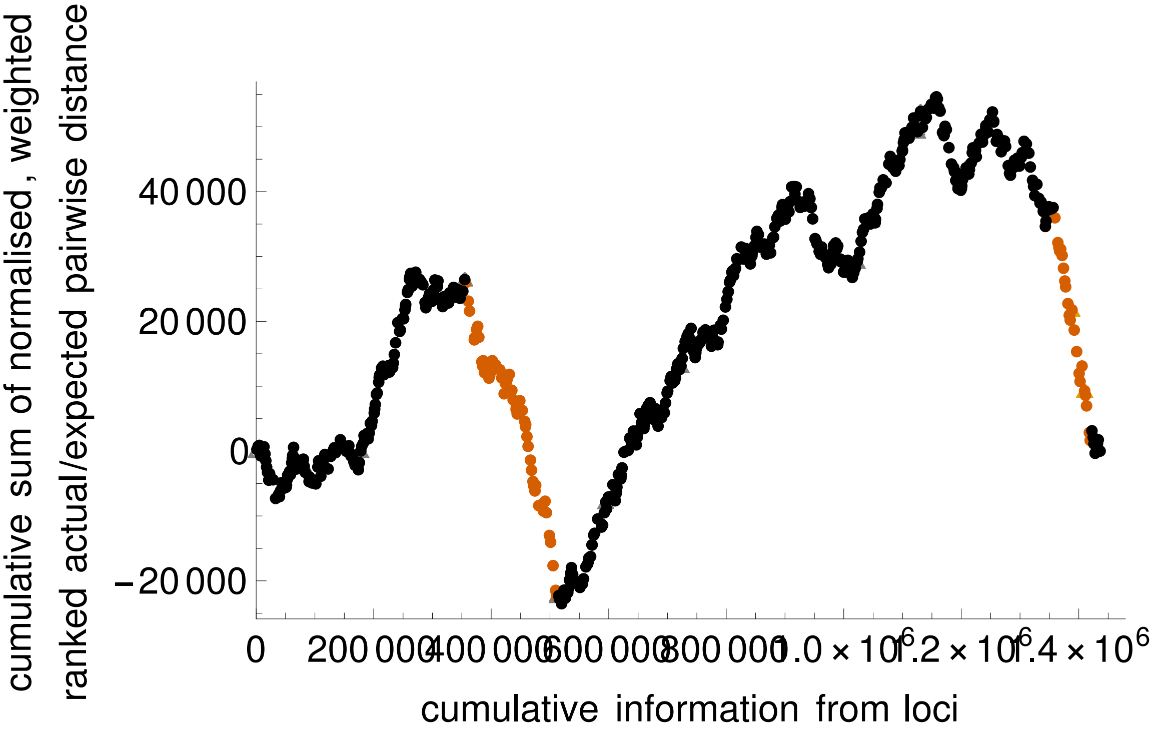
cumulative sum of normalised, weighted ranked actual/expected pairwise distance
40 000
20 000
0
−20 000
0
200 000
400 000
600 000
800 000
1.0 × 10^6
1.2 × 10^6
1.4 × 10^6
cumulative information from loci

PA

PA-X

Gene length histogram

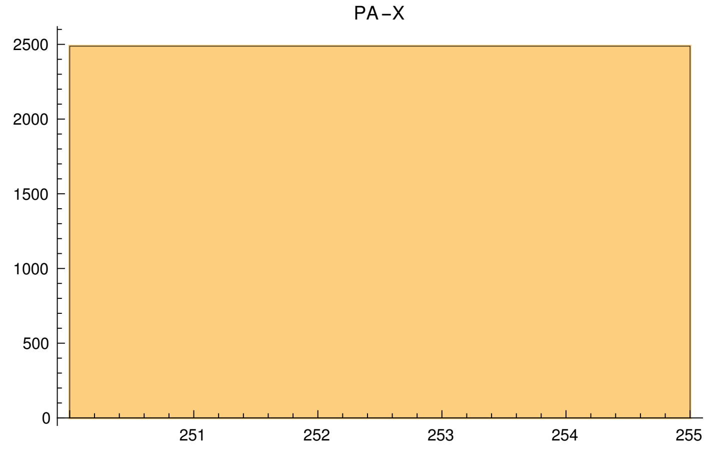

Information vs. nPD

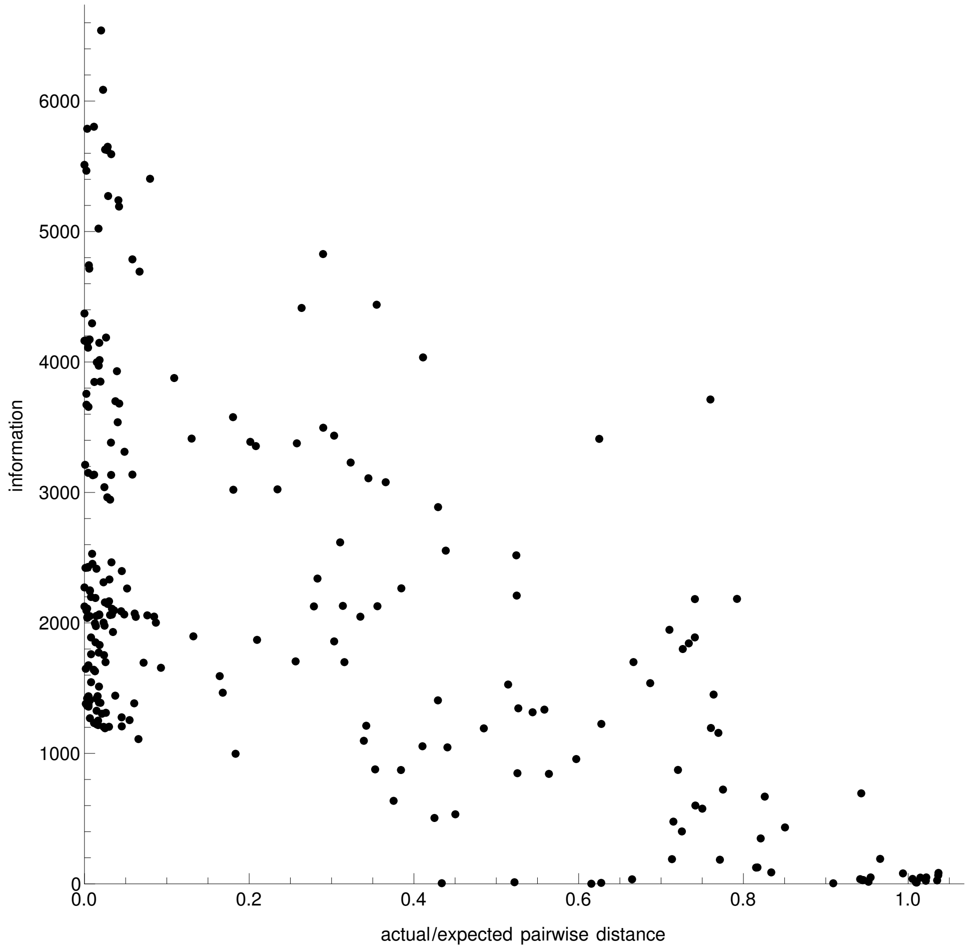

PA–X

Example sequences highlighted by regions found in analyses to be conserved

Interesting points (by weighted raw PD) highlighted for gene PA-X:

ATGGAAGACTTTGTGCGACAATGCTTCAATCCAATGATTGTCGAGCTTGCGGAAAAGGCAATGAAAGAATATGGGGAG⋱
GATCCGAAAATCGAAACGAACAAATTTGCAGCAATATGCACACACCTAGAAGTCTGTTTCATGTATTCGGATTTTCA⋱
TTTTATTGATGAACGAGGTGGATCAATAATTGTAGAATCTGGCGACCCGAATGCATTATTGAAACACCGATTTGAGA⋱
TAATTGAAGGGAGAGACCGAACAATGGCCTGGACAGTGGTAAATAGTATTTGCCACACCACAGGGGTCGAGAAACCT⋱
AAATTCCTCCCAGATCTGTATGACTACAAAGAGAACCGATTCATCGAAATTGGAGTAACACGAAGGGAAGTTCACAT⋱
ATACTATCTAGAAAAAGCCAACAAGATAAAATCAGAGAAGACACACATTCACATATTCTCATTCACTGGAGAGGAGA⋱
TGGCCACCAAAGCGGACTACACCCTTGACGAGGAGAGCAGAGCAAGAATCAAAACCAGGCTGTTCACTATAAGGCAA⋱
GAAATGGCCAATAGGGGTCTATGGGATTCCTTTGTCAGTCCG
AGAGAGGCGAAGAGACAATTGAAGAAAGATTTGAAATTACAGGAACCATGCGCAG
GCTTGCCGACCAAAGTCTCCCACCGAACTTCTCCAGCCTTGAAAACTTTAGA
GCCTATGTGGATGGATTCGAACCGAACGGCTGCATTGAGGGCAAGCTTTCTC
AAATGTCAAAAGAAGTGA

Interesting points (by weighted ranked PD) highlighted for gene PA-X:

ATGGAAGACTTTGTGCGACAATGCTTCAATCCAATGATTGTCGAGCTTGCGGAAAAGGCAATGAAAGAATATGGGGAG⋱
GATCCGAAAATCGAAACGAACAAATTTGCAGCAATATGCACACACCTAGAAGTCTGTTTCATGTATTCGGATTTTCA⋱
TTTTATTGATGAACGAGGTGGATCAATAATTGTAGAATCTGGCGACCCGAATGCATTATTGAAACACCGATTTGAGA⋱
TAATTGAAGGGAGAGACCGAACAATGGCCTGGACAGTGGTAAATAGTATTTGCCACACCACAGGGGTCGAGAAACCT⋱
AAATTCCTCCCAGATCTGTATGACTACAAAGAGAACCGATTCATCGAAATTGGAGTAACACGAAGGGAAGTTCACAT⋱
ATACTATCTAGAAAAAGCCAACAAGATAAAATCAGAGAAGACACACATTCACATATTCTCATTCACTGGAGAGGAGA⋱
TGGCCACCAAAGCGGACTACACCCTTGACGAGGAGAGCAGAGCAAGAATCAAAACCAGGCTGTTCACTATAAGGCAA⋱
GAAATGGCCAATAGGGGTCTATGGGATTCCTTTGTCAGTCCGAGAGAGGCGAAGAGACAATTGAAGAAAGATTTGAA⋱
ATTACAGGAACCATGCGCAGGCTTGCCGACCAAAGTCTCCCACCGAACTTCTCCAGCCTTGAAAACTTTAGAGCCTA⋱
TGTGGATGGATTCGAACCGAACGGCTGCA **TTGAGGGCAAGCTTTCTCAAA**TGTCAAAAGAAGTGA

Per codon conservation report

{

PA-X			
Pos .	1	obs :	exp :
atg	M	2488	2488.00
---	--	------	------
mPD		0	0
		nPD :	1.
		N. weight :	0.
		Sc. PD :	0
		Sc. rank :	0

,

PA-X			
Pos .	2	obs :	exp :
caa	Q	0	5.60
cag	Q	11	5.40
gtt	V	0	0.22
gtc	V	0	0.21
gta	V	1	0.20
gtg	V	0	0.37
gaa	E	2465	1478.00
gag	E	4	990.70
ggt	G	0	1.01
ggc	G	0	0.92
gga	G	7	3.06
ggg	G	0	2.01
---	--	------	------
mPD		0.027	0.50
		nPD :	0.05
		N. weight :	0.59
		Sc. PD :	-0.045
		Sc. rank :	327.2

,

PA-X			
Pos .	3	obs :	exp :
cgt	R	0	0.04
cgc	R	0	0.05
cga	R	0	0.10
cgg	R	0	0.08
aat	N	0	1.59
aac	N	3	1.41
aaa	K	9	5.05
aag	K	0	3.95
aga	R	1	0.46
agg	R	0	0.27
gct	A	0	0.51
gcc	A	0	0.39
gca	A	2	0.88
gcg	A	0	0.22
gat	D	11	1417.00
gac	D	2452	1046.00
gaa	E	6	3.59
gag	E	0	2.41
ggt	G	0	0.58
ggc	G	4	0.53
gga	G	0	1.75
ggg	G	0	1.15
---	--	------	------
mPD		0.039	0.51
		nPD :	0.08
		N. weight :	0.96
		Sc. PD :	-0.053
		Sc. rank :	688.1

,

PA-X			
Pos .	4	obs :	exp :
ttt	F	2469	1052.00
ttc	F	3	1420.00
tta	L	0	1.31
ttg	L	2	2.25
ctt	L	9	2.25
ctc	L	0	2.07
cta	L	1	1.60
ctg	L	0	2.52
att	I	2	0.74
atc	I	0	0.50
ata	I	0	0.76
act	T	1	0.26
acc	T	0	0.22
aca	T	0	0.43
acg	T	0	0.08
gtt	V	1	0.22
gtc	V	0	0.21
gta	V	0	0.20
gtg	V	0	0.37
---	--	------	------
mPD		0.017	0.50
		nPD :	0.03
		N. weight :	0.99
		Sc. PD :	-0.096
		Sc. rank :	234.6

,

PA-X			
Pos .	5	obs :	exp :
tta	L	0	0.11
ttg	L	0	0.19
ctt	L	0	0.19
ctc	L	0	0.17
cta	L	0	0.13
ctg	L	1	0.21
atg	M	1	1.00
gtt	V	1	540.70
gtc	V	1	525.30
gta	V	0	489.10
gtg	V	2484	930.90
---	--	------	------
mPD		0.0032	0.73
		nPD :	0.
		N. weight :	1.1
		Sc. PD :	-0.14
		Sc. rank :	-1491.6

,

PA-X			
Pos .	6	obs :	exp :
cgt	R	0	94.32
cgc	R	0	132.50
cga	R	2390	240.80
cgg	R	94	194.80
aga	R	3	1141.00
agg	R	0	683.80
ggt	G	0	0.14
ggc	G	0	0.13
gga	G	1	0.44
ggg	G	0	0.29
---	--	------	------
mPD		0.076	0.95
		nPD :	0.08
		N. weight :	2.5
		Sc. PD :	-0.13
		Sc. rank :	1900.3

,

PA-X			
Pos .	7	obs :	exp :
caa	Q	2486	1265.00
cag	Q	1	1222.00
cgt	R	0	0.04
cgc	R	0	0.05
cga	R	0	0.10
cgg	R	1	0.08
aga	R	0	0.46
agg	R	0	0.27
---	--	------	------
mPD		0.0024	0.50
		nPD :	0.
		N. weight :	0.79
		Sc. PD :	-0.098
		Sc. rank :	-1002.8

,

PA-X			
Pos .	8	obs :	exp :
tat	Y	0	0.49
tac	Y	1	0.51
tgt	C	0	1081.00
tgc	C	2486	1405.00
ggt	G	0	0.14
ggc	G	1	0.13
gga	G	0	0.44
ggg	G	0	0.29
---	--	------	------
mPD		0.0016	0.49
		nPD :	0.
		N. weight :	0.67
		Sc. PD :	-0.084
		Sc. rank :	-999.6

,

PA-X			
Pos .	9	obs :	exp :
ttt	F	1	1058.00
ttc	F	2486	1429.00
tgt	C	0	0.43
tgc	C	1	0.57
---	--	------	------
mPD		0.0016	0.49
		nPD :	0.
		N. weight :	0.64
		Sc. PD :	-0.081
		Sc. rank :	-958.6

,

PA-X			
Pos .	10	obs :	exp :
att	I	1	0.37
atc	I	0	0.25
ata	I	0	0.38
aat	N	2483	1322.00
aac	N	4	1165.00
---	--	------	------
mPD		0.0040	0.50
		nPD :	0.01
		N. weight :	0.72
		Sc. PD :	-0.088
		Sc. rank :	-722.6

,

PA-X			
Pos .	11	obs :	exp :
cct	P	0	701.30
ccc	P	1	450.00
cca	P	2482	1015.00
ccg	P	4	320.20
act	T	0	0.26
acc	T	1	0.22
aca	T	0	0.43
acg	T	0	0.08
---	--	------	------
mPD		0.0056	0.71
		nPD :	0.01
		N. weight :	1.
		Sc. PD :	-0.13
		Sc. rank :	-1064.5

,

PA-X			
Pos .	12	obs :	exp :
tta	L	0	0.11
ttg	L	0	0.19
tgg	W	1	1.00
ctt	L	0	0.19
ctc	L	0	0.17
cta	L	0	0.13
ctg	L	1	0.21
att	I	0	1.11
atc	I	0	0.75
ata	I	3	1.13
atg	M	2483	2483.00
---	--	------	------
mPD		0.0048	0.0053
		nPD :	0.91
		N. weight :	0.0021
		Sc. PD :	0.0016
		Sc. rank :	3.8

,

PA-X			
Pos .	13	obs :	exp :
tta	L	0	0.11
ttg	L	0	0.19
ctt	L	1	0.19
ctc	L	0	0.17
cta	L	0	0.13
ctg	L	0	0.21
att	I	1887	922.40
atc	I	597	623.00
ata	I	0	938.60
act	T	0	0.26
acc	T	1	0.22
aca	T	0	0.43
acg	T	0	0.08
gtt	V	2	0.43
gtc	V	0	0.42
gta	V	0	0.39
gtg	V	0	0.75
---	--	------	------
mPD		0.37	0.66
		nPD :	0.56
		N. weight :	0.63
		Sc. PD :	0.26
		Sc. rank :	994.0

,

PA-X			
Pos .	14	obs :	exp :
ttt	F	0	0.43
ttc	F	1	0.57
att	I	0	3.71
atc	I	10	2.51
ata	I	0	3.78
gtt	V	1	537.60
gtc	V	2470	522.30
gta	V	1	486.40
gtg	V	0	925.70
gct	A	0	1.27
gcc	A	5	0.98
gca	A	0	2.20
gcg	A	0	0.56
---	--	------	------
mPD		0.014	0.74
		nPD :	0.02
		N. weight :	1.8
		Sc. PD :	-0.2
		Sc. rank :	-725.3

,

PA-X			
Pos .	15	obs :	exp :
aaa	K	0	0.56
aag	K	1	0.44
gaa	E	17	1489.00
gag	E	2470	997.90
---	--	------	------
mPD		0.014	0.48
		nPD :	0.03
		N. weight :	1.
		Sc. PD :	-0.1
		Sc. rank :	59.9

,

PA-X			
Pos.	16	obs:	exp:
ttt	F	1	0.43
ttc	F	0	0.57
tta	L	0	271.30
ttg	L	0	466.50
ctt	L	2484	466.20
ctc	L	1	428.90
cta	L	0	330.80
ctg	L	1	522.30
cgt	R	0	0.04
cgc	R	0	0.05
cga	R	0	0.10
cgg	R	1	0.08
aga	R	0	0.46
agg	R	0	0.27
---	--	------	------
mPD		0.0040	1.1
		nPD:	0.
		N. weight:	1.9
		Sc. PD:	-0.24
		Sc. rank:	-2667.0

,

PA-X			
Pos.	17	obs:	exp:
gtt	V	0	0.22
gtc	V	0	0.21
gta	V	0	0.20
gtg	V	1	0.37
gct	A	1	632.40
gcc	A	0	484.80
gca	A	33	1093.00
gcg	A	2452	276.00
ggt	G	0	0.14
ggc	G	0	0.13
gga	G	0	0.44
ggg	G	1	0.29
---	--	------	------
mPD		0.029	0.69
		nPD:	0.04
		N. weight:	2.5
		Sc. PD:	-0.22
		Sc. rank:	950.4

,

PA-X			
Pos.	18	obs:	exp:
aaa	K	2	1.12
aag	K	0	0.88
gtt	V	0	0.22
gtc	V	0	0.21
gta	V	0	0.20
gtg	V	1	0.37
gat	D	0	0.58
gac	D	1	0.42
gaa	E	2479	1487.00
gag	E	4	996.30
ggt	G	0	0.14
ggc	G	0	0.13
gga	G	1	0.44
ggg	G	0	0.29
---	--	------	------
mPD		0.0080	0.48
		nPD:	0.02
		N. weight:	0.59
		Sc. PD:	-0.066
		Sc. rank:	-372.9

,

PA-X			
Pos.	19	obs:	exp:
cgt	R	0	11.00
cgc	R	0	15.45
cga	R	0	28.08
cgg	R	0	22.71
aat	N	1	0.53
aac	N	0	0.47
aaa	K	9	1233.00
aag	K	2188	963.80
aga	R	0	133.00
agg	R	290	79.74
---	--	------	------
mPD		0.21	0.77
		nPD:	0.28
		N. weight:	1.
		Sc. PD:	0.14
		Sc. rank:	1080.4

,

PA-X			
Pos.	20	obs:	exp:
tct	S	0	0.17
tcc	S	0	0.15
tca	S	0	0.23
tcg	S	1	0.06
act	T	0	145.40
acc	T	0	121.30
aca	T	549	235.90
acg	T	0	46.37
agt	S	0	0.20
agc	S	0	0.19
gct	A	0	493.00
gcc	A	0	377.90
gca	A	1936	851.90
gcg	A	2	215.10
---	--	------	------
mPD		0.35	1.0
		nPD:	0.33
		N. weight:	0.96
		Sc. PD:	0.19
		Sc. rank:	1222.0

,

PA-X			
Pos.	21	obs:	exp:
atg	M	2487	2487.00
gtt	V	0	0.22
gtc	V	0	0.21
gta	V	0	0.20
gtg	V	1	0.37
---	--	------	------
mPD		0.00080	0.0013
		nPD:	0.62
		N. weight:	0.00046
		Sc. PD:	0.00022
		Sc. rank:	0.7

,

PA-X			
Pos.	22	obs:	exp:
cgt	R	0	0.11
cgc	R	0	0.16
cga	R	0	0.29
cgg	R	0	0.23
aat	N	1	0.53
aac	N	0	0.47
aaa	K	2476	1394.00
aag	K	8	1090.00
aga	R	3	1.38
agg	R	0	0.82
---	--	------	------
mPD		0.0096	0.50
		nPD:	0.02
		N. weight:	0.65
		Sc. PD:	-0.072
		Sc. rank:	-278.5

,

PA-X			
Pos.	23	obs:	exp:
gtt	V	0	0.22
gtc	V	0	0.21
gta	V	1	0.20
gtg	V	0	0.37
gaa	E	2474	1489.00
gag	E	13	997.90
---	--	------	------
mPD		0.011	0.48
		nPD:	0.02
		N. weight:	0.56
		Sc. PD:	-0.06
		Sc. rank:	-151.7

,

PA-X			
Pos.	24	obs:	exp:
ttt	F	2	0.85
ttc	F	0	1.15
tat	Y	2473	1205.00
tac	Y	6	1274.00
tgt	C	1	0.43
tgc	C	0	0.57
cat	H	6	3.57
cac	H	0	2.43
---	--	------	------
mPD		0.012	0.51
		nPD:	0.02
		N. weight:	0.82
		Sc. PD:	-0.087
		Sc. rank:	-211.2

,

PA-X			
Pos.	25	obs:	exp:
gtt	V	0	0.22
gtc	V	0	0.21
gta	V	0	0.20
gtg	V	1	0.37
ggt	G	1	357.80
ggc	G	0	328.30
gga	G	25	1086.00
ggg	G	2461	715.30
---	--	------	------
mPD		0.022	0.69
		nPD:	0.03
		N. weight:	1.4
		Sc. PD:	-0.14
		Sc. rank:	150.8

,

PA-X			
Pos.	26	obs:	exp:
gaa	E	2201	1490.00
gag	E	287	998.30
---	--	------	------
mPD		0.20	0.48
		nPD:	0.42
		N. weight:	0.24
		Sc. PD:	0.067
		Sc. rank:	342.3

,

PA-X			
Pos.	27	obs:	exp:
tct	S	0	0.17
tcc	S	0	0.15
tca	S	0	0.23
tcg	S	0	0.06
tat	Y	2	0.97
tac	Y	0	1.03
aat	N	586	311.40
aac	N	0	274.60
aaa	K	1	0.56
aag	K	0	0.44
agt	S	1	0.20
agc	S	0	0.19
gat	D	1893	1091.00
gac	D	3	805.40
ggt	G	2	0.29
ggc	G	0	0.26
gga	G	0	0.87
ggg	G	0	0.58
---	--	------	------
mPD		0.37	0.86
		nPD:	0.43
		N. weight:	0.66
		Sc. PD:	0.19
		Sc. rank:	959.8

,

PA-X			
Pos.	28	obs:	exp:
tta	L	0	0.44
ttg	L	0	0.75
tct	S	2	0.34
tcc	S	0	0.29
tca	S	0	0.45
tcg	S	0	0.13
ctt	L	0	0.75
ctc	L	0	0.69
cta	L	0	0.53
ctg	L	4	0.84
cct	P	909	699.60
ccc	P	4	448.90
cca	P	828	1013.00
ccg	P	740	319.50
caa	Q	1	0.51
cag	Q	0	0.49
agt	S	0	0.41
agc	S	0	0.38
---	--	------	------
mPD		0.67	0.71
		nPD:	0.94
		N. weight:	0.33
		Sc. PD:	0.26
		Sc. rank:	595.7

,

PA-X			
Pos.	29	obs:	exp:
cat	H	1	0.59
cac	H	0	0.41
cgt	R	0	0.27
cgc	R	0	0.37
cga	R	0	0.68
cgg	R	0	0.55
aat	N	1	0.53
aac	N	0	0.47
aaa	K	1566	1391.00
aag	K	913	1088.00
aga	R	7	3.21
agg	R	0	1.92
---	--	------	------
mPD		0.47	0.50
		nPD:	0.94
		N. weight:	0.017
		Sc. PD:	0.013
		Sc. rank:	30.9

,

PA-X			
Pos.	30	obs:	exp:
tta	L	0	0.11
ttg	L	0	0.19
ctt	L	0	0.19
ctc	L	1	0.17
cta	L	0	0.13
ctg	L	0	0.21
att	I	22	916.80
atc	I	2447	619.20
ata	I	0	933.00
act	T	0	0.79
acc	T	3	0.66
aca	T	0	1.29
acg	T	0	0.25
aat	N	0	0.53
aac	N	1	0.47
gtt	V	0	3.04
gtc	V	14	2.96
gta	V	0	2.75
gtg	V	0	5.24
---	--	------	------
mPD		0.033	0.67
		nPD:	0.05
		N. weight:	1.6
		Sc. PD:	-0.13
		Sc. rank:	825.9

,

PA-X			
Pos.	31	obs:	exp:
gaa	E	2481	1490.00
gag	E	7	998.30
---	--	------	------
mPD		0.0056	0.48
		nPD:	0.01
		N. weight:	0.58
		Sc. PD:	-0.068
		Sc. rank:	-492.9

,

PA-X			
Pos.	32	obs:	exp:
att	I	0	6.68
atc	I	0	4.51
ata	I	18	6.80
atg	M	2	2.00
act	T	7	576.90
acc	T	1	481.20
aca	T	1209	935.90
acg	T	961	184.00
gct	A	0	73.77
gcc	A	0	56.55
gca	A	109	127.50
gcg	A	181	32.19
---	--	------	------
mPD		0.72	0.91
		nPD:	0.79
		N. weight:	1.
		Sc. PD:	0.66
		Sc. rank:	1857.5

,

PA-X			
Pos.	33	obs:	exp:
aat	N	229	1322.00
aac	N	2258	1165.00
gct	A	0	0.25
gcc	A	0	0.20
gca	A	1	0.44
gcg	A	0	0.11
---	--	------	------
mPD		0.17	0.50
		nPD:	0.34
		N. weight:	0.51
		Sc. PD:	0.1
		Sc. rank:	658.2

,

PA-X			
Pos.	34	obs:	exp:
cgt	R	0	0.04
cgc	R	0	0.05
cga	R	0	0.10
cgg	R	0	0.08
aat	N	1	0.53
aac	N	0	0.47
aaa	K	1328	1395.00
aag	K	1157	1090.00
aga	R	1	0.46
agg	R	0	0.27
gaa	E	0	0.60
gag	E	1	0.40
---	--	------	------
mPD		0.50	0.49
		nPD:	1.01
		N. weight:	0.0047
		Sc. PD:	0.004
		Sc. rank:	8.7

,

PA-X			
Pos.	35	obs:	exp:
ttt	F	1890	1054.00
ttc	F	588	1424.00
tta	L	2	0.98
ttg	L	0	1.69
tgt	C	1	0.43
tgc	C	0	0.57
ctt	L	7	1.69
ctc	L	0	1.55
cta	L	0	1.20
ctg	L	0	1.89
---	--	------	------
mPD		0.37	0.50
		nPD:	0.74
		N. weight:	0.28
		Sc. PD:	0.17
		Sc. rank:	489.1

,

PA-X			
Pos.	36	obs:	exp:
cct	P	0	0.28
ccc	P	1	0.18
cca	P	0	0.41
ccg	P	0	0.13
act	T	3	9.80
acc	T	34	8.18
aca	T	0	15.90
acg	T	0	3.13
gct	A	566	623.30
gcc	A	1557	477.80
gca	A	327	1077.00
gcg	A	0	272.00
---	--	------	------
mPD		0.55	0.72
		nPD:	0.76
		N. weight:	0.68
		Sc. PD:	0.42
		Sc. rank:	1214.1

,

PA-X			
Pos.	37	obs:	exp:
att	I	0	0.37
atc	I	0	0.25
ata	I	1	0.38
gct	A	0	632.70
gcc	A	0	485.00
gca	A	2486	1093.00
gcg	A	1	276.10
---	--	------	------
mPD		0.0024	0.69
		nPD:	0.
		N. weight:	0.96
		Sc. PD:	-0.12
		Sc. rank:	-1337.6

,

PA-X			
Pos.	38	obs:	exp:
att	I	0	922.80
atc	I	0	623.20
ata	I	2485	939.00
gtt	V	0	0.65
gtc	V	0	0.63
gta	V	3	0.59
gtg	V	0	1.12
---	--	------	------
mPD		0.0024	0.66
		nPD:	0.
		N. weight:	1.1
		Sc. PD:	-0.14
		Sc. rank:	-1538.9

,

PA-X			
Pos.	39	obs:	exp:
tgt	C	911	1082.00
tgc	C	1577	1406.00
---	--	------	------
mPD		0.46	0.49
		nPD:	0.94
		N. weight:	0.013
		Sc. PD:	0.011
		Sc. rank:	24.3

,

PA-X			
Pos.	40	obs:	exp:
act	T	579	659.00
acc	T	14	549.70
aca	T	1857	1069.00
acg	T	38	210.20
---	--	------	------
mPD		0.39	0.69
		nPD:	0.56
		N. weight:	0.39
		Sc. PD:	0.17
		Sc. rank:	629.7

,

PA-X			
Pos.	41	obs:	exp:
cat	H	27	1479.00
cac	H	2461	1009.00
---	--	------	------
mPD		0.021	0.48
		nPD:	0.04
		N. weight:	0.98
		Sc. PD:	-0.084
		Sc. rank:	452.4

,

PA-X			
Pos.	42	obs:	exp:
tta	L	2447	271.50
ttg	L	13	466.70
ctt	L	0	466.30
ctc	L	0	429.10
cta	L	27	330.90
ctg	L	0	522.50
att	I	0	0.37
atc	I	0	0.25
ata	I	1	0.38
---	--	------	------
mPD		0.033	1.1
		nPD:	0.03
		N. weight:	2.5
		Sc. PD:	-0.25
		Sc. rank:	89.0

,

PA-X			
Pos.	43	obs:	exp:
gaa	E	1942	1490.00
gag	E	546	998.30
---	--	------	------
mPD		0.34	0.48
		nPD:	0.71
		N. weight:	0.089
		Sc. PD:	0.05
		Sc. rank:	148.1

,

PA-X			
Pos.	44	obs:	exp:
att	I	0	2.60
atc	I	7	1.76
ata	I	0	2.65
gtt	V	8	538.90
gtc	V	2449	523.60
gta	V	14	487.60
gtg	V	7	927.90
gct	A	0	0.76
gcc	A	3	0.59
gca	A	0	1.32
gcg	A	0	0.33
---	--	------	------
mPD		0.031	0.74
		nPD:	0.04
		N. weight:	1.7
		Sc. PD:	-0.15
		Sc. rank:	772.4

,

PA-X			
Pos.	45	obs:	exp:
tct	S	0	0.34
tcc	S	2	0.29
tca	S	0	0.45
tcg	S	0	0.13
tgt	C	1303	1081.00
tgc	C	1183	1405.00
agt	S	0	0.41
agc	S	0	0.38
---	--	------	------
mPD		0.50	0.49
		nPD:	1.01
		N. weight:	0.022
		Sc. PD:	0.019
		Sc. rank:	41.2

,

PA-X			
Pos.	46	obs:	exp:
ttt	F	0	1058.00
ttc	F	2487	1429.00
gtt	V	0	0.22
gtc	V	1	0.21
gta	V	0	0.20
gtg	V	0	0.37
---	--	------	------
mPD		0.00080	0.49
		nPD:	0.
		N. weight:	0.65
		Sc. PD:	-0.083
		Sc. rank:	-1080.0

,

PA-X			
Pos.	47	obs:	exp:
atg	M	2488	2488.00
---	--	------	------
mPD		0	0
		nPD:	1.
		N. weight:	0.
		Sc. PD:	0
		Sc. rank:	0

,

PA-X			
Pos.	48	obs:	exp:
tat	Y	2483	1209.00
tac	Y	5	1279.00
---	--	------	------
mPD		0.0040	0.50
		nPD:	0.01
		N. weight:	0.82
		Sc. PD:	-0.1
		Sc. rank:	-831.5

,

PA-X			
Pos.	49	obs:	exp:
tct	S	5	424.90
tcc	S	0	366.80
tca	S	37	560.80
tcg	S	2446	160.70
agt	S	0	508.00
agc	S	0	466.80
---	--	------	------
mPD		0.033	1.6
		nPD:	0.02
		N. weight:	3.1
		Sc. PD:	-0.34
		Sc. rank:	-1095.2

,

PA-X			
Pos.	50	obs:	exp:
gat	D	2175	1431.00
gac	D	313	1057.00
---	--	------	------
mPD		0.22	0.49
		nPD:	0.45
		N. weight:	0.25
		Sc. PD:	0.077
		Sc. rank:	375.9

,

PA-X			
Pos.	51	obs:	exp:
ttt	F	686	1058.00
ttc	F	1801	1429.00
tta	L	1	0.11
ttg	L	0	0.19
ctt	L	0	0.19
ctc	L	0	0.17
cta	L	0	0.13
ctg	L	0	0.21
---	--	------	------
mPD		0.40	0.49
		nPD:	0.82
		N. weight:	0.059
		Sc. PD:	0.039
		Sc. rank:	107.2

,

PA-X			
Pos.	52	obs:	exp:
cat	H	1205	1479.00
cac	H	1283	1009.00
---	--	------	------
mPD		0.50	0.48
		nPD:	1.04
		N. weight:	0.031
		Sc. PD:	0.027
		Sc. rank:	56.5

,

PA-X			
Pos.	53	obs:	exp:
ttt	F	1538	1058.00
ttc	F	949	1429.00
tat	Y	0	0.49
tac	Y	1	0.51
---	--	------	------
mPD		0.47	0.49
		nPD:	0.97
		N. weight:	0.09
		Sc. PD:	0.073
		Sc. rank:	164.6

,

PA-X			
Pos.	54	obs:	exp:
tta	L	0	0.11
ttg	L	0	0.19
ctt	L	0	0.19
ctc	L	1	0.17
cta	L	0	0.13
ctg	L	0	0.21
att	I	2477	922.80
atc	I	7	623.20
ata	I	1	939.00
gtt	V	2	0.43
gtc	V	0	0.42
gta	V	0	0.39
gtg	V	0	0.75
---	--	------	------
mPD		0.0096	0.66
		nPD:	0.01
		N. weight:	1.1
		Sc. PD:	-0.13
		Sc. rank:	-791.6

,

PA-X			
Pos.	55	obs:	exp:
aat	N	5	2.66
aac	N	0	2.34
gat	D	2472	1428.00
gac	D	11	1055.00
---	--	------	------
mPD		0.013	0.49
		nPD:	0.03
		N. weight:	0.61
		Sc. PD:	-0.064
		Sc. rank:	-86.5

,

PA-X

Pos.	56	obs:	exp:
gat	D	0	2.30
gac	D	4	1.70
gaa	E	2470	1487.00
gag	E	13	996.30
ggt	G	0	0.14
ggc	G	0	0.13
gga	G	1	0.44
ggg	G	0	0.29
---	--	------	------
mPD		0.014	0.48
		nPD:	0.03
		N. weight:	0.56
		Sc. PD:	-0.057
		Sc. rank:	25.0

PA-X

Pos.	57	obs:	exp:
caa	Q	10	5.09
cag	Q	0	4.91
cgt	R	1	93.97
cgc	R	0	132.00
cga	R	2454	239.90
cgg	R	20	194.10
aga	R	3	1137.00
agg	R	0	681.40
---	--	------	------
mPD		0.027	0.96
		nPD:	0.03
		N. weight:	2.6
		Sc. PD:	-0.27
		Sc. rank:	-0.1

PA-X

Pos.	58	obs:	exp:
tct	S	0	0.34
tcc	S	0	0.29
tca	S	0	0.45
tcg	S	0	0.13
agt	S	2	0.41
agc	S	0	0.38
ggt	G	772	357.60
ggc	G	1711	328.10
gga	G	3	1085.00
ggg	G	0	715.00
---	--	------	------
mPD		0.43	0.69
		nPD:	0.62
		N. weight:	1.6
		Sc. PD:	0.77
		Sc. rank:	2595.7

PA-X

Pos.	59	obs:	exp:
att	I	0	2.60
atc	I	0	1.76
ata	I	7	2.65
aat	N	0	1.59
aac	N	3	1.41
aaa	K	530	300.90
aag	K	6	235.10
gat	D	0	0.58
gac	D	1	0.42
gaa	E	1650	991.00
gag	E	5	664.00
ggt	G	0	41.14
ggc	G	0	37.75
gga	G	286	124.80
ggg	G	0	82.26
---	--	------	------
mPD		0.56	1.1
		nPD:	0.53
		N. weight:	0.63
		Sc. PD:	0.24
		Sc. rank:	990.1

PA-X

Pos.	60	obs:	exp:
tct	S	0	424.90
tcc	S	0	366.80
tca	S	2478	560.80
tcg	S	10	160.70
agt	S	0	508.00
agc	S	0	466.80
---	--	------	------
mPD		0.0080	1.6
		nPD:	0.
		N. weight:	1.7
		Sc. PD:	-0.21
		Sc. rank:	-2128.9

PA-X

Pos.	61	obs:	exp:
att	I	0	793.50
atc	I	0	535.90
ata	I	2137	807.50
atg	M	45	45.00
act	T	0	79.73
acc	T	0	66.51
aca	T	301	129.30
acg	T	0	25.42
aaa	K	2	1.12
aag	K	0	0.88
gtt	V	0	0.65
gtc	V	0	0.63
gta	V	3	0.59
gtg	V	0	1.12
---	--	------	------
mPD		0.25	0.89
		nPD:	0.28
		N. weight:	1.1
		Sc. PD:	0.16
		Sc. rank:	1206.4

PA-X

Pos.	62	obs:	exp:
att	I	1323	922.80
atc	I	1160	623.20
ata	I	2	939.00
act	T	2	0.53
acc	T	0	0.44
aca	T	0	0.86
acg	T	0	0.17
gtt	V	1	0.22
gtc	V	0	0.21
gta	V	0	0.20
gtg	V	0	0.37
---	--	------	------
mPD		0.50	0.66
		nPD:	0.76
		N. weight:	0.56
		Sc. PD:	0.34
		Sc. rank:	994.1

PA-X

Pos.	63	obs:	exp:
tta	L	3	0.33
ttg	L	0	0.56
ctt	L	0	0.56
ctc	L	0	0.52
cta	L	0	0.40
ctg	L	0	0.63
att	I	1	7.43
atc	I	0	5.02
ata	I	19	7.56
atg	M	19	19.00
gtt	V	0	531.70
gtc	V	0	516.60
gta	V	2299	481.10
gtg	V	146	915.60
gct	A	0	0.25
gcc	A	0	0.20
gca	A	1	0.44
gcg	A	0	0.11
---	--	------	------
mPD		0.16	0.76
		nPD:	0.21
		N. weight:	1.6
		Sc. PD:	0.12
		Sc. rank:	1527.1

PA-X

Pos.	64	obs:	exp:
gaa	E	2473	1490.00
gag	E	15	998.30
---	--	------	------
mPD		0.012	0.48
		nPD:	0.02
		N. weight:	0.56
		Sc. PD:	-0.059
		Sc. rank:	-111.7

PA-X

Pos.	65	obs:	exp:
ttt	F	2	0.85
ttc	F	0	1.15
tct	S	2469	424.20
tcc	S	9	366.20
tca	S	3	559.90
tcg	S	3	160.50
tat	Y	1	0.49
tac	Y	0	0.51
cct	P	1	0.28
ccc	P	0	0.18
cca	P	0	0.41
ccg	P	0	0.13
agt	S	0	507.20
agc	S	0	466.00
---	--	------	------
mPD		0.015	1.6
		nPD:	0.01
		N. weight:	2.
		Sc. PD:	-0.24
		Sc. rank:	-1949.3

PA-X

Pos.	66	obs:	exp:
tct	S	0	0.85
tcc	S	0	0.74
tca	S	0	1.13
tcg	S	0	0.32
agt	S	0	1.02
agc	S	5	0.94
gat	D	0	167.40
gac	D	291	123.60
ggt	G	17	315.30
ggc	G	2147	289.30
gga	G	27	956.90
ggg	G	1	630.50
---	--	------	------
mPD		0.25	0.93
		nPD:	0.26
		N. weight:	2.1
		Sc. PD:	0.26
		Sc. rank:	2212.5

PA-X

Pos.	67	obs:	exp:
aat	N	1	0.53
aac	N	0	0.47
gat	D	1288	1429.00
gac	D	1197	1056.00
gaa	E	1	0.60
gag	E	0	0.40
ggt	G	0	0.14
ggc	G	1	0.13
gga	G	0	0.44
ggg	G	0	0.29
---	--	------	------
mPD		0.50	0.49
		nPD:	1.02
		N. weight:	0.011
		Sc. PD:	0.0096
		Sc. rank:	20.3

PA-X

Pos.	68	obs:	exp:
tta	L	0	0.33
ttg	L	0	0.56
ctt	L	0	0.56
ctc	L	0	0.52
cta	L	0	0.40
ctg	L	3	0.63
cct	P	23	700.50
ccc	P	0	449.50
cca	P	25	1014.00
ccg	P	2436	319.80
act	T	0	0.26
acc	T	0	0.22
aca	T	0	0.43
acg	T	1	0.08
---	--	------	------
mPD		0.041	0.71
		nPD:	0.06
		N. weight:	2.2
		Sc. PD:	-0.16
		Sc. rank:	1369.3

PA-X

Pos.	69	obs:	exp:
aat	N	2450	1322.00
aac	N	38	1166.00
---	--	------	------
mPD		0.030	0.50
		nPD:	0.06
		N. weight:	0.65
		Sc. PD:	-0.046
		Sc. rank:	402.0

PA-X

Pos.	70	obs:	exp:
gtt	V	0	0.43
gtc	V	0	0.42
gta	V	2	0.39
gtg	V	0	0.75
gct	A	2	632.20
gcc	A	2	484.60
gca	A	2477	1092.00
gcg	A	4	275.90
gaa	E	1	0.60
gag	E	0	0.40
---	--	------	------
mPD		0.0088	0.69
		nPD:	0.01
		N. weight:	0.94
		Sc. PD:	-0.11
		Sc. rank:	-740.9

PA-X

Pos.	71	obs:	exp:
tta	L	2435	271.60
ttg	L	18	466.90
ctt	L	0	466.50
ctc	L	7	429.30
cta	L	28	331.00
ctg	L	0	522.80
---	--	------	------
mPD		0.048	1.1
		nPD:	0.04
		N. weight:	2.4
		Sc. PD:	-0.22
		Sc. rank:	1028.0

PA-X

Pos.	72	obs:	exp:
tta	L	0	271.60
ttg	L	2108	466.90
ctt	L	0	466.50
ctc	L	0	429.30
cta	L	9	331.00
ctg	L	371	522.80
---	--	------	------
mPD		0.27	1.1
		nPD:	0.23
		N. weight:	1.4
		Sc. PD:	0.14
		Sc. rank:	1423.7

PA-X

Pos.	73	obs:	exp:
aaa	K	1571	1397.00
aag	K	917	1091.00
---	--	------	------
mPD		0.47	0.49
		nPD:	0.95
		N. weight:	0.014
		Sc. PD:	0.011
		Sc. rank:	25.2

PA-X

Pos.	74	obs:	exp:
cat	H	8	1479.00
cac	H	2480	1009.00
---	--	------	------
mPD		0.0064	0.48
		nPD:	0.01
		N. weight:	1.
		Sc. PD:	-0.12
		Sc. rank:	-786.4

PA-X

Pos.	75	obs:	exp:
cgt	R	17	94.35
cgc	R	1	132.50
cga	R	2455	240.90
cgg	R	13	194.90
aga	R	2	1141.00
agg	R	0	684.10
---	--	------	------
mPD		0.026	0.95
		nPD:	0.03
		N. weight:	2.6
		Sc. PD:	-0.27
		Sc. rank:	-194.0

PA-X			
Pos.	76	obs:	exp:
ttt	F	2477	1058.00
ttc	F	10	1429.00
tgt	C	1	0.43
tgc	C	0	0.57
---	--	------	------
mPD		0.0088	0.49
		nPD:	0.02
		N. weight:	0.97
		Sc. PD:	-0.11
		Sc. rank:	-488.6

PA-X			
Pos.	77	obs:	exp:
gat	D	0	0.58
gac	D	1	0.42
gaa	E	36	1489.00
gag	E	2451	997.90
---	--	------	------
mPD		0.029	0.48
		nPD:	0.06
		N. weight:	0.97
		Sc. PD:	-0.068
		Sc. rank:	615.7

PA-X			
Pos.	78	obs:	exp:
tta	L	0	0.98
ttg	L	0	1.69
ctt	L	0	1.69
ctc	L	0	1.55
cta	L	9	1.20
ctg	L	0	1.89
att	I	4	912.00
atc	I	3	616.00
ata	I	2449	928.00
gtt	V	0	5.00
gtc	V	0	4.86
gta	V	23	4.53
gtg	V	0	8.61
---	--	------	------
mPD		0.031	0.69
		nPD:	0.05
		N. weight:	1.1
		Sc. PD:	-0.096
		Sc. rank:	556.6

PA-X			
Pos.	79	obs:	exp:
att	I	2480	921.70
atc	I	2	622.50
ata	I	0	937.90
aat	N	1	0.53
aac	N	0	0.47
gtt	V	5	1.09
gtc	V	0	1.06
gta	V	0	0.98
gtg	V	0	1.87
---	--	------	------
mPD		0.0064	0.66
		nPD:	0.01
		N. weight:	1.1
		Sc. PD:	-0.14
		Sc. rank:	-1073.7

PA-X			
Pos.	80	obs:	exp:
gaa	E	2479	1490.00
gag	E	9	998.30
---	--	------	------
mPD		0.0072	0.48
		nPD:	0.02
		N. weight:	0.57
		Sc. PD:	-0.066
		Sc. rank:	-390.5

PA-X			
Pos.	81	obs:	exp:
cgt	R	0	0.04
cgc	R	0	0.05
cga	R	0	0.10
cgg	R	0	0.08
aga	R	0	0.46
agg	R	1	0.27
ggt	G	0	357.80
ggc	G	0	328.30
gga	G	23	1086.00
ggg	G	2464	715.30
---	--	------	------
mPD		0.019	0.69
		nPD:	0.03
		N. weight:	1.4
		Sc. PD:	-0.14
		Sc. rank:	-74.1

PA-X			
Pos.	82	obs:	exp:
cgt	R	0	94.35
cgc	R	0	132.50
cga	R	1	240.90
cgg	R	0	194.90
aga	R	2472	1141.00
agg	R	15	684.10
---	--	------	------
mPD		0.013	0.95
		nPD:	0.01
		N. weight:	0.87
		Sc. PD:	-0.1
		Sc. rank:	-653.3

PA-X			
Pos.	83	obs:	exp:
gat	D	10	1431.00
gac	D	2477	1056.00
ggt	G	0	0.14
ggc	G	1	0.13
gga	G	0	0.44
ggg	G	0	0.29
---	--	------	------
mPD		0.0088	0.49
		nPD:	0.02
		N. weight:	0.97
		Sc. PD:	-0.11
		Sc. rank:	-503.5

PA-X			
Pos.	84	obs:	exp:
tta	L	0	0.22
ttg	L	0	0.38
ctt	L	0	0.38
ctc	L	0	0.35
cta	L	2	0.27
ctg	L	0	0.42
caa	Q	1	0.51
cag	Q	0	0.49
cgt	R	3	94.24
cgc	R	5	132.40
cga	R	2458	240.60
cgg	R	12	194.60
aga	R	7	1140.00
agg	R	0	683.30
---	--	------	------
mPD		0.024	0.96
		nPD:	0.03
		N. weight:	2.6
		Sc. PD:	-0.28
		Sc. rank:	-425.4

PA-X			
Pos.	85	obs:	exp:
cct	P	0	0.28
ccc	P	0	0.18
cca	P	1	0.41
ccg	P	0	0.13
att	I	0	1.11
atc	I	0	0.75
ata	I	3	1.13
act	T	27	658.00
acc	T	4	548.80
aca	T	1927	1067.00
acg	T	526	209.80
---	--	------	------
mPD		0.36	0.69
		nPD:	0.51
		N. weight:	0.72
		Sc. PD:	0.27
		Sc. rank:	1090.3

PA-X			
Pos.	86	obs:	exp:
tta	L	0	0.11
ttg	L	1	0.19
tct	S	0	0.17
tcc	S	0	0.15
tca	S	0	0.23
tcg	S	0	0.06
ctt	L	0	0.19
ctc	L	0	0.17
cta	L	0	0.13
ctg	L	0	0.21
att	I	1	2.23
atc	I	0	1.50
ata	I	5	2.27
atg	M	2452	2452.00
agt	S	1	0.20
agc	S	0	0.19
gtt	V	0	6.09
gtc	V	0	5.92
gta	V	0	5.51
gtg	V	28	10.49
---	--	------	------
mPD		0.029	0.044
		nPD:	0.66
		N. weight:	0.016
		Sc. PD:	0.0085
		Sc. rank:	26.6

PA-X			
Pos.	87	obs:	exp:
gct	A	579	632.20
gcc	A	1899	484.60
gca	A	7	1092.00
gcg	A	0	275.90
gat	D	0	0.58
gac	D	1	0.42
ggt	G	0	0.29
ggc	G	2	0.26
gga	G	0	0.87
ggg	G	0	0.58
---	--	------	------
mPD		0.36	0.69
		nPD:	0.52
		N. weight:	1.2
		Sc. PD:	0.45
		Sc. rank:	1817.4

PA-X			
Pos.	88	obs:	exp:
tgg	W	2488	2488.00
---	--	------	------
mPD		0	0
		nPD:	1.
		N. weight:	0.
		Sc. PD:	0
		Sc. rank:	0

PA-X			
Pos.	89	obs:	exp:
atg	M	3	3.00
act	T	0	658.20
acc	T	0	549.10
aca	T	2247	1068.00
acg	T	238	209.90
---	--	------	------
mPD		0.18	0.69
		nPD:	0.26
		N. weight:	0.8
		Sc. PD:	0.096
		Sc. rank:	812.2

PA-X			
Pos.	90	obs:	exp:
tta	L	0	0.11
ttg	L	1	0.19
ctt	L	0	0.19
ctc	L	0	0.17
cta	L	0	0.13
ctg	L	0	0.21
atg	M	8	8.00
gtt	V	0	539.10
gtc	V	0	523.80
gta	V	39	487.80
gtg	V	2440	928.30
---	--	------	------
mPD		0.038	0.74
		nPD:	0.05
		N. weight:	1.1
		Sc. PD:	-0.084
		Sc. rank:	581.0

PA-X			
Pos.	91	obs:	exp:
att	I	0	0.37
atc	I	0	0.25
ata	I	1	0.38
gtt	V	0	540.40
gtc	V	0	525.10
gta	V	1259	489.00
gtg	V	1226	930.60
gct	A	0	0.51
gcc	A	0	0.39
gca	A	0	0.88
gcg	A	2	0.22
---	--	------	------
mPD		0.50	0.73
		nPD:	0.69
		N. weight:	0.72
		Sc. PD:	0.39
		Sc. rank:	1192.9

PA-X			
Pos.	92	obs:	exp:
tct	S	0	0.17
tcc	S	0	0.15
tca	S	0	0.23
tcg	S	0	0.06
aat	N	2477	1322.00
aac	N	10	1165.00
agt	S	1	0.20
agc	S	0	0.19
---	--	------	------
mPD		0.0088	0.50
		nPD:	0.02
		N. weight:	0.71
		Sc. PD:	-0.079
		Sc. rank:	-378.7

PA-X			
Pos.	93	obs:	exp:
tct	S	0	421.10
tcc	S	0	363.50
tca	S	0	555.90
tcg	S	0	159.30
agt	S	2411	503.50
agc	S	55	462.70
ggt	G	20	3.16
ggc	G	2	2.90
gga	G	0	9.60
ggg	G	0	6.33
---	--	------	------
mPD		0.062	1.7
		nPD:	0.04
		N. weight:	1.7
		Sc. PD:	-0.16
		Sc. rank:	520.3

PA-X			
Pos.	94	obs:	exp:
att	I	292	923.50
atc	I	752	623.70
ata	I	1443	939.80
gtt	V	0	0.22
gtc	V	0	0.21
gta	V	1	0.20
gtg	V	0	0.37
---	--	------	------
mPD		0.56	0.66
		nPD:	0.85
		N. weight:	0.2
		Sc. PD:	0.14
		Sc. rank:	370.4

PA-X			
Pos.	95	obs:	exp:
tgt	C	2	1081.00
tgc	C	2485	1406.00
cat	H	0	0.59
cac	H	1	0.41
---	--	------	------
mPD		0.0032	0.49
		nPD:	0.01
		N. weight:	0.66
		Sc. PD:	-0.081
		Sc. rank:	-716.4

PA-X

Pos.	96	obs:	exp:
cat	H	2	170.10
cac	H	284	115.90
aat	N	9	1170.00
aac	N	2193	1032.00
---	--	------	------
mPD		0.21	0.70
		nPD:	0.3
		N. weight:	0.87
		Sc. PD:	0.14
		Sc. rank:	1039.2

PA-X

Pos.	97	obs:	exp:
tct	S	0	0.17
tcc	S	0	0.15
tca	S	0	0.23
tcg	S	0	0.06
act	T	1172	658.50
acc	T	1310	549.30
aca	T	4	1068.00
acg	T	0	210.00
aat	N	1	0.53
aac	N	0	0.47
agt	S	0	0.20
agc	S	1	0.19
---	--	------	------
mPD		0.50	0.69
		nPD:	0.73
		N. weight:	0.84
		Sc. PD:	0.49
		Sc. rank:	1428.7

PA-X

Pos.	98	obs:	exp:
act	T	3	659.00
acc	T	2	549.70
aca	T	2468	1069.00
acg	T	15	210.20
---	--	------	------
mPD		0.016	0.69
		nPD:	0.02
		N. weight:	0.94
		Sc. PD:	-0.1
		Sc. rank:	-260.2

PA-X

Pos.	99	obs:	exp:
cgt	R	0	0.04
cgc	R	0	0.05
cga	R	0	0.10
cgg	R	0	0.08
aga	R	1	0.46
agg	R	0	0.27
gaa	E	8	4.79
gag	E	0	3.21
ggt	G	22	356.60
ggc	G	0	327.20
gga	G	2069	1082.00
ggg	G	388	713.00
---	--	------	------
mPD		0.29	0.70
		nPD:	0.41
		N. weight:	0.49
		Sc. PD:	0.13
		Sc. rank:	699.6

PA-X

Pos.	100	obs:	exp:
att	I	0	9.28
atc	I	25	6.27
ata	I	0	9.45
act	T	0	0.26
acc	T	1	0.22
aca	T	0	0.43
acg	T	0	0.08
gtt	V	57	512.40
gtc	V	2295	497.80
gta	V	4	463.60
gtg	V	0	882.20
gct	A	0	26.97
gcc	A	106	20.67
gca	A	0	46.60
gcg	A	0	11.77
---	--	------	------
mPD		0.15	0.84
		nPD:	0.18
		N. weight:	1.7
		Sc. PD:	0.078
		Sc. rank:	1504.7

PA-X

Pos.	101	obs:	exp:
gat	D	1067	640.80
gac	D	47	473.20
gaa	E	1216	822.10
gag	E	157	550.90
ggt	G	1	0.14
ggc	G	0	0.13
gga	G	0	0.44
ggg	G	0	0.29
---	--	------	------
mPD		0.57	0.74
		nPD:	0.78
		N. weight:	0.34
		Sc. PD:	0.21
		Sc. rank:	609.5

PA-X

Pos.	102	obs:	exp:
aaa	K	308	1397.00
aag	K	2180	1091.00
---	--	------	------
mPD		0.22	0.49
		nPD:	0.44
		N. weight:	0.49
		Sc. PD:	0.15
		Sc. rank:	735.7

PA-X

Pos.	103	obs:	exp:
cct	P	1290	701.60
ccc	P	1196	450.20
cca	P	2	1016.00
ccg	P	0	320.40
---	--	------	------
mPD		0.50	0.70
		nPD:	0.71
		N. weight:	0.91
		Sc. PD:	0.51
		Sc. rank:	1522.2

PA-X

Pos.	104	obs:	exp:
caa	Q	1	0.51
cag	Q	0	0.49
cgt	R	0	0.08
cgc	R	0	0.11
cga	R	0	0.19
cgg	R	0	0.16
aat	N	3	1.59
aac	N	0	1.41
aaa	K	1328	1393.00
aag	K	1154	1089.00
aga	R	2	0.92
agg	R	0	0.55
---	--	------	------
mPD		0.50	0.50
		nPD:	1.01
		N. weight:	0.0055
		Sc. PD:	0.0047
		Sc. rank:	10.1

PA-X

Pos.	105	obs:	exp:
ttt	F	20	1056.00
ttc	F	2461	1425.00
tta	L	2	0.65
ttg	L	0	1.13
tct	S	0	0.17
tcc	S	1	0.15
tca	S	0	0.23
tcg	S	0	0.06
ctt	L	1	1.13
ctc	L	3	1.04
cta	L	0	0.80
ctg	L	0	1.26
agt	S	0	0.20
agc	S	0	0.19
---	--	------	------
mPD		0.022	0.50
		nPD:	0.05
		N. weight:	0.6
		Sc. PD:	-0.051
		Sc. rank:	281.6

PA-X

Pos.	106	obs:	exp:
tta	L	0	271.60
ttg	L	0	466.90
ctt	L	1156	466.50
ctc	L	1331	429.30
cta	L	0	331.00
ctg	L	1	522.80
---	--	------	------
mPD		0.50	1.1
		nPD:	0.44
		N. weight:	1.2
		Sc. PD:	0.36
		Sc. rank:	1787.4

PA-X

Pos.	107	obs:	exp:
cct	P	1211	701.60
ccc	P	4	450.20
cca	P	1258	1016.00
ccg	P	15	320.40
---	--	------	------
mPD		0.51	0.70
		nPD:	0.72
		N. weight:	0.41
		Sc. PD:	0.23
		Sc. rank:	686.9

PA-X

Pos.	108	obs:	exp:
gat	D	2234	1431.00
gac	D	254	1057.00
---	--	------	------
mPD		0.18	0.49
		nPD:	0.38
		N. weight:	0.3
		Sc. PD:	0.07
		Sc. rank:	413.6

PA-X

Pos.	109	obs:	exp:
tta	L	16	271.60
ttg	L	2291	466.90
ctt	L	0	466.50
ctc	L	0	429.30
cta	L	0	331.00
ctg	L	181	522.80
---	--	------	------
mPD		0.15	1.1
		nPD:	0.13
		N. weight:	1.6
		Sc. PD:	-0.0042
		Sc. rank:	1342.2

PA-X

Pos.	110	obs:	exp:
tat	Y	2472	1209.00
tac	Y	16	1279.00
---	--	------	------
mPD		0.013	0.50
		nPD:	0.03
		N. weight:	0.8
		Sc. PD:	-0.083
		Sc. rank:	-119.2

PA-X

Pos.	111	obs:	exp:
gat	D	601	1431.00
gac	D	1887	1057.00
---	--	------	------
mPD		0.37	0.49
		nPD:	0.75
		N. weight:	0.27
		Sc. PD:	0.16
		Sc. rank:	470.6

PA-X

Pos.	112	obs:	exp:
tat	Y	1	1209.00
tac	Y	2487	1279.00
---	--	------	------
mPD		0.00080	0.50
		nPD:	0.
		N. weight:	0.77
		Sc. PD:	-0.099
		Sc. rank:	-1297.3

PA-X

Pos.	113	obs:	exp:
cgt	R	0	41.45
cgc	R	0	58.22
cga	R	68	105.80
cgg	R	15	85.60
aaa	K	555	781.30
aag	K	837	610.70
aga	R	995	501.40
agg	R	15	300.50
ggt	G	0	0.43
ggc	G	0	0.40
gga	G	3	1.31
ggg	G	0	0.86
---	--	------	------
mPD		1.0	1.2
		nPD:	0.83
		N. weight:	0.31
		Sc. PD:	0.21
		Sc. rank:	571.4

PA-X

Pos.	114	obs:	exp:
aaa	K	1	0.56
aag	K	0	0.44
gat	D	0	0.58
gac	D	1	0.42
gaa	E	1188	1484.00
gag	E	1291	994.70
ggt	G	0	1.01
ggc	G	0	0.92
gga	G	1	3.06
ggg	G	6	2.01
---	--	------	------
mPD		0.51	0.49
		nPD:	1.04
		N. weight:	0.039
		Sc. PD:	0.035
		Sc. rank:	72.3

PA-X

Pos.	115	obs:	exp:
tct	S	0	0.85
tcc	S	0	0.74
tca	S	0	1.13
tcg	S	0	0.32
act	T	0	0.26
acc	T	1	0.22
aca	T	0	0.43
acg	T	0	0.08
aat	N	47	1315.00
aac	N	2428	1160.00
agt	S	0	1.02
agc	S	5	0.94
gat	D	0	4.03
gac	D	7	2.97
---	--	------	------
mPD		0.048	0.51
		nPD:	0.09
		N. weight:	0.78
		Sc. PD:	-0.03
		Sc. rank:	612.6

PA-X

Pos.	116	obs:	exp:
caa	Q	2	1.02
cag	Q	0	0.98
cgt	R	3	94.28
cgc	R	5	132.40
cga	R	2449	240.70
cgg	R	26	194.70
aga	R	3	1140.00
agg	R	0	683.60
mPD		0.031	0.96
		nPD:	0.03
		N. weight:	2.6
		Sc. PD:	-0.26
		Sc. rank:	503.4

PA-X

Pos.	117	obs:	exp:
ttt	F	2	1059.00
ttc	F	2486	1429.00
mPD		0.0016	0.49
		nPD:	0.
		N. weight:	0.64
		Sc. PD:	-0.081
		Sc. rank:	-946.2

PA-X

Pos.	118	obs:	exp:
tta	L	0	0.11
ttg	L	0	0.19
ctt	L	1	0.19
ctc	L	0	0.17
cta	L	0	0.13
ctg	L	0	0.21
att	I	2195	920.50
atc	I	284	621.70
ata	I	0	936.70
atg	M	2	2.00
gtt	V	6	1.30
gtc	V	0	1.27
gta	V	0	1.18
gtg	V	0	2.25
mPD		0.21	0.66
		nPD:	0.32
		N. weight:	0.8
		Sc. PD:	0.14
		Sc. rank:	985.4

PA-X

Pos.	119	obs:	exp:
gaa	E	2478	1490.00
gag	E	10	998.30
mPD		0.0080	0.48
		nPD:	0.02
		N. weight:	0.57
		Sc. PD:	-0.064
		Sc. rank:	-358.1

PA-X

Pos.	120	obs:	exp:
att	I	2460	914.20
atc	I	2	617.50
ata	I	0	930.30
gtt	V	26	5.65
gtc	V	0	5.49
gta	V	0	5.12
gtg	V	0	9.74
mPD		0.022	0.68
		nPD:	0.03
		N. weight:	1.2
		Sc. PD:	-0.11
		Sc. rank:	241.3

PA-X

Pos.	121	obs:	exp:
ggt	G	0	357.90
ggc	G	1	328.40
gga	G	2149	1086.00
ggg	G	338	715.60
mPD		0.24	0.69
		nPD:	0.34
		N. weight:	0.57
		Sc. PD:	0.12
		Sc. rank:	732.2

PA-X

Pos.	122	obs:	exp:
att	I	0	5.94
atc	I	0	4.01
ata	I	16	6.05
gtt	V	2	537.60
gtc	V	3	522.30
gta	V	1474	486.40
gtg	V	993	925.70
mPD		0.50	0.74
		nPD:	0.67
		N. weight:	0.8
		Sc. PD:	0.41
		Sc. rank:	1309.8

PA-X

Pos.	123	obs:	exp:
tct	S	0	0.17
tcc	S	0	0.15
tca	S	0	0.23
tcg	S	1	0.06
act	T	24	658.80
acc	T	4	549.50
aca	T	1276	1069.00
acg	T	1183	210.10
agt	S	0	0.20
agc	S	0	0.19
mPD		0.51	0.69
		nPD:	0.74
		N. weight:	1.
		Sc. PD:	0.61
		Sc. rank:	1773.8

PA-X

Pos.	124	obs:	exp:
cgt	R	0	94.35
cgc	R	0	132.50
cga	R	1887	240.90
cgg	R	8	194.90
aga	R	13	1141.00
agg	R	580	684.10
mPD		0.72	0.95
		nPD:	0.76
		N. weight:	1.7
		Sc. PD:	1.1
		Sc. rank:	3075.3

PA-X

Pos.	125	obs:	exp:
cgt	R	0	94.35
cgc	R	0	132.50
cga	R	0	240.90
cgg	R	0	194.90
aga	R	12	1141.00
agg	R	2476	684.10
mPD		0.0096	0.95
		nPD:	0.01
		N. weight:	1.5
		Sc. PD:	-0.18
		Sc. rank:	-1339.7

PA-X

Pos.	126	obs:	exp:
gaa	E	2460	1481.00
gag	E	13	992.30
ggt	G	0	2.16
ggc	G	0	1.98
gga	G	15	6.55
ggg	G	0	4.31
mPD		0.022	0.49
		nPD:	0.05
		N. weight:	0.57
		Sc. PD:	-0.048
		Sc. rank:	270.9

PA-X

Pos.	127	obs:	exp:
att	I	392	145.60
atc	I	0	98.31
ata	I	0	148.10
gtt	V	2035	448.00
gtc	V	22	435.30
gta	V	1	405.30
gtg	V	2	771.40
gct	A	35	9.16
gcc	A	1	7.02
gca	A	0	15.83
gcg	A	0	4.00
mPD		0.31	1.0
		nPD:	0.3
		N. weight:	1.6
		Sc. PD:	0.27
		Sc. rank:	1900.5

PA-X

Pos.	128	obs:	exp:
cat	H	16	1479.00
cac	H	2471	1008.00
caa	Q	1	0.51
cag	Q	0	0.49
mPD		0.014	0.48
		nPD:	0.03
		N. weight:	1.
		Sc. PD:	-0.1
		Sc. rank:	-38.9

PA-X

Pos.	129	obs:	exp:
att	I	0	698.10
atc	I	0	471.50
ata	I	1880	710.40
atg	M	26	26.00
act	T	0	153.90
acc	T	0	128.40
aca	T	558	249.70
acg	T	23	49.08
gtt	V	0	0.22
gtc	V	0	0.21
gta	V	1	0.20
gtg	V	0	0.37
mPD		0.40	1.0
		nPD:	0.38
		N. weight:	1.1
		Sc. PD:	0.26
		Sc. rank:	1494.8

PA-X

Pos.	130	obs:	exp:
tat	Y	243	1209.00
tac	Y	2245	1279.00
mPD		0.18	0.50
		nPD:	0.35
		N. weight:	0.41
		Sc. PD:	0.088
		Sc. rank:	541.3

PA-X

Pos.	131	obs:	exp:
tat	Y	1897	1209.00
tac	Y	591	1279.00
mPD		0.36	0.50
		nPD:	0.73
		N. weight:	0.19
		Sc. PD:	0.11
		Sc. rank:	316.6

PA-X

Pos.	132	obs:	exp:
tta	L	16	271.50
ttg	L	0	466.70
ctt	L	916	466.30
ctc	L	0	429.10
cta	L	1538	330.90
ctg	L	17	522.50
att	I	1	0.37
atc	I	0	0.25
ata	I	0	0.38
mPD		0.49	1.1
		nPD:	0.43
		N. weight:	1.4
		Sc. PD:	0.39
		Sc. rank:	1997.2

PA-X

Pos.	133	obs:	exp:
aaa	K	2	1.12
aag	K	0	0.88
gct	A	0	0.25
gcc	A	0	0.20
gca	A	1	0.44
gcg	A	0	0.11
gat	D	1	0.58
gac	D	0	0.42
gaa	E	2448	1487.00
gag	E	35	996.30
ggt	G	0	0.14
ggc	G	0	0.13
gga	G	1	0.44
ggg	G	0	0.29
mPD		0.032	0.48
		nPD:	0.07
		N. weight:	0.52
		Sc. PD:	-0.034
		Sc. rank:	340.9

PA-X

Pos.	134	obs:	exp:
aaa	K	1854	1397.00
aag	K	634	1091.00
mPD		0.38	0.49
		nPD:	0.77
		N. weight:	0.087
		Sc. PD:	0.054
		Sc. rank:	155.5

PA-X

Pos.	135	obs:	exp:
act	T	0	0.26
acc	T	0	0.22
aca	T	1	0.43
acg	T	0	0.08
gtt	V	0	0.65
gtc	V	1	0.63
gta	V	2	0.59
gtg	V	0	1.12
gct	A	7	631.90
gcc	A	1901	484.40
gca	A	576	1092.00
gcg	A	0	275.80
mPD		0.36	0.70
		nPD:	0.52
		N. weight:	1.
		Sc. PD:	0.4
		Sc. rank:	1610.4

PA-X

Pos.	136	obs:	exp:
tct	S	0	1.54
tcc	S	0	1.33
tca	S	0	2.03
tcg	S	0	0.58
aat	N	580	1317.00
aac	N	1898	1161.00
agt	S	0	1.84
agc	S	9	1.69
gat	D	0	0.58
gac	D	1	0.42
---	--	------	------
mPD		0.37	0.51
		nPD:	0.71
		N. weight:	0.22
		Sc. PD:	0.13
		Sc. rank:	373.9

,

PA-X

Pos.	137	obs:	exp:
aaa	K	105	1397.00
aag	K	2383	1091.00
---	--	------	------
mPD		0.081	0.49
		nPD:	0.16
		N. weight:	0.75
		Sc. PD:	0.023
		Sc. rank:	644.0

,

PA-X

Pos.	138	obs:	exp:
att	I	0	923.50
atc	I	0	623.70
ata	I	2487	939.80
gtt	V	0	0.22
gtc	V	0	0.21
gta	V	1	0.20
gtg	V	0	0.37
---	--	------	------
mPD		0.00080	0.66
		nPD:	0.
		N. weight:	1.1
		Sc. PD:	-0.15
		Sc. rank:	-1918.8

,

PA-X

Pos.	139	obs:	exp:
aat	N	1	0.53
aac	N	0	0.47
aaa	K	2370	1396.00
aag	K	117	1091.00
---	--	------	------
mPD		0.090	0.49
		nPD:	0.18
		N. weight:	0.47
		Sc. PD:	0.023
		Sc. rank:	432.3

,

PA-X

Pos.	140	obs:	exp:
tta	L	23	2.51
ttg	L	0	4.32
tct	S	9	420.60
tcc	S	11	363.10
tca	S	2050	555.20
tcg	S	393	159.10
ctt	L	0	4.31
ctc	L	0	3.97
cta	L	0	3.06
ctg	L	0	4.83
cct	P	0	0.28
ccc	P	0	0.18
cca	P	1	0.41
ccg	P	0	0.13
caa	Q	0	0.51
cag	Q	1	0.49
agt	S	0	502.90
agc	S	0	462.10
---	--	------	------
mPD		0.30	1.7
		nPD:	0.18
		N. weight:	1.4
		Sc. PD:	0.066
		Sc. rank:	1299.8

,

PA-X

Pos.	141	obs:	exp:
aaa	K	0	1.68
aag	K	3	1.32
gaa	E	78	1487.00
gag	E	2406	996.70
ggt	G	0	0.14
ggc	G	0	0.13
gga	G	0	0.44
ggg	G	1	0.29
---	--	------	------
mPD		0.064	0.48
		nPD:	0.13
		N. weight:	0.89
		Sc. PD:	-0.00051
		Sc. rank:	757.7

,

PA-X

Pos.	142	obs:	exp:
cgt	R	0	1.33
cgc	R	0	1.86
cga	R	0	3.39
cgg	R	0	2.74
act	T	0	0.53
acc	T	0	0.44
aca	T	2	0.86
acg	T	0	0.17
aaa	K	1157	1375.00
aag	K	1293	1075.00
aga	R	3	16.05
agg	R	32	9.62
gaa	E	1	0.60
gag	E	0	0.40
---	--	------	------
mPD		0.53	0.53
		nPD:	0.99
		N. weight:	0.038
		Sc. PD:	0.032
		Sc. rank:	69.0

,

PA-X

Pos.	143	obs:	exp:
act	T	0	658.80
acc	T	0	549.50
aca	T	2486	1069.00
acg	T	1	210.10
gct	A	0	0.25
gcc	A	0	0.20
gca	A	1	0.44
gcg	A	0	0.11
---	--	------	------
mPD		0.0016	0.69
		nPD:	0.
		N. weight:	0.98
		Sc. PD:	-0.12
		Sc. rank:	-1535.7

,

PA-X

Pos.	144	obs:	exp:
tat	Y	0	0.49
tac	Y	1	0.51
cat	H	36	1478.00
cac	H	2450	1008.00
caa	Q	1	0.51
cag	Q	0	0.49
---	--	------	------
mPD		0.030	0.48
		nPD:	0.06
		N. weight:	0.96
		Sc. PD:	-0.066
		Sc. rank:	621.5

,

PA-X

Pos.	145	obs:	exp:
att	I	2463	923.50
atc	I	21	623.70
ata	I	3	939.80
gtt	V	1	0.22
gtc	V	0	0.21
gta	V	0	0.20
gtg	V	0	0.37
---	--	------	------
mPD		0.020	0.66
		nPD:	0.03
		N. weight:	1.1
		Sc. PD:	-0.11
		Sc. rank:	82.0

,

PA-X

Pos.	146	obs:	exp:
tta	L	0	0.11
ttg	L	0	0.19
ctt	L	0	0.19
ctc	L	1	0.17
cta	L	0	0.13
ctg	L	0	0.21
cat	H	14	1479.00
cac	H	2473	1008.00
---	--	------	------
mPD		0.012	0.48
		nPD:	0.02
		N. weight:	1.
		Sc. PD:	-0.11
		Sc. rank:	-210.2

,

PA-X

Pos.	147	obs:	exp:
att	I	6	923.50
atc	I	12	623.70
ata	I	2469	939.80
atg	M	1	1.00
---	--	------	------
mPD		0.015	0.66
		nPD:	0.02
		N. weight:	1.1
		Sc. PD:	-0.12
		Sc. rank:	-315.1

,

PA-X

Pos.	148	obs:	exp:
ttt	F	13	1059.00
ttc	F	2475	1429.00
---	--	------	------
mPD		0.010	0.49
		nPD:	0.02
		N. weight:	0.61
		Sc. PD:	-0.066
		Sc. rank:	-212.9

,

PA-X

Pos.	149	obs:	exp:
tct	S	2	424.90
tcc	S	1	366.80
tca	S	2483	560.80
tcg	S	2	160.70
agt	S	0	508.00
agc	S	0	466.80
---	--	------	------
mPD		0.0040	1.6
		nPD:	0.
		N. weight:	1.7
		Sc. PD:	-0.22
		Sc. rank:	-2599.4

,

PA-X

Pos.	150	obs:	exp:
ttt	F	3	1059.00
ttc	F	2485	1429.00
---	--	------	------
mPD		0.0024	0.49
		nPD:	0.
		N. weight:	0.64
		Sc. PD:	-0.079
		Sc. rank:	-766.6

,

PA-X

Pos.	151	obs:	exp:
att	I	3	1.11
atc	I	0	0.75
ata	I	0	1.13
act	T	2436	651.90
acc	T	18	543.80
aca	T	6	1057.00
acg	T	1	207.90
aat	N	24	12.75
aac	N	0	11.25
---	--	------	------
mPD		0.041	0.71
		nPD:	0.06
		N. weight:	1.5
		Sc. PD:	-0.11
		Sc. rank:	849.2

,

PA-X

Pos.	152	obs:	exp:
ggt	G	0	357.90
ggc	G	0	328.40
gga	G	785	1086.00
ggg	G	1703	715.60
---	--	------	------
mPD		0.43	0.69
		nPD:	0.63
		N. weight:	0.57
		Sc. PD:	0.28
		Sc. rank:	937.7

,

PA-X

Pos.	153	obs:	exp:
gaa	E	52	1489.00
gag	E	2435	997.90
ggt	G	0	0.14
ggc	G	0	0.13
gga	G	0	0.44
ggg	G	1	0.29
---	--	------	------
mPD		0.042	0.48
		nPD:	0.09
		N. weight:	0.94
		Sc. PD:	-0.042
		Sc. rank:	729.9

,

PA-X

Pos.	154	obs:	exp:
gaa	E	1608	1490.00
gag	E	880	998.30
---	--	------	------
mPD		0.46	0.48
		nPD:	0.95
		N. weight:	0.0075
		Sc. PD:	0.006
		Sc. rank:	13.7

,

PA-X			
Pos.	155	obs:	exp:
atg	M	2488	2488.00
---	--	------	------
mPD		0	0
		nPD:	1.
		N. weight:	0.
		Sc. PD:	0
		Sc. rank:	0

,

PA-X			
Pos.	156	obs:	exp:
act	T	0	0.26
acc	T	0	0.22
aca	T	1	0.43
acg	T	0	0.08
gtt	V	0	0.22
gtc	V	1	0.21
gta	V	0	0.20
gtg	V	0	0.37
gct	A	9	632.40
gcc	A	2476	484.80
gca	A	1	1093.00
gcg	A	0	276.00
---	--	------	------
mPD		0.010	0.69
		nPD:	0.02
		N. weight:	1.9
		Sc. PD:	-0.21
		Sc. rank:	-1226.3

,

PA-X			
Pos.	157	obs:	exp:
tct	S	0	0.17
tcc	S	1	0.15
tca	S	0	0.23
tcg	S	0	0.06
act	T	8	658.20
acc	T	2186	549.10
aca	T	291	1068.00
acg	T	0	209.90
agt	S	0	0.20
agc	S	0	0.19
gct	A	0	0.51
gcc	A	2	0.39
gca	A	0	0.88
gcg	A	0	0.22
---	--	------	------
mPD		0.21	0.69
		nPD:	0.31
		N. weight:	1.2
		Sc. PD:	0.21
		Sc. rank:	1485.6

,

PA-X			
Pos.	158	obs:	exp:
caa	Q	4	2.04
cag	Q	0	1.96
cgt	R	0	0.72
cgc	R	0	1.01
cga	R	0	1.84
cgg	R	0	1.49
aaa	K	1265	1382.00
aag	K	1197	1080.00
aga	R	2	8.72
agg	R	17	5.22
gaa	E	3	1.80
gag	E	0	1.20
---	--	------	------
mPD		0.52	0.52
		nPD:	1.01
		N. weight:	0.018
		Sc. PD:	0.015
		Sc. rank:	32.6

,

PA-X			
Pos.	159	obs:	exp:
tct	S	1	0.17
tcc	S	0	0.15
tca	S	0	0.23
tcg	S	0	0.06
agt	S	0	0.20
agc	S	0	0.19
gtt	V	0	0.22
gtc	V	0	0.21
gta	V	1	0.20
gtg	V	0	0.37
gct	A	593	632.20
gcc	A	78	484.60
gca	A	1485	1092.00
gcg	A	329	275.90
gat	D	1	0.58
gac	D	0	0.42
---	--	------	------
mPD		0.57	0.69
		nPD:	0.82
		N. weight:	0.16
		Sc. PD:	0.11
		Sc. rank:	296.8

,

PA-X			
Pos.	160	obs:	exp:
aat	N	1	1.59
aac	N	2	1.41
gat	D	1197	1429.00
gac	D	1287	1055.00
gaa	E	1	0.60
gag	E	0	0.40
---	--	------	------
mPD		0.50	0.49
		nPD:	1.02
		N. weight:	0.023
		Sc. PD:	0.02
		Sc. rank:	42.5

,

PA-X			
Pos.	161	obs:	exp:
tat	Y	974	1209.00
tac	Y	1513	1278.00
cat	H	1	0.59
cac	H	0	0.41
---	--	------	------
mPD		0.48	0.50
		nPD:	0.95
		N. weight:	0.023
		Sc. PD:	0.019
		Sc. rank:	42.5

,

PA-X			
Pos.	162	obs:	exp:
att	I	1	12.25
atc	I	32	8.28
ata	I	0	12.47
act	T	1165	650.00
acc	T	1289	542.20
aca	T	0	1054.00
acg	T	0	207.30
gtt	V	0	0.22
gtc	V	1	0.21
gta	V	0	0.20
gtg	V	0	0.37
---	--	------	------
mPD		0.53	0.72
		nPD:	0.73
		N. weight:	0.86
		Sc. PD:	0.51
		Sc. rank:	1474.0

,

PA-X			
Pos.	163	obs:	exp:
ttt	F	1	0.43
ttc	F	0	0.57
tta	L	0	268.70
ttg	L	0	462.00
ctt	L	2462	461.70
ctc	L	0	424.80
cta	L	0	327.60
ctg	L	0	517.30
att	I	25	9.28
atc	I	0	6.27
ata	I	0	9.45
---	--	------	------
mPD		0.021	1.1
		nPD:	0.02
		N. weight:	1.9
		Sc. PD:	-0.22
		Sc. rank:	-928.5

,

PA-X			
Pos.	164	obs:	exp:
gat	D	1805	1431.00
gac	D	683	1057.00
---	--	------	------
mPD		0.40	0.49
		nPD:	0.82
		N. weight:	0.058
		Sc. PD:	0.039
		Sc. rank:	106.2

,

PA-X			
Pos.	165	obs:	exp:
gaa	E	1799	1490.00
gag	E	689	998.30
---	--	------	------
mPD		0.40	0.48
		nPD:	0.83
		N. weight:	0.041
		Sc. PD:	0.028
		Sc. rank:	75.1

,

PA-X			
Pos.	166	obs:	exp:
aaa	K	0	0.56
aag	K	1	0.44
gaa	E	431	1489.00
gag	E	2056	997.90
---	--	------	------
mPD		0.29	0.48
		nPD:	0.6
		N. weight:	0.45
		Sc. PD:	0.2
		Sc. rank:	717.5

,

PA-X			
Pos.	167	obs:	exp:
tct	S	0	424.70
tcc	S	0	366.60
tca	S	0	560.60
tcg	S	0	160.60
aat	N	0	0.53
aac	N	1	0.47
agt	S	8	507.80
agc	S	2479	466.60
---	--	------	------
mPD		0.0072	1.6
		nPD:	0.
		N. weight:	1.9
		Sc. PD:	-0.24
		Sc. rank:	-2555.7

,

PA-X			
Pos.	168	obs:	exp:
cgt	R	0	94.32
cgc	R	0	132.50
cga	R	0	240.80
cgg	R	0	194.80
aaa	K	1	0.56
aag	K	0	0.44
aga	R	2466	1141.00
agg	R	21	683.80
---	--	------	------
mPD		0.018	0.95
		nPD:	0.02
		N. weight:	0.86
		Sc. PD:	-0.096
		Sc. rank:	-375.7

,

PA-X			
Pos.	169	obs:	exp:
gct	A	4	632.90
gcc	A	1	485.20
gca	A	2476	1094.00
gcg	A	7	276.20
---	--	------	------
mPD		0.0096	0.69
		nPD:	0.01
		N. weight:	0.93
		Sc. PD:	-0.11
		Sc. rank:	-684.7

,

PA-X			
Pos.	170	obs:	exp:
cgt	R	0	94.32
cgc	R	0	132.50
cga	R	1	240.80
cgg	R	0	194.80
aaa	K	0	0.56
aag	K	1	0.44
aga	R	1890	1141.00
agg	R	596	683.80
---	--	------	------
mPD		0.37	0.95
		nPD:	0.38
		N. weight:	0.41
		Sc. PD:	0.1
		Sc. rank:	569.5

,

PA-X			
Pos.	171	obs:	exp:
att	I	11	923.10
atc	I	1258	623.50
ata	I	1217	939.40
act	T	0	0.26
acc	T	0	0.22
aca	T	1	0.43
acg	T	0	0.08
aat	N	1	0.53
aac	N	0	0.47
---	--	------	------
mPD		0.51	0.66
		nPD:	0.77
		N. weight:	0.54
		Sc. PD:	0.34
		Sc. rank:	972.6

,

PA-X			
Pos.	172	obs:	exp:
aaa	K	2485	1397.00
aag	K	3	1091.00
---	--	------	------
mPD		0.0024	0.49
		nPD:	0.
		N. weight:	0.66
		Sc. PD:	-0.083
		Sc. rank:	-811.5

,

PA-X			
Pos.	173	obs:	exp:
act	T	9	659.00
acc	T	2453	549.70
aca	T	26	1069.00
acg	T	0	210.20
---	--	------	------
mPD		0.028	0.69
		nPD:	0.04
		N. weight:	1.7
		Sc. PD:	-0.15
		Sc. rank:	582.3

,

PA-X			
Pos.	174	obs:	exp:
cgt	R	0	94.35
cgc	R	0	132.50
cga	R	0	240.90
cgg	R	1	194.90
aga	R	28	1141.00
agg	R	2459	684.10
---	--	------	------
mPD		0.023	0.95
		nPD:	0.02
		N. weight:	1.4
		Sc. PD:	-0.15
		Sc. rank:	-336.6

,

PA-X

Pos. 175		obs:	exp:
tta	L	1	271.60
ttg	L	541	466.90
ctt	L	2	466.50
ctc	L	0	429.30
cta	L	1208	331.00
ctg	L	736	522.80
mPD		0.84	1.1
		nPD:	0.74
		N. weight:	0.89
		Sc. PD:	0.52
		Sc. rank:	1521.9

,

PA-X

Pos. 176		obs:	exp:
ttt	F	1	1058.00
ttc	F	2485	1428.00
tta	L	0	0.11
ttg	L	0	0.19
ctt	L	0	0.19
ctc	L	1	0.17
cta	L	0	0.13
ctg	L	0	0.21
gtt	V	0	0.22
gtc	V	1	0.21
gta	V	0	0.20
gtg	V	0	0.37
mPD		0.0024	0.49
		nPD:	0.
		N. weight:	0.64
		Sc. PD:	-0.08
		Sc. rank:	-781.3

,

PA-X

Pos. 177		obs:	exp:
act	T	2460	659.00
acc	T	16	549.70
aca	T	12	1069.00
acg	T	0	210.20
mPD		0.022	0.69
		nPD:	0.03
		N. weight:	1.5
		Sc. PD:	-0.14
		Sc. rank:	225.9

,

PA-X

Pos. 178		obs:	exp:
att	I	0	923.50
atc	I	577	623.70
ata	I	1910	939.80
gtt	V	0	0.22
gtc	V	1	0.21
gta	V	0	0.20
gtg	V	0	0.37
mPD		0.36	0.66
		nPD:	0.54
		N. weight:	0.62
		Sc. PD:	0.25
		Sc. rank:	973.3

,

PA-X

Pos. 179		obs:	exp:
cgt	R	0	94.35
cgc	R	0	132.50
cga	R	1	240.90
cgg	R	0	194.90
aga	R	1246	1141.00
agg	R	1241	684.10
mPD		0.50	0.95
		nPD:	0.53
		N. weight:	0.4
		Sc. PD:	0.15
		Sc. rank:	620.5

,

PA-X

Pos. 180		obs:	exp:
caa	Q	2480	1266.00
cag	Q	8	1222.00
mPD		0.0064	0.50
		nPD:	0.01
		N. weight:	0.76
		Sc. PD:	-0.089
		Sc. rank:	-596.2

,

PA-X

Pos. 181		obs:	exp:
gat	D	5	2.88
gac	D	0	2.12
gaa	E	1332	1487.00
gag	E	1151	996.30
mPD		0.50	0.48
		nPD:	1.04
		N. weight:	0.013
		Sc. PD:	0.011
		Sc. rank:	23.0

,

PA-X

Pos. 182		obs:	exp:
tta	L	0	0.44
ttg	L	4	0.75
ctt	L	0	0.75
ctc	L	0	0.69
cta	L	0	0.53
ctg	L	0	0.84
atg	M	2483	2483.00
aaa	K	0	0.56
aag	K	1	0.44
mPD		0.0040	0.0064
		nPD:	0.63
		N. weight:	0.0035
		Sc. PD:	0.0017
		Sc. rank:	5.7

,

PA-X

Pos. 183		obs:	exp:
gtt	V	0	1.96
gtc	V	9	1.90
gta	V	0	1.77
gtg	V	0	3.37
gct	A	0	630.60
gcc	A	2472	483.40
gca	A	7	1090.00
gcg	A	0	275.20
mPD		0.013	0.70
		nPD:	0.02
		N. weight:	1.9
		Sc. PD:	-0.21
		Sc. rank:	-847.3

,

PA-X

Pos. 184		obs:	exp:
tct	S	0	109.00
tcc	S	0	94.05
tca	S	0	143.80
tcg	S	0	41.21
tat	Y	1	0.49
tac	Y	0	0.51
act	T	1	0.26
acc	T	0	0.22
aca	T	0	0.43
acg	T	0	0.08
aat	N	1844	982.10
aac	N	4	865.90
agt	S	633	130.30
agc	S	5	119.70
mPD		0.39	1.2
		nPD:	0.31
		N. weight:	1.
		Sc. PD:	0.18
		Sc. rank:	1223.8

,

PA-X

Pos. 185		obs:	exp:
cgt	R	0	94.28
cgc	R	0	132.40
cga	R	0	240.70
cgg	R	0	194.70
aaa	K	1	1.12
aag	K	1	0.88
aga	R	11	1140.00
agg	R	2475	683.60
mPD		0.011	0.95
		nPD:	0.01
		N. weight:	1.5
		Sc. PD:	-0.17
		Sc. rank:	-1222.7

,

PA-X

Pos. 186		obs:	exp:
tct	S	0	7.34
tcc	S	0	6.34
tca	S	0	9.69
tcg	S	0	2.78
agt	S	39	8.78
agc	S	4	8.07
ggt	G	2429	351.70
ggc	G	11	322.70
gga	G	0	1067.00
ggg	G	5	703.20
mPD		0.050	0.75
		nPD:	0.07
		N. weight:	2.2
		Sc. PD:	-0.14
		Sc. rank:	1512.4

,

PA-X

Pos. 187		obs:	exp:
tta	L	6	271.20
ttg	L	0	466.30
ctt	L	33	466.00
ctc	L	7	428.70
cta	L	1909	330.60
ctg	L	530	522.10
att	I	0	1.11
atc	I	0	0.75
ata	I	3	1.13
mPD		0.37	1.1
		nPD:	0.32
		N. weight:	1.5
		Sc. PD:	0.28
		Sc. rank:	1905.9

,

PA-X

Pos. 188		obs:	exp:
tgg	W	2488	2488.00
mPD		0	0
		nPD:	1.
		N. weight:	0.
		Sc. PD:	0
		Sc. rank:	0

,

PA-X

Pos. 189		obs:	exp:
gat	D	2479	1431.00
gac	D	8	1056.00
gaa	E	0	0.60
gag	E	1	0.40
mPD		0.0072	0.49
		nPD:	0.01
		N. weight:	0.62
		Sc. PD:	-0.071
		Sc. rank:	-429.2

,

PA-X

Pos. 190		obs:	exp:
ttt	F	0	0.85
ttc	F	2	1.15
tct	S	1	423.90
tcc	S	2476	365.90
tca	S	5	559.50
tcg	S	0	160.30
cct	P	0	1.13
ccc	P	4	0.72
cca	P	0	1.63
ccg	P	0	0.52
agt	S	0	506.80
agc	S	0	465.70
mPD		0.0096	1.6
		nPD:	0.01
		N. weight:	2.2
		Sc. PD:	-0.27
		Sc. rank:	-2518.4

,

PA-X

Pos. 191		obs:	exp:
ttt	F	2486	1059.00
ttc	F	2	1429.00
mPD		0.0016	0.49
		nPD:	0.
		N. weight:	0.99
		Sc. PD:	-0.12
		Sc. rank:	-1463.1

,

PA-X

Pos. 192		obs:	exp:
gtt	V	0	539.40
gtc	V	2477	524.00
gta	V	3	488.00
gtg	V	0	928.70
gct	A	0	2.04
gcc	A	8	1.56
gca	A	0	3.52
gcg	A	0	0.89
mPD		0.0088	0.74
		nPD:	0.01
		N. weight:	1.8
		Sc. PD:	-0.21
		Sc. rank:	-1452.2

,

PA-X

Pos. 193		obs:	exp:
tct	S	0	324.00
tcc	S	0	279.70
tca	S	0	427.60
tcg	S	0	122.50
att	I	1	0.37
atc	I	0	0.25
ata	I	0	0.38
aat	N	590	313.50
aac	N	0	276.50
agt	S	1897	387.30
agc	S	0	355.90
mPD		0.36	1.8
		nPD:	0.2
		N. weight:	1.6
		Sc. PD:	0.11
		Sc. rank:	1505.5

,

PA-X

Pos. 194		obs:	exp:
tta	L	0	60.14
ttg	L	0	103.40
ctt	L	0	103.30
ctc	L	0	95.06
cta	L	0	73.31
ctg	L	551	115.80
cct	P	0	545.60
ccc	P	0	350.10
cca	P	0	790.10
ccg	P	1935	249.20
caa	Q	0	1.02
cag	Q	2	0.98
mPD		0.35	1.2
		nPD:	0.29
		N. weight:	2.3
		Sc. PD:	0.35
		Sc. rank:	2563.4

PA-X

Pos.	195	obs:	exp:
cgt	R	0	92.72
cgc	R	0	130.20
cga	R	0	236.80
cgg	R	0	191.50
aaa	K	43	24.14
aag	K	0	18.86
aga	R	2445	1122.00
agg	R	0	672.30
mPD		0.034	0.98
		nPD:	0.03
		N. weight:	0.9
		Sc. PD:	-0.086
		Sc. rank:	226.7

PA-X

Pos.	196	obs:	exp:
gaa	E	0	1490.00
gag	E	2488	998.30
mPD		0	0.48
		nPD:	0.
		N. weight:	1.1
		Sc. PD:	-0.14
		Sc. rank:	-1948.5

PA-X

Pos.	197	obs:	exp:
gct	A	0	632.40
gcc	A	0	484.80
gca	A	0	1093.00
gcg	A	2486	276.00
gaa	E	0	1.20
gag	E	2	0.80
mPD		0.0016	0.69
		nPD:	0.
		N. weight:	2.6
		Sc. PD:	-0.33
		Sc. rank:	-4108.8

PA-X

Pos.	198	obs:	exp:
cgt	R	0	0.15
cgc	R	0	0.21
cga	R	0	0.39
cgg	R	0	0.31
aaa	K	0	1394.00
aag	K	2484	1090.00
aga	R	0	1.83
agg	R	4	1.10
mPD		0.0032	0.50
		nPD:	0.01
		N. weight:	0.96
		Sc. PD:	-0.12
		Sc. rank:	-1054.9

PA-X

Pos.	199	obs:	exp:
cgt	R	0	81.19
cgc	R	0	114.00
cga	R	0	207.30
cgg	R	0	167.70
att	I	0	0.74
atc	I	0	0.50
ata	I	2	0.76
aaa	K	344	193.10
aag	K	0	150.90
aga	R	2141	982.10
agg	R	0	588.70
ggt	G	0	0.14
ggc	G	0	0.13
gga	G	1	0.44
ggg	G	0	0.29
mPD		0.24	1.1
		nPD:	0.21
		N. weight:	0.88
		Sc. PD:	0.065
		Sc. rank:	862.3

PA-X

Pos.	200	obs:	exp:
cat	H	1	0.59
cac	H	0	0.41
caa	Q	2481	1265.00
cag	Q	6	1222.00
mPD		0.0056	0.50
		nPD:	0.01
		N. weight:	0.77
		Sc. PD:	-0.091
		Sc. rank:	-693.2

PA-X

Pos.	201	obs:	exp:
tta	L	0	267.50
ttg	L	2449	459.90
tct	S	0	6.32
tcc	S	0	5.45
tca	S	0	8.34
tcg	S	37	2.39
ctt	L	0	459.60
ctc	L	0	422.90
cta	L	0	326.10
ctg	L	2	515.00
agt	S	0	7.55
agc	S	0	6.94
mPD		0.031	1.2
		nPD:	0.03
		N. weight:	2.
		Sc. PD:	-0.2
		Sc. rank:	-219.9

PA-X

Pos.	202	obs:	exp:
cgt	R	0	1.21
cgc	R	0	1.70
cga	R	0	3.10
cgg	R	0	2.51
aaa	K	0	1379.00
aag	K	2456	1077.00
aga	R	0	14.68
agg	R	32	8.80
mPD		0.025	0.53
		nPD:	0.05
		N. weight:	0.97
		Sc. PD:	-0.08
		Sc. rank:	493.1

PA-X

Pos.	203	obs:	exp:
cgt	R	0	0.27
cgc	R	0	0.37
cga	R	0	0.68
cgg	R	0	0.55
aat	N	0	1.59
aac	N	3	1.41
aaa	K	2478	1391.00
aag	K	0	1087.00
aga	R	7	3.21
agg	R	0	1.92
mPD		0.0080	0.50
		nPD:	0.02
		N. weight:	0.67
		Sc. PD:	-0.077
		Sc. rank:	-434.7

PA-X

Pos.	204	obs:	exp:
aat	N	1	0.53
aac	N	0	0.47
gat	D	1939	1115.00
gac	D	0	823.70
ggt	G	547	78.83
ggc	G	0	72.33
gga	G	1	239.20
ggg	G	0	157.60
mPD		0.35	0.97
		nPD:	0.36
		N. weight:	1.
		Sc. PD:	0.22
		Sc. rank:	1357.7

PA-X

Pos.	205	obs:	exp:
tta	L	56	271.50
ttg	L	2431	466.70
tct	S	0	0.17
tcc	S	0	0.15
tca	S	0	0.23
tcg	S	1	0.06
ctt	L	0	466.30
ctc	L	0	429.10
cta	L	0	330.90
ctg	L	0	522.50
agt	S	0	0.20
agc	S	0	0.19
mPD		0.045	1.1
		nPD:	0.04
		N. weight:	1.8
		Sc. PD:	-0.17
		Sc. rank:	602.2

PA-X

Pos.	206	obs:	exp:
cgt	R	0	0.23
cgc	R	0	0.32
cga	R	0	0.58
cgg	R	0	0.47
act	T	0	3.44
acc	T	0	2.87
aca	T	13	5.59
acg	T	0	1.10
aat	N	0	2.66
aac	N	5	2.34
aaa	K	2464	1383.00
aag	K	0	1081.00
aga	R	6	2.75
agg	R	0	1.65
mPD		0.019	0.51
		nPD:	0.04
		N. weight:	0.68
		Sc. PD:	-0.063
		Sc. rank:	185.8

PA-X

Pos.	207	obs:	exp:
tta	L	298	32.64
ttg	L	1	56.11
tct	S	1	373.80
tcc	S	0	322.70
tca	S	1802	493.40
tcg	S	386	141.40
ctt	L	0	56.07
ctc	L	0	51.59
cta	L	0	39.78
ctg	L	0	62.82
agt	S	0	446.90
agc	S	0	410.70
mPD		0.48	1.8
		nPD:	0.26
		N. weight:	1.6
		Sc. PD:	0.19
		Sc. rank:	1644.7

PA-X

Pos.	208	obs:	exp:
tta	L	0	0.11
ttg	L	0	0.19
ctt	L	0	0.19
ctc	L	0	0.17
cta	L	0	0.13
ctg	L	1	0.21
cct	P	0	2.26
ccc	P	0	1.45
cca	P	0	3.27
ccg	P	8	1.03
caa	Q	1	1261.00
cag	Q	2477	1217.00
aaa	K	0	0.56
aag	K	1	0.44
mPD		0.0088	0.51
		nPD:	0.02
		N. weight:	0.83
		Sc. PD:	-0.093
		Sc. rank:	-482.9

PA-X

Pos.	209	obs:	exp:
gaa	E	2484	1488.00
gag	E	1	997.10
ggt	G	0	0.43
ggc	G	0	0.40
gga	G	3	1.31
ggg	G	0	0.86
mPD		0.0032	0.48
		nPD:	0.01
		N. weight:	0.59
		Sc. PD:	-0.073
		Sc. rank:	-632.7

PA-X

Pos.	210	obs:	exp:
tct	S	0	0.17
tcc	S	0	0.15
tca	S	1	0.23
tcg	S	0	0.06
cct	P	36	689.50
ccc	P	0	442.40
cca	P	2409	998.30
ccg	P	0	314.80
caa	Q	42	21.37
cag	Q	0	20.63
agt	S	0	0.20
agc	S	0	0.19
mPD		0.063	0.74
		nPD:	0.08
		N. weight:	0.96
		Sc. PD:	-0.045
		Sc. rank:	733.7

PA-X

Pos.	211	obs:	exp:
ttt	F	0	2.13
ttc	F	5	2.87
tat	Y	0	1.94
tac	Y	4	2.06
tgt	C	0	1078.00
tgc	C	2479	1401.00
mPD		0.0072	0.50
		nPD:	0.01
		N. weight:	0.67
		Sc. PD:	-0.077
		Sc. rank:	-476.4

PA-X

Pos.	212	obs:	exp:
act	T	0	1.06
acc	T	0	0.88
aca	T	4	1.72
acg	T	0	0.34
gtt	V	0	5.22
gtc	V	0	5.07
gta	V	24	4.72
gtg	V	0	8.99
gct	A	0	625.30
gcc	A	0	479.30
gca	A	2458	1080.00
gcg	A	0	272.90
gaa	E	2	1.20
gag	E	0	0.80
mPD		0.024	0.72
		nPD:	0.03
		N. weight:	0.97
		Sc. PD:	-0.094
		Sc. rank:	215.7

PA-X

Pos.	213	obs:	exp:
tct	S	0	0.34
tcc	S	0	0.29
tca	S	0	0.45
tcg	S	0	0.13
agt	S	0	0.41
agc	S	2	0.38
gat	D	0	7.48
gac	D	13	5.52
ggt	G	0	355.70
ggc	G	2473	326.40
gga	G	0	1080.00
ggg	G	0	711.30
mPD		0.012	0.70
		nPD:	0.02
		N. weight:	2.4
		Sc. PD:	-0.27
		Sc. rank:	-1396.9

PA-X

Pos.	214	obs:	exp:
tta	L	0	270.60
ttg	L	2479	465.20
tct	S	0	1.54
tcc	S	0	1.33
tca	S	0	2.03
tcg	S	9	0.58
ctt	L	0	464.80
ctc	L	0	427.70
cta	L	0	329.80
ctg	L	0	520.90
agt	S	0	1.84
agc	S	0	1.69
mPD		0.0072	1.1
		nPD:	0.01
		N. weight:	2.
		Sc. PD:	-0.24
		Sc. rank:	-2172.7

PA-X			
Pos.	215	obs:	exp:
tta	L	0	105.00
ttg	L	0	180.50
ctt	L	1	180.40
ctc	L	0	166.00
cta	L	1	128.00
ctg	L	960	202.10
cct	P	7	423.50
ccc	P	0	271.80
cca	P	14	613.30
ccg	P	1481	193.40
caa	Q	1	12.21
cag	Q	23	11.79
---	--	------	------
mPD		0.51	1.4
		nPD:	0.35
		N. weight:	2.1
		Sc. PD:	0.45
		Sc. rank:	2801.7

PA-X			
Pos.	216	obs:	exp:
att	I	0	0.74
atc	I	2	0.50
ata	I	0	0.76
act	T	0	658.50
acc	T	2486	549.30
aca	T	0	1068.00
acg	T	0	210.00
---	--	------	------
mPD		0.0016	0.69
		nPD:	0.
		N. weight:	1.8
		Sc. PD:	-0.22
		Sc. rank:	-2778.2

PA-X			
Pos.	217	obs:	exp:
cgt	R	0	5.31
cgc	R	0	7.46
cga	R	0	13.56
cgg	R	0	10.96
aaa	K	2348	1318.00
aag	K	0	1030.00
aga	R	140	64.22
agg	R	0	38.50
---	--	------	------
mPD		0.11	0.63
		nPD:	0.17
		N. weight:	0.69
		Sc. PD:	0.023
		Sc. rank:	599.5

PA-X			
Pos.	218	obs:	exp:
gtt	V	0	515.60
gtc	V	2371	501.00
gta	V	0	466.50
gtg	V	0	887.90
gct	A	0	29.76
gcc	A	117	22.82
gca	A	0	51.43
gcg	A	0	12.99
---	--	------	------
mPD		0.090	0.82
		nPD:	0.11
		N. weight:	1.8
		Sc. PD:	-0.042
		Sc. rank:	1482.4

PA-X			
Pos.	219	obs:	exp:
ttt	F	0	4.26
ttc	F	10	5.74
tct	S	0	423.20
tcc	S	2477	365.30
tca	S	1	558.60
tcg	S	0	160.10
agt	S	0	505.90
agc	S	0	464.90
---	--	------	------
mPD		0.0088	1.6
		nPD:	0.01
		N. weight:	2.2
		Sc. PD:	-0.28
		Sc. rank:	-2604.0

PA-X			
Pos.	220	obs:	exp:
cat	H	1	1478.00
cac	H	2484	1007.00
cgt	R	0	0.11
cgc	R	3	0.16
cga	R	0	0.29
cgg	R	0	0.23
aga	R	0	1.38
agg	R	0	0.82
---	--	------	------
mPD		0.0032	0.49
		nPD:	0.01
		N. weight:	1.1
		Sc. PD:	-0.13
		Sc. rank:	-1127.6

PA-X			
Pos.	221	obs:	exp:
tta	L	0	1.42
ttg	L	0	2.44
ctt	L	0	2.44
ctc	L	0	2.24
cta	L	13	1.73
ctg	L	0	2.73
caa	Q	1	0.51
cag	Q	0	0.49
cgt	R	0	93.82
cgc	R	0	131.80
cga	R	2474	239.60
cgg	R	0	193.80
aga	R	0	1135.00
agg	R	0	680.30
---	--	------	------
mPD		0.011	0.97
		nPD:	0.01
		N. weight:	2.7
		Sc. PD:	-0.32
		Sc. rank:	-2343.5

PA-X			
Pos.	222	obs:	exp:
att	I	582	216.10
atc	I	0	146.00
ata	I	0	219.90
act	T	1904	504.30
acc	T	0	420.70
aca	T	0	818.10
acg	T	0	160.80
gtt	V	1	0.22
gtc	V	0	0.21
gta	V	0	0.20
gtg	V	0	0.37
gct	A	1	0.25
gcc	A	0	0.20
gca	A	0	0.44
gcg	A	0	0.11
---	--	------	------
mPD		0.36	1.0
		nPD:	0.34
		N. weight:	1.5
		Sc. PD:	0.3
		Sc. rank:	1909.2

PA-X			
Pos.	223	obs:	exp:
ttt	F	1	0.43
ttc	F	0	0.57
tct	S	1557	424.70
tcc	S	13	366.60
tca	S	14	560.60
tcg	S	903	160.60
agt	S	0	507.80
agc	S	0	466.60
---	--	------	------
mPD		0.48	1.6
		nPD:	0.29
		N. weight:	1.6
		Sc. PD:	0.25
		Sc. rank:	1895.6

PA-X			
Pos.	224	obs:	exp:
cct	P	1	700.50
ccc	P	27	449.50
cca	P	2456	1014.00
ccg	P	0	319.80
caa	Q	4	2.04
cag	Q	0	1.96
---	--	------	------
mPD		0.025	0.71
		nPD:	0.04
		N. weight:	0.98
		Sc. PD:	-0.093
		Sc. rank:	260.3

PA-X			
Pos.	225	obs:	exp:
gtt	V	0	0.22
gtc	V	1	0.21
gta	V	0	0.20
gtg	V	0	0.37
gct	A	11	632.70
gcc	A	2473	485.00
gca	A	3	1093.00
gcg	A	0	276.10
---	--	------	------
mPD		0.012	0.69
		nPD:	0.02
		N. weight:	1.9
		Sc. PD:	-0.21
		Sc. rank:	-1031.5

PA-X			
Pos.	226	obs:	exp:
tta	L	0	271.20
ttg	L	2485	466.30
tct	S	0	0.34
tcc	S	0	0.29
tca	S	0	0.45
tcg	S	2	0.13
ctt	L	0	466.00
ctc	L	0	428.70
cta	L	0	330.60
ctg	L	0	522.10
agt	S	0	0.41
agc	S	0	0.38
gtt	V	0	0.22
gtc	V	0	0.21
gta	V	0	0.20
gtg	V	1	0.37
---	--	------	------
mPD		0.0024	1.1
		nPD:	0.
		N. weight:	2.
		Sc. PD:	-0.25
		Sc. rank:	-3203.2

PA-X			
Pos.	227	obs:	exp:
cgt	R	0	0.04
cgc	R	0	0.05
cga	R	0	0.10
cgg	R	0	0.08
aaa	K	2485	1395.00
aag	K	0	1090.00
aga	R	1	0.46
agg	R	0	0.27
gaa	E	2	1.20
gag	E	0	0.80
---	--	------	------
mPD		0.0024	0.50
		nPD:	0.
		N. weight:	0.67
		Sc. PD:	-0.084
		Sc. rank:	-830.1

PA-X			
Pos.	228	obs:	exp:
att	I	669	248.40
atc	I	0	167.80
ata	I	0	252.80
act	T	1817	481.30
acc	T	0	401.50
aca	T	0	780.80
acg	T	0	153.50
aat	N	1	0.53
aac	N	0	0.47
gct	A	1	0.25
gcc	A	0	0.20
gca	A	0	0.44
gcg	A	0	0.11
---	--	------	------
mPD		0.39	1.1
		nPD:	0.37
		N. weight:	1.4
		Sc. PD:	0.33
		Sc. rank:	1994.4

PA-X			
Pos.	229	obs:	exp:
tta	L	2488	271.60
ttg	L	0	466.90
ctt	L	0	466.50
ctc	L	0	429.30
cta	L	0	331.00
ctg	L	0	522.80
---	--	------	------
mPD		0	1.1
		nPD:	0.
		N. weight:	2.6
		Sc. PD:	-0.33
		Sc. rank:	-4726.2

PA-X			
Pos.	230	obs:	exp:
gaa	E	3	1489.00
gag	E	2484	997.90
ggt	G	0	0.14
ggc	G	0	0.13
gga	G	0	0.44
ggg	G	1	0.29
---	--	------	------
mPD		0.0032	0.48
		nPD:	0.01
		N. weight:	1.1
		Sc. PD:	-0.13
		Sc. rank:	-1104.9

PA-X			
Pos.	231	obs:	exp:
tct	S	3	0.51
tcc	S	0	0.44
tca	S	0	0.68
tcg	S	0	0.19
cct	P	2484	700.50
ccc	P	0	449.50
cca	P	0	1014.00
ccg	P	0	319.80
cat	H	1	0.59
cac	H	0	0.41
agt	S	0	0.61
agc	S	0	0.56
---	--	------	------
mPD		0.0032	0.71
		nPD:	0.
		N. weight:	1.5
		Sc. PD:	-0.18
		Sc. rank:	-1902.7

PA-X			
Pos.	232	obs:	exp:
atg	M	2488	2488.00
---	--	------	------
mPD		0	0
		nPD:	1.
		N. weight:	0.
		Sc. PD:	0
		Sc. rank:	0

PA-X			
Pos.	233	obs:	exp:
tta	L	0	0.33
ttg	L	3	0.56
tgg	W	2485	2485.00
ctt	L	0	0.56
ctc	L	0	0.52
cta	L	0	0.40
ctg	L	0	0.63
---	--	------	------
mPD		0.0024	0.0056
		nPD:	0.43
		N. weight:	0.0024
		Sc. PD:	0.00069
		Sc. rank:	3.5

PA-X

Pos.	234	obs:	exp:
atg	M	2483	2483.00
act	T	0	1.32
acc	T	0	1.10
aca	T	0	2.15
acg	T	5	0.42
---	--	------	------
mPD		0.0040	0.0077
		nPD:	0.52
		N. weight:	0.0058
		Sc. PD:	0.0022
		Sc. rank:	8.8

,

PA-X

Pos.	235	obs:	exp:
gtt	V	2	0.43
gtc	V	0	0.42
gta	V	0	0.39
gtg	V	0	0.75
gat	D	2477	1425.00
gac	D	0	1052.00
ggt	G	9	1.29
ggc	G	0	1.19
gga	G	0	3.93
ggg	G	0	2.59
---	--	------	------
mPD		0.0088	0.50
		nPD:	0.02
		N. weight:	0.65
		Sc. PD:	-0.073
		Sc. rank:	-354.9

,

PA-X

Pos.	236	obs:	exp:
tta	L	2	99.87
ttg	L	913	171.70
tct	S	0	268.60
tcc	S	0	231.90
tca	S	601	354.60
tcg	S	972	101.60
ctt	L	0	171.60
ctc	L	0	157.90
cta	L	0	121.70
ctg	L	0	192.30
agt	S	0	321.20
agc	S	0	295.10
---	--	------	------
mPD		0.83	2.0
		nPD:	0.41
		N. weight:	1.9
		Sc. PD:	0.51
		Sc. rank:	2728.8

,

PA-X

Pos.	237	obs:	exp:
tct	S	0	0.51
tcc	S	0	0.44
tca	S	0	0.68
tcg	S	0	0.19
aat	N	0	1320.00
aac	N	2483	1163.00
aaa	K	1	0.56
aag	K	0	0.44
agt	S	0	0.61
agc	S	3	0.56
gat	D	0	0.58
gac	D	1	0.42
---	--	------	------
mPD		0.0040	0.50
		nPD:	0.01
		N. weight:	0.89
		Sc. PD:	-0.11
		Sc. rank:	-902.9

,

PA-X

Pos.	238	obs:	exp:
caa	Q	2	1.02
cag	Q	0	0.98
cgt	R	0	94.28
cgc	R	0	132.40
cga	R	2484	240.70
cgg	R	0	194.70
aga	R	2	1140.00
agg	R	0	683.60
---	--	------	------
mPD		0.0032	0.96
		nPD:	0.
		N. weight:	2.7
		Sc. PD:	-0.34
		Sc. rank:	-3873.0

,

PA-X

Pos.	239	obs:	exp:
atg	M	18	18.00
act	T	0	654.30
acc	T	0	545.70
aca	T	2	1061.00
acg	T	2468	208.60
---	--	------	------
mPD		0.016	0.71
		nPD:	0.02
		N. weight:	2.9
		Sc. PD:	-0.31
		Sc. rank:	-874.9

,

PA-X

Pos.	240	obs:	exp:
gct	A	2459	625.60
gcc	A	0	479.50
gca	A	0	1081.00
gcg	A	0	273.00
gat	D	29	16.68
gac	D	0	12.32
---	--	------	------
mPD		0.023	0.72
		nPD:	0.03
		N. weight:	1.6
		Sc. PD:	-0.16
		Sc. rank:	209.8

,

PA-X

Pos.	241	obs:	exp:
act	T	0	0.26
acc	T	0	0.22
aca	T	1	0.43
acg	T	0	0.08
gct	A	0	632.70
gcc	A	529	485.00
gca	A	1958	1093.00
gcg	A	0	276.10
---	--	------	------
mPD		0.34	0.69
		nPD:	0.48
		N. weight:	0.56
		Sc. PD:	0.19
		Sc. rank:	844.7

,

PA-X

Pos.	242	obs:	exp:
tta	L	0	271.60
ttg	L	2488	466.90
ctt	L	0	466.50
ctc	L	0	429.30
cta	L	0	331.00
ctg	L	0	522.80
---	--	------	------
mPD		0	1.1
		nPD:	0.
		N. weight:	2.
		Sc. PD:	-0.25
		Sc. rank:	-3570.1

,

PA-X

Pos.	243	obs:	exp:
cgt	R	0	94.32
cgc	R	0	132.50
cga	R	0	240.80
cgg	R	0	194.80
aaa	K	0	0.56
aag	K	1	0.44
aga	R	0	1141.00
agg	R	2487	683.80
---	--	------	------
mPD		0.00080	0.95
		nPD:	0.
		N. weight:	1.5
		Sc. PD:	-0.19
		Sc. rank:	-2569.5

,

PA-X

Pos.	244	obs:	exp:
gct	A	0	632.20
gcc	A	0	484.60
gca	A	2485	1092.00
gcg	A	0	275.90
gaa	E	3	1.80
gag	E	0	1.20
---	--	------	------
mPD		0.0024	0.69
		nPD:	0.
		N. weight:	0.96
		Sc. PD:	-0.12
		Sc. rank:	-1354.4

,

PA-X

Pos.	245	obs:	exp:
tct	S	0	424.00
tcc	S	0	366.00
tca	S	0	559.70
tcg	S	0	160.40
aat	N	0	2.66
aac	N	5	2.34
agt	S	0	507.00
agc	S	2483	465.90
---	--	------	------
mPD		0.0040	1.6
		nPD:	0.
		N. weight:	1.9
		Sc. PD:	-0.25
		Sc. rank:	-2992.6

,

PA-X

Pos.	246	obs:	exp:
ttt	F	2488	1059.00
ttc	F	0	1429.00
---	--	------	------
mPD		0	0.49
		nPD:	0.
		N. weight:	1.
		Sc. PD:	-0.13
		Sc. rank:	-1823.1

,

PA-X

Pos.	247	obs:	exp:
tta	L	0	271.60
ttg	L	0	466.90
ctt	L	0	466.50
ctc	L	2488	429.30
cta	L	0	331.00
ctg	L	0	522.80
---	--	------	------
mPD		0	1.1
		nPD:	0.
		N. weight:	2.
		Sc. PD:	-0.26
		Sc. rank:	-3749.2

,

PA-X

Pos.	248	obs:	exp:
cgt	R	0	0.11
cgc	R	0	0.16
cga	R	0	0.29
cgg	R	0	0.23
aaa	K	2485	1395.00
aag	K	0	1090.00
aga	R	3	1.38
agg	R	0	0.82
---	--	------	------
mPD		0.0024	0.50
		nPD:	0.
		N. weight:	0.67
		Sc. PD:	-0.084
		Sc. rank:	-853.8

,

PA-X

Pos.	249	obs:	exp:
tgt	C	2473	1082.00
tgc	C	15	1406.00
---	--	------	------
mPD		0.012	0.49
		nPD:	0.02
		N. weight:	0.93
		Sc. PD:	-0.098
		Sc. rank:	-206.5

,

PA-X

Pos.	250	obs:	exp:
cct	P	0	13.54
ccc	P	0	8.69
cca	P	48	19.60
ccg	P	0	6.18
caa	Q	2438	1240.00
cag	Q	0	1198.00
cgt	R	0	0.08
cgc	R	0	0.11
cga	R	2	0.19
cgg	R	0	0.16
aga	R	0	0.92
agg	R	0	0.55
---	--	------	------
mPD		0.039	0.55
		nPD:	0.07
		N. weight:	0.79
		Sc. PD:	-0.047
		Sc. rank:	555.5

,

PA-X

Pos.	251	obs:	exp:
cgt	R	0	0.27
cgc	R	0	0.37
cga	R	0	0.68
cgg	R	0	0.55
aaa	K	0	1391.00
aag	K	2479	1088.00
aga	R	0	3.21
agg	R	7	1.92
gaa	E	0	1.20
gag	E	2	0.80
---	--	------	------
mPD		0.0072	0.50
		nPD:	0.01
		N. weight:	0.96
		Sc. PD:	-0.11
		Sc. rank:	-698.2

,

PA-X

Pos.	252	obs:	exp:
cgt	R	0	0.72
cgc	R	0	1.01
cga	R	0	1.84
cgg	R	0	1.49
act	T	0	0.26
acc	T	0	0.22
aca	T	0	0.43
acg	T	1	0.08
aaa	K	0	1385.00
aag	K	2468	1083.00
aga	R	0	8.72
agg	R	19	5.22
---	--	------	------
mPD		0.016	0.51
		nPD:	0.03
		N. weight:	0.97
		Sc. PD:	-0.096
		Sc. rank:	86.0

,

PA-X

Pos.	253	obs:	exp:
taa	.	10	912.40
tag	.	0	698.30
tga	.	2478	877.40
---	--	------	------
mPD		0.0080	0.86
		nPD:	0.01
		N. weight:	1.2
		Sc. PD:	-0.14
		Sc. rank:	-1127.3

}

Amino acid level conservation per codon

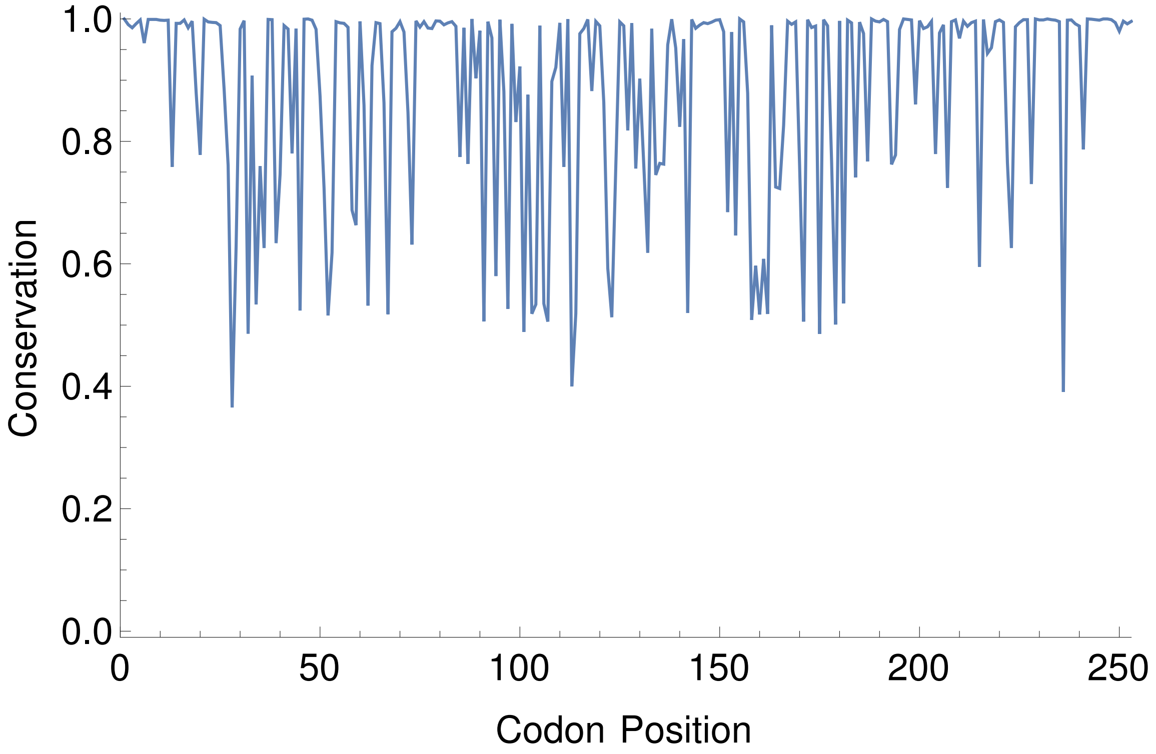

Conservation analysis plots

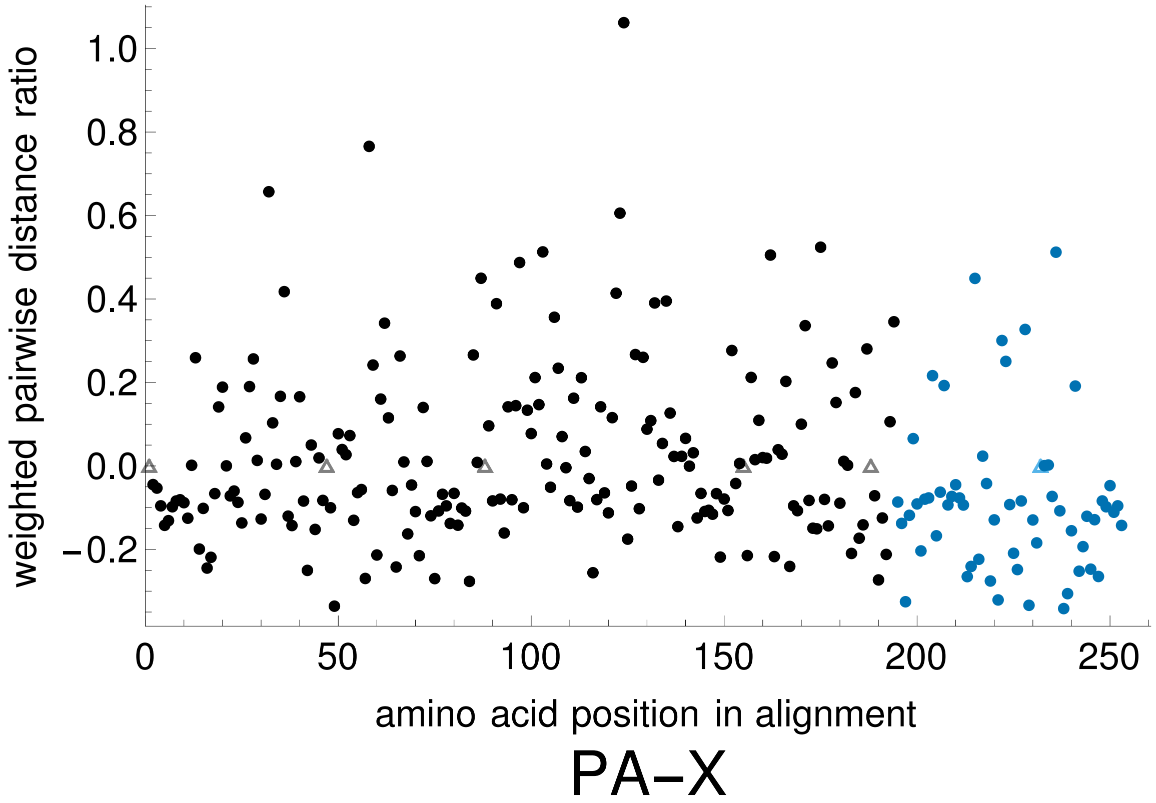

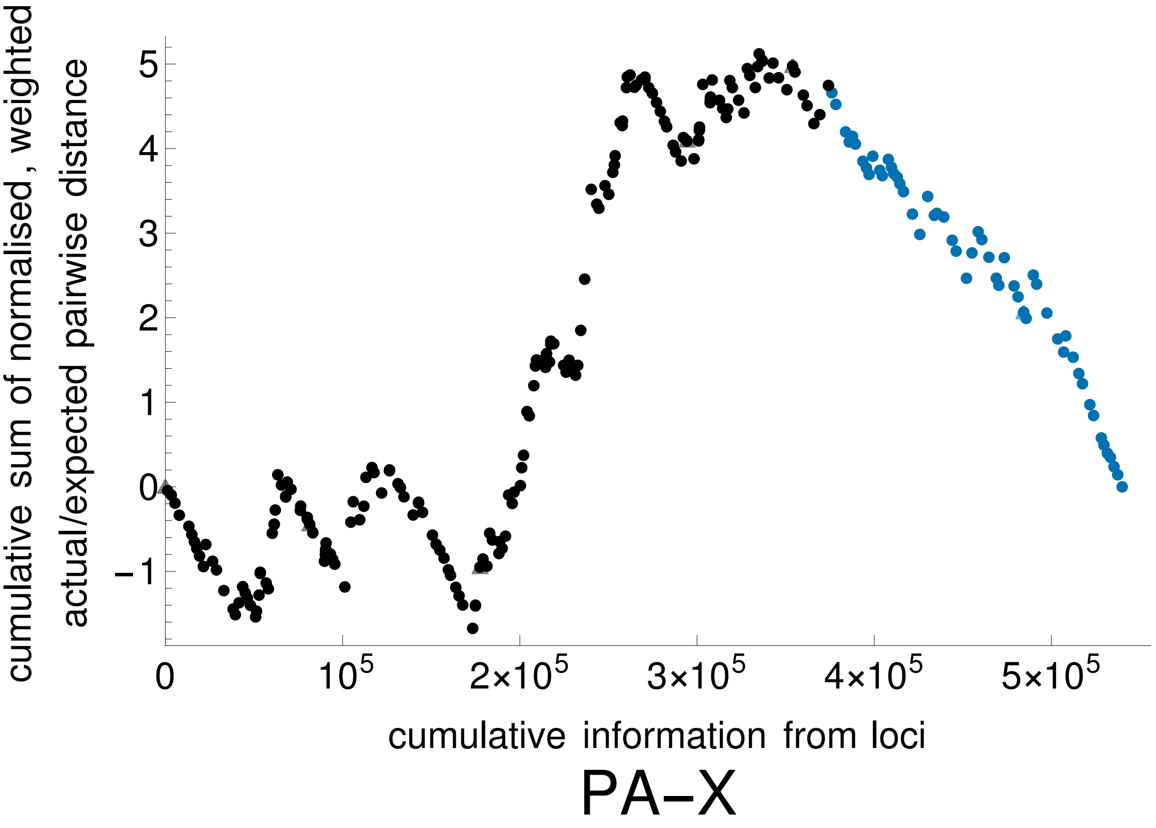

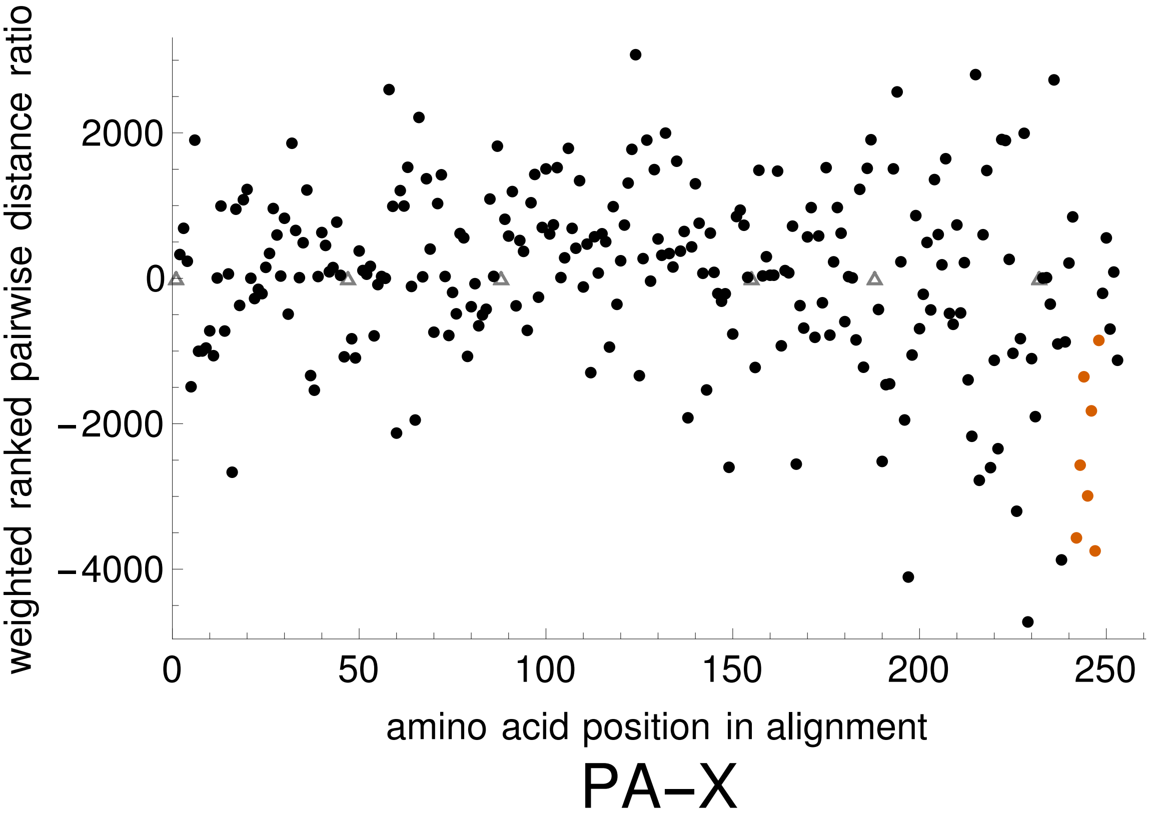
weighted ranked pairwise distance ratio
2000
0
−2000
−4000
0
50
100
150
200
250
amino acid position in alignment
PA−X

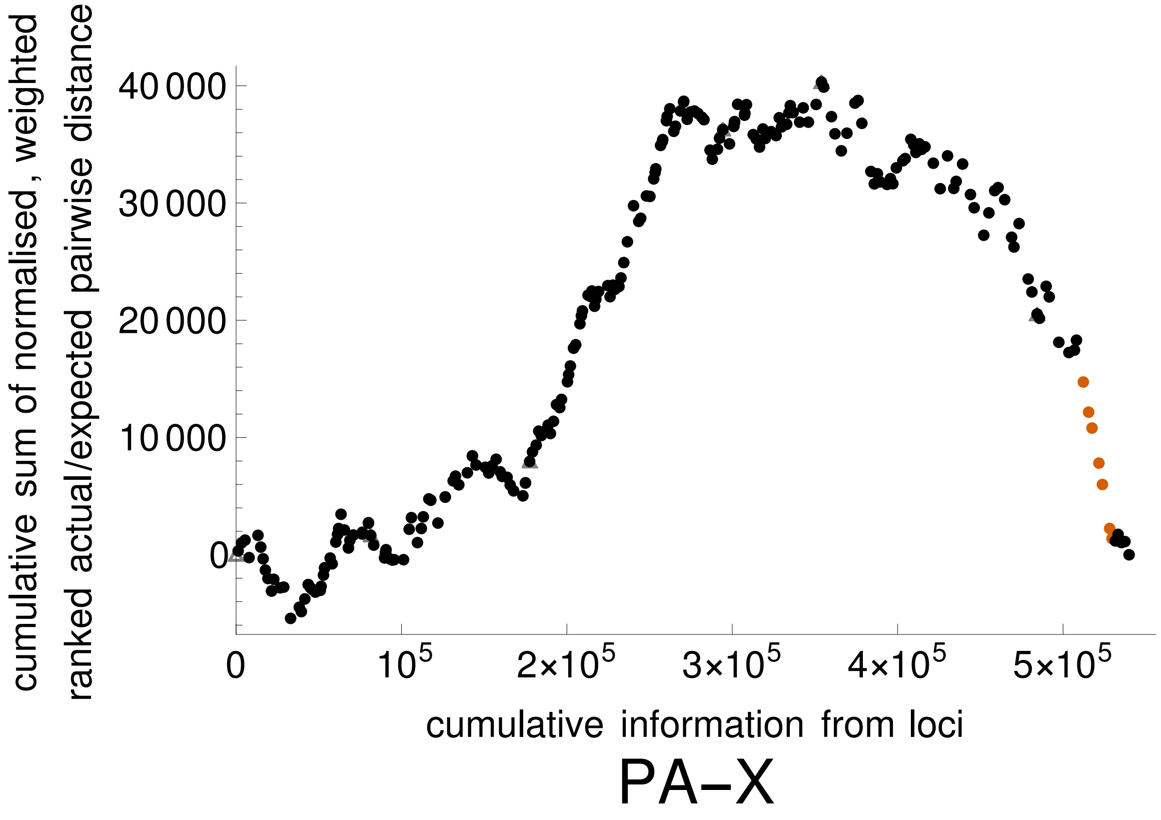
cumulative sum of normalised, weighted
ranked actual/expected pairwise distance
40 000
30 000
20 000
10 000
0
0
10^5
2×10^5
3×10^5
4×10^5
5×10^5
cumulative information from loci
PA−X

HA

Gene length histogram

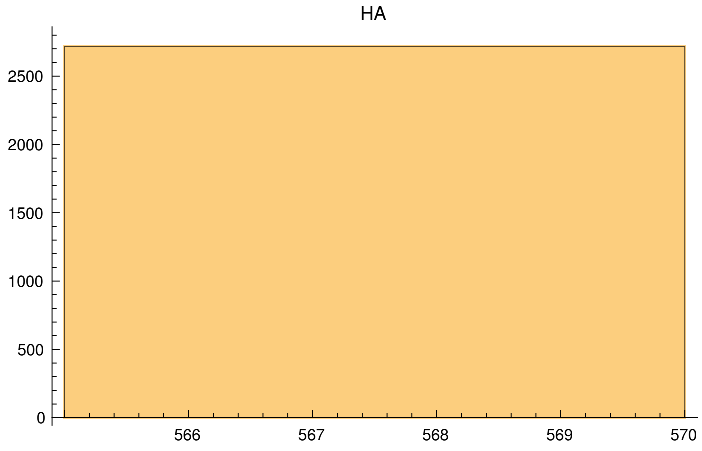

Information vs. nPD

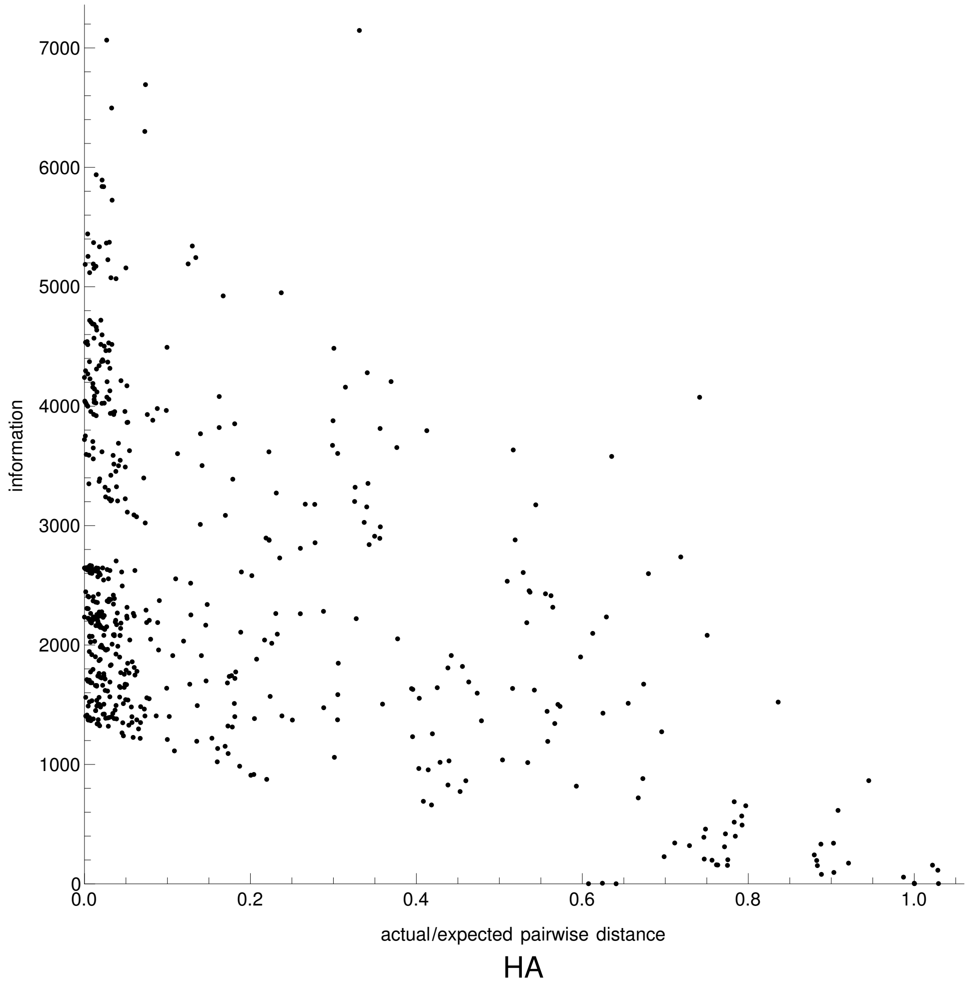

Example sequences highlighted by regions found in analyses to be conserved

Interesting points (by weighted raw PD) highlighted for gene HA:

------ATGGAGAACATAGTGCTTCTTCTTGCAATAGTTAGCCTTGTTAAAAGTGATCAGATTTGCATTGGTTACC⋱
ATGCAAACAACTCGACAGAGCAAGTTGACACGATAATGGAAAAGAACGTCACTGTTACACATGCCCAAGACATAC⋱
TGGAAAAAACACACAACGGGAAGCTCTGCGATCTAAATGGGGTGAAGCCTCTGATTTTAAAGGATTGTAGTGTAG⋱
CTGGATGGCTCCTCGGAAACCCAATGTGCGACGAATTCATCAGAGTGCCGGAATGGTCTTACATAGTGGAGAGGG⋱
CTAACCCAGCTAATGACCTCTGTTACCCAGGGAGCCTCAATGACTATGAAGAACTGAAACACCTGTTGAGCAGAA⋱
TAAATCATTTTGAGAAGATTCTGATCATCCCCAAGAGTTCTTGGCCCAATCATGAAACATCATTAGGGGTGAGCG⋱
CAGCTTGTCCATACCAGGGAACGCCCTCCTTTTTCAGAAATGTGGTATGGCTTATCAAAAAGAACGATGCATACC⋱
CAACAATAAAGATAAGCTACAATAATACCAATCGGGAAGATCTCTTGATACTGTGGGGGATTCATCATTCCAACA⋱
ATGCAGAAGAGCAGACAAATCTCTATAAAAACCCAACCACCTATATTTCAGTTGGAACATCAACATTAAACCAGA⋱
GATTGGTACCAAAAATAGCTACTAGATCCCAAGTAAACGGGCAACGTGGAAGAATGGACTTCTTCTGGACAATTT⋱
TAAAACCGAATGATGCAATCCACTTCGAGAGTAATGGAAATTTCATTGCTCCAGAATATGCATACAAAATTGTCA⋱
AGAAAGGGGACTCAACAATTATGAAAAGTGGAGTGGAATATGGCCACTGCAACACCAAATGTCAAACCCCAGTAG⋱
GAGCGATAAACTCTAGTATGCCGTTCCACAATATACATCCTCTCACCATCGGGGAATGCCCCAAATACGTGAAGT⋱
CAAACAAGTTGGTCCTTGCGACTGGGCTCAGAAATAGTCCTCTAAGAGAAAAGAGAAGA ---AAA---AGAGGGCT⋱
GTTTGGGGCTATAGCAGGTTTTATAGAGGGAGGATGGCAGGGAATGGTTGATGGTTGGTATGGGTACCACCATAG⋱
CAATGAGCAGGGGAGTGGGTACGCTGCAGACAAAGAATCCACCCAAAAGGCAATAGATGGAGTTACCAATAAGGT⋱
CAACTCGATCATTGACAAAATGAACACTCAATTTGAGGCAGTTGGAAGGGAGTTTAATAACTTAGAAAGGAGGAT⋱
AGAGAATTTGAACAAGAAAATGGAAGACGGATTCCTAGATGTCTGGACCTATAATGCTGAACTTCTAGTTCTCAT⋱
GGAAAACGAGAGGACTCTAGATTTCCATGACTCAAATGTCAAGAACCTTTACGACAAAGTCAGACTGCAGCTTAG⋱
GGATAATGCAAAGGAGCTGGGTAACGGTTGTTTCGAGTTCTATCACAAATGTGATAATGAATGTATGGAAAGTGT⋱
GAGAAATGGGACGTATGACTACCCTCAGTATTCAGAAGAAGCAAGATTAAAAAGAGAAGAAATAAGCGGAGTGAA⋱
ATTAGAGTCAATAGGAACTTACCAAATACTGTCAATTTATTCAACAGTGGCGAGTTCCCTAGCACTGGCAATCAT⋱
GGTGGCTGGTCTATCTTTATGGATGTGCTCCAATGGGTCGTTACAGTGCAGAATTTGCATTTAA

Interesting points (by weighted ranked PD) highlighted for gene HA:

------ATGGAGAACATAGTGCTTCTTCTTGCAATAGTTAGCCTTGTTAAAAGTGATCAGATTTGCATTGGTTACCAT⋱
GCAAACAACTCGACAGAGCAAGTTGACACGATAATGGAAAAGAACGTCACTGTTACACATGCCCAAGACATACTG⋱
GAAAAAACACACAACGGGAAGCTCTGCGATCTAAATGGGGTGAAGCCTCTGATTTTAAAGGATTGTAGTGTAGCT⋱
GGATGGCTCCTCGGAAACCCAATGTGCGACGAATTCATCAGAGTGCCGGAATGGTCTTACATAGTGGAGAGGGCT⋱
AACCCAGCTAATGACCTCTGTTACCCAGGGAGCCTCAATGACTATGAAGAACTGAAACACCTGTTGAGCAGAATA⋱
AATCATTTTGAGAAGATTCTGATCATCCCCAAGAGTTCTTGGCCCAATCATGAAACATCATTAGGGGTGAGCGCA⋱
GCTTGTCCATACCAGGGAACGCCCTCCTTTTTCAGAAATGTGGTATGGCTTATCAAAAAGAACGATGCATACCCA⋱
ACAATAAAGATAAGCTACAATAATACCAATCGGGAAGATCTCTTGATACTGTGGGGGATTCATCATTCCAACAAT⋱
GCAGAAGAGCAGACAAATCTCTATAAAAACCCAACCACCTATATTTCAGTTGGAACATCAACATTAAACCAGAGA⋱
TTGGTACCAAAAATAGCTACTAGATCCCAAGTAAACGGGCAACGTGGAAGAATGGACTTCTTCTGGACAATTTTA⋱
AAACCGAATGATGCA
ATCCACTTCGAGAGTAATGGAAATTTCATTGCTCCAGAATATGCATACAAAATTG
TCAAGAAAGGGGACTCAACAATTATGAAAAGTGGAGTGGAATATGGCCACTGCAACACC⋱
AAATGTCAAACCCCAGTAGGAGCGATAAACTCTAGTATGCCGTTCCACAATATACATCCTCTCACCATCG⋱
GGGAATGCCCCAAATACGTGAAGTCAAACAAGTTGGTCCTTGCGACTGGGCTCAGAAATAGTCCTCTAAG⋱
AGAAAAGAGAAGA ---AAA---AGAGGGCTGTTTGGGGCTATAGCAGGTTTTATAGAGGGAGGATGGCAGGG⋱
AATGGTTGATGGTTGGTATGGGTACCACCATAGCAATGAGCAGGGGAGTGGGTACGCTGCAGACAAAGAATCCAC⋱
CCAAAAGGCAATAGATGGAGTTACCAATAAGGTCAACTCGATCATTGACAAAATGAACACTCAATTTGAGGCAGT⋱

TGGAAGGGAGTTTAATAACTTAGAAAGGAGGATAGAGAATTTGAACAAGAAAATGGAAGACGGATTCCTAGATGT∵.
CTGGACCTATAATGCTGAACTTCTAGTTCTCATGGAAAACGAGAGGACTCTAGATTTCCATGACTCAAATGTCAA∵.
GAACCTTTACGACAAAGTCAGACTGCAGCTTAGGGATAATGCAAAGGAGCTGGGTAACGGTTGTTTCGAGTTCTA∵.
TCACAAATGTGATAATGAATGTATGGAAAGTGTGAGAAATGGGACGTATGACTACCCTCAGTATTCAGAAGAAGC∵.
AAGATTAAAAAGAGAAGAAATAAGCGGAGTGAAATTAGAGTCAATAGGAACTTACCAAATACTGTCAATTTATTC∵.
AACAGTGGCG **AGTTCCCTAGCACTGGCAATCATGGTGGCTGGTCTATCTTTA**
TGGATGTGCTCCAATGGGTCGTTACAGTGCAGAATTTGCATTTAA

Per codon conservation report

HA

Pos.	1	obs:	exp:
atg	M	1	1.00
---	-	2717	2717.00
---	--	------	------
mPD		0	0
		nPD:	1.
		N. weight:	0.
		Sc. PD:	0
		Sc. rank:	0

HA

Pos.	2	obs:	exp:
aaa	K	1	0.56
aag	K	0	0.44
---	-	2717	2717.00
---	--	------	------
mPD		0	6.7×10^{-8}
		nPD:	0.
		N. weight:	0.
		Sc. PD:	0
		Sc. rank:	0

HA

Pos.	3	obs:	exp:
atg	M	2718	2718.00
---	--	------	------
mPD		0	0
		nPD:	1.
		N. weight:	0.
		Sc. PD:	0
		Sc. rank:	0

HA

Pos.	4	obs:	exp:
aaa	K	0	4.49
aag	K	8	3.51
gaa	E	14	1620.00
gag	E	2692	1086.00
ggt	G	0	0.29
ggc	G	0	0.26
gga	G	0	0.87
ggg	G	2	0.58
---	-	2	2.00
---	--	------	------
mPD		0.018	0.49
		nPD:	0.04
		N. weight:	1.
		Sc. PD:	-0.072
		Sc. rank:	226.7

HA

Pos.	5	obs:	exp:
cgt	R	0	0.34
cgc	R	0	0.48
cga	R	0	0.87
cgg	R	0	0.70
act	T	0	0.26
acc	T	0	0.22
aca	T	1	0.43
acg	T	0	0.08
aat	N	2	1010.00
aac	N	1899	890.80
aaa	K	802	450.20
aag	K	0	351.80
aga	R	9	4.13
agg	R	0	2.47
gat	D	0	1.73
gac	D	3	1.27
---	-	2	2.00
---	--	------	------
mPD		0.43	0.72
		nPD:	0.6
		N. weight:	0.8
		Sc. PD:	0.38
		Sc. rank:	1547.6

HA

Pos.	6	obs:	exp:
tta	L	1	0.11
ttg	L	0	0.19
ctt	L	0	0.19
ctc	L	0	0.17
cta	L	0	0.13
ctg	L	0	0.21
att	I	0	969.20
atc	I	0	654.60
ata	I	2610	986.20
atg	M	91	91.00
act	T	0	2.91
acc	T	0	2.43
aca	T	11	4.73
acg	T	0	0.93
aaa	K	2	1.12
aag	K	0	0.88
gtt	V	0	0.22
gtc	V	0	0.21
gta	V	1	0.20
gtg	V	0	0.37
---	-	2	2.00
---	--	------	------
mPD		0.076	0.69
		nPD:	0.11
		N. weight:	1.1
		Sc. PD:	-0.00012
		Sc. rank:	1045.0

HA

Pos.	7	obs:	exp:
tta	L	1	0.33
ttg	L	1	0.56
ctt	L	0	0.56
ctc	L	0	0.52
cta	L	0	0.40
ctg	L	1	0.63
att	I	0	0.74
atc	I	0	0.50
ata	I	2	0.76
atg	M	27	27.00
gtt	V	1	580.90
gtc	V	0	564.40
gta	V	980	525.50
gtg	V	1690	1000.00
gct	A	0	3.82
gcc	A	0	2.93
gca	A	13	6.59
gcg	A	2	1.67
---	--	------	------
mPD		0.50	0.76
		nPD:	0.66
		N. weight:	0.64
		Sc. PD:	0.34
		Sc. rank:	1252.5

HA

Pos.	8	obs:	exp:
ttt	F	29	12.34
ttc	F	0	16.66
tta	L	0	292.30
ttg	L	0	502.50
ctt	L	2671	502.20
ctc	L	3	462.00
cta	L	2	356.30
ctg	L	2	562.70
cct	P	1	0.28
ccc	P	0	0.18
cca	P	0	0.41
ccg	P	0	0.13
att	I	10	3.71
atc	I	0	2.51
ata	I	0	3.78
---	--	------	------
mPD		0.034	1.1
		nPD:	0.03
		N. weight:	1.9
		Sc. PD:	-0.15
		Sc. rank:	-131.9

HA

Pos.	9	obs:	exp:
ttt	F	13	5.53
ttc	F	0	7.47
tta	L	0	293.70
ttg	L	0	504.90
ctt	L	2690	504.60
ctc	L	1	464.30
cta	L	0	358.00
ctg	L	0	565.40
cct	P	1	0.28
ccc	P	0	0.18
cca	P	0	0.41
ccg	P	0	0.13
cgt	R	1	0.04
cgc	R	0	0.05
cga	R	0	0.10
cgg	R	0	0.08
att	I	3	1.11
atc	I	0	0.75
ata	I	0	1.13
aga	R	0	0.46
agg	R	0	0.27
gct	A	0	2.29
gcc	A	9	1.76
gca	A	0	3.96
gcg	A	0	1.00
---	--	------	------
mPD		0.034	1.2
		nPD:	0.03
		N. weight:	1.9
		Sc. PD:	-0.15
		Sc. rank:	-215.6

HA

Pos.	10	obs:	exp:
ttt	F	5	2.13
ttc	F	0	2.87
tta	L	0	295.80
ttg	L	0	508.50
ctt	L	2684	508.20
ctc	L	21	467.60
cta	L	0	360.60
ctg	L	5	569.40
cat	H	2	1.19
cac	H	0	0.81
att	I	1	0.37
atc	I	0	0.25
ata	I	0	0.38
---	--	------	------
mPD		0.025	1.1
		nPD:	0.02
		N. weight:	1.9
		Sc. PD:	-0.16
		Sc. rank:	-921.3

HA

Pos.	11	obs:	exp:
tct	S	0	0.34
tcc	S	0	0.29
tca	S	2	0.45
tcg	S	0	0.13
act	T	0	1.85
acc	T	0	1.55
aca	T	7	3.01
acg	T	0	0.59
agt	S	0	0.41
agc	S	0	0.38
gtt	V	0	0.22
gtc	V	0	0.21
gta	V	1	0.20
gtg	V	0	0.37
gct	A	1	688.60
gcc	A	0	527.90
gca	A	2704	1190.00
gcg	A	2	300.50
gaa	E	1	0.60
gag	E	0	0.40
---	--	------	------
mPD		0.010	0.70
		nPD:	0.01
		N. weight:	0.94
		Sc. PD:	-0.086
		Sc. rank:	-893.3

HA

Pos.	12	obs:	exp:
tta	L	2	0.33
ttg	L	0	0.56
ctt	L	0	0.56
ctc	L	0	0.52
cta	L	0	0.40
ctg	L	1	0.63
att	I	0	760.90
atc	I	0	513.90
ata	I	2049	774.30
atg	M	4	4.00
act	T	0	1.59
acc	T	0	1.33
aca	T	6	2.58
acg	T	0	0.51
aaa	K	1	0.56
aag	K	0	0.44
gtt	V	0	141.40
gtc	V	0	137.30
gta	V	14	127.90
gtg	V	636	243.40
gct	A	0	1.27
gcc	A	0	0.98
gca	A	0	2.20
gcg	A	5	0.56
---	--	------	------
mPD		0.74	1.1
		nPD:	0.68
		N. weight:	1.1
		Sc. PD:	0.6
		Sc. rank:	2171.5

HA

Pos.	13	obs:	exp:
att	I	135	53.10
atc	I	8	35.86
ata	I	0	54.04
act	T	0	0.26
acc	T	1	0.22
aca	T	0	0.43
acg	T	0	0.08
gtt	V	2536	559.40
gtc	V	15	543.40
gta	V	1	506.10
gtg	V	20	963.10
gct	A	1	0.51
gcc	A	1	0.39
gca	A	0	0.88
gcg	A	0	0.22
---	--	------	------
mPD		0.14	0.84
		nPD:	0.16
		N. weight:	1.6
		Sc. PD:	0.081
		Sc. rank:	1837.4

HA

Pos.	14	obs:	exp:
tct	S	0	456.50
tcc	S	0	394.00
tca	S	0	602.50
tcg	S	0	172.70
act	T	0	0.53
acc	T	2	0.44
aca	T	0	0.86
acg	T	0	0.17
aat	N	0	4.78
aac	N	9	4.22
agt	S	14	545.80
agc	S	2659	501.50
ggt	G	0	4.89
ggc	G	34	4.49
gga	G	0	14.84
ggg	G	0	9.78
---	--	------	------
mPD		0.043	1.7
		nPD:	0.03
		N. weight:	1.9
		Sc. PD:	-0.15
		Sc. rank:	-559.5

HA

Pos.	15	obs:	exp:
ttt	F	1	0.43
ttc	F	0	0.57
tta	L	0	295.50
ttg	L	0	508.00
ctt	L	2674	507.60
ctc	L	33	467.00
cta	L	0	360.20
ctg	L	0	568.80
cct	P	1	0.28
ccc	P	0	0.18
cca	P	0	0.41
ccg	P	0	0.13
att	I	9	3.34
atc	I	0	2.26
ata	I	0	3.40
---	--	------	------
mPD		0.032	1.1
		nPD:	0.03
		N. weight:	1.8
		Sc. PD:	-0.15
		Sc. rank:	-336.6

HA

Pos.	16	obs:	exp:
tta	L	0	0.11
ttg	L	0	0.19
ctt	L	1	0.19
ctc	L	0	0.17
cta	L	0	0.13
ctg	L	0	0.21
act	T	5	1.32
acc	T	0	1.10
aca	T	0	2.15
acg	T	0	0.42
gtt	V	2646	583.70
gtc	V	34	567.10
gta	V	4	528.10
gtg	V	0	1005.00
gct	A	25	6.87
gcc	A	2	5.27
gca	A	0	11.87
gcg	A	0	3.00
ggt	G	1	0.14
ggc	G	0	0.13
gga	G	0	0.44
ggg	G	0	0.29
---	--	------	------
mPD		0.057	0.76
		nPD:	0.08
		N. weight:	1.7
		Sc. PD:	-0.055
		Sc. rank:	1407.6

,

HA

Pos.	17	obs:	exp:
caa	Q	8	4.07
cag	Q	0	3.93
cgt	R	0	0.04
cgc	R	0	0.05
cga	R	0	0.10
cgg	R	0	0.08
att	I	0	0.37
atc	I	0	0.25
ata	I	1	0.38
aaa	K	2667	1508.00
aag	K	19	1178.00
aga	R	1	0.46
agg	R	0	0.27
gaa	E	22	13.17
gag	E	0	8.83
---	--	------	------
mPD		0.037	0.52
		nPD:	0.07
		N. weight:	0.62
		Sc. PD:	-0.023
		Sc. rank:	473.0

,

HA

Pos.	18	obs:	exp:
tct	S	0	459.20
tcc	S	0	396.40
tca	S	0	606.10
tcg	S	0	173.70
cgt	R	0	0.04
cgc	R	0	0.05
cga	R	0	0.10
cgg	R	0	0.08
aat	N	1	0.53
aac	N	0	0.47
agt	S	2687	549.00
agc	S	2	504.50
aga	R	0	0.46
agg	R	1	0.27
gct	A	0	0.25
gcc	A	1	0.20
gca	A	0	0.44
gcg	A	0	0.11
ggt	G	26	3.74
ggc	G	0	3.43
gga	G	0	11.35
ggg	G	0	7.48
---	--	------	------
mPD		0.024	1.7
		nPD:	0.01
		N. weight:	1.8
		Sc. PD:	-0.17
		Sc. rank:	-1750.0

,

HA

Pos.	19	obs:	exp:
aat	N	1	0.53
aac	N	0	0.47
gct	A	2	0.51
gcc	A	0	0.39
gca	A	0	0.88
gcg	A	0	0.22
gat	D	2692	1562.00
gac	D	23	1153.00
---	--	------	------
mPD		0.019	0.49
		nPD:	0.04
		N. weight:	0.58
		Sc. PD:	-0.04
		Sc. rank:	182.6

,

HA

Pos.	20	obs:	exp:
cct	P	0	0.28
ccc	P	0	0.18
cca	P	0	0.41
ccg	P	1	0.13
caa	Q	308	1382.00
cag	Q	2409	1335.00
---	--	------	------
mPD		0.20	0.50
		nPD:	0.4
		N. weight:	0.41
		Sc. PD:	0.12
		Sc. rank:	701.3

,

HA

Pos.	21	obs:	exp:
att	I	2702	1009.00
atc	I	16	681.70
ata	I	0	1027.00
---	--	------	------
mPD		0.012	0.66
		nPD:	0.02
		N. weight:	1.1
		Sc. PD:	-0.098
		Sc. rank:	-836.9

,

HA

Pos.	22	obs:	exp:
tgt	C	3	1181.00
tgc	C	2713	1535.00
cgt	R	0	0.04
cgc	R	1	0.05
cga	R	0	0.10
cgg	R	0	0.08
aga	R	0	0.46
agg	R	0	0.27
gct	A	0	0.25
gcc	A	1	0.20
gca	A	0	0.44
gcg	A	0	0.11
---	--	------	------
mPD		0.0044	0.49
		nPD:	0.01
		N. weight:	0.65
		Sc. PD:	-0.063
		Sc. rank:	-900.4

,

HA

Pos.	23	obs:	exp:
tta	L	0	0.11
ttg	L	0	0.19
ctt	L	1	0.19
ctc	L	0	0.17
cta	L	0	0.13
ctg	L	0	0.21
att	I	2712	1009.00
atc	I	4	681.20
ata	I	0	1026.00
aat	N	1	0.53
aac	N	0	0.47
---	--	------	------
mPD		0.0044	0.66
		nPD:	0.01
		N. weight:	1.1
		Sc. PD:	-0.11
		Sc. rank:	-1713.4

,

HA

Pos.	24	obs:	exp:
ggt	G	2705	391.00
ggc	G	3	358.80
gga	G	9	1186.00
ggg	G	1	781.80
---	--	------	------
mPD		0.0095	0.69
		nPD:	0.01
		N. weight:	2.2
		Sc. PD:	-0.2
		Sc. rank:	-2241.2

,

HA

Pos.	25	obs:	exp:
tct	S	0	0.17
tcc	S	1	0.15
tca	S	0	0.23
tcg	S	0	0.06
tat	Y	16	1321.00
tac	Y	2701	1396.00
agt	S	0	0.20
agc	S	0	0.19
---	--	------	------
mPD		0.012	0.50
		nPD:	0.02
		N. weight:	0.72
		Sc. PD:	-0.06
		Sc. rank:	-242.8

,

HA

Pos.	26	obs:	exp:
cat	H	2716	1615.00
cac	H	0	1101.00
caa	Q	2	1.02
cag	Q	0	0.98
---	--	------	------
mPD		0.0015	0.48
		nPD:	0.
		N. weight:	0.6
		Sc. PD:	-0.062
		Sc. rank:	-1101.5

,

HA

Pos.	27	obs:	exp:
tct	S	0	0.17
tcc	S	0	0.15
tca	S	1	0.23
tcg	S	0	0.06
agt	S	0	0.20
agc	S	0	0.19
gct	A	0	691.20
gcc	A	1	529.80
gca	A	2677	1194.00
gcg	A	39	301.60
---	--	------	------
mPD		0.030	0.69
		nPD:	0.04
		N. weight:	0.88
		Sc. PD:	-0.057
		Sc. rank:	344.2

,

HA

Pos.	28	obs:	exp:
aat	N	29	1444.00
aac	N	2689	1274.00
---	--	------	------
mPD		0.021	0.50
		nPD:	0.04
		N. weight:	0.8
		Sc. PD:	-0.052
		Sc. rank:	299.9

,

HA

Pos.	29	obs:	exp:
aat	N	967	1444.00
aac	N	1751	1274.00
---	--	------	------
mPD		0.46	0.50
		nPD:	0.92
		N. weight:	0.073
		Sc. PD:	0.057
		Sc. rank:	149.7

,

HA

Pos.	30	obs:	exp:
tta	L	0	0.44
ttg	L	4	0.75
tct	S	8	463.30
tcc	S	2	399.90
tca	S	698	611.50
tcg	S	2005	175.20
tgg	W	1	1.00
ctt	L	0	0.75
ctc	L	0	0.69
cta	L	0	0.53
ctg	L	0	0.84
agt	S	0	553.90
agc	S	0	509.00
---	--	------	------
mPD		0.39	1.6
		nPD:	0.24
		N. weight:	2.1
		Sc. PD:	0.26
		Sc. rank:	2906.7

,

HA			
Pos.	31	obs:	exp:
cct	P	0	0.28
ccc	P	0	0.18
cca	P	1	0.41
ccg	P	0	0.13
act	T	2	719.40
acc	T	0	600.10
aca	T	2711	1167.00
acg	T	3	229.40
gct	A	0	0.25
gcc	A	0	0.20
gca	A	1	0.44
gcg	A	0	0.11
---	--	------	------
mPD		0.0051	0.69
		nPD:	0.01
		N. weight:	0.96
		Sc. PD:	-0.095
		Sc. rank:	-1399.3

,

HA			
Pos.	32	obs:	exp:
cgt	R	0	0.04
cgc	R	0	0.05
cga	R	0	0.10
cgg	R	0	0.08
atg	M	36	36.00
act	T	0	0.26
acc	T	0	0.22
aca	T	0	0.43
acg	T	1	0.08
aaa	K	32	340.20
aag	K	574	265.80
aga	R	0	0.46
agg	R	1	0.27
gat	D	0	0.58
gac	D	1	0.42
gaa	E	309	1241.00
gag	E	1764	831.80
---	--	------	------
mPD		0.61	0.88
		nPD:	0.7
		N. weight:	0.54
		Sc. PD:	0.3
		Sc. rank:	1066.7

,

HA			
Pos.	33	obs:	exp:
cat	H	1	0.59
cac	H	0	0.41
caa	Q	2007	1380.00
cag	Q	706	1333.00
cgt	R	0	0.04
cgc	R	0	0.05
cga	R	1	0.10
cgg	R	0	0.08
aaa	K	2	1.68
aag	K	1	1.32
aga	R	0	0.46
agg	R	0	0.27
---	--	------	------
mPD		0.39	0.50
		nPD:	0.77
		N. weight:	0.13
		Sc. PD:	0.084
		Sc. rank:	264.7

,

HA			
Pos.	34	obs:	exp:
att	I	3	1.11
atc	I	0	0.75
ata	I	0	1.13
gtt	V	2408	590.50
gtc	V	303	573.70
gta	V	0	534.20
gtg	V	4	1017.00
---	--	------	------
mPD		0.20	0.73
		nPD:	0.28
		N. weight:	1.3
		Sc. PD:	0.22
		Sc. rank:	1923.7

,

HA			
Pos.	35	obs:	exp:
gat	D	13	1562.00
gac	D	2702	1153.00
ggt	G	0	0.43
ggc	G	3	0.40
gga	G	0	1.31
ggg	G	0	0.86
---	--	------	------
mPD		0.012	0.49
		nPD:	0.02
		N. weight:	0.95
		Sc. PD:	-0.079
		Sc. rank:	-342.6

,

HA			
Pos.	36	obs:	exp:
cct	P	0	1.13
ccc	P	0	0.72
cca	P	0	1.63
ccg	P	4	0.52
act	T	0	718.90
acc	T	0	599.60
aca	T	66	1166.00
acg	T	2648	229.20
---	--	------	------
mPD		0.050	0.69
		nPD:	0.07
		N. weight:	2.7
		Sc. PD:	-0.096
		Sc. rank:	2086.8

,

HA			
Pos.	37	obs:	exp:
att	I	0	1009.00
atc	I	7	681.40
ata	I	2710	1027.00
atg	M	1	1.00
---	--	------	------
mPD		0.0059	0.66
		nPD:	0.01
		N. weight:	1.1
		Sc. PD:	-0.11
		Sc. rank:	-1518.2

,

HA			
Pos.	38	obs:	exp:
att	I	1	0.37
atc	I	0	0.25
ata	I	0	0.38
atg	M	2716	2716.00
act	T	1	0.26
acc	T	0	0.22
aca	T	0	0.43
acg	T	0	0.08
---	--	------	------
mPD		0.0022	0.0021
		nPD:	1.03
		N. weight:	0.00098
		Sc. PD:	0.00087
		Sc. rank:	2.0

,

HA			
Pos.	39	obs:	exp:
aaa	K	1	0.56
aag	K	0	0.44
gaa	E	2580	1627.00
gag	E	137	1090.00
---	--	------	------
mPD		0.096	0.48
		nPD:	0.2
		N. weight:	0.38
		Sc. PD:	0.034
		Sc. rank:	486.6

,

HA			
Pos.	40	obs:	exp:
cgt	R	0	0.04
cgc	R	0	0.05
cga	R	0	0.10
cgg	R	0	0.08
aaa	K	562	1525.00
aag	K	2155	1192.00
aga	R	1	0.46
agg	R	0	0.27
---	--	------	------
mPD		0.33	0.49
		nPD:	0.67
		N. weight:	0.3
		Sc. PD:	0.16
		Sc. rank:	597.1

,

HA			
Pos.	41	obs:	exp:
aat	N	16	1444.00
aac	N	2701	1273.00
gat	D	0	0.58
gac	D	1	0.42
---	--	------	------
mPD		0.012	0.50
		nPD:	0.02
		N. weight:	0.83
		Sc. PD:	-0.068
		Sc. rank:	-272.8

,

HA			
Pos.	42	obs:	exp:
gtt	V	5	591.10
gtc	V	2703	574.30
gta	V	1	534.80
gtg	V	9	1018.00
---	--	------	------
mPD		0.011	0.73
		nPD:	0.02
		N. weight:	1.7
		Sc. PD:	-0.16
		Sc. rank:	-1557.2

,

HA			
Pos.	43	obs:	exp:
act	T	2708	719.70
acc	T	0	600.30
aca	T	1	1167.00
acg	T	8	229.50
gct	A	1	0.25
gcc	A	0	0.20
gca	A	0	0.44
gcg	A	0	0.11
---	--	------	------
mPD		0.0073	0.69
		nPD:	0.01
		N. weight:	1.5
		Sc. PD:	-0.14
		Sc. rank:	-1912.4

,

HA			
Pos.	44	obs:	exp:
gtt	V	2697	591.10
gtc	V	20	574.30
gta	V	1	534.80
gtg	V	0	1018.00
---	--	------	------
mPD		0.015	0.73
		nPD:	0.02
		N. weight:	1.7
		Sc. PD:	-0.15
		Sc. rank:	-969.9

,

HA			
Pos.	45	obs:	exp:
cct	P	0	0.28
ccc	P	1	0.18
cca	P	0	0.41
ccg	P	0	0.13
act	T	0	719.40
acc	T	3	600.10
aca	T	2705	1167.00
acg	T	8	229.40
gct	A	0	0.25
gcc	A	0	0.20
gca	A	1	0.44
gcg	A	0	0.11
---	--	------	------
mPD		0.010	0.69
		nPD:	0.01
		N. weight:	0.94
		Sc. PD:	-0.087
		Sc. rank:	-868.2

,

HA			
Pos.	46	obs:	exp:
cct	P	0	0.28
ccc	P	1	0.18
cca	P	0	0.41
ccg	P	0	0.13
cat	H	2700	1615.00
cac	H	17	1102.00
---	--	------	------
mPD		0.014	0.48
		nPD:	0.03
		N. weight:	0.56
		Sc. PD:	-0.044
		Sc. rank:	-88.3

,

HA			
Pos.	47	obs:	exp:
gct	A	41	691.40
gcc	A	2676	530.00
gca	A	1	1195.00
gcg	A	0	301.70
---	--	------	------
mPD		0.030	0.69
		nPD:	0.04
		N. weight:	1.8
		Sc. PD:	-0.11
		Sc. rank:	780.8

,

HA			
Pos.	48	obs:	exp:
caa	Q	2702	1381.00
cag	Q	13	1334.00
cgt	R	0	0.08
cgc	R	0	0.11
cga	R	2	0.19
cgg	R	0	0.16
aaa	K	1	0.56
aag	K	0	0.44
aga	R	0	0.92
agg	R	0	0.55
---	--	------	------
mPD		0.012	0.50
		nPD:	0.02
		N. weight:	0.74
		Sc. PD:	-0.062
		Sc. rank:	-304.6

,

HA			
Pos.	49	obs:	exp:
aat	N	0	3.19
aac	N	6	2.81
gct	A	0	0.25
gcc	A	1	0.20
gca	A	0	0.44
gcg	A	0	0.11
gat	D	7	1559.00
gac	D	2704	1152.00
---	--	------	------
mPD		0.010	0.49
		nPD:	0.02
		N. weight:	0.96
		Sc. PD:	-0.083
		Sc. rank:	-560.9

,

HA			
Pos.	50	obs:	exp:
att	I	1	1009.00
atc	I	0	681.70
ata	I	2717	1027.00
---	--	------	------
mPD		0.00074	0.66
		nPD:	0.
		N. weight:	1.1
		Sc. PD:	-0.12
		Sc. rank:	-2190.0

,

HA			
Pos.	51	obs:	exp:
tta	L	0	296.70
ttg	L	10	510.00
ctt	L	3	509.70
ctc	L	0	468.90
cta	L	44	361.60
ctg	L	2661	571.10
mPD		0.041	1.1
		nPD:	0.04
		N. weight:	1.7
		Sc. PD:	-0.12
		Sc. rank:	395.1

HA			
Pos.	52	obs:	exp:
gaa	E	2566	1627.00
gag	E	151	1090.00
ggt	G	0	0.14
ggc	G	0	0.13
gga	G	1	0.44
ggg	G	0	0.29
mPD		0.11	0.48
		nPD:	0.22
		N. weight:	0.37
		Sc. PD:	0.039
		Sc. rank:	482.3

HA			
Pos.	53	obs:	exp:
cgt	R	0	0.15
cgc	R	0	0.21
cga	R	0	0.39
cgg	R	0	0.31
aaa	K	2019	1523.00
aag	K	695	1191.00
aga	R	1	1.83
agg	R	3	1.10
mPD		0.38	0.50
		nPD:	0.78
		N. weight:	0.085
		Sc. PD:	0.055
		Sc. rank:	172.3

HA			
Pos.	54	obs:	exp:
tat	Y	1	0.49
tac	Y	0	0.51
act	T	0	625.90
acc	T	3	522.10
aca	T	2360	1015.00
acg	T	0	199.60
gct	A	0	87.77
gcc	A	0	67.28
gca	A	345	151.70
gcg	A	0	38.30
gaa	E	5	5.39
gag	E	4	3.61
mPD		0.24	0.92
		nPD:	0.26
		N. weight:	0.95
		Sc. PD:	0.14
		Sc. rank:	1343.0

HA			
Pos.	55	obs:	exp:
cct	P	0	0.56
ccc	P	2	0.36
cca	P	0	0.82
ccg	P	0	0.26
cat	H	38	1615.00
cac	H	2678	1101.00
mPD		0.029	0.48
		nPD:	0.06
		N. weight:	0.95
		Sc. PD:	-0.046
		Sc. rank:	665.4

HA			
Pos.	56	obs:	exp:
act	T	0	0.53
acc	T	2	0.44
aca	T	0	0.86
acg	T	0	0.17
aat	N	24	1443.00
aac	N	2692	1273.00
mPD		0.019	0.50
		nPD:	0.04
		N. weight:	0.81
		Sc. PD:	-0.057
		Sc. rank:	219.9

HA			
Pos.	57	obs:	exp:
cgt	R	0	0.19
cgc	R	0	0.27
cga	R	0	0.48
cgg	R	0	0.39
aga	R	0	2.29
agg	R	5	1.37
gaa	E	0	1.20
gag	E	2	0.80
ggt	G	0	390.00
ggc	G	0	357.80
gga	G	31	1183.00
ggg	G	2680	779.70
mPD		0.028	0.69
		nPD:	0.04
		N. weight:	1.4
		Sc. PD:	-0.092
		Sc. rank:	455.1

HA			
Pos.	58	obs:	exp:
cgt	R	0	0.04
cgc	R	0	0.05
cga	R	0	0.10
cgg	R	0	0.08
aaa	K	28	1525.00
aag	K	2689	1192.00
aga	R	0	0.46
agg	R	1	0.27
mPD		0.021	0.49
		nPD:	0.04
		N. weight:	0.88
		Sc. PD:	-0.057
		Sc. rank:	338.7

HA			
Pos.	59	obs:	exp:
tta	L	0	296.60
ttg	L	0	509.80
ctt	L	14	509.50
ctc	L	2703	468.80
cta	L	0	361.50
ctg	L	0	570.90
att	I	0	0.37
atc	I	1	0.25
ata	I	0	0.38
mPD		0.011	1.1
		nPD:	0.01
		N. weight:	2.
		Sc. PD:	-0.19
		Sc. rank:	-2697.3

HA			
Pos.	60	obs:	exp:
tgt	C	875	1181.00
tgc	C	1842	1536.00
cgt	R	0	0.04
cgc	R	1	0.05
cga	R	0	0.10
cgg	R	0	0.08
aga	R	0	0.46
agg	R	0	0.27
mPD		0.44	0.49
		nPD:	0.89
		N. weight:	0.034
		Sc. PD:	0.025
		Sc. rank:	68.4

HA			
Pos.	61	obs:	exp:
tct	S	0	2.39
tcc	S	0	2.06
tca	S	0	3.16
tcg	S	0	0.90
aat	N	10	5.31
aac	N	0	4.69
agt	S	8	2.86
agc	S	6	2.63
gat	D	2690	1550.00
gac	D	4	1144.00
mPD		0.035	0.52
		nPD:	0.07
		N. weight:	0.63
		Sc. PD:	-0.026
		Sc. rank:	470.2

HA			
Pos.	62	obs:	exp:
tta	L	343	296.20
ttg	L	0	509.30
ctt	L	648	508.90
ctc	L	2	468.20
cta	L	1719	361.10
ctg	L	2	570.20
att	I	3	1.49
atc	I	1	1.00
ata	I	0	1.51
mPD		0.59	1.1
		nPD:	0.52
		N. weight:	1.2
		Sc. PD:	0.48
		Sc. rank:	2227.0

HA			
Pos.	63	obs:	exp:
tct	S	0	0.51
tcc	S	0	0.44
tca	S	0	0.68
tcg	S	0	0.19
aat	N	2688	1434.00
aac	N	11	1265.00
aaa	K	9	8.98
aag	K	7	7.02
agt	S	3	0.61
agc	S	0	0.56
mPD		0.022	0.51
		nPD:	0.04
		N. weight:	0.69
		Sc. PD:	-0.045
		Sc. rank:	283.1

HA			
Pos.	64	obs:	exp:
gaa	E	0	0.60
gag	E	1	0.40
ggt	G	1	390.90
ggc	G	0	358.60
gga	G	727	1186.00
ggg	G	1989	781.50
mPD		0.39	0.69
		nPD:	0.57
		N. weight:	0.63
		Sc. PD:	0.28
		Sc. rank:	1215.6

HA			
Pos.	65	obs:	exp:
gtt	V	3	590.90
gtc	V	0	574.10
gta	V	3	534.60
gtg	V	2711	1017.00
ggt	G	0	0.14
ggc	G	0	0.13
gga	G	0	0.44
ggg	G	1	0.29
mPD		0.0051	0.73
		nPD:	0.01
		N. weight:	1.1
		Sc. PD:	-0.11
		Sc. rank:	-1672.5

HA			
Pos.	66	obs:	exp:
cgt	R	0	0.42
cgc	R	0	0.59
cga	R	0	1.07
cgg	R	0	0.86
aaa	K	386	1519.00
aag	K	2321	1188.00
aga	R	3	5.05
agg	R	8	3.02
mPD		0.25	0.50
		nPD:	0.5
		N. weight:	0.44
		Sc. PD:	0.17
		Sc. rank:	788.2

HA			
Pos.	67	obs:	exp:
cct	P	2040	766.40
ccc	P	673	491.80
cca	P	5	1110.00
ccg	P	0	350.00
mPD		0.38	0.70
		nPD:	0.53
		N. weight:	0.92
		Sc. PD:	0.38
		Sc. rank:	1704.3

HA			
Pos.	68	obs:	exp:
tta	L	0	296.70
ttg	L	19	510.00
ctt	L	1	509.70
ctc	L	14	468.90
cta	L	21	361.60
ctg	L	2663	571.10
mPD		0.040	1.1
		nPD:	0.04
		N. weight:	1.7
		Sc. PD:	-0.12
		Sc. rank:	315.4

HA			
Pos.	69	obs:	exp:
att	I	2347	876.00
atc	I	12	591.60
ata	I	0	891.40
gtt	V	358	78.08
gtc	V	0	75.85
gta	V	0	70.64
gtg	V	1	134.40
mPD		0.24	0.92
		nPD:	0.26
		N. weight:	1.2
		Sc. PD:	0.17
		Sc. rank:	1678.2

HA			
Pos.	70	obs:	exp:
tta	L	2025	296.70
ttg	L	3	510.00
ctt	L	0	509.70
ctc	L	0	468.90
cta	L	682	361.60
ctg	L	8	571.10
mPD		0.39	1.1
		nPD:	0.34
		N. weight:	1.8
		Sc. PD:	0.4
		Sc. rank:	2911.8

HA			
Pos.	71	obs:	exp:
cgt	R	0	0.27
cgc	R	0	0.37
cga	R	0	0.68
cgg	R	0	0.55
aat	N	307	163.10
aac	N	0	143.90
aaa	K	117	1349.00
aag	K	2287	1055.00
aga	R	0	3.21
agg	R	7	1.92
mPD		0.28	0.60
		nPD:	0.46
		N. weight:	0.71
		Sc. PD:	0.24
		Sc. rank:	1275.4

HA			
Pos.	72	obs:	exp:
aat	N	16	8.50
aac	N	0	7.50
gat	D	2702	1554.00
gac	D	0	1148.00
mPD		0.012	0.50
		nPD:	0.02
		N. weight:	0.64
		Sc. PD:	-0.053
		Sc. rank:	-253.5

HA			
Pos.	73	obs:	exp:
tgt	C	2707	1182.00
tgc	C	11	1536.00
mPD		0.0081	0.49
		nPD:	0.02
		N. weight:	0.92
		Sc. PD:	-0.084
		Sc. rank:	-789.4

HA			
Pos.	74	obs:	exp:
tct	S	0	464.00
tcc	S	0	400.50
tca	S	0	612.50
tcg	S	0	175.50
agt	S	2079	554.70
agc	S	638	509.80
ggt	G	1	0.14
ggc	G	0	0.13
gga	G	0	0.44
ggg	G	0	0.29
mPD		0.36	1.6
		nPD:	0.22
		N. weight:	1.2
		Sc. PD:	0.13
		Sc. rank:	1592.7

HA			
Pos.	75	obs:	exp:
gtt	V	1	591.10
gtc	V	0	574.30
gta	V	2712	534.80
gtg	V	5	1018.00
mPD		0.0044	0.73
		nPD:	0.01
		N. weight:	1.8
		Sc. PD:	-0.19
		Sc. rank:	-2961.8

HA

Pos.	76	obs:	exp:
gct	A	2679	691.40
gcc	A	7	530.00
gca	A	9	1195.00
gcg	A	23	301.70
---	--	------	------
mPD		0.028	0.69
		nPD:	0.04
		N. weight:	1.5
		Sc. PD:	-0.098
		Sc. rank:	538.0

HA

Pos.	77	obs:	exp:
ggt	G	2	391.00
ggc	G	0	358.80
gga	G	2059	1186.00
ggg	G	657	781.80
---	--	------	------
mPD		0.37	0.69
		nPD:	0.53
		N. weight:	0.43
		Sc. PD:	0.18
		Sc. rank:	792.7

HA

Pos.	78	obs:	exp:
tgg	W	2718	2718.00
---	--	------	------
mPD		0	0
		nPD:	1.
		N. weight:	0.
		Sc. PD:	0
		Sc. rank:	0

HA

Pos.	79	obs:	exp:
tta	L	0	296.70
ttg	L	0	510.00
ctt	L	52	509.70
ctc	L	2666	468.90
cta	L	0	361.60
ctg	L	0	571.10
---	--	------	------
mPD		0.038	1.1
		nPD:	0.03
		N. weight:	1.9
		Sc. PD:	-0.14
		Sc. rank:	203.8

HA

Pos.	80	obs:	exp:
tta	L	0	296.70
ttg	L	0	510.00
ctt	L	662	509.70
ctc	L	2054	468.90
cta	L	2	361.60
ctg	L	0	571.10
---	--	------	------
mPD		0.37	1.1
		nPD:	0.33
		N. weight:	1.4
		Sc. PD:	0.28
		Sc. rank:	2083.6

HA

Pos.	81	obs:	exp:
ggt	G	0	391.00
ggc	G	0	358.80
gga	G	2684	1186.00
ggg	G	34	781.80
---	--	------	------
mPD		0.025	0.69
		nPD:	0.04
		N. weight:	0.88
		Sc. PD:	-0.063
		Sc. rank:	186.1

HA

Pos.	82	obs:	exp:
aat	N	673	1444.00
aac	N	2045	1274.00
---	--	------	------
mPD		0.37	0.50
		nPD:	0.75
		N. weight:	0.19
		Sc. PD:	0.12
		Sc. rank:	389.1

HA

Pos.	83	obs:	exp:
cct	P	4	766.40
ccc	P	4	491.80
cca	P	2708	1110.00
ccg	P	2	350.00
---	--	------	------
mPD		0.0073	0.70
		nPD:	0.01
		N. weight:	1.
		Sc. PD:	-0.096
		Sc. rank:	-1299.3

HA

Pos.	84	obs:	exp:
tta	L	0	0.44
ttg	L	3	0.75
ctt	L	0	0.75
ctc	L	0	0.69
cta	L	0	0.53
ctg	L	1	0.84
atg	M	2714	2714.00
---	--	------	------
mPD		0.0029	0.0047
		nPD:	0.62
		N. weight:	0.0022
		Sc. PD:	0.0011
		Sc. rank:	4.2

HA

Pos.	85	obs:	exp:
tgt	C	121	1182.00
tgc	C	2597	1536.00
---	--	------	------
mPD		0.085	0.49
		nPD:	0.17
		N. weight:	0.46
		Sc. PD:	0.028
		Sc. rank:	548.9

HA

Pos.	86	obs:	exp:
gat	D	54	1563.00
gac	D	2664	1155.00
---	--	------	------
mPD		0.039	0.49
		nPD:	0.08
		N. weight:	0.86
		Sc. PD:	-0.025
		Sc. rank:	748.9

HA

Pos.	87	obs:	exp:
gaa	E	2063	1627.00
gag	E	655	1091.00
---	--	------	------
mPD		0.37	0.48
		nPD:	0.76
		N. weight:	0.067
		Sc. PD:	0.042
		Sc. rank:	135.7

HA

Pos.	88	obs:	exp:
ttt	F	9	1157.00
ttc	F	2709	1561.00
---	--	------	------
mPD		0.0066	0.49
		nPD:	0.01
		N. weight:	0.61
		Sc. PD:	-0.057
		Sc. rank:	-656.9

HA

Pos.	89	obs:	exp:
tta	L	0	1.64
ttg	L	0	2.81
ctt	L	1	2.81
ctc	L	5	2.59
cta	L	0	2.00
ctg	L	9	3.15
att	I	3	1001.00
atc	I	2678	676.40
ata	I	16	1019.00
act	T	0	0.53
acc	T	2	0.44
aca	T	0	0.86
acg	T	0	0.17
aat	N	0	0.53
aac	N	1	0.47
gtt	V	0	0.65
gtc	V	3	0.63
gta	V	0	0.59
gtg	V	0	1.12
---	--	------	------
mPD		0.037	0.67
		nPD:	0.05
		N. weight:	1.5
		Sc. PD:	-0.082
		Sc. rank:	988.7

HA

Pos.	90	obs:	exp:
tct	S	0	5.64
tcc	S	0	4.86
tca	S	0	7.44
tcg	S	0	2.13
cgt	R	0	101.10
cgc	R	0	142.00
cga	R	2	258.20
cgg	R	0	208.80
att	I	0	0.37
atc	I	0	0.25
ata	I	1	0.38
aat	N	13	6.91
aac	N	0	6.09
aaa	K	2	2.25
aag	K	2	1.75
agt	S	13	6.74
agc	S	20	6.19
aga	R	1993	1223.00
agg	R	671	733.10
ggt	G	0	0.14
ggc	G	0	0.13
gga	G	1	0.44
ggg	G	0	0.29
---	--	------	------
mPD		0.41	1.0
		nPD:	0.41
		N. weight:	0.4
		Sc. PD:	0.12
		Sc. rank:	701.6

HA

Pos.	91	obs:	exp:
atg	M	10	10.00
gtt	V	0	588.90
gtc	V	0	572.20
gta	V	36	532.80
gtg	V	2672	1014.00
---	--	------	------
mPD		0.033	0.74
		nPD:	0.05
		N. weight:	1.1
		Sc. PD:	-0.066
		Sc. rank:	482.5

HA

Pos.	92	obs:	exp:
cct	P	3	766.40
ccc	P	0	491.80
cca	P	26	1110.00
ccg	P	2689	350.00
---	--	------	------
mPD		0.021	0.70
		nPD:	0.03
		N. weight:	2.3
		Sc. PD:	-0.18
		Sc. rank:	-112.2

HA

Pos.	93	obs:	exp:
gaa	E	2715	1627.00
gag	E	3	1091.00
---	--	------	------
mPD		0.0022	0.48
		nPD:	0.
		N. weight:	0.58
		Sc. PD:	-0.059
		Sc. rank:	-976.7

HA

Pos.	94	obs:	exp:
tgg	W	2718	2718.00
---	--	------	------
mPD		0	0
		nPD:	1.
		N. weight:	0.
		Sc. PD:	0
		Sc. rank:	0

HA

Pos.	95	obs:	exp:
tct	S	1847	464.20
tcc	S	862	400.70
tca	S	9	612.70
tcg	S	0	175.60
agt	S	0	554.90
agc	S	0	510.00
---	--	------	------
mPD		0.44	1.6
		nPD:	0.27
		N. weight:	1.3
		Sc. PD:	0.2
		Sc. rank:	1911.8

HA

Pos.	96	obs:	exp:
tat	Y	21	1321.00
tac	Y	2697	1397.00
---	--	------	------
mPD		0.015	0.50
		nPD:	0.03
		N. weight:	0.71
		Sc. PD:	-0.055
		Sc. rank:	-10.0

HA

Pos.	97	obs:	exp:
att	I	13	1009.00
atc	I	652	681.70
ata	I	2053	1027.00
---	--	------	------
mPD		0.37	0.66
		nPD:	0.57
		N. weight:	0.57
		Sc. PD:	0.25
		Sc. rank:	1083.8

HA

Pos.	98	obs:	exp:
gtt	V	2	591.10
gtc	V	0	574.30
gta	V	345	534.80
gtg	V	2371	1018.00
---	--	------	------
mPD		0.22	0.73
		nPD:	0.31
		N. weight:	0.78
		Sc. PD:	0.15
		Sc. rank:	1184.9

HA

Pos.	99	obs:	exp:
gaa	E	18	1627.00
gag	E	2700	1091.00
---	--	------	------
mPD		0.013	0.48
		nPD:	0.03
		N. weight:	1.
		Sc. PD:	-0.08
		Sc. rank:	-214.5

HA

Pos.	100	obs:	exp:
caa	Q	0	0.51
cag	Q	1	0.49
cgt	R	0	101.60
cgc	R	0	142.80
cga	R	0	259.50
cgg	R	126	209.90
aaa	K	9	20.77
aag	K	28	16.23
aga	R	18	1229.00
agg	R	2536	736.90
---	--	------	------
mPD		0.14	0.98
		nPD:	0.14
		N. weight:	1.3
		Sc. PD:	0.036
		Sc. rank:	1360.5

HA

Pos.	101	obs:	exp:
act	T	403	107.50
acc	T	3	89.70
aca	T	0	174.50
acg	T	0	34.29
gtt	V	1	0.22
gtc	V	0	0.21
gta	V	0	0.20
gtg	V	0	0.37
gct	A	2265	577.50
gcc	A	5	442.70
gca	A	0	997.90
gcg	A	0	252.00
gat	D	30	23.58
gac	D	11	17.42
---	--	------	------
mPD		0.30	0.98
		nPD:	0.31
		N. weight:	1.5
		Sc. PD:	0.29
		Sc. rank:	2295.7

,

HA

Pos.	102	obs:	exp:
tct	S	0	0.34
tcc	S	0	0.29
tca	S	0	0.45
tcg	S	0	0.13
aat	N	1186	1439.00
aac	N	1522	1269.00
agt	S	0	0.41
agc	S	2	0.38
gat	D	5	4.60
gac	D	3	3.40
---	--	------	------
mPD		0.50	0.51
		nPD:	0.99
		N. weight:	0.024
		Sc. PD:	0.02
		Sc. rank:	48.6

,

HA

Pos.	103	obs:	exp:
cct	P	0	766.40
ccc	P	2	491.80
cca	P	2703	1110.00
ccg	P	13	350.00
---	--	------	------
mPD		0.011	0.70
		nPD:	0.02
		N. weight:	0.99
		Sc. PD:	-0.091
		Sc. rank:	-879.5

,

HA

Pos.	104	obs:	exp:
tct	S	28	4.78
tcc	S	0	4.13
tca	S	0	6.31
tcg	S	0	1.81
cct	P	1	0.28
ccc	P	0	0.18
cca	P	0	0.41
ccg	P	0	0.13
att	I	0	0.37
atc	I	1	0.25
ata	I	0	0.38
act	T	5	1.59
acc	T	1	1.33
aca	T	0	2.58
acg	T	0	0.51
agt	S	0	5.72
agc	S	0	5.25
gtt	V	19	6.31
gtc	V	10	6.13
gta	V	0	5.71
gtg	V	0	10.86
gct	A	2039	674.90
gcc	A	613	517.40
gca	A	1	1166.00
gcg	A	0	294.50
---	--	------	------
mPD		0.40	0.75
		nPD:	0.54
		N. weight:	1.
		Sc. PD:	0.43
		Sc. rank:	1922.0

,

HA

Pos.	105	obs:	exp:
cat	H	1	0.59
cac	H	0	0.41
aat	N	2090	1440.00
aac	N	620	1270.00
aaa	K	3	1.68
aag	K	0	1.32
gat	D	4	2.30
gac	D	0	1.70
---	--	------	------
mPD		0.36	0.50
		nPD:	0.71
		N. weight:	0.14
		Sc. PD:	0.084
		Sc. rank:	287.0

,

HA

Pos.	106	obs:	exp:
aat	N	0	0.53
aac	N	1	0.47
gat	D	11	1540.00
gac	D	2666	1137.00
ggt	G	0	5.75
ggc	G	38	5.28
gga	G	2	17.46
ggg	G	0	11.50
---	--	------	------
mPD		0.039	0.53
		nPD:	0.07
		N. weight:	0.97
		Sc. PD:	-0.033
		Sc. rank:	798.4

,

HA

Pos.	107	obs:	exp:
tta	L	1	296.60
ttg	L	1	509.80
ctt	L	1	509.50
ctc	L	2688	468.80
cta	L	19	361.50
ctg	L	7	570.90
caa	Q	0	0.51
cag	Q	1	0.49
---	--	------	------
mPD		0.024	1.1
		nPD:	0.02
		N. weight:	1.9
		Sc. PD:	-0.17
		Sc. rank:	-1059.9

,

HA

Pos.	108	obs:	exp:
tgt	C	2657	1182.00
tgc	C	61	1536.00
---	--	------	------
mPD		0.044	0.49
		nPD:	0.09
		N. weight:	0.83
		Sc. PD:	-0.017
		Sc. rank:	749.6

,

HA

Pos.	109	obs:	exp:
tat	Y	11	1321.00
tac	Y	2707	1397.00
---	--	------	------
mPD		0.0081	0.50
		nPD:	0.02
		N. weight:	0.73
		Sc. PD:	-0.067
		Sc. rank:	-635.3

,

HA

Pos.	110	obs:	exp:
cct	P	1	766.40
ccc	P	0	491.80
cca	P	2714	1110.00
ccg	P	3	350.00
---	--	------	------
mPD		0.0029	0.70
		nPD:	0.
		N. weight:	1.
		Sc. PD:	-0.1
		Sc. rank:	-1774.8

,

HA

Pos.	111	obs:	exp:
ggt	G	3	391.00
ggc	G	0	358.80
gga	G	27	1186.00
ggg	G	2688	781.80
---	--	------	------
mPD		0.022	0.69
		nPD:	0.03
		N. weight:	1.4
		Sc. PD:	-0.1
		Sc. rank:	38.3

,

HA

Pos.	112	obs:	exp:
tct	S	0	348.60
tcc	S	0	300.90
tca	S	0	460.10
tcg	S	0	131.80
cgt	R	0	0.19
cgc	R	5	0.27
cga	R	0	0.48
cgg	R	0	0.39
att	I	0	0.37
atc	I	1	0.25
ata	I	0	0.38
act	T	13	170.60
acc	T	631	142.30
aca	T	0	276.70
acg	T	0	54.40
aat	N	0	6.91
aac	N	13	6.09
agt	S	126	416.70
agc	S	1915	382.90
aga	R	0	2.29
agg	R	0	1.37
gct	A	0	0.51
gcc	A	2	0.39
gca	A	0	0.88
gcg	A	0	0.22
gat	D	3	5.75
gac	D	7	4.25
gaa	E	1	0.60
gag	E	0	0.40
ggt	G	0	0.14
ggc	G	1	0.13
gga	G	0	0.44
ggg	G	0	0.29
---	--	------	------
mPD		0.49	1.6
		nPD:	0.3
		N. weight:	1.6
		Sc. PD:	0.3
		Sc. rank:	2418.0

,

HA

Pos.	113	obs:	exp:
ttt	F	0	11.91
ttc	F	28	16.09
tta	L	0	293.60
ttg	L	0	504.80
ctt	L	3	504.40
ctc	L	2687	464.10
cta	L	0	357.90
ctg	L	0	565.20
---	--	------	------
mPD		0.023	1.1
		nPD:	0.02
		N. weight:	2.
		Sc. PD:	-0.17
		Sc. rank:	-1236.6

,

HA

Pos.	114	obs:	exp:
tct	S	0	0.34
tcc	S	0	0.29
tca	S	0	0.45
tcg	S	0	0.13
aat	N	2608	1389.00
aac	N	6	1225.00
aaa	K	0	57.25
aag	K	102	44.75
agt	S	1	0.41
agc	S	1	0.38
---	--	------	------
mPD		0.079	0.54
		nPD:	0.15
		N. weight:	0.72
		Sc. PD:	0.025
		Sc. rank:	796.4

,

HA

Pos.	115	obs:	exp:
tat	Y	0	0.49
tac	Y	1	0.51
aat	N	1	63.77
aac	N	119	56.23
gat	D	52	1493.00
gac	D	2543	1102.00
gaa	E	2	1.20
gag	E	0	0.80
---	--	------	------
mPD		0.12	0.58
		nPD:	0.22
		N. weight:	0.86
		Sc. PD:	0.089
		Sc. rank:	1115.4

,

HA

Pos.	116	obs:	exp:
tat	Y	2696	1321.00
tac	Y	22	1397.00
---	--	------	------
mPD		0.016	0.50
		nPD:	0.03
		N. weight:	0.77
		Sc. PD:	-0.058
		Sc. rank:	46.1

,

HA

Pos.	117	obs:	exp:
gaa	E	2061	1627.00
gag	E	657	1091.00
---	--	------	------
mPD		0.37	0.48
		nPD:	0.76
		N. weight:	0.067
		Sc. PD:	0.042
		Sc. rank:	134.5

,

HA

Pos.	118	obs:	exp:
aaa	K	1	0.56
aag	K	0	0.44
gaa	E	2713	1627.00
gag	E	4	1090.00
---	--	------	------
mPD		0.0037	0.48
		nPD:	0.01
		N. weight:	0.58
		Sc. PD:	-0.057
		Sc. rank:	-835.5

,

HA

Pos.	119	obs:	exp:
tta	L	0	296.70
ttg	L	4	510.00
ctt	L	2	509.70
ctc	L	0	468.90
cta	L	304	361.60
ctg	L	2408	571.10
---	--	------	------
mPD		0.20	1.1
		nPD:	0.18
		N. weight:	1.4
		Sc. PD:	0.095
		Sc. rank:	1740.0

,

HA

Pos.	120	obs:	exp:
aaa	K	2706	1526.00
aag	K	12	1192.00
---	--	------	------
mPD		0.0088	0.49
		nPD:	0.02
		N. weight:	0.63
		Sc. PD:	-0.056
		Sc. rank:	-475.8

,

HA

Pos.	121	obs:	exp:
cat	H	50	1616.00
cac	H	2668	1102.00
---	--	------	------
mPD		0.036	0.48
		nPD:	0.07
		N. weight:	0.92
		Sc. PD:	-0.031
		Sc. rank:	768.4

,

HA

Pos.	122	obs:	exp:
tta	L	9	296.70
ttg	L	4	510.00
ctt	L	4	509.70
ctc	L	0	468.90
cta	L	699	361.60
ctg	L	2002	571.10
---	--	------	------
mPD		0.40	1.1
		nPD:	0.35
		N. weight:	1.2
		Sc. PD:	0.28
		Sc. rank:	2014.3

,

HA

Pos.	123	obs:	exp:
tta	L	5	295.70
ttg	L	2670	508.30
ctt	L	0	508.00
ctc	L	0	467.40
cta	L	0	360.40
ctg	L	34	569.20
atg	M	9	9.00
---	--	------	------
mPD		0.035	1.1
		nPD:	0.03
		N. weight:	1.8
		Sc. PD:	-0.14
		Sc. rank:	-34.9

,

HA

Pos.	124	obs:	exp:
tct	S	0	463.80
tcc	S	0	400.40
tca	S	0	612.20
tcg	S	0	175.40
cgt	R	0	0.04
cgc	R	1	0.05
cga	R	0	0.10
cgg	R	0	0.08
agt	S	3	554.50
agc	S	2713	509.60
aga	R	0	0.46
agg	R	0	0.27
ggt	G	1	0.14
ggc	G	0	0.13
gga	G	0	0.44
ggg	G	0	0.29
---	--	------	------
mPD		0.0044	1.6
		nPD:	0.
		N. weight:	1.9
		Sc. PD:	-0.2
		Sc. rank:	-3573.0

,

HA

Pos.	125	obs:	exp:
tct	S	0	1.88
tcc	S	0	1.62
tca	S	0	2.48
tcg	S	0	0.71
tgt	C	1	0.87
tgc	C	1	1.13
cgt	R	0	102.50
cgc	R	0	144.00
cga	R	24	261.80
cgg	R	0	211.80
aat	N	0	0.53
aac	N	1	0.47
agt	S	1	2.25
agc	S	10	2.06
aga	R	2677	1240.00
agg	R	3	743.50
---	--	------	------
mPD		0.032	0.97
		nPD:	0.03
		N. weight:	0.85
		Sc. PD:	-0.063
		Sc. rank:	110.4

,

HA

Pos.	126	obs:	exp:
att	I	0	1004.00
atc	I	0	678.10
ata	I	2704	1022.00
act	T	0	3.71
acc	T	0	3.09
aca	T	13	6.02
acg	T	1	1.18
---	--	------	------
mPD		0.011	0.67
		nPD:	0.02
		N. weight:	1.1
		Sc. PD:	-0.1
		Sc. rank:	-943.1

,

HA

Pos.	127	obs:	exp:
aat	N	2454	1316.00
aac	N	22	1160.00
aaa	K	242	135.80
aag	K	0	106.20
---	--	------	------
mPD		0.18	0.58
		nPD:	0.31
		N. weight:	0.67
		Sc. PD:	0.13
		Sc. rank:	1012.4

,

HA

Pos.	128	obs:	exp:
cat	H	2716	1616.00
cac	H	2	1102.00
---	--	------	------
mPD		0.0015	0.48
		nPD:	0.
		N. weight:	0.59
		Sc. PD:	-0.061
		Sc. rank:	-1087.5

,

HA

Pos.	129	obs:	exp:
ttt	F	2716	1157.00
ttc	F	2	1561.00
---	--	------	------
mPD		0.0015	0.49
		nPD:	0.
		N. weight:	0.97
		Sc. PD:	-0.1
		Sc. rank:	-1808.5

,

HA

Pos.	130	obs:	exp:
aaa	K	0	0.56
aag	K	1	0.44
gat	D	5	2.88
gac	D	0	2.12
gaa	E	28	1624.00
gag	E	2684	1088.00
---	--	------	------
mPD		0.025	0.48
		nPD:	0.05
		N. weight:	0.98
		Sc. PD:	-0.055
		Sc. rank:	562.6

,

HA

Pos.	131	obs:	exp:
aaa	K	722	1526.00
aag	K	1996	1192.00
---	--	------	------
mPD		0.39	0.49
		nPD:	0.79
		N. weight:	0.21
		Sc. PD:	0.14
		Sc. rank:	421.8

,

HA

Pos.	132	obs:	exp:
tta	L	0	0.11
ttg	L	0	0.19
ctt	L	1	0.19
ctc	L	0	0.17
cta	L	0	0.13
ctg	L	0	0.21
att	I	2063	767.20
atc	I	3	518.10
ata	I	0	780.70
act	T	650	172.20
acc	T	0	143.60
aca	T	0	279.30
acg	T	0	54.90
gct	A	1	0.25
gcc	A	0	0.20
gca	A	0	0.44
gcg	A	0	0.11
---	--	------	------
mPD		0.37	1.0
		nPD:	0.36
		N. weight:	1.2
		Sc. PD:	0.29
		Sc. rank:	2012.7

,

HA

Pos.	133	obs:	exp:
tta	L	0	281.00
ttg	L	3	483.00
ctt	L	7	482.70
ctc	L	0	444.10
cta	L	7	342.50
ctg	L	2557	540.80
cct	P	0	0.28
ccc	P	0	0.18
cca	P	0	0.41
ccg	P	1	0.13
caa	Q	3	43.25
cag	Q	82	41.75
cgt	R	0	0.15
cgc	R	0	0.21
cga	R	2	0.39
cgg	R	2	0.31
atg	M	54	54.00
aga	R	0	1.83
agg	R	0	1.10
---	--	------	------
mPD		0.12	1.2
		nPD:	0.1
		N. weight:	1.7
		Sc. PD:	-0.018
		Sc. rank:	1549.3

,

HA

Pos.	134	obs:	exp:
att	I	11	1007.00
atc	I	2679	680.40
ata	I	23	1025.00
gtt	V	0	1.09
gtc	V	5	1.06
gta	V	0	0.98
gtg	V	0	1.87
---	--	------	------
mPD		0.028	0.66
		nPD:	0.04
		N. weight:	1.5
		Sc. PD:	-0.097
		Sc. rank:	603.1

,

HA

Pos.	135	obs:	exp:
ttt	F	0	1.28
ttc	F	3	1.72
att	I	30	1008.00
atc	I	2653	680.90
ata	I	32	1026.00
---	--	------	------
mPD		0.047	0.66
		nPD:	0.07
		N. weight:	1.4
		Sc. PD:	-0.053
		Sc. rank:	1091.9

,

HA			
Pos.	136	obs:	exp:
cct	P	10	766.40
ccc	P	2707	491.80
cca	P	1	1110.00
ccg	P	0	350.00
---	--	------	------
mPD		0.0081	0.70
		nPD:	0.01
		N. weight:	1.9
		Sc. PD:	-0.18
		Sc. rank:	-2301.6

,

HA			
Pos.	137	obs:	exp:
cgt	R	0	4.74
cgc	R	0	6.66
cga	R	0	12.10
cgg	R	0	9.79
aat	N	2	1.06
aac	N	0	0.94
aaa	K	30	1454.00
aag	K	2561	1137.00
aga	R	5	57.34
agg	R	120	34.37
---	--	------	------
mPD		0.11	0.61
		nPD:	0.19
		N. weight:	0.89
		Sc. PD:	0.067
		Sc. rank:	1118.0

,

HA			
Pos.	138	obs:	exp:
tct	S	0	462.30
tcc	S	0	399.10
tca	S	0	610.20
tcg	S	0	174.90
aat	N	5	2.66
aac	N	0	2.34
agt	S	2654	552.70
agc	S	53	507.90
gat	D	3	1.73
gac	D	0	1.27
ggt	G	3	0.43
ggc	G	0	0.40
gga	G	0	1.31
ggg	G	0	0.86
---	--	------	------
mPD		0.049	1.7
		nPD:	0.03
		N. weight:	1.7
		Sc. PD:	-0.13
		Sc. rank:	-172.1

,

HA			
Pos.	139	obs:	exp:
tct	S	1852	464.20
tcc	S	754	400.70
tca	S	112	612.70
tcg	S	0	175.60
agt	S	0	554.90
agc	S	0	510.00
---	--	------	------
mPD		0.46	1.6
		nPD:	0.28
		N. weight:	1.2
		Sc. PD:	0.2
		Sc. rank:	1739.7

,

HA			
Pos.	140	obs:	exp:
tgg	W	2718	2718.00
---	--	------	------
mPD		0	0
		nPD:	1.
		N. weight:	0.
		Sc. PD:	0
		Sc. rank:	0

,

HA			
Pos.	141	obs:	exp:
tta	L	0	0.98
ttg	L	0	1.69
tct	S	0	3.76
tcc	S	11	3.24
tca	S	11	4.96
tcg	S	0	1.42
ctt	L	0	1.69
ctc	L	9	1.55
cta	L	0	1.20
ctg	L	0	1.89
cct	P	1	755.20
ccc	P	1815	484.60
cca	P	861	1093.00
ccg	P	1	344.80
caa	Q	4	2.04
cag	Q	0	1.96
act	T	0	0.79
acc	T	3	0.66
aca	T	0	1.29
acg	T	0	0.25
agt	S	0	4.49
agc	S	0	4.13
gct	A	0	0.51
gcc	A	2	0.39
gca	A	0	0.88
gcg	A	0	0.22
---	--	------	------
mPD		0.47	0.74
		nPD:	0.63
		N. weight:	0.94
		Sc. PD:	0.47
		Sc. rank:	1837.0

,

HA			
Pos.	142	obs:	exp:
tct	S	0	0.51
tcc	S	0	0.44
tca	S	0	0.68
tcg	S	0	0.19
aat	N	2358	1424.00
aac	N	321	1255.00
aaa	K	1	0.56
aag	K	0	0.44
agt	S	3	0.61
agc	S	0	0.56
gat	D	31	19.56
gac	D	3	14.44
gaa	E	0	0.60
gag	E	1	0.40
---	--	------	------
mPD		0.24	0.53
		nPD:	0.45
		N. weight:	0.33
		Sc. PD:	0.11
		Sc. rank:	578.9

,

HA			
Pos.	143	obs:	exp:
cat	H	2715	1616.00
cac	H	3	1102.00
---	--	------	------
mPD		0.0022	0.48
		nPD:	0.
		N. weight:	0.59
		Sc. PD:	-0.06
		Sc. rank:	-992.7

,

HA			
Pos.	144	obs:	exp:
tat	Y	1	0.49
tac	Y	0	0.51
caa	Q	1	0.51
cag	Q	0	0.49
aat	N	1	0.53
aac	N	0	0.47
gat	D	11	6.33
gac	D	0	4.67
gaa	E	2702	1618.00
gag	E	1	1085.00
---	-	1	1.00
---	--	------	------
mPD		0.012	0.49
		nPD:	0.03
		N. weight:	0.59
		Sc. PD:	-0.048
		Sc. rank:	-181.7

,

HA			
Pos.	145	obs:	exp:
att	I	1	0.37
atc	I	0	0.25
ata	I	0	0.38
act	T	1	715.20
acc	T	0	596.60
aca	T	2695	1160.00
acg	T	4	228.10
gct	A	1	4.32
gcc	A	13	3.32
gca	A	3	7.47
gcg	A	0	1.89
---	--	------	------
mPD		0.028	0.70
		nPD:	0.04
		N. weight:	0.96
		Sc. PD:	-0.065
		Sc. rank:	312.6

,

HA			
Pos.	146	obs:	exp:
tct	S	4	453.90
tcc	S	0	391.80
tca	S	2646	599.20
tcg	S	8	171.70
agt	S	0	542.70
agc	S	0	498.70
gct	A	0	15.26
gcc	A	0	11.70
gca	A	60	26.38
gcg	A	0	6.66
---	--	------	------
mPD		0.052	1.7
		nPD:	0.03
		N. weight:	1.7
		Sc. PD:	-0.13
		Sc. rank:	18.9

,

HA			
Pos.	147	obs:	exp:
tta	L	1584	294.70
ttg	L	243	506.60
tct	S	0	2.73
tcc	S	0	2.36
tca	S	15	3.61
tcg	S	1	1.03
ctt	L	0	506.30
ctc	L	0	465.80
cta	L	849	359.20
ctg	L	24	567.30
cct	P	0	0.28
ccc	P	0	0.18
cca	P	1	0.41
ccg	P	0	0.13
caa	Q	1	0.51
cag	Q	0	0.49
agt	S	0	3.27
agc	S	0	3.00
---	--	------	------
mPD		0.63	1.2
		nPD:	0.54
		N. weight:	1.3
		Sc. PD:	0.56
		Sc. rank:	2516.8

,

HA			
Pos.	148	obs:	exp:
ggt	G	0	391.00
ggc	G	0	358.80
gga	G	57	1186.00
ggg	G	2661	781.80
---	--	------	------
mPD		0.041	0.69
		nPD:	0.06
		N. weight:	1.3
		Sc. PD:	-0.063
		Sc. rank:	900.1

,

HA			
Pos.	149	obs:	exp:
att	I	0	0.37
atc	I	0	0.25
ata	I	1	0.38
act	T	0	0.26
acc	T	0	0.22
aca	T	0	0.43
acg	T	1	0.08
gtt	V	0	590.70
gtc	V	0	573.90
gta	V	242	534.40
gtg	V	2474	1017.00
---	--	------	------
mPD		0.17	0.73
		nPD:	0.23
		N. weight:	0.85
		Sc. PD:	0.095
		Sc. rank:	1143.6

,

HA			
Pos.	150	obs:	exp:
tct	S	0	464.20
tcc	S	0	400.70
tca	S	0	612.70
tcg	S	0	175.60
agt	S	40	554.90
agc	S	2678	510.00
---	--	------	------
mPD		0.029	1.6
		nPD:	0.02
		N. weight:	1.8
		Sc. PD:	-0.16
		Sc. rank:	-1440.8

,

HA

Pos.	151	obs:	exp:
tct	S	4	2.56
tcc	S	1	2.21
tca	S	5	3.38
tcg	S	5	0.97
act	T	0	0.53
acc	T	0	0.44
aca	T	2	0.86
acg	T	0	0.17
agt	S	0	3.06
agc	S	0	2.81
gtt	V	0	0.22
gtc	V	0	0.21
gta	V	1	0.20
gtg	V	0	0.37
gct	A	1	686.90
gcc	A	0	526.50
gca	A	2692	1187.00
gcg	A	7	299.70
---	--	------	------
mPD		0.026	0.71
		nPD:	0.04
		N. weight:	0.93
		Sc. PD:	-0.065
		Sc. rank:	229.4

HA

Pos.	152	obs:	exp:
act	T	1	0.26
acc	T	0	0.22
aca	T	0	0.43
acg	T	0	0.08
gct	A	1924	691.20
gcc	A	96	529.80
gca	A	695	1194.00
gcg	A	2	301.60
---	--	------	------
mPD		0.43	0.69
		nPD:	0.62
		N. weight:	0.6
		Sc. PD:	0.3
		Sc. rank:	1170.6

HA

Pos.	153	obs:	exp:
tgt	C	2591	1182.00
tgc	C	127	1536.00
---	--	------	------
mPD		0.089	0.49
		nPD:	0.18
		N. weight:	0.73
		Sc. PD:	0.05
		Sc. rank:	902.1

HA

Pos.	154	obs:	exp:
tta	L	0	0.11
ttg	L	0	0.19
tct	S	0	4.61
tcc	S	0	3.98
tca	S	27	6.09
tcg	S	0	1.74
ctt	L	0	0.19
ctc	L	0	0.17
cta	L	1	0.13
ctg	L	0	0.21
cct	P	0	758.30
ccc	P	0	486.60
cca	P	2683	1098.00
ccg	P	6	346.20
caa	Q	1	0.51
cag	Q	0	0.49
agt	S	0	5.51
agc	S	0	5.07
---	--	------	------
mPD		0.026	0.73
		nPD:	0.03
		N. weight:	1.
		Sc. PD:	-0.074
		Sc. rank:	168.1

HA

Pos.	155	obs:	exp:
tat	Y	221	1320.00
tac	Y	2495	1396.00
cat	H	0	1.19
cac	H	2	0.81
---	--	------	------
mPD		0.15	0.50
		nPD:	0.3
		N. weight:	0.45
		Sc. PD:	0.083
		Sc. rank:	668.1

HA

Pos.	156	obs:	exp:
tta	L	0	1.09
ttg	L	0	1.88
ctt	L	0	1.88
ctc	L	0	1.73
cta	L	0	1.33
ctg	L	10	2.10
cat	H	5	2.97
cac	H	0	2.03
caa	Q	146	1368.00
cag	Q	2543	1321.00
cgt	R	0	0.04
cgc	R	0	0.05
cga	R	0	0.10
cgg	R	1	0.08
att	I	1	0.37
atc	I	0	0.25
ata	I	0	0.38
aat	N	12	6.38
aac	N	0	5.62
aga	R	0	0.46
agg	R	0	0.27
---	--	------	------
mPD		0.13	0.53
		nPD:	0.25
		N. weight:	0.58
		Sc. PD:	0.079
		Sc. rank:	810.5

HA

Pos.	157	obs:	exp:
gaa	E	2	1.20
gag	E	0	0.80
ggt	G	3	390.70
ggc	G	4	358.50
gga	G	2695	1186.00
ggg	G	14	781.20
---	--	------	------
mPD		0.017	0.69
		nPD:	0.02
		N. weight:	0.9
		Sc. PD:	-0.074
		Sc. rank:	-315.2

HA

Pos.	158	obs:	exp:
tct	S	0	0.17
tcc	S	0	0.15
tca	S	1	0.23
tcg	S	0	0.06
cgt	R	0	0.38
cgc	R	0	0.53
cga	R	0	0.97
cgg	R	0	0.78
atg	M	8	8.00
act	T	3	313.40
acc	T	0	261.40
aca	T	42	508.30
acg	T	1138	99.92
aaa	K	1	3.37
aag	K	5	2.63
agt	S	0	0.20
agc	S	0	0.19
aga	R	6	4.59
agg	R	4	2.75
gct	A	0	383.40
gcc	A	0	293.90
gca	A	639	662.50
gcg	A	868	167.30
gaa	E	1	0.60
gag	E	0	0.40
ggt	G	0	0.29
ggc	G	0	0.26
gga	G	1	0.87
ggg	G	1	0.58
---	--	------	------
mPD		0.90	1.2
		nPD:	0.74
		N. weight:	1.7
		Sc. PD:	1.
		Sc. rank:	3452.8

HA

Pos.	159	obs:	exp:
tct	S	9	95.64
tcc	S	550	82.55
tca	S	1	126.20
tcg	S	0	36.17
cct	P	37	608.50
ccc	P	2118	390.50
cca	P	3	881.10
ccg	P	0	277.90
agt	S	0	114.30
agc	S	0	105.10
---	--	------	------
mPD		0.36	1.2
		nPD:	0.3
		N. weight:	1.9
		Sc. PD:	0.35
		Sc. rank:	2821.9

HA

Pos.	160	obs:	exp:
tct	S	33	464.20
tcc	S	2040	400.70
tca	S	645	612.70
tcg	S	0	175.60
agt	S	0	554.90
agc	S	0	510.00
---	--	------	------
mPD		0.38	1.6
		nPD:	0.23
		N. weight:	1.4
		Sc. PD:	0.16
		Sc. rank:	1881.0

HA

Pos.	161	obs:	exp:
ttt	F	2716	1157.00
ttc	F	2	1561.00
---	--	------	------
mPD		0.0015	0.49
		nPD:	0.
		N. weight:	0.97
		Sc. PD:	-0.1
		Sc. rank:	-1808.5

HA

Pos.	162	obs:	exp:
ttt	F	3	1112.00
ttc	F	2611	1502.00
tat	Y	1	50.55
tac	Y	103	53.45
---	--	------	------
mPD		0.077	0.56
		nPD:	0.14
		N. weight:	0.63
		Sc. PD:	0.016
		Sc. rank:	668.9

HA

Pos.	163	obs:	exp:
cgt	R	0	103.00
cgc	R	0	144.70
cga	R	3	263.00
cgg	R	0	212.70
aaa	K	2	1.12
aag	K	0	0.88
aga	R	2684	1246.00
agg	R	29	746.80
---	--	------	------
mPD		0.025	0.95
		nPD:	0.03
		N. weight:	0.83
		Sc. PD:	-0.067
		Sc. rank:	-240.4

HA

Pos.	164	obs:	exp:
tct	S	0	0.17
tcc	S	0	0.15
tca	S	0	0.23
tcg	S	0	0.06
aat	N	2682	1444.00
aac	N	35	1273.00
agt	S	1	0.20
agc	S	0	0.19
---	--	------	------
mPD		0.026	0.50
		nPD:	0.05
		N. weight:	0.65
		Sc. PD:	-0.036
		Sc. rank:	406.3

HA

Pos.	165	obs:	exp:
tta	L	0	0.22
ttg	L	2	0.38
ctt	L	0	0.38
ctc	L	0	0.35
cta	L	0	0.27
ctg	L	0	0.42
atg	M	2	2.00
gtt	V	0	590.20
gtc	V	0	573.40
gta	V	15	534.00
gtg	V	2699	1016.00
---	--	------	------
mPD		0.014	0.73
		nPD:	0.02
		N. weight:	1.1
		Sc. PD:	-0.096
		Sc. rank:	-758.3

HA

Pos.	166	obs:	exp:
att	I	0	1.11
atc	I	0	0.75
ata	I	3	1.13
gtt	V	1	590.50
gtc	V	0	573.70
gta	V	1805	534.20
gtg	V	909	1017.00
---	--	------	------
mPD		0.45	0.73
		nPD:	0.61
		N. weight:	0.89
		Sc. PD:	0.43
		Sc. rank:	1714.1

,

HA

Pos.	167	obs:	exp:
tgg	W	2718	2718.00
---	--	------	------
mPD		0	0
		nPD:	1.
		N. weight:	0.
		Sc. PD:	0
		Sc. rank:	0

,

HA

Pos.	168	obs:	exp:
tta	L	2	296.70
ttg	L	5	510.00
ctt	L	2036	509.70
ctc	L	673	468.90
cta	L	0	361.60
ctg	L	2	571.10
---	--	------	------
mPD		0.38	1.1
		nPD:	0.34
		N. weight:	1.3
		Sc. PD:	0.28
		Sc. rank:	2030.5

,

HA

Pos.	169	obs:	exp:
att	I	11	999.30
atc	I	2680	674.90
ata	I	0	1017.00
act	T	0	0.53
acc	T	2	0.44
aca	T	0	0.86
acg	T	0	0.17
aat	N	0	0.53
aac	N	1	0.47
gtt	V	0	5.22
gtc	V	24	5.07
gta	V	0	4.72
gtg	V	0	8.99
---	--	------	------
mPD		0.028	0.68
		nPD:	0.04
		N. weight:	1.6
		Sc. PD:	-0.1
		Sc. rank:	550.3

,

HA

Pos.	170	obs:	exp:
aaa	K	2705	1526.00
aag	K	13	1192.00
---	--	------	------
mPD		0.0095	0.49
		nPD:	0.02
		N. weight:	0.63
		Sc. PD:	-0.055
		Sc. rank:	-418.9

,

HA

Pos.	171	obs:	exp:
cgt	R	0	0.04
cgc	R	0	0.05
cga	R	0	0.10
cgg	R	0	0.08
aaa	K	2	1525.00
aag	K	2715	1192.00
aga	R	0	0.46
agg	R	1	0.27
---	--	------	------
mPD		0.0022	0.49
		nPD:	0.
		N. weight:	0.94
		Sc. PD:	-0.096
		Sc. rank:	-1600.6

,

HA

Pos.	172	obs:	exp:
tct	S	0	0.17
tcc	S	0	0.15
tca	S	0	0.23
tcg	S	0	0.06
aat	N	327	1435.00
aac	N	2374	1266.00
agt	S	0	0.20
agc	S	1	0.19
gat	D	0	8.63
gac	D	15	6.37
ggt	G	0	0.14
ggc	G	1	0.13
gga	G	0	0.44
ggg	G	0	0.29
---	--	------	------
mPD		0.22	0.51
		nPD:	0.44
		N. weight:	0.43
		Sc. PD:	0.14
		Sc. rank:	767.6

,

HA

Pos.	173	obs:	exp:
aat	N	29	15.41
aac	N	0	13.59
gat	D	2685	1544.00
gac	D	0	1141.00
gaa	E	1	0.60
gag	E	0	0.40
ggt	G	3	0.43
ggc	G	0	0.40
gga	G	0	1.31
ggg	G	0	0.86
---	--	------	------
mPD		0.024	0.51
		nPD:	0.05
		N. weight:	0.64
		Sc. PD:	-0.039
		Sc. rank:	297.5

,

HA

Pos.	174	obs:	exp:
tct	S	0	0.17
tcc	S	0	0.15
tca	S	1	0.23
tcg	S	0	0.06
act	T	0	0.79
acc	T	0	0.66
aca	T	3	1.29
acg	T	0	0.25
agt	S	0	0.20
agc	S	0	0.19
gtt	V	0	4.35
gtc	V	0	4.23
gta	V	20	3.94
gtg	V	0	7.49
gct	A	1	683.80
gcc	A	0	524.20
gca	A	2675	1182.00
gcg	A	12	298.40
gaa	E	6	3.59
gag	E	0	2.41
---	--	------	------
mPD		0.031	0.72
		nPD:	0.04
		N. weight:	0.92
		Sc. PD:	-0.059
		Sc. rank:	392.8

,

HA

Pos.	175	obs:	exp:
tat	Y	54	1321.00
tac	Y	2664	1397.00
---	--	------	------
mPD		0.039	0.50
		nPD:	0.08
		N. weight:	0.65
		Sc. PD:	-0.02
		Sc. rank:	558.3

,

HA

Pos.	176	obs:	exp:
cct	P	10	766.40
ccc	P	0	491.80
cca	P	2074	1110.00
ccg	P	634	350.00
---	--	------	------
mPD		0.36	0.70
		nPD:	0.52
		N. weight:	0.69
		Sc. PD:	0.27
		Sc. rank:	1251.6

,

HA

Pos.	177	obs:	exp:
cct	P	0	0.28
ccc	P	0	0.18
cca	P	1	0.41
ccg	P	0	0.13
att	I	0	0.74
atc	I	0	0.50
ata	I	2	0.76
act	T	0	718.40
acc	T	1	599.20
aca	T	2701	1165.00
acg	T	10	229.10
aaa	K	1	0.56
aag	K	0	0.44
gct	A	0	0.51
gcc	A	0	0.39
gca	A	2	0.88
gcg	A	0	0.22
---	--	------	------
mPD		0.012	0.69
		nPD:	0.02
		N. weight:	0.95
		Sc. PD:	-0.084
		Sc. rank:	-705.5

,

HA

Pos.	178	obs:	exp:
att	I	1	1009.00
atc	I	5	681.70
ata	I	2712	1027.00
---	--	------	------
mPD		0.0044	0.66
		nPD:	0.01
		N. weight:	1.1
		Sc. PD:	-0.11
		Sc. rank:	-1665.3

,

HA

Pos.	179	obs:	exp:
cgt	R	0	0.08
cgc	R	0	0.11
cga	R	0	0.19
cgg	R	0	0.16
aat	N	1	0.53
aac	N	0	0.47
aaa	K	13	1468.00
aag	K	2602	1147.00
aga	R	0	0.92
agg	R	2	0.55
gaa	E	0	59.88
gag	E	100	40.12
---	--	------	------
mPD		0.083	0.57
		nPD:	0.15
		N. weight:	0.91
		Sc. PD:	0.032
		Sc. rank:	1010.6

,

HA

Pos.	180	obs:	exp:
cgt	R	0	0.46
cgc	R	0	0.64
cga	R	0	1.16
cgg	R	0	0.94
att	I	0	992.60
atc	I	0	670.40
ata	I	2673	1010.00
atg	M	19	19.00
act	T	0	2.91
acc	T	0	2.43
aca	T	11	4.73
acg	T	0	0.93
aaa	K	2	1.68
aag	K	1	1.32
aga	R	1	5.50
agg	R	11	3.30
---	--	------	------
mPD		0.042	0.68
		nPD:	0.06
		N. weight:	1.1
		Sc. PD:	-0.053
		Sc. rank:	786.9

,

HA

Pos.	181	obs:	exp:
tct	S	0	385.30
tcc	S	0	332.60
tca	S	0	508.50
tcg	S	0	145.70
act	T	0	2.65
acc	T	10	2.21
aca	T	0	4.30
acg	T	0	0.84
aat	N	0	240.20
aac	N	452	211.80
agt	S	8	460.60
agc	S	2248	423.30
---	--	------	------
mPD		0.29	1.8
		nPD:	0.16
		N. weight:	1.7
		Sc. PD:	0.087
		Sc. rank:	1983.7

,

HA

Pos.	182	obs:	exp:
tat	Y	13	1321.00
tac	Y	2705	1397.00
---	--	------	------
mPD		0.0095	0.50
		nPD:	0.02
		N. weight:	0.73
		Sc. PD:	-0.064
		Sc. rank:	-502.9

,

HA

Pos.	183	obs:	exp:
tct	S	0	0.34
tcc	S	0	0.29
tca	S	0	0.45
tcg	S	0	0.13
aat	N	2705	1442.00
aac	N	9	1272.00
aaa	K	1	1.12
aag	K	1	0.88
agt	S	0	0.41
agc	S	2	0.38
---	--	------	------
mPD		0.011	0.50
		nPD:	0.02
		N. weight:	0.7
		Sc. PD:	-0.06
		Sc. rank:	-339.9

,

HA

Pos.	184	obs:	exp:
aat	N	2684	1444.00
aac	N	34	1274.00
---	--	------	------
mPD		0.025	0.50
		nPD:	0.05
		N. weight:	0.65
		Sc. PD:	-0.038
		Sc. rank:	340.5

,

HA

Pos.	185	obs:	exp:
act	T	12	719.70
acc	T	2705	600.30
aca	T	0	1167.00
acg	T	0	229.50
gct	A	0	0.25
gcc	A	1	0.20
gca	A	0	0.44
gcg	A	0	0.11
---	--	------	------
mPD		0.0095	0.69
		nPD:	0.01
		N. weight:	1.7
		Sc. PD:	-0.16
		Sc. rank:	-1771.7

,

HA

Pos.	186	obs:	exp:
aat	N	2712	1444.00
aac	N	5	1273.00
aaa	K	1	0.56
aag	K	0	0.44
---	--	------	------
mPD		0.0044	0.50
		nPD:	0.01
		N. weight:	0.71
		Sc. PD:	-0.069
		Sc. rank:	-992.2

,

HA

Pos.	187	obs:	exp:
tta	L	0	0.11
ttg	L	0	0.19
tgg	W	2	2.00
ctt	L	0	0.19
ctc	L	0	0.17
cta	L	0	0.13
ctg	L	1	0.21
caa	Q	4	25.95
cag	Q	47	25.05
cgt	R	0	100.60
cgc	R	0	141.40
cga	R	10	257.00
cgg	R	2639	207.90
att	I	0	0.37
atc	I	0	0.25
ata	I	1	0.38
aaa	K	1	0.56
aag	K	0	0.44
aga	R	0	1217.00
agg	R	5	729.80
gtt	V	0	1.74
gtc	V	0	1.69
gta	V	8	1.57
gtg	V	0	3.00
---	--	------	------
mPD		0.075	1.0
		nPD:	0.07
		N. weight:	2.8
		Sc. PD:	-0.1
		Sc. rank:	2311.7

,

HA

Pos.	188	obs:	exp:
aaa	K	2	1.12
aag	K	0	0.88
gaa	E	2707	1626.00
gag	E	9	1090.00
---	--	------	------
mPD		0.0081	0.48
		nPD:	0.02
		N. weight:	0.57
		Sc. PD:	-0.051
		Sc. rank:	-464.8

,

HA

Pos.	189	obs:	exp:
gat	D	2698	1563.00
gac	D	20	1155.00
---	--	------	------
mPD		0.015	0.49
		nPD:	0.03
		N. weight:	0.59
		Sc. PD:	-0.046
		Sc. rank:	-38.7

,

HA

Pos.	190	obs:	exp:
tta	L	0	296.20
ttg	L	0	509.30
ctt	L	698	508.90
ctc	L	2014	468.20
cta	L	2	361.10
ctg	L	0	570.20
att	I	3	1.49
atc	I	1	1.00
ata	I	0	1.51
---	--	------	------
mPD		0.39	1.1
		nPD:	0.34
		N. weight:	1.3
		Sc. PD:	0.3
		Sc. rank:	2130.3

,

HA

Pos.	191	obs:	exp:
tta	L	20	296.70
ttg	L	2688	510.00
ctt	L	1	509.70
ctc	L	0	468.90
cta	L	1	361.60
ctg	L	8	571.10
---	--	------	------
mPD		0.023	1.1
		nPD:	0.02
		N. weight:	1.8
		Sc. PD:	-0.16
		Sc. rank:	-1100.6

,

HA

Pos.	192	obs:	exp:
att	I	0	1006.00
atc	I	0	679.20
ata	I	2708	1023.00
gtt	V	0	2.17
gtc	V	0	2.11
gta	V	7	1.97
gtg	V	3	3.74
---	--	------	------
mPD		0.0095	0.66
		nPD:	0.01
		N. weight:	1.1
		Sc. PD:	-0.1
		Sc. rank:	-1088.2

,

HA

Pos.	193	obs:	exp:
tta	L	2	292.20
ttg	L	34	502.30
ctt	L	0	502.00
ctc	L	0	461.90
cta	L	145	356.20
ctg	L	2496	562.50
att	I	0	2.97
atc	I	0	2.01
ata	I	8	3.02
atg	M	33	33.00
---	--	------	------
mPD		0.16	1.2
		nPD:	0.14
		N. weight:	1.5
		Sc. PD:	0.045
		Sc. rank:	1624.3

,

HA

Pos.	194	obs:	exp:
tgg	W	2718	2718.00
---	--	------	------
mPD		0	0
		nPD:	1.
		N. weight:	0.
		Sc. PD:	0
		Sc. rank:	0

,

HA

Pos.	195	obs:	exp:
ggt	G	0	391.00
ggc	G	0	358.80
gga	G	204	1186.00
ggg	G	2514	781.80
---	--	------	------
mPD		0.14	0.69
		nPD:	0.2
		N. weight:	1.1
		Sc. PD:	0.096
		Sc. rank:	1389.4

,

HA

Pos.	196	obs:	exp:
att	I	2598	1005.00
atc	I	99	678.90
ata	I	10	1023.00
gtt	V	11	2.39
gtc	V	0	2.32
gta	V	0	2.16
gtg	V	0	4.12
---	--	------	------
mPD		0.085	0.67
		nPD:	0.13
		N. weight:	0.95
		Sc. PD:	0.017
		Sc. rank:	971.1

,

HA

Pos.	197	obs:	exp:
cat	H	2577	1616.00
cac	H	141	1102.00
---	--	------	------
mPD		0.098	0.48
		nPD:	0.2
		N. weight:	0.39
		Sc. PD:	0.035
		Sc. rank:	494.2

,

HA

Pos.	198	obs:	exp:
cat	H	2679	1616.00
cac	H	39	1102.00
---	--	------	------
mPD		0.028	0.48
		nPD:	0.06
		N. weight:	0.52
		Sc. PD:	-0.026
		Sc. rank:	349.0

,

HA

Pos.	199	obs:	exp:
tct	S	9	456.20
tcc	S	2659	393.80
tca	S	3	602.10
tcg	S	0	172.50
cct	P	13	12.97
ccc	P	32	8.32
cca	P	1	18.78
ccg	P	0	5.92
act	T	0	0.26
acc	T	1	0.22
aca	T	0	0.43
acg	T	0	0.08
agt	S	0	545.30
agc	S	0	501.10
---	--	------	------
mPD		0.053	1.7
		nPD:	0.03
		N. weight:	2.1
		Sc. PD:	-0.16
		Sc. rank:	115.8

,

HA

Pos.	200	obs:	exp:
tct	S	0	0.17
tcc	S	0	0.15
tca	S	0	0.23
tcg	S	0	0.06
act	T	1	0.26
acc	T	0	0.22
aca	T	0	0.43
acg	T	0	0.08
aat	N	16	1443.00
aac	N	2700	1273.00
agt	S	0	0.20
agc	S	1	0.19
---	--	------	------
mPD		0.014	0.50
		nPD:	0.03
		N. weight:	0.83
		Sc. PD:	-0.066
		Sc. rank:	-166.3

,

HA

Pos.	201	obs:	exp:
tct	S	0	2.05
tcc	S	0	1.77
tca	S	0	2.70
tcg	S	0	0.78
aat	N	2683	1431.00
aac	N	9	1261.00
agt	S	12	2.45
agc	S	0	2.25
gat	D	14	8.05
gac	D	0	5.95
---	--	------	------
mPD		0.026	0.52
		nPD:	0.05
		N. weight:	0.71
		Sc. PD:	-0.042
		Sc. rank:	351.4

,

HA

Pos.	202	obs:	exp:
gtt	V	0	0.87
gtc	V	0	0.85
gta	V	4	0.79
gtg	V	0	1.50
gct	A	3	614.10
gcc	A	0	470.70
gca	A	2409	1061.00
gcg	A	2	268.00
gaa	E	294	179.00
gag	E	5	120.00
ggt	G	0	0.14
ggc	G	0	0.13
gga	G	1	0.44
ggg	G	0	0.29
---	--	------	------
mPD		0.21	0.89
		nPD:	0.23
		N. weight:	0.88
		Sc. PD:	0.1
		Sc. rank:	1207.4

,

HA

Pos.	203	obs:	exp:
aaa	K	5	2.81
aag	K	0	2.19
gct	A	5	168.70
gcc	A	0	129.30
gca	A	655	291.40
gcg	A	3	73.60
gat	D	2	1.15
gac	D	0	0.85
gaa	E	2046	1226.00
gag	E	1	821.30
ggt	G	0	0.14
ggc	G	0	0.13
gga	G	1	0.44
ggg	G	0	0.29
---	--	------	------
mPD		0.38	0.94
		nPD:	0.4
		N. weight:	0.66
		Sc. PD:	0.19
		Sc. rank:	1131.1

,

HA

Pos.	204	obs:	exp:
gaa	E	19	1627.00
gag	E	2699	1091.00
---	--	------	------
mPD		0.014	0.48
		nPD:	0.03
		N. weight:	1.
		Sc. PD:	-0.078
		Sc. rank:	-143.3

,

HA

Pos.	205	obs:	exp:
caa	Q	30	1383.00
cag	Q	2688	1335.00
---	--	------	------
mPD		0.022	0.50
		nPD:	0.04
		N. weight:	0.75
		Sc. PD:	-0.048
		Sc. rank:	313.3

,

HA			
Pos.	206	obs:	exp:
att	I	0	98.03
atc	I	0	66.21
ata	I	264	99.76
atg	M	2	2.00
act	T	0	643.90
acc	T	2	537.10
aca	T	2418	1045.00
acg	T	11	205.30
gct	A	0	5.34
gcc	A	0	4.10
gca	A	21	9.23
gcg	A	0	2.33
---	--	------	------
mPD		0.20	0.88
		nPD:	0.23
		N. weight:	0.96
		Sc. PD:	0.11
		Sc. rank:	1291.6

HA			
Pos.	207	obs:	exp:
tct	S	0	1.88
tcc	S	0	1.62
tca	S	0	2.48
tcg	S	0	0.71
act	T	1	0.26
acc	T	0	0.22
aca	T	0	0.43
acg	T	0	0.08
aat	N	2682	1430.00
aac	N	9	1261.00
aaa	K	3	6.74
aag	K	9	5.26
agt	S	11	2.25
agc	S	0	2.06
gat	D	3	1.73
gac	D	0	1.27
---	--	------	------
mPD		0.026	0.52
		nPD:	0.05
		N. weight:	0.7
		Sc. PD:	-0.04
		Sc. rank:	386.7

HA			
Pos.	208	obs:	exp:
tta	L	0	296.70
ttg	L	0	510.00
ctt	L	7	509.70
ctc	L	2403	468.90
cta	L	308	361.60
ctg	L	0	571.10
---	--	------	------
mPD		0.21	1.1
		nPD:	0.18
		N. weight:	1.6
		Sc. PD:	0.11
		Sc. rank:	2004.7

HA			
Pos.	209	obs:	exp:
tat	Y	2571	1321.00
tac	Y	147	1397.00
---	--	------	------
mPD		0.10	0.50
		nPD:	0.2
		N. weight:	0.58
		Sc. PD:	0.053
		Sc. rank:	749.2

HA			
Pos.	210	obs:	exp:
caa	Q	6	7.63
cag	Q	9	7.37
act	T	0	0.26
acc	T	0	0.22
aca	T	1	0.43
acg	T	0	0.08
aaa	K	2688	1517.00
aag	K	14	1185.00
---	--	------	------
mPD		0.029	0.50
		nPD:	0.06
		N. weight:	0.63
		Sc. PD:	-0.032
		Sc. rank:	410.4

HA			
Pos.	211	obs:	exp:
act	T	0	1.06
acc	T	4	0.88
aca	T	0	1.72
acg	T	0	0.34
aat	N	10	1442.00
aac	N	2704	1272.00
---	--	------	------
mPD		0.010	0.50
		nPD:	0.02
		N. weight:	0.84
		Sc. PD:	-0.073
		Sc. rank:	-518.4

HA			
Pos.	212	obs:	exp:
tta	L	0	0.22
ttg	L	0	0.38
tct	S	0	1.37
tcc	S	0	1.18
tca	S	2	1.80
tcg	S	6	0.52
ctt	L	0	0.38
ctc	L	0	0.35
cta	L	2	0.27
ctg	L	0	0.42
cct	P	8	763.10
ccc	P	0	489.60
cca	P	2696	1105.00
ccg	P	2	348.40
act	T	0	0.53
acc	T	0	0.44
aca	T	1	0.86
acg	T	1	0.17
agt	S	0	1.63
agc	S	0	1.50
---	--	------	------
mPD		0.021	0.72
		nPD:	0.03
		N. weight:	1.
		Sc. PD:	-0.078
		Sc. rank:	-84.0

HA			
Pos.	213	obs:	exp:
att	I	0	2.23
atc	I	6	1.50
ata	I	0	2.27
act	T	1	542.50
acc	T	2046	452.50
aca	T	1	880.00
acg	T	0	173.00
aat	N	1	7.44
aac	N	13	6.56
gct	A	0	1.53
gcc	A	6	1.17
gca	A	0	2.64
gcg	A	0	0.67
gat	D	3	368.10
gac	D	637	271.90
gaa	E	4	2.40
gag	E	0	1.60
---	--	------	------
mPD		0.74	1.4
		nPD:	0.52
		N. weight:	1.5
		Sc. PD:	0.6
		Sc. rank:	2796.0

HA			
Pos.	214	obs:	exp:
act	T	658	720.00
acc	T	2056	600.50
aca	T	4	1168.00
acg	T	0	229.60
---	--	------	------
mPD		0.37	0.69
		nPD:	0.54
		N. weight:	1.
		Sc. PD:	0.43
		Sc. rank:	1923.4

HA			
Pos.	215	obs:	exp:
tat	Y	1827	1321.00
tac	Y	891	1397.00
---	--	------	------
mPD		0.44	0.50
		nPD:	0.88
		N. weight:	0.083
		Sc. PD:	0.062
		Sc. rank:	168.4

HA			
Pos.	216	obs:	exp:
att	I	2020	751.20
atc	I	3	507.40
ata	I	0	764.40
aat	N	1	0.53
aac	N	0	0.47
gtt	V	685	150.90
gtc	V	1	146.60
gta	V	1	136.60
gtg	V	7	259.90
---	--	------	------
mPD		0.39	1.1
		nPD:	0.36
		N. weight:	1.3
		Sc. PD:	0.3
		Sc. rank:	2105.8

HA			
Pos.	217	obs:	exp:
tct	S	13	463.70
tcc	S	686	400.20
tca	S	2015	612.00
tcg	S	1	175.40
cct	P	0	0.28
ccc	P	0	0.18
cca	P	1	0.41
ccg	P	0	0.13
agt	S	0	554.30
agc	S	0	509.40
gct	A	0	0.51
gcc	A	0	0.39
gca	A	2	0.88
gcg	A	0	0.22
---	--	------	------
mPD		0.39	1.6
		nPD:	0.24
		N. weight:	1.2
		Sc. PD:	0.14
		Sc. rank:	1585.3

HA			
Pos.	218	obs:	exp:
att	I	8	2.97
atc	I	0	2.01
ata	I	0	3.02
gtt	V	2602	589.20
gtc	V	3	572.40
gta	V	9	533.00
gtg	V	95	1014.00
gat	D	1	0.58
gac	D	0	0.42
---	--	------	------
mPD		0.083	0.74
		nPD:	0.11
		N. weight:	1.5
		Sc. PD:	0.0031
		Sc. rank:	1490.5

HA			
Pos.	219	obs:	exp:
ggt	G	1	391.00
ggc	G	0	358.80
gga	G	1729	1186.00
ggg	G	988	781.80
---	--	------	------
mPD		0.46	0.69
		nPD:	0.67
		N. weight:	0.37
		Sc. PD:	0.2
		Sc. rank:	732.1

HA			
Pos.	220	obs:	exp:
act	T	4	720.00
acc	T	1	600.50
aca	T	2708	1168.00
acg	T	5	229.60
---	--	------	------
mPD		0.0073	0.69
		nPD:	0.01
		N. weight:	0.94
		Sc. PD:	-0.091
		Sc. rank:	-1194.8

HA			
Pos.	221	obs:	exp:
tct	S	0	464.00
tcc	S	3	400.50
tca	S	2709	612.50
tcg	S	5	175.50
cct	P	0	0.28
ccc	P	0	0.18
cca	P	1	0.41
ccg	P	0	0.13
agt	S	0	554.70
agc	S	0	509.80
---	--	------	------
mPD		0.0066	1.6
		nPD:	0.
		N. weight:	1.7
		Sc. PD:	-0.17
		Sc. rank:	-2959.4

HA			
Pos.	222	obs:	exp:
att	I	1	0.74
atc	I	0	0.50
ata	I	1	0.76
act	T	894	719.40
acc	T	2	600.10
aca	T	1597	1167.00
acg	T	223	229.40
---	--	------	------
mPD		0.54	0.69
		nPD:	0.78
		N. weight:	0.29
		Sc. PD:	0.19
		Sc. rank:	587.9

HA			
Pos.	223	obs:	exp:
tta	L	2699	296.60
ttg	L	3	509.80
ctt	L	0	509.50
ctc	L	0	468.80
cta	L	1	361.50
ctg	L	14	570.90
gtt	V	0	0.22
gtc	V	0	0.21
gta	V	1	0.20
gtg	V	0	0.37
---	--	------	------
mPD		0.024	1.1
		nPD:	0.02
		N. weight:	2.5
		Sc. PD:	-0.21
		Sc. rank:	-1314.3

HA			
Pos.	224	obs:	exp:
aat	N	12	1444.00
aac	N	2705	1273.00
aaa	K	1	0.56
aag	K	0	0.44
---	--	------	------
mPD		0.0095	0.50
		nPD:	0.02
		N. weight:	0.84
		Sc. PD:	-0.074
		Sc. rank:	-567.1

HA			
Pos.	225	obs:	exp:
tta	L	0	0.87
ttg	L	0	1.50
ctt	L	0	1.50
ctc	L	0	1.38
cta	L	0	1.06
ctg	L	8	1.68
caa	Q	27	1379.00
cag	Q	2683	1331.00
---	--	------	------
mPD		0.026	0.51
		nPD:	0.05
		N. weight:	0.76
		Sc. PD:	-0.044
		Sc. rank:	410.9

HA

Pos.	226	obs:	exp:
cgt	R	0	102.80
cgc	R	0	144.40
cga	R	0	262.50
cgg	R	0	212.30
aaa	K	5	3.93
aag	K	2	3.07
aga	R	1845	1244.00
agg	R	866	745.40
---	--	------	------
mPD		0.44	0.96
		nPD:	0.46
		N. weight:	0.36
		Sc. PD:	0.12
		Sc. rank:	649.5

,

HA

Pos.	227	obs:	exp:
tta	L	0	294.80
ttg	L	2698	506.80
tct	S	0	2.39
tcc	S	0	2.06
tca	S	11	3.16
tcg	S	3	0.90
tgg	W	1	1.00
ctt	L	0	506.50
ctc	L	0	466.00
cta	L	0	359.40
ctg	L	3	567.50
agt	S	0	2.86
agc	S	0	2.63
gtt	V	0	0.43
gtc	V	0	0.42
gta	V	0	0.39
gtg	V	2	0.75
---	--	------	------
mPD		0.023	1.2
		nPD:	0.02
		N. weight:	1.9
		Sc. PD:	-0.17
		Sc. rank:	-1210.8

,

HA

Pos.	228	obs:	exp:
tta	L	0	0.22
ttg	L	0	0.38
ctt	L	0	0.38
ctc	L	0	0.35
cta	L	2	0.27
ctg	L	0	0.42
att	I	2	9.28
atc	I	8	6.27
ata	I	15	9.45
atg	M	1	1.00
gtt	V	2	583.90
gtc	V	1	567.30
gta	V	1948	528.30
gtg	V	734	1005.00
gct	A	0	0.76
gcc	A	0	0.59
gca	A	3	1.32
gcg	A	0	0.33
gaa	E	1	0.60
gag	E	0	0.40
ggt	G	0	0.14
ggc	G	0	0.13
gga	G	1	0.44
ggg	G	0	0.29
---	--	------	------
mPD		0.43	0.75
		nPD:	0.56
		N. weight:	0.98
		Sc. PD:	0.43
		Sc. rank:	1865.8

,

HA

Pos.	229	obs:	exp:
cct	P	0	766.40
ccc	P	8	491.80
cca	P	2705	1110.00
ccg	P	5	350.00
---	--	------	------
mPD		0.0095	0.70
		nPD:	0.01
		N. weight:	1.
		Sc. PD:	-0.093
		Sc. rank:	-1056.8

,

HA

Pos.	230	obs:	exp:
aaa	K	2599	1518.00
aag	K	106	1187.00
gtt	V	0	0.22
gtc	V	0	0.21
gta	V	1	0.20
gtg	V	0	0.37
gaa	E	12	7.19
gag	E	0	4.81
---	--	------	------
mPD		0.085	0.50
		nPD:	0.17
		N. weight:	0.49
		Sc. PD:	0.028
		Sc. rank:	568.9

,

HA

Pos.	231	obs:	exp:
att	I	0	1007.00
atc	I	0	679.90
ata	I	2711	1024.00
atg	M	7	7.00
---	--	------	------
mPD		0.0051	0.66
		nPD:	0.01
		N. weight:	1.1
		Sc. PD:	-0.11
		Sc. rank:	-1598.8

,

HA

Pos.	232	obs:	exp:
act	T	5	1.32
acc	T	0	1.10
aca	T	0	2.15
acg	T	0	0.42
gtt	V	4	1.09
gtc	V	1	1.06
gta	V	0	0.98
gtg	V	0	1.87
gct	A	2686	688.90
gcc	A	18	528.10
gca	A	4	1190.00
gcg	A	0	300.60
---	--	------	------
mPD		0.024	0.70
		nPD:	0.03
		N. weight:	1.5
		Sc. PD:	-0.11
		Sc. rank:	238.5

,

HA

Pos.	233	obs:	exp:
cct	P	2	0.56
ccc	P	0	0.36
cca	P	0	0.82
ccg	P	0	0.26
att	I	1	0.37
atc	I	0	0.25
ata	I	0	0.38
act	T	2682	718.10
acc	T	28	599.00
aca	T	0	1165.00
acg	T	1	229.00
aat	N	2	1.06
aac	N	0	0.94
gct	A	2	0.51
gcc	A	0	0.39
gca	A	0	0.88
gcg	A	0	0.22
---	--	------	------
mPD		0.026	0.69
		nPD:	0.04
		N. weight:	1.5
		Sc. PD:	-0.1
		Sc. rank:	386.2

,

HA

Pos.	234	obs:	exp:
cgt	R	0	103.00
cgc	R	0	144.70
cga	R	0	263.10
cgg	R	0	212.80
act	T	0	0.26
acc	T	0	0.22
aca	T	1	0.43
acg	T	0	0.08
aga	R	2691	1246.00
agg	R	26	747.10
---	--	------	------
mPD		0.020	0.95
		nPD:	0.02
		N. weight:	0.84
		Sc. PD:	-0.072
		Sc. rank:	-495.1

,

HA

Pos.	235	obs:	exp:
tct	S	2	460.80
tcc	S	2692	397.70
tca	S	4	608.20
tcg	S	0	174.30
cct	P	0	5.64
ccc	P	20	3.62
cca	P	0	8.17
ccg	P	0	2.58
agt	S	0	550.90
agc	S	0	506.20
---	--	------	------
mPD		0.019	1.7
		nPD:	0.01
		N. weight:	2.2
		Sc. PD:	-0.21
		Sc. rank:	-2562.1

,

HA

Pos.	236	obs:	exp:
tta	L	0	0.11
ttg	L	0	0.19
ctt	L	0	0.19
ctc	L	0	0.17
cta	L	1	0.13
ctg	L	0	0.21
caa	Q	2696	1373.00
cag	Q	2	1325.00
cgt	R	0	0.15
cgc	R	0	0.21
cga	R	4	0.39
cgg	R	0	0.31
aaa	K	13	7.30
aag	K	0	5.70
aga	R	0	1.83
agg	R	0	1.10
gaa	E	2	1.20
gag	E	0	0.80
---	--	------	------
mPD		0.016	0.52
		nPD:	0.03
		N. weight:	0.77
		Sc. PD:	-0.059
		Sc. rank:	-0.4

,

HA

Pos.	237	obs:	exp:
tta	L	1	0.11
ttg	L	0	0.19
ctt	L	0	0.19
ctc	L	0	0.17
cta	L	0	0.13
ctg	L	0	0.21
att	I	0	0.74
atc	I	0	0.50
ata	I	2	0.76
gtt	V	0	590.50
gtc	V	0	573.70
gta	V	2628	534.20
gtg	V	87	1017.00
---	--	------	------
mPD		0.064	0.73
		nPD:	0.09
		N. weight:	1.7
		Sc. PD:	-0.036
		Sc. rank:	1502.2

,

HA

Pos.	238	obs:	exp:
aat	N	330	1444.00
aac	N	2388	1274.00
---	--	------	------
mPD		0.21	0.50
		nPD:	0.43
		N. weight:	0.43
		Sc. PD:	0.13
		Sc. rank:	753.8

,

HA

Pos.	239	obs:	exp:
ggt	G	0	391.00
ggc	G	11	358.80
gga	G	13	1186.00
ggg	G	2694	781.80
---	--	------	------
mPD		0.018	0.69
		nPD:	0.03
		N. weight:	1.4
		Sc. PD:	-0.11
		Sc. rank:	-430.3

,

HA

Pos.	240	obs:	exp:
caa	Q	2378	1383.00
cag	Q	340	1335.00
---	--	------	------
mPD		0.22	0.50
		nPD:	0.44
		N. weight:	0.35
		Sc. PD:	0.11
		Sc. rank:	615.9

,

HA			
Pos.	241	obs:	exp:
tct	S	0	19.64
tcc	S	0	16.95
tca	S	0	25.92
tcg	S	0	7.43
cgt	R	2198	98.71
cgc	R	3	138.70
cga	R	6	252.10
cgg	R	380	203.90
agt	S	115	23.48
agc	S	0	21.58
aga	R	16	1194.00
agg	R	0	715.70
---	--	------	------
mPD		0.35	1.1
		nPD:	0.33
		N. weight:	3.
		Sc. PD:	0.64
		Sc. rank:	4766.3

,

HA			
Pos.	242	obs:	exp:
ggt	G	1	391.00
ggc	G	0	358.80
gga	G	2546	1186.00
ggg	G	171	781.80
---	--	------	------
mPD		0.12	0.69
		nPD:	0.17
		N. weight:	0.71
		Sc. PD:	0.043
		Sc. rank:	841.1

,

HA			
Pos.	243	obs:	exp:
cgt	R	0	103.00
cgc	R	0	144.70
cga	R	1	263.10
cgg	R	0	212.80
att	I	0	0.37
atc	I	0	0.25
ata	I	1	0.38
aga	R	2710	1246.00
agg	R	6	747.10
---	--	------	------
mPD		0.0059	0.95
		nPD:	0.01
		N. weight:	0.88
		Sc. PD:	-0.088
		Sc. rank:	-1395.8

,

HA			
Pos.	244	obs:	exp:
att	I	1	1.11
atc	I	0	0.75
ata	I	2	1.13
atg	M	2715	2715.00
---	--	------	------
mPD		0.0022	0.0022
		nPD:	1.
		N. weight:	0.00078
		Sc. PD:	0.00067
		Sc. rank:	1.6

,

HA			
Pos.	245	obs:	exp:
act	T	0	0.53
acc	T	0	0.44
aca	T	2	0.86
acg	T	0	0.17
gat	D	697	1553.00
gac	D	2002	1146.00
gaa	E	15	10.18
gag	E	2	6.82
---	--	------	------
mPD		0.39	0.50
		nPD:	0.79
		N. weight:	0.24
		Sc. PD:	0.16
		Sc. rank:	486.5

,

HA			
Pos.	246	obs:	exp:
ttt	F	31	1157.00
ttc	F	2687	1561.00
---	--	------	------
mPD		0.023	0.49
		nPD:	0.05
		N. weight:	0.57
		Sc. PD:	-0.035
		Sc. rank:	263.3

,

HA			
Pos.	247	obs:	exp:
ttt	F	15	1157.00
ttc	F	2703	1561.00
---	--	------	------
mPD		0.011	0.49
		nPD:	0.02
		N. weight:	0.6
		Sc. PD:	-0.051
		Sc. rank:	-276.2

,

HA			
Pos.	248	obs:	exp:
tgg	W	2718	2718.00
---	--	------	------
mPD		0	0
		nPD:	1.
		N. weight:	0.
		Sc. PD:	0
		Sc. rank:	0

,

HA			
Pos.	249	obs:	exp:
act	T	3	716.80
acc	T	0	597.90
aca	T	2700	1163.00
acg	T	3	228.60
gct	A	0	3.05
gcc	A	0	2.34
gca	A	12	5.28
gcg	A	0	1.33
---	--	------	------
mPD		0.013	0.70
		nPD:	0.02
		N. weight:	0.95
		Sc. PD:	-0.084
		Sc. rank:	-669.1

,

HA			
Pos.	250	obs:	exp:
att	I	2706	1009.00
atc	I	2	681.40
ata	I	9	1027.00
gtt	V	1	0.22
gtc	V	0	0.21
gta	V	0	0.20
gtg	V	0	0.37
---	--	------	------
mPD		0.0088	0.66
		nPD:	0.01
		N. weight:	1.1
		Sc. PD:	-0.1
		Sc. rank:	-1191.2

,

HA			
Pos.	251	obs:	exp:
tta	L	2670	296.70
ttg	L	34	510.00
ctt	L	0	509.70
ctc	L	0	468.90
cta	L	10	361.60
ctg	L	4	571.10
---	--	------	------
mPD		0.038	1.1
		nPD:	0.03
		N. weight:	2.4
		Sc. PD:	-0.18
		Sc. rank:	300.2

,

HA			
Pos.	252	obs:	exp:
cgt	R	0	0.27
cgc	R	0	0.37
cga	R	0	0.68
cgg	R	0	0.55
act	T	0	0.26
acc	T	0	0.22
aca	T	1	0.43
acg	T	0	0.08
aaa	K	2697	1521.00
aag	K	13	1189.00
aga	R	2	3.21
agg	R	5	1.92
---	--	------	------
mPD		0.019	0.50
		nPD:	0.04
		N. weight:	0.63
		Sc. PD:	-0.044
		Sc. rank:	183.2

,

HA			
Pos.	253	obs:	exp:
tct	S	0	0.85
tcc	S	0	0.74
tca	S	0	1.13
tcg	S	5	0.32
cct	P	0	765.00
ccc	P	0	490.90
cca	P	341	1108.00
ccg	P	2372	349.30
agt	S	0	1.02
agc	S	0	0.94
---	--	------	------
mPD		0.22	0.71
		nPD:	0.31
		N. weight:	1.8
		Sc. PD:	0.35
		Sc. rank:	2689.5

,

HA			
Pos.	254	obs:	exp:
tct	S	0	0.34
tcc	S	0	0.29
tca	S	0	0.45
tcg	S	0	0.13
aat	N	1851	987.40
aac	N	7	870.60
agt	S	2	0.41
agc	S	0	0.38
gat	D	856	493.50
gac	D	2	364.50
---	--	------	------
mPD		0.44	0.93
		nPD:	0.47
		N. weight:	0.67
		Sc. PD:	0.24
		Sc. rank:	1207.9

,

HA			
Pos.	255	obs:	exp:
aat	N	1	0.53
aac	N	0	0.47
gat	D	2707	1557.00
gac	D	0	1150.00
gaa	E	10	5.99
gag	E	0	4.01
---	--	------	------
mPD		0.0081	0.49
		nPD:	0.02
		N. weight:	0.63
		Sc. PD:	-0.057
		Sc. rank:	-545.6

,

HA			
Pos.	256	obs:	exp:
tct	S	0	0.17
tcc	S	0	0.15
tca	S	1	0.23
tcg	S	0	0.06
act	T	0	1.85
acc	T	1	1.55
aca	T	6	3.01
acg	T	0	0.59
agt	S	0	0.20
agc	S	0	0.19
gtt	V	0	8.05
gtc	V	0	7.82
gta	V	37	7.28
gtg	V	0	13.86
gct	A	1	679.50
gcc	A	2	520.90
gca	A	2668	1174.00
gcg	A	0	296.50
gaa	E	2	1.20
gag	E	0	0.80
---	--	------	------
mPD		0.037	0.73
		nPD:	0.05
		N. weight:	0.95
		Sc. PD:	-0.054
		Sc. rank:	526.1

,

HA			
Pos.	257	obs:	exp:
tta	L	0	0.11
ttg	L	0	0.19
ctt	L	0	0.19
ctc	L	1	0.17
cta	L	0	0.13
ctg	L	0	0.21
att	I	8	1009.00
atc	I	2709	681.40
ata	I	0	1027.00
---	--	------	------
mPD		0.0066	0.66
		nPD:	0.01
		N. weight:	1.6
		Sc. PD:	-0.15
		Sc. rank:	-2113.1

,

HA			
Pos.	258	obs:	exp:
tct	S	0	1.54
tcc	S	0	1.33
tca	S	0	2.03
tcg	S	0	0.58
cat	H	1250	1607.00
cac	H	1453	1096.00
caa	Q	1	0.51
cag	Q	0	0.49
aat	N	5	2.66
aac	N	0	2.34
agt	S	3	1.84
agc	S	6	1.69
---	--	------	------
mPD		0.51	0.50
		nPD:	1.03
		N. weight:	0.048
		Sc. PD:	0.043
		Sc. rank:	98.6

,

HA			
Pos.	259	obs:	exp:
ttt	F	665	1157.00
ttc	F	2053	1561.00
---	--	------	------
mPD		0.37	0.49
		nPD:	0.76
		N. weight:	0.084
		Sc. PD:	0.052
		Sc. rank:	168.5

,

HA			
Pos.	260	obs:	exp:
caa	Q	0	0.51
cag	Q	1	0.49
aaa	K	0	1.12
aag	K	2	0.88
gaa	E	8	1626.00
gag	E	2707	1089.00
---	--	------	------
mPD		0.0081	0.48
		nPD:	0.02
		N. weight:	1.
		Sc. PD:	-0.092
		Sc. rank:	-845.9

,

HA

Pos.	261	obs:	exp:
tct	S	0	464.20
tcc	S	0	400.70
tca	S	0	612.70
tcg	S	0	175.60
agt	S	2703	554.90
agc	S	15	510.00
---	--	------	------
mPD		0.011	1.6
		nPD:	0.01
		N. weight:	1.8
		Sc. PD:	-0.18
		Sc. rank:	-2746.4

,

HA

Pos.	262	obs:	exp:
tct	S	0	0.17
tcc	S	0	0.15
tca	S	0	0.23
tcg	S	0	0.06
act	T	1	0.26
acc	T	0	0.22
aca	T	0	0.43
acg	T	0	0.08
aat	N	2714	1443.00
aac	N	2	1273.00
agt	S	1	0.20
agc	S	0	0.19
---	--	------	------
mPD		0.0029	0.50
		nPD:	0.01
		N. weight:	0.72
		Sc. PD:	-0.072
		Sc. rank:	-1167.8

,

HA

Pos.	263	obs:	exp:
ggt	G	0	391.00
ggc	G	0	358.80
gga	G	2697	1186.00
ggg	G	21	781.80
---	--	------	------
mPD		0.015	0.69
		nPD:	0.02
		N. weight:	0.9
		Sc. PD:	-0.077
		Sc. rank:	-426.4

,

HA

Pos.	264	obs:	exp:
aat	N	2699	1444.00
aac	N	18	1273.00
gat	D	1	0.58
gac	D	0	0.42
---	--	------	------
mPD		0.014	0.50
		nPD:	0.03
		N. weight:	0.68
		Sc. PD:	-0.054
		Sc. rank:	-133.4

,

HA

Pos.	265	obs:	exp:
ttt	F	15	1156.00
ttc	F	2702	1561.00
tta	L	0	0.11
ttg	L	0	0.19
ctt	L	0	0.19
ctc	L	1	0.17
cta	L	0	0.13
ctg	L	0	0.21
---	--	------	------
mPD		0.012	0.49
		nPD:	0.02
		N. weight:	0.6
		Sc. PD:	-0.05
		Sc. rank:	-214.2

,

HA

Pos.	266	obs:	exp:
att	I	2709	1009.00
atc	I	0	681.70
ata	I	9	1027.00
---	--	------	------
mPD		0.0066	0.66
		nPD:	0.01
		N. weight:	1.1
		Sc. PD:	-0.11
		Sc. rank:	-1493.9

,

HA

Pos.	267	obs:	exp:
tct	S	1	0.17
tcc	S	0	0.15
tca	S	0	0.23
tcg	S	0	0.06
cct	P	1	0.28
ccc	P	0	0.18
cca	P	0	0.41
ccg	P	0	0.13
agt	S	0	0.20
agc	S	0	0.19
gtt	V	1	0.22
gtc	V	0	0.21
gta	V	0	0.20
gtg	V	0	0.37
gct	A	2698	690.70
gcc	A	15	529.40
gca	A	2	1193.00
gcg	A	0	301.40
---	--	------	------
mPD		0.015	0.69
		nPD:	0.02
		N. weight:	1.5
		Sc. PD:	-0.13
		Sc. rank:	-855.4

,

HA

Pos.	268	obs:	exp:
cct	P	9	766.40
ccc	P	4	491.80
cca	P	2599	1110.00
ccg	P	106	350.00
---	--	------	------
mPD		0.084	0.70
		nPD:	0.12
		N. weight:	0.86
		Sc. PD:	0.0078
		Sc. rank:	846.3

,

HA

Pos.	269	obs:	exp:
aaa	K	3	1.68
aag	K	0	1.32
gtt	V	0	0.22
gtc	V	0	0.21
gta	V	1	0.20
gtg	V	0	0.37
gaa	E	2711	1624.00
gag	E	2	1089.00
ggt	G	0	0.14
ggc	G	0	0.13
gga	G	1	0.44
ggg	G	0	0.29
---	--	------	------
mPD		0.0051	0.48
		nPD:	0.01
		N. weight:	0.58
		Sc. PD:	-0.056
		Sc. rank:	-748.5

,

HA

Pos.	270	obs:	exp:
tat	Y	2714	1320.00
tac	Y	2	1396.00
aat	N	2	1.06
aac	N	0	0.94
---	--	------	------
mPD		0.0029	0.50
		nPD:	0.01
		N. weight:	0.82
		Sc. PD:	-0.083
		Sc. rank:	-1336.4

,

HA

Pos.	271	obs:	exp:
tct	S	0	0.17
tcc	S	0	0.15
tca	S	1	0.23
tcg	S	0	0.06
agt	S	0	0.20
agc	S	0	0.19
gct	A	0	691.20
gcc	A	192	529.80
gca	A	2491	1194.00
gcg	A	34	301.60
---	--	------	------
mPD		0.16	0.69
		nPD:	0.22
		N. weight:	0.66
		Sc. PD:	0.073
		Sc. rank:	887.0

,

HA

Pos.	272	obs:	exp:
tat	Y	5	1321.00
tac	Y	2713	1397.00
---	--	------	------
mPD		0.0037	0.50
		nPD:	0.01
		N. weight:	0.75
		Sc. PD:	-0.074
		Sc. rank:	-1124.8

,

HA

Pos.	273	obs:	exp:
aaa	K	2606	1526.00
aag	K	112	1192.00
---	--	------	------
mPD		0.079	0.49
		nPD:	0.16
		N. weight:	0.48
		Sc. PD:	0.023
		Sc. rank:	539.6

,

HA

Pos.	274	obs:	exp:
att	I	2708	1009.00
atc	I	8	681.70
ata	I	2	1027.00
---	--	------	------
mPD		0.0073	0.66
		nPD:	0.01
		N. weight:	1.1
		Sc. PD:	-0.11
		Sc. rank:	-1339.0

,

HA

Pos.	275	obs:	exp:
att	I	0	9.65
atc	I	26	6.52
ata	I	0	9.82
gtt	V	1	585.50
gtc	V	2690	568.80
gta	V	1	529.70
gtg	V	0	1008.00
---	--	------	------
mPD		0.020	0.75
		nPD:	0.03
		N. weight:	1.8
		Sc. PD:	-0.14
		Sc. rank:	-393.5

,

HA

Pos.	276	obs:	exp:
aaa	K	13	1526.00
aag	K	2705	1192.00
---	--	------	------
mPD		0.0095	0.49
		nPD:	0.02
		N. weight:	0.91
		Sc. PD:	-0.08
		Sc. rank:	-605.1

,

HA

Pos.	277	obs:	exp:
cgt	R	0	0.04
cgc	R	0	0.05
cga	R	0	0.10
cgg	R	0	0.08
att	I	0	0.37
atc	I	0	0.25
ata	I	1	0.38
act	T	0	0.53
acc	T	0	0.44
aca	T	2	0.86
acg	T	0	0.17
aaa	K	2680	1523.00
aag	K	34	1191.00
aga	R	0	0.46
agg	R	1	0.27
---	--	------	------
mPD		0.028	0.50
		nPD:	0.06
		N. weight:	0.59
		Sc. PD:	-0.03
		Sc. rank:	390.3

,

HA

Pos.	278	obs:	exp:
cgt	R	0	0.08
cgc	R	0	0.11
cga	R	0	0.19
cgg	R	0	0.16
aga	R	0	0.92
agg	R	2	0.55
ggt	G	1	390.70
ggc	G	0	358.50
gga	G	28	1186.00
ggg	G	2687	781.20
---	--	------	------
mPD		0.023	0.69
		nPD:	0.03
		N. weight:	1.4
		Sc. PD:	-0.1
		Sc. rank:	120.8

,

HA

Pos.	279	obs:	exp:
aat	N	1	0.53
aac	N	0	0.47
gct	A	0	0.25
gcc	A	1	0.20
gca	A	0	0.44
gcg	A	0	0.11
gat	D	12	1562.00
gac	D	2704	1154.00
---	--	------	------
mPD		0.011	0.49
		nPD:	0.02
		N. weight:	0.95
		Sc. PD:	-0.08
		Sc. rank:	-441.5

,

HA

Pos.	280	obs:	exp:
tct	S	0	464.20
tcc	S	0	400.70
tca	S	2714	612.70
tcg	S	4	175.60
agt	S	0	554.90
agc	S	0	510.00
---	--	------	------
mPD		0.0029	1.6
		nPD:	0.
		N. weight:	1.7
		Sc. PD:	-0.18
		Sc. rank:	-3232.6

,

HA	Pos. 281	obs:	exp:
act	T	0	716.30
acc	T	1	597.40
aca	T	2701	1162.00
acg	T	2	228.40
gct	A	0	3.56
gcc	A	0	2.73
gca	A	14	6.15
gcg	A	0	1.55
---	--	------	------
mPD		0.012	0.70
		nPD:	0.02
		N. weight:	0.96
		Sc. PD:	-0.086
		Sc. rank:	-739.4

HA	Pos. 282	obs:	exp:
att	I	2692	1009.00
atc	I	25	681.70
ata	I	1	1027.00
---	--	------	------
mPD		0.019	0.66
		nPD:	0.03
		N. weight:	1.1
		Sc. PD:	-0.085
		Sc. rank:	-162.6

HA	Pos. 283	obs:	exp:
att	I	0	0.74
atc	I	0	0.50
ata	I	2	0.76
atg	M	2716	2716.00
---	--	------	------
mPD		0.0015	0.0015
		nPD:	1.
		N. weight:	0.00082
		Sc. PD:	0.00071
		Sc. rank:	1.7

HA	Pos. 284	obs:	exp:
cgt	R	0	0.30
cgc	R	0	0.43
cga	R	0	0.77
cgg	R	0	0.63
aat	N	0	0.53
aac	N	1	0.47
aaa	K	2708	1521.00
aag	K	1	1188.00
aga	R	6	3.67
agg	R	2	2.20
---	--	------	------
mPD		0.0088	0.50
		nPD:	0.02
		N. weight:	0.66
		Sc. PD:	-0.059
		Sc. rank:	-527.1

HA	Pos. 285	obs:	exp:
tct	S	0	464.20
tcc	S	0	400.70
tca	S	0	612.70
tcg	S	0	175.60
agt	S	2715	554.90
agc	S	3	510.00
---	--	------	------
mPD		0.0022	1.6
		nPD:	0.
		N. weight:	1.8
		Sc. PD:	-0.19
		Sc. rank:	-3501.8

HA	Pos. 286	obs:	exp:
cgt	R	0	0.04
cgc	R	0	0.05
cga	R	0	0.10
cgg	R	0	0.08
aaa	K	3	1.68
aag	K	0	1.32
aga	R	1	0.46
agg	R	0	0.27
gat	D	0	1.15
gac	D	2	0.85
gaa	E	1283	768.80
gag	E	1	515.20
ggt	G	1	205.40
ggc	G	1	188.50
gga	G	1425	623.40
ggg	G	1	410.70
---	--	------	------
mPD		0.51	1.1
		nPD:	0.46
		N. weight:	0.77
		Sc. PD:	0.26
		Sc. rank:	1367.6

HA	Pos. 287	obs:	exp:
tta	L	1	2.07
ttg	L	13	3.57
ctt	L	0	3.56
ctc	L	0	3.28
cta	L	0	2.53
ctg	L	5	3.99
atg	M	124	124.00
gtt	V	0	560.00
gtc	V	0	544.10
gta	V	5	506.70
gtg	V	2570	964.30
---	--	------	------
mPD		0.10	0.82
		nPD:	0.13
		N. weight:	1.1
		Sc. PD:	0.018
		Sc. rank:	1078.4

HA	Pos. 288	obs:	exp:
aaa	K	0	0.56
aag	K	1	0.44
gaa	E	2139	1625.00
gag	E	575	1089.00
ggt	G	0	0.43
ggc	G	0	0.40
gga	G	1	1.31
ggg	G	2	0.86
---	--	------	------
mPD		0.34	0.48
		nPD:	0.7
		N. weight:	0.096
		Sc. PD:	0.055
		Sc. rank:	190.7

HA	Pos. 289	obs:	exp:
tat	Y	2712	1321.00
tac	Y	6	1397.00
---	--	------	------
mPD		0.0044	0.50
		nPD:	0.01
		N. weight:	0.81
		Sc. PD:	-0.079
		Sc. rank:	-1135.0

HA	Pos. 290	obs:	exp:
tct	S	0	0.17
tcc	S	0	0.15
tca	S	0	0.23
tcg	S	0	0.06
agt	S	0	0.20
agc	S	1	0.19
gat	D	0	0.58
gac	D	1	0.42
ggt	G	22	390.70
ggc	G	2693	358.50
gga	G	1	1186.00
ggg	G	0	781.20
---	--	------	------
mPD		0.018	0.69
		nPD:	0.03
		N. weight:	2.3
		Sc. PD:	-0.18
		Sc. rank:	-622.2

HA	Pos. 291	obs:	exp:
tat	Y	0	0.97
tac	Y	2	1.03
cat	H	9	1586.00
cac	H	2659	1082.00
caa	Q	8	4.07
cag	Q	0	3.93
aat	N	4	20.73
aac	N	35	18.27
gat	D	1	0.58
gac	D	0	0.42
---	--	------	------
mPD		0.047	0.52
		nPD:	0.09
		N. weight:	1.
		Sc. PD:	-0.019
		Sc. rank:	915.1

HA	Pos. 292	obs:	exp:
tgt	C	23	1182.00
tgc	C	2695	1536.00
---	--	------	------
mPD		0.017	0.49
		nPD:	0.03
		N. weight:	0.6
		Sc. PD:	-0.044
		Sc. rank:	88.3

HA	Pos. 293	obs:	exp:
tct	S	0	0.51
tcc	S	0	0.44
tca	S	0	0.68
tcg	S	0	0.19
act	T	0	0.26
acc	T	1	0.22
aca	T	0	0.43
acg	T	0	0.08
aat	N	11	1436.00
aac	N	2692	1267.00
agt	S	0	0.61
agc	S	3	0.56
gat	D	2	6.33
gac	D	9	4.67
---	--	------	------
mPD		0.021	0.51
		nPD:	0.04
		N. weight:	0.84
		Sc. PD:	-0.057
		Sc. rank:	287.4

HA	Pos. 294	obs:	exp:
cct	P	0	0.56
ccc	P	2	0.36
cca	P	0	0.82
ccg	P	0	0.26
act	T	41	718.60
acc	T	2669	599.40
aca	T	3	1166.00
acg	T	0	229.20
gct	A	0	0.76
gcc	A	3	0.59
gca	A	0	1.32
gcg	A	0	0.33
---	--	------	------
mPD		0.036	0.69
		nPD:	0.05
		N. weight:	1.6
		Sc. PD:	-0.092
		Sc. rank:	957.6

HA	Pos. 295	obs:	exp:
cgt	R	0	0.68
cgc	R	0	0.96
cga	R	0	1.74
cgg	R	0	1.41
aaa	K	2667	1516.00
aag	K	33	1184.00
aga	R	18	8.26
agg	R	0	4.95
---	--	------	------
mPD		0.037	0.51
		nPD:	0.07
		N. weight:	0.59
		Sc. PD:	-0.021
		Sc. rank:	468.2

HA	Pos. 296	obs:	exp:
tgt	C	2645	1182.00
tgc	C	73	1536.00
---	--	------	------
mPD		0.052	0.49
		nPD:	0.11
		N. weight:	0.81
		Sc. PD:	-0.0028
		Sc. rank:	773.0

HA	Pos. 297	obs:	exp:
caa	Q	2702	1383.00
cag	Q	16	1335.00
---	--	------	------
mPD		0.012	0.50
		nPD:	0.02
		N. weight:	0.74
		Sc. PD:	-0.061
		Sc. rank:	-289.5

HA	Pos. 298	obs:	exp:
act	T	714	720.00
acc	T	2004	600.50
aca	T	0	1168.00
acg	T	0	229.60
---	--	------	------
mPD		0.39	0.69
		nPD:	0.56
		N. weight:	1.
		Sc. PD:	0.44
		Sc. rank:	1936.7

HA	Pos. 299	obs:	exp:
cct	P	0	766.40
ccc	P	2	491.80
cca	P	2713	1110.00
ccg	P	3	350.00
---	--	------	------
mPD		0.0037	0.70
		nPD:	0.01
		N. weight:	1.
		Sc. PD:	-0.1
		Sc. rank:	-1693.0

HA	Pos. 300	obs:	exp:
tta	L	1	0.33
ttg	L	2	0.56
ctt	L	0	0.56
ctc	L	0	0.52
cta	L	0	0.40
ctg	L	0	0.63
att	I	0	250.70
atc	I	0	169.30
ata	I	675	255.10
atg	M	4	4.00
gtt	V	0	442.80
gtc	V	0	430.20
gta	V	2009	400.60
gtg	V	27	762.40
---	--	------	------
mPD		0.40	1.1
		nPD:	0.36
		N. weight:	1.6
		Sc. PD:	0.38
		Sc. rank:	2671.2

HA			
Pos.	301	obs:	exp:
ggt	G	54	391.00
ggc	G	0	358.80
gga	G	1973	1186.00
ggg	G	691	781.80
---	--	------	------
mPD		0.41	0.69
		nPD:	0.59
		N. weight:	0.35
		Sc. PD:	0.16
		Sc. rank:	664.4

,

HA			
Pos.	302	obs:	exp:
tct	S	0	0.17
tcc	S	0	0.15
tca	S	0	0.23
tcg	S	1	0.06
act	T	0	0.26
acc	T	0	0.22
aca	T	1	0.43
acg	T	0	0.08
agt	S	0	0.20
agc	S	0	0.19
gct	A	15	690.90
gcc	A	0	529.60
gca	A	114	1194.00
gcg	A	2587	301.50
---	--	------	------
mPD		0.093	0.69
		nPD:	0.13
		N. weight:	2.2
		Sc. PD:	0.052
		Sc. rank:	2332.3

,

HA			
Pos.	303	obs:	exp:
tta	L	1	0.11
ttg	L	0	0.19
ctt	L	0	0.19
ctc	L	0	0.17
cta	L	0	0.13
ctg	L	0	0.21
att	I	2	1009.00
atc	I	0	681.20
ata	I	2714	1026.00
gtt	V	0	0.22
gtc	V	0	0.21
gta	V	1	0.20
gtg	V	0	0.37
---	--	------	------
mPD		0.0029	0.66
		nPD:	0.
		N. weight:	1.1
		Sc. PD:	-0.11
		Sc. rank:	-1901.4

,

HA			
Pos.	304	obs:	exp:
tct	S	0	0.34
tcc	S	0	0.29
tca	S	0	0.45
tcg	S	0	0.13
aat	N	882	1443.00
aac	N	1834	1273.00
agt	S	1	0.41
agc	S	1	0.38
---	--	------	------
mPD		0.44	0.50
		nPD:	0.88
		N. weight:	0.1
		Sc. PD:	0.076
		Sc. rank:	208.5

,

HA			
Pos.	305	obs:	exp:
tct	S	2700	464.00
tcc	S	16	400.50
tca	S	0	612.50
tcg	S	1	175.50
act	T	1	0.26
acc	T	0	0.22
aca	T	0	0.43
acg	T	0	0.08
agt	S	0	554.70
agc	S	0	509.80
---	--	------	------
mPD		0.013	1.6
		nPD:	0.01
		N. weight:	2.
		Sc. PD:	-0.2
		Sc. rank:	-2821.6

,

HA			
Pos.	306	obs:	exp:
tct	S	0	464.00
tcc	S	0	400.50
tca	S	0	612.50
tcg	S	0	175.50
aat	N	1	0.53
aac	N	0	0.47
agt	S	2063	554.70
agc	S	654	509.80
---	--	------	------
mPD		0.37	1.6
		nPD:	0.22
		N. weight:	1.2
		Sc. PD:	0.13
		Sc. rank:	1620.1

,

HA			
Pos.	307	obs:	exp:
atg	M	2718	2718.00
---	--	------	------
mPD		0	0
		nPD:	1.
		N. weight:	0.
		Sc. PD:	0
		Sc. rank:	0

,

HA			
Pos.	308	obs:	exp:
cct	P	3	766.40
ccc	P	12	491.80
cca	P	2224	1110.00
ccg	P	479	350.00
---	--	------	------
mPD		0.30	0.70
		nPD:	0.43
		N. weight:	0.69
		Sc. PD:	0.21
		Sc. rank:	1214.7

,

HA			
Pos.	309	obs:	exp:
ttt	F	20	1156.00
ttc	F	2696	1560.00
tta	L	0	0.22
ttg	L	0	0.38
ctt	L	0	0.38
ctc	L	2	0.35
cta	L	0	0.27
ctg	L	0	0.42
---	--	------	------
mPD		0.016	0.49
		nPD:	0.03
		N. weight:	0.59
		Sc. PD:	-0.044
		Sc. rank:	55.1

,

HA			
Pos.	310	obs:	exp:
cat	H	7	1616.00
cac	H	2711	1102.00
---	--	------	------
mPD		0.0051	0.48
		nPD:	0.01
		N. weight:	1.
		Sc. PD:	-0.097
		Sc. rank:	-1277.7

,

HA			
Pos.	311	obs:	exp:
act	T	1	0.26
acc	T	0	0.22
aca	T	0	0.43
acg	T	0	0.08
aat	N	1789	1444.00
aac	N	928	1273.00
---	--	------	------
mPD		0.45	0.50
		nPD:	0.9
		N. weight:	0.04
		Sc. PD:	0.031
		Sc. rank:	82.2

,

HA			
Pos.	312	obs:	exp:
tta	L	1	0.11
ttg	L	0	0.19
ctt	L	0	0.19
ctc	L	0	0.17
cta	L	0	0.13
ctg	L	0	0.21
att	I	1	1004.00
atc	I	1	678.10
ata	I	2702	1022.00
gtt	V	9	2.83
gtc	V	0	2.75
gta	V	4	2.56
gtg	V	0	4.87
---	--	------	------
mPD		0.018	0.67
		nPD:	0.03
		N. weight:	1.1
		Sc. PD:	-0.089
		Sc. rank:	-229.5

,

HA			
Pos.	313	obs:	exp:
cat	H	2043	1614.00
cac	H	672	1101.00
aat	N	1	1.59
aac	N	2	1.41
---	--	------	------
mPD		0.38	0.48
		nPD:	0.77
		N. weight:	0.065
		Sc. PD:	0.042
		Sc. rank:	132.0

,

HA			
Pos.	314	obs:	exp:
cct	P	2690	766.40
ccc	P	27	491.80
cca	P	1	1110.00
ccg	P	0	350.00
---	--	------	------
mPD		0.020	0.70
		nPD:	0.03
		N. weight:	1.4
		Sc. PD:	-0.11
		Sc. rank:	-175.9

,

HA			
Pos.	315	obs:	exp:
tta	L	0	296.20
ttg	L	0	509.30
ctt	L	16	508.90
ctc	L	2696	468.20
cta	L	2	361.10
ctg	L	0	570.20
att	I	0	1.49
atc	I	4	1.00
ata	I	0	1.51
---	--	------	------
mPD		0.016	1.1
		nPD:	0.01
		N. weight:	2.
		Sc. PD:	-0.18
		Sc. rank:	-1934.2

,

HA			
Pos.	316	obs:	exp:
tct	S	0	0.34
tcc	S	0	0.29
tca	S	0	0.45
tcg	S	0	0.13
cct	P	0	0.28
ccc	P	1	0.18
cca	P	0	0.41
ccg	P	0	0.13
act	T	7	718.90
acc	T	2707	599.60
aca	T	0	1166.00
acg	T	0	229.20
agt	S	0	0.41
agc	S	2	0.38
gct	A	0	0.25
gcc	A	1	0.20
gca	A	0	0.44
gcg	A	0	0.11
---	--	------	------
mPD		0.0081	0.69
		nPD:	0.01
		N. weight:	1.7
		Sc. PD:	-0.16
		Sc. rank:	-1971.1

,

HA			
Pos.	317	obs:	exp:
att	I	891	1009.00
atc	I	1824	681.70
ata	I	3	1027.00
---	--	------	------
mPD		0.44	0.66
		nPD:	0.67
		N. weight:	0.71
		Sc. PD:	0.38
		Sc. rank:	1392.4

,

HA			
Pos.	318	obs:	exp:
ggt	G	0	391.00
ggc	G	0	358.80
gga	G	5	1186.00
ggg	G	2713	781.80
---	--	------	------
mPD		0.0037	0.69
		nPD:	0.01
		N. weight:	1.4
		Sc. PD:	-0.14
		Sc. rank:	-2333.3

,

HA			
Pos.	319	obs:	exp:
aaa	K	2	1.68
aag	K	1	1.32
gct	A	0	0.25
gcc	A	0	0.20
gca	A	1	0.44
gcg	A	0	0.11
gaa	E	2689	1625.00
gag	E	25	1089.00
---	--	------	------
mPD		0.022	0.48
		nPD:	0.05
		N. weight:	0.53
		Sc. PD:	-0.033
		Sc. rank:	240.5

,

HA			
Pos.	320	obs:	exp:
tgt	C	10	1182.00
tgc	C	2708	1536.00
---	--	------	------
mPD		0.0073	0.49
		nPD:	0.01
		N. weight:	0.63
		Sc. PD:	-0.058
		Sc. rank:	-575.3

,

HA			
Pos.	321	obs:	exp:
cct	P	22	766.40
ccc	P	2695	491.80
cca	P	1	1110.00
ccg	P	0	350.00
---	--	------	------
mPD		0.017	0.70
		nPD:	0.02
		N. weight:	1.9
		Sc. PD:	-0.16
		Sc. rank:	-722.7

,

HA			
Pos.	322	obs:	exp:
aaa	K	2706	1525.00
aag	K	11	1192.00
gaa	E	1	0.60
gag	E	0	0.40
---	--	------	------
mPD		0.0088	0.49
		nPD:	0.02
		N. weight:	0.63
		Sc. PD:	-0.056
		Sc. rank:	-480.3

,

HA			
Pos.	323	obs:	exp:
tat	Y	29	1321.00
tac	Y	2689	1397.00
---	--	------	------
mPD		0.021	0.50
		nPD:	0.04
		N. weight:	0.7
		Sc. PD:	-0.046
		Sc. rank:	257.5

,

HA			
Pos.	324	obs:	exp:
gtt	V	6	591.10
gtc	V	9	574.30
gta	V	1	534.80
gtg	V	2702	1018.00
---	--	------	------
mPD		0.012	0.73
		nPD:	0.02
		N. weight:	1.1
		Sc. PD:	-0.099
		Sc. rank:	-944.4

,

HA			
Pos.	325	obs:	exp:
aaa	K	297	1526.00
aag	K	2421	1192.00
---	--	------	------
mPD		0.19	0.49
		nPD:	0.4
		N. weight:	0.52
		Sc. PD:	0.14
		Sc. rank:	890.5

,

HA			
Pos.	326	obs:	exp:
tct	S	0	464.20
tcc	S	6	400.70
tca	S	2701	612.70
tcg	S	11	175.60
agt	S	0	554.90
agc	S	0	510.00
---	--	------	------
mPD		0.012	1.6
		nPD:	0.01
		N. weight:	1.7
		Sc. PD:	-0.17
		Sc. rank:	-2424.0

,

HA			
Pos.	327	obs:	exp:
tct	S	0	0.51
tcc	S	0	0.44
tca	S	0	0.68
tcg	S	0	0.19
act	T	0	0.79
acc	T	3	0.66
aca	T	0	1.29
acg	T	0	0.25
aat	N	2	1427.00
aac	N	2684	1259.00
aaa	K	0	2.25
aag	K	4	1.75
agt	S	0	0.61
agc	S	3	0.56
gat	D	4	12.08
gac	D	17	8.92
gaa	E	1	0.60
gag	E	0	0.40
---	--	------	------
mPD		0.029	0.52
		nPD:	0.05
		N. weight:	0.86
		Sc. PD:	-0.046
		Sc. rank:	561.9

,

HA			
Pos.	328	obs:	exp:
cgt	R	0	0.27
cgc	R	0	0.37
cga	R	0	0.68
cgg	R	0	0.55
att	I	0	0.37
atc	I	0	0.25
ata	I	1	0.38
aaa	K	703	1521.00
aag	K	2007	1189.00
aga	R	5	3.21
agg	R	2	1.92
---	--	------	------
mPD		0.39	0.50
		nPD:	0.78
		N. weight:	0.22
		Sc. PD:	0.14
		Sc. rank:	441.4

,

HA			
Pos.	329	obs:	exp:
ttt	F	0	0.43
ttc	F	1	0.57
tta	L	622	296.50
ttg	L	2077	509.60
tgg	W	1	1.00
ctt	L	0	509.30
ctc	L	0	468.60
cta	L	10	361.40
ctg	L	7	570.70
---	--	------	------
mPD		0.37	1.1
		nPD:	0.33
		N. weight:	1.4
		Sc. PD:	0.29
		Sc. rank:	2175.2

,

HA			
Pos.	330	obs:	exp:
att	I	2	5.57
atc	I	13	3.76
ata	I	0	5.67
gtt	V	13	587.80
gtc	V	2689	571.10
gta	V	1	531.80
gtg	V	0	1012.00
---	--	------	------
mPD		0.023	0.74
		nPD:	0.03
		N. weight:	1.7
		Sc. PD:	-0.13
		Sc. rank:	-56.1

,

HA			
Pos.	331	obs:	exp:
ttt	F	1	0.43
ttc	F	0	0.57
tta	L	0	296.50
ttg	L	1	509.60
tct	S	1	0.17
tcc	S	0	0.15
tca	S	0	0.23
tcg	S	0	0.06
ctt	L	2715	509.30
ctc	L	0	468.60
cta	L	0	361.40
ctg	L	0	570.70
agt	S	0	0.20
agc	S	0	0.19
---	--	------	------
mPD		0.0037	1.1
		nPD:	0.
		N. weight:	1.9
		Sc. PD:	-0.2
		Sc. rank:	-3481.1

,

HA			
Pos.	332	obs:	exp:
tct	S	0	0.17
tcc	S	0	0.15
tca	S	0	0.23
tcg	S	1	0.06
agt	S	0	0.20
agc	S	0	0.19
gtt	V	0	0.22
gtc	V	0	0.21
gta	V	0	0.20
gtg	V	1	0.37
gct	A	26	690.90
gcc	A	0	529.60
gca	A	376	1194.00
gcg	A	2314	301.50
---	--	------	------
mPD		0.26	0.69
		nPD:	0.37
		N. weight:	1.8
		Sc. PD:	0.44
		Sc. rank:	2993.3

,

HA			
Pos.	333	obs:	exp:
act	T	2688	720.00
acc	T	11	600.50
aca	T	17	1168.00
acg	T	2	229.60
---	--	------	------
mPD		0.022	0.69
		nPD:	0.03
		N. weight:	1.4
		Sc. PD:	-0.11
		Sc. rank:	55.8

,

HA			
Pos.	334	obs:	exp:
cgt	R	0	0.04
cgc	R	0	0.05
cga	R	0	0.10
cgg	R	0	0.08
aga	R	0	0.46
agg	R	1	0.27
ggt	G	0	390.90
ggc	G	3	358.60
gga	G	56	1186.00
ggg	G	2658	781.50
---	--	------	------
mPD		0.043	0.69
		nPD:	0.06
		N. weight:	1.3
		Sc. PD:	-0.059
		Sc. rank:	940.9

,

HA			
Pos.	335	obs:	exp:
tta	L	1	296.60
ttg	L	0	509.80
ctt	L	0	509.50
ctc	L	2702	468.80
cta	L	14	361.50
ctg	L	0	570.90
cct	P	0	0.28
ccc	P	0	0.18
cca	P	1	0.41
ccg	P	0	0.13
---	--	------	------
mPD		0.013	1.1
		nPD:	0.01
		N. weight:	2.
		Sc. PD:	-0.19
		Sc. rank:	-2301.3

,

HA			
Pos.	336	obs:	exp:
cgt	R	0	103.10
cgc	R	0	144.80
cga	R	0	263.20
cgg	R	0	212.90
aga	R	2702	1247.00
agg	R	16	747.40
---	--	------	------
mPD		0.012	0.95
		nPD:	0.01
		N. weight:	0.86
		Sc. PD:	-0.081
		Sc. rank:	-949.7

,

HA			
Pos.	337	obs:	exp:
aat	N	2708	1444.00
aac	N	10	1274.00
---	--	------	------
mPD		0.0073	0.50
		nPD:	0.01
		N. weight:	0.7
		Sc. PD:	-0.064
		Sc. rank:	-660.3

,

HA			
Pos.	338	obs:	exp:
tct	S	0	417.70
tcc	S	0	360.60
tca	S	0	551.40
tcg	S	0	158.00
act	T	1	0.53
acc	T	1	0.44
aca	T	0	0.86
acg	T	0	0.17
aat	N	255	136.60
aac	N	2	120.40
agt	S	2384	499.40
agc	S	62	458.90
gtt	V	3	2.83
gtc	V	4	2.75
gta	V	6	2.56
gtg	V	0	4.87
---	--	------	------
mPD		0.24	1.7
		nPD:	0.14
		N. weight:	1.6
		Sc. PD:	0.046
		Sc. rank:	1721.9

,

HA			
Pos.	339	obs:	exp:
tta	L	0	0.33
ttg	L	0	0.56
tct	S	1	0.17
tcc	S	0	0.15
tca	S	0	0.23
tcg	S	0	0.06
ctt	L	3	0.56
ctc	L	0	0.52
cta	L	0	0.40
ctg	L	0	0.63
cct	P	2694	765.30
ccc	P	8	491.10
cca	P	2	1108.00
ccg	P	10	349.50
agt	S	0	0.20
agc	S	0	0.19
---	--	------	------
mPD		0.018	0.71
		nPD:	0.02
		N. weight:	1.4
		Sc. PD:	-0.12
		Sc. rank:	-477.3

,

HA			
Pos.	340	obs:	exp:
tta	L	12	293.20
ttg	L	0	504.00
ctt	L	26	503.70
ctc	L	7	463.40
cta	L	2640	357.40
ctg	L	1	564.40
cct	P	0	0.85
ccc	P	0	0.54
cca	P	3	1.22
ccg	P	0	0.39
caa	Q	19	10.18
cag	Q	1	9.82
att	I	0	3.34
atc	I	0	2.26
ata	I	9	3.40
---	--	------	------
mPD		0.058	1.2
		nPD:	0.05
		N. weight:	2.2
		Sc. PD:	-0.13
		Sc. rank:	1172.7

,

HA			
Pos.	341	obs:	exp:
cgt	R	0	101.80
cgc	R	0	143.00
cga	R	12	259.90
cgg	R	0	210.20
att	I	2	1.86
atc	I	0	1.25
ata	I	3	1.89
aaa	K	15	8.42
aag	K	0	6.58
aga	R	2631	1231.00
agg	R	41	738.00
ggt	G	0	0.14
ggc	G	0	0.13
gga	G	1	0.44
ggg	G	0	0.29
---	-	13	13.00
---	--	------	------
mPD		0.055	0.96
		nPD:	0.06
		N. weight:	0.79
		Sc. PD:	-0.04
		Sc. rank:	526.2

HA			
Pos.	342	obs:	exp:
gat	D	0	0.58
gac	D	1	0.42
gaa	E	2594	1610.00
gag	E	95	1079.00
ggt	G	0	2.01
ggc	G	0	1.85
gga	G	14	6.11
ggg	G	0	4.03
---	-	14	14.00
---	--	------	------
mPD		0.078	0.49
		nPD:	0.16
		N. weight:	0.43
		Sc. PD:	0.021
		Sc. rank:	484.9

HA			
Pos.	343	obs:	exp:
cgt	R	0	24.65
cgc	R	0	34.62
cga	R	22	62.94
cgg	R	0	50.91
aaa	K	5	1150.00
aag	K	2044	898.90
aga	R	624	298.20
agg	R	4	178.70
gaa	E	0	0.60
gag	E	1	0.40
---	-	18	18.00
---	--	------	------
mPD		0.74	0.98
		nPD:	0.75
		N. weight:	0.88
		Sc. PD:	0.54
		Sc. rank:	1772.0

HA			
Pos.	344	obs:	exp:
cgt	R	0	102.40
cgc	R	0	143.80
cga	R	0	261.40
cgg	R	0	211.50
aaa	K	1	0.56
aag	K	0	0.44
aga	R	2617	1238.00
agg	R	83	742.40
---	-	17	17.00
---	--	------	------
mPD		0.060	0.94
		nPD:	0.06
		N. weight:	0.75
		Sc. PD:	-0.034
		Sc. rank:	551.9

HA			
Pos.	345	obs:	exp:
cgt	R	0	102.70
cgc	R	0	144.20
cga	R	1	262.20
cgg	R	0	212.10
aaa	K	6	3.37
aag	K	0	2.63
aga	R	2645	1242.00
agg	R	62	744.60
---	-	4	4.00
---	--	------	------
mPD		0.050	0.95
		nPD:	0.05
		N. weight:	0.78
		Sc. PD:	-0.044
		Sc. rank:	474.8

HA			
Pos.	346	obs:	exp:
aaa	K	1	0.56
aag	K	0	0.44
---	-	2717	2717.00
---	--	------	------
mPD		0	6.7×10^{-8}
		nPD:	0.
		N. weight:	0.
		Sc. PD:	0
		Sc. rank:	0

HA			
Pos.	347	obs:	exp:
cgt	R	0	0.08
cgc	R	0	0.11
cga	R	0	0.19
cgg	R	0	0.16
act	T	2	1.06
acc	T	0	0.88
aca	T	0	1.72
acg	T	2	0.34
aaa	K	2614	1515.00
aag	K	85	1184.00
aga	R	2	0.92
agg	R	0	0.55
gaa	E	12	7.19
gag	E	0	4.81
ggt	G	0	0.14
ggc	G	0	0.13
gga	G	1	0.44
ggg	G	0	0.29
---	--	------	------
mPD		0.078	0.51
		nPD:	0.15
		N. weight:	0.51
		Sc. PD:	0.022
		Sc. rank:	577.1

HA			
Pos.	348	obs:	exp:
act	T	0	3.18
acc	T	0	2.65
aca	T	11	5.16
acg	T	1	1.01
aat	N	0	0.53
aac	N	1	0.47
aaa	K	0	0.56
aag	K	1	0.44
gct	A	0	0.25
gcc	A	0	0.20
gca	A	0	0.44
gcg	A	1	0.11
---	-	2703	2703.00
---	--	------	------
mPD		0.000024	0.000032
		nPD:	0.73
		N. weight:	0.
		Sc. PD:	0
		Sc. rank:	0

HA			
Pos.	349	obs:	exp:
cgt	R	0	103.10
cgc	R	0	144.80
cga	R	1	263.20
cgg	R	0	212.90
aga	R	2710	1247.00
agg	R	7	747.40
---	--	------	------
mPD		0.0059	0.95
		nPD:	0.01
		N. weight:	0.87
		Sc. PD:	-0.088
		Sc. rank:	-1386.6

HA			
Pos.	350	obs:	exp:
ggt	G	4	391.00
ggc	G	880	358.80
gga	G	667	1186.00
ggg	G	1167	781.80
---	--	------	------
mPD		0.65	0.69
		nPD:	0.95
		N. weight:	0.36
		Sc. PD:	0.29
		Sc. rank:	746.4

HA			
Pos.	351	obs:	exp:
tta	L	6	296.70
ttg	L	1	510.00
ctt	L	33	509.70
ctc	L	0	468.90
cta	L	650	361.60
ctg	L	2028	571.10
---	--	------	------
mPD		0.39	1.1
		nPD:	0.34
		N. weight:	1.2
		Sc. PD:	0.27
		Sc. rank:	1955.1

HA			
Pos.	352	obs:	exp:
ttt	F	2591	1157.00
ttc	F	127	1561.00
---	--	------	------
mPD		0.089	0.49
		nPD:	0.18
		N. weight:	0.75
		Sc. PD:	0.052
		Sc. rank:	934.4

HA			
Pos.	353	obs:	exp:
ggt	G	3	391.00
ggc	G	0	358.80
gga	G	730	1186.00
ggg	G	1985	781.80
---	--	------	------
mPD		0.39	0.69
		nPD:	0.57
		N. weight:	0.63
		Sc. PD:	0.28
		Sc. rank:	1205.3

HA			
Pos.	354	obs:	exp:
gct	A	1533	691.40
gcc	A	3	530.00
gca	A	13	1195.00
gcg	A	1169	301.70
---	--	------	------
mPD		0.50	0.69
		nPD:	0.72
		N. weight:	1.2
		Sc. PD:	0.68
		Sc. rank:	2304.2

HA			
Pos.	355	obs:	exp:
att	I	4	1009.00
atc	I	0	681.20
ata	I	2712	1026.00
aaa	K	2	1.12
aag	K	0	0.88
---	--	------	------
mPD		0.0044	0.66
		nPD:	0.01
		N. weight:	1.1
		Sc. PD:	-0.11
		Sc. rank:	-1690.2

HA			
Pos.	356	obs:	exp:
gct	A	0	691.40
gcc	A	3	530.00
gca	A	2711	1195.00
gcg	A	4	301.70
---	--	------	------
mPD		0.0051	0.69
		nPD:	0.01
		N. weight:	0.93
		Sc. PD:	-0.092
		Sc. rank:	-1360.5

HA			
Pos.	357	obs:	exp:
ggt	G	1127	391.00
ggc	G	7	358.80
gga	G	239	1186.00
ggg	G	1345	781.80
---	--	------	------
mPD		0.58	0.69
		nPD:	0.84
		N. weight:	0.64
		Sc. PD:	0.45
		Sc. rank:	1308.4

HA			
Pos.	358	obs:	exp:
ttt	F	2403	1157.00
ttc	F	315	1561.00
---	--	------	------
mPD		0.21	0.49
		nPD:	0.42
		N. weight:	0.53
		Sc. PD:	0.16
		Sc. rank:	926.6

HA			
Pos.	359	obs:	exp:
att	I	0	1009.00
atc	I	0	681.70
ata	I	2718	1027.00
---	--	------	------
mPD		0	0.66
		nPD:	0.
		N. weight:	1.1
		Sc. PD:	-0.12
		Sc. rank:	-2284.7

HA			
Pos.	360	obs:	exp:
gaa	E	39	1627.00
gag	E	2679	1091.00
---	--	------	------
mPD		0.028	0.48
		nPD:	0.06
		N. weight:	0.96
		Sc. PD:	-0.047
		Sc. rank:	651.0

HA			
Pos.	361	obs:	exp:
ggt	G	0	391.00
ggc	G	0	358.80
gga	G	2713	1186.00
ggg	G	5	781.80
---	--	------	------
mPD		0.0037	0.69
		nPD:	0.01
		N. weight:	0.94
		Sc. PD:	-0.095
		Sc. rank:	-1546.4

HA			
Pos.	362	obs:	exp:
ggt	G	0	391.00
ggc	G	2	358.80
gga	G	2712	1186.00
ggg	G	4	781.80
---	--	------	------
mPD		0.0044	0.69
		nPD:	0.01
		N. weight:	0.93
		Sc. PD:	-0.094
		Sc. rank:	-1449.4

HA			
Pos.	363	obs:	exp:
tgg	W	2718	2718.00
---	--	------	------
mPD		0	0
		nPD:	1.
		N. weight:	0.
		Sc. PD:	0
		Sc. rank:	0

HA			
Pos.	364	obs:	exp:
caa	Q	33	1383.00
cag	Q	2685	1335.00
---	--	------	------
mPD		0.024	0.50
		nPD:	0.05
		N. weight:	0.74
		Sc. PD:	-0.044
		Sc. rank:	356.4

HA			
Pos.	365	obs:	exp:
ggt	G	0	391.00
ggc	G	3	358.80
gga	G	2702	1186.00
ggg	G	13	781.80
---	--	------	------
mPD		0.012	0.69
		nPD:	0.02
		N. weight:	0.91
		Sc. PD:	-0.082
		Sc. rank:	-743.5

,

HA			
Pos.	366	obs:	exp:
atg	M	2718	2718.00
---	--	------	------
mPD		0	0
		nPD:	1.
		N. weight:	0.
		Sc. PD:	0
		Sc. rank:	0

,

HA			
Pos.	367	obs:	exp:
att	I	4	1.86
atc	I	0	1.25
ata	I	1	1.89
gtt	V	2016	590.00
gtc	V	1	573.20
gta	V	686	533.80
gtg	V	10	1016.00
---	--	------	------
mPD		0.39	0.73
		nPD:	0.53
		N. weight:	1.1
		Sc. PD:	0.44
		Sc. rank:	2024.4

,

HA			
Pos.	368	obs:	exp:
tat	Y	1	0.49
tac	Y	0	0.51
gat	D	2065	1563.00
gac	D	652	1154.00
---	--	------	------
mPD		0.37	0.49
		nPD:	0.75
		N. weight:	0.088
		Sc. PD:	0.054
		Sc. rank:	176.2

,

HA			
Pos.	369	obs:	exp:
ggt	G	2708	391.00
ggc	G	1	358.80
gga	G	6	1186.00
ggg	G	3	781.80
---	--	------	------
mPD		0.0073	0.69
		nPD:	0.01
		N. weight:	2.2
		Sc. PD:	-0.21
		Sc. rank:	-2724.0

,

HA			
Pos.	370	obs:	exp:
tgg	W	2718	2718.00
---	--	------	------
mPD		0	0
		nPD:	1.
		N. weight:	0.
		Sc. PD:	0
		Sc. rank:	0

,

HA			
Pos.	371	obs:	exp:
tat	Y	2681	1321.00
tac	Y	37	1397.00
---	--	------	------
mPD		0.027	0.50
		nPD:	0.05
		N. weight:	0.75
		Sc. PD:	-0.041
		Sc. rank:	473.0

,

HA			
Pos.	372	obs:	exp:
ggt	G	3	391.00
ggc	G	0	358.80
gga	G	187	1186.00
ggg	G	2528	781.80
---	--	------	------
mPD		0.13	0.69
		nPD:	0.19
		N. weight:	1.1
		Sc. PD:	0.084
		Sc. rank:	1394.4

,

HA			
Pos.	373	obs:	exp:
ttt	F	0	0.43
ttc	F	1	0.57
tat	Y	122	1321.00
tac	Y	2595	1396.00
---	--	------	------
mPD		0.087	0.50
		nPD:	0.17
		N. weight:	0.56
		Sc. PD:	0.034
		Sc. rank:	662.9

,

HA			
Pos.	374	obs:	exp:
tta	L	0	0.11
ttg	L	0	0.19
ctt	L	0	0.19
ctc	L	1	0.17
cta	L	0	0.13
ctg	L	0	0.21
cct	P	0	0.28
ccc	P	1	0.18
cca	P	0	0.41
ccg	P	0	0.13
cat	H	259	1615.00
cac	H	2457	1101.00
---	--	------	------
mPD		0.17	0.48
		nPD:	0.36
		N. weight:	0.64
		Sc. PD:	0.15
		Sc. rank:	1063.0

,

HA			
Pos.	375	obs:	exp:
tat	Y	1	0.49
tac	Y	0	0.51
cat	H	2714	1615.00
cac	H	3	1102.00
---	--	------	------
mPD		0.0029	0.48
		nPD:	0.01
		N. weight:	0.59
		Sc. PD:	-0.059
		Sc. rank:	-940.7

,

HA			
Pos.	376	obs:	exp:
tct	S	0	463.30
tcc	S	0	399.90
tca	S	0	611.50
tcg	S	0	175.20
cgt	R	0	0.04
cgc	R	0	0.05
cga	R	0	0.10
cgg	R	0	0.08
act	T	0	0.26
acc	T	1	0.22
aca	T	0	0.43
acg	T	0	0.08
aat	N	0	1.59
aac	N	3	1.41
agt	S	4	553.90
agc	S	2709	509.00
aga	R	1	0.46
agg	R	0	0.27
---	--	------	------
mPD		0.0066	1.6
		nPD:	0.
		N. weight:	1.9
		Sc. PD:	-0.2
		Sc. rank:	-3364.2

,

HA			
Pos.	377	obs:	exp:
aat	N	2708	1444.00
aac	N	9	1273.00
aaa	K	1	0.56
aag	K	0	0.44
---	--	------	------
mPD		0.0073	0.50
		nPD:	0.01
		N. weight:	0.7
		Sc. PD:	-0.065
		Sc. rank:	-666.0

,

HA			
Pos.	378	obs:	exp:
gaa	E	16	1624.00
gag	E	2697	1089.00
ggt	G	0	0.72
ggc	G	0	0.66
gga	G	0	2.18
ggg	G	5	1.44
---	--	------	------
mPD		0.015	0.48
		nPD:	0.03
		N. weight:	1.
		Sc. PD:	-0.076
		Sc. rank:	18.7

,

HA			
Pos.	379	obs:	exp:
tta	L	0	0.22
ttg	L	0	0.38
ctt	L	0	0.38
ctc	L	0	0.35
cta	L	0	0.27
ctg	L	2	0.42
caa	Q	10	1382.00
cag	Q	2706	1334.00
---	--	------	------
mPD		0.0088	0.50
		nPD:	0.02
		N. weight:	0.79
		Sc. PD:	-0.071
		Sc. rank:	-639.1

,

HA			
Pos.	380	obs:	exp:
ggt	G	0	391.00
ggc	G	0	358.80
gga	G	417	1186.00
ggg	G	2301	781.80
---	--	------	------
mPD		0.26	0.69
		nPD:	0.38
		N. weight:	0.87
		Sc. PD:	0.22
		Sc. rank:	1475.2

,

HA			
Pos.	381	obs:	exp:
tct	S	0	463.80
tcc	S	0	400.40
tca	S	0	612.20
tcg	S	0	175.40
aat	N	2	1.06
aac	N	0	0.94
agt	S	2709	554.50
agc	S	7	509.60
---	--	------	------
mPD		0.0066	1.6
		nPD:	0.
		N. weight:	1.8
		Sc. PD:	-0.18
		Sc. rank:	-3206.1

,

HA			
Pos.	382	obs:	exp:
cgt	R	0	0.04
cgc	R	0	0.05
cga	R	0	0.10
cgg	R	0	0.08
aga	R	0	0.46
agg	R	1	0.27
ggt	G	17	390.90
ggc	G	0	358.60
gga	G	52	1186.00
ggg	G	2648	781.50
---	--	------	------
mPD		0.050	0.69
		nPD:	0.07
		N. weight:	1.3
		Sc. PD:	-0.045
		Sc. rank:	1018.0

,

HA			
Pos.	383	obs:	exp:
tat	Y	31	1321.00
tac	Y	2686	1396.00
gtt	V	0	0.22
gtc	V	1	0.21
gta	V	0	0.20
gtg	V	0	0.37
---	--	------	------
mPD		0.024	0.50
		nPD:	0.05
		N. weight:	0.69
		Sc. PD:	-0.042
		Sc. rank:	330.3

,

HA			
Pos.	384	obs:	exp:
gct	A	2671	691.40
gcc	A	45	530.00
gca	A	1	1195.00
gcg	A	1	301.70
---	--	------	------
mPD		0.034	0.69
		nPD:	0.05
		N. weight:	1.5
		Sc. PD:	-0.087
		Sc. rank:	763.8

,

HA Pos. 385		obs:	exp:
gct	A	4	691.40
gcc	A	0	530.00
gca	A	2710	1195.00
gcg	A	4	301.70
mPD		0.0059	0.69
		nPD:	0.01
		N. weight:	0.92
		Sc. PD:	-0.09
		Sc. rank:	-1304.7

HA Pos. 386		obs:	exp:
gat	D	3	1563.00
gac	D	2715	1155.00
mPD		0.0022	0.49
		nPD:	0.
		N. weight:	0.97
		Sc. PD:	-0.099
		Sc. rank:	-1646.9

HA Pos. 387		obs:	exp:
cgt	R	0	0.08
cgc	R	0	0.11
cga	R	0	0.19
cgg	R	0	0.16
aaa	K	2409	1525.00
aag	K	307	1191.00
aga	R	2	0.92
agg	R	0	0.55
mPD		0.20	0.49
		nPD:	0.41
		N. weight:	0.29
		Sc. PD:	0.084
		Sc. rank:	503.7

HA Pos. 388		obs:	exp:
aaa	K	0	0.56
aag	K	1	0.44
gtt	V	0	0.22
gtc	V	0	0.21
gta	V	1	0.20
gtg	V	0	0.37
gat	D	3	2.30
gac	D	1	1.70
gaa	E	2646	1624.00
gag	E	66	1088.00
mPD		0.052	0.48
		nPD:	0.11
		N. weight:	0.47
		Sc. PD:	-0.00074
		Sc. rank:	451.9

HA Pos. 389		obs:	exp:
tct	S	12	464.00
tcc	S	2704	400.50
tca	S	1	612.50
tcg	S	0	175.50
act	T	0	0.26
acc	T	1	0.22
aca	T	0	0.43
acg	T	0	0.08
agt	S	0	554.70
agc	S	0	509.80
mPD		0.010	1.6
		nPD:	0.01
		N. weight:	2.2
		Sc. PD:	-0.22
		Sc. rank:	-3366.1

HA Pos. 390		obs:	exp:
act	T	11	720.00
acc	T	2707	600.50
aca	T	0	1168.00
acg	T	0	229.60
mPD		0.0081	0.69
		nPD:	0.01
		N. weight:	1.7
		Sc. PD:	-0.16
		Sc. rank:	-1938.6

HA Pos. 391		obs:	exp:
caa	Q	2706	1383.00
cag	Q	12	1335.00
mPD		0.0088	0.50
		nPD:	0.02
		N. weight:	0.74
		Sc. PD:	-0.066
		Sc. rank:	-598.4

HA Pos. 392		obs:	exp:
cgt	R	0	0.38
cgc	R	0	0.53
cga	R	0	0.97
cgg	R	0	0.78
aaa	K	17	1518.00
aag	K	2688	1187.00
aga	R	0	4.59
agg	R	10	2.75
gaa	E	0	1.80
gag	E	3	1.20
mPD		0.022	0.50
		nPD:	0.04
		N. weight:	0.9
		Sc. PD:	-0.058
		Sc. rank:	374.1

HA Pos. 393		obs:	exp:
act	T	0	0.26
acc	T	0	0.22
aca	T	1	0.43
acg	T	0	0.08
gct	A	2	691.20
gcc	A	9	529.80
gca	A	2705	1194.00
gcg	A	1	301.60
mPD		0.0095	0.69
		nPD:	0.01
		N. weight:	0.91
		Sc. PD:	-0.085
		Sc. rank:	-962.6

HA Pos. 394		obs:	exp:
tta	L	0	0.11
ttg	L	1	0.19
ctt	L	0	0.19
ctc	L	0	0.17
cta	L	0	0.13
ctg	L	0	0.21
att	I	0	997.40
atc	I	9	673.60
ata	I	2677	1015.00
gtt	V	0	6.74
gtc	V	0	6.55
gta	V	31	6.10
gtg	V	0	11.61
mPD		0.031	0.68
		nPD:	0.04
		N. weight:	1.1
		Sc. PD:	-0.069
		Sc. rank:	492.6

HA Pos. 395		obs:	exp:
aat	N	152	80.78
aac	N	0	71.22
gat	D	2563	1475.00
gac	D	1	1089.00
ggt	G	2	0.29
ggc	G	0	0.26
gga	G	0	0.87
ggg	G	0	0.58
mPD		0.11	0.60
		nPD:	0.18
		N. weight:	0.64
		Sc. PD:	0.043
		Sc. rank:	778.4

HA Pos. 396		obs:	exp:
ggt	G	18	391.00
ggc	G	0	358.80
gga	G	2684	1186.00
ggg	G	16	781.80
mPD		0.025	0.69
		nPD:	0.04
		N. weight:	0.88
		Sc. PD:	-0.063
		Sc. rank:	190.8

HA Pos. 397		obs:	exp:
att	I	6	7.43
atc	I	14	5.02
ata	I	0	7.56
gtt	V	2647	586.80
gtc	V	51	570.10
gta	V	0	530.90
gtg	V	0	1010.00
mPD		0.061	0.74
		nPD:	0.08
		N. weight:	1.6
		Sc. PD:	-0.044
		Sc. rank:	1438.5

HA Pos. 398		obs:	exp:
cct	P	0	0.56
ccc	P	2	0.36
cca	P	0	0.82
ccg	P	0	0.26
act	T	7	719.40
acc	T	2706	600.10
aca	T	3	1167.00
acg	T	0	229.40
mPD		0.0088	0.69
		nPD:	0.01
		N. weight:	1.7
		Sc. PD:	-0.16
		Sc. rank:	-1864.9

HA Pos. 399		obs:	exp:
aat	N	2709	1444.00
aac	N	9	1274.00
mPD		0.0066	0.50
		nPD:	0.01
		N. weight:	0.7
		Sc. PD:	-0.066
		Sc. rank:	-760.7

HA Pos. 400		obs:	exp:
aaa	K	13	1526.00
aag	K	2705	1192.00
mPD		0.0095	0.49
		nPD:	0.02
		N. weight:	0.91
		Sc. PD:	-0.08
		Sc. rank:	-605.1

HA Pos. 401		obs:	exp:
att	I	0	0.37
atc	I	1	0.25
ata	I	0	0.38
gtt	V	1	590.90
gtc	V	2691	574.10
gta	V	24	534.60
gtg	V	1	1017.00
mPD		0.020	0.73
		nPD:	0.03
		N. weight:	1.7
		Sc. PD:	-0.14
		Sc. rank:	-403.3

HA Pos. 402		obs:	exp:
aat	N	5	1444.00
aac	N	2713	1274.00
mPD		0.0037	0.50
		nPD:	0.01
		N. weight:	0.85
		Sc. PD:	-0.085
		Sc. rank:	-1278.4

HA Pos. 403		obs:	exp:
tct	S	0	464.20
tcc	S	0	400.70
tca	S	1534	612.70
tcg	S	1184	175.60
agt	S	0	554.90
agc	S	0	510.00
mPD		0.49	1.6
		nPD:	0.3
		N. weight:	1.5
		Sc. PD:	0.28
		Sc. rank:	2271.2

HA Pos. 404		obs:	exp:
att	I	0	1009.00
atc	I	2557	681.70
ata	I	161	1027.00
mPD		0.11	0.66
		nPD:	0.17
		N. weight:	1.3
		Sc. PD:	0.075
		Sc. rank:	1536.0

HA Pos. 405		obs:	exp:
att	I	2713	1009.00
atc	I	4	681.70
ata	I	1	1027.00
mPD		0.0037	0.66
		nPD:	0.01
		N. weight:	1.1
		Sc. PD:	-0.11
		Sc. rank:	-1832.6

HA Pos. 406		obs:	exp:
aat	N	0	158.40
aac	N	298	139.60
gat	D	2	1391.00
gac	D	2417	1028.00
gaa	E	1	0.60
gag	E	0	0.40
mPD		0.20	0.69
		nPD:	0.29
		N. weight:	0.96
		Sc. PD:	0.17
		Sc. rank:	1396.5

HA Pos. 407		obs:	exp:
aaa	K	2717	1526.00
aag	K	1	1192.00
mPD		0.00074	0.49
		nPD:	0.
		N. weight:	0.66
		Sc. PD:	-0.069
		Sc. rank:	-1269.9

HA Pos. 408		obs:	exp:
atg	M	2718	2718.00
mPD		0	0
		nPD:	1.
		N. weight:	0.
		Sc. PD:	0
		Sc. rank:	0

HA

Pos.	409	obs:	exp:
aat	N	5	1444.00
aac	N	2713	1274.00
---	--	------	------
mPD		0.0037	0.50
		nPD:	0.01
		N. weight:	0.85
		Sc. PD:	-0.085
		Sc. rank:	-1278.4

,

HA

Pos.	410	obs:	exp:
tct	S	0	0.17
tcc	S	0	0.15
tca	S	0	0.23
tcg	S	0	0.06
act	T	2713	719.40
acc	T	2	600.10
aca	T	0	1167.00
acg	T	1	229.40
agt	S	1	0.20
agc	S	0	0.19
gct	A	1	0.25
gcc	A	0	0.20
gca	A	0	0.44
gcg	A	0	0.11
---	--	------	------
mPD		0.0037	0.69
		nPD:	0.01
		N. weight:	1.5
		Sc. PD:	-0.15
		Sc. rank:	-2512.8

,

HA

Pos.	411	obs:	exp:
cat	H	1	0.59
cac	H	0	0.41
caa	Q	2711	1382.00
cag	Q	6	1335.00
---	--	------	------
mPD		0.0051	0.50
		nPD:	0.01
		N. weight:	0.76
		Sc. PD:	-0.073
		Sc. rank:	-995.2

,

HA

Pos.	412	obs:	exp:
ttt	F	2708	1157.00
ttc	F	10	1561.00
---	--	------	------
mPD		0.0073	0.49
		nPD:	0.02
		N. weight:	0.95
		Sc. PD:	-0.087
		Sc. rank:	-864.5

,

HA

Pos.	413	obs:	exp:
gaa	E	19	1627.00
gag	E	2699	1091.00
---	--	------	------
mPD		0.014	0.48
		nPD:	0.03
		N. weight:	1.
		Sc. PD:	-0.078
		Sc. rank:	-143.3

,

HA

Pos.	414	obs:	exp:
tct	S	0	0.17
tcc	S	1	0.15
tca	S	0	0.23
tcg	S	0	0.06
act	T	0	0.26
acc	T	1	0.22
aca	T	0	0.43
acg	T	0	0.08
agt	S	0	0.20
agc	S	0	0.19
gct	A	13	690.90
gcc	A	687	529.60
gca	A	2016	1194.00
gcg	A	0	301.50
---	--	------	------
mPD		0.39	0.69
		nPD:	0.56
		N. weight:	0.5
		Sc. PD:	0.22
		Sc. rank:	953.7

,

HA

Pos.	415	obs:	exp:
att	I	2	0.74
atc	I	0	0.50
ata	I	0	0.76
gtt	V	2706	590.50
gtc	V	8	573.70
gta	V	0	534.20
gtg	V	1	1017.00
gct	A	1	0.25
gcc	A	0	0.20
gca	A	0	0.44
gcg	A	0	0.11
---	--	------	------
mPD		0.0088	0.73
		nPD:	0.01
		N. weight:	1.7
		Sc. PD:	-0.16
		Sc. rank:	-1942.5

,

HA

Pos.	416	obs:	exp:
gtt	V	0	0.43
gtc	V	0	0.42
gta	V	2	0.39
gtg	V	0	0.75
ggt	G	0	390.70
ggc	G	0	358.50
gga	G	2701	1186.00
ggg	G	15	781.20
---	--	------	------
mPD		0.012	0.69
		nPD:	0.02
		N. weight:	0.92
		Sc. PD:	-0.081
		Sc. rank:	-677.1

,

HA

Pos.	417	obs:	exp:
cgt	R	0	102.50
cgc	R	0	144.00
cga	R	0	261.80
cgg	R	0	211.80
aaa	K	9	7.86
aag	K	5	6.14
aga	R	28	1240.00
agg	R	2676	743.50
---	--	------	------
mPD		0.037	0.96
		nPD:	0.04
		N. weight:	1.4
		Sc. PD:	-0.097
		Sc. rank:	433.5

,

HA

Pos.	418	obs:	exp:
gaa	E	701	1627.00
gag	E	2017	1091.00
---	--	------	------
mPD		0.38	0.48
		nPD:	0.8
		N. weight:	0.28
		Sc. PD:	0.18
		Sc. rank:	560.8

,

HA

Pos.	419	obs:	exp:
ttt	F	2692	1157.00
ttc	F	26	1561.00
---	--	------	------
mPD		0.019	0.49
		nPD:	0.04
		N. weight:	0.92
		Sc. PD:	-0.063
		Sc. rank:	292.8

,

HA

Pos.	420	obs:	exp:
aat	N	2701	1444.00
aac	N	17	1274.00
---	--	------	------
mPD		0.012	0.50
		nPD:	0.02
		N. weight:	0.68
		Sc. PD:	-0.056
		Sc. rank:	-221.8

,

HA

Pos.	421	obs:	exp:
aat	N	170	1443.00
aac	N	2546	1273.00
aaa	K	2	1.12
aag	K	0	0.88
---	--	------	------
mPD		0.12	0.50
		nPD:	0.24
		N. weight:	0.59
		Sc. PD:	0.073
		Sc. rank:	828.4

,

HA

Pos.	422	obs:	exp:
tta	L	2692	296.70
ttg	L	12	510.00
ctt	L	0	509.70
ctc	L	0	468.90
cta	L	7	361.60
ctg	L	7	571.10
---	--	------	------
mPD		0.024	1.1
		nPD:	0.02
		N. weight:	2.5
		Sc. PD:	-0.21
		Sc. rank:	-1258.7

,

HA

Pos.	423	obs:	exp:
gaa	E	2687	1627.00
gag	E	31	1091.00
---	--	------	------
mPD		0.023	0.48
		nPD:	0.05
		N. weight:	0.52
		Sc. PD:	-0.032
		Sc. rank:	246.3

,

HA

Pos.	424	obs:	exp:
cgt	R	0	103.00
cgc	R	0	144.70
cga	R	0	263.10
cgg	R	4	212.80
aaa	K	0	0.56
aag	K	1	0.44
aga	R	18	1246.00
agg	R	2695	747.10
---	--	------	------
mPD		0.017	0.95
		nPD:	0.02
		N. weight:	1.4
		Sc. PD:	-0.13
		Sc. rank:	-1104.6

,

HA

Pos.	425	obs:	exp:
cgt	R	0	103.10
cgc	R	0	144.80
cga	R	0	263.20
cgg	R	0	212.90
aga	R	681	1247.00
agg	R	2037	747.40
---	--	------	------
mPD		0.38	0.95
		nPD:	0.39
		N. weight:	0.69
		Sc. PD:	0.19
		Sc. rank:	1178.7

,

HA

Pos.	426	obs:	exp:
tta	L	0	1.42
ttg	L	0	2.44
ctt	L	0	2.44
ctc	L	0	2.24
cta	L	13	1.73
ctg	L	0	2.73
att	I	0	1002.00
atc	I	1	676.90
ata	I	2698	1020.00
atg	M	6	6.00
---	--	------	------
mPD		0.015	0.67
		nPD:	0.02
		N. weight:	1.1
		Sc. PD:	-0.095
		Sc. rank:	-546.4

,

HA

Pos.	427	obs:	exp:
gct	A	0	0.51
gcc	A	0	0.39
gca	A	0	0.88
gcg	A	2	0.22
gaa	E	57	1626.00
gag	E	2659	1090.00
---	--	------	------
mPD		0.043	0.48
		nPD:	0.09
		N. weight:	0.92
		Sc. PD:	-0.019
		Sc. rank:	832.0

,

HA

Pos.	428	obs:	exp:
aat	N	2713	1444.00
aac	N	5	1274.00
---	--	------	------
mPD		0.0037	0.50
		nPD:	0.01
		N. weight:	0.71
		Sc. PD:	-0.07
		Sc. rank:	-1063.2

,

HA

Pos.	429	obs:	exp:
tta	L	703	296.70
ttg	L	2011	510.00
ctt	L	0	509.70
ctc	L	0	468.90
cta	L	4	361.60
ctg	L	0	571.10
---	--	------	------
mPD		0.39	1.1
		nPD:	0.34
		N. weight:	1.4
		Sc. PD:	0.32
		Sc. rank:	2295.6

,

HA

Pos.	430	obs:	exp:
aat	N	13	1443.00
aac	N	2703	1273.00
aaa	K	1	0.56
aag	K	0	0.44
gat	D	0	0.58
gac	D	1	0.42
---	--	------	------
mPD		0.011	0.50
		nPD:	0.02
		N. weight:	0.84
		Sc. PD:	-0.071
		Sc. rank:	-399.5

,

HA

Pos.	431	obs:	exp:
cgt	R	0	0.11
cgc	R	0	0.16
cga	R	0	0.29
cgg	R	0	0.23
aaa	K	4	1524.00
aag	K	2711	1191.00
aga	R	0	1.38
agg	R	3	0.82
---	--	------	------
mPD		0.0051	0.50
		nPD:	0.01
		N. weight:	0.93
		Sc. PD:	-0.09
		Sc. rank:	-1219.3

,

HA

Pos.	432	obs:	exp:
aaa	K	2672	1526.00
aag	K	46	1192.00
---	--	------	------
mPD		0.033	0.49
		nPD:	0.07
		N. weight:	0.57
		Sc. PD:	-0.023
		Sc. rank:	425.5

,

HA

Pos.	433	obs:	exp:
atg	M	2718	2718.00
---	--	------	------
mPD		0	0
		nPD:	1.
		N. weight:	0.
		Sc. PD:	0
		Sc. rank:	0

,

HA			
Pos.	434	obs:	exp:
gaa	E	2706	1627.00
gag	E	12	1091.00
---	--	------	------
mPD		0.0088	0.48
		nPD:	0.02
		N. weight:	0.56
		Sc. PD:	-0.05
		Sc. rank:	-401.9

,

HA			
Pos.	435	obs:	exp:
gat	D	15	1563.00
gac	D	2703	1155.00
---	--	------	------
mPD		0.011	0.49
		nPD:	0.02
		N. weight:	0.94
		Sc. PD:	-0.08
		Sc. rank:	-427.7

,

HA			
Pos.	436	obs:	exp:
ggt	G	3	391.00
ggc	G	3	358.80
gga	G	2395	1186.00
ggg	G	317	781.80
---	--	------	------
mPD		0.21	0.69
		nPD:	0.31
		N. weight:	0.58
		Sc. PD:	0.11
		Sc. rank:	868.7

,

HA			
Pos.	437	obs:	exp:
ttt	F	36	1157.00
ttc	F	2682	1561.00
---	--	------	------
mPD		0.026	0.49
		nPD:	0.05
		N. weight:	0.56
		Sc. PD:	-0.031
		Sc. rank:	353.0

,

HA			
Pos.	438	obs:	exp:
tta	L	11	296.70
ttg	L	0	510.00
ctt	L	0	509.70
ctc	L	1	468.90
cta	L	2701	361.60
ctg	L	5	571.10
---	--	------	------
mPD		0.012	1.1
		nPD:	0.01
		N. weight:	2.3
		Sc. PD:	-0.22
		Sc. rank:	-2781.1

,

HA			
Pos.	439	obs:	exp:
gat	D	2715	1563.00
gac	D	2	1154.00
gaa	E	0	0.60
gag	E	1	0.40
---	--	------	------
mPD		0.0022	0.49
		nPD:	0.
		N. weight:	0.63
		Sc. PD:	-0.064
		Sc. rank:	-1068.6

,

HA			
Pos.	440	obs:	exp:
gtt	V	16	591.10
gtc	V	2669	574.30
gta	V	33	534.80
gtg	V	0	1018.00
---	--	------	------
mPD		0.036	0.73
		nPD:	0.05
		N. weight:	1.7
		Sc. PD:	-0.099
		Sc. rank:	823.1

,

HA			
Pos.	441	obs:	exp:
tgg	W	2718	2718.00
---	--	------	------
mPD		0	0
		nPD:	1.
		N. weight:	0.
		Sc. PD:	0
		Sc. rank:	0

,

HA			
Pos.	442	obs:	exp:
act	T	701	720.00
acc	T	2017	600.50
aca	T	0	1168.00
acg	T	0	229.60
---	--	------	------
mPD		0.38	0.69
		nPD:	0.56
		N. weight:	1.
		Sc. PD:	0.44
		Sc. rank:	1933.8

,

HA			
Pos.	443	obs:	exp:
tat	Y	2649	1321.00
tac	Y	69	1397.00
---	--	------	------
mPD		0.050	0.50
		nPD:	0.1
		N. weight:	0.69
		Sc. PD:	-0.0073
		Sc. rank:	643.7

,

HA			
Pos.	444	obs:	exp:
aat	N	2715	1444.00
aac	N	3	1274.00
---	--	------	------
mPD		0.0022	0.50
		nPD:	0.
		N. weight:	0.72
		Sc. PD:	-0.073
		Sc. rank:	-1235.2

,

HA			
Pos.	445	obs:	exp:
gct	A	2708	691.40
gcc	A	0	530.00
gca	A	9	1195.00
gcg	A	1	301.70
---	--	------	------
mPD		0.0073	0.69
		nPD:	0.01
		N. weight:	1.5
		Sc. PD:	-0.15
		Sc. rank:	-1984.5

,

HA			
Pos.	446	obs:	exp:
gaa	E	2715	1627.00
gag	E	3	1091.00
---	--	------	------
mPD		0.0022	0.48
		nPD:	0.
		N. weight:	0.58
		Sc. PD:	-0.059
		Sc. rank:	-976.7

,

HA			
Pos.	447	obs:	exp:
tta	L	0	296.70
ttg	L	0	510.00
ctt	L	2716	509.70
ctc	L	2	468.90
cta	L	0	361.60
ctg	L	0	571.10
---	--	------	------
mPD		0.0015	1.1
		nPD:	0.
		N. weight:	1.9
		Sc. PD:	-0.2
		Sc. rank:	-3720.7

,

HA			
Pos.	448	obs:	exp:
tta	L	951	296.70
ttg	L	1	510.00
ctt	L	6	509.70
ctc	L	1	468.90
cta	L	1749	361.60
ctg	L	10	571.10
---	--	------	------
mPD		0.47	1.1
		nPD:	0.41
		N. weight:	1.6
		Sc. PD:	0.47
		Sc. rank:	2784.3

,

HA			
Pos.	449	obs:	exp:
att	I	1	0.37
atc	I	0	0.25
ata	I	0	0.38
gtt	V	2684	590.90
gtc	V	23	574.10
gta	V	2	534.60
gtg	V	8	1017.00
---	--	------	------
mPD		0.025	0.73
		nPD:	0.03
		N. weight:	1.7
		Sc. PD:	-0.12
		Sc. rank:	236.9

,

HA			
Pos.	450	obs:	exp:
tta	L	0	296.70
ttg	L	0	510.00
ctt	L	20	509.70
ctc	L	2695	468.90
cta	L	3	361.60
ctg	L	0	571.10
---	--	------	------
mPD		0.017	1.1
		nPD:	0.01
		N. weight:	2.
		Sc. PD:	-0.18
		Sc. rank:	-1822.2

,

HA			
Pos.	451	obs:	exp:
atg	M	2718	2718.00
---	--	------	------
mPD		0	0
		nPD:	1.
		N. weight:	0.
		Sc. PD:	0
		Sc. rank:	0

,

HA			
Pos.	452	obs:	exp:
aaa	K	1	0.56
aag	K	0	0.44
gaa	E	2713	1627.00
gag	E	4	1090.00
---	--	------	------
mPD		0.0037	0.48
		nPD:	0.01
		N. weight:	0.58
		Sc. PD:	-0.057
		Sc. rank:	-835.5

,

HA			
Pos.	453	obs:	exp:
aat	N	706	1444.00
aac	N	2012	1274.00
---	--	------	------
mPD		0.38	0.50
		nPD:	0.77
		N. weight:	0.18
		Sc. PD:	0.11
		Sc. rank:	357.2

,

HA			
Pos.	454	obs:	exp:
gaa	E	52	1627.00
gag	E	2666	1091.00
---	--	------	------
mPD		0.038	0.48
		nPD:	0.08
		N. weight:	0.93
		Sc. PD:	-0.029
		Sc. rank:	801.0

,

HA			
Pos.	455	obs:	exp:
cgt	R	0	103.10
cgc	R	0	144.80
cga	R	0	263.20
cgg	R	0	212.90
aga	R	685	1247.00
agg	R	2033	747.40
---	--	------	------
mPD		0.38	0.95
		nPD:	0.4
		N. weight:	0.69
		Sc. PD:	0.19
		Sc. rank:	1180.0

,

HA			
Pos.	456	obs:	exp:
act	T	2716	720.00
acc	T	2	600.50
aca	T	0	1168.00
acg	T	0	229.60
---	--	------	------
mPD		0.0015	0.69
		nPD:	0.
		N. weight:	1.5
		Sc. PD:	-0.16
		Sc. rank:	-2870.9

,

HA			
Pos.	457	obs:	exp:
tta	L	11	296.70
ttg	L	6	510.00
ctt	L	0	509.70
ctc	L	0	468.90
cta	L	2696	361.60
ctg	L	5	571.10
---	--	------	------
mPD		0.020	1.1
		nPD:	0.02
		N. weight:	2.3
		Sc. PD:	-0.2
		Sc. rank:	-1628.4

,

HA			
Pos.	458	obs:	exp:
gat	D	2650	1563.00
gac	D	68	1155.00
---	--	------	------
mPD		0.049	0.49
		nPD:	0.1
		N. weight:	0.51
		Sc. PD:	-0.005
		Sc. rank:	483.7

,

HA			
Pos.	459	obs:	exp:
ttt	F	44	1157.00
ttc	F	2674	1561.00
---	--	------	------
mPD		0.032	0.49
		nPD:	0.07
		N. weight:	0.55
		Sc. PD:	-0.024
		Sc. rank:	404.5

,

HA			
Pos.	460	obs:	exp:
cat	H	2717	1616.00
cac	H	1	1102.00
---	--	------	------
mPD		0.00074	0.48
		nPD:	0.
		N. weight:	0.59
		Sc. PD:	-0.062
		Sc. rank:	-1139.3

,

HA			
Pos.	461	obs:	exp:
gat	D	892	1563.00
gac	D	1826	1155.00
---	--	------	------
mPD		0.44	0.49
		nPD:	0.9
		N. weight:	0.14
		Sc. PD:	0.11
		Sc. rank:	293.4

,

HA			
Pos.	462	obs:	exp:
tct	S	0	464.00
tcc	S	4	400.50
tca	S	2693	612.50
tcg	S	20	175.50
agt	S	0	554.70
agc	S	0	509.80
gct	A	0	0.25
gcc	A	0	0.20
gca	A	1	0.44
gcg	A	0	0.11
---	--	------	------
mPD		0.018	1.6
		nPD:	0.01
		N. weight:	1.7
		Sc. PD:	-0.16
		Sc. rank:	-2016.3

,

HA			
Pos.	463	obs:	exp:
aat	N	2647	1444.00
aac	N	71	1274.00
---	--	------	------
mPD		0.051	0.50
		nPD:	0.1
		N. weight:	0.59
		Sc. PD:	-0.0045
		Sc. rank:	563.4

,

HA			
Pos.	464	obs:	exp:
gtt	V	5	591.10
gtc	V	2706	574.30
gta	V	7	534.80
gtg	V	0	1018.00
mPD		0.0088	0.73
		nPD:	0.01
		N. weight:	1.7
		Sc. PD:	-0.17
		Sc. rank:	-1948.8

HA			
Pos.	465	obs:	exp:
caa	Q	0	0.51
cag	Q	1	0.49
cgt	R	0	0.27
cgc	R	0	0.37
cga	R	0	0.68
cgg	R	0	0.55
aaa	K	203	1521.00
aag	K	2507	1189.00
aga	R	0	3.21
agg	R	7	1.92
mPD		0.14	0.50
		nPD:	0.29
		N. weight:	0.62
		Sc. PD:	0.11
		Sc. rank:	905.4

HA			
Pos.	466	obs:	exp:
tct	S	0	0.17
tcc	S	0	0.15
tca	S	0	0.23
tcg	S	0	0.06
aat	N	86	1443.00
aac	N	2629	1272.00
aaa	K	2	1.12
aag	K	0	0.88
agt	S	0	0.20
agc	S	1	0.19
mPD		0.063	0.50
		nPD:	0.13
		N. weight:	0.71
		Sc. PD:	0.011
		Sc. rank:	710.6

HA			
Pos.	467	obs:	exp:
tta	L	1	295.90
ttg	L	0	508.70
ctt	L	2684	508.40
ctc	L	14	467.70
cta	L	10	360.70
ctg	L	2	569.60
att	I	7	2.60
atc	I	0	1.76
ata	I	0	2.65
mPD		0.026	1.1
		nPD:	0.02
		N. weight:	1.8
		Sc. PD:	-0.16
		Sc. rank:	-814.3

HA			
Pos.	468	obs:	exp:
tat	Y	52	1321.00
tac	Y	2666	1397.00
mPD		0.038	0.50
		nPD:	0.08
		N. weight:	0.66
		Sc. PD:	-0.022
		Sc. rank:	550.2

HA			
Pos.	469	obs:	exp:
aat	N	0	2.13
aac	N	4	1.87
gat	D	15	1552.00
gac	D	2683	1146.00
gaa	E	15	9.58
gag	E	1	6.42
mPD		0.026	0.50
		nPD:	0.05
		N. weight:	0.94
		Sc. PD:	-0.053
		Sc. rank:	557.5

HA			
Pos.	470	obs:	exp:
cgt	R	0	0.08
cgc	R	0	0.11
cga	R	0	0.19
cgg	R	0	0.16
aaa	K	2624	1525.00
aag	K	92	1191.00
aga	R	2	0.92
agg	R	0	0.55
mPD		0.067	0.49
		nPD:	0.14
		N. weight:	0.5
		Sc. PD:	0.012
		Sc. rank:	532.8

HA			
Pos.	471	obs:	exp:
gtt	V	9	591.10
gtc	V	2708	574.30
gta	V	1	534.80
gtg	V	0	1018.00
mPD		0.0073	0.73
		nPD:	0.01
		N. weight:	1.8
		Sc. PD:	-0.17
		Sc. rank:	-2336.5

HA			
Pos.	472	obs:	exp:
cgt	R	0	103.00
cgc	R	0	144.70
cga	R	607	263.10
cgg	R	95	212.80
aaa	K	1	0.56
aag	K	0	0.44
aga	R	2008	1246.00
agg	R	7	747.10
mPD		0.46	0.95
		nPD:	0.48
		N. weight:	0.58
		Sc. PD:	0.21
		Sc. rank:	1035.4

HA			
Pos.	473	obs:	exp:
tta	L	107	296.70
ttg	L	1	510.00
ctt	L	35	509.70
ctc	L	9	468.90
cta	L	1456	361.60
ctg	L	1110	571.10
mPD		0.58	1.1
		nPD:	0.51
		N. weight:	1.1
		Sc. PD:	0.41
		Sc. rank:	1932.9

HA			
Pos.	474	obs:	exp:
caa	Q	4	1327.00
cag	Q	2604	1281.00
aaa	K	1	61.74
aag	K	109	48.26
mPD		0.081	0.58
		nPD:	0.14
		N. weight:	0.81
		Sc. PD:	0.024
		Sc. rank:	877.8

HA			
Pos.	475	obs:	exp:
tta	L	0	296.70
ttg	L	2	510.00
ctt	L	2639	509.70
ctc	L	70	468.90
cta	L	0	361.60
ctg	L	7	571.10
mPD		0.058	1.1
		nPD:	0.05
		N. weight:	1.8
		Sc. PD:	-0.1
		Sc. rank:	1000.9

HA			
Pos.	476	obs:	exp:
cgt	R	0	102.80
cgc	R	0	144.50
cga	R	0	262.60
cgg	R	0	212.40
aaa	K	0	3.37
aag	K	6	2.63
aga	R	18	1244.00
agg	R	2694	745.70
mPD		0.018	0.96
		nPD:	0.02
		N. weight:	1.4
		Sc. PD:	-0.13
		Sc. rank:	-1014.7

HA			
Pos.	477	obs:	exp:
gat	D	2679	1559.00
gac	D	32	1152.00
ggt	G	7	1.01
ggc	G	0	0.92
gga	G	0	3.06
ggg	G	0	2.01
mPD		0.028	0.50
		nPD:	0.06
		N. weight:	0.57
		Sc. PD:	-0.029
		Sc. rank:	379.1

HA			
Pos.	478	obs:	exp:
aat	N	2715	1444.00
aac	N	3	1274.00
mPD		0.0022	0.50
		nPD:	0.
		N. weight:	0.72
		Sc. PD:	-0.073
		Sc. rank:	-1235.2

HA			
Pos.	479	obs:	exp:
gct	A	1	691.40
gcc	A	0	530.00
gca	A	2711	1195.00
gcg	A	6	301.70
mPD		0.0051	0.69
		nPD:	0.01
		N. weight:	0.93
		Sc. PD:	-0.092
		Sc. rank:	-1368.2

HA			
Pos.	480	obs:	exp:
cgt	R	0	0.38
cgc	R	0	0.53
cga	R	0	0.97
cgg	R	0	0.78
aaa	K	115	1519.00
aag	K	2592	1188.00
aga	R	0	4.59
agg	R	10	2.75
gaa	E	0	0.60
gag	E	1	0.40
mPD		0.089	0.50
		nPD:	0.18
		N. weight:	0.74
		Sc. PD:	0.048
		Sc. rank:	884.7

HA			
Pos.	481	obs:	exp:
gaa	E	23	1627.00
gag	E	2695	1091.00
mPD		0.017	0.48
		nPD:	0.03
		N. weight:	0.99
		Sc. PD:	-0.072
		Sc. rank:	176.2

HA			
Pos.	482	obs:	exp:
tta	L	2	296.70
ttg	L	11	510.00
ctt	L	12	509.70
ctc	L	0	468.90
cta	L	10	361.60
ctg	L	2683	571.10
mPD		0.027	1.1
		nPD:	0.02
		N. weight:	1.7
		Sc. PD:	-0.14
		Sc. rank:	-625.4

HA			
Pos.	483	obs:	exp:
ggt	G	2682	391.00
ggc	G	31	358.80
gga	G	0	1186.00
ggg	G	5	781.80
mPD		0.026	0.69
		nPD:	0.04
		N. weight:	2.1
		Sc. PD:	-0.15
		Sc. rank:	612.0

HA			
Pos.	484	obs:	exp:
aat	N	723	1444.00
aac	N	1995	1274.00
mPD		0.39	0.50
		nPD:	0.78
		N. weight:	0.17
		Sc. PD:	0.11
		Sc. rank:	341.4

HA			
Pos.	485	obs:	exp:
ggt	G	1849	391.00
ggc	G	859	358.80
gga	G	9	1186.00
ggg	G	1	781.80
mPD		0.44	0.69
		nPD:	0.64
		N. weight:	1.5
		Sc. PD:	0.77
		Sc. rank:	2958.3

HA			
Pos.	486	obs:	exp:
tgt	C	2700	1182.00
tgc	C	18	1536.00
mPD		0.013	0.49
		nPD:	0.03
		N. weight:	0.91
		Sc. PD:	-0.073
		Sc. rank:	-243.7

HA			
Pos.	487	obs:	exp:
ttt	F	23	1156.00
ttc	F	2694	1561.00
tta	L	0	0.11
ttg	L	0	0.19
ctt	L	0	0.19
ctc	L	1	0.17
cta	L	0	0.13
ctg	L	0	0.21
mPD		0.018	0.49
		nPD:	0.04
		N. weight:	0.58
		Sc. PD:	-0.042
		Sc. rank:	119.8

HA			
Pos.	488	obs:	exp:
gaa	E	1175	1627.00
gag	E	1542	1090.00
---	-	1	1.00
mPD		0.49	0.48
		nPD:	1.02
		N. weight:	0.066
		Sc. PD:	0.058
		Sc. rank:	135.2

HA

Pos.	489	obs:	exp:
ttt	F	9	1157.00
ttc	F	2709	1561.00
---	--	------	------
mPD		0.0066	0.49
		nPD:	0.01
		N. weight:	0.61
		Sc. PD:	-0.057
		Sc. rank:	-656.9

,

HA

Pos.	490	obs:	exp:
tat	Y	2676	1313.00
tac	Y	26	1389.00
cat	H	15	8.92
cac	H	0	6.08
aat	N	1	0.53
aac	N	0	0.47
---	--	------	------
mPD		0.031	0.51
		nPD:	0.06
		N. weight:	0.76
		Sc. PD:	-0.037
		Sc. rank:	532.1

,

HA

Pos.	491	obs:	exp:
cat	H	1	1616.00
cac	H	2717	1102.00
---	--	------	------
mPD		0.00074	0.48
		nPD:	0.
		N. weight:	1.
		Sc. PD:	-0.11
		Sc. rank:	-1982.3

,

HA

Pos.	492	obs:	exp:
caa	Q	2	1.02
cag	Q	0	0.98
cgt	R	0	0.04
cgc	R	0	0.05
cga	R	0	0.10
cgg	R	0	0.08
act	T	0	0.26
acc	T	0	0.22
aca	T	1	0.43
acg	T	0	0.08
aaa	K	2693	1523.00
aag	K	21	1191.00
aga	R	1	0.46
agg	R	0	0.27
---	--	------	------
mPD		0.018	0.50
		nPD:	0.04
		N. weight:	0.61
		Sc. PD:	-0.043
		Sc. rank:	148.1

,

HA

Pos.	493	obs:	exp:
tgt	C	1845	1182.00
tgc	C	873	1536.00
---	--	------	------
mPD		0.44	0.49
		nPD:	0.89
		N. weight:	0.14
		Sc. PD:	0.11
		Sc. rank:	286.2

,

HA

Pos.	494	obs:	exp:
aat	N	44	23.38
aac	N	0	20.62
gat	D	2656	1538.00
gac	D	18	1136.00
---	--	------	------
mPD		0.045	0.52
		nPD:	0.09
		N. weight:	0.59
		Sc. PD:	-0.014
		Sc. rank:	523.7

,

HA

Pos.	495	obs:	exp:
act	T	1	0.26
acc	T	0	0.22
aca	T	0	0.43
acg	T	0	0.08
aat	N	2065	1439.00
aac	N	642	1268.00
aaa	K	1	0.56
aag	K	0	0.44
gat	D	9	5.18
gac	D	0	3.82
---	--	------	------
mPD		0.37	0.51
		nPD:	0.73
		N. weight:	0.14
		Sc. PD:	0.081
		Sc. rank:	269.7

,

HA

Pos.	496	obs:	exp:
aaa	K	27	15.16
aag	K	0	11.84
gtt	V	0	0.22
gtc	V	1	0.21
gta	V	0	0.20
gtg	V	0	0.37
gat	D	0	0.58
gac	D	1	0.42
gaa	E	2675	1604.00
gag	E	4	1075.00
ggt	G	0	1.44
ggc	G	0	1.32
gga	G	10	4.37
ggg	G	0	2.88
---	--	------	------
mPD		0.032	0.51
		nPD:	0.06
		N. weight:	0.58
		Sc. PD:	-0.026
		Sc. rank:	422.8

,

HA

Pos.	497	obs:	exp:
tgt	C	2596	1182.00
tgc	C	122	1536.00
---	--	------	------
mPD		0.086	0.49
		nPD:	0.17
		N. weight:	0.73
		Sc. PD:	0.046
		Sc. rank:	877.6

,

HA

Pos.	498	obs:	exp:
att	I	7	4.83
atc	I	0	3.26
ata	I	6	4.91
atg	M	2705	2705.00
---	--	------	------
mPD		0.0095	0.0095
		nPD:	1.
		N. weight:	0.0022
		Sc. PD:	0.0019
		Sc. rank:	4.6

,

HA

Pos.	499	obs:	exp:
gaa	E	2587	1615.00
gag	E	111	1083.00
ggt	G	0	2.88
ggc	G	0	2.64
gga	G	20	8.73
ggg	G	0	5.75
---	--	------	------
mPD		0.093	0.50
		nPD:	0.19
		N. weight:	0.42
		Sc. PD:	0.031
		Sc. rank:	520.0

,

HA

Pos.	500	obs:	exp:
tct	S	0	463.50
tcc	S	0	400.10
tca	S	0	611.80
tcg	S	0	175.30
aat	N	3	2.13
aac	N	1	1.87
agt	S	2067	554.10
agc	S	647	509.20
---	--	------	------
mPD		0.37	1.6
		nPD:	0.22
		N. weight:	1.2
		Sc. PD:	0.13
		Sc. rank:	1611.9

,

HA

Pos.	501	obs:	exp:
att	I	0	22.28
atc	I	1	15.05
ata	I	59	22.67
gtt	V	0	578.10
gtc	V	0	561.60
gta	V	624	523.00
gtg	V	2034	995.30
---	--	------	------
mPD		0.42	0.77
		nPD:	0.54
		N. weight:	0.68
		Sc. PD:	0.29
		Sc. rank:	1280.1

,

HA

Pos.	502	obs:	exp:
cgt	R	0	102.10
cgc	R	0	143.40
cga	R	0	260.70
cgg	R	0	210.80
aaa	K	26	14.59
aag	K	0	11.41
aga	R	2687	1235.00
agg	R	5	740.20
---	--	------	------
mPD		0.023	0.97
		nPD:	0.02
		N. weight:	0.88
		Sc. PD:	-0.074
		Sc. rank:	-354.6

,

HA

Pos.	503	obs:	exp:
aat	N	2689	1444.00
aac	N	29	1274.00
---	--	------	------
mPD		0.021	0.50
		nPD:	0.04
		N. weight:	0.66
		Sc. PD:	-0.043
		Sc. rank:	247.1

,

HA

Pos.	504	obs:	exp:
ggt	G	16	391.00
ggc	G	0	358.80
gga	G	33	1186.00
ggg	G	2669	781.80
---	--	------	------
mPD		0.036	0.69
		nPD:	0.05
		N. weight:	1.3
		Sc. PD:	-0.074
		Sc. rank:	792.9

,

HA

Pos.	505	obs:	exp:
act	T	1	720.00
acc	T	0	600.50
aca	T	30	1168.00
acg	T	2687	229.60
---	--	------	------
mPD		0.023	0.69
		nPD:	0.03
		N. weight:	2.7
		Sc. PD:	-0.2
		Sc. rank:	217.4

,

HA

Pos.	506	obs:	exp:
tat	Y	2709	1321.00
tac	Y	9	1397.00
---	--	------	------
mPD		0.0066	0.50
		nPD:	0.01
		N. weight:	0.8
		Sc. PD:	-0.075
		Sc. rank:	-875.0

,

HA

Pos.	507	obs:	exp:
tat	Y	0	174.50
tac	Y	359	184.50
aat	N	2	2.13
aac	N	2	1.87
gat	D	5	1355.00
gac	D	2350	1000.00
---	--	------	------
mPD		0.24	0.72
		nPD:	0.33
		N. weight:	0.94
		Sc. PD:	0.2
		Sc. rank:	1460.8

,

HA

Pos.	508	obs:	exp:
tct	S	0	0.17
tcc	S	0	0.15
tca	S	1	0.23
tcg	S	0	0.06
tat	Y	125	1321.00
tac	Y	2592	1396.00
agt	S	0	0.20
agc	S	0	0.19
---	--	------	------
mPD		0.089	0.50
		nPD:	0.18
		N. weight:	0.55
		Sc. PD:	0.036
		Sc. rank:	668.8

,

HA			
Pos.	509	obs:	exp:
tta	L	0	0.22
ttg	L	0	0.38
tct	S	4	0.68
tcc	S	0	0.59
tca	S	0	0.90
tcg	S	0	0.26
ctt	L	2	0.38
ctc	L	0	0.35
cta	L	0	0.27
ctg	L	0	0.42
cct	P	2670	764.20
ccc	P	25	490.40
cca	P	4	1106.00
ccg	P	11	348.90
act	T	2	0.53
acc	T	0	0.44
aca	T	0	0.86
acg	T	0	0.17
agt	S	0	0.82
agc	S	0	0.75
---	--	------	------
mPD		0.035	0.71
		nPD:	0.05
		N. weight:	1.4
		Sc. PD:	-0.08
		Sc. rank:	691.2

,

HA			
Pos.	510	obs:	exp:
tta	L	0	0.22
ttg	L	0	0.38
ctt	L	0	0.38
ctc	L	0	0.35
cta	L	0	0.27
ctg	L	2	0.42
cat	H	9	5.35
cac	H	0	3.65
caa	Q	14	1048.00
cag	Q	2045	1011.00
cgt	R	0	0.08
cgc	R	0	0.11
cga	R	0	0.19
cgg	R	1	0.16
aaa	K	2	362.60
aag	K	644	283.40
aga	R	0	0.92
agg	R	1	0.55
---	--	------	------
mPD		0.38	0.87
		nPD:	0.44
		N. weight:	0.81
		Sc. PD:	0.26
		Sc. rank:	1430.0

,

HA			
Pos.	511	obs:	exp:
tat	Y	2676	1321.00
tac	Y	41	1396.00
cat	H	1	0.59
cac	H	0	0.41
---	--	------	------
mPD		0.030	0.50
		nPD:	0.06
		N. weight:	0.74
		Sc. PD:	-0.035
		Sc. rank:	528.2

,

HA			
Pos.	512	obs:	exp:
tct	S	1	464.20
tcc	S	0	400.70
tca	S	2711	612.70
tcg	S	6	175.60
agt	S	0	554.90
agc	S	0	510.00
---	--	------	------
mPD		0.0051	1.6
		nPD:	0.
		N. weight:	1.7
		Sc. PD:	-0.17
		Sc. rank:	-3096.0

,

HA			
Pos.	513	obs:	exp:
aaa	K	4	2.25
aag	K	0	1.75
gtt	V	0	1.52
gtc	V	0	1.48
gta	V	7	1.38
gtg	V	0	2.62
gaa	E	2699	1617.00
gag	E	2	1084.00
ggt	G	0	0.86
ggc	G	0	0.79
gga	G	6	2.62
ggg	G	0	1.73
---	--	------	------
mPD		0.014	0.49
		nPD:	0.03
		N. weight:	0.59
		Sc. PD:	-0.046
		Sc. rank:	-104.6

,

HA			
Pos.	514	obs:	exp:
gat	D	0	0.58
gac	D	1	0.42
gaa	E	2708	1627.00
gag	E	9	1090.00
---	--	------	------
mPD		0.0073	0.48
		nPD:	0.02
		N. weight:	0.57
		Sc. PD:	-0.052
		Sc. rank:	-504.2

,

HA			
Pos.	515	obs:	exp:
tct	S	0	1.37
tcc	S	0	1.18
tca	S	8	1.80
tcg	S	0	0.52
act	T	0	6.36
acc	T	0	5.30
aca	T	24	10.31
acg	T	0	2.03
agt	S	0	1.63
agc	S	0	1.50
gct	A	4	683.30
gcc	A	0	523.80
gca	A	2681	1181.00
gcg	A	1	298.20
---	--	------	------
mPD		0.027	0.72
		nPD:	0.04
		N. weight:	0.93
		Sc. PD:	-0.065
		Sc. rank:	237.1

,

HA			
Pos.	516	obs:	exp:
cgt	R	0	93.37
cgc	R	0	131.10
cga	R	0	238.40
cgg	R	0	192.80
att	I	0	43.07
atc	I	0	29.09
ata	I	116	43.83
atg	M	1	1.00
aaa	K	25	14.03
aag	K	0	10.97
aga	R	2411	1129.00
agg	R	51	677.00
ggt	G	0	16.40
ggc	G	0	15.05
gga	G	96	49.76
ggg	G	18	32.79
---	--	------	------
mPD		0.23	1.1
		nPD:	0.21
		N. weight:	0.79
		Sc. PD:	0.075
		Sc. rank:	1022.9

,

HA			
Pos.	517	obs:	exp:
tta	L	2691	296.60
ttg	L	12	509.80
tct	S	0	0.17
tcc	S	0	0.15
tca	S	1	0.23
tcg	S	0	0.06
ctt	L	0	509.50
ctc	L	0	468.80
cta	L	5	361.50
ctg	L	9	570.90
agt	S	0	0.20
agc	S	0	0.19
---	--	------	------
mPD		0.026	1.1
		nPD:	0.02
		N. weight:	2.5
		Sc. PD:	-0.21
		Sc. rank:	-1023.6

,

HA			
Pos.	518	obs:	exp:
cgt	R	0	0.08
cgc	R	0	0.11
cga	R	0	0.19
cgg	R	0	0.16
att	I	0	0.37
atc	I	0	0.25
ata	I	1	0.38
aat	N	3	8.50
aac	N	13	7.50
aaa	K	2697	1514.00
aag	K	0	1183.00
aga	R	2	0.92
agg	R	0	0.55
gaa	E	2	1.20
gag	E	0	0.80
---	--	------	------
mPD		0.015	0.50
		nPD:	0.03
		N. weight:	0.66
		Sc. PD:	-0.051
		Sc. rank:	-29.6

,

HA			
Pos.	519	obs:	exp:
cgt	R	0	103.00
cgc	R	0	144.60
cga	R	1	262.90
cgg	R	0	212.60
att	I	0	0.37
atc	I	0	0.25
ata	I	1	0.38
aaa	K	2	1.12
aag	K	0	0.88
aga	R	2459	1245.00
agg	R	255	746.50
---	--	------	------
mPD		0.17	0.96
		nPD:	0.18
		N. weight:	0.59
		Sc. PD:	0.041
		Sc. rank:	731.1

,

HA			
Pos.	520	obs:	exp:
gaa	E	2673	1623.00
gag	E	37	1087.00
ggt	G	0	1.15
ggc	G	0	1.06
gga	G	8	3.49
ggg	G	0	2.30
---	--	------	------
mPD		0.033	0.49
		nPD:	0.07
		N. weight:	0.51
		Sc. PD:	-0.021
		Sc. rank:	381.8

,

HA			
Pos.	521	obs:	exp:
aaa	K	7	3.93
aag	K	0	3.07
gtt	V	0	0.22
gtc	V	0	0.21
gta	V	1	0.20
gtg	V	0	0.37
gaa	E	2707	1623.00
gag	E	3	1087.00
---	--	------	------
mPD		0.0081	0.49
		nPD:	0.02
		N. weight:	0.58
		Sc. PD:	-0.052
		Sc. rank:	-488.3

,

HA			
Pos.	522	obs:	exp:
att	I	1	1009.00
atc	I	1	681.40
ata	I	2715	1027.00
act	T	0	0.26
acc	T	0	0.22
aca	T	1	0.43
acg	T	0	0.08
---	--	------	------
mPD		0.0022	0.66
		nPD:	0.
		N. weight:	1.1
		Sc. PD:	-0.11
		Sc. rank:	-2007.0

,

HA			
Pos.	523	obs:	exp:
tct	S	0	425.80
tcc	S	0	367.50
tca	S	0	562.00
tcg	S	0	161.00
cgt	R	0	0.08
cgc	R	0	0.11
cga	R	0	0.19
cgg	R	0	0.16
att	I	0	0.37
atc	I	1	0.25
ata	I	0	0.38
aat	N	0	1.59
aac	N	3	1.41
agt	S	20	509.00
agc	S	2473	467.70
aga	R	2	0.92
agg	R	0	0.55
gat	D	0	5.75
gac	D	10	4.25
ggt	G	0	30.07
ggc	G	209	27.59
gga	G	0	91.24
ggg	G	0	60.11
---	--	------	------
mPD		0.17	1.8
		nPD:	0.1
		N. weight:	1.9
		Sc. PD:	-0.019
		Sc. rank:	1791.1

,

HA			
Pos.	524	obs:	exp:
ggt	G	1	391.00
ggc	G	1	358.80
gga	G	2685	1186.00
ggg	G	31	781.80
---	--	------	------
mPD		0.024	0.69
		nPD:	0.03
		N. weight:	0.88
		Sc. PD:	-0.064
		Sc. rank:	150.6

,

HA			
Pos.	525	obs:	exp:
tta	L	0	0.22
ttg	L	1	0.38
ctt	L	0	0.38
ctc	L	0	0.35
cta	L	0	0.27
ctg	L	1	0.42
gtt	V	3	590.70
gtc	V	10	573.90
gta	V	8	534.40
gtg	V	2695	1017.00
---	--	------	------
mPD		0.017	0.73
		nPD:	0.02
		N. weight:	1.1
		Sc. PD:	-0.09
		Sc. rank:	-465.1

,

HA			
Pos.	526	obs:	exp:
cgt	R	0	0.04
cgc	R	0	0.05
cga	R	0	0.10
cgg	R	0	0.08
aaa	K	2708	1523.00
aag	K	5	1190.00
aga	R	1	0.46
agg	R	0	0.27
gaa	E	4	2.40
gag	E	0	1.60
---	--	------	------
mPD		0.0073	0.50
		nPD:	0.01
		N. weight:	0.65
		Sc. PD:	-0.06
		Sc. rank:	-600.5

,

HA			
Pos.	527	obs:	exp:
ttt	F	1	0.85
ttc	F	1	1.15
tta	L	2433	296.30
ttg	L	273	509.50
ctt	L	0	509.10
ctc	L	0	468.40
cta	L	8	361.20
ctg	L	1	570.40
att	I	0	0.37
atc	I	0	0.25
ata	I	1	0.38
---	--	------	------
mPD		0.19	1.1
		nPD:	0.17
		N. weight:	2.1
		Sc. PD:	0.11
		Sc. rank:	2424.2

,

HA			
Pos.	528	obs:	exp:
gaa	E	2715	1627.00
gag	E	3	1091.00
---	--	------	------
mPD		0.0022	0.48
		nPD:	0.
		N. weight:	0.58
		Sc. PD:	-0.059
		Sc. rank:	-976.7

,

HA			
Pos.	529	obs:	exp:
tct	S	0	464.20
tcc	S	0	400.70
tca	S	2717	612.70
tcg	S	1	175.60
agt	S	0	554.90
agc	S	0	510.00
---	--	------	------
mPD		0.00074	1.6
		nPD:	0.
		N. weight:	1.7
		Sc. PD:	-0.18
		Sc. rank:	-3421.1

,

HA			
Pos.	530	obs:	exp:
tta	L	0	0.22
ttg	L	1	0.38
ctt	L	0	0.38
ctc	L	0	0.35
cta	L	1	0.27
ctg	L	0	0.42
att	I	1	999.60
atc	I	0	675.10
ata	I	2691	1017.00
atg	M	18	18.00
act	T	0	0.26
acc	T	0	0.22
aca	T	1	0.43
acg	T	0	0.08
gtt	V	0	1.09
gtc	V	0	1.06
gta	V	5	0.98
gtg	V	0	1.87
---	--	------	------
mPD		0.020	0.67
		nPD:	0.03
		N. weight:	1.1
		Sc. PD:	-0.085
		Sc. rank:	-6.7

,

HA			
Pos.	531	obs:	exp:
ggt	G	2	391.00
ggc	G	8	358.80
gga	G	2700	1186.00
ggg	G	8	781.80
---	--	------	------
mPD		0.013	0.69
		nPD:	0.02
		N. weight:	0.91
		Sc. PD:	-0.08
		Sc. rank:	-608.0

,

HA			
Pos.	532	obs:	exp:
tta	L	0	0.11
ttg	L	0	0.19
ctt	L	1	0.19
ctc	L	0	0.17
cta	L	0	0.13
ctg	L	0	0.21
cct	P	3	0.85
ccc	P	0	0.54
cca	P	0	1.22
ccg	P	0	0.39
att	I	12	4.46
atc	I	0	3.01
ata	I	0	4.53
act	T	2685	715.20
acc	T	11	596.60
aca	T	0	1160.00
acg	T	4	228.10
aat	N	1	0.53
aac	N	0	0.47
gct	A	1	0.25
gcc	A	0	0.20
gca	A	0	0.44
gcg	A	0	0.11
---	--	------	------
mPD		0.025	0.70
		nPD:	0.04
		N. weight:	1.5
		Sc. PD:	-0.11
		Sc. rank:	297.8

,

HA			
Pos.	533	obs:	exp:
tat	Y	14	1321.00
tac	Y	2704	1397.00
---	--	------	------
mPD		0.010	0.50
		nPD:	0.02
		N. weight:	0.73
		Sc. PD:	-0.063
		Sc. rank:	-443.6

,

HA			
Pos.	534	obs:	exp:
tta	L	0	0.11
ttg	L	0	0.19
ctt	L	0	0.19
ctc	L	0	0.17
cta	L	0	0.13
ctg	L	1	0.21
caa	Q	1823	1382.00
cag	Q	893	1334.00
aaa	K	1	0.56
aag	K	0	0.44
---	--	------	------
mPD		0.44	0.50
		nPD:	0.88
		N. weight:	0.065
		Sc. PD:	0.048
		Sc. rank:	131.5

,

HA			
Pos.	535	obs:	exp:
tta	L	2	0.22
ttg	L	0	0.38
ctt	L	0	0.38
ctc	L	0	0.35
cta	L	0	0.27
ctg	L	0	0.42
att	I	0	1009.00
atc	I	0	681.20
ata	I	2716	1026.00
---	--	------	------
mPD		0.0015	0.66
		nPD:	0.
		N. weight:	1.1
		Sc. PD:	-0.12
		Sc. rank:	-2104.8

,

HA			
Pos.	536	obs:	exp:
tta	L	1	295.40
ttg	L	38	507.80
ctt	L	1	507.40
ctc	L	2	466.90
cta	L	27	360.00
ctg	L	2637	568.60
cct	P	0	0.28
ccc	P	0	0.18
cca	P	0	0.41
ccg	P	1	0.13
atg	M	11	11.00
---	--	------	------
mPD		0.060	1.1
		nPD:	0.05
		N. weight:	1.6
		Sc. PD:	-0.091
		Sc. rank:	1012.7

,

HA			
Pos.	537	obs:	exp:
tct	S	0	464.20
tcc	S	2	400.70
tca	S	2716	612.70
tcg	S	0	175.60
agt	S	0	554.90
agc	S	0	510.00
---	--	------	------
mPD		0.0015	1.6
		nPD:	0.
		N. weight:	1.7
		Sc. PD:	-0.18
		Sc. rank:	-3393.9

,

HA			
Pos.	538	obs:	exp:
att	I	2703	1009.00
atc	I	13	681.40
ata	I	1	1027.00
gtt	V	1	0.22
gtc	V	0	0.21
gta	V	0	0.20
gtg	V	0	0.37
---	--	------	------
mPD		0.011	0.66
		nPD:	0.02
		N. weight:	1.1
		Sc. PD:	-0.099
		Sc. rank:	-918.2

HA			
Pos.	539	obs:	exp:
tat	Y	2705	1321.00
tac	Y	13	1397.00
---	--	------	------
mPD		0.0095	0.50
		nPD:	0.02
		N. weight:	0.79
		Sc. PD:	-0.07
		Sc. rank:	-546.4

HA			
Pos.	540	obs:	exp:
tct	S	0	464.00
tcc	S	0	400.50
tca	S	2686	612.50
tcg	S	31	175.50
cct	P	0	0.28
ccc	P	0	0.18
cca	P	1	0.41
ccg	P	0	0.13
agt	S	0	554.70
agc	S	0	509.80
---	--	------	------
mPD		0.023	1.6
		nPD:	0.01
		N. weight:	1.7
		Sc. PD:	-0.15
		Sc. rank:	-1649.7

HA			
Pos.	541	obs:	exp:
act	T	7	720.00
acc	T	4	600.50
aca	T	2703	1168.00
acg	T	4	229.60
---	--	------	------
mPD		0.011	0.69
		nPD:	0.02
		N. weight:	0.93
		Sc. PD:	-0.085
		Sc. rank:	-816.8

HA			
Pos.	542	obs:	exp:
atg	M	1	1.00
gtt	V	1	401.30
gtc	V	0	389.80
gta	V	3	363.00
gtg	V	1841	690.90
gct	A	0	221.60
gcc	A	0	169.90
gca	A	7	382.90
gcg	A	864	96.69
ggt	G	0	0.14
ggc	G	0	0.13
gga	G	0	0.44
ggg	G	1	0.29
---	--	------	------
mPD		0.44	1.2
		nPD:	0.38
		N. weight:	1.5
		Sc. PD:	0.4
		Sc. rank:	2617.3

HA			
Pos.	543	obs:	exp:
tct	S	0	0.17
tcc	S	0	0.15
tca	S	0	0.23
tcg	S	1	0.06
act	T	0	1.06
acc	T	0	0.88
aca	T	2	1.72
acg	T	2	0.34
agt	S	0	0.20
agc	S	0	0.19
gtt	V	0	1.96
gtc	V	0	1.90
gta	V	1	1.77
gtg	V	8	3.37
gct	A	0	687.90
gcc	A	0	527.30
gca	A	112	1189.00
gcg	A	2592	300.20
---	--	------	------
mPD		0.091	0.70
		nPD:	0.13
		N. weight:	2.3
		Sc. PD:	0.043
		Sc. rank:	2339.6

HA			
Pos.	544	obs:	exp:
tct	S	0	464.20
tcc	S	0	400.70
tca	S	0	612.70
tcg	S	0	175.60
agt	S	2695	554.90
agc	S	23	510.00
---	--	------	------
mPD		0.017	1.6
		nPD:	0.01
		N. weight:	1.8
		Sc. PD:	-0.17
		Sc. rank:	-2331.7

HA			
Pos.	545	obs:	exp:
tct	S	1	464.20
tcc	S	2716	400.70
tca	S	1	612.70
tcg	S	0	175.60
agt	S	0	554.90
agc	S	0	510.00
---	--	------	------
mPD		0.0015	1.6
		nPD:	0.
		N. weight:	2.2
		Sc. PD:	-0.23
		Sc. rank:	-4328.5

HA			
Pos.	546	obs:	exp:
tta	L	6	296.70
ttg	L	0	510.00
ctt	L	0	509.70
ctc	L	0	468.90
cta	L	2712	361.60
ctg	L	0	571.10
---	--	------	------
mPD		0.0044	1.1
		nPD:	0.
		N. weight:	2.3
		Sc. PD:	-0.24
		Sc. rank:	-4116.0

HA			
Pos.	547	obs:	exp:
tct	S	0	0.34
tcc	S	0	0.29
tca	S	2	0.45
tcg	S	0	0.13
act	T	0	2.65
acc	T	0	2.21
aca	T	10	4.30
acg	T	0	0.84
agt	S	0	0.41
agc	S	0	0.38
gtt	V	0	34.36
gtc	V	0	33.38
gta	V	157	31.09
gtg	V	1	59.17
gct	A	0	648.20
gcc	A	0	496.90
gca	A	2545	1120.00
gcg	A	3	282.90
---	--	------	------
mPD		0.12	0.82
		nPD:	0.15
		N. weight:	0.99
		Sc. PD:	0.036
		Sc. rank:	1103.3

HA			
Pos.	548	obs:	exp:
tta	L	0	296.70
ttg	L	0	510.00
ctt	L	0	509.70
ctc	L	0	468.90
cta	L	0	361.60
ctg	L	2718	571.10
---	--	------	------
mPD		0	1.1
		nPD:	0.
		N. weight:	1.8
		Sc. PD:	-0.19
		Sc. rank:	-3662.6

HA			
Pos.	549	obs:	exp:
gct	A	0	691.40
gcc	A	0	530.00
gca	A	2718	1195.00
gcg	A	0	301.70
---	--	------	------
mPD		0	0.69
		nPD:	0.
		N. weight:	0.94
		Sc. PD:	-0.1
		Sc. rank:	-1929.6

HA			
Pos.	550	obs:	exp:
att	I	0	1009.00
atc	I	2717	681.70
ata	I	1	1027.00
---	--	------	------
mPD		0.00074	0.66
		nPD:	0.
		N. weight:	1.6
		Sc. PD:	-0.17
		Sc. rank:	-3114.8

HA			
Pos.	551	obs:	exp:
att	I	3	241.40
atc	I	0	163.00
ata	I	647	245.60
atg	M	2068	2068.00
---	--	------	------
mPD		0.36	0.40
		nPD:	0.91
		N. weight:	0.26
		Sc. PD:	0.2
		Sc. rank:	530.3

HA			
Pos.	552	obs:	exp:
tta	L	0	0.33
ttg	L	3	0.56
ctt	L	0	0.56
ctc	L	0	0.52
cta	L	0	0.40
ctg	L	0	0.63
att	I	0	7.06
atc	I	0	4.77
ata	I	19	7.18
atg	M	872	872.00
act	T	0	0.53
acc	T	0	0.44
aca	T	0	0.86
acg	T	2	0.17
gtt	V	0	395.60
gtc	V	0	384.30
gta	V	2	357.90
gtg	V	1817	681.20
gct	A	0	0.25
gcc	A	0	0.20
gca	A	0	0.44
gcg	A	1	0.11
ggt	G	2	0.29
ggc	G	0	0.26
gga	G	0	0.87
ggg	G	0	0.58
---	--	------	------
mPD		0.46	1.1
		nPD:	0.44
		N. weight:	0.76
		Sc. PD:	0.24
		Sc. rank:	1343.5

HA			
Pos.	553	obs:	exp:
gct	A	2718	691.40
gcc	A	0	530.00
gca	A	0	1195.00
gcg	A	0	301.70
---	--	------	------
mPD		0	0.69
		nPD:	0.
		N. weight:	1.6
		Sc. PD:	-0.17
		Sc. rank:	-3213.6

,

HA			
Pos.	554	obs:	exp:
tct	S	0	0.34
tcc	S	0	0.29
tca	S	0	0.45
tcg	S	0	0.13
agt	S	2	0.41
agc	S	0	0.38
ggt	G	2714	390.70
ggc	G	2	358.50
gga	G	0	1186.00
ggg	G	0	781.20
---	--	------	------
mPD		0.0029	0.69
		nPD:	0.
		N. weight:	2.2
		Sc. PD:	-0.23
		Sc. rank:	-3836.4

,

HA			
Pos.	555	obs:	exp:
tta	L	8	296.60
ttg	L	0	509.80
ctt	L	1	509.50
ctc	L	0	468.80
cta	L	2674	361.50
ctg	L	34	570.90
att	I	0	0.37
atc	I	0	0.25
ata	I	1	0.38
---	--	------	------
mPD		0.032	1.1
		nPD:	0.03
		N. weight:	2.2
		Sc. PD:	-0.17
		Sc. rank:	-358.4

,

HA			
Pos.	556	obs:	exp:
tct	S	2704	464.20
tcc	S	13	400.70
tca	S	0	612.70
tcg	S	1	175.60
agt	S	0	554.90
agc	S	0	510.00
---	--	------	------
mPD		0.010	1.6
		nPD:	0.01
		N. weight:	2.
		Sc. PD:	-0.2
		Sc. rank:	-3134.1

,

HA			
Pos.	557	obs:	exp:
ttt	F	9	5.96
ttc	F	5	8.04
tta	L	2696	295.00
ttg	L	5	507.20
ctt	L	0	506.90
ctc	L	0	466.40
cta	L	2	359.60
ctg	L	0	567.90
att	I	0	0.37
atc	I	0	0.25
ata	I	1	0.38
---	--	------	------
mPD		0.016	1.1
		nPD:	0.01
		N. weight:	2.5
		Sc. PD:	-0.23
		Sc. rank:	-2528.3

,

HA			
Pos.	558	obs:	exp:
tgg	W	2717	2717.00
cgt	R	0	0.04
cgc	R	0	0.05
cga	R	0	0.10
cgg	R	0	0.08
aga	R	0	0.46
agg	R	1	0.27
---	--	------	------
mPD		0.00074	0.0012
		nPD:	0.61
		N. weight:	0.00055
		Sc. PD:	0.00026
		Sc. rank:	1.1

,

HA			
Pos.	559	obs:	exp:
atg	M	2717	2717.00
aaa	K	0	0.56
aag	K	1	0.44
---	--	------	------
mPD		0.00074	0.0011
		nPD:	0.64
		N. weight:	0.00035
		Sc. PD:	0.00018
		Sc. rank:	0.7

,

HA			
Pos.	560	obs:	exp:
tgt	C	316	1182.00
tgc	C	2402	1536.00
---	--	------	------
mPD		0.21	0.49
		nPD:	0.42
		N. weight:	0.28
		Sc. PD:	0.083
		Sc. rank:	486.1

,

HA			
Pos.	561	obs:	exp:
ttt	F	1	1.28
ttc	F	2	1.72
tct	S	640	463.00
tcc	S	2070	399.70
tca	S	1	611.10
tcg	S	0	175.10
cct	P	0	0.28
ccc	P	1	0.18
cca	P	0	0.41
ccg	P	0	0.13
agt	S	0	553.50
agc	S	0	508.60
gct	A	0	0.76
gcc	A	3	0.59
gca	A	0	1.32
gcg	A	0	0.33
---	--	------	------
mPD		0.37	1.6
		nPD:	0.22
		N. weight:	1.5
		Sc. PD:	0.17
		Sc. rank:	2010.6

,

HA			
Pos.	562	obs:	exp:
aat	N	2716	1444.00
aac	N	1	1273.00
gat	D	1	0.58
gac	D	0	0.42
---	--	------	------
mPD		0.0015	0.50
		nPD:	0.
		N. weight:	0.72
		Sc. PD:	-0.075
		Sc. rank:	-1346.1

,

HA			
Pos.	563	obs:	exp:
tgg	W	1	1.00
ggt	G	0	390.90
ggc	G	0	358.60
gga	G	27	1186.00
ggg	G	2690	781.50
---	--	------	------
mPD		0.020	0.69
		nPD:	0.03
		N. weight:	1.4
		Sc. PD:	-0.11
		Sc. rank:	-123.5

,

HA			
Pos.	564	obs:	exp:
tta	L	0	0.11
ttg	L	1	0.19
tct	S	9	463.80
tcc	S	1	400.40
tca	S	49	612.20
tcg	S	2657	175.40
ctt	L	0	0.19
ctc	L	0	0.17
cta	L	0	0.13
ctg	L	0	0.21
cct	P	0	0.28
ccc	P	0	0.18
cca	P	0	0.41
ccg	P	1	0.13
agt	S	0	554.50
agc	S	0	509.60
---	--	------	------
mPD		0.044	1.6
		nPD:	0.03
		N. weight:	3.
		Sc. PD:	-0.24
		Sc. rank:	-736.0

,

HA			
Pos.	565	obs:	exp:
tta	L	2512	296.70
ttg	L	17	510.00
ctt	L	0	509.70
ctc	L	0	468.90
cta	L	189	361.60
ctg	L	0	571.10
---	--	------	------
mPD		0.14	1.1
		nPD:	0.12
		N. weight:	2.2
		Sc. PD:	0.032
		Sc. rank:	2195.8

,

HA			
Pos.	566	obs:	exp:
caa	Q	674	1383.00
cag	Q	2044	1335.00
---	--	------	------
mPD		0.37	0.50
		nPD:	0.75
		N. weight:	0.16
		Sc. PD:	0.1
		Sc. rank:	330.8

,

HA			
Pos.	567	obs:	exp:
tgt	C	4	1182.00
tgc	C	2714	1536.00
---	--	------	------
mPD		0.0029	0.49
		nPD:	0.01
		N. weight:	0.64
		Sc. PD:	-0.065
		Sc. rank:	-1040.5

,

HA			
Pos.	568	obs:	exp:
cct	P	0	0.28
ccc	P	0	0.18
cca	P	1	0.41
ccg	P	0	0.13
cgt	R	0	103.00
cgc	R	0	144.60
cga	R	6	262.90
cgg	R	0	212.60
aaa	K	2	1.12
aag	K	0	0.88
aga	R	2707	1245.00
agg	R	2	746.50
---	--	------	------
mPD		0.0088	0.96
		nPD:	0.01
		N. weight:	0.87
		Sc. PD:	-0.085
		Sc. rank:	-1204.6

,

HA			
Pos.	569	obs:	exp:
att	I	2682	996.30
atc	I	1	672.90
ata	I	0	1014.00
gtt	V	35	7.61
gtc	V	0	7.40
gta	V	0	6.89
gtg	V	0	13.11
---	--	------	------
mPD		0.026	0.69
		nPD:	0.04
		N. weight:	1.1
		Sc. PD:	-0.079
		Sc. rank:	341.0

,

HA			
Pos.	570	obs:	exp:
tgt	C	12	1181.00
tgc	C	2705	1536.00
ggt	G	0	0.14
ggc	G	1	0.13
gga	G	0	0.44
ggg	G	0	0.29
---	--	------	------
mPD		0.0095	0.49
		nPD:	0.02
		N. weight:	0.63
		Sc. PD:	-0.055
		Sc. rank:	-405.4

,

HA			
Pos.	571	obs:	exp:
tta	L	0	0.11
ttg	L	0	0.19
ctt	L	1	0.19
ctc	L	0	0.17
cta	L	0	0.13
ctg	L	0	0.21
att	I	2063	1008.00
atc	I	650	680.90
ata	I	2	1026.00
gtt	V	2	0.43
gtc	V	0	0.42
gta	V	0	0.39
gtg	V	0	0.75
---	--	------	------
mPD		0.37	0.66
		nPD:	0.56
		N. weight:	0.61
		Sc. PD:	0.26
		Sc. rank:	1152.7

,

HA			
Pos.	572	obs:	exp:
taa	.	2708	996.70
tag	.	9	762.80
tga	.	1	958.50
---	--	------	------
mPD		0.0073	0.86
		nPD:	0.01
		N. weight:	1.1
		Sc. PD:	-0.11
		Sc. rank:	-1581.1

}

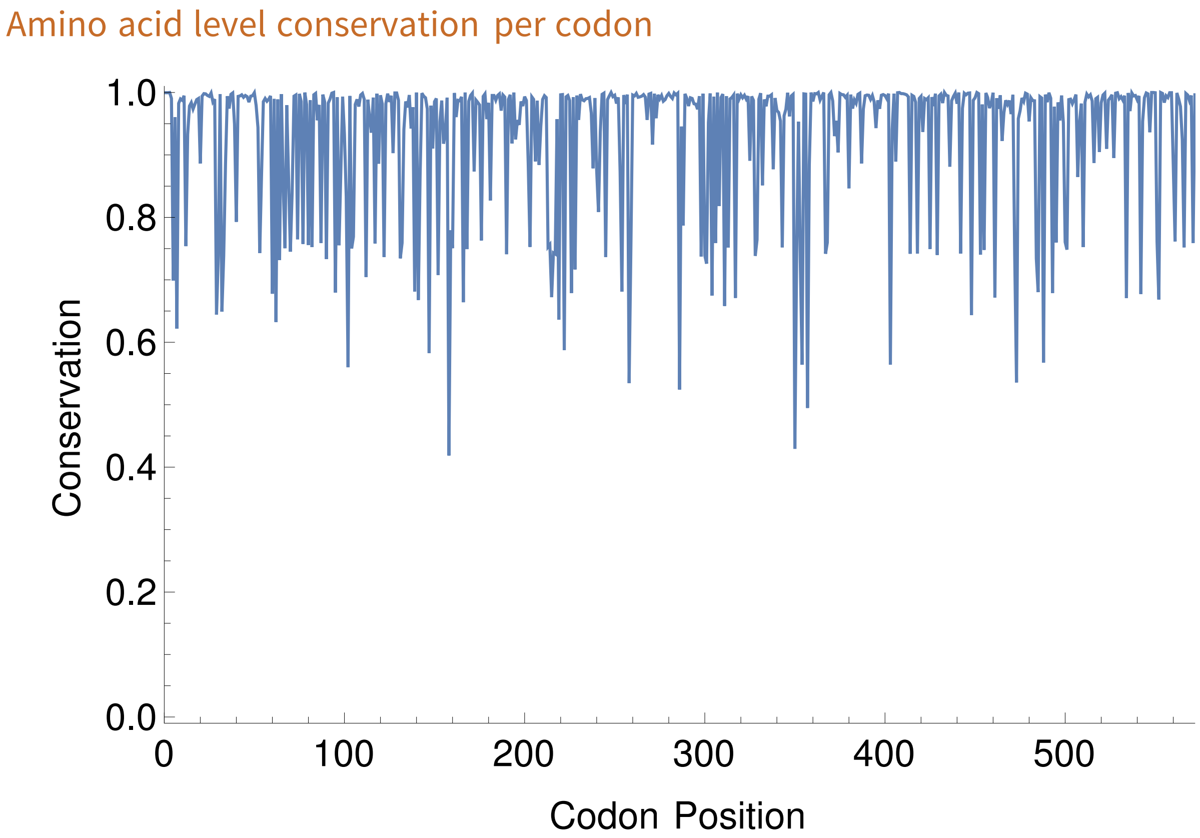
Amino acid level conservation per codon
Conservation
1.0
0.8
0.6
0.4
0.2
0.0
0
100
200
300
400
500
Codon Position

Conservation analysis plots

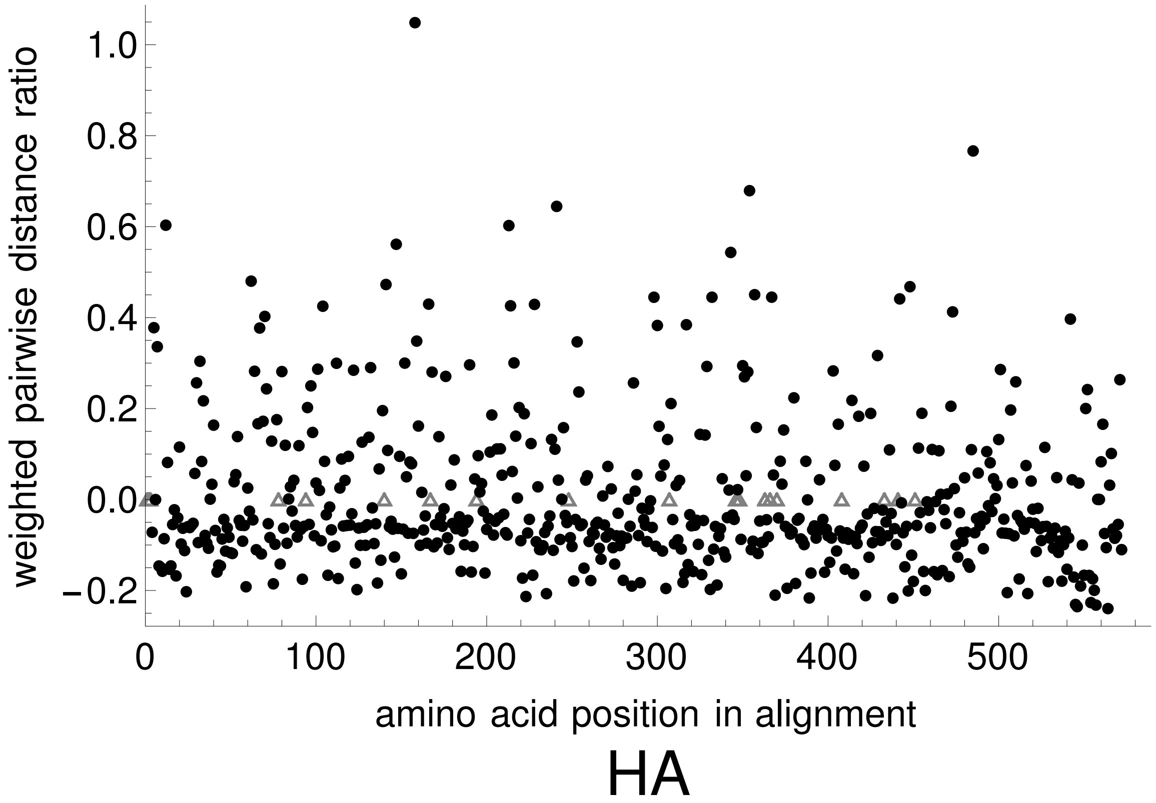

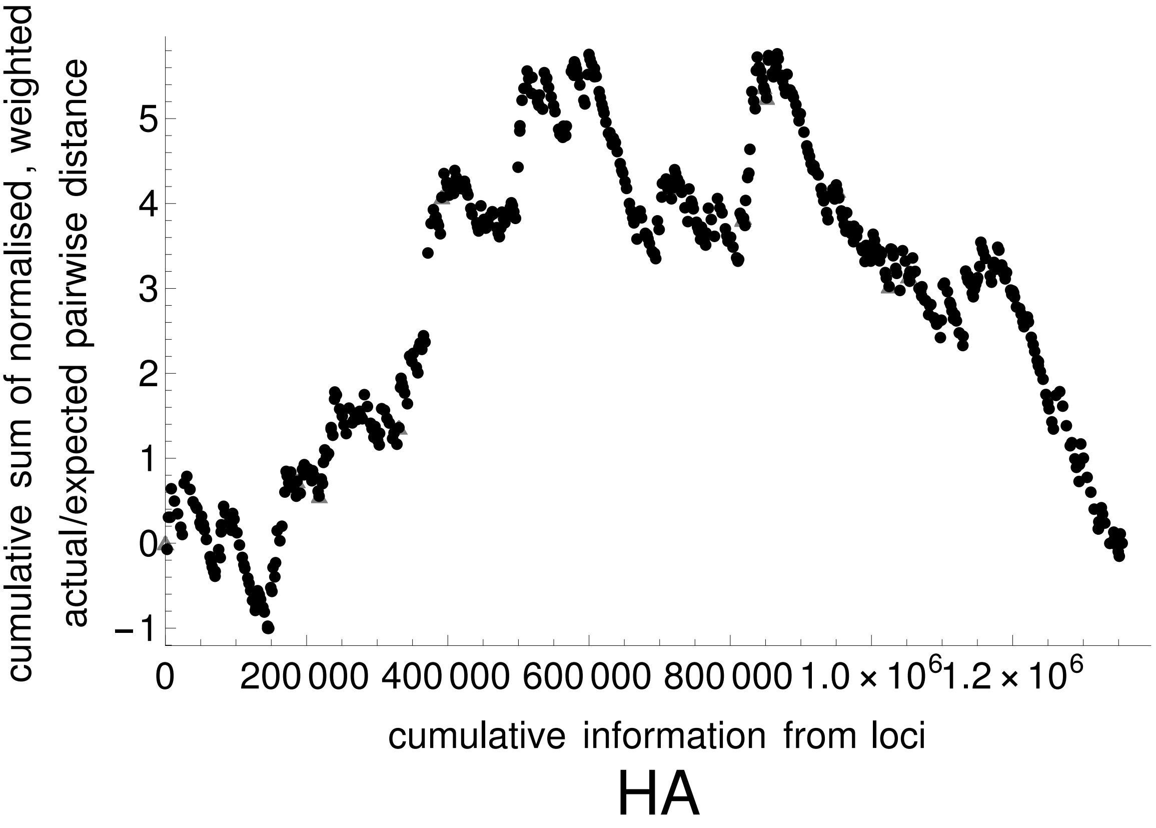

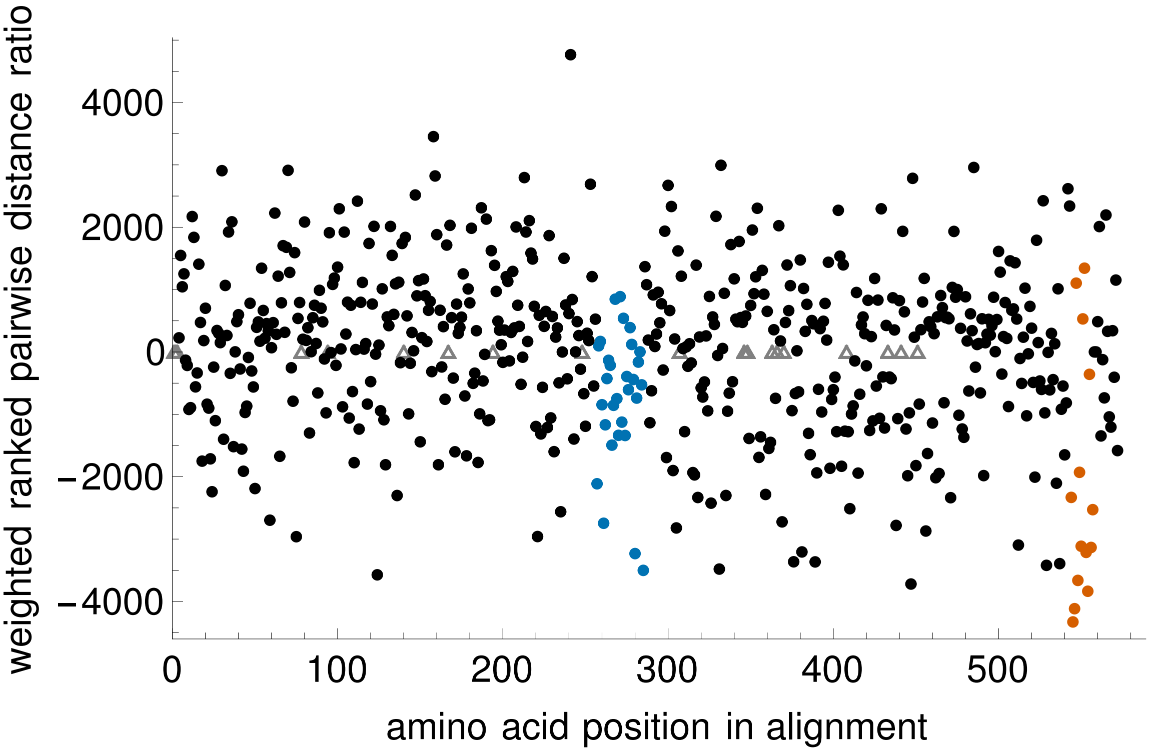
weighted ranked pairwise distance ratio
4000
2000
0
−2000
−4000
0
100
200
300
400
500
amino acid position in alignment

HA

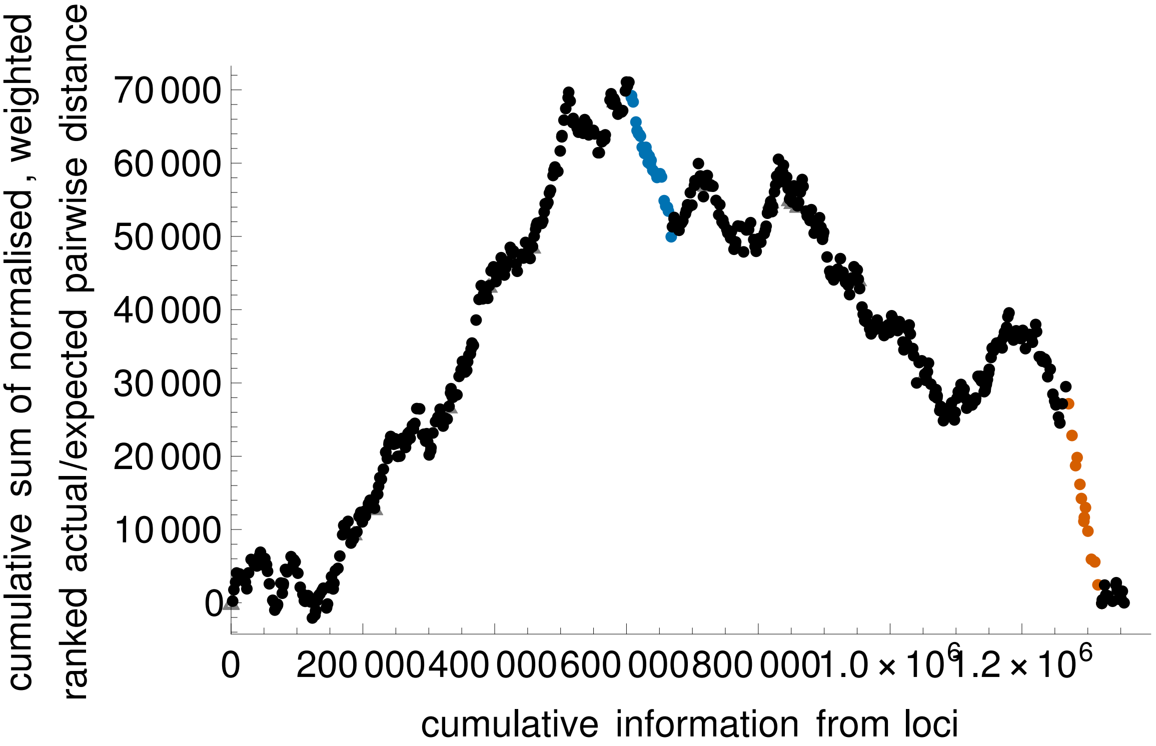
cumulative sum of normalised, weighted ranked actual/expected pairwise distance
70 000
60 000
50 000
40 000
30 000
20 000
10 000
0
0
200 000
400 000
600 000
800 000
1.0 × 10^6
1.2 × 10^6
cumulative information from loci

HA

NP

Gene length histogram

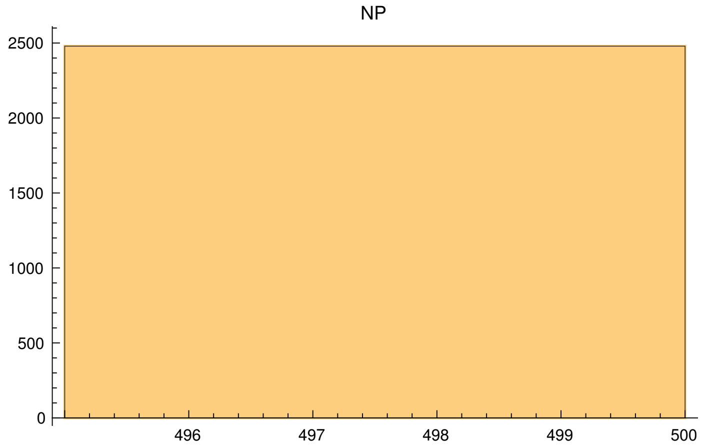

Information vs. nPD

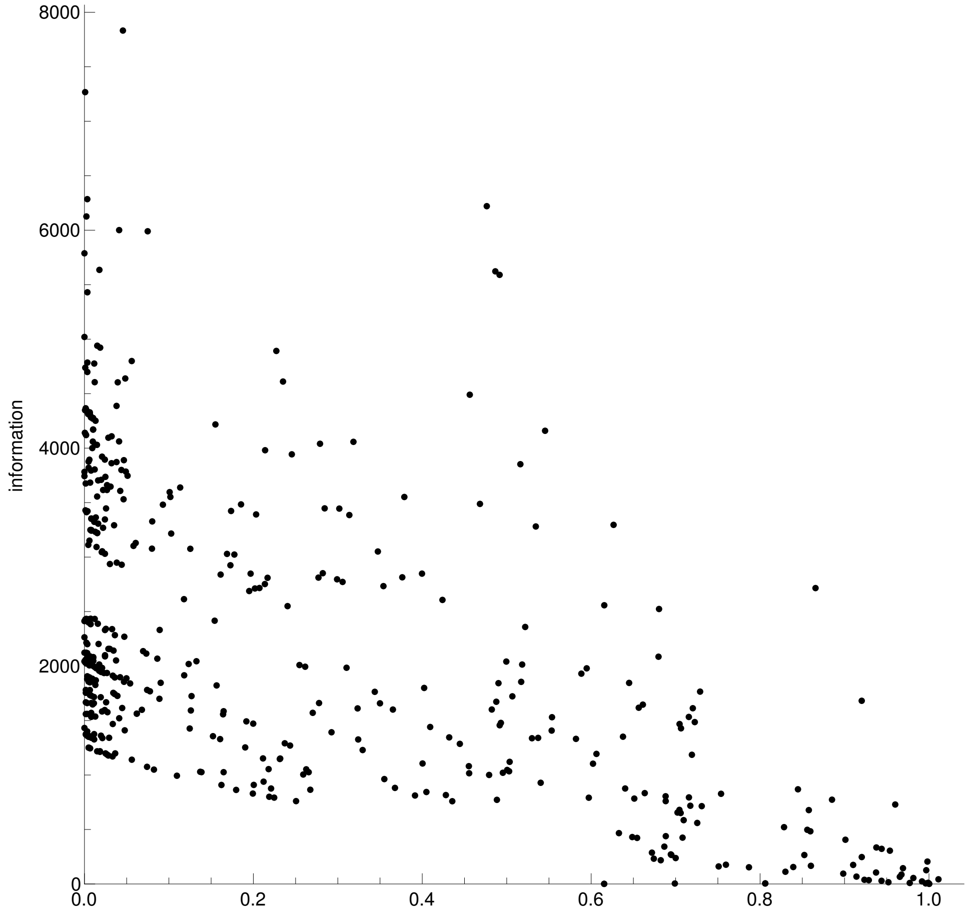

NP

Example sequences highlighted by regions found in analyses to be conserved

Interesting points (by weighted raw PD) highlighted for gene NP:

ATG

GCGTCTCAAGGCACCAAACGATCTTATGAACAGATGGAAACTGGTGGAGAGCGCC

AGAATGCCACTGAGATCAGAGCATCTGTTGGAAGAATGGTTGGTGGAATTGG

AAGGTTTTACATACAGATGTGCACTGAACTCAAACTCAGCGACTATGAAGGA

AGGCTGATTCAAAACAGCATAACAATAGAGAGAATGGTTCTCTCTGCATTTG

ACGAAAGGAGGAACAAATACCTGGAAGAACATCCCAGTGCGGGGAAGGACCCGAAGA⋱

AAACTGGAGGTCCAATTTATCGAAGGAGAGATGGGAAATGGGTGAGGGAACTGATCCTGTATGACAAAGA⋱

GGAGATCAGGAGGATCTGGCGTCAAGCGAACAATGGAGAAGACGCAACTGCTGGTCTCACTCACCTGATG⋱

ATCTGGCATTCCAATCTAAATGATGCCACATACCAGAGGACAAGAGCTCTCGTGCGTACTGGGATGGACC⋱

CCAGAATGTGCTCTCTTATGCAAGGATCAACTCTCCCAAGGAGATCTGGAGCTGCTGGTGCAGCAGTAAA⋱

GGGAGTCGGAACGATGGTGATGGAACTAATTCGGATGATAAAGCGAGGAATTAATGATCGGAACTTCTGG⋱

AGAGGCGAGAATGGACGAAGGACAAGGATTGCATATGAGAGAATGTGCAACATCCTCAAGGGGAAATTCC⋱

AAACAGCAGCACAAAGAGCAATGATGGACCAGGTTCGTGAAAGCAGGAATCCTGGCAATGCTGAAATTGA⋱

AGATCTCATTTTTCTAGCACGGTCTGCACTCATCCTGAGAGGGTCAGTGGCCCATAAGTCCTGCTTGCCT⋱

GCTTGTGTGTACGGACTTGCTGTGGCCAGTGGGTATGACTTTGAGAGAGAAGGGTACTCTCTAGTTGGAA⋱

TAGATCCTTTCCGTCTGCTTCAAAACAGCCAGGTCTTCAGTCTCATTAGACCAAATGAGAATCCAGCACA⋱

CAAGAGTCAATTGGTGTGGATGGCATGTCATTCTGCAGCATTCGAGGATCTGAGAGTCTCAAGTTTCATC⋱

AGAGGGTCAAGAGTAGTCCCAAGAGGACAACTATCCACCAGAGGAGTTCAAATTGCTTCAAATGAGAACA⋱

TG

GAAACGATGGACTCCAGCACTCTTGAACTAAGAAGCAGATACTGGGCTATAAGAA

CCAGGAGTGGAGGAAACACCAACCAACAGAGAGCATCTGCAGGACAAATCAG

TGTACAGCCCACTTTCTCAGTACAGAGAAATCTTCCCTTTGAGAGAGCGACCATTATGGCGGCGTTTACA⋱

GGGAATACTGAGGGCAGAACATCT

GACATGAGGACTGAAATCATAAGAATGATGGAAAGTGCCAGACCAGAAGATGTGT

CTTTCCAGGGGCGGGGAGTCTTCGAGCTCTCAGACGAAAAGGCAACGAACCC

GATCGTGCCTTCCTTTGACATGAGTAATGAAGGATCTTATTTCTTCGGAGAC

AATGCAGAGGAGTATGACAATTAA

Interesting points (by weighted ranked PD) highlighted for gene NP:

ATG

GCGTCTCAAGGCACCAAACGATCTTATGAACAGATGGAAACTGGTGGAGAGCGCCAGAATGCCACTGA⋱

GATCAGAGCATCTGTTGGAAGAATGGTTGGTGGAATTGGAAGGTTTTACATACAGATGTGCACTGAACTC⋱

AAACTCAGCGACTATGAAGGAAGGCTGATTCAAAACAGCATAACAATAGAGAGAATGGTTCTCTCTGCAT⋱

TTGACGAAAGGAGGAACAAATACCTGGAAGAACATCCCAGTGCGGGGAAGGACCCGAAGAAAACTGGAGG⋱

TCCAATTTATCGAAGGAGAGATGGGAAATGGGTGAGGGAACTGATCCTGTATGACAAAGAGGAGATCAGG⋱

AGGATCTGGCGTCAAGCGAACAATGGAGAAGACGCAACTGCTGGTCTCACTCACCTGATGATCTGGCATT⋱

CCAATCTAAATGATGCCACATACCAGAGGACAAGAGCTCTCGTGCGTACTGGGATGGACCCCAGAATGTG⋱

CTCTCTTATGCAAGGATCAACTCTCCCAAGGAGATCTGGAGCTGCTGGTGCAGCAGTAAAGGGAGTCGGA⋱

ACGATGGTGATGGAACTAATTCGGATGATAAAGCGAGGAATTAATGATCGGAACTTCTGGAGAGGCGAGA⋱

ATGGACGAAGGACAAGGATTGCATATGAGAGAATGTGCAACATCCTCAAGGGGAAATTCCAAACAGCAGC⋱

ACAAAGAGCAATGATGGACCAGGTTCGTGAAAGCAGGAATCCTGGCAATGCTGAAATTGAAGATCTCATT⋱

TTTCTAGCACGGTCTGCACTCATCCTGAGAGGGTCAGTGGCCCATAAGTCCTGCTTGCCTGCTTGTGTGT⋱

ACGGACTTGCTGTGGCCAGTGGGTATGACTTTGAGAGAGAAGGGTACTCTCTAGTTGGAATAGATCCTTT⋱

CCGTCTGCTTCAAAACAGCCAGGTCTTCAGTCTCATTAGACCAAATGAGAATCCAGCACACAAGAGTCAA⋱
TTGGTGTGGATGGCATGTCATTCTGCAGCATTCGAGGATCTGAGAGTCTCAAGTTTCATCAGAGGGTCAA⋱
GAGTAGTCCCAAGAGGACAACTATCCACCAGAGGAGTTCAAATTGCTTCAAATGAGAACATGGAAACGAT⋱
GGACTCCAGCACTCTTGAACTAAGAAGCAGATACTGGGCTATAAGAACCAGGAGTGGAGGAAACACCAAC⋱
CAACAGAGAGCATCTGCAGGACAAATCAGTGTACAGCCCACTTTCTCAGTACAGAGAAATCTTCCCTTTG⋱
AGAGAGCGACCATTATGGCGGCGTTTACAGGGAATACTGAGGGCAGAACATCTGACATGAGGACTGAAAT⋱
CATAAGAATGATGGAAAGTGCCAGACCAGAAGATGTGTCTTTCCAGGGGCGGGGAGTCTTCGAGCTCTCA
GACGAAAAGGCAACGAACCCGATCGTGCCTTCCTTTGACATGAGTAATGAAGGAT
CTTATTTCTTCGGAGACAATGCAGAGGAGTATGACAATTAA

Per codon conservation report

NP

Pos.	1	obs:	exp:
atg	M	2479	2479.00
---	--	------	------
mPD		0	0
		nPD:	1.
		N. weight:	0.
		Sc. PD:	0
		Sc. rank:	0

NP

Pos.	2	obs:	exp:
gct	A	1	630.40
gcc	A	0	483.20
gca	A	1	1089.00
gcg	A	2476	275.10
ggt	G	0	0.14
ggc	G	0	0.13
gga	G	0	0.44
ggg	G	1	0.29
---	--	------	------
mPD		0.0024	0.69
		nPD:	0.
		N. weight:	2.6
		Sc. PD:	-0.34
		Sc. rank:	-3384.3

NP

Pos.	3	obs:	exp:
ttt	F	1	0.43
ttc	F	0	0.57
tct	S	2458	422.90
tcc	S	18	365.00
tca	S	0	558.10
tcg	S	0	159.90
tat	Y	2	0.97
tac	Y	0	1.03
agt	S	0	505.50
agc	S	0	464.50
---	--	------	------
mPD		0.017	1.6
		nPD:	0.01
		N. weight:	2.1
		Sc. PD:	-0.25
		Sc. rank:	-1435.9

NP

Pos.	4	obs:	exp:
cat	H	0	1.19
cac	H	2	0.81
caa	Q	2473	1260.00
cag	Q	4	1217.00
---	--	------	------
mPD		0.0048	0.50
		nPD:	0.01
		N. weight:	0.79
		Sc. PD:	-0.098
		Sc. rank:	-598.7

NP

Pos.	5	obs:	exp:
ggt	G	0	356.60
ggc	G	2479	327.20
gga	G	0	1082.00
ggg	G	0	713.00
---	--	------	------
mPD		0	0.69
		nPD:	0.
		N. weight:	2.4
		Sc. PD:	-0.32
		Sc. rank:	-4328.5

NP

Pos.	6	obs:	exp:
act	T	0	656.70
acc	T	2479	547.70
aca	T	0	1065.00
acg	T	0	209.40
---	--	------	------
mPD		0	0.69
		nPD:	0.
		N. weight:	1.8
		Sc. PD:	-0.24
		Sc. rank:	-3227.3

NP

Pos.	7	obs:	exp:
aaa	K	2479	1391.00
aag	K	0	1088.00
---	--	------	------
mPD		0	0.49
		nPD:	0.
		N. weight:	0.69
		Sc. PD:	-0.091
		Sc. rank:	-1234.4

NP

Pos.	8	obs:	exp:
cgt	R	0	94.01
cgc	R	0	132.00
cga	R	2479	240.00
cgg	R	0	194.20
aga	R	0	1137.00
agg	R	0	681.60
---	--	------	------
mPD		0	0.95
		nPD:	0.
		N. weight:	2.8
		Sc. PD:	-0.37
		Sc. rank:	-4990.6

NP

Pos.	9	obs:	exp:
tct	S	2452	422.70
tcc	S	22	364.90
tca	S	1	557.90
tcg	S	0	159.90
cct	P	4	1.13
ccc	P	0	0.72
cca	P	0	1.63
ccg	P	0	0.52
agt	S	0	505.30
agc	S	0	464.40
---	--	------	------
mPD		0.022	1.6
		nPD:	0.01
		N. weight:	2.
		Sc. PD:	-0.25
		Sc. rank:	-999.8

NP

Pos.	10	obs:	exp:
tat	Y	2475	1204.00
tac	Y	1	1272.00
cat	H	3	1.78
cac	H	0	1.22
---	--	------	------
mPD		0.0032	0.50
		nPD:	0.01
		N. weight:	0.86
		Sc. PD:	-0.11
		Sc. rank:	-900.2

NP

Pos.	11	obs:	exp:
gat	D	0	0.58
gac	D	1	0.42
gaa	E	2459	1484.00
gag	E	19	994.30
---	--	------	------
mPD		0.016	0.48
		nPD:	0.03
		N. weight:	0.56
		Sc. PD:	-0.057
		Sc. rank:	51.1

NP

Pos.	12	obs:	exp:
caa	Q	39	1261.00
cag	Q	2440	1218.00
---	--	------	------
mPD		0.031	0.50
		nPD:	0.06
		N. weight:	0.75
		Sc. PD:	-0.055
		Sc. rank:	363.1

NP

Pos.	13	obs:	exp:
atg	M	2479	2479.00
---	--	------	------
mPD		0	0
		nPD:	1.
		N. weight:	0.
		Sc. PD:	0
		Sc. rank:	0

NP

Pos.	14	obs:	exp:
gat	D	1	1.15
gac	D	1	0.85
gaa	E	2337	1483.00
gag	E	140	993.90
---	--	------	------
mPD		0.11	0.48
		nPD:	0.22
		N. weight:	0.38
		Sc. PD:	0.032
		Sc. rank:	371.2

NP

Pos.	15	obs:	exp:
act	T	2383	634.90
acc	T	14	529.60
aca	T	0	1030.00
acg	T	0	202.50
gtt	V	3	0.65
gtc	V	0	0.63
gta	V	0	0.59
gtg	V	0	1.12
gct	A	79	20.10
gcc	A	0	15.41
gca	A	0	34.73
gcg	A	0	8.77
---	--	------	------
mPD		0.078	0.76
		nPD:	0.1
		N. weight:	1.5
		Sc. PD:	-0.052
		Sc. rank:	1000.5

NP

Pos.	16	obs:	exp:
tct	S	0	54.99
tcc	S	0	47.47
tca	S	0	72.58
tcg	S	0	20.80
aat	N	1	0.53
aac	N	0	0.47
agt	S	322	65.74
agc	S	0	60.41
gat	D	46	26.46
gac	D	0	19.54
ggt	G	2107	303.40
ggc	G	0	278.40
gga	G	0	920.60
ggg	G	2	606.60
---	-	1	1.00
---	--	------	------
mPD		0.27	1.1
		nPD:	0.24
		N. weight:	2.2
		Sc. PD:	0.21
		Sc. rank:	2250.8

NP

Pos.	17	obs:	exp:
ggt	G	3	356.60
ggc	G	0	327.20
gga	G	1400	1082.00
ggg	G	1076	713.00
---	--	------	------
mPD		0.49	0.69
		nPD:	0.72
		N. weight:	0.38
		Sc. PD:	0.21
		Sc. rank:	657.3

NP

Pos.	18	obs:	exp:
gat	D	0	1.73
gac	D	3	1.27
gaa	E	879	1483.00
gag	E	1597	993.50
---	--	------	------
mPD		0.46	0.48
		nPD:	0.95
		N. weight:	0.15
		Sc. PD:	0.12
		Sc. rank:	262.9

NP

Pos.	19	obs:	exp:
tgt	C	0	0.43
tgc	C	1	0.57
cgt	R	0	93.97
cgc	R	2478	132.00
cga	R	0	239.90
cgg	R	0	194.10
aga	R	0	1137.00
agg	R	0	681.40
---	--	------	------
mPD		0.00081	0.95
		nPD:	0.
		N. weight:	3.5
		Sc. PD:	-0.46
		Sc. rank:	-5767.1

NP

Pos.	20	obs:	exp:
caa	Q	4	1261.00
cag	Q	2475	1218.00
---	--	------	------
mPD		0.0032	0.50
		nPD:	0.01
		N. weight:	0.83
		Sc. PD:	-0.11
		Sc. rank:	-872.1

NP	Pos. 21	obs:	exp:
tct	S	0	0.17
tcc	S	0	0.15
tca	S	0	0.23
tcg	S	0	0.06
aat	N	2477	1317.00
aac	N	1	1161.00
agt	S	1	0.20
agc	S	0	0.19
mPD		0.0016	0.50
		nPD:	0.
		N. weight:	0.75
		Sc. PD:	-0.097
		Sc. rank:	-1049.9

NP	Pos. 22	obs:	exp:
gct	A	159	630.60
gcc	A	2320	483.40
gca	A	0	1090.00
gcg	A	0	275.20
mPD		0.12	0.69
		nPD:	0.17
		N. weight:	1.6
		Sc. PD:	0.057
		Sc. rank:	1359.1

NP	Pos. 23	obs:	exp:
act	T	2466	656.70
acc	T	3	547.70
aca	T	0	1065.00
acg	T	10	209.40
mPD		0.010	0.69
		nPD:	0.02
		N. weight:	1.5
		Sc. PD:	-0.18
		Sc. rank:	-583.1

NP	Pos. 24	obs:	exp:
gaa	E	861	1484.00
gag	E	1618	994.70
mPD		0.45	0.48
		nPD:	0.94
		N. weight:	0.15
		Sc. PD:	0.12
		Sc. rank:	277.5

NP	Pos. 25	obs:	exp:
att	I	893	917.20
atc	I	1577	619.50
ata	I	0	933.30
gtt	V	0	1.96
gtc	V	9	1.90
gta	V	0	1.77
gtg	V	0	3.37
mPD		0.47	0.66
		nPD:	0.7
		N. weight:	0.71
		Sc. PD:	0.38
		Sc. rank:	1201.0

NP	Pos. 26	obs:	exp:
cgt	R	0	93.90
cgc	R	0	131.90
cga	R	0	239.80
cgg	R	0	193.90
aaa	K	3	1.68
aag	K	0	1.32
aga	R	2468	1136.00
agg	R	8	680.80
mPD		0.0089	0.96
		nPD:	0.01
		N. weight:	0.91
		Sc. PD:	-0.11
		Sc. rank:	-739.2

NP	Pos. 27	obs:	exp:
gtt	V	0	0.43
gtc	V	0	0.42
gta	V	2	0.39
gtg	V	0	0.75
gct	A	2	630.10
gcc	A	2	483.00
gca	A	2467	1089.00
gcg	A	6	275.00
mPD		0.0097	0.69
		nPD:	0.01
		N. weight:	0.95
		Sc. PD:	-0.11
		Sc. rank:	-441.9

NP	Pos. 28	obs:	exp:
tct	S	1596	423.40
tcc	S	1	365.50
tca	S	32	558.80
tcg	S	850	160.10
agt	S	0	506.10
agc	S	0	465.10
mPD		0.47	1.6
		nPD:	0.28
		N. weight:	1.7
		Sc. PD:	0.23
		Sc. rank:	1879.0

NP	Pos. 29	obs:	exp:
gtt	V	2479	539.10
gtc	V	0	523.80
gta	V	0	487.80
gtg	V	0	928.30
mPD		0	0.73
		nPD:	0.
		N. weight:	1.8
		Sc. PD:	-0.24
		Sc. rank:	-3261.1

NP	Pos. 30	obs:	exp:
ggt	G	0	356.60
ggc	G	0	327.20
gga	G	2450	1082.00
ggg	G	29	713.00
mPD		0.023	0.69
		nPD:	0.03
		N. weight:	0.92
		Sc. PD:	-0.092
		Sc. rank:	94.2

NP	Pos. 31	obs:	exp:
cgt	R	0	94.01
cgc	R	0	132.00
cga	R	1	240.00
cgg	R	0	194.20
aga	R	2166	1137.00
agg	R	312	681.60
mPD		0.22	0.95
		nPD:	0.23
		N. weight:	0.55
		Sc. PD:	0.05
		Sc. rank:	553.1

NP	Pos. 32	obs:	exp:
atg	M	2479	2479.00
mPD		0	0
		nPD:	1.
		N. weight:	0.
		Sc. PD:	0
		Sc. rank:	0

NP	Pos. 33	obs:	exp:
att	I	6	2.23
atc	I	0	1.50
ata	I	0	2.27
gtt	V	2440	537.80
gtc	V	33	522.50
gta	V	0	486.60
gtg	V	0	926.10
mPD		0.031	0.73
		nPD:	0.04
		N. weight:	1.7
		Sc. PD:	-0.16
		Sc. rank:	484.5

NP	Pos. 34	obs:	exp:
tct	S	0	0.34
tcc	S	0	0.29
tca	S	0	0.45
tcg	S	0	0.13
agt	S	1	0.41
agc	S	1	0.38
gat	D	4	2.30
gac	D	0	1.70
ggt	G	2470	355.70
ggc	G	2	326.40
gga	G	1	1080.00
ggg	G	0	711.30
mPD		0.0081	0.70
		nPD:	0.01
		N. weight:	2.3
		Sc. PD:	-0.28
		Sc. rank:	-1386.4

NP	Pos. 35	obs:	exp:
ggt	G	0	356.60
ggc	G	2	327.20
gga	G	2469	1082.00
ggg	G	8	713.00
mPD		0.0080	0.69
		nPD:	0.01
		N. weight:	0.96
		Sc. PD:	-0.12
		Sc. rank:	-561.0

NP	Pos. 36	obs:	exp:
att	I	2366	920.50
atc	I	113	621.70
ata	I	0	936.70
mPD		0.087	0.66
		nPD:	0.13
		N. weight:	0.98
		Sc. PD:	-0.005
		Sc. rank:	707.7

NP	Pos. 37	obs:	exp:
ggt	G	0	356.60
ggc	G	0	327.20
gga	G	521	1082.00
ggg	G	1958	713.00
mPD		0.33	0.69
		nPD:	0.48
		N. weight:	0.77
		Sc. PD:	0.26
		Sc. rank:	1079.8

NP	Pos. 38	obs:	exp:
cgt	R	0	94.01
cgc	R	0	132.00
cga	R	0	240.00
cgg	R	0	194.20
aga	R	29	1137.00
agg	R	2450	681.60
mPD		0.023	0.95
		nPD:	0.02
		N. weight:	1.5
		Sc. PD:	-0.16
		Sc. rank:	-170.3

NP	Pos. 39	obs:	exp:
ttt	F	977	1055.00
ttc	F	1502	1424.00
mPD		0.48	0.49
		nPD:	0.98
		N. weight:	0.0044
		Sc. PD:	0.0036
		Sc. rank:	7.9

NP	Pos. 40	obs:	exp:
tat	Y	504	1205.00
tac	Y	1975	1274.00
mPD		0.32	0.50
		nPD:	0.65
		N. weight:	0.21
		Sc. PD:	0.1
		Sc. rank:	340.3

NP	Pos. 41	obs:	exp:
att	I	0	918.70
atc	I	0	620.50
ata	I	2474	934.80
gtt	V	0	1.09
gtc	V	0	1.06
gta	V	5	0.98
gtg	V	0	1.87
mPD		0.0040	0.66
		nPD:	0.01
		N. weight:	1.2
		Sc. PD:	-0.15
		Sc. rank:	-1263.9

NP	Pos. 42	obs:	exp:
caa	Q	5	1261.00
cag	Q	2474	1218.00
mPD		0.0040	0.50
		nPD:	0.01
		N. weight:	0.83
		Sc. PD:	-0.1
		Sc. rank:	-734.3

NP	Pos. 43	obs:	exp:
atg	M	2479	2479.00
mPD		0	0
		nPD:	1.
		N. weight:	0.
		Sc. PD:	0
		Sc. rank:	0

NP	Pos. 44	obs:	exp:
tgt	C	13	1078.00
tgc	C	2466	1401.00
mPD		0.010	0.49
		nPD:	0.02
		N. weight:	0.64
		Sc. PD:	-0.072
		Sc. rank:	-121.4

NP	Pos. 45	obs:	exp:
act	T	2473	656.70
acc	T	3	547.70
aca	T	3	1065.00
acg	T	0	209.40
mPD		0.0048	0.69
		nPD:	0.01
		N. weight:	1.6
		Sc. PD:	-0.2
		Sc. rank:	-1547.1

NP			
Pos.	46	obs:	exp:
gaa	E	988	1484.00
gag	E	1491	994.70
---	--	------	------
mPD		0.48	0.48
		nPD:	1.
		N. weight:	0.099
		Sc. PD:	0.082
		Sc. rank:	177.5

NP			
Pos.	47	obs:	exp:
tta	L	0	270.60
ttg	L	0	465.20
ctt	L	4	464.80
ctc	L	2473	427.70
cta	L	2	329.80
ctg	L	0	520.90
---	--	------	------
mPD		0.0048	1.1
		nPD:	0.
		N. weight:	2.1
		Sc. PD:	-0.27
		Sc. rank:	-2583.1

NP			
Pos.	48	obs:	exp:
caa	Q	1	0.51
cag	Q	0	0.49
cgt	R	0	0.34
cgc	R	0	0.48
cga	R	0	0.87
cgg	R	0	0.70
aaa	K	2301	1386.00
aag	K	168	1083.00
aga	R	9	4.13
agg	R	0	2.47
---	--	------	------
mPD		0.13	0.50
		nPD:	0.27
		N. weight:	0.42
		Sc. PD:	0.052
		Sc. rank:	447.7

NP			
Pos.	49	obs:	exp:
ttt	F	0	1.28
ttc	F	3	1.72
tta	L	0	270.30
ttg	L	0	464.60
ctt	L	0	464.30
ctc	L	2476	427.20
cta	L	0	329.40
ctg	L	0	520.20
---	--	------	------
mPD		0.0024	1.1
		nPD:	0.
		N. weight:	2.1
		Sc. PD:	-0.27
		Sc. rank:	-3083.0

NP			
Pos.	50	obs:	exp:
tct	S	0	422.00
tcc	S	0	364.30
tca	S	0	557.00
tcg	S	0	159.60
cgt	R	0	0.15
cgc	R	0	0.21
cga	R	0	0.39
cgg	R	0	0.31
aat	N	0	1.59
aac	N	3	1.41
agt	S	59	504.50
agc	S	2412	463.60
aga	R	4	1.83
agg	R	0	1.10
ggt	G	0	0.14
ggc	G	1	0.13
gga	G	0	0.44
ggg	G	0	0.29
---	--	------	------
mPD		0.053	1.6
		nPD:	0.03
		N. weight:	1.9
		Sc. PD:	-0.19
		Sc. rank:	119.5

NP			
Pos.	51	obs:	exp:
aat	N	44	53.14
aac	N	56	46.86
gat	D	611	1321.00
gac	D	1686	975.70
gaa	E	81	49.10
gag	E	1	32.90
---	--	------	------
mPD		0.51	0.60
		nPD:	0.86
		N. weight:	0.24
		Sc. PD:	0.17
		Sc. rank:	421.0

NP			
Pos.	52	obs:	exp:
tat	Y	2419	1182.00
tac	Y	13	1250.00
cat	H	46	27.35
cac	H	0	18.65
aat	N	1	0.53
aac	N	0	0.47
---	--	------	------
mPD		0.048	0.54
		nPD:	0.09
		N. weight:	0.82
		Sc. PD:	-0.039
		Sc. rank:	477.6

NP			
Pos.	53	obs:	exp:
gaa	E	1967	1484.00
gag	E	512	994.70
---	--	------	------
mPD		0.33	0.48
		nPD:	0.68
		N. weight:	0.1
		Sc. PD:	0.055
		Sc. rank:	176.0

NP			
Pos.	54	obs:	exp:
ggt	G	0	356.60
ggc	G	0	327.20
gga	G	1867	1082.00
ggg	G	612	713.00
---	--	------	------
mPD		0.37	0.69
		nPD:	0.54
		N. weight:	0.45
		Sc. PD:	0.17
		Sc. rank:	693.5

NP			
Pos.	55	obs:	exp:
cgt	R	0	94.01
cgc	R	0	132.00
cga	R	0	240.00
cgg	R	0	194.20
aga	R	1566	1137.00
agg	R	913	681.60
---	--	------	------
mPD		0.47	0.95
		nPD:	0.49
		N. weight:	0.37
		Sc. PD:	0.13
		Sc. rank:	531.1

NP			
Pos.	56	obs:	exp:
tta	L	0	270.60
ttg	L	26	465.20
ctt	L	0	464.80
ctc	L	0	427.70
cta	L	12	329.80
ctg	L	2441	520.90
---	--	------	------
mPD		0.030	1.1
		nPD:	0.03
		N. weight:	1.8
		Sc. PD:	-0.19
		Sc. rank:	-26.8

NP			
Pos.	57	obs:	exp:
att	I	133	919.40
atc	I	2340	621.00
ata	I	3	935.60
gtt	V	0	0.65
gtc	V	3	0.63
gta	V	0	0.59
gtg	V	0	1.12
---	--	------	------
mPD		0.11	0.66
		nPD:	0.16
		N. weight:	1.4
		Sc. PD:	0.031
		Sc. rank:	1058.9

NP			
Pos.	58	obs:	exp:
cat	H	0	0.59
cac	H	1	0.41
caa	Q	243	1261.00
cag	Q	2235	1217.00
---	--	------	------
mPD		0.18	0.50
		nPD:	0.36
		N. weight:	0.46
		Sc. PD:	0.097
		Sc. rank:	579.5

NP			
Pos.	59	obs:	exp:
aat	N	3	1317.00
aac	N	2476	1162.00
---	--	------	------
mPD		0.0024	0.50
		nPD:	0.
		N. weight:	0.89
		Sc. PD:	-0.11
		Sc. rank:	-1090.6

NP			
Pos.	60	obs:	exp:
tct	S	0	423.40
tcc	S	0	365.50
tca	S	0	558.80
tcg	S	0	160.10
agt	S	3	506.10
agc	S	2476	465.10
---	--	------	------
mPD		0.0024	1.6
		nPD:	0.
		N. weight:	2.
		Sc. PD:	-0.26
		Sc. rank:	-3146.7

NP			
Pos.	61	obs:	exp:
att	I	0	892.30
atc	I	1	602.70
ata	I	2402	908.00
atg	M	76	76.00
---	--	------	------
mPD		0.060	0.68
		nPD:	0.09
		N. weight:	1.1
		Sc. PD:	-0.053
		Sc. rank:	664.3

NP			
Pos.	62	obs:	exp:
act	T	0	656.70
acc	T	6	547.70
aca	T	2471	1065.00
acg	T	2	209.40
---	--	------	------
mPD		0.0064	0.69
		nPD:	0.01
		N. weight:	0.98
		Sc. PD:	-0.12
		Sc. rank:	-762.7

NP			
Pos.	63	obs:	exp:
att	I	4	919.80
atc	I	0	621.20
ata	I	2473	936.00
atg	M	1	1.00
gtt	V	0	0.22
gtc	V	0	0.21
gta	V	1	0.20
gtg	V	0	0.37
---	--	------	------
mPD		0.0048	0.66
		nPD:	0.01
		N. weight:	1.1
		Sc. PD:	-0.14
		Sc. rank:	-1099.1

NP			
Pos.	64	obs:	exp:
gaa	E	493	1484.00
gag	E	1986	994.70
---	--	------	------
mPD		0.32	0.48
		nPD:	0.66
		N. weight:	0.4
		Sc. PD:	0.2
		Sc. rank:	666.5

NP			
Pos.	65	obs:	exp:
cgt	R	0	94.01
cgc	R	0	132.00
cga	R	0	240.00
cgg	R	0	194.20
aga	R	2471	1137.00
agg	R	8	681.60
---	--	------	------
mPD		0.0064	0.95
		nPD:	0.01
		N. weight:	0.91
		Sc. PD:	-0.11
		Sc. rank:	-919.0

NP			
Pos.	66	obs:	exp:
atg	M	2479	2479.00
---	--	------	------
mPD		0	0
		nPD:	1.
		N. weight:	0.
		Sc. PD:	0
		Sc. rank:	0

NP			
Pos.	67	obs:	exp:
att	I	105	38.99
atc	I	0	26.33
ata	I	0	39.68
gtt	V	2360	516.30
gtc	V	12	501.60
gta	V	2	467.10
gtg	V	0	889.00
---	--	------	------
mPD		0.092	0.82
		nPD:	0.11
		N. weight:	1.7
		Sc. PD:	-0.041
		Sc. rank:	1160.0

NP			
Pos.	68	obs:	exp:
tta	L	0	270.60
ttg	L	0	465.20
ctt	L	37	464.80
ctc	L	2433	427.70
cta	L	9	329.80
ctg	L	0	520.90
---	--	------	------
mPD		0.037	1.1
		nPD:	0.03
		N. weight:	2.
		Sc. PD:	-0.2
		Sc. rank:	155.3

NP			
Pos.	69	obs:	exp:
tct	S	1314	423.40
tcc	S	1153	365.50
tca	S	11	558.80
tcg	S	1	160.10
agt	S	0	506.10
agc	S	0	465.10
---	--	------	------
mPD		0.50	1.6
		nPD:	0.31
		N. weight:	1.3
		Sc. PD:	0.22
		Sc. rank:	1559.1

NP			
Pos.	70	obs:	exp:
act	T	0	0.53
acc	T	0	0.44
aca	T	2	0.86
acg	T	0	0.17
gct	A	3	629.90
gcc	A	7	482.80
gca	A	2466	1088.00
gcg	A	0	274.90
ggt	G	0	0.14
ggc	G	0	0.13
gga	G	1	0.44
ggg	G	0	0.29
---	--	------	------
mPD		0.010	0.69
		nPD:	0.02
		N. weight:	0.95
		Sc. PD:	-0.11
		Sc. rank:	-395.5

NP			
Pos.	71	obs :	exp :
ttt	F	2264	1055.00
ttc	F	215	1424.00
---	--	------	------
mPD		0.16	0.49
		nPD :	0.32
		N. weight :	0.64
		Sc. PD :	0.11
		Sc. rank :	773.2

,

NP			
Pos.	72	obs :	exp :
gat	D	2351	1426.00
gac	D	128	1053.00
---	--	------	------
mPD		0.098	0.49
		nPD :	0.2
		N. weight :	0.44
		Sc. PD :	0.026
		Sc. rank :	389.5

,

NP			
Pos.	73	obs :	exp :
gct	A	0	0.51
gcc	A	0	0.39
gca	A	2	0.88
gcg	A	0	0.22
gaa	E	2464	1483.00
gag	E	13	993.90
---	--	------	------
mPD		0.012	0.48
		nPD :	0.02
		N. weight :	0.58
		Sc. PD :	-0.063
		Sc. rank :	-44.5

,

NP			
Pos.	74	obs :	exp :
cgt	R	0	94.01
cgc	R	0	132.00
cga	R	0	240.00
cgg	R	0	194.20
aga	R	7	1137.00
agg	R	2472	681.60
---	--	------	------
mPD		0.0056	0.95
		nPD :	0.01
		N. weight :	1.5
		Sc. PD :	-0.19
		Sc. rank :	-1684.4

,

NP			
Pos.	75	obs :	exp :
cgt	R	0	94.01
cgc	R	0	132.00
cga	R	0	240.00
cgg	R	0	194.20
aga	R	973	1137.00
agg	R	1506	681.60
---	--	------	------
mPD		0.48	0.95
		nPD :	0.5
		N. weight :	0.5
		Sc. PD :	0.18
		Sc. rank :	745.0

,

NP			
Pos.	76	obs :	exp :
aat	N	8	1317.00
aac	N	2471	1162.00
---	--	------	------
mPD		0.0064	0.50
		nPD :	0.01
		N. weight :	0.88
		Sc. PD :	-0.11
		Sc. rank :	-442.5

,

NP			
Pos.	77	obs :	exp :
cgt	R	0	0.30
cgc	R	0	0.43
cga	R	0	0.77
cgg	R	0	0.63
aaa	K	1931	1387.00
aag	K	540	1084.00
aga	R	7	3.67
agg	R	1	2.20
---	--	------	------
mPD		0.35	0.50
		nPD :	0.69
		N. weight :	0.13
		Sc. PD :	0.07
		Sc. rank :	220.5

,

NP			
Pos.	78	obs :	exp :
tat	Y	1360	1205.00
tac	Y	1119	1274.00
---	--	------	------
mPD		0.50	0.50
		nPD :	0.99
		N. weight :	0.011
		Sc. PD :	0.0093
		Sc. rank :	20.3

,

NP			
Pos.	79	obs :	exp :
tta	L	0	270.60
ttg	L	256	465.20
ctt	L	0	464.80
ctc	L	0	427.70
cta	L	8	329.80
ctg	L	2215	520.90
---	--	------	------
mPD		0.19	1.1
		nPD :	0.17
		N. weight :	1.5
		Sc. PD :	0.043
		Sc. rank :	1170.7

,

NP			
Pos.	80	obs :	exp :
gaa	E	2470	1484.00
gag	E	9	994.70
---	--	------	------
mPD		0.0072	0.48
		nPD :	0.02
		N. weight :	0.59
		Sc. PD :	-0.069
		Sc. rank :	-247.6

,

NP			
Pos.	81	obs :	exp :
gaa	E	2412	1484.00
gag	E	67	994.70
---	--	------	------
mPD		0.053	0.48
		nPD :	0.11
		N. weight :	0.48
		Sc. PD :	-0.013
		Sc. rank :	310.7

,

NP			
Pos.	82	obs :	exp :
cat	H	2378	1474.00
cac	H	101	1005.00
---	--	------	------
mPD		0.078	0.48
		nPD :	0.16
		N. weight :	0.44
		Sc. PD :	0.01
		Sc. rank :	340.2

,

NP			
Pos.	83	obs :	exp :
cct	P	6	699.10
ccc	P	2470	448.60
cca	P	3	1012.00
ccg	P	0	319.20
---	--	------	------
mPD		0.0072	0.70
		nPD :	0.01
		N. weight :	2.
		Sc. PD :	-0.25
		Sc. rank :	-1371.3

,

NP			
Pos.	84	obs :	exp :
tct	S	0	422.30
tcc	S	0	364.60
tca	S	0	557.40
tcg	S	0	159.70
cgt	R	0	0.15
cgc	R	0	0.21
cga	R	0	0.39
cgg	R	0	0.31
aat	N	2	1.06
aac	N	0	0.94
agt	S	2467	504.90
agc	S	6	464.00
aga	R	4	1.83
agg	R	0	1.10
---	--	------	------
mPD		0.0097	1.6
		nPD :	0.01
		N. weight :	1.9
		Sc. PD :	-0.24
		Sc. rank :	-2097.8

,

NP			
Pos.	85	obs :	exp :
tct	S	1	0.17
tcc	S	0	0.15
tca	S	0	0.23
tcg	S	0	0.06
act	T	1	6.89
acc	T	0	5.74
aca	T	1	11.17
acg	T	24	2.20
agt	S	0	0.20
agc	S	0	0.19
gct	A	347	623.80
gcc	A	1	478.20
gca	A	184	1078.00
gcg	A	1920	272.20
---	--	------	------
mPD		0.38	0.71
		nPD :	0.53
		N. weight :	1.6
		Sc. PD :	0.6
		Sc. rank :	2437.7

,

NP			
Pos.	86	obs :	exp :
ggt	G	0	356.60
ggc	G	0	327.20
gga	G	105	1082.00
ggg	G	2374	713.00
---	--	------	------
mPD		0.081	0.69
		nPD :	0.12
		N. weight :	1.3
		Sc. PD :	-0.024
		Sc. rank :	844.8

,

NP			
Pos.	87	obs :	exp :
cgt	R	0	0.15
cgc	R	0	0.21
cga	R	0	0.39
cgg	R	0	0.31
aaa	K	1178	1389.00
aag	K	1297	1086.00
aga	R	2	1.83
agg	R	2	1.10
---	--	------	------
mPD		0.50	0.50
		nPD :	1.01
		N. weight :	0.021
		Sc. PD :	0.017
		Sc. rank :	36.9

,

NP			
Pos.	88	obs :	exp :
gat	D	192	1426.00
gac	D	2287	1053.00
---	--	------	------
mPD		0.14	0.49
		nPD :	0.29
		N. weight :	0.67
		Sc. PD :	0.1
		Sc. rank :	762.1

,

NP			
Pos.	89	obs :	exp :
cct	P	16	699.10
ccc	P	9	448.60
cca	P	742	1012.00
ccg	P	1712	319.20
---	--	------	------
mPD		0.43	0.70
		nPD :	0.62
		N. weight :	1.2
		Sc. PD :	0.57
		Sc. rank :	1986.9

,

NP			
Pos.	90	obs :	exp :
aat	N	1	0.53
aac	N	0	0.47
aaa	K	0	1391.00
aag	K	2478	1087.00
---	--	------	------
mPD		0.00081	0.49
		nPD :	0.
		N. weight :	0.98
		Sc. PD :	-0.13
		Sc. rank :	-1499.4

,

NP			
Pos.	91	obs :	exp :
cgt	R	0	0.08
cgc	R	0	0.11
cga	R	0	0.19
cgg	R	0	0.16
aaa	K	2461	1390.00
aag	K	16	1087.00
aga	R	2	0.92
agg	R	0	0.55
---	--	------	------
mPD		0.014	0.49
		nPD :	0.03
		N. weight :	0.64
		Sc. PD :	-0.067
		Sc. rank :	13.3

,

NP			
Pos.	92	obs :	exp :
act	T	1466	656.70
acc	T	120	547.70
aca	T	889	1065.00
acg	T	4	209.40
---	--	------	------
mPD		0.52	0.69
		nPD :	0.75
		N. weight :	0.4
		Sc. PD :	0.24
		Sc. rank :	697.1

,

NP			
Pos.	93	obs :	exp :
ggt	G	0	356.60
ggc	G	0	327.20
gga	G	2471	1082.00
ggg	G	8	713.00
---	--	------	------
mPD		0.0064	0.69
		nPD :	0.01
		N. weight :	0.96
		Sc. PD :	-0.12
		Sc. rank :	-741.2

,

NP			
Pos.	94	obs :	exp :
ggt	G	2445	356.60
ggc	G	26	327.20
gga	G	6	1082.00
ggg	G	2	713.00
---	--	------	------
mPD		0.027	0.69
		nPD :	0.04
		N. weight :	2.2
		Sc. PD :	-0.21
		Sc. rank :	487.1

,

NP			
Pos.	95	obs :	exp :
cct	P	0	699.10
ccc	P	1	448.60
cca	P	2476	1012.00
ccg	P	2	319.20
---	--	------	------
mPD		0.0024	0.70
		nPD :	0.
		N. weight :	1.1
		Sc. PD :	-0.14
		Sc. rank :	-1391.6

,

NP			
Pos.	96	obs:	exp:
ttt	F	1	0.43
ttc	F	0	0.57
att	I	1925	920.20
atc	I	549	621.50
ata	I	4	936.40
---	--	------	------
mPD		0.35	0.66
		nPD:	0.53
		N. weight:	0.64
		Sc. PD:	0.24
		Sc. rank:	986.4

NP			
Pos.	97	obs:	exp:
tat	Y	1910	1205.00
tac	Y	569	1274.00
---	--	------	------
mPD		0.35	0.50
		nPD:	0.71
		N. weight:	0.2
		Sc. PD:	0.11
		Sc. rank:	349.6

NP			
Pos.	98	obs:	exp:
cgt	R	0	93.82
cgc	R	1	131.80
cga	R	1927	239.60
cgg	R	8	193.80
aaa	K	4	2.81
aag	K	1	2.19
aga	R	528	1135.00
agg	R	10	680.30
---	--	------	------
mPD		0.36	0.96
		nPD:	0.38
		N. weight:	1.7
		Sc. PD:	0.4
		Sc. rank:	2190.7

NP			
Pos.	99	obs:	exp:
cgt	R	0	94.01
cgc	R	0	132.00
cga	R	0	240.00
cgg	R	5	194.20
aga	R	742	1137.00
agg	R	1732	681.60
---	--	------	------
mPD		0.42	0.95
		nPD:	0.44
		N. weight:	0.62
		Sc. PD:	0.18
		Sc. rank:	826.7

NP			
Pos.	100	obs:	exp:
cgt	R	0	94.01
cgc	R	0	132.00
cga	R	0	240.00
cgg	R	8	194.20
aga	R	2177	1137.00
agg	R	294	681.60
---	--	------	------
mPD		0.22	0.95
		nPD:	0.23
		N. weight:	0.55
		Sc. PD:	0.05
		Sc. rank:	548.8

NP			
Pos.	101	obs:	exp:
aat	N	0	1.59
aac	N	3	1.41
gct	A	0	1.02
gcc	A	4	0.78
gca	A	0	1.76
gcg	A	0	0.44
gat	D	1628	1400.00
gac	D	806	1034.00
gaa	E	34	22.75
gag	E	4	15.25
---	--	------	------
mPD		0.47	0.51
		nPD:	0.91
		N. weight:	0.032
		Sc. PD:	0.024
		Sc. rank:	57.9

NP			
Pos.	102	obs:	exp:
ggt	G	3	356.60
ggc	G	0	327.20
gga	G	682	1082.00
ggg	G	1794	713.00
---	--	------	------
mPD		0.40	0.69
		nPD:	0.58
		N. weight:	0.64
		Sc. PD:	0.27
		Sc. rank:	1013.2

NP			
Pos.	103	obs:	exp:
aaa	K	1936	1391.00
aag	K	543	1088.00
---	--	------	------
mPD		0.34	0.49
		nPD:	0.69
		N. weight:	0.13
		Sc. PD:	0.069
		Sc. rank:	216.4

NP			
Pos.	104	obs:	exp:
tgg	W	2479	2479.00
---	--	------	------
mPD		0	0
		nPD:	1.
		N. weight:	0.
		Sc. PD:	0
		Sc. rank:	0

NP			
Pos.	105	obs:	exp:
tta	L	0	14.95
ttg	L	0	25.71
ctt	L	0	25.69
ctc	L	0	23.64
cta	L	0	18.23
ctg	L	137	28.79
caa	Q	0	0.51
cag	Q	1	0.49
att	I	1	11.88
atc	I	0	8.03
ata	I	31	12.09
atg	M	806	806.00
aaa	K	0	0.56
aag	K	1	0.44
gtt	V	0	326.40
gtc	V	0	317.10
gta	V	9	295.30
gtg	V	1492	562.10
gct	A	0	0.25
gcc	A	0	0.20
gca	A	0	0.44
gcg	A	1	0.11
---	--	------	------
mPD		0.55	1.1
		nPD:	0.49
		N. weight:	0.8
		Sc. PD:	0.27
		Sc. rank:	1148.2

NP			
Pos.	106	obs:	exp:
cgt	R	0	93.97
cgc	R	0	132.00
cga	R	19	239.90
cgg	R	0	194.10
aaa	K	1	0.56
aag	K	0	0.44
aga	R	1882	1137.00
agg	R	577	681.40
---	--	------	------
mPD		0.37	0.95
		nPD:	0.39
		N. weight:	0.39
		Sc. PD:	0.095
		Sc. rank:	501.9

NP			
Pos.	107	obs:	exp:
gaa	E	470	1484.00
gag	E	2008	994.30
ggt	G	0	0.14
ggc	G	0	0.13
gga	G	0	0.44
ggg	G	1	0.29
---	--	------	------
mPD		0.31	0.48
		nPD:	0.64
		N. weight:	0.42
		Sc. PD:	0.2
		Sc. rank:	689.4

NP			
Pos.	108	obs:	exp:
tta	L	2	270.40
ttg	L	17	464.80
ctt	L	16	464.50
ctc	L	0	427.40
cta	L	27	329.60
ctg	L	2415	520.40
cct	P	0	0.28
ccc	P	0	0.18
cca	P	0	0.41
ccg	P	1	0.13
gtt	V	0	0.22
gtc	V	0	0.21
gta	V	0	0.20
gtg	V	1	0.37
---	--	------	------
mPD		0.053	1.1
		nPD:	0.05
		N. weight:	1.7
		Sc. PD:	-0.15
		Sc. rank:	590.2

NP			
Pos.	109	obs:	exp:
att	I	844	887.10
atc	I	1543	599.10
ata	I	2	902.70
atg	M	1	1.00
act	T	3	23.57
acc	T	86	19.66
aca	T	0	38.24
acg	T	0	7.52
---	--	------	------
mPD		0.52	0.73
		nPD:	0.72
		N. weight:	0.74
		Sc. PD:	0.41
		Sc. rank:	1266.1

NP			
Pos.	110	obs:	exp:
tta	L	0	270.50
ttg	L	10	465.00
ctt	L	0	464.70
ctc	L	0	427.50
cta	L	33	329.70
ctg	L	2435	520.70
atg	M	1	1.00
---	--	------	------
mPD		0.035	1.1
		nPD:	0.03
		N. weight:	1.8
		Sc. PD:	-0.18
		Sc. rank:	89.4

NP			
Pos.	111	obs:	exp:
tat	Y	2467	1205.00
tac	Y	12	1274.00
---	--	------	------
mPD		0.0096	0.50
		nPD:	0.02
		N. weight:	0.82
		Sc. PD:	-0.094
		Sc. rank:	-218.1

NP			
Pos.	112	obs:	exp:
gat	D	55	1425.00
gac	D	2423	1053.00
ggt	G	0	0.14
ggc	G	1	0.13
gga	G	0	0.44
ggg	G	0	0.29
---	--	------	------
mPD		0.044	0.49
		nPD:	0.09
		N. weight:	0.89
		Sc. PD:	-0.041
		Sc. rank:	531.7

NP			
Pos.	113	obs:	exp:
aaa	K	2467	1391.00
aag	K	12	1088.00
---	--	------	------
mPD		0.0096	0.49
		nPD:	0.02
		N. weight:	0.65
		Sc. PD:	-0.074
		Sc. rank:	-170.1

NP			
Pos.	114	obs:	exp:
gaa	E	722	1484.00
gag	E	1757	994.70
---	--	------	------
mPD		0.41	0.48
		nPD:	0.86
		N. weight:	0.23
		Sc. PD:	0.16
		Sc. rank:	410.3

NP			
Pos.	115	obs:	exp:
gat	D	21	12.08
gac	D	0	8.92
gaa	E	24	1472.00
gag	E	2434	986.20
---	--	------	------
mPD		0.036	0.49
		nPD:	0.07
		N. weight:	1.
		Sc. PD:	-0.063
		Sc. rank:	517.2

NP			
Pos.	116	obs:	exp:
att	I	1	920.50
atc	I	2477	621.70
ata	I	1	936.70
---	--	------	------
mPD		0.0016	0.66
		nPD:	0.
		N. weight:	1.6
		Sc. PD:	-0.21
		Sc. rank:	-2324.9

NP			
Pos.	117	obs:	exp:
cgt	R	0	93.90
cgc	R	0	131.90
cga	R	0	239.80
cgg	R	0	193.90
aaa	K	2	1.68
aag	K	1	1.32
aga	R	652	1136.00
agg	R	1824	680.80
---	--	------	------
mPD		0.39	0.96
		nPD:	0.41
		N. weight:	0.69
		Sc. PD:	0.18
		Sc. rank:	909.8

NP			
Pos.	118	obs:	exp:
tct	S	0	0.17
tcc	S	0	0.15
tca	S	0	0.23
tcg	S	0	0.06
cgt	R	0	93.97
cgc	R	0	132.00
cga	R	0	239.90
cgg	R	0	194.10
agt	S	0	0.20
agc	S	1	0.19
aga	R	197	1137.00
agg	R	2281	681.40
---	--	------	------
mPD		0.15	0.95
		nPD:	0.15
		N. weight:	1.2
		Sc. PD:	0.018
		Sc. rank:	859.9

NP			
Pos.	119	obs:	exp:
att	I	15	917.60
atc	I	2450	619.70
ata	I	6	933.70
act	T	0	1.59
acc	T	6	1.33
aca	T	0	2.58
acg	T	0	0.51
gtt	V	0	0.43
gtc	V	2	0.42
gta	V	0	0.39
gtg	V	0	0.75
---	--	------	------
mPD		0.023	0.66
		nPD:	0.04
		N. weight:	1.6
		Sc. PD:	-0.16
		Sc. rank:	201.7

NP			
Pos.	120	obs:	exp:
tgg	W	2479	2479.00
---	--	------	------
mPD		0	0
		nPD:	1.
		N. weight:	0.
		Sc. PD:	0
		Sc. rank:	0

NP			
Pos.	121	obs:	exp:
cgt	R	2424	94.01
cgc	R	55	132.00
cga	R	0	240.00
cgg	R	0	194.20
aga	R	0	1137.00
agg	R	0	681.60
---	--	------	------
mPD		0.043	0.95
		nPD:	0.05
		N. weight:	3.8
		Sc. PD:	-0.33
		Sc. rank:	1263.1

NP			
Pos.	122	obs:	exp:
caa	Q	2340	1261.00
cag	Q	138	1217.00
cgt	R	0	0.04
cgc	R	0	0.05
cga	R	1	0.10
cgg	R	0	0.08
aga	R	0	0.46
agg	R	0	0.27
---	--	------	------
mPD		0.11	0.50
		nPD:	0.21
		N. weight:	0.55
		Sc. PD:	0.039
		Sc. rank:	512.9

NP			
Pos.	123	obs:	exp:
gct	A	2	630.60
gcc	A	3	483.40
gca	A	497	1090.00
gcg	A	1977	275.20
---	--	------	------
mPD		0.32	0.69
		nPD:	0.47
		N. weight:	1.7
		Sc. PD:	0.53
		Sc. rank:	2301.2

NP			
Pos.	124	obs:	exp:
aat	N	1352	1317.00
aac	N	1127	1162.00
---	--	------	------
mPD		0.50	0.50
		nPD:	1.
		N. weight:	0.0025
		Sc. PD:	0.002
		Sc. rank:	4.4

NP			
Pos.	125	obs:	exp:
tct	S	0	1.37
tcc	S	0	1.18
tca	S	0	1.80
tcg	S	0	0.52
cat	H	1	0.59
cac	H	0	0.41
act	T	5	1.32
acc	T	0	1.10
aca	T	0	2.15
acg	T	0	0.42
aat	N	2303	1310.00
aac	N	162	1155.00
agt	S	8	1.63
agc	S	0	1.50
---	--	------	------
mPD		0.13	0.52
		nPD:	0.26
		N. weight:	0.48
		Sc. PD:	0.056
		Sc. rank:	511.8

NP			
Pos.	126	obs:	exp:
cgt	R	0	0.04
cgc	R	0	0.05
cga	R	0	0.10
cgg	R	0	0.08
aga	R	1	0.46
agg	R	0	0.27
ggt	G	3	356.50
ggc	G	0	327.10
gga	G	2432	1082.00
ggg	G	43	712.70
---	--	------	------
mPD		0.037	0.69
		nPD:	0.05
		N. weight:	0.88
		Sc. PD:	-0.071
		Sc. rank:	385.3

NP			
Pos.	127	obs:	exp:
caa	Q	1	0.51
cag	Q	0	0.49
gat	D	0	0.58
gac	D	1	0.42
gaa	E	2434	1483.00
gag	E	43	993.90
---	--	------	------
mPD		0.036	0.48
		nPD:	0.07
		N. weight:	0.52
		Sc. PD:	-0.032
		Sc. rank:	264.9

NP			
Pos.	128	obs:	exp:
cat	H	0	0.59
cac	H	1	0.41
aat	N	0	0.53
aac	N	1	0.47
gat	D	548	1425.00
gac	D	1929	1052.00
---	--	------	------
mPD		0.35	0.49
		nPD:	0.71
		N. weight:	0.31
		Sc. PD:	0.17
		Sc. rank:	533.5

NP			
Pos.	129	obs:	exp:
tct	S	0	1.37
tcc	S	1	1.18
tca	S	7	1.80
tcg	S	0	0.52
act	T	0	0.53
acc	T	0	0.44
aca	T	2	0.86
acg	T	0	0.17
agt	S	0	1.63
agc	S	0	1.50
gct	A	11	628.10
gcc	A	8	481.50
gca	A	2443	1085.00
gcg	A	7	274.10
---	--	------	------
mPD		0.030	0.70
		nPD:	0.04
		N. weight:	0.91
		Sc. PD:	-0.084
		Sc. rank:	243.4

NP			
Pos.	130	obs:	exp:
act	T	2468	656.40
acc	T	10	547.50
aca	T	0	1065.00
acg	T	0	209.30
gct	A	1	0.25
gcc	A	0	0.20
gca	A	0	0.44
gcg	A	0	0.11
---	--	------	------
mPD		0.0088	0.69
		nPD:	0.01
		N. weight:	1.6
		Sc. PD:	-0.19
		Sc. rank:	-801.2

NP			
Pos.	131	obs:	exp:
act	T	1	0.26
acc	T	0	0.22
aca	T	0	0.43
acg	T	0	0.08
gct	A	2472	630.40
gcc	A	2	483.20
gca	A	0	1089.00
gcg	A	4	275.10
---	--	------	------
mPD		0.0056	0.69
		nPD:	0.01
		N. weight:	1.6
		Sc. PD:	-0.2
		Sc. rank:	-1370.7

NP			
Pos.	132	obs:	exp:
ggt	G	2476	356.60
ggc	G	3	327.20
gga	G	0	1082.00
ggg	G	0	713.00
---	--	------	------
mPD		0.0024	0.69
		nPD:	0.
		N. weight:	2.3
		Sc. PD:	-0.3
		Sc. rank:	-2927.5

NP			
Pos.	133	obs:	exp:
tta	L	0	270.30
ttg	L	0	464.60
ctt	L	3	464.30
ctc	L	2470	427.20
cta	L	3	329.40
ctg	L	0	520.20
cgt	R	0	0.04
cgc	R	1	0.05
cga	R	0	0.10
cgg	R	0	0.08
att	I	0	0.74
atc	I	2	0.50
ata	I	0	0.76
aga	R	0	0.46
agg	R	0	0.27
---	--	------	------
mPD		0.0073	1.1
		nPD:	0.01
		N. weight:	2.1
		Sc. PD:	-0.26
		Sc. rank:	-2194.7

NP			
Pos.	134	obs:	exp:
act	T	1906	656.70
acc	T	564	547.70
aca	T	4	1065.00
acg	T	5	209.40
---	--	------	------
mPD		0.36	0.69
		nPD:	0.52
		N. weight:	0.97
		Sc. PD:	0.35
		Sc. rank:	1472.9

NP			
Pos.	135	obs:	exp:
cat	H	683	1474.00
cac	H	1796	1005.00
---	--	------	------
mPD		0.40	0.48
		nPD:	0.83
		N. weight:	0.25
		Sc. PD:	0.17
		Sc. rank:	439.8

NP

Pos.	136	obs:	exp:
tta	L	3	270.40
ttg	L	207	464.80
ctt	L	1	464.50
ctc	L	0	427.40
cta	L	80	329.60
ctg	L	2186	520.40
att	I	0	0.74
atc	I	0	0.50
ata	I	2	0.76
---	--	------	------
mPD		0.22	1.1
		nPD:	0.2
		N. weight:	1.4
		Sc. PD:	0.078
		Sc. rank:	1205.1

,

NP

Pos.	137	obs:	exp:
atg	M	2478	2478.00
gtt	V	0	0.22
gtc	V	0	0.21
gta	V	0	0.20
gtg	V	1	0.37
---	--	------	------
mPD		0.00081	0.0013
		nPD:	0.62
		N. weight:	0.00047
		Sc. PD:	0.00022
		Sc. rank:	0.8

,

NP

Pos.	138	obs:	exp:
att	I	24	919.40
atc	I	2310	621.00
ata	I	142	935.60
atg	M	1	1.00
gtt	V	0	0.43
gtc	V	2	0.42
gta	V	0	0.39
gtg	V	0	0.75
---	--	------	------
mPD		0.13	0.66
		nPD:	0.2
		N. weight:	1.3
		Sc. PD:	0.071
		Sc. rank:	1124.8

,

NP

Pos.	139	obs:	exp:
tgg	W	2479	2479.00
---	--	------	------
mPD		0	0
		nPD:	1.
		N. weight:	0.
		Sc. PD:	0
		Sc. rank:	0

,

NP

Pos.	140	obs:	exp:
cct	P	1	0.28
ccc	P	0	0.18
cca	P	0	0.41
ccg	P	0	0.13
cat	H	1890	1473.00
cac	H	588	1005.00
---	--	------	------
mPD		0.36	0.48
		nPD:	0.75
		N. weight:	0.077
		Sc. PD:	0.046
		Sc. rank:	135.3

,

NP

Pos.	141	obs:	exp:
tct	S	7	423.40
tcc	S	2472	365.50
tca	S	0	558.80
tcg	S	0	160.10
agt	S	0	506.10
agc	S	0	465.10
---	--	------	------
mPD		0.0056	1.6
		nPD:	0.
		N. weight:	2.3
		Sc. PD:	-0.29
		Sc. rank:	-3022.2

,

NP

Pos.	142	obs:	exp:
aat	N	1742	1317.00
aac	N	737	1162.00
---	--	------	------
mPD		0.42	0.50
		nPD:	0.84
		N. weight:	0.075
		Sc. PD:	0.05
		Sc. rank:	131.2

,

NP

Pos.	143	obs:	exp:
tta	L	4	270.60
ttg	L	0	465.20
ctt	L	0	464.80
ctc	L	0	427.70
cta	L	1908	329.80
ctg	L	567	520.90
---	--	------	------
mPD		0.36	1.1
		nPD:	0.31
		N. weight:	1.6
		Sc. PD:	0.28
		Sc. rank:	1934.6

,

NP

Pos.	144	obs:	exp:
aat	N	2474	1317.00
aac	N	5	1162.00
---	--	------	------
mPD		0.0040	0.50
		nPD:	0.01
		N. weight:	0.74
		Sc. PD:	-0.092
		Sc. rank:	-643.8

,

NP

Pos.	145	obs:	exp:
gat	D	2476	1425.00
gac	D	2	1053.00
ggt	G	1	0.14
ggc	G	0	0.13
gga	G	0	0.44
ggg	G	0	0.29
---	--	------	------
mPD		0.0024	0.49
		nPD:	0.
		N. weight:	0.65
		Sc. PD:	-0.084
		Sc. rank:	-771.6

,

NP

Pos.	146	obs:	exp:
tct	S	0	0.17
tcc	S	1	0.15
tca	S	0	0.23
tcg	S	0	0.06
act	T	0	3.18
acc	T	12	2.65
aca	T	0	5.16
acg	T	0	1.01
agt	S	0	0.20
agc	S	0	0.19
gct	A	27	627.30
gcc	A	2438	480.90
gca	A	1	1084.00
gcg	A	0	273.80
---	--	------	------
mPD		0.033	0.70
		nPD:	0.05
		N. weight:	1.9
		Sc. PD:	-0.16
		Sc. rank:	675.4

,

NP

Pos.	147	obs:	exp:
act	T	1	656.70
acc	T	4	547.70
aca	T	2464	1065.00
acg	T	10	209.40
---	--	------	------
mPD		0.012	0.69
		nPD:	0.02
		N. weight:	0.97
		Sc. PD:	-0.11
		Sc. rank:	-305.8

,

NP

Pos.	148	obs:	exp:
tat	Y	510	1205.00
tac	Y	1969	1274.00
---	--	------	------
mPD		0.33	0.50
		nPD:	0.65
		N. weight:	0.2
		Sc. PD:	0.1
		Sc. rank:	335.0

,

NP

Pos.	149	obs:	exp:
caa	Q	1	1261.00
cag	Q	2478	1218.00
---	--	------	------
mPD		0.00081	0.50
		nPD:	0.
		N. weight:	0.84
		Sc. PD:	-0.11
		Sc. rank:	-1308.7

,

NP

Pos.	150	obs:	exp:
cgt	R	0	94.01
cgc	R	0	132.00
cga	R	0	240.00
cgg	R	0	194.20
aga	R	718	1137.00
agg	R	1761	681.60
---	--	------	------
mPD		0.41	0.95
		nPD:	0.43
		N. weight:	0.65
		Sc. PD:	0.18
		Sc. rank:	860.4

,

NP

Pos.	151	obs:	exp:
act	T	12	656.70
acc	T	296	547.70
aca	T	2087	1065.00
acg	T	84	209.40
---	--	------	------
mPD		0.28	0.69
		nPD:	0.4
		N. weight:	0.53
		Sc. PD:	0.13
		Sc. rank:	690.8

,

NP

Pos.	152	obs:	exp:
cgt	R	0	94.01
cgc	R	0	132.00
cga	R	0	240.00
cgg	R	0	194.20
aga	R	1832	1137.00
agg	R	647	681.60
---	--	------	------
mPD		0.39	0.95
		nPD:	0.4
		N. weight:	0.41
		Sc. PD:	0.1
		Sc. rank:	531.0

,

NP

Pos.	153	obs:	exp:
gct	A	2460	630.60
gcc	A	5	483.40
gca	A	14	1090.00
gcg	A	0	275.20
---	--	------	------
mPD		0.015	0.69
		nPD:	0.02
		N. weight:	1.6
		Sc. PD:	-0.18
		Sc. rank:	-278.6

,

NP

Pos.	154	obs:	exp:
tta	L	0	270.60
ttg	L	0	465.20
ctt	L	54	464.80
ctc	L	2420	427.70
cta	L	5	329.80
ctg	L	0	520.90
---	--	------	------
mPD		0.047	1.1
		nPD:	0.04
		N. weight:	2.
		Sc. PD:	-0.18
		Sc. rank:	457.3

,

NP

Pos.	155	obs:	exp:
gtt	V	25	539.10
gtc	V	0	523.80
gta	V	255	487.80
gtg	V	2199	928.30
---	--	------	------
mPD		0.20	0.73
		nPD:	0.28
		N. weight:	0.8
		Sc. PD:	0.11
		Sc. rank:	876.4

,

NP

Pos.	156	obs:	exp:
cgt	R	1616	94.01
cgc	R	863	132.00
cga	R	0	240.00
cgg	R	0	194.20
aga	R	0	1137.00
agg	R	0	681.60
---	--	------	------
mPD		0.45	0.95
		nPD:	0.48
		N. weight:	3.
		Sc. PD:	0.97
		Sc. rank:	4168.4

,

NP

Pos.	157	obs:	exp:
act	T	1657	656.70
acc	T	818	547.70
aca	T	4	1065.00
acg	T	0	209.40
---	--	------	------
mPD		0.44	0.69
		nPD:	0.64
		N. weight:	0.89
		Sc. PD:	0.43
		Sc. rank:	1457.4

,

NP

Pos.	158	obs:	exp:
cgt	R	0	0.04
cgc	R	0	0.05
cga	R	0	0.10
cgg	R	0	0.08
aga	R	0	0.46
agg	R	1	0.27
ggt	G	888	356.50
ggc	G	1	327.10
gga	G	668	1082.00
ggg	G	921	712.70
---	--	------	------
mPD		0.66	0.69
		nPD:	0.96
		N. weight:	0.35
		Sc. PD:	0.28
		Sc. rank:	628.9

,

NP

Pos.	159	obs:	exp:
att	I	0	4.08
atc	I	0	2.76
ata	I	11	4.16
atg	M	2468	2468.00
---	--	------	------
mPD		0.0088	0.0088
		nPD:	1.
		N. weight:	0.0051
		Sc. PD:	0.0043
		Sc. rank:	9.2

,

NP

Pos.	160	obs:	exp:
gat	D	545	1426.00
gac	D	1934	1053.00
---	--	------	------
mPD		0.34	0.49
		nPD:	0.7
		N. weight:	0.31
		Sc. PD:	0.17
		Sc. rank:	533.9

,

NP			
Pos.	161	obs:	exp:
cct	P	3	699.10
ccc	P	2470	448.60
cca	P	6	1012.00
ccg	P	0	319.20
---	--	------	------
mPD		0.0072	0.70
		nPD:	0.01
		N. weight:	2.
		Sc. PD:	-0.25
		Sc. rank:	-1371.0

,

NP			
Pos.	162	obs:	exp:
cgt	R	0	94.01
cgc	R	0	132.00
cga	R	0	240.00
cgg	R	0	194.20
aga	R	986	1137.00
agg	R	1493	681.60
---	--	------	------
mPD		0.48	0.95
		nPD:	0.5
		N. weight:	0.5
		Sc. PD:	0.17
		Sc. rank:	738.1

,

NP			
Pos.	163	obs:	exp:
att	I	0	0.37
atc	I	0	0.25
ata	I	1	0.38
atg	M	2478	2478.00
---	--	------	------
mPD		0.00081	0.00081
		nPD:	1.
		N. weight:	0.00047
		Sc. PD:	0.00039
		Sc. rank:	0.8

,

NP			
Pos.	164	obs:	exp:
tgt	C	6	1078.00
tgc	C	2472	1400.00
tgg	W	1	1.00
---	--	------	------
mPD		0.0056	0.49
		nPD:	0.01
		N. weight:	0.66
		Sc. PD:	-0.08
		Sc. rank:	-413.2

,

NP			
Pos.	165	obs:	exp:
tct	S	1582	423.40
tcc	S	889	365.50
tca	S	2	558.80
tcg	S	6	160.10
agt	S	0	506.10
agc	S	0	465.10
---	--	------	------
mPD		0.46	1.6
		nPD:	0.28
		N. weight:	1.4
		Sc. PD:	0.19
		Sc. rank:	1538.2

,

NP			
Pos.	166	obs:	exp:
tta	L	0	270.50
ttg	L	0	465.00
ctt	L	439	464.70
ctc	L	1166	427.50
cta	L	17	329.70
ctg	L	856	520.70
atg	M	1	1.00
---	--	------	------
mPD		0.63	1.1
		nPD:	0.55
		N. weight:	0.74
		Sc. PD:	0.29
		Sc. rank:	1160.9

,

NP			
Pos.	167	obs:	exp:
atg	M	2479	2479.00
---	--	------	------
mPD		0	0
		nPD:	1.
		N. weight:	0.
		Sc. PD:	0
		Sc. rank:	0

,

NP			
Pos.	168	obs:	exp:
caa	Q	2477	1261.00
cag	Q	2	1218.00
---	--	------	------
mPD		0.0016	0.50
		nPD:	0.
		N. weight:	0.8
		Sc. PD:	-0.1
		Sc. rank:	-1121.3

,

NP			
Pos.	169	obs:	exp:
ggt	G	2	356.60
ggc	G	7	327.20
gga	G	1538	1082.00
ggg	G	932	713.00
---	--	------	------
mPD		0.47	0.69
		nPD:	0.69
		N. weight:	0.37
		Sc. PD:	0.19
		Sc. rank:	617.0

,

NP			
Pos.	170	obs:	exp:
tct	S	0	423.40
tcc	S	0	365.50
tca	S	2476	558.80
tcg	S	3	160.10
agt	S	0	506.10
agc	S	0	465.10
---	--	------	------
mPD		0.0024	1.6
		nPD:	0.
		N. weight:	1.8
		Sc. PD:	-0.23
		Sc. rank:	-2802.8

,

NP			
Pos.	171	obs:	exp:
act	T	2472	656.70
acc	T	7	547.70
aca	T	0	1065.00
acg	T	0	209.40
---	--	------	------
mPD		0.0056	0.69
		nPD:	0.01
		N. weight:	1.6
		Sc. PD:	-0.19
		Sc. rank:	-1309.8

,

NP			
Pos.	172	obs:	exp:
tta	L	0	270.60
ttg	L	0	465.20
ctt	L	9	464.80
ctc	L	2468	427.70
cta	L	1	329.80
ctg	L	1	520.90
---	--	------	------
mPD		0.0088	1.1
		nPD:	0.01
		N. weight:	2.1
		Sc. PD:	-0.26
		Sc. rank:	-1943.4

,

NP			
Pos.	173	obs:	exp:
cct	P	0	699.10
ccc	P	5	448.60
cca	P	2155	1012.00
ccg	P	319	319.20
---	--	------	------
mPD		0.23	0.70
		nPD:	0.32
		N. weight:	0.77
		Sc. PD:	0.14
		Sc. rank:	935.9

,

NP			
Pos.	174	obs:	exp:
cgt	R	0	94.01
cgc	R	0	132.00
cga	R	0	240.00
cgg	R	0	194.20
aga	R	524	1137.00
agg	R	1955	681.60
---	--	------	------
mPD		0.33	0.95
		nPD:	0.35
		N. weight:	0.8
		Sc. PD:	0.16
		Sc. rank:	987.9

,

NP			
Pos.	175	obs:	exp:
cgt	R	0	94.01
cgc	R	0	132.00
cga	R	0	240.00
cgg	R	0	194.20
aga	R	556	1137.00
agg	R	1923	681.60
---	--	------	------
mPD		0.35	0.95
		nPD:	0.37
		N. weight:	0.77
		Sc. PD:	0.17
		Sc. rank:	967.9

,

NP			
Pos.	176	obs:	exp:
tct	S	2472	423.40
tcc	S	0	365.50
tca	S	4	558.80
tcg	S	3	160.10
agt	S	0	506.10
agc	S	0	465.10
---	--	------	------
mPD		0.0056	1.6
		nPD:	0.
		N. weight:	2.1
		Sc. PD:	-0.27
		Sc. rank:	-2755.8

,

NP			
Pos.	177	obs:	exp:
ggt	G	0	356.60
ggc	G	0	327.20
gga	G	2330	1082.00
ggg	G	149	713.00
---	--	------	------
mPD		0.11	0.69
		nPD:	0.16
		N. weight:	0.75
		Sc. PD:	0.019
		Sc. rank:	587.1

,

NP			
Pos.	178	obs:	exp:
gct	A	2469	630.60
gcc	A	5	483.40
gca	A	4	1090.00
gcg	A	1	275.20
---	--	------	------
mPD		0.0080	0.69
		nPD:	0.01
		N. weight:	1.6
		Sc. PD:	-0.19
		Sc. rank:	-946.2

,

NP			
Pos.	179	obs:	exp:
gct	A	2321	630.60
gcc	A	3	483.40
gca	A	0	1090.00
gcg	A	155	275.20
---	--	------	------
mPD		0.12	0.69
		nPD:	0.17
		N. weight:	1.4
		Sc. PD:	0.047
		Sc. rank:	1144.6

,

NP			
Pos.	180	obs:	exp:
ggt	G	2284	356.60
ggc	G	147	327.20
gga	G	48	1082.00
ggg	G	0	713.00
---	--	------	------
mPD		0.15	0.69
		nPD:	0.21
		N. weight:	1.9
		Sc. PD:	0.14
		Sc. rank:	1822.7

,

NP			
Pos.	181	obs:	exp:
gct	A	5	630.60
gcc	A	1	483.40
gca	A	2462	1090.00
gcg	A	11	275.20
---	--	------	------
mPD		0.014	0.69
		nPD:	0.02
		N. weight:	0.94
		Sc. PD:	-0.11
		Sc. rank:	-236.9

,

NP			
Pos.	182	obs:	exp:
tct	S	0	0.17
tcc	S	0	0.15
tca	S	1	0.23
tcg	S	0	0.06
agt	S	0	0.20
agc	S	0	0.19
gct	A	17	630.40
gcc	A	0	483.20
gca	A	2459	1089.00
gcg	A	2	275.10
---	--	------	------
mPD		0.016	0.69
		nPD:	0.02
		N. weight:	0.93
		Sc. PD:	-0.1
		Sc. rank:	-147.1

,

NP			
Pos.	183	obs:	exp:
att	I	0	4.08
atc	I	0	2.76
ata	I	11	4.16
gtt	V	55	536.70
gtc	V	0	521.50
gta	V	1561	485.60
gtg	V	852	924.20
---	--	------	------
mPD		0.49	0.74
		nPD:	0.66
		N. weight:	0.79
		Sc. PD:	0.4
		Sc. rank:	1313.4

,

NP			
Pos.	184	obs:	exp:
aaa	K	559	1391.00
aag	K	1920	1088.00
---	--	------	------
mPD		0.35	0.49
		nPD:	0.71
		N. weight:	0.28
		Sc. PD:	0.15
		Sc. rank:	482.1

,

NP			
Pos.	185	obs:	exp:
ggt	G	23	356.60
ggc	G	0	327.20
gga	G	1777	1082.00
ggg	G	679	713.00
---	--	------	------
mPD		0.41	0.69
		nPD:	0.6
		N. weight:	0.38
		Sc. PD:	0.17
		Sc. rank:	608.5

,

NP			
Pos.	186	obs:	exp:
att	I	5	4.08
atc	I	5	2.76
ata	I	1	4.16
gtt	V	1049	536.70
gtc	V	1411	521.50
gta	V	8	485.60
gtg	V	0	924.20
mPD		0.50	0.74
		nPD:	0.68
		N. weight:	1.
		Sc. PD:	0.52
		Sc. rank:	1676.3

NP			
Pos.	187	obs:	exp:
ggt	G	1	356.60
ggc	G	0	327.20
gga	G	1963	1082.00
ggg	G	515	713.00
mPD		0.33	0.69
		nPD:	0.48
		N. weight:	0.48
		Sc. PD:	0.16
		Sc. rank:	672.1

NP			
Pos.	188	obs:	exp:
act	T	1	656.70
acc	T	0	547.70
aca	T	572	1065.00
acg	T	1906	209.40
mPD		0.36	0.69
		nPD:	0.52
		N. weight:	1.9
		Sc. PD:	0.67
		Sc. rank:	2792.6

NP			
Pos.	189	obs:	exp:
atg	M	2479	2479.00
mPD		0	0
		nPD:	1.
		N. weight:	0.
		Sc. PD:	0
		Sc. rank:	0

NP			
Pos.	190	obs:	exp:
att	I	0	0.37
atc	I	0	0.25
ata	I	1	0.38
gtt	V	2	538.00
gtc	V	0	522.70
gta	V	681	486.80
gtg	V	1791	926.40
ggt	G	0	0.58
ggc	G	0	0.53
gga	G	4	1.75
ggg	G	0	1.15
mPD		0.41	0.73
		nPD:	0.55
		N. weight:	0.68
		Sc. PD:	0.27
		Sc. rank:	1064.3

NP			
Pos.	191	obs:	exp:
tta	L	0	0.22
ttg	L	1	0.38
ctt	L	0	0.38
ctc	L	0	0.35
cta	L	0	0.27
ctg	L	1	0.42
att	I	0	1.11
atc	I	0	0.75
ata	I	3	1.13
atg	M	2474	2474.00
mPD		0.0040	0.0050
		nPD:	0.81
		N. weight:	0.0026
		Sc. PD:	0.0017
		Sc. rank:	4.6

NP			
Pos.	192	obs:	exp:
gaa	E	2476	1484.00
gag	E	3	994.70
mPD		0.0024	0.48
		nPD:	0.01
		N. weight:	0.6
		Sc. PD:	-0.077
		Sc. rank:	-697.4

NP			
Pos.	193	obs:	exp:
tta	L	13	268.30
ttg	L	550	461.20
ctt	L	0	460.90
ctc	L	0	424.10
cta	L	1875	327.00
ctg	L	20	516.40
att	I	0	7.80
atc	I	0	5.27
ata	I	21	7.94
mPD		0.72	1.1
		nPD:	0.63
		N. weight:	1.6
		Sc. PD:	0.74
		Sc. rank:	2578.2

NP			
Pos.	194	obs:	exp:
att	I	2452	919.10
atc	I	23	620.70
ata	I	0	935.20
act	T	1	0.26
acc	T	0	0.22
aca	T	0	0.43
acg	T	0	0.08
gtt	V	3	0.65
gtc	V	0	0.63
gta	V	0	0.59
gtg	V	0	1.12
mPD		0.022	0.66
		nPD:	0.03
		N. weight:	1.1
		Sc. PD:	-0.11
		Sc. rank:	97.5

NP			
Pos.	195	obs:	exp:
cgt	R	0	94.01
cgc	R	0	132.00
cga	R	664	240.00
cgg	R	1778	194.20
aga	R	36	1137.00
agg	R	1	681.60
mPD		0.43	0.95
		nPD:	0.46
		N. weight:	2.2
		Sc. PD:	0.66
		Sc. rank:	2936.1

NP			
Pos.	196	obs:	exp:
atg	M	2479	2479.00
mPD		0	0
		nPD:	1.
		N. weight:	0.
		Sc. PD:	0
		Sc. rank:	0

NP			
Pos.	197	obs:	exp:
att	I	1	917.60
atc	I	12	619.70
ata	I	2458	933.70
gtt	V	0	1.74
gtc	V	0	1.69
gta	V	8	1.57
gtg	V	0	3.00
mPD		0.017	0.66
		nPD:	0.03
		N. weight:	1.1
		Sc. PD:	-0.12
		Sc. rank:	-77.7

NP			
Pos.	198	obs:	exp:
aaa	K	319	1391.00
aag	K	2160	1088.00
mPD		0.22	0.49
		nPD:	0.46
		N. weight:	0.49
		Sc. PD:	0.15
		Sc. rank:	656.8

NP			
Pos.	199	obs:	exp:
cgt	R	0	94.01
cgc	R	1	132.00
cga	R	2458	240.00
cgg	R	6	194.20
aga	R	14	1137.00
agg	R	0	681.60
mPD		0.017	0.95
		nPD:	0.02
		N. weight:	2.7
		Sc. PD:	-0.31
		Sc. rank:	-802.5

NP			
Pos.	200	obs:	exp:
cgt	R	0	0.04
cgc	R	0	0.05
cga	R	0	0.10
cgg	R	0	0.08
aga	R	1	0.46
agg	R	0	0.27
ggt	G	9	356.50
ggc	G	0	327.10
gga	G	1648	1082.00
ggg	G	821	712.70
mPD		0.45	0.69
		nPD:	0.65
		N. weight:	0.38
		Sc. PD:	0.19
		Sc. rank:	620.6

NP			
Pos.	201	obs:	exp:
att	I	1564	587.10
atc	I	16	396.50
ata	I	1	597.40
gtt	V	898	195.30
gtc	V	0	189.70
gta	V	0	176.70
gtg	V	0	336.30
mPD		0.48	1.2
		nPD:	0.4
		N. weight:	1.4
		Sc. PD:	0.34
		Sc. rank:	1774.1

NP			
Pos.	202	obs:	exp:
aat	N	2474	1317.00
aac	N	4	1161.00
aaa	K	0	0.56
aag	K	1	0.44
mPD		0.0040	0.50
		nPD:	0.01
		N. weight:	0.74
		Sc. PD:	-0.092
		Sc. rank:	-650.4

NP			
Pos.	203	obs:	exp:
gat	D	2476	1426.00
gac	D	3	1053.00
mPD		0.0024	0.49
		nPD:	0.
		N. weight:	0.65
		Sc. PD:	-0.083
		Sc. rank:	-764.1

NP			
Pos.	204	obs:	exp:
cgt	R	9	94.01
cgc	R	0	132.00
cga	R	1152	240.00
cgg	R	1296	194.20
aga	R	14	1137.00
agg	R	8	681.60
mPD		0.52	0.95
		nPD:	0.55
		N. weight:	2.
		Sc. PD:	0.78
		Sc. rank:	3134.9

NP			
Pos.	205	obs:	exp:
aat	N	860	1317.00
aac	N	1619	1162.00
mPD		0.45	0.50
		nPD:	0.91
		N. weight:	0.084
		Sc. PD:	0.062
		Sc. rank:	149.7

NP			
Pos.	206	obs:	exp:
ttt	F	2	1055.00
ttc	F	2477	1424.00
mPD		0.0016	0.49
		nPD:	0.
		N. weight:	0.65
		Sc. PD:	-0.085
		Sc. rank:	-903.8

NP			
Pos.	207	obs:	exp:
tgg	W	2479	2479.00
mPD		0	0
		nPD:	1.
		N. weight:	0.
		Sc. PD:	0
		Sc. rank:	0

NP			
Pos.	208	obs:	exp:
cgt	R	0	94.01
cgc	R	0	132.00
cga	R	1	240.00
cgg	R	0	194.20
aga	R	2468	1137.00
agg	R	10	681.60
mPD		0.0088	0.95
		nPD:	0.01
		N. weight:	0.9
		Sc. PD:	-0.11
		Sc. rank:	-721.4

NP			
Pos.	209	obs:	exp:
gct	A	0	0.25
gcc	A	1	0.20
gca	A	0	0.44
gcg	A	0	0.11
ggt	G	13	356.50
ggc	G	2463	327.10
gga	G	2	1082.00
ggg	G	0	712.70
mPD		0.013	0.69
		nPD:	0.02
		N. weight:	2.4
		Sc. PD:	-0.27
		Sc. rank:	-640.5

NP			
Pos.	210	obs:	exp:
gaa	E	849	1484.00
gag	E	1630	994.70
mPD		0.45	0.48
		nPD:	0.94
		N. weight:	0.16
		Sc. PD:	0.12
		Sc. rank:	288.1

NP			
Pos.	211	obs:	exp:
tct	S	0	0.68
tcc	S	0	0.59
tca	S	0	0.90
tcg	S	0	0.26
aat	N	2335	1315.00
aac	N	140	1160.00
agt	S	4	0.82
agc	S	0	0.75
---	--	------	------
mPD		0.11	0.50
		nPD:	0.22
		N. weight:	0.51
		Sc. PD:	0.039
		Sc. rank:	489.4

NP			
Pos.	212	obs:	exp:
ggt	G	0	356.60
ggc	G	0	327.20
gga	G	2465	1082.00
ggg	G	14	713.00
---	--	------	------
mPD		0.011	0.69
		nPD:	0.02
		N. weight:	0.95
		Sc. PD:	-0.11
		Sc. rank:	-320.4

NP			
Pos.	213	obs:	exp:
cgt	R	0	94.01
cgc	R	0	132.00
cga	R	713	240.00
cgg	R	1260	194.20
aga	R	503	1137.00
agg	R	3	681.60
---	--	------	------
mPD		0.83	0.95
		nPD:	0.87
		N. weight:	1.3
		Sc. PD:	0.91
		Sc. rank:	2318.1

NP			
Pos.	214	obs:	exp:
cgt	R	0	93.94
cgc	R	0	131.90
cga	R	0	239.90
cgg	R	5	194.00
aaa	K	0	1.12
aag	K	2	0.88
aga	R	341	1136.00
agg	R	2131	681.10
---	--	------	------
mPD		0.24	0.95
		nPD:	0.25
		N. weight:	0.97
		Sc. PD:	0.11
		Sc. rank:	1020.4

NP			
Pos.	215	obs:	exp:
act	T	3	656.40
acc	T	0	547.50
aca	T	2470	1065.00
acg	T	5	209.30
gct	A	0	0.25
gcc	A	0	0.20
gca	A	1	0.44
gcg	A	0	0.11
---	--	------	------
mPD		0.0072	0.69
		nPD:	0.01
		N. weight:	0.98
		Sc. PD:	-0.12
		Sc. rank:	-637.8

NP			
Pos.	216	obs:	exp:
cgt	R	0	93.97
cgc	R	0	132.00
cga	R	0	239.90
cgg	R	4	194.10
aaa	K	0	0.56
aag	K	1	0.44
aga	R	20	1137.00
agg	R	2454	681.40
---	--	------	------
mPD		0.020	0.95
		nPD:	0.02
		N. weight:	1.5
		Sc. PD:	-0.17
		Sc. rank:	-283.7

NP			
Pos.	217	obs:	exp:
att	I	2469	918.30
atc	I	4	620.20
ata	I	0	934.50
gtt	V	6	1.30
gtc	V	0	1.27
gta	V	0	1.18
gtg	V	0	2.25
---	--	------	------
mPD		0.0081	0.66
		nPD:	0.01
		N. weight:	1.2
		Sc. PD:	-0.14
		Sc. rank:	-640.5

NP			
Pos.	218	obs:	exp:
gct	A	11	630.60
gcc	A	541	483.40
gca	A	1927	1090.00
gcg	A	0	275.20
---	--	------	------
mPD		0.35	0.69
		nPD:	0.5
		N. weight:	0.54
		Sc. PD:	0.19
		Sc. rank:	802.1

NP			
Pos.	219	obs:	exp:
ttt	F	892	379.60
ttc	F	0	512.40
tat	Y	1576	771.40
tac	Y	11	815.60
---	--	------	------
mPD		0.47	0.96
		nPD:	0.49
		N. weight:	0.89
		Sc. PD:	0.3
		Sc. rank:	1273.3

NP			
Pos.	220	obs:	exp:
gat	D	1	0.58
gac	D	0	0.42
gaa	E	17	1484.00
gag	E	2461	994.30
---	--	------	------
mPD		0.014	0.48
		nPD:	0.03
		N. weight:	1.
		Sc. PD:	-0.11
		Sc. rank:	29.2

NP			
Pos.	221	obs:	exp:
cgt	R	0	94.01
cgc	R	0	132.00
cga	R	0	240.00
cgg	R	0	194.20
aga	R	2272	1137.00
agg	R	207	681.60
---	--	------	------
mPD		0.15	0.95
		nPD:	0.16
		N. weight:	0.64
		Sc. PD:	0.014
		Sc. rank:	489.5

NP			
Pos.	222	obs:	exp:
tta	L	0	0.22
ttg	L	2	0.38
tgg	W	1	1.00
ctt	L	0	0.38
ctc	L	0	0.35
cta	L	0	0.27
ctg	L	0	0.42
att	I	0	0.37
atc	I	0	0.25
ata	I	1	0.38
atg	M	2475	2475.00
---	--	------	------
mPD		0.0040	0.0050
		nPD:	0.81
		N. weight:	0.0021
		Sc. PD:	0.0013
		Sc. rank:	3.6

NP			
Pos.	223	obs:	exp:
tgt	C	2	1078.00
tgc	C	2477	1401.00
---	--	------	------
mPD		0.0016	0.49
		nPD:	0.
		N. weight:	0.67
		Sc. PD:	-0.087
		Sc. rank:	-938.8

NP			
Pos.	224	obs:	exp:
att	I	0	0.37
atc	I	1	0.25
ata	I	0	0.38
aat	N	1002	1316.00
aac	N	1475	1161.00
aaa	K	1	0.56
aag	K	0	0.44
---	--	------	------
mPD		0.48	0.50
		nPD:	0.97
		N. weight:	0.041
		Sc. PD:	0.033
		Sc. rank:	74.4

NP			
Pos.	225	obs:	exp:
tta	L	0	0.11
ttg	L	0	0.19
ctt	L	1	0.19
ctc	L	0	0.17
cta	L	0	0.13
ctg	L	0	0.21
att	I	887	919.80
atc	I	1584	621.20
ata	I	6	936.00
aat	N	1	0.53
aac	N	0	0.47
---	--	------	------
mPD		0.46	0.66
		nPD:	0.71
		N. weight:	0.69
		Sc. PD:	0.38
		Sc. rank:	1173.7

NP			
Pos.	226	obs:	exp:
tta	L	0	270.60
ttg	L	0	465.20
ctt	L	0	464.80
ctc	L	2469	427.70
cta	L	10	329.80
ctg	L	0	520.90
---	--	------	------
mPD		0.0080	1.1
		nPD:	0.01
		N. weight:	2.1
		Sc. PD:	-0.26
		Sc. rank:	-2014.7

NP			
Pos.	227	obs:	exp:
aaa	K	2342	1391.00
aag	K	137	1088.00
---	--	------	------
mPD		0.10	0.49
		nPD:	0.21
		N. weight:	0.45
		Sc. PD:	0.032
		Sc. rank:	419.4

NP			
Pos.	228	obs:	exp:
ggt	G	0	356.60
ggc	G	0	327.20
gga	G	26	1082.00
ggg	G	2453	713.00
---	--	------	------
mPD		0.021	0.69
		nPD:	0.03
		N. weight:	1.4
		Sc. PD:	-0.15
		Sc. rank:	54.1

NP			
Pos.	229	obs:	exp:
aaa	K	2374	1391.00
aag	K	105	1088.00
---	--	------	------
mPD		0.081	0.49
		nPD:	0.16
		N. weight:	0.49
		Sc. PD:	0.013
		Sc. rank:	389.1

NP			
Pos.	230	obs:	exp:
ttt	F	543	1055.00
ttc	F	1936	1424.00
---	--	------	------
mPD		0.34	0.49
		nPD:	0.7
		N. weight:	0.11
		Sc. PD:	0.062
		Sc. rank:	193.5

NP			
Pos.	231	obs:	exp:
caa	Q	2478	1261.00
cag	Q	1	1218.00
---	--	------	------
mPD		0.00081	0.50
		nPD:	0.
		N. weight:	0.8
		Sc. PD:	-0.11
		Sc. rank:	-1243.7

NP			
Pos.	232	obs:	exp:
tct	S	0	0.17
tcc	S	0	0.15
tca	S	1	0.23
tcg	S	0	0.06
act	T	2	656.40
acc	T	0	547.50
aca	T	2476	1065.00
acg	T	0	209.30
agt	S	0	0.20
agc	S	0	0.19
---	--	------	------
mPD		0.0024	0.69
		nPD:	0.
		N. weight:	1.
		Sc. PD:	-0.13
		Sc. rank:	-1289.5

NP			
Pos.	233	obs:	exp:
gct	A	1	630.60
gcc	A	1	483.40
gca	A	2477	1090.00
gcg	A	0	275.20
---	--	------	------
mPD		0.0016	0.69
		nPD:	0.
		N. weight:	0.97
		Sc. PD:	-0.13
		Sc. rank:	-1410.7

NP			
Pos.	234	obs:	exp:
tct	S	0	0.68
tcc	S	0	0.59
tca	S	4	0.90
tcg	S	0	0.26
agt	S	0	0.82
agc	S	0	0.75
gct	A	0	629.60
gcc	A	1	482.60
gca	A	2474	1088.00
gcg	A	0	274.80
---	--	------	------
mPD		0.0040	0.70
		nPD:	0.01
		N. weight:	0.98
		Sc. PD:	-0.12
		Sc. rank:	-1122.3

NP			
Pos.	235	obs:	exp:
cct	P	0	0.28
ccc	P	0	0.18
cca	P	1	0.41
ccg	P	0	0.13
caa	Q	2475	1260.00
cag	Q	2	1217.00
act	T	0	0.26
acc	T	0	0.22
aca	T	1	0.43
acg	T	0	0.08
---	--	------	------
mPD		0.0040	0.50
		nPD:	0.01
		N. weight:	0.8
		Sc. PD:	-0.1
		Sc. rank:	-710.5

NP			
Pos.	236	obs:	exp:
cgt	R	0	93.94
cgc	R	0	131.90
cga	R	12	239.90
cgg	R	0	194.00
aaa	K	1	1.12
aag	K	1	0.88
aga	R	2464	1136.00
agg	R	1	681.10
---	--	------	------
mPD		0.013	0.95
		nPD:	0.01
		N. weight:	0.9
		Sc. PD:	-0.11
		Sc. rank:	-423.2

NP			
Pos.	237	obs:	exp:
tct	S	0	0.17
tcc	S	0	0.15
tca	S	0	0.23
tcg	S	0	0.06
agt	S	0	0.20
agc	S	1	0.19
gct	A	4	630.40
gcc	A	0	483.20
gca	A	2473	1089.00
gcg	A	1	275.10
---	--	------	------
mPD		0.0064	0.69
		nPD:	0.01
		N. weight:	0.96
		Sc. PD:	-0.12
		Sc. rank:	-754.7

NP			
Pos.	238	obs:	exp:
atg	M	2479	2479.00
---	--	------	------
mPD		0	0
		nPD:	1.
		N. weight:	0.
		Sc. PD:	0
		Sc. rank:	0

NP			
Pos.	239	obs:	exp:
atg	M	2478	2478.00
gtt	V	0	0.22
gtc	V	0	0.21
gta	V	0	0.20
gtg	V	1	0.37
---	--	------	------
mPD		0.00081	0.0013
		nPD:	0.62
		N. weight:	0.00047
		Sc. PD:	0.00022
		Sc. rank:	0.8

NP			
Pos.	240	obs:	exp:
gat	D	1869	1426.00
gac	D	610	1053.00
---	--	------	------
mPD		0.37	0.49
		nPD:	0.76
		N. weight:	0.085
		Sc. PD:	0.051
		Sc. rank:	148.8

NP			
Pos.	241	obs:	exp:
caa	Q	1704	1261.00
cag	Q	775	1218.00
---	--	------	------
mPD		0.43	0.50
		nPD:	0.86
		N. weight:	0.08
		Sc. PD:	0.056
		Sc. rank:	141.4

NP			
Pos.	242	obs:	exp:
gtt	V	33	539.10
gtc	V	0	523.80
gta	V	31	487.80
gtg	V	2415	928.30
---	--	------	------
mPD		0.051	0.73
		nPD:	0.07
		N. weight:	1.
		Sc. PD:	-0.068
		Sc. rank:	515.2

NP			
Pos.	243	obs:	exp:
cgt	R	1604	94.01
cgc	R	1	132.00
cga	R	861	240.00
cgg	R	0	194.20
aga	R	10	1137.00
agg	R	3	681.60
---	--	------	------
mPD		0.47	0.95
		nPD:	0.49
		N. weight:	2.7
		Sc. PD:	0.91
		Sc. rank:	3914.8

NP			
Pos.	244	obs:	exp:
gaa	E	2462	1484.00
gag	E	17	994.70
---	--	------	------
mPD		0.014	0.48
		nPD:	0.03
		N. weight:	0.57
		Sc. PD:	-0.06
		Sc. rank:	5.8

NP			
Pos.	245	obs:	exp:
tct	S	0	423.40
tcc	S	0	365.50
tca	S	0	558.80
tcg	S	0	160.10
agt	S	1	506.10
agc	S	2478	465.10
---	--	------	------
mPD		0.00081	1.6
		nPD:	0.
		N. weight:	2.
		Sc. PD:	-0.26
		Sc. rank:	-3365.8

NP			
Pos.	246	obs:	exp:
cgt	R	2	93.71
cgc	R	0	131.60
cga	R	2	239.30
cgg	R	7	193.50
aaa	K	0	4.49
aag	K	8	3.51
aga	R	333	1133.00
agg	R	2127	679.40
---	--	------	------
mPD		0.25	0.96
		nPD:	0.26
		N. weight:	0.96
		Sc. PD:	0.11
		Sc. rank:	1022.6

NP			
Pos.	247	obs:	exp:
tct	S	0	0.51
tcc	S	0	0.44
tca	S	0	0.68
tcg	S	0	0.19
aat	N	2458	1316.00
aac	N	18	1160.00
agt	S	3	0.61
agc	S	0	0.56
---	--	------	------
mPD		0.017	0.50
		nPD:	0.03
		N. weight:	0.71
		Sc. PD:	-0.071
		Sc. rank:	67.7

NP			
Pos.	248	obs:	exp:
cct	P	2440	699.10
ccc	P	30	448.60
cca	P	7	1012.00
ccg	P	2	319.20
---	--	------	------
mPD		0.031	0.70
		nPD:	0.04
		N. weight:	1.4
		Sc. PD:	-0.13
		Sc. rank:	426.4

NP			
Pos.	249	obs:	exp:
ggt	G	0	356.60
ggc	G	1617	327.20
gga	G	56	1082.00
ggg	G	806	713.00
---	--	------	------
mPD		0.47	0.69
		nPD:	0.68
		N. weight:	1.2
		Sc. PD:	0.63
		Sc. rank:	2038.9

NP			
Pos.	250	obs:	exp:
aat	N	2478	1317.00
aac	N	1	1162.00
---	--	------	------
mPD		0.00081	0.50
		nPD:	0.
		N. weight:	0.75
		Sc. PD:	-0.098
		Sc. rank:	-1150.2

NP			
Pos.	251	obs:	exp:
gtt	V	1	0.22
gtc	V	0	0.21
gta	V	0	0.20
gtg	V	0	0.37
gct	A	2472	630.40
gcc	A	4	483.20
gca	A	1	1089.00
gcg	A	1	275.10
---	--	------	------
mPD		0.0056	0.69
		nPD:	0.01
		N. weight:	1.6
		Sc. PD:	-0.2
		Sc. rank:	-1388.2

NP			
Pos.	252	obs:	exp:
aaa	K	1	0.56
aag	K	0	0.44
gaa	E	2468	1484.00
gag	E	10	994.30
---	--	------	------
mPD		0.0088	0.48
		nPD:	0.02
		N. weight:	0.58
		Sc. PD:	-0.067
		Sc. rank:	-167.7

NP			
Pos.	253	obs:	exp:
att	I	2473	919.40
atc	I	1	621.00
ata	I	2	935.60
act	T	3	0.79
acc	T	0	0.66
aca	T	0	1.29
acg	T	0	0.25
---	--	------	------
mPD		0.0048	0.66
		nPD:	0.01
		N. weight:	1.2
		Sc. PD:	-0.15
		Sc. rank:	-1131.9

NP			
Pos.	254	obs:	exp:
gaa	E	2367	1484.00
gag	E	112	994.70
---	--	------	------
mPD		0.086	0.48
		nPD:	0.18
		N. weight:	0.41
		Sc. PD:	0.017
		Sc. rank:	348.3

NP			
Pos.	255	obs:	exp:
gat	D	2472	1426.00
gac	D	7	1053.00
---	--	------	------
mPD		0.0056	0.49
		nPD:	0.01
		N. weight:	0.64
		Sc. PD:	-0.078
		Sc. rank:	-395.7

NP			
Pos.	256	obs:	exp:
tta	L	0	270.60
ttg	L	0	465.20
ctt	L	7	464.80
ctc	L	2468	427.70
cta	L	4	329.80
ctg	L	0	520.90
---	--	------	------
mPD		0.0088	1.1
		nPD:	0.01
		N. weight:	2.1
		Sc. PD:	-0.26
		Sc. rank:	-1912.9

NP			
Pos.	257	obs:	exp:
att	I	1535	919.40
atc	I	941	621.00
ata	I	0	935.60
act	T	1	0.53
acc	T	1	0.44
aca	T	0	0.86
acg	T	0	0.17
gtt	V	0	0.22
gtc	V	1	0.21
gta	V	0	0.20
gtg	V	0	0.37
---	--	------	------
mPD		0.47	0.66
		nPD:	0.72
		N. weight:	0.57
		Sc. PD:	0.32
		Sc. rank:	984.6

NP			
Pos.	258	obs:	exp:
ttt	F	2163	1055.00
ttc	F	316	1424.00
---	--	------	------
mPD		0.22	0.49
		nPD:	0.46
		N. weight:	0.52
		Sc. PD:	0.16
		Sc. rank:	697.1

NP			
Pos.	259	obs:	exp:
tta	L	0	270.60
ttg	L	3	465.20
ctt	L	19	464.80
ctc	L	3	427.70
cta	L	253	329.80
ctg	L	2201	520.90
---	--	------	------
mPD		0.20	1.1
		nPD:	0.18
		N. weight:	1.5
		Sc. PD:	0.055
		Sc. rank:	1215.6

NP			
Pos.	260	obs:	exp:
tct	S	0	0.34
tcc	S	0	0.29
tca	S	2	0.45
tcg	S	0	0.13
act	T	0	0.79
acc	T	0	0.66
aca	T	2	1.29
acg	T	1	0.25
agt	S	0	0.41
agc	S	0	0.38
gtt	V	0	0.43
gtc	V	0	0.42
gta	V	0	0.39
gtg	V	2	0.75
gct	A	1	628.90
gcc	A	0	482.10
gca	A	1619	1087.00
gcg	A	852	274.40
---	--	------	------
mPD		0.46	0.70
		nPD:	0.66
		N. weight:	0.78
		Sc. PD:	0.39
		Sc. rank:	1286.3

NP			
Pos.	261	obs:	exp:
cgt	R	5	94.01
cgc	R	0	132.00
cga	R	14	240.00
cgg	R	2431	194.20
aga	R	1	1137.00
agg	R	28	681.60
---	--	------	------
mPD		0.039	0.95
		nPD:	0.04
		N. weight:	2.9
		Sc. PD:	-0.27
		Sc. rank:	735.6

NP			
Pos.	262	obs:	exp:
ttt	F	1	0.43
ttc	F	0	0.57
tct	S	2421	423.00
tcc	S	1	365.20
tca	S	55	558.40
tcg	S	0	160.00
tgt	C	0	0.43
tgc	C	1	0.57
agt	S	0	505.70
agc	S	0	464.70
---	--	------	------
mPD		0.047	1.6
		nPD:	0.03
		N. weight:	2.
		Sc. PD:	-0.21
		Sc. rank:	8.7

NP			
Pos.	263	obs:	exp:
act	T	0	0.26
acc	T	0	0.22
aca	T	1	0.43
acg	T	0	0.08
gct	A	1170	630.40
gcc	A	0	483.20
gca	A	1025	1089.00
gcg	A	283	275.10
---	--	------	------
mPD		0.59	0.69
		nPD:	0.86
		N. weight:	0.33
		Sc. PD:	0.23
		Sc. rank:	574.8

NP			
Pos.	264	obs:	exp:
tta	L	0	270.60
ttg	L	0	465.20
ctt	L	1	464.80
ctc	L	2478	427.70
cta	L	0	329.80
ctg	L	0	520.90
---	--	------	------
mPD		0.00081	1.1
		nPD:	0.
		N. weight:	2.1
		Sc. PD:	-0.28
		Sc. rank:	-3503.3

NP			
Pos.	265	obs:	exp:
att	I	853	899.40
atc	I	1564	607.40
ata	I	5	915.20
gtt	V	52	12.40
gtc	V	5	12.04
gta	V	0	11.22
gtg	V	0	21.34
---	--	------	------
mPD		0.51	0.71
		nPD:	0.72
		N. weight:	0.71
		Sc. PD:	0.4
		Sc. rank:	1241.4

NP			
Pos.	266	obs:	exp:
tta	L	0	270.60
ttg	L	4	465.20
ctt	L	0	464.80
ctc	L	0	427.70
cta	L	3	329.80
ctg	L	2472	520.90
---	--	------	------
mPD		0.0056	1.1
		nPD:	0.
		N. weight:	1.8
		Sc. PD:	-0.23
		Sc. rank:	-2137.3

NP			
Pos.	267	obs:	exp:
cgt	R	0	93.94
cgc	R	0	131.90
cga	R	1	239.90
cgg	R	0	194.00
aaa	K	2	1.12
aag	K	0	0.88
aga	R	2467	1136.00
agg	R	9	681.10
---	--	------	------
mPD		0.0097	0.95
		nPD:	0.01
		N. weight:	0.9
		Sc. PD:	-0.11
		Sc. rank:	-642.0

NP			
Pos.	268	obs:	exp:
ggt	G	0	356.60
ggc	G	1	327.20
gga	G	2156	1082.00
ggg	G	322	713.00
---	--	------	------
mPD		0.23	0.69
		nPD:	0.33
		N. weight:	0.59
		Sc. PD:	0.11
		Sc. rank:	719.1

NP			
Pos.	269	obs:	exp:
tct	S	2	423.40
tcc	S	1	365.50
tca	S	2448	558.80
tcg	S	28	160.10
agt	S	0	506.10
agc	S	0	465.10
---	--	------	------
mPD		0.025	1.6
		nPD:	0.02
		N. weight:	1.7
		Sc. PD:	-0.2
		Sc. rank:	-730.2

NP			
Pos.	270	obs:	exp:
gtt	V	0	539.10
gtc	V	0	523.80
gta	V	33	487.80
gtg	V	2446	928.30
---	--	------	------
mPD		0.026	0.73
		nPD:	0.04
		N. weight:	1.1
		Sc. PD:	-0.11
		Sc. rank:	162.5

NP			
Pos.	271	obs:	exp:
gct	A	31	630.40
gcc	A	2446	483.20
gca	A	1	1089.00
gcg	A	0	275.10
ggt	G	0	0.14
ggc	G	1	0.13
gga	G	0	0.44
ggg	G	0	0.29
---	--	------	------
mPD		0.026	0.69
		nPD:	0.04
		N. weight:	1.9
		Sc. PD:	-0.18
		Sc. rank:	321.5

NP			
Pos.	272	obs:	exp:
cat	H	1973	1474.00
cac	H	506	1005.00
---	--	------	------
mPD		0.33	0.48
		nPD:	0.67
		N. weight:	0.11
		Sc. PD:	0.058
		Sc. rank:	185.7

NP			
Pos.	273	obs:	exp:
aaa	K	105	1391.00
aag	K	2374	1088.00
---	--	------	------
mPD		0.081	0.49
		nPD:	0.16
		N. weight:	0.76
		Sc. PD:	0.02
		Sc. rank:	601.2

NP			
Pos.	274	obs:	exp:
tct	S	526	423.40
tcc	S	1953	365.50
tca	S	0	558.80
tcg	S	0	160.10
agt	S	0	506.10
agc	S	0	465.10
---	--	------	------
mPD		0.33	1.6
		nPD:	0.2
		N. weight:	1.6
		Sc. PD:	0.1
		Sc. rank:	1487.7

NP			
Pos.	275	obs:	exp:
tgt	C	517	1078.00
tgc	C	1961	1400.00
cgt	R	0	0.04
cgc	R	1	0.05
cga	R	0	0.10
cgg	R	0	0.08
aga	R	0	0.46
agg	R	0	0.27
---	--	------	------
mPD		0.33	0.49
		nPD:	0.67
		N. weight:	0.14
		Sc. PD:	0.071
		Sc. rank:	229.8

NP			
Pos.	276	obs:	exp:
ttt	F	4	1.70
ttc	F	0	2.30
tta	L	5	270.10
ttg	L	1642	464.40
ctt	L	306	464.10
ctc	L	0	427.00
cta	L	2	329.30
ctg	L	520	520.00
---	--	------	------
mPD		0.67	1.1
		nPD:	0.59
		N. weight:	0.93
		Sc. PD:	0.4
		Sc. rank:	1474.7

NP			
Pos.	277	obs:	exp:
tta	L	0	0.11
ttg	L	0	0.19
ctt	L	1	0.19
ctc	L	0	0.17
cta	L	0	0.13
ctg	L	0	0.21
cct	P	1445	698.80
ccc	P	966	448.40
cca	P	65	1012.00
ccg	P	2	319.10
---	--	------	------
mPD		0.51	0.71
		nPD:	0.72
		N. weight:	0.78
		Sc. PD:	0.43
		Sc. rank:	1343.6

NP			
Pos.	278	obs:	exp:
gct	A	2280	630.60
gcc	A	198	483.40
gca	A	0	1090.00
gcg	A	1	275.20
---	--	------	------
mPD		0.15	0.69
		nPD:	0.21
		N. weight:	1.3
		Sc. PD:	0.096
		Sc. rank:	1242.1

NP			
Pos.	279	obs:	exp:
tgt	C	2475	1078.00
tgc	C	4	1401.00
---	--	------	------
mPD		0.0032	0.49
		nPD:	0.01
		N. weight:	0.98
		Sc. PD:	-0.12
		Sc. rank:	-1010.4

NP			
Pos.	280	obs:	exp:
att	I	2	0.74
atc	I	0	0.50
ata	I	0	0.76
gtt	V	540	538.70
gtc	V	0	523.40
gta	V	1045	487.40
gtg	V	892	927.60
---	--	------	------
mPD		0.65	0.73
		nPD:	0.89
		N. weight:	0.37
		Sc. PD:	0.27
		Sc. rank:	661.2

NP			
Pos.	281	obs:	exp:
tat	Y	177	1205.00
tac	Y	2302	1274.00
---	--	------	------
mPD		0.13	0.50
		nPD:	0.27
		N. weight:	0.49
		Sc. PD:	0.061
		Sc. rank:	529.7

NP			
Pos.	282	obs:	exp:
ggt	G	0	356.60
ggc	G	37	327.20
gga	G	1523	1082.00
ggg	G	919	713.00
---	--	------	------
mPD		0.49	0.69
		nPD:	0.7
		N. weight:	0.33
		Sc. PD:	0.18
		Sc. rank:	554.6

NP			
Pos.	283	obs:	exp:
ttt	F	0	0.43
ttc	F	1	0.57
tta	L	0	270.50
ttg	L	0	465.00
ctt	L	995	464.70
ctc	L	1483	427.50
cta	L	0	329.70
ctg	L	0	520.70
---	--	------	------
mPD		0.48	1.1
		nPD:	0.42
		N. weight:	1.3
		Sc. PD:	0.34
		Sc. rank:	1657.7

NP			
Pos.	284	obs:	exp:
act	T	5	1.32
acc	T	0	1.10
aca	T	0	2.15
acg	T	0	0.42
gct	A	2426	629.40
gcc	A	30	482.40
gca	A	9	1088.00
gcg	A	9	274.70
---	--	------	------
mPD		0.042	0.70
		nPD:	0.06
		N. weight:	1.5
		Sc. PD:	-0.11
		Sc. rank:	718.8

NP			
Pos.	285	obs:	exp:
gtt	V	2	539.10
gtc	V	5	523.80
gta	V	894	487.80
gtg	V	1578	928.30
---	--	------	------
mPD		0.46	0.73
		nPD:	0.64
		N. weight:	0.65
		Sc. PD:	0.31
		Sc. rank:	1061.4

NP			
Pos.	286	obs:	exp:
gtt	V	1	66.98
gtc	V	307	65.08
gta	V	0	60.60
gtg	V	0	115.30
gct	A	5	552.00
gcc	A	2159	423.20
gca	A	6	953.90
gcg	A	0	240.90
ggt	G	0	0.14
ggc	G	1	0.13
gga	G	0	0.44
ggg	G	0	0.29
---	--	------	------
mPD		0.23	0.93
		nPD:	0.25
		N. weight:	1.9
		Sc. PD:	0.2
		Sc. rank:	1984.4

NP			
Pos.	287	obs:	exp:
tct	S	0	407.10
tcc	S	0	351.40
tca	S	0	537.40
tcg	S	0	154.00
aat	N	92	48.89
aac	N	0	43.11
agt	S	2373	486.80
agc	S	11	447.30
ggt	G	3	0.43
ggc	G	0	0.40
gga	G	0	1.31
ggg	G	0	0.86
---	--	------	------
mPD		0.083	1.7
		nPD:	0.05
		N. weight:	1.8
		Sc. PD:	-0.16
		Sc. rank:	745.3

NP			
Pos.	288	obs:	exp:
ggt	G	0	356.60
ggc	G	2	327.20
gga	G	2295	1082.00
ggg	G	182	713.00
---	--	------	------
mPD		0.14	0.69
		nPD:	0.2
		N. weight:	0.71
		Sc. PD:	0.042
		Sc. rank:	628.2

NP			
Pos.	289	obs:	exp:
ttt	F	0	0.43
ttc	F	1	0.57
tat	Y	1010	1180.00
tac	Y	1418	1248.00
cat	H	50	29.73
cac	H	0	20.27
---	--	------	------
mPD		0.53	0.54
		nPD:	0.98
		N. weight:	0.026
		Sc. PD:	0.021
		Sc. rank:	47.2

NP			
Pos.	290	obs:	exp:
aat	N	0	2.66
aac	N	5	2.34
gat	D	38	1404.00
gac	D	2403	1037.00
gaa	E	27	16.17
gag	E	0	10.83
ggt	G	0	0.86
ggc	G	6	0.79
gga	G	0	2.62
ggg	G	0	1.73
---	--	------	------
mPD		0.060	0.51
		nPD:	0.12
		N. weight:	0.92
		Sc. PD:	-0.017
		Sc. rank:	625.0

NP			
Pos.	291	obs:	exp:
ttt	F	2475	1055.00
ttc	F	4	1424.00
---	--	------	------
mPD		0.0032	0.49
		nPD:	0.01
		N. weight:	1.
		Sc. PD:	-0.13
		Sc. rank:	-1029.7

NP			
Pos.	292	obs:	exp:
gaa	E	17	1484.00
gag	E	2462	994.70
---	--	------	------
mPD		0.014	0.48
		nPD:	0.03
		N. weight:	1.
		Sc. PD:	-0.11
		Sc. rank:	10.6

NP			
Pos.	293	obs:	exp:
cgt	R	0	93.94
cgc	R	0	131.90
cga	R	0	239.90
cgg	R	0	194.00
aaa	K	2	1.12
aag	K	0	0.88
aga	R	2466	1136.00
agg	R	11	681.10
---	--	------	------
mPD		0.010	0.95
		nPD:	0.01
		N. weight:	0.9
		Sc. PD:	-0.11
		Sc. rank:	-576.3

NP			
Pos.	294	obs:	exp:
gat	D	1	0.58
gac	D	0	0.42
gaa	E	2463	1484.00
gag	E	15	994.30
---	--	------	------
mPD		0.013	0.48
		nPD:	0.03
		N. weight:	0.57
		Sc. PD:	-0.061
		Sc. rank:	-19.8

NP			
Pos.	295	obs:	exp:
ggt	G	0	356.60
ggc	G	121	327.20
gga	G	552	1082.00
ggg	G	1806	713.00
---	--	------	------
mPD		0.42	0.69
		nPD:	0.61
		N. weight:	0.57
		Sc. PD:	0.26
		Sc. rank:	922.2

NP			
Pos.	296	obs:	exp:
tat	Y	11	1204.00
tac	Y	2466	1273.00
cat	H	0	1.19
cac	H	2	0.81
---	--	------	------
mPD		0.010	0.50
		nPD:	0.02
		N. weight:	0.76
		Sc. PD:	-0.086
		Sc. rank:	-160.4

NP			
Pos.	297	obs:	exp:
tct	S	2463	423.40
tcc	S	15	365.50
tca	S	1	558.80
tcg	S	0	160.10
agt	S	0	506.10
agc	S	0	465.10
---	--	------	------
mPD		0.013	1.6
		nPD:	0.01
		N. weight:	2.1
		Sc. PD:	-0.26
		Sc. rank:	-1884.0

NP			
Pos.	298	obs:	exp:
tta	L	2	270.50
ttg	L	0	465.00
ctt	L	0	464.70
ctc	L	0	427.50
cta	L	1937	329.70
ctg	L	539	520.70
att	I	0	0.37
atc	I	0	0.25
ata	I	1	0.38
---	--	------	------
mPD		0.34	1.1
		nPD:	0.3
		N. weight:	1.7
		Sc. PD:	0.26
		Sc. rank:	1921.7

NP			
Pos.	299	obs:	exp:
gtt	V	2102	539.10
gtc	V	376	523.80
gta	V	0	487.80
gtg	V	1	928.30
---	--	------	------
mPD		0.26	0.73
		nPD:	0.35
		N. weight:	1.3
		Sc. PD:	0.27
		Sc. rank:	1641.3

NP			
Pos.	300	obs:	exp:
ggt	G	0	356.60
ggc	G	0	327.20
gga	G	2456	1082.00
ggg	G	23	713.00
---	--	------	------
mPD		0.018	0.69
		nPD:	0.03
		N. weight:	0.93
		Sc. PD:	-0.1
		Sc. rank:	-26.0

NP			
Pos.	301	obs:	exp:
tta	L	0	0.11
ttg	L	0	0.19
ctt	L	0	0.19
ctc	L	0	0.17
cta	L	1	0.13
ctg	L	0	0.21
att	I	9	917.90
atc	I	4	620.00
ata	I	2459	934.10
atg	M	2	2.00
gtt	V	0	0.87
gtc	V	0	0.85
gta	V	4	0.79
gtg	V	0	1.50
---	--	------	------
mPD		0.016	0.66
		nPD:	0.02
		N. weight:	1.1
		Sc. PD:	-0.12
		Sc. rank:	-121.9

NP			
Pos.	302	obs:	exp:
tat	Y	0	0.49
tac	Y	1	0.51
gat	D	1039	1425.00
gac	D	1439	1053.00
---	--	------	------
mPD		0.49	0.49
		nPD:	1.
		N. weight:	0.061
		Sc. PD:	0.05
		Sc. rank:	109.0

NP			
Pos.	303	obs:	exp:
cct	P	2461	698.80
ccc	P	17	448.40
cca	P	0	1012.00
ccg	P	0	319.10
cat	H	1	0.59
cac	H	0	0.41
---	--	------	------
mPD		0.014	0.71
		nPD:	0.02
		N. weight:	1.5
		Sc. PD:	-0.17
		Sc. rank:	-336.5

NP			
Pos.	304	obs:	exp:
ttt	F	0	1054.00
ttc	F	2478	1424.00
tta	L	1	0.11
ttg	L	0	0.19
ctt	L	0	0.19
ctc	L	0	0.17
cta	L	0	0.13
ctg	L	0	0.21
---	--	------	------
mPD		0.00081	0.49
		nPD:	0.
		N. weight:	0.66
		Sc. PD:	-0.087
		Sc. rank:	-1006.8

NP			
Pos.	305	obs:	exp:
caa	Q	2	1.02
cag	Q	0	0.98
cgt	R	1587	93.94
cgc	R	0	131.90
cga	R	884	239.90
cgg	R	5	194.00
aga	R	1	1136.00
agg	R	0	681.10
---	--	------	------
mPD		0.46	0.96
		nPD:	0.49
		N. weight:	2.7
		Sc. PD:	0.91
		Sc. rank:	3844.7

NP			
Pos.	306	obs:	exp:
tta	L	0	270.60
ttg	L	11	465.20
ctt	L	0	464.80
ctc	L	0	427.70
cta	L	17	329.80
ctg	L	2451	520.90
---	--	------	------
mPD		0.022	1.1
		nPD:	0.02
		N. weight:	1.8
		Sc. PD:	-0.2
		Sc. rank:	-428.7

,

NP			
Pos.	307	obs:	exp:
tta	L	0	270.60
ttg	L	0	465.20
ctt	L	2458	464.80
ctc	L	20	427.70
cta	L	1	329.80
ctg	L	0	520.90
---	--	------	------
mPD		0.017	1.1
		nPD:	0.01
		N. weight:	1.9
		Sc. PD:	-0.23
		Sc. rank:	-851.5

,

NP			
Pos.	308	obs:	exp:
caa	Q	2304	1261.00
cag	Q	175	1218.00
---	--	------	------
mPD		0.13	0.50
		nPD:	0.26
		N. weight:	0.51
		Sc. PD:	0.061
		Sc. rank:	541.5

,

NP			
Pos.	309	obs:	exp:
aat	N	4	1317.00
aac	N	2475	1162.00
---	--	------	------
mPD		0.0032	0.50
		nPD:	0.01
		N. weight:	0.89
		Sc. PD:	-0.11
		Sc. rank:	-925.0

,

NP			
Pos.	310	obs:	exp:
tct	S	0	422.20
tcc	S	0	364.40
tca	S	0	557.20
tcg	S	0	159.70
tgt	C	0	0.87
tgc	C	2	1.13
att	I	0	0.37
atc	I	1	0.25
ata	I	0	0.38
act	T	0	0.53
acc	T	2	0.44
aca	T	0	0.86
acg	T	0	0.17
aat	N	0	1.06
aac	N	2	0.94
agt	S	19	504.70
agc	S	2453	463.80
---	--	------	------
mPD		0.021	1.6
		nPD:	0.01
		N. weight:	1.9
		Sc. PD:	-0.23
		Sc. rank:	-1034.8

,

NP			
Pos.	311	obs:	exp:
cat	H	1	0.59
cac	H	0	0.41
caa	Q	27	1261.00
cag	Q	2451	1217.00
---	--	------	------
mPD		0.022	0.50
		nPD:	0.04
		N. weight:	0.78
		Sc. PD:	-0.07
		Sc. rank:	239.3

,

NP			
Pos.	312	obs:	exp:
att	I	0	0.37
atc	I	1	0.25
ata	I	0	0.38
gtt	V	4	538.30
gtc	V	2468	522.90
gta	V	3	487.00
gtg	V	0	926.80
gct	A	0	0.76
gcc	A	3	0.59
gca	A	0	1.32
gcg	A	0	0.33
---	--	------	------
mPD		0.0089	0.73
		nPD:	0.01
		N. weight:	1.8
		Sc. PD:	-0.22
		Sc. rank:	-1046.3

,

NP			
Pos.	313	obs:	exp:
ttt	F	894	1054.00
ttc	F	1583	1423.00
tta	L	0	0.11
ttg	L	0	0.19
tct	S	1	0.17
tcc	S	0	0.15
tca	S	0	0.23
tcg	S	0	0.06
ctt	L	1	0.19
ctc	L	0	0.17
cta	L	0	0.13
ctg	L	0	0.21
agt	S	0	0.20
agc	S	0	0.19
---	--	------	------
mPD		0.46	0.49
		nPD:	0.94
		N. weight:	0.014
		Sc. PD:	0.011
		Sc. rank:	25.0

,

NP			
Pos.	314	obs:	exp:
tct	S	0	423.20
tcc	S	0	365.30
tca	S	0	558.60
tcg	S	0	160.10
act	T	1	0.26
acc	T	0	0.22
aca	T	0	0.43
acg	T	0	0.08
agt	S	2190	505.90
agc	S	288	464.90
---	--	------	------
mPD		0.21	1.6
		nPD:	0.13
		N. weight:	1.5
		Sc. PD:	-0.018
		Sc. rank:	1037.4

,

NP			
Pos.	315	obs:	exp:
tta	L	0	270.60
ttg	L	0	465.20
ctt	L	14	464.80
ctc	L	2465	427.70
cta	L	0	329.80
ctg	L	0	520.90
---	--	------	------
mPD		0.011	1.1
		nPD:	0.01
		N. weight:	2.1
		Sc. PD:	-0.25
		Sc. rank:	-1506.5

,

NP			
Pos.	316	obs:	exp:
att	I	2440	919.80
atc	I	33	621.20
ata	I	4	936.00
act	T	1	0.26
acc	T	0	0.22
aca	T	0	0.43
acg	T	0	0.08
gtt	V	1	0.22
gtc	V	0	0.21
gta	V	0	0.20
gtg	V	0	0.37
---	--	------	------
mPD		0.031	0.66
		nPD:	0.05
		N. weight:	1.1
		Sc. PD:	-0.095
		Sc. rank:	409.6

,

NP			
Pos.	317	obs:	exp:
cgt	R	0	93.97
cgc	R	0	132.00
cga	R	1	239.90
cgg	R	0	194.10
att	I	0	0.37
atc	I	0	0.25
ata	I	1	0.38
aga	R	2468	1137.00
agg	R	9	681.40
---	--	------	------
mPD		0.0089	0.95
		nPD:	0.01
		N. weight:	0.9
		Sc. PD:	-0.11
		Sc. rank:	-729.2

,

NP			
Pos.	318	obs:	exp:
tct	S	0	6.15
tcc	S	0	5.31
tca	S	36	8.11
tcg	S	0	2.33
cct	P	1	688.90
ccc	P	0	442.10
cca	P	2398	997.50
ccg	P	44	314.60
agt	S	0	7.35
agc	S	0	6.75
---	--	------	------
mPD		0.064	0.75
		nPD:	0.09
		N. weight:	0.99
		Sc. PD:	-0.049
		Sc. rank:	575.3

,

NP			
Pos.	319	obs:	exp:
aat	N	2453	1307.00
aac	N	7	1153.00
aaa	K	19	10.66
aag	K	0	8.34
---	--	------	------
mPD		0.021	0.51
		nPD:	0.04
		N. weight:	0.73
		Sc. PD:	-0.068
		Sc. rank:	190.3

,

NP			
Pos.	320	obs:	exp:
gaa	E	563	1484.00
gag	E	1916	994.70
---	--	------	------
mPD		0.35	0.48
		nPD:	0.73
		N. weight:	0.34
		Sc. PD:	0.2
		Sc. rank:	600.5

,

NP			
Pos.	321	obs:	exp:
tct	S	0	0.85
tcc	S	0	0.74
tca	S	0	1.13
tcg	S	0	0.32
aat	N	1627	1314.00
aac	N	845	1158.00
aaa	K	1	0.56
aag	K	0	0.44
agt	S	5	1.02
agc	S	0	0.94
gat	D	1	0.58
gac	D	0	0.42
---	--	------	------
mPD		0.45	0.51
		nPD:	0.9
		N. weight:	0.045
		Sc. PD:	0.033
		Sc. rank:	81.0

,

NP			
Pos.	322	obs:	exp:
cct	P	14	699.10
ccc	P	1	448.60
cca	P	2446	1012.00
ccg	P	18	319.20
---	--	------	------
mPD		0.026	0.70
		nPD:	0.04
		N. weight:	0.99
		Sc. PD:	-0.095
		Sc. rank:	157.1

,

NP			
Pos.	323	obs:	exp:
act	T	0	32.05
acc	T	0	26.73
aca	T	121	51.99
acg	T	0	10.22
gtt	V	0	0.22
gtc	V	0	0.21
gta	V	1	0.20
gtg	V	0	0.37
gct	A	0	599.60
gcc	A	0	459.60
gca	A	2353	1036.00
gcg	A	4	261.70
---	--	------	------
mPD		0.097	0.79
		nPD:	0.12
		N. weight:	0.97
		Sc. PD:	-0.014
		Sc. rank:	665.7

,

NP			
Pos.	324	obs:	exp:
cat	H	551	1474.00
cac	H	1928	1005.00
---	--	------	------
mPD		0.35	0.48
		nPD:	0.72
		N. weight:	0.34
		Sc. PD:	0.19
		Sc. rank:	594.7

,

NP			
Pos.	325	obs:	exp:
cgt	R	0	0.04
cgc	R	0	0.05
cga	R	0	0.10
cgg	R	0	0.08
aaa	K	577	1391.00
aag	K	1901	1087.00
aga	R	0	0.46
agg	R	1	0.27
---	--	------	------
mPD		0.36	0.49
		nPD:	0.73
		N. weight:	0.27
		Sc. PD:	0.15
		Sc. rank:	468.5

,

NP			
Pos.	326	obs:	exp:
tct	S	0	423.40
tcc	S	0	365.50
tca	S	0	558.80
tcg	S	0	160.10
agt	S	1958	506.10
agc	S	521	465.10
mPD		0.33	1.6
		nPD:	0.2
		N. weight:	1.3
		Sc. PD:	0.08
		Sc. rank:	1174.3

NP			
Pos.	327	obs:	exp:
caa	Q	1991	1261.00
cag	Q	488	1218.00
mPD		0.32	0.50
		nPD:	0.63
		N. weight:	0.22
		Sc. PD:	0.11
		Sc. rank:	365.3

NP			
Pos.	328	obs:	exp:
tta	L	2	270.60
ttg	L	2329	465.20
ctt	L	0	464.80
ctc	L	0	427.70
cta	L	2	329.80
ctg	L	146	520.90
mPD		0.12	1.1
		nPD:	0.1
		N. weight:	1.7
		Sc. PD:	-0.059
		Sc. rank:	1086.0

NP			
Pos.	329	obs:	exp:
atg	M	8	8.00
gtt	V	1	537.40
gtc	V	0	522.10
gta	V	570	486.20
gtg	V	1900	925.30
mPD		0.36	0.73
		nPD:	0.49
		N. weight:	0.7
		Sc. PD:	0.24
		Sc. rank:	1023.4

NP			
Pos.	330	obs:	exp:
tgg	W	2479	2479.00
mPD		0	0
		nPD:	1.
		N. weight:	0.
		Sc. PD:	0
		Sc. rank:	0

NP			
Pos.	331	obs:	exp:
att	I	0	2.60
atc	I	0	1.76
ata	I	7	2.65
atg	M	2472	2472.00
mPD		0.0056	0.0056
		nPD:	1.
		N. weight:	0.0033
		Sc. PD:	0.0027
		Sc. rank:	5.9

NP			
Pos.	332	obs:	exp:
gct	A	0	630.40
gcc	A	0	483.20
gca	A	2475	1089.00
gcg	A	3	275.10
gaa	E	1	0.60
gag	E	0	0.40
mPD		0.0032	0.69
		nPD:	0.
		N. weight:	0.97
		Sc. PD:	-0.12
		Sc. rank:	-1192.3

NP			
Pos.	333	obs:	exp:
ttt	F	1	0.43
ttc	F	0	0.57
tgt	C	1622	1078.00
tgc	C	856	1400.00
mPD		0.45	0.49
		nPD:	0.92
		N. weight:	0.12
		Sc. PD:	0.089
		Sc. rank:	212.2

NP			
Pos.	334	obs:	exp:
cat	H	2319	1474.00
cac	H	160	1005.00
mPD		0.12	0.48
		nPD:	0.25
		N. weight:	0.37
		Sc. PD:	0.04
		Sc. rank:	383.6

NP			
Pos.	335	obs:	exp:
ttt	F	1	0.43
ttc	F	0	0.57
tct	S	1913	423.20
tcc	S	16	365.30
tca	S	543	558.60
tcg	S	6	160.10
agt	S	0	505.90
agc	S	0	464.90
mPD		0.36	1.6
		nPD:	0.22
		N. weight:	1.4
		Sc. PD:	0.1
		Sc. rank:	1299.8

NP			
Pos.	336	obs:	exp:
gct	A	5	630.60
gcc	A	0	483.40
gca	A	2464	1090.00
gcg	A	10	275.20
mPD		0.012	0.69
		nPD:	0.02
		N. weight:	0.94
		Sc. PD:	-0.11
		Sc. rank:	-303.6

NP			
Pos.	337	obs:	exp:
gct	A	1	630.60
gcc	A	0	483.40
gca	A	1939	1090.00
gcg	A	539	275.20
mPD		0.34	0.69
		nPD:	0.49
		N. weight:	0.71
		Sc. PD:	0.24
		Sc. rank:	1042.4

NP			
Pos.	338	obs:	exp:
ttt	F	863	1055.00
ttc	F	1616	1424.00
mPD		0.45	0.49
		nPD:	0.93
		N. weight:	0.017
		Sc. PD:	0.013
		Sc. rank:	30.2

NP			
Pos.	339	obs:	exp:
gaa	E	173	1484.00
gag	E	2306	994.70
mPD		0.13	0.48
		nPD:	0.27
		N. weight:	0.75
		Sc. PD:	0.096
		Sc. rank:	817.3

NP			
Pos.	340	obs:	exp:
gat	D	1627	1426.00
gac	D	852	1053.00
mPD		0.45	0.49
		nPD:	0.92
		N. weight:	0.018
		Sc. PD:	0.014
		Sc. rank:	32.8

NP			
Pos.	341	obs:	exp:
tta	L	0	270.60
ttg	L	6	465.20
ctt	L	0	464.80
ctc	L	1	427.70
cta	L	4	329.80
ctg	L	2468	520.90
mPD		0.0089	1.1
		nPD:	0.01
		N. weight:	1.8
		Sc. PD:	-0.23
		Sc. rank:	-1643.7

NP			
Pos.	342	obs:	exp:
cgt	R	0	94.01
cgc	R	0	132.00
cga	R	4	240.00
cgg	R	0	194.20
aga	R	1921	1137.00
agg	R	554	681.60
mPD		0.35	0.95
		nPD:	0.37
		N. weight:	0.42
		Sc. PD:	0.094
		Sc. rank:	534.5

NP			
Pos.	343	obs:	exp:
tat	Y	0	0.49
tac	Y	1	0.51
att	I	0	7.80
atc	I	10	5.27
ata	I	11	7.94
gtt	V	31	534.30
gtc	V	1873	519.10
gta	V	544	483.40
gtg	V	9	920.10
mPD		0.39	0.75
		nPD:	0.52
		N. weight:	1.1
		Sc. PD:	0.42
		Sc. rank:	1734.3

NP			
Pos.	344	obs:	exp:
tta	L	11	1.20
ttg	L	0	2.06
tct	S	0	421.50
tcc	S	0	363.80
tca	S	2465	556.30
tcg	S	3	159.40
ctt	L	0	2.06
ctc	L	0	1.90
cta	L	0	1.46
ctg	L	0	2.31
agt	S	0	503.90
agc	S	0	463.00
mPD		0.011	1.7
		nPD:	0.01
		N. weight:	1.8
		Sc. PD:	-0.22
		Sc. rank:	-1773.9

NP			
Pos.	345	obs:	exp:
tct	S	0	423.20
tcc	S	0	365.30
tca	S	0	558.60
tcg	S	0	160.10
agt	S	2304	505.90
agc	S	174	464.90
ggt	G	0	0.14
ggc	G	1	0.13
gga	G	0	0.44
ggg	G	0	0.29
mPD		0.13	1.6
		nPD:	0.08
		N. weight:	1.6
		Sc. PD:	-0.089
		Sc. rank:	908.4

NP			
Pos.	346	obs:	exp:
ttt	F	86	1055.00
ttc	F	2393	1424.00
mPD		0.067	0.49
		nPD:	0.14
		N. weight:	0.49
		Sc. PD:	-0.00039
		Sc. rank:	358.2

NP			
Pos.	347	obs:	exp:
tta	L	0	0.11
ttg	L	0	0.19
ctt	L	0	0.19
ctc	L	1	0.17
cta	L	0	0.13
ctg	L	0	0.21
att	I	0	919.80
atc	I	2476	621.20
ata	I	1	936.00
gtt	V	0	0.22
gtc	V	1	0.21
gta	V	0	0.20
gtg	V	0	0.37
mPD		0.0024	0.66
		nPD:	0.
		N. weight:	1.6
		Sc. PD:	-0.21
		Sc. rank:	-2073.4

NP			
Pos.	348	obs:	exp:
cgt	R	0	94.01
cgc	R	0	132.00
cga	R	0	240.00
cgg	R	0	194.20
aga	R	2475	1137.00
agg	R	4	681.60
mPD		0.0032	0.95
		nPD:	0.
		N. weight:	0.92
		Sc. PD:	-0.12
		Sc. rank:	-1253.9

NP			
Pos.	349	obs:	exp:
ggt	G	0	356.60
ggc	G	0	327.20
gga	G	1524	1082.00
ggg	G	955	713.00
mPD		0.47	0.69
		nPD:	0.69
		N. weight:	0.39
		Sc. PD:	0.2
		Sc. rank:	652.2

NP

Pos.	350	obs:	exp:
tct	S	0	0.17
tcc	S	0	0.15
tca	S	1	0.23
tcg	S	0	0.06
att	I	0	0.37
atc	I	0	0.25
ata	I	1	0.38
act	T	1	655.10
acc	T	0	546.40
aca	T	2470	1063.00
acg	T	2	208.90
agt	S	0	0.20
agc	S	0	0.19
gct	A	0	1.02
gcc	A	0	0.78
gca	A	4	1.76
gcg	A	0	0.44
---	--	------	------
mPD		0.0073	0.69
		nPD:	0.01
		N. weight:	1.
		Sc. PD:	-0.12
		Sc. rank:	-653.6

,

NP

Pos.	351	obs:	exp:
cgt	R	0	92.27
cgc	R	0	129.60
cga	R	0	235.60
cgg	R	0	190.60
aaa	K	46	25.82
aag	K	0	20.18
aga	R	2421	1116.00
agg	R	12	669.00
---	--	------	------
mPD		0.046	0.98
		nPD:	0.05
		N. weight:	0.89
		Sc. PD:	-0.078
		Sc. rank:	327.9

,

NP

Pos.	352	obs:	exp:
tta	L	2	0.55
ttg	L	0	0.94
ctt	L	0	0.94
ctc	L	0	0.86
cta	L	3	0.67
ctg	L	0	1.05
gtt	V	2	538.00
gtc	V	0	522.70
gta	V	1694	486.80
gtg	V	778	926.40
---	--	------	------
mPD		0.44	0.73
		nPD:	0.59
		N. weight:	0.95
		Sc. PD:	0.42
		Sc. rank:	1518.6

,

NP

Pos.	353	obs:	exp:
ttt	F	0	28.94
ttc	F	68	39.06
att	I	4	2.97
atc	I	4	2.01
ata	I	0	3.02
gtt	V	326	519.60
gtc	V	2062	504.80
gta	V	1	470.10
gtg	V	0	894.60
gct	A	0	3.56
gcc	A	14	2.73
gca	A	0	6.15
gcg	A	0	1.55
---	--	------	------
mPD		0.30	0.80
		nPD:	0.38
		N. weight:	1.4
		Sc. PD:	0.31
		Sc. rank:	1719.3

,

NP

Pos.	354	obs:	exp:
tct	S	0	0.17
tcc	S	0	0.15
tca	S	1	0.23
tcg	S	0	0.06
cct	P	0	698.80
ccc	P	1	448.40
cca	P	2477	1012.00
ccg	P	0	319.10
agt	S	0	0.20
agc	S	0	0.19
---	--	------	------
mPD		0.0016	0.71
		nPD:	0.
		N. weight:	1.1
		Sc. PD:	-0.14
		Sc. rank:	-1551.2

,

NP

Pos.	355	obs:	exp:
cgt	R	0	94.01
cgc	R	0	132.00
cga	R	1	240.00
cgg	R	0	194.20
aga	R	2322	1137.00
agg	R	156	681.60
---	--	------	------
mPD		0.12	0.95
		nPD:	0.12
		N. weight:	0.69
		Sc. PD:	-0.0087
		Sc. rank:	473.8

,

NP

Pos.	356	obs:	exp:
ggt	G	1	356.60
ggc	G	0	327.20
gga	G	2303	1082.00
ggg	G	175	713.00
---	--	------	------
mPD		0.13	0.69
		nPD:	0.19
		N. weight:	0.72
		Sc. PD:	0.037
		Sc. rank:	617.6

,

NP

Pos.	357	obs:	exp:
tta	L	0	0.11
ttg	L	0	0.19
ctt	L	0	0.19
ctc	L	0	0.17
cta	L	1	0.13
ctg	L	0	0.21
cat	H	0	0.59
cac	H	1	0.41
caa	Q	2472	1259.00
cag	Q	3	1216.00
cgt	R	0	0.08
cgc	R	0	0.11
cga	R	2	0.19
cgg	R	0	0.16
aga	R	0	0.92
agg	R	0	0.55
---	--	------	------
mPD		0.0056	0.50
		nPD:	0.01
		N. weight:	0.8
		Sc. PD:	-0.097
		Sc. rank:	-506.8

,

NP

Pos.	358	obs:	exp:
tta	L	41	270.60
ttg	L	0	465.20
ctt	L	0	464.80
ctc	L	0	427.70
cta	L	2410	329.80
ctg	L	28	520.90
---	--	------	------
mPD		0.055	1.1
		nPD:	0.05
		N. weight:	2.2
		Sc. PD:	-0.19
		Sc. rank:	884.3

,

NP

Pos.	359	obs:	exp:
tct	S	23	423.20
tcc	S	2454	365.30
tca	S	1	558.60
tcg	S	0	160.10
tat	Y	0	0.49
tac	Y	1	0.51
agt	S	0	505.90
agc	S	0	464.90
---	--	------	------
mPD		0.020	1.6
		nPD:	0.01
		N. weight:	2.2
		Sc. PD:	-0.27
		Sc. rank:	-1231.3

,

NP

Pos.	360	obs:	exp:
act	T	1181	656.70
acc	T	1290	547.70
aca	T	8	1065.00
acg	T	0	209.40
---	--	------	------
mPD		0.50	0.69
		nPD:	0.73
		N. weight:	0.85
		Sc. PD:	0.48
		Sc. rank:	1481.8

,

NP

Pos.	361	obs:	exp:
cgt	R	0	94.01
cgc	R	0	132.00
cga	R	159	240.00
cgg	R	0	194.20
aga	R	2320	1137.00
agg	R	0	681.60
---	--	------	------
mPD		0.12	0.95
		nPD:	0.13
		N. weight:	0.77
		Sc. PD:	-0.0087
		Sc. rank:	541.2

,

NP

Pos.	362	obs:	exp:
ggt	G	2	356.60
ggc	G	0	327.20
gga	G	2411	1082.00
ggg	G	66	713.00
---	--	------	------
mPD		0.053	0.69
		nPD:	0.08
		N. weight:	0.85
		Sc. PD:	-0.049
		Sc. rank:	463.9

,

NP

Pos.	363	obs:	exp:
att	I	18	6.68
atc	I	0	4.51
ata	I	0	6.80
gtt	V	2456	535.20
gtc	V	2	520.00
gta	V	3	484.20
gtg	V	0	921.60
---	--	------	------
mPD		0.018	0.74
		nPD:	0.02
		N. weight:	1.8
		Sc. PD:	-0.2
		Sc. rank:	-146.1

,

NP

Pos.	364	obs:	exp:
caa	Q	2462	1261.00
cag	Q	17	1218.00
---	--	------	------
mPD		0.014	0.50
		nPD:	0.03
		N. weight:	0.76
		Sc. PD:	-0.081
		Sc. rank:	-7.4

,

NP

Pos.	365	obs:	exp:
att	I	2475	920.50
atc	I	4	621.70
ata	I	0	936.70
---	--	------	------
mPD		0.0032	0.66
		nPD:	0.
		N. weight:	1.2
		Sc. PD:	-0.15
		Sc. rank:	-1391.3

,

NP

Pos.	366	obs:	exp:
tct	S	1	0.17
tcc	S	0	0.15
tca	S	0	0.23
tcg	S	0	0.06
agt	S	0	0.20
agc	S	0	0.19
gct	A	1923	630.10
gcc	A	5	483.00
gca	A	539	1089.00
gcg	A	10	275.00
ggt	G	1	0.14
ggc	G	0	0.13
gga	G	0	0.44
ggg	G	0	0.29
---	--	------	------
mPD		0.35	0.69
		nPD:	0.51
		N. weight:	0.83
		Sc. PD:	0.29
		Sc. rank:	1237.2

,

NP

Pos.	367	obs:	exp:
tct	S	26	423.40
tcc	S	0	365.50
tca	S	2426	558.80
tcg	S	27	160.10
agt	S	0	506.10
agc	S	0	465.10
---	--	------	------
mPD		0.042	1.6
		nPD:	0.03
		N. weight:	1.7
		Sc. PD:	-0.18
		Sc. rank:	-94.6

,

NP

Pos.	368	obs:	exp:
tct	S	0	0.51
tcc	S	0	0.44
tca	S	0	0.68
tcg	S	0	0.19
aat	N	2471	1316.00
aac	N	5	1160.00
agt	S	3	0.61
agc	S	0	0.56
---	--	------	------
mPD		0.0064	0.50
		nPD:	0.01
		N. weight:	0.74
		Sc. PD:	-0.089
		Sc. rank:	-376.9

,

NP

Pos.	369	obs:	exp:
gaa	E	785	1484.00
gag	E	1694	994.70
---	--	------	------
mPD		0.43	0.48
		nPD:	0.9
		N. weight:	0.2
		Sc. PD:	0.14
		Sc. rank:	347.4

,

NP

Pos. 370		obs:	exp:
tct	S	0	0.17
tcc	S	0	0.15
tca	S	0	0.23
tcg	S	0	0.06
aat	N	160	1317.00
aac	N	2318	1161.00
agt	S	0	0.20
agc	S	1	0.19
mPD		0.12	0.50
		nPD:	0.24
		N. weight:	0.61
		Sc. PD:	0.062
		Sc. rank:	630.9

NP

Pos. 371		obs:	exp:
att	I	0	329.70
atc	I	0	222.70
ata	I	888	335.50
atg	M	1587	1587.00
act	T	0	0.53
acc	T	0	0.44
aca	T	1	0.86
acg	T	1	0.17
gtt	V	0	0.43
gtc	V	0	0.42
gta	V	0	0.39
gtg	V	2	0.75
mPD		0.46	0.55
		nPD:	0.84
		N. weight:	0.42
		Sc. PD:	0.28
		Sc. rank:	734.7

NP

Pos. 372		obs:	exp:
gat	D	8	4.60
gac	D	0	3.40
gaa	E	2457	1479.00
gag	E	13	991.10
ggt	G	0	0.14
ggc	G	0	0.13
gga	G	1	0.44
ggg	G	0	0.29
mPD		0.018	0.48
		nPD:	0.04
		N. weight:	0.58
		Sc. PD:	-0.056
		Sc. rank:	87.7

NP

Pos. 373		obs:	exp:
cct	P	0	0.28
ccc	P	0	0.18
cca	P	1	0.41
ccg	P	0	0.13
act	T	0	654.80
acc	T	0	546.20
aca	T	2470	1062.00
acg	T	2	208.80
gct	A	0	1.53
gcc	A	0	1.17
gca	A	6	2.64
gcg	A	0	0.67
mPD		0.0072	0.69
		nPD:	0.01
		N. weight:	1.
		Sc. PD:	-0.12
		Sc. rank:	-662.9

NP

Pos. 374		obs:	exp:
tta	L	0	0.11
ttg	L	0	0.19
ctt	L	0	0.19
ctc	L	0	0.17
cta	L	0	0.13
ctg	L	1	0.21
att	I	0	53.84
atc	I	0	36.37
ata	I	145	54.79
atg	M	2331	2331.00
gtt	V	0	0.43
gtc	V	0	0.42
gta	V	0	0.39
gtg	V	2	0.75
mPD		0.11	0.12
		nPD:	0.97
		N. weight:	0.07
		Sc. PD:	0.056
		Sc. rank:	124.8

NP

Pos. 375		obs:	exp:
aat	N	0	1.59
aac	N	3	1.41
gat	D	5	1420.00
gac	D	2464	1049.00
gaa	E	4	2.40
gag	E	0	1.60
ggt	G	0	0.43
ggc	G	3	0.40
gga	G	0	1.31
ggg	G	0	0.86
mPD		0.012	0.50
		nPD:	0.02
		N. weight:	1.
		Sc. PD:	-0.11
		Sc. rank:	-102.0

NP

Pos. 376		obs:	exp:
tct	S	79	423.20
tcc	S	2399	365.30
tca	S	0	558.60
tcg	S	0	160.10
cct	P	0	0.28
ccc	P	1	0.18
cca	P	0	0.41
ccg	P	0	0.13
agt	S	0	505.90
agc	S	0	464.90
mPD		0.063	1.6
		nPD:	0.04
		N. weight:	2.1
		Sc. PD:	-0.2
		Sc. rank:	396.3

NP

Pos. 377		obs:	exp:
tct	S	0	420.10
tcc	S	0	362.60
tca	S	0	554.50
tcg	S	0	158.90
cgt	R	0	0.04
cgc	R	0	0.05
cga	R	0	0.10
cgg	R	0	0.08
aat	N	2	9.03
aac	N	15	7.97
agt	S	87	502.30
agc	S	2373	461.50
aga	R	1	0.46
agg	R	0	0.27
ggt	G	0	0.14
ggc	G	1	0.13
gga	G	0	0.44
ggg	G	0	0.29
mPD		0.084	1.7
		nPD:	0.05
		N. weight:	1.8
		Sc. PD:	-0.15
		Sc. rank:	773.0

NP

Pos. 378		obs:	exp:
cct	P	1	0.28
ccc	P	0	0.18
cca	P	0	0.41
ccg	P	0	0.13
act	T	2466	656.40
acc	T	12	547.50
aca	T	0	1065.00
acg	T	0	209.30
mPD		0.010	0.69
		nPD:	0.02
		N. weight:	1.5
		Sc. PD:	-0.18
		Sc. rank:	-627.1

NP

Pos. 379		obs:	exp:
tta	L	0	270.60
ttg	L	0	465.20
ctt	L	2465	464.80
ctc	L	2	427.70
cta	L	10	329.80
ctg	L	2	520.90
mPD		0.011	1.1
		nPD:	0.01
		N. weight:	2.
		Sc. PD:	-0.24
		Sc. rank:	-1404.7

NP

Pos. 380		obs:	exp:
gaa	E	2354	1484.00
gag	E	125	994.70
mPD		0.096	0.48
		nPD:	0.2
		N. weight:	0.4
		Sc. PD:	0.024
		Sc. rank:	352.2

NP

Pos. 381		obs:	exp:
tta	L	3	270.10
ttg	L	657	464.40
ctt	L	0	464.10
ctc	L	0	427.00
cta	L	268	329.30
ctg	L	1547	520.00
atg	M	4	4.00
mPD		0.59	1.1
		nPD:	0.52
		N. weight:	0.89
		Sc. PD:	0.33
		Sc. rank:	1350.4

NP

Pos. 382		obs:	exp:
cgt	R	0	94.01
cgc	R	0	132.00
cga	R	0	240.00
cgg	R	0	194.20
aga	R	2229	1137.00
agg	R	250	681.60
mPD		0.18	0.95
		nPD:	0.19
		N. weight:	0.6
		Sc. PD:	0.03
		Sc. rank:	515.6

NP

Pos. 383		obs:	exp:
tct	S	0	423.40
tcc	S	0	365.50
tca	S	0	558.80
tcg	S	0	160.10
agt	S	4	506.10
agc	S	2475	465.10
mPD		0.0032	1.6
		nPD:	0.
		N. weight:	2.
		Sc. PD:	-0.26
		Sc. rank:	-2948.1

NP

Pos. 384		obs:	exp:
cgt	R	0	93.94
cgc	R	0	131.90
cga	R	0	239.90
cgg	R	0	194.00
aaa	K	2	1.12
aag	K	0	0.88
aga	R	2438	1136.00
agg	R	39	681.10
mPD		0.033	0.95
		nPD:	0.03
		N. weight:	0.84
		Sc. PD:	-0.084
		Sc. rank:	91.6

NP

Pos. 385		obs:	exp:
tat	Y	1553	1204.00
tac	Y	924	1273.00
cat	H	0	1.19
cac	H	2	0.81
mPD		0.47	0.50
		nPD:	0.94
		N. weight:	0.051
		Sc. PD:	0.039
		Sc. rank:	90.4

NP

Pos. 386		obs:	exp:
tgg	W	2479	2479.00
mPD		0	0
		nPD:	1.
		N. weight:	0.
		Sc. PD:	0
		Sc. rank:	0

NP

Pos. 387		obs:	exp:
gct	A	2465	630.60
gcc	A	12	483.40
gca	A	2	1090.00
gcg	A	0	275.20
mPD		0.011	0.69
		nPD:	0.02
		N. weight:	1.6
		Sc. PD:	-0.19
		Sc. rank:	-546.9

NP

Pos. 388		obs:	exp:
att	I	0	920.50
atc	I	0	621.70
ata	I	2479	936.70
mPD		0	0.66
		nPD:	0.
		N. weight:	1.2
		Sc. PD:	-0.15
		Sc. rank:	-2080.2

NP

Pos. 389		obs:	exp:
cgt	R	0	93.94
cgc	R	0	131.90
cga	R	0	239.90
cgg	R	0	194.00
aaa	K	2	1.12
aag	K	0	0.88
aga	R	2284	1136.00
agg	R	193	681.10
mPD		0.15	0.95
		nPD:	0.15
		N. weight:	0.65
		Sc. PD:	0.009
		Sc. rank:	477.2

NP

Pos. 390		obs:	exp:
act	T	2	656.70
acc	T	2460	547.70
aca	T	16	1065.00
acg	T	1	209.40
mPD		0.015	0.69
		nPD:	0.02
		N. weight:	1.7
		Sc. PD:	-0.19
		Sc. rank:	-286.4

NP

Pos. 391		obs:	exp:
cgt	R	0	94.01
cgc	R	0	132.00
cga	R	0	240.00
cgg	R	1	194.20
aga	R	45	1137.00
agg	R	2433	681.60
mPD		0.036	0.95
		nPD:	0.04
		N. weight:	1.4
		Sc. PD:	-0.14
		Sc. rank:	280.9

NP

Pos. 392		obs:	exp:
tct	S	0	423.40
tcc	S	0	365.50
tca	S	0	558.80
tcg	S	0	160.10
agt	S	2469	506.10
agc	S	10	465.10
mPD		0.0080	1.6
		nPD:	0.
		N. weight:	1.9
		Sc. PD:	-0.24
		Sc. rank:	-2251.3

NP

Pos. 393		obs:	exp:
ggt	G	0	356.60
ggc	G	1	327.20
gga	G	2414	1082.00
ggg	G	64	713.00
mPD		0.051	0.69
		nPD:	0.07
		N. weight:	0.86
		Sc. PD:	-0.052
		Sc. rank:	444.1

NP

Pos. 394		obs:	exp:
ggt	G	5	356.60
ggc	G	0	327.20
gga	G	2448	1082.00
ggg	G	26	713.00
mPD		0.025	0.69
		nPD:	0.04
		N. weight:	0.91
		Sc. PD:	-0.089
		Sc. rank:	127.6

NP

Pos.	395	obs:	exp:
act	T	0	8.21
acc	T	31	6.85
aca	T	0	13.32
acg	T	0	2.62
aat	N	2	1301.00
aac	N	2446	1147.00
---	--	------	------
mPD		0.026	0.53
		nPD:	0.05
		N. weight:	0.91
		Sc. PD:	-0.077
		Sc. rank:	377.6

NP

Pos.	396	obs:	exp:
cct	P	0	0.28
ccc	P	1	0.18
cca	P	0	0.41
ccg	P	0	0.13
act	T	12	655.60
acc	T	2456	546.80
aca	T	7	1064.00
acg	T	0	209.10
aat	N	0	0.53
aac	N	1	0.47
gct	A	0	0.51
gcc	A	2	0.39
gca	A	0	0.88
gcg	A	0	0.22
---	--	------	------
mPD		0.018	0.69
		nPD:	0.03
		N. weight:	1.7
		Sc. PD:	-0.19
		Sc. rank:	-67.4

NP

Pos.	397	obs:	exp:
tct	S	0	0.34
tcc	S	0	0.29
tca	S	0	0.45
tcg	S	0	0.13
tat	Y	1	0.49
tac	Y	0	0.51
aat	N	153	1316.00
aac	N	2323	1160.00
agt	S	0	0.41
agc	S	2	0.38
---	--	------	------
mPD		0.12	0.50
		nPD:	0.24
		N. weight:	0.62
		Sc. PD:	0.059
		Sc. rank:	633.0

NP

Pos.	398	obs:	exp:
cat	H	1	0.59
cac	H	0	0.41
caa	Q	2464	1260.00
cag	Q	13	1217.00
aaa	K	1	0.56
aag	K	0	0.44
---	--	------	------
mPD		0.012	0.50
		nPD:	0.02
		N. weight:	0.77
		Sc. PD:	-0.084
		Sc. rank:	-117.0

NP

Pos.	399	obs:	exp:
caa	Q	39	1261.00
cag	Q	2440	1218.00
---	--	------	------
mPD		0.031	0.50
		nPD:	0.06
		N. weight:	0.75
		Sc. PD:	-0.055
		Sc. rank:	363.1

NP

Pos.	400	obs:	exp:
cgt	R	0	93.97
cgc	R	0	132.00
cga	R	4	239.90
cgg	R	0	194.10
aaa	K	1	0.56
aag	K	0	0.44
aga	R	2472	1137.00
agg	R	2	681.40
---	--	------	------
mPD		0.0056	0.95
		nPD:	0.01
		N. weight:	0.91
		Sc. PD:	-0.12
		Sc. rank:	-999.7

NP

Pos.	401	obs:	exp:
aaa	K	1	0.56
aag	K	0	0.44
gct	A	538	630.40
gcc	A	1	483.20
gca	A	1937	1089.00
gcg	A	2	275.10
---	--	------	------
mPD		0.34	0.69
		nPD:	0.5
		N. weight:	0.49
		Sc. PD:	0.17
		Sc. rank:	721.4

NP

Pos.	402	obs:	exp:
ttt	F	6	2.55
ttc	F	0	3.45
tct	S	2466	422.30
tcc	S	6	364.60
tca	S	1	557.40
tcg	S	0	159.70
agt	S	0	504.90
agc	S	0	464.00
---	--	------	------
mPD		0.010	1.6
		nPD:	0.01
		N. weight:	2.1
		Sc. PD:	-0.26
		Sc. rank:	-2233.3

NP

Pos.	403	obs:	exp:
tct	S	0	0.17
tcc	S	0	0.15
tca	S	1	0.23
tcg	S	0	0.06
agt	S	0	0.20
agc	S	0	0.19
gct	A	0	630.40
gcc	A	0	483.20
gca	A	2468	1089.00
gcg	A	10	275.10
---	--	------	------
mPD		0.0088	0.69
		nPD:	0.01
		N. weight:	0.96
		Sc. PD:	-0.12
		Sc. rank:	-503.8

NP

Pos.	404	obs:	exp:
ggt	G	0	356.60
ggc	G	0	327.20
gga	G	2471	1082.00
ggg	G	8	713.00
---	--	------	------
mPD		0.0064	0.69
		nPD:	0.01
		N. weight:	0.96
		Sc. PD:	-0.12
		Sc. rank:	-741.2

NP

Pos.	405	obs:	exp:
caa	Q	2171	1261.00
cag	Q	308	1218.00
---	--	------	------
mPD		0.22	0.50
		nPD:	0.44
		N. weight:	0.36
		Sc. PD:	0.1
		Sc. rank:	486.5

NP

Pos.	406	obs:	exp:
att	I	3	919.80
atc	I	2468	621.20
ata	I	6	936.00
gtt	V	0	0.43
gtc	V	2	0.42
gta	V	0	0.39
gtg	V	0	0.75
---	--	------	------
mPD		0.0089	0.66
		nPD:	0.01
		N. weight:	1.6
		Sc. PD:	-0.19
		Sc. rank:	-772.2

NP

Pos.	407	obs:	exp:
tct	S	0	423.40
tcc	S	0	365.50
tca	S	0	558.80
tcg	S	0	160.10
agt	S	1806	506.10
agc	S	673	465.10
---	--	------	------
mPD		0.40	1.6
		nPD:	0.24
		N. weight:	1.2
		Sc. PD:	0.12
		Sc. rank:	1261.1

NP

Pos.	408	obs:	exp:
att	I	0	0.74
atc	I	0	0.50
ata	I	2	0.76
gtt	V	3	538.70
gtc	V	1	523.40
gta	V	2457	487.40
gtg	V	16	927.60
---	--	------	------
mPD		0.018	0.73
		nPD:	0.02
		N. weight:	1.9
		Sc. PD:	-0.2
		Sc. rank:	-260.3

NP

Pos.	409	obs:	exp:
caa	Q	547	1261.00
cag	Q	1932	1218.00
---	--	------	------
mPD		0.34	0.50
		nPD:	0.69
		N. weight:	0.21
		Sc. PD:	0.11
		Sc. rank:	356.4

NP

Pos.	410	obs:	exp:
cct	P	348	698.80
ccc	P	2126	448.40
cca	P	4	1012.00
ccg	P	0	319.10
gct	A	0	0.25
gcc	A	1	0.20
gca	A	0	0.44
gcg	A	0	0.11
---	--	------	------
mPD		0.25	0.71
		nPD:	0.35
		N. weight:	1.5
		Sc. PD:	0.3
		Sc. rank:	1809.7

NP

Pos.	411	obs:	exp:
act	T	1935	656.70
acc	T	535	547.70
aca	T	8	1065.00
acg	T	1	209.40
---	--	------	------
mPD		0.34	0.69
		nPD:	0.5
		N. weight:	0.98
		Sc. PD:	0.34
		Sc. rank:	1449.7

NP

Pos.	412	obs:	exp:
ttt	F	142	1055.00
ttc	F	2337	1424.00
---	--	------	------
mPD		0.11	0.49
		nPD:	0.22
		N. weight:	0.42
		Sc. PD:	0.034
		Sc. rank:	409.3

NP

Pos.	413	obs:	exp:
tct	S	15	423.40
tcc	S	0	365.50
tca	S	851	558.80
tcg	S	1613	160.10
agt	S	0	506.10
agc	S	0	465.10
---	--	------	------
mPD		0.46	1.6
		nPD:	0.28
		N. weight:	1.9
		Sc. PD:	0.26
		Sc. rank:	2159.7

NP

Pos.	414	obs:	exp:
att	I	0	1.11
atc	I	0	0.75
ata	I	3	1.13
gtt	V	0	538.50
gtc	V	1	523.20
gta	V	2460	487.20
gtg	V	15	927.20
---	--	------	------
mPD		0.015	0.73
		nPD:	0.02
		N. weight:	1.9
		Sc. PD:	-0.21
		Sc. rank:	-407.7

NP

Pos.	415	obs:	exp:
caa	Q	16	1261.00
cag	Q	2463	1218.00
---	--	------	------
mPD		0.013	0.50
		nPD:	0.03
		N. weight:	0.8
		Sc. PD:	-0.087
		Sc. rank:	-41.1

NP

Pos.	416	obs:	exp:
cgt	R	0	94.01
cgc	R	0	132.00
cga	R	0	240.00
cgg	R	0	194.20
aga	R	2472	1137.00
agg	R	7	681.60
---	--	------	------
mPD		0.0056	0.95
		nPD:	0.01
		N. weight:	0.91
		Sc. PD:	-0.12
		Sc. rank:	-1010.1

NP

Pos.	417	obs:	exp:
tct	S	0	5.81
tcc	S	0	5.01
tca	S	0	7.66
tcg	S	0	2.20
act	T	1	0.53
acc	T	1	0.44
aca	T	0	0.86
acg	T	0	0.17
aat	N	1222	1298.00
aac	N	1220	1144.00
aaa	K	1	0.56
aag	K	0	0.44
agt	S	34	6.94
agc	S	0	6.38
---	--	------	------
mPD		0.53	0.55
		nPD:	0.97
		N. weight:	0.032
		Sc. PD:	0.025
		Sc. rank:	56.6

NP

Pos.	418	obs:	exp:
ttt	F	0	0.43
ttc	F	1	0.57
tta	L	0	270.50
ttg	L	0	465.00
ctt	L	2329	464.70
ctc	L	148	427.50
cta	L	1	329.70
ctg	L	0	520.70
---	--	------	------
mPD		0.11	1.1
		nPD:	0.1
		N. weight:	1.7
		Sc. PD:	-0.062
		Sc. rank:	1078.2

NP

Pos.	419	obs:	exp:
cct	P	159	699.10
ccc	P	2306	448.60
cca	P	12	1012.00
ccg	P	2	319.20
---	--	------	------
mPD		0.13	0.70
		nPD:	0.19
		N. weight:	1.7
		Sc. PD:	0.077
		Sc. rank:	1425.9

NP

Pos.	420	obs:	exp:
ttt	F	644	1054.00
ttc	F	1834	1424.00
tta	L	1	0.11
ttg	L	0	0.19
ctt	L	0	0.19
ctc	L	0	0.17
cta	L	0	0.13
ctg	L	0	0.21
---	--	------	------
mPD		0.39	0.49
		nPD:	0.79
		N. weight:	0.074
		Sc. PD:	0.046
		Sc. rank:	129.2

NP

Pos.	421	obs:	exp:
gat	D	0	0.58
gac	D	1	0.42
gaa	E	2341	1484.00
gag	E	137	994.30
---	--	------	------
mPD		0.11	0.48
		nPD:	0.22
		N. weight:	0.38
		Sc. PD:	0.03
		Sc. rank:	372.6

NP

Pos.	422	obs:	exp:
cgt	R	0	94.01
cgc	R	0	132.00
cga	R	0	240.00
cgg	R	0	194.20
aga	R	2432	1137.00
agg	R	47	681.60
---	--	------	------
mPD		0.037	0.95
		nPD:	0.04
		N. weight:	0.83
		Sc. PD:	-0.079
		Sc. rank:	169.4

NP

Pos.	423	obs:	exp:
act	T	26	29.93
acc	T	0	24.97
aca	T	0	48.56
acg	T	87	9.54
gtt	V	0	0.22
gtc	V	0	0.21
gta	V	0	0.20
gtg	V	1	0.37
gct	A	0	601.60
gcc	A	0	461.20
gca	A	89	1040.00
gcg	A	2276	262.60
---	--	------	------
mPD		0.18	0.78
		nPD:	0.23
		N. weight:	2.4
		Sc. PD:	0.2
		Sc. rank:	2331.6

NP

Pos.	424	obs:	exp:
att	I	0	2.60
atc	I	7	1.76
ata	I	0	2.65
act	T	1	654.30
acc	T	2465	545.70
aca	T	4	1061.00
acg	T	0	208.60
gct	A	0	0.51
gcc	A	2	0.39
gca	A	0	0.88
gcg	A	0	0.22
---	--	------	------
mPD		0.011	0.70
		nPD:	0.02
		N. weight:	1.8
		Sc. PD:	-0.21
		Sc. rank:	-632.9

NP

Pos.	425	obs:	exp:
att	I	1913	919.80
atc	I	562	621.20
ata	I	2	936.00
gtt	V	2	0.43
gtc	V	0	0.42
gta	V	0	0.39
gtg	V	0	0.75
---	--	------	------
mPD		0.35	0.66
		nPD:	0.54
		N. weight:	0.64
		Sc. PD:	0.25
		Sc. rank:	999.0

NP

Pos.	426	obs:	exp:
atg	M	2479	2479.00
---	--	------	------
mPD		0	0
		nPD:	1.
		N. weight:	0.
		Sc. PD:	0
		Sc. rank:	0

NP

Pos.	427	obs:	exp:
gct	A	8	630.60
gcc	A	0	483.40
gca	A	303	1090.00
gcg	A	2168	275.20
---	--	------	------
mPD		0.22	0.69
		nPD:	0.32
		N. weight:	2.
		Sc. PD:	0.34
		Sc. rank:	2346.0

NP

Pos.	428	obs:	exp:
gtt	V	0	0.22
gtc	V	0	0.21
gta	V	0	0.20
gtg	V	1	0.37
gct	A	121	630.40
gcc	A	0	483.20
gca	A	1809	1089.00
gcg	A	548	275.10
---	--	------	------
mPD		0.42	0.69
		nPD:	0.6
		N. weight:	0.53
		Sc. PD:	0.24
		Sc. rank:	850.5

NP

Pos.	429	obs:	exp:
ttt	F	701	1055.00
ttc	F	1778	1424.00
---	--	------	------
mPD		0.41	0.49
		nPD:	0.83
		N. weight:	0.054
		Sc. PD:	0.036
		Sc. rank:	94.6

NP

Pos.	430	obs:	exp:
act	T	3	651.60
acc	T	81	543.50
aca	T	2365	1057.00
acg	T	11	207.80
gct	A	0	4.83
gcc	A	0	3.71
gca	A	19	8.35
gcg	A	0	2.11
---	--	------	------
mPD		0.089	0.70
		nPD:	0.13
		N. weight:	0.83
		Sc. PD:	-0.0088
		Sc. rank:	591.0

NP

Pos.	431	obs:	exp:
ggt	G	0	356.60
ggc	G	2	327.20
gga	G	409	1082.00
ggg	G	2068	713.00
---	--	------	------
mPD		0.28	0.69
		nPD:	0.4
		N. weight:	0.86
		Sc. PD:	0.22
		Sc. rank:	1129.4

NP

Pos.	432	obs:	exp:
tct	S	0	0.17
tcc	S	0	0.15
tca	S	0	0.23
tcg	S	0	0.06
act	T	2	0.53
acc	T	0	0.44
aca	T	0	0.86
acg	T	0	0.17
aat	N	757	1316.00
aac	N	1719	1160.00
agt	S	0	0.20
agc	S	1	0.19
---	--	------	------
mPD		0.43	0.50
		nPD:	0.85
		N. weight:	0.13
		Sc. PD:	0.088
		Sc. rank:	224.8

NP

Pos.	433	obs:	exp:
tct	S	5	0.85
tcc	S	0	0.74
tca	S	0	1.13
tcg	S	0	0.32
act	T	2428	652.90
acc	T	35	544.60
aca	T	0	1059.00
acg	T	2	208.20
aat	N	8	4.25
aac	N	0	3.75
agt	S	0	1.02
agc	S	0	0.94
gct	A	1	0.25
gcc	A	0	0.20
gca	A	0	0.44
gcg	A	0	0.11
---	--	------	------
mPD		0.041	0.70
		nPD:	0.06
		N. weight:	1.5
		Sc. PD:	-0.11
		Sc. rank:	696.4

NP

Pos.	434	obs:	exp:
gaa	E	1602	1484.00
gag	E	877	994.70
---	--	------	------
mPD		0.46	0.48
		nPD:	0.95
		N. weight:	0.0076
		Sc. PD:	0.006
		Sc. rank:	13.7

NP

Pos.	435	obs:	exp:
ggt	G	13	356.60
ggc	G	2466	327.20
gga	G	0	1082.00
ggg	G	0	713.00
---	--	------	------
mPD		0.010	0.69
		nPD:	0.02
		N. weight:	2.4
		Sc. PD:	-0.28
		Sc. rank:	-920.9

NP

Pos.	436	obs:	exp:
cgt	R	0	94.01
cgc	R	0	132.00
cga	R	0	240.00
cgg	R	0	194.20
aga	R	1772	1137.00
agg	R	707	681.60
---	--	------	------
mPD		0.41	0.95
		nPD:	0.43
		N. weight:	0.39
		Sc. PD:	0.11
		Sc. rank:	520.0

NP

Pos.	437	obs:	exp:
att	I	0	0.37
atc	I	1	0.25
ata	I	0	0.38
act	T	11	656.40
acc	T	497	547.50
aca	T	811	1065.00
acg	T	1159	209.30
---	--	------	------
mPD		0.64	0.69
		nPD:	0.92
		N. weight:	0.81
		Sc. PD:	0.61
		Sc. rank:	1444.1

NP

Pos.	438	obs:	exp:
tct	S	992	423.40
tcc	S	1462	365.50
tca	S	20	558.80
tcg	S	5	160.10
agt	S	0	506.10
agc	S	0	465.10
---	--	------	------
mPD		0.49	1.6
		nPD:	0.3
		N. weight:	1.3
		Sc. PD:	0.21
		Sc. rank:	1543.6

NP

Pos.	439	obs:	exp:
gat	D	6	1426.00
gac	D	2473	1053.00
---	--	------	------
mPD		0.0048	0.49
		nPD:	0.01
		N. weight:	1.
		Sc. PD:	-0.12
		Sc. rank:	-748.7

NP

Pos.	440	obs:	exp:
atg	M	2479	2479.00
---	--	------	------
mPD		0	0
		nPD:	1.
		N. weight:	0.
		Sc. PD:	0
		Sc. rank:	0

NP

Pos.	441	obs:	exp:
cgt	R	0	94.01
cgc	R	0	132.00
cga	R	0	240.00
cgg	R	0	194.20
aga	R	17	1137.00
agg	R	2462	681.60
---	--	------	------
mPD		0.014	0.95
		nPD:	0.01
		N. weight:	1.5
		Sc. PD:	-0.18
		Sc. rank:	-674.2

NP

Pos.	442	obs:	exp:
act	T	2472	656.70
acc	T	1	547.70
aca	T	6	1065.00
acg	T	0	209.40
---	--	------	------
mPD		0.0056	0.69
		nPD:	0.01
		N. weight:	1.6
		Sc. PD:	-0.19
		Sc. rank:	-1289.9

NP

Pos.	443	obs:	exp:
gaa	E	2429	1484.00
gag	E	50	994.70
---	--	------	------
mPD		0.040	0.48
		nPD:	0.08
		N. weight:	0.5
		Sc. PD:	-0.027
		Sc. rank:	288.4

NP

Pos.	444	obs:	exp:
att	I	8	917.60
atc	I	2459	619.70
ata	I	4	933.70
gtt	V	0	1.74
gtc	V	8	1.69
gta	V	0	1.57
gtg	V	0	3.00
---	--	------	------
mPD		0.016	0.66
		nPD:	0.02
		N. weight:	1.6
		Sc. PD:	-0.18
		Sc. rank:	-204.9

NP			
Pos.	445	obs:	exp:
att	I	0	919.80
atc	I	2	621.20
ata	I	2475	936.00
gtt	V	0	0.43
gtc	V	0	0.42
gta	V	2	0.39
gtg	V	0	0.75
---	--	------	------
mPD		0.0032	0.66
		nPD:	0.
		N. weight:	1.2
		Sc. PD:	-0.15
		Sc. rank:	-1384.1

NP			
Pos.	446	obs:	exp:
cgt	R	0	93.97
cgc	R	0	132.00
cga	R	0	239.90
cgg	R	0	194.10
aaa	K	1	0.56
aag	K	0	0.44
aga	R	2436	1137.00
agg	R	42	681.40
---	--	------	------
mPD		0.034	0.95
		nPD:	0.04
		N. weight:	0.84
		Sc. PD:	-0.082
		Sc. rank:	111.9

NP			
Pos.	447	obs:	exp:
att	I	2	0.74
atc	I	0	0.50
ata	I	0	0.76
atg	M	2477	2477.00
---	--	------	------
mPD		0.0016	0.0016
		nPD:	1.
		N. weight:	0.00095
		Sc. PD:	0.00079
		Sc. rank:	1.7

NP			
Pos.	448	obs:	exp:
atg	M	2479	2479.00
---	--	------	------
mPD		0	0
		nPD:	1.
		N. weight:	0.
		Sc. PD:	0
		Sc. rank:	0

NP			
Pos.	449	obs:	exp:
gaa	E	2475	1484.00
gag	E	4	994.70
---	--	------	------
mPD		0.0032	0.48
		nPD:	0.01
		N. weight:	0.6
		Sc. PD:	-0.076
		Sc. rank:	-611.1

NP			
Pos.	450	obs:	exp:
tct	S	0	409.00
tcc	S	0	353.10
tca	S	0	539.90
tcg	S	0	154.70
att	I	1	0.37
atc	I	0	0.25
ata	I	0	0.38
aat	N	81	43.05
aac	N	0	37.95
agt	S	2385	489.00
agc	S	10	449.30
ggt	G	2	0.29
ggc	G	0	0.26
gga	G	0	0.87
ggg	G	0	0.58
---	--	------	------
mPD		0.074	1.7
		nPD:	0.04
		N. weight:	1.8
		Sc. PD:	-0.17
		Sc. rank:	534.3

NP			
Pos.	451	obs:	exp:
act	T	0	0.26
acc	T	1	0.22
aca	T	0	0.43
acg	T	0	0.08
gct	A	0	630.40
gcc	A	2471	483.20
gca	A	7	1089.00
gcg	A	0	275.10
---	--	------	------
mPD		0.0064	0.69
		nPD:	0.01
		N. weight:	1.9
		Sc. PD:	-0.24
		Sc. rank:	-1518.4

NP			
Pos.	452	obs:	exp:
cgt	R	0	63.64
cgc	R	0	89.38
cga	R	0	162.50
cgg	R	0	131.40
aaa	K	801	449.60
aag	K	0	351.40
aga	R	1677	769.70
agg	R	1	461.40
---	--	------	------
mPD		0.44	1.3
		nPD:	0.34
		N. weight:	0.85
		Sc. PD:	0.17
		Sc. rank:	1037.0

NP			
Pos.	453	obs:	exp:
tta	L	0	0.11
ttg	L	0	0.19
tct	S	0	2.56
tcc	S	0	2.21
tca	S	15	3.38
tcg	S	0	0.97
ctt	L	0	0.19
ctc	L	0	0.17
cta	L	1	0.13
ctg	L	0	0.21
cct	P	12	694.50
ccc	P	1	445.70
cca	P	2448	1006.00
ccg	P	2	317.10
agt	S	0	3.06
agc	S	0	2.81
---	--	------	------
mPD		0.025	0.72
		nPD:	0.03
		N. weight:	1.
		Sc. PD:	-0.1
		Sc. rank:	119.5

NP			
Pos.	454	obs:	exp:
gat	D	8	4.60
gac	D	0	3.40
gaa	E	2445	1480.00
gag	E	26	991.50
---	--	------	------
mPD		0.027	0.48
		nPD:	0.06
		N. weight:	0.55
		Sc. PD:	-0.043
		Sc. rank:	250.1

NP			
Pos.	455	obs:	exp:
aat	N	1	0.53
aac	N	0	0.47
gat	D	2478	1425.00
gac	D	0	1053.00
---	--	------	------
mPD		0.00081	0.49
		nPD:	0.
		N. weight:	0.66
		Sc. PD:	-0.086
		Sc. rank:	-1000.2

NP			
Pos.	456	obs:	exp:
atg	M	2	2.00
gtt	V	0	538.70
gtc	V	0	523.40
gta	V	0	487.40
gtg	V	2477	927.60
---	--	------	------
mPD		0.0016	0.73
		nPD:	0.
		N. weight:	1.2
		Sc. PD:	-0.15
		Sc. rank:	-1713.5

NP			
Pos.	457	obs:	exp:
ttt	F	1	0.43
ttc	F	0	0.57
tct	S	1633	423.20
tcc	S	824	365.30
tca	S	13	558.60
tcg	S	8	160.10
agt	S	0	505.90
agc	S	0	464.90
---	--	------	------
mPD		0.46	1.6
		nPD:	0.28
		N. weight:	1.4
		Sc. PD:	0.18
		Sc. rank:	1476.3

NP			
Pos.	458	obs:	exp:
ttt	F	87	1055.00
ttc	F	2392	1424.00
---	--	------	------
mPD		0.068	0.49
		nPD:	0.14
		N. weight:	0.49
		Sc. PD:	0.00034
		Sc. rank:	358.9

NP			
Pos.	459	obs:	exp:
caa	Q	7	1261.00
cag	Q	2472	1218.00
---	--	------	------
mPD		0.0056	0.50
		nPD:	0.01
		N. weight:	0.82
		Sc. PD:	-0.1
		Sc. rank:	-519.5

NP			
Pos.	460	obs:	exp:
tct	S	0	5.81
tcc	S	0	5.01
tca	S	0	7.66
tcg	S	0	2.20
aat	N	1	0.53
aac	N	0	0.47
agt	S	34	6.94
agc	S	0	6.38
ggt	G	0	351.60
ggc	G	0	322.60
gga	G	3	1067.00
ggg	G	2441	702.90
---	--	------	------
mPD		0.059	0.74
		nPD:	0.08
		N. weight:	1.5
		Sc. PD:	-0.082
		Sc. rank:	823.1

NP			
Pos.	461	obs:	exp:
cgt	R	0	94.01
cgc	R	0	132.00
cga	R	4	240.00
cgg	R	2475	194.20
aga	R	0	1137.00
agg	R	0	681.60
---	--	------	------
mPD		0.0032	0.95
		nPD:	0.
		N. weight:	3.
		Sc. PD:	-0.39
		Sc. rank:	-4134.5

NP			
Pos.	462	obs:	exp:
ggt	G	1	356.60
ggc	G	1	327.20
gga	G	2474	1082.00
ggg	G	3	713.00
---	--	------	------
mPD		0.0040	0.69
		nPD:	0.01
		N. weight:	0.97
		Sc. PD:	-0.12
		Sc. rank:	-1102.1

NP			
Pos.	463	obs:	exp:
gtt	V	86	538.90
gtc	V	2392	523.60
gta	V	0	487.60
gtg	V	0	927.90
gct	A	0	0.25
gcc	A	1	0.20
gca	A	0	0.44
gcg	A	0	0.11
---	--	------	------
mPD		0.068	0.73
		nPD:	0.09
		N. weight:	1.7
		Sc. PD:	-0.072
		Sc. rank:	1022.8

NP			
Pos.	464	obs:	exp:
ttt	F	2	1054.00
ttc	F	2476	1424.00
tta	L	0	0.11
ttg	L	1	0.19
ctt	L	0	0.19
ctc	L	0	0.17
cta	L	0	0.13
ctg	L	0	0.21
---	--	------	------
mPD		0.0024	0.49
		nPD:	0.
		N. weight:	0.65
		Sc. PD:	-0.084
		Sc. rank:	-776.3

NP			
Pos.	465	obs:	exp:
gaa	E	0	1484.00
gag	E	2479	994.70
---	--	------	------
mPD		0	0.48
		nPD:	0.
		N. weight:	1.1
		Sc. PD:	-0.14
		Sc. rank:	-1952.0

NP			
Pos.	466	obs:	exp:
ttt	F	0	0.43
ttc	F	1	0.57
tta	L	0	241.90
ttg	L	0	415.80
ctt	L	2	415.50
ctc	L	2213	382.30
cta	L	1	294.80
ctg	L	0	465.60
att	I	2	97.29
atc	I	259	65.71
ata	I	1	99.00
---	--	------	------
mPD		0.19	1.3
		nPD:	0.16
		N. weight:	2.
		Sc. PD:	0.034
		Sc. rank:	1530.7

NP			
Pos.	467	obs:	exp:
tct	S	2	423.00
tcc	S	0	365.20
tca	S	158	558.40
tcg	S	2317	160.00
act	T	0	0.53
acc	T	0	0.44
aca	T	1	0.86
acg	T	1	0.17
agt	S	0	505.70
agc	S	0	464.70
---	--	------	------
mPD		0.12	1.6
		nPD:	0.07
		N. weight:	2.9
		Sc. PD:	-0.17
		Sc. rank:	1553.9

NP			
Pos.	468	obs:	exp:
gat	D	0	1426.00
gac	D	2479	1053.00
---	--	------	------
mPD		0	0.49
		nPD:	0.
		N. weight:	1.
		Sc. PD:	-0.14
		Sc. rank:	-1830.1

NP			
Pos.	469	obs:	exp:
gaa	E	2476	1484.00
gag	E	3	994.70
---	--	------	------
mPD		0.0024	0.48
		nPD:	0.01
		N. weight:	0.6
		Sc. PD:	-0.077
		Sc. rank:	-697.4

NP			
Pos.	470	obs:	exp:
aaa	K	0	1391.00
aag	K	2479	1088.00
---	--	------	------
mPD		0	0.49
		nPD:	0.
		N. weight:	0.98
		Sc. PD:	-0.13
		Sc. rank:	-1761.2

NP			
Pos.	471	obs:	exp:
gct	A	0	630.60
gcc	A	0	483.40
gca	A	2478	1090.00
gcg	A	1	275.20
---	--	------	------
mPD		0.00081	0.69
		nPD:	0.
		N. weight:	0.98
		Sc. PD:	-0.13
		Sc. rank:	-1580.7

NP			
Pos.	472	obs:	exp:
act	T	0	656.10
acc	T	0	547.30
aca	T	0	1064.00
acg	T	2477	209.20
gct	A	0	0.51
gcc	A	0	0.39
gca	A	0	0.88
gcg	A	2	0.22
---	--	------	------
mPD		0.0016	0.69
		nPD:	0.
		N. weight:	2.9
		Sc. PD:	-0.38
		Sc. rank:	-4208.6

NP			
Pos.	473	obs:	exp:
tct	S	0	0.34
tcc	S	0	0.29
tca	S	0	0.45
tcg	S	0	0.13
aat	N	0	1316.00
aac	N	2477	1161.00
agt	S	0	0.41
agc	S	2	0.38
---	--	------	------
mPD		0.0016	0.50
		nPD:	0.
		N. weight:	0.9
		Sc. PD:	-0.12
		Sc. rank:	-1275.7

NP			
Pos.	474	obs:	exp:
tct	S	0	0.51
tcc	S	0	0.44
tca	S	0	0.68
tcg	S	3	0.19
cct	P	0	698.20
ccc	P	0	448.00
cca	P	47	1011.00
ccg	P	2429	318.80
agt	S	0	0.61
agc	S	0	0.56
---	--	------	------
mPD		0.040	0.71
		nPD:	0.06
		N. weight:	2.3
		Sc. PD:	-0.18
		Sc. rank:	1043.3

NP			
Pos.	475	obs:	exp:
att	I	0	920.20
atc	I	2478	621.50
ata	I	0	936.40
gtt	V	0	0.22
gtc	V	1	0.21
gta	V	0	0.20
gtg	V	0	0.37
---	--	------	------
mPD		0.00081	0.66
		nPD:	0.
		N. weight:	1.6
		Sc. PD:	-0.22
		Sc. rank:	-2637.2

NP			
Pos.	476	obs:	exp:
gtt	V	0	539.10
gtc	V	0	523.80
gta	V	1	487.80
gtg	V	2478	928.30
---	--	------	------
mPD		0.00081	0.73
		nPD:	0.
		N. weight:	1.2
		Sc. PD:	-0.15
		Sc. rank:	-1897.7

NP			
Pos.	477	obs:	exp:
cct	P	2475	699.10
ccc	P	4	448.60
cca	P	0	1012.00
ccg	P	0	319.20
---	--	------	------
mPD		0.0032	0.70
		nPD:	0.
		N. weight:	1.5
		Sc. PD:	-0.19
		Sc. rank:	-1847.5

NP			
Pos.	478	obs:	exp:
tct	S	1	423.20
tcc	S	2477	365.30
tca	S	0	558.60
tcg	S	0	160.10
cct	P	0	0.28
ccc	P	1	0.18
cca	P	0	0.41
ccg	P	0	0.13
agt	S	0	505.90
agc	S	0	464.90
---	--	------	------
mPD		0.0016	1.6
		nPD:	0.
		N. weight:	2.3
		Sc. PD:	-0.3
		Sc. rank:	-3722.4

NP			
Pos.	479	obs:	exp:
ttt	F	2477	1054.00
ttc	F	0	1423.00
tct	S	1	0.17
tcc	S	0	0.15
tca	S	0	0.23
tcg	S	0	0.06
tgt	C	1	0.43
tgc	C	0	0.57
agt	S	0	0.20
agc	S	0	0.19
---	--	------	------
mPD		0.0016	0.49
		nPD:	0.
		N. weight:	1.
		Sc. PD:	-0.13
		Sc. rank:	-1413.3

NP			
Pos.	480	obs:	exp:
cat	H	0	0.59
cac	H	1	0.41
gat	D	0	1425.00
gac	D	2478	1053.00
---	--	------	------
mPD		0.00081	0.49
		nPD:	0.
		N. weight:	1.
		Sc. PD:	-0.13
		Sc. rank:	-1540.9

NP			
Pos.	481	obs:	exp:
tta	L	0	0.11
ttg	L	0	0.19
ctt	L	0	0.19
ctc	L	0	0.17
cta	L	0	0.13
ctg	L	1	0.21
att	I	0	0.37
atc	I	0	0.25
ata	I	1	0.38
atg	M	2475	2475.00
aaa	K	0	1.12
aag	K	2	0.88
---	--	------	------
mPD		0.0032	0.0046
		nPD:	0.7
		N. weight:	0.002
		Sc. PD:	0.0011
		Sc. rank:	3.4

NP			
Pos.	482	obs:	exp:
tct	S	0	419.10
tcc	S	0	361.80
tca	S	0	553.20
tcg	S	0	158.50
cgt	R	0	0.04
cgc	R	0	0.05
cga	R	0	0.10
cgg	R	0	0.08
att	I	2	0.74
atc	I	0	0.50
ata	I	0	0.76
aat	N	21	11.69
aac	N	1	10.31
agt	S	1952	501.00
agc	S	502	460.40
aga	R	0	0.46
agg	R	1	0.27
---	--	------	------
mPD		0.34	1.7
		nPD:	0.21
		N. weight:	1.3
		Sc. PD:	0.087
		Sc. rank:	1203.8

NP			
Pos.	483	obs:	exp:
act	T	1	0.26
acc	T	0	0.22
aca	T	0	0.43
acg	T	0	0.08
aat	N	1935	1315.00
aac	N	539	1159.00
aaa	K	4	2.25
aag	K	0	1.75
---	--	------	------
mPD		0.34	0.50
		nPD:	0.69
		N. weight:	0.16
		Sc. PD:	0.087
		Sc. rank:	277.1

NP			
Pos.	484	obs:	exp:
gat	D	0	1.15
gac	D	2	0.85
gaa	E	2468	1483.00
gag	E	9	993.90
---	--	------	------
mPD		0.0088	0.48
		nPD:	0.02
		N. weight:	0.59
		Sc. PD:	-0.067
		Sc. rank:	-171.0

NP

Pos.	485	obs:	exp:
cgt	R	0	0.19
cgc	R	0	0.27
cga	R	0	0.48
cgg	R	0	0.39
aga	R	5	2.29
agg	R	0	1.37
gaa	E	1	0.60
gag	E	0	0.40
ggt	G	2	355.70
ggc	G	0	326.40
gga	G	2461	1080.00
ggg	G	10	711.30
---	--	------	------
mPD		0.014	0.69
		nPD:	0.02
		N. weight:	0.95
		Sc. PD:	-0.11
		Sc. rank:	-194.3

NP

Pos.	486	obs:	exp:
ttt	F	1	0.43
ttc	F	0	0.57
tct	S	2476	423.20
tcc	S	2	365.30
tca	S	0	558.60
tcg	S	0	160.10
agt	S	0	505.90
agc	S	0	464.90
---	--	------	------
mPD		0.0024	1.6
		nPD:	0.
		N. weight:	2.1
		Sc. PD:	-0.28
		Sc. rank:	-3269.1

NP

Pos.	487	obs:	exp:
tat	Y	2478	1205.00
tac	Y	1	1274.00
---	--	------	------
mPD		0.00081	0.50
		nPD:	0.
		N. weight:	0.86
		Sc. PD:	-0.11
		Sc. rank:	-1317.7

NP

Pos.	488	obs:	exp:
ttt	F	0	1042.00
ttc	F	2449	1407.00
tta	L	0	1.31
ttg	L	0	2.25
tct	S	1	3.07
tcc	S	17	2.65
tca	S	0	4.06
tcg	S	0	1.16
ctt	L	0	2.25
ctc	L	12	2.07
cta	L	0	1.60
ctg	L	0	2.52
agt	S	0	3.68
agc	S	0	3.38
---	--	------	------
mPD		0.025	0.52
		nPD:	0.05
		N. weight:	0.68
		Sc. PD:	-0.059
		Sc. rank:	257.9

NP

Pos.	489	obs:	exp:
ttt	F	5	1055.00
ttc	F	2474	1424.00
---	--	------	------
mPD		0.0040	0.49
		nPD:	0.01
		N. weight:	0.64
		Sc. PD:	-0.08
		Sc. rank:	-530.6

NP

Pos.	490	obs:	exp:
cgt	R	0	0.04
cgc	R	0	0.05
cga	R	1	0.10
cgg	R	0	0.08
aga	R	0	0.46
agg	R	0	0.27
ggt	G	0	356.50
ggc	G	0	327.10
gga	G	2478	1082.00
ggg	G	0	712.70
---	--	------	------
mPD		0.00081	0.69
		nPD:	0.
		N. weight:	0.99
		Sc. PD:	-0.13
		Sc. rank:	-1593.3

NP

Pos.	491	obs:	exp:
gat	D	0	1425.00
gac	D	2478	1053.00
gaa	E	0	0.60
gag	E	1	0.40
---	--	------	------
mPD		0.00081	0.49
		nPD:	0.
		N. weight:	1.
		Sc. PD:	-0.13
		Sc. rank:	-1533.4

NP

Pos.	492	obs:	exp:
tct	S	0	70.19
tcc	S	0	60.59
tca	S	0	92.65
tcg	S	0	26.55
att	I	89	33.05
atc	I	0	22.32
ata	I	0	33.63
aat	N	1976	1051.00
aac	N	1	926.40
aaa	K	1	1.12
aag	K	1	0.88
agt	S	411	83.92
agc	S	0	77.11
---	--	------	------
mPD		0.34	1.1
		nPD:	0.31
		N. weight:	0.95
		Sc. PD:	0.16
		Sc. rank:	1122.6

NP

Pos.	493	obs:	exp:
gct	A	5	630.60
gcc	A	0	483.40
gca	A	2474	1090.00
gcg	A	0	275.20
---	--	------	------
mPD		0.0040	0.69
		nPD:	0.01
		N. weight:	0.96
		Sc. PD:	-0.12
		Sc. rank:	-1100.6

NP

Pos.	494	obs:	exp:
aaa	K	0	0.56
aag	K	1	0.44
gaa	E	9	1484.00
gag	E	2469	994.30
---	--	------	------
mPD		0.0080	0.48
		nPD:	0.02
		N. weight:	1.1
		Sc. PD:	-0.12
		Sc. rank:	-348.9

NP

Pos.	495	obs:	exp:
gat	D	1	0.58
gac	D	0	0.42
gaa	E	96	1484.00
gag	E	2382	994.30
---	--	------	------
mPD		0.075	0.48
		nPD:	0.16
		N. weight:	0.88
		Sc. PD:	0.016
		Sc. rank:	666.8

NP

Pos.	496	obs:	exp:
tat	Y	2440	1204.00
tac	Y	37	1273.00
gtt	V	0	0.43
gtc	V	0	0.42
gta	V	2	0.39
gtg	V	0	0.75
---	--	------	------
mPD		0.034	0.50
		nPD:	0.07
		N. weight:	0.77
		Sc. PD:	-0.052
		Sc. rank:	379.7

NP

Pos.	497	obs:	exp:
caa	Q	1	0.51
cag	Q	0	0.49
gat	D	3	1420.00
gac	D	2465	1048.00
gaa	E	8	4.79
gag	E	0	3.21
ggt	G	0	0.29
ggc	G	2	0.26
gga	G	0	0.87
ggg	G	0	0.58
---	--	------	------
mPD		0.012	0.50
		nPD:	0.02
		N. weight:	1.
		Sc. PD:	-0.11
		Sc. rank:	-95.2

NP

Pos.	498	obs:	exp:
tct	S	0	0.51
tcc	S	0	0.44
tca	S	0	0.68
tcg	S	0	0.19
caa	Q	1	0.51
cag	Q	0	0.49
aat	N	2475	1315.00
aac	N	0	1160.00
agt	S	3	0.61
agc	S	0	0.56
---	--	------	------
mPD		0.0040	0.50
		nPD:	0.01
		N. weight:	0.75
		Sc. PD:	-0.094
		Sc. rank:	-676.1

NP

Pos.	499	obs:	exp:
taa	.	2462	909.10
tag	.	0	695.70
tga	.	17	874.20
---	--	------	------
mPD		0.014	0.86
		nPD:	0.02
		N. weight:	1.1
		Sc. PD:	-0.13
		Sc. rank:	-422.9

Amino acid level conservation per codon

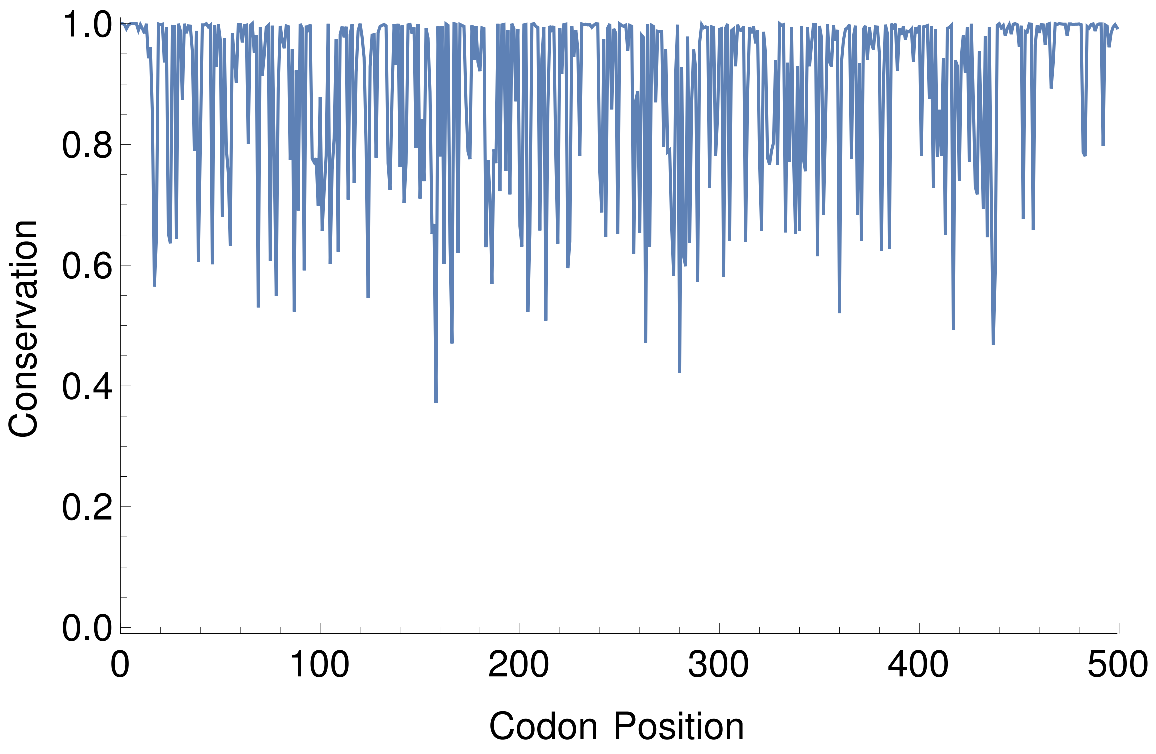

Conservation analysis plots

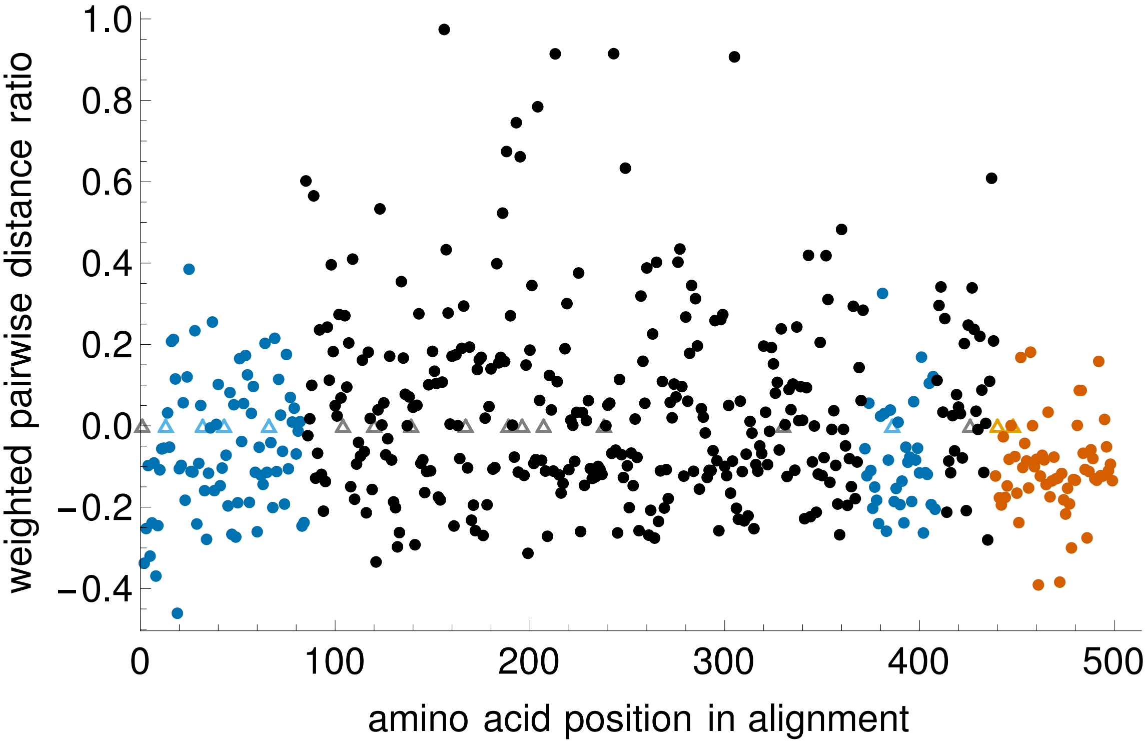

NP

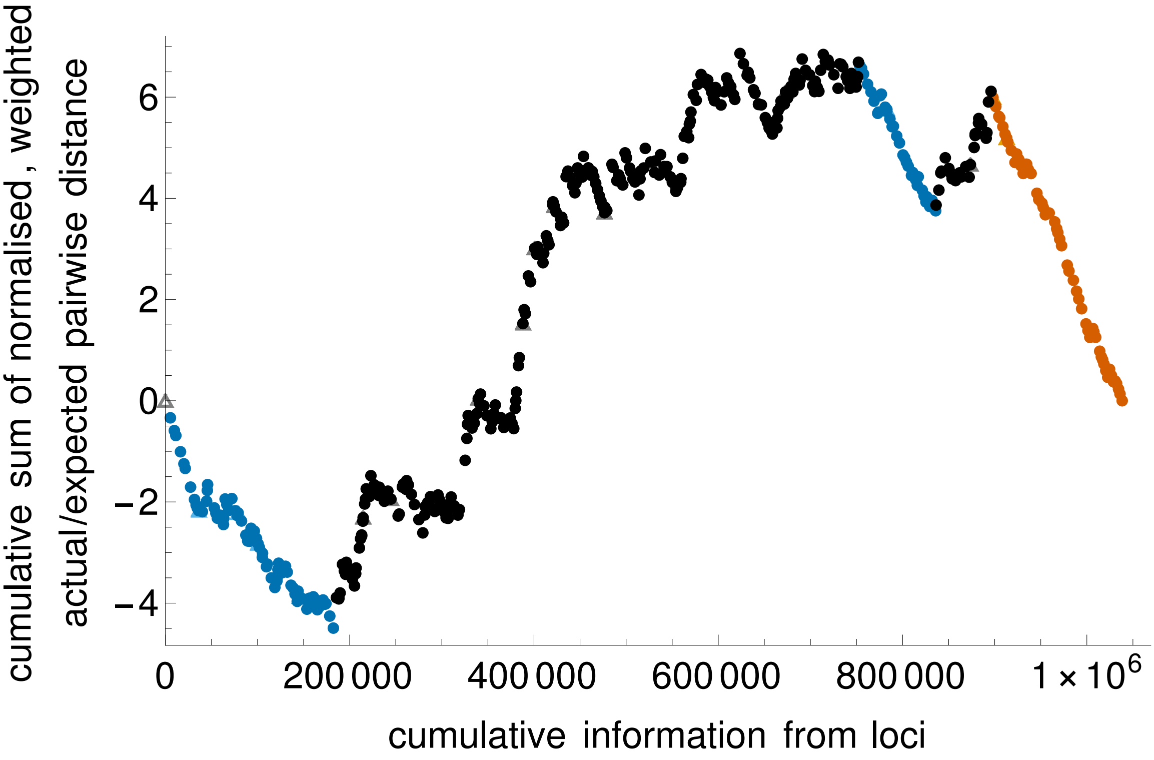

NP

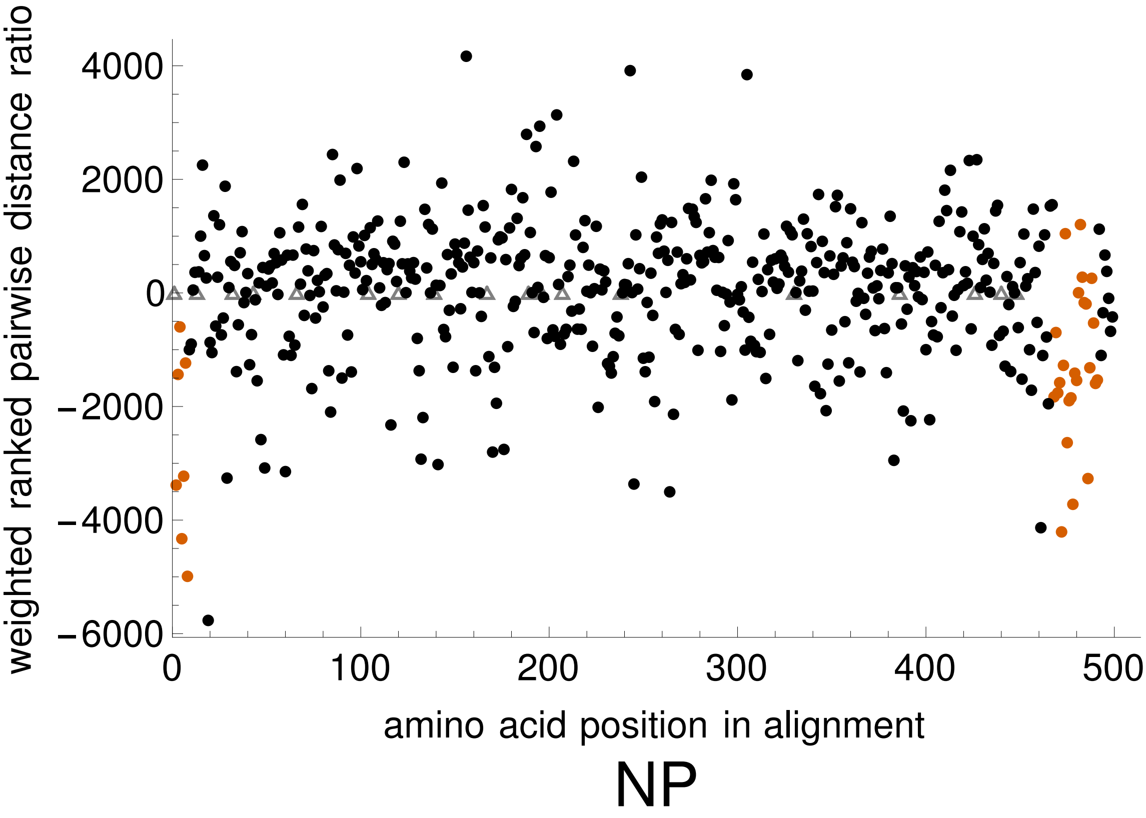
weighted ranked pairwise distance ratio
4000
2000
0
−2000
−4000
−6000
0
100
200
300
400
500
amino acid position in alignment
NP

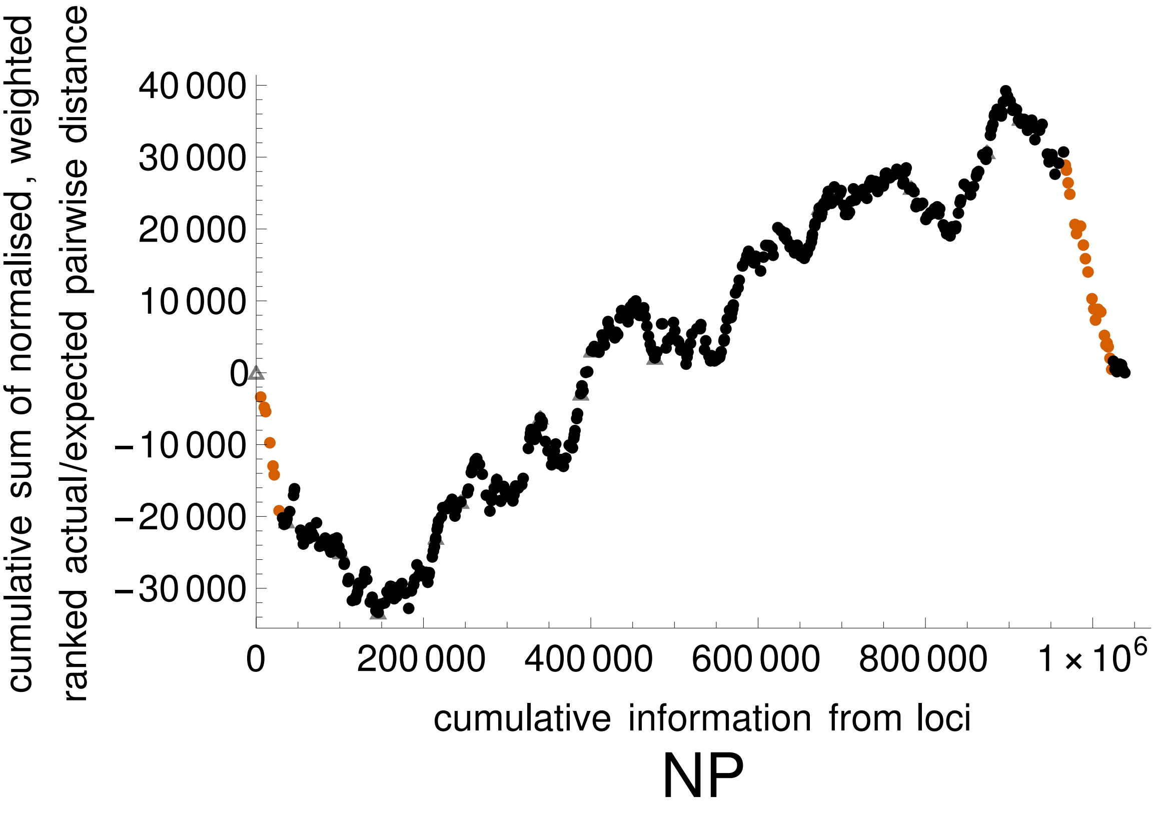
cumulative sum of normalised, weighted ranked actual/expected pairwise distance
40 000
30 000
20 000
10 000
0
−10 000
−20 000
−30 000
0
200 000
400 000
600 000
800 000
1 × 10^6
cumulative information from loci
NP

NA

Gene length histogram

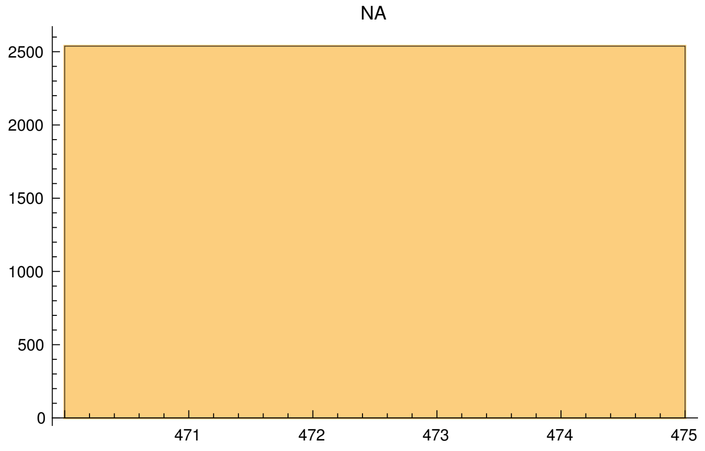

Information vs. nPD

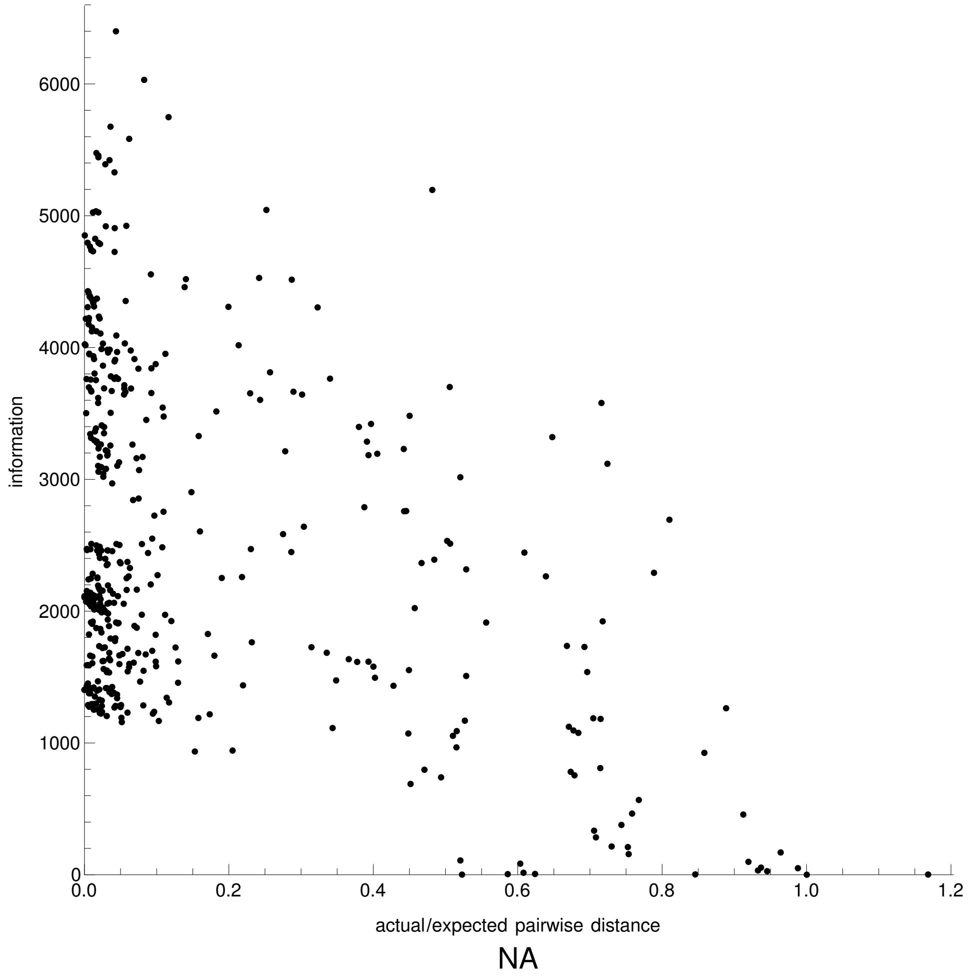

Example sequences highlighted by regions found in analyses to be conserved

Interesting points (by weighted raw PD) highlighted for gene NA:

ATGAATCCAAATCAGAAAATAGCGACCATTGGCTCCATTTCATTAGGGCTGGCTGTATTCAATGTTCTACTGCATG⋱
CCGTGAGCATCATATTAATGGTGTTAGCCCTGGGGAAAAGTGAAAACAATGGAATCTGCAAGGGAACTATAGTAA⋱
GAGAATATAATGAAACAGTTAGGATAGAGAAAGTGACACAATGGTACAATACTAGTGTGGTCGAATATGTACCGC⋱
ATTGGAACGAGGGCACT
TATATAAACAACACCGAACCAATATGTGATGTCAAGGGCTTTGCACCTTTTTCCA
AGGACAACGGGATAAGAGTTGGCTCCAGAGGACATATTTTTGTCATAAGGGA
GCCTTTCGTCTCTTGTTCACCTGTAGAGTGCAGAACTTTCTTCCTCACTCAG
GGAGCTCTACTCAATGACAAACACTCAAATGGAACAGTGAAGGATAGAAGCC
CATTCAGAACTCTCATGAGTGTCGAAGTGGGTCAATCACCCAATGTGTATCAAGCAAGGT⋱
TTGAAGCTGTAGCATGGTCAGCAACAGCCTGTCATGATGGCAAGAAATGGATGACGATTGGTGTAACAGG⋱
GCCAGATTCTAAAGCAGTAGCAGTAGTCCATTACGGAGGGGTGCCTACTGATGTTGTTAACTCCTGGGCG⋱
GGAGATATATTAAGG
ACTCAGGAGTCATCTTGTACTTGCATTCAAGGTAATTGTTATTGGGTAATGACTG
ACGGTCCAGCCAATAGACAGGCGCAGTATAGAATATACAAAGCAAATCAAGG
CAAAATAATTGACCAAACAGATGTCAGCTTTAGTGGAGGACATATTGAGGAA
TGTTCTTGTTATCCAAATGATGGTAAAGTGGAATGCGTGTGTAGAGACAACT
GGACGGGAACTAACAGGCCTGTGCTAGTTATTTCGCCTGATCTCTCTTACAG
GGTTGGGTATTTATGTGCAGGATTGCCCAGTGACACTCCAAGAGGGGAAGAT
GCTCAATTTGTCGGTTCATGCACTAGTCCCATGGGAAATCAGGGATATGGCG
TAAAAGGTTTCGGGTTTCGACAGGGAACTGATGTGTGGGTGGGGCGGACAAT
TAGTCGAACCTCCAGGTCAGGGTTTGAAATAATAAGGATAAAGAATGGTTGG
ACGCAGACAAGCAAAGAACAGATTAGAAGGCAGGTGGTTGTTGATAATTTGA
ATTGGTCGGGATACAGTGGGTCTTTCACTTTACCAGTAGAATTGTCTGGGAG
GGAATGTTTAGTCCCCTGTTTTTGGGTCGAAATGATCAGAGGC
AGGCCAGAAGAAAGAACA---ATCTGGACCTCTAGTAGCTCTATTGTAATGTGTG
GAGTTGATCATGAAATTGCCGATTGGTCATGGCACGATGGAGCTGTTCTTCC
CTTTGACATCGATAAGATGTAA

Interesting points (by weighted ranked PD) highlighted for gene NA:

ATGAATCCAAATCAGAAAATAGCGACCATTGGCTCCATTTCATTAGGGCTGGCTGTATTCAATGTTCTACTGCATGCC⋱
GTGAGCATCATATTAATGGTGTTAGCCCTGGGGAAAAGTGAAAACAATGGAATCTGCAAGGGAACTATAGTAAGAGA⋱
ATATAATGAAACAGTTAGGATAGAGAAAGTGACACAATGGTACAATACTAGTGTGGTCGAATATGTACCGCATTGGA⋱
ACGAGGGCACTTATATAAACAACACCGAACCAATA
TGTGATGTCAAGGGCTTTGCACCTTTTTCCAAGGACAACGGGATAAGAGTTGGCT
CCAGAGGACATATTTTTGTCATAAGGGAGCCTTTCGTCTCTTGTTCACCTGT
AGAGTGCAGAACTTTCTTCCTCACTCAGGGAGCTCTACTCAATGACAAACAC
TCAAATGGAACAGTGAAGGATAGAAGCCCATTCAGAACTCTCATGAGTGTCG
AAGTGGGTCAATCACCCAATGTGTATCAAGCAAGGTTTGAAGCTGTAGCATG
GTCAGCAACAGCCTGTCATGATGGCAAGAAATGGATGACGATTGGTGTAACAGGGCCAGATTC⋱
TAAAGCAGTAGCAGTAGTCCATTACGGAGGGGTGCCTACTGATGTTGTTAACTCCTGGGCGGGAGATATA⋱
TTAAGGACTCAGGAGTCATCTTGTACTTGCATTCAAGGTAATTGTTATTGGGTAATGACTGACGGTCCAG⋱
CCAATAGACAGGCGCAGTATAGAATATACAAAGCAAATCAAGGCAAAATAATTGACCAAACAGATGTCAG⋱
CTTTAGTGGAGGACATATTGAGGAATGTTCTTGTTATCCAAATGATGGTAAAGTGGAATGCGTGTGTAGA⋱

GACAACTGGACGGGAACTAACAGGCCTGTGCTAGTTATTTCGCCTGATCTCTCTTACAGGGTTGGGTATT∵
TATGTGCAGGATTGCCCAGTGACACTCCAAGAGGGGAAGATGCTCAATTTGTCGGTTCATGCACTAGTCC∵
CATGGGAAATCAGGGATATGGCGTAAAAGGTTTCGGGTTTCGACAGGGAACTGATGTGTGGGTGGGGCGG∵
ACAATTAGTCGAACCTCCAGGTCAGGGTTTGAAATAATAAGGATAAAGAATGGTTGGACGCAGACAAGCA∵
AAGAACAGATTAGAAGGCAGGTGGTTGTTGATAATTTGAATTGGTCGGGATACAGTGGGTCTTTCACTTT∵
ACCAGTAGAATTGTCTGGGAGGGAATGTTTAGTCCCCTGTTTTTGGGTCGAAATGATCAGAGGCAGGCCA∵
GAAGAAAGAACA –––ATCTGG
ACCTCTAGTAGCTCTATTGTAATGTGTGGAGTTGATCATGAAATTGCCGATTGGT
CATGGCACGATGGAGCTGTTCTTCCCTTTGACATCGATAAGATGTAA

Per codon conservation report

NA			
Pos.	1	obs:	exp:
atg	M	2537	2537.00
---	-	1	1.00
---	--	------	------
mPD		0	0
		nPD:	1.
		N. weight:	0.
		Sc. PD:	0
		Sc. rank:	0

NA			
Pos.	2	obs:	exp:
aat	N	2533	1347.00
aac	N	2	1188.00
aaa	K	2	1.12
aag	K	0	0.88
---	-	1	1.00
---	--	------	------
mPD		0.0031	0.50
		nPD:	0.01
		N. weight:	0.66
		Sc. PD:	-0.063
		Sc. rank:	-1148.9

NA			
Pos.	3	obs:	exp:
tta	L	0	0.11
ttg	L	0	0.19
tct	S	0	0.17
tcc	S	0	0.15
tca	S	1	0.23
tcg	S	0	0.06
ctt	L	0	0.19
ctc	L	0	0.17
cta	L	1	0.13
ctg	L	0	0.21
cct	P	1	714.30
ccc	P	0	458.30
cca	P	2529	1034.00
ccg	P	3	326.20
caa	Q	2	1.02
cag	Q	0	0.98
agt	S	0	0.20
agc	S	0	0.19
---	-	1	1.00
---	--	------	------
mPD		0.0063	0.71
		nPD:	0.01
		N. weight:	0.93
		Sc. PD:	-0.087
		Sc. rank:	-1427.9

NA			
Pos.	4	obs:	exp:
tct	S	0	0.34
tcc	S	0	0.29
tca	S	0	0.45
tcg	S	0	0.13
act	T	1	0.26
acc	T	0	0.22
aca	T	0	0.43
acg	T	0	0.08
aat	N	2517	1342.00
aac	N	9	1184.00
aaa	K	0	0.56
aag	K	1	0.44
agt	S	2	0.41
agc	S	0	0.38
gat	D	7	4.03
gac	D	0	2.97
---	-	1	1.00
---	--	------	------
mPD		0.016	0.51
		nPD:	0.03
		N. weight:	0.64
		Sc. PD:	-0.046
		Sc. rank:	-78.4

NA			
Pos.	5	obs:	exp:
tta	L	0	0.33
ttg	L	0	0.56
ctt	L	0	0.56
ctc	L	0	0.52
cta	L	0	0.40
ctg	L	3	0.63
cat	H	1	0.59
cac	H	0	0.41
caa	Q	221	1271.00
cag	Q	2278	1228.00
cgt	R	0	1.29
cgc	R	0	1.81
cga	R	1	3.29
cgg	R	33	2.66
aga	R	0	15.60
agg	R	0	9.35
---	-	1	1.00
---	--	------	------
mPD		0.19	0.55
		nPD:	0.34
		N. weight:	0.46
		Sc. PD:	0.11
		Sc. rank:	755.9

NA			
Pos.	6	obs:	exp:
cgt	R	0	0.30
cgc	R	0	0.43
cga	R	0	0.77
cgg	R	0	0.63
aaa	K	2518	1418.00
aag	K	8	1108.00
aga	R	8	3.67
agg	R	0	2.20
gaa	E	3	1.80
gag	E	0	1.20
---	-	1	1.00
---	--	------	------
mPD		0.015	0.50
		nPD:	0.03
		N. weight:	0.59
		Sc. PD:	-0.043
		Sc. rank:	-93.6

NA			
Pos.	7	obs:	exp:
tta	L	1	0.11
ttg	L	0	0.19
ctt	L	0	0.19
ctc	L	0	0.17
cta	L	0	0.13
ctg	L	0	0.21
cgt	R	0	0.04
cgc	R	0	0.05
cga	R	0	0.10
cgg	R	0	0.08
att	I	1	936.90
atc	I	0	632.80
ata	I	2522	953.40
act	T	0	2.65
acc	T	0	2.21
aca	T	10	4.30
acg	T	0	0.84
aaa	K	2	1.12
aag	K	0	0.88
aga	R	1	0.46
agg	R	0	0.27
---	-	1	1.00
---	--	------	------
mPD		0.012	0.67
		nPD:	0.02
		N. weight:	1.
		Sc. PD:	-0.087
		Sc. rank:	-900.0

NA			
Pos.	8	obs:	exp:
tct	S	0	0.17
tcc	S	0	0.15
tca	S	0	0.23
tcg	S	1	0.06
att	I	0	10.03
atc	I	0	6.77
ata	I	27	10.20
atg	M	24	24.00
act	T	0	4.50
acc	T	0	3.76
aca	T	0	7.30
acg	T	17	1.44
agt	S	0	0.20
agc	S	0	0.19
gtt	V	0	252.90
gtc	V	0	245.70
gta	V	593	228.80
gtg	V	570	435.50
gct	A	0	332.00
gcc	A	0	254.50
gca	A	12	573.70
gcg	A	1293	144.90
gaa	E	0	0.60
gag	E	1	0.40
---	--	------	------
mPD		0.93	1.3
		nPD:	0.72
		N. weight:	1.5
		Sc. PD:	0.88
		Sc. rank:	3009.3

NA			
Pos.	9	obs:	exp:
tct	S	0	0.17
tcc	S	0	0.15
tca	S	0	0.23
tcg	S	0	0.06
att	I	4	92.46
atc	I	245	62.45
ata	I	0	94.09
act	T	20	602.90
acc	T	2247	502.90
aca	T	9	978.00
acg	T	0	192.20
agt	S	0	0.20
agc	S	1	0.19
gct	A	0	3.05
gcc	A	12	2.34
gca	A	0	5.28
gcg	A	0	1.33
---	--	------	------
mPD		0.21	0.88
		nPD:	0.24
		N. weight:	1.5
		Sc. PD:	0.2
		Sc. rank:	2204.5

NA			
Pos.	10	obs:	exp:
tgg	W	1	1.00
att	I	2131	798.40
atc	I	15	539.20
ata	I	4	812.40
gtt	V	146	84.16
gtc	V	241	81.77
gta	V	0	76.15
gtg	V	0	144.90
---	--	------	------
mPD		0.45	0.95
		nPD:	0.47
		N. weight:	0.98
		Sc. PD:	0.34
		Sc. rank:	1785.7

NA			
Pos.	11	obs:	exp:
gtt	V	1	0.22
gtc	V	0	0.21
gta	V	0	0.20
gtg	V	0	0.37
ggt	G	17	365.00
ggc	G	2502	334.90
gga	G	7	1107.00
ggg	G	11	729.70
---	--	------	------
mPD		0.029	0.69
		nPD:	0.04
		N. weight:	2.
		Sc. PD:	-0.13
		Sc. rank:	636.6

NA			
Pos.	12	obs:	exp:
ttt	F	0	0.43
ttc	F	1	0.57
tct	S	4	433.10
tcc	S	2518	373.90
tca	S	14	571.70
tcg	S	0	163.80
act	T	0	0.26
acc	T	1	0.22
aca	T	0	0.43
acg	T	0	0.08
agt	S	0	517.80
agc	S	0	475.80
---	--	------	------
mPD		0.016	1.6
		nPD:	0.01
		N. weight:	2.
		Sc. PD:	-0.18
		Sc. rank:	-2829.4

NA			
Pos.	13	obs:	exp:
tta	L	0	0.55
ttg	L	0	0.94
ctt	L	3	0.94
ctc	L	0	0.86
cta	L	2	0.67
ctg	L	0	1.05
att	I	1612	934.70
atc	I	898	631.30
ata	I	7	951.10
atg	M	3	3.00
act	T	2	0.53
acc	T	0	0.44
aca	T	0	0.86
acg	T	0	0.17
gtt	V	4	2.39
gtc	V	0	2.32
gta	V	4	2.16
gtg	V	3	4.12
---	--	------	------
mPD		0.48	0.67
		nPD:	0.71
		N. weight:	0.49
		Sc. PD:	0.29
		Sc. rank:	987.5

NA			
Pos.	14	obs:	exp:
tta	L	1	0.11
ttg	L	0	0.19
tct	S	2	432.40
tcc	S	11	373.30
tca	S	2501	570.70
tcg	S	18	163.60
ctt	L	0	0.19
ctc	L	0	0.17
cta	L	0	0.13
ctg	L	0	0.21
cct	P	0	0.56
ccc	P	0	0.36
cca	P	2	0.82
ccg	P	0	0.26
caa	Q	3	1.53
cag	Q	0	1.47
agt	S	0	517.00
agc	S	0	475.10
---	--	------	------
mPD		0.031	1.6
		nPD:	0.02
		N. weight:	1.5
		Sc. PD:	-0.13
		Sc. rank:	-1222.0

NA			
Pos.	15	obs:	exp:
ttt	F	1	1.70
ttc	F	3	2.30
tta	L	1555	276.60
ttg	L	933	475.50
ctt	L	0	475.20
ctc	L	0	437.20
cta	L	46	337.20
ctg	L	0	532.40
---	--	------	------
mPD		0.50	1.1
		nPD:	0.44
		N. weight:	1.3
		Sc. PD:	0.44
		Sc. rank:	2356.9

NA

Pos.	16	obs:	exp:
gtt	V	0	0.65
gtc	V	0	0.63
gta	V	0	0.59
gtg	V	3	1.12
ggt	G	0	364.70
ggc	G	2	334.60
gga	G	954	1107.00
ggg	G	1579	729.10
mPD		0.47	0.69
		nPD:	0.68
		N. weight:	0.45
		Sc. PD:	0.25
		Sc. rank:	887.7

,

NA

Pos.	17	obs:	exp:
ttt	F	2	0.85
ttc	F	0	1.15
tta	L	22	276.80
ttg	L	1606	475.90
ctt	L	0	475.50
ctc	L	0	437.50
cta	L	886	337.40
ctg	L	22	532.80
mPD		0.92	1.1
		nPD:	0.81
		N. weight:	1.1
		Sc. PD:	0.76
		Sc. rank:	2313.7

,

NA

Pos.	18	obs:	exp:
gtt	V	2498	546.30
gtc	V	0	530.80
gta	V	12	494.30
gtg	V	2	940.70
gct	A	24	6.61
gcc	A	2	5.07
gca	A	0	11.43
gcg	A	0	2.89
mPD		0.033	0.75
		nPD:	0.04
		N. weight:	1.6
		Sc. PD:	-0.094
		Sc. rank:	598.3

,

NA

Pos.	19	obs:	exp:
tta	L	2	0.22
ttg	L	0	0.38
ctt	L	0	0.38
ctc	L	0	0.35
cta	L	0	0.27
ctg	L	0	0.42
att	I	0	2.97
atc	I	1	2.01
ata	I	7	3.02
gtt	V	5	549.80
gtc	V	7	534.10
gta	V	2515	497.40
gtg	V	1	946.70
mPD		0.019	0.74
		nPD:	0.03
		N. weight:	1.7
		Sc. PD:	-0.13
		Sc. rank:	-583.0

,

NA

Pos.	20	obs:	exp:
ttt	F	5	1074.00
ttc	F	2519	1450.00
tta	L	2	0.87
ttg	L	0	1.50
tct	S	0	0.17
tcc	S	1	0.15
tca	S	0	0.23
tcg	S	0	0.06
ctt	L	5	1.50
ctc	L	1	1.38
cta	L	0	1.06
ctg	L	0	1.68
att	I	1	1.86
atc	I	3	1.25
ata	I	1	1.89
agt	S	0	0.20
agc	S	0	0.19
mPD		0.020	0.50
		nPD:	0.04
		N. weight:	0.57
		Sc. PD:	-0.036
		Sc. rank:	124.7

,

NA

Pos.	21	obs:	exp:
tct	S	0	0.17
tcc	S	0	0.15
tca	S	0	0.23
tcg	S	0	0.06
aat	N	2535	1348.00
aac	N	2	1189.00
agt	S	1	0.20
agc	S	0	0.19
mPD		0.0024	0.50
		nPD:	0.
		N. weight:	0.66
		Sc. PD:	-0.064
		Sc. rank:	-1234.6

,

NA

Pos.	22	obs:	exp:
att	I	37	14.11
atc	I	1	9.53
ata	I	0	14.36
gtt	V	2432	543.50
gtc	V	67	528.00
gta	V	0	491.70
gtg	V	0	935.80
gct	A	1	0.25
gcc	A	0	0.20
gca	A	0	0.44
gcg	A	0	0.11
mPD		0.082	0.76
		nPD:	0.11
		N. weight:	1.5
		Sc. PD:	0.0037
		Sc. rank:	1701.8

,

NA

Pos.	23	obs:	exp:
tta	L	0	269.60
ttg	L	0	463.50
ctt	L	2	463.20
ctc	L	7	426.20
cta	L	2454	328.60
ctg	L	7	519.00
att	I	0	25.25
atc	I	4	17.05
ata	I	64	25.70
mPD		0.068	1.2
		nPD:	0.06
		N. weight:	2.
		Sc. PD:	-0.094
		Sc. rank:	1375.7

,

NA

Pos.	24	obs:	exp:
tta	L	0	277.00
ttg	L	33	476.20
ctt	L	2	475.90
ctc	L	0	437.90
cta	L	20	337.70
ctg	L	2483	533.30
mPD		0.043	1.1
		nPD:	0.04
		N. weight:	1.5
		Sc. PD:	-0.1
		Sc. rank:	266.0

,

NA

Pos.	25	obs:	exp:
tta	L	0	0.11
ttg	L	0	0.19
ctt	L	1	0.19
ctc	L	0	0.17
cta	L	0	0.13
ctg	L	0	0.21
cat	H	2526	1508.00
cac	H	11	1029.00
mPD		0.0094	0.48
		nPD:	0.02
		N. weight:	0.52
		Sc. PD:	-0.043
		Sc. rank:	-366.5

,

NA

Pos.	26	obs:	exp:
tct	S	0	0.34
tcc	S	2	0.29
tca	S	0	0.45
tcg	S	0	0.13
att	I	0	4.08
atc	I	0	2.76
ata	I	11	4.16
act	T	0	0.26
acc	T	1	0.22
aca	T	0	0.43
acg	T	0	0.08
agt	S	0	0.41
agc	S	0	0.38
gtt	V	0	1.09
gtc	V	5	1.06
gta	V	0	0.98
gtg	V	0	1.87
gct	A	47	640.80
gcc	A	2469	491.20
gca	A	2	1107.00
gcg	A	1	279.60
mPD		0.071	0.72
		nPD:	0.1
		N. weight:	1.6
		Sc. PD:	-0.011
		Sc. rank:	1785.6

,

NA

Pos.	27	obs:	exp:
ttt	F	2	0.85
ttc	F	0	1.15
tta	L	1	98.45
ttg	L	899	169.30
ctt	L	0	169.10
ctc	L	0	155.60
cta	L	0	120.00
ctg	L	2	189.50
att	I	1	0.74
atc	I	1	0.50
ata	I	0	0.76
atg	M	279	279.00
gtt	V	11	294.30
gtc	V	0	285.90
gta	V	1	266.20
gtg	V	1341	506.70
mPD		0.59	1.3
		nPD:	0.45
		N. weight:	1.1
		Sc. PD:	0.38
		Sc. rank:	2036.8

,

NA

Pos.	28	obs:	exp:
tct	S	0	432.20
tcc	S	0	373.10
tca	S	0	570.50
tcg	S	0	163.50
aat	N	0	2.66
aac	N	5	2.34
agt	S	5	516.80
agc	S	2526	474.90
ggt	G	0	0.29
ggc	G	2	0.26
gga	G	0	0.87
ggg	G	0	0.58
mPD		0.0094	1.6
		nPD:	0.01
		N. weight:	1.7
		Sc. PD:	-0.17
		Sc. rank:	-3115.2

,

NA

Pos.	29	obs:	exp:
att	I	47	939.50
atc	I	2410	634.50
ata	I	73	956.00
act	T	0	2.12
acc	T	7	1.77
aca	T	1	3.44
acg	T	0	0.68
mPD		0.098	0.66
		nPD:	0.15
		N. weight:	1.2
		Sc. PD:	0.049
		Sc. rank:	1566.4

,

NA

Pos.	30	obs:	exp:
att	I	1	935.40
atc	I	0	631.80
ata	I	2518	951.90
atg	M	2	2.00
act	T	0	3.97
acc	T	0	3.31
aca	T	15	6.45
acg	T	0	1.27
gtt	V	0	0.43
gtc	V	0	0.42
gta	V	2	0.39
gtg	V	0	0.75
mPD		0.016	0.67
		nPD:	0.02
		N. weight:	1.
		Sc. PD:	-0.081
		Sc. rank:	-462.9

,

NA			
Pos.	31	obs:	exp:
tta	L	2489	275.30
ttg	L	1	473.20
ctt	L	0	472.90
ctc	L	0	435.10
cta	L	32	335.60
ctg	L	0	529.90
att	I	0	0.74
atc	I	0	0.50
ata	I	2	0.76
gtt	V	0	3.04
gtc	V	0	2.96
gta	V	12	2.75
gtg	V	2	5.24
---	--	------	------
mPD		0.040	1.1
		nPD:	0.03
		N. weight:	2.2
		Sc. PD:	-0.15
		Sc. rank:	150.3

,

NA			
Pos.	32	obs:	exp:
att	I	1	0.74
atc	I	0	0.50
ata	I	1	0.76
atg	M	1816	1816.00
act	T	6	190.70
acc	T	0	159.10
aca	T	575	309.40
acg	T	139	60.82
---	--	------	------
mPD		0.76	0.84
		nPD:	0.91
		N. weight:	0.19
		Sc. PD:	0.15
		Sc. rank:	394.3

,

NA			
Pos.	33	obs:	exp:
tta	L	0	0.44
ttg	L	4	0.75
ctt	L	0	0.75
ctc	L	0	0.69
cta	L	0	0.53
ctg	L	0	0.84
atg	M	24	24.00
gtt	V	3	545.70
gtc	V	0	530.10
gta	V	15	493.70
gtg	V	2491	939.50
ggt	G	0	0.14
ggc	G	0	0.13
gga	G	0	0.44
ggg	G	1	0.29
---	--	------	------
mPD		0.037	0.75
		nPD:	0.05
		N. weight:	0.98
		Sc. PD:	-0.054
		Sc. rank:	522.2

,

NA			
Pos.	34	obs:	exp:
ttt	F	1	0.85
ttc	F	1	1.15
tta	L	2510	276.80
ttg	L	24	475.90
ctt	L	0	475.50
ctc	L	0	437.50
cta	L	2	337.40
ctg	L	0	532.80
---	--	------	------
mPD		0.022	1.1
		nPD:	0.02
		N. weight:	2.3
		Sc. PD:	-0.19
		Sc. rank:	-1781.0

,

NA			
Pos.	35	obs:	exp:
tct	S	0	0.17
tcc	S	1	0.15
tca	S	0	0.23
tcg	S	0	0.06
att	I	0	0.37
atc	I	1	0.25
ata	I	0	0.38
act	T	0	0.26
acc	T	1	0.22
aca	T	0	0.43
acg	T	0	0.08
agt	S	0	0.20
agc	S	0	0.19
gtt	V	8	3.04
gtc	V	6	2.96
gta	V	0	2.75
gtg	V	0	5.24
gct	A	58	641.30
gcc	A	2462	491.60
gca	A	1	1108.00
gcg	A	0	279.90
---	--	------	------
mPD		0.066	0.71
		nPD:	0.09
		N. weight:	1.6
		Sc. PD:	-0.02
		Sc. rank:	1682.4

,

NA			
Pos.	36	obs:	exp:
ttt	F	1	0.43
ttc	F	0	0.57
tta	L	0	271.30
ttg	L	5	466.50
ctt	L	4	466.20
ctc	L	9	428.90
cta	L	11	330.80
ctg	L	2457	522.30
cct	P	0	0.56
ccc	P	0	0.36
cca	P	0	0.82
ccg	P	2	0.26
caa	Q	0	8.14
cag	Q	16	7.86
atg	M	33	33.00
---	--	------	------
mPD		0.064	1.2
		nPD:	0.06
		N. weight:	1.5
		Sc. PD:	-0.075
		Sc. rank:	942.1

,

NA			
Pos.	37	obs:	exp:
tgg	W	6	6.00
cct	P	11	3.10
ccc	P	0	1.99
cca	P	0	4.49
ccg	P	0	1.42
cgt	R	0	0.53
cgc	R	0	0.75
cga	R	0	1.36
cgg	R	0	1.10
aga	R	0	6.42
agg	R	14	3.85
gaa	E	0	2.40
gag	E	4	1.60
ggt	G	0	360.10
ggc	G	0	330.40
gga	G	13	1093.00
ggg	G	2490	719.90
---	--	------	------
mPD		0.055	0.72
		nPD:	0.08
		N. weight:	1.3
		Sc. PD:	-0.037
		Sc. rank:	1158.6

,

NA			
Pos.	38	obs:	exp:
cgt	R	0	0.72
cgc	R	0	1.01
cga	R	0	1.84
cgg	R	0	1.49
aaa	K	1950	1407.00
aag	K	557	1100.00
aga	R	19	8.72
agg	R	0	5.22
ggt	G	0	1.73
ggc	G	0	1.58
gga	G	11	5.24
ggg	G	1	3.45
---	--	------	------
mPD		0.38	0.53
		nPD:	0.71
		N. weight:	0.12
		Sc. PD:	0.069
		Sc. rank:	236.2

,

NA			
Pos.	39	obs:	exp:
tct	S	0	412.10
tcc	S	0	355.70
tca	S	0	543.90
tcg	S	0	155.90
cgt	R	5	0.19
cgc	R	0	0.27
cga	R	0	0.48
cgg	R	0	0.39
act	T	1	0.26
acc	T	0	0.22
aca	T	0	0.43
acg	T	0	0.08
aat	N	19	10.10
aac	N	0	8.90
agt	S	2388	492.70
agc	S	25	452.70
aga	R	0	2.29
agg	R	0	1.37
gat	D	4	2.30
gac	D	0	1.70
ggt	G	95	13.81
ggc	G	1	12.67
gga	G	0	41.91
ggg	G	0	27.61
---	--	------	------
mPD		0.12	1.7
		nPD:	0.07
		N. weight:	1.6
		Sc. PD:	-0.057
		Sc. rank:	1354.5

,

NA			
Pos.	40	obs:	exp:
aaa	K	1	0.56
aag	K	0	0.44
gat	D	0	0.58
gac	D	1	0.42
gaa	E	2508	1505.00
gag	E	5	1008.00
ggt	G	0	3.31
ggc	G	1	3.04
gga	G	21	10.04
ggg	G	1	6.62
---	--	------	------
mPD		0.025	0.50
		nPD:	0.05
		N. weight:	0.53
		Sc. PD:	-0.029
		Sc. rank:	282.6

,

NA

Pos.	41	obs:	exp:
tat	Y	0	0.49
tac	Y	1	0.51
cat	H	1	0.59
cac	H	0	0.41
act	T	0	0.26
acc	T	1	0.22
aca	T	0	0.43
acg	T	0	0.08
aat	N	9	1347.00
aac	N	2525	1187.00
gat	D	0	0.58
gac	D	1	0.42
---	--	------	------
mPD		0.011	0.50
		nPD:	0.02
		N. weight:	0.77
		Sc. PD:	-0.063
		Sc. rank:	-409.4

,

NA

Pos.	42	obs:	exp:
tct	S	0	42.87
tcc	S	0	37.00
tca	S	0	56.58
tcg	S	0	16.21
act	T	1	0.26
acc	T	0	0.22
aca	T	0	0.43
acg	T	0	0.08
aat	N	2271	1214.00
aac	N	14	1071.00
aaa	K	1	0.56
aag	K	0	0.44
agt	S	251	51.25
agc	S	0	47.09
---	--	------	------
mPD		0.19	0.82
		nPD:	0.23
		N. weight:	0.73
		Sc. PD:	0.089
		Sc. rank:	1057.4

,

NA

Pos.	43	obs:	exp:
cgt	R	0	0.15
cgc	R	0	0.21
cga	R	0	0.39
cgg	R	0	0.31
aga	R	4	1.83
agg	R	0	1.10
gct	A	0	0.25
gcc	A	0	0.20
gca	A	1	0.44
gcg	A	0	0.11
gaa	E	6	5.39
gag	E	3	3.61
ggt	G	0	363.10
ggc	G	0	333.10
gga	G	2425	1102.00
ggg	G	99	726.00
---	--	------	------
mPD		0.088	0.70
		nPD:	0.13
		N. weight:	0.71
		Sc. PD:	0.014
		Sc. rank:	891.3

,

NA

Pos.	44	obs:	exp:
tta	L	0	0.11
ttg	L	0	0.19
tct	S	0	1.54
tcc	S	0	1.33
tca	S	0	2.03
tcg	S	0	0.58
ctt	L	0	0.19
ctc	L	1	0.17
cta	L	0	0.13
ctg	L	0	0.21
att	I	4	907.50
atc	I	2439	612.90
ata	I	1	923.50
act	T	0	18.54
acc	T	70	15.47
aca	T	0	30.08
acg	T	0	5.91
aat	N	4	3.19
aac	N	2	2.81
agt	S	1	1.84
agc	S	8	1.69
gtt	V	0	1.52
gtc	V	7	1.48
gta	V	0	1.38
gtg	V	0	2.62
gct	A	0	0.25
gcc	A	1	0.20
gca	A	0	0.44
gcg	A	0	0.11
---	--	------	------
mPD		0.081	0.74
		nPD:	0.11
		N. weight:	1.4
		Sc. PD:	0.0056
		Sc. rank:	1702.0

,

NA

Pos.	45	obs:	exp:
tgt	C	9	1103.00
tgc	C	2527	1433.00
cgt	R	0	0.04
cgc	R	1	0.05
cga	R	0	0.10
cgg	R	0	0.08
aga	R	0	0.46
agg	R	0	0.27
ggt	G	0	0.14
ggc	G	1	0.13
gga	G	0	0.44
ggg	G	0	0.29
---	--	------	------
mPD		0.0086	0.49
		nPD:	0.02
		N. weight:	0.58
		Sc. PD:	-0.049
		Sc. rank:	-516.2

,

NA

Pos.	46	obs:	exp:
tct	S	0	0.85
tcc	S	0	0.74
tca	S	0	1.13
tcg	S	0	0.32
cgt	R	0	0.11
cgc	R	0	0.16
cga	R	0	0.29
cgg	R	0	0.23
atg	M	14	14.00
act	T	0	3.44
acc	T	0	2.87
aca	T	0	5.59
acg	T	13	1.10
aat	N	586	313.50
aac	N	4	276.50
aaa	K	9	1073.00
aag	K	1903	838.80
agt	S	5	1.02
agc	S	0	0.94
aga	R	0	1.38
agg	R	3	0.82
gat	D	1	0.58
gac	D	0	0.42
---	--	------	------
mPD		0.39	0.71
		nPD:	0.56
		N. weight:	0.79
		Sc. PD:	0.35
		Sc. rank:	1535.0

,

NA

Pos.	47	obs:	exp:
cgt	R	0	0.08
cgc	R	0	0.11
cga	R	0	0.19
cgg	R	0	0.16
aga	R	2	0.92
agg	R	0	0.55
gaa	E	17	12.57
gag	E	4	8.43
ggt	G	0	361.80
ggc	G	0	332.00
gga	G	2511	1098.00
ggg	G	4	723.40
---	--	------	------
mPD		0.024	0.71
		nPD:	0.03
		N. weight:	0.85
		Sc. PD:	-0.059
		Sc. rank:	35.9

,

NA

Pos.	48	obs:	exp:
att	I	22	8.17
atc	I	0	5.52
ata	I	0	8.31
act	T	2472	663.50
acc	T	10	553.50
aca	T	21	1076.00
acg	T	2	211.60
aat	N	2	1.06
aac	N	0	0.94
gct	A	9	2.29
gcc	A	0	1.76
gca	A	0	3.96
gcg	A	0	1.00
---	--	------	------
mPD		0.052	0.72
		nPD:	0.07
		N. weight:	1.3
		Sc. PD:	-0.043
		Sc. rank:	1118.2

,

NA

Pos.	49	obs:	exp:
att	I	0	620.90
atc	I	6	419.30
ata	I	1666	631.80
atg	M	4	4.00
act	T	0	65.69
acc	T	0	54.79
aca	T	248	106.60
acg	T	0	20.95
gtt	V	0	133.50
gtc	V	5	129.70
gta	V	607	120.80
gtg	V	2	229.90
---	--	------	------
mPD		0.56	1.3
		nPD:	0.44
		N. weight:	1.1
		Sc. PD:	0.37
		Sc. rank:	2024.1

,

NA

Pos.	50	obs:	exp:
tta	L	1	0.65
ttg	L	5	1.13
ctt	L	0	1.13
ctc	L	0	1.04
cta	L	0	0.80
ctg	L	0	1.26
att	I	9	8.17
atc	I	2	5.52
ata	I	11	8.31
gtt	V	0	543.50
gtc	V	0	528.00
gta	V	1894	491.70
gtg	V	605	935.80
gct	A	0	2.80
gcc	A	0	2.15
gca	A	11	4.84
gcg	A	0	1.22
---	--	------	------
mPD		0.40	0.76
		nPD:	0.53
		N. weight:	0.96
		Sc. PD:	0.39
		Sc. rank:	1851.8

,

NA

Pos.	51	obs:	exp:
tct	S	0	0.17
tcc	S	0	0.15
tca	S	0	0.23
tcg	S	0	0.06
cgt	R	0	94.77
cgc	R	0	133.10
cga	R	0	242.00
cgg	R	0	195.70
atg	M	5	5.00
aaa	K	0	13.47
aag	K	24	10.53
agt	S	1	0.20
agc	S	0	0.19
aga	R	21	1146.00
agg	R	2478	687.10
ggt	G	0	1.29
ggc	G	0	1.19
gga	G	0	3.93
ggg	G	9	2.59
---	--	------	------
mPD		0.047	0.98
		nPD:	0.05
		N. weight:	1.3
		Sc. PD:	-0.073
		Sc. rank:	644.1

,

NA

Pos.	52	obs:	exp:
aaa	K	2	1.12
aag	K	0	0.88
gtt	V	0	0.65
gtc	V	0	0.63
gta	V	3	0.59
gtg	V	0	1.12
gat	D	0	0.58
gac	D	1	0.42
gaa	E	2523	1513.00
gag	E	4	1014.00
ggt	G	0	0.72
ggc	G	0	0.66
gga	G	5	2.18
ggg	G	0	1.44
---	--	------	------
mPD		0.012	0.49
		nPD:	0.02
		N. weight:	0.53
		Sc. PD:	-0.042
		Sc. rank:	-210.1

,

NA

Pos.	53	obs:	exp:
tat	Y	1916	1182.00
tac	Y	516	1250.00
tgt	C	13	5.65
tgc	C	0	7.35
cat	H	0	55.30
cac	H	93	37.70
---	--	------	------
mPD		0.45	0.58
		nPD:	0.77
		N. weight:	0.24
		Sc. PD:	0.15
		Sc. rank:	483.1

,

NA

Pos.	54	obs:	exp:
aat	N	2521	1343.00
aac	N	7	1185.00
aaa	K	0	5.61
aag	K	10	4.39
---	--	------	------
mPD		0.013	0.50
		nPD:	0.03
		N. weight:	0.65
		Sc. PD:	-0.05
		Sc. rank:	-198.6

,

NA

Pos.	55	obs:	exp:
gaa	E	2519	1519.00
gag	E	18	1018.00
ggt	G	0	0.14
ggc	G	0	0.13
gga	G	1	0.44
ggg	G	0	0.29
---	--	------	------
mPD		0.015	0.48
		nPD:	0.03
		N. weight:	0.5
		Sc. PD:	-0.036
		Sc. rank:	-63.8

,

NA			
Pos.	56	obs:	exp:
att	I	0	0.37
atc	I	0	0.25
ata	I	1	0.38
act	T	6	671.80
acc	T	2	560.30
aca	T	2528	1090.00
acg	T	0	214.20
aaa	K	1	0.56
aag	K	0	0.44
---	--	------	------
mPD		0.0079	0.69
		nPD:	0.01
		N. weight:	0.87
		Sc. PD:	-0.079
		Sc. rank:	-1143.8

,

NA			
Pos.	57	obs:	exp:
att	I	11	4.08
atc	I	0	2.76
ata	I	0	4.16
gtt	V	2487	549.10
gtc	V	27	533.50
gta	V	7	496.80
gtg	V	4	945.50
gct	A	1	0.51
gcc	A	1	0.39
gca	A	0	0.88
gcg	A	0	0.22
---	--	------	------
mPD		0.041	0.74
		nPD:	0.05
		N. weight:	1.5
		Sc. PD:	-0.074
		Sc. rank:	883.1

,

NA			
Pos.	58	obs:	exp:
cgt	R	0	95.72
cgc	R	0	134.40
cga	R	0	244.40
cgg	R	4	197.70
aaa	K	0	1.68
aag	K	3	1.32
aga	R	571	1158.00
agg	R	1949	694.00
ggt	G	0	1.58
ggc	G	0	1.45
gga	G	0	4.80
ggg	G	11	3.16
---	--	------	------
mPD		0.36	0.96
		nPD:	0.38
		N. weight:	0.67
		Sc. PD:	0.18
		Sc. rank:	1108.1

,

NA			
Pos.	59	obs:	exp:
cgt	R	0	0.19
cgc	R	0	0.27
cga	R	0	0.48
cgg	R	0	0.39
att	I	17	930.20
atc	I	0	628.20
ata	I	2488	946.60
atg	M	5	5.00
act	T	0	0.26
acc	T	0	0.22
aca	T	1	0.43
acg	T	0	0.08
aga	R	5	2.29
agg	R	0	1.37
gtt	V	2	4.78
gtc	V	0	4.65
gta	V	20	4.33
gtg	V	0	8.24
---	--	------	------
mPD		0.041	0.68
		nPD:	0.06
		N. weight:	0.98
		Sc. PD:	-0.044
		Sc. rank:	687.7

,

NA			
Pos.	60	obs:	exp:
gat	D	2	1.15
gac	D	0	0.85
gaa	E	9	1518.00
gag	E	2527	1018.00
---	--	------	------
mPD		0.0086	0.48
		nPD:	0.02
		N. weight:	0.93
		Sc. PD:	-0.079
		Sc. rank:	-817.5

,

NA			
Pos.	61	obs:	exp:
cgt	R	0	0.38
cgc	R	0	0.53
cga	R	0	0.97
cgg	R	0	0.78
aaa	K	2509	1418.00
aag	K	18	1109.00
aga	R	10	4.59
agg	R	0	2.75
gaa	E	1	0.60
gag	E	0	0.40
---	--	------	------
mPD		0.023	0.50
		nPD:	0.05
		N. weight:	0.57
		Sc. PD:	-0.033
		Sc. rank:	237.3

,

NA			
Pos.	62	obs:	exp:
att	I	0	1.11
atc	I	0	0.75
ata	I	3	1.13
atg	M	3	3.00
gtt	V	2	550.40
gtc	V	0	534.80
gta	V	15	498.00
gtg	V	2514	947.80
gct	A	0	0.25
gcc	A	0	0.20
gca	A	0	0.44
gcg	A	1	0.11
---	--	------	------
mPD		0.021	0.73
		nPD:	0.03
		N. weight:	0.99
		Sc. PD:	-0.074
		Sc. rank:	-224.1

,

NA			
Pos.	63	obs:	exp:
att	I	10	3.71
atc	I	0	2.51
ata	I	0	3.78
act	T	1299	669.40
acc	T	905	558.30
aca	T	323	1086.00
acg	T	0	213.40
aat	N	1	0.53
aac	N	0	0.47
---	--	------	------
mPD		0.60	0.70
		nPD:	0.86
		N. weight:	0.38
		Sc. PD:	0.28
		Sc. rank:	795.9

,

NA			
Pos.	64	obs:	exp:
cct	P	0	0.28
ccc	P	0	0.18
cca	P	0	0.41
ccg	P	1	0.13
caa	Q	1630	1291.00
cag	Q	907	1246.00
---	--	------	------
mPD		0.46	0.50
		nPD:	0.92
		N. weight:	0.041
		Sc. PD:	0.032
		Sc. rank:	84.8

,

NA			
Pos.	65	obs:	exp:
tgg	W	2538	2538.00
---	--	------	------
mPD		0	0
		nPD:	1.
		N. weight:	0.
		Sc. PD:	0
		Sc. rank:	0

,

NA			
Pos.	66	obs:	exp:
tat	Y	0	1202.00
tac	Y	2473	1271.00
tgt	C	0	1.74
tgc	C	4	2.26
cat	H	1	35.67
cac	H	59	24.33
caa	Q	1	0.51
cag	Q	0	0.49
---	--	------	------
mPD		0.052	0.55
		nPD:	0.09
		N. weight:	0.7
		Sc. PD:	-0.0081
		Sc. rank:	754.9

,

NA			
Pos.	67	obs:	exp:
aat	N	1597	1348.00
aac	N	940	1189.00
gat	D	1	0.58
gac	D	0	0.42
---	--	------	------
mPD		0.47	0.50
		nPD:	0.94
		N. weight:	0.023
		Sc. PD:	0.018
		Sc. rank:	47.0

,

NA			
Pos.	68	obs:	exp:
act	T	2518	672.00
acc	T	19	560.50
aca	T	0	1090.00
acg	T	0	214.30
aat	N	1	0.53
aac	N	0	0.47
---	--	------	------
mPD		0.016	0.69
		nPD:	0.02
		N. weight:	1.4
		Sc. PD:	-0.11
		Sc. rank:	-658.7

,

NA			
Pos.	69	obs:	exp:
tct	S	0	406.60
tcc	S	0	351.00
tca	S	0	536.70
tcg	S	0	153.80
act	T	1	0.26
acc	T	0	0.22
aca	T	0	0.43
acg	T	0	0.08
aat	N	94	82.90
aac	N	62	73.10
agt	S	1859	486.10
agc	S	522	446.70
---	--	------	------
mPD		0.47	1.7
		nPD:	0.28
		N. weight:	1.1
		Sc. PD:	0.18
		Sc. rank:	1626.1

,

NA			
Pos.	70	obs:	exp:
att	I	0	2.23
atc	I	0	1.50
ata	I	6	2.27
act	T	0	0.26
acc	T	0	0.22
aca	T	1	0.43
acg	T	0	0.08
gtt	V	3	542.20
gtc	V	9	526.70
gta	V	2477	490.50
gtg	V	4	933.60
gct	A	0	9.67
gcc	A	0	7.41
gca	A	37	16.70
gcg	A	1	4.22
---	--	------	------
mPD		0.049	0.77
		nPD:	0.06
		N. weight:	1.6
		Sc. PD:	-0.067
		Sc. rank:	1284.4

,

NA

Pos.	71	obs:	exp:
ttt	F	0	0.43
ttc	F	1	0.57
att	I	11	9.28
atc	I	14	6.27
ata	I	0	9.45
gtt	V	50	546.30
gtc	V	2459	530.80
gta	V	3	494.30
gtg	V	0	940.70
---	--	------	------
mPD		0.070	0.75
		nPD:	0.09
		N. weight:	1.5
		Sc. PD:	-0.019
		Sc. rank:	1579.1

,

NA

Pos.	72	obs:	exp:
aaa	K	51	28.63
aag	K	0	22.37
gaa	E	2473	1488.00
gag	E	12	997.10
ggt	G	0	0.29
ggc	G	0	0.26
gga	G	2	0.87
ggg	G	0	0.58
---	--	------	------
mPD		0.050	0.52
		nPD:	0.1
		N. weight:	0.51
		Sc. PD:	-0.0046
		Sc. rank:	554.1

,

NA

Pos.	73	obs:	exp:
tat	Y	2210	1077.00
tac	Y	5	1138.00
cat	H	321	191.50
cac	H	1	130.50
aat	N	1	0.53
aac	N	0	0.47
---	--	------	------
mPD		0.23	0.72
		nPD:	0.31
		N. weight:	0.72
		Sc. PD:	0.14
		Sc. rank:	1143.6

,

NA

Pos.	74	obs:	exp:
att	I	0	13.74
atc	I	0	9.28
ata	I	37	13.98
gtt	V	0	542.00
gtc	V	0	526.50
gta	V	2483	490.30
gtg	V	9	933.20
gct	A	0	2.29
gcc	A	0	1.76
gca	A	9	3.96
gcg	A	0	1.00
---	--	------	------
mPD		0.043	0.77
		nPD:	0.06
		N. weight:	1.7
		Sc. PD:	-0.081
		Sc. rank:	1047.2

,

NA

Pos.	75	obs:	exp:
tta	L	0	0.55
ttg	L	0	0.94
tct	S	0	1.37
tcc	S	0	1.18
tca	S	0	1.80
tcg	S	8	0.52
ctt	L	0	0.94
ctc	L	0	0.86
cta	L	0	0.67
ctg	L	5	1.05
cct	P	5	622.90
ccc	P	0	399.70
cca	P	38	901.90
ccg	P	2166	284.40
caa	Q	0	153.10
cag	Q	301	147.90
cgt	R	0	0.15
cgc	R	0	0.21
cga	R	0	0.39
cgg	R	4	0.31
agt	S	0	1.63
agc	S	0	1.50
aga	R	0	1.83
agg	R	0	1.10
gaa	E	3	6.59
gag	E	8	4.41
---	--	------	------
mPD		0.27	0.95
		nPD:	0.29
		N. weight:	1.9
		Sc. PD:	0.33
		Sc. rank:	2910.6

,

NA

Pos.	76	obs:	exp:
tta	L	0	0.87
ttg	L	0	1.50
tat	Y	9	4.37
tac	Y	0	4.63
ctt	L	8	1.50
ctc	L	0	1.38
cta	L	0	1.06
ctg	L	0	1.68
cct	P	19	5.36
ccc	P	0	3.44
cca	P	0	7.76
ccg	P	0	2.45
cat	H	2479	1475.00
cac	H	1	1005.00
caa	Q	1	0.51
cag	Q	0	0.49
cgt	R	4	0.53
cgc	R	0	0.75
cga	R	0	1.36
cgg	R	0	1.10
aat	N	6	3.19
aac	N	0	2.81
aaa	K	1	0.56
aag	K	0	0.44
aga	R	9	6.42
agg	R	1	3.85
---	--	-------	-------
mPD		0.063	0.55
		nPD:	0.11
		N. weight:	0.56
		Sc. PD:	0.0044
		Sc. rank:	672.4

,

NA

Pos.	77	obs:	exp:
tta	L	0	3.38
ttg	L	31	5.82
tct	S	0	0.85
tcc	S	0	0.74
tca	S	5	1.13
tcg	S	0	0.32
tgt	C	3	1.30
tgc	C	0	1.70
tgg	W	2486	2486.00
ctt	L	0	5.81
ctc	L	0	5.35
cta	L	0	4.12
ctg	L	0	6.51
cct	P	0	1.69
ccc	P	0	1.09
cca	P	5	2.45
ccg	P	1	0.77
cgt	R	0	0.27
cgc	R	0	0.37
cga	R	0	0.68
cgg	R	7	0.55
agt	S	0	1.02
agc	S	0	0.94
aga	R	0	3.21
agg	R	0	1.92
---	--	------	------
mPD		0.053	0.088
		nPD:	0.6
		N. weight:	0.035
		Sc. PD:	0.017
		Sc. rank:	67.7

,

NA

Pos.	78	obs:	exp:
tct	S	0	0.34
tcc	S	0	0.29
tca	S	0	0.45
tcg	S	0	0.13
act	T	0	0.26
acc	T	1	0.22
aca	T	0	0.43
acg	T	0	0.08
aat	N	608	1328.00
aac	N	1891	1171.00
aaa	K	20	11.23
aag	K	0	8.77
agt	S	2	0.41
agc	S	0	0.38
gat	D	1	2.88
gac	D	4	2.12
gaa	E	0	5.99
gag	E	10	4.01
ggt	G	0	0.14
ggc	G	0	0.13
gga	G	0	0.44
ggg	G	1	0.29
---	--	------	------
mPD		0.40	0.53
		nPD:	0.76
		N. weight:	0.19
		Sc. PD:	0.12
		Sc. rank:	394.8

,

NA

Pos.	79	obs:	exp:
tct	S	0	1.02
tcc	S	0	0.88
tca	S	0	1.35
tcg	S	0	0.39
aat	N	4	2.13
aac	N	0	1.87
aaa	K	0	5.61
aag	K	10	4.39
agt	S	6	1.23
agc	S	0	1.13
gat	D	113	65.00
gac	D	0	48.00
gaa	E	3	1435.00
gag	E	2394	961.80
ggt	G	0	1.15
ggc	G	0	1.06
gga	G	0	3.49
ggg	G	8	2.30
---	--	------	------
mPD		0.12	0.56
		nPD:	0.22
		N. weight:	0.94
		Sc. PD:	0.1
		Sc. rank:	1322.1

,

NA

Pos.	80	obs:	exp:
tct	S	0	1.20
tcc	S	0	1.03
tca	S	0	1.58
tcg	S	0	0.45
aat	N	0	0.53
aac	N	1	0.47
agt	S	0	1.43
agc	S	7	1.31
gtt	V	0	0.87
gtc	V	4	0.85
gta	V	0	0.79
gtg	V	0	1.50
gat	D	9	9.78
gac	D	8	7.22
ggt	G	33	360.90
ggc	G	2358	331.20
gga	G	117	1095.00
ggg	G	1	721.60
---	--	------	------
mPD		0.14	0.72
		nPD:	0.2
		N. weight:	1.8
		Sc. PD:	0.16
		Sc. rank:	2474.6

,

NA			
Pos.	81	obs:	exp:
tct	S	2	0.34
tcc	S	0	0.29
tca	S	0	0.45
tcg	S	0	0.13
cct	P	1	0.28
ccc	P	0	0.18
cca	P	0	0.41
ccg	P	0	0.13
cat	H	3	1.78
cac	H	0	1.22
caa	Q	3	3.05
cag	Q	3	2.95
att	I	2	0.74
atc	I	0	0.50
ata	I	0	0.76
act	T	675	179.10
acc	T	1	149.40
aca	T	0	290.50
acg	T	0	57.10
aat	N	1	0.53
aac	N	0	0.47
agt	S	0	0.41
agc	S	0	0.38
gct	A	1841	469.60
gcc	A	5	360.00
gca	A	0	811.50
gcg	A	0	204.90
gat	D	1	0.58
gac	D	0	0.42
---	--	------	------
mPD		0.42	1.1
		nPD:	0.38
		N. weight:	1.4
		Sc. PD:	0.37
		Sc. rank:	2348.8

NA			
Pos.	82	obs:	exp:
ttt	F	5	5.11
ttc	F	7	6.89
tat	Y	2517	1225.00
tac	Y	4	1296.00
cat	H	5	2.97
cac	H	0	2.03
---	--	------	------
mPD		0.022	0.51
		nPD:	0.04
		N. weight:	0.74
		Sc. PD:	-0.045
		Sc. rank:	253.2

NA			
Pos.	83	obs:	exp:
att	I	0	913.10
atc	I	0	616.70
ata	I	2459	929.20
atg	M	26	26.00
act	T	0	12.18
acc	T	0	10.16
aca	T	45	19.77
acg	T	1	3.89
gtt	V	0	1.52
gtc	V	0	1.48
gta	V	7	1.38
gtg	V	0	2.62
---	--	------	------
mPD		0.062	0.71
		nPD:	0.09
		N. weight:	1.
		Sc. PD:	-0.017
		Sc. rank:	1016.0

NA			
Pos.	84	obs:	exp:
tct	S	0	0.34
tcc	S	0	0.29
tca	S	0	0.45
tcg	S	0	0.13
aat	N	1118	1347.00
aac	N	1416	1187.00
aaa	K	2	1.12
aag	K	0	0.88
agt	S	0	0.41
agc	S	2	0.38
---	--	------	------
mPD		0.50	0.50
		nPD:	0.99
		N. weight:	0.021
		Sc. PD:	0.018
		Sc. rank:	43.2

NA			
Pos.	85	obs:	exp:
aat	N	23	1349.00
aac	N	2515	1189.00
---	--	------	------
mPD		0.018	0.50
		nPD:	0.04
		N. weight:	0.74
		Sc. PD:	-0.05
		Sc. rank:	100.1

NA			
Pos.	86	obs:	exp:
tct	S	0	0.17
tcc	S	0	0.15
tca	S	0	0.23
tcg	S	0	0.06
att	I	0	0.74
atc	I	2	0.50
ata	I	0	0.76
act	T	4	671.50
acc	T	2514	560.10
aca	T	15	1089.00
acg	T	2	214.10
agt	S	0	0.20
agc	S	1	0.19
---	--	------	------
mPD		0.019	0.69
		nPD:	0.03
		N. weight:	1.5
		Sc. PD:	-0.12
		Sc. rank:	-409.6

NA			
Pos.	87	obs:	exp:
aaa	K	5	2.81
aag	K	0	2.19
gaa	E	2530	1515.00
gag	E	1	1016.00
ggt	G	0	0.29
ggc	G	0	0.26
gga	G	2	0.87
ggg	G	0	0.58
---	--	------	------
mPD		0.0063	0.49
		nPD:	0.01
		N. weight:	0.54
		Sc. PD:	-0.048
		Sc. rank:	-643.8

NA			
Pos.	88	obs:	exp:
tta	L	1	0.22
ttg	L	0	0.38
tct	S	0	3.07
tcc	S	0	2.65
tca	S	18	4.06
tcg	S	0	1.16
ctt	L	0	0.38
ctc	L	0	0.35
cta	L	1	0.27
ctg	L	0	0.42
cct	P	0	687.80
ccc	P	0	441.30
cca	P	2438	995.80
ccg	P	1	314.10
caa	Q	3	1.53
cag	Q	0	1.47
act	T	0	16.69
acc	T	0	13.92
aca	T	63	27.07
acg	T	0	5.32
agt	S	0	3.68
agc	S	0	3.38
gct	A	0	2.54
gcc	A	0	1.95
gca	A	10	4.40
gcg	A	0	1.11
gaa	E	3	1.80
gag	E	0	1.20
---	--	------	------
mPD		0.080	0.79
		nPD:	0.1
		N. weight:	0.94
		Sc. PD:	-0.0041
		Sc. rank:	1066.6

NA			
Pos.	89	obs:	exp:
tta	L	0	1.20
ttg	L	11	2.06
ctt	L	0	2.06
ctc	L	0	1.90
cta	L	0	1.46
ctg	L	0	2.31
att	I	0	919.40
atc	I	0	621.00
ata	I	2476	935.60
atg	M	1	1.00
gtt	V	0	10.87
gtc	V	0	10.56
gta	V	50	9.84
gtg	V	0	18.72
---	--	------	------
mPD		0.057	0.71
		nPD:	0.08
		N. weight:	1.
		Sc. PD:	-0.026
		Sc. rank:	969.6

NA			
Pos.	90	obs:	exp:
tgt	C	2537	1104.00
tgc	C	1	1434.00
---	--	------	------
mPD		0.00079	0.49
		nPD:	0.
		N. weight:	0.87
		Sc. PD:	-0.088
		Sc. rank:	-1757.6

NA			
Pos.	91	obs:	exp:
aat	N	2	1.06
aac	N	0	0.94
gat	D	2535	1458.00
gac	D	0	1077.00
gaa	E	1	0.60
gag	E	0	0.40
---	--	------	------
mPD		0.0024	0.49
		nPD:	0.
		N. weight:	0.58
		Sc. PD:	-0.057
		Sc. rank:	-1073.9

NA			
Pos.	92	obs:	exp:
gtt	V	3	550.40
gtc	V	2519	534.80
gta	V	9	498.00
gtg	V	0	947.80
gct	A	5	1.78
gcc	A	2	1.37
gca	A	0	3.08
gcg	A	0	0.78
---	--	------	------
mPD		0.019	0.73
		nPD:	0.03
		N. weight:	1.6
		Sc. PD:	-0.12
		Sc. rank:	-536.1

NA			
Pos.	93	obs:	exp:
cgt	R	0	0.08
cgc	R	0	0.11
cga	R	0	0.19
cgg	R	0	0.16
aaa	K	9	1423.00
aag	K	2526	1112.00
aga	R	0	0.92
agg	R	2	0.55
gaa	E	0	0.60
gag	E	1	0.40
---	--	------	------
mPD		0.0094	0.50
		nPD:	0.02
		N. weight:	0.84
		Sc. PD:	-0.071
		Sc. rank:	-680.2

NA			
Pos.	94	obs:	exp:
ggt	G	13	365.10
ggc	G	2521	335.00
gga	G	2	1108.00
ggg	G	2	730.00
---	--	------	------
mPD		0.013	0.69
		nPD:	0.02
		N. weight:	2.1
		Sc. PD:	-0.17
		Sc. rank:	-1560.2

NA			
Pos.	95	obs:	exp:
ttt	F	2536	1080.00
ttc	F	2	1458.00
---	--	------	------
mPD		0.0016	0.49
		nPD:	0.
		N. weight:	0.89
		Sc. PD:	-0.089
		Sc. rank:	-1729.1

NA

Pos.	96	obs:	exp:
gtt	V	0	0.22
gtc	V	0	0.21
gta	V	1	0.20
gtg	V	0	0.37
gct	A	4	645.40
gcc	A	0	494.70
gca	A	2532	1115.00
gcg	A	1	281.60
---	--	------	------
mPD		0.0047	0.69
		nPD:	0.01
		N. weight:	0.85
		Sc. PD:	-0.082
		Sc. rank:	-1426.3

,

NA

Pos.	97	obs:	exp:
cct	P	2512	715.70
ccc	P	24	459.20
cca	P	0	1036.00
ccg	P	2	326.80
---	--	------	------
mPD		0.020	0.70
		nPD:	0.03
		N. weight:	1.3
		Sc. PD:	-0.095
		Sc. rank:	-273.2

,

NA

Pos.	98	obs:	exp:
ttt	F	2528	1080.00
ttc	F	10	1458.00
---	--	------	------
mPD		0.0079	0.49
		nPD:	0.02
		N. weight:	0.87
		Sc. PD:	-0.076
		Sc. rank:	-865.2

,

NA

Pos.	99	obs:	exp:
tct	S	20	433.40
tcc	S	2518	374.10
tca	S	0	572.10
tcg	S	0	163.90
agt	S	0	518.20
agc	S	0	476.20
---	--	------	------
mPD		0.016	1.6
		nPD:	0.01
		N. weight:	2.
		Sc. PD:	-0.18
		Sc. rank:	-2865.7

,

NA

Pos.	100	obs:	exp:
aat	N	3	1.59
aac	N	0	1.41
aaa	K	16	1423.00
aag	K	2519	1112.00
---	--	------	------
mPD		0.015	0.49
		nPD:	0.03
		N. weight:	0.83
		Sc. PD:	-0.06
		Sc. rank:	-116.3

,

NA

Pos.	101	obs:	exp:
gat	D	5	1460.00
gac	D	2533	1078.00
---	--	------	------
mPD		0.0039	0.49
		nPD:	0.01
		N. weight:	0.89
		Sc. PD:	-0.084
		Sc. rank:	-1384.9

,

NA

Pos.	102	obs:	exp:
aat	N	48	1349.00
aac	N	2490	1189.00
---	--	------	------
mPD		0.037	0.50
		nPD:	0.07
		N. weight:	0.7
		Sc. PD:	-0.021
		Sc. rank:	610.0

,

NA

Pos.	103	obs:	exp:
ggt	G	0	365.10
ggc	G	2	335.00
gga	G	934	1108.00
ggg	G	1602	730.00
---	--	------	------
mPD		0.47	0.69
		nPD:	0.68
		N. weight:	0.45
		Sc. PD:	0.25
		Sc. rank:	899.6

,

NA

Pos.	104	obs:	exp:
tta	L	42	4.69
ttg	L	0	8.07
ctt	L	0	8.06
ctc	L	0	7.42
cta	L	1	5.72
ctg	L	0	9.03
att	I	0	884.90
atc	I	1	597.60
ata	I	2382	900.50
gtt	V	0	24.36
gtc	V	0	23.66
gta	V	27	22.04
gtg	V	85	41.94
---	--	------	------
mPD		0.18	0.79
		nPD:	0.23
		N. weight:	1.
		Sc. PD:	0.12
		Sc. rank:	1474.7

,

NA

Pos.	105	obs:	exp:
cgt	R	0	96.25
cgc	R	0	135.20
cga	R	0	245.80
cgg	R	0	198.80
aga	R	2526	1164.00
agg	R	12	697.90
---	--	------	------
mPD		0.0094	0.95
		nPD:	0.01
		N. weight:	0.79
		Sc. PD:	-0.073
		Sc. rank:	-1102.0

,

NA

Pos.	106	obs:	exp:
tta	L	0	0.11
ttg	L	0	0.19
ctt	L	1	0.19
ctc	L	0	0.17
cta	L	0	0.13
ctg	L	0	0.21
att	I	994	369.10
atc	I	0	249.30
ata	I	0	375.60
gtt	V	1518	335.60
gtc	V	4	326.00
gta	V	0	303.60
gtg	V	21	577.80
---	--	------	------
mPD		0.50	1.2
		nPD:	0.41
		N. weight:	1.3
		Sc. PD:	0.38
		Sc. rank:	2308.0

,

NA

Pos.	107	obs:	exp:
ggt	G	97	365.10
ggc	G	2410	335.00
gga	G	30	1108.00
ggg	G	1	730.00
---	--	------	------
mPD		0.097	0.69
		nPD:	0.14
		N. weight:	1.9
		Sc. PD:	0.063
		Sc. rank:	2418.5

,

NA

Pos.	108	obs:	exp:
tct	S	4	433.30
tcc	S	2522	374.00
tca	S	1	571.90
tcg	S	10	163.90
cct	P	0	0.28
ccc	P	1	0.18
cca	P	0	0.41
ccg	P	0	0.13
agt	S	0	518.00
agc	S	0	476.00
---	--	------	------
mPD		0.013	1.6
		nPD:	0.01
		N. weight:	2.
		Sc. PD:	-0.19
		Sc. rank:	-3196.3

,

NA

Pos.	109	obs:	exp:
tct	S	0	1.54
tcc	S	0	1.33
tca	S	0	2.03
tcg	S	0	0.58
tgg	W	1	1.00
cgt	R	0	95.42
cgc	R	0	134.00
cga	R	2	243.60
cgg	R	1	197.10
aaa	K	2	6.17
aag	K	9	4.83
agt	S	9	1.84
agc	S	0	1.69
aga	R	998	1154.00
agg	R	1515	691.80
ggt	G	0	0.14
ggc	G	0	0.13
gga	G	0	0.44
ggg	G	1	0.29
---	--	------	------
mPD		0.49	0.97
		nPD:	0.51
		N. weight:	0.44
		Sc. PD:	0.17
		Sc. rank:	826.2

,

NA

Pos.	110	obs:	exp:
ggt	G	9	365.10
ggc	G	2	335.00
gga	G	2330	1108.00
ggg	G	197	730.00
---	--	------	------
mPD		0.15	0.69
		nPD:	0.22
		N. weight:	0.6
		Sc. PD:	0.066
		Sc. rank:	844.4

,

NA

Pos.	111	obs:	exp:
cat	H	2525	1509.00
cac	H	13	1029.00
---	--	------	------
mPD		0.010	0.48
		nPD:	0.02
		N. weight:	0.52
		Sc. PD:	-0.042
		Sc. rank:	-307.3

,

NA

Pos.	112	obs:	exp:
att	I	2502	929.80
atc	I	2	628.00
ata	I	0	946.20
gtt	V	31	7.39
gtc	V	3	7.18
gta	V	0	6.69
gtg	V	0	12.73
---	--	------	------
mPD		0.030	0.69
		nPD:	0.04
		N. weight:	1.
		Sc. PD:	-0.062
		Sc. rank:	430.3

,

NA

Pos.	113	obs:	exp:
ttt	F	2524	1074.00
ttc	F	1	1451.00
tat	Y	13	6.32
tac	Y	0	6.68
---	--	------	------
mPD		0.011	0.50
		nPD:	0.02
		N. weight:	0.89
		Sc. PD:	-0.073
		Sc. rank:	-466.6

,

NA

Pos.	114	obs:	exp:
att	I	0	2.97
atc	I	8	2.01
ata	I	0	3.02
gtt	V	22	550.20
gtc	V	2499	534.60
gta	V	9	497.80
gtg	V	0	947.40
---	--	------	------
mPD		0.030	0.74
		nPD:	0.04
		N. weight:	1.6
		Sc. PD:	-0.097
		Sc. rank:	373.1

,

NA

Pos.	115	obs:	exp:
att	I	1	941.70
atc	I	0	636.00
ata	I	2535	958.30
gtt	V	0	0.43
gtc	V	0	0.42
gta	V	2	0.39
gtg	V	0	0.75
---	--	------	------
mPD		0.0024	0.66
		nPD:	0.
		N. weight:	1.
		Sc. PD:	-0.1
		Sc. rank:	-1960.0

,

NA			
Pos.	116	obs:	exp:
cgt	R	0	96.25
cgc	R	0	135.20
cga	R	0	245.80
cgg	R	0	198.80
aga	R	571	1164.00
agg	R	1967	697.90
---	--	------	------
mPD		0.35	0.95
		nPD:	0.37
		N. weight:	0.68
		Sc. PD:	0.17
		Sc. rank:	1118.1

,

NA			
Pos.	117	obs:	exp:
aaa	K	0	0.56
aag	K	1	0.44
gaa	E	10	1519.00
gag	E	2527	1018.00
---	--	------	------
mPD		0.0086	0.48
		nPD:	0.02
		N. weight:	0.93
		Sc. PD:	-0.079
		Sc. rank:	-807.9

,

NA			
Pos.	118	obs:	exp:
tct	S	1	0.17
tcc	S	0	0.15
tca	S	0	0.23
tcg	S	0	0.06
cct	P	2517	715.10
ccc	P	8	458.90
cca	P	10	1035.00
ccg	P	1	326.50
cgt	R	1	0.04
cgc	R	0	0.05
cga	R	0	0.10
cgg	R	0	0.08
agt	S	0	0.20
agc	S	0	0.19
aga	R	0	0.46
agg	R	0	0.27
---	--	------	------
mPD		0.016	0.71
		nPD:	0.02
		N. weight:	1.3
		Sc. PD:	-0.1
		Sc. rank:	-603.7

,

NA			
Pos.	119	obs:	exp:
ttt	F	24	1080.00
ttc	F	2513	1457.00
caa	Q	1	0.51
cag	Q	0	0.49
---	--	------	------
mPD		0.021	0.49
		nPD:	0.04
		N. weight:	0.53
		Sc. PD:	-0.032
		Sc. rank:	183.2

,

NA			
Pos.	120	obs:	exp:
att	I	0	0.74
atc	I	2	0.50
ata	I	0	0.76
gtt	V	17	551.50
gtc	V	2383	535.80
gta	V	136	499.00
gtg	V	0	949.70
---	--	------	------
mPD		0.12	0.73
		nPD:	0.16
		N. weight:	1.4
		Sc. PD:	0.07
		Sc. rank:	1823.8

,

NA			
Pos.	121	obs:	exp:
tct	S	2528	433.10
tcc	S	3	373.90
tca	S	5	571.70
tcg	S	0	163.80
cct	P	1	0.28
ccc	P	0	0.18
cca	P	0	0.41
ccg	P	0	0.13
agt	S	0	517.80
agc	S	0	475.80
gct	A	1	0.25
gcc	A	0	0.20
gca	A	0	0.44
gcg	A	0	0.11
---	--	------	------
mPD		0.0079	1.6
		nPD:	0.
		N. weight:	1.8
		Sc. PD:	-0.18
		Sc. rank:	-3406.7

,

NA			
Pos.	122	obs:	exp:
tgt	C	2494	1103.00
tgc	C	43	1434.00
ggt	G	1	0.14
ggc	G	0	0.13
gga	G	0	0.44
ggg	G	0	0.29
---	--	------	------
mPD		0.034	0.49
		nPD:	0.07
		N. weight:	0.78
		Sc. PD:	-0.028
		Sc. rank:	659.1

,

NA			
Pos.	123	obs:	exp:
tct	S	6	433.30
tcc	S	6	374.00
tca	S	2518	571.90
tcg	S	7	163.90
agt	S	0	518.00
agc	S	0	476.00
gct	A	0	0.25
gcc	A	0	0.20
gca	A	1	0.44
gcg	A	0	0.11
---	--	------	------
mPD		0.016	1.6
		nPD:	0.01
		N. weight:	1.5
		Sc. PD:	-0.14
		Sc. rank:	-2168.9

,

NA			
Pos.	124	obs:	exp:
tta	L	0	0.65
ttg	L	0	1.13
tct	S	5	0.85
tcc	S	0	0.74
tca	S	0	1.13
tcg	S	0	0.32
ctt	L	6	1.13
ctc	L	0	1.04
cta	L	0	0.80
ctg	L	0	1.26
cct	P	2386	712.00
ccc	P	10	456.90
cca	P	129	1031.00
ccg	P	0	325.10
cat	H	1	0.59
cac	H	0	0.41
att	I	1	0.37
atc	I	0	0.25
ata	I	0	0.38
agt	S	0	1.02
agc	S	0	0.94
---	--	------	------
mPD		0.11	0.72
		nPD:	0.16
		N. weight:	1.1
		Sc. PD:	0.057
		Sc. rank:	1437.4

,

NA			
Pos.	125	obs:	exp:
tta	L	3	0.33
ttg	L	0	0.56
ctt	L	0	0.56
ctc	L	0	0.52
cta	L	0	0.40
ctg	L	0	0.63
cgt	R	0	0.04
cgc	R	0	0.05
cga	R	0	0.10
cgg	R	0	0.08
att	I	0	115.50
atc	I	0	78.00
ata	I	311	117.50
act	T	0	3.18
acc	T	0	2.65
aca	T	10	5.16
acg	T	2	1.01
aga	R	1	0.46
agg	R	0	0.27
gtt	V	1	479.50
gtc	V	2	465.90
gta	V	2191	433.90
gtg	V	11	825.70
gct	A	0	1.53
gcc	A	0	1.17
gca	A	6	2.64
gcg	A	0	0.67
---	--	------	------
mPD		0.25	0.98
		nPD:	0.26
		N. weight:	1.6
		Sc. PD:	0.23
		Sc. rank:	2383.7

,

NA			
Pos.	126	obs:	exp:
aaa	K	0	0.56
aag	K	1	0.44
gct	A	0	0.25
gcc	A	0	0.20
gca	A	0	0.44
gcg	A	1	0.11
gat	D	4	2.30
gac	D	0	1.70
gaa	E	14	1441.00
gag	E	2393	965.80
ggt	G	0	17.98
ggc	G	0	16.50
gga	G	4	54.57
ggg	G	121	35.95
---	--	------	------
mPD		0.11	0.59
		nPD:	0.19
		N. weight:	0.93
		Sc. PD:	0.076
		Sc. rank:	1278.6

,

NA			
Pos.	127	obs:	exp:
tct	S	0	0.17
tcc	S	0	0.15
tca	S	0	0.23
tcg	S	0	0.06
tgt	C	65	1103.00
tgc	C	2472	1434.00
agt	S	0	0.20
agc	S	1	0.19
---	--	------	------
mPD		0.051	0.49
		nPD:	0.1
		N. weight:	0.48
		Sc. PD:	-0.0013
		Sc. rank:	549.6

,

NA			
Pos.	128	obs:	exp:
cgt	R	0	96.25
cgc	R	0	135.20
cga	R	0	245.80
cgg	R	0	198.80
aga	R	1676	1164.00
agg	R	862	697.90
---	--	------	------
mPD		0.45	0.95
		nPD:	0.47
		N. weight:	0.33
		Sc. PD:	0.12
		Sc. rank:	602.6

,

NA			
Pos.	129	obs:	exp:
act	T	2523	672.00
acc	T	3	560.50
aca	T	2	1090.00
acg	T	9	214.30
gct	A	1	0.25
gcc	A	0	0.20
gca	A	0	0.44
gcg	A	0	0.11
---	--	------	------
mPD		0.012	0.69
		nPD:	0.02
		N. weight:	1.4
		Sc. PD:	-0.12
		Sc. rank:	-1243.7

,

NA			
Pos.	130	obs:	exp:
ttt	F	4	1080.00
ttc	F	2534	1458.00
---	--	------	------
mPD		0.0031	0.49
		nPD:	0.01
		N. weight:	0.57
		Sc. PD:	-0.055
		Sc. rank:	-991.2

,

NA			
Pos.	131	obs:	exp:
ttt	F	9	1080.00
ttc	F	2529	1458.00
---	--	------	------
mPD		0.0071	0.49
		nPD:	0.01
		N. weight:	0.56
		Sc. PD:	-0.05
		Sc. rank:	-606.0

,

NA			
Pos.	132	obs:	exp:
tta	L	0	277.00
ttg	L	0	476.20
ctt	L	12	475.90
ctc	L	2524	437.90
cta	L	2	337.70
ctg	L	0	533.30
---	--	------	------
mPD		0.011	1.1
		nPD:	0.01
		N. weight:	1.8
		Sc. PD:	-0.17
		Sc. rank:	-2542.6

,

NA			
Pos.	133	obs:	exp:
act	T	2526	672.30
acc	T	11	560.80
aca	T	1	1091.00
acg	T	0	214.40
---	--	------	------
mPD		0.0094	0.69
		nPD:	0.01
		N. weight:	1.4
		Sc. PD:	-0.12
		Sc. rank:	-1526.3

,

NA			
Pos.	134	obs:	exp:
cat	H	2	1.19
cac	H	0	0.81
caa	Q	11	1290.00
cag	Q	2525	1246.00
---	--	------	------
mPD		0.010	0.50
		nPD:	0.02
		N. weight:	0.72
		Sc. PD:	-0.059
		Sc. rank:	-474.9

,

NA			
Pos.	135	obs:	exp:
gaa	E	1	0.60
gag	E	0	0.40
ggt	G	22	365.00
ggc	G	0	334.90
gga	G	2497	1107.00
ggg	G	18	729.70
---	--	------	------
mPD		0.032	0.69
		nPD:	0.05
		N. weight:	0.79
		Sc. PD:	-0.045
		Sc. rank:	360.8

,

NA

Pos.	136	obs:	exp:
tct	S	579	100.80
tcc	S	11	86.98
tca	S	0	133.00
tcg	S	0	38.11
act	T	25	6.62
acc	T	0	5.52
aca	T	0	10.74
acg	T	0	2.11
agt	S	0	120.50
agc	S	0	110.70
gct	A	1921	489.20
gcc	A	2	375.00
gca	A	0	845.30
gcg	A	0	213.50
---	--	------	------
mPD		0.38	1.3
		nPD:	0.3
		N. weight:	1.5
		Sc. PD:	0.29
		Sc. rank:	2389.3

,

NA

Pos.	137	obs:	exp:
tta	L	5	276.80
ttg	L	2	475.90
ctt	L	0	475.50
ctc	L	0	437.50
cta	L	2523	337.40
ctg	L	6	532.80
att	I	0	0.37
atc	I	0	0.25
ata	I	1	0.38
gtt	V	0	0.22
gtc	V	0	0.21
gta	V	1	0.20
gtg	V	0	0.37
---	--	------	------
mPD		0.013	1.1
		nPD:	0.01
		N. weight:	2.1
		Sc. PD:	-0.19
		Sc. rank:	-2694.1

,

NA

Pos.	138	obs:	exp:
tta	L	0	277.00
ttg	L	0	476.20
ctt	L	297	475.90
ctc	L	2240	437.90
cta	L	1	337.70
ctg	L	0	533.30
---	--	------	------
mPD		0.21	1.1
		nPD:	0.18
		N. weight:	1.5
		Sc. PD:	0.11
		Sc. rank:	1983.8

,

NA

Pos.	139	obs:	exp:
tct	S	0	0.17
tcc	S	0	0.15
tca	S	0	0.23
tcg	S	0	0.06
aat	N	2531	1345.00
aac	N	0	1186.00
agt	S	1	0.20
agc	S	0	0.19
gat	D	6	3.45
gac	D	0	2.55
---	--	------	------
mPD		0.0055	0.50
		nPD:	0.01
		N. weight:	0.66
		Sc. PD:	-0.061
		Sc. rank:	-897.2

,

NA

Pos.	140	obs:	exp:
gat	D	7	1460.00
gac	D	2531	1078.00
---	--	------	------
mPD		0.0055	0.49
		nPD:	0.01
		N. weight:	0.88
		Sc. PD:	-0.081
		Sc. rank:	-1175.4

,

NA

Pos.	141	obs:	exp:
caa	Q	1	0.51
cag	Q	0	0.49
cgt	R	0	0.64
cgc	R	0	0.91
cga	R	0	1.65
cgg	R	0	1.33
aaa	K	2513	1414.00
aag	K	7	1106.00
aga	R	17	7.80
agg	R	0	4.67
---	--	------	------
mPD		0.020	0.51
		nPD:	0.04
		N. weight:	0.59
		Sc. PD:	-0.039
		Sc. rank:	109.3

,

NA

Pos.	142	obs:	exp:
cat	H	22	1509.00
cac	H	2516	1029.00
---	--	------	------
mPD		0.017	0.48
		nPD:	0.04
		N. weight:	0.89
		Sc. PD:	-0.061
		Sc. rank:	85.4

,

NA

Pos.	143	obs:	exp:
tct	S	8	433.40
tcc	S	3	374.10
tca	S	2525	572.10
tcg	S	2	163.90
agt	S	0	518.20
agc	S	0	476.20
---	--	------	------
mPD		0.010	1.6
		nPD:	0.01
		N. weight:	1.5
		Sc. PD:	-0.15
		Sc. rank:	-2716.9

,

NA

Pos.	144	obs:	exp:
aat	N	2434	1348.00
aac	N	103	1189.00
gat	D	1	0.58
gac	D	0	0.42
---	--	------	------
mPD		0.079	0.50
		nPD:	0.16
		N. weight:	0.49
		Sc. PD:	0.025
		Sc. rank:	645.8

,

NA

Pos.	145	obs:	exp:
cgt	R	0	0.04
cgc	R	0	0.05
cga	R	0	0.10
cgg	R	0	0.08
aga	R	1	0.46
agg	R	0	0.27
gaa	E	6	3.59
gag	E	0	2.41
ggt	G	0	364.10
ggc	G	11	334.10
gga	G	2509	1105.00
ggg	G	11	728.00
---	--	------	------
mPD		0.023	0.69
		nPD:	0.03
		N. weight:	0.82
		Sc. PD:	-0.058
		Sc. rank:	7.9

,

NA

Pos.	146	obs:	exp:
act	T	0	672.30
acc	T	0	560.80
aca	T	2534	1091.00
acg	T	4	214.40
---	--	------	------
mPD		0.0031	0.69
		nPD:	0.
		N. weight:	0.88
		Sc. PD:	-0.086
		Sc. rank:	-1652.2

,

NA

Pos.	147	obs:	exp:
tta	L	0	0.11
ttg	L	1	0.19
ctt	L	0	0.19
ctc	L	0	0.17
cta	L	0	0.13
ctg	L	0	0.21
atg	M	7	7.00
gtt	V	3	549.80
gtc	V	0	534.10
gta	V	6	497.40
gtg	V	2519	946.70
gct	A	0	0.51
gcc	A	0	0.39
gca	A	0	0.88
gcg	A	2	0.22
---	--	------	------
mPD		0.015	0.74
		nPD:	0.02
		N. weight:	1.
		Sc. PD:	-0.083
		Sc. rank:	-672.3

,

NA

Pos.	148	obs:	exp:
caa	Q	0	0.51
cag	Q	1	0.49
cgt	R	0	0.87
cgc	R	0	1.23
cga	R	0	2.23
cgg	R	0	1.80
aat	N	1	0.53
aac	N	0	0.47
aaa	K	22	1407.00
aag	K	2485	1100.00
aga	R	0	10.55
agg	R	23	6.32
gaa	E	0	3.59
gag	E	6	2.41
---	--	------	------
mPD		0.041	0.52
		nPD:	0.08
		N. weight:	0.82
		Sc. PD:	-0.021
		Sc. rank:	754.9

,

NA

Pos.	149	obs:	exp:
gat	D	2512	1460.00
gac	D	26	1078.00
---	--	------	------
mPD		0.020	0.49
		nPD:	0.04
		N. weight:	0.53
		Sc. PD:	-0.033
		Sc. rank:	128.3

,

NA

Pos.	150	obs:	exp:
cgt	R	0	96.17
cgc	R	0	135.10
cga	R	3	245.60
cgg	R	9	198.60
aaa	K	2	1.12
aag	K	0	0.88
aga	R	1622	1163.00
agg	R	902	697.30
---	--	------	------
mPD		0.47	0.95
		nPD:	0.49
		N. weight:	0.31
		Sc. PD:	0.12
		Sc. rank:	568.3

,

NA

Pos.	151	obs:	exp:
tct	S	0	433.40
tcc	S	0	374.10
tca	S	0	572.10
tcg	S	0	163.90
agt	S	4	518.20
agc	S	2534	476.20
---	--	------	------
mPD		0.0031	1.6
		nPD:	0.
		N. weight:	1.7
		Sc. PD:	-0.18
		Sc. rank:	-3494.4

,

NA

Pos.	152	obs:	exp:
tct	S	0	0.17
tcc	S	0	0.15
tca	S	1	0.23
tcg	S	0	0.06
cct	P	2	715.40
ccc	P	10	459.10
cca	P	2520	1036.00
ccg	P	5	326.70
agt	S	0	0.20
agc	S	0	0.19
---	--	------	------
mPD		0.014	0.71
		nPD:	0.02
		N. weight:	0.9
		Sc. PD:	-0.075
		Sc. rank:	-609.5

,

NA

Pos.	153	obs:	exp:
ttt	F	38	1074.00
ttc	F	2487	1451.00
tat	Y	10	5.83
tac	Y	2	6.17
gtt	V	0	0.22
gtc	V	1	0.21
gta	V	0	0.20
gtg	V	0	0.37
---	--	------	------
mPD		0.047	0.50
		nPD:	0.09
		N. weight:	0.51
		Sc. PD:	-0.0054
		Sc. rank:	545.4

,

NA

Pos.	154	obs:	exp:
cgt	R	0	96.25
cgc	R	0	135.20
cga	R	0	245.80
cgg	R	0	198.80
aga	R	2527	1164.00
agg	R	11	697.90
---	--	------	------
mPD		0.0086	0.95
		nPD:	0.01
		N. weight:	0.79
		Sc. PD:	-0.074
		Sc. rank:	-1200.1

,

NA

Pos.	155	obs:	exp:
act	T	2510	672.30
acc	T	24	560.80
aca	T	4	1091.00
acg	T	0	214.40
---	--	------	------
mPD		0.022	0.69
		nPD:	0.03
		N. weight:	1.3
		Sc. PD:	-0.095
		Sc. rank:	-117.0

,

NA

Pos.	156	obs:	exp:
tta	L	1	277.00
ttg	L	0	476.20
ctt	L	0	475.90
ctc	L	2520	437.90
cta	L	16	337.70
ctg	L	1	533.30
---	--	------	------
mPD		0.015	1.1
		nPD:	0.01
		N. weight:	1.8
		Sc. PD:	-0.16
		Sc. rank:	-2118.8

NA

Pos.	157	obs:	exp:
atg	M	2538	2538.00
---	--	------	------
mPD		0	0
		nPD:	1.
		N. weight:	0.
		Sc. PD:	0
		Sc. rank:	0

NA

Pos.	158	obs:	exp:
tct	S	0	433.30
tcc	S	0	374.00
tca	S	0	571.90
tcg	S	0	163.90
agt	S	2535	518.00
agc	S	2	476.00
ggt	G	1	0.14
ggc	G	0	0.13
gga	G	0	0.44
ggg	G	0	0.29
---	--	------	------
mPD		0.0024	1.6
		nPD:	0.
		N. weight:	1.7
		Sc. PD:	-0.17
		Sc. rank:	-3366.9

NA

Pos.	159	obs:	exp:
gtt	V	1	552.00
gtc	V	2486	536.30
gta	V	50	499.40
gtg	V	1	950.40
---	--	------	------
mPD		0.040	0.73
		nPD:	0.06
		N. weight:	1.5
		Sc. PD:	-0.075
		Sc. rank:	915.0

NA

Pos.	160	obs:	exp:
aaa	K	3	1.68
aag	K	0	1.32
gaa	E	2532	1517.00
gag	E	1	1016.00
ggt	G	0	0.29
ggc	G	0	0.26
gga	G	2	0.87
ggg	G	0	0.58
---	--	------	------
mPD		0.0047	0.48
		nPD:	0.01
		N. weight:	0.54
		Sc. PD:	-0.05
		Sc. rank:	-751.0

NA

Pos.	161	obs:	exp:
att	I	0	4.08
atc	I	0	2.76
ata	I	11	4.16
gtt	V	0	549.40
gtc	V	0	533.70
gta	V	37	497.00
gtg	V	2489	945.90
gct	A	0	0.25
gcc	A	0	0.20
gca	A	0	0.44
gcg	A	1	0.11
---	--	------	------
mPD		0.047	0.74
		nPD:	0.06
		N. weight:	0.96
		Sc. PD:	-0.04
		Sc. rank:	737.5

NA

Pos.	162	obs:	exp:
ggt	G	1697	365.10
ggc	G	830	335.00
gga	G	0	1108.00
ggg	G	11	730.00
---	--	------	------
mPD		0.45	0.69
		nPD:	0.65
		N. weight:	1.4
		Sc. PD:	0.72
		Sc. rank:	2704.5

NA

Pos.	163	obs:	exp:
caa	Q	2521	1291.00
cag	Q	16	1246.00
cgt	R	0	0.04
cgc	R	0	0.05
cga	R	1	0.10
cgg	R	0	0.08
aga	R	0	0.46
agg	R	0	0.27
---	--	------	------
mPD		0.013	0.50
		nPD:	0.03
		N. weight:	0.67
		Sc. PD:	-0.052
		Sc. rank:	-202.4

NA

Pos.	164	obs:	exp:
tct	S	0	432.90
tcc	S	0	373.70
tca	S	2532	571.40
tcg	S	3	163.70
cct	P	0	0.85
ccc	P	0	0.54
cca	P	3	1.22
ccg	P	0	0.39
agt	S	0	517.60
agc	S	0	475.60
---	--	------	------
mPD		0.0047	1.6
		nPD:	0.
		N. weight:	1.6
		Sc. PD:	-0.16
		Sc. rank:	-3062.5

NA

Pos.	165	obs:	exp:
cct	P	1	715.70
ccc	P	2534	459.20
cca	P	3	1036.00
ccg	P	0	326.80
---	--	------	------
mPD		0.0031	0.70
		nPD:	0.
		N. weight:	1.8
		Sc. PD:	-0.18
		Sc. rank:	-3366.7

NA

Pos.	166	obs:	exp:
tct	S	0	5.46
tcc	S	0	4.72
tca	S	0	7.21
tcg	S	0	2.07
act	T	1	0.26
acc	T	0	0.22
aca	T	0	0.43
acg	T	0	0.08
aat	N	2495	1331.00
aac	N	10	1174.00
agt	S	32	6.53
agc	S	0	6.00
---	--	------	------
mPD		0.034	0.54
		nPD:	0.06
		N. weight:	0.65
		Sc. PD:	-0.028
		Sc. rank:	475.9

NA

Pos.	167	obs:	exp:
tta	L	0	3.49
ttg	L	32	6.00
ctt	L	0	6.00
ctc	L	0	5.52
cta	L	0	4.26
ctg	L	0	6.72
gtt	V	2	544.80
gtc	V	1	529.30
gta	V	1198	492.90
gtg	V	1304	938.00
gct	A	0	0.25
gcc	A	0	0.20
gca	A	0	0.44
gcg	A	1	0.11
---	--	------	------
mPD		0.53	0.75
		nPD:	0.7
		N. weight:	0.64
		Sc. PD:	0.36
		Sc. rank:	1275.8

NA

Pos.	168	obs:	exp:
tat	Y	2520	1234.00
tac	Y	18	1304.00
---	--	------	------
mPD		0.014	0.50
		nPD:	0.03
		N. weight:	0.71
		Sc. PD:	-0.054
		Sc. rank:	-172.8

NA

Pos.	169	obs:	exp:
caa	Q	2522	1291.00
cag	Q	16	1247.00
---	--	------	------
mPD		0.013	0.50
		nPD:	0.03
		N. weight:	0.67
		Sc. PD:	-0.052
		Sc. rank:	-254.7

NA

Pos.	170	obs:	exp:
tct	S	0	0.34
tcc	S	2	0.29
tca	S	0	0.45
tcg	S	0	0.13
agt	S	0	0.41
agc	S	0	0.38
gct	A	1	645.10
gcc	A	599	494.50
gca	A	1933	1115.00
gcg	A	3	281.50
---	--	------	------
mPD		0.37	0.69
		nPD:	0.53
		N. weight:	0.48
		Sc. PD:	0.2
		Sc. rank:	927.4

NA

Pos.	171	obs:	exp:
cgt	R	0	95.15
cgc	R	0	133.60
cga	R	0	243.00
cgg	R	0	196.50
aaa	K	0	16.28
aag	K	29	12.72
aga	R	11	1151.00
agg	R	2498	689.90
---	--	------	------
mPD		0.031	0.97
		nPD:	0.03
		N. weight:	1.3
		Sc. PD:	-0.094
		Sc. rank:	-59.4

NA

Pos.	172	obs:	exp:
ttt	F	2529	1080.00
ttc	F	9	1458.00
---	--	------	------
mPD		0.0071	0.49
		nPD:	0.01
		N. weight:	0.87
		Sc. PD:	-0.077
		Sc. rank:	-945.1

NA

Pos.	173	obs:	exp:
gaa	E	2530	1520.00
gag	E	8	1018.00
---	--	------	------
mPD		0.0063	0.48
		nPD:	0.01
		N. weight:	0.52
		Sc. PD:	-0.047
		Sc. rank:	-620.4

NA

Pos.	174	obs:	exp:
tct	S	1	0.17
tcc	S	0	0.15
tca	S	0	0.23
tcg	S	0	0.06
agt	S	0	0.20
agc	S	0	0.19
gct	A	2524	645.40
gcc	A	0	494.70
gca	A	11	1115.00
gcg	A	2	281.60
---	--	------	------
mPD		0.011	0.69
		nPD:	0.02
		N. weight:	1.4
		Sc. PD:	-0.12
		Sc. rank:	-1449.7

NA

Pos.	175	obs:	exp:
tta	L	1	0.11
ttg	L	0	0.19
ctt	L	0	0.19
ctc	L	0	0.17
cta	L	0	0.13
ctg	L	0	0.21
att	I	0	0.74
atc	I	0	0.50
ata	I	2	0.76
gtt	V	1	551.30
gtc	V	0	535.60
gta	V	1957	498.80
gtg	V	577	949.30
---	--	------	------
mPD		0.35	0.73
		nPD:	0.48
		N. weight:	0.99
		Sc. PD:	0.36
		Sc. rank:	1835.5

NA	Pos. 176	obs:	exp:
gct	A	1	645.60
gcc	A	0	494.90
gca	A	2526	1116.00
gcg	A	11	281.80
---	--	------	------
mPD		0.0094	0.69
		nPD:	0.01
		N. weight:	0.84
		Sc. PD:	-0.075
		Sc. rank:	-954.0

NA	Pos. 177	obs:	exp:
tgg	W	2538	2538.00
---	--	------	------
mPD		0	0
		nPD:	1.
		N. weight:	0.
		Sc. PD:	0
		Sc. rank:	0

NA	Pos. 178	obs:	exp:
tct	S	25	433.40
tcc	S	0	374.10
tca	S	2498	572.10
tcg	S	15	163.90
agt	S	0	518.20
agc	S	0	476.20
---	--	------	------
mPD		0.031	1.6
		nPD:	0.02
		N. weight:	1.5
		Sc. PD:	-0.12
		Sc. rank:	-1228.7

NA	Pos. 179	obs:	exp:
gct	A	5	645.60
gcc	A	3	494.90
gca	A	2526	1116.00
gcg	A	4	281.80
---	--	------	------
mPD		0.0094	0.69
		nPD:	0.01
		N. weight:	0.83
		Sc. PD:	-0.074
		Sc. rank:	-941.9

NA	Pos. 180	obs:	exp:
act	T	12	663.00
acc	T	1	553.00
aca	T	2488	1076.00
acg	T	2	211.40
gct	A	0	8.90
gcc	A	0	6.83
gca	A	35	15.39
gcg	A	0	3.89
---	--	------	------
mPD		0.039	0.72
		nPD:	0.05
		N. weight:	0.85
		Sc. PD:	-0.042
		Sc. rank:	487.0

NA	Pos. 181	obs:	exp:
gct	A	19	645.60
gcc	A	2509	494.90
gca	A	9	1116.00
gcg	A	1	281.80
---	--	------	------
mPD		0.023	0.69
		nPD:	0.03
		N. weight:	1.6
		Sc. PD:	-0.12
		Sc. rank:	3.9

NA	Pos. 182	obs:	exp:
tgt	C	2523	1103.00
tgc	C	14	1434.00
cgt	R	0	0.04
cgc	R	1	0.05
cga	R	0	0.10
cgg	R	0	0.08
aga	R	0	0.46
agg	R	0	0.27
---	--	------	------
mPD		0.013	0.49
		nPD:	0.03
		N. weight:	0.84
		Sc. PD:	-0.065
		Sc. rank:	-305.7

NA	Pos. 183	obs:	exp:
cat	H	2526	1509.00
cac	H	12	1029.00
---	--	------	------
mPD		0.0094	0.48
		nPD:	0.02
		N. weight:	0.52
		Sc. PD:	-0.043
		Sc. rank:	-362.3

NA	Pos. 184	obs:	exp:
gat	D	2534	1460.00
gac	D	4	1078.00
---	--	------	------
mPD		0.0031	0.49
		nPD:	0.01
		N. weight:	0.57
		Sc. PD:	-0.055
		Sc. rank:	-979.2

NA	Pos. 185	obs:	exp:
ggt	G	1452	365.10
ggc	G	1059	335.00
gga	G	14	1108.00
ggg	G	13	730.00
---	--	------	------
mPD		0.50	0.69
		nPD:	0.72
		N. weight:	1.3
		Sc. PD:	0.77
		Sc. rank:	2645.4

NA	Pos. 186	obs:	exp:
cgt	R	0	0.04
cgc	R	0	0.05
cga	R	0	0.10
cgg	R	0	0.08
aat	N	3	1.59
aac	N	0	1.41
aaa	K	4	1422.00
aag	K	2530	1112.00
aga	R	0	0.46
agg	R	1	0.27
---	--	------	------
mPD		0.0063	0.49
		nPD:	0.01
		N. weight:	0.85
		Sc. PD:	-0.077
		Sc. rank:	-1044.5

NA	Pos. 187	obs:	exp:
cgt	R	0	0.11
cgc	R	0	0.16
cga	R	0	0.29
cgg	R	0	0.23
aaa	K	1912	1423.00
aag	K	623	1112.00
aga	R	3	1.38
agg	R	0	0.82
---	--	------	------
mPD		0.37	0.50
		nPD:	0.75
		N. weight:	0.087
		Sc. PD:	0.055
		Sc. rank:	178.6

NA	Pos. 188	obs:	exp:
tgg	W	2538	2538.00
---	--	------	------
mPD		0	0
		nPD:	1.
		N. weight:	0.
		Sc. PD:	0
		Sc. rank:	0

NA	Pos. 189	obs:	exp:
tta	L	0	0.22
ttg	L	1	0.38
ctt	L	0	0.38
ctc	L	0	0.35
cta	L	0	0.27
ctg	L	1	0.42
atg	M	2535	2535.00
act	T	0	0.26
acc	T	0	0.22
aca	T	0	0.43
acg	T	1	0.08
---	--	------	------
mPD		0.0024	0.0040
		nPD:	0.59
		N. weight:	0.0021
		Sc. PD:	0.00097
		Sc. rank:	4.0

NA	Pos. 190	obs:	exp:
act	T	0	531.90
acc	T	0	443.70
aca	T	66	862.80
acg	T	1942	169.60
gct	A	0	134.80
gcc	A	0	103.40
gca	A	158	233.00
gcg	A	372	58.84
---	--	------	------
mPD		0.49	1.0
		nPD:	0.48
		N. weight:	2.2
		Sc. PD:	0.78
		Sc. rank:	3970.1

NA	Pos. 191	obs:	exp:
att	I	2520	937.60
atc	I	5	633.30
ata	I	0	954.10
gtt	V	13	2.83
gtc	V	0	2.75
gta	V	0	2.56
gtg	V	0	4.87
---	--	------	------
mPD		0.014	0.67
		nPD:	0.02
		N. weight:	1.
		Sc. PD:	-0.084
		Sc. rank:	-604.8

NA	Pos. 192	obs:	exp:
ggt	G	2525	365.10
ggc	G	2	335.00
gga	G	11	1108.00
ggg	G	0	730.00
---	--	------	------
mPD		0.010	0.69
		nPD:	0.01
		N. weight:	2.
		Sc. PD:	-0.18
		Sc. rank:	-2090.5

NA	Pos. 193	obs:	exp:
att	I	0	8.91
atc	I	0	6.02
ata	I	24	9.07
gtt	V	6	546.70
gtc	V	0	531.20
gta	V	1501	494.70
gtg	V	1007	941.40
---	--	------	------
mPD		0.50	0.75
		nPD:	0.67
		N. weight:	0.72
		Sc. PD:	0.39
		Sc. rank:	1418.7

NA	Pos. 194	obs:	exp:
tct	S	0	0.17
tcc	S	0	0.15
tca	S	0	0.23
tcg	S	1	0.06
act	T	6	672.00
acc	T	0	560.50
aca	T	2520	1090.00
acg	T	11	214.30
agt	S	0	0.20
agc	S	0	0.19
---	--	------	------
mPD		0.015	0.69
		nPD:	0.02
		N. weight:	0.85
		Sc. PD:	-0.069
		Sc. rank:	-472.2

NA	Pos. 195	obs:	exp:
ggt	G	0	365.10
ggc	G	15	335.00
gga	G	45	1108.00
ggg	G	2478	730.00
---	--	------	------
mPD		0.046	0.69
		nPD:	0.07
		N. weight:	1.2
		Sc. PD:	-0.044
		Sc. rank:	960.3

NA	Pos. 196	obs:	exp:
tct	S	0	0.51
tcc	S	0	0.44
tca	S	3	0.68
tcg	S	0	0.19
cct	P	13	714.80
ccc	P	0	458.70
cca	P	2521	1035.00
ccg	P	1	326.40
agt	S	0	0.61
agc	S	0	0.56
---	--	------	------
mPD		0.013	0.71
		nPD:	0.02
		N. weight:	0.91
		Sc. PD:	-0.077
		Sc. rank:	-775.4

NA	Pos. 197	obs:	exp:
gat	D	2525	1460.00
gac	D	13	1078.00
---	--	------	------
mPD		0.010	0.49
		nPD:	0.02
		N. weight:	0.55
		Sc. PD:	-0.045
		Sc. rank:	-343.2

NA	Pos. 198	obs:	exp:
tct	S	1622	431.00
tcc	S	7	372.10
tca	S	1	568.90
tcg	S	894	163.00
cct	P	1	0.85
ccc	P	0	0.54
cca	P	0	1.22
ccg	P	2	0.39
agt	S	0	515.30
agc	S	0	473.60
gct	A	0	2.54
gcc	A	10	1.95
gca	A	0	4.40
gcg	A	0	1.11
gat	D	1	0.58
gac	D	0	0.42
---	--	------	------
mPD		0.48	1.7
		nPD:	0.29
		N. weight:	1.5
		Sc. PD:	0.27
		Sc. rank:	2383.0

NA	Pos. 199	obs:	exp:
caa	Q	1	0.51
cag	Q	0	0.49
cgt	R	0	0.19
cgc	R	0	0.27
cga	R	0	0.48
cgg	R	0	0.39
aat	N	0	1.59
aac	N	3	1.41
aaa	K	2519	1418.00
aag	K	8	1109.00
aga	R	4	2.29
agg	R	1	1.37
gaa	E	2	1.20
gag	E	0	0.80
---	--	------	------
mPD		0.016	0.50
		nPD:	0.03
		N. weight:	0.59
		Sc. PD:	-0.042
		Sc. rank:	-65.3

NA	Pos. 200	obs:	exp:
gct	A	2	645.60
gcc	A	0	494.90
gca	A	2536	1116.00
gcg	A	0	281.80
---	--	------	------
mPD		0.0016	0.69
		nPD:	0.
		N. weight:	0.86
		Sc. PD:	-0.086
		Sc. rank:	-1710.0

NA

Pos.	201	obs:	exp:
att	I	0	367.30
atc	I	0	248.00
ata	I	989	373.70
act	T	0	1.59
acc	T	0	1.33
aca	T	6	2.58
acg	T	0	0.51
gtt	V	3	335.60
gtc	V	0	326.00
gta	V	1535	303.60
gtg	V	5	577.80
---	--	------	------
mPD		0.49	1.2
		nPD:	0.4
		N. weight:	1.4
		Sc. PD:	0.4
		Sc. rank:	2438.8

,

NA

Pos.	202	obs:	exp:
gct	A	1	645.60
gcc	A	3	494.90
gca	A	2530	1116.00
gcg	A	4	281.80
---	--	------	------
mPD		0.0063	0.69
		nPD:	0.01
		N. weight:	0.84
		Sc. PD:	-0.079
		Sc. rank:	-1270.1

,

NA

Pos.	203	obs:	exp:
gtt	V	27	552.00
gtc	V	0	536.30
gta	V	2499	499.40
gtg	V	12	950.40
---	--	------	------
mPD		0.030	0.73
		nPD:	0.04
		N. weight:	1.6
		Sc. PD:	-0.1
		Sc. rank:	448.4

,

NA

Pos.	204	obs:	exp:
ttt	F	0	0.43
ttc	F	1	0.57
att	I	61	23.77
atc	I	3	16.05
ata	I	0	24.18
gtt	V	783	537.80
gtc	V	1679	522.50
gta	V	1	486.60
gtg	V	10	926.10
---	--	------	------
mPD		0.50	0.78
		nPD:	0.64
		N. weight:	0.94
		Sc. PD:	0.48
		Sc. rank:	1832.7

,

NA

Pos.	205	obs:	exp:
tta	L	0	0.55
ttg	L	0	0.94
ctt	L	5	0.94
ctc	L	0	0.86
cta	L	0	0.67
ctg	L	0	1.05
cat	H	2528	1505.00
cac	H	4	1027.00
caa	Q	0	0.51
cag	Q	1	0.49
---	--	------	------
mPD		0.0079	0.49
		nPD:	0.02
		N. weight:	0.54
		Sc. PD:	-0.047
		Sc. rank:	-531.9

,

NA

Pos.	206	obs:	exp:
tat	Y	46	1232.00
tac	Y	2488	1302.00
aat	N	0	2.13
aac	N	4	1.87
---	--	------	------
mPD		0.039	0.50
		nPD:	0.08
		N. weight:	0.61
		Sc. PD:	-0.017
		Sc. rank:	556.2

,

NA

Pos.	207	obs:	exp:
ggt	G	1	365.10
ggc	G	0	335.00
gga	G	2517	1108.00
ggg	G	20	730.00
---	--	------	------
mPD		0.016	0.69
		nPD:	0.02
		N. weight:	0.82
		Sc. PD:	-0.065
		Sc. rank:	-340.7

,

NA

Pos.	208	obs:	exp:
ggt	G	0	365.10
ggc	G	0	335.00
gga	G	918	1108.00
ggg	G	1620	730.00
---	--	------	------
mPD		0.46	0.69
		nPD:	0.67
		N. weight:	0.47
		Sc. PD:	0.25
		Sc. rank:	919.5

,

NA

Pos.	209	obs:	exp:
tta	L	0	0.11
ttg	L	1	0.19
ctt	L	0	0.19
ctc	L	0	0.17
cta	L	0	0.13
ctg	L	0	0.21
att	I	10	4.08
atc	I	1	2.76
ata	I	0	4.16
gtt	V	1	548.90
gtc	V	0	533.30
gta	V	4	496.60
gtg	V	2519	945.20
gct	A	0	0.51
gcc	A	0	0.39
gca	A	0	0.88
gcg	A	2	0.22
---	--	------	------
mPD		0.024	0.74
		nPD:	0.03
		N. weight:	1.
		Sc. PD:	-0.073
		Sc. rank:	-80.4

,

NA

Pos.	210	obs:	exp:
cct	P	2250	715.40
ccc	P	287	459.10
cca	P	0	1036.00
ccg	P	0	326.70
gtt	V	1	0.22
gtc	V	0	0.21
gta	V	0	0.20
gtg	V	0	0.37
---	--	------	------
mPD		0.20	0.71
		nPD:	0.29
		N. weight:	1.
		Sc. PD:	0.18
		Sc. rank:	1561.7

,

NA

Pos.	211	obs:	exp:
att	I	2	0.74
atc	I	0	0.50
ata	I	0	0.76
act	T	2531	671.50
acc	T	4	560.10
aca	T	0	1089.00
acg	T	0	214.10
gct	A	1	0.25
gcc	A	0	0.20
gca	A	0	0.44
gcg	A	0	0.11
---	--	------	------
mPD		0.0055	0.69
		nPD:	0.01
		N. weight:	1.4
		Sc. PD:	-0.13
		Sc. rank:	-2216.5

,

NA

Pos.	212	obs:	exp:
aat	N	1	0.53
aac	N	0	0.47
gat	D	1946	1458.00
gac	D	589	1077.00
ggt	G	2	0.29
ggc	G	0	0.26
gga	G	0	0.87
ggg	G	0	0.58
---	--	------	------
mPD		0.36	0.49
		nPD:	0.73
		N. weight:	0.089
		Sc. PD:	0.054
		Sc. rank:	182.2

,

NA

Pos.	213	obs:	exp:
att	I	902	334.90
atc	I	0	226.20
ata	I	0	340.80
act	T	2	0.53
acc	T	0	0.44
aca	T	0	0.86
acg	T	0	0.17
gtt	V	1618	355.40
gtc	V	5	345.20
gta	V	7	321.50
gtg	V	4	611.90
---	--	------	------
mPD		0.47	1.2
		nPD:	0.39
		N. weight:	1.4
		Sc. PD:	0.38
		Sc. rank:	2301.8

,

NA

Pos.	214	obs:	exp:
att	I	12	8.54
atc	I	11	5.77
ata	I	0	8.69
gtt	V	2487	547.00
gtc	V	25	531.40
gta	V	1	494.90
gtg	V	2	941.80
---	--	------	------
mPD		0.048	0.75
		nPD:	0.06
		N. weight:	1.5
		Sc. PD:	-0.061
		Sc. rank:	1211.9

,

NA

Pos.	215	obs:	exp:
tct	S	0	0.17
tcc	S	0	0.15
tca	S	0	0.23
tcg	S	0	0.06
aat	N	51	1347.00
aac	N	2483	1187.00
agt	S	0	0.20
agc	S	1	0.19
gat	D	0	1.73
gac	D	3	1.27
---	--	------	------
mPD		0.043	0.50
		nPD:	0.08
		N. weight:	0.69
		Sc. PD:	-0.014
		Sc. rank:	680.9

,

NA

Pos.	216	obs:	exp:
tct	S	6	433.40
tcc	S	2529	374.10
tca	S	3	572.10
tcg	S	0	163.90
agt	S	0	518.20
agc	S	0	476.20
---	--	------	------
mPD		0.0071	1.6
		nPD:	0.
		N. weight:	2.
		Sc. PD:	-0.2
		Sc. rank:	-3779.7

,

NA

Pos.	217	obs:	exp:
tgg	W	2538	2538.00
---	--	------	------
mPD		0	0
		nPD:	1.
		N. weight:	0.
		Sc. PD:	0
		Sc. rank:	0

,

NA

Pos.	218	obs:	exp:
tct	S	0	0.17
tcc	S	0	0.15
tca	S	1	0.23
tcg	S	0	0.06
agt	S	0	0.20
agc	S	0	0.19
gct	A	4	645.40
gcc	A	1	494.70
gca	A	1539	1115.00
gcg	A	993	281.60
---	--	------	------
mPD		0.48	0.69
		nPD:	0.69
		N. weight:	0.72
		Sc. PD:	0.41
		Sc. rank:	1429.8

,

NA

Pos.	219	obs:	exp:
gaa	E	1	0.60
gag	E	0	0.40
ggt	G	0	365.00
ggc	G	1	334.90
gga	G	2520	1107.00
ggg	G	16	729.70
---	--	------	------
mPD		0.014	0.69
		nPD:	0.02
		N. weight:	0.83
		Sc. PD:	-0.069
		Sc. rank:	-543.8

,

NA

Pos.	220	obs:	exp:
aat	N	2	1.06
aac	N	0	0.94
gat	D	1617	1458.00
gac	D	917	1076.00
gaa	E	2	1.20
gag	E	0	0.80
---	--	------	------
mPD		0.46	0.49
		nPD:	0.95
		N. weight:	0.011
		Sc. PD:	0.0092
		Sc. rank:	23.5

,

NA			
Pos.	221	obs:	exp:
att	I	17	940.20
atc	I	3	635.00
ata	I	2512	956.80
atg	M	3	3.00
gtt	V	0	0.65
gtc	V	0	0.63
gta	V	3	0.59
gtg	V	0	1.12
---	--	------	------
mPD		0.020	0.66
		nPD:	0.03
		N. weight:	0.97
		Sc. PD:	-0.071
		Sc. rank:	-128.7

NA			
Pos.	222	obs:	exp:
tta	L	2517	277.00
ttg	L	10	476.20
ctt	L	0	475.90
ctc	L	0	437.90
cta	L	8	337.70
ctg	L	3	533.30
---	--	------	------
mPD		0.019	1.1
		nPD:	0.02
		N. weight:	2.3
		Sc. PD:	-0.2
		Sc. rank:	-2126.4

NA			
Pos.	223	obs:	exp:
cgt	R	0	96.25
cgc	R	0	135.20
cga	R	10	245.80
cgg	R	888	198.80
aga	R	662	1164.00
agg	R	978	697.90
---	--	------	------
mPD		0.85	0.95
		nPD:	0.89
		N. weight:	0.52
		Sc. PD:	0.4
		Sc. rank:	1089.5

NA			
Pos.	224	obs:	exp:
act	T	2530	672.30
acc	T	3	560.80
aca	T	3	1091.00
acg	T	2	214.40
---	--	------	------
mPD		0.0063	0.69
		nPD:	0.01
		N. weight:	1.4
		Sc. PD:	-0.13
		Sc. rank:	-2039.8

NA			
Pos.	225	obs:	exp:
cat	H	1	0.59
cac	H	0	0.41
caa	Q	21	1291.00
cag	Q	2516	1246.00
---	--	------	------
mPD		0.017	0.50
		nPD:	0.03
		N. weight:	0.7
		Sc. PD:	-0.048
		Sc. rank:	24.0

NA			
Pos.	226	obs:	exp:
gaa	E	70	1520.00
gag	E	2468	1018.00
---	--	------	------
mPD		0.054	0.48
		nPD:	0.11
		N. weight:	0.82
		Sc. PD:	0.0047
		Sc. rank:	971.5

NA			
Pos.	227	obs:	exp:
tct	S	7	433.40
tcc	S	0	374.10
tca	S	2460	572.10
tcg	S	71	163.90
agt	S	0	518.20
agc	S	0	476.20
---	--	------	------
mPD		0.060	1.6
		nPD:	0.04
		N. weight:	1.5
		Sc. PD:	-0.098
		Sc. rank:	214.4

NA			
Pos.	228	obs:	exp:
tct	S	2510	433.40
tcc	S	11	374.10
tca	S	13	572.10
tcg	S	4	163.90
agt	S	0	518.20
agc	S	0	476.20
---	--	------	------
mPD		0.022	1.6
		nPD:	0.01
		N. weight:	1.8
		Sc. PD:	-0.16
		Sc. rank:	-2044.3

NA			
Pos.	229	obs:	exp:
tgt	C	2218	1104.00
tgc	C	320	1434.00
---	--	------	------
mPD		0.22	0.49
		nPD:	0.45
		N. weight:	0.44
		Sc. PD:	0.15
		Sc. rank:	792.8

NA			
Pos.	230	obs:	exp:
act	T	2466	664.30
acc	T	42	554.10
aca	T	0	1078.00
acg	T	0	211.80
gtt	V	2	0.43
gtc	V	0	0.42
gta	V	0	0.39
gtg	V	0	0.75
gct	A	28	7.12
gcc	A	0	5.46
gca	A	0	12.31
gcg	A	0	3.11
---	--	------	------
mPD		0.058	0.71
		nPD:	0.08
		N. weight:	1.3
		Sc. PD:	-0.032
		Sc. rank:	1240.2

NA			
Pos.	231	obs:	exp:
tgt	C	4	1104.00
tgc	C	2534	1434.00
---	--	------	------
mPD		0.0031	0.49
		nPD:	0.01
		N. weight:	0.59
		Sc. PD:	-0.057
		Sc. rank:	-1024.5

NA			
Pos.	232	obs:	exp:
tta	L	0	0.33
ttg	L	0	0.56
ctt	L	3	0.56
ctc	L	0	0.52
cta	L	0	0.40
ctg	L	0	0.63
att	I	2218	940.60
atc	I	314	635.30
ata	I	1	957.10
atg	M	2	2.00
---	--	------	------
mPD		0.22	0.66
		nPD:	0.34
		N. weight:	0.7
		Sc. PD:	0.16
		Sc. rank:	1131.1

NA			
Pos.	233	obs:	exp:
cat	H	0	0.59
cac	H	1	0.41
caa	Q	2462	1253.00
cag	Q	1	1210.00
cgt	R	0	2.69
cgc	R	0	3.78
cga	R	70	6.88
cgg	R	1	5.56
aaa	K	3	1.68
aag	K	0	1.32
aga	R	0	32.57
agg	R	0	19.52
---	--	------	------
mPD		0.059	0.60
		nPD:	0.1
		N. weight:	0.75
		Sc. PD:	-0.0052
		Sc. rank:	828.4

NA			
Pos.	234	obs:	exp:
ggt	G	2455	365.10
ggc	G	81	335.00
gga	G	2	1108.00
ggg	G	0	730.00
---	--	------	------
mPD		0.063	0.69
		nPD:	0.09
		N. weight:	1.9
		Sc. PD:	-0.025
		Sc. rank:	1942.7

NA			
Pos.	235	obs:	exp:
tct	S	0	0.17
tcc	S	0	0.15
tca	S	0	0.23
tcg	S	0	0.06
aat	N	2519	1339.00
aac	N	0	1180.00
agt	S	1	0.20
agc	S	0	0.19
gat	D	5	2.88
gac	D	0	2.12
gaa	E	9	7.78
gag	E	4	5.22
---	--	------	------
mPD		0.025	0.52
		nPD:	0.05
		N. weight:	0.66
		Sc. PD:	-0.037
		Sc. rank:	336.9

NA			
Pos.	236	obs:	exp:
tgt	C	2532	1104.00
tgc	C	6	1434.00
---	--	------	------
mPD		0.0047	0.49
		nPD:	0.01
		N. weight:	0.86
		Sc. PD:	-0.08
		Sc. rank:	-1219.0

NA			
Pos.	237	obs:	exp:
ttt	F	3	1.28
ttc	F	0	1.72
tat	Y	2534	1232.00
tac	Y	1	1303.00
---	--	------	------
mPD		0.0032	0.50
		nPD:	0.01
		N. weight:	0.76
		Sc. PD:	-0.073
		Sc. rank:	-1322.7

NA			
Pos.	238	obs:	exp:
tgg	W	2537	2537.00
ggt	G	1	0.14
ggc	G	0	0.13
gga	G	0	0.44
ggg	G	0	0.29
---	--	------	------
mPD		0.0016	0.0013
		nPD:	1.17
		N. weight:	0.0008
		Sc. PD:	0.00083
		Sc. rank:	1.7

NA			
Pos.	239	obs:	exp:
att	I	0	0.37
atc	I	0	0.25
ata	I	1	0.38
gtt	V	2	551.70
gtc	V	0	536.00
gta	V	2516	499.20
gtg	V	19	950.00
---	--	------	------
mPD		0.017	0.73
		nPD:	0.02
		N. weight:	1.7
		Sc. PD:	-0.13
		Sc. rank:	-715.3

NA			
Pos.	240	obs:	exp:
cgt	R	0	0.04
cgc	R	0	0.05
cga	R	0	0.10
cgg	R	0	0.08
atg	M	2537	2537.00
aga	R	0	0.46
agg	R	1	0.27
---	--	------	------
mPD		0.00079	0.0015
		nPD:	0.52
		N. weight:	0.00053
		Sc. PD:	0.00022
		Sc. rank:	1.0

NA

Pos.	241	obs:	exp:
act	T	2520	672.30
acc	T	1	560.80
aca	T	11	1091.00
acg	T	6	214.40
---	--	------	------
mPD		0.014	0.69
		nPD:	0.02
		N. weight:	1.3
		Sc. PD:	-0.11
		Sc. rank:	-865.4

,

NA

Pos.	242	obs:	exp:
gat	D	19	1459.00
gac	D	2518	1078.00
gaa	E	1	0.60
gag	E	0	0.40
---	--	------	------
mPD		0.016	0.49
		nPD:	0.03
		N. weight:	0.85
		Sc. PD:	-0.061
		Sc. rank:	-48.3

,

NA

Pos.	243	obs:	exp:
ggt	G	2519	365.10
ggc	G	7	335.00
gga	G	11	1108.00
ggg	G	1	730.00
---	--	------	------
mPD		0.015	0.69
		nPD:	0.02
		N. weight:	2.
		Sc. PD:	-0.16
		Sc. rank:	-1063.7

,

NA

Pos.	244	obs:	exp:
tta	L	0	0.11
ttg	L	0	0.19
ctt	L	0	0.19
ctc	L	1	0.17
cta	L	0	0.13
ctg	L	0	0.21
cct	P	597	715.40
ccc	P	3	459.10
cca	P	1936	1036.00
ccg	P	1	326.70
---	--	------	------
mPD		0.36	0.71
		nPD:	0.52
		N. weight:	0.45
		Sc. PD:	0.18
		Sc. rank:	857.8

,

NA

Pos.	245	obs:	exp:
ttt	F	0	0.43
ttc	F	1	0.57
tct	S	0	153.50
tcc	S	899	132.50
tca	S	0	202.60
tcg	S	0	58.07
cct	P	0	1.13
ccc	P	4	0.72
cca	P	0	1.63
ccg	P	0	0.52
act	T	0	0.53
acc	T	2	0.44
aca	T	0	0.86
acg	T	0	0.17
agt	S	0	183.60
agc	S	0	168.70
gct	A	1	414.20
gcc	A	1614	317.50
gca	A	13	715.60
gcg	A	0	180.70
gat	D	0	2.30
gac	D	4	1.70
---	--	------	------
mPD		0.48	1.5
		nPD:	0.32
		N. weight:	1.8
		Sc. PD:	0.38
		Sc. rank:	2879.5

,

NA

Pos.	246	obs:	exp:
tct	S	0	0.17
tcc	S	0	0.15
tca	S	0	0.23
tcg	S	0	0.06
cat	H	7	4.16
cac	H	0	2.84
aat	N	2518	1344.00
aac	N	11	1185.00
agt	S	1	0.20
agc	S	0	0.19
gat	D	1	0.58
gac	D	0	0.42
---	--	------	------
mPD		0.016	0.51
		nPD:	0.03
		N. weight:	0.64
		Sc. PD:	-0.046
		Sc. rank:	-74.2

,

NA

Pos.	247	obs:	exp:
tct	S	0	0.17
tcc	S	0	0.15
tca	S	0	0.23
tcg	S	0	0.06
cgt	R	0	95.30
cgc	R	0	133.90
cga	R	0	243.30
cgg	R	0	196.80
att	I	0	0.37
atc	I	0	0.25
ata	I	1	0.38
aaa	K	12	6.74
aag	K	0	5.26
agt	S	0	0.20
agc	S	1	0.19
aga	R	2496	1153.00
agg	R	17	691.00
ggt	G	0	1.58
ggc	G	0	1.45
gga	G	11	4.80
ggg	G	0	3.16
---	--	------	------
mPD		0.033	0.97
		nPD:	0.03
		N. weight:	0.78
		Sc. PD:	-0.054
		Sc. rank:	12.9

,

NA

Pos.	248	obs:	exp:
tta	L	0	0.11
ttg	L	0	0.19
ctt	L	0	0.19
ctc	L	0	0.17
cta	L	0	0.13
ctg	L	1	0.21
caa	Q	14	1290.00
cag	Q	2522	1246.00
aaa	K	0	0.56
aag	K	1	0.44
---	--	------	------
mPD		0.013	0.50
		nPD:	0.03
		N. weight:	0.71
		Sc. PD:	-0.056
		Sc. rank:	-274.5

,

NA

Pos.	249	obs:	exp:
gct	A	2	645.60
gcc	A	1	494.90
gca	A	34	1116.00
gcg	A	2501	281.80
---	--	------	------
mPD		0.029	0.69
		nPD:	0.04
		N. weight:	2.2
		Sc. PD:	-0.14
		Sc. rank:	582.0

,

NA

Pos.	250	obs:	exp:
cat	H	25	14.86
cac	H	0	10.14
caa	Q	14	1279.00
cag	Q	2499	1234.00
---	--	------	------
mPD		0.030	0.51
		nPD:	0.06
		N. weight:	0.71
		Sc. PD:	-0.032
		Sc. rank:	501.2

,

NA

Pos.	251	obs:	exp:
tat	Y	2453	1234.00
tac	Y	85	1304.00
---	--	------	------
mPD		0.065	0.50
		nPD:	0.13
		N. weight:	0.6
		Sc. PD:	0.014
		Sc. rank:	756.3

,

NA

Pos.	252	obs:	exp:
cgt	R	0	96.14
cgc	R	0	135.00
cga	R	0	245.50
cgg	R	0	198.50
aaa	K	2	1.12
aag	K	0	0.88
aga	R	2525	1163.00
agg	R	10	697.00
ggt	G	1	0.14
ggc	G	0	0.13
gga	G	0	0.44
ggg	G	0	0.29
---	--	------	------
mPD		0.011	0.95
		nPD:	0.01
		N. weight:	0.8
		Sc. PD:	-0.073
		Sc. rank:	-1044.3

,

NA

Pos.	253	obs:	exp:
tta	L	1	0.11
ttg	L	0	0.19
ctt	L	0	0.19
ctc	L	0	0.17
cta	L	0	0.13
ctg	L	0	0.21
att	I	1	937.30
atc	I	0	633.00
ata	I	2523	953.70
gtt	V	1	0.87
gtc	V	0	0.85
gta	V	2	0.79
gtg	V	1	1.50
gct	A	0	2.29
gcc	A	0	1.76
gca	A	7	3.96
gcg	A	2	1.00
---	--	------	------
mPD		0.022	0.68
		nPD:	0.03
		N. weight:	1.
		Sc. PD:	-0.072
		Sc. rank:	-12.4

,

NA

Pos.	254	obs:	exp:
ttt	F	0	5.11
ttc	F	12	6.89
tat	Y	27	1228.00
tac	Y	2499	1298.00
---	--	------	------
mPD		0.030	0.51
		nPD:	0.06
		N. weight:	0.64
		Sc. PD:	-0.028
		Sc. rank:	457.6

,

NA

Pos.	255	obs:	exp:
aaa	K	2530	1424.00
aag	K	7	1113.00
gaa	E	1	0.60
gag	E	0	0.40
---	--	------	------
mPD		0.0063	0.49
		nPD:	0.01
		N. weight:	0.59
		Sc. PD:	-0.053
		Sc. rank:	-716.8

,

NA			
Pos.	256	obs:	exp:
tct	S	0	0.34
tcc	S	0	0.29
tca	S	2	0.45
tcg	S	0	0.13
agt	S	0	0.41
agc	S	0	0.38
gct	A	4	645.10
gcc	A	7	494.50
gca	A	2499	1115.00
gcg	A	26	281.50
---	--	------	------
mPD		0.030	0.69
		nPD:	0.04
		N. weight:	0.79
		Sc. PD:	-0.048
		Sc. rank:	309.0

,

NA			
Pos.	257	obs:	exp:
tct	S	0	0.51
tcc	S	0	0.44
tca	S	0	0.68
tcg	S	0	0.19
tat	Y	7	3.40
tac	Y	0	3.60
cgt	R	0	0.04
cgc	R	0	0.05
cga	R	0	0.10
cgg	R	0	0.08
aat	N	2422	1337.00
aac	N	94	1179.00
aaa	K	5	5.61
aag	K	5	4.39
agt	S	3	0.61
agc	S	0	0.56
aga	R	0	0.46
agg	R	1	0.27
gat	D	1	0.58
gac	D	0	0.42
---	--	------	------
mPD		0.089	0.51
		nPD:	0.17
		N. weight:	0.5
		Sc. PD:	0.033
		Sc. rank:	677.5

,

NA			
Pos.	258	obs:	exp:
cat	H	4	2.38
cac	H	0	1.62
caa	Q	2516	1287.00
cag	Q	13	1242.00
cgt	R	0	0.11
cgc	R	0	0.16
cga	R	3	0.29
cgg	R	0	0.23
aaa	K	2	1.12
aag	K	0	0.88
aga	R	0	1.38
agg	R	0	0.82
---	--	------	------
mPD		0.017	0.51
		nPD:	0.03
		N. weight:	0.68
		Sc. PD:	-0.047
		Sc. rank:	15.3

,

NA			
Pos.	259	obs:	exp:
ggt	G	3	365.10
ggc	G	2524	335.00
gga	G	3	1108.00
ggg	G	8	730.00
---	--	------	------
mPD		0.011	0.69
		nPD:	0.02
		N. weight:	2.1
		Sc. PD:	-0.18
		Sc. rank:	-2087.7

,

NA			
Pos.	260	obs:	exp:
cgt	R	0	0.11
cgc	R	0	0.16
cga	R	0	0.29
cgg	R	0	0.23
aaa	K	2525	1423.00
aag	K	10	1112.00
aga	R	3	1.38
agg	R	0	0.82
---	--	------	------
mPD		0.010	0.50
		nPD:	0.02
		N. weight:	0.58
		Sc. PD:	-0.048
		Sc. rank:	-370.5

,

NA			
Pos.	261	obs:	exp:
att	I	0	939.90
atc	I	0	634.80
ata	I	2531	956.40
atg	M	3	3.00
gtt	V	0	0.87
gtc	V	0	0.85
gta	V	4	0.79
gtg	V	0	1.50
---	--	------	------
mPD		0.0055	0.66
		nPD:	0.01
		N. weight:	1.
		Sc. PD:	-0.097
		Sc. rank:	-1591.2

,

NA			
Pos.	262	obs:	exp:
att	I	2530	939.90
atc	I	1	634.80
ata	I	0	956.40
act	T	1	0.26
acc	T	0	0.22
aca	T	0	0.43
acg	T	0	0.08
gtt	V	6	1.30
gtc	V	0	1.27
gta	V	0	1.18
gtg	V	0	2.25
---	--	------	------
mPD		0.0063	0.66
		nPD:	0.01
		N. weight:	1.
		Sc. PD:	-0.097
		Sc. rank:	-1535.3

,

NA			
Pos.	263	obs:	exp:
aat	N	0	2.13
aac	N	4	1.87
gat	D	9	1092.00
gac	D	1889	806.20
ggt	G	0	91.49
ggc	G	624	83.95
gga	G	1	277.60
ggg	G	11	182.90
---	--	------	------
mPD		0.40	1.0
		nPD:	0.39
		N. weight:	1.2
		Sc. PD:	0.32
		Sc. rank:	1939.4

,

NA			
Pos.	264	obs:	exp:
cat	H	1	0.59
cac	H	0	0.41
caa	Q	1979	1007.00
cag	Q	0	972.10
cgt	R	0	21.16
cgc	R	0	29.72
cga	R	558	54.03
cgg	R	0	43.70
aga	R	0	256.00
agg	R	0	153.40
---	--	------	------
mPD		0.34	1.1
		nPD:	0.3
		N. weight:	1.1
		Sc. PD:	0.21
		Sc. rank:	1742.2

,

NA			
Pos.	265	obs:	exp:
att	I	1	0.74
atc	I	0	0.50
ata	I	1	0.76
act	T	3	395.70
acc	T	0	330.10
aca	T	1487	642.00
acg	T	4	126.20
aaa	K	116	65.11
aag	K	0	50.89
gtt	V	4	1.52
gtc	V	0	1.48
gta	V	3	1.38
gtg	V	0	2.62
gct	A	3	233.80
gcc	A	0	179.20
gca	A	913	404.00
gcg	A	3	102.00
---	--	------	------
mPD		0.57	1.3
		nPD:	0.46
		N. weight:	0.84
		Sc. PD:	0.29
		Sc. rank:	1520.2

,

NA			
Pos.	266	obs:	exp:
aat	N	17	9.57
aac	N	1	8.43
gat	D	2487	1443.00
gac	D	22	1066.00
gaa	E	10	5.99
gag	E	0	4.01
ggt	G	0	0.14
ggc	G	0	0.13
gga	G	1	0.44
ggg	G	0	0.29
---	--	------	------
mPD		0.041	0.51
		nPD:	0.08
		N. weight:	0.53
		Sc. PD:	-0.012
		Sc. rank:	505.3

,

NA			
Pos.	267	obs:	exp:
att	I	1	5.20
atc	I	13	3.51
ata	I	0	5.29
gtt	V	596	548.90
gtc	V	1926	533.30
gta	V	2	496.60
gtg	V	0	945.20
---	--	------	------
mPD		0.37	0.74
		nPD:	0.5
		N. weight:	1.
		Sc. PD:	0.4
		Sc. rank:	1957.2

,

NA			
Pos.	268	obs:	exp:
tct	S	0	429.30
tcc	S	0	370.60
tca	S	0	566.70
tcg	S	0	162.40
cgt	R	0	0.04
cgc	R	0	0.05
cga	R	0	0.10
cgg	R	0	0.08
att	I	0	5.57
atc	I	15	3.76
ata	I	0	5.67
aat	N	1	4.25
aac	N	7	3.75
agt	S	22	513.30
agc	S	2492	471.70
aga	R	1	0.46
agg	R	0	0.27
---	--	------	------
mPD		0.037	1.7
		nPD:	0.02
		N. weight:	1.7
		Sc. PD:	-0.14
		Sc. rank:	-862.0

,

NA			
Pos.	269	obs:	exp:
ttt	F	2525	1078.00
ttc	F	8	1455.00
tta	L	0	0.55
ttg	L	3	0.94
ctt	L	2	0.94
ctc	L	0	0.86
cta	L	0	0.67
ctg	L	0	1.05
---	--	------	------
mPD		0.010	0.49
		nPD:	0.02
		N. weight:	0.88
		Sc. PD:	-0.072
		Sc. rank:	-551.0

,

NA			
Pos.	270	obs:	exp:
tct	S	0	422.50
tcc	S	0	364.70
tca	S	0	557.70
tcg	S	0	159.80
att	I	2	0.74
atc	I	0	0.50
ata	I	0	0.76
act	T	27	7.15
acc	T	0	5.97
aca	T	0	11.60
acg	T	0	2.28
aat	N	21	11.69
aac	N	1	10.31
agt	S	2471	505.10
agc	S	3	464.20
ggt	G	13	1.87
ggc	G	0	1.72
gga	G	0	5.67
ggg	G	0	3.74
---	--	------	------
mPD		0.053	1.7
		nPD:	0.03
		N. weight:	1.6
		Sc. PD:	-0.12
		Sc. rank:	-106.0

,

NA

Pos.	271	obs:	exp:
cgt	R	0	0.04
cgc	R	0	0.05
cga	R	0	0.10
cgg	R	0	0.08
aga	R	1	0.46
agg	R	0	0.27
gaa	E	2	1.20
gag	E	0	0.80
ggt	G	0	364.70
ggc	G	0	334.60
gga	G	2530	1107.00
ggg	G	5	729.10
---	--	------	------
mPD		0.0063	0.69
		nPD:	0.01
		N. weight:	0.86
		Sc. PD:	-0.08
		Sc. rank:	-1284.7

,

NA

Pos.	272	obs:	exp:
ggt	G	5	365.10
ggc	G	11	335.00
gga	G	1616	1108.00
ggg	G	906	730.00
---	--	------	------
mPD		0.47	0.69
		nPD:	0.68
		N. weight:	0.31
		Sc. PD:	0.17
		Sc. rank:	620.9

,

NA

Pos.	273	obs:	exp:
cat	H	2523	1509.00
cac	H	15	1029.00
---	--	------	------
mPD		0.012	0.48
		nPD:	0.02
		N. weight:	0.51
		Sc. PD:	-0.04
		Sc. rank:	-200.4

,

NA

Pos.	274	obs:	exp:
att	I	2511	942.50
atc	I	16	636.50
ata	I	11	959.00
---	--	------	------
mPD		0.021	0.66
		nPD:	0.03
		N. weight:	0.98
		Sc. PD:	-0.07
		Sc. rank:	-35.5

,

NA

Pos.	275	obs:	exp:
gaa	E	20	1520.00
gag	E	2518	1018.00
---	--	------	------
mPD		0.016	0.48
		nPD:	0.03
		N. weight:	0.91
		Sc. PD:	-0.064
		Sc. rank:	-19.3

,

NA

Pos.	276	obs:	exp:
gaa	E	2525	1520.00
gag	E	13	1018.00
---	--	------	------
mPD		0.010	0.48
		nPD:	0.02
		N. weight:	0.51
		Sc. PD:	-0.042
		Sc. rank:	-296.0

,

NA

Pos.	277	obs:	exp:
tgt	C	1560	1104.00
tgc	C	978	1434.00
---	--	------	------
mPD		0.47	0.49
		nPD:	0.96
		N. weight:	0.07
		Sc. PD:	0.058
		Sc. rank:	146.3

,

NA

Pos.	278	obs:	exp:
tct	S	2523	433.40
tcc	S	4	374.10
tca	S	11	572.10
tcg	S	0	163.90
agt	S	0	518.20
agc	S	0	476.20
---	--	------	------
mPD		0.012	1.6
		nPD:	0.01
		N. weight:	1.8
		Sc. PD:	-0.17
		Sc. rank:	-3002.2

,

NA

Pos.	279	obs:	exp:
tgt	C	2521	1104.00
tgc	C	17	1434.00
---	--	------	------
mPD		0.013	0.49
		nPD:	0.03
		N. weight:	0.83
		Sc. PD:	-0.063
		Sc. rank:	-234.3

,

NA

Pos.	280	obs:	exp:
tat	Y	2507	1234.00
tac	Y	31	1304.00
---	--	------	------
mPD		0.024	0.50
		nPD:	0.05
		N. weight:	0.69
		Sc. PD:	-0.038
		Sc. rank:	346.7

,

NA

Pos.	281	obs:	exp:
cct	P	6	715.70
ccc	P	12	459.20
cca	P	2519	1036.00
ccg	P	1	326.80
---	--	------	------
mPD		0.015	0.70
		nPD:	0.02
		N. weight:	0.9
		Sc. PD:	-0.073
		Sc. rank:	-538.2

,

NA

Pos.	282	obs:	exp:
aat	N	2536	1349.00
aac	N	2	1189.00
---	--	------	------
mPD		0.0016	0.50
		nPD:	0.
		N. weight:	0.66
		Sc. PD:	-0.065
		Sc. rank:	-1290.5

,

NA

Pos.	283	obs:	exp:
tat	Y	1	0.49
tac	Y	0	0.51
aat	N	4	2.13
aac	N	0	1.87
gct	A	1	0.25
gcc	A	0	0.20
gca	A	0	0.44
gcg	A	0	0.11
gat	D	2516	1448.00
gac	D	2	1070.00
gaa	E	10	7.78
gag	E	3	5.22
ggt	G	1	0.14
ggc	G	0	0.13
gga	G	0	0.44
ggg	G	0	0.29
---	--	------	------
mPD		0.017	0.50
		nPD:	0.03
		N. weight:	0.57
		Sc. PD:	-0.04
		Sc. rank:	27.0

,

NA

Pos.	284	obs:	exp:
ggt	G	2501	365.10
ggc	G	35	335.00
gga	G	0	1108.00
ggg	G	2	730.00
---	--	------	------
mPD		0.029	0.69
		nPD:	0.04
		N. weight:	2.
		Sc. PD:	-0.12
		Sc. rank:	578.0

,

NA

Pos.	285	obs:	exp:
cgt	R	0	0.04
cgc	R	0	0.05
cga	R	0	0.10
cgg	R	0	0.08
aaa	K	2535	1424.00
aag	K	2	1113.00
aga	R	1	0.46
agg	R	0	0.27
---	--	------	------
mPD		0.0024	0.49
		nPD:	0.
		N. weight:	0.6
		Sc. PD:	-0.059
		Sc. rank:	-1113.5

,

NA

Pos.	286	obs:	exp:
gtt	V	0	552.00
gtc	V	1	536.30
gta	V	2	499.40
gtg	V	2535	950.40
---	--	------	------
mPD		0.0024	0.73
		nPD:	0.
		N. weight:	1.
		Sc. PD:	-0.1
		Sc. rank:	-1976.0

,

NA

Pos.	287	obs:	exp:
gaa	E	2530	1520.00
gag	E	8	1018.00
---	--	------	------
mPD		0.0063	0.48
		nPD:	0.01
		N. weight:	0.52
		Sc. PD:	-0.047
		Sc. rank:	-620.4

,

NA

Pos.	288	obs:	exp:
tgt	C	32	1104.00
tgc	C	2506	1434.00
---	--	------	------
mPD		0.025	0.49
		nPD:	0.05
		N. weight:	0.53
		Sc. PD:	-0.028
		Sc. rank:	293.1

,

NA

Pos.	289	obs:	exp:
tta	L	0	0.55
ttg	L	5	0.94
ctt	L	0	0.94
ctc	L	0	0.86
cta	L	0	0.67
ctg	L	0	1.05
att	I	1	0.37
atc	I	0	0.25
ata	I	0	0.38
atg	M	5	5.00
gtt	V	13	549.60
gtc	V	1	533.90
gta	V	32	497.20
gtg	V	2481	946.30
---	--	------	------
mPD		0.045	0.74
		nPD:	0.06
		N. weight:	0.94
		Sc. PD:	-0.04
		Sc. rank:	679.1

,

NA

Pos.	290	obs:	exp:
tgt	C	2523	1103.00
tgc	C	14	1434.00
cgt	R	1	0.04
cgc	R	0	0.05
cga	R	0	0.10
cgg	R	0	0.08
aga	R	0	0.46
agg	R	0	0.27
---	--	------	------
mPD		0.012	0.49
		nPD:	0.02
		N. weight:	0.84
		Sc. PD:	-0.067
		Sc. rank:	-340.8

,

NA

Pos.	291	obs:	exp:
cgt	R	0	96.25
cgc	R	0	135.20
cga	R	1	245.80
cgg	R	0	198.80
aga	R	2518	1164.00
agg	R	19	697.90
---	--	------	------
mPD		0.016	0.95
		nPD:	0.02
		N. weight:	0.78
		Sc. PD:	-0.067
		Sc. rank:	-761.3

,

NA

Pos.	292	obs:	exp:
gat	D	8	1460.00
gac	D	2530	1078.00
---	--	------	------
mPD		0.0063	0.49
		nPD:	0.01
		N. weight:	0.88
		Sc. PD:	-0.079
		Sc. rank:	-1057.2

,

NA

Pos.	293	obs:	exp:
tct	S	0	0.17
tcc	S	0	0.15
tca	S	0	0.23
tcg	S	0	0.06
aat	N	14	1348.00
aac	N	2523	1189.00
agt	S	0	0.20
agc	S	1	0.19
---	--	------	------
mPD		0.012	0.50
		nPD:	0.02
		N. weight:	0.76
		Sc. PD:	-0.061
		Sc. rank:	-340.9

,

NA

Pos.	294	obs:	exp:
tgg	W	2538	2538.00
---	--	------	------
mPD		0	0
		nPD:	1.
		N. weight:	0.
		Sc. PD:	0
		Sc. rank:	0

,

NA

Pos.	295	obs:	exp:
cgt	R	0	0.83
cgc	R	0	1.17
cga	R	0	2.13
cgg	R	0	1.72
att	I	1	2.60
atc	I	0	1.76
ata	I	6	2.65
atg	M	983	983.00
act	T	9	401.30
acc	T	2	334.70
aca	T	36	651.00
acg	T	1468	128.00
aaa	K	0	3.93
aag	K	7	3.07
aga	R	0	10.09
agg	R	22	6.05
gtt	V	0	0.87
gtc	V	0	0.85
gta	V	0	0.79
gtg	V	4	1.50
---	--	------	------
mPD		0.54	1.2
		nPD:	0.45
		N. weight:	1.4
		Sc. PD:	0.48
		Sc. rank:	2602.1

,

NA

Pos.	296	obs:	exp:
ggt	G	2	365.10
ggc	G	0	335.00
gga	G	2531	1108.00
ggg	G	5	730.00
---	--	------	------
mPD		0.0055	0.69
		nPD:	0.01
		N. weight:	0.85
		Sc. PD:	-0.081
		Sc. rank:	-1351.8

,

NA

Pos.	297	obs:	exp:
act	T	2506	669.40
acc	T	21	558.30
aca	T	0	1086.00
acg	T	0	213.40
gct	A	11	2.80
gcc	A	0	2.15
gca	A	0	4.84
gcg	A	0	1.22
---	--	------	------
mPD		0.025	0.70
		nPD:	0.04
		N. weight:	1.3
		Sc. PD:	-0.091
		Sc. rank:	144.9

,

NA

Pos.	298	obs:	exp:
aat	N	27	1349.00
aac	N	2511	1189.00
---	--	------	------
mPD		0.021	0.50
		nPD:	0.04
		N. weight:	0.73
		Sc. PD:	-0.045
		Sc. rank:	234.9

,

NA

Pos.	299	obs:	exp:
cgt	R	0	96.25
cgc	R	0	135.20
cga	R	0	245.80
cgg	R	0	198.80
aga	R	123	1164.00
agg	R	2415	697.90
---	--	------	------
mPD		0.092	0.95
		nPD:	0.1
		N. weight:	1.1
		Sc. PD:	-0.0098
		Sc. rank:	1232.2

,

NA

Pos.	300	obs:	exp:
cct	P	2521	715.70
ccc	P	1	459.20
cca	P	11	1036.00
ccg	P	5	326.80
---	--	------	------
mPD		0.013	0.70
		nPD:	0.02
		N. weight:	1.3
		Sc. PD:	-0.11
		Sc. rank:	-1081.8

,

NA

Pos.	301	obs:	exp:
att	I	1	0.37
atc	I	0	0.25
ata	I	0	0.38
gtt	V	299	551.50
gtc	V	4	535.80
gta	V	62	499.00
gtg	V	2171	949.70
gct	A	0	0.25
gcc	A	0	0.20
gca	A	0	0.44
gcg	A	1	0.11
---	--	------	------
mPD		0.25	0.73
		nPD:	0.35
		N. weight:	0.61
		Sc. PD:	0.14
		Sc. rank:	1004.5

,

NA

Pos.	302	obs:	exp:
tta	L	1	276.90
ttg	L	11	476.10
ctt	L	0	475.70
ctc	L	0	437.70
cta	L	2506	337.50
ctg	L	19	533.00
att	I	0	0.37
atc	I	0	0.25
ata	I	1	0.38
---	--	------	------
mPD		0.034	1.1
		nPD:	0.03
		N. weight:	2.
		Sc. PD:	-0.15
		Sc. rank:	-335.7

,

NA

Pos.	303	obs:	exp:
att	I	709	263.30
atc	I	0	177.80
ata	I	0	267.90
gtt	V	1764	397.80
gtc	V	55	386.40
gta	V	1	359.90
gtg	V	9	684.90
---	--	------	------
mPD		0.45	1.2
		nPD:	0.39
		N. weight:	1.3
		Sc. PD:	0.37
		Sc. rank:	2245.5

,

NA

Pos.	304	obs:	exp:
ttt	F	0	0.43
ttc	F	1	0.57
att	I	1625	939.90
atc	I	900	634.80
ata	I	6	956.40
gtt	V	5	1.30
gtc	V	1	1.27
gta	V	0	1.18
gtg	V	0	2.25
---	--	------	------
mPD		0.47	0.66
		nPD:	0.7
		N. weight:	0.49
		Sc. PD:	0.29
		Sc. rank:	987.0

,

NA

Pos.	305	obs:	exp:
tct	S	17	433.40
tcc	S	0	374.10
tca	S	77	572.10
tcg	S	2444	163.90
agt	S	0	518.20
agc	S	0	476.20
---	--	------	------
mPD		0.072	1.6
		nPD:	0.04
		N. weight:	2.7
		Sc. PD:	-0.16
		Sc. rank:	977.7

,

NA

Pos.	306	obs:	exp:
tct	S	4	0.85
tcc	S	0	0.74
tca	S	0	1.13
tcg	S	1	0.32
cct	P	2512	714.30
ccc	P	5	458.30
cca	P	10	1034.00
ccg	P	6	326.20
agt	S	0	1.02
agc	S	0	0.94
---	--	------	------
mPD		0.021	0.71
		nPD:	0.03
		N. weight:	1.3
		Sc. PD:	-0.094
		Sc. rank:	-189.1

,

NA

Pos.	307	obs:	exp:
gat	D	1647	1460.00
gac	D	891	1078.00
---	--	------	------
mPD		0.46	0.49
		nPD:	0.93
		N. weight:	0.014
		Sc. PD:	0.011
		Sc. rank:	28.4

,

NA

Pos.	308	obs:	exp:
tta	L	0	276.90
ttg	L	10	476.10
ctt	L	1	475.70
ctc	L	2524	437.70
cta	L	2	337.50
ctg	L	0	533.00
atg	M	1	1.00
---	--	------	------
mPD		0.020	1.1
		nPD:	0.02
		N. weight:	1.8
		Sc. PD:	-0.16
		Sc. rank:	-1624.9

,

NA

Pos.	309	obs:	exp:
ttt	F	1	0.43
ttc	F	0	0.57
tct	S	2526	433.30
tcc	S	11	374.00
tca	S	0	571.90
tcg	S	0	163.90
agt	S	0	518.00
agc	S	0	476.00
---	--	------	------
mPD		0.0094	1.6
		nPD:	0.01
		N. weight:	1.8
		Sc. PD:	-0.18
		Sc. rank:	-3326.4

,

NA

Pos.	310	obs:	exp:
tat	Y	5	1233.00
tac	Y	2531	1303.00
cat	H	0	1.19
cac	H	2	0.81
---	--	-----	-----
mPD		0.0055	0.50
		nPD:	0.01
		N. weight:	0.69
		Sc. PD:	-0.063
		Sc. rank:	-921.5

,

NA

Pos.	311	obs:	exp:
cgt	R	0	95.19
cgc	R	0	133.70
cga	R	0	243.00
cgg	R	2	196.60
atg	M	1	1.00
aaa	K	2	14.03
aag	K	23	10.97
aga	R	106	1151.00
agg	R	2402	690.20
gaa	E	0	1.20
gag	E	2	0.80
---	--	-----	-----
mPD		0.11	0.97
		nPD:	0.11
		N. weight:	1.1
		Sc. PD:	0.0042
		Sc. rank:	1333.9

,

NA

Pos.	312	obs:	exp:
att	I	12	4.46
atc	I	0	3.01
ata	I	0	4.53
gtt	V	2484	548.70
gtc	V	38	533.10
gta	V	1	496.40
gtg	V	0	944.80
gct	A	2	0.51
gcc	A	0	0.39
gca	A	0	0.88
gcg	A	0	0.22
gat	D	1	0.58
gac	D	0	0.42
---	--	-----	-----
mPD		0.042	0.74
		nPD:	0.06
		N. weight:	1.5
		Sc. PD:	-0.072
		Sc. rank:	972.0

,

NA

Pos.	313	obs:	exp:
ggt	G	53	365.10
ggc	G	0	335.00
gga	G	14	1108.00
ggg	G	2471	730.00
---	--	-----	-----
mPD		0.052	0.69
		nPD:	0.08
		N. weight:	1.2
		Sc. PD:	-0.035
		Sc. rank:	1063.9

,

NA

Pos.	314	obs:	exp:
tat	Y	2494	1234.00
tac	Y	44	1304.00
---	--	-----	-----
mPD		0.034	0.50
		nPD:	0.07
		N. weight:	0.67
		Sc. PD:	-0.024
		Sc. rank:	547.5

,

NA

Pos.	315	obs:	exp:
tta	L	2510	277.00
ttg	L	21	476.20
ctt	L	0	475.90
ctc	L	0	437.90
cta	L	7	337.70
ctg	L	0	533.30
---	--	-----	-----
mPD		0.022	1.1
		nPD:	0.02
		N. weight:	2.3
		Sc. PD:	-0.19
		Sc. rank:	-1731.7

,

NA

Pos.	316	obs:	exp:
tgt	C	2538	1104.00
tgc	C	0	1434.00
---	--	-----	-----
mPD		0	0.49
		nPD:	0.
		N. weight:	0.88
		Sc. PD:	-0.09
		Sc. rank:	-1829.5

,

NA

Pos.	317	obs:	exp:
tct	S	0	0.17
tcc	S	0	0.15
tca	S	1	0.23
tcg	S	0	0.06
agt	S	0	0.20
agc	S	0	0.19
gct	A	1	645.40
gcc	A	0	494.70
gca	A	1383	1115.00
gcg	A	1153	281.60
---	--	-----	-----
mPD		0.50	0.69
		nPD:	0.72
		N. weight:	0.8
		Sc. PD:	0.47
		Sc. rank:	1622.1

,

NA

Pos.	318	obs:	exp:
ggt	G	11	365.10
ggc	G	0	335.00
gga	G	1626	1108.00
ggg	G	901	730.00
---	--	-----	-----
mPD		0.46	0.69
		nPD:	0.67
		N. weight:	0.32
		Sc. PD:	0.18
		Sc. rank:	640.6

,

NA

Pos.	319	obs:	exp:
tta	L	36	276.80
ttg	L	2483	475.90
ctt	L	1	475.50
ctc	L	8	437.50
cta	L	0	337.40
ctg	L	8	532.80
att	I	0	0.74
atc	I	1	0.50
ata	I	1	0.76
---	--	-----	-----
mPD		0.051	1.1
		nPD:	0.05
		N. weight:	1.6
		Sc. PD:	-0.096
		Sc. rank:	712.2

,

NA

Pos.	320	obs:	exp:
cct	P	0	712.60
ccc	P	2527	457.30
cca	P	0	1032.00
ccg	P	0	325.40
gct	A	0	2.80
gcc	A	11	2.15
gca	A	0	4.84
gcg	A	0	1.22
---	--	-----	-----
mPD		0.0086	0.71
		nPD:	0.01
		N. weight:	1.8
		Sc. PD:	-0.16
		Sc. rank:	-2237.1

,

NA

Pos.	321	obs:	exp:
tct	S	0	433.40
tcc	S	0	374.10
tca	S	0	572.10
tcg	S	0	163.90
agt	S	2537	518.20
agc	S	1	476.20
---	--	-----	-----
mPD		0.00079	1.6
		nPD:	0.
		N. weight:	1.7
		Sc. PD:	-0.17
		Sc. rank:	-3424.2

,

NA

Pos.	322	obs:	exp:
gtt	V	0	0.22
gtc	V	1	0.21
gta	V	0	0.20
gtg	V	0	0.37
gat	D	13	1459.00
gac	D	2524	1078.00
---	--	-----	-----
mPD		0.011	0.49
		nPD:	0.02
		N. weight:	0.87
		Sc. PD:	-0.07
		Sc. rank:	-432.1

,

NA

Pos.	323	obs:	exp:
act	T	2523	672.30
acc	T	13	560.80
aca	T	2	1091.00
acg	T	0	214.40
---	--	-----	-----
mPD		0.012	0.69
		nPD:	0.02
		N. weight:	1.4
		Sc. PD:	-0.12
		Sc. rank:	-1256.9

,

NA

Pos.	324	obs:	exp:
cct	P	5	715.70
ccc	P	0	459.20
cca	P	2533	1036.00
ccg	P	0	326.80
---	--	-----	-----
mPD		0.0039	0.70
		nPD:	0.01
		N. weight:	0.93
		Sc. PD:	-0.09
		Sc. rank:	-1702.4

,

NA

Pos.	325	obs:	exp:
cgt	R	0	96.25
cgc	R	0	135.20
cga	R	1	245.80
cgg	R	0	198.80
aga	R	2525	1164.00
agg	R	12	697.90
---	--	-----	-----
mPD		0.010	0.95
		nPD:	0.01
		N. weight:	0.79
		Sc. PD:	-0.073
		Sc. rank:	-1068.9

,

NA

Pos.	326	obs:	exp:
gtt	V	0	0.87
gtc	V	0	0.85
gta	V	0	0.79
gtg	V	4	1.50
ggt	G	1	364.50
ggc	G	0	334.50
gga	G	18	1106.00
ggg	G	2515	728.80
---	--	-----	-----
mPD		0.018	0.69
		nPD:	0.03
		N. weight:	1.3
		Sc. PD:	-0.097
		Sc. rank:	-408.2

,

NA

Pos.	327	obs:	exp:
gaa	E	2441	1519.00
gag	E	96	1018.00
ggt	G	0	0.14
ggc	G	0	0.13
gga	G	1	0.44
ggg	G	0	0.29
---	--	-----	-----
mPD		0.074	0.48
		nPD:	0.15
		N. weight:	0.39
		Sc. PD:	0.018
		Sc. rank:	505.9

,

NA

Pos.	328	obs:	exp:
gat	D	2538	1460.00
gac	D	0	1078.00
---	--	-----	-----
mPD		0	0.49
		nPD:	0.
		N. weight:	0.58
		Sc. PD:	-0.06
		Sc. rank:	-1214.9

,

NA

Pos.	329	obs:	exp:
tct	S	0	1.71
tcc	S	0	1.47
tca	S	0	2.25
tcg	S	0	0.65
att	I	69	25.62
atc	I	0	17.30
ata	I	0	26.07
act	T	464	123.40
acc	T	2	103.00
aca	T	0	200.20
acg	T	0	39.36
aat	N	1	0.53
aac	N	0	0.47
agt	S	10	2.04
agc	S	0	1.88
gtt	V	0	0.22
gtc	V	1	0.21
gta	V	0	0.20
gtg	V	0	0.37
gct	A	1093	506.00
gcc	A	896	387.90
gca	A	0	874.30
gcg	A	0	220.80
gat	D	1	1.15
gac	D	1	0.85
---	--	-----	-----
mPD		0.86	1.1
		nPD:	0.79
		N. weight:	0.95
		Sc. PD:	0.63
		Sc. rank:	1958.4

,

NA			
Pos.	330	obs:	exp:
cct	P	0	1.13
ccc	P	0	0.72
cca	P	4	1.63
ccg	P	0	0.52
cat	H	1	1.19
cac	H	1	0.81
caa	Q	2515	1287.00
cag	Q	15	1243.00
cgt	R	0	0.04
cgc	R	0	0.05
cga	R	1	0.10
cgg	R	0	0.08
aaa	K	1	0.56
aag	K	0	0.44
aga	R	0	0.46
agg	R	0	0.27
---	--	------	------
mPD		0.018	0.51
		nPD:	0.04
		N. weight:	0.67
		Sc. PD:	-0.046
		Sc. rank:	59.0

,

NA			
Pos.	331	obs:	exp:
ttt	F	2529	1080.00
ttc	F	8	1457.00
tta	L	0	0.11
ttg	L	1	0.19
ctt	L	0	0.19
ctc	L	0	0.17
cta	L	0	0.13
ctg	L	0	0.21
---	--	------	------
mPD		0.0071	0.49
		nPD:	0.01
		N. weight:	0.88
		Sc. PD:	-0.077
		Sc. rank:	-953.7

,

NA			
Pos.	332	obs:	exp:
att	I	0	3.34
atc	I	9	2.26
ata	I	0	3.40
act	T	4	3.97
acc	T	0	3.31
aca	T	4	6.45
acg	T	7	1.27
gtt	V	13	545.90
gtc	V	2495	530.30
gta	V	2	493.90
gtg	V	0	939.90
gct	A	0	1.02
gcc	A	4	0.78
gca	A	0	1.76
gcg	A	0	0.44
---	--	------	------
mPD		0.057	0.76
		nPD:	0.07
		N. weight:	1.6
		Sc. PD:	-0.048
		Sc. rank:	1414.3

,

NA			
Pos.	333	obs:	exp:
ggt	G	2521	365.10
ggc	G	4	335.00
gga	G	6	1108.00
ggg	G	7	730.00
---	--	------	------
mPD		0.013	0.69
		nPD:	0.02
		N. weight:	2.
		Sc. PD:	-0.17
		Sc. rank:	-1431.5

,

NA			
Pos.	334	obs:	exp:
tct	S	1	433.40
tcc	S	5	374.10
tca	S	1647	572.10
tcg	S	885	163.90
agt	S	0	518.20
agc	S	0	476.20
---	--	------	------
mPD		0.46	1.6
		nPD:	0.28
		N. weight:	1.3
		Sc. PD:	0.22
		Sc. rank:	2037.3

,

NA			
Pos.	335	obs:	exp:
tgt	C	2	1104.00
tgc	C	2536	1434.00
---	--	------	------
mPD		0.0016	0.49
		nPD:	0.
		N. weight:	0.59
		Sc. PD:	-0.059
		Sc. rank:	-1156.2

,

NA			
Pos.	336	obs:	exp:
att	I	2	0.74
atc	I	0	0.50
ata	I	0	0.76
act	T	2525	668.80
acc	T	0	557.90
aca	T	0	1085.00
acg	T	0	213.30
aat	N	11	5.85
aac	N	0	5.15
---	--	------	------
mPD		0.010	0.70
		nPD:	0.01
		N. weight:	1.4
		Sc. PD:	-0.12
		Sc. rank:	-1481.7

,

NA			
Pos.	337	obs:	exp:
tct	S	0	432.10
tcc	S	0	373.00
tca	S	0	570.30
tcg	S	0	163.40
cgt	R	0	0.08
cgc	R	0	0.11
cga	R	0	0.19
cgg	R	0	0.16
att	I	2	0.74
atc	I	0	0.50
ata	I	0	0.76
aat	N	4	2.13
aac	N	0	1.87
agt	S	2510	516.60
agc	S	20	474.70
aga	R	1	0.92
agg	R	1	0.55
---	--	------	------
mPD		0.022	1.6
		nPD:	0.01
		N. weight:	1.6
		Sc. PD:	-0.15
		Sc. rank:	-1880.9

,

NA			
Pos.	338	obs:	exp:
tct	S	0	0.51
tcc	S	3	0.44
tca	S	0	0.68
tcg	S	0	0.19
cct	P	11	714.80
ccc	P	2520	458.70
cca	P	2	1035.00
ccg	P	2	326.40
agt	S	0	0.61
agc	S	0	0.56
---	--	------	------
mPD		0.014	0.71
		nPD:	0.02
		N. weight:	1.8
		Sc. PD:	-0.15
		Sc. rank:	-1200.2

,

NA			
Pos.	339	obs:	exp:
tta	L	0	0.22
ttg	L	0	0.38
ctt	L	0	0.38
ctc	L	0	0.35
cta	L	0	0.27
ctg	L	2	0.42
atg	M	2525	2525.00
act	T	0	0.26
acc	T	0	0.22
aca	T	0	0.43
acg	T	1	0.08
gtt	V	0	2.17
gtc	V	0	2.11
gta	V	0	1.97
gtg	V	10	3.74
---	--	------	------
mPD		0.010	0.017
		nPD:	0.61
		N. weight:	0.0064
		Sc. PD:	0.0031
		Sc. rank:	12.4

,

NA			
Pos.	340	obs:	exp:
ggt	G	3	365.10
ggc	G	0	335.00
gga	G	2526	1108.00
ggg	G	9	730.00
---	--	------	------
mPD		0.0094	0.69
		nPD:	0.01
		N. weight:	0.84
		Sc. PD:	-0.075
		Sc. rank:	-934.3

,

NA			
Pos.	341	obs:	exp:
tct	S	0	0.34
tcc	S	0	0.29
tca	S	0	0.45
tcg	S	0	0.13
cat	H	5	2.97
cac	H	0	2.03
aat	N	2208	1343.00
aac	N	320	1185.00
aaa	K	0	0.56
aag	K	1	0.44
agt	S	1	0.41
agc	S	1	0.38
gat	D	2	1.15
gac	D	0	0.85
---	--	------	------
mPD		0.23	0.51
		nPD:	0.45
		N. weight:	0.29
		Sc. PD:	0.096
		Sc. rank:	515.7

,

NA			
Pos.	342	obs:	exp:
tta	L	0	11.68
ttg	L	27	20.08
ctt	L	0	20.06
ctc	L	0	18.46
cta	L	0	14.24
ctg	L	80	22.48
cat	H	2	1.19
cac	H	0	0.81
caa	Q	2	1235.00
cag	Q	2425	1192.00
cgt	R	0	0.04
cgc	R	0	0.05
cga	R	0	0.10
cgg	R	1	0.08
aaa	K	0	0.56
aag	K	1	0.44
aga	R	0	0.46
agg	R	0	0.27
---	--	------	------
mPD		0.11	0.62
		nPD:	0.17
		N. weight:	0.76
		Sc. PD:	0.048
		Sc. rank:	1013.2

,

NA			
Pos.	343	obs:	exp:
cgt	R	0	0.04
cgc	R	0	0.05
cga	R	0	0.10
cgg	R	0	0.08
aga	R	0	0.46
agg	R	1	0.27
gaa	E	17	10.78
gag	E	1	7.22
ggt	G	2	362.40
ggc	G	1	332.50
gga	G	1466	1100.00
ggg	G	1050	724.50
---	--	------	------
mPD		0.50	0.70
		nPD:	0.71
		N. weight:	0.34
		Sc. PD:	0.2
		Sc. rank:	674.6

,

NA			
Pos.	344	obs:	exp:
ttt	F	1	0.43
ttc	F	0	0.57
tct	S	2	0.34
tcc	S	0	0.29
tca	S	0	0.45
tcg	S	0	0.13
tat	Y	2504	1231.00
tac	Y	29	1302.00
cat	H	2	1.19
cac	H	0	0.81
agt	S	0	0.41
agc	S	0	0.38
---	--	------	------
mPD		0.027	0.50
		nPD:	0.05
		N. weight:	0.69
		Sc. PD:	-0.036
		Sc. rank:	391.5

,

NA			
Pos.	345	obs:	exp:
ggt	G	25	365.10
ggc	G	2412	335.00
gga	G	13	1108.00
ggg	G	88	730.00
---	--	------	------
mPD		0.096	0.69
		nPD:	0.14
		N. weight:	1.8
		Sc. PD:	0.059
		Sc. rank:	2355.5

,

NA			
Pos.	346	obs:	exp:
att	I	0	2.23
atc	I	0	1.50
ata	I	6	2.27
gtt	V	11	550.70
gtc	V	1	535.00
gta	V	2498	498.20
gtg	V	22	948.20
---	--	------	------
mPD		0.031	0.73
		nPD:	0.04
		N. weight:	1.6
		Sc. PD:	-0.099
		Sc. rank:	540.6

,

NA			
Pos.	347	obs:	exp:
aaa	K	2530	1425.00
aag	K	8	1113.00
---	--	------	------
mPD		0.0063	0.49
		nPD:	0.01
		N. weight:	0.59
		Sc. PD:	-0.053
		Sc. rank:	-711.0

,

NA			
Pos.	348	obs:	exp:
ggt	G	1958	365.10
ggc	G	12	335.00
gga	G	21	1108.00
ggg	G	547	730.00
---	--	------	------
mPD		0.36	0.69
		nPD:	0.52
		N. weight:	1.2
		Sc. PD:	0.5
		Sc. rank:	2387.0

,

NA			
Pos.	349	obs:	exp:
ttt	F	15	1080.00
ttc	F	2523	1458.00
---	--	------	------
mPD		0.012	0.49
		nPD:	0.02
		N. weight:	0.55
		Sc. PD:	-0.043
		Sc. rank:	-219.4

,

NA			
Pos.	350	obs:	exp:
cgt	R	0	0.04
cgc	R	0	0.05
cga	R	0	0.10
cgg	R	0	0.08
aga	R	0	0.46
agg	R	1	0.27
ggt	G	4	365.00
ggc	G	0	334.90
gga	G	29	1107.00
ggg	G	2504	729.70
---	--	------	------
mPD		0.027	0.69
		nPD:	0.04
		N. weight:	1.2
		Sc. PD:	-0.08
		Sc. rank:	241.3

NA			
Pos.	351	obs:	exp:
ttt	F	2421	1080.00
ttc	F	117	1458.00
---	--	------	------
mPD		0.088	0.49
		nPD:	0.18
		N. weight:	0.69
		Sc. PD:	0.05
		Sc. rank:	929.3

NA			
Pos.	352	obs:	exp:
cgt	R	0	96.25
cgc	R	0	135.20
cga	R	2503	245.80
cgg	R	8	198.80
aga	R	18	1164.00
agg	R	9	697.90
---	--	------	------
mPD		0.034	0.95
		nPD:	0.04
		N. weight:	2.4
		Sc. PD:	-0.16
		Sc. rank:	301.5

NA			
Pos.	353	obs:	exp:
caa	Q	40	1291.00
cag	Q	2498	1247.00
---	--	------	------
mPD		0.031	0.50
		nPD:	0.06
		N. weight:	0.66
		Sc. PD:	-0.028
		Sc. rank:	501.3

NA			
Pos.	354	obs:	exp:
gaa	E	7	4.19
gag	E	0	2.81
ggt	G	0	364.10
ggc	G	11	334.10
gga	G	2513	1105.00
ggg	G	7	728.00
---	--	------	------
mPD		0.020	0.69
		nPD:	0.03
		N. weight:	0.83
		Sc. PD:	-0.062
		Sc. rank:	-194.5

NA			
Pos.	355	obs:	exp:
tct	S	1	0.34
tcc	S	0	0.29
tca	S	0	0.45
tcg	S	0	0.13
att	I	4	1.49
atc	I	0	1.00
ata	I	0	1.51
act	T	2477	659.80
acc	T	5	550.40
aca	T	0	1070.00
acg	T	9	210.40
aat	N	35	18.60
aac	N	0	16.40
agt	S	1	0.41
agc	S	0	0.38
gct	A	5	1.53
gcc	A	1	1.17
gca	A	0	2.64
gcg	A	0	0.67
---	--	------	------
mPD		0.048	0.73
		nPD:	0.07
		N. weight:	1.4
		Sc. PD:	-0.051
		Sc. rank:	1088.4

NA			
Pos.	356	obs:	exp:
aat	N	1	0.53
aac	N	0	0.47
gat	D	2533	1459.00
gac	D	4	1078.00
---	--	------	------
mPD		0.0039	0.49
		nPD:	0.01
		N. weight:	0.57
		Sc. PD:	-0.054
		Sc. rank:	-896.6

NA			
Pos.	357	obs:	exp:
gtt	V	14	551.30
gtc	V	0	535.60
gta	V	38	498.80
gtg	V	2483	949.30
gct	A	0	0.76
gcc	A	0	0.59
gca	A	0	1.32
gcg	A	3	0.33
---	--	------	------
mPD		0.043	0.73
		nPD:	0.06
		N. weight:	0.93
		Sc. PD:	-0.043
		Sc. rank:	636.3

NA			
Pos.	358	obs:	exp:
tgg	W	2538	2538.00
---	--	------	------
mPD		0	0
		nPD:	1.
		N. weight:	0.
		Sc. PD:	0
		Sc. rank:	0

NA			
Pos.	359	obs:	exp:
att	I	0	1.49
atc	I	0	1.00
ata	I	4	1.51
atg	M	925	925.00
gtt	V	3	349.90
gtc	V	0	340.00
gta	V	2	316.60
gtg	V	1604	602.50
---	--	------	------
mPD		0.47	1.0
		nPD:	0.45
		N. weight:	0.64
		Sc. PD:	0.21
		Sc. rank:	1152.2

NA			
Pos.	360	obs:	exp:
ggt	G	1	365.10
ggc	G	2	335.00
gga	G	20	1108.00
ggg	G	2515	730.00
---	--	------	------
mPD		0.018	0.69
		nPD:	0.03
		N. weight:	1.3
		Sc. PD:	-0.096
		Sc. rank:	-391.2

NA			
Pos.	361	obs:	exp:
cgt	R	15	96.25
cgc	R	0	135.20
cga	R	3	245.80
cgg	R	2260	198.80
aga	R	55	1164.00
agg	R	205	697.90
---	--	------	------
mPD		0.24	0.95
		nPD:	0.25
		N. weight:	2.1
		Sc. PD:	0.3
		Sc. rank:	3124.7

NA			
Pos.	362	obs:	exp:
act	T	11	672.30
acc	T	11	560.80
aca	T	2514	1091.00
acg	T	2	214.40
---	--	------	------
mPD		0.019	0.69
		nPD:	0.03
		N. weight:	0.83
		Sc. PD:	-0.063
		Sc. rank:	-216.7

NA			
Pos.	363	obs:	exp:
tta	L	0	0.11
ttg	L	0	0.19
ctt	L	1	0.19
ctc	L	0	0.17
cta	L	0	0.13
ctg	L	0	0.21
att	I	2459	941.30
atc	I	75	635.80
ata	I	1	957.90
gtt	V	2	0.43
gtc	V	0	0.42
gta	V	0	0.39
gtg	V	0	0.75
---	--	------	------
mPD		0.061	0.66
		nPD:	0.09
		N. weight:	0.91
		Sc. PD:	-0.012
		Sc. rank:	924.1

NA			
Pos.	364	obs:	exp:
tct	S	0	431.90
tcc	S	0	372.80
tca	S	0	570.10
tcg	S	0	163.40
aat	N	4	2.66
aac	N	1	2.34
agt	S	2513	516.40
agc	S	16	474.50
ggt	G	4	0.58
ggc	G	0	0.53
gga	G	0	1.75
ggg	G	0	1.15
---	--	------	------
mPD		0.020	1.7
		nPD:	0.01
		N. weight:	1.6
		Sc. PD:	-0.15
		Sc. rank:	-2005.3

NA			
Pos.	365	obs:	exp:
tta	L	0	0.22
ttg	L	0	0.38
ctt	L	0	0.38
ctc	L	0	0.35
cta	L	2	0.27
ctg	L	0	0.42
caa	Q	8	4.07
cag	Q	0	3.93
cgt	R	0	95.87
cgc	R	0	134.70
cga	R	2467	244.80
cgg	R	48	198.00
aga	R	7	1160.00
agg	R	6	695.10
---	--	------	------
mPD		0.060	0.96
		nPD:	0.06
		N. weight:	2.3
		Sc. PD:	-0.098
		Sc. rank:	1735.7

NA			
Pos.	366	obs:	exp:
att	I	0	0.37
atc	I	1	0.25
ata	I	0	0.38
act	T	60	671.20
acc	T	2461	559.90
aca	T	12	1089.00
acg	T	1	214.00
aat	N	0	0.53
aac	N	1	0.47
gct	A	0	0.51
gcc	A	2	0.39
gca	A	0	0.88
gcg	A	0	0.22
---	--	------	------
mPD		0.059	0.69
		nPD:	0.09
		N. weight:	1.4
		Sc. PD:	-0.028
		Sc. rank:	1423.4

NA			
Pos.	367	obs:	exp:
ttt	F	0	0.43
ttc	F	1	0.57
tct	S	2	432.40
tcc	S	2513	373.30
tca	S	17	570.70
tcg	S	0	163.60
tat	Y	0	1.94
tac	Y	4	2.06
agt	S	0	517.00
agc	S	0	475.10
gct	A	0	0.25
gcc	A	1	0.20
gca	A	0	0.44
gcg	A	0	0.11
---	--	------	------
mPD		0.020	1.6
		nPD:	0.01
		N. weight:	2.
		Sc. PD:	-0.18
		Sc. rank:	-2501.7

NA			
Pos.	368	obs:	exp:
cgt	R	0	96.25
cgc	R	0	135.20
cga	R	0	245.80
cgg	R	0	198.80
aga	R	656	1164.00
agg	R	1882	697.90
---	--	------	------
mPD		0.38	0.95
		nPD:	0.4
		N. weight:	0.62
		Sc. PD:	0.18
		Sc. rank:	1072.8

NA			
Pos.	369	obs:	exp:
tta	L	4	0.44
ttg	L	0	0.75
tct	S	1	430.90
tcc	S	0	371.90
tca	S	2520	568.70
tcg	S	2	163.00
ctt	L	0	0.75
ctc	L	0	0.69
cta	L	0	0.53
ctg	L	0	0.84
act	T	0	0.53
acc	T	0	0.44
aca	T	2	0.86
acg	T	0	0.17
agt	S	0	515.10
agc	S	0	473.40
gct	A	0	2.29
gcc	A	0	1.76
gca	A	9	3.96
gcg	A	0	1.00
---	--	------	------
mPD		0.014	1.7
		nPD:	0.01
		N. weight:	1.6
		Sc. PD:	-0.15
		Sc. rank:	-2399.0

NA			
Pos.	370	obs:	exp:
ggt	G	1	365.10
ggc	G	1	335.00
gga	G	604	1108.00
ggg	G	1932	730.00
---	--	------	------
mPD		0.36	0.69
		nPD:	0.53
		N. weight:	0.62
		Sc. PD:	0.26
		Sc. rank:	1200.1

NA			
Pos.	371	obs:	exp:
ttt	F	2533	1080.00
ttc	F	4	1457.00
tta	L	0	0.11
ttg	L	0	0.19
ctt	L	1	0.19
ctc	L	0	0.17
cta	L	0	0.13
ctg	L	0	0.21
---	--	------	------
mPD		0.0039	0.49
		nPD:	0.01
		N. weight:	0.89
		Sc. PD:	-0.084
		Sc. rank:	-1398.3

NA			
Pos.	372	obs:	exp:
gaa	E	2534	1520.00
gag	E	4	1018.00
---	--	------	------
mPD		0.0031	0.48
		nPD:	0.01
		N. weight:	0.53
		Sc. PD:	-0.051
		Sc. rank:	-904.6

NA			
Pos.	373	obs:	exp:
att	I	0	939.90
atc	I	11	634.80
ata	I	2520	956.40
atg	M	3	3.00
act	T	0	0.53
acc	T	0	0.44
aca	T	2	0.86
acg	T	0	0.17
gtt	V	0	0.43
gtc	V	0	0.42
gta	V	2	0.39
gtg	V	0	0.75
---	--	------	------
mPD		0.014	0.66
		nPD:	0.02
		N. weight:	1.
		Sc. PD:	-0.081
		Sc. rank:	-559.3

NA			
Pos.	374	obs:	exp:
tta	L	0	1.64
ttg	L	0	2.81
ctt	L	0	2.81
ctc	L	0	2.59
cta	L	3	2.00
ctg	L	12	3.15
att	I	2	935.40
atc	I	0	631.80
ata	I	2517	951.90
atg	M	4	4.00
---	--	------	------
mPD		0.026	0.67
		nPD:	0.04
		N. weight:	1.
		Sc. PD:	-0.066
		Sc. rank:	208.6

NA			
Pos.	375	obs:	exp:
tct	S	0	0.17
tcc	S	0	0.15
tca	S	0	0.23
tcg	S	0	0.06
cgt	R	0	95.53
cgc	R	0	134.20
cga	R	0	243.90
cgg	R	2	197.30
aaa	K	9	10.10
aag	K	9	7.90
agt	S	1	0.20
agc	S	0	0.19
aga	R	9	1155.00
agg	R	2508	692.60
---	--	------	------
mPD		0.031	0.97
		nPD:	0.03
		N. weight:	1.3
		Sc. PD:	-0.095
		Sc. rank:	-131.3

NA			
Pos.	376	obs:	exp:
att	I	1	908.30
atc	I	1	613.40
ata	I	2444	924.30
gtt	V	0	20.01
gtc	V	10	19.44
gta	V	82	18.10
gtg	V	0	34.45
---	--	------	------
mPD		0.079	0.74
		nPD:	0.11
		N. weight:	1.
		Sc. PD:	0.0021
		Sc. rank:	1179.4

NA			
Pos.	377	obs:	exp:
cgt	R	0	0.46
cgc	R	0	0.64
cga	R	0	1.16
cgg	R	0	0.94
atg	M	1	1.00
aaa	K	361	1417.00
aag	K	2164	1108.00
aga	R	11	5.50
agg	R	1	3.30
---	--	------	------
mPD		0.26	0.51
		nPD:	0.52
		N. weight:	0.4
		Sc. PD:	0.16
		Sc. rank:	759.0

NA			
Pos.	378	obs:	exp:
tct	S	0	0.17
tcc	S	0	0.15
tca	S	0	0.23
tcg	S	0	0.06
aat	N	2468	1313.00
aac	N	2	1157.00
aaa	K	0	0.56
aag	K	1	0.44
agt	S	1	0.20
agc	S	0	0.19
gat	D	65	37.96
gac	D	1	28.04
---	--	------	------
mPD		0.055	0.55
		nPD:	0.1
		N. weight:	0.66
		Sc. PD:	-0.0041
		Sc. rank:	736.7

NA			
Pos.	379	obs:	exp:
ggt	G	2339	365.10
ggc	G	55	335.00
gga	G	0	1108.00
ggg	G	144	730.00
---	--	------	------
mPD		0.15	0.69
		nPD:	0.21
		N. weight:	1.7
		Sc. PD:	0.17
		Sc. rank:	2337.6

NA			
Pos.	380	obs:	exp:
tgg	W	2538	2538.00
---	--	------	------
mPD		0	1
		nPD:	1.
		N. weight:	0.
		Sc. PD:	0
		Sc. rank:	0

NA			
Pos.	381	obs:	exp:
cgt	R	0	0.04
cgc	R	0	0.05
cga	R	0	0.10
cgg	R	0	0.08
att	I	0	0.37
atc	I	0	0.25
ata	I	1	0.38
atg	M	17	17.00
act	T	2	663.50
acc	T	0	553.50
aca	T	23	1076.00
acg	T	2480	211.60
aaa	K	0	1.12
aag	K	2	0.88
aga	R	0	0.46
agg	R	1	0.27
gtt	V	1	1.96
gtc	V	0	1.90
gta	V	8	1.77
gtg	V	0	3.37
gct	A	0	0.76
gcc	A	0	0.59
gca	A	0	1.32
gcg	A	3	0.33
---	--	------	------
mPD		0.060	0.73
		nPD:	0.08
		N. weight:	2.5
		Sc. PD:	-0.056
		Sc. rank:	2440.6

NA			
Pos.	382	obs:	exp:
tta	L	0	0.44
ttg	L	0	0.75
ctt	L	0	0.75
ctc	L	0	0.69
cta	L	0	0.53
ctg	L	4	0.84
cat	H	2	1.19
cac	H	0	0.81
caa	Q	627	1287.00
cag	Q	1902	1242.00
cgt	R	0	0.11
cgc	R	0	0.16
cga	R	0	0.29
cgg	R	3	0.23
aga	R	0	1.38
agg	R	0	0.82
---	--	------	------
mPD		0.38	0.51
		nPD:	0.74
		N. weight:	0.16
		Sc. PD:	0.097
		Sc. rank:	321.3

NA			
Pos.	383	obs:	exp:
cct	P	0	0.28
ccc	P	0	0.18
cca	P	1	0.41
ccg	P	0	0.13
att	I	0	0.74
atc	I	0	0.50
ata	I	2	0.76
act	T	1	665.40
acc	T	1	555.00
aca	T	2509	1079.00
acg	T	1	212.20
aat	N	4	6.91
aac	N	9	6.09
aaa	K	4	2.25
aag	K	0	1.75
gct	A	0	1.53
gcc	A	0	1.17
gca	A	6	2.64
gcg	A	0	0.67
---	--	------	------
mPD		0.033	0.71
		nPD:	0.05
		N. weight:	0.88
		Sc. PD:	-0.05
		Sc. rank:	406.8

NA			
Pos.	384	obs:	exp:
tct	S	0	432.20
tcc	S	0	373.10
tca	S	0	570.50
tcg	S	0	163.50
aat	N	0	2.66
aac	N	5	2.34
agt	S	15	516.80
agc	S	2516	474.90
ggt	G	0	0.29
ggc	G	2	0.26
gga	G	0	0.87
ggg	G	0	0.58
---	--	------	------
mPD		0.017	1.6
		nPD:	0.01
		N. weight:	1.7
		Sc. PD:	-0.16
		Sc. rank:	-2343.3

NA

Pos.	385	obs:	exp:
caa	Q	1	0.51
cag	Q	0	0.49
cgt	R	0	0.42
cgc	R	0	0.59
cga	R	0	1.07
cgg	R	0	0.86
aaa	K	2526	1418.00
aag	K	0	1108.00
aga	R	11	5.05
agg	R	0	3.02
---	--	------	------
mPD		0.0094	0.50
		nPD:	0.02
		N. weight:	0.61
		Sc. PD:	-0.051
		Sc. rank:	-523.7

,

NA

Pos.	386	obs:	exp:
aaa	K	3	1.68
aag	K	0	1.32
gtt	V	0	1.30
gtc	V	0	1.27
gta	V	5	1.18
gtg	V	1	2.25
gaa	E	2507	1514.00
gag	E	22	1015.00
---	--	------	------
mPD		0.025	0.49
		nPD:	0.05
		N. weight:	0.49
		Sc. PD:	-0.026
		Sc. rank:	273.3

,

NA

Pos.	387	obs:	exp:
tta	L	0	0.11
ttg	L	0	0.19
ctt	L	0	0.19
ctc	L	0	0.17
cta	L	0	0.13
ctg	L	1	0.21
caa	Q	6	1245.00
cag	Q	2440	1201.00
cgt	R	0	3.30
cgc	R	0	4.63
cga	R	0	8.42
cgg	R	87	6.81
aaa	K	0	2.25
aag	K	4	1.75
aga	R	0	39.91
agg	R	0	23.92
---	--	------	------
mPD		0.075	0.62
		nPD:	0.12
		N. weight:	0.8
		Sc. PD:	0.011
		Sc. rank:	989.9

,

NA

Pos.	388	obs:	exp:
tta	L	0	0.22
ttg	L	0	0.38
ctt	L	0	0.38
ctc	L	2	0.35
cta	L	0	0.27
ctg	L	0	0.42
att	I	2153	936.10
atc	I	368	632.30
ata	I	0	952.60
atg	M	1	1.00
act	T	8	2.38
acc	T	1	1.99
aca	T	0	3.87
acg	T	0	0.76
gtt	V	4	1.09
gtc	V	0	1.06
gta	V	0	0.98
gtg	V	1	1.87
---	--	------	------
mPD		0.26	0.67
		nPD:	0.39
		N. weight:	0.67
		Sc. PD:	0.19
		Sc. rank:	1144.3

,

NA

Pos.	389	obs:	exp:
cgt	R	0	95.57
cgc	R	0	134.20
cga	R	140	244.00
cgg	R	1	197.40
att	I	0	0.37
atc	I	0	0.25
ata	I	1	0.38
aaa	K	12	8.42
aag	K	3	6.58
aga	R	2375	1156.00
agg	R	4	692.90
ggt	G	0	0.29
ggc	G	0	0.26
gga	G	2	0.87
ggg	G	0	0.58
---	--	------	------
mPD		0.13	0.96
		nPD:	0.13
		N. weight:	0.67
		Sc. PD:	0.016
		Sc. rank:	844.1

,

NA

Pos.	390	obs:	exp:
tct	S	0	0.17
tcc	S	0	0.15
tca	S	0	0.23
tcg	S	0	0.06
cgt	R	0	94.58
cgc	R	0	132.80
cga	R	0	241.50
cgg	R	0	195.30
aaa	K	8	24.14
aag	K	35	18.86
agt	S	0	0.20
agc	S	1	0.19
aga	R	587	1144.00
agg	R	1907	685.80
---	--	------	------
mPD		0.39	0.98
		nPD:	0.4
		N. weight:	0.65
		Sc. PD:	0.19
		Sc. rank:	1129.6

,

NA

Pos.	391	obs:	exp:
caa	Q	53	1291.00
cag	Q	2485	1247.00
---	--	------	------
mPD		0.041	0.50
		nPD:	0.08
		N. weight:	0.64
		Sc. PD:	-0.015
		Sc. rank:	612.2

,

NA

Pos.	392	obs:	exp:
att	I	0	0.74
atc	I	0	0.50
ata	I	2	0.76
atg	M	26	26.00
gtt	V	11	545.90
gtc	V	0	530.30
gta	V	7	493.90
gtg	V	2492	939.90
---	--	------	------
mPD		0.038	0.75
		nPD:	0.05
		N. weight:	0.98
		Sc. PD:	-0.053
		Sc. rank:	533.2

,

NA

Pos.	393	obs:	exp:
att	I	24	8.91
atc	I	0	6.02
ata	I	0	9.07
gtt	V	2503	546.70
gtc	V	0	531.20
gta	V	4	494.70
gtg	V	7	941.40
---	--	------	------
mPD		0.027	0.75
		nPD:	0.04
		N. weight:	1.6
		Sc. PD:	-0.1
		Sc. rank:	253.0

,

NA

Pos.	394	obs:	exp:
att	I	304	113.30
atc	I	1	76.49
ata	I	0	115.30
gtt	V	2225	485.60
gtc	V	8	471.80
gta	V	0	439.40
gtg	V	0	836.20
---	--	------	------
mPD		0.22	0.95
		nPD:	0.23
		N. weight:	1.5
		Sc. PD:	0.18
		Sc. rank:	2166.2

,

NA

Pos.	395	obs:	exp:
gat	D	2507	1456.00
gac	D	25	1076.00
ggt	G	6	0.86
ggc	G	0	0.79
gga	G	0	2.62
ggg	G	0	1.73
---	--	------	------
mPD		0.024	0.50
		nPD:	0.05
		N. weight:	0.53
		Sc. PD:	-0.029
		Sc. rank:	277.5

,

NA

Pos.	396	obs:	exp:
tct	S	0	0.17
tcc	S	0	0.15
tca	S	0	0.23
tcg	S	0	0.06
att	I	2	0.74
atc	I	0	0.50
ata	I	0	0.76
aat	N	1958	1341.00
aac	N	565	1182.00
aaa	K	2	1.12
aag	K	0	0.88
agt	S	1	0.20
agc	S	0	0.19
gat	D	5	5.75
gac	D	5	4.25
---	--	------	------
mPD		0.36	0.51
		nPD:	0.71
		N. weight:	0.14
		Sc. PD:	0.081
		Sc. rank:	278.2

,

NA

Pos.	397	obs:	exp:
tta	L	6	214.00
ttg	L	1902	368.00
tct	S	1	95.98
tcc	S	0	82.85
tca	S	2	126.70
tcg	S	559	36.30
ctt	L	0	367.70
ctc	L	0	338.30
cta	L	1	260.90
ctg	L	52	412.00
agt	S	0	114.70
agc	S	0	105.40
gtt	V	0	3.26
gtc	V	0	3.17
gta	V	0	2.95
gtg	V	15	5.62
---	--	------	------
mPD		0.41	1.7
		nPD:	0.24
		N. weight:	1.9
		Sc. PD:	0.25
		Sc. rank:	2745.5

,

NA

Pos.	398	obs:	exp:
tct	S	0	0.34
tcc	S	0	0.29
tca	S	0	0.45
tcg	S	0	0.13
tat	Y	1	0.49
tac	Y	0	0.51
cat	H	1	0.59
cac	H	0	0.41
aat	N	2516	1343.00
aac	N	12	1185.00
aaa	K	0	2.25
aag	K	4	1.75
agt	S	2	0.41
agc	S	0	0.38
gat	D	2	1.15
gac	D	0	0.85
---	--	------	------
mPD		0.017	0.51
		nPD:	0.03
		N. weight:	0.64
		Sc. PD:	-0.044
		Sc. rank:	17.9

,

NA

Pos.	399	obs:	exp:
tgg	W	2538	2538.00
---	--	------	------
mPD		0	0
		nPD:	1.
		N. weight:	0.
		Sc. PD:	0
		Sc. rank:	0

,

NA

Pos.	400	obs:	exp:
tct	S	1	433.30
tcc	S	0	374.00
tca	S	270	571.90
tcg	S	2266	163.90
tgg	W	1	1.00
agt	S	0	518.00
agc	S	0	476.00
---	--	------	------
mPD		0.19	1.6
		nPD:	0.12
		N. weight:	2.4
		Sc. PD:	0.025
		Sc. rank:	2927.7

,

NA

Pos.	401	obs:	exp:
ggt	G	0	365.10
ggc	G	0	335.00
gga	G	2496	1108.00
ggg	G	42	730.00
---	--	------	------
mPD		0.033	0.69
		nPD:	0.05
		N. weight:	0.79
		Sc. PD:	-0.045
		Sc. rank:	384.0

,

NA

Pos.	402	obs:	exp:
tct	S	0	0.17
tcc	S	1	0.15
tca	S	0	0.23
tcg	S	0	0.06
tat	Y	4	1233.00
tac	Y	2533	1304.00
agt	S	0	0.20
agc	S	0	0.19
---	--	------	------
mPD		0.0039	0.50
		nPD:	0.01
		N. weight:	0.69
		Sc. PD:	-0.065
		Sc. rank:	-1110.8

,

NA

Pos.	403	obs:	exp:
tct	S	0	433.30
tcc	S	0	374.00
tca	S	0	571.90
tcg	S	0	163.90
cgt	R	0	0.04
cgc	R	0	0.05
cga	R	0	0.10
cgg	R	0	0.08
agt	S	2524	518.00
agc	S	13	476.00
aga	R	0	0.46
agg	R	1	0.27
---	--	------	------
mPD		0.011	1.6
		nPD:	0.01
		N. weight:	1.6
		Sc. PD:	-0.16
		Sc. rank:	-2757.3

,

NA

Pos.	404	obs:	exp:
ggt	G	11	365.10
ggc	G	1	335.00
gga	G	5	1108.00
ggg	G	2521	730.00
---	--	------	------
mPD		0.013	0.69
		nPD:	0.02
		N. weight:	1.3
		Sc. PD:	-0.11
		Sc. rank:	-935.3

,

NA			
Pos.	405	obs:	exp:
ttt	F	1	0.43
ttc	F	0	0.57
tct	S	2522	433.30
tcc	S	11	374.00
tca	S	1	571.90
tcg	S	3	163.90
agt	S	0	518.00
agc	S	0	476.00
---	--	------	------
mPD		0.013	1.6
		nPD:	0.01
		N. weight:	1.8
		Sc. PD:	-0.17
		Sc. rank:	-2976.0

,

NA			
Pos.	406	obs:	exp:
ttt	F	4	1080.00
ttc	F	2534	1458.00
---	--	------	------
mPD		0.0031	0.49
		nPD:	0.01
		N. weight:	0.57
		Sc. PD:	-0.055
		Sc. rank:	-991.2

,

NA			
Pos.	407	obs:	exp:
att	I	1	0.37
atc	I	0	0.25
ata	I	0	0.38
act	T	2512	671.80
acc	T	13	560.30
aca	T	11	1090.00
acg	T	0	214.20
gct	A	1	0.25
gcc	A	0	0.20
gca	A	0	0.44
gcg	A	0	0.11
---	--	------	------
mPD		0.020	0.69
		nPD:	0.03
		N. weight:	1.3
		Sc. PD:	-0.099
		Sc. rank:	-243.8

,

NA			
Pos.	408	obs:	exp:
ttt	F	1	0.85
ttc	F	1	1.15
tta	L	2501	276.80
ttg	L	14	475.90
ctt	L	0	475.50
ctc	L	0	437.50
cta	L	16	337.40
ctg	L	5	532.80
---	--	------	------
mPD		0.033	1.1
		nPD:	0.03
		N. weight:	2.2
		Sc. PD:	-0.17
		Sc. rank:	-434.5

,

NA			
Pos.	409	obs:	exp:
tct	S	0	0.17
tcc	S	0	0.15
tca	S	1	0.23
tcg	S	0	0.06
cct	P	0	715.40
ccc	P	0	459.10
cca	P	2502	1036.00
ccg	P	35	326.70
agt	S	0	0.20
agc	S	0	0.19
---	--	------	------
mPD		0.028	0.71
		nPD:	0.04
		N. weight:	0.88
		Sc. PD:	-0.057
		Sc. rank:	188.1

,

NA			
Pos.	410	obs:	exp:
tta	L	4	0.44
ttg	L	0	0.75
ctt	L	0	0.75
ctc	L	0	0.69
cta	L	0	0.53
ctg	L	0	0.84
att	I	1	2.60
atc	I	0	1.76
ata	I	6	2.65
gtt	V	1	529.30
gtc	V	0	514.30
gta	V	2420	478.90
gtg	V	13	911.50
gct	A	0	23.66
gcc	A	0	18.14
gca	A	93	40.88
gcg	A	0	10.32
---	--	------	------
mPD		0.091	0.81
		nPD:	0.11
		N. weight:	1.6
		Sc. PD:	0.01
		Sc. rank:	1970.5

,

NA			
Pos.	411	obs:	exp:
aat	N	2	1.06
aac	N	0	0.94
aaa	K	8	4.49
aag	K	0	3.51
gaa	E	2503	1506.00
gag	E	13	1010.00
ggt	G	0	1.73
ggc	G	0	1.58
gga	G	11	5.24
ggg	G	1	3.45
---	--	------	------
mPD		0.030	0.50
		nPD:	0.06
		N. weight:	0.51
		Sc. PD:	-0.023
		Sc. rank:	361.9

,

NA			
Pos.	412	obs:	exp:
tta	L	12	277.00
ttg	L	2518	476.20
ctt	L	0	475.90
ctc	L	0	437.90
cta	L	4	337.70
ctg	L	4	533.30
---	--	------	------
mPD		0.019	1.1
		nPD:	0.02
		N. weight:	1.7
		Sc. PD:	-0.15
		Sc. rank:	-1635.6

,

NA			
Pos.	413	obs:	exp:
tct	S	2425	415.30
tcc	S	6	358.50
tca	S	1	548.20
tcg	S	0	157.10
act	T	95	28.08
acc	T	0	23.42
aca	T	11	45.55
acg	T	0	8.95
agt	S	0	496.60
agc	S	0	456.30
---	--	------	------
mPD		0.094	1.6
		nPD:	0.06
		N. weight:	1.8
		Sc. PD:	-0.085
		Sc. rank:	1183.8

,

NA			
Pos.	414	obs:	exp:
cgt	R	0	0.08
cgc	R	0	0.11
cga	R	0	0.19
cgg	R	0	0.16
aaa	K	10	6.17
aag	K	1	4.83
aga	R	0	0.92
agg	R	2	0.55
ggt	G	1	363.20
ggc	G	0	333.30
gga	G	6	1102.00
ggg	G	2518	726.20
---	--	------	------
mPD		0.032	0.71
		nPD:	0.05
		N. weight:	1.3
		Sc. PD:	-0.075
		Sc. rank:	578.2

,

NA			
Pos.	415	obs:	exp:
cgt	R	0	96.02
cgc	R	0	134.90
cga	R	0	245.20
cgg	R	0	198.30
aaa	K	4	2.25
aag	K	0	1.75
aga	R	16	1161.00
agg	R	2516	696.20
ggt	G	0	0.29
ggc	G	0	0.26
gga	G	0	0.87
ggg	G	2	0.58
---	--	------	------
mPD		0.020	0.96
		nPD:	0.02
		N. weight:	1.3
		Sc. PD:	-0.11
		Sc. rank:	-749.4

,

NA			
Pos.	416	obs:	exp:
cgt	R	0	0.11
cgc	R	0	0.16
cga	R	0	0.29
cgg	R	0	0.23
aat	N	6	3.19
aac	N	0	2.81
aaa	K	15	8.42
aag	K	0	6.58
aga	R	3	1.38
agg	R	0	0.82
gat	D	5	8.05
gac	D	9	5.95
gaa	E	2467	1477.00
gag	E	0	989.90
ggt	G	0	4.75
ggc	G	0	4.36
gga	G	32	14.41
ggg	G	1	9.49
---	--	------	------
mPD		0.063	0.54
		nPD:	0.12
		N. weight:	0.54
		Sc. PD:	0.006
		Sc. rank:	668.4

,

NA			
Pos.	417	obs:	exp:
tgt	C	2533	1103.00
tgc	C	4	1434.00
ggt	G	1	0.14
ggc	G	0	0.13
gga	G	0	0.44
ggg	G	0	0.29
---	--	------	------
mPD		0.0039	0.49
		nPD:	0.01
		N. weight:	0.86
		Sc. PD:	-0.082
		Sc. rank:	-1375.9

,

NA			
Pos.	418	obs:	exp:
tta	L	1907	277.00
ttg	L	611	476.20
ctt	L	0	475.90
ctc	L	0	437.90
cta	L	8	337.70
ctg	L	12	533.30
---	--	------	------
mPD		0.39	1.1
		nPD:	0.34
		N. weight:	1.6
		Sc. PD:	0.35
		Sc. rank:	2549.0

,

NA			
Pos.	419	obs:	exp:
tta	L	0	0.11
ttg	L	0	0.19
ctt	L	0	0.19
ctc	L	1	0.17
cta	L	0	0.13
ctg	L	0	0.21
att	I	0	0.74
atc	I	2	0.50
ata	I	0	0.76
gtt	V	614	551.30
gtc	V	1919	535.60
gta	V	1	498.80
gtg	V	1	949.30
---	--	------	------
mPD		0.37	0.73
		nPD:	0.51
		N. weight:	1.
		Sc. PD:	0.4
		Sc. rank:	1965.1

,

NA			
Pos.	420	obs:	exp:
tct	S	0	0.17
tcc	S	1	0.15
tca	S	0	0.23
tcg	S	0	0.06
cct	P	11	715.40
ccc	P	2498	459.10
cca	P	28	1036.00
ccg	P	0	326.70
agt	S	0	0.20
agc	S	0	0.19
---	--	------	------
mPD		0.031	0.71
		nPD:	0.04
		N. weight:	1.7
		Sc. PD:	-0.1
		Sc. rank:	685.9

,

NA			
Pos.	421	obs:	exp:
tgt	C	2492	1104.00
tgc	C	46	1434.00
---	--	------	------
mPD		0.036	0.49
		nPD:	0.07
		N. weight:	0.78
		Sc. PD:	-0.025
		Sc. rank:	668.5

,

NA			
Pos.	422	obs:	exp:
ttt	F	2534	1080.00
ttc	F	4	1458.00
---	--	------	------
mPD		0.0031	0.49
		nPD:	0.01
		N. weight:	0.89
		Sc. PD:	-0.085
		Sc. rank:	-1536.8

,

NA			
Pos.	423	obs:	exp:
tgg	W	2538	2538.00
---	--	------	------
mPD		0	0
		nPD:	1.
		N. weight:	0.
		Sc. PD:	0
		Sc. rank:	0

,

NA			
Pos.	424	obs:	exp:
tta	L	0	0.11
ttg	L	1	0.19
ctt	L	0	0.19
ctc	L	0	0.17
cta	L	0	0.13
ctg	L	0	0.21
att	I	0	2.97
atc	I	8	2.01
ata	I	0	3.02
gtt	V	33	550.00
gtc	V	2495	534.40
gta	V	1	497.60
gtg	V	0	947.00
---	--	------	------
mPD		0.034	0.74
		nPD:	0.05
		N. weight:	1.6
		Sc. PD:	-0.089
		Sc. rank:	745.5

,

NA			
Pos.	425	obs:	exp:
gaa	E	2524	1520.00
gag	E	14	1018.00
---	--	------	------
mPD		0.011	0.48
		nPD:	0.02
		N. weight:	0.51
		Sc. PD:	-0.041
		Sc. rank:	-244.5

,

NA			
Pos.	426	obs:	exp:
tta	L	0	0.44
ttg	L	0	0.75
ctt	L	0	0.75
ctc	L	0	0.69
cta	L	0	0.53
ctg	L	4	0.84
atg	M	2534	2534.00
---	--	------	------
mPD		0.0031	0.0050
		nPD:	0.62
		N. weight:	0.0026
		Sc. PD:	0.0013
		Sc. rank:	5.0

,

NA			
Pos.	427	obs:	exp:
att	I	0	941.00
atc	I	2518	635.50
ata	I	16	957.50
gtt	V	0	0.87
gtc	V	4	0.85
gta	V	0	0.79
gtg	V	0	1.50
---	--	------	------
mPD		0.016	0.66
		nPD:	0.02
		N. weight:	1.4
		Sc. PD:	-0.11
		Sc. rank:	-593.8

,

NA			
Pos.	428	obs:	exp:
cgt	R	0	96.25
cgc	R	0	135.20
cga	R	108	245.80
cgg	R	0	198.80
aga	R	2414	1164.00
agg	R	16	697.90
---	--	------	------
mPD		0.094	0.95
		nPD:	0.1
		N. weight:	0.67
		Sc. PD:	-0.0046
		Sc. rank:	749.1

,

NA			
Pos.	429	obs:	exp:
ggt	G	560	365.10
ggc	G	1971	335.00
gga	G	4	1108.00
ggg	G	3	730.00
---	--	------	------
mPD		0.35	0.69
		nPD:	0.51
		N. weight:	1.5
		Sc. PD:	0.59
		Sc. rank:	2880.3

,

NA			
Pos.	430	obs:	exp:
cgt	R	0	95.53
cgc	R	0	134.20
cga	R	0	243.90
cgg	R	0	197.30
aaa	K	1	10.66
aag	K	18	8.34
aga	R	4	1155.00
agg	R	2515	692.60
---	--	------	------
mPD		0.019	0.97
		nPD:	0.02
		N. weight:	1.3
		Sc. PD:	-0.11
		Sc. rank:	-949.8

,

NA			
Pos.	431	obs:	exp:
cct	P	10	715.70
ccc	P	0	459.20
cca	P	2517	1036.00
ccg	P	11	326.80
---	--	------	------
mPD		0.016	0.70
		nPD:	0.02
		N. weight:	0.89
		Sc. PD:	-0.071
		Sc. rank:	-414.2

,

NA			
Pos.	432	obs:	exp:
gat	D	1	0.58
gac	D	0	0.42
gaa	E	2535	1519.00
gag	E	2	1018.00
---	--	------	------
mPD		0.0024	0.48
		nPD:	0.
		N. weight:	0.53
		Sc. PD:	-0.052
		Sc. rank:	-982.6

,

NA			
Pos.	433	obs:	exp:
gaa	E	1935	1519.00
gag	E	602	1018.00
ggt	G	0	0.14
ggc	G	0	0.13
gga	G	1	0.44
ggg	G	0	0.29
---	--	------	------
mPD		0.36	0.48
		nPD:	0.75
		N. weight:	0.065
		Sc. PD:	0.041
		Sc. rank:	133.3

,

NA			
Pos.	434	obs:	exp:
cgt	R	0	95.30
cgc	R	0	133.90
cga	R	0	243.30
cgg	R	0	196.80
att	I	0	0.74
atc	I	0	0.50
ata	I	2	0.76
aat	N	3	2.13
aac	N	1	1.87
aaa	K	8	7.86
aag	K	6	6.14
aga	R	2508	1153.00
agg	R	5	691.00
ggt	G	0	0.72
ggc	G	0	0.66
gga	G	5	2.18
ggg	G	0	1.44
---	--	------	------
mPD		0.031	0.97
		nPD:	0.03
		N. weight:	0.8
		Sc. PD:	-0.057
		Sc. rank:	-23.4

,

NA			
Pos.	435	obs:	exp:
cct	P	0	0.28
ccc	P	0	0.18
cca	P	1	0.41
ccg	P	0	0.13
att	I	0	1.11
atc	I	0	0.75
ata	I	3	1.13
act	T	6	666.50
acc	T	0	555.90
aca	T	2501	1081.00
acg	T	9	212.50
gct	A	0	4.58
gcc	A	0	3.51
gca	A	18	7.91
gcg	A	0	2.00
---	--	------	------
mPD		0.029	0.71
		nPD:	0.04
		N. weight:	0.85
		Sc. PD:	-0.054
		Sc. rank:	192.8

,

NA			
Pos.	436	obs:	exp:
tct	S	0	0.34
tcc	S	0	0.29
tca	S	0	0.45
tcg	S	0	0.13
agt	S	0	0.41
agc	S	2	0.38
---	-	2536	2536.00
---	--	------	------
mPD		0	1.0×10^{-6}
		nPD:	0.
		N. weight:	0.
		Sc. PD:	0
		Sc. rank:	0

,

NA			
Pos.	437	obs:	exp:
att	I	4	940.20
atc	I	2515	635.00
ata	I	13	956.80
act	T	0	0.79
acc	T	3	0.66
aca	T	0	1.29
acg	T	0	0.25
gtt	V	0	0.65
gtc	V	3	0.63
gta	V	0	0.59
gtg	V	0	1.12
---	--	------	------
mPD		0.018	0.66
		nPD:	0.03
		N. weight:	1.4
		Sc. PD:	-0.11
		Sc. rank:	-349.1

,

NA			
Pos.	438	obs:	exp:
tgg	W	2538	2538.00
---	--	------	------
mPD		0	0
		nPD:	1.
		N. weight:	0.
		Sc. PD:	0
		Sc. rank:	0

,

NA			
Pos.	439	obs:	exp:
act	T	11	671.80
acc	T	2524	560.30
aca	T	1	1090.00
acg	T	0	214.20
gct	A	0	0.51
gcc	A	2	0.39
gca	A	0	0.88
gcg	A	0	0.22
---	--	------	------
mPD		0.011	0.69
		nPD:	0.02
		N. weight:	1.6
		Sc. PD:	-0.14
		Sc. rank:	-1585.2

,

NA			
Pos.	440	obs:	exp:
ttt	F	4	1.70
ttc	F	0	2.30
tct	S	2513	432.80
tcc	S	8	373.60
tca	S	13	571.20
tcg	S	0	163.70
agt	S	0	517.40
agc	S	0	475.40
---	--	------	------
mPD		0.020	1.6
		nPD:	0.01
		N. weight:	1.8
		Sc. PD:	-0.16
		Sc. rank:	-2271.0

,

NA			
Pos.	441	obs:	exp:
tct	S	0	433.40
tcc	S	0	374.10
tca	S	0	572.10
tcg	S	0	163.90
agt	S	2524	518.20
agc	S	14	476.20
---	--	------	------
mPD		0.011	1.6
		nPD:	0.01
		N. weight:	1.6
		Sc. PD:	-0.16
		Sc. rank:	-2777.7

,

NA			
Pos.	442	obs:	exp:
tct	S	0	433.30
tcc	S	0	374.00
tca	S	0	571.90
tcg	S	0	163.90
act	T	0	0.26
acc	T	1	0.22
aca	T	0	0.43
acg	T	0	0.08
agt	S	11	518.00
agc	S	2526	476.00
---	--	------	------
mPD		0.0094	1.6
		nPD:	0.01
		N. weight:	1.7
		Sc. PD:	-0.17
		Sc. rank:	-3118.8

,

NA			
Pos.	443	obs:	exp:
tct	S	1	433.40
tcc	S	2537	374.10
tca	S	0	572.10
tcg	S	0	163.90
agt	S	0	518.20
agc	S	0	476.20
---	--	------	------
mPD		0.00079	1.6
		nPD:	0.
		N. weight:	2.
		Sc. PD:	-0.2
		Sc. rank:	-4127.3

,

NA			
Pos.	444	obs:	exp:
tta	L	0	0.33
ttg	L	0	0.56
ctt	L	3	0.56
ctc	L	0	0.52
cta	L	0	0.40
ctg	L	0	0.63
att	I	2524	938.40
atc	I	3	633.80
ata	I	0	954.90
act	T	2	0.53
acc	T	0	0.44
aca	T	0	0.86
acg	T	0	0.17
gtt	V	6	1.30
gtc	V	0	1.27
gta	V	0	1.18
gtg	V	0	2.25
---	--	------	------
mPD		0.011	0.67
		nPD:	0.02
		N. weight:	1.
		Sc. PD:	-0.089
		Sc. rank:	-1007.1

,

NA			
Pos.	445	obs:	exp:
att	I	0	0.37
atc	I	0	0.25
ata	I	1	0.38
gtt	V	0	549.60
gtc	V	0	533.90
gta	V	2506	497.20
gtg	V	21	946.30
gct	A	0	2.54
gcc	A	0	1.95
gca	A	9	4.40
gcg	A	1	1.11
---	--	------	------
mPD		0.026	0.74
		nPD:	0.03
		N. weight:	1.7
		Sc. PD:	-0.11
		Sc. rank:	134.6

,

NA			
Pos.	446	obs:	exp:
ttt	F	2	0.85
ttc	F	0	1.15
cgt	R	0	0.04
cgc	R	0	0.05
cga	R	0	0.10
cgg	R	0	0.08
atg	M	2535	2535.00
aga	R	0	0.46
agg	R	1	0.27
---	--	------	------
mPD		0.0039	0.0047
		nPD:	0.85
		N. weight:	0.0012
		Sc. PD:	0.00089
		Sc. rank:	2.6

,

NA			
Pos.	447	obs:	exp:
tgt	C	2536	1103.00
tgc	C	1	1434.00
ggt	G	1	0.14
ggc	G	0	0.13
gga	G	0	0.44
ggg	G	0	0.29
---	--	------	------
mPD		0.0016	0.49
		nPD:	0.
		N. weight:	0.87
		Sc. PD:	-0.087
		Sc. rank:	-1703.3

,

NA			
Pos.	448	obs:	exp:
ggt	G	3	365.10
ggc	G	0	335.00
gga	G	2532	1108.00
ggg	G	3	730.00
---	--	------	------
mPD		0.0047	0.69
		nPD:	0.01
		N. weight:	0.86
		Sc. PD:	-0.082
		Sc. rank:	-1427.1

,

NA			
Pos.	449	obs:	exp:
ttt	F	2	0.85
ttc	F	0	1.15
gtt	V	2525	551.50
gtc	V	0	535.80
gta	V	11	499.00
gtg	V	0	949.70
---	--	------	------
mPD		0.010	0.73
		nPD:	0.01
		N. weight:	1.6
		Sc. PD:	-0.14
		Sc. rank:	-1727.2

,

NA			
Pos.	450	obs:	exp:
tct	S	2	0.34
tcc	S	0	0.29
tca	S	0	0.45
tcg	S	0	0.13
aat	N	5	2.66
aac	N	0	2.34
agt	S	0	0.41
agc	S	0	0.38
gat	D	2511	1452.00
gac	D	13	1072.00
gaa	E	0	0.60
gag	E	1	0.40
ggt	G	6	0.86
ggc	G	0	0.79
gga	G	0	2.62
ggg	G	0	1.73
---	--	------	------
mPD		0.023	0.50
		nPD:	0.05
		N. weight:	0.55
		Sc. PD:	-0.032
		Sc. rank:	243.3

,

NA			
Pos.	451	obs:	exp:
tct	S	0	0.34
tcc	S	0	0.29
tca	S	0	0.45
tcg	S	0	0.13
tat	Y	597	290.70
tac	Y	1	307.30
cat	H	1934	1151.00
cac	H	1	784.50
cgt	R	2	0.08
cgc	R	0	0.11
cga	R	0	0.19
cgg	R	0	0.16
agt	S	2	0.41
agc	S	0	0.38
aga	R	0	0.92
agg	R	0	0.55
gat	D	1	0.58
gac	D	0	0.42
---	--	------	------
mPD		0.37	0.86
		nPD:	0.43
		N. weight:	0.59
		Sc. PD:	0.19
		Sc. rank:	1039.2

,

NA			
Pos.	452	obs:	exp:
aaa	K	1	0.56
aag	K	0	0.44
gaa	E	2507	1518.00
gag	E	29	1018.00
ggt	G	0	0.14
ggc	G	0	0.13
gga	G	0	0.44
ggg	G	1	0.29
---	--	------	------
mPD		0.025	0.48
		nPD:	0.05
		N. weight:	0.48
		Sc. PD:	-0.025
		Sc. rank:	267.8

,

NA			
Pos.	453	obs:	exp:
att	I	2450	909.80
atc	I	0	614.40
ata	I	0	925.80
atg	M	2	2.00
gtt	V	84	18.70
gtc	V	2	18.17
gta	V	0	16.92
gtg	V	0	32.20
---	--	------	------
mPD		0.069	0.73
		nPD:	0.09
		N. weight:	1.1
		Sc. PD:	-0.012
		Sc. rank:	1126.5

,

NA			
Pos.	454	obs:	exp:
cct	P	0	1.13
ccc	P	2	0.72
cca	P	2	1.63
ccg	P	0	0.52
gct	A	2	644.60
gcc	A	2531	494.10
gca	A	0	1114.00
gcg	A	1	281.30
---	--	------	------
mPD		0.0071	0.69
		nPD:	0.01
		N. weight:	1.7
		Sc. PD:	-0.16
		Sc. rank:	-2352.9

,

NA			
Pos.	455	obs:	exp:
aat	N	4	2.13
aac	N	0	1.87
gat	D	2405	1456.00
gac	D	127	1076.00
ggt	G	0	0.29
ggc	G	0	0.26
gga	G	2	0.87
ggg	G	0	0.58
---	--	------	------
mPD		0.10	0.49
		nPD:	0.21
		N. weight:	0.39
		Sc. PD:	0.038
		Sc. rank:	542.6

,

NA			
Pos.	456	obs:	exp:
tgt	C	0	0.43
tgc	C	1	0.57
tgg	W	2537	2537.00
---	--	------	------
mPD		0.00079	0.00079
		nPD:	1.
		N. weight:	0.00024
		Sc. PD:	0.0002
		Sc. rank:	0.5

,

NA			
Pos.	457	obs:	exp:
tct	S	5	433.40
tcc	S	2	374.10
tca	S	2519	572.10
tcg	S	12	163.90
agt	S	0	518.20
agc	S	0	476.20
---	--	------	------
mPD		0.015	1.6
		nPD:	0.01
		N. weight:	1.5
		Sc. PD:	-0.14
		Sc. rank:	-2313.7

,

NA			
Pos.	458	obs:	exp:
tgg	W	2538	2538.00
---	--	------	------
mPD		0	0
		nPD:	1.
		N. weight:	0.
		Sc. PD:	0
		Sc. rank:	0

,

NA			
Pos.	459	obs:	exp:
cat	H	1	1507.00
cac	H	2534	1028.00
cgt	R	0	0.04
cgc	R	0	0.05
cga	R	0	0.10
cgg	R	1	0.08
aga	R	0	0.46
agg	R	0	0.27
gat	D	2	1.15
gac	D	0	0.85
---	--	------	------
mPD		0.0055	0.49
		nPD:	0.01
		N. weight:	0.95
		Sc. PD:	-0.087
		Sc. rank:	-1255.8

,

NA			
Pos.	460	obs:	exp:
gat	D	2538	1460.00
gac	D	0	1078.00
---	--	------	------
mPD		0	0.49
		nPD:	0.
		N. weight:	0.58
		Sc. PD:	-0.06
		Sc. rank:	-1214.9

,

NA			
Pos.	461	obs:	exp:
ggt	G	0	365.10
ggc	G	0	335.00
gga	G	2538	1108.00
ggg	G	0	730.00
---	--	------	------
mPD		0	0.69
		nPD:	0.
		N. weight:	0.87
		Sc. PD:	-0.089
		Sc. rank:	-1820.9

,

NA			
Pos.	462	obs:	exp:
tct	S	1	0.17
tcc	S	0	0.15
tca	S	0	0.23
tcg	S	0	0.06
agt	S	0	0.20
agc	S	0	0.19
gtt	V	1	0.22
gtc	V	0	0.21
gta	V	0	0.20
gtg	V	0	0.37
gct	A	2514	645.10
gcc	A	20	494.50
gca	A	2	1115.00
gcg	A	0	281.50
---	--	------	------
mPD		0.019	0.69
		nPD:	0.03
		N. weight:	1.4
		Sc. PD:	-0.11
		Sc. rank:	-400.8

,

NA

Pos.	463	obs:	exp:
tct	S	0	0.17
tcc	S	0	0.15
tca	S	0	0.23
tcg	S	0	0.06
att	I	2495	928.30
atc	I	5	627.00
ata	I	0	944.70
act	T	1	0.26
acc	T	0	0.22
aca	T	0	0.43
acg	T	0	0.08
agt	S	1	0.20
agc	S	0	0.19
gtt	V	36	7.83
gtc	V	0	7.61
gta	V	0	7.08
gtg	V	0	13.48
---	--	------	------
mPD		0.033	0.69
		nPD:	0.05
		N. weight:	1.
		Sc. PD:	-0.057
		Sc. rank:	536.9

,

NA

Pos.	464	obs:	exp:
ttt	F	1	0.43
ttc	F	0	0.57
tta	L	0	276.50
ttg	L	0	475.30
ctt	L	2530	475.00
ctc	L	1	437.00
cta	L	2	337.00
ctg	L	0	532.20
cct	P	0	0.28
ccc	P	1	0.18
cca	P	0	0.41
ccg	P	0	0.13
att	I	2	0.74
atc	I	0	0.50
ata	I	0	0.76
gtt	V	1	0.22
gtc	V	0	0.21
gta	V	0	0.20
gtg	V	0	0.37
---	--	------	------
mPD		0.0071	1.1
		nPD:	0.01
		N. weight:	1.7
		Sc. PD:	-0.17
		Sc. rank:	-3076.0

,

NA

Pos.	465	obs:	exp:
tct	S	0	0.17
tcc	S	1	0.15
tca	S	0	0.23
tcg	S	0	0.06
cct	P	16	715.40
ccc	P	2519	459.10
cca	P	2	1036.00
ccg	P	0	326.70
agt	S	0	0.20
agc	S	0	0.19
---	--	------	------
mPD		0.015	0.71
		nPD:	0.02
		N. weight:	1.7
		Sc. PD:	-0.14
		Sc. rank:	-1063.5

,

NA

Pos.	466	obs:	exp:
ttt	F	2522	1074.00
ttc	F	2	1450.00
tta	L	0	1.20
ttg	L	6	2.06
tat	Y	1	0.49
tac	Y	0	0.51
tgt	C	1	0.43
tgc	C	0	0.57
ctt	L	5	2.06
ctc	L	0	1.90
cta	L	0	1.46
ctg	L	0	2.31
att	I	1	0.37
atc	I	0	0.25
ata	I	0	0.38
---	--	------	------
mPD		0.013	0.50
		nPD:	0.03
		N. weight:	0.89
		Sc. PD:	-0.07
		Sc. rank:	-331.7

,

NA

Pos.	467	obs:	exp:
tat	Y	0	0.97
tac	Y	2	1.03
aat	N	0	19.13
aac	N	36	16.87
gat	D	1	1438.00
gac	D	2499	1062.00
---	--	------	------
mPD		0.030	0.52
		nPD:	0.06
		N. weight:	0.9
		Sc. PD:	-0.041
		Sc. rank:	618.3

,

NA

Pos.	468	obs:	exp:
att	I	0	942.10
atc	I	2536	636.30
ata	I	1	958.70
act	T	0	0.26
acc	T	1	0.22
aca	T	0	0.43
acg	T	0	0.08
---	--	------	------
mPD		0.0016	0.66
		nPD:	0.
		N. weight:	1.5
		Sc. PD:	-0.15
		Sc. rank:	-2871.5

,

NA

Pos.	469	obs:	exp:
tta	L	4	0.44
ttg	L	0	0.75
ctt	L	0	0.75
ctc	L	0	0.69
cta	L	0	0.53
ctg	L	0	0.84
cat	H	1	0.59
cac	H	0	0.41
aaa	K	1	0.56
aag	K	0	0.44
gat	D	2523	1455.00
gac	D	6	1074.00
gaa	E	3	1.80
gag	E	0	1.20
---	--	------	------
mPD		0.019	0.50
		nPD:	0.04
		N. weight:	0.57
		Sc. PD:	-0.037
		Sc. rank:	102.3

,

NA

Pos.	470	obs:	exp:
tct	S	0	0.68
tcc	S	0	0.59
tca	S	0	0.90
tcg	S	4	0.26
cgt	R	0	0.23
cgc	R	0	0.32
cga	R	0	0.58
cgg	R	0	0.47
aaa	K	1	945.80
aag	K	1684	739.20
agt	S	0	0.82
agc	S	0	0.75
aga	R	0	2.75
agg	R	6	1.65
gct	A	0	0.25
gcc	A	0	0.20
gca	A	0	0.44
gcg	A	1	0.11
gaa	E	1	6.59
gag	E	10	4.41
ggt	G	0	119.50
ggc	G	0	109.70
gga	G	0	362.80
ggg	G	831	239.00
---	--	------	------
mPD		0.89	1.5
		nPD:	0.61
		N. weight:	1.
		Sc. PD:	0.49
		Sc. rank:	1970.1

,

NA

Pos.	471	obs:	exp:
atg	M	2494	2494.00
act	T	0	11.65
acc	T	0	9.72
aca	T	0	18.91
acg	T	44	3.72
---	--	------	------
mPD		0.034	0.065
		nPD:	0.52
		N. weight:	0.045
		Sc. PD:	0.018
		Sc. rank:	86.1

,

NA

Pos.	472	obs:	exp:
taa	.	2457	930.70
tag	.	13	712.30
tga	.	68	895.00
---	--	------	------
mPD		0.062	0.86
		nPD:	0.07
		N. weight:	0.9
		Sc. PD:	-0.029
		Sc. rank:	778.6

}

Amino acid level conservation per codon

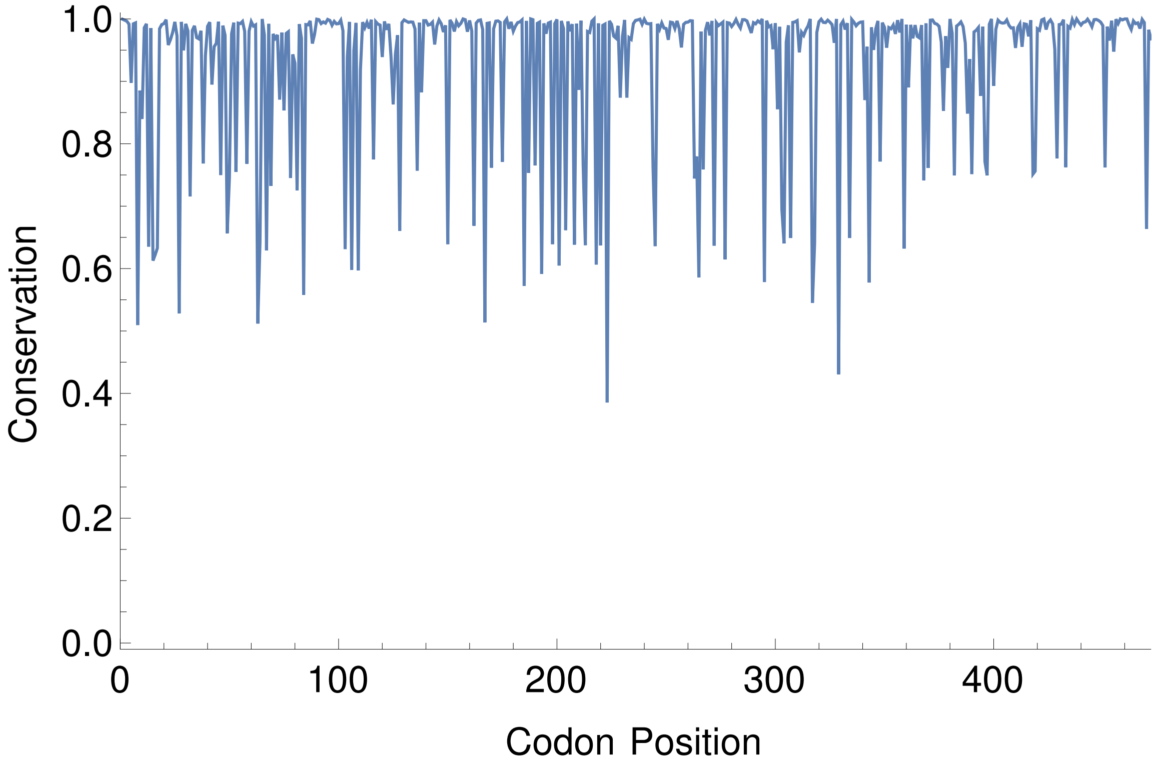

Conservation analysis plots

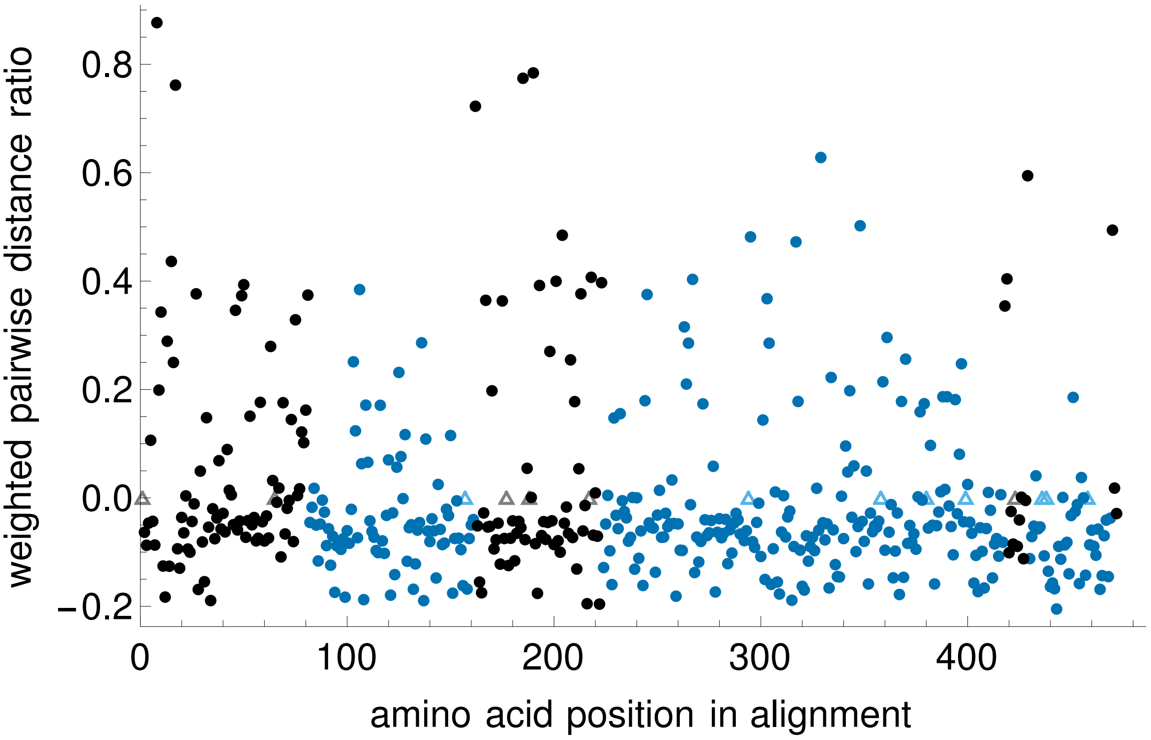

NA

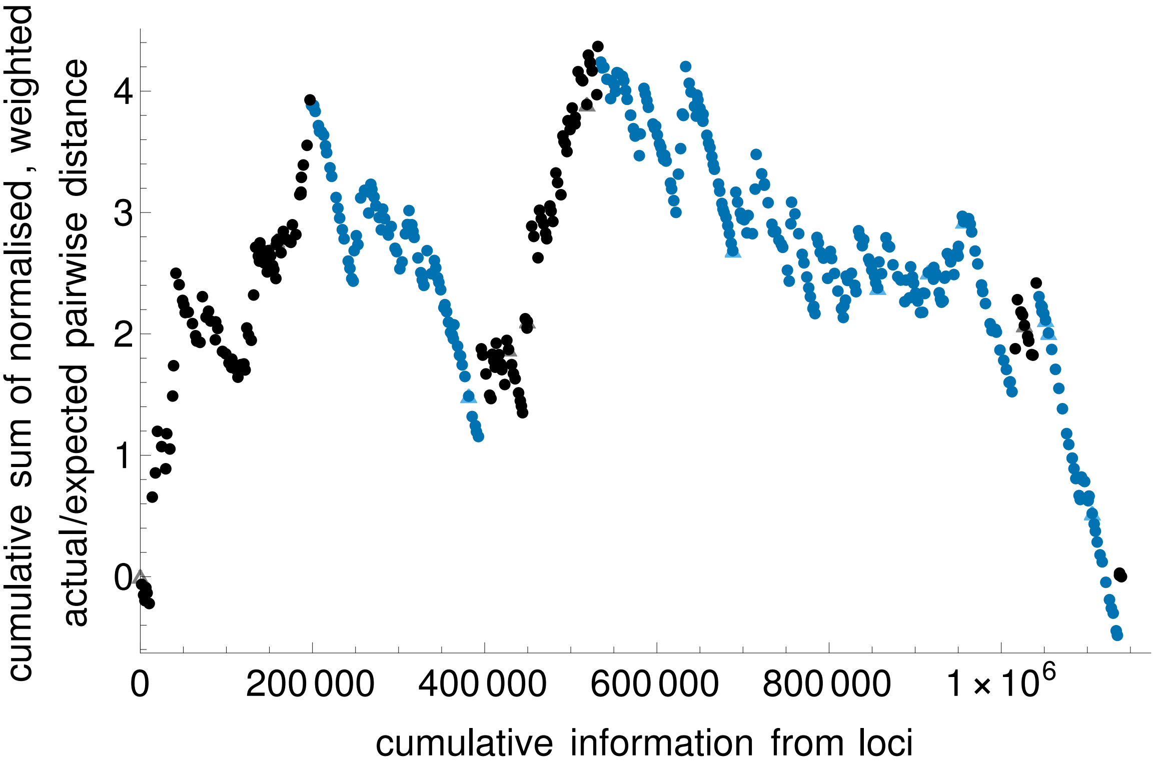

NA

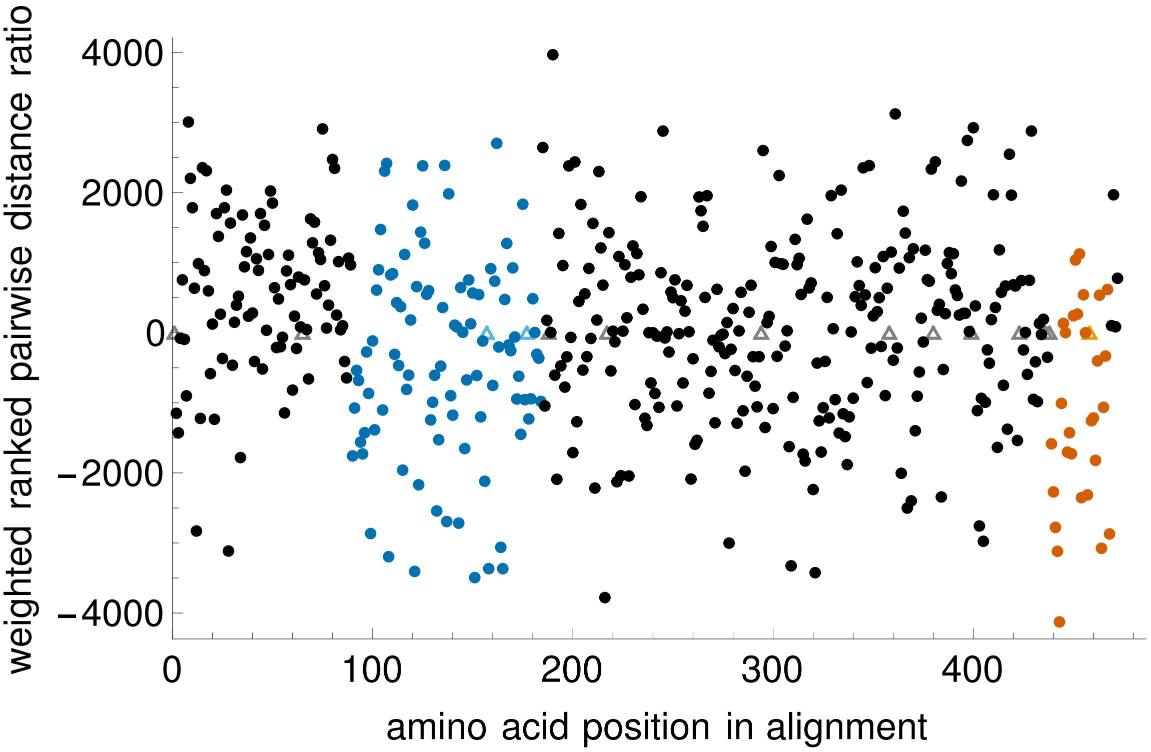
weighted ranked pairwise distance ratio
4000
2000
0
−2000
−4000
0
100
200
300
400
amino acid position in alignment

NA

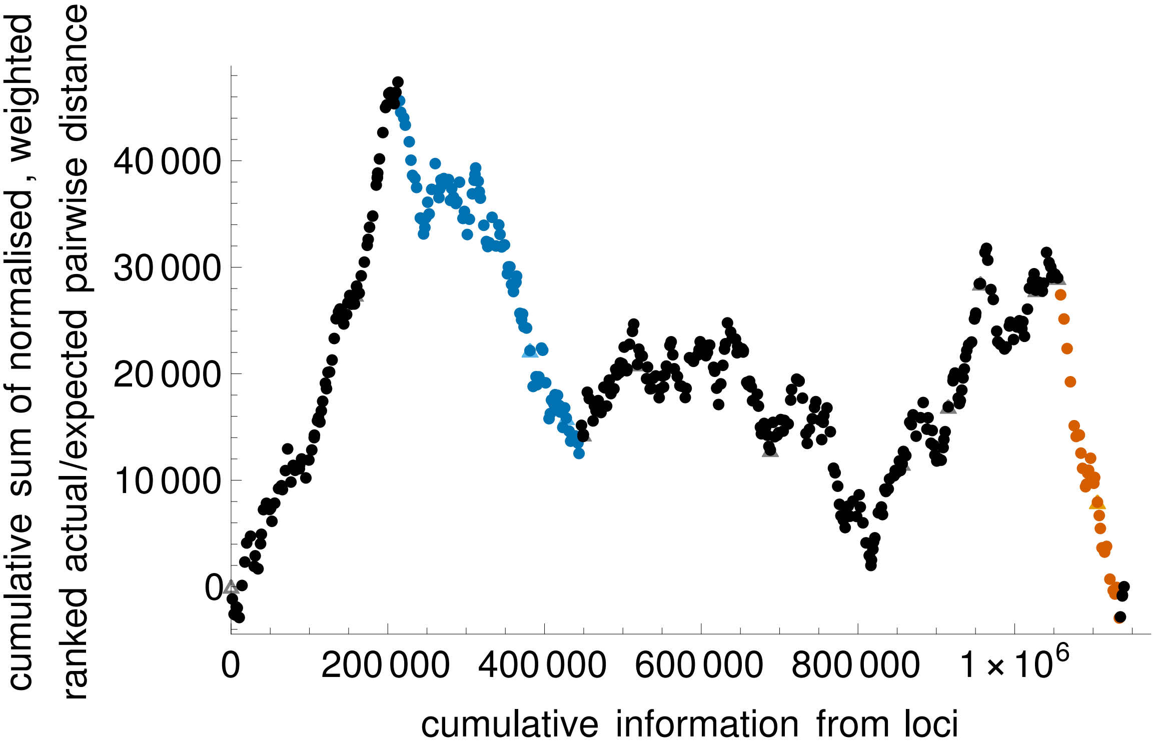
cumulative sum of normalised, weighted ranked actual/expected pairwise distance
40 000
30 000
20 000
10 000
0
0
200 000
400 000
600 000
800 000
1 × 10^6
cumulative information from loci

NA

M1

Gene length histogram

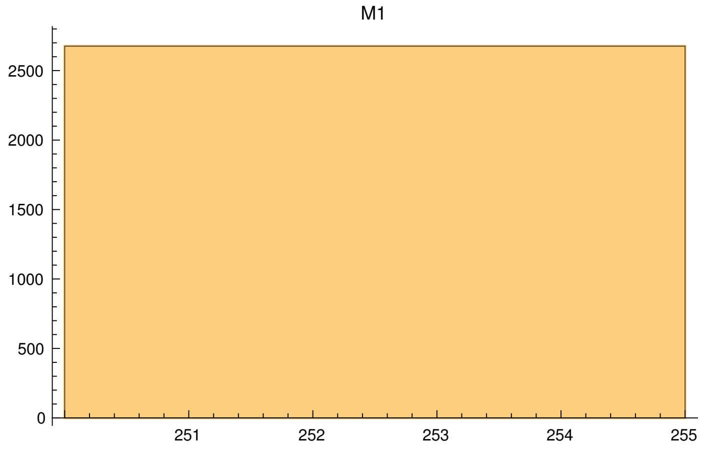

Information vs. nPD

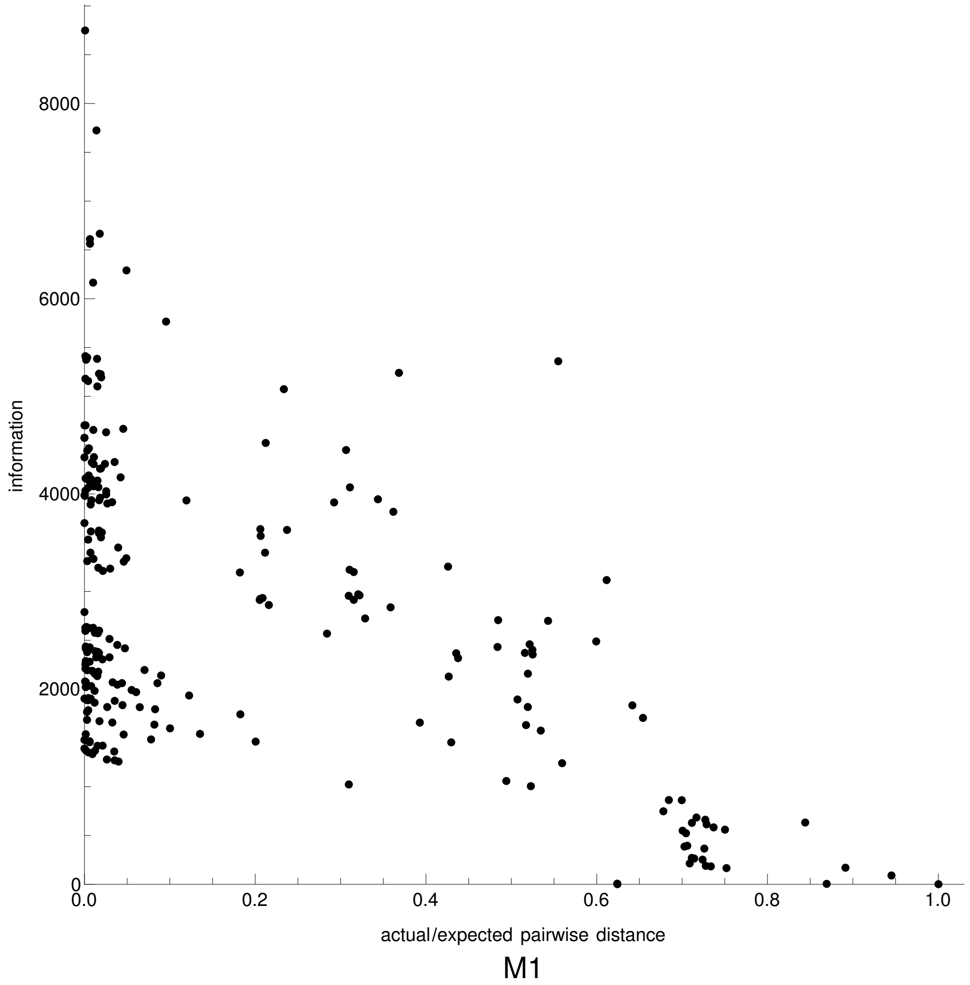

Example sequences highlighted by regions found in analyses to be conserved

Interesting points (by weighted raw PD) highlighted for gene M1:

ATG
AGTCTTCTAACCGAGGTCGAAACGTACGTTCTCTCTATCGTCCCGTCAGGCCCCC
TCAAAGCCGAGATCGCGCAGAGACTTGAAGATGTCTTTGTAGGGAAGAACAC
CGATCTTGAGGCTCTCATGGAATGGCTAAAGACAAGACCAATCCTGTCACCT
CTGACTAAGGGGATTTTAGGGTTTGTGTTCACGCTCACCGTGCCCAGTGAGC
GAGGACTGCAGCGTAGACGCTTTGTCCAAAATGCTCTAAATGGAAATGGAGACCCAAACAACAT⋱
GGACAGGGCAGTCAAACTGTACAGGAAATTAAAGAGAGAGATAACATTCCATGGGGCTAAAGAAGTTGCA⋱
CTCAGTTATTCAACCGGTGCACTTGCCAGTTGTATGGGTCTCATATACAACAGGATGGGGACGGTGACCA⋱
CAGAAGTGGCGTTGGGCCTAGTGTGTGCCACCTGTGAGCAGATTGCTGATTCACAGCATCGGTCTCACAG⋱
ACAGATAGCAACCACCACCAACCCACTAATCAGACATGAAAACAGAATGGTGCTGGCCAGTACTACAGCT⋱
AAGGCTATGGAGCAGATGGCTGGGTCGAGTGAGCAGGCAGCGGAAGCCATGGAGGTTGCTAGTCAGGCTA⋱
GGCAGATGGTGCAGGCGATGAGGACCATTGGAACTCATCCTAGCTCCAGTGCCGGTCTGAGAGATGATCT⋱
CCTTGAAAATTTGCAGGCCTACCAGAAACGGATGGGAGTGCAAATGCAGCGATTCAAGTGA

Interesting points (by weighted ranked PD) highlighted for gene M1:

ATGAGTCTTCTAACCGAGGTCGAAACGTACGTTCTCTCTATCGTCCCG
TCAGGCCCCCTCAAAGCCGAGATCGCGCAGAGACTTGAAGATGTCTTTGTAGGGA
AGAACACCGATCTTGAGGCTCTCATGGAATGGCTAAAGACAAGACCAATCCT
GTCACCTCTGACTAAGGGGATTTTAGGGTTTGTGTTCACGCTCACCGTGCCC
AGTGAGCGAGGACTGCAGCGTAGACGCTTTGTCCAAAATGCTCTAAATGGAAATGGAGACCC⋱
AAACAACATGGACAGGGCAGTCAAACTGTACAGGAAATTAAAGAGAGAGATAACATTCCATGGGGCTAAA⋱
GAAGTTGCACTCAGTTATTCAACCGGTGCACTTGCCAGTTGTATGGGTCTCATATACAACAGGATGGGGA⋱
CGGTGACCACAGAAGTGGCGTTGGGCCTAGTGTGTGCCACCTGTGAGCAGATTGCTGATTCACAGCATCG⋱
GTCTCACAGACAGATAGCAACCACCACCAACCCACTAATCAGACATGAAAACAGAATGGTGCTGGCCAGT⋱
ACTACAGCTAAGGCTATGGAGCAGATGGCTGGGTCGAGTGAGCAGGCAGCGGAAGCCATGGAGGTTGCTA⋱
GTCAGGCTAGGCAGATGGTGCAGGCGATGAGGACCATTGGAACTCATCCTAGCTCCAGTGCCGGTCTGAG⋱
AGATGATCTCCTTGAAAATTTGCAGGCCTACCAGAAACGGATGGGAGTGCAAATGCAGCGATTCAAGTGA

Per codon conservation report

M1			
Pos.	1	obs:	exp:
atg	M	2676	2676.00
---	--	------	------
mPD		0	0
		nPD:	1.
		N. weight:	0.
		Sc. PD:	0
		Sc. rank:	0

M1			
Pos.	2	obs:	exp:
tct	S	0	454.80
tcc	S	0	392.60
tca	S	0	600.30
tcg	S	0	172.00
cgt	R	1	0.04
cgc	R	0	0.05
cga	R	0	0.10
cgg	R	0	0.08
att	I	2	0.74
atc	I	0	0.50
ata	I	0	0.76
act	T	1	0.26
acc	T	0	0.22
aca	T	0	0.43
acg	T	0	0.08
agt	S	2642	543.70
agc	S	21	499.60
aga	R	0	0.46
agg	R	0	0.27
ggt	G	9	1.29
ggc	G	0	1.19
gga	G	0	3.93
ggg	G	0	2.59
---	--	------	------
mPD		0.025	1.7
		nPD:	0.02
		N. weight:	1.6
		Sc. PD:	-0.14
		Sc. rank:	-330.4

M1			
Pos.	3	obs:	exp:
ttt	F	4	1.70
ttc	F	0	2.30
tta	L	0	289.70
ttg	L	0	498.00
ctt	L	2621	497.70
ctc	L	33	457.90
cta	L	0	353.10
ctg	L	0	557.60
cct	P	1	0.28
ccc	P	0	0.18
cca	P	0	0.41
ccg	P	0	0.13
cgt	R	15	0.57
cgc	R	0	0.80
cga	R	0	1.45
cgg	R	0	1.17
att	I	1	0.37
atc	I	0	0.25
ata	I	0	0.38
aga	R	0	6.88
agg	R	0	4.12
gtt	V	1	0.22
gtc	V	0	0.21
gta	V	0	0.20
gtg	V	0	0.37
---	--	------	------
mPD		0.041	1.2
		nPD:	0.04
		N. weight:	1.6
		Sc. PD:	-0.12
		Sc. rank:	1075.7

M1			
Pos.	4	obs:	exp:
ttt	F	1	0.43
ttc	F	0	0.57
tta	L	6	291.60
ttg	L	0	501.40
ctt	L	11	501.00
ctc	L	3	461.00
cta	L	2648	355.50
ctg	L	4	561.40
caa	Q	2	1.02
cag	Q	0	0.98
att	I	0	0.37
atc	I	0	0.25
ata	I	1	0.38
---	--	------	------
mPD		0.022	1.1
		nPD:	0.02
		N. weight:	2.
		Sc. PD:	-0.17
		Sc. rank:	364.2

M1			
Pos.	5	obs:	exp:
tct	S	0	2.56
tcc	S	0	2.21
tca	S	0	3.38
tcg	S	0	0.97
att	I	0	5.20
atc	I	14	3.51
ata	I	0	5.29
act	T	4	675.70
acc	T	2546	563.60
aca	T	0	1096.00
acg	T	1	215.50
aat	N	0	51.02
aac	N	96	44.98
agt	S	0	3.06
agc	S	15	2.81
---	--	------	------
mPD		0.094	0.78
		nPD:	0.12
		N. weight:	1.5
		Sc. PD:	0.015
		Sc. rank:	1696.5

M1			
Pos.	6	obs:	exp:
aaa	K	0	0.56
aag	K	1	0.44
gaa	E	9	1602.00
gag	E	2666	1073.00
---	--	------	------
mPD		0.0075	0.48
		nPD:	0.02
		N. weight:	0.9
		Sc. PD:	-0.081
		Sc. rank:	-175.5

M1			
Pos.	7	obs:	exp:
gtt	V	15	581.50
gtc	V	2653	565.00
gta	V	5	526.10
gtg	V	1	1001.00
ggt	G	2	0.29
ggc	G	0	0.26
gga	G	0	0.87
ggg	G	0	0.58
---	--	------	------
mPD		0.019	0.73
		nPD:	0.03
		N. weight:	1.5
		Sc. PD:	-0.12
		Sc. rank:	662.5

M1			
Pos.	8	obs:	exp:
cgt	R	0	0.11
cgc	R	0	0.16
cga	R	2	0.29
cgg	R	0	0.23
aga	R	1	1.38
agg	R	0	0.82
gaa	E	2671	1600.00
gag	E	1	1072.00
ggt	G	0	0.14
ggc	G	0	0.13
gga	G	1	0.44
ggg	G	0	0.29
---	--	------	------
mPD		0.0060	0.49
		nPD:	0.01
		N. weight:	0.52
		Sc. PD:	-0.048
		Sc. rank:	-233.3

M1			
Pos.	9	obs:	exp:
act	T	0	707.20
acc	T	0	589.90
aca	T	0	1147.00
acg	T	2670	225.50
aaa	K	0	0.56
aag	K	1	0.44
gct	A	0	1.27
gcc	A	0	0.98
gca	A	0	2.20
gcg	A	5	0.56
---	--	------	------
mPD		0.0045	0.69
		nPD:	0.01
		N. weight:	2.5
		Sc. PD:	-0.25
		Sc. rank:	-2611.6

M1			
Pos.	10	obs:	exp:
tct	S	0	0.17
tcc	S	1	0.15
tca	S	0	0.23
tcg	S	0	0.06
tat	Y	0	1300.00
tac	Y	2674	1374.00
cct	P	0	0.28
ccc	P	1	0.18
cca	P	0	0.41
ccg	P	0	0.13
agt	S	0	0.20
agc	S	0	0.19
---	--	------	------
mPD		0.0022	0.50
		nPD:	0.
		N. weight:	0.68
		Sc. PD:	-0.068
		Sc. rank:	-849.1

M1			
Pos.	11	obs:	exp:
gtt	V	2672	581.80
gtc	V	0	565.20
gta	V	3	526.30
gtg	V	0	1002.00
ggt	G	1	0.14
ggc	G	0	0.13
gga	G	0	0.44
ggg	G	0	0.29
---	--	------	------
mPD		0.0030	0.73
		nPD:	0.
		N. weight:	1.5
		Sc. PD:	-0.16
		Sc. rank:	-2064.0

M1			
Pos.	12	obs:	exp:
ttt	F	13	8.08
ttc	F	6	10.92
tta	L	0	289.90
ttg	L	0	498.40
ctt	L	6	498.00
ctc	L	2650	458.20
cta	L	0	353.40
ctg	L	0	558.10
att	I	0	0.37
atc	I	1	0.25
ata	I	0	0.38
---	--	------	------
mPD		0.029	1.1
		nPD:	0.03
		N. weight:	1.8
		Sc. PD:	-0.14
		Sc. rank:	713.5

M1			
Pos.	13	obs:	exp:
ttt	F	13	5.53
ttc	F	0	7.47
tct	S	2653	454.80
tcc	S	10	392.60
tca	S	0	600.30
tcg	S	0	172.00
agt	S	0	543.70
agc	S	0	499.60
---	--	------	------
mPD		0.017	1.6
		nPD:	0.01
		N. weight:	1.8
		Sc. PD:	-0.17
		Sc. rank:	-1108.7

M1			
Pos.	14	obs:	exp:
ttt	F	1	0.85
ttc	F	1	1.15
att	I	11	992.60
atc	I	2662	670.40
ata	I	0	1010.00
atg	M	1	1.00
---	--	------	------
mPD		0.011	0.66
		nPD:	0.02
		N. weight:	1.4
		Sc. PD:	-0.12
		Sc. rank:	-119.4

M1			
Pos.	15	obs:	exp:
att	I	10	245.80
atc	I	652	166.00
ata	I	0	250.10
gtt	V	5	438.00
gtc	V	2007	425.50
gta	V	2	396.30
gtg	V	0	754.20
---	--	------	------
mPD		0.39	1.1
		nPD:	0.34
		N. weight:	1.5
		Sc. PD:	0.34
		Sc. rank:	2585.3

M1			
Pos.	16	obs:	exp:
cct	P	0	754.60
ccc	P	0	484.20
cca	P	14	1093.00
ccg	P	2662	344.60
---	--	------	------
mPD		0.010	0.70
		nPD:	0.01
		N. weight:	2.
		Sc. PD:	-0.19
		Sc. rank:	-608.8

M1			
Pos.	17	obs:	exp:
tct	S	0	457.00
tcc	S	0	394.50
tca	S	2675	603.20
tcg	S	1	172.90
agt	S	0	546.40
agc	S	0	502.10
---	--	------	------
mPD		0.00075	1.6
		nPD:	0.
		N. weight:	1.5
		Sc. PD:	-0.16
		Sc. rank:	-3067.6

M1			
Pos.	18	obs:	exp:
ggt	G	1	385.00
ggc	G	2675	353.20
gga	G	0	1168.00
ggg	G	0	769.70
---	--	------	------
mPD		0.00075	0.69
		nPD:	0.
		N. weight:	2.1
		Sc. PD:	-0.21
		Sc. rank:	-3833.1

M1			
Pos.	19	obs:	exp:
cct	P	0	754.60
ccc	P	2676	484.20
cca	P	0	1093.00
ccg	P	0	344.60
---	--	------	------
mPD		0	0.70
		nPD:	0.
		N. weight:	1.7
		Sc. PD:	-0.18
		Sc. rank:	-3931.2

M1			
Pos.	20	obs:	exp:
tta	L	0	292.00
ttg	L	0	501.90
ctt	L	0	501.60
ctc	L	2675	461.50
cta	L	0	355.90
ctg	L	0	562.00
ggt	G	0	0.14
ggc	G	1	0.13
gga	G	0	0.44
ggg	G	0	0.29
---	--	------	------
mPD		0.0015	1.1
		nPD:	0.
		N. weight:	1.8
		Sc. PD:	-0.19
		Sc. rank:	-3137.9

M1			
Pos.	21	obs:	exp:
aaa	K	2675	1502.00
aag	K	1	1174.00
---	--	------	------
mPD		0.00075	0.49
		nPD:	0.
		N. weight:	0.58
		Sc. PD:	-0.06
		Sc. rank:	-995.3

M1			
Pos.	22	obs:	exp:
gct	A	0	680.80
gcc	A	2676	521.80
gca	A	0	1176.00
gcg	A	0	297.10
---	--	------	------
mPD		0	0.69
		nPD:	0.
		N. weight:	1.7
		Sc. PD:	-0.17
		Sc. rank:	-3759.1

M1			
Pos.	23	obs:	exp:
gaa	E	3	1602.00
gag	E	2673	1074.00
---	--	------	------
mPD		0.0022	0.48
		nPD:	0.
		N. weight:	0.92
		Sc. PD:	-0.092
		Sc. rank:	-1128.6

M1			
Pos.	24	obs:	exp:
att	I	3	992.20
atc	I	2658	670.10
ata	I	11	1010.00
gtt	V	0	0.87
gtc	V	4	0.85
gta	V	0	0.79
gtg	V	0	1.50
---	--	------	------
mPD		0.013	0.66
		nPD:	0.02
		N. weight:	1.4
		Sc. PD:	-0.12
		Sc. rank:	406.7

M1			
Pos.	25	obs:	exp:
gct	A	2	680.80
gcc	A	0	521.80
gca	A	320	1176.00
gcg	A	2354	297.10
---	--	------	------
mPD		0.21	0.69
		nPD:	0.31
		N. weight:	1.7
		Sc. PD:	0.32
		Sc. rank:	2569.3

M1			
Pos.	26	obs:	exp:
caa	Q	0	1360.00
cag	Q	2673	1313.00
cgt	R	0	0.11
cgc	R	0	0.16
cga	R	0	0.29
cgg	R	3	0.23
aga	R	0	1.38
agg	R	0	0.82
---	--	------	------
mPD		0.0022	0.50
		nPD:	0.
		N. weight:	0.72
		Sc. PD:	-0.073
		Sc. rank:	-917.1

M1			
Pos.	27	obs:	exp:
cgt	R	0	99.85
cgc	R	0	140.20
cga	R	0	255.00
cgg	R	0	206.20
aaa	K	43	24.14
aag	K	0	18.86
aga	R	2632	1208.00
agg	R	1	724.00
---	--	------	------
mPD		0.032	0.98
		nPD:	0.03
		N. weight:	0.79
		Sc. PD:	-0.057
		Sc. rank:	484.0

M1			
Pos.	28	obs:	exp:
tta	L	0	292.10
ttg	L	0	502.10
ctt	L	2659	501.80
ctc	L	0	461.70
cta	L	0	356.00
ctg	L	17	562.30
---	--	------	------
mPD		0.013	1.1
		nPD:	0.01
		N. weight:	1.7
		Sc. PD:	-0.16
		Sc. rank:	-859.5

M1			
Pos.	29	obs:	exp:
gaa	E	2670	1602.00
gag	E	6	1074.00
---	--	------	------
mPD		0.0045	0.48
		nPD:	0.01
		N. weight:	0.51
		Sc. PD:	-0.049
		Sc. rank:	-375.9

M1			
Pos.	30	obs:	exp:
gat	D	2676	1539.00
gac	D	0	1137.00
---	--	------	------
mPD		0	0.49
		nPD:	0.
		N. weight:	0.56
		Sc. PD:	-0.059
		Sc. rank:	-1271.7

M1			
Pos.	31	obs:	exp:
gtt	V	10	582.00
gtc	V	2651	565.40
gta	V	1	526.50
gtg	V	14	1002.00
---	--	------	------
mPD		0.019	0.73
		nPD:	0.03
		N. weight:	1.5
		Sc. PD:	-0.12
		Sc. rank:	698.6

M1			
Pos.	32	obs:	exp:
ttt	F	2675	1138.00
ttc	F	0	1537.00
tta	L	0	0.11
ttg	L	0	0.19
ctt	L	1	0.19
ctc	L	0	0.17
cta	L	0	0.13
ctg	L	0	0.21
---	--	------	------
mPD		0.00075	0.49
		nPD:	0.
		N. weight:	0.87
		Sc. PD:	-0.09
		Sc. rank:	-1466.9

M1			
Pos.	33	obs:	exp:
gtt	V	0	99.82
gtc	V	0	96.98
gta	V	459	90.31
gtg	V	0	171.90
gct	A	0	564.00
gcc	A	0	432.30
gca	A	2217	974.60
gcg	A	0	246.10
---	--	------	------
mPD		0.28	1.0
		nPD:	0.28
		N. weight:	0.97
		Sc. PD:	0.16
		Sc. rank:	1427.4

M1			
Pos.	34	obs:	exp:
ggt	G	0	385.00
ggc	G	0	353.20
gga	G	3	1168.00
ggg	G	2673	769.70
---	--	------	------
mPD		0.0022	0.69
		nPD:	0.
		N. weight:	1.3
		Sc. PD:	-0.13
		Sc. rank:	-1793.4

M1			
Pos.	35	obs:	exp:
aaa	K	654	1502.00
aag	K	2022	1174.00
---	--	------	------
mPD		0.37	0.49
		nPD:	0.75
		N. weight:	0.21
		Sc. PD:	0.13
		Sc. rank:	481.8

M1			
Pos.	36	obs:	exp:
tct	S	0	0.17
tcc	S	0	0.15
tca	S	0	0.23
tcg	S	0	0.06
aat	N	7	1422.00
aac	N	2668	1253.00
agt	S	0	0.20
agc	S	1	0.19
---	--	------	------
mPD		0.0060	0.50
		nPD:	0.01
		N. weight:	0.75
		Sc. PD:	-0.07
		Sc. rank:	-369.4

M1			
Pos.	37	obs:	exp:
act	T	7	708.00
acc	T	2662	590.60
aca	T	4	1149.00
acg	T	0	225.80
gtt	V	0	0.22
gtc	V	0	0.21
gta	V	1	0.20
gtg	V	0	0.37
gct	A	1	0.51
gcc	A	1	0.39
gca	A	0	0.88
gcg	A	0	0.22
---	--	------	------
mPD		0.013	0.69
		nPD:	0.02
		N. weight:	1.5
		Sc. PD:	-0.13
		Sc. rank:	222.2

M1			
Pos.	38	obs:	exp:
gat	D	2666	1539.00
gac	D	10	1137.00
---	--	------	------
mPD		0.0074	0.49
		nPD:	0.02
		N. weight:	0.54
		Sc. PD:	-0.049
		Sc. rank:	-128.6

M1			
Pos.	39	obs:	exp:
tta	L	0	291.90
ttg	L	0	501.80
ctt	L	2043	501.40
ctc	L	607	461.30
cta	L	11	355.80
ctg	L	13	561.80
gtt	V	2	0.43
gtc	V	0	0.42
gta	V	0	0.39
gtg	V	0	0.75
---	--	------	------
mPD		0.37	1.1
		nPD:	0.32
		N. weight:	1.1
		Sc. PD:	0.23
		Sc. rank:	1888.9

M1			
Pos.	40	obs:	exp:
gaa	E	2	1602.00
gag	E	2673	1073.00
ggt	G	0	0.14
ggc	G	0	0.13
gga	G	1	0.44
ggg	G	0	0.29
---	--	------	------
mPD		0.0030	0.48
		nPD:	0.01
		N. weight:	0.92
		Sc. PD:	-0.091
		Sc. rank:	-1000.8

M1			
Pos.	41	obs:	exp:
act	T	1	0.26
acc	T	0	0.22
aca	T	0	0.43
acg	T	0	0.08
gct	A	2658	680.50
gcc	A	4	521.60
gca	A	4	1176.00
gcg	A	9	297.00
---	--	------	------
mPD		0.013	0.69
		nPD:	0.02
		N. weight:	1.3
		Sc. PD:	-0.12
		Sc. rank:	319.8

M1			
Pos.	42	obs:	exp:
ttt	F	0	0.43
ttc	F	1	0.57
tta	L	0	284.20
ttg	L	0	488.60
ctt	L	1	488.30
ctc	L	2603	449.30
cta	L	0	346.50
ctg	L	0	547.10
att	I	0	26.36
atc	I	71	17.81
ata	I	0	26.83
---	--	------	------
mPD		0.053	1.2
		nPD:	0.05
		N. weight:	1.8
		Sc. PD:	-0.11
		Sc. rank:	1464.3

M1			
Pos.	43	obs:	exp:
tta	L	0	0.44
ttg	L	4	0.75
ctt	L	0	0.75
ctc	L	0	0.69
cta	L	0	0.53
ctg	L	0	0.84
atg	M	2672	2672.00
---	--	------	------
mPD		0.0030	0.0048
		nPD:	0.62
		N. weight:	0.0025
		Sc. PD:	0.0013
		Sc. rank:	5.5

M1			
Pos.	44	obs:	exp:
gaa	E	2072	1602.00
gag	E	604	1074.00
---	--	------	------
mPD		0.35	0.48
		nPD:	0.73
		N. weight:	0.072
		Sc. PD:	0.043
		Sc. rank:	161.9

M1			
Pos.	45	obs:	exp:
tgg	W	2676	2676.00
---	--	------	------
mPD		0	0
		nPD:	1.
		N. weight:	0.
		Sc. PD:	0
		Sc. rank:	0

M1

Pos .	46	obs :	exp :
tta	L	2	292.10
ttg	L	0	502.10
ctt	L	0	501.80
ctc	L	0	461.70
cta	L	2673	356.00
ctg	L	1	562.30
---	--	------	------
mPD		0.0022	1.1
		nPD :	0.
		N. weight :	2.
		Sc. PD :	-0.21
		Sc. rank :	-3235.3

M1

Pos .	47	obs :	exp :
cgt	R	0	0.04
cgc	R	0	0.05
cga	R	0	0.10
cgg	R	0	0.08
aaa	K	1	1501.00
aag	K	2674	1174.00
aga	R	0	0.46
agg	R	1	0.27
---	--	------	------
mPD		0.0015	0.49
		nPD :	0.
		N. weight :	0.83
		Sc. PD :	-0.085
		Sc. rank :	-1240.9

M1

Pos .	48	obs :	exp :
act	T	0	708.80
acc	T	0	591.30
aca	T	2675	1150.00
acg	T	1	226.00
---	--	------	------
mPD		0.00075	0.69
		nPD :	0.
		N. weight :	0.86
		Sc. PD :	-0.089
		Sc. rank :	-1640.2

M1

Pos .	49	obs :	exp :
cgt	R	0	101.50
cgc	R	0	142.50
cga	R	0	259.10
cgg	R	0	209.60
aga	R	2674	1227.00
agg	R	2	735.80
---	--	------	------
mPD		0.0015	0.95
		nPD :	0.
		N. weight :	0.79
		Sc. PD :	-0.081
		Sc. rank :	-1275.9

M1

Pos .	50	obs :	exp :
cct	P	0	754.60
ccc	P	0	484.20
cca	P	2673	1093.00
ccg	P	3	344.60
---	--	------	------
mPD		0.0022	0.70
		nPD :	0.
		N. weight :	0.9
		Sc. PD :	-0.092
		Sc. rank :	-1328.7

M1

Pos .	51	obs :	exp :
att	I	0	993.70
atc	I	2676	671.10
ata	I	0	1011.00
---	--	------	------
mPD		0	0.66
		nPD :	0.
		N. weight :	1.4
		Sc. PD :	-0.15
		Sc. rank :	-3180.6

M1

Pos .	52	obs :	exp :
tta	L	0	292.00
ttg	L	3	501.90
ctt	L	0	501.60
ctc	L	0	461.50
cta	L	0	355.90
ctg	L	2672	562.00
cct	P	0	0.28
ccc	P	0	0.18
cca	P	0	0.41
ccg	P	1	0.13
---	--	------	------
mPD		0.0030	1.1
		nPD :	0.
		N. weight :	1.6
		Sc. PD :	-0.16
		Sc. rank :	-2427.2

M1

Pos .	53	obs :	exp :
tct	S	0	457.00
tcc	S	0	394.50
tca	S	2676	603.20
tcg	S	0	172.90
agt	S	0	546.40
agc	S	0	502.10
---	--	------	------
mPD		0	1.6
		nPD :	0.
		N. weight :	1.5
		Sc. PD :	-0.16
		Sc. rank :	-3425.9

M1

Pos .	54	obs :	exp :
tct	S	1	0.17
tcc	S	0	0.15
tca	S	0	0.23
tcg	S	0	0.06
cct	P	2667	754.30
ccc	P	8	484.00
cca	P	0	1092.00
ccg	P	0	344.40
agt	S	0	0.20
agc	S	0	0.19
---	--	------	------
mPD		0.0067	0.71
		nPD :	0.01
		N. weight :	1.3
		Sc. PD :	-0.12
		Sc. rank :	-911.8

M1

Pos .	55	obs :	exp :
tta	L	0	292.10
ttg	L	1	502.10
ctt	L	0	501.80
ctc	L	0	461.70
cta	L	1	356.00
ctg	L	2674	562.30
---	--	------	------
mPD		0.0015	1.1
		nPD :	0.
		N. weight :	1.6
		Sc. PD :	-0.16
		Sc. rank :	-2730.8

M1

Pos .	56	obs :	exp :
att	I	2	0.74
atc	I	0	0.50
ata	I	0	0.76
act	T	2630	708.30
acc	T	44	590.80
aca	T	0	1149.00
acg	T	0	225.90
---	--	------	------
mPD		0.034	0.69
		nPD :	0.05
		N. weight :	1.3
		Sc. PD :	-0.073
		Sc. rank :	1140.2

M1

Pos .	57	obs :	exp :
aat	N	0	0.53
aac	N	1	0.47
aaa	K	605	1501.00
aag	K	2070	1174.00
---	--	------	------
mPD		0.35	0.49
		nPD :	0.71
		N. weight :	0.24
		Sc. PD :	0.14
		Sc. rank :	535.0

M1

Pos .	58	obs :	exp :
ggt	G	0	385.00
ggc	G	0	353.20
gga	G	20	1168.00
ggg	G	2656	769.70
---	--	------	------
mPD		0.015	0.69
		nPD :	0.02
		N. weight :	1.2
		Sc. PD :	-0.1
		Sc. rank :	420.1

M1

Pos .	59	obs :	exp :
att	I	2668	991.80
atc	I	2	669.90
ata	I	1	1009.00
atg	M	1	1.00
gtt	V	3	0.87
gtc	V	0	0.85
gta	V	0	0.79
gtg	V	1	1.50
---	--	------	------
mPD		0.0067	0.66
		nPD :	0.01
		N. weight :	1.
		Sc. PD :	-0.095
		Sc. rank :	-657.7

M1

Pos .	60	obs :	exp :
tta	L	1080	291.90
ttg	L	1593	501.80
ctt	L	0	501.40
ctc	L	0	461.30
cta	L	1	355.80
ctg	L	0	561.80
gtt	V	0	0.43
gtc	V	0	0.42
gta	V	0	0.39
gtg	V	2	0.75
---	--	------	------
mPD		0.48	1.1
		nPD :	0.43
		N. weight :	1.2
		Sc. PD :	0.38
		Sc. rank :	2274.9

M1

Pos .	61	obs :	exp :
ggt	G	0	385.00
ggc	G	1	353.20
gga	G	1661	1168.00
ggg	G	1014	769.70
---	--	------	------
mPD		0.47	0.69
		nPD :	0.68
		N. weight :	0.33
		Sc. PD :	0.18
		Sc. rank :	725.1

M1

Pos .	62	obs :	exp :
ttt	F	2672	1137.00
ttc	F	1	1536.00
tta	L	0	0.11
ttg	L	0	0.19
ctt	L	1	0.19
ctc	L	0	0.17
cta	L	0	0.13
ctg	L	0	0.21
gtt	V	2	0.43
gtc	V	0	0.42
gta	V	0	0.39
gtg	V	0	0.75
---	--	------	------
mPD		0.0030	0.49
		nPD :	0.01
		N. weight :	0.87
		Sc. PD :	-0.086
		Sc. rank :	-971.8

M1

Pos .	63	obs :	exp :
gtt	V	2	582.00
gtc	V	0	565.40
gta	V	45	526.50
gtg	V	2629	1002.00
---	--	------	------
mPD		0.035	0.73
		nPD :	0.05
		N. weight :	0.92
		Sc. PD :	-0.054
		Sc. rank :	804.2

M1

Pos .	64	obs :	exp :
ttt	F	4	1139.00
ttc	F	2672	1537.00
---	--	------	------
mPD		0.0030	0.49
		nPD :	0.01
		N. weight :	0.55
		Sc. PD :	-0.055
		Sc. rank :	-609.4

M1

Pos .	65	obs :	exp :
act	T	0	708.80
acc	T	0	591.30
aca	T	6	1150.00
acg	T	2670	226.00
---	--	------	------
mPD		0.0045	0.69
		nPD :	0.01
		N. weight :	2.5
		Sc. PD :	-0.25
		Sc. rank :	-2481.0

M1

Pos .	66	obs :	exp :
tta	L	0	292.10
ttg	L	0	502.10
ctt	L	0	501.80
ctc	L	2676	461.70
cta	L	0	356.00
ctg	L	0	562.30
---	--	------	------
mPD		0	1.1
		nPD :	0.
		N. weight :	1.8
		Sc. PD :	-0.19
		Sc. rank :	-4040.7

M1

Pos .	67	obs :	exp :
act	T	1	708.80
acc	T	2675	591.30
aca	T	0	1150.00
acg	T	0	226.00
---	--	------	------
mPD		0.00075	0.69
		nPD :	0.
		N. weight :	1.5
		Sc. PD :	-0.16
		Sc. rank :	-2934.0

M1

Pos .	68	obs :	exp :
gtt	V	0	581.80
gtc	V	0	565.20
gta	V	0	526.30
gtg	V	2675	1002.00
gaa	E	0	0.60
gag	E	1	0.40
---	--	------	------
mPD		0.00075	0.73
		nPD :	0.
		N. weight :	1.
		Sc. PD :	-0.1
		Sc. rank :	-1934.0

M1

Pos .	69	obs :	exp :
cct	P	0	754.60
ccc	P	2676	484.20
cca	P	0	1093.00
ccg	P	0	344.60
---	--	------	------
mPD		0	0.70
		nPD :	0.
		N. weight :	1.7
		Sc. PD :	-0.18
		Sc. rank :	-3931.2

M1

Pos .	70	obs :	exp :
tct	S	0	457.00
tcc	S	0	394.50
tca	S	0	603.20
tcg	S	0	172.90
agt	S	2658	546.40
agc	S	18	502.10
---	--	------	------
mPD		0.013	1.6
		nPD :	0.01
		N. weight :	1.6
		Sc. PD :	-0.15
		Sc. rank :	-1319.0

M1

Pos.	71	obs:	exp:
gaa	E	1	1602.00
gag	E	2675	1074.00
---	--	------	------
mPD		0.00075	0.48
		nPD:	0.
		N. weight:	0.92
		Sc. PD:	-0.096
		Sc. rank:	-1513.1

M1

Pos.	72	obs:	exp:
caa	Q	1	0.51
cag	Q	0	0.49
cgt	R	0	101.40
cgc	R	0	142.50
cga	R	2548	259.00
cgg	R	127	209.50
aga	R	0	1227.00
agg	R	0	735.50
---	--	------	------
mPD		0.091	0.95
		nPD:	0.1
		N. weight:	2.2
		Sc. PD:	-0.029
		Sc. rank:	2404.1

M1

Pos.	73	obs:	exp:
ggt	G	0	385.00
ggc	G	0	353.20
gga	G	2619	1168.00
ggg	G	57	769.70
---	--	------	------
mPD		0.042	0.69
		nPD:	0.06
		N. weight:	0.75
		Sc. PD:	-0.035
		Sc. rank:	724.5

M1

Pos.	74	obs:	exp:
tta	L	0	292.00
ttg	L	1	501.90
ctt	L	12	501.60
ctc	L	1	461.50
cta	L	1	355.90
ctg	L	2660	562.00
caa	Q	0	0.51
cag	Q	1	0.49
---	--	------	------
mPD		0.012	1.1
		nPD:	0.01
		N. weight:	1.5
		Sc. PD:	-0.15
		Sc. rank:	-928.0

M1

Pos.	75	obs:	exp:
caa	Q	2	1362.00
cag	Q	2674	1314.00
---	--	------	------
mPD		0.0015	0.50
		nPD:	0.
		N. weight:	0.72
		Sc. PD:	-0.073
		Sc. rank:	-1093.5

M1

Pos.	76	obs:	exp:
cgt	R	2675	101.50
cgc	R	1	142.50
cga	R	0	259.10
cgg	R	0	209.60
aga	R	0	1227.00
agg	R	0	735.80
---	--	------	------
mPD		0.00075	0.95
		nPD:	0.
		N. weight:	3.3
		Sc. PD:	-0.35
		Sc. rank:	-6619.6

M1

Pos.	77	obs:	exp:
cgt	R	0	101.50
cgc	R	0	142.50
cga	R	0	259.10
cgg	R	0	209.60
aga	R	2675	1227.00
agg	R	1	735.80
---	--	------	------
mPD		0.00075	0.95
		nPD:	0.
		N. weight:	0.79
		Sc. PD:	-0.082
		Sc. rank:	-1572.6

M1

Pos.	78	obs:	exp:
cgt	R	4	101.50
cgc	R	2658	142.50
cga	R	13	259.10
cgg	R	1	209.60
aga	R	0	1227.00
agg	R	0	735.80
---	--	------	------
mPD		0.013	0.95
		nPD:	0.01
		N. weight:	2.9
		Sc. PD:	-0.27
		Sc. rank:	-981.4

M1

Pos.	79	obs:	exp:
ttt	F	2115	1139.00
ttc	F	561	1537.00
---	--	------	------
mPD		0.33	0.49
		nPD:	0.68
		N. weight:	0.28
		Sc. PD:	0.16
		Sc. rank:	626.4

M1

Pos.	80	obs:	exp:
att	I	0	0.37
atc	I	1	0.25
ata	I	0	0.38
gtt	V	15	581.80
gtc	V	2660	565.20
gta	V	0	526.30
gtg	V	0	1002.00
---	--	------	------
mPD		0.012	0.73
		nPD:	0.02
		N. weight:	1.5
		Sc. PD:	-0.14
		Sc. rank:	-172.3

M1

Pos.	81	obs:	exp:
caa	Q	2064	1362.00
cag	Q	612	1314.00
---	--	------	------
mPD		0.35	0.50
		nPD:	0.71
		N. weight:	0.15
		Sc. PD:	0.086
		Sc. rank:	334.3

M1

Pos.	82	obs:	exp:
tct	S	0	0.17
tcc	S	0	0.15
tca	S	0	0.23
tcg	S	0	0.06
aat	N	2645	1422.00
aac	N	30	1253.00
agt	S	1	0.20
agc	S	0	0.19
---	--	------	------
mPD		0.023	0.50
		nPD:	0.05
		N. weight:	0.58
		Sc. PD:	-0.035
		Sc. rank:	487.6

M1

Pos.	83	obs:	exp:
tct	S	5	1.20
tcc	S	2	1.03
tca	S	0	1.58
tcg	S	0	0.45
act	T	1	0.26
acc	T	0	0.22
aca	T	0	0.43
acg	T	0	0.08
agt	S	0	1.43
agc	S	0	1.31
gtt	V	2	0.43
gtc	V	0	0.42
gta	V	0	0.39
gtg	V	0	0.75
gct	A	2038	678.20
gcc	A	624	519.90
gca	A	2	1172.00
gcg	A	2	296.00
---	--	------	------
mPD		0.37	0.70
		nPD:	0.52
		N. weight:	0.89
		Sc. PD:	0.36
		Sc. rank:	1844.5

M1

Pos.	84	obs:	exp:
tta	L	4	292.10
ttg	L	0	502.10
ctt	L	13	501.80
ctc	L	8	461.70
cta	L	2646	356.00
ctg	L	5	562.30
---	--	------	------
mPD		0.022	1.1
		nPD:	0.02
		N. weight:	2.
		Sc. PD:	-0.17
		Sc. rank:	537.4

M1

Pos.	85	obs:	exp:
tct	S	0	1.37
tcc	S	0	1.18
tca	S	0	1.80
tcg	S	0	0.52
aat	N	2664	1418.00
aac	N	4	1250.00
agt	S	8	1.63
agc	S	0	1.50
---	--	------	------
mPD		0.0089	0.51
		nPD:	0.02
		N. weight:	0.63
		Sc. PD:	-0.056
		Sc. rank:	29.2

M1

Pos.	86	obs:	exp:
ggt	G	0	385.00
ggc	G	0	353.20
gga	G	2640	1168.00
ggg	G	36	769.70
---	--	------	------
mPD		0.027	0.69
		nPD:	0.04
		N. weight:	0.78
		Sc. PD:	-0.053
		Sc. rank:	549.1

M1

Pos.	87	obs:	exp:
tct	S	0	1.37
tcc	S	0	1.18
tca	S	0	1.80
tcg	S	0	0.52
aat	N	2623	1417.00
aac	N	44	1250.00
agt	S	8	1.63
agc	S	0	1.50
gct	A	1	0.25
gcc	A	0	0.20
gca	A	0	0.44
gcg	A	0	0.11
---	--	------	------
mPD		0.040	0.51
		nPD:	0.08
		N. weight:	0.56
		Sc. PD:	-0.017
		Sc. rank:	567.3

M1

Pos.	88	obs:	exp:
ggt	G	1	385.00
ggc	G	0	353.20
gga	G	2095	1168.00
ggg	G	580	769.70
---	--	------	------
mPD		0.34	0.69
		nPD:	0.49
		N. weight:	0.4
		Sc. PD:	0.15
		Sc. rank:	779.7

M1

Pos.	89	obs:	exp:
gat	D	618	1539.00
gac	D	2058	1137.00
---	--	------	------
mPD		0.36	0.49
		nPD:	0.73
		N. weight:	0.25
		Sc. PD:	0.15
		Sc. rank:	566.0

M1

Pos.	90	obs:	exp:
cct	P	0	754.60
ccc	P	0	484.20
cca	P	2656	1093.00
ccg	P	20	344.60
---	--	------	------
mPD		0.015	0.70
		nPD:	0.02
		N. weight:	0.87
		Sc. PD:	-0.074
		Sc. rank:	273.8

M1			
Pos.	91	obs:	exp:
tct	S	0	1.02
tcc	S	0	0.88
tca	S	0	1.35
tcg	S	0	0.39
att	I	0	0.37
atc	I	1	0.25
ata	I	0	0.38
act	T	0	2.91
acc	T	11	2.43
aca	T	0	4.73
acg	T	0	0.93
aat	N	602	1413.00
aac	N	2056	1245.00
agt	S	0	1.23
agc	S	6	1.13
---	--	------	------
mPD		0.36	0.52
		nPD:	0.7
		N. weight:	0.21
		Sc. PD:	0.12
		Sc. rank:	463.1

,

M1			
Pos.	92	obs:	exp:
aat	N	607	1422.00
aac	N	2069	1254.00
---	--	------	------
mPD		0.35	0.50
		nPD:	0.7
		N. weight:	0.2
		Sc. PD:	0.11
		Sc. rank:	442.6

,

M1			
Pos.	93	obs:	exp:
atg	M	2676	2676.00
---	--	------	------
mPD		0	0
		nPD:	1.
		N. weight:	0.
		Sc. PD:	0
		Sc. rank:	0

,

M1			
Pos.	94	obs:	exp:
aat	N	0	0.53
aac	N	1	0.47
gat	D	605	1538.00
gac	D	2068	1135.00
ggt	G	0	0.29
ggc	G	2	0.26
gga	G	0	0.87
ggg	G	0	0.58
---	--	------	------
mPD		0.35	0.49
		nPD:	0.72
		N. weight:	0.26
		Sc. PD:	0.15
		Sc. rank:	583.2

,

M1			
Pos.	95	obs:	exp:
cgt	R	0	78.39
cgc	R	0	110.10
cga	R	0	200.20
cgg	R	1	161.90
aaa	K	16	341.80
aag	K	593	267.20
aga	R	31	948.10
agg	R	2035	568.40
---	--	------	------
mPD		0.39	1.2
		nPD:	0.32
		N. weight:	1.1
		Sc. PD:	0.22
		Sc. rank:	1814.5

,

M1			
Pos.	96	obs:	exp:
gct	A	0	680.80
gcc	A	0	521.80
gca	A	2662	1176.00
gcg	A	14	297.10
---	--	------	------
mPD		0.010	0.69
		nPD:	0.02
		N. weight:	0.81
		Sc. PD:	-0.073
		Sc. rank:	-229.4

,

M1			
Pos.	97	obs:	exp:
att	I	0	1.86
atc	I	5	1.25
ata	I	0	1.89
gtt	V	607	580.90
gtc	V	2063	564.40
gta	V	1	525.50
gtg	V	0	1000.00
---	--	------	------
mPD		0.36	0.73
		nPD:	0.48
		N. weight:	1.
		Sc. PD:	0.37
		Sc. rank:	1986.0

,

M1			
Pos.	98	obs:	exp:
cgt	R	0	0.19
cgc	R	0	0.27
cga	R	0	0.48
cgg	R	0	0.39
aaa	K	2066	1499.00
aag	K	605	1172.00
aga	R	5	2.29
agg	R	0	1.37
---	--	------	------
mPD		0.35	0.50
		nPD:	0.71
		N. weight:	0.1
		Sc. PD:	0.059
		Sc. rank:	229.3

,

M1			
Pos.	99	obs:	exp:
tta	L	0	292.10
ttg	L	4	502.10
ctt	L	1	501.80
ctc	L	0	461.70
cta	L	631	356.00
ctg	L	2040	562.30
---	--	------	------
mPD		0.36	1.1
		nPD:	0.32
		N. weight:	1.1
		Sc. PD:	0.23
		Sc. rank:	1872.8

,

M1			
Pos.	100	obs:	exp:
ttt	F	0	0.43
ttc	F	1	0.57
tat	Y	607	1300.00
tac	Y	2068	1375.00
---	--	------	------
mPD		0.35	0.50
		nPD:	0.7
		N. weight:	0.15
		Sc. PD:	0.084
		Sc. rank:	326.4

,

M1			
Pos.	101	obs:	exp:
cgt	R	0	78.39
cgc	R	0	110.10
cga	R	0	200.20
cgg	R	2	161.90
aaa	K	4	341.80
aag	K	605	267.20
aga	R	32	948.10
agg	R	2033	568.40
---	--	------	------
mPD		0.38	1.2
		nPD:	0.31
		N. weight:	1.1
		Sc. PD:	0.22
		Sc. rank:	1729.8

,

M1			
Pos.	102	obs:	exp:
aaa	K	2055	1502.00
aag	K	621	1174.00
---	--	------	------
mPD		0.36	0.49
		nPD:	0.72
		N. weight:	0.096
		Sc. PD:	0.057
		Sc. rank:	215.5

,

M1			
Pos.	103	obs:	exp:
tta	L	439	291.80
ttg	L	1343	501.60
ctt	L	4	501.20
ctc	L	6	461.20
cta	L	6	355.60
ctg	L	875	561.60
atg	M	3	3.00
---	--	------	------
mPD		0.73	1.1
		nPD:	0.64
		N. weight:	0.7
		Sc. PD:	0.36
		Sc. rank:	1523.7

,

M1			
Pos.	104	obs:	exp:
aaa	K	637	1502.00
aag	K	2039	1174.00
---	--	------	------
mPD		0.36	0.49
		nPD:	0.74
		N. weight:	0.22
		Sc. PD:	0.13
		Sc. rank:	501.3

,

M1			
Pos.	105	obs:	exp:
cgt	R	0	101.50
cgc	R	0	142.50
cga	R	1	259.10
cgg	R	0	209.60
aga	R	2618	1227.00
agg	R	57	735.80
---	--	------	------
mPD		0.042	0.95
		nPD:	0.04
		N. weight:	0.7
		Sc. PD:	-0.043
		Sc. rank:	553.7

,

M1			
Pos.	106	obs:	exp:
gaa	E	59	1602.00
gag	E	2617	1074.00
---	--	------	------
mPD		0.043	0.48
		nPD:	0.09
		N. weight:	0.81
		Sc. PD:	-0.015
		Sc. rank:	860.1

,

M1			
Pos.	107	obs:	exp:
att	I	1	990.40
atc	I	4	668.90
ata	I	2662	1008.00
atg	M	2	2.00
gtt	V	0	1.52
gtc	V	0	1.48
gta	V	7	1.38
gtg	V	0	2.62
---	--	------	------
mPD		0.010	0.66
		nPD:	0.02
		N. weight:	0.98
		Sc. PD:	-0.088
		Sc. rank:	-172.3

,

M1			
Pos.	108	obs:	exp:
act	T	0	708.80
acc	T	14	591.30
aca	T	2661	1150.00
acg	T	1	226.00
---	--	------	------
mPD		0.011	0.69
		nPD:	0.02
		N. weight:	0.83
		Sc. PD:	-0.074
		Sc. rank:	-133.6

,

M1			
Pos.	109	obs:	exp:
ttt	F	23	1139.00
ttc	F	2653	1537.00
---	--	------	------
mPD		0.017	0.49
		nPD:	0.03
		N. weight:	0.52
		Sc. PD:	-0.037
		Sc. rank:	322.9

,

M1			
Pos.	110	obs:	exp:
cat	H	2676	1591.00
cac	H	0	1085.00
---	--	------	------
mPD		0	0.48
		nPD:	0.
		N. weight:	0.53
		Sc. PD:	-0.055
		Sc. rank:	-1195.5

,

M1			
Pos.	111	obs:	exp:
gtt	V	0	0.22
gtc	V	0	0.21
gta	V	0	0.20
gtg	V	1	0.37
ggt	G	0	384.80
ggc	G	0	353.10
gga	G	14	1168.00
ggg	G	2661	769.40
---	--	------	------
mPD		0.011	0.69
		nPD:	0.02
		N. weight:	1.2
		Sc. PD:	-0.11
		Sc. rank:	-171.6

,

M1			
Pos.	112	obs:	exp:
tct	S	2	0.34
tcc	S	0	0.29
tca	S	0	0.45
tcg	S	0	0.13
agt	S	0	0.41
agc	S	0	0.38
gct	A	2639	680.20
gcc	A	16	521.40
gca	A	18	1175.00
gcg	A	1	296.90
---	--	------	------
mPD		0.027	0.69
		nPD:	0.04
		N. weight:	1.3
		Sc. PD:	-0.088
		Sc. rank:	957.3

,

M1			
Pos.	113	obs:	exp:
aaa	K	2067	1502.00
aag	K	609	1174.00
---	--	------	------
mPD		0.35	0.49
		nPD:	0.71
		N. weight:	0.1
		Sc. PD:	0.058
		Sc. rank:	224.7

,

M1			
Pos.	114	obs:	exp:
gaa	E	2065	1602.00
gag	E	611	1074.00
---	--	------	------
mPD		0.35	0.48
		nPD:	0.73
		N. weight:	0.07
		Sc. PD:	0.042
		Sc. rank:	157.4

,

M1			
Pos.	115	obs:	exp:
att	I	323	119.90
atc	I	0	81.01
ata	I	0	122.10
gtt	V	1732	511.70
gtc	V	605	497.20
gta	V	3	463.00
gtg	V	13	881.10
---	--	------	------
mPD		0.57	0.95
		nPD:	0.6
		N. weight:	0.94
		Sc. PD:	0.45
		Sc. rank:	2035.9

,

M1

Pos.	116	obs:	exp:
tct	S	0	0.34
tcc	S	0	0.29
tca	S	2	0.45
tcg	S	0	0.13
act	T	0	0.26
acc	T	0	0.22
aca	T	1	0.43
acg	T	0	0.08
agt	S	0	0.41
agc	S	0	0.38
gct	A	0	680.00
gcc	A	0	521.30
gca	A	2664	1175.00
gcg	A	9	296.70
---	--	------	------
mPD		0.0089	0.69
		nPD:	0.01
		N. weight:	0.82
		Sc. PD:	-0.076
		Sc. rank:	-342.1

,

M1

Pos.	117	obs:	exp:
ttt	F	0	1.28
ttc	F	3	1.72
tta	L	0	291.60
ttg	L	0	501.40
ctt	L	605	501.00
ctc	L	2067	461.00
cta	L	0	355.50
ctg	L	0	561.40
att	I	0	0.37
atc	I	1	0.25
ata	I	0	0.38
---	--	------	------
mPD		0.35	1.1
		nPD:	0.31
		N. weight:	1.2
		Sc. PD:	0.24
		Sc. rank:	1921.2

,

M1

Pos.	118	obs:	exp:
tct	S	0	454.30
tcc	S	0	392.10
tca	S	0	599.60
tcg	S	0	171.80
agt	S	2095	543.10
agc	S	565	499.10
ggt	G	16	2.30
ggc	G	0	2.11
gga	G	0	6.98
ggg	G	0	4.60
---	--	------	------
mPD		0.35	1.7
		nPD:	0.21
		N. weight:	1.1
		Sc. PD:	0.11
		Sc. rank:	1462.2

,

M1

Pos.	119	obs:	exp:
tat	Y	226	1301.00
tac	Y	2450	1375.00
---	--	------	------
mPD		0.15	0.50
		nPD:	0.31
		N. weight:	0.39
		Sc. PD:	0.075
		Sc. rank:	601.2

,

M1

Pos.	120	obs:	exp:
tct	S	0	455.10
tcc	S	5	392.90
tca	S	2658	600.70
tcg	S	2	172.10
cct	P	0	3.10
ccc	P	0	1.99
cca	P	11	4.49
ccg	P	0	1.42
agt	S	0	544.10
agc	S	0	500.00
---	--	------	------
mPD		0.013	1.6
		nPD:	0.01
		N. weight:	1.5
		Sc. PD:	-0.15
		Sc. rank:	-1211.2

,

M1

Pos.	121	obs:	exp:
act	T	377	708.60
acc	T	2291	591.00
aca	T	7	1149.00
acg	T	0	225.90
gct	A	0	0.25
gcc	A	1	0.20
gca	A	0	0.44
gcg	A	0	0.11
---	--	------	------
mPD		0.25	0.69
		nPD:	0.36
		N. weight:	1.1
		Sc. PD:	0.26
		Sc. rank:	1880.6

,

M1

Pos.	122	obs:	exp:
ggt	G	2672	385.00
ggc	G	2	353.20
gga	G	2	1168.00
ggg	G	0	769.70
---	--	------	------
mPD		0.0030	0.69
		nPD:	0.
		N. weight:	2.
		Sc. PD:	-0.2
		Sc. rank:	-2504.1

,

M1

Pos.	123	obs:	exp:
gct	A	8	680.80
gcc	A	0	521.80
gca	A	2624	1176.00
gcg	A	44	297.10
---	--	------	------
mPD		0.038	0.69
		nPD:	0.06
		N. weight:	0.76
		Sc. PD:	-0.039
		Sc. rank:	722.1

,

M1

Pos.	124	obs:	exp:
tta	L	0	292.10
ttg	L	0	502.10
ctt	L	2671	501.80
ctc	L	4	461.70
cta	L	1	356.00
ctg	L	0	562.30
---	--	------	------
mPD		0.0037	1.1
		nPD:	0.
		N. weight:	1.7
		Sc. PD:	-0.17
		Sc. rank:	-2305.1

,

M1

Pos.	125	obs:	exp:
gct	A	9	680.80
gcc	A	2666	521.80
gca	A	1	1176.00
gcg	A	0	297.10
---	--	------	------
mPD		0.0075	0.69
		nPD:	0.01
		N. weight:	1.6
		Sc. PD:	-0.15
		Sc. rank:	-894.3

,

M1

Pos.	126	obs:	exp:
tct	S	0	456.80
tcc	S	0	394.30
tca	S	0	603.00
tcg	S	0	172.80
agt	S	2101	546.20
agc	S	574	501.90
ggt	G	1	0.14
ggc	G	0	0.13
gga	G	0	0.44
ggg	G	0	0.29
---	--	------	------
mPD		0.34	1.6
		nPD:	0.21
		N. weight:	1.1
		Sc. PD:	0.1
		Sc. rank:	1353.5

,

M1

Pos.	127	obs:	exp:
tgt	C	2051	1164.00
tgc	C	625	1512.00
---	--	------	------
mPD		0.36	0.49
		nPD:	0.73
		N. weight:	0.23
		Sc. PD:	0.14
		Sc. rank:	526.7

,

M1

Pos.	128	obs:	exp:
tta	L	0	0.11
ttg	L	0	0.19
ctt	L	0	0.19
ctc	L	0	0.17
cta	L	0	0.13
ctg	L	1	0.21
att	I	0	1.11
atc	I	0	0.75
ata	I	3	1.13
atg	M	2672	2672.00
---	--	------	------
mPD		0.0030	0.0034
		nPD:	0.87
		N. weight:	0.0017
		Sc. PD:	0.0012
		Sc. rank:	3.9

,

M1

Pos.	129	obs:	exp:
ggt	G	2662	385.00
ggc	G	13	353.20
gga	G	0	1168.00
ggg	G	1	769.70
---	--	------	------
mPD		0.010	0.69
		nPD:	0.02
		N. weight:	1.9
		Sc. PD:	-0.18
		Sc. rank:	-481.0

,

M1

Pos.	130	obs:	exp:
tta	L	0	243.90
ttg	L	0	419.40
ctt	L	1	419.10
ctc	L	2233	385.60
cta	L	1	297.40
ctg	L	0	469.60
att	I	0	163.80
atc	I	441	110.60
ata	I	0	166.60
---	--	------	------
mPD		0.28	1.3
		nPD:	0.21
		N. weight:	1.7
		Sc. PD:	0.17
		Sc. rank:	2347.1

,

M1

Pos.	131	obs:	exp:
att	I	1	993.70
atc	I	0	671.10
ata	I	2675	1011.00
---	--	------	------
mPD		0.00075	0.66
		nPD:	0.
		N. weight:	0.99
		Sc. PD:	-0.1
		Sc. rank:	-1764.3

,

M1

Pos.	132	obs:	exp:
tat	Y	2	1301.00
tac	Y	2674	1375.00
---	--	------	------
mPD		0.0015	0.50
		nPD:	0.
		N. weight:	0.67
		Sc. PD:	-0.068
		Sc. rank:	-1015.5

,

M1

Pos.	133	obs:	exp:
tct	S	0	0.17
tcc	S	0	0.15
tca	S	0	0.23
tcg	S	0	0.06
cat	H	1	0.59
cac	H	0	0.41
aat	N	41	1421.00
aac	N	2633	1253.00
agt	S	0	0.20
agc	S	1	0.19
---	--	------	------
mPD		0.032	0.50
		nPD:	0.06
		N. weight:	0.69
		Sc. PD:	-0.029
		Sc. rank:	676.3

,

M1

Pos.	134	obs:	exp:
cgt	R	0	91.09
cgc	R	0	127.90
cga	R	2	232.60
cgg	R	1	188.10
aaa	K	0	153.80
aag	K	274	120.20
aga	R	18	1102.00
agg	R	2381	660.50
---	--	------	------
mPD		0.20	1.1
		nPD:	0.18
		N. weight:	1.2
		Sc. PD:	0.085
		Sc. rank:	1433.4

,

M1

Pos.	135	obs:	exp:
atg	M	2676	2676.00
---	--	------	------
mPD		0	0
		nPD:	1.
		N. weight:	0.
		Sc. PD:	0
		Sc. rank:	0

,

M1

Pos.	136	obs:	exp:
ggt	G	0	385.00
ggc	G	0	353.20
gga	G	620	1168.00
ggg	G	2056	769.70
---	--	------	------
mPD		0.36	0.69
		nPD:	0.52
		N. weight:	0.62
		Sc. PD:	0.24
		Sc. rank:	1226.5

,

M1

Pos.	137	obs:	exp:
act	T	13	708.80
acc	T	0	591.30
aca	T	33	1150.00
acg	T	2630	226.00
---	--	------	------
mPD		0.034	0.69
		nPD:	0.05
		N. weight:	2.4
		Sc. PD:	-0.14
		Sc. rank:	2249.5

,

M1

Pos.	138	obs:	exp:
atg	M	2	2.00
gtt	V	9	581.50
gtc	V	0	565.00
gta	V	450	526.10
gtg	V	2215	1001.00
---	--	------	------
mPD		0.29	0.73
		nPD:	0.39
		N. weight:	0.63
		Sc. PD:	0.17
		Sc. rank:	1143.9

,

M1

Pos.	139	obs:	exp:
act	T	613	706.50
acc	T	2049	589.30
aca	T	5	1146.00
acg	T	0	225.30
aat	N	0	0.53
aac	N	1	0.47
gct	A	0	2.04
gcc	A	8	1.56
gca	A	0	3.52
gcg	A	0	0.89
---	--	------	------
mPD		0.36	0.70
		nPD:	0.52
		N. weight:	0.93
		Sc. PD:	0.37
		Sc. rank:	1889.8

,

M1

Pos.	140	obs:	exp:
tct	S	0	4.44
tcc	S	0	3.83
tca	S	26	5.86
tcg	S	0	1.68
act	T	13	437.10
acc	T	0	364.60
aca	T	1622	709.00
acg	T	15	139.40
agt	S	0	5.31
agc	S	0	4.88
gct	A	0	254.40
gcc	A	0	195.00
gca	A	1000	439.60
gcg	A	0	111.00
---	--	------	------
mPD		0.50	1.2
		nPD:	0.43
		N. weight:	0.81
		Sc. PD:	0.25
		Sc. rank:	1499.4

,

M1

Pos.	141	obs:	exp:
gaa	E	2673	1602.00
gag	E	3	1074.00
---	--	------	------
mPD		0.0022	0.48
		nPD:	0.
		N. weight:	0.51
		Sc. PD:	-0.052
		Sc. rank:	-630.3

,

M1

Pos.	142	obs:	exp:
gtt	V	4	579.60
gtc	V	0	563.10
gta	V	2	524.40
gtg	V	2659	998.00
gct	A	0	2.04
gcc	A	0	1.56
gca	A	0	3.52
gcg	A	8	0.89
ggt	G	0	0.43
ggc	G	0	0.40
gga	G	0	1.31
ggg	G	3	0.86
---	--	------	------
mPD		0.013	0.74
		nPD:	0.02
		N. weight:	0.99
		Sc. PD:	-0.087
		Sc. rank:	7.5

,

M1

Pos.	143	obs:	exp:
tct	S	0	0.51
tcc	S	0	0.44
tca	S	3	0.68
tcg	S	0	0.19
agt	S	0	0.61
agc	S	0	0.56
gct	A	608	680.00
gcc	A	6	521.30
gca	A	1519	1175.00
gcg	A	540	296.70
---	--	------	------
mPD		0.59	0.69
		nPD:	0.84
		N. weight:	0.24
		Sc. PD:	0.17
		Sc. rank:	546.1

,

M1

Pos.	144	obs:	exp:
ttt	F	621	264.70
ttc	F	1	357.30
tta	L	2	224.20
ttg	L	2047	385.40
ctt	L	1	385.20
ctc	L	0	354.40
cta	L	0	273.30
ctg	L	4	431.60
---	--	------	------
mPD		0.36	1.2
		nPD:	0.29
		N. weight:	1.5
		Sc. PD:	0.26
		Sc. rank:	2214.2

,

M1

Pos.	145	obs:	exp:
ggt	G	3	385.00
ggc	G	2673	353.20
gga	G	0	1168.00
ggg	G	0	769.70
---	--	------	------
mPD		0.0022	0.69
		nPD:	0.
		N. weight:	2.
		Sc. PD:	-0.21
		Sc. rank:	-2922.8

,

M1

Pos.	146	obs:	exp:
tta	L	14	292.10
ttg	L	0	502.10
ctt	L	0	501.80
ctc	L	2	461.70
cta	L	2650	356.00
ctg	L	10	562.30
---	--	------	------
mPD		0.019	1.1
		nPD:	0.02
		N. weight:	2.
		Sc. PD:	-0.18
		Sc. rank:	-52.4

,

M1

Pos.	147	obs:	exp:
atg	M	8	8.00
gtt	V	0	580.20
gtc	V	0	563.70
gta	V	21	525.00
gtg	V	2647	999.10
---	--	------	------
mPD		0.022	0.73
		nPD:	0.03
		N. weight:	0.95
		Sc. PD:	-0.073
		Sc. rank:	501.7

,

M1

Pos.	148	obs:	exp:
tgt	C	2670	1164.00
tgc	C	6	1512.00
---	--	------	------
mPD		0.0045	0.49
		nPD:	0.01
		N. weight:	0.83
		Sc. PD:	-0.08
		Sc. rank:	-636.0

,

M1

Pos.	149	obs:	exp:
tct	S	0	1.20
tcc	S	7	1.03
tca	S	0	1.58
tcg	S	0	0.45
agt	S	0	1.43
agc	S	0	1.31
gct	A	1	679.00
gcc	A	2636	520.50
gca	A	31	1173.00
gcg	A	1	296.30
---	--	------	------
mPD		0.030	0.70
		nPD:	0.04
		N. weight:	1.6
		Sc. PD:	-0.1
		Sc. rank:	1215.6

,

M1

Pos.	150	obs:	exp:
att	I	0	0.74
atc	I	2	0.50
ata	I	0	0.76
act	T	609	708.30
acc	T	2049	590.80
aca	T	7	1149.00
acg	T	9	225.90
---	--	------	------
mPD		0.36	0.69
		nPD:	0.52
		N. weight:	0.91
		Sc. PD:	0.36
		Sc. rank:	1867.4

,

M1

Pos.	151	obs:	exp:
tgt	C	2670	1164.00
tgc	C	6	1512.00
---	--	------	------
mPD		0.0045	0.49
		nPD:	0.01
		N. weight:	0.83
		Sc. PD:	-0.08
		Sc. rank:	-636.0

,

M1

Pos.	152	obs:	exp:
gaa	E	8	1602.00
gag	E	2668	1074.00
---	--	------	------
mPD		0.0060	0.48
		nPD:	0.01
		N. weight:	0.91
		Sc. PD:	-0.084
		Sc. rank:	-392.4

,

M1

Pos.	153	obs:	exp:
cat	H	113	67.19
cac	H	0	45.81
caa	Q	25	1304.00
cag	Q	2538	1259.00
---	--	------	------
mPD		0.099	0.54
		nPD:	0.18
		N. weight:	0.66
		Sc. PD:	0.047
		Sc. rank:	789.3

,

M1

Pos.	154	obs:	exp:
att	I	2674	993.70
atc	I	2	671.10
ata	I	0	1011.00
---	--	------	------
mPD		0.0015	0.66
		nPD:	0.
		N. weight:	1.
		Sc. PD:	-0.1
		Sc. rank:	-1569.1

,

M1

Pos.	155	obs:	exp:
gct	A	2070	680.80
gcc	A	1	521.80
gca	A	604	1176.00
gcg	A	1	297.10
---	--	------	------
mPD		0.35	0.69
		nPD:	0.51
		N. weight:	0.72
		Sc. PD:	0.28
		Sc. rank:	1403.9

,

M1

Pos.	156	obs:	exp:
gat	D	2662	1537.00
gac	D	10	1135.00
gaa	E	0	2.40
gag	E	4	1.60
---	--	------	------
mPD		0.010	0.49
		nPD:	0.02
		N. weight:	0.54
		Sc. PD:	-0.046
		Sc. rank:	174.0

,

M1

Pos.	157	obs:	exp:
tct	S	2	457.00
tcc	S	13	394.50
tca	S	2660	603.20
tcg	S	1	172.90
agt	S	0	546.40
agc	S	0	502.10
---	--	------	------
mPD		0.012	1.6
		nPD:	0.01
		N. weight:	1.5
		Sc. PD:	-0.14
		Sc. rank:	-1355.6

,

M1

Pos.	158	obs:	exp:
cat	H	2	1.19
cac	H	0	0.81
caa	Q	6	1361.00
cag	Q	2668	1313.00
---	--	------	------
mPD		0.0060	0.50
		nPD:	0.01
		N. weight:	0.71
		Sc. PD:	-0.066
		Sc. rank:	-356.4

,

M1

Pos.	159	obs:	exp:
cat	H	2653	1591.00
cac	H	23	1085.00
---	--	------	------
mPD		0.017	0.48
		nPD:	0.04
		N. weight:	0.48
		Sc. PD:	-0.034
		Sc. rank:	320.0

,

M1

Pos.	160	obs:	exp:
cgt	R	1	101.50
cgc	R	0	142.50
cga	R	18	259.10
cgg	R	2653	209.60
aga	R	0	1227.00
agg	R	4	735.80
---	--	------	------
mPD		0.017	0.95
		nPD:	0.02
		N. weight:	2.5
		Sc. PD:	-0.22
		Sc. rank:	231.7

,

M1

Pos.	161	obs:	exp:
tct	S	2076	457.00
tcc	S	600	394.50
tca	S	0	603.20
tcg	S	0	172.90
agt	S	0	546.40
agc	S	0	502.10
---	--	------	------
mPD		0.35	1.6
		nPD:	0.21
		N. weight:	1.3
		Sc. PD:	0.13
		Sc. rank:	1723.6

M1

Pos.	162	obs:	exp:
cat	H	0	1591.00
cac	H	2675	1084.00
caa	Q	1	0.51
cag	Q	0	0.49
---	--	------	------
mPD		0.00075	0.48
		nPD:	0.
		N. weight:	0.92
		Sc. PD:	-0.095
		Sc. rank:	-1524.8

M1

Pos.	163	obs:	exp:
cgt	R	0	101.50
cgc	R	0	142.50
cga	R	0	259.10
cgg	R	1	209.60
aga	R	2667	1227.00
agg	R	8	735.80
---	--	------	------
mPD		0.0075	0.95
		nPD:	0.01
		N. weight:	0.77
		Sc. PD:	-0.075
		Sc. rank:	-667.8

M1

Pos.	164	obs:	exp:
tta	L	0	0.11
ttg	L	0	0.19
ctt	L	0	0.19
ctc	L	0	0.17
cta	L	0	0.13
ctg	L	1	0.21
caa	Q	55	1361.00
cag	Q	2620	1314.00
---	--	------	------
mPD		0.041	0.50
		nPD:	0.08
		N. weight:	0.62
		Sc. PD:	-0.016
		Sc. rank:	632.3

M1

Pos.	165	obs:	exp:
att	I	129	666.50
atc	I	0	450.20
ata	I	1666	678.30
atg	M	625	625.00
gtt	V	0	55.67
gtc	V	0	54.09
gta	V	256	50.37
gtg	V	0	95.86
---	--	------	------
mPD		0.60	0.92
		nPD:	0.65
		N. weight:	0.65
		Sc. PD:	0.34
		Sc. rank:	1424.4

M1

Pos.	166	obs:	exp:
gtt	V	0	4.78
gtc	V	0	4.65
gta	V	7	4.33
gtg	V	15	8.24
gct	A	0	675.20
gcc	A	2	517.50
gca	A	2571	1167.00
gcg	A	81	294.60
---	--	------	------
mPD		0.087	0.71
		nPD:	0.12
		N. weight:	0.73
		Sc. PD:	0.0095
		Sc. rank:	844.0

M1

Pos.	167	obs:	exp:
tct	S	0	0.17
tcc	S	0	0.15
tca	S	0	0.23
tcg	S	0	0.06
act	T	5	707.50
acc	T	2651	590.10
aca	T	14	1148.00
acg	T	1	225.60
aat	N	0	1.59
aac	N	3	1.41
agt	S	0	0.20
agc	S	1	0.19
gct	A	0	0.25
gcc	A	1	0.20
gca	A	0	0.44
gcg	A	0	0.11
---	--	------	------
mPD		0.019	0.69
		nPD:	0.03
		N. weight:	1.5
		Sc. PD:	-0.12
		Sc. rank:	753.1

M1

Pos.	168	obs:	exp:
att	I	4	236.50
atc	I	633	159.80
ata	I	0	240.70
act	T	4	540.10
acc	T	2021	450.50
aca	T	14	876.20
acg	T	0	172.20
---	--	------	------
mPD		0.38	1.0
		nPD:	0.36
		N. weight:	1.4
		Sc. PD:	0.35
		Sc. rank:	2567.1

M1

Pos.	169	obs:	exp:
act	T	3	708.60
acc	T	2660	591.00
aca	T	12	1149.00
acg	T	0	225.90
gct	A	0	0.25
gcc	A	1	0.20
gca	A	0	0.44
gcg	A	0	0.11
---	--	------	------
mPD		0.012	0.69
		nPD:	0.02
		N. weight:	1.5
		Sc. PD:	-0.13
		Sc. rank:	51.6

M1

Pos.	170	obs:	exp:
aat	N	141	1422.00
aac	N	2535	1254.00
---	--	------	------
mPD		0.10	0.50
		nPD:	0.2
		N. weight:	0.55
		Sc. PD:	0.049
		Sc. rank:	668.1

M1

Pos.	171	obs:	exp:
cct	P	0	754.60
ccc	P	3	484.20
cca	P	2663	1093.00
ccg	P	10	344.60
---	--	------	------
mPD		0.0097	0.70
		nPD:	0.01
		N. weight:	0.88
		Sc. PD:	-0.081
		Sc. rank:	-342.1

M1

Pos.	172	obs:	exp:
tta	L	605	292.00
ttg	L	1	501.90
ctt	L	0	501.60
ctc	L	0	461.50
cta	L	2067	355.90
ctg	L	2	562.00
att	I	0	0.37
atc	I	0	0.25
ata	I	1	0.38
---	--	------	------
mPD		0.35	1.1
		nPD:	0.31
		N. weight:	1.5
		Sc. PD:	0.3
		Sc. rank:	2467.4

M1

Pos.	173	obs:	exp:
att	I	4	993.70
atc	I	2661	671.10
ata	I	11	1011.00
---	--	------	------
mPD		0.011	0.66
		nPD:	0.02
		N. weight:	1.4
		Sc. PD:	-0.12
		Sc. rank:	-84.9

M1

Pos.	174	obs:	exp:
cgt	R	0	100.80
cgc	R	0	141.60
cga	R	1	257.50
cgg	R	0	208.30
aaa	K	17	9.54
aag	K	0	7.46
aga	R	2491	1220.00
agg	R	167	731.10
---	--	------	------
mPD		0.13	0.96
		nPD:	0.14
		N. weight:	0.58
		Sc. PD:	0.015
		Sc. rank:	678.6

M1

Pos.	175	obs:	exp:
cat	H	2650	1591.00
cac	H	26	1085.00
---	--	------	------
mPD		0.019	0.48
		nPD:	0.04
		N. weight:	0.48
		Sc. PD:	-0.032
		Sc. rank:	353.0

M1

Pos.	176	obs:	exp:
gaa	E	2043	1602.00
gag	E	633	1074.00
---	--	------	------
mPD		0.36	0.48
		nPD:	0.75
		N. weight:	0.063
		Sc. PD:	0.039
		Sc. rank:	142.9

M1

Pos.	177	obs:	exp:
aat	N	1	1422.00
aac	N	2675	1254.00
---	--	------	------
mPD		0.00075	0.50
		nPD:	0.
		N. weight:	0.77
		Sc. PD:	-0.079
		Sc. rank:	-1315.8

M1

Pos.	178	obs:	exp:
cgt	R	0	101.50
cgc	R	0	142.50
cga	R	0	259.10
cgg	R	0	209.60
aga	R	2630	1227.00
agg	R	46	735.80
---	--	------	------
mPD		0.034	0.95
		nPD:	0.04
		N. weight:	0.71
		Sc. PD:	-0.051
		Sc. rank:	482.7

M1

Pos.	179	obs:	exp:
atg	M	2676	2676.00
---	--	------	------
mPD		0	0
		nPD:	1.
		N. weight:	0.
		Sc. PD:	0
		Sc. rank:	0

M1

Pos.	180	obs:	exp:
gtt	V	1	582.00
gtc	V	0	565.40
gta	V	37	526.50
gtg	V	2638	1002.00
---	--	------	------
mPD		0.028	0.73
		nPD:	0.04
		N. weight:	0.93
		Sc. PD:	-0.063
		Sc. rank:	645.5

M1

Pos.	181	obs:	exp:
tta	L	1	291.80
ttg	L	996	501.60
ctt	L	0	501.20
ctc	L	0	461.20
cta	L	34	355.60
ctg	L	1642	561.60
atg	M	3	3.00
---	--	------	------
mPD		0.49	1.1
		nPD:	0.44
		N. weight:	0.9
		Sc. PD:	0.28
		Sc. rank:	1691.2

M1

Pos.	182	obs:	exp:
gct	A	1	680.80
gcc	A	2659	521.80
gca	A	16	1176.00
gcg	A	0	297.10
---	--	------	------
mPD		0.013	0.69
		nPD:	0.02
		N. weight:	1.6
		Sc. PD:	-0.14
		Sc. rank:	195.1

M1

Pos.	183	obs:	exp:
tct	S	0	457.00
tcc	S	0	394.50
tca	S	0	603.20
tcg	S	0	172.90
agt	S	2058	546.40
agc	S	618	502.10
---	--	------	------
mPD		0.36	1.6
		nPD:	0.22
		N. weight:	1.1
		Sc. PD:	0.11
		Sc. rank:	1506.6

M1

Pos.	184	obs:	exp:
act	T	2633	708.60
acc	T	11	591.00
aca	T	31	1149.00
acg	T	0	225.90
aat	N	1	0.53
aac	N	0	0.47
---	--	------	------
mPD		0.032	0.69
		nPD:	0.05
		N. weight:	1.3
		Sc. PD:	-0.076
		Sc. rank:	1078.8

M1

Pos.	185	obs:	exp:
act	T	20	708.80
acc	T	2	591.30
aca	T	2635	1150.00
acg	T	19	226.00
---	--	------	------
mPD		0.030	0.69
		nPD:	0.04
		N. weight:	0.78
		Sc. PD:	-0.049
		Sc. rank:	612.0

M1			
Pos.	186	obs:	exp:
gct	A	2669	680.80
gcc	A	6	521.80
gca	A	1	1176.00
gcg	A	0	297.10
---	--	------	------
mPD		0.0052	0.69
		nPD:	0.01
		N. weight:	1.4
		Sc. PD:	-0.13
		Sc. rank:	-1207.8

M1			
Pos.	187	obs:	exp:
act	T	0	0.26
acc	T	0	0.22
aca	T	0	0.43
acg	T	1	0.08
aaa	K	7	1501.00
aag	K	2668	1174.00
---	--	------	------
mPD		0.0060	0.49
		nPD:	0.01
		N. weight:	0.82
		Sc. PD:	-0.076
		Sc. rank:	-390.2

M1			
Pos.	188	obs:	exp:
gtt	V	0	0.22
gtc	V	1	0.21
gta	V	0	0.20
gtg	V	0	0.37
gct	A	2056	680.50
gcc	A	617	521.60
gca	A	2	1176.00
gcg	A	0	297.00
---	--	------	------
mPD		0.36	0.69
		nPD:	0.52
		N. weight:	0.9
		Sc. PD:	0.35
		Sc. rank:	1772.6

M1			
Pos.	189	obs:	exp:
atg	M	2676	2676.00
---	--	------	------
mPD		0	0
		nPD:	1.
		N. weight:	0.
		Sc. PD:	0
		Sc. rank:	0

M1			
Pos.	190	obs:	exp:
gaa	E	11	1602.00
gag	E	2665	1074.00
---	--	------	------
mPD		0.0082	0.48
		nPD:	0.02
		N. weight:	0.9
		Sc. PD:	-0.08
		Sc. rank:	-9.1

M1			
Pos.	191	obs:	exp:
cat	H	4	2.38
cac	H	0	1.62
caa	Q	14	1359.00
cag	Q	2658	1313.00
---	--	------	------
mPD		0.013	0.50
		nPD:	0.03
		N. weight:	0.69
		Sc. PD:	-0.055
		Sc. rank:	332.1

M1			
Pos.	192	obs:	exp:
att	I	0	0.37
atc	I	0	0.25
ata	I	1	0.38
atg	M	2675	2675.00
---	--	------	------
mPD		0.00075	0.00075
		nPD:	1.
		N. weight:	0.00037
		Sc. PD:	0.00032
		Sc. rank:	0.8

M1			
Pos.	193	obs:	exp:
gct	A	2056	680.80
gcc	A	3	521.80
gca	A	602	1176.00
gcg	A	15	297.10
---	--	------	------
mPD		0.36	0.69
		nPD:	0.52
		N. weight:	0.69
		Sc. PD:	0.27
		Sc. rank:	1374.5

M1			
Pos.	194	obs:	exp:
ggt	G	0	385.00
ggc	G	1	353.20
gga	G	1597	1168.00
ggg	G	1078	769.70
---	--	------	------
mPD		0.48	0.69
		nPD:	0.7
		N. weight:	0.33
		Sc. PD:	0.19
		Sc. rank:	725.6

M1			
Pos.	195	obs:	exp:
tct	S	0	451.90
tcc	S	0	390.10
tca	S	636	596.40
tcg	S	2010	170.90
act	T	0	7.95
acc	T	0	6.63
aca	T	0	12.89
acg	T	30	2.53
agt	S	0	540.20
agc	S	0	496.40
---	--	------	------
mPD		0.38	1.6
		nPD:	0.23
		N. weight:	1.9
		Sc. PD:	0.23
		Sc. rank:	2737.7

M1			
Pos.	196	obs:	exp:
tct	S	0	455.60
tcc	S	0	393.30
tca	S	0	601.40
tcg	S	0	172.30
act	T	8	2.12
acc	T	0	1.77
aca	T	0	3.44
acg	T	0	0.68
agt	S	2104	544.70
agc	S	564	500.60
---	--	------	------
mPD		0.34	1.6
		nPD:	0.21
		N. weight:	1.1
		Sc. PD:	0.1
		Sc. rank:	1381.0

M1			
Pos.	197	obs:	exp:
gaa	E	19	1602.00
gag	E	2657	1074.00
---	--	------	------
mPD		0.014	0.48
		nPD:	0.03
		N. weight:	0.88
		Sc. PD:	-0.068
		Sc. rank:	478.2

M1			
Pos.	198	obs:	exp:
cat	H	1	0.59
cac	H	0	0.41
caa	Q	895	1361.00
cag	Q	1780	1314.00
---	--	------	------
mPD		0.45	0.50
		nPD:	0.89
		N. weight:	0.064
		Sc. PD:	0.048
		Sc. rank:	146.6

M1			
Pos.	199	obs:	exp:
act	T	0	0.26
acc	T	0	0.22
aca	T	1	0.43
acg	T	0	0.08
gtt	V	0	0.22
gtc	V	0	0.21
gta	V	1	0.20
gtg	V	0	0.37
gct	A	0	680.20
gcc	A	0	521.40
gca	A	2673	1175.00
gcg	A	1	296.90
---	--	------	------
mPD		0.0022	0.69
		nPD:	0.
		N. weight:	0.83
		Sc. PD:	-0.085
		Sc. rank:	-1212.9

M1			
Pos.	200	obs:	exp:
tct	S	0	95.98
tcc	S	2	82.85
tca	S	560	126.70
tcg	S	0	36.30
agt	S	0	114.70
agc	S	0	105.40
gct	A	0	537.80
gcc	A	0	412.20
gca	A	16	929.30
gcg	A	2098	234.70
---	--	------	------
mPD		0.67	1.2
		nPD:	0.55
		N. weight:	2.
		Sc. PD:	0.87
		Sc. rank:	4333.0

M1			
Pos.	201	obs:	exp:
gaa	E	2659	1602.00
gag	E	17	1074.00
---	--	------	------
mPD		0.013	0.48
		nPD:	0.03
		N. weight:	0.49
		Sc. PD:	-0.039
		Sc. rank:	227.9

M1			
Pos.	202	obs:	exp:
gct	A	17	680.80
gcc	A	2658	521.80
gca	A	1	1176.00
gcg	A	0	297.10
---	--	------	------
mPD		0.013	0.69
		nPD:	0.02
		N. weight:	1.6
		Sc. PD:	-0.14
		Sc. rank:	344.0

M1			
Pos.	203	obs:	exp:
atg	M	2676	2676.00
---	--	------	------
mPD		0	0
		nPD:	1.
		N. weight:	0.
		Sc. PD:	0
		Sc. rank:	0

M1			
Pos.	204	obs:	exp:
gaa	E	46	1602.00
gag	E	2630	1074.00
---	--	------	------
mPD		0.034	0.48
		nPD:	0.07
		N. weight:	0.83
		Sc. PD:	-0.031
		Sc. rank:	830.7

M1			
Pos.	205	obs:	exp:
att	I	1	0.37
atc	I	0	0.25
ata	I	0	0.38
gtt	V	2644	581.80
gtc	V	1	565.20
gta	V	30	526.30
gtg	V	0	1002.00
---	--	------	------
mPD		0.024	0.73
		nPD:	0.03
		N. weight:	1.5
		Sc. PD:	-0.11
		Sc. rank:	877.7

M1			
Pos.	206	obs:	exp:
gtt	V	1	0.22
gtc	V	0	0.21
gta	V	0	0.20
gtg	V	0	0.37
gct	A	2326	680.50
gcc	A	349	521.60
gca	A	0	1176.00
gcg	A	0	297.00
---	--	------	------
mPD		0.23	0.69
		nPD:	0.33
		N. weight:	1.
		Sc. PD:	0.22
		Sc. rank:	1757.1

M1			
Pos.	207	obs:	exp:
tct	S	0	351.10
tcc	S	0	303.10
tca	S	0	463.50
tcg	S	0	132.80
aat	N	612	325.20
aac	N	0	286.80
agt	S	2038	419.80
agc	S	18	385.70
ggt	G	8	1.15
ggc	G	0	1.06
gga	G	0	3.49
ggg	G	0	2.30
---	--	------	------
mPD		0.37	1.8
		nPD:	0.21
		N. weight:	1.4
		Sc. PD:	0.13
		Sc. rank:	1752.2

M1			
Pos.	208	obs:	exp:
caa	Q	65	1359.00
cag	Q	2607	1313.00
aaa	K	0	2.25
aag	K	4	1.75
---	--	------	------
mPD		0.050	0.50
		nPD:	0.1
		N. weight:	0.61
		Sc. PD:	-0.0052
		Sc. rank:	672.9

M1			
Pos.	209	obs:	exp:
act	T	0	0.26
acc	T	1	0.22
aca	T	0	0.43
acg	T	0	0.08
gct	A	2109	680.50
gcc	A	565	521.60
gca	A	0	1176.00
gcg	A	1	297.00
---	--	------	------
mPD		0.34	0.69
		nPD:	0.48
		N. weight:	0.92
		Sc. PD:	0.33
		Sc. rank:	1767.1

M1			
Pos.	210	obs:	exp:
cgt	R	0	101.40
cgc	R	0	142.50
cga	R	0	259.00
cgg	R	0	209.50
aaa	K	0	0.56
aag	K	1	0.44
aga	R	8	1227.00
agg	R	2667	735.50
---	--	------	------
mPD		0.0067	0.95
		nPD:	0.01
		N. weight:	1.3
		Sc. PD:	-0.13
		Sc. rank:	-1218.2

M1

Pos.	211	obs:	exp:
cat	H	1	0.59
cac	H	0	0.41
caa	Q	2	1361.00
cag	Q	2673	1314.00
---	--	------	------
mPD		0.0022	0.50
		nPD:	0.
		N. weight:	0.72
		Sc. PD:	-0.072
		Sc. rank:	-889.0

M1

Pos.	212	obs:	exp:
atg	M	2676	2676.00
---	--	------	------
mPD		0	0
		nPD:	1.
		N. weight:	0.
		Sc. PD:	0
		Sc. rank:	0

M1

Pos.	213	obs:	exp:
att	I	1	0.37
atc	I	0	0.25
ata	I	0	0.38
gtt	V	103	581.80
gtc	V	0	565.20
gta	V	606	526.30
gtg	V	1966	1002.00
---	--	------	------
mPD		0.41	0.73
		nPD:	0.56
		N. weight:	0.47
		Sc. PD:	0.2
		Sc. rank:	1007.3

M1

Pos.	214	obs:	exp:
cat	H	41	24.38
cac	H	0	16.62
caa	Q	17	1341.00
cag	Q	2618	1294.00
---	--	------	------
mPD		0.043	0.51
		nPD:	0.08
		N. weight:	0.68
		Sc. PD:	-0.017
		Sc. rank:	701.5

M1

Pos.	215	obs:	exp:
gct	A	2	680.80
gcc	A	0	521.80
gca	A	809	1176.00
gcg	A	1865	297.10
---	--	------	------
mPD		0.42	0.69
		nPD:	0.61
		N. weight:	1.2
		Sc. PD:	0.57
		Sc. rank:	2575.5

M1

Pos.	216	obs:	exp:
tta	L	0	0.11
ttg	L	0	0.19
ctt	L	0	0.19
ctc	L	0	0.17
cta	L	0	0.13
ctg	L	1	0.21
atg	M	2675	2675.00
---	--	------	------
mPD		0.00075	0.0012
		nPD:	0.62
		N. weight:	0.00059
		Sc. PD:	0.00029
		Sc. rank:	1.3

M1

Pos.	217	obs:	exp:
cgt	R	0	101.50
cgc	R	0	142.50
cga	R	0	259.10
cgg	R	0	209.60
aga	R	39	1227.00
agg	R	2637	735.80
---	--	------	------
mPD		0.029	0.95
		nPD:	0.03
		N. weight:	1.2
		Sc. PD:	-0.093
		Sc. rank:	692.2

M1

Pos.	218	obs:	exp:
att	I	0	0.74
atc	I	0	0.50
ata	I	2	0.76
act	T	7	707.80
acc	T	2056	590.40
aca	T	609	1148.00
acg	T	0	225.70
gct	A	0	0.51
gcc	A	2	0.39
gca	A	0	0.88
gcg	A	0	0.22
---	--	------	------
mPD		0.36	0.69
		nPD:	0.52
		N. weight:	0.82
		Sc. PD:	0.32
		Sc. rank:	1644.8

M1

Pos.	219	obs:	exp:
tta	L	0	0.11
ttg	L	0	0.19
ctt	L	1	0.19
ctc	L	0	0.17
cta	L	0	0.13
ctg	L	0	0.21
att	I	2671	993.30
atc	I	4	670.90
ata	I	0	1011.00
---	--	------	------
mPD		0.0037	0.66
		nPD:	0.01
		N. weight:	1.
		Sc. PD:	-0.099
		Sc. rank:	-1134.1

M1

Pos.	220	obs:	exp:
gaa	E	1	0.60
gag	E	0	0.40
ggt	G	2	384.80
ggc	G	2	353.10
gga	G	2048	1168.00
ggg	G	623	769.40
---	--	------	------
mPD		0.36	0.69
		nPD:	0.52
		N. weight:	0.38
		Sc. PD:	0.15
		Sc. rank:	775.3

M1

Pos.	221	obs:	exp:
att	I	1	0.37
atc	I	0	0.25
ata	I	0	0.38
act	T	2672	708.60
acc	T	1	591.00
aca	T	1	1149.00
acg	T	1	225.90
---	--	------	------
mPD		0.0030	0.69
		nPD:	0.
		N. weight:	1.3
		Sc. PD:	-0.14
		Sc. rank:	-1762.4

M1

Pos.	222	obs:	exp:
cat	H	2091	1591.00
cac	H	585	1085.00
---	--	------	------
mPD		0.34	0.48
		nPD:	0.71
		N. weight:	0.081
		Sc. PD:	0.047
		Sc. rank:	181.3

M1

Pos.	223	obs:	exp:
tct	S	1	0.17
tcc	S	0	0.15
tca	S	0	0.23
tcg	S	0	0.06
cct	P	2666	754.30
ccc	P	9	484.00
cca	P	0	1092.00
ccg	P	0	344.40
agt	S	0	0.20
agc	S	0	0.19
---	--	------	------
mPD		0.0075	0.71
		nPD:	0.01
		N. weight:	1.3
		Sc. PD:	-0.12
		Sc. rank:	-729.8

M1

Pos.	224	obs:	exp:
tct	S	0	350.40
tcc	S	0	302.50
tca	S	0	462.60
tcg	S	0	132.50
att	I	0	0.37
atc	I	1	0.25
ata	I	0	0.38
aat	N	96	331.10
aac	N	527	291.90
agt	S	0	419.00
agc	S	2052	385.00
---	--	------	------
mPD		0.43	1.8
		nPD:	0.24
		N. weight:	1.4
		Sc. PD:	0.17
		Sc. rank:	1993.5

M1

Pos.	225	obs:	exp:
tct	S	577	457.00
tcc	S	2098	394.50
tca	S	1	603.20
tcg	S	0	172.90
agt	S	0	546.40
agc	S	0	502.10
---	--	------	------
mPD		0.34	1.6
		nPD:	0.21
		N. weight:	1.4
		Sc. PD:	0.13
		Sc. rank:	1752.6

M1

Pos.	226	obs:	exp:
tct	S	0	456.80
tcc	S	0	394.30
tca	S	0	603.00
tcg	S	0	172.80
agt	S	2665	546.20
agc	S	10	501.90
ggt	G	1	0.14
ggc	G	0	0.13
gga	G	0	0.44
ggg	G	0	0.29
---	--	------	------
mPD		0.0082	1.6
		nPD:	0.
		N. weight:	1.6
		Sc. PD:	-0.16
		Sc. rank:	-1887.0

M1

Pos.	227	obs:	exp:
tct	S	0	0.51
tcc	S	3	0.44
tca	S	0	0.68
tcg	S	0	0.19
act	T	0	0.26
acc	T	1	0.22
aca	T	0	0.43
acg	T	0	0.08
agt	S	0	0.61
agc	S	0	0.56
gct	A	668	679.70
gcc	A	2004	521.10
gca	A	0	1175.00
gcg	A	0	296.60
---	--	------	------
mPD		0.38	0.70
		nPD:	0.54
		N. weight:	1.
		Sc. PD:	0.43
		Sc. rank:	2144.7

M1

Pos.	228	obs:	exp:
ggt	G	2675	385.00
ggc	G	0	353.20
gga	G	1	1168.00
ggg	G	0	769.70
---	--	------	------
mPD		0.00075	0.69
		nPD:	0.
		N. weight:	2.
		Sc. PD:	-0.2
		Sc. rank:	-3669.3

M1

Pos.	229	obs:	exp:
tta	L	0	291.90
ttg	L	2	501.80
ctt	L	0	501.40
ctc	L	0	461.30
cta	L	6	355.80
ctg	L	2666	561.80
cct	P	0	0.28
ccc	P	0	0.18
cca	P	0	0.41
ccg	P	1	0.13
gtt	V	0	0.22
gtc	V	0	0.21
gta	V	0	0.20
gtg	V	1	0.37
---	--	------	------
mPD		0.0075	1.1
		nPD:	0.01
		N. weight:	1.6
		Sc. PD:	-0.15
		Sc. rank:	-1514.1

M1

Pos.	230	obs:	exp:
cgt	R	0	96.82
cgc	R	0	136.00
cga	R	0	247.20
cgg	R	0	200.00
aaa	K	123	69.04
aag	K	0	53.96
aga	R	2553	1171.00
agg	R	0	702.00
---	--	------	------
mPD		0.088	1.0
		nPD:	0.09
		N. weight:	0.78
		Sc. PD:	-0.018
		Sc. rank:	817.0

M1

Pos.	231	obs:	exp:
gat	D	2672	1538.00
gac	D	2	1136.00
gaa	E	2	1.20
gag	E	0	0.80
---	--	------	------
mPD		0.0030	0.49
		nPD:	0.01
		N. weight:	0.56
		Sc. PD:	-0.055
		Sc. rank:	-618.8

M1

Pos.	232	obs:	exp:
act	T	1	0.26
acc	T	0	0.22
aca	T	0	0.43
acg	T	0	0.08
aat	N	604	321.50
aac	N	1	283.50
gat	D	2056	1190.00
gac	D	12	878.50
gaa	E	2	1.20
gag	E	0	0.80
---	--	------	------
mPD		0.36	0.84
		nPD:	0.43
		N. weight:	0.55
		Sc. PD:	0.17
		Sc. rank:	1030.0

M1

Pos.	233	obs:	exp:
tta	L	0	292.10
ttg	L	0	502.10
ctt	L	625	501.80
ctc	L	2051	461.70
cta	L	0	356.00
ctg	L	0	562.30
---	--	------	------
mPD		0.36	1.1
		nPD:	0.32
		N. weight:	1.2
		Sc. PD:	0.24
		Sc. rank:	1967.9

M1

Pos.	234	obs:	exp:
ttt	F	1	0.43
ttc	F	0	0.57
tta	L	0	291.30
ttg	L	0	500.80
ctt	L	2668	500.50
ctc	L	1	460.50
cta	L	0	355.10
ctg	L	0	560.80
att	I	6	2.23
atc	I	0	1.50
ata	I	0	2.27
---	--	------	------
mPD		0.0060	1.1
		nPD:	0.01
		N. weight:	1.7
		Sc. PD:	-0.17
		Sc. rank:	-1960.2

M1

Pos.	235	obs:	exp:
gat	D	0	0.58
gac	D	1	0.42
gaa	E	2675	1602.00
gag	E	0	1073.00
---	--	------	------
mPD		0.00075	0.48
		nPD:	0.
		N. weight:	0.52
		Sc. PD:	-0.054
		Sc. rank:	-861.6

M1			
Pos.	236	obs:	exp:
act	T	1	0.26
acc	T	0	0.22
aca	T	0	0.43
acg	T	0	0.08
aat	N	2674	1422.00
aac	N	1	1253.00
---	--	------	------
mPD		0.0015	0.50
		nPD:	0.
		N. weight:	0.64
		Sc. PD:	-0.065
		Sc. rank:	-961.1

,

M1			
Pos.	237	obs:	exp:
tta	L	36	292.00
ttg	L	2639	501.90
tgg	W	1	1.00
ctt	L	0	501.60
ctc	L	0	461.50
cta	L	0	355.90
ctg	L	0	562.00
---	--	------	------
mPD		0.027	1.1
		nPD:	0.02
		N. weight:	1.6
		Sc. PD:	-0.13
		Sc. rank:	611.7

,

M1			
Pos.	238	obs:	exp:
caa	Q	0	1362.00
cag	Q	2676	1314.00
---	--	------	------
mPD		0	0.50
		nPD:	0.
		N. weight:	0.72
		Sc. PD:	-0.076
		Sc. rank:	-1634.7

,

M1			
Pos.	239	obs:	exp:
act	T	0	0.26
acc	T	1	0.22
aca	T	0	0.43
acg	T	0	0.08
gct	A	6	680.50
gcc	A	2668	521.60
gca	A	1	1176.00
gcg	A	0	297.00
---	--	------	------
mPD		0.0060	0.69
		nPD:	0.01
		N. weight:	1.6
		Sc. PD:	-0.16
		Sc. rank:	-1282.2

,

M1			
Pos.	240	obs:	exp:
tat	Y	22	1301.00
tac	Y	2654	1375.00
---	--	------	------
mPD		0.016	0.50
		nPD:	0.03
		N. weight:	0.63
		Sc. PD:	-0.046
		Sc. rank:	378.7

,

M1			
Pos.	241	obs:	exp:
caa	Q	1023	1362.00
cag	Q	1653	1314.00
---	--	------	------
mPD		0.47	0.50
		nPD:	0.95
		N. weight:	0.034
		Sc. PD:	0.028
		Sc. rank:	78.2

,

M1			
Pos.	242	obs:	exp:
tct	S	0	0.17
tcc	S	0	0.15
tca	S	0	0.23
tcg	S	0	0.06
cgt	R	0	0.11
cgc	R	0	0.16
cga	R	0	0.29
cgg	R	0	0.23
aat	N	4	321.50
aac	N	601	283.50
aaa	K	2056	1160.00
aag	K	10	906.30
agt	S	0	0.20
agc	S	1	0.19
aga	R	3	1.38
agg	R	0	0.82
gat	D	0	0.58
gac	D	1	0.42
---	--	------	------
mPD		0.36	0.68
		nPD:	0.53
		N. weight:	0.6
		Sc. PD:	0.24
		Sc. rank:	1240.4

,

M1			
Pos.	243	obs:	exp:
cgt	R	0	101.50
cgc	R	0	142.50
cga	R	601	259.10
cgg	R	2072	209.60
aga	R	1	1227.00
agg	R	2	735.80
---	--	------	------
mPD		0.35	0.95
		nPD:	0.37
		N. weight:	2.
		Sc. PD:	0.5
		Sc. rank:	3595.9

,

M1			
Pos.	244	obs:	exp:
atg	M	2676	2676.00
---	--	------	------
mPD		0	0
		nPD:	1.
		N. weight:	0.
		Sc. PD:	0
		Sc. rank:	0

,

M1			
Pos.	245	obs:	exp:
ggt	G	0	385.00
ggc	G	0	353.20
gga	G	2675	1168.00
ggg	G	1	769.70
---	--	------	------
mPD		0.00075	0.69
		nPD:	0.
		N. weight:	0.84
		Sc. PD:	-0.087
		Sc. rank:	-1566.0

,

M1			
Pos.	246	obs:	exp:
tta	L	0	0.11
ttg	L	0	0.19
ctt	L	0	0.19
ctc	L	0	0.17
cta	L	0	0.13
ctg	L	1	0.21
att	I	0	0.37
atc	I	0	0.25
ata	I	1	0.38
gtt	V	0	581.50
gtc	V	0	565.00
gta	V	9	526.10
gtg	V	2665	1001.00
---	--	------	------
mPD		0.0089	0.73
		nPD:	0.01
		N. weight:	0.98
		Sc. PD:	-0.091
		Sc. rank:	-448.6

,

M1			
Pos.	247	obs:	exp:
caa	Q	2039	1362.00
cag	Q	637	1314.00
---	--	------	------
mPD		0.36	0.50
		nPD:	0.73
		N. weight:	0.14
		Sc. PD:	0.082
		Sc. rank:	312.5

,

M1			
Pos.	248	obs:	exp:
tta	L	0	165.00
ttg	L	2	283.70
ctt	L	4	283.50
ctc	L	0	260.90
cta	L	2	201.20
ctg	L	1504	317.70
att	I	0	4.08
atc	I	0	2.76
ata	I	11	4.16
atg	M	1153	1153.00
---	--	------	------
mPD		0.51	1.2
		nPD:	0.44
		N. weight:	0.88
		Sc. PD:	0.28
		Sc. rank:	1669.4

,

M1			
Pos.	249	obs:	exp:
caa	Q	1	1359.00
cag	Q	2671	1313.00
cgt	R	0	0.15
cgc	R	0	0.21
cga	R	0	0.39
cgg	R	4	0.31
aga	R	0	1.83
agg	R	0	1.10
---	--	------	------
mPD		0.0037	0.51
		nPD:	0.01
		N. weight:	0.72
		Sc. PD:	-0.071
		Sc. rank:	-653.2

,

M1			
Pos.	250	obs:	exp:
cgt	R	1	101.50
cgc	R	0	142.50
cga	R	2663	259.10
cgg	R	11	209.60
aga	R	1	1227.00
agg	R	0	735.80
---	--	------	------
mPD		0.0097	0.95
		nPD:	0.01
		N. weight:	2.3
		Sc. PD:	-0.22
		Sc. rank:	-1584.7

,

M1			
Pos.	251	obs:	exp:
ttt	F	1	1139.00
ttc	F	2675	1537.00
---	--	------	------
mPD		0.00075	0.49
		nPD:	0.
		N. weight:	0.56
		Sc. PD:	-0.058
		Sc. rank:	-940.7

,

M1			
Pos.	252	obs:	exp:
cgt	R	0	0.04
cgc	R	0	0.05
cga	R	0	0.10
cgg	R	0	0.08
aaa	K	8	1501.00
aag	K	2667	1174.00
aga	R	0	0.46
agg	R	1	0.27
---	--	------	------
mPD		0.0067	0.49
		nPD:	0.01
		N. weight:	0.82
		Sc. PD:	-0.075
		Sc. rank:	-329.4

,

M1			
Pos.	253	obs:	exp:
taa	.	0	981.30
tag	.	0	751.00
tga	.	2676	943.70
---	--	------	------
mPD		0	0.86
		nPD:	0.
		N. weight:	1.1
		Sc. PD:	-0.11
		Sc. rank:	-2396.8

}

Amino acid level conservation per codon

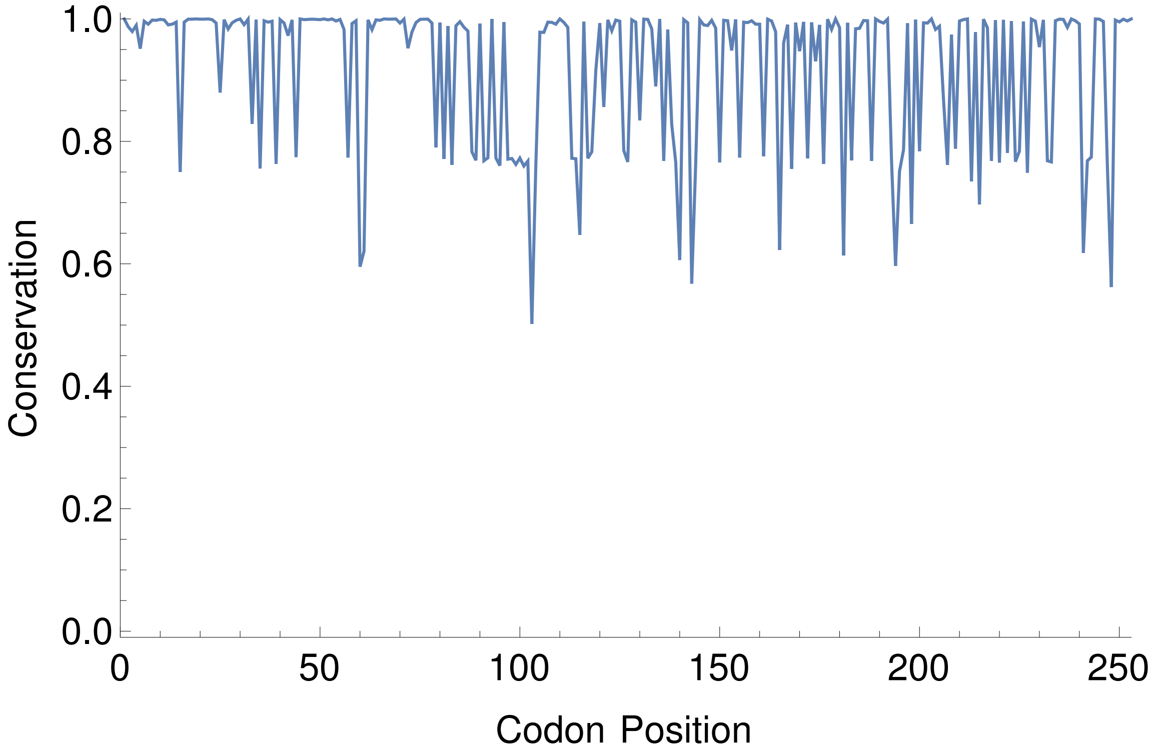

Conservation analysis plots

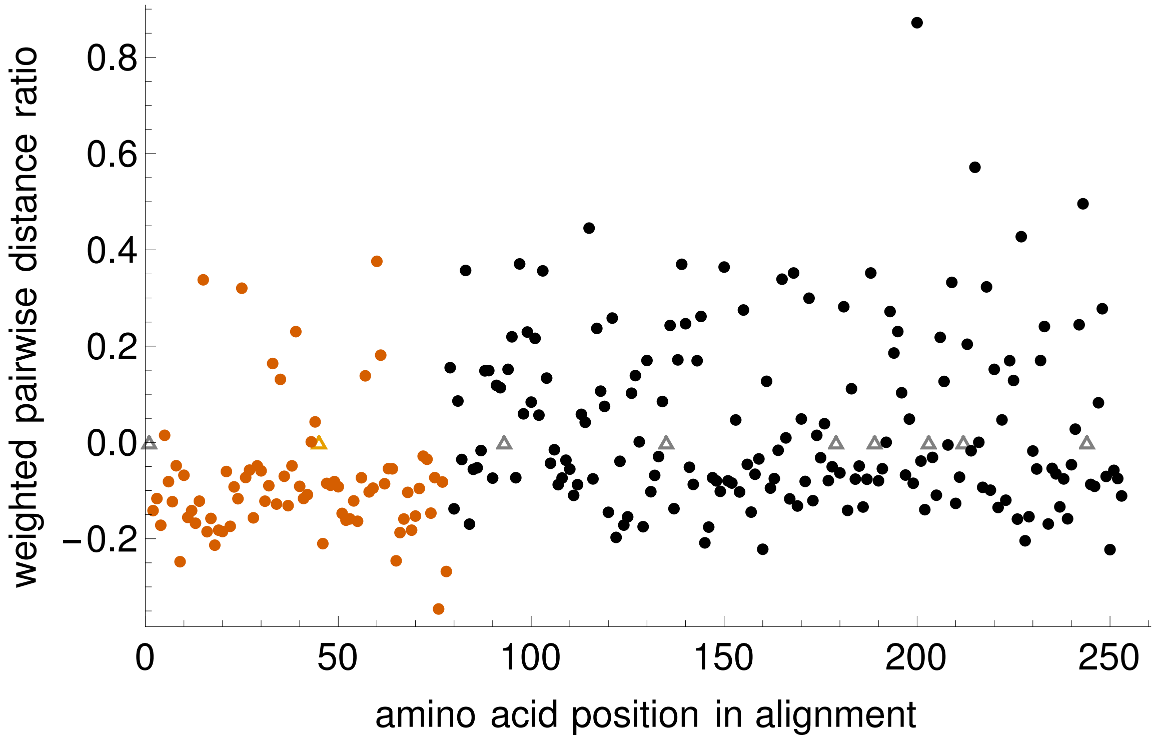

M1

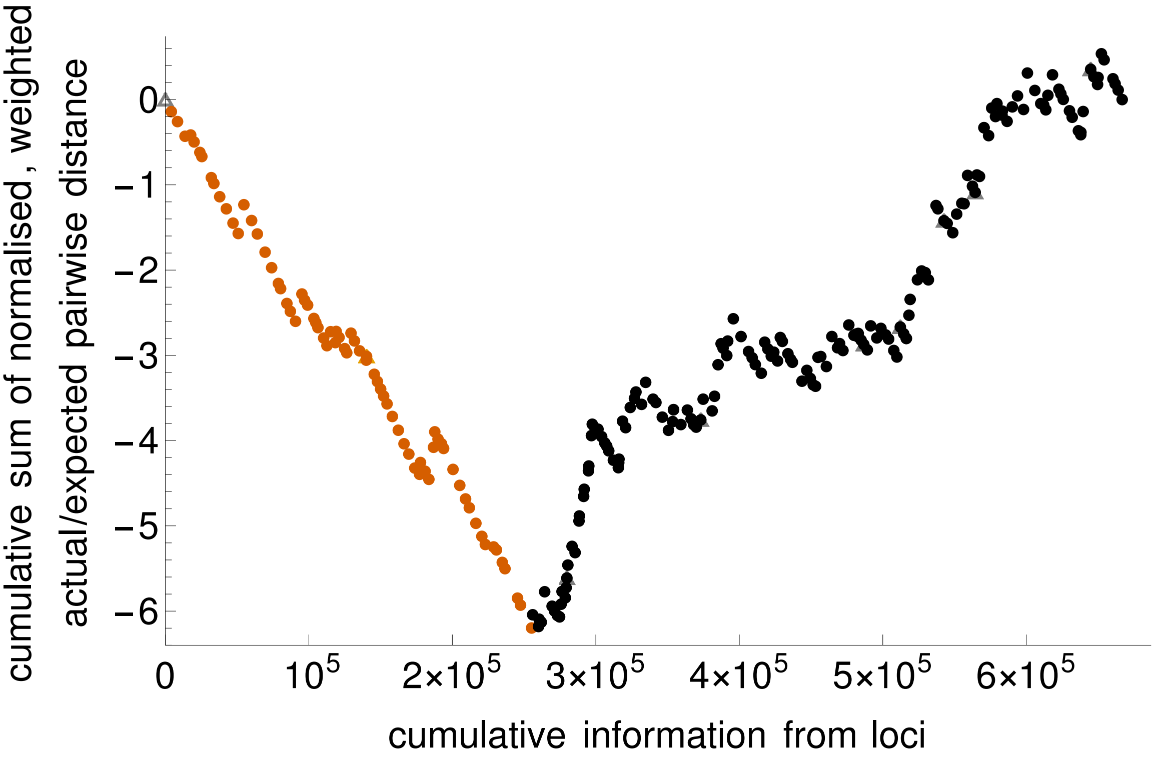

M1

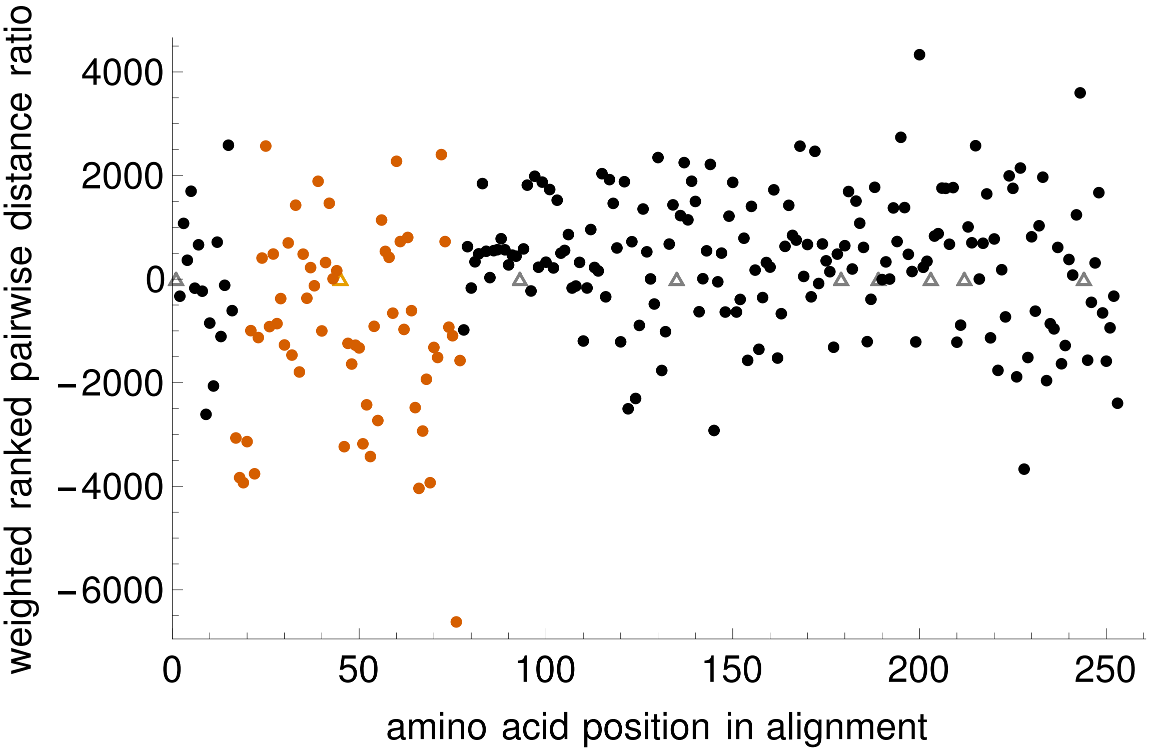
weighted ranked pairwise distance ratio
4000
2000
0
−2000
−4000
−6000
0
50
100
150
200
250
amino acid position in alignment

M1

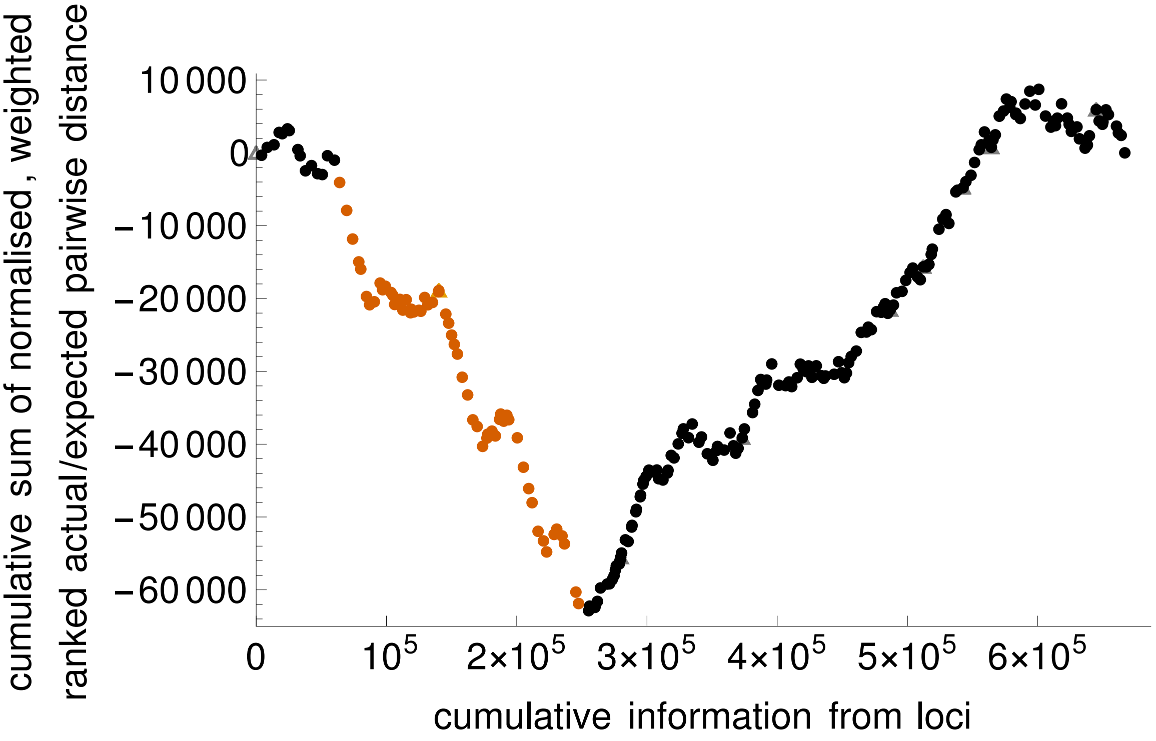
cumulative sum of normalised, weighted ranked actual/expected pairwise distance
10 000
0
−10 000
−20 000
−30 000
−40 000
−50 000
−60 000
0
10^5
2×10^5
3×10^5
4×10^5
5×10^5
6×10^5
cumulative information from loci

M1

M2

Gene length histogram

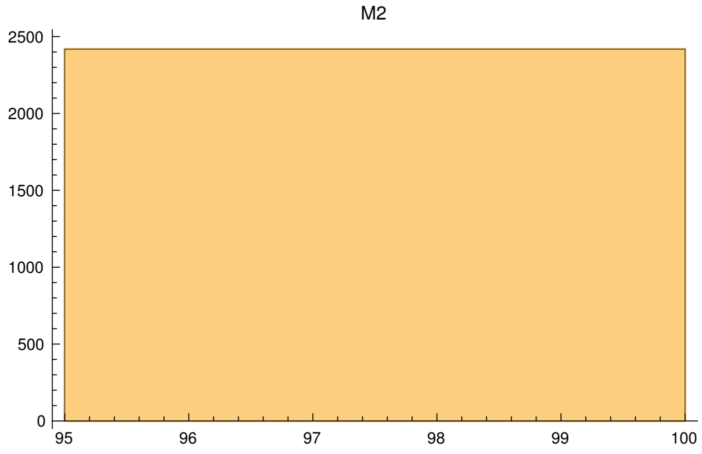

Information vs. nPD

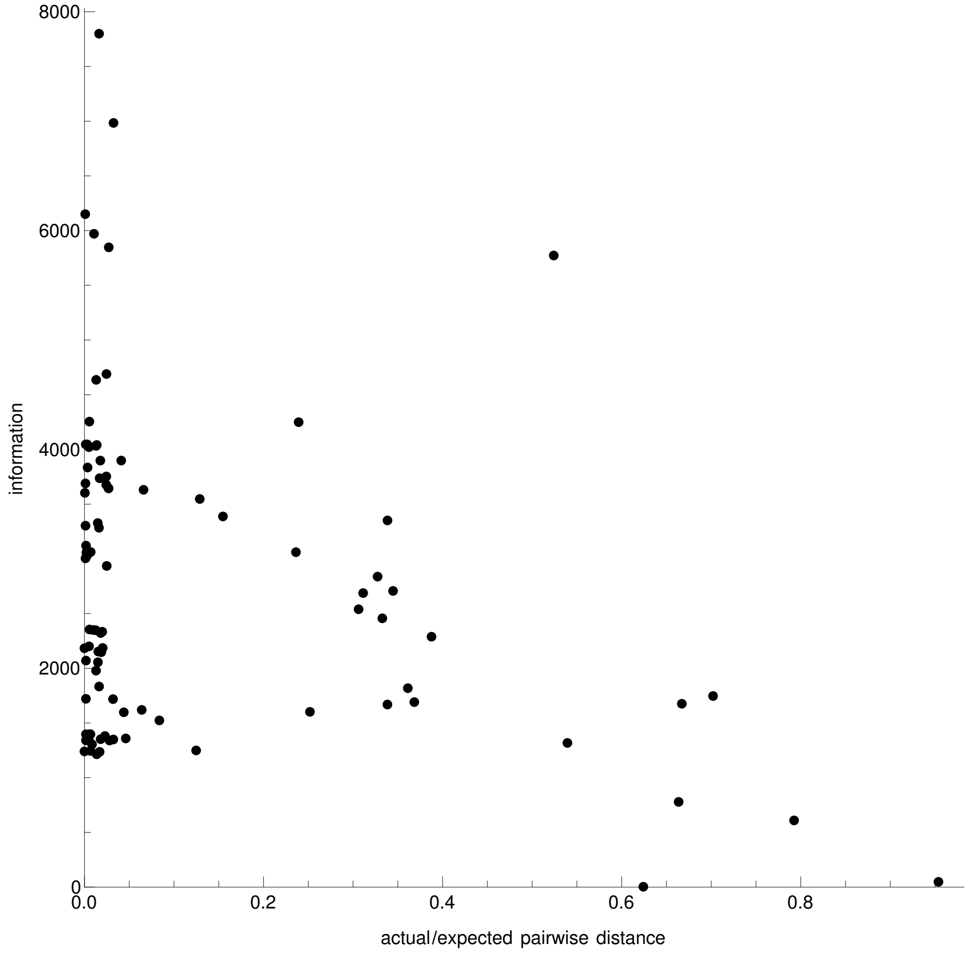

M2

Example sequences highlighted by regions found in analyses to be conserved

```
Interesting points (by weighted raw PD) highlighted for gene M2:
ATGAGTCTTCTAACCGAGGTCGAAACGCCTACCAGAAACGGATGGGAGTGCAAATGCAGCGATTCAAGTGATCCTC⋱
    TCGTTATTGCCGCAAGTATCATTGGGATCTTGCACTTGATATTGTGGATTCTTGATCGTCTTTTCTTCAAATGCG⋱
    TTTATCGTCGCCTTAAATACGGTTTGAAAAGAGGGCCTTCTACGGAAGGAGTGCCTGAGTCTATGAGGGAAGAGT⋱
    ATCGGCAGGAACAGCAGAGTGCTGTGGATGTTGACGATGGTCATTTTGTCAACATAGAGCTGGAGTAA

Interesting points (by weighted ranked PD) highlighted for gene M2:
ATGAGTCTTCTAACCGAGGTCGAAACGCCTACCAGAAACGGATGGGAGTGCAAATGCAGCGATTCAAGTGATCCTCTC⋱
    GTTATTGCCGCAAGTATCATTGGGATCTTGCACTTGATATTGTGGATTCTTGATCGTCTTTTCTTCAAATGCGTTTA⋱
    TCGTCGCCTTAAATACGGTTTGAAAAGAGGGCCTTCTACGGAAGGAGTGCCTGAGTCTATGAGGGAAGAGTATCGGC⋱
    AGGAACAGCAGAGTGCTGTGGATGTTGACGATGGTCATTTTGTCAACATAGAGCTGGAGTAA
```

Per codon conservation report

M2

Pos.	1	obs:	exp:
atg	M	2418	2418.00
---	--	------	------
mPD		0	0
		nPD:	1.
		N. weight:	0.
		Sc. PD:	0
		Sc. rank:	0

M2

Pos.	2	obs:	exp:
tct	S	0	410.70
tcc	S	0	354.50
tca	S	0	542.10
tcg	S	0	155.30
cgt	R	1	0.04
cgc	R	0	0.05
cga	R	0	0.10
cgg	R	0	0.08
att	I	2	0.74
atc	I	0	0.50
ata	I	0	0.76
act	T	1	0.26
acc	T	0	0.22
aca	T	0	0.43
acg	T	0	0.08
agt	S	2386	491.00
agc	S	19	451.20
aga	R	0	0.46
agg	R	0	0.27
gat	D	0	0.58
gac	D	1	0.42
ggt	G	8	1.15
ggc	G	0	1.06
gga	G	0	3.49
ggg	G	0	2.30
---	--	------	------
mPD		0.028	1.7
		nPD:	0.02
		N. weight:	1.4
		Sc. PD:	-0.093
		Sc. rank:	92.7

M2

Pos.	3	obs:	exp:
ttt	F	5	2.13
ttc	F	0	2.87
tta	L	0	261.30
ttg	L	0	449.20
ctt	L	2362	448.90
ctc	L	32	413.00
cta	L	0	318.50
ctg	L	0	503.00
cct	P	1	0.28
ccc	P	0	0.18
cca	P	0	0.41
ccg	P	0	0.13
cgt	R	15	0.61
cgc	R	0	0.85
cga	R	0	1.55
cgg	R	0	1.25
att	I	1	0.37
atc	I	0	0.25
ata	I	0	0.38
aga	R	0	7.34
agg	R	1	4.40
gtt	V	1	0.22
gtc	V	0	0.21
gta	V	0	0.20
gtg	V	0	0.37
---	--	------	------
mPD		0.048	1.2
		nPD:	0.04
		N. weight:	1.5
		Sc. PD:	-0.062
		Sc. rank:	1679.4

M2

Pos.	4	obs:	exp:
ttt	F	1	0.43
ttc	F	0	0.57
tta	L	7	263.40
ttg	L	0	452.80
ctt	L	11	452.50
ctc	L	4	416.30
cta	L	2386	321.10
ctg	L	5	507.00
caa	Q	2	1.02
cag	Q	0	0.98
att	I	0	0.37
atc	I	0	0.25
ata	I	1	0.38
gct	A	0	0.25
gcc	A	0	0.20
gca	A	1	0.44
gcg	A	0	0.11
---	--	------	------
mPD		0.028	1.1
		nPD:	0.02
		N. weight:	1.8
		Sc. PD:	-0.1
		Sc. rank:	1030.5

M2

Pos.	5	obs:	exp:
tct	S	0	2.56
tcc	S	0	2.21
tca	S	0	3.38
tcg	S	0	0.97
att	I	0	5.20
atc	I	14	3.51
ata	I	0	5.29
act	T	4	608.40
acc	T	2292	507.50
aca	T	0	987.00
acg	T	1	194.00
aat	N	0	48.36
aac	N	91	42.64
agt	S	0	3.06
agc	S	15	2.81
gtt	V	0	0.22
gtc	V	0	0.21
gta	V	0	0.20
gtg	V	1	0.37
---	--	------	------
mPD		0.10	0.79
		nPD:	0.13
		N. weight:	1.4
		Sc. PD:	0.058
		Sc. rank:	1874.8

M2

Pos.	6	obs:	exp:
aaa	K	0	1.12
aag	K	2	0.88
gaa	E	9	1447.00
gag	E	2407	969.40
---	--	------	------
mPD		0.0091	0.48
		nPD:	0.02
		N. weight:	0.82
		Sc. PD:	-0.052
		Sc. rank:	211.5

M2

Pos.	7	obs:	exp:
tta	L	0	0.11
ttg	L	0	0.19
ctt	L	1	0.19
ctc	L	0	0.17
cta	L	0	0.13
ctg	L	0	0.21
gtt	V	12	525.20
gtc	V	2397	510.30
gta	V	5	475.20
gtg	V	1	904.30
ggt	G	2	0.29
ggc	G	0	0.26
gga	G	0	0.87
ggg	G	0	0.58
---	--	------	------
mPD		0.020	0.73
		nPD:	0.03
		N. weight:	1.4
		Sc. PD:	-0.077
		Sc. rank:	1051.5

M2

Pos.	8	obs:	exp:
tat	Y	0	0.49
tac	Y	1	0.51
cgt	R	0	0.11
cgc	R	0	0.16
cga	R	2	0.29
cgg	R	0	0.23
aga	R	1	1.38
agg	R	0	0.82
gaa	E	2412	1445.00
gag	E	1	968.20
ggt	G	0	0.14
ggc	G	0	0.13
gga	G	1	0.44
ggg	G	0	0.29
---	--	------	------
mPD		0.0083	0.49
		nPD:	0.02
		N. weight:	0.47
		Sc. PD:	-0.031
		Sc. rank:	41.0

M2

Pos.	9	obs:	exp:
cgt	R	0	0.04
cgc	R	0	0.05
cga	R	0	0.10
cgg	R	0	0.08
act	T	0	638.40
acc	T	0	532.50
aca	T	0	1036.00
acg	T	2410	203.60
aaa	K	0	0.56
aag	K	1	0.44
aga	R	1	0.46
agg	R	0	0.27
gct	A	0	1.53
gcc	A	0	1.17
gca	A	0	2.64
gcg	A	6	0.67
---	--	------	------
mPD		0.0074	0.70
		nPD:	0.01
		N. weight:	2.3
		Sc. PD:	-0.16
		Sc. rank:	-1556.8

M2

Pos.	10	obs:	exp:
tta	L	0	0.55
ttg	L	0	0.94
ctt	L	5	0.94
ctc	L	0	0.86
cta	L	0	0.67
ctg	L	0	1.05
cct	P	2412	680.20
ccc	P	0	436.40
cca	P	0	984.80
ccg	P	0	310.60
cat	H	1	0.59
cac	H	0	0.41
---	--	------	------
mPD		0.0050	0.71
		nPD:	0.01
		N. weight:	1.2
		Sc. PD:	-0.087
		Sc. rank:	-996.7

M2

Pos.	11	obs:	exp:
att	I	0	8.54
atc	I	23	5.77
ata	I	0	8.69
act	T	0	634.40
acc	T	2395	529.20
aca	T	0	1029.00
acg	T	0	202.30
---	--	------	------
mPD		0.019	0.71
		nPD:	0.03
		N. weight:	1.4
		Sc. PD:	-0.077
		Sc. rank:	963.3

M2

Pos.	12	obs:	exp:
cgt	R	0	56.58
cgc	R	0	79.47
cga	R	0	144.50
cgg	R	0	116.90
aaa	K	926	519.80
aag	K	0	406.20
aga	R	1491	684.40
agg	R	1	410.20
---	--	------	------
mPD		0.47	1.3
		nPD:	0.37
		N. weight:	0.64
		Sc. PD:	0.18
		Sc. rank:	1281.1

M2

Pos.	13	obs:	exp:
tct	S	0	1.37
tcc	S	0	1.18
tca	S	0	1.80
tcg	S	0	0.52
att	I	0	1.49
atc	I	4	1.00
ata	I	0	1.51
act	T	0	156.80
acc	T	592	130.80
aca	T	0	254.40
acg	T	0	50.00
aat	N	0	959.70
aac	N	1806	846.30
aaa	K	3	1.68
aag	K	0	1.32
agt	S	0	1.63
agc	S	8	1.50
gct	A	0	0.25
gcc	A	1	0.20
gca	A	0	0.44
gcg	A	0	0.11
gat	D	0	2.30
gac	D	4	1.70
---	--	------	------
mPD		0.38	0.99
		nPD:	0.39
		N. weight:	0.87
		Sc. PD:	0.25
		Sc. rank:	1769.2

M2

Pos.	14	obs:	exp:
gaa	E	589	352.70
gag	E	0	236.30
ggt	G	0	263.10
ggc	G	0	241.40
gga	G	1829	798.40
ggg	G	0	526.10
---	--	------	------
mPD		0.37	1.0
		nPD:	0.36
		N. weight:	0.69
		Sc. PD:	0.18
		Sc. rank:	1356.9

M2

Pos.	15	obs:	exp:
tgg	W	2418	2418.00
---	--	------	------
mPD		0	0
		nPD:	1.
		N. weight:	0.
		Sc. PD:	0
		Sc. rank:	0

M2

Pos.	16	obs:	exp:
gat	D	0	0.58
gac	D	1	0.42
gaa	E	1	1447.00
gag	E	2415	969.40
ggt	G	0	0.14
ggc	G	0	0.13
gga	G	0	0.44
ggg	G	1	0.29
---	--	------	------
mPD		0.0025	0.48
		nPD:	0.01
		N. weight:	0.84
		Sc. PD:	-0.064
		Sc. rank:	-898.4

,

M2

Pos.	17	obs:	exp:
tat	Y	0	0.97
tac	Y	2	1.03
tgt	C	0	1051.00
tgc	C	2416	1365.00
---	--	------	------
mPD		0.0017	0.49
		nPD:	0.
		N. weight:	0.53
		Sc. PD:	-0.041
		Sc. rank:	-760.4

,

M2

Pos.	18	obs:	exp:
tct	S	0	0.34
tcc	S	0	0.29
tca	S	0	0.45
tcg	S	0	0.13
cgt	R	0	23.36
cgc	R	0	32.81
cga	R	0	59.65
cgg	R	0	48.25
aat	N	2	699.30
aac	N	1314	616.70
aaa	K	484	271.70
aag	K	0	212.30
agt	S	0	0.41
agc	S	2	0.38
aga	R	616	282.60
agg	R	0	169.40
---	--	------	------
mPD		0.88	1.3
		nPD:	0.7
		N. weight:	0.67
		Sc. PD:	0.4
		Sc. rank:	1483.0

,

M2

Pos.	19	obs:	exp:
ttt	F	0	1.70
ttc	F	4	2.30
tat	Y	0	4.86
tac	Y	10	5.14
tgt	C	0	1045.00
tgc	C	2404	1359.00
---	--	------	------
mPD		0.012	0.50
		nPD:	0.02
		N. weight:	0.53
		Sc. PD:	-0.031
		Sc. rank:	190.8

,

M2

Pos.	20	obs:	exp:
tct	S	0	411.90
tcc	S	0	355.60
tca	S	0	543.70
tcg	S	0	155.80
cgt	R	0	0.04
cgc	R	0	0.05
cga	R	0	0.10
cgg	R	0	0.08
aat	N	0	0.53
aac	N	1	0.47
agt	S	0	492.50
agc	S	2412	452.50
aga	R	1	0.46
agg	R	0	0.27
ggt	G	0	0.58
ggc	G	4	0.53
gga	G	0	1.75
ggg	G	0	1.15
---	--	------	------
mPD		0.0050	1.6
		nPD:	0.
		N. weight:	1.5
		Sc. PD:	-0.12
		Sc. rank:	-2266.9

,

M2

Pos.	21	obs:	exp:
gtt	V	1	0.22
gtc	V	0	0.21
gta	V	0	0.20
gtg	V	0	0.37
gat	D	2407	1385.00
gac	D	0	1022.00
ggt	G	10	1.44
ggc	G	0	1.32
gga	G	0	4.37
ggg	G	0	2.88
---	--	------	------
mPD		0.0091	0.50
		nPD:	0.02
		N. weight:	0.52
		Sc. PD:	-0.033
		Sc. rank:	92.6

,

M2

Pos.	22	obs:	exp:
tta	L	1	0.11
ttg	L	0	0.19
tct	S	0	412.80
tcc	S	0	356.30
tca	S	2417	544.80
tcg	S	0	156.10
ctt	L	0	0.19
ctc	L	0	0.17
cta	L	0	0.13
ctg	L	0	0.21
agt	S	0	493.50
agc	S	0	453.50
---	--	------	------
mPD		0.00083	1.6
		nPD:	0.
		N. weight:	1.4
		Sc. PD:	-0.11
		Sc. rank:	-2988.7

,

M2

Pos.	23	obs:	exp:
tct	S	0	411.80
tcc	S	0	355.40
tca	S	0	543.50
tcg	S	0	155.70
aat	N	7	3.72
aac	N	0	3.28
agt	S	2411	492.30
agc	S	0	452.40
---	--	------	------
mPD		0.0058	1.6
		nPD:	0.
		N. weight:	1.5
		Sc. PD:	-0.11
		Sc. rank:	-2014.3

,

M2

Pos.	24	obs:	exp:
gat	D	2413	1391.00
gac	D	5	1027.00
---	--	------	------
mPD		0.0041	0.49
		nPD:	0.01
		N. weight:	0.5
		Sc. PD:	-0.036
		Sc. rank:	-413.2

,

M2

Pos.	25	obs:	exp:
cct	P	2415	681.80
ccc	P	2	437.50
cca	P	1	987.30
ccg	P	0	311.30
---	--	------	------
mPD		0.0025	0.70
		nPD:	0.
		N. weight:	1.2
		Sc. PD:	-0.09
		Sc. rank:	-1534.0

,

M2

Pos.	26	obs:	exp:
tta	L	0	263.90
ttg	L	0	453.70
ctt	L	592	453.40
ctc	L	1823	417.20
cta	L	3	321.70
ctg	L	0	508.00
---	--	------	------
mPD		0.37	1.1
		nPD:	0.33
		N. weight:	1.1
		Sc. PD:	0.25
		Sc. rank:	1888.7

,

M2

Pos.	27	obs:	exp:
tct	S	4	0.68
tcc	S	0	0.59
tca	S	0	0.90
tcg	S	0	0.26
att	I	8	2.97
atc	I	0	2.01
ata	I	0	3.02
agt	S	0	0.82
agc	S	0	0.75
gtt	V	2400	522.20
gtc	V	1	507.30
gta	V	0	472.40
gtg	V	0	899.10
gct	A	4	1.02
gcc	A	0	0.78
gca	A	0	1.76
gcg	A	0	0.44
ggt	G	1	0.14
ggc	G	0	0.13
gga	G	0	0.44
ggg	G	0	0.29
---	--	------	------
mPD		0.018	0.75
		nPD:	0.02
		N. weight:	1.4
		Sc. PD:	-0.081
		Sc. rank:	599.4

,

M2

Pos.	28	obs:	exp:
ttt	F	1	0.43
ttc	F	0	0.57
att	I	1822	676.60
atc	I	0	456.90
ata	I	0	688.50
gtt	V	593	129.20
gtc	V	1	125.50
gta	V	0	116.90
gtg	V	0	222.40
gct	A	1	0.25
gcc	A	0	0.20
gca	A	0	0.44
gcg	A	0	0.11
---	--	------	------
mPD		0.37	1.1
		nPD:	0.34
		N. weight:	1.
		Sc. PD:	0.26
		Sc. rank:	1987.2

,

M2

Pos.	29	obs:	exp:
act	T	0	0.53
acc	T	2	0.44
aca	T	0	0.86
acg	T	0	0.17
gtt	V	0	1.09
gtc	V	5	1.06
gta	V	0	0.98
gtg	V	0	1.87
gct	A	8	613.30
gcc	A	2403	470.20
gca	A	0	1060.00
gcg	A	0	267.70
---	--	------	------
mPD		0.012	0.70
		nPD:	0.02
		N. weight:	1.5
		Sc. PD:	-0.095
		Sc. rank:	231.4

,

M2

Pos.	30	obs:	exp:
tct	S	0	1.20
tcc	S	0	1.03
tca	S	7	1.58
tcg	S	0	0.45
cct	P	0	0.28
ccc	P	0	0.18
cca	P	1	0.41
ccg	P	0	0.13
act	T	0	0.26
acc	T	0	0.22
aca	T	1	0.43
acg	T	0	0.08
agt	S	0	1.43
agc	S	0	1.31
gct	A	1	612.80
gcc	A	0	469.80
gca	A	2407	1059.00
gcg	A	1	267.40
---	--	------	------
mPD		0.0091	0.70
		nPD:	0.01
		N. weight:	0.75
		Sc. PD:	-0.052
		Sc. rank:	-458.3

,

M2

Pos.	31	obs:	exp:
tct	S	0	310.80
tcc	S	0	268.30
tca	S	0	410.30
tcg	S	0	117.60
cgt	R	0	0.08
cgc	R	0	0.11
cga	R	0	0.19
cgg	R	0	0.16
aat	N	534	316.70
aac	N	62	279.30
agt	S	1817	371.60
agc	S	3	341.50
aga	R	2	0.92
agg	R	0	0.55
mPD		0.43	1.8
		nPD:	0.24
		N. weight:	1.2
		Sc. PD:	0.17
		Sc. rank:	1750.8

M2

Pos.	32	obs:	exp:
ttt	F	0	1.28
ttc	F	3	1.72
att	I	0	894.20
atc	I	2406	603.90
ata	I	2	909.90
act	T	0	0.53
acc	T	2	0.44
aca	T	0	0.86
acg	T	0	0.17
gtt	V	0	1.09
gtc	V	5	1.06
gta	V	0	0.98
gtg	V	0	1.87
mPD		0.0099	0.67
		nPD:	0.01
		N. weight:	1.3
		Sc. PD:	-0.085
		Sc. rank:	-384.5

M2

Pos.	33	obs:	exp:
att	I	2408	897.10
atc	I	8	605.90
ata	I	0	912.90
gtt	V	2	0.43
gtc	V	0	0.42
gta	V	0	0.39
gtg	V	0	0.75
mPD		0.0083	0.66
		nPD:	0.01
		N. weight:	0.89
		Sc. PD:	-0.062
		Sc. rank:	-575.1

M2

Pos.	34	obs:	exp:
ggt	G	0	347.80
ggc	G	0	319.20
gga	G	1	1056.00
ggg	G	2417	695.50
mPD		0.00083	0.69
		nPD:	0.
		N. weight:	1.1
		Sc. PD:	-0.092
		Sc. rank:	-2166.0

M2

Pos.	35	obs:	exp:
att	I	9	896.40
atc	I	2405	605.40
ata	I	0	912.20
gtt	V	0	0.87
gtc	V	4	0.85
gta	V	0	0.79
gtg	V	0	1.50
mPD		0.011	0.66
		nPD:	0.02
		N. weight:	1.3
		Sc. PD:	-0.082
		Sc. rank:	-213.9

M2

Pos.	36	obs:	exp:
tta	L	0	263.70
ttg	L	1864	453.30
ctt	L	0	453.00
ctc	L	0	416.80
cta	L	0	321.50
ctg	L	552	507.60
gtt	V	0	0.43
gtc	V	0	0.42
gta	V	0	0.39
gtg	V	2	0.75
mPD		0.35	1.1
		nPD:	0.31
		N. weight:	1.
		Sc. PD:	0.22
		Sc. rank:	1737.4

M2

Pos.	37	obs:	exp:
cat	H	0	1438.00
cac	H	2418	980.30
mPD		0	0.48
		nPD:	0.
		N. weight:	0.83
		Sc. PD:	-0.068
		Sc. rank:	-1888.9

M2

Pos.	38	obs:	exp:
tta	L	0	263.40
ttg	L	2413	452.80
tct	S	0	0.17
tcc	S	0	0.15
tca	S	0	0.23
tcg	S	1	0.06
ctt	L	0	452.50
ctc	L	0	416.30
cta	L	0	321.10
ctg	L	0	507.00
atg	M	4	4.00
agt	S	0	0.20
agc	S	0	0.19
mPD		0.0041	1.1
		nPD:	0.
		N. weight:	1.5
		Sc. PD:	-0.12
		Sc. rank:	-1929.7

M2

Pos.	39	obs:	exp:
tta	L	0	0.11
ttg	L	0	0.19
ctt	L	0	0.19
ctc	L	0	0.17
cta	L	1	0.13
ctg	L	0	0.21
att	I	0	894.90
atc	I	0	604.40
ata	I	2410	910.70
atg	M	4	4.00
act	T	0	0.79
acc	T	0	0.66
aca	T	3	1.29
acg	T	0	0.25
mPD		0.0066	0.66
		nPD:	0.01
		N. weight:	0.9
		Sc. PD:	-0.064
		Sc. rank:	-707.1

M2

Pos.	40	obs:	exp:
tta	L	3	263.90
ttg	L	2324	453.70
ctt	L	0	453.40
ctc	L	0	417.20
cta	L	0	321.70
ctg	L	91	508.00
mPD		0.075	1.1
		nPD:	0.07
		N. weight:	1.4
		Sc. PD:	-0.025
		Sc. rank:	1764.8

M2

Pos.	41	obs:	exp:
tgg	W	2418	2418.00
mPD		0	0
		nPD:	1.
		N. weight:	0.
		Sc. PD:	0
		Sc. rank:	0

M2

Pos.	42	obs:	exp:
att	I	1863	897.10
atc	I	553	605.90
ata	I	0	912.90
act	T	0	0.26
acc	T	1	0.22
aca	T	0	0.43
acg	T	0	0.08
gtt	V	1	0.22
gtc	V	0	0.21
gta	V	0	0.20
gtg	V	0	0.37
mPD		0.36	0.66
		nPD:	0.54
		N. weight:	0.5
		Sc. PD:	0.22
		Sc. rank:	1081.6

M2

Pos.	43	obs:	exp:
ttt	F	2	0.85
ttc	F	0	1.15
tta	L	0	263.70
ttg	L	0	453.30
ctt	L	2416	453.00
ctc	L	0	416.80
cta	L	0	321.50
ctg	L	0	507.60
mPD		0.0017	1.1
		nPD:	0.
		N. weight:	1.5
		Sc. PD:	-0.12
		Sc. rank:	-2806.3

M2

Pos.	44	obs:	exp:
tat	Y	1	0.49
tac	Y	0	0.51
gat	D	2415	1389.00
gac	D	0	1026.00
ggt	G	2	0.29
ggc	G	0	0.26
gga	G	0	0.87
ggg	G	0	0.58
mPD		0.0025	0.49
		nPD:	0.01
		N. weight:	0.51
		Sc. PD:	-0.039
		Sc. rank:	-615.4

M2

Pos.	45	obs:	exp:
cat	H	18	10.70
cac	H	0	7.30
cgt	R	1115	91.02
cgc	R	1285	127.80
cga	R	0	232.40
cgg	R	0	188.00
aga	R	0	1101.00
agg	R	0	659.90
mPD		0.51	0.98
		nPD:	0.52
		N. weight:	2.2
		Sc. PD:	0.93
		Sc. rank:	4686.9

M2

Pos.	46	obs:	exp:
ttt	F	18	7.66
ttc	F	0	10.34
tta	L	0	262.00
ttg	L	0	450.30
ctt	L	2400	450.00
ctc	L	0	414.10
cta	L	0	319.30
ctg	L	0	504.30
mPD		0.015	1.1
		nPD:	0.01
		N. weight:	1.5
		Sc. PD:	-0.11
		Sc. rank:	-824.6

M2

Pos.	47	obs:	exp:
ttt	F	0	1028.00
ttc	F	2417	1389.00
tta	L	0	0.11
ttg	L	0	0.19
ctt	L	0	0.19
ctc	L	1	0.17
cta	L	0	0.13
ctg	L	0	0.21
mPD		0.00083	0.49
		nPD:	0.
		N. weight:	0.51
		Sc. PD:	-0.041
		Sc. rank:	-890.2

M2

Pos.	48	obs:	exp:
ttt	F	2	1021.00
ttc	F	2398	1379.00
tta	L	1	0.33
ttg	L	0	0.56
tct	S	0	2.56
tcc	S	15	2.21
tca	S	0	3.38
tcg	S	0	0.97
ctt	L	0	0.56
ctc	L	2	0.52
cta	L	0	0.40
ctg	L	0	0.63
agt	S	0	3.06
agc	S	0	2.81
mPD		0.016	0.51
		nPD:	0.03
		N. weight:	0.51
		Sc. PD:	-0.026
		Sc. rank:	482.5

M2

Pos.	49	obs:	exp:
cgt	R	0	0.11
cgc	R	0	0.16
cga	R	0	0.29
cgg	R	0	0.23
aat	N	1	0.53
aac	N	0	0.47
aaa	K	2414	1355.00
aag	K	0	1059.00
aga	R	3	1.38
agg	R	0	0.82
mPD		0.0033	0.50
		nPD:	0.01
		N. weight:	0.53
		Sc. PD:	-0.04
		Sc. rank:	-495.2

M2

Pos.	50	obs:	exp:
tat	Y	1	4.86
tac	Y	9	5.14
tgt	C	6	1047.00
tgc	C	2402	1361.00
mPD		0.014	0.50
		nPD:	0.03
		N. weight:	0.51
		Sc. PD:	-0.028
		Sc. rank:	451.5

M2			
Pos.	51	obs:	exp:
att	I	619	230.20
atc	I	1	155.50
ata	I	0	234.30
gtt	V	1798	391.00
gtc	V	0	379.90
gta	V	0	353.80
gtg	V	0	673.30
---	--	------	------
mPD		0.38	1.1
		nPD:	0.34
		N. weight:	1.3
		Sc. PD:	0.31
		Sc. rank:	2399.0

,

M2			
Pos.	52	obs:	exp:
tat	Y	2366	1171.00
tac	Y	44	1239.00
tgt	C	1	0.43
tgc	C	0	0.57
cat	H	7	4.16
cac	H	0	2.84
---	--	------	------
mPD		0.042	0.51
		nPD:	0.08
		N. weight:	0.58
		Sc. PD:	-0.00041
		Sc. rank:	756.1

,

M2			
Pos.	53	obs:	exp:
cat	H	1	0.59
cac	H	0	0.41
cgt	R	2399	91.66
cgc	R	18	128.70
cga	R	0	234.00
cgg	R	0	189.30
aga	R	0	1109.00
agg	R	0	664.60
---	--	------	------
mPD		0.016	0.95
		nPD:	0.02
		N. weight:	3.
		Sc. PD:	-0.19
		Sc. rank:	-2.6

,

M2			
Pos.	54	obs:	exp:
tta	L	0	0.11
ttg	L	0	0.19
tgt	C	0	12.61
tgc	C	29	16.39
ctt	L	0	0.19
ctc	L	1	0.17
cta	L	0	0.13
ctg	L	0	0.21
cat	H	0	2.97
cac	H	5	2.03
cgt	R	4	90.37
cgc	R	2379	126.90
cga	R	0	230.80
cgg	R	0	186.60
aga	R	0	1093.00
agg	R	0	655.20
---	--	------	------
mPD		0.032	0.99
		nPD:	0.03
		N. weight:	2.7
		Sc. PD:	-0.13
		Sc. rank:	2825.6

,

M2			
Pos.	55	obs:	exp:
ttt	F	2	0.85
ttc	F	0	1.15
tta	L	0	262.70
ttg	L	0	451.70
ctt	L	2407	451.30
ctc	L	0	415.30
cta	L	0	320.30
ctg	L	0	505.70
att	I	1	3.34
atc	I	8	2.26
ata	I	0	3.40
---	--	------	------
mPD		0.016	1.1
		nPD:	0.01
		N. weight:	1.5
		Sc. PD:	-0.1
		Sc. rank:	-557.4

,

M2			
Pos.	56	obs:	exp:
cgt	R	0	0.04
cgc	R	0	0.05
cga	R	0	0.10
cgg	R	0	0.08
aaa	K	2417	1357.00
aag	K	0	1060.00
aga	R	1	0.46
agg	R	0	0.27
---	--	------	------
mPD		0.00083	0.49
		nPD:	0.
		N. weight:	0.53
		Sc. PD:	-0.042
		Sc. rank:	-939.4

,

M2			
Pos.	57	obs:	exp:
tat	Y	0	1155.00
tac	Y	2376	1221.00
cat	H	0	24.97
cac	H	42	17.03
---	--	------	------
mPD		0.034	0.53
		nPD:	0.06
		N. weight:	0.62
		Sc. PD:	-0.012
		Sc. rank:	747.6

,

M2			
Pos.	58	obs:	exp:
tct	S	0	0.34
tcc	S	0	0.29
tca	S	0	0.45
tcg	S	0	0.13
agt	S	2	0.41
agc	S	0	0.38
gtt	V	2	0.43
gtc	V	0	0.42
gta	V	0	0.39
gtg	V	0	0.75
ggt	G	2407	347.30
ggc	G	0	318.60
gga	G	0	1054.00
ggg	G	7	694.30
---	--	------	------
mPD		0.0091	0.69
		nPD:	0.01
		N. weight:	1.8
		Sc. PD:	-0.12
		Sc. rank:	-803.4

,

M2			
Pos.	59	obs:	exp:
ttt	F	2	0.85
ttc	F	0	1.15
tta	L	3	263.50
ttg	L	2411	453.00
ctt	L	0	452.70
ctc	L	0	416.50
cta	L	0	321.20
ctg	L	0	507.20
atg	M	2	2.00
---	--	------	------
mPD		0.0058	1.1
		nPD:	0.01
		N. weight:	1.5
		Sc. PD:	-0.12
		Sc. rank:	-1701.4

,

M2			
Pos.	60	obs:	exp:
aat	N	0	1.59
aac	N	3	1.41
aaa	K	2415	1356.00
aag	K	0	1059.00
---	--	------	------
mPD		0.0025	0.49
		nPD:	0.01
		N. weight:	0.53
		Sc. PD:	-0.041
		Sc. rank:	-654.1

,

M2			
Pos.	61	obs:	exp:
cgt	R	0	69.10
cgc	R	0	97.05
cga	R	0	176.40
cgg	R	0	142.70
att	I	0	219.50
atc	I	0	148.20
ata	I	591	223.30
atg	M	2	2.00
aaa	K	2	1.12
aag	K	0	0.88
aga	R	1724	835.70
agg	R	98	501.00
ggt	G	0	0.14
ggc	G	0	0.13
gga	G	1	0.44
ggg	G	0	0.29
---	--	------	------
mPD		0.45	1.3
		nPD:	0.34
		N. weight:	0.64
		Sc. PD:	0.16
		Sc. rank:	1157.0

,

M2			
Pos.	62	obs:	exp:
tgg	W	1	1.00
cgt	R	0	0.30
cgc	R	0	0.43
cga	R	0	0.77
cgg	R	0	0.63
aga	R	0	3.67
agg	R	8	2.20
ggt	G	0	346.50
ggc	G	7	318.00
gga	G	5	1052.00
ggg	G	2397	692.90
---	--	------	------
mPD		0.017	0.70
		nPD:	0.02
		N. weight:	1.1
		Sc. PD:	-0.064
		Sc. rank:	703.1

,

M2			
Pos.	63	obs:	exp:
tta	L	0	0.22
ttg	L	0	0.38
ctt	L	2	0.38
ctc	L	0	0.35
cta	L	0	0.27
ctg	L	0	0.42
cct	P	2416	681.30
ccc	P	0	437.20
cca	P	0	986.50
ccg	P	0	311.10
---	--	------	------
mPD		0.0017	0.71
		nPD:	0.
		N. weight:	1.2
		Sc. PD:	-0.092
		Sc. rank:	-1861.8

,

M2			
Pos.	64	obs:	exp:
tct	S	2407	411.40
tcc	S	2	355.10
tca	S	0	543.00
tcg	S	0	155.60
cct	P	9	2.54
ccc	P	0	1.63
cca	P	0	3.67
ccg	P	0	1.16
agt	S	0	491.90
agc	S	0	452.00
---	--	------	------
mPD		0.0091	1.6
		nPD:	0.01
		N. weight:	1.6
		Sc. PD:	-0.12
		Sc. rank:	-1615.4

,

M2			
Pos.	65	obs:	exp:
atg	M	6	6.00
act	T	0	638.40
acc	T	0	532.50
aca	T	15	1036.00
acg	T	2395	203.60
gct	A	0	0.51
gcc	A	0	0.39
gca	A	0	0.88
gcg	A	2	0.22
---	--	------	------
mPD		0.019	0.70
		nPD:	0.03
		N. weight:	2.2
		Sc. PD:	-0.12
		Sc. rank:	1917.4

,

M2			
Pos.	66	obs:	exp:
aaa	K	14	7.86
aag	K	0	6.14
gaa	E	2332	1398.00
gag	E	2	936.50
ggt	G	0	10.07
ggc	G	0	9.24
gga	G	70	30.56
ggg	G	0	20.13
---	--	------	------
mPD		0.069	0.56
		nPD:	0.12
		N. weight:	0.48
		Sc. PD:	0.018
		Sc. rank:	630.4

,

M2			
Pos.	67	obs:	exp:
ggt	G	3	347.80
ggc	G	0	319.20
gga	G	1568	1056.00
ggg	G	847	695.50
---	--	------	------
mPD		0.46	0.69
		nPD:	0.66
		N. weight:	0.3
		Sc. PD:	0.17
		Sc. rank:	643.3

,

M2			
Pos.	68	obs:	exp:
tta	L	9	0.98
ttg	L	0	1.69
ctt	L	0	1.69
ctc	L	0	1.55
cta	L	0	1.20
ctg	L	0	1.89
atg	M	8	8.00
gtt	V	2	522.20
gtc	V	1	507.30
gta	V	1441	472.40
gtg	V	957	899.10
---	--	------	------
mPD		0.50	0.74
		nPD:	0.67
		N. weight:	0.64
		Sc. PD:	0.36
		Sc. rank:	1403.1

,

M2			
Pos.	69	obs:	exp:
tta	L	0	0.11
ttg	L	0	0.19
ctt	L	1	0.19
ctc	L	0	0.17
cta	L	0	0.13
ctg	L	0	0.21
cct	P	2416	681.60
ccc	P	0	437.30
cca	P	1	986.90
ccg	P	0	311.20
---	--	------	------
mPD		0.0017	0.71
		nPD:	0.
		N. weight:	1.2
		Sc. PD:	-0.092
		Sc. rank:	-1793.9

,

M2			
Pos.	70	obs:	exp:
aaa	K	0	0.56
aag	K	1	0.44
gaa	E	8	1447.00
gag	E	2409	969.80
---	--	------	------
mPD		0.0074	0.48
		nPD:	0.02
		N. weight:	0.82
		Sc. PD:	-0.054
		Sc. rank:	-187.8

,

M2

Pos.	71	obs:	exp:
tct	S	1421	412.90
tcc	S	938	356.50
tca	S	59	545.10
tcg	S	0	156.20
agt	S	0	493.70
agc	S	0	453.70
mPD		0.50	1.6
		nPD:	0.31
		N. weight:	0.97
		Sc. PD:	0.21
		Sc. rank:	1595.7

M2

Pos.	72	obs:	exp:
tta	L	0	0.22
ttg	L	2	0.38
ctt	L	0	0.38
ctc	L	0	0.35
cta	L	0	0.27
ctg	L	0	0.42
atg	M	2416	2416.00
mPD		0.0017	0.0026
		nPD:	0.62
		N. weight:	0.0013
		Sc. PD:	0.00066
		Sc. rank:	2.7

M2

Pos.	73	obs:	exp:
cgt	R	0	91.62
cgc	R	0	128.70
cga	R	0	233.90
cgg	R	0	189.20
aaa	K	0	1.12
aag	K	2	0.88
aga	R	0	1108.00
agg	R	2416	664.30
mPD		0.0017	0.95
		nPD:	0.
		N. weight:	1.2
		Sc. PD:	-0.095
		Sc. rank:	-1962.1

M2

Pos.	74	obs:	exp:
gaa	E	2418	1448.00
gag	E	0	970.20
mPD		0	0.48
		nPD:	0.
		N. weight:	0.47
		Sc. PD:	-0.038
		Sc. rank:	-1073.0

M2

Pos.	75	obs:	exp:
aaa	K	3	6.17
aag	K	8	4.83
gaa	E	144	1441.00
gag	E	2263	965.80
mPD		0.12	0.49
		nPD:	0.25
		N. weight:	0.61
		Sc. PD:	0.098
		Sc. rank:	979.2

M2

Pos.	76	obs:	exp:
tat	Y	946	1175.00
tac	Y	1472	1243.00
mPD		0.48	0.50
		nPD:	0.95
		N. weight:	0.018
		Sc. PD:	0.015
		Sc. rank:	41.0

M2

Pos.	77	obs:	exp:
cgt	R	0	91.70
cgc	R	0	128.80
cga	R	1	234.10
cgg	R	2417	189.40
aga	R	0	1109.00
agg	R	0	664.90
mPD		0.00083	0.95
		nPD:	0.
		N. weight:	2.3
		Sc. PD:	-0.19
		Sc. rank:	-4847.2

M2

Pos.	78	obs:	exp:
caa	Q	0	1230.00
cag	Q	2417	1187.00
cgt	R	0	0.04
cgc	R	0	0.05
cga	R	0	0.10
cgg	R	1	0.08
aga	R	0	0.46
agg	R	0	0.27
mPD		0.00083	0.50
		nPD:	0.
		N. weight:	0.66
		Sc. PD:	-0.052
		Sc. rank:	-1173.6

M2

Pos.	79	obs:	exp:
cgt	R	0	0.04
cgc	R	0	0.05
cga	R	0	0.10
cgg	R	0	0.08
aaa	K	1	0.56
aag	K	0	0.44
aga	R	1	0.46
agg	R	0	0.27
gat	D	1	0.58
gac	D	0	0.42
gaa	E	2411	1446.00
gag	E	4	969.00
mPD		0.0066	0.48
		nPD:	0.01
		N. weight:	0.46
		Sc. PD:	-0.032
		Sc. rank:	-200.5

M2

Pos.	80	obs:	exp:
tta	L	0	0.11
ttg	L	0	0.19
ctt	L	0	0.19
ctc	L	0	0.17
cta	L	0	0.13
ctg	L	1	0.21
cat	H	0	1.19
cac	H	2	0.81
caa	Q	0	1223.00
cag	Q	2404	1181.00
cgt	R	0	0.04
cgc	R	0	0.05
cga	R	0	0.10
cgg	R	1	0.08
atg	M	6	6.00
aaa	K	0	2.25
aag	K	4	1.75
aga	R	0	0.46
agg	R	0	0.27
mPD		0.016	0.52
		nPD:	0.03
		N. weight:	0.65
		Sc. PD:	-0.033
		Sc. rank:	598.8

M2

Pos.	81	obs:	exp:
cat	H	2	1.78
cac	H	1	1.22
caa	Q	22	1228.00
cag	Q	2391	1185.00
cgt	R	0	0.08
cgc	R	0	0.11
cga	R	0	0.19
cgg	R	2	0.16
aga	R	0	0.92
agg	R	0	0.55
mPD		0.022	0.50
		nPD:	0.04
		N. weight:	0.61
		Sc. PD:	-0.024
		Sc. rank:	705.5

M2

Pos.	82	obs:	exp:
tct	S	0	349.60
tcc	S	0	301.80
tca	S	0	461.40
tcg	S	0	132.20
aat	N	371	197.20
aac	N	0	173.80
agt	S	2029	417.90
agc	S	18	384.10
mPD		0.27	1.8
		nPD:	0.15
		N. weight:	1.3
		Sc. PD:	0.087
		Sc. rank:	1867.6

M2

Pos.	83	obs:	exp:
gct	A	2417	615.10
gcc	A	0	471.50
gca	A	0	1063.00
gcg	A	1	268.40
mPD		0.00083	0.69
		nPD:	0.
		N. weight:	1.3
		Sc. PD:	-0.1
		Sc. rank:	-2447.7

M2

Pos.	84	obs:	exp:
gtt	V	2	525.40
gtc	V	0	510.50
gta	V	1	475.40
gtg	V	2413	904.70
ggt	G	0	0.29
ggc	G	0	0.26
gga	G	0	0.87
ggg	G	2	0.58
mPD		0.0041	0.73
		nPD:	0.01
		N. weight:	0.9
		Sc. PD:	-0.068
		Sc. rank:	-856.8

M2

Pos.	85	obs:	exp:
aat	N	1	0.53
aac	N	0	0.47
gat	D	2415	1389.00
gac	D	0	1026.00
ggt	G	2	0.29
ggc	G	0	0.26
gga	G	0	0.87
ggg	G	0	0.58
mPD		0.0025	0.49
		nPD:	0.01
		N. weight:	0.51
		Sc. PD:	-0.039
		Sc. rank:	-603.3

M2

Pos.	86	obs:	exp:
gtt	V	2417	525.60
gtc	V	0	510.70
gta	V	0	475.60
gtg	V	0	905.10
ggt	G	1	0.14
ggc	G	0	0.13
gga	G	0	0.44
ggg	G	0	0.29
mPD		0.00083	0.73
		nPD:	0.
		N. weight:	1.4
		Sc. PD:	-0.11
		Sc. rank:	-2816.1

M2

Pos.	87	obs:	exp:
aat	N	0	0.53
aac	N	1	0.47
gat	D	0	1390.00
gac	D	2417	1027.00
mPD		0.00083	0.49
		nPD:	0.
		N. weight:	0.79
		Sc. PD:	-0.063
		Sc. rank:	-1344.9

M2

Pos.	88	obs:	exp:
tct	S	0	0.17
tcc	S	0	0.15
tca	S	0	0.23
tcg	S	0	0.06
tat	Y	1	0.49
tac	Y	0	0.51
aat	N	12	6.38
aac	N	0	5.62
agt	S	1	0.20
agc	S	0	0.19
gat	D	2390	1375.00
gac	D	0	1015.00
ggt	G	14	2.01
ggc	G	0	1.85
gga	G	0	6.11
ggg	G	0	4.03
mPD		0.024	0.52
		nPD:	0.05
		N. weight:	0.52
		Sc. PD:	-0.019
		Sc. rank:	612.4

M2

Pos.	89	obs:	exp:
tct	S	0	0.68
tcc	S	0	0.59
tca	S	0	0.90
tcg	S	0	0.26
tgt	C	1	0.43
tgc	C	0	0.57
agt	S	4	0.82
agc	S	0	0.75
gat	D	309	177.70
gac	D	0	131.30
ggt	G	2103	302.70
ggc	G	0	277.70
gga	G	0	918.50
ggg	G	1	605.20
---	--	------	------
mPD		0.23	0.95
		nPD:	0.24
		N. weight:	1.6
		Sc. PD:	0.24
		Sc. rank:	2551.8

,

M2

Pos.	90	obs:	exp:
tta	L	0	0.11
ttg	L	0	0.19
ctt	L	1	0.19
ctc	L	0	0.17
cta	L	0	0.13
ctg	L	0	0.21
cat	H	2414	1437.00
cac	H	2	979.50
caa	Q	0	0.51
cag	Q	1	0.49
---	--	------	------
mPD		0.0033	0.48
		nPD:	0.01
		N. weight:	0.47
		Sc. PD:	-0.035
		Sc. rank:	-430.7

,

M2

Pos.	91	obs:	exp:
ttt	F	2410	1026.00
ttc	F	2	1386.00
tta	L	4	0.55
ttg	L	0	0.94
ctt	L	0	0.94
ctc	L	1	0.86
cta	L	0	0.67
ctg	L	0	1.05
att	I	1	0.37
atc	I	0	0.25
ata	I	0	0.38
---	--	------	------
mPD		0.0074	0.49
		nPD:	0.02
		N. weight:	0.78
		Sc. PD:	-0.052
		Sc. rank:	-209.1

,

M2

Pos.	92	obs:	exp:
tta	L	0	0.22
ttg	L	0	0.38
tgt	C	0	0.87
tgc	C	2	1.13
ctt	L	0	0.38
ctc	L	2	0.35
cta	L	0	0.27
ctg	L	0	0.42
cct	P	0	0.28
ccc	P	0	0.18
cca	P	1	0.41
ccg	P	0	0.13
cat	H	0	0.59
cac	H	1	0.41
act	T	0	0.79
acc	T	0	0.66
aca	T	3	1.29
acg	T	0	0.25
aat	N	0	0.53
aac	N	1	0.47
gtt	V	0	523.70
gtc	V	2408	508.80
gta	V	0	473.80
gtg	V	0	901.70
---	--	------	------
mPD		0.018	0.74
		nPD:	0.02
		N. weight:	1.4
		Sc. PD:	-0.083
		Sc. rank:	706.4

,

M2

Pos.	93	obs:	exp:
cgt	R	0	0.04
cgc	R	0	0.05
cga	R	0	0.10
cgg	R	0	0.08
act	T	0	1.32
acc	T	3	1.10
aca	T	2	2.15
acg	T	0	0.42
aat	N	0	1281.00
aac	N	2411	1130.00
aaa	K	1	0.56
aag	K	0	0.44
aga	R	0	0.46
agg	R	1	0.27
---	--	------	------
mPD		0.0083	0.51
		nPD:	0.02
		N. weight:	0.7
		Sc. PD:	-0.046
		Sc. rank:	-96.8

,

M2

Pos.	94	obs:	exp:
tta	L	0	0.22
ttg	L	0	0.38
ctt	L	0	0.38
ctc	L	0	0.35
cta	L	2	0.27
ctg	L	0	0.42
cct	P	0	0.28
ccc	P	0	0.18
cca	P	1	0.41
ccg	P	0	0.13
att	I	2	893.10
atc	I	0	603.20
ata	I	2403	908.80
atg	M	6	6.00
aaa	K	3	1.68
aag	K	0	1.32
gtt	V	0	0.22
gtc	V	0	0.21
gta	V	1	0.20
gtg	V	0	0.37
---	--	------	------
mPD		0.013	0.67
		nPD:	0.02
		N. weight:	0.89
		Sc. PD:	-0.055
		Sc. rank:	266.6

,

M2

Pos.	95	obs:	exp:
caa	Q	0	1.02
cag	Q	2	0.98
aaa	K	9	8.98
aag	K	7	7.02
gat	D	0	1.73
gac	D	3	1.27
gaa	E	600	1435.00
gag	E	1797	961.80
---	--	------	------
mPD		0.39	0.50
		nPD:	0.79
		N. weight:	0.23
		Sc. PD:	0.16
		Sc. rank:	520.1

,

M2

Pos.	96	obs:	exp:
tta	L	1	262.70
ttg	L	587	451.70
ctt	L	0	451.30
ctc	L	0	415.30
cta	L	4	320.30
ctg	L	1815	505.70
atg	M	11	11.00
---	--	------	------
mPD		0.38	1.1
		nPD:	0.33
		N. weight:	0.94
		Sc. PD:	0.22
		Sc. rank:	1675.6

,

M2

Pos.	97	obs:	exp:
tct	S	0	0.17
tcc	S	0	0.15
tca	S	0	0.23
tcg	S	0	0.06
aaa	K	0	2.25
aag	K	4	1.75
agt	S	1	0.20
agc	S	0	0.19
gat	D	1	0.58
gac	D	0	0.42
gaa	E	3	1444.00
gag	E	2408	967.40
ggt	G	0	0.14
ggc	G	0	0.13
gga	G	0	0.44
ggg	G	1	0.29
---	--	------	------
mPD		0.0099	0.49
		nPD:	0.02
		N. weight:	0.83
		Sc. PD:	-0.051
		Sc. rank:	281.8

,

M2

Pos.	98	obs:	exp:
taa	.	2399	886.70
tag	.	19	678.60
tga	.	0	852.70
---	--	------	------
mPD		0.016	0.86
		nPD:	0.02
		N. weight:	0.88
		Sc. PD:	-0.056
		Sc. rank:	195.3

}

Amino acid level conservation per codon

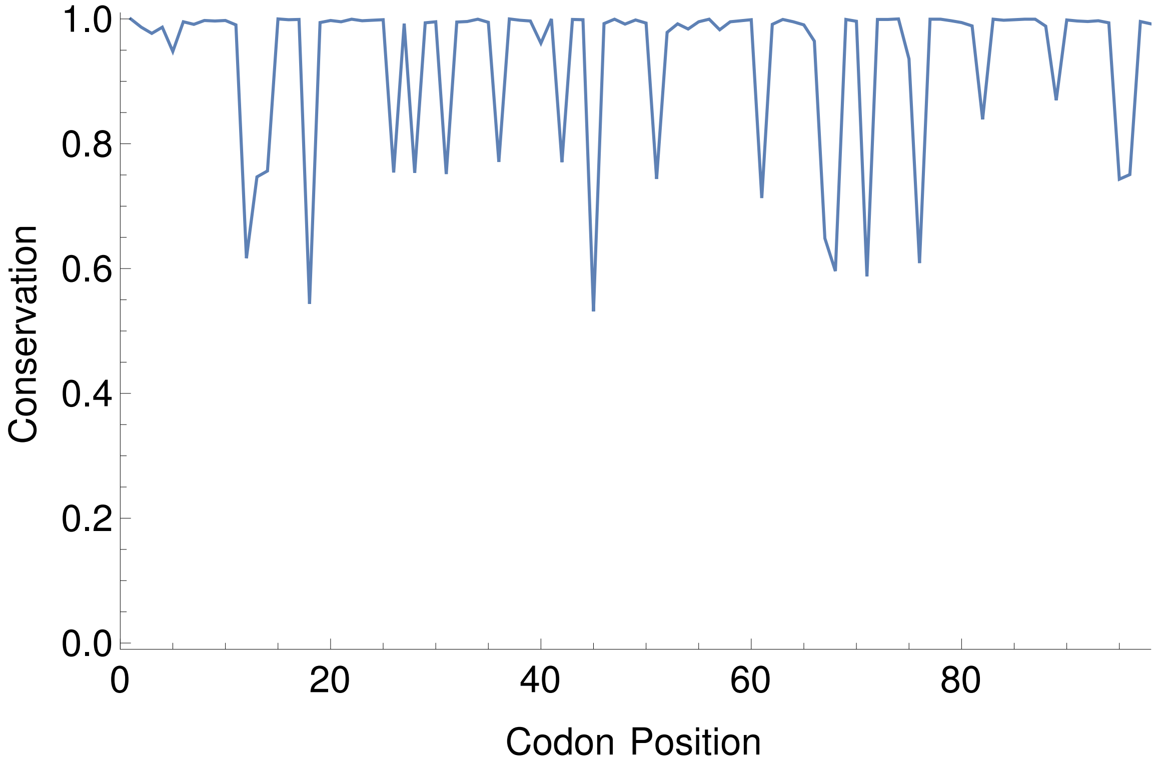

Conservation analysis plots

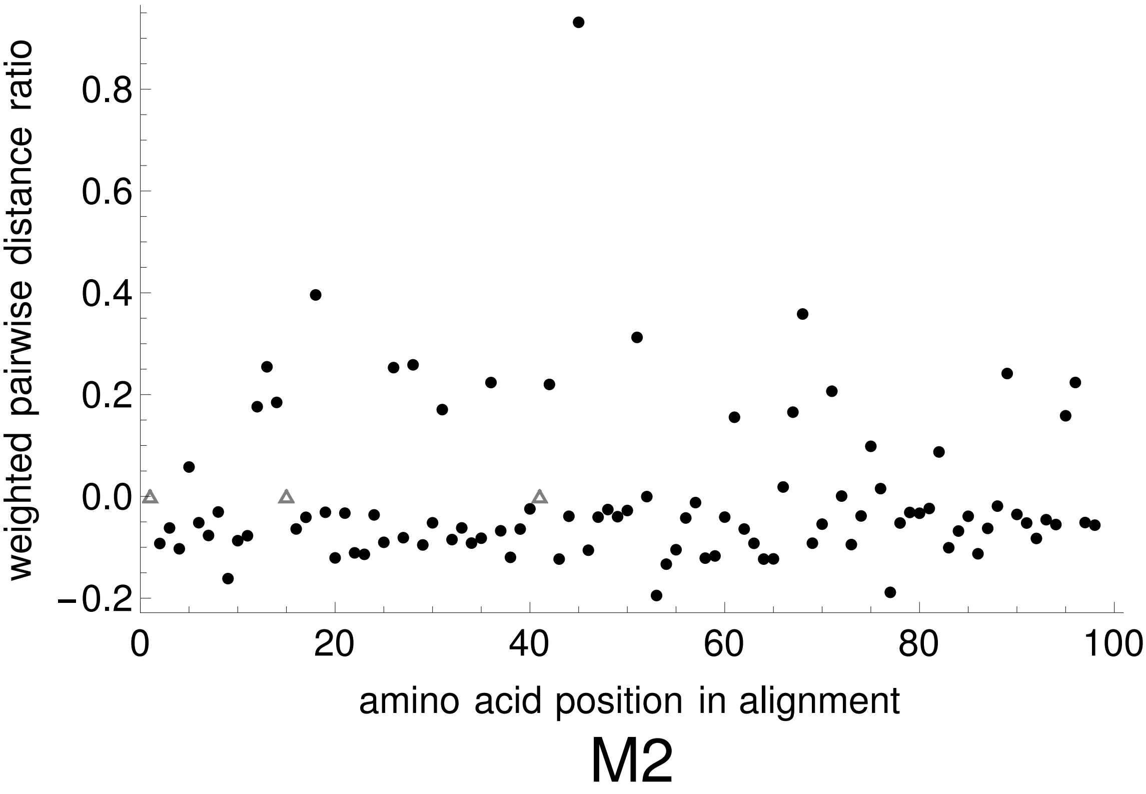

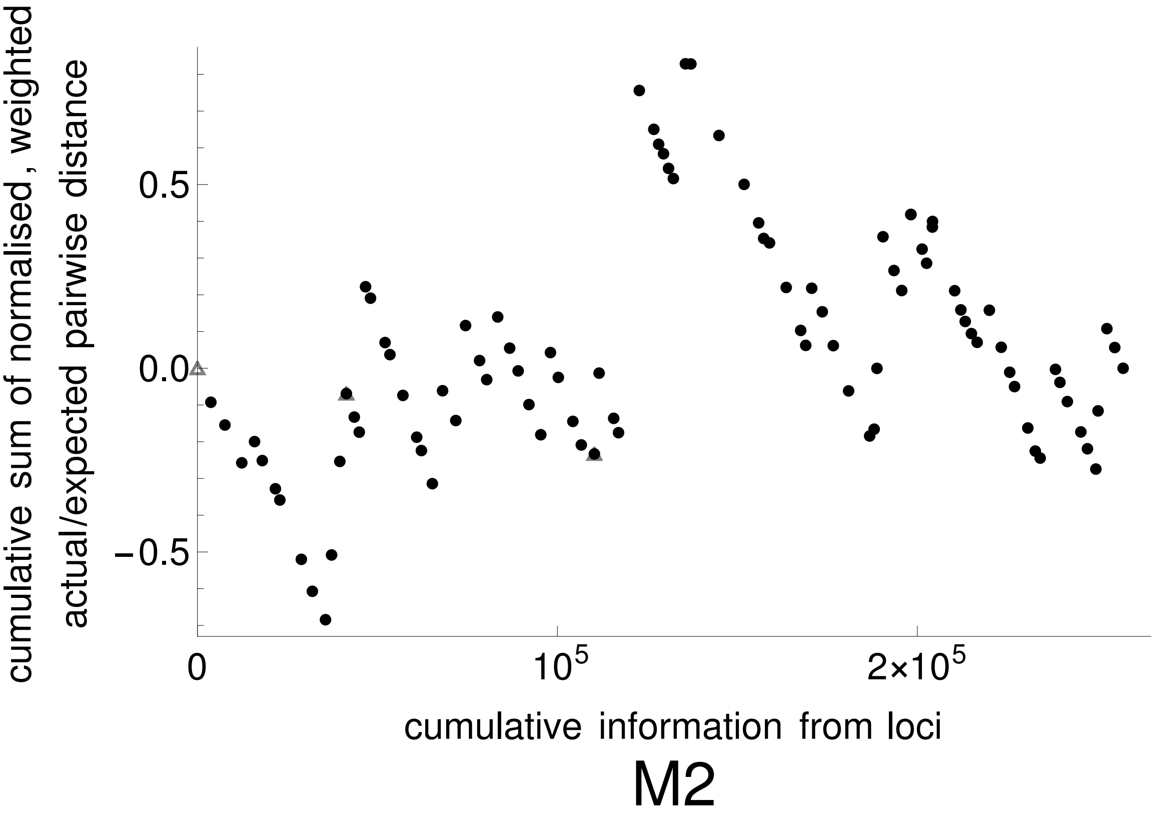

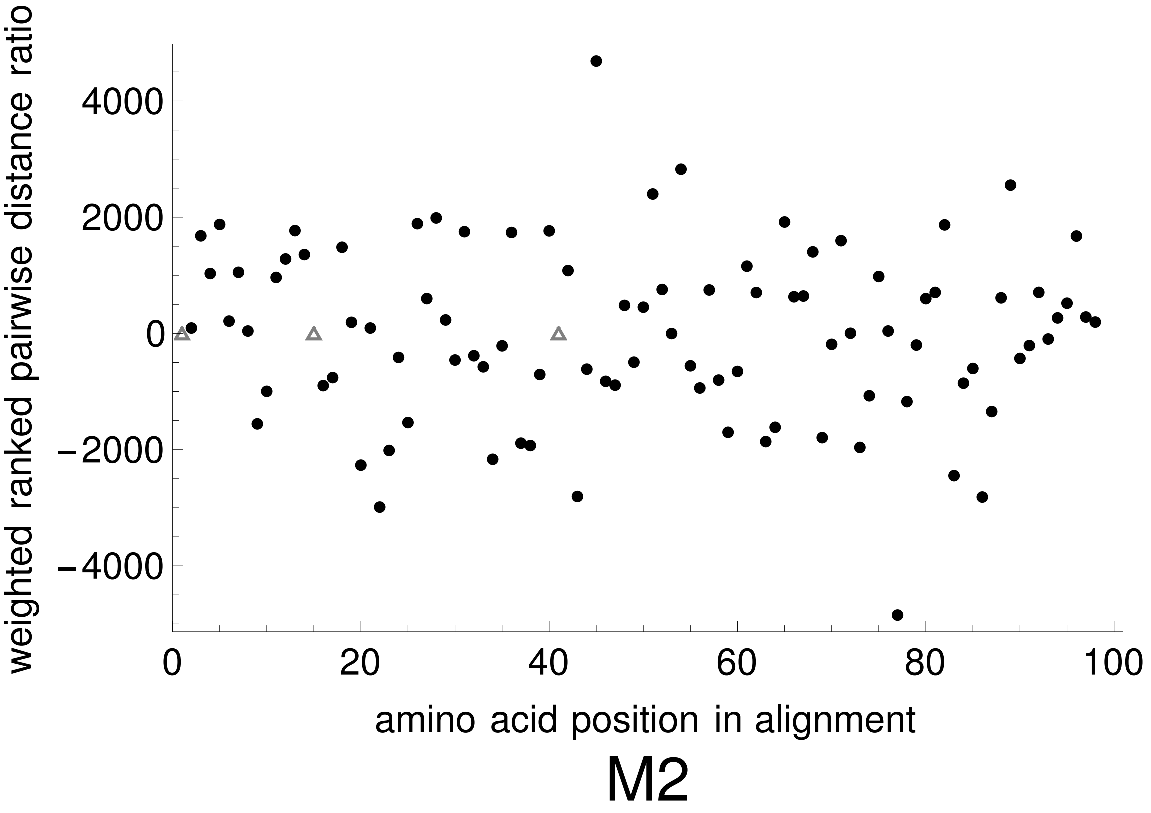
weighted ranked pairwise distance ratio
4000
2000
0
−2000
−4000
0
20
40
60
80
100
amino acid position in alignment
M2

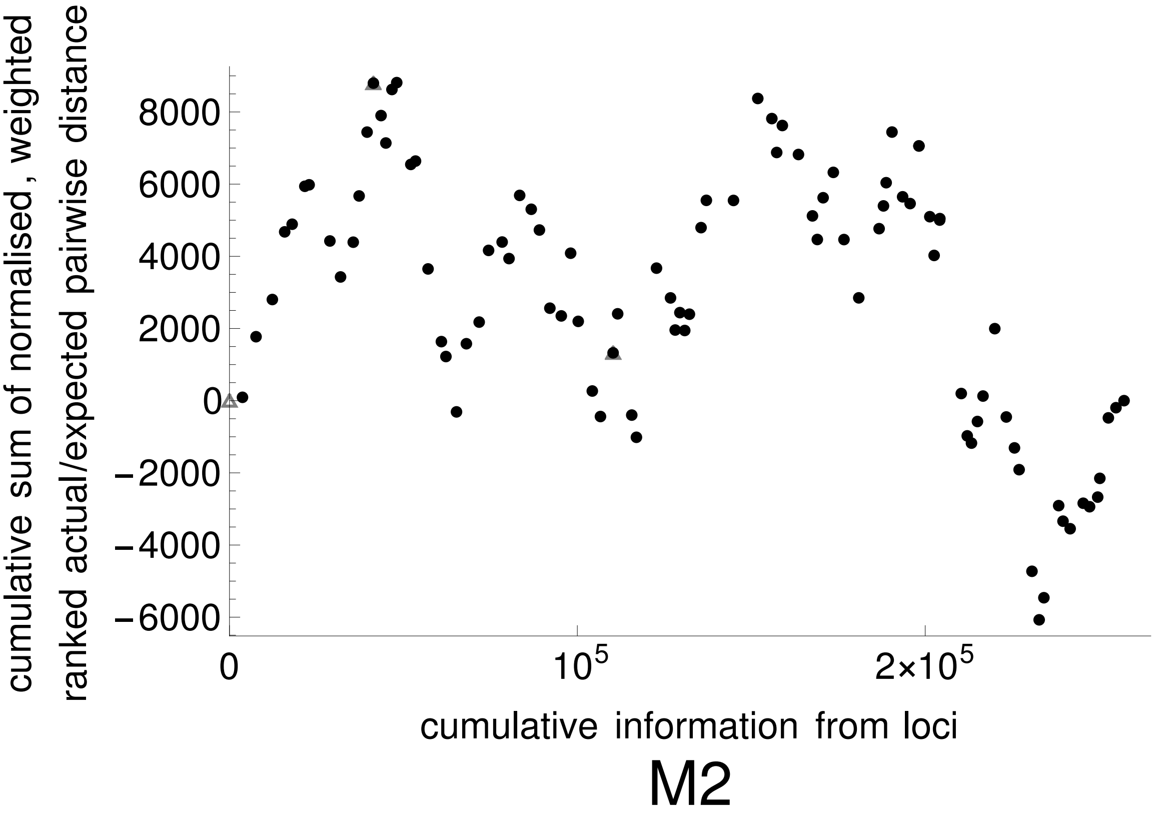
cumulative sum of normalised, weighted
ranked actual/expected pairwise distance
8000
6000
4000
2000
0
−2000
−4000
−6000
0
10^5
2×10^5
cumulative information from loci
M2

NS1

Gene length histogram

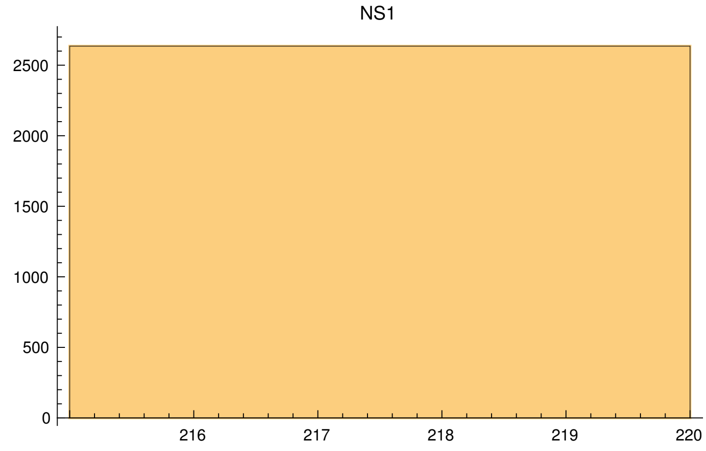

Information vs. nPD

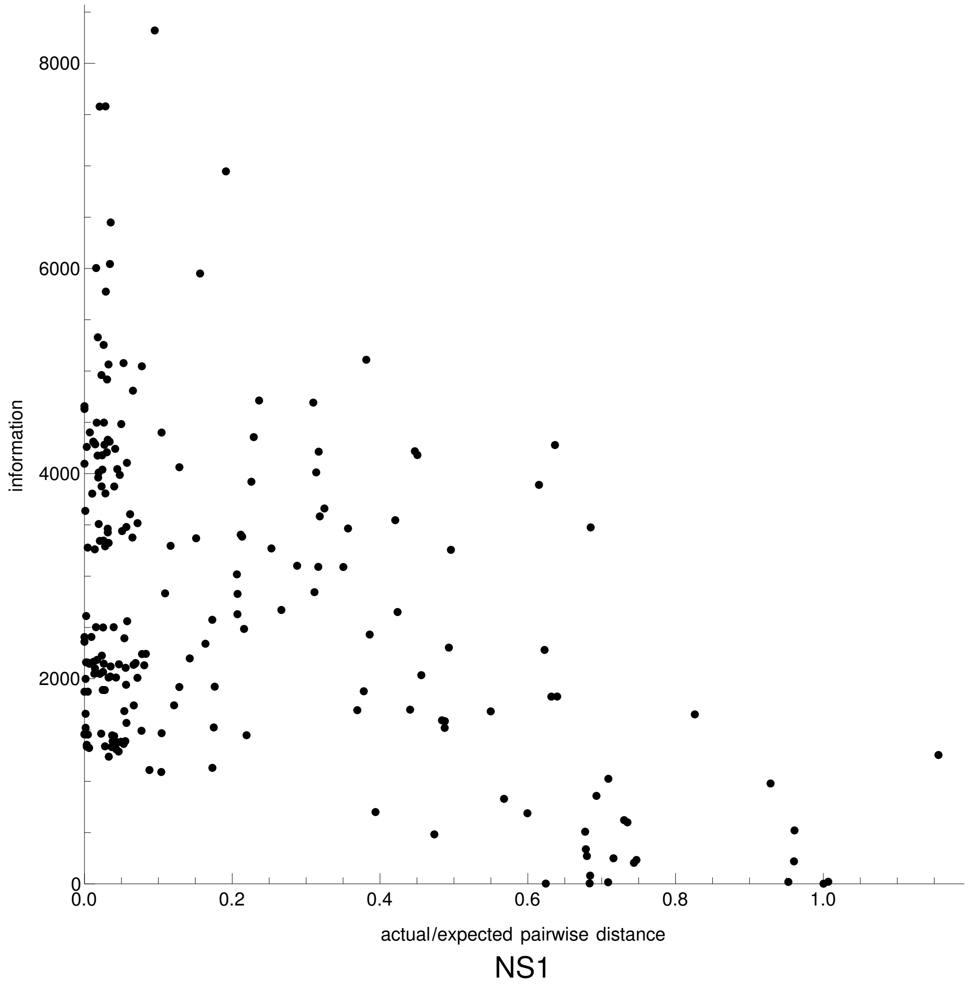

Example sequences highlighted by regions found in analyses to be conserved

Interesting points (by weighted raw PD) highlighted for gene NS1:

ATGGATTCCAACACTATGTTA
AGCTTTCAGGTAGACTGTTTTCTTTGGTATGTCCGCAAACGATTCGCAGACCAAG
AAATGGGTGATGCCCCGTTCCTTGACCGGCTTCGCCGAGATCAGAAGTCTTTAAGAG
GAAGAGGCAACACTCTTGGTCTGGGCATCGAAACAGCTACTCGTGCGGGAAAGCAGATAGTGGAGCGAAT
TCTGGAGGAAGAACCTGATGAGGCACTTAAAATGACTGTTGCTTCAAGTCCGTCTTCACGCTACCTAACT
GACATGACTCTTGAAGAAATGTCAAGGGACTGGTTCATGCTCATGCCCAAACAGAAAGTGGCAGGTTCAC
TTTGCATCAAAATGGACCAGGCAATAATGGATAAAAACATCATATTGAAAGCAAACTTCAGTGTAATTTT
TAACCGGCTGGAAGCTCTAATACTACTTAGA
GCTTTCACAGAAGAAGGAGCAATTGTGGGAGAAATCTCACCGTTACCTTCTTTTC
CAGGACATACTGATGAGGATGTCAAAAATGCAATTGGGGTCCTCATCGGAGG
ACTTGAATGGAATGATAACACAGTTCGGGTCTCTGAAACTCTACAGAGATTC
GCTTGGAGAAACAGTAATGAGGATGGGAGACCTTCACTCCCTCCAAAG--------
--TAG

Interesting points (by weighted ranked PD) highlighted for gene NS1:

ATGGATTCCAACACTATGTTA
AGCTTTCAGGTAGACTGTTTTCTTTGGTATGTCCGCAAACGATTCGCAGACCAAG
AAATGGGTGATGCCCCGTTCCTTGACCGGCTTCGCCGAGATCAGAAGTCTTT
AAGAGGAAGAGGCAACACTCTTGGTCTGGGCATCGAAACAGCTACTCGTGCGGGAAAGCAG
ATAGTGGAGCGAATTCTGGAGGAAGAACCTGATGAGGCACTTAAAATGACTGTTGCTTCAAGTCCGTCTT
CACGCTACCTAACTGACATGACTCTTGAAGAAATGTCAAGGGACTGGTTCATGCTCATGCCCAAACAGAA
AGTGGCAGGTTCACTTTGCATCAAAATGGACCAGGCAATAATGGATAAAAACATCATATTGAAAGCAAAC
TTCAGTGTAATTTTTAACCGGCTGGAAGCTCTAATACTACTTAGA
GCTTTCACAGAAGAAGGAGCAATTGTGGGAGAAATCTCACCGTTACCTTCTTTTC
CAGGACATACTGATGAGGATGTCAAAAATGCAATTGGGGTCCTCATCGGAGG
ACTTGAATGGAATGATAACACAGTTCGGGTCTCTGAAACTCTACAGAGATTC
GCTTGGAGAAACAGTAATGAGGATGGGAGACCTTCACTCCCTCCAAAG--------
--TAG

Per codon conservation report

NS1			
Pos.	1	obs:	exp:
atg	M	2570	2570.00
---	-	65	65.00
---	--	------	------
mPD		0	0
		nPD:	1.
		N. weight:	0.
		Sc. PD:	0
		Sc. rank:	0

NS1			
Pos.	2	obs:	exp:
gct	A	1	0.25
gcc	A	0	0.20
gca	A	0	0.44
gcg	A	0	0.11
gat	D	2028	1476.00
gac	D	538	1090.00
gaa	E	3	1.80
gag	E	0	1.20
---	-	65	65.00
---	--	------	------
mPD		0.32	0.47
		nPD:	0.68
		N. weight:	0.11
		Sc. PD:	0.054
		Sc. rank:	223.9

NS1			
Pos.	3	obs:	exp:
tta	L	0	0.11
ttg	L	0	0.19
tct	S	3	312.70
tcc	S	1823	269.90
tca	S	5	412.70
tcg	S	0	118.30
ctt	L	0	0.19
ctc	L	1	0.17
cta	L	0	0.13
ctg	L	0	0.21
cct	P	0	207.80
ccc	P	737	133.40
cca	P	0	300.90
ccg	P	0	94.90
act	T	0	0.26
acc	T	1	0.22
aca	T	0	0.43
acg	T	0	0.08
agt	S	0	373.80
agc	S	0	343.50
---	-	65	65.00
---	--	------	------
mPD		0.40	1.7
		nPD:	0.24
		N. weight:	1.9
		Sc. PD:	0.17
		Sc. rank:	2458.6

NS1			
Pos.	4	obs:	exp:
act	T	0	0.26
acc	T	1	0.22
aca	T	0	0.43
acg	T	0	0.08
aat	N	9	1362.00
aac	N	2554	1201.00
aaa	K	0	3.37
aag	K	6	2.63
---	-	65	65.00
---	--	------	------
mPD		0.012	0.48
		nPD:	0.02
		N. weight:	0.78
		Sc. PD:	-0.077
		Sc. rank:	-712.3

NS1			
Pos.	5	obs:	exp:
tct	S	1	0.17
tcc	S	0	0.15
tca	S	0	0.23
tcg	S	0	0.06
act	T	2545	680.50
acc	T	6	567.60
aca	T	0	1104.00
acg	T	18	217.00
agt	S	0	0.20
agc	S	0	0.19
---	-	65	65.00
---	--	------	------
mPD		0.018	0.66
		nPD:	0.03
		N. weight:	1.4
		Sc. PD:	-0.13
		Sc. rank:	-937.3

NS1			
Pos.	6	obs:	exp:
att	I	0	6.31
atc	I	0	4.26
ata	I	17	6.42
atg	M	1791	1791.00
gtt	V	0	179.90
gtc	V	0	174.70
gta	V	0	162.70
gtg	V	827	309.70
---	--	------	------
mPD		0.44	0.78
		nPD:	0.57
		N. weight:	0.34
		Sc. PD:	0.13
		Sc. rank:	644.3

NS1			
Pos.	7	obs:	exp:
ttt	F	2	0.85
ttc	F	0	1.15
tta	L	1915	220.90
ttg	L	13	379.80
tct	S	84	101.10
tcc	S	0	87.27
tca	S	508	133.40
tcg	S	0	38.24
ctt	L	0	379.50
ctc	L	0	349.20
cta	L	96	269.30
ctg	L	0	425.30
act	T	0	4.50
acc	T	17	3.76
aca	T	0	7.30
acg	T	0	1.44
agt	S	0	120.90
agc	S	0	111.10
---	--	------	------
mPD		0.52	1.7
		nPD:	0.31
		N. weight:	1.9
		Sc. PD:	0.3
		Sc. rank:	2641.6

NS1			
Pos.	8	obs:	exp:
tct	S	0	449.80
tcc	S	0	388.30
tca	S	0	593.70
tcg	S	17	170.10
cgt	R	0	0.04
cgc	R	1	0.05
cga	R	0	0.10
cgg	R	0	0.08
agt	S	7	537.80
agc	S	2610	494.20
aga	R	0	0.46
agg	R	0	0.27
---	--	------	------
mPD		0.044	1.6
		nPD:	0.03
		N. weight:	1.8
		Sc. PD:	-0.17
		Sc. rank:	-1303.2

NS1			
Pos.	9	obs:	exp:
ttt	F	2618	1121.00
ttc	F	17	1514.00
---	--	------	------
mPD		0.013	0.49
		nPD:	0.03
		N. weight:	0.89
		Sc. PD:	-0.086
		Sc. rank:	-709.9

NS1			
Pos.	10	obs:	exp:
caa	Q	0	1339.00
cag	Q	2632	1293.00
aaa	K	0	1.68
aag	K	3	1.32
---	--	------	------
mPD		0.0023	0.50
		nPD:	0.
		N. weight:	0.77
		Sc. PD:	-0.09
		Sc. rank:	-1335.3

NS1			
Pos.	11	obs:	exp:
gtt	V	0	573.10
gtc	V	0	556.70
gta	V	2632	518.50
gtg	V	3	986.70
---	--	------	------
mPD		0.0023	0.73
		nPD:	0.
		N. weight:	1.8
		Sc. PD:	-0.21
		Sc. rank:	-3164.4

NS1			
Pos.	12	obs:	exp:
gat	D	23	1516.00
gac	D	2612	1119.00
---	--	------	------
mPD		0.017	0.49
		nPD:	0.04
		N. weight:	0.88
		Sc. PD:	-0.078
		Sc. rank:	-198.5

NS1			
Pos.	13	obs:	exp:
tgt	C	2012	1145.00
tgc	C	621	1488.00
ggt	G	0	0.29
ggc	G	2	0.26
gga	G	0	0.87
ggg	G	0	0.58
---	--	------	------
mPD		0.36	0.49
		nPD:	0.73
		N. weight:	0.25
		Sc. PD:	0.13
		Sc. rank:	507.3

NS1			
Pos.	14	obs:	exp:
ttt	F	2550	1113.00
ttc	F	66	1503.00
tat	Y	16	8.75
tac	Y	2	9.25
tgt	C	1	0.43
tgc	C	0	0.57
---	--	------	------
mPD		0.065	0.50
		nPD:	0.13
		N. weight:	0.79
		Sc. PD:	-0.005
		Sc. rank:	631.1

NS1			
Pos.	15	obs:	exp:
tta	L	0	287.60
ttg	L	0	494.40
ctt	L	2617	494.10
ctc	L	0	454.60
cta	L	18	350.60
ctg	L	0	553.60
---	--	------	------
mPD		0.014	1.1
		nPD:	0.01
		N. weight:	1.8
		Sc. PD:	-0.2
		Sc. rank:	-2815.9

NS1			
Pos.	16	obs:	exp:
tgt	C	2	0.87
tgc	C	0	1.13
tgg	W	2633	2633.00
---	--	------	------
mPD		0.0015	0.0015
		nPD:	1.
		N. weight:	0.00069
		Sc. PD:	0.00053
		Sc. rank:	1.4

NS1			
Pos.	17	obs:	exp:
tat	Y	2001	973.20
tac	Y	1	1029.00
tgt	C	7	3.04
tgc	C	0	3.96
cat	H	607	371.60
cac	H	18	253.40
cgt	R	1	0.04
cgc	R	0	0.05
cga	R	0	0.10
cgg	R	0	0.08
aga	R	0	0.46
agg	R	0	0.27
---	--	------	------
mPD		0.38	0.87
		nPD:	0.44
		N. weight:	0.7
		Sc. PD:	0.19
		Sc. rank:	1199.2

NS1			
Pos.	18	obs:	exp:
att	I	0	7.80
atc	I	3	5.27
ata	I	18	7.94
gtt	V	0	568.50
gtc	V	2609	552.30
gta	V	5	514.30
gtg	V	0	978.90
---	--	------	------
mPD		0.033	0.75
		nPD:	0.04
		N. weight:	1.7
		Sc. PD:	-0.14
		Sc. rank:	-13.7

NS1			
Pos.	19	obs:	exp:
cgt	R	0	99.93
cgc	R	2617	140.40
cga	R	0	255.20
cgg	R	0	206.40
aga	R	17	1209.00
agg	R	1	724.50
---	--	------	------
mPD		0.027	0.95
		nPD:	0.03
		N. weight:	3.1
		Sc. PD:	-0.3
		Sc. rank:	-1899.8

NS1			
Pos.	20	obs:	exp:
cgt	R	0	0.08
cgc	R	0	0.11
cga	R	0	0.19
cgg	R	0	0.16
aaa	K	2601	1477.00
aag	K	31	1155.00
aga	R	2	0.92
agg	R	0	0.55
gaa	E	0	0.60
gag	E	1	0.40
---	--	------	------
mPD		0.026	0.50
		nPD:	0.05
		N. weight:	0.56
		Sc. PD:	-0.041
		Sc. rank:	96.0

NS1

Pos. 21		obs:	exp:
tta	L	0	1.96
ttg	L	0	3.38
ctt	L	0	3.38
ctc	L	0	3.11
cta	L	12	2.39
ctg	L	6	3.78
caa	Q	8	4.07
cag	Q	0	3.93
cgt	R	0	98.94
cgc	R	0	139.00
cga	R	2596	252.60
cgg	R	13	204.30
aga	R	0	1197.00
agg	R	0	717.40
---	--	------	------
mPD		0.034	0.98
		nPD:	0.03
		N. weight:	2.5
		Sc. PD:	-0.22
		Sc. rank:	-641.1

NS1

Pos. 22		obs:	exp:
ttt	F	623	1113.00
ttc	F	1993	1503.00
tta	L	0	2.07
ttg	L	0	3.57
ctt	L	2	3.56
ctc	L	17	3.28
cta	L	0	2.53
ctg	L	0	3.99
---	--	------	------
mPD		0.38	0.50
		nPD:	0.75
		N. weight:	0.096
		Sc. PD:	0.052
		Sc. rank:	197.5

NS1

Pos. 23		obs:	exp:
tct	S	0	3.07
tcc	S	0	2.65
tca	S	0	4.06
tcg	S	0	1.16
act	T	0	1.85
acc	T	0	1.55
aca	T	7	3.01
acg	T	0	0.59
agt	S	12	3.68
agc	S	6	3.38
gct	A	0	664.00
gcc	A	1	509.00
gca	A	2606	1147.00
gcg	A	3	289.80
---	--	------	------
mPD		0.049	0.72
		nPD:	0.07
		N. weight:	0.89
		Sc. PD:	-0.053
		Sc. rank:	402.6

NS1

Pos. 24		obs:	exp:
atg	M	18	18.00
gat	D	0	1505.00
gac	D	2617	1112.00
---	--	------	------
mPD		0.041	0.52
		nPD:	0.08
		N. weight:	0.93
		Sc. PD:	-0.047
		Sc. rank:	531.2

NS1

Pos. 25		obs:	exp:
caa	Q	2606	1329.00
cag	Q	6	1283.00
cgt	R	0	0.87
cgc	R	0	1.23
cga	R	5	2.23
cgg	R	0	1.80
aga	R	18	10.55
agg	R	0	6.32
---	--	------	------
mPD		0.035	0.53
		nPD:	0.07
		N. weight:	0.72
		Sc. PD:	-0.044
		Sc. rank:	315.2

NS1

Pos. 26		obs:	exp:
gat	D	1	10.93
gac	D	18	8.07
gaa	E	2605	1566.00
gag	E	11	1050.00
---	--	------	------
mPD		0.023	0.49
		nPD:	0.05
		N. weight:	0.53
		Sc. PD:	-0.042
		Sc. rank:	6.0

NS1

Pos. 27		obs:	exp:
tta	L	0	279.60
ttg	L	2	480.70
ctt	L	0	480.40
ctc	L	0	442.00
cta	L	0	340.90
ctg	L	2560	538.30
caa	Q	0	1.53
cag	Q	3	1.47
cgt	R	0	0.04
cgc	R	0	0.05
cga	R	0	0.10
cgg	R	1	0.08
atg	M	69	69.00
aga	R	0	0.46
agg	R	0	0.27
---	--	------	------
mPD		0.056	1.2
		nPD:	0.05
		N. weight:	1.6
		Sc. PD:	-0.13
		Sc. rank:	91.8

NS1

Pos. 28		obs:	exp:
tct	S	0	1.71
tcc	S	0	1.47
tca	S	0	2.25
tcg	S	0	0.65
tgt	C	18	7.83
tgc	C	0	10.17
agt	S	10	2.04
agc	S	0	1.88
ggt	G	2604	375.00
ggc	G	3	344.10
gga	G	0	1138.00
ggg	G	0	749.80
---	--	------	------
mPD		0.023	0.72
		nPD:	0.03
		N. weight:	2.1
		Sc. PD:	-0.19
		Sc. rank:	-794.8

NS1

Pos. 29		obs:	exp:
gat	D	2635	1516.00
gac	D	0	1119.00
---	--	------	------
mPD		0	0.49
		nPD:	0.
		N. weight:	0.6
		Sc. PD:	-0.072
		Sc. rank:	-1254.7

NS1

Pos. 30		obs:	exp:
tct	S	0	0.17
tcc	S	1	0.15
tca	S	0	0.23
tcg	S	0	0.06
agt	S	0	0.20
agc	S	0	0.19
gct	A	20	670.10
gcc	A	2613	513.60
gca	A	1	1158.00
gcg	A	0	292.40
---	--	------	------
mPD		0.017	0.69
		nPD:	0.02
		N. weight:	1.7
		Sc. PD:	-0.17
		Sc. rank:	-1648.9

NS1

Pos. 31		obs:	exp:
tta	L	0	0.11
ttg	L	0	0.19
ctt	L	0	0.19
ctc	L	0	0.17
cta	L	0	0.13
ctg	L	1	0.21
cct	P	1057	742.80
ccc	P	22	476.60
cca	P	586	1075.00
ccg	P	969	339.20
---	--	------	------
mPD		0.66	0.71
		nPD:	0.93
		N. weight:	0.4
		Sc. PD:	0.28
		Sc. rank:	836.9

NS1

Pos. 32		obs:	exp:
ttt	F	24	1121.00
ttc	F	2611	1514.00
---	--	------	------
mPD		0.018	0.49
		nPD:	0.04
		N. weight:	0.55
		Sc. PD:	-0.048
		Sc. rank:	-93.7

NS1

Pos. 33		obs:	exp:
tta	L	0	285.50
ttg	L	0	490.90
ctt	L	2589	490.50
ctc	L	26	451.30
cta	L	1	348.10
ctg	L	0	549.60
att	I	1	0.37
atc	I	0	0.25
ata	I	0	0.38
gat	D	18	10.35
gac	D	0	7.65
---	--	------	------
mPD		0.048	1.2
		nPD:	0.04
		N. weight:	1.8
		Sc. PD:	-0.15
		Sc. rank:	-108.3

NS1

Pos. 34		obs:	exp:
gat	D	47	1516.00
gac	D	2588	1119.00
---	--	------	------
mPD		0.035	0.49
		nPD:	0.07
		N. weight:	0.83
		Sc. PD:	-0.047
		Sc. rank:	402.0

NS1

Pos. 35		obs:	exp:
tta	L	0	0.11
ttg	L	0	0.19
ctt	L	0	0.19
ctc	L	0	0.17
cta	L	0	0.13
ctg	L	1	0.21
cgt	R	0	99.89
cgc	R	0	140.30
cga	R	19	255.10
cgg	R	2596	206.30
aga	R	6	1208.00
agg	R	13	724.30
---	--	------	------
mPD		0.034	0.95
		nPD:	0.04
		N. weight:	2.7
		Sc. PD:	-0.24
		Sc. rank:	-479.0

NS1

Pos. 36		obs:	exp:
tta	L	0	287.60
ttg	L	0	494.40
ctt	L	2615	494.10
ctc	L	20	454.60
cta	L	0	350.60
ctg	L	0	553.60
---	--	------	------
mPD		0.015	1.1
		nPD:	0.01
		N. weight:	1.8
		Sc. PD:	-0.19
		Sc. rank:	-2723.2

NS1

Pos. 37		obs:	exp:
cat	H	0	0.59
cac	H	1	0.41
cgt	R	1	99.89
cgc	R	2615	140.30
cga	R	11	255.10
cgg	R	1	206.30
aga	R	4	1208.00
agg	R	2	724.30
---	--	------	------
mPD		0.020	0.95
		nPD:	0.02
		N. weight:	3.1
		Sc. PD:	-0.32
		Sc. rank:	-3493.3

NS1

Pos. 38		obs:	exp:
cgt	R	1	99.93
cgc	R	0	140.40
cga	R	2615	255.20
cgg	R	1	206.40
aga	R	18	1209.00
agg	R	0	724.50
---	--	------	------
mPD		0.015	0.95
		nPD:	0.02
		N. weight:	2.5
		Sc. PD:	-0.26
		Sc. rank:	-3403.4

NS1

Pos. 39		obs:	exp:
aat	N	1	0.53
aac	N	0	0.47
gat	D	2006	1515.00
gac	D	628	1119.00
---	--	------	------
mPD		0.36	0.49
		nPD:	0.74
		N. weight:	0.085
		Sc. PD:	0.046
		Sc. rank:	173.5

NS1

Pos. 40		obs:	exp:
cat	H	65	38.65
cac	H	0	26.35
caa	Q	21	1308.00
cag	Q	2549	1262.00
---	--	------	------
mPD		0.064	0.52
		nPD:	0.12
		N. weight:	0.72
		Sc. PD:	-0.0091
		Sc. rank:	562.4

NS1			
Pos.	41	obs:	exp:
cgt	R	0	1.48
cgc	R	0	2.08
cga	R	0	3.78
cgg	R	0	3.05
aaa	K	8	1457.00
aag	K	2588	1139.00
aga	R	0	17.89
agg	R	39	10.72
---	--	------	------
mPD		0.035	0.53
		nPD:	0.07
		N. weight:	0.88
		Sc. PD:	-0.054
		Sc. rank:	375.7

,

NS1			
Pos.	42	obs:	exp:
tct	S	2072	446.90
tcc	S	545	385.80
tca	S	0	589.90
tcg	S	0	169.00
agt	S	0	534.30
agc	S	0	491.00
gct	A	0	4.58
gcc	A	0	3.51
gca	A	18	7.91
gcg	A	0	2.00
---	--	------	------
mPD		0.35	1.7
		nPD:	0.21
		N. weight:	1.4
		Sc. PD:	0.097
		Sc. rank:	1594.0

,

NS1			
Pos.	43	obs:	exp:
tta	L	2033	287.60
ttg	L	15	494.40
ctt	L	0	494.10
ctc	L	0	454.60
cta	L	584	350.60
ctg	L	3	553.60
---	--	------	------
mPD		0.36	1.1
		nPD:	0.32
		N. weight:	1.7
		Sc. PD:	0.28
		Sc. rank:	2551.3

,

NS1			
Pos.	44	obs:	exp:
cgt	R	0	90.07
cgc	R	0	126.50
cga	R	0	230.00
cgg	R	0	186.00
aaa	K	242	145.90
aag	K	18	114.10
aga	R	2372	1089.00
agg	R	3	653.00
---	--	------	------
mPD		0.19	1.1
		nPD:	0.18
		N. weight:	0.79
		Sc. PD:	0.029
		Sc. rank:	777.0

,

NS1			
Pos.	45	obs:	exp:
ggt	G	0	379.10
ggc	G	0	347.80
gga	G	2629	1150.00
ggg	G	6	757.90
---	--	------	------
mPD		0.0045	0.69
		nPD:	0.01
		N. weight:	0.89
		Sc. PD:	-0.1
		Sc. rank:	-1497.8

,

NS1			
Pos.	46	obs:	exp:
cgt	R	0	99.93
cgc	R	0	140.40
cga	R	0	255.20
cgg	R	1	206.40
aga	R	2601	1209.00
agg	R	33	724.50
---	--	------	------
mPD		0.026	0.95
		nPD:	0.03
		N. weight:	0.78
		Sc. PD:	-0.075
		Sc. rank:	-564.5

,

NS1			
Pos.	47	obs:	exp:
tct	S	0	2.73
tcc	S	0	2.36
tca	S	0	3.61
tcg	S	0	1.03
tgt	C	0	0.43
tgc	C	1	0.57
agt	S	0	3.27
agc	S	16	3.00
ggt	G	0	376.60
ggc	G	2618	345.60
gga	G	0	1143.00
ggg	G	0	753.00
---	--	------	------
mPD		0.013	0.71
		nPD:	0.02
		N. weight:	2.2
		Sc. PD:	-0.23
		Sc. rank:	-2761.5

,

NS1			
Pos.	48	obs:	exp:
tct	S	0	151.50
tcc	S	0	130.80
tca	S	0	199.90
tcg	S	0	57.29
att	I	0	0.74
atc	I	2	0.50
ata	I	0	0.76
act	T	0	259.10
acc	T	978	216.10
aca	T	0	420.20
acg	T	0	82.61
aat	N	0	408.10
aac	N	768	359.90
agt	S	0	181.10
agc	S	887	166.40
---	--	------	------
mPD		0.66	1.6
		nPD:	0.42
		N. weight:	1.5
		Sc. PD:	0.37
		Sc. rank:	2457.6

,

NS1			
Pos.	49	obs:	exp:
att	I	2	0.74
atc	I	0	0.50
ata	I	0	0.76
act	T	2605	697.40
acc	T	10	581.80
aca	T	18	1131.00
acg	T	0	222.40
---	--	------	------
mPD		0.023	0.69
		nPD:	0.03
		N. weight:	1.4
		Sc. PD:	-0.13
		Sc. rank:	-468.4

,

NS1			
Pos.	50	obs:	exp:
tta	L	0	287.40
ttg	L	0	494.10
ctt	L	2613	493.70
ctc	L	15	454.30
cta	L	4	350.30
ctg	L	1	553.20
att	I	2	0.74
atc	I	0	0.50
ata	I	0	0.76
---	--	------	------
mPD		0.017	1.1
		nPD:	0.01
		N. weight:	1.8
		Sc. PD:	-0.19
		Sc. rank:	-2538.7

,

NS1			
Pos.	51	obs:	exp:
gat	D	1	0.58
gac	D	0	0.42
ggt	G	2614	378.90
ggc	G	1	347.70
gga	G	12	1150.00
ggg	G	7	757.60
---	--	------	------
mPD		0.016	0.69
		nPD:	0.02
		N. weight:	2.
		Sc. PD:	-0.2
		Sc. rank:	-2110.7

,

NS1			
Pos.	52	obs:	exp:
tta	L	0	287.40
ttg	L	1	494.10
ctt	L	0	493.70
ctc	L	18	454.30
cta	L	6	350.30
ctg	L	2608	553.20
att	I	0	0.37
atc	I	1	0.25
ata	I	0	0.38
atg	M	1	1.00
---	--	------	------
mPD		0.021	1.1
		nPD:	0.02
		N. weight:	1.6
		Sc. PD:	-0.17
		Sc. rank:	-2005.9

,

NS1			
Pos.	53	obs:	exp:
tct	S	0	169.20
tcc	S	0	146.10
tca	S	0	223.40
tcg	S	0	64.01
agt	S	1	202.30
agc	S	990	185.90
gat	D	12	363.50
gac	D	620	268.50
ggt	G	0	145.60
ggc	G	1012	133.60
gga	G	0	441.80
ggg	G	0	291.10
---	--	------	------
mPD		0.84	1.9
		nPD:	0.45
		N. weight:	1.7
		Sc. PD:	0.48
		Sc. rank:	3057.8

,

NS1			
Pos.	54	obs:	exp:
ttt	F	0	3.40
ttc	F	8	4.60
tta	L	0	1.96
ttg	L	0	3.38
ctt	L	0	3.38
ctc	L	0	3.11
cta	L	14	2.39
ctg	L	4	3.78
att	I	5	966.20
atc	I	2597	652.60
ata	I	0	983.20
act	T	0	1.32
acc	T	5	1.10
aca	T	0	2.15
acg	T	0	0.42
gtt	V	0	0.43
gtc	V	2	0.42
gta	V	0	0.39
gtg	V	0	0.75
---	--	------	------
mPD		0.042	0.68
		nPD:	0.06
		N. weight:	1.5
		Sc. PD:	-0.097
		Sc. rank:	522.4

,

NS1			
Pos.	55	obs:	exp:
cgt	R	0	0.64
cgc	R	0	0.91
cga	R	6	1.65
cgg	R	0	1.33
att	I	0	0.74
atc	I	0	0.50
ata	I	2	0.76
aaa	K	13	8.42
aag	K	2	6.58
aga	R	11	7.80
agg	R	0	4.67
gat	D	0	0.58
gac	D	1	0.42
gaa	E	2032	1545.00
gag	E	548	1035.00
ggt	G	0	2.88
ggc	G	0	2.64
gga	G	20	8.73
ggg	G	0	5.75
---	--	------	------
mPD		0.39	0.54
		nPD:	0.72
		N. weight:	0.1
		Sc. PD:	0.053
		Sc. rank:	210.1

,

NS1

Pos.	56	obs :	exp :
att	I	0	3.34
atc	I	8	2.26
ata	I	1	3.40
act	T	2	430.70
acc	T	2	359.30
aca	T	1588	698.70
acg	T	34	137.30
aaa	K	9	5.05
aag	K	0	3.95
gtt	V	0	3.91
gtc	V	0	3.80
gta	V	10	3.54
gtg	V	8	6.74
gct	A	3	247.30
gcc	A	1	189.50
gca	A	966	427.30
gcg	A	2	107.90
gaa	E	1	0.60
gag	E	0	0.40
---	--	------	------
mPD		0.54	1.2
		nPD :	0.46
		N. weight :	0.84
		Sc. PD :	0.24
		Sc. rank :	1500.7

,

NS1

Pos.	57	obs :	exp :
tct	S	1	0.17
tcc	S	0	0.15
tca	S	0	0.23
tcg	S	0	0.06
agt	S	0	0.20
agc	S	0	0.19
gtt	V	2	0.43
gtc	V	0	0.42
gta	V	0	0.39
gtg	V	0	0.75
gct	A	2606	669.60
gcc	A	21	513.30
gca	A	0	1157.00
gcg	A	5	292.20
---	--	------	------
mPD		0.022	0.69
		nPD :	0.03
		N. weight :	1.4
		Sc. PD :	-0.13
		Sc. rank :	-676.8

,

NS1

Pos.	58	obs :	exp :
att	I	3	1.11
atc	I	0	0.75
ata	I	0	1.13
act	T	2312	623.50
acc	T	24	520.10
aca	T	18	1012.00
acg	T	0	198.80
aat	N	2	1.06
aac	N	0	0.94
gct	A	276	70.21
gcc	A	0	53.82
gca	A	0	121.30
gcg	A	0	30.64
---	--	------	------
mPD		0.22	0.88
		nPD :	0.25
		N. weight :	1.4
		Sc. PD :	0.14
		Sc. rank :	1738.3

,

NS1

Pos.	59	obs :	exp :
tct	S	0	0.17
tcc	S	0	0.15
tca	S	0	0.23
tcg	S	0	0.06
tgt	C	31	13.48
tgc	C	0	17.52
cct	P	1	0.28
ccc	P	0	0.18
cca	P	0	0.41
ccg	P	0	0.13
cat	H	37	22.00
cac	H	0	15.00
cgt	R	2539	96.59
cgc	R	5	135.70
cga	R	2	246.60
cgg	R	1	199.50
atg	M	18	18.00
agt	S	1	0.20
agc	S	0	0.19
aga	R	0	1168.00
agg	R	0	700.30
---	--	------	------
mPD		0.099	1.0
		nPD :	0.1
		N. weight :	3.4
		Sc. PD :	-0.12
		Sc. rank :	2317.5

,

NS1

Pos.	60	obs :	exp :
tct	S	0	0.68
tcc	S	1	0.59
tca	S	0	0.90
tcg	S	3	0.26
act	T	0	0.26
acc	T	0	0.22
aca	T	0	0.43
acg	T	1	0.08
agt	S	0	0.82
agc	S	0	0.75
gtt	V	0	0.22
gtc	V	0	0.21
gta	V	0	0.20
gtg	V	1	0.37
gct	A	18	406.00
gcc	A	0	311.20
gca	A	14	701.60
gcg	A	1564	177.20
gaa	E	6	618.50
gag	E	1027	414.50
---	--	------	------
mPD		0.51	1.1
		nPD :	0.45
		N. weight :	1.7
		Sc. PD :	0.48
		Sc. rank :	3031.9

,

NS1

Pos.	61	obs :	exp :
ggt	G	0	379.10
ggc	G	6	347.80
gga	G	2583	1150.00
ggg	G	46	757.90
---	--	------	------
mPD		0.039	0.69
		nPD :	0.06
		N. weight :	0.8
		Sc. PD :	-0.056
		Sc. rank :	192.0

,

NS1

Pos.	62	obs :	exp :
aaa	K	28	1479.00
aag	K	2607	1156.00
---	--	------	------
mPD		0.021	0.49
		nPD :	0.04
		N. weight :	0.83
		Sc. PD :	-0.068
		Sc. rank :	-39.2

,

NS1

Pos.	63	obs :	exp :
cat	H	1	0.59
cac	H	0	0.41
caa	Q	559	1329.00
cag	Q	2054	1284.00
cgt	R	0	0.11
cgc	R	0	0.16
cga	R	0	0.29
cgg	R	3	0.23
aaa	K	0	10.10
aag	K	18	7.90
aga	R	0	1.38
agg	R	0	0.82
---	--	------	------
mPD		0.35	0.52
		nPD :	0.68
		N. weight :	0.21
		Sc. PD :	0.1
		Sc. rank :	418.5

,

NS1

Pos.	64	obs :	exp :
att	I	7	972.90
atc	I	25	657.10
ata	I	2588	990.00
act	T	0	0.79
acc	T	0	0.66
aca	T	3	1.29
acg	T	0	0.25
gtt	V	1	2.61
gtc	V	0	2.54
gta	V	11	2.36
gtg	V	0	4.49
---	--	------	------
mPD		0.036	0.67
		nPD :	0.05
		N. weight :	0.99
		Sc. PD :	-0.072
		Sc. rank :	197.8

,

NS1

Pos.	65	obs :	exp :
tta	L	0	3.93
ttg	L	36	6.76
ctt	L	0	6.75
ctc	L	0	6.21
cta	L	0	4.79
ctg	L	0	7.56
att	I	0	0.37
atc	I	0	0.25
ata	I	1	0.38
atg	M	7	7.00
gtt	V	18	563.50
gtc	V	0	547.50
gta	V	84	509.80
gtg	V	2489	970.30
---	--	------	------
mPD		0.11	0.76
		nPD :	0.14
		N. weight :	0.91
		Sc. PD :	0.0056
		Sc. rank :	764.1

,

NS1

Pos.	66	obs :	exp :
aaa	K	1	549.50
aag	K	978	429.50
gat	D	2	1.15
gac	D	0	0.85
gaa	E	1	990.40
gag	E	1653	663.60
---	--	------	------
mPD		0.47	0.95
		nPD :	0.49
		N. weight :	0.95
		Sc. PD :	0.3
		Sc. rank :	1750.4

,

NS1

Pos.	67	obs :	exp :
tgg	W	4	4.00
caa	Q	1	1.02
cag	Q	1	0.98
cgt	R	145	99.02
cgc	R	0	139.10
cga	R	1668	252.80
cgg	R	700	204.50
aga	R	1	1198.00
agg	R	97	717.90
gat	D	0	10.35
gac	D	18	7.65
---	--	------	------
mPD		0.61	0.98
		nPD :	0.62
		N. weight :	1.6
		Sc. PD :	0.68
		Sc. rank :	3077.3

,

NS1

Pos.	68	obs :	exp :
tta	L	0	1.20
ttg	L	0	2.06
ctt	L	11	2.06
ctc	L	0	1.90
cta	L	0	1.46
ctg	L	0	2.31
att	I	2601	974.00
atc	I	22	657.80
ata	I	0	991.20
gtt	V	0	0.22
gtc	V	1	0.21
gta	V	0	0.20
gtg	V	0	0.37
---	--	------	------
mPD		0.026	0.67
		nPD :	0.04
		N. weight :	1.
		Sc. PD :	-0.088
		Sc. rank :	-135.7

,

NS1

Pos.	69	obs :	exp :
tta	L	0	287.40
ttg	L	5	494.10
ctt	L	3	493.70
ctc	L	0	454.30
cta	L	237	350.30
ctg	L	2388	553.20
att	I	0	0.37
atc	I	0	0.25
ata	I	1	0.38
atg	M	1	1.00
---	--	------	------
mPD		0.17	1.1
		nPD :	0.15
		N. weight :	1.4
		Sc. PD :	0.019
		Sc. rank :	1205.4

,

NS1

Pos.	70	obs :	exp :
caa	Q	2	1.02
cag	Q	0	0.98
cgt	R	0	0.04
cgc	R	0	0.05
cga	R	0	0.10
cgg	R	0	0.08
aaa	K	0	559.60
aag	K	997	437.40
aga	R	0	0.46
agg	R	1	0.27
gat	D	4	2.30
gac	D	0	1.70
gaa	E	46	667.00
gag	E	1068	447.00
ggt	G	0	74.37
ggc	G	0	68.24
gga	G	0	225.70
ggg	G	517	148.70
---	--	------	------
mPD		0.83	1.3
		nPD :	0.62
		N. weight :	0.94
		Sc. PD :	0.41
		Sc. rank :	1819.8

,

NS1			
Pos.	71	obs:	exp:
tct	S	0	3.07
tcc	S	0	2.65
tca	S	0	4.06
tcg	S	0	1.16
aaa	K	2	1.12
aag	K	0	0.88
agt	S	16	3.68
agc	S	2	3.38
gat	D	0	2.30
gac	D	4	1.70
gaa	E	2549	1559.00
gag	E	54	1044.00
ggt	G	0	1.01
ggc	G	0	0.92
gga	G	7	3.06
ggg	G	0	2.01
---	-	1	1.00
---	--	------	------
mPD		0.090	0.52
		nPD:	0.17
		N. weight:	0.47
		Sc. PD:	0.016
		Sc. rank:	445.4

NS1			
Pos.	72	obs:	exp:
aaa	K	25	14.03
aag	K	0	10.97
gat	D	0	0.58
gac	D	1	0.42
gaa	E	1529	1562.00
gag	E	1079	1046.00
---	-	1	1.00
---	--	------	------
mPD		0.50	0.50
		nPD:	1.01
		N. weight:	0.0084
		Sc. PD:	0.0065
		Sc. rank:	17.4

NS1			
Pos.	73	obs:	exp:
ttt	F	1	0.43
ttc	F	0	0.57
tct	S	2543	437.90
tcc	S	19	378.00
tca	S	2	578.00
tcg	S	0	165.60
tat	Y	1	0.49
tac	Y	0	0.51
cct	P	48	14.10
ccc	P	2	9.05
cca	P	0	20.42
ccg	P	0	6.44
act	T	0	4.77
acc	T	0	3.98
aca	T	12	7.73
acg	T	6	1.52
agt	S	0	523.50
agc	S	0	481.10
---	-	1	1.00
---	--	------	------
mPD		0.083	1.7
		nPD:	0.05
		N. weight:	1.9
		Sc. PD:	-0.14
		Sc. rank:	182.3

NS1			
Pos.	74	obs:	exp:
aat	N	17	9.03
aac	N	0	7.97
gtt	V	1	0.22
gtc	V	0	0.21
gta	V	0	0.20
gtg	V	0	0.37
gat	D	2601	1504.00
gac	D	13	1110.00
gaa	E	0	0.60
gag	E	1	0.40
ggt	G	1	0.14
ggc	G	0	0.13
gga	G	0	0.44
ggg	G	0	0.29
---	-	1	1.00
---	--	------	------
mPD		0.025	0.50
		nPD:	0.05
		N. weight:	0.57
		Sc. PD:	-0.044
		Sc. rank:	37.7

NS1			
Pos.	75	obs:	exp:
tgg	W	2	2.00
cgt	R	0	0.08
cgc	R	0	0.11
cga	R	0	0.19
cgg	R	0	0.16
aaa	K	0	2.25
aag	K	4	1.75
aga	R	0	0.92
agg	R	2	0.55
gtt	V	0	4.13
gtc	V	0	4.01
gta	V	0	3.74
gtg	V	19	7.11
gat	D	1	0.58
gac	D	0	0.42
gaa	E	544	1560.00
gag	E	2061	1045.00
ggt	G	0	0.29
ggc	G	0	0.26
gga	G	0	0.87
ggg	G	2	0.58
---	--	------	------
mPD		0.35	0.51
		nPD:	0.69
		N. weight:	0.35
		Sc. PD:	0.18
		Sc. rank:	719.2

NS1			
Pos.	76	obs:	exp:
act	T	0	3.18
acc	T	0	2.65
aca	T	12	5.16
acg	T	0	1.01
aat	N	2	9.57
aac	N	16	8.43
gct	A	0	662.70
gcc	A	0	508.00
gca	A	2604	1145.00
gcg	A	1	289.20
---	--	------	------
mPD		0.050	0.73
		nPD:	0.07
		N. weight:	0.89
		Sc. PD:	-0.052
		Sc. rank:	417.9

NS1			
Pos.	77	obs:	exp:
ttt	F	372	158.30
ttc	F	0	213.70
tta	L	0	246.50
ttg	L	0	423.70
tct	S	5	0.85
tcc	S	0	0.74
tca	S	0	1.13
tcg	S	0	0.32
ctt	L	2222	423.40
ctc	L	34	389.60
cta	L	0	300.40
ctg	L	2	474.40
agt	S	0	1.02
agc	S	0	0.94
---	--	------	------
mPD		0.28	1.2
		nPD:	0.23
		N. weight:	1.6
		Sc. PD:	0.13
		Sc. rank:	1938.6

NS1			
Pos.	78	obs:	exp:
cgt	R	0	0.19
cgc	R	0	0.27
cga	R	0	0.48
cgg	R	0	0.39
aaa	K	2346	1475.00
aag	K	282	1153.00
aga	R	5	2.29
agg	R	0	1.37
gaa	E	2	1.20
gag	E	0	0.80
---	--	------	------
mPD		0.20	0.50
		nPD:	0.39
		N. weight:	0.29
		Sc. PD:	0.066
		Sc. rank:	477.7

NS1			
Pos.	79	obs:	exp:
att	I	14	10.03
atc	I	0	6.77
ata	I	13	10.20
atg	M	2577	2577.00
act	T	0	7.15
acc	T	0	5.97
aca	T	0	11.60
acg	T	27	2.28
aaa	K	0	1.12
aag	K	2	0.88
gtt	V	0	0.43
gtc	V	0	0.42
gta	V	0	0.39
gtg	V	2	0.75
---	--	------	------
mPD		0.044	0.064
		nPD:	0.68
		N. weight:	0.033
		Sc. PD:	0.016
		Sc. rank:	66.1

NS1			
Pos.	80	obs:	exp:
tct	S	0	11.78
tcc	S	0	10.17
tca	S	0	15.55
tcg	S	0	4.46
att	I	2	0.74
atc	I	0	0.50
ata	I	0	0.76
act	T	2063	549.10
acc	T	0	458.00
aca	T	10	890.80
acg	T	0	175.10
aat	N	448	238.60
aac	N	1	210.40
agt	S	69	14.09
agc	S	0	12.95
gct	A	8	6.61
gcc	A	12	5.07
gca	A	6	11.43
gcg	A	0	2.89
gat	D	1	0.58
gac	D	0	0.42
---	-	15	15.00
---	--	------	------
mPD		0.37	1.0
		nPD:	0.35
		N. weight:	1.3
		Sc. PD:	0.24
		Sc. rank:	1966.5

NS1

Pos.	81	obs:	exp:
att	I	587	218.70
atc	I	2	147.70
ata	I	0	222.60
atg	M	1	1.00
gtt	V	2023	440.20
gtc	V	1	427.70
gta	V	0	398.20
gtg	V	0	757.90
gct	A	4	1.27
gcc	A	1	0.98
gca	A	0	2.20
gcg	A	0	0.56
ggt	G	1	0.14
ggc	G	0	0.13
gga	G	0	0.44
ggg	G	0	0.29
---	-	15	15.00
---	--	------	------
mPD		0.35	1.1
		nPD:	0.32
		N. weight:	1.5
		Sc. PD:	0.25
		Sc. rank:	2295.8

,

NS1

Pos.	82	obs:	exp:
tct	S	2	0.34
tcc	S	0	0.29
tca	S	0	0.45
tcg	S	0	0.13
act	T	21	5.56
acc	T	0	4.64
aca	T	0	9.02
acg	T	0	1.77
agt	S	0	0.41
agc	S	0	0.38
gtt	V	6	1.30
gtc	V	0	1.27
gta	V	0	1.18
gtg	V	0	2.25
gct	A	2566	656.60
gcc	A	8	503.30
gca	A	4	1135.00
gcg	A	3	286.50
gat	D	10	5.75
gac	D	0	4.25
---	-	15	15.00
---	--	------	------
mPD		0.041	0.71
		nPD:	0.06
		N. weight:	1.4
		Sc. PD:	-0.1
		Sc. rank:	380.6

,

NS1

Pos.	83	obs:	exp:
tta	L	3	0.33
ttg	L	0	0.56
tct	S	524	445.60
tcc	S	18	384.60
tca	S	2064	588.10
tcg	S	3	168.50
ctt	L	0	0.56
ctc	L	0	0.52
cta	L	0	0.40
ctg	L	0	0.63
cct	P	1	2.26
ccc	P	0	1.45
cca	P	7	3.27
ccg	P	0	1.03
agt	S	0	532.70
agc	S	0	489.50
---	-	15	15.00
---	--	------	------
mPD		0.34	1.6
		nPD:	0.21
		N. weight:	1.1
		Sc. PD:	0.069
		Sc. rank:	1162.0

,

NS1

Pos.	84	obs:	exp:
tta	L	0	3.38
ttg	L	31	5.82
tct	S	0	175.70
tcc	S	0	151.70
tca	S	0	232.00
tcg	S	0	66.47
tgt	C	2	0.87
tgc	C	0	1.13
ctt	L	0	5.81
ctc	L	0	5.35
cta	L	0	4.12
ctg	L	0	6.51
cgt	R	4	0.15
cgc	R	0	0.21
cga	R	0	0.39
cgg	R	0	0.31
att	I	1	0.37
atc	I	0	0.25
ata	I	0	0.38
aat	N	1	0.53
aac	N	0	0.47
agt	S	1023	210.10
agc	S	6	193.10
aga	R	0	1.83
agg	R	0	1.10
gtt	V	0	124.40
gtc	V	0	120.90
gta	V	525	112.50
gtg	V	47	214.20
gat	D	3	1.73
gac	D	0	1.27
ggt	G	973	140.50
ggc	G	3	129.00
gga	G	0	426.50
ggg	G	1	281.00
---	-	15	15.00
---	--	------	------
mPD		1.2	1.9
		nPD:	0.64
		N. weight:	1.8
		Sc. PD:	0.79
		Sc. rank:	3492.3

,

NS1

Pos.	85	obs:	exp:
tta	L	0	0.87
ttg	L	0	1.50
tct	S	0	1.37
tcc	S	0	1.18
tca	S	0	1.80
tcg	S	8	0.52
ctt	L	0	1.50
ctc	L	0	1.38
cta	L	0	1.06
ctg	L	8	1.68
cct	P	28	737.40
ccc	P	0	473.20
cca	P	27	1068.00
ccg	P	2560	336.70
caa	Q	0	1.53
cag	Q	3	1.47
act	T	0	0.26
acc	T	0	0.22
aca	T	0	0.43
acg	T	1	0.08
agt	S	0	1.63
agc	S	0	1.50
---	--	------	------
mPD		0.056	0.72
		nPD:	0.08
		N. weight:	2.1
		Sc. PD:	-0.11
		Sc. rank:	1162.3

,

NS1

Pos.	86	obs:	exp:
ttt	F	7	2.98
ttc	F	0	4.02
tct	S	2015	344.30
tcc	S	1	297.20
tca	S	0	454.40
tcg	S	0	130.20
cct	P	9	2.54
ccc	P	0	1.63
cca	P	0	3.67
ccg	P	0	1.16
act	T	532	141.70
acc	T	3	118.20
aca	T	0	229.90
acg	T	0	45.19
agt	S	0	411.60
agc	S	0	378.20
gtt	V	2	0.43
gtc	V	0	0.42
gta	V	0	0.39
gtg	V	0	0.75
gct	A	65	16.79
gcc	A	0	12.87
gca	A	1	29.01
gcg	A	0	7.33
---	--	------	------
mPD		0.38	1.7
		nPD:	0.23
		N. weight:	1.8
		Sc. PD:	0.15
		Sc. rank:	2210.9

,

NS1

Pos.	87	obs:	exp:
tta	L	2	0.22
ttg	L	0	0.38
tct	S	0	445.10
tcc	S	3	384.20
tca	S	2602	587.40
tcg	S	1	168.30
ctt	L	0	0.38
ctc	L	0	0.35
cta	L	0	0.27
ctg	L	0	0.42
cct	P	18	7.61
ccc	P	0	4.89
cca	P	9	11.02
ccg	P	0	3.48
agt	S	0	532.10
agc	S	0	488.90
---	--	------	------
mPD		0.038	1.7
		nPD:	0.02
		N. weight:	1.6
		Sc. PD:	-0.16
		Sc. rank:	-1603.7

,

NS1

Pos.	88	obs:	exp:
tta	L	0	0.33
ttg	L	0	0.56
tgt	C	25	12.18
tgc	C	3	15.82
ctt	L	0	0.56
ctc	L	3	0.52
cta	L	0	0.40
ctg	L	0	0.63
cat	H	0	167.10
cac	H	281	113.90
cgt	R	3	88.10
cgc	R	2301	123.70
cga	R	1	224.90
cgg	R	18	181.90
aga	R	0	1066.00
agg	R	0	638.70
---	--	------	------
mPD		0.25	1.3
		nPD:	0.19
		N. weight:	2.9
		Sc. PD:	0.14
		Sc. rank:	2952.6

,

NS1

Pos.	89	obs:	exp:
tat	Y	36	1280.00
tac	Y	2597	1353.00
cat	H	0	1.19
cac	H	2	0.81
---	--	------	------
mPD		0.028	0.50
		nPD:	0.06
		N. weight:	0.65
		Sc. PD:	-0.045
		Sc. rank:	179.0

,

NS1

Pos.	90	obs:	exp:
tta	L	38	285.20
ttg	L	0	490.30
ctt	L	3	490.00
ctc	L	0	450.80
cta	L	2572	347.70
ctg	L	0	549.00
caa	Q	2	1.02
cag	Q	0	0.98
att	I	3	7.06
atc	I	15	4.77
ata	I	1	7.18
gtt	V	0	0.22
gtc	V	0	0.21
gta	V	1	0.20
gtg	V	0	0.37
---	--	------	------
mPD		0.061	1.1
		nPD:	0.05
		N. weight:	2.1
		Sc. PD:	-0.15
		Sc. rank:	336.4

,

NS1			
Pos.	91	obs:	exp:
att	I	6	2.23
atc	I	0	1.50
ata	I	0	2.27
act	T	2587	686.60
acc	T	4	572.70
aca	T	1	1114.00
acg	T	0	218.90
aat	N	32	17.01
aac	N	0	14.99
aaa	K	1	0.56
aag	K	0	0.44
gct	A	4	1.02
gcc	A	0	0.78
gca	A	0	1.76
gcg	A	0	0.44
mPD		0.037	0.72
		nPD:	0.05
		N. weight:	1.4
		Sc. PD:	-0.11
		Sc. rank:	175.4

,

NS1			
Pos.	92	obs:	exp:
aat	N	0	0.53
aac	N	1	0.47
gat	D	987	1512.00
gac	D	1642	1117.00
gaa	E	5	2.99
gag	E	0	2.01
mPD		0.47	0.49
		nPD:	0.96
		N. weight:	0.091
		Sc. PD:	0.066
		Sc. rank:	187.7

,

NS1			
Pos.	93	obs:	exp:
atg	M	2635	2635.00
mPD		0	0
		nPD:	1.
		N. weight:	0.
		Sc. PD:	0
		Sc. rank:	0

,

NS1			
Pos.	94	obs:	exp:
tct	S	0	3.07
tcc	S	0	2.65
tca	S	0	4.06
tcg	S	0	1.16
act	T	2592	692.90
acc	T	24	578.00
aca	T	0	1124.00
acg	T	0	221.00
aat	N	1	0.53
aac	N	0	0.47
agt	S	0	3.68
agc	S	18	3.38
mPD		0.046	0.70
		nPD:	0.06
		N. weight:	1.4
		Sc. PD:	-0.087
		Sc. rank:	523.7

,

NS1			
Pos.	95	obs:	exp:
tta	L	0	285.50
ttg	L	0	490.90
ctt	L	2605	490.50
ctc	L	11	451.30
cta	L	0	348.10
ctg	L	0	549.60
cct	P	1	0.28
ccc	P	0	0.18
cca	P	0	0.41
ccg	P	0	0.13
att	I	0	6.68
atc	I	0	4.51
ata	I	18	6.80
mPD		0.036	1.1
		nPD:	0.03
		N. weight:	1.8
		Sc. PD:	-0.17
		Sc. rank:	-789.9

,

NS1			
Pos.	96	obs:	exp:
gaa	E	2578	1577.00
gag	E	55	1056.00
ggt	G	0	0.29
ggc	G	0	0.26
gga	G	2	0.87
ggg	G	0	0.58
mPD		0.042	0.48
		nPD:	0.09
		N. weight:	0.46
		Sc. PD:	-0.019
		Sc. rank:	281.4

,

NS1			
Pos.	97	obs:	exp:
gat	D	8	4.60
gac	D	0	3.40
gaa	E	2289	1573.00
gag	E	338	1054.00
mPD		0.23	0.48
		nPD:	0.47
		N. weight:	0.2
		Sc. PD:	0.06
		Sc. rank:	356.5

,

NS1			
Pos.	98	obs:	exp:
cgt	R	0	0.04
cgc	R	0	0.05
cga	R	0	0.10
cgg	R	0	0.08
att	I	0	6.68
atc	I	0	4.51
ata	I	18	6.80
atg	M	2616	2616.00
aga	R	0	0.46
agg	R	1	0.27
mPD		0.014	0.015
		nPD:	0.95
		N. weight:	0.0078
		Sc. PD:	0.0056
		Sc. rank:	16.1

,

NS1			
Pos.	99	obs:	exp:
tta	L	3	0.33
ttg	L	0	0.56
tct	S	0	449.20
tcc	S	0	387.70
tca	S	2609	592.80
tcg	S	3	169.90
ctt	L	0	0.56
ctc	L	0	0.52
cta	L	0	0.40
ctg	L	0	0.63
act	T	0	0.53
acc	T	0	0.44
aca	T	2	0.86
acg	T	0	0.17
agt	S	0	537.00
agc	S	18	493.40
mPD		0.047	1.6
		nPD:	0.03
		N. weight:	1.6
		Sc. PD:	-0.15
		Sc. rank:	-1040.6

,

NS1			
Pos.	100	obs:	exp:
cgt	R	0	99.81
cgc	R	0	140.20
cga	R	12	254.90
cgg	R	0	206.10
aaa	K	2	1.68
aag	K	1	1.32
aga	R	561	1207.00
agg	R	2059	723.70
mPD		0.35	0.96
		nPD:	0.37
		N. weight:	0.7
		Sc. PD:	0.15
		Sc. rank:	1104.1

,

NS1			
Pos.	101	obs:	exp:
tat	Y	0	0.49
tac	Y	1	0.51
aat	N	0	11.16
aac	N	21	9.84
gat	D	1	1483.00
gac	D	2578	1096.00
gaa	E	19	13.17
gag	E	3	8.83
ggt	G	0	1.73
ggc	G	11	1.58
gga	G	1	5.24
ggg	G	0	3.45
mPD		0.044	0.53
		nPD:	0.08
		N. weight:	0.93
		Sc. PD:	-0.043
		Sc. rank:	561.0

,

NS1			
Pos.	102	obs:	exp:
tgg	W	2635	2635.00
mPD		0	0
		nPD:	1.
		N. weight:	0.
		Sc. PD:	0
		Sc. rank:	0

,

NS1			
Pos.	103	obs:	exp:
ttt	F	4	1111.00
ttc	F	2608	1501.00
tat	Y	0	8.75
tac	Y	18	9.25
gtt	V	0	1.09
gtc	V	5	1.06
gta	V	0	0.98
gtg	V	0	1.87
mPD		0.020	0.51
		nPD:	0.04
		N. weight:	0.59
		Sc. PD:	-0.05
		Sc. rank:	-71.9

,

NS1			
Pos.	104	obs:	exp:
atg	M	2635	2635.00
mPD		0	0
		nPD:	1.
		N. weight:	0.
		Sc. PD:	0
		Sc. rank:	0

,

NS1			
Pos.	105	obs:	exp:
ttt	F	0	1.28
ttc	F	3	1.72
tta	L	0	287.10
ttg	L	0	493.50
ctt	L	8	493.20
ctc	L	2610	453.80
cta	L	0	349.90
ctg	L	12	552.60
cct	P	0	0.28
ccc	P	1	0.18
cca	P	0	0.41
ccg	P	0	0.13
att	I	0	0.37
atc	I	1	0.25
ata	I	0	0.38
mPD		0.019	1.1
		nPD:	0.02
		N. weight:	1.9
		Sc. PD:	-0.2
		Sc. rank:	-2487.9

,

NS1			
Pos.	106	obs:	exp:
tta	L	0	0.22
ttg	L	1	0.38
ctt	L	0	0.38
ctc	L	0	0.35
cta	L	0	0.27
ctg	L	1	0.42
atg	M	2633	2633.00
mPD		0.0015	0.0024
		nPD:	0.62
		N. weight:	0.001
		Sc. PD:	0.00046
		Sc. rank:	2.0

,

NS1			
Pos.	107	obs:	exp:
cct	P	26	742.80
ccc	P	2603	476.60
cca	P	5	1075.00
ccg	P	0	339.20
gct	A	0	0.25
gcc	A	1	0.20
gca	A	0	0.44
gcg	A	0	0.11
mPD		0.024	0.71
		nPD:	0.03
		N. weight:	1.8
		Sc. PD:	-0.16
		Sc. rank:	-535.6

,

NS1			
Pos.	108	obs:	exp:
cgt	R	0	0.72
cgc	R	0	1.01
cga	R	0	1.84
cgg	R	0	1.49
aat	N	1	0.53
aac	N	0	0.47
aaa	K	2078	1468.00
aag	K	537	1147.00
aga	R	2	8.72
agg	R	17	5.22
mPD		0.35	0.51
		nPD:	0.68
		N. weight:	0.14
		Sc. PD:	0.067
		Sc. rank:	278.4

,

NS1			
Pos.	109	obs:	exp:
tta	L	0	0.11
ttg	L	0	0.19
tgg	W	3	3.00
ctt	L	0	0.19
ctc	L	0	0.17
cta	L	0	0.13
ctg	L	1	0.21
caa	Q	5	848.20
cag	Q	1662	818.80
cgt	R	0	36.56
cgc	R	0	51.35
cga	R	32	93.35
cgg	R	932	75.50
aga	R	0	442.20
agg	R	0	265.10
mPD		0.50	1.4
		nPD:	0.36
		N. weight:	1.4
		Sc. PD:	0.28
		Sc. rank:	2241.6

,

NS1			
Pos.	110	obs:	exp:
cgt	R	0	0.42
cgc	R	0	0.59
cga	R	0	1.07
cgg	R	0	0.86
att	I	0	0.37
atc	I	0	0.25
ata	I	1	0.38
aaa	K	2610	1472.00
aag	K	13	1151.00
aga	R	11	5.05
agg	R	0	3.02
mPD		0.019	0.50
		nPD:	0.04
		N. weight:	0.6
		Sc. PD:	-0.052
		Sc. rank:	-95.4

,

NS1

Pos.	111	obs:	exp:
ttt	F	1	0.43
ttc	F	0	0.57
tta	L	0	0.11
ttg	L	1	0.19
ctt	L	0	0.19
ctc	L	0	0.17
cta	L	0	0.13
ctg	L	0	0.21
att	I	0	6.31
atc	I	0	4.26
ata	I	17	6.42
atg	M	40	40.00
gtt	V	0	559.40
gtc	V	0	543.40
gta	V	525	506.10
gtg	V	2047	963.10
gct	A	0	1.02
gcc	A	0	0.78
gca	A	0	1.76
gcg	A	4	0.44
---	--	------	------
mPD		0.37	0.77
		nPD:	0.48
		N. weight:	0.66
		Sc. PD:	0.2
		Sc. rank:	1185.8

,

NS1

Pos.	112	obs:	exp:
tct	S	0	0.17
tcc	S	0	0.15
tca	S	0	0.23
tcg	S	0	0.06
act	T	12	5.56
acc	T	0	4.64
aca	T	9	9.02
acg	T	0	1.77
agt	S	1	0.20
agc	S	0	0.19
gtt	V	0	0.65
gtc	V	0	0.63
gta	V	1	0.59
gtg	V	2	1.12
gct	A	1	664.00
gcc	A	0	509.00
gca	A	2607	1147.00
gcg	A	2	289.80
---	--	------	------
mPD		0.033	0.71
		nPD:	0.05
		N. weight:	0.88
		Sc. PD:	-0.07
		Sc. rank:	23.7

,

NS1

Pos.	113	obs:	exp:
gat	D	1	0.58
gac	D	0	0.42
ggt	G	2607	378.90
ggc	G	9	347.70
gga	G	12	1150.00
ggg	G	6	757.60
---	--	------	------
mPD		0.021	0.69
		nPD:	0.03
		N. weight:	2.
		Sc. PD:	-0.19
		Sc. rank:	-1012.4

,

NS1

Pos.	114	obs:	exp:
tct	S	518	271.90
tcc	S	632	234.70
tca	S	442	358.90
tcg	S	0	102.80
cct	P	28	288.80
ccc	P	15	185.30
cca	P	981	418.10
ccg	P	0	131.90
act	T	1	0.26
acc	T	0	0.22
aca	T	0	0.43
acg	T	0	0.08
agt	S	0	325.00
agc	S	0	298.70
ggt	G	6	2.59
ggc	G	12	2.38
gga	G	0	7.86
ggg	G	0	5.18
---	--	------	------
mPD		1.1	1.7
		nPD:	0.63
		N. weight:	0.75
		Sc. PD:	0.33
		Sc. rank:	1466.6

,

NS1

Pos.	115	obs:	exp:
ttt	F	5	2.13
ttc	F	0	2.87
tta	L	0	287.00
ttg	L	0	493.30
ctt	L	2099	493.00
ctc	L	524	453.60
cta	L	5	349.80
ctg	L	1	552.40
att	I	0	0.37
atc	I	1	0.25
ata	I	0	0.38
---	--	------	------
mPD		0.33	1.1
		nPD:	0.29
		N. weight:	1.3
		Sc. PD:	0.17
		Sc. rank:	1702.3

,

NS1

Pos.	116	obs:	exp:
ttt	F	0	1.28
ttc	F	3	1.72
tct	S	0	12.64
tcc	S	0	10.91
tca	S	0	16.68
tcg	S	0	4.78
tat	Y	0	0.49
tac	Y	1	0.51
tgt	C	8	1104.00
tgc	C	2531	1435.00
atg	M	18	18.00
agt	S	0	15.11
agc	S	74	13.88
---	--	------	------
mPD		0.10	0.59
		nPD:	0.17
		N. weight:	0.63
		Sc. PD:	0.022
		Sc. rank:	607.5

,

NS1

Pos.	117	obs:	exp:
att	I	67	970.70
atc	I	2547	655.60
ata	I	0	987.80
gtt	V	0	4.57
gtc	V	3	4.44
gta	V	0	4.13
gtg	V	18	7.86
---	--	------	------
mPD		0.079	0.67
		nPD:	0.12
		N. weight:	1.4
		Sc. PD:	-0.023
		Sc. rank:	1047.5

,

NS1

Pos.	118	obs:	exp:
cgt	R	0	1.90
cgc	R	0	2.66
cga	R	0	4.84
cgg	R	0	3.92
aaa	K	2579	1451.00
aag	K	6	1134.00
aga	R	50	22.93
agg	R	0	13.75
---	--	------	------
mPD		0.042	0.54
		nPD:	0.08
		N. weight:	0.62
		Sc. PD:	-0.032
		Sc. rank:	321.2

,

NS1

Pos.	119	obs:	exp:
tta	L	0	0.11
ttg	L	0	0.19
ctt	L	0	0.19
ctc	L	0	0.17
cta	L	0	0.13
ctg	L	1	0.21
att	I	0	0.37
atc	I	0	0.25
ata	I	1	0.38
atg	M	2631	2631.00
gtt	V	0	0.43
gtc	V	0	0.42
gta	V	0	0.39
gtg	V	2	0.75
---	--	------	------
mPD		0.0030	0.0044
		nPD:	0.68
		N. weight:	0.0019
		Sc. PD:	0.0009
		Sc. rank:	3.7

,

NS1

Pos.	120	obs:	exp:
gat	D	11	1516.00
gac	D	2624	1119.00
---	--	------	------
mPD		0.0083	0.49
		nPD:	0.02
		N. weight:	0.9
		Sc. PD:	-0.095
		Sc. rank:	-1193.9

,

NS1

Pos.	121	obs:	exp:
caa	Q	36	1341.00
cag	Q	2599	1294.00
---	--	------	------
mPD		0.027	0.50
		nPD:	0.05
		N. weight:	0.7
		Sc. PD:	-0.05
		Sc. rank:	127.0

,

NS1

Pos.	122	obs:	exp:
gct	A	2	670.30
gcc	A	18	513.80
gca	A	2605	1158.00
gcg	A	10	292.50
---	--	------	------
mPD		0.023	0.69
		nPD:	0.03
		N. weight:	0.83
		Sc. PD:	-0.076
		Sc. rank:	-303.5

,

NS1

Pos.	123	obs:	exp:
att	I	6	917.20
atc	I	6	619.50
ata	I	2458	933.30
atg	M	1	1.00
act	T	0	5.56
acc	T	0	4.64
aca	T	11	9.02
acg	T	10	1.77
gtt	V	0	31.10
gtc	V	1	30.21
gta	V	142	28.14
gtg	V	0	53.55
---	--	------	------
mPD		0.14	0.79
		nPD:	0.17
		N. weight:	1.1
		Sc. PD:	0.035
		Sc. rank:	1005.1

,

NS1

Pos.	124	obs:	exp:
tta	L	0	0.65
ttg	L	0	1.13
ctt	L	0	1.13
ctc	L	0	1.04
cta	L	0	0.80
ctg	L	6	1.26
att	I	0	1.49
atc	I	0	1.00
ata	I	4	1.51
atg	M	2624	2624.00
act	T	0	0.26
acc	T	0	0.22
aca	T	0	0.43
acg	T	1	0.08
---	--	------	------
mPD		0.0083	0.012
		nPD:	0.71
		N. weight:	0.0065
		Sc. PD:	0.0033
		Sc. rank:	13.2

,

NS1

Pos.	125	obs:	exp:
aat	N	12	7.44
aac	N	2	6.56
gat	D	2607	1507.00
gac	D	13	1113.00
ggt	G	1	0.14
ggc	G	0	0.13
gga	G	0	0.44
ggg	G	0	0.29
---	--	------	------
mPD		0.023	0.50
		nPD:	0.05
		N. weight:	0.57
		Sc. PD:	-0.046
		Sc. rank:	1.0

,

NS1			
Pos.	126	obs:	exp:
cgt	R	0	0.42
cgc	R	0	0.59
cga	R	0	1.07
cgg	R	0	0.86
aaa	K	2598	1472.00
aag	K	25	1151.00
aga	R	11	5.05
agg	R	0	3.02
gaa	E	1	0.60
gag	E	0	0.40
---	--	------	------
mPD		0.028	0.50
		nPD:	0.06
		N. weight:	0.57
		Sc. PD:	-0.041
		Sc. rank:	120.7

NS1			
Pos.	127	obs:	exp:
tct	S	0	1.20
tcc	S	0	1.03
tca	S	0	1.58
tcg	S	0	0.45
cat	H	0	0.59
cac	H	1	0.41
cgt	R	0	0.57
cgc	R	0	0.80
cga	R	0	1.45
cgg	R	0	1.17
att	I	0	0.37
atc	I	1	0.25
ata	I	0	0.38
act	T	0	152.60
acc	T	576	127.30
aca	T	0	247.50
acg	T	0	48.65
aat	N	0	1078.00
aac	N	2029	950.70
aaa	K	3	2.81
aag	K	2	2.19
agt	S	0	1.43
agc	S	7	1.31
aga	R	12	6.88
agg	R	3	4.12
gat	D	0	0.58
gac	D	1	0.42
---	--	------	------
mPD		0.37	0.96
		nPD:	0.39
		N. weight:	1.
		Sc. PD:	0.22
		Sc. rank:	1653.8

NS1			
Pos.	128	obs:	exp:
tta	L	0	0.22
ttg	L	0	0.38
ctt	L	0	0.38
ctc	L	2	0.35
cta	L	0	0.27
ctg	L	0	0.42
att	I	4	964.00
atc	I	2579	651.10
ata	I	13	980.90
gtt	V	10	8.05
gtc	V	27	7.82
gta	V	0	7.28
gtg	V	0	13.86
---	--	------	------
mPD		0.050	0.69
		nPD:	0.07
		N. weight:	1.5
		Sc. PD:	-0.082
		Sc. rank:	741.0

NS1			
Pos.	129	obs:	exp:
att	I	2	889.30
atc	I	11	600.70
ata	I	2382	905.00
atg	M	1	1.00
act	T	0	52.71
acc	T	0	43.97
aca	T	199	85.51
acg	T	0	16.81
gtt	V	0	8.70
gtc	V	0	8.45
gta	V	40	7.87
gtg	V	0	14.98
---	--	------	------
mPD		0.18	0.83
		nPD:	0.22
		N. weight:	1.
		Sc. PD:	0.073
		Sc. rank:	1189.0

NS1			
Pos.	130	obs:	exp:
tta	L	15	287.60
ttg	L	2074	494.40
ctt	L	12	494.10
ctc	L	6	454.60
cta	L	0	350.60
ctg	L	528	553.60
---	--	------	------
mPD		0.35	1.1
		nPD:	0.31
		N. weight:	1.2
		Sc. PD:	0.18
		Sc. rank:	1625.4

NS1			
Pos.	131	obs:	exp:
aaa	K	2610	1479.00
aag	K	25	1156.00
---	--	------	------
mPD		0.019	0.49
		nPD:	0.04
		N. weight:	0.57
		Sc. PD:	-0.05
		Sc. rank:	-85.8

NS1			
Pos.	132	obs:	exp:
gtt	V	0	0.22
gtc	V	0	0.21
gta	V	1	0.20
gtg	V	0	0.37
gct	A	3	670.10
gcc	A	0	513.60
gca	A	2622	1158.00
gcg	A	9	292.40
---	--	------	------
mPD		0.0098	0.69
		nPD:	0.01
		N. weight:	0.87
		Sc. PD:	-0.093
		Sc. rank:	-1271.5

NS1			
Pos.	133	obs:	exp:
aat	N	481	1400.00
aac	N	2154	1235.00
---	--	------	------
mPD		0.30	0.50
		nPD:	0.6
		N. weight:	0.28
		Sc. PD:	0.12
		Sc. rank:	536.7

NS1			
Pos.	134	obs:	exp:
ttt	F	1	1120.00
ttc	F	2632	1513.00
tta	L	0	0.22
ttg	L	0	0.38
ctt	L	0	0.38
ctc	L	2	0.35
cta	L	0	0.27
ctg	L	0	0.42
---	--	------	------
mPD		0.0023	0.49
		nPD:	0.
		N. weight:	0.6
		Sc. PD:	-0.07
		Sc. rank:	-1030.9

NS1			
Pos.	135	obs:	exp:
tct	S	7	449.80
tcc	S	0	388.30
tca	S	11	593.70
tcg	S	0	170.10
cgt	R	1	0.04
cgc	R	0	0.05
cga	R	0	0.10
cgg	R	0	0.08
agt	S	2611	537.80
agc	S	5	494.20
aga	R	0	0.46
agg	R	0	0.27
---	--	------	------
mPD		0.040	1.6
		nPD:	0.02
		N. weight:	1.7
		Sc. PD:	-0.16
		Sc. rank:	-1544.6

NS1			
Pos.	136	obs:	exp:
tta	L	1	0.11
ttg	L	0	0.19
ctt	L	0	0.19
ctc	L	0	0.17
cta	L	0	0.13
ctg	L	0	0.21
att	I	0	2.97
atc	I	0	2.01
ata	I	8	3.02
atg	M	4	4.00
gtt	V	47	569.80
gtc	V	8	553.60
gta	V	1728	515.50
gtg	V	837	981.10
gct	A	0	0.51
gcc	A	0	0.39
gca	A	2	0.88
gcg	A	0	0.22
---	--	------	------
mPD		0.47	0.74
		nPD:	0.64
		N. weight:	0.75
		Sc. PD:	0.34
		Sc. rank:	1500.9

NS1			
Pos.	137	obs:	exp:
ttt	F	15	6.38
ttc	F	0	8.62
tta	L	2	1.96
ttg	L	0	3.38
ctt	L	0	3.38
ctc	L	0	3.11
cta	L	16	2.39
ctg	L	0	3.78
att	I	2496	952.10
atc	I	66	643.00
ata	I	2	968.90
act	T	17	4.50
acc	T	0	3.76
aca	T	0	7.30
acg	T	0	1.44
aat	N	6	3.19
aac	N	0	2.81
gtt	V	15	3.26
gtc	V	0	3.17
gta	V	0	2.95
gtg	V	0	5.62
---	--	------	------
mPD		0.12	0.71
		nPD:	0.16
		N. weight:	0.97
		Sc. PD:	0.024
		Sc. rank:	895.8

NS1			
Pos.	138	obs:	exp:
ttt	F	1367	999.10
ttc	F	981	1349.00
tta	L	7	0.98
ttg	L	1	1.69
tct	S	2	47.14
tcc	S	1	40.69
tca	S	270	62.21
tcg	S	3	17.83
tat	Y	0	0.97
tac	Y	2	1.03
ctt	L	0	1.69
ctc	L	1	1.55
cta	L	0	1.20
ctg	L	0	1.89
agt	S	0	56.35
agc	S	0	51.78
---	--	------	------
mPD		0.77	0.80
		nPD:	0.96
		N. weight:	0.22
		Sc. PD:	0.16
		Sc. rank:	446.7

NS1			
Pos.	139	obs:	exp:
tct	S	0	1.20
tcc	S	0	1.03
tca	S	0	1.58
tcg	S	0	0.45
cat	H	1	0.59
cac	H	0	0.41
aat	N	25	1077.00
aac	N	2002	949.80
aaa	K	0	0.56
aag	K	1	0.44
agt	S	0	1.43
agc	S	7	1.31
gat	D	570	342.80
gac	D	26	253.20
ggt	G	1	0.43
ggc	G	2	0.40
gga	G	0	1.31
ggg	G	0	0.86
---	--	------	------
mPD		0.71	0.86
		nPD:	0.83
		N. weight:	0.68
		Sc. PD:	0.42
		Sc. rank:	1408.1

NS1			
Pos.	140	obs:	exp:
caa	Q	16	9.67
cag	Q	3	9.33
cgt	R	0	99.21
cgc	R	0	139.30
cga	R	173	253.30
cgg	R	2437	204.90
aga	R	0	1200.00
agg	R	6	719.30
---	--	------	------
mPD		0.15	0.97
		nPD:	0.16
		N. weight:	2.5
		Sc. PD:	0.046
		Sc. rank:	2235.4

NS1

Pos. 141		obs:	exp:
tta	L	16	287.00
ttg	L	7	493.30
ctt	L	275	493.00
ctc	L	0	453.60
cta	L	23	349.80
ctg	L	2308	552.40
cct	P	0	0.85
ccc	P	0	0.54
cca	P	0	1.22
ccg	P	3	0.39
atg	M	3	3.00
mPD		0.23	1.1
		nPD:	0.21
		N. weight:	1.2
		Sc. PD:	0.078
		Sc. rank:	1309.9

NS1

Pos. 142		obs:	exp:
aaa	K	3	1.68
aag	K	0	1.32
gaa	E	2567	1574.00
gag	E	61	1054.00
ggt	G	0	0.58
ggc	G	0	0.53
gga	G	4	1.75
ggg	G	0	1.15
mPD		0.051	0.49
		nPD:	0.1
		N. weight:	0.45
		Sc. PD:	-0.013
		Sc. rank:	307.3

NS1

Pos. 143		obs:	exp:
tct	S	8	1.37
tcc	S	0	1.18
tca	S	0	1.80
tcg	S	0	0.52
act	T	6	160.50
acc	T	577	133.90
aca	T	11	260.40
acg	T	12	51.19
agt	S	0	1.63
agc	S	0	1.50
gct	A	2006	514.10
gcc	A	15	394.10
gca	A	0	888.40
gcg	A	0	224.40
mPD		0.72	1.1
		nPD:	0.69
		N. weight:	1.4
		Sc. PD:	0.7
		Sc. rank:	2904.8

NS1

Pos. 144		obs:	exp:
tta	L	21	287.60
ttg	L	9	494.40
ctt	L	54	494.10
ctc	L	1	454.60
cta	L	2544	350.60
ctg	L	6	553.60
mPD		0.075	1.1
		nPD:	0.07
		N. weight:	2.
		Sc. PD:	-0.12
		Sc. rank:	815.2

NS1

Pos. 145		obs:	exp:
att	I	0	966.20
atc	I	0	652.60
ata	I	2602	983.20
atg	M	11	11.00
act	T	0	0.79
acc	T	0	0.66
aca	T	3	1.29
acg	T	0	0.25
gtt	V	0	4.13
gtc	V	18	4.01
gta	V	0	3.74
gtg	V	1	7.11
mPD		0.039	0.68
		nPD:	0.06
		N. weight:	1.1
		Sc. PD:	-0.073
		Sc. rank:	343.5

NS1

Pos. 146		obs:	exp:
tta	L	525	285.20
ttg	L	1	490.30
tct	S	12	3.59
tcc	S	0	3.10
tca	S	9	4.73
tcg	S	0	1.36
ctt	L	0	490.00
ctc	L	0	450.80
cta	L	2076	347.70
ctg	L	11	549.00
agt	S	0	4.29
agc	S	0	3.94
gtt	V	0	0.22
gtc	V	0	0.21
gta	V	1	0.20
gtg	V	0	0.37
mPD		0.36	1.2
		nPD:	0.31
		N. weight:	1.7
		Sc. PD:	0.26
		Sc. rank:	2341.0

NS1

Pos. 147		obs:	exp:
tta	L	0	286.10
ttg	L	0	491.80
ctt	L	2589	491.50
ctc	L	13	452.20
cta	L	1	348.70
ctg	L	18	550.70
att	I	14	5.20
atc	I	0	3.51
ata	I	0	5.29
mPD		0.035	1.1
		nPD:	0.03
		N. weight:	1.7
		Sc. PD:	-0.16
		Sc. rank:	-928.4

NS1

Pos. 148		obs:	exp:
cgt	R	0	99.93
cgc	R	0	140.40
cga	R	1043	255.20
cgg	R	2	206.40
aga	R	1531	1209.00
agg	R	59	724.50
mPD		0.52	0.95
		nPD:	0.55
		N. weight:	0.69
		Sc. PD:	0.26
		Sc. rank:	1303.0

NS1

Pos. 149		obs:	exp:
gct	A	2606	670.30
gcc	A	15	513.80
gca	A	12	1158.00
gcg	A	2	292.50
mPD		0.022	0.69
		nPD:	0.03
		N. weight:	1.4
		Sc. PD:	-0.13
		Sc. rank:	-589.9

NS1

Pos. 150		obs:	exp:
ttt	F	0	1121.00
ttc	F	2635	1514.00
mPD		0	0.49
		nPD:	0.
		N. weight:	0.6
		Sc. PD:	-0.073
		Sc. rank:	-1257.6

NS1

Pos. 151		obs:	exp:
act	T	5	697.70
acc	T	1	582.00
aca	T	2624	1132.00
acg	T	4	222.50
gct	A	0	0.25
gcc	A	0	0.20
gca	A	1	0.44
gcg	A	0	0.11
mPD		0.0083	0.69
		nPD:	0.01
		N. weight:	0.89
		Sc. PD:	-0.098
		Sc. rank:	-1399.6

NS1

Pos. 152		obs:	exp:
aat	N	1	0.53
aac	N	0	0.47
aaa	K	2	1.12
aag	K	0	0.88
gat	D	5	6.33
gac	D	6	4.67
gaa	E	2618	1568.00
gag	E	1	1051.00
ggt	G	0	0.29
ggc	G	0	0.26
gga	G	2	0.87
ggg	G	0	0.58
mPD		0.014	0.49
		nPD:	0.03
		N. weight:	0.55
		Sc. PD:	-0.053
		Sc. rank:	-395.2

NS1

Pos. 153		obs:	exp:
aaa	K	3	1.68
aag	K	0	1.32
gat	D	18	10.93
gac	D	1	8.07
gaa	E	2607	1565.00
gag	E	6	1048.00
mPD		0.021	0.49
		nPD:	0.04
		N. weight:	0.54
		Sc. PD:	-0.044
		Sc. rank:	-20.4

NS1

Pos. 154		obs:	exp:
gaa	E	2	1.20
gag	E	0	0.80
ggt	G	14	378.80
ggc	G	4	347.50
gga	G	2603	1149.00
ggg	G	12	757.30
mPD		0.024	0.69
		nPD:	0.03
		N. weight:	0.83
		Sc. PD:	-0.075
		Sc. rank:	-202.1

NS1

Pos. 155		obs:	exp:
tct	S	6	1.54
tcc	S	0	1.33
tca	S	3	2.03
tcg	S	0	0.58
act	T	0	5.83
acc	T	0	4.86
aca	T	21	9.45
acg	T	1	1.86
agt	S	0	1.84
agc	S	0	1.69
gtt	V	0	6.74
gtc	V	0	6.55
gta	V	31	6.10
gtg	V	0	11.61
gct	A	4	654.30
gcc	A	6	501.60
gca	A	2562	1131.00
gcg	A	0	285.50
gaa	E	1	0.60
gag	E	0	0.40
mPD		0.060	0.74
		nPD:	0.08
		N. weight:	0.88
		Sc. PD:	-0.043
		Sc. rank:	518.8

NS1

Pos. 156		obs:	exp:
att	I	2614	977.70
atc	I	16	660.30
ata	I	3	994.90
gtt	V	1	0.43
gtc	V	0	0.42
gta	V	1	0.39
gtg	V	0	0.75
mPD		0.017	0.66
		nPD:	0.03
		N. weight:	1.
		Sc. PD:	-0.1
		Sc. rank:	-907.6

NS1

Pos. 157		obs:	exp:
gtt	V	1	572.80
gtc	V	6	556.50
gta	V	7	518.30
gtg	V	2620	986.40
gct	A	0	0.25
gcc	A	0	0.20
gca	A	0	0.44
gcg	A	1	0.11
mPD		0.011	0.73
		nPD:	0.02
		N. weight:	1.
		Sc. PD:	-0.11
		Sc. rank:	-1463.8

NS1

Pos. 158		obs:	exp:
gct	A	18	4.58
gcc	A	0	3.51
gca	A	0	7.91
gcg	A	0	2.00
ggt	G	1	376.50
ggc	G	12	345.40
gga	G	2601	1142.00
ggg	G	3	752.70
mPD		0.039	0.70
		nPD:	0.06
		N. weight:	0.87
		Sc. PD:	-0.062
		Sc. rank:	196.1

NS1

Pos. 159		obs:	exp:
gaa	E	2614	1578.00
gag	E	21	1057.00
mPD		0.016	0.48
		nPD:	0.03
		N. weight:	0.51
		Sc. PD:	-0.047
		Sc. rank:	-170.1

NS1

Pos. 160		obs:	exp:
att	I	20	977.70
atc	I	2443	660.30
ata	I	170	994.90
gtt	V	0	0.43
gtc	V	2	0.42
gta	V	0	0.39
gtg	V	0	0.75
mPD		0.14	0.66
		nPD:	0.21
		N. weight:	1.2
		Sc. PD:	0.074
		Sc. rank:	1273.4

NS1

Pos.	161	obs:	exp:
tta	L	1	0.11
ttg	L	0	0.19
tct	S	18	449.70
tcc	S	2	388.20
tca	S	2612	593.50
tcg	S	1	170.10
ctt	L	0	0.19
ctc	L	0	0.17
cta	L	0	0.13
ctg	L	0	0.21
act	T	0	0.26
acc	T	0	0.22
aca	T	1	0.43
acg	T	0	0.08
agt	S	0	537.60
agc	S	0	494.00
---	--	------	------
mPD		0.017	1.6
		nPD:	0.01
		N. weight:	1.6
		Sc. PD:	-0.17
		Sc. rank:	-2534.1

,

NS1

Pos.	162	obs:	exp:
tct	S	0	0.51
tcc	S	0	0.44
tca	S	2	0.68
tcg	S	1	0.19
cct	P	7	741.90
ccc	P	15	476.10
cca	P	558	1074.00
ccg	P	2051	338.80
caa	Q	1	0.51
cag	Q	0	0.49
agt	S	0	0.61
agc	S	0	0.56
---	--	------	------
mPD		0.35	0.71
		nPD:	0.5
		N. weight:	1.3
		Sc. PD:	0.43
		Sc. rank:	2507.0

,

NS1

Pos.	163	obs:	exp:
ttt	F	0	0.43
ttc	F	1	0.57
tta	L	2609	285.30
ttg	L	5	490.50
tct	S	0	0.34
tcc	S	0	0.29
tca	S	2	0.45
tcg	S	0	0.13
ctt	L	0	490.20
ctc	L	0	451.00
cta	L	0	347.80
ctg	L	0	549.20
att	I	18	6.68
atc	I	0	4.51
ata	I	0	6.80
agt	S	0	0.41
agc	S	0	0.38
---	--	------	------
mPD		0.033	1.1
		nPD:	0.03
		N. weight:	2.4
		Sc. PD:	-0.23
		Sc. rank:	-1346.9

,

NS1

Pos.	164	obs:	exp:
cct	P	2622	743.00
ccc	P	13	476.80
cca	P	0	1076.00
ccg	P	0	339.30
---	--	------	------
mPD		0.0098	0.70
		nPD:	0.01
		N. weight:	1.3
		Sc. PD:	-0.15
		Sc. rank:	-2014.8

,

NS1

Pos.	165	obs:	exp:
ttt	F	14	5.96
ttc	F	0	8.04
tct	S	2577	444.00
tcc	S	23	383.30
tca	S	0	586.10
tcg	S	0	167.90
tat	Y	20	9.72
tac	Y	0	10.28
cct	P	1	0.28
ccc	P	0	0.18
cca	P	0	0.41
ccg	P	0	0.13
agt	S	0	530.90
agc	S	0	487.80
---	--	------	------
mPD		0.044	1.7
		nPD:	0.03
		N. weight:	1.9
		Sc. PD:	-0.18
		Sc. rank:	-1426.8

,

NS1

Pos.	166	obs:	exp:
ttt	F	2016	862.10
ttc	F	10	1164.00
tta	L	0	64.51
ttg	L	0	110.90
ctt	L	590	110.80
ctc	L	0	102.00
cta	L	1	78.63
ctg	L	0	124.20
atg	M	18	18.00
---	--	------	------
mPD		0.38	0.90
		nPD:	0.42
		N. weight:	1.1
		Sc. PD:	0.28
		Sc. rank:	1858.0

,

NS1

Pos.	167	obs:	exp:
cct	P	0	743.00
ccc	P	0	476.80
cca	P	2635	1076.00
ccg	P	0	339.30
---	--	------	------
mPD		0	0.70
		nPD:	0.
		N. weight:	0.97
		Sc. PD:	-0.12
		Sc. rank:	-2032.4

,

NS1

Pos.	168	obs:	exp:
ggt	G	0	379.10
ggc	G	0	347.80
gga	G	2631	1150.00
ggg	G	4	757.90
---	--	------	------
mPD		0.0030	0.69
		nPD:	0.
		N. weight:	0.89
		Sc. PD:	-0.1
		Sc. rank:	-1549.9

,

NS1

Pos.	169	obs:	exp:
cat	H	2633	1567.00
cac	H	2	1068.00
---	--	------	------
mPD		0.0015	0.48
		nPD:	0.
		N. weight:	0.56
		Sc. PD:	-0.066
		Sc. rank:	-1001.4

,

NS1

Pos.	170	obs:	exp:
tct	S	18	3.07
tcc	S	0	2.65
tca	S	0	4.06
tcg	S	0	1.16
act	T	2617	693.20
acc	T	0	578.20
aca	T	0	1125.00
acg	T	0	221.00
agt	S	0	3.68
agc	S	0	3.38
---	--	------	------
mPD		0.014	0.70
		nPD:	0.02
		N. weight:	1.4
		Sc. PD:	-0.15
		Sc. rank:	-1697.1

,

NS1

Pos.	171	obs:	exp:
tct	S	0	0.34
tcc	S	0	0.29
tca	S	0	0.45
tcg	S	0	0.13
tat	Y	1	0.49
tac	Y	0	0.51
act	T	0	4.77
acc	T	0	3.98
aca	T	18	7.73
acg	T	0	1.52
aat	N	25	13.29
aac	N	0	11.71
agt	S	1	0.41
agc	S	1	0.38
gtt	V	1	0.22
gtc	V	0	0.21
gta	V	0	0.20
gtg	V	0	0.37
gct	A	3	0.76
gcc	A	0	0.59
gca	A	0	1.32
gcg	A	0	0.33
gat	D	2503	1443.00
gac	D	5	1065.00
gaa	E	59	35.93
gag	E	1	24.07
ggt	G	15	2.45
ggc	G	2	2.24
gga	G	0	7.42
ggg	G	0	4.89
---	--	------	------
mPD		0.13	0.59
		nPD:	0.22
		N. weight:	0.6
		Sc. PD:	0.045
		Sc. rank:	699.6

,

NS1

Pos.	172	obs:	exp:
aaa	K	0	2.81
aag	K	5	2.19
gct	A	0	0.25
gcc	A	0	0.20
gca	A	0	0.44
gcg	A	1	0.11
gaa	E	0	1574.00
gag	E	2629	1055.00
---	--	------	------
mPD		0.0045	0.49
		nPD:	0.01
		N. weight:	0.99
		Sc. PD:	-0.11
		Sc. rank:	-1630.8

,

NS1

Pos.	173	obs:	exp:
aat	N	1	0.53
aac	N	0	0.47
gat	D	2634	1515.00
gac	D	0	1119.00
---	--	------	------
mPD		0.00076	0.49
		nPD:	0.
		N. weight:	0.6
		Sc. PD:	-0.072
		Sc. rank:	-1122.0

,

NS1

Pos.	174	obs:	exp:
gtt	V	0	573.10
gtc	V	2635	556.70
gta	V	0	518.50
gtg	V	0	986.70
---	--	------	------
mPD		0	0.73
		nPD:	0.
		N. weight:	1.7
		Sc. PD:	-0.2
		Sc. rank:	-3527.1

,

NS1

Pos.	175	obs:	exp:
cgt	R	0	0.04
cgc	R	0	0.05
cga	R	0	0.10
cgg	R	0	0.08
aaa	K	2634	1478.00
aag	K	0	1156.00
aga	R	1	0.46
agg	R	0	0.27
---	--	------	------
mPD		0.00076	0.49
		nPD:	0.
		N. weight:	0.63
		Sc. PD:	-0.075
		Sc. rank:	-1178.4

,

NS1

Pos.	176	obs:	exp:
att	I	553	205.30
atc	I	0	138.70
ata	I	0	209.00
act	T	16	4.24
acc	T	0	3.54
aca	T	0	6.88
acg	T	0	1.35
aat	N	2061	1095.00
aac	N	0	965.70
gtt	V	3	0.65
gtc	V	0	0.63
gta	V	0	0.59
gtg	V	0	1.12
gat	D	2	1.15
gac	D	0	0.85
---	--	------	------
mPD		0.35	0.92
		nPD:	0.38
		N. weight:	0.78
		Sc. PD:	0.17
		Sc. rank:	1235.0

NS1

Pos.	177	obs:	exp:
tct	S	0	0.17
tcc	S	0	0.15
tca	S	1	0.23
tcg	S	0	0.06
agt	S	0	0.20
agc	S	0	0.19
gct	A	0	670.10
gcc	A	0	513.60
gca	A	2633	1158.00
gcg	A	1	292.40
---	--	------	------
mPD		0.0015	0.69
		nPD:	0.
		N. weight:	0.89
		Sc. PD:	-0.11
		Sc. rank:	-1649.0

NS1

Pos.	178	obs:	exp:
att	I	2633	977.70
atc	I	0	660.30
ata	I	0	994.90
gtt	V	2	0.43
gtc	V	0	0.42
gta	V	0	0.39
gtg	V	0	0.75
---	--	------	------
mPD		0.0015	0.66
		nPD:	0.
		N. weight:	1.1
		Sc. PD:	-0.13
		Sc. rank:	-1973.2

NS1

Pos.	179	obs:	exp:
gaa	E	0	7.78
gag	E	13	5.22
ggt	G	0	377.20
ggc	G	15	346.10
gga	G	75	1145.00
ggg	G	2532	754.10
---	--	------	------
mPD		0.076	0.70
		nPD:	0.11
		N. weight:	1.2
		Sc. PD:	-0.027
		Sc. rank:	872.2

NS1

Pos.	180	obs:	exp:
ttt	F	0	0.43
ttc	F	1	0.57
att	I	0	54.96
atc	I	148	37.12
ata	I	0	55.92
gtt	V	1	540.40
gtc	V	2484	525.10
gta	V	0	489.00
gtg	V	0	930.60
gct	A	0	0.25
gcc	A	1	0.20
gca	A	0	0.44
gcg	A	0	0.11
---	--	------	------
mPD		0.11	0.84
		nPD:	0.13
		N. weight:	1.7
		Sc. PD:	-0.011
		Sc. rank:	1385.3

NS1

Pos.	181	obs:	exp:
tta	L	0	287.60
ttg	L	0	494.40
ctt	L	0	494.10
ctc	L	2635	454.60
cta	L	0	350.60
ctg	L	0	553.60
---	--	------	------
mPD		0	1.1
		nPD:	0.
		N. weight:	1.9
		Sc. PD:	-0.23
		Sc. rank:	-3986.9

NS1

Pos.	182	obs:	exp:
att	I	1	978.50
atc	I	2634	660.80
ata	I	0	995.70
---	--	------	------
mPD		0.00076	0.66
		nPD:	0.
		N. weight:	1.5
		Sc. PD:	-0.18
		Sc. rank:	-2872.1

NS1

Pos.	183	obs:	exp:
ggt	G	18	379.10
ggc	G	0	347.80
gga	G	2612	1150.00
ggg	G	5	757.90
---	--	------	------
mPD		0.017	0.69
		nPD:	0.03
		N. weight:	0.85
		Sc. PD:	-0.084
		Sc. rank:	-765.7

NS1

Pos.	184	obs:	exp:
ggt	G	1	379.10
ggc	G	0	347.80
gga	G	1110	1150.00
ggg	G	1524	757.90
---	--	------	------
mPD		0.49	0.69
		nPD:	0.71
		N. weight:	0.42
		Sc. PD:	0.22
		Sc. rank:	861.9

NS1

Pos.	185	obs:	exp:
ttt	F	11	4.68
ttc	F	0	6.32
tta	L	0	286.40
ttg	L	0	492.40
ctt	L	2624	492.00
ctc	L	0	452.70
cta	L	0	349.10
ctg	L	0	551.30
---	--	------	------
mPD		0.0083	1.1
		nPD:	0.01
		N. weight:	1.8
		Sc. PD:	-0.21
		Sc. rank:	-3013.9

NS1

Pos.	186	obs:	exp:
gaa	E	2631	1578.00
gag	E	4	1057.00
---	--	------	------
mPD		0.0030	0.48
		nPD:	0.01
		N. weight:	0.55
		Sc. PD:	-0.063
		Sc. rank:	-933.1

NS1

Pos.	187	obs:	exp:
tgg	W	2635	2635.00
---	--	------	------
mPD		0	0
		nPD:	1.
		N. weight:	0.
		Sc. PD:	0
		Sc. rank:	0

NS1

Pos.	188	obs:	exp:
aat	N	2634	1400.00
aac	N	1	1235.00
---	--	------	------
mPD		0.00076	0.50
		nPD:	0.
		N. weight:	0.68
		Sc. PD:	-0.081
		Sc. rank:	-1291.4

NS1

Pos.	189	obs:	exp:
aat	N	972	516.50
aac	N	0	455.50
gat	D	1661	956.60
gac	D	2	706.40
---	--	------	------
mPD		0.47	0.96
		nPD:	0.49
		N. weight:	0.63
		Sc. PD:	0.2
		Sc. rank:	1137.9

NS1

Pos.	190	obs:	exp:
tct	S	0	0.17
tcc	S	0	0.15
tca	S	0	0.23
tcg	S	0	0.06
aat	N	0	1400.00
aac	N	2634	1234.00
agt	S	0	0.20
agc	S	1	0.19
---	--	------	------
mPD		0.00076	0.50
		nPD:	0.
		N. weight:	0.83
		Sc. PD:	-0.098
		Sc. rank:	-1566.4

NS1

Pos.	191	obs:	exp:
tct	S	0	3.07
tcc	S	0	2.65
tca	S	18	4.06
tcg	S	0	1.16
act	T	0	692.70
acc	T	0	577.80
aca	T	2613	1124.00
acg	T	2	220.90
aaa	K	2	1.12
aag	K	0	0.88
agt	S	0	3.68
agc	S	0	3.38
---	--	------	------
mPD		0.017	0.70
		nPD:	0.02
		N. weight:	0.92
		Sc. PD:	-0.091
		Sc. rank:	-905.9

NS1

Pos.	192	obs:	exp:
att	I	19	7.06
atc	I	0	4.77
ata	I	0	7.18
gtt	V	2616	568.90
gtc	V	0	552.70
gta	V	0	514.70
gtg	V	0	979.60
---	--	------	------
mPD		0.014	0.74
		nPD:	0.02
		N. weight:	1.7
		Sc. PD:	-0.17
		Sc. rank:	-1982.0

NS1

Pos.	193	obs:	exp:
tgg	W	1	1.00
caa	Q	1	17.81
cag	Q	34	17.19
cgt	R	0	98.52
cgc	R	0	138.40
cga	R	583	251.60
cgg	R	2012	203.50
aaa	K	0	0.56
aag	K	1	0.44
aga	R	3	1192.00
agg	R	0	714.40
---	--	------	------
mPD		0.38	0.99
		nPD:	0.38
		N. weight:	2.1
		Sc. PD:	0.46
		Sc. rank:	3439.1

NS1

Pos.	194	obs:	exp:
tta	L	0	0.11
ttg	L	0	0.19
ctt	L	0	0.19
ctc	L	1	0.17
cta	L	0	0.13
ctg	L	0	0.21
att	I	0	7.43
atc	I	20	5.02
ata	I	0	7.56
gtt	V	0	564.40
gtc	V	2595	548.30
gta	V	0	510.60
gtg	V	0	971.70
gct	A	0	4.58
gcc	A	0	3.51
gca	A	0	7.91
gcg	A	18	2.00
ggt	G	0	0.14
ggc	G	1	0.13
gga	G	0	0.44
ggg	G	0	0.29
---	--	------	------
mPD		0.044	0.76
		nPD:	0.06
		N. weight:	1.7
		Sc. PD:	-0.12
		Sc. rank:	519.0

NS1

Pos.	195	obs:	exp:
tct	S	2635	450.00
tcc	S	0	388.40
tca	S	0	594.00
tcg	S	0	170.20
agt	S	0	538.00
agc	S	0	494.40
---	--	------	------
mPD		0	1.6
		nPD:	0.
		N. weight:	1.9
		Sc. PD:	-0.23
		Sc. rank:	-4010.1

NS1

Pos.	196	obs:	exp:
gaa	E	2633	1578.00
gag	E	2	1057.00
---	--	------	------
mPD		0.0015	0.48
		nPD:	0.
		N. weight:	0.55
		Sc. PD:	-0.065
		Sc. rank:	-982.4

,

NS1

Pos.	197	obs:	exp:
att	I	237	88.01
atc	I	0	59.44
ata	I	0	89.56
act	T	2370	627.80
acc	T	0	523.60
aca	T	0	1018.00
acg	T	0	200.20
aat	N	23	12.22
aac	N	0	10.78
gtt	V	1	0.22
gtc	V	0	0.21
gta	V	0	0.20
gtg	V	0	0.37
gct	A	3	1.02
gcc	A	0	0.78
gca	A	0	1.76
gcg	A	1	0.44
---	--	------	------
mPD		0.18	0.87
		nPD:	0.21
		N. weight:	1.4
		Sc. PD:	0.095
		Sc. rank:	1568.4

,

NS1

Pos.	198	obs:	exp:
tta	L	2	283.60
ttg	L	0	487.50
ctt	L	0	487.20
ctc	L	0	448.20
cta	L	2595	345.70
ctg	L	1	545.90
att	I	0	13.00
atc	I	0	8.78
ata	I	35	13.23
gtt	V	0	0.43
gtc	V	0	0.42
gta	V	2	0.39
gtg	V	0	0.75
---	--	------	------
mPD		0.030	1.2
		nPD:	0.03
		N. weight:	2.2
		Sc. PD:	-0.21
		Sc. rank:	-1772.0

,

NS1

Pos.	199	obs:	exp:
caa	Q	0	1341.00
cag	Q	2635	1294.00
---	--	------	------
mPD		0	0.50
		nPD:	0.
		N. weight:	0.77
		Sc. PD:	-0.093
		Sc. rank:	-1613.0

,

NS1

Pos.	200	obs:	exp:
cgt	R	0	99.32
cgc	R	0	139.50
cga	R	0	253.60
cgg	R	0	205.10
aaa	K	14	8.42
aag	K	1	6.58
aga	R	2619	1201.00
agg	R	0	720.10
ggt	G	0	0.14
ggc	G	0	0.13
gga	G	1	0.44
ggg	G	0	0.29
---	--	------	------
mPD		0.013	0.96
		nPD:	0.01
		N. weight:	0.85
		Sc. PD:	-0.092
		Sc. rank:	-1285.3

,

NS1

Pos.	201	obs:	exp:
ttt	F	0	1115.00
ttc	F	2620	1505.00
tta	L	0	0.22
ttg	L	0	0.38
tat	Y	0	6.32
tac	Y	13	6.68
ctt	L	0	0.38
ctc	L	2	0.35
cta	L	0	0.27
ctg	L	0	0.42
---	--	------	------
mPD		0.011	0.50
		nPD:	0.02
		N. weight:	0.6
		Sc. PD:	-0.061
		Sc. rank:	-644.9

,

NS1

Pos.	202	obs:	exp:
act	T	523	138.50
acc	T	0	115.60
aca	T	0	224.70
acg	T	0	44.18
aat	N	4	2.13
aac	N	0	1.87
gct	A	2108	536.30
gcc	A	0	411.10
gca	A	0	926.60
gcg	A	0	234.00
---	--	------	------
mPD		0.32	1.0
		nPD:	0.32
		N. weight:	1.5
		Sc. PD:	0.24
		Sc. rank:	2208.0

,

NS1

Pos.	203	obs:	exp:
tgg	W	2635	2635.00
---	--	------	------
mPD		0	0
		nPD:	1.
		N. weight:	0.
		Sc. PD:	0
		Sc. rank:	0

,

NS1

Pos.	204	obs:	exp:
cgt	R	0	98.94
cgc	R	0	139.00
cga	R	0	252.60
cgg	R	0	204.30
aga	R	2608	1197.00
agg	R	1	717.40
ggt	G	0	3.74
ggc	G	0	3.43
gga	G	26	11.35
ggg	G	0	7.48
---	--	------	------
mPD		0.020	0.97
		nPD:	0.02
		N. weight:	0.85
		Sc. PD:	-0.086
		Sc. rank:	-910.7

,

NS1

Pos.	205	obs:	exp:
tct	S	0	202.00
tcc	S	0	174.40
tca	S	0	266.70
tcg	S	0	76.41
att	I	0	8.17
atc	I	22	5.52
ata	I	0	8.31
act	T	0	0.26
acc	T	1	0.22
aca	T	0	0.43
acg	T	0	0.08
aat	N	1	756.70
aac	N	1423	667.30
agt	S	0	241.50
agc	S	1183	222.00
gtt	V	0	0.87
gtc	V	4	0.85
gta	V	0	0.79
gtg	V	0	1.50
gat	D	0	0.58
gac	D	1	0.42
---	--	------	------
mPD		0.51	1.6
		nPD:	0.32
		N. weight:	1.3
		Sc. PD:	0.21
		Sc. rank:	1832.3

,

NS1

Pos.	206	obs:	exp:
tct	S	0	444.00
tcc	S	0	383.30
tca	S	0	586.10
tcg	S	0	167.90
tgt	C	3	1.30
tgc	C	0	1.70
cgt	R	18	0.72
cgc	R	0	1.01
cga	R	0	1.84
cgg	R	0	1.49
aat	N	9	4.78
aac	N	0	4.22
agt	S	2596	530.90
agc	S	4	487.80
aga	R	1	8.72
agg	R	0	5.22
ggt	G	4	0.58
ggc	G	0	0.53
gga	G	0	1.75
ggg	G	0	1.15
---	--	------	------
mPD		0.029	1.7
		nPD:	0.02
		N. weight:	1.7
		Sc. PD:	-0.18
		Sc. rank:	-2230.7

,

NS1

Pos.	207	obs:	exp:
aat	N	1633	867.80
aac	N	0	765.20
gat	D	1002	576.40
gac	D	0	425.60
---	--	------	------
mPD		0.47	0.97
		nPD:	0.49
		N. weight:	0.66
		Sc. PD:	0.2
		Sc. rank:	1194.7

,

NS1

Pos.	208	obs:	exp:
gaa	E	0	1578.00
gag	E	2635	1057.00
---	--	------	------
mPD		0	0.48
		nPD:	0.
		N. weight:	0.99
		Sc. PD:	-0.12
		Sc. rank:	-2072.0

,

NS1

Pos.	209	obs:	exp:
aat	N	65	34.54
aac	N	0	30.46
gat	D	2558	1472.00
gac	D	1	1087.00
gaa	E	0	0.60
gag	E	1	0.40
ggt	G	10	1.44
ggc	G	0	1.32
gga	G	0	4.37
ggg	G	0	2.88
---	--	------	------
mPD		0.057	0.55
		nPD:	0.1
		N. weight:	0.61
		Sc. PD:	-0.017
		Sc. rank:	439.9

,

NS1

Pos.	210	obs:	exp:
tgg	W	4	4.00
ggt	G	0	378.50
ggc	G	0	347.30
gga	G	0	1149.00
ggg	G	2631	756.70
---	--	------	------
mPD		0.0030	0.69
		nPD:	0.
		N. weight:	1.4
		Sc. PD:	-0.16
		Sc. rank:	-2376.4

,

NS1

Pos.	211	obs:	exp:
cgt	R	0	99.25
cgc	R	0	139.40
cga	R	0	253.40
cgg	R	0	205.00
aga	R	2617	1200.00
agg	R	0	719.60
ggt	G	0	2.59
ggc	G	0	2.38
gga	G	18	7.86
ggg	G	0	5.18
---	--	------	------
mPD		0.014	0.96
		nPD:	0.01
		N. weight:	0.85
		Sc. PD:	-0.091
		Sc. rank:	-1256.5

,

NS1

Pos.	212	obs:	exp:
tta	L	0	1.75
ttg	L	0	3.00
tct	S	9	1.54
tcc	S	0	1.33
tca	S	0	2.03
tcg	S	0	0.58
ctt	L	16	3.00
ctc	L	0	2.76
cta	L	0	2.13
ctg	L	0	3.36
cct	P	2610	736.00
ccc	P	0	472.30
cca	P	0	1066.00
ccg	P	0	336.10
agt	S	0	1.84
agc	S	0	1.69
---	--	------	------
mPD		0.019	0.73
		nPD:	0.03
		N. weight:	1.4
		Sc. PD:	-0.13
		Sc. rank:	-1181.4

,

NS1

Pos.	213	obs:	exp:
tta	L	2	0.33
ttg	L	0	0.56
tct	S	0	434.50
tcc	S	0	375.00
tca	S	2544	573.50
tcg	S	0	164.30
ctt	L	0	0.56
ctc	L	0	0.52
cta	L	1	0.40
ctg	L	0	0.63
cct	P	0	24.82
ccc	P	0	15.92
cca	P	88	35.93
ccg	P	0	11.33
agt	S	0	519.40
agc	S	0	477.30
---	--	------	------
mPD		0.068	1.7
		nPD:	0.04
		N. weight:	1.6
		Sc. PD:	-0.14
		Sc. rank:	-148.2

,

NS1

Pos.	214	obs:	exp:
ttt	F	1	71.91
ttc	F	168	97.09
tta	L	0	269.20
ttg	L	0	462.70
ctt	L	3	462.40
ctc	L	2463	425.50
cta	L	0	328.10
ctg	L	0	518.10
---	--	------	------
mPD		0.12	1.2
		nPD:	0.1
		N. weight:	1.8
		Sc. PD:	-0.05
		Sc. rank:	1298.2

,

NS1

Pos.	215	obs:	exp:
tta	L	0	1.53
ttg	L	0	2.63
tct	S	5	0.85
tcc	S	0	0.74
tca	S	0	1.13
tcg	S	0	0.32
ctt	L	14	2.63
ctc	L	0	2.42
cta	L	0	1.86
ctg	L	0	2.94
cct	P	2615	737.40
ccc	P	0	473.20
cca	P	0	1068.00
ccg	P	0	336.70
agt	S	0	1.02
agc	S	0	0.94
gct	A	1	0.25
gcc	A	0	0.20
gca	A	0	0.44
gcg	A	0	0.11
---	--	------	------
mPD		0.015	0.72
		nPD:	0.02
		N. weight:	1.4
		Sc. PD:	-0.14
		Sc. rank:	-1507.9

,

NS1

Pos.	216	obs:	exp:
tct	S	0	89.15
tcc	S	0	76.95
tca	S	522	117.70
tcg	S	0	33.72
cct	P	0	595.80
ccc	P	0	382.30
cca	P	2113	862.70
ccg	P	0	272.10
agt	S	0	106.60
agc	S	0	97.94
---	--	------	------
mPD		0.32	1.2
		nPD:	0.27
		N. weight:	1.1
		Sc. PD:	0.13
		Sc. rank:	1440.9

,

NS1

Pos.	217	obs:	exp:
cgt	R	0	0.04
cgc	R	0	0.05
cga	R	0	0.10
cgg	R	0	0.08
aat	N	15	13.29
aac	N	10	11.71
aaa	K	581	1453.00
aag	K	2007	1135.00
aga	R	0	0.46
agg	R	1	0.27
gaa	E	9	12.57
gag	E	12	8.43
---	--	------	------
mPD		0.38	0.52
		nPD:	0.73
		N. weight:	0.26
		Sc. PD:	0.14
		Sc. rank:	524.4

,

NS1

Pos.	218	obs:	exp:
cat	H	0	0.59
cac	H	1	0.41
caa	Q	0	617.70
cag	Q	1214	596.30
---	-	1420	1420.00
---	--	------	------
mPD		0.00035	0.11
		nPD:	0.
		N. weight:	0.
		Sc. PD:	0
		Sc. rank:	0

,

NS1

Pos.	219	obs:	exp:
aaa	K	1215	682.00
aag	K	0	533.00
---	-	1420	1420.00
---	--	------	------
mPD		0	0.10
		nPD:	0.
		N. weight:	0.
		Sc. PD:	0
		Sc. rank:	0

,

NS1

Pos.	220	obs:	exp:
tgt	C	0	0.43
tgc	C	1	0.57
tgg	W	15	15.00
cct	P	0	0.28
ccc	P	0	0.18
cca	P	0	0.41
ccg	P	1	0.13
caa	Q	0	0.51
cag	Q	1	0.49
cgt	R	0	45.39
cgc	R	17	63.76
cga	R	69	115.90
cgg	R	1111	93.75
aga	R	0	549.10
agg	R	0	329.10
---	-	1420	1420.00
---	--	------	------
mPD		0.035	0.21
		nPD:	0.17
		N. weight:	0.
		Sc. PD:	0
		Sc. rank:	0

,

NS1

Pos.	221	obs:	exp:
tat	Y	0	8.75
tac	Y	18	9.25
aat	N	0	0.53
aac	N	1	0.47
aaa	K	1196	671.30
aag	K	0	524.70
---	-	1420	1420.00
---	--	------	------
mPD		0.013	0.11
		nPD:	0.11
		N. weight:	0.
		Sc. PD:	0
		Sc. rank:	0

,

NS1

Pos.	222	obs:	exp:
tta	L	0	0.33
ttg	L	0	0.56
ctt	L	0	0.56
ctc	L	0	0.52
cta	L	0	0.40
ctg	L	3	0.63
atg	M	1210	1210.00
gtt	V	0	0.43
gtc	V	0	0.42
gta	V	0	0.39
gtg	V	2	0.75
---	-	1420	1420.00
---	--	------	------
mPD		0.0017	0.0028
		nPD:	0.62
		N. weight:	0.
		Sc. PD:	0
		Sc. rank:	0

,

NS1

Pos.	223	obs:	exp:
gct	A	0	309.10
gcc	A	0	236.90
gca	A	0	534.10
gcg	A	1215	134.90
---	-	1420	1420.00
---	--	------	------
mPD		0	0.15
		nPD:	0.
		N. weight:	0.
		Sc. PD:	0
		Sc. rank:	0

,

NS1

Pos.	224	obs:	exp:
cgt	R	0	45.13
cgc	R	0	63.39
cga	R	0	115.20
cgg	R	0	93.20
aga	R	1190	545.80
agg	R	0	327.20
ggt	G	0	3.60
ggc	G	0	3.30
gga	G	25	10.91
ggg	G	0	7.19
---	-	1420	1420.00
---	--	------	------
mPD		0.0086	0.21
		nPD:	0.04
		N. weight:	0.
		Sc. PD:	0
		Sc. rank:	0

,

NS1

Pos.	225	obs:	exp:
cgt	R	0	0.68
cgc	R	0	0.96
cga	R	12	1.74
cgg	R	0	1.41
act	T	0	316.50
acc	T	0	264.00
aca	T	1195	513.50
acg	T	0	100.90
aga	R	6	8.26
agg	R	0	4.95
gct	A	0	0.51
gcc	A	0	0.39
gca	A	2	0.88
gcg	A	0	0.22
---	-	1420	1420.00
---	--	------	------
mPD		0.011	0.16
		nPD:	0.07
		N. weight:	0.
		Sc. PD:	0
		Sc. rank:	0

,

NS1

Pos.	226	obs:	exp:
att	I	1190	441.90
atc	I	0	298.40
ata	I	0	449.70
act	T	5	1.32
acc	T	0	1.10
aca	T	0	2.15
acg	T	0	0.42
gtt	V	20	4.35
gtc	V	0	4.23
gta	V	0	3.94
gtg	V	0	7.49
---	-	1420	1420.00
---	--	------	------
mPD		0.0086	0.15
		nPD:	0.06
		N. weight:	0.
		Sc. PD:	0
		Sc. rank:	0

,

NS1

Pos.	227	obs:	exp:
gaa	E	0	370.00
gag	E	618	248.00
ggt	G	0	85.88
ggc	G	0	78.80
gga	G	0	260.60
ggg	G	597	171.70
---	-	1420	1420.00
---	--	------	------
mPD		0.11	0.23
		nPD:	0.45
		N. weight:	0.
		Sc. PD:	0
		Sc. rank:	0

,

NS1

Pos.	228	obs:	exp:
tct	S	0	204.80
tcc	S	0	176.80
tca	S	1199	270.30
tcg	S	0	77.45
cct	P	0	4.51
ccc	P	0	2.90
cca	P	16	6.53
ccg	P	0	2.06
agt	S	0	244.80
agc	S	0	225.00
---	-	1420	1420.00
---	--	------	------
mPD		0.0055	0.35
		nPD:	0.02
		N. weight:	0.
		Sc. PD:	0
		Sc. rank:	0

,

NS1

Pos.	229	obs:	exp:
gaa	E	1215	727.50
gag	E	0	487.50
---	-	1420	1420.00
---	--	------	------
mPD		0	0.10
		nPD:	0.
		N. weight:	0.
		Sc. PD:	0
		Sc. rank:	0

,

NS1			
Pos.	230	obs:	exp:
att	I	4	1.49
atc	I	0	1.00
ata	I	0	1.51
gtt	V	1211	263.40
gtc	V	0	255.90
gta	V	0	238.30
gtg	V	0	453.50
---	-	1420	1420.00
---	--	------	------
mPD		0.0014	0.16
		nPD:	0.01
		N. weight:	0.
		Sc. PD:	0
		Sc. rank:	0

,

NS1			
Pos.	231	obs:	exp:
cgt	R	0	19.11
cgc	R	0	26.85
cga	R	504	48.80
cgg	R	0	39.47
aga	R	0	231.20
agg	R	0	138.60
---	-	2131	2131.00
---	--	------	------
mPD		0	0.035
		nPD:	0.
		N. weight:	0.
		Sc. PD:	0
		Sc. rank:	0

,

NS1			
Pos.	232	obs:	exp:
cgt	R	0	0.15
cgc	R	0	0.21
cga	R	0	0.39
cgg	R	0	0.31
aga	R	4	1.83
agg	R	0	1.10
ggt	G	0	71.93
ggc	G	0	66.00
gga	G	500	218.30
ggg	G	0	143.80
---	-	2131	2131.00
---	--	------	------
mPD		0.00058	0.026
		nPD:	0.02
		N. weight:	0.
		Sc. PD:	0
		Sc. rank:	0

,

NS1			
Pos.	233	obs:	exp:
aat	N	504	267.80
aac	N	0	236.20
---	-	2131	2131.00
---	--	------	------
mPD		0	0.018
		nPD:	0.
		N. weight:	0.
		Sc. PD:	0
		Sc. rank:	0

,

NS1			
Pos.	234	obs:	exp:
act	T	0	0.53
acc	T	0	0.44
aca	T	0	0.86
acg	T	2	0.17
aaa	K	0	281.80
aag	K	502	220.20
---	-	2131	2131.00
---	--	------	------
mPD		0.00029	0.018
		nPD:	0.02
		N. weight:	0.
		Sc. PD:	0
		Sc. rank:	0

,

NS1			
Pos.	235	obs:	exp:
atg	M	503	503.00
gtt	V	0	0.22
gtc	V	0	0.21
gta	V	0	0.20
gtg	V	1	0.37
---	-	2131	2131.00
---	--	------	------
mPD		0.00014	0.00024
		nPD:	0.62
		N. weight:	0.
		Sc. PD:	0
		Sc. rank:	0

,

NS1			
Pos.	236	obs:	exp:
tct	S	1	0.17
tcc	S	0	0.15
tca	S	0	0.23
tcg	S	0	0.06
agt	S	0	0.20
agc	S	0	0.19
gct	A	503	128.00
gcc	A	0	98.09
gca	A	0	221.10
gcg	A	0	55.84
---	-	2131	2131.00
---	--	------	------
mPD		0.00014	0.026
		nPD:	0.01
		N. weight:	0.
		Sc. PD:	0
		Sc. rank:	0

,

NS1			
Pos.	237	obs:	exp:
aat	N	2	1.06
aac	N	0	0.94
gat	D	501	288.20
gac	D	0	212.80
ggt	G	1	0.14
ggc	G	0	0.13
gga	G	0	0.44
ggg	G	0	0.29
---	-	2131	2131.00
---	--	------	------
mPD		0.00043	0.018
		nPD:	0.02
		N. weight:	0.
		Sc. PD:	0
		Sc. rank:	0

,

NS1			
Pos.	238	obs:	exp:
taa	.	0	966.30
tag	.	1420	739.50
tga	.	1215	929.20
---	--	------	------
mPD		0.99	0.86
		nPD:	1.16
		N. weight:	0.52
		Sc. PD:	0.47
		Sc. rank:	1082.1

}

Amino acid level conservation per codon

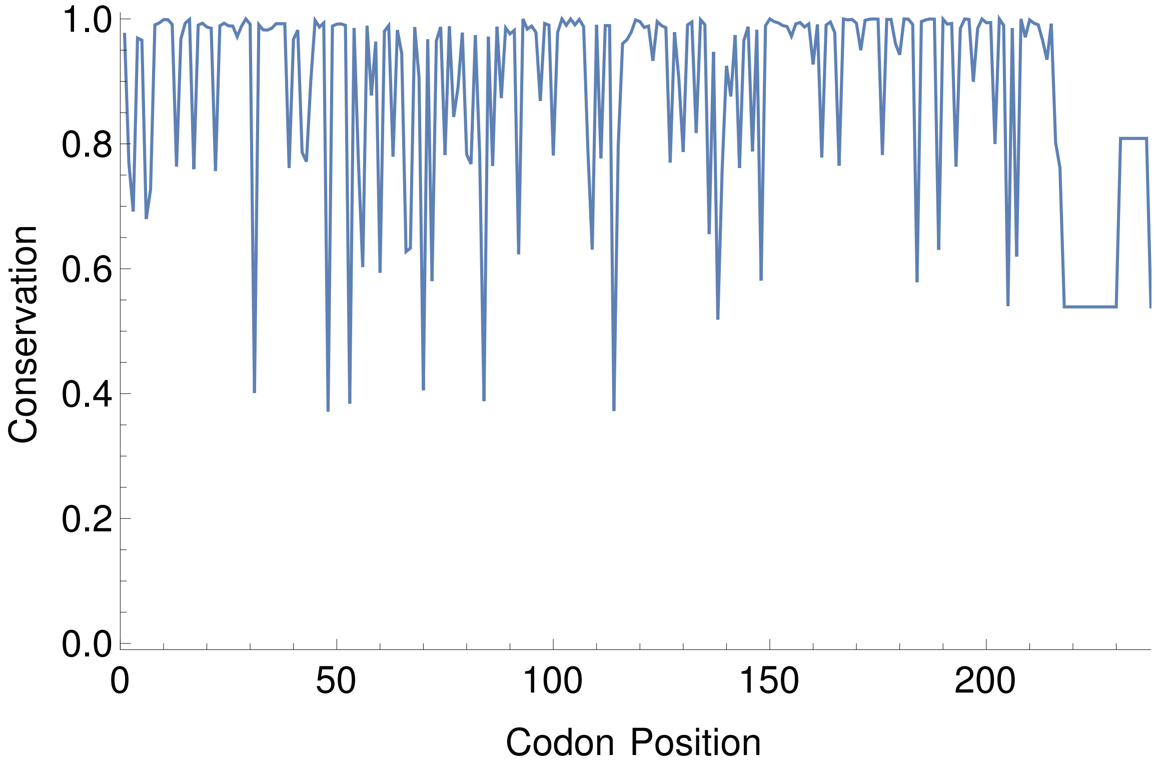

Conservation analysis plots

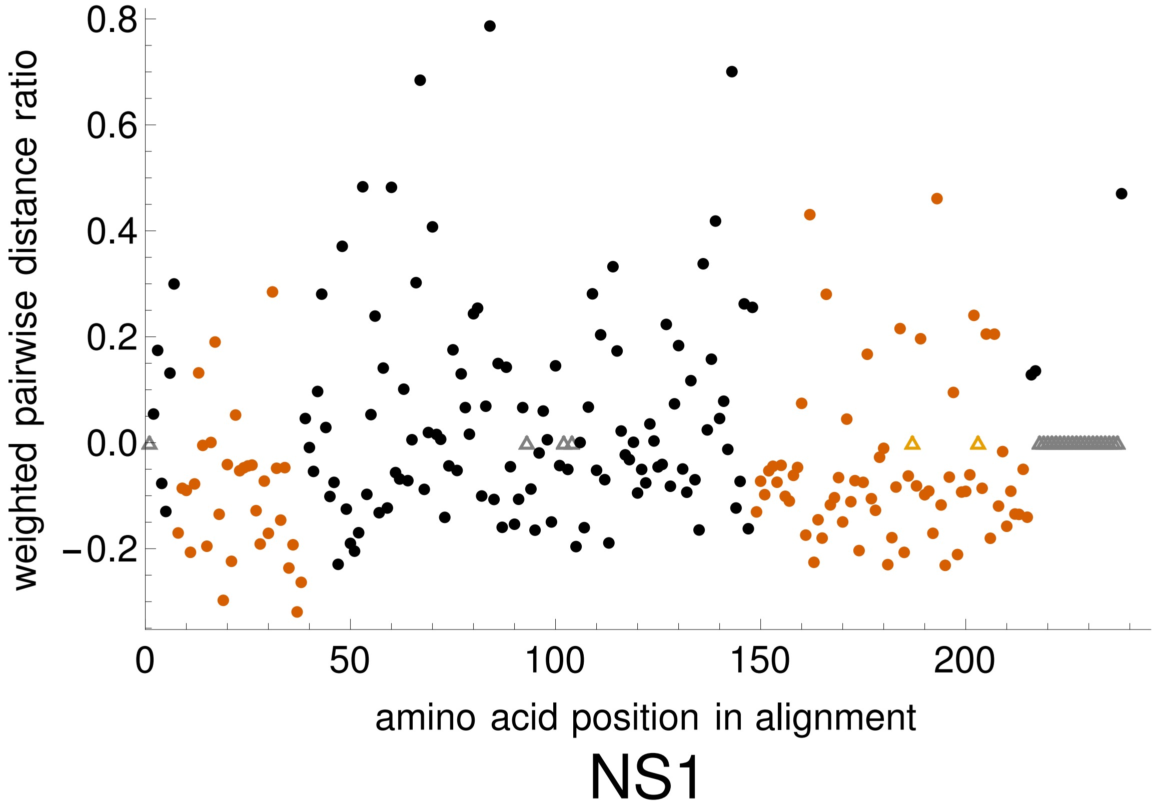

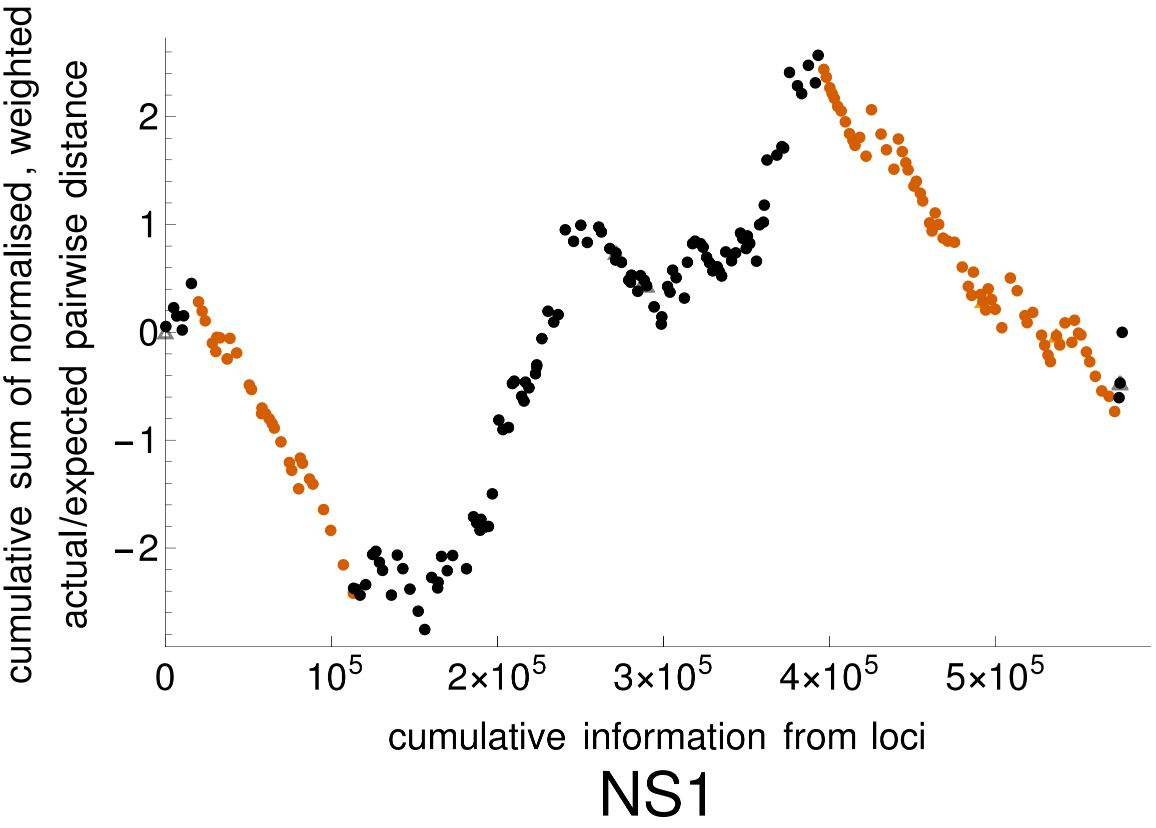

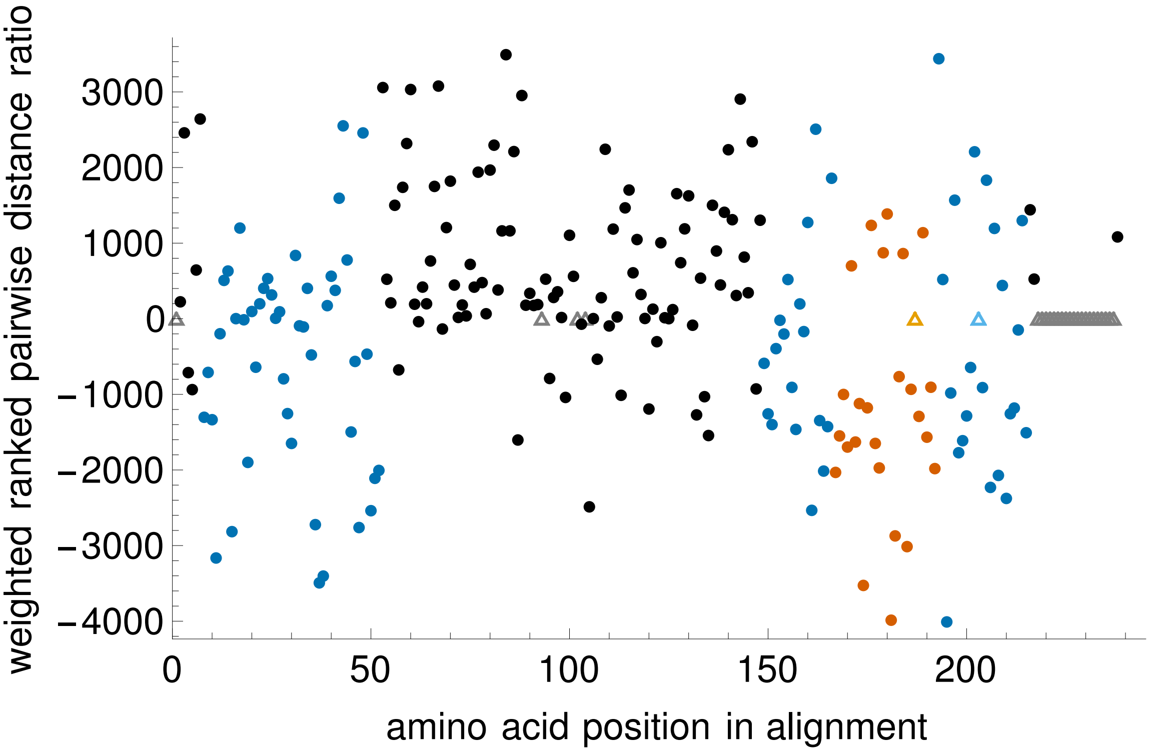
weighted ranked pairwise distance ratio
3000
2000
1000
0
−1000
−2000
−3000
−4000
0
50
100
150
200
amino acid position in alignment

NS1

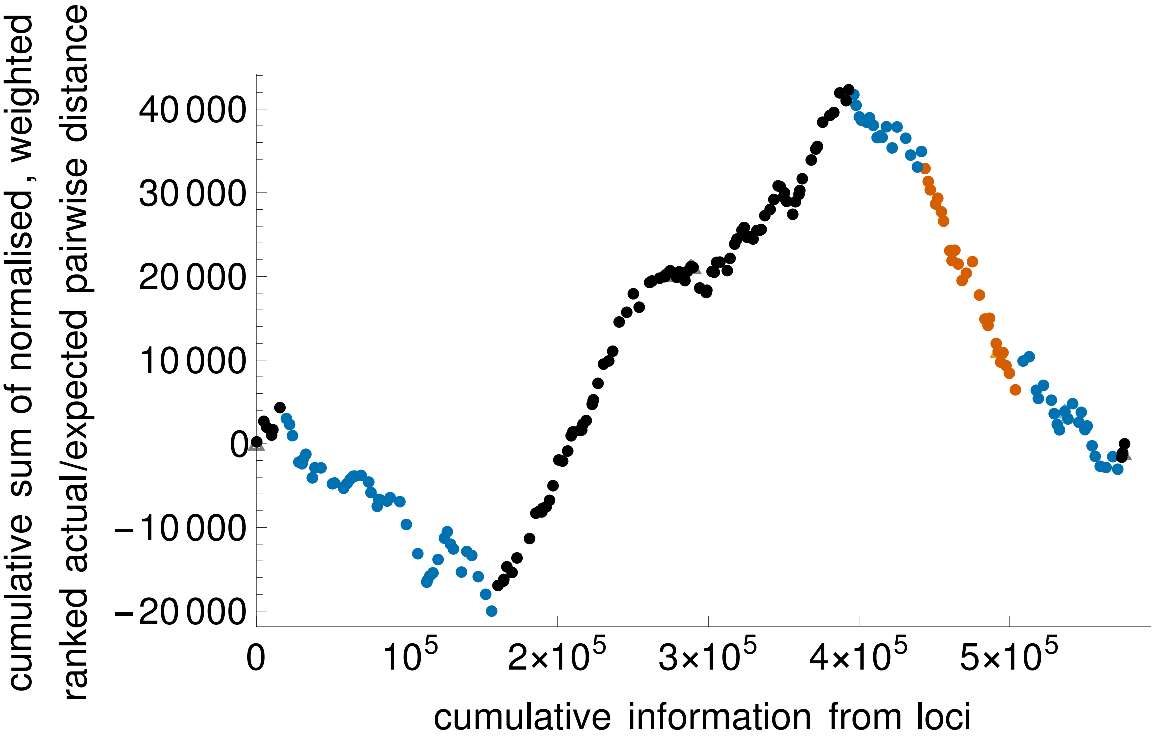
cumulative sum of normalised, weighted ranked actual/expected pairwise distance
40 000
30 000
20 000
10 000
0
−10 000
−20 000
0
10^5
2×10^5
3×10^5
4×10^5
5×10^5
cumulative information from loci

NS1

NS2

Gene length histogram

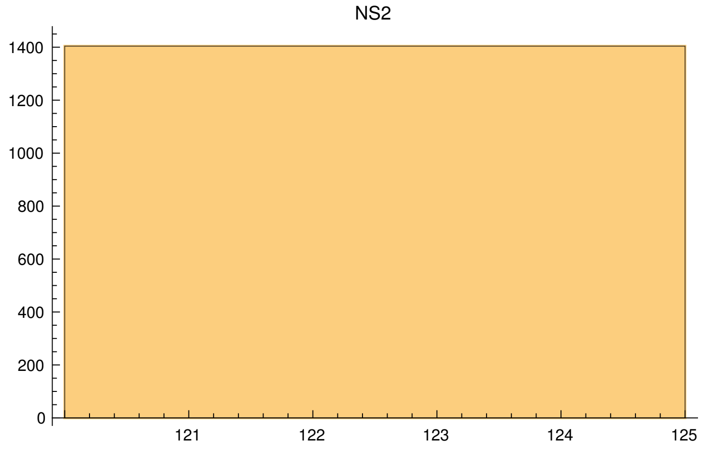

Information vs. nPD

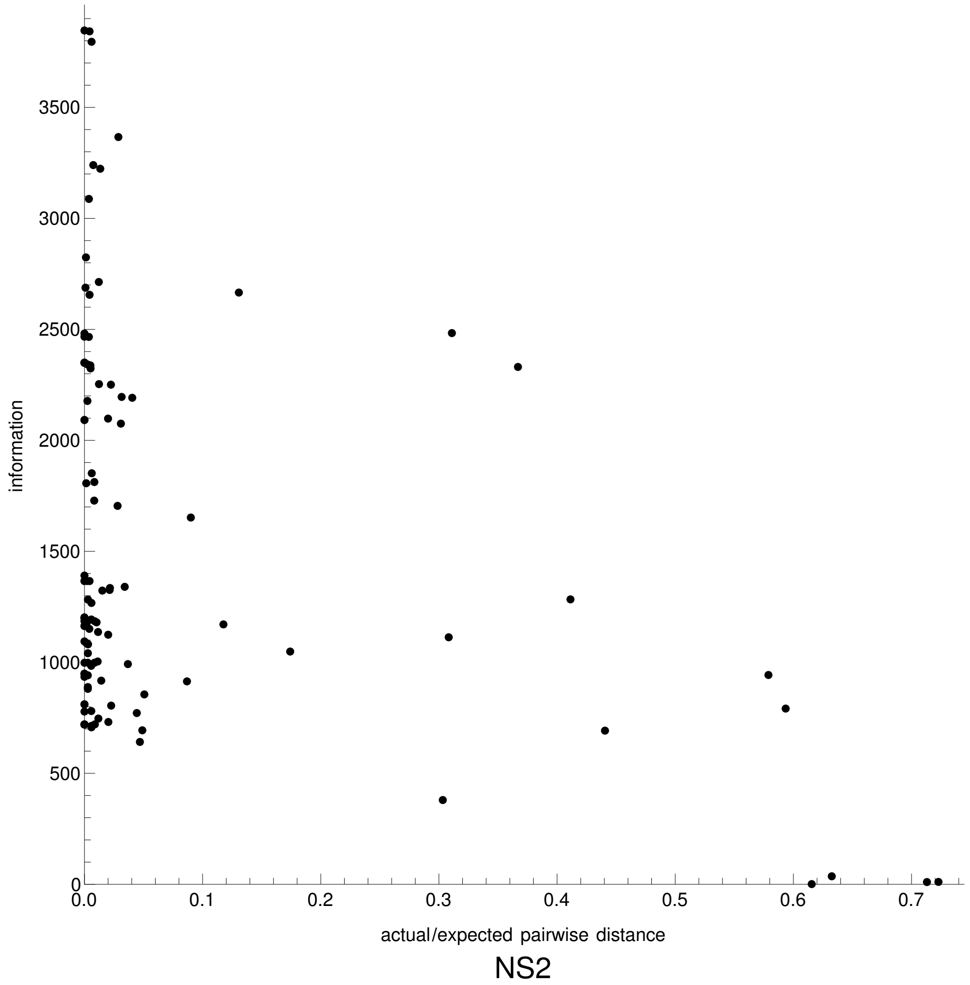

Example sequences highlighted by regions found in analyses to be conserved

Interesting points (by weighted raw PD) highlighted for gene NS2:

ATGGATTCCAACACTATGTTAAGCTTTCAG ---------------GACATACTGATGAGGATGTCAAAAATGCAATT⋱
GGGGTCCTCATCGGAGGACTTGAATGGAATGATAACACAGTTCGGGTCTCTGAAACTCTACAGAGATTCGCTTGG⋱
AGAAACAGTAATGAGGATGGGAGACCTTCACTCCCTCCAAAGTAGAAACGGAAAATGGCGAGAACAATTGGGTCA⋱
GAAGTTTGAAGAAATAAGATGGCTGATTGAAGAAGTGCGACATAGATTGAAGATTACAGAAAATAGCTTCGAACA⋱
GATAACGTTTATGCAAGCCTTACAACTATTGCTTGAAGTGGAACAAGAGATAAGAACTTTCTCGTTTCAGCTTAT⋱
TTGA

Interesting points (by weighted ranked PD) highlighted for gene NS2:

ATGGATTCCAACACTATGTTA
AGCTTTCAG---------------GACATACTGATGAGGATGTCAAAAATGCA
ATTGGGGTCCTCATCGGAGGACTTGAATGGAATGATAACACAGTTCGGGTCT
CTGAAACTCTACAGAGATTCGCTTGGAGAAACAGTAATGAGGATGGGAGACCTTCACTCCCTCCAA⋱
AGTAGAAACGGAAAATGGCGAGAACAATTGGGTCAGAAGTTTGAAGAAATAAGATGGCTGATTGAAGAAG⋱
TGCGACATAGATTGAAGATTACAGAAAATAGCTTCGAACAGATAACGTTTATGCAAGCCTTACAACTATT⋱
GCTTGAAGTGGAACAAGAGATAAGAACTTTCTCGTTTCAGCTTATTTGA

Per codon conservation report

NS2			
Pos.	1	obs:	exp:
atg	M	1404	1404.00
---	--	------	------
mPD		0	0
		nPD:	1.
		N. weight:	0.
		Sc. PD:	0
		Sc. rank:	0

NS2			
Pos.	2	obs:	exp:
gct	A	1	0.25
gcc	A	0	0.20
gca	A	0	0.44
gcg	A	0	0.11
gat	D	1379	794.90
gac	D	3	587.10
---	-	21	21.00
---	--	------	------
mPD		0.0056	0.48
		nPD:	0.01
		N. weight:	0.55
		Sc. PD:	-0.012
		Sc. rank:	283.4

NS2			
Pos.	3	obs:	exp:
tta	L	0	0.11
ttg	L	0	0.19
tct	S	0	110.20
tcc	S	643	95.08
tca	S	2	145.40
tcg	S	0	41.66
ctt	L	0	0.19
ctc	L	1	0.17
cta	L	0	0.13
ctg	L	0	0.21
cct	P	0	207.50
ccc	P	736	133.20
cca	P	0	300.50
ccg	P	0	94.77
act	T	0	0.26
acc	T	1	0.22
aca	T	0	0.43
acg	T	0	0.08
agt	S	0	131.70
agc	S	0	121.00
---	-	21	21.00
---	--	------	------
mPD		0.49	1.6
		nPD:	0.31
		N. weight:	1.8
		Sc. PD:	0.46
		Sc. rank:	2168.5

NS2			
Pos.	4	obs:	exp:
aat	N	1	735.00
aac	N	1382	648.00
---	-	21	21.00
---	--	------	------
mPD		0.0014	0.48
		nPD:	0.
		N. weight:	0.77
		Sc. PD:	-0.023
		Sc. rank:	-112.1

NS2			
Pos.	5	obs:	exp:
act	T	1379	366.30
acc	T	4	305.60
aca	T	0	594.30
acg	T	0	116.80
---	-	21	21.00
---	--	------	------
mPD		0.0056	0.67
		nPD:	0.01
		N. weight:	1.3
		Sc. PD:	-0.033
		Sc. rank:	558.9

NS2			
Pos.	6	obs:	exp:
atg	M	1382	1382.00
gtt	V	0	0.22
gtc	V	0	0.21
gta	V	0	0.20
gtg	V	1	0.37
---	-	21	21.00
---	--	------	------
mPD		0.0014	0.0023
		nPD:	0.62
		N. weight:	0.00073
		Sc. PD:	0.00038
		Sc. rank:	0.9

NS2			
Pos.	7	obs:	exp:
tta	L	1294	153.10
ttg	L	13	263.30
tct	S	1	0.17
tcc	S	0	0.15
tca	S	0	0.23
tcg	S	0	0.06
ctt	L	0	263.10
ctc	L	0	242.10
cta	L	96	186.70
ctg	L	0	294.80
agt	S	0	0.20
agc	S	0	0.19
---	--	------	------
mPD		0.15	1.1
		nPD:	0.13
		N. weight:	2.
		Sc. PD:	0.17
		Sc. rank:	2191.2

NS2			
Pos.	8	obs:	exp:
tct	S	0	239.80
tcc	S	0	207.00
tca	S	0	316.50
tcg	S	0	90.69
agt	S	0	286.70
agc	S	1404	263.40
---	--	------	------
mPD		0	1.6
		nPD:	0.
		N. weight:	1.7
		Sc. PD:	-0.056
		Sc. rank:	-1879.6

NS2			
Pos.	9	obs:	exp:
ttt	F	1404	597.40
ttc	F	0	806.60
---	--	------	------
mPD		0	0.49
		nPD:	0.
		N. weight:	0.89
		Sc. PD:	-0.029
		Sc. rank:	-959.7

NS2			
Pos.	10	obs:	exp:
caa	Q	0	714.30
cag	Q	1404	689.70
---	--	------	------
mPD		0	0.50
		nPD:	0.
		N. weight:	0.74
		Sc. PD:	-0.024
		Sc. rank:	-798.5

NS2			
Pos.	11	obs:	exp:
gtt	V	0	4.57
gtc	V	0	4.44
gta	V	21	4.13
gtg	V	0	7.86
---	-	1383	1383.00
---	--	------	------
mPD		0	0.00016
		nPD:	0.
		N. weight:	0.
		Sc. PD:	0
		Sc. rank:	0

NS2			
Pos.	12	obs:	exp:
gat	D	0	12.08
gac	D	21	8.92
---	-	1383	1383.00
---	--	------	------
mPD		0	0.00011
		nPD:	0.
		N. weight:	0.
		Sc. PD:	0
		Sc. rank:	0

NS2			
Pos.	13	obs:	exp:
tgt	C	21	9.13
tgc	C	0	11.87
---	-	1383	1383.00
---	--	------	------
mPD		0	0.00011
		nPD:	0.
		N. weight:	0.
		Sc. PD:	0
		Sc. rank:	0

NS2			
Pos.	14	obs:	exp:
ttt	F	21	8.94
ttc	F	0	12.06
---	-	1383	1383.00
---	--	------	------
mPD		0	0.00011
		nPD:	0.
		N. weight:	0.
		Sc. PD:	0
		Sc. rank:	0

NS2			
Pos.	15	obs:	exp:
tta	L	0	2.29
ttg	L	0	3.94
ctt	L	21	3.94
ctc	L	0	3.62
cta	L	0	2.79
ctg	L	0	4.41
---	-	1383	1383.00
---	--	------	------
mPD		0	0.00025
		nPD:	0.
		N. weight:	0.
		Sc. PD:	0
		Sc. rank:	0

NS2			
Pos.	16	obs:	exp:
gat	D	0	807.60
gac	D	1404	596.40
---	--	------	------
mPD		0	0.49
		nPD:	0.
		N. weight:	0.89
		Sc. PD:	-0.029
		Sc. rank:	-961.7

NS2			
Pos.	17	obs:	exp:
att	I	0	520.60
atc	I	0	351.60
ata	I	1402	529.80
act	T	0	0.53
acc	T	0	0.44
aca	T	2	0.86
acg	T	0	0.17
---	--	------	------
mPD		0.0028	0.66
		nPD:	0.
		N. weight:	1.
		Sc. PD:	-0.029
		Sc. rank:	104.2

NS2			
Pos.	18	obs:	exp:
tta	L	0	153.20
ttg	L	0	263.50
ctt	L	1	263.30
ctc	L	0	242.20
cta	L	24	186.80
ctg	L	1379	295.00
---	--	------	------
mPD		0.035	1.1
		nPD:	0.03
		N. weight:	1.5
		Sc. PD:	-0.0068
		Sc. rank:	1365.0

NS2			
Pos.	19	obs:	exp:
att	I	0	1.49
atc	I	0	1.00
ata	I	4	1.51
atg	M	1396	1396.00
act	T	0	0.53
acc	T	0	0.44
aca	T	0	0.86
acg	T	2	0.17
gtt	V	0	0.43
gtc	V	0	0.42
gta	V	0	0.39
gtg	V	2	0.75
---	--	------	------
mPD		0.011	0.016
		nPD:	0.72
		N. weight:	0.008
		Sc. PD:	0.005
		Sc. rank:	10.1

NS2			
Pos.	20	obs:	exp:
cgt	R	0	53.24
cgc	R	0	74.79
cga	R	0	136.00
cgg	R	1	110.00
aga	R	0	644.00
agg	R	1403	386.10
---	--	------	------
mPD		0.0014	0.95
		nPD:	0.
		N. weight:	1.3
		Sc. PD:	-0.042
		Sc. rank:	-439.1

NS2			
Pos.	21	obs:	exp:
atg	M	1404	1404.00
---	--	------	------
mPD		0	0
		nPD:	1.
		N. weight:	0.
		Sc. PD:	0
		Sc. rank:	0

,

NS2			
Pos.	22	obs:	exp:
tct	S	0	239.80
tcc	S	0	207.00
tca	S	1404	316.50
tcg	S	0	90.69
agt	S	0	286.70
agc	S	0	263.40
---	--	------	------
mPD		0	1.6
		nPD:	0.
		N. weight:	1.5
		Sc. PD:	-0.05
		Sc. rank:	-1673.4

,

NS2			
Pos.	23	obs:	exp:
aaa	K	1404	788.10
aag	K	0	615.90
---	--	------	------
mPD		0	0.49
		nPD:	0.
		N. weight:	0.6
		Sc. PD:	-0.019
		Sc. rank:	-648.7

,

NS2			
Pos.	24	obs:	exp:
tta	L	0	2.29
ttg	L	21	3.94
ctt	L	0	3.94
ctc	L	0	3.62
cta	L	0	2.79
ctg	L	0	4.41
att	I	1	0.37
atc	I	0	0.25
ata	I	0	0.38
atg	M	1382	1382.00
---	--	------	------
mPD		0.031	0.049
		nPD:	0.63
		N. weight:	0.027
		Sc. PD:	0.015
		Sc. rank:	33.8

,

NS2			
Pos.	25	obs:	exp:
caa	Q	1404	714.30
cag	Q	0	689.70
---	--	------	------
mPD		0	0.50
		nPD:	0.
		N. weight:	0.7
		Sc. PD:	-0.023
		Sc. rank:	-759.0

,

NS2			
Pos.	26	obs:	exp:
tta	L	0	153.20
ttg	L	1404	263.50
ctt	L	0	263.30
ctc	L	0	242.20
cta	L	0	186.80
ctg	L	0	295.00
---	--	------	------
mPD		0	1.1
		nPD:	0.
		N. weight:	1.7
		Sc. PD:	-0.056
		Sc. rank:	-1879.4

,

NS2			
Pos.	27	obs:	exp:
gaa	E	0	19.16
gag	E	32	12.84
ggt	G	0	197.40
ggc	G	0	181.10
gga	G	15	598.90
ggg	G	1357	394.60
---	--	------	------
mPD		0.066	0.73
		nPD:	0.09
		N. weight:	1.2
		Sc. PD:	0.06
		Sc. rank:	1293.4

,

NS2			
Pos.	28	obs:	exp:
tct	S	0	239.60
tcc	S	1403	206.80
tca	S	0	316.30
tcg	S	0	90.63
cct	P	0	0.28
ccc	P	1	0.18
cca	P	0	0.41
ccg	P	0	0.13
agt	S	0	286.50
agc	S	0	263.20
---	--	------	------
mPD		0.0014	1.6
		nPD:	0.
		N. weight:	2.
		Sc. PD:	-0.063
		Sc. rank:	-770.4

,

NS2			
Pos.	29	obs:	exp:
tct	S	0	239.80
tcc	S	0	207.00
tca	S	1404	316.50
tcg	S	0	90.69
agt	S	0	286.70
agc	S	0	263.40
---	--	------	------
mPD		0	1.6
		nPD:	0.
		N. weight:	1.5
		Sc. PD:	-0.05
		Sc. rank:	-1673.4

,

NS2			
Pos.	30	obs:	exp:
tct	S	0	239.80
tcc	S	0	207.00
tca	S	0	316.50
tcg	S	1404	90.69
agt	S	0	286.70
agc	S	0	263.40
---	--	------	------
mPD		0	1.6
		nPD:	0.
		N. weight:	2.8
		Sc. PD:	-0.092
		Sc. rank:	-3077.3

,

NS2			
Pos.	31	obs:	exp:
gaa	E	0	840.10
gag	E	1403	562.90
ggt	G	0	0.14
ggc	G	0	0.13
gga	G	0	0.44
ggg	G	1	0.29
---	--	------	------
mPD		0.0014	0.48
		nPD:	0.
		N. weight:	0.95
		Sc. PD:	-0.028
		Sc. rank:	-121.2

,

NS2			
Pos.	32	obs:	exp:
gtt	V	0	0.22
gtc	V	1	0.21
gta	V	0	0.20
gtg	V	0	0.37
gat	D	0	251.90
gac	D	438	186.10
ggt	G	0	138.80
ggc	G	965	127.40
gga	G	0	421.30
ggg	G	0	277.60
---	--	------	------
mPD		0.43	1.2
		nPD:	0.37
		N. weight:	1.7
		Sc. PD:	0.52
		Sc. rank:	2090.8

,

NS2			
Pos.	33	obs:	exp:
tta	L	0	153.20
ttg	L	1404	263.50
ctt	L	0	263.30
ctc	L	0	242.20
cta	L	0	186.80
ctg	L	0	295.00
---	--	------	------
mPD		0	1.1
		nPD:	0.
		N. weight:	1.7
		Sc. PD:	-0.056
		Sc. rank:	-1879.4

,

NS2			
Pos.	34	obs:	exp:
tct	S	0	0.17
tcc	S	0	0.15
tca	S	0	0.23
tcg	S	0	0.06
aat	N	1403	745.60
aac	N	0	657.40
agt	S	1	0.20
agc	S	0	0.19
---	--	------	------
mPD		0.0014	0.50
		nPD:	0.
		N. weight:	0.66
		Sc. PD:	-0.02
		Sc. rank:	-121.6

,

NS2			
Pos.	35	obs:	exp:
ggt	G	0	202.00
ggc	G	0	185.30
gga	G	1404	612.90
ggg	G	0	403.80
---	--	------	------
mPD		0	0.69
		nPD:	0.
		N. weight:	0.86
		Sc. PD:	-0.028
		Sc. rank:	-931.0

,

NS2			
Pos.	36	obs:	exp:
att	I	0	359.50
atc	I	0	242.80
ata	I	968	365.80
atg	M	435	435.00
act	T	0	0.26
acc	T	0	0.22
aca	T	1	0.43
acg	T	0	0.08
---	--	------	------
mPD		0.43	0.74
		nPD:	0.58
		N. weight:	0.7
		Sc. PD:	0.34
		Sc. rank:	873.9

,

NS2			
Pos.	37	obs:	exp:
att	I	0	521.00
atc	I	0	351.90
ata	I	1403	530.20
act	T	0	0.26
acc	T	0	0.22
aca	T	1	0.43
acg	T	0	0.08
---	--	------	------
mPD		0.0014	0.66
		nPD:	0.
		N. weight:	1.
		Sc. PD:	-0.031
		Sc. rank:	-276.4

,

NS2			
Pos.	38	obs:	exp:
act	T	0	371.90
acc	T	0	310.20
aca	T	1404	603.30
acg	T	0	118.60
---	--	------	------
mPD		0	0.69
		nPD:	0.
		N. weight:	0.88
		Sc. PD:	-0.029
		Sc. rank:	-948.8

,

NS2			
Pos.	39	obs:	exp:
caa	Q	0	712.30
cag	Q	1400	687.70
cgt	R	0	0.11
cgc	R	0	0.16
cga	R	0	0.29
cgg	R	3	0.23
aaa	K	0	0.56
aag	K	1	0.44
aga	R	0	1.38
agg	R	0	0.82
---	--	------	------
mPD		0.0057	0.51
		nPD:	0.01
		N. weight:	0.74
		Sc. PD:	-0.017
		Sc. rank:	361.7

,

NS2			
Pos.	40	obs:	exp:
ttt	F	0	597.40
ttc	F	1404	806.60
---	--	------	------
mPD		0	0.49
		nPD:	0.
		N. weight:	0.58
		Sc. PD:	-0.019
		Sc. rank:	-622.6

,

NS2			
Pos.	41	obs:	exp:
cgt	R	0	0.04
cgc	R	0	0.05
cga	R	0	0.10
cgg	R	0	0.08
aga	R	0	0.46
agg	R	1	0.27
ggt	G	0	201.80
ggc	G	1	185.20
gga	G	2	612.50
ggg	G	1400	403.50
---	--	------	------
mPD		0.0057	0.69
		nPD:	0.01
		N. weight:	1.3
		Sc. PD:	-0.032
		Sc. rank:	501.1

,

NS2			
Pos.	42	obs:	exp:
tct	S	1404	239.80
tcc	S	0	207.00
tca	S	0	316.50
tcg	S	0	90.69
agt	S	0	286.70
agc	S	0	263.40
---	--	------	------
mPD		0	1.6
		nPD:	0.
		N. weight:	1.8
		Sc. PD:	-0.06
		Sc. rank:	-1985.2

,

NS2			
Pos.	43	obs:	exp:
tta	L	0	153.20
ttg	L	0	263.50
ctt	L	0	263.30
ctc	L	0	242.20
cta	L	2	186.80
ctg	L	1402	295.00
---	--	------	------
mPD		0.0028	1.1
		nPD:	0.
		N. weight:	1.6
		Sc. PD:	-0.049
		Sc. rank:	-392.0

,

NS2			
Pos.	44	obs:	exp:
aaa	K	1404	788.10
aag	K	0	615.90
---	--	------	------
mPD		0	0.49
		nPD:	0.
		N. weight:	0.6
		Sc. PD:	-0.019
		Sc. rank:	-648.7

,

NS2			
Pos.	45	obs:	exp:
tta	L	0	152.90
ttg	L	0	262.90
ctt	L	0	262.70
ctc	L	1401	241.70
cta	L	0	186.40
ctg	L	0	294.40
att	I	0	1.11
atc	I	3	0.75
ata	I	0	1.13
---	--	------	------
mPD		0.0043	1.1
		nPD:	0.
		N. weight:	1.8
		Sc. PD:	-0.053
		Sc. rank:	-116.6

,

NS2			
Pos.	46	obs:	exp:
tat	Y	0	682.50
tac	Y	1404	721.50
---	--	------	------
mPD		0	0.50
		nPD:	0.
		N. weight:	0.69
		Sc. PD:	-0.022
		Sc. rank:	-747.8

,

NS2			
Pos.	47	obs:	exp:
cgt	R	0	53.24
cgc	R	0	74.79
cga	R	0	136.00
cgg	R	0	110.00
aga	R	1404	644.00
agg	R	0	386.10
---	--	------	------
mPD		0	0.95
		nPD:	0.
		N. weight:	0.81
		Sc. PD:	-0.026
		Sc. rank:	-875.4

,

NS2			
Pos.	48	obs:	exp:
tct	S	0	0.17
tcc	S	0	0.15
tca	S	0	0.23
tcg	S	0	0.06
aat	N	13	6.91
aac	N	0	6.09
agt	S	1	0.20
agc	S	0	0.19
gat	D	1389	799.50
gac	D	1	590.50
---	--	------	------
mPD		0.023	0.51
		nPD:	0.04
		N. weight:	0.57
		Sc. PD:	0.0044
		Sc. rank:	566.5

,

NS2			
Pos.	49	obs:	exp:
tct	S	0	239.80
tcc	S	0	207.00
tca	S	7	316.50
tcg	S	1397	90.69
agt	S	0	286.70
agc	S	0	263.40
---	--	------	------
mPD		0.0099	1.6
		nPD:	0.01
		N. weight:	2.8
		Sc. PD:	-0.076
		Sc. rank:	836.0

,

NS2			
Pos.	50	obs:	exp:
tta	L	0	153.20
ttg	L	0	263.50
ctt	L	1404	263.30
ctc	L	0	242.20
cta	L	0	186.80
ctg	L	0	295.00
---	--	------	------
mPD		0	1.1
		nPD:	0.
		N. weight:	1.7
		Sc. PD:	-0.057
		Sc. rank:	-1880.2

,

NS2			
Pos.	51	obs:	exp:
ggt	G	0	202.00
ggc	G	0	185.30
gga	G	1402	612.90
ggg	G	2	403.80
---	--	------	------
mPD		0.0028	0.69
		nPD:	0.
		N. weight:	0.85
		Sc. PD:	-0.024
		Sc. rank:	-4.6

,

NS2			
Pos.	52	obs:	exp:
gaa	E	1402	840.10
gag	E	1	562.90
ggt	G	0	0.14
ggc	G	0	0.13
gga	G	1	0.44
ggg	G	0	0.29
---	--	------	------
mPD		0.0028	0.48
		nPD:	0.01
		N. weight:	0.53
		Sc. PD:	-0.014
		Sc. rank:	115.1

,

NS2			
Pos.	53	obs:	exp:
tct	S	0	0.17
tcc	S	0	0.15
tca	S	1	0.23
tcg	S	0	0.06
atg	M	1	1.00
act	T	0	371.10
acc	T	0	309.50
aca	T	1400	602.00
acg	T	1	118.30
agt	S	0	0.20
agc	S	0	0.19
gct	A	0	0.25
gcc	A	0	0.20
gca	A	1	0.44
gcg	A	0	0.11
---	--	------	------
mPD		0.0071	0.69
		nPD:	0.01
		N. weight:	0.87
		Sc. PD:	-0.02
		Sc. rank:	413.1

,

NS2			
Pos.	54	obs:	exp:
atg	M	3	3.00
gtt	V	0	304.50
gtc	V	0	295.80
gta	V	439	275.50
gtg	V	961	524.30
gaa	E	0	0.60
gag	E	1	0.40
---	--	------	------
mPD		0.44	0.73
		nPD:	0.59
		N. weight:	0.59
		Sc. PD:	0.3
		Sc. rank:	740.1

,

NS2			
Pos.	55	obs:	exp:
atg	M	1404	1404.00
---	--	------	------
mPD		0	0
		nPD:	1.
		N. weight:	0.
		Sc. PD:	0
		Sc. rank:	0

,

NS2			
Pos.	56	obs:	exp:
cgt	R	0	53.24
cgc	R	0	74.79
cga	R	0	136.00
cgg	R	0	110.00
aga	R	19	644.00
agg	R	1385	386.10
---	--	------	------
mPD		0.027	0.95
		nPD:	0.03
		N. weight:	1.3
		Sc. PD:	-0.0088
		Sc. rank:	1026.6

,

NS2			
Pos.	57	obs:	exp:
cgt	R	0	0.04
cgc	R	0	0.05
cga	R	0	0.10
cgg	R	0	0.08
att	I	4	1.49
atc	I	0	1.00
ata	I	0	1.51
atg	M	1394	1394.00
aga	R	0	0.46
agg	R	1	0.27
gtt	V	0	1.09
gtc	V	0	1.06
gta	V	0	0.98
gtg	V	5	1.87
---	--	------	------
mPD		0.014	0.020
		nPD:	0.71
		N. weight:	0.0075
		Sc. PD:	0.0046
		Sc. rank:	9.5

,

NS2			
Pos.	58	obs:	exp:
ggt	G	0	202.00
ggc	G	0	185.30
gga	G	1404	612.90
ggg	G	0	403.80
---	--	------	------
mPD		0	0.69
		nPD:	0.
		N. weight:	0.86
		Sc. PD:	-0.028
		Sc. rank:	-931.0

,

NS2			
Pos.	59	obs:	exp:
gat	D	0	807.60
gac	D	1404	596.40
---	--	------	------
mPD		0	0.49
		nPD:	0.
		N. weight:	0.89
		Sc. PD:	-0.029
		Sc. rank:	-961.7

,

NS2			
Pos.	60	obs:	exp:
ttt	F	1	0.43
ttc	F	0	0.57
tta	L	0	153.10
ttg	L	0	263.30
ctt	L	1402	263.10
ctc	L	1	242.10
cta	L	0	186.70
ctg	L	0	294.80
---	--	------	------
mPD		0.0028	1.1
		nPD:	0.
		N. weight:	1.7
		Sc. PD:	-0.052
		Sc. rank:	-365.7

,

NS2			
Pos.	61	obs:	exp:
tat	Y	0	0.97
tac	Y	2	1.03
cat	H	168	833.60
cac	H	1234	568.40
---	--	------	------
mPD		0.21	0.49
		nPD:	0.44
		N. weight:	0.51
		Sc. PD:	0.19
		Sc. rank:	634.9

,

NS2			
Pos.	62	obs:	exp:
tct	S	5	239.80
tcc	S	1399	207.00
tca	S	0	316.50
tcg	S	0	90.69
agt	S	0	286.70
agc	S	0	263.40
---	--	------	------
mPD		0.0071	1.6
		nPD:	0.
		N. weight:	2.
		Sc. PD:	-0.056
		Sc. rank:	165.5

,

NS2			
Pos.	63	obs:	exp:
tta	L	0	153.20
ttg	L	0	263.50
ctt	L	0	263.30
ctc	L	1404	242.20
cta	L	0	186.80
ctg	L	0	295.00
---	--	------	------
mPD		0	1.1
		nPD:	0.
		N. weight:	1.8
		Sc. PD:	-0.059
		Sc. rank:	-1973.7

,

NS2			
Pos.	64	obs:	exp:
caa	Q	1386	714.30
cag	Q	18	689.70
---	--	------	------
mPD		0.025	0.50
		nPD:	0.05
		N. weight:	0.63
		Sc. PD:	0.0086
		Sc. rank:	647.2

,

NS2			
Pos.	65	obs:	exp:
tct	S	0	233.30
tcc	S	0	201.40
tca	S	0	307.90
tcg	S	0	88.24
aat	N	37	19.66
aac	N	0	17.34
agt	S	1366	278.90
agc	S	0	256.30
ggt	G	1	0.14
ggc	G	0	0.13
gga	G	0	0.44
ggg	G	0	0.29
---	--	------	------
mPD		0.053	1.7
		nPD:	0.03
		N. weight:	1.6
		Sc. PD:	-0.0063
		Sc. rank:	1493.4

,

NS2

Pos.	66	obs:	exp:
cgt	R	0	53.24
cgc	R	0	74.79
cga	R	0	136.00
cgg	R	0	110.00
aga	R	1403	644.00
agg	R	1	386.10
mPD		0.0014	0.95
		nPD:	0.
		N. weight:	0.81
		Sc. PD:	-0.025
		Sc. rank:	-264.3

NS2

Pos.	67	obs:	exp:
aat	N	31	746.10
aac	N	1373	657.90
mPD		0.043	0.50
		nPD:	0.09
		N. weight:	0.68
		Sc. PD:	0.031
		Sc. rank:	700.2

NS2

Pos.	68	obs:	exp:
cgt	R	0	0.04
cgc	R	0	0.05
cga	R	0	0.10
cgg	R	0	0.08
aga	R	1	0.46
agg	R	0	0.27
gaa	E	83	49.70
gag	E	0	33.30
ggt	G	2	189.90
ggc	G	0	174.20
gga	G	1302	576.20
ggg	G	16	379.70
mPD		0.14	0.79
		nPD:	0.17
		N. weight:	0.78
		Sc. PD:	0.098
		Sc. rank:	873.1

NS2

Pos.	69	obs:	exp:
caa	Q	2	1.02
cag	Q	0	0.98
cgt	R	0	0.15
cgc	R	0	0.21
cga	R	0	0.39
cgg	R	0	0.31
aat	N	1	0.53
aac	N	0	0.47
aaa	K	1396	784.10
aag	K	1	612.90
aga	R	4	1.83
agg	R	0	1.10
mPD		0.011	0.50
		nPD:	0.02
		N. weight:	0.6
		Sc. PD:	-0.0071
		Sc. rank:	470.7

NS2

Pos.	70	obs:	exp:
tgg	W	1404	1404.00
mPD		0	0
		nPD:	1.
		N. weight:	0.
		Sc. PD:	0
		Sc. rank:	0

NS2

Pos.	71	obs:	exp:
cgt	R	0	53.24
cgc	R	0	74.79
cga	R	1399	136.00
cgg	R	2	110.00
aga	R	3	644.00
agg	R	0	386.10
mPD		0.0071	0.95
		nPD:	0.01
		N. weight:	2.4
		Sc. PD:	-0.062
		Sc. rank:	882.1

NS2

Pos.	72	obs:	exp:
aaa	K	2	1.12
aag	K	0	0.88
gaa	E	1401	838.90
gag	E	0	562.10
ggt	G	0	0.14
ggc	G	0	0.13
gga	G	1	0.44
ggg	G	0	0.29
mPD		0.0043	0.48
		nPD:	0.01
		N. weight:	0.53
		Sc. PD:	-0.013
		Sc. rank:	243.6

NS2

Pos.	73	obs:	exp:
caa	Q	1399	714.30
cag	Q	5	689.70
mPD		0.0071	0.50
		nPD:	0.01
		N. weight:	0.68
		Sc. PD:	-0.013
		Sc. rank:	433.5

NS2

Pos.	74	obs:	exp:
tta	L	4	153.10
ttg	L	1386	263.30
ctt	L	0	263.10
ctc	L	0	242.10
cta	L	0	186.70
ctg	L	13	294.80
atg	M	1	1.00
mPD		0.025	1.1
		nPD:	0.02
		N. weight:	1.7
		Sc. PD:	-0.02
		Sc. rank:	1297.8

NS2

Pos.	75	obs:	exp:
tct	S	0	0.85
tcc	S	0	0.74
tca	S	0	1.13
tcg	S	0	0.32
agt	S	5	1.02
agc	S	0	0.94
ggt	G	1398	201.30
ggc	G	1	184.70
gga	G	0	610.70
ggg	G	0	402.40
mPD		0.0085	0.70
		nPD:	0.01
		N. weight:	2.
		Sc. PD:	-0.043
		Sc. rank:	1105.1

NS2

Pos.	76	obs:	exp:
caa	Q	0	713.80
cag	Q	1403	689.20
aaa	K	0	0.56
aag	K	1	0.44
mPD		0.0014	0.50
		nPD:	0.
		N. weight:	0.74
		Sc. PD:	-0.022
		Sc. rank:	-145.7

NS2

Pos.	77	obs:	exp:
cgt	R	0	0.04
cgc	R	0	0.05
cga	R	0	0.10
cgg	R	0	0.08
aaa	K	3	787.50
aag	K	1400	615.50
aga	R	0	0.46
agg	R	1	0.27
mPD		0.0057	0.49
		nPD:	0.01
		N. weight:	0.84
		Sc. PD:	-0.019
		Sc. rank:	422.8

NS2

Pos.	78	obs:	exp:
ttt	F	1402	597.00
ttc	F	1	806.00
tta	L	0	0.11
ttg	L	0	0.19
ctt	L	1	0.19
ctc	L	0	0.17
cta	L	0	0.13
ctg	L	0	0.21
mPD		0.0028	0.49
		nPD:	0.01
		N. weight:	0.88
		Sc. PD:	-0.024
		Sc. rank:	183.8

NS2

Pos.	79	obs:	exp:
gaa	E	1402	840.70
gag	E	2	563.30
mPD		0.0028	0.48
		nPD:	0.01
		N. weight:	0.52
		Sc. PD:	-0.014
		Sc. rank:	121.2

NS2

Pos.	80	obs:	exp:
gaa	E	1404	840.70
gag	E	0	563.30
mPD		0	0.48
		nPD:	0.
		N. weight:	0.53
		Sc. PD:	-0.017
		Sc. rank:	-576.1

NS2

Pos.	81	obs:	exp:
att	I	0	521.40
atc	I	0	352.10
ata	I	1404	530.50
mPD		0	0.66
		nPD:	0.
		N. weight:	1.
		Sc. PD:	-0.033
		Sc. rank:	-1093.1

NS2

Pos.	82	obs:	exp:
cgt	R	0	53.24
cgc	R	0	74.79
cga	R	0	136.00
cgg	R	0	110.00
aga	R	1402	644.00
agg	R	2	386.10
mPD		0.0028	0.95
		nPD:	0.
		N. weight:	0.8
		Sc. PD:	-0.024
		Sc. rank:	-90.3

NS2

Pos.	83	obs:	exp:
tgg	W	1404	1404.00
mPD		0	0
		nPD:	1.
		N. weight:	0.
		Sc. PD:	0
		Sc. rank:	0

NS2

Pos.	84	obs:	exp:
tta	L	0	153.20
ttg	L	10	263.50
ctt	L	1	263.30
ctc	L	0	242.20
cta	L	5	186.80
ctg	L	1388	295.00
mPD		0.023	1.1
		nPD:	0.02
		N. weight:	1.6
		Sc. PD:	-0.022
		Sc. rank:	1064.7

NS2

Pos.	85	obs:	exp:
att	I	1404	521.40
atc	I	0	352.10
ata	I	0	530.50
mPD		0	0.66
		nPD:	0.
		N. weight:	1.
		Sc. PD:	-0.033
		Sc. rank:	-1112.7

NS2

Pos.	86	obs:	exp:
gaa	E	1404	840.70
gag	E	0	563.30
mPD		0	0.48
		nPD:	0.
		N. weight:	0.53
		Sc. PD:	-0.017
		Sc. rank:	-576.1

NS2

Pos.	87	obs:	exp:
gaa	E	1404	840.70
gag	E	0	563.30
mPD		0	0.48
		nPD:	0.
		N. weight:	0.53
		Sc. PD:	-0.017
		Sc. rank:	-576.1

NS2

Pos.	88	obs:	exp:
att	I	0	0.37
atc	I	0	0.25
ata	I	1	0.38
atg	M	243	243.00
act	T	0	0.26
acc	T	0	0.22
aca	T	0	0.43
acg	T	1	0.08
gtt	V	1	252.10
gtc	V	0	244.90
gta	V	4	228.00
gtg	V	1154	434.00
mPD		0.30	0.97
		nPD:	0.31
		N. weight:	0.82
		Sc. PD:	0.2
		Sc. rank:	943.6

NS2

Pos.	89	obs:	exp:
cgt	R	0	53.24
cgc	R	0	74.79
cga	R	1395	136.00
cgg	R	8	110.00
aga	R	1	644.00
agg	R	0	386.10
mPD		0.013	0.95
		nPD:	0.01
		N. weight:	2.4
		Sc. PD:	-0.048
		Sc. rank:	1493.1

NS2

Pos.	90	obs:	exp:
tat	Y	5	2.43
tac	Y	0	2.57
cat	H	1397	830.60
cac	H	0	566.40
caa	Q	0	0.51
cag	Q	1	0.49
aat	N	1	0.53
aac	N	0	0.47
mPD		0.0099	0.49
		nPD:	0.02
		N. weight:	0.54
		Sc. PD:	-0.0076
		Sc. rank:	384.9

NS2			
Pos.	91	obs:	exp:
cgt	R	0	53.13
cgc	R	0	74.63
cga	R	1	135.70
cgg	R	0	109.70
aaa	K	3	1.68
aag	K	0	1.32
aga	R	1379	642.60
agg	R	21	385.20
---	--	------	------
mPD		0.035	0.96
		nPD:	0.04
		N. weight:	0.73
		Sc. PD:	0.00069
		Sc. rank:	698.4

,

NS2			
Pos.	92	obs:	exp:
tta	L	27	153.20
ttg	L	408	263.50
ctt	L	1	263.30
ctc	L	0	242.20
cta	L	0	186.80
ctg	L	968	295.00
---	--	------	------
mPD		0.47	1.1
		nPD:	0.41
		N. weight:	0.95
		Sc. PD:	0.32
		Sc. rank:	1168.3

,

NS2			
Pos.	93	obs:	exp:
aaa	K	5	786.90
aag	K	1397	615.10
gaa	E	0	1.20
gag	E	2	0.80
---	--	------	------
mPD		0.0099	0.50
		nPD:	0.02
		N. weight:	0.83
		Sc. PD:	-0.012
		Sc. rank:	583.5

,

NS2			
Pos.	94	obs:	exp:
att	I	1388	519.10
atc	I	10	350.60
ata	I	0	528.30
act	T	3	0.79
acc	T	0	0.66
aca	T	0	1.29
acg	T	0	0.25
gtt	V	3	0.65
gtc	V	0	0.63
gta	V	0	0.59
gtg	V	0	1.12
---	--	------	------
mPD		0.023	0.67
		nPD:	0.03
		N. weight:	0.99
		Sc. PD:	-0.0015
		Sc. rank:	929.8

,

NS2			
Pos.	95	obs:	exp:
act	T	1	371.90
acc	T	0	310.20
aca	T	1403	603.30
acg	T	0	118.60
---	--	------	------
mPD		0.0014	0.69
		nPD:	0.
		N. weight:	0.87
		Sc. PD:	-0.027
		Sc. rank:	-255.0

,

NS2			
Pos.	96	obs:	exp:
gaa	E	1404	840.70
gag	E	0	563.30
---	--	------	------
mPD		0	0.48
		nPD:	0.
		N. weight:	0.53
		Sc. PD:	-0.017
		Sc. rank:	-576.1

,

NS2			
Pos.	97	obs:	exp:
aat	N	1403	746.10
aac	N	1	657.90
---	--	------	------
mPD		0.0014	0.50
		nPD:	0.
		N. weight:	0.65
		Sc. PD:	-0.019
		Sc. rank:	-104.1

,

NS2			
Pos.	98	obs:	exp:
tct	S	0	239.40
tcc	S	0	206.70
tca	S	0	316.00
tcg	S	0	90.56
agt	S	4	286.30
agc	S	1398	263.00
ggt	G	0	0.29
ggc	G	2	0.26
gga	G	0	0.87
ggg	G	0	0.58
---	--	------	------
mPD		0.0085	1.6
		nPD:	0.01
		N. weight:	1.7
		Sc. PD:	-0.048
		Sc. rank:	287.9

,

NS2			
Pos.	99	obs:	exp:
ttt	F	17	597.40
ttc	F	1387	806.60
---	--	------	------
mPD		0.024	0.49
		nPD:	0.05
		N. weight:	0.51
		Sc. PD:	0.0062
		Sc. rank:	518.8

,

NS2			
Pos.	100	obs:	exp:
gaa	E	1388	840.70
gag	E	16	563.30
---	--	------	------
mPD		0.023	0.48
		nPD:	0.05
		N. weight:	0.48
		Sc. PD:	0.0048
		Sc. rank:	475.1

,

NS2			
Pos.	101	obs:	exp:
caa	Q	2	714.30
cag	Q	1402	689.70
---	--	------	------
mPD		0.0028	0.50
		nPD:	0.01
		N. weight:	0.73
		Sc. PD:	-0.02
		Sc. rank:	131.8

,

NS2			
Pos.	102	obs:	exp:
att	I	2	521.40
atc	I	5	352.10
ata	I	1397	530.50
---	--	------	------
mPD		0.0099	0.66
		nPD:	0.02
		N. weight:	0.98
		Sc. PD:	-0.018
		Sc. rank:	643.2

,

NS2			
Pos.	103	obs:	exp:
act	T	3	371.90
acc	T	0	310.20
aca	T	11	603.30
acg	T	1390	118.60
---	--	------	------
mPD		0.020	0.69
		nPD:	0.03
		N. weight:	2.5
		Sc. PD:	-0.016
		Sc. rank:	2142.9

,

NS2			
Pos.	104	obs:	exp:
ttt	F	1404	597.40
ttc	F	0	806.60
---	--	------	------
mPD		0	0.49
		nPD:	0.
		N. weight:	0.89
		Sc. PD:	-0.029
		Sc. rank:	-959.7

,

NS2			
Pos.	105	obs:	exp:
atg	M	1404	1404.00
---	--	------	------
mPD		0	0
		nPD:	1.
		N. weight:	0.
		Sc. PD:	0
		Sc. rank:	0

,

NS2			
Pos.	106	obs:	exp:
caa	Q	1404	714.30
cag	Q	0	689.70
---	--	------	------
mPD		0	0.50
		nPD:	0.
		N. weight:	0.7
		Sc. PD:	-0.023
		Sc. rank:	-759.0

,

NS2			
Pos.	107	obs:	exp:
gct	A	0	357.20
gcc	A	1398	273.80
gca	A	6	617.20
gcg	A	0	155.90
---	--	------	------
mPD		0.0085	0.69
		nPD:	0.01
		N. weight:	1.7
		Sc. PD:	-0.036
		Sc. rank:	969.6

,

NS2			
Pos.	108	obs:	exp:
tta	L	1401	153.20
ttg	L	2	263.50
ctt	L	0	263.30
ctc	L	0	242.20
cta	L	1	186.80
ctg	L	0	295.00
---	--	------	------
mPD		0.0043	1.1
		nPD:	0.
		N. weight:	2.3
		Sc. PD:	-0.066
		Sc. rank:	-48.7

,

NS2			
Pos.	109	obs:	exp:
caa	Q	1404	714.30
cag	Q	0	689.70
---	--	------	------
mPD		0	0.50
		nPD:	0.
		N. weight:	0.7
		Sc. PD:	-0.023
		Sc. rank:	-759.0

,

NS2			
Pos.	110	obs:	exp:
tta	L	0	153.20
ttg	L	0	263.50
ctt	L	0	263.30
ctc	L	0	242.20
cta	L	1403	186.80
ctg	L	1	295.00
---	--	------	------
mPD		0.0014	1.1
		nPD:	0.
		N. weight:	2.1
		Sc. PD:	-0.066
		Sc. rank:	-728.1

,

NS2			
Pos.	111	obs:	exp:
tta	L	0	153.20
ttg	L	1371	263.50
ctt	L	0	263.30
ctc	L	0	242.20
cta	L	0	186.80
ctg	L	33	295.00
---	--	------	------
mPD		0.046	1.1
		nPD:	0.04
		N. weight:	1.6
		Sc. PD:	0.0069
		Sc. rank:	1592.0

,

NS2			
Pos.	112	obs:	exp:
tta	L	0	153.20
ttg	L	0	263.50
ctt	L	1404	263.30
ctc	L	0	242.20
cta	L	0	186.80
ctg	L	0	295.00
---	--	------	------
mPD		0	1.1
		nPD:	0.
		N. weight:	1.7
		Sc. PD:	-0.057
		Sc. rank:	-1880.2

,

NS2			
Pos.	113	obs:	exp:
gaa	E	1404	840.70
gag	E	0	563.30
---	--	------	------
mPD		0	0.48
		nPD:	0.
		N. weight:	0.53
		Sc. PD:	-0.017
		Sc. rank:	-576.1

NS2			
Pos.	114	obs:	exp:
gtt	V	1	305.10
gtc	V	0	296.40
gta	V	9	276.10
gtg	V	1393	525.40
ggt	G	0	0.14
ggc	G	0	0.13
gga	G	0	0.44
ggg	G	1	0.29
---	--	------	------
mPD		0.016	0.73
		nPD:	0.02
		N. weight:	0.98
		Sc. PD:	-0.013
		Sc. rank:	716.0

NS2			
Pos.	115	obs:	exp:
gaa	E	1293	840.70
gag	E	111	563.30
---	--	------	------
mPD		0.15	0.48
		nPD:	0.3
		N. weight:	0.28
		Sc. PD:	0.068
		Sc. rank:	317.6

NS2			
Pos.	116	obs:	exp:
caa	Q	1403	714.30
cag	Q	1	689.70
---	--	------	------
mPD		0.0014	0.50
		nPD:	0.
		N. weight:	0.7
		Sc. PD:	-0.021
		Sc. rank:	-119.8

NS2			
Pos.	117	obs:	exp:
gaa	E	2	840.70
gag	E	1402	563.30
---	--	------	------
mPD		0.0028	0.48
		nPD:	0.01
		N. weight:	0.94
		Sc. PD:	-0.025
		Sc. rank:	217.3

NS2			
Pos.	118	obs:	exp:
att	I	0	521.40
atc	I	0	352.10
ata	I	1404	530.50
---	--	------	------
mPD		0	0.66
		nPD:	0.
		N. weight:	1.
		Sc. PD:	-0.033
		Sc. rank:	-1093.1

NS2			
Pos.	119	obs:	exp:
cgt	R	0	53.24
cgc	R	0	74.79
cga	R	0	136.00
cgg	R	0	110.00
aga	R	1402	644.00
agg	R	2	386.10
---	--	------	------
mPD		0.0028	0.95
		nPD:	0.
		N. weight:	0.8
		Sc. PD:	-0.024
		Sc. rank:	-90.3

NS2			
Pos.	120	obs:	exp:
act	T	1401	371.60
acc	T	2	310.00
aca	T	0	602.90
acg	T	0	118.50
gct	A	1	0.25
gcc	A	0	0.20
gca	A	0	0.44
gcg	A	0	0.11
---	--	------	------
mPD		0.0043	0.69
		nPD:	0.01
		N. weight:	1.4
		Sc. PD:	-0.037
		Sc. rank:	442.9

NS2			
Pos.	121	obs:	exp:
ttt	F	0	596.60
ttc	F	1402	805.40
tta	L	0	0.22
ttg	L	0	0.38
ctt	L	0	0.38
ctc	L	2	0.35
cta	L	0	0.27
ctg	L	0	0.42
---	--	------	------
mPD		0.0028	0.49
		nPD:	0.01
		N. weight:	0.58
		Sc. PD:	-0.016
		Sc. rank:	110.8

NS2			
Pos.	122	obs:	exp:
tta	L	0	0.11
ttg	L	1	0.19
tct	S	0	239.10
tcc	S	0	206.40
tca	S	0	315.60
tcg	S	1400	90.43
ctt	L	0	0.19
ctc	L	0	0.17
cta	L	0	0.13
ctg	L	0	0.21
agt	S	0	285.80
agc	S	0	262.70
gtt	V	0	0.22
gtc	V	0	0.21
gta	V	0	0.20
gtg	V	1	0.37
gct	A	0	0.51
gcc	A	0	0.39
gca	A	0	0.88
gcg	A	2	0.22
---	--	------	------
mPD		0.0071	1.6
		nPD:	0.
		N. weight:	2.8
		Sc. PD:	-0.081
		Sc. rank:	135.3

NS2			
Pos.	123	obs:	exp:
ttt	F	1401	597.00
ttc	F	2	806.00
tat	Y	1	0.49
tac	Y	0	0.51
---	--	------	------
mPD		0.0043	0.49
		nPD:	0.01
		N. weight:	0.88
		Sc. PD:	-0.022
		Sc. rank:	391.9

NS2			
Pos.	124	obs:	exp:
cat	H	1	0.59
cac	H	0	0.41
caa	Q	0	712.80
cag	Q	1401	688.20
aaa	K	0	1.12
aag	K	2	0.88
---	--	------	------
mPD		0.0043	0.50
		nPD:	0.01
		N. weight:	0.74
		Sc. PD:	-0.018
		Sc. rank:	318.0

NS2			
Pos.	125	obs:	exp:
tta	L	0	153.00
ttg	L	0	263.10
ctt	L	1400	262.90
ctc	L	2	241.90
cta	L	0	186.50
ctg	L	0	294.60
cct	P	1	0.28
ccc	P	0	0.18
cca	P	0	0.41
ccg	P	0	0.13
cgt	R	1	0.04
cgc	R	0	0.05
cga	R	0	0.10
cgg	R	0	0.08
aga	R	0	0.46
agg	R	0	0.27
---	--	------	------
mPD		0.0057	1.1
		nPD:	0.01
		N. weight:	1.7
		Sc. PD:	-0.048
		Sc. rank:	234.1

NS2			
Pos.	126	obs:	exp:
att	I	1394	521.40
atc	I	9	352.10
ata	I	1	530.50
---	--	------	------
mPD		0.014	0.66
		nPD:	0.02
		N. weight:	0.99
		Sc. PD:	-0.013
		Sc. rank:	738.9

NS2			
Pos.	127	obs:	exp:
taa	.	75	514.90
tag	.	0	394.00
tga	.	1329	495.10
---	--	------	------
mPD		0.10	0.86
		nPD:	0.12
		N. weight:	0.87
		Sc. PD:	0.065
		Sc. rank:	930.5

}

Amino acid level conservation per codon

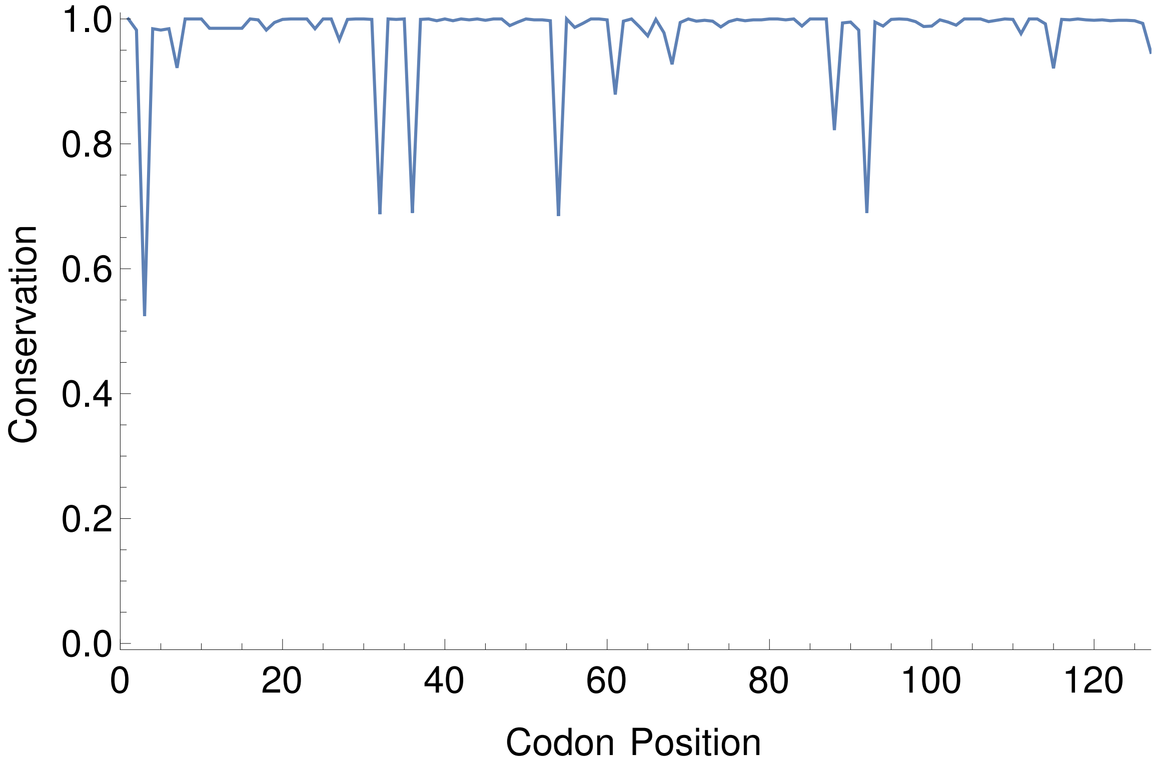

Conservation analysis plots

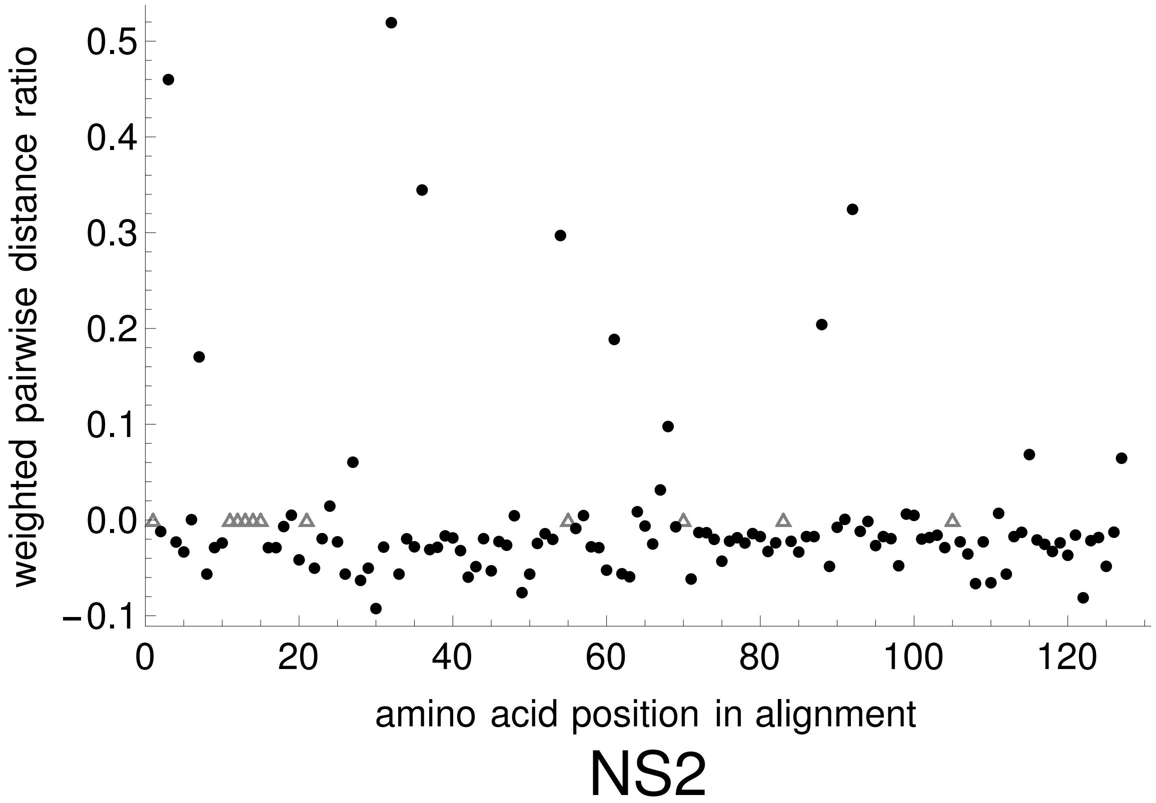

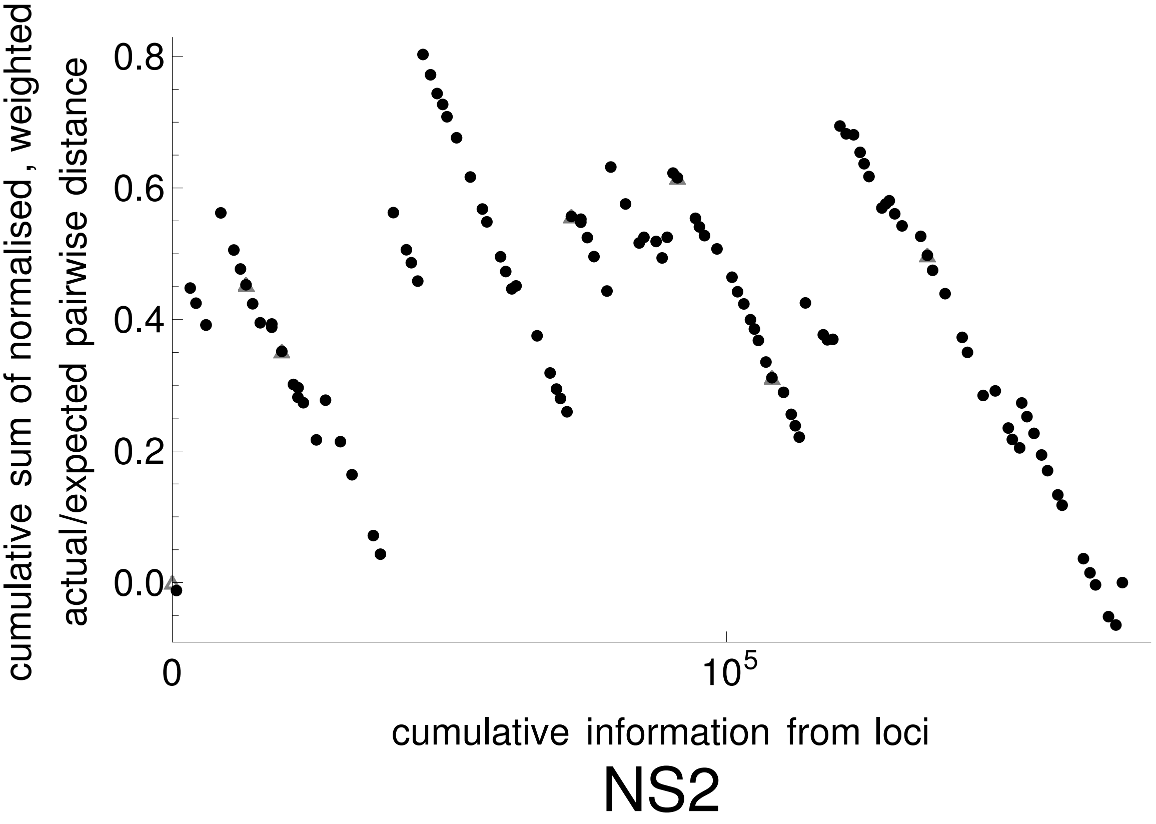

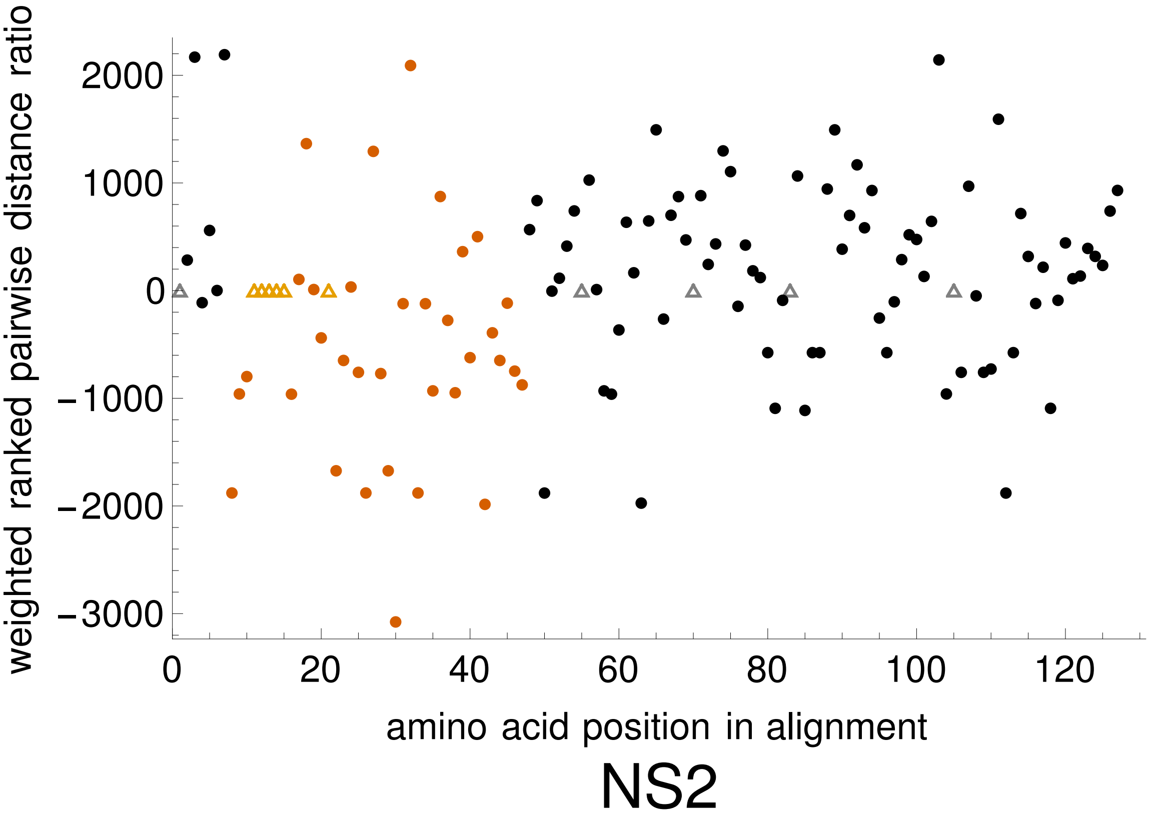
weighted ranked pairwise distance ratio
2000
1000
0
−1000
−2000
−3000
0
20
40
60
80
100
120
amino acid position in alignment
NS2

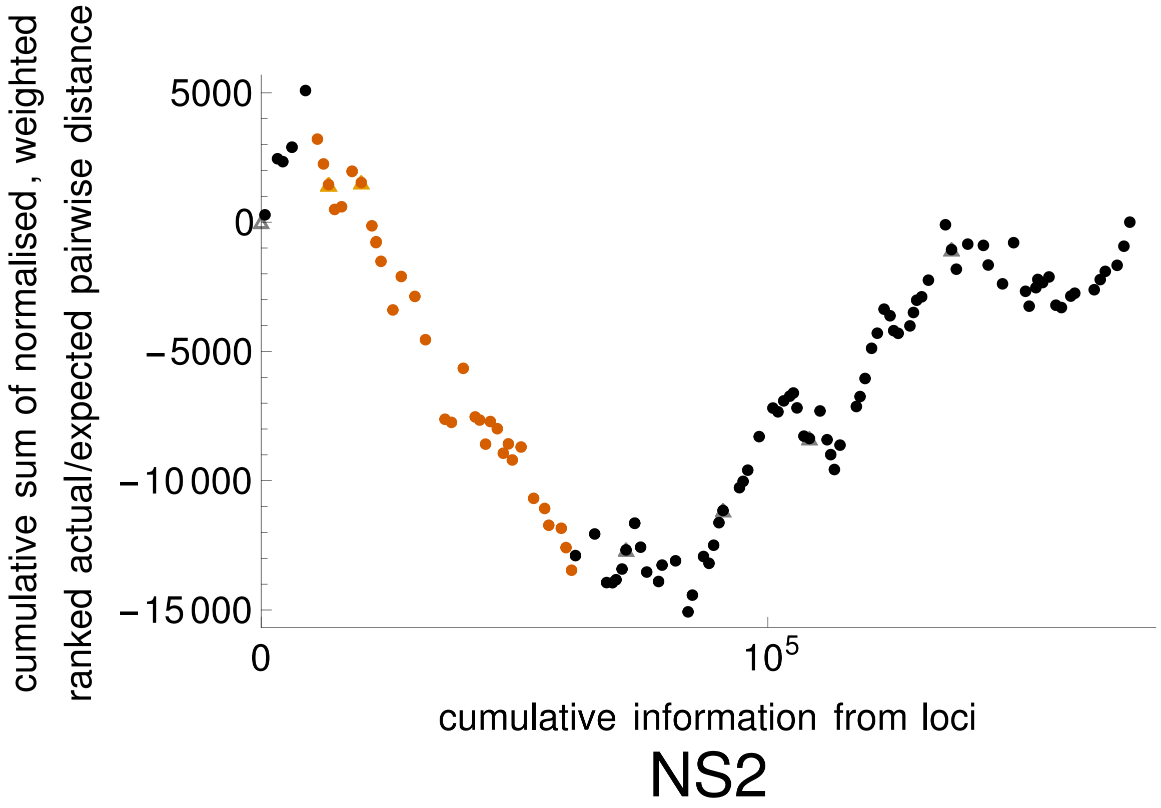
cumulative sum of normalised, weighted ranked actual/expected pairwise distance
5000
0
−5000
−10 000
−15 000
0
10^5
cumulative information from loci
NS2
