# Supplementary material for: Locations and structures of influenza A virus packaging-associated signals and other functional elements via an in silico pipeline for predicting constrained features in RNA viruses
Source: PLoS Comput Biol. 2024 Apr 22;20(4):e1012009. doi: 10.1371/journal.pcbi.1012009 (PMC11034665; doi:10.1371/journal.pcbi.1012009)
Supplement: S6 Code — The content of the notebook follows the same pattern as that in S1 Code. (ZIP) [file pcbi.1012009.s119.zip › S6_Code.pdf]

# H7N7 avian hosts

## PB2

### Gene length histogram

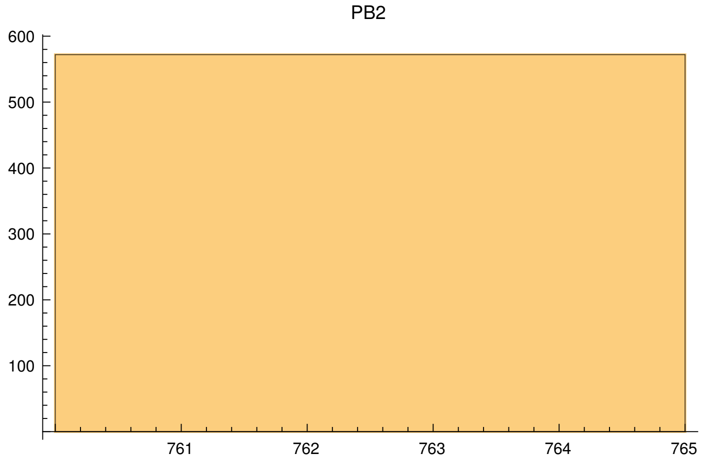

## Information vs. nPD

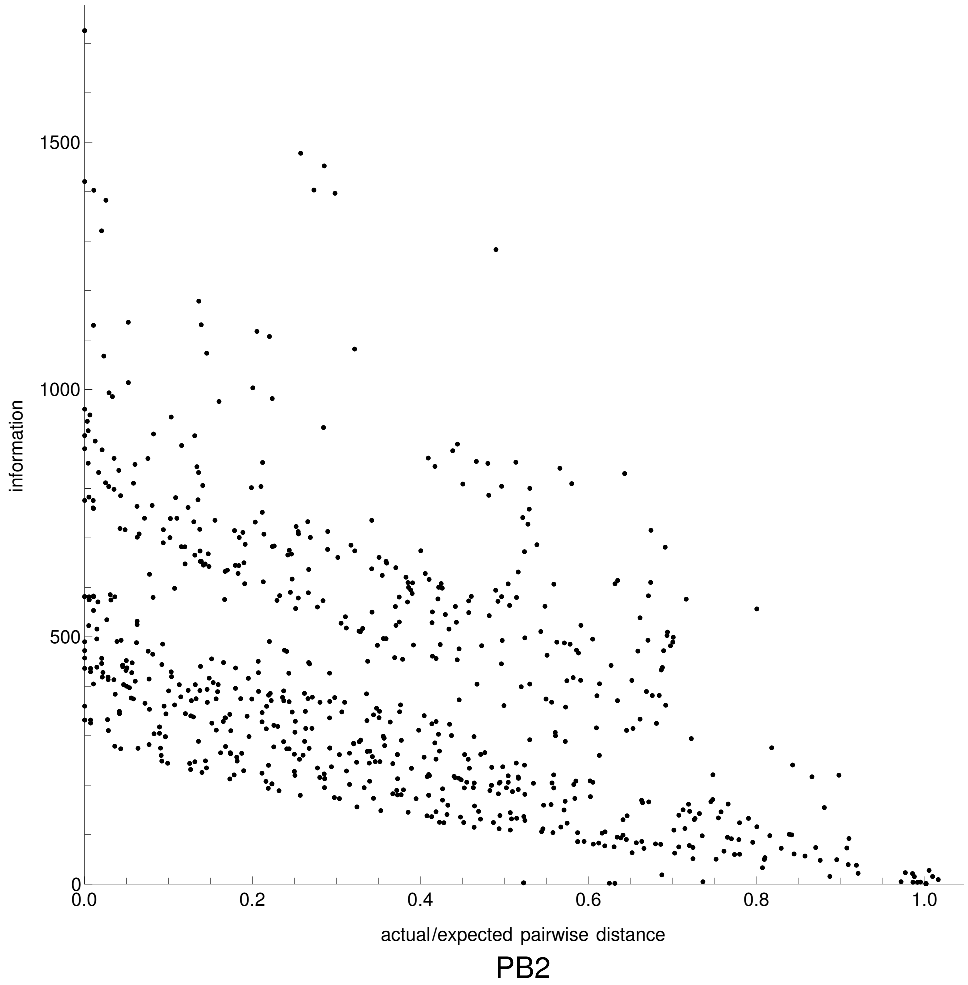

## Example sequences highlighted by regions found in analyses to be conserved

Interesting points (by weighted raw PD) highlighted for gene PB2:

ATGGAG

**AGAATAAAAGAACTAAGATATTTGATGTCGCAGTCTCGCACTCGCGAGATACTGA**
**CAAAAACCACTGTGGACCATATGGCCATA**ATCAAGAAATATACGTCAGGAAGACAGGAGA
AGAATCCTGCTCTTAGGATGAAATGGATGATGGCAATGAAATATCCGATTACAGCAGACAAAAGGATAAT
GGAGATGATCCCTGAAAGAAATGAGCAAGGTCAGACTCTTTGGATCAAAACAAATGATGCTGGATCAGAC
AGAGTGATGGTGTCACCTCTGGCTGTGACGTGGTGGAATAGAAATGGACCAACGACAAGTACAGTTCATT
ATCCAAAGGTCTATAAAACCTACTTTGAAAAGGTCGAAAGGTTAAAGCATGGAACCTTCGGTCCCGTTCA
CTTTCGAAATCAGGTTAAAATACGCCGCAGGGTTGACATAAACCCGGGCCATGCAGATCTCAGTGCTAAA
GAAGCACAAGATGTCATCATGGAGGTCGTTTTCCCAAATGAAGTCGGCGCCAGGATATTGACATCAGAGT
CACAGTTAACAATAACAAAAGAAAAGAAGGAGGAGCTTCAGGACTGCAAGATTGCTCCTTTAATGGTGGC
ATACATGTTGGAGAGAGAACTGGTCCGCAAAACCAGATTCCTACCAGTAGCTGGCGGGACAAGCAGCGTG
TATATCGAGGTGTTGCACTTGACTCAAGGAACCTGCTGGGAACAAATGTACACACCGGGAGGGGAGGTGA
GAAATGATGATGTTGATCAGAGTTTAATTATTGCTGCTAGAAATATTGTTAGGAGAGCAACAGTATCAGC
AGACCCGTTAGCTTCGCTCTTGGAGATGTGCCATAGTACACAAATTGGCGGAATAAGGATGGTAGACATC
CTTAGACAAAACCCAACAGAAGAGCAAGCTGTGGATATATGCAAAGCAGCAATGGGTCTAAGGATC
**AGTTCATCCTTCAGCTTTGGAGGTTTCACTTTCAAAAGGACAAGTGGGTCATCTG**
**TCAAAAGAGAAGAGGAAGTGCTCACAGGCAACCTCCAAACATTGAAAATA**AA
AGTGCATGAAGGATATGAGGAATTCACAATGGTCGGGCGAAGAGCAACAGCCATTCTAAGAAAGGCAACC
AGAAGGCTGATCCAATTGATAGTGAGTGGGAGAGACGAGCAGTCAATCGCCGAAGCAATTATAGTGGCAA
TGGTGTTCTCACAAGAGGATTGCATGATAAAAGCTGTACGAGGTGATTTGAATTTTGTCAACAGAGCGAA
CCAGCGGCTAAATCCTATGCATCAGCTCCTGAGGCATTTCCAAAAGGATGCAAAGGTGCTGTTCCAAAAC
TGGGGAATTGAACCCATTGACAATGTTATGGGGATGATCGGAATATTACCTGACATGACCCCCAGCACAG
AGATGTCACTAAGAGGTGTGAGAGTCAGTAAAATGGGAGTGGATGAATATTCCAGTACTGAGAGAGTGGT
CGTGAGCATTGATCGTTTCTTGAGGGTCCGAGACCAGAGGGGAAACGTGCTCTTGTCTCCTGAAGAGGTT
AGTGAAACACAGGGAACAGAAAAGCTGACGATAACATATTCATCGTCCATGATGTGGGAAATCAATGGTC
CGGAATCAGTGTTAGTTAATACATATCAATGGATCATCAGAAACTGGGAAACTGTGAAGATTCAGTGGTC
CCAAGACCCTACAATGCTATACAATAAGATGGAGTTTGAGCCCTTTCAATCCTTGGTGCCTAAGGCTGCC
AGAGGCCAGTATAGTGGATTTGTAAGGACGCTATTCCAGCAGATGCGTGATGTGCTGGGGACATTTGACA
CTGTCCAGATAATAAAGCTGCTACCATTTGCAGCAGCCCCACCAGAACAGAGTAGGATGCAGTTCTCTTC
TCTAACTGTGAACGTAAGGGGTTCAGGAATGAGAATACTTGTGAGAGGCAACTCCCCTGTGTTCAACTAT
AACAAGGCAACCAAGAGGCTTACAGTCCTTGGGAAGGATGCAGGTGCATTGACAGAAGATCCAGATGAGG
GAACAGCAGGAGTGGAATCTGCGGTATTAAGAGGATTTCTAATTCTGGGCAAAGAAGACAAAAGATATGG
ACCAGCATTGAGCATCAACGAATTGAGCAATCTTGCGAAAGGGGAGAAGGCTAATGTATTGATA
**GGGCAAGGAGACGTGGTGTTGGTAATGAAACGGAAACGGGACTCTAGCATACTTA**
**CTGACAGCCAGACAGCGACCAAAAGAATTCGGATGGCCATCAGTTAG**

Interesting points (by weighted ranked PD) highlighted for gene PB2:

ATGGAG

**AGAATAAAAGAACTAAGATATTTGATGTCGCAGTCTCGCACTCGCGAGATACTGA**
**CAAAAACCACTGTGGACCATATGGCCATA**ATCAAGAAATATACGTCAGGAAGACAGGAGA
AGAATCCTGCTCTTAGGATGAAATGGATGATGGCAATGAAATATCCGATTACAGCAGACAAAAGGATAAT
GGAGATGATCCCTGAAAGAAATGAGCAAGGTCAGACTCTTTGGATCAAAACAAATGATGCTGGATCAGAC
AGAGTGATGGTGTCACCTCTGGCTGTGACGTGGTGGAATAGAAATGGACCAACGACAAGTACAGTTCATT

ATCCAAAGGTCTATAAAACCTACTTTGAAAAGGTCGAAAGGTTAAAGCATGGAACCTTCGGTCCCGTTCA⋱
CTTTCGAAATCAGGTTAAAATACGCCGCAGGGTTGACATAAACCCGGGCCATGCAGATCTCAGTGCTAAA⋱
GAAGCACAAGATGTCATCATGGAGGTCGTTTTCCCAAATGAAGTCGGCGCCAGGATATTGACATCAGAGT⋱
CACAGTTAACAATAACAAAAGAAAAGAAGGAGGAGCTTCAGGACTGCAAGATTGCTCCTTTAATGGTGGC⋱
ATACATGTTGGAGAGAGAACTGGTCCGCAAAACCAGATTCCTACCAGTAGCTGGCGGGACAAGCAGCGTG⋱
TATATCGAGGTGTTGCACTTGACTCAAGGAACCTGCTGGGAACAAATGTACACACCGGGAGGGGAGGTGA⋱
GAAATGATGATGTTGATCAGAGTTTAATTATTGCTGCTAGAAATATTGTTAGGAGAGCAACAGTATCAGC⋱
AGACCCGTTAGCTTCGCTCTTGGAGATGTGCCATAGTACACAAATTGGCGGAATAAGGATGGTAGACATC⋱
CTTAGACAAAACCCAACAGAAGAGCAAGCTGTGGATATATGCAAAGCAGCAATGGGTCTAAGGATCAGTT⋱
CATCCTTCAGCTTTGGAGGTTTCACTTTCAAAAGGACAAGTGGGTCATCTGTCAAAAGAGAAGAGGAAGT⋱
GCTCACAGGCAACCTCCAAACATTGAAAATAAAAGTGCATGAAGGATATGAGGAATTCACAATGGTCGGG⋱
CGAAGAGCAACAGCCATTCTAAGAAAGGCAACCAGAAGGCTGATCCAATTGATAGTGAGTGGGAGAGACG⋱
AGCAGTCAATCGCCGAAGCAATTATAGTGGCAATGGTGTTCTCACAAGAGGATTGCATGATAAAAGCTGT⋱
ACGAGGTGATTTGAATTTTGTCAACAGAGCGAACCAGCGGCTAAATCCTATGCATCAGCTCCTGAGGCAT⋱
TTCCAAAAGGATGCAAAGGTGCTGTTCCAAAACTGGGGAATTGAACCCATTGACAATGTTATGGGGATGA⋱
TCGGAATATTACCTGACATGACCCCCAGCACAGAGATGTCACTAAGAGGTGTGAGAGTCAGTAAAATGGG⋱
AGTGGATGAATATTCCAGTACTGAGAGAGTGGTCGTGAGCATTGATCGTTTCTTGAGGGTCCGAGACCAG⋱
AGGGGAAACGTGCTCTTGTCTCCTGAAGAGGTTAGTGAAACACAGGGAACAGAAAAGCTGACGATAACAT⋱
ATTCATCGTCCATGATGTGGGAAATCAATGGTCCGGAATCAGTGTTAGTTAATACATATCAATGGATCAT⋱
CAGAAACTGGGAAACTGTGAAGATTCAGTGGTCCCAAGACCCTACAATGCTATACAATAAGATGGAGTTT⋱
GAGCCCTTTCAATCCTTGGTGCCTAAGGCTGCCAGAGGCCAGTATAGTGGATTTGTAAGGACGCTATTCC⋱
AGCAGATGCGTGATGTGCTGGGGACATTTGACACTGTCCAGATAATAAAGCTGCTACCATTTGCAGCAGC⋱
CCCACCAGAACAGAGTAGGATGCAGTTCTCTTCTCTAACTGTGAACGTAAGGGGTTCAGGAATGAGAATA⋱
CTTGTGAGAGGCAACTCCCCTGTGTTCAACTATAACAAGGCAACCAAGAGGCTTACAGTCCTTGGGAAGG⋱
ATGCAGGTGCATTGACAGAAGATCCAGATGAGGGAACAGCAGGAGTGGAATCTGCGGTATTAAGAGGATT⋱
TCTAATTCTGGGCAAAGAAGACAAAAGATATGGACCAGCATTGAGCATCAACGAATTGAGCAATCTTGCG⋱
AAAGGGGAGAAGGCTAATGTATTGATA

**GGGCAAGGAGACGTGGTGTTGGTAATGAAACGGAAACGGGACTCTAGCATACTTA**
**CTGACAGCCAGACAGCGACCAAAAGAATTCGGATGGCCATCAGTTAG**

# Per codon conservation report

PB2

| Pos. | 1 | obs: | exp: |
|---|---|---|---|
| atg | M | 572 | 572.00 |
| --- | -- | ------ | ------ |
| mPD | | 0 | 0 |
| | | nPD: | 1. |
| | | N. weight: | 0. |
| | | Sc. PD: | 0 |
| | | Sc. rank: | 0 |

PB2

| Pos. | 2 | obs: | exp: |
|---|---|---|---|
| gaa | E | 36 | 328.60 |
| gag | E | 535 | 242.40 |
| ggt | G | 0 | 0.12 |
| ggc | G | 0 | 0.14 |
| gga | G | 0 | 0.48 |
| ggg | G | 1 | 0.26 |
| --- | -- | ------ | ------ |
| mPD | | 0.12 | 0.49 |
| | | nPD: | 0.25 |
| | | N. weight: | 0.87 |
| | | Sc. PD: | -0.02 |
| | | Sc. rank: | 10.8 |

PB2

| Pos. | 3 | obs: | exp: |
|---|---|---|---|
| cgt | R | 0 | 23.75 |
| cgc | R | 0 | 28.01 |
| cga | R | 0 | 54.97 |
| cgg | R | 0 | 47.73 |
| aga | R | 572 | 257.20 |
| agg | R | 0 | 160.30 |
| --- | -- | ------ | ------ |
| mPD | | 0 | 0.96 |
| | | nPD: | 0. |
| | | N. weight: | 1.1 |
| | | Sc. PD: | -0.3 |
| | | Sc. rank: | -393.9 |

PB2

| Pos. | 4 | obs: | exp: |
|---|---|---|---|
| att | I | 0 | 214.30 |
| atc | I | 1 | 149.40 |
| ata | I | 569 | 206.30 |
| aat | N | 1 | 0.52 |
| aac | N | 0 | 0.48 |
| gat | D | 1 | 0.53 |
| gac | D | 0 | 0.47 |
| --- | -- | ------ | ------ |
| mPD | | 0.021 | 0.67 |
| | | nPD: | 0.03 |
| | | N. weight: | 1.4 |
| | | Sc. PD: | -0.33 |
| | | Sc. rank: | -348.9 |

PB2

| Pos. | 5 | obs: | exp: |
|---|---|---|---|
| aaa | K | 571 | 320.30 |
| aag | K | 1 | 251.70 |
| --- | -- | ------ | ------ |
| mPD | | 0.0035 | 0.49 |
| | | nPD: | 0.01 |
| | | N. weight: | 0.81 |
| | | Sc. PD: | -0.21 |
| | | Sc. rank: | -242.7 |

PB2

| Pos. | 6 | obs: | exp: |
|---|---|---|---|
| aaa | K | 1 | 0.56 |
| aag | K | 0 | 0.44 |
| gaa | E | 540 | 328.60 |
| gag | E | 31 | 242.40 |
| --- | -- | ------ | ------ |
| mPD | | 0.11 | 0.49 |
| | | nPD: | 0.22 |
| | | N. weight: | 0.52 |
| | | Sc. PD: | -0.028 |
| | | Sc. rank: | -12.6 |

PB2

| Pos. | 7 | obs: | exp: |
|---|---|---|---|
| tta | L | 16 | 49.97 |
| ttg | L | 0 | 110.20 |
| ctt | L | 0 | 106.70 |
| ctc | L | 0 | 92.53 |
| cta | L | 555 | 86.17 |
| ctg | L | 1 | 126.40 |
| --- | -- | ------ | ------ |
| mPD | | 0.058 | 1.1 |
| | | nPD: | 0.05 |
| | | N. weight: | 2.5 |
| | | Sc. PD: | -0.53 |
| | | Sc. rank: | -529.8 |

PB2

| Pos. | 8 | obs: | exp: |
|---|---|---|---|
| cgt | R | 0 | 23.75 |
| cgc | R | 0 | 28.01 |
| cga | R | 3 | 54.97 |
| cgg | R | 0 | 47.73 |
| aga | R | 568 | 257.20 |
| agg | R | 1 | 160.30 |
| --- | -- | ------ | ------ |
| mPD | | 0.014 | 0.96 |
| | | nPD: | 0.01 |
| | | N. weight: | 1.1 |
| | | Sc. PD: | -0.27 |
| | | Sc. rank: | -303.6 |

PB2

| Pos. | 9 | obs: | exp: |
|---|---|---|---|
| tat | Y | 238 | 123.20 |
| tac | Y | 0 | 114.80 |
| aat | N | 1 | 0.52 |
| aac | N | 0 | 0.48 |
| gat | D | 332 | 177.70 |
| gac | D | 1 | 155.30 |
| --- | -- | ------ | ------ |
| mPD | | 0.49 | 0.99 |
| | | nPD: | 0.5 |
| | | N. weight: | 0.9 |
| | | Sc. PD: | 0.2 |
| | | Sc. rank: | 207.6 |

PB2

| Pos. | 10 | obs: | exp: |
|---|---|---|---|
| tta | L | 20 | 49.97 |
| ttg | L | 460 | 110.20 |
| ctt | L | 0 | 106.70 |
| ctc | L | 0 | 92.53 |
| cta | L | 87 | 86.17 |
| ctg | L | 5 | 126.40 |
| --- | -- | ------ | ------ |
| mPD | | 0.58 | 1.1 |
| | | nPD: | 0.52 |
| | | N. weight: | 1.6 |
| | | Sc. PD: | 0.37 |
| | | Sc. rank: | 380.3 |

PB2

| Pos. | 11 | obs: | exp: |
|---|---|---|---|
| tta | L | 0 | 0.09 |
| ttg | L | 0 | 0.19 |
| ctt | L | 0 | 0.19 |
| ctc | L | 0 | 0.16 |
| cta | L | 0 | 0.15 |
| ctg | L | 1 | 0.22 |
| atg | M | 571 | 571.00 |
| --- | -- | ------ | ------ |
| mPD | | 0.0035 | 0.0055 |
| | | nPD: | 0.63 |
| | | N. weight: | 0.0038 |
| | | Sc. PD: | 0.0013 |
| | | Sc. rank: | 1.1 |

PB2

| Pos. | 12 | obs: | exp: |
|---|---|---|---|
| tct | S | 1 | 90.93 |
| tcc | S | 0 | 79.68 |
| tca | S | 136 | 126.70 |
| tcg | S | 435 | 34.87 |
| agt | S | 0 | 125.80 |
| agc | S | 0 | 114.00 |
| --- | -- | ------ | ------ |
| mPD | | 0.37 | 1.7 |
| | | nPD: | 0.22 |
| | | N. weight: | 2.8 |
| | | Sc. PD: | -0.14 |
| | | Sc. rank: | -47.9 |

PB2

| Pos. | 13 | obs: | exp: |
|---|---|---|---|
| tta | L | 0 | 0.09 |
| ttg | L | 0 | 0.19 |
| ctt | L | 0 | 0.19 |
| ctc | L | 0 | 0.16 |
| cta | L | 0 | 0.15 |
| ctg | L | 1 | 0.22 |
| caa | Q | 24 | 304.40 |
| cag | Q | 547 | 266.60 |
| --- | -- | ------ | ------ |
| mPD | | 0.084 | 0.50 |
| | | nPD: | 0.17 |
| | | N. weight: | 0.84 |
| | | Sc. PD: | -0.084 |
| | | Sc. rank: | -64.2 |

PB2

| Pos. | 14 | obs: | exp: |
|---|---|---|---|
| tct | S | 530 | 90.93 |
| tcc | S | 42 | 79.68 |
| tca | S | 0 | 126.70 |
| tcg | S | 0 | 34.87 |
| agt | S | 0 | 125.80 |
| agc | S | 0 | 114.00 |
| --- | -- | ------ | ------ |
| mPD | | 0.14 | 1.7 |
| | | nPD: | 0.08 |
| | | N. weight: | 2.3 |
| | | Sc. PD: | -0.41 |
| | | Sc. rank: | -398.2 |

PB2

| Pos. | 15 | obs: | exp: |
|---|---|---|---|
| cgt | R | 0 | 23.75 |
| cgc | R | 572 | 28.01 |
| cga | R | 0 | 54.97 |
| cgg | R | 0 | 47.73 |
| aga | R | 0 | 257.20 |
| agg | R | 0 | 160.30 |
| --- | -- | ------ | ------ |
| mPD | | 0 | 0.96 |
| | | nPD: | 0. |
| | | N. weight: | 4.3 |
| | | Sc. PD: | -1.1 |
| | | Sc. rank: | -1486.9 |

PB2

| Pos. | 16 | obs: | exp: |
|---|---|---|---|
| tct | S | 0 | 0.16 |
| tcc | S | 0 | 0.14 |
| tca | S | 0 | 0.22 |
| tcg | S | 0 | 0.06 |
| act | T | 559 | 150.90 |
| acc | T | 12 | 122.50 |
| aca | T | 0 | 250.20 |
| acg | T | 0 | 47.47 |
| agt | S | 1 | 0.22 |
| agc | S | 0 | 0.20 |
| --- | -- | ------ | ------ |
| mPD | | 0.045 | 0.69 |
| | | nPD: | 0.06 |
| | | N. weight: | 1.8 |
| | | Sc. PD: | -0.35 |
| | | Sc. rank: | -337.3 |

PB2

| Pos. | 17 | obs: | exp: |
|---|---|---|---|
| cgt | R | 0 | 23.75 |
| cgc | R | 572 | 28.01 |
| cga | R | 0 | 54.97 |
| cgg | R | 0 | 47.73 |
| aga | R | 0 | 257.20 |
| agg | R | 0 | 160.30 |
| --- | -- | ------ | ------ |
| mPD | | 0 | 0.96 |
| | | nPD: | 0. |
| | | N. weight: | 4.3 |
| | | Sc. PD: | -1.1 |
| | | Sc. rank: | -1486.9 |

PB2

| Pos. | 18 | obs: | exp: |
|---|---|---|---|
| gaa | E | 0 | 329.10 |
| gag | E | 572 | 242.90 |
| --- | -- | ------ | ------ |
| mPD | | 0 | 0.49 |
| | | nPD: | 0. |
| | | N. weight: | 1.2 |
| | | Sc. PD: | -0.32 |
| | | Sc. rank: | -422.3 |

PB2

| Pos. | 19 | obs: | exp: |
|---|---|---|---|
| att | I | 1 | 215.00 |
| atc | I | 0 | 149.90 |
| ata | I | 571 | 207.10 |
| --- | -- | ------ | ------ |
| mPD | | 0.0035 | 0.66 |
| | | nPD: | 0.01 |
| | | N. weight: | 1.4 |
| | | Sc. PD: | -0.36 |
| | | Sc. rank: | -440.3 |

PB2

| Pos. | 20 | obs: | exp: |
|---|---|---|---|
| tta | L | 0 | 49.97 |
| ttg | L | 0 | 110.20 |
| ctt | L | 3 | 106.70 |
| ctc | L | 33 | 92.53 |
| cta | L | 56 | 86.17 |
| ctg | L | 480 | 126.40 |
| --- | -- | ------ | ------ |
| mPD | | 0.28 | 1.1 |
| | | nPD: | 0.25 |
| | | N. weight: | 1.4 |
| | | Sc. PD: | -0.024 |
| | | Sc. rank: | 30.8 |

PB2

| Pos. | 21 | obs: | exp: |
|---|---|---|---|
| act | T | 3 | 151.10 |
| acc | T | 68 | 122.70 |
| aca | T | 499 | 250.60 |
| acg | T | 2 | 47.55 |
| --- | -- | ------ | ------ |
| mPD | | 0.23 | 0.69 |
| | | nPD: | 0.33 |
| | | N. weight: | 0.73 |
| | | Sc. PD: | 0.039 |
| | | Sc. rank: | 65.3 |

PB2

| Pos. | 22 | obs: | exp: |
|---|---|---|---|
| aaa | K | 559 | 320.30 |
| aag | K | 13 | 251.70 |
| --- | -- | ------ | ------ |
| mPD | | 0.044 | 0.49 |
| | | nPD: | 0.09 |
| | | N. weight: | 0.69 |
| | | Sc. PD: | -0.12 |
| | | Sc. rank: | -117.9 |

PB2

| Pos. | 23 | obs: | exp: |
|---|---|---|---|
| act | T | 11 | 151.10 |
| acc | T | 534 | 122.70 |
| aca | T | 27 | 250.60 |
| acg | T | 0 | 47.55 |
| --- | -- | ------ | ------ |
| mPD | | 0.13 | 0.69 |
| | | nPD: | 0.18 |
| | | N. weight: | 1.8 |
| | | Sc. PD: | -0.15 |
| | | Sc. rank: | -101.3 |

PB2

| Pos. | 24 | obs: | exp: |
|---|---|---|---|
| act | T | 543 | 151.10 |
| acc | T | 26 | 122.70 |
| aca | T | 3 | 250.60 |
| acg | T | 0 | 47.55 |
| --- | -- | ------ | ------ |
| mPD | | 0.097 | 0.69 |
| | | nPD: | 0.14 |
| | | N. weight: | 1.6 |
| | | Sc. PD: | -0.2 |
| | | Sc. rank: | -163.6 |

PB2

| Pos. | 25 | obs: | exp: |
|---|---|---|---|
| gtt | V | 42 | 117.60 |
| gtc | V | 0 | 120.10 |
| gta | V | 4 | 107.20 |
| gtg | V | 526 | 227.20 |
| --- | -- | ------ | ------ |
| mPD | | 0.15 | 0.72 |
| | | nPD: | 0.21 |
| | | N. weight: | 0.97 |
| | | Sc. PD: | -0.06 |
| | | Sc. rank: | -34.4 |

PB2

| Pos. | 26 | obs: | exp: |
| --- | --- | --- | --- |
| aat | N | 0 | 0.52 |
| aac | N | 1 | 0.48 |
| gat | D | 3 | 304.70 |
| gac | D | 568 | 266.30 |
| mPD | | 0.014 | 0.50 |
| | | nPD: | 0.03 |
| | | N. weight: | 1. |
| | | Sc. PD: | -0.24 |
| | | Sc. rank: | -264.9 |

,

PB2

| Pos. | 27 | obs: | exp: |
| --- | --- | --- | --- |
| cct | P | 0 | 0.26 |
| ccc | P | 1 | 0.18 |
| cca | P | 0 | 0.39 |
| ccg | P | 0 | 0.16 |
| cat | H | 488 | 309.40 |
| cac | H | 83 | 261.60 |
| mPD | | 0.25 | 0.50 |
| | | nPD: | 0.51 |
| | | N. weight: | 0.33 |
| | | Sc. PD: | 0.074 |
| | | Sc. rank: | 77.5 |

,

PB2

| Pos. | 28 | obs: | exp: |
| --- | --- | --- | --- |
| atg | M | 571 | 571.00 |
| aat | N | 1 | 0.52 |
| aac | N | 0 | 0.48 |
| mPD | | 0.0070 | 0.0070 |
| | | nPD: | 1. |
| | | N. weight: | 0.0016 |
| | | Sc. PD: | 0.0011 |
| | | Sc. rank: | 0.6 |

,

PB2

| Pos. | 29 | obs: | exp: |
| --- | --- | --- | --- |
| gct | A | 10 | 143.80 |
| gcc | A | 560 | 117.00 |
| gca | A | 1 | 254.50 |
| gcg | A | 0 | 55.72 |
| gat | D | 0 | 0.53 |
| gac | D | 1 | 0.47 |
| mPD | | 0.041 | 0.69 |
| | | nPD: | 0.06 |
| | | N. weight: | 2.1 |
| | | Sc. PD: | -0.43 |
| | | Sc. rank: | -425.6 |

,

PB2

| Pos. | 30 | obs: | exp: |
| --- | --- | --- | --- |
| att | I | 0 | 212.80 |
| atc | I | 0 | 148.30 |
| ata | I | 566 | 204.90 |
| gtt | V | 0 | 1.23 |
| gtc | V | 0 | 1.26 |
| gta | V | 6 | 1.12 |
| gtg | V | 0 | 2.38 |
| mPD | | 0.021 | 0.68 |
| | | nPD: | 0.03 |
| | | N. weight: | 1.5 |
| | | Sc. PD: | -0.34 |
| | | Sc. rank: | -357.3 |

,

PB2

| Pos. | 31 | obs: | exp: |
| --- | --- | --- | --- |
| att | I | 34 | 215.00 |
| atc | I | 470 | 149.90 |
| ata | I | 68 | 207.10 |
| mPD | | 0.31 | 0.66 |
| | | nPD: | 0.47 |
| | | N. weight: | 1. |
| | | Sc. PD: | 0.19 |
| | | Sc. rank: | 208.1 |

,

PB2

| Pos. | 32 | obs: | exp: |
| --- | --- | --- | --- |
| cgt | R | 0 | 0.04 |
| cgc | R | 0 | 0.05 |
| cga | R | 0 | 0.10 |
| cgg | R | 0 | 0.08 |
| aaa | K | 66 | 319.70 |
| aag | K | 505 | 251.30 |
| aga | R | 0 | 0.45 |
| agg | R | 1 | 0.28 |
| mPD | | 0.21 | 0.50 |
| | | nPD: | 0.42 |
| | | N. weight: | 0.63 |
| | | Sc. PD: | 0.088 |
| | | Sc. rank: | 103.9 |

,

PB2

| Pos. | 33 | obs: | exp: |
| --- | --- | --- | --- |
| aaa | K | 503 | 320.30 |
| aag | K | 69 | 251.70 |
| mPD | | 0.21 | 0.49 |
| | | nPD: | 0.43 |
| | | N. weight: | 0.35 |
| | | Sc. PD: | 0.053 |
| | | Sc. rank: | 62.5 |

,

PB2

| Pos. | 34 | obs: | exp: |
| --- | --- | --- | --- |
| tat | Y | 368 | 296.10 |
| tac | Y | 204 | 275.90 |
| mPD | | 0.46 | 0.50 |
| | | nPD: | 0.92 |
| | | N. weight: | 0.054 |
| | | Sc. PD: | 0.034 |
| | | Sc. rank: | 18.8 |

,

PB2

| Pos. | 35 | obs: | exp: |
| --- | --- | --- | --- |
| act | T | 0 | 150.90 |
| acc | T | 0 | 122.50 |
| aca | T | 203 | 250.20 |
| acg | T | 368 | 47.47 |
| aat | N | 0 | 0.52 |
| aac | N | 1 | 0.48 |
| mPD | | 0.46 | 0.69 |
| | | nPD: | 0.67 |
| | | N. weight: | 1.8 |
| | | Sc. PD: | 0.69 |
| | | Sc. rank: | 564.2 |

,

PB2

| Pos. | 36 | obs: | exp: |
| --- | --- | --- | --- |
| tct | S | 0 | 90.78 |
| tcc | S | 0 | 79.54 |
| tca | S | 488 | 126.50 |
| tcg | S | 83 | 34.80 |
| agt | S | 0 | 125.60 |
| agc | S | 0 | 113.80 |
| gtt | V | 0 | 0.21 |
| gtc | V | 1 | 0.21 |
| gta | V | 0 | 0.19 |
| gtg | V | 0 | 0.40 |
| mPD | | 0.26 | 1.7 |
| | | nPD: | 0.16 |
| | | N. weight: | 1.8 |
| | | Sc. PD: | -0.2 |
| | | Sc. rank: | -158.9 |

,

PB2

| Pos. | 37 | obs: | exp: |
| --- | --- | --- | --- |
| cgt | R | 0 | 0.04 |
| cgc | R | 0 | 0.05 |
| cga | R | 0 | 0.10 |
| cgg | R | 0 | 0.08 |
| aga | R | 0 | 0.45 |
| agg | R | 1 | 0.28 |
| ggt | G | 0 | 70.45 |
| ggc | G | 1 | 77.22 |
| gga | G | 559 | 276.20 |
| ggg | G | 11 | 147.10 |
| mPD | | 0.048 | 0.67 |
| | | nPD: | 0.07 |
| | | N. weight: | 0.91 |
| | | Sc. PD: | -0.17 |
| | | Sc. rank: | -171.9 |

,

PB2

| Pos. | 38 | obs: | exp: |
| --- | --- | --- | --- |
| cgt | R | 0 | 23.71 |
| cgc | R | 0 | 27.96 |
| cga | R | 0 | 54.88 |
| cgg | R | 0 | 47.65 |
| aaa | K | 0 | 0.56 |
| aag | K | 1 | 0.44 |
| aga | R | 496 | 256.80 |
| agg | R | 75 | 160.00 |
| mPD | | 0.23 | 0.96 |
| | | nPD: | 0.24 |
| | | N. weight: | 0.68 |
| | | Sc. PD: | -0.018 |
| | | Sc. rank: | 4.0 |

,

PB2

| Pos. | 39 | obs: | exp: |
| --- | --- | --- | --- |
| caa | Q | 97 | 304.40 |
| cag | Q | 474 | 266.60 |
| act | T | 0 | 0.26 |
| acc | T | 0 | 0.21 |
| aca | T | 0 | 0.44 |
| acg | T | 1 | 0.08 |
| mPD | | 0.29 | 0.51 |
| | | nPD: | 0.57 |
| | | N. weight: | 0.42 |
| | | Sc. PD: | 0.12 |
| | | Sc. rank: | 114.7 |

,

PB2

| Pos. | 40 | obs: | exp: |
| --- | --- | --- | --- |
| gaa | E | 8 | 329.10 |
| gag | E | 564 | 242.90 |
| mPD | | 0.028 | 0.49 |
| | | nPD: | 0.06 |
| | | N. weight: | 1.1 |
| | | Sc. PD: | -0.23 |
| | | Sc. rank: | -229.6 |

,

PB2

| Pos. | 41 | obs: | exp: |
| --- | --- | --- | --- |
| aaa | K | 8 | 320.30 |
| aag | K | 564 | 251.70 |
| mPD | | 0.028 | 0.49 |
| | | nPD: | 0.06 |
| | | N. weight: | 1.1 |
| | | Sc. PD: | -0.22 |
| | | Sc. rank: | -220.4 |

,

PB2

| Pos. | 42 | obs: | exp: |
| --- | --- | --- | --- |
| aat | N | 412 | 299.00 |
| aac | N | 160 | 273.00 |
| mPD | | 0.40 | 0.50 |
| | | nPD: | 0.81 |
| | | N. weight: | 0.12 |
| | | Sc. PD: | 0.064 |
| | | Sc. rank: | 42.6 |

,

PB2

| Pos. | 43 | obs: | exp: |
| --- | --- | --- | --- |
| cct | P | 532 | 150.60 |
| ccc | P | 38 | 103.20 |
| cca | P | 1 | 225.70 |
| ccg | P | 1 | 92.49 |
| mPD | | 0.13 | 0.72 |
| | | nPD: | 0.18 |
| | | N. weight: | 1.6 |
| | | Sc. PD: | -0.13 |
| | | Sc. rank: | -95.6 |

,

PB2

| Pos. | 44 | obs: | exp: |
| --- | --- | --- | --- |
| gct | A | 306 | 144.10 |
| gcc | A | 113 | 117.20 |
| gca | A | 153 | 254.90 |
| gcg | A | 0 | 55.82 |
| mPD | | 0.60 | 0.69 |
| | | nPD: | 0.88 |
| | | N. weight: | 0.39 |
| | | Sc. PD: | 0.22 |
| | | Sc. rank: | 133.3 |

,

PB2

| Pos. | 45 | obs: | exp: |
| --- | --- | --- | --- |
| tta | L | 0 | 49.97 |
| ttg | L | 0 | 110.20 |
| ctt | L | 414 | 106.70 |
| ctc | L | 158 | 92.53 |
| cta | L | 0 | 86.17 |
| ctg | L | 0 | 126.40 |
| mPD | | 0.40 | 1.1 |
| | | nPD: | 0.36 |
| | | N. weight: | 1.6 |
| | | Sc. PD: | 0.14 |
| | | Sc. rank: | 191.7 |

,

PB2

| Pos. | 46 | obs: | exp: |
| --- | --- | --- | --- |
| cgt | R | 0 | 23.75 |
| cgc | R | 0 | 28.01 |
| cga | R | 0 | 54.97 |
| cgg | R | 0 | 47.73 |
| aga | R | 75 | 257.20 |
| agg | R | 497 | 160.30 |
| mPD | | 0.23 | 0.96 |
| | | nPD: | 0.24 |
| | | N. weight: | 1.2 |
| | | Sc. PD: | -0.038 |
| | | Sc. rank: | 0.2 |

,

PB2

| Pos. | 47 | obs: | exp: |
| --- | --- | --- | --- |
| atg | M | 572 | 572.00 |
| mPD | | 0 | 0 |
| | | nPD: | 1. |
| | | N. weight: | 0. |
| | | Sc. PD: | 0 |
| | | Sc. rank: | 0 |

,

PB2

| Pos. | 48 | obs: | exp: |
| --- | --- | --- | --- |
| aaa | K | 500 | 320.30 |
| aag | K | 72 | 251.70 |
| mPD | | 0.22 | 0.49 |
| | | nPD: | 0.45 |
| | | N. weight: | 0.34 |
| | | Sc. PD: | 0.057 |
| | | Sc. rank: | 65.0 |

,

PB2

| Pos. | 49 | obs: | exp: |
| --- | --- | --- | --- |
| tgg | W | 572 | 572.00 |
| mPD | | 0 | 0 |
| | | nPD: | 1. |
| | | N. weight: | 0. |
| | | Sc. PD: | 0 |
| | | Sc. rank: | 0 |

,

PB2

| Pos. | 50 | obs: | exp: |
| --- | --- | --- | --- |
| atg | M | 572 | 572.00 |
| mPD | | 0 | 0 |
| | | nPD: | 1. |
| | | N. weight: | 0. |
| | | Sc. PD: | 0 |
| | | Sc. rank: | 0 |

,

| PB2 | | | |
|---|---|---|---|
| Pos. | 51 | obs: | exp: |
| atg | M | 572 | 572.00 |
| --- | -- | ------ | ------ |
| mPD | | 0 | 0 |
| | | nPD: | 1. |
| | | N. weight: | 0. |
| | | Sc. PD: | 0 |
| | | Sc. rank: | 0 |

| PB2 | | | |
|---|---|---|---|
| Pos. | 52 | obs: | exp: |
| gtt | V | 0 | 0.21 |
| gtc | V | 0 | 0.21 |
| gta | V | 1 | 0.19 |
| gtg | V | 0 | 0.40 |
| gct | A | 1 | 143.80 |
| gcc | A | 1 | 117.00 |
| gca | A | 533 | 254.50 |
| gcg | A | 36 | 55.72 |
| --- | -- | ------ | ------ |
| mPD | | 0.13 | 0.69 |
| | | nPD: | 0.19 |
| | | N. weight: | 0.94 |
| | | Sc. PD: | -0.077 |
| | | Sc. rank: | -53.4 |

| PB2 | | | |
|---|---|---|---|
| Pos. | 53 | obs: | exp: |
| atg | M | 572 | 572.00 |
| --- | -- | ------ | ------ |
| mPD | | 0 | 0 |
| | | nPD: | 1. |
| | | N. weight: | 0. |
| | | Sc. PD: | 0 |
| | | Sc. rank: | 0 |

| PB2 | | | |
|---|---|---|---|
| Pos. | 54 | obs: | exp: |
| aaa | K | 561 | 320.30 |
| aag | K | 11 | 251.70 |
| --- | -- | ------ | ------ |
| mPD | | 0.038 | 0.49 |
| | | nPD: | 0.08 |
| | | N. weight: | 0.7 |
| | | Sc. PD: | -0.13 |
| | | Sc. rank: | -129.9 |

| PB2 | | | |
|---|---|---|---|
| Pos. | 55 | obs: | exp: |
| tat | Y | 547 | 296.10 |
| tac | Y | 25 | 275.90 |
| --- | -- | ------ | ------ |
| mPD | | 0.084 | 0.50 |
| | | nPD: | 0.17 |
| | | N. weight: | 0.69 |
| | | Sc. PD: | -0.069 |
| | | Sc. rank: | -51.2 |

| PB2 | | | |
|---|---|---|---|
| Pos. | 56 | obs: | exp: |
| cct | P | 0 | 150.60 |
| ccc | P | 2 | 103.20 |
| cca | P | 132 | 225.70 |
| ccg | P | 438 | 92.49 |
| --- | -- | ------ | ------ |
| mPD | | 0.36 | 0.72 |
| | | nPD: | 0.5 |
| | | N. weight: | 1.5 |
| | | Sc. PD: | 0.34 |
| | | Sc. rank: | 352.0 |

| PB2 | | | |
|---|---|---|---|
| Pos. | 57 | obs: | exp: |
| att | I | 474 | 215.00 |
| atc | I | 95 | 149.90 |
| ata | I | 3 | 207.10 |
| --- | -- | ------ | ------ |
| mPD | | 0.29 | 0.66 |
| | | nPD: | 0.43 |
| | | N. weight: | 0.81 |
| | | Sc. PD: | 0.13 |
| | | Sc. rank: | 145.3 |

| PB2 | | | |
|---|---|---|---|
| Pos. | 58 | obs: | exp: |
| act | T | 0 | 151.10 |
| acc | T | 0 | 122.70 |
| aca | T | 563 | 250.60 |
| acg | T | 9 | 47.55 |
| --- | -- | ------ | ------ |
| mPD | | 0.031 | 0.69 |
| | | nPD: | 0.05 |
| | | N. weight: | 1.1 |
| | | Sc. PD: | -0.24 |
| | | Sc. rank: | -246.1 |

| PB2 | | | |
|---|---|---|---|
| Pos. | 59 | obs: | exp: |
| gct | A | 31 | 144.10 |
| gcc | A | 0 | 117.20 |
| gca | A | 503 | 254.90 |
| gcg | A | 38 | 55.82 |
| --- | -- | ------ | ------ |
| mPD | | 0.22 | 0.69 |
| | | nPD: | 0.32 |
| | | N. weight: | 0.71 |
| | | Sc. PD: | 0.033 |
| | | Sc. rank: | 57.4 |

| PB2 | | | |
|---|---|---|---|
| Pos. | 60 | obs: | exp: |
| aat | N | 0 | 0.52 |
| aac | N | 1 | 0.48 |
| gat | D | 24 | 304.70 |
| gac | D | 547 | 266.30 |
| --- | -- | ------ | ------ |
| mPD | | 0.084 | 0.50 |
| | | nPD: | 0.17 |
| | | N. weight: | 0.84 |
| | | Sc. PD: | -0.083 |
| | | Sc. rank: | -62.3 |

| PB2 | | | |
|---|---|---|---|
| Pos. | 61 | obs: | exp: |
| cgt | R | 0 | 0.08 |
| cgc | R | 0 | 0.10 |
| cga | R | 0 | 0.19 |
| cgg | R | 0 | 0.17 |
| aaa | K | 477 | 319.20 |
| aag | K | 93 | 250.80 |
| aga | R | 2 | 0.90 |
| agg | R | 0 | 0.56 |
| --- | -- | ------ | ------ |
| mPD | | 0.28 | 0.50 |
| | | nPD: | 0.56 |
| | | N. weight: | 0.26 |
| | | Sc. PD: | 0.071 |
| | | Sc. rank: | 69.0 |

| PB2 | | | |
|---|---|---|---|
| Pos. | 62 | obs: | exp: |
| caa | Q | 1 | 0.53 |
| cag | Q | 0 | 0.47 |
| cgt | R | 0 | 23.41 |
| cgc | R | 0 | 27.62 |
| cga | R | 40 | 54.20 |
| cgg | R | 2 | 47.06 |
| aaa | K | 1 | 1.68 |
| aag | K | 2 | 1.32 |
| aga | R | 104 | 253.60 |
| agg | R | 418 | 158.10 |
| ggt | G | 0 | 0.49 |
| ggc | G | 0 | 0.54 |
| gga | G | 2 | 1.93 |
| ggg | G | 2 | 1.03 |
| --- | -- | ------ | ------ |
| mPD | | 0.55 | 0.98 |
| | | nPD: | 0.56 |
| | | N. weight: | 0.77 |
| | | Sc. PD: | 0.21 |
| | | Sc. rank: | 205.6 |

| PB2 | | | |
|---|---|---|---|
| Pos. | 63 | obs: | exp: |
| att | I | 0 | 215.00 |
| atc | I | 1 | 149.90 |
| ata | I | 571 | 207.10 |
| --- | -- | ------ | ------ |
| mPD | | 0.0035 | 0.66 |
| | | nPD: | 0.01 |
| | | N. weight: | 1.4 |
| | | Sc. PD: | -0.36 |
| | | Sc. rank: | -440.6 |

| PB2 | | | |
|---|---|---|---|
| Pos. | 64 | obs: | exp: |
| atg | M | 571 | 571.00 |
| act | T | 0 | 0.26 |
| acc | T | 0 | 0.21 |
| aca | T | 0 | 0.44 |
| acg | T | 1 | 0.08 |
| --- | -- | ------ | ------ |
| mPD | | 0.0035 | 0.0067 |
| | | nPD: | 0.52 |
| | | N. weight: | 0.0062 |
| | | Sc. PD: | 0.0015 |
| | | Sc. rank: | 1.5 |

| PB2 | | | |
|---|---|---|---|
| Pos. | 65 | obs: | exp: |
| gaa | E | 139 | 329.10 |
| gag | E | 433 | 242.90 |
| --- | -- | ------ | ------ |
| mPD | | 0.37 | 0.49 |
| | | nPD: | 0.75 |
| | | N. weight: | 0.33 |
| | | Sc. PD: | 0.15 |
| | | Sc. rank: | 112.7 |

| PB2 | | | |
|---|---|---|---|
| Pos. | 66 | obs: | exp: |
| att | I | 2 | 0.75 |
| atc | I | 0 | 0.52 |
| ata | I | 0 | 0.72 |
| atg | M | 567 | 567.00 |
| gtt | V | 0 | 0.62 |
| gtc | V | 0 | 0.63 |
| gta | V | 0 | 0.56 |
| gtg | V | 3 | 1.19 |
| --- | -- | ------ | ------ |
| mPD | | 0.017 | 0.024 |
| | | nPD: | 0.74 |
| | | N. weight: | 0.012 |
| | | Sc. PD: | 0.0052 |
| | | Sc. rank: | 4.0 |

| PB2 | | | |
|---|---|---|---|
| Pos. | 67 | obs: | exp: |
| att | I | 66 | 215.00 |
| atc | I | 506 | 149.90 |
| ata | I | 0 | 207.10 |
| --- | -- | ------ | ------ |
| mPD | | 0.20 | 0.66 |
| | | nPD: | 0.31 |
| | | N. weight: | 1.4 |
| | | Sc. PD: | 0.05 |
| | | Sc. rank: | 102.1 |

| PB2 | | | |
|---|---|---|---|
| Pos. | 68 | obs: | exp: |
| cct | P | 522 | 150.60 |
| ccc | P | 15 | 103.20 |
| cca | P | 5 | 225.70 |
| ccg | P | 30 | 92.49 |
| --- | -- | ------ | ------ |
| mPD | | 0.16 | 0.72 |
| | | nPD: | 0.23 |
| | | N. weight: | 1.4 |
| | | Sc. PD: | -0.058 |
| | | Sc. rank: | -9.5 |

| PB2 | | | |
|---|---|---|---|
| Pos. | 69 | obs: | exp: |
| gaa | E | 551 | 329.10 |
| gag | E | 21 | 242.90 |
| --- | -- | ------ | ------ |
| mPD | | 0.071 | 0.49 |
| | | nPD: | 0.15 |
| | | N. weight: | 0.59 |
| | | Sc. PD: | -0.071 |
| | | Sc. rank: | -58.1 |

| PB2 | | | |
|---|---|---|---|
| Pos. | 70 | obs: | exp: |
| cgt | R | 0 | 23.75 |
| cgc | R | 0 | 28.01 |
| cga | R | 11 | 54.97 |
| cgg | R | 1 | 47.73 |
| aga | R | 463 | 257.20 |
| agg | R | 97 | 160.30 |
| --- | -- | ------ | ------ |
| mPD | | 0.33 | 0.96 |
| | | nPD: | 0.34 |
| | | N. weight: | 0.52 |
| | | Sc. PD: | 0.033 |
| | | Sc. rank: | 49.7 |

| PB2 | | | |
|---|---|---|---|
| Pos. | 71 | obs: | exp: |
| aat | N | 566 | 298.40 |
| aac | N | 5 | 272.60 |
| gat | D | 1 | 0.53 |
| gac | D | 0 | 0.47 |
| --- | -- | ------ | ------ |
| mPD | | 0.021 | 0.50 |
| | | nPD: | 0.04 |
| | | N. weight: | 0.86 |
| | | Sc. PD: | -0.19 |
| | | Sc. rank: | -196.9 |

| PB2 | | | |
|---|---|---|---|
| Pos. | 72 | obs: | exp: |
| gaa | E | 128 | 329.10 |
| gag | E | 444 | 242.90 |
| --- | -- | ------ | ------ |
| mPD | | 0.35 | 0.49 |
| | | nPD: | 0.71 |
| | | N. weight: | 0.38 |
| | | Sc. PD: | 0.16 |
| | | Sc. rank: | 124.2 |

| PB2 | | | |
|---|---|---|---|
| Pos. | 73 | obs: | exp: |
| caa | Q | 512 | 304.40 |
| cag | Q | 59 | 266.60 |
| aaa | K | 1 | 0.56 |
| aag | K | 0 | 0.44 |
| --- | -- | ------ | ------ |
| mPD | | 0.19 | 0.50 |
| | | nPD: | 0.38 |
| | | N. weight: | 0.45 |
| | | Sc. PD: | 0.045 |
| | | Sc. rank: | 59.6 |

| PB2 | | | |
|---|---|---|---|
| Pos. | 74 | obs: | exp: |
| ggt | G | 401 | 70.58 |
| ggc | G | 71 | 77.35 |
| gga | G | 33 | 276.70 |
| ggg | G | 67 | 147.40 |
| --- | -- | ------ | ------ |
| mPD | | 0.48 | 0.67 |
| | | nPD: | 0.72 |
| | | N. weight: | 1.4 |
| | | Sc. PD: | 0.61 |
| | | Sc. rank: | 478.5 |

| PB2 | | | |
|---|---|---|---|
| Pos. | 75 | obs: | exp: |
| caa | Q | 146 | 304.90 |
| cag | Q | 426 | 267.10 |
| --- | -- | ------ | ------ |
| mPD | | 0.38 | 0.50 |
| | | nPD: | 0.77 |
| | | N. weight: | 0.24 |
| | | Sc. PD: | 0.11 |
| | | Sc. rank: | 79.9 |

PB2

| Pos. | 76 | obs: | exp: |
|---|---|---|---|
| att | I | 2 | 2.26 |
| atc | I | 4 | 1.57 |
| ata | I | 0 | 2.17 |
| act | T | 423 | 149.50 |
| acc | T | 98 | 121.50 |
| aca | T | 5 | 248.00 |
| acg | T | 40 | 47.05 |
| --- | -- | ------ | ------ |
| mPD | | 0.43 | 0.71 |
| | | nPD: | 0.61 |
| | | N. weight: | 1. |
| | | Sc. PD: | 0.33 |
| | | Sc. rank: | 296.7 |

PB2

| Pos. | 77 | obs: | exp: |
|---|---|---|---|
| tta | L | 0 | 49.97 |
| ttg | L | 0 | 110.20 |
| ctt | L | 429 | 106.70 |
| ctc | L | 137 | 92.53 |
| cta | L | 5 | 86.17 |
| ctg | L | 1 | 126.40 |
| --- | -- | ------ | ------ |
| mPD | | 0.38 | 1.1 |
| | | nPD: | 0.34 |
| | | N. weight: | 1.6 |
| | | Sc. PD: | 0.11 |
| | | Sc. rank: | 155.7 |

PB2

| Pos. | 78 | obs: | exp: |
|---|---|---|---|
| tgg | W | 572 | 572.00 |
| --- | -- | ------ | ------ |
| mPD | | 0 | 0 |
| | | nPD: | 1. |
| | | N. weight: | 0. |
| | | Sc. PD: | 0 |
| | | Sc. rank: | 0 |

PB2

| Pos. | 79 | obs: | exp: |
|---|---|---|---|
| tct | S | 0 | 69.31 |
| tcc | S | 0 | 60.74 |
| tca | S | 0 | 96.59 |
| tcg | S | 0 | 26.58 |
| cgt | R | 0 | 0.04 |
| cgc | R | 0 | 0.05 |
| cga | R | 0 | 0.10 |
| cgg | R | 0 | 0.08 |
| att | I | 1 | 50.00 |
| atc | I | 132 | 34.86 |
| ata | I | 0 | 48.14 |
| aat | N | 0 | 1.05 |
| aac | N | 2 | 0.95 |
| agt | S | 5 | 95.92 |
| agc | S | 431 | 86.86 |
| aga | R | 1 | 0.45 |
| agg | R | 0 | 0.28 |
| --- | -- | ------ | ------ |
| mPD | | 0.39 | 1.8 |
| | | nPD: | 0.21 |
| | | N. weight: | 2.1 |
| | | Sc. PD: | -0.12 |
| | | Sc. rank: | -59.2 |

PB2

| Pos. | 80 | obs: | exp: |
|---|---|---|---|
| cgt | R | 0 | 0.04 |
| cgc | R | 0 | 0.05 |
| cga | R | 0 | 0.10 |
| cgg | R | 0 | 0.08 |
| aaa | K | 505 | 319.70 |
| aag | K | 66 | 251.30 |
| aga | R | 1 | 0.45 |
| agg | R | 0 | 0.28 |
| --- | -- | ------ | ------ |
| mPD | | 0.21 | 0.50 |
| | | nPD: | 0.42 |
| | | N. weight: | 0.37 |
| | | Sc. PD: | 0.051 |
| | | Sc. rank: | 60.2 |

PB2

| Pos. | 81 | obs: | exp: |
|---|---|---|---|
| act | T | 0 | 151.10 |
| acc | T | 0 | 122.70 |
| aca | T | 563 | 250.60 |
| acg | T | 9 | 47.55 |
| --- | -- | ------ | ------ |
| mPD | | 0.031 | 0.69 |
| | | nPD: | 0.05 |
| | | N. weight: | 1.1 |
| | | Sc. PD: | -0.24 |
| | | Sc. rank: | -246.1 |

PB2

| Pos. | 82 | obs: | exp: |
|---|---|---|---|
| tct | S | 0 | 0.16 |
| tcc | S | 0 | 0.14 |
| tca | S | 0 | 0.22 |
| tcg | S | 0 | 0.06 |
| aat | N | 512 | 298.40 |
| aac | N | 59 | 272.60 |
| agt | S | 0 | 0.22 |
| agc | S | 1 | 0.20 |
| --- | -- | ------ | ------ |
| mPD | | 0.19 | 0.50 |
| | | nPD: | 0.38 |
| | | N. weight: | 0.48 |
| | | Sc. PD: | 0.049 |
| | | Sc. rank: | 63.9 |

PB2

| Pos. | 83 | obs: | exp: |
|---|---|---|---|
| gtt | V | 1 | 0.21 |
| gtc | V | 0 | 0.21 |
| gta | V | 0 | 0.19 |
| gtg | V | 0 | 0.40 |
| gat | D | 546 | 304.70 |
| gac | D | 25 | 266.30 |
| --- | -- | ------ | ------ |
| mPD | | 0.087 | 0.50 |
| | | nPD: | 0.17 |
| | | N. weight: | 0.66 |
| | | Sc. PD: | -0.062 |
| | | Sc. rank: | -45.9 |

PB2

| Pos. | 84 | obs: | exp: |
|---|---|---|---|
| gct | A | 470 | 144.10 |
| gcc | A | 95 | 117.20 |
| gca | A | 7 | 254.90 |
| gcg | A | 0 | 55.82 |
| --- | -- | ------ | ------ |
| mPD | | 0.30 | 0.69 |
| | | nPD: | 0.43 |
| | | N. weight: | 1.3 |
| | | Sc. PD: | 0.2 |
| | | Sc. rank: | 230.9 |

PB2

| Pos. | 85 | obs: | exp: |
|---|---|---|---|
| ggt | G | 1 | 70.58 |
| ggc | G | 20 | 77.35 |
| gga | G | 507 | 276.70 |
| ggg | G | 44 | 147.40 |
| --- | -- | ------ | ------ |
| mPD | | 0.21 | 0.67 |
| | | nPD: | 0.31 |
| | | N. weight: | 0.57 |
| | | Sc. PD: | 0.022 |
| | | Sc. rank: | 44.2 |

PB2

| Pos. | 86 | obs: | exp: |
|---|---|---|---|
| tct | S | 3 | 90.93 |
| tcc | S | 1 | 79.68 |
| tca | S | 519 | 126.70 |
| tcg | S | 49 | 34.87 |
| agt | S | 0 | 125.80 |
| agc | S | 0 | 114.00 |
| --- | -- | ------ | ------ |
| mPD | | 0.17 | 1.7 |
| | | nPD: | 0.1 |
| | | N. weight: | 1.8 |
| | | Sc. PD: | -0.3 |
| | | Sc. rank: | -290.9 |

PB2

| Pos. | 87 | obs: | exp: |
|---|---|---|---|
| gat | D | 4 | 304.70 |
| gac | D | 567 | 266.30 |
| gaa | E | 1 | 0.58 |
| gag | E | 0 | 0.42 |
| --- | -- | ------ | ------ |
| mPD | | 0.017 | 0.50 |
| | | nPD: | 0.03 |
| | | N. weight: | 1. |
| | | Sc. PD: | -0.23 |
| | | Sc. rank: | -247.7 |

PB2

| Pos. | 88 | obs: | exp: |
|---|---|---|---|
| cgt | R | 0 | 23.75 |
| cgc | R | 1 | 28.01 |
| cga | R | 66 | 54.97 |
| cgg | R | 5 | 47.73 |
| aga | R | 446 | 257.20 |
| agg | R | 54 | 160.30 |
| --- | -- | ------ | ------ |
| mPD | | 0.41 | 0.96 |
| | | nPD: | 0.43 |
| | | N. weight: | 0.48 |
| | | Sc. PD: | 0.071 |
| | | Sc. rank: | 84.6 |

PB2

| Pos. | 89 | obs: | exp: |
|---|---|---|---|
| tta | L | 0 | 0.17 |
| ttg | L | 1 | 0.39 |
| ctt | L | 0 | 0.37 |
| ctc | L | 0 | 0.32 |
| cta | L | 0 | 0.30 |
| ctg | L | 1 | 0.44 |
| gtt | V | 18 | 117.20 |
| gtc | V | 0 | 119.60 |
| gta | V | 12 | 106.80 |
| gtg | V | 540 | 226.40 |
| --- | -- | ------ | ------ |
| mPD | | 0.11 | 0.73 |
| | | nPD: | 0.15 |
| | | N. weight: | 1. |
| | | Sc. PD: | -0.12 |
| | | Sc. rank: | -94.5 |

PB2

| Pos. | 90 | obs: | exp: |
|---|---|---|---|
| att | I | 1 | 0.75 |
| atc | I | 0 | 0.52 |
| ata | I | 1 | 0.72 |
| atg | M | 570 | 570.00 |
| --- | -- | ------ | ------ |
| mPD | | 0.0070 | 0.0070 |
| | | nPD: | 1. |
| | | N. weight: | 0.0033 |
| | | Sc. PD: | 0.0023 |
| | | Sc. rank: | 1.1 |

PB2

| Pos. | 91 | obs: | exp: |
|---|---|---|---|
| att | I | 0 | 0.38 |
| atc | I | 0 | 0.26 |
| ata | I | 1 | 0.36 |
| gtt | V | 6 | 117.40 |
| gtc | V | 4 | 119.80 |
| gta | V | 92 | 107.00 |
| gtg | V | 469 | 226.80 |
| --- | -- | ------ | ------ |
| mPD | | 0.31 | 0.72 |
| | | nPD: | 0.42 |
| | | N. weight: | 0.76 |
| | | Sc. PD: | 0.11 |
| | | Sc. rank: | 127.8 |

PB2

| Pos. | 92 | obs: | exp: |
|---|---|---|---|
| tct | S | 25 | 90.78 |
| tcc | S | 0 | 79.54 |
| tca | S | 494 | 126.50 |
| tcg | S | 52 | 34.80 |
| agt | S | 0 | 125.60 |
| agc | S | 0 | 113.80 |
| gct | A | 0 | 0.25 |
| gcc | A | 0 | 0.20 |
| gca | A | 1 | 0.45 |
| gcg | A | 0 | 0.10 |
| --- | -- | ------ | ------ |
| mPD | | 0.24 | 1.7 |
| | | nPD: | 0.15 |
| | | N. weight: | 1.7 |
| | | Sc. PD: | -0.2 |
| | | Sc. rank: | -155.6 |

PB2

| Pos. | 93 | obs: | exp: |
|---|---|---|---|
| tct | S | 68 | 10.81 |
| tcc | S | 0 | 9.47 |
| tca | S | 0 | 15.06 |
| tcg | S | 0 | 4.14 |
| cct | P | 435 | 132.70 |
| ccc | P | 67 | 90.91 |
| cca | P | 2 | 198.90 |
| ccg | P | 0 | 81.49 |
| agt | S | 0 | 14.96 |
| agc | S | 0 | 13.55 |
| --- | -- | ------ | ------ |
| mPD | | 0.42 | 1.0 |
| | | nPD: | 0.41 |
| | | N. weight: | 1.5 |
| | | Sc. PD: | 0.2 |
| | | Sc. rank: | 241.0 |

PB2

| Pos. | 94 | obs: | exp: |
|---|---|---|---|
| ttt | F | 1 | 0.41 |
| ttc | F | 0 | 0.59 |
| tta | L | 0 | 49.88 |
| ttg | L | 1 | 110.00 |
| ctt | L | 1 | 106.50 |
| ctc | L | 0 | 92.36 |
| cta | L | 33 | 86.02 |
| ctg | L | 536 | 126.20 |
| --- | -- | ------ | ------ |
| mPD | | 0.12 | 1.1 |
| | | nPD: | 0.11 |
| | | N. weight: | 1.8 |
| | | Sc. PD: | -0.29 |
| | | Sc. rank: | -273.1 |

PB2

| Pos. | 95 | obs: | exp: |
|---|---|---|---|
| gct | A | 544 | 144.10 |
| gcc | A | 8 | 117.20 |
| gca | A | 18 | 254.90 |
| gcg | A | 2 | 55.82 |
| --- | -- | ------ | ------ |
| mPD | | 0.094 | 0.69 |
| | | nPD: | 0.14 |
| | | N. weight: | 1.6 |
| | | Sc. PD: | -0.21 |
| | | Sc. rank: | -180.1 |

PB2

| Pos. | 96 | obs: | exp: |
|---|---|---|---|
| att | I | 0 | 0.38 |
| atc | I | 0 | 0.26 |
| ata | I | 1 | 0.36 |
| gtt | V | 0 | 117.40 |
| gtc | V | 0 | 119.80 |
| gta | V | 28 | 107.00 |
| gtg | V | 543 | 226.80 |
| --- | -- | ------ | ------ |
| mPD | | 0.10 | 0.72 |
| | | nPD: | 0.14 |
| | | N. weight: | 1.1 |
| | | Sc. PD: | -0.14 |
| | | Sc. rank: | -120.3 |

PB2

| Pos. | 97 | obs: | exp: |
|---|---|---|---|
| cct | P | 0 | 0.26 |
| ccc | P | 0 | 0.18 |
| cca | P | 1 | 0.39 |
| ccg | P | 0 | 0.16 |
| act | T | 24 | 150.90 |
| acc | T | 1 | 122.50 |
| aca | T | 166 | 250.20 |
| acg | T | 380 | 47.47 |
| --- | -- | ------ | ------ |
| mPD | | 0.48 | 0.69 |
| | | nPD: | 0.69 |
| | | N. weight: | 1.7 |
| | | Sc. PD: | 0.68 |
| | | Sc. rank: | 550.0 |

PB2

| Pos. | 98 | obs: | exp: |
|---|---|---|---|
| tgg | W | 572 | 572.00 |
| --- | -- | ------ | ------ |
| mPD | | 0 | 0 |
| | | nPD: | 1. |
| | | N. weight: | 0. |
| | | Sc. PD: | 0 |
| | | Sc. rank: | 0 |

PB2

| Pos. | 99 | obs: | exp: |
|---|---|---|---|
| tgg | W | 572 | 572.00 |
| --- | -- | ------ | ------ |
| mPD | | 0 | 0 |
| | | nPD: | 1. |
| | | N. weight: | 0. |
| | | Sc. PD: | 0 |
| | | Sc. rank: | 0 |

PB2

| Pos. | 100 | obs: | exp: |
|---|---|---|---|
| aat | N | 458 | 299.00 |
| aac | N | 114 | 273.00 |
| --- | -- | ------ | ------ |
| mPD | | 0.32 | 0.50 |
| | | nPD: | 0.64 |
| | | N. weight: | 0.25 |
| | | Sc. PD: | 0.087 |
| | | Sc. rank: | 74.0 |

| | | | |
|---|---|---|---|
| PB2 | | | |
| Pos. | 101 | obs: | exp: |
| cgt | R | 0 | 23.75 |
| cgc | R | 0 | 28.01 |
| cga | R | 0 | 54.97 |
| cgg | R | 0 | 47.73 |
| aga | R | 439 | 257.20 |
| agg | R | 133 | 160.30 |
| --- | -- | ------ | ------ |
| mPD | | 0.36 | 0.96 |
| | | nPD: | 0.37 |
| | | N. weight: | 0.53 |
| | | Sc. PD: | 0.051 |
| | | Sc. rank: | 67.8 |

,

| | | | |
|---|---|---|---|
| PB2 | | | |
| Pos. | 102 | obs: | exp: |
| aat | N | 552 | 299.00 |
| aac | N | 20 | 273.00 |
| --- | -- | ------ | ------ |
| mPD | | 0.068 | 0.50 |
| | | nPD: | 0.14 |
| | | N. weight: | 0.72 |
| | | Sc. PD: | -0.094 |
| | | Sc. rank: | -86.9 |

,

| | | | |
|---|---|---|---|
| PB2 | | | |
| Pos. | 103 | obs: | exp: |
| ggt | G | 0 | 70.58 |
| ggc | G | 1 | 77.35 |
| gga | G | 457 | 276.70 |
| ggg | G | 114 | 147.40 |
| --- | -- | ------ | ------ |
| mPD | | 0.32 | 0.67 |
| | | nPD: | 0.48 |
| | | N. weight: | 0.5 |
| | | Sc. PD: | 0.1 |
| | | Sc. rank: | 107.3 |

,

| | | | |
|---|---|---|---|
| PB2 | | | |
| Pos. | 104 | obs: | exp: |
| cct | P | 6 | 150.60 |
| ccc | P | 0 | 103.20 |
| cca | P | 489 | 225.70 |
| ccg | P | 77 | 92.49 |
| --- | -- | ------ | ------ |
| mPD | | 0.25 | 0.72 |
| | | nPD: | 0.35 |
| | | N. weight: | 0.87 |
| | | Sc. PD: | 0.066 |
| | | Sc. rank: | 94.3 |

,

| | | | |
|---|---|---|---|
| PB2 | | | |
| Pos. | 105 | obs: | exp: |
| atg | M | 4 | 4.00 |
| act | T | 0 | 149.30 |
| acc | T | 6 | 121.20 |
| aca | T | 136 | 247.50 |
| acg | T | 423 | 46.97 |
| aaa | K | 0 | 0.56 |
| aag | K | 1 | 0.44 |
| gct | A | 0 | 0.50 |
| gcc | A | 0 | 0.41 |
| gca | A | 0 | 0.89 |
| gcg | A | 2 | 0.20 |
| --- | -- | ------ | ------ |
| mPD | | 0.40 | 0.71 |
| | | nPD: | 0.57 |
| | | N. weight: | 2.1 |
| | | Sc. PD: | 0.59 |
| | | Sc. rank: | 571.7 |

,

| | | | |
|---|---|---|---|
| PB2 | | | |
| Pos. | 106 | obs: | exp: |
| act | T | 19 | 143.50 |
| acc | T | 0 | 116.50 |
| aca | T | 518 | 237.90 |
| acg | T | 6 | 45.14 |
| gct | A | 1 | 7.30 |
| gcc | A | 0 | 5.94 |
| gca | A | 28 | 12.93 |
| gcg | A | 0 | 2.83 |
| --- | -- | ------ | ------ |
| mPD | | 0.18 | 0.78 |
| | | nPD: | 0.24 |
| | | N. weight: | 0.94 |
| | | Sc. PD: | -0.033 |
| | | Sc. rank: | -3.1 |

,

| | | | |
|---|---|---|---|
| PB2 | | | |
| Pos. | 107 | obs: | exp: |
| tct | S | 0 | 90.62 |
| tcc | S | 0 | 79.40 |
| tca | S | 0 | 126.30 |
| tcg | S | 0 | 34.74 |
| aat | N | 2 | 1.05 |
| aac | N | 0 | 0.95 |
| agt | S | 551 | 125.40 |
| agc | S | 19 | 113.60 |
| --- | -- | ------ | ------ |
| mPD | | 0.071 | 1.7 |
| | | nPD: | 0.04 |
| | | N. weight: | 2. |
| | | Sc. PD: | -0.43 |
| | | Sc. rank: | -442.0 |

,

| | | | |
|---|---|---|---|
| PB2 | | | |
| Pos. | 108 | obs: | exp: |
| act | T | 1 | 151.10 |
| acc | T | 4 | 122.70 |
| aca | T | 544 | 250.60 |
| acg | T | 23 | 47.55 |
| --- | -- | ------ | ------ |
| mPD | | 0.094 | 0.69 |
| | | nPD: | 0.14 |
| | | N. weight: | 0.98 |
| | | Sc. PD: | -0.13 |
| | | Sc. rank: | -110.9 |

,

| | | | |
|---|---|---|---|
| PB2 | | | |
| Pos. | 109 | obs: | exp: |
| att | I | 7 | 3.01 |
| atc | I | 1 | 2.10 |
| ata | I | 0 | 2.90 |
| gtt | V | 311 | 115.70 |
| gtc | V | 250 | 118.20 |
| gta | V | 2 | 105.50 |
| gtg | V | 0 | 223.60 |
| gct | A | 1 | 0.25 |
| gcc | A | 0 | 0.20 |
| gca | A | 0 | 0.45 |
| gcg | A | 0 | 0.10 |
| --- | -- | ------ | ------ |
| mPD | | 0.53 | 0.75 |
| | | nPD: | 0.7 |
| | | N. weight: | 1.2 |
| | | Sc. PD: | 0.51 |
| | | Sc. rank: | 411.0 |

,

| | | | |
|---|---|---|---|
| PB2 | | | |
| Pos. | 110 | obs: | exp: |
| cat | H | 471 | 310.00 |
| cac | H | 101 | 262.00 |
| --- | -- | ------ | ------ |
| mPD | | 0.29 | 0.50 |
| | | nPD: | 0.59 |
| | | N. weight: | 0.26 |
| | | Sc. PD: | 0.078 |
| | | Sc. rank: | 73.6 |

,

| | | | |
|---|---|---|---|
| PB2 | | | |
| Pos. | 111 | obs: | exp: |
| tat | Y | 506 | 296.10 |
| tac | Y | 66 | 275.90 |
| --- | -- | ------ | ------ |
| mPD | | 0.20 | 0.50 |
| | | nPD: | 0.41 |
| | | N. weight: | 0.45 |
| | | Sc. PD: | 0.059 |
| | | Sc. rank: | 69.5 |

,

| | | | |
|---|---|---|---|
| PB2 | | | |
| Pos. | 112 | obs: | exp: |
| tct | S | 0 | 0.16 |
| tcc | S | 0 | 0.14 |
| tca | S | 1 | 0.22 |
| tcg | S | 0 | 0.06 |
| cct | P | 0 | 150.40 |
| ccc | P | 27 | 103.00 |
| cca | P | 524 | 225.30 |
| ccg | P | 20 | 92.32 |
| agt | S | 0 | 0.22 |
| agc | S | 0 | 0.20 |
| --- | -- | ------ | ------ |
| mPD | | 0.16 | 0.72 |
| | | nPD: | 0.22 |
| | | N. weight: | 0.95 |
| | | Sc. PD: | -0.048 |
| | | Sc. rank: | -20.0 |

,

| | | | |
|---|---|---|---|
| PB2 | | | |
| Pos. | 113 | obs: | exp: |
| aaa | K | 7 | 320.30 |
| aag | K | 565 | 251.70 |
| --- | -- | ------ | ------ |
| mPD | | 0.024 | 0.49 |
| | | nPD: | 0.05 |
| | | N. weight: | 1.1 |
| | | Sc. PD: | -0.23 |
| | | Sc. rank: | -234.1 |

,

| | | | |
|---|---|---|---|
| PB2 | | | |
| Pos. | 114 | obs: | exp: |
| att | I | 0 | 0.75 |
| atc | I | 2 | 0.52 |
| ata | I | 0 | 0.72 |
| gtt | V | 52 | 117.20 |
| gtc | V | 420 | 119.60 |
| gta | V | 94 | 106.80 |
| gtg | V | 4 | 226.40 |
| --- | -- | ------ | ------ |
| mPD | | 0.43 | 0.73 |
| | | nPD: | 0.59 |
| | | N. weight: | 1.2 |
| | | Sc. PD: | 0.35 |
| | | Sc. rank: | 332.7 |

,

| | | | |
|---|---|---|---|
| PB2 | | | |
| Pos. | 115 | obs: | exp: |
| tat | Y | 412 | 295.60 |
| tac | Y | 159 | 275.40 |
| aat | N | 0 | 0.52 |
| aac | N | 1 | 0.48 |
| --- | -- | ------ | ------ |
| mPD | | 0.41 | 0.50 |
| | | nPD: | 0.81 |
| | | N. weight: | 0.13 |
| | | Sc. PD: | 0.069 |
| | | Sc. rank: | 45.6 |

,

| | | | |
|---|---|---|---|
| PB2 | | | |
| Pos. | 116 | obs: | exp: |
| aaa | K | 568 | 320.30 |
| aag | K | 4 | 251.70 |
| --- | -- | ------ | ------ |
| mPD | | 0.014 | 0.49 |
| | | nPD: | 0.03 |
| | | N. weight: | 0.78 |
| | | Sc. PD: | -0.18 |
| | | Sc. rank: | -195.2 |

,

| | | | |
|---|---|---|---|
| PB2 | | | |
| Pos. | 117 | obs: | exp: |
| act | T | 73 | 150.90 |
| acc | T | 472 | 122.50 |
| aca | T | 25 | 250.20 |
| acg | T | 1 | 47.47 |
| gct | A | 0 | 0.25 |
| gcc | A | 0 | 0.20 |
| gca | A | 1 | 0.45 |
| gcg | A | 0 | 0.10 |
| --- | -- | ------ | ------ |
| mPD | | 0.30 | 0.69 |
| | | nPD: | 0.44 |
| | | N. weight: | 1.3 |
| | | Sc. PD: | 0.22 |
| | | Sc. rank: | 246.2 |

,

| | | | |
|---|---|---|---|
| PB2 | | | |
| Pos. | 118 | obs: | exp: |
| tat | Y | 184 | 296.10 |
| tac | Y | 388 | 275.90 |
| --- | -- | ------ | ------ |
| mPD | | 0.44 | 0.50 |
| | | nPD: | 0.88 |
| | | N. weight: | 0.12 |
| | | Sc. PD: | 0.069 |
| | | Sc. rank: | 41.4 |

,

| | | | |
|---|---|---|---|
| PB2 | | | |
| Pos. | 119 | obs: | exp: |
| ttt | F | 487 | 235.20 |
| ttc | F | 85 | 336.80 |
| --- | -- | ------ | ------ |
| mPD | | 0.25 | 0.48 |
| | | nPD: | 0.52 |
| | | N. weight: | 0.6 |
| | | Sc. PD: | 0.14 |
| | | Sc. rank: | 148.8 |

,

| | | | |
|---|---|---|---|
| PB2 | | | |
| Pos. | 120 | obs: | exp: |
| gaa | E | 527 | 329.10 |
| gag | E | 45 | 242.90 |
| --- | -- | ------ | ------ |
| mPD | | 0.15 | 0.49 |
| | | nPD: | 0.3 |
| | | N. weight: | 0.44 |
| | | Sc. PD: | 0.011 |
| | | Sc. rank: | 28.4 |

,

| | | | |
|---|---|---|---|
| PB2 | | | |
| Pos. | 121 | obs: | exp: |
| aaa | K | 133 | 320.30 |
| aag | K | 439 | 251.70 |
| --- | -- | ------ | ------ |
| mPD | | 0.36 | 0.49 |
| | | nPD: | 0.73 |
| | | N. weight: | 0.33 |
| | | Sc. PD: | 0.14 |
| | | Sc. rank: | 109.1 |

,

| | | | |
|---|---|---|---|
| PB2 | | | |
| Pos. | 122 | obs: | exp: |
| att | I | 2 | 17.67 |
| atc | I | 45 | 12.32 |
| ata | I | 0 | 17.01 |
| gtt | V | 173 | 107.70 |
| gtc | V | 349 | 110.00 |
| gta | V | 2 | 98.21 |
| gtg | V | 0 | 208.10 |
| gct | A | 1 | 0.25 |
| gcc | A | 0 | 0.20 |
| gca | A | 0 | 0.45 |
| gcg | A | 0 | 0.10 |
| --- | -- | ------ | ------ |
| mPD | | 0.59 | 0.89 |
| | | nPD: | 0.66 |
| | | N. weight: | 1.3 |
| | | Sc. PD: | 0.5 |
| | | Sc. rank: | 413.0 |

,

| | | | |
|---|---|---|---|
| PB2 | | | |
| Pos. | 123 | obs: | exp: |
| caa | Q | 1 | 0.53 |
| cag | Q | 0 | 0.47 |
| aaa | K | 5 | 2.80 |
| aag | K | 0 | 2.20 |
| gat | D | 0 | 0.53 |
| gac | D | 1 | 0.47 |
| gaa | E | 540 | 325.10 |
| gag | E | 25 | 239.90 |
| --- | -- | ------ | ------ |
| mPD | | 0.11 | 0.51 |
| | | nPD: | 0.21 |
| | | N. weight: | 0.56 |
| | | Sc. PD: | -0.032 |
| | | Sc. rank: | -18.5 |

,

| | | | |
|---|---|---|---|
| PB2 | | | |
| Pos. | 124 | obs: | exp: |
| cgt | R | 0 | 23.75 |
| cgc | R | 0 | 28.01 |
| cga | R | 0 | 54.97 |
| cgg | R | 0 | 47.73 |
| aga | R | 22 | 257.20 |
| agg | R | 550 | 160.30 |
| --- | -- | ------ | ------ |
| mPD | | 0.074 | 0.96 |
| | | nPD: | 0.08 |
| | | N. weight: | 1.6 |
| | | Sc. PD: | -0.29 |
| | | Sc. rank: | -283.8 |

,

| | | | |
|---|---|---|---|
| PB2 | | | |
| Pos. | 125 | obs: | exp: |
| tta | L | 384 | 49.97 |
| ttg | L | 159 | 110.20 |
| ctt | L | 0 | 106.70 |
| ctc | L | 0 | 92.53 |
| cta | L | 27 | 86.17 |
| ctg | L | 2 | 126.40 |
| --- | -- | ------ | ------ |
| mPD | | 0.50 | 1.1 |
| | | nPD: | 0.45 |
| | | N. weight: | 2. |
| | | Sc. PD: | 0.34 |
| | | Sc. rank: | 392.5 |

,

| PB2 | | | |
|---|---|---|---|
| Pos. | 126 | obs: | exp: |
| aaa | K | 280 | 320.30 |
| aag | K | 292 | 251.70 |
| --- | -- | ------ | ------ |
| mPD | | 0.50 | 0.49 |
| | | nPD: | 1.02 |
| | | N. weight: | 0.023 |
| | | Sc. PD: | 0.016 |
| | | Sc. rank: | 7.9 |

| PB2 | | | |
|---|---|---|---|
| Pos. | 127 | obs: | exp: |
| tat | Y | 3 | 1.55 |
| tac | Y | 0 | 1.45 |
| cat | H | 485 | 307.20 |
| cac | H | 82 | 259.80 |
| caa | Q | 2 | 1.07 |
| cag | Q | 0 | 0.93 |
| --- | -- | ------ | ------ |
| mPD | | 0.26 | 0.51 |
| | | nPD: | 0.51 |
| | | N. weight: | 0.33 |
| | | Sc. PD: | 0.077 |
| | | Sc. rank: | 79.8 |

| PB2 | | | |
|---|---|---|---|
| Pos. | 128 | obs: | exp: |
| ggt | G | 1 | 70.58 |
| ggc | G | 0 | 77.35 |
| gga | G | 502 | 276.70 |
| ggg | G | 69 | 147.40 |
| --- | -- | ------ | ------ |
| mPD | | 0.22 | 0.67 |
| | | nPD: | 0.32 |
| | | N. weight: | 0.62 |
| | | Sc. PD: | 0.031 |
| | | Sc. rank: | 52.8 |

| PB2 | | | |
|---|---|---|---|
| Pos. | 129 | obs: | exp: |
| act | T | 20 | 151.10 |
| acc | T | 547 | 122.70 |
| aca | T | 5 | 250.60 |
| acg | T | 0 | 47.55 |
| --- | -- | ------ | ------ |
| mPD | | 0.084 | 0.69 |
| | | nPD: | 0.12 |
| | | N. weight: | 1.9 |
| | | Sc. PD: | -0.27 |
| | | Sc. rank: | -260.4 |

| PB2 | | | |
|---|---|---|---|
| Pos. | 130 | obs: | exp: |
| ttt | F | 178 | 235.20 |
| ttc | F | 394 | 336.80 |
| --- | -- | ------ | ------ |
| mPD | | 0.43 | 0.48 |
| | | nPD: | 0.89 |
| | | N. weight: | 0.039 |
| | | Sc. PD: | 0.023 |
| | | Sc. rank: | 13.4 |

| PB2 | | | |
|---|---|---|---|
| Pos. | 131 | obs: | exp: |
| ggt | G | 395 | 70.58 |
| ggc | G | 177 | 77.35 |
| gga | G | 0 | 276.70 |
| ggg | G | 0 | 147.40 |
| --- | -- | ------ | ------ |
| mPD | | 0.43 | 0.67 |
| | | nPD: | 0.64 |
| | | N. weight: | 2.1 |
| | | Sc. PD: | 0.73 |
| | | Sc. rank: | 625.3 |

| PB2 | | | |
|---|---|---|---|
| Pos. | 132 | obs: | exp: |
| cct | P | 253 | 150.40 |
| ccc | P | 316 | 103.00 |
| cca | P | 2 | 225.30 |
| ccg | P | 0 | 92.32 |
| cat | H | 1 | 0.54 |
| cac | H | 0 | 0.46 |
| --- | -- | ------ | ------ |
| mPD | | 0.50 | 0.72 |
| | | nPD: | 0.7 |
| | | N. weight: | 1.2 |
| | | Sc. PD: | 0.49 |
| | | Sc. rank: | 394.1 |

| PB2 | | | |
|---|---|---|---|
| Pos. | 133 | obs: | exp: |
| att | I | 0 | 0.38 |
| atc | I | 1 | 0.26 |
| ata | I | 0 | 0.36 |
| gtt | V | 434 | 117.40 |
| gtc | V | 92 | 119.80 |
| gta | V | 17 | 107.00 |
| gtg | V | 28 | 226.80 |
| --- | -- | ------ | ------ |
| mPD | | 0.40 | 0.72 |
| | | nPD: | 0.55 |
| | | N. weight: | 1.2 |
| | | Sc. PD: | 0.31 |
| | | Sc. rank: | 304.6 |

| PB2 | | | |
|---|---|---|---|
| Pos. | 134 | obs: | exp: |
| cat | H | 82 | 308.90 |
| cac | H | 488 | 261.10 |
| cgt | R | 2 | 0.08 |
| cgc | R | 0 | 0.10 |
| cga | R | 0 | 0.19 |
| cgg | R | 0 | 0.17 |
| aga | R | 0 | 0.90 |
| agg | R | 0 | 0.56 |
| --- | -- | ------ | ------ |
| mPD | | 0.26 | 0.51 |
| | | nPD: | 0.5 |
| | | N. weight: | 0.51 |
| | | Sc. PD: | 0.11 |
| | | Sc. rank: | 119.8 |

| PB2 | | | |
|---|---|---|---|
| Pos. | 135 | obs: | exp: |
| ttt | F | 444 | 235.20 |
| ttc | F | 128 | 336.80 |
| --- | -- | ------ | ------ |
| mPD | | 0.35 | 0.48 |
| | | nPD: | 0.72 |
| | | N. weight: | 0.4 |
| | | Sc. PD: | 0.17 |
| | | Sc. rank: | 134.3 |

| PB2 | | | |
|---|---|---|---|
| Pos. | 136 | obs: | exp: |
| cgt | R | 0 | 23.75 |
| cgc | R | 0 | 28.01 |
| cga | R | 447 | 54.97 |
| cgg | R | 53 | 47.73 |
| aga | R | 68 | 257.20 |
| agg | R | 4 | 160.30 |
| --- | -- | ------ | ------ |
| mPD | | 0.40 | 0.96 |
| | | nPD: | 0.42 |
| | | N. weight: | 2.1 |
| | | Sc. PD: | 0.29 |
| | | Sc. rank: | 346.4 |

| PB2 | | | |
|---|---|---|---|
| Pos. | 137 | obs: | exp: |
| tct | S | 0 | 0.16 |
| tcc | S | 0 | 0.14 |
| tca | S | 0 | 0.22 |
| tcg | S | 0 | 0.06 |
| aat | N | 495 | 298.40 |
| aac | N | 76 | 272.60 |
| agt | S | 1 | 0.22 |
| agc | S | 0 | 0.20 |
| --- | -- | ------ | ------ |
| mPD | | 0.23 | 0.50 |
| | | nPD: | 0.46 |
| | | N. weight: | 0.39 |
| | | Sc. PD: | 0.072 |
| | | Sc. rank: | 80.2 |

| PB2 | | | |
|---|---|---|---|
| Pos. | 138 | obs: | exp: |
| caa | Q | 132 | 304.90 |
| cag | Q | 440 | 267.10 |
| --- | -- | ------ | ------ |
| mPD | | 0.36 | 0.50 |
| | | nPD: | 0.71 |
| | | N. weight: | 0.28 |
| | | Sc. PD: | 0.12 |
| | | Sc. rank: | 93.0 |

| PB2 | | | |
|---|---|---|---|
| Pos. | 139 | obs: | exp: |
| att | I | 5 | 1.88 |
| atc | I | 0 | 1.31 |
| ata | I | 0 | 1.81 |
| gtt | V | 514 | 116.50 |
| gtc | V | 52 | 119.00 |
| gta | V | 0 | 106.30 |
| gtg | V | 1 | 225.20 |
| --- | -- | ------ | ------ |
| mPD | | 0.19 | 0.74 |
| | | nPD: | 0.25 |
| | | N. weight: | 1.8 |
| | | Sc. PD: | -0.034 |
| | | Sc. rank: | 33.2 |

| PB2 | | | |
|---|---|---|---|
| Pos. | 140 | obs: | exp: |
| aaa | K | 445 | 320.30 |
| aag | K | 127 | 251.70 |
| --- | -- | ------ | ------ |
| mPD | | 0.35 | 0.49 |
| | | nPD: | 0.7 |
| | | N. weight: | 0.16 |
| | | Sc. PD: | 0.064 |
| | | Sc. rank: | 51.7 |

| PB2 | | | |
|---|---|---|---|
| Pos. | 141 | obs: | exp: |
| att | I | 0 | 214.70 |
| atc | I | 0 | 149.60 |
| ata | I | 571 | 206.70 |
| aaa | K | 1 | 0.56 |
| aag | K | 0 | 0.44 |
| --- | -- | ------ | ------ |
| mPD | | 0.0035 | 0.66 |
| | | nPD: | 0.01 |
| | | N. weight: | 1.5 |
| | | Sc. PD: | -0.37 |
| | | Sc. rank: | -446.8 |

| PB2 | | | |
|---|---|---|---|
| Pos. | 142 | obs: | exp: |
| cgt | R | 9 | 23.75 |
| cgc | R | 492 | 28.01 |
| cga | R | 0 | 54.97 |
| cgg | R | 0 | 47.73 |
| aga | R | 45 | 257.20 |
| agg | R | 26 | 160.30 |
| --- | -- | ------ | ------ |
| mPD | | 0.47 | 0.96 |
| | | nPD: | 0.49 |
| | | N. weight: | 3.2 |
| | | Sc. PD: | 0.67 |
| | | Sc. rank: | 706.5 |

| PB2 | | | |
|---|---|---|---|
| Pos. | 143 | obs: | exp: |
| cgt | R | 5 | 23.75 |
| cgc | R | 491 | 28.01 |
| cga | R | 4 | 54.97 |
| cgg | R | 68 | 47.73 |
| aga | R | 1 | 257.20 |
| agg | R | 3 | 160.30 |
| --- | -- | ------ | ------ |
| mPD | | 0.26 | 0.96 |
| | | nPD: | 0.27 |
| | | N. weight: | 3.5 |
| | | Sc. PD: | 0.0041 |
| | | Sc. rank: | 158.8 |

| PB2 | | | |
|---|---|---|---|
| Pos. | 144 | obs: | exp: |
| cgt | R | 1 | 23.75 |
| cgc | R | 0 | 28.01 |
| cga | R | 24 | 54.97 |
| cgg | R | 1 | 47.73 |
| aga | R | 53 | 257.20 |
| agg | R | 493 | 160.30 |
| --- | -- | ------ | ------ |
| mPD | | 0.32 | 0.96 |
| | | nPD: | 0.34 |
| | | N. weight: | 1.1 |
| | | Sc. PD: | 0.07 |
| | | Sc. rank: | 106.6 |

| PB2 | | | |
|---|---|---|---|
| Pos. | 145 | obs: | exp: |
| gtt | V | 470 | 117.60 |
| gtc | V | 84 | 120.10 |
| gta | V | 3 | 107.20 |
| gtg | V | 15 | 227.20 |
| --- | -- | ------ | ------ |
| mPD | | 0.30 | 0.72 |
| | | nPD: | 0.42 |
| | | N. weight: | 1.4 |
| | | Sc. PD: | 0.2 |
| | | Sc. rank: | 242.3 |

| PB2 | | | |
|---|---|---|---|
| Pos. | 146 | obs: | exp: |
| gat | D | 40 | 305.20 |
| gac | D | 532 | 266.80 |
| --- | -- | ------ | ------ |
| mPD | | 0.13 | 0.50 |
| | | nPD: | 0.26 |
| | | N. weight: | 0.72 |
| | | Sc. PD: | -0.0068 |
| | | Sc. rank: | 21.2 |

| PB2 | | | |
|---|---|---|---|
| Pos. | 147 | obs: | exp: |
| att | I | 0 | 208.60 |
| atc | I | 0 | 145.50 |
| ata | I | 555 | 200.90 |
| atg | M | 1 | 1.00 |
| act | T | 0 | 3.43 |
| acc | T | 0 | 2.79 |
| aca | T | 13 | 5.70 |
| acg | T | 0 | 1.08 |
| gtt | V | 0 | 0.62 |
| gtc | V | 0 | 0.63 |
| gta | V | 3 | 0.56 |
| gtg | V | 0 | 1.19 |
| --- | -- | ------ | ------ |
| mPD | | 0.058 | 0.72 |
| | | nPD: | 0.08 |
| | | N. weight: | 1.4 |
| | | Sc. PD: | -0.26 |
| | | Sc. rank: | -256.7 |

| PB2 | | | |
|---|---|---|---|
| Pos. | 148 | obs: | exp: |
| tct | S | 0 | 0.32 |
| tcc | S | 0 | 0.28 |
| tca | S | 0 | 0.44 |
| tcg | S | 0 | 0.12 |
| aat | N | 23 | 297.90 |
| aac | N | 547 | 272.10 |
| agt | S | 0 | 0.44 |
| agc | S | 2 | 0.40 |
| --- | -- | ------ | ------ |
| mPD | | 0.084 | 0.51 |
| | | nPD: | 0.16 |
| | | N. weight: | 0.82 |
| | | Sc. PD: | -0.084 |
| | | Sc. rank: | -65.3 |

| PB2 | | | |
|---|---|---|---|
| Pos. | 149 | obs: | exp: |
| cct | P | 29 | 150.60 |
| ccc | P | 1 | 103.20 |
| cca | P | 93 | 225.70 |
| ccg | P | 449 | 92.49 |
| --- | -- | ------ | ------ |
| mPD | | 0.36 | 0.72 |
| | | nPD: | 0.5 |
| | | N. weight: | 1.5 |
| | | Sc. PD: | 0.31 |
| | | Sc. rank: | 328.1 |

| PB2 | | | |
|---|---|---|---|
| Pos. | 150 | obs: | exp: |
| ggt | G | 41 | 70.58 |
| ggc | G | 531 | 77.35 |
| gga | G | 0 | 276.70 |
| ggg | G | 0 | 147.40 |
| --- | -- | ------ | ------ |
| mPD | | 0.13 | 0.67 |
| | | nPD: | 0.2 |
| | | N. weight: | 2.5 |
| | | Sc. PD: | -0.17 |
| | | Sc. rank: | -106.4 |

| PB2 | | | |
|---|---|---|---|
| Pos. | 151 | obs: | exp: |
| cat | H | 545 | 310.00 |
| cac | H | 27 | 262.00 |
| --- | -- | ------ | ------ |
| mPD | | 0.090 | 0.50 |
| | | nPD: | 0.18 |
| | | N. weight: | 0.62 |
| | | Sc. PD: | -0.053 |
| | | Sc. rank: | -38.7 |

| PB2 | | | |
|---|---|---|---|
| Pos. | 152 | obs: | exp: |
| tct | S | 0 | 0.48 |
| tcc | S | 0 | 0.42 |
| tca | S | 3 | 0.66 |
| tcg | S | 0 | 0.18 |
| act | T | 0 | 0.26 |
| acc | T | 0 | 0.21 |
| aca | T | 1 | 0.44 |
| acg | T | 0 | 0.08 |
| agt | S | 0 | 0.66 |
| agc | S | 0 | 0.60 |
| gct | A | 39 | 143.10 |
| gcc | A | 2 | 116.40 |
| gca | A | 518 | 253.20 |
| gcg | A | 9 | 55.43 |
| --- | -- | ------ | ------ |
| mPD | | 0.18 | 0.71 |
| | | nPD: | 0.25 |
| | | N. weight: | 0.77 |
| | | Sc. PD: | -0.016 |
| | | Sc. rank: | 11.3 |

| PB2 | | | |
|---|---|---|---|
| Pos. | 153 | obs: | exp: |
| gat | D | 449 | 305.20 |
| gac | D | 123 | 266.80 |
| --- | -- | ------ | ------ |
| mPD | | 0.34 | 0.50 |
| | | nPD: | 0.68 |
| | | N. weight: | 0.2 |
| | | Sc. PD: | 0.079 |
| | | Sc. rank: | 64.3 |

| PB2 | | | |
|---|---|---|---|
| Pos. | 154 | obs: | exp: |
| tta | L | 1 | 49.97 |
| ttg | L | 0 | 110.20 |
| ctt | L | 34 | 106.70 |
| ctc | L | 534 | 92.53 |
| cta | L | 2 | 86.17 |
| ctg | L | 1 | 126.40 |
| --- | -- | ------ | ------ |
| mPD | | 0.13 | 1.1 |
| | | nPD: | 0.12 |
| | | N. weight: | 2.2 |
| | | Sc. PD: | -0.33 |
| | | Sc. rank: | -315.5 |

| PB2 | | | |
|---|---|---|---|
| Pos. | 155 | obs: | exp: |
| tct | S | 0 | 90.14 |
| tcc | S | 0 | 78.99 |
| tca | S | 0 | 125.60 |
| tcg | S | 0 | 34.56 |
| aat | N | 5 | 2.61 |
| aac | N | 0 | 2.39 |
| agt | S | 524 | 124.70 |
| agc | S | 43 | 113.00 |
| --- | -- | ------ | ------ |
| mPD | | 0.16 | 1.7 |
| | | nPD: | 0.09 |
| | | N. weight: | 1.8 |
| | | Sc. PD: | -0.3 |
| | | Sc. rank: | -295.7 |

| PB2 | | | |
|---|---|---|---|
| Pos. | 156 | obs: | exp: |
| tct | S | 17 | 2.70 |
| tcc | S | 0 | 2.37 |
| tca | S | 0 | 3.77 |
| tcg | S | 0 | 1.04 |
| act | T | 0 | 0.53 |
| acc | T | 2 | 0.43 |
| aca | T | 0 | 0.88 |
| acg | T | 0 | 0.17 |
| agt | S | 0 | 3.74 |
| agc | S | 0 | 3.39 |
| gct | A | 423 | 139.30 |
| gcc | A | 127 | 113.30 |
| gca | A | 2 | 246.50 |
| gcg | A | 1 | 53.96 |
| --- | -- | ------ | ------ |
| mPD | | 0.42 | 0.78 |
| | | nPD: | 0.54 |
| | | N. weight: | 1.3 |
| | | Sc. PD: | 0.33 |
| | | Sc. rank: | 331.0 |

| PB2 | | | |
|---|---|---|---|
| Pos. | 157 | obs: | exp: |
| aaa | K | 544 | 320.30 |
| aag | K | 28 | 251.70 |
| --- | -- | ------ | ------ |
| mPD | | 0.093 | 0.49 |
| | | nPD: | 0.19 |
| | | N. weight: | 0.57 |
| | | Sc. PD: | -0.045 |
| | | Sc. rank: | -31.1 |

| PB2 | | | |
|---|---|---|---|
| Pos. | 158 | obs: | exp: |
| gaa | E | 492 | 329.10 |
| gag | E | 80 | 242.90 |
| --- | -- | ------ | ------ |
| mPD | | 0.24 | 0.49 |
| | | nPD: | 0.49 |
| | | N. weight: | 0.28 |
| | | Sc. PD: | 0.059 |
| | | Sc. rank: | 62.2 |

| PB2 | | | |
|---|---|---|---|
| Pos. | 159 | obs: | exp: |
| gct | A | 0 | 144.10 |
| gcc | A | 0 | 117.20 |
| gca | A | 521 | 254.90 |
| gcg | A | 51 | 55.82 |
| --- | -- | ------ | ------ |
| mPD | | 0.16 | 0.69 |
| | | nPD: | 0.24 |
| | | N. weight: | 0.93 |
| | | Sc. PD: | -0.031 |
| | | Sc. rank: | -0.8 |

| PB2 | | | |
|---|---|---|---|
| Pos. | 160 | obs: | exp: |
| caa | Q | 475 | 304.90 |
| cag | Q | 97 | 267.10 |
| --- | -- | ------ | ------ |
| mPD | | 0.28 | 0.50 |
| | | nPD: | 0.57 |
| | | N. weight: | 0.29 |
| | | Sc. PD: | 0.081 |
| | | Sc. rank: | 78.5 |

| PB2 | | | |
|---|---|---|---|
| Pos. | 161 | obs: | exp: |
| gat | D | 523 | 305.20 |
| gac | D | 49 | 266.80 |
| --- | -- | ------ | ------ |
| mPD | | 0.16 | 0.50 |
| | | nPD: | 0.32 |
| | | N. weight: | 0.5 |
| | | Sc. PD: | 0.021 |
| | | Sc. rank: | 39.5 |

| PB2 | | | |
|---|---|---|---|
| Pos. | 162 | obs: | exp: |
| gtt | V | 19 | 117.60 |
| gtc | V | 480 | 120.10 |
| gta | V | 68 | 107.20 |
| gtg | V | 5 | 227.20 |
| --- | -- | ------ | ------ |
| mPD | | 0.28 | 0.72 |
| | | nPD: | 0.39 |
| | | N. weight: | 1.5 |
| | | Sc. PD: | 0.17 |
| | | Sc. rank: | 210.1 |

| PB2 | | | |
|---|---|---|---|
| Pos. | 163 | obs: | exp: |
| att | I | 5 | 215.00 |
| atc | I | 564 | 149.90 |
| ata | I | 3 | 207.10 |
| --- | -- | ------ | ------ |
| mPD | | 0.028 | 0.66 |
| | | nPD: | 0.04 |
| | | N. weight: | 1.8 |
| | | Sc. PD: | -0.39 |
| | | Sc. rank: | -407.9 |

| PB2 | | | |
|---|---|---|---|
| Pos. | 164 | obs: | exp: |
| att | I | 0 | 0.38 |
| atc | I | 0 | 0.26 |
| ata | I | 1 | 0.36 |
| atg | M | 571 | 571.00 |
| --- | -- | ------ | ------ |
| mPD | | 0.0035 | 0.0035 |
| | | nPD: | 1. |
| | | N. weight: | 0.0025 |
| | | Sc. PD: | 0.0018 |
| | | Sc. rank: | 0.9 |

| PB2 | | | |
|---|---|---|---|
| Pos. | 165 | obs: | exp: |
| gaa | E | 73 | 329.10 |
| gag | E | 499 | 242.90 |
| --- | -- | ------ | ------ |
| mPD | | 0.22 | 0.49 |
| | | nPD: | 0.46 |
| | | N. weight: | 0.63 |
| | | Sc. PD: | 0.11 |
| | | Sc. rank: | 124.0 |

| PB2 | | | |
|---|---|---|---|
| Pos. | 166 | obs: | exp: |
| att | I | 0 | 0.38 |
| atc | I | 1 | 0.26 |
| ata | I | 0 | 0.36 |
| gtt | V | 152 | 117.40 |
| gtc | V | 418 | 119.80 |
| gta | V | 0 | 107.00 |
| gtg | V | 1 | 226.80 |
| --- | -- | ------ | ------ |
| mPD | | 0.40 | 0.72 |
| | | nPD: | 0.55 |
| | | N. weight: | 1.4 |
| | | Sc. PD: | 0.37 |
| | | Sc. rank: | 367.1 |

| PB2 | | | |
|---|---|---|---|
| Pos. | 167 | obs: | exp: |
| att | I | 1 | 0.38 |
| atc | I | 0 | 0.26 |
| ata | I | 0 | 0.36 |
| gtt | V | 490 | 117.40 |
| gtc | V | 49 | 119.80 |
| gta | V | 32 | 107.00 |
| gtg | V | 0 | 226.80 |
| --- | -- | ------ | ------ |
| mPD | | 0.26 | 0.72 |
| | | nPD: | 0.35 |
| | | N. weight: | 1.6 |
| | | Sc. PD: | 0.12 |
| | | Sc. rank: | 172.1 |

| PB2 | | | |
|---|---|---|---|
| Pos. | 168 | obs: | exp: |
| ttt | F | 53 | 234.70 |
| ttc | F | 518 | 336.30 |
| tat | Y | 0 | 0.52 |
| tac | Y | 1 | 0.48 |
| --- | -- | ------ | ------ |
| mPD | | 0.17 | 0.49 |
| | | nPD: | 0.35 |
| | | N. weight: | 0.37 |
| | | Sc. PD: | 0.029 |
| | | Sc. rank: | 40.4 |

| PB2 | | | |
|---|---|---|---|
| Pos. | 169 | obs: | exp: |
| tct | S | 0 | 0.32 |
| tcc | S | 0 | 0.28 |
| tca | S | 2 | 0.44 |
| tcg | S | 0 | 0.12 |
| cct | P | 0 | 150.10 |
| ccc | P | 1 | 102.80 |
| cca | P | 559 | 224.90 |
| ccg | P | 10 | 92.16 |
| agt | S | 0 | 0.44 |
| agc | S | 0 | 0.40 |
| --- | -- | ------ | ------ |
| mPD | | 0.045 | 0.73 |
| | | nPD: | 0.06 |
| | | N. weight: | 1.2 |
| | | Sc. PD: | -0.24 |
| | | Sc. rank: | -242.3 |

| PB2 | | | |
|---|---|---|---|
| Pos. | 170 | obs: | exp: |
| aat | N | 437 | 299.00 |
| aac | N | 135 | 273.00 |
| --- | -- | ------ | ------ |
| mPD | | 0.36 | 0.50 |
| | | nPD: | 0.72 |
| | | N. weight: | 0.18 |
| | | Sc. PD: | 0.08 |
| | | Sc. rank: | 61.8 |

| PB2 | | | |
|---|---|---|---|
| Pos. | 171 | obs: | exp: |
| gaa | E | 563 | 329.10 |
| gag | E | 9 | 242.90 |
| --- | -- | ------ | ------ |
| mPD | | 0.031 | 0.49 |
| | | nPD: | 0.06 |
| | | N. weight: | 0.69 |
| | | Sc. PD: | -0.14 |
| | | Sc. rank: | -131.9 |

| PB2 | | | |
|---|---|---|---|
| Pos. | 172 | obs: | exp: |
| att | I | 1 | 0.38 |
| atc | I | 0 | 0.26 |
| ata | I | 0 | 0.36 |
| atg | M | 1 | 1.00 |
| gtt | V | 156 | 117.20 |
| gtc | V | 308 | 119.60 |
| gta | V | 9 | 106.80 |
| gtg | V | 97 | 226.40 |
| --- | -- | ------ | ------ |
| mPD | | 0.61 | 0.73 |
| | | nPD: | 0.84 |
| | | N. weight: | 0.6 |
| | | Sc. PD: | 0.33 |
| | | Sc. rank: | 206.7 |

| PB2 | | | |
|---|---|---|---|
| Pos. | 173 | obs: | exp: |
| ggt | G | 2 | 70.58 |
| ggc | G | 236 | 77.35 |
| gga | G | 284 | 276.70 |
| ggg | G | 50 | 147.40 |
| --- | -- | ------ | ------ |
| mPD | | 0.58 | 0.67 |
| | | nPD: | 0.87 |
| | | N. weight: | 0.54 |
| | | Sc. PD: | 0.31 |
| | | Sc. rank: | 186.6 |

| PB2 | | | |
|---|---|---|---|
| Pos. | 174 | obs: | exp: |
| gct | A | 30 | 144.10 |
| gcc | A | 468 | 117.20 |
| gca | A | 11 | 254.90 |
| gcg | A | 63 | 55.82 |
| --- | -- | ------ | ------ |
| mPD | | 0.32 | 0.69 |
| | | nPD: | 0.46 |
| | | N. weight: | 1.5 |
| | | Sc. PD: | 0.26 |
| | | Sc. rank: | 292.1 |

| PB2 | | | |
|---|---|---|---|
| Pos. | 175 | obs: | exp: |
| cgt | R | 0 | 23.62 |
| cgc | R | 0 | 27.86 |
| cga | R | 0 | 54.68 |
| cgg | R | 0 | 47.48 |
| aaa | K | 2 | 1.68 |
| aag | K | 1 | 1.32 |
| aga | R | 129 | 255.90 |
| agg | R | 440 | 159.50 |
| --- | -- | ------ | ------ |
| mPD | | 0.36 | 0.97 |
| | | nPD: | 0.38 |
| | | N. weight: | 0.91 |
| | | Sc. PD: | 0.09 |
| | | Sc. rank: | 119.1 |

PB2

| Pos. | 176 | obs: | exp: |
|---|---|---|---|
| att | I | 0 | 214.30 |
| atc | I | 0 | 149.40 |
| ata | I | 570 | 206.30 |
| gtt | V | 0 | 0.41 |
| gtc | V | 0 | 0.42 |
| gta | V | 2 | 0.37 |
| gtg | V | 0 | 0.79 |
| --- | -- | ------ | ------ |
| mPD | | 0.0070 | 0.67 |
| | | nPD: | 0.01 |
| | | N. weight: | 1.5 |
| | | Sc. PD: | -0.36 |
| | | Sc. rank: | -427.2 |

,

PB2

| Pos. | 177 | obs: | exp: |
|---|---|---|---|
| tta | L | 38 | 49.97 |
| ttg | L | 460 | 110.20 |
| ctt | L | 1 | 106.70 |
| ctc | L | 1 | 92.53 |
| cta | L | 25 | 86.17 |
| ctg | L | 47 | 126.40 |
| --- | -- | ------ | ------ |
| mPD | | 0.43 | 1.1 |
| | | nPD: | 0.38 |
| | | N. weight: | 1.4 |
| | | Sc. PD: | 0.15 |
| | | Sc. rank: | 195.0 |

,

PB2

| Pos. | 178 | obs: | exp: |
|---|---|---|---|
| act | T | 1 | 151.10 |
| acc | T | 0 | 122.70 |
| aca | T | 525 | 250.60 |
| acg | T | 46 | 47.55 |
| --- | -- | ------ | ------ |
| mPD | | 0.15 | 0.69 |
| | | nPD: | 0.22 |
| | | N. weight: | 0.96 |
| | | Sc. PD: | -0.047 |
| | | Sc. rank: | -15.8 |

,

PB2

| Pos. | 179 | obs: | exp: |
|---|---|---|---|
| tct | S | 1 | 90.93 |
| tcc | S | 1 | 79.68 |
| tca | S | 502 | 126.70 |
| tcg | S | 68 | 34.87 |
| agt | S | 0 | 125.80 |
| agc | S | 0 | 114.00 |
| --- | -- | ------ | ------ |
| mPD | | 0.22 | 1.7 |
| | | nPD: | 0.13 |
| | | N. weight: | 1.8 |
| | | Sc. PD: | -0.25 |
| | | Sc. rank: | -239.5 |

,

PB2

| Pos. | 180 | obs: | exp: |
|---|---|---|---|
| gaa | E | 55 | 329.10 |
| gag | E | 517 | 242.90 |
| --- | -- | ------ | ------ |
| mPD | | 0.17 | 0.49 |
| | | nPD: | 0.36 |
| | | N. weight: | 0.74 |
| | | Sc. PD: | 0.06 |
| | | Sc. rank: | 83.2 |

,

PB2

| Pos. | 181 | obs: | exp: |
|---|---|---|---|
| tct | S | 1 | 90.93 |
| tcc | S | 5 | 79.68 |
| tca | S | 541 | 126.70 |
| tcg | S | 25 | 34.87 |
| agt | S | 0 | 125.80 |
| agc | S | 0 | 114.00 |
| --- | -- | ------ | ------ |
| mPD | | 0.10 | 1.7 |
| | | nPD: | 0.06 |
| | | N. weight: | 1.9 |
| | | Sc. PD: | -0.38 |
| | | Sc. rank: | -375.8 |

,

PB2

| Pos. | 182 | obs: | exp: |
|---|---|---|---|
| caa | Q | 191 | 304.90 |
| cag | Q | 381 | 267.10 |
| --- | -- | ------ | ------ |
| mPD | | 0.45 | 0.50 |
| | | nPD: | 0.9 |
| | | N. weight: | 0.12 |
| | | Sc. PD: | 0.073 |
| | | Sc. rank: | 42.6 |

,

PB2

| Pos. | 183 | obs: | exp: |
|---|---|---|---|
| tta | L | 417 | 49.97 |
| ttg | L | 95 | 110.20 |
| ctt | L | 0 | 106.70 |
| ctc | L | 0 | 92.53 |
| cta | L | 18 | 86.17 |
| ctg | L | 42 | 126.40 |
| --- | -- | ------ | ------ |
| mPD | | 0.55 | 1.1 |
| | | nPD: | 0.5 |
| | | N. weight: | 2. |
| | | Sc. PD: | 0.43 |
| | | Sc. rank: | 457.8 |

,

PB2

| Pos. | 184 | obs: | exp: |
|---|---|---|---|
| att | I | 0 | 2.63 |
| atc | I | 0 | 1.83 |
| ata | I | 7 | 2.53 |
| act | T | 1 | 147.40 |
| acc | T | 0 | 119.70 |
| aca | T | 465 | 244.50 |
| acg | T | 92 | 46.39 |
| aaa | K | 1 | 0.56 |
| aag | K | 0 | 0.44 |
| gct | A | 0 | 1.51 |
| gcc | A | 1 | 1.23 |
| gca | A | 4 | 2.67 |
| gcg | A | 1 | 0.59 |
| --- | -- | ------ | ------ |
| mPD | | 0.33 | 0.73 |
| | | nPD: | 0.45 |
| | | N. weight: | 0.93 |
| | | Sc. PD: | 0.15 |
| | | Sc. rank: | 178.3 |

,

PB2

| Pos. | 185 | obs: | exp: |
|---|---|---|---|
| att | I | 0 | 214.30 |
| atc | I | 0 | 149.40 |
| ata | I | 570 | 206.30 |
| atg | M | 1 | 1.00 |
| gtt | V | 0 | 0.21 |
| gtc | V | 0 | 0.21 |
| gta | V | 1 | 0.19 |
| gtg | V | 0 | 0.40 |
| --- | -- | ------ | ------ |
| mPD | | 0.0070 | 0.66 |
| | | nPD: | 0.01 |
| | | N. weight: | 1.5 |
| | | Sc. PD: | -0.36 |
| | | Sc. rank: | -419.0 |

,

PB2

| Pos. | 186 | obs: | exp: |
|---|---|---|---|
| act | T | 5 | 150.90 |
| acc | T | 30 | 122.50 |
| aca | T | 528 | 250.20 |
| acg | T | 8 | 47.47 |
| gct | A | 0 | 0.25 |
| gcc | A | 0 | 0.20 |
| gca | A | 1 | 0.45 |
| gcg | A | 0 | 0.10 |
| --- | -- | ------ | ------ |
| mPD | | 0.15 | 0.69 |
| | | nPD: | 0.21 |
| | | N. weight: | 0.82 |
| | | Sc. PD: | -0.047 |
| | | Sc. rank: | -26.4 |

,

PB2

| Pos. | 187 | obs: | exp: |
|---|---|---|---|
| cgt | R | 0 | 0.42 |
| cgc | R | 0 | 0.49 |
| cga | R | 0 | 0.96 |
| cgg | R | 0 | 0.83 |
| aat | N | 8 | 4.18 |
| aac | N | 0 | 3.82 |
| aaa | K | 311 | 310.20 |
| aag | K | 243 | 243.80 |
| aga | R | 0 | 4.50 |
| agg | R | 10 | 2.80 |
| --- | -- | ------ | ------ |
| mPD | | 0.54 | 0.55 |
| | | nPD: | 0.99 |
| | | N. weight: | 0.053 |
| | | Sc. PD: | 0.036 |
| | | Sc. rank: | 18.4 |

,

PB2

| Pos. | 188 | obs: | exp: |
|---|---|---|---|
| gaa | E | 489 | 328.60 |
| gag | E | 82 | 242.40 |
| ggt | G | 0 | 0.12 |
| ggc | G | 0 | 0.14 |
| gga | G | 1 | 0.48 |
| ggg | G | 0 | 0.26 |
| --- | -- | ------ | ------ |
| mPD | | 0.25 | 0.49 |
| | | nPD: | 0.51 |
| | | N. weight: | 0.27 |
| | | Sc. PD: | 0.061 |
| | | Sc. rank: | 64.2 |

,

PB2

| Pos. | 189 | obs: | exp: |
|---|---|---|---|
| cgt | R | 0 | 0.04 |
| cgc | R | 0 | 0.05 |
| cga | R | 0 | 0.10 |
| cgg | R | 0 | 0.08 |
| aaa | K | 17 | 319.70 |
| aag | K | 554 | 251.30 |
| aga | R | 0 | 0.45 |
| agg | R | 1 | 0.28 |
| --- | -- | ------ | ------ |
| mPD | | 0.061 | 0.50 |
| | | nPD: | 0.12 |
| | | N. weight: | 0.98 |
| | | Sc. PD: | -0.14 |
| | | Sc. rank: | -133.1 |

,

PB2

| Pos. | 190 | obs: | exp: |
|---|---|---|---|
| aaa | K | 74 | 320.30 |
| aag | K | 498 | 251.70 |
| --- | -- | ------ | ------ |
| mPD | | 0.23 | 0.49 |
| | | nPD: | 0.46 |
| | | N. weight: | 0.59 |
| | | Sc. PD: | 0.1 |
| | | Sc. rank: | 116.9 |

,

PB2

| Pos. | 191 | obs: | exp: |
|---|---|---|---|
| gaa | E | 116 | 329.10 |
| gag | E | 456 | 242.90 |
| --- | -- | ------ | ------ |
| mPD | | 0.32 | 0.49 |
| | | nPD: | 0.66 |
| | | N. weight: | 0.42 |
| | | Sc. PD: | 0.16 |
| | | Sc. rank: | 130.5 |

,

PB2

| Pos. | 192 | obs: | exp: |
|---|---|---|---|
| gaa | E | 152 | 329.10 |
| gag | E | 420 | 242.90 |
| --- | -- | ------ | ------ |
| mPD | | 0.39 | 0.49 |
| | | nPD: | 0.8 |
| | | N. weight: | 0.29 |
| | | Sc. PD: | 0.15 |
| | | Sc. rank: | 98.8 |

,

PB2

| Pos. | 193 | obs: | exp: |
|---|---|---|---|
| tta | L | 0 | 49.97 |
| ttg | L | 1 | 110.20 |
| ctt | L | 409 | 106.70 |
| ctc | L | 162 | 92.53 |
| cta | L | 0 | 86.17 |
| ctg | L | 0 | 126.40 |
| --- | -- | ------ | ------ |
| mPD | | 0.41 | 1.1 |
| | | nPD: | 0.37 |
| | | N. weight: | 1.6 |
| | | Sc. PD: | 0.15 |
| | | Sc. rank: | 197.7 |

,

PB2

| Pos. | 194 | obs: | exp: |
|---|---|---|---|
| cat | H | 5 | 2.71 |
| cac | H | 0 | 2.29 |
| caa | Q | 15 | 301.70 |
| cag | Q | 551 | 264.30 |
| cgt | R | 0 | 0.04 |
| cgc | R | 0 | 0.05 |
| cga | R | 1 | 0.10 |
| cgg | R | 0 | 0.08 |
| aga | R | 0 | 0.45 |
| agg | R | 0 | 0.28 |
| --- | -- | ------ | ------ |
| mPD | | 0.075 | 0.51 |
| | | nPD: | 0.15 |
| | | N. weight: | 0.92 |
| | | Sc. PD: | -0.11 |
| | | Sc. rank: | -87.0 |

,

PB2

| Pos. | 195 | obs: | exp: |
|---|---|---|---|
| aat | N | 1 | 3.66 |
| aac | N | 6 | 3.34 |
| gat | D | 31 | 299.90 |
| gac | D | 531 | 262.10 |
| gaa | E | 1 | 0.58 |
| gag | E | 0 | 0.42 |
| ggt | G | 0 | 0.25 |
| ggc | G | 2 | 0.27 |
| gga | G | 0 | 0.97 |
| ggg | G | 0 | 0.52 |
| --- | -- | ------ | ------ |
| mPD | | 0.14 | 0.53 |
| | | nPD: | 0.26 |
| | | N. weight: | 0.79 |
| | | Sc. PD: | -0.0064 |
| | | Sc. rank: | 24.3 |

,

PB2

| Pos. | 196 | obs: | exp: |
|---|---|---|---|
| tgt | C | 216 | 235.80 |
| tgc | C | 356 | 336.20 |
| --- | -- | ------ | ------ |
| mPD | | 0.47 | 0.48 |
| | | nPD: | 0.97 |
| | | N. weight: | 0.012 |
| | | Sc. PD: | 0.008 |
| | | Sc. rank: | 4.1 |

,

PB2

| Pos. | 197 | obs: | exp: |
|---|---|---|---|
| cgt | R | 0 | 0.12 |
| cgc | R | 0 | 0.15 |
| cga | R | 0 | 0.29 |
| cgg | R | 0 | 0.25 |
| aaa | K | 133 | 318.60 |
| aag | K | 436 | 250.40 |
| aga | R | 0 | 1.35 |
| agg | R | 3 | 0.84 |
| --- | -- | ------ | ------ |
| mPD | | 0.37 | 0.51 |
| | | nPD: | 0.73 |
| | | N. weight: | 0.33 |
| | | Sc. PD: | 0.14 |
| | | Sc. rank: | 111.0 |

,

PB2

| Pos. | 198 | obs: | exp: |
|---|---|---|---|
| att | I | 490 | 215.00 |
| atc | I | 80 | 149.90 |
| ata | I | 2 | 207.10 |
| --- | -- | ------ | ------ |
| mPD | | 0.25 | 0.66 |
| | | nPD: | 0.37 |
| | | N. weight: | 0.87 |
| | | Sc. PD: | 0.086 |
| | | Sc. rank: | 113.8 |

,

PB2

| Pos. | 199 | obs: | exp: |
|---|---|---|---|
| act | T | 1 | 0.26 |
| acc | T | 0 | 0.21 |
| aca | T | 0 | 0.44 |
| acg | T | 0 | 0.08 |
| gct | A | 417 | 143.80 |
| gcc | A | 153 | 117.00 |
| gca | A | 1 | 254.50 |
| gcg | A | 0 | 55.72 |
| --- | -- | ------ | ------ |
| mPD | | 0.40 | 0.69 |
| | | nPD: | 0.58 |
| | | N. weight: | 1.2 |
| | | Sc. PD: | 0.35 |
| | | Sc. rank: | 337.0 |

,

PB2

| Pos. | 200 | obs: | exp: |
|---|---|---|---|
| cct | P | 562 | 150.60 |
| ccc | P | 9 | 103.20 |
| cca | P | 0 | 225.70 |
| ccg | P | 1 | 92.49 |
| --- | -- | ------ | ------ |
| mPD | | 0.034 | 0.72 |
| | | nPD: | 0.05 |
| | | N. weight: | 1.8 |
| | | Sc. PD: | -0.38 |
| | | Sc. rank: | -390.2 |

,

| PB2 | | | |
|---|---|---|---|
| Pos. | 201 | obs: | exp: |
| tta | L | 389 | 49.97 |
| ttg | L | 116 | 110.20 |
| ctt | L | 0 | 106.70 |
| ctc | L | 0 | 92.53 |
| cta | L | 41 | 86.17 |
| ctg | L | 26 | 126.40 |
| --- | -- | ------ | ------ |
| mPD | | 0.58 | 1.1 |
| | | nPD: | 0.52 |
| | | N. weight: | 1.9 |
| | | Sc. PD: | 0.44 |
| | | Sc. rank: | 454.1 |

,

| PB2 | | | |
|---|---|---|---|
| Pos. | 202 | obs: | exp: |
| atg | M | 572 | 572.00 |
| --- | -- | ------ | ------ |
| mPD | | 0 | 0 |
| | | nPD: | 1. |
| | | N. weight: | 0. |
| | | Sc. PD: | 0 |
| | | Sc. rank: | 0 |

,

| PB2 | | | |
|---|---|---|---|
| Pos. | 203 | obs: | exp: |
| gtt | V | 65 | 117.60 |
| gtc | V | 6 | 120.10 |
| gta | V | 12 | 107.20 |
| gtg | V | 489 | 227.20 |
| --- | -- | ------ | ------ |
| mPD | | 0.26 | 0.72 |
| | | nPD: | 0.36 |
| | | N. weight: | 0.75 |
| | | Sc. PD: | 0.06 |
| | | Sc. rank: | 82.9 |

,

| PB2 | | | |
|---|---|---|---|
| Pos. | 204 | obs: | exp: |
| gct | A | 5 | 144.10 |
| gcc | A | 29 | 117.20 |
| gca | A | 527 | 254.90 |
| gcg | A | 11 | 55.82 |
| --- | -- | ------ | ------ |
| mPD | | 0.15 | 0.69 |
| | | nPD: | 0.22 |
| | | N. weight: | 0.78 |
| | | Sc. PD: | -0.041 |
| | | Sc. rank: | -18.5 |

,

| PB2 | | | |
|---|---|---|---|
| Pos. | 205 | obs: | exp: |
| tat | Y | 8 | 296.10 |
| tac | Y | 564 | 275.90 |
| --- | -- | ------ | ------ |
| mPD | | 0.028 | 0.50 |
| | | nPD: | 0.06 |
| | | N. weight: | 0.94 |
| | | Sc. PD: | -0.19 |
| | | Sc. rank: | -194.9 |

,

| PB2 | | | |
|---|---|---|---|
| Pos. | 206 | obs: | exp: |
| atg | M | 572 | 572.00 |
| --- | -- | ------ | ------ |
| mPD | | 0 | 0 |
| | | nPD: | 1. |
| | | N. weight: | 0. |
| | | Sc. PD: | 0 |
| | | Sc. rank: | 0 |

,

| PB2 | | | |
|---|---|---|---|
| Pos. | 207 | obs: | exp: |
| tta | L | 10 | 49.97 |
| ttg | L | 450 | 110.20 |
| ctt | L | 0 | 106.70 |
| ctc | L | 0 | 92.53 |
| cta | L | 98 | 86.17 |
| ctg | L | 14 | 126.40 |
| --- | -- | ------ | ------ |
| mPD | | 0.62 | 1.1 |
| | | nPD: | 0.56 |
| | | N. weight: | 1.5 |
| | | Sc. PD: | 0.41 |
| | | Sc. rank: | 405.6 |

,

| PB2 | | | |
|---|---|---|---|
| Pos. | 208 | obs: | exp: |
| gaa | E | 130 | 329.10 |
| gag | E | 442 | 242.90 |
| --- | -- | ------ | ------ |
| mPD | | 0.35 | 0.49 |
| | | nPD: | 0.72 |
| | | N. weight: | 0.37 |
| | | Sc. PD: | 0.16 |
| | | Sc. rank: | 122.5 |

,

| PB2 | | | |
|---|---|---|---|
| Pos. | 209 | obs: | exp: |
| cgt | R | 0 | 23.75 |
| cgc | R | 0 | 28.01 |
| cga | R | 0 | 54.97 |
| cgg | R | 0 | 47.73 |
| aga | R | 537 | 257.20 |
| agg | R | 35 | 160.30 |
| --- | -- | ------ | ------ |
| mPD | | 0.12 | 0.96 |
| | | nPD: | 0.12 |
| | | N. weight: | 0.86 |
| | | Sc. PD: | -0.12 |
| | | Sc. rank: | -119.3 |

,

| PB2 | | | |
|---|---|---|---|
| Pos. | 210 | obs: | exp: |
| gaa | E | 469 | 329.10 |
| gag | E | 103 | 242.90 |
| --- | -- | ------ | ------ |
| mPD | | 0.30 | 0.49 |
| | | nPD: | 0.61 |
| | | N. weight: | 0.2 |
| | | Sc. PD: | 0.064 |
| | | Sc. rank: | 58.6 |

,

| PB2 | | | |
|---|---|---|---|
| Pos. | 211 | obs: | exp: |
| tta | L | 6 | 49.97 |
| ttg | L | 81 | 110.20 |
| ctt | L | 0 | 106.70 |
| ctc | L | 0 | 92.53 |
| cta | L | 11 | 86.17 |
| ctg | L | 474 | 126.40 |
| --- | -- | ------ | ------ |
| mPD | | 0.32 | 1.1 |
| | | nPD: | 0.28 |
| | | N. weight: | 1.4 |
| | | Sc. PD: | 0.016 |
| | | Sc. rank: | 71.2 |

,

| PB2 | | | |
|---|---|---|---|
| Pos. | 212 | obs: | exp: |
| gtt | V | 218 | 117.60 |
| gtc | V | 349 | 120.10 |
| gta | V | 3 | 107.20 |
| gtg | V | 2 | 227.20 |
| --- | -- | ------ | ------ |
| mPD | | 0.48 | 0.72 |
| | | nPD: | 0.67 |
| | | N. weight: | 1.2 |
| | | Sc. PD: | 0.47 |
| | | Sc. rank: | 383.0 |

,

| PB2 | | | |
|---|---|---|---|
| Pos. | 213 | obs: | exp: |
| cgt | R | 60 | 23.75 |
| cgc | R | 480 | 28.01 |
| cga | R | 29 | 54.97 |
| cgg | R | 2 | 47.73 |
| aga | R | 1 | 257.20 |
| agg | R | 0 | 160.30 |
| --- | -- | ------ | ------ |
| mPD | | 0.29 | 0.96 |
| | | nPD: | 0.3 |
| | | N. weight: | 3.5 |
| | | Sc. PD: | 0.088 |
| | | Sc. rank: | 241.0 |

,

| PB2 | | | |
|---|---|---|---|
| Pos. | 214 | obs: | exp: |
| cgt | R | 0 | 0.04 |
| cgc | R | 0 | 0.05 |
| cga | R | 0 | 0.10 |
| cgg | R | 0 | 0.08 |
| aaa | K | 496 | 319.70 |
| aag | K | 75 | 251.30 |
| aga | R | 0 | 0.45 |
| agg | R | 1 | 0.28 |
| --- | -- | ------ | ------ |
| mPD | | 0.23 | 0.50 |
| | | nPD: | 0.47 |
| | | N. weight: | 0.33 |
| | | Sc. PD: | 0.062 |
| | | Sc. rank: | 67.9 |

,

| PB2 | | | |
|---|---|---|---|
| Pos. | 215 | obs: | exp: |
| act | T | 13 | 151.10 |
| acc | T | 459 | 122.70 |
| aca | T | 57 | 250.60 |
| acg | T | 43 | 47.55 |
| --- | -- | ------ | ------ |
| mPD | | 0.34 | 0.69 |
| | | nPD: | 0.5 |
| | | N. weight: | 1.2 |
| | | Sc. PD: | 0.26 |
| | | Sc. rank: | 281.8 |

,

| PB2 | | | |
|---|---|---|---|
| Pos. | 216 | obs: | exp: |
| cgt | R | 0 | 23.75 |
| cgc | R | 0 | 28.01 |
| cga | R | 3 | 54.97 |
| cgg | R | 1 | 47.73 |
| aga | R | 478 | 257.20 |
| agg | R | 90 | 160.30 |
| --- | -- | ------ | ------ |
| mPD | | 0.28 | 0.96 |
| | | nPD: | 0.29 |
| | | N. weight: | 0.59 |
| | | Sc. PD: | 0.012 |
| | | Sc. rank: | 38.3 |

,

| PB2 | | | |
|---|---|---|---|
| Pos. | 217 | obs: | exp: |
| ttt | F | 112 | 235.20 |
| ttc | F | 460 | 336.80 |
| --- | -- | ------ | ------ |
| mPD | | 0.32 | 0.48 |
| | | nPD: | 0.65 |
| | | N. weight: | 0.16 |
| | | Sc. PD: | 0.057 |
| | | Sc. rank: | 48.2 |

,

| PB2 | | | |
|---|---|---|---|
| Pos. | 218 | obs: | exp: |
| tta | L | 9 | 49.97 |
| ttg | L | 2 | 110.20 |
| ctt | L | 3 | 106.70 |
| ctc | L | 72 | 92.53 |
| cta | L | 426 | 86.17 |
| ctg | L | 60 | 126.40 |
| --- | -- | ------ | ------ |
| mPD | | 0.43 | 1.1 |
| | | nPD: | 0.39 |
| | | N. weight: | 1.5 |
| | | Sc. PD: | 0.16 |
| | | Sc. rank: | 209.8 |

,

| PB2 | | | |
|---|---|---|---|
| Pos. | 219 | obs: | exp: |
| cct | P | 1 | 150.60 |
| ccc | P | 3 | 103.20 |
| cca | P | 519 | 225.70 |
| ccg | P | 49 | 92.49 |
| --- | -- | ------ | ------ |
| mPD | | 0.17 | 0.72 |
| | | nPD: | 0.24 |
| | | N. weight: | 0.98 |
| | | Sc. PD: | -0.032 |
| | | Sc. rank: | -1.7 |

,

| PB2 | | | |
|---|---|---|---|
| Pos. | 220 | obs: | exp: |
| gtt | V | 26 | 117.60 |
| gtc | V | 1 | 120.10 |
| gta | V | 465 | 107.20 |
| gtg | V | 80 | 227.20 |
| --- | -- | ------ | ------ |
| mPD | | 0.32 | 0.72 |
| | | nPD: | 0.44 |
| | | N. weight: | 1.4 |
| | | Sc. PD: | 0.23 |
| | | Sc. rank: | 259.3 |

,

| PB2 | | | |
|---|---|---|---|
| Pos. | 221 | obs: | exp: |
| gtt | V | 1 | 0.21 |
| gtc | V | 0 | 0.21 |
| gta | V | 0 | 0.19 |
| gtg | V | 0 | 0.40 |
| gct | A | 501 | 143.80 |
| gcc | A | 9 | 117.00 |
| gca | A | 50 | 254.50 |
| gcg | A | 11 | 55.72 |
| --- | -- | ------ | ------ |
| mPD | | 0.23 | 0.69 |
| | | nPD: | 0.33 |
| | | N. weight: | 1.3 |
| | | Sc. PD: | 0.067 |
| | | Sc. rank: | 111.6 |

,

| PB2 | | | |
|---|---|---|---|
| Pos. | 222 | obs: | exp: |
| ggt | G | 94 | 70.58 |
| ggc | G | 450 | 77.35 |
| gga | G | 27 | 276.70 |
| ggg | G | 1 | 147.40 |
| --- | -- | ------ | ------ |
| mPD | | 0.35 | 0.67 |
| | | nPD: | 0.53 |
| | | N. weight: | 1.9 |
| | | Sc. PD: | 0.46 |
| | | Sc. rank: | 479.0 |

,

| PB2 | | | |
|---|---|---|---|
| Pos. | 223 | obs: | exp: |
| ggt | G | 8 | 70.58 |
| ggc | G | 0 | 77.35 |
| gga | G | 309 | 276.70 |
| ggg | G | 255 | 147.40 |
| --- | -- | ------ | ------ |
| mPD | | 0.51 | 0.67 |
| | | nPD: | 0.77 |
| | | N. weight: | 0.4 |
| | | Sc. PD: | 0.19 |
| | | Sc. rank: | 136.8 |

,

| PB2 | | | |
|---|---|---|---|
| Pos. | 224 | obs: | exp: |
| act | T | 0 | 151.10 |
| acc | T | 1 | 122.70 |
| aca | T | 562 | 250.60 |
| acg | T | 9 | 47.55 |
| --- | -- | ------ | ------ |
| mPD | | 0.034 | 0.69 |
| | | nPD: | 0.05 |
| | | N. weight: | 1.1 |
| | | Sc. PD: | -0.23 |
| | | Sc. rank: | -233.7 |

,

| PB2 | | | |
|---|---|---|---|
| Pos. | 225 | obs: | exp: |
| tct | S | 0 | 90.78 |
| tcc | S | 0 | 79.54 |
| tca | S | 0 | 126.50 |
| tcg | S | 0 | 34.80 |
| agt | S | 1 | 125.60 |
| agc | S | 570 | 113.80 |
| ggt | G | 0 | 0.12 |
| ggc | G | 1 | 0.14 |
| gga | G | 0 | 0.48 |
| ggg | G | 0 | 0.26 |
| --- | -- | ------ | ------ |
| mPD | | 0.0070 | 1.7 |
| | | nPD: | 0. |
| | | N. weight: | 2.3 |
| | | Sc. PD: | -0.58 |
| | | Sc. rank: | -719.5 |

,

| PB2 | | | |
|---|---|---|---|
| Pos. | 226 | obs: | exp: |
| tct | S | 0 | 90.93 |
| tcc | S | 0 | 79.68 |
| tca | S | 0 | 126.70 |
| tcg | S | 0 | 34.87 |
| agt | S | 149 | 125.80 |
| agc | S | 423 | 114.00 |
| --- | -- | ------ | ------ |
| mPD | | 0.39 | 1.7 |
| | | nPD: | 0.23 |
| | | N. weight: | 1.5 |
| | | Sc. PD: | -0.055 |
| | | Sc. rank: | -6.1 |

,

| PB2 | | | |
|---|---|---|---|
| Pos. | 227 | obs: | exp: |
| gtt | V | 9 | 117.60 |
| gtc | V | 64 | 120.10 |
| gta | V | 81 | 107.20 |
| gtg | V | 418 | 227.20 |
| --- | -- | ------ | ------ |
| mPD | | 0.43 | 0.72 |
| | | nPD: | 0.6 |
| | | N. weight: | 0.44 |
| | | Sc. PD: | 0.14 |
| | | Sc. rank: | 127.3 |

,

| PB2 | | | |
|---|---|---|---|
| Pos. | 228 | obs: | exp: |
| tat | Y | 438 | 296.10 |
| tac | Y | 134 | 275.90 |
| --- | -- | ------ | ------ |
| mPD | | 0.36 | 0.50 |
| | | nPD: | 0.72 |
| | | N. weight: | 0.19 |
| | | Sc. PD: | 0.083 |
| | | Sc. rank: | 64.9 |

,

| PB2 | | | |
|---|---|---|---|
| Pos. | 229 | obs: | exp: |
| att | I | 123 | 215.00 |
| atc | I | 445 | 149.90 |
| ata | I | 4 | 207.10 |
| --- | -- | ------ | ------ |
| mPD | | 0.35 | 0.66 |
| | | nPD: | 0.53 |
| | | N. weight: | 1. |
| | | Sc. PD: | 0.25 |
| | | Sc. rank: | 257.2 |

,

| PB2 | | | |
|---|---|---|---|
| Pos. | 230 | obs: | exp: |
| gaa | E | 98 | 329.10 |
| gag | E | 474 | 242.90 |
| --- | -- | ------ | ------ |
| mPD | | 0.28 | 0.49 |
| | | nPD: | 0.58 |
| | | N. weight: | 0.5 |
| | | Sc. PD: | 0.15 |
| | | Sc. rank: | 141.5 |

,

PB2

| Pos. | 231 | obs: | exp: |
|---|---|---|---|
| gtt | V | 0 | 117.60 |
| gtc | V | 0 | 120.10 |
| gta | V | 101 | 107.20 |
| gtg | V | 471 | 227.20 |
| --- | -- | ------ | ------ |
| mPD | | 0.29 | 0.72 |
| | | nPD: | 0.4 |
| | | N. weight: | 0.85 |
| | | Sc. PD: | 0.11 |
| | | Sc. rank: | 127.3 |

PB2

| Pos. | 232 | obs: | exp: |
|---|---|---|---|
| tta | L | 9 | 49.97 |
| ttg | L | 488 | 110.20 |
| ctt | L | 1 | 106.70 |
| ctc | L | 0 | 92.53 |
| cta | L | 2 | 86.17 |
| ctg | L | 72 | 126.40 |
| --- | -- | ------ | ------ |
| mPD | | 0.27 | 1.1 |
| | | nPD: | 0.24 |
| | | N. weight: | 1.7 |
| | | Sc. PD: | -0.047 |
| | | Sc. rank: | 5.6 |

PB2

| Pos. | 233 | obs: | exp: |
|---|---|---|---|
| cat | H | 235 | 310.00 |
| cac | H | 337 | 262.00 |
| --- | -- | ------ | ------ |
| mPD | | 0.48 | 0.50 |
| | | nPD: | 0.98 |
| | | N. weight: | 0.058 |
| | | Sc. PD: | 0.039 |
| | | Sc. rank: | 20.0 |

PB2

| Pos. | 234 | obs: | exp: |
|---|---|---|---|
| tta | L | 39 | 49.97 |
| ttg | L | 528 | 110.20 |
| ctt | L | 0 | 106.70 |
| ctc | L | 0 | 92.53 |
| cta | L | 4 | 86.17 |
| ctg | L | 1 | 126.40 |
| --- | -- | ------ | ------ |
| mPD | | 0.16 | 1.1 |
| | | nPD: | 0.14 |
| | | N. weight: | 2. |
| | | Sc. PD: | -0.25 |
| | | Sc. rank: | -207.5 |

PB2

| Pos. | 235 | obs: | exp: |
|---|---|---|---|
| act | T | 488 | 151.10 |
| acc | T | 78 | 122.70 |
| aca | T | 2 | 250.60 |
| acg | T | 4 | 47.55 |
| --- | -- | ------ | ------ |
| mPD | | 0.25 | 0.69 |
| | | nPD: | 0.37 |
| | | N. weight: | 1.3 |
| | | Sc. PD: | 0.12 |
| | | Sc. rank: | 163.2 |

PB2

| Pos. | 236 | obs: | exp: |
|---|---|---|---|
| caa | Q | 495 | 304.90 |
| cag | Q | 77 | 267.10 |
| --- | -- | ------ | ------ |
| mPD | | 0.23 | 0.50 |
| | | nPD: | 0.47 |
| | | N. weight: | 0.37 |
| | | Sc. PD: | 0.069 |
| | | Sc. rank: | 75.8 |

PB2

| Pos. | 237 | obs: | exp: |
|---|---|---|---|
| ggt | G | 4 | 70.58 |
| ggc | G | 0 | 77.35 |
| gga | G | 327 | 276.70 |
| ggg | G | 241 | 147.40 |
| --- | -- | ------ | ------ |
| mPD | | 0.50 | 0.67 |
| | | nPD: | 0.75 |
| | | N. weight: | 0.42 |
| | | Sc. PD: | 0.19 |
| | | Sc. rank: | 139.8 |

PB2

| Pos. | 238 | obs: | exp: |
|---|---|---|---|
| act | T | 10 | 151.10 |
| acc | T | 489 | 122.70 |
| aca | T | 72 | 250.60 |
| acg | T | 1 | 47.55 |
| --- | -- | ------ | ------ |
| mPD | | 0.25 | 0.69 |
| | | nPD: | 0.37 |
| | | N. weight: | 1.4 |
| | | Sc. PD: | 0.13 |
| | | Sc. rank: | 171.4 |

PB2

| Pos. | 239 | obs: | exp: |
|---|---|---|---|
| tgt | C | 13 | 235.80 |
| tgc | C | 559 | 336.20 |
| --- | -- | ------ | ------ |
| mPD | | 0.044 | 0.48 |
| | | nPD: | 0.09 |
| | | N. weight: | 0.62 |
| | | Sc. PD: | -0.11 |
| | | Sc. rank: | -106.0 |

PB2

| Pos. | 240 | obs: | exp: |
|---|---|---|---|
| tgg | W | 572 | 572.00 |
| --- | -- | ------ | ------ |
| mPD | | 0 | 0 |
| | | nPD: | 1. |
| | | N. weight: | 0. |
| | | Sc. PD: | 0 |
| | | Sc. rank: | 0 |

PB2

| Pos. | 241 | obs: | exp: |
|---|---|---|---|
| gaa | E | 473 | 329.10 |
| gag | E | 99 | 242.90 |
| --- | -- | ------ | ------ |
| mPD | | 0.29 | 0.49 |
| | | nPD: | 0.59 |
| | | N. weight: | 0.21 |
| | | Sc. PD: | 0.064 |
| | | Sc. rank: | 60.8 |

PB2

| Pos. | 242 | obs: | exp: |
|---|---|---|---|
| caa | Q | 528 | 304.90 |
| cag | Q | 44 | 267.10 |
| --- | -- | ------ | ------ |
| mPD | | 0.14 | 0.50 |
| | | nPD: | 0.29 |
| | | N. weight: | 0.53 |
| | | Sc. PD: | 0.0072 |
| | | Sc. rank: | 30.2 |

PB2

| Pos. | 243 | obs: | exp: |
|---|---|---|---|
| atg | M | 572 | 572.00 |
| --- | -- | ------ | ------ |
| mPD | | 0 | 0 |
| | | nPD: | 1. |
| | | N. weight: | 0. |
| | | Sc. PD: | 0 |
| | | Sc. rank: | 0 |

PB2

| Pos. | 244 | obs: | exp: |
|---|---|---|---|
| tat | Y | 93 | 296.10 |
| tac | Y | 479 | 275.90 |
| --- | -- | ------ | ------ |
| mPD | | 0.27 | 0.50 |
| | | nPD: | 0.55 |
| | | N. weight: | 0.4 |
| | | Sc. PD: | 0.1 |
| | | Sc. rank: | 103.8 |

PB2

| Pos. | 245 | obs: | exp: |
|---|---|---|---|
| cct | P | 0 | 0.26 |
| ccc | P | 1 | 0.18 |
| cca | P | 0 | 0.39 |
| ccg | P | 0 | 0.16 |
| act | T | 95 | 150.90 |
| acc | T | 38 | 122.50 |
| aca | T | 375 | 250.20 |
| acg | T | 63 | 47.47 |
| --- | -- | ------ | ------ |
| mPD | | 0.53 | 0.69 |
| | | nPD: | 0.77 |
| | | N. weight: | 0.23 |
| | | Sc. PD: | 0.11 |
| | | Sc. rank: | 77.5 |

PB2

| Pos. | 246 | obs: | exp: |
|---|---|---|---|
| cct | P | 28 | 150.60 |
| ccc | P | 0 | 103.20 |
| cca | P | 136 | 225.70 |
| ccg | P | 408 | 92.49 |
| --- | -- | ------ | ------ |
| mPD | | 0.43 | 0.72 |
| | | nPD: | 0.6 |
| | | N. weight: | 1.2 |
| | | Sc. PD: | 0.39 |
| | | Sc. rank: | 358.0 |

PB2

| Pos. | 247 | obs: | exp: |
|---|---|---|---|
| ggt | G | 17 | 70.58 |
| ggc | G | 11 | 77.35 |
| gga | G | 541 | 276.70 |
| ggg | G | 3 | 147.40 |
| --- | -- | ------ | ------ |
| mPD | | 0.10 | 0.67 |
| | | nPD: | 0.16 |
| | | N. weight: | 0.78 |
| | | Sc. PD: | -0.085 |
| | | Sc. rank: | -66.1 |

PB2

| Pos. | 248 | obs: | exp: |
|---|---|---|---|
| ggt | G | 1 | 70.58 |
| ggc | G | 0 | 77.35 |
| gga | G | 136 | 276.70 |
| ggg | G | 435 | 147.40 |
| --- | -- | ------ | ------ |
| mPD | | 0.37 | 0.67 |
| | | nPD: | 0.55 |
| | | N. weight: | 0.93 |
| | | Sc. PD: | 0.25 |
| | | Sc. rank: | 245.4 |

PB2

| Pos. | 249 | obs: | exp: |
|---|---|---|---|
| gaa | E | 189 | 329.10 |
| gag | E | 383 | 242.90 |
| --- | -- | ------ | ------ |
| mPD | | 0.44 | 0.49 |
| | | nPD: | 0.91 |
| | | N. weight: | 0.18 |
| | | Sc. PD: | 0.11 |
| | | Sc. rank: | 63.0 |

PB2

| Pos. | 250 | obs: | exp: |
|---|---|---|---|
| gtt | V | 1 | 117.40 |
| gtc | V | 0 | 119.80 |
| gta | V | 14 | 107.00 |
| gtg | V | 556 | 226.80 |
| gct | A | 0 | 0.25 |
| gcc | A | 0 | 0.20 |
| gca | A | 0 | 0.45 |
| gcg | A | 1 | 0.10 |
| --- | -- | ------ | ------ |
| mPD | | 0.055 | 0.72 |
| | | nPD: | 0.08 |
| | | N. weight: | 1.2 |
| | | Sc. PD: | -0.22 |
| | | Sc. rank: | -217.8 |

PB2

| Pos. | 251 | obs: | exp: |
|---|---|---|---|
| cgt | R | 0 | 23.62 |
| cgc | R | 0 | 27.86 |
| cga | R | 0 | 54.68 |
| cgg | R | 0 | 47.48 |
| aaa | K | 2 | 1.68 |
| aag | K | 1 | 1.32 |
| aga | R | 542 | 255.90 |
| agg | R | 27 | 159.50 |
| --- | -- | ------ | ------ |
| mPD | | 0.10 | 0.97 |
| | | nPD: | 0.11 |
| | | N. weight: | 0.9 |
| | | Sc. PD: | -0.14 |
| | | Sc. rank: | -136.9 |

PB2

| Pos. | 252 | obs: | exp: |
|---|---|---|---|
| aat | N | 528 | 299.00 |
| aac | N | 44 | 273.00 |
| --- | -- | ------ | ------ |
| mPD | | 0.14 | 0.50 |
| | | nPD: | 0.29 |
| | | N. weight: | 0.56 |
| | | Sc. PD: | 0.0071 |
| | | Sc. rank: | 29.1 |

PB2

| Pos. | 253 | obs: | exp: |
|---|---|---|---|
| aat | N | 1 | 0.52 |
| aac | N | 0 | 0.48 |
| gat | D | 559 | 304.70 |
| gac | D | 12 | 266.30 |
| --- | -- | ------ | ------ |
| mPD | | 0.045 | 0.50 |
| | | nPD: | 0.09 |
| | | N. weight: | 0.76 |
| | | Sc. PD: | -0.13 |
| | | Sc. rank: | -132.4 |

PB2

| Pos. | 254 | obs: | exp: |
|---|---|---|---|
| gat | D | 520 | 305.20 |
| gac | D | 52 | 266.80 |
| --- | -- | ------ | ------ |
| mPD | | 0.17 | 0.50 |
| | | nPD: | 0.33 |
| | | N. weight: | 0.49 |
| | | Sc. PD: | 0.028 |
| | | Sc. rank: | 44.5 |

PB2

| Pos. | 255 | obs: | exp: |
|---|---|---|---|
| tta | L | 1 | 0.17 |
| ttg | L | 0 | 0.39 |
| ctt | L | 1 | 0.37 |
| ctc | L | 0 | 0.32 |
| cta | L | 0 | 0.30 |
| ctg | L | 0 | 0.44 |
| att | I | 15 | 6.39 |
| atc | I | 2 | 4.46 |
| ata | I | 0 | 6.15 |
| gtt | V | 468 | 113.30 |
| gtc | V | 33 | 115.60 |
| gta | V | 47 | 103.30 |
| gtg | V | 3 | 218.80 |
| gct | A | 2 | 0.50 |
| gcc | A | 0 | 0.41 |
| gca | A | 0 | 0.89 |
| gcg | A | 0 | 0.20 |
| --- | -- | ------ | ------ |
| mPD | | 0.34 | 0.80 |
| | | nPD: | 0.43 |
| | | N. weight: | 1.5 |
| | | Sc. PD: | 0.22 |
| | | Sc. rank: | 261.6 |

| | | | |
|---|---|---|---|
| PB2 | | | |
| Pos. | 256 | obs: | exp: |
| aat | N | 1 | 0.52 |
| aac | N | 0 | 0.48 |
| gat | D | 447 | 304.70 |
| gac | D | 124 | 266.30 |
| --- | -- | ------ | ------ |
| mPD | | 0.34 | 0.50 |
| | | nPD: | 0.69 |
| | | N. weight: | 0.2 |
| | | Sc. PD: | 0.079 |
| | | Sc. rank: | 64.0 |

| | | | |
|---|---|---|---|
| PB2 | | | |
| Pos. | 257 | obs: | exp: |
| caa | Q | 49 | 304.90 |
| cag | Q | 523 | 267.10 |
| --- | -- | ------ | ------ |
| mPD | | 0.16 | 0.50 |
| | | nPD: | 0.32 |
| | | N. weight: | 0.66 |
| | | Sc. PD: | 0.027 |
| | | Sc. rank: | 51.5 |

| | | | |
|---|---|---|---|
| PB2 | | | |
| Pos. | 258 | obs: | exp: |
| tct | S | 0 | 90.93 |
| tcc | S | 0 | 79.68 |
| tca | S | 0 | 126.70 |
| tcg | S | 0 | 34.87 |
| agt | S | 493 | 125.80 |
| agc | S | 79 | 114.00 |
| --- | -- | ------ | ------ |
| mPD | | 0.24 | 1.7 |
| | | nPD: | 0.14 |
| | | N. weight: | 1.6 |
| | | Sc. PD: | -0.2 |
| | | Sc. rank: | -161.8 |

| | | | |
|---|---|---|---|
| PB2 | | | |
| Pos. | 259 | obs: | exp: |
| tta | L | 389 | 49.97 |
| ttg | L | 175 | 110.20 |
| ctt | L | 0 | 106.70 |
| ctc | L | 0 | 92.53 |
| cta | L | 6 | 86.17 |
| ctg | L | 2 | 126.40 |
| --- | -- | ------ | ------ |
| mPD | | 0.46 | 1.1 |
| | | nPD: | 0.41 |
| | | N. weight: | 2.2 |
| | | Sc. PD: | 0.28 |
| | | Sc. rank: | 331.1 |

| | | | |
|---|---|---|---|
| PB2 | | | |
| Pos. | 260 | obs: | exp: |
| att | I | 434 | 214.30 |
| atc | I | 136 | 149.40 |
| ata | I | 0 | 206.30 |
| gtt | V | 0 | 0.41 |
| gtc | V | 2 | 0.42 |
| gta | V | 0 | 0.37 |
| gtg | V | 0 | 0.79 |
| --- | -- | ------ | ------ |
| mPD | | 0.37 | 0.67 |
| | | nPD: | 0.56 |
| | | N. weight: | 0.75 |
| | | Sc. PD: | 0.21 |
| | | Sc. rank: | 201.6 |

| | | | |
|---|---|---|---|
| PB2 | | | |
| Pos. | 261 | obs: | exp: |
| att | I | 486 | 214.70 |
| atc | I | 70 | 149.60 |
| ata | I | 15 | 206.70 |
| act | T | 1 | 0.26 |
| acc | T | 0 | 0.21 |
| aca | T | 0 | 0.44 |
| acg | T | 0 | 0.08 |
| --- | -- | ------ | ------ |
| mPD | | 0.26 | 0.66 |
| | | nPD: | 0.4 |
| | | N. weight: | 0.78 |
| | | Sc. PD: | 0.093 |
| | | Sc. rank: | 114.1 |

| | | | |
|---|---|---|---|
| PB2 | | | |
| Pos. | 262 | obs: | exp: |
| gct | A | 570 | 144.10 |
| gcc | A | 1 | 117.20 |
| gca | A | 1 | 254.90 |
| gcg | A | 0 | 55.82 |
| --- | -- | ------ | ------ |
| mPD | | 0.0070 | 0.69 |
| | | nPD: | 0.01 |
| | | N. weight: | 1.9 |
| | | Sc. PD: | -0.48 |
| | | Sc. rank: | -571.3 |

| | | | |
|---|---|---|---|
| PB2 | | | |
| Pos. | 263 | obs: | exp: |
| gct | A | 415 | 144.10 |
| gcc | A | 154 | 117.20 |
| gca | A | 2 | 254.90 |
| gcg | A | 1 | 55.82 |
| --- | -- | ------ | ------ |
| mPD | | 0.40 | 0.69 |
| | | nPD: | 0.59 |
| | | N. weight: | 1.2 |
| | | Sc. PD: | 0.35 |
| | | Sc. rank: | 334.7 |

| | | | |
|---|---|---|---|
| PB2 | | | |
| Pos. | 264 | obs: | exp: |
| cgt | R | 0 | 23.75 |
| cgc | R | 0 | 28.01 |
| cga | R | 8 | 54.97 |
| cgg | R | 0 | 47.73 |
| aga | R | 493 | 257.20 |
| agg | R | 71 | 160.30 |
| --- | -- | ------ | ------ |
| mPD | | 0.25 | 0.96 |
| | | nPD: | 0.26 |
| | | N. weight: | 0.63 |
| | | Sc. PD: | -0.0096 |
| | | Sc. rank: | 14.8 |

| | | | |
|---|---|---|---|
| PB2 | | | |
| Pos. | 265 | obs: | exp: |
| aat | N | 423 | 299.00 |
| aac | N | 149 | 273.00 |
| --- | -- | ------ | ------ |
| mPD | | 0.39 | 0.50 |
| | | nPD: | 0.77 |
| | | N. weight: | 0.15 |
| | | Sc. PD: | 0.072 |
| | | Sc. rank: | 50.6 |

| | | | |
|---|---|---|---|
| PB2 | | | |
| Pos. | 266 | obs: | exp: |
| att | I | 447 | 214.70 |
| atc | I | 51 | 149.60 |
| ata | I | 73 | 206.70 |
| gtt | V | 0 | 0.21 |
| gtc | V | 0 | 0.21 |
| gta | V | 1 | 0.19 |
| gtg | V | 0 | 0.40 |
| --- | -- | ------ | ------ |
| mPD | | 0.37 | 0.66 |
| | | nPD: | 0.56 |
| | | N. weight: | 0.51 |
| | | Sc. PD: | 0.14 |
| | | Sc. rank: | 135.5 |

| | | | |
|---|---|---|---|
| PB2 | | | |
| Pos. | 267 | obs: | exp: |
| att | I | 2 | 0.75 |
| atc | I | 0 | 0.52 |
| ata | I | 0 | 0.72 |
| gtt | V | 479 | 117.20 |
| gtc | V | 19 | 119.60 |
| gta | V | 70 | 106.80 |
| gtg | V | 2 | 226.40 |
| --- | -- | ------ | ------ |
| mPD | | 0.28 | 0.73 |
| | | nPD: | 0.39 |
| | | N. weight: | 1.5 |
| | | Sc. PD: | 0.17 |
| | | Sc. rank: | 220.0 |

| | | | |
|---|---|---|---|
| PB2 | | | |
| Pos. | 268 | obs: | exp: |
| cgt | R | 0 | 23.75 |
| cgc | R | 0 | 28.01 |
| cga | R | 0 | 54.97 |
| cgg | R | 5 | 47.73 |
| aga | R | 107 | 257.20 |
| agg | R | 460 | 160.30 |
| --- | -- | ------ | ------ |
| mPD | | 0.32 | 0.96 |
| | | nPD: | 0.34 |
| | | N. weight: | 0.96 |
| | | Sc. PD: | 0.058 |
| | | Sc. rank: | 88.7 |

| | | | |
|---|---|---|---|
| PB2 | | | |
| Pos. | 269 | obs: | exp: |
| cgt | R | 0 | 23.75 |
| cgc | R | 0 | 28.01 |
| cga | R | 0 | 54.97 |
| cgg | R | 0 | 47.73 |
| aga | R | 559 | 257.20 |
| agg | R | 13 | 160.30 |
| --- | -- | ------ | ------ |
| mPD | | 0.044 | 0.96 |
| | | nPD: | 0.05 |
| | | N. weight: | 1. |
| | | Sc. PD: | -0.22 |
| | | Sc. rank: | -221.3 |

| | | | |
|---|---|---|---|
| PB2 | | | |
| Pos. | 270 | obs: | exp: |
| gct | A | 0 | 144.10 |
| gcc | A | 0 | 117.20 |
| gca | A | 549 | 254.90 |
| gcg | A | 23 | 55.82 |
| --- | -- | ------ | ------ |
| mPD | | 0.077 | 0.69 |
| | | nPD: | 0.11 |
| | | N. weight: | 1. |
| | | Sc. PD: | -0.15 |
| | | Sc. rank: | -147.9 |

| | | | |
|---|---|---|---|
| PB2 | | | |
| Pos. | 271 | obs: | exp: |
| act | T | 0 | 150.60 |
| acc | T | 0 | 122.30 |
| aca | T | 553 | 249.70 |
| acg | T | 17 | 47.38 |
| gct | A | 0 | 0.50 |
| gcc | A | 0 | 0.41 |
| gca | A | 0 | 0.89 |
| gcg | A | 2 | 0.20 |
| --- | -- | ------ | ------ |
| mPD | | 0.071 | 0.69 |
| | | nPD: | 0.1 |
| | | N. weight: | 1.1 |
| | | Sc. PD: | -0.17 |
| | | Sc. rank: | -165.5 |

| | | | |
|---|---|---|---|
| PB2 | | | |
| Pos. | 272 | obs: | exp: |
| gtt | V | 0 | 117.60 |
| gtc | V | 5 | 120.10 |
| gta | V | 525 | 107.20 |
| gtg | V | 42 | 227.20 |
| --- | -- | ------ | ------ |
| mPD | | 0.15 | 0.72 |
| | | nPD: | 0.21 |
| | | N. weight: | 1.9 |
| | | Sc. PD: | -0.11 |
| | | Sc. rank: | -55.9 |

| | | | |
|---|---|---|---|
| PB2 | | | |
| Pos. | 273 | obs: | exp: |
| tct | S | 4 | 90.93 |
| tcc | S | 0 | 79.68 |
| tca | S | 560 | 126.70 |
| tcg | S | 8 | 34.87 |
| agt | S | 0 | 125.80 |
| agc | S | 0 | 114.00 |
| --- | -- | ------ | ------ |
| mPD | | 0.041 | 1.7 |
| | | nPD: | 0.02 |
| | | N. weight: | 2. |
| | | Sc. PD: | -0.48 |
| | | Sc. rank: | -525.8 |

| | | | |
|---|---|---|---|
| PB2 | | | |
| Pos. | 274 | obs: | exp: |
| gct | A | 8 | 144.10 |
| gcc | A | 0 | 117.20 |
| gca | A | 545 | 254.90 |
| gcg | A | 19 | 55.82 |
| --- | -- | ------ | ------ |
| mPD | | 0.091 | 0.69 |
| | | nPD: | 0.13 |
| | | N. weight: | 0.94 |
| | | Sc. PD: | -0.12 |
| | | Sc. rank: | -117.0 |

| | | | |
|---|---|---|---|
| PB2 | | | |
| Pos. | 275 | obs: | exp: |
| gat | D | 45 | 305.20 |
| gac | D | 527 | 266.80 |
| --- | -- | ------ | ------ |
| mPD | | 0.15 | 0.50 |
| | | nPD: | 0.29 |
| | | N. weight: | 0.69 |
| | | Sc. PD: | 0.013 |
| | | Sc. rank: | 44.1 |

| | | | |
|---|---|---|---|
| PB2 | | | |
| Pos. | 276 | obs: | exp: |
| cct | P | 0 | 150.60 |
| ccc | P | 1 | 103.20 |
| cca | P | 243 | 225.70 |
| ccg | P | 328 | 92.49 |
| --- | -- | ------ | ------ |
| mPD | | 0.49 | 0.72 |
| | | nPD: | 0.69 |
| | | N. weight: | 1.1 |
| | | Sc. PD: | 0.43 |
| | | Sc. rank: | 345.6 |

| | | | |
|---|---|---|---|
| PB2 | | | |
| Pos. | 277 | obs: | exp: |
| tta | L | 245 | 49.97 |
| ttg | L | 233 | 110.20 |
| ctt | L | 0 | 106.70 |
| ctc | L | 0 | 92.53 |
| cta | L | 63 | 86.17 |
| ctg | L | 31 | 126.40 |
| --- | -- | ------ | ------ |
| mPD | | 0.77 | 1.1 |
| | | nPD: | 0.69 |
| | | N. weight: | 1.3 |
| | | Sc. PD: | 0.51 |
| | | Sc. rank: | 415.4 |

| | | | |
|---|---|---|---|
| PB2 | | | |
| Pos. | 278 | obs: | exp: |
| act | T | 0 | 0.26 |
| acc | T | 0 | 0.21 |
| aca | T | 1 | 0.44 |
| acg | T | 0 | 0.08 |
| gtt | V | 3 | 1.23 |
| gtc | V | 2 | 1.26 |
| gta | V | 1 | 1.12 |
| gtg | V | 0 | 2.38 |
| gct | A | 448 | 142.10 |
| gcc | A | 17 | 115.50 |
| gca | A | 98 | 251.40 |
| gcg | A | 1 | 55.04 |
| gat | D | 1 | 0.53 |
| gac | D | 0 | 0.47 |
| --- | -- | ------ | ------ |
| mPD | | 0.37 | 0.72 |
| | | nPD: | 0.52 |
| | | N. weight: | 1. |
| | | Sc. PD: | 0.24 |
| | | Sc. rank: | 242.8 |

| | | | |
|---|---|---|---|
| PB2 | | | |
| Pos. | 279 | obs: | exp: |
| tct | S | 71 | 90.93 |
| tcc | S | 0 | 79.68 |
| tca | S | 44 | 126.70 |
| tcg | S | 457 | 34.87 |
| agt | S | 0 | 125.80 |
| agc | S | 0 | 114.00 |
| --- | -- | ------ | ------ |
| mPD | | 0.34 | 1.7 |
| | | nPD: | 0.21 |
| | | N. weight: | 2.8 |
| | | Sc. PD: | -0.18 |
| | | Sc. rank: | -106.8 |

| | | | |
|---|---|---|---|
| PB2 | | | |
| Pos. | 280 | obs: | exp: |
| tta | L | 6 | 49.97 |
| ttg | L | 2 | 110.20 |
| ctt | L | 41 | 106.70 |
| ctc | L | 385 | 92.53 |
| cta | L | 135 | 86.17 |
| ctg | L | 3 | 126.40 |
| --- | -- | ------ | ------ |
| mPD | | 0.51 | 1.1 |
| | | nPD: | 0.46 |
| | | N. weight: | 1.4 |
| | | Sc. PD: | 0.24 |
| | | Sc. rank: | 273.1 |

| PB2 | | | |
|---|---|---|---|
| Pos. | 281 | obs: | exp: |
| tta | L | 8 | 49.88 |
| ttg | L | 492 | 110.00 |
| ctt | L | 0 | 106.50 |
| ctc | L | 0 | 92.36 |
| cta | L | 1 | 86.02 |
| ctg | L | 70 | 126.20 |
| gtt | V | 0 | 0.21 |
| gtc | V | 0 | 0.21 |
| gta | V | 0 | 0.19 |
| gtg | V | 1 | 0.40 |
| mPD | | 0.25 | 1.1 |
| | | nPD: | 0.23 |
| | | N. weight: | 1.7 |
| | | Sc. PD: | -0.075 |
| | | Sc. rank: | -13.6 |

| PB2 | | | |
|---|---|---|---|
| Pos. | 282 | obs: | exp: |
| gaa | E | 23 | 329.10 |
| gag | E | 549 | 242.90 |
| mPD | | 0.077 | 0.49 |
| | | nPD: | 0.16 |
| | | N. weight: | 0.97 |
| | | Sc. PD: | -0.11 |
| | | Sc. rank: | -81.7 |

| PB2 | | | |
|---|---|---|---|
| Pos. | 283 | obs: | exp: |
| atg | M | 571 | 571.00 |
| act | T | 0 | 0.26 |
| acc | T | 0 | 0.21 |
| aca | T | 0 | 0.44 |
| acg | T | 1 | 0.08 |
| mPD | | 0.0035 | 0.0067 |
| | | nPD: | 0.52 |
| | | N. weight: | 0.0062 |
| | | Sc. PD: | 0.0015 |
| | | Sc. rank: | 1.5 |

| PB2 | | | |
|---|---|---|---|
| Pos. | 284 | obs: | exp: |
| tgt | C | 25 | 235.80 |
| tgc | C | 547 | 336.20 |
| mPD | | 0.084 | 0.48 |
| | | nPD: | 0.17 |
| | | N. weight: | 0.53 |
| | | Sc. PD: | -0.05 |
| | | Sc. rank: | -37.7 |

| PB2 | | | |
|---|---|---|---|
| Pos. | 285 | obs: | exp: |
| tat | Y | 0 | 0.52 |
| tac | Y | 1 | 0.48 |
| cat | H | 458 | 308.90 |
| cac | H | 112 | 261.10 |
| aat | N | 1 | 0.52 |
| aac | N | 0 | 0.48 |
| mPD | | 0.32 | 0.50 |
| | | nPD: | 0.64 |
| | | N. weight: | 0.23 |
| | | Sc. PD: | 0.08 |
| | | Sc. rank: | 68.0 |

| PB2 | | | |
|---|---|---|---|
| Pos. | 286 | obs: | exp: |
| tct | S | 0 | 90.30 |
| tcc | S | 0 | 79.13 |
| tca | S | 0 | 125.80 |
| tcg | S | 0 | 34.62 |
| agt | S | 461 | 125.00 |
| agc | S | 107 | 113.20 |
| ggt | G | 4 | 0.49 |
| ggc | G | 0 | 0.54 |
| gga | G | 0 | 1.93 |
| ggg | G | 0 | 1.03 |
| mPD | | 0.32 | 1.7 |
| | | nPD: | 0.19 |
| | | N. weight: | 1.5 |
| | | Sc. PD: | -0.12 |
| | | Sc. rank: | -78.0 |

| PB2 | | | |
|---|---|---|---|
| Pos. | 287 | obs: | exp: |
| act | T | 1 | 151.10 |
| acc | T | 9 | 122.70 |
| aca | T | 540 | 250.60 |
| acg | T | 22 | 47.55 |
| mPD | | 0.11 | 0.69 |
| | | nPD: | 0.16 |
| | | N. weight: | 0.94 |
| | | Sc. PD: | -0.1 |
| | | Sc. rank: | -80.1 |

| PB2 | | | |
|---|---|---|---|
| Pos. | 288 | obs: | exp: |
| caa | Q | 514 | 304.90 |
| cag | Q | 58 | 267.10 |
| mPD | | 0.18 | 0.50 |
| | | nPD: | 0.37 |
| | | N. weight: | 0.46 |
| | | Sc. PD: | 0.041 |
| | | Sc. rank: | 54.5 |

| PB2 | | | |
|---|---|---|---|
| Pos. | 289 | obs: | exp: |
| att | I | 554 | 215.00 |
| atc | I | 17 | 149.90 |
| ata | I | 1 | 207.10 |
| mPD | | 0.061 | 0.66 |
| | | nPD: | 0.09 |
| | | N. weight: | 1.2 |
| | | Sc. PD: | -0.21 |
| | | Sc. rank: | -204.2 |

| PB2 | | | |
|---|---|---|---|
| Pos. | 290 | obs: | exp: |
| ggt | G | 38 | 70.58 |
| ggc | G | 457 | 77.35 |
| gga | G | 34 | 276.70 |
| ggg | G | 43 | 147.40 |
| mPD | | 0.35 | 0.67 |
| | | nPD: | 0.52 |
| | | N. weight: | 1.7 |
| | | Sc. PD: | 0.4 |
| | | Sc. rank: | 414.9 |

| PB2 | | | |
|---|---|---|---|
| Pos. | 291 | obs: | exp: |
| ggt | G | 2 | 70.58 |
| ggc | G | 1 | 77.35 |
| gga | G | 321 | 276.70 |
| ggg | G | 248 | 147.40 |
| mPD | | 0.50 | 0.67 |
| | | nPD: | 0.75 |
| | | N. weight: | 0.43 |
| | | Sc. PD: | 0.19 |
| | | Sc. rank: | 143.6 |

| PB2 | | | |
|---|---|---|---|
| Pos. | 292 | obs: | exp: |
| att | I | 22 | 188.30 |
| atc | I | 0 | 131.30 |
| ata | I | 479 | 181.40 |
| atg | M | 6 | 6.00 |
| act | T | 0 | 0.53 |
| acc | T | 0 | 0.43 |
| aca | T | 2 | 0.88 |
| acg | T | 0 | 0.17 |
| gtt | V | 27 | 12.95 |
| gtc | V | 0 | 13.22 |
| gta | V | 36 | 11.81 |
| gtg | V | 0 | 25.02 |
| mPD | | 0.38 | 0.90 |
| | | nPD: | 0.42 |
| | | N. weight: | 1.2 |
| | | Sc. PD: | 0.17 |
| | | Sc. rank: | 208.4 |

| PB2 | | | |
|---|---|---|---|
| Pos. | 293 | obs: | exp: |
| cgt | R | 0 | 23.71 |
| cgc | R | 0 | 27.96 |
| cga | R | 0 | 54.88 |
| cgg | R | 0 | 47.65 |
| aaa | K | 0 | 0.56 |
| aag | K | 1 | 0.44 |
| aga | R | 30 | 256.80 |
| agg | R | 541 | 160.00 |
| mPD | | 0.10 | 0.96 |
| | | nPD: | 0.11 |
| | | N. weight: | 1.5 |
| | | Sc. PD: | -0.23 |
| | | Sc. rank: | -227.2 |

| PB2 | | | |
|---|---|---|---|
| Pos. | 294 | obs: | exp: |
| atg | M | 572 | 572.00 |
| mPD | | 0 | 0 |
| | | nPD: | 1. |
| | | N. weight: | 0. |
| | | Sc. PD: | 0 |
| | | Sc. rank: | 0 |

| PB2 | | | |
|---|---|---|---|
| Pos. | 295 | obs: | exp: |
| att | I | 0 | 8.27 |
| atc | I | 0 | 5.77 |
| ata | I | 22 | 7.96 |
| atg | M | 1 | 1.00 |
| gtt | V | 34 | 112.80 |
| gtc | V | 3 | 115.20 |
| gta | V | 382 | 102.90 |
| gtg | V | 130 | 218.00 |
| mPD | | 0.52 | 0.80 |
| | | nPD: | 0.65 |
| | | N. weight: | 1. |
| | | Sc. PD: | 0.37 |
| | | Sc. rank: | 312.5 |

| PB2 | | | |
|---|---|---|---|
| Pos. | 296 | obs: | exp: |
| gat | D | 4 | 305.20 |
| gac | D | 568 | 266.80 |
| mPD | | 0.014 | 0.50 |
| | | nPD: | 0.03 |
| | | N. weight: | 1. |
| | | Sc. PD: | -0.24 |
| | | Sc. rank: | -260.7 |

| PB2 | | | |
|---|---|---|---|
| Pos. | 297 | obs: | exp: |
| att | I | 148 | 174.80 |
| atc | I | 316 | 121.90 |
| ata | I | 1 | 168.30 |
| gtt | V | 1 | 21.99 |
| gtc | V | 106 | 22.46 |
| gta | V | 0 | 20.05 |
| gtg | V | 0 | 42.50 |
| mPD | | 0.69 | 1.0 |
| | | nPD: | 0.69 |
| | | N. weight: | 1.1 |
| | | Sc. PD: | 0.43 |
| | | Sc. rank: | 350.7 |

| PB2 | | | |
|---|---|---|---|
| Pos. | 298 | obs: | exp: |
| tta | L | 0 | 49.97 |
| ttg | L | 0 | 110.20 |
| ctt | L | 547 | 106.70 |
| ctc | L | 24 | 92.53 |
| cta | L | 1 | 86.17 |
| ctg | L | 0 | 126.40 |
| mPD | | 0.084 | 1.1 |
| | | nPD: | 0.08 |
| | | N. weight: | 2.1 |
| | | Sc. PD: | -0.4 |
| | | Sc. rank: | -400.4 |

| PB2 | | | |
|---|---|---|---|
| Pos. | 299 | obs: | exp: |
| caa | Q | 0 | 0.53 |
| cag | Q | 1 | 0.47 |
| cgt | R | 0 | 23.54 |
| cgc | R | 0 | 27.76 |
| cga | R | 3 | 54.49 |
| cgg | R | 66 | 47.32 |
| aaa | K | 4 | 2.24 |
| aag | K | 0 | 1.76 |
| aga | R | 442 | 255.00 |
| agg | R | 56 | 158.90 |
| mPD | | 0.57 | 0.98 |
| | | nPD: | 0.58 |
| | | N. weight: | 0.52 |
| | | Sc. PD: | 0.16 |
| | | Sc. rank: | 146.9 |

| PB2 | | | |
|---|---|---|---|
| Pos. | 300 | obs: | exp: |
| caa | Q | 460 | 304.90 |
| cag | Q | 112 | 267.10 |
| mPD | | 0.32 | 0.50 |
| | | nPD: | 0.63 |
| | | N. weight: | 0.24 |
| | | Sc. PD: | 0.082 |
| | | Sc. rank: | 70.4 |

| PB2 | | | |
|---|---|---|---|
| Pos. | 301 | obs: | exp: |
| aat | N | 114 | 299.00 |
| aac | N | 458 | 273.00 |
| mPD | | 0.32 | 0.50 |
| | | nPD: | 0.64 |
| | | N. weight: | 0.33 |
| | | Sc. PD: | 0.11 |
| | | Sc. rank: | 97.4 |

| PB2 | | | |
|---|---|---|---|
| Pos. | 302 | obs: | exp: |
| cct | P | 0 | 150.60 |
| ccc | P | 14 | 103.20 |
| cca | P | 555 | 225.70 |
| ccg | P | 3 | 92.49 |
| mPD | | 0.058 | 0.72 |
| | | nPD: | 0.08 |
| | | N. weight: | 1.2 |
| | | Sc. PD: | -0.21 |
| | | Sc. rank: | -207.3 |

| PB2 | | | |
|---|---|---|---|
| Pos. | 303 | obs: | exp: |
| act | T | 21 | 150.60 |
| acc | T | 6 | 122.30 |
| aca | T | 525 | 249.70 |
| acg | T | 18 | 47.38 |
| aaa | K | 2 | 1.12 |
| aag | K | 0 | 0.88 |
| mPD | | 0.16 | 0.69 |
| | | nPD: | 0.23 |
| | | N. weight: | 0.8 |
| | | Sc. PD: | -0.036 |
| | | Sc. rank: | -7.6 |

| PB2 | | | |
|---|---|---|---|
| Pos. | 304 | obs: | exp: |
| gaa | E | 473 | 329.10 |
| gag | E | 99 | 242.90 |
| mPD | | 0.29 | 0.49 |
| | | nPD: | 0.59 |
| | | N. weight: | 0.21 |
| | | Sc. PD: | 0.064 |
| | | Sc. rank: | 60.8 |

| PB2 | | | |
|---|---|---|---|
| Pos. | 305 | obs: | exp: |
| gaa | E | 133 | 329.10 |
| gag | E | 439 | 242.90 |
| mPD | | 0.36 | 0.49 |
| | | nPD: | 0.73 |
| | | N. weight: | 0.36 |
| | | Sc. PD: | 0.16 |
| | | Sc. rank: | 119.4 |

PB2

| Pos. | 306 | obs: | exp: |
|---|---|---|---|
| caa | Q | 529 | 304.90 |
| cag | Q | 43 | 267.10 |
| mPD | | 0.14 | 0.50 |
| | | nPD: | 0.28 |
| | | N. weight: | 0.54 |
| | | Sc. PD: | 0.0042 |
| | | Sc. rank: | 25.6 |

PB2

| Pos. | 307 | obs: | exp: |
|---|---|---|---|
| gct | A | 455 | 144.10 |
| gcc | A | 92 | 117.20 |
| gca | A | 22 | 254.90 |
| gcg | A | 3 | 55.82 |
| mPD | | 0.34 | 0.69 |
| | | nPD: | 0.5 |
| | | N. weight: | 1.1 |
| | | Sc. PD: | 0.24 |
| | | Sc. rank: | 250.0 |

PB2

| Pos. | 308 | obs: | exp: |
|---|---|---|---|
| gtt | V | 2 | 117.60 |
| gtc | V | 0 | 120.10 |
| gta | V | 6 | 107.20 |
| gtg | V | 564 | 227.20 |
| mPD | | 0.028 | 0.72 |
| | | nPD: | 0.04 |
| | | N. weight: | 1.2 |
| | | Sc. PD: | -0.27 |
| | | Sc. rank: | -284.5 |

PB2

| Pos. | 309 | obs: | exp: |
|---|---|---|---|
| aat | N | 1 | 0.52 |
| aac | N | 0 | 0.48 |
| gat | D | 463 | 304.10 |
| gac | D | 107 | 265.90 |
| ggt | G | 1 | 0.12 |
| ggc | G | 0 | 0.14 |
| gga | G | 0 | 0.48 |
| ggg | G | 0 | 0.26 |
| mPD | | 0.31 | 0.51 |
| | | nPD: | 0.62 |
| | | N. weight: | 0.26 |
| | | Sc. PD: | 0.085 |
| | | Sc. rank: | 75.6 |

PB2

| Pos. | 310 | obs: | exp: |
|---|---|---|---|
| att | I | 0 | 215.00 |
| atc | I | 0 | 149.90 |
| ata | I | 572 | 207.10 |
| mPD | | 0 | 0.66 |
| | | nPD: | 0. |
| | | N. weight: | 1.5 |
| | | Sc. PD: | -0.38 |
| | | Sc. rank: | -500.9 |

PB2

| Pos. | 311 | obs: | exp: |
|---|---|---|---|
| tgt | C | 5 | 235.80 |
| tgc | C | 567 | 336.20 |
| mPD | | 0.017 | 0.48 |
| | | nPD: | 0.04 |
| | | N. weight: | 0.7 |
| | | Sc. PD: | -0.16 |
| | | Sc. rank: | -164.0 |

PB2

| Pos. | 312 | obs: | exp: |
|---|---|---|---|
| aaa | K | 454 | 320.30 |
| aag | K | 118 | 251.70 |
| mPD | | 0.33 | 0.49 |
| | | nPD: | 0.67 |
| | | N. weight: | 0.18 |
| | | Sc. PD: | 0.068 |
| | | Sc. rank: | 55.7 |

PB2

| Pos. | 313 | obs: | exp: |
|---|---|---|---|
| gct | A | 17 | 144.10 |
| gcc | A | 0 | 117.20 |
| gca | A | 532 | 254.90 |
| gcg | A | 23 | 55.82 |
| mPD | | 0.13 | 0.69 |
| | | nPD: | 0.19 |
| | | N. weight: | 0.85 |
| | | Sc. PD: | -0.063 |
| | | Sc. rank: | -41.6 |

PB2

| Pos. | 314 | obs: | exp: |
|---|---|---|---|
| gct | A | 0 | 144.10 |
| gcc | A | 1 | 117.20 |
| gca | A | 530 | 254.90 |
| gcg | A | 41 | 55.82 |
| mPD | | 0.14 | 0.69 |
| | | nPD: | 0.2 |
| | | N. weight: | 0.93 |
| | | Sc. PD: | -0.065 |
| | | Sc. rank: | -41.8 |

PB2

| Pos. | 315 | obs: | exp: |
|---|---|---|---|
| atg | M | 572 | 572.00 |
| mPD | | 0 | 0 |
| | | nPD: | 1. |
| | | N. weight: | 0. |
| | | Sc. PD: | 0 |
| | | Sc. rank: | 0 |

PB2

| Pos. | 316 | obs: | exp: |
|---|---|---|---|
| ggt | G | 450 | 70.58 |
| ggc | G | 114 | 77.35 |
| gga | G | 4 | 276.70 |
| ggg | G | 4 | 147.40 |
| mPD | | 0.34 | 0.67 |
| | | nPD: | 0.51 |
| | | N. weight: | 2.1 |
| | | Sc. PD: | 0.49 |
| | | Sc. rank: | 506.3 |

PB2

| Pos. | 317 | obs: | exp: |
|---|---|---|---|
| tta | L | 54 | 49.97 |
| ttg | L | 101 | 110.20 |
| ctt | L | 0 | 106.70 |
| ctc | L | 0 | 92.53 |
| cta | L | 408 | 86.17 |
| ctg | L | 9 | 126.40 |
| mPD | | 0.71 | 1.1 |
| | | nPD: | 0.63 |
| | | N. weight: | 1.5 |
| | | Sc. PD: | 0.53 |
| | | Sc. rank: | 458.6 |

PB2

| Pos. | 318 | obs: | exp: |
|---|---|---|---|
| cgt | R | 0 | 23.75 |
| cgc | R | 0 | 28.01 |
| cga | R | 3 | 54.97 |
| cgg | R | 0 | 47.73 |
| aga | R | 189 | 257.20 |
| agg | R | 380 | 160.30 |
| mPD | | 0.46 | 0.96 |
| | | nPD: | 0.48 |
| | | N. weight: | 0.66 |
| | | Sc. PD: | 0.13 |
| | | Sc. rank: | 138.8 |

PB2

| Pos. | 319 | obs: | exp: |
|---|---|---|---|
| att | I | 58 | 215.00 |
| atc | I | 514 | 149.90 |
| ata | I | 0 | 207.10 |
| mPD | | 0.18 | 0.66 |
| | | nPD: | 0.28 |
| | | N. weight: | 1.4 |
| | | Sc. PD: | 0.0072 |
| | | Sc. rank: | 65.2 |

PB2

| Pos. | 320 | obs: | exp: |
|---|---|---|---|
| tct | S | 0 | 90.93 |
| tcc | S | 0 | 79.68 |
| tca | S | 0 | 126.70 |
| tcg | S | 0 | 34.87 |
| agt | S | 490 | 125.80 |
| agc | S | 82 | 114.00 |
| mPD | | 0.25 | 1.7 |
| | | nPD: | 0.15 |
| | | N. weight: | 1.6 |
| | | Sc. PD: | -0.19 |
| | | Sc. rank: | -147.3 |

PB2

| Pos. | 321 | obs: | exp: |
|---|---|---|---|
| tct | S | 4 | 90.93 |
| tcc | S | 0 | 79.68 |
| tca | S | 555 | 126.70 |
| tcg | S | 13 | 34.87 |
| agt | S | 0 | 125.80 |
| agc | S | 0 | 114.00 |
| mPD | | 0.058 | 1.7 |
| | | nPD: | 0.03 |
| | | N. weight: | 2. |
| | | Sc. PD: | -0.45 |
| | | Sc. rank: | -474.8 |

PB2

| Pos. | 322 | obs: | exp: |
|---|---|---|---|
| tct | S | 71 | 90.93 |
| tcc | S | 501 | 79.68 |
| tca | S | 0 | 126.70 |
| tcg | S | 0 | 34.87 |
| agt | S | 0 | 125.80 |
| agc | S | 0 | 114.00 |
| mPD | | 0.22 | 1.7 |
| | | nPD: | 0.13 |
| | | N. weight: | 2.3 |
| | | Sc. PD: | -0.3 |
| | | Sc. rank: | -291.6 |

PB2

| Pos. | 323 | obs: | exp: |
|---|---|---|---|
| ttt | F | 38 | 235.20 |
| ttc | F | 534 | 336.80 |
| mPD | | 0.12 | 0.48 |
| | | nPD: | 0.26 |
| | | N. weight: | 0.45 |
| | | Sc. PD: | -0.0064 |
| | | Sc. rank: | 10.7 |

PB2

| Pos. | 324 | obs: | exp: |
|---|---|---|---|
| tct | S | 0 | 90.93 |
| tcc | S | 0 | 79.68 |
| tca | S | 0 | 126.70 |
| tcg | S | 0 | 34.87 |
| agt | S | 20 | 125.80 |
| agc | S | 552 | 114.00 |
| mPD | | 0.068 | 1.7 |
| | | nPD: | 0.04 |
| | | N. weight: | 2.1 |
| | | Sc. PD: | -0.46 |
| | | Sc. rank: | -481.2 |

PB2

| Pos. | 325 | obs: | exp: |
|---|---|---|---|
| ttt | F | 559 | 235.20 |
| ttc | F | 13 | 336.80 |
| mPD | | 0.044 | 0.48 |
| | | nPD: | 0.09 |
| | | N. weight: | 1.1 |
| | | Sc. PD: | -0.19 |
| | | Sc. rank: | -188.0 |

PB2

| Pos. | 326 | obs: | exp: |
|---|---|---|---|
| ggt | G | 50 | 70.58 |
| ggc | G | 21 | 77.35 |
| gga | G | 499 | 276.70 |
| ggg | G | 2 | 147.40 |
| mPD | | 0.23 | 0.67 |
| | | nPD: | 0.35 |
| | | N. weight: | 0.62 |
| | | Sc. PD: | 0.044 |
| | | Sc. rank: | 63.7 |

PB2

| Pos. | 327 | obs: | exp: |
|---|---|---|---|
| ggt | G | 451 | 70.58 |
| ggc | G | 34 | 77.35 |
| gga | G | 73 | 276.70 |
| ggg | G | 14 | 147.40 |
| mPD | | 0.36 | 0.67 |
| | | nPD: | 0.54 |
| | | N. weight: | 1.7 |
| | | Sc. PD: | 0.44 |
| | | Sc. rank: | 442.8 |

PB2

| Pos. | 328 | obs: | exp: |
|---|---|---|---|
| ttt | F | 14 | 235.20 |
| ttc | F | 558 | 336.80 |
| mPD | | 0.048 | 0.48 |
| | | nPD: | 0.1 |
| | | N. weight: | 0.61 |
| | | Sc. PD: | -0.1 |
| | | Sc. rank: | -98.7 |

PB2

| Pos. | 329 | obs: | exp: |
|---|---|---|---|
| att | I | 2 | 0.75 |
| atc | I | 0 | 0.52 |
| ata | I | 0 | 0.72 |
| act | T | 553 | 150.60 |
| acc | T | 17 | 122.30 |
| aca | T | 0 | 249.70 |
| acg | T | 0 | 47.38 |
| mPD | | 0.065 | 0.69 |
| | | nPD: | 0.09 |
| | | N. weight: | 1.7 |
| | | Sc. PD: | -0.29 |
| | | Sc. rank: | -287.6 |

PB2

| Pos. | 330 | obs: | exp: |
|---|---|---|---|
| ttt | F | 49 | 235.20 |
| ttc | F | 523 | 336.80 |
| mPD | | 0.16 | 0.48 |
| | | nPD: | 0.32 |
| | | N. weight: | 0.39 |
| | | Sc. PD: | 0.019 |
| | | Sc. rank: | 33.6 |

PB2

| Pos. | 331 | obs: | exp: |
|---|---|---|---|
| aaa | K | 551 | 320.30 |
| aag | K | 21 | 251.70 |
| mPD | | 0.071 | 0.49 |
| | | nPD: | 0.14 |
| | | N. weight: | 0.62 |
| | | Sc. PD: | -0.076 |
| | | Sc. rank: | -62.0 |

PB2

| Pos. | 332 | obs: | exp: |
|---|---|---|---|
| cgt | R | 0 | 23.66 |
| cgc | R | 0 | 27.91 |
| cga | R | 31 | 54.78 |
| cgg | R | 14 | 47.57 |
| aaa | K | 1 | 1.12 |
| aag | K | 1 | 0.88 |
| aga | R | 102 | 256.30 |
| agg | R | 423 | 159.70 |
| mPD | | 0.51 | 0.97 |
| | | nPD: | 0.53 |
| | | N. weight: | 0.73 |
| | | Sc. PD: | 0.18 |
| | | Sc. rank: | 184.9 |

PB2

| Pos. | 333 | obs: | exp: |
|---|---|---|---|
| act | T | 0 | 151.10 |
| acc | T | 0 | 122.70 |
| aca | T | 539 | 250.60 |
| acg | T | 33 | 47.55 |
| mPD | | 0.11 | 0.69 |
| | | nPD: | 0.16 |
| | | N. weight: | 1. |
| | | Sc. PD: | -0.11 |
| | | Sc. rank: | -83.8 |

PB2

| Pos. | 334 | obs: | exp: |
|---|---|---|---|
| tct | S | 0 | 90.78 |
| tcc | S | 0 | 79.54 |
| tca | S | 0 | 126.50 |
| tcg | S | 0 | 34.80 |
| aat | N | 1 | 0.52 |
| aac | N | 0 | 0.48 |
| agt | S | 511 | 125.60 |
| agc | S | 60 | 113.80 |
| mPD | | 0.19 | 1.7 |
| | | nPD: | 0.12 |
| | | N. weight: | 1.7 |
| | | Sc. PD: | -0.25 |
| | | Sc. rank: | -246.2 |

PB2

| Pos. | 335 | obs: | exp: |
|---|---|---|---|
| ggt | G | 0 | 70.58 |
| ggc | G | 1 | 77.35 |
| gga | G | 78 | 276.70 |
| ggg | G | 493 | 147.40 |
| mPD | | 0.24 | 0.67 |
| | | nPD: | 0.36 |
| | | N. weight: | 1.2 |
| | | Sc. PD: | 0.1 |
| | | Sc. rank: | 144.7 |

| PB2 | | | |
|---|---|---|---|
| Pos. | 336 | obs: | exp: |
| tct | S | 27 | 90.78 |
| tcc | S | 0 | 79.54 |
| tca | S | 501 | 126.50 |
| tcg | S | 43 | 34.80 |
| agt | S | 0 | 125.60 |
| agc | S | 0 | 113.80 |
| gct | A | 0 | 0.25 |
| gcc | A | 0 | 0.20 |
| gca | A | 0 | 0.45 |
| gcg | A | 1 | 0.10 |
| --- | -- | ------ | ------ |
| mPD | | 0.23 | 1.7 |
| | | nPD: | 0.14 |
| | | N. weight: | 1.7 |
| | | Sc. PD: | -0.22 |
| | | Sc. rank: | -188.3 |

| PB2 | | | |
|---|---|---|---|
| Pos. | 337 | obs: | exp: |
| tct | S | 474 | 90.93 |
| tcc | S | 27 | 79.68 |
| tca | S | 70 | 126.70 |
| tcg | S | 1 | 34.87 |
| agt | S | 0 | 125.80 |
| agc | S | 0 | 114.00 |
| --- | -- | ------ | ------ |
| mPD | | 0.30 | 1.7 |
| | | nPD: | 0.18 |
| | | N. weight: | 1.8 |
| | | Sc. PD: | -0.16 |
| | | Sc. rank: | -119.4 |

| PB2 | | | |
|---|---|---|---|
| Pos. | 338 | obs: | exp: |
| att | I | 0 | 0.38 |
| atc | I | 1 | 0.26 |
| ata | I | 0 | 0.36 |
| gtt | V | 31 | 117.40 |
| gtc | V | 525 | 119.80 |
| gta | V | 14 | 107.00 |
| gtg | V | 1 | 226.80 |
| --- | -- | ------ | ------ |
| mPD | | 0.15 | 0.72 |
| | | nPD: | 0.21 |
| | | N. weight: | 1.8 |
| | | Sc. PD: | -0.098 |
| | | Sc. rank: | -43.9 |

| PB2 | | | |
|---|---|---|---|
| Pos. | 339 | obs: | exp: |
| cgt | R | 0 | 0.08 |
| cgc | R | 0 | 0.10 |
| cga | R | 0 | 0.19 |
| cgg | R | 0 | 0.17 |
| aaa | K | 518 | 319.20 |
| aag | K | 52 | 250.80 |
| aga | R | 2 | 0.90 |
| agg | R | 0 | 0.56 |
| --- | -- | ------ | ------ |
| mPD | | 0.17 | 0.50 |
| | | nPD: | 0.34 |
| | | N. weight: | 0.43 |
| | | Sc. PD: | 0.03 |
| | | Sc. rank: | 44.1 |

| PB2 | | | |
|---|---|---|---|
| Pos. | 340 | obs: | exp: |
| cgt | R | 0 | 22.00 |
| cgc | R | 0 | 25.95 |
| cga | R | 0 | 50.94 |
| cgg | R | 0 | 44.23 |
| aaa | K | 15 | 22.96 |
| aag | K | 26 | 18.04 |
| aga | R | 474 | 238.40 |
| agg | R | 56 | 148.50 |
| ggt | G | 0 | 0.12 |
| ggc | G | 0 | 0.14 |
| gga | G | 1 | 0.48 |
| ggg | G | 0 | 0.26 |
| --- | -- | ------ | ------ |
| mPD | | 0.38 | 1.1 |
| | | nPD: | 0.36 |
| | | N. weight: | 0.7 |
| | | Sc. PD: | 0.057 |
| | | Sc. rank: | 79.8 |

| PB2 | | | |
|---|---|---|---|
| Pos. | 341 | obs: | exp: |
| aaa | K | 0 | 0.56 |
| aag | K | 1 | 0.44 |
| gaa | E | 554 | 328.60 |
| gag | E | 17 | 242.40 |
| --- | -- | ------ | ------ |
| mPD | | 0.065 | 0.49 |
| | | nPD: | 0.13 |
| | | N. weight: | 0.62 |
| | | Sc. PD: | -0.083 |
| | | Sc. rank: | -78.3 |

| PB2 | | | |
|---|---|---|---|
| Pos. | 342 | obs: | exp: |
| gaa | E | 246 | 329.10 |
| gag | E | 326 | 242.90 |
| --- | -- | ------ | ------ |
| mPD | | 0.49 | 0.49 |
| | | nPD: | 1.01 |
| | | N. weight: | 0.069 |
| | | Sc. PD: | 0.048 |
| | | Sc. rank: | 24.0 |

| PB2 | | | |
|---|---|---|---|
| Pos. | 343 | obs: | exp: |
| gaa | E | 559 | 329.10 |
| gag | E | 13 | 242.90 |
| --- | -- | ------ | ------ |
| mPD | | 0.044 | 0.49 |
| | | nPD: | 0.09 |
| | | N. weight: | 0.65 |
| | | Sc. PD: | -0.11 |
| | | Sc. rank: | -111.2 |

| PB2 | | | |
|---|---|---|---|
| Pos. | 344 | obs: | exp: |
| atg | M | 47 | 47.00 |
| gtt | V | 5 | 107.90 |
| gtc | V | 1 | 110.20 |
| gta | V | 1 | 98.40 |
| gtg | V | 518 | 208.50 |
| --- | -- | ------ | ------ |
| mPD | | 0.18 | 0.85 |
| | | nPD: | 0.21 |
| | | N. weight: | 1.1 |
| | | Sc. PD: | -0.07 |
| | | Sc. rank: | -41.9 |

| PB2 | | | |
|---|---|---|---|
| Pos. | 345 | obs: | exp: |
| ttt | F | 0 | 0.41 |
| ttc | F | 1 | 0.59 |
| tta | L | 0 | 49.88 |
| ttg | L | 0 | 110.00 |
| ctt | L | 135 | 106.50 |
| ctc | L | 426 | 92.36 |
| cta | L | 2 | 86.02 |
| ctg | L | 8 | 126.20 |
| --- | -- | ------ | ------ |
| mPD | | 0.39 | 1.1 |
| | | nPD: | 0.35 |
| | | N. weight: | 1.6 |
| | | Sc. PD: | 0.12 |
| | | Sc. rank: | 176.9 |

| PB2 | | | |
|---|---|---|---|
| Pos. | 346 | obs: | exp: |
| act | T | 2 | 151.10 |
| acc | T | 50 | 122.70 |
| aca | T | 453 | 250.60 |
| acg | T | 67 | 47.55 |
| --- | -- | ------ | ------ |
| mPD | | 0.35 | 0.69 |
| | | nPD: | 0.51 |
| | | N. weight: | 0.61 |
| | | Sc. PD: | 0.14 |
| | | Sc. rank: | 145.6 |

| PB2 | | | |
|---|---|---|---|
| Pos. | 347 | obs: | exp: |
| ggt | G | 0 | 70.58 |
| ggc | G | 570 | 77.35 |
| gga | G | 1 | 276.70 |
| ggg | G | 1 | 147.40 |
| --- | -- | ------ | ------ |
| mPD | | 0.0070 | 0.67 |
| | | nPD: | 0.01 |
| | | N. weight: | 2.8 |
| | | Sc. PD: | -0.7 |
| | | Sc. rank: | -818.6 |

| PB2 | | | |
|---|---|---|---|
| Pos. | 348 | obs: | exp: |
| aat | N | 36 | 299.00 |
| aac | N | 536 | 273.00 |
| --- | -- | ------ | ------ |
| mPD | | 0.12 | 0.50 |
| | | nPD: | 0.24 |
| | | N. weight: | 0.72 |
| | | Sc. PD: | -0.024 |
| | | Sc. rank: | -1.9 |

| PB2 | | | |
|---|---|---|---|
| Pos. | 349 | obs: | exp: |
| tta | L | 0 | 49.97 |
| ttg | L | 0 | 110.20 |
| ctt | L | 76 | 106.70 |
| ctc | L | 495 | 92.53 |
| cta | L | 1 | 86.17 |
| ctg | L | 0 | 126.40 |
| --- | -- | ------ | ------ |
| mPD | | 0.23 | 1.1 |
| | | nPD: | 0.21 |
| | | N. weight: | 2. |
| | | Sc. PD: | -0.12 |
| | | Sc. rank: | -67.3 |

| PB2 | | | |
|---|---|---|---|
| Pos. | 350 | obs: | exp: |
| caa | Q | 558 | 304.90 |
| cag | Q | 14 | 267.10 |
| --- | -- | ------ | ------ |
| mPD | | 0.048 | 0.50 |
| | | nPD: | 0.1 |
| | | N. weight: | 0.74 |
| | | Sc. PD: | -0.12 |
| | | Sc. rank: | -121.9 |

| PB2 | | | |
|---|---|---|---|
| Pos. | 351 | obs: | exp: |
| act | T | 0 | 151.10 |
| acc | T | 1 | 122.70 |
| aca | T | 563 | 250.60 |
| acg | T | 8 | 47.55 |
| --- | -- | ------ | ------ |
| mPD | | 0.031 | 0.69 |
| | | nPD: | 0.05 |
| | | N. weight: | 1.1 |
| | | Sc. PD: | -0.24 |
| | | Sc. rank: | -242.2 |

| PB2 | | | |
|---|---|---|---|
| Pos. | 352 | obs: | exp: |
| tta | L | 12 | 49.97 |
| ttg | L | 494 | 110.20 |
| ctt | L | 0 | 106.70 |
| ctc | L | 0 | 92.53 |
| cta | L | 1 | 86.17 |
| ctg | L | 65 | 126.40 |
| --- | -- | ------ | ------ |
| mPD | | 0.25 | 1.1 |
| | | nPD: | 0.22 |
| | | N. weight: | 1.7 |
| | | Sc. PD: | -0.078 |
| | | Sc. rank: | -18.5 |

| PB2 | | | |
|---|---|---|---|
| Pos. | 353 | obs: | exp: |
| cgt | R | 0 | 0.17 |
| cgc | R | 0 | 0.20 |
| cga | R | 0 | 0.38 |
| cgg | R | 0 | 0.33 |
| aaa | K | 568 | 318.10 |
| aag | K | 0 | 249.90 |
| aga | R | 4 | 1.80 |
| agg | R | 0 | 1.12 |
| --- | -- | ------ | ------ |
| mPD | | 0.014 | 0.51 |
| | | nPD: | 0.03 |
| | | N. weight: | 0.83 |
| | | Sc. PD: | -0.19 |
| | | Sc. rank: | -211.3 |

| PB2 | | | |
|---|---|---|---|
| Pos. | 354 | obs: | exp: |
| att | I | 0 | 214.30 |
| atc | I | 0 | 149.40 |
| ata | I | 570 | 206.30 |
| atg | M | 1 | 1.00 |
| gtt | V | 0 | 0.21 |
| gtc | V | 0 | 0.21 |
| gta | V | 1 | 0.19 |
| gtg | V | 0 | 0.40 |
| --- | -- | ------ | ------ |
| mPD | | 0.0070 | 0.66 |
| | | nPD: | 0.01 |
| | | N. weight: | 1.5 |
| | | Sc. PD: | -0.36 |
| | | Sc. rank: | -419.0 |

| PB2 | | | |
|---|---|---|---|
| Pos. | 355 | obs: | exp: |
| cgt | R | 0 | 18.47 |
| cgc | R | 0 | 21.79 |
| cga | R | 3 | 42.77 |
| cgg | R | 0 | 37.13 |
| aaa | K | 125 | 71.11 |
| aag | K | 2 | 55.89 |
| aga | R | 380 | 200.10 |
| agg | R | 62 | 124.70 |
| --- | -- | ------ | ------ |
| mPD | | 0.56 | 1.2 |
| | | nPD: | 0.45 |
| | | N. weight: | 0.65 |
| | | Sc. PD: | 0.11 |
| | | Sc. rank: | 127.9 |

| PB2 | | | |
|---|---|---|---|
| Pos. | 356 | obs: | exp: |
| att | I | 0 | 1.13 |
| atc | I | 0 | 0.79 |
| ata | I | 3 | 1.09 |
| gtt | V | 0 | 117.00 |
| gtc | V | 0 | 119.40 |
| gta | V | 209 | 106.60 |
| gtg | V | 360 | 226.00 |
| --- | -- | ------ | ------ |
| mPD | | 0.48 | 0.73 |
| | | nPD: | 0.65 |
| | | N. weight: | 0.79 |
| | | Sc. PD: | 0.29 |
| | | Sc. rank: | 239.4 |

| PB2 | | | |
|---|---|---|---|
| Pos. | 357 | obs: | exp: |
| tat | Y | 0 | 0.52 |
| tac | Y | 1 | 0.48 |
| cat | H | 561 | 309.40 |
| cac | H | 10 | 261.60 |
| --- | -- | ------ | ------ |
| mPD | | 0.041 | 0.50 |
| | | nPD: | 0.08 |
| | | N. weight: | 0.76 |
| | | Sc. PD: | -0.14 |
| | | Sc. rank: | -132.5 |

| PB2 | | | |
|---|---|---|---|
| Pos. | 358 | obs: | exp: |
| aaa | K | 0 | 0.56 |
| aag | K | 1 | 0.44 |
| gaa | E | 472 | 328.60 |
| gag | E | 99 | 242.40 |
| --- | -- | ------ | ------ |
| mPD | | 0.29 | 0.49 |
| | | nPD: | 0.59 |
| | | N. weight: | 0.22 |
| | | Sc. PD: | 0.066 |
| | | Sc. rank: | 61.9 |

| PB2 | | | |
|---|---|---|---|
| Pos. | 359 | obs: | exp: |
| ggt | G | 0 | 70.58 |
| ggc | G | 3 | 77.35 |
| gga | G | 423 | 276.70 |
| ggg | G | 146 | 147.40 |
| --- | -- | ------ | ------ |
| mPD | | 0.39 | 0.67 |
| | | nPD: | 0.58 |
| | | N. weight: | 0.43 |
| | | Sc. PD: | 0.13 |
| | | Sc. rank: | 121.9 |

| PB2 | | | |
|---|---|---|---|
| Pos. | 360 | obs: | exp: |
| tat | Y | 529 | 295.10 |
| tac | Y | 41 | 274.90 |
| cat | H | 2 | 1.08 |
| cac | H | 0 | 0.92 |
| --- | -- | ------ | ------ |
| mPD | | 0.14 | 0.51 |
| | | nPD: | 0.28 |
| | | N. weight: | 0.59 |
| | | Sc. PD: | 0.003 |
| | | Sc. rank: | 27.6 |

| | | | |
|---|---|---|---|
| PB2 | | | |
| Pos. | 361 | obs: | exp: |
| gaa | E | 146 | 329.10 |
| gag | E | 426 | 242.90 |
| --- | -- | ------ | ------ |
| mPD | | 0.38 | 0.49 |
| | | nPD: | 0.78 |
| | | N. weight: | 0.31 |
| | | Sc. PD: | 0.15 |
| | | Sc. rank: | 105.0 |

| | | | |
|---|---|---|---|
| PB2 | | | |
| Pos. | 362 | obs: | exp: |
| gaa | E | 441 | 329.10 |
| gag | E | 131 | 242.90 |
| --- | -- | ------ | ------ |
| mPD | | 0.35 | 0.49 |
| | | nPD: | 0.72 |
| | | N. weight: | 0.13 |
| | | Sc. PD: | 0.055 |
| | | Sc. rank: | 42.9 |

| | | | |
|---|---|---|---|
| PB2 | | | |
| Pos. | 363 | obs: | exp: |
| ttt | F | 6 | 235.20 |
| ttc | F | 566 | 336.80 |
| --- | -- | ------ | ------ |
| mPD | | 0.021 | 0.48 |
| | | nPD: | 0.04 |
| | | N. weight: | 0.68 |
| | | Sc. PD: | -0.15 |
| | | Sc. rank: | -153.5 |

| | | | |
|---|---|---|---|
| PB2 | | | |
| Pos. | 364 | obs: | exp: |
| act | T | 0 | 151.10 |
| acc | T | 0 | 122.70 |
| aca | T | 568 | 250.60 |
| acg | T | 4 | 47.55 |
| --- | -- | ------ | ------ |
| mPD | | 0.014 | 0.69 |
| | | nPD: | 0.02 |
| | | N. weight: | 1.1 |
| | | Sc. PD: | -0.27 |
| | | Sc. rank: | -305.2 |

| | | | |
|---|---|---|---|
| PB2 | | | |
| Pos. | 365 | obs: | exp: |
| atg | M | 572 | 572.00 |
| --- | -- | ------ | ------ |
| mPD | | 0 | 0 |
| | | nPD: | 1. |
| | | N. weight: | 0. |
| | | Sc. PD: | 0 |
| | | Sc. rank: | 0 |

| | | | |
|---|---|---|---|
| PB2 | | | |
| Pos. | 366 | obs: | exp: |
| gtt | V | 269 | 117.60 |
| gtc | V | 300 | 120.10 |
| gta | V | 0 | 107.20 |
| gtg | V | 3 | 227.20 |
| --- | -- | ------ | ------ |
| mPD | | 0.50 | 0.72 |
| | | nPD: | 0.7 |
| | | N. weight: | 1.2 |
| | | Sc. PD: | 0.5 |
| | | Sc. rank: | 401.6 |

| | | | |
|---|---|---|---|
| PB2 | | | |
| Pos. | 367 | obs: | exp: |
| ggt | G | 2 | 70.58 |
| ggc | G | 0 | 77.35 |
| gga | G | 76 | 276.70 |
| ggg | G | 494 | 147.40 |
| --- | -- | ------ | ------ |
| mPD | | 0.24 | 0.67 |
| | | nPD: | 0.36 |
| | | N. weight: | 1.2 |
| | | Sc. PD: | 0.099 |
| | | Sc. rank: | 139.1 |

| | | | |
|---|---|---|---|
| PB2 | | | |
| Pos. | 368 | obs: | exp: |
| caa | Q | 1 | 0.53 |
| cag | Q | 0 | 0.47 |
| cgt | R | 0 | 23.66 |
| cgc | R | 0 | 27.91 |
| cga | R | 407 | 54.78 |
| cgg | R | 62 | 47.57 |
| aaa | K | 1 | 0.56 |
| aag | K | 0 | 0.44 |
| aga | R | 95 | 256.30 |
| agg | R | 6 | 159.70 |
| --- | -- | ------ | ------ |
| mPD | | 0.51 | 0.97 |
| | | nPD: | 0.53 |
| | | N. weight: | 1.8 |
| | | Sc. PD: | 0.44 |
| | | Sc. rank: | 456.6 |

| | | | |
|---|---|---|---|
| PB2 | | | |
| Pos. | 369 | obs: | exp: |
| cgt | R | 0 | 23.66 |
| cgc | R | 0 | 27.91 |
| cga | R | 1 | 54.78 |
| cgg | R | 1 | 47.57 |
| aaa | K | 2 | 1.12 |
| aag | K | 0 | 0.88 |
| aga | R | 521 | 256.30 |
| agg | R | 47 | 159.70 |
| --- | -- | ------ | ------ |
| mPD | | 0.17 | 0.97 |
| | | nPD: | 0.17 |
| | | N. weight: | 0.77 |
| | | Sc. PD: | -0.072 |
| | | Sc. rank: | -53.4 |

| | | | |
|---|---|---|---|
| PB2 | | | |
| Pos. | 370 | obs: | exp: |
| gct | A | 44 | 144.10 |
| gcc | A | 9 | 117.20 |
| gca | A | 507 | 254.90 |
| gcg | A | 12 | 55.82 |
| --- | -- | ------ | ------ |
| mPD | | 0.21 | 0.69 |
| | | nPD: | 0.3 |
| | | N. weight: | 0.65 |
| | | Sc. PD: | 0.02 |
| | | Sc. rank: | 46.5 |

| | | | |
|---|---|---|---|
| PB2 | | | |
| Pos. | 371 | obs: | exp: |
| act | T | 3 | 151.10 |
| acc | T | 1 | 122.70 |
| aca | T | 520 | 250.60 |
| acg | T | 48 | 47.55 |
| --- | -- | ------ | ------ |
| mPD | | 0.17 | 0.69 |
| | | nPD: | 0.24 |
| | | N. weight: | 0.92 |
| | | Sc. PD: | -0.025 |
| | | Sc. rank: | 4.8 |

| | | | |
|---|---|---|---|
| PB2 | | | |
| Pos. | 372 | obs: | exp: |
| gct | A | 54 | 144.10 |
| gcc | A | 517 | 117.20 |
| gca | A | 1 | 254.90 |
| gcg | A | 0 | 55.82 |
| --- | -- | ------ | ------ |
| mPD | | 0.17 | 0.69 |
| | | nPD: | 0.25 |
| | | N. weight: | 1.8 |
| | | Sc. PD: | -0.03 |
| | | Sc. rank: | 35.7 |

| | | | |
|---|---|---|---|
| PB2 | | | |
| Pos. | 373 | obs: | exp: |
| att | I | 536 | 215.00 |
| atc | I | 35 | 149.90 |
| ata | I | 1 | 207.10 |
| --- | -- | ------ | ------ |
| mPD | | 0.12 | 0.66 |
| | | nPD: | 0.18 |
| | | N. weight: | 1.1 |
| | | Sc. PD: | -0.096 |
| | | Sc. rank: | -69.3 |

| | | | |
|---|---|---|---|
| PB2 | | | |
| Pos. | 374 | obs: | exp: |
| tta | L | 10 | 49.97 |
| ttg | L | 10 | 110.20 |
| ctt | L | 0 | 106.70 |
| ctc | L | 69 | 92.53 |
| cta | L | 440 | 86.17 |
| ctg | L | 43 | 126.40 |
| --- | -- | ------ | ------ |
| mPD | | 0.43 | 1.1 |
| | | nPD: | 0.38 |
| | | N. weight: | 1.5 |
| | | Sc. PD: | 0.16 |
| | | Sc. rank: | 210.1 |

| | | | |
|---|---|---|---|
| PB2 | | | |
| Pos. | 375 | obs: | exp: |
| cgt | R | 0 | 23.75 |
| cgc | R | 0 | 28.01 |
| cga | R | 0 | 54.97 |
| cgg | R | 0 | 47.73 |
| aga | R | 256 | 257.20 |
| agg | R | 316 | 160.30 |
| --- | -- | ------ | ------ |
| mPD | | 0.50 | 0.96 |
| | | nPD: | 0.52 |
| | | N. weight: | 0.54 |
| | | Sc. PD: | 0.13 |
| | | Sc. rank: | 131.1 |

| | | | |
|---|---|---|---|
| PB2 | | | |
| Pos. | 376 | obs: | exp: |
| aaa | K | 262 | 320.30 |
| aag | K | 310 | 251.70 |
| --- | -- | ------ | ------ |
| mPD | | 0.50 | 0.49 |
| | | nPD: | 1.01 |
| | | N. weight: | 0.038 |
| | | Sc. PD: | 0.027 |
| | | Sc. rank: | 13.3 |

| | | | |
|---|---|---|---|
| PB2 | | | |
| Pos. | 377 | obs: | exp: |
| gct | A | 0 | 144.10 |
| gcc | A | 0 | 117.20 |
| gca | A | 568 | 254.90 |
| gcg | A | 4 | 55.82 |
| --- | -- | ------ | ------ |
| mPD | | 0.014 | 0.69 |
| | | nPD: | 0.02 |
| | | N. weight: | 1.1 |
| | | Sc. PD: | -0.27 |
| | | Sc. rank: | -299.4 |

| | | | |
|---|---|---|---|
| PB2 | | | |
| Pos. | 378 | obs: | exp: |
| act | T | 21 | 151.10 |
| acc | T | 550 | 122.70 |
| aca | T | 1 | 250.60 |
| acg | T | 0 | 47.55 |
| --- | -- | ------ | ------ |
| mPD | | 0.074 | 0.69 |
| | | nPD: | 0.11 |
| | | N. weight: | 2. |
| | | Sc. PD: | -0.3 |
| | | Sc. rank: | -291.8 |

| | | | |
|---|---|---|---|
| PB2 | | | |
| Pos. | 379 | obs: | exp: |
| cgt | R | 0 | 23.75 |
| cgc | R | 0 | 28.01 |
| cga | R | 0 | 54.97 |
| cgg | R | 0 | 47.73 |
| aga | R | 558 | 257.20 |
| agg | R | 14 | 160.30 |
| --- | -- | ------ | ------ |
| mPD | | 0.048 | 0.96 |
| | | nPD: | 0.05 |
| | | N. weight: | 1. |
| | | Sc. PD: | -0.21 |
| | | Sc. rank: | -215.0 |

| | | | |
|---|---|---|---|
| PB2 | | | |
| Pos. | 380 | obs: | exp: |
| cgt | R | 0 | 23.75 |
| cgc | R | 0 | 28.01 |
| cga | R | 0 | 54.97 |
| cgg | R | 0 | 47.73 |
| aga | R | 76 | 257.20 |
| agg | R | 496 | 160.30 |
| --- | -- | ------ | ------ |
| mPD | | 0.23 | 0.96 |
| | | nPD: | 0.24 |
| | | N. weight: | 1.2 |
| | | Sc. PD: | -0.035 |
| | | Sc. rank: | 1.5 |

| | | | |
|---|---|---|---|
| PB2 | | | |
| Pos. | 381 | obs: | exp: |
| tta | L | 2 | 49.97 |
| ttg | L | 50 | 110.20 |
| ctt | L | 5 | 106.70 |
| ctc | L | 0 | 92.53 |
| cta | L | 14 | 86.17 |
| ctg | L | 501 | 126.40 |
| --- | -- | ------ | ------ |
| mPD | | 0.24 | 1.1 |
| | | nPD: | 0.21 |
| | | N. weight: | 1.5 |
| | | Sc. PD: | -0.086 |
| | | Sc. rank: | -40.3 |

| | | | |
|---|---|---|---|
| PB2 | | | |
| Pos. | 382 | obs: | exp: |
| att | I | 53 | 214.30 |
| atc | I | 508 | 149.40 |
| ata | I | 9 | 206.30 |
| gtt | V | 0 | 0.41 |
| gtc | V | 2 | 0.42 |
| gta | V | 0 | 0.37 |
| gtg | V | 0 | 0.79 |
| --- | -- | ------ | ------ |
| mPD | | 0.20 | 0.67 |
| | | nPD: | 0.31 |
| | | N. weight: | 1.3 |
| | | Sc. PD: | 0.042 |
| | | Sc. rank: | 95.9 |

| | | | |
|---|---|---|---|
| PB2 | | | |
| Pos. | 383 | obs: | exp: |
| caa | Q | 502 | 304.90 |
| cag | Q | 70 | 267.10 |
| --- | -- | ------ | ------ |
| mPD | | 0.22 | 0.50 |
| | | nPD: | 0.43 |
| | | N. weight: | 0.4 |
| | | Sc. PD: | 0.061 |
| | | Sc. rank: | 70.9 |

| | | | |
|---|---|---|---|
| PB2 | | | |
| Pos. | 384 | obs: | exp: |
| tta | L | 98 | 49.97 |
| ttg | L | 335 | 110.20 |
| ctt | L | 1 | 106.70 |
| ctc | L | 1 | 92.53 |
| cta | L | 47 | 86.17 |
| ctg | L | 90 | 126.40 |
| --- | -- | ------ | ------ |
| mPD | | 0.75 | 1.1 |
| | | nPD: | 0.68 |
| | | N. weight: | 0.95 |
| | | Sc. PD: | 0.37 |
| | | Sc. rank: | 301.5 |

| | | | |
|---|---|---|---|
| PB2 | | | |
| Pos. | 385 | obs: | exp: |
| att | I | 12 | 215.00 |
| atc | I | 0 | 149.90 |
| ata | I | 560 | 207.10 |
| --- | -- | ------ | ------ |
| mPD | | 0.041 | 0.66 |
| | | nPD: | 0.06 |
| | | N. weight: | 1.3 |
| | | Sc. PD: | -0.26 |
| | | Sc. rank: | -255.0 |

| | | | |
|---|---|---|---|
| PB2 | | | |
| Pos. | 386 | obs: | exp: |
| gtt | V | 15 | 117.60 |
| gtc | V | 0 | 120.10 |
| gta | V | 161 | 107.20 |
| gtg | V | 396 | 227.20 |
| --- | -- | ------ | ------ |
| mPD | | 0.44 | 0.72 |
| | | nPD: | 0.61 |
| | | N. weight: | 0.65 |
| | | Sc. PD: | 0.21 |
| | | Sc. rank: | 190.0 |

| | | | |
|---|---|---|---|
| PB2 | | | |
| Pos. | 387 | obs: | exp: |
| tct | S | 0 | 90.78 |
| tcc | S | 0 | 79.54 |
| tca | S | 0 | 126.50 |
| tcg | S | 0 | 34.80 |
| agt | S | 519 | 125.60 |
| agc | S | 52 | 113.80 |
| ggt | G | 1 | 0.12 |
| ggc | G | 0 | 0.14 |
| gga | G | 0 | 0.48 |
| ggg | G | 0 | 0.26 |
| --- | -- | ------ | ------ |
| mPD | | 0.17 | 1.7 |
| | | nPD: | 0.1 |
| | | N. weight: | 1.7 |
| | | Sc. PD: | -0.28 |
| | | Sc. rank: | -278.6 |

| | | | |
|---|---|---|---|
| PB2 | | | |
| Pos. | 388 | obs: | exp: |
| ggt | G | 2 | 70.58 |
| ggc | G | 0 | 77.35 |
| gga | G | 137 | 276.70 |
| ggg | G | 433 | 147.40 |
| --- | -- | ------ | ------ |
| mPD | | 0.37 | 0.67 |
| | | nPD: | 0.56 |
| | | N. weight: | 0.92 |
| | | Sc. PD: | 0.25 |
| | | Sc. rank: | 243.3 |

| | | | |
|---|---|---|---|
| PB2 | | | |
| Pos. | 389 | obs: | exp: |
| cgt | R | 0 | 22.21 |
| cgc | R | 0 | 26.20 |
| cga | R | 0 | 51.42 |
| cgg | R | 0 | 44.64 |
| aaa | K | 36 | 20.72 |
| aag | K | 1 | 16.28 |
| aga | R | 505 | 240.60 |
| agg | R | 30 | 149.90 |
| --- | -- | ------ | ------ |
| mPD | | 0.22 | 1.1 |
| | | nPD: | 0.21 |
| | | N. weight: | 0.87 |
| | | Sc. PD: | -0.05 |
| | | Sc. rank: | -27.3 |

| | | | |
|---|---|---|---|
| PB2 | | | |
| Pos. | 390 | obs: | exp: |
| aat | N | 2 | 1.05 |
| aac | N | 0 | 0.95 |
| gat | D | 83 | 304.10 |
| gac | D | 487 | 265.90 |
| --- | -- | ------ | ------ |
| mPD | | 0.26 | 0.50 |
| | | nPD: | 0.52 |
| | | N. weight: | 0.48 |
| | | Sc. PD: | 0.11 |
| | | Sc. rank: | 115.5 |

PB2

| Pos. 391 | | obs: | exp: |
|---|---|---|---|
| gaa | E | 32 | 329.10 |
| gag | E | 540 | 242.90 |
| --- | -- | ------ | ------ |
| mPD | | 0.11 | 0.49 |
| | | nPD: | 0.22 |
| | | N. weight: | 0.9 |
| | | Sc. PD: | -0.047 |
| | | Sc. rank: | -20.5 |

PB2

| Pos. 392 | | obs: | exp: |
|---|---|---|---|
| cat | H | 1 | 0.54 |
| cac | H | 0 | 0.46 |
| caa | Q | 114 | 304.40 |
| cag | Q | 457 | 266.60 |
| --- | -- | ------ | ------ |
| mPD | | 0.32 | 0.50 |
| | | nPD: | 0.65 |
| | | N. weight: | 0.34 |
| | | Sc. PD: | 0.12 |
| | | Sc. rank: | 104.5 |

PB2

| Pos. 393 | | obs: | exp: |
|---|---|---|---|
| tct | S | 0 | 90.93 |
| tcc | S | 0 | 79.68 |
| tca | S | 564 | 126.70 |
| tcg | S | 8 | 34.87 |
| agt | S | 0 | 125.80 |
| agc | S | 0 | 114.00 |
| --- | -- | ------ | ------ |
| mPD | | 0.028 | 1.7 |
| | | nPD: | 0.02 |
| | | N. weight: | 2.1 |
| | | Sc. PD: | -0.51 |
| | | Sc. rank: | -566.9 |

PB2

| Pos. 394 | | obs: | exp: |
|---|---|---|---|
| att | I | 79 | 214.70 |
| atc | I | 487 | 149.60 |
| ata | I | 5 | 206.70 |
| gtt | V | 1 | 0.21 |
| gtc | V | 0 | 0.21 |
| gta | V | 0 | 0.19 |
| gtg | V | 0 | 0.40 |
| --- | -- | ------ | ------ |
| mPD | | 0.26 | 0.66 |
| | | nPD: | 0.39 |
| | | N. weight: | 1.2 |
| | | Sc. PD: | 0.14 |
| | | Sc. rank: | 175.9 |

PB2

| Pos. 395 | | obs: | exp: |
|---|---|---|---|
| gct | A | 211 | 144.10 |
| gcc | A | 358 | 117.20 |
| gca | A | 3 | 254.90 |
| gcg | A | 0 | 55.82 |
| --- | -- | ------ | ------ |
| mPD | | 0.47 | 0.69 |
| | | nPD: | 0.69 |
| | | N. weight: | 1.2 |
| | | Sc. PD: | 0.47 |
| | | Sc. rank: | 379.2 |

PB2

| Pos. 396 | | obs: | exp: |
|---|---|---|---|
| gaa | E | 508 | 329.10 |
| gag | E | 64 | 242.90 |
| --- | -- | ------ | ------ |
| mPD | | 0.20 | 0.49 |
| | | nPD: | 0.41 |
| | | N. weight: | 0.34 |
| | | Sc. PD: | 0.045 |
| | | Sc. rank: | 52.2 |

PB2

| Pos. 397 | | obs: | exp: |
|---|---|---|---|
| gtt | V | 0 | 0.21 |
| gtc | V | 0 | 0.21 |
| gta | V | 0 | 0.19 |
| gtg | V | 1 | 0.40 |
| gct | A | 2 | 143.80 |
| gcc | A | 0 | 117.00 |
| gca | A | 392 | 254.50 |
| gcg | A | 177 | 55.72 |
| --- | -- | ------ | ------ |
| mPD | | 0.44 | 0.69 |
| | | nPD: | 0.63 |
| | | N. weight: | 0.93 |
| | | Sc. PD: | 0.32 |
| | | Sc. rank: | 275.7 |

PB2

| Pos. 398 | | obs: | exp: |
|---|---|---|---|
| att | I | 271 | 214.30 |
| atc | I | 228 | 149.40 |
| ata | I | 71 | 206.30 |
| atg | M | 1 | 1.00 |
| gtt | V | 1 | 0.21 |
| gtc | V | 0 | 0.21 |
| gta | V | 0 | 0.19 |
| gtg | V | 0 | 0.40 |
| --- | -- | ------ | ------ |
| mPD | | 0.60 | 0.66 |
| | | nPD: | 0.91 |
| | | N. weight: | 0.23 |
| | | Sc. PD: | 0.14 |
| | | Sc. rank: | 79.7 |

PB2

| Pos. 399 | | obs: | exp: |
|---|---|---|---|
| att | I | 130 | 215.00 |
| atc | I | 18 | 149.90 |
| ata | I | 424 | 207.10 |
| --- | -- | ------ | ------ |
| mPD | | 0.40 | 0.66 |
| | | nPD: | 0.6 |
| | | N. weight: | 0.51 |
| | | Sc. PD: | 0.16 |
| | | Sc. rank: | 149.0 |

PB2

| Pos. 400 | | obs: | exp: |
|---|---|---|---|
| gtt | V | 0 | 117.60 |
| gtc | V | 0 | 120.10 |
| gta | V | 73 | 107.20 |
| gtg | V | 499 | 227.20 |
| --- | -- | ------ | ------ |
| mPD | | 0.22 | 0.72 |
| | | nPD: | 0.31 |
| | | N. weight: | 0.92 |
| | | Sc. PD: | 0.033 |
| | | Sc. rank: | 68.3 |

PB2

| Pos. 401 | | obs: | exp: |
|---|---|---|---|
| tct | S | 0 | 0.16 |
| tcc | S | 0 | 0.14 |
| tca | S | 1 | 0.22 |
| tcg | S | 0 | 0.06 |
| agt | S | 0 | 0.22 |
| agc | S | 0 | 0.20 |
| gct | A | 3 | 143.80 |
| gcc | A | 67 | 117.00 |
| gca | A | 499 | 254.50 |
| gcg | A | 2 | 55.72 |
| --- | -- | ------ | ------ |
| mPD | | 0.23 | 0.69 |
| | | nPD: | 0.33 |
| | | N. weight: | 0.72 |
| | | Sc. PD: | 0.038 |
| | | Sc. rank: | 63.2 |

PB2

| Pos. 402 | | obs: | exp: |
|---|---|---|---|
| atg | M | 572 | 572.00 |
| --- | -- | ------ | ------ |
| mPD | | 0 | 0 |
| | | nPD: | 1. |
| | | N. weight: | 0. |
| | | Sc. PD: | 0 |
| | | Sc. rank: | 0 |

PB2

| Pos. 403 | | obs: | exp: |
|---|---|---|---|
| gtt | V | 87 | 117.60 |
| gtc | V | 5 | 120.10 |
| gta | V | 85 | 107.20 |
| gtg | V | 395 | 227.20 |
| --- | -- | ------ | ------ |
| mPD | | 0.48 | 0.72 |
| | | nPD: | 0.66 |
| | | N. weight: | 0.41 |
| | | Sc. PD: | 0.15 |
| | | Sc. rank: | 126.9 |

PB2

| Pos. 404 | | obs: | exp: |
|---|---|---|---|
| ttt | F | 32 | 235.20 |
| ttc | F | 540 | 336.80 |
| --- | -- | ------ | ------ |
| mPD | | 0.11 | 0.48 |
| | | nPD: | 0.22 |
| | | N. weight: | 0.48 |
| | | Sc. PD: | -0.025 |
| | | Sc. rank: | -10.8 |

PB2

| Pos. 405 | | obs: | exp: |
|---|---|---|---|
| tct | S | 0 | 90.93 |
| tcc | S | 49 | 79.68 |
| tca | S | 482 | 126.70 |
| tcg | S | 41 | 34.87 |
| agt | S | 0 | 125.80 |
| agc | S | 0 | 114.00 |
| --- | -- | ------ | ------ |
| mPD | | 0.28 | 1.7 |
| | | nPD: | 0.17 |
| | | N. weight: | 1.6 |
| | | Sc. PD: | -0.16 |
| | | Sc. rank: | -118.5 |

PB2

| Pos. 406 | | obs: | exp: |
|---|---|---|---|
| caa | Q | 534 | 304.90 |
| cag | Q | 38 | 267.10 |
| --- | -- | ------ | ------ |
| mPD | | 0.12 | 0.50 |
| | | nPD: | 0.25 |
| | | N. weight: | 0.57 |
| | | Sc. PD: | -0.012 |
| | | Sc. rank: | 7.7 |

PB2

| Pos. 407 | | obs: | exp: |
|---|---|---|---|
| gaa | E | 19 | 329.10 |
| gag | E | 553 | 242.90 |
| --- | -- | ------ | ------ |
| mPD | | 0.064 | 0.49 |
| | | nPD: | 0.13 |
| | | N. weight: | 1. |
| | | Sc. PD: | -0.13 |
| | | Sc. rank: | -126.7 |

PB2

| Pos. 408 | | obs: | exp: |
|---|---|---|---|
| gat | D | 484 | 305.20 |
| gac | D | 88 | 266.80 |
| --- | -- | ------ | ------ |
| mPD | | 0.26 | 0.50 |
| | | nPD: | 0.52 |
| | | N. weight: | 0.32 |
| | | Sc. PD: | 0.077 |
| | | Sc. rank: | 80.2 |

PB2

| Pos. 409 | | obs: | exp: |
|---|---|---|---|
| tgt | C | 67 | 235.80 |
| tgc | C | 505 | 336.20 |
| --- | -- | ------ | ------ |
| mPD | | 0.21 | 0.48 |
| | | nPD: | 0.43 |
| | | N. weight: | 0.31 |
| | | Sc. PD: | 0.046 |
| | | Sc. rank: | 54.6 |

PB2

| Pos. 410 | | obs: | exp: |
|---|---|---|---|
| atg | M | 572 | 572.00 |
| --- | -- | ------ | ------ |
| mPD | | 0 | 0 |
| | | nPD: | 1. |
| | | N. weight: | 0. |
| | | Sc. PD: | 0 |
| | | Sc. rank: | 0 |

PB2

| Pos. 411 | | obs: | exp: |
|---|---|---|---|
| att | I | 46 | 213.50 |
| atc | I | 24 | 148.90 |
| ata | I | 498 | 205.60 |
| atg | M | 1 | 1.00 |
| gtt | V | 0 | 0.62 |
| gtc | V | 0 | 0.63 |
| gta | V | 3 | 0.56 |
| gtg | V | 0 | 1.19 |
| --- | -- | ------ | ------ |
| mPD | | 0.24 | 0.67 |
| | | nPD: | 0.35 |
| | | N. weight: | 0.84 |
| | | Sc. PD: | 0.063 |
| | | Sc. rank: | 88.8 |

PB2

| Pos. 412 | | obs: | exp: |
|---|---|---|---|
| aaa | K | 432 | 320.30 |
| aag | K | 140 | 251.70 |
| --- | -- | ------ | ------ |
| mPD | | 0.37 | 0.49 |
| | | nPD: | 0.75 |
| | | N. weight: | 0.13 |
| | | Sc. PD: | 0.058 |
| | | Sc. rank: | 42.4 |

PB2

| Pos. 413 | | obs: | exp: |
|---|---|---|---|
| tct | S | 0 | 0.16 |
| tcc | S | 0 | 0.14 |
| tca | S | 1 | 0.22 |
| tcg | S | 0 | 0.06 |
| agt | S | 0 | 0.22 |
| agc | S | 0 | 0.20 |
| gct | A | 20 | 143.80 |
| gcc | A | 0 | 117.00 |
| gca | A | 546 | 254.50 |
| gcg | A | 5 | 55.72 |
| --- | -- | ------ | ------ |
| mPD | | 0.088 | 0.69 |
| | | nPD: | 0.13 |
| | | N. weight: | 0.93 |
| | | Sc. PD: | -0.13 |
| | | Sc. rank: | -123.5 |

PB2

| Pos. 414 | | obs: | exp: |
|---|---|---|---|
| gtt | V | 87 | 117.60 |
| gtc | V | 45 | 120.10 |
| gta | V | 398 | 107.20 |
| gtg | V | 42 | 227.20 |
| --- | -- | ------ | ------ |
| mPD | | 0.48 | 0.72 |
| | | nPD: | 0.67 |
| | | N. weight: | 0.97 |
| | | Sc. PD: | 0.37 |
| | | Sc. rank: | 301.4 |

PB2

| Pos. 415 | | obs: | exp: |
|---|---|---|---|
| cgt | R | 1 | 23.75 |
| cgc | R | 0 | 28.01 |
| cga | R | 533 | 54.97 |
| cgg | R | 5 | 47.73 |
| aga | R | 32 | 257.20 |
| agg | R | 1 | 160.30 |
| --- | -- | ------ | ------ |
| mPD | | 0.13 | 0.96 |
| | | nPD: | 0.14 |
| | | N. weight: | 2.8 |
| | | Sc. PD: | -0.36 |
| | | Sc. rank: | -301.9 |

PB2

| Pos. 416 | | obs: | exp: |
|---|---|---|---|
| ggt | G | 543 | 70.58 |
| ggc | G | 27 | 77.35 |
| gga | G | 1 | 276.70 |
| ggg | G | 1 | 147.40 |
| --- | -- | ------ | ------ |
| mPD | | 0.097 | 0.67 |
| | | nPD: | 0.15 |
| | | N. weight: | 2.7 |
| | | Sc. PD: | -0.32 |
| | | Sc. rank: | -258.9 |

PB2

| Pos. 417 | | obs: | exp: |
|---|---|---|---|
| gat | D | 539 | 305.20 |
| gac | D | 33 | 266.80 |
| --- | -- | ------ | ------ |
| mPD | | 0.11 | 0.50 |
| | | nPD: | 0.22 |
| | | N. weight: | 0.6 |
| | | Sc. PD: | -0.03 |
| | | Sc. rank: | -13.1 |

PB2

| Pos. 418 | | obs: | exp: |
|---|---|---|---|
| tta | L | 41 | 49.97 |
| ttg | L | 393 | 110.20 |
| ctt | L | 0 | 106.70 |
| ctc | L | 0 | 92.53 |
| cta | L | 9 | 86.17 |
| ctg | L | 129 | 126.40 |
| --- | -- | ------ | ------ |
| mPD | | 0.53 | 1.1 |
| | | nPD: | 0.47 |
| | | N. weight: | 1.2 |
| | | Sc. PD: | 0.23 |
| | | Sc. rank: | 250.9 |

PB2

| Pos. 419 | | obs: | exp: |
|---|---|---|---|
| aat | N | 503 | 299.00 |
| aac | N | 69 | 273.00 |
| --- | -- | ------ | ------ |
| mPD | | 0.21 | 0.50 |
| | | nPD: | 0.43 |
| | | N. weight: | 0.42 |
| | | Sc. PD: | 0.062 |
| | | Sc. rank: | 74.4 |

PB2

| Pos. 420 | | obs: | exp: |
|---|---|---|---|
| ttt | F | 507 | 235.20 |
| ttc | F | 65 | 336.80 |
| --- | -- | ------ | ------ |
| mPD | | 0.20 | 0.48 |
| | | nPD: | 0.42 |
| | | N. weight: | 0.71 |
| | | Sc. PD: | 0.099 |
| | | Sc. rank: | 115.7 |

PB2

| Pos. | 421 | obs : | exp : |
|---|---|---|---|
| gtt | V | 85 | 117.60 |
| gtc | V | 423 | 120.10 |
| gta | V | 32 | 107.20 |
| gtg | V | 32 | 227.20 |
| --- | -- | ------ | ------ |
| mPD | | 0.43 | 0.72 |
| | | nPD : | 0.59 |
| | | N. weight : | 1. |
| | | Sc. PD : | 0.31 |
| | | Sc. rank : | 294.3 |

PB2

| Pos. | 422 | obs : | exp : |
|---|---|---|---|
| aat | N | 129 | 299.00 |
| aac | N | 443 | 273.00 |
| --- | -- | ------ | ------ |
| mPD | | 0.35 | 0.50 |
| | | nPD : | 0.7 |
| | | N. weight : | 0.27 |
| | | Sc. PD : | 0.11 |
| | | Sc. rank : | 90.0 |

PB2

| Pos. | 423 | obs : | exp : |
|---|---|---|---|
| cgt | R | 0 | 23.71 |
| cgc | R | 0 | 27.96 |
| cga | R | 0 | 54.88 |
| cgg | R | 0 | 47.65 |
| act | T | 0 | 0.26 |
| acc | T | 0 | 0.21 |
| aca | T | 1 | 0.44 |
| acg | T | 0 | 0.08 |
| aga | R | 496 | 256.80 |
| agg | R | 75 | 160.00 |
| --- | -- | ------ | ------ |
| mPD | | 0.23 | 0.96 |
| | | nPD : | 0.24 |
| | | N. weight : | 0.68 |
| | | Sc. PD : | -0.02 |
| | | Sc. rank : | 1.3 |

PB2

| Pos. | 424 | obs : | exp : |
|---|---|---|---|
| gct | A | 5 | 144.10 |
| gcc | A | 0 | 117.20 |
| gca | A | 112 | 254.90 |
| gcg | A | 455 | 55.82 |
| --- | -- | ------ | ------ |
| mPD | | 0.33 | 0.69 |
| | | nPD : | 0.48 |
| | | N. weight : | 2.1 |
| | | Sc. PD : | 0.42 |
| | | Sc. rank : | 448.3 |

PB2

| Pos. | 425 | obs : | exp : |
|---|---|---|---|
| aat | N | 138 | 299.00 |
| aac | N | 434 | 273.00 |
| --- | -- | ------ | ------ |
| mPD | | 0.37 | 0.50 |
| | | nPD : | 0.74 |
| | | N. weight : | 0.24 |
| | | Sc. PD : | 0.11 |
| | | Sc. rank : | 81.9 |

PB2

| Pos. | 426 | obs : | exp : |
|---|---|---|---|
| caa | Q | 62 | 304.90 |
| cag | Q | 510 | 267.10 |
| --- | -- | ------ | ------ |
| mPD | | 0.19 | 0.50 |
| | | nPD : | 0.39 |
| | | N. weight : | 0.58 |
| | | Sc. PD : | 0.065 |
| | | Sc. rank : | 82.9 |

PB2

| Pos. | 427 | obs : | exp : |
|---|---|---|---|
| cgt | R | 0 | 23.75 |
| cgc | R | 0 | 28.01 |
| cga | R | 50 | 54.97 |
| cgg | R | 494 | 47.73 |
| aga | R | 20 | 257.20 |
| agg | R | 8 | 160.30 |
| --- | -- | ------ | ------ |
| mPD | | 0.31 | 0.96 |
| | | nPD : | 0.32 |
| | | N. weight : | 2.7 |
| | | Sc. PD : | 0.13 |
| | | Sc. rank : | 226.2 |

PB2

| Pos. | 428 | obs : | exp : |
|---|---|---|---|
| tta | L | 46 | 49.97 |
| ttg | L | 16 | 110.20 |
| ctt | L | 17 | 106.70 |
| ctc | L | 29 | 92.53 |
| cta | L | 362 | 86.17 |
| ctg | L | 102 | 126.40 |
| --- | -- | ------ | ------ |
| mPD | | 0.64 | 1.1 |
| | | nPD : | 0.57 |
| | | N. weight : | 1. |
| | | Sc. PD : | 0.3 |
| | | Sc. rank : | 284.2 |

PB2

| Pos. | 429 | obs : | exp : |
|---|---|---|---|
| aat | N | 463 | 299.00 |
| aac | N | 109 | 273.00 |
| --- | -- | ------ | ------ |
| mPD | | 0.31 | 0.50 |
| | | nPD : | 0.62 |
| | | N. weight : | 0.26 |
| | | Sc. PD : | 0.087 |
| | | Sc. rank : | 77.5 |

PB2

| Pos. | 430 | obs : | exp : |
|---|---|---|---|
| cct | P | 402 | 150.60 |
| ccc | P | 101 | 103.20 |
| cca | P | 67 | 225.70 |
| ccg | P | 2 | 92.49 |
| --- | -- | ------ | ------ |
| mPD | | 0.46 | 0.72 |
| | | nPD : | 0.64 |
| | | N. weight : | 0.78 |
| | | Sc. PD : | 0.28 |
| | | Sc. rank : | 234.9 |

PB2

| Pos. | 431 | obs : | exp : |
|---|---|---|---|
| atg | M | 572 | 572.00 |
| --- | -- | ------ | ------ |
| mPD | | 0 | 0 |
| | | nPD : | 1. |
| | | N. weight : | 0. |
| | | Sc. PD : | 0 |
| | | Sc. rank : | 0 |

PB2

| Pos. | 432 | obs : | exp : |
|---|---|---|---|
| cat | H | 534 | 310.00 |
| cac | H | 38 | 262.00 |
| --- | -- | ------ | ------ |
| mPD | | 0.12 | 0.50 |
| | | nPD : | 0.25 |
| | | N. weight : | 0.55 |
| | | Sc. PD : | -0.011 |
| | | Sc. rank : | 7.7 |

PB2

| Pos. | 433 | obs : | exp : |
|---|---|---|---|
| caa | Q | 247 | 304.90 |
| cag | Q | 325 | 267.10 |
| --- | -- | ------ | ------ |
| mPD | | 0.49 | 0.50 |
| | | nPD : | 0.99 |
| | | N. weight : | 0.038 |
| | | Sc. PD : | 0.026 |
| | | Sc. rank : | 13.1 |

PB2

| Pos. | 434 | obs : | exp : |
|---|---|---|---|
| tta | L | 0 | 49.97 |
| ttg | L | 1 | 110.20 |
| ctt | L | 72 | 106.70 |
| ctc | L | 461 | 92.53 |
| cta | L | 29 | 86.17 |
| ctg | L | 9 | 126.40 |
| --- | -- | ------ | ------ |
| mPD | | 0.34 | 1.1 |
| | | nPD : | 0.3 |
| | | N. weight : | 1.6 |
| | | Sc. PD : | 0.047 |
| | | Sc. rank : | 116.4 |

PB2

| Pos. | 435 | obs : | exp : |
|---|---|---|---|
| tta | L | 2 | 49.97 |
| ttg | L | 88 | 110.20 |
| ctt | L | 1 | 106.70 |
| ctc | L | 0 | 92.53 |
| cta | L | 6 | 86.17 |
| ctg | L | 475 | 126.40 |
| --- | -- | ------ | ------ |
| mPD | | 0.30 | 1.1 |
| | | nPD : | 0.27 |
| | | N. weight : | 1.5 |
| | | Sc. PD : | -0.0076 |
| | | Sc. rank : | 51.2 |

PB2

| Pos. | 436 | obs : | exp : |
|---|---|---|---|
| cgt | R | 0 | 23.75 |
| cgc | R | 0 | 28.01 |
| cga | R | 0 | 54.97 |
| cgg | R | 1 | 47.73 |
| aga | R | 85 | 257.20 |
| agg | R | 486 | 160.30 |
| --- | -- | ------ | ------ |
| mPD | | 0.26 | 0.96 |
| | | nPD : | 0.27 |
| | | N. weight : | 1.1 |
| | | Sc. PD : | -0.0042 |
| | | Sc. rank : | 43.3 |

PB2

| Pos. | 437 | obs : | exp : |
|---|---|---|---|
| cat | H | 455 | 310.00 |
| cac | H | 117 | 262.00 |
| --- | -- | ------ | ------ |
| mPD | | 0.33 | 0.50 |
| | | nPD : | 0.66 |
| | | N. weight : | 0.21 |
| | | Sc. PD : | 0.077 |
| | | Sc. rank : | 63.6 |

PB2

| Pos. | 438 | obs : | exp : |
|---|---|---|---|
| ttt | F | 18 | 235.20 |
| ttc | F | 554 | 336.80 |
| --- | -- | ------ | ------ |
| mPD | | 0.061 | 0.48 |
| | | nPD : | 0.13 |
| | | N. weight : | 0.58 |
| | | Sc. PD : | -0.08 |
| | | Sc. rank : | -78.1 |

PB2

| Pos. | 439 | obs : | exp : |
|---|---|---|---|
| caa | Q | 528 | 304.90 |
| cag | Q | 44 | 267.10 |
| --- | -- | ------ | ------ |
| mPD | | 0.14 | 0.50 |
| | | nPD : | 0.29 |
| | | N. weight : | 0.53 |
| | | Sc. PD : | 0.0072 |
| | | Sc. rank : | 30.2 |

PB2

| Pos. | 440 | obs : | exp : |
|---|---|---|---|
| aaa | K | 41 | 320.30 |
| aag | K | 531 | 251.70 |
| --- | -- | ------ | ------ |
| mPD | | 0.13 | 0.49 |
| | | nPD : | 0.27 |
| | | N. weight : | 0.79 |
| | | Sc. PD : | -0.00087 |
| | | Sc. rank : | 32.4 |

PB2

| Pos. | 441 | obs : | exp : |
|---|---|---|---|
| gat | D | 558 | 305.20 |
| gac | D | 14 | 266.80 |
| --- | -- | ------ | ------ |
| mPD | | 0.048 | 0.50 |
| | | nPD : | 0.1 |
| | | N. weight : | 0.74 |
| | | Sc. PD : | -0.12 |
| | | Sc. rank : | -121.2 |

PB2

| Pos. | 442 | obs : | exp : |
|---|---|---|---|
| gct | A | 1 | 144.10 |
| gcc | A | 9 | 117.20 |
| gca | A | 560 | 254.90 |
| gcg | A | 2 | 55.82 |
| --- | -- | ------ | ------ |
| mPD | | 0.041 | 0.69 |
| | | nPD : | 0.06 |
| | | N. weight : | 1. |
| | | Sc. PD : | -0.21 |
| | | Sc. rank : | -204.8 |

PB2

| Pos. | 443 | obs : | exp : |
|---|---|---|---|
| cgt | R | 0 | 0.04 |
| cgc | R | 0 | 0.05 |
| cga | R | 0 | 0.10 |
| cgg | R | 0 | 0.08 |
| aaa | K | 158 | 319.70 |
| aag | K | 413 | 251.30 |
| aga | R | 1 | 0.45 |
| agg | R | 0 | 0.28 |
| --- | -- | ------ | ------ |
| mPD | | 0.41 | 0.50 |
| | | nPD : | 0.82 |
| | | N. weight : | 0.24 |
| | | Sc. PD : | 0.13 |
| | | Sc. rank : | 83.6 |

PB2

| Pos. | 444 | obs : | exp : |
|---|---|---|---|
| atg | M | 1 | 1.00 |
| gtt | V | 31 | 117.40 |
| gtc | V | 1 | 119.80 |
| gta | V | 55 | 107.00 |
| gtg | V | 484 | 226.80 |
| --- | -- | ------ | ------ |
| mPD | | 0.27 | 0.72 |
| | | nPD : | 0.38 |
| | | N. weight : | 0.73 |
| | | Sc. PD : | 0.073 |
| | | Sc. rank : | 96.4 |

PB2

| Pos. | 445 | obs : | exp : |
|---|---|---|---|
| tta | L | 2 | 49.97 |
| ttg | L | 40 | 110.20 |
| ctt | L | 77 | 106.70 |
| ctc | L | 4 | 92.53 |
| cta | L | 8 | 86.17 |
| ctg | L | 441 | 126.40 |
| --- | -- | ------ | ------ |
| mPD | | 0.41 | 1.1 |
| | | nPD : | 0.37 |
| | | N. weight : | 1.1 |
| | | Sc. PD : | 0.11 |
| | | Sc. rank : | 138.4 |

PB2

| Pos. | 446 | obs: | exp: |
|---|---|---|---|
| ttt | F | 224 | 235.20 |
| ttc | F | 348 | 336.80 |
| mPD | | 0.48 | 0.48 |
| | | nPD: | 0.99 |
| | | N. weight: | 0.0096 |
| | | Sc. PD: | 0.0065 |
| | | Sc. rank: | 3.3 |

PB2

| Pos. | 447 | obs: | exp: |
|---|---|---|---|
| caa | Q | 485 | 300.70 |
| cag | Q | 79 | 263.30 |
| aaa | K | 8 | 4.48 |
| aag | K | 0 | 3.52 |
| mPD | | 0.27 | 0.53 |
| | | nPD: | 0.51 |
| | | N. weight: | 0.36 |
| | | Sc. PD: | 0.081 |
| | | Sc. rank: | 84.7 |

PB2

| Pos. | 448 | obs: | exp: |
|---|---|---|---|
| tct | S | 0 | 0.48 |
| tcc | S | 0 | 0.42 |
| tca | S | 0 | 0.66 |
| tcg | S | 0 | 0.18 |
| act | T | 0 | 0.79 |
| acc | T | 3 | 0.64 |
| aca | T | 0 | 1.31 |
| acg | T | 0 | 0.25 |
| aat | N | 67 | 295.30 |
| aac | N | 498 | 269.70 |
| aaa | K | 1 | 0.56 |
| aag | K | 0 | 0.44 |
| agt | S | 1 | 0.66 |
| agc | S | 2 | 0.60 |
| mPD | | 0.23 | 0.53 |
| | | nPD: | 0.44 |
| | | N. weight: | 0.54 |
| | | Sc. PD: | 0.087 |
| | | Sc. rank: | 99.7 |

PB2

| Pos. | 449 | obs: | exp: |
|---|---|---|---|
| tgg | W | 572 | 572.00 |
| mPD | | 0 | 0 |
| | | nPD: | 1. |
| | | N. weight: | 0. |
| | | Sc. PD: | 0 |
| | | Sc. rank: | 0 |

PB2

| Pos. | 450 | obs: | exp: |
|---|---|---|---|
| ggt | G | 1 | 70.58 |
| ggc | G | 0 | 77.35 |
| gga | G | 553 | 276.70 |
| ggg | G | 18 | 147.40 |
| mPD | | 0.064 | 0.67 |
| | | nPD: | 0.1 |
| | | N. weight: | 0.86 |
| | | Sc. PD: | -0.14 |
| | | Sc. rank: | -139.5 |

PB2

| Pos. | 451 | obs: | exp: |
|---|---|---|---|
| tta | L | 0 | 0.09 |
| ttg | L | 1 | 0.19 |
| ctt | L | 0 | 0.19 |
| ctc | L | 0 | 0.16 |
| cta | L | 0 | 0.15 |
| ctg | L | 0 | 0.22 |
| att | I | 533 | 200.70 |
| atc | I | 1 | 140.00 |
| ata | I | 0 | 193.30 |
| act | T | 5 | 1.32 |
| acc | T | 0 | 1.07 |
| aca | T | 0 | 2.19 |
| acg | T | 0 | 0.42 |
| gtt | V | 32 | 6.58 |
| gtc | V | 0 | 6.72 |
| gta | V | 0 | 6.00 |
| gtg | V | 0 | 12.71 |
| mPD | | 0.13 | 0.80 |
| | | nPD: | 0.17 |
| | | N. weight: | 1.4 |
| | | Sc. PD: | -0.14 |
| | | Sc. rank: | -111.0 |

PB2

| Pos. | 452 | obs: | exp: |
|---|---|---|---|
| gaa | E | 546 | 329.10 |
| gag | E | 26 | 242.90 |
| mPD | | 0.087 | 0.49 |
| | | nPD: | 0.18 |
| | | N. weight: | 0.55 |
| | | Sc. PD: | -0.049 |
| | | Sc. rank: | -37.8 |

PB2

| Pos. | 453 | obs: | exp: |
|---|---|---|---|
| tta | L | 0 | 0.17 |
| ttg | L | 0 | 0.39 |
| tct | S | 0 | 4.45 |
| tcc | S | 28 | 3.90 |
| tca | S | 0 | 6.20 |
| tcg | S | 0 | 1.71 |
| ctt | L | 0 | 0.37 |
| ctc | L | 2 | 0.32 |
| cta | L | 0 | 0.30 |
| ctg | L | 0 | 0.44 |
| cct | P | 16 | 142.20 |
| ccc | P | 464 | 97.40 |
| cca | P | 58 | 213.10 |
| ccg | P | 2 | 87.31 |
| caa | Q | 0 | 0.53 |
| cag | Q | 1 | 0.47 |
| act | T | 0 | 0.26 |
| acc | T | 1 | 0.21 |
| aca | T | 0 | 0.44 |
| acg | T | 0 | 0.08 |
| agt | S | 0 | 6.16 |
| agc | S | 0 | 5.58 |
| mPD | | 0.35 | 0.87 |
| | | nPD: | 0.4 |
| | | N. weight: | 1.7 |
| | | Sc. PD: | 0.21 |
| | | Sc. rank: | 249.7 |

PB2

| Pos. | 454 | obs: | exp: |
|---|---|---|---|
| att | I | 530 | 215.00 |
| atc | I | 42 | 149.90 |
| ata | I | 0 | 207.10 |
| mPD | | 0.14 | 0.66 |
| | | nPD: | 0.21 |
| | | N. weight: | 1.1 |
| | | Sc. PD: | -0.066 |
| | | Sc. rank: | -38.7 |

PB2

| Pos. | 455 | obs: | exp: |
|---|---|---|---|
| gat | D | 73 | 305.20 |
| gac | D | 499 | 266.80 |
| mPD | | 0.22 | 0.50 |
| | | nPD: | 0.45 |
| | | N. weight: | 0.53 |
| | | Sc. PD: | 0.089 |
| | | Sc. rank: | 101.4 |

PB2

| Pos. | 456 | obs: | exp: |
|---|---|---|---|
| cat | H | 1 | 0.54 |
| cac | H | 0 | 0.46 |
| aat | N | 566 | 297.90 |
| aac | N | 4 | 272.10 |
| gat | D | 1 | 0.53 |
| gac | D | 0 | 0.47 |
| mPD | | 0.021 | 0.51 |
| | | nPD: | 0.04 |
| | | N. weight: | 0.87 |
| | | Sc. PD: | -0.19 |
| | | Sc. rank: | -200.2 |

PB2

| Pos. | 457 | obs: | exp: |
|---|---|---|---|
| gtt | V | 243 | 117.60 |
| gtc | V | 227 | 120.10 |
| gta | V | 56 | 107.20 |
| gtg | V | 46 | 227.20 |
| mPD | | 0.65 | 0.72 |
| | | nPD: | 0.9 |
| | | N. weight: | 0.55 |
| | | Sc. PD: | 0.33 |
| | | Sc. rank: | 190.1 |

PB2

| Pos. | 458 | obs: | exp: |
|---|---|---|---|
| att | I | 0 | 0.38 |
| atc | I | 0 | 0.26 |
| ata | I | 1 | 0.36 |
| atg | M | 571 | 571.00 |
| mPD | | 0.0035 | 0.0035 |
| | | nPD: | 1. |
| | | N. weight: | 0.0025 |
| | | Sc. PD: | 0.0018 |
| | | Sc. rank: | 0.9 |

PB2

| Pos. | 459 | obs: | exp: |
|---|---|---|---|
| ggt | G | 2 | 70.58 |
| ggc | G | 0 | 77.35 |
| gga | G | 93 | 276.70 |
| ggg | G | 477 | 147.40 |
| mPD | | 0.28 | 0.67 |
| | | nPD: | 0.42 |
| | | N. weight: | 1.1 |
| | | Sc. PD: | 0.16 |
| | | Sc. rank: | 189.3 |

PB2

| Pos. | 460 | obs: | exp: |
|---|---|---|---|
| atg | M | 572 | 572.00 |
| mPD | | 0 | 0 |
| | | nPD: | 1. |
| | | N. weight: | 0. |
| | | Sc. PD: | 0 |
| | | Sc. rank: | 0 |

PB2

| Pos. | 461 | obs: | exp: |
|---|---|---|---|
| att | I | 83 | 206.80 |
| atc | I | 436 | 144.10 |
| ata | I | 31 | 199.10 |
| atg | M | 1 | 1.00 |
| gtt | V | 0 | 4.32 |
| gtc | V | 4 | 4.41 |
| gta | V | 17 | 3.94 |
| gtg | V | 0 | 8.34 |
| mPD | | 0.45 | 0.74 |
| | | nPD: | 0.61 |
| | | N. weight: | 0.95 |
| | | Sc. PD: | 0.31 |
| | | Sc. rank: | 277.4 |

PB2

| Pos. | 462 | obs: | exp: |
|---|---|---|---|
| ggt | G | 2 | 70.58 |
| ggc | G | 0 | 77.35 |
| gga | G | 480 | 276.70 |
| ggg | G | 90 | 147.40 |
| mPD | | 0.27 | 0.67 |
| | | nPD: | 0.41 |
| | | N. weight: | 0.54 |
| | | Sc. PD: | 0.07 |
| | | Sc. rank: | 82.4 |

PB2

| Pos. | 463 | obs: | exp: |
|---|---|---|---|
| att | I | 0 | 212.40 |
| atc | I | 0 | 148.10 |
| ata | I | 565 | 204.50 |
| atg | M | 3 | 3.00 |
| gtt | V | 0 | 0.82 |
| gtc | V | 0 | 0.84 |
| gta | V | 4 | 0.75 |
| gtg | V | 0 | 1.59 |
| mPD | | 0.024 | 0.68 |
| | | nPD: | 0.04 |
| | | N. weight: | 1.5 |
| | | Sc. PD: | -0.33 |
| | | Sc. rank: | -339.8 |

PB2

| Pos. | 464 | obs: | exp: |
|---|---|---|---|
| tta | L | 305 | 49.88 |
| ttg | L | 199 | 110.00 |
| ctt | L | 0 | 106.50 |
| ctc | L | 0 | 92.36 |
| cta | L | 14 | 86.02 |
| ctg | L | 53 | 126.20 |
| atg | M | 1 | 1.00 |
| mPD | | 0.70 | 1.1 |
| | | nPD: | 0.63 |
| | | N. weight: | 1.5 |
| | | Sc. PD: | 0.52 |
| | | Sc. rank: | 449.8 |

PB2

| Pos. | 465 | obs: | exp: |
|---|---|---|---|
| cct | P | 535 | 150.60 |
| ccc | P | 35 | 103.20 |
| cca | P | 2 | 225.70 |
| ccg | P | 0 | 92.49 |
| mPD | | 0.12 | 0.72 |
| | | nPD: | 0.17 |
| | | N. weight: | 1.6 |
| | | Sc. PD: | -0.15 |
| | | Sc. rank: | -114.4 |

| | | | |
|---|---|---|---|
| PB2 | | | |
| Pos. | 466 | obs: | exp: |
| gat | D | 58 | 305.20 |
| gac | D | 514 | 266.80 |
| --- | -- | ------ | ------ |
| mPD | | 0.18 | 0.50 |
| | | nPD: | 0.37 |
| | | N. weight: | 0.61 |
| | | Sc. PD: | 0.055 |
| | | Sc. rank: | 73.0 |

| | | | |
|---|---|---|---|
| PB2 | | | |
| Pos. | 467 | obs: | exp: |
| atg | M | 572 | 572.00 |
| --- | -- | ------ | ------ |
| mPD | | 0 | 0 |
| | | nPD: | 1. |
| | | N. weight: | 0. |
| | | Sc. PD: | 0 |
| | | Sc. rank: | 0 |

| | | | |
|---|---|---|---|
| PB2 | | | |
| Pos. | 468 | obs: | exp: |
| act | T | 48 | 151.10 |
| acc | T | 480 | 122.70 |
| aca | T | 44 | 250.60 |
| acg | T | 0 | 47.55 |
| --- | -- | ------ | ------ |
| mPD | | 0.28 | 0.69 |
| | | nPD: | 0.41 |
| | | N. weight: | 1.3 |
| | | Sc. PD: | 0.18 |
| | | Sc. rank: | 210.4 |

| | | | |
|---|---|---|---|
| PB2 | | | |
| Pos. | 469 | obs: | exp: |
| cct | P | 9 | 150.60 |
| ccc | P | 458 | 103.20 |
| cca | P | 102 | 225.70 |
| ccg | P | 3 | 92.49 |
| --- | -- | ------ | ------ |
| mPD | | 0.33 | 0.72 |
| | | nPD: | 0.46 |
| | | N. weight: | 1.4 |
| | | Sc. PD: | 0.25 |
| | | Sc. rank: | 283.1 |

| | | | |
|---|---|---|---|
| PB2 | | | |
| Pos. | 470 | obs: | exp: |
| tct | S | 0 | 90.46 |
| tcc | S | 0 | 79.26 |
| tca | S | 0 | 126.10 |
| tcg | S | 0 | 34.68 |
| aat | N | 0 | 1.57 |
| aac | N | 3 | 1.43 |
| agt | S | 103 | 125.20 |
| agc | S | 466 | 113.40 |
| --- | -- | ------ | ------ |
| mPD | | 0.31 | 1.7 |
| | | nPD: | 0.18 |
| | | N. weight: | 1.6 |
| | | Sc. PD: | -0.14 |
| | | Sc. rank: | -95.6 |

| | | | |
|---|---|---|---|
| PB2 | | | |
| Pos. | 471 | obs: | exp: |
| att | I | 1 | 0.38 |
| atc | I | 0 | 0.26 |
| ata | I | 0 | 0.36 |
| act | T | 67 | 150.30 |
| acc | T | 4 | 122.10 |
| aca | T | 491 | 249.30 |
| acg | T | 7 | 47.30 |
| gct | A | 2 | 0.50 |
| gcc | A | 0 | 0.41 |
| gca | A | 0 | 0.89 |
| gcg | A | 0 | 0.20 |
| --- | -- | ------ | ------ |
| mPD | | 0.26 | 0.70 |
| | | nPD: | 0.37 |
| | | N. weight: | 0.65 |
| | | Sc. PD: | 0.063 |
| | | Sc. rank: | 82.4 |

| | | | |
|---|---|---|---|
| PB2 | | | |
| Pos. | 472 | obs: | exp: |
| gaa | E | 55 | 329.10 |
| gag | E | 517 | 242.90 |
| --- | -- | ------ | ------ |
| mPD | | 0.17 | 0.49 |
| | | nPD: | 0.36 |
| | | N. weight: | 0.74 |
| | | Sc. PD: | 0.06 |
| | | Sc. rank: | 83.2 |

| | | | |
|---|---|---|---|
| PB2 | | | |
| Pos. | 473 | obs: | exp: |
| tta | L | 0 | 0.09 |
| ttg | L | 1 | 0.19 |
| ctt | L | 0 | 0.19 |
| ctc | L | 0 | 0.16 |
| cta | L | 0 | 0.15 |
| ctg | L | 0 | 0.22 |
| att | I | 0 | 1.50 |
| atc | I | 0 | 1.05 |
| ata | I | 4 | 1.45 |
| atg | M | 558 | 558.00 |
| act | T | 0 | 0.79 |
| acc | T | 0 | 0.64 |
| aca | T | 0 | 1.31 |
| acg | T | 3 | 0.25 |
| gtt | V | 0 | 1.03 |
| gtc | V | 0 | 1.05 |
| gta | V | 0 | 0.94 |
| gtg | V | 5 | 1.99 |
| gaa | E | 0 | 0.58 |
| gag | E | 1 | 0.42 |
| --- | -- | ------ | ------ |
| mPD | | 0.052 | 0.076 |
| | | nPD: | 0.69 |
| | | N. weight: | 0.047 |
| | | Sc. PD: | 0.018 |
| | | Sc. rank: | 14.9 |

| | | | |
|---|---|---|---|
| PB2 | | | |
| Pos. | 474 | obs: | exp: |
| tct | S | 0 | 90.93 |
| tcc | S | 1 | 79.68 |
| tca | S | 531 | 126.70 |
| tcg | S | 40 | 34.87 |
| agt | S | 0 | 125.80 |
| agc | S | 0 | 114.00 |
| --- | -- | ------ | ------ |
| mPD | | 0.13 | 1.7 |
| | | nPD: | 0.08 |
| | | N. weight: | 1.9 |
| | | Sc. PD: | -0.35 |
| | | Sc. rank: | -343.5 |

| | | | |
|---|---|---|---|
| PB2 | | | |
| Pos. | 475 | obs: | exp: |
| tta | L | 15 | 49.97 |
| ttg | L | 35 | 110.20 |
| ctt | L | 0 | 106.70 |
| ctc | L | 0 | 92.53 |
| cta | L | 346 | 86.17 |
| ctg | L | 176 | 126.40 |
| --- | -- | ------ | ------ |
| mPD | | 0.63 | 1.1 |
| | | nPD: | 0.56 |
| | | N. weight: | 1.2 |
| | | Sc. PD: | 0.34 |
| | | Sc. rank: | 330.3 |

| | | | |
|---|---|---|---|
| PB2 | | | |
| Pos. | 476 | obs: | exp: |
| cgt | R | 0 | 23.75 |
| cgc | R | 0 | 28.01 |
| cga | R | 1 | 54.97 |
| cgg | R | 3 | 47.73 |
| aga | R | 497 | 257.20 |
| agg | R | 71 | 160.30 |
| --- | -- | ------ | ------ |
| mPD | | 0.24 | 0.96 |
| | | nPD: | 0.25 |
| | | N. weight: | 0.66 |
| | | Sc. PD: | -0.014 |
| | | Sc. rank: | 8.5 |

| | | | |
|---|---|---|---|
| PB2 | | | |
| Pos. | 477 | obs: | exp: |
| ggt | G | 239 | 70.58 |
| ggc | G | 0 | 77.35 |
| gga | G | 302 | 276.70 |
| ggg | G | 31 | 147.40 |
| --- | -- | ------ | ------ |
| mPD | | 0.54 | 0.67 |
| | | nPD: | 0.82 |
| | | N. weight: | 0.69 |
| | | Sc. PD: | 0.36 |
| | | Sc. rank: | 235.7 |

| | | | |
|---|---|---|---|
| PB2 | | | |
| Pos. | 478 | obs: | exp: |
| att | I | 0 | 27.82 |
| atc | I | 0 | 19.39 |
| ata | I | 74 | 26.79 |
| gtt | V | 0 | 102.40 |
| gtc | V | 0 | 104.50 |
| gta | V | 47 | 93.34 |
| gtg | V | 451 | 197.80 |
| --- | -- | ------ | ------ |
| mPD | | 0.56 | 0.96 |
| | | nPD: | 0.58 |
| | | N. weight: | 1. |
| | | Sc. PD: | 0.31 |
| | | Sc. rank: | 292.9 |

| | | | |
|---|---|---|---|
| PB2 | | | |
| Pos. | 479 | obs: | exp: |
| cgt | R | 0 | 23.75 |
| cgc | R | 0 | 28.01 |
| cga | R | 0 | 54.97 |
| cgg | R | 0 | 47.73 |
| aga | R | 535 | 257.20 |
| agg | R | 37 | 160.30 |
| --- | -- | ------ | ------ |
| mPD | | 0.12 | 0.96 |
| | | nPD: | 0.13 |
| | | N. weight: | 0.85 |
| | | Sc. PD: | -0.12 |
| | | Sc. rank: | -114.0 |

| | | | |
|---|---|---|---|
| PB2 | | | |
| Pos. | 480 | obs: | exp: |
| att | I | 0 | 1.13 |
| atc | I | 3 | 0.79 |
| ata | I | 0 | 1.09 |
| gtt | V | 56 | 117.00 |
| gtc | V | 481 | 119.40 |
| gta | V | 32 | 106.60 |
| gtg | V | 0 | 226.00 |
| --- | -- | ------ | ------ |
| mPD | | 0.28 | 0.73 |
| | | nPD: | 0.39 |
| | | N. weight: | 1.5 |
| | | Sc. PD: | 0.16 |
| | | Sc. rank: | 209.7 |

| | | | |
|---|---|---|---|
| PB2 | | | |
| Pos. | 481 | obs: | exp: |
| tct | S | 0 | 90.93 |
| tcc | S | 0 | 79.68 |
| tca | S | 0 | 126.70 |
| tcg | S | 0 | 34.87 |
| agt | S | 558 | 125.80 |
| agc | S | 14 | 114.00 |
| --- | -- | ------ | ------ |
| mPD | | 0.048 | 1.7 |
| | | nPD: | 0.03 |
| | | N. weight: | 2. |
| | | Sc. PD: | -0.47 |
| | | Sc. rank: | -501.8 |

| | | | |
|---|---|---|---|
| PB2 | | | |
| Pos. | 482 | obs: | exp: |
| cgt | R | 0 | 0.08 |
| cgc | R | 0 | 0.10 |
| cga | R | 0 | 0.19 |
| cgg | R | 0 | 0.17 |
| act | T | 0 | 0.26 |
| acc | T | 0 | 0.21 |
| aca | T | 0 | 0.44 |
| acg | T | 1 | 0.08 |
| aaa | K | 480 | 318.60 |
| aag | K | 89 | 250.40 |
| aga | R | 2 | 0.90 |
| agg | R | 0 | 0.56 |
| --- | -- | ------ | ------ |
| mPD | | 0.28 | 0.51 |
| | | nPD: | 0.55 |
| | | N. weight: | 0.28 |
| | | Sc. PD: | 0.073 |
| | | Sc. rank: | 72.6 |

| | | | |
|---|---|---|---|
| PB2 | | | |
| Pos. | 483 | obs: | exp: |
| atg | M | 572 | 572.00 |
| --- | -- | ------ | ------ |
| mPD | | 0 | 0 |
| | | nPD: | 1. |
| | | N. weight: | 0. |
| | | Sc. PD: | 0 |
| | | Sc. rank: | 0 |

| | | | |
|---|---|---|---|
| PB2 | | | |
| Pos. | 484 | obs: | exp: |
| ggt | G | 0 | 70.58 |
| ggc | G | 0 | 77.35 |
| gga | G | 565 | 276.70 |
| ggg | G | 7 | 147.40 |
| --- | -- | ------ | ------ |
| mPD | | 0.024 | 0.67 |
| | | nPD: | 0.04 |
| | | N. weight: | 0.96 |
| | | Sc. PD: | -0.22 |
| | | Sc. rank: | -223.8 |

| | | | |
|---|---|---|---|
| PB2 | | | |
| Pos. | 485 | obs: | exp: |
| gtt | V | 9 | 117.60 |
| gtc | V | 0 | 120.10 |
| gta | V | 109 | 107.20 |
| gtg | V | 454 | 227.20 |
| --- | -- | ------ | ------ |
| mPD | | 0.33 | 0.72 |
| | | nPD: | 0.46 |
| | | N. weight: | 0.74 |
| | | Sc. PD: | 0.14 |
| | | Sc. rank: | 150.6 |

| PB2 | | | |
|---|---|---|---|
| Pos. | 486 | obs: | exp: |
| gat | D | 548 | 305.20 |
| gac | D | 24 | 266.80 |
| --- | -- | ------ | ------ |
| mPD | | 0.081 | 0.50 |
| | | nPD: | 0.16 |
| | | N. weight: | 0.66 |
| | | Sc. PD: | -0.069 |
| | | Sc. rank: | -53.2 |

| PB2 | | | |
|---|---|---|---|
| Pos. | 487 | obs: | exp: |
| gaa | E | 554 | 329.10 |
| gag | E | 18 | 242.90 |
| --- | -- | ------ | ------ |
| mPD | | 0.061 | 0.49 |
| | | nPD: | 0.12 |
| | | N. weight: | 0.61 |
| | | Sc. PD: | -0.085 |
| | | Sc. rank: | -82.5 |

| PB2 | | | |
|---|---|---|---|
| Pos. | 488 | obs: | exp: |
| tat | Y | 473 | 296.10 |
| tac | Y | 99 | 275.90 |
| --- | -- | ------ | ------ |
| mPD | | 0.29 | 0.50 |
| | | nPD: | 0.57 |
| | | N. weight: | 0.31 |
| | | Sc. PD: | 0.089 |
| | | Sc. rank: | 85.3 |

| PB2 | | | |
|---|---|---|---|
| Pos. | 489 | obs: | exp: |
| tct | S | 8 | 90.93 |
| tcc | S | 561 | 79.68 |
| tca | S | 3 | 126.70 |
| tcg | S | 0 | 34.87 |
| agt | S | 0 | 125.80 |
| agc | S | 0 | 114.00 |
| --- | -- | ------ | ------ |
| mPD | | 0.038 | 1.7 |
| | | nPD: | 0.02 |
| | | N. weight: | 2.7 |
| | | Sc. PD: | -0.63 |
| | | Sc. rank: | -696.8 |

| PB2 | | | |
|---|---|---|---|
| Pos. | 490 | obs: | exp: |
| tct | S | 0 | 90.30 |
| tcc | S | 0 | 79.13 |
| tca | S | 0 | 125.80 |
| tcg | S | 0 | 34.62 |
| aat | N | 4 | 2.09 |
| aac | N | 0 | 1.91 |
| agt | S | 509 | 125.00 |
| agc | S | 59 | 113.20 |
| --- | -- | ------ | ------ |
| mPD | | 0.20 | 1.7 |
| | | nPD: | 0.12 |
| | | N. weight: | 1.7 |
| | | Sc. PD: | -0.25 |
| | | Sc. rank: | -240.0 |

| PB2 | | | |
|---|---|---|---|
| Pos. | 491 | obs: | exp: |
| act | T | 494 | 150.60 |
| acc | T | 6 | 122.30 |
| aca | T | 68 | 249.70 |
| acg | T | 2 | 47.38 |
| gct | A | 2 | 0.50 |
| gcc | A | 0 | 0.41 |
| gca | A | 0 | 0.89 |
| gcg | A | 0 | 0.20 |
| --- | -- | ------ | ------ |
| mPD | | 0.24 | 0.69 |
| | | nPD: | 0.35 |
| | | N. weight: | 1.2 |
| | | Sc. PD: | 0.088 |
| | | Sc. rank: | 126.6 |

| PB2 | | | |
|---|---|---|---|
| Pos. | 492 | obs: | exp: |
| gaa | E | 7 | 329.10 |
| gag | E | 565 | 242.90 |
| --- | -- | ------ | ------ |
| mPD | | 0.024 | 0.49 |
| | | nPD: | 0.05 |
| | | N. weight: | 1.1 |
| | | Sc. PD: | -0.24 |
| | | Sc. rank: | -243.8 |

| PB2 | | | |
|---|---|---|---|
| Pos. | 493 | obs: | exp: |
| cgt | R | 0 | 23.75 |
| cgc | R | 0 | 28.01 |
| cga | R | 1 | 54.97 |
| cgg | R | 38 | 47.73 |
| aga | R | 468 | 257.20 |
| agg | R | 65 | 160.30 |
| --- | -- | ------ | ------ |
| mPD | | 0.42 | 0.96 |
| | | nPD: | 0.44 |
| | | N. weight: | 0.54 |
| | | Sc. PD: | 0.087 |
| | | Sc. rank: | 98.8 |

| PB2 | | | |
|---|---|---|---|
| Pos. | 494 | obs: | exp: |
| att | I | 0 | 0.38 |
| atc | I | 0 | 0.26 |
| ata | I | 1 | 0.36 |
| atg | M | 1 | 1.00 |
| gtt | V | 9 | 117.20 |
| gtc | V | 0 | 119.60 |
| gta | V | 75 | 106.80 |
| gtg | V | 486 | 226.40 |
| --- | -- | ------ | ------ |
| mPD | | 0.26 | 0.73 |
| | | nPD: | 0.36 |
| | | N. weight: | 0.82 |
| | | Sc. PD: | 0.072 |
| | | Sc. rank: | 97.4 |

| PB2 | | | |
|---|---|---|---|
| Pos. | 495 | obs: | exp: |
| att | I | 1 | 0.75 |
| atc | I | 1 | 0.52 |
| ata | I | 0 | 0.72 |
| gtt | V | 53 | 117.20 |
| gtc | V | 443 | 119.60 |
| gta | V | 69 | 106.80 |
| gtg | V | 5 | 226.40 |
| --- | -- | ------ | ------ |
| mPD | | 0.38 | 0.73 |
| | | nPD: | 0.52 |
| | | N. weight: | 1.2 |
| | | Sc. PD: | 0.3 |
| | | Sc. rank: | 309.4 |

| PB2 | | | |
|---|---|---|---|
| Pos. | 496 | obs: | exp: |
| gtt | V | 0 | 117.60 |
| gtc | V | 2 | 120.10 |
| gta | V | 59 | 107.20 |
| gtg | V | 511 | 227.20 |
| --- | -- | ------ | ------ |
| mPD | | 0.19 | 0.72 |
| | | nPD: | 0.27 |
| | | N. weight: | 0.94 |
| | | Sc. PD: | -0.0052 |
| | | Sc. rank: | 31.4 |

| PB2 | | | |
|---|---|---|---|
| Pos. | 497 | obs: | exp: |
| tct | S | 0 | 89.34 |
| tcc | S | 0 | 78.29 |
| tca | S | 0 | 124.50 |
| tcg | S | 0 | 34.26 |
| aat | N | 2 | 4.18 |
| aac | N | 6 | 3.82 |
| agt | S | 96 | 123.60 |
| agc | S | 466 | 112.00 |
| ggt | G | 0 | 0.25 |
| ggc | G | 2 | 0.27 |
| gga | G | 0 | 0.97 |
| ggg | G | 0 | 0.52 |
| --- | -- | ------ | ------ |
| mPD | | 0.32 | 1.7 |
| | | nPD: | 0.19 |
| | | N. weight: | 1.6 |
| | | Sc. PD: | -0.13 |
| | | Sc. rank: | -85.6 |

| PB2 | | | |
|---|---|---|---|
| Pos. | 498 | obs: | exp: |
| att | I | 539 | 215.00 |
| atc | I | 33 | 149.90 |
| ata | I | 0 | 207.10 |
| --- | -- | ------ | ------ |
| mPD | | 0.11 | 0.66 |
| | | nPD: | 0.17 |
| | | N. weight: | 1.1 |
| | | Sc. PD: | -0.11 |
| | | Sc. rank: | -87.9 |

| PB2 | | | |
|---|---|---|---|
| Pos. | 499 | obs: | exp: |
| gat | D | 459 | 305.20 |
| gac | D | 113 | 266.80 |
| --- | -- | ------ | ------ |
| mPD | | 0.32 | 0.50 |
| | | nPD: | 0.64 |
| | | N. weight: | 0.23 |
| | | Sc. PD: | 0.082 |
| | | Sc. rank: | 69.8 |

| PB2 | | | |
|---|---|---|---|
| Pos. | 500 | obs: | exp: |
| cgt | R | 483 | 23.75 |
| cgc | R | 19 | 28.01 |
| cga | R | 64 | 54.97 |
| cgg | R | 6 | 47.73 |
| aga | R | 0 | 257.20 |
| agg | R | 0 | 160.30 |
| --- | -- | ------ | ------ |
| mPD | | 0.27 | 0.96 |
| | | nPD: | 0.29 |
| | | N. weight: | 3.6 |
| | | Sc. PD: | 0.047 |
| | | Sc. rank: | 201.9 |

| PB2 | | | |
|---|---|---|---|
| Pos. | 501 | obs: | exp: |
| ttt | F | 73 | 234.70 |
| ttc | F | 498 | 336.30 |
| gtt | V | 0 | 0.21 |
| gtc | V | 1 | 0.21 |
| gta | V | 0 | 0.19 |
| gtg | V | 0 | 0.40 |
| --- | -- | ------ | ------ |
| mPD | | 0.23 | 0.49 |
| | | nPD: | 0.46 |
| | | N. weight: | 0.29 |
| | | Sc. PD: | 0.052 |
| | | Sc. rank: | 58.1 |

| PB2 | | | |
|---|---|---|---|
| Pos. | 502 | obs: | exp: |
| tta | L | 94 | 49.97 |
| ttg | L | 472 | 110.20 |
| ctt | L | 0 | 106.70 |
| ctc | L | 0 | 92.53 |
| cta | L | 0 | 86.17 |
| ctg | L | 6 | 126.40 |
| --- | -- | ------ | ------ |
| mPD | | 0.30 | 1.1 |
| | | nPD: | 0.27 |
| | | N. weight: | 1.8 |
| | | Sc. PD: | -0.011 |
| | | Sc. rank: | 59.7 |

| PB2 | | | |
|---|---|---|---|
| Pos. | 503 | obs: | exp: |
| cgt | R | 0 | 23.71 |
| cgc | R | 0 | 27.96 |
| cga | R | 0 | 54.88 |
| cgg | R | 1 | 47.65 |
| aaa | K | 1 | 0.56 |
| aag | K | 0 | 0.44 |
| aga | R | 151 | 256.80 |
| agg | R | 419 | 160.00 |
| --- | -- | ------ | ------ |
| mPD | | 0.40 | 0.96 |
| | | nPD: | 0.41 |
| | | N. weight: | 0.81 |
| | | Sc. PD: | 0.11 |
| | | Sc. rank: | 128.0 |

| PB2 | | | |
|---|---|---|---|
| Pos. | 504 | obs: | exp: |
| gtt | V | 62 | 117.60 |
| gtc | V | 510 | 120.10 |
| gta | V | 0 | 107.20 |
| gtg | V | 0 | 227.20 |
| --- | -- | ------ | ------ |
| mPD | | 0.19 | 0.72 |
| | | nPD: | 0.27 |
| | | N. weight: | 1.8 |
| | | Sc. PD: | -0.0051 |
| | | Sc. rank: | 71.0 |

| PB2 | | | |
|---|---|---|---|
| Pos. | 505 | obs: | exp: |
| cgt | R | 2 | 23.75 |
| cgc | R | 0 | 28.01 |
| cga | R | 536 | 54.97 |
| cgg | R | 27 | 47.73 |
| aga | R | 4 | 257.20 |
| agg | R | 3 | 160.30 |
| --- | -- | ------ | ------ |
| mPD | | 0.13 | 0.96 |
| | | nPD: | 0.14 |
| | | N. weight: | 2.9 |
| | | Sc. PD: | -0.38 |
| | | Sc. rank: | -341.6 |

| PB2 | | | |
|---|---|---|---|
| Pos. | 506 | obs: | exp: |
| gat | D | 155 | 305.20 |
| gac | D | 417 | 266.80 |
| --- | -- | ------ | ------ |
| mPD | | 0.40 | 0.50 |
| | | nPD: | 0.8 |
| | | N. weight: | 0.21 |
| | | Sc. PD: | 0.11 |
| | | Sc. rank: | 71.7 |

| PB2 | | | |
|---|---|---|---|
| Pos. | 507 | obs: | exp: |
| cat | H | 3 | 1.63 |
| cac | H | 0 | 1.37 |
| caa | Q | 81 | 303.30 |
| cag | Q | 488 | 265.70 |
| --- | -- | ------ | ------ |
| mPD | | 0.25 | 0.50 |
| | | nPD: | 0.5 |
| | | N. weight: | 0.49 |
| | | Sc. PD: | 0.11 |
| | | Sc. rank: | 112.2 |

| PB2 | | | |
|---|---|---|---|
| Pos. | 508 | obs: | exp: |
| caa | Q | 1 | 1.07 |
| cag | Q | 1 | 0.93 |
| cgt | R | 2 | 22.50 |
| cgc | R | 0 | 26.54 |
| cga | R | 26 | 52.09 |
| cgg | R | 103 | 45.23 |
| aaa | K | 0 | 15.68 |
| aag | K | 28 | 12.32 |
| aga | R | 48 | 243.80 |
| agg | R | 363 | 151.90 |
| --- | -- | ------ | ------ |
| mPD | | 0.69 | 1.0 |
| | | nPD: | 0.66 |
| | | N. weight: | 0.83 |
| | | Sc. PD: | 0.31 |
| | | Sc. rank: | 256.5 |

| PB2 | | | |
|---|---|---|---|
| Pos. | 509 | obs: | exp: |
| ggt | G | 1 | 70.58 |
| ggc | G | 0 | 77.35 |
| gga | G | 457 | 276.70 |
| ggg | G | 114 | 147.40 |
| --- | -- | ------ | ------ |
| mPD | | 0.32 | 0.67 |
| | | nPD: | 0.48 |
| | | N. weight: | 0.5 |
| | | Sc. PD: | 0.1 |
| | | Sc. rank: | 107.3 |

| PB2 | | | |
|---|---|---|---|
| Pos. | 510 | obs: | exp: |
| aat | N | 76 | 299.00 |
| aac | N | 496 | 273.00 |
| --- | -- | ------ | ------ |
| mPD | | 0.23 | 0.50 |
| | | nPD: | 0.46 |
| | | N. weight: | 0.49 |
| | | Sc. PD: | 0.089 |
| | | Sc. rank: | 98.1 |

PB2

| Pos. | 511 | obs : | exp : |
|---|---|---|---|
| att | I | 0 | 4.89 |
| atc | I | 1 | 3.41 |
| ata | I | 12 | 4.71 |
| gtt | V | 0 | 114.90 |
| gtc | V | 0 | 117.30 |
| gta | V | 160 | 104.80 |
| gtg | V | 399 | 222.00 |
| --- | -- | ------ | ------ |
| mPD | | 0.47 | 0.77 |
| | | nPD : | 0.61 |
| | | N. weight : | 0.79 |
| | | Sc. PD : | 0.25 |
| | | Sc. rank : | 229.4 |

PB2

| Pos. | 512 | obs : | exp : |
|---|---|---|---|
| tta | L | 8 | 49.97 |
| ttg | L | 0 | 110.20 |
| ctt | L | 21 | 106.70 |
| ctc | L | 474 | 92.53 |
| cta | L | 68 | 86.17 |
| ctg | L | 1 | 126.40 |
| --- | -- | ------ | ------ |
| mPD | | 0.32 | 1.1 |
| | | nPD : | 0.29 |
| | | N. weight : | 1.8 |
| | | Sc. PD : | 0.03 |
| | | Sc. rank : | 107.3 |

PB2

| Pos. | 513 | obs : | exp : |
|---|---|---|---|
| tta | L | 36 | 49.97 |
| ttg | L | 447 | 110.20 |
| ctt | L | 0 | 106.70 |
| ctc | L | 0 | 92.53 |
| cta | L | 34 | 86.17 |
| ctg | L | 55 | 126.40 |
| --- | -- | ------ | ------ |
| mPD | | 0.48 | 1.1 |
| | | nPD : | 0.43 |
| | | N. weight : | 1.4 |
| | | Sc. PD : | 0.2 |
| | | Sc. rank : | 241.5 |

PB2

| Pos. | 514 | obs : | exp : |
|---|---|---|---|
| tct | S | 468 | 90.93 |
| tcc | S | 34 | 79.68 |
| tca | S | 55 | 126.70 |
| tcg | S | 15 | 34.87 |
| agt | S | 0 | 125.80 |
| agc | S | 0 | 114.00 |
| --- | -- | ------ | ------ |
| mPD | | 0.32 | 1.7 |
| | | nPD : | 0.19 |
| | | N. weight : | 1.7 |
| | | Sc. PD : | -0.13 |
| | | Sc. rank : | -85.5 |

PB2

| Pos. | 515 | obs : | exp : |
|---|---|---|---|
| cct | P | 517 | 150.60 |
| ccc | P | 51 | 103.20 |
| cca | P | 4 | 225.70 |
| ccg | P | 0 | 92.49 |
| --- | -- | ------ | ------ |
| mPD | | 0.18 | 0.72 |
| | | nPD : | 0.24 |
| | | N. weight : | 1.5 |
| | | Sc. PD : | -0.038 |
| | | Sc. rank : | 12.8 |

PB2

| Pos. | 516 | obs : | exp : |
|---|---|---|---|
| gaa | E | 539 | 329.10 |
| gag | E | 33 | 242.90 |
| --- | -- | ------ | ------ |
| mPD | | 0.11 | 0.49 |
| | | nPD : | 0.22 |
| | | N. weight : | 0.51 |
| | | Sc. PD : | -0.024 |
| | | Sc. rank : | -7.7 |

PB2

| Pos. | 517 | obs : | exp : |
|---|---|---|---|
| gaa | E | 164 | 329.10 |
| gag | E | 408 | 242.90 |
| --- | -- | ------ | ------ |
| mPD | | 0.41 | 0.49 |
| | | nPD : | 0.84 |
| | | N. weight : | 0.25 |
| | | Sc. PD : | 0.14 |
| | | Sc. rank : | 86.2 |

PB2

| Pos. | 518 | obs : | exp : |
|---|---|---|---|
| gtt | V | 442 | 117.60 |
| gtc | V | 129 | 120.10 |
| gta | V | 1 | 107.20 |
| gtg | V | 0 | 227.20 |
| --- | -- | ------ | ------ |
| mPD | | 0.35 | 0.72 |
| | | nPD : | 0.49 |
| | | N. weight : | 1.5 |
| | | Sc. PD : | 0.31 |
| | | Sc. rank : | 322.8 |

PB2

| Pos. | 519 | obs : | exp : |
|---|---|---|---|
| tct | S | 0 | 90.93 |
| tcc | S | 0 | 79.68 |
| tca | S | 0 | 126.70 |
| tcg | S | 0 | 34.87 |
| agt | S | 496 | 125.80 |
| agc | S | 76 | 114.00 |
| --- | -- | ------ | ------ |
| mPD | | 0.23 | 1.7 |
| | | nPD : | 0.14 |
| | | N. weight : | 1.6 |
| | | Sc. PD : | -0.21 |
| | | Sc. rank : | -171.8 |

PB2

| Pos. | 520 | obs : | exp : |
|---|---|---|---|
| gaa | E | 512 | 329.10 |
| gag | E | 60 | 242.90 |
| --- | -- | ------ | ------ |
| mPD | | 0.19 | 0.49 |
| | | nPD : | 0.38 |
| | | N. weight : | 0.36 |
| | | Sc. PD : | 0.039 |
| | | Sc. rank : | 50.3 |

PB2

| Pos. | 521 | obs : | exp : |
|---|---|---|---|
| act | T | 0 | 151.10 |
| acc | T | 0 | 122.70 |
| aca | T | 551 | 250.60 |
| acg | T | 21 | 47.55 |
| --- | -- | ------ | ------ |
| mPD | | 0.071 | 0.69 |
| | | nPD : | 0.1 |
| | | N. weight : | 1. |
| | | Sc. PD : | -0.17 |
| | | Sc. rank : | -160.8 |

PB2

| Pos. | 522 | obs : | exp : |
|---|---|---|---|
| caa | Q | 85 | 304.90 |
| cag | Q | 487 | 267.10 |
| --- | -- | ------ | ------ |
| mPD | | 0.25 | 0.50 |
| | | nPD : | 0.51 |
| | | N. weight : | 0.47 |
| | | Sc. PD : | 0.11 |
| | | Sc. rank : | 110.1 |

PB2

| Pos. | 523 | obs : | exp : |
|---|---|---|---|
| ggt | G | 0 | 70.58 |
| ggc | G | 0 | 77.35 |
| gga | G | 498 | 276.70 |
| ggg | G | 74 | 147.40 |
| --- | -- | ------ | ------ |
| mPD | | 0.23 | 0.67 |
| | | nPD : | 0.34 |
| | | N. weight : | 0.61 |
| | | Sc. PD : | 0.039 |
| | | Sc. rank : | 58.2 |

PB2

| Pos. | 524 | obs : | exp : |
|---|---|---|---|
| att | I | 0 | 0.38 |
| atc | I | 0 | 0.26 |
| ata | I | 1 | 0.36 |
| act | T | 0 | 150.60 |
| acc | T | 0 | 122.30 |
| aca | T | 515 | 249.70 |
| acg | T | 55 | 47.38 |
| gct | A | 0 | 0.25 |
| gcc | A | 0 | 0.20 |
| gca | A | 1 | 0.45 |
| gcg | A | 0 | 0.10 |
| --- | -- | ------ | ------ |
| mPD | | 0.18 | 0.69 |
| | | nPD : | 0.26 |
| | | N. weight : | 0.96 |
| | | Sc. PD : | -0.0092 |
| | | Sc. rank : | 27.7 |

PB2

| Pos. | 525 | obs : | exp : |
|---|---|---|---|
| gaa | E | 338 | 329.10 |
| gag | E | 234 | 242.90 |
| --- | -- | ------ | ------ |
| mPD | | 0.48 | 0.49 |
| | | nPD : | 0.99 |
| | | N. weight : | 0.0092 |
| | | Sc. PD : | 0.0063 |
| | | Sc. rank : | 3.2 |

PB2

| Pos. | 526 | obs : | exp : |
|---|---|---|---|
| cgt | R | 0 | 0.08 |
| cgc | R | 0 | 0.10 |
| cga | R | 0 | 0.19 |
| cgg | R | 0 | 0.17 |
| aaa | K | 128 | 319.20 |
| aag | K | 442 | 250.80 |
| aga | R | 0 | 0.90 |
| agg | R | 2 | 0.56 |
| --- | -- | ------ | ------ |
| mPD | | 0.35 | 0.50 |
| | | nPD : | 0.71 |
| | | N. weight : | 0.35 |
| | | Sc. PD : | 0.14 |
| | | Sc. rank : | 115.0 |

PB2

| Pos. | 527 | obs : | exp : |
|---|---|---|---|
| tta | L | 12 | 49.97 |
| ttg | L | 60 | 110.20 |
| ctt | L | 3 | 106.70 |
| ctc | L | 0 | 92.53 |
| cta | L | 1 | 86.17 |
| ctg | L | 496 | 126.40 |
| --- | -- | ------ | ------ |
| mPD | | 0.28 | 1.1 |
| | | nPD : | 0.25 |
| | | N. weight : | 1.5 |
| | | Sc. PD : | -0.036 |
| | | Sc. rank : | 17.9 |

PB2

| Pos. | 528 | obs : | exp : |
|---|---|---|---|
| act | T | 28 | 151.10 |
| acc | T | 0 | 122.70 |
| aca | T | 116 | 250.60 |
| acg | T | 428 | 47.55 |
| --- | -- | ------ | ------ |
| mPD | | 0.40 | 0.69 |
| | | nPD : | 0.58 |
| | | N. weight : | 2. |
| | | Sc. PD : | 0.59 |
| | | Sc. rank : | 566.2 |

PB2

| Pos. | 529 | obs : | exp : |
|---|---|---|---|
| att | I | 1 | 215.00 |
| atc | I | 49 | 149.90 |
| ata | I | 522 | 207.10 |
| --- | -- | ------ | ------ |
| mPD | | 0.16 | 0.66 |
| | | nPD : | 0.24 |
| | | N. weight : | 1.1 |
| | | Sc. PD : | -0.029 |
| | | Sc. rank : | 4.6 |

PB2

| Pos. | 530 | obs : | exp : |
|---|---|---|---|
| act | T | 67 | 151.10 |
| acc | T | 4 | 122.70 |
| aca | T | 501 | 250.60 |
| acg | T | 0 | 47.55 |
| --- | -- | ------ | ------ |
| mPD | | 0.22 | 0.69 |
| | | nPD : | 0.32 |
| | | N. weight : | 0.71 |
| | | Sc. PD : | 0.033 |
| | | Sc. rank : | 57.5 |

PB2

| Pos. | 531 | obs : | exp : |
|---|---|---|---|
| tat | Y | 559 | 296.10 |
| tac | Y | 13 | 275.90 |
| --- | -- | ------ | ------ |
| mPD | | 0.044 | 0.50 |
| | | nPD : | 0.09 |
| | | N. weight : | 0.79 |
| | | Sc. PD : | -0.14 |
| | | Sc. rank : | -137.3 |

PB2

| Pos. | 532 | obs : | exp : |
|---|---|---|---|
| tct | S | 4 | 90.93 |
| tcc | S | 1 | 79.68 |
| tca | S | 498 | 126.70 |
| tcg | S | 69 | 34.87 |
| agt | S | 0 | 125.80 |
| agc | S | 0 | 114.00 |
| --- | -- | ------ | ------ |
| mPD | | 0.23 | 1.7 |
| | | nPD : | 0.14 |
| | | N. weight : | 1.8 |
| | | Sc. PD : | -0.23 |
| | | Sc. rank : | -205.0 |

PB2

| Pos. | 533 | obs : | exp : |
|---|---|---|---|
| tct | S | 4 | 90.93 |
| tcc | S | 12 | 79.68 |
| tca | S | 183 | 126.70 |
| tcg | S | 373 | 34.87 |
| agt | S | 0 | 125.80 |
| agc | S | 0 | 114.00 |
| --- | -- | ------ | ------ |
| mPD | | 0.47 | 1.7 |
| | | nPD : | 0.28 |
| | | N. weight : | 2.3 |
| | | Sc. PD : | 0.028 |
| | | Sc. rank : | 119.6 |

PB2

| Pos. | 534 | obs : | exp : |
|---|---|---|---|
| ttt | F | 0 | 0.82 |
| ttc | F | 2 | 1.18 |
| tct | S | 65 | 90.46 |
| tcc | S | 413 | 79.26 |
| tca | S | 90 | 126.10 |
| tcg | S | 1 | 34.68 |
| cct | P | 0 | 0.26 |
| ccc | P | 1 | 0.18 |
| cca | P | 0 | 0.39 |
| ccg | P | 0 | 0.16 |
| agt | S | 0 | 125.20 |
| agc | S | 0 | 113.40 |
| --- | -- | ------ | ------ |
| mPD | | 0.44 | 1.7 |
| | | nPD : | 0.27 |
| | | N. weight : | 1.6 |
| | | Sc. PD : | -0.0076 |
| | | Sc. rank : | 59.3 |

PB2

| Pos. | 535 | obs : | exp : |
|---|---|---|---|
| atg | M | 572 | 572.00 |
| --- | -- | ------ | ------ |
| mPD | | 0 | 0 |
| | | nPD : | 1. |
| | | N. weight : | 0. |
| | | Sc. PD : | 0 |
| | | Sc. rank : | 0 |

| | | | |
|---|---|---|---|
| PB2 | | | |
| Pos. | 536 | obs: | exp: |
| atg | M | 572 | 572.00 |
| --- | -- | ------ | ------ |
| mPD | | 0 | 0 |
| | | nPD: | 1. |
| | | N. weight: | 0. |
| | | Sc. PD: | 0 |
| | | Sc. rank: | 0 |

| | | | |
|---|---|---|---|
| PB2 | | | |
| Pos. | 537 | obs: | exp: |
| tgg | W | 572 | 572.00 |
| --- | -- | ------ | ------ |
| mPD | | 0 | 0 |
| | | nPD: | 1. |
| | | N. weight: | 0. |
| | | Sc. PD: | 0 |
| | | Sc. rank: | 0 |

| | | | |
|---|---|---|---|
| PB2 | | | |
| Pos. | 538 | obs: | exp: |
| gaa | E | 466 | 329.10 |
| gag | E | 106 | 242.90 |
| --- | -- | ------ | ------ |
| mPD | | 0.30 | 0.49 |
| | | nPD: | 0.62 |
| | | N. weight: | 0.19 |
| | | Sc. PD: | 0.064 |
| | | Sc. rank: | 56.7 |

| | | | |
|---|---|---|---|
| PB2 | | | |
| Pos. | 539 | obs: | exp: |
| att | I | 82 | 212.00 |
| atc | I | 471 | 147.80 |
| ata | I | 11 | 204.20 |
| act | T | 0 | 0.26 |
| acc | T | 1 | 0.21 |
| aca | T | 0 | 0.44 |
| acg | T | 0 | 0.08 |
| gtt | V | 0 | 1.44 |
| gtc | V | 7 | 1.47 |
| gta | V | 0 | 1.31 |
| gtg | V | 0 | 2.78 |
| --- | -- | ------ | ------ |
| mPD | | 0.31 | 0.69 |
| | | nPD: | 0.44 |
| | | N. weight: | 1.1 |
| | | Sc. PD: | 0.19 |
| | | Sc. rank: | 212.0 |

| | | | |
|---|---|---|---|
| PB2 | | | |
| Pos. | 540 | obs: | exp: |
| aat | N | 513 | 299.00 |
| aac | N | 59 | 273.00 |
| --- | -- | ------ | ------ |
| mPD | | 0.19 | 0.50 |
| | | nPD: | 0.37 |
| | | N. weight: | 0.47 |
| | | Sc. PD: | 0.045 |
| | | Sc. rank: | 59.3 |

| | | | |
|---|---|---|---|
| PB2 | | | |
| Pos. | 541 | obs: | exp: |
| ggt | G | 474 | 70.58 |
| ggc | G | 84 | 77.35 |
| gga | G | 2 | 276.70 |
| ggg | G | 12 | 147.40 |
| --- | -- | ------ | ------ |
| mPD | | 0.29 | 0.67 |
| | | nPD: | 0.44 |
| | | N. weight: | 2.2 |
| | | Sc. PD: | 0.35 |
| | | Sc. rank: | 399.8 |

| | | | |
|---|---|---|---|
| PB2 | | | |
| Pos. | 542 | obs: | exp: |
| cct | P | 99 | 150.60 |
| ccc | P | 0 | 103.20 |
| cca | P | 53 | 225.70 |
| ccg | P | 420 | 92.49 |
| --- | -- | ------ | ------ |
| mPD | | 0.42 | 0.72 |
| | | nPD: | 0.59 |
| | | N. weight: | 1.3 |
| | | Sc. PD: | 0.4 |
| | | Sc. rank: | 375.2 |

| | | | |
|---|---|---|---|
| PB2 | | | |
| Pos. | 543 | obs: | exp: |
| gaa | E | 463 | 328.60 |
| gag | E | 108 | 242.40 |
| ggt | G | 0 | 0.12 |
| ggc | G | 0 | 0.14 |
| gga | G | 1 | 0.48 |
| ggg | G | 0 | 0.26 |
| --- | -- | ------ | ------ |
| mPD | | 0.31 | 0.49 |
| | | nPD: | 0.63 |
| | | N. weight: | 0.19 |
| | | Sc. PD: | 0.064 |
| | | Sc. rank: | 55.5 |

| | | | |
|---|---|---|---|
| PB2 | | | |
| Pos. | 544 | obs: | exp: |
| tct | S | 2 | 90.93 |
| tcc | S | 1 | 79.68 |
| tca | S | 462 | 126.70 |
| tcg | S | 107 | 34.87 |
| agt | S | 0 | 125.80 |
| agc | S | 0 | 114.00 |
| --- | -- | ------ | ------ |
| mPD | | 0.31 | 1.7 |
| | | nPD: | 0.19 |
| | | N. weight: | 1.8 |
| | | Sc. PD: | -0.14 |
| | | Sc. rank: | -97.3 |

| | | | |
|---|---|---|---|
| PB2 | | | |
| Pos. | 545 | obs: | exp: |
| gtt | V | 20 | 117.60 |
| gtc | V | 0 | 120.10 |
| gta | V | 46 | 107.20 |
| gtg | V | 506 | 227.20 |
| --- | -- | ------ | ------ |
| mPD | | 0.21 | 0.72 |
| | | nPD: | 0.29 |
| | | N. weight: | 0.84 |
| | | Sc. PD: | 0.016 |
| | | Sc. rank: | 52.0 |

| | | | |
|---|---|---|---|
| PB2 | | | |
| Pos. | 546 | obs: | exp: |
| tta | L | 336 | 49.97 |
| ttg | L | 69 | 110.20 |
| ctt | L | 23 | 106.70 |
| ctc | L | 2 | 92.53 |
| cta | L | 122 | 86.17 |
| ctg | L | 20 | 126.40 |
| --- | -- | ------ | ------ |
| mPD | | 0.75 | 1.1 |
| | | nPD: | 0.67 |
| | | N. weight: | 1.5 |
| | | Sc. PD: | 0.55 |
| | | Sc. rank: | 454.8 |

| | | | |
|---|---|---|---|
| PB2 | | | |
| Pos. | 547 | obs: | exp: |
| att | I | 0 | 0.38 |
| atc | I | 1 | 0.26 |
| ata | I | 0 | 0.36 |
| gtt | V | 372 | 117.40 |
| gtc | V | 183 | 119.80 |
| gta | V | 0 | 107.00 |
| gtg | V | 16 | 226.80 |
| --- | -- | ------ | ------ |
| mPD | | 0.48 | 0.72 |
| | | nPD: | 0.66 |
| | | N. weight: | 1.2 |
| | | Sc. PD: | 0.43 |
| | | Sc. rank: | 360.0 |

| | | | |
|---|---|---|---|
| PB2 | | | |
| Pos. | 548 | obs: | exp: |
| aat | N | 313 | 299.00 |
| aac | N | 259 | 273.00 |
| --- | -- | ------ | ------ |
| mPD | | 0.50 | 0.50 |
| | | nPD: | 0.99 |
| | | N. weight: | 0.01 |
| | | Sc. PD: | 0.007 |
| | | Sc. rank: | 3.5 |

| | | | |
|---|---|---|---|
| PB2 | | | |
| Pos. | 549 | obs: | exp: |
| act | T | 86 | 151.10 |
| acc | T | 15 | 122.70 |
| aca | T | 469 | 250.60 |
| acg | T | 2 | 47.55 |
| --- | -- | ------ | ------ |
| mPD | | 0.30 | 0.69 |
| | | nPD: | 0.44 |
| | | N. weight: | 0.54 |
| | | Sc. PD: | 0.088 |
| | | Sc. rank: | 101.5 |

| | | | |
|---|---|---|---|
| PB2 | | | |
| Pos. | 550 | obs: | exp: |
| tat | Y | 506 | 296.10 |
| tac | Y | 66 | 275.90 |
| --- | -- | ------ | ------ |
| mPD | | 0.20 | 0.50 |
| | | nPD: | 0.41 |
| | | N. weight: | 0.45 |
| | | Sc. PD: | 0.059 |
| | | Sc. rank: | 69.5 |

| | | | |
|---|---|---|---|
| PB2 | | | |
| Pos. | 551 | obs: | exp: |
| caa | Q | 490 | 304.90 |
| cag | Q | 82 | 267.10 |
| --- | -- | ------ | ------ |
| mPD | | 0.25 | 0.50 |
| | | nPD: | 0.49 |
| | | N. weight: | 0.35 |
| | | Sc. PD: | 0.073 |
| | | Sc. rank: | 77.3 |

| | | | |
|---|---|---|---|
| PB2 | | | |
| Pos. | 552 | obs: | exp: |
| tgg | W | 572 | 572.00 |
| --- | -- | ------ | ------ |
| mPD | | 0 | 0 |
| | | nPD: | 1. |
| | | N. weight: | 0. |
| | | Sc. PD: | 0 |
| | | Sc. rank: | 0 |

| | | | |
|---|---|---|---|
| PB2 | | | |
| Pos. | 553 | obs: | exp: |
| att | I | 18 | 213.90 |
| atc | I | 507 | 149.10 |
| ata | I | 44 | 206.00 |
| gtt | V | 0 | 0.62 |
| gtc | V | 3 | 0.63 |
| gta | V | 0 | 0.56 |
| gtg | V | 0 | 1.19 |
| --- | -- | ------ | ------ |
| mPD | | 0.21 | 0.67 |
| | | nPD: | 0.31 |
| | | N. weight: | 1.3 |
| | | Sc. PD: | 0.049 |
| | | Sc. rank: | 99.3 |

| | | | |
|---|---|---|---|
| PB2 | | | |
| Pos. | 554 | obs: | exp: |
| att | I | 203 | 214.30 |
| atc | I | 361 | 149.40 |
| ata | I | 6 | 206.30 |
| act | T | 2 | 0.53 |
| acc | T | 0 | 0.43 |
| aca | T | 0 | 0.88 |
| acg | T | 0 | 0.17 |
| --- | -- | ------ | ------ |
| mPD | | 0.48 | 0.67 |
| | | nPD: | 0.72 |
| | | N. weight: | 0.73 |
| | | Sc. PD: | 0.32 |
| | | Sc. rank: | 245.4 |

| | | | |
|---|---|---|---|
| PB2 | | | |
| Pos. | 555 | obs: | exp: |
| cgt | R | 0 | 23.71 |
| cgc | R | 0 | 27.96 |
| cga | R | 0 | 54.88 |
| cgg | R | 0 | 47.65 |
| aaa | K | 1 | 0.56 |
| aag | K | 0 | 0.44 |
| aga | R | 527 | 256.80 |
| agg | R | 44 | 160.00 |
| --- | -- | ------ | ------ |
| mPD | | 0.15 | 0.96 |
| | | nPD: | 0.15 |
| | | N. weight: | 0.81 |
| | | Sc. PD: | -0.093 |
| | | Sc. rank: | -72.4 |

| | | | |
|---|---|---|---|
| PB2 | | | |
| Pos. | 556 | obs: | exp: |
| tct | S | 0 | 0.64 |
| tcc | S | 0 | 0.56 |
| tca | S | 0 | 0.89 |
| tcg | S | 0 | 0.24 |
| aat | N | 207 | 296.90 |
| aac | N | 361 | 271.10 |
| agt | S | 4 | 0.88 |
| agc | S | 0 | 0.80 |
| --- | -- | ------ | ------ |
| mPD | | 0.48 | 0.52 |
| | | nPD: | 0.92 |
| | | N. weight: | 0.095 |
| | | Sc. PD: | 0.059 |
| | | Sc. rank: | 33.0 |

| | | | |
|---|---|---|---|
| PB2 | | | |
| Pos. | 557 | obs: | exp: |
| tgg | W | 572 | 572.00 |
| --- | -- | ------ | ------ |
| mPD | | 0 | 0 |
| | | nPD: | 1. |
| | | N. weight: | 0. |
| | | Sc. PD: | 0 |
| | | Sc. rank: | 0 |

| | | | |
|---|---|---|---|
| PB2 | | | |
| Pos. | 558 | obs: | exp: |
| gaa | E | 500 | 329.10 |
| gag | E | 72 | 242.90 |
| --- | -- | ------ | ------ |
| mPD | | 0.22 | 0.49 |
| | | nPD: | 0.45 |
| | | N. weight: | 0.31 |
| | | Sc. PD: | 0.053 |
| | | Sc. rank: | 60.5 |

| | | | |
|---|---|---|---|
| PB2 | | | |
| Pos. | 559 | obs: | exp: |
| att | I | 5 | 1.88 |
| atc | I | 0 | 1.31 |
| ata | I | 0 | 1.81 |
| act | T | 534 | 149.30 |
| acc | T | 30 | 121.20 |
| aca | T | 1 | 247.50 |
| acg | T | 0 | 46.97 |
| gct | A | 2 | 0.50 |
| gcc | A | 0 | 0.41 |
| gca | A | 0 | 0.89 |
| gcg | A | 0 | 0.20 |
| --- | -- | ------ | ------ |
| mPD | | 0.13 | 0.71 |
| | | nPD: | 0.18 |
| | | N. weight: | 1.6 |
| | | Sc. PD: | -0.14 |
| | | Sc. rank: | -105.3 |

| | | | |
|---|---|---|---|
| PB2 | | | |
| Pos. | 560 | obs: | exp: |
| att | I | 0 | 0.38 |
| atc | I | 0 | 0.26 |
| ata | I | 1 | 0.36 |
| atg | M | 1 | 1.00 |
| gtt | V | 0 | 117.00 |
| gtc | V | 0 | 119.40 |
| gta | V | 66 | 106.60 |
| gtg | V | 503 | 226.00 |
| gct | A | 0 | 0.25 |
| gcc | A | 0 | 0.20 |
| gca | A | 0 | 0.45 |
| gcg | A | 1 | 0.10 |
| --- | -- | ------ | ------ |
| mPD | | 0.22 | 0.73 |
| | | nPD: | 0.3 |
| | | N. weight: | 0.94 |
| | | Sc. PD: | 0.023 |
| | | Sc. rank: | 62.1 |

| | | | |
|---|---|---|---|
| PB2 | | | |
| Pos. | 561 | obs: | exp: |
| aaa | K | 94 | 320.30 |
| aag | K | 478 | 251.70 |
| --- | -- | ------ | ------ |
| mPD | | 0.28 | 0.49 |
| | | nPD: | 0.56 |
| | | N. weight: | 0.49 |
| | | Sc. PD: | 0.13 |
| | | Sc. rank: | 129.4 |

| | | | |
|---|---|---|---|
| PB2 | | | |
| Pos. | 562 | obs: | exp: |
| att | I | 567 | 215.00 |
| atc | I | 5 | 149.90 |
| ata | I | 0 | 207.10 |
| --- | -- | ------ | ------ |
| mPD | | 0.017 | 0.66 |
| | | nPD: | 0.03 |
| | | N. weight: | 1.3 |
| | | Sc. PD: | -0.31 |
| | | Sc. rank: | -340.5 |

| | | | |
|---|---|---|---|
| PB2 | | | |
| Pos. | 563 | obs: | exp: |
| caa | Q | 211 | 295.30 |
| cag | Q | 343 | 258.70 |
| cgt | R | 0 | 0.75 |
| cgc | R | 0 | 0.88 |
| cga | R | 0 | 1.73 |
| cgg | R | 18 | 1.50 |
| aga | R | 0 | 8.10 |
| agg | R | 0 | 5.04 |
| --- | -- | ------ | ------ |
| mPD | | 0.53 | 0.61 |
| | | nPD: | 0.87 |
| | | N. weight: | 0.18 |
| | | Sc. PD: | 0.11 |
| | | Sc. rank: | 63.6 |

| | | | |
|---|---|---|---|
| PB2 | | | |
| Pos. | 564 | obs: | exp: |
| tgg | W | 572 | 572.00 |
| --- | -- | ------ | ------ |
| mPD | | 0 | 0 |
| | | nPD: | 1. |
| | | N. weight: | 0. |
| | | Sc. PD: | 0 |
| | | Sc. rank: | 0 |

| | | | |
|---|---|---|---|
| PB2 | | | |
| Pos. | 565 | obs: | exp: |
| tct | S | 37 | 90.93 |
| tcc | S | 458 | 79.68 |
| tca | S | 76 | 126.70 |
| tcg | S | 1 | 34.87 |
| agt | S | 0 | 125.80 |
| agc | S | 0 | 114.00 |
| --- | -- | ------ | ------ |
| mPD | | 0.34 | 1.7 |
| | | nPD: | 0.2 |
| | | N. weight: | 1.8 |
| | | Sc. PD: | -0.12 |
| | | Sc. rank: | -74.6 |

| | | | |
|---|---|---|---|
| PB2 | | | |
| Pos. | 566 | obs: | exp: |
| caa | Q | 509 | 304.90 |
| cag | Q | 63 | 267.10 |
| --- | -- | ------ | ------ |
| mPD | | 0.20 | 0.50 |
| | | nPD: | 0.39 |
| | | N. weight: | 0.43 |
| | | Sc. PD: | 0.051 |
| | | Sc. rank: | 63.0 |

| | | | |
|---|---|---|---|
| PB2 | | | |
| Pos. | 567 | obs: | exp: |
| tat | Y | 0 | 0.52 |
| tac | Y | 1 | 0.48 |
| gat | D | 52 | 304.10 |
| gac | D | 518 | 265.90 |
| gaa | E | 1 | 0.58 |
| gag | E | 0 | 0.42 |
| --- | -- | ------ | ------ |
| mPD | | 0.17 | 0.50 |
| | | nPD: | 0.34 |
| | | N. weight: | 0.64 |
| | | Sc. PD: | 0.043 |
| | | Sc. rank: | 65.2 |

| | | | |
|---|---|---|---|
| PB2 | | | |
| Pos. | 568 | obs: | exp: |
| cct | P | 482 | 150.60 |
| ccc | P | 85 | 103.20 |
| cca | P | 5 | 225.70 |
| ccg | P | 0 | 92.49 |
| --- | -- | ------ | ------ |
| mPD | | 0.27 | 0.72 |
| | | nPD: | 0.37 |
| | | N. weight: | 1.3 |
| | | Sc. PD: | 0.13 |
| | | Sc. rank: | 172.1 |

| | | | |
|---|---|---|---|
| PB2 | | | |
| Pos. | 569 | obs: | exp: |
| act | T | 1 | 151.10 |
| acc | T | 0 | 122.70 |
| aca | T | 513 | 250.60 |
| acg | T | 58 | 47.55 |
| --- | -- | ------ | ------ |
| mPD | | 0.19 | 0.69 |
| | | nPD: | 0.27 |
| | | N. weight: | 0.94 |
| | | Sc. PD: | -0.00064 |
| | | Sc. rank: | 39.7 |

| | | | |
|---|---|---|---|
| PB2 | | | |
| Pos. | 570 | obs: | exp: |
| atg | M | 570 | 570.00 |
| gtt | V | 0 | 0.41 |
| gtc | V | 0 | 0.42 |
| gta | V | 0 | 0.37 |
| gtg | V | 2 | 0.79 |
| --- | -- | ------ | ------ |
| mPD | | 0.0070 | 0.011 |
| | | nPD: | 0.62 |
| | | N. weight: | 0.0046 |
| | | Sc. PD: | 0.0016 |
| | | Sc. rank: | 1.4 |

| | | | |
|---|---|---|---|
| PB2 | | | |
| Pos. | 571 | obs: | exp: |
| tta | L | 58 | 49.97 |
| ttg | L | 100 | 110.20 |
| ctt | L | 4 | 106.70 |
| ctc | L | 0 | 92.53 |
| cta | L | 375 | 86.17 |
| ctg | L | 35 | 126.40 |
| --- | -- | ------ | ------ |
| mPD | | 0.77 | 1.1 |
| | | nPD: | 0.69 |
| | | N. weight: | 1.3 |
| | | Sc. PD: | 0.5 |
| | | Sc. rank: | 408.3 |

| | | | |
|---|---|---|---|
| PB2 | | | |
| Pos. | 572 | obs: | exp: |
| tat | Y | 74 | 296.10 |
| tac | Y | 498 | 275.90 |
| --- | -- | ------ | ------ |
| mPD | | 0.23 | 0.50 |
| | | nPD: | 0.45 |
| | | N. weight: | 0.49 |
| | | Sc. PD: | 0.083 |
| | | Sc. rank: | 94.7 |

| | | | |
|---|---|---|---|
| PB2 | | | |
| Pos. | 573 | obs: | exp: |
| aat | N | 484 | 299.00 |
| aac | N | 88 | 273.00 |
| --- | -- | ------ | ------ |
| mPD | | 0.26 | 0.50 |
| | | nPD: | 0.52 |
| | | N. weight: | 0.34 |
| | | Sc. PD: | 0.082 |
| | | Sc. rank: | 83.8 |

| | | | |
|---|---|---|---|
| PB2 | | | |
| Pos. | 574 | obs: | exp: |
| cgt | R | 0 | 0.21 |
| cgc | R | 0 | 0.24 |
| cga | R | 0 | 0.48 |
| cgg | R | 0 | 0.42 |
| aaa | K | 17 | 317.50 |
| aag | K | 550 | 249.50 |
| aga | R | 0 | 2.25 |
| agg | R | 5 | 1.40 |
| --- | -- | ------ | ------ |
| mPD | | 0.075 | 0.52 |
| | | nPD: | 0.15 |
| | | N. weight: | 0.98 |
| | | Sc. PD: | -0.12 |
| | | Sc. rank: | -94.1 |

| | | | |
|---|---|---|---|
| PB2 | | | |
| Pos. | 575 | obs: | exp: |
| atg | M | 572 | 572.00 |
| --- | -- | ------ | ------ |
| mPD | | 0 | 0 |
| | | nPD: | 1. |
| | | N. weight: | 0. |
| | | Sc. PD: | 0 |
| | | Sc. rank: | 0 |

| | | | |
|---|---|---|---|
| PB2 | | | |
| Pos. | 576 | obs: | exp: |
| gaa | E | 165 | 329.10 |
| gag | E | 407 | 242.90 |
| --- | -- | ------ | ------ |
| mPD | | 0.41 | 0.49 |
| | | nPD: | 0.84 |
| | | N. weight: | 0.25 |
| | | Sc. PD: | 0.14 |
| | | Sc. rank: | 85.3 |

| | | | |
|---|---|---|---|
| PB2 | | | |
| Pos. | 577 | obs: | exp: |
| ttt | F | 497 | 235.20 |
| ttc | F | 75 | 336.80 |
| --- | -- | ------ | ------ |
| mPD | | 0.23 | 0.48 |
| | | nPD: | 0.47 |
| | | N. weight: | 0.66 |
| | | Sc. PD: | 0.12 |
| | | Sc. rank: | 135.8 |

| | | | |
|---|---|---|---|
| PB2 | | | |
| Pos. | 578 | obs: | exp: |
| gaa | E | 118 | 329.10 |
| gag | E | 454 | 242.90 |
| --- | -- | ------ | ------ |
| mPD | | 0.33 | 0.49 |
| | | nPD: | 0.67 |
| | | N. weight: | 0.42 |
| | | Sc. PD: | 0.16 |
| | | Sc. rank: | 129.8 |

| | | | |
|---|---|---|---|
| PB2 | | | |
| Pos. | 579 | obs: | exp: |
| cct | P | 9 | 150.60 |
| ccc | P | 453 | 103.20 |
| cca | P | 95 | 225.70 |
| ccg | P | 15 | 92.49 |
| --- | -- | ------ | ------ |
| mPD | | 0.34 | 0.72 |
| | | nPD: | 0.48 |
| | | N. weight: | 1.4 |
| | | Sc. PD: | 0.27 |
| | | Sc. rank: | 290.1 |

| | | | |
|---|---|---|---|
| PB2 | | | |
| Pos. | 580 | obs: | exp: |
| ttt | F | 425 | 235.20 |
| ttc | F | 147 | 336.80 |
| --- | -- | ------ | ------ |
| mPD | | 0.38 | 0.48 |
| | | nPD: | 0.79 |
| | | N. weight: | 0.33 |
| | | Sc. PD: | 0.16 |
| | | Sc. rank: | 112.7 |

| | | | |
|---|---|---|---|
| PB2 | | | |
| Pos. | 581 | obs: | exp: |
| caa | Q | 453 | 304.90 |
| cag | Q | 119 | 267.10 |
| --- | -- | ------ | ------ |
| mPD | | 0.33 | 0.50 |
| | | nPD: | 0.66 |
| | | N. weight: | 0.22 |
| | | Sc. PD: | 0.08 |
| | | Sc. rank: | 66.5 |

| | | | |
|---|---|---|---|
| PB2 | | | |
| Pos. | 582 | obs: | exp: |
| tct | S | 111 | 90.93 |
| tcc | S | 455 | 79.68 |
| tca | S | 6 | 126.70 |
| tcg | S | 0 | 34.87 |
| agt | S | 0 | 125.80 |
| agc | S | 0 | 114.00 |
| --- | -- | ------ | ------ |
| mPD | | 0.33 | 1.7 |
| | | nPD: | 0.2 |
| | | N. weight: | 2. |
| | | Sc. PD: | -0.14 |
| | | Sc. rank: | -91.3 |

| | | | |
|---|---|---|---|
| PB2 | | | |
| Pos. | 583 | obs: | exp: |
| tta | L | 12 | 49.97 |
| ttg | L | 455 | 110.20 |
| ctt | L | 4 | 106.70 |
| ctc | L | 0 | 92.53 |
| cta | L | 34 | 86.17 |
| ctg | L | 67 | 126.40 |
| --- | -- | ------ | ------ |
| mPD | | 0.46 | 1.1 |
| | | nPD: | 0.41 |
| | | N. weight: | 1.4 |
| | | Sc. PD: | 0.19 |
| | | Sc. rank: | 220.8 |

| | | | |
|---|---|---|---|
| PB2 | | | |
| Pos. | 584 | obs: | exp: |
| att | I | 0 | 0.38 |
| atc | I | 1 | 0.26 |
| ata | I | 0 | 0.36 |
| gtt | V | 7 | 117.40 |
| gtc | V | 65 | 119.80 |
| gta | V | 53 | 107.00 |
| gtg | V | 446 | 226.80 |
| --- | -- | ------ | ------ |
| mPD | | 0.37 | 0.72 |
| | | nPD: | 0.52 |
| | | N. weight: | 0.53 |
| | | Sc. PD: | 0.12 |
| | | Sc. rank: | 129.7 |

| | | | |
|---|---|---|---|
| PB2 | | | |
| Pos. | 585 | obs: | exp: |
| cct | P | 491 | 150.60 |
| ccc | P | 78 | 103.20 |
| cca | P | 3 | 225.70 |
| ccg | P | 0 | 92.49 |
| --- | -- | ------ | ------ |
| mPD | | 0.24 | 0.72 |
| | | nPD: | 0.34 |
| | | N. weight: | 1.4 |
| | | Sc. PD: | 0.092 |
| | | Sc. rank: | 138.4 |

| | | | |
|---|---|---|---|
| PB2 | | | |
| Pos. | 586 | obs: | exp: |
| cgt | R | 0 | 0.08 |
| cgc | R | 0 | 0.10 |
| cga | R | 0 | 0.19 |
| cgg | R | 0 | 0.17 |
| aaa | K | 82 | 319.20 |
| aag | K | 488 | 250.80 |
| aga | R | 0 | 0.90 |
| agg | R | 2 | 0.56 |
| --- | -- | ------ | ------ |
| mPD | | 0.25 | 0.50 |
| | | nPD: | 0.5 |
| | | N. weight: | 0.55 |
| | | Sc. PD: | 0.12 |
| | | Sc. rank: | 127.2 |

| | | | |
|---|---|---|---|
| PB2 | | | |
| Pos. | 587 | obs: | exp: |
| gct | A | 437 | 144.10 |
| gcc | A | 16 | 117.20 |
| gca | A | 76 | 254.90 |
| gcg | A | 43 | 55.82 |
| --- | -- | ------ | ------ |
| mPD | | 0.39 | 0.69 |
| | | nPD: | 0.57 |
| | | N. weight: | 0.89 |
| | | Sc. PD: | 0.26 |
| | | Sc. rank: | 246.6 |

| | | | |
|---|---|---|---|
| PB2 | | | |
| Pos. | 588 | obs: | exp: |
| gtt | V | 0 | 0.21 |
| gtc | V | 1 | 0.21 |
| gta | V | 0 | 0.19 |
| gtg | V | 0 | 0.40 |
| gct | A | 51 | 143.80 |
| gcc | A | 517 | 117.00 |
| gca | A | 3 | 254.50 |
| gcg | A | 0 | 55.72 |
| --- | -- | ------ | ------ |
| mPD | | 0.18 | 0.69 |
| | | nPD: | 0.25 |
| | | N. weight: | 1.8 |
| | | Sc. PD: | -0.029 |
| | | Sc. rank: | 40.6 |

| | | | |
|---|---|---|---|
| PB2 | | | |
| Pos. | 589 | obs: | exp: |
| cgt | R | 0 | 23.75 |
| cgc | R | 0 | 28.01 |
| cga | R | 2 | 54.97 |
| cgg | R | 0 | 47.73 |
| aga | R | 566 | 257.20 |
| agg | R | 4 | 160.30 |
| --- | -- | ------ | ------ |
| mPD | | 0.021 | 0.96 |
| | | nPD: | 0.02 |
| | | N. weight: | 1.1 |
| | | Sc. PD: | -0.25 |
| | | Sc. rank: | -281.8 |

| | | | |
|---|---|---|---|
| PB2 | | | |
| Pos. | 590 | obs: | exp: |
| tct | S | 0 | 0.79 |
| tcc | S | 0 | 0.70 |
| tca | S | 0 | 1.11 |
| tcg | S | 0 | 0.30 |
| agt | S | 2 | 1.10 |
| agc | S | 3 | 1.00 |
| ggt | G | 99 | 69.96 |
| ggc | G | 468 | 76.68 |
| gga | G | 0 | 274.30 |
| ggg | G | 0 | 146.10 |
| --- | -- | ------ | ------ |
| mPD | | 0.31 | 0.70 |
| | | nPD: | 0.44 |
| | | N. weight: | 2.2 |
| | | Sc. PD: | 0.36 |
| | | Sc. rank: | 420.4 |

| PB2 | | | |
|---|---|---|---|
| Pos. | 591 | obs: | exp: |
| caa | Q | 150 | 304.90 |
| cag | Q | 422 | 267.10 |
| --- | -- | ------ | ------ |
| mPD | | 0.39 | 0.50 |
| | | nPD: | 0.78 |
| | | N. weight: | 0.22 |
| | | Sc. PD: | 0.11 |
| | | Sc. rank: | 76.1 |

| PB2 | | | |
|---|---|---|---|
| Pos. | 592 | obs: | exp: |
| tat | Y | 421 | 296.10 |
| tac | Y | 151 | 275.90 |
| --- | -- | ------ | ------ |
| mPD | | 0.39 | 0.50 |
| | | nPD: | 0.78 |
| | | N. weight: | 0.15 |
| | | Sc. PD: | 0.073 |
| | | Sc. rank: | 51.2 |

| PB2 | | | |
|---|---|---|---|
| Pos. | 593 | obs: | exp: |
| tct | S | 0 | 90.93 |
| tcc | S | 0 | 79.68 |
| tca | S | 0 | 126.70 |
| tcg | S | 0 | 34.87 |
| agt | S | 558 | 125.80 |
| agc | S | 14 | 114.00 |
| --- | -- | ------ | ------ |
| mPD | | 0.048 | 1.7 |
| | | nPD: | 0.03 |
| | | N. weight: | 2. |
| | | Sc. PD: | -0.47 |
| | | Sc. rank: | -501.8 |

| PB2 | | | |
|---|---|---|---|
| Pos. | 594 | obs: | exp: |
| ggt | G | 3 | 70.58 |
| ggc | G | 3 | 77.35 |
| gga | G | 456 | 276.70 |
| ggg | G | 110 | 147.40 |
| --- | -- | ------ | ------ |
| mPD | | 0.33 | 0.67 |
| | | nPD: | 0.49 |
| | | N. weight: | 0.46 |
| | | Sc. PD: | 0.096 |
| | | Sc. rank: | 101.3 |

| PB2 | | | |
|---|---|---|---|
| Pos. | 595 | obs: | exp: |
| ttt | F | 471 | 235.20 |
| ttc | F | 101 | 336.80 |
| --- | -- | ------ | ------ |
| mPD | | 0.29 | 0.48 |
| | | nPD: | 0.6 |
| | | N. weight: | 0.52 |
| | | Sc. PD: | 0.16 |
| | | Sc. rank: | 150.0 |

| PB2 | | | |
|---|---|---|---|
| Pos. | 596 | obs: | exp: |
| gtt | V | 0 | 117.60 |
| gtc | V | 0 | 120.10 |
| gta | V | 322 | 107.20 |
| gtg | V | 250 | 227.20 |
| --- | -- | ------ | ------ |
| mPD | | 0.49 | 0.72 |
| | | nPD: | 0.68 |
| | | N. weight: | 0.95 |
| | | Sc. PD: | 0.37 |
| | | Sc. rank: | 303.4 |

| PB2 | | | |
|---|---|---|---|
| Pos. | 597 | obs: | exp: |
| cgt | R | 0 | 23.75 |
| cgc | R | 0 | 28.01 |
| cga | R | 0 | 54.97 |
| cgg | R | 0 | 47.73 |
| aga | R | 86 | 257.20 |
| agg | R | 486 | 160.30 |
| --- | -- | ------ | ------ |
| mPD | | 0.26 | 0.96 |
| | | nPD: | 0.27 |
| | | N. weight: | 1.1 |
| | | Sc. PD: | -0.0054 |
| | | Sc. rank: | 40.1 |

| PB2 | | | |
|---|---|---|---|
| Pos. | 598 | obs: | exp: |
| att | I | 0 | 0.38 |
| atc | I | 0 | 0.26 |
| ata | I | 1 | 0.36 |
| atg | M | 1 | 1.00 |
| act | T | 31 | 143.50 |
| acc | T | 21 | 116.50 |
| aca | T | 156 | 237.90 |
| acg | T | 335 | 45.14 |
| gtt | V | 0 | 5.55 |
| gtc | V | 0 | 5.67 |
| gta | V | 0 | 5.06 |
| gtg | V | 27 | 10.72 |
| --- | -- | ------ | ------ |
| mPD | | 0.71 | 0.88 |
| | | nPD: | 0.8 |
| | | N. weight: | 1.4 |
| | | Sc. PD: | 0.7 |
| | | Sc. rank: | 473.9 |

| PB2 | | | |
|---|---|---|---|
| Pos. | 599 | obs: | exp: |
| tta | L | 45 | 49.97 |
| ttg | L | 26 | 110.20 |
| ctt | L | 1 | 106.70 |
| ctc | L | 0 | 92.53 |
| cta | L | 476 | 86.17 |
| ctg | L | 24 | 126.40 |
| --- | -- | ------ | ------ |
| mPD | | 0.38 | 1.1 |
| | | nPD: | 0.34 |
| | | N. weight: | 1.8 |
| | | Sc. PD: | 0.12 |
| | | Sc. rank: | 182.7 |

| PB2 | | | |
|---|---|---|---|
| Pos. | 600 | obs: | exp: |
| ttt | F | 20 | 235.20 |
| ttc | F | 552 | 336.80 |
| --- | -- | ------ | ------ |
| mPD | | 0.068 | 0.48 |
| | | nPD: | 0.14 |
| | | N. weight: | 0.56 |
| | | Sc. PD: | -0.071 |
| | | Sc. rank: | -59.1 |

| PB2 | | | |
|---|---|---|---|
| Pos. | 601 | obs: | exp: |
| caa | Q | 45 | 304.90 |
| cag | Q | 527 | 267.10 |
| --- | -- | ------ | ------ |
| mPD | | 0.15 | 0.50 |
| | | nPD: | 0.29 |
| | | N. weight: | 0.69 |
| | | Sc. PD: | 0.013 |
| | | Sc. rank: | 43.6 |

| PB2 | | | |
|---|---|---|---|
| Pos. | 602 | obs: | exp: |
| caa | Q | 88 | 304.90 |
| cag | Q | 484 | 267.10 |
| --- | -- | ------ | ------ |
| mPD | | 0.26 | 0.50 |
| | | nPD: | 0.52 |
| | | N. weight: | 0.45 |
| | | Sc. PD: | 0.11 |
| | | Sc. rank: | 113.0 |

| PB2 | | | |
|---|---|---|---|
| Pos. | 603 | obs: | exp: |
| atg | M | 572 | 572.00 |
| --- | -- | ------ | ------ |
| mPD | | 0 | 0 |
| | | nPD: | 1. |
| | | N. weight: | 0. |
| | | Sc. PD: | 0 |
| | | Sc. rank: | 0 |

| PB2 | | | |
|---|---|---|---|
| Pos. | 604 | obs: | exp: |
| cgt | R | 497 | 23.75 |
| cgc | R | 1 | 28.01 |
| cga | R | 45 | 54.97 |
| cgg | R | 26 | 47.73 |
| aga | R | 3 | 257.20 |
| agg | R | 0 | 160.30 |
| --- | -- | ------ | ------ |
| mPD | | 0.25 | 0.96 |
| | | nPD: | 0.26 |
| | | N. weight: | 3.7 |
| | | Sc. PD: | -0.051 |
| | | Sc. rank: | 100.8 |

| PB2 | | | |
|---|---|---|---|
| Pos. | 605 | obs: | exp: |
| gat | D | 513 | 305.20 |
| gac | D | 59 | 266.80 |
| --- | -- | ------ | ------ |
| mPD | | 0.19 | 0.50 |
| | | nPD: | 0.37 |
| | | N. weight: | 0.45 |
| | | Sc. PD: | 0.043 |
| | | Sc. rank: | 57.2 |

| PB2 | | | |
|---|---|---|---|
| Pos. | 606 | obs: | exp: |
| gtt | V | 2 | 117.60 |
| gtc | V | 12 | 120.10 |
| gta | V | 45 | 107.20 |
| gtg | V | 513 | 227.20 |
| --- | -- | ------ | ------ |
| mPD | | 0.19 | 0.72 |
| | | nPD: | 0.26 |
| | | N. weight: | 0.87 |
| | | Sc. PD: | -0.0075 |
| | | Sc. rank: | 26.4 |

| PB2 | | | |
|---|---|---|---|
| Pos. | 607 | obs: | exp: |
| tta | L | 2 | 49.88 |
| ttg | L | 37 | 110.00 |
| ctt | L | 72 | 106.50 |
| ctc | L | 2 | 92.36 |
| cta | L | 1 | 86.02 |
| ctg | L | 457 | 126.20 |
| atg | M | 1 | 1.00 |
| --- | -- | ------ | ------ |
| mPD | | 0.37 | 1.1 |
| | | nPD: | 0.33 |
| | | N. weight: | 1.3 |
| | | Sc. PD: | 0.069 |
| | | Sc. rank: | 113.6 |

| PB2 | | | |
|---|---|---|---|
| Pos. | 608 | obs: | exp: |
| ggt | G | 1 | 70.58 |
| ggc | G | 0 | 77.35 |
| gga | G | 93 | 276.70 |
| ggg | G | 478 | 147.40 |
| --- | -- | ------ | ------ |
| mPD | | 0.28 | 0.67 |
| | | nPD: | 0.41 |
| | | N. weight: | 1.2 |
| | | Sc. PD: | 0.16 |
| | | Sc. rank: | 186.1 |

| PB2 | | | |
|---|---|---|---|
| Pos. | 609 | obs: | exp: |
| act | T | 16 | 151.10 |
| acc | T | 45 | 122.70 |
| aca | T | 496 | 250.60 |
| acg | T | 15 | 47.55 |
| --- | -- | ------ | ------ |
| mPD | | 0.24 | 0.69 |
| | | nPD: | 0.35 |
| | | N. weight: | 0.62 |
| | | Sc. PD: | 0.047 |
| | | Sc. rank: | 67.2 |

| PB2 | | | |
|---|---|---|---|
| Pos. | 610 | obs: | exp: |
| ttt | F | 546 | 235.20 |
| ttc | F | 26 | 336.80 |
| --- | -- | ------ | ------ |
| mPD | | 0.087 | 0.48 |
| | | nPD: | 0.18 |
| | | N. weight: | 0.99 |
| | | Sc. PD: | -0.087 |
| | | Sc. rank: | -63.8 |

| PB2 | | | |
|---|---|---|---|
| Pos. | 611 | obs: | exp: |
| gat | D | 77 | 302.50 |
| gac | D | 490 | 264.50 |
| gaa | E | 4 | 2.30 |
| gag | E | 0 | 1.70 |
| ggt | G | 1 | 0.12 |
| ggc | G | 0 | 0.14 |
| gga | G | 0 | 0.48 |
| ggg | G | 0 | 0.26 |
| --- | -- | ------ | ------ |
| mPD | | 0.25 | 0.51 |
| | | nPD: | 0.49 |
| | | N. weight: | 0.51 |
| | | Sc. PD: | 0.11 |
| | | Sc. rank: | 113.6 |

| PB2 | | | |
|---|---|---|---|
| Pos. | 612 | obs: | exp: |
| act | T | 519 | 151.10 |
| acc | T | 38 | 122.70 |
| aca | T | 13 | 250.60 |
| acg | T | 2 | 47.55 |
| --- | -- | ------ | ------ |
| mPD | | 0.17 | 0.69 |
| | | nPD: | 0.25 |
| | | N. weight: | 1.4 |
| | | Sc. PD: | -0.027 |
| | | Sc. rank: | 23.3 |

| PB2 | | | |
|---|---|---|---|
| Pos. | 613 | obs: | exp: |
| gtt | V | 136 | 117.00 |
| gtc | V | 432 | 119.40 |
| gta | V | 0 | 106.60 |
| gtg | V | 1 | 226.00 |
| gct | A | 0 | 0.76 |
| gcc | A | 3 | 0.61 |
| gca | A | 0 | 1.34 |
| gcg | A | 0 | 0.29 |
| --- | -- | ------ | ------ |
| mPD | | 0.38 | 0.73 |
| | | nPD: | 0.51 |
| | | N. weight: | 1.4 |
| | | Sc. PD: | 0.33 |
| | | Sc. rank: | 346.8 |

| PB2 | | | |
|---|---|---|---|
| Pos. | 614 | obs: | exp: |
| caa | Q | 166 | 304.90 |
| cag | Q | 406 | 267.10 |
| --- | -- | ------ | ------ |
| mPD | | 0.41 | 0.50 |
| | | nPD: | 0.83 |
| | | N. weight: | 0.18 |
| | | Sc. PD: | 0.096 |
| | | Sc. rank: | 62.0 |

| PB2 | | | |
|---|---|---|---|
| Pos. | 615 | obs: | exp: |
| att | I | 0 | 214.70 |
| atc | I | 2 | 149.60 |
| ata | I | 569 | 206.70 |
| gtt | V | 0 | 0.21 |
| gtc | V | 0 | 0.21 |
| gta | V | 1 | 0.19 |
| gtg | V | 0 | 0.40 |
| --- | -- | ------ | ------ |
| mPD | | 0.010 | 0.66 |
| | | nPD: | 0.02 |
| | | N. weight: | 1.4 |
| | | Sc. PD: | -0.35 |
| | | Sc. rank: | -391.3 |

| PB2 | | | |
|---|---|---|---|
| Pos. | 616 | obs: | exp: |
| att | I | 0 | 215.00 |
| atc | I | 1 | 149.90 |
| ata | I | 571 | 207.10 |
| --- | -- | ------ | ------ |
| mPD | | 0.0035 | 0.66 |
| | | nPD: | 0.01 |
| | | N. weight: | 1.4 |
| | | Sc. PD: | -0.36 |
| | | Sc. rank: | -440.6 |

| PB2 | | | |
|---|---|---|---|
| Pos. | 617 | obs: | exp: |
| aaa | K | 89 | 320.30 |
| aag | K | 483 | 251.70 |
| --- | -- | ------ | ------ |
| mPD | | 0.26 | 0.49 |
| | | nPD: | 0.53 |
| | | N. weight: | 0.51 |
| | | Sc. PD: | 0.13 |
| | | Sc. rank: | 130.8 |

| PB2 | | | |
|---|---|---|---|
| Pos. | 618 | obs: | exp: |
| tta | L | 1 | 49.97 |
| ttg | L | 5 | 110.20 |
| ctt | L | 28 | 106.70 |
| ctc | L | 135 | 92.53 |
| cta | L | 96 | 86.17 |
| ctg | L | 307 | 126.40 |
| --- | -- | ------ | ------ |
| mPD | | 0.64 | 1.1 |
| | | nPD: | 0.57 |
| | | N. weight: | 0.72 |
| | | Sc. PD: | 0.21 |
| | | Sc. rank: | 198.1 |

| PB2 | | | |
|---|---|---|---|
| Pos. | 619 | obs: | exp: |
| tta | L | 31 | 49.97 |
| ttg | L | 0 | 110.20 |
| ctt | L | 9 | 106.70 |
| ctc | L | 107 | 92.53 |
| cta | L | 418 | 86.17 |
| ctg | L | 7 | 126.40 |
| --- | -- | ------ | ------ |
| mPD | | 0.45 | 1.1 |
| | | nPD: | 0.41 |
| | | N. weight: | 1.6 |
| | | Sc. PD: | 0.2 |
| | | Sc. rank: | 237.0 |

| PB2 | | | |
|---|---|---|---|
| Pos. | 620 | obs: | exp: |
| cct | P | 6 | 150.60 |
| ccc | P | 67 | 103.20 |
| cca | P | 494 | 225.70 |
| ccg | P | 5 | 92.49 |
| --- | -- | ------ | ------ |
| mPD | | 0.24 | 0.72 |
| | | nPD: | 0.34 |
| | | N. weight: | 0.83 |
| | | Sc. PD: | 0.051 |
| | | Sc. rank: | 76.8 |

| PB2 | | | |
|---|---|---|---|
| Pos. | 621 | obs: | exp: |
| ttt | F | 570 | 235.20 |
| ttc | F | 2 | 336.80 |
| --- | -- | ------ | ------ |
| mPD | | 0.0070 | 0.48 |
| | | nPD: | 0.01 |
| | | N. weight: | 1.2 |
| | | Sc. PD: | -0.3 |
| | | Sc. rank: | -344.3 |

| PB2 | | | |
|---|---|---|---|
| Pos. | 622 | obs: | exp: |
| gct | A | 71 | 144.10 |
| gcc | A | 5 | 117.20 |
| gca | A | 496 | 254.90 |
| gcg | A | 0 | 55.82 |
| --- | -- | ------ | ------ |
| mPD | | 0.23 | 0.69 |
| | | nPD: | 0.34 |
| | | N. weight: | 0.67 |
| | | Sc. PD: | 0.043 |
| | | Sc. rank: | 64.7 |

| PB2 | | | |
|---|---|---|---|
| Pos. | 623 | obs: | exp: |
| gct | A | 69 | 144.10 |
| gcc | A | 2 | 117.20 |
| gca | A | 497 | 254.90 |
| gcg | A | 4 | 55.82 |
| --- | -- | ------ | ------ |
| mPD | | 0.23 | 0.69 |
| | | nPD: | 0.34 |
| | | N. weight: | 0.67 |
| | | Sc. PD: | 0.041 |
| | | Sc. rank: | 62.8 |

| PB2 | | | |
|---|---|---|---|
| Pos. | 624 | obs: | exp: |
| gct | A | 57 | 144.10 |
| gcc | A | 509 | 117.20 |
| gca | A | 5 | 254.90 |
| gcg | A | 1 | 55.82 |
| --- | -- | ------ | ------ |
| mPD | | 0.20 | 0.69 |
| | | nPD: | 0.29 |
| | | N. weight: | 1.7 |
| | | Sc. PD: | 0.028 |
| | | Sc. rank: | 99.1 |

| PB2 | | | |
|---|---|---|---|
| Pos. | 625 | obs: | exp: |
| cct | P | 1 | 150.60 |
| ccc | P | 0 | 103.20 |
| cca | P | 529 | 225.70 |
| ccg | P | 42 | 92.49 |
| --- | -- | ------ | ------ |
| mPD | | 0.14 | 0.72 |
| | | nPD: | 0.19 |
| | | N. weight: | 1. |
| | | Sc. PD: | -0.076 |
| | | Sc. rank: | -50.0 |

| PB2 | | | |
|---|---|---|---|
| Pos. | 626 | obs: | exp: |
| cct | P | 1 | 150.60 |
| ccc | P | 0 | 103.20 |
| cca | P | 320 | 225.70 |
| ccg | P | 251 | 92.49 |
| --- | -- | ------ | ------ |
| mPD | | 0.50 | 0.72 |
| | | nPD: | 0.69 |
| | | N. weight: | 0.9 |
| | | Sc. PD: | 0.36 |
| | | Sc. rank: | 292.7 |

| PB2 | | | |
|---|---|---|---|
| Pos. | 627 | obs: | exp: |
| aaa | K | 4 | 2.24 |
| aag | K | 0 | 1.76 |
| gaa | E | 466 | 326.80 |
| gag | E | 102 | 241.20 |
| --- | -- | ------ | ------ |
| mPD | | 0.31 | 0.50 |
| | | nPD: | 0.61 |
| | | N. weight: | 0.21 |
| | | Sc. PD: | 0.067 |
| | | Sc. rank: | 60.5 |

| PB2 | | | |
|---|---|---|---|
| Pos. | 628 | obs: | exp: |
| caa | Q | 81 | 301.70 |
| cag | Q | 485 | 264.30 |
| cgt | R | 0 | 0.25 |
| cgc | R | 0 | 0.29 |
| cga | R | 0 | 0.58 |
| cgg | R | 6 | 0.50 |
| aga | R | 0 | 2.70 |
| agg | R | 0 | 1.68 |
| --- | -- | ------ | ------ |
| mPD | | 0.26 | 0.53 |
| | | nPD: | 0.49 |
| | | N. weight: | 0.51 |
| | | Sc. PD: | 0.11 |
| | | Sc. rank: | 115.0 |

| PB2 | | | |
|---|---|---|---|
| Pos. | 629 | obs: | exp: |
| tct | S | 0 | 90.78 |
| tcc | S | 0 | 79.54 |
| tca | S | 0 | 126.50 |
| tcg | S | 0 | 34.80 |
| aat | N | 1 | 0.52 |
| aac | N | 0 | 0.48 |
| agt | S | 536 | 125.60 |
| agc | S | 35 | 113.80 |
| --- | -- | ------ | ------ |
| mPD | | 0.12 | 1.7 |
| | | nPD: | 0.07 |
| | | N. weight: | 1.8 |
| | | Sc. PD: | -0.35 |
| | | Sc. rank: | -349.3 |

| PB2 | | | |
|---|---|---|---|
| Pos. | 630 | obs: | exp: |
| cgt | R | 0 | 23.75 |
| cgc | R | 0 | 28.01 |
| cga | R | 0 | 54.97 |
| cgg | R | 0 | 47.73 |
| aga | R | 96 | 257.20 |
| agg | R | 476 | 160.30 |
| --- | -- | ------ | ------ |
| mPD | | 0.28 | 0.96 |
| | | nPD: | 0.29 |
| | | N. weight: | 1.1 |
| | | Sc. PD: | 0.02 |
| | | Sc. rank: | 65.2 |

| PB2 | | | |
|---|---|---|---|
| Pos. | 631 | obs: | exp: |
| atg | M | 572 | 572.00 |
| --- | -- | ------ | ------ |
| mPD | | 0 | 0 |
| | | nPD: | 1. |
| | | N. weight: | 0. |
| | | Sc. PD: | 0 |
| | | Sc. rank: | 0 |

| PB2 | | | |
|---|---|---|---|
| Pos. | 632 | obs: | exp: |
| caa | Q | 76 | 304.90 |
| cag | Q | 496 | 267.10 |
| --- | -- | ------ | ------ |
| mPD | | 0.23 | 0.50 |
| | | nPD: | 0.46 |
| | | N. weight: | 0.51 |
| | | Sc. PD: | 0.094 |
| | | Sc. rank: | 103.6 |

| PB2 | | | |
|---|---|---|---|
| Pos. | 633 | obs: | exp: |
| ttt | F | 66 | 235.20 |
| ttc | F | 506 | 336.80 |
| --- | -- | ------ | ------ |
| mPD | | 0.20 | 0.48 |
| | | nPD: | 0.42 |
| | | N. weight: | 0.31 |
| | | Sc. PD: | 0.045 |
| | | Sc. rank: | 53.5 |

| PB2 | | | |
|---|---|---|---|
| Pos. | 634 | obs: | exp: |
| tct | S | 502 | 89.82 |
| tcc | S | 58 | 78.71 |
| tca | S | 5 | 125.20 |
| tcg | S | 0 | 34.44 |
| act | T | 7 | 1.85 |
| acc | T | 0 | 1.50 |
| aca | T | 0 | 3.07 |
| acg | T | 0 | 0.58 |
| agt | S | 0 | 124.30 |
| agc | S | 0 | 112.60 |
| --- | -- | ------ | ------ |
| mPD | | 0.22 | 1.7 |
| | | nPD: | 0.13 |
| | | N. weight: | 2.1 |
| | | Sc. PD: | -0.28 |
| | | Sc. rank: | -259.3 |

| PB2 | | | |
|---|---|---|---|
| Pos. | 635 | obs: | exp: |
| ttt | F | 1 | 0.41 |
| ttc | F | 0 | 0.59 |
| tct | S | 499 | 90.78 |
| tcc | S | 68 | 79.54 |
| tca | S | 2 | 126.50 |
| tcg | S | 2 | 34.80 |
| agt | S | 0 | 125.60 |
| agc | S | 0 | 113.80 |
| --- | -- | ------ | ------ |
| mPD | | 0.23 | 1.7 |
| | | nPD: | 0.14 |
| | | N. weight: | 2.1 |
| | | Sc. PD: | -0.27 |
| | | Sc. rank: | -246.7 |

| PB2 | | | |
|---|---|---|---|
| Pos. | 636 | obs: | exp: |
| tta | L | 12 | 49.97 |
| ttg | L | 36 | 110.20 |
| ctt | L | 0 | 106.70 |
| ctc | L | 0 | 92.53 |
| cta | L | 406 | 86.17 |
| ctg | L | 118 | 126.40 |
| --- | -- | ------ | ------ |
| mPD | | 0.55 | 1.1 |
| | | nPD: | 0.49 |
| | | N. weight: | 1.4 |
| | | Sc. PD: | 0.3 |
| | | Sc. rank: | 316.7 |

| PB2 | | | |
|---|---|---|---|
| Pos. | 637 | obs: | exp: |
| att | I | 1 | 0.38 |
| atc | I | 0 | 0.26 |
| ata | I | 0 | 0.36 |
| act | T | 547 | 150.60 |
| acc | T | 21 | 122.30 |
| aca | T | 2 | 249.70 |
| acg | T | 0 | 47.38 |
| gtt | V | 0 | 0.21 |
| gtc | V | 1 | 0.21 |
| gta | V | 0 | 0.19 |
| gtg | V | 0 | 0.40 |
| --- | -- | ------ | ------ |
| mPD | | 0.091 | 0.70 |
| | | nPD: | 0.13 |
| | | N. weight: | 1.7 |
| | | Sc. PD: | -0.22 |
| | | Sc. rank: | -211.5 |

| PB2 | | | |
|---|---|---|---|
| Pos. | 638 | obs: | exp: |
| gtt | V | 2 | 117.60 |
| gtc | V | 0 | 120.10 |
| gta | V | 103 | 107.20 |
| gtg | V | 467 | 227.20 |
| --- | -- | ------ | ------ |
| mPD | | 0.30 | 0.72 |
| | | nPD: | 0.42 |
| | | N. weight: | 0.82 |
| | | Sc. PD: | 0.11 |
| | | Sc. rank: | 137.0 |

| PB2 | | | |
|---|---|---|---|
| Pos. | 639 | obs: | exp: |
| aat | N | 198 | 299.00 |
| aac | N | 374 | 273.00 |
| --- | -- | ------ | ------ |
| mPD | | 0.45 | 0.50 |
| | | nPD: | 0.91 |
| | | N. weight: | 0.098 |
| | | Sc. PD: | 0.06 |
| | | Sc. rank: | 34.1 |

| PB2 | | | |
|---|---|---|---|
| Pos. | 640 | obs: | exp: |
| att | I | 0 | 0.38 |
| atc | I | 0 | 0.26 |
| ata | I | 1 | 0.36 |
| gtt | V | 1 | 117.40 |
| gtc | V | 1 | 119.80 |
| gta | V | 408 | 107.00 |
| gtg | V | 161 | 226.80 |
| --- | -- | ------ | ------ |
| mPD | | 0.41 | 0.72 |
| | | nPD: | 0.57 |
| | | N. weight: | 1.2 |
| | | Sc. PD: | 0.35 |
| | | Sc. rank: | 333.7 |

| | | | |
|---|---|---|---|
| PB2 | | | |
| Pos. | 641 | obs: | exp: |
| cgt | R | 0 | 23.75 |
| cgc | R | 0 | 28.01 |
| cga | R | 0 | 54.97 |
| cgg | R | 1 | 47.73 |
| aga | R | 168 | 257.20 |
| agg | R | 403 | 160.30 |
| --- | -- | ------ | ------ |
| mPD | | 0.42 | 0.96 |
| | | nPD: | 0.44 |
| | | N. weight: | 0.75 |
| | | Sc. PD: | 0.12 |
| | | Sc. rank: | 135.6 |

,

| | | | |
|---|---|---|---|
| PB2 | | | |
| Pos. | 642 | obs: | exp: |
| ggt | G | 413 | 70.58 |
| ggc | G | 81 | 77.35 |
| gga | G | 54 | 276.70 |
| ggg | G | 24 | 147.40 |
| --- | -- | ------ | ------ |
| mPD | | 0.45 | 0.67 |
| | | nPD: | 0.67 |
| | | N. weight: | 1.5 |
| | | Sc. PD: | 0.58 |
| | | Sc. rank: | 478.6 |

,

| | | | |
|---|---|---|---|
| PB2 | | | |
| Pos. | 643 | obs: | exp: |
| tct | S | 1 | 90.93 |
| tcc | S | 0 | 79.68 |
| tca | S | 570 | 126.70 |
| tcg | S | 1 | 34.87 |
| agt | S | 0 | 125.80 |
| agc | S | 0 | 114.00 |
| --- | -- | ------ | ------ |
| mPD | | 0.0070 | 1.7 |
| | | nPD: | 0. |
| | | N. weight: | 2.1 |
| | | Sc. PD: | -0.54 |
| | | Sc. rank: | -663.8 |

,

| | | | |
|---|---|---|---|
| PB2 | | | |
| Pos. | 644 | obs: | exp: |
| ggt | G | 0 | 70.58 |
| ggc | G | 3 | 77.35 |
| gga | G | 557 | 276.70 |
| ggg | G | 12 | 147.40 |
| --- | -- | ------ | ------ |
| mPD | | 0.051 | 0.67 |
| | | nPD: | 0.08 |
| | | N. weight: | 0.88 |
| | | Sc. PD: | -0.16 |
| | | Sc. rank: | -161.4 |

,

| | | | |
|---|---|---|---|
| PB2 | | | |
| Pos. | 645 | obs: | exp: |
| atg | M | 572 | 572.00 |
| --- | -- | ------ | ------ |
| mPD | | 0 | 0 |
| | | nPD: | 1. |
| | | N. weight: | 0. |
| | | Sc. PD: | 0 |
| | | Sc. rank: | 0 |

,

| | | | |
|---|---|---|---|
| PB2 | | | |
| Pos. | 646 | obs: | exp: |
| cgt | R | 0 | 23.75 |
| cgc | R | 0 | 28.01 |
| cga | R | 0 | 54.97 |
| cgg | R | 0 | 47.73 |
| aga | R | 557 | 257.20 |
| agg | R | 15 | 160.30 |
| --- | -- | ------ | ------ |
| mPD | | 0.051 | 0.96 |
| | | nPD: | 0.05 |
| | | N. weight: | 0.99 |
| | | Sc. PD: | -0.21 |
| | | Sc. rank: | -206.5 |

,

| | | | |
|---|---|---|---|
| PB2 | | | |
| Pos. | 647 | obs: | exp: |
| att | I | 0 | 214.70 |
| atc | I | 11 | 149.60 |
| ata | I | 560 | 206.70 |
| atg | M | 1 | 1.00 |
| --- | -- | ------ | ------ |
| mPD | | 0.041 | 0.66 |
| | | nPD: | 0.06 |
| | | N. weight: | 1.3 |
| | | Sc. PD: | -0.26 |
| | | Sc. rank: | -259.9 |

,

| | | | |
|---|---|---|---|
| PB2 | | | |
| Pos. | 648 | obs: | exp: |
| tta | L | 3 | 47.61 |
| ttg | L | 0 | 105.00 |
| ctt | L | 445 | 101.70 |
| ctc | L | 29 | 88.16 |
| cta | L | 18 | 82.10 |
| ctg | L | 50 | 120.50 |
| gtt | V | 16 | 5.55 |
| gtc | V | 11 | 5.67 |
| gta | V | 0 | 5.06 |
| gtg | V | 0 | 10.72 |
| --- | -- | ------ | ------ |
| mPD | | 0.44 | 1.2 |
| | | nPD: | 0.37 |
| | | N. weight: | 1.4 |
| | | Sc. PD: | 0.14 |
| | | Sc. rank: | 186.7 |

,

| | | | |
|---|---|---|---|
| PB2 | | | |
| Pos. | 649 | obs: | exp: |
| gtt | V | 1 | 117.60 |
| gtc | V | 0 | 120.10 |
| gta | V | 67 | 107.20 |
| gtg | V | 504 | 227.20 |
| --- | -- | ------ | ------ |
| mPD | | 0.21 | 0.72 |
| | | nPD: | 0.29 |
| | | N. weight: | 0.92 |
| | | Sc. PD: | 0.018 |
| | | Sc. rank: | 58.0 |

,

| | | | |
|---|---|---|---|
| PB2 | | | |
| Pos. | 650 | obs: | exp: |
| cgt | R | 0 | 23.75 |
| cgc | R | 0 | 28.01 |
| cga | R | 1 | 54.97 |
| cgg | R | 0 | 47.73 |
| aga | R | 502 | 257.20 |
| agg | R | 69 | 160.30 |
| --- | -- | ------ | ------ |
| mPD | | 0.22 | 0.96 |
| | | nPD: | 0.23 |
| | | N. weight: | 0.69 |
| | | Sc. PD: | -0.031 |
| | | Sc. rank: | -7.1 |

,

| | | | |
|---|---|---|---|
| PB2 | | | |
| Pos. | 651 | obs: | exp: |
| ggt | G | 44 | 70.58 |
| ggc | G | 526 | 77.35 |
| gga | G | 2 | 276.70 |
| ggg | G | 0 | 147.40 |
| --- | -- | ------ | ------ |
| mPD | | 0.15 | 0.67 |
| | | nPD: | 0.22 |
| | | N. weight: | 2.5 |
| | | Sc. PD: | -0.11 |
| | | Sc. rank: | -30.5 |

,

| | | | |
|---|---|---|---|
| PB2 | | | |
| Pos. | 652 | obs: | exp: |
| tct | S | 0 | 0.16 |
| tcc | S | 0 | 0.14 |
| tca | S | 0 | 0.22 |
| tcg | S | 0 | 0.06 |
| aat | N | 178 | 298.40 |
| aac | N | 393 | 272.60 |
| agt | S | 0 | 0.22 |
| agc | S | 1 | 0.20 |
| --- | -- | ------ | ------ |
| mPD | | 0.43 | 0.50 |
| | | nPD: | 0.86 |
| | | N. weight: | 0.14 |
| | | Sc. PD: | 0.079 |
| | | Sc. rank: | 48.7 |

,

| | | | |
|---|---|---|---|
| PB2 | | | |
| Pos. | 653 | obs: | exp: |
| tct | S | 54 | 90.93 |
| tcc | S | 518 | 79.68 |
| tca | S | 0 | 126.70 |
| tcg | S | 0 | 34.87 |
| agt | S | 0 | 125.80 |
| agc | S | 0 | 114.00 |
| --- | -- | ------ | ------ |
| mPD | | 0.17 | 1.7 |
| | | nPD: | 0.1 |
| | | N. weight: | 2.4 |
| | | Sc. PD: | -0.38 |
| | | Sc. rank: | -366.5 |

,

| | | | |
|---|---|---|---|
| PB2 | | | |
| Pos. | 654 | obs: | exp: |
| cct | P | 483 | 150.60 |
| ccc | P | 18 | 103.20 |
| cca | P | 71 | 225.70 |
| ccg | P | 0 | 92.49 |
| --- | -- | ------ | ------ |
| mPD | | 0.27 | 0.72 |
| | | nPD: | 0.38 |
| | | N. weight: | 1.1 |
| | | Sc. PD: | 0.12 |
| | | Sc. rank: | 151.8 |

,

| | | | |
|---|---|---|---|
| PB2 | | | |
| Pos. | 655 | obs: | exp: |
| gtt | V | 24 | 117.60 |
| gtc | V | 1 | 120.10 |
| gta | V | 8 | 107.20 |
| gtg | V | 539 | 227.20 |
| --- | -- | ------ | ------ |
| mPD | | 0.11 | 0.72 |
| | | nPD: | 0.15 |
| | | N. weight: | 1. |
| | | Sc. PD: | -0.12 |
| | | Sc. rank: | -89.7 |

,

| | | | |
|---|---|---|---|
| PB2 | | | |
| Pos. | 656 | obs: | exp: |
| ttt | F | 34 | 235.20 |
| ttc | F | 538 | 336.80 |
| --- | -- | ------ | ------ |
| mPD | | 0.11 | 0.48 |
| | | nPD: | 0.23 |
| | | N. weight: | 0.47 |
| | | Sc. PD: | -0.018 |
| | | Sc. rank: | -2.6 |

,

| | | | |
|---|---|---|---|
| PB2 | | | |
| Pos. | 657 | obs: | exp: |
| aat | N | 79 | 298.40 |
| aac | N | 492 | 272.60 |
| aaa | K | 1 | 0.56 |
| aag | K | 0 | 0.44 |
| --- | -- | ------ | ------ |
| mPD | | 0.24 | 0.50 |
| | | nPD: | 0.48 |
| | | N. weight: | 0.47 |
| | | Sc. PD: | 0.095 |
| | | Sc. rank: | 101.3 |

,

| | | | |
|---|---|---|---|
| PB2 | | | |
| Pos. | 658 | obs: | exp: |
| tat | Y | 426 | 295.60 |
| tac | Y | 145 | 275.40 |
| cat | H | 1 | 0.54 |
| cac | H | 0 | 0.46 |
| --- | -- | ------ | ------ |
| mPD | | 0.38 | 0.50 |
| | | nPD: | 0.76 |
| | | N. weight: | 0.17 |
| | | Sc. PD: | 0.078 |
| | | Sc. rank: | 56.1 |

,

| | | | |
|---|---|---|---|
| PB2 | | | |
| Pos. | 659 | obs: | exp: |
| aat | N | 41 | 299.00 |
| aac | N | 531 | 273.00 |
| --- | -- | ------ | ------ |
| mPD | | 0.13 | 0.50 |
| | | nPD: | 0.27 |
| | | N. weight: | 0.69 |
| | | Sc. PD: | -0.0029 |
| | | Sc. rank: | 26.0 |

,

| | | | |
|---|---|---|---|
| PB2 | | | |
| Pos. | 660 | obs: | exp: |
| aaa | K | 11 | 320.30 |
| aag | K | 561 | 251.70 |
| --- | -- | ------ | ------ |
| mPD | | 0.038 | 0.49 |
| | | nPD: | 0.08 |
| | | N. weight: | 1. |
| | | Sc. PD: | -0.19 |
| | | Sc. rank: | -191.0 |

,

| | | | |
|---|---|---|---|
| PB2 | | | |
| Pos. | 661 | obs: | exp: |
| gct | A | 1 | 144.10 |
| gcc | A | 66 | 117.20 |
| gca | A | 489 | 254.90 |
| gcg | A | 16 | 55.82 |
| --- | -- | ------ | ------ |
| mPD | | 0.26 | 0.69 |
| | | nPD: | 0.37 |
| | | N. weight: | 0.65 |
| | | Sc. PD: | 0.063 |
| | | Sc. rank: | 82.8 |

,

| | | | |
|---|---|---|---|
| PB2 | | | |
| Pos. | 662 | obs: | exp: |
| tct | S | 0 | 0.32 |
| tcc | S | 0 | 0.28 |
| tca | S | 0 | 0.44 |
| tcg | S | 0 | 0.12 |
| att | I | 0 | 0.75 |
| atc | I | 2 | 0.52 |
| ata | I | 0 | 0.72 |
| act | T | 22 | 149.80 |
| acc | T | 544 | 121.70 |
| aca | T | 1 | 248.40 |
| acg | T | 0 | 47.14 |
| aat | N | 0 | 0.52 |
| aac | N | 1 | 0.48 |
| agt | S | 0 | 0.44 |
| agc | S | 2 | 0.40 |
| --- | -- | ------ | ------ |
| mPD | | 0.095 | 0.70 |
| | | nPD: | 0.13 |
| | | N. weight: | 1.9 |
| | | Sc. PD: | -0.25 |
| | | Sc. rank: | -235.4 |

,

| | | | |
|---|---|---|---|
| PB2 | | | |
| Pos. | 663 | obs: | exp: |
| cgt | R | 0 | 0.04 |
| cgc | R | 0 | 0.05 |
| cga | R | 0 | 0.10 |
| cgg | R | 0 | 0.08 |
| aaa | K | 36 | 319.70 |
| aag | K | 535 | 251.30 |
| aga | R | 1 | 0.45 |
| agg | R | 0 | 0.28 |
| --- | -- | ------ | ------ |
| mPD | | 0.12 | 0.50 |
| | | nPD: | 0.25 |
| | | N. weight: | 0.82 |
| | | Sc. PD: | -0.016 |
| | | Sc. rank: | 12.7 |

,

| | | | |
|---|---|---|---|
| PB2 | | | |
| Pos. | 664 | obs: | exp: |
| cgt | R | 1 | 23.75 |
| cgc | R | 0 | 28.01 |
| cga | R | 0 | 54.97 |
| cgg | R | 0 | 47.73 |
| aga | R | 66 | 257.20 |
| agg | R | 505 | 160.30 |
| --- | -- | ------ | ------ |
| mPD | | 0.21 | 0.96 |
| | | nPD: | 0.22 |
| | | N. weight: | 1.2 |
| | | Sc. PD: | -0.06 |
| | | Sc. rank: | -24.3 |

,

| | | | |
|---|---|---|---|
| PB2 | | | |
| Pos. | 665 | obs: | exp: |
| tta | L | 0 | 49.97 |
| ttg | L | 0 | 110.20 |
| ctt | L | 363 | 106.70 |
| ctc | L | 205 | 92.53 |
| cta | L | 4 | 86.17 |
| ctg | L | 0 | 126.40 |
| --- | -- | ------ | ------ |
| mPD | | 0.47 | 1.1 |
| | | nPD: | 0.42 |
| | | N. weight: | 1.5 |
| | | Sc. PD: | 0.21 |
| | | Sc. rank: | 255.3 |

,

| PB2 | | | |
|---|---|---|---|
| Pos. | 666 | obs: | exp: |
| act | T | 8 | 151.10 |
| acc | T | 7 | 122.70 |
| aca | T | 549 | 250.60 |
| acg | T | 8 | 47.55 |
| --- | -- | ------ | ------ |
| mPD | | 0.078 | 0.69 |
| | | nPD: | 0.11 |
| | | N. weight: | 0.95 |
| | | Sc. PD: | -0.14 |
| | | Sc. rank: | -138.1 |

,

| PB2 | | | |
|---|---|---|---|
| Pos. | 667 | obs: | exp: |
| att | I | 1 | 2.63 |
| atc | I | 6 | 1.83 |
| ata | I | 0 | 2.53 |
| gtt | V | 134 | 116.10 |
| gtc | V | 398 | 118.60 |
| gta | V | 5 | 105.90 |
| gtg | V | 28 | 224.40 |
| --- | -- | ------ | ------ |
| mPD | | 0.47 | 0.75 |
| | | nPD: | 0.63 |
| | | N. weight: | 1.1 |
| | | Sc. PD: | 0.37 |
| | | Sc. rank: | 325.7 |

,

| PB2 | | | |
|---|---|---|---|
| Pos. | 668 | obs: | exp: |
| ttt | F | 3 | 1.23 |
| ttc | F | 0 | 1.77 |
| tta | L | 0 | 49.71 |
| ttg | L | 0 | 109.60 |
| ctt | L | 417 | 106.20 |
| ctc | L | 152 | 92.04 |
| cta | L | 0 | 85.72 |
| ctg | L | 0 | 125.80 |
| --- | -- | ------ | ------ |
| mPD | | 0.40 | 1.1 |
| | | nPD: | 0.36 |
| | | N. weight: | 1.6 |
| | | Sc. PD: | 0.14 |
| | | Sc. rank: | 188.5 |

,

| PB2 | | | |
|---|---|---|---|
| Pos. | 669 | obs: | exp: |
| gaa | E | 1 | 0.58 |
| gag | E | 0 | 0.42 |
| ggt | G | 0 | 70.45 |
| ggc | G | 0 | 77.22 |
| gga | G | 260 | 276.20 |
| ggg | G | 311 | 147.10 |
| --- | -- | ------ | ------ |
| mPD | | 0.50 | 0.67 |
| | | nPD: | 0.75 |
| | | N. weight: | 0.55 |
| | | Sc. PD: | 0.25 |
| | | Sc. rank: | 185.9 |

,

| PB2 | | | |
|---|---|---|---|
| Pos. | 670 | obs: | exp: |
| cgt | R | 0 | 0.08 |
| cgc | R | 0 | 0.10 |
| cga | R | 0 | 0.19 |
| cgg | R | 0 | 0.17 |
| act | T | 0 | 0.53 |
| acc | T | 0 | 0.43 |
| aca | T | 0 | 0.88 |
| acg | T | 2 | 0.17 |
| aaa | K | 75 | 318.10 |
| aag | K | 493 | 249.90 |
| aga | R | 2 | 0.90 |
| agg | R | 0 | 0.56 |
| --- | -- | ------ | ------ |
| mPD | | 0.25 | 0.51 |
| | | nPD: | 0.48 |
| | | N. weight: | 0.59 |
| | | Sc. PD: | 0.12 |
| | | Sc. rank: | 127.3 |

,

| PB2 | | | |
|---|---|---|---|
| Pos. | 671 | obs: | exp: |
| gat | D | 545 | 305.20 |
| gac | D | 27 | 266.80 |
| --- | -- | ------ | ------ |
| mPD | | 0.090 | 0.50 |
| | | nPD: | 0.18 |
| | | N. weight: | 0.64 |
| | | Sc. PD: | -0.055 |
| | | Sc. rank: | -40.3 |

,

| PB2 | | | |
|---|---|---|---|
| Pos. | 672 | obs: | exp: |
| gct | A | 46 | 144.10 |
| gcc | A | 0 | 117.20 |
| gca | A | 523 | 254.90 |
| gcg | A | 3 | 55.82 |
| --- | -- | ------ | ------ |
| mPD | | 0.16 | 0.69 |
| | | nPD: | 0.23 |
| | | N. weight: | 0.8 |
| | | Sc. PD: | -0.032 |
| | | Sc. rank: | -4.7 |

,

| PB2 | | | |
|---|---|---|---|
| Pos. | 673 | obs: | exp: |
| ggt | G | 562 | 70.58 |
| ggc | G | 4 | 77.35 |
| gga | G | 1 | 276.70 |
| ggg | G | 5 | 147.40 |
| --- | -- | ------ | ------ |
| mPD | | 0.035 | 0.67 |
| | | nPD: | 0.05 |
| | | N. weight: | 2.8 |
| | | Sc. PD: | -0.59 |
| | | Sc. rank: | -600.1 |

,

| PB2 | | | |
|---|---|---|---|
| Pos. | 674 | obs: | exp: |
| gct | A | 21 | 143.80 |
| gcc | A | 65 | 117.00 |
| gca | A | 480 | 254.50 |
| gcg | A | 5 | 55.72 |
| gaa | E | 1 | 0.58 |
| gag | E | 0 | 0.42 |
| --- | -- | ------ | ------ |
| mPD | | 0.28 | 0.69 |
| | | nPD: | 0.41 |
| | | N. weight: | 0.55 |
| | | Sc. PD: | 0.073 |
| | | Sc. rank: | 85.4 |

,

| PB2 | | | |
|---|---|---|---|
| Pos. | 675 | obs: | exp: |
| tta | L | 41 | 49.97 |
| ttg | L | 493 | 110.20 |
| ctt | L | 30 | 106.70 |
| ctc | L | 1 | 92.53 |
| cta | L | 2 | 86.17 |
| ctg | L | 5 | 126.40 |
| --- | -- | ------ | ------ |
| mPD | | 0.36 | 1.1 |
| | | nPD: | 0.32 |
| | | N. weight: | 1.7 |
| | | Sc. PD: | 0.08 |
| | | Sc. rank: | 143.5 |

,

| PB2 | | | |
|---|---|---|---|
| Pos. | 676 | obs: | exp: |
| att | I | 0 | 2.63 |
| atc | I | 1 | 1.83 |
| ata | I | 6 | 2.53 |
| atg | M | 27 | 27.00 |
| act | T | 29 | 140.00 |
| acc | T | 41 | 113.70 |
| aca | T | 453 | 232.20 |
| acg | T | 7 | 44.06 |
| gtt | V | 1 | 0.21 |
| gtc | V | 0 | 0.21 |
| gta | V | 0 | 0.19 |
| gtg | V | 0 | 0.40 |
| gct | A | 0 | 1.76 |
| gcc | A | 2 | 1.43 |
| gca | A | 5 | 3.12 |
| gcg | A | 0 | 0.68 |
| --- | -- | ------ | ------ |
| mPD | | 0.47 | 0.85 |
| | | nPD: | 0.56 |
| | | N. weight: | 0.55 |
| | | Sc. PD: | 0.15 |
| | | Sc. rank: | 146.7 |

,

| PB2 | | | |
|---|---|---|---|
| Pos. | 677 | obs: | exp: |
| gaa | E | 526 | 329.10 |
| gag | E | 46 | 242.90 |
| --- | -- | ------ | ------ |
| mPD | | 0.15 | 0.49 |
| | | nPD: | 0.3 |
| | | N. weight: | 0.43 |
| | | Sc. PD: | 0.013 |
| | | Sc. rank: | 30.9 |

,

| PB2 | | | |
|---|---|---|---|
| Pos. | 678 | obs: | exp: |
| gat | D | 441 | 304.70 |
| gac | D | 130 | 266.30 |
| ggt | G | 1 | 0.12 |
| ggc | G | 0 | 0.14 |
| gga | G | 0 | 0.48 |
| ggg | G | 0 | 0.26 |
| --- | -- | ------ | ------ |
| mPD | | 0.36 | 0.50 |
| | | nPD: | 0.71 |
| | | N. weight: | 0.19 |
| | | Sc. PD: | 0.078 |
| | | Sc. rank: | 62.0 |

,

| PB2 | | | |
|---|---|---|---|
| Pos. | 679 | obs: | exp: |
| cct | P | 1 | 150.60 |
| ccc | P | 0 | 103.20 |
| cca | P | 563 | 225.70 |
| ccg | P | 8 | 92.49 |
| --- | -- | ------ | ------ |
| mPD | | 0.031 | 0.72 |
| | | nPD: | 0.04 |
| | | N. weight: | 1.2 |
| | | Sc. PD: | -0.27 |
| | | Sc. rank: | -275.3 |

,

| PB2 | | | |
|---|---|---|---|
| Pos. | 680 | obs: | exp: |
| aat | N | 2 | 1.05 |
| aac | N | 0 | 0.95 |
| gat | D | 546 | 304.10 |
| gac | D | 24 | 265.90 |
| --- | -- | ------ | ------ |
| mPD | | 0.088 | 0.50 |
| | | nPD: | 0.17 |
| | | N. weight: | 0.66 |
| | | Sc. PD: | -0.062 |
| | | Sc. rank: | -46.6 |

,

| PB2 | | | |
|---|---|---|---|
| Pos. | 681 | obs: | exp: |
| gat | D | 1 | 0.53 |
| gac | D | 0 | 0.47 |
| gaa | E | 80 | 328.60 |
| gag | E | 491 | 242.40 |
| --- | -- | ------ | ------ |
| mPD | | 0.24 | 0.49 |
| | | nPD: | 0.5 |
| | | N. weight: | 0.59 |
| | | Sc. PD: | 0.13 |
| | | Sc. rank: | 136.0 |

,

| PB2 | | | |
|---|---|---|---|
| Pos. | 682 | obs: | exp: |
| ggt | G | 1 | 70.58 |
| ggc | G | 73 | 77.35 |
| gga | G | 441 | 276.70 |
| ggg | G | 57 | 147.40 |
| --- | -- | ------ | ------ |
| mPD | | 0.38 | 0.67 |
| | | nPD: | 0.57 |
| | | N. weight: | 0.37 |
| | | Sc. PD: | 0.11 |
| | | Sc. rank: | 102.1 |

,

| PB2 | | | |
|---|---|---|---|
| Pos. | 683 | obs: | exp: |
| act | T | 1 | 151.10 |
| acc | T | 2 | 122.70 |
| aca | T | 511 | 250.60 |
| acg | T | 58 | 47.55 |
| --- | -- | ------ | ------ |
| mPD | | 0.19 | 0.69 |
| | | nPD: | 0.28 |
| | | N. weight: | 0.92 |
| | | Sc. PD: | 0.0074 |
| | | Sc. rank: | 44.5 |

,

| PB2 | | | |
|---|---|---|---|
| Pos. | 684 | obs: | exp: |
| act | T | 0 | 0.53 |
| acc | T | 2 | 0.43 |
| aca | T | 0 | 0.88 |
| acg | T | 0 | 0.17 |
| gct | A | 77 | 143.60 |
| gcc | A | 22 | 116.80 |
| gca | A | 469 | 254.00 |
| gcg | A | 2 | 55.62 |
| --- | -- | ------ | ------ |
| mPD | | 0.32 | 0.69 |
| | | nPD: | 0.45 |
| | | N. weight: | 0.51 |
| | | Sc. PD: | 0.09 |
| | | Sc. rank: | 100.9 |

,

| PB2 | | | |
|---|---|---|---|
| Pos. | 685 | obs: | exp: |
| ggt | G | 0 | 70.58 |
| ggc | G | 0 | 77.35 |
| gga | G | 532 | 276.70 |
| ggg | G | 40 | 147.40 |
| --- | -- | ------ | ------ |
| mPD | | 0.13 | 0.67 |
| | | nPD: | 0.2 |
| | | N. weight: | 0.75 |
| | | Sc. PD: | -0.054 |
| | | Sc. rank: | -35.3 |

,

| PB2 | | | |
|---|---|---|---|
| Pos. | 686 | obs: | exp: |
| gtt | V | 15 | 117.60 |
| gtc | V | 0 | 120.10 |
| gta | V | 34 | 107.20 |
| gtg | V | 523 | 227.20 |
| --- | -- | ------ | ------ |
| mPD | | 0.16 | 0.72 |
| | | nPD: | 0.22 |
| | | N. weight: | 0.93 |
| | | Sc. PD: | -0.044 |
| | | Sc. rank: | -14.4 |

,

| PB2 | | | |
|---|---|---|---|
| Pos. | 687 | obs: | exp: |
| gaa | E | 418 | 329.10 |
| gag | E | 154 | 242.90 |
| --- | -- | ------ | ------ |
| mPD | | 0.39 | 0.49 |
| | | nPD: | 0.81 |
| | | N. weight: | 0.082 |
| | | Sc. PD: | 0.042 |
| | | Sc. rank: | 28.2 |

,

| PB2 | | | |
|---|---|---|---|
| Pos. | 688 | obs: | exp: |
| ttt | F | 3 | 1.23 |
| ttc | F | 0 | 1.77 |
| tct | S | 558 | 90.46 |
| tcc | S | 9 | 79.26 |
| tca | S | 2 | 126.10 |
| tcg | S | 0 | 34.68 |
| agt | S | 0 | 125.20 |
| agc | S | 0 | 113.40 |
| --- | -- | ------ | ------ |
| mPD | | 0.048 | 1.7 |
| | | nPD: | 0.03 |
| | | N. weight: | 2.5 |
| | | Sc. PD: | -0.57 |
| | | Sc. rank: | -609.8 |

,

| PB2 | | | |
|---|---|---|---|
| Pos. | 689 | obs: | exp: |
| gct | A | 52 | 144.10 |
| gcc | A | 1 | 117.20 |
| gca | A | 53 | 254.90 |
| gcg | A | 466 | 55.82 |
| --- | -- | ------ | ------ |
| mPD | | 0.32 | 0.69 |
| | | nPD: | 0.47 |
| | | N. weight: | 2.1 |
| | | Sc. PD: | 0.4 |
| | | Sc. rank: | 438.0 |

,

| PB2 | | | |
|---|---|---|---|
| Pos. | 690 | obs: | exp: |
| att | I | 0 | 0.38 |
| atc | I | 0 | 0.26 |
| ata | I | 1 | 0.36 |
| gtt | V | 45 | 117.40 |
| gtc | V | 26 | 119.80 |
| gta | V | 483 | 107.00 |
| gtg | V | 17 | 226.80 |
| --- | -- | ------ | ------ |
| mPD | | 0.28 | 0.72 |
| | | nPD: | 0.38 |
| | | N. weight: | 1.5 |
| | | Sc. PD: | 0.16 |
| | | Sc. rank: | 210.5 |

,

| PB2 | | | |
|---|---|---|---|
| Pos. | 691 | obs: | exp: |
| tta | L | 410 | 49.97 |
| ttg | L | 98 | 110.20 |
| ctt | L | 1 | 106.70 |
| ctc | L | 0 | 92.53 |
| cta | L | 40 | 86.17 |
| ctg | L | 23 | 126.40 |
| --- | -- | ------ | ------ |
| mPD | | 0.54 | 1.1 |
| | | nPD: | 0.48 |
| | | N. weight: | 2. |
| | | Sc. PD: | 0.39 |
| | | Sc. rank: | 417.9 |

| PB2 | | | |
|---|---|---|---|
| Pos. | 692 | obs: | exp: |
| cgt | R | 0 | 23.75 |
| cgc | R | 0 | 28.01 |
| cga | R | 2 | 54.97 |
| cgg | R | 0 | 47.73 |
| aga | R | 545 | 257.20 |
| agg | R | 25 | 160.30 |
| --- | -- | ------ | ------ |
| mPD | | 0.091 | 0.96 |
| | | nPD: | 0.09 |
| | | N. weight: | 0.9 |
| | | Sc. PD: | -0.15 |
| | | Sc. rank: | -147.8 |

| PB2 | | | |
|---|---|---|---|
| Pos. | 693 | obs: | exp: |
| ggt | G | 1 | 70.58 |
| ggc | G | 0 | 77.35 |
| gga | G | 479 | 276.70 |
| ggg | G | 92 | 147.40 |
| --- | -- | ------ | ------ |
| mPD | | 0.27 | 0.67 |
| | | nPD: | 0.41 |
| | | N. weight: | 0.55 |
| | | Sc. PD: | 0.072 |
| | | Sc. rank: | 86.1 |

| PB2 | | | |
|---|---|---|---|
| Pos. | 694 | obs: | exp: |
| ttt | F | 434 | 235.20 |
| ttc | F | 138 | 336.80 |
| --- | -- | ------ | ------ |
| mPD | | 0.37 | 0.48 |
| | | nPD: | 0.76 |
| | | N. weight: | 0.36 |
| | | Sc. PD: | 0.17 |
| | | Sc. rank: | 123.1 |

| PB2 | | | |
|---|---|---|---|
| Pos. | 695 | obs: | exp: |
| tta | L | 5 | 49.97 |
| ttg | L | 0 | 110.20 |
| ctt | L | 0 | 106.70 |
| ctc | L | 71 | 92.53 |
| cta | L | 454 | 86.17 |
| ctg | L | 42 | 126.40 |
| --- | -- | ------ | ------ |
| mPD | | 0.35 | 1.1 |
| | | nPD: | 0.32 |
| | | N. weight: | 1.7 |
| | | Sc. PD: | 0.074 |
| | | Sc. rank: | 136.9 |

| PB2 | | | |
|---|---|---|---|
| Pos. | 696 | obs: | exp: |
| att | I | 542 | 215.00 |
| atc | I | 30 | 149.90 |
| ata | I | 0 | 207.10 |
| --- | -- | ------ | ------ |
| mPD | | 0.10 | 0.66 |
| | | nPD: | 0.15 |
| | | N. weight: | 1.1 |
| | | Sc. PD: | -0.13 |
| | | Sc. rank: | -102.2 |

| PB2 | | | |
|---|---|---|---|
| Pos. | 697 | obs: | exp: |
| tta | L | 34 | 49.97 |
| ttg | L | 65 | 110.20 |
| ctt | L | 0 | 106.70 |
| ctc | L | 0 | 92.53 |
| cta | L | 34 | 86.17 |
| ctg | L | 439 | 126.40 |
| --- | -- | ------ | ------ |
| mPD | | 0.50 | 1.1 |
| | | nPD: | 0.45 |
| | | N. weight: | 1.2 |
| | | Sc. PD: | 0.2 |
| | | Sc. rank: | 226.7 |

| PB2 | | | |
|---|---|---|---|
| Pos. | 698 | obs: | exp: |
| ggt | G | 3 | 70.58 |
| ggc | G | 540 | 77.35 |
| gga | G | 28 | 276.70 |
| ggg | G | 1 | 147.40 |
| --- | -- | ------ | ------ |
| mPD | | 0.11 | 0.67 |
| | | nPD: | 0.16 |
| | | N. weight: | 2.4 |
| | | Sc. PD: | -0.26 |
| | | Sc. rank: | -197.2 |

| PB2 | | | |
|---|---|---|---|
| Pos. | 699 | obs: | exp: |
| cgt | R | 0 | 0.08 |
| cgc | R | 0 | 0.10 |
| cga | R | 0 | 0.19 |
| cgg | R | 0 | 0.17 |
| aaa | K | 528 | 319.20 |
| aag | K | 42 | 250.80 |
| aga | R | 2 | 0.90 |
| agg | R | 0 | 0.56 |
| --- | -- | ------ | ------ |
| mPD | | 0.14 | 0.50 |
| | | nPD: | 0.29 |
| | | N. weight: | 0.49 |
| | | Sc. PD: | 0.0064 |
| | | Sc. rank: | 27.3 |

| PB2 | | | |
|---|---|---|---|
| Pos. | 700 | obs: | exp: |
| gaa | E | 539 | 329.10 |
| gag | E | 33 | 242.90 |
| --- | -- | ------ | ------ |
| mPD | | 0.11 | 0.49 |
| | | nPD: | 0.22 |
| | | N. weight: | 0.51 |
| | | Sc. PD: | -0.024 |
| | | Sc. rank: | -7.7 |

| PB2 | | | |
|---|---|---|---|
| Pos. | 701 | obs: | exp: |
| aat | N | 0 | 3.14 |
| aac | N | 6 | 2.86 |
| gat | D | 9 | 302.00 |
| gac | D | 557 | 264.00 |
| --- | -- | ------ | ------ |
| mPD | | 0.052 | 0.52 |
| | | nPD: | 0.1 |
| | | N. weight: | 0.98 |
| | | Sc. PD: | -0.16 |
| | | Sc. rank: | -156.9 |

| PB2 | | | |
|---|---|---|---|
| Pos. | 702 | obs: | exp: |
| cgt | R | 0 | 0.04 |
| cgc | R | 0 | 0.05 |
| cga | R | 0 | 0.10 |
| cgg | R | 0 | 0.08 |
| aaa | K | 492 | 319.70 |
| aag | K | 79 | 251.30 |
| aga | R | 1 | 0.45 |
| agg | R | 0 | 0.28 |
| --- | -- | ------ | ------ |
| mPD | | 0.24 | 0.50 |
| | | nPD: | 0.49 |
| | | N. weight: | 0.31 |
| | | Sc. PD: | 0.064 |
| | | Sc. rank: | 67.2 |

| PB2 | | | |
|---|---|---|---|
| Pos. | 703 | obs: | exp: |
| cgt | R | 0 | 23.75 |
| cgc | R | 0 | 28.01 |
| cga | R | 0 | 54.97 |
| cgg | R | 0 | 47.73 |
| aga | R | 534 | 257.20 |
| agg | R | 38 | 160.30 |
| --- | -- | ------ | ------ |
| mPD | | 0.12 | 0.96 |
| | | nPD: | 0.13 |
| | | N. weight: | 0.84 |
| | | Sc. PD: | -0.11 |
| | | Sc. rank: | -111.9 |

| PB2 | | | |
|---|---|---|---|
| Pos. | 704 | obs: | exp: |
| tat | Y | 559 | 296.10 |
| tac | Y | 13 | 275.90 |
| --- | -- | ------ | ------ |
| mPD | | 0.044 | 0.50 |
| | | nPD: | 0.09 |
| | | N. weight: | 0.79 |
| | | Sc. PD: | -0.14 |
| | | Sc. rank: | -137.3 |

| PB2 | | | |
|---|---|---|---|
| Pos. | 705 | obs: | exp: |
| ggt | G | 1 | 70.58 |
| ggc | G | 78 | 77.35 |
| gga | G | 445 | 276.70 |
| ggg | G | 48 | 147.40 |
| --- | -- | ------ | ------ |
| mPD | | 0.37 | 0.67 |
| | | nPD: | 0.56 |
| | | N. weight: | 0.4 |
| | | Sc. PD: | 0.11 |
| | | Sc. rank: | 106.0 |

| PB2 | | | |
|---|---|---|---|
| Pos. | 706 | obs: | exp: |
| cct | P | 1 | 150.60 |
| ccc | P | 0 | 103.20 |
| cca | P | 569 | 225.70 |
| ccg | P | 2 | 92.49 |
| --- | -- | ------ | ------ |
| mPD | | 0.010 | 0.72 |
| | | nPD: | 0.01 |
| | | N. weight: | 1.3 |
| | | Sc. PD: | -0.32 |
| | | Sc. rank: | -355.4 |

| PB2 | | | |
|---|---|---|---|
| Pos. | 707 | obs: | exp: |
| gct | A | 0 | 144.10 |
| gcc | A | 0 | 117.20 |
| gca | A | 493 | 254.90 |
| gcg | A | 79 | 55.82 |
| --- | -- | ------ | ------ |
| mPD | | 0.24 | 0.69 |
| | | nPD: | 0.35 |
| | | N. weight: | 0.89 |
| | | Sc. PD: | 0.064 |
| | | Sc. rank: | 92.2 |

| PB2 | | | |
|---|---|---|---|
| Pos. | 708 | obs: | exp: |
| tta | L | 6 | 49.97 |
| ttg | L | 481 | 110.20 |
| ctt | L | 0 | 106.70 |
| ctc | L | 0 | 92.53 |
| cta | L | 0 | 86.17 |
| ctg | L | 85 | 126.40 |
| --- | -- | ------ | ------ |
| mPD | | 0.27 | 1.1 |
| | | nPD: | 0.25 |
| | | N. weight: | 1.7 |
| | | Sc. PD: | -0.041 |
| | | Sc. rank: | 17.0 |

| PB2 | | | |
|---|---|---|---|
| Pos. | 709 | obs: | exp: |
| tct | S | 0 | 90.78 |
| tcc | S | 0 | 79.54 |
| tca | S | 0 | 126.50 |
| tcg | S | 0 | 34.80 |
| agt | S | 9 | 125.60 |
| agc | S | 562 | 113.80 |
| ggt | G | 0 | 0.12 |
| ggc | G | 1 | 0.14 |
| gga | G | 0 | 0.48 |
| ggg | G | 0 | 0.26 |
| --- | -- | ------ | ------ |
| mPD | | 0.035 | 1.7 |
| | | nPD: | 0.02 |
| | | N. weight: | 2.2 |
| | | Sc. PD: | -0.52 |
| | | Sc. rank: | -582.6 |

| PB2 | | | |
|---|---|---|---|
| Pos. | 710 | obs: | exp: |
| att | I | 10 | 215.00 |
| atc | I | 560 | 149.90 |
| ata | I | 2 | 207.10 |
| --- | -- | ------ | ------ |
| mPD | | 0.041 | 0.66 |
| | | nPD: | 0.06 |
| | | N. weight: | 1.8 |
| | | Sc. PD: | -0.35 |
| | | Sc. rank: | -338.1 |

| PB2 | | | |
|---|---|---|---|
| Pos. | 711 | obs: | exp: |
| aat | N | 172 | 299.00 |
| aac | N | 400 | 273.00 |
| --- | -- | ------ | ------ |
| mPD | | 0.42 | 0.50 |
| | | nPD: | 0.84 |
| | | N. weight: | 0.15 |
| | | Sc. PD: | 0.083 |
| | | Sc. rank: | 52.3 |

| PB2 | | | |
|---|---|---|---|
| Pos. | 712 | obs: | exp: |
| gaa | E | 507 | 329.10 |
| gag | E | 65 | 242.90 |
| --- | -- | ------ | ------ |
| mPD | | 0.20 | 0.49 |
| | | nPD: | 0.41 |
| | | N. weight: | 0.34 |
| | | Sc. PD: | 0.046 |
| | | Sc. rank: | 53.6 |

| PB2 | | | |
|---|---|---|---|
| Pos. | 713 | obs: | exp: |
| tta | L | 1 | 49.88 |
| ttg | L | 483 | 110.00 |
| ctt | L | 0 | 106.50 |
| ctc | L | 0 | 92.36 |
| cta | L | 2 | 86.02 |
| ctg | L | 85 | 126.20 |
| atg | M | 1 | 1.00 |
| --- | -- | ------ | ------ |
| mPD | | 0.27 | 1.1 |
| | | nPD: | 0.24 |
| | | N. weight: | 1.7 |
| | | Sc. PD: | -0.045 |
| | | Sc. rank: | 12.4 |

| PB2 | | | |
|---|---|---|---|
| Pos. | 714 | obs: | exp: |
| tct | S | 0 | 90.30 |
| tcc | S | 0 | 79.13 |
| tca | S | 0 | 125.80 |
| tcg | S | 0 | 34.62 |
| att | I | 0 | 1.13 |
| atc | I | 3 | 0.79 |
| ata | I | 0 | 1.09 |
| act | T | 0 | 0.26 |
| acc | T | 1 | 0.21 |
| aca | T | 0 | 0.44 |
| acg | T | 0 | 0.08 |
| agt | S | 13 | 125.00 |
| agc | S | 555 | 113.20 |
| --- | -- | ------ | ------ |
| mPD | | 0.058 | 1.7 |
| | | nPD: | 0.04 |
| | | N. weight: | 2.2 |
| | | Sc. PD: | -0.49 |
| | | Sc. rank: | -507.8 |

| PB2 | | | |
|---|---|---|---|
| Pos. | 715 | obs: | exp: |
| tct | S | 0 | 3.50 |
| tcc | S | 0 | 3.06 |
| tca | S | 0 | 4.87 |
| tcg | S | 0 | 1.34 |
| aat | N | 540 | 287.50 |
| aac | N | 10 | 262.50 |
| agt | S | 22 | 4.84 |
| agc | S | 0 | 4.38 |
| --- | -- | ------ | ------ |
| mPD | | 0.11 | 0.63 |
| | | nPD: | 0.17 |
| | | N. weight: | 0.86 |
| | | Sc. PD: | -0.081 |
| | | Sc. rank: | -61.2 |

| PB2 | | | |
|---|---|---|---|
| Pos. | 716 | obs: | exp: |
| tta | L | 0 | 49.97 |
| ttg | L | 0 | 110.20 |
| ctt | L | 570 | 106.70 |
| ctc | L | 2 | 92.53 |
| cta | L | 0 | 86.17 |
| ctg | L | 0 | 126.40 |
| --- | -- | ------ | ------ |
| mPD | | 0.0070 | 1.1 |
| | | nPD: | 0.01 |
| | | N. weight: | 2.4 |
| | | Sc. PD: | -0.6 |
| | | Sc. rank: | -715.3 |

| PB2 | | | |
|---|---|---|---|
| Pos. | 717 | obs: | exp: |
| gtt | V | 0 | 0.21 |
| gtc | V | 0 | 0.21 |
| gta | V | 0 | 0.19 |
| gtg | V | 1 | 0.40 |
| gct | A | 5 | 143.80 |
| gcc | A | 1 | 117.00 |
| gca | A | 127 | 254.50 |
| gcg | A | 438 | 55.72 |
| --- | -- | ------ | ------ |
| mPD | | 0.37 | 0.69 |
| | | nPD: | 0.53 |
| | | N. weight: | 2. |
| | | Sc. PD: | 0.49 |
| | | Sc. rank: | 512.3 |

| PB2 | | | |
|---|---|---|---|
| Pos. | 718 | obs: | exp: |
| aaa | K | 481 | 320.30 |
| aag | K | 91 | 251.70 |
| --- | -- | ------ | ------ |
| mPD | | 0.27 | 0.49 |
| | | nPD: | 0.54 |
| | | N. weight: | 0.26 |
| | | Sc. PD: | 0.069 |
| | | Sc. rank: | 68.8 |

| PB2 | | | |
|---|---|---|---|
| Pos. | 719 | obs: | exp: |
| ggt | G | 0 | 70.58 |
| ggc | G | 0 | 77.35 |
| gga | G | 72 | 276.70 |
| ggg | G | 500 | 147.40 |
| --- | -- | ------ | ------ |
| mPD | | 0.22 | 0.67 |
| | | nPD: | 0.33 |
| | | N. weight: | 1.3 |
| | | Sc. PD: | 0.073 |
| | | Sc. rank: | 117.5 |

| PB2 | | | |
|---|---|---|---|
| Pos. | 720 | obs: | exp: |
| gaa | E | 66 | 328.60 |
| gag | E | 505 | 242.40 |
| ggt | G | 0 | 0.12 |
| ggc | G | 0 | 0.14 |
| gga | G | 0 | 0.48 |
| ggg | G | 1 | 0.26 |
| --- | -- | ------ | ------ |
| mPD | | 0.21 | 0.49 |
| | | nPD: | 0.42 |
| | | N. weight: | 0.67 |
| | | Sc. PD: | 0.096 |
| | | Sc. rank: | 114.8 |

| PB2 | | | |
|---|---|---|---|
| Pos. | 721 | obs: | exp: |
| aaa | K | 64 | 320.30 |
| aag | K | 508 | 251.70 |
| --- | -- | ------ | ------ |
| mPD | | 0.20 | 0.49 |
| | | nPD: | 0.4 |
| | | N. weight: | 0.64 |
| | | Sc. PD: | 0.081 |
| | | Sc. rank: | 95.4 |

| PB2 | | | |
|---|---|---|---|
| Pos. | 722 | obs: | exp: |
| gct | A | 571 | 144.10 |
| gcc | A | 1 | 117.20 |
| gca | A | 0 | 254.90 |
| gcg | A | 0 | 55.82 |
| --- | -- | ------ | ------ |
| mPD | | 0.0035 | 0.69 |
| | | nPD: | 0.01 |
| | | N. weight: | 2. |
| | | Sc. PD: | -0.5 |
| | | Sc. rank: | -604.6 |

| PB2 | | | |
|---|---|---|---|
| Pos. | 723 | obs: | exp: |
| aat | N | 544 | 299.00 |
| aac | N | 28 | 273.00 |
| --- | -- | ------ | ------ |
| mPD | | 0.093 | 0.50 |
| | | nPD: | 0.19 |
| | | N. weight: | 0.66 |
| | | Sc. PD: | -0.053 |
| | | Sc. rank: | -37.3 |

| PB2 | | | |
|---|---|---|---|
| Pos. | 724 | obs: | exp: |
| gtt | V | 0 | 117.60 |
| gtc | V | 0 | 120.10 |
| gta | V | 245 | 107.20 |
| gtg | V | 327 | 227.20 |
| --- | -- | ------ | ------ |
| mPD | | 0.49 | 0.72 |
| | | nPD: | 0.68 |
| | | N. weight: | 0.81 |
| | | Sc. PD: | 0.32 |
| | | Sc. rank: | 257.8 |

| PB2 | | | |
|---|---|---|---|
| Pos. | 725 | obs: | exp: |
| tta | L | 10 | 49.97 |
| ttg | L | 449 | 110.20 |
| ctt | L | 0 | 106.70 |
| ctc | L | 0 | 92.53 |
| cta | L | 72 | 86.17 |
| ctg | L | 41 | 126.40 |
| --- | -- | ------ | ------ |
| mPD | | 0.56 | 1.1 |
| | | nPD: | 0.51 |
| | | N. weight: | 1.4 |
| | | Sc. PD: | 0.31 |
| | | Sc. rank: | 329.9 |

| PB2 | | | |
|---|---|---|---|
| Pos. | 726 | obs: | exp: |
| att | I | 63 | 214.70 |
| atc | I | 9 | 149.60 |
| ata | I | 499 | 206.70 |
| atg | M | 1 | 1.00 |
| --- | -- | ------ | ------ |
| mPD | | 0.23 | 0.66 |
| | | nPD: | 0.34 |
| | | N. weight: | 0.85 |
| | | Sc. PD: | 0.059 |
| | | Sc. rank: | 87.6 |

| PB2 | | | |
|---|---|---|---|
| Pos. | 727 | obs: | exp: |
| ggt | G | 0 | 70.58 |
| ggc | G | 0 | 77.35 |
| gga | G | 0 | 276.70 |
| ggg | G | 572 | 147.40 |
| --- | -- | ------ | ------ |
| mPD | | 0 | 0.67 |
| | | nPD: | 0. |
| | | N. weight: | 1.9 |
| | | Sc. PD: | -0.5 |
| | | Sc. rank: | -668.5 |

| PB2 | | | |
|---|---|---|---|
| Pos. | 728 | obs: | exp: |
| caa | Q | 572 | 304.90 |
| cag | Q | 0 | 267.10 |
| --- | -- | ------ | ------ |
| mPD | | 0 | 0.50 |
| | | nPD: | 0. |
| | | N. weight: | 0.9 |
| | | Sc. PD: | -0.23 |
| | | Sc. rank: | -310.1 |

| PB2 | | | |
|---|---|---|---|
| Pos. | 729 | obs: | exp: |
| ggt | G | 0 | 70.58 |
| ggc | G | 0 | 77.35 |
| gga | G | 570 | 276.70 |
| ggg | G | 2 | 147.40 |
| --- | -- | ------ | ------ |
| mPD | | 0.0070 | 0.67 |
| | | nPD: | 0.01 |
| | | N. weight: | 1. |
| | | Sc. PD: | -0.25 |
| | | Sc. rank: | -295.8 |

| PB2 | | | |
|---|---|---|---|
| Pos. | 730 | obs: | exp: |
| gat | D | 0 | 304.70 |
| gac | D | 571 | 266.30 |
| gaa | E | 1 | 0.58 |
| gag | E | 0 | 0.42 |
| --- | -- | ------ | ------ |
| mPD | | 0.0035 | 0.50 |
| | | nPD: | 0.01 |
| | | N. weight: | 1.1 |
| | | Sc. PD: | -0.27 |
| | | Sc. rank: | -327.7 |

| PB2 | | | |
|---|---|---|---|
| Pos. | 731 | obs: | exp: |
| tta | L | 0 | 0.09 |
| ttg | L | 0 | 0.19 |
| ctt | L | 0 | 0.19 |
| ctc | L | 0 | 0.16 |
| cta | L | 0 | 0.15 |
| ctg | L | 1 | 0.22 |
| gtt | V | 8 | 117.40 |
| gtc | V | 0 | 119.80 |
| gta | V | 62 | 107.00 |
| gtg | V | 501 | 226.80 |
| --- | -- | ------ | ------ |
| mPD | | 0.22 | 0.72 |
| | | nPD: | 0.31 |
| | | N. weight: | 0.87 |
| | | Sc. PD: | 0.029 |
| | | Sc. rank: | 64.0 |

| PB2 | | | |
|---|---|---|---|
| Pos. | 732 | obs: | exp: |
| gtt | V | 0 | 117.60 |
| gtc | V | 0 | 120.10 |
| gta | V | 1 | 107.20 |
| gtg | V | 571 | 227.20 |
| --- | -- | ------ | ------ |
| mPD | | 0.0035 | 0.72 |
| | | nPD: | 0. |
| | | N. weight: | 1.3 |
| | | Sc. PD: | -0.33 |
| | | Sc. rank: | -406.1 |

| PB2 | | | |
|---|---|---|---|
| Pos. | 733 | obs: | exp: |
| tta | L | 0 | 49.97 |
| ttg | L | 571 | 110.20 |
| ctt | L | 0 | 106.70 |
| ctc | L | 0 | 92.53 |
| cta | L | 0 | 86.17 |
| ctg | L | 1 | 126.40 |
| --- | -- | ------ | ------ |
| mPD | | 0.0035 | 1.1 |
| | | nPD: | 0. |
| | | N. weight: | 2.3 |
| | | Sc. PD: | -0.6 |
| | | Sc. rank: | -739.2 |

| PB2 | | | |
|---|---|---|---|
| Pos. | 734 | obs: | exp: |
| att | I | 0 | 1.88 |
| atc | I | 0 | 1.31 |
| ata | I | 5 | 1.81 |
| gtt | V | 1 | 116.50 |
| gtc | V | 0 | 119.00 |
| gta | V | 464 | 106.30 |
| gtg | V | 102 | 225.20 |
| --- | -- | ------ | ------ |
| mPD | | 0.31 | 0.74 |
| | | nPD: | 0.42 |
| | | N. weight: | 1.5 |
| | | Sc. PD: | 0.22 |
| | | Sc. rank: | 263.6 |

| PB2 | | | |
|---|---|---|---|
| Pos. | 735 | obs: | exp: |
| atg | M | 572 | 572.00 |
| --- | -- | ------ | ------ |
| mPD | | 0 | 0 |
| | | nPD: | 1. |
| | | N. weight: | 0. |
| | | Sc. PD: | 0 |
| | | Sc. rank: | 0 |

| PB2 | | | |
|---|---|---|---|
| Pos. | 736 | obs: | exp: |
| aaa | K | 572 | 320.30 |
| aag | K | 0 | 251.70 |
| --- | -- | ------ | ------ |
| mPD | | 0 | 0.49 |
| | | nPD: | 0. |
| | | N. weight: | 0.83 |
| | | Sc. PD: | -0.21 |
| | | Sc. rank: | -285.8 |

| PB2 | | | |
|---|---|---|---|
| Pos. | 737 | obs: | exp: |
| cgt | R | 0 | 23.75 |
| cgc | R | 0 | 28.01 |
| cga | R | 0 | 54.97 |
| cgg | R | 572 | 47.73 |
| aga | R | 0 | 257.20 |
| agg | R | 0 | 160.30 |
| --- | -- | ------ | ------ |
| mPD | | 0 | 0.96 |
| | | nPD: | 0. |
| | | N. weight: | 3.5 |
| | | Sc. PD: | -0.92 |
| | | Sc. rank: | -1224.1 |

| PB2 | | | |
|---|---|---|---|
| Pos. | 738 | obs: | exp: |
| aaa | K | 572 | 320.30 |
| aag | K | 0 | 251.70 |
| --- | -- | ------ | ------ |
| mPD | | 0 | 0.49 |
| | | nPD: | 0. |
| | | N. weight: | 0.83 |
| | | Sc. PD: | -0.21 |
| | | Sc. rank: | -285.8 |

| PB2 | | | |
|---|---|---|---|
| Pos. | 739 | obs: | exp: |
| cgt | R | 3 | 23.75 |
| cgc | R | 0 | 28.01 |
| cga | R | 4 | 54.97 |
| cgg | R | 565 | 47.73 |
| aga | R | 0 | 257.20 |
| agg | R | 0 | 160.30 |
| --- | -- | ------ | ------ |
| mPD | | 0.024 | 0.96 |
| | | nPD: | 0.03 |
| | | N. weight: | 3.5 |
| | | Sc. PD: | -0.81 |
| | | Sc. rank: | -885.1 |

| PB2 | | | |
|---|---|---|---|
| Pos. | 740 | obs: | exp: |
| gat | D | 0 | 305.20 |
| gac | D | 572 | 266.80 |
| --- | -- | ------ | ------ |
| mPD | | 0 | 0.50 |
| | | nPD: | 0. |
| | | N. weight: | 1.1 |
| | | Sc. PD: | -0.28 |
| | | Sc. rank: | -375.9 |

| PB2 | | | |
|---|---|---|---|
| Pos. | 741 | obs: | exp: |
| ttt | F | 3 | 1.23 |
| ttc | F | 0 | 1.77 |
| tct | S | 556 | 90.46 |
| tcc | S | 11 | 79.26 |
| tca | S | 2 | 126.10 |
| tcg | S | 0 | 34.68 |
| agt | S | 0 | 125.20 |
| agc | S | 0 | 113.40 |
| --- | -- | ------ | ------ |
| mPD | | 0.055 | 1.7 |
| | | nPD: | 0.03 |
| | | N. weight: | 2.5 |
| | | Sc. PD: | -0.56 |
| | | Sc. rank: | -593.1 |

| PB2 | | | |
|---|---|---|---|
| Pos. | 742 | obs: | exp: |
| tct | S | 0 | 90.78 |
| tcc | S | 0 | 79.54 |
| tca | S | 0 | 126.50 |
| tcg | S | 0 | 34.80 |
| agt | S | 5 | 125.60 |
| agc | S | 566 | 113.80 |
| ggt | G | 0 | 0.12 |
| ggc | G | 1 | 0.14 |
| gga | G | 0 | 0.48 |
| ggg | G | 0 | 0.26 |
| --- | -- | ------ | ------ |
| mPD | | 0.021 | 1.7 |
| | | nPD: | 0.01 |
| | | N. weight: | 2.2 |
| | | Sc. PD: | -0.55 |
| | | Sc. rank: | -624.7 |

| PB2 | | | |
|---|---|---|---|
| Pos. | 743 | obs: | exp: |
| att | I | 0 | 215.00 |
| atc | I | 0 | 149.90 |
| ata | I | 572 | 207.10 |
| --- | -- | ------ | ------ |
| mPD | | 0 | 0.66 |
| | | nPD: | 0. |
| | | N. weight: | 1.5 |
| | | Sc. PD: | -0.38 |
| | | Sc. rank: | -500.9 |

| PB2 | | | |
|---|---|---|---|
| Pos. | 744 | obs: | exp: |
| tta | L | 0 | 49.97 |
| ttg | L | 0 | 110.20 |
| ctt | L | 572 | 106.70 |
| ctc | L | 0 | 92.53 |
| cta | L | 0 | 86.17 |
| ctg | L | 0 | 126.40 |
| --- | -- | ------ | ------ |
| mPD | | 0 | 1.1 |
| | | nPD: | 0. |
| | | N. weight: | 2.4 |
| | | Sc. PD: | -0.62 |
| | | Sc. rank: | -827.5 |

| PB2 | | | |
|---|---|---|---|
| Pos. | 745 | obs: | exp: |
| att | I | 2 | 0.75 |
| atc | I | 0 | 0.52 |
| ata | I | 0 | 0.72 |
| act | T | 570 | 150.60 |
| acc | T | 0 | 122.30 |
| aca | T | 0 | 249.70 |
| acg | T | 0 | 47.38 |
| --- | -- | ------ | ------ |
| mPD | | 0.0070 | 0.69 |
| | | nPD: | 0.01 |
| | | N. weight: | 1.9 |
| | | Sc. PD: | -0.47 |
| | | Sc. rank: | -563.7 |

| PB2 | | | |
|---|---|---|---|
| Pos. | 746 | obs: | exp: |
| gat | D | 3 | 305.20 |
| gac | D | 569 | 266.80 |
| --- | -- | ------ | ------ |
| mPD | | 0.010 | 0.50 |
| | | nPD: | 0.02 |
| | | N. weight: | 1. |
| | | Sc. PD: | -0.25 |
| | | Sc. rank: | -276.7 |

| PB2 | | | |
|---|---|---|---|
| Pos. | 747 | obs: | exp: |
| tct | S | 0 | 90.78 |
| tcc | S | 0 | 79.54 |
| tca | S | 0 | 126.50 |
| tcg | S | 0 | 34.80 |
| att | I | 0 | 0.38 |
| atc | I | 1 | 0.26 |
| ata | I | 0 | 0.36 |
| agt | S | 28 | 125.60 |
| agc | S | 543 | 113.80 |
| --- | -- | ------ | ------ |
| mPD | | 0.097 | 1.7 |
| | | nPD: | 0.06 |
| | | N. weight: | 2. |
| | | Sc. PD: | -0.41 |
| | | Sc. rank: | -410.4 |

| PB2 | | | |
|---|---|---|---|
| Pos. | 748 | obs: | exp: |
| caa | Q | 1 | 304.90 |
| cag | Q | 571 | 267.10 |
| --- | -- | ------ | ------ |
| mPD | | 0.0035 | 0.50 |
| | | nPD: | 0.01 |
| | | N. weight: | 1.1 |
| | | Sc. PD: | -0.27 |
| | | Sc. rank: | -321.5 |

| PB2 | | | |
|---|---|---|---|
| Pos. | 749 | obs: | exp: |
| act | T | 0 | 151.10 |
| acc | T | 0 | 122.70 |
| aca | T | 572 | 250.60 |
| acg | T | 0 | 47.55 |
| --- | -- | ------ | ------ |
| mPD | | 0 | 0.69 |
| | | nPD: | 0. |
| | | N. weight: | 1.2 |
| | | Sc. PD: | -0.31 |
| | | Sc. rank: | -406.8 |

| PB2 | | | |
|---|---|---|---|
| Pos. | 750 | obs: | exp: |
| cct | P | 0 | 0.26 |
| ccc | P | 0 | 0.18 |
| cca | P | 1 | 0.39 |
| ccg | P | 0 | 0.16 |
| gct | A | 0 | 143.60 |
| gcc | A | 0 | 116.80 |
| gca | A | 1 | 254.00 |
| gcg | A | 569 | 55.62 |
| ggt | G | 0 | 0.12 |
| ggc | G | 0 | 0.14 |
| gga | G | 0 | 0.48 |
| ggg | G | 1 | 0.26 |
| --- | -- | ------ | ------ |
| mPD | | 0.014 | 0.69 |
| | | nPD: | 0.02 |
| | | N. weight: | 3.3 |
| | | Sc. PD: | -0.79 |
| | | Sc. rank: | -889.8 |

| PB2 | | | |
|---|---|---|---|
| Pos. | 751 | obs: | exp: |
| act | T | 0 | 151.10 |
| acc | T | 572 | 122.70 |
| aca | T | 0 | 250.60 |
| acg | T | 0 | 47.55 |
| --- | -- | ------ | ------ |
| mPD | | 0 | 0.69 |
| | | nPD: | 0. |
| | | N. weight: | 2.2 |
| | | Sc. PD: | -0.57 |
| | | Sc. rank: | -758.6 |

| PB2 | | | |
|---|---|---|---|
| Pos. | 752 | obs: | exp: |
| caa | Q | 1 | 0.53 |
| cag | Q | 0 | 0.47 |
| aaa | K | 571 | 319.70 |
| aag | K | 0 | 251.30 |
| --- | -- | ------ | ------ |
| mPD | | 0.0035 | 0.50 |
| | | nPD: | 0.01 |
| | | N. weight: | 0.83 |
| | | Sc. PD: | -0.21 |
| | | Sc. rank: | -247.9 |

| PB2 | | | |
|---|---|---|---|
| Pos. | 753 | obs: | exp: |
| cgt | R | 0 | 23.75 |
| cgc | R | 0 | 28.01 |
| cga | R | 0 | 54.97 |
| cgg | R | 1 | 47.73 |
| aga | R | 490 | 257.20 |
| agg | R | 81 | 160.30 |
| --- | -- | ------ | ------ |
| mPD | | 0.25 | 0.96 |
| | | nPD: | 0.26 |
| | | N. weight: | 0.65 |
| | | Sc. PD: | -0.0073 |
| | | Sc. rank: | 18.2 |

| PB2 | | | |
|---|---|---|---|
| Pos. | 754 | obs: | exp: |
| att | I | 570 | 214.70 |
| atc | I | 1 | 149.60 |
| ata | I | 0 | 206.70 |
| aat | N | 1 | 0.52 |
| aac | N | 0 | 0.48 |
| --- | -- | ------ | ------ |
| mPD | | 0.0070 | 0.66 |
| | | nPD: | 0.01 |
| | | N. weight: | 1.4 |
| | | Sc. PD: | -0.34 |
| | | Sc. rank: | -393.1 |

| PB2 | | | |
|---|---|---|---|
| Pos. | 755 | obs: | exp: |
| cgt | R | 0 | 23.75 |
| cgc | R | 0 | 28.01 |
| cga | R | 3 | 54.97 |
| cgg | R | 569 | 47.73 |
| aga | R | 0 | 257.20 |
| agg | R | 0 | 160.30 |
| --- | -- | ------ | ------ |
| mPD | | 0.010 | 0.96 |
| | | nPD: | 0.01 |
| | | N. weight: | 3.5 |
| | | Sc. PD: | -0.87 |
| | | Sc. rank: | -985.6 |

| PB2 | | | |
|---|---|---|---|
| Pos. | 756 | obs: | exp: |
| atg | M | 572 | 572.00 |
| --- | -- | ------ | ------ |
| mPD | | 0 | 0 |
| | | nPD: | 1. |
| | | N. weight: | 0. |
| | | Sc. PD: | 0 |
| | | Sc. rank: | 0 |

| PB2 | | | |
|---|---|---|---|
| Pos. | 757 | obs: | exp: |
| gct | A | 0 | 144.10 |
| gcc | A | 572 | 117.20 |
| gca | A | 0 | 254.90 |
| gcg | A | 0 | 55.82 |
| --- | -- | ------ | ------ |
| mPD | | 0 | 0.69 |
| | | nPD: | 0. |
| | | N. weight: | 2.3 |
| | | Sc. PD: | -0.59 |
| | | Sc. rank: | -781.5 |

| PB2 | | | |
|---|---|---|---|
| Pos. | 758 | obs: | exp: |
| att | I | 1 | 214.70 |
| atc | I | 570 | 149.60 |
| ata | I | 0 | 206.70 |
| act | T | 0 | 0.26 |
| acc | T | 1 | 0.21 |
| aca | T | 0 | 0.44 |
| acg | T | 0 | 0.08 |
| --- | -- | ------ | ------ |
| mPD | | 0.0070 | 0.66 |
| | | nPD: | 0.01 |
| | | N. weight: | 1.9 |
| | | Sc. PD: | -0.47 |
| | | Sc. rank: | -542.0 |

| PB2 | | | |
|---|---|---|---|
| Pos. | 759 | obs: | exp: |
| tct | S | 0 | 0.64 |
| tcc | S | 0 | 0.56 |
| tca | S | 0 | 0.89 |
| tcg | S | 0 | 0.24 |
| cat | H | 1 | 0.54 |
| cac | H | 0 | 0.46 |
| att | I | 2 | 1.13 |
| atc | I | 0 | 0.79 |
| ata | I | 1 | 1.09 |
| aat | N | 564 | 294.80 |
| aac | N | 0 | 269.20 |
| agt | S | 4 | 0.88 |
| agc | S | 0 | 0.80 |
| --- | -- | ------ | ------ |
| mPD | | 0.031 | 0.54 |
| | | nPD: | 0.06 |
| | | N. weight: | 0.94 |
| | | Sc. PD: | -0.19 |
| | | Sc. rank: | -191.3 |

| PB2 | | | |
|---|---|---|---|
| Pos. | 760 | obs: | exp: |
| taa | . | 28 | 267.90 |
| tag | . | 544 | 148.00 |
| tga | . | 0 | 156.10 |
| --- | -- | ------ | ------ |
| mPD | | 0.093 | 0.78 |
| | | nPD: | 0.12 |
| | | N. weight: | 1.6 |
| | | Sc. PD: | -0.23 |
| | | Sc. rank: | -225.5 |

## Amino acid level conservation per codon

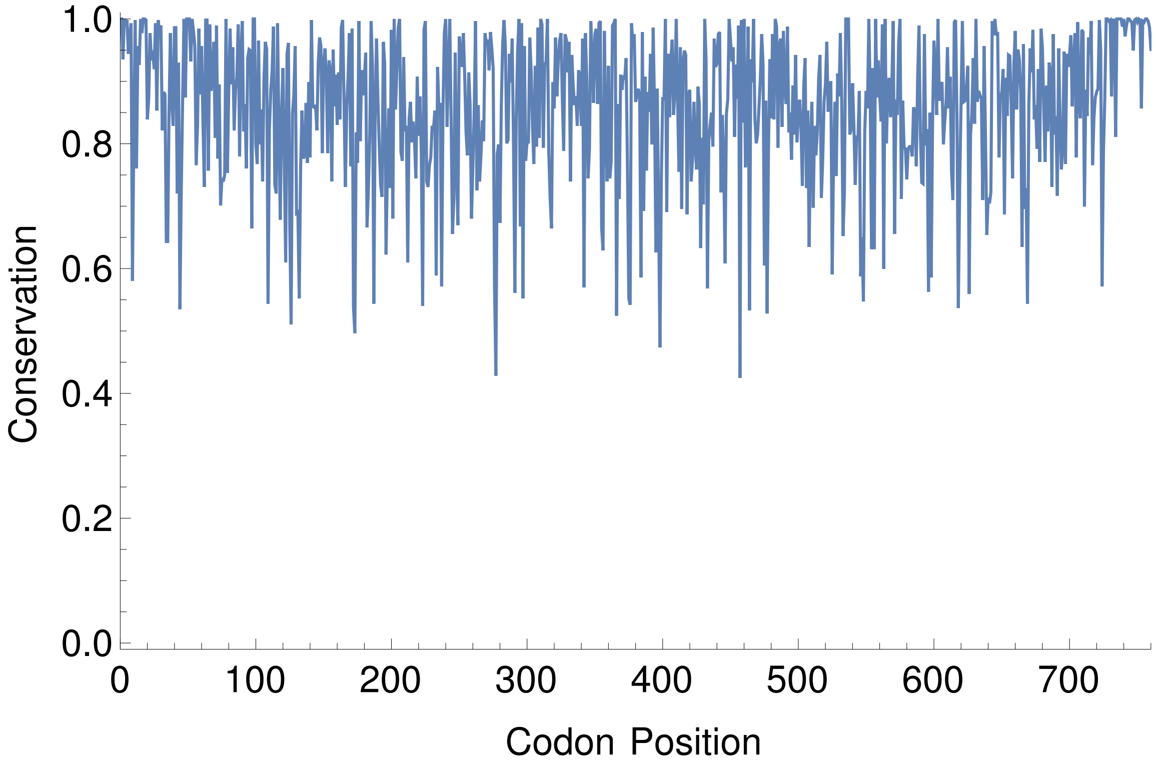

## Conservation analysis plots

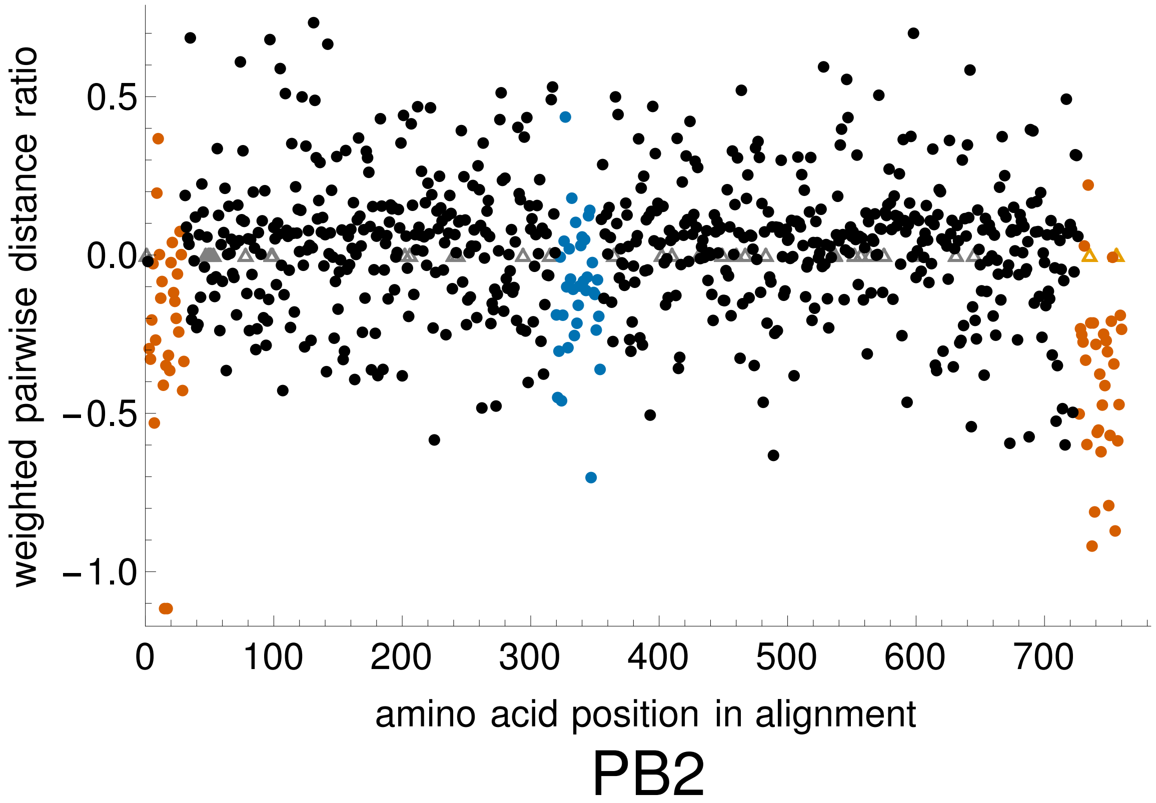


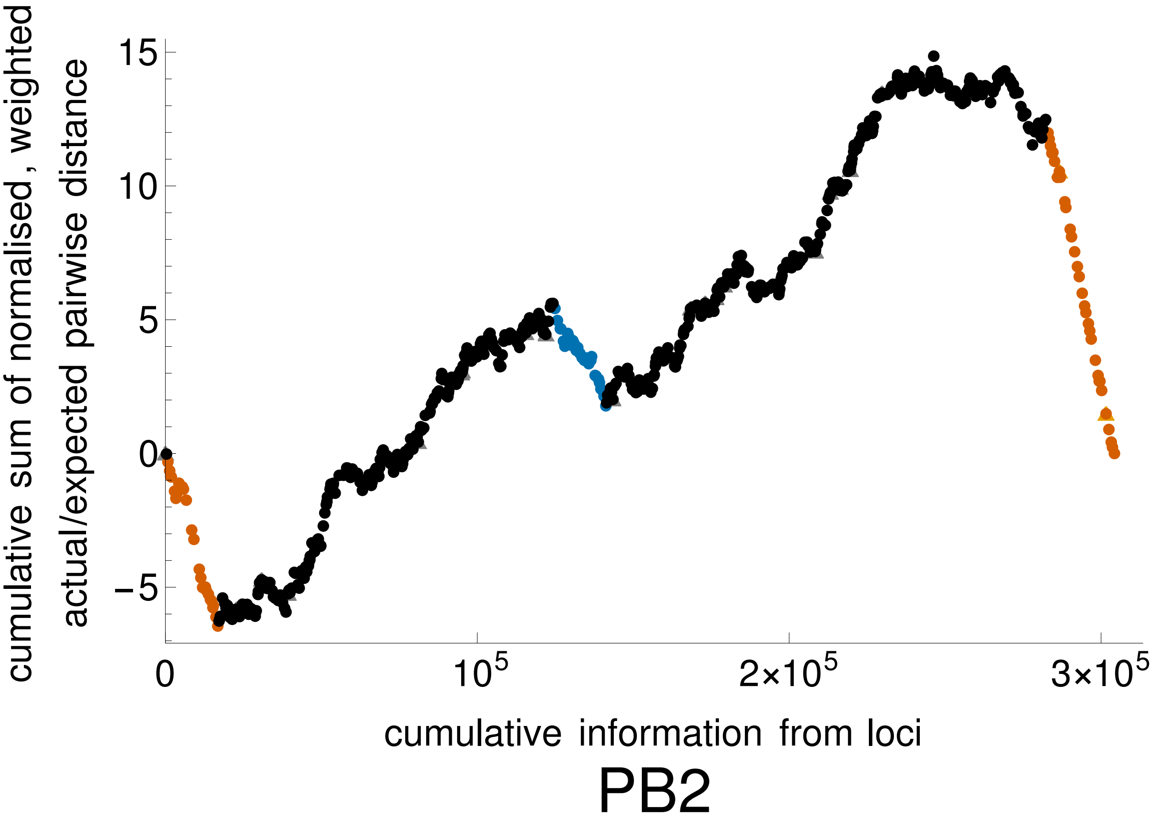

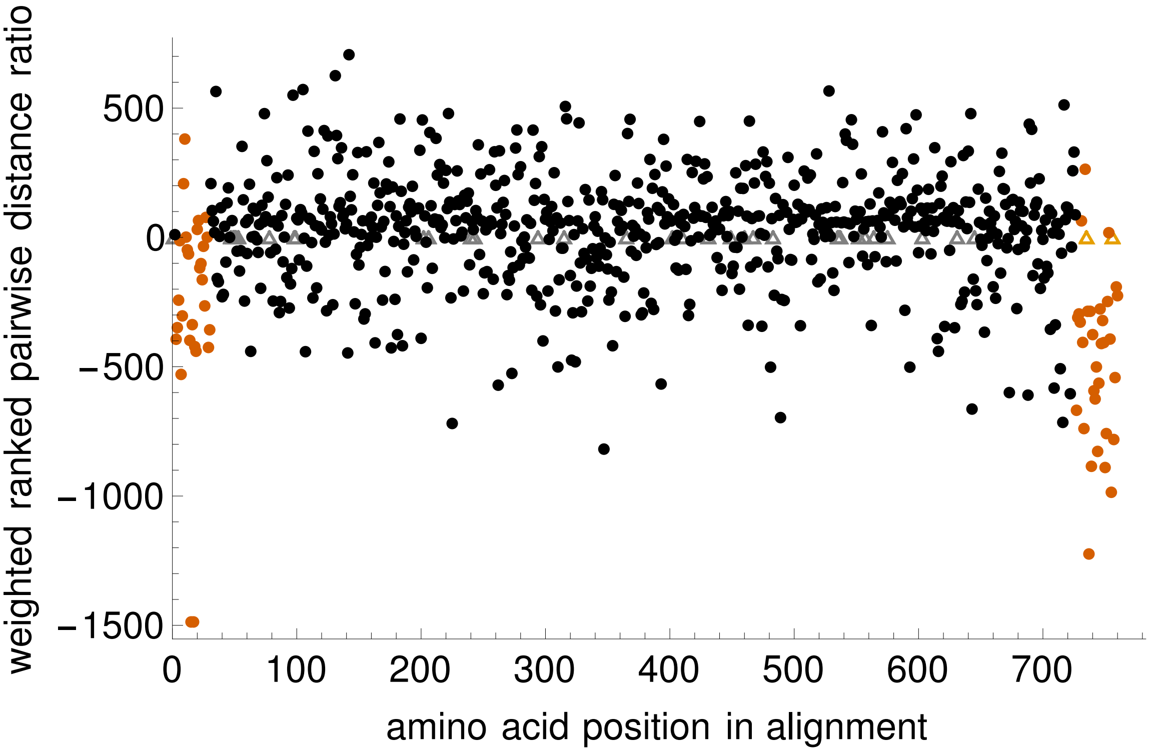
weighted ranked pairwise distance ratio
500
0
−500
−1000
−1500
0
100
200
300
400
500
600
700
amino acid position in alignment

PB2

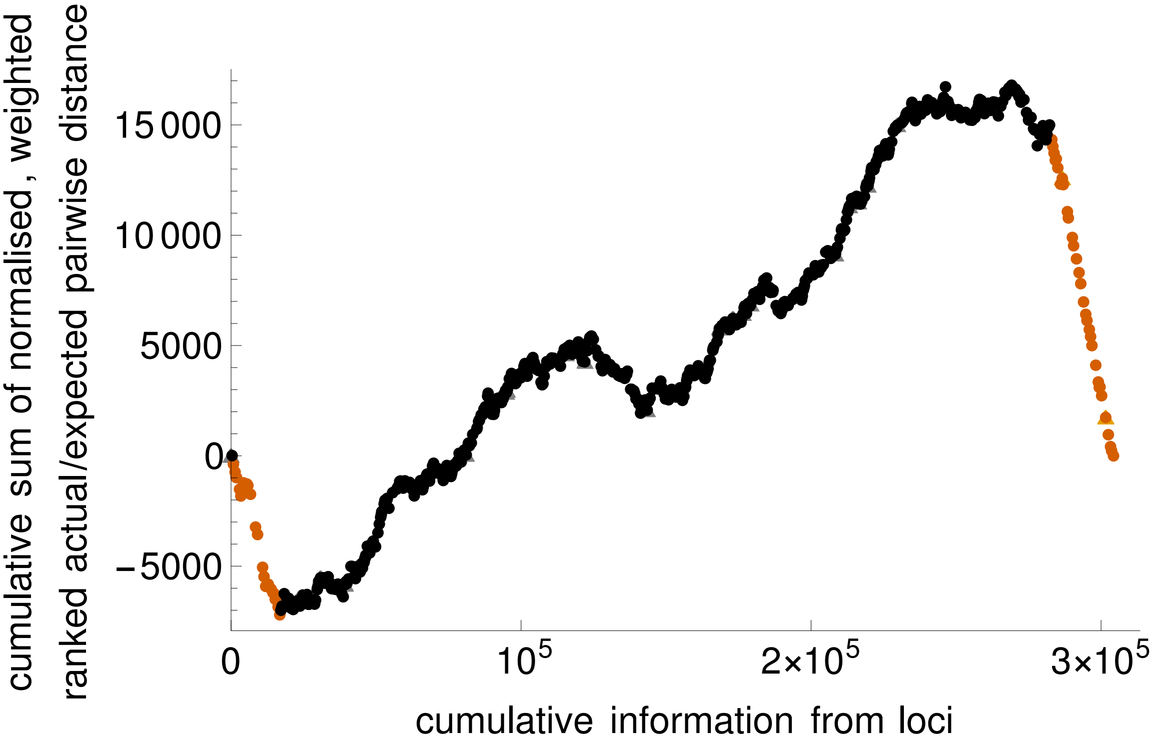
cumulative sum of normalised, weighted ranked actual/expected pairwise distance
15 000
10 000
5000
0
−5000
0
$10^5$
$2\times10^5$
$3\times10^5$
cumulative information from loci

PB2

# PB1

## Gene length histogram

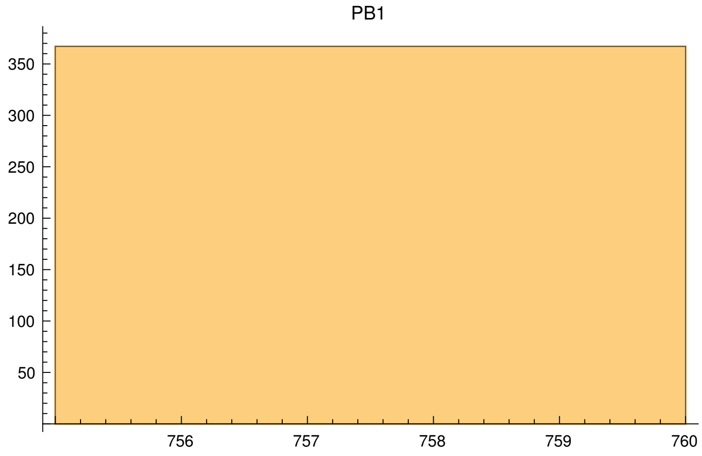

## Information vs. nPD

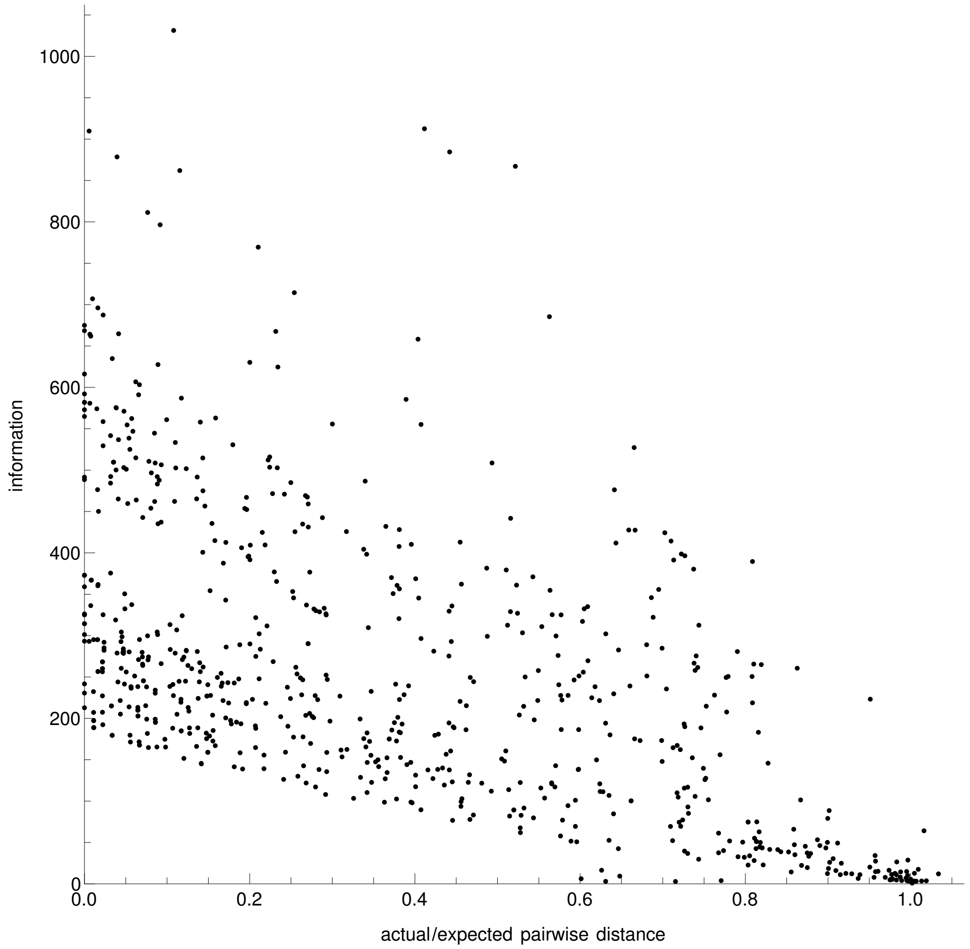


PB1

## Example sequences highlighted by regions found in analyses to be conserved

Interesting points (by weighted raw PD) highlighted for gene PB1:

ATGGATGTCAATCCGACTTTACTTTTCTTAAAAGTGCCAGCGCAAAATGCCATAAGTACTACGTTCCCTTATACTG⋱
GAGATCCTCCATACAGCCATGGAACAGGAACAGGATACACCATGGACACAGTCAACAGAACACATCAATACTCAG⋱
AAAAGGGGAAATGGACAACAAACACAGAGACCGGAGCACCCCAACTCAACCCAATTGATGGACCACTACCTGAGG⋱
ACAACGAACCAAGCGGATATGCACAAACAGATTGCGTGTTGGAAGCAATGGCTTTCCTTGAAGAGTCCCACCCAG⋱
GGATCTTTGAAAACTCTTGTCTTGAAACGATGGAAATCGTTCAGCAAACAAGAGTGGACAAACTAACTCAGGGCC⋱
GCCAGACTTATGACTGGACACTGAATAGAAACCAACCAGCTGCAACTGCCCTGGCCAACACTATAGAGGTCTTCA⋱
GATCAAACGGTTTGACAGCCAATGAATCAGGGAGACTAATAGATTTCCTCAAGGATGTGATGGATTCAATGGACA⋱
AAGAAGAAATGGAAATAACAACACATTTCCAGAGAAAGAGAAGAGTAAGGGACAACATGACCAAGAAAATGGTCA⋱
CACAAAGAACAATAGGAAAGAAGAAACAAAGGCTGAACAAGAGGAGCTACTTAATAAGAGCACTGACACTGAATA⋱
CAATGACAAAAGACGCAGAAAGAGGCAAATTGAAAAGGCGGGCAATTGCAACACCAGGGATGCAGATTAGAGGAT⋱
TTGTGTACTTTGTCGAAACACTGGCAAGGAGCATCTGTGAGAAACTTGAGCAATCTGGACTCCCCGTTGGAGGGA⋱
ATGAGAAAAAGGCTAAATTGGCAAATGTTGTGAGAAAAATGATGACTAACTCACAAGATACAGAGCTCTCCTTCA⋱
CAATTACTGGAGATAACACCAAATGGAATGAGAATCAAAATCCTCGGATGTTTCTGGCAATGATAACATACATCA⋱
CAAGAAACCAACCTGAATGGTTCAGAAATGTCTTGAGCATTGCCCCTATAATGTTCTCGAACAAAATGGCGAGAT⋱
TGGGAAAAGGATACATGTTTGAAAGTAAGAGCATGAAGCTACGGACACAAATACCTGCAGAAATGCTTGCAAACA⋱
TTGACTTAAAATACTTCAATGAATCGACAAGAAAGAAAATCGAGAAAATAAGACCTCTATTAATAGATGGCACTG⋱
CCTCGTTGAGTCCTGGAATGATGATGGGCATGTTCAATATGCTGAGTACAGTATTAGGAGTTTCAATCCTGAATC⋱
TTGGGCAAAAGAGGTACACCAAAACCACATACTGGTGGGATGGACTCCAATCCTCTGATGATTTCGCCCTCATAG⋱
TAAATGCACCGAATCATGAGGGAATACAAGCGGGAGTGGATAGGTTCTATAGGACCTGCAAACTGGTTGGAATCA⋱
ACATGAGCAAAAAGAAGTCTTACATAAACCGAACAGGAACATTTGAGTTCACAAGCTTTTTCTATCGCTATGGAT⋱
TTGTGGCTAACTTCAGTATGGAGCTGCCCAGCTTTGGAGTGTCTGGAATCAATGAATCAGCTGACATGAGCATTG⋱
GAATTACAGTGATAAAGAACAACATGATAAACAATGACCTTGGACCAGCAACAGCTCAAATGGCCCTTCAGCTAT⋱
TCATCAAGGACTACAGGTACACGTACCGATGCCACAGGGGTGACACACAAATTCAAACGAGGAGATCATTCGAGC⋱
TGAAGAAGCTGTGGGAGCAGACCCGTTCAAAGGCAGGGCTGTTGGTGTCAGATGGAGGACCAAACCTATACAACA⋱
TTCGGAATCTCCACATCCCAGAGGTCTGCCTGAAGTGGGAGCTGATGGACGAAGATTACCAGGGCAGGTTGTGTA⋱
ATCCTCTGAACCCATTTGTCAGTCATAAGGAAATTGAGTCCGTAAACAATGCTGTAGTAATGCCAGCTCATGGCC⋱
CCGCCAAGAGCATGGAATATGATGCTGTTGCGACTACACACTCATGGATTCCTAAGAGGAATCGTTCCATTCTCA⋱
ATACCAGCCAAAGGGGAATTCTTGAGGATGAGCAGATGTACCAGAAGTGCTGCAGTCTATTCGAGAAATTCTTCC⋱
CCAGTAGTTCATATAGGAGGCCA
**GTTGGAATTTCCAGCATGGTGGAGGCCATGGTATCTAGGGCCCGAATTGATGCAC**
**GCATTGATTTCGAATCTGGAAGGATTAAGAAGGAAGAGTTTGCTGAGATCAT**
**GAAGATCTGTTCCACCATTGAAGAGCTCAGACGG**CAAAAA---TAG

Interesting points (by weighted ranked PD) highlighted for gene PB1:

ATGGATGTCAATCCGACTTTACTTTTCTTAAAAGTGCCAGCGCAAAATGCCATAAGTACTACGTTCCCTTAT
**ACTGGAGATCCTCCATACAGCCATGGA**ACAGGAACAGGATACACCATGGACACAGTCAACAGAAC⋱
ACATCAATACTCAGAAAAGGGGAAATGGACAACAAACACAGAGACCGGAGCACCCCAACTCAACCCAATT⋱
GATGGACCACTACCTGAGGACAACGAACCAAGCGGATATGCACAAACAGATTGCGTGTTGGAAGCAATGG⋱
CTTTCCTTGAAGAGTCCCACCCAGGGATCTTTGAAAACTCTTGTCTTGAAACGATGGAAATCGTTCAGCA⋱
AACAAGAGTGGACAAACTAACTCAGGGCCGCCAGACTTATGACTGGACACTGAATAGAAACCAACCAGCT⋱
GCAACTGCCCTGGCCAACACTATAGAGGTCTTCAGATCAAACGGTTTGACAGCCAATGAATCAGGGAGAC⋱
TAATAGATTTCCTCAAGGATGTGATGGATTCAATGGACAAAGAAGAAATGGAAATAACAACACATTTCCA⋱
GAGAAAGAGAAGAGTAAGGGACAACATGACCAAGAAAATGGTCACACAAAGAACAATAGGAAAGAAGAAA⋱

CAAAGGCTGAACAAGAGGAGCTACTTAATAAGAGCACTGACACTGAATACAATGACAAAAGACGCAGAAA⋱

GAGGCAAATTGAAAAGGCGGGCAATTGCAACACCAGGGATGCAGATTAGAGGATTTGTGTACTTTGTCGA⋱

AACACTGGCAAGGAGCATCTGTGAGAAACTTGAGCAATCTGGACTCCCCGTTGGAGGGAATGAGAAAAAG⋱

GCTAAATTGGCAAATGTTGTGAGAAAAATGATGACTAACTCACAAGATACAGAGCTCTCCTTCACAATTA⋱

CTGGAGATAACACCAAATGGAATGAGAATCAAAATCCTCGGATGTTTCTGGCAATGATAACATACATCAC⋱

AAGAAACCAACCTGAATGGTTCAGAAATGTCTTGAGCATTGCCCCTATAATGTTCTCGAACAAAATGGCG⋱

AGATTGGGAAAAGGATACATGTTTGAAAGTAAGAGCATGAAGCTACGGACACAAATACCTGCAGAAATGC⋱

TTGCAAACATTGACTTAAAATACTTCAATGAATCGACAAGAAAGAAAATCGAGAAAATAAGACCTCTATT⋱

AATAGATGGCACTGCCTCGTTGAGTCCTGGAATGATGATGGGCATGTTCAATATGCTGAGTACAGTATTA⋱

GGAGTTTCAATCCTGAATCTTGGGCAAAAGAGGTACACCAAAACCACATACTGGTGGGATGGACTCCAAT⋱

CCTCTGATGATTTCGCCCTCATAGTAAATGCACCGAATCATGAGGGAATACAAGCGGGAGTGGATAGGTT⋱

CTATAGGACCTGCAAACTGGTTGGAATCAACATGAGCAAAAAGAAGTCTTACATAAACCGAACAGGAACA⋱

TTTGAGTTCACAAGCTTTTTCTATCGCTATGGATTTGTGGCTAACTTCAGTATGGAGCTGCCCAGCTTTG⋱

GAGTGTCTGGAATCAATGAATCAGCTGACATGAGCATTGGAATTACAGTGATAAAGAACAACATGATAAA⋱

CAATGACCTTGGACCAGCAACAGCTCAAATGGCCCTTCAGCTATTCATCAAGGACTACAGGTACACGTAC⋱

CGATGCCACAGGGGTGACACACAAATTCAAACGAGGAGATCATTCGAGCTGAAGAAGCTGTGGGAGCAGA⋱

CCCGTTCAAAGGCAGGGCTGTTGGTGTCAGATGGAGGACCAAACCTATACAACATTCGGAATCTCCACAT⋱

CCCAGAGGTCTGCCTGAAGTGGGAGCTGATGGACGAAGATTACCAGGGCAGGTTGTGTAATCCTCTGAAC⋱

CCATTTGTCAGTCATAAGGAAATTGAGTCCGTAAACAATGCTGTAGTAATGCCAGCTCATGGCCCCGCCA⋱

AGAGCATGGAATATGATGCTGTTGCGACTACACACTCATGGATTCCTAAGAGGAATCGTTCCATTCTCAA⋱

TACCAGCCAAAGGGGAATTCTTGAGGATGAGCAGATGTACCAGAAGTGCTGCAGTCTATTCGAGAAATTC⋱

TTCCCCAGTAGTTCATATAGGAGGCCA

**GTTGGAATTTCCAGCATGGTGGAGGCCATGGTATCTAGGGCCCGAATTGATGCAC**

**GCATTGATTTCGAATCTGGAAGGATTAAGAAGGAAGAGTTTGCTGAGATCAT**

**GAAGATCTGTTCCACCATTGAAGAGCTCAGACGG**CAAAAA---TAG

# Per codon conservation report

PB1

| Pos. | 1 | obs: | exp: |
|---|---|---|---|
| atg | M | 367 | 367.00 |
| --- | -- | ------ | ------ |
| mPD | | 0 | 0 |
| | | nPD: | 1. |
| | | N. weight: | 0. |
| | | Sc. PD: | 0 |
| | | Sc. rank: | 0 |

PB1

| Pos. | 2 | obs: | exp: |
|---|---|---|---|
| gat | D | 367 | 195.80 |
| gac | D | 0 | 171.20 |
| --- | -- | ------ | ------ |
| mPD | | 0 | 0.50 |
| | | nPD: | 0. |
| | | N. weight: | 0.96 |
| | | Sc. PD: | -0.23 |
| | | Sc. rank: | -198.5 |

PB1

| Pos. | 3 | obs: | exp: |
|---|---|---|---|
| gtt | V | 0 | 75.43 |
| gtc | V | 367 | 77.03 |
| gta | V | 0 | 68.78 |
| gtg | V | 0 | 145.80 |
| --- | -- | ------ | ------ |
| mPD | | 0 | 0.72 |
| | | nPD: | 0. |
| | | N. weight: | 2.4 |
| | | Sc. PD: | -0.58 |
| | | Sc. rank: | -493.2 |

PB1

| Pos. | 4 | obs: | exp: |
|---|---|---|---|
| att | I | 0 | 2.63 |
| atc | I | 7 | 1.83 |
| ata | I | 0 | 2.53 |
| aat | N | 327 | 188.20 |
| aac | N | 33 | 171.80 |
| --- | -- | ------ | ------ |
| mPD | | 0.23 | 0.54 |
| | | nPD: | 0.43 |
| | | N. weight: | 0.57 |
| | | Sc. PD: | 0.093 |
| | | Sc. rank: | 63.7 |

PB1

| Pos. | 5 | obs: | exp: |
|---|---|---|---|
| cct | P | 0 | 96.64 |
| ccc | P | 0 | 66.20 |
| cca | P | 1 | 144.80 |
| ccg | P | 366 | 59.34 |
| --- | -- | ------ | ------ |
| mPD | | 0.0054 | 0.72 |
| | | nPD: | 0.01 |
| | | N. weight: | 2.7 |
| | | Sc. PD: | -0.65 |
| | | Sc. rank: | -490.1 |

PB1

| Pos. | 6 | obs: | exp: |
|---|---|---|---|
| act | T | 346 | 96.96 |
| acc | T | 21 | 78.75 |
| aca | T | 0 | 160.80 |
| acg | T | 0 | 30.51 |
| --- | -- | ------ | ------ |
| mPD | | 0.11 | 0.69 |
| | | nPD: | 0.16 |
| | | N. weight: | 1.7 |
| | | Sc. PD: | -0.16 |
| | | Sc. rank: | -18.0 |

PB1

| Pos. | 7 | obs: | exp: |
|---|---|---|---|
| tta | L | 351 | 32.06 |
| ttg | L | 11 | 70.68 |
| ctt | L | 0 | 68.48 |
| ctc | L | 0 | 59.37 |
| cta | L | 5 | 55.29 |
| ctg | L | 0 | 81.13 |
| --- | -- | ------ | ------ |
| mPD | | 0.085 | 1.1 |
| | | nPD: | 0.08 |
| | | N. weight: | 3.4 |
| | | Sc. PD: | -0.58 |
| | | Sc. rank: | -273.1 |

PB1

| Pos. | 8 | obs: | exp: |
|---|---|---|---|
| tta | L | 0 | 32.06 |
| ttg | L | 0 | 70.68 |
| ctt | L | 355 | 68.48 |
| ctc | L | 10 | 59.37 |
| cta | L | 0 | 55.29 |
| ctg | L | 2 | 81.13 |
| --- | -- | ------ | ------ |
| mPD | | 0.064 | 1.1 |
| | | nPD: | 0.06 |
| | | N. weight: | 2.3 |
| | | Sc. PD: | -0.44 |
| | | Sc. rank: | -244.8 |

PB1

| Pos. | 9 | obs: | exp: |
|---|---|---|---|
| ttt | F | 3 | 150.90 |
| ttc | F | 364 | 216.10 |
| --- | -- | ------ | ------ |
| mPD | | 0.016 | 0.48 |
| | | nPD: | 0.03 |
| | | N. weight: | 0.74 |
| | | Sc. PD: | -0.16 |
| | | Sc. rank: | -106.7 |

PB1

| Pos. | 10 | obs: | exp: |
|---|---|---|---|
| tta | L | 167 | 32.06 |
| ttg | L | 186 | 70.68 |
| ctt | L | 0 | 68.48 |
| ctc | L | 0 | 59.37 |
| cta | L | 14 | 55.29 |
| ctg | L | 0 | 81.13 |
| --- | -- | ------ | ------ |
| mPD | | 0.57 | 1.1 |
| | | nPD: | 0.52 |
| | | N. weight: | 1.8 |
| | | Sc. PD: | 0.45 |
| | | Sc. rank: | 244.9 |

PB1

| Pos. | 11 | obs: | exp: |
|---|---|---|---|
| aaa | K | 366 | 205.50 |
| aag | K | 1 | 161.50 |
| --- | -- | ------ | ------ |
| mPD | | 0.0054 | 0.49 |
| | | nPD: | 0.01 |
| | | N. weight: | 0.86 |
| | | Sc. PD: | -0.2 |
| | | Sc. rank: | -147.9 |

PB1

| Pos. | 12 | obs: | exp: |
|---|---|---|---|
| gtt | V | 75 | 75.43 |
| gtc | V | 20 | 77.03 |
| gta | V | 1 | 68.78 |
| gtg | V | 271 | 145.80 |
| --- | -- | ------ | ------ |
| mPD | | 0.41 | 0.72 |
| | | nPD: | 0.57 |
| | | N. weight: | 0.59 |
| | | Sc. PD: | 0.18 |
| | | Sc. rank: | 88.2 |

PB1

| Pos. | 13 | obs: | exp: |
|---|---|---|---|
| tct | S | 0 | 0.16 |
| tcc | S | 0 | 0.14 |
| tca | S | 0 | 0.22 |
| tcg | S | 1 | 0.06 |
| cct | P | 3 | 96.37 |
| ccc | P | 0 | 66.02 |
| cca | P | 292 | 144.40 |
| ccg | P | 71 | 59.18 |
| agt | S | 0 | 0.22 |
| agc | S | 0 | 0.20 |
| --- | -- | ------ | ------ |
| mPD | | 0.33 | 0.72 |
| | | nPD: | 0.46 |
| | | N. weight: | 0.89 |
| | | Sc. PD: | 0.17 |
| | | Sc. rank: | 110.3 |

PB1

| Pos. | 14 | obs: | exp: |
|---|---|---|---|
| gtt | V | 0 | 5.34 |
| gtc | V | 0 | 5.46 |
| gta | V | 0 | 4.87 |
| gtg | V | 26 | 10.33 |
| gct | A | 0 | 85.89 |
| gcc | A | 0 | 69.85 |
| gca | A | 8 | 152.00 |
| gcg | A | 333 | 33.28 |
| --- | -- | ------ | ------ |
| mPD | | 0.17 | 0.83 |
| | | nPD: | 0.21 |
| | | N. weight: | 3.2 |
| | | Sc. PD: | -0.14 |
| | | Sc. rank: | 64.4 |

PB1

| Pos. | 15 | obs: | exp: |
|---|---|---|---|
| caa | Q | 360 | 195.60 |
| cag | Q | 7 | 171.40 |
| --- | -- | ------ | ------ |
| mPD | | 0.038 | 0.50 |
| | | nPD: | 0.08 |
| | | N. weight: | 0.83 |
| | | Sc. PD: | -0.14 |
| | | Sc. rank: | -69.4 |

PB1

| Pos. | 16 | obs: | exp: |
|---|---|---|---|
| aat | N | 361 | 191.80 |
| aac | N | 6 | 175.20 |
| --- | -- | ------ | ------ |
| mPD | | 0.032 | 0.50 |
| | | nPD: | 0.06 |
| | | N. weight: | 0.87 |
| | | Sc. PD: | -0.16 |
| | | Sc. rank: | -84.7 |

PB1

| Pos. | 17 | obs: | exp: |
|---|---|---|---|
| gct | A | 191 | 92.44 |
| gcc | A | 173 | 75.18 |
| gca | A | 3 | 163.60 |
| gcg | A | 0 | 35.81 |
| --- | -- | ------ | ------ |
| mPD | | 0.51 | 0.69 |
| | | nPD: | 0.74 |
| | | N. weight: | 1.1 |
| | | Sc. PD: | 0.53 |
| | | Sc. rank: | 218.4 |

PB1

| Pos. | 18 | obs: | exp: |
|---|---|---|---|
| att | I | 1 | 138.00 |
| atc | I | 0 | 96.18 |
| ata | I | 366 | 132.80 |
| --- | -- | ------ | ------ |
| mPD | | 0.0054 | 0.66 |
| | | nPD: | 0.01 |
| | | N. weight: | 1.5 |
| | | Sc. PD: | -0.36 |
| | | Sc. rank: | -268.7 |

PB1

| Pos. | 19 | obs: | exp: |
|---|---|---|---|
| tct | S | 0 | 58.34 |
| tcc | S | 0 | 51.12 |
| tca | S | 0 | 81.31 |
| tcg | S | 0 | 22.37 |
| agt | S | 235 | 80.74 |
| agc | S | 132 | 73.12 |
| --- | -- | ------ | ------ |
| mPD | | 0.46 | 1.7 |
| | | nPD: | 0.28 |
| | | N. weight: | 1.4 |
| | | Sc. PD: | 0.028 |
| | | Sc. rank: | 84.7 |

PB1

| Pos. | 20 | obs: | exp: |
|---|---|---|---|
| act | T | 174 | 96.96 |
| acc | T | 191 | 78.75 |
| aca | T | 2 | 160.80 |
| acg | T | 0 | 30.51 |
| --- | -- | ------ | ------ |
| mPD | | 0.51 | 0.69 |
| | | nPD: | 0.74 |
| | | N. weight: | 1.1 |
| | | Sc. PD: | 0.51 |
| | | Sc. rank: | 209.8 |

PB1

| Pos. | 21 | obs: | exp: |
|---|---|---|---|
| act | T | 0 | 96.96 |
| acc | T | 0 | 78.75 |
| aca | T | 293 | 160.80 |
| acg | T | 74 | 30.51 |
| --- | -- | ------ | ------ |
| mPD | | 0.32 | 0.69 |
| | | nPD: | 0.47 |
| | | N. weight: | 1. |
| | | Sc. PD: | 0.21 |
| | | Sc. rank: | 127.3 |

PB1

| Pos. | 22 | obs: | exp: |
|---|---|---|---|
| ttt | F | 6 | 150.90 |
| ttc | F | 361 | 216.10 |
| --- | -- | ------ | ------ |
| mPD | | 0.032 | 0.48 |
| | | nPD: | 0.07 |
| | | N. weight: | 0.7 |
| | | Sc. PD: | -0.13 |
| | | Sc. rank: | -63.4 |

PB1

| Pos. | 23 | obs: | exp: |
|---|---|---|---|
| cct | P | 248 | 96.64 |
| ccc | P | 2 | 66.20 |
| cca | P | 21 | 144.80 |
| ccg | P | 96 | 59.34 |
| --- | -- | ------ | ------ |
| mPD | | 0.47 | 0.72 |
| | | nPD: | 0.66 |
| | | N. weight: | 0.99 |
| | | Sc. PD: | 0.38 |
| | | Sc. rank: | 168.5 |

PB1

| Pos. | 24 | obs: | exp: |
|---|---|---|---|
| tat | Y | 213 | 190.00 |
| tac | Y | 154 | 177.00 |
| --- | -- | ------ | ------ |
| mPD | | 0.49 | 0.50 |
| | | nPD: | 0.98 |
| | | N. weight: | 0.025 |
| | | Sc. PD: | 0.017 |
| | | Sc. rank: | 5.3 |

PB1

| Pos. | 25 | obs: | exp: |
|---|---|---|---|
| act | T | 367 | 96.96 |
| acc | T | 0 | 78.75 |
| aca | T | 0 | 160.80 |
| acg | T | 0 | 30.51 |
| --- | -- | ------ | ------ |
| mPD | | 0 | 0.69 |
| | | nPD: | 0. |
| | | N. weight: | 2. |
| | | Sc. PD: | -0.49 |
| | | Sc. rank: | -420.5 |

PB1

| Pos. | 26 | obs: | exp: |
|---|---|---|---|
| ggt | G | 0 | 45.28 |
| ggc | G | 0 | 49.63 |
| gga | G | 347 | 177.50 |
| ggg | G | 20 | 94.55 |
| --- | -- | ------ | ------ |
| mPD | | 0.10 | 0.67 |
| | | nPD: | 0.16 |
| | | N. weight: | 0.85 |
| | | Sc. PD: | -0.081 |
| | | Sc. rank: | -10.3 |

PB1

| Pos. | 27 | obs: | exp: |
|---|---|---|---|
| aat | N | 1 | 0.52 |
| aac | N | 0 | 0.48 |
| gat | D | 319 | 195.30 |
| gac | D | 47 | 170.70 |
| --- | -- | ------ | ------ |
| mPD | | 0.23 | 0.50 |
| | | nPD: | 0.46 |
| | | N. weight: | 0.41 |
| | | Sc. PD: | 0.078 |
| | | Sc. rank: | 50.1 |

PB1

| Pos. | 28 | obs: | exp: |
|---|---|---|---|
| cct | P | 360 | 96.64 |
| ccc | P | 7 | 66.20 |
| cca | P | 0 | 144.80 |
| ccg | P | 0 | 59.34 |
| --- | -- | ------ | ------ |
| mPD | | 0.038 | 0.72 |
| | | nPD: | 0.05 |
| | | N. weight: | 1.9 |
| | | Sc. PD: | -0.37 |
| | | Sc. rank: | -214.0 |

PB1

| Pos. | 29 | obs: | exp: |
|---|---|---|---|
| cct | P | 0 | 96.64 |
| ccc | P | 0 | 66.20 |
| cca | P | 366 | 144.80 |
| ccg | P | 1 | 59.34 |
| --- | -- | ------ | ------ |
| mPD | | 0.0054 | 0.72 |
| | | nPD: | 0.01 |
| | | N. weight: | 1.4 |
| | | Sc. PD: | -0.33 |
| | | Sc. rank: | -249.0 |

PB1

| Pos. | 30 | obs: | exp: |
|---|---|---|---|
| tat | Y | 2 | 190.00 |
| tac | Y | 365 | 177.00 |
| --- | -- | ------ | ------ |
| mPD | | 0.011 | 0.50 |
| | | nPD: | 0.02 |
| | | N. weight: | 1.1 |
| | | Sc. PD: | -0.24 |
| | | Sc. rank: | -169.7 |

PB1

| Pos. | 31 | obs: | exp: |
|---|---|---|---|
| tct | S | 0 | 58.34 |
| tcc | S | 0 | 51.12 |
| tca | S | 0 | 81.31 |
| tcg | S | 0 | 22.37 |
| agt | S | 0 | 80.74 |
| agc | S | 367 | 73.12 |
| --- | -- | ------ | ------ |
| mPD | | 0 | 1.7 |
| | | nPD: | 0. |
| | | N. weight: | 2.5 |
| | | Sc. PD: | -0.6 |
| | | Sc. rank: | -509.7 |

PB1

| Pos. | 32 | obs: | exp: |
|---|---|---|---|
| cat | H | 363 | 198.90 |
| cac | H | 4 | 168.10 |
| --- | -- | ------ | ------ |
| mPD | | 0.022 | 0.50 |
| | | nPD: | 0.04 |
| | | N. weight: | 0.85 |
| | | Sc. PD: | -0.17 |
| | | Sc. rank: | -107.8 |

PB1

| Pos. | 33 | obs: | exp: |
|---|---|---|---|
| ggt | G | 0 | 45.28 |
| ggc | G | 0 | 49.63 |
| gga | G | 365 | 177.50 |
| ggg | G | 2 | 94.55 |
| --- | -- | ------ | ------ |
| mPD | | 0.011 | 0.67 |
| | | nPD: | 0.02 |
| | | N. weight: | 1.1 |
| | | Sc. PD: | -0.24 |
| | | Sc. rank: | -173.2 |

PB1

| Pos. | 34 | obs: | exp: |
|---|---|---|---|
| act | T | 0 | 96.96 |
| acc | T | 15 | 78.75 |
| aca | T | 340 | 160.80 |
| acg | T | 12 | 30.51 |
| --- | -- | ------ | ------ |
| mPD | | 0.14 | 0.69 |
| | | nPD: | 0.2 |
| | | N. weight: | 0.92 |
| | | Sc. PD: | -0.047 |
| | | Sc. rank: | 14.6 |

PB1

| Pos. | 35 | obs: | exp: |
|---|---|---|---|
| ggt | G | 0 | 45.28 |
| ggc | G | 0 | 49.63 |
| gga | G | 361 | 177.50 |
| ggg | G | 6 | 94.55 |
| --- | -- | ------ | ------ |
| mPD | | 0.032 | 0.67 |
| | | nPD: | 0.05 |
| | | N. weight: | 1. |
| | | Sc. PD: | -0.2 |
| | | Sc. rank: | -118.0 |

PB1

| Pos. | 36 | obs: | exp: |
|---|---|---|---|
| act | T | 0 | 96.96 |
| acc | T | 0 | 78.75 |
| aca | T | 348 | 160.80 |
| acg | T | 19 | 30.51 |
| --- | -- | ------ | ------ |
| mPD | | 0.098 | 0.69 |
| | | nPD: | 0.14 |
| | | N. weight: | 1.1 |
| | | Sc. PD: | -0.12 |
| | | Sc. rank: | -21.3 |

PB1

| Pos. | 37 | obs: | exp: |
|---|---|---|---|
| ggt | G | 0 | 45.28 |
| ggc | G | 0 | 49.63 |
| gga | G | 344 | 177.50 |
| ggg | G | 23 | 94.55 |
| --- | -- | ------ | ------ |
| mPD | | 0.12 | 0.67 |
| | | nPD: | 0.18 |
| | | N. weight: | 0.82 |
| | | Sc. PD: | -0.062 |
| | | Sc. rank: | -0.7 |

PB1

| Pos. | 38 | obs: | exp: |
|---|---|---|---|
| tat | Y | 65 | 190.00 |
| tac | Y | 302 | 177.00 |
| --- | -- | ------ | ------ |
| mPD | | 0.29 | 0.50 |
| | | nPD: | 0.59 |
| | | N. weight: | 0.39 |
| | | Sc. PD: | 0.12 |
| | | Sc. rank: | 60.1 |

PB1

| Pos. | 39 | obs: | exp: |
|---|---|---|---|
| act | T | 10 | 96.96 |
| acc | T | 355 | 78.75 |
| aca | T | 2 | 160.80 |
| acg | T | 0 | 30.51 |
| --- | -- | ------ | ------ |
| mPD | | 0.064 | 0.69 |
| | | nPD: | 0.09 |
| | | N. weight: | 2.1 |
| | | Sc. PD: | -0.33 |
| | | Sc. rank: | -122.3 |

PB1

| Pos. | 40 | obs: | exp: |
|---|---|---|---|
| atg | M | 367 | 367.00 |
| --- | -- | ------ | ------ |
| mPD | | 0 | 0 |
| | | nPD: | 1. |
| | | N. weight: | 0. |
| | | Sc. PD: | 0 |
| | | Sc. rank: | 0 |

PB1

| Pos. | 41 | obs: | exp: |
|---|---|---|---|
| gat | D | 2 | 195.80 |
| gac | D | 365 | 171.20 |
| --- | -- | ------ | ------ |
| mPD | | 0.011 | 0.50 |
| | | nPD: | 0.02 |
| | | N. weight: | 1.1 |
| | | Sc. PD: | -0.25 |
| | | Sc. rank: | -174.0 |

PB1

| Pos. | 42 | obs: | exp: |
|---|---|---|---|
| act | T | 17 | 96.96 |
| acc | T | 2 | 78.75 |
| aca | T | 313 | 160.80 |
| acg | T | 35 | 30.51 |
| --- | -- | ------ | ------ |
| mPD | | 0.26 | 0.69 |
| | | nPD: | 0.38 |
| | | N. weight: | 0.76 |
| | | Sc. PD: | 0.091 |
| | | Sc. rank: | 72.6 |

PB1

| Pos. | 43 | obs: | exp: |
|---|---|---|---|
| gtt | V | 83 | 75.43 |
| gtc | V | 284 | 77.03 |
| gta | V | 0 | 68.78 |
| gtg | V | 0 | 145.80 |
| --- | -- | ------ | ------ |
| mPD | | 0.35 | 0.72 |
| | | nPD: | 0.49 |
| | | N. weight: | 1.6 |
| | | Sc. PD: | 0.35 |
| | | Sc. rank: | 200.6 |

PB1

| Pos. | 44 | obs: | exp: |
|---|---|---|---|
| aat | N | 43 | 191.80 |
| aac | N | 324 | 175.20 |
| --- | -- | ------ | ------ |
| mPD | | 0.21 | 0.50 |
| | | nPD: | 0.42 |
| | | N. weight: | 0.57 |
| | | Sc. PD: | 0.086 |
| | | Sc. rank: | 62.4 |

PB1

| Pos. | 45 | obs: | exp: |
|---|---|---|---|
| cgt | R | 0 | 15.24 |
| cgc | R | 0 | 17.97 |
| cga | R | 0 | 35.27 |
| cgg | R | 0 | 30.63 |
| aga | R | 347 | 165.00 |
| agg | R | 20 | 102.80 |
| --- | -- | ------ | ------ |
| mPD | | 0.10 | 0.96 |
| | | nPD: | 0.11 |
| | | N. weight: | 0.94 |
| | | Sc. PD: | -0.13 |
| | | Sc. rank: | -49.8 |

PB1

| Pos. | 46 | obs: | exp: |
|---|---|---|---|
| att | I | 0 | 0.38 |
| atc | I | 0 | 0.26 |
| ata | I | 1 | 0.36 |
| act | T | 0 | 96.69 |
| acc | T | 0 | 78.54 |
| aca | T | 358 | 160.30 |
| acg | T | 8 | 30.43 |
| --- | -- | ------ | ------ |
| mPD | | 0.048 | 0.69 |
| | | nPD: | 0.07 |
| | | N. weight: | 1.2 |
| | | Sc. PD: | -0.21 |
| | | Sc. rank: | -104.0 |

PB1

| Pos. | 47 | obs: | exp: |
|---|---|---|---|
| cat | H | 312 | 198.90 |
| cac | H | 55 | 168.10 |
| --- | -- | ------ | ------ |
| mPD | | 0.26 | 0.50 |
| | | nPD: | 0.51 |
| | | N. weight: | 0.34 |
| | | Sc. PD: | 0.083 |
| | | Sc. rank: | 44.8 |

PB1

| Pos. | 48 | obs: | exp: |
|---|---|---|---|
| caa | Q | 335 | 181.80 |
| cag | Q | 6 | 159.20 |
| aat | N | 0 | 1.05 |
| aac | N | 2 | 0.95 |
| aaa | K | 24 | 13.44 |
| aag | K | 0 | 10.56 |
| --- | -- | ------ | ------ |
| mPD | | 0.17 | 0.63 |
| | | nPD: | 0.28 |
| | | N. weight: | 0.84 |
| | | Sc. PD: | 0.015 |
| | | Sc. rank: | 50.6 |

PB1

| Pos. | 49 | obs: | exp: |
|---|---|---|---|
| tat | Y | 97 | 190.00 |
| tac | Y | 270 | 177.00 |
| --- | -- | ------ | ------ |
| mPD | | 0.39 | 0.50 |
| | | nPD: | 0.78 |
| | | N. weight: | 0.21 |
| | | Sc. PD: | 0.11 |
| | | Sc. rank: | 42.4 |

PB1

| Pos. | 50 | obs: | exp: |
|---|---|---|---|
| tct | S | 3 | 58.34 |
| tcc | S | 0 | 51.12 |
| tca | S | 352 | 81.31 |
| tcg | S | 12 | 22.37 |
| agt | S | 0 | 80.74 |
| agc | S | 0 | 73.12 |
| --- | -- | ------ | ------ |
| mPD | | 0.079 | 1.7 |
| | | nPD: | 0.05 |
| | | N. weight: | 2.1 |
| | | Sc. PD: | -0.41 |
| | | Sc. rank: | -249.8 |

PB1

| Pos. | 51 | obs: | exp: |
|---|---|---|---|
| gct | A | 0 | 0.25 |
| gcc | A | 0 | 0.20 |
| gca | A | 1 | 0.45 |
| gcg | A | 0 | 0.10 |
| gaa | E | 213 | 210.60 |
| gag | E | 153 | 155.40 |
| --- | -- | ------ | ------ |
| mPD | | 0.49 | 0.50 |
| | | nPD: | 1. |
| | | N. weight: | 0.017 |
| | | Sc. PD: | 0.012 |
| | | Sc. rank: | 3.5 |

PB1

| Pos. | 52 | obs: | exp: |
|---|---|---|---|
| caa | Q | 1 | 1.07 |
| cag | Q | 1 | 0.93 |
| aaa | K | 117 | 204.40 |
| aag | K | 248 | 160.60 |
| --- | -- | ------ | ------ |
| mPD | | 0.45 | 0.50 |
| | | nPD: | 0.89 |
| | | N. weight: | 0.19 |
| | | Sc. PD: | 0.12 |
| | | Sc. rank: | 39.6 |

PB1

| Pos. | 53 | obs: | exp: |
|---|---|---|---|
| ggt | G | 0 | 45.28 |
| ggc | G | 0 | 49.63 |
| gga | G | 171 | 177.50 |
| ggg | G | 196 | 94.55 |
| --- | -- | ------ | ------ |
| mPD | | 0.50 | 0.67 |
| | | nPD: | 0.75 |
| | | N. weight: | 0.58 |
| | | Sc. PD: | 0.27 |
| | | Sc. rank: | 111.9 |

PB1

| Pos. | 54 | obs: | exp: |
|---|---|---|---|
| cgt | R | 0 | 0.50 |
| cgc | R | 0 | 0.59 |
| cga | R | 0 | 1.15 |
| cgg | R | 0 | 1.00 |
| aaa | K | 162 | 198.80 |
| aag | K | 193 | 156.20 |
| aga | R | 6 | 5.40 |
| agg | R | 6 | 3.36 |
| --- | -- | ------ | ------ |
| mPD | | 0.56 | 0.58 |
| | | nPD: | 0.97 |
| | | N. weight: | 0.068 |
| | | Sc. PD: | 0.046 |
| | | Sc. rank: | 14.2 |

PB1

| Pos. | 55 | obs: | exp: |
|---|---|---|---|
| tgg | W | 367 | 367.00 |
| --- | -- | ------ | ------ |
| mPD | | 0 | 0 |
| | | nPD: | 1. |
| | | N. weight: | 0. |
| | | Sc. PD: | 0 |
| | | Sc. rank: | 0 |

| PB1 | | | |
|---|---|---|---|
| Pos. | 56 | obs: | exp: |
| act | T | 0 | 95.64 |
| acc | T | 0 | 77.68 |
| aca | T | 361 | 158.60 |
| acg | T | 1 | 30.09 |
| gct | A | 0 | 1.26 |
| gcc | A | 0 | 1.02 |
| gca | A | 5 | 2.23 |
| gcg | A | 0 | 0.49 |
| --- | -- | ------ | ------ |
| mPD | | 0.032 | 0.71 |
| | | nPD: | 0.05 |
| | | N. weight: | 1.2 |
| | | Sc. PD: | -0.25 |
| | | Sc. rank: | -153.6 |

| PB1 | | | |
|---|---|---|---|
| Pos. | 57 | obs: | exp: |
| atg | M | 2 | 2.00 |
| act | T | 1 | 96.43 |
| acc | T | 4 | 78.32 |
| aca | T | 330 | 159.90 |
| acg | T | 30 | 30.34 |
| --- | -- | ------ | ------ |
| mPD | | 0.20 | 0.70 |
| | | nPD: | 0.28 |
| | | N. weight: | 0.94 |
| | | Sc. PD: | 0.02 |
| | | Sc. rank: | 58.5 |

| PB1 | | | |
|---|---|---|---|
| Pos. | 58 | obs: | exp: |
| aat | N | 40 | 191.80 |
| aac | N | 327 | 175.20 |
| --- | -- | ------ | ------ |
| mPD | | 0.19 | 0.50 |
| | | nPD: | 0.39 |
| | | N. weight: | 0.6 |
| | | Sc. PD: | 0.076 |
| | | Sc. rank: | 59.1 |

| PB1 | | | |
|---|---|---|---|
| Pos. | 59 | obs: | exp: |
| tct | S | 0 | 12.72 |
| tcc | S | 0 | 11.14 |
| tca | S | 80 | 17.72 |
| tcg | S | 0 | 4.88 |
| cct | P | 0 | 0.26 |
| ccc | P | 0 | 0.18 |
| cca | P | 1 | 0.39 |
| ccg | P | 0 | 0.16 |
| act | T | 7 | 75.56 |
| acc | T | 11 | 61.37 |
| aca | T | 264 | 125.30 |
| acg | T | 4 | 23.78 |
| agt | S | 0 | 17.60 |
| agc | S | 0 | 15.94 |
| --- | -- | ------ | ------ |
| mPD | | 0.46 | 1.1 |
| | | nPD: | 0.42 |
| | | N. weight: | 1.2 |
| | | Sc. PD: | 0.18 |
| | | Sc. rank: | 128.6 |

| PB1 | | | |
|---|---|---|---|
| Pos. | 60 | obs: | exp: |
| aaa | K | 0 | 0.56 |
| aag | K | 1 | 0.44 |
| gaa | E | 68 | 210.60 |
| gag | E | 298 | 155.40 |
| --- | -- | ------ | ------ |
| mPD | | 0.31 | 0.49 |
| | | nPD: | 0.62 |
| | | N. weight: | 0.5 |
| | | Sc. PD: | 0.17 |
| | | Sc. rank: | 81.5 |

| PB1 | | | |
|---|---|---|---|
| Pos. | 61 | obs: | exp: |
| act | T | 196 | 96.96 |
| acc | T | 168 | 78.75 |
| aca | T | 3 | 160.80 |
| acg | T | 0 | 30.51 |
| --- | -- | ------ | ------ |
| mPD | | 0.51 | 0.69 |
| | | nPD: | 0.74 |
| | | N. weight: | 1.1 |
| | | Sc. PD: | 0.49 |
| | | Sc. rank: | 203.6 |

| PB1 | | | |
|---|---|---|---|
| Pos. | 62 | obs: | exp: |
| cgt | R | 0 | 0.08 |
| cgc | R | 0 | 0.10 |
| cga | R | 0 | 0.19 |
| cgg | R | 0 | 0.17 |
| aga | R | 2 | 0.90 |
| agg | R | 0 | 0.56 |
| gaa | E | 1 | 0.58 |
| gag | E | 0 | 0.42 |
| ggt | G | 7 | 44.91 |
| ggc | G | 0 | 49.22 |
| gga | G | 318 | 176.10 |
| ggg | G | 39 | 93.78 |
| --- | -- | ------ | ------ |
| mPD | | 0.24 | 0.68 |
| | | nPD: | 0.35 |
| | | N. weight: | 0.61 |
| | | Sc. PD: | 0.056 |
| | | Sc. rank: | 50.5 |

| PB1 | | | |
|---|---|---|---|
| Pos. | 63 | obs: | exp: |
| tct | S | 0 | 0.16 |
| tcc | S | 0 | 0.14 |
| tca | S | 1 | 0.22 |
| tcg | S | 0 | 0.06 |
| agt | S | 0 | 0.22 |
| agc | S | 0 | 0.20 |
| gct | A | 0 | 92.19 |
| gcc | A | 0 | 74.97 |
| gca | A | 357 | 163.10 |
| gcg | A | 9 | 35.71 |
| --- | -- | ------ | ------ |
| mPD | | 0.053 | 0.69 |
| | | nPD: | 0.08 |
| | | N. weight: | 1.1 |
| | | Sc. PD: | -0.19 |
| | | Sc. rank: | -90.4 |

| PB1 | | | |
|---|---|---|---|
| Pos. | 64 | obs: | exp: |
| tta | L | 0 | 0.09 |
| ttg | L | 0 | 0.19 |
| tct | S | 0 | 0.16 |
| tcc | S | 1 | 0.14 |
| tca | S | 0 | 0.22 |
| tcg | S | 0 | 0.06 |
| ctt | L | 0 | 0.19 |
| ctc | L | 1 | 0.16 |
| cta | L | 0 | 0.15 |
| ctg | L | 0 | 0.22 |
| cct | P | 71 | 96.11 |
| ccc | P | 292 | 65.84 |
| cca | P | 2 | 144.00 |
| ccg | P | 0 | 59.02 |
| agt | S | 0 | 0.22 |
| agc | S | 0 | 0.20 |
| --- | -- | ------ | ------ |
| mPD | | 0.33 | 0.73 |
| | | nPD: | 0.45 |
| | | N. weight: | 1.7 |
| | | Sc. PD: | 0.32 |
| | | Sc. rank: | 207.4 |

| PB1 | | | |
|---|---|---|---|
| Pos. | 65 | obs: | exp: |
| caa | Q | 346 | 195.60 |
| cag | Q | 21 | 171.40 |
| --- | -- | ------ | ------ |
| mPD | | 0.11 | 0.50 |
| | | nPD: | 0.22 |
| | | N. weight: | 0.65 |
| | | Sc. PD: | -0.024 |
| | | Sc. rank: | 15.3 |

| PB1 | | | |
|---|---|---|---|
| Pos. | 66 | obs: | exp: |
| tta | L | 0 | 32.06 |
| ttg | L | 0 | 70.68 |
| ctt | L | 53 | 68.48 |
| ctc | L | 314 | 59.37 |
| cta | L | 0 | 55.29 |
| ctg | L | 0 | 81.13 |
| --- | -- | ------ | ------ |
| mPD | | 0.25 | 1.1 |
| | | nPD: | 0.22 |
| | | N. weight: | 2.1 |
| | | Sc. PD: | -0.069 |
| | | Sc. rank: | 57.4 |

| PB1 | | | |
|---|---|---|---|
| Pos. | 67 | obs: | exp: |
| aat | N | 153 | 191.80 |
| aac | N | 214 | 175.20 |
| --- | -- | ------ | ------ |
| mPD | | 0.49 | 0.50 |
| | | nPD: | 0.98 |
| | | N. weight: | 0.047 |
| | | Sc. PD: | 0.032 |
| | | Sc. rank: | 9.8 |

| PB1 | | | |
|---|---|---|---|
| Pos. | 68 | obs: | exp: |
| cct | P | 1 | 96.64 |
| ccc | P | 2 | 66.20 |
| cca | P | 308 | 144.80 |
| ccg | P | 56 | 59.34 |
| --- | -- | ------ | ------ |
| mPD | | 0.27 | 0.72 |
| | | nPD: | 0.38 |
| | | N. weight: | 0.92 |
| | | Sc. PD: | 0.11 |
| | | Sc. rank: | 88.2 |

| PB1 | | | |
|---|---|---|---|
| Pos. | 69 | obs: | exp: |
| att | I | 354 | 137.60 |
| atc | I | 12 | 95.92 |
| ata | I | 0 | 132.50 |
| gtt | V | 1 | 0.21 |
| gtc | V | 0 | 0.21 |
| gta | V | 0 | 0.19 |
| gtg | V | 0 | 0.40 |
| --- | -- | ------ | ------ |
| mPD | | 0.069 | 0.67 |
| | | nPD: | 0.1 |
| | | N. weight: | 1.3 |
| | | Sc. PD: | -0.19 |
| | | Sc. rank: | -71.4 |

| PB1 | | | |
|---|---|---|---|
| Pos. | 70 | obs: | exp: |
| gat | D | 356 | 195.80 |
| gac | D | 11 | 171.20 |
| --- | -- | ------ | ------ |
| mPD | | 0.058 | 0.50 |
| | | nPD: | 0.12 |
| | | N. weight: | 0.77 |
| | | Sc. PD: | -0.1 |
| | | Sc. rank: | -31.3 |

| PB1 | | | |
|---|---|---|---|
| Pos. | 71 | obs: | exp: |
| ggt | G | 0 | 45.28 |
| ggc | G | 2 | 49.63 |
| gga | G | 360 | 177.50 |
| ggg | G | 5 | 94.55 |
| --- | -- | ------ | ------ |
| mPD | | 0.038 | 0.67 |
| | | nPD: | 0.06 |
| | | N. weight: | 0.98 |
| | | Sc. PD: | -0.19 |
| | | Sc. rank: | -104.2 |

| PB1 | | | |
|---|---|---|---|
| Pos. | 72 | obs: | exp: |
| cct | P | 0 | 96.64 |
| ccc | P | 0 | 66.20 |
| cca | P | 355 | 144.80 |
| ccg | P | 12 | 59.34 |
| --- | -- | ------ | ------ |
| mPD | | 0.063 | 0.72 |
| | | nPD: | 0.09 |
| | | N. weight: | 1.2 |
| | | Sc. PD: | -0.2 |
| | | Sc. rank: | -82.9 |

| PB1 | | | |
|---|---|---|---|
| Pos. | 73 | obs: | exp: |
| tta | L | 146 | 32.06 |
| ttg | L | 108 | 70.68 |
| ctt | L | 0 | 68.48 |
| ctc | L | 0 | 59.37 |
| cta | L | 70 | 55.29 |
| ctg | L | 43 | 81.13 |
| --- | -- | ------ | ------ |
| mPD | | 0.91 | 1.1 |
| | | nPD: | 0.82 |
| | | N. weight: | 1.1 |
| | | Sc. PD: | 0.59 |
| | | Sc. rank: | 223.7 |

| PB1 | | | |
|---|---|---|---|
| Pos. | 74 | obs: | exp: |
| cct | P | 244 | 96.64 |
| ccc | P | 33 | 66.20 |
| cca | P | 82 | 144.80 |
| ccg | P | 8 | 59.34 |
| --- | -- | ------ | ------ |
| mPD | | 0.50 | 0.72 |
| | | nPD: | 0.7 |
| | | N. weight: | 0.61 |
| | | Sc. PD: | 0.26 |
| | | Sc. rank: | 109.4 |

| PB1 | | | |
|---|---|---|---|
| Pos. | 75 | obs: | exp: |
| gat | D | 28 | 14.94 |
| gac | D | 0 | 13.06 |
| gaa | E | 23 | 193.90 |
| gag | E | 314 | 143.10 |
| ggt | G | 0 | 0.25 |
| ggc | G | 0 | 0.27 |
| gga | G | 0 | 0.97 |
| ggg | G | 2 | 0.52 |
| --- | -- | ------ | ------ |
| mPD | | 0.26 | 0.57 |
| | | nPD: | 0.45 |
| | | N. weight: | 0.91 |
| | | Sc. PD: | 0.17 |
| | | Sc. rank: | 109.9 |

| PB1 | | | |
|---|---|---|---|
| Pos. | 76 | obs: | exp: |
| gat | D | 66 | 195.80 |
| gac | D | 301 | 171.20 |
| --- | -- | ------ | ------ |
| mPD | | 0.30 | 0.50 |
| | | nPD: | 0.59 |
| | | N. weight: | 0.42 |
| | | Sc. PD: | 0.13 |
| | | Sc. rank: | 64.9 |

| PB1 | | | |
|---|---|---|---|
| Pos. | 77 | obs: | exp: |
| aat | N | 145 | 191.80 |
| aac | N | 222 | 175.20 |
| --- | -- | ------ | ------ |
| mPD | | 0.48 | 0.50 |
| | | nPD: | 0.96 |
| | | N. weight: | 0.063 |
| | | Sc. PD: | 0.042 |
| | | Sc. rank: | 13.1 |

| PB1 | | | |
|---|---|---|---|
| Pos. | 78 | obs: | exp: |
| gat | D | 3 | 3.73 |
| gac | D | 4 | 3.27 |
| gaa | E | 118 | 207.20 |
| gag | E | 242 | 152.80 |
| --- | -- | ------ | ------ |
| mPD | | 0.46 | 0.51 |
| | | nPD: | 0.91 |
| | | N. weight: | 0.2 |
| | | Sc. PD: | 0.13 |
| | | Sc. rank: | 42.4 |

| PB1 | | | |
|---|---|---|---|
| Pos. | 79 | obs: | exp: |
| cct | P | 0 | 96.64 |
| ccc | P | 2 | 66.20 |
| cca | P | 329 | 144.80 |
| ccg | P | 36 | 59.34 |
| --- | -- | ------ | ------ |
| mPD | | 0.19 | 0.72 |
| | | nPD: | 0.26 |
| | | N. weight: | 1. |
| | | Sc. PD: | 0.0048 |
| | | Sc. rank: | 49.8 |

| PB1 | | | |
|---|---|---|---|
| Pos. | 80 | obs: | exp: |
| tct | S | 0 | 58.34 |
| tcc | S | 0 | 51.12 |
| tca | S | 0 | 81.31 |
| tcg | S | 0 | 22.37 |
| agt | S | 148 | 80.74 |
| agc | S | 219 | 73.12 |
| --- | -- | ------ | ------ |
| mPD | | 0.48 | 1.7 |
| | | nPD: | 0.29 |
| | | N. weight: | 1.4 |
| | | Sc. PD: | 0.044 |
| | | Sc. rank: | 91.7 |

| | | | |
|---|---|---|---|
| PB1 | | | |
| Pos. | 81 | obs: | exp: |
| ggt | G | 0 | 45.28 |
| ggc | G | 0 | 49.63 |
| gga | G | 275 | 177.50 |
| ggg | G | 92 | 94.55 |
| --- | -- | ------ | ------ |
| mPD | | 0.38 | 0.67 |
| | | nPD: | 0.57 |
| | | N. weight: | 0.5 |
| | | Sc. PD: | 0.15 |
| | | Sc. rank: | 74.0 |

| | | | |
|---|---|---|---|
| PB1 | | | |
| Pos. | 82 | obs: | exp: |
| tat | Y | 361 | 190.00 |
| tac | Y | 6 | 177.00 |
| --- | -- | ------ | ------ |
| mPD | | 0.032 | 0.50 |
| | | nPD: | 0.06 |
| | | N. weight: | 0.88 |
| | | Sc. PD: | -0.16 |
| | | Sc. rank: | -86.9 |

| | | | |
|---|---|---|---|
| PB1 | | | |
| Pos. | 83 | obs: | exp: |
| gct | A | 2 | 92.44 |
| gcc | A | 15 | 75.18 |
| gca | A | 344 | 163.60 |
| gcg | A | 6 | 35.81 |
| --- | -- | ------ | ------ |
| mPD | | 0.12 | 0.69 |
| | | nPD: | 0.17 |
| | | N. weight: | 0.91 |
| | | Sc. PD: | -0.071 |
| | | Sc. rank: | -1.1 |

| | | | |
|---|---|---|---|
| PB1 | | | |
| Pos. | 84 | obs: | exp: |
| caa | Q | 361 | 195.60 |
| cag | Q | 6 | 171.40 |
| --- | -- | ------ | ------ |
| mPD | | 0.032 | 0.50 |
| | | nPD: | 0.06 |
| | | N. weight: | 0.84 |
| | | Sc. PD: | -0.15 |
| | | Sc. rank: | -80.6 |

| | | | |
|---|---|---|---|
| PB1 | | | |
| Pos. | 85 | obs: | exp: |
| act | T | 3 | 96.96 |
| acc | T | 0 | 78.75 |
| aca | T | 151 | 160.80 |
| acg | T | 213 | 30.51 |
| --- | -- | ------ | ------ |
| mPD | | 0.50 | 0.69 |
| | | nPD: | 0.72 |
| | | N. weight: | 1.7 |
| | | Sc. PD: | 0.73 |
| | | Sc. rank: | 305.5 |

| | | | |
|---|---|---|---|
| PB1 | | | |
| Pos. | 86 | obs: | exp: |
| gat | D | 267 | 195.80 |
| gac | D | 100 | 171.20 |
| --- | -- | ------ | ------ |
| mPD | | 0.40 | 0.50 |
| | | nPD: | 0.8 |
| | | N. weight: | 0.13 |
| | | Sc. PD: | 0.069 |
| | | Sc. rank: | 26.4 |

| | | | |
|---|---|---|---|
| PB1 | | | |
| Pos. | 87 | obs: | exp: |
| tgt | C | 161 | 151.30 |
| tgc | C | 206 | 215.70 |
| --- | -- | ------ | ------ |
| mPD | | 0.49 | 0.48 |
| | | nPD: | 1.02 |
| | | N. weight: | 0.015 |
| | | Sc. PD: | 0.011 |
| | | Sc. rank: | 3.2 |

| | | | |
|---|---|---|---|
| PB1 | | | |
| Pos. | 88 | obs: | exp: |
| att | I | 0 | 0.38 |
| atc | I | 1 | 0.26 |
| ata | I | 0 | 0.36 |
| gtt | V | 43 | 75.23 |
| gtc | V | 96 | 76.82 |
| gta | V | 43 | 68.60 |
| gtg | V | 184 | 145.40 |
| --- | -- | ------ | ------ |
| mPD | | 0.66 | 0.73 |
| | | nPD: | 0.91 |
| | | N. weight: | 0.13 |
| | | Sc. PD: | 0.078 |
| | | Sc. rank: | 26.1 |

| | | | |
|---|---|---|---|
| PB1 | | | |
| Pos. | 89 | obs: | exp: |
| tta | L | 0 | 31.97 |
| ttg | L | 264 | 70.49 |
| tgg | W | 1 | 1.00 |
| ctt | L | 90 | 68.29 |
| ctc | L | 0 | 59.20 |
| cta | L | 0 | 55.14 |
| ctg | L | 12 | 80.90 |
| --- | -- | ------ | ------ |
| mPD | | 0.78 | 1.1 |
| | | nPD: | 0.7 |
| | | N. weight: | 1.5 |
| | | Sc. PD: | 0.61 |
| | | Sc. rank: | 261.0 |

| | | | |
|---|---|---|---|
| PB1 | | | |
| Pos. | 90 | obs: | exp: |
| gaa | E | 362 | 211.20 |
| gag | E | 5 | 155.80 |
| --- | -- | ------ | ------ |
| mPD | | 0.027 | 0.49 |
| | | nPD: | 0.06 |
| | | N. weight: | 0.75 |
| | | Sc. PD: | -0.14 |
| | | Sc. rank: | -80.7 |

| | | | |
|---|---|---|---|
| PB1 | | | |
| Pos. | 91 | obs: | exp: |
| gct | A | 15 | 92.44 |
| gcc | A | 0 | 75.18 |
| gca | A | 352 | 163.60 |
| gcg | A | 0 | 35.81 |
| --- | -- | ------ | ------ |
| mPD | | 0.079 | 0.69 |
| | | nPD: | 0.11 |
| | | N. weight: | 1. |
| | | Sc. PD: | -0.14 |
| | | Sc. rank: | -45.3 |

| | | | |
|---|---|---|---|
| PB1 | | | |
| Pos. | 92 | obs: | exp: |
| atg | M | 367 | 367.00 |
| --- | -- | ------ | ------ |
| mPD | | 0 | 0 |
| | | nPD: | 1. |
| | | N. weight: | 0. |
| | | Sc. PD: | 0 |
| | | Sc. rank: | 0 |

| | | | |
|---|---|---|---|
| PB1 | | | |
| Pos. | 93 | obs: | exp: |
| gct | A | 340 | 92.44 |
| gcc | A | 24 | 75.18 |
| gca | A | 1 | 163.60 |
| gcg | A | 2 | 35.81 |
| --- | -- | ------ | ------ |
| mPD | | 0.14 | 0.69 |
| | | nPD: | 0.2 |
| | | N. weight: | 1.7 |
| | | Sc. PD: | -0.09 |
| | | Sc. rank: | 26.0 |

| | | | |
|---|---|---|---|
| PB1 | | | |
| Pos. | 94 | obs: | exp: |
| ttt | F | 4 | 143.10 |
| ttc | F | 344 | 204.90 |
| tta | L | 5 | 0.44 |
| ttg | L | 0 | 0.96 |
| tat | Y | 0 | 7.25 |
| tac | Y | 14 | 6.75 |
| ctt | L | 0 | 0.93 |
| ctc | L | 0 | 0.81 |
| cta | L | 0 | 0.75 |
| ctg | L | 0 | 1.11 |
| --- | -- | ------ | ------ |
| mPD | | 0.12 | 0.59 |
| | | nPD: | 0.21 |
| | | N. weight: | 0.78 |
| | | Sc. PD: | -0.036 |
| | | Sc. rank: | 13.9 |

| | | | |
|---|---|---|---|
| PB1 | | | |
| Pos. | 95 | obs: | exp: |
| tta | L | 0 | 32.06 |
| ttg | L | 0 | 70.68 |
| ctt | L | 367 | 68.48 |
| ctc | L | 0 | 59.37 |
| cta | L | 0 | 55.29 |
| ctg | L | 0 | 81.13 |
| --- | -- | ------ | ------ |
| mPD | | 0 | 1.1 |
| | | nPD: | 0. |
| | | N. weight: | 2.6 |
| | | Sc. PD: | -0.62 |
| | | Sc. rank: | -530.4 |

| | | | |
|---|---|---|---|
| PB1 | | | |
| Pos. | 96 | obs: | exp: |
| gaa | E | 349 | 211.20 |
| gag | E | 18 | 155.80 |
| --- | -- | ------ | ------ |
| mPD | | 0.094 | 0.49 |
| | | nPD: | 0.19 |
| | | N. weight: | 0.58 |
| | | Sc. PD: | -0.036 |
| | | Sc. rank: | 3.1 |

| | | | |
|---|---|---|---|
| PB1 | | | |
| Pos. | 97 | obs: | exp: |
| gaa | E | 204 | 211.20 |
| gag | E | 163 | 155.80 |
| --- | -- | ------ | ------ |
| mPD | | 0.50 | 0.49 |
| | | nPD: | 1.01 |
| | | N. weight: | 0.014 |
| | | Sc. PD: | 0.01 |
| | | Sc. rank: | 3.0 |

| | | | |
|---|---|---|---|
| PB1 | | | |
| Pos. | 98 | obs: | exp: |
| tct | S | 26 | 57.87 |
| tcc | S | 338 | 50.71 |
| tca | S | 0 | 80.64 |
| tcg | S | 0 | 22.19 |
| act | T | 0 | 0.53 |
| acc | T | 2 | 0.43 |
| aca | T | 0 | 0.88 |
| acg | T | 0 | 0.17 |
| agt | S | 0 | 80.08 |
| agc | S | 0 | 72.52 |
| gct | A | 0 | 0.25 |
| gcc | A | 1 | 0.20 |
| gca | A | 0 | 0.45 |
| gcg | A | 0 | 0.10 |
| --- | -- | ------ | ------ |
| mPD | | 0.15 | 1.7 |
| | | nPD: | 0.09 |
| | | N. weight: | 2.6 |
| | | Sc. PD: | -0.41 |
| | | Sc. rank: | -169.0 |

| | | | |
|---|---|---|---|
| PB1 | | | |
| Pos. | 99 | obs: | exp: |
| cat | H | 10 | 198.90 |
| cac | H | 357 | 168.10 |
| --- | -- | ------ | ------ |
| mPD | | 0.053 | 0.50 |
| | | nPD: | 0.11 |
| | | N. weight: | 1. |
| | | Sc. PD: | -0.14 |
| | | Sc. rank: | -53.3 |

| | | | |
|---|---|---|---|
| PB1 | | | |
| Pos. | 100 | obs: | exp: |
| tct | S | 0 | 0.16 |
| tcc | S | 0 | 0.14 |
| tca | S | 1 | 0.22 |
| tcg | S | 0 | 0.06 |
| cct | P | 0 | 96.37 |
| ccc | P | 0 | 66.02 |
| cca | P | 355 | 144.40 |
| ccg | P | 11 | 59.18 |
| agt | S | 0 | 0.22 |
| agc | S | 0 | 0.20 |
| --- | -- | ------ | ------ |
| mPD | | 0.064 | 0.72 |
| | | nPD: | 0.09 |
| | | N. weight: | 1.3 |
| | | Sc. PD: | -0.2 |
| | | Sc. rank: | -87.4 |

| | | | |
|---|---|---|---|
| PB1 | | | |
| Pos. | 101 | obs: | exp: |
| ggt | G | 0 | 45.28 |
| ggc | G | 0 | 49.63 |
| gga | G | 97 | 177.50 |
| ggg | G | 270 | 94.55 |
| --- | -- | ------ | ------ |
| mPD | | 0.39 | 0.67 |
| | | nPD: | 0.59 |
| | | N. weight: | 0.94 |
| | | Sc. PD: | 0.3 |
| | | Sc. rank: | 145.4 |

| | | | |
|---|---|---|---|
| PB1 | | | |
| Pos. | 102 | obs: | exp: |
| att | I | 0 | 138.00 |
| atc | I | 362 | 96.18 |
| ata | I | 5 | 132.80 |
| --- | -- | ------ | ------ |
| mPD | | 0.027 | 0.66 |
| | | nPD: | 0.04 |
| | | N. weight: | 1.9 |
| | | Sc. PD: | -0.4 |
| | | Sc. rank: | -251.6 |

| | | | |
|---|---|---|---|
| PB1 | | | |
| Pos. | 103 | obs: | exp: |
| ttt | F | 367 | 150.90 |
| ttc | F | 0 | 216.10 |
| --- | -- | ------ | ------ |
| mPD | | 0 | 0.48 |
| | | nPD: | 0. |
| | | N. weight: | 1.4 |
| | | Sc. PD: | -0.33 |
| | | Sc. rank: | -280.8 |

| | | | |
|---|---|---|---|
| PB1 | | | |
| Pos. | 104 | obs: | exp: |
| gaa | E | 366 | 211.20 |
| gag | E | 1 | 155.80 |
| --- | -- | ------ | ------ |
| mPD | | 0.0054 | 0.49 |
| | | nPD: | 0.01 |
| | | N. weight: | 0.82 |
| | | Sc. PD: | -0.19 |
| | | Sc. rank: | -140.0 |

| | | | |
|---|---|---|---|
| PB1 | | | |
| Pos. | 105 | obs: | exp: |
| aat | N | 2 | 191.80 |
| aac | N | 365 | 175.20 |
| --- | -- | ------ | ------ |
| mPD | | 0.011 | 0.50 |
| | | nPD: | 0.02 |
| | | N. weight: | 1.1 |
| | | Sc. PD: | -0.24 |
| | | Sc. rank: | -171.6 |

| PB1 | | | |
|---|---|---|---|
| Pos. | 106 | obs: | exp: |
| tct | S | 189 | 58.34 |
| tcc | S | 0 | 51.12 |
| tca | S | 81 | 81.31 |
| tcg | S | 97 | 22.37 |
| agt | S | 0 | 80.74 |
| agc | S | 0 | 73.12 |
| --- | -- | ------ | ------ |
| mPD | | 0.62 | 1.7 |
| | | nPD: | 0.37 |
| | | N. weight: | 1.5 |
| | | Sc. PD: | 0.17 |
| | | Sc. rank: | 133.5 |

| PB1 | | | |
|---|---|---|---|
| Pos. | 107 | obs: | exp: |
| tgt | C | 367 | 151.30 |
| tgc | C | 0 | 215.70 |
| --- | -- | ------ | ------ |
| mPD | | 0 | 0.48 |
| | | nPD: | 0. |
| | | N. weight: | 1.3 |
| | | Sc. PD: | -0.33 |
| | | Sc. rank: | -280.0 |

| PB1 | | | |
|---|---|---|---|
| Pos. | 108 | obs: | exp: |
| tta | L | 0 | 32.06 |
| ttg | L | 0 | 70.68 |
| ctt | L | 335 | 68.48 |
| ctc | L | 32 | 59.37 |
| cta | L | 0 | 55.29 |
| ctg | L | 0 | 81.13 |
| --- | -- | ------ | ------ |
| mPD | | 0.16 | 1.1 |
| | | nPD: | 0.14 |
| | | N. weight: | 2.1 |
| | | Sc. PD: | -0.23 |
| | | Sc. rank: | -45.4 |

| PB1 | | | |
|---|---|---|---|
| Pos. | 109 | obs: | exp: |
| gaa | E | 340 | 211.20 |
| gag | E | 27 | 155.80 |
| --- | -- | ------ | ------ |
| mPD | | 0.14 | 0.49 |
| | | nPD: | 0.28 |
| | | N. weight: | 0.49 |
| | | Sc. PD: | 0.011 |
| | | Sc. rank: | 30.2 |

| PB1 | | | |
|---|---|---|---|
| Pos. | 110 | obs: | exp: |
| act | T | 2 | 96.96 |
| acc | T | 0 | 78.75 |
| aca | T | 3 | 160.80 |
| acg | T | 362 | 30.51 |
| --- | -- | ------ | ------ |
| mPD | | 0.027 | 0.69 |
| | | nPD: | 0.04 |
| | | N. weight: | 3.6 |
| | | Sc. PD: | -0.75 |
| | | Sc. rank: | -483.4 |

| PB1 | | | |
|---|---|---|---|
| Pos. | 111 | obs: | exp: |
| att | I | 0 | 0.38 |
| atc | I | 0 | 0.26 |
| ata | I | 1 | 0.36 |
| atg | M | 366 | 366.00 |
| --- | -- | ------ | ------ |
| mPD | | 0.0054 | 0.0054 |
| | | nPD: | 1. |
| | | N. weight: | 0.0042 |
| | | Sc. PD: | 0.003 |
| | | Sc. rank: | 0.9 |

| PB1 | | | |
|---|---|---|---|
| Pos. | 112 | obs: | exp: |
| gaa | E | 344 | 211.20 |
| gag | E | 23 | 155.80 |
| --- | -- | ------ | ------ |
| mPD | | 0.12 | 0.49 |
| | | nPD: | 0.24 |
| | | N. weight: | 0.52 |
| | | Sc. PD: | -0.0076 |
| | | Sc. rank: | 19.7 |

| PB1 | | | |
|---|---|---|---|
| Pos. | 113 | obs: | exp: |
| ttt | F | 0 | 2.06 |
| ttc | F | 5 | 2.94 |
| att | I | 37 | 18.04 |
| atc | I | 11 | 12.58 |
| ata | I | 0 | 17.38 |
| gtt | V | 180 | 64.54 |
| gtc | V | 134 | 65.90 |
| gta | V | 0 | 58.85 |
| gtg | V | 0 | 124.70 |
| --- | -- | ------ | ------ |
| mPD | | 0.74 | 0.99 |
| | | nPD: | 0.74 |
| | | N. weight: | 1.3 |
| | | Sc. PD: | 0.6 |
| | | Sc. rank: | 249.5 |

| PB1 | | | |
|---|---|---|---|
| Pos. | 114 | obs: | exp: |
| att | I | 2 | 0.75 |
| atc | I | 0 | 0.52 |
| ata | I | 0 | 0.72 |
| gtt | V | 360 | 75.02 |
| gtc | V | 5 | 76.61 |
| gta | V | 0 | 68.41 |
| gtg | V | 0 | 145.00 |
| --- | -- | ------ | ------ |
| mPD | | 0.038 | 0.73 |
| | | nPD: | 0.05 |
| | | N. weight: | 2.3 |
| | | Sc. PD: | -0.45 |
| | | Sc. rank: | -260.6 |

| PB1 | | | |
|---|---|---|---|
| Pos. | 115 | obs: | exp: |
| caa | Q | 103 | 195.60 |
| cag | Q | 264 | 171.40 |
| --- | -- | ------ | ------ |
| mPD | | 0.40 | 0.50 |
| | | nPD: | 0.81 |
| | | N. weight: | 0.21 |
| | | Sc. PD: | 0.11 |
| | | Sc. rank: | 42.8 |

| PB1 | | | |
|---|---|---|---|
| Pos. | 116 | obs: | exp: |
| caa | Q | 361 | 195.60 |
| cag | Q | 6 | 171.40 |
| --- | -- | ------ | ------ |
| mPD | | 0.032 | 0.50 |
| | | nPD: | 0.06 |
| | | N. weight: | 0.84 |
| | | Sc. PD: | -0.15 |
| | | Sc. rank: | -80.6 |

| PB1 | | | |
|---|---|---|---|
| Pos. | 117 | obs: | exp: |
| act | T | 0 | 96.96 |
| acc | T | 0 | 78.75 |
| aca | T | 361 | 160.80 |
| acg | T | 6 | 30.51 |
| --- | -- | ------ | ------ |
| mPD | | 0.032 | 0.69 |
| | | nPD: | 0.05 |
| | | N. weight: | 1.2 |
| | | Sc. PD: | -0.23 |
| | | Sc. rank: | -144.6 |

| PB1 | | | |
|---|---|---|---|
| Pos. | 118 | obs: | exp: |
| cgt | R | 0 | 15.24 |
| cgc | R | 0 | 17.97 |
| cga | R | 0 | 35.27 |
| cgg | R | 0 | 30.63 |
| aga | R | 354 | 165.00 |
| agg | R | 13 | 102.80 |
| --- | -- | ------ | ------ |
| mPD | | 0.069 | 0.96 |
| | | nPD: | 0.07 |
| | | N. weight: | 1. |
| | | Sc. PD: | -0.18 |
| | | Sc. rank: | -87.7 |

| PB1 | | | |
|---|---|---|---|
| Pos. | 119 | obs: | exp: |
| atg | M | 1 | 1.00 |
| gtt | V | 2 | 75.23 |
| gtc | V | 0 | 76.82 |
| gta | V | 14 | 68.60 |
| gtg | V | 350 | 145.40 |
| --- | -- | ------ | ------ |
| mPD | | 0.089 | 0.73 |
| | | nPD: | 0.12 |
| | | N. weight: | 1.2 |
| | | Sc. PD: | -0.15 |
| | | Sc. rank: | -39.9 |

| PB1 | | | |
|---|---|---|---|
| Pos. | 120 | obs: | exp: |
| gat | D | 49 | 195.80 |
| gac | D | 318 | 171.20 |
| --- | -- | ------ | ------ |
| mPD | | 0.23 | 0.50 |
| | | nPD: | 0.47 |
| | | N. weight: | 0.55 |
| | | Sc. PD: | 0.11 |
| | | Sc. rank: | 67.9 |

| PB1 | | | |
|---|---|---|---|
| Pos. | 121 | obs: | exp: |
| aaa | K | 353 | 205.50 |
| aag | K | 14 | 161.50 |
| --- | -- | ------ | ------ |
| mPD | | 0.074 | 0.49 |
| | | nPD: | 0.15 |
| | | N. weight: | 0.66 |
| | | Sc. PD: | -0.067 |
| | | Sc. rank: | -10.7 |

| PB1 | | | |
|---|---|---|---|
| Pos. | 122 | obs: | exp: |
| tta | L | 6 | 32.06 |
| ttg | L | 82 | 70.68 |
| ctt | L | 0 | 68.48 |
| ctc | L | 0 | 59.37 |
| cta | L | 187 | 55.29 |
| ctg | L | 92 | 81.13 |
| --- | -- | ------ | ------ |
| mPD | | 0.87 | 1.1 |
| | | nPD: | 0.78 |
| | | N. weight: | 1. |
| | | Sc. PD: | 0.51 |
| | | Sc. rank: | 203.0 |

| PB1 | | | |
|---|---|---|---|
| Pos. | 123 | obs: | exp: |
| act | T | 96 | 96.96 |
| acc | T | 269 | 78.75 |
| aca | T | 2 | 160.80 |
| acg | T | 0 | 30.51 |
| --- | -- | ------ | ------ |
| mPD | | 0.40 | 0.69 |
| | | nPD: | 0.58 |
| | | N. weight: | 1.3 |
| | | Sc. PD: | 0.41 |
| | | Sc. rank: | 205.1 |

| PB1 | | | |
|---|---|---|---|
| Pos. | 124 | obs: | exp: |
| caa | Q | 353 | 195.60 |
| cag | Q | 14 | 171.40 |
| --- | -- | ------ | ------ |
| mPD | | 0.074 | 0.50 |
| | | nPD: | 0.15 |
| | | N. weight: | 0.73 |
| | | Sc. PD: | -0.075 |
| | | Sc. rank: | -12.9 |

| PB1 | | | |
|---|---|---|---|
| Pos. | 125 | obs: | exp: |
| ggt | G | 246 | 45.28 |
| ggc | G | 121 | 49.63 |
| gga | G | 0 | 177.50 |
| ggg | G | 0 | 94.55 |
| --- | -- | ------ | ------ |
| mPD | | 0.44 | 0.67 |
| | | nPD: | 0.67 |
| | | N. weight: | 2.2 |
| | | Sc. PD: | 0.85 |
| | | Sc. rank: | 375.2 |

| PB1 | | | |
|---|---|---|---|
| Pos. | 126 | obs: | exp: |
| cgt | R | 99 | 15.24 |
| cgc | R | 268 | 17.97 |
| cga | R | 0 | 35.27 |
| cgg | R | 0 | 30.63 |
| aga | R | 0 | 165.00 |
| agg | R | 0 | 102.80 |
| --- | -- | ------ | ------ |
| mPD | | 0.40 | 0.96 |
| | | nPD: | 0.41 |
| | | N. weight: | 3.8 |
| | | Sc. PD: | 0.56 |
| | | Sc. rank: | 412.7 |

| PB1 | | | |
|---|---|---|---|
| Pos. | 127 | obs: | exp: |
| caa | Q | 2 | 195.60 |
| cag | Q | 365 | 171.40 |
| --- | -- | ------ | ------ |
| mPD | | 0.011 | 0.50 |
| | | nPD: | 0.02 |
| | | N. weight: | 1.1 |
| | | Sc. PD: | -0.25 |
| | | Sc. rank: | -174.4 |

| PB1 | | | |
|---|---|---|---|
| Pos. | 128 | obs: | exp: |
| act | T | 175 | 96.96 |
| acc | T | 189 | 78.75 |
| aca | T | 3 | 160.80 |
| acg | T | 0 | 30.51 |
| --- | -- | ------ | ------ |
| mPD | | 0.51 | 0.69 |
| | | nPD: | 0.74 |
| | | N. weight: | 1.1 |
| | | Sc. PD: | 0.5 |
| | | Sc. rank: | 208.0 |

| PB1 | | | |
|---|---|---|---|
| Pos. | 129 | obs: | exp: |
| tat | Y | 363 | 190.00 |
| tac | Y | 4 | 177.00 |
| --- | -- | ------ | ------ |
| mPD | | 0.022 | 0.50 |
| | | nPD: | 0.04 |
| | | N. weight: | 0.92 |
| | | Sc. PD: | -0.19 |
| | | Sc. rank: | -116.8 |

| PB1 | | | |
|---|---|---|---|
| Pos. | 130 | obs: | exp: |
| gat | D | 140 | 195.80 |
| gac | D | 227 | 171.20 |
| --- | -- | ------ | ------ |
| mPD | | 0.47 | 0.50 |
| | | nPD: | 0.95 |
| | | N. weight: | 0.084 |
| | | Sc. PD: | 0.055 |
| | | Sc. rank: | 17.4 |

| PB1 | | | |
|---|---|---|---|
| Pos. | 131 | obs: | exp: |
| tgg | W | 367 | 367.00 |
| --- | -- | ------ | ------ |
| mPD | | 0 | 0 |
| | | nPD: | 1. |
| | | N. weight: | 0. |
| | | Sc. PD: | 0 |
| | | Sc. rank: | 0 |

| PB1 | | | |
|---|---|---|---|
| Pos. | 132 | obs: | exp: |
| act | T | 2 | 96.96 |
| acc | T | 0 | 78.75 |
| aca | T | 342 | 160.80 |
| acg | T | 23 | 30.51 |
| --- | -- | ------ | ------ |
| mPD | | 0.13 | 0.69 |
| | | nPD: | 0.19 |
| | | N. weight: | 1. |
| | | Sc. PD: | -0.068 |
| | | Sc. rank: | 2.8 |

| PB1 | | | |
|---|---|---|---|
| Pos. | 133 | obs: | exp: |
| tta | L | 91 | 32.06 |
| ttg | L | 73 | 70.68 |
| ctt | L | 0 | 68.48 |
| ctc | L | 0 | 59.37 |
| cta | L | 12 | 55.29 |
| ctg | L | 191 | 81.13 |
| --- | -- | ------ | ------ |
| mPD | | 0.90 | 1.1 |
| | | nPD: | 0.81 |
| | | N. weight: | 1. |
| | | Sc. PD: | 0.54 |
| | | Sc. rank: | 207.3 |

| PB1 | | | |
|---|---|---|---|
| Pos. | 134 | obs: | exp: |
| aat | N | 240 | 191.80 |
| aac | N | 127 | 175.20 |
| --- | -- | ------ | ------ |
| mPD | | 0.45 | 0.50 |
| | | nPD: | 0.91 |
| | | N. weight: | 0.067 |
| | | Sc. PD: | 0.041 |
| | | Sc. rank: | 13.8 |

| PB1 | | | |
|---|---|---|---|
| Pos. | 135 | obs: | exp: |
| cgt | R | 0 | 15.24 |
| cgc | R | 0 | 17.97 |
| cga | R | 0 | 35.27 |
| cgg | R | 0 | 30.63 |
| aga | R | 272 | 165.00 |
| agg | R | 95 | 102.80 |
| --- | -- | ------ | ------ |
| mPD | | 0.38 | 0.96 |
| | | nPD: | 0.4 |
| | | N. weight: | 0.54 |
| | | Sc. PD: | 0.075 |
| | | Sc. rank: | 55.3 |

PB1

| Pos. | 136 | obs: | exp: |
|---|---|---|---|
| act | T | 1 | 0.26 |
| acc | T | 0 | 0.21 |
| aca | T | 0 | 0.44 |
| acg | T | 0 | 0.08 |
| aat | N | 157 | 191.30 |
| aac | N | 209 | 174.70 |
| --- | -- | ------ | ------ |
| mPD | | 0.50 | 0.51 |
| | | nPD: | 0.98 |
| | | N. weight: | 0.045 |
| | | Sc. PD: | 0.031 |
| | | Sc. rank: | 9.5 |

,

PB1

| Pos. | 137 | obs: | exp: |
|---|---|---|---|
| caa | Q | 208 | 195.60 |
| cag | Q | 159 | 171.40 |
| --- | -- | ------ | ------ |
| mPD | | 0.49 | 0.50 |
| | | nPD: | 0.99 |
| | | N. weight: | 0.017 |
| | | Sc. PD: | 0.012 |
| | | Sc. rank: | 3.5 |

,

PB1

| Pos. | 138 | obs: | exp: |
|---|---|---|---|
| tct | S | 0 | 0.48 |
| tcc | S | 0 | 0.42 |
| tca | S | 3 | 0.66 |
| tcg | S | 0 | 0.18 |
| cct | P | 2 | 95.58 |
| ccc | P | 0 | 65.48 |
| cca | P | 194 | 143.20 |
| ccg | P | 167 | 58.69 |
| act | T | 0 | 0.26 |
| acc | T | 0 | 0.21 |
| aca | T | 1 | 0.44 |
| acg | T | 0 | 0.08 |
| agt | S | 0 | 0.66 |
| agc | S | 0 | 0.60 |
| --- | -- | ------ | ------ |
| mPD | | 0.52 | 0.75 |
| | | nPD: | 0.7 |
| | | N. weight: | 0.98 |
| | | Sc. PD: | 0.42 |
| | | Sc. rank: | 175.7 |

,

PB1

| Pos. | 139 | obs: | exp: |
|---|---|---|---|
| gct | A | 363 | 92.44 |
| gcc | A | 3 | 75.18 |
| gca | A | 1 | 163.60 |
| gcg | A | 0 | 35.81 |
| --- | -- | ------ | ------ |
| mPD | | 0.022 | 0.69 |
| | | nPD: | 0.03 |
| | | N. weight: | 2. |
| | | Sc. PD: | -0.43 |
| | | Sc. rank: | -297.2 |

,

PB1

| Pos. | 140 | obs: | exp: |
|---|---|---|---|
| gct | A | 26 | 92.44 |
| gcc | A | 0 | 75.18 |
| gca | A | 320 | 163.60 |
| gcg | A | 21 | 35.81 |
| --- | -- | ------ | ------ |
| mPD | | 0.23 | 0.69 |
| | | nPD: | 0.34 |
| | | N. weight: | 0.73 |
| | | Sc. PD: | 0.056 |
| | | Sc. rank: | 55.7 |

,

PB1

| Pos. | 141 | obs: | exp: |
|---|---|---|---|
| act | T | 365 | 96.96 |
| acc | T | 0 | 78.75 |
| aca | T | 2 | 160.80 |
| acg | T | 0 | 30.51 |
| --- | -- | ------ | ------ |
| mPD | | 0.011 | 0.69 |
| | | nPD: | 0.02 |
| | | N. weight: | 2. |
| | | Sc. PD: | -0.45 |
| | | Sc. rank: | -330.5 |

,

PB1

| Pos. | 142 | obs: | exp: |
|---|---|---|---|
| gct | A | 182 | 92.44 |
| gcc | A | 63 | 75.18 |
| gca | A | 121 | 163.60 |
| gcg | A | 1 | 35.81 |
| --- | -- | ------ | ------ |
| mPD | | 0.62 | 0.69 |
| | | nPD: | 0.9 |
| | | N. weight: | 0.33 |
| | | Sc. PD: | 0.2 |
| | | Sc. rank: | 67.8 |

,

PB1

| Pos. | 143 | obs: | exp: |
|---|---|---|---|
| tta | L | 98 | 32.06 |
| ttg | L | 209 | 70.68 |
| ctt | L | 0 | 68.48 |
| ctc | L | 0 | 59.37 |
| cta | L | 2 | 55.29 |
| ctg | L | 58 | 81.13 |
| --- | -- | ------ | ------ |
| mPD | | 0.67 | 1.1 |
| | | nPD: | 0.6 |
| | | N. weight: | 1.3 |
| | | Sc. PD: | 0.43 |
| | | Sc. rank: | 207.2 |

,

PB1

| Pos. | 144 | obs: | exp: |
|---|---|---|---|
| act | T | 0 | 0.26 |
| acc | T | 1 | 0.21 |
| aca | T | 0 | 0.44 |
| acg | T | 0 | 0.08 |
| gct | A | 82 | 92.19 |
| gcc | A | 282 | 74.97 |
| gca | A | 2 | 163.10 |
| gcg | A | 0 | 35.71 |
| --- | -- | ------ | ------ |
| mPD | | 0.36 | 0.69 |
| | | nPD: | 0.52 |
| | | N. weight: | 1.5 |
| | | Sc. PD: | 0.38 |
| | | Sc. rank: | 205.0 |

,

PB1

| Pos. | 145 | obs: | exp: |
|---|---|---|---|
| aat | N | 109 | 191.80 |
| aac | N | 258 | 175.20 |
| --- | -- | ------ | ------ |
| mPD | | 0.42 | 0.50 |
| | | nPD: | 0.84 |
| | | N. weight: | 0.17 |
| | | Sc. PD: | 0.095 |
| | | Sc. rank: | 35.0 |

,

PB1

| Pos. | 146 | obs: | exp: |
|---|---|---|---|
| act | T | 355 | 96.96 |
| acc | T | 11 | 78.75 |
| aca | T | 1 | 160.80 |
| acg | T | 0 | 30.51 |
| --- | -- | ------ | ------ |
| mPD | | 0.064 | 0.69 |
| | | nPD: | 0.09 |
| | | N. weight: | 1.8 |
| | | Sc. PD: | -0.28 |
| | | Sc. rank: | -108.8 |

,

PB1

| Pos. | 147 | obs: | exp: |
|---|---|---|---|
| att | I | 1 | 138.00 |
| atc | I | 0 | 96.18 |
| ata | I | 366 | 132.80 |
| --- | -- | ------ | ------ |
| mPD | | 0.0054 | 0.66 |
| | | nPD: | 0.01 |
| | | N. weight: | 1.5 |
| | | Sc. PD: | -0.36 |
| | | Sc. rank: | -268.7 |

,

PB1

| Pos. | 148 | obs: | exp: |
|---|---|---|---|
| gaa | E | 17 | 211.20 |
| gag | E | 350 | 155.80 |
| --- | -- | ------ | ------ |
| mPD | | 0.089 | 0.49 |
| | | nPD: | 0.18 |
| | | N. weight: | 1. |
| | | Sc. PD: | -0.072 |
| | | Sc. rank: | 1.3 |

,

PB1

| Pos. | 149 | obs: | exp: |
|---|---|---|---|
| att | I | 0 | 2.63 |
| atc | I | 7 | 1.83 |
| ata | I | 0 | 2.53 |
| gtt | V | 35 | 73.99 |
| gtc | V | 267 | 75.56 |
| gta | V | 27 | 67.47 |
| gtg | V | 31 | 143.00 |
| --- | -- | ------ | ------ |
| mPD | | 0.46 | 0.76 |
| | | nPD: | 0.6 |
| | | N. weight: | 1.1 |
| | | Sc. PD: | 0.35 |
| | | Sc. rank: | 167.8 |

,

PB1

| Pos. | 150 | obs: | exp: |
|---|---|---|---|
| ttt | F | 5 | 150.50 |
| ttc | F | 361 | 215.50 |
| tat | Y | 0 | 0.52 |
| tac | Y | 1 | 0.48 |
| --- | -- | ------ | ------ |
| mPD | | 0.032 | 0.49 |
| | | nPD: | 0.07 |
| | | N. weight: | 0.71 |
| | | Sc. PD: | -0.13 |
| | | Sc. rank: | -66.2 |

,

PB1

| Pos. | 151 | obs: | exp: |
|---|---|---|---|
| cgt | R | 0 | 15.24 |
| cgc | R | 0 | 17.97 |
| cga | R | 0 | 35.27 |
| cgg | R | 0 | 30.63 |
| aga | R | 359 | 165.00 |
| agg | R | 8 | 102.80 |
| --- | -- | ------ | ------ |
| mPD | | 0.043 | 0.96 |
| | | nPD: | 0.04 |
| | | N. weight: | 1.1 |
| | | Sc. PD: | -0.22 |
| | | Sc. rank: | -135.5 |

,

PB1

| Pos. | 152 | obs: | exp: |
|---|---|---|---|
| tta | L | 0 | 0.26 |
| ttg | L | 3 | 0.58 |
| tct | S | 0 | 57.87 |
| tcc | S | 0 | 50.71 |
| tca | S | 36 | 80.64 |
| tcg | S | 328 | 22.19 |
| ctt | L | 0 | 0.56 |
| ctc | L | 0 | 0.49 |
| cta | L | 0 | 0.45 |
| ctg | L | 0 | 0.66 |
| agt | S | 0 | 80.08 |
| agc | S | 0 | 72.52 |
| --- | -- | ------ | ------ |
| mPD | | 0.19 | 1.7 |
| | | nPD: | 0.12 |
| | | N. weight: | 3.6 |
| | | Sc. PD: | -0.48 |
| | | Sc. rank: | -152.6 |

,

PB1

| Pos. | 153 | obs: | exp: |
|---|---|---|---|
| aat | N | 51 | 191.80 |
| aac | N | 316 | 175.20 |
| --- | -- | ------ | ------ |
| mPD | | 0.24 | 0.50 |
| | | nPD: | 0.48 |
| | | N. weight: | 0.5 |
| | | Sc. PD: | 0.11 |
| | | Sc. rank: | 63.5 |

,

PB1

| Pos. | 154 | obs: | exp: |
|---|---|---|---|
| tct | S | 0 | 0.32 |
| tcc | S | 0 | 0.28 |
| tca | S | 0 | 0.44 |
| tcg | S | 0 | 0.12 |
| agt | S | 2 | 0.44 |
| agc | S | 0 | 0.40 |
| gat | D | 4 | 2.13 |
| gac | D | 0 | 1.87 |
| ggt | G | 335 | 44.54 |
| ggc | G | 26 | 48.82 |
| gga | G | 0 | 174.60 |
| ggg | G | 0 | 93.01 |
| --- | -- | ------ | ------ |
| mPD | | 0.16 | 0.71 |
| | | nPD: | 0.23 |
| | | N. weight: | 2.8 |
| | | Sc. PD: | -0.066 |
| | | Sc. rank: | 92.6 |

,

PB1

| Pos. | 155 | obs: | exp: |
|---|---|---|---|
| tta | L | 1 | 32.06 |
| ttg | L | 14 | 70.68 |
| ctt | L | 57 | 68.48 |
| ctc | L | 4 | 59.37 |
| cta | L | 99 | 55.29 |
| ctg | L | 192 | 81.13 |
| --- | -- | ------ | ------ |
| mPD | | 0.67 | 1.1 |
| | | nPD: | 0.6 |
| | | N. weight: | 0.77 |
| | | Sc. PD: | 0.25 |
| | | Sc. rank: | 120.8 |

,

| PB1 | | | |
|---|---|---|---|
| Pos. | 156 | obs: | exp: |
| act | T | 1 | 96.17 |
| acc | T | 9 | 78.11 |
| aca | T | 345 | 159.50 |
| acg | T | 9 | 30.26 |
| gct | A | 0 | 0.76 |
| gcc | A | 0 | 0.61 |
| gca | A | 3 | 1.34 |
| gcg | A | 0 | 0.29 |
| --- | -- | ------ | ------ |
| mPD | | 0.12 | 0.70 |
| | | nPD: | 0.17 |
| | | N. weight: | 0.99 |
| | | Sc. PD: | -0.085 |
| | | Sc. rank: | -7.0 |

,

| PB1 | | | |
|---|---|---|---|
| Pos. | 157 | obs: | exp: |
| tct | S | 1 | 0.16 |
| tcc | S | 0 | 0.14 |
| tca | S | 0 | 0.22 |
| tcg | S | 0 | 0.06 |
| act | T | 1 | 0.26 |
| acc | T | 0 | 0.21 |
| aca | T | 0 | 0.44 |
| acg | T | 0 | 0.08 |
| agt | S | 0 | 0.22 |
| agc | S | 0 | 0.20 |
| gct | A | 91 | 91.93 |
| gcc | A | 273 | 74.77 |
| gca | A | 1 | 162.70 |
| gcg | A | 0 | 35.62 |
| --- | -- | ------ | ------ |
| mPD | | 0.39 | 0.70 |
| | | nPD: | 0.56 |
| | | N. weight: | 1.5 |
| | | Sc. PD: | 0.43 |
| | | Sc. rank: | 216.3 |

,

| PB1 | | | |
|---|---|---|---|
| Pos. | 158 | obs: | exp: |
| aat | N | 362 | 191.80 |
| aac | N | 5 | 175.20 |
| --- | -- | ------ | ------ |
| mPD | | 0.027 | 0.50 |
| | | nPD: | 0.05 |
| | | N. weight: | 0.89 |
| | | Sc. PD: | -0.17 |
| | | Sc. rank: | -98.0 |

,

| PB1 | | | |
|---|---|---|---|
| Pos. | 159 | obs: | exp: |
| gat | D | 25 | 13.34 |
| gac | D | 0 | 11.66 |
| gaa | E | 258 | 196.80 |
| gag | E | 84 | 145.20 |
| --- | -- | ------ | ------ |
| mPD | | 0.45 | 0.55 |
| | | nPD: | 0.81 |
| | | N. weight: | 0.18 |
| | | Sc. PD: | 0.093 |
| | | Sc. rank: | 35.6 |

,

| PB1 | | | |
|---|---|---|---|
| Pos. | 160 | obs: | exp: |
| tct | S | 0 | 58.34 |
| tcc | S | 0 | 51.12 |
| tca | S | 171 | 81.31 |
| tcg | S | 196 | 22.37 |
| agt | S | 0 | 80.74 |
| agc | S | 0 | 73.12 |
| --- | -- | ------ | ------ |
| mPD | | 0.50 | 1.7 |
| | | nPD: | 0.3 |
| | | N. weight: | 2.3 |
| | | Sc. PD: | 0.096 |
| | | Sc. rank: | 165.0 |

,

| PB1 | | | |
|---|---|---|---|
| Pos. | 161 | obs: | exp: |
| ggt | G | 1 | 45.28 |
| ggc | G | 0 | 49.63 |
| gga | G | 132 | 177.50 |
| ggg | G | 234 | 94.55 |
| --- | -- | ------ | ------ |
| mPD | | 0.47 | 0.67 |
| | | nPD: | 0.7 |
| | | N. weight: | 0.72 |
| | | Sc. PD: | 0.3 |
| | | Sc. rank: | 127.4 |

,

| PB1 | | | |
|---|---|---|---|
| Pos. | 162 | obs: | exp: |
| cgt | R | 0 | 15.24 |
| cgc | R | 0 | 17.97 |
| cga | R | 0 | 35.27 |
| cgg | R | 0 | 30.63 |
| aga | R | 254 | 165.00 |
| agg | R | 113 | 102.80 |
| --- | -- | ------ | ------ |
| mPD | | 0.43 | 0.96 |
| | | nPD: | 0.45 |
| | | N. weight: | 0.51 |
| | | Sc. PD: | 0.092 |
| | | Sc. rank: | 60.6 |

,

| PB1 | | | |
|---|---|---|---|
| Pos. | 163 | obs: | exp: |
| tta | L | 10 | 32.06 |
| ttg | L | 26 | 70.68 |
| ctt | L | 2 | 68.48 |
| ctc | L | 2 | 59.37 |
| cta | L | 316 | 55.29 |
| ctg | L | 11 | 81.13 |
| --- | -- | ------ | ------ |
| mPD | | 0.38 | 1.1 |
| | | nPD: | 0.34 |
| | | N. weight: | 2. |
| | | Sc. PD: | 0.16 |
| | | Sc. rank: | 156.6 |

,

| PB1 | | | |
|---|---|---|---|
| Pos. | 164 | obs: | exp: |
| att | I | 0 | 138.00 |
| atc | I | 26 | 96.18 |
| ata | I | 341 | 132.80 |
| --- | -- | ------ | ------ |
| mPD | | 0.13 | 0.66 |
| | | nPD: | 0.2 |
| | | N. weight: | 1.2 |
| | | Sc. PD: | -0.064 |
| | | Sc. rank: | 16.4 |

,

| PB1 | | | |
|---|---|---|---|
| Pos. | 165 | obs: | exp: |
| gat | D | 359 | 195.30 |
| gac | D | 7 | 170.70 |
| gaa | E | 0 | 0.58 |
| gag | E | 1 | 0.42 |
| --- | -- | ------ | ------ |
| mPD | | 0.043 | 0.50 |
| | | nPD: | 0.09 |
| | | N. weight: | 0.83 |
| | | Sc. PD: | -0.13 |
| | | Sc. rank: | -59.2 |

,

| PB1 | | | |
|---|---|---|---|
| Pos. | 166 | obs: | exp: |
| ttt | F | 86 | 150.50 |
| ttc | F | 280 | 215.50 |
| gtt | V | 0 | 0.21 |
| gtc | V | 1 | 0.21 |
| gta | V | 0 | 0.19 |
| gtg | V | 0 | 0.40 |
| --- | -- | ------ | ------ |
| mPD | | 0.37 | 0.49 |
| | | nPD: | 0.74 |
| | | N. weight: | 0.12 |
| | | Sc. PD: | 0.057 |
| | | Sc. rank: | 23.7 |

,

| PB1 | | | |
|---|---|---|---|
| Pos. | 167 | obs: | exp: |
| tta | L | 0 | 32.06 |
| ttg | L | 0 | 70.68 |
| ctt | L | 6 | 68.48 |
| ctc | L | 354 | 59.37 |
| cta | L | 7 | 55.29 |
| ctg | L | 0 | 81.13 |
| --- | -- | ------ | ------ |
| mPD | | 0.069 | 1.1 |
| | | nPD: | 0.06 |
| | | N. weight: | 2.5 |
| | | Sc. PD: | -0.46 |
| | | Sc. rank: | -254.2 |

,

| PB1 | | | |
|---|---|---|---|
| Pos. | 168 | obs: | exp: |
| aaa | K | 21 | 205.50 |
| aag | K | 346 | 161.50 |
| --- | -- | ------ | ------ |
| mPD | | 0.11 | 0.49 |
| | | nPD: | 0.22 |
| | | N. weight: | 0.9 |
| | | Sc. PD: | -0.032 |
| | | Sc. rank: | 22.8 |

,

| PB1 | | | |
|---|---|---|---|
| Pos. | 169 | obs: | exp: |
| gat | D | 338 | 195.80 |
| gac | D | 29 | 171.20 |
| --- | -- | ------ | ------ |
| mPD | | 0.15 | 0.50 |
| | | nPD: | 0.29 |
| | | N. weight: | 0.56 |
| | | Sc. PD: | 0.02 |
| | | Sc. rank: | 38.8 |

,

| PB1 | | | |
|---|---|---|---|
| Pos. | 170 | obs: | exp: |
| gtt | V | 5 | 75.43 |
| gtc | V | 0 | 77.03 |
| gta | V | 25 | 68.78 |
| gtg | V | 337 | 145.80 |
| --- | -- | ------ | ------ |
| mPD | | 0.15 | 0.72 |
| | | nPD: | 0.21 |
| | | N. weight: | 1. |
| | | Sc. PD: | -0.044 |
| | | Sc. rank: | 21.3 |

,

| PB1 | | | |
|---|---|---|---|
| Pos. | 171 | obs: | exp: |
| att | I | 0 | 0.38 |
| atc | I | 0 | 0.26 |
| ata | I | 1 | 0.36 |
| atg | M | 357 | 357.00 |
| gtt | V | 0 | 1.85 |
| gtc | V | 0 | 1.89 |
| gta | V | 0 | 1.69 |
| gtg | V | 9 | 3.57 |
| --- | -- | ------ | ------ |
| mPD | | 0.053 | 0.082 |
| | | nPD: | 0.65 |
| | | N. weight: | 0.039 |
| | | Sc. PD: | 0.014 |
| | | Sc. rank: | 6.5 |

,

| PB1 | | | |
|---|---|---|---|
| Pos. | 172 | obs: | exp: |
| gat | D | 31 | 38.42 |
| gac | D | 41 | 33.58 |
| gaa | E | 259 | 168.00 |
| gag | E | 33 | 124.00 |
| ggt | G | 0 | 0.37 |
| ggc | G | 1 | 0.41 |
| gga | G | 2 | 1.45 |
| ggg | G | 0 | 0.77 |
| --- | -- | ------ | ------ |
| mPD | | 0.48 | 0.67 |
| | | nPD: | 0.72 |
| | | N. weight: | 0.32 |
| | | Sc. PD: | 0.14 |
| | | Sc. rank: | 59.3 |

,

| PB1 | | | |
|---|---|---|---|
| Pos. | 173 | obs: | exp: |
| tct | S | 0 | 58.34 |
| tcc | S | 0 | 51.12 |
| tca | S | 360 | 81.31 |
| tcg | S | 7 | 22.37 |
| agt | S | 0 | 80.74 |
| agc | S | 0 | 73.12 |
| --- | -- | ------ | ------ |
| mPD | | 0.038 | 1.7 |
| | | nPD: | 0.02 |
| | | N. weight: | 2.2 |
| | | Sc. PD: | -0.49 |
| | | Sc. rank: | -337.7 |

,

| PB1 | | | |
|---|---|---|---|
| Pos. | 174 | obs: | exp: |
| att | I | 0 | 0.38 |
| atc | I | 0 | 0.26 |
| ata | I | 1 | 0.36 |
| atg | M | 366 | 366.00 |
| --- | -- | ------ | ------ |
| mPD | | 0.0054 | 0.0054 |
| | | nPD: | 1. |
| | | N. weight: | 0.0042 |
| | | Sc. PD: | 0.003 |
| | | Sc. rank: | 0.9 |

,

| PB1 | | | |
|---|---|---|---|
| Pos. | 175 | obs: | exp: |
| aat | N | 3 | 2.09 |
| aac | N | 1 | 1.91 |
| gat | D | 281 | 193.10 |
| gac | D | 81 | 168.90 |
| ggt | G | 1 | 0.12 |
| ggc | G | 0 | 0.14 |
| gga | G | 0 | 0.48 |
| ggg | G | 0 | 0.26 |
| --- | -- | ------ | ------ |
| mPD | | 0.38 | 0.53 |
| | | nPD: | 0.71 |
| | | N. weight: | 0.22 |
| | | Sc. PD: | 0.094 |
| | | Sc. rank: | 39.2 |

,

| PB1 | | | |
|---|---|---|---|
| Pos. | 176 | obs: | exp: |
| cgt | R | 0 | 0.25 |
| cgc | R | 0 | 0.29 |
| cga | R | 0 | 0.58 |
| cgg | R | 0 | 0.50 |
| aaa | K | 317 | 202.10 |
| aag | K | 44 | 158.90 |
| aga | R | 6 | 2.70 |
| agg | R | 0 | 1.68 |
| --- | -- | ------ | ------ |
| mPD | | 0.24 | 0.54 |
| | | nPD: | 0.46 |
| | | N. weight: | 0.39 |
| | | Sc. PD: | 0.074 |
| | | Sc. rank: | 47.1 |

,

| PB1 | | | |
|---|---|---|---|
| Pos. | 177 | obs: | exp: |
| cgt | R | 0 | 0.04 |
| cgc | R | 0 | 0.05 |
| cga | R | 0 | 0.10 |
| cgg | R | 0 | 0.08 |
| aaa | K | 3 | 2.24 |
| aag | K | 1 | 1.76 |
| aga | R | 1 | 0.45 |
| agg | R | 0 | 0.28 |
| gat | D | 1 | 1.60 |
| gac | D | 2 | 1.40 |
| gaa | E | 265 | 203.70 |
| gag | E | 89 | 150.30 |
| ggt | G | 0 | 0.62 |
| ggc | G | 0 | 0.68 |
| gga | G | 5 | 2.42 |
| ggg | G | 0 | 1.29 |
| --- | -- | ------ | ------ |
| mPD | | 0.44 | 0.56 |
| | | nPD: | 0.79 |
| | | N. weight: | 0.14 |
| | | Sc. PD: | 0.069 |
| | | Sc. rank: | 26.9 |

,

| PB1 | | | |
|---|---|---|---|
| Pos. | 178 | obs: | exp: |
| aaa | K | 2 | 1.12 |
| aag | K | 0 | 0.88 |
| gaa | E | 346 | 209.50 |
| gag | E | 18 | 154.50 |
| ggt | G | 0 | 0.12 |
| ggc | G | 0 | 0.14 |
| gga | G | 1 | 0.48 |
| ggg | G | 0 | 0.26 |
| --- | -- | ------ | ------ |
| mPD | | 0.11 | 0.51 |
| | | nPD: | 0.22 |
| | | N. weight: | 0.58 |
| | | Sc. PD: | -0.021 |
| | | Sc. rank: | 13.5 |

,

| PB1 | | | |
|---|---|---|---|
| Pos. | 179 | obs: | exp: |
| att | I | 0 | 13.16 |
| atc | I | 0 | 9.17 |
| ata | I | 35 | 12.67 |
| atg | M | 321 | 321.00 |
| act | T | 0 | 0.26 |
| acc | T | 0 | 0.21 |
| aca | T | 0 | 0.44 |
| acg | T | 1 | 0.08 |
| gtt | V | 0 | 2.06 |
| gtc | V | 0 | 2.10 |
| gta | V | 0 | 1.87 |
| gtg | V | 10 | 3.97 |
| --- | -- | ------ | ------ |
| mPD | | 0.23 | 0.27 |
| | | nPD: | 0.86 |
| | | N. weight: | 0.2 |
| | | Sc. PD: | 0.11 |
| | | Sc. rank: | 40.2 |

,

| PB1 | | | |
|---|---|---|---|
| Pos. | 180 | obs: | exp: |
| gaa | E | 326 | 208.90 |
| gag | E | 37 | 154.10 |
| ggt | G | 0 | 0.49 |
| ggc | G | 0 | 0.54 |
| gga | G | 4 | 1.93 |
| ggg | G | 0 | 1.03 |
| --- | -- | ------ | ------ |
| mPD | | 0.20 | 0.51 |
| | | nPD: | 0.4 |
| | | N. weight: | 0.41 |
| | | Sc. PD: | 0.054 |
| | | Sc. rank: | 41.1 |

,

| PB1 | | | |
|---|---|---|---|
| Pos. | 181 | obs: | exp: |
| tta | L | 2 | 0.17 |
| ttg | L | 0 | 0.39 |
| ctt | L | 0 | 0.37 |
| ctc | L | 0 | 0.32 |
| cta | L | 0 | 0.30 |
| ctg | L | 0 | 0.44 |
| att | I | 26 | 137.20 |
| atc | I | 0 | 95.66 |
| ata | I | 339 | 132.10 |
| --- | -- | ------ | ------ |
| mPD | | 0.14 | 0.67 |
| | | nPD: | 0.21 |
| | | N. weight: | 1.2 |
| | | Sc. PD: | -0.049 |
| | | Sc. rank: | 26.0 |

| PB1 | | | |
|---|---|---|---|
| Pos. | 182 | obs: | exp: |
| att | I | 0 | 2.63 |
| atc | I | 0 | 1.83 |
| ata | I | 7 | 2.53 |
| act | T | 0 | 95.11 |
| acc | T | 0 | 77.25 |
| aca | T | 353 | 157.70 |
| acg | T | 7 | 29.93 |
| --- | -- | ------ | ------ |
| mPD | | 0.075 | 0.72 |
| | | nPD: | 0.1 |
| | | N. weight: | 1.2 |
| | | Sc. PD: | -0.17 |
| | | Sc. rank: | -63.8 |

| PB1 | | | |
|---|---|---|---|
| Pos. | 183 | obs: | exp: |
| act | T | 0 | 96.96 |
| acc | T | 0 | 78.75 |
| aca | T | 279 | 160.80 |
| acg | T | 88 | 30.51 |
| --- | -- | ------ | ------ |
| mPD | | 0.37 | 0.69 |
| | | nPD: | 0.53 |
| | | N. weight: | 1. |
| | | Sc. PD: | 0.27 |
| | | Sc. rank: | 146.0 |

| PB1 | | | |
|---|---|---|---|
| Pos. | 184 | obs: | exp: |
| cat | H | 349 | 198.90 |
| cac | H | 18 | 168.10 |
| --- | -- | ------ | ------ |
| mPD | | 0.094 | 0.50 |
| | | nPD: | 0.19 |
| | | N. weight: | 0.66 |
| | | Sc. PD: | -0.042 |
| | | Sc. rank: | 2.5 |

| PB1 | | | |
|---|---|---|---|
| Pos. | 185 | obs: | exp: |
| ttt | F | 1 | 150.90 |
| ttc | F | 366 | 216.10 |
| --- | -- | ------ | ------ |
| mPD | | 0.0054 | 0.48 |
| | | nPD: | 0.01 |
| | | N. weight: | 0.78 |
| | | Sc. PD: | -0.18 |
| | | Sc. rank: | -133.3 |

| PB1 | | | |
|---|---|---|---|
| Pos. | 186 | obs: | exp: |
| caa | Q | 104 | 195.60 |
| cag | Q | 263 | 171.40 |
| --- | -- | ------ | ------ |
| mPD | | 0.41 | 0.50 |
| | | nPD: | 0.82 |
| | | N. weight: | 0.21 |
| | | Sc. PD: | 0.11 |
| | | Sc. rank: | 42.1 |

| PB1 | | | |
|---|---|---|---|
| Pos. | 187 | obs: | exp: |
| cgt | R | 0 | 15.24 |
| cgc | R | 0 | 17.97 |
| cga | R | 2 | 35.27 |
| cgg | R | 0 | 30.63 |
| aga | R | 233 | 165.00 |
| agg | R | 132 | 102.80 |
| --- | -- | ------ | ------ |
| mPD | | 0.47 | 0.96 |
| | | nPD: | 0.49 |
| | | N. weight: | 0.46 |
| | | Sc. PD: | 0.1 |
| | | Sc. rank: | 59.3 |

| PB1 | | | |
|---|---|---|---|
| Pos. | 188 | obs: | exp: |
| aaa | K | 102 | 205.50 |
| aag | K | 265 | 161.50 |
| --- | -- | ------ | ------ |
| mPD | | 0.40 | 0.49 |
| | | nPD: | 0.82 |
| | | N. weight: | 0.26 |
| | | Sc. PD: | 0.14 |
| | | Sc. rank: | 52.8 |

| PB1 | | | |
|---|---|---|---|
| Pos. | 189 | obs: | exp: |
| cgt | R | 0 | 15.24 |
| cgc | R | 0 | 17.97 |
| cga | R | 0 | 35.27 |
| cgg | R | 0 | 30.63 |
| aga | R | 330 | 165.00 |
| agg | R | 37 | 102.80 |
| --- | -- | ------ | ------ |
| mPD | | 0.18 | 0.96 |
| | | nPD: | 0.19 |
| | | N. weight: | 0.8 |
| | | Sc. PD: | -0.051 |
| | | Sc. rank: | 3.4 |

| PB1 | | | |
|---|---|---|---|
| Pos. | 190 | obs: | exp: |
| cgt | R | 0 | 15.24 |
| cgc | R | 0 | 17.97 |
| cga | R | 0 | 35.27 |
| cgg | R | 0 | 30.63 |
| aga | R | 367 | 165.00 |
| agg | R | 0 | 102.80 |
| --- | -- | ------ | ------ |
| mPD | | 0 | 0.96 |
| | | nPD: | 0. |
| | | N. weight: | 1.2 |
| | | Sc. PD: | -0.3 |
| | | Sc. rank: | -252.5 |

| PB1 | | | |
|---|---|---|---|
| Pos. | 191 | obs: | exp: |
| att | I | 0 | 5.64 |
| atc | I | 0 | 3.93 |
| ata | I | 15 | 5.43 |
| atg | M | 1 | 1.00 |
| gtt | V | 0 | 72.14 |
| gtc | V | 0 | 73.67 |
| gta | V | 239 | 65.78 |
| gtg | V | 112 | 139.40 |
| --- | -- | ------ | ------ |
| mPD | | 0.51 | 0.81 |
| | | nPD: | 0.63 |
| | | N. weight: | 1.3 |
| | | Sc. PD: | 0.45 |
| | | Sc. rank: | 205.7 |

| PB1 | | | |
|---|---|---|---|
| Pos. | 192 | obs: | exp: |
| cgt | R | 0 | 15.24 |
| cgc | R | 0 | 17.97 |
| cga | R | 0 | 35.27 |
| cgg | R | 0 | 30.63 |
| aga | R | 114 | 165.00 |
| agg | R | 253 | 102.80 |
| --- | -- | ------ | ------ |
| mPD | | 0.43 | 0.96 |
| | | nPD: | 0.45 |
| | | N. weight: | 0.78 |
| | | Sc. PD: | 0.14 |
| | | Sc. rank: | 93.6 |

| PB1 | | | |
|---|---|---|---|
| Pos. | 193 | obs: | exp: |
| gat | D | 13 | 195.80 |
| gac | D | 354 | 171.20 |
| --- | -- | ------ | ------ |
| mPD | | 0.069 | 0.50 |
| | | nPD: | 0.14 |
| | | N. weight: | 0.93 |
| | | Sc. PD: | -0.1 |
| | | Sc. rank: | -25.1 |

| PB1 | | | |
|---|---|---|---|
| Pos. | 194 | obs: | exp: |
| aat | N | 8 | 191.80 |
| aac | N | 359 | 175.20 |
| --- | -- | ------ | ------ |
| mPD | | 0.043 | 0.50 |
| | | nPD: | 0.09 |
| | | N. weight: | 0.97 |
| | | Sc. PD: | -0.16 |
| | | Sc. rank: | -69.2 |

| PB1 | | | |
|---|---|---|---|
| Pos. | 195 | obs: | exp: |
| atg | M | 367 | 367.00 |
| --- | -- | ------ | ------ |
| mPD | | 0 | 0 |
| | | nPD: | 1. |
| | | N. weight: | 0. |
| | | Sc. PD: | 0 |
| | | Sc. rank: | 0 |

| PB1 | | | |
|---|---|---|---|
| Pos. | 196 | obs: | exp: |
| act | T | 2 | 96.96 |
| acc | T | 360 | 78.75 |
| aca | T | 5 | 160.80 |
| acg | T | 0 | 30.51 |
| --- | -- | ------ | ------ |
| mPD | | 0.038 | 0.69 |
| | | nPD: | 0.05 |
| | | N. weight: | 2.2 |
| | | Sc. PD: | -0.42 |
| | | Sc. rank: | -236.7 |

| PB1 | | | |
|---|---|---|---|
| Pos. | 197 | obs: | exp: |
| cgt | R | 0 | 0.08 |
| cgc | R | 0 | 0.10 |
| cga | R | 0 | 0.19 |
| cgg | R | 0 | 0.17 |
| att | I | 1 | 0.38 |
| atc | I | 0 | 0.26 |
| ata | I | 0 | 0.36 |
| aaa | K | 8 | 203.80 |
| aag | K | 356 | 160.20 |
| aga | R | 0 | 0.90 |
| agg | R | 2 | 0.56 |
| --- | -- | ------ | ------ |
| mPD | | 0.064 | 0.51 |
| | | nPD: | 0.13 |
| | | N. weight: | 1.1 |
| | | Sc. PD: | -0.14 |
| | | Sc. rank: | -36.7 |

| PB1 | | | |
|---|---|---|---|
| Pos. | 198 | obs: | exp: |
| caa | Q | 1 | 0.53 |
| cag | Q | 0 | 0.47 |
| aaa | K | 241 | 204.90 |
| aag | K | 125 | 161.10 |
| --- | -- | ------ | ------ |
| mPD | | 0.46 | 0.50 |
| | | nPD: | 0.91 |
| | | N. weight: | 0.046 |
| | | Sc. PD: | 0.029 |
| | | Sc. rank: | 9.6 |

| PB1 | | | |
|---|---|---|---|
| Pos. | 199 | obs: | exp: |
| tta | L | 0 | 0.17 |
| ttg | L | 0 | 0.39 |
| ctt | L | 0 | 0.37 |
| ctc | L | 0 | 0.32 |
| cta | L | 0 | 0.30 |
| ctg | L | 2 | 0.44 |
| atg | M | 365 | 365.00 |
| --- | -- | ------ | ------ |
| mPD | | 0.011 | 0.017 |
| | | nPD: | 0.63 |
| | | N. weight: | 0.013 |
| | | Sc. PD: | 0.0045 |
| | | Sc. rank: | 2.0 |

| PB1 | | | |
|---|---|---|---|
| Pos. | 200 | obs: | exp: |
| att | I | 0 | 2.63 |
| atc | I | 7 | 1.83 |
| ata | I | 0 | 2.53 |
| gtt | V | 4 | 73.99 |
| gtc | V | 355 | 75.56 |
| gta | V | 0 | 67.47 |
| gtg | V | 1 | 143.00 |
| --- | -- | ------ | ------ |
| mPD | | 0.065 | 0.76 |
| | | nPD: | 0.08 |
| | | N. weight: | 2.3 |
| | | Sc. PD: | -0.37 |
| | | Sc. rank: | -169.3 |

| PB1 | | | |
|---|---|---|---|
| Pos. | 201 | obs: | exp: |
| act | T | 1 | 96.96 |
| acc | T | 7 | 78.75 |
| aca | T | 358 | 160.80 |
| acg | T | 1 | 30.51 |
| --- | -- | ------ | ------ |
| mPD | | 0.048 | 0.69 |
| | | nPD: | 0.07 |
| | | N. weight: | 1.1 |
| | | Sc. PD: | -0.19 |
| | | Sc. rank: | -97.3 |

| PB1 | | | |
|---|---|---|---|
| Pos. | 202 | obs: | exp: |
| caa | Q | 332 | 195.60 |
| cag | Q | 35 | 171.40 |
| --- | -- | ------ | ------ |
| mPD | | 0.17 | 0.50 |
| | | nPD: | 0.35 |
| | | N. weight: | 0.51 |
| | | Sc. PD: | 0.044 |
| | | Sc. rank: | 41.8 |

| PB1 | | | |
|---|---|---|---|
| Pos. | 203 | obs: | exp: |
| cgt | R | 0 | 15.24 |
| cgc | R | 0 | 17.97 |
| cga | R | 15 | 35.27 |
| cgg | R | 0 | 30.63 |
| aga | R | 283 | 165.00 |
| agg | R | 69 | 102.80 |
| --- | -- | ------ | ------ |
| mPD | | 0.38 | 0.96 |
| | | nPD: | 0.4 |
| | | N. weight: | 0.49 |
| | | Sc. PD: | 0.067 |
| | | Sc. rank: | 49.5 |

| PB1 | | | |
|---|---|---|---|
| Pos. | 204 | obs: | exp: |
| act | T | 1 | 96.96 |
| acc | T | 0 | 78.75 |
| aca | T | 361 | 160.80 |
| acg | T | 5 | 30.51 |
| --- | -- | ------ | ------ |
| mPD | | 0.032 | 0.69 |
| | | nPD: | 0.05 |
| | | N. weight: | 1.2 |
| | | Sc. PD: | -0.23 |
| | | Sc. rank: | -142.4 |

| PB1 | | | |
|---|---|---|---|
| Pos. | 205 | obs: | exp: |
| att | I | 0 | 138.00 |
| atc | I | 0 | 96.18 |
| ata | I | 367 | 132.80 |
| --- | -- | ------ | ------ |
| mPD | | 0 | 0.66 |
| | | nPD: | 0. |
| | | N. weight: | 1.5 |
| | | Sc. PD: | -0.38 |
| | | Sc. rank: | -321.0 |

PB1

| Pos. | 206 | obs: | exp: |
|---|---|---|---|
| ggt | G | 5 | 45.28 |
| ggc | G | 0 | 49.63 |
| gga | G | 209 | 177.50 |
| ggg | G | 153 | 94.55 |
| --- | -- | ------ | ------ |
| mPD | | 0.50 | 0.67 |
| | | nPD: | 0.76 |
| | | N. weight: | 0.42 |
| | | Sc. PD: | 0.2 |
| | | Sc. rank: | 82.0 |

PB1

| Pos. | 207 | obs: | exp: |
|---|---|---|---|
| aaa | K | 6 | 205.50 |
| aag | K | 361 | 161.50 |
| --- | -- | ------ | ------ |
| mPD | | 0.032 | 0.49 |
| | | nPD: | 0.07 |
| | | N. weight: | 1.1 |
| | | Sc. PD: | -0.2 |
| | | Sc. rank: | -106.5 |

PB1

| Pos. | 208 | obs: | exp: |
|---|---|---|---|
| aaa | K | 75 | 205.50 |
| aag | K | 292 | 161.50 |
| --- | -- | ------ | ------ |
| mPD | | 0.33 | 0.49 |
| | | nPD: | 0.66 |
| | | N. weight: | 0.42 |
| | | Sc. PD: | 0.16 |
| | | Sc. rank: | 70.8 |

PB1

| Pos. | 209 | obs: | exp: |
|---|---|---|---|
| cgt | R | 0 | 0.04 |
| cgc | R | 0 | 0.05 |
| cga | R | 0 | 0.10 |
| cgg | R | 0 | 0.08 |
| aaa | K | 166 | 204.90 |
| aag | K | 200 | 161.10 |
| aga | R | 0 | 0.45 |
| agg | R | 1 | 0.28 |
| --- | -- | ------ | ------ |
| mPD | | 0.50 | 0.50 |
| | | nPD: | 1. |
| | | N. weight: | 0.053 |
| | | Sc. PD: | 0.038 |
| | | Sc. rank: | 11.1 |

PB1

| Pos. | 210 | obs: | exp: |
|---|---|---|---|
| caa | Q | 117 | 195.60 |
| cag | Q | 250 | 171.40 |
| --- | -- | ------ | ------ |
| mPD | | 0.44 | 0.50 |
| | | nPD: | 0.87 |
| | | N. weight: | 0.16 |
| | | Sc. PD: | 0.091 |
| | | Sc. rank: | 31.9 |

PB1

| Pos. | 211 | obs: | exp: |
|---|---|---|---|
| cgt | R | 0 | 15.15 |
| cgc | R | 0 | 17.87 |
| cga | R | 0 | 35.08 |
| cgg | R | 0 | 30.46 |
| aaa | K | 0 | 1.12 |
| aag | K | 2 | 0.88 |
| aga | R | 54 | 164.10 |
| agg | R | 311 | 102.30 |
| --- | -- | ------ | ------ |
| mPD | | 0.26 | 0.97 |
| | | nPD: | 0.27 |
| | | N. weight: | 1.2 |
| | | Sc. PD: | 0.017 |
| | | Sc. rank: | 68.7 |

PB1

| Pos. | 212 | obs: | exp: |
|---|---|---|---|
| tta | L | 10 | 32.06 |
| ttg | L | 38 | 70.68 |
| ctt | L | 18 | 68.48 |
| ctc | L | 0 | 59.37 |
| cta | L | 95 | 55.29 |
| ctg | L | 206 | 81.13 |
| --- | -- | ------ | ------ |
| mPD | | 0.70 | 1.1 |
| | | nPD: | 0.63 |
| | | N. weight: | 0.81 |
| | | Sc. PD: | 0.29 |
| | | Sc. rank: | 131.6 |

PB1

| Pos. | 213 | obs: | exp: |
|---|---|---|---|
| tct | S | 0 | 1.27 |
| tcc | S | 0 | 1.11 |
| tca | S | 0 | 1.77 |
| tcg | S | 0 | 0.49 |
| act | T | 1 | 0.26 |
| acc | T | 0 | 0.21 |
| aca | T | 0 | 0.44 |
| acg | T | 0 | 0.08 |
| aat | N | 107 | 185.00 |
| aac | N | 247 | 169.00 |
| aaa | K | 2 | 1.12 |
| aag | K | 0 | 0.88 |
| agt | S | 0 | 1.76 |
| agc | S | 8 | 1.59 |
| gat | D | 0 | 1.07 |
| gac | D | 2 | 0.93 |
| --- | -- | ------ | ------ |
| mPD | | 0.48 | 0.60 |
| | | nPD: | 0.81 |
| | | N. weight: | 0.23 |
| | | Sc. PD: | 0.12 |
| | | Sc. rank: | 46.1 |

PB1

| Pos. | 214 | obs: | exp: |
|---|---|---|---|
| cgt | R | 0 | 0.25 |
| cgc | R | 0 | 0.29 |
| cga | R | 0 | 0.58 |
| cgg | R | 0 | 0.50 |
| aaa | K | 175 | 202.10 |
| aag | K | 186 | 158.90 |
| aga | R | 0 | 2.70 |
| agg | R | 6 | 1.68 |
| --- | -- | ------ | ------ |
| mPD | | 0.53 | 0.54 |
| | | nPD: | 1. |
| | | N. weight: | 0.062 |
| | | Sc. PD: | 0.043 |
| | | Sc. rank: | 12.9 |

PB1

| Pos. | 215 | obs: | exp: |
|---|---|---|---|
| cgt | R | 0 | 12.70 |
| cgc | R | 0 | 14.98 |
| cga | R | 0 | 29.41 |
| cgg | R | 0 | 25.54 |
| aaa | K | 2 | 34.16 |
| aag | K | 59 | 26.84 |
| aga | R | 40 | 137.60 |
| agg | R | 266 | 85.75 |
| --- | -- | ------ | ------ |
| mPD | | 0.48 | 1.2 |
| | | nPD: | 0.41 |
| | | N. weight: | 1.2 |
| | | Sc. PD: | 0.18 |
| | | Sc. rank: | 131.5 |

PB1

| Pos. | 216 | obs: | exp: |
|---|---|---|---|
| tct | S | 0 | 57.07 |
| tcc | S | 0 | 50.01 |
| tca | S | 0 | 79.53 |
| tcg | S | 0 | 21.88 |
| aat | N | 0 | 3.14 |
| aac | N | 6 | 2.86 |
| agt | S | 38 | 78.98 |
| agc | S | 321 | 71.52 |
| ggt | G | 0 | 0.25 |
| ggc | G | 2 | 0.27 |
| gga | G | 0 | 0.97 |
| ggg | G | 0 | 0.52 |
| --- | -- | ------ | ------ |
| mPD | | 0.23 | 1.7 |
| | | nPD: | 0.14 |
| | | N. weight: | 1.9 |
| | | Sc. PD: | -0.22 |
| | | Sc. rank: | -57.4 |

PB1

| Pos. | 217 | obs: | exp: |
|---|---|---|---|
| tat | Y | 105 | 190.00 |
| tac | Y | 262 | 177.00 |
| --- | -- | ------ | ------ |
| mPD | | 0.41 | 0.50 |
| | | nPD: | 0.82 |
| | | N. weight: | 0.18 |
| | | Sc. PD: | 0.097 |
| | | Sc. rank: | 36.8 |

PB1

| Pos. | 218 | obs: | exp: |
|---|---|---|---|
| tta | L | 233 | 32.06 |
| ttg | L | 14 | 70.68 |
| ctt | L | 0 | 68.48 |
| ctc | L | 1 | 59.37 |
| cta | L | 113 | 55.29 |
| ctg | L | 6 | 81.13 |
| --- | -- | ------ | ------ |
| mPD | | 0.55 | 1.1 |
| | | nPD: | 0.49 |
| | | N. weight: | 2.1 |
| | | Sc. PD: | 0.47 |
| | | Sc. rank: | 271.8 |

PB1

| Pos. | 219 | obs: | exp: |
|---|---|---|---|
| att | I | 2 | 138.00 |
| atc | I | 0 | 96.18 |
| ata | I | 365 | 132.80 |
| --- | -- | ------ | ------ |
| mPD | | 0.011 | 0.66 |
| | | nPD: | 0.02 |
| | | N. weight: | 1.5 |
| | | Sc. PD: | -0.34 |
| | | Sc. rank: | -241.9 |

PB1

| Pos. | 220 | obs: | exp: |
|---|---|---|---|
| cgt | R | 0 | 15.24 |
| cgc | R | 0 | 17.97 |
| cga | R | 0 | 35.27 |
| cgg | R | 0 | 30.63 |
| aga | R | 334 | 165.00 |
| agg | R | 33 | 102.80 |
| --- | -- | ------ | ------ |
| mPD | | 0.16 | 0.96 |
| | | nPD: | 0.17 |
| | | N. weight: | 0.83 |
| | | Sc. PD: | -0.068 |
| | | Sc. rank: | -3.8 |

PB1

| Pos. | 221 | obs: | exp: |
|---|---|---|---|
| gct | A | 24 | 92.44 |
| gcc | A | 1 | 75.18 |
| gca | A | 319 | 163.60 |
| gcg | A | 23 | 35.81 |
| --- | -- | ------ | ------ |
| mPD | | 0.24 | 0.69 |
| | | nPD: | 0.35 |
| | | N. weight: | 0.71 |
| | | Sc. PD: | 0.06 |
| | | Sc. rank: | 57.8 |

PB1

| Pos. | 222 | obs: | exp: |
|---|---|---|---|
| tta | L | 7 | 31.97 |
| ttg | L | 127 | 70.49 |
| ctt | L | 8 | 68.29 |
| ctc | L | 0 | 59.20 |
| cta | L | 19 | 55.14 |
| ctg | L | 205 | 80.90 |
| att | I | 0 | 0.38 |
| atc | I | 0 | 0.26 |
| ata | I | 1 | 0.36 |
| --- | -- | ------ | ------ |
| mPD | | 0.64 | 1.1 |
| | | nPD: | 0.58 |
| | | N. weight: | 0.94 |
| | | Sc. PD: | 0.29 |
| | | Sc. rank: | 143.0 |

PB1

| Pos. | 223 | obs: | exp: |
|---|---|---|---|
| act | T | 0 | 96.96 |
| acc | T | 0 | 78.75 |
| aca | T | 365 | 160.80 |
| acg | T | 2 | 30.51 |
| --- | -- | ------ | ------ |
| mPD | | 0.011 | 0.69 |
| | | nPD: | 0.02 |
| | | N. weight: | 1.2 |
| | | Sc. PD: | -0.28 |
| | | Sc. rank: | -204.8 |

PB1

| Pos. | 224 | obs: | exp: |
|---|---|---|---|
| tta | L | 4 | 32.06 |
| ttg | L | 131 | 70.68 |
| ctt | L | 1 | 68.48 |
| ctc | L | 0 | 59.37 |
| cta | L | 30 | 55.29 |
| ctg | L | 201 | 81.13 |
| --- | -- | ------ | ------ |
| mPD | | 0.64 | 1.1 |
| | | nPD: | 0.57 |
| | | N. weight: | 1. |
| | | Sc. PD: | 0.3 |
| | | Sc. rank: | 150.4 |

PB1

| Pos. | 225 | obs: | exp: |
|---|---|---|---|
| aat | N | 26 | 191.80 |
| aac | N | 341 | 175.20 |
| --- | -- | ------ | ------ |
| mPD | | 0.13 | 0.50 |
| | | nPD: | 0.26 |
| | | N. weight: | 0.74 |
| | | Sc. PD: | 0.0057 |
| | | Sc. rank: | 37.4 |

PB1

| Pos. | 226 | obs: | exp: |
|---|---|---|---|
| act | T | 4 | 96.96 |
| acc | T | 2 | 78.75 |
| aca | T | 358 | 160.80 |
| acg | T | 3 | 30.51 |
| --- | -- | ------ | ------ |
| mPD | | 0.048 | 0.69 |
| | | nPD: | 0.07 |
| | | N. weight: | 1.1 |
| | | Sc. PD: | -0.19 |
| | | Sc. rank: | -95.8 |

PB1

| Pos. | 227 | obs: | exp: |
|---|---|---|---|
| atg | M | 367 | 367.00 |
| --- | -- | ------ | ------ |
| mPD | | 0 | 0 |
| | | nPD: | 1. |
| | | N. weight: | 0. |
| | | Sc. PD: | 0 |
| | | Sc. rank: | 0 |

PB1

| Pos. | 228 | obs: | exp: |
|---|---|---|---|
| act | T | 5 | 96.69 |
| acc | T | 92 | 78.54 |
| aca | T | 267 | 160.30 |
| acg | T | 2 | 30.43 |
| gct | A | 0 | 0.25 |
| gcc | A | 1 | 0.20 |
| gca | A | 0 | 0.45 |
| gcg | A | 0 | 0.10 |
| --- | -- | ------ | ------ |
| mPD | | 0.41 | 0.69 |
| | | nPD: | 0.6 |
| | | N. weight: | 0.57 |
| | | Sc. PD: | 0.19 |
| | | Sc. rank: | 89.1 |

PB1

| Pos. | 229 | obs: | exp: |
|---|---|---|---|
| aaa | K | 318 | 205.50 |
| aag | K | 49 | 161.50 |
| --- | -- | ------ | ------ |
| mPD | | 0.23 | 0.49 |
| | | nPD: | 0.47 |
| | | N. weight: | 0.35 |
| | | Sc. PD: | 0.07 |
| | | Sc. rank: | 43.1 |

PB1

| Pos. | 230 | obs: | exp: |
|---|---|---|---|
| gat | D | 250 | 195.80 |
| gac | D | 117 | 171.20 |
| --- | -- | ------ | ------ |
| mPD | | 0.44 | 0.50 |
| | | nPD: | 0.87 |
| | | N. weight: | 0.082 |
| | | Sc. PD: | 0.048 |
| | | Sc. rank: | 16.8 |

PB1

| Pos. | 231 | obs : | exp : |
| --- | --- | --- | --- |
| gct | A | 43 | 92.44 |
| gcc | A | 76 | 75.18 |
| gca | A | 246 | 163.60 |
| gcg | A | 2 | 35.81 |
| --- | -- | ------ | ------ |
| mPD | | 0.50 | 0.69 |
| | | nPD : | 0.72 |
| | | N. weight : | 0.29 |
| | | Sc. PD : | 0.13 |
| | | Sc. rank : | 53.0 |

PB1

| Pos. | 232 | obs : | exp : |
| --- | --- | --- | --- |
| gaa | E | 365 | 211.20 |
| gag | E | 2 | 155.80 |
| --- | -- | ------ | ------ |
| mPD | | 0.011 | 0.49 |
| | | nPD : | 0.02 |
| | | N. weight : | 0.8 |
| | | Sc. PD : | -0.18 |
| | | Sc. rank : | -124.3 |

PB1

| Pos. | 233 | obs : | exp : |
| --- | --- | --- | --- |
| cgt | R | 0 | 15.24 |
| cgc | R | 0 | 17.97 |
| cga | R | 3 | 35.27 |
| cgg | R | 0 | 30.63 |
| aga | R | 358 | 165.00 |
| agg | R | 6 | 102.80 |
| --- | -- | ------ | ------ |
| mPD | | 0.049 | 0.96 |
| | | nPD : | 0.05 |
| | | N. weight : | 1.1 |
| | | Sc. PD : | -0.21 |
| | | Sc. rank : | -122.9 |

PB1

| Pos. | 234 | obs : | exp : |
| --- | --- | --- | --- |
| ggt | G | 29 | 45.28 |
| ggc | G | 336 | 49.63 |
| gga | G | 2 | 177.50 |
| ggg | G | 0 | 94.55 |
| --- | -- | ------ | ------ |
| mPD | | 0.16 | 0.67 |
| | | nPD : | 0.23 |
| | | N. weight : | 2.6 |
| | | Sc. PD : | -0.055 |
| | | Sc. rank : | 95.4 |

PB1

| Pos. | 235 | obs : | exp : |
| --- | --- | --- | --- |
| aaa | K | 327 | 205.50 |
| aag | K | 40 | 161.50 |
| --- | -- | ------ | ------ |
| mPD | | 0.19 | 0.49 |
| | | nPD : | 0.4 |
| | | N. weight : | 0.41 |
| | | Sc. PD : | 0.054 |
| | | Sc. rank : | 41.0 |

PB1

| Pos. | 236 | obs : | exp : |
| --- | --- | --- | --- |
| tta | L | 80 | 32.06 |
| ttg | L | 272 | 70.68 |
| ctt | L | 2 | 68.48 |
| ctc | L | 0 | 59.37 |
| cta | L | 4 | 55.29 |
| ctg | L | 9 | 81.13 |
| --- | -- | ------ | ------ |
| mPD | | 0.44 | 1.1 |
| | | nPD : | 0.4 |
| | | N. weight : | 1.7 |
| | | Sc. PD : | 0.23 |
| | | Sc. rank : | 171.7 |

PB1

| Pos. | 237 | obs : | exp : |
| --- | --- | --- | --- |
| cgt | R | 0 | 0.08 |
| cgc | R | 0 | 0.10 |
| cga | R | 0 | 0.19 |
| cgg | R | 0 | 0.17 |
| aaa | K | 86 | 204.40 |
| aag | K | 279 | 160.60 |
| aga | R | 0 | 0.90 |
| agg | R | 2 | 0.56 |
| --- | -- | ------ | ------ |
| mPD | | 0.37 | 0.51 |
| | | nPD : | 0.73 |
| | | N. weight : | 0.35 |
| | | Sc. PD : | 0.16 |
| | | Sc. rank : | 66.4 |

PB1

| Pos. | 238 | obs : | exp : |
| --- | --- | --- | --- |
| cgt | R | 0 | 15.24 |
| cgc | R | 0 | 17.97 |
| cga | R | 0 | 35.27 |
| cgg | R | 1 | 30.63 |
| aga | R | 148 | 165.00 |
| agg | R | 218 | 102.80 |
| --- | -- | ------ | ------ |
| mPD | | 0.49 | 0.96 |
| | | nPD : | 0.51 |
| | | N. weight : | 0.62 |
| | | Sc. PD : | 0.15 |
| | | Sc. rank : | 79.7 |

PB1

| Pos. | 239 | obs : | exp : |
| --- | --- | --- | --- |
| cgt | R | 1 | 15.24 |
| cgc | R | 0 | 17.97 |
| cga | R | 9 | 35.27 |
| cgg | R | 237 | 30.63 |
| aga | R | 45 | 165.00 |
| agg | R | 75 | 102.80 |
| --- | -- | ------ | ------ |
| mPD | | 0.70 | 0.96 |
| | | nPD : | 0.73 |
| | | N. weight : | 1.6 |
| | | Sc. PD : | 0.73 |
| | | Sc. rank : | 307.7 |

PB1

| Pos. | 240 | obs : | exp : |
| --- | --- | --- | --- |
| gct | A | 0 | 92.44 |
| gcc | A | 0 | 75.18 |
| gca | A | 333 | 163.60 |
| gcg | A | 34 | 35.81 |
| --- | -- | ------ | ------ |
| mPD | | 0.17 | 0.69 |
| | | nPD : | 0.25 |
| | | N. weight : | 0.99 |
| | | Sc. PD : | -0.01 |
| | | Sc. rank : | 39.0 |

PB1

| Pos. | 241 | obs : | exp : |
| --- | --- | --- | --- |
| att | I | 340 | 138.00 |
| atc | I | 27 | 96.18 |
| ata | I | 0 | 132.80 |
| --- | -- | ------ | ------ |
| mPD | | 0.14 | 0.66 |
| | | nPD : | 0.21 |
| | | N. weight : | 1.1 |
| | | Sc. PD : | -0.053 |
| | | Sc. rank : | 21.0 |

PB1

| Pos. | 242 | obs : | exp : |
| --- | --- | --- | --- |
| gct | A | 1 | 92.44 |
| gcc | A | 0 | 75.18 |
| gca | A | 323 | 163.60 |
| gcg | A | 43 | 35.81 |
| --- | -- | ------ | ------ |
| mPD | | 0.21 | 0.69 |
| | | nPD : | 0.31 |
| | | N. weight : | 0.94 |
| | | Sc. PD : | 0.047 |
| | | Sc. rank : | 67.9 |

PB1

| Pos. | 243 | obs : | exp : |
| --- | --- | --- | --- |
| act | T | 13 | 96.96 |
| acc | T | 1 | 78.75 |
| aca | T | 330 | 160.80 |
| acg | T | 23 | 30.51 |
| --- | -- | ------ | ------ |
| mPD | | 0.19 | 0.69 |
| | | nPD : | 0.27 |
| | | N. weight : | 0.85 |
| | | Sc. PD : | 0.013 |
| | | Sc. rank : | 50.0 |

PB1

| Pos. | 244 | obs : | exp : |
| --- | --- | --- | --- |
| cct | P | 8 | 96.64 |
| ccc | P | 309 | 66.20 |
| cca | P | 50 | 144.80 |
| ccg | P | 0 | 59.34 |
| --- | -- | ------ | ------ |
| mPD | | 0.27 | 0.72 |
| | | nPD : | 0.38 |
| | | N. weight : | 1.7 |
| | | Sc. PD : | 0.2 |
| | | Sc. rank : | 156.8 |

PB1

| Pos. | 245 | obs : | exp : |
| --- | --- | --- | --- |
| ggt | G | 0 | 45.28 |
| ggc | G | 0 | 49.63 |
| gga | G | 156 | 177.50 |
| ggg | G | 211 | 94.55 |
| --- | -- | ------ | ------ |
| mPD | | 0.49 | 0.67 |
| | | nPD : | 0.74 |
| | | N. weight : | 0.63 |
| | | Sc. PD : | 0.29 |
| | | Sc. rank : | 118.9 |

PB1

| Pos. | 246 | obs : | exp : |
| --- | --- | --- | --- |
| atg | M | 367 | 367.00 |
| --- | -- | ------ | ------ |
| mPD | | 0 | 0 |
| | | nPD : | 1. |
| | | N. weight : | 0. |
| | | Sc. PD : | 0 |
| | | Sc. rank : | 0 |

PB1

| Pos. | 247 | obs : | exp : |
| --- | --- | --- | --- |
| caa | Q | 154 | 195.60 |
| cag | Q | 213 | 171.40 |
| --- | -- | ------ | ------ |
| mPD | | 0.49 | 0.50 |
| | | nPD : | 0.98 |
| | | N. weight : | 0.052 |
| | | Sc. PD : | 0.036 |
| | | Sc. rank : | 10.9 |

PB1

| Pos. | 248 | obs : | exp : |
| --- | --- | --- | --- |
| att | I | 195 | 138.00 |
| atc | I | 169 | 96.18 |
| ata | I | 3 | 132.80 |
| --- | -- | ------ | ------ |
| mPD | | 0.51 | 0.66 |
| | | nPD : | 0.77 |
| | | N. weight : | 0.65 |
| | | Sc. PD : | 0.32 |
| | | Sc. rank : | 126.6 |

PB1

| Pos. | 249 | obs : | exp : |
| --- | --- | --- | --- |
| cgt | R | 0 | 15.24 |
| cgc | R | 0 | 17.97 |
| cga | R | 0 | 35.27 |
| cgg | R | 0 | 30.63 |
| aga | R | 356 | 165.00 |
| agg | R | 11 | 102.80 |
| --- | -- | ------ | ------ |
| mPD | | 0.058 | 0.96 |
| | | nPD : | 0.06 |
| | | N. weight : | 1. |
| | | Sc. PD : | -0.19 |
| | | Sc. rank : | -106.7 |

PB1

| Pos. | 250 | obs : | exp : |
| --- | --- | --- | --- |
| ggt | G | 1 | 45.28 |
| ggc | G | 0 | 49.63 |
| gga | G | 259 | 177.50 |
| ggg | G | 107 | 94.55 |
| --- | -- | ------ | ------ |
| mPD | | 0.42 | 0.67 |
| | | nPD : | 0.63 |
| | | N. weight : | 0.46 |
| | | Sc. PD : | 0.16 |
| | | Sc. rank : | 75.2 |

PB1

| Pos. | 251 | obs : | exp : |
| --- | --- | --- | --- |
| ttt | F | 153 | 150.90 |
| ttc | F | 214 | 216.10 |
| --- | -- | ------ | ------ |
| mPD | | 0.49 | 0.48 |
| | | nPD : | 1.01 |
| | | N. weight : | 0.013 |
| | | Sc. PD : | 0.0094 |
| | | Sc. rank : | 2.8 |

PB1

| Pos. | 252 | obs : | exp : |
| --- | --- | --- | --- |
| gtt | V | 0 | 75.43 |
| gtc | V | 22 | 77.03 |
| gta | V | 60 | 68.78 |
| gtg | V | 285 | 145.80 |
| --- | -- | ------ | ------ |
| mPD | | 0.37 | 0.72 |
| | | nPD : | 0.51 |
| | | N. weight : | 0.67 |
| | | Sc. PD : | 0.16 |
| | | Sc. rank : | 86.5 |

PB1

| Pos. | 253 | obs : | exp : |
| --- | --- | --- | --- |
| tat | Y | 49 | 190.00 |
| tac | Y | 318 | 177.00 |
| --- | -- | ------ | ------ |
| mPD | | 0.23 | 0.50 |
| | | nPD : | 0.46 |
| | | N. weight : | 0.51 |
| | | Sc. PD : | 0.1 |
| | | Sc. rank : | 63.0 |

PB1

| Pos. | 254 | obs : | exp : |
| --- | --- | --- | --- |
| ttt | F | 360 | 150.10 |
| ttc | F | 5 | 214.90 |
| tgt | C | 2 | 0.82 |
| tgc | C | 0 | 1.18 |
| --- | -- | ------ | ------ |
| mPD | | 0.038 | 0.50 |
| | | nPD : | 0.08 |
| | | N. weight : | 1.2 |
| | | Sc. PD : | -0.21 |
| | | Sc. rank : | -103.2 |

PB1

| Pos. | 255 | obs : | exp : |
| --- | --- | --- | --- |
| gtt | V | 135 | 75.43 |
| gtc | V | 200 | 77.03 |
| gta | V | 32 | 68.78 |
| gtg | V | 0 | 145.80 |
| --- | -- | ------ | ------ |
| mPD | | 0.56 | 0.72 |
| | | nPD : | 0.78 |
| | | N. weight : | 1. |
| | | Sc. PD : | 0.52 |
| | | Sc. rank : | 205.2 |

PB1

| Pos. | 256 | obs : | exp : |
| --- | --- | --- | --- |
| gaa | E | 358 | 211.20 |
| gag | E | 9 | 155.80 |
| --- | -- | ------ | ------ |
| mPD | | 0.048 | 0.49 |
| | | nPD : | 0.1 |
| | | N. weight : | 0.69 |
| | | Sc. PD : | -0.1 |
| | | Sc. rank : | -39.4 |

PB1

| Pos. | 257 | obs : | exp : |
| --- | --- | --- | --- |
| act | T | 0 | 90.09 |
| acc | T | 0 | 73.17 |
| aca | T | 337 | 149.40 |
| acg | T | 4 | 28.35 |
| gct | A | 0 | 6.55 |
| gcc | A | 0 | 5.33 |
| gca | A | 26 | 11.59 |
| gcg | A | 0 | 2.54 |
| --- | -- | ------ | ------ |
| mPD | | 0.15 | 0.82 |
| | | nPD : | 0.19 |
| | | N. weight : | 1.2 |
| | | Sc. PD : | -0.078 |
| | | Sc. rank : | 4.1 |

PB1

| Pos. | 258 | obs : | exp : |
| --- | --- | --- | --- |
| tta | L | 114 | 32.06 |
| ttg | L | 14 | 70.68 |
| ctt | L | 0 | 68.48 |
| ctc | L | 0 | 59.37 |
| cta | L | 141 | 55.29 |
| ctg | L | 98 | 81.13 |
| --- | -- | ------ | ------ |
| mPD | | 0.88 | 1.1 |
| | | nPD : | 0.79 |
| | | N. weight : | 1.2 |
| | | Sc. PD : | 0.59 |
| | | Sc. rank : | 230.7 |

PB1

| Pos. | 259 | obs : | exp : |
| --- | --- | --- | --- |
| gct | A | 1 | 92.44 |
| gcc | A | 0 | 75.18 |
| gca | A | 124 | 163.60 |
| gcg | A | 242 | 35.81 |
| --- | -- | ------ | ------ |
| mPD | | 0.45 | 0.69 |
| | | nPD : | 0.66 |
| | | N. weight : | 1.8 |
| | | Sc. PD : | 0.68 |
| | | Sc. rank : | 300.4 |

PB1

| Pos. | 260 | obs : | exp : |
| --- | --- | --- | --- |
| cgt | R | 0 | 15.24 |
| cgc | R | 0 | 17.97 |
| cga | R | 0 | 35.27 |
| cgg | R | 0 | 30.63 |
| aga | R | 41 | 165.00 |
| agg | R | 326 | 102.80 |
| --- | -- | ------ | ------ |
| mPD | | 0.20 | 0.96 |
| | | nPD : | 0.21 |
| | | N. weight : | 1.3 |
| | | Sc. PD : | -0.062 |
| | | Sc. rank : | 23.2 |

| PB1 | | | |
|---|---|---|---|
| Pos. | 261 | obs: | exp: |
| tct | S | 0 | 58.34 |
| tcc | S | 0 | 51.12 |
| tca | S | 0 | 81.31 |
| tcg | S | 0 | 22.37 |
| agt | S | 2 | 80.74 |
| agc | S | 365 | 73.12 |
| --- | -- | ------ | ------ |
| mPD | | 0.011 | 1.7 |
| | | nPD: | 0.01 |
| | | N. weight: | 2.4 |
| | | Sc. PD: | -0.57 |
| | | Sc. rank: | -436.5 |

| PB1 | | | |
|---|---|---|---|
| Pos. | 262 | obs: | exp: |
| att | I | 94 | 137.60 |
| atc | I | 271 | 95.92 |
| ata | I | 1 | 132.50 |
| gtt | V | 1 | 0.21 |
| gtc | V | 0 | 0.21 |
| gta | V | 0 | 0.19 |
| gtg | V | 0 | 0.40 |
| --- | -- | ------ | ------ |
| mPD | | 0.39 | 0.67 |
| | | nPD: | 0.59 |
| | | N. weight: | 1. |
| | | Sc. PD: | 0.33 |
| | | Sc. rank: | 158.0 |

| PB1 | | | |
|---|---|---|---|
| Pos. | 263 | obs: | exp: |
| tgt | C | 356 | 151.30 |
| tgc | C | 11 | 215.70 |
| --- | -- | ------ | ------ |
| mPD | | 0.058 | 0.48 |
| | | nPD: | 0.12 |
| | | N. weight: | 1.1 |
| | | Sc. PD: | -0.15 |
| | | Sc. rank: | -42.8 |

| PB1 | | | |
|---|---|---|---|
| Pos. | 264 | obs: | exp: |
| gaa | E | 32 | 210.60 |
| gag | E | 334 | 155.40 |
| ggt | G | 0 | 0.12 |
| ggc | G | 0 | 0.14 |
| gga | G | 0 | 0.48 |
| ggg | G | 1 | 0.26 |
| --- | -- | ------ | ------ |
| mPD | | 0.17 | 0.49 |
| | | nPD: | 0.33 |
| | | N. weight: | 0.83 |
| | | Sc. PD: | 0.061 |
| | | Sc. rank: | 61.9 |

| PB1 | | | |
|---|---|---|---|
| Pos. | 265 | obs: | exp: |
| aaa | K | 264 | 205.50 |
| aag | K | 103 | 161.50 |
| --- | -- | ------ | ------ |
| mPD | | 0.40 | 0.49 |
| | | nPD: | 0.82 |
| | | N. weight: | 0.095 |
| | | Sc. PD: | 0.051 |
| | | Sc. rank: | 19.3 |

| PB1 | | | |
|---|---|---|---|
| Pos. | 266 | obs: | exp: |
| tta | L | 0 | 32.06 |
| ttg | L | 0 | 70.68 |
| ctt | L | 284 | 68.48 |
| ctc | L | 81 | 59.37 |
| cta | L | 1 | 55.29 |
| ctg | L | 1 | 81.13 |
| --- | -- | ------ | ------ |
| mPD | | 0.35 | 1.1 |
| | | nPD: | 0.32 |
| | | N. weight: | 1.8 |
| | | Sc. PD: | 0.1 |
| | | Sc. rank: | 130.3 |

| PB1 | | | |
|---|---|---|---|
| Pos. | 267 | obs: | exp: |
| gaa | E | 119 | 211.20 |
| gag | E | 248 | 155.80 |
| --- | -- | ------ | ------ |
| mPD | | 0.44 | 0.49 |
| | | nPD: | 0.9 |
| | | N. weight: | 0.21 |
| | | Sc. PD: | 0.13 |
| | | Sc. rank: | 42.9 |

| PB1 | | | |
|---|---|---|---|
| Pos. | 268 | obs: | exp: |
| caa | Q | 308 | 195.60 |
| cag | Q | 59 | 171.40 |
| --- | -- | ------ | ------ |
| mPD | | 0.27 | 0.50 |
| | | nPD: | 0.54 |
| | | N. weight: | 0.33 |
| | | Sc. PD: | 0.09 |
| | | Sc. rank: | 46.9 |

| PB1 | | | |
|---|---|---|---|
| Pos. | 269 | obs: | exp: |
| tct | S | 365 | 58.34 |
| tcc | S | 1 | 51.12 |
| tca | S | 0 | 81.31 |
| tcg | S | 1 | 22.37 |
| agt | S | 0 | 80.74 |
| agc | S | 0 | 73.12 |
| --- | -- | ------ | ------ |
| mPD | | 0.011 | 1.7 |
| | | nPD: | 0.01 |
| | | N. weight: | 2.8 |
| | | Sc. PD: | -0.65 |
| | | Sc. rank: | -495.0 |

| PB1 | | | |
|---|---|---|---|
| Pos. | 270 | obs: | exp: |
| ggt | G | 3 | 45.28 |
| ggc | G | 26 | 49.63 |
| gga | G | 290 | 177.50 |
| ggg | G | 48 | 94.55 |
| --- | -- | ------ | ------ |
| mPD | | 0.35 | 0.67 |
| | | nPD: | 0.53 |
| | | N. weight: | 0.38 |
| | | Sc. PD: | 0.099 |
| | | Sc. rank: | 53.3 |

| PB1 | | | |
|---|---|---|---|
| Pos. | 271 | obs: | exp: |
| tta | L | 0 | 32.06 |
| ttg | L | 0 | 70.68 |
| ctt | L | 10 | 68.48 |
| ctc | L | 353 | 59.37 |
| cta | L | 4 | 55.29 |
| ctg | L | 0 | 81.13 |
| --- | -- | ------ | ------ |
| mPD | | 0.074 | 1.1 |
| | | nPD: | 0.07 |
| | | N. weight: | 2.5 |
| | | Sc. PD: | -0.45 |
| | | Sc. rank: | -229.3 |

| PB1 | | | |
|---|---|---|---|
| Pos. | 272 | obs: | exp: |
| cct | P | 176 | 96.64 |
| ccc | P | 94 | 66.20 |
| cca | P | 90 | 144.80 |
| ccg | P | 7 | 59.34 |
| --- | -- | ------ | ------ |
| mPD | | 0.65 | 0.72 |
| | | nPD: | 0.9 |
| | | N. weight: | 0.37 |
| | | Sc. PD: | 0.23 |
| | | Sc. rank: | 76.1 |

| PB1 | | | |
|---|---|---|---|
| Pos. | 273 | obs: | exp: |
| gtt | V | 334 | 75.43 |
| gtc | V | 33 | 77.03 |
| gta | V | 0 | 68.78 |
| gtg | V | 0 | 145.80 |
| --- | -- | ------ | ------ |
| mPD | | 0.16 | 0.72 |
| | | nPD: | 0.23 |
| | | N. weight: | 2. |
| | | Sc. PD: | -0.054 |
| | | Sc. rank: | 59.5 |

| PB1 | | | |
|---|---|---|---|
| Pos. | 274 | obs: | exp: |
| ggt | G | 0 | 45.28 |
| ggc | G | 0 | 49.63 |
| gga | G | 323 | 177.50 |
| ggg | G | 44 | 94.55 |
| --- | -- | ------ | ------ |
| mPD | | 0.21 | 0.67 |
| | | nPD: | 0.32 |
| | | N. weight: | 0.67 |
| | | Sc. PD: | 0.039 |
| | | Sc. rank: | 50.0 |

| PB1 | | | |
|---|---|---|---|
| Pos. | 275 | obs: | exp: |
| ggt | G | 7 | 45.28 |
| ggc | G | 85 | 49.63 |
| gga | G | 32 | 177.50 |
| ggg | G | 243 | 94.55 |
| --- | -- | ------ | ------ |
| mPD | | 0.50 | 0.67 |
| | | nPD: | 0.75 |
| | | N. weight: | 0.89 |
| | | Sc. PD: | 0.42 |
| | | Sc. rank: | 173.0 |

| PB1 | | | |
|---|---|---|---|
| Pos. | 276 | obs: | exp: |
| aat | N | 355 | 191.80 |
| aac | N | 12 | 175.20 |
| --- | -- | ------ | ------ |
| mPD | | 0.063 | 0.50 |
| | | nPD: | 0.13 |
| | | N. weight: | 0.78 |
| | | Sc. PD: | -0.096 |
| | | Sc. rank: | -25.2 |

| PB1 | | | |
|---|---|---|---|
| Pos. | 277 | obs: | exp: |
| gaa | E | 98 | 211.20 |
| gag | E | 269 | 155.80 |
| --- | -- | ------ | ------ |
| mPD | | 0.39 | 0.49 |
| | | nPD: | 0.8 |
| | | N. weight: | 0.31 |
| | | Sc. PD: | 0.16 |
| | | Sc. rank: | 61.5 |

| PB1 | | | |
|---|---|---|---|
| Pos. | 278 | obs: | exp: |
| aaa | K | 27 | 205.50 |
| aag | K | 340 | 161.50 |
| --- | -- | ------ | ------ |
| mPD | | 0.14 | 0.49 |
| | | nPD: | 0.28 |
| | | N. weight: | 0.83 |
| | | Sc. PD: | 0.017 |
| | | Sc. rank: | 50.6 |

| PB1 | | | |
|---|---|---|---|
| Pos. | 279 | obs: | exp: |
| aaa | K | 75 | 205.50 |
| aag | K | 292 | 161.50 |
| --- | -- | ------ | ------ |
| mPD | | 0.33 | 0.49 |
| | | nPD: | 0.66 |
| | | N. weight: | 0.42 |
| | | Sc. PD: | 0.16 |
| | | Sc. rank: | 70.8 |

| PB1 | | | |
|---|---|---|---|
| Pos. | 280 | obs: | exp: |
| tct | S | 1 | 0.16 |
| tcc | S | 0 | 0.14 |
| tca | S | 0 | 0.22 |
| tcg | S | 0 | 0.06 |
| agt | S | 0 | 0.22 |
| agc | S | 0 | 0.20 |
| gct | A | 337 | 92.19 |
| gcc | A | 28 | 74.97 |
| gca | A | 1 | 163.10 |
| gcg | A | 0 | 35.71 |
| --- | -- | ------ | ------ |
| mPD | | 0.15 | 0.69 |
| | | nPD: | 0.22 |
| | | N. weight: | 1.7 |
| | | Sc. PD: | -0.061 |
| | | Sc. rank: | 41.9 |

| PB1 | | | |
|---|---|---|---|
| Pos. | 281 | obs: | exp: |
| aaa | K | 342 | 205.50 |
| aag | K | 25 | 161.50 |
| --- | -- | ------ | ------ |
| mPD | | 0.13 | 0.49 |
| | | nPD: | 0.26 |
| | | N. weight: | 0.54 |
| | | Sc. PD: | 0.00095 |
| | | Sc. rank: | 25.7 |

| PB1 | | | |
|---|---|---|---|
| Pos. | 282 | obs: | exp: |
| tta | L | 3 | 32.06 |
| ttg | L | 263 | 70.68 |
| ctt | L | 1 | 68.48 |
| ctc | L | 0 | 59.37 |
| cta | L | 0 | 55.29 |
| ctg | L | 100 | 81.13 |
| --- | -- | ------ | ------ |
| mPD | | 0.42 | 1.1 |
| | | nPD: | 0.38 |
| | | N. weight: | 1.5 |
| | | Sc. PD: | 0.17 |
| | | Sc. rank: | 135.6 |

| PB1 | | | |
|---|---|---|---|
| Pos. | 283 | obs: | exp: |
| gct | A | 0 | 92.44 |
| gcc | A | 15 | 75.18 |
| gca | A | 341 | 163.60 |
| gcg | A | 11 | 35.81 |
| --- | -- | ------ | ------ |
| mPD | | 0.13 | 0.69 |
| | | nPD: | 0.2 |
| | | N. weight: | 0.9 |
| | | Sc. PD: | -0.052 |
| | | Sc. rank: | 6.3 |

| PB1 | | | |
|---|---|---|---|
| Pos. | 284 | obs: | exp: |
| aat | N | 361 | 191.80 |
| aac | N | 6 | 175.20 |
| --- | -- | ------ | ------ |
| mPD | | 0.032 | 0.50 |
| | | nPD: | 0.06 |
| | | N. weight: | 0.87 |
| | | Sc. PD: | -0.16 |
| | | Sc. rank: | -84.7 |

| PB1 | | | |
|---|---|---|---|
| Pos. | 285 | obs: | exp: |
| gtt | V | 160 | 75.43 |
| gtc | V | 206 | 77.03 |
| gta | V | 0 | 68.78 |
| gtg | V | 1 | 145.80 |
| --- | -- | ------ | ------ |
| mPD | | 0.50 | 0.72 |
| | | nPD: | 0.69 |
| | | N. weight: | 1.3 |
| | | Sc. PD: | 0.55 |
| | | Sc. rank: | 235.3 |

| PB1 | | | |
|---|---|---|---|
| Pos. | 286 | obs: | exp: |
| gtt | V | 20 | 75.43 |
| gtc | V | 0 | 77.03 |
| gta | V | 45 | 68.78 |
| gtg | V | 302 | 145.80 |
| --- | -- | ------ | ------ |
| mPD | | 0.31 | 0.72 |
| | | nPD: | 0.42 |
| | | N. weight: | 0.74 |
| | | Sc. PD: | 0.12 |
| | | Sc. rank: | 82.4 |

| PB1 | | | |
|---|---|---|---|
| Pos. | 287 | obs: | exp: |
| cgt | R | 0 | 15.24 |
| cgc | R | 0 | 17.97 |
| cga | R | 0 | 35.27 |
| cgg | R | 0 | 30.63 |
| aga | R | 287 | 165.00 |
| agg | R | 80 | 102.80 |
| --- | -- | ------ | ------ |
| mPD | | 0.34 | 0.96 |
| | | nPD: | 0.36 |
| | | N. weight: | 0.59 |
| | | Sc. PD: | 0.056 |
| | | Sc. rank: | 48.9 |

| PB1 | | | |
|---|---|---|---|
| Pos. | 288 | obs: | exp: |
| aaa | K | 281 | 205.50 |
| aag | K | 86 | 161.50 |
| --- | -- | ------ | ------ |
| mPD | | 0.36 | 0.49 |
| | | nPD: | 0.73 |
| | | N. weight: | 0.15 |
| | | Sc. PD: | 0.069 |
| | | Sc. rank: | 28.5 |

| PB1 | | | |
|---|---|---|---|
| Pos. | 289 | obs: | exp: |
| atg | M | 367 | 367.00 |
| --- | -- | ------ | ------ |
| mPD | | 0 | 0 |
| | | nPD: | 1. |
| | | N. weight: | 0. |
| | | Sc. PD: | 0 |
| | | Sc. rank: | 0 |

| PB1 | | | |
|---|---|---|---|
| Pos. | 290 | obs: | exp: |
| atg | M | 367 | 367.00 |
| --- | -- | ------ | ------ |
| mPD | | 0 | 0 |
| | | nPD: | 1. |
| | | N. weight: | 0. |
| | | Sc. PD: | 0 |
| | | Sc. rank: | 0 |

PB1

| Pos. | 291 | obs: | exp: |
|---|---|---|---|
| act | T | 336 | 96.96 |
| acc | T | 17 | 78.75 |
| aca | T | 7 | 160.80 |
| acg | T | 7 | 30.51 |
| --- | -- | ------ | ------ |
| mPD | | 0.16 | 0.69 |
| | | nPD: | 0.23 |
| | | N. weight: | 1.5 |
| | | Sc. PD: | -0.034 |
| | | Sc. rank: | 51.9 |

,

PB1

| Pos. | 292 | obs: | exp: |
|---|---|---|---|
| aat | N | 129 | 191.80 |
| aac | N | 238 | 175.20 |
| --- | -- | ------ | ------ |
| mPD | | 0.46 | 0.50 |
| | | nPD: | 0.92 |
| | | N. weight: | 0.1 |
| | | Sc. PD: | 0.065 |
| | | Sc. rank: | 21.4 |

,

PB1

| Pos. | 293 | obs: | exp: |
|---|---|---|---|
| tct | S | 0 | 58.34 |
| tcc | S | 0 | 51.12 |
| tca | S | 292 | 81.31 |
| tcg | S | 75 | 22.37 |
| agt | S | 0 | 80.74 |
| agc | S | 0 | 73.12 |
| --- | -- | ------ | ------ |
| mPD | | 0.33 | 1.7 |
| | | nPD: | 0.2 |
| | | N. weight: | 1.9 |
| | | Sc. PD: | -0.11 |
| | | Sc. rank: | 16.5 |

,

PB1

| Pos. | 294 | obs: | exp: |
|---|---|---|---|
| cat | H | 4 | 2.17 |
| cac | H | 0 | 1.83 |
| caa | Q | 360 | 193.50 |
| cag | Q | 3 | 169.50 |
| --- | -- | ------ | ------ |
| mPD | | 0.038 | 0.51 |
| | | nPD: | 0.07 |
| | | N. weight: | 0.89 |
| | | Sc. PD: | -0.15 |
| | | Sc. rank: | -76.0 |

,

PB1

| Pos. | 295 | obs: | exp: |
|---|---|---|---|
| gat | D | 237 | 195.80 |
| gac | D | 130 | 171.20 |
| --- | -- | ------ | ------ |
| mPD | | 0.46 | 0.50 |
| | | nPD: | 0.92 |
| | | N. weight: | 0.052 |
| | | Sc. PD: | 0.033 |
| | | Sc. rank: | 10.8 |

,

PB1

| Pos. | 296 | obs: | exp: |
|---|---|---|---|
| act | T | 2 | 96.96 |
| acc | T | 2 | 78.75 |
| aca | T | 362 | 160.80 |
| acg | T | 1 | 30.51 |
| --- | -- | ------ | ------ |
| mPD | | 0.027 | 0.69 |
| | | nPD: | 0.04 |
| | | N. weight: | 1.2 |
| | | Sc. PD: | -0.24 |
| | | Sc. rank: | -152.7 |

,

PB1

| Pos. | 297 | obs: | exp: |
|---|---|---|---|
| gaa | E | 38 | 211.20 |
| gag | E | 329 | 155.80 |
| --- | -- | ------ | ------ |
| mPD | | 0.19 | 0.49 |
| | | nPD: | 0.38 |
| | | N. weight: | 0.76 |
| | | Sc. PD: | 0.09 |
| | | Sc. rank: | 70.9 |

,

PB1

| Pos. | 298 | obs: | exp: |
|---|---|---|---|
| tta | L | 0 | 32.06 |
| ttg | L | 0 | 70.68 |
| ctt | L | 55 | 68.48 |
| ctc | L | 311 | 59.37 |
| cta | L | 1 | 55.29 |
| ctg | L | 0 | 81.13 |
| --- | -- | ------ | ------ |
| mPD | | 0.26 | 1.1 |
| | | nPD: | 0.23 |
| | | N. weight: | 2.1 |
| | | Sc. PD: | -0.046 |
| | | Sc. rank: | 73.8 |

,

PB1

| Pos. | 299 | obs: | exp: |
|---|---|---|---|
| tct | S | 118 | 58.34 |
| tcc | S | 247 | 51.12 |
| tca | S | 2 | 81.31 |
| tcg | S | 0 | 22.37 |
| agt | S | 0 | 80.74 |
| agc | S | 0 | 73.12 |
| --- | -- | ------ | ------ |
| mPD | | 0.44 | 1.7 |
| | | nPD: | 0.27 |
| | | N. weight: | 1.9 |
| | | Sc. PD: | 0.02 |
| | | Sc. rank: | 101.4 |

,

PB1

| Pos. | 300 | obs: | exp: |
|---|---|---|---|
| ttt | F | 191 | 150.90 |
| ttc | F | 176 | 216.10 |
| --- | -- | ------ | ------ |
| mPD | | 0.50 | 0.48 |
| | | nPD: | 1.03 |
| | | N. weight: | 0.05 |
| | | Sc. PD: | 0.037 |
| | | Sc. rank: | 10.5 |

,

PB1

| Pos. | 301 | obs: | exp: |
|---|---|---|---|
| act | T | 1 | 96.96 |
| acc | T | 3 | 78.75 |
| aca | T | 344 | 160.80 |
| acg | T | 19 | 30.51 |
| --- | -- | ------ | ------ |
| mPD | | 0.12 | 0.69 |
| | | nPD: | 0.17 |
| | | N. weight: | 1. |
| | | Sc. PD: | -0.079 |
| | | Sc. rank: | -1.8 |

,

PB1

| Pos. | 302 | obs: | exp: |
|---|---|---|---|
| att | I | 248 | 137.60 |
| atc | I | 118 | 95.92 |
| ata | I | 0 | 132.50 |
| gtt | V | 1 | 0.21 |
| gtc | V | 0 | 0.21 |
| gta | V | 0 | 0.19 |
| gtg | V | 0 | 0.40 |
| --- | -- | ------ | ------ |
| mPD | | 0.44 | 0.67 |
| | | nPD: | 0.67 |
| | | N. weight: | 0.73 |
| | | Sc. PD: | 0.28 |
| | | Sc. rank: | 125.0 |

,

PB1

| Pos. | 303 | obs: | exp: |
|---|---|---|---|
| act | T | 358 | 96.96 |
| acc | T | 4 | 78.75 |
| aca | T | 3 | 160.80 |
| acg | T | 2 | 30.51 |
| --- | -- | ------ | ------ |
| mPD | | 0.048 | 0.69 |
| | | nPD: | 0.07 |
| | | N. weight: | 1.8 |
| | | Sc. PD: | -0.32 |
| | | Sc. rank: | -160.7 |

,

PB1

| Pos. | 304 | obs: | exp: |
|---|---|---|---|
| ggt | G | 0 | 45.28 |
| ggc | G | 0 | 49.63 |
| gga | G | 343 | 177.50 |
| ggg | G | 24 | 94.55 |
| --- | -- | ------ | ------ |
| mPD | | 0.12 | 0.67 |
| | | nPD: | 0.18 |
| | | N. weight: | 0.81 |
| | | Sc. PD: | -0.056 |
| | | Sc. rank: | 1.8 |

,

PB1

| Pos. | 305 | obs: | exp: |
|---|---|---|---|
| gat | D | 45 | 195.80 |
| gac | D | 322 | 171.20 |
| --- | -- | ------ | ------ |
| mPD | | 0.22 | 0.50 |
| | | nPD: | 0.43 |
| | | N. weight: | 0.58 |
| | | Sc. PD: | 0.098 |
| | | Sc. rank: | 64.9 |

,

PB1

| Pos. | 306 | obs: | exp: |
|---|---|---|---|
| aat | N | 35 | 191.80 |
| aac | N | 332 | 175.20 |
| --- | -- | ------ | ------ |
| mPD | | 0.17 | 0.50 |
| | | nPD: | 0.35 |
| | | N. weight: | 0.64 |
| | | Sc. PD: | 0.055 |
| | | Sc. rank: | 52.4 |

,

PB1

| Pos. | 307 | obs: | exp: |
|---|---|---|---|
| act | T | 3 | 96.96 |
| acc | T | 363 | 78.75 |
| aca | T | 1 | 160.80 |
| acg | T | 0 | 30.51 |
| --- | -- | ------ | ------ |
| mPD | | 0.022 | 0.69 |
| | | nPD: | 0.03 |
| | | N. weight: | 2.2 |
| | | Sc. PD: | -0.48 |
| | | Sc. rank: | -329.6 |

,

PB1

| Pos. | 308 | obs: | exp: |
|---|---|---|---|
| aaa | K | 353 | 205.50 |
| aag | K | 14 | 161.50 |
| --- | -- | ------ | ------ |
| mPD | | 0.074 | 0.49 |
| | | nPD: | 0.15 |
| | | N. weight: | 0.66 |
| | | Sc. PD: | -0.067 |
| | | Sc. rank: | -10.7 |

,

PB1

| Pos. | 309 | obs: | exp: |
|---|---|---|---|
| tgg | W | 367 | 367.00 |
| --- | -- | ------ | ------ |
| mPD | | 0 | 0 |
| | | nPD: | 1. |
| | | N. weight: | 0. |
| | | Sc. PD: | 0 |
| | | Sc. rank: | 0 |

,

PB1

| Pos. | 310 | obs: | exp: |
|---|---|---|---|
| aat | N | 361 | 191.80 |
| aac | N | 6 | 175.20 |
| --- | -- | ------ | ------ |
| mPD | | 0.032 | 0.50 |
| | | nPD: | 0.06 |
| | | N. weight: | 0.87 |
| | | Sc. PD: | -0.16 |
| | | Sc. rank: | -84.7 |

,

PB1

| Pos. | 311 | obs: | exp: |
|---|---|---|---|
| gaa | E | 98 | 211.20 |
| gag | E | 269 | 155.80 |
| --- | -- | ------ | ------ |
| mPD | | 0.39 | 0.49 |
| | | nPD: | 0.8 |
| | | N. weight: | 0.31 |
| | | Sc. PD: | 0.16 |
| | | Sc. rank: | 61.5 |

,

PB1

| Pos. | 312 | obs: | exp: |
|---|---|---|---|
| aat | N | 347 | 191.80 |
| aac | N | 20 | 175.20 |
| --- | -- | ------ | ------ |
| mPD | | 0.10 | 0.50 |
| | | nPD: | 0.21 |
| | | N. weight: | 0.68 |
| | | Sc. PD: | -0.032 |
| | | Sc. rank: | 11.4 |

,

PB1

| Pos. | 313 | obs: | exp: |
|---|---|---|---|
| caa | Q | 215 | 195.60 |
| cag | Q | 152 | 171.40 |
| --- | -- | ------ | ------ |
| mPD | | 0.49 | 0.50 |
| | | nPD: | 0.98 |
| | | N. weight: | 0.022 |
| | | Sc. PD: | 0.015 |
| | | Sc. rank: | 4.5 |

,

PB1

| Pos. | 314 | obs: | exp: |
|---|---|---|---|
| tct | S | 0 | 0.32 |
| tcc | S | 0 | 0.28 |
| tca | S | 0 | 0.44 |
| tcg | S | 0 | 0.12 |
| aat | N | 208 | 190.80 |
| aac | N | 157 | 174.20 |
| agt | S | 2 | 0.44 |
| agc | S | 0 | 0.40 |
| --- | -- | ------ | ------ |
| mPD | | 0.50 | 0.52 |
| | | nPD: | 0.97 |
| | | N. weight: | 0.032 |
| | | Sc. PD: | 0.022 |
| | | Sc. rank: | 6.8 |

,

PB1

| Pos. | 315 | obs: | exp: |
|---|---|---|---|
| cct | P | 327 | 96.64 |
| ccc | P | 39 | 66.20 |
| cca | P | 1 | 144.80 |
| ccg | P | 0 | 59.34 |
| --- | -- | ------ | ------ |
| mPD | | 0.20 | 0.72 |
| | | nPD: | 0.27 |
| | | N. weight: | 1.6 |
| | | Sc. PD: | 0.024 |
| | | Sc. rank: | 92.8 |

,

PB1

| Pos. | 316 | obs: | exp: |
|---|---|---|---|
| cgt | R | 10 | 15.24 |
| cgc | R | 5 | 17.97 |
| cga | R | 41 | 35.27 |
| cgg | R | 219 | 30.63 |
| aga | R | 10 | 165.00 |
| agg | R | 82 | 102.80 |
| --- | -- | ------ | ------ |
| mPD | | 0.68 | 0.96 |
| | | nPD: | 0.71 |
| | | N. weight: | 1.6 |
| | | Sc. PD: | 0.7 |
| | | Sc. rank: | 295.9 |

,

PB1

| Pos. | 317 | obs: | exp: |
|---|---|---|---|
| att | I | 0 | 0.38 |
| atc | I | 0 | 0.26 |
| ata | I | 1 | 0.36 |
| atg | M | 364 | 364.00 |
| gtt | V | 0 | 0.41 |
| gtc | V | 0 | 0.42 |
| gta | V | 0 | 0.37 |
| gtg | V | 2 | 0.79 |
| --- | -- | ------ | ------ |
| mPD | | 0.016 | 0.023 |
| | | nPD: | 0.72 |
| | | N. weight: | 0.012 |
| | | Sc. PD: | 0.0052 |
| | | Sc. rank: | 2.2 |

,

PB1

| Pos. | 318 | obs: | exp: |
|---|---|---|---|
| ttt | F | 342 | 150.90 |
| ttc | F | 25 | 216.10 |
| --- | -- | ------ | ------ |
| mPD | | 0.13 | 0.48 |
| | | nPD: | 0.26 |
| | | N. weight: | 0.95 |
| | | Sc. PD: | 0.0058 |
| | | Sc. rank: | 46.2 |

,

PB1

| Pos. | 319 | obs: | exp: |
|---|---|---|---|
| tta | L | 27 | 32.06 |
| ttg | L | 82 | 70.68 |
| ctt | L | 2 | 68.48 |
| ctc | L | 0 | 59.37 |
| cta | L | 31 | 55.29 |
| ctg | L | 225 | 81.13 |
| --- | -- | ------ | ------ |
| mPD | | 0.69 | 1.1 |
| | | nPD: | 0.62 |
| | | N. weight: | 0.92 |
| | | Sc. PD: | 0.32 |
| | | Sc. rank: | 148.9 |

,

PB1

| Pos. | 320 | obs: | exp: |
|---|---|---|---|
| gct | A | 2 | 92.44 |
| gcc | A | 0 | 75.18 |
| gca | A | 364 | 163.60 |
| gcg | A | 1 | 35.81 |
| --- | -- | ------ | ------ |
| mPD | | 0.016 | 0.69 |
| | | nPD: | 0.02 |
| | | N. weight: | 1.2 |
| | | Sc. PD: | -0.26 |
| | | Sc. rank: | -174.5 |

,

| | | | |
|---|---|---|---|
| PB1 | | | |
| Pos. | 321 | obs: | exp: |
| atg | M | 367 | 367.00 |
| --- | -- | ------ | ------ |
| mPD | | 0 | 0 |
| | | nPD: | 1. |
| | | N. weight: | 0. |
| | | Sc. PD: | 0 |
| | | Sc. rank: | 0 |

| | | | |
|---|---|---|---|
| PB1 | | | |
| Pos. | 322 | obs: | exp: |
| att | I | 1 | 138.00 |
| atc | I | 0 | 96.18 |
| ata | I | 366 | 132.80 |
| --- | -- | ------ | ------ |
| mPD | | 0.0054 | 0.66 |
| | | nPD: | 0.01 |
| | | N. weight: | 1.5 |
| | | Sc. PD: | -0.36 |
| | | Sc. rank: | -268.7 |

| | | | |
|---|---|---|---|
| PB1 | | | |
| Pos. | 323 | obs: | exp: |
| act | T | 5 | 96.96 |
| acc | T | 0 | 78.75 |
| aca | T | 345 | 160.80 |
| acg | T | 17 | 30.51 |
| --- | -- | ------ | ------ |
| mPD | | 0.11 | 0.69 |
| | | nPD: | 0.17 |
| | | N. weight: | 1. |
| | | Sc. PD: | -0.086 |
| | | Sc. rank: | -6.6 |

| | | | |
|---|---|---|---|
| PB1 | | | |
| Pos. | 324 | obs: | exp: |
| tat | Y | 105 | 190.00 |
| tac | Y | 262 | 177.00 |
| --- | -- | ------ | ------ |
| mPD | | 0.41 | 0.50 |
| | | nPD: | 0.82 |
| | | N. weight: | 0.18 |
| | | Sc. PD: | 0.097 |
| | | Sc. rank: | 36.8 |

| | | | |
|---|---|---|---|
| PB1 | | | |
| Pos. | 325 | obs: | exp: |
| att | I | 7 | 138.00 |
| atc | I | 268 | 96.18 |
| ata | I | 92 | 132.80 |
| --- | -- | ------ | ------ |
| mPD | | 0.40 | 0.66 |
| | | nPD: | 0.61 |
| | | N. weight: | 0.93 |
| | | Sc. PD: | 0.32 |
| | | Sc. rank: | 149.9 |

| | | | |
|---|---|---|---|
| PB1 | | | |
| Pos. | 326 | obs: | exp: |
| act | T | 0 | 96.96 |
| acc | T | 0 | 78.75 |
| aca | T | 365 | 160.80 |
| acg | T | 2 | 30.51 |
| --- | -- | ------ | ------ |
| mPD | | 0.011 | 0.69 |
| | | nPD: | 0.02 |
| | | N. weight: | 1.2 |
| | | Sc. PD: | -0.28 |
| | | Sc. rank: | -204.8 |

| | | | |
|---|---|---|---|
| PB1 | | | |
| Pos. | 327 | obs: | exp: |
| cgt | R | 0 | 15.24 |
| cgc | R | 0 | 17.97 |
| cga | R | 0 | 35.27 |
| cgg | R | 0 | 30.63 |
| aga | R | 300 | 165.00 |
| agg | R | 67 | 102.80 |
| --- | -- | ------ | ------ |
| mPD | | 0.30 | 0.96 |
| | | nPD: | 0.31 |
| | | N. weight: | 0.64 |
| | | Sc. PD: | 0.033 |
| | | Sc. rank: | 46.4 |

| | | | |
|---|---|---|---|
| PB1 | | | |
| Pos. | 328 | obs: | exp: |
| tct | S | 0 | 0.79 |
| tcc | S | 0 | 0.70 |
| tca | S | 0 | 1.11 |
| tcg | S | 0 | 0.30 |
| aat | N | 19 | 188.70 |
| aac | N | 342 | 172.30 |
| agt | S | 0 | 1.10 |
| agc | S | 5 | 1.00 |
| gat | D | 0 | 0.53 |
| gac | D | 1 | 0.47 |
| --- | -- | ------ | ------ |
| mPD | | 0.13 | 0.55 |
| | | nPD: | 0.24 |
| | | N. weight: | 0.84 |
| | | Sc. PD: | -0.015 |
| | | Sc. rank: | 31.2 |

| | | | |
|---|---|---|---|
| PB1 | | | |
| Pos. | 329 | obs: | exp: |
| caa | Q | 355 | 195.60 |
| cag | Q | 12 | 171.40 |
| --- | -- | ------ | ------ |
| mPD | | 0.063 | 0.50 |
| | | nPD: | 0.13 |
| | | N. weight: | 0.75 |
| | | Sc. PD: | -0.092 |
| | | Sc. rank: | -23.9 |

| | | | |
|---|---|---|---|
| PB1 | | | |
| Pos. | 330 | obs: | exp: |
| cct | P | 327 | 96.64 |
| ccc | P | 6 | 66.20 |
| cca | P | 27 | 144.80 |
| ccg | P | 7 | 59.34 |
| --- | -- | ------ | ------ |
| mPD | | 0.20 | 0.72 |
| | | nPD: | 0.28 |
| | | N. weight: | 1.4 |
| | | Sc. PD: | 0.031 |
| | | Sc. rank: | 86.3 |

| | | | |
|---|---|---|---|
| PB1 | | | |
| Pos. | 331 | obs: | exp: |
| gaa | E | 354 | 208.90 |
| gag | E | 9 | 154.10 |
| ggt | G | 0 | 0.49 |
| ggc | G | 0 | 0.54 |
| gga | G | 4 | 1.93 |
| ggg | G | 0 | 1.03 |
| --- | -- | ------ | ------ |
| mPD | | 0.070 | 0.51 |
| | | nPD: | 0.14 |
| | | N. weight: | 0.69 |
| | | Sc. PD: | -0.079 |
| | | Sc. rank: | -21.2 |

| | | | |
|---|---|---|---|
| PB1 | | | |
| Pos. | 332 | obs: | exp: |
| tgg | W | 367 | 367.00 |
| --- | -- | ------ | ------ |
| mPD | | 0 | 0 |
| | | nPD: | 1. |
| | | N. weight: | 0. |
| | | Sc. PD: | 0 |
| | | Sc. rank: | 0 |

| | | | |
|---|---|---|---|
| PB1 | | | |
| Pos. | 333 | obs: | exp: |
| ttt | F | 285 | 150.90 |
| ttc | F | 82 | 216.10 |
| --- | -- | ------ | ------ |
| mPD | | 0.35 | 0.48 |
| | | nPD: | 0.72 |
| | | N. weight: | 0.43 |
| | | Sc. PD: | 0.19 |
| | | Sc. rank: | 79.6 |

| | | | |
|---|---|---|---|
| PB1 | | | |
| Pos. | 334 | obs: | exp: |
| cgt | R | 0 | 15.24 |
| cgc | R | 0 | 17.97 |
| cga | R | 0 | 35.27 |
| cgg | R | 0 | 30.63 |
| aga | R | 264 | 165.00 |
| agg | R | 103 | 102.80 |
| --- | -- | ------ | ------ |
| mPD | | 0.40 | 0.96 |
| | | nPD: | 0.42 |
| | | N. weight: | 0.53 |
| | | Sc. PD: | 0.083 |
| | | Sc. rank: | 57.9 |

| | | | |
|---|---|---|---|
| PB1 | | | |
| Pos. | 335 | obs: | exp: |
| aat | N | 365 | 191.80 |
| aac | N | 2 | 175.20 |
| --- | -- | ------ | ------ |
| mPD | | 0.011 | 0.50 |
| | | nPD: | 0.02 |
| | | N. weight: | 0.94 |
| | | Sc. PD: | -0.21 |
| | | Sc. rank: | -149.9 |

| | | | |
|---|---|---|---|
| PB1 | | | |
| Pos. | 336 | obs: | exp: |
| gtt | V | 41 | 75.23 |
| gtc | V | 324 | 76.82 |
| gta | V | 1 | 68.60 |
| gtg | V | 0 | 145.40 |
| gct | A | 0 | 0.25 |
| gcc | A | 1 | 0.20 |
| gca | A | 0 | 0.45 |
| gcg | A | 0 | 0.10 |
| --- | -- | ------ | ------ |
| mPD | | 0.21 | 0.73 |
| | | nPD: | 0.29 |
| | | N. weight: | 1.8 |
| | | Sc. PD: | 0.055 |
| | | Sc. rank: | 120.4 |

| | | | |
|---|---|---|---|
| PB1 | | | |
| Pos. | 337 | obs: | exp: |
| tta | L | 34 | 32.06 |
| ttg | L | 312 | 70.68 |
| ctt | L | 0 | 68.48 |
| ctc | L | 0 | 59.37 |
| cta | L | 4 | 55.29 |
| ctg | L | 17 | 81.13 |
| --- | -- | ------ | ------ |
| mPD | | 0.29 | 1.1 |
| | | nPD: | 0.26 |
| | | N. weight: | 1.8 |
| | | Sc. PD: | 0.013 |
| | | Sc. rank: | 89.7 |

| | | | |
|---|---|---|---|
| PB1 | | | |
| Pos. | 338 | obs: | exp: |
| tct | S | 0 | 58.34 |
| tcc | S | 0 | 51.12 |
| tca | S | 0 | 81.31 |
| tcg | S | 0 | 22.37 |
| agt | S | 20 | 80.74 |
| agc | S | 347 | 73.12 |
| --- | -- | ------ | ------ |
| mPD | | 0.10 | 1.7 |
| | | nPD: | 0.06 |
| | | N. weight: | 2.1 |
| | | Sc. PD: | -0.39 |
| | | Sc. rank: | -213.2 |

| | | | |
|---|---|---|---|
| PB1 | | | |
| Pos. | 339 | obs: | exp: |
| att | I | 333 | 138.00 |
| atc | I | 32 | 96.18 |
| ata | I | 2 | 132.80 |
| --- | -- | ------ | ------ |
| mPD | | 0.17 | 0.66 |
| | | nPD: | 0.26 |
| | | N. weight: | 1.1 |
| | | Sc. PD: | 0.00081 |
| | | Sc. rank: | 49.9 |

| | | | |
|---|---|---|---|
| PB1 | | | |
| Pos. | 340 | obs: | exp: |
| gct | A | 54 | 92.44 |
| gcc | A | 218 | 75.18 |
| gca | A | 89 | 163.60 |
| gcg | A | 6 | 35.81 |
| --- | -- | ------ | ------ |
| mPD | | 0.57 | 0.69 |
| | | nPD: | 0.83 |
| | | N. weight: | 0.6 |
| | | Sc. PD: | 0.33 |
| | | Sc. rank: | 123.4 |

| | | | |
|---|---|---|---|
| PB1 | | | |
| Pos. | 341 | obs: | exp: |
| cct | P | 339 | 96.64 |
| ccc | P | 25 | 66.20 |
| cca | P | 0 | 144.80 |
| ccg | P | 3 | 59.34 |
| --- | -- | ------ | ------ |
| mPD | | 0.14 | 0.72 |
| | | nPD: | 0.2 |
| | | N. weight: | 1.6 |
| | | Sc. PD: | -0.09 |
| | | Sc. rank: | 18.7 |

| | | | |
|---|---|---|---|
| PB1 | | | |
| Pos. | 342 | obs: | exp: |
| att | I | 0 | 138.00 |
| atc | I | 0 | 96.18 |
| ata | I | 367 | 132.80 |
| --- | -- | ------ | ------ |
| mPD | | 0 | 0.66 |
| | | nPD: | 0. |
| | | N. weight: | 1.5 |
| | | Sc. PD: | -0.38 |
| | | Sc. rank: | -321.0 |

| | | | |
|---|---|---|---|
| PB1 | | | |
| Pos. | 343 | obs: | exp: |
| atg | M | 367 | 367.00 |
| --- | -- | ------ | ------ |
| mPD | | 0 | 0 |
| | | nPD: | 1. |
| | | N. weight: | 0. |
| | | Sc. PD: | 0 |
| | | Sc. rank: | 0 |

| | | | |
|---|---|---|---|
| PB1 | | | |
| Pos. | 344 | obs: | exp: |
| ttt | F | 5 | 150.90 |
| ttc | F | 362 | 216.10 |
| --- | -- | ------ | ------ |
| mPD | | 0.027 | 0.48 |
| | | nPD: | 0.06 |
| | | N. weight: | 0.71 |
| | | Sc. PD: | -0.14 |
| | | Sc. rank: | -76.7 |

| | | | |
|---|---|---|---|
| PB1 | | | |
| Pos. | 345 | obs: | exp: |
| tct | S | 0 | 58.34 |
| tcc | S | 3 | 51.12 |
| tca | S | 294 | 81.31 |
| tcg | S | 70 | 22.37 |
| agt | S | 0 | 80.74 |
| agc | S | 0 | 73.12 |
| --- | -- | ------ | ------ |
| mPD | | 0.32 | 1.7 |
| | | nPD: | 0.19 |
| | | N. weight: | 1.9 |
| | | Sc. PD: | -0.11 |
| | | Sc. rank: | 12.2 |

| | | | |
|---|---|---|---|
| PB1 | | | |
| Pos. | 346 | obs: | exp: |
| aat | N | 118 | 191.80 |
| aac | N | 249 | 175.20 |
| --- | -- | ------ | ------ |
| mPD | | 0.44 | 0.50 |
| | | nPD: | 0.88 |
| | | N. weight: | 0.14 |
| | | Sc. PD: | 0.081 |
| | | Sc. rank: | 28.5 |

| | | | |
|---|---|---|---|
| PB1 | | | |
| Pos. | 347 | obs: | exp: |
| aaa | K | 333 | 205.50 |
| aag | K | 34 | 161.50 |
| --- | -- | ------ | ------ |
| mPD | | 0.17 | 0.49 |
| | | nPD: | 0.34 |
| | | N. weight: | 0.46 |
| | | Sc. PD: | 0.037 |
| | | Sc. rank: | 36.3 |

| | | | |
|---|---|---|---|
| PB1 | | | |
| Pos. | 348 | obs: | exp: |
| atg | M | 367 | 367.00 |
| --- | -- | ------ | ------ |
| mPD | | 0 | 0 |
| | | nPD: | 1. |
| | | N. weight: | 0. |
| | | Sc. PD: | 0 |
| | | Sc. rank: | 0 |

| | | | |
|---|---|---|---|
| PB1 | | | |
| Pos. | 349 | obs: | exp: |
| gct | A | 1 | 91.93 |
| gcc | A | 0 | 74.77 |
| gca | A | 123 | 162.70 |
| gcg | A | 241 | 35.62 |
| ggt | G | 0 | 0.25 |
| ggc | G | 0 | 0.27 |
| gga | G | 2 | 0.97 |
| ggg | G | 0 | 0.52 |
| --- | -- | ------ | ------ |
| mPD | | 0.46 | 0.70 |
| | | nPD: | 0.67 |
| | | N. weight: | 1.8 |
| | | Sc. PD: | 0.69 |
| | | Sc. rank: | 306.6 |

| | | | |
|---|---|---|---|
| PB1 | | | |
| Pos. | 350 | obs: | exp: |
| cgt | R | 0 | 15.24 |
| cgc | R | 0 | 17.97 |
| cga | R | 0 | 35.27 |
| cgg | R | 0 | 30.63 |
| aga | R | 354 | 165.00 |
| agg | R | 13 | 102.80 |
| --- | -- | ------ | ------ |
| mPD | | 0.069 | 0.96 |
| | | nPD: | 0.07 |
| | | N. weight: | 1. |
| | | Sc. PD: | -0.18 |
| | | Sc. rank: | -87.7 |

PB1

| Pos. | 351 | obs: | exp: |
|---|---|---|---|
| tta | L | 194 | 32.06 |
| ttg | L | 66 | 70.68 |
| ctt | L | 0 | 68.48 |
| ctc | L | 0 | 59.37 |
| cta | L | 105 | 55.29 |
| ctg | L | 2 | 81.13 |
| --- | -- | ------ | ------ |
| mPD | | 0.72 | 1.1 |
| | | nPD: | 0.64 |
| | | N. weight: | 1.7 |
| | | Sc. PD: | 0.63 |
| | | Sc. rank: | 286.4 |

PB1

| Pos. | 352 | obs: | exp: |
|---|---|---|---|
| ggt | G | 0 | 45.28 |
| ggc | G | 0 | 49.63 |
| gga | G | 189 | 177.50 |
| ggg | G | 178 | 94.55 |
| --- | -- | ------ | ------ |
| mPD | | 0.50 | 0.67 |
| | | nPD: | 0.75 |
| | | N. weight: | 0.53 |
| | | Sc. PD: | 0.25 |
| | | Sc. rank: | 102.6 |

PB1

| Pos. | 353 | obs: | exp: |
|---|---|---|---|
| aaa | K | 367 | 205.50 |
| aag | K | 0 | 161.50 |
| --- | -- | ------ | ------ |
| mPD | | 0 | 0.49 |
| | | nPD: | 0. |
| | | N. weight: | 0.88 |
| | | Sc. PD: | -0.22 |
| | | Sc. rank: | -183.2 |

PB1

| Pos. | 354 | obs: | exp: |
|---|---|---|---|
| ggt | G | 0 | 45.28 |
| ggc | G | 3 | 49.63 |
| gga | G | 351 | 177.50 |
| ggg | G | 13 | 94.55 |
| --- | -- | ------ | ------ |
| mPD | | 0.084 | 0.67 |
| | | nPD: | 0.13 |
| | | N. weight: | 0.87 |
| | | Sc. PD: | -0.11 |
| | | Sc. rank: | -28.2 |

PB1

| Pos. | 355 | obs: | exp: |
|---|---|---|---|
| tat | Y | 20 | 190.00 |
| tac | Y | 347 | 177.00 |
| --- | -- | ------ | ------ |
| mPD | | 0.10 | 0.50 |
| | | nPD: | 0.21 |
| | | N. weight: | 0.79 |
| | | Sc. PD: | -0.037 |
| | | Sc. rank: | 12.9 |

PB1

| Pos. | 356 | obs: | exp: |
|---|---|---|---|
| atg | M | 367 | 367.00 |
| --- | -- | ------ | ------ |
| mPD | | 0 | 0 |
| | | nPD: | 1. |
| | | N. weight: | 0. |
| | | Sc. PD: | 0 |
| | | Sc. rank: | 0 |

PB1

| Pos. | 357 | obs: | exp: |
|---|---|---|---|
| ttt | F | 219 | 150.90 |
| ttc | F | 148 | 216.10 |
| --- | -- | ------ | ------ |
| mPD | | 0.48 | 0.48 |
| | | nPD: | 1. |
| | | N. weight: | 0.12 |
| | | Sc. PD: | 0.084 |
| | | Sc. rank: | 24.9 |

PB1

| Pos. | 358 | obs: | exp: |
|---|---|---|---|
| gaa | E | 319 | 211.20 |
| gag | E | 48 | 155.80 |
| --- | -- | ------ | ------ |
| mPD | | 0.23 | 0.49 |
| | | nPD: | 0.47 |
| | | N. weight: | 0.32 |
| | | Sc. PD: | 0.064 |
| | | Sc. rank: | 40.1 |

PB1

| Pos. | 359 | obs: | exp: |
|---|---|---|---|
| tct | S | 0 | 58.34 |
| tcc | S | 0 | 51.12 |
| tca | S | 0 | 81.31 |
| tcg | S | 0 | 22.37 |
| agt | S | 356 | 80.74 |
| agc | S | 11 | 73.12 |
| --- | -- | ------ | ------ |
| mPD | | 0.058 | 1.7 |
| | | nPD: | 0.04 |
| | | N. weight: | 2.1 |
| | | Sc. PD: | -0.44 |
| | | Sc. rank: | -296.5 |

PB1

| Pos. | 360 | obs: | exp: |
|---|---|---|---|
| aaa | K | 14 | 205.50 |
| aag | K | 353 | 161.50 |
| --- | -- | ------ | ------ |
| mPD | | 0.074 | 0.49 |
| | | nPD: | 0.15 |
| | | N. weight: | 1. |
| | | Sc. PD: | -0.1 |
| | | Sc. rank: | -16.2 |

PB1

| Pos. | 361 | obs: | exp: |
|---|---|---|---|
| tct | S | 0 | 58.34 |
| tcc | S | 0 | 51.12 |
| tca | S | 0 | 81.31 |
| tcg | S | 0 | 22.37 |
| agt | S | 30 | 80.74 |
| agc | S | 337 | 73.12 |
| --- | -- | ------ | ------ |
| mPD | | 0.15 | 1.7 |
| | | nPD: | 0.09 |
| | | N. weight: | 2. |
| | | Sc. PD: | -0.32 |
| | | Sc. rank: | -127.1 |

PB1

| Pos. | 362 | obs: | exp: |
|---|---|---|---|
| atg | M | 367 | 367.00 |
| --- | -- | ------ | ------ |
| mPD | | 0 | 0 |
| | | nPD: | 1. |
| | | N. weight: | 0. |
| | | Sc. PD: | 0 |
| | | Sc. rank: | 0 |

PB1

| Pos. | 363 | obs: | exp: |
|---|---|---|---|
| aaa | K | 45 | 205.50 |
| aag | K | 322 | 161.50 |
| --- | -- | ------ | ------ |
| mPD | | 0.22 | 0.49 |
| | | nPD: | 0.44 |
| | | N. weight: | 0.65 |
| | | Sc. PD: | 0.11 |
| | | Sc. rank: | 73.0 |

PB1

| Pos. | 364 | obs: | exp: |
|---|---|---|---|
| tta | L | 32 | 32.06 |
| ttg | L | 7 | 70.68 |
| ctt | L | 91 | 68.48 |
| ctc | L | 4 | 59.37 |
| cta | L | 213 | 55.29 |
| ctg | L | 20 | 81.13 |
| --- | -- | ------ | ------ |
| mPD | | 0.68 | 1.1 |
| | | nPD: | 0.61 |
| | | N. weight: | 1.1 |
| | | Sc. PD: | 0.37 |
| | | Sc. rank: | 179.2 |

PB1

| Pos. | 365 | obs: | exp: |
|---|---|---|---|
| cgt | R | 0 | 15.24 |
| cgc | R | 0 | 17.97 |
| cga | R | 109 | 35.27 |
| cgg | R | 182 | 30.63 |
| aga | R | 14 | 165.00 |
| agg | R | 62 | 102.80 |
| --- | -- | ------ | ------ |
| mPD | | 0.78 | 0.96 |
| | | nPD: | 0.81 |
| | | N. weight: | 1.6 |
| | | Sc. PD: | 0.85 |
| | | Sc. rank: | 323.7 |

PB1

| Pos. | 366 | obs: | exp: |
|---|---|---|---|
| act | T | 1 | 96.96 |
| acc | T | 0 | 78.75 |
| aca | T | 345 | 160.80 |
| acg | T | 21 | 30.51 |
| --- | -- | ------ | ------ |
| mPD | | 0.11 | 0.69 |
| | | nPD: | 0.17 |
| | | N. weight: | 1.1 |
| | | Sc. PD: | -0.091 |
| | | Sc. rank: | -8.0 |

PB1

| Pos. | 367 | obs: | exp: |
|---|---|---|---|
| caa | Q | 359 | 195.60 |
| cag | Q | 8 | 171.40 |
| --- | -- | ------ | ------ |
| mPD | | 0.043 | 0.50 |
| | | nPD: | 0.09 |
| | | N. weight: | 0.81 |
| | | Sc. PD: | -0.13 |
| | | Sc. rank: | -57.4 |

PB1

| Pos. | 368 | obs: | exp: |
|---|---|---|---|
| att | I | 0 | 125.20 |
| atc | I | 0 | 87.27 |
| ata | I | 333 | 120.50 |
| gtt | V | 0 | 6.99 |
| gtc | V | 0 | 7.14 |
| gta | V | 34 | 6.37 |
| gtg | V | 0 | 13.50 |
| --- | -- | ------ | ------ |
| mPD | | 0.17 | 0.85 |
| | | nPD: | 0.2 |
| | | N. weight: | 1.6 |
| | | Sc. PD: | -0.091 |
| | | Sc. rank: | 17.2 |

PB1

| Pos. | 369 | obs: | exp: |
|---|---|---|---|
| tct | S | 0 | 0.79 |
| tcc | S | 0 | 0.70 |
| tca | S | 0 | 1.11 |
| tcg | S | 5 | 0.30 |
| cct | P | 31 | 95.32 |
| ccc | P | 17 | 65.30 |
| cca | P | 99 | 142.90 |
| ccg | P | 215 | 58.53 |
| agt | S | 0 | 1.10 |
| agc | S | 0 | 1.00 |
| --- | -- | ------ | ------ |
| mPD | | 0.59 | 0.76 |
| | | nPD: | 0.78 |
| | | N. weight: | 0.86 |
| | | Sc. PD: | 0.43 |
| | | Sc. rank: | 169.5 |

PB1

| Pos. | 370 | obs: | exp: |
|---|---|---|---|
| act | T | 0 | 0.26 |
| acc | T | 0 | 0.21 |
| aca | T | 1 | 0.44 |
| acg | T | 0 | 0.08 |
| gct | A | 14 | 92.19 |
| gcc | A | 0 | 74.97 |
| gca | A | 326 | 163.10 |
| gcg | A | 26 | 35.71 |
| --- | -- | ------ | ------ |
| mPD | | 0.21 | 0.69 |
| | | nPD: | 0.3 |
| | | N. weight: | 0.82 |
| | | Sc. PD: | 0.032 |
| | | Sc. rank: | 57.4 |

PB1

| Pos. | 371 | obs: | exp: |
|---|---|---|---|
| gaa | E | 341 | 210.60 |
| gag | E | 25 | 155.40 |
| ggt | G | 0 | 0.12 |
| ggc | G | 0 | 0.14 |
| gga | G | 1 | 0.48 |
| ggg | G | 0 | 0.26 |
| --- | -- | ------ | ------ |
| mPD | | 0.13 | 0.49 |
| | | nPD: | 0.27 |
| | | N. weight: | 0.51 |
| | | Sc. PD: | 0.0057 |
| | | Sc. rank: | 26.7 |

PB1

| Pos. | 372 | obs: | exp: |
|---|---|---|---|
| atg | M | 367 | 367.00 |
| --- | -- | ------ | ------ |
| mPD | | 0 | 0 |
| | | nPD: | 1. |
| | | N. weight: | 0. |
| | | Sc. PD: | 0 |
| | | Sc. rank: | 0 |

PB1

| Pos. | 373 | obs: | exp: |
|---|---|---|---|
| tta | L | 0 | 32.06 |
| ttg | L | 0 | 70.68 |
| ctt | L | 244 | 68.48 |
| ctc | L | 28 | 59.37 |
| cta | L | 91 | 55.29 |
| ctg | L | 4 | 81.13 |
| --- | -- | ------ | ------ |
| mPD | | 0.49 | 1.1 |
| | | nPD: | 0.44 |
| | | N. weight: | 1.4 |
| | | Sc. PD: | 0.24 |
| | | Sc. rank: | 156.1 |

PB1

| Pos. | 374 | obs: | exp: |
|---|---|---|---|
| tct | S | 0 | 0.16 |
| tcc | S | 0 | 0.14 |
| tca | S | 1 | 0.22 |
| tcg | S | 0 | 0.06 |
| act | T | 0 | 0.26 |
| acc | T | 0 | 0.21 |
| aca | T | 1 | 0.44 |
| acg | T | 0 | 0.08 |
| agt | S | 0 | 0.22 |
| agc | S | 0 | 0.20 |
| gct | A | 0 | 91.93 |
| gcc | A | 3 | 74.77 |
| gca | A | 361 | 162.70 |
| gcg | A | 1 | 35.62 |
| --- | -- | ------ | ------ |
| mPD | | 0.033 | 0.70 |
| | | nPD: | 0.05 |
| | | N. weight: | 1.2 |
| | | Sc. PD: | -0.23 |
| | | Sc. rank: | -143.0 |

PB1

| Pos. | 375 | obs: | exp: |
|---|---|---|---|
| tct | S | 0 | 16.37 |
| tcc | S | 0 | 14.35 |
| tca | S | 0 | 22.82 |
| tcg | S | 0 | 6.28 |
| act | T | 0 | 0.26 |
| acc | T | 1 | 0.21 |
| aca | T | 0 | 0.44 |
| acg | T | 0 | 0.08 |
| aat | N | 39 | 133.80 |
| aac | N | 217 | 122.20 |
| agt | S | 29 | 22.66 |
| agc | S | 74 | 20.52 |
| gat | D | 4 | 3.73 |
| gac | D | 3 | 3.27 |
| --- | -- | ------ | ------ |
| mPD | | 0.76 | 1.3 |
| | | nPD: | 0.58 |
| | | N. weight: | 0.77 |
| | | Sc. PD: | 0.24 |
| | | Sc. rank: | 118.0 |

| | | | |
|---|---|---|---|
| PB1 | | | |
| Pos. | 376 | obs: | exp: |
| att | I | 345 | 138.00 |
| atc | I | 22 | 96.18 |
| ata | I | 0 | 132.80 |
| --- | -- | ------ | ------ |
| mPD | | 0.11 | 0.66 |
| | | nPD: | 0.17 |
| | | N. weight: | 1.2 |
| | | Sc. PD: | -0.096 |
| | | Sc. rank: | -2.7 |

| | | | |
|---|---|---|---|
| PB1 | | | |
| Pos. | 377 | obs: | exp: |
| gat | D | 118 | 195.80 |
| gac | D | 249 | 171.20 |
| --- | -- | ------ | ------ |
| mPD | | 0.44 | 0.50 |
| | | nPD: | 0.88 |
| | | N. weight: | 0.15 |
| | | Sc. PD: | 0.09 |
| | | Sc. rank: | 31.3 |

| | | | |
|---|---|---|---|
| PB1 | | | |
| Pos. | 378 | obs: | exp: |
| tta | L | 29 | 32.06 |
| ttg | L | 178 | 70.68 |
| ctt | L | 2 | 68.48 |
| ctc | L | 0 | 59.37 |
| cta | L | 4 | 55.29 |
| ctg | L | 154 | 81.13 |
| --- | -- | ------ | ------ |
| mPD | | 0.67 | 1.1 |
| | | nPD: | 0.6 |
| | | N. weight: | 1. |
| | | Sc. PD: | 0.34 |
| | | Sc. rank: | 163.4 |

| | | | |
|---|---|---|---|
| PB1 | | | |
| Pos. | 379 | obs: | exp: |
| aaa | K | 296 | 205.50 |
| aag | K | 71 | 161.50 |
| --- | -- | ------ | ------ |
| mPD | | 0.31 | 0.49 |
| | | nPD: | 0.63 |
| | | N. weight: | 0.22 |
| | | Sc. PD: | 0.078 |
| | | Sc. rank: | 35.8 |

| | | | |
|---|---|---|---|
| PB1 | | | |
| Pos. | 380 | obs: | exp: |
| tat | Y | 160 | 190.00 |
| tac | Y | 207 | 177.00 |
| --- | -- | ------ | ------ |
| mPD | | 0.49 | 0.50 |
| | | nPD: | 0.99 |
| | | N. weight: | 0.033 |
| | | Sc. PD: | 0.023 |
| | | Sc. rank: | 7.0 |

| | | | |
|---|---|---|---|
| PB1 | | | |
| Pos. | 381 | obs: | exp: |
| ttt | F | 7 | 150.50 |
| ttc | F | 359 | 215.50 |
| tta | L | 0 | 0.09 |
| ttg | L | 0 | 0.19 |
| ctt | L | 0 | 0.19 |
| ctc | L | 1 | 0.16 |
| cta | L | 0 | 0.15 |
| ctg | L | 0 | 0.22 |
| --- | -- | ------ | ------ |
| mPD | | 0.043 | 0.49 |
| | | nPD: | 0.09 |
| | | N. weight: | 0.69 |
| | | Sc. PD: | -0.11 |
| | | Sc. rank: | -48.0 |

| | | | |
|---|---|---|---|
| PB1 | | | |
| Pos. | 382 | obs: | exp: |
| aat | N | 165 | 191.80 |
| aac | N | 202 | 175.20 |
| --- | -- | ------ | ------ |
| mPD | | 0.50 | 0.50 |
| | | nPD: | 0.99 |
| | | N. weight: | 0.029 |
| | | Sc. PD: | 0.021 |
| | | Sc. rank: | 6.1 |

| | | | |
|---|---|---|---|
| PB1 | | | |
| Pos. | 383 | obs: | exp: |
| aaa | K | 26 | 14.56 |
| aag | K | 0 | 11.44 |
| gat | D | 0 | 1.07 |
| gac | D | 2 | 0.93 |
| gaa | E | 227 | 195.10 |
| gag | E | 112 | 143.90 |
| --- | -- | ------ | ------ |
| mPD | | 0.56 | 0.63 |
| | | nPD: | 0.9 |
| | | N. weight: | 0.11 |
| | | Sc. PD: | 0.066 |
| | | Sc. rank: | 22.3 |

| | | | |
|---|---|---|---|
| PB1 | | | |
| Pos. | 384 | obs: | exp: |
| tta | L | 2 | 0.17 |
| ttg | L | 0 | 0.39 |
| tct | S | 0 | 57.39 |
| tcc | S | 2 | 50.29 |
| tca | S | 341 | 79.98 |
| tcg | S | 18 | 22.00 |
| ctt | L | 0 | 0.37 |
| ctc | L | 0 | 0.32 |
| cta | L | 0 | 0.30 |
| ctg | L | 0 | 0.44 |
| cct | P | 0 | 1.05 |
| ccc | P | 0 | 0.72 |
| cca | P | 4 | 1.58 |
| ccg | P | 0 | 0.65 |
| agt | S | 0 | 79.42 |
| agc | S | 0 | 71.92 |
| --- | -- | ------ | ------ |
| mPD | | 0.14 | 1.7 |
| | | nPD: | 0.08 |
| | | N. weight: | 2.1 |
| | | Sc. PD: | -0.34 |
| | | Sc. rank: | -157.0 |

| | | | |
|---|---|---|---|
| PB1 | | | |
| Pos. | 385 | obs: | exp: |
| act | T | 0 | 96.96 |
| acc | T | 0 | 78.75 |
| aca | T | 232 | 160.80 |
| acg | T | 135 | 30.51 |
| --- | -- | ------ | ------ |
| mPD | | 0.47 | 0.69 |
| | | nPD: | 0.68 |
| | | N. weight: | 1.2 |
| | | Sc. PD: | 0.48 |
| | | Sc. rank: | 208.6 |

| | | | |
|---|---|---|---|
| PB1 | | | |
| Pos. | 386 | obs: | exp: |
| cgt | R | 0 | 14.36 |
| cgc | R | 0 | 16.94 |
| cga | R | 0 | 33.25 |
| cgg | R | 1 | 28.87 |
| aaa | K | 15 | 11.76 |
| aag | K | 6 | 9.24 |
| aga | R | 300 | 155.60 |
| agg | R | 45 | 96.96 |
| --- | -- | ------ | ------ |
| mPD | | 0.36 | 1.0 |
| | | nPD: | 0.34 |
| | | N. weight: | 0.69 |
| | | Sc. PD: | 0.055 |
| | | Sc. rank: | 53.5 |

| | | | |
|---|---|---|---|
| PB1 | | | |
| Pos. | 387 | obs: | exp: |
| aaa | K | 0 | 190.90 |
| aag | K | 341 | 150.10 |
| gaa | E | 0 | 14.96 |
| gag | E | 26 | 11.04 |
| --- | -- | ------ | ------ |
| mPD | | 0.13 | 0.62 |
| | | nPD: | 0.21 |
| | | N. weight: | 1.3 |
| | | Sc. PD: | -0.054 |
| | | Sc. rank: | 26.9 |

| | | | |
|---|---|---|---|
| PB1 | | | |
| Pos. | 388 | obs: | exp: |
| cgt | R | 0 | 0.04 |
| cgc | R | 0 | 0.05 |
| cga | R | 0 | 0.10 |
| cgg | R | 0 | 0.08 |
| aaa | K | 365 | 204.90 |
| aag | K | 1 | 161.10 |
| aga | R | 1 | 0.45 |
| agg | R | 0 | 0.28 |
| --- | -- | ------ | ------ |
| mPD | | 0.011 | 0.50 |
| | | nPD: | 0.02 |
| | | N. weight: | 0.86 |
| | | Sc. PD: | -0.19 |
| | | Sc. rank: | -135.4 |

| | | | |
|---|---|---|---|
| PB1 | | | |
| Pos. | 389 | obs: | exp: |
| att | I | 30 | 138.00 |
| atc | I | 252 | 96.18 |
| ata | I | 85 | 132.80 |
| --- | -- | ------ | ------ |
| mPD | | 0.47 | 0.66 |
| | | nPD: | 0.71 |
| | | N. weight: | 0.68 |
| | | Sc. PD: | 0.3 |
| | | Sc. rank: | 123.8 |

| | | | |
|---|---|---|---|
| PB1 | | | |
| Pos. | 390 | obs: | exp: |
| gaa | E | 15 | 211.20 |
| gag | E | 352 | 155.80 |
| --- | -- | ------ | ------ |
| mPD | | 0.079 | 0.49 |
| | | nPD: | 0.16 |
| | | N. weight: | 1. |
| | | Sc. PD: | -0.094 |
| | | Sc. rank: | -8.5 |

| | | | |
|---|---|---|---|
| PB1 | | | |
| Pos. | 391 | obs: | exp: |
| cgt | R | 0 | 0.17 |
| cgc | R | 0 | 0.20 |
| cga | R | 0 | 0.38 |
| cgg | R | 0 | 0.33 |
| aaa | K | 278 | 203.30 |
| aag | K | 85 | 159.70 |
| aga | R | 4 | 1.80 |
| agg | R | 0 | 1.12 |
| --- | -- | ------ | ------ |
| mPD | | 0.38 | 0.52 |
| | | nPD: | 0.73 |
| | | N. weight: | 0.16 |
| | | Sc. PD: | 0.073 |
| | | Sc. rank: | 30.5 |

| | | | |
|---|---|---|---|
| PB1 | | | |
| Pos. | 392 | obs: | exp: |
| att | I | 8 | 136.10 |
| atc | I | 2 | 94.87 |
| ata | I | 352 | 131.00 |
| atg | M | 3 | 3.00 |
| gtt | V | 0 | 0.41 |
| gtc | V | 0 | 0.42 |
| gta | V | 2 | 0.37 |
| gtg | V | 0 | 0.79 |
| --- | -- | ------ | ------ |
| mPD | | 0.080 | 0.68 |
| | | nPD: | 0.12 |
| | | N. weight: | 1.3 |
| | | Sc. PD: | -0.18 |
| | | Sc. rank: | -52.1 |

| | | | |
|---|---|---|---|
| PB1 | | | |
| Pos. | 393 | obs: | exp: |
| cgt | R | 0 | 15.24 |
| cgc | R | 0 | 17.97 |
| cga | R | 0 | 35.27 |
| cgg | R | 0 | 30.63 |
| aga | R | 305 | 165.00 |
| agg | R | 62 | 102.80 |
| --- | -- | ------ | ------ |
| mPD | | 0.28 | 0.96 |
| | | nPD: | 0.29 |
| | | N. weight: | 0.66 |
| | | Sc. PD: | 0.023 |
| | | Sc. rank: | 45.7 |

| | | | |
|---|---|---|---|
| PB1 | | | |
| Pos. | 394 | obs: | exp: |
| tct | S | 2 | 0.32 |
| tcc | S | 0 | 0.28 |
| tca | S | 0 | 0.44 |
| tcg | S | 0 | 0.12 |
| cct | P | 356 | 96.11 |
| ccc | P | 9 | 65.84 |
| cca | P | 0 | 144.00 |
| ccg | P | 0 | 59.02 |
| agt | S | 0 | 0.44 |
| agc | S | 0 | 0.40 |
| --- | -- | ------ | ------ |
| mPD | | 0.059 | 0.73 |
| | | nPD: | 0.08 |
| | | N. weight: | 1.9 |
| | | Sc. PD: | -0.31 |
| | | Sc. rank: | -145.6 |

| | | | |
|---|---|---|---|
| PB1 | | | |
| Pos. | 395 | obs: | exp: |
| tta | L | 2 | 32.06 |
| ttg | L | 0 | 70.68 |
| ctt | L | 88 | 68.48 |
| ctc | L | 41 | 59.37 |
| cta | L | 64 | 55.29 |
| ctg | L | 172 | 81.13 |
| --- | -- | ------ | ------ |
| mPD | | 0.69 | 1.1 |
| | | nPD: | 0.62 |
| | | N. weight: | 0.62 |
| | | Sc. PD: | 0.21 |
| | | Sc. rank: | 100.5 |

| | | | |
|---|---|---|---|
| PB1 | | | |
| Pos. | 396 | obs: | exp: |
| tta | L | 43 | 31.89 |
| ttg | L | 3 | 70.30 |
| ctt | L | 0 | 68.10 |
| ctc | L | 4 | 59.04 |
| cta | L | 221 | 54.99 |
| ctg | L | 94 | 80.68 |
| att | I | 0 | 0.75 |
| atc | I | 0 | 0.52 |
| ata | I | 2 | 0.72 |
| --- | -- | ------ | ------ |
| mPD | | 0.64 | 1.1 |
| | | nPD: | 0.57 |
| | | N. weight: | 1.3 |
| | | Sc. PD: | 0.4 |
| | | Sc. rank: | 200.1 |

| | | | |
|---|---|---|---|
| PB1 | | | |
| Pos. | 397 | obs: | exp: |
| att | I | 141 | 138.00 |
| atc | I | 2 | 96.18 |
| ata | I | 224 | 132.80 |
| --- | -- | ------ | ------ |
| mPD | | 0.48 | 0.66 |
| | | nPD: | 0.73 |
| | | N. weight: | 0.48 |
| | | Sc. PD: | 0.22 |
| | | Sc. rank: | 90.8 |

| | | | |
|---|---|---|---|
| PB1 | | | |
| Pos. | 398 | obs: | exp: |
| gat | D | 364 | 195.80 |
| gac | D | 3 | 171.20 |
| --- | -- | ------ | ------ |
| mPD | | 0.016 | 0.50 |
| | | nPD: | 0.03 |
| | | N. weight: | 0.89 |
| | | Sc. PD: | -0.19 |
| | | Sc. rank: | -128.6 |

| | | | |
|---|---|---|---|
| PB1 | | | |
| Pos. | 399 | obs: | exp: |
| tct | S | 0 | 0.16 |
| tcc | S | 0 | 0.14 |
| tca | S | 0 | 0.22 |
| tcg | S | 0 | 0.06 |
| agt | S | 1 | 0.22 |
| agc | S | 0 | 0.20 |
| ggt | G | 94 | 45.16 |
| ggc | G | 261 | 49.50 |
| gga | G | 9 | 177.00 |
| ggg | G | 2 | 94.30 |
| --- | -- | ------ | ------ |
| mPD | | 0.43 | 0.68 |
| | | nPD: | 0.64 |
| | | N. weight: | 2. |
| | | Sc. PD: | 0.72 |
| | | Sc. rank: | 329.2 |

| | | | |
|---|---|---|---|
| PB1 | | | |
| Pos. | 400 | obs: | exp: |
| act | T | 7 | 96.96 |
| acc | T | 13 | 78.75 |
| aca | T | 345 | 160.80 |
| acg | T | 2 | 30.51 |
| --- | -- | ------ | ------ |
| mPD | | 0.11 | 0.69 |
| | | nPD: | 0.17 |
| | | N. weight: | 0.92 |
| | | Sc. PD: | -0.077 |
| | | Sc. rank: | -5.5 |

| PB1 | | | |
|---|---|---|---|
| Pos. | 401 | obs: | exp: |
| gct | A | 9 | 92.44 |
| gcc | A | 351 | 75.18 |
| gca | A | 7 | 163.60 |
| gcg | A | 0 | 35.81 |
| --- | -- | ------ | ------ |
| mPD | | 0.085 | 0.69 |
| | | nPD: | 0.12 |
| | | N. weight: | 2.1 |
| | | Sc. PD: | -0.26 |
| | | Sc. rank: | -73.8 |

| PB1 | | | |
|---|---|---|---|
| Pos. | 402 | obs: | exp: |
| tct | S | 0 | 58.34 |
| tcc | S | 5 | 51.12 |
| tca | S | 356 | 81.31 |
| tcg | S | 6 | 22.37 |
| agt | S | 0 | 80.74 |
| agc | S | 0 | 73.12 |
| --- | -- | ------ | ------ |
| mPD | | 0.059 | 1.7 |
| | | nPD: | 0.04 |
| | | N. weight: | 2.1 |
| | | Sc. PD: | -0.44 |
| | | Sc. rank: | -294.2 |

| PB1 | | | |
|---|---|---|---|
| Pos. | 403 | obs: | exp: |
| tta | L | 50 | 32.06 |
| ttg | L | 290 | 70.68 |
| ctt | L | 0 | 68.48 |
| ctc | L | 0 | 59.37 |
| cta | L | 1 | 55.29 |
| ctg | L | 26 | 81.13 |
| --- | -- | ------ | ------ |
| mPD | | 0.38 | 1.1 |
| | | nPD: | 0.34 |
| | | N. weight: | 1.7 |
| | | Sc. PD: | 0.13 |
| | | Sc. rank: | 127.6 |

| PB1 | | | |
|---|---|---|---|
| Pos. | 404 | obs: | exp: |
| tct | S | 0 | 58.34 |
| tcc | S | 0 | 51.12 |
| tca | S | 0 | 81.31 |
| tcg | S | 0 | 22.37 |
| agt | S | 215 | 80.74 |
| agc | S | 152 | 73.12 |
| --- | -- | ------ | ------ |
| mPD | | 0.49 | 1.7 |
| | | nPD: | 0.29 |
| | | N. weight: | 1.3 |
| | | Sc. PD: | 0.046 |
| | | Sc. rank: | 92.5 |

| PB1 | | | |
|---|---|---|---|
| Pos. | 405 | obs: | exp: |
| cct | P | 344 | 96.64 |
| ccc | P | 8 | 66.20 |
| cca | P | 8 | 144.80 |
| ccg | P | 7 | 59.34 |
| --- | -- | ------ | ------ |
| mPD | | 0.12 | 0.72 |
| | | nPD: | 0.17 |
| | | N. weight: | 1.6 |
| | | Sc. PD: | -0.13 |
| | | Sc. rank: | -8.3 |

| PB1 | | | |
|---|---|---|---|
| Pos. | 406 | obs: | exp: |
| ggt | G | 0 | 45.28 |
| ggc | G | 0 | 49.63 |
| gga | G | 360 | 177.50 |
| ggg | G | 7 | 94.55 |
| --- | -- | ------ | ------ |
| mPD | | 0.038 | 0.67 |
| | | nPD: | 0.06 |
| | | N. weight: | 0.99 |
| | | Sc. PD: | -0.19 |
| | | Sc. rank: | -106.0 |

| PB1 | | | |
|---|---|---|---|
| Pos. | 407 | obs: | exp: |
| atg | M | 367 | 367.00 |
| --- | -- | ------ | ------ |
| mPD | | 0 | 0 |
| | | nPD: | 1. |
| | | N. weight: | 0. |
| | | Sc. PD: | 0 |
| | | Sc. rank: | 0 |

| PB1 | | | |
|---|---|---|---|
| Pos. | 408 | obs: | exp: |
| atg | M | 367 | 367.00 |
| --- | -- | ------ | ------ |
| mPD | | 0 | 0 |
| | | nPD: | 1. |
| | | N. weight: | 0. |
| | | Sc. PD: | 0 |
| | | Sc. rank: | 0 |

| PB1 | | | |
|---|---|---|---|
| Pos. | 409 | obs: | exp: |
| atg | M | 367 | 367.00 |
| --- | -- | ------ | ------ |
| mPD | | 0 | 0 |
| | | nPD: | 1. |
| | | N. weight: | 0. |
| | | Sc. PD: | 0 |
| | | Sc. rank: | 0 |

| PB1 | | | |
|---|---|---|---|
| Pos. | 410 | obs: | exp: |
| ggt | G | 22 | 45.28 |
| ggc | G | 341 | 49.63 |
| gga | G | 4 | 177.50 |
| ggg | G | 0 | 94.55 |
| --- | -- | ------ | ------ |
| mPD | | 0.13 | 0.67 |
| | | nPD: | 0.2 |
| | | N. weight: | 2.6 |
| | | Sc. PD: | -0.14 |
| | | Sc. rank: | 33.5 |

| PB1 | | | |
|---|---|---|---|
| Pos. | 411 | obs: | exp: |
| atg | M | 367 | 367.00 |
| --- | -- | ------ | ------ |
| mPD | | 0 | 0 |
| | | nPD: | 1. |
| | | N. weight: | 0. |
| | | Sc. PD: | 0 |
| | | Sc. rank: | 0 |

| PB1 | | | |
|---|---|---|---|
| Pos. | 412 | obs: | exp: |
| ttt | F | 3 | 150.90 |
| ttc | F | 364 | 216.10 |
| --- | -- | ------ | ------ |
| mPD | | 0.016 | 0.48 |
| | | nPD: | 0.03 |
| | | N. weight: | 0.74 |
| | | Sc. PD: | -0.16 |
| | | Sc. rank: | -106.7 |

| PB1 | | | |
|---|---|---|---|
| Pos. | 413 | obs: | exp: |
| aat | N | 200 | 191.80 |
| aac | N | 167 | 175.20 |
| --- | -- | ------ | ------ |
| mPD | | 0.50 | 0.50 |
| | | nPD: | 1. |
| | | N. weight: | 0.015 |
| | | Sc. PD: | 0.01 |
| | | Sc. rank: | 3.1 |

| PB1 | | | |
|---|---|---|---|
| Pos. | 414 | obs: | exp: |
| atg | M | 367 | 367.00 |
| --- | -- | ------ | ------ |
| mPD | | 0 | 0 |
| | | nPD: | 1. |
| | | N. weight: | 0. |
| | | Sc. PD: | 0 |
| | | Sc. rank: | 0 |

| PB1 | | | |
|---|---|---|---|
| Pos. | 415 | obs: | exp: |
| tta | L | 1 | 32.06 |
| ttg | L | 47 | 70.68 |
| ctt | L | 0 | 68.48 |
| ctc | L | 0 | 59.37 |
| cta | L | 57 | 55.29 |
| ctg | L | 262 | 81.13 |
| --- | -- | ------ | ------ |
| mPD | | 0.49 | 1.1 |
| | | nPD: | 0.44 |
| | | N. weight: | 1.2 |
| | | Sc. PD: | 0.22 |
| | | Sc. rank: | 142.8 |

| PB1 | | | |
|---|---|---|---|
| Pos. | 416 | obs: | exp: |
| tct | S | 0 | 58.34 |
| tcc | S | 0 | 51.12 |
| tca | S | 0 | 81.31 |
| tcg | S | 0 | 22.37 |
| agt | S | 227 | 80.74 |
| agc | S | 140 | 73.12 |
| --- | -- | ------ | ------ |
| mPD | | 0.47 | 1.7 |
| | | nPD: | 0.28 |
| | | N. weight: | 1.4 |
| | | Sc. PD: | 0.036 |
| | | Sc. rank: | 88.0 |

| PB1 | | | |
|---|---|---|---|
| Pos. | 417 | obs: | exp: |
| act | T | 1 | 96.96 |
| acc | T | 1 | 78.75 |
| aca | T | 361 | 160.80 |
| acg | T | 4 | 30.51 |
| --- | -- | ------ | ------ |
| mPD | | 0.032 | 0.69 |
| | | nPD: | 0.05 |
| | | N. weight: | 1.2 |
| | | Sc. PD: | -0.23 |
| | | Sc. rank: | -139.6 |

| PB1 | | | |
|---|---|---|---|
| Pos. | 418 | obs: | exp: |
| gtt | V | 93 | 75.43 |
| gtc | V | 52 | 77.03 |
| gta | V | 212 | 68.78 |
| gtg | V | 10 | 145.80 |
| --- | -- | ------ | ------ |
| mPD | | 0.58 | 0.72 |
| | | nPD: | 0.81 |
| | | N. weight: | 0.91 |
| | | Sc. PD: | 0.48 |
| | | Sc. rank: | 182.2 |

| PB1 | | | |
|---|---|---|---|
| Pos. | 419 | obs: | exp: |
| tta | L | 253 | 32.06 |
| ttg | L | 107 | 70.68 |
| ctt | L | 0 | 68.48 |
| ctc | L | 0 | 59.37 |
| cta | L | 6 | 55.29 |
| ctg | L | 1 | 81.13 |
| --- | -- | ------ | ------ |
| mPD | | 0.45 | 1.1 |
| | | nPD: | 0.41 |
| | | N. weight: | 2.3 |
| | | Sc. PD: | 0.33 |
| | | Sc. rank: | 244.7 |

| PB1 | | | |
|---|---|---|---|
| Pos. | 420 | obs: | exp: |
| ggt | G | 1 | 45.28 |
| ggc | G | 0 | 49.63 |
| gga | G | 260 | 177.50 |
| ggg | G | 106 | 94.55 |
| --- | -- | ------ | ------ |
| mPD | | 0.42 | 0.67 |
| | | nPD: | 0.62 |
| | | N. weight: | 0.46 |
| | | Sc. PD: | 0.16 |
| | | Sc. rank: | 75.3 |

| PB1 | | | |
|---|---|---|---|
| Pos. | 421 | obs: | exp: |
| gtt | V | 110 | 75.43 |
| gtc | V | 253 | 77.03 |
| gta | V | 2 | 68.78 |
| gtg | V | 2 | 145.80 |
| --- | -- | ------ | ------ |
| mPD | | 0.44 | 0.72 |
| | | nPD: | 0.6 |
| | | N. weight: | 1.4 |
| | | Sc. PD: | 0.46 |
| | | Sc. rank: | 219.0 |

| PB1 | | | |
|---|---|---|---|
| Pos. | 422 | obs: | exp: |
| tct | S | 0 | 58.34 |
| tcc | S | 0 | 51.12 |
| tca | S | 265 | 81.31 |
| tcg | S | 102 | 22.37 |
| agt | S | 0 | 80.74 |
| agc | S | 0 | 73.12 |
| --- | -- | ------ | ------ |
| mPD | | 0.40 | 1.7 |
| | | nPD: | 0.24 |
| | | N. weight: | 2. |
| | | Sc. PD: | -0.027 |
| | | Sc. rank: | 75.5 |

| PB1 | | | |
|---|---|---|---|
| Pos. | 423 | obs: | exp: |
| att | I | 137 | 137.20 |
| atc | I | 226 | 95.66 |
| ata | I | 2 | 132.10 |
| act | T | 0 | 0.26 |
| acc | T | 1 | 0.21 |
| aca | T | 0 | 0.44 |
| acg | T | 0 | 0.08 |
| gtt | V | 0 | 0.21 |
| gtc | V | 1 | 0.21 |
| gta | V | 0 | 0.19 |
| gtg | V | 0 | 0.40 |
| --- | -- | ------ | ------ |
| mPD | | 0.49 | 0.67 |
| | | nPD: | 0.73 |
| | | N. weight: | 0.8 |
| | | Sc. PD: | 0.36 |
| | | Sc. rank: | 148.6 |

| PB1 | | | |
|---|---|---|---|
| Pos. | 424 | obs: | exp: |
| tta | L | 2 | 32.06 |
| ttg | L | 7 | 70.68 |
| ctt | L | 0 | 68.48 |
| ctc | L | 0 | 59.37 |
| cta | L | 87 | 55.29 |
| ctg | L | 271 | 81.13 |
| --- | -- | ------ | ------ |
| mPD | | 0.42 | 1.1 |
| | | nPD: | 0.37 |
| | | N. weight: | 1.5 |
| | | Sc. PD: | 0.16 |
| | | Sc. rank: | 128.3 |

| PB1 | | | |
|---|---|---|---|
| Pos. | 425 | obs: | exp: |
| aat | N | 353 | 191.80 |
| aac | N | 14 | 175.20 |
| --- | -- | ------ | ------ |
| mPD | | 0.074 | 0.50 |
| | | nPD: | 0.15 |
| | | N. weight: | 0.76 |
| | | Sc. PD: | -0.078 |
| | | Sc. rank: | -13.7 |

| PB1 | | | |
|---|---|---|---|
| Pos. | 426 | obs: | exp: |
| tta | L | 2 | 31.97 |
| ttg | L | 0 | 70.49 |
| ctt | L | 246 | 68.29 |
| ctc | L | 28 | 59.20 |
| cta | L | 89 | 55.14 |
| ctg | L | 1 | 80.90 |
| att | I | 1 | 0.38 |
| atc | I | 0 | 0.26 |
| ata | I | 0 | 0.36 |
| --- | -- | ------ | ------ |
| mPD | | 0.50 | 1.1 |
| | | nPD: | 0.44 |
| | | N. weight: | 1.4 |
| | | Sc. PD: | 0.25 |
| | | Sc. rank: | 164.8 |

| PB1 | | | |
|---|---|---|---|
| Pos. | 427 | obs: | exp: |
| ggt | G | 0 | 45.28 |
| ggc | G | 41 | 49.63 |
| gga | G | 129 | 177.50 |
| ggg | G | 197 | 94.55 |
| --- | -- | ------ | ------ |
| mPD | | 0.58 | 0.67 |
| | | nPD: | 0.87 |
| | | N. weight: | 0.42 |
| | | Sc. PD: | 0.24 |
| | | Sc. rank: | 86.5 |

| PB1 | | | |
|---|---|---|---|
| Pos. | 428 | obs: | exp: |
| caa | Q | 310 | 195.60 |
| cag | Q | 57 | 171.40 |
| --- | -- | ------ | ------ |
| mPD | | 0.26 | 0.50 |
| | | nPD: | 0.53 |
| | | N. weight: | 0.34 |
| | | Sc. PD: | 0.089 |
| | | Sc. rank: | 47.7 |

| PB1 | | | |
|---|---|---|---|
| Pos. | 429 | obs: | exp: |
| aaa | K | 13 | 205.50 |
| aag | K | 354 | 161.50 |
| --- | -- | ------ | ------ |
| mPD | | 0.069 | 0.49 |
| | | nPD: | 0.14 |
| | | N. weight: | 1. |
| | | Sc. PD: | -0.11 |
| | | Sc. rank: | -26.8 |

| PB1 | | | |
|---|---|---|---|
| Pos. | 430 | obs: | exp: |
| cgt | R | 0 | 13.70 |
| cgc | R | 0 | 16.16 |
| cga | R | 0 | 31.71 |
| cgg | R | 0 | 27.54 |
| aaa | K | 0 | 20.72 |
| aag | K | 37 | 16.28 |
| aga | R | 41 | 148.40 |
| agg | R | 289 | 92.48 |
| --- | -- | ------ | ------ |
| mPD | | 0.38 | 1.1 |
| | | nPD: | 0.34 |
| | | N. weight: | 1.3 |
| | | Sc. PD: | 0.11 |
| | | Sc. rank: | 103.5 |

| PB1 | | | |
|---|---|---|---|
| Pos. | 431 | obs: | exp: |
| tat | Y | 6 | 187.90 |
| tac | Y | 357 | 175.10 |
| cat | H | 0 | 2.17 |
| cac | H | 4 | 1.83 |
| --- | -- | ------ | ------ |
| mPD | | 0.054 | 0.52 |
| | | nPD: | 0.1 |
| | | N. weight: | 0.99 |
| | | Sc. PD: | -0.14 |
| | | Sc. rank: | -55.1 |

,

| PB1 | | | |
|---|---|---|---|
| Pos. | 432 | obs: | exp: |
| act | T | 3 | 96.96 |
| acc | T | 357 | 78.75 |
| aca | T | 7 | 160.80 |
| acg | T | 0 | 30.51 |
| --- | -- | ------ | ------ |
| mPD | | 0.053 | 0.69 |
| | | nPD: | 0.08 |
| | | N. weight: | 2.1 |
| | | Sc. PD: | -0.36 |
| | | Sc. rank: | -166.0 |

,

| PB1 | | | |
|---|---|---|---|
| Pos. | 433 | obs: | exp: |
| aaa | K | 360 | 205.50 |
| aag | K | 7 | 161.50 |
| --- | -- | ------ | ------ |
| mPD | | 0.038 | 0.49 |
| | | nPD: | 0.08 |
| | | N. weight: | 0.75 |
| | | Sc. PD: | -0.13 |
| | | Sc. rank: | -63.1 |

,

| PB1 | | | |
|---|---|---|---|
| Pos. | 434 | obs: | exp: |
| act | T | 2 | 96.96 |
| acc | T | 271 | 78.75 |
| aca | T | 92 | 160.80 |
| acg | T | 2 | 30.51 |
| --- | -- | ------ | ------ |
| mPD | | 0.39 | 0.69 |
| | | nPD: | 0.57 |
| | | N. weight: | 1.1 |
| | | Sc. PD: | 0.34 |
| | | Sc. rank: | 171.9 |

,

| PB1 | | | |
|---|---|---|---|
| Pos. | 435 | obs: | exp: |
| att | I | 0 | 0.38 |
| atc | I | 0 | 0.26 |
| ata | I | 1 | 0.36 |
| act | T | 0 | 96.69 |
| acc | T | 3 | 78.54 |
| aca | T | 355 | 160.30 |
| acg | T | 8 | 30.43 |
| --- | -- | ------ | ------ |
| mPD | | 0.064 | 0.69 |
| | | nPD: | 0.09 |
| | | N. weight: | 1.1 |
| | | Sc. PD: | -0.17 |
| | | Sc. rank: | -65.6 |

,

| PB1 | | | |
|---|---|---|---|
| Pos. | 436 | obs: | exp: |
| tat | Y | 36 | 190.00 |
| tac | Y | 331 | 177.00 |
| --- | -- | ------ | ------ |
| mPD | | 0.18 | 0.50 |
| | | nPD: | 0.36 |
| | | N. weight: | 0.62 |
| | | Sc. PD: | 0.058 |
| | | Sc. rank: | 51.5 |

,

| PB1 | | | |
|---|---|---|---|
| Pos. | 437 | obs: | exp: |
| tgg | W | 367 | 367.00 |
| --- | -- | ------ | ------ |
| mPD | | 0 | 0 |
| | | nPD: | 1. |
| | | N. weight: | 0. |
| | | Sc. PD: | 0 |
| | | Sc. rank: | 0 |

,

| PB1 | | | |
|---|---|---|---|
| Pos. | 438 | obs: | exp: |
| tgg | W | 367 | 367.00 |
| --- | -- | ------ | ------ |
| mPD | | 0 | 0 |
| | | nPD: | 1. |
| | | N. weight: | 0. |
| | | Sc. PD: | 0 |
| | | Sc. rank: | 0 |

,

| PB1 | | | |
|---|---|---|---|
| Pos. | 439 | obs: | exp: |
| gat | D | 231 | 195.30 |
| gac | D | 135 | 170.70 |
| gaa | E | 0 | 0.58 |
| gag | E | 1 | 0.42 |
| --- | -- | ------ | ------ |
| mPD | | 0.47 | 0.50 |
| | | nPD: | 0.94 |
| | | N. weight: | 0.046 |
| | | Sc. PD: | 0.03 |
| | | Sc. rank: | 9.6 |

,

| PB1 | | | |
|---|---|---|---|
| Pos. | 440 | obs: | exp: |
| ggt | G | 0 | 45.28 |
| ggc | G | 0 | 49.63 |
| gga | G | 351 | 177.50 |
| ggg | G | 16 | 94.55 |
| --- | -- | ------ | ------ |
| mPD | | 0.084 | 0.67 |
| | | nPD: | 0.13 |
| | | N. weight: | 0.88 |
| | | Sc. PD: | -0.11 |
| | | Sc. rank: | -29.2 |

,

| PB1 | | | |
|---|---|---|---|
| Pos. | 441 | obs: | exp: |
| tta | L | 0 | 32.06 |
| ttg | L | 0 | 70.68 |
| ctt | L | 39 | 68.48 |
| ctc | L | 326 | 59.37 |
| cta | L | 2 | 55.29 |
| ctg | L | 0 | 81.13 |
| --- | -- | ------ | ------ |
| mPD | | 0.20 | 1.1 |
| | | nPD: | 0.18 |
| | | N. weight: | 2.2 |
| | | Sc. PD: | -0.16 |
| | | Sc. rank: | 1.9 |

,

| PB1 | | | |
|---|---|---|---|
| Pos. | 442 | obs: | exp: |
| caa | Q | 339 | 195.60 |
| cag | Q | 28 | 171.40 |
| --- | -- | ------ | ------ |
| mPD | | 0.14 | 0.50 |
| | | nPD: | 0.28 |
| | | N. weight: | 0.57 |
| | | Sc. PD: | 0.015 |
| | | Sc. rank: | 36.6 |

,

| PB1 | | | |
|---|---|---|---|
| Pos. | 443 | obs: | exp: |
| tct | S | 4 | 58.34 |
| tcc | S | 362 | 51.12 |
| tca | S | 1 | 81.31 |
| tcg | S | 0 | 22.37 |
| agt | S | 0 | 80.74 |
| agc | S | 0 | 73.12 |
| --- | -- | ------ | ------ |
| mPD | | 0.027 | 1.7 |
| | | nPD: | 0.02 |
| | | N. weight: | 2.9 |
| | | Sc. PD: | -0.66 |
| | | Sc. rank: | -471.3 |

,

| PB1 | | | |
|---|---|---|---|
| Pos. | 444 | obs: | exp: |
| tct | S | 297 | 58.34 |
| tcc | S | 44 | 51.12 |
| tca | S | 26 | 81.31 |
| tcg | S | 0 | 22.37 |
| agt | S | 0 | 80.74 |
| agc | S | 0 | 73.12 |
| --- | -- | ------ | ------ |
| mPD | | 0.33 | 1.7 |
| | | nPD: | 0.2 |
| | | N. weight: | 1.9 |
| | | Sc. PD: | -0.11 |
| | | Sc. rank: | 18.0 |

,

| PB1 | | | |
|---|---|---|---|
| Pos. | 445 | obs: | exp: |
| gat | D | 357 | 195.80 |
| gac | D | 10 | 171.20 |
| --- | -- | ------ | ------ |
| mPD | | 0.053 | 0.50 |
| | | nPD: | 0.11 |
| | | N. weight: | 0.78 |
| | | Sc. PD: | -0.11 |
| | | Sc. rank: | -42.0 |

,

| PB1 | | | |
|---|---|---|---|
| Pos. | 446 | obs: | exp: |
| gat | D | 225 | 195.80 |
| gac | D | 142 | 171.20 |
| --- | -- | ------ | ------ |
| mPD | | 0.48 | 0.50 |
| | | nPD: | 0.96 |
| | | N. weight: | 0.033 |
| | | Sc. PD: | 0.022 |
| | | Sc. rank: | 6.8 |

,

| PB1 | | | |
|---|---|---|---|
| Pos. | 447 | obs: | exp: |
| ttt | F | 107 | 150.90 |
| ttc | F | 260 | 216.10 |
| --- | -- | ------ | ------ |
| mPD | | 0.41 | 0.48 |
| | | nPD: | 0.86 |
| | | N. weight: | 0.06 |
| | | Sc. PD: | 0.034 |
| | | Sc. rank: | 12.2 |

,

| PB1 | | | |
|---|---|---|---|
| Pos. | 448 | obs: | exp: |
| gct | A | 281 | 92.44 |
| gcc | A | 86 | 75.18 |
| gca | A | 0 | 163.60 |
| gcg | A | 0 | 35.81 |
| --- | -- | ------ | ------ |
| mPD | | 0.36 | 0.69 |
| | | nPD: | 0.52 |
| | | N. weight: | 1.4 |
| | | Sc. PD: | 0.34 |
| | | Sc. rank: | 186.7 |

,

| PB1 | | | |
|---|---|---|---|
| Pos. | 449 | obs: | exp: |
| tta | L | 0 | 32.06 |
| ttg | L | 0 | 70.68 |
| ctt | L | 3 | 68.48 |
| ctc | L | 336 | 59.37 |
| cta | L | 28 | 55.29 |
| ctg | L | 0 | 81.13 |
| --- | -- | ------ | ------ |
| mPD | | 0.16 | 1.1 |
| | | nPD: | 0.14 |
| | | N. weight: | 2.3 |
| | | Sc. PD: | -0.26 |
| | | Sc. rank: | -55.5 |

,

| PB1 | | | |
|---|---|---|---|
| Pos. | 450 | obs: | exp: |
| att | I | 4 | 138.00 |
| atc | I | 3 | 96.18 |
| ata | I | 360 | 132.80 |
| --- | -- | ------ | ------ |
| mPD | | 0.038 | 0.66 |
| | | nPD: | 0.06 |
| | | N. weight: | 1.4 |
| | | Sc. PD: | -0.26 |
| | | Sc. rank: | -145.8 |

,

| PB1 | | | |
|---|---|---|---|
| Pos. | 451 | obs: | exp: |
| gtt | V | 0 | 75.43 |
| gtc | V | 0 | 77.03 |
| gta | V | 19 | 68.78 |
| gtg | V | 348 | 145.80 |
| --- | -- | ------ | ------ |
| mPD | | 0.098 | 0.72 |
| | | nPD: | 0.14 |
| | | N. weight: | 1.2 |
| | | Sc. PD: | -0.13 |
| | | Sc. rank: | -32.6 |

,

| PB1 | | | |
|---|---|---|---|
| Pos. | 452 | obs: | exp: |
| aat | N | 366 | 191.80 |
| aac | N | 1 | 175.20 |
| --- | -- | ------ | ------ |
| mPD | | 0.0054 | 0.50 |
| | | nPD: | 0.01 |
| | | N. weight: | 0.96 |
| | | Sc. PD: | -0.22 |
| | | Sc. rank: | -166.4 |

,

| PB1 | | | |
|---|---|---|---|
| Pos. | 453 | obs: | exp: |
| gct | A | 84 | 92.44 |
| gcc | A | 7 | 75.18 |
| gca | A | 274 | 163.60 |
| gcg | A | 2 | 35.81 |
| --- | -- | ------ | ------ |
| mPD | | 0.39 | 0.69 |
| | | nPD: | 0.57 |
| | | N. weight: | 0.49 |
| | | Sc. PD: | 0.14 |
| | | Sc. rank: | 72.2 |

,

| PB1 | | | |
|---|---|---|---|
| Pos. | 454 | obs: | exp: |
| cct | P | 2 | 96.64 |
| ccc | P | 0 | 66.20 |
| cca | P | 14 | 144.80 |
| ccg | P | 351 | 59.34 |
| --- | -- | ------ | ------ |
| mPD | | 0.084 | 0.72 |
| | | nPD: | 0.12 |
| | | N. weight: | 2.4 |
| | | Sc. PD: | -0.32 |
| | | Sc. rank: | -96.1 |

,

| PB1 | | | |
|---|---|---|---|
| Pos. | 455 | obs: | exp: |
| aat | N | 352 | 190.80 |
| aac | N | 13 | 174.20 |
| gat | D | 2 | 1.07 |
| gac | D | 0 | 0.93 |
| --- | -- | ------ | ------ |
| mPD | | 0.079 | 0.51 |
| | | nPD: | 0.16 |
| | | N. weight: | 0.77 |
| | | Sc. PD: | -0.074 |
| | | Sc. rank: | -8.8 |

,

| PB1 | | | |
|---|---|---|---|
| Pos. | 456 | obs: | exp: |
| cat | H | 352 | 198.30 |
| cac | H | 14 | 167.70 |
| cgt | R | 1 | 0.04 |
| cgc | R | 0 | 0.05 |
| cga | R | 0 | 0.10 |
| cgg | R | 0 | 0.08 |
| aga | R | 0 | 0.45 |
| agg | R | 0 | 0.28 |
| --- | -- | ------ | ------ |
| mPD | | 0.079 | 0.51 |
| | | nPD: | 0.16 |
| | | N. weight: | 0.72 |
| | | Sc. PD: | -0.069 |
| | | Sc. rank: | -8.5 |

,

| PB1 | | | |
|---|---|---|---|
| Pos. | 457 | obs: | exp: |
| gaa | E | 4 | 211.20 |
| gag | E | 363 | 155.80 |
| --- | -- | ------ | ------ |
| mPD | | 0.022 | 0.49 |
| | | nPD: | 0.04 |
| | | N. weight: | 1.2 |
| | | Sc. PD: | -0.24 |
| | | Sc. rank: | -153.0 |

,

| PB1 | | | |
|---|---|---|---|
| Pos. | 458 | obs: | exp: |
| ggt | G | 0 | 45.28 |
| ggc | G | 0 | 49.63 |
| gga | G | 192 | 177.50 |
| ggg | G | 175 | 94.55 |
| --- | -- | ------ | ------ |
| mPD | | 0.50 | 0.67 |
| | | nPD: | 0.75 |
| | | N. weight: | 0.52 |
| | | Sc. PD: | 0.25 |
| | | Sc. rank: | 101.1 |

,

| PB1 | | | |
|---|---|---|---|
| Pos. | 459 | obs: | exp: |
| att | I | 0 | 136.50 |
| atc | I | 0 | 95.14 |
| ata | I | 363 | 131.40 |
| gtt | V | 0 | 0.82 |
| gtc | V | 0 | 0.84 |
| gta | V | 4 | 0.75 |
| gtg | V | 0 | 1.59 |
| --- | -- | ------ | ------ |
| mPD | | 0.022 | 0.68 |
| | | nPD: | 0.03 |
| | | N. weight: | 1.6 |
| | | Sc. PD: | -0.33 |
| | | Sc. rank: | -227.2 |

,

| PB1 | | | |
|---|---|---|---|
| Pos. | 460 | obs: | exp: |
| caa | Q | 333 | 195.10 |
| cag | Q | 33 | 170.90 |
| cgt | R | 0 | 0.04 |
| cgc | R | 0 | 0.05 |
| cga | R | 1 | 0.10 |
| cgg | R | 0 | 0.08 |
| aga | R | 0 | 0.45 |
| agg | R | 0 | 0.28 |
| --- | -- | ------ | ------ |
| mPD | | 0.17 | 0.51 |
| | | nPD: | 0.33 |
| | | N. weight: | 0.53 |
| | | Sc. PD: | 0.039 |
| | | Sc. rank: | 40.1 |

,

| | | | |
|---|---|---|---|
| PB1 | | | |
| Pos. | 461 | obs: | exp: |
| gct | A | 2 | 92.44 |
| gcc | A | 7 | 75.18 |
| gca | A | 341 | 163.60 |
| gcg | A | 17 | 35.81 |
| --- | -- | ------ | ------ |
| mPD | | 0.13 | 0.69 |
| | | nPD: | 0.2 |
| | | N. weight: | 0.91 |
| | | Sc. PD: | -0.052 |
| | | Sc. rank: | 6.8 |

,

| | | | |
|---|---|---|---|
| PB1 | | | |
| Pos. | 462 | obs: | exp: |
| ggt | G | 6 | 45.28 |
| ggc | G | 0 | 49.63 |
| gga | G | 289 | 177.50 |
| ggg | G | 72 | 94.55 |
| --- | -- | ------ | ------ |
| mPD | | 0.34 | 0.67 |
| | | nPD: | 0.51 |
| | | N. weight: | 0.47 |
| | | Sc. PD: | 0.12 |
| | | Sc. rank: | 62.2 |

,

| | | | |
|---|---|---|---|
| PB1 | | | |
| Pos. | 463 | obs: | exp: |
| gtt | V | 0 | 75.43 |
| gtc | V | 0 | 77.03 |
| gta | V | 93 | 68.78 |
| gtg | V | 274 | 145.80 |
| --- | -- | ------ | ------ |
| mPD | | 0.38 | 0.72 |
| | | nPD: | 0.53 |
| | | N. weight: | 0.85 |
| | | Sc. PD: | 0.22 |
| | | Sc. rank: | 116.9 |

,

| | | | |
|---|---|---|---|
| PB1 | | | |
| Pos. | 464 | obs: | exp: |
| aat | N | 1 | 1.57 |
| aac | N | 2 | 1.43 |
| gat | D | 266 | 194.20 |
| gac | D | 98 | 169.80 |
| --- | -- | ------ | ------ |
| mPD | | 0.41 | 0.51 |
| | | nPD: | 0.81 |
| | | N. weight: | 0.14 |
| | | Sc. PD: | 0.073 |
| | | Sc. rank: | 28.0 |

,

| | | | |
|---|---|---|---|
| PB1 | | | |
| Pos. | 465 | obs: | exp: |
| cgt | R | 0 | 15.24 |
| cgc | R | 0 | 17.97 |
| cga | R | 3 | 35.27 |
| cgg | R | 0 | 30.63 |
| aga | R | 101 | 165.00 |
| agg | R | 263 | 102.80 |
| --- | -- | ------ | ------ |
| mPD | | 0.42 | 0.96 |
| | | nPD: | 0.44 |
| | | N. weight: | 0.81 |
| | | Sc. PD: | 0.14 |
| | | Sc. rank: | 91.5 |

,

| | | | |
|---|---|---|---|
| PB1 | | | |
| Pos. | 466 | obs: | exp: |
| ttt | F | 6 | 150.90 |
| ttc | F | 361 | 216.10 |
| --- | -- | ------ | ------ |
| mPD | | 0.032 | 0.48 |
| | | nPD: | 0.07 |
| | | N. weight: | 0.7 |
| | | Sc. PD: | -0.13 |
| | | Sc. rank: | -63.4 |

,

| | | | |
|---|---|---|---|
| PB1 | | | |
| Pos. | 467 | obs: | exp: |
| tat | Y | 330 | 190.00 |
| tac | Y | 37 | 177.00 |
| --- | -- | ------ | ------ |
| mPD | | 0.18 | 0.50 |
| | | nPD: | 0.36 |
| | | N. weight: | 0.53 |
| | | Sc. PD: | 0.054 |
| | | Sc. rank: | 44.1 |

,

| | | | |
|---|---|---|---|
| PB1 | | | |
| Pos. | 468 | obs: | exp: |
| cgt | R | 0 | 15.24 |
| cgc | R | 0 | 17.97 |
| cga | R | 0 | 35.27 |
| cgg | R | 1 | 30.63 |
| aga | R | 145 | 165.00 |
| agg | R | 221 | 102.80 |
| --- | -- | ------ | ------ |
| mPD | | 0.48 | 0.96 |
| | | nPD: | 0.5 |
| | | N. weight: | 0.63 |
| | | Sc. PD: | 0.15 |
| | | Sc. rank: | 80.9 |

,

| | | | |
|---|---|---|---|
| PB1 | | | |
| Pos. | 469 | obs: | exp: |
| act | T | 8 | 96.96 |
| acc | T | 356 | 78.75 |
| aca | T | 3 | 160.80 |
| acg | T | 0 | 30.51 |
| --- | -- | ------ | ------ |
| mPD | | 0.059 | 0.69 |
| | | nPD: | 0.09 |
| | | N. weight: | 2.1 |
| | | Sc. PD: | -0.34 |
| | | Sc. rank: | -153.5 |

,

| | | | |
|---|---|---|---|
| PB1 | | | |
| Pos. | 470 | obs: | exp: |
| tgt | C | 11 | 151.30 |
| tgc | C | 356 | 215.70 |
| --- | -- | ------ | ------ |
| mPD | | 0.058 | 0.48 |
| | | nPD: | 0.12 |
| | | N. weight: | 0.63 |
| | | Sc. PD: | -0.081 |
| | | Sc. rank: | -23.7 |

,

| | | | |
|---|---|---|---|
| PB1 | | | |
| Pos. | 471 | obs: | exp: |
| aaa | K | 246 | 205.50 |
| aag | K | 121 | 161.50 |
| --- | -- | ------ | ------ |
| mPD | | 0.44 | 0.49 |
| | | nPD: | 0.9 |
| | | N. weight: | 0.052 |
| | | Sc. PD: | 0.031 |
| | | Sc. rank: | 10.7 |

,

| | | | |
|---|---|---|---|
| PB1 | | | |
| Pos. | 472 | obs: | exp: |
| tta | L | 28 | 32.06 |
| ttg | L | 8 | 70.68 |
| ctt | L | 0 | 68.48 |
| ctc | L | 0 | 59.37 |
| cta | L | 14 | 55.29 |
| ctg | L | 317 | 81.13 |
| --- | -- | ------ | ------ |
| mPD | | 0.38 | 1.1 |
| | | nPD: | 0.34 |
| | | N. weight: | 1.7 |
| | | Sc. PD: | 0.13 |
| | | Sc. rank: | 130.3 |

,

| | | | |
|---|---|---|---|
| PB1 | | | |
| Pos. | 473 | obs: | exp: |
| att | I | 1 | 0.38 |
| atc | I | 0 | 0.26 |
| ata | I | 0 | 0.36 |
| gtt | V | 278 | 75.23 |
| gtc | V | 88 | 76.82 |
| gta | V | 0 | 68.60 |
| gtg | V | 0 | 145.40 |
| --- | -- | ------ | ------ |
| mPD | | 0.37 | 0.73 |
| | | nPD: | 0.51 |
| | | N. weight: | 1.6 |
| | | Sc. PD: | 0.38 |
| | | Sc. rank: | 205.7 |

,

| | | | |
|---|---|---|---|
| PB1 | | | |
| Pos. | 474 | obs: | exp: |
| ggt | G | 0 | 45.28 |
| ggc | G | 0 | 49.63 |
| gga | G | 146 | 177.50 |
| ggg | G | 221 | 94.55 |
| --- | -- | ------ | ------ |
| mPD | | 0.48 | 0.67 |
| | | nPD: | 0.72 |
| | | N. weight: | 0.67 |
| | | Sc. PD: | 0.3 |
| | | Sc. rank: | 123.6 |

,

| | | | |
|---|---|---|---|
| PB1 | | | |
| Pos. | 475 | obs: | exp: |
| att | I | 0 | 138.00 |
| atc | I | 341 | 96.18 |
| ata | I | 26 | 132.80 |
| --- | -- | ------ | ------ |
| mPD | | 0.13 | 0.66 |
| | | nPD: | 0.2 |
| | | N. weight: | 1.6 |
| | | Sc. PD: | -0.087 |
| | | Sc. rank: | 22.1 |

,

| | | | |
|---|---|---|---|
| PB1 | | | |
| Pos. | 476 | obs: | exp: |
| aat | N | 61 | 191.80 |
| aac | N | 306 | 175.20 |
| --- | -- | ------ | ------ |
| mPD | | 0.28 | 0.50 |
| | | nPD: | 0.56 |
| | | N. weight: | 0.43 |
| | | Sc. PD: | 0.12 |
| | | Sc. rank: | 62.2 |

,

| | | | |
|---|---|---|---|
| PB1 | | | |
| Pos. | 477 | obs: | exp: |
| atg | M | 367 | 367.00 |
| --- | -- | ------ | ------ |
| mPD | | 0 | 0 |
| | | nPD: | 1. |
| | | N. weight: | 0. |
| | | Sc. PD: | 0 |
| | | Sc. rank: | 0 |

,

| | | | |
|---|---|---|---|
| PB1 | | | |
| Pos. | 478 | obs: | exp: |
| tct | S | 0 | 58.34 |
| tcc | S | 0 | 51.12 |
| tca | S | 0 | 81.31 |
| tcg | S | 0 | 22.37 |
| agt | S | 7 | 80.74 |
| agc | S | 360 | 73.12 |
| --- | -- | ------ | ------ |
| mPD | | 0.038 | 1.7 |
| | | nPD: | 0.02 |
| | | N. weight: | 2.3 |
| | | Sc. PD: | -0.51 |
| | | Sc. rank: | -356.4 |

,

| | | | |
|---|---|---|---|
| PB1 | | | |
| Pos. | 479 | obs: | exp: |
| aaa | K | 358 | 205.50 |
| aag | K | 9 | 161.50 |
| --- | -- | ------ | ------ |
| mPD | | 0.048 | 0.49 |
| | | nPD: | 0.1 |
| | | N. weight: | 0.72 |
| | | Sc. PD: | -0.11 |
| | | Sc. rank: | -41.9 |

,

| | | | |
|---|---|---|---|
| PB1 | | | |
| Pos. | 480 | obs: | exp: |
| cgt | R | 0 | 0.04 |
| cgc | R | 0 | 0.05 |
| cga | R | 0 | 0.10 |
| cgg | R | 0 | 0.08 |
| aaa | K | 4 | 204.90 |
| aag | K | 362 | 161.10 |
| aga | R | 0 | 0.45 |
| agg | R | 1 | 0.28 |
| --- | -- | ------ | ------ |
| mPD | | 0.027 | 0.50 |
| | | nPD: | 0.05 |
| | | N. weight: | 1.2 |
| | | Sc. PD: | -0.22 |
| | | Sc. rank: | -127.8 |

,

| | | | |
|---|---|---|---|
| PB1 | | | |
| Pos. | 481 | obs: | exp: |
| aaa | K | 37 | 205.50 |
| aag | K | 330 | 161.50 |
| --- | -- | ------ | ------ |
| mPD | | 0.18 | 0.49 |
| | | nPD: | 0.37 |
| | | N. weight: | 0.73 |
| | | Sc. PD: | 0.078 |
| | | Sc. rank: | 62.2 |

,

| | | | |
|---|---|---|---|
| PB1 | | | |
| Pos. | 482 | obs: | exp: |
| tct | S | 242 | 58.34 |
| tcc | S | 125 | 51.12 |
| tca | S | 0 | 81.31 |
| tcg | S | 0 | 22.37 |
| agt | S | 0 | 80.74 |
| agc | S | 0 | 73.12 |
| --- | -- | ------ | ------ |
| mPD | | 0.45 | 1.7 |
| | | nPD: | 0.27 |
| | | N. weight: | 1.9 |
| | | Sc. PD: | 0.026 |
| | | Sc. rank: | 107.4 |

,

| | | | |
|---|---|---|---|
| PB1 | | | |
| Pos. | 483 | obs: | exp: |
| tat | Y | 11 | 190.00 |
| tac | Y | 356 | 177.00 |
| --- | -- | ------ | ------ |
| mPD | | 0.058 | 0.50 |
| | | nPD: | 0.12 |
| | | N. weight: | 0.91 |
| | | Sc. PD: | -0.12 |
| | | Sc. rank: | -38.4 |

,

| | | | |
|---|---|---|---|
| PB1 | | | |
| Pos. | 484 | obs: | exp: |
| att | I | 0 | 138.00 |
| atc | I | 0 | 96.18 |
| ata | I | 367 | 132.80 |
| --- | -- | ------ | ------ |
| mPD | | 0 | 0.66 |
| | | nPD: | 0. |
| | | N. weight: | 1.5 |
| | | Sc. PD: | -0.38 |
| | | Sc. rank: | -321.0 |

,

| | | | |
|---|---|---|---|
| PB1 | | | |
| Pos. | 485 | obs: | exp: |
| aat | N | 100 | 191.80 |
| aac | N | 267 | 175.20 |
| --- | -- | ------ | ------ |
| mPD | | 0.40 | 0.50 |
| | | nPD: | 0.8 |
| | | N. weight: | 0.21 |
| | | Sc. PD: | 0.11 |
| | | Sc. rank: | 41.5 |

,

| | | | |
|---|---|---|---|
| PB1 | | | |
| Pos. | 486 | obs: | exp: |
| cgt | R | 0 | 15.24 |
| cgc | R | 0 | 17.97 |
| cga | R | 58 | 35.27 |
| cgg | R | 208 | 30.63 |
| aga | R | 4 | 165.00 |
| agg | R | 97 | 102.80 |
| --- | -- | ------ | ------ |
| mPD | | 0.68 | 0.96 |
| | | nPD: | 0.71 |
| | | N. weight: | 1.7 |
| | | Sc. PD: | 0.74 |
| | | Sc. rank: | 310.8 |

,

| | | | |
|---|---|---|---|
| PB1 | | | |
| Pos. | 487 | obs: | exp: |
| act | T | 0 | 96.96 |
| acc | T | 0 | 78.75 |
| aca | T | 364 | 160.80 |
| acg | T | 3 | 30.51 |
| --- | -- | ------ | ------ |
| mPD | | 0.016 | 0.69 |
| | | nPD: | 0.02 |
| | | N. weight: | 1.2 |
| | | Sc. PD: | -0.27 |
| | | Sc. rank: | -181.3 |

,

| | | | |
|---|---|---|---|
| PB1 | | | |
| Pos. | 488 | obs: | exp: |
| ggt | G | 0 | 45.28 |
| ggc | G | 0 | 49.63 |
| gga | G | 333 | 177.50 |
| ggg | G | 34 | 94.55 |
| --- | -- | ------ | ------ |
| mPD | | 0.17 | 0.67 |
| | | nPD: | 0.25 |
| | | N. weight: | 0.74 |
| | | Sc. PD: | -0.0024 |
| | | Sc. rank: | 31.5 |

,

| | | | |
|---|---|---|---|
| PB1 | | | |
| Pos. | 489 | obs: | exp: |
| act | T | 15 | 96.96 |
| acc | T | 1 | 78.75 |
| aca | T | 351 | 160.80 |
| acg | T | 0 | 30.51 |
| --- | -- | ------ | ------ |
| mPD | | 0.084 | 0.69 |
| | | nPD: | 0.12 |
| | | N. weight: | 1. |
| | | Sc. PD: | -0.13 |
| | | Sc. rank: | -37.2 |

,

| | | | |
|---|---|---|---|
| PB1 | | | |
| Pos. | 490 | obs: | exp: |
| ttt | F | 329 | 150.90 |
| ttc | F | 38 | 216.10 |
| --- | -- | ------ | ------ |
| mPD | | 0.19 | 0.48 |
| | | nPD: | 0.38 |
| | | N. weight: | 0.8 |
| | | Sc. PD: | 0.097 |
| | | Sc. rank: | 77.4 |

| | | | |
|---|---|---|---|
| PB1 | | | |
| Pos. | 491 | obs: | exp: |
| gaa | E | 160 | 211.20 |
| gag | E | 207 | 155.80 |
| --- | -- | ------ | ------ |
| mPD | | 0.49 | 0.49 |
| | | nPD: | 1.01 |
| | | N. weight: | 0.073 |
| | | Sc. PD: | 0.052 |
| | | Sc. rank: | 15.2 |

| | | | |
|---|---|---|---|
| PB1 | | | |
| Pos. | 492 | obs: | exp: |
| ttt | F | 1 | 150.90 |
| ttc | F | 366 | 216.10 |
| --- | -- | ------ | ------ |
| mPD | | 0.0054 | 0.48 |
| | | nPD: | 0.01 |
| | | N. weight: | 0.78 |
| | | Sc. PD: | -0.18 |
| | | Sc. rank: | -133.3 |

| | | | |
|---|---|---|---|
| PB1 | | | |
| Pos. | 493 | obs: | exp: |
| act | T | 1 | 96.96 |
| acc | T | 0 | 78.75 |
| aca | T | 358 | 160.80 |
| acg | T | 8 | 30.51 |
| --- | -- | ------ | ------ |
| mPD | | 0.048 | 0.69 |
| | | nPD: | 0.07 |
| | | N. weight: | 1.1 |
| | | Sc. PD: | -0.2 |
| | | Sc. rank: | -101.2 |

| | | | |
|---|---|---|---|
| PB1 | | | |
| Pos. | 494 | obs: | exp: |
| tct | S | 0 | 58.34 |
| tcc | S | 0 | 51.12 |
| tca | S | 0 | 81.31 |
| tcg | S | 0 | 22.37 |
| agt | S | 94 | 80.74 |
| agc | S | 273 | 73.12 |
| --- | -- | ------ | ------ |
| mPD | | 0.38 | 1.7 |
| | | nPD: | 0.23 |
| | | N. weight: | 1.6 |
| | | Sc. PD: | -0.04 |
| | | Sc. rank: | 49.9 |

| | | | |
|---|---|---|---|
| PB1 | | | |
| Pos. | 495 | obs: | exp: |
| ttt | F | 340 | 150.90 |
| ttc | F | 27 | 216.10 |
| --- | -- | ------ | ------ |
| mPD | | 0.14 | 0.48 |
| | | nPD: | 0.28 |
| | | N. weight: | 0.92 |
| | | Sc. PD: | 0.023 |
| | | Sc. rank: | 58.6 |

| | | | |
|---|---|---|---|
| PB1 | | | |
| Pos. | 496 | obs: | exp: |
| ttt | F | 1 | 150.90 |
| ttc | F | 366 | 216.10 |
| --- | -- | ------ | ------ |
| mPD | | 0.0054 | 0.48 |
| | | nPD: | 0.01 |
| | | N. weight: | 0.78 |
| | | Sc. PD: | -0.18 |
| | | Sc. rank: | -133.3 |

| | | | |
|---|---|---|---|
| PB1 | | | |
| Pos. | 497 | obs: | exp: |
| ttt | F | 0 | 0.41 |
| ttc | F | 1 | 0.59 |
| tat | Y | 104 | 189.50 |
| tac | Y | 262 | 176.50 |
| --- | -- | ------ | ------ |
| mPD | | 0.41 | 0.50 |
| | | nPD: | 0.82 |
| | | N. weight: | 0.19 |
| | | Sc. PD: | 0.099 |
| | | Sc. rank: | 37.6 |

| | | | |
|---|---|---|---|
| PB1 | | | |
| Pos. | 498 | obs: | exp: |
| cgt | R | 19 | 15.24 |
| cgc | R | 347 | 17.97 |
| cga | R | 1 | 35.27 |
| cgg | R | 0 | 30.63 |
| aga | R | 0 | 165.00 |
| agg | R | 0 | 102.80 |
| --- | -- | ------ | ------ |
| mPD | | 0.10 | 0.96 |
| | | nPD: | 0.11 |
| | | N. weight: | 4.3 |
| | | Sc. PD: | -0.6 |
| | | Sc. rank: | -215.7 |

| | | | |
|---|---|---|---|
| PB1 | | | |
| Pos. | 499 | obs: | exp: |
| tat | Y | 354 | 190.00 |
| tac | Y | 13 | 177.00 |
| --- | -- | ------ | ------ |
| mPD | | 0.069 | 0.50 |
| | | nPD: | 0.14 |
| | | N. weight: | 0.78 |
| | | Sc. PD: | -0.089 |
| | | Sc. rank: | -21.5 |

| | | | |
|---|---|---|---|
| PB1 | | | |
| Pos. | 500 | obs: | exp: |
| ggt | G | 0 | 45.28 |
| ggc | G | 0 | 49.63 |
| gga | G | 310 | 177.50 |
| ggg | G | 57 | 94.55 |
| --- | -- | ------ | ------ |
| mPD | | 0.26 | 0.67 |
| | | nPD: | 0.39 |
| | | N. weight: | 0.61 |
| | | Sc. PD: | 0.08 |
| | | Sc. rank: | 60.7 |

| | | | |
|---|---|---|---|
| PB1 | | | |
| Pos. | 501 | obs: | exp: |
| ttt | F | 324 | 150.90 |
| ttc | F | 43 | 216.10 |
| --- | -- | ------ | ------ |
| mPD | | 0.21 | 0.48 |
| | | nPD: | 0.43 |
| | | N. weight: | 0.75 |
| | | Sc. PD: | 0.12 |
| | | Sc. rank: | 83.6 |

| | | | |
|---|---|---|---|
| PB1 | | | |
| Pos. | 502 | obs: | exp: |
| gtt | V | 0 | 75.43 |
| gtc | V | 6 | 77.03 |
| gta | V | 293 | 68.78 |
| gtg | V | 68 | 145.80 |
| --- | -- | ------ | ------ |
| mPD | | 0.33 | 0.72 |
| | | nPD: | 0.46 |
| | | N. weight: | 1.5 |
| | | Sc. PD: | 0.29 |
| | | Sc. rank: | 183.8 |

| | | | |
|---|---|---|---|
| PB1 | | | |
| Pos. | 503 | obs: | exp: |
| gct | A | 107 | 92.44 |
| gcc | A | 259 | 75.18 |
| gca | A | 1 | 163.60 |
| gcg | A | 0 | 35.81 |
| --- | -- | ------ | ------ |
| mPD | | 0.42 | 0.69 |
| | | nPD: | 0.61 |
| | | N. weight: | 1.4 |
| | | Sc. PD: | 0.47 |
| | | Sc. rank: | 221.8 |

| | | | |
|---|---|---|---|
| PB1 | | | |
| Pos. | 504 | obs: | exp: |
| aat | N | 73 | 191.80 |
| aac | N | 294 | 175.20 |
| --- | -- | ------ | ------ |
| mPD | | 0.32 | 0.50 |
| | | nPD: | 0.64 |
| | | N. weight: | 0.35 |
| | | Sc. PD: | 0.13 |
| | | Sc. rank: | 57.9 |

| | | | |
|---|---|---|---|
| PB1 | | | |
| Pos. | 505 | obs: | exp: |
| ttt | F | 71 | 150.90 |
| ttc | F | 296 | 216.10 |
| --- | -- | ------ | ------ |
| mPD | | 0.31 | 0.48 |
| | | nPD: | 0.65 |
| | | N. weight: | 0.18 |
| | | Sc. PD: | 0.065 |
| | | Sc. rank: | 29.6 |

| | | | |
|---|---|---|---|
| PB1 | | | |
| Pos. | 506 | obs: | exp: |
| tct | S | 0 | 58.34 |
| tcc | S | 0 | 51.12 |
| tca | S | 0 | 81.31 |
| tcg | S | 0 | 22.37 |
| agt | S | 257 | 80.74 |
| agc | S | 110 | 73.12 |
| --- | -- | ------ | ------ |
| mPD | | 0.42 | 1.7 |
| | | nPD: | 0.25 |
| | | N. weight: | 1.4 |
| | | Sc. PD: | -0.0045 |
| | | Sc. rank: | 62.5 |

| | | | |
|---|---|---|---|
| PB1 | | | |
| Pos. | 507 | obs: | exp: |
| atg | M | 367 | 367.00 |
| --- | -- | ------ | ------ |
| mPD | | 0 | 0 |
| | | nPD: | 1. |
| | | N. weight: | 0. |
| | | Sc. PD: | 0 |
| | | Sc. rank: | 0 |

| | | | |
|---|---|---|---|
| PB1 | | | |
| Pos. | 508 | obs: | exp: |
| gaa | E | 37 | 211.20 |
| gag | E | 330 | 155.80 |
| --- | -- | ------ | ------ |
| mPD | | 0.18 | 0.49 |
| | | nPD: | 0.37 |
| | | N. weight: | 0.77 |
| | | Sc. PD: | 0.085 |
| | | Sc. rank: | 67.4 |

| | | | |
|---|---|---|---|
| PB1 | | | |
| Pos. | 509 | obs: | exp: |
| tta | L | 125 | 32.06 |
| ttg | L | 123 | 70.68 |
| ctt | L | 0 | 68.48 |
| ctc | L | 0 | 59.37 |
| cta | L | 8 | 55.29 |
| ctg | L | 111 | 81.13 |
| --- | -- | ------ | ------ |
| mPD | | 0.90 | 1.1 |
| | | nPD: | 0.81 |
| | | N. weight: | 1.1 |
| | | Sc. PD: | 0.58 |
| | | Sc. rank: | 221.9 |

| | | | |
|---|---|---|---|
| PB1 | | | |
| Pos. | 510 | obs: | exp: |
| cct | P | 28 | 96.37 |
| ccc | P | 335 | 66.02 |
| cca | P | 3 | 144.40 |
| ccg | P | 0 | 59.18 |
| gct | A | 0 | 0.25 |
| gcc | A | 1 | 0.20 |
| gca | A | 0 | 0.45 |
| gcg | A | 0 | 0.10 |
| --- | -- | ------ | ------ |
| mPD | | 0.16 | 0.72 |
| | | nPD: | 0.22 |
| | | N. weight: | 2.1 |
| | | Sc. PD: | -0.064 |
| | | Sc. rank: | 58.8 |

| | | | |
|---|---|---|---|
| PB1 | | | |
| Pos. | 511 | obs: | exp: |
| tct | S | 0 | 58.19 |
| tcc | S | 0 | 50.99 |
| tca | S | 0 | 81.08 |
| tcg | S | 0 | 22.31 |
| aat | N | 0 | 0.52 |
| aac | N | 1 | 0.48 |
| agt | S | 54 | 80.52 |
| agc | S | 312 | 72.92 |
| --- | -- | ------ | ------ |
| mPD | | 0.26 | 1.7 |
| | | nPD: | 0.15 |
| | | N. weight: | 1.8 |
| | | Sc. PD: | -0.18 |
| | | Sc. rank: | -22.9 |

| | | | |
|---|---|---|---|
| PB1 | | | |
| Pos. | 512 | obs: | exp: |
| ttt | F | 361 | 150.90 |
| ttc | F | 6 | 216.10 |
| --- | -- | ------ | ------ |
| mPD | | 0.032 | 0.48 |
| | | nPD: | 0.07 |
| | | N. weight: | 1.2 |
| | | Sc. PD: | -0.22 |
| | | Sc. rank: | -111.6 |

| | | | |
|---|---|---|---|
| PB1 | | | |
| Pos. | 513 | obs: | exp: |
| ggt | G | 1 | 45.28 |
| ggc | G | 0 | 49.63 |
| gga | G | 330 | 177.50 |
| ggg | G | 36 | 94.55 |
| --- | -- | ------ | ------ |
| mPD | | 0.18 | 0.67 |
| | | nPD: | 0.27 |
| | | N. weight: | 0.7 |
| | | Sc. PD: | 0.012 |
| | | Sc. rank: | 42.1 |

| | | | |
|---|---|---|---|
| PB1 | | | |
| Pos. | 514 | obs: | exp: |
| gtt | V | 1 | 75.43 |
| gtc | V | 0 | 77.03 |
| gta | V | 16 | 68.78 |
| gtg | V | 350 | 145.80 |
| --- | -- | ------ | ------ |
| mPD | | 0.089 | 0.72 |
| | | nPD: | 0.12 |
| | | N. weight: | 1.2 |
| | | Sc. PD: | -0.15 |
| | | Sc. rank: | -40.8 |

| | | | |
|---|---|---|---|
| PB1 | | | |
| Pos. | 515 | obs: | exp: |
| tct | S | 334 | 58.34 |
| tcc | S | 30 | 51.12 |
| tca | S | 3 | 81.31 |
| tcg | S | 0 | 22.37 |
| agt | S | 0 | 80.74 |
| agc | S | 0 | 73.12 |
| --- | -- | ------ | ------ |
| mPD | | 0.17 | 1.7 |
| | | nPD: | 0.1 |
| | | N. weight: | 2.3 |
| | | Sc. PD: | -0.35 |
| | | Sc. rank: | -130.7 |

| | | | |
|---|---|---|---|
| PB1 | | | |
| Pos. | 516 | obs: | exp: |
| ggt | G | 1 | 45.28 |
| ggc | G | 0 | 49.63 |
| gga | G | 225 | 177.50 |
| ggg | G | 141 | 94.55 |
| --- | -- | ------ | ------ |
| mPD | | 0.48 | 0.67 |
| | | nPD: | 0.72 |
| | | N. weight: | 0.46 |
| | | Sc. PD: | 0.2 |
| | | Sc. rank: | 83.3 |

| | | | |
|---|---|---|---|
| PB1 | | | |
| Pos. | 517 | obs: | exp: |
| att | I | 104 | 138.00 |
| atc | I | 263 | 96.18 |
| ata | I | 0 | 132.80 |
| --- | -- | ------ | ------ |
| mPD | | 0.41 | 0.66 |
| | | nPD: | 0.62 |
| | | N. weight: | 0.99 |
| | | Sc. PD: | 0.34 |
| | | Sc. rank: | 159.4 |

| | | | |
|---|---|---|---|
| PB1 | | | |
| Pos. | 518 | obs: | exp: |
| aat | N | 311 | 191.80 |
| aac | N | 56 | 175.20 |
| --- | -- | ------ | ------ |
| mPD | | 0.26 | 0.50 |
| | | nPD: | 0.52 |
| | | N. weight: | 0.37 |
| | | Sc. PD: | 0.093 |
| | | Sc. rank: | 49.6 |

| | | | |
|---|---|---|---|
| PB1 | | | |
| Pos. | 519 | obs: | exp: |
| gaa | E | 331 | 211.20 |
| gag | E | 36 | 155.80 |
| --- | -- | ------ | ------ |
| mPD | | 0.18 | 0.49 |
| | | nPD: | 0.36 |
| | | N. weight: | 0.41 |
| | | Sc. PD: | 0.041 |
| | | Sc. rank: | 34.1 |

PB1

| Pos. | 520 | obs: | exp: |
|---|---|---|---|
| tct | S | 87 | 58.34 |
| tcc | S | 3 | 51.12 |
| tca | S | 96 | 81.31 |
| tcg | S | 181 | 22.37 |
| agt | S | 0 | 80.74 |
| agc | S | 0 | 73.12 |
| --- | -- | ------ | ------ |
| mPD | | 0.63 | 1.7 |
| | | nPD: | 0.38 |
| | | N. weight: | 1.8 |
| | | Sc. PD: | 0.21 |
| | | Sc. rank: | 167.2 |

PB1

| Pos. | 521 | obs: | exp: |
|---|---|---|---|
| gct | A | 273 | 92.44 |
| gcc | A | 12 | 75.18 |
| gca | A | 82 | 163.60 |
| gcg | A | 0 | 35.81 |
| --- | -- | ------ | ------ |
| mPD | | 0.40 | 0.69 |
| | | nPD: | 0.58 |
| | | N. weight: | 0.92 |
| | | Sc. PD: | 0.28 |
| | | Sc. rank: | 141.0 |

PB1

| Pos. | 522 | obs: | exp: |
|---|---|---|---|
| gat | D | 24 | 195.80 |
| gac | D | 343 | 171.20 |
| --- | -- | ------ | ------ |
| mPD | | 0.12 | 0.50 |
| | | nPD: | 0.25 |
| | | N. weight: | 0.79 |
| | | Sc. PD: | -0.0077 |
| | | Sc. rank: | 31.6 |

PB1

| Pos. | 523 | obs: | exp: |
|---|---|---|---|
| tta | L | 0 | 0.87 |
| ttg | L | 10 | 1.93 |
| ctt | L | 0 | 1.87 |
| ctc | L | 0 | 1.62 |
| cta | L | 0 | 1.51 |
| ctg | L | 0 | 2.21 |
| atg | M | 357 | 357.00 |
| --- | -- | ------ | ------ |
| mPD | | 0.053 | 0.085 |
| | | nPD: | 0.63 |
| | | N. weight: | 0.068 |
| | | Sc. PD: | 0.024 |
| | | Sc. rank: | 11.1 |

PB1

| Pos. | 524 | obs: | exp: |
|---|---|---|---|
| tct | S | 0 | 58.34 |
| tcc | S | 0 | 51.12 |
| tca | S | 0 | 81.31 |
| tcg | S | 0 | 22.37 |
| agt | S | 13 | 80.74 |
| agc | S | 354 | 73.12 |
| --- | -- | ------ | ------ |
| mPD | | 0.069 | 1.7 |
| | | nPD: | 0.04 |
| | | N. weight: | 2.2 |
| | | Sc. PD: | -0.46 |
| | | Sc. rank: | -287.6 |

PB1

| Pos. | 525 | obs: | exp: |
|---|---|---|---|
| att | I | 361 | 138.00 |
| atc | I | 6 | 96.18 |
| ata | I | 0 | 132.80 |
| --- | -- | ------ | ------ |
| mPD | | 0.032 | 0.66 |
| | | nPD: | 0.05 |
| | | N. weight: | 1.4 |
| | | Sc. PD: | -0.27 |
| | | Sc. rank: | -160.3 |

PB1

| Pos. | 526 | obs: | exp: |
|---|---|---|---|
| ggt | G | 27 | 45.28 |
| ggc | G | 2 | 49.63 |
| gga | G | 321 | 177.50 |
| ggg | G | 17 | 94.55 |
| --- | -- | ------ | ------ |
| mPD | | 0.23 | 0.67 |
| | | nPD: | 0.34 |
| | | N. weight: | 0.61 |
| | | Sc. PD: | 0.05 |
| | | Sc. rank: | 48.6 |

PB1

| Pos. | 527 | obs: | exp: |
|---|---|---|---|
| att | I | 3 | 1.13 |
| atc | I | 0 | 0.79 |
| ata | I | 0 | 1.09 |
| gtt | V | 252 | 74.82 |
| gtc | V | 14 | 76.40 |
| gta | V | 86 | 68.22 |
| gtg | V | 12 | 144.60 |
| --- | -- | ------ | ------ |
| mPD | | 0.48 | 0.74 |
| | | nPD: | 0.65 |
| | | N. weight: | 1.2 |
| | | Sc. PD: | 0.43 |
| | | Sc. rank: | 197.4 |

PB1

| Pos. | 528 | obs: | exp: |
|---|---|---|---|
| act | T | 1 | 96.69 |
| acc | T | 0 | 78.54 |
| aca | T | 357 | 160.30 |
| acg | T | 8 | 30.43 |
| gct | A | 0 | 0.25 |
| gcc | A | 0 | 0.20 |
| gca | A | 1 | 0.45 |
| gcg | A | 0 | 0.10 |
| --- | -- | ------ | ------ |
| mPD | | 0.054 | 0.69 |
| | | nPD: | 0.08 |
| | | N. weight: | 1.1 |
| | | Sc. PD: | -0.19 |
| | | Sc. rank: | -90.5 |

PB1

| Pos. | 529 | obs: | exp: |
|---|---|---|---|
| gtt | V | 0 | 75.43 |
| gtc | V | 0 | 77.03 |
| gta | V | 39 | 68.78 |
| gtg | V | 328 | 145.80 |
| --- | -- | ------ | ------ |
| mPD | | 0.19 | 0.72 |
| | | nPD: | 0.26 |
| | | N. weight: | 1. |
| | | Sc. PD: | 0.0075 |
| | | Sc. rank: | 51.5 |

PB1

| Pos. | 530 | obs: | exp: |
|---|---|---|---|
| att | I | 0 | 137.60 |
| atc | I | 5 | 95.92 |
| ata | I | 361 | 132.50 |
| gtt | V | 0 | 0.21 |
| gtc | V | 0 | 0.21 |
| gta | V | 1 | 0.19 |
| gtg | V | 0 | 0.40 |
| --- | -- | ------ | ------ |
| mPD | | 0.032 | 0.67 |
| | | nPD: | 0.05 |
| | | N. weight: | 1.5 |
| | | Sc. PD: | -0.29 |
| | | Sc. rank: | -170.2 |

PB1

| Pos. | 531 | obs: | exp: |
|---|---|---|---|
| aaa | K | 37 | 205.50 |
| aag | K | 330 | 161.50 |
| --- | -- | ------ | ------ |
| mPD | | 0.18 | 0.49 |
| | | nPD: | 0.37 |
| | | N. weight: | 0.73 |
| | | Sc. PD: | 0.078 |
| | | Sc. rank: | 62.2 |

PB1

| Pos. | 532 | obs: | exp: |
|---|---|---|---|
| aat | N | 11 | 191.80 |
| aac | N | 356 | 175.20 |
| --- | -- | ------ | ------ |
| mPD | | 0.058 | 0.50 |
| | | nPD: | 0.12 |
| | | N. weight: | 0.93 |
| | | Sc. PD: | -0.12 |
| | | Sc. rank: | -38.6 |

PB1

| Pos. | 533 | obs: | exp: |
|---|---|---|---|
| aat | N | 197 | 191.80 |
| aac | N | 170 | 175.20 |
| --- | -- | ------ | ------ |
| mPD | | 0.50 | 0.50 |
| | | nPD: | 1. |
| | | N. weight: | 0.014 |
| | | Sc. PD: | 0.0097 |
| | | Sc. rank: | 2.9 |

PB1

| Pos. | 534 | obs: | exp: |
|---|---|---|---|
| atg | M | 367 | 367.00 |
| --- | -- | ------ | ------ |
| mPD | | 0 | 0 |
| | | nPD: | 1. |
| | | N. weight: | 0. |
| | | Sc. PD: | 0 |
| | | Sc. rank: | 0 |

PB1

| Pos. | 535 | obs: | exp: |
|---|---|---|---|
| att | I | 0 | 137.60 |
| atc | I | 55 | 95.92 |
| ata | I | 311 | 132.50 |
| gtt | V | 0 | 0.21 |
| gtc | V | 0 | 0.21 |
| gta | V | 1 | 0.19 |
| gtg | V | 0 | 0.40 |
| --- | -- | ------ | ------ |
| mPD | | 0.26 | 0.67 |
| | | nPD: | 0.39 |
| | | N. weight: | 0.99 |
| | | Sc. PD: | 0.13 |
| | | Sc. rank: | 98.7 |

PB1

| Pos. | 536 | obs: | exp: |
|---|---|---|---|
| aat | N | 7 | 191.80 |
| aac | N | 360 | 175.20 |
| --- | -- | ------ | ------ |
| mPD | | 0.038 | 0.50 |
| | | nPD: | 0.08 |
| | | N. weight: | 0.99 |
| | | Sc. PD: | -0.17 |
| | | Sc. rank: | -83.5 |

PB1

| Pos. | 537 | obs: | exp: |
|---|---|---|---|
| aat | N | 303 | 191.80 |
| aac | N | 64 | 175.20 |
| --- | -- | ------ | ------ |
| mPD | | 0.29 | 0.50 |
| | | nPD: | 0.58 |
| | | N. weight: | 0.32 |
| | | Sc. PD: | 0.098 |
| | | Sc. rank: | 48.9 |

PB1

| Pos. | 538 | obs: | exp: |
|---|---|---|---|
| gat | D | 163 | 194.20 |
| gac | D | 201 | 169.80 |
| gaa | E | 3 | 1.73 |
| gag | E | 0 | 1.27 |
| --- | -- | ------ | ------ |
| mPD | | 0.50 | 0.51 |
| | | nPD: | 1. |
| | | N. weight: | 0.042 |
| | | Sc. PD: | 0.03 |
| | | Sc. rank: | 8.8 |

PB1

| Pos. | 539 | obs: | exp: |
|---|---|---|---|
| tta | L | 0 | 32.06 |
| ttg | L | 0 | 70.68 |
| ctt | L | 359 | 68.48 |
| ctc | L | 3 | 59.37 |
| cta | L | 0 | 55.29 |
| ctg | L | 5 | 81.13 |
| --- | -- | ------ | ------ |
| mPD | | 0.043 | 1.1 |
| | | nPD: | 0.04 |
| | | N. weight: | 2.4 |
| | | Sc. PD: | -0.49 |
| | | Sc. rank: | -321.2 |

PB1

| Pos. | 540 | obs: | exp: |
|---|---|---|---|
| ggt | G | 0 | 45.28 |
| ggc | G | 2 | 49.63 |
| gga | G | 344 | 177.50 |
| ggg | G | 21 | 94.55 |
| --- | -- | ------ | ------ |
| mPD | | 0.12 | 0.67 |
| | | nPD: | 0.18 |
| | | N. weight: | 0.8 |
| | | Sc. PD: | -0.06 |
| | | Sc. rank: | -0.3 |

PB1

| Pos. | 541 | obs: | exp: |
|---|---|---|---|
| cct | P | 10 | 96.64 |
| ccc | P | 1 | 66.20 |
| cca | P | 348 | 144.80 |
| ccg | P | 8 | 59.34 |
| --- | -- | ------ | ------ |
| mPD | | 0.10 | 0.72 |
| | | nPD: | 0.14 |
| | | N. weight: | 1.1 |
| | | Sc. PD: | -0.12 |
| | | Sc. rank: | -28.7 |

PB1

| Pos. | 542 | obs: | exp: |
|---|---|---|---|
| gct | A | 0 | 92.44 |
| gcc | A | 0 | 75.18 |
| gca | A | 364 | 163.60 |
| gcg | A | 3 | 35.81 |
| --- | -- | ------ | ------ |
| mPD | | 0.016 | 0.69 |
| | | nPD: | 0.02 |
| | | N. weight: | 1.2 |
| | | Sc. PD: | -0.26 |
| | | Sc. rank: | -177.9 |

PB1

| Pos. | 543 | obs: | exp: |
|---|---|---|---|
| act | T | 1 | 96.96 |
| acc | T | 0 | 78.75 |
| aca | T | 361 | 160.80 |
| acg | T | 5 | 30.51 |
| --- | -- | ------ | ------ |
| mPD | | 0.032 | 0.69 |
| | | nPD: | 0.05 |
| | | N. weight: | 1.2 |
| | | Sc. PD: | -0.23 |
| | | Sc. rank: | -142.4 |

PB1

| Pos. | 544 | obs: | exp: |
|---|---|---|---|
| gct | A | 282 | 92.44 |
| gcc | A | 79 | 75.18 |
| gca | A | 6 | 163.60 |
| gcg | A | 0 | 35.81 |
| --- | -- | ------ | ------ |
| mPD | | 0.36 | 0.69 |
| | | nPD: | 0.53 |
| | | N. weight: | 1.3 |
| | | Sc. PD: | 0.33 |
| | | Sc. rank: | 175.6 |

PB1

| Pos. | 545 | obs: | exp: |
|---|---|---|---|
| caa | Q | 212 | 195.60 |
| cag | Q | 155 | 171.40 |
| --- | -- | ------ | ------ |
| mPD | | 0.49 | 0.50 |
| | | nPD: | 0.98 |
| | | N. weight: | 0.019 |
| | | Sc. PD: | 0.013 |
| | | Sc. rank: | 4.0 |

PB1

| Pos. | 546 | obs: | exp: |
|---|---|---|---|
| atg | M | 367 | 367.00 |
| --- | -- | ------ | ------ |
| mPD | | 0 | 0 |
| | | nPD: | 1. |
| | | N. weight: | 0. |
| | | Sc. PD: | 0 |
| | | Sc. rank: | 0 |

PB1

| Pos. | 547 | obs: | exp: |
|---|---|---|---|
| gct | A | 283 | 92.44 |
| gcc | A | 84 | 75.18 |
| gca | A | 0 | 163.60 |
| gcg | A | 0 | 35.81 |
| --- | -- | ------ | ------ |
| mPD | | 0.35 | 0.69 |
| | | nPD: | 0.52 |
| | | N. weight: | 1.4 |
| | | Sc. PD: | 0.34 |
| | | Sc. rank: | 181.0 |

PB1

| Pos. | 548 | obs: | exp: |
|---|---|---|---|
| tta | L | 0 | 32.06 |
| ttg | L | 0 | 70.68 |
| ctt | L | 343 | 68.48 |
| ctc | L | 24 | 59.37 |
| cta | L | 0 | 55.29 |
| ctg | L | 0 | 81.13 |
| --- | -- | ------ | ------ |
| mPD | | 0.12 | 1.1 |
| | | nPD: | 0.11 |
| | | N. weight: | 2.2 |
| | | Sc. PD: | -0.31 |
| | | Sc. rank: | -105.1 |

PB1

| Pos. | 549 | obs: | exp: |
|---|---|---|---|
| caa | Q | 39 | 195.60 |
| cag | Q | 328 | 171.40 |
| --- | -- | ------ | ------ |
| mPD | | 0.19 | 0.50 |
| | | nPD: | 0.38 |
| | | N. weight: | 0.63 |
| | | Sc. PD: | 0.076 |
| | | Sc. rank: | 61.0 |

| | | | |
|---|---|---|---|
| PB1 | | | |
| Pos. | 550 | obs: | exp: |
| tta | L | 4 | 31.97 |
| ttg | L | 2 | 70.49 |
| ctt | L | 2 | 68.29 |
| ctc | L | 0 | 59.20 |
| cta | L | 319 | 55.14 |
| ctg | L | 39 | 80.90 |
| atg | M | 1 | 1.00 |
| --- | -- | ------ | ------ |
| mPD | | 0.25 | 1.1 |
| | | nPD: | 0.22 |
| | | N. weight: | 2.1 |
| | | Sc. PD: | -0.066 |
| | | Sc. rank: | 62.8 |

,

| | | | |
|---|---|---|---|
| PB1 | | | |
| Pos. | 551 | obs: | exp: |
| ttt | F | 63 | 150.90 |
| ttc | F | 304 | 216.10 |
| --- | -- | ------ | ------ |
| mPD | | 0.29 | 0.48 |
| | | nPD: | 0.59 |
| | | N. weight: | 0.21 |
| | | Sc. PD: | 0.068 |
| | | Sc. rank: | 33.0 |

,

| | | | |
|---|---|---|---|
| PB1 | | | |
| Pos. | 552 | obs: | exp: |
| att | I | 8 | 138.00 |
| atc | I | 356 | 96.18 |
| ata | I | 3 | 132.80 |
| --- | -- | ------ | ------ |
| mPD | | 0.059 | 0.66 |
| | | nPD: | 0.09 |
| | | N. weight: | 1.8 |
| | | Sc. PD: | -0.29 |
| | | Sc. rank: | -115.4 |

,

| | | | |
|---|---|---|---|
| PB1 | | | |
| Pos. | 553 | obs: | exp: |
| aaa | K | 71 | 205.50 |
| aag | K | 296 | 161.50 |
| --- | -- | ------ | ------ |
| mPD | | 0.31 | 0.49 |
| | | nPD: | 0.63 |
| | | N. weight: | 0.44 |
| | | Sc. PD: | 0.16 |
| | | Sc. rank: | 72.8 |

,

| | | | |
|---|---|---|---|
| PB1 | | | |
| Pos. | 554 | obs: | exp: |
| gat | D | 103 | 195.80 |
| gac | D | 264 | 171.20 |
| --- | -- | ------ | ------ |
| mPD | | 0.40 | 0.50 |
| | | nPD: | 0.81 |
| | | N. weight: | 0.21 |
| | | Sc. PD: | 0.11 |
| | | Sc. rank: | 42.9 |

,

| | | | |
|---|---|---|---|
| PB1 | | | |
| Pos. | 555 | obs: | exp: |
| tat | Y | 144 | 190.00 |
| tac | Y | 223 | 177.00 |
| --- | -- | ------ | ------ |
| mPD | | 0.48 | 0.50 |
| | | nPD: | 0.96 |
| | | N. weight: | 0.061 |
| | | Sc. PD: | 0.041 |
| | | Sc. rank: | 12.7 |

,

| | | | |
|---|---|---|---|
| PB1 | | | |
| Pos. | 556 | obs: | exp: |
| cgt | R | 0 | 15.24 |
| cgc | R | 0 | 17.97 |
| cga | R | 0 | 35.27 |
| cgg | R | 1 | 30.63 |
| aga | R | 189 | 165.00 |
| agg | R | 177 | 102.80 |
| --- | -- | ------ | ------ |
| mPD | | 0.51 | 0.96 |
| | | nPD: | 0.53 |
| | | N. weight: | 0.51 |
| | | Sc. PD: | 0.13 |
| | | Sc. rank: | 70.4 |

,

| | | | |
|---|---|---|---|
| PB1 | | | |
| Pos. | 557 | obs: | exp: |
| tat | Y | 6 | 190.00 |
| tac | Y | 361 | 177.00 |
| --- | -- | ------ | ------ |
| mPD | | 0.032 | 0.50 |
| | | nPD: | 0.06 |
| | | N. weight: | 0.99 |
| | | Sc. PD: | -0.18 |
| | | Sc. rank: | -97.1 |

,

| | | | |
|---|---|---|---|
| PB1 | | | |
| Pos. | 558 | obs: | exp: |
| act | T | 5 | 96.96 |
| acc | T | 1 | 78.75 |
| aca | T | 152 | 160.80 |
| acg | T | 209 | 30.51 |
| --- | -- | ------ | ------ |
| mPD | | 0.51 | 0.69 |
| | | nPD: | 0.74 |
| | | N. weight: | 1.6 |
| | | Sc. PD: | 0.72 |
| | | Sc. rank: | 298.2 |

,

| | | | |
|---|---|---|---|
| PB1 | | | |
| Pos. | 559 | obs: | exp: |
| tat | Y | 110 | 190.00 |
| tac | Y | 257 | 177.00 |
| --- | -- | ------ | ------ |
| mPD | | 0.42 | 0.50 |
| | | nPD: | 0.84 |
| | | N. weight: | 0.16 |
| | | Sc. PD: | 0.09 |
| | | Sc. rank: | 32.9 |

,

| | | | |
|---|---|---|---|
| PB1 | | | |
| Pos. | 560 | obs: | exp: |
| cgt | R | 0 | 15.24 |
| cgc | R | 0 | 17.97 |
| cga | R | 275 | 35.27 |
| cgg | R | 90 | 30.63 |
| aga | R | 0 | 165.00 |
| agg | R | 2 | 102.80 |
| --- | -- | ------ | ------ |
| mPD | | 0.39 | 0.96 |
| | | nPD: | 0.4 |
| | | N. weight: | 2.7 |
| | | Sc. PD: | 0.38 |
| | | Sc. rank: | 284.0 |

,

| | | | |
|---|---|---|---|
| PB1 | | | |
| Pos. | 561 | obs: | exp: |
| tgt | C | 127 | 151.30 |
| tgc | C | 240 | 215.70 |
| --- | -- | ------ | ------ |
| mPD | | 0.45 | 0.48 |
| | | nPD: | 0.94 |
| | | N. weight: | 0.027 |
| | | Sc. PD: | 0.017 |
| | | Sc. rank: | 5.6 |

,

| | | | |
|---|---|---|---|
| PB1 | | | |
| Pos. | 562 | obs: | exp: |
| cat | H | 14 | 198.90 |
| cac | H | 353 | 168.10 |
| --- | -- | ------ | ------ |
| mPD | | 0.074 | 0.50 |
| | | nPD: | 0.15 |
| | | N. weight: | 0.94 |
| | | Sc. PD: | -0.097 |
| | | Sc. rank: | -16.2 |

,

| | | | |
|---|---|---|---|
| PB1 | | | |
| Pos. | 563 | obs: | exp: |
| cgt | R | 0 | 15.19 |
| cgc | R | 0 | 17.92 |
| cga | R | 0 | 35.17 |
| cgg | R | 2 | 30.54 |
| aaa | K | 0 | 0.56 |
| aag | K | 1 | 0.44 |
| aga | R | 107 | 164.60 |
| agg | R | 257 | 102.60 |
| --- | -- | ------ | ------ |
| mPD | | 0.43 | 0.96 |
| | | nPD: | 0.45 |
| | | N. weight: | 0.79 |
| | | Sc. PD: | 0.14 |
| | | Sc. rank: | 93.8 |

,

| | | | |
|---|---|---|---|
| PB1 | | | |
| Pos. | 564 | obs: | exp: |
| ggt | G | 259 | 45.28 |
| ggc | G | 12 | 49.63 |
| gga | G | 75 | 177.50 |
| ggg | G | 21 | 94.55 |
| --- | -- | ------ | ------ |
| mPD | | 0.46 | 0.67 |
| | | nPD: | 0.69 |
| | | N. weight: | 1.4 |
| | | Sc. PD: | 0.59 |
| | | Sc. rank: | 251.6 |

,

| | | | |
|---|---|---|---|
| PB1 | | | |
| Pos. | 565 | obs: | exp: |
| gat | D | 216 | 195.80 |
| gac | D | 151 | 171.20 |
| --- | -- | ------ | ------ |
| mPD | | 0.49 | 0.50 |
| | | nPD: | 0.98 |
| | | N. weight: | 0.022 |
| | | Sc. PD: | 0.015 |
| | | Sc. rank: | 4.7 |

,

| | | | |
|---|---|---|---|
| PB1 | | | |
| Pos. | 566 | obs: | exp: |
| act | T | 0 | 95.90 |
| acc | T | 0 | 77.89 |
| aca | T | 325 | 159.00 |
| acg | T | 38 | 30.18 |
| gct | A | 0 | 1.01 |
| gcc | A | 0 | 0.82 |
| gca | A | 4 | 1.78 |
| gcg | A | 0 | 0.39 |
| --- | -- | ------ | ------ |
| mPD | | 0.21 | 0.71 |
| | | nPD: | 0.29 |
| | | N. weight: | 1. |
| | | Sc. PD: | 0.036 |
| | | Sc. rank: | 71.6 |

,

| | | | |
|---|---|---|---|
| PB1 | | | |
| Pos. | 567 | obs: | exp: |
| caa | Q | 356 | 195.60 |
| cag | Q | 11 | 171.40 |
| --- | -- | ------ | ------ |
| mPD | | 0.058 | 0.50 |
| | | nPD: | 0.12 |
| | | N. weight: | 0.77 |
| | | Sc. PD: | -0.1 |
| | | Sc. rank: | -31.7 |

,

| | | | |
|---|---|---|---|
| PB1 | | | |
| Pos. | 568 | obs: | exp: |
| att | I | 312 | 137.60 |
| atc | I | 54 | 95.92 |
| ata | I | 0 | 132.50 |
| act | T | 1 | 0.26 |
| acc | T | 0 | 0.21 |
| aca | T | 0 | 0.44 |
| acg | T | 0 | 0.08 |
| --- | -- | ------ | ------ |
| mPD | | 0.26 | 0.66 |
| | | nPD: | 0.39 |
| | | N. weight: | 0.95 |
| | | Sc. PD: | 0.12 |
| | | Sc. rank: | 92.1 |

,

| | | | |
|---|---|---|---|
| PB1 | | | |
| Pos. | 569 | obs: | exp: |
| caa | Q | 273 | 195.60 |
| cag | Q | 94 | 171.40 |
| --- | -- | ------ | ------ |
| mPD | | 0.38 | 0.50 |
| | | nPD: | 0.77 |
| | | N. weight: | 0.16 |
| | | Sc. PD: | 0.076 |
| | | Sc. rank: | 30.4 |

,

| | | | |
|---|---|---|---|
| PB1 | | | |
| Pos. | 570 | obs: | exp: |
| act | T | 1 | 96.96 |
| acc | T | 0 | 78.75 |
| aca | T | 144 | 160.80 |
| acg | T | 222 | 30.51 |
| --- | -- | ------ | ------ |
| mPD | | 0.48 | 0.69 |
| | | nPD: | 0.7 |
| | | N. weight: | 1.8 |
| | | Sc. PD: | 0.75 |
| | | Sc. rank: | 315.5 |

,

| | | | |
|---|---|---|---|
| PB1 | | | |
| Pos. | 571 | obs: | exp: |
| cgt | R | 0 | 15.24 |
| cgc | R | 0 | 17.97 |
| cga | R | 0 | 35.27 |
| cgg | R | 0 | 30.63 |
| aga | R | 29 | 165.00 |
| agg | R | 338 | 102.80 |
| --- | -- | ------ | ------ |
| mPD | | 0.15 | 0.96 |
| | | nPD: | 0.15 |
| | | N. weight: | 1.5 |
| | | Sc. PD: | -0.15 |
| | | Sc. rank: | -20.8 |

,

| | | | |
|---|---|---|---|
| PB1 | | | |
| Pos. | 572 | obs: | exp: |
| cgt | R | 0 | 15.24 |
| cgc | R | 0 | 17.97 |
| cga | R | 2 | 35.27 |
| cgg | R | 0 | 30.63 |
| aga | R | 261 | 165.00 |
| agg | R | 104 | 102.80 |
| --- | -- | ------ | ------ |
| mPD | | 0.42 | 0.96 |
| | | nPD: | 0.44 |
| | | N. weight: | 0.5 |
| | | Sc. PD: | 0.084 |
| | | Sc. rank: | 55.5 |

,

| | | | |
|---|---|---|---|
| PB1 | | | |
| Pos. | 573 | obs: | exp: |
| tct | S | 0 | 54.21 |
| tcc | S | 0 | 47.50 |
| tca | S | 330 | 75.55 |
| tcg | S | 11 | 20.79 |
| agt | S | 0 | 75.02 |
| agc | S | 0 | 67.94 |
| gct | A | 0 | 6.55 |
| gcc | A | 0 | 5.33 |
| gca | A | 26 | 11.59 |
| gcg | A | 0 | 2.54 |
| --- | -- | ------ | ------ |
| mPD | | 0.19 | 1.7 |
| | | nPD: | 0.11 |
| | | N. weight: | 2.1 |
| | | Sc. PD: | -0.29 |
| | | Sc. rank: | -96.7 |

,

| | | | |
|---|---|---|---|
| PB1 | | | |
| Pos. | 574 | obs: | exp: |
| ttt | F | 45 | 150.90 |
| ttc | F | 322 | 216.10 |
| --- | -- | ------ | ------ |
| mPD | | 0.22 | 0.48 |
| | | nPD: | 0.45 |
| | | N. weight: | 0.32 |
| | | Sc. PD: | 0.057 |
| | | Sc. rank: | 37.8 |

,

PB1

| Pos. | 575 | obs: | exp: |
|---|---|---|---|
| gaa | E | 12 | 211.20 |
| gag | E | 355 | 155.80 |
| --- | -- | ------ | ------ |
| mPD | | 0.063 | 0.49 |
| | | nPD: | 0.13 |
| | | N. weight: | 1.1 |
| | | Sc. PD: | -0.13 |
| | | Sc. rank: | -33.6 |

PB1

| Pos. | 576 | obs: | exp: |
|---|---|---|---|
| tta | L | 1 | 31.97 |
| ttg | L | 2 | 70.49 |
| ctt | L | 0 | 68.29 |
| ctc | L | 0 | 59.20 |
| cta | L | 26 | 55.14 |
| ctg | L | 337 | 80.90 |
| att | I | 0 | 0.38 |
| atc | I | 0 | 0.26 |
| ata | I | 1 | 0.36 |
| --- | -- | ------ | ------ |
| mPD | | 0.16 | 1.1 |
| | | nPD: | 0.15 |
| | | N. weight: | 1.9 |
| | | Sc. PD: | -0.2 |
| | | Sc. rank: | -35.1 |

PB1

| Pos. | 577 | obs: | exp: |
|---|---|---|---|
| cgt | R | 0 | 0.95 |
| cgc | R | 0 | 1.13 |
| cga | R | 0 | 2.21 |
| cgg | R | 0 | 1.92 |
| aaa | K | 35 | 192.60 |
| aag | K | 309 | 151.40 |
| aga | R | 2 | 10.34 |
| agg | R | 21 | 6.45 |
| --- | -- | ------ | ------ |
| mPD | | 0.30 | 0.65 |
| | | nPD: | 0.46 |
| | | N. weight: | 0.77 |
| | | Sc. PD: | 0.15 |
| | | Sc. rank: | 95.0 |

PB1

| Pos. | 578 | obs: | exp: |
|---|---|---|---|
| cgt | R | 0 | 0.12 |
| cgc | R | 0 | 0.15 |
| cga | R | 0 | 0.29 |
| cgg | R | 0 | 0.25 |
| aaa | K | 44 | 203.80 |
| aag | K | 320 | 160.20 |
| aga | R | 0 | 1.35 |
| agg | R | 3 | 0.84 |
| --- | -- | ------ | ------ |
| mPD | | 0.23 | 0.51 |
| | | nPD: | 0.44 |
| | | N. weight: | 0.67 |
| | | Sc. PD: | 0.12 |
| | | Sc. rank: | 77.9 |

PB1

| Pos. | 579 | obs: | exp: |
|---|---|---|---|
| tta | L | 0 | 32.06 |
| ttg | L | 138 | 70.68 |
| ctt | L | 1 | 68.48 |
| ctc | L | 0 | 59.37 |
| cta | L | 27 | 55.29 |
| ctg | L | 201 | 81.13 |
| --- | -- | ------ | ------ |
| mPD | | 0.61 | 1.1 |
| | | nPD: | 0.55 |
| | | N. weight: | 1.1 |
| | | Sc. PD: | 0.3 |
| | | Sc. rank: | 153.3 |

PB1

| Pos. | 580 | obs: | exp: |
|---|---|---|---|
| tgg | W | 367 | 367.00 |
| --- | -- | ------ | ------ |
| mPD | | 0 | 0 |
| | | nPD: | 1. |
| | | N. weight: | 0. |
| | | Sc. PD: | 0 |
| | | Sc. rank: | 0 |

PB1

| Pos. | 581 | obs: | exp: |
|---|---|---|---|
| gat | D | 3 | 1.60 |
| gac | D | 0 | 1.40 |
| gaa | E | 98 | 209.50 |
| gag | E | 266 | 154.50 |
| --- | -- | ------ | ------ |
| mPD | | 0.40 | 0.50 |
| | | nPD: | 0.81 |
| | | N. weight: | 0.31 |
| | | Sc. PD: | 0.16 |
| | | Sc. rank: | 62.8 |

PB1

| Pos. | 582 | obs: | exp: |
|---|---|---|---|
| caa | Q | 94 | 195.60 |
| cag | Q | 273 | 171.40 |
| --- | -- | ------ | ------ |
| mPD | | 0.38 | 0.50 |
| | | nPD: | 0.77 |
| | | N. weight: | 0.25 |
| | | Sc. PD: | 0.12 |
| | | Sc. rank: | 49.6 |

PB1

| Pos. | 583 | obs: | exp: |
|---|---|---|---|
| act | T | 18 | 96.96 |
| acc | T | 349 | 78.75 |
| aca | T | 0 | 160.80 |
| acg | T | 0 | 30.51 |
| --- | -- | ------ | ------ |
| mPD | | 0.094 | 0.69 |
| | | nPD: | 0.14 |
| | | N. weight: | 2. |
| | | Sc. PD: | -0.23 |
| | | Sc. rank: | -58.3 |

PB1

| Pos. | 584 | obs: | exp: |
|---|---|---|---|
| cat | H | 13 | 11.92 |
| cac | H | 9 | 10.08 |
| cgt | R | 255 | 14.32 |
| cgc | R | 89 | 16.89 |
| cga | R | 1 | 33.16 |
| cgg | R | 0 | 28.79 |
| aga | R | 0 | 155.20 |
| agg | R | 0 | 96.68 |
| --- | -- | ------ | ------ |
| mPD | | 0.51 | 1.2 |
| | | nPD: | 0.44 |
| | | N. weight: | 3.7 |
| | | Sc. PD: | 0.65 |
| | | Sc. rank: | 427.5 |

PB1

| Pos. | 585 | obs: | exp: |
|---|---|---|---|
| tct | S | 1 | 58.34 |
| tcc | S | 1 | 51.12 |
| tca | S | 338 | 81.31 |
| tcg | S | 27 | 22.37 |
| agt | S | 0 | 80.74 |
| agc | S | 0 | 73.12 |
| --- | -- | ------ | ------ |
| mPD | | 0.15 | 1.7 |
| | | nPD: | 0.09 |
| | | N. weight: | 2. |
| | | Sc. PD: | -0.32 |
| | | Sc. rank: | -134.4 |

PB1

| Pos. | 586 | obs: | exp: |
|---|---|---|---|
| cgt | R | 0 | 0.04 |
| cgc | R | 0 | 0.05 |
| cga | R | 0 | 0.10 |
| cgg | R | 0 | 0.08 |
| aaa | K | 121 | 204.90 |
| aag | K | 245 | 161.10 |
| aga | R | 0 | 0.45 |
| agg | R | 1 | 0.28 |
| --- | -- | ------ | ------ |
| mPD | | 0.45 | 0.50 |
| | | nPD: | 0.9 |
| | | N. weight: | 0.18 |
| | | Sc. PD: | 0.11 |
| | | Sc. rank: | 37.2 |

PB1

| Pos. | 587 | obs: | exp: |
|---|---|---|---|
| act | T | 0 | 0.26 |
| acc | T | 0 | 0.21 |
| aca | T | 1 | 0.44 |
| acg | T | 0 | 0.08 |
| gct | A | 6 | 92.19 |
| gcc | A | 4 | 74.97 |
| gca | A | 347 | 163.10 |
| gcg | A | 9 | 35.71 |
| --- | -- | ------ | ------ |
| mPD | | 0.11 | 0.69 |
| | | nPD: | 0.15 |
| | | N. weight: | 0.94 |
| | | Sc. PD: | -0.093 |
| | | Sc. rank: | -12.9 |

PB1

| Pos. | 588 | obs: | exp: |
|---|---|---|---|
| ggt | G | 0 | 45.28 |
| ggc | G | 0 | 49.63 |
| gga | G | 218 | 177.50 |
| ggg | G | 149 | 94.55 |
| --- | -- | ------ | ------ |
| mPD | | 0.48 | 0.67 |
| | | nPD: | 0.73 |
| | | N. weight: | 0.48 |
| | | Sc. PD: | 0.21 |
| | | Sc. rank: | 89.1 |

PB1

| Pos. | 589 | obs: | exp: |
|---|---|---|---|
| tta | L | 0 | 32.06 |
| ttg | L | 14 | 70.68 |
| ctt | L | 0 | 68.48 |
| ctc | L | 0 | 59.37 |
| cta | L | 9 | 55.29 |
| ctg | L | 344 | 81.13 |
| --- | -- | ------ | ------ |
| mPD | | 0.12 | 1.1 |
| | | nPD: | 0.11 |
| | | N. weight: | 1.9 |
| | | Sc. PD: | -0.27 |
| | | Sc. rank: | -94.7 |

PB1

| Pos. | 590 | obs: | exp: |
|---|---|---|---|
| tta | L | 7 | 32.06 |
| ttg | L | 266 | 70.68 |
| ctt | L | 0 | 68.48 |
| ctc | L | 0 | 59.37 |
| cta | L | 1 | 55.29 |
| ctg | L | 93 | 81.13 |
| --- | -- | ------ | ------ |
| mPD | | 0.42 | 1.1 |
| | | nPD: | 0.38 |
| | | N. weight: | 1.5 |
| | | Sc. PD: | 0.18 |
| | | Sc. rank: | 140.4 |

PB1

| Pos. | 591 | obs: | exp: |
|---|---|---|---|
| gtt | V | 92 | 75.43 |
| gtc | V | 81 | 77.03 |
| gta | V | 132 | 68.78 |
| gtg | V | 62 | 145.80 |
| --- | -- | ------ | ------ |
| mPD | | 0.73 | 0.72 |
| | | nPD: | 1.02 |
| | | N. weight: | 0.27 |
| | | Sc. PD: | 0.19 |
| | | Sc. rank: | 55.6 |

PB1

| Pos. | 592 | obs: | exp: |
|---|---|---|---|
| tct | S | 0 | 55.64 |
| tcc | S | 1 | 48.76 |
| tca | S | 319 | 77.54 |
| tcg | S | 30 | 21.33 |
| cct | P | 0 | 0.26 |
| ccc | P | 0 | 0.18 |
| cca | P | 1 | 0.39 |
| ccg | P | 0 | 0.16 |
| agt | S | 0 | 77.00 |
| agc | S | 0 | 69.73 |
| gct | A | 0 | 4.03 |
| gcc | A | 0 | 3.28 |
| gca | A | 16 | 7.13 |
| gcg | A | 0 | 1.56 |
| --- | -- | ------ | ------ |
| mPD | | 0.24 | 1.7 |
| | | nPD: | 0.14 |
| | | N. weight: | 2. |
| | | Sc. PD: | -0.21 |
| | | Sc. rank: | -39.7 |

PB1

| Pos. | 593 | obs: | exp: |
|---|---|---|---|
| gat | D | 359 | 195.80 |
| gac | D | 8 | 171.20 |
| --- | -- | ------ | ------ |
| mPD | | 0.043 | 0.50 |
| | | nPD: | 0.09 |
| | | N. weight: | 0.81 |
| | | Sc. PD: | -0.13 |
| | | Sc. rank: | -56.9 |

PB1

| Pos. | 594 | obs: | exp: |
|---|---|---|---|
| ggt | G | 0 | 45.28 |
| ggc | G | 1 | 49.63 |
| gga | G | 362 | 177.50 |
| ggg | G | 4 | 94.55 |
| --- | -- | ------ | ------ |
| mPD | | 0.027 | 0.67 |
| | | nPD: | 0.04 |
| | | N. weight: | 1. |
| | | Sc. PD: | -0.21 |
| | | Sc. rank: | -133.0 |

PB1

| Pos. | 595 | obs: | exp: |
|---|---|---|---|
| ggt | G | 0 | 45.28 |
| ggc | G | 0 | 49.63 |
| gga | G | 315 | 177.50 |
| ggg | G | 52 | 94.55 |
| --- | -- | ------ | ------ |
| mPD | | 0.24 | 0.67 |
| | | nPD: | 0.37 |
| | | N. weight: | 0.63 |
| | | Sc. PD: | 0.066 |
| | | Sc. rank: | 53.7 |

PB1

| Pos. | 596 | obs: | exp: |
|---|---|---|---|
| tct | S | 0 | 0.32 |
| tcc | S | 0 | 0.28 |
| tca | S | 2 | 0.44 |
| tcg | S | 0 | 0.12 |
| cct | P | 0 | 96.11 |
| ccc | P | 0 | 65.84 |
| cca | P | 323 | 144.00 |
| ccg | P | 42 | 59.02 |
| agt | S | 0 | 0.44 |
| agc | S | 0 | 0.40 |
| --- | -- | ------ | ------ |
| mPD | | 0.21 | 0.73 |
| | | nPD: | 0.29 |
| | | N. weight: | 1. |
| | | Sc. PD: | 0.036 |
| | | Sc. rank: | 71.1 |

PB1

| Pos. | 597 | obs: | exp: |
|---|---|---|---|
| aat | N | 112 | 191.80 |
| aac | N | 255 | 175.20 |
| --- | -- | ------ | ------ |
| mPD | | 0.43 | 0.50 |
| | | nPD: | 0.85 |
| | | N. weight: | 0.16 |
| | | Sc. PD: | 0.091 |
| | | Sc. rank: | 32.7 |

PB1

| Pos. | 598 | obs: | exp: |
|---|---|---|---|
| tta | L | 17 | 32.06 |
| ttg | L | 6 | 70.68 |
| ctt | L | 0 | 68.48 |
| ctc | L | 0 | 59.37 |
| cta | L | 286 | 55.29 |
| ctg | L | 58 | 81.13 |
| --- | -- | ------ | ------ |
| mPD | | 0.41 | 1.1 |
| | | nPD: | 0.36 |
| | | N. weight: | 1.8 |
| | | Sc. PD: | 0.18 |
| | | Sc. rank: | 151.7 |

PB1

| Pos. | 599 | obs: | exp: |
|---|---|---|---|
| tat | Y | 8 | 190.00 |
| tac | Y | 359 | 177.00 |
| --- | -- | ------ | ------ |
| mPD | | 0.043 | 0.50 |
| | | nPD: | 0.09 |
| | | N. weight: | 0.96 |
| | | Sc. PD: | -0.16 |
| | | Sc. rank: | -69.0 |

| PB1 | | | |
|---|---|---|---|
| Pos. | 600 | obs: | exp: |
| aat | N | 171 | 191.80 |
| aac | N | 196 | 175.20 |
| --- | -- | ------ | ------ |
| mPD | | 0.50 | 0.50 |
| | | nPD: | 1. |
| | | N. weight: | 0.023 |
| | | Sc. PD: | 0.016 |
| | | Sc. rank: | 4.8 |

| PB1 | | | |
|---|---|---|---|
| Pos. | 601 | obs: | exp: |
| att | I | 246 | 138.00 |
| atc | I | 121 | 96.18 |
| ata | I | 0 | 132.80 |
| --- | -- | ------ | ------ |
| mPD | | 0.44 | 0.66 |
| | | nPD: | 0.67 |
| | | N. weight: | 0.72 |
| | | Sc. PD: | 0.28 |
| | | Sc. rank: | 124.5 |

| PB1 | | | |
|---|---|---|---|
| Pos. | 602 | obs: | exp: |
| tgg | W | 1 | 1.00 |
| cgt | R | 2 | 15.19 |
| cgc | R | 0 | 17.92 |
| cga | R | 28 | 35.17 |
| cgg | R | 320 | 30.54 |
| aga | R | 1 | 164.60 |
| agg | R | 15 | 102.60 |
| --- | -- | ------ | ------ |
| mPD | | 0.24 | 0.96 |
| | | nPD: | 0.25 |
| | | N. weight: | 3. |
| | | Sc. PD: | -0.0068 |
| | | Sc. rank: | 134.1 |

| PB1 | | | |
|---|---|---|---|
| Pos. | 603 | obs: | exp: |
| aat | N | 319 | 191.80 |
| aac | N | 48 | 175.20 |
| --- | -- | ------ | ------ |
| mPD | | 0.23 | 0.50 |
| | | nPD: | 0.46 |
| | | N. weight: | 0.43 |
| | | Sc. PD: | 0.081 |
| | | Sc. rank: | 52.3 |

| PB1 | | | |
|---|---|---|---|
| Pos. | 604 | obs: | exp: |
| tta | L | 0 | 32.06 |
| ttg | L | 0 | 70.68 |
| ctt | L | 55 | 68.48 |
| ctc | L | 307 | 59.37 |
| cta | L | 5 | 55.29 |
| ctg | L | 0 | 81.13 |
| --- | -- | ------ | ------ |
| mPD | | 0.28 | 1.1 |
| | | nPD: | 0.25 |
| | | N. weight: | 2. |
| | | Sc. PD: | -0.013 |
| | | Sc. rank: | 83.7 |

| PB1 | | | |
|---|---|---|---|
| Pos. | 605 | obs: | exp: |
| cat | H | 14 | 198.90 |
| cac | H | 353 | 168.10 |
| --- | -- | ------ | ------ |
| mPD | | 0.074 | 0.50 |
| | | nPD: | 0.15 |
| | | N. weight: | 0.94 |
| | | Sc. PD: | -0.097 |
| | | Sc. rank: | -16.2 |

| PB1 | | | |
|---|---|---|---|
| Pos. | 606 | obs: | exp: |
| tta | L | 0 | 0.09 |
| ttg | L | 0 | 0.19 |
| ctt | L | 0 | 0.19 |
| ctc | L | 1 | 0.16 |
| cta | L | 0 | 0.15 |
| ctg | L | 0 | 0.22 |
| att | I | 157 | 137.60 |
| atc | I | 209 | 95.92 |
| ata | I | 0 | 132.50 |
| --- | -- | ------ | ------ |
| mPD | | 0.50 | 0.67 |
| | | nPD: | 0.75 |
| | | N. weight: | 0.78 |
| | | Sc. PD: | 0.36 |
| | | Sc. rank: | 150.8 |

| PB1 | | | |
|---|---|---|---|
| Pos. | 607 | obs: | exp: |
| cct | P | 0 | 96.64 |
| ccc | P | 6 | 66.20 |
| cca | P | 273 | 144.80 |
| ccg | P | 88 | 59.34 |
| --- | -- | ------ | ------ |
| mPD | | 0.39 | 0.72 |
| | | nPD: | 0.54 |
| | | N. weight: | 0.82 |
| | | Sc. PD: | 0.22 |
| | | Sc. rank: | 117.0 |

| PB1 | | | |
|---|---|---|---|
| Pos. | 608 | obs: | exp: |
| gaa | E | 116 | 211.20 |
| gag | E | 251 | 155.80 |
| --- | -- | ------ | ------ |
| mPD | | 0.43 | 0.49 |
| | | nPD: | 0.89 |
| | | N. weight: | 0.22 |
| | | Sc. PD: | 0.13 |
| | | Sc. rank: | 45.6 |

| PB1 | | | |
|---|---|---|---|
| Pos. | 609 | obs: | exp: |
| gtt | V | 27 | 75.43 |
| gtc | V | 328 | 77.03 |
| gta | V | 12 | 68.78 |
| gtg | V | 0 | 145.80 |
| --- | -- | ------ | ------ |
| mPD | | 0.20 | 0.72 |
| | | nPD: | 0.27 |
| | | N. weight: | 1.8 |
| | | Sc. PD: | 0.025 |
| | | Sc. rank: | 103.9 |

| PB1 | | | |
|---|---|---|---|
| Pos. | 610 | obs: | exp: |
| tgt | C | 7 | 151.30 |
| tgc | C | 360 | 215.70 |
| --- | -- | ------ | ------ |
| mPD | | 0.038 | 0.48 |
| | | nPD: | 0.08 |
| | | N. weight: | 0.68 |
| | | Sc. PD: | -0.12 |
| | | Sc. rank: | -54.8 |

| PB1 | | | |
|---|---|---|---|
| Pos. | 611 | obs: | exp: |
| tta | L | 17 | 32.06 |
| ttg | L | 280 | 70.68 |
| ctt | L | 0 | 68.48 |
| ctc | L | 0 | 59.37 |
| cta | L | 11 | 55.29 |
| ctg | L | 59 | 81.13 |
| --- | -- | ------ | ------ |
| mPD | | 0.45 | 1.1 |
| | | nPD: | 0.4 |
| | | N. weight: | 1.4 |
| | | Sc. PD: | 0.2 |
| | | Sc. rank: | 150.2 |

| PB1 | | | |
|---|---|---|---|
| Pos. | 612 | obs: | exp: |
| aaa | K | 139 | 205.50 |
| aag | K | 228 | 161.50 |
| --- | -- | ------ | ------ |
| mPD | | 0.47 | 0.49 |
| | | nPD: | 0.96 |
| | | N. weight: | 0.11 |
| | | Sc. PD: | 0.076 |
| | | Sc. rank: | 23.7 |

| PB1 | | | |
|---|---|---|---|
| Pos. | 613 | obs: | exp: |
| tgg | W | 367 | 367.00 |
| --- | -- | ------ | ------ |
| mPD | | 0 | 0 |
| | | nPD: | 1. |
| | | N. weight: | 0. |
| | | Sc. PD: | 0 |
| | | Sc. rank: | 0 |

| PB1 | | | |
|---|---|---|---|
| Pos. | 614 | obs: | exp: |
| gaa | E | 223 | 210.60 |
| gag | E | 143 | 155.40 |
| ggt | G | 0 | 0.12 |
| ggc | G | 0 | 0.14 |
| gga | G | 1 | 0.48 |
| ggg | G | 0 | 0.26 |
| --- | -- | ------ | ------ |
| mPD | | 0.48 | 0.49 |
| | | nPD: | 0.98 |
| | | N. weight: | 0.02 |
| | | Sc. PD: | 0.013 |
| | | Sc. rank: | 4.1 |

| PB1 | | | |
|---|---|---|---|
| Pos. | 615 | obs: | exp: |
| tta | L | 10 | 31.89 |
| ttg | L | 49 | 70.30 |
| ctt | L | 14 | 68.10 |
| ctc | L | 0 | 59.04 |
| cta | L | 151 | 54.99 |
| ctg | L | 141 | 80.68 |
| atg | M | 2 | 2.00 |
| --- | -- | ------ | ------ |
| mPD | | 0.81 | 1.1 |
| | | nPD: | 0.73 |
| | | N. weight: | 0.79 |
| | | Sc. PD: | 0.35 |
| | | Sc. rank: | 146.7 |

| PB1 | | | |
|---|---|---|---|
| Pos. | 616 | obs: | exp: |
| atg | M | 367 | 367.00 |
| --- | -- | ------ | ------ |
| mPD | | 0 | 0 |
| | | nPD: | 1. |
| | | N. weight: | 0. |
| | | Sc. PD: | 0 |
| | | Sc. rank: | 0 |

| PB1 | | | |
|---|---|---|---|
| Pos. | 617 | obs: | exp: |
| aat | N | 1 | 0.52 |
| aac | N | 0 | 0.48 |
| gat | D | 234 | 195.30 |
| gac | D | 132 | 170.70 |
| --- | -- | ------ | ------ |
| mPD | | 0.47 | 0.50 |
| | | nPD: | 0.93 |
| | | N. weight: | 0.05 |
| | | Sc. PD: | 0.032 |
| | | Sc. rank: | 10.5 |

| PB1 | | | |
|---|---|---|---|
| Pos. | 618 | obs: | exp: |
| gaa | E | 350 | 211.20 |
| gag | E | 17 | 155.80 |
| --- | -- | ------ | ------ |
| mPD | | 0.089 | 0.49 |
| | | nPD: | 0.18 |
| | | N. weight: | 0.59 |
| | | Sc. PD: | -0.042 |
| | | Sc. rank: | 0.8 |

| PB1 | | | |
|---|---|---|---|
| Pos. | 619 | obs: | exp: |
| aat | N | 1 | 0.52 |
| aac | N | 0 | 0.48 |
| gat | D | 169 | 192.60 |
| gac | D | 192 | 168.40 |
| gaa | E | 0 | 0.58 |
| gag | E | 1 | 0.42 |
| ggt | G | 1 | 0.49 |
| ggc | G | 3 | 0.54 |
| gga | G | 0 | 1.93 |
| ggg | G | 0 | 1.03 |
| --- | -- | ------ | ------ |
| mPD | | 0.53 | 0.54 |
| | | nPD: | 0.99 |
| | | N. weight: | 0.06 |
| | | Sc. PD: | 0.042 |
| | | Sc. rank: | 12.5 |

| PB1 | | | |
|---|---|---|---|
| Pos. | 620 | obs: | exp: |
| tat | Y | 107 | 190.00 |
| tac | Y | 260 | 177.00 |
| --- | -- | ------ | ------ |
| mPD | | 0.41 | 0.50 |
| | | nPD: | 0.83 |
| | | N. weight: | 0.17 |
| | | Sc. PD: | 0.094 |
| | | Sc. rank: | 35.2 |

| PB1 | | | |
|---|---|---|---|
| Pos. | 621 | obs: | exp: |
| caa | Q | 85 | 195.10 |
| cag | Q | 281 | 170.90 |
| cgt | R | 0 | 0.04 |
| cgc | R | 0 | 0.05 |
| cga | R | 1 | 0.10 |
| cgg | R | 0 | 0.08 |
| aga | R | 0 | 0.45 |
| agg | R | 0 | 0.28 |
| --- | -- | ------ | ------ |
| mPD | | 0.37 | 0.51 |
| | | nPD: | 0.72 |
| | | N. weight: | 0.31 |
| | | Sc. PD: | 0.14 |
| | | Sc. rank: | 56.6 |

| PB1 | | | |
|---|---|---|---|
| Pos. | 622 | obs: | exp: |
| ggt | G | 56 | 45.28 |
| ggc | G | 212 | 49.63 |
| gga | G | 97 | 177.50 |
| ggg | G | 2 | 94.55 |
| --- | -- | ------ | ------ |
| mPD | | 0.57 | 0.67 |
| | | nPD: | 0.86 |
| | | N. weight: | 1.1 |
| | | Sc. PD: | 0.62 |
| | | Sc. rank: | 222.0 |

| PB1 | | | |
|---|---|---|---|
| Pos. | 623 | obs: | exp: |
| cgt | R | 0 | 15.24 |
| cgc | R | 0 | 17.97 |
| cga | R | 0 | 35.27 |
| cgg | R | 4 | 30.63 |
| aga | R | 39 | 165.00 |
| agg | R | 324 | 102.80 |
| --- | -- | ------ | ------ |
| mPD | | 0.21 | 0.96 |
| | | nPD: | 0.22 |
| | | N. weight: | 1.3 |
| | | Sc. PD: | -0.044 |
| | | Sc. rank: | 33.4 |

| PB1 | | | |
|---|---|---|---|
| Pos. | 624 | obs: | exp: |
| tta | L | 6 | 32.06 |
| ttg | L | 48 | 70.68 |
| ctt | L | 98 | 68.48 |
| ctc | L | 6 | 59.37 |
| cta | L | 5 | 55.29 |
| ctg | L | 204 | 81.13 |
| --- | -- | ------ | ------ |
| mPD | | 0.71 | 1.1 |
| | | nPD: | 0.64 |
| | | N. weight: | 0.75 |
| | | Sc. PD: | 0.27 |
| | | Sc. rank: | 123.1 |

| | | | |
|---|---|---|---|
| PB1 | | | |
| Pos. | 625 | obs: | exp: |
| tgt | C | 330 | 151.30 |
| tgc | C | 37 | 215.70 |
| --- | -- | ------ | ------ |
| mPD | | 0.18 | 0.48 |
| | | nPD: | 0.38 |
| | | N. weight: | 0.81 |
| | | Sc. PD: | 0.091 |
| | | Sc. rank: | 71.6 |

,

| | | | |
|---|---|---|---|
| PB1 | | | |
| Pos. | 626 | obs: | exp: |
| aat | N | 271 | 191.30 |
| aac | N | 95 | 174.70 |
| gat | D | 1 | 0.53 |
| gac | D | 0 | 0.47 |
| --- | -- | ------ | ------ |
| mPD | | 0.39 | 0.50 |
| | | nPD: | 0.77 |
| | | N. weight: | 0.17 |
| | | Sc. PD: | 0.082 |
| | | Sc. rank: | 32.6 |

,

| | | | |
|---|---|---|---|
| PB1 | | | |
| Pos. | 627 | obs: | exp: |
| cct | P | 268 | 96.64 |
| ccc | P | 98 | 66.20 |
| cca | P | 1 | 144.80 |
| ccg | P | 0 | 59.34 |
| --- | -- | ------ | ------ |
| mPD | | 0.40 | 0.72 |
| | | nPD: | 0.55 |
| | | N. weight: | 1.3 |
| | | Sc. PD: | 0.36 |
| | | Sc. rank: | 186.2 |

,

| | | | |
|---|---|---|---|
| PB1 | | | |
| Pos. | 628 | obs: | exp: |
| tta | L | 0 | 29.53 |
| ttg | L | 11 | 65.10 |
| ctt | L | 1 | 63.07 |
| ctc | L | 0 | 54.67 |
| cta | L | 9 | 50.92 |
| ctg | L | 317 | 74.72 |
| caa | Q | 0 | 0.53 |
| cag | Q | 1 | 0.47 |
| atg | M | 28 | 28.00 |
| --- | -- | ------ | ------ |
| mPD | | 0.25 | 1.2 |
| | | nPD: | 0.22 |
| | | N. weight: | 1.8 |
| | | Sc. PD: | -0.069 |
| | | Sc. rank: | 40.6 |

,

| | | | |
|---|---|---|---|
| PB1 | | | |
| Pos. | 629 | obs: | exp: |
| aat | N | 188 | 191.80 |
| aac | N | 179 | 175.20 |
| --- | -- | ------ | ------ |
| mPD | | 0.50 | 0.50 |
| | | nPD: | 1. |
| | | N. weight: | 0.014 |
| | | Sc. PD: | 0.0096 |
| | | Sc. rank: | 2.8 |

,

| | | | |
|---|---|---|---|
| PB1 | | | |
| Pos. | 630 | obs: | exp: |
| cct | P | 1 | 96.64 |
| ccc | P | 0 | 66.20 |
| cca | P | 95 | 144.80 |
| ccg | P | 271 | 59.34 |
| --- | -- | ------ | ------ |
| mPD | | 0.39 | 0.72 |
| | | nPD: | 0.54 |
| | | N. weight: | 1.5 |
| | | Sc. PD: | 0.42 |
| | | Sc. rank: | 218.0 |

,

| | | | |
|---|---|---|---|
| PB1 | | | |
| Pos. | 631 | obs: | exp: |
| ttt | F | 363 | 150.90 |
| ttc | F | 4 | 216.10 |
| --- | -- | ------ | ------ |
| mPD | | 0.022 | 0.48 |
| | | nPD: | 0.04 |
| | | N. weight: | 1.3 |
| | | Sc. PD: | -0.25 |
| | | Sc. rank: | -157.4 |

,

| | | | |
|---|---|---|---|
| PB1 | | | |
| Pos. | 632 | obs: | exp: |
| gtt | V | 4 | 74.82 |
| gtc | V | 359 | 76.40 |
| gta | V | 1 | 68.22 |
| gtg | V | 0 | 144.60 |
| gct | A | 0 | 0.76 |
| gcc | A | 3 | 0.61 |
| gca | A | 0 | 1.34 |
| gcg | A | 0 | 0.29 |
| --- | -- | ------ | ------ |
| mPD | | 0.043 | 0.74 |
| | | nPD: | 0.06 |
| | | N. weight: | 2.3 |
| | | Sc. PD: | -0.43 |
| | | Sc. rank: | -233.6 |

,

| | | | |
|---|---|---|---|
| PB1 | | | |
| Pos. | 633 | obs: | exp: |
| tct | S | 0 | 58.34 |
| tcc | S | 0 | 51.12 |
| tca | S | 0 | 81.31 |
| tcg | S | 0 | 22.37 |
| agt | S | 244 | 80.74 |
| agc | S | 123 | 73.12 |
| --- | -- | ------ | ------ |
| mPD | | 0.45 | 1.7 |
| | | nPD: | 0.27 |
| | | N. weight: | 1.4 |
| | | Sc. PD: | 0.016 |
| | | Sc. rank: | 75.0 |

,

| | | | |
|---|---|---|---|
| PB1 | | | |
| Pos. | 634 | obs: | exp: |
| cat | H | 265 | 198.90 |
| cac | H | 102 | 168.10 |
| --- | -- | ------ | ------ |
| mPD | | 0.40 | 0.50 |
| | | nPD: | 0.81 |
| | | N. weight: | 0.12 |
| | | Sc. PD: | 0.062 |
| | | Sc. rank: | 23.5 |

,

| | | | |
|---|---|---|---|
| PB1 | | | |
| Pos. | 635 | obs: | exp: |
| cgt | R | 0 | 1.08 |
| cgc | R | 0 | 1.27 |
| cga | R | 0 | 2.50 |
| cgg | R | 0 | 2.17 |
| aaa | K | 120 | 190.90 |
| aag | K | 221 | 150.10 |
| aga | R | 0 | 11.69 |
| agg | R | 26 | 7.29 |
| --- | -- | ------ | ------ |
| mPD | | 0.57 | 0.67 |
| | | nPD: | 0.86 |
| | | N. weight: | 0.27 |
| | | Sc. PD: | 0.16 |
| | | Sc. rank: | 56.0 |

,

| | | | |
|---|---|---|---|
| PB1 | | | |
| Pos. | 636 | obs: | exp: |
| gaa | E | 311 | 210.60 |
| gag | E | 55 | 155.40 |
| ggt | G | 0 | 0.12 |
| ggc | G | 0 | 0.14 |
| gga | G | 1 | 0.48 |
| ggg | G | 0 | 0.26 |
| --- | -- | ------ | ------ |
| mPD | | 0.26 | 0.49 |
| | | nPD: | 0.53 |
| | | N. weight: | 0.28 |
| | | Sc. PD: | 0.072 |
| | | Sc. rank: | 38.8 |

,

| | | | |
|---|---|---|---|
| PB1 | | | |
| Pos. | 637 | obs: | exp: |
| att | I | 271 | 138.00 |
| atc | I | 5 | 96.18 |
| ata | I | 91 | 132.80 |
| --- | -- | ------ | ------ |
| mPD | | 0.39 | 0.66 |
| | | nPD: | 0.6 |
| | | N. weight: | 0.57 |
| | | Sc. PD: | 0.19 |
| | | Sc. rank: | 89.5 |

,

| | | | |
|---|---|---|---|
| PB1 | | | |
| Pos. | 638 | obs: | exp: |
| gat | D | 26 | 13.87 |
| gac | D | 0 | 12.13 |
| gaa | E | 16 | 196.20 |
| gag | E | 325 | 144.80 |
| --- | -- | ------ | ------ |
| mPD | | 0.21 | 0.56 |
| | | nPD: | 0.38 |
| | | N. weight: | 1. |
| | | Sc. PD: | 0.11 |
| | | Sc. rank: | 89.3 |

,

| | | | |
|---|---|---|---|
| PB1 | | | |
| Pos. | 639 | obs: | exp: |
| tct | S | 120 | 58.34 |
| tcc | S | 245 | 51.12 |
| tca | S | 2 | 81.31 |
| tcg | S | 0 | 22.37 |
| agt | S | 0 | 80.74 |
| agc | S | 0 | 73.12 |
| --- | -- | ------ | ------ |
| mPD | | 0.45 | 1.7 |
| | | nPD: | 0.27 |
| | | N. weight: | 1.9 |
| | | Sc. PD: | 0.025 |
| | | Sc. rank: | 106.1 |

,

| | | | |
|---|---|---|---|
| PB1 | | | |
| Pos. | 640 | obs: | exp: |
| gtt | V | 0 | 75.43 |
| gtc | V | 26 | 77.03 |
| gta | V | 272 | 68.78 |
| gtg | V | 69 | 145.80 |
| --- | -- | ------ | ------ |
| mPD | | 0.41 | 0.72 |
| | | nPD: | 0.57 |
| | | N. weight: | 1.2 |
| | | Sc. PD: | 0.37 |
| | | Sc. rank: | 185.8 |

,

| | | | |
|---|---|---|---|
| PB1 | | | |
| Pos. | 641 | obs: | exp: |
| aat | N | 2 | 191.80 |
| aac | N | 365 | 175.20 |
| --- | -- | ------ | ------ |
| mPD | | 0.011 | 0.50 |
| | | nPD: | 0.02 |
| | | N. weight: | 1.1 |
| | | Sc. PD: | -0.24 |
| | | Sc. rank: | -171.6 |

,

| | | | |
|---|---|---|---|
| PB1 | | | |
| Pos. | 642 | obs: | exp: |
| tct | S | 0 | 0.48 |
| tcc | S | 0 | 0.42 |
| tca | S | 0 | 0.66 |
| tcg | S | 0 | 0.18 |
| aat | N | 356 | 190.20 |
| aac | N | 8 | 173.80 |
| agt | S | 3 | 0.66 |
| agc | S | 0 | 0.60 |
| --- | -- | ------ | ------ |
| mPD | | 0.059 | 0.53 |
| | | nPD: | 0.11 |
| | | N. weight: | 0.85 |
| | | Sc. PD: | -0.12 |
| | | Sc. rank: | -38.4 |

,

| | | | |
|---|---|---|---|
| PB1 | | | |
| Pos. | 643 | obs: | exp: |
| gct | A | 359 | 92.44 |
| gcc | A | 4 | 75.18 |
| gca | A | 3 | 163.60 |
| gcg | A | 1 | 35.81 |
| --- | -- | ------ | ------ |
| mPD | | 0.043 | 0.69 |
| | | nPD: | 0.06 |
| | | N. weight: | 1.9 |
| | | Sc. PD: | -0.35 |
| | | Sc. rank: | -190.0 |

,

| | | | |
|---|---|---|---|
| PB1 | | | |
| Pos. | 644 | obs: | exp: |
| gtt | V | 9 | 75.43 |
| gtc | V | 0 | 77.03 |
| gta | V | 49 | 68.78 |
| gtg | V | 309 | 145.80 |
| --- | -- | ------ | ------ |
| mPD | | 0.27 | 0.72 |
| | | nPD: | 0.38 |
| | | N. weight: | 0.83 |
| | | Sc. PD: | 0.097 |
| | | Sc. rank: | 76.0 |

,

| | | | |
|---|---|---|---|
| PB1 | | | |
| Pos. | 645 | obs: | exp: |
| att | I | 0 | 0.38 |
| atc | I | 0 | 0.26 |
| ata | I | 1 | 0.36 |
| gtt | V | 0 | 75.23 |
| gtc | V | 0 | 76.82 |
| gta | V | 211 | 68.60 |
| gtg | V | 155 | 145.40 |
| --- | -- | ------ | ------ |
| mPD | | 0.49 | 0.73 |
| | | nPD: | 0.68 |
| | | N. weight: | 1. |
| | | Sc. PD: | 0.42 |
| | | Sc. rank: | 181.9 |

,

| | | | |
|---|---|---|---|
| PB1 | | | |
| Pos. | 646 | obs: | exp: |
| atg | M | 367 | 367.00 |
| --- | -- | ------ | ------ |
| mPD | | 0 | 0 |
| | | nPD: | 1. |
| | | N. weight: | 0. |
| | | Sc. PD: | 0 |
| | | Sc. rank: | 0 |

,

| | | | |
|---|---|---|---|
| PB1 | | | |
| Pos. | 647 | obs: | exp: |
| cct | P | 1 | 96.64 |
| ccc | P | 0 | 66.20 |
| cca | P | 362 | 144.80 |
| ccg | P | 4 | 59.34 |
| --- | -- | ------ | ------ |
| mPD | | 0.027 | 0.72 |
| | | nPD: | 0.04 |
| | | N. weight: | 1.3 |
| | | Sc. PD: | -0.27 |
| | | Sc. rank: | -183.1 |

,

| | | | |
|---|---|---|---|
| PB1 | | | |
| Pos. | 648 | obs: | exp: |
| act | T | 0 | 0.26 |
| acc | T | 1 | 0.21 |
| aca | T | 0 | 0.44 |
| acg | T | 0 | 0.08 |
| gct | A | 125 | 92.19 |
| gcc | A | 233 | 74.97 |
| gca | A | 8 | 163.10 |
| gcg | A | 0 | 35.71 |
| --- | -- | ------ | ------ |
| mPD | | 0.48 | 0.69 |
| | | nPD: | 0.7 |
| | | N. weight: | 1.2 |
| | | Sc. PD: | 0.5 |
| | | Sc. rank: | 210.2 |

,

| | | | |
|---|---|---|---|
| PB1 | | | |
| Pos. | 649 | obs: | exp: |
| cat | H | 352 | 198.90 |
| cac | H | 15 | 168.10 |
| --- | -- | ------ | ------ |
| mPD | | 0.079 | 0.50 |
| | | nPD: | 0.16 |
| | | N. weight: | 0.69 |
| | | Sc. PD: | -0.065 |
| | | Sc. rank: | -7.0 |

,

| | | | |
|---|---|---|---|
| PB1 | | | |
| Pos. | 650 | obs: | exp: |
| ggt | G | 56 | 45.28 |
| ggc | G | 311 | 49.63 |
| gga | G | 0 | 177.50 |
| ggg | G | 0 | 94.55 |
| --- | -- | ------ | ------ |
| mPD | | 0.26 | 0.67 |
| | | nPD: | 0.39 |
| | | N. weight: | 2.4 |
| | | Sc. PD: | 0.31 |
| | | Sc. rank: | 239.3 |

| | | | |
|---|---|---|---|
| PB1 | | | |
| Pos. | 651 | obs: | exp: |
| cct | P | 5 | 96.64 |
| ccc | P | 15 | 66.20 |
| cca | P | 332 | 144.80 |
| ccg | P | 15 | 59.34 |
| --- | -- | ------ | ------ |
| mPD | | 0.18 | 0.72 |
| | | nPD: | 0.25 |
| | | N. weight: | 0.93 |
| | | Sc. PD: | -0.0063 |
| | | Sc. rank: | 37.6 |

| | | | |
|---|---|---|---|
| PB1 | | | |
| Pos. | 652 | obs: | exp: |
| act | T | 0 | 1.06 |
| acc | T | 4 | 0.86 |
| aca | T | 0 | 1.75 |
| acg | T | 0 | 0.33 |
| gct | A | 1 | 91.43 |
| gcc | A | 362 | 74.36 |
| gca | A | 0 | 161.80 |
| gcg | A | 0 | 35.42 |
| --- | -- | ------ | ------ |
| mPD | | 0.027 | 0.71 |
| | | nPD: | 0.04 |
| | | N. weight: | 2.4 |
| | | Sc. PD: | -0.49 |
| | | Sc. rank: | -327.5 |

| | | | |
|---|---|---|---|
| PB1 | | | |
| Pos. | 653 | obs: | exp: |
| aaa | K | 34 | 205.50 |
| aag | K | 333 | 161.50 |
| --- | -- | ------ | ------ |
| mPD | | 0.17 | 0.49 |
| | | nPD: | 0.34 |
| | | N. weight: | 0.76 |
| | | Sc. PD: | 0.062 |
| | | Sc. rank: | 59.9 |

| | | | |
|---|---|---|---|
| PB1 | | | |
| Pos. | 654 | obs: | exp: |
| tct | S | 0 | 57.39 |
| tcc | S | 0 | 50.29 |
| tca | S | 0 | 79.98 |
| tcg | S | 0 | 22.00 |
| aat | N | 0 | 3.14 |
| aac | N | 6 | 2.86 |
| agt | S | 11 | 79.42 |
| agc | S | 350 | 71.92 |
| --- | -- | ------ | ------ |
| mPD | | 0.091 | 1.7 |
| | | nPD: | 0.05 |
| | | N. weight: | 2.2 |
| | | Sc. PD: | -0.43 |
| | | Sc. rank: | -248.0 |

| | | | |
|---|---|---|---|
| PB1 | | | |
| Pos. | 655 | obs: | exp: |
| att | I | 0 | 0.75 |
| atc | I | 0 | 0.52 |
| ata | I | 2 | 0.72 |
| atg | M | 363 | 363.00 |
| gtt | V | 0 | 0.41 |
| gtc | V | 0 | 0.42 |
| gta | V | 0 | 0.37 |
| gtg | V | 2 | 0.79 |
| --- | -- | ------ | ------ |
| mPD | | 0.022 | 0.028 |
| | | nPD: | 0.77 |
| | | N. weight: | 0.016 |
| | | Sc. PD: | 0.0079 |
| | | Sc. rank: | 3.1 |

| | | | |
|---|---|---|---|
| PB1 | | | |
| Pos. | 656 | obs: | exp: |
| gaa | E | 326 | 211.20 |
| gag | E | 41 | 155.80 |
| --- | -- | ------ | ------ |
| mPD | | 0.20 | 0.49 |
| | | nPD: | 0.41 |
| | | N. weight: | 0.37 |
| | | Sc. PD: | 0.053 |
| | | Sc. rank: | 39.0 |

| | | | |
|---|---|---|---|
| PB1 | | | |
| Pos. | 657 | obs: | exp: |
| tat | Y | 367 | 190.00 |
| tac | Y | 0 | 177.00 |
| --- | -- | ------ | ------ |
| mPD | | 0 | 0.50 |
| | | nPD: | 0. |
| | | N. weight: | 1. |
| | | Sc. PD: | -0.24 |
| | | Sc. rank: | -208.0 |

| | | | |
|---|---|---|---|
| PB1 | | | |
| Pos. | 658 | obs: | exp: |
| gat | D | 364 | 195.80 |
| gac | D | 3 | 171.20 |
| --- | -- | ------ | ------ |
| mPD | | 0.016 | 0.50 |
| | | nPD: | 0.03 |
| | | N. weight: | 0.89 |
| | | Sc. PD: | -0.19 |
| | | Sc. rank: | -128.6 |

| | | | |
|---|---|---|---|
| PB1 | | | |
| Pos. | 659 | obs: | exp: |
| gct | A | 313 | 92.44 |
| gcc | A | 36 | 75.18 |
| gca | A | 18 | 163.60 |
| gcg | A | 0 | 35.81 |
| --- | -- | ------ | ------ |
| mPD | | 0.26 | 0.69 |
| | | nPD: | 0.38 |
| | | N. weight: | 1.3 |
| | | Sc. PD: | 0.16 |
| | | Sc. rank: | 122.1 |

| | | | |
|---|---|---|---|
| PB1 | | | |
| Pos. | 660 | obs: | exp: |
| gtt | V | 331 | 75.43 |
| gtc | V | 16 | 77.03 |
| gta | V | 11 | 68.78 |
| gtg | V | 9 | 145.80 |
| --- | -- | ------ | ------ |
| mPD | | 0.18 | 0.72 |
| | | nPD: | 0.25 |
| | | N. weight: | 1.8 |
| | | Sc. PD: | -0.0028 |
| | | Sc. rank: | 81.6 |

| | | | |
|---|---|---|---|
| PB1 | | | |
| Pos. | 661 | obs: | exp: |
| gct | A | 49 | 92.44 |
| gcc | A | 59 | 75.18 |
| gca | A | 68 | 163.60 |
| gcg | A | 191 | 35.81 |
| --- | -- | ------ | ------ |
| mPD | | 0.65 | 0.69 |
| | | nPD: | 0.95 |
| | | N. weight: | 0.93 |
| | | Sc. PD: | 0.61 |
| | | Sc. rank: | 192.5 |

| | | | |
|---|---|---|---|
| PB1 | | | |
| Pos. | 662 | obs: | exp: |
| act | T | 284 | 96.96 |
| acc | T | 83 | 78.75 |
| aca | T | 0 | 160.80 |
| acg | T | 0 | 30.51 |
| --- | -- | ------ | ------ |
| mPD | | 0.35 | 0.69 |
| | | nPD: | 0.51 |
| | | N. weight: | 1.3 |
| | | Sc. PD: | 0.31 |
| | | Sc. rank: | 170.4 |

| | | | |
|---|---|---|---|
| PB1 | | | |
| Pos. | 663 | obs: | exp: |
| act | T | 52 | 96.96 |
| acc | T | 3 | 78.75 |
| aca | T | 281 | 160.80 |
| acg | T | 31 | 30.51 |
| --- | -- | ------ | ------ |
| mPD | | 0.39 | 0.69 |
| | | nPD: | 0.57 |
| | | N. weight: | 0.51 |
| | | Sc. PD: | 0.15 |
| | | Sc. rank: | 74.6 |

| | | | |
|---|---|---|---|
| PB1 | | | |
| Pos. | 664 | obs: | exp: |
| cat | H | 60 | 198.90 |
| cac | H | 307 | 168.10 |
| --- | -- | ------ | ------ |
| mPD | | 0.27 | 0.50 |
| | | nPD: | 0.55 |
| | | N. weight: | 0.48 |
| | | Sc. PD: | 0.14 |
| | | Sc. rank: | 69.0 |

| | | | |
|---|---|---|---|
| PB1 | | | |
| Pos. | 665 | obs: | exp: |
| tct | S | 5 | 58.34 |
| tcc | S | 93 | 51.12 |
| tca | S | 264 | 81.31 |
| tcg | S | 5 | 22.37 |
| agt | S | 0 | 80.74 |
| agc | S | 0 | 73.12 |
| --- | -- | ------ | ------ |
| mPD | | 0.42 | 1.7 |
| | | nPD: | 0.25 |
| | | N. weight: | 1.5 |
| | | Sc. PD: | -0.0062 |
| | | Sc. rank: | 62.2 |

| | | | |
|---|---|---|---|
| PB1 | | | |
| Pos. | 666 | obs: | exp: |
| tgg | W | 367 | 367.00 |
| --- | -- | ------ | ------ |
| mPD | | 0 | 0 |
| | | nPD: | 1. |
| | | N. weight: | 0. |
| | | Sc. PD: | 0 |
| | | Sc. rank: | 0 |

| | | | |
|---|---|---|---|
| PB1 | | | |
| Pos. | 667 | obs: | exp: |
| att | I | 167 | 121.10 |
| atc | I | 148 | 84.39 |
| ata | I | 7 | 116.60 |
| act | T | 0 | 2.11 |
| acc | T | 8 | 1.72 |
| aca | T | 0 | 3.50 |
| acg | T | 0 | 0.67 |
| gtt | V | 34 | 7.60 |
| gtc | V | 1 | 7.77 |
| gta | V | 2 | 6.93 |
| gtg | V | 0 | 14.69 |
| --- | -- | ------ | ------ |
| mPD | | 0.74 | 0.91 |
| | | nPD: | 0.82 |
| | | N. weight: | 0.76 |
| | | Sc. PD: | 0.4 |
| | | Sc. rank: | 153.8 |

| | | | |
|---|---|---|---|
| PB1 | | | |
| Pos. | 668 | obs: | exp: |
| cct | P | 343 | 96.64 |
| ccc | P | 24 | 66.20 |
| cca | P | 0 | 144.80 |
| ccg | P | 0 | 59.34 |
| --- | -- | ------ | ------ |
| mPD | | 0.12 | 0.72 |
| | | nPD: | 0.17 |
| | | N. weight: | 1.7 |
| | | Sc. PD: | -0.14 |
| | | Sc. rank: | -5.1 |

| | | | |
|---|---|---|---|
| PB1 | | | |
| Pos. | 669 | obs: | exp: |
| aaa | K | 38 | 205.50 |
| aag | K | 329 | 161.50 |
| --- | -- | ------ | ------ |
| mPD | | 0.19 | 0.49 |
| | | nPD: | 0.38 |
| | | N. weight: | 0.72 |
| | | Sc. PD: | 0.082 |
| | | Sc. rank: | 64.1 |

| | | | |
|---|---|---|---|
| PB1 | | | |
| Pos. | 670 | obs: | exp: |
| cgt | R | 0 | 15.24 |
| cgc | R | 0 | 17.97 |
| cga | R | 0 | 35.27 |
| cgg | R | 0 | 30.63 |
| aga | R | 33 | 165.00 |
| agg | R | 334 | 102.80 |
| --- | -- | ------ | ------ |
| mPD | | 0.16 | 0.96 |
| | | nPD: | 0.17 |
| | | N. weight: | 1.4 |
| | | Sc. PD: | -0.12 |
| | | Sc. rank: | -6.4 |

| | | | |
|---|---|---|---|
| PB1 | | | |
| Pos. | 671 | obs: | exp: |
| aat | N | 84 | 191.80 |
| aac | N | 283 | 175.20 |
| --- | -- | ------ | ------ |
| mPD | | 0.35 | 0.50 |
| | | nPD: | 0.71 |
| | | N. weight: | 0.29 |
| | | Sc. PD: | 0.12 |
| | | Sc. rank: | 51.8 |

| | | | |
|---|---|---|---|
| PB1 | | | |
| Pos. | 672 | obs: | exp: |
| cgt | R | 216 | 15.24 |
| cgc | R | 144 | 17.97 |
| cga | R | 0 | 35.27 |
| cgg | R | 7 | 30.63 |
| aga | R | 0 | 165.00 |
| agg | R | 0 | 102.80 |
| --- | -- | ------ | ------ |
| mPD | | 0.50 | 0.96 |
| | | nPD: | 0.52 |
| | | N. weight: | 3.6 |
| | | Sc. PD: | 0.91 |
| | | Sc. rank: | 489.4 |

| | | | |
|---|---|---|---|
| PB1 | | | |
| Pos. | 673 | obs: | exp: |
| tct | S | 12 | 58.34 |
| tcc | S | 354 | 51.12 |
| tca | S | 1 | 81.31 |
| tcg | S | 0 | 22.37 |
| agt | S | 0 | 80.74 |
| agc | S | 0 | 73.12 |
| --- | -- | ------ | ------ |
| mPD | | 0.069 | 1.7 |
| | | nPD: | 0.04 |
| | | N. weight: | 2.8 |
| | | Sc. PD: | -0.56 |
| | | Sc. rank: | -352.0 |

| | | | |
|---|---|---|---|
| PB1 | | | |
| Pos. | 674 | obs: | exp: |
| att | I | 319 | 138.00 |
| atc | I | 47 | 96.18 |
| ata | I | 1 | 132.80 |
| --- | -- | ------ | ------ |
| mPD | | 0.23 | 0.66 |
| | | nPD: | 0.35 |
| | | N. weight: | 0.96 |
| | | Sc. PD: | 0.083 |
| | | Sc. rank: | 79.0 |

| PB1 | | | |
|---|---|---|---|
| Pos. | 675 | obs: | exp: |
| tta | L | 0 | 32.06 |
| ttg | L | 0 | 70.68 |
| ctt | L | 7 | 68.48 |
| ctc | L | 360 | 59.37 |
| cta | L | 0 | 55.29 |
| ctg | L | 0 | 81.13 |
| --- | -- | ------ | ------ |
| mPD | | 0.038 | 1.1 |
| | | nPD: | 0.03 |
| | | N. weight: | 2.6 |
| | | Sc. PD: | -0.56 |
| | | Sc. rank: | -372.5 |

| PB1 | | | |
|---|---|---|---|
| Pos. | 676 | obs: | exp: |
| aat | N | 311 | 191.80 |
| aac | N | 56 | 175.20 |
| --- | -- | ------ | ------ |
| mPD | | 0.26 | 0.50 |
| | | nPD: | 0.52 |
| | | N. weight: | 0.37 |
| | | Sc. PD: | 0.093 |
| | | Sc. rank: | 49.6 |

| PB1 | | | |
|---|---|---|---|
| Pos. | 677 | obs: | exp: |
| act | T | 13 | 96.96 |
| acc | T | 260 | 78.75 |
| aca | T | 89 | 160.80 |
| acg | T | 5 | 30.51 |
| --- | -- | ------ | ------ |
| mPD | | 0.44 | 0.69 |
| | | nPD: | 0.64 |
| | | N. weight: | 0.95 |
| | | Sc. PD: | 0.35 |
| | | Sc. rank: | 157.8 |

| PB1 | | | |
|---|---|---|---|
| Pos. | 678 | obs: | exp: |
| tct | S | 0 | 58.19 |
| tcc | S | 0 | 50.99 |
| tca | S | 0 | 81.08 |
| tcg | S | 0 | 22.31 |
| aat | N | 0 | 0.52 |
| aac | N | 1 | 0.48 |
| agt | S | 28 | 80.52 |
| agc | S | 338 | 72.92 |
| --- | -- | ------ | ------ |
| mPD | | 0.15 | 1.7 |
| | | nPD: | 0.09 |
| | | N. weight: | 2. |
| | | Sc. PD: | -0.33 |
| | | Sc. rank: | -139.1 |

| PB1 | | | |
|---|---|---|---|
| Pos. | 679 | obs: | exp: |
| cat | H | 0 | 2.17 |
| cac | H | 4 | 1.83 |
| caa | Q | 343 | 193.50 |
| cag | Q | 20 | 169.50 |
| --- | -- | ------ | ------ |
| mPD | | 0.12 | 0.51 |
| | | nPD: | 0.24 |
| | | N. weight: | 0.66 |
| | | Sc. PD: | -0.0082 |
| | | Sc. rank: | 25.7 |

| PB1 | | | |
|---|---|---|---|
| Pos. | 680 | obs: | exp: |
| cgt | R | 0 | 15.24 |
| cgc | R | 0 | 17.97 |
| cga | R | 0 | 35.27 |
| cgg | R | 1 | 30.63 |
| aga | R | 2 | 165.00 |
| agg | R | 364 | 102.80 |
| --- | -- | ------ | ------ |
| mPD | | 0.016 | 0.96 |
| | | nPD: | 0.02 |
| | | N. weight: | 1.9 |
| | | Sc. PD: | -0.42 |
| | | Sc. rank: | -299.1 |

| PB1 | | | |
|---|---|---|---|
| Pos. | 681 | obs: | exp: |
| ggt | G | 14 | 45.28 |
| ggc | G | 1 | 49.63 |
| gga | G | 317 | 177.50 |
| ggg | G | 35 | 94.55 |
| --- | -- | ------ | ------ |
| mPD | | 0.24 | 0.67 |
| | | nPD: | 0.37 |
| | | N. weight: | 0.56 |
| | | Sc. PD: | 0.058 |
| | | Sc. rank: | 47.6 |

| PB1 | | | |
|---|---|---|---|
| Pos. | 682 | obs: | exp: |
| att | I | 228 | 136.80 |
| atc | I | 135 | 95.40 |
| ata | I | 1 | 131.80 |
| gtt | V | 3 | 0.62 |
| gtc | V | 0 | 0.63 |
| gta | V | 0 | 0.56 |
| gtg | V | 0 | 1.19 |
| --- | -- | ------ | ------ |
| mPD | | 0.49 | 0.68 |
| | | nPD: | 0.72 |
| | | N. weight: | 0.69 |
| | | Sc. PD: | 0.3 |
| | | Sc. rank: | 126.9 |

| PB1 | | | |
|---|---|---|---|
| Pos. | 683 | obs: | exp: |
| tta | L | 0 | 32.06 |
| ttg | L | 0 | 70.68 |
| ctt | L | 357 | 68.48 |
| ctc | L | 9 | 59.37 |
| cta | L | 0 | 55.29 |
| ctg | L | 1 | 81.13 |
| --- | -- | ------ | ------ |
| mPD | | 0.053 | 1.1 |
| | | nPD: | 0.05 |
| | | N. weight: | 2.4 |
| | | Sc. PD: | -0.47 |
| | | Sc. rank: | -280.4 |

| PB1 | | | |
|---|---|---|---|
| Pos. | 684 | obs: | exp: |
| gtt | V | 0 | 0.62 |
| gtc | V | 0 | 0.63 |
| gta | V | 0 | 0.56 |
| gtg | V | 3 | 1.19 |
| gaa | E | 138 | 209.50 |
| gag | E | 226 | 154.50 |
| --- | -- | ------ | ------ |
| mPD | | 0.49 | 0.51 |
| | | nPD: | 0.96 |
| | | N. weight: | 0.14 |
| | | Sc. PD: | 0.094 |
| | | Sc. rank: | 29.5 |

| PB1 | | | |
|---|---|---|---|
| Pos. | 685 | obs: | exp: |
| gat | D | 301 | 195.80 |
| gac | D | 66 | 171.20 |
| --- | -- | ------ | ------ |
| mPD | | 0.30 | 0.50 |
| | | nPD: | 0.59 |
| | | N. weight: | 0.29 |
| | | Sc. PD: | 0.092 |
| | | Sc. rank: | 44.6 |

| PB1 | | | |
|---|---|---|---|
| Pos. | 686 | obs: | exp: |
| gaa | E | 146 | 211.20 |
| gag | E | 221 | 155.80 |
| --- | -- | ------ | ------ |
| mPD | | 0.48 | 0.49 |
| | | nPD: | 0.98 |
| | | N. weight: | 0.11 |
| | | Sc. PD: | 0.076 |
| | | Sc. rank: | 22.9 |

| PB1 | | | |
|---|---|---|---|
| Pos. | 687 | obs: | exp: |
| caa | Q | 46 | 195.60 |
| cag | Q | 321 | 171.40 |
| --- | -- | ------ | ------ |
| mPD | | 0.22 | 0.50 |
| | | nPD: | 0.44 |
| | | N. weight: | 0.57 |
| | | Sc. PD: | 0.1 |
| | | Sc. rank: | 65.4 |

| PB1 | | | |
|---|---|---|---|
| Pos. | 688 | obs: | exp: |
| atg | M | 367 | 367.00 |
| --- | -- | ------ | ------ |
| mPD | | 0 | 0 |
| | | nPD: | 1. |
| | | N. weight: | 0. |
| | | Sc. PD: | 0 |
| | | Sc. rank: | 0 |

| PB1 | | | |
|---|---|---|---|
| Pos. | 689 | obs: | exp: |
| tat | Y | 114 | 190.00 |
| tac | Y | 253 | 177.00 |
| --- | -- | ------ | ------ |
| mPD | | 0.43 | 0.50 |
| | | nPD: | 0.86 |
| | | N. weight: | 0.15 |
| | | Sc. PD: | 0.084 |
| | | Sc. rank: | 29.9 |

| PB1 | | | |
|---|---|---|---|
| Pos. | 690 | obs: | exp: |
| caa | Q | 113 | 190.30 |
| cag | Q | 244 | 166.70 |
| aaa | K | 0 | 5.60 |
| aag | K | 10 | 4.40 |
| --- | -- | ------ | ------ |
| mPD | | 0.48 | 0.55 |
| | | nPD: | 0.87 |
| | | N. weight: | 0.19 |
| | | Sc. PD: | 0.11 |
| | | Sc. rank: | 38.7 |

| PB1 | | | |
|---|---|---|---|
| Pos. | 691 | obs: | exp: |
| aaa | K | 26 | 205.50 |
| aag | K | 341 | 161.50 |
| --- | -- | ------ | ------ |
| mPD | | 0.13 | 0.49 |
| | | nPD: | 0.27 |
| | | N. weight: | 0.84 |
| | | Sc. PD: | 0.0092 |
| | | Sc. rank: | 44.4 |

| PB1 | | | |
|---|---|---|---|
| Pos. | 692 | obs: | exp: |
| tgt | C | 13 | 151.30 |
| tgc | C | 354 | 215.70 |
| --- | -- | ------ | ------ |
| mPD | | 0.069 | 0.48 |
| | | nPD: | 0.14 |
| | | N. weight: | 0.6 |
| | | Sc. PD: | -0.066 |
| | | Sc. rank: | -14.3 |

| PB1 | | | |
|---|---|---|---|
| Pos. | 693 | obs: | exp: |
| tgt | C | 64 | 151.30 |
| tgc | C | 303 | 215.70 |
| --- | -- | ------ | ------ |
| mPD | | 0.29 | 0.48 |
| | | nPD: | 0.6 |
| | | N. weight: | 0.21 |
| | | Sc. PD: | 0.068 |
| | | Sc. rank: | 32.7 |

| PB1 | | | |
|---|---|---|---|
| Pos. | 694 | obs: | exp: |
| tct | S | 0 | 25.91 |
| tcc | S | 0 | 22.71 |
| tca | S | 0 | 36.11 |
| tcg | S | 0 | 9.94 |
| tgt | C | 1 | 0.41 |
| tgc | C | 0 | 0.59 |
| aat | N | 166 | 105.60 |
| aac | N | 36 | 96.43 |
| agt | S | 159 | 35.86 |
| agc | S | 4 | 32.47 |
| ggt | G | 1 | 0.12 |
| ggc | G | 0 | 0.14 |
| gga | G | 0 | 0.48 |
| ggg | G | 0 | 0.26 |
| --- | -- | ------ | ------ |
| mPD | | 0.70 | 1.6 |
| | | nPD: | 0.44 |
| | | N. weight: | 1.1 |
| | | Sc. PD: | 0.2 |
| | | Sc. rank: | 129.0 |

| PB1 | | | |
|---|---|---|---|
| Pos. | 695 | obs: | exp: |
| tta | L | 14 | 32.06 |
| ttg | L | 0 | 70.68 |
| ctt | L | 0 | 68.48 |
| ctc | L | 0 | 59.37 |
| cta | L | 333 | 55.29 |
| ctg | L | 20 | 81.13 |
| --- | -- | ------ | ------ |
| mPD | | 0.18 | 1.1 |
| | | nPD: | 0.16 |
| | | N. weight: | 2.3 |
| | | Sc. PD: | -0.22 |
| | | Sc. rank: | -20.5 |

| PB1 | | | |
|---|---|---|---|
| Pos. | 696 | obs: | exp: |
| ttt | F | 55 | 150.90 |
| ttc | F | 312 | 216.10 |
| --- | -- | ------ | ------ |
| mPD | | 0.26 | 0.48 |
| | | nPD: | 0.53 |
| | | N. weight: | 0.26 |
| | | Sc. PD: | 0.066 |
| | | Sc. rank: | 35.6 |

| PB1 | | | |
|---|---|---|---|
| Pos. | 697 | obs: | exp: |
| gaa | E | 85 | 211.20 |
| gag | E | 282 | 155.80 |
| --- | -- | ------ | ------ |
| mPD | | 0.36 | 0.49 |
| | | nPD: | 0.73 |
| | | N. weight: | 0.39 |
| | | Sc. PD: | 0.17 |
| | | Sc. rank: | 72.3 |

| PB1 | | | |
|---|---|---|---|
| Pos. | 698 | obs: | exp: |
| aaa | K | 329 | 205.50 |
| aag | K | 38 | 161.50 |
| --- | -- | ------ | ------ |
| mPD | | 0.19 | 0.49 |
| | | nPD: | 0.38 |
| | | N. weight: | 0.43 |
| | | Sc. PD: | 0.049 |
| | | Sc. rank: | 38.1 |

| PB1 | | | |
|---|---|---|---|
| Pos. | 699 | obs: | exp: |
| ttt | F | 13 | 150.90 |
| ttc | F | 354 | 216.10 |
| --- | -- | ------ | ------ |
| mPD | | 0.069 | 0.48 |
| | | nPD: | 0.14 |
| | | N. weight: | 0.6 |
| | | Sc. PD: | -0.066 |
| | | Sc. rank: | -14.0 |

PB1

| Pos. | 700 | obs: | exp: |
|---|---|---|---|
| ttt | F | 28 | 150.90 |
| ttc | F | 339 | 216.10 |
| --- | -- | ------ | ------ |
| mPD | | 0.14 | 0.48 |
| | | nPD: | 0.29 |
| | | N. weight: | 0.45 |
| | | Sc. PD: | 0.015 |
| | | Sc. rank: | 29.8 |

PB1

| Pos. | 701 | obs: | exp: |
|---|---|---|---|
| cct | P | 106 | 96.64 |
| ccc | P | 220 | 66.20 |
| cca | P | 41 | 144.80 |
| ccg | P | 0 | 59.34 |
| --- | -- | ------ | ------ |
| mPD | | 0.55 | 0.72 |
| | | nPD: | 0.76 |
| | | N. weight: | 0.95 |
| | | Sc. PD: | 0.45 |
| | | Sc. rank: | 184.5 |

PB1

| Pos. | 702 | obs: | exp: |
|---|---|---|---|
| tct | S | 0 | 58.19 |
| tcc | S | 0 | 50.99 |
| tca | S | 0 | 81.08 |
| tcg | S | 0 | 22.31 |
| aat | N | 0 | 0.52 |
| aac | N | 1 | 0.48 |
| agt | S | 220 | 80.52 |
| agc | S | 146 | 72.92 |
| --- | -- | ------ | ------ |
| mPD | | 0.49 | 1.7 |
| | | nPD: | 0.29 |
| | | N. weight: | 1.4 |
| | | Sc. PD: | 0.046 |
| | | Sc. rank: | 91.2 |

PB1

| Pos. | 703 | obs: | exp: |
|---|---|---|---|
| tct | S | 0 | 58.34 |
| tcc | S | 0 | 51.12 |
| tca | S | 0 | 81.31 |
| tcg | S | 0 | 22.37 |
| agt | S | 339 | 80.74 |
| agc | S | 28 | 73.12 |
| --- | -- | ------ | ------ |
| mPD | | 0.14 | 1.7 |
| | | nPD: | 0.08 |
| | | N. weight: | 1.9 |
| | | Sc. PD: | -0.31 |
| | | Sc. rank: | -141.6 |

PB1

| Pos. | 704 | obs: | exp: |
|---|---|---|---|
| tct | S | 3 | 58.34 |
| tcc | S | 0 | 51.12 |
| tca | S | 351 | 81.31 |
| tcg | S | 13 | 22.37 |
| agt | S | 0 | 80.74 |
| agc | S | 0 | 73.12 |
| --- | -- | ------ | ------ |
| mPD | | 0.084 | 1.7 |
| | | nPD: | 0.05 |
| | | N. weight: | 2.1 |
| | | Sc. PD: | -0.41 |
| | | Sc. rank: | -238.1 |

PB1

| Pos. | 705 | obs: | exp: |
|---|---|---|---|
| tat | Y | 31 | 190.00 |
| tac | Y | 336 | 177.00 |
| --- | -- | ------ | ------ |
| mPD | | 0.16 | 0.50 |
| | | nPD: | 0.31 |
| | | N. weight: | 0.67 |
| | | Sc. PD: | 0.034 |
| | | Sc. rank: | 48.6 |

PB1

| Pos. | 706 | obs: | exp: |
|---|---|---|---|
| cgt | R | 0 | 15.24 |
| cgc | R | 0 | 17.97 |
| cga | R | 26 | 35.27 |
| cgg | R | 0 | 30.63 |
| aga | R | 46 | 165.00 |
| agg | R | 295 | 102.80 |
| --- | -- | ------ | ------ |
| mPD | | 0.45 | 0.96 |
| | | nPD: | 0.47 |
| | | N. weight: | 1. |
| | | Sc. PD: | 0.21 |
| | | Sc. rank: | 129.2 |

PB1

| Pos. | 707 | obs: | exp: |
|---|---|---|---|
| cgt | R | 0 | 15.19 |
| cgc | R | 0 | 17.92 |
| cga | R | 0 | 35.17 |
| cgg | R | 0 | 30.54 |
| aaa | K | 1 | 0.56 |
| aag | K | 0 | 0.44 |
| aga | R | 54 | 164.60 |
| agg | R | 312 | 102.60 |
| --- | -- | ------ | ------ |
| mPD | | 0.26 | 0.96 |
| | | nPD: | 0.27 |
| | | N. weight: | 1.2 |
| | | Sc. PD: | 0.016 |
| | | Sc. rank: | 66.7 |

PB1

| Pos. | 708 | obs: | exp: |
|---|---|---|---|
| cct | P | 1 | 96.64 |
| ccc | P | 0 | 66.20 |
| cca | P | 274 | 144.80 |
| ccg | P | 92 | 59.34 |
| --- | -- | ------ | ------ |
| mPD | | 0.38 | 0.72 |
| | | nPD: | 0.53 |
| | | N. weight: | 0.89 |
| | | Sc. PD: | 0.23 |
| | | Sc. rank: | 124.6 |

PB1

| Pos. | 709 | obs: | exp: |
|---|---|---|---|
| att | I | 1 | 0.38 |
| atc | I | 0 | 0.26 |
| ata | I | 0 | 0.36 |
| gtt | V | 365 | 75.23 |
| gtc | V | 1 | 76.82 |
| gta | V | 0 | 68.60 |
| gtg | V | 0 | 145.40 |
| --- | -- | ------ | ------ |
| mPD | | 0.011 | 0.73 |
| | | nPD: | 0.01 |
| | | N. weight: | 2.4 |
| | | Sc. PD: | -0.55 |
| | | Sc. rank: | -400.3 |

PB1

| Pos. | 710 | obs: | exp: |
|---|---|---|---|
| ggt | G | 0 | 45.28 |
| ggc | G | 0 | 49.63 |
| gga | G | 360 | 177.50 |
| ggg | G | 7 | 94.55 |
| --- | -- | ------ | ------ |
| mPD | | 0.038 | 0.67 |
| | | nPD: | 0.06 |
| | | N. weight: | 0.99 |
| | | Sc. PD: | -0.19 |
| | | Sc. rank: | -106.0 |

PB1

| Pos. | 711 | obs: | exp: |
|---|---|---|---|
| att | I | 353 | 138.00 |
| atc | I | 14 | 96.18 |
| ata | I | 0 | 132.80 |
| --- | -- | ------ | ------ |
| mPD | | 0.074 | 0.66 |
| | | nPD: | 0.11 |
| | | N. weight: | 1.3 |
| | | Sc. PD: | -0.18 |
| | | Sc. rank: | -58.1 |

PB1

| Pos. | 712 | obs: | exp: |
|---|---|---|---|
| tct | S | 6 | 58.34 |
| tcc | S | 360 | 51.12 |
| tca | S | 1 | 81.31 |
| tcg | S | 0 | 22.37 |
| agt | S | 0 | 80.74 |
| agc | S | 0 | 73.12 |
| --- | -- | ------ | ------ |
| mPD | | 0.038 | 1.7 |
| | | nPD: | 0.02 |
| | | N. weight: | 2.9 |
| | | Sc. PD: | -0.63 |
| | | Sc. rank: | -430.6 |

PB1

| Pos. | 713 | obs: | exp: |
|---|---|---|---|
| tct | S | 0 | 58.34 |
| tcc | S | 0 | 51.12 |
| tca | S | 0 | 81.31 |
| tcg | S | 0 | 22.37 |
| agt | S | 72 | 80.74 |
| agc | S | 295 | 73.12 |
| --- | -- | ------ | ------ |
| mPD | | 0.32 | 1.7 |
| | | nPD: | 0.19 |
| | | N. weight: | 1.7 |
| | | Sc. PD: | -0.11 |
| | | Sc. rank: | 8.6 |

PB1

| Pos. | 714 | obs: | exp: |
|---|---|---|---|
| atg | M | 367 | 367.00 |
| --- | -- | ------ | ------ |
| mPD | | 0 | 0 |
| | | nPD: | 1. |
| | | N. weight: | 0. |
| | | Sc. PD: | 0 |
| | | Sc. rank: | 0 |

PB1

| Pos. | 715 | obs: | exp: |
|---|---|---|---|
| gtt | V | 0 | 75.43 |
| gtc | V | 0 | 77.03 |
| gta | V | 3 | 68.78 |
| gtg | V | 364 | 145.80 |
| --- | -- | ------ | ------ |
| mPD | | 0.016 | 0.72 |
| | | nPD: | 0.02 |
| | | N. weight: | 1.3 |
| | | Sc. PD: | -0.3 |
| | | Sc. rank: | -209.2 |

PB1

| Pos. | 716 | obs: | exp: |
|---|---|---|---|
| gaa | E | 0 | 211.20 |
| gag | E | 367 | 155.80 |
| --- | -- | ------ | ------ |
| mPD | | 0 | 0.49 |
| | | nPD: | 0. |
| | | N. weight: | 1.3 |
| | | Sc. PD: | -0.32 |
| | | Sc. rank: | -270.7 |

PB1

| Pos. | 717 | obs: | exp: |
|---|---|---|---|
| gct | A | 0 | 92.44 |
| gcc | A | 367 | 75.18 |
| gca | A | 0 | 163.60 |
| gcg | A | 0 | 35.81 |
| --- | -- | ------ | ------ |
| mPD | | 0 | 0.69 |
| | | nPD: | 0. |
| | | N. weight: | 2.4 |
| | | Sc. PD: | -0.59 |
| | | Sc. rank: | -500.9 |

PB1

| Pos. | 718 | obs: | exp: |
|---|---|---|---|
| atg | M | 367 | 367.00 |
| --- | -- | ------ | ------ |
| mPD | | 0 | 0 |
| | | nPD: | 1. |
| | | N. weight: | 0. |
| | | Sc. PD: | 0 |
| | | Sc. rank: | 0 |

PB1

| Pos. | 719 | obs: | exp: |
|---|---|---|---|
| atg | M | 4 | 4.00 |
| gtt | V | 97 | 74.61 |
| gtc | V | 2 | 76.19 |
| gta | V | 37 | 68.03 |
| gtg | V | 227 | 144.20 |
| --- | -- | ------ | ------ |
| mPD | | 0.55 | 0.74 |
| | | nPD: | 0.74 |
| | | N. weight: | 0.44 |
| | | Sc. PD: | 0.2 |
| | | Sc. rank: | 83.5 |

PB1

| Pos. | 720 | obs: | exp: |
|---|---|---|---|
| tct | S | 346 | 58.34 |
| tcc | S | 18 | 51.12 |
| tca | S | 3 | 81.31 |
| tcg | S | 0 | 22.37 |
| agt | S | 0 | 80.74 |
| agc | S | 0 | 73.12 |
| --- | -- | ------ | ------ |
| mPD | | 0.11 | 1.7 |
| | | nPD: | 0.07 |
| | | N. weight: | 2.5 |
| | | Sc. PD: | -0.44 |
| | | Sc. rank: | -229.0 |

PB1

| Pos. | 721 | obs: | exp: |
|---|---|---|---|
| cgt | R | 0 | 15.24 |
| cgc | R | 0 | 17.97 |
| cga | R | 0 | 35.27 |
| cgg | R | 26 | 30.63 |
| aga | R | 1 | 165.00 |
| agg | R | 340 | 102.80 |
| --- | -- | ------ | ------ |
| mPD | | 0.14 | 0.96 |
| | | nPD: | 0.14 |
| | | N. weight: | 1.7 |
| | | Sc. PD: | -0.18 |
| | | Sc. rank: | -37.3 |

PB1

| Pos. | 722 | obs: | exp: |
|---|---|---|---|
| gct | A | 0 | 92.44 |
| gcc | A | 367 | 75.18 |
| gca | A | 0 | 163.60 |
| gcg | A | 0 | 35.81 |
| --- | -- | ------ | ------ |
| mPD | | 0 | 0.69 |
| | | nPD: | 0. |
| | | N. weight: | 2.4 |
| | | Sc. PD: | -0.59 |
| | | Sc. rank: | -500.9 |

PB1

| Pos. | 723 | obs: | exp: |
|---|---|---|---|
| caa | Q | 4 | 2.13 |
| cag | Q | 0 | 1.87 |
| cgt | R | 0 | 15.07 |
| cgc | R | 0 | 17.77 |
| cga | R | 350 | 34.89 |
| cgg | R | 12 | 30.29 |
| aga | R | 1 | 163.30 |
| agg | R | 0 | 101.70 |
| --- | -- | ------ | ------ |
| mPD | | 0.090 | 0.99 |
| | | nPD: | 0.09 |
| | | N. weight: | 3.3 |
| | | Sc. PD: | -0.52 |
| | | Sc. rank: | -201.5 |

PB1

| Pos. | 724 | obs: | exp: |
|---|---|---|---|
| att | I | 337 | 138.00 |
| atc | I | 30 | 96.18 |
| ata | I | 0 | 132.80 |
| --- | -- | ------ | ------ |
| mPD | | 0.15 | 0.66 |
| | | nPD: | 0.23 |
| | | N. weight: | 1.1 |
| | | Sc. PD: | -0.03 |
| | | Sc. rank: | 34.6 |

PB1

| Pos. | 725 | obs: | exp: |
|---|---|---|---|
| gat | D | 341 | 195.80 |
| gac | D | 26 | 171.20 |
| --- | -- | ------ | ------ |
| mPD | | 0.13 | 0.50 |
| | | nPD: | 0.27 |
| | | N. weight: | 0.59 |
| | | Sc. PD: | 0.0049 |
| | | Sc. rank: | 30.2 |

PB1

| Pos. | 726 | obs: | exp: |
|---|---|---|---|
| gct | A | 1 | 92.44 |
| gcc | A | 1 | 75.18 |
| gca | A | 276 | 163.60 |
| gcg | A | 89 | 35.81 |
| --- | -- | ------ | ------ |
| mPD | | 0.38 | 0.69 |
| | | nPD: | 0.55 |
| | | N. weight: | 0.92 |
| | | Sc. PD: | 0.26 |
| | | Sc. rank: | 131.2 |

PB1

| Pos. | 727 | obs: | exp: |
|---|---|---|---|
| cgt | R | 3 | 15.24 |
| cgc | R | 191 | 17.97 |
| cga | R | 162 | 35.27 |
| cgg | R | 10 | 30.63 |
| aga | R | 0 | 165.00 |
| agg | R | 1 | 102.80 |
| --- | -- | ------ | ------ |
| mPD | | 0.54 | 0.96 |
| | | nPD: | 0.56 |
| | | N. weight: | 2.8 |
| | | Sc. PD: | 0.83 |
| | | Sc. rank: | 415.5 |

PB1

| Pos. | 728 | obs: | exp: |
|---|---|---|---|
| att | I | 365 | 137.20 |
| atc | I | 0 | 95.66 |
| ata | I | 0 | 132.10 |
| gtt | V | 2 | 0.41 |
| gtc | V | 0 | 0.42 |
| gta | V | 0 | 0.37 |
| gtg | V | 0 | 0.79 |
| --- | -- | ------ | ------ |
| mPD | | 0.011 | 0.67 |
| | | nPD: | 0.02 |
| | | N. weight: | 1.5 |
| | | Sc. PD: | -0.34 |
| | | Sc. rank: | -246.3 |

PB1

| Pos. | 729 | obs: | exp: |
|---|---|---|---|
| gat | D | 193 | 195.80 |
| gac | D | 174 | 171.20 |
| --- | -- | ------ | ------ |
| mPD | | 0.50 | 0.50 |
| | | nPD: | 1. |
| | | N. weight: | 0.013 |
| | | Sc. PD: | 0.0095 |
| | | Sc. rank: | 2.8 |

PB1

| Pos. | 730 | obs: | exp: |
|---|---|---|---|
| ttt | F | 31 | 150.50 |
| ttc | F | 335 | 215.50 |
| tct | S | 0 | 0.16 |
| tcc | S | 1 | 0.14 |
| tca | S | 0 | 0.22 |
| tcg | S | 0 | 0.06 |
| agt | S | 0 | 0.22 |
| agc | S | 0 | 0.20 |
| --- | -- | ------ | ------ |
| mPD | | 0.16 | 0.49 |
| | | nPD: | 0.33 |
| | | N. weight: | 0.43 |
| | | Sc. PD: | 0.028 |
| | | Sc. rank: | 31.9 |

PB1

| Pos. | 731 | obs: | exp: |
|---|---|---|---|
| gaa | E | 305 | 211.20 |
| gag | E | 62 | 155.80 |
| --- | -- | ------ | ------ |
| mPD | | 0.28 | 0.49 |
| | | nPD: | 0.58 |
| | | N. weight: | 0.24 |
| | | Sc. PD: | 0.073 |
| | | Sc. rank: | 36.2 |

PB1

| Pos. | 732 | obs: | exp: |
|---|---|---|---|
| tct | S | 367 | 58.34 |
| tcc | S | 0 | 51.12 |
| tca | S | 0 | 81.31 |
| tcg | S | 0 | 22.37 |
| agt | S | 0 | 80.74 |
| agc | S | 0 | 73.12 |
| --- | -- | ------ | ------ |
| mPD | | 0 | 1.7 |
| | | nPD: | 0. |
| | | N. weight: | 2.8 |
| | | Sc. PD: | -0.68 |
| | | Sc. rank: | -581.0 |

PB1

| Pos. | 733 | obs: | exp: |
|---|---|---|---|
| ggt | G | 0 | 45.28 |
| ggc | G | 1 | 49.63 |
| gga | G | 365 | 177.50 |
| ggg | G | 1 | 94.55 |
| --- | -- | ------ | ------ |
| mPD | | 0.011 | 0.67 |
| | | nPD: | 0.02 |
| | | N. weight: | 1.1 |
| | | Sc. PD: | -0.24 |
| | | Sc. rank: | -172.5 |

PB1

| Pos. | 734 | obs: | exp: |
|---|---|---|---|
| cgt | R | 0 | 15.24 |
| cgc | R | 0 | 17.97 |
| cga | R | 0 | 35.27 |
| cgg | R | 93 | 30.63 |
| aga | R | 1 | 165.00 |
| agg | R | 273 | 102.80 |
| --- | -- | ------ | ------ |
| mPD | | 0.38 | 0.96 |
| | | nPD: | 0.4 |
| | | N. weight: | 1.5 |
| | | Sc. PD: | 0.21 |
| | | Sc. rank: | 156.8 |

PB1

| Pos. | 735 | obs: | exp: |
|---|---|---|---|
| att | I | 333 | 137.60 |
| atc | I | 33 | 95.92 |
| ata | I | 0 | 132.50 |
| atg | M | 1 | 1.00 |
| --- | -- | ------ | ------ |
| mPD | | 0.17 | 0.66 |
| | | nPD: | 0.26 |
| | | N. weight: | 1.1 |
| | | Sc. PD: | -0.00063 |
| | | Sc. rank: | 50.8 |

PB1

| Pos. | 736 | obs: | exp: |
|---|---|---|---|
| aaa | K | 1 | 205.50 |
| aag | K | 366 | 161.50 |
| --- | -- | ------ | ------ |
| mPD | | 0.0054 | 0.49 |
| | | nPD: | 0.01 |
| | | N. weight: | 1.2 |
| | | Sc. PD: | -0.29 |
| | | Sc. rank: | -210.7 |

PB1

| Pos. | 737 | obs: | exp: |
|---|---|---|---|
| caa | Q | 5 | 2.67 |
| cag | Q | 0 | 2.33 |
| cgt | R | 0 | 0.33 |
| cgc | R | 0 | 0.39 |
| cga | R | 0 | 0.77 |
| cgg | R | 0 | 0.67 |
| aaa | K | 230 | 198.20 |
| aag | K | 124 | 155.80 |
| aga | R | 8 | 3.60 |
| agg | R | 0 | 2.24 |
| --- | -- | ------ | ------ |
| mPD | | 0.52 | 0.58 |
| | | nPD: | 0.9 |
| | | N. weight: | 0.077 |
| | | Sc. PD: | 0.047 |
| | | Sc. rank: | 15.9 |

PB1

| Pos. | 738 | obs: | exp: |
|---|---|---|---|
| gaa | E | 269 | 211.20 |
| gag | E | 98 | 155.80 |
| --- | -- | ------ | ------ |
| mPD | | 0.39 | 0.49 |
| | | nPD: | 0.8 |
| | | N. weight: | 0.094 |
| | | Sc. PD: | 0.049 |
| | | Sc. rank: | 18.7 |

PB1

| Pos. | 739 | obs: | exp: |
|---|---|---|---|
| gaa | E | 13 | 211.20 |
| gag | E | 354 | 155.80 |
| --- | -- | ------ | ------ |
| mPD | | 0.069 | 0.49 |
| | | nPD: | 0.14 |
| | | N. weight: | 1.1 |
| | | Sc. PD: | -0.12 |
| | | Sc. rank: | -26.9 |

PB1

| Pos. | 740 | obs: | exp: |
|---|---|---|---|
| ttt | F | 357 | 150.90 |
| ttc | F | 10 | 216.10 |
| --- | -- | ------ | ------ |
| mPD | | 0.053 | 0.48 |
| | | nPD: | 0.11 |
| | | N. weight: | 1.2 |
| | | Sc. PD: | -0.16 |
| | | Sc. rank: | -56.4 |

PB1

| Pos. | 741 | obs: | exp: |
|---|---|---|---|
| act | T | 4 | 1.06 |
| acc | T | 0 | 0.86 |
| aca | T | 0 | 1.75 |
| acg | T | 0 | 0.33 |
| gct | A | 362 | 91.43 |
| gcc | A | 1 | 74.36 |
| gca | A | 0 | 161.80 |
| gcg | A | 0 | 35.42 |
| --- | -- | ------ | ------ |
| mPD | | 0.027 | 0.71 |
| | | nPD: | 0.04 |
| | | N. weight: | 2.1 |
| | | Sc. PD: | -0.43 |
| | | Sc. rank: | -284.6 |

PB1

| Pos. | 742 | obs: | exp: |
|---|---|---|---|
| gaa | E | 0 | 211.20 |
| gag | E | 367 | 155.80 |
| --- | -- | ------ | ------ |
| mPD | | 0 | 0.49 |
| | | nPD: | 0. |
| | | N. weight: | 1.3 |
| | | Sc. PD: | -0.32 |
| | | Sc. rank: | -270.7 |

PB1

| Pos. | 743 | obs: | exp: |
|---|---|---|---|
| att | I | 0 | 138.00 |
| atc | I | 367 | 96.18 |
| ata | I | 0 | 132.80 |
| --- | -- | ------ | ------ |
| mPD | | 0 | 0.66 |
| | | nPD: | 0. |
| | | N. weight: | 2. |
| | | Sc. PD: | -0.5 |
| | | Sc. rank: | -423.1 |

PB1

| Pos. | 744 | obs: | exp: |
|---|---|---|---|
| atg | M | 362 | 362.00 |
| act | T | 0 | 0.26 |
| acc | T | 0 | 0.21 |
| aca | T | 0 | 0.44 |
| acg | T | 1 | 0.08 |
| gtt | V | 0 | 0.82 |
| gtc | V | 0 | 0.84 |
| gta | V | 0 | 0.75 |
| gtg | V | 4 | 1.59 |
| --- | -- | ------ | ------ |
| mPD | | 0.027 | 0.045 |
| | | nPD: | 0.6 |
| | | N. weight: | 0.026 |
| | | Sc. PD: | 0.0084 |
| | | Sc. rank: | 4.0 |

PB1

| Pos. | 745 | obs: | exp: |
|---|---|---|---|
| aaa | K | 0 | 205.50 |
| aag | K | 367 | 161.50 |
| --- | -- | ------ | ------ |
| mPD | | 0 | 0.49 |
| | | nPD: | 0. |
| | | N. weight: | 1.2 |
| | | Sc. PD: | -0.3 |
| | | Sc. rank: | -259.3 |

PB1

| Pos. | 746 | obs: | exp: |
|---|---|---|---|
| att | I | 0 | 136.50 |
| atc | I | 363 | 95.14 |
| ata | I | 0 | 131.40 |
| act | T | 0 | 1.06 |
| acc | T | 4 | 0.86 |
| aca | T | 0 | 1.75 |
| acg | T | 0 | 0.33 |
| --- | -- | ------ | ------ |
| mPD | | 0.022 | 0.68 |
| | | nPD: | 0.03 |
| | | N. weight: | 2. |
| | | Sc. PD: | -0.44 |
| | | Sc. rank: | -295.5 |

PB1

| Pos. | 747 | obs: | exp: |
|---|---|---|---|
| tgt | C | 367 | 151.30 |
| tgc | C | 0 | 215.70 |
| --- | -- | ------ | ------ |
| mPD | | 0 | 0.48 |
| | | nPD: | 0. |
| | | N. weight: | 1.3 |
| | | Sc. PD: | -0.33 |
| | | Sc. rank: | -280.0 |

PB1

| Pos. | 748 | obs: | exp: |
|---|---|---|---|
| tct | S | 3 | 58.34 |
| tcc | S | 364 | 51.12 |
| tca | S | 0 | 81.31 |
| tcg | S | 0 | 22.37 |
| agt | S | 0 | 80.74 |
| agc | S | 0 | 73.12 |
| --- | -- | ------ | ------ |
| mPD | | 0.016 | 1.7 |
| | | nPD: | 0.01 |
| | | N. weight: | 2.9 |
| | | Sc. PD: | -0.69 |
| | | Sc. rank: | -508.2 |

PB1

| Pos. | 749 | obs: | exp: |
|---|---|---|---|
| act | T | 0 | 96.96 |
| acc | T | 367 | 78.75 |
| aca | T | 0 | 160.80 |
| acg | T | 0 | 30.51 |
| --- | -- | ------ | ------ |
| mPD | | 0 | 0.69 |
| | | nPD: | 0. |
| | | N. weight: | 2.3 |
| | | Sc. PD: | -0.57 |
| | | Sc. rank: | -486.2 |

PB1

| Pos. | 750 | obs: | exp: |
|---|---|---|---|
| att | I | 367 | 138.00 |
| atc | I | 0 | 96.18 |
| ata | I | 0 | 132.80 |
| --- | -- | ------ | ------ |
| mPD | | 0 | 0.66 |
| | | nPD: | 0. |
| | | N. weight: | 1.5 |
| | | Sc. PD: | -0.36 |
| | | Sc. rank: | -309.1 |

PB1

| Pos. | 751 | obs: | exp: |
|---|---|---|---|
| gaa | E | 366 | 211.20 |
| gag | E | 1 | 155.80 |
| --- | -- | ------ | ------ |
| mPD | | 0.0054 | 0.49 |
| | | nPD: | 0.01 |
| | | N. weight: | 0.82 |
| | | Sc. PD: | -0.19 |
| | | Sc. rank: | -140.0 |

PB1

| Pos. | 752 | obs: | exp: |
|---|---|---|---|
| gat | D | 2 | 1.07 |
| gac | D | 0 | 0.93 |
| gaa | E | 9 | 210.00 |
| gag | E | 356 | 155.00 |
| --- | -- | ------ | ------ |
| mPD | | 0.059 | 0.49 |
| | | nPD: | 0.12 |
| | | N. weight: | 1.1 |
| | | Sc. PD: | -0.15 |
| | | Sc. rank: | -42.9 |

PB1

| Pos. | 753 | obs: | exp: |
|---|---|---|---|
| tta | L | 0 | 32.06 |
| ttg | L | 0 | 70.68 |
| ctt | L | 0 | 68.48 |
| ctc | L | 367 | 59.37 |
| cta | L | 0 | 55.29 |
| ctg | L | 0 | 81.13 |
| --- | -- | ------ | ------ |
| mPD | | 0 | 1.1 |
| | | nPD: | 0. |
| | | N. weight: | 2.8 |
| | | Sc. PD: | -0.68 |
| | | Sc. rank: | -575.5 |

PB1

| Pos. | 754 | obs: | exp: |
|---|---|---|---|
| cgt | R | 0 | 15.19 |
| cgc | R | 0 | 17.92 |
| cga | R | 0 | 35.17 |
| cgg | R | 0 | 30.54 |
| aaa | K | 1 | 0.56 |
| aag | K | 0 | 0.44 |
| aga | R | 366 | 164.60 |
| agg | R | 0 | 102.60 |
| --- | -- | ------ | ------ |
| mPD | | 0.0054 | 0.96 |
| | | nPD: | 0.01 |
| | | N. weight: | 1.2 |
| | | Sc. PD: | -0.29 |
| | | Sc. rank: | -221.8 |

| PB1 | | | |
|---|---|---|---|
| Pos. | 755 | obs: | exp: |
| caa | Q | 0 | 0.53 |
| cag | Q | 1 | 0.47 |
| cgt | R | 0 | 15.19 |
| cgc | R | 0 | 17.92 |
| cga | R | 0 | 35.17 |
| cgg | R | 366 | 30.54 |
| aga | R | 0 | 164.60 |
| agg | R | 0 | 102.60 |
| --- | -- | ------ | ------ |
| mPD | | 0.0054 | 0.97 |
| | | nPD: | 0.01 |
| | | N. weight: | 3.8 |
| | | Sc. PD: | -0.9 |
| | | Sc. rank: | -691.2 |

,

| PB1 | | | |
|---|---|---|---|
| Pos. | 756 | obs: | exp: |
| tta | L | 0 | 0.09 |
| ttg | L | 0 | 0.19 |
| ctt | L | 0 | 0.19 |
| ctc | L | 0 | 0.16 |
| cta | L | 0 | 0.15 |
| ctg | L | 1 | 0.22 |
| caa | Q | 251 | 195.10 |
| cag | Q | 115 | 170.90 |
| --- | -- | ------ | ------ |
| mPD | | 0.44 | 0.51 |
| | | nPD: | 0.87 |
| | | N. weight: | 0.092 |
| | | Sc. PD: | 0.054 |
| | | Sc. rank: | 19.0 |

,

| PB1 | | | |
|---|---|---|---|
| Pos. | 757 | obs: | exp: |
| caa | Q | 7 | 3.73 |
| cag | Q | 0 | 3.27 |
| aaa | K | 352 | 201.60 |
| aag | K | 8 | 158.40 |
| --- | -- | ------ | ------ |
| mPD | | 0.080 | 0.53 |
| | | nPD: | 0.15 |
| | | N. weight: | 0.74 |
| | | Sc. PD: | -0.074 |
| | | Sc. rank: | -11.1 |

,

| PB1 | | | |
|---|---|---|---|
| Pos. | 758 | obs: | exp: |
| caa | Q | 0 | 13.86 |
| cag | Q | 26 | 12.14 |
| --- | - | 341 | 341.00 |
| --- | -- | ------ | ------ |
| mPD | | 0 | 0.0025 |
| | | nPD: | 0. |
| | | N. weight: | 0. |
| | | Sc. PD: | 0 |
| | | Sc. rank: | 0 |

,

| PB1 | | | |
|---|---|---|---|
| Pos. | 759 | obs: | exp: |
| taa | . | 25 | 171.90 |
| tag | . | 315 | 94.97 |
| tga | . | 27 | 100.10 |
| --- | -- | ------ | ------ |
| mPD | | 0.38 | 0.78 |
| | | nPD: | 0.49 |
| | | N. weight: | 1.2 |
| | | Sc. PD: | 0.27 |
| | | Sc. rank: | 158.1 |

}

Amino acid level conservation per codon

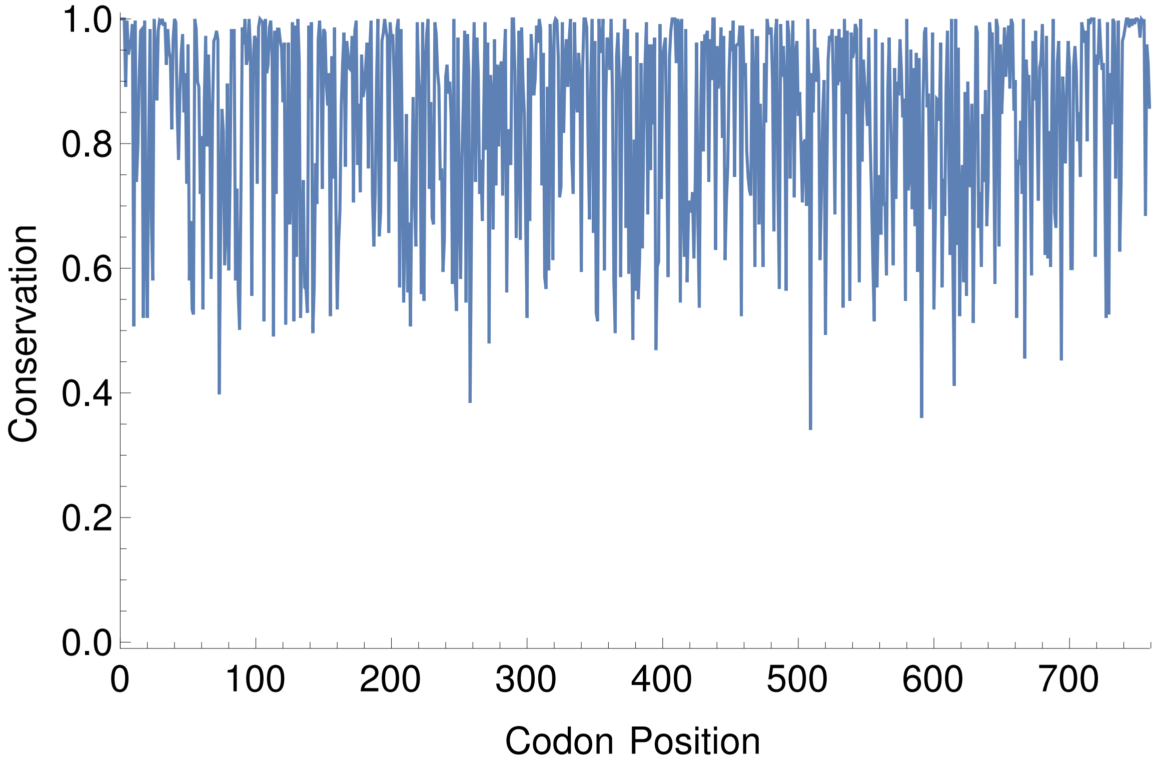

## Conservation analysis plots

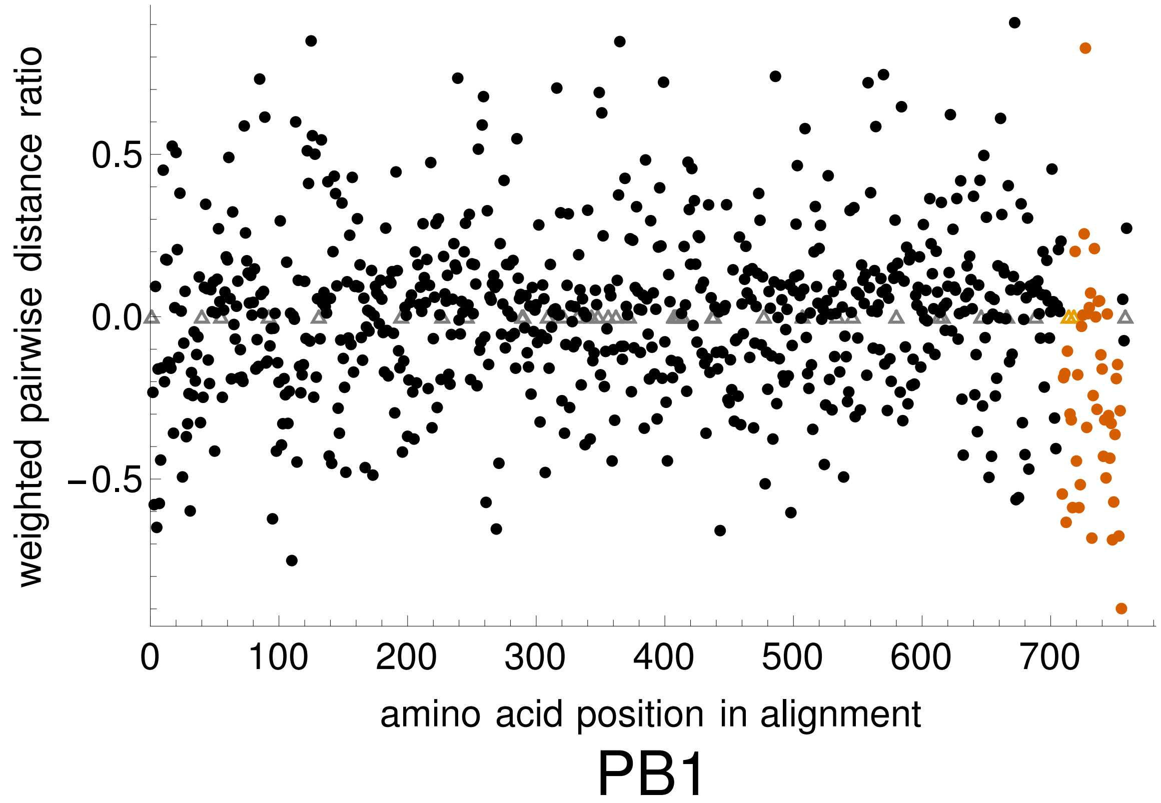


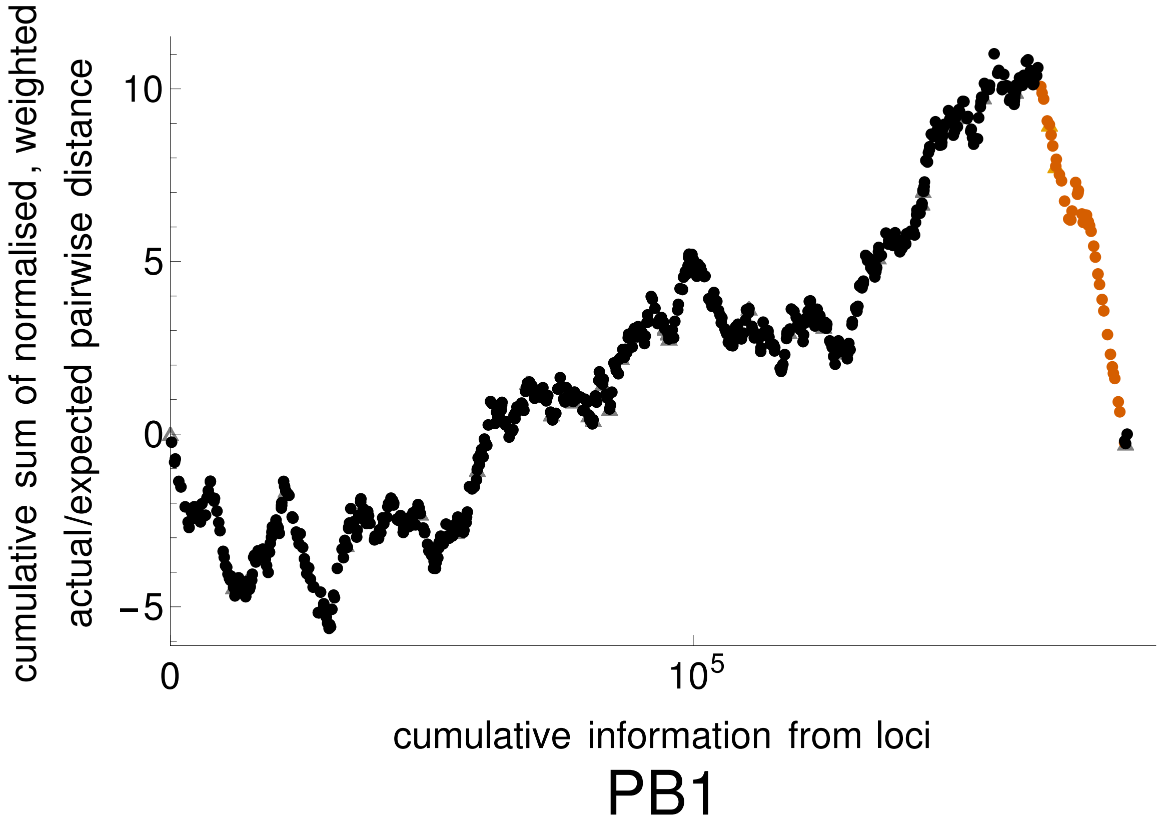

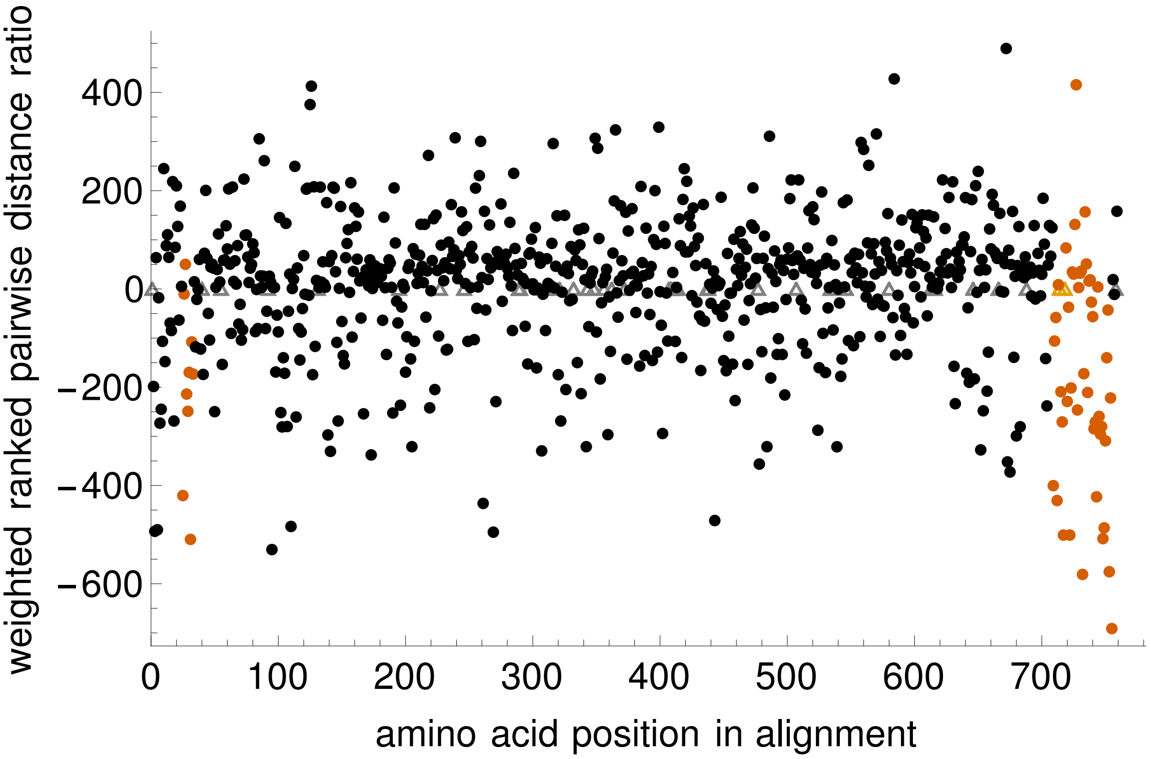
weighted ranked pairwise distance ratio
400
200
0
−200
−400
−600
0
100
200
300
400
500
600
700
amino acid position in alignment

PB1

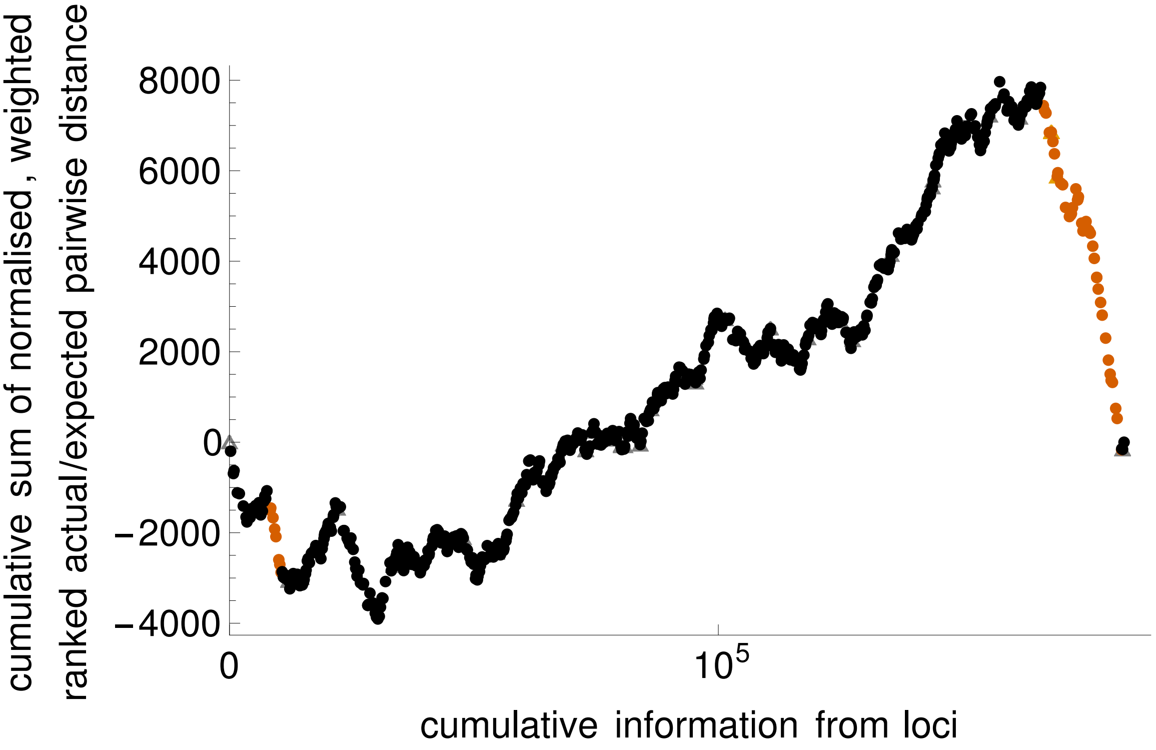
cumulative sum of normalised, weighted ranked actual/expected pairwise distance
8000
6000
4000
2000
0
−2000
−4000
0
$10^5$
cumulative information from loci

PB1

# PB1-F2

## Gene length histogram

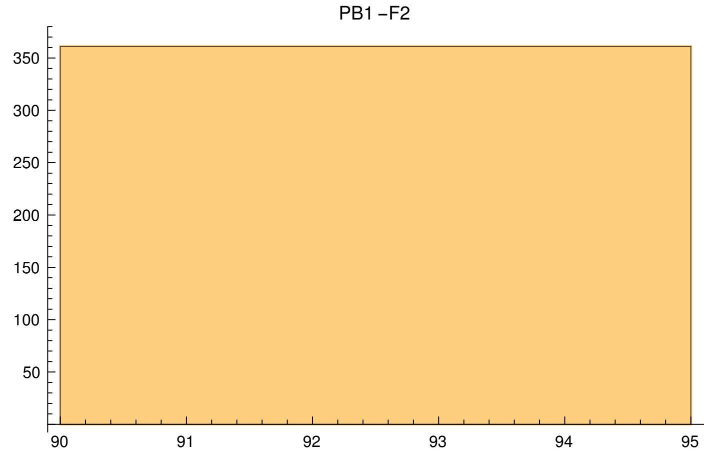

## Information vs. nPD

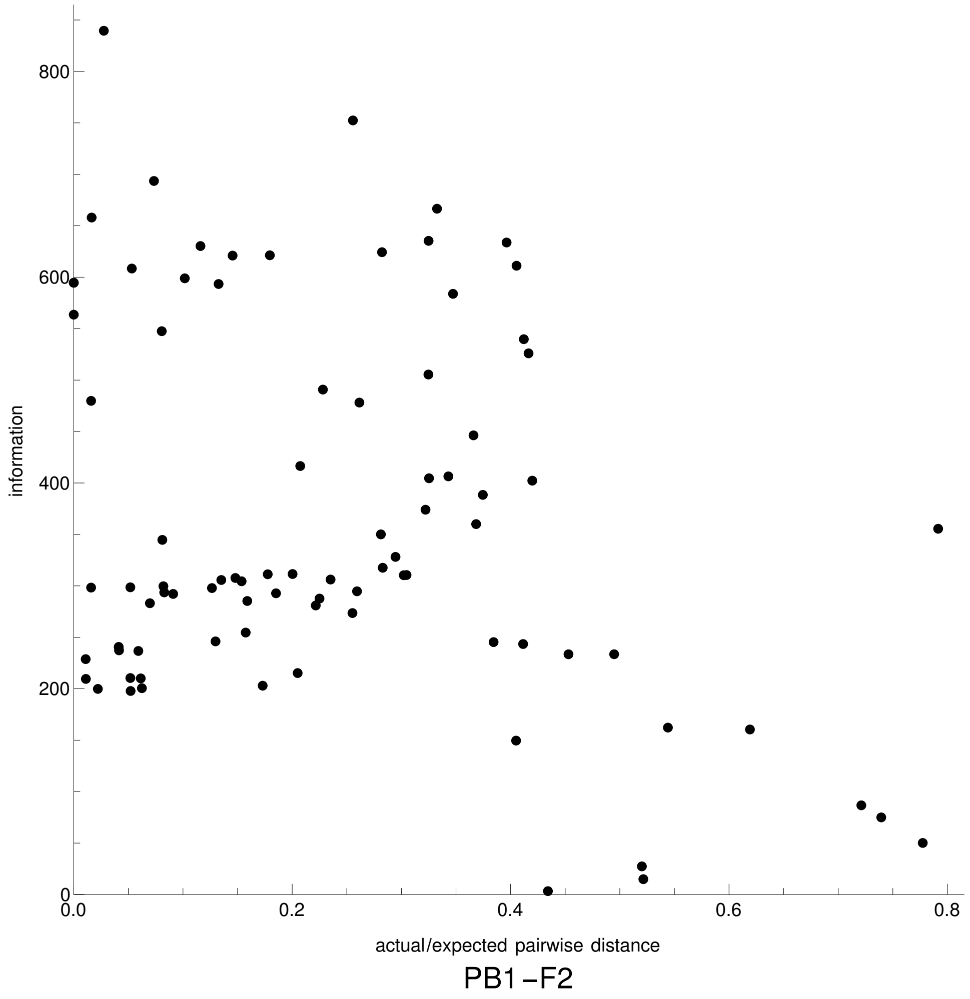

## Example sequences highlighted by regions found in analyses to be conserved

Interesting points (by weighted raw PD) highlighted for gene PB1-F2:

ATG
**GAACAGGAACAGGATACACCATGGACACAGTCAACAGAACAC**ATCAATACTCAGAAAAG
GGGAAATGGACAACAAACACAGAGACCGGAGCACCCCAACTCAACCCAATTGATGGACCACTACCTGAGG
ACAACGAACCAAGCGGATATGCACAAACAGATTGCGTGTTGG
**AAGCAATGGCTTTCCTTGAAGAGTCCCACCCAGGGATCTTTGAAAACTCTTGTCTTGAAACGA**TGGAAATCGTTCAGCAAACAAGAGTGGACAAACTAA

Interesting points (by weighted ranked PD) highlighted for gene PB1-F2:

ATGGAACAGGAACAGGATACACCATGGACACAGTCAACAGAACACATCAATACTCAGAAAAGGGGAAATGGACAACAA
ACACAGAGACCGGAGCACCCCAACTCAACCCAATTGATGGACCACTACCTGAGGACAACGAACCAAGCGGATATGCA
CAAACAGATTGCGTGTTGG
**AAGCAATGGCTTTCCTTGAAGAGTCCCACCCAGGGATCTTTGAAAACTCTTGTCTTGAAACGA**TGGAAATCGTTCAGCAAACAAGAGTGGACAAACTAA

# Per codon conservation report

PB1-F2

| Pos. | 1 | obs: | exp: |
|---|---|---|---|
| atg | M | 361 | 361.00 |
| mPD | | 0 | 0 |
| | | nPD: | 1. |
| | | N. weight: | 0. |
| | | Sc. PD: | 0 |
| | | Sc. rank: | 0 |

PB1-F2

| Pos. | 2 | obs: | exp: |
|---|---|---|---|
| gaa | E | 359 | 206.60 |
| gag | E | 0 | 152.40 |
| ggt | G | 0 | 0.25 |
| ggc | G | 0 | 0.27 |
| gga | G | 2 | 0.97 |
| ggg | G | 0 | 0.52 |
| mPD | | 0.011 | 0.50 |
| | | nPD: | 0.02 |
| | | N. weight: | 0.58 |
| | | Sc. PD: | -0.1 |
| | | Sc. rank: | -133.2 |

PB1-F2

| Pos. | 3 | obs: | exp: |
|---|---|---|---|
| cct | P | 0 | 3.95 |
| ccc | P | 0 | 2.71 |
| cca | P | 0 | 5.92 |
| ccg | P | 15 | 2.43 |
| caa | Q | 0 | 178.10 |
| cag | Q | 334 | 155.90 |
| cgt | R | 0 | 0.50 |
| cgc | R | 0 | 0.59 |
| cga | R | 0 | 1.15 |
| cgg | R | 12 | 1.00 |
| aga | R | 0 | 5.40 |
| agg | R | 0 | 3.36 |
| mPD | | 0.14 | 0.71 |
| | | nPD: | 0.2 |
| | | N. weight: | 0.9 |
| | | Sc. PD: | -0.011 |
| | | Sc. rank: | 0.9 |

PB1-F2

| Pos. | 4 | obs: | exp: |
|---|---|---|---|
| gaa | E | 355 | 204.30 |
| gag | E | 0 | 150.70 |
| ggt | G | 0 | 0.74 |
| ggc | G | 0 | 0.81 |
| gga | G | 6 | 2.90 |
| ggg | G | 0 | 1.55 |
| mPD | | 0.033 | 0.53 |
| | | nPD: | 0.06 |
| | | N. weight: | 0.58 |
| | | Sc. PD: | -0.083 |
| | | Sc. rank: | -97.6 |

PB1-F2

| Pos. | 5 | obs: | exp: |
|---|---|---|---|
| caa | Q | 0 | 182.30 |
| cag | Q | 342 | 159.70 |
| cgt | R | 0 | 0.79 |
| cgc | R | 0 | 0.93 |
| cga | R | 0 | 1.83 |
| cgg | R | 19 | 1.59 |
| aga | R | 0 | 8.54 |
| agg | R | 0 | 5.32 |
| mPD | | 0.10 | 0.68 |
| | | nPD: | 0.15 |
| | | N. weight: | 0.89 |
| | | Sc. PD: | -0.055 |
| | | Sc. rank: | -42.6 |

PB1-F2

| Pos. | 6 | obs: | exp: |
|---|---|---|---|
| gat | D | 342 | 182.50 |
| gac | D | 0 | 159.50 |
| ggt | G | 19 | 2.34 |
| ggc | G | 0 | 2.57 |
| gga | G | 0 | 9.19 |
| ggg | G | 0 | 4.90 |
| mPD | | 0.10 | 0.64 |
| | | nPD: | 0.16 |
| | | N. weight: | 0.73 |
| | | Sc. PD: | -0.039 |
| | | Sc. rank: | -27.5 |

PB1-F2

| Pos. | 7 | obs: | exp: |
|---|---|---|---|
| att | I | 0 | 24.44 |
| atc | I | 0 | 17.04 |
| ata | I | 65 | 23.53 |
| act | T | 0 | 78.20 |
| acc | T | 0 | 63.52 |
| aca | T | 296 | 129.70 |
| acg | T | 0 | 24.61 |
| mPD | | 0.30 | 0.98 |
| | | nPD: | 0.3 |
| | | N. weight: | 0.89 |
| | | Sc. PD: | 0.076 |
| | | Sc. rank: | 98.3 |

PB1-F2

| Pos. | 8 | obs: | exp: |
|---|---|---|---|
| tta | L | 0 | 0.87 |
| ttg | L | 0 | 1.93 |
| ctt | L | 0 | 1.87 |
| ctc | L | 0 | 1.62 |
| cta | L | 10 | 1.51 |
| ctg | L | 0 | 2.21 |
| cct | P | 0 | 91.90 |
| ccc | P | 0 | 62.95 |
| cca | P | 349 | 137.70 |
| ccg | P | 0 | 56.43 |
| caa | Q | 2 | 1.07 |
| cag | Q | 0 | 0.93 |
| mPD | | 0.065 | 0.80 |
| | | nPD: | 0.08 |
| | | N. weight: | 0.99 |
| | | Sc. PD: | -0.12 |
| | | Sc. rank: | -132.5 |

PB1-F2

| Pos. | 9 | obs: | exp: |
|---|---|---|---|
| tgg | W | 361 | 361.00 |
| mPD | | 0 | 0 |
| | | nPD: | 1. |
| | | N. weight: | 0. |
| | | Sc. PD: | 0 |
| | | Sc. rank: | 0 |

PB1-F2

| Pos. | 10 | obs: | exp: |
|---|---|---|---|
| att | I | 0 | 0.75 |
| atc | I | 0 | 0.52 |
| ata | I | 2 | 0.72 |
| act | T | 0 | 94.84 |
| acc | T | 0 | 77.04 |
| aca | T | 359 | 157.30 |
| acg | T | 0 | 29.84 |
| mPD | | 0.011 | 0.70 |
| | | nPD: | 0.02 |
| | | N. weight: | 0.86 |
| | | Sc. PD: | -0.16 |
| | | Sc. rank: | -219.2 |

PB1-F2

| Pos. | 11 | obs: | exp: |
|---|---|---|---|
| tta | L | 0 | 1.49 |
| ttg | L | 0 | 3.27 |
| ctt | L | 0 | 3.17 |
| ctc | L | 0 | 2.75 |
| cta | L | 0 | 2.56 |
| ctg | L | 17 | 3.76 |
| cct | P | 0 | 0.53 |
| ccc | P | 0 | 0.36 |
| cca | P | 0 | 0.79 |
| ccg | P | 2 | 0.32 |
| caa | Q | 0 | 163.70 |
| cag | Q | 307 | 143.30 |
| cgt | R | 0 | 1.45 |
| cgc | R | 0 | 1.71 |
| cga | R | 0 | 3.36 |
| cgg | R | 35 | 2.92 |
| aga | R | 0 | 15.74 |
| agg | R | 0 | 9.81 |
| mPD | | 0.27 | 0.95 |
| | | nPD: | 0.28 |
| | | N. weight: | 1. |
| | | Sc. PD: | 0.065 |
| | | Sc. rank: | 80.6 |

PB1-F2

| Pos. | 12 | obs: | exp: |
|---|---|---|---|
| tta | L | 83 | 7.25 |
| ttg | L | 0 | 15.99 |
| tct | S | 0 | 44.20 |
| tcc | S | 0 | 38.73 |
| tca | S | 278 | 61.59 |
| tcg | S | 0 | 16.95 |
| ctt | L | 0 | 15.49 |
| ctc | L | 0 | 13.43 |
| cta | L | 0 | 12.50 |
| ctg | L | 0 | 18.35 |
| agt | S | 0 | 61.16 |
| agc | S | 0 | 55.39 |
| mPD | | 0.36 | 2.0 |
| | | nPD: | 0.18 |
| | | N. weight: | 1.8 |
| | | Sc. PD: | -0.057 |
| | | Sc. rank: | -18.7 |

PB1-F2

| Pos. | 13 | obs: | exp: |
|---|---|---|---|
| att | I | 0 | 16.17 |
| atc | I | 0 | 11.27 |
| ata | I | 43 | 15.57 |
| act | T | 0 | 84.01 |
| acc | T | 0 | 68.24 |
| aca | T | 318 | 139.30 |
| acg | T | 0 | 26.44 |
| mPD | | 0.21 | 0.89 |
| | | nPD: | 0.24 |
| | | N. weight: | 0.88 |
| | | Sc. PD: | 0.019 |
| | | Sc. rank: | 34.4 |

PB1-F2

| Pos. | 14 | obs: | exp: |
|---|---|---|---|
| gaa | E | 341 | 196.20 |
| gag | E | 0 | 144.80 |
| ggt | G | 0 | 2.47 |
| ggc | G | 0 | 2.70 |
| gga | G | 20 | 9.67 |
| ggg | G | 0 | 5.15 |
| mPD | | 0.10 | 0.61 |
| | | nPD: | 0.17 |
| | | N. weight: | 0.58 |
| | | Sc. PD: | -0.022 |
| | | Sc. rank: | -16.5 |

PB1-F2

| Pos. | 15 | obs: | exp: |
|---|---|---|---|
| tat | Y | 0 | 0.52 |
| tac | Y | 1 | 0.48 |
| cat | H | 0 | 190.70 |
| cac | H | 352 | 161.30 |
| cgt | R | 0 | 0.33 |
| cgc | R | 8 | 0.39 |
| cga | R | 0 | 0.77 |
| cgg | R | 0 | 0.67 |
| aga | R | 0 | 3.60 |
| agg | R | 0 | 2.24 |
| mPD | | 0.049 | 0.60 |
| | | nPD: | 0.08 |
| | | N. weight: | 0.86 |
| | | Sc. PD: | -0.11 |
| | | Sc. rank: | -110.2 |

PB1-F2

| Pos. | 16 | obs: | exp: |
|---|---|---|---|
| att | I | 0 | 115.00 |
| atc | I | 281 | 80.20 |
| ata | I | 25 | 110.80 |
| act | T | 0 | 14.53 |
| acc | T | 55 | 11.80 |
| aca | T | 0 | 24.10 |
| acg | T | 0 | 4.57 |
| mPD | | 0.39 | 0.92 |
| | | nPD: | 0.42 |
| | | N. weight: | 1.2 |
| | | Sc. PD: | 0.23 |
| | | Sc. rank: | 313.2 |

PB1-F2

| Pos. | 17 | obs: | exp: |
|---|---|---|---|
| tct | S | 0 | 0.32 |
| tcc | S | 0 | 0.28 |
| tca | S | 0 | 0.44 |
| tcg | S | 0 | 0.12 |
| act | T | 2 | 0.53 |
| acc | T | 0 | 0.43 |
| aca | T | 0 | 0.88 |
| acg | T | 0 | 0.17 |
| aat | N | 357 | 186.60 |
| aac | N | 0 | 170.40 |
| agt | S | 2 | 0.44 |
| agc | S | 0 | 0.40 |
| mPD | | 0.022 | 0.53 |
| | | nPD: | 0.04 |
| | | N. weight: | 0.68 |
| | | Sc. PD: | -0.11 |
| | | Sc. rank: | -141.0 |

PB1-F2

| Pos. | 18 | obs: | exp: |
|---|---|---|---|
| att | I | 97 | 36.47 |
| atc | I | 0 | 25.42 |
| ata | I | 0 | 35.11 |
| act | T | 264 | 69.75 |
| acc | T | 0 | 56.65 |
| aca | T | 0 | 115.70 |
| acg | T | 0 | 21.95 |
| mPD | | 0.39 | 1.1 |
| | | nPD: | 0.37 |
| | | N. weight: | 1.3 |
| | | Sc. PD: | 0.19 |
| | | Sc. rank: | 247.3 |

PB1-F2

| Pos. | 19 | obs: | exp: |
|---|---|---|---|
| tta | L | 0 | 0.26 |
| ttg | L | 0 | 0.58 |
| ctt | L | 0 | 0.56 |
| ctc | L | 0 | 0.49 |
| cta | L | 0 | 0.45 |
| ctg | L | 3 | 0.66 |
| caa | Q | 0 | 184.50 |
| cag | Q | 346 | 161.50 |
| cgt | R | 0 | 0.50 |
| cgc | R | 0 | 0.59 |
| cga | R | 0 | 1.15 |
| cgg | R | 12 | 1.00 |
| aga | R | 0 | 5.40 |
| agg | R | 0 | 3.36 |
| mPD | | 0.080 | 0.64 |
| | | nPD: | 0.13 |
| | | N. weight: | 0.86 |
| | | Sc. PD: | -0.07 |
| | | Sc. rank: | -75.1 |

PB1-F2

| Pos. | 20 | obs: | exp: |
|---|---|---|---|
| tct | S | 0 | 0.32 |
| tcc | S | 0 | 0.28 |
| tca | S | 0 | 0.44 |
| tcg | S | 0 | 0.12 |
| caa | Q | 1 | 0.53 |
| cag | Q | 0 | 0.47 |
| cgt | R | 0 | 6.06 |
| cgc | R | 0 | 7.15 |
| cga | R | 0 | 14.03 |
| cgg | R | 0 | 12.18 |
| aaa | K | 212 | 118.70 |
| aag | K | 0 | 93.29 |
| agt | S | 0 | 0.44 |
| agc | S | 2 | 0.40 |
| aga | R | 146 | 65.66 |
| agg | R | 0 | 40.92 |
| mPD | | 0.50 | 1.2 |
| | | nPD: | 0.41 |
| | | N. weight: | 0.7 |
| | | Sc. PD: | 0.13 |
| | | Sc. rank: | 170.0 |

| PB1-F2 | | | |
|---|---|---|---|
| Pos. | 21 | obs: | exp: |
| cgt | R | 0 | 10.13 |
| cgc | R | 0 | 11.95 |
| cga | R | 0 | 23.45 |
| cgg | R | 0 | 20.36 |
| aaa | K | 0 | 65.52 |
| aag | K | 117 | 51.48 |
| aga | R | 0 | 109.70 |
| agg | R | 244 | 68.38 |
| --- | -- | ------ | ------ |
| mPD | | 0.44 | 1.3 |
| | | nPD: | 0.34 |
| | | N. weight: | 1.2 |
| | | Sc. PD: | 0.14 |
| | | Sc. rank: | 202.3 |

,

| PB1-F2 | | | |
|---|---|---|---|
| Pos. | 22 | obs: | exp: |
| gaa | E | 165 | 94.95 |
| gag | E | 0 | 70.05 |
| ggt | G | 0 | 24.18 |
| ggc | G | 0 | 26.51 |
| gga | G | 196 | 94.81 |
| ggg | G | 0 | 50.50 |
| --- | -- | ------ | ------ |
| mPD | | 0.50 | 1.1 |
| | | nPD: | 0.45 |
| | | N. weight: | 0.67 |
| | | Sc. PD: | 0.15 |
| | | Sc. rank: | 184.9 |

,

| PB1-F2 | | | |
|---|---|---|---|
| Pos. | 23 | obs: | exp: |
| tct | S | 0 | 29.73 |
| tcc | S | 0 | 26.05 |
| tca | S | 0 | 41.43 |
| tcg | S | 0 | 11.40 |
| aat | N | 162 | 84.67 |
| aac | N | 0 | 77.33 |
| agt | S | 187 | 41.14 |
| agc | S | 0 | 37.26 |
| gat | D | 6 | 3.20 |
| gac | D | 0 | 2.80 |
| ggt | G | 6 | 0.74 |
| ggc | G | 0 | 0.81 |
| gga | G | 0 | 2.90 |
| ggg | G | 0 | 1.55 |
| --- | -- | ------ | ------ |
| mPD | | 0.56 | 1.7 |
| | | nPD: | 0.33 |
| | | N. weight: | 1.2 |
| | | Sc. PD: | 0.12 |
| | | Sc. rank: | 177.6 |

,

| PB1-F2 | | | |
|---|---|---|---|
| Pos. | 24 | obs: | exp: |
| ggt | G | 0 | 44.54 |
| ggc | G | 0 | 48.82 |
| gga | G | 356 | 174.60 |
| ggg | G | 5 | 93.01 |
| --- | -- | ------ | ------ |
| mPD | | 0.027 | 0.67 |
| | | nPD: | 0.04 |
| | | N. weight: | 0.69 |
| | | Sc. PD: | -0.11 |
| | | Sc. rank: | -146.1 |

,

| PB1-F2 | | | |
|---|---|---|---|
| Pos. | 25 | obs: | exp: |
| caa | Q | 360 | 191.90 |
| cag | Q | 0 | 168.10 |
| cgt | R | 0 | 0.04 |
| cgc | R | 0 | 0.05 |
| cga | R | 1 | 0.10 |
| cgg | R | 0 | 0.08 |
| aga | R | 0 | 0.45 |
| agg | R | 0 | 0.28 |
| --- | -- | ------ | ------ |
| mPD | | 0.0055 | 0.51 |
| | | nPD: | 0.01 |
| | | N. weight: | 0.66 |
| | | Sc. PD: | -0.13 |
| | | Sc. rank: | -175.6 |

,

| PB1-F2 | | | |
|---|---|---|---|
| Pos. | 26 | obs: | exp: |
| tta | L | 0 | 0.09 |
| ttg | L | 0 | 0.19 |
| ctt | L | 0 | 0.19 |
| ctc | L | 0 | 0.16 |
| cta | L | 1 | 0.15 |
| ctg | L | 0 | 0.22 |
| cct | P | 0 | 1.05 |
| ccc | P | 0 | 0.72 |
| cca | P | 4 | 1.58 |
| ccg | P | 0 | 0.65 |
| caa | Q | 326 | 173.80 |
| cag | Q | 0 | 152.20 |
| cgt | R | 0 | 1.25 |
| cgc | R | 0 | 1.47 |
| cga | R | 30 | 2.88 |
| cgg | R | 0 | 2.50 |
| aga | R | 0 | 13.49 |
| agg | R | 0 | 8.41 |
| --- | -- | ------ | ------ |
| mPD | | 0.18 | 0.80 |
| | | nPD: | 0.22 |
| | | N. weight: | 0.81 |
| | | Sc. PD: | 0.0067 |
| | | Sc. rank: | 14.9 |

,

| PB1-F2 | | | |
|---|---|---|---|
| Pos. | 27 | obs: | exp: |
| att | I | 1 | 14.66 |
| atc | I | 0 | 10.22 |
| ata | I | 38 | 14.12 |
| act | T | 79 | 85.07 |
| acc | T | 1 | 69.10 |
| aca | T | 242 | 141.10 |
| acg | T | 0 | 26.77 |
| --- | -- | ------ | ------ |
| mPD | | 0.54 | 0.88 |
| | | nPD: | 0.62 |
| | | N. weight: | 0.46 |
| | | Sc. PD: | 0.18 |
| | | Sc. rank: | 132.2 |

,

| PB1-F2 | | | |
|---|---|---|---|
| Pos. | 28 | obs: | exp: |
| tta | L | 0 | 0.61 |
| ttg | L | 0 | 1.35 |
| ctt | L | 0 | 1.31 |
| ctc | L | 0 | 1.13 |
| cta | L | 0 | 1.05 |
| ctg | L | 7 | 1.55 |
| cct | P | 0 | 2.90 |
| ccc | P | 0 | 1.98 |
| cca | P | 0 | 4.34 |
| ccg | P | 11 | 1.78 |
| caa | Q | 1 | 180.70 |
| cag | Q | 338 | 158.30 |
| cgt | R | 0 | 0.17 |
| cgc | R | 0 | 0.20 |
| cga | R | 0 | 0.38 |
| cgg | R | 4 | 0.33 |
| aga | R | 0 | 1.80 |
| agg | R | 0 | 1.12 |
| --- | -- | ------ | ------ |
| mPD | | 0.12 | 0.66 |
| | | nPD: | 0.19 |
| | | N. weight: | 0.84 |
| | | Sc. PD: | -0.022 |
| | | Sc. rank: | -4.1 |

,

| PB1-F2 | | | |
|---|---|---|---|
| Pos. | 29 | obs: | exp: |
| cgt | R | 0 | 12.16 |
| cgc | R | 0 | 14.35 |
| cga | R | 0 | 28.16 |
| cgg | R | 0 | 24.45 |
| aaa | K | 68 | 38.08 |
| aag | K | 0 | 29.92 |
| aga | R | 293 | 131.80 |
| agg | R | 0 | 82.11 |
| --- | -- | ------ | ------ |
| mPD | | 0.31 | 1.2 |
| | | nPD: | 0.26 |
| | | N. weight: | 0.79 |
| | | Sc. PD: | 0.032 |
| | | Sc. rank: | 34.9 |

,

| PB1-F2 | | | |
|---|---|---|---|
| Pos. | 30 | obs: | exp: |
| tta | L | 0 | 16.60 |
| ttg | L | 0 | 36.59 |
| ctt | L | 0 | 35.45 |
| ctc | L | 0 | 30.73 |
| cta | L | 0 | 28.62 |
| ctg | L | 190 | 42.00 |
| cct | P | 0 | 44.24 |
| ccc | P | 0 | 30.30 |
| cca | P | 2 | 66.30 |
| ccg | P | 166 | 27.16 |
| caa | Q | 0 | 1.60 |
| cag | Q | 3 | 1.40 |
| --- | -- | ------ | ------ |
| mPD | | 0.52 | 1.5 |
| | | nPD: | 0.35 |
| | | N. weight: | 1.7 |
| | | Sc. PD: | 0.21 |
| | | Sc. rank: | 309.2 |

,

| PB1-F2 | | | |
|---|---|---|---|
| Pos. | 31 | obs: | exp: |
| aaa | K | 0 | 0.56 |
| aag | K | 1 | 0.44 |
| gtt | V | 0 | 0.62 |
| gtc | V | 0 | 0.63 |
| gta | V | 0 | 0.56 |
| gtg | V | 3 | 1.19 |
| gat | D | 1 | 0.53 |
| gac | D | 0 | 0.47 |
| gaa | E | 0 | 183.00 |
| gag | E | 318 | 135.00 |
| ggt | G | 0 | 4.69 |
| ggc | G | 0 | 5.14 |
| gga | G | 0 | 18.38 |
| ggg | G | 38 | 9.79 |
| --- | -- | ------ | ------ |
| mPD | | 0.21 | 0.73 |
| | | nPD: | 0.29 |
| | | N. weight: | 0.94 |
| | | Sc. PD: | 0.073 |
| | | Sc. rank: | 98.4 |

,

| PB1-F2 | | | |
|---|---|---|---|
| Pos. | 32 | obs: | exp: |
| cat | H | 1 | 191.30 |
| cac | H | 352 | 161.70 |
| cgt | R | 0 | 0.33 |
| cgc | R | 8 | 0.39 |
| cga | R | 0 | 0.77 |
| cgg | R | 0 | 0.67 |
| aga | R | 0 | 3.60 |
| agg | R | 0 | 2.24 |
| --- | -- | ------ | ------ |
| mPD | | 0.049 | 0.59 |
| | | nPD: | 0.08 |
| | | N. weight: | 0.85 |
| | | Sc. PD: | -0.1 |
| | | Sc. rank: | -103.3 |

,

| PB1-F2 | | | |
|---|---|---|---|
| Pos. | 33 | obs: | exp: |
| tta | L | 0 | 6.12 |
| ttg | L | 0 | 13.48 |
| tct | S | 0 | 0.16 |
| tcc | S | 1 | 0.14 |
| tca | S | 0 | 0.22 |
| tcg | S | 0 | 0.06 |
| ctt | L | 0 | 13.06 |
| ctc | L | 70 | 11.32 |
| cta | L | 0 | 10.55 |
| ctg | L | 0 | 15.47 |
| cct | P | 0 | 75.84 |
| ccc | P | 288 | 51.95 |
| cca | P | 0 | 113.70 |
| ccg | P | 0 | 46.57 |
| cat | H | 0 | 1.08 |
| cac | H | 2 | 0.92 |
| agt | S | 0 | 0.22 |
| agc | S | 0 | 0.20 |
| --- | -- | ------ | ------ |
| mPD | | 0.33 | 1.2 |
| | | nPD: | 0.28 |
| | | N. weight: | 1.8 |
| | | Sc. PD: | 0.12 |
| | | Sc. rank: | 165.0 |

,

| PB1-F2 | | | |
|---|---|---|---|
| Pos. | 34 | obs: | exp: |
| tct | S | 0 | 3.34 |
| tcc | S | 0 | 2.93 |
| tca | S | 0 | 4.65 |
| tcg | S | 0 | 1.28 |
| aat | N | 0 | 177.70 |
| aac | N | 340 | 162.30 |
| agt | S | 0 | 4.62 |
| agc | S | 21 | 4.18 |
| --- | -- | ------ | ------ |
| mPD | | 0.11 | 0.69 |
| | | nPD: | 0.16 |
| | | N. weight: | 0.82 |
| | | Sc. PD: | -0.042 |
| | | Sc. rank: | -26.3 |

,

| PB1-F2 | | | |
|---|---|---|---|
| Pos. | 35 | obs: | exp: |
| tta | L | 53 | 4.63 |
| ttg | L | 0 | 10.21 |
| tct | S | 0 | 48.96 |
| tcc | S | 0 | 42.91 |
| tca | S | 308 | 68.23 |
| tcg | S | 0 | 18.77 |
| ctt | L | 0 | 9.89 |
| ctc | L | 0 | 8.57 |
| cta | L | 0 | 7.98 |
| ctg | L | 0 | 11.72 |
| agt | S | 0 | 67.76 |
| agc | S | 0 | 61.36 |
| --- | -- | ------ | ------ |
| mPD | | 0.25 | 1.9 |
| | | nPD: | 0.13 |
| | | N. weight: | 1.7 |
| | | Sc. PD: | -0.13 |
| | | Sc. rank: | -122.3 |

,

PB1-F2

| Pos. | 36 | obs: | exp: |
|---|---|---|---|
| att | I | 0 | 55.64 |
| atc | I | 148 | 38.79 |
| ata | I | 0 | 53.57 |
| act | T | 0 | 56.27 |
| acc | T | 213 | 45.71 |
| aca | T | 0 | 93.31 |
| acg | T | 0 | 17.71 |
| --- | -- | ------ | ------ |
| mPD | | 0.49 | 1.2 |
| | | nPD: | 0.42 |
| | | N. weight: | 1.5 |
| | | Sc. PD: | 0.29 |
| | | Sc. rank: | 398.0 |

,

PB1-F2

| Pos. | 37 | obs: | exp: |
|---|---|---|---|
| tta | L | 0 | 0.09 |
| ttg | L | 0 | 0.19 |
| ctt | L | 0 | 0.19 |
| ctc | L | 0 | 0.16 |
| cta | L | 1 | 0.15 |
| ctg | L | 0 | 0.22 |
| cct | P | 0 | 0.53 |
| ccc | P | 0 | 0.36 |
| cca | P | 2 | 0.79 |
| ccg | P | 0 | 0.32 |
| caa | Q | 302 | 161.50 |
| cag | Q | 1 | 141.50 |
| cgt | R | 0 | 2.28 |
| cgc | R | 0 | 2.69 |
| cga | R | 55 | 5.29 |
| cgg | R | 0 | 4.59 |
| aga | R | 0 | 24.73 |
| agg | R | 0 | 15.41 |
| --- | -- | ------ | ------ |
| mPD | | 0.28 | 0.98 |
| | | nPD: | 0.28 |
| | | N. weight: | 0.91 |
| | | Sc. PD: | 0.061 |
| | | Sc. rank: | 89.5 |

,

PB1-F2

| Pos. | 38 | obs: | exp: |
|---|---|---|---|
| tta | L | 0 | 30.49 |
| ttg | L | 349 | 67.21 |
| tct | S | 0 | 1.91 |
| tcc | S | 0 | 1.67 |
| tca | S | 0 | 2.66 |
| tcg | S | 12 | 0.73 |
| ctt | L | 0 | 65.12 |
| ctc | L | 0 | 56.45 |
| cta | L | 0 | 52.58 |
| ctg | L | 0 | 77.15 |
| agt | S | 0 | 2.64 |
| agc | S | 0 | 2.39 |
| --- | -- | ------ | ------ |
| mPD | | 0.064 | 1.2 |
| | | nPD: | 0.05 |
| | | N. weight: | 1.8 |
| | | Sc. PD: | -0.27 |
| | | Sc. rank: | -317.7 |

,

PB1-F2

| Pos. | 39 | obs: | exp: |
|---|---|---|---|
| atg | M | 350 | 350.00 |
| act | T | 0 | 2.91 |
| acc | T | 0 | 2.36 |
| aca | T | 0 | 4.82 |
| acg | T | 11 | 0.91 |
| --- | -- | ------ | ------ |
| mPD | | 0.059 | 0.11 |
| | | nPD: | 0.52 |
| | | N. weight: | 0.079 |
| | | Sc. PD: | 0.023 |
| | | Sc. rank: | 22.1 |

,

PB1-F2

| Pos. | 40 | obs: | exp: |
|---|---|---|---|
| gct | A | 0 | 0.50 |
| gcc | A | 2 | 0.41 |
| gca | A | 0 | 0.89 |
| gcg | A | 0 | 0.20 |
| gat | D | 0 | 188.90 |
| gac | D | 354 | 165.10 |
| ggt | G | 0 | 0.62 |
| ggc | G | 5 | 0.68 |
| gga | G | 0 | 2.42 |
| ggg | G | 0 | 1.29 |
| --- | -- | ------ | ------ |
| mPD | | 0.038 | 0.55 |
| | | nPD: | 0.07 |
| | | N. weight: | 0.82 |
| | | Sc. PD: | -0.11 |
| | | Sc. rank: | -133.4 |

,

PB1-F2

| Pos. | 41 | obs: | exp: |
|---|---|---|---|
| cat | H | 242 | 189.10 |
| cac | H | 107 | 159.90 |
| cgt | R | 6 | 0.50 |
| cgc | R | 6 | 0.59 |
| cga | R | 0 | 1.15 |
| cgg | R | 0 | 1.00 |
| aga | R | 0 | 5.40 |
| agg | R | 0 | 3.36 |
| --- | -- | ------ | ------ |
| mPD | | 0.50 | 0.64 |
| | | nPD: | 0.78 |
| | | N. weight: | 0.14 |
| | | Sc. PD: | 0.077 |
| | | Sc. rank: | 41.9 |

,

PB1-F2

| Pos. | 42 | obs: | exp: |
|---|---|---|---|
| tat | Y | 0 | 109.20 |
| tac | Y | 211 | 101.80 |
| tgt | C | 0 | 61.83 |
| tgc | C | 150 | 88.17 |
| --- | -- | ------ | ------ |
| mPD | | 0.49 | 0.98 |
| | | nPD: | 0.49 |
| | | N. weight: | 0.67 |
| | | Sc. PD: | 0.18 |
| | | Sc. rank: | 187.9 |

,

PB1-F2

| Pos. | 43 | obs: | exp: |
|---|---|---|---|
| tta | L | 0 | 21.23 |
| ttg | L | 0 | 46.80 |
| ctt | L | 0 | 45.34 |
| ctc | L | 0 | 39.31 |
| cta | L | 0 | 36.61 |
| ctg | L | 243 | 53.72 |
| cct | P | 0 | 8.69 |
| ccc | P | 0 | 5.95 |
| cca | P | 0 | 13.02 |
| ccg | P | 33 | 5.34 |
| caa | Q | 0 | 41.05 |
| cag | Q | 77 | 35.95 |
| cgt | R | 0 | 0.33 |
| cgc | R | 0 | 0.39 |
| cga | R | 0 | 0.77 |
| cgg | R | 8 | 0.67 |
| aga | R | 0 | 3.60 |
| agg | R | 0 | 2.24 |
| --- | -- | ------ | ------ |
| mPD | | 0.49 | 1.5 |
| | | nPD: | 0.32 |
| | | N. weight: | 1.5 |
| | | Sc. PD: | 0.15 |
| | | Sc. rank: | 193.0 |

,

PB1-F2

| Pos. | 44 | obs: | exp: |
|---|---|---|---|
| cgt | R | 0 | 12.99 |
| cgc | R | 0 | 15.33 |
| cga | R | 0 | 30.08 |
| cgg | R | 0 | 26.12 |
| atg | M | 27 | 27.00 |
| aaa | K | 0 | 10.64 |
| aag | K | 19 | 8.36 |
| aga | R | 0 | 140.80 |
| agg | R | 313 | 87.72 |
| ggt | G | 0 | 0.25 |
| ggc | G | 0 | 0.27 |
| gga | G | 0 | 0.97 |
| ggg | G | 2 | 0.52 |
| --- | -- | ------ | ------ |
| mPD | | 0.24 | 1.2 |
| | | nPD: | 0.21 |
| | | N. weight: | 1.2 |
| | | Sc. PD: | -0.0063 |
| | | Sc. rank: | 15.7 |

,

PB1-F2

| Pos. | 45 | obs: | exp: |
|---|---|---|---|
| att | I | 0 | 24.81 |
| atc | I | 0 | 17.30 |
| ata | I | 66 | 23.89 |
| act | T | 0 | 77.94 |
| acc | T | 0 | 63.30 |
| aca | T | 295 | 129.20 |
| acg | T | 0 | 24.52 |
| --- | -- | ------ | ------ |
| mPD | | 0.30 | 0.98 |
| | | nPD: | 0.3 |
| | | N. weight: | 0.89 |
| | | Sc. PD: | 0.078 |
| | | Sc. rank: | 103.6 |

,

PB1-F2

| Pos. | 46 | obs: | exp: |
|---|---|---|---|
| atg | M | 144 | 144.00 |
| act | T | 0 | 57.33 |
| acc | T | 0 | 46.57 |
| aca | T | 0 | 95.07 |
| acg | T | 217 | 18.04 |
| --- | -- | ------ | ------ |
| mPD | | 0.48 | 1.2 |
| | | nPD: | 0.41 |
| | | N. weight: | 1.6 |
| | | Sc. PD: | 0.29 |
| | | Sc. rank: | 392.8 |

,

PB1-F2

| Pos. | 47 | obs: | exp: |
|---|---|---|---|
| tct | S | 0 | 37.52 |
| tcc | S | 0 | 32.88 |
| tca | S | 0 | 52.28 |
| tcg | S | 0 | 14.39 |
| att | I | 0 | 1.13 |
| atc | I | 3 | 0.79 |
| ata | I | 0 | 1.09 |
| act | T | 0 | 1.06 |
| acc | T | 4 | 0.86 |
| aca | T | 0 | 1.75 |
| acg | T | 0 | 0.33 |
| aat | N | 0 | 61.67 |
| aac | N | 118 | 56.33 |
| agt | S | 0 | 51.92 |
| agc | S | 236 | 47.02 |
| --- | -- | ------ | ------ |
| mPD | | 0.47 | 1.8 |
| | | nPD: | 0.26 |
| | | N. weight: | 1.4 |
| | | Sc. PD: | 0.064 |
| | | Sc. rank: | 100.9 |

,

PB1-F2

| Pos. | 48 | obs: | exp: |
|---|---|---|---|
| cct | P | 0 | 0.53 |
| ccc | P | 0 | 0.36 |
| cca | P | 2 | 0.79 |
| ccg | P | 0 | 0.32 |
| caa | Q | 324 | 172.70 |
| cag | Q | 0 | 151.30 |
| cgt | R | 0 | 1.45 |
| cgc | R | 0 | 1.71 |
| cga | R | 35 | 3.36 |
| cgg | R | 0 | 2.92 |
| aga | R | 0 | 15.74 |
| agg | R | 0 | 9.81 |
| --- | -- | ------ | ------ |
| mPD | | 0.19 | 0.82 |
| | | nPD: | 0.23 |
| | | N. weight: | 0.83 |
| | | Sc. PD: | 0.0095 |
| | | Sc. rank: | 19.8 |

,

PB1-F2

| Pos. | 49 | obs: | exp: |
|---|---|---|---|
| gtt | V | 0 | 30.21 |
| gtc | V | 0 | 30.85 |
| gta | V | 0 | 27.55 |
| gtg | V | 147 | 58.38 |
| gct | A | 0 | 53.90 |
| gcc | A | 0 | 43.84 |
| gca | A | 0 | 95.38 |
| gcg | A | 214 | 20.88 |
| --- | -- | ------ | ------ |
| mPD | | 0.48 | 1.2 |
| | | nPD: | 0.4 |
| | | N. weight: | 1.8 |
| | | Sc. PD: | 0.32 |
| | | Sc. rank: | 407.6 |

,

PB1-F2

| Pos. | 50 | obs: | exp: |
|---|---|---|---|
| gat | D | 270 | 144.10 |
| gac | D | 0 | 125.90 |
| ggt | G | 91 | 11.23 |
| ggc | G | 0 | 12.31 |
| gga | G | 0 | 44.02 |
| ggg | G | 0 | 23.45 |
| --- | -- | ------ | ------ |
| mPD | | 0.38 | 1.0 |
| | | nPD: | 0.37 |
| | | N. weight: | 1. |
| | | Sc. PD: | 0.15 |
| | | Sc. rank: | 206.6 |

,

PB1-F2

| Pos. | 51 | obs: | exp: |
|---|---|---|---|
| atg | M | 355 | 355.00 |
| act | T | 0 | 1.59 |
| acc | T | 0 | 1.29 |
| aca | T | 0 | 2.63 |
| acg | T | 6 | 0.50 |
| --- | -- | ------ | ------ |
| mPD | | 0.033 | 0.063 |
| | | nPD: | 0.52 |
| | | N. weight: | 0.043 |
| | | Sc. PD: | 0.013 |
| | | Sc. rank: | 12.0 |

,

PB1-F2

| Pos. | 52 | obs: | exp: |
|---|---|---|---|
| tta | L | 0 | 0.17 |
| ttg | L | 0 | 0.39 |
| ctt | L | 0 | 0.37 |
| ctc | L | 2 | 0.32 |
| cta | L | 0 | 0.30 |
| ctg | L | 0 | 0.44 |
| cct | P | 0 | 3.95 |
| ccc | P | 15 | 2.71 |
| cca | P | 0 | 5.92 |
| ccg | P | 0 | 2.43 |
| cat | H | 0 | 183.20 |
| cac | H | 338 | 154.80 |
| cgt | R | 0 | 0.25 |
| cgc | R | 6 | 0.29 |
| cga | R | 0 | 0.58 |
| cgg | R | 0 | 0.50 |
| aga | R | 0 | 2.70 |
| agg | R | 0 | 1.68 |
| --- | -- | ------ | ------ |
| mPD | | 0.12 | 0.69 |
| | | nPD: | 0.18 |
| | | N. weight: | 0.9 |
| | | Sc. PD: | -0.03 |
| | | Sc. rank: | -20.0 |

,

PB1-F2

| Pos. | 53 | obs: | exp: |
|---|---|---|---|
| cgt | R | 0 | 0.21 |
| cgc | R | 0 | 0.24 |
| cga | R | 0 | 0.48 |
| cgg | R | 0 | 0.42 |
| aaa | K | 356 | 199.30 |
| aag | K | 0 | 156.70 |
| aga | R | 5 | 2.25 |
| agg | R | 0 | 1.40 |
| --- | -- | ------ | ------ |
| mPD | | 0.027 | 0.53 |
| | | nPD: | 0.05 |
| | | N. weight: | 0.61 |
| | | Sc. PD: | -0.093 |
| | | Sc. rank: | -122.1 |

,

PB1-F2

| Pos. | 54 | obs: | exp: |
|---|---|---|---|
| tta | L | 0 | 0.26 |
| ttg | L | 0 | 0.58 |
| ctt | L | 0 | 0.56 |
| ctc | L | 0 | 0.49 |
| cta | L | 0 | 0.45 |
| ctg | L | 3 | 0.66 |
| caa | Q | 0 | 79.97 |
| cag | Q | 150 | 70.03 |
| cgt | R | 0 | 8.64 |
| cgc | R | 0 | 10.18 |
| cga | R | 0 | 19.99 |
| cgg | R | 208 | 17.36 |
| aga | R | 0 | 93.54 |
| agg | R | 0 | 58.29 |
| --- | -- | ------ | ------ |
| mPD | | 0.50 | 1.5 |
| | | nPD: | 0.32 |
| | | N. weight: | 1.8 |
| | | Sc. PD: | 0.19 |
| | | Sc. rank: | 264.7 |

,

PB1-F2

| Pos. | 55 | obs: | exp: |
|---|---|---|---|
| att | I | 261 | 98.12 |
| atc | I | 0 | 68.40 |
| ata | I | 0 | 94.48 |
| act | T | 100 | 26.42 |
| acc | T | 0 | 21.46 |
| aca | T | 0 | 43.81 |
| acg | T | 0 | 8.31 |
| --- | -- | ------ | ------ |
| mPD | | 0.40 | 1.1 |
| | | nPD: | 0.37 |
| | | N. weight: | 1.1 |
| | | Sc. PD: | 0.17 |
| | | Sc. rank: | 231.2 |

,

PB1-F2

| Pos. | 56 | obs: | exp: |
|---|---|---|---|
| gtt | V | 0 | 32.89 |
| gtc | V | 0 | 33.58 |
| gta | V | 1 | 29.99 |
| gtg | V | 159 | 63.55 |
| gct | A | 0 | 50.63 |
| gcc | A | 0 | 41.17 |
| gca | A | 0 | 89.59 |
| gcg | A | 201 | 19.61 |
| --- | -- | ------ | ------ |
| mPD | | 0.50 | 1.2 |
| | | nPD: | 0.41 |
| | | N. weight: | 1.8 |
| | | Sc. PD: | 0.32 |
| | | Sc. rank: | 418.6 |

,

PB1-F2

| Pos. | 57 | obs: | exp: |
|---|---|---|---|
| ttt | F | 40 | 17.27 |
| ttc | F | 2 | 24.73 |
| tct | S | 5 | 15.42 |
| tcc | S | 92 | 13.51 |
| tca | S | 0 | 21.49 |
| tcg | S | 0 | 5.91 |
| tat | Y | 42 | 22.26 |
| tac | Y | 1 | 20.74 |
| tgt | C | 172 | 73.79 |
| tgc | C | 7 | 105.20 |
| agt | S | 0 | 21.34 |
| agc | S | 0 | 19.33 |
| --- | -- | ------ | ------ |
| mPD | | 1.1 | 1.3 |
| | | nPD: | 0.79 |
| | | N. weight: | 1. |
| | | Sc. PD: | 0.56 |
| | | Sc. rank: | 304.1 |

,

PB1-F2

| Pos. | 58 | obs: | exp: |
|---|---|---|---|
| tta | L | 0 | 7.86 |
| ttg | L | 90 | 17.33 |
| tgg | W | 270 | 270.00 |
| ctt | L | 0 | 16.79 |
| ctc | L | 0 | 14.56 |
| cta | L | 0 | 13.56 |
| ctg | L | 0 | 19.89 |
| ggt | G | 0 | 0.12 |
| ggc | G | 0 | 0.14 |
| gga | G | 0 | 0.48 |
| ggg | G | 1 | 0.26 |
| --- | -- | ------ | ------ |
| mPD | | 0.38 | 0.94 |
| | | nPD: | 0.41 |
| | | N. weight: | 0.43 |
| | | Sc. PD: | 0.079 |
| | | Sc. rank: | 97.5 |

,

PB1-F2

| Pos. | 59 | obs: | exp: |
|---|---|---|---|
| cgt | R | 0 | 0.21 |
| cgc | R | 0 | 0.24 |
| cga | R | 0 | 0.48 |
| cgg | R | 0 | 0.42 |
| aaa | K | 0 | 199.30 |
| aag | K | 356 | 156.70 |
| aga | R | 0 | 2.25 |
| agg | R | 5 | 1.40 |
| --- | -- | ------ | ------ |
| mPD | | 0.027 | 0.53 |
| | | nPD: | 0.05 |
| | | N. weight: | 0.86 |
| | | Sc. PD: | -0.13 |
| | | Sc. rank: | -173.3 |

,

PB1-F2

| Pos. | 60 | obs: | exp: |
|---|---|---|---|
| tta | L | 0 | 1.31 |
| ttg | L | 0 | 2.89 |
| ctt | L | 0 | 2.80 |
| ctc | L | 0 | 2.43 |
| cta | L | 15 | 2.26 |
| ctg | L | 0 | 3.32 |
| caa | Q | 346 | 184.50 |
| cag | Q | 0 | 161.50 |
| --- | -- | ------ | ------ |
| mPD | | 0.080 | 0.62 |
| | | nPD: | 0.13 |
| | | N. weight: | 0.71 |
| | | Sc. PD: | -0.056 |
| | | Sc. rank: | -58.7 |

,

PB1-F2

| Pos. | 61 | obs: | exp: |
|---|---|---|---|
| tgg | W | 361 | 361.00 |
| --- | -- | ------ | ------ |
| mPD | | 0 | 0 |
| | | nPD: | 1. |
| | | N. weight: | 0. |
| | | Sc. PD: | 0 |
| | | Sc. rank: | 0 |

,

PB1-F2

| Pos. | 62 | obs: | exp: |
|---|---|---|---|
| tta | L | 0 | 29.44 |
| ttg | L | 0 | 64.90 |
| ctt | L | 337 | 62.88 |
| ctc | L | 0 | 54.51 |
| cta | L | 0 | 50.77 |
| ctg | L | 0 | 74.49 |
| cct | P | 22 | 5.79 |
| ccc | P | 0 | 3.97 |
| cca | P | 0 | 8.68 |
| ccg | P | 0 | 3.56 |
| cat | H | 1 | 0.54 |
| cac | H | 0 | 0.46 |
| cgt | R | 1 | 0.04 |
| cgc | R | 0 | 0.05 |
| cga | R | 0 | 0.10 |
| cgg | R | 0 | 0.08 |
| aga | R | 0 | 0.45 |
| agg | R | 0 | 0.28 |
| --- | -- | ------ | ------ |
| mPD | | 0.13 | 1.2 |
| | | nPD: | 0.1 |
| | | N. weight: | 1.7 |
| | | Sc. PD: | -0.18 |
| | | Sc. rank: | -181.5 |

,

PB1-F2

| Pos. | 63 | obs: | exp: |
|---|---|---|---|
| ttt | F | 0 | 1.64 |
| ttc | F | 4 | 2.36 |
| tct | S | 0 | 53.73 |
| tcc | S | 338 | 47.09 |
| tca | S | 0 | 74.88 |
| tcg | S | 0 | 20.60 |
| tat | Y | 0 | 2.59 |
| tac | Y | 5 | 2.41 |
| act | T | 0 | 3.70 |
| acc | T | 14 | 3.00 |
| aca | T | 0 | 6.13 |
| acg | T | 0 | 1.16 |
| agt | S | 0 | 74.36 |
| agc | S | 0 | 67.34 |
| --- | -- | ------ | ------ |
| mPD | | 0.12 | 1.7 |
| | | nPD: | 0.07 |
| | | N. weight: | 2. |
| | | Sc. PD: | -0.26 |
| | | Sc. rank: | -300.4 |

,

PB1-F2

| Pos. | 64 | obs: | exp: |
|---|---|---|---|
| tta | L | 0 | 31.54 |
| ttg | L | 361 | 69.53 |
| ctt | L | 0 | 67.36 |
| ctc | L | 0 | 58.40 |
| cta | L | 0 | 54.38 |
| ctg | L | 0 | 79.80 |
| --- | -- | ------ | ------ |
| mPD | | 0 | 1.1 |
| | | nPD: | 0. |
| | | N. weight: | 1.7 |
| | | Sc. PD: | -0.35 |
| | | Sc. rank: | -501.9 |

PB1-F2

| Pos. | 65 | obs: | exp: |
|---|---|---|---|
| cgt | R | 0 | 0.75 |
| cgc | R | 0 | 0.88 |
| cga | R | 0 | 1.73 |
| cgg | R | 0 | 1.50 |
| aaa | K | 0 | 192.10 |
| aag | K | 343 | 150.90 |
| aga | R | 0 | 8.10 |
| agg | R | 18 | 5.04 |
| --- | -- | ------ | ------ |
| mPD | | 0.095 | 0.62 |
| | | nPD: | 0.15 |
| | | N. weight: | 0.88 |
| | | Sc. PD: | -0.049 |
| | | Sc. rank: | -37.1 |

PB1-F2

| Pos. | 66 | obs: | exp: |
|---|---|---|---|
| tct | S | 0 | 25.60 |
| tcc | S | 0 | 22.43 |
| tca | S | 0 | 35.67 |
| tcg | S | 0 | 9.81 |
| cgt | R | 0 | 0.08 |
| cgc | R | 0 | 0.10 |
| cga | R | 0 | 0.19 |
| cgg | R | 0 | 0.17 |
| aat | N | 197 | 103.00 |
| aac | N | 0 | 94.04 |
| aaa | K | 0 | 0.56 |
| aag | K | 1 | 0.44 |
| agt | S | 161 | 35.42 |
| agc | S | 0 | 32.08 |
| aga | R | 2 | 0.90 |
| agg | R | 0 | 0.56 |
| --- | -- | ------ | ------ |
| mPD | | 0.51 | 1.6 |
| | | nPD: | 0.32 |
| | | N. weight: | 1.1 |
| | | Sc. PD: | 0.11 |
| | | Sc. rank: | 132.5 |

PB1-F2

| Pos. | 67 | obs: | exp: |
|---|---|---|---|
| tta | L | 0 | 2.18 |
| ttg | L | 0 | 4.81 |
| ctt | L | 0 | 4.66 |
| ctc | L | 25 | 4.04 |
| cta | L | 0 | 3.77 |
| ctg | L | 0 | 5.53 |
| cct | P | 0 | 88.48 |
| ccc | P | 336 | 60.61 |
| cca | P | 0 | 132.60 |
| ccg | P | 0 | 54.33 |
| --- | -- | ------ | ------ |
| mPD | | 0.13 | 0.89 |
| | | nPD: | 0.15 |
| | | N. weight: | 1.8 |
| | | Sc. PD: | -0.11 |
| | | Sc. rank: | -96.5 |

PB1-F2

| Pos. | 68 | obs: | exp: |
|---|---|---|---|
| att | I | 0 | 3.76 |
| atc | I | 10 | 2.62 |
| ata | I | 0 | 3.62 |
| act | T | 1 | 92.73 |
| acc | T | 350 | 75.32 |
| aca | T | 0 | 153.80 |
| acg | T | 0 | 29.18 |
| --- | -- | ------ | ------ |
| mPD | | 0.060 | 0.74 |
| | | nPD: | 0.08 |
| | | N. weight: | 1.6 |
| | | Sc. PD: | -0.2 |
| | | Sc. rank: | -220.8 |

PB1-F2

| Pos. | 69 | obs: | exp: |
|---|---|---|---|
| caa | Q | 0 | 187.10 |
| cag | Q | 351 | 163.90 |
| cgt | R | 0 | 0.42 |
| cgc | R | 0 | 0.49 |
| cga | R | 0 | 0.96 |
| cgg | R | 10 | 0.83 |
| aga | R | 0 | 4.50 |
| agg | R | 0 | 2.80 |
| --- | -- | ------ | ------ |
| mPD | | 0.054 | 0.59 |
| | | nPD: | 0.09 |
| | | N. weight: | 0.84 |
| | | Sc. PD: | -0.097 |
| | | Sc. rank: | -98.1 |

PB1-F2

| Pos. | 70 | obs: | exp: |
|---|---|---|---|
| gaa | E | 97 | 55.82 |
| gag | E | 0 | 41.18 |
| ggt | G | 0 | 32.57 |
| ggc | G | 0 | 35.70 |
| gga | G | 264 | 127.70 |
| ggg | G | 0 | 68.02 |
| --- | -- | ------ | ------ |
| mPD | | 0.39 | 1.0 |
| | | nPD: | 0.38 |
| | | N. weight: | 0.71 |
| | | Sc. PD: | 0.12 |
| | | Sc. rank: | 149.3 |

PB1-F2

| Pos. | 71 | obs: | exp: |
|---|---|---|---|
| tct | S | 356 | 56.60 |
| tcc | S | 0 | 49.59 |
| tca | S | 0 | 78.87 |
| tcg | S | 0 | 21.70 |
| tat | Y | 5 | 2.59 |
| tac | Y | 0 | 2.41 |
| agt | S | 0 | 78.32 |
| agc | S | 0 | 70.93 |
| --- | -- | ------ | ------ |
| mPD | | 0.027 | 1.7 |
| | | nPD: | 0.02 |
| | | N. weight: | 1.9 |
| | | Sc. PD: | -0.35 |
| | | Sc. rank: | -445.7 |

PB1-F2

| Pos. | 72 | obs: | exp: |
|---|---|---|---|
| tta | L | 0 | 31.54 |
| ttg | L | 361 | 69.53 |
| ctt | L | 0 | 67.36 |
| ctc | L | 0 | 58.40 |
| cta | L | 0 | 54.38 |
| ctg | L | 0 | 79.80 |
| --- | -- | ------ | ------ |
| mPD | | 0 | 1.1 |
| | | nPD: | 0. |
| | | N. weight: | 1.7 |
| | | Sc. PD: | -0.35 |
| | | Sc. rank: | -501.9 |

PB1-F2

| Pos. | 73 | obs: | exp: |
|---|---|---|---|
| cgt | R | 0 | 0.04 |
| cgc | R | 0 | 0.05 |
| cga | R | 0 | 0.10 |
| cgg | R | 0 | 0.08 |
| aaa | K | 360 | 201.60 |
| aag | K | 0 | 158.40 |
| aga | R | 1 | 0.45 |
| agg | R | 0 | 0.28 |
| --- | -- | ------ | ------ |
| mPD | | 0.0055 | 0.50 |
| | | nPD: | 0.01 |
| | | N. weight: | 0.6 |
| | | Sc. PD: | -0.12 |
| | | Sc. rank: | -158.4 |

PB1-F2

| Pos. | 74 | obs: | exp: |
|---|---|---|---|
| att | I | 2 | 0.75 |
| atc | I | 0 | 0.52 |
| ata | I | 0 | 0.72 |
| act | T | 359 | 94.84 |
| acc | T | 0 | 77.04 |
| aca | T | 0 | 157.30 |
| acg | T | 0 | 29.84 |
| --- | -- | ------ | ------ |
| mPD | | 0.011 | 0.70 |
| | | nPD: | 0.02 |
| | | N. weight: | 1.4 |
| | | Sc. PD: | -0.26 |
| | | Sc. rank: | -352.5 |

PB1-F2

| Pos. | 75 | obs: | exp: |
|---|---|---|---|
| tta | L | 0 | 16.07 |
| ttg | L | 0 | 35.44 |
| ctt | L | 184 | 34.33 |
| ctc | L | 0 | 29.76 |
| cta | L | 0 | 27.72 |
| ctg | L | 0 | 40.67 |
| cat | H | 80 | 43.35 |
| cac | H | 0 | 36.65 |
| cgt | R | 97 | 4.03 |
| cgc | R | 0 | 4.75 |
| cga | R | 0 | 9.32 |
| cgg | R | 0 | 8.09 |
| aga | R | 0 | 43.62 |
| agg | R | 0 | 27.18 |
| --- | -- | ------ | ------ |
| mPD | | 0.62 | 1.9 |
| | | nPD: | 0.33 |
| | | N. weight: | 1.9 |
| | | Sc. PD: | 0.22 |
| | | Sc. rank: | 316.9 |

PB1-F2

| Pos. | 76 | obs: | exp: |
|---|---|---|---|
| gtt | V | 0 | 74.20 |
| gtc | V | 361 | 75.77 |
| gta | V | 0 | 67.66 |
| gtg | V | 0 | 143.40 |
| --- | -- | ------ | ------ |
| mPD | | 0 | 0.72 |
| | | nPD: | 0. |
| | | N. weight: | 1.6 |
| | | Sc. PD: | -0.33 |
| | | Sc. rank: | -475.7 |

PB1-F2

| Pos. | 77 | obs: | exp: |
|---|---|---|---|
| tta | L | 0 | 28.83 |
| ttg | L | 330 | 63.56 |
| tct | S | 0 | 4.93 |
| tcc | S | 0 | 4.32 |
| tca | S | 0 | 6.87 |
| tcg | S | 31 | 1.89 |
| ctt | L | 0 | 61.57 |
| ctc | L | 0 | 53.38 |
| cta | L | 0 | 49.71 |
| ctg | L | 0 | 72.95 |
| agt | S | 0 | 6.82 |
| agc | S | 0 | 6.18 |
| --- | -- | ------ | ------ |
| mPD | | 0.16 | 1.4 |
| | | nPD: | 0.12 |
| | | N. weight: | 1.8 |
| | | Sc. PD: | -0.17 |
| | | Sc. rank: | -169.2 |

PB1-F2

| Pos. | 78 | obs: | exp: |
|---|---|---|---|
| cgt | R | 0 | 1.12 |
| cgc | R | 0 | 1.32 |
| cga | R | 0 | 2.59 |
| cgg | R | 0 | 2.25 |
| aaa | K | 334 | 187.00 |
| aag | K | 0 | 147.00 |
| aga | R | 27 | 12.14 |
| agg | R | 0 | 7.57 |
| --- | -- | ------ | ------ |
| mPD | | 0.14 | 0.68 |
| | | nPD: | 0.21 |
| | | N. weight: | 0.62 |
| | | Sc. PD: | -0.0046 |
| | | Sc. rank: | 3.2 |

PB1-F2

| Pos. | 79 | obs: | exp: |
|---|---|---|---|
| tta | L | 0 | 0.17 |
| ttg | L | 0 | 0.39 |
| ctt | L | 0 | 0.37 |
| ctc | L | 0 | 0.32 |
| cta | L | 2 | 0.30 |
| ctg | L | 0 | 0.44 |
| caa | Q | 3 | 1.60 |
| cag | Q | 0 | 1.40 |
| cgt | R | 0 | 14.78 |
| cgc | R | 0 | 17.43 |
| cga | R | 356 | 34.21 |
| cgg | R | 0 | 29.71 |
| aga | R | 0 | 160.10 |
| agg | R | 0 | 99.77 |
| --- | -- | ------ | ------ |
| mPD | | 0.027 | 1.0 |
| | | nPD: | 0.03 |
| | | N. weight: | 2.4 |
| | | Sc. PD: | -0.43 |
| | | Sc. rank: | -520.8 |

PB1-F2

| Pos. | 80 | obs: | exp: |
|---|---|---|---|
| tgg | W | 360 | 360.00 |
| --- | - | 1 | 1.00 |
| --- | -- | ------ | ------ |
| mPD | | 0 | 0 |
| | | nPD: | 1. |
| | | N. weight: | 0. |
| | | Sc. PD: | 0 |
| | | Sc. rank: | 0 |

PB1-F2

| Pos. | 81 | obs: | exp: |
|---|---|---|---|
| cgt | R | 0 | 0.91 |
| cgc | R | 0 | 1.08 |
| cga | R | 0 | 2.11 |
| cgg | R | 0 | 1.84 |
| aat | N | 5 | 2.61 |
| aac | N | 0 | 2.39 |
| aaa | K | 42 | 186.50 |
| aag | K | 291 | 146.50 |
| aga | R | 3 | 9.89 |
| agg | R | 19 | 6.17 |
| --- | - | 1 | 1.00 |
| --- | -- | ------ | ------ |
| mPD | | 0.36 | 0.66 |
| | | nPD: | 0.54 |
| | | N. weight: | 0.47 |
| | | Sc. PD: | 0.15 |
| | | Sc. rank: | 132.3 |

PB1-F2

| Pos. | 82 | obs: | exp: |
|---|---|---|---|
| tta | L | 1 | 18.52 |
| ttg | L | 211 | 40.83 |
| tct | S | 0 | 23.53 |
| tcc | S | 0 | 20.62 |
| tca | S | 1 | 32.79 |
| tcg | S | 147 | 9.02 |
| ctt | L | 0 | 39.56 |
| ctc | L | 0 | 34.29 |
| cta | L | 0 | 31.94 |
| ctg | L | 0 | 46.86 |
| agt | S | 0 | 32.56 |
| agc | S | 0 | 29.49 |
| --- | - | 1 | 1.00 |
| --- | -- | ------ | ------ |
| mPD | | 0.49 | 1.9 |
| | | nPD: | 0.26 |
| | | N. weight: | 2.2 |
| | | Sc. PD: | 0.088 |
| | | Sc. rank: | 126.9 |

,

PB1-F2

| Pos. | 83 | obs: | exp: |
|---|---|---|---|
| ttt | F | 0 | 145.90 |
| ttc | F | 355 | 209.10 |
| tct | S | 0 | 0.79 |
| tcc | S | 5 | 0.70 |
| tca | S | 0 | 1.11 |
| tcg | S | 0 | 0.30 |
| agt | S | 0 | 1.10 |
| agc | S | 0 | 1.00 |
| --- | - | 1 | 1.00 |
| --- | -- | ------ | ------ |
| mPD | | 0.027 | 0.52 |
| | | nPD: | 0.05 |
| | | N. weight: | 0.57 |
| | | Sc. PD: | -0.087 |
| | | Sc. rank: | -109.9 |

,

PB1-F2

| Pos. | 84 | obs: | exp: |
|---|---|---|---|
| tct | S | 0 | 40.86 |
| tcc | S | 0 | 35.80 |
| tca | S | 0 | 56.94 |
| tcg | S | 0 | 15.67 |
| aat | N | 0 | 53.83 |
| aac | N | 103 | 49.17 |
| agt | S | 0 | 56.54 |
| agc | S | 257 | 51.20 |
| --- | - | 1 | 1.00 |
| --- | -- | ------ | ------ |
| mPD | | 0.41 | 1.8 |
| | | nPD: | 0.23 |
| | | N. weight: | 1.4 |
| | | Sc. PD: | 0.02 |
| | | Sc. rank: | 46.9 |

,

PB1-F2

| Pos. | 85 | obs: | exp: |
|---|---|---|---|
| cgt | R | 0 | 0.25 |
| cgc | R | 0 | 0.29 |
| cga | R | 0 | 0.58 |
| cgg | R | 0 | 0.50 |
| aaa | K | 354 | 198.20 |
| aag | K | 0 | 155.80 |
| aga | R | 6 | 2.70 |
| agg | R | 0 | 1.68 |
| --- | - | 1 | 1.00 |
| --- | -- | ------ | ------ |
| mPD | | 0.033 | 0.53 |
| | | nPD: | 0.06 |
| | | N. weight: | 0.6 |
| | | Sc. PD: | -0.087 |
| | | Sc. rank: | -104.6 |

,

PB1-F2

| Pos. | 86 | obs: | exp: |
|---|---|---|---|
| caa | Q | 354 | 188.70 |
| cag | Q | 0 | 165.30 |
| cgt | R | 0 | 0.25 |
| cgc | R | 0 | 0.29 |
| cga | R | 6 | 0.58 |
| cgg | R | 0 | 0.50 |
| aga | R | 0 | 2.70 |
| agg | R | 0 | 1.68 |
| --- | - | 1 | 1.00 |
| --- | -- | ------ | ------ |
| mPD | | 0.033 | 0.55 |
| | | nPD: | 0.06 |
| | | N. weight: | 0.68 |
| | | Sc. PD: | -0.1 |
| | | Sc. rank: | -120.5 |

,

PB1-F2

| Pos. | 87 | obs: | exp: |
|---|---|---|---|
| gaa | E | 0 | 195.60 |
| gag | E | 340 | 144.40 |
| ggt | G | 0 | 1.60 |
| ggc | G | 0 | 1.76 |
| gga | G | 1 | 6.29 |
| ggg | G | 12 | 3.35 |
| --- | - | 8 | 8.00 |
| --- | -- | ------ | ------ |
| mPD | | 0.073 | 0.54 |
| | | nPD: | 0.14 |
| | | N. weight: | 0.88 |
| | | Sc. PD: | -0.065 |
| | | Sc. rank: | -57.9 |

,

PB1-F2

| Pos. | 88 | obs: | exp: |
|---|---|---|---|
| tta | L | 0 | 0.17 |
| ttg | L | 2 | 0.39 |
| tgg | W | 344 | 344.00 |
| ctt | L | 0 | 0.37 |
| ctc | L | 0 | 0.32 |
| cta | L | 0 | 0.30 |
| ctg | L | 0 | 0.44 |
| --- | - | 15 | 15.00 |
| --- | -- | ------ | ------ |
| mPD | | 0.011 | 0.024 |
| | | nPD: | 0.43 |
| | | N. weight: | 0.0095 |
| | | Sc. PD: | 0.002 |
| | | Sc. rank: | 2.6 |

,

PB1-F2

| Pos. | 89 | obs: | exp: |
|---|---|---|---|
| att | I | 0 | 18.04 |
| atc | I | 0 | 12.58 |
| ata | I | 48 | 17.38 |
| act | T | 0 | 78.73 |
| acc | T | 0 | 63.95 |
| aca | T | 298 | 130.60 |
| acg | T | 0 | 24.77 |
| --- | - | 15 | 15.00 |
| --- | -- | ------ | ------ |
| mPD | | 0.22 | 0.85 |
| | | nPD: | 0.26 |
| | | N. weight: | 0.85 |
| | | Sc. PD: | 0.037 |
| | | Sc. rank: | 54.5 |

,

PB1-F2

| Pos. | 90 | obs: | exp: |
|---|---|---|---|
| tct | S | 0 | 2.23 |
| tcc | S | 0 | 1.95 |
| tca | S | 0 | 3.10 |
| tcg | S | 0 | 0.85 |
| aat | N | 73 | 173.50 |
| aac | N | 259 | 158.50 |
| agt | S | 7 | 3.08 |
| agc | S | 7 | 2.79 |
| gaa | E | 0 | 4.03 |
| gag | E | 7 | 2.97 |
| --- | - | 8 | 8.00 |
| --- | -- | ------ | ------ |
| mPD | | 0.48 | 0.66 |
| | | nPD: | 0.72 |
| | | N. weight: | 0.25 |
| | | Sc. PD: | 0.12 |
| | | Sc. rank: | 71.9 |

,

PB1-F2

| Pos. | 91 | obs: | exp: |
|---|---|---|---|
| taa | . | 182 | 169.10 |
| tag | . | 15 | 93.42 |
| tga | . | 164 | 98.51 |
| --- | -- | ------ | ------ |
| mPD | | 0.58 | 0.78 |
| | | nPD: | 0.74 |
| | | N. weight: | 0.22 |
| | | Sc. PD: | 0.11 |
| | | Sc. rank: | 62.5 |

}

Amino acid level conservation per codon

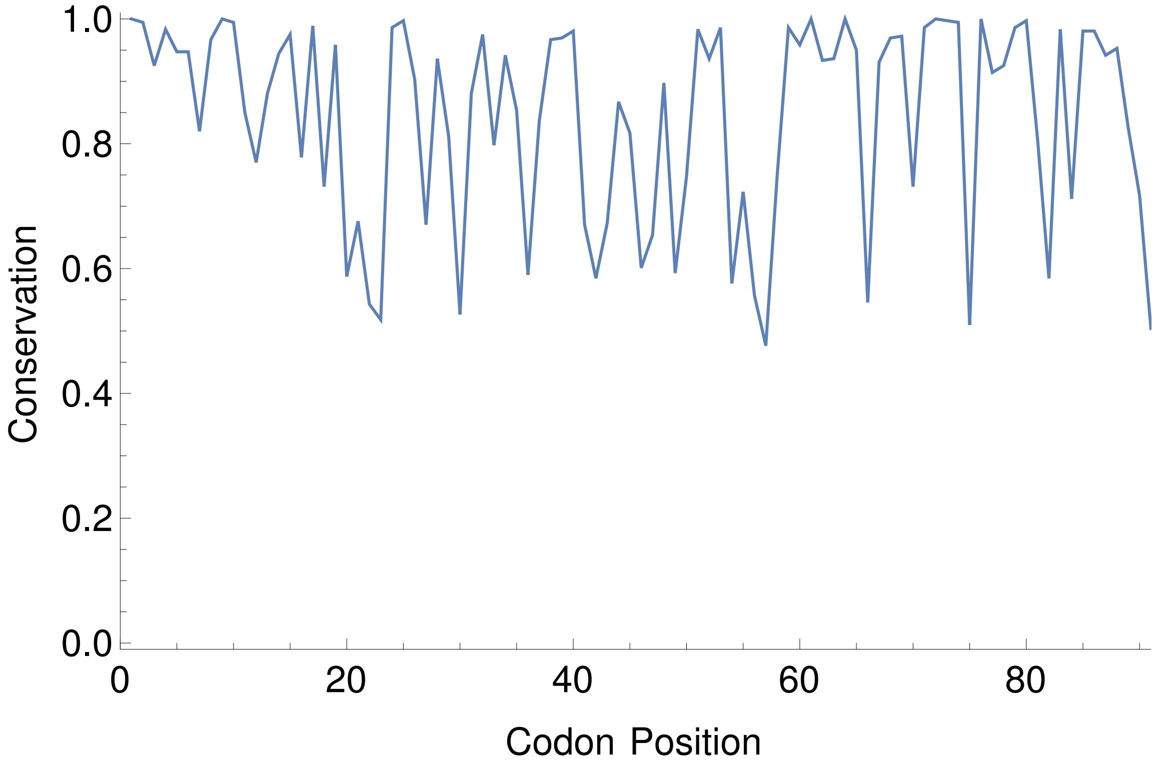

Conservation analysis plots

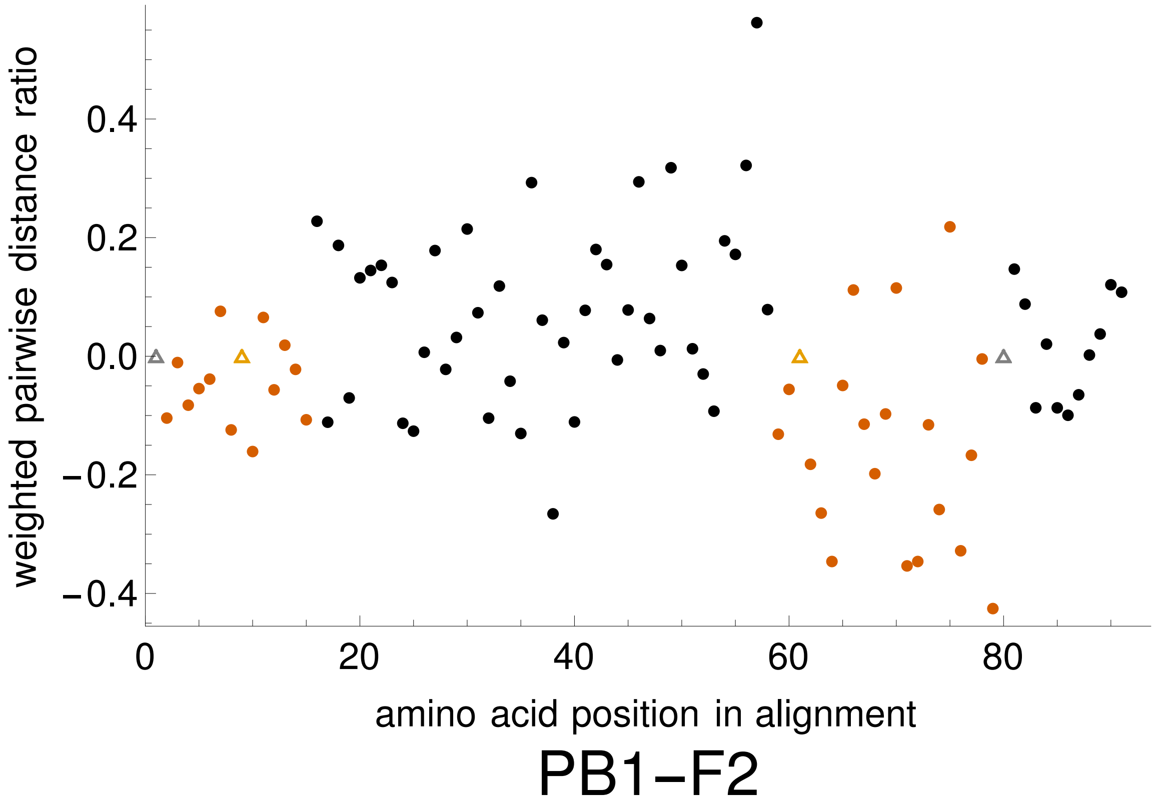


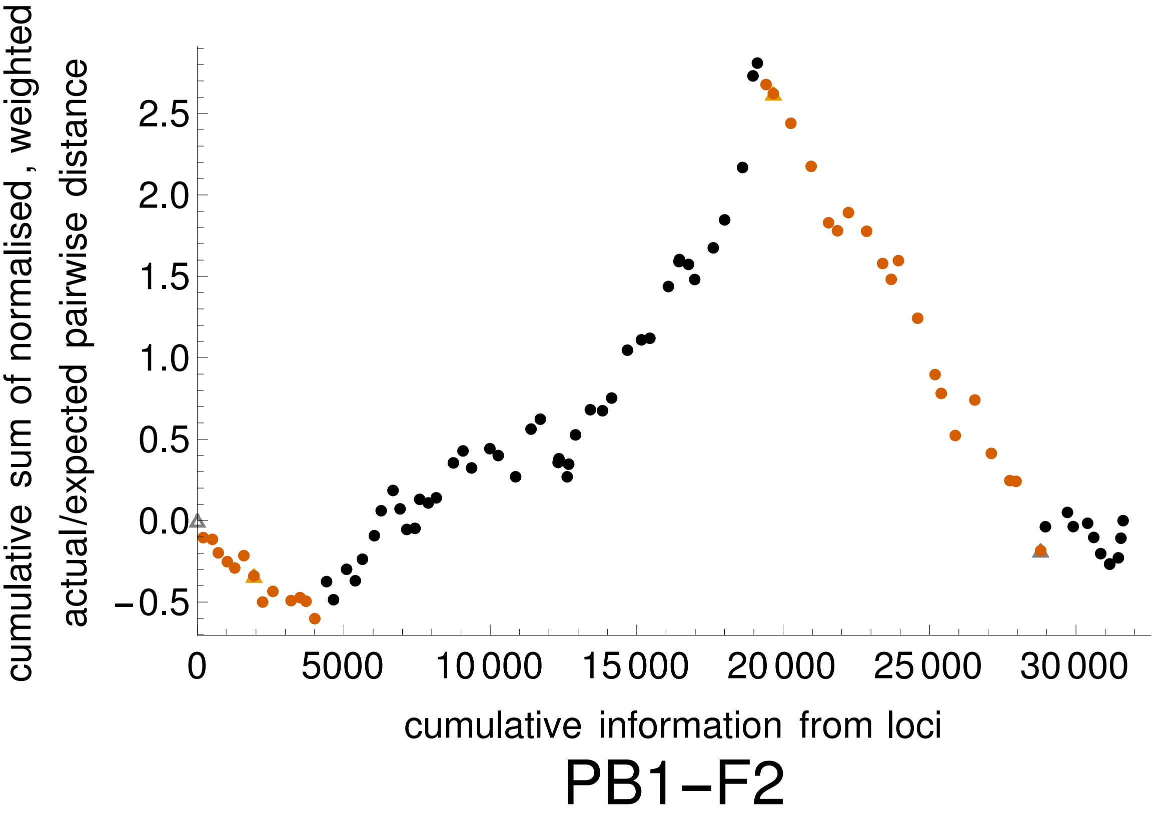

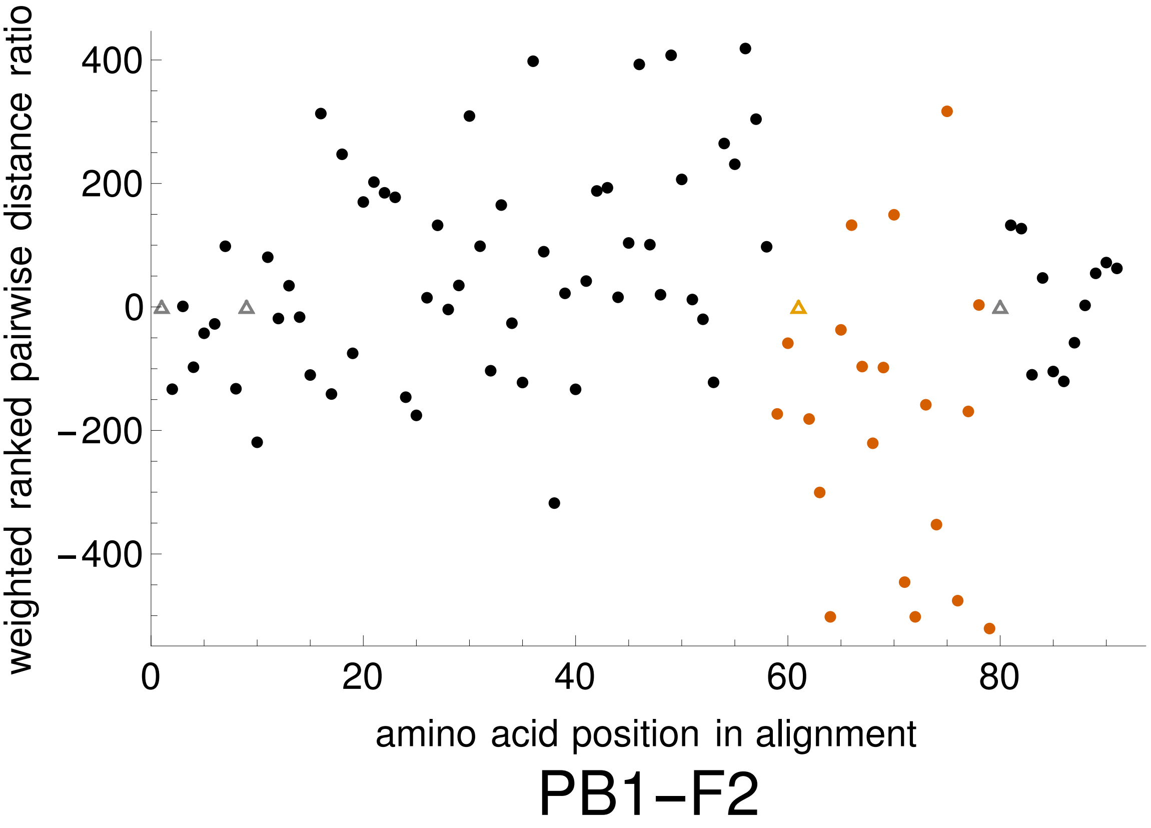
weighted ranked pairwise distance ratio
400
200
0
−200
−400
0
20
40
60
80
amino acid position in alignment
PB1−F2

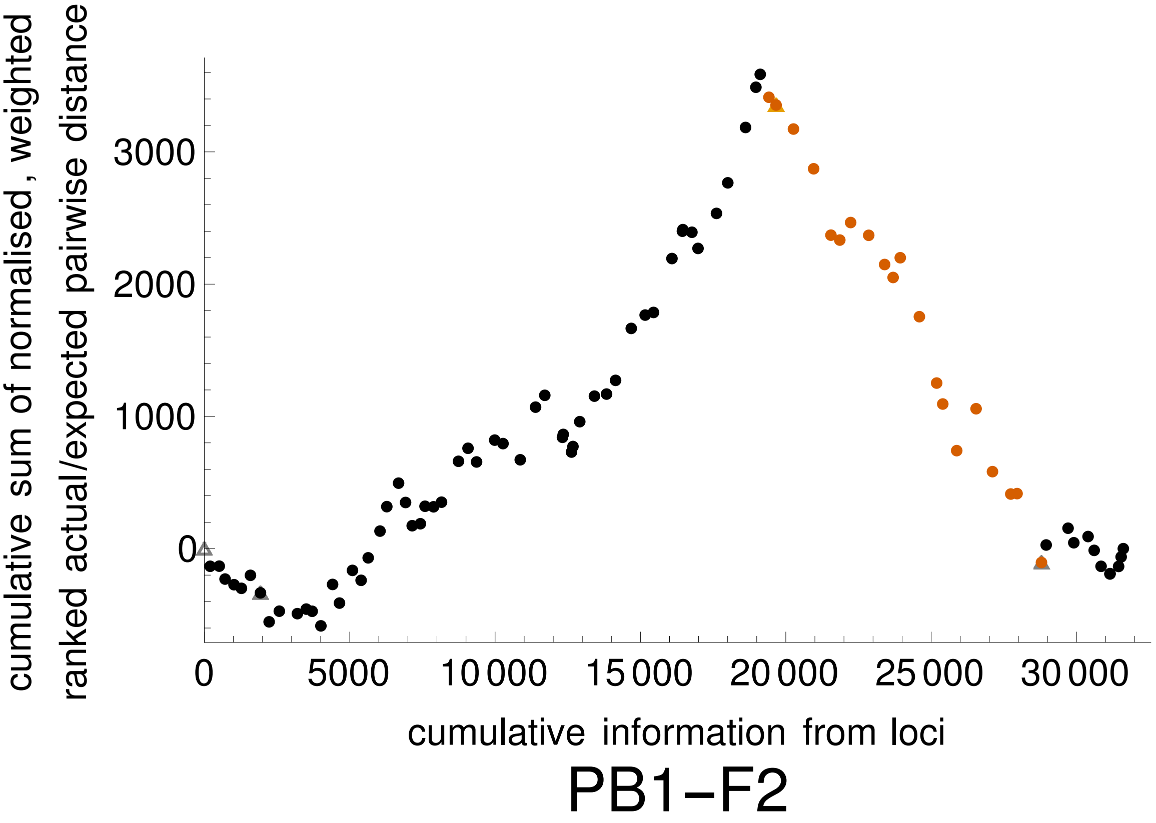
cumulative sum of normalised, weighted
ranked actual/expected pairwise distance
3000
2000
1000
0
0
5000
10 000
15 000
20 000
25 000
30 000
cumulative information from loci
PB1−F2

# PA

## Gene length histogram

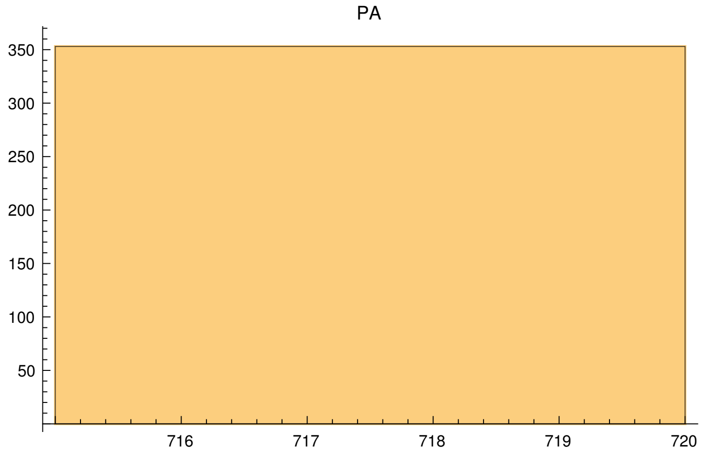

## Information vs. nPD

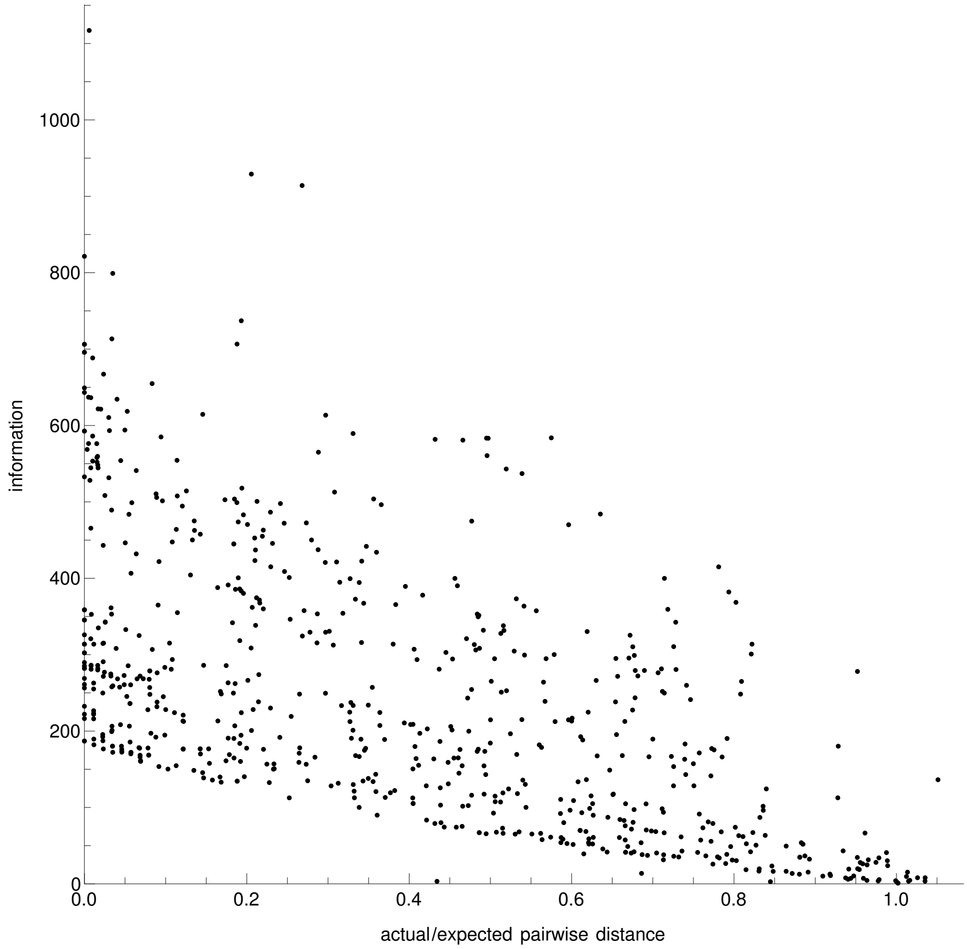


PA

## Example sequences highlighted by regions found in analyses to be conserved

Interesting points (by weighted raw PD) highlighted for gene PA:

ATGGAAGAT
**TTTGTGCGACAATGCTTCAATCCAATGATTGTCGAGCTTGCGGAAAAGGCAATGA AAGAATATGGGGAA**GATCCGAAAATCGAAACGAACAAATTTGCCGCAATATGCACACACTTAGAGG⋱
TCTGTTTCATGTATTCAGATTTCCACTTTATTGATGAACGGGGCGAGTCAATAATTGTGGAATCTGGCGA⋱
TCCAAATGCATTATTGAAACACCGATTTGAAATAATTGAAGGGAGAGACCGAACGATGGCTTGGACAGTG⋱
GTGAATAGCATCTGCAACACCACAGGAGTCGAGAAGCCCAAGTTCCTCCCAGATTTGTATGACTACAAAG⋱
AGAATCGATTCATTGAAATTGGAGTAACACGGAGGGAAGTTCATATATATTATCTAGAAAAGGCCAACAA⋱
GATAAAATCAGAGAAAACACACATTCACATATTCTCATTCACTGGAGAGGAAATGGCCACCAAAGCGGAC⋱
TACACTCTTGACGAAGAGAGTAGGGCAAGGATCAAAACCAGGCTGTTCACTATAAGGCAGGAAATGGCCA⋱
GTAGGGGCCTATGG
**GATTCCTTTCGCCAGTCCGAGAGAGGCGAAGAGACAGTTGAAGAAAGATTTGAAA TCACAGGAACCATGCGCAGGCTTGCCGACCAAAGTCTCCCACCGAACTTCTC CAGCCTTGAAAACTTTAGAGCCTATGTGGATGGATTCGAACCGAACGGCTGC ATTGAGGGCAAGCTTTCT**CAAATGTCAAAAGAAGTGAACGCCCGAATTGAGCCATTTCTAAAGA⋱
CAACACCACGCCCTCTCAAACTACCTGATGGGCCTCCCTGCTCTCAACGGTCGAAGTTCTTGCTGATGGA⋱
TGCCCTTAAATTAAGCATCGAAGACCCGAGTCATGAGGGGGAGGGTATACCACTATATGATGCAATCAAA⋱
TGCATGAAGACATTTTTCGGCTGGAAAGAGCCCAACATTGTAAAACCACATGCAAGGGGCATAAACCCCA⋱
ATTATCTCCTGGCTTGGAAGCAGGTGCTGGCAGAACTCCAAGATATTGAAAATGAGGAGAAAATCCCAAA⋱
AACAAAGAACATGAAGAAAACGAGTCAGTTGAAGTGGGCACTTGGTGAGAATATGGCACCAGAGAAAGTA⋱
GACTTTGGGGATTGCAAAGATGTTAGCGATCTGAGACAATATGACAGTGATGAACCAGAGTCCAGATCGC⋱
TAGCAAGCTGGATCCAGAGTGAATTCAACAAGGCATGTGAATTGACAGATTCAAGTTGGATTGAGCTTGA⋱
TGAAATAGGAGAAGATATTGCTCCAATTGAGCACATTGCGAGCATGAGAAGGAACTATTTCACAGCGGAA⋱
GTATCCCATTGCAGAGCTACTGAATACATAATGAAGGGAGTGTACATAAACACAGCTTTGTTGAATGCTT⋱
CCTGTGCAGCCATGGATGACTTCCAACTGATTCCAATGATAAGCAAATGCAGGACCAAAGAAGGGAGGCG⋱
GAAGACTAATCTGTACGGATTCATTGTAAAAGGGAGGTCCCATTTGAGAAATGACACCGATGTGGTAAAC⋱
TTTGTGAGCATGGAATTCTCTCTTACTGACCCGAGGTTGGAGCCACACAAGTGGGAAAAGTACTGTGTTC⋱
TCGAGGTAGGAGACATGCTCCTACGGACTGCAATAGGCCAAGTGTCAAGGCCCATGTTCCTGTATGTGAG⋱
AACTAATGGAACTTCCAAGATCAAAATGAAGTGGGGAATGGAAATGAGGCGATGCCTCCTTCAATCCCTC⋱
CAACAGATTGAGAGTATGATTGAAGCAGAGTCTTCTGTCAAAGAGAAAGACATGACCAAAGAATTCTTTG⋱
AAAACAAATCAGAAACATGGCCAATTGGAGAGTCACCCAAAGGGGTGGAGGAAGGCTCCATTGGGAAGGT⋱
GTGCAGAACCTTACTGGCAAAATCTGTGTTCAACAGCCTATATGCATCTCCACAACTCGAGGGATTTTCA⋱
GCTGAATCAAGAAAATTGCTT
**CTCATTGTTCAGGCACTTAGGGACAACCTGGAACCTGGGACCTTCGATCTCGGGG GGCTATATGAAGCAATTGAGGAGTGCCTGATTAATGATCCCTGGGTTTTGCT TAATGCATCTTGGTTCAACTCCTTCCTCACACATGCACTGAAATAG**

Interesting points (by weighted ranked PD) highlighted for gene PA:

ATG
**GAAGATTTTGTGCGACAATGCTTCAATCCAATGATTGTCGAGCTTGCGGAAAAGG CAATGAAAGAATATGGG**GAAGATCCGAAAATCGAAACGAACAAATTTGCCGCAATATGCACACAC⋱
TTAGAGGTCTGTTTCATGTATTCAGATTTCCACTTTATTGATGAACGGGGCGAGTCAATAATTGTGGAAT⋱
CTGGCGATCCAAATGCATTATTGAAACACCGATTTGAAATAATTGAAGGGAGAGACCGAACGATGGCTTG⋱
GACAGTGGTGAATAGCATCTGCAACACCACAGGAGTCGAGAAGCCCAAGTTCCTCCCAGATTTGTATGAC⋱

TACAAAGAGAATCGATTCATTGAAATTGGAGTAACACGGAGGGAAGTTCATATATATTATCTAGAAAAGG⋱
CCAACAAGATAAAATCAGAGAAAACACACATTCACATATTCTCATTCACTGGAGAGGAAATGGCCACCAA⋱
AGCGGACTACACTCTTGACGAAGAGAGTAGGGCAAGGATCAAAACCAGGCTGTTCACTATAAGGCAGGAA⋱
ATGGCCAGTAGGGGCCTATGGGAT
**TCCTTTCGCCAGTCCGAGAGAGGCGAAGAGACAGTTGAAGAAAGATTTGAAATCA**
**CAGGAACCATGCGCAGGCTTGCCGACCAAAGTCTCCCACCGAACTTCTCCAG**
**CCTTGAAAACTTTAGAGCCTATGTGGATGGATTCGAACCGAACGGCTGCATT**
**GAGGGCAAGCTTTCT**CAAATGTCAAAAGAAGTGAACGCCCGAATTGAGCCATTTCTAAAGACAACA⋱
CCACGCCCTCTCAAACTACCTGATGGGCCTCCCTGCTCTCAACGGTCGAAGTTCTTGCTGATGGATGCCC⋱
TTAAATTAAGCATCGAAGACCCGAGTCATGAGGGGGAGGGTATACCACTATATGATGCAATCAAATGCAT⋱
GAAGACATTTTTCGGCTGGAAAGAGCCCAACATTGTAAAACCACATGCAAGGGGCATAAACCCCAATTAT⋱
CTCCTGGCTTGGAAGCAGGTGCTGGCAGAACTCCAAGATATTGAAAATGAGGAGAAAATCCCAAAAACAA⋱
AGAACATGAAGAAAACGAGTCAGTTGAAGTGGGCACTTGGTGAGAATATGGCACCAGAGAAAGTAGACTT⋱
TGGGGATTGCAAAGATGTTAGCGATCTGAGACAATATGACAGTGATGAACCAGAGTCCAGATCGCTAGCA⋱
AGCTGGATCCAGAGTGAATTCAACAAGGCATGTGAATTGACAGATTCAAGTTGGATTGAGCTTGATGAAA⋱
TAGGAGAAGATATTGCTCCAATTGAGCACATTGCGAGCATGAGAAGGAACTATTTCACAGCGGAAGTATC⋱
CCATTGCAGAGCTACTGAATACATAATGAAGGGAGTGTACATAAACACAGCTTTGTTGAATGCTTCCTGT⋱
GCAGCCATGGATGACTTCCAACTGATTCCAATGATAAGCAAATGCAGGACCAAAGAAGGGAGGCGGAAGA⋱
CTAATCTGTACGGATTCATTGTAAAAGGGAGGTCCCATTTGAGAAATGACACCGATGTGGTAAACTTTGT⋱
GAGCATGGAATTCTCTCTTACTGACCCGAGGTTGGAGCCACACAAGTGGGAAAAGTACTGTGTTCTCGAG⋱
GTAGGAGACATGCTCCTACGGACTGCAATAGGCCAAGTGTCAAGGCCCATGTTCCTGTATGTGAGAACTA⋱
ATGGAACTTCCAAGATCAAAATGAAGTGGGGAATGGAAATGAGGCGATGCCTCCTTCAATCCCTCCAACA⋱
GATTGAGAGTATGATTGAAGCAGAGTCTTCTGTCAAAGAGAAAGACATGACCAAAGAATTCTTTGAAAAC⋱
AAATCAGAAACATGGCCAATTGGAGAGTCACCCAAAGGGGTGGAGGAAGGCTCCATTGGGAAGGTGTGCA⋱
GAACCTTACTGGCAAAATCTGTGTTCAACAGCCTATATGCATCTCCACAACTCGAGGGATTTTCAGCTGA⋱
ATCAAGAAAATTGCTT
**CTCATTGTTCAGGCACTTAGGGACAACCTGGAACCTGGGACCTTCGATCTCGGGG**
**GGCTATATGAAGCAATTGAGGAGTGCCTGATTAATGATCCCTGGGTTTTGCT**
**TAATGCATCTTGGTTCAACTCCTTCCTCACACATGCACTG**AAATAG

# Per codon conservation report

```
PA
Pos.  1   obs:    exp:
atg   M   353     353.00
---   --  ------  ------
mPD       0       0
          nPD:    1.
     N. weight:   0.
       Sc. PD:    0
     Sc. rank:    0

PA
Pos.  2   obs:    exp:
gct   A   0       0.25
gcc   A   0       0.20
gca   A   1       0.45
gcg   A   0       0.10
gaa   E   351     202.00
gag   E   0       149.00
ggt   G   0       0.12
ggc   G   0       0.14
gga   G   1       0.48
ggg   G   0       0.26
---   --  ------  ------
mPD       0.011   0.50
          nPD:    0.02
     N. weight:   0.84
       Sc. PD:    -0.19
     Sc. rank:    -108.7

PA
Pos.  3   obs:    exp:
gat   D   72      187.80
gac   D   280     164.20
gaa   E   0       0.58
gag   E   1       0.42
---   --  ------  ------
mPD       0.33    0.50
          nPD:    0.66
     N. weight:   0.36
       Sc. PD:    0.14
     Sc. rank:    58.9

PA
Pos.  4   obs:    exp:
ttt   F   351     144.30
ttc   F   0       206.70
tta   L   0       0.17
ttg   L   1       0.39
ctt   L   1       0.37
ctc   L   0       0.32
cta   L   0       0.30
ctg   L   0       0.44
---   --  ------  ------
mPD       0.011   0.50
          nPD:    0.02
     N. weight:   1.3
       Sc. PD:    -0.31
     Sc. rank:    -172.5

PA
Pos.  5   obs:    exp:
gtt   V   0       72.56
gtc   V   0       74.09
gta   V   1       66.16
gtg   V   352     140.20
---   --  ------  ------
mPD       0.0057  0.72
          nPD:    0.01
     N. weight:   1.4
       Sc. PD:    -0.34
     Sc. rank:    -221.0

PA
Pos.  6   obs:    exp:
cgt   R   0       14.65
cgc   R   0       17.28
cga   R   347     33.93
cgg   R   6       29.46
aga   R   0       158.80
agg   R   0       98.92
---   --  ------  ------
mPD       0.034   0.96
          nPD:    0.03
     N. weight:   3.4
       Sc. PD:    -0.76
     Sc. rank:    -355.2

PA
Pos.  7   obs:    exp:
caa   Q   349     187.70
cag   Q   3       164.30
cgt   R   0       0.04
cgc   R   0       0.05
cga   R   1       0.10
cgg   R   0       0.08
aga   R   0       0.45
agg   R   0       0.28
---   --  ------  ------
mPD       0.023   0.51
          nPD:    0.04
     N. weight:   0.89
       Sc. PD:    -0.19
     Sc. rank:    -88.5

PA
Pos.  8   obs:    exp:
tgt   C   4       145.10
tgc   C   348     206.90
ggt   G   0       0.12
ggc   G   1       0.14
gga   G   0       0.48
ggg   G   0       0.26
---   --  ------  ------
mPD       0.028   0.49
          nPD:    0.06
     N. weight:   0.73
       Sc. PD:    -0.15
     Sc. rank:    -61.5

PA
Pos.  9   obs:    exp:
ttt   F   3       144.70
ttc   F   349     207.30
tat   Y   0       0.52
tac   Y   1       0.48
---   --  ------  ------
mPD       0.023   0.49
          nPD:    0.05
     N. weight:   0.74
       Sc. PD:    -0.16
     Sc. rank:    -70.9

PA
Pos.  10  obs:    exp:
aat   N   352     184.50
aac   N   1       168.50
---   --  ------  ------
mPD       0.0057  0.50
          nPD:    0.01
     N. weight:   0.96
       Sc. PD:    -0.23
     Sc. rank:    -141.6

PA
Pos.  11  obs:    exp:
cct   P   0       92.95
ccc   P   0       63.67
cca   P   348     139.30
ccg   P   5       57.08
---   --  ------  ------
mPD       0.028   0.72
          nPD:    0.04
     N. weight:   1.3
       Sc. PD:    -0.29
     Sc. rank:    -135.2

PA
Pos.  12  obs:    exp:
atg   M   353     353.00
---   --  ------  ------
mPD       0       0
          nPD:    1.
     N. weight:   0.
       Sc. PD:    0
     Sc. rank:    0

PA
Pos.  13  obs:    exp:
att   I   215     131.20
atc   I   134     91.47
ata   I   0       126.30
gtt   V   4       0.82
gtc   V   0       0.84
gta   V   0       0.75
gtg   V   0       1.59
---   --  ------  ------
mPD       0.49    0.68
          nPD:    0.72
     N. weight:   0.72
       Sc. PD:    0.31
     Sc. rank:    128.5

PA
Pos.  14  obs:    exp:
gtt   V   0       72.56
gtc   V   352     74.09
gta   V   0       66.16
gtg   V   1       140.20
---   --  ------  ------
mPD       0.0057  0.72
          nPD:    0.01
     N. weight:   2.3
       Sc. PD:    -0.58
     Sc. rank:    -375.1

PA
Pos.  15  obs:    exp:
gaa   E   6       203.10
gag   E   347     149.90
---   --  ------  ------
mPD       0.034   0.49
          nPD:    0.07
     N. weight:   1.2
       Sc. PD:    -0.22
     Sc. rank:    -88.3

PA
Pos.  16  obs:    exp:
tta   L   0       30.84
ttg   L   0       67.98
ctt   L   353     65.86
ctc   L   0       57.10
cta   L   0       53.18
ctg   L   0       78.03
---   --  ------  ------
mPD       0       1.1
          nPD:    0.
     N. weight:   2.5
       Sc. PD:    -0.65
     Sc. rank:    -508.8

PA
Pos.  17  obs:    exp:
gct   A   0       88.91
gcc   A   0       72.31
gca   A   0       157.30
gcg   A   353     34.45
---   --  ------  ------
mPD       0       0.69
          nPD:    0.
     N. weight:   3.5
       Sc. PD:    -0.9
     Sc. rank:    -705.2

PA
Pos.  18  obs:    exp:
gaa   E   350     203.10
gag   E   3       149.90
---   --  ------  ------
mPD       0.017   0.49
          nPD:    0.03
     N. weight:   0.77
       Sc. PD:    -0.17
     Sc. rank:    -82.3

PA
Pos.  19  obs:    exp:
cgt   R   0       0.08
cgc   R   0       0.10
cga   R   0       0.19
cgg   R   0       0.17
aaa   K   38      196.00
aag   K   312     154.00
aga   R   0       0.90
agg   R   2       0.56
gaa   E   0       0.58
gag   E   1       0.42
---   --  ------  ------
mPD       0.21    0.51
          nPD:    0.41
     N. weight:   0.7
       Sc. PD:    0.097
     Sc. rank:    63.2

PA
Pos.  20  obs:    exp:
act   T   0       0.26
acc   T   0       0.21
aca   T   1       0.44
acg   T   0       0.08
gct   A   1       88.66
gcc   A   0       72.11
gca   A   351     156.90
gcg   A   0       34.35
---   --  ------  ------
mPD       0.011   0.69
          nPD:    0.02
     N. weight:   1.2
       Sc. PD:    -0.29
     Sc. rank:    -166.2

PA
Pos.  21  obs:    exp:
atg   M   353     353.00
---   --  ------  ------
mPD       0       0
          nPD:    1.
     N. weight:   0.
       Sc. PD:    0
     Sc. rank:    0

PA
Pos.  22  obs:    exp:
aaa   K   291     197.70
aag   K   62      155.30
---   --  ------  ------
mPD       0.29    0.49
          nPD:    0.59
     N. weight:   0.25
       Sc. PD:    0.078
     Sc. rank:    37.0

PA
Pos.  23  obs:    exp:
gaa   E   340     203.10
gag   E   13      149.90
---   --  ------  ------
mPD       0.071   0.49
          nPD:    0.15
     N. weight:   0.62
       Sc. PD:    -0.071
     Sc. rank:    -19.7

PA
Pos.  24  obs:    exp:
tat   Y   352     182.70
tac   Y   1       170.30
---   --  ------  ------
mPD       0.0057  0.50
          nPD:    0.01
     N. weight:   0.97
       Sc. PD:    -0.24
     Sc. rank:    -144.4

PA
Pos.  25  obs:    exp:
ggt   G   0       43.56
ggc   G   0       47.74
gga   G   6       170.80
ggg   G   347     90.95
---   --  ------  ------
mPD       0.034   0.67
          nPD:    0.05
     N. weight:   1.9
       Sc. PD:    -0.39
     Sc. rank:    -176.4
```

| | | | |
|---|---|---|---|
| PA | | | |
| Pos. | 26 | obs: | exp: |
| gaa | E | 341 | 203.10 |
| gag | E | 12 | 149.90 |
| mPD | | 0.066 | 0.49 |
| | | nPD: | 0.13 |
| | | N. weight: | 0.64 |
| | | Sc. PD: | -0.079 |
| | | Sc. rank: | -23.2 |

| | | | |
|---|---|---|---|
| PA | | | |
| Pos. | 27 | obs: | exp: |
| aat | N | 2 | 1.05 |
| aac | N | 0 | 0.95 |
| gat | D | 331 | 187.30 |
| gac | D | 20 | 163.70 |
| mPD | | 0.12 | 0.51 |
| | | nPD: | 0.23 |
| | | N. weight: | 0.64 |
| | | Sc. PD: | -0.02 |
| | | Sc. rank: | 18.1 |

| | | | |
|---|---|---|---|
| PA | | | |
| Pos. | 28 | obs: | exp: |
| tct | S | 0 | 0.16 |
| tcc | S | 0 | 0.14 |
| tca | S | 1 | 0.22 |
| tcg | S | 0 | 0.06 |
| cct | P | 0 | 92.69 |
| ccc | P | 0 | 63.49 |
| cca | P | 91 | 138.90 |
| ccg | P | 261 | 56.91 |
| agt | S | 0 | 0.22 |
| agc | S | 0 | 0.20 |
| mPD | | 0.39 | 0.72 |
| | | nPD: | 0.54 |
| | | N. weight: | 1.6 |
| | | Sc. PD: | 0.41 |
| | | Sc. rank: | 215.8 |

| | | | |
|---|---|---|---|
| PA | | | |
| Pos. | 29 | obs: | exp: |
| cgt | R | 0 | 0.04 |
| cgc | R | 0 | 0.05 |
| cga | R | 0 | 0.10 |
| cgg | R | 0 | 0.08 |
| aaa | K | 350 | 196.50 |
| aag | K | 1 | 154.50 |
| aga | R | 1 | 0.45 |
| agg | R | 0 | 0.28 |
| gaa | E | 1 | 0.58 |
| gag | E | 0 | 0.42 |
| mPD | | 0.017 | 0.51 |
| | | nPD: | 0.03 |
| | | N. weight: | 0.85 |
| | | Sc. PD: | -0.19 |
| | | Sc. rank: | -94.2 |

| | | | |
|---|---|---|---|
| PA | | | |
| Pos. | 30 | obs: | exp: |
| tta | L | 0 | 0.17 |
| ttg | L | 0 | 0.39 |
| ctt | L | 0 | 0.37 |
| ctc | L | 2 | 0.32 |
| cta | L | 0 | 0.30 |
| ctg | L | 0 | 0.44 |
| att | I | 14 | 131.20 |
| atc | I | 330 | 91.47 |
| ata | I | 5 | 126.30 |
| gtt | V | 1 | 0.41 |
| gtc | V | 1 | 0.42 |
| gta | V | 0 | 0.37 |
| gtg | V | 0 | 0.79 |
| mPD | | 0.13 | 0.68 |
| | | nPD: | 0.19 |
| | | N. weight: | 1.7 |
| | | Sc. PD: | -0.12 |
| | | Sc. rank: | -9.1 |

| | | | |
|---|---|---|---|
| PA | | | |
| Pos. | 31 | obs: | exp: |
| gaa | E | 281 | 203.10 |
| gag | E | 72 | 149.90 |
| mPD | | 0.33 | 0.49 |
| | | nPD: | 0.67 |
| | | N. weight: | 0.18 |
| | | Sc. PD: | 0.068 |
| | | Sc. rank: | 29.2 |

| | | | |
|---|---|---|---|
| PA | | | |
| Pos. | 32 | obs: | exp: |
| act | T | 15 | 93.26 |
| acc | T | 5 | 75.75 |
| aca | T | 168 | 154.60 |
| acg | T | 165 | 29.35 |
| mPD | | 0.55 | 0.69 |
| | | nPD: | 0.81 |
| | | N. weight: | 1.1 |
| | | Sc. PD: | 0.59 |
| | | Sc. rank: | 221.6 |

| | | | |
|---|---|---|---|
| PA | | | |
| Pos. | 33 | obs: | exp: |
| aat | N | 27 | 184.50 |
| aac | N | 326 | 168.50 |
| mPD | | 0.14 | 0.50 |
| | | nPD: | 0.28 |
| | | N. weight: | 0.71 |
| | | Sc. PD: | 0.013 |
| | | Sc. rank: | 32.0 |

| | | | |
|---|---|---|---|
| PA | | | |
| Pos. | 34 | obs: | exp: |
| aaa | K | 335 | 197.70 |
| aag | K | 18 | 155.30 |
| mPD | | 0.097 | 0.49 |
| | | nPD: | 0.2 |
| | | N. weight: | 0.6 |
| | | Sc. PD: | -0.039 |
| | | Sc. rank: | 2.1 |

| | | | |
|---|---|---|---|
| PA | | | |
| Pos. | 35 | obs: | exp: |
| ttt | F | 270 | 134.80 |
| ttc | F | 58 | 193.20 |
| tta | L | 0 | 2.18 |
| ttg | L | 0 | 4.81 |
| ctt | L | 0 | 4.66 |
| ctc | L | 25 | 4.04 |
| cta | L | 0 | 3.77 |
| ctg | L | 0 | 5.53 |
| mPD | | 0.49 | 0.63 |
| | | nPD: | 0.79 |
| | | N. weight: | 0.71 |
| | | Sc. PD: | 0.36 |
| | | Sc. rank: | 135.8 |

| | | | |
|---|---|---|---|
| PA | | | |
| Pos. | 36 | obs: | exp: |
| act | T | 1 | 0.26 |
| acc | T | 0 | 0.21 |
| aca | T | 0 | 0.44 |
| acg | T | 0 | 0.08 |
| gct | A | 150 | 88.66 |
| gcc | A | 136 | 72.11 |
| gca | A | 65 | 156.90 |
| gcg | A | 1 | 34.35 |
| mPD | | 0.64 | 0.69 |
| | | nPD: | 0.93 |
| | | N. weight: | 0.48 |
| | | Sc. PD: | 0.31 |
| | | Sc. rank: | 96.5 |

| | | | |
|---|---|---|---|
| PA | | | |
| Pos. | 37 | obs: | exp: |
| tct | S | 0 | 4.13 |
| tcc | S | 0 | 3.62 |
| tca | S | 26 | 5.76 |
| tcg | S | 0 | 1.58 |
| agt | S | 0 | 5.72 |
| agc | S | 0 | 5.18 |
| gct | A | 0 | 82.36 |
| gcc | A | 2 | 66.99 |
| gca | A | 303 | 145.70 |
| gcg | A | 22 | 31.91 |
| mPD | | 0.26 | 0.89 |
| | | nPD: | 0.3 |
| | | N. weight: | 1.1 |
| | | Sc. PD: | 0.033 |
| | | Sc. rank: | 54.2 |

| | | | |
|---|---|---|---|
| PA | | | |
| Pos. | 38 | obs: | exp: |
| att | I | 0 | 128.20 |
| atc | I | 0 | 89.37 |
| ata | I | 341 | 123.40 |
| atg | M | 1 | 1.00 |
| gtt | V | 0 | 2.26 |
| gtc | V | 0 | 2.31 |
| gta | V | 11 | 2.06 |
| gtg | V | 0 | 4.37 |
| mPD | | 0.066 | 0.73 |
| | | nPD: | 0.09 |
| | | N. weight: | 1.6 |
| | | Sc. PD: | -0.26 |
| | | Sc. rank: | -93.2 |

| | | | |
|---|---|---|---|
| PA | | | |
| Pos. | 39 | obs: | exp: |
| tct | S | 0 | 0.16 |
| tcc | S | 0 | 0.14 |
| tca | S | 0 | 0.22 |
| tcg | S | 0 | 0.06 |
| tgt | C | 5 | 145.10 |
| tgc | C | 347 | 206.90 |
| agt | S | 0 | 0.22 |
| agc | S | 1 | 0.20 |
| mPD | | 0.034 | 0.49 |
| | | nPD: | 0.07 |
| | | N. weight: | 0.71 |
| | | Sc. PD: | -0.13 |
| | | Sc. rank: | -54.2 |

| | | | |
|---|---|---|---|
| PA | | | |
| Pos. | 40 | obs: | exp: |
| act | T | 25 | 93.26 |
| acc | T | 3 | 75.75 |
| aca | T | 308 | 154.60 |
| acg | T | 17 | 29.35 |
| mPD | | 0.23 | 0.69 |
| | | nPD: | 0.34 |
| | | N. weight: | 0.71 |
| | | Sc. PD: | 0.051 |
| | | Sc. rank: | 49.4 |

| | | | |
|---|---|---|---|
| PA | | | |
| Pos. | 41 | obs: | exp: |
| cat | H | 15 | 191.30 |
| cac | H | 338 | 161.70 |
| mPD | | 0.082 | 0.50 |
| | | nPD: | 0.16 |
| | | N. weight: | 0.91 |
| | | Sc. PD: | -0.088 |
| | | Sc. rank: | -24.2 |

| | | | |
|---|---|---|---|
| PA | | | |
| Pos. | 42 | obs: | exp: |
| tta | L | 276 | 30.84 |
| ttg | L | 68 | 67.98 |
| ctt | L | 0 | 65.86 |
| ctc | L | 0 | 57.10 |
| cta | L | 7 | 53.18 |
| ctg | L | 2 | 78.03 |
| mPD | | 0.37 | 1.1 |
| | | nPD: | 0.33 |
| | | N. weight: | 2.5 |
| | | Sc. PD: | 0.16 |
| | | Sc. rank: | 163.7 |

| | | | |
|---|---|---|---|
| PA | | | |
| Pos. | 43 | obs: | exp: |
| gaa | E | 338 | 203.10 |
| gag | E | 15 | 149.90 |
| mPD | | 0.082 | 0.49 |
| | | nPD: | 0.17 |
| | | N. weight: | 0.6 |
| | | Sc. PD: | -0.056 |
| | | Sc. rank: | -15.6 |

| | | | |
|---|---|---|---|
| PA | | | |
| Pos. | 44 | obs: | exp: |
| att | I | 0 | 1.50 |
| atc | I | 3 | 1.05 |
| ata | I | 1 | 1.45 |
| gtt | V | 8 | 71.53 |
| gtc | V | 265 | 73.04 |
| gta | V | 33 | 65.22 |
| gtg | V | 42 | 138.20 |
| gct | A | 0 | 0.25 |
| gcc | A | 1 | 0.20 |
| gca | A | 0 | 0.45 |
| gcg | A | 0 | 0.10 |
| mPD | | 0.42 | 0.75 |
| | | nPD: | 0.57 |
| | | N. weight: | 1.1 |
| | | Sc. PD: | 0.33 |
| | | Sc. rank: | 161.4 |

| | | | |
|---|---|---|---|
| PA | | | |
| Pos. | 45 | obs: | exp: |
| tgt | C | 284 | 145.50 |
| tgc | C | 69 | 207.50 |
| mPD | | 0.32 | 0.48 |
| | | nPD: | 0.65 |
| | | N. weight: | 0.5 |
| | | Sc. PD: | 0.19 |
| | | Sc. rank: | 80.2 |

| | | | |
|---|---|---|---|
| PA | | | |
| Pos. | 46 | obs: | exp: |
| ttt | F | 3 | 144.70 |
| ttc | F | 349 | 207.30 |
| tta | L | 0 | 0.09 |
| ttg | L | 0 | 0.19 |
| ctt | L | 0 | 0.19 |
| ctc | L | 1 | 0.16 |
| cta | L | 0 | 0.15 |
| ctg | L | 0 | 0.22 |
| mPD | | 0.023 | 0.49 |
| | | nPD: | 0.05 |
| | | N. weight: | 0.74 |
| | | Sc. PD: | -0.16 |
| | | Sc. rank: | -71.7 |

| | | | |
|---|---|---|---|
| PA | | | |
| Pos. | 47 | obs: | exp: |
| atg | M | 353 | 353.00 |
| mPD | | 0 | 0 |
| | | nPD: | 1. |
| | | N. weight: | 0. |
| | | Sc. PD: | 0 |
| | | Sc. rank: | 0 |

| | | | |
|---|---|---|---|
| PA | | | |
| Pos. | 48 | obs: | exp: |
| tat | Y | 286 | 182.70 |
| tac | Y | 67 | 170.30 |
| mPD | | 0.31 | 0.50 |
| | | nPD: | 0.62 |
| | | N. weight: | 0.29 |
| | | Sc. PD: | 0.1 |
| | | Sc. rank: | 45.1 |

| | | | |
|---|---|---|---|
| PA | | | |
| Pos. | 49 | obs: | exp: |
| tct | S | 26 | 56.12 |
| tcc | S | 1 | 49.17 |
| tca | S | 88 | 78.20 |
| tcg | S | 238 | 21.52 |
| agt | S | 0 | 77.66 |
| agc | S | 0 | 70.33 |
| mPD | | 0.48 | 1.7 |
| | | nPD: | 0.29 |
| | | N. weight: | 2.4 |
| | | Sc. PD: | 0.055 |
| | | Sc. rank: | 118.9 |

| | | | |
|---|---|---|---|
| PA | | | |
| Pos. | 50 | obs: | exp: |
| gat | D | 327 | 188.30 |
| gac | D | 26 | 164.70 |
| mPD | | 0.14 | 0.50 |
| | | nPD: | 0.27 |
| | | N. weight: | 0.58 |
| | | Sc. PD: | 0.0058 |
| | | Sc. rank: | 24.7 |

| | | | |
|---|---|---|---|
| PA | | | |
| Pos. | 51 | obs: | exp: |
| ttt | F | 44 | 144.70 |
| ttc | F | 308 | 207.30 |
| tta | L | 0 | 0.09 |
| ttg | L | 0 | 0.19 |
| ctt | L | 0 | 0.19 |
| ctc | L | 1 | 0.16 |
| cta | L | 0 | 0.15 |
| ctg | L | 0 | 0.22 |
| --- | -- | ------ | ------ |
| mPD | | 0.22 | 0.49 |
| | | nPD: | 0.46 |
| | | N. weight: | 0.32 |
| | | Sc. PD: | 0.059 |
| | | Sc. rank: | 32.4 |

| | | | |
|---|---|---|---|
| PA | | | |
| Pos. | 52 | obs: | exp: |
| cat | H | 99 | 191.30 |
| cac | H | 254 | 161.70 |
| --- | -- | ------ | ------ |
| mPD | | 0.40 | 0.50 |
| | | nPD: | 0.82 |
| | | N. weight: | 0.23 |
| | | Sc. PD: | 0.12 |
| | | Sc. rank: | 44.0 |

| | | | |
|---|---|---|---|
| PA | | | |
| Pos. | 53 | obs: | exp: |
| ttt | F | 214 | 145.10 |
| ttc | F | 139 | 207.90 |
| --- | -- | ------ | ------ |
| mPD | | 0.48 | 0.48 |
| | | nPD: | 0.99 |
| | | N. weight: | 0.13 |
| | | Sc. PD: | 0.09 |
| | | Sc. rank: | 26.3 |

| | | | |
|---|---|---|---|
| PA | | | |
| Pos. | 54 | obs: | exp: |
| att | I | 335 | 132.30 |
| atc | I | 17 | 92.25 |
| ata | I | 0 | 127.40 |
| act | T | 1 | 0.26 |
| acc | T | 0 | 0.21 |
| aca | T | 0 | 0.44 |
| acg | T | 0 | 0.08 |
| --- | -- | ------ | ------ |
| mPD | | 0.098 | 0.66 |
| | | nPD: | 0.15 |
| | | N. weight: | 1.2 |
| | | Sc. PD: | -0.14 |
| | | Sc. rank: | -35.6 |

| | | | |
|---|---|---|---|
| PA | | | |
| Pos. | 55 | obs: | exp: |
| aat | N | 19 | 9.93 |
| aac | N | 0 | 9.07 |
| gat | D | 303 | 178.20 |
| gac | D | 31 | 155.80 |
| --- | -- | ------ | ------ |
| mPD | | 0.26 | 0.60 |
| | | nPD: | 0.44 |
| | | N. weight: | 0.54 |
| | | Sc. PD: | 0.09 |
| | | Sc. rank: | 52.1 |

| | | | |
|---|---|---|---|
| PA | | | |
| Pos. | 56 | obs: | exp: |
| gaa | E | 336 | 203.10 |
| gag | E | 17 | 149.90 |
| --- | -- | ------ | ------ |
| mPD | | 0.092 | 0.49 |
| | | nPD: | 0.19 |
| | | N. weight: | 0.58 |
| | | Sc. PD: | -0.042 |
| | | Sc. rank: | -5.1 |

| | | | |
|---|---|---|---|
| PA | | | |
| Pos. | 57 | obs: | exp: |
| caa | Q | 2 | 1.07 |
| cag | Q | 0 | 0.93 |
| cgt | R | 0 | 14.57 |
| cgc | R | 0 | 17.19 |
| cga | R | 237 | 33.73 |
| cgg | R | 107 | 29.29 |
| aga | R | 3 | 157.90 |
| agg | R | 4 | 98.36 |
| --- | -- | ------ | ------ |
| mPD | | 0.48 | 0.97 |
| | | nPD: | 0.5 |
| | | N. weight: | 2.5 |
| | | Sc. PD: | 0.55 |
| | | Sc. rank: | 300.7 |

| | | | |
|---|---|---|---|
| PA | | | |
| Pos. | 58 | obs: | exp: |
| ggt | G | 100 | 43.56 |
| ggc | G | 249 | 47.74 |
| gga | G | 4 | 170.80 |
| ggg | G | 0 | 90.95 |
| --- | -- | ------ | ------ |
| mPD | | 0.42 | 0.67 |
| | | nPD: | 0.64 |
| | | N. weight: | 2.1 |
| | | Sc. PD: | 0.74 |
| | | Sc. rank: | 330.0 |

| | | | |
|---|---|---|---|
| PA | | | |
| Pos. | 59 | obs: | exp: |
| gat | D | 2 | 1.07 |
| gac | D | 0 | 0.93 |
| gaa | E | 279 | 202.00 |
| gag | E | 72 | 149.00 |
| --- | -- | ------ | ------ |
| mPD | | 0.33 | 0.49 |
| | | nPD: | 0.68 |
| | | N. weight: | 0.18 |
| | | Sc. PD: | 0.071 |
| | | Sc. rank: | 30.3 |

| | | | |
|---|---|---|---|
| PA | | | |
| Pos. | 60 | obs: | exp: |
| tct | S | 1 | 56.12 |
| tcc | S | 0 | 49.17 |
| tca | S | 287 | 78.20 |
| tcg | S | 65 | 21.52 |
| agt | S | 0 | 77.66 |
| agc | S | 0 | 70.33 |
| --- | -- | ------ | ------ |
| mPD | | 0.31 | 1.7 |
| | | nPD: | 0.18 |
| | | N. weight: | 1.9 |
| | | Sc. PD: | -0.15 |
| | | Sc. rank: | -33.0 |

| | | | |
|---|---|---|---|
| PA | | | |
| Pos. | 61 | obs: | exp: |
| att | I | 0 | 119.50 |
| atc | I | 0 | 83.34 |
| ata | I | 318 | 115.10 |
| atg | M | 3 | 3.00 |
| act | T | 1 | 7.13 |
| acc | T | 25 | 5.79 |
| aca | T | 1 | 11.83 |
| acg | T | 0 | 2.24 |
| gtt | V | 0 | 1.03 |
| gtc | V | 0 | 1.05 |
| gta | V | 5 | 0.94 |
| gtg | V | 0 | 1.99 |
| --- | -- | ------ | ------ |
| mPD | | 0.32 | 0.84 |
| | | nPD: | 0.38 |
| | | N. weight: | 1.6 |
| | | Sc. PD: | 0.18 |
| | | Sc. rank: | 132.7 |

| | | | |
|---|---|---|---|
| PA | | | |
| Pos. | 62 | obs: | exp: |
| att | I | 253 | 123.70 |
| atc | I | 76 | 86.23 |
| ata | I | 0 | 119.10 |
| gtt | V | 24 | 4.93 |
| gtc | V | 0 | 5.04 |
| gta | V | 0 | 4.50 |
| gtg | V | 0 | 9.53 |
| --- | -- | ------ | ------ |
| mPD | | 0.47 | 0.80 |
| | | nPD: | 0.58 |
| | | N. weight: | 0.91 |
| | | Sc. PD: | 0.28 |
| | | Sc. rank: | 133.6 |

| | | | |
|---|---|---|---|
| PA | | | |
| Pos. | 63 | obs: | exp: |
| tta | L | 8 | 0.96 |
| ttg | L | 2 | 2.12 |
| ctt | L | 0 | 2.05 |
| ctc | L | 0 | 1.78 |
| cta | L | 1 | 1.66 |
| ctg | L | 0 | 2.43 |
| att | I | 0 | 10.15 |
| atc | I | 0 | 7.08 |
| ata | I | 27 | 9.77 |
| gtt | V | 0 | 63.92 |
| gtc | V | 1 | 65.27 |
| gta | V | 209 | 58.29 |
| gtg | V | 101 | 123.50 |
| gct | A | 0 | 1.01 |
| gcc | A | 0 | 0.82 |
| gca | A | 4 | 1.78 |
| gcg | A | 0 | 0.39 |
| --- | -- | ------ | ------ |
| mPD | | 0.64 | 0.95 |
| | | nPD: | 0.67 |
| | | N. weight: | 1.3 |
| | | Sc. PD: | 0.49 |
| | | Sc. rank: | 210.1 |

| | | | |
|---|---|---|---|
| PA | | | |
| Pos. | 64 | obs: | exp: |
| gat | D | 0 | 0.53 |
| gac | D | 1 | 0.47 |
| gaa | E | 299 | 202.60 |
| gag | E | 53 | 149.40 |
| --- | -- | ------ | ------ |
| mPD | | 0.26 | 0.49 |
| | | nPD: | 0.53 |
| | | N. weight: | 0.28 |
| | | Sc. PD: | 0.071 |
| | | Sc. rank: | 37.3 |

| | | | |
|---|---|---|---|
| PA | | | |
| Pos. | 65 | obs: | exp: |
| tct | S | 282 | 56.12 |
| tcc | S | 22 | 49.17 |
| tca | S | 22 | 78.20 |
| tcg | S | 27 | 21.52 |
| agt | S | 0 | 77.66 |
| agc | S | 0 | 70.33 |
| --- | -- | ------ | ------ |
| mPD | | 0.35 | 1.7 |
| | | nPD: | 0.21 |
| | | N. weight: | 1.8 |
| | | Sc. PD: | -0.095 |
| | | Sc. rank: | 23.1 |

| | | | |
|---|---|---|---|
| PA | | | |
| Pos. | 66 | obs: | exp: |
| tct | S | 0 | 0.79 |
| tcc | S | 0 | 0.70 |
| tca | S | 0 | 1.11 |
| tcg | S | 0 | 0.30 |
| agt | S | 3 | 1.10 |
| agc | S | 2 | 1.00 |
| gat | D | 0 | 0.53 |
| gac | D | 1 | 0.47 |
| ggt | G | 38 | 42.82 |
| ggc | G | 287 | 46.93 |
| gga | G | 17 | 167.90 |
| ggg | G | 5 | 89.40 |
| --- | -- | ------ | ------ |
| mPD | | 0.34 | 0.72 |
| | | nPD: | 0.48 |
| | | N. weight: | 2. |
| | | Sc. PD: | 0.42 |
| | | Sc. rank: | 224.9 |

| | | | |
|---|---|---|---|
| PA | | | |
| Pos. | 67 | obs: | exp: |
| gat | D | 321 | 188.30 |
| gac | D | 32 | 164.70 |
| --- | -- | ------ | ------ |
| mPD | | 0.17 | 0.50 |
| | | nPD: | 0.33 |
| | | N. weight: | 0.52 |
| | | Sc. PD: | 0.034 |
| | | Sc. rank: | 34.3 |

| | | | |
|---|---|---|---|
| PA | | | |
| Pos. | 68 | obs: | exp: |
| tct | S | 0 | 0.32 |
| tcc | S | 0 | 0.28 |
| tca | S | 0 | 0.44 |
| tcg | S | 2 | 0.12 |
| cct | P | 0 | 92.42 |
| ccc | P | 1 | 63.31 |
| cca | P | 142 | 138.50 |
| ccg | P | 208 | 56.75 |
| agt | S | 0 | 0.44 |
| agc | S | 0 | 0.40 |
| --- | -- | ------ | ------ |
| mPD | | 0.50 | 0.73 |
| | | nPD: | 0.68 |
| | | N. weight: | 1.2 |
| | | Sc. PD: | 0.48 |
| | | Sc. rank: | 204.4 |

| | | | |
|---|---|---|---|
| PA | | | |
| Pos. | 69 | obs: | exp: |
| aat | N | 318 | 184.50 |
| aac | N | 35 | 168.50 |
| --- | -- | ------ | ------ |
| mPD | | 0.18 | 0.50 |
| | | nPD: | 0.36 |
| | | N. weight: | 0.52 |
| | | Sc. PD: | 0.047 |
| | | Sc. rank: | 40.5 |

| | | | |
|---|---|---|---|
| PA | | | |
| Pos. | 70 | obs: | exp: |
| gtt | V | 0 | 3.49 |
| gtc | V | 0 | 3.57 |
| gta | V | 2 | 3.19 |
| gtg | V | 15 | 6.75 |
| gct | A | 1 | 84.63 |
| gcc | A | 1 | 68.83 |
| gca | A | 319 | 149.80 |
| gcg | A | 15 | 32.79 |
| --- | -- | ------ | ------ |
| mPD | | 0.26 | 0.79 |
| | | nPD: | 0.33 |
| | | N. weight: | 1. |
| | | Sc. PD: | 0.062 |
| | | Sc. rank: | 63.5 |

| | | | |
|---|---|---|---|
| PA | | | |
| Pos. | 71 | obs: | exp: |
| tta | L | 216 | 30.84 |
| ttg | L | 40 | 67.98 |
| ctt | L | 2 | 65.86 |
| ctc | L | 66 | 57.10 |
| cta | L | 25 | 53.18 |
| ctg | L | 4 | 78.03 |
| --- | -- | ------ | ------ |
| mPD | | 0.88 | 1.1 |
| | | nPD: | 0.79 |
| | | N. weight: | 1.6 |
| | | Sc. PD: | 0.83 |
| | | Sc. rank: | 315.0 |

| | | | |
|---|---|---|---|
| PA | | | |
| Pos. | 72 | obs: | exp: |
| tta | L | 10 | 30.84 |
| ttg | L | 219 | 67.98 |
| ctt | L | 0 | 65.86 |
| ctc | L | 0 | 57.10 |
| cta | L | 66 | 53.18 |
| ctg | L | 58 | 78.03 |
| --- | -- | ------ | ------ |
| mPD | | 0.80 | 1.1 |
| | | nPD: | 0.71 |
| | | N. weight: | 1.1 |
| | | Sc. PD: | 0.46 |
| | | Sc. rank: | 190.0 |

| | | | |
|---|---|---|---|
| PA | | | |
| Pos. | 73 | obs: | exp: |
| aaa | K | 298 | 197.70 |
| aag | K | 55 | 155.30 |
| --- | -- | ------ | ------ |
| mPD | | 0.26 | 0.49 |
| | | nPD: | 0.54 |
| | | N. weight: | 0.29 |
| | | Sc. PD: | 0.076 |
| | | Sc. rank: | 39.5 |

| | | | |
|---|---|---|---|
| PA | | | |
| Pos. | 74 | obs: | exp: |
| cat | H | 17 | 191.30 |
| cac | H | 336 | 161.70 |
| --- | -- | ------ | ------ |
| mPD | | 0.092 | 0.50 |
| | | nPD: | 0.19 |
| | | N. weight: | 0.89 |
| | | Sc. PD: | -0.068 |
| | | Sc. rank: | -12.1 |

| | | | |
|---|---|---|---|
| PA | | | |
| Pos. | 75 | obs: | exp: |
| cgt | R | 22 | 14.65 |
| cgc | R | 5 | 17.28 |
| cga | R | 237 | 33.93 |
| cgg | R | 84 | 29.46 |
| aga | R | 5 | 158.80 |
| agg | R | 0 | 98.92 |
| --- | -- | ------ | ------ |
| mPD | | 0.50 | 0.96 |
| | | nPD: | 0.52 |
| | | N. weight: | 2.3 |
| | | Sc. PD: | 0.57 |
| | | Sc. rank: | 306.0 |

| PA | | | |
|---|---|---|---|
| Pos. | 76 | obs: | exp: |
| ttt | F | 346 | 145.10 |
| ttc | F | 7 | 207.90 |
| mPD | | 0.039 | 0.48 |
| | | nPD: | 0.08 |
| | | N. weight: | 1.2 |
| | | Sc. PD: | -0.21 |
| | | Sc. rank: | -82.8 |

,

| PA | | | |
|---|---|---|---|
| Pos. | 77 | obs: | exp: |
| gaa | E | 174 | 203.10 |
| gag | E | 179 | 149.90 |
| mPD | | 0.50 | 0.49 |
| | | nPD: | 1.03 |
| | | N. weight: | 0.034 |
| | | Sc. PD: | 0.025 |
| | | Sc. rank: | 7.0 |

,

| PA | | | |
|---|---|---|---|
| Pos. | 78 | obs: | exp: |
| att | I | 0 | 132.70 |
| atc | I | 0 | 92.52 |
| ata | I | 353 | 127.80 |
| mPD | | 0 | 0.66 |
| | | nPD: | 0. |
| | | N. weight: | 1.5 |
| | | Sc. PD: | -0.39 |
| | | Sc. rank: | -308.0 |

,

| PA | | | |
|---|---|---|---|
| Pos. | 79 | obs: | exp: |
| att | I | 267 | 132.70 |
| atc | I | 85 | 92.52 |
| ata | I | 1 | 127.80 |
| mPD | | 0.37 | 0.66 |
| | | nPD: | 0.56 |
| | | N. weight: | 0.77 |
| | | Sc. PD: | 0.22 |
| | | Sc. rank: | 108.6 |

,

| PA | | | |
|---|---|---|---|
| Pos. | 80 | obs: | exp: |
| gaa | E | 348 | 203.10 |
| gag | E | 5 | 149.90 |
| mPD | | 0.028 | 0.49 |
| | | nPD: | 0.06 |
| | | N. weight: | 0.74 |
| | | Sc. PD: | -0.15 |
| | | Sc. rank: | -61.9 |

,

| PA | | | |
|---|---|---|---|
| Pos. | 81 | obs: | exp: |
| ggt | G | 0 | 43.56 |
| ggc | G | 0 | 47.74 |
| gga | G | 46 | 170.80 |
| ggg | G | 307 | 90.95 |
| mPD | | 0.23 | 0.67 |
| | | nPD: | 0.34 |
| | | N. weight: | 1.4 |
| | | Sc. PD: | 0.1 |
| | | Sc. rank: | 95.3 |

,

| PA | | | |
|---|---|---|---|
| Pos. | 82 | obs: | exp: |
| cgt | R | 0 | 14.61 |
| cgc | R | 0 | 17.24 |
| cga | R | 1 | 33.83 |
| cgg | R | 0 | 29.37 |
| aaa | K | 1 | 0.56 |
| aag | K | 0 | 0.44 |
| aga | R | 347 | 158.30 |
| agg | R | 4 | 98.64 |
| mPD | | 0.034 | 0.96 |
| | | nPD: | 0.04 |
| | | N. weight: | 1.1 |
| | | Sc. PD: | -0.25 |
| | | Sc. rank: | -114.5 |

,

| PA | | | |
|---|---|---|---|
| Pos. | 83 | obs: | exp: |
| tat | Y | 0 | 0.52 |
| tac | Y | 1 | 0.48 |
| gat | D | 70 | 187.80 |
| gac | D | 282 | 164.20 |
| mPD | | 0.32 | 0.50 |
| | | nPD: | 0.64 |
| | | N. weight: | 0.37 |
| | | Sc. PD: | 0.14 |
| | | Sc. rank: | 59.5 |

,

| PA | | | |
|---|---|---|---|
| Pos. | 84 | obs: | exp: |
| cgt | R | 27 | 14.65 |
| cgc | R | 42 | 17.28 |
| cga | R | 269 | 33.93 |
| cgg | R | 1 | 29.46 |
| aga | R | 14 | 158.80 |
| agg | R | 0 | 98.92 |
| mPD | | 0.41 | 0.96 |
| | | nPD: | 0.43 |
| | | N. weight: | 2.5 |
| | | Sc. PD: | 0.4 |
| | | Sc. rank: | 237.7 |

,

| PA | | | |
|---|---|---|---|
| Pos. | 85 | obs: | exp: |
| cct | P | 0 | 0.26 |
| ccc | P | 0 | 0.18 |
| cca | P | 1 | 0.39 |
| ccg | P | 0 | 0.16 |
| att | I | 0 | 0.38 |
| atc | I | 0 | 0.26 |
| ata | I | 1 | 0.36 |
| act | T | 72 | 91.94 |
| acc | T | 8 | 74.68 |
| aca | T | 225 | 152.50 |
| acg | T | 43 | 28.93 |
| gct | A | 0 | 0.76 |
| gcc | A | 0 | 0.61 |
| gca | A | 3 | 1.34 |
| gcg | A | 0 | 0.29 |
| mPD | | 0.55 | 0.71 |
| | | nPD: | 0.77 |
| | | N. weight: | 0.35 |
| | | Sc. PD: | 0.17 |
| | | Sc. rank: | 65.2 |

,

| PA | | | |
|---|---|---|---|
| Pos. | 86 | obs: | exp: |
| atg | M | 334 | 334.00 |
| gtt | V | 2 | 3.91 |
| gtc | V | 0 | 3.99 |
| gta | V | 0 | 3.56 |
| gtg | V | 17 | 7.55 |
| mPD | | 0.11 | 0.17 |
| | | nPD: | 0.69 |
| | | N. weight: | 0.059 |
| | | Sc. PD: | 0.024 |
| | | Sc. rank: | 10.1 |

,

| PA | | | |
|---|---|---|---|
| Pos. | 87 | obs: | exp: |
| gct | A | 14 | 88.91 |
| gcc | A | 326 | 72.31 |
| gca | A | 13 | 157.30 |
| gcg | A | 0 | 34.45 |
| mPD | | 0.14 | 0.69 |
| | | nPD: | 0.21 |
| | | N. weight: | 1.9 |
| | | Sc. PD: | -0.097 |
| | | Sc. rank: | 25.8 |

,

| PA | | | |
|---|---|---|---|
| Pos. | 88 | obs: | exp: |
| tgg | W | 353 | 353.00 |
| mPD | | 0 | 0 |
| | | nPD: | 1. |
| | | N. weight: | 0. |
| | | Sc. PD: | 0 |
| | | Sc. rank: | 0 |

,

| PA | | | |
|---|---|---|---|
| Pos. | 89 | obs: | exp: |
| act | T | 0 | 93.26 |
| acc | T | 1 | 75.75 |
| aca | T | 325 | 154.60 |
| acg | T | 27 | 29.35 |
| mPD | | 0.15 | 0.69 |
| | | nPD: | 0.21 |
| | | N. weight: | 1. |
| | | Sc. PD: | -0.049 |
| | | Sc. rank: | 17.7 |

,

| PA | | | |
|---|---|---|---|
| Pos. | 90 | obs: | exp: |
| att | I | 0 | 0.38 |
| atc | I | 0 | 0.26 |
| ata | I | 1 | 0.36 |
| gtt | V | 0 | 72.35 |
| gtc | V | 0 | 73.88 |
| gta | V | 70 | 65.97 |
| gtg | V | 282 | 139.80 |
| mPD | | 0.33 | 0.73 |
| | | nPD: | 0.45 |
| | | N. weight: | 0.88 |
| | | Sc. PD: | 0.16 |
| | | Sc. rank: | 87.7 |

,

| PA | | | |
|---|---|---|---|
| Pos. | 91 | obs: | exp: |
| atg | M | 1 | 1.00 |
| gtt | V | 4 | 72.35 |
| gtc | V | 0 | 73.88 |
| gta | V | 58 | 65.97 |
| gtg | V | 290 | 139.80 |
| mPD | | 0.30 | 0.73 |
| | | nPD: | 0.41 |
| | | N. weight: | 0.84 |
| | | Sc. PD: | 0.12 |
| | | Sc. rank: | 77.2 |

,

| PA | | | |
|---|---|---|---|
| Pos. | 92 | obs: | exp: |
| aat | N | 279 | 184.50 |
| aac | N | 74 | 168.50 |
| mPD | | 0.33 | 0.50 |
| | | nPD: | 0.67 |
| | | N. weight: | 0.25 |
| | | Sc. PD: | 0.095 |
| | | Sc. rank: | 40.5 |

,

| PA | | | |
|---|---|---|---|
| Pos. | 93 | obs: | exp: |
| tct | S | 0 | 56.12 |
| tcc | S | 0 | 49.17 |
| tca | S | 0 | 78.20 |
| tcg | S | 0 | 21.52 |
| agt | S | 235 | 77.66 |
| agc | S | 118 | 70.33 |
| mPD | | 0.45 | 1.7 |
| | | nPD: | 0.27 |
| | | N. weight: | 1.4 |
| | | Sc. PD: | 0.0052 |
| | | Sc. rank: | 55.7 |

,

| PA | | | |
|---|---|---|---|
| Pos. | 94 | obs: | exp: |
| att | I | 72 | 132.70 |
| atc | I | 280 | 92.52 |
| ata | I | 1 | 127.80 |
| mPD | | 0.33 | 0.66 |
| | | nPD: | 0.5 |
| | | N. weight: | 1.1 |
| | | Sc. PD: | 0.26 |
| | | Sc. rank: | 141.6 |

,

| PA | | | |
|---|---|---|---|
| Pos. | 95 | obs: | exp: |
| tgt | C | 14 | 145.50 |
| tgc | C | 339 | 207.50 |
| mPD | | 0.076 | 0.48 |
| | | nPD: | 0.16 |
| | | N. weight: | 0.58 |
| | | Sc. PD: | -0.06 |
| | | Sc. rank: | -16.2 |

,

| PA | | | |
|---|---|---|---|
| Pos. | 96 | obs: | exp: |
| aat | N | 5 | 184.50 |
| aac | N | 348 | 168.50 |
| mPD | | 0.028 | 0.50 |
| | | nPD: | 0.06 |
| | | N. weight: | 1. |
| | | Sc. PD: | -0.2 |
| | | Sc. rank: | -87.4 |

,

| PA | | | |
|---|---|---|---|
| Pos. | 97 | obs: | exp: |
| act | T | 43 | 92.73 |
| acc | T | 306 | 75.32 |
| aca | T | 2 | 153.80 |
| acg | T | 0 | 29.18 |
| gct | A | 0 | 0.50 |
| gcc | A | 2 | 0.41 |
| gca | A | 0 | 0.89 |
| gcg | A | 0 | 0.20 |
| mPD | | 0.24 | 0.70 |
| | | nPD: | 0.34 |
| | | N. weight: | 1.7 |
| | | Sc. PD: | 0.12 |
| | | Sc. rank: | 116.2 |

,

| PA | | | |
|---|---|---|---|
| Pos. | 98 | obs: | exp: |
| act | T | 5 | 92.47 |
| acc | T | 4 | 75.10 |
| aca | T | 308 | 153.30 |
| acg | T | 33 | 29.10 |
| gct | A | 0 | 0.76 |
| gcc | A | 0 | 0.61 |
| gca | A | 3 | 1.34 |
| gcg | A | 0 | 0.29 |
| mPD | | 0.23 | 0.70 |
| | | nPD: | 0.33 |
| | | N. weight: | 0.86 |
| | | Sc. PD: | 0.055 |
| | | Sc. rank: | 54.6 |

,

| PA | | | |
|---|---|---|---|
| Pos. | 99 | obs: | exp: |
| cgt | R | 0 | 0.08 |
| cgc | R | 0 | 0.10 |
| cga | R | 0 | 0.19 |
| cgg | R | 0 | 0.17 |
| aga | R | 2 | 0.90 |
| agg | R | 0 | 0.56 |
| ggt | G | 7 | 43.31 |
| ggc | G | 0 | 47.47 |
| gga | G | 285 | 169.80 |
| ggg | G | 59 | 90.43 |
| mPD | | 0.32 | 0.68 |
| | | nPD: | 0.48 |
| | | N. weight: | 0.5 |
| | | Sc. PD: | 0.1 |
| | | Sc. rank: | 54.4 |

,

| PA | | | |
|---|---|---|---|
| Pos. | 100 | obs: | exp: |
| att | I | 0 | 1.13 |
| atc | I | 3 | 0.79 |
| ata | I | 0 | 1.09 |
| gtt | V | 64 | 71.12 |
| gtc | V | 275 | 72.62 |
| gta | V | 4 | 64.85 |
| gtg | V | 3 | 137.40 |
| gct | A | 0 | 1.01 |
| gcc | A | 4 | 0.82 |
| gca | A | 0 | 1.78 |
| gcg | A | 0 | 0.39 |
| mPD | | 0.37 | 0.76 |
| | | nPD: | 0.48 |
| | | N. weight: | 1.5 |
| | | Sc. PD: | 0.32 |
| | | Sc. rank: | 171.5 |

,

| PA | | | |
|---|---|---|---|
| Pos. | 101 | obs: | exp: |
| aat | N | 0 | 1.05 |
| aac | N | 2 | 0.95 |
| gat | D | 99 | 65.09 |
| gac | D | 23 | 56.91 |
| gaa | E | 107 | 131.20 |
| gag | E | 121 | 96.80 |
| ggt | G | 0 | 0.12 |
| ggc | G | 0 | 0.14 |
| gga | G | 0 | 0.48 |
| ggg | G | 1 | 0.26 |
| --- | -- | ------ | ------ |
| mPD | | 0.72 | 0.74 |
| | | nPD: | 0.98 |
| | | N. weight: | 0.15 |
| | | Sc. PD: | 0.1 |
| | | Sc. rank: | 29.5 |

,

| PA | | | |
|---|---|---|---|
| Pos. | 102 | obs: | exp: |
| aaa | K | 136 | 197.70 |
| aag | K | 217 | 155.30 |
| --- | -- | ------ | ------ |
| mPD | | 0.48 | 0.49 |
| | | nPD: | 0.96 |
| | | N. weight: | 0.11 |
| | | Sc. PD: | 0.072 |
| | | Sc. rank: | 21.5 |

,

| PA | | | |
|---|---|---|---|
| Pos. | 103 | obs: | exp: |
| cct | P | 204 | 92.95 |
| ccc | P | 147 | 63.67 |
| cca | P | 2 | 139.30 |
| ccg | P | 0 | 57.08 |
| --- | -- | ------ | ------ |
| mPD | | 0.49 | 0.72 |
| | | nPD: | 0.69 |
| | | N. weight: | 1.2 |
| | | Sc. PD: | 0.49 |
| | | Sc. rank: | 206.3 |

,

| PA | | | |
|---|---|---|---|
| Pos. | 104 | obs: | exp: |
| cgt | R | 0 | 0.46 |
| cgc | R | 0 | 0.54 |
| cga | R | 0 | 1.06 |
| cgg | R | 0 | 0.92 |
| aaa | K | 265 | 191.50 |
| aag | K | 77 | 150.50 |
| aga | R | 5 | 4.95 |
| agg | R | 6 | 3.08 |
| --- | -- | ------ | ------ |
| mPD | | 0.42 | 0.57 |
| | | nPD: | 0.74 |
| | | N. weight: | 0.18 |
| | | Sc. PD: | 0.083 |
| | | Sc. rank: | 33.7 |

,

| PA | | | |
|---|---|---|---|
| Pos. | 105 | obs: | exp: |
| ttt | F | 174 | 145.10 |
| ttc | F | 179 | 207.90 |
| --- | -- | ------ | ------ |
| mPD | | 0.50 | 0.48 |
| | | nPD: | 1.04 |
| | | N. weight: | 0.034 |
| | | Sc. PD: | 0.025 |
| | | Sc. rank: | 6.9 |

,

| PA | | | |
|---|---|---|---|
| Pos. | 106 | obs: | exp: |
| tta | L | 0 | 30.84 |
| ttg | L | 0 | 67.98 |
| ctt | L | 54 | 65.86 |
| ctc | L | 296 | 57.10 |
| cta | L | 3 | 53.18 |
| ctg | L | 0 | 78.03 |
| --- | -- | ------ | ------ |
| mPD | | 0.27 | 1.1 |
| | | nPD: | 0.25 |
| | | N. weight: | 2. |
| | | Sc. PD: | -0.036 |
| | | Sc. rank: | 64.1 |

,

| PA | | | |
|---|---|---|---|
| Pos. | 107 | obs: | exp: |
| cct | P | 58 | 92.95 |
| ccc | P | 46 | 63.67 |
| cca | P | 135 | 139.30 |
| ccg | P | 114 | 57.08 |
| --- | -- | ------ | ------ |
| mPD | | 0.71 | 0.72 |
| | | nPD: | 0.99 |
| | | N. weight: | 0.18 |
| | | Sc. PD: | 0.12 |
| | | Sc. rank: | 35.5 |

,

| PA | | | |
|---|---|---|---|
| Pos. | 108 | obs: | exp: |
| gat | D | 340 | 188.30 |
| gac | D | 13 | 164.70 |
| --- | -- | ------ | ------ |
| mPD | | 0.071 | 0.50 |
| | | nPD: | 0.14 |
| | | N. weight: | 0.73 |
| | | Sc. PD: | -0.085 |
| | | Sc. rank: | -23.2 |

,

| PA | | | |
|---|---|---|---|
| Pos. | 109 | obs: | exp: |
| tta | L | 48 | 30.84 |
| ttg | L | 246 | 67.98 |
| ctt | L | 0 | 65.86 |
| ctc | L | 0 | 57.10 |
| cta | L | 5 | 53.18 |
| ctg | L | 54 | 78.03 |
| --- | -- | ------ | ------ |
| mPD | | 0.54 | 1.1 |
| | | nPD: | 0.48 |
| | | N. weight: | 1.3 |
| | | Sc. PD: | 0.28 |
| | | Sc. rank: | 149.4 |

,

| PA | | | |
|---|---|---|---|
| Pos. | 110 | obs: | exp: |
| tat | Y | 287 | 182.70 |
| tac | Y | 66 | 170.30 |
| --- | -- | ------ | ------ |
| mPD | | 0.30 | 0.50 |
| | | nPD: | 0.61 |
| | | N. weight: | 0.3 |
| | | Sc. PD: | 0.1 |
| | | Sc. rank: | 45.5 |

,

| PA | | | |
|---|---|---|---|
| Pos. | 111 | obs: | exp: |
| gat | D | 114 | 188.30 |
| gac | D | 239 | 164.70 |
| --- | -- | ------ | ------ |
| mPD | | 0.44 | 0.50 |
| | | nPD: | 0.88 |
| | | N. weight: | 0.15 |
| | | Sc. PD: | 0.089 |
| | | Sc. rank: | 29.8 |

,

| PA | | | |
|---|---|---|---|
| Pos. | 112 | obs: | exp: |
| tat | Y | 53 | 182.70 |
| tac | Y | 300 | 170.30 |
| --- | -- | ------ | ------ |
| mPD | | 0.26 | 0.50 |
| | | nPD: | 0.51 |
| | | N. weight: | 0.46 |
| | | Sc. PD: | 0.11 |
| | | Sc. rank: | 58.1 |

,

| PA | | | |
|---|---|---|---|
| Pos. | 113 | obs: | exp: |
| cgt | R | 0 | 0.08 |
| cgc | R | 0 | 0.10 |
| cga | R | 0 | 0.19 |
| cgg | R | 0 | 0.17 |
| aaa | K | 290 | 196.50 |
| aag | K | 61 | 154.50 |
| aga | R | 2 | 0.90 |
| agg | R | 0 | 0.56 |
| --- | -- | ------ | ------ |
| mPD | | 0.30 | 0.51 |
| | | nPD: | 0.59 |
| | | N. weight: | 0.26 |
| | | Sc. PD: | 0.081 |
| | | Sc. rank: | 38.3 |

,

| PA | | | |
|---|---|---|---|
| Pos. | 114 | obs: | exp: |
| gaa | E | 42 | 203.10 |
| gag | E | 311 | 149.90 |
| --- | -- | ------ | ------ |
| mPD | | 0.21 | 0.49 |
| | | nPD: | 0.43 |
| | | N. weight: | 0.7 |
| | | Sc. PD: | 0.11 |
| | | Sc. rank: | 65.7 |

,

| PA | | | |
|---|---|---|---|
| Pos. | 115 | obs: | exp: |
| aat | N | 81 | 184.00 |
| aac | N | 271 | 168.00 |
| gat | D | 0 | 0.53 |
| gac | D | 1 | 0.47 |
| --- | -- | ------ | ------ |
| mPD | | 0.36 | 0.50 |
| | | nPD: | 0.71 |
| | | N. weight: | 0.29 |
| | | Sc. PD: | 0.12 |
| | | Sc. rank: | 50.7 |

,

| PA | | | |
|---|---|---|---|
| Pos. | 116 | obs: | exp: |
| caa | Q | 0 | 1.07 |
| cag | Q | 2 | 0.93 |
| cgt | R | 64 | 14.57 |
| cgc | R | 13 | 17.19 |
| cga | R | 250 | 33.73 |
| cgg | R | 21 | 29.29 |
| aga | R | 1 | 157.90 |
| agg | R | 2 | 98.36 |
| --- | -- | ------ | ------ |
| mPD | | 0.48 | 0.97 |
| | | nPD: | 0.5 |
| | | N. weight: | 2.5 |
| | | Sc. PD: | 0.56 |
| | | Sc. rank: | 307.5 |

,

| PA | | | |
|---|---|---|---|
| Pos. | 117 | obs: | exp: |
| ttt | F | 8 | 145.10 |
| ttc | F | 345 | 207.90 |
| --- | -- | ------ | ------ |
| mPD | | 0.044 | 0.48 |
| | | nPD: | 0.09 |
| | | N. weight: | 0.66 |
| | | Sc. PD: | -0.11 |
| | | Sc. rank: | -39.0 |

,

| PA | | | |
|---|---|---|---|
| Pos. | 118 | obs: | exp: |
| att | I | 331 | 131.20 |
| atc | I | 18 | 91.47 |
| ata | I | 0 | 126.30 |
| gtt | V | 4 | 0.82 |
| gtc | V | 0 | 0.84 |
| gta | V | 0 | 0.75 |
| gtg | V | 0 | 1.59 |
| --- | -- | ------ | ------ |
| mPD | | 0.12 | 0.68 |
| | | nPD: | 0.17 |
| | | N. weight: | 1.2 |
| | | Sc. PD: | -0.11 |
| | | Sc. rank: | -27.3 |

,

| PA | | | |
|---|---|---|---|
| Pos. | 119 | obs: | exp: |
| gaa | E | 336 | 203.10 |
| gag | E | 17 | 149.90 |
| --- | -- | ------ | ------ |
| mPD | | 0.092 | 0.49 |
| | | nPD: | 0.19 |
| | | N. weight: | 0.58 |
| | | Sc. PD: | -0.042 |
| | | Sc. rank: | -5.1 |

,

| PA | | | |
|---|---|---|---|
| Pos. | 120 | obs: | exp: |
| att | I | 340 | 130.80 |
| atc | I | 8 | 91.20 |
| ata | I | 0 | 126.00 |
| gtt | V | 5 | 1.03 |
| gtc | V | 0 | 1.05 |
| gta | V | 0 | 0.94 |
| gtg | V | 0 | 1.99 |
| --- | -- | ------ | ------ |
| mPD | | 0.072 | 0.69 |
| | | nPD: | 0.1 |
| | | N. weight: | 1.4 |
| | | Sc. PD: | -0.21 |
| | | Sc. rank: | -70.4 |

,

| PA | | | |
|---|---|---|---|
| Pos. | 121 | obs: | exp: |
| ggt | G | 1 | 43.56 |
| ggc | G | 0 | 47.74 |
| gga | G | 325 | 170.80 |
| ggg | G | 27 | 90.95 |
| --- | -- | ------ | ------ |
| mPD | | 0.15 | 0.67 |
| | | nPD: | 0.22 |
| | | N. weight: | 0.75 |
| | | Sc. PD: | -0.032 |
| | | Sc. rank: | 17.5 |

,

| PA | | | |
|---|---|---|---|
| Pos. | 122 | obs: | exp: |
| gtt | V | 0 | 72.56 |
| gtc | V | 0 | 74.09 |
| gta | V | 115 | 66.16 |
| gtg | V | 238 | 140.20 |
| --- | -- | ------ | ------ |
| mPD | | 0.44 | 0.72 |
| | | nPD: | 0.61 |
| | | N. weight: | 0.83 |
| | | Sc. PD: | 0.28 |
| | | Sc. rank: | 125.9 |

,

| PA | | | |
|---|---|---|---|
| Pos. | 123 | obs: | exp: |
| act | T | 1 | 93.26 |
| acc | T | 74 | 75.75 |
| aca | T | 206 | 154.60 |
| acg | T | 72 | 29.35 |
| --- | -- | ------ | ------ |
| mPD | | 0.58 | 0.69 |
| | | nPD: | 0.84 |
| | | N. weight: | 0.53 |
| | | Sc. PD: | 0.29 |
| | | Sc. rank: | 105.7 |

,

| PA | | | |
|---|---|---|---|
| Pos. | 124 | obs: | exp: |
| cgt | R | 0 | 14.65 |
| cgc | R | 0 | 17.28 |
| cga | R | 159 | 33.93 |
| cgg | R | 90 | 29.46 |
| aga | R | 34 | 158.80 |
| agg | R | 70 | 98.92 |
| --- | -- | ------ | ------ |
| mPD | | 0.91 | 0.96 |
| | | nPD: | 0.95 |
| | | N. weight: | 1.2 |
| | | Sc. PD: | 0.79 |
| | | Sc. rank: | 240.0 |

,

| PA | | | |
|---|---|---|---|
| Pos. | 125 | obs: | exp: |
| cgt | R | 0 | 14.65 |
| cgc | R | 0 | 17.28 |
| cga | R | 0 | 33.93 |
| cgg | R | 0 | 29.46 |
| aga | R | 36 | 158.80 |
| agg | R | 317 | 98.92 |
| --- | -- | ------ | ------ |
| mPD | | 0.18 | 0.96 |
| | | nPD: | 0.19 |
| | | N. weight: | 1.4 |
| | | Sc. PD: | -0.096 |
| | | Sc. rank: | -5.8 |

,

| PA | | | |
|---|---|---|---|
| Pos. | 126 | obs: | exp: |
| gaa | E | 277 | 203.10 |
| gag | E | 76 | 149.90 |
| --- | -- | ------ | ------ |
| mPD | | 0.34 | 0.49 |
| | | nPD: | 0.69 |
| | | N. weight: | 0.16 |
| | | Sc. PD: | 0.066 |
| | | Sc. rank: | 27.6 |

,

| PA | | | |
|---|---|---|---|
| Pos. | 127 | obs: | exp: |
| gtt | V | 211 | 72.56 |
| gtc | V | 140 | 74.09 |
| gta | V | 1 | 66.16 |
| gtg | V | 1 | 140.20 |
| --- | -- | ------ | ------ |
| mPD | | 0.49 | 0.72 |
| | | nPD: | 0.68 |
| | | N. weight: | 1.3 |
| | | Sc. PD: | 0.53 |
| | | Sc. rank: | 223.8 |

,

| PA | | | |
|---|---|---|---|
| Pos. | 128 | obs: | exp: |
| cat | H | 115 | 191.30 |
| cac | H | 238 | 161.70 |
| --- | -- | ------ | ------ |
| mPD | | 0.44 | 0.50 |
| | | nPD: | 0.89 |
| | | N. weight: | 0.16 |
| | | Sc. PD: | 0.094 |
| | | Sc. rank: | 31.2 |

,

| PA | | | |
|---|---|---|---|
| Pos. | 129 | obs: | exp: |
| att | I | 1 | 132.30 |
| atc | I | 4 | 92.25 |
| ata | I | 347 | 127.40 |
| atg | M | 1 | 1.00 |
| --- | -- | ------ | ------ |
| mPD | | 0.034 | 0.66 |
| | | nPD: | 0.05 |
| | | N. weight: | 1.4 |
| | | Sc. PD: | -0.29 |
| | | Sc. rank: | -130.4 |

,

| PA | | | |
|---|---|---|---|
| Pos. | 130 | obs: | exp: |
| tat | Y | 42 | 182.70 |
| tac | Y | 311 | 170.30 |
| --- | -- | ------ | ------ |
| mPD | | 0.21 | 0.50 |
| | | nPD: | 0.42 |
| | | N. weight: | 0.55 |
| | | Sc. PD: | 0.083 |
| | | Sc. rank: | 50.9 |

,

| PA | | | |
|---|---|---|---|
| Pos. | 131 | obs: | exp: |
| tat | Y | 287 | 182.70 |
| tac | Y | 66 | 170.30 |
| --- | -- | ------ | ------ |
| mPD | | 0.30 | 0.50 |
| | | nPD: | 0.61 |
| | | N. weight: | 0.3 |
| | | Sc. PD: | 0.1 |
| | | Sc. rank: | 45.5 |

| PA | | | |
|---|---|---|---|
| Pos. | 132 | obs: | exp: |
| tta | L | 76 | 30.75 |
| ttg | L | 44 | 67.79 |
| ctt | L | 0 | 65.68 |
| ctc | L | 0 | 56.94 |
| cta | L | 212 | 53.03 |
| ctg | L | 20 | 77.81 |
| att | I | 0 | 0.38 |
| atc | I | 0 | 0.26 |
| ata | I | 1 | 0.36 |
| --- | -- | ------ | ------ |
| mPD | | 0.75 | 1.1 |
| | | nPD: | 0.67 |
| | | N. weight: | 1.4 |
| | | Sc. PD: | 0.55 |
| | | Sc. rank: | 232.5 |

| PA | | | |
|---|---|---|---|
| Pos. | 133 | obs: | exp: |
| gaa | E | 285 | 203.10 |
| gag | E | 68 | 149.90 |
| --- | -- | ------ | ------ |
| mPD | | 0.31 | 0.49 |
| | | nPD: | 0.64 |
| | | N. weight: | 0.2 |
| | | Sc. PD: | 0.07 |
| | | Sc. rank: | 31.2 |

| PA | | | |
|---|---|---|---|
| Pos. | 134 | obs: | exp: |
| aaa | K | 260 | 197.70 |
| aag | K | 93 | 155.30 |
| --- | -- | ------ | ------ |
| mPD | | 0.39 | 0.49 |
| | | nPD: | 0.79 |
| | | N. weight: | 0.11 |
| | | Sc. PD: | 0.058 |
| | | Sc. rank: | 21.8 |

| PA | | | |
|---|---|---|---|
| Pos. | 135 | obs: | exp: |
| gct | A | 11 | 88.91 |
| gcc | A | 342 | 72.31 |
| gca | A | 0 | 157.30 |
| gcg | A | 0 | 34.45 |
| --- | -- | ------ | ------ |
| mPD | | 0.061 | 0.69 |
| | | nPD: | 0.09 |
| | | N. weight: | 2.2 |
| | | Sc. PD: | -0.37 |
| | | Sc. rank: | -138.9 |

| PA | | | |
|---|---|---|---|
| Pos. | 136 | obs: | exp: |
| aat | N | 96 | 184.50 |
| aac | N | 257 | 168.50 |
| --- | -- | ------ | ------ |
| mPD | | 0.40 | 0.50 |
| | | nPD: | 0.8 |
| | | N. weight: | 0.21 |
| | | Sc. PD: | 0.11 |
| | | Sc. rank: | 40.3 |

| PA | | | |
|---|---|---|---|
| Pos. | 137 | obs: | exp: |
| aaa | K | 97 | 197.70 |
| aag | K | 256 | 155.30 |
| --- | -- | ------ | ------ |
| mPD | | 0.40 | 0.49 |
| | | nPD: | 0.81 |
| | | N. weight: | 0.27 |
| | | Sc. PD: | 0.14 |
| | | Sc. rank: | 51.8 |

| PA | | | |
|---|---|---|---|
| Pos. | 138 | obs: | exp: |
| att | I | 0 | 132.70 |
| atc | I | 0 | 92.52 |
| ata | I | 353 | 127.80 |
| --- | -- | ------ | ------ |
| mPD | | 0 | 0.66 |
| | | nPD: | 0. |
| | | N. weight: | 1.5 |
| | | Sc. PD: | -0.39 |
| | | Sc. rank: | -308.0 |

| PA | | | |
|---|---|---|---|
| Pos. | 139 | obs: | exp: |
| aaa | K | 296 | 197.70 |
| aag | K | 57 | 155.30 |
| --- | -- | ------ | ------ |
| mPD | | 0.27 | 0.49 |
| | | nPD: | 0.55 |
| | | N. weight: | 0.28 |
| | | Sc. PD: | 0.077 |
| | | Sc. rank: | 39.1 |

| PA | | | |
|---|---|---|---|
| Pos. | 140 | obs: | exp: |
| tct | S | 98 | 56.12 |
| tcc | S | 60 | 49.17 |
| tca | S | 166 | 78.20 |
| tcg | S | 29 | 21.52 |
| agt | S | 0 | 77.66 |
| agc | S | 0 | 70.33 |
| --- | -- | ------ | ------ |
| mPD | | 0.67 | 1.7 |
| | | nPD: | 0.4 |
| | | N. weight: | 0.89 |
| | | Sc. PD: | 0.12 |
| | | Sc. rank: | 77.4 |

| PA | | | |
|---|---|---|---|
| Pos. | 141 | obs: | exp: |
| aaa | K | 0 | 0.56 |
| aag | K | 1 | 0.44 |
| gaa | E | 18 | 202.60 |
| gag | E | 334 | 149.40 |
| --- | -- | ------ | ------ |
| mPD | | 0.10 | 0.49 |
| | | nPD: | 0.21 |
| | | N. weight: | 0.98 |
| | | Sc. PD: | -0.053 |
| | | Sc. rank: | 10.4 |

| PA | | | |
|---|---|---|---|
| Pos. | 142 | obs: | exp: |
| cgt | R | 0 | 0.17 |
| cgc | R | 0 | 0.20 |
| cga | R | 0 | 0.38 |
| cgg | R | 0 | 0.33 |
| aaa | K | 48 | 195.40 |
| aag | K | 301 | 153.60 |
| aga | R | 0 | 1.80 |
| agg | R | 4 | 1.12 |
| --- | -- | ------ | ------ |
| mPD | | 0.26 | 0.52 |
| | | nPD: | 0.49 |
| | | N. weight: | 0.61 |
| | | Sc. PD: | 0.14 |
| | | Sc. rank: | 72.8 |

| PA | | | |
|---|---|---|---|
| Pos. | 143 | obs: | exp: |
| act | T | 0 | 93.26 |
| acc | T | 1 | 75.75 |
| aca | T | 351 | 154.60 |
| acg | T | 1 | 29.35 |
| --- | -- | ------ | ------ |
| mPD | | 0.011 | 0.69 |
| | | nPD: | 0.02 |
| | | N. weight: | 1.2 |
| | | Sc. PD: | -0.29 |
| | | Sc. rank: | -164.2 |

| PA | | | |
|---|---|---|---|
| Pos. | 144 | obs: | exp: |
| cat | H | 8 | 191.30 |
| cac | H | 345 | 161.70 |
| --- | -- | ------ | ------ |
| mPD | | 0.044 | 0.50 |
| | | nPD: | 0.09 |
| | | N. weight: | 1. |
| | | Sc. PD: | -0.17 |
| | | Sc. rank: | -62.4 |

| PA | | | |
|---|---|---|---|
| Pos. | 145 | obs: | exp: |
| att | I | 240 | 132.70 |
| atc | I | 113 | 92.52 |
| ata | I | 0 | 127.80 |
| --- | -- | ------ | ------ |
| mPD | | 0.44 | 0.66 |
| | | nPD: | 0.66 |
| | | N. weight: | 0.72 |
| | | Sc. PD: | 0.28 |
| | | Sc. rank: | 117.7 |

| PA | | | |
|---|---|---|---|
| Pos. | 146 | obs: | exp: |
| cat | H | 19 | 191.30 |
| cac | H | 334 | 161.70 |
| --- | -- | ------ | ------ |
| mPD | | 0.10 | 0.50 |
| | | nPD: | 0.21 |
| | | N. weight: | 0.86 |
| | | Sc. PD: | -0.049 |
| | | Sc. rank: | 7.9 |

| PA | | | |
|---|---|---|---|
| Pos. | 147 | obs: | exp: |
| att | I | 45 | 132.70 |
| atc | I | 85 | 92.52 |
| ata | I | 223 | 127.80 |
| --- | -- | ------ | ------ |
| mPD | | 0.53 | 0.66 |
| | | nPD: | 0.8 |
| | | N. weight: | 0.32 |
| | | Sc. PD: | 0.16 |
| | | Sc. rank: | 61.2 |

| PA | | | |
|---|---|---|---|
| Pos. | 148 | obs: | exp: |
| ttt | F | 95 | 145.10 |
| ttc | F | 258 | 207.90 |
| --- | -- | ------ | ------ |
| mPD | | 0.39 | 0.48 |
| | | nPD: | 0.81 |
| | | N. weight: | 0.079 |
| | | Sc. PD: | 0.042 |
| | | Sc. rank: | 15.5 |

| PA | | | |
|---|---|---|---|
| Pos. | 149 | obs: | exp: |
| tct | S | 2 | 56.12 |
| tcc | S | 3 | 49.17 |
| tca | S | 343 | 78.20 |
| tcg | S | 5 | 21.52 |
| agt | S | 0 | 77.66 |
| agc | S | 0 | 70.33 |
| --- | -- | ------ | ------ |
| mPD | | 0.056 | 1.7 |
| | | nPD: | 0.03 |
| | | N. weight: | 2.1 |
| | | Sc. PD: | -0.47 |
| | | Sc. rank: | -232.2 |

| PA | | | |
|---|---|---|---|
| Pos. | 150 | obs: | exp: |
| ttt | F | 34 | 145.10 |
| ttc | F | 319 | 207.90 |
| --- | -- | ------ | ------ |
| mPD | | 0.17 | 0.48 |
| | | nPD: | 0.36 |
| | | N. weight: | 0.39 |
| | | Sc. PD: | 0.036 |
| | | Sc. rank: | 30.6 |

| PA | | | |
|---|---|---|---|
| Pos. | 151 | obs: | exp: |
| act | T | 325 | 93.26 |
| acc | T | 24 | 75.75 |
| aca | T | 4 | 154.60 |
| acg | T | 0 | 29.35 |
| --- | -- | ------ | ------ |
| mPD | | 0.15 | 0.69 |
| | | nPD: | 0.22 |
| | | N. weight: | 1.6 |
| | | Sc. PD: | -0.074 |
| | | Sc. rank: | 31.1 |

| PA | | | |
|---|---|---|---|
| Pos. | 152 | obs: | exp: |
| ggt | G | 1 | 43.56 |
| ggc | G | 0 | 47.74 |
| gga | G | 276 | 170.80 |
| ggg | G | 76 | 90.95 |
| --- | -- | ------ | ------ |
| mPD | | 0.34 | 0.67 |
| | | nPD: | 0.52 |
| | | N. weight: | 0.51 |
| | | Sc. PD: | 0.12 |
| | | Sc. rank: | 65.5 |

| PA | | | |
|---|---|---|---|
| Pos. | 153 | obs: | exp: |
| gaa | E | 157 | 203.10 |
| gag | E | 196 | 149.90 |
| --- | -- | ------ | ------ |
| mPD | | 0.50 | 0.49 |
| | | nPD: | 1.01 |
| | | N. weight: | 0.066 |
| | | Sc. PD: | 0.047 |
| | | Sc. rank: | 13.3 |

| PA | | | |
|---|---|---|---|
| Pos. | 154 | obs: | exp: |
| gaa | E | 310 | 203.10 |
| gag | E | 43 | 149.90 |
| --- | -- | ------ | ------ |
| mPD | | 0.21 | 0.49 |
| | | nPD: | 0.44 |
| | | N. weight: | 0.34 |
| | | Sc. PD: | 0.058 |
| | | Sc. rank: | 33.4 |

| PA | | | |
|---|---|---|---|
| Pos. | 155 | obs: | exp: |
| atg | M | 353 | 353.00 |
| --- | -- | ------ | ------ |
| mPD | | 0 | 0 |
| | | nPD: | 1. |
| | | N. weight: | 0. |
| | | Sc. PD: | 0 |
| | | Sc. rank: | 0 |

| PA | | | |
|---|---|---|---|
| Pos. | 156 | obs: | exp: |
| gct | A | 69 | 88.91 |
| gcc | A | 280 | 72.31 |
| gca | A | 4 | 157.30 |
| gcg | A | 0 | 34.45 |
| --- | -- | ------ | ------ |
| mPD | | 0.33 | 0.69 |
| | | nPD: | 0.49 |
| | | N. weight: | 1.5 |
| | | Sc. PD: | 0.32 |
| | | Sc. rank: | 173.8 |

| PA | | | |
|---|---|---|---|
| Pos. | 157 | obs: | exp: |
| act | T | 78 | 93.26 |
| acc | T | 273 | 75.75 |
| aca | T | 2 | 154.60 |
| acg | T | 0 | 29.35 |
| --- | -- | ------ | ------ |
| mPD | | 0.35 | 0.69 |
| | | nPD: | 0.52 |
| | | N. weight: | 1.4 |
| | | Sc. PD: | 0.34 |
| | | Sc. rank: | 185.0 |

| PA | | | |
|---|---|---|---|
| Pos. | 158 | obs: | exp: |
| cgt | R | 0 | 0.04 |
| cgc | R | 0 | 0.05 |
| cga | R | 0 | 0.10 |
| cgg | R | 0 | 0.08 |
| aaa | K | 286 | 197.10 |
| aag | K | 66 | 154.90 |
| aga | R | 1 | 0.45 |
| agg | R | 0 | 0.28 |
| --- | -- | ------ | ------ |
| mPD | | 0.31 | 0.50 |
| | | nPD: | 0.62 |
| | | N. weight: | 0.23 |
| | | Sc. PD: | 0.079 |
| | | Sc. rank: | 35.9 |

| PA | | | |
|---|---|---|---|
| Pos. | 159 | obs: | exp: |
| tct | S | 0 | 0.48 |
| tcc | S | 0 | 0.42 |
| tca | S | 2 | 0.66 |
| tcg | S | 1 | 0.18 |
| agt | S | 0 | 0.66 |
| agc | S | 0 | 0.60 |
| gtt | V | 0 | 0.21 |
| gtc | V | 0 | 0.21 |
| gta | V | 0 | 0.19 |
| gtg | V | 1 | 0.40 |
| gct | A | 2 | 87.40 |
| gcc | A | 5 | 71.08 |
| gca | A | 127 | 154.70 |
| gcg | A | 213 | 33.86 |
| gaa | E | 0 | 1.15 |
| gag | E | 2 | 0.85 |
| --- | -- | ------ | ------ |
| mPD | | 0.52 | 0.73 |
| | | nPD: | 0.72 |
| | | N. weight: | 1.5 |
| | | Sc. PD: | 0.67 |
| | | Sc. rank: | 276.2 |

| PA | | | |
|---|---|---|---|
| Pos. | 160 | obs: | exp: |
| gat | D | 25 | 188.30 |
| gac | D | 328 | 164.70 |
| --- | -- | ------ | ------ |
| mPD | | 0.13 | 0.50 |
| | | nPD: | 0.27 |
| | | N. weight: | 0.76 |
| | | Sc. PD: | 0.00045 |
| | | Sc. rank: | 28.3 |

| PA | | | |
|---|---|---|---|
| Pos. | 161 | obs: | exp: |
| tat | Y | 62 | 182.20 |
| tac | Y | 290 | 169.80 |
| cat | H | 0 | 0.54 |
| cac | H | 1 | 0.46 |
| --- | -- | ------ | ------ |
| mPD | | 0.30 | 0.51 |
| | | nPD: | 0.59 |
| | | N. weight: | 0.39 |
| | | Sc. PD: | 0.12 |
| | | Sc. rank: | 58.0 |

| PA | | | |
|---|---|---|---|
| Pos. | 162 | obs: | exp: |
| tct | S | 0 | 0.16 |
| tcc | S | 0 | 0.14 |
| tca | S | 0 | 0.22 |
| tcg | S | 0 | 0.06 |
| att | I | 0 | 0.38 |
| atc | I | 1 | 0.26 |
| ata | I | 0 | 0.36 |
| act | T | 138 | 92.73 |
| acc | T | 212 | 75.32 |
| aca | T | 1 | 153.80 |
| acg | T | 0 | 29.18 |
| agt | S | 0 | 0.22 |
| agc | S | 1 | 0.20 |
| --- | -- | ------ | ------ |
| mPD | | 0.49 | 0.70 |
| | | nPD: | 0.71 |
| | | N. weight: | 1.2 |
| | | Sc. PD: | 0.5 |
| | | Sc. rank: | 207.0 |

| PA | | | |
|---|---|---|---|
| Pos. | 163 | obs: | exp: |
| tta | L | 0 | 30.84 |
| ttg | L | 0 | 67.98 |
| ctt | L | 328 | 65.86 |
| ctc | L | 10 | 57.10 |
| cta | L | 15 | 53.18 |
| ctg | L | 0 | 78.03 |
| --- | -- | ------ | ------ |
| mPD | | 0.13 | 1.1 |
| | | nPD: | 0.12 |
| | | N. weight: | 2.1 |
| | | Sc. PD: | -0.29 |
| | | Sc. rank: | -91.2 |

| PA | | | |
|---|---|---|---|
| Pos. | 164 | obs: | exp: |
| gat | D | 257 | 188.30 |
| gac | D | 96 | 164.70 |
| --- | -- | ------ | ------ |
| mPD | | 0.40 | 0.50 |
| | | nPD: | 0.8 |
| | | N. weight: | 0.13 |
| | | Sc. PD: | 0.068 |
| | | Sc. rank: | 25.7 |

| PA | | | |
|---|---|---|---|
| Pos. | 165 | obs: | exp: |
| gaa | E | 292 | 203.10 |
| gag | E | 61 | 149.90 |
| --- | -- | ------ | ------ |
| mPD | | 0.29 | 0.49 |
| | | nPD: | 0.59 |
| | | N. weight: | 0.23 |
| | | Sc. PD: | 0.072 |
| | | Sc. rank: | 34.1 |

| PA | | | |
|---|---|---|---|
| Pos. | 166 | obs: | exp: |
| gaa | E | 101 | 203.10 |
| gag | E | 252 | 149.90 |
| --- | -- | ------ | ------ |
| mPD | | 0.41 | 0.49 |
| | | nPD: | 0.84 |
| | | N. weight: | 0.27 |
| | | Sc. PD: | 0.15 |
| | | Sc. rank: | 53.9 |

| PA | | | |
|---|---|---|---|
| Pos. | 167 | obs: | exp: |
| tct | S | 0 | 56.12 |
| tcc | S | 0 | 49.17 |
| tca | S | 0 | 78.20 |
| tcg | S | 0 | 21.52 |
| agt | S | 127 | 77.66 |
| agc | S | 226 | 70.33 |
| --- | -- | ------ | ------ |
| mPD | | 0.46 | 1.7 |
| | | nPD: | 0.28 |
| | | N. weight: | 1.4 |
| | | Sc. PD: | 0.018 |
| | | Sc. rank: | 61.5 |

| PA | | | |
|---|---|---|---|
| Pos. | 168 | obs: | exp: |
| cgt | R | 0 | 14.65 |
| cgc | R | 0 | 17.28 |
| cga | R | 0 | 33.93 |
| cgg | R | 0 | 29.46 |
| aga | R | 278 | 158.80 |
| agg | R | 75 | 98.92 |
| --- | -- | ------ | ------ |
| mPD | | 0.34 | 0.96 |
| | | nPD: | 0.35 |
| | | N. weight: | 0.59 |
| | | Sc. PD: | 0.048 |
| | | Sc. rank: | 44.3 |

| PA | | | |
|---|---|---|---|
| Pos. | 169 | obs: | exp: |
| gct | A | 1 | 88.91 |
| gcc | A | 1 | 72.31 |
| gca | A | 308 | 157.30 |
| gcg | A | 43 | 34.45 |
| --- | -- | ------ | ------ |
| mPD | | 0.22 | 0.69 |
| | | nPD: | 0.33 |
| | | N. weight: | 0.91 |
| | | Sc. PD: | 0.055 |
| | | Sc. rank: | 55.4 |

| PA | | | |
|---|---|---|---|
| Pos. | 170 | obs: | exp: |
| cgt | R | 0 | 14.65 |
| cgc | R | 0 | 17.28 |
| cga | R | 1 | 33.93 |
| cgg | R | 0 | 29.46 |
| aga | R | 277 | 158.80 |
| agg | R | 75 | 98.92 |
| --- | -- | ------ | ------ |
| mPD | | 0.34 | 0.96 |
| | | nPD: | 0.36 |
| | | N. weight: | 0.57 |
| | | Sc. PD: | 0.05 |
| | | Sc. rank: | 43.8 |

| PA | | | |
|---|---|---|---|
| Pos. | 171 | obs: | exp: |
| att | I | 19 | 132.00 |
| atc | I | 228 | 91.99 |
| ata | I | 104 | 127.10 |
| gtt | V | 1 | 0.41 |
| gtc | V | 0 | 0.42 |
| gta | V | 1 | 0.37 |
| gtg | V | 0 | 0.79 |
| --- | -- | ------ | ------ |
| mPD | | 0.50 | 0.67 |
| | | nPD: | 0.75 |
| | | N. weight: | 0.67 |
| | | Sc. PD: | 0.31 |
| | | Sc. rank: | 125.3 |

| PA | | | |
|---|---|---|---|
| Pos. | 172 | obs: | exp: |
| aat | N | 0 | 1.05 |
| aac | N | 2 | 0.95 |
| aaa | K | 339 | 196.50 |
| aag | K | 12 | 154.50 |
| --- | -- | ------ | ------ |
| mPD | | 0.077 | 0.50 |
| | | nPD: | 0.15 |
| | | N. weight: | 0.68 |
| | | Sc. PD: | -0.072 |
| | | Sc. rank: | -19.1 |

| PA | | | |
|---|---|---|---|
| Pos. | 173 | obs: | exp: |
| act | T | 74 | 93.26 |
| acc | T | 279 | 75.75 |
| aca | T | 0 | 154.60 |
| acg | T | 0 | 29.35 |
| --- | -- | ------ | ------ |
| mPD | | 0.33 | 0.69 |
| | | nPD: | 0.48 |
| | | N. weight: | 1.5 |
| | | Sc. PD: | 0.32 |
| | | Sc. rank: | 171.0 |

| PA | | | |
|---|---|---|---|
| Pos. | 174 | obs: | exp: |
| cgt | R | 0 | 14.65 |
| cgc | R | 0 | 17.28 |
| cga | R | 0 | 33.93 |
| cgg | R | 0 | 29.46 |
| aga | R | 89 | 158.80 |
| agg | R | 264 | 98.92 |
| --- | -- | ------ | ------ |
| mPD | | 0.38 | 0.96 |
| | | nPD: | 0.39 |
| | | N. weight: | 0.9 |
| | | Sc. PD: | 0.11 |
| | | Sc. rank: | 76.9 |

| PA | | | |
|---|---|---|---|
| Pos. | 175 | obs: | exp: |
| tta | L | 5 | 30.84 |
| ttg | L | 6 | 67.98 |
| ctt | L | 6 | 65.86 |
| ctc | L | 0 | 57.10 |
| cta | L | 80 | 53.18 |
| ctg | L | 256 | 78.03 |
| --- | -- | ------ | ------ |
| mPD | | 0.45 | 1.1 |
| | | nPD: | 0.41 |
| | | N. weight: | 1.3 |
| | | Sc. PD: | 0.18 |
| | | Sc. rank: | 117.2 |

| PA | | | |
|---|---|---|---|
| Pos. | 176 | obs: | exp: |
| ttt | F | 0 | 144.30 |
| ttc | F | 351 | 206.70 |
| tat | Y | 0 | 1.04 |
| tac | Y | 2 | 0.96 |
| --- | -- | ------ | ------ |
| mPD | | 0.011 | 0.50 |
| | | nPD: | 0.02 |
| | | N. weight: | 0.8 |
| | | Sc. PD: | -0.19 |
| | | Sc. rank: | -103.3 |

| PA | | | |
|---|---|---|---|
| Pos. | 177 | obs: | exp: |
| act | T | 233 | 93.26 |
| acc | T | 120 | 75.75 |
| aca | T | 0 | 154.60 |
| acg | T | 0 | 29.35 |
| --- | -- | ------ | ------ |
| mPD | | 0.45 | 0.69 |
| | | nPD: | 0.66 |
| | | N. weight: | 1.2 |
| | | Sc. PD: | 0.44 |
| | | Sc. rank: | 189.6 |

| PA | | | |
|---|---|---|---|
| Pos. | 178 | obs: | exp: |
| att | I | 1 | 132.70 |
| atc | I | 0 | 92.52 |
| ata | I | 352 | 127.80 |
| --- | -- | ------ | ------ |
| mPD | | 0.0057 | 0.66 |
| | | nPD: | 0.01 |
| | | N. weight: | 1.5 |
| | | Sc. PD: | -0.37 |
| | | Sc. rank: | -233.0 |

| PA | | | |
|---|---|---|---|
| Pos. | 179 | obs: | exp: |
| cgt | R | 0 | 14.65 |
| cgc | R | 0 | 17.28 |
| cga | R | 0 | 33.93 |
| cgg | R | 0 | 29.46 |
| aga | R | 208 | 158.80 |
| agg | R | 145 | 98.92 |
| --- | -- | ------ | ------ |
| mPD | | 0.49 | 0.96 |
| | | nPD: | 0.51 |
| | | N. weight: | 0.49 |
| | | Sc. PD: | 0.11 |
| | | Sc. rank: | 61.9 |

| PA | | | |
|---|---|---|---|
| Pos. | 180 | obs: | exp: |
| caa | Q | 313 | 188.20 |
| cag | Q | 40 | 164.80 |
| --- | -- | ------ | ------ |
| mPD | | 0.20 | 0.50 |
| | | nPD: | 0.4 |
| | | N. weight: | 0.45 |
| | | Sc. PD: | 0.061 |
| | | Sc. rank: | 39.7 |

PA

| Pos. | 181 | obs: | exp: |
|---|---|---|---|
| gaa | E | 301 | 203.10 |
| gag | E | 52 | 149.90 |
| --- | -- | ------ | ------ |
| mPD | | 0.25 | 0.49 |
| | | nPD: | 0.52 |
| | | N. weight: | 0.28 |
| | | Sc. PD: | 0.068 |
| | | Sc. rank: | 36.4 |

PA

| Pos. | 182 | obs: | exp: |
|---|---|---|---|
| atg | M | 353 | 353.00 |
| --- | -- | ------ | ------ |
| mPD | | 0 | 0 |
| | | nPD: | 1. |
| | | N. weight: | 0. |
| | | Sc. PD: | 0 |
| | | Sc. rank: | 0 |

PA

| Pos. | 183 | obs: | exp: |
|---|---|---|---|
| act | T | 0 | 0.26 |
| acc | T | 1 | 0.21 |
| aca | T | 0 | 0.44 |
| acg | T | 0 | 0.08 |
| gct | A | 2 | 88.66 |
| gcc | A | 341 | 72.11 |
| gca | A | 9 | 156.90 |
| gcg | A | 0 | 34.35 |
| --- | -- | ------ | ------ |
| mPD | | 0.067 | 0.69 |
| | | nPD: | 0.1 |
| | | N. weight: | 2.1 |
| | | Sc. PD: | -0.35 |
| | | Sc. rank: | -119.4 |

PA

| Pos. | 184 | obs: | exp: |
|---|---|---|---|
| tct | S | 0 | 55.01 |
| tcc | S | 0 | 48.20 |
| tca | S | 0 | 76.65 |
| tcg | S | 0 | 21.09 |
| act | T | 5 | 1.32 |
| acc | T | 0 | 1.07 |
| aca | T | 0 | 2.19 |
| acg | T | 0 | 0.42 |
| aat | N | 2 | 1.05 |
| aac | N | 0 | 0.95 |
| agt | S | 289 | 76.12 |
| agc | S | 57 | 68.93 |
| --- | -- | ------ | ------ |
| mPD | | 0.31 | 1.7 |
| | | nPD: | 0.19 |
| | | N. weight: | 1.7 |
| | | Sc. PD: | -0.12 |
| | | Sc. rank: | -20.0 |

PA

| Pos. | 185 | obs: | exp: |
|---|---|---|---|
| cgt | R | 0 | 14.57 |
| cgc | R | 0 | 17.19 |
| cga | R | 0 | 33.73 |
| cgg | R | 0 | 29.29 |
| aaa | K | 0 | 1.12 |
| aag | K | 2 | 0.88 |
| aga | R | 90 | 157.90 |
| agg | R | 261 | 98.36 |
| --- | -- | ------ | ------ |
| mPD | | 0.39 | 0.97 |
| | | nPD: | 0.4 |
| | | N. weight: | 0.9 |
| | | Sc. PD: | 0.12 |
| | | Sc. rank: | 78.5 |

PA

| Pos. | 186 | obs: | exp: |
|---|---|---|---|
| ggt | G | 271 | 43.56 |
| ggc | G | 81 | 47.74 |
| gga | G | 1 | 170.80 |
| ggg | G | 0 | 90.95 |
| --- | -- | ------ | ------ |
| mPD | | 0.36 | 0.67 |
| | | nPD: | 0.54 |
| | | N. weight: | 2.3 |
| | | Sc. PD: | 0.61 |
| | | Sc. rank: | 316.1 |

PA

| Pos. | 187 | obs: | exp: |
|---|---|---|---|
| tta | L | 14 | 30.84 |
| ttg | L | 0 | 67.98 |
| ctt | L | 1 | 65.86 |
| ctc | L | 69 | 57.10 |
| cta | L | 268 | 53.18 |
| ctg | L | 1 | 78.03 |
| --- | -- | ------ | ------ |
| mPD | | 0.40 | 1.1 |
| | | nPD: | 0.36 |
| | | N. weight: | 1.9 |
| | | Sc. PD: | 0.17 |
| | | Sc. rank: | 147.4 |

PA

| Pos. | 188 | obs: | exp: |
|---|---|---|---|
| tgg | W | 353 | 353.00 |
| --- | -- | ------ | ------ |
| mPD | | 0 | 0 |
| | | nPD: | 1. |
| | | N. weight: | 0. |
| | | Sc. PD: | 0 |
| | | Sc. rank: | 0 |

PA

| Pos. | 189 | obs: | exp: |
|---|---|---|---|
| gat | D | 342 | 188.30 |
| gac | D | 11 | 164.70 |
| --- | -- | ------ | ------ |
| mPD | | 0.061 | 0.50 |
| | | nPD: | 0.12 |
| | | N. weight: | 0.76 |
| | | Sc. PD: | -0.1 |
| | | Sc. rank: | -31.5 |

PA

| Pos. | 190 | obs: | exp: |
|---|---|---|---|
| tct | S | 1 | 55.80 |
| tcc | S | 346 | 48.90 |
| tca | S | 4 | 77.76 |
| tcg | S | 0 | 21.39 |
| cct | P | 0 | 0.53 |
| ccc | P | 2 | 0.36 |
| cca | P | 0 | 0.79 |
| ccg | P | 0 | 0.32 |
| agt | S | 0 | 77.22 |
| agc | S | 0 | 69.93 |
| --- | -- | ------ | ------ |
| mPD | | 0.039 | 1.7 |
| | | nPD: | 0.02 |
| | | N. weight: | 2.9 |
| | | Sc. PD: | -0.66 |
| | | Sc. rank: | -350.9 |

PA

| Pos. | 191 | obs: | exp: |
|---|---|---|---|
| ttt | F | 353 | 145.10 |
| ttc | F | 0 | 207.90 |
| --- | -- | ------ | ------ |
| mPD | | 0 | 0.48 |
| | | nPD: | 0. |
| | | N. weight: | 1.3 |
| | | Sc. PD: | -0.34 |
| | | Sc. rank: | -269.4 |

PA

| Pos. | 192 | obs: | exp: |
|---|---|---|---|
| cgt | R | 352 | 14.65 |
| cgc | R | 1 | 17.28 |
| cga | R | 0 | 33.93 |
| cgg | R | 0 | 29.46 |
| aga | R | 0 | 158.80 |
| agg | R | 0 | 98.92 |
| --- | -- | ------ | ------ |
| mPD | | 0.0057 | 0.96 |
| | | nPD: | 0.01 |
| | | N. weight: | 4.8 |
| | | Sc. PD: | -1.2 |
| | | Sc. rank: | -780.6 |

PA

| Pos. | 193 | obs: | exp: |
|---|---|---|---|
| caa | Q | 1 | 188.20 |
| cag | Q | 352 | 164.80 |
| --- | -- | ------ | ------ |
| mPD | | 0.0057 | 0.50 |
| | | nPD: | 0.01 |
| | | N. weight: | 1.1 |
| | | Sc. PD: | -0.27 |
| | | Sc. rank: | -165.4 |

PA

| Pos. | 194 | obs: | exp: |
|---|---|---|---|
| tct | S | 0 | 56.12 |
| tcc | S | 353 | 49.17 |
| tca | S | 0 | 78.20 |
| tcg | S | 0 | 21.52 |
| agt | S | 0 | 77.66 |
| agc | S | 0 | 70.33 |
| --- | -- | ------ | ------ |
| mPD | | 0 | 1.7 |
| | | nPD: | 0. |
| | | N. weight: | 3. |
| | | Sc. PD: | -0.76 |
| | | Sc. rank: | -597.4 |

PA

| Pos. | 195 | obs: | exp: |
|---|---|---|---|
| gaa | E | 66 | 203.10 |
| gag | E | 287 | 149.90 |
| --- | -- | ------ | ------ |
| mPD | | 0.30 | 0.49 |
| | | nPD: | 0.62 |
| | | N. weight: | 0.49 |
| | | Sc. PD: | 0.17 |
| | | Sc. rank: | 77.0 |

PA

| Pos. | 196 | obs: | exp: |
|---|---|---|---|
| cgt | R | 0 | 14.65 |
| cgc | R | 0 | 17.28 |
| cga | R | 0 | 33.93 |
| cgg | R | 0 | 29.46 |
| aga | R | 353 | 158.80 |
| agg | R | 0 | 98.92 |
| --- | -- | ------ | ------ |
| mPD | | 0 | 0.96 |
| | | nPD: | 0. |
| | | N. weight: | 1.2 |
| | | Sc. PD: | -0.31 |
| | | Sc. rank: | -242.2 |

PA

| Pos. | 197 | obs: | exp: |
|---|---|---|---|
| ggt | G | 0 | 43.56 |
| ggc | G | 353 | 47.74 |
| gga | G | 0 | 170.80 |
| ggg | G | 0 | 90.95 |
| --- | -- | ------ | ------ |
| mPD | | 0 | 0.67 |
| | | nPD: | 0. |
| | | N. weight: | 3. |
| | | Sc. PD: | -0.77 |
| | | Sc. rank: | -606.3 |

PA

| Pos. | 198 | obs: | exp: |
|---|---|---|---|
| gaa | E | 347 | 203.10 |
| gag | E | 6 | 149.90 |
| --- | -- | ------ | ------ |
| mPD | | 0.034 | 0.49 |
| | | nPD: | 0.07 |
| | | N. weight: | 0.72 |
| | | Sc. PD: | -0.14 |
| | | Sc. rank: | -54.6 |

PA

| Pos. | 199 | obs: | exp: |
|---|---|---|---|
| gaa | E | 46 | 203.10 |
| gag | E | 307 | 149.90 |
| --- | -- | ------ | ------ |
| mPD | | 0.23 | 0.49 |
| | | nPD: | 0.47 |
| | | N. weight: | 0.66 |
| | | Sc. PD: | 0.13 |
| | | Sc. rank: | 69.2 |

PA

| Pos. | 200 | obs: | exp: |
|---|---|---|---|
| act | T | 0 | 93.26 |
| acc | T | 1 | 75.75 |
| aca | T | 352 | 154.60 |
| acg | T | 0 | 29.35 |
| --- | -- | ------ | ------ |
| mPD | | 0.0057 | 0.69 |
| | | nPD: | 0.01 |
| | | N. weight: | 1.2 |
| | | Sc. PD: | -0.3 |
| | | Sc. rank: | -190.1 |

PA

| Pos. | 201 | obs: | exp: |
|---|---|---|---|
| att | I | 337 | 126.70 |
| atc | I | 0 | 88.32 |
| ata | I | 0 | 122.00 |
| gtt | V | 16 | 3.29 |
| gtc | V | 0 | 3.36 |
| gta | V | 0 | 3.00 |
| gtg | V | 0 | 6.35 |
| --- | -- | ------ | ------ |
| mPD | | 0.087 | 0.76 |
| | | nPD: | 0.11 |
| | | N. weight: | 1.5 |
| | | Sc. PD: | -0.22 |
| | | Sc. rank: | -67.3 |

PA

| Pos. | 202 | obs: | exp: |
|---|---|---|---|
| gaa | E | 352 | 203.10 |
| gag | E | 1 | 149.90 |
| --- | -- | ------ | ------ |
| mPD | | 0.0057 | 0.49 |
| | | nPD: | 0.01 |
| | | N. weight: | 0.81 |
| | | Sc. PD: | -0.2 |
| | | Sc. rank: | -117.8 |

PA

| Pos. | 203 | obs: | exp: |
|---|---|---|---|
| gaa | E | 350 | 203.10 |
| gag | E | 3 | 149.90 |
| --- | -- | ------ | ------ |
| mPD | | 0.017 | 0.49 |
| | | nPD: | 0.03 |
| | | N. weight: | 0.77 |
| | | Sc. PD: | -0.17 |
| | | Sc. rank: | -82.3 |

PA

| Pos. | 204 | obs: | exp: |
|---|---|---|---|
| cgt | R | 0 | 14.57 |
| cgc | R | 0 | 17.19 |
| cga | R | 0 | 33.73 |
| cgg | R | 0 | 29.29 |
| aaa | K | 2 | 1.12 |
| aag | K | 0 | 0.88 |
| aga | R | 349 | 157.90 |
| agg | R | 2 | 98.36 |
| --- | -- | ------ | ------ |
| mPD | | 0.023 | 0.97 |
| | | nPD: | 0.02 |
| | | N. weight: | 1.2 |
| | | Sc. PD: | -0.27 |
| | | Sc. rank: | -146.1 |

PA

| Pos. | 205 | obs: | exp: |
|---|---|---|---|
| ttt | F | 352 | 144.70 |
| ttc | F | 0 | 207.30 |
| tgt | C | 1 | 0.41 |
| tgc | C | 0 | 0.59 |
| --- | -- | ------ | ------ |
| mPD | | 0.0057 | 0.49 |
| | | nPD: | 0.01 |
| | | N. weight: | 1.3 |
| | | Sc. PD: | -0.33 |
| | | Sc. rank: | -195.7 |

PA

| Pos. | 206 | obs: | exp: |
|---|---|---|---|
| gaa | E | 348 | 203.10 |
| gag | E | 5 | 149.90 |
| --- | -- | ------ | ------ |
| mPD | | 0.028 | 0.49 |
| | | nPD: | 0.06 |
| | | N. weight: | 0.74 |
| | | Sc. PD: | -0.15 |
| | | Sc. rank: | -61.9 |

,

PA

| Pos. | 207 | obs: | exp: |
|---|---|---|---|
| att | I | 114 | 132.70 |
| atc | I | 239 | 92.52 |
| ata | I | 0 | 127.80 |
| --- | -- | ------ | ------ |
| mPD | | 0.44 | 0.66 |
| | | nPD: | 0.67 |
| | | N. weight: | 0.91 |
| | | Sc. PD: | 0.35 |
| | | Sc. rank: | 149.7 |

,

PA

| Pos. | 208 | obs: | exp: |
|---|---|---|---|
| act | T | 1 | 93.00 |
| acc | T | 0 | 75.53 |
| aca | T | 351 | 154.20 |
| acg | T | 0 | 29.26 |
| gct | A | 0 | 0.25 |
| gcc | A | 0 | 0.20 |
| gca | A | 1 | 0.45 |
| gcg | A | 0 | 0.10 |
| --- | -- | ------ | ------ |
| mPD | | 0.011 | 0.69 |
| | | nPD: | 0.02 |
| | | N. weight: | 1.2 |
| | | Sc. PD: | -0.29 |
| | | Sc. rank: | -168.9 |

,

PA

| Pos. | 209 | obs: | exp: |
|---|---|---|---|
| ggt | G | 1 | 43.56 |
| ggc | G | 0 | 47.74 |
| gga | G | 329 | 170.80 |
| ggg | G | 23 | 90.95 |
| --- | -- | ------ | ------ |
| mPD | | 0.13 | 0.67 |
| | | nPD: | 0.19 |
| | | N. weight: | 0.79 |
| | | Sc. PD: | -0.055 |
| | | Sc. rank: | -4.0 |

,

PA

| Pos. | 210 | obs: | exp: |
|---|---|---|---|
| act | T | 3 | 93.26 |
| acc | T | 323 | 75.75 |
| aca | T | 27 | 154.60 |
| acg | T | 0 | 29.35 |
| --- | -- | ------ | ------ |
| mPD | | 0.16 | 0.69 |
| | | nPD: | 0.23 |
| | | N. weight: | 1.8 |
| | | Sc. PD: | -0.06 |
| | | Sc. rank: | 47.5 |

,

PA

| Pos. | 211 | obs: | exp: |
|---|---|---|---|
| att | I | 3 | 1.13 |
| atc | I | 0 | 0.79 |
| ata | I | 0 | 1.09 |
| atg | M | 350 | 350.00 |
| --- | -- | ------ | ------ |
| mPD | | 0.017 | 0.017 |
| | | nPD: | 1. |
| | | N. weight: | 0.013 |
| | | Sc. PD: | 0.0089 |
| | | Sc. rank: | 2.5 |

,

PA

| Pos. | 212 | obs: | exp: |
|---|---|---|---|
| cat | H | 0 | 0.54 |
| cac | H | 1 | 0.46 |
| cgt | R | 49 | 14.61 |
| cgc | R | 300 | 17.24 |
| cga | R | 3 | 33.83 |
| cgg | R | 0 | 29.37 |
| aga | R | 0 | 158.30 |
| agg | R | 0 | 98.64 |
| --- | -- | ------ | ------ |
| mPD | | 0.26 | 0.97 |
| | | nPD: | 0.27 |
| | | N. weight: | 3.9 |
| | | Sc. PD: | 0.013 |
| | | Sc. rank: | 154.0 |

,

PA

| Pos. | 213 | obs: | exp: |
|---|---|---|---|
| cgt | R | 0 | 14.53 |
| cgc | R | 0 | 17.14 |
| cga | R | 0 | 33.64 |
| cgg | R | 1 | 29.21 |
| aaa | K | 0 | 1.68 |
| aag | K | 3 | 1.32 |
| aga | R | 0 | 157.40 |
| agg | R | 349 | 98.08 |
| --- | -- | ------ | ------ |
| mPD | | 0.023 | 0.97 |
| | | nPD: | 0.02 |
| | | N. weight: | 1.9 |
| | | Sc. PD: | -0.44 |
| | | Sc. rank: | -239.6 |

,

PA

| Pos. | 214 | obs: | exp: |
|---|---|---|---|
| tta | L | 0 | 30.75 |
| ttg | L | 0 | 67.79 |
| ctt | L | 275 | 65.68 |
| ctc | L | 77 | 56.94 |
| cta | L | 0 | 53.03 |
| ctg | L | 0 | 77.81 |
| cct | P | 1 | 0.26 |
| ccc | P | 0 | 0.18 |
| cca | P | 0 | 0.39 |
| ccg | P | 0 | 0.16 |
| --- | -- | ------ | ------ |
| mPD | | 0.35 | 1.1 |
| | | nPD: | 0.31 |
| | | N. weight: | 1.8 |
| | | Sc. PD: | 0.08 |
| | | Sc. rank: | 103.2 |

,

PA

| Pos. | 215 | obs: | exp: |
|---|---|---|---|
| gct | A | 2 | 88.91 |
| gcc | A | 351 | 72.31 |
| gca | A | 0 | 157.30 |
| gcg | A | 0 | 34.45 |
| --- | -- | ------ | ------ |
| mPD | | 0.011 | 0.69 |
| | | nPD: | 0.02 |
| | | N. weight: | 2.4 |
| | | Sc. PD: | -0.56 |
| | | Sc. rank: | -320.8 |

,

PA

| Pos. | 216 | obs: | exp: |
|---|---|---|---|
| aat | N | 0 | 0.52 |
| aac | N | 1 | 0.48 |
| gat | D | 2 | 187.80 |
| gac | D | 350 | 164.20 |
| --- | -- | ------ | ------ |
| mPD | | 0.017 | 0.50 |
| | | nPD: | 0.03 |
| | | N. weight: | 1.1 |
| | | Sc. PD: | -0.25 |
| | | Sc. rank: | -121.2 |

,

PA

| Pos. | 217 | obs: | exp: |
|---|---|---|---|
| caa | Q | 353 | 188.20 |
| cag | Q | 0 | 164.80 |
| --- | -- | ------ | ------ |
| mPD | | 0 | 0.50 |
| | | nPD: | 0. |
| | | N. weight: | 0.95 |
| | | Sc. PD: | -0.24 |
| | | Sc. rank: | -190.6 |

,

PA

| Pos. | 218 | obs: | exp: |
|---|---|---|---|
| tct | S | 0 | 55.96 |
| tcc | S | 0 | 49.04 |
| tca | S | 0 | 77.98 |
| tcg | S | 0 | 21.46 |
| att | I | 1 | 0.38 |
| atc | I | 0 | 0.26 |
| ata | I | 0 | 0.36 |
| agt | S | 351 | 77.44 |
| agc | S | 1 | 70.13 |
| --- | -- | ------ | ------ |
| mPD | | 0.011 | 1.7 |
| | | nPD: | 0.01 |
| | | N. weight: | 2.3 |
| | | Sc. PD: | -0.56 |
| | | Sc. rank: | -366.2 |

,

PA

| Pos. | 219 | obs: | exp: |
|---|---|---|---|
| tta | L | 0 | 30.84 |
| ttg | L | 0 | 67.98 |
| ctt | L | 1 | 65.86 |
| ctc | L | 352 | 57.10 |
| cta | L | 0 | 53.18 |
| ctg | L | 0 | 78.03 |
| --- | -- | ------ | ------ |
| mPD | | 0.0057 | 1.1 |
| | | nPD: | 0.01 |
| | | N. weight: | 2.7 |
| | | Sc. PD: | -0.68 |
| | | Sc. rank: | -456.5 |

,

PA

| Pos. | 220 | obs: | exp: |
|---|---|---|---|
| cct | P | 0 | 92.95 |
| ccc | P | 0 | 63.67 |
| cca | P | 350 | 139.30 |
| ccg | P | 3 | 57.08 |
| --- | -- | ------ | ------ |
| mPD | | 0.017 | 0.72 |
| | | nPD: | 0.02 |
| | | N. weight: | 1.4 |
| | | Sc. PD: | -0.31 |
| | | Sc. rank: | -167.9 |

,

PA

| Pos. | 221 | obs: | exp: |
|---|---|---|---|
| cct | P | 0 | 92.95 |
| ccc | P | 0 | 63.67 |
| cca | P | 0 | 139.30 |
| ccg | P | 353 | 57.08 |
| --- | -- | ------ | ------ |
| mPD | | 0 | 0.72 |
| | | nPD: | 0. |
| | | N. weight: | 2.8 |
| | | Sc. PD: | -0.7 |
| | | Sc. rank: | -552.2 |

,

PA

| Pos. | 222 | obs: | exp: |
|---|---|---|---|
| aat | N | 1 | 184.50 |
| aac | N | 352 | 168.50 |
| --- | -- | ------ | ------ |
| mPD | | 0.0057 | 0.50 |
| | | nPD: | 0.01 |
| | | N. weight: | 1.1 |
| | | Sc. PD: | -0.27 |
| | | Sc. rank: | -161.8 |

,

PA

| Pos. | 223 | obs: | exp: |
|---|---|---|---|
| ttt | F | 0 | 145.10 |
| ttc | F | 353 | 207.90 |
| --- | -- | ------ | ------ |
| mPD | | 0 | 0.48 |
| | | nPD: | 0. |
| | | N. weight: | 0.8 |
| | | Sc. PD: | -0.2 |
| | | Sc. rank: | -160.5 |

,

PA

| Pos. | 224 | obs: | exp: |
|---|---|---|---|
| tct | S | 0 | 55.96 |
| tcc | S | 351 | 49.04 |
| tca | S | 1 | 77.98 |
| tcg | S | 0 | 21.46 |
| cct | P | 0 | 0.26 |
| ccc | P | 0 | 0.18 |
| cca | P | 1 | 0.39 |
| ccg | P | 0 | 0.16 |
| agt | S | 0 | 77.44 |
| agc | S | 0 | 70.13 |
| --- | -- | ------ | ------ |
| mPD | | 0.017 | 1.7 |
| | | nPD: | 0.01 |
| | | N. weight: | 3. |
| | | Sc. PD: | -0.72 |
| | | Sc. rank: | -441.7 |

,

PA

| Pos. | 225 | obs: | exp: |
|---|---|---|---|
| tct | S | 0 | 55.96 |
| tcc | S | 0 | 49.04 |
| tca | S | 0 | 77.98 |
| tcg | S | 0 | 21.46 |
| aat | N | 0 | 0.52 |
| aac | N | 1 | 0.48 |
| agt | S | 0 | 77.44 |
| agc | S | 352 | 70.13 |
| --- | -- | ------ | ------ |
| mPD | | 0.0057 | 1.7 |
| | | nPD: | 0. |
| | | N. weight: | 2.4 |
| | | Sc. PD: | -0.61 |
| | | Sc. rank: | -411.1 |

,

PA

| Pos. | 226 | obs: | exp: |
|---|---|---|---|
| tta | L | 0 | 30.84 |
| ttg | L | 0 | 67.98 |
| ctt | L | 353 | 65.86 |
| ctc | L | 0 | 57.10 |
| cta | L | 0 | 53.18 |
| ctg | L | 0 | 78.03 |
| --- | -- | ------ | ------ |
| mPD | | 0 | 1.1 |
| | | nPD: | 0. |
| | | N. weight: | 2.5 |
| | | Sc. PD: | -0.65 |
| | | Sc. rank: | -508.8 |

,

PA

| Pos. | 227 | obs: | exp: |
|---|---|---|---|
| gct | A | 0 | 2.01 |
| gcc | A | 0 | 1.64 |
| gca | A | 8 | 3.57 |
| gcg | A | 0 | 0.78 |
| gaa | E | 345 | 198.50 |
| gag | E | 0 | 146.50 |
| --- | -- | ------ | ------ |
| mPD | | 0.044 | 0.54 |
| | | nPD: | 0.08 |
| | | N. weight: | 0.85 |
| | | Sc. PD: | -0.15 |
| | | Sc. rank: | -57.0 |

,

PA

| Pos. | 228 | obs: | exp: |
|---|---|---|---|
| act | T | 0 | 0.79 |
| acc | T | 3 | 0.64 |
| aca | T | 0 | 1.31 |
| acg | T | 0 | 0.25 |
| aat | N | 1 | 182.90 |
| aac | N | 349 | 167.10 |
| --- | -- | ------ | ------ |
| mPD | | 0.023 | 0.52 |
| | | nPD: | 0.04 |
| | | N. weight: | 1.1 |
| | | Sc. PD: | -0.23 |
| | | Sc. rank: | -109.9 |

,

PA

| Pos. | 229 | obs: | exp: |
|---|---|---|---|
| ttt | F | 353 | 145.10 |
| ttc | F | 0 | 207.90 |
| --- | -- | ------ | ------ |
| mPD | | 0 | 0.48 |
| | | nPD: | 0. |
| | | N. weight: | 1.3 |
| | | Sc. PD: | -0.34 |
| | | Sc. rank: | -269.4 |

,

PA

| Pos. | 230 | obs: | exp: |
|---|---|---|---|
| cgt | R | 0 | 14.65 |
| cgc | R | 0 | 17.28 |
| cga | R | 0 | 33.93 |
| cgg | R | 0 | 29.46 |
| aga | R | 353 | 158.80 |
| agg | R | 0 | 98.92 |
| --- | -- | ------ | ------ |
| mPD | | 0 | 0.96 |
| | | nPD: | 0. |
| | | N. weight: | 1.2 |
| | | Sc. PD: | -0.31 |
| | | Sc. rank: | -242.2 |

,

| PA | | | |
|---|---|---|---|
| Pos. | 231 | obs: | exp: |
| act | T | 0 | 0.53 |
| acc | T | 2 | 0.43 |
| aca | T | 0 | 0.88 |
| acg | T | 0 | 0.17 |
| gct | A | 0 | 88.41 |
| gcc | A | 351 | 71.90 |
| gca | A | 0 | 156.40 |
| gcg | A | 0 | 34.25 |
| mPD | | 0.011 | 0.70 |
| | | nPD: | 0.02 |
| | | N. weight: | 2.4 |
| | | Sc. PD: | -0.57 |
| | | Sc. rank: | -333.9 |

| PA | | | |
|---|---|---|---|
| Pos. | 232 | obs: | exp: |
| tat | Y | 353 | 182.70 |
| tac | Y | 0 | 170.30 |
| mPD | | 0 | 0.50 |
| | | nPD: | 0. |
| | | N. weight: | 1. |
| | | Sc. PD: | -0.25 |
| | | Sc. rank: | -199.6 |

| PA | | | |
|---|---|---|---|
| Pos. | 233 | obs: | exp: |
| gtt | V | 0 | 72.56 |
| gtc | V | 0 | 74.09 |
| gta | V | 0 | 66.16 |
| gtg | V | 353 | 140.20 |
| mPD | | 0 | 0.72 |
| | | nPD: | 0. |
| | | N. weight: | 1.4 |
| | | Sc. PD: | -0.36 |
| | | Sc. rank: | -279.8 |

| PA | | | |
|---|---|---|---|
| Pos. | 234 | obs: | exp: |
| gat | D | 352 | 188.30 |
| gac | D | 1 | 164.70 |
| mPD | | 0.0057 | 0.50 |
| | | nPD: | 0.01 |
| | | N. weight: | 0.93 |
| | | Sc. PD: | -0.23 |
| | | Sc. rank: | -135.4 |

| PA | | | |
|---|---|---|---|
| Pos. | 235 | obs: | exp: |
| ggt | G | 0 | 43.56 |
| ggc | G | 0 | 47.74 |
| gga | G | 353 | 170.80 |
| ggg | G | 0 | 90.95 |
| mPD | | 0 | 0.67 |
| | | nPD: | 0. |
| | | N. weight: | 1.1 |
| | | Sc. PD: | -0.28 |
| | | Sc. rank: | -220.1 |

| PA | | | |
|---|---|---|---|
| Pos. | 236 | obs: | exp: |
| ttt | F | 3 | 145.10 |
| ttc | F | 350 | 207.90 |
| mPD | | 0.017 | 0.48 |
| | | nPD: | 0.03 |
| | | N. weight: | 0.74 |
| | | Sc. PD: | -0.16 |
| | | Sc. rank: | -78.0 |

| PA | | | |
|---|---|---|---|
| Pos. | 237 | obs: | exp: |
| aaa | K | 1 | 0.56 |
| aag | K | 0 | 0.44 |
| gaa | E | 349 | 202.60 |
| gag | E | 3 | 149.40 |
| mPD | | 0.023 | 0.49 |
| | | nPD: | 0.05 |
| | | N. weight: | 0.77 |
| | | Sc. PD: | -0.16 |
| | | Sc. rank: | -75.1 |

| PA | | | |
|---|---|---|---|
| Pos. | 238 | obs: | exp: |
| cct | P | 0 | 92.95 |
| ccc | P | 0 | 63.67 |
| cca | P | 1 | 139.30 |
| ccg | P | 352 | 57.08 |
| mPD | | 0.0057 | 0.72 |
| | | nPD: | 0.01 |
| | | N. weight: | 2.7 |
| | | Sc. PD: | -0.67 |
| | | Sc. rank: | -431.9 |

| PA | | | |
|---|---|---|---|
| Pos. | 239 | obs: | exp: |
| aat | N | 5 | 184.50 |
| aac | N | 348 | 168.50 |
| mPD | | 0.028 | 0.50 |
| | | nPD: | 0.06 |
| | | N. weight: | 1. |
| | | Sc. PD: | -0.2 |
| | | Sc. rank: | -87.4 |

| PA | | | |
|---|---|---|---|
| Pos. | 240 | obs: | exp: |
| ggt | G | 0 | 43.56 |
| ggc | G | 353 | 47.74 |
| gga | G | 0 | 170.80 |
| ggg | G | 0 | 90.95 |
| mPD | | 0 | 0.67 |
| | | nPD: | 0. |
| | | N. weight: | 3. |
| | | Sc. PD: | -0.77 |
| | | Sc. rank: | -606.3 |

| PA | | | |
|---|---|---|---|
| Pos. | 241 | obs: | exp: |
| tgt | C | 0 | 144.70 |
| tgc | C | 351 | 206.30 |
| cgt | R | 0 | 0.08 |
| cgc | R | 2 | 0.10 |
| cga | R | 0 | 0.19 |
| cgg | R | 0 | 0.17 |
| aga | R | 0 | 0.90 |
| agg | R | 0 | 0.56 |
| mPD | | 0.011 | 0.50 |
| | | nPD: | 0.02 |
| | | N. weight: | 0.83 |
| | | Sc. PD: | -0.19 |
| | | Sc. rank: | -107.5 |

| PA | | | |
|---|---|---|---|
| Pos. | 242 | obs: | exp: |
| att | I | 353 | 132.70 |
| atc | I | 0 | 92.52 |
| ata | I | 0 | 127.80 |
| mPD | | 0 | 0.66 |
| | | nPD: | 0. |
| | | N. weight: | 1.5 |
| | | Sc. PD: | -0.38 |
| | | Sc. rank: | -296.5 |

| PA | | | |
|---|---|---|---|
| Pos. | 243 | obs: | exp: |
| gaa | E | 2 | 203.10 |
| gag | E | 351 | 149.90 |
| mPD | | 0.011 | 0.49 |
| | | nPD: | 0.02 |
| | | N. weight: | 1.2 |
| | | Sc. PD: | -0.29 |
| | | Sc. rank: | -158.6 |

| PA | | | |
|---|---|---|---|
| Pos. | 244 | obs: | exp: |
| ggt | G | 0 | 43.56 |
| ggc | G | 335 | 47.74 |
| gga | G | 18 | 170.80 |
| ggg | G | 0 | 90.95 |
| mPD | | 0.097 | 0.67 |
| | | nPD: | 0.15 |
| | | N. weight: | 2.6 |
| | | Sc. PD: | -0.3 |
| | | Sc. rank: | -79.1 |

| PA | | | |
|---|---|---|---|
| Pos. | 245 | obs: | exp: |
| aaa | K | 0 | 197.70 |
| aag | K | 353 | 155.30 |
| mPD | | 0 | 0.49 |
| | | nPD: | 0. |
| | | N. weight: | 1.2 |
| | | Sc. PD: | -0.32 |
| | | Sc. rank: | -248.8 |

| PA | | | |
|---|---|---|---|
| Pos. | 246 | obs: | exp: |
| tta | L | 0 | 30.84 |
| ttg | L | 0 | 67.98 |
| ctt | L | 350 | 65.86 |
| ctc | L | 2 | 57.10 |
| cta | L | 1 | 53.18 |
| ctg | L | 0 | 78.03 |
| mPD | | 0.017 | 1.1 |
| | | nPD: | 0.02 |
| | | N. weight: | 2.5 |
| | | Sc. PD: | -0.59 |
| | | Sc. rank: | -353.8 |

| PA | | | |
|---|---|---|---|
| Pos. | 247 | obs: | exp: |
| tct | S | 353 | 56.12 |
| tcc | S | 0 | 49.17 |
| tca | S | 0 | 78.20 |
| tcg | S | 0 | 21.52 |
| agt | S | 0 | 77.66 |
| agc | S | 0 | 70.33 |
| mPD | | 0 | 1.7 |
| | | nPD: | 0. |
| | | N. weight: | 2.8 |
| | | Sc. PD: | -0.71 |
| | | Sc. rank: | -557.3 |

| PA | | | |
|---|---|---|---|
| Pos. | 248 | obs: | exp: |
| caa | Q | 324 | 188.20 |
| cag | Q | 29 | 164.80 |
| mPD | | 0.15 | 0.50 |
| | | nPD: | 0.3 |
| | | N. weight: | 0.55 |
| | | Sc. PD: | 0.021 |
| | | Sc. rank: | 29.7 |

| PA | | | |
|---|---|---|---|
| Pos. | 249 | obs: | exp: |
| atg | M | 353 | 353.00 |
| mPD | | 0 | 0 |
| | | nPD: | 1. |
| | | N. weight: | 0. |
| | | Sc. PD: | 0 |
| | | Sc. rank: | 0 |

| PA | | | |
|---|---|---|---|
| Pos. | 250 | obs: | exp: |
| tct | S | 1 | 56.12 |
| tcc | S | 78 | 49.17 |
| tca | S | 272 | 78.20 |
| tcg | S | 2 | 21.52 |
| agt | S | 0 | 77.66 |
| agc | S | 0 | 70.33 |
| mPD | | 0.36 | 1.7 |
| | | nPD: | 0.22 |
| | | N. weight: | 1.6 |
| | | Sc. PD: | -0.075 |
| | | Sc. rank: | 30.0 |

| PA | | | |
|---|---|---|---|
| Pos. | 251 | obs: | exp: |
| cgt | R | 0 | 0.04 |
| cgc | R | 0 | 0.05 |
| cga | R | 0 | 0.10 |
| cgg | R | 0 | 0.08 |
| aaa | K | 322 | 197.10 |
| aag | K | 30 | 154.90 |
| aga | R | 0 | 0.45 |
| agg | R | 1 | 0.28 |
| mPD | | 0.17 | 0.50 |
| | | nPD: | 0.33 |
| | | N. weight: | 0.48 |
| | | Sc. PD: | 0.032 |
| | | Sc. rank: | 32.0 |

| PA | | | |
|---|---|---|---|
| Pos. | 252 | obs: | exp: |
| aaa | K | 1 | 0.56 |
| aag | K | 0 | 0.44 |
| gaa | E | 346 | 202.60 |
| gag | E | 6 | 149.40 |
| mPD | | 0.039 | 0.49 |
| | | nPD: | 0.08 |
| | | N. weight: | 0.72 |
| | | Sc. PD: | -0.13 |
| | | Sc. rank: | -51.4 |

| PA | | | |
|---|---|---|---|
| Pos. | 253 | obs: | exp: |
| gtt | V | 0 | 72.56 |
| gtc | V | 0 | 74.09 |
| gta | V | 89 | 66.16 |
| gtg | V | 264 | 140.20 |
| mPD | | 0.38 | 0.72 |
| | | nPD: | 0.52 |
| | | N. weight: | 0.84 |
| | | Sc. PD: | 0.21 |
| | | Sc. rank: | 111.9 |

| PA | | | |
|---|---|---|---|
| Pos. | 254 | obs: | exp: |
| tct | S | 0 | 0.16 |
| tcc | S | 0 | 0.14 |
| tca | S | 0 | 0.22 |
| tcg | S | 0 | 0.06 |
| aat | N | 134 | 183.40 |
| aac | N | 217 | 167.60 |
| aaa | K | 1 | 0.56 |
| aag | K | 0 | 0.44 |
| agt | S | 0 | 0.22 |
| agc | S | 1 | 0.20 |
| mPD | | 0.48 | 0.51 |
| | | nPD: | 0.94 |
| | | N. weight: | 0.083 |
| | | Sc. PD: | 0.054 |
| | | Sc. rank: | 16.6 |

| PA | | | |
|---|---|---|---|
| Pos. | 255 | obs: | exp: |
| gct | A | 55 | 88.91 |
| gcc | A | 248 | 72.31 |
| gca | A | 50 | 157.30 |
| gcg | A | 0 | 34.45 |
| mPD | | 0.46 | 0.69 |
| | | nPD: | 0.68 |
| | | N. weight: | 0.98 |
| | | Sc. PD: | 0.38 |
| | | Sc. rank: | 163.3 |

PA

| Pos. | 256 | obs: | exp: |
|---|---|---|---|
| cgt | R | 0 | 14.61 |
| cgc | R | 0 | 17.24 |
| cga | R | 3 | 33.83 |
| cgg | R | 0 | 29.37 |
| aaa | K | 1 | 0.56 |
| aag | K | 0 | 0.44 |
| aga | R | 282 | 158.30 |
| agg | R | 67 | 98.64 |
| --- | -- | ------ | ------ |
| mPD | | 0.33 | 0.96 |
| | | nPD: | 0.34 |
| | | N. weight: | 0.58 |
| | | Sc. PD: | 0.044 |
| | | Sc. rank: | 41.4 |

,

PA

| Pos. | 257 | obs: | exp: |
|---|---|---|---|
| att | I | 319 | 132.70 |
| atc | I | 34 | 92.52 |
| ata | I | 0 | 127.80 |
| --- | -- | ------ | ------ |
| mPD | | 0.17 | 0.66 |
| | | nPD: | 0.26 |
| | | N. weight: | 1.1 |
| | | Sc. PD: | 0.00038 |
| | | Sc. rank: | 39.0 |

,

PA

| Pos. | 258 | obs: | exp: |
|---|---|---|---|
| gaa | E | 111 | 203.10 |
| gag | E | 242 | 149.90 |
| --- | -- | ------ | ------ |
| mPD | | 0.43 | 0.49 |
| | | nPD: | 0.88 |
| | | N. weight: | 0.22 |
| | | Sc. PD: | 0.13 |
| | | Sc. rank: | 44.4 |

,

PA

| Pos. | 259 | obs: | exp: |
|---|---|---|---|
| tct | S | 0 | 0.16 |
| tcc | S | 0 | 0.14 |
| tca | S | 1 | 0.22 |
| tcg | S | 0 | 0.06 |
| cct | P | 1 | 92.69 |
| ccc | P | 0 | 63.49 |
| cca | P | 329 | 138.90 |
| ccg | P | 22 | 56.91 |
| agt | S | 0 | 0.22 |
| agc | S | 0 | 0.20 |
| --- | -- | ------ | ------ |
| mPD | | 0.13 | 0.72 |
| | | nPD: | 0.18 |
| | | N. weight: | 1.1 |
| | | Sc. PD: | -0.095 |
| | | Sc. rank: | -24.4 |

,

PA

| Pos. | 260 | obs: | exp: |
|---|---|---|---|
| ttt | F | 314 | 145.10 |
| ttc | F | 39 | 207.90 |
| --- | -- | ------ | ------ |
| mPD | | 0.20 | 0.48 |
| | | nPD: | 0.41 |
| | | N. weight: | 0.77 |
| | | Sc. PD: | 0.11 |
| | | Sc. rank: | 68.9 |

,

PA

| Pos. | 261 | obs: | exp: |
|---|---|---|---|
| tta | L | 1 | 30.66 |
| ttg | L | 51 | 67.60 |
| ctt | L | 0 | 65.49 |
| ctc | L | 0 | 56.78 |
| cta | L | 38 | 52.88 |
| ctg | L | 261 | 77.59 |
| caa | Q | 0 | 0.53 |
| cag | Q | 1 | 0.47 |
| atg | M | 1 | 1.00 |
| --- | -- | ------ | ------ |
| mPD | | 0.46 | 1.1 |
| | | nPD: | 0.41 |
| | | N. weight: | 1.3 |
| | | Sc. PD: | 0.18 |
| | | Sc. rank: | 113.9 |

,

PA

| Pos. | 262 | obs: | exp: |
|---|---|---|---|
| cgt | R | 0 | 1.12 |
| cgc | R | 0 | 1.32 |
| cga | R | 0 | 2.59 |
| cgg | R | 0 | 2.25 |
| aaa | K | 59 | 182.50 |
| aag | K | 267 | 143.50 |
| aga | R | 0 | 12.14 |
| agg | R | 27 | 7.57 |
| --- | -- | ------ | ------ |
| mPD | | 0.42 | 0.68 |
| | | nPD: | 0.62 |
| | | N. weight: | 0.58 |
| | | Sc. PD: | 0.2 |
| | | Sc. rank: | 90.0 |

,

PA

| Pos. | 263 | obs: | exp: |
|---|---|---|---|
| act | T | 0 | 93.26 |
| acc | T | 0 | 75.75 |
| aca | T | 345 | 154.60 |
| acg | T | 8 | 29.35 |
| --- | -- | ------ | ------ |
| mPD | | 0.044 | 0.69 |
| | | nPD: | 0.06 |
| | | N. weight: | 1.2 |
| | | Sc. PD: | -0.22 |
| | | Sc. rank: | -90.1 |

,

PA

| Pos. | 264 | obs: | exp: |
|---|---|---|---|
| act | T | 4 | 93.26 |
| acc | T | 2 | 75.75 |
| aca | T | 341 | 154.60 |
| acg | T | 6 | 29.35 |
| --- | -- | ------ | ------ |
| mPD | | 0.067 | 0.69 |
| | | nPD: | 0.1 |
| | | N. weight: | 1.1 |
| | | Sc. PD: | -0.17 |
| | | Sc. rank: | -57.7 |

,

PA

| Pos. | 265 | obs: | exp: |
|---|---|---|---|
| cct | P | 3 | 92.95 |
| ccc | P | 3 | 63.67 |
| cca | P | 300 | 139.30 |
| ccg | P | 47 | 57.08 |
| --- | -- | ------ | ------ |
| mPD | | 0.26 | 0.72 |
| | | nPD: | 0.36 |
| | | N. weight: | 0.89 |
| | | Sc. PD: | 0.085 |
| | | Sc. rank: | 71.5 |

,

PA

| Pos. | 266 | obs: | exp: |
|---|---|---|---|
| cgt | R | 30 | 14.65 |
| cgc | R | 316 | 17.28 |
| cga | R | 1 | 33.93 |
| cgg | R | 5 | 29.46 |
| aga | R | 0 | 158.80 |
| agg | R | 1 | 98.92 |
| --- | -- | ------ | ------ |
| mPD | | 0.20 | 0.96 |
| | | nPD: | 0.21 |
| | | N. weight: | 4. |
| | | Sc. PD: | -0.23 |
| | | Sc. rank: | 34.6 |

,

PA

| Pos. | 267 | obs: | exp: |
|---|---|---|---|
| tct | S | 0 | 0.16 |
| tcc | S | 1 | 0.14 |
| tca | S | 0 | 0.22 |
| tcg | S | 0 | 0.06 |
| cct | P | 316 | 92.69 |
| ccc | P | 32 | 63.49 |
| cca | P | 4 | 138.90 |
| ccg | P | 0 | 56.91 |
| agt | S | 0 | 0.22 |
| agc | S | 0 | 0.20 |
| --- | -- | ------ | ------ |
| mPD | | 0.20 | 0.72 |
| | | nPD: | 0.27 |
| | | N. weight: | 1.5 |
| | | Sc. PD: | 0.0088 |
| | | Sc. rank: | 62.8 |

,

PA

| Pos. | 268 | obs: | exp: |
|---|---|---|---|
| tta | L | 0 | 30.84 |
| ttg | L | 1 | 67.98 |
| ctt | L | 3 | 65.86 |
| ctc | L | 285 | 57.10 |
| cta | L | 0 | 53.18 |
| ctg | L | 64 | 78.03 |
| --- | -- | ------ | ------ |
| mPD | | 0.32 | 1.1 |
| | | nPD: | 0.29 |
| | | N. weight: | 1.9 |
| | | Sc. PD: | 0.042 |
| | | Sc. rank: | 89.5 |

,

PA

| Pos. | 269 | obs: | exp: |
|---|---|---|---|
| caa | Q | 8 | 4.26 |
| cag | Q | 0 | 3.74 |
| cgt | R | 0 | 13.37 |
| cgc | R | 0 | 15.77 |
| cga | R | 1 | 30.95 |
| cgg | R | 0 | 26.87 |
| aaa | K | 21 | 12.88 |
| aag | K | 2 | 10.12 |
| aga | R | 272 | 144.80 |
| agg | R | 49 | 90.24 |
| --- | -- | ------ | ------ |
| mPD | | 0.46 | 1.1 |
| | | nPD: | 0.41 |
| | | N. weight: | 0.67 |
| | | Sc. PD: | 0.094 |
| | | Sc. rank: | 60.5 |

,

PA

| Pos. | 270 | obs: | exp: |
|---|---|---|---|
| tta | L | 285 | 30.84 |
| ttg | L | 60 | 67.98 |
| ctt | L | 0 | 65.86 |
| ctc | L | 0 | 57.10 |
| cta | L | 7 | 53.18 |
| ctg | L | 1 | 78.03 |
| --- | -- | ------ | ------ |
| mPD | | 0.33 | 1.1 |
| | | nPD: | 0.3 |
| | | N. weight: | 2.6 |
| | | Sc. PD: | 0.082 |
| | | Sc. rank: | 139.4 |

,

PA

| Pos. | 271 | obs: | exp: |
|---|---|---|---|
| cct | P | 283 | 92.95 |
| ccc | P | 2 | 63.67 |
| cca | P | 44 | 139.30 |
| ccg | P | 24 | 57.08 |
| --- | -- | ------ | ------ |
| mPD | | 0.34 | 0.72 |
| | | nPD: | 0.47 |
| | | N. weight: | 1. |
| | | Sc. PD: | 0.21 |
| | | Sc. rank: | 112.3 |

,

PA

| Pos. | 272 | obs: | exp: |
|---|---|---|---|
| aat | N | 27 | 14.11 |
| aac | N | 0 | 12.89 |
| gat | D | 250 | 133.40 |
| gac | D | 0 | 116.60 |
| gaa | E | 74 | 42.58 |
| gag | E | 0 | 31.42 |
| ggt | G | 0 | 0.25 |
| ggc | G | 0 | 0.27 |
| gga | G | 2 | 0.97 |
| ggg | G | 0 | 0.52 |
| --- | -- | ------ | ------ |
| mPD | | 0.49 | 0.82 |
| | | nPD: | 0.6 |
| | | N. weight: | 0.93 |
| | | Sc. PD: | 0.3 |
| | | Sc. rank: | 140.3 |

,

PA

| Pos. | 273 | obs: | exp: |
|---|---|---|---|
| ggt | G | 0 | 43.56 |
| ggc | G | 10 | 47.74 |
| gga | G | 57 | 170.80 |
| ggg | G | 286 | 90.95 |
| --- | -- | ------ | ------ |
| mPD | | 0.32 | 0.67 |
| | | nPD: | 0.48 |
| | | N. weight: | 1.1 |
| | | Sc. PD: | 0.22 |
| | | Sc. rank: | 118.9 |

,

PA

| Pos. | 274 | obs: | exp: |
|---|---|---|---|
| tta | L | 0 | 0.17 |
| ttg | L | 0 | 0.39 |
| ctt | L | 2 | 0.37 |
| ctc | L | 0 | 0.32 |
| cta | L | 0 | 0.30 |
| ctg | L | 0 | 0.44 |
| cct | P | 329 | 92.42 |
| ccc | P | 19 | 63.31 |
| cca | P | 1 | 138.50 |
| ccg | P | 2 | 56.75 |
| --- | -- | ------ | ------ |
| mPD | | 0.13 | 0.73 |
| | | nPD: | 0.18 |
| | | N. weight: | 1.7 |
| | | Sc. PD: | -0.14 |
| | | Sc. rank: | -34.7 |

,

PA

| Pos. | 275 | obs: | exp: |
|---|---|---|---|
| tta | L | 0 | 0.26 |
| ttg | L | 0 | 0.58 |
| ctt | L | 1 | 0.56 |
| ctc | L | 2 | 0.49 |
| cta | L | 0 | 0.45 |
| ctg | L | 0 | 0.66 |
| cct | P | 126 | 92.16 |
| ccc | P | 218 | 63.13 |
| cca | P | 5 | 138.10 |
| ccg | P | 1 | 56.59 |
| --- | -- | ------ | ------ |
| mPD | | 0.50 | 0.74 |
| | | nPD: | 0.68 |
| | | N. weight: | 1.3 |
| | | Sc. PD: | 0.51 |
| | | Sc. rank: | 217.2 |

,

PA

| Pos. | 276 | obs: | exp: |
|---|---|---|---|
| tgt | C | 48 | 145.50 |
| tgc | C | 305 | 207.50 |
| --- | -- | ------ | ------ |
| mPD | | 0.24 | 0.48 |
| | | nPD: | 0.49 |
| | | N. weight: | 0.29 |
| | | Sc. PD: | 0.061 |
| | | Sc. rank: | 33.2 |

,

PA

| Pos. | 277 | obs: | exp: |
|---|---|---|---|
| ttt | F | 21 | 9.04 |
| ttc | F | 1 | 12.96 |
| tct | S | 291 | 51.83 |
| tcc | S | 35 | 45.41 |
| tca | S | 0 | 72.22 |
| tcg | S | 0 | 19.87 |
| tat | Y | 0 | 0.52 |
| tac | Y | 1 | 0.48 |
| agt | S | 0 | 71.72 |
| agc | S | 0 | 64.95 |
| gct | A | 4 | 1.01 |
| gcc | A | 0 | 0.82 |
| gca | A | 0 | 1.78 |
| gcg | A | 0 | 0.39 |
| --- | -- | ------ | ------ |
| mPD | | 0.33 | 1.7 |
| | | nPD: | 0.19 |
| | | N. weight: | 2.2 |
| | | Sc. PD: | -0.15 |
| | | Sc. rank: | 2.4 |

,

PA

| Pos. | 278 | obs: | exp: |
|---|---|---|---|
| caa | Q | 10 | 188.20 |
| cag | Q | 343 | 164.80 |
| --- | -- | ------ | ------ |
| mPD | | 0.055 | 0.50 |
| | | nPD: | 0.11 |
| | | N. weight: | 0.96 |
| | | Sc. PD: | -0.14 |
| | | Sc. rank: | -47.2 |

,

PA

| Pos. | 279 | obs: | exp: |
|---|---|---|---|
| cgt | R | 0 | 14.65 |
| cgc | R | 22 | 17.28 |
| cga | R | 60 | 33.93 |
| cgg | R | 253 | 29.46 |
| aga | R | 0 | 158.80 |
| agg | R | 18 | 98.92 |
| --- | -- | ------ | ------ |
| mPD | | 0.48 | 0.96 |
| | | nPD: | 0.5 |
| | | N. weight: | 2.4 |
| | | Sc. PD: | 0.53 |
| | | Sc. rank: | 292.1 |

,

| | | | |
|---|---|---|---|
| PA | | | |
| Pos. | 280 | obs: | exp: |
| tct | S | 20 | 56.12 |
| tcc | S | 1 | 49.17 |
| tca | S | 43 | 78.20 |
| tcg | S | 289 | 21.52 |
| agt | S | 0 | 77.66 |
| agc | S | 0 | 70.33 |
| --- | -- | ------ | ------ |
| mPD | | 0.31 | 1.7 |
| | | nPD: | 0.19 |
| | | N. weight: | 3. |
| | | Sc. PD: | -0.22 |
| | | Sc. rank: | -27.8 |

,

| | | | |
|---|---|---|---|
| PA | | | |
| Pos. | 281 | obs: | exp: |
| aaa | K | 164 | 197.70 |
| aag | K | 189 | 155.30 |
| --- | -- | ------ | ------ |
| mPD | | 0.50 | 0.49 |
| | | nPD: | 1.01 |
| | | N. weight: | 0.041 |
| | | Sc. PD: | 0.03 |
| | | Sc. rank: | 8.3 |

,

| | | | |
|---|---|---|---|
| PA | | | |
| Pos. | 282 | obs: | exp: |
| ttt | F | 78 | 145.10 |
| ttc | F | 275 | 207.90 |
| --- | -- | ------ | ------ |
| mPD | | 0.35 | 0.48 |
| | | nPD: | 0.71 |
| | | N. weight: | 0.14 |
| | | Sc. PD: | 0.058 |
| | | Sc. rank: | 23.8 |

,

| | | | |
|---|---|---|---|
| PA | | | |
| Pos. | 283 | obs: | exp: |
| tta | L | 38 | 30.84 |
| ttg | L | 240 | 67.98 |
| ctt | L | 0 | 65.86 |
| ctc | L | 0 | 57.10 |
| cta | L | 2 | 53.18 |
| ctg | L | 73 | 78.03 |
| --- | -- | ------ | ------ |
| mPD | | 0.54 | 1.1 |
| | | nPD: | 0.48 |
| | | N. weight: | 1.3 |
| | | Sc. PD: | 0.27 |
| | | Sc. rank: | 147.0 |

,

| | | | |
|---|---|---|---|
| PA | | | |
| Pos. | 284 | obs: | exp: |
| tta | L | 0 | 30.84 |
| ttg | L | 7 | 67.98 |
| ctt | L | 0 | 65.86 |
| ctc | L | 0 | 57.10 |
| cta | L | 15 | 53.18 |
| ctg | L | 331 | 78.03 |
| --- | -- | ------ | ------ |
| mPD | | 0.12 | 1.1 |
| | | nPD: | 0.11 |
| | | N. weight: | 1.9 |
| | | Sc. PD: | -0.29 |
| | | Sc. rank: | -96.7 |

,

| | | | |
|---|---|---|---|
| PA | | | |
| Pos. | 285 | obs: | exp: |
| atg | M | 353 | 353.00 |
| --- | -- | ------ | ------ |
| mPD | | 0 | 0 |
| | | nPD: | 1. |
| | | N. weight: | 0. |
| | | Sc. PD: | 0 |
| | | Sc. rank: | 0 |

,

| | | | |
|---|---|---|---|
| PA | | | |
| Pos. | 286 | obs: | exp: |
| gat | D | 337 | 188.30 |
| gac | D | 16 | 164.70 |
| --- | -- | ------ | ------ |
| mPD | | 0.087 | 0.50 |
| | | nPD: | 0.17 |
| | | N. weight: | 0.69 |
| | | Sc. PD: | -0.06 |
| | | Sc. rank: | -15.9 |

,

| | | | |
|---|---|---|---|
| PA | | | |
| Pos. | 287 | obs: | exp: |
| gct | A | 138 | 88.91 |
| gcc | A | 212 | 72.31 |
| gca | A | 3 | 157.30 |
| gcg | A | 0 | 34.45 |
| --- | -- | ------ | ------ |
| mPD | | 0.49 | 0.69 |
| | | nPD: | 0.71 |
| | | N. weight: | 1.2 |
| | | Sc. PD: | 0.52 |
| | | Sc. rank: | 211.7 |

,

| | | | |
|---|---|---|---|
| PA | | | |
| Pos. | 288 | obs: | exp: |
| tta | L | 0 | 30.84 |
| ttg | L | 16 | 67.98 |
| ctt | L | 181 | 65.86 |
| ctc | L | 45 | 57.10 |
| cta | L | 2 | 53.18 |
| ctg | L | 109 | 78.03 |
| --- | -- | ------ | ------ |
| mPD | | 0.68 | 1.1 |
| | | nPD: | 0.61 |
| | | N. weight: | 0.81 |
| | | Sc. PD: | 0.27 |
| | | Sc. rank: | 123.7 |

,

| | | | |
|---|---|---|---|
| PA | | | |
| Pos. | 289 | obs: | exp: |
| aaa | K | 293 | 197.70 |
| aag | K | 60 | 155.30 |
| --- | -- | ------ | ------ |
| mPD | | 0.28 | 0.49 |
| | | nPD: | 0.57 |
| | | N. weight: | 0.26 |
| | | Sc. PD: | 0.078 |
| | | Sc. rank: | 37.7 |

,

| | | | |
|---|---|---|---|
| PA | | | |
| Pos. | 290 | obs: | exp: |
| tta | L | 216 | 30.84 |
| ttg | L | 48 | 67.98 |
| ctt | L | 64 | 65.86 |
| ctc | L | 3 | 57.10 |
| cta | L | 15 | 53.18 |
| ctg | L | 7 | 78.03 |
| --- | -- | ------ | ------ |
| mPD | | 0.89 | 1.1 |
| | | nPD: | 0.8 |
| | | N. weight: | 1.6 |
| | | Sc. PD: | 0.82 |
| | | Sc. rank: | 305.8 |

,

| | | | |
|---|---|---|---|
| PA | | | |
| Pos. | 291 | obs: | exp: |
| tct | S | 0 | 55.96 |
| tcc | S | 0 | 49.04 |
| tca | S | 0 | 77.98 |
| tcg | S | 0 | 21.46 |
| aat | N | 0 | 0.52 |
| aac | N | 1 | 0.48 |
| agt | S | 8 | 77.44 |
| agc | S | 344 | 70.13 |
| --- | -- | ------ | ------ |
| mPD | | 0.050 | 1.7 |
| | | nPD: | 0.03 |
| | | N. weight: | 2.3 |
| | | Sc. PD: | -0.51 |
| | | Sc. rank: | -265.2 |

,

| | | | |
|---|---|---|---|
| PA | | | |
| Pos. | 292 | obs: | exp: |
| att | I | 150 | 132.00 |
| atc | I | 199 | 91.99 |
| ata | I | 2 | 127.10 |
| gtt | V | 1 | 0.41 |
| gtc | V | 1 | 0.42 |
| gta | V | 0 | 0.37 |
| gtg | V | 0 | 0.79 |
| --- | -- | ------ | ------ |
| mPD | | 0.51 | 0.67 |
| | | nPD: | 0.76 |
| | | N. weight: | 0.73 |
| | | Sc. PD: | 0.35 |
| | | Sc. rank: | 137.1 |

,

| | | | |
|---|---|---|---|
| PA | | | |
| Pos. | 293 | obs: | exp: |
| gaa | E | 250 | 203.10 |
| gag | E | 103 | 149.90 |
| --- | -- | ------ | ------ |
| mPD | | 0.41 | 0.49 |
| | | nPD: | 0.85 |
| | | N. weight: | 0.07 |
| | | Sc. PD: | 0.039 |
| | | Sc. rank: | 13.9 |

,

| | | | |
|---|---|---|---|
| PA | | | |
| Pos. | 294 | obs: | exp: |
| gat | D | 11 | 188.30 |
| gac | D | 342 | 164.70 |
| --- | -- | ------ | ------ |
| mPD | | 0.061 | 0.50 |
| | | nPD: | 0.12 |
| | | N. weight: | 0.95 |
| | | Sc. PD: | -0.13 |
| | | Sc. rank: | -39.4 |

,

| | | | |
|---|---|---|---|
| PA | | | |
| Pos. | 295 | obs: | exp: |
| cct | P | 7 | 92.95 |
| ccc | P | 0 | 63.67 |
| cca | P | 43 | 139.30 |
| ccg | P | 303 | 57.08 |
| --- | -- | ------ | ------ |
| mPD | | 0.25 | 0.72 |
| | | nPD: | 0.35 |
| | | N. weight: | 1.9 |
| | | Sc. PD: | 0.15 |
| | | Sc. rank: | 141.1 |

,

| | | | |
|---|---|---|---|
| PA | | | |
| Pos. | 296 | obs: | exp: |
| tct | S | 0 | 55.80 |
| tcc | S | 0 | 48.90 |
| tca | S | 0 | 77.76 |
| tcg | S | 0 | 21.39 |
| agt | S | 195 | 77.22 |
| agc | S | 156 | 69.93 |
| gtt | V | 0 | 0.21 |
| gtc | V | 1 | 0.21 |
| gta | V | 0 | 0.19 |
| gtg | V | 0 | 0.40 |
| ggt | G | 0 | 0.12 |
| ggc | G | 1 | 0.14 |
| gga | G | 0 | 0.48 |
| ggg | G | 0 | 0.26 |
| --- | -- | ------ | ------ |
| mPD | | 0.51 | 1.7 |
| | | nPD: | 0.31 |
| | | N. weight: | 1.3 |
| | | Sc. PD: | 0.054 |
| | | Sc. rank: | 73.5 |

,

| | | | |
|---|---|---|---|
| PA | | | |
| Pos. | 297 | obs: | exp: |
| cat | H | 347 | 190.70 |
| cac | H | 5 | 161.30 |
| atg | M | 1 | 1.00 |
| --- | -- | ------ | ------ |
| mPD | | 0.045 | 0.51 |
| | | nPD: | 0.09 |
| | | N. weight: | 0.82 |
| | | Sc. PD: | -0.14 |
| | | Sc. rank: | -53.3 |

,

| | | | |
|---|---|---|---|
| PA | | | |
| Pos. | 298 | obs: | exp: |
| cgt | R | 0 | 0.04 |
| cgc | R | 0 | 0.05 |
| cga | R | 0 | 0.10 |
| cgg | R | 0 | 0.08 |
| aga | R | 0 | 0.45 |
| agg | R | 1 | 0.28 |
| gaa | E | 110 | 202.60 |
| gag | E | 242 | 149.40 |
| --- | -- | ------ | ------ |
| mPD | | 0.44 | 0.50 |
| | | nPD: | 0.88 |
| | | N. weight: | 0.23 |
| | | Sc. PD: | 0.14 |
| | | Sc. rank: | 46.0 |

,

| | | | |
|---|---|---|---|
| PA | | | |
| Pos. | 299 | obs: | exp: |
| ggt | G | 0 | 43.56 |
| ggc | G | 67 | 47.74 |
| gga | G | 31 | 170.80 |
| ggg | G | 255 | 90.95 |
| --- | -- | ------ | ------ |
| mPD | | 0.44 | 0.67 |
| | | nPD: | 0.65 |
| | | N. weight: | 1. |
| | | Sc. PD: | 0.38 |
| | | Sc. rank: | 164.3 |

,

| | | | |
|---|---|---|---|
| PA | | | |
| Pos. | 300 | obs: | exp: |
| gaa | E | 129 | 203.10 |
| gag | E | 224 | 149.90 |
| --- | -- | ------ | ------ |
| mPD | | 0.47 | 0.49 |
| | | nPD: | 0.95 |
| | | N. weight: | 0.15 |
| | | Sc. PD: | 0.098 |
| | | Sc. rank: | 29.7 |

,

| | | | |
|---|---|---|---|
| PA | | | |
| Pos. | 301 | obs: | exp: |
| ggt | G | 114 | 43.56 |
| ggc | G | 62 | 47.74 |
| gga | G | 38 | 170.80 |
| ggg | G | 139 | 90.95 |
| --- | -- | ------ | ------ |
| mPD | | 0.70 | 0.67 |
| | | nPD: | 1.05 |
| | | N. weight: | 0.58 |
| | | Sc. PD: | 0.44 |
| | | Sc. rank: | 118.5 |

,

| | | | |
|---|---|---|---|
| PA | | | |
| Pos. | 302 | obs: | exp: |
| att | I | 1 | 132.70 |
| atc | I | 2 | 92.52 |
| ata | I | 350 | 127.80 |
| --- | -- | ------ | ------ |
| mPD | | 0.017 | 0.66 |
| | | nPD: | 0.03 |
| | | N. weight: | 1.5 |
| | | Sc. PD: | -0.34 |
| | | Sc. rank: | -174.9 |

,

| | | | |
|---|---|---|---|
| PA | | | |
| Pos. | 303 | obs: | exp: |
| cct | P | 0 | 92.95 |
| ccc | P | 1 | 63.67 |
| cca | P | 71 | 139.30 |
| ccg | P | 281 | 57.08 |
| --- | -- | ------ | ------ |
| mPD | | 0.33 | 0.72 |
| | | nPD: | 0.46 |
| | | N. weight: | 1.7 |
| | | Sc. PD: | 0.32 |
| | | Sc. rank: | 174.6 |

,

| | | | |
|---|---|---|---|
| PA | | | |
| Pos. | 304 | obs: | exp: |
| tta | L | 31 | 30.84 |
| ttg | L | 4 | 67.98 |
| ctt | L | 0 | 65.86 |
| ctc | L | 1 | 57.10 |
| cta | L | 194 | 53.18 |
| ctg | L | 123 | 78.03 |
| --- | -- | ------ | ------ |
| mPD | | 0.64 | 1.1 |
| | | nPD: | 0.58 |
| | | N. weight: | 1.3 |
| | | Sc. PD: | 0.39 |
| | | Sc. rank: | 188.1 |

,

PA

| Pos. | 305 | obs: | exp: |
|---|---|---|---|
| tat | Y | 331 | 182.20 |
| tac | Y | 21 | 169.80 |
| tgt | C | 1 | 0.41 |
| tgc | C | 0 | 0.59 |
| --- | -- | ------ | ------ |
| mPD | | 0.12 | 0.51 |
| | | nPD: | 0.23 |
| | | N. weight: | 0.67 |
| | | Sc. PD: | -0.02 |
| | | Sc. rank: | 19.2 |

,

PA

| Pos. | 306 | obs: | exp: |
|---|---|---|---|
| gat | D | 352 | 187.80 |
| gac | D | 0 | 164.20 |
| gaa | E | 0 | 0.58 |
| gag | E | 1 | 0.42 |
| --- | -- | ------ | ------ |
| mPD | | 0.0057 | 0.50 |
| | | nPD: | 0.01 |
| | | N. weight: | 0.95 |
| | | Sc. PD: | -0.23 |
| | | Sc. rank: | -141.9 |

,

PA

| Pos. | 307 | obs: | exp: |
|---|---|---|---|
| gct | A | 1 | 88.91 |
| gcc | A | 0 | 72.31 |
| gca | A | 276 | 157.30 |
| gcg | A | 76 | 34.45 |
| --- | -- | ------ | ------ |
| mPD | | 0.34 | 0.69 |
| | | nPD: | 0.5 |
| | | N. weight: | 0.92 |
| | | Sc. PD: | 0.21 |
| | | Sc. rank: | 114.1 |

,

PA

| Pos. | 308 | obs: | exp: |
|---|---|---|---|
| att | I | 15 | 131.20 |
| atc | I | 315 | 91.47 |
| ata | I | 19 | 126.30 |
| gtt | V | 0 | 0.82 |
| gtc | V | 3 | 0.84 |
| gta | V | 1 | 0.75 |
| gtg | V | 0 | 1.59 |
| --- | -- | ------ | ------ |
| mPD | | 0.21 | 0.68 |
| | | nPD: | 0.3 |
| | | N. weight: | 1.4 |
| | | Sc. PD: | 0.05 |
| | | Sc. rank: | 76.2 |

,

PA

| Pos. | 309 | obs: | exp: |
|---|---|---|---|
| cgt | R | 0 | 0.04 |
| cgc | R | 0 | 0.05 |
| cga | R | 0 | 0.10 |
| cgg | R | 0 | 0.08 |
| aaa | K | 348 | 197.10 |
| aag | K | 4 | 154.90 |
| aga | R | 1 | 0.45 |
| agg | R | 0 | 0.28 |
| --- | -- | ------ | ------ |
| mPD | | 0.028 | 0.50 |
| | | nPD: | 0.06 |
| | | N. weight: | 0.8 |
| | | Sc. PD: | -0.16 |
| | | Sc. rank: | -67.9 |

,

PA

| Pos. | 310 | obs: | exp: |
|---|---|---|---|
| tgt | C | 13 | 145.50 |
| tgc | C | 340 | 207.50 |
| --- | -- | ------ | ------ |
| mPD | | 0.071 | 0.48 |
| | | nPD: | 0.15 |
| | | N. weight: | 0.59 |
| | | Sc. PD: | -0.067 |
| | | Sc. rank: | -17.7 |

,

PA

| Pos. | 311 | obs: | exp: |
|---|---|---|---|
| atg | M | 353 | 353.00 |
| --- | -- | ------ | ------ |
| mPD | | 0 | 0 |
| | | nPD: | 1. |
| | | N. weight: | 0. |
| | | Sc. PD: | 0 |
| | | Sc. rank: | 0 |

,

PA

| Pos. | 312 | obs: | exp: |
|---|---|---|---|
| aaa | K | 46 | 197.70 |
| aag | K | 307 | 155.30 |
| --- | -- | ------ | ------ |
| mPD | | 0.23 | 0.49 |
| | | nPD: | 0.46 |
| | | N. weight: | 0.62 |
| | | Sc. PD: | 0.12 |
| | | Sc. rank: | 64.4 |

,

PA

| Pos. | 313 | obs: | exp: |
|---|---|---|---|
| act | T | 22 | 93.26 |
| acc | T | 46 | 75.75 |
| aca | T | 283 | 154.60 |
| acg | T | 2 | 29.35 |
| --- | -- | ------ | ------ |
| mPD | | 0.34 | 0.69 |
| | | nPD: | 0.49 |
| | | N. weight: | 0.5 |
| | | Sc. PD: | 0.11 |
| | | Sc. rank: | 59.3 |

,

PA

| Pos. | 314 | obs: | exp: |
|---|---|---|---|
| ttt | F | 284 | 145.10 |
| ttc | F | 69 | 207.90 |
| --- | -- | ------ | ------ |
| mPD | | 0.32 | 0.48 |
| | | nPD: | 0.65 |
| | | N. weight: | 0.5 |
| | | Sc. PD: | 0.19 |
| | | Sc. rank: | 80.8 |

,

PA

| Pos. | 315 | obs: | exp: |
|---|---|---|---|
| ttt | F | 98 | 145.10 |
| ttc | F | 255 | 207.90 |
| --- | -- | ------ | ------ |
| mPD | | 0.40 | 0.48 |
| | | nPD: | 0.83 |
| | | N. weight: | 0.071 |
| | | Sc. PD: | 0.039 |
| | | Sc. rank: | 14.1 |

,

PA

| Pos. | 316 | obs: | exp: |
|---|---|---|---|
| ggt | G | 11 | 43.56 |
| ggc | G | 307 | 47.74 |
| gga | G | 9 | 170.80 |
| ggg | G | 26 | 90.95 |
| --- | -- | ------ | ------ |
| mPD | | 0.24 | 0.67 |
| | | nPD: | 0.36 |
| | | N. weight: | 2.2 |
| | | Sc. PD: | 0.19 |
| | | Sc. rank: | 167.5 |

,

PA

| Pos. | 317 | obs: | exp: |
|---|---|---|---|
| tta | L | 0 | 0.17 |
| ttg | L | 2 | 0.39 |
| tgg | W | 351 | 351.00 |
| ctt | L | 0 | 0.37 |
| ctc | L | 0 | 0.32 |
| cta | L | 0 | 0.30 |
| ctg | L | 0 | 0.44 |
| --- | -- | ------ | ------ |
| mPD | | 0.011 | 0.026 |
| | | nPD: | 0.43 |
| | | N. weight: | 0.014 |
| | | Sc. PD: | 0.0023 |
| | | Sc. rank: | 1.3 |

,

PA

| Pos. | 318 | obs: | exp: |
|---|---|---|---|
| cgt | R | 0 | 0.04 |
| cgc | R | 0 | 0.05 |
| cga | R | 0 | 0.10 |
| cgg | R | 0 | 0.08 |
| aaa | K | 346 | 197.10 |
| aag | K | 6 | 154.90 |
| aga | R | 1 | 0.45 |
| agg | R | 0 | 0.28 |
| --- | -- | ------ | ------ |
| mPD | | 0.039 | 0.50 |
| | | nPD: | 0.08 |
| | | N. weight: | 0.76 |
| | | Sc. PD: | -0.14 |
| | | Sc. rank: | -54.9 |

,

PA

| Pos. | 319 | obs: | exp: |
|---|---|---|---|
| gaa | E | 7 | 203.10 |
| gag | E | 346 | 149.90 |
| --- | -- | ------ | ------ |
| mPD | | 0.039 | 0.49 |
| | | nPD: | 0.08 |
| | | N. weight: | 1.1 |
| | | Sc. PD: | -0.2 |
| | | Sc. rank: | -81.0 |

,

PA

| Pos. | 320 | obs: | exp: |
|---|---|---|---|
| cct | P | 69 | 92.95 |
| ccc | P | 281 | 63.67 |
| cca | P | 1 | 139.30 |
| ccg | P | 2 | 57.08 |
| --- | -- | ------ | ------ |
| mPD | | 0.33 | 0.72 |
| | | nPD: | 0.46 |
| | | N. weight: | 1.7 |
| | | Sc. PD: | 0.31 |
| | | Sc. rank: | 172.2 |

,

PA

| Pos. | 321 | obs: | exp: |
|---|---|---|---|
| tct | S | 0 | 1.27 |
| tcc | S | 0 | 1.11 |
| tca | S | 0 | 1.77 |
| tcg | S | 0 | 0.49 |
| att | I | 0 | 7.89 |
| atc | I | 21 | 5.50 |
| ata | I | 0 | 7.60 |
| act | T | 0 | 0.53 |
| acc | T | 2 | 0.43 |
| aca | T | 0 | 0.88 |
| acg | T | 0 | 0.17 |
| aat | N | 31 | 167.80 |
| aac | N | 290 | 153.20 |
| agt | S | 4 | 1.76 |
| agc | S | 4 | 1.59 |
| gct | A | 0 | 0.25 |
| gcc | A | 1 | 0.20 |
| gca | A | 0 | 0.45 |
| gcg | A | 0 | 0.10 |
| --- | -- | ------ | ------ |
| mPD | | 0.35 | 0.73 |
| | | nPD: | 0.49 |
| | | N. weight: | 0.76 |
| | | Sc. PD: | 0.16 |
| | | Sc. rank: | 86.5 |

,

PA

| Pos. | 322 | obs: | exp: |
|---|---|---|---|
| att | I | 179 | 132.30 |
| atc | I | 153 | 92.25 |
| ata | I | 20 | 127.40 |
| gtt | V | 1 | 0.21 |
| gtc | V | 0 | 0.21 |
| gta | V | 0 | 0.19 |
| gtg | V | 0 | 0.40 |
| --- | -- | ------ | ------ |
| mPD | | 0.56 | 0.67 |
| | | nPD: | 0.84 |
| | | N. weight: | 0.44 |
| | | Sc. PD: | 0.24 |
| | | Sc. rank: | 86.1 |

,

PA

| Pos. | 323 | obs: | exp: |
|---|---|---|---|
| att | I | 0 | 35.71 |
| atc | I | 55 | 24.90 |
| ata | I | 40 | 34.39 |
| gtt | V | 5 | 52.82 |
| gtc | V | 77 | 53.94 |
| gta | V | 163 | 48.17 |
| gtg | V | 12 | 102.10 |
| gct | A | 0 | 0.25 |
| gcc | A | 0 | 0.20 |
| gca | A | 1 | 0.45 |
| gcg | A | 0 | 0.10 |
| --- | -- | ------ | ------ |
| mPD | | 0.93 | 1.1 |
| | | nPD: | 0.81 |
| | | N. weight: | 1.1 |
| | | Sc. PD: | 0.56 |
| | | Sc. rank: | 207.0 |

,

PA

| Pos. | 324 | obs: | exp: |
|---|---|---|---|
| aaa | K | 230 | 196.50 |
| aag | K | 121 | 154.50 |
| gaa | E | 2 | 1.15 |
| gag | E | 0 | 0.85 |
| --- | -- | ------ | ------ |
| mPD | | 0.46 | 0.50 |
| | | nPD: | 0.92 |
| | | N. weight: | 0.046 |
| | | Sc. PD: | 0.029 |
| | | Sc. rank: | 9.3 |

,

| PA | | | |
|---|---|---|---|
| Pos. | 325 | obs: | exp: |
| tta | L | 0 | 0.09 |
| ttg | L | 0 | 0.19 |
| tct | S | 0 | 0.32 |
| tcc | S | 0 | 0.28 |
| tca | S | 2 | 0.44 |
| tcg | S | 0 | 0.12 |
| ctt | L | 0 | 0.19 |
| ctc | L | 0 | 0.16 |
| cta | L | 0 | 0.15 |
| ctg | L | 1 | 0.22 |
| cct | P | 0 | 92.16 |
| ccc | P | 0 | 63.13 |
| cca | P | 326 | 138.10 |
| ccg | P | 24 | 56.59 |
| agt | S | 0 | 0.44 |
| agc | S | 0 | 0.40 |
| --- | -- | ------ | ------ |
| mPD | | 0.15 | 0.74 |
| | | nPD: | 0.2 |
| | | N. weight: | 1.1 |
| | | Sc. PD: | -0.069 |
| | | Sc. rank: | 6.5 |

,

| PA | | | |
|---|---|---|---|
| Pos. | 326 | obs: | exp: |
| cat | H | 350 | 191.30 |
| cac | H | 3 | 161.70 |
| --- | -- | ------ | ------ |
| mPD | | 0.017 | 0.50 |
| | | nPD: | 0.03 |
| | | N. weight: | 0.86 |
| | | Sc. PD: | -0.19 |
| | | Sc. rank: | -92.1 |

,

| PA | | | |
|---|---|---|---|
| Pos. | 327 | obs: | exp: |
| gct | A | 0 | 0.76 |
| gcc | A | 0 | 0.61 |
| gca | A | 3 | 1.34 |
| gcg | A | 0 | 0.29 |
| gaa | E | 285 | 200.80 |
| gag | E | 64 | 148.20 |
| ggt | G | 0 | 0.12 |
| ggc | G | 0 | 0.14 |
| gga | G | 1 | 0.48 |
| ggg | G | 0 | 0.26 |
| --- | -- | ------ | ------ |
| mPD | | 0.32 | 0.52 |
| | | nPD: | 0.62 |
| | | N. weight: | 0.22 |
| | | Sc. PD: | 0.077 |
| | | Sc. rank: | 34.7 |

,

| PA | | | |
|---|---|---|---|
| Pos. | 328 | obs: | exp: |
| cgt | R | 0 | 0.12 |
| cgc | R | 0 | 0.15 |
| cga | R | 0 | 0.29 |
| cgg | R | 0 | 0.25 |
| aaa | K | 220 | 196.00 |
| aag | K | 130 | 154.00 |
| aga | R | 0 | 1.35 |
| agg | R | 3 | 0.84 |
| --- | -- | ------ | ------ |
| mPD | | 0.49 | 0.51 |
| | | nPD: | 0.95 |
| | | N. weight: | 0.044 |
| | | Sc. PD: | 0.029 |
| | | Sc. rank: | 8.9 |

,

| PA | | | |
|---|---|---|---|
| Pos. | 329 | obs: | exp: |
| ggt | G | 13 | 43.56 |
| ggc | G | 305 | 47.74 |
| gga | G | 34 | 170.80 |
| ggg | G | 1 | 90.95 |
| --- | -- | ------ | ------ |
| mPD | | 0.24 | 0.67 |
| | | nPD: | 0.37 |
| | | N. weight: | 2.1 |
| | | Sc. PD: | 0.21 |
| | | Sc. rank: | 173.8 |

,

| PA | | | |
|---|---|---|---|
| Pos. | 330 | obs: | exp: |
| att | I | 1 | 132.30 |
| atc | I | 25 | 92.25 |
| ata | I | 326 | 127.40 |
| gtt | V | 0 | 0.21 |
| gtc | V | 0 | 0.21 |
| gta | V | 1 | 0.19 |
| gtg | V | 0 | 0.40 |
| --- | -- | ------ | ------ |
| mPD | | 0.14 | 0.67 |
| | | nPD: | 0.21 |
| | | N. weight: | 1.2 |
| | | Sc. PD: | -0.056 |
| | | Sc. rank: | 21.1 |

,

| PA | | | |
|---|---|---|---|
| Pos. | 331 | obs: | exp: |
| aat | N | 74 | 184.50 |
| aac | N | 279 | 168.50 |
| --- | -- | ------ | ------ |
| mPD | | 0.33 | 0.50 |
| | | nPD: | 0.67 |
| | | N. weight: | 0.33 |
| | | Sc. PD: | 0.13 |
| | | Sc. rank: | 53.6 |

,

| PA | | | |
|---|---|---|---|
| Pos. | 332 | obs: | exp: |
| tct | S | 0 | 0.16 |
| tcc | S | 1 | 0.14 |
| tca | S | 0 | 0.22 |
| tcg | S | 0 | 0.06 |
| cct | P | 93 | 92.69 |
| ccc | P | 257 | 63.49 |
| cca | P | 2 | 138.90 |
| ccg | P | 0 | 56.91 |
| agt | S | 0 | 0.22 |
| agc | S | 0 | 0.20 |
| --- | -- | ------ | ------ |
| mPD | | 0.40 | 0.72 |
| | | nPD: | 0.56 |
| | | N. weight: | 1.5 |
| | | Sc. PD: | 0.43 |
| | | Sc. rank: | 215.8 |

,

| PA | | | |
|---|---|---|---|
| Pos. | 333 | obs: | exp: |
| aat | N | 323 | 184.50 |
| aac | N | 30 | 168.50 |
| --- | -- | ------ | ------ |
| mPD | | 0.16 | 0.50 |
| | | nPD: | 0.31 |
| | | N. weight: | 0.56 |
| | | Sc. PD: | 0.026 |
| | | Sc. rank: | 32.4 |

,

| PA | | | |
|---|---|---|---|
| Pos. | 334 | obs: | exp: |
| tat | Y | 101 | 182.70 |
| tac | Y | 252 | 170.30 |
| --- | -- | ------ | ------ |
| mPD | | 0.41 | 0.50 |
| | | nPD: | 0.82 |
| | | N. weight: | 0.18 |
| | | Sc. PD: | 0.096 |
| | | Sc. rank: | 35.2 |

,

| PA | | | |
|---|---|---|---|
| Pos. | 335 | obs: | exp: |
| tta | L | 0 | 30.84 |
| ttg | L | 0 | 67.98 |
| ctt | L | 35 | 65.86 |
| ctc | L | 312 | 57.10 |
| cta | L | 6 | 53.18 |
| ctg | L | 0 | 78.03 |
| --- | -- | ------ | ------ |
| mPD | | 0.21 | 1.1 |
| | | nPD: | 0.19 |
| | | N. weight: | 2.1 |
| | | Sc. PD: | -0.16 |
| | | Sc. rank: | -23.3 |

,

| PA | | | |
|---|---|---|---|
| Pos. | 336 | obs: | exp: |
| tta | L | 0 | 30.66 |
| ttg | L | 28 | 67.60 |
| ctt | L | 0 | 65.49 |
| ctc | L | 0 | 56.78 |
| cta | L | 10 | 52.88 |
| ctg | L | 313 | 77.59 |
| caa | Q | 0 | 0.53 |
| cag | Q | 1 | 0.47 |
| atg | M | 1 | 1.00 |
| --- | -- | ------ | ------ |
| mPD | | 0.21 | 1.1 |
| | | nPD: | 0.19 |
| | | N. weight: | 1.7 |
| | | Sc. PD: | -0.12 |
| | | Sc. rank: | -11.0 |

,

| PA | | | |
|---|---|---|---|
| Pos. | 337 | obs: | exp: |
| act | T | 29 | 7.66 |
| acc | T | 0 | 6.22 |
| aca | T | 0 | 12.70 |
| acg | T | 0 | 2.41 |
| gct | A | 321 | 81.61 |
| gcc | A | 3 | 66.37 |
| gca | A | 0 | 144.40 |
| gcg | A | 0 | 31.62 |
| --- | -- | ------ | ------ |
| mPD | | 0.17 | 0.84 |
| | | nPD: | 0.2 |
| | | N. weight: | 2. |
| | | Sc. PD: | -0.12 |
| | | Sc. rank: | 10.2 |

,

| PA | | | |
|---|---|---|---|
| Pos. | 338 | obs: | exp: |
| tgg | W | 353 | 353.00 |
| --- | -- | ------ | ------ |
| mPD | | 0 | 0 |
| | | nPD: | 1. |
| | | N. weight: | 0. |
| | | Sc. PD: | 0 |
| | | Sc. rank: | 0 |

,

| PA | | | |
|---|---|---|---|
| Pos. | 339 | obs: | exp: |
| cgt | R | 0 | 0.08 |
| cgc | R | 0 | 0.10 |
| cga | R | 0 | 0.19 |
| cgg | R | 0 | 0.17 |
| aaa | K | 16 | 196.50 |
| aag | K | 335 | 154.50 |
| aga | R | 0 | 0.90 |
| agg | R | 2 | 0.56 |
| --- | -- | ------ | ------ |
| mPD | | 0.098 | 0.51 |
| | | nPD: | 0.19 |
| | | N. weight: | 0.96 |
| | | Sc. PD: | -0.066 |
| | | Sc. rank: | -0.2 |

,

| PA | | | |
|---|---|---|---|
| Pos. | 340 | obs: | exp: |
| caa | Q | 114 | 188.20 |
| cag | Q | 239 | 164.80 |
| --- | -- | ------ | ------ |
| mPD | | 0.44 | 0.50 |
| | | nPD: | 0.88 |
| | | N. weight: | 0.15 |
| | | Sc. PD: | 0.088 |
| | | Sc. rank: | 29.6 |

,

| PA | | | |
|---|---|---|---|
| Pos. | 341 | obs: | exp: |
| tta | L | 0 | 0.70 |
| ttg | L | 0 | 1.54 |
| ctt | L | 0 | 1.49 |
| ctc | L | 0 | 1.29 |
| cta | L | 8 | 1.21 |
| ctg | L | 0 | 1.77 |
| atg | M | 25 | 25.00 |
| gtt | V | 0 | 65.77 |
| gtc | V | 0 | 67.16 |
| gta | V | 18 | 59.97 |
| gtg | V | 302 | 127.10 |
| --- | -- | ------ | ------ |
| mPD | | 0.31 | 0.87 |
| | | nPD: | 0.35 |
| | | N. weight: | 1.1 |
| | | Sc. PD: | 0.095 |
| | | Sc. rank: | 83.8 |

,

| PA | | | |
|---|---|---|---|
| Pos. | 342 | obs: | exp: |
| tta | L | 38 | 30.75 |
| ttg | L | 5 | 67.79 |
| ctt | L | 1 | 65.68 |
| ctc | L | 0 | 56.94 |
| cta | L | 103 | 53.03 |
| ctg | L | 205 | 77.81 |
| atg | M | 1 | 1.00 |
| --- | -- | ------ | ------ |
| mPD | | 0.70 | 1.1 |
| | | nPD: | 0.63 |
| | | N. weight: | 1.1 |
| | | Sc. PD: | 0.4 |
| | | Sc. rank: | 179.7 |

,

| PA | | | |
|---|---|---|---|
| Pos. | 343 | obs: | exp: |
| gct | A | 0 | 88.91 |
| gcc | A | 0 | 72.31 |
| gca | A | 348 | 157.30 |
| gcg | A | 5 | 34.45 |
| --- | -- | ------ | ------ |
| mPD | | 0.028 | 0.69 |
| | | nPD: | 0.04 |
| | | N. weight: | 1.2 |
| | | Sc. PD: | -0.25 |
| | | Sc. rank: | -115.2 |

,

| PA | | | |
|---|---|---|---|
| Pos. | 344 | obs: | exp: |
| gaa | E | 280 | 203.10 |
| gag | E | 73 | 149.90 |
| --- | -- | ------ | ------ |
| mPD | | 0.33 | 0.49 |
| | | nPD: | 0.67 |
| | | N. weight: | 0.17 |
| | | Sc. PD: | 0.068 |
| | | Sc. rank: | 28.8 |

,

| PA | | | |
|---|---|---|---|
| Pos. | 345 | obs: | exp: |
| ttt | F | 1 | 0.82 |
| ttc | F | 1 | 1.18 |
| tta | L | 2 | 30.66 |
| ttg | L | 2 | 67.60 |
| ctt | L | 40 | 65.49 |
| ctc | L | 238 | 56.78 |
| cta | L | 65 | 52.88 |
| ctg | L | 4 | 77.59 |
| --- | -- | ------ | ------ |
| mPD | | 0.53 | 1.1 |
| | | nPD: | 0.47 |
| | | N. weight: | 1.4 |
| | | Sc. PD: | 0.27 |
| | | Sc. rank: | 147.4 |

,

| PA | | | |
|---|---|---|---|
| Pos. | 346 | obs: | exp: |
| caa | Q | 229 | 188.20 |
| cag | Q | 124 | 164.80 |
| --- | -- | ------ | ------ |
| mPD | | 0.46 | 0.50 |
| | | nPD: | 0.92 |
| | | N. weight: | 0.055 |
| | | Sc. PD: | 0.034 |
| | | Sc. rank: | 10.9 |

,

| PA | | | |
|---|---|---|---|
| Pos. | 347 | obs: | exp: |
| aat | N | 2 | 1.05 |
| aac | N | 0 | 0.95 |
| aaa | K | 1 | 0.56 |
| aag | K | 0 | 0.44 |
| gat | D | 264 | 186.70 |
| gac | D | 86 | 163.30 |
| --- | -- | ------ | ------ |
| mPD | | 0.39 | 0.52 |
| | | nPD: | 0.76 |
| | | N. weight: | 0.18 |
| | | Sc. PD: | 0.083 |
| | | Sc. rank: | 32.9 |

,

| PA | | | |
|---|---|---|---|
| Pos. | 348 | obs: | exp: |
| ttt | F | 1 | 0.41 |
| ttc | F | 0 | 0.59 |
| tta | L | 0 | 1.66 |
| ttg | L | 0 | 3.66 |
| ctt | L | 19 | 3.55 |
| ctc | L | 0 | 3.07 |
| cta | L | 0 | 2.86 |
| ctg | L | 0 | 4.20 |
| att | I | 326 | 124.10 |
| atc | I | 4 | 86.49 |
| ata | I | 0 | 119.50 |
| gtt | V | 3 | 0.62 |
| gtc | V | 0 | 0.63 |
| gta | V | 0 | 0.56 |
| gtg | V | 0 | 1.19 |
| --- | -- | ------ | ------ |
| mPD | | 0.15 | 0.80 |
| | | nPD: | 0.18 |
| | | N. weight: | 1.5 |
| | | Sc. PD: | -0.12 |
| | | Sc. rank: | -27.8 |

,

| PA | | | |
|---|---|---|---|
| Pos. | 349 | obs: | exp: |
| gaa | E | 302 | 203.10 |
| gag | E | 51 | 149.90 |
| --- | -- | ------ | ------ |
| mPD | | 0.25 | 0.49 |
| | | nPD: | 0.51 |
| | | N. weight: | 0.29 |
| | | Sc. PD: | 0.068 |
| | | Sc. rank: | 36.6 |

,

| PA | | | |
|---|---|---|---|
| Pos. | 350 | obs: | exp: |
| tct | S | 0 | 0.32 |
| tcc | S | 0 | 0.28 |
| tca | S | 0 | 0.44 |
| tcg | S | 0 | 0.12 |
| act | T | 0 | 0.26 |
| acc | T | 1 | 0.21 |
| aca | T | 0 | 0.44 |
| acg | T | 0 | 0.08 |
| aat | N | 304 | 182.90 |
| aac | N | 46 | 167.10 |
| agt | S | 2 | 0.44 |
| agc | S | 0 | 0.40 |
| --- | -- | ------ | ------ |
| mPD | | 0.25 | 0.53 |
| | | nPD: | 0.47 |
| | | N. weight: | 0.44 |
| | | Sc. PD: | 0.088 |
| | | Sc. rank: | 47.4 |

,

| PA | | | |
|---|---|---|---|
| Pos. | 351 | obs: | exp: |
| gaa | E | 77 | 202.60 |
| gag | E | 275 | 149.40 |
| ggt | G | 1 | 0.12 |
| ggc | G | 0 | 0.14 |
| gga | G | 0 | 0.48 |
| ggg | G | 0 | 0.26 |
| --- | -- | ------ | ------ |
| mPD | | 0.35 | 0.49 |
| | | nPD: | 0.71 |
| | | N. weight: | 0.42 |
| | | Sc. PD: | 0.18 |
| | | Sc. rank: | 74.0 |

,

| PA | | | |
|---|---|---|---|
| Pos. | 352 | obs: | exp: |
| aaa | K | 0 | 0.56 |
| aag | K | 1 | 0.44 |
| gaa | E | 5 | 202.00 |
| gag | E | 346 | 149.00 |
| ggt | G | 0 | 0.12 |
| ggc | G | 0 | 0.14 |
| gga | G | 1 | 0.48 |
| ggg | G | 0 | 0.26 |
| --- | -- | ------ | ------ |
| mPD | | 0.045 | 0.50 |
| | | nPD: | 0.09 |
| | | N. weight: | 1.2 |
| | | Sc. PD: | -0.2 |
| | | Sc. rank: | -71.6 |

,

| PA | | | |
|---|---|---|---|
| Pos. | 353 | obs: | exp: |
| aaa | K | 273 | 197.70 |
| aag | K | 80 | 155.30 |
| --- | -- | ------ | ------ |
| mPD | | 0.35 | 0.49 |
| | | nPD: | 0.71 |
| | | N. weight: | 0.16 |
| | | Sc. PD: | 0.07 |
| | | Sc. rank: | 28.8 |

,

| PA | | | |
|---|---|---|---|
| Pos. | 354 | obs: | exp: |
| att | I | 147 | 132.70 |
| atc | I | 206 | 92.52 |
| ata | I | 0 | 127.80 |
| --- | -- | ------ | ------ |
| mPD | | 0.49 | 0.66 |
| | | nPD: | 0.74 |
| | | N. weight: | 0.78 |
| | | Sc. PD: | 0.36 |
| | | Sc. rank: | 144.3 |

,

| PA | | | |
|---|---|---|---|
| Pos. | 355 | obs: | exp: |
| cct | P | 3 | 92.95 |
| ccc | P | 6 | 63.67 |
| cca | P | 322 | 139.30 |
| ccg | P | 22 | 57.08 |
| --- | -- | ------ | ------ |
| mPD | | 0.16 | 0.72 |
| | | nPD: | 0.23 |
| | | N. weight: | 0.99 |
| | | Sc. PD: | -0.034 |
| | | Sc. rank: | 25.3 |

,

| PA | | | |
|---|---|---|---|
| Pos. | 356 | obs: | exp: |
| cgt | R | 0 | 0.25 |
| cgc | R | 0 | 0.29 |
| cga | R | 0 | 0.58 |
| cgg | R | 0 | 0.50 |
| aaa | K | 276 | 194.30 |
| aag | K | 71 | 152.70 |
| aga | R | 5 | 2.70 |
| agg | R | 1 | 1.68 |
| --- | -- | ------ | ------ |
| mPD | | 0.36 | 0.54 |
| | | nPD: | 0.67 |
| | | N. weight: | 0.21 |
| | | Sc. PD: | 0.082 |
| | | Sc. rank: | 34.6 |

,

| PA | | | |
|---|---|---|---|
| Pos. | 357 | obs: | exp: |
| tta | L | 1 | 0.09 |
| ttg | L | 0 | 0.19 |
| ctt | L | 0 | 0.19 |
| ctc | L | 0 | 0.16 |
| cta | L | 0 | 0.15 |
| ctg | L | 0 | 0.22 |
| atg | M | 1 | 1.00 |
| act | T | 0 | 92.73 |
| acc | T | 1 | 75.32 |
| aca | T | 262 | 153.80 |
| acg | T | 88 | 29.18 |
| --- | -- | ------ | ------ |
| mPD | | 0.40 | 0.70 |
| | | nPD: | 0.57 |
| | | N. weight: | 1. |
| | | Sc. PD: | 0.3 |
| | | Sc. rank: | 146.6 |

,

| PA | | | |
|---|---|---|---|
| Pos. | 358 | obs: | exp: |
| aaa | K | 67 | 197.70 |
| aag | K | 286 | 155.30 |
| --- | -- | ------ | ------ |
| mPD | | 0.31 | 0.49 |
| | | nPD: | 0.63 |
| | | N. weight: | 0.45 |
| | | Sc. PD: | 0.16 |
| | | Sc. rank: | 70.3 |

,

| PA | | | |
|---|---|---|---|
| Pos. | 359 | obs: | exp: |
| act | T | 0 | 0.26 |
| acc | T | 1 | 0.21 |
| aca | T | 0 | 0.44 |
| acg | T | 0 | 0.08 |
| aat | N | 17 | 184.00 |
| aac | N | 335 | 168.00 |
| --- | -- | ------ | ------ |
| mPD | | 0.098 | 0.51 |
| | | nPD: | 0.19 |
| | | N. weight: | 0.83 |
| | | Sc. PD: | -0.057 |
| | | Sc. rank: | -2.9 |

,

| PA | | | |
|---|---|---|---|
| Pos. | 360 | obs: | exp: |
| cgt | R | 0 | 0.04 |
| cgc | R | 0 | 0.05 |
| cga | R | 0 | 0.10 |
| cgg | R | 1 | 0.08 |
| att | I | 0 | 0.38 |
| atc | I | 0 | 0.26 |
| ata | I | 1 | 0.36 |
| atg | M | 351 | 351.00 |
| aga | R | 0 | 0.45 |
| agg | R | 0 | 0.28 |
| --- | -- | ------ | ------ |
| mPD | | 0.017 | 0.016 |
| | | nPD: | 1.04 |
| | | N. weight: | 0.015 |
| | | Sc. PD: | 0.011 |
| | | Sc. rank: | 3.0 |

,

| PA | | | |
|---|---|---|---|
| Pos. | 361 | obs: | exp: |
| cgt | R | 0 | 0.17 |
| cgc | R | 0 | 0.20 |
| cga | R | 0 | 0.38 |
| cgg | R | 0 | 0.33 |
| aaa | K | 1 | 195.40 |
| aag | K | 348 | 153.60 |
| aga | R | 0 | 1.80 |
| agg | R | 4 | 1.12 |
| --- | -- | ------ | ------ |
| mPD | | 0.028 | 0.52 |
| | | nPD: | 0.05 |
| | | N. weight: | 1.2 |
| | | Sc. PD: | -0.25 |
| | | Sc. rank: | -108.4 |

,

| PA | | | |
|---|---|---|---|
| Pos. | 362 | obs: | exp: |
| aaa | K | 332 | 197.70 |
| aag | K | 21 | 155.30 |
| --- | -- | ------ | ------ |
| mPD | | 0.11 | 0.49 |
| | | nPD: | 0.23 |
| | | N. weight: | 0.57 |
| | | Sc. PD: | -0.02 |
| | | Sc. rank: | 13.6 |

,

| PA | | | |
|---|---|---|---|
| Pos. | 363 | obs: | exp: |
| cct | P | 0 | 0.26 |
| ccc | P | 0 | 0.18 |
| cca | P | 1 | 0.39 |
| ccg | P | 0 | 0.16 |
| act | T | 0 | 93.00 |
| acc | T | 0 | 75.53 |
| aca | T | 344 | 154.20 |
| acg | T | 8 | 29.26 |
| --- | -- | ------ | ------ |
| mPD | | 0.050 | 0.69 |
| | | nPD: | 0.07 |
| | | N. weight: | 1.2 |
| | | Sc. PD: | -0.21 |
| | | Sc. rank: | -84.0 |

,

| PA | | | |
|---|---|---|---|
| Pos. | 364 | obs: | exp: |
| tct | S | 0 | 56.12 |
| tcc | S | 0 | 49.17 |
| tca | S | 0 | 78.20 |
| tcg | S | 0 | 21.52 |
| agt | S | 18 | 77.66 |
| agc | S | 335 | 70.33 |
| --- | -- | ------ | ------ |
| mPD | | 0.097 | 1.7 |
| | | nPD: | 0.06 |
| | | N. weight: | 2.1 |
| | | Sc. PD: | -0.42 |
| | | Sc. rank: | -173.9 |

,

| PA | | | |
|---|---|---|---|
| Pos. | 365 | obs: | exp: |
| cat | H | 0 | 14.09 |
| cac | H | 26 | 11.91 |
| caa | Q | 279 | 174.30 |
| cag | Q | 48 | 152.70 |
| --- | -- | ------ | ------ |
| mPD | | 0.35 | 0.57 |
| | | nPD: | 0.62 |
| | | N. weight: | 0.42 |
| | | Sc. PD: | 0.15 |
| | | Sc. rank: | 65.9 |

| PA | | | |
|---|---|---|---|
| Pos. | 366 | obs: | exp: |
| tta | L | 77 | 30.84 |
| ttg | L | 231 | 67.98 |
| ctt | L | 0 | 65.86 |
| ctc | L | 0 | 57.10 |
| cta | L | 2 | 53.18 |
| ctg | L | 43 | 78.03 |
| --- | -- | ------ | ------ |
| mPD | | 0.57 | 1.1 |
| | | nPD: | 0.51 |
| | | N. weight: | 1.4 |
| | | Sc. PD: | 0.34 |
| | | Sc. rank: | 178.9 |

| PA | | | |
|---|---|---|---|
| Pos. | 367 | obs: | exp: |
| aaa | K | 88 | 197.70 |
| aag | K | 265 | 155.30 |
| --- | -- | ------ | ------ |
| mPD | | 0.38 | 0.49 |
| | | nPD: | 0.76 |
| | | N. weight: | 0.31 |
| | | Sc. PD: | 0.15 |
| | | Sc. rank: | 58.9 |

| PA | | | |
|---|---|---|---|
| Pos. | 368 | obs: | exp: |
| tgg | W | 353 | 353.00 |
| --- | -- | ------ | ------ |
| mPD | | 0 | 0 |
| | | nPD: | 1. |
| | | N. weight: | 0. |
| | | Sc. PD: | 0 |
| | | Sc. rank: | 0 |

| PA | | | |
|---|---|---|---|
| Pos. | 369 | obs: | exp: |
| act | T | 0 | 0.26 |
| acc | T | 0 | 0.21 |
| aca | T | 1 | 0.44 |
| acg | T | 0 | 0.08 |
| gct | A | 1 | 88.66 |
| gcc | A | 0 | 72.11 |
| gca | A | 349 | 156.90 |
| gcg | A | 2 | 34.35 |
| --- | -- | ------ | ------ |
| mPD | | 0.023 | 0.69 |
| | | nPD: | 0.03 |
| | | N. weight: | 1.2 |
| | | Sc. PD: | -0.26 |
| | | Sc. rank: | -132.5 |

| PA | | | |
|---|---|---|---|
| Pos. | 370 | obs: | exp: |
| tta | L | 0 | 30.84 |
| ttg | L | 0 | 67.98 |
| ctt | L | 306 | 65.86 |
| ctc | L | 44 | 57.10 |
| cta | L | 2 | 53.18 |
| ctg | L | 1 | 78.03 |
| --- | -- | ------ | ------ |
| mPD | | 0.23 | 1.1 |
| | | nPD: | 0.21 |
| | | N. weight: | 1.9 |
| | | Sc. PD: | -0.1 |
| | | Sc. rank: | 22.7 |

| PA | | | |
|---|---|---|---|
| Pos. | 371 | obs: | exp: |
| ggt | G | 349 | 43.56 |
| ggc | G | 0 | 47.74 |
| gga | G | 1 | 170.80 |
| ggg | G | 3 | 90.95 |
| --- | -- | ------ | ------ |
| mPD | | 0.023 | 0.67 |
| | | nPD: | 0.03 |
| | | N. weight: | 3.1 |
| | | Sc. PD: | -0.68 |
| | | Sc. rank: | -330.0 |

| PA | | | |
|---|---|---|---|
| Pos. | 372 | obs: | exp: |
| gaa | E | 137 | 203.10 |
| gag | E | 216 | 149.90 |
| --- | -- | ------ | ------ |
| mPD | | 0.48 | 0.49 |
| | | nPD: | 0.97 |
| | | N. weight: | 0.12 |
| | | Sc. PD: | 0.082 |
| | | Sc. rank: | 24.3 |

| PA | | | |
|---|---|---|---|
| Pos. | 373 | obs: | exp: |
| aat | N | 78 | 184.50 |
| aac | N | 275 | 168.50 |
| --- | -- | ------ | ------ |
| mPD | | 0.35 | 0.50 |
| | | nPD: | 0.69 |
| | | N. weight: | 0.3 |
| | | Sc. PD: | 0.12 |
| | | Sc. rank: | 52.2 |

| PA | | | |
|---|---|---|---|
| Pos. | 374 | obs: | exp: |
| att | I | 0 | 0.75 |
| atc | I | 0 | 0.52 |
| ata | I | 2 | 0.72 |
| atg | M | 350 | 350.00 |
| aaa | K | 0 | 0.56 |
| aag | K | 1 | 0.44 |
| --- | -- | ------ | ------ |
| mPD | | 0.017 | 0.020 |
| | | nPD: | 0.84 |
| | | N. weight: | 0.012 |
| | | Sc. PD: | 0.0068 |
| | | Sc. rank: | 2.4 |

| PA | | | |
|---|---|---|---|
| Pos. | 375 | obs: | exp: |
| gct | A | 0 | 88.91 |
| gcc | A | 9 | 72.31 |
| gca | A | 343 | 157.30 |
| gcg | A | 1 | 34.45 |
| --- | -- | ------ | ------ |
| mPD | | 0.055 | 0.69 |
| | | nPD: | 0.08 |
| | | N. weight: | 1.1 |
| | | Sc. PD: | -0.19 |
| | | Sc. rank: | -72.3 |

| PA | | | |
|---|---|---|---|
| Pos. | 376 | obs: | exp: |
| tct | S | 0 | 0.16 |
| tcc | S | 0 | 0.14 |
| tca | S | 1 | 0.22 |
| tcg | S | 0 | 0.06 |
| cct | P | 12 | 92.69 |
| ccc | P | 0 | 63.49 |
| cca | P | 287 | 138.90 |
| ccg | P | 53 | 56.91 |
| agt | S | 0 | 0.22 |
| agc | S | 0 | 0.20 |
| --- | -- | ------ | ------ |
| mPD | | 0.32 | 0.72 |
| | | nPD: | 0.44 |
| | | N. weight: | 0.8 |
| | | Sc. PD: | 0.13 |
| | | Sc. rank: | 77.1 |

| PA | | | |
|---|---|---|---|
| Pos. | 377 | obs: | exp: |
| gaa | E | 72 | 203.10 |
| gag | E | 281 | 149.90 |
| --- | -- | ------ | ------ |
| mPD | | 0.33 | 0.49 |
| | | nPD: | 0.67 |
| | | N. weight: | 0.45 |
| | | Sc. PD: | 0.17 |
| | | Sc. rank: | 74.1 |

| PA | | | |
|---|---|---|---|
| Pos. | 378 | obs: | exp: |
| cgt | R | 0 | 0.04 |
| cgc | R | 0 | 0.05 |
| cga | R | 0 | 0.10 |
| cgg | R | 0 | 0.08 |
| aaa | K | 295 | 197.10 |
| aag | K | 57 | 154.90 |
| aga | R | 0 | 0.45 |
| agg | R | 1 | 0.28 |
| --- | -- | ------ | ------ |
| mPD | | 0.28 | 0.50 |
| | | nPD: | 0.56 |
| | | N. weight: | 0.28 |
| | | Sc. PD: | 0.081 |
| | | Sc. rank: | 40.1 |

| PA | | | |
|---|---|---|---|
| Pos. | 379 | obs: | exp: |
| gtt | V | 1 | 72.14 |
| gtc | V | 0 | 73.67 |
| gta | V | 208 | 65.78 |
| gtg | V | 142 | 139.40 |
| gct | A | 0 | 0.50 |
| gcc | A | 0 | 0.41 |
| gca | A | 2 | 0.89 |
| gcg | A | 0 | 0.20 |
| --- | -- | ------ | ------ |
| mPD | | 0.50 | 0.73 |
| | | nPD: | 0.68 |
| | | N. weight: | 1. |
| | | Sc. PD: | 0.41 |
| | | Sc. rank: | 177.5 |

| PA | | | |
|---|---|---|---|
| Pos. | 380 | obs: | exp: |
| gat | D | 9 | 188.30 |
| gac | D | 344 | 164.70 |
| --- | -- | ------ | ------ |
| mPD | | 0.050 | 0.50 |
| | | nPD: | 0.1 |
| | | N. weight: | 0.98 |
| | | Sc. PD: | -0.15 |
| | | Sc. rank: | -52.1 |

| PA | | | |
|---|---|---|---|
| Pos. | 381 | obs: | exp: |
| ttt | F | 346 | 145.10 |
| ttc | F | 7 | 207.90 |
| --- | -- | ------ | ------ |
| mPD | | 0.039 | 0.48 |
| | | nPD: | 0.08 |
| | | N. weight: | 1.2 |
| | | Sc. PD: | -0.21 |
| | | Sc. rank: | -82.8 |

| PA | | | |
|---|---|---|---|
| Pos. | 382 | obs: | exp: |
| gat | D | 21 | 11.20 |
| gac | D | 0 | 9.80 |
| gaa | E | 86 | 189.30 |
| gag | E | 243 | 139.70 |
| ggt | G | 0 | 0.37 |
| ggc | G | 0 | 0.41 |
| gga | G | 0 | 1.45 |
| ggg | G | 3 | 0.77 |
| --- | -- | ------ | ------ |
| mPD | | 0.47 | 0.56 |
| | | nPD: | 0.83 |
| | | N. weight: | 0.37 |
| | | Sc. PD: | 0.2 |
| | | Sc. rank: | 73.6 |

| PA | | | |
|---|---|---|---|
| Pos. | 383 | obs: | exp: |
| gtt | V | 0 | 0.21 |
| gtc | V | 1 | 0.21 |
| gta | V | 0 | 0.19 |
| gtg | V | 0 | 0.40 |
| gat | D | 75 | 187.80 |
| gac | D | 277 | 164.20 |
| --- | -- | ------ | ------ |
| mPD | | 0.34 | 0.51 |
| | | nPD: | 0.68 |
| | | N. weight: | 0.35 |
| | | Sc. PD: | 0.14 |
| | | Sc. rank: | 58.1 |

| PA | | | |
|---|---|---|---|
| Pos. | 384 | obs: | exp: |
| tgt | C | 49 | 145.50 |
| tgc | C | 304 | 207.50 |
| --- | -- | ------ | ------ |
| mPD | | 0.24 | 0.48 |
| | | nPD: | 0.49 |
| | | N. weight: | 0.28 |
| | | Sc. PD: | 0.062 |
| | | Sc. rank: | 33.4 |

| PA | | | |
|---|---|---|---|
| Pos. | 385 | obs: | exp: |
| aaa | K | 286 | 197.70 |
| aag | K | 67 | 155.30 |
| --- | -- | ------ | ------ |
| mPD | | 0.31 | 0.49 |
| | | nPD: | 0.63 |
| | | N. weight: | 0.22 |
| | | Sc. PD: | 0.078 |
| | | Sc. rank: | 35.0 |

| PA | | | |
|---|---|---|---|
| Pos. | 386 | obs: | exp: |
| gat | D | 305 | 185.10 |
| gac | D | 42 | 161.90 |
| gaa | E | 6 | 3.45 |
| gag | E | 0 | 2.55 |
| --- | -- | ------ | ------ |
| mPD | | 0.24 | 0.51 |
| | | nPD: | 0.47 |
| | | N. weight: | 0.44 |
| | | Sc. PD: | 0.084 |
| | | Sc. rank: | 45.7 |

| PA | | | |
|---|---|---|---|
| Pos. | 387 | obs: | exp: |
| att | I | 14 | 5.64 |
| atc | I | 0 | 3.93 |
| ata | I | 1 | 5.43 |
| gtt | V | 227 | 69.47 |
| gtc | V | 88 | 70.94 |
| gta | V | 20 | 63.35 |
| gtg | V | 3 | 134.20 |
| --- | -- | ------ | ------ |
| mPD | | 0.55 | 0.81 |
| | | nPD: | 0.68 |
| | | N. weight: | 1.2 |
| | | Sc. PD: | 0.47 |
| | | Sc. rank: | 199.9 |

| PA | | | |
|---|---|---|---|
| Pos. | 388 | obs: | exp: |
| tct | S | 0 | 48.81 |
| tcc | S | 0 | 42.77 |
| tca | S | 0 | 68.01 |
| tcg | S | 0 | 18.71 |
| att | I | 0 | 0.38 |
| atc | I | 1 | 0.26 |
| ata | I | 0 | 0.36 |
| aat | N | 0 | 14.63 |
| aac | N | 28 | 13.37 |
| agt | S | 29 | 67.54 |
| agc | S | 278 | 61.16 |
| ggt | G | 0 | 2.10 |
| ggc | G | 17 | 2.30 |
| gga | G | 0 | 8.22 |
| ggg | G | 0 | 4.38 |
| --- | -- | ------ | ------ |
| mPD | | 0.39 | 1.8 |
| | | nPD: | 0.22 |
| | | N. weight: | 2. |
| | | Sc. PD: | -0.085 |
| | | Sc. rank: | 40.6 |

| PA | | | |
|---|---|---|---|
| Pos. | 389 | obs: | exp: |
| gat | D | 285 | 188.30 |
| gac | D | 68 | 164.70 |
| --- | -- | ------ | ------ |
| mPD | | 0.31 | 0.50 |
| | | nPD: | 0.63 |
| | | N. weight: | 0.26 |
| | | Sc. PD: | 0.091 |
| | | Sc. rank: | 40.9 |

PA

| Pos. | 390 | obs: | exp: |
|---|---|---|---|
| tta | L | 49 | 30.75 |
| ttg | L | 45 | 67.79 |
| ctt | L | 27 | 65.68 |
| ctc | L | 4 | 56.94 |
| cta | L | 186 | 53.03 |
| ctg | L | 41 | 77.81 |
| att | I | 0 | 0.38 |
| atc | I | 0 | 0.26 |
| ata | I | 1 | 0.36 |
| --- | -- | ------ | ------ |
| mPD | | 0.89 | 1.1 |
| | | nPD: | 0.79 |
| | | N. weight: | 0.82 |
| | | Sc. PD: | 0.41 |
| | | Sc. rank: | 156.1 |

,

PA

| Pos. | 391 | obs: | exp: |
|---|---|---|---|
| cgt | R | 0 | 11.62 |
| cgc | R | 0 | 13.71 |
| cga | R | 14 | 26.91 |
| cgg | R | 4 | 23.37 |
| aaa | K | 59 | 40.88 |
| aag | K | 14 | 32.12 |
| aga | R | 170 | 125.90 |
| agg | R | 92 | 78.47 |
| --- | -- | ------ | ------ |
| mPD | | 0.86 | 1.2 |
| | | nPD: | 0.7 |
| | | N. weight: | 0.29 |
| | | Sc. PD: | 0.12 |
| | | Sc. rank: | 51.0 |

,

PA

| Pos. | 392 | obs: | exp: |
|---|---|---|---|
| caa | Q | 45 | 188.20 |
| cag | Q | 308 | 164.80 |
| --- | -- | ------ | ------ |
| mPD | | 0.22 | 0.50 |
| | | nPD: | 0.45 |
| | | N. weight: | 0.56 |
| | | Sc. PD: | 0.099 |
| | | Sc. rank: | 55.5 |

,

PA

| Pos. | 393 | obs: | exp: |
|---|---|---|---|
| tat | Y | 287 | 182.70 |
| tac | Y | 66 | 170.30 |
| --- | -- | ------ | ------ |
| mPD | | 0.30 | 0.50 |
| | | nPD: | 0.61 |
| | | N. weight: | 0.3 |
| | | Sc. PD: | 0.1 |
| | | Sc. rank: | 45.5 |

,

PA

| Pos. | 394 | obs: | exp: |
|---|---|---|---|
| gat | D | 21 | 187.30 |
| gac | D | 330 | 163.70 |
| ggt | G | 0 | 0.25 |
| ggc | G | 2 | 0.27 |
| gga | G | 0 | 0.97 |
| ggg | G | 0 | 0.52 |
| --- | -- | ------ | ------ |
| mPD | | 0.12 | 0.51 |
| | | nPD: | 0.24 |
| | | N. weight: | 0.82 |
| | | Sc. PD: | -0.019 |
| | | Sc. rank: | 24.1 |

,

PA

| Pos. | 395 | obs: | exp: |
|---|---|---|---|
| tct | S | 0 | 55.96 |
| tcc | S | 0 | 49.04 |
| tca | S | 0 | 77.98 |
| tcg | S | 0 | 21.46 |
| aat | N | 1 | 0.52 |
| aac | N | 0 | 0.48 |
| agt | S | 296 | 77.44 |
| agc | S | 56 | 70.13 |
| --- | -- | ------ | ------ |
| mPD | | 0.27 | 1.7 |
| | | nPD: | 0.16 |
| | | N. weight: | 1.7 |
| | | Sc. PD: | -0.16 |
| | | Sc. rank: | -44.8 |

,

PA

| Pos. | 396 | obs: | exp: |
|---|---|---|---|
| aat | N | 3 | 1.57 |
| aac | N | 0 | 1.43 |
| gat | D | 255 | 185.10 |
| gac | D | 92 | 161.90 |
| gaa | E | 1 | 0.58 |
| gag | E | 0 | 0.42 |
| ggt | G | 1 | 0.25 |
| ggc | G | 1 | 0.27 |
| gga | G | 0 | 0.97 |
| ggg | G | 0 | 0.52 |
| --- | -- | ------ | ------ |
| mPD | | 0.42 | 0.53 |
| | | nPD: | 0.79 |
| | | N. weight: | 0.17 |
| | | Sc. PD: | 0.084 |
| | | Sc. rank: | 31.6 |

,

PA

| Pos. | 397 | obs: | exp: |
|---|---|---|---|
| gaa | E | 235 | 202.60 |
| gag | E | 117 | 149.40 |
| ggt | G | 0 | 0.12 |
| ggc | G | 0 | 0.14 |
| gga | G | 1 | 0.48 |
| ggg | G | 0 | 0.26 |
| --- | -- | ------ | ------ |
| mPD | | 0.45 | 0.49 |
| | | nPD: | 0.91 |
| | | N. weight: | 0.043 |
| | | Sc. PD: | 0.027 |
| | | Sc. rank: | 8.6 |

,

PA

| Pos. | 398 | obs: | exp: |
|---|---|---|---|
| cct | P | 64 | 92.95 |
| ccc | P | 2 | 63.67 |
| cca | P | 268 | 139.30 |
| ccg | P | 19 | 57.08 |
| --- | -- | ------ | ------ |
| mPD | | 0.39 | 0.72 |
| | | nPD: | 0.54 |
| | | N. weight: | 0.56 |
| | | Sc. PD: | 0.15 |
| | | Sc. rank: | 77.9 |

,

PA

| Pos. | 399 | obs: | exp: |
|---|---|---|---|
| gaa | E | 19 | 194.50 |
| gag | E | 319 | 143.50 |
| ggt | G | 0 | 1.85 |
| ggc | G | 0 | 2.03 |
| gga | G | 0 | 7.26 |
| ggg | G | 15 | 3.86 |
| --- | -- | ------ | ------ |
| mPD | | 0.18 | 0.58 |
| | | nPD: | 0.32 |
| | | N. weight: | 1. |
| | | Sc. PD: | 0.05 |
| | | Sc. rank: | 59.0 |

,

PA

| Pos. | 400 | obs: | exp: |
|---|---|---|---|
| ttt | F | 1 | 0.41 |
| ttc | F | 0 | 0.59 |
| tct | S | 143 | 24.48 |
| tcc | S | 11 | 21.45 |
| tca | S | 0 | 34.12 |
| tcg | S | 0 | 9.39 |
| cct | P | 85 | 33.70 |
| ccc | P | 42 | 23.09 |
| cca | P | 1 | 50.51 |
| ccg | P | 0 | 20.70 |
| caa | Q | 67 | 35.72 |
| cag | Q | 0 | 31.28 |
| cgt | R | 0 | 0.08 |
| cgc | R | 0 | 0.10 |
| cga | R | 0 | 0.19 |
| cgg | R | 2 | 0.17 |
| act | T | 1 | 0.26 |
| acc | T | 0 | 0.21 |
| aca | T | 0 | 0.44 |
| acg | T | 0 | 0.08 |
| agt | S | 0 | 33.88 |
| agc | S | 0 | 30.68 |
| aga | R | 0 | 0.90 |
| agg | R | 0 | 0.56 |
| --- | -- | ------ | ------ |
| mPD | | 1.3 | 1.9 |
| | | nPD: | 0.71 |
| | | N. weight: | 1.7 |
| | | Sc. PD: | 0.74 |
| | | Sc. rank: | 305.9 |

,

PA

| Pos. | 401 | obs: | exp: |
|---|---|---|---|
| cgt | R | 0 | 14.65 |
| cgc | R | 0 | 17.28 |
| cga | R | 1 | 33.93 |
| cgg | R | 0 | 29.46 |
| aga | R | 344 | 158.80 |
| agg | R | 8 | 98.92 |
| --- | -- | ------ | ------ |
| mPD | | 0.050 | 0.96 |
| | | nPD: | 0.05 |
| | | N. weight: | 1.1 |
| | | Sc. PD: | -0.21 |
| | | Sc. rank: | -95.5 |

,

PA

| Pos. | 402 | obs: | exp: |
|---|---|---|---|
| tct | S | 3 | 56.12 |
| tcc | S | 2 | 49.17 |
| tca | S | 160 | 78.20 |
| tcg | S | 188 | 21.52 |
| agt | S | 0 | 77.66 |
| agc | S | 0 | 70.33 |
| --- | -- | ------ | ------ |
| mPD | | 0.51 | 1.7 |
| | | nPD: | 0.31 |
| | | N. weight: | 2.2 |
| | | Sc. PD: | 0.092 |
| | | Sc. rank: | 123.3 |

,

PA

| Pos. | 403 | obs: | exp: |
|---|---|---|---|
| tta | L | 7 | 30.40 |
| ttg | L | 0 | 67.02 |
| ctt | L | 1 | 64.93 |
| ctc | L | 0 | 56.29 |
| cta | L | 291 | 52.43 |
| ctg | L | 49 | 76.93 |
| cct | P | 0 | 1.32 |
| ccc | P | 0 | 0.90 |
| cca | P | 5 | 1.97 |
| ccg | P | 0 | 0.81 |
| --- | -- | ------ | ------ |
| mPD | | 0.31 | 1.1 |
| | | nPD: | 0.27 |
| | | N. weight: | 2. |
| | | Sc. PD: | 0.017 |
| | | Sc. rank: | 86.0 |

,

PA

| Pos. | 404 | obs: | exp: |
|---|---|---|---|
| tct | S | 0 | 0.16 |
| tcc | S | 0 | 0.14 |
| tca | S | 1 | 0.22 |
| tcg | S | 0 | 0.06 |
| agt | S | 0 | 0.22 |
| agc | S | 0 | 0.20 |
| gct | A | 0 | 88.66 |
| gcc | A | 0 | 72.11 |
| gca | A | 349 | 156.90 |
| gcg | A | 3 | 34.35 |
| --- | -- | ------ | ------ |
| mPD | | 0.023 | 0.69 |
| | | nPD: | 0.03 |
| | | N. weight: | 1.2 |
| | | Sc. PD: | -0.26 |
| | | Sc. rank: | -134.6 |

,

PA

| Pos. | 405 | obs: | exp: |
|---|---|---|---|
| tct | S | 0 | 51.67 |
| tcc | S | 0 | 45.27 |
| tca | S | 0 | 72.00 |
| tcg | S | 0 | 19.81 |
| tgt | C | 26 | 10.72 |
| tgc | C | 0 | 15.28 |
| agt | S | 74 | 71.50 |
| agc | S | 251 | 64.75 |
| ggt | G | 0 | 0.25 |
| ggc | G | 2 | 0.27 |
| gga | G | 0 | 0.97 |
| ggg | G | 0 | 0.52 |
| --- | -- | ------ | ------ |
| mPD | | 0.55 | 1.7 |
| | | nPD: | 0.33 |
| | | N. weight: | 1.6 |
| | | Sc. PD: | 0.11 |
| | | Sc. rank: | 107.2 |

,

PA

| Pos. | 406 | obs: | exp: |
|---|---|---|---|
| tgg | W | 353 | 353.00 |
| --- | -- | ------ | ------ |
| mPD | | 0 | 0 |
| | | nPD: | 1. |
| | | N. weight: | 0. |
| | | Sc. PD: | 0 |
| | | Sc. rank: | 0 |

,

PA

| Pos. | 407 | obs: | exp: |
|---|---|---|---|
| att | I | 99 | 132.70 |
| atc | I | 253 | 92.52 |
| ata | I | 1 | 127.80 |
| --- | -- | ------ | ------ |
| mPD | | 0.41 | 0.66 |
| | | nPD: | 0.62 |
| | | N. weight: | 0.96 |
| | | Sc. PD: | 0.33 |
| | | Sc. rank: | 149.4 |

,

PA

| Pos. | 408 | obs: | exp: |
|---|---|---|---|
| caa | Q | 68 | 188.20 |
| cag | Q | 285 | 164.80 |
| --- | -- | ------ | ------ |
| mPD | | 0.31 | 0.50 |
| | | nPD: | 0.63 |
| | | N. weight: | 0.38 |
| | | Sc. PD: | 0.13 |
| | | Sc. rank: | 60.3 |

,

PA

| Pos. | 409 | obs: | exp: |
|---|---|---|---|
| tct | S | 0 | 53.58 |
| tcc | S | 0 | 46.95 |
| tca | S | 0 | 74.66 |
| tcg | S | 0 | 20.54 |
| aat | N | 16 | 8.36 |
| aac | N | 0 | 7.64 |
| agt | S | 279 | 74.14 |
| agc | S | 58 | 67.14 |
| --- | -- | ------ | ------ |
| mPD | | 0.36 | 1.7 |
| | | nPD: | 0.21 |
| | | N. weight: | 1.6 |
| | | Sc. PD: | -0.081 |
| | | Sc. rank: | 24.9 |

,

| | | | |
|---|---|---|---|
| PA | | | |
| Pos. | 410 | obs: | exp: |
| gaa | E | 243 | 203.10 |
| gag | E | 110 | 149.90 |
| --- | -- | ------ | ------ |
| mPD | | 0.43 | 0.49 |
| | | nPD: | 0.88 |
| | | N. weight: | 0.054 |
| | | Sc. PD: | 0.032 |
| | | Sc. rank: | 10.7 |

,

| | | | |
|---|---|---|---|
| PA | | | |
| Pos. | 411 | obs: | exp: |
| ttt | F | 6 | 145.10 |
| ttc | F | 347 | 207.90 |
| --- | -- | ------ | ------ |
| mPD | | 0.034 | 0.48 |
| | | nPD: | 0.07 |
| | | N. weight: | 0.69 |
| | | Sc. PD: | -0.13 |
| | | Sc. rank: | -51.2 |

,

| | | | |
|---|---|---|---|
| PA | | | |
| Pos. | 412 | obs: | exp: |
| aat | N | 25 | 184.50 |
| aac | N | 328 | 168.50 |
| --- | -- | ------ | ------ |
| mPD | | 0.13 | 0.50 |
| | | nPD: | 0.26 |
| | | N. weight: | 0.73 |
| | | Sc. PD: | -0.00003 |
| | | Sc. rank: | 26.4 |

,

| | | | |
|---|---|---|---|
| PA | | | |
| Pos. | 413 | obs: | exp: |
| aaa | K | 92 | 197.70 |
| aag | K | 261 | 155.30 |
| --- | -- | ------ | ------ |
| mPD | | 0.39 | 0.49 |
| | | nPD: | 0.78 |
| | | N. weight: | 0.29 |
| | | Sc. PD: | 0.15 |
| | | Sc. rank: | 55.6 |

,

| | | | |
|---|---|---|---|
| PA | | | |
| Pos. | 414 | obs: | exp: |
| gct | A | 53 | 88.91 |
| gcc | A | 15 | 72.31 |
| gca | A | 280 | 157.30 |
| gcg | A | 5 | 34.45 |
| --- | -- | ------ | ------ |
| mPD | | 0.35 | 0.69 |
| | | nPD: | 0.51 |
| | | N. weight: | 0.46 |
| | | Sc. PD: | 0.11 |
| | | Sc. rank: | 58.1 |

,

| | | | |
|---|---|---|---|
| PA | | | |
| Pos. | 415 | obs: | exp: |
| tgt | C | 135 | 145.50 |
| tgc | C | 218 | 207.50 |
| --- | -- | ------ | ------ |
| mPD | | 0.47 | 0.48 |
| | | nPD: | 0.98 |
| | | N. weight: | 0.016 |
| | | Sc. PD: | 0.011 |
| | | Sc. rank: | 3.3 |

,

| | | | |
|---|---|---|---|
| PA | | | |
| Pos. | 416 | obs: | exp: |
| gaa | E | 312 | 203.10 |
| gag | E | 41 | 149.90 |
| --- | -- | ------ | ------ |
| mPD | | 0.21 | 0.49 |
| | | nPD: | 0.42 |
| | | N. weight: | 0.36 |
| | | Sc. PD: | 0.054 |
| | | Sc. rank: | 33.2 |

,

| | | | |
|---|---|---|---|
| PA | | | |
| Pos. | 417 | obs: | exp: |
| tta | L | 5 | 30.84 |
| ttg | L | 335 | 67.98 |
| ctt | L | 0 | 65.86 |
| ctc | L | 0 | 57.10 |
| cta | L | 0 | 53.18 |
| ctg | L | 13 | 78.03 |
| --- | -- | ------ | ------ |
| mPD | | 0.099 | 1.1 |
| | | nPD: | 0.09 |
| | | N. weight: | 2.2 |
| | | Sc. PD: | -0.37 |
| | | Sc. rank: | -135.0 |

,

| | | | |
|---|---|---|---|
| PA | | | |
| Pos. | 418 | obs: | exp: |
| act | T | 79 | 93.26 |
| acc | T | 47 | 75.75 |
| aca | T | 227 | 154.60 |
| acg | T | 0 | 29.35 |
| --- | -- | ------ | ------ |
| mPD | | 0.52 | 0.69 |
| | | nPD: | 0.76 |
| | | N. weight: | 0.25 |
| | | Sc. PD: | 0.12 |
| | | Sc. rank: | 45.9 |

,

| | | | |
|---|---|---|---|
| PA | | | |
| Pos. | 419 | obs: | exp: |
| gat | D | 247 | 188.30 |
| gac | D | 106 | 164.70 |
| --- | -- | ------ | ------ |
| mPD | | 0.42 | 0.50 |
| | | nPD: | 0.85 |
| | | N. weight: | 0.1 |
| | | Sc. PD: | 0.056 |
| | | Sc. rank: | 19.9 |

,

| | | | |
|---|---|---|---|
| PA | | | |
| Pos. | 420 | obs: | exp: |
| tct | S | 0 | 56.12 |
| tcc | S | 1 | 49.17 |
| tca | S | 262 | 78.20 |
| tcg | S | 90 | 21.52 |
| agt | S | 0 | 77.66 |
| agc | S | 0 | 70.33 |
| --- | -- | ------ | ------ |
| mPD | | 0.39 | 1.7 |
| | | nPD: | 0.23 |
| | | N. weight: | 1.9 |
| | | Sc. PD: | -0.06 |
| | | Sc. rank: | 53.0 |

,

| | | | |
|---|---|---|---|
| PA | | | |
| Pos. | 421 | obs: | exp: |
| tct | S | 0 | 55.96 |
| tcc | S | 0 | 49.04 |
| tca | S | 0 | 77.98 |
| tcg | S | 0 | 21.46 |
| agt | S | 276 | 77.44 |
| agc | S | 76 | 70.13 |
| ggt | G | 1 | 0.12 |
| ggc | G | 0 | 0.14 |
| gga | G | 0 | 0.48 |
| ggg | G | 0 | 0.26 |
| --- | -- | ------ | ------ |
| mPD | | 0.34 | 1.7 |
| | | nPD: | 0.21 |
| | | N. weight: | 1.6 |
| | | Sc. PD: | -0.086 |
| | | Sc. rank: | 15.6 |

,

| | | | |
|---|---|---|---|
| PA | | | |
| Pos. | 422 | obs: | exp: |
| tgg | W | 353 | 353.00 |
| --- | -- | ------ | ------ |
| mPD | | 0 | 0 |
| | | nPD: | 1. |
| | | N. weight: | 0. |
| | | Sc. PD: | 0 |
| | | Sc. rank: | 0 |

,

| | | | |
|---|---|---|---|
| PA | | | |
| Pos. | 423 | obs: | exp: |
| att | I | 225 | 122.90 |
| atc | I | 1 | 85.70 |
| ata | I | 101 | 118.40 |
| gtt | V | 0 | 5.34 |
| gtc | V | 0 | 5.46 |
| gta | V | 26 | 4.87 |
| gtg | V | 0 | 10.33 |
| --- | -- | ------ | ------ |
| mPD | | 0.60 | 0.81 |
| | | nPD: | 0.74 |
| | | N. weight: | 0.7 |
| | | Sc. PD: | 0.32 |
| | | Sc. rank: | 128.3 |

,

| | | | |
|---|---|---|---|
| PA | | | |
| Pos. | 424 | obs: | exp: |
| gaa | E | 321 | 203.10 |
| gag | E | 32 | 149.90 |
| --- | -- | ------ | ------ |
| mPD | | 0.17 | 0.49 |
| | | nPD: | 0.34 |
| | | N. weight: | 0.43 |
| | | Sc. PD: | 0.03 |
| | | Sc. rank: | 29.1 |

,

| | | | |
|---|---|---|---|
| PA | | | |
| Pos. | 425 | obs: | exp: |
| tta | L | 1 | 30.84 |
| ttg | L | 1 | 67.98 |
| ctt | L | 212 | 65.86 |
| ctc | L | 81 | 57.10 |
| cta | L | 48 | 53.18 |
| ctg | L | 10 | 78.03 |
| --- | -- | ------ | ------ |
| mPD | | 0.58 | 1.1 |
| | | nPD: | 0.52 |
| | | N. weight: | 1.1 |
| | | Sc. PD: | 0.27 |
| | | Sc. rank: | 143.1 |

,

| | | | |
|---|---|---|---|
| PA | | | |
| Pos. | 426 | obs: | exp: |
| gat | D | 344 | 186.70 |
| gac | D | 6 | 163.30 |
| gaa | E | 0 | 1.73 |
| gag | E | 3 | 1.27 |
| --- | -- | ------ | ------ |
| mPD | | 0.050 | 0.51 |
| | | nPD: | 0.1 |
| | | N. weight: | 0.83 |
| | | Sc. PD: | -0.13 |
| | | Sc. rank: | -44.9 |

,

| | | | |
|---|---|---|---|
| PA | | | |
| Pos. | 427 | obs: | exp: |
| gaa | E | 291 | 203.10 |
| gag | E | 62 | 149.90 |
| --- | -- | ------ | ------ |
| mPD | | 0.29 | 0.49 |
| | | nPD: | 0.59 |
| | | N. weight: | 0.23 |
| | | Sc. PD: | 0.072 |
| | | Sc. rank: | 33.5 |

,

| | | | |
|---|---|---|---|
| PA | | | |
| Pos. | 428 | obs: | exp: |
| att | I | 0 | 132.70 |
| atc | I | 8 | 92.52 |
| ata | I | 345 | 127.80 |
| --- | -- | ------ | ------ |
| mPD | | 0.044 | 0.66 |
| | | nPD: | 0.07 |
| | | N. weight: | 1.4 |
| | | Sc. PD: | -0.26 |
| | | Sc. rank: | -107.4 |

,

| | | | |
|---|---|---|---|
| PA | | | |
| Pos. | 429 | obs: | exp: |
| ggt | G | 0 | 43.56 |
| ggc | G | 0 | 47.74 |
| gga | G | 96 | 170.80 |
| ggg | G | 257 | 90.95 |
| --- | -- | ------ | ------ |
| mPD | | 0.40 | 0.67 |
| | | nPD: | 0.6 |
| | | N. weight: | 0.92 |
| | | Sc. PD: | 0.29 |
| | | Sc. rank: | 136.6 |

,

| | | | |
|---|---|---|---|
| PA | | | |
| Pos. | 430 | obs: | exp: |
| gtt | V | 0 | 0.62 |
| gtc | V | 0 | 0.63 |
| gta | V | 3 | 0.56 |
| gtg | V | 0 | 1.19 |
| gaa | E | 273 | 201.40 |
| gag | E | 77 | 148.60 |
| --- | -- | ------ | ------ |
| mPD | | 0.36 | 0.51 |
| | | nPD: | 0.7 |
| | | N. weight: | 0.17 |
| | | Sc. PD: | 0.073 |
| | | Sc. rank: | 30.2 |

,

| | | | |
|---|---|---|---|
| PA | | | |
| Pos. | 431 | obs: | exp: |
| gat | D | 117 | 188.30 |
| gac | D | 236 | 164.70 |
| --- | -- | ------ | ------ |
| mPD | | 0.44 | 0.50 |
| | | nPD: | 0.89 |
| | | N. weight: | 0.14 |
| | | Sc. PD: | 0.084 |
| | | Sc. rank: | 27.7 |

,

| | | | |
|---|---|---|---|
| PA | | | |
| Pos. | 432 | obs: | exp: |
| att | I | 34 | 12.78 |
| atc | I | 0 | 8.91 |
| ata | I | 0 | 12.31 |
| gtt | V | 311 | 65.57 |
| gtc | V | 6 | 66.95 |
| gta | V | 1 | 59.79 |
| gtg | V | 1 | 126.70 |
| --- | -- | ------ | ------ |
| mPD | | 0.22 | 0.91 |
| | | nPD: | 0.24 |
| | | N. weight: | 2.1 |
| | | Sc. PD: | -0.048 |
| | | Sc. rank: | 65.1 |

,

| | | | |
|---|---|---|---|
| PA | | | |
| Pos. | 433 | obs: | exp: |
| gct | A | 270 | 88.91 |
| gcc | A | 81 | 72.31 |
| gca | A | 2 | 157.30 |
| gcg | A | 0 | 34.45 |
| --- | -- | ------ | ------ |
| mPD | | 0.36 | 0.69 |
| | | nPD: | 0.53 |
| | | N. weight: | 1.3 |
| | | Sc. PD: | 0.33 |
| | | Sc. rank: | 174.4 |

,

| | | | |
|---|---|---|---|
| PA | | | |
| Pos. | 434 | obs: | exp: |
| cct | P | 5 | 92.95 |
| ccc | P | 0 | 63.67 |
| cca | P | 289 | 139.30 |
| ccg | P | 59 | 57.08 |
| --- | -- | ------ | ------ |
| mPD | | 0.30 | 0.72 |
| | | nPD: | 0.42 |
| | | N. weight: | 0.87 |
| | | Sc. PD: | 0.13 |
| | | Sc. rank: | 81.1 |

,

| | | | |
|---|---|---|---|
| PA | | | |
| Pos. | 435 | obs: | exp: |
| att | I | 249 | 132.70 |
| atc | I | 98 | 92.52 |
| ata | I | 6 | 127.80 |
| --- | -- | ------ | ------ |
| mPD | | 0.43 | 0.66 |
| | | nPD: | 0.65 |
| | | N. weight: | 0.64 |
| | | Sc. PD: | 0.23 |
| | | Sc. rank: | 102.0 |

,

| | | | |
|---|---|---|---|
| PA | | | |
| Pos. | 436 | obs: | exp: |
| gaa | E | 143 | 203.10 |
| gag | E | 210 | 149.90 |
| --- | -- | ------ | ------ |
| mPD | | 0.48 | 0.49 |
| | | nPD: | 0.99 |
| | | N. weight: | 0.1 |
| | | Sc. PD: | 0.071 |
| | | Sc. rank: | 20.6 |

,

| | | | |
|---|---|---|---|
| PA | | | |
| Pos. | 437 | obs: | exp: |
| tat | Y | 0 | 6.73 |
| tac | Y | 13 | 6.27 |
| cat | H | 23 | 184.20 |
| cac | H | 317 | 155.80 |
| --- | -- | ------ | ------ |
| mPD | | 0.19 | 0.57 |
| | | nPD: | 0.34 |
| | | N. weight: | 0.81 |
| | | Sc. PD: | 0.059 |
| | | Sc. rank: | 56.5 |

,

| | | | |
|---|---|---|---|
| PA | | | |
| Pos. | 438 | obs: | exp: |
| att | I | 351 | 132.70 |
| atc | I | 2 | 92.52 |
| ata | I | 0 | 127.80 |
| --- | -- | ------ | ------ |
| mPD | | 0.011 | 0.66 |
| | | nPD: | 0.02 |
| | | N. weight: | 1.4 |
| | | Sc. PD: | -0.34 |
| | | Sc. rank: | -189.8 |

,

| PA | | | |
|---|---|---|---|
| Pos. | 439 | obs: | exp: |
| gct | A | 3 | 88.91 |
| gcc | A | 2 | 72.31 |
| gca | A | 194 | 157.30 |
| gcg | A | 154 | 34.45 |
| --- | -- | ------ | ------ |
| mPD | | 0.51 | 0.69 |
| | | nPD: | 0.74 |
| | | N. weight: | 1.1 |
| | | Sc. PD: | 0.51 |
| | | Sc. rank: | 205.9 |

| PA | | | |
|---|---|---|---|
| Pos. | 440 | obs: | exp: |
| tct | S | 0 | 56.12 |
| tcc | S | 0 | 49.17 |
| tca | S | 0 | 78.20 |
| tcg | S | 0 | 21.52 |
| agt | S | 216 | 77.66 |
| agc | S | 137 | 70.33 |
| --- | -- | ------ | ------ |
| mPD | | 0.48 | 1.7 |
| | | nPD: | 0.29 |
| | | N. weight: | 1.4 |
| | | Sc. PD: | 0.028 |
| | | Sc. rank: | 62.0 |

| PA | | | |
|---|---|---|---|
| Pos. | 441 | obs: | exp: |
| att | I | 0 | 0.38 |
| atc | I | 0 | 0.26 |
| ata | I | 1 | 0.36 |
| atg | M | 352 | 352.00 |
| --- | -- | ------ | ------ |
| mPD | | 0.0057 | 0.0057 |
| | | nPD: | 1. |
| | | N. weight: | 0.0044 |
| | | Sc. PD: | 0.0031 |
| | | Sc. rank: | 0.9 |

| PA | | | |
|---|---|---|---|
| Pos. | 442 | obs: | exp: |
| cgt | R | 0 | 14.65 |
| cgc | R | 0 | 17.28 |
| cga | R | 0 | 33.93 |
| cgg | R | 0 | 29.46 |
| aga | R | 176 | 158.80 |
| agg | R | 177 | 98.92 |
| --- | -- | ------ | ------ |
| mPD | | 0.50 | 0.96 |
| | | nPD: | 0.52 |
| | | N. weight: | 0.53 |
| | | Sc. PD: | 0.13 |
| | | Sc. rank: | 70.5 |

| PA | | | |
|---|---|---|---|
| Pos. | 443 | obs: | exp: |
| cgt | R | 0 | 14.65 |
| cgc | R | 0 | 17.28 |
| cga | R | 0 | 33.93 |
| cgg | R | 26 | 29.46 |
| aga | R | 181 | 158.80 |
| agg | R | 146 | 98.92 |
| --- | -- | ------ | ------ |
| mPD | | 0.64 | 0.96 |
| | | nPD: | 0.66 |
| | | N. weight: | 0.36 |
| | | Sc. PD: | 0.14 |
| | | Sc. rank: | 58.2 |

| PA | | | |
|---|---|---|---|
| Pos. | 444 | obs: | exp: |
| aat | N | 90 | 184.00 |
| aac | N | 262 | 168.00 |
| gat | D | 1 | 0.53 |
| gac | D | 0 | 0.47 |
| --- | -- | ------ | ------ |
| mPD | | 0.39 | 0.50 |
| | | nPD: | 0.77 |
| | | N. weight: | 0.24 |
| | | Sc. PD: | 0.12 |
| | | Sc. rank: | 44.9 |

| PA | | | |
|---|---|---|---|
| Pos. | 445 | obs: | exp: |
| tat | Y | 280 | 182.70 |
| tac | Y | 73 | 170.30 |
| --- | -- | ------ | ------ |
| mPD | | 0.33 | 0.50 |
| | | nPD: | 0.66 |
| | | N. weight: | 0.26 |
| | | Sc. PD: | 0.098 |
| | | Sc. rank: | 42.3 |

| PA | | | |
|---|---|---|---|
| Pos. | 446 | obs: | exp: |
| ttt | F | 130 | 145.10 |
| ttc | F | 223 | 207.90 |
| --- | -- | ------ | ------ |
| mPD | | 0.47 | 0.48 |
| | | nPD: | 0.96 |
| | | N. weight: | 0.019 |
| | | Sc. PD: | 0.013 |
| | | Sc. rank: | 3.9 |

| PA | | | |
|---|---|---|---|
| Pos. | 447 | obs: | exp: |
| act | T | 7 | 93.00 |
| acc | T | 27 | 75.53 |
| aca | T | 298 | 154.20 |
| acg | T | 20 | 29.26 |
| gct | A | 0 | 0.25 |
| gcc | A | 0 | 0.20 |
| gca | A | 1 | 0.45 |
| gcg | A | 0 | 0.10 |
| --- | -- | ------ | ------ |
| mPD | | 0.28 | 0.69 |
| | | nPD: | 0.4 |
| | | N. weight: | 0.64 |
| | | Sc. PD: | 0.087 |
| | | Sc. rank: | 56.3 |

| PA | | | |
|---|---|---|---|
| Pos. | 448 | obs: | exp: |
| gct | A | 50 | 88.91 |
| gcc | A | 0 | 72.31 |
| gca | A | 96 | 157.30 |
| gcg | A | 207 | 34.45 |
| --- | -- | ------ | ------ |
| mPD | | 0.56 | 0.69 |
| | | nPD: | 0.82 |
| | | N. weight: | 1.3 |
| | | Sc. PD: | 0.69 |
| | | Sc. rank: | 253.0 |

| PA | | | |
|---|---|---|---|
| Pos. | 449 | obs: | exp: |
| gaa | E | 278 | 203.10 |
| gag | E | 75 | 149.90 |
| --- | -- | ------ | ------ |
| mPD | | 0.34 | 0.49 |
| | | nPD: | 0.69 |
| | | N. weight: | 0.16 |
| | | Sc. PD: | 0.067 |
| | | Sc. rank: | 28.2 |

| PA | | | |
|---|---|---|---|
| Pos. | 450 | obs: | exp: |
| att | I | 0 | 1.13 |
| atc | I | 0 | 0.79 |
| ata | I | 3 | 1.09 |
| gtt | V | 25 | 71.94 |
| gtc | V | 0 | 73.46 |
| gta | V | 187 | 65.60 |
| gtg | V | 138 | 139.00 |
| --- | -- | ------ | ------ |
| mPD | | 0.57 | 0.74 |
| | | nPD: | 0.77 |
| | | N. weight: | 0.76 |
| | | Sc. PD: | 0.37 |
| | | Sc. rank: | 143.0 |

| PA | | | |
|---|---|---|---|
| Pos. | 451 | obs: | exp: |
| tct | S | 90 | 56.12 |
| tcc | S | 263 | 49.17 |
| tca | S | 0 | 78.20 |
| tcg | S | 0 | 21.52 |
| agt | S | 0 | 77.66 |
| agc | S | 0 | 70.33 |
| --- | -- | ------ | ------ |
| mPD | | 0.38 | 1.7 |
| | | nPD: | 0.23 |
| | | N. weight: | 2.1 |
| | | Sc. PD: | -0.071 |
| | | Sc. rank: | 52.4 |

| PA | | | |
|---|---|---|---|
| Pos. | 452 | obs: | exp: |
| cat | H | 237 | 191.30 |
| cac | H | 116 | 161.70 |
| --- | -- | ------ | ------ |
| mPD | | 0.44 | 0.50 |
| | | nPD: | 0.89 |
| | | N. weight: | 0.066 |
| | | Sc. PD: | 0.04 |
| | | Sc. rank: | 13.1 |

| PA | | | |
|---|---|---|---|
| Pos. | 453 | obs: | exp: |
| tgt | C | 15 | 145.50 |
| tgc | C | 338 | 207.50 |
| --- | -- | ------ | ------ |
| mPD | | 0.082 | 0.48 |
| | | nPD: | 0.17 |
| | | N. weight: | 0.57 |
| | | Sc. PD: | -0.053 |
| | | Sc. rank: | -14.4 |

| PA | | | |
|---|---|---|---|
| Pos. | 454 | obs: | exp: |
| cgt | R | 0 | 14.65 |
| cgc | R | 0 | 17.28 |
| cga | R | 0 | 33.93 |
| cgg | R | 4 | 29.46 |
| aga | R | 34 | 158.80 |
| agg | R | 315 | 98.92 |
| --- | -- | ------ | ------ |
| mPD | | 0.20 | 0.96 |
| | | nPD: | 0.21 |
| | | N. weight: | 1.3 |
| | | Sc. PD: | -0.075 |
| | | Sc. rank: | 8.5 |

| PA | | | |
|---|---|---|---|
| Pos. | 455 | obs: | exp: |
| gct | A | 225 | 88.91 |
| gcc | A | 56 | 72.31 |
| gca | A | 71 | 157.30 |
| gcg | A | 1 | 34.45 |
| --- | -- | ------ | ------ |
| mPD | | 0.53 | 0.69 |
| | | nPD: | 0.77 |
| | | N. weight: | 0.61 |
| | | Sc. PD: | 0.3 |
| | | Sc. rank: | 113.8 |

| PA | | | |
|---|---|---|---|
| Pos. | 456 | obs: | exp: |
| act | T | 272 | 93.26 |
| acc | T | 11 | 75.75 |
| aca | T | 68 | 154.60 |
| acg | T | 2 | 29.35 |
| --- | -- | ------ | ------ |
| mPD | | 0.37 | 0.69 |
| | | nPD: | 0.54 |
| | | N. weight: | 0.92 |
| | | Sc. PD: | 0.24 |
| | | Sc. rank: | 125.3 |

| PA | | | |
|---|---|---|---|
| Pos. | 457 | obs: | exp: |
| gaa | E | 307 | 203.10 |
| gag | E | 46 | 149.90 |
| --- | -- | ------ | ------ |
| mPD | | 0.23 | 0.49 |
| | | nPD: | 0.47 |
| | | N. weight: | 0.32 |
| | | Sc. PD: | 0.062 |
| | | Sc. rank: | 33.7 |

| PA | | | |
|---|---|---|---|
| Pos. | 458 | obs: | exp: |
| tat | Y | 35 | 182.70 |
| tac | Y | 318 | 170.30 |
| --- | -- | ------ | ------ |
| mPD | | 0.18 | 0.50 |
| | | nPD: | 0.36 |
| | | N. weight: | 0.61 |
| | | Sc. PD: | 0.056 |
| | | Sc. rank: | 47.9 |

| PA | | | |
|---|---|---|---|
| Pos. | 459 | obs: | exp: |
| att | I | 3 | 132.70 |
| atc | I | 0 | 92.52 |
| ata | I | 350 | 127.80 |
| --- | -- | ------ | ------ |
| mPD | | 0.017 | 0.66 |
| | | nPD: | 0.03 |
| | | N. weight: | 1.5 |
| | | Sc. PD: | -0.34 |
| | | Sc. rank: | -176.3 |

| PA | | | |
|---|---|---|---|
| Pos. | 460 | obs: | exp: |
| atg | M | 353 | 353.00 |
| --- | -- | ------ | ------ |
| mPD | | 0 | 0 |
| | | nPD: | 1. |
| | | N. weight: | 0. |
| | | Sc. PD: | 0 |
| | | Sc. rank: | 0 |

| PA | | | |
|---|---|---|---|
| Pos. | 461 | obs: | exp: |
| aaa | K | 96 | 197.70 |
| aag | K | 257 | 155.30 |
| --- | -- | ------ | ------ |
| mPD | | 0.40 | 0.49 |
| | | nPD: | 0.81 |
| | | N. weight: | 0.27 |
| | | Sc. PD: | 0.14 |
| | | Sc. rank: | 52.4 |

| PA | | | |
|---|---|---|---|
| Pos. | 462 | obs: | exp: |
| ggt | G | 2 | 43.56 |
| ggc | G | 1 | 47.74 |
| gga | G | 328 | 170.80 |
| ggg | G | 22 | 90.95 |
| --- | -- | ------ | ------ |
| mPD | | 0.13 | 0.67 |
| | | nPD: | 0.2 |
| | | N. weight: | 0.76 |
| | | Sc. PD: | -0.047 |
| | | Sc. rank: | 3.0 |

| PA | | | |
|---|---|---|---|
| Pos. | 463 | obs: | exp: |
| gtt | V | 5 | 72.56 |
| gtc | V | 16 | 74.09 |
| gta | V | 120 | 66.16 |
| gtg | V | 212 | 140.20 |
| --- | -- | ------ | ------ |
| mPD | | 0.52 | 0.72 |
| | | nPD: | 0.73 |
| | | N. weight: | 0.55 |
| | | Sc. PD: | 0.24 |
| | | Sc. rank: | 99.0 |

| PA | | | |
|---|---|---|---|
| Pos. | 464 | obs: | exp: |
| tat | Y | 17 | 182.70 |
| tac | Y | 336 | 170.30 |
| --- | -- | ------ | ------ |
| mPD | | 0.092 | 0.50 |
| | | nPD: | 0.18 |
| | | N. weight: | 0.82 |
| | | Sc. PD: | -0.063 |
| | | Sc. rank: | -12.9 |

| PA | | | |
|---|---|---|---|
| Pos. | 465 | obs: | exp: |
| att | I | 1 | 131.60 |
| atc | I | 0 | 91.73 |
| ata | I | 349 | 126.70 |
| act | T | 0 | 0.53 |
| acc | T | 0 | 0.43 |
| aca | T | 2 | 0.88 |
| acg | T | 0 | 0.17 |
| gtt | V | 0 | 0.21 |
| gtc | V | 0 | 0.21 |
| gta | V | 1 | 0.19 |
| gtg | V | 0 | 0.40 |
| --- | -- | ------ | ------ |
| mPD | | 0.023 | 0.68 |
| | | nPD: | 0.03 |
| | | N. weight: | 1.5 |
| | | Sc. PD: | -0.34 |
| | | Sc. rank: | -169.4 |

| PA | | | |
|---|---|---|---|
| Pos. | 466 | obs: | exp: |
| aat | N | 79 | 184.50 |
| aac | N | 274 | 168.50 |
| --- | -- | ------ | ------ |
| mPD | | 0.35 | 0.50 |
| | | nPD: | 0.7 |
| | | N. weight: | 0.3 |
| | | Sc. PD: | 0.12 |
| | | Sc. rank: | 51.4 |

| PA | | | |
|---|---|---|---|
| Pos. | 467 | obs: | exp: |
| act | T | 3 | 93.26 |
| acc | T | 0 | 75.75 |
| aca | T | 350 | 154.60 |
| acg | T | 0 | 29.35 |
| --- | -- | ------ | ------ |
| mPD | | 0.017 | 0.69 |
| | | nPD: | 0.02 |
| | | N. weight: | 1.2 |
| | | Sc. PD: | -0.27 |
| | | Sc. rank: | -144.9 |

| PA | | | |
|---|---|---|---|
| Pos. | 468 | obs: | exp: |
| gct | A | 160 | 88.91 |
| gcc | A | 192 | 72.31 |
| gca | A | 1 | 157.30 |
| gcg | A | 0 | 34.45 |
| --- | -- | ------ | ------ |
| mPD | | 0.50 | 0.69 |
| | | nPD: | 0.73 |
| | | N. weight: | 1.2 |
| | | Sc. PD: | 0.54 |
| | | Sc. rank: | 219.8 |

PA

| Pos. | 469 | obs: | exp: |
|---|---|---|---|
| tta | L | 23 | 30.84 |
| ttg | L | 116 | 67.98 |
| ctt | L | 0 | 65.86 |
| ctc | L | 0 | 57.10 |
| cta | L | 42 | 53.18 |
| ctg | L | 172 | 78.03 |
| --- | -- | ------ | ------ |
| mPD | | 0.78 | 1.1 |
| | | nPD: | 0.7 |
| | | N. weight: | 0.81 |
| | | Sc. PD: | 0.34 |
| | | Sc. rank: | 141.2 |

,

PA

| Pos. | 470 | obs: | exp: |
|---|---|---|---|
| tta | L | 9 | 30.84 |
| ttg | L | 180 | 67.98 |
| ctt | L | 26 | 65.86 |
| ctc | L | 101 | 57.10 |
| cta | L | 0 | 53.18 |
| ctg | L | 37 | 78.03 |
| --- | -- | ------ | ------ |
| mPD | | 1.0 | 1.1 |
| | | nPD: | 0.93 |
| | | N. weight: | 0.77 |
| | | Sc. PD: | 0.49 |
| | | Sc. rank: | 154.8 |

,

PA

| Pos. | 471 | obs: | exp: |
|---|---|---|---|
| aat | N | 336 | 184.50 |
| aac | N | 17 | 168.50 |
| --- | -- | ------ | ------ |
| mPD | | 0.092 | 0.50 |
| | | nPD: | 0.18 |
| | | N. weight: | 0.71 |
| | | Sc. PD: | -0.054 |
| | | Sc. rank: | -10.9 |

,

PA

| Pos. | 472 | obs: | exp: |
|---|---|---|---|
| gct | A | 71 | 88.91 |
| gcc | A | 10 | 72.31 |
| gca | A | 272 | 157.30 |
| gcg | A | 0 | 34.45 |
| --- | -- | ------ | ------ |
| mPD | | 0.37 | 0.69 |
| | | nPD: | 0.53 |
| | | N. weight: | 0.51 |
| | | Sc. PD: | 0.13 |
| | | Sc. rank: | 68.5 |

,

PA

| Pos. | 473 | obs: | exp: |
|---|---|---|---|
| tct | S | 137 | 56.12 |
| tcc | S | 211 | 49.17 |
| tca | S | 5 | 78.20 |
| tcg | S | 0 | 21.52 |
| agt | S | 0 | 77.66 |
| agc | S | 0 | 70.33 |
| --- | -- | ------ | ------ |
| mPD | | 0.49 | 1.7 |
| | | nPD: | 0.3 |
| | | N. weight: | 1.8 |
| | | Sc. PD: | 0.056 |
| | | Sc. rank: | 90.4 |

,

PA

| Pos. | 474 | obs: | exp: |
|---|---|---|---|
| tgt | C | 318 | 145.50 |
| tgc | C | 35 | 207.50 |
| --- | -- | ------ | ------ |
| mPD | | 0.18 | 0.48 |
| | | nPD: | 0.37 |
| | | N. weight: | 0.81 |
| | | Sc. PD: | 0.082 |
| | | Sc. rank: | 66.6 |

,

PA

| Pos. | 475 | obs: | exp: |
|---|---|---|---|
| act | T | 0 | 0.26 |
| acc | T | 0 | 0.21 |
| aca | T | 1 | 0.44 |
| acg | T | 0 | 0.08 |
| gct | A | 32 | 88.66 |
| gcc | A | 1 | 72.11 |
| gca | A | 261 | 156.90 |
| gcg | A | 58 | 34.35 |
| --- | -- | ------ | ------ |
| mPD | | 0.42 | 0.69 |
| | | nPD: | 0.61 |
| | | N. weight: | 0.57 |
| | | Sc. PD: | 0.19 |
| | | Sc. rank: | 86.8 |

,

PA

| Pos. | 476 | obs: | exp: |
|---|---|---|---|
| gct | A | 7 | 88.91 |
| gcc | A | 281 | 72.31 |
| gca | A | 64 | 157.30 |
| gcg | A | 1 | 34.45 |
| --- | -- | ------ | ------ |
| mPD | | 0.33 | 0.69 |
| | | nPD: | 0.49 |
| | | N. weight: | 1.3 |
| | | Sc. PD: | 0.28 |
| | | Sc. rank: | 153.9 |

,

PA

| Pos. | 477 | obs: | exp: |
|---|---|---|---|
| atg | M | 353 | 353.00 |
| --- | -- | ------ | ------ |
| mPD | | 0 | 0 |
| | | nPD: | 1. |
| | | N. weight: | 0. |
| | | Sc. PD: | 0 |
| | | Sc. rank: | 0 |

,

PA

| Pos. | 478 | obs: | exp: |
|---|---|---|---|
| gat | D | 342 | 188.30 |
| gac | D | 11 | 164.70 |
| --- | -- | ------ | ------ |
| mPD | | 0.061 | 0.50 |
| | | nPD: | 0.12 |
| | | N. weight: | 0.76 |
| | | Sc. PD: | -0.1 |
| | | Sc. rank: | -31.5 |

,

PA

| Pos. | 479 | obs: | exp: |
|---|---|---|---|
| aat | N | 0 | 0.52 |
| aac | N | 1 | 0.48 |
| gat | D | 135 | 187.80 |
| gac | D | 217 | 164.20 |
| --- | -- | ------ | ------ |
| mPD | | 0.48 | 0.50 |
| | | nPD: | 0.95 |
| | | N. weight: | 0.085 |
| | | Sc. PD: | 0.056 |
| | | Sc. rank: | 17.1 |

,

PA

| Pos. | 480 | obs: | exp: |
|---|---|---|---|
| ttt | F | 41 | 144.70 |
| ttc | F | 311 | 207.30 |
| tta | L | 0 | 0.09 |
| ttg | L | 0 | 0.19 |
| ctt | L | 0 | 0.19 |
| ctc | L | 1 | 0.16 |
| cta | L | 0 | 0.15 |
| ctg | L | 0 | 0.22 |
| --- | -- | ------ | ------ |
| mPD | | 0.21 | 0.49 |
| | | nPD: | 0.43 |
| | | N. weight: | 0.34 |
| | | Sc. PD: | 0.054 |
| | | Sc. rank: | 31.8 |

,

PA

| Pos. | 481 | obs: | exp: |
|---|---|---|---|
| caa | Q | 260 | 188.20 |
| cag | Q | 93 | 164.80 |
| --- | -- | ------ | ------ |
| mPD | | 0.39 | 0.50 |
| | | nPD: | 0.78 |
| | | N. weight: | 0.15 |
| | | Sc. PD: | 0.072 |
| | | Sc. rank: | 27.6 |

,

PA

| Pos. | 482 | obs: | exp: |
|---|---|---|---|
| tta | L | 0 | 30.84 |
| ttg | L | 135 | 67.98 |
| ctt | L | 0 | 65.86 |
| ctc | L | 0 | 57.10 |
| cta | L | 4 | 53.18 |
| ctg | L | 214 | 78.03 |
| --- | -- | ------ | ------ |
| mPD | | 0.50 | 1.1 |
| | | nPD: | 0.45 |
| | | N. weight: | 1.3 |
| | | Sc. PD: | 0.23 |
| | | Sc. rank: | 127.4 |

,

PA

| Pos. | 483 | obs: | exp: |
|---|---|---|---|
| att | I | 285 | 132.70 |
| atc | I | 68 | 92.52 |
| ata | I | 0 | 127.80 |
| --- | -- | ------ | ------ |
| mPD | | 0.31 | 0.66 |
| | | nPD: | 0.47 |
| | | N. weight: | 0.86 |
| | | Sc. PD: | 0.17 |
| | | Sc. rank: | 92.9 |

,

PA

| Pos. | 484 | obs: | exp: |
|---|---|---|---|
| tct | S | 0 | 0.16 |
| tcc | S | 0 | 0.14 |
| tca | S | 1 | 0.22 |
| tcg | S | 0 | 0.06 |
| cct | P | 22 | 92.69 |
| ccc | P | 0 | 63.49 |
| cca | P | 318 | 138.90 |
| ccg | P | 12 | 56.91 |
| agt | S | 0 | 0.22 |
| agc | S | 0 | 0.20 |
| --- | -- | ------ | ------ |
| mPD | | 0.18 | 0.72 |
| | | nPD: | 0.25 |
| | | N. weight: | 0.94 |
| | | Sc. PD: | -0.0089 |
| | | Sc. rank: | 33.1 |

,

PA

| Pos. | 485 | obs: | exp: |
|---|---|---|---|
| atg | M | 353 | 353.00 |
| --- | -- | ------ | ------ |
| mPD | | 0 | 0 |
| | | nPD: | 1. |
| | | N. weight: | 0. |
| | | Sc. PD: | 0 |
| | | Sc. rank: | 0 |

,

PA

| Pos. | 486 | obs: | exp: |
|---|---|---|---|
| att | I | 0 | 132.70 |
| atc | I | 0 | 92.52 |
| ata | I | 353 | 127.80 |
| --- | -- | ------ | ------ |
| mPD | | 0 | 0.66 |
| | | nPD: | 0. |
| | | N. weight: | 1.5 |
| | | Sc. PD: | -0.39 |
| | | Sc. rank: | -308.0 |

,

PA

| Pos. | 487 | obs: | exp: |
|---|---|---|---|
| tct | S | 0 | 56.12 |
| tcc | S | 0 | 49.17 |
| tca | S | 0 | 78.20 |
| tcg | S | 0 | 21.52 |
| agt | S | 3 | 77.66 |
| agc | S | 350 | 70.33 |
| --- | -- | ------ | ------ |
| mPD | | 0.017 | 1.7 |
| | | nPD: | 0.01 |
| | | N. weight: | 2.4 |
| | | Sc. PD: | -0.58 |
| | | Sc. rank: | -359.0 |

,

PA

| Pos. | 488 | obs: | exp: |
|---|---|---|---|
| aaa | K | 226 | 197.70 |
| aag | K | 127 | 155.30 |
| --- | -- | ------ | ------ |
| mPD | | 0.46 | 0.49 |
| | | nPD: | 0.94 |
| | | N. weight: | 0.034 |
| | | Sc. PD: | 0.022 |
| | | Sc. rank: | 6.7 |

,

PA

| Pos. | 489 | obs: | exp: |
|---|---|---|---|
| tgt | C | 48 | 145.50 |
| tgc | C | 305 | 207.50 |
| --- | -- | ------ | ------ |
| mPD | | 0.24 | 0.48 |
| | | nPD: | 0.49 |
| | | N. weight: | 0.29 |
| | | Sc. PD: | 0.061 |
| | | Sc. rank: | 33.2 |

,

PA

| Pos. | 490 | obs: | exp: |
|---|---|---|---|
| cgt | R | 0 | 14.65 |
| cgc | R | 0 | 17.28 |
| cga | R | 0 | 33.93 |
| cgg | R | 0 | 29.46 |
| aga | R | 320 | 158.80 |
| agg | R | 33 | 98.92 |
| --- | -- | ------ | ------ |
| mPD | | 0.17 | 0.96 |
| | | nPD: | 0.18 |
| | | N. weight: | 0.82 |
| | | Sc. PD: | -0.069 |
| | | Sc. rank: | -16.5 |

,

PA

| Pos. | 491 | obs: | exp: |
|---|---|---|---|
| act | T | 1 | 93.26 |
| acc | T | 335 | 75.75 |
| aca | T | 15 | 154.60 |
| acg | T | 2 | 29.35 |
| --- | -- | ------ | ------ |
| mPD | | 0.098 | 0.69 |
| | | nPD: | 0.14 |
| | | N. weight: | 2. |
| | | Sc. PD: | -0.23 |
| | | Sc. rank: | -63.4 |

,

PA

| Pos. | 492 | obs: | exp: |
|---|---|---|---|
| cgt | R | 0 | 0.17 |
| cgc | R | 0 | 0.20 |
| cga | R | 1 | 0.38 |
| cgg | R | 0 | 0.33 |
| aaa | K | 319 | 195.40 |
| aag | K | 30 | 153.60 |
| aga | R | 1 | 1.80 |
| agg | R | 2 | 1.12 |
| --- | -- | ------ | ------ |
| mPD | | 0.19 | 0.52 |
| | | nPD: | 0.37 |
| | | N. weight: | 0.48 |
| | | Sc. PD: | 0.049 |
| | | Sc. rank: | 40.0 |

,

PA

| Pos. | 493 | obs: | exp: |
|---|---|---|---|
| gaa | E | 343 | 203.10 |
| gag | E | 10 | 149.90 |
| --- | -- | ------ | ------ |
| mPD | | 0.055 | 0.49 |
| | | nPD: | 0.11 |
| | | N. weight: | 0.66 |
| | | Sc. PD: | -0.097 |
| | | Sc. rank: | -32.3 |

,

PA

| Pos. | 494 | obs: | exp: |
|---|---|---|---|
| ggt | G | 0 | 43.56 |
| ggc | G | 1 | 47.74 |
| gga | G | 260 | 170.80 |
| ggg | G | 92 | 90.95 |
| --- | -- | ------ | ------ |
| mPD | | 0.39 | 0.67 |
| | | nPD: | 0.59 |
| | | N. weight: | 0.47 |
| | | Sc. PD: | 0.15 |
| | | Sc. rank: | 69.7 |

,

PA

| Pos. | 495 | obs: | exp: |
|---|---|---|---|
| cgt | R | 0 | 14.65 |
| cgc | R | 0 | 17.28 |
| cga | R | 2 | 33.93 |
| cgg | R | 65 | 29.46 |
| aga | R | 249 | 158.80 |
| agg | R | 37 | 98.92 |
| --- | -- | ------ | ------ |
| mPD | | 0.72 | 0.96 |
| | | nPD: | 0.75 |
| | | N. weight: | 0.55 |
| | | Sc. PD: | 0.26 |
| | | Sc. rank: | 102.4 |

,

PA

| Pos. | 496 | obs: | exp: |
|---|---|---|---|
| cgt | R | 0 | 14.65 |
| cgc | R | 0 | 17.28 |
| cga | R | 28 | 33.93 |
| cgg | R | 318 | 29.46 |
| aga | R | 0 | 158.80 |
| agg | R | 7 | 98.92 |
| --- | -- | ------ | ------ |
| mPD | | 0.19 | 0.96 |
| | | nPD: | 0.19 |
| | | N. weight: | 3.2 |
| | | Sc. PD: | -0.22 |
| | | Sc. rank: | -2.3 |

,

PA

| Pos. | 497 | obs: | exp: |
|---|---|---|---|
| aaa | K | 134 | 197.70 |
| aag | K | 219 | 155.30 |
| --- | -- | ------ | ------ |
| mPD | | 0.47 | 0.49 |
| | | nPD: | 0.96 |
| | | N. weight: | 0.11 |
| | | Sc. PD: | 0.075 |
| | | Sc. rank: | 22.7 |

,

PA

| Pos. | 498 | obs: | exp: |
|---|---|---|---|
| act | T | 78 | 93.26 |
| acc | T | 25 | 75.75 |
| aca | T | 246 | 154.60 |
| acg | T | 4 | 29.35 |
| --- | -- | ------ | ------ |
| mPD | | 0.46 | 0.69 |
| | | nPD: | 0.67 |
| | | N. weight: | 0.31 |
| | | Sc. PD: | 0.12 |
| | | Sc. rank: | 51.3 |

,

PA

| Pos. | 499 | obs: | exp: |
|---|---|---|---|
| aat | N | 276 | 184.50 |
| aac | N | 77 | 168.50 |
| --- | -- | ------ | ------ |
| mPD | | 0.34 | 0.50 |
| | | nPD: | 0.69 |
| | | N. weight: | 0.23 |
| | | Sc. PD: | 0.093 |
| | | Sc. rank: | 39.6 |

,

PA

| Pos. | 500 | obs: | exp: |
|---|---|---|---|
| tta | L | 0 | 30.84 |
| ttg | L | 9 | 67.98 |
| ctt | L | 0 | 65.86 |
| ctc | L | 0 | 57.10 |
| cta | L | 66 | 53.18 |
| ctg | L | 278 | 78.03 |
| --- | -- | ------ | ------ |
| mPD | | 0.35 | 1.1 |
| | | nPD: | 0.32 |
| | | N. weight: | 1.5 |
| | | Sc. PD: | 0.078 |
| | | Sc. rank: | 90.8 |

,

PA

| Pos. | 501 | obs: | exp: |
|---|---|---|---|
| tat | Y | 332 | 182.70 |
| tac | Y | 21 | 170.30 |
| --- | -- | ------ | ------ |
| mPD | | 0.11 | 0.50 |
| | | nPD: | 0.22 |
| | | N. weight: | 0.67 |
| | | Sc. PD: | -0.026 |
| | | Sc. rank: | 15.9 |

,

PA

| Pos. | 502 | obs: | exp: |
|---|---|---|---|
| ggt | G | 1 | 43.56 |
| ggc | G | 0 | 47.74 |
| gga | G | 228 | 170.80 |
| ggg | G | 124 | 90.95 |
| --- | -- | ------ | ------ |
| mPD | | 0.46 | 0.67 |
| | | nPD: | 0.69 |
| | | N. weight: | 0.45 |
| | | Sc. PD: | 0.18 |
| | | Sc. rank: | 77.4 |

,

PA

| Pos. | 503 | obs: | exp: |
|---|---|---|---|
| ttt | F | 2 | 145.10 |
| ttc | F | 351 | 207.90 |
| --- | -- | ------ | ------ |
| mPD | | 0.011 | 0.48 |
| | | nPD: | 0.02 |
| | | N. weight: | 0.76 |
| | | Sc. PD: | -0.18 |
| | | Sc. rank: | -94.7 |

,

PA

| Pos. | 504 | obs: | exp: |
|---|---|---|---|
| tta | L | 0 | 0.26 |
| ttg | L | 0 | 0.58 |
| ctt | L | 0 | 0.56 |
| ctc | L | 3 | 0.49 |
| cta | L | 0 | 0.45 |
| ctg | L | 0 | 0.66 |
| att | I | 191 | 122.60 |
| atc | I | 91 | 85.44 |
| ata | I | 44 | 118.00 |
| gtt | V | 24 | 4.93 |
| gtc | V | 0 | 5.04 |
| gta | V | 0 | 4.50 |
| gtg | V | 0 | 9.53 |
| --- | -- | ------ | ------ |
| mPD | | 0.69 | 0.82 |
| | | nPD: | 0.84 |
| | | N. weight: | 0.41 |
| | | Sc. PD: | 0.23 |
| | | Sc. rank: | 81.7 |

,

PA

| Pos. | 505 | obs: | exp: |
|---|---|---|---|
| att | I | 0 | 131.20 |
| atc | I | 0 | 91.47 |
| ata | I | 349 | 126.30 |
| gtt | V | 0 | 0.82 |
| gtc | V | 0 | 0.84 |
| gta | V | 4 | 0.75 |
| gtg | V | 0 | 1.59 |
| --- | -- | ------ | ------ |
| mPD | | 0.022 | 0.68 |
| | | nPD: | 0.03 |
| | | N. weight: | 1.5 |
| | | Sc. PD: | -0.34 |
| | | Sc. rank: | -174.7 |

,

PA

| Pos. | 506 | obs: | exp: |
|---|---|---|---|
| aaa | K | 271 | 197.70 |
| aag | K | 82 | 155.30 |
| --- | -- | ------ | ------ |
| mPD | | 0.36 | 0.49 |
| | | nPD: | 0.73 |
| | | N. weight: | 0.15 |
| | | Sc. PD: | 0.069 |
| | | Sc. rank: | 28.1 |

,

PA

| Pos. | 507 | obs: | exp: |
|---|---|---|---|
| ggt | G | 0 | 43.56 |
| ggc | G | 6 | 47.74 |
| gga | G | 318 | 170.80 |
| ggg | G | 29 | 90.95 |
| --- | -- | ------ | ------ |
| mPD | | 0.18 | 0.67 |
| | | nPD: | 0.27 |
| | | N. weight: | 0.67 |
| | | Sc. PD: | 0.0055 |
| | | Sc. rank: | 27.7 |

,

PA

| Pos. | 508 | obs: | exp: |
|---|---|---|---|
| cgt | R | 0 | 14.65 |
| cgc | R | 0 | 17.28 |
| cga | R | 12 | 33.93 |
| cgg | R | 0 | 29.46 |
| aga | R | 270 | 158.80 |
| agg | R | 71 | 98.92 |
| --- | -- | ------ | ------ |
| mPD | | 0.39 | 0.96 |
| | | nPD: | 0.4 |
| | | N. weight: | 0.48 |
| | | Sc. PD: | 0.065 |
| | | Sc. rank: | 41.9 |

,

PA

| Pos. | 509 | obs: | exp: |
|---|---|---|---|
| tct | S | 129 | 56.12 |
| tcc | S | 224 | 49.17 |
| tca | S | 0 | 78.20 |
| tcg | S | 0 | 21.52 |
| agt | S | 0 | 77.66 |
| agc | S | 0 | 70.33 |
| --- | -- | ------ | ------ |
| mPD | | 0.47 | 1.7 |
| | | nPD: | 0.28 |
| | | N. weight: | 1.9 |
| | | Sc. PD: | 0.028 |
| | | Sc. rank: | 86.2 |

,

PA

| Pos. | 510 | obs: | exp: |
|---|---|---|---|
| cat | H | 286 | 191.30 |
| cac | H | 67 | 161.70 |
| --- | -- | ------ | ------ |
| mPD | | 0.31 | 0.50 |
| | | nPD: | 0.62 |
| | | N. weight: | 0.25 |
| | | Sc. PD: | 0.087 |
| | | Sc. rank: | 39.3 |

,

PA

| Pos. | 511 | obs: | exp: |
|---|---|---|---|
| tta | L | 6 | 30.84 |
| ttg | L | 325 | 67.98 |
| ctt | L | 0 | 65.86 |
| ctc | L | 0 | 57.10 |
| cta | L | 0 | 53.18 |
| ctg | L | 22 | 78.03 |
| --- | -- | ------ | ------ |
| mPD | | 0.15 | 1.1 |
| | | nPD: | 0.14 |
| | | N. weight: | 2. |
| | | Sc. PD: | -0.25 |
| | | Sc. rank: | -72.0 |

,

PA

| Pos. | 512 | obs: | exp: |
|---|---|---|---|
| cgt | R | 0 | 14.65 |
| cgc | R | 0 | 17.28 |
| cga | R | 0 | 33.93 |
| cgg | R | 0 | 29.46 |
| aga | R | 134 | 158.80 |
| agg | R | 219 | 98.92 |
| --- | -- | ------ | ------ |
| mPD | | 0.47 | 0.96 |
| | | nPD: | 0.49 |
| | | N. weight: | 0.66 |
| | | Sc. PD: | 0.14 |
| | | Sc. rank: | 77.9 |

,

PA

| Pos. | 513 | obs: | exp: |
|---|---|---|---|
| aat | N | 309 | 184.50 |
| aac | N | 44 | 168.50 |
| --- | -- | ------ | ------ |
| mPD | | 0.22 | 0.50 |
| | | nPD: | 0.44 |
| | | N. weight: | 0.44 |
| | | Sc. PD: | 0.074 |
| | | Sc. rank: | 42.8 |

,

PA

| Pos. | 514 | obs: | exp: |
|---|---|---|---|
| gat | D | 136 | 188.30 |
| gac | D | 217 | 164.70 |
| --- | -- | ------ | ------ |
| mPD | | 0.48 | 0.50 |
| | | nPD: | 0.95 |
| | | N. weight: | 0.08 |
| | | Sc. PD: | 0.053 |
| | | Sc. rank: | 16.2 |

,

PA

| Pos. | 515 | obs: | exp: |
|---|---|---|---|
| act | T | 149 | 93.26 |
| acc | T | 197 | 75.75 |
| aca | T | 6 | 154.60 |
| acg | T | 1 | 29.35 |
| --- | -- | ------ | ------ |
| mPD | | 0.51 | 0.69 |
| | | nPD: | 0.75 |
| | | N. weight: | 1. |
| | | Sc. PD: | 0.48 |
| | | Sc. rank: | 191.7 |

,

PA

| Pos. | 516 | obs: | exp: |
|---|---|---|---|
| gat | D | 313 | 188.30 |
| gac | D | 40 | 164.70 |
| --- | -- | ------ | ------ |
| mPD | | 0.20 | 0.50 |
| | | nPD: | 0.4 |
| | | N. weight: | 0.45 |
| | | Sc. PD: | 0.061 |
| | | Sc. rank: | 39.8 |

,

PA

| Pos. | 517 | obs: | exp: |
|---|---|---|---|
| gtt | V | 8 | 72.56 |
| gtc | V | 1 | 74.09 |
| gta | V | 67 | 66.16 |
| gtg | V | 277 | 140.20 |
| --- | -- | ------ | ------ |
| mPD | | 0.35 | 0.72 |
| | | nPD: | 0.48 |
| | | N. weight: | 0.74 |
| | | Sc. PD: | 0.16 |
| | | Sc. rank: | 83.5 |

,

PA

| Pos. | 518 | obs: | exp: |
|---|---|---|---|
| att | I | 0 | 0.38 |
| atc | I | 0 | 0.26 |
| ata | I | 1 | 0.36 |
| gtt | V | 10 | 72.14 |
| gtc | V | 0 | 73.67 |
| gta | V | 257 | 65.78 |
| gtg | V | 84 | 139.40 |
| gct | A | 0 | 0.25 |
| gcc | A | 0 | 0.20 |
| gca | A | 1 | 0.45 |
| gcg | A | 0 | 0.10 |
| --- | -- | ------ | ------ |
| mPD | | 0.42 | 0.73 |
| | | nPD: | 0.57 |
| | | N. weight: | 1.3 |
| | | Sc. PD: | 0.37 |
| | | Sc. rank: | 181.8 |

,

PA

| Pos. | 519 | obs: | exp: |
|---|---|---|---|
| aat | N | 85 | 184.50 |
| aac | N | 268 | 168.50 |
| --- | -- | ------ | ------ |
| mPD | | 0.37 | 0.50 |
| | | nPD: | 0.73 |
| | | N. weight: | 0.26 |
| | | Sc. PD: | 0.12 |
| | | Sc. rank: | 48.2 |

PA

| Pos. | 520 | obs: | exp: |
|---|---|---|---|
| ttt | F | 338 | 144.70 |
| ttc | F | 14 | 207.30 |
| tat | Y | 1 | 0.52 |
| tac | Y | 0 | 0.48 |
| --- | -- | ------ | ------ |
| mPD | | 0.082 | 0.49 |
| | | nPD: | 0.17 |
| | | N. weight: | 1.1 |
| | | Sc. PD: | -0.1 |
| | | Sc. rank: | -27.5 |

PA

| Pos. | 521 | obs: | exp: |
|---|---|---|---|
| atg | M | 1 | 1.00 |
| gtt | V | 0 | 72.35 |
| gtc | V | 0 | 73.88 |
| gta | V | 47 | 65.97 |
| gtg | V | 305 | 139.80 |
| --- | -- | ------ | ------ |
| mPD | | 0.24 | 0.73 |
| | | nPD: | 0.33 |
| | | N. weight: | 0.96 |
| | | Sc. PD: | 0.058 |
| | | Sc. rank: | 58.2 |

PA

| Pos. | 522 | obs: | exp: |
|---|---|---|---|
| tct | S | 0 | 56.12 |
| tcc | S | 0 | 49.17 |
| tca | S | 0 | 78.20 |
| tcg | S | 0 | 21.52 |
| agt | S | 106 | 77.66 |
| agc | S | 247 | 70.33 |
| --- | -- | ------ | ------ |
| mPD | | 0.42 | 1.7 |
| | | nPD: | 0.25 |
| | | N. weight: | 1.5 |
| | | Sc. PD: | -0.016 |
| | | Sc. rank: | 51.6 |

PA

| Pos. | 523 | obs: | exp: |
|---|---|---|---|
| atg | M | 353 | 353.00 |
| --- | -- | ------ | ------ |
| mPD | | 0 | 0 |
| | | nPD: | 1. |
| | | N. weight: | 0. |
| | | Sc. PD: | 0 |
| | | Sc. rank: | 0 |

PA

| Pos. | 524 | obs: | exp: |
|---|---|---|---|
| gaa | E | 245 | 203.10 |
| gag | E | 108 | 149.90 |
| --- | -- | ------ | ------ |
| mPD | | 0.43 | 0.49 |
| | | nPD: | 0.87 |
| | | N. weight: | 0.058 |
| | | Sc. PD: | 0.034 |
| | | Sc. rank: | 11.6 |

PA

| Pos. | 525 | obs: | exp: |
|---|---|---|---|
| ttt | F | 123 | 145.10 |
| ttc | F | 230 | 207.90 |
| --- | -- | ------ | ------ |
| mPD | | 0.46 | 0.48 |
| | | nPD: | 0.94 |
| | | N. weight: | 0.026 |
| | | Sc. PD: | 0.017 |
| | | Sc. rank: | 5.2 |

PA

| Pos. | 526 | obs: | exp: |
|---|---|---|---|
| tct | S | 281 | 56.12 |
| tcc | S | 72 | 49.17 |
| tca | S | 0 | 78.20 |
| tcg | S | 0 | 21.52 |
| agt | S | 0 | 77.66 |
| agc | S | 0 | 70.33 |
| --- | -- | ------ | ------ |
| mPD | | 0.33 | 1.7 |
| | | nPD: | 0.2 |
| | | N. weight: | 2.1 |
| | | Sc. PD: | -0.14 |
| | | Sc. rank: | 4.6 |

PA

| Pos. | 527 | obs: | exp: |
|---|---|---|---|
| tta | L | 0 | 30.84 |
| ttg | L | 0 | 67.98 |
| ctt | L | 238 | 65.86 |
| ctc | L | 115 | 57.10 |
| cta | L | 0 | 53.18 |
| ctg | L | 0 | 78.03 |
| --- | -- | ------ | ------ |
| mPD | | 0.44 | 1.1 |
| | | nPD: | 0.4 |
| | | N. weight: | 1.7 |
| | | Sc. PD: | 0.21 |
| | | Sc. rank: | 143.7 |

PA

| Pos. | 528 | obs: | exp: |
|---|---|---|---|
| act | T | 300 | 93.26 |
| acc | T | 47 | 75.75 |
| aca | T | 4 | 154.60 |
| acg | T | 2 | 29.35 |
| --- | -- | ------ | ------ |
| mPD | | 0.26 | 0.69 |
| | | nPD: | 0.38 |
| | | N. weight: | 1.3 |
| | | Sc. PD: | 0.15 |
| | | Sc. rank: | 112.3 |

PA

| Pos. | 529 | obs: | exp: |
|---|---|---|---|
| gat | D | 64 | 188.30 |
| gac | D | 289 | 164.70 |
| --- | -- | ------ | ------ |
| mPD | | 0.30 | 0.50 |
| | | nPD: | 0.6 |
| | | N. weight: | 0.41 |
| | | Sc. PD: | 0.13 |
| | | Sc. rank: | 61.9 |

PA

| Pos. | 530 | obs: | exp: |
|---|---|---|---|
| cct | P | 67 | 92.69 |
| ccc | P | 0 | 63.49 |
| cca | P | 39 | 138.90 |
| ccg | P | 246 | 56.91 |
| caa | Q | 1 | 0.53 |
| cag | Q | 0 | 0.47 |
| --- | -- | ------ | ------ |
| mPD | | 0.47 | 0.72 |
| | | nPD: | 0.65 |
| | | N. weight: | 1.3 |
| | | Sc. PD: | 0.47 |
| | | Sc. rank: | 204.4 |

PA

| Pos. | 531 | obs: | exp: |
|---|---|---|---|
| cgt | R | 0 | 14.61 |
| cgc | R | 0 | 17.24 |
| cga | R | 0 | 33.83 |
| cgg | R | 0 | 29.37 |
| aaa | K | 1 | 0.56 |
| aag | K | 0 | 0.44 |
| aga | R | 114 | 158.30 |
| agg | R | 238 | 98.64 |
| --- | -- | ------ | ------ |
| mPD | | 0.45 | 0.96 |
| | | nPD: | 0.46 |
| | | N. weight: | 0.75 |
| | | Sc. PD: | 0.14 |
| | | Sc. rank: | 78.5 |

PA

| Pos. | 532 | obs: | exp: |
|---|---|---|---|
| tta | L | 1 | 30.84 |
| ttg | L | 68 | 67.98 |
| ctt | L | 0 | 65.86 |
| ctc | L | 1 | 57.10 |
| cta | L | 51 | 53.18 |
| ctg | L | 232 | 78.03 |
| --- | -- | ------ | ------ |
| mPD | | 0.57 | 1.1 |
| | | nPD: | 0.51 |
| | | N. weight: | 1.1 |
| | | Sc. PD: | 0.26 |
| | | Sc. rank: | 137.5 |

PA

| Pos. | 533 | obs: | exp: |
|---|---|---|---|
| gaa | E | 55 | 203.10 |
| gag | E | 298 | 149.90 |
| --- | -- | ------ | ------ |
| mPD | | 0.26 | 0.49 |
| | | nPD: | 0.54 |
| | | N. weight: | 0.58 |
| | | Sc. PD: | 0.15 |
| | | Sc. rank: | 80.1 |

PA

| Pos. | 534 | obs: | exp: |
|---|---|---|---|
| tta | L | 0 | 0.17 |
| ttg | L | 0 | 0.39 |
| ctt | L | 0 | 0.37 |
| ctc | L | 0 | 0.32 |
| cta | L | 2 | 0.30 |
| ctg | L | 0 | 0.44 |
| cct | P | 4 | 92.42 |
| ccc | P | 1 | 63.31 |
| cca | P | 340 | 138.50 |
| ccg | P | 6 | 56.75 |
| --- | -- | ------ | ------ |
| mPD | | 0.072 | 0.73 |
| | | nPD: | 0.1 |
| | | N. weight: | 1.2 |
| | | Sc. PD: | -0.19 |
| | | Sc. rank: | -65.9 |

PA

| Pos. | 535 | obs: | exp: |
|---|---|---|---|
| tat | Y | 0 | 0.52 |
| tac | Y | 1 | 0.48 |
| cat | H | 101 | 190.70 |
| cac | H | 251 | 161.30 |
| --- | -- | ------ | ------ |
| mPD | | 0.42 | 0.50 |
| | | nPD: | 0.83 |
| | | N. weight: | 0.22 |
| | | Sc. PD: | 0.12 |
| | | Sc. rank: | 42.8 |

PA

| Pos. | 536 | obs: | exp: |
|---|---|---|---|
| aaa | K | 224 | 197.70 |
| aag | K | 129 | 155.30 |
| --- | -- | ------ | ------ |
| mPD | | 0.47 | 0.49 |
| | | nPD: | 0.94 |
| | | N. weight: | 0.031 |
| | | Sc. PD: | 0.02 |
| | | Sc. rank: | 6.2 |

PA

| Pos. | 537 | obs: | exp: |
|---|---|---|---|
| tgg | W | 353 | 353.00 |
| --- | -- | ------ | ------ |
| mPD | | 0 | 0 |
| | | nPD: | 1. |
| | | N. weight: | 0. |
| | | Sc. PD: | 0 |
| | | Sc. rank: | 0 |

PA

| Pos. | 538 | obs: | exp: |
|---|---|---|---|
| aaa | K | 1 | 0.56 |
| aag | K | 0 | 0.44 |
| gaa | E | 252 | 202.00 |
| gag | E | 99 | 149.00 |
| ggt | G | 0 | 0.12 |
| ggc | G | 0 | 0.14 |
| gga | G | 1 | 0.48 |
| ggg | G | 0 | 0.26 |
| --- | -- | ------ | ------ |
| mPD | | 0.42 | 0.50 |
| | | nPD: | 0.83 |
| | | N. weight: | 0.084 |
| | | Sc. PD: | 0.046 |
| | | Sc. rank: | 16.6 |

PA

| Pos. | 539 | obs: | exp: |
|---|---|---|---|
| cgt | R | 0 | 0.08 |
| cgc | R | 0 | 0.10 |
| cga | R | 0 | 0.19 |
| cgg | R | 0 | 0.17 |
| aaa | K | 72 | 196.50 |
| aag | K | 279 | 154.50 |
| aga | R | 2 | 0.90 |
| agg | R | 0 | 0.56 |
| --- | -- | ------ | ------ |
| mPD | | 0.34 | 0.51 |
| | | nPD: | 0.68 |
| | | N. weight: | 0.42 |
| | | Sc. PD: | 0.17 |
| | | Sc. rank: | 70.3 |

PA

| Pos. | 540 | obs: | exp: |
|---|---|---|---|
| tat | Y | 57 | 182.70 |
| tac | Y | 296 | 170.30 |
| --- | -- | ------ | ------ |
| mPD | | 0.27 | 0.50 |
| | | nPD: | 0.54 |
| | | N. weight: | 0.43 |
| | | Sc. PD: | 0.12 |
| | | Sc. rank: | 60.0 |

PA

| Pos. | 541 | obs: | exp: |
|---|---|---|---|
| tgt | C | 308 | 145.50 |
| tgc | C | 45 | 207.50 |
| --- | -- | ------ | ------ |
| mPD | | 0.22 | 0.48 |
| | | nPD: | 0.46 |
| | | N. weight: | 0.71 |
| | | Sc. PD: | 0.13 |
| | | Sc. rank: | 73.1 |

PA

| Pos. | 542 | obs: | exp: |
|---|---|---|---|
| gtt | V | 236 | 72.56 |
| gtc | V | 116 | 74.09 |
| gta | V | 1 | 66.16 |
| gtg | V | 0 | 140.20 |
| --- | -- | ------ | ------ |
| mPD | | 0.45 | 0.72 |
| | | nPD: | 0.62 |
| | | N. weight: | 1.4 |
| | | Sc. PD: | 0.48 |
| | | Sc. rank: | 218.9 |

PA

| Pos. | 543 | obs: | exp: |
|---|---|---|---|
| tta | L | 0 | 30.49 |
| ttg | L | 0 | 67.21 |
| ctt | L | 233 | 65.12 |
| ctc | L | 114 | 56.45 |
| cta | L | 2 | 52.58 |
| ctg | L | 0 | 77.15 |
| att | I | 3 | 1.50 |
| atc | I | 1 | 1.05 |
| ata | I | 0 | 1.45 |
| --- | -- | ------ | ------ |
| mPD | | 0.47 | 1.1 |
| | | nPD: | 0.42 |
| | | N. weight: | 1.6 |
| | | Sc. PD: | 0.24 |
| | | Sc. rank: | 149.5 |

| PA | | | |
|---|---|---|---|
| Pos. | 544 | obs: | exp: |
| gat | D | 1 | 0.53 |
| gac | D | 0 | 0.47 |
| gaa | E | 133 | 202.60 |
| gag | E | 219 | 149.40 |
| --- | -- | ------ | ------ |
| mPD | | 0.47 | 0.49 |
| | | nPD: | 0.97 |
| | | N. weight: | 0.14 |
| | | Sc. PD: | 0.091 |
| | | Sc. rank: | 27.3 |

| PA | | | |
|---|---|---|---|
| Pos. | 545 | obs: | exp: |
| att | I | 0 | 127.10 |
| atc | I | 3 | 88.58 |
| ata | I | 335 | 122.30 |
| gtt | V | 0 | 3.08 |
| gtc | V | 0 | 3.15 |
| gta | V | 4 | 2.81 |
| gtg | V | 11 | 5.96 |
| --- | -- | ------ | ------ |
| mPD | | 0.16 | 0.75 |
| | | nPD: | 0.21 |
| | | N. weight: | 1.5 |
| | | Sc. PD: | -0.075 |
| | | Sc. rank: | 21.2 |

| PA | | | |
|---|---|---|---|
| Pos. | 546 | obs: | exp: |
| ggt | G | 0 | 43.56 |
| ggc | G | 1 | 47.74 |
| gga | G | 256 | 170.80 |
| ggg | G | 96 | 90.95 |
| --- | -- | ------ | ------ |
| mPD | | 0.40 | 0.67 |
| | | nPD: | 0.6 |
| | | N. weight: | 0.47 |
| | | Sc. PD: | 0.15 |
| | | Sc. rank: | 70.7 |

| PA | | | |
|---|---|---|---|
| Pos. | 547 | obs: | exp: |
| aat | N | 0 | 2.09 |
| aac | N | 4 | 1.91 |
| gat | D | 29 | 186.20 |
| gac | D | 320 | 162.80 |
| --- | -- | ------ | ------ |
| mPD | | 0.17 | 0.52 |
| | | nPD: | 0.33 |
| | | N. weight: | 0.72 |
| | | Sc. PD: | 0.048 |
| | | Sc. rank: | 48.6 |

| PA | | | |
|---|---|---|---|
| Pos. | 548 | obs: | exp: |
| att | I | 0 | 1.50 |
| atc | I | 0 | 1.05 |
| ata | I | 4 | 1.45 |
| atg | M | 349 | 349.00 |
| --- | -- | ------ | ------ |
| mPD | | 0.022 | 0.022 |
| | | nPD: | 1. |
| | | N. weight: | 0.017 |
| | | Sc. PD: | 0.012 |
| | | Sc. rank: | 3.5 |

| PA | | | |
|---|---|---|---|
| Pos. | 549 | obs: | exp: |
| tta | L | 0 | 30.84 |
| ttg | L | 0 | 67.98 |
| ctt | L | 7 | 65.86 |
| ctc | L | 316 | 57.10 |
| cta | L | 27 | 53.18 |
| ctg | L | 3 | 78.03 |
| --- | -- | ------ | ------ |
| mPD | | 0.19 | 1.1 |
| | | nPD: | 0.17 |
| | | N. weight: | 2.2 |
| | | Sc. PD: | -0.19 |
| | | Sc. rank: | -50.3 |

| PA | | | |
|---|---|---|---|
| Pos. | 550 | obs: | exp: |
| tta | L | 21 | 30.84 |
| ttg | L | 4 | 67.98 |
| ctt | L | 0 | 65.86 |
| ctc | L | 7 | 57.10 |
| cta | L | 207 | 53.18 |
| ctg | L | 114 | 78.03 |
| --- | -- | ------ | ------ |
| mPD | | 0.60 | 1.1 |
| | | nPD: | 0.54 |
| | | N. weight: | 1.3 |
| | | Sc. PD: | 0.34 |
| | | Sc. rank: | 178.7 |

| PA | | | |
|---|---|---|---|
| Pos. | 551 | obs: | exp: |
| cgt | R | 0 | 14.65 |
| cgc | R | 0 | 17.28 |
| cga | R | 68 | 33.93 |
| cgg | R | 260 | 29.46 |
| aga | R | 1 | 158.80 |
| agg | R | 24 | 98.92 |
| --- | -- | ------ | ------ |
| mPD | | 0.45 | 0.96 |
| | | nPD: | 0.47 |
| | | N. weight: | 2.5 |
| | | Sc. PD: | 0.48 |
| | | Sc. rank: | 264.8 |

| PA | | | |
|---|---|---|---|
| Pos. | 552 | obs: | exp: |
| act | T | 328 | 93.26 |
| acc | T | 24 | 75.75 |
| aca | T | 1 | 154.60 |
| acg | T | 0 | 29.35 |
| --- | -- | ------ | ------ |
| mPD | | 0.13 | 0.69 |
| | | nPD: | 0.19 |
| | | N. weight: | 1.6 |
| | | Sc. PD: | -0.11 |
| | | Sc. rank: | -4.1 |

| PA | | | |
|---|---|---|---|
| Pos. | 553 | obs: | exp: |
| gct | A | 1 | 88.91 |
| gcc | A | 0 | 72.31 |
| gca | A | 346 | 157.30 |
| gcg | A | 6 | 34.45 |
| --- | -- | ------ | ------ |
| mPD | | 0.039 | 0.69 |
| | | nPD: | 0.06 |
| | | N. weight: | 1.1 |
| | | Sc. PD: | -0.22 |
| | | Sc. rank: | -94.6 |

| PA | | | |
|---|---|---|---|
| Pos. | 554 | obs: | exp: |
| att | I | 12 | 118.80 |
| atc | I | 0 | 82.82 |
| ata | I | 304 | 114.40 |
| gtt | V | 0 | 7.60 |
| gtc | V | 0 | 7.77 |
| gta | V | 36 | 6.93 |
| gtg | V | 1 | 14.69 |
| --- | -- | ------ | ------ |
| mPD | | 0.26 | 0.87 |
| | | nPD: | 0.3 |
| | | N. weight: | 1.4 |
| | | Sc. PD: | 0.044 |
| | | Sc. rank: | 72.7 |

| PA | | | |
|---|---|---|---|
| Pos. | 555 | obs: | exp: |
| ggt | G | 7 | 43.56 |
| ggc | G | 343 | 47.74 |
| gga | G | 3 | 170.80 |
| ggg | G | 0 | 90.95 |
| --- | -- | ------ | ------ |
| mPD | | 0.056 | 0.67 |
| | | nPD: | 0.08 |
| | | N. weight: | 2.8 |
| | | Sc. PD: | -0.49 |
| | | Sc. rank: | -185.1 |

| PA | | | |
|---|---|---|---|
| Pos. | 556 | obs: | exp: |
| caa | Q | 335 | 187.70 |
| cag | Q | 17 | 164.30 |
| cgt | R | 0 | 0.04 |
| cgc | R | 0 | 0.05 |
| cga | R | 1 | 0.10 |
| cgg | R | 0 | 0.08 |
| aga | R | 0 | 0.45 |
| agg | R | 0 | 0.28 |
| --- | -- | ------ | ------ |
| mPD | | 0.098 | 0.51 |
| | | nPD: | 0.19 |
| | | N. weight: | 0.69 |
| | | Sc. PD: | -0.048 |
| | | Sc. rank: | -2.7 |

| PA | | | |
|---|---|---|---|
| Pos. | 557 | obs: | exp: |
| gtt | V | 0 | 72.35 |
| gtc | V | 1 | 73.88 |
| gta | V | 69 | 65.97 |
| gtg | V | 282 | 139.80 |
| gct | A | 0 | 0.25 |
| gcc | A | 0 | 0.20 |
| gca | A | 1 | 0.45 |
| gcg | A | 0 | 0.10 |
| --- | -- | ------ | ------ |
| mPD | | 0.33 | 0.73 |
| | | nPD: | 0.45 |
| | | N. weight: | 0.86 |
| | | Sc. PD: | 0.16 |
| | | Sc. rank: | 86.1 |

| PA | | | |
|---|---|---|---|
| Pos. | 558 | obs: | exp: |
| tct | S | 0 | 56.12 |
| tcc | S | 1 | 49.17 |
| tca | S | 287 | 78.20 |
| tcg | S | 65 | 21.52 |
| agt | S | 0 | 77.66 |
| agc | S | 0 | 70.33 |
| --- | -- | ------ | ------ |
| mPD | | 0.31 | 1.7 |
| | | nPD: | 0.18 |
| | | N. weight: | 1.9 |
| | | Sc. PD: | -0.15 |
| | | Sc. rank: | -33.0 |

| PA | | | |
|---|---|---|---|
| Pos. | 559 | obs: | exp: |
| cgt | R | 0 | 14.65 |
| cgc | R | 0 | 17.28 |
| cga | R | 0 | 33.93 |
| cgg | R | 3 | 29.46 |
| aga | R | 74 | 158.80 |
| agg | R | 276 | 98.92 |
| --- | -- | ------ | ------ |
| mPD | | 0.35 | 0.96 |
| | | nPD: | 0.36 |
| | | N. weight: | 0.96 |
| | | Sc. PD: | 0.092 |
| | | Sc. rank: | 76.9 |

| PA | | | |
|---|---|---|---|
| Pos. | 560 | obs: | exp: |
| cct | P | 24 | 92.95 |
| ccc | P | 328 | 63.67 |
| cca | P | 1 | 139.30 |
| ccg | P | 0 | 57.08 |
| --- | -- | ------ | ------ |
| mPD | | 0.13 | 0.72 |
| | | nPD: | 0.18 |
| | | N. weight: | 2.2 |
| | | Sc. PD: | -0.17 |
| | | Sc. rank: | -30.6 |

| PA | | | |
|---|---|---|---|
| Pos. | 561 | obs: | exp: |
| att | I | 0 | 0.38 |
| atc | I | 0 | 0.26 |
| ata | I | 1 | 0.36 |
| atg | M | 352 | 352.00 |
| --- | -- | ------ | ------ |
| mPD | | 0.0057 | 0.0057 |
| | | nPD: | 1. |
| | | N. weight: | 0.0044 |
| | | Sc. PD: | 0.0031 |
| | | Sc. rank: | 0.9 |

| PA | | | |
|---|---|---|---|
| Pos. | 562 | obs: | exp: |
| ttt | F | 43 | 145.10 |
| ttc | F | 310 | 207.90 |
| --- | -- | ------ | ------ |
| mPD | | 0.21 | 0.48 |
| | | nPD: | 0.44 |
| | | N. weight: | 0.32 |
| | | Sc. PD: | 0.055 |
| | | Sc. rank: | 31.0 |

| PA | | | |
|---|---|---|---|
| Pos. | 563 | obs: | exp: |
| tta | L | 2 | 30.84 |
| ttg | L | 53 | 67.98 |
| ctt | L | 0 | 65.86 |
| ctc | L | 0 | 57.10 |
| cta | L | 43 | 53.18 |
| ctg | L | 255 | 78.03 |
| --- | -- | ------ | ------ |
| mPD | | 0.49 | 1.1 |
| | | nPD: | 0.44 |
| | | N. weight: | 1.2 |
| | | Sc. PD: | 0.2 |
| | | Sc. rank: | 115.6 |

| PA | | | |
|---|---|---|---|
| Pos. | 564 | obs: | exp: |
| tat | Y | 316 | 182.70 |
| tac | Y | 37 | 170.30 |
| --- | -- | ------ | ------ |
| mPD | | 0.19 | 0.50 |
| | | nPD: | 0.38 |
| | | N. weight: | 0.51 |
| | | Sc. PD: | 0.055 |
| | | Sc. rank: | 42.3 |

| PA | | | |
|---|---|---|---|
| Pos. | 565 | obs: | exp: |
| att | I | 1 | 0.75 |
| atc | I | 0 | 0.52 |
| ata | I | 1 | 0.72 |
| gtt | V | 35 | 72.14 |
| gtc | V | 0 | 73.67 |
| gta | V | 29 | 65.78 |
| gtg | V | 287 | 139.40 |
| --- | -- | ------ | ------ |
| mPD | | 0.33 | 0.73 |
| | | nPD: | 0.46 |
| | | N. weight: | 0.71 |
| | | Sc. PD: | 0.13 |
| | | Sc. rank: | 71.2 |

| PA | | | |
|---|---|---|---|
| Pos. | 566 | obs: | exp: |
| cgt | R | 0 | 14.65 |
| cgc | R | 0 | 17.28 |
| cga | R | 1 | 33.93 |
| cgg | R | 0 | 29.46 |
| aga | R | 301 | 158.80 |
| agg | R | 51 | 98.92 |
| --- | -- | ------ | ------ |
| mPD | | 0.25 | 0.96 |
| | | nPD: | 0.26 |
| | | N. weight: | 0.68 |
| | | Sc. PD: | -0.00026 |
| | | Sc. rank: | 24.3 |

| PA | | | |
|---|---|---|---|
| Pos. | 567 | obs: | exp: |
| act | T | 12 | 93.26 |
| acc | T | 336 | 75.75 |
| aca | T | 5 | 154.60 |
| acg | T | 0 | 29.35 |
| --- | -- | ------ | ------ |
| mPD | | 0.093 | 0.69 |
| | | nPD: | 0.14 |
| | | N. weight: | 2. |
| | | Sc. PD: | -0.25 |
| | | Sc. rank: | -67.9 |

| PA | | | |
|---|---|---|---|
| Pos. | 568 | obs: | exp: |
| aat | N | 340 | 184.50 |
| aac | N | 13 | 168.50 |
| --- | -- | ------ | ------ |
| mPD | | 0.071 | 0.50 |
| | | nPD: | 0.14 |
| | | N. weight: | 0.76 |
| | | Sc. PD: | -0.089 |
| | | Sc. rank: | -25.6 |

| PA | | | |
|---|---|---|---|
| Pos. | 569 | obs: | exp: |
| ggt | G | 0 | 43.56 |
| ggc | G | 0 | 47.74 |
| gga | G | 143 | 170.80 |
| ggg | G | 210 | 90.95 |
| --- | -- | ------ | ------ |
| mPD | | 0.48 | 0.67 |
| | | nPD: | 0.73 |
| | | N. weight: | 0.66 |
| | | Sc. PD: | 0.29 |
| | | Sc. rank: | 118.7 |

| PA | | | |
|---|---|---|---|
| Pos. | 570 | obs: | exp: |
| act | T | 212 | 93.26 |
| acc | T | 138 | 75.75 |
| aca | T | 1 | 154.60 |
| acg | T | 2 | 29.35 |
| --- | -- | ------ | ------ |
| mPD | | 0.49 | 0.69 |
| | | nPD: | 0.71 |
| | | N. weight: | 1.1 |
| | | Sc. PD: | 0.46 |
| | | Sc. rank: | 190.4 |

| PA | | | |
|---|---|---|---|
| Pos. | 571 | obs: | exp: |
| tct | S | 6 | 56.12 |
| tcc | S | 337 | 49.17 |
| tca | S | 10 | 78.20 |
| tcg | S | 0 | 21.52 |
| agt | S | 0 | 77.66 |
| agc | S | 0 | 70.33 |
| --- | -- | ------ | ------ |
| mPD | | 0.088 | 1.7 |
| | | nPD: | 0.05 |
| | | N. weight: | 2.7 |
| | | Sc. PD: | -0.54 |
| | | Sc. rank: | -236.8 |

| PA | | | |
|---|---|---|---|
| Pos. | 572 | obs: | exp: |
| aaa | K | 108 | 197.70 |
| aag | K | 245 | 155.30 |
| --- | -- | ------ | ------ |
| mPD | | 0.43 | 0.49 |
| | | nPD: | 0.86 |
| | | N. weight: | 0.21 |
| | | Sc. PD: | 0.12 |
| | | Sc. rank: | 42.1 |

| | | | |
|---|---|---|---|
| PA | | | |
| Pos. | 573 | obs: | exp: |
| att | I | 11 | 132.70 |
| atc | I | 342 | 92.52 |
| ata | I | 0 | 127.80 |
| --- | -- | ------ | ------ |
| mPD | | 0.061 | 0.66 |
| | | nPD: | 0.09 |
| | | N. weight: | 1.8 |
| | | Sc. PD: | -0.3 |
| | | Sc. rank: | -105.2 |

,

| | | | |
|---|---|---|---|
| PA | | | |
| Pos. | 574 | obs: | exp: |
| aaa | K | 182 | 197.70 |
| aag | K | 171 | 155.30 |
| --- | -- | ------ | ------ |
| mPD | | 0.50 | 0.49 |
| | | nPD: | 1.02 |
| | | N. weight: | 0.02 |
| | | Sc. PD: | 0.014 |
| | | Sc. rank: | 4.0 |

,

| | | | |
|---|---|---|---|
| PA | | | |
| Pos. | 575 | obs: | exp: |
| atg | M | 353 | 353.00 |
| --- | -- | ------ | ------ |
| mPD | | 0 | 0 |
| | | nPD: | 1. |
| | | N. weight: | 0. |
| | | Sc. PD: | 0 |
| | | Sc. rank: | 0 |

,

| | | | |
|---|---|---|---|
| PA | | | |
| Pos. | 576 | obs: | exp: |
| aaa | K | 347 | 197.70 |
| aag | K | 6 | 155.30 |
| --- | -- | ------ | ------ |
| mPD | | 0.034 | 0.49 |
| | | nPD: | 0.07 |
| | | N. weight: | 0.76 |
| | | Sc. PD: | -0.14 |
| | | Sc. rank: | -58.3 |

,

| | | | |
|---|---|---|---|
| PA | | | |
| Pos. | 577 | obs: | exp: |
| tgg | W | 353 | 353.00 |
| --- | -- | ------ | ------ |
| mPD | | 0 | 0 |
| | | nPD: | 1. |
| | | N. weight: | 0. |
| | | Sc. PD: | 0 |
| | | Sc. rank: | 0 |

,

| | | | |
|---|---|---|---|
| PA | | | |
| Pos. | 578 | obs: | exp: |
| ggt | G | 135 | 43.56 |
| ggc | G | 204 | 47.74 |
| gga | G | 10 | 170.80 |
| ggg | G | 4 | 90.95 |
| --- | -- | ------ | ------ |
| mPD | | 0.52 | 0.67 |
| | | nPD: | 0.78 |
| | | N. weight: | 1.8 |
| | | Sc. PD: | 0.88 |
| | | Sc. rank: | 338.2 |

,

| | | | |
|---|---|---|---|
| PA | | | |
| Pos. | 579 | obs: | exp: |
| atg | M | 353 | 353.00 |
| --- | -- | ------ | ------ |
| mPD | | 0 | 0 |
| | | nPD: | 1. |
| | | N. weight: | 0. |
| | | Sc. PD: | 0 |
| | | Sc. rank: | 0 |

,

| | | | |
|---|---|---|---|
| PA | | | |
| Pos. | 580 | obs: | exp: |
| gaa | E | 195 | 203.10 |
| gag | E | 158 | 149.90 |
| --- | -- | ------ | ------ |
| mPD | | 0.50 | 0.49 |
| | | nPD: | 1.01 |
| | | N. weight: | 0.015 |
| | | Sc. PD: | 0.011 |
| | | Sc. rank: | 3.1 |

,

| | | | |
|---|---|---|---|
| PA | | | |
| Pos. | 581 | obs: | exp: |
| atg | M | 353 | 353.00 |
| --- | -- | ------ | ------ |
| mPD | | 0 | 0 |
| | | nPD: | 1. |
| | | N. weight: | 0. |
| | | Sc. PD: | 0 |
| | | Sc. rank: | 0 |

,

| | | | |
|---|---|---|---|
| PA | | | |
| Pos. | 582 | obs: | exp: |
| cgt | R | 0 | 14.65 |
| cgc | R | 0 | 17.28 |
| cga | R | 0 | 33.93 |
| cgg | R | 0 | 29.46 |
| aga | R | 75 | 158.80 |
| agg | R | 278 | 98.92 |
| --- | -- | ------ | ------ |
| mPD | | 0.34 | 0.96 |
| | | nPD: | 0.35 |
| | | N. weight: | 1. |
| | | Sc. PD: | 0.082 |
| | | Sc. rank: | 75.2 |

,

| | | | |
|---|---|---|---|
| PA | | | |
| Pos. | 583 | obs: | exp: |
| cgt | R | 56 | 14.65 |
| cgc | R | 73 | 17.28 |
| cga | R | 218 | 33.93 |
| cgg | R | 6 | 29.46 |
| aga | R | 0 | 158.80 |
| agg | R | 0 | 98.92 |
| --- | -- | ------ | ------ |
| mPD | | 0.55 | 0.96 |
| | | nPD: | 0.58 |
| | | N. weight: | 2.5 |
| | | Sc. PD: | 0.75 |
| | | Sc. rank: | 364.0 |

,

| | | | |
|---|---|---|---|
| PA | | | |
| Pos. | 584 | obs: | exp: |
| tgt | C | 68 | 145.50 |
| tgc | C | 285 | 207.50 |
| --- | -- | ------ | ------ |
| mPD | | 0.31 | 0.48 |
| | | nPD: | 0.64 |
| | | N. weight: | 0.18 |
| | | Sc. PD: | 0.065 |
| | | Sc. rank: | 28.4 |

,

| | | | |
|---|---|---|---|
| PA | | | |
| Pos. | 585 | obs: | exp: |
| ttt | F | 1 | 0.41 |
| ttc | F | 0 | 0.59 |
| tta | L | 0 | 30.75 |
| ttg | L | 0 | 67.79 |
| ctt | L | 311 | 65.68 |
| ctc | L | 41 | 56.94 |
| cta | L | 0 | 53.03 |
| ctg | L | 0 | 77.81 |
| --- | -- | ------ | ------ |
| mPD | | 0.21 | 1.1 |
| | | nPD: | 0.19 |
| | | N. weight: | 2. |
| | | Sc. PD: | -0.15 |
| | | Sc. rank: | -15.0 |

,

| | | | |
|---|---|---|---|
| PA | | | |
| Pos. | 586 | obs: | exp: |
| tta | L | 0 | 30.84 |
| ttg | L | 0 | 67.98 |
| ctt | L | 329 | 65.86 |
| ctc | L | 23 | 57.10 |
| cta | L | 1 | 53.18 |
| ctg | L | 0 | 78.03 |
| --- | -- | ------ | ------ |
| mPD | | 0.13 | 1.1 |
| | | nPD: | 0.11 |
| | | N. weight: | 2.2 |
| | | Sc. PD: | -0.31 |
| | | Sc. rank: | -98.1 |

,

| | | | |
|---|---|---|---|
| PA | | | |
| Pos. | 587 | obs: | exp: |
| caa | Q | 256 | 188.20 |
| cag | Q | 97 | 164.80 |
| --- | -- | ------ | ------ |
| mPD | | 0.40 | 0.50 |
| | | nPD: | 0.8 |
| | | N. weight: | 0.13 |
| | | Sc. PD: | 0.067 |
| | | Sc. rank: | 25.2 |

,

| | | | |
|---|---|---|---|
| PA | | | |
| Pos. | 588 | obs: | exp: |
| tct | S | 79 | 55.96 |
| tcc | S | 273 | 49.04 |
| tca | S | 0 | 77.98 |
| tcg | S | 0 | 21.46 |
| cct | P | 0 | 0.26 |
| ccc | P | 1 | 0.18 |
| cca | P | 0 | 0.39 |
| ccg | P | 0 | 0.16 |
| agt | S | 0 | 77.44 |
| agc | S | 0 | 70.13 |
| --- | -- | ------ | ------ |
| mPD | | 0.35 | 1.7 |
| | | nPD: | 0.21 |
| | | N. weight: | 2.1 |
| | | Sc. PD: | -0.11 |
| | | Sc. rank: | 35.9 |

,

| | | | |
|---|---|---|---|
| PA | | | |
| Pos. | 589 | obs: | exp: |
| tta | L | 0 | 30.84 |
| ttg | L | 0 | 67.98 |
| ctt | L | 268 | 65.86 |
| ctc | L | 71 | 57.10 |
| cta | L | 4 | 53.18 |
| ctg | L | 10 | 78.03 |
| --- | -- | ------ | ------ |
| mPD | | 0.38 | 1.1 |
| | | nPD: | 0.34 |
| | | N. weight: | 1.6 |
| | | Sc. PD: | 0.12 |
| | | Sc. rank: | 114.3 |

,

| | | | |
|---|---|---|---|
| PA | | | |
| Pos. | 590 | obs: | exp: |
| caa | Q | 215 | 188.20 |
| cag | Q | 138 | 164.80 |
| --- | -- | ------ | ------ |
| mPD | | 0.48 | 0.50 |
| | | nPD: | 0.96 |
| | | N. weight: | 0.031 |
| | | Sc. PD: | 0.021 |
| | | Sc. rank: | 6.3 |

,

| | | | |
|---|---|---|---|
| PA | | | |
| Pos. | 591 | obs: | exp: |
| caa | Q | 263 | 188.20 |
| cag | Q | 90 | 164.80 |
| --- | -- | ------ | ------ |
| mPD | | 0.38 | 0.50 |
| | | nPD: | 0.77 |
| | | N. weight: | 0.16 |
| | | Sc. PD: | 0.076 |
| | | Sc. rank: | 29.4 |

,

| | | | |
|---|---|---|---|
| PA | | | |
| Pos. | 592 | obs: | exp: |
| att | I | 340 | 132.70 |
| atc | I | 12 | 92.52 |
| ata | I | 1 | 127.80 |
| --- | -- | ------ | ------ |
| mPD | | 0.071 | 0.66 |
| | | nPD: | 0.11 |
| | | N. weight: | 1.3 |
| | | Sc. PD: | -0.19 |
| | | Sc. rank: | -62.5 |

,

| | | | |
|---|---|---|---|
| PA | | | |
| Pos. | 593 | obs: | exp: |
| gaa | E | 111 | 203.10 |
| gag | E | 242 | 149.90 |
| --- | -- | ------ | ------ |
| mPD | | 0.43 | 0.49 |
| | | nPD: | 0.88 |
| | | N. weight: | 0.22 |
| | | Sc. PD: | 0.13 |
| | | Sc. rank: | 44.4 |

,

| | | | |
|---|---|---|---|
| PA | | | |
| Pos. | 594 | obs: | exp: |
| tct | S | 0 | 56.12 |
| tcc | S | 0 | 49.17 |
| tca | S | 0 | 78.20 |
| tcg | S | 0 | 21.52 |
| agt | S | 5 | 77.66 |
| agc | S | 348 | 70.33 |
| --- | -- | ------ | ------ |
| mPD | | 0.028 | 1.7 |
| | | nPD: | 0.02 |
| | | N. weight: | 2.3 |
| | | Sc. PD: | -0.56 |
| | | Sc. rank: | -313.9 |

,

| | | | |
|---|---|---|---|
| PA | | | |
| Pos. | 595 | obs: | exp: |
| atg | M | 353 | 353.00 |
| --- | -- | ------ | ------ |
| mPD | | 0 | 0 |
| | | nPD: | 1. |
| | | N. weight: | 0. |
| | | Sc. PD: | 0 |
| | | Sc. rank: | 0 |

,

| | | | |
|---|---|---|---|
| PA | | | |
| Pos. | 596 | obs: | exp: |
| att | I | 343 | 132.30 |
| atc | I | 8 | 92.25 |
| ata | I | 1 | 127.40 |
| gtt | V | 1 | 0.21 |
| gtc | V | 0 | 0.21 |
| gta | V | 0 | 0.19 |
| gtg | V | 0 | 0.40 |
| --- | -- | ------ | ------ |
| mPD | | 0.056 | 0.67 |
| | | nPD: | 0.08 |
| | | N. weight: | 1.3 |
| | | Sc. PD: | -0.23 |
| | | Sc. rank: | -85.7 |

,

| | | | |
|---|---|---|---|
| PA | | | |
| Pos. | 597 | obs: | exp: |
| gaa | E | 44 | 203.10 |
| gag | E | 309 | 149.90 |
| --- | -- | ------ | ------ |
| mPD | | 0.22 | 0.49 |
| | | nPD: | 0.45 |
| | | N. weight: | 0.68 |
| | | Sc. PD: | 0.12 |
| | | Sc. rank: | 67.1 |

,

| | | | |
|---|---|---|---|
| PA | | | |
| Pos. | 598 | obs: | exp: |
| gct | A | 60 | 88.91 |
| gcc | A | 281 | 72.31 |
| gca | A | 12 | 157.30 |
| gcg | A | 0 | 34.45 |
| --- | -- | ------ | ------ |
| mPD | | 0.34 | 0.69 |
| | | nPD: | 0.49 |
| | | N. weight: | 1.4 |
| | | Sc. PD: | 0.31 |
| | | Sc. rank: | 166.8 |

,

| | | | |
|---|---|---|---|
| PA | | | |
| Pos. | 599 | obs: | exp: |
| gaa | E | 98 | 203.10 |
| gag | E | 255 | 149.90 |
| --- | -- | ------ | ------ |
| mPD | | 0.40 | 0.49 |
| | | nPD: | 0.82 |
| | | N. weight: | 0.29 |
| | | Sc. PD: | 0.15 |
| | | Sc. rank: | 56.7 |

,

| | | | |
|---|---|---|---|
| PA | | | |
| Pos. | 600 | obs: | exp: |
| tct | S | 269 | 56.12 |
| tcc | S | 82 | 49.17 |
| tca | S | 0 | 78.20 |
| tcg | S | 2 | 21.52 |
| agt | S | 0 | 77.66 |
| agc | S | 0 | 70.33 |
| --- | -- | ------ | ------ |
| mPD | | 0.37 | 1.7 |
| | | nPD: | 0.22 |
| | | N. weight: | 2. |
| | | Sc. PD: | -0.084 |
| | | Sc. rank: | 43.6 |

,

| | | | |
|---|---|---|---|
| PA | | | |
| Pos. | 601 | obs: | exp: |
| tct | S | 312 | 56.12 |
| tcc | S | 36 | 49.17 |
| tca | S | 4 | 78.20 |
| tcg | S | 1 | 21.52 |
| agt | S | 0 | 77.66 |
| agc | S | 0 | 70.33 |
| --- | -- | ------ | ------ |
| mPD | | 0.21 | 1.7 |
| | | nPD: | 0.13 |
| | | N. weight: | 2.2 |
| | | Sc. PD: | -0.29 |
| | | Sc. rank: | -85.8 |

,

| | | | |
|---|---|---|---|
| PA | | | |
| Pos. | 602 | obs: | exp: |
| att | I | 0 | 0.38 |
| atc | I | 1 | 0.26 |
| ata | I | 0 | 0.36 |
| gtt | V | 38 | 72.35 |
| gtc | V | 306 | 73.88 |
| gta | V | 8 | 65.97 |
| gtg | V | 0 | 139.80 |
| --- | -- | ------ | ------ |
| mPD | | 0.24 | 0.73 |
| | | nPD: | 0.33 |
| | | N. weight: | 1.7 |
| | | Sc. PD: | 0.1 |
| | | Sc. rank: | 105.9 |

,

| PA | | | |
|---|---|---|---|
| Pos. | 603 | obs: | exp: |
| cgt | R | 0 | 0.37 |
| cgc | R | 0 | 0.44 |
| cga | R | 0 | 0.86 |
| cgg | R | 0 | 0.75 |
| aaa | K | 335 | 192.60 |
| aag | K | 9 | 151.40 |
| aga | R | 9 | 4.05 |
| agg | R | 0 | 2.52 |
| --- | -- | ------ | ------ |
| mPD | | 0.10 | 0.56 |
| | | nPD: | 0.18 |
| | | N. weight: | 0.73 |
| | | Sc. PD: | -0.06 |
| | | Sc. rank: | -14.4 |

| PA | | | |
|---|---|---|---|
| Pos. | 604 | obs: | exp: |
| gaa | E | 79 | 203.10 |
| gag | E | 274 | 149.90 |
| --- | -- | ------ | ------ |
| mPD | | 0.35 | 0.49 |
| | | nPD: | 0.71 |
| | | N. weight: | 0.4 |
| | | Sc. PD: | 0.17 |
| | | Sc. rank: | 70.9 |

| PA | | | |
|---|---|---|---|
| Pos. | 605 | obs: | exp: |
| cgt | R | 0 | 0.08 |
| cgc | R | 0 | 0.10 |
| cga | R | 0 | 0.19 |
| cgg | R | 0 | 0.17 |
| aaa | K | 133 | 196.50 |
| aag | K | 218 | 154.50 |
| aga | R | 2 | 0.90 |
| agg | R | 0 | 0.56 |
| --- | -- | ------ | ------ |
| mPD | | 0.48 | 0.51 |
| | | nPD: | 0.96 |
| | | N. weight: | 0.12 |
| | | Sc. PD: | 0.079 |
| | | Sc. rank: | 24.1 |

| PA | | | |
|---|---|---|---|
| Pos. | 606 | obs: | exp: |
| gat | D | 8 | 188.30 |
| gac | D | 345 | 164.70 |
| --- | -- | ------ | ------ |
| mPD | | 0.044 | 0.50 |
| | | nPD: | 0.09 |
| | | N. weight: | 0.99 |
| | | Sc. PD: | -0.17 |
| | | Sc. rank: | -61.3 |

| PA | | | |
|---|---|---|---|
| Pos. | 607 | obs: | exp: |
| tta | L | 0 | 2.27 |
| ttg | L | 0 | 5.01 |
| ctt | L | 0 | 4.85 |
| ctc | L | 0 | 4.21 |
| cta | L | 0 | 3.92 |
| ctg | L | 26 | 5.75 |
| atg | M | 327 | 327.00 |
| --- | -- | ------ | ------ |
| mPD | | 0.14 | 0.22 |
| | | nPD: | 0.61 |
| | | N. weight: | 0.17 |
| | | Sc. PD: | 0.057 |
| | | Sc. rank: | 25.8 |

| PA | | | |
|---|---|---|---|
| Pos. | 608 | obs: | exp: |
| act | T | 22 | 93.26 |
| acc | T | 321 | 75.75 |
| aca | T | 10 | 154.60 |
| acg | T | 0 | 29.35 |
| --- | -- | ------ | ------ |
| mPD | | 0.17 | 0.69 |
| | | nPD: | 0.25 |
| | | N. weight: | 1.8 |
| | | Sc. PD: | -0.031 |
| | | Sc. rank: | 57.2 |

| PA | | | |
|---|---|---|---|
| Pos. | 609 | obs: | exp: |
| cgt | R | 0 | 0.62 |
| cgc | R | 0 | 0.73 |
| cga | R | 0 | 1.44 |
| cgg | R | 0 | 1.25 |
| aaa | K | 246 | 189.30 |
| aag | K | 92 | 148.70 |
| aga | R | 15 | 6.75 |
| agg | R | 0 | 4.20 |
| --- | -- | ------ | ------ |
| mPD | | 0.47 | 0.60 |
| | | nPD: | 0.78 |
| | | N. weight: | 0.15 |
| | | Sc. PD: | 0.075 |
| | | Sc. rank: | 28.6 |

| PA | | | |
|---|---|---|---|
| Pos. | 610 | obs: | exp: |
| aaa | K | 2 | 1.12 |
| aag | K | 0 | 0.88 |
| gat | D | 0 | 2.13 |
| gac | D | 4 | 1.87 |
| gaa | E | 330 | 196.80 |
| gag | E | 12 | 145.20 |
| ggt | G | 0 | 0.62 |
| ggc | G | 0 | 0.68 |
| gga | G | 5 | 2.42 |
| ggg | G | 0 | 1.29 |
| --- | -- | ------ | ------ |
| mPD | | 0.13 | 0.54 |
| | | nPD: | 0.23 |
| | | N. weight: | 0.65 |
| | | Sc. PD: | -0.019 |
| | | Sc. rank: | 18.6 |

| PA | | | |
|---|---|---|---|
| Pos. | 611 | obs: | exp: |
| ttt | F | 6 | 145.10 |
| ttc | F | 347 | 207.90 |
| --- | -- | ------ | ------ |
| mPD | | 0.034 | 0.48 |
| | | nPD: | 0.07 |
| | | N. weight: | 0.69 |
| | | Sc. PD: | -0.13 |
| | | Sc. rank: | -51.2 |

| PA | | | |
|---|---|---|---|
| Pos. | 612 | obs: | exp: |
| ttt | F | 338 | 145.10 |
| ttc | F | 15 | 207.90 |
| --- | -- | ------ | ------ |
| mPD | | 0.082 | 0.48 |
| | | nPD: | 0.17 |
| | | N. weight: | 1.1 |
| | | Sc. PD: | -0.098 |
| | | Sc. rank: | -26.2 |

| PA | | | |
|---|---|---|---|
| Pos. | 613 | obs: | exp: |
| gaa | E | 290 | 203.10 |
| gag | E | 63 | 149.90 |
| --- | -- | ------ | ------ |
| mPD | | 0.29 | 0.49 |
| | | nPD: | 0.6 |
| | | N. weight: | 0.22 |
| | | Sc. PD: | 0.072 |
| | | Sc. rank: | 33.4 |

| PA | | | |
|---|---|---|---|
| Pos. | 614 | obs: | exp: |
| aat | N | 7 | 184.50 |
| aac | N | 346 | 168.50 |
| --- | -- | ------ | ------ |
| mPD | | 0.039 | 0.50 |
| | | nPD: | 0.08 |
| | | N. weight: | 0.98 |
| | | Sc. PD: | -0.18 |
| | | Sc. rank: | -70.8 |

| PA | | | |
|---|---|---|---|
| Pos. | 615 | obs: | exp: |
| cgt | R | 0 | 0.04 |
| cgc | R | 0 | 0.05 |
| cga | R | 0 | 0.10 |
| cgg | R | 0 | 0.08 |
| aaa | K | 300 | 197.10 |
| aag | K | 52 | 154.90 |
| aga | R | 1 | 0.45 |
| agg | R | 0 | 0.28 |
| --- | -- | ------ | ------ |
| mPD | | 0.26 | 0.50 |
| | | nPD: | 0.51 |
| | | N. weight: | 0.31 |
| | | Sc. PD: | 0.075 |
| | | Sc. rank: | 40.0 |

| PA | | | |
|---|---|---|---|
| Pos. | 616 | obs: | exp: |
| tct | S | 2 | 55.96 |
| tcc | S | 0 | 49.04 |
| tca | S | 309 | 77.98 |
| tcg | S | 41 | 21.46 |
| agt | S | 0 | 77.44 |
| agc | S | 0 | 70.13 |
| gct | A | 0 | 0.25 |
| gcc | A | 0 | 0.20 |
| gca | A | 1 | 0.45 |
| gcg | A | 0 | 0.10 |
| --- | -- | ------ | ------ |
| mPD | | 0.22 | 1.7 |
| | | nPD: | 0.13 |
| | | N. weight: | 1.9 |
| | | Sc. PD: | -0.24 |
| | | Sc. rank: | -71.2 |

| PA | | | |
|---|---|---|---|
| Pos. | 617 | obs: | exp: |
| gaa | E | 295 | 203.10 |
| gag | E | 58 | 149.90 |
| --- | -- | ------ | ------ |
| mPD | | 0.28 | 0.49 |
| | | nPD: | 0.56 |
| | | N. weight: | 0.25 |
| | | Sc. PD: | 0.071 |
| | | Sc. rank: | 35.2 |

| PA | | | |
|---|---|---|---|
| Pos. | 618 | obs: | exp: |
| atg | M | 1 | 1.00 |
| act | T | 0 | 92.47 |
| acc | T | 0 | 75.10 |
| aca | T | 331 | 153.30 |
| acg | T | 19 | 29.10 |
| aaa | K | 1 | 1.12 |
| aag | K | 1 | 0.88 |
| --- | -- | ------ | ------ |
| mPD | | 0.13 | 0.70 |
| | | nPD: | 0.18 |
| | | N. weight: | 1.1 |
| | | Sc. PD: | -0.083 |
| | | Sc. rank: | -19.7 |

| PA | | | |
|---|---|---|---|
| Pos. | 619 | obs: | exp: |
| tgg | W | 353 | 353.00 |
| --- | -- | ------ | ------ |
| mPD | | 0 | 0 |
| | | nPD: | 1. |
| | | N. weight: | 0. |
| | | Sc. PD: | 0 |
| | | Sc. rank: | 0 |

| PA | | | |
|---|---|---|---|
| Pos. | 620 | obs: | exp: |
| cct | P | 3 | 92.95 |
| ccc | P | 67 | 63.67 |
| cca | P | 276 | 139.30 |
| ccg | P | 7 | 57.08 |
| --- | -- | ------ | ------ |
| mPD | | 0.35 | 0.72 |
| | | nPD: | 0.49 |
| | | N. weight: | 0.74 |
| | | Sc. PD: | 0.16 |
| | | Sc. rank: | 88.0 |

| PA | | | |
|---|---|---|---|
| Pos. | 621 | obs: | exp: |
| att | I | 267 | 132.00 |
| atc | I | 83 | 91.99 |
| ata | I | 1 | 127.10 |
| gtt | V | 2 | 0.41 |
| gtc | V | 0 | 0.42 |
| gta | V | 0 | 0.37 |
| gtg | V | 0 | 0.79 |
| --- | -- | ------ | ------ |
| mPD | | 0.38 | 0.67 |
| | | nPD: | 0.56 |
| | | N. weight: | 0.78 |
| | | Sc. PD: | 0.22 |
| | | Sc. rank: | 110.2 |

| PA | | | |
|---|---|---|---|
| Pos. | 622 | obs: | exp: |
| ggt | G | 3 | 43.56 |
| ggc | G | 15 | 47.74 |
| gga | G | 266 | 170.80 |
| ggg | G | 69 | 90.95 |
| --- | -- | ------ | ------ |
| mPD | | 0.39 | 0.67 |
| | | nPD: | 0.59 |
| | | N. weight: | 0.34 |
| | | Sc. PD: | 0.11 |
| | | Sc. rank: | 50.8 |

| PA | | | |
|---|---|---|---|
| Pos. | 623 | obs: | exp: |
| gaa | E | 264 | 203.10 |
| gag | E | 89 | 149.90 |
| --- | -- | ------ | ------ |
| mPD | | 0.38 | 0.49 |
| | | nPD: | 0.77 |
| | | N. weight: | 0.11 |
| | | Sc. PD: | 0.054 |
| | | Sc. rank: | 20.9 |

| PA | | | |
|---|---|---|---|
| Pos. | 624 | obs: | exp: |
| tct | S | 1 | 56.12 |
| tcc | S | 41 | 49.17 |
| tca | S | 276 | 78.20 |
| tcg | S | 35 | 21.52 |
| agt | S | 0 | 77.66 |
| agc | S | 0 | 70.33 |
| --- | -- | ------ | ------ |
| mPD | | 0.37 | 1.7 |
| | | nPD: | 0.22 |
| | | N. weight: | 1.5 |
| | | Sc. PD: | -0.066 |
| | | Sc. rank: | 35.2 |

| PA | | | |
|---|---|---|---|
| Pos. | 625 | obs: | exp: |
| cct | P | 90 | 92.95 |
| ccc | P | 263 | 63.67 |
| cca | P | 0 | 139.30 |
| ccg | P | 0 | 57.08 |
| --- | -- | ------ | ------ |
| mPD | | 0.38 | 0.72 |
| | | nPD: | 0.53 |
| | | N. weight: | 1.6 |
| | | Sc. PD: | 0.41 |
| | | Sc. rank: | 215.3 |

| PA | | | |
|---|---|---|---|
| Pos. | 626 | obs: | exp: |
| caa | Q | 1 | 0.53 |
| cag | Q | 0 | 0.47 |
| cgt | R | 0 | 0.71 |
| cgc | R | 0 | 0.83 |
| cga | R | 0 | 1.63 |
| cgg | R | 0 | 1.42 |
| aaa | K | 294 | 187.60 |
| aag | K | 41 | 147.40 |
| aga | R | 15 | 7.65 |
| agg | R | 2 | 4.76 |
| --- | -- | ------ | ------ |
| mPD | | 0.31 | 0.62 |
| | | nPD: | 0.5 |
| | | N. weight: | 0.4 |
| | | Sc. PD: | 0.091 |
| | | Sc. rank: | 49.5 |

| PA | | | |
|---|---|---|---|
| Pos. | 627 | obs: | exp: |
| ggt | G | 0 | 43.56 |
| ggc | G | 0 | 47.74 |
| gga | G | 154 | 170.80 |
| ggg | G | 199 | 90.95 |
| --- | -- | ------ | ------ |
| mPD | | 0.49 | 0.67 |
| | | nPD: | 0.74 |
| | | N. weight: | 0.61 |
| | | Sc. PD: | 0.28 |
| | | Sc. rank: | 113.0 |

| | | | |
|---|---|---|---|
| PA | | | |
| Pos. | 628 | obs: | exp: |
| gtt | V | 4 | 72.35 |
| gtc | V | 0 | 73.88 |
| gta | V | 77 | 65.97 |
| gtg | V | 271 | 139.80 |
| --- | - | 1 | 1.00 |
| mPD | | 0.36 | 0.72 |
| | | nPD: | 0.5 |
| | | N. weight: | 0.79 |
| | | Sc. PD: | 0.18 |
| | | Sc. rank: | 97.5 |

| | | | |
|---|---|---|---|
| PA | | | |
| Pos. | 629 | obs: | exp: |
| gaa | E | 89 | 203.10 |
| gag | E | 264 | 149.90 |
| mPD | | 0.38 | 0.49 |
| | | nPD: | 0.77 |
| | | N. weight: | 0.34 |
| | | Sc. PD: | 0.17 |
| | | Sc. rank: | 63.9 |

| | | | |
|---|---|---|---|
| PA | | | |
| Pos. | 630 | obs: | exp: |
| gtt | V | 0 | 0.41 |
| gtc | V | 0 | 0.42 |
| gta | V | 2 | 0.37 |
| gtg | V | 0 | 0.79 |
| gaa | E | 347 | 202.00 |
| gag | E | 4 | 149.00 |
| mPD | | 0.034 | 0.50 |
| | | nPD: | 0.07 |
| | | N. weight: | 0.76 |
| | | Sc. PD: | -0.14 |
| | | Sc. rank: | -59.5 |

| | | | |
|---|---|---|---|
| PA | | | |
| Pos. | 631 | obs: | exp: |
| tgt | C | 0 | 0.82 |
| tgc | C | 2 | 1.18 |
| ggt | G | 80 | 43.31 |
| ggc | G | 261 | 47.47 |
| gga | G | 9 | 169.80 |
| ggg | G | 1 | 90.43 |
| mPD | | 0.41 | 0.68 |
| | | nPD: | 0.6 |
| | | N. weight: | 2. |
| | | Sc. PD: | 0.64 |
| | | Sc. rank: | 301.4 |

| | | | |
|---|---|---|---|
| PA | | | |
| Pos. | 632 | obs: | exp: |
| tct | S | 28 | 56.12 |
| tcc | S | 323 | 49.17 |
| tca | S | 2 | 78.20 |
| tcg | S | 0 | 21.52 |
| agt | S | 0 | 77.66 |
| agc | S | 0 | 70.33 |
| mPD | | 0.16 | 1.7 |
| | | nPD: | 0.09 |
| | | N. weight: | 2.5 |
| | | Sc. PD: | -0.41 |
| | | Sc. rank: | -142.4 |

| | | | |
|---|---|---|---|
| PA | | | |
| Pos. | 633 | obs: | exp: |
| att | I | 250 | 132.70 |
| atc | I | 102 | 92.52 |
| ata | I | 1 | 127.80 |
| mPD | | 0.42 | 0.66 |
| | | nPD: | 0.63 |
| | | N. weight: | 0.72 |
| | | Sc. PD: | 0.25 |
| | | Sc. rank: | 113.3 |

| | | | |
|---|---|---|---|
| PA | | | |
| Pos. | 634 | obs: | exp: |
| ggt | G | 0 | 43.56 |
| ggc | G | 0 | 47.74 |
| gga | G | 97 | 170.80 |
| ggg | G | 256 | 90.95 |
| mPD | | 0.40 | 0.67 |
| | | nPD: | 0.6 |
| | | N. weight: | 0.91 |
| | | Sc. PD: | 0.29 |
| | | Sc. rank: | 137.3 |

| | | | |
|---|---|---|---|
| PA | | | |
| Pos. | 635 | obs: | exp: |
| aaa | K | 33 | 197.70 |
| aag | K | 320 | 155.30 |
| mPD | | 0.17 | 0.49 |
| | | nPD: | 0.34 |
| | | N. weight: | 0.75 |
| | | Sc. PD: | 0.058 |
| | | Sc. rank: | 54.7 |

| | | | |
|---|---|---|---|
| PA | | | |
| Pos. | 636 | obs: | exp: |
| gtt | V | 9 | 72.56 |
| gtc | V | 0 | 74.09 |
| gta | V | 78 | 66.16 |
| gtg | V | 266 | 140.20 |
| mPD | | 0.38 | 0.72 |
| | | nPD: | 0.53 |
| | | N. weight: | 0.73 |
| | | Sc. PD: | 0.19 |
| | | Sc. rank: | 98.1 |

| | | | |
|---|---|---|---|
| PA | | | |
| Pos. | 637 | obs: | exp: |
| tgt | C | 6 | 145.50 |
| tgc | C | 347 | 207.50 |
| mPD | | 0.034 | 0.48 |
| | | nPD: | 0.07 |
| | | N. weight: | 0.69 |
| | | Sc. PD: | -0.13 |
| | | Sc. rank: | -51.7 |

| | | | |
|---|---|---|---|
| PA | | | |
| Pos. | 638 | obs: | exp: |
| cgt | R | 0 | 14.65 |
| cgc | R | 0 | 17.28 |
| cga | R | 2 | 33.93 |
| cgg | R | 3 | 29.46 |
| aga | R | 275 | 158.80 |
| agg | R | 73 | 98.92 |
| mPD | | 0.37 | 0.96 |
| | | nPD: | 0.38 |
| | | N. weight: | 0.52 |
| | | Sc. PD: | 0.059 |
| | | Sc. rank: | 43.9 |

| | | | |
|---|---|---|---|
| PA | | | |
| Pos. | 639 | obs: | exp: |
| act | T | 68 | 93.26 |
| acc | T | 123 | 75.75 |
| aca | T | 159 | 154.60 |
| acg | T | 3 | 29.35 |
| mPD | | 0.64 | 0.69 |
| | | nPD: | 0.93 |
| | | N. weight: | 0.19 |
| | | Sc. PD: | 0.12 |
| | | Sc. rank: | 37.1 |

| | | | |
|---|---|---|---|
| PA | | | |
| Pos. | 640 | obs: | exp: |
| tta | L | 180 | 30.84 |
| ttg | L | 66 | 67.98 |
| ctt | L | 0 | 65.86 |
| ctc | L | 0 | 57.10 |
| cta | L | 20 | 53.18 |
| ctg | L | 87 | 78.03 |
| mPD | | 0.92 | 1.1 |
| | | nPD: | 0.82 |
| | | N. weight: | 1.3 |
| | | Sc. PD: | 0.72 |
| | | Sc. rank: | 264.9 |

| | | | |
|---|---|---|---|
| PA | | | |
| Pos. | 641 | obs: | exp: |
| tta | L | 29 | 30.84 |
| ttg | L | 3 | 67.98 |
| ctt | L | 0 | 65.86 |
| ctc | L | 0 | 57.10 |
| cta | L | 223 | 53.18 |
| ctg | L | 98 | 78.03 |
| mPD | | 0.58 | 1.1 |
| | | nPD: | 0.52 |
| | | N. weight: | 1.4 |
| | | Sc. PD: | 0.35 |
| | | Sc. rank: | 187.3 |

| | | | |
|---|---|---|---|
| PA | | | |
| Pos. | 642 | obs: | exp: |
| gct | A | 0 | 88.91 |
| gcc | A | 0 | 72.31 |
| gca | A | 343 | 157.30 |
| gcg | A | 10 | 34.45 |
| mPD | | 0.055 | 0.69 |
| | | nPD: | 0.08 |
| | | N. weight: | 1.1 |
| | | Sc. PD: | -0.19 |
| | | Sc. rank: | -77.0 |

| | | | |
|---|---|---|---|
| PA | | | |
| Pos. | 643 | obs: | exp: |
| aaa | K | 245 | 197.70 |
| aag | K | 108 | 155.30 |
| mPD | | 0.43 | 0.49 |
| | | nPD: | 0.86 |
| | | N. weight: | 0.07 |
| | | Sc. PD: | 0.041 |
| | | Sc. rank: | 14.0 |

| | | | |
|---|---|---|---|
| PA | | | |
| Pos. | 644 | obs: | exp: |
| tct | S | 348 | 56.12 |
| tcc | S | 1 | 49.17 |
| tca | S | 3 | 78.20 |
| tcg | S | 1 | 21.52 |
| agt | S | 0 | 77.66 |
| agc | S | 0 | 70.33 |
| mPD | | 0.028 | 1.7 |
| | | nPD: | 0.02 |
| | | N. weight: | 2.7 |
| | | Sc. PD: | -0.63 |
| | | Sc. rank: | -354.5 |

| | | | |
|---|---|---|---|
| PA | | | |
| Pos. | 645 | obs: | exp: |
| gtt | V | 0 | 72.56 |
| gtc | V | 0 | 74.09 |
| gta | V | 134 | 66.16 |
| gtg | V | 219 | 140.20 |
| mPD | | 0.47 | 0.72 |
| | | nPD: | 0.66 |
| | | N. weight: | 0.84 |
| | | Sc. PD: | 0.31 |
| | | Sc. rank: | 135.8 |

| | | | |
|---|---|---|---|
| PA | | | |
| Pos. | 646 | obs: | exp: |
| ttt | F | 9 | 145.10 |
| ttc | F | 344 | 207.90 |
| mPD | | 0.050 | 0.48 |
| | | nPD: | 0.1 |
| | | N. weight: | 0.64 |
| | | Sc. PD: | -0.1 |
| | | Sc. rank: | -34.1 |

| | | | |
|---|---|---|---|
| PA | | | |
| Pos. | 647 | obs: | exp: |
| aat | N | 2 | 184.50 |
| aac | N | 351 | 168.50 |
| mPD | | 0.011 | 0.50 |
| | | nPD: | 0.02 |
| | | N. weight: | 1.1 |
| | | Sc. PD: | -0.25 |
| | | Sc. rank: | -138.3 |

| | | | |
|---|---|---|---|
| PA | | | |
| Pos. | 648 | obs: | exp: |
| tct | S | 0 | 54.05 |
| tcc | S | 0 | 47.36 |
| tca | S | 0 | 75.32 |
| tcg | S | 0 | 20.72 |
| aat | N | 13 | 6.79 |
| aac | N | 0 | 6.21 |
| agt | S | 91 | 74.80 |
| agc | S | 249 | 67.74 |
| mPD | | 0.49 | 1.7 |
| | | nPD: | 0.29 |
| | | N. weight: | 1.5 |
| | | Sc. PD: | 0.033 |
| | | Sc. rank: | 70.7 |

| | | | |
|---|---|---|---|
| PA | | | |
| Pos. | 649 | obs: | exp: |
| tta | L | 11 | 30.84 |
| ttg | L | 76 | 67.98 |
| ctt | L | 0 | 65.86 |
| ctc | L | 1 | 57.10 |
| cta | L | 230 | 53.18 |
| ctg | L | 35 | 78.03 |
| mPD | | 0.81 | 1.1 |
| | | nPD: | 0.73 |
| | | N. weight: | 1.3 |
| | | Sc. PD: | 0.59 |
| | | Sc. rank: | 241.1 |

| | | | |
|---|---|---|---|
| PA | | | |
| Pos. | 650 | obs: | exp: |
| tat | Y | 321 | 182.70 |
| tac | Y | 32 | 170.30 |
| mPD | | 0.17 | 0.50 |
| | | nPD: | 0.33 |
| | | N. weight: | 0.56 |
| | | Sc. PD: | 0.036 |
| | | Sc. rank: | 36.6 |

| | | | |
|---|---|---|---|
| PA | | | |
| Pos. | 651 | obs: | exp: |
| gct | A | 0 | 88.91 |
| gcc | A | 0 | 72.31 |
| gca | A | 352 | 157.30 |
| gcg | A | 1 | 34.45 |
| mPD | | 0.0057 | 0.69 |
| | | nPD: | 0.01 |
| | | N. weight: | 1.2 |
| | | Sc. PD: | -0.3 |
| | | Sc. rank: | -187.4 |

| | | | |
|---|---|---|---|
| PA | | | |
| Pos. | 652 | obs: | exp: |
| tct | S | 344 | 56.12 |
| tcc | S | 9 | 49.17 |
| tca | S | 0 | 78.20 |
| tcg | S | 0 | 21.52 |
| agt | S | 0 | 77.66 |
| agc | S | 0 | 70.33 |
| mPD | | 0.050 | 1.7 |
| | | nPD: | 0.03 |
| | | N. weight: | 2.6 |
| | | Sc. PD: | -0.59 |
| | | Sc. rank: | -307.9 |

| | | | |
|---|---|---|---|
| PA | | | |
| Pos. | 653 | obs: | exp: |
| cct | P | 1 | 92.95 |
| ccc | P | 0 | 63.67 |
| cca | P | 339 | 139.30 |
| ccg | P | 13 | 57.08 |
| mPD | | 0.077 | 0.72 |
| | | nPD: | 0.11 |
| | | N. weight: | 1.2 |
| | | Sc. PD: | -0.18 |
| | | Sc. rank: | -62.0 |

| | | | |
|---|---|---|---|
| PA | | | |
| Pos. | 654 | obs: | exp: |
| caa | Q | 342 | 188.20 |
| cag | Q | 11 | 164.80 |
| mPD | | 0.061 | 0.50 |
| | | nPD: | 0.12 |
| | | N. weight: | 0.76 |
| | | Sc. PD: | -0.1 |
| | | Sc. rank: | -31.9 |

| | | | |
|---|---|---|---|
| PA | | | |
| Pos. | 655 | obs: | exp: |
| tta | L | 0 | 30.84 |
| ttg | L | 1 | 67.98 |
| ctt | L | 82 | 65.86 |
| ctc | L | 267 | 57.10 |
| cta | L | 3 | 53.18 |
| ctg | L | 0 | 78.03 |
| mPD | | 0.38 | 1.1 |
| | | nPD: | 0.34 |
| | | N. weight: | 1.8 |
| | | Sc. PD: | 0.13 |
| | | Sc. rank: | 129.2 |

| | | | |
|---|---|---|---|
| PA | | | |
| Pos. | 656 | obs: | exp: |
| gaa | E | 31 | 203.10 |
| gag | E | 322 | 149.90 |
| mPD | | 0.16 | 0.49 |
| | | nPD: | 0.33 |
| | | N. weight: | 0.82 |
| | | Sc. PD: | 0.05 |
| | | Sc. rank: | 51.4 |

| | | | |
|---|---|---|---|
| PA | | | |
| Pos. | 657 | obs: | exp: |
| gaa | E | 0 | 0.58 |
| gag | E | 1 | 0.42 |
| ggt | G | 5 | 43.43 |
| ggc | G | 0 | 47.60 |
| gga | G | 116 | 170.30 |
| ggg | G | 231 | 90.69 |
| mPD | | 0.47 | 0.67 |
| | | nPD: | 0.7 |
| | | N. weight: | 0.71 |
| | | Sc. PD: | 0.3 |
| | | Sc. rank: | 123.5 |

| PA | | | |
|---|---|---|---|
| Pos. | 658 | obs: | exp: |
| ttt | F | 268 | 145.10 |
| ttc | F | 85 | 207.90 |
| --- | -- | ------ | ------ |
| mPD | | 0.37 | 0.48 |
| | | nPD: | 0.76 |
| | | N. weight: | 0.39 |
| | | Sc. PD: | 0.19 |
| | | Sc. rank: | 73.2 |

| PA | | | |
|---|---|---|---|
| Pos. | 659 | obs: | exp: |
| tct | S | 0 | 56.12 |
| tcc | S | 0 | 49.17 |
| tca | S | 316 | 78.20 |
| tcg | S | 37 | 21.52 |
| agt | S | 0 | 77.66 |
| agc | S | 0 | 70.33 |
| --- | -- | ------ | ------ |
| mPD | | 0.19 | 1.7 |
| | | nPD: | 0.11 |
| | | N. weight: | 2. |
| | | Sc. PD: | -0.29 |
| | | Sc. rank: | -94.8 |

| PA | | | |
|---|---|---|---|
| Pos. | 660 | obs: | exp: |
| gct | A | 328 | 88.91 |
| gcc | A | 14 | 72.31 |
| gca | A | 11 | 157.30 |
| gcg | A | 0 | 34.45 |
| --- | -- | ------ | ------ |
| mPD | | 0.13 | 0.69 |
| | | nPD: | 0.2 |
| | | N. weight: | 1.6 |
| | | Sc. PD: | -0.11 |
| | | Sc. rank: | 5.1 |

| PA | | | |
|---|---|---|---|
| Pos. | 661 | obs: | exp: |
| gaa | E | 281 | 203.10 |
| gag | E | 72 | 149.90 |
| --- | -- | ------ | ------ |
| mPD | | 0.33 | 0.49 |
| | | nPD: | 0.67 |
| | | N. weight: | 0.18 |
| | | Sc. PD: | 0.068 |
| | | Sc. rank: | 29.2 |

| PA | | | |
|---|---|---|---|
| Pos. | 662 | obs: | exp: |
| tta | L | 0 | 0.09 |
| ttg | L | 1 | 0.19 |
| tct | S | 21 | 55.96 |
| tcc | S | 1 | 49.04 |
| tca | S | 215 | 77.98 |
| tcg | S | 115 | 21.46 |
| ctt | L | 0 | 0.19 |
| ctc | L | 0 | 0.16 |
| cta | L | 0 | 0.15 |
| ctg | L | 0 | 0.22 |
| agt | S | 0 | 77.44 |
| agc | S | 0 | 70.13 |
| --- | -- | ------ | ------ |
| mPD | | 0.52 | 1.7 |
| | | nPD: | 0.31 |
| | | N. weight: | 1.7 |
| | | Sc. PD: | 0.081 |
| | | Sc. rank: | 98.8 |

| PA | | | |
|---|---|---|---|
| Pos. | 663 | obs: | exp: |
| cgt | R | 0 | 14.65 |
| cgc | R | 0 | 17.28 |
| cga | R | 0 | 33.93 |
| cgg | R | 0 | 29.46 |
| aga | R | 331 | 158.80 |
| agg | R | 22 | 98.92 |
| --- | -- | ------ | ------ |
| mPD | | 0.12 | 0.96 |
| | | nPD: | 0.12 |
| | | N. weight: | 0.91 |
| | | Sc. PD: | -0.12 |
| | | Sc. rank: | -36.6 |

| PA | | | |
|---|---|---|---|
| Pos. | 664 | obs: | exp: |
| aaa | K | 270 | 197.70 |
| aag | K | 83 | 155.30 |
| --- | -- | ------ | ------ |
| mPD | | 0.36 | 0.49 |
| | | nPD: | 0.73 |
| | | N. weight: | 0.15 |
| | | Sc. PD: | 0.068 |
| | | Sc. rank: | 27.6 |

| PA | | | |
|---|---|---|---|
| Pos. | 665 | obs: | exp: |
| tta | L | 34 | 30.84 |
| ttg | L | 228 | 67.98 |
| ctt | L | 0 | 65.86 |
| ctc | L | 0 | 57.10 |
| cta | L | 1 | 53.18 |
| ctg | L | 90 | 78.03 |
| --- | -- | ------ | ------ |
| mPD | | 0.56 | 1.1 |
| | | nPD: | 0.51 |
| | | N. weight: | 1.3 |
| | | Sc. PD: | 0.29 |
| | | Sc. rank: | 158.6 |

| PA | | | |
|---|---|---|---|
| Pos. | 666 | obs: | exp: |
| tta | L | 0 | 30.84 |
| ttg | L | 0 | 67.98 |
| ctt | L | 232 | 65.86 |
| ctc | L | 85 | 57.10 |
| cta | L | 26 | 53.18 |
| ctg | L | 10 | 78.03 |
| --- | -- | ------ | ------ |
| mPD | | 0.51 | 1.1 |
| | | nPD: | 0.45 |
| | | N. weight: | 1.3 |
| | | Sc. PD: | 0.23 |
| | | Sc. rank: | 126.8 |

| PA | | | |
|---|---|---|---|
| Pos. | 667 | obs: | exp: |
| tta | L | 0 | 30.84 |
| ttg | L | 0 | 67.98 |
| ctt | L | 7 | 65.86 |
| ctc | L | 343 | 57.10 |
| cta | L | 3 | 53.18 |
| ctg | L | 0 | 78.03 |
| --- | -- | ------ | ------ |
| mPD | | 0.056 | 1.1 |
| | | nPD: | 0.05 |
| | | N. weight: | 2.5 |
| | | Sc. PD: | -0.53 |
| | | Sc. rank: | -237.5 |

| PA | | | |
|---|---|---|---|
| Pos. | 668 | obs: | exp: |
| att | I | 330 | 132.30 |
| atc | I | 2 | 92.25 |
| ata | I | 20 | 127.40 |
| gtt | V | 1 | 0.21 |
| gtc | V | 0 | 0.21 |
| gta | V | 0 | 0.19 |
| gtg | V | 0 | 0.40 |
| --- | -- | ------ | ------ |
| mPD | | 0.12 | 0.67 |
| | | nPD: | 0.19 |
| | | N. weight: | 1.1 |
| | | Sc. PD: | -0.085 |
| | | Sc. rank: | -14.6 |

| PA | | | |
|---|---|---|---|
| Pos. | 669 | obs: | exp: |
| att | I | 1 | 0.38 |
| atc | I | 0 | 0.26 |
| ata | I | 0 | 0.36 |
| gtt | V | 351 | 72.35 |
| gtc | V | 1 | 73.88 |
| gta | V | 0 | 65.97 |
| gtg | V | 0 | 139.80 |
| --- | -- | ------ | ------ |
| mPD | | 0.011 | 0.73 |
| | | nPD: | 0.02 |
| | | N. weight: | 2.4 |
| | | Sc. PD: | -0.57 |
| | | Sc. rank: | -332.5 |

| PA | | | |
|---|---|---|---|
| Pos. | 670 | obs: | exp: |
| caa | Q | 0 | 188.20 |
| cag | Q | 353 | 164.80 |
| --- | -- | ------ | ------ |
| mPD | | 0 | 0.50 |
| | | nPD: | 0. |
| | | N. weight: | 1.2 |
| | | Sc. PD: | -0.29 |
| | | Sc. rank: | -230.8 |

| PA | | | |
|---|---|---|---|
| Pos. | 671 | obs: | exp: |
| gct | A | 1 | 88.91 |
| gcc | A | 1 | 72.31 |
| gca | A | 347 | 157.30 |
| gcg | A | 4 | 34.45 |
| --- | -- | ------ | ------ |
| mPD | | 0.034 | 0.69 |
| | | nPD: | 0.05 |
| | | N. weight: | 1.1 |
| | | Sc. PD: | -0.23 |
| | | Sc. rank: | -105.8 |

| PA | | | |
|---|---|---|---|
| Pos. | 672 | obs: | exp: |
| ttt | F | 1 | 0.41 |
| ttc | F | 0 | 0.59 |
| tta | L | 0 | 30.75 |
| ttg | L | 0 | 67.79 |
| ctt | L | 351 | 65.68 |
| ctc | L | 1 | 56.94 |
| cta | L | 0 | 53.03 |
| ctg | L | 0 | 77.81 |
| --- | -- | ------ | ------ |
| mPD | | 0.011 | 1.1 |
| | | nPD: | 0.01 |
| | | N. weight: | 2.5 |
| | | Sc. PD: | -0.61 |
| | | Sc. rank: | -383.6 |

| PA | | | |
|---|---|---|---|
| Pos. | 673 | obs: | exp: |
| cgt | R | 0 | 14.57 |
| cgc | R | 0 | 17.19 |
| cga | R | 0 | 33.73 |
| cgg | R | 0 | 29.29 |
| atg | M | 2 | 2.00 |
| aga | R | 8 | 157.90 |
| agg | R | 343 | 98.36 |
| --- | -- | ------ | ------ |
| mPD | | 0.056 | 0.97 |
| | | nPD: | 0.06 |
| | | N. weight: | 1.7 |
| | | Sc. PD: | -0.35 |
| | | Sc. rank: | -143.8 |

| PA | | | |
|---|---|---|---|
| Pos. | 674 | obs: | exp: |
| gat | D | 66 | 188.30 |
| gac | D | 287 | 164.70 |
| --- | -- | ------ | ------ |
| mPD | | 0.30 | 0.50 |
| | | nPD: | 0.61 |
| | | N. weight: | 0.4 |
| | | Sc. PD: | 0.13 |
| | | Sc. rank: | 61.0 |

| PA | | | |
|---|---|---|---|
| Pos. | 675 | obs: | exp: |
| aat | N | 11 | 184.50 |
| aac | N | 342 | 168.50 |
| --- | -- | ------ | ------ |
| mPD | | 0.061 | 0.50 |
| | | nPD: | 0.12 |
| | | N. weight: | 0.91 |
| | | Sc. PD: | -0.13 |
| | | Sc. rank: | -38.8 |

| PA | | | |
|---|---|---|---|
| Pos. | 676 | obs: | exp: |
| tta | L | 0 | 30.84 |
| ttg | L | 2 | 67.98 |
| ctt | L | 9 | 65.86 |
| ctc | L | 0 | 57.10 |
| cta | L | 0 | 53.18 |
| ctg | L | 342 | 78.03 |
| --- | -- | ------ | ------ |
| mPD | | 0.061 | 1.1 |
| | | nPD: | 0.05 |
| | | N. weight: | 2.1 |
| | | Sc. PD: | -0.42 |
| | | Sc. rank: | -181.3 |

| PA | | | |
|---|---|---|---|
| Pos. | 677 | obs: | exp: |
| gaa | E | 349 | 203.10 |
| gag | E | 4 | 149.90 |
| --- | -- | ------ | ------ |
| mPD | | 0.022 | 0.49 |
| | | nPD: | 0.05 |
| | | N. weight: | 0.75 |
| | | Sc. PD: | -0.16 |
| | | Sc. rank: | -73.1 |

| PA | | | |
|---|---|---|---|
| Pos. | 678 | obs: | exp: |
| cct | P | 352 | 92.95 |
| ccc | P | 1 | 63.67 |
| cca | P | 0 | 139.30 |
| ccg | P | 0 | 57.08 |
| --- | -- | ------ | ------ |
| mPD | | 0.0057 | 0.72 |
| | | nPD: | 0.01 |
| | | N. weight: | 2. |
| | | Sc. PD: | -0.49 |
| | | Sc. rank: | -315.9 |

| PA | | | |
|---|---|---|---|
| Pos. | 679 | obs: | exp: |
| ggt | G | 63 | 43.56 |
| ggc | G | 4 | 47.74 |
| gga | G | 134 | 170.80 |
| ggg | G | 152 | 90.95 |
| --- | -- | ------ | ------ |
| mPD | | 0.64 | 0.67 |
| | | nPD: | 0.96 |
| | | N. weight: | 0.29 |
| | | Sc. PD: | 0.19 |
| | | Sc. rank: | 57.5 |

| PA | | | |
|---|---|---|---|
| Pos. | 680 | obs: | exp: |
| act | T | 7 | 93.26 |
| acc | T | 320 | 75.75 |
| aca | T | 26 | 154.60 |
| acg | T | 0 | 29.35 |
| --- | -- | ------ | ------ |
| mPD | | 0.17 | 0.69 |
| | | nPD: | 0.25 |
| | | N. weight: | 1.7 |
| | | Sc. PD: | -0.02 |
| | | Sc. rank: | 57.8 |

| PA | | | |
|---|---|---|---|
| Pos. | 681 | obs: | exp: |
| ttt | F | 23 | 145.10 |
| ttc | F | 330 | 207.90 |
| --- | -- | ------ | ------ |
| mPD | | 0.12 | 0.48 |
| | | nPD: | 0.25 |
| | | N. weight: | 0.48 |
| | | Sc. PD: | -0.0057 |
| | | Sc. rank: | 16.4 |

| PA | | | |
|---|---|---|---|
| Pos. | 682 | obs: | exp: |
| aat | N | 2 | 1.05 |
| aac | N | 0 | 0.95 |
| gat | D | 348 | 187.30 |
| gac | D | 3 | 163.70 |
| --- | -- | ------ | ------ |
| mPD | | 0.028 | 0.51 |
| | | nPD: | 0.06 |
| | | N. weight: | 0.88 |
| | | Sc. PD: | -0.18 |
| | | Sc. rank: | -76.9 |

| | | | |
|---|---|---|---|
| PA | | | |
| Pos. | 683 | obs: | exp: |
| tta | L | 0 | 30.84 |
| ttg | L | 0 | 67.98 |
| ctt | L | 340 | 65.86 |
| ctc | L | 13 | 57.10 |
| cta | L | 0 | 53.18 |
| ctg | L | 0 | 78.03 |
| --- | -- | ------ | ------ |
| mPD | | 0.071 | 1.1 |
| | | nPD: | 0.06 |
| | | N. weight: | 2.3 |
| | | Sc. PD: | -0.45 |
| | | Sc. rank: | -185.5 |

| | | | |
|---|---|---|---|
| PA | | | |
| Pos. | 684 | obs: | exp: |
| gaa | E | 26 | 16.11 |
| gag | E | 2 | 11.89 |
| ggt | G | 0 | 40.10 |
| ggc | G | 0 | 43.95 |
| gga | G | 111 | 157.20 |
| ggg | G | 214 | 83.73 |
| --- | -- | ------ | ------ |
| mPD | | 0.62 | 0.80 |
| | | nPD: | 0.78 |
| | | N. weight: | 0.75 |
| | | Sc. PD: | 0.37 |
| | | Sc. rank: | 142.1 |

| | | | |
|---|---|---|---|
| PA | | | |
| Pos. | 685 | obs: | exp: |
| ggt | G | 0 | 43.56 |
| ggc | G | 1 | 47.74 |
| gga | G | 15 | 170.80 |
| ggg | G | 337 | 90.95 |
| --- | -- | ------ | ------ |
| mPD | | 0.087 | 0.67 |
| | | nPD: | 0.13 |
| | | N. weight: | 1.7 |
| | | Sc. PD: | -0.22 |
| | | Sc. rank: | -65.8 |

| | | | |
|---|---|---|---|
| PA | | | |
| Pos. | 686 | obs: | exp: |
| tta | L | 5 | 30.75 |
| ttg | L | 0 | 67.79 |
| ctt | L | 0 | 65.68 |
| ctc | L | 0 | 56.94 |
| cta | L | 345 | 53.03 |
| ctg | L | 2 | 77.81 |
| gtt | V | 0 | 0.21 |
| gtc | V | 0 | 0.21 |
| gta | V | 1 | 0.19 |
| gtg | V | 0 | 0.40 |
| --- | -- | ------ | ------ |
| mPD | | 0.045 | 1.1 |
| | | nPD: | 0.04 |
| | | N. weight: | 2.7 |
| | | Sc. PD: | -0.59 |
| | | Sc. rank: | -274.1 |

| | | | |
|---|---|---|---|
| PA | | | |
| Pos. | 687 | obs: | exp: |
| tat | Y | 339 | 182.70 |
| tac | Y | 14 | 170.30 |
| --- | -- | ------ | ------ |
| mPD | | 0.076 | 0.50 |
| | | nPD: | 0.15 |
| | | N. weight: | 0.76 |
| | | Sc. PD: | -0.081 |
| | | Sc. rank: | -21.7 |

| | | | |
|---|---|---|---|
| PA | | | |
| Pos. | 688 | obs: | exp: |
| gaa | E | 346 | 202.60 |
| gag | E | 6 | 149.40 |
| ggt | G | 0 | 0.12 |
| ggc | G | 0 | 0.14 |
| gga | G | 1 | 0.48 |
| ggg | G | 0 | 0.26 |
| --- | -- | ------ | ------ |
| mPD | | 0.039 | 0.49 |
| | | nPD: | 0.08 |
| | | N. weight: | 0.72 |
| | | Sc. PD: | -0.13 |
| | | Sc. rank: | -51.7 |

| | | | |
|---|---|---|---|
| PA | | | |
| Pos. | 689 | obs: | exp: |
| gct | A | 0 | 88.91 |
| gcc | A | 0 | 72.31 |
| gca | A | 353 | 157.30 |
| gcg | A | 0 | 34.45 |
| --- | -- | ------ | ------ |
| mPD | | 0 | 0.69 |
| | | nPD: | 0. |
| | | N. weight: | 1.2 |
| | | Sc. PD: | -0.31 |
| | | Sc. rank: | -244.9 |

| | | | |
|---|---|---|---|
| PA | | | |
| Pos. | 690 | obs: | exp: |
| att | I | 309 | 132.30 |
| atc | I | 43 | 92.25 |
| ata | I | 0 | 127.40 |
| gtt | V | 1 | 0.21 |
| gtc | V | 0 | 0.21 |
| gta | V | 0 | 0.19 |
| gtg | V | 0 | 0.40 |
| --- | -- | ------ | ------ |
| mPD | | 0.22 | 0.67 |
| | | nPD: | 0.33 |
| | | N. weight: | 1. |
| | | Sc. PD: | 0.064 |
| | | Sc. rank: | 65.4 |

| | | | |
|---|---|---|---|
| PA | | | |
| Pos. | 691 | obs: | exp: |
| gaa | E | 0 | 203.10 |
| gag | E | 353 | 149.90 |
| --- | -- | ------ | ------ |
| mPD | | 0 | 0.49 |
| | | nPD: | 0. |
| | | N. weight: | 1.3 |
| | | Sc. PD: | -0.33 |
| | | Sc. rank: | -259.6 |

| | | | |
|---|---|---|---|
| PA | | | |
| Pos. | 692 | obs: | exp: |
| gaa | E | 0 | 203.10 |
| gag | E | 353 | 149.90 |
| --- | -- | ------ | ------ |
| mPD | | 0 | 0.49 |
| | | nPD: | 0. |
| | | N. weight: | 1.3 |
| | | Sc. PD: | -0.33 |
| | | Sc. rank: | -259.6 |

| | | | |
|---|---|---|---|
| PA | | | |
| Pos. | 693 | obs: | exp: |
| tgt | C | 1 | 145.50 |
| tgc | C | 352 | 207.50 |
| --- | -- | ------ | ------ |
| mPD | | 0.0057 | 0.48 |
| | | nPD: | 0.01 |
| | | N. weight: | 0.78 |
| | | Sc. PD: | -0.19 |
| | | Sc. rank: | -112.9 |

| | | | |
|---|---|---|---|
| PA | | | |
| Pos. | 694 | obs: | exp: |
| tta | L | 0 | 30.84 |
| ttg | L | 0 | 67.98 |
| ctt | L | 0 | 65.86 |
| ctc | L | 0 | 57.10 |
| cta | L | 0 | 53.18 |
| ctg | L | 353 | 78.03 |
| --- | -- | ------ | ------ |
| mPD | | 0 | 1.1 |
| | | nPD: | 0. |
| | | N. weight: | 2.3 |
| | | Sc. PD: | -0.58 |
| | | Sc. rank: | -457.4 |

| | | | |
|---|---|---|---|
| PA | | | |
| Pos. | 695 | obs: | exp: |
| att | I | 353 | 132.70 |
| atc | I | 0 | 92.52 |
| ata | I | 0 | 127.80 |
| --- | -- | ------ | ------ |
| mPD | | 0 | 0.66 |
| | | nPD: | 0. |
| | | N. weight: | 1.5 |
| | | Sc. PD: | -0.38 |
| | | Sc. rank: | -296.5 |

| | | | |
|---|---|---|---|
| PA | | | |
| Pos. | 696 | obs: | exp: |
| aat | N | 205 | 184.50 |
| aac | N | 148 | 168.50 |
| --- | -- | ------ | ------ |
| mPD | | 0.49 | 0.50 |
| | | nPD: | 0.98 |
| | | N. weight: | 0.024 |
| | | Sc. PD: | 0.016 |
| | | Sc. rank: | 4.8 |

| | | | |
|---|---|---|---|
| PA | | | |
| Pos. | 697 | obs: | exp: |
| gat | D | 350 | 188.30 |
| gac | D | 3 | 164.70 |
| --- | -- | ------ | ------ |
| mPD | | 0.017 | 0.50 |
| | | nPD: | 0.03 |
| | | N. weight: | 0.88 |
| | | Sc. PD: | -0.2 |
| | | Sc. rank: | -95.0 |

| | | | |
|---|---|---|---|
| PA | | | |
| Pos. | 698 | obs: | exp: |
| tct | S | 0 | 0.32 |
| tcc | S | 2 | 0.28 |
| tca | S | 0 | 0.44 |
| tcg | S | 0 | 0.12 |
| cct | P | 2 | 92.42 |
| ccc | P | 349 | 63.31 |
| cca | P | 0 | 138.50 |
| ccg | P | 0 | 56.75 |
| agt | S | 0 | 0.44 |
| agc | S | 0 | 0.40 |
| --- | -- | ------ | ------ |
| mPD | | 0.023 | 0.73 |
| | | nPD: | 0.03 |
| | | N. weight: | 2.5 |
| | | Sc. PD: | -0.57 |
| | | Sc. rank: | -292.4 |

| | | | |
|---|---|---|---|
| PA | | | |
| Pos. | 699 | obs: | exp: |
| tgg | W | 353 | 353.00 |
| --- | -- | ------ | ------ |
| mPD | | 0 | 0 |
| | | nPD: | 1. |
| | | N. weight: | 0. |
| | | Sc. PD: | 0 |
| | | Sc. rank: | 0 |

| | | | |
|---|---|---|---|
| PA | | | |
| Pos. | 700 | obs: | exp: |
| gtt | V | 351 | 72.14 |
| gtc | V | 0 | 73.67 |
| gta | V | 0 | 65.78 |
| gtg | V | 0 | 139.40 |
| gct | A | 2 | 0.50 |
| gcc | A | 0 | 0.41 |
| gca | A | 0 | 0.89 |
| gcg | A | 0 | 0.20 |
| --- | -- | ------ | ------ |
| mPD | | 0.011 | 0.73 |
| | | nPD: | 0.02 |
| | | N. weight: | 2.4 |
| | | Sc. PD: | -0.57 |
| | | Sc. rank: | -339.4 |

| | | | |
|---|---|---|---|
| PA | | | |
| Pos. | 701 | obs: | exp: |
| tta | L | 1 | 30.84 |
| ttg | L | 352 | 67.98 |
| ctt | L | 0 | 65.86 |
| ctc | L | 0 | 57.10 |
| cta | L | 0 | 53.18 |
| ctg | L | 0 | 78.03 |
| --- | -- | ------ | ------ |
| mPD | | 0.0057 | 1.1 |
| | | nPD: | 0.01 |
| | | N. weight: | 2.5 |
| | | Sc. PD: | -0.62 |
| | | Sc. rank: | -413.1 |

| | | | |
|---|---|---|---|
| PA | | | |
| Pos. | 702 | obs: | exp: |
| tta | L | 0 | 30.84 |
| ttg | L | 0 | 67.98 |
| ctt | L | 344 | 65.86 |
| ctc | L | 9 | 57.10 |
| cta | L | 0 | 53.18 |
| ctg | L | 0 | 78.03 |
| --- | -- | ------ | ------ |
| mPD | | 0.050 | 1.1 |
| | | nPD: | 0.04 |
| | | N. weight: | 2.4 |
| | | Sc. PD: | -0.5 |
| | | Sc. rank: | -232.0 |

| | | | |
|---|---|---|---|
| PA | | | |
| Pos. | 703 | obs: | exp: |
| aat | N | 340 | 184.50 |
| aac | N | 13 | 168.50 |
| --- | -- | ------ | ------ |
| mPD | | 0.071 | 0.50 |
| | | nPD: | 0.14 |
| | | N. weight: | 0.76 |
| | | Sc. PD: | -0.089 |
| | | Sc. rank: | -25.6 |

| | | | |
|---|---|---|---|
| PA | | | |
| Pos. | 704 | obs: | exp: |
| gct | A | 5 | 88.91 |
| gcc | A | 0 | 72.31 |
| gca | A | 143 | 157.30 |
| gcg | A | 205 | 34.45 |
| --- | -- | ------ | ------ |
| mPD | | 0.50 | 0.69 |
| | | nPD: | 0.73 |
| | | N. weight: | 1.5 |
| | | Sc. PD: | 0.65 |
| | | Sc. rank: | 267.3 |

| | | | |
|---|---|---|---|
| PA | | | |
| Pos. | 705 | obs: | exp: |
| tct | S | 347 | 56.12 |
| tcc | S | 6 | 49.17 |
| tca | S | 0 | 78.20 |
| tcg | S | 0 | 21.52 |
| agt | S | 0 | 77.66 |
| agc | S | 0 | 70.33 |
| --- | -- | ------ | ------ |
| mPD | | 0.034 | 1.7 |
| | | nPD: | 0.02 |
| | | N. weight: | 2.7 |
| | | Sc. PD: | -0.63 |
| | | Sc. rank: | -348.1 |

| | | | |
|---|---|---|---|
| PA | | | |
| Pos. | 706 | obs: | exp: |
| tgg | W | 353 | 353.00 |
| --- | -- | ------ | ------ |
| mPD | | 0 | 0 |
| | | nPD: | 1. |
| | | N. weight: | 0. |
| | | Sc. PD: | 0 |
| | | Sc. rank: | 0 |

| | | | |
|---|---|---|---|
| PA | | | |
| Pos. | 707 | obs: | exp: |
| ttt | F | 6 | 145.10 |
| ttc | F | 347 | 207.90 |
| --- | -- | ------ | ------ |
| mPD | | 0.034 | 0.48 |
| | | nPD: | 0.07 |
| | | N. weight: | 0.69 |
| | | Sc. PD: | -0.13 |
| | | Sc. rank: | -51.2 |

PA

| Pos. | 708 | obs: | exp: |
|---|---|---|---|
| aat | N | 0 | 184.50 |
| aac | N | 353 | 168.50 |
| --- | -- | ------ | ------ |
| mPD | | 0 | 0.50 |
| | | nPD: | 0. |
| | | N. weight: | 1.1 |
| | | Sc. PD: | -0.28 |
| | | Sc. rank: | -224.1 |

,

PA

| Pos. | 709 | obs: | exp: |
|---|---|---|---|
| tct | S | 0 | 56.12 |
| tcc | S | 353 | 49.17 |
| tca | S | 0 | 78.20 |
| tcg | S | 0 | 21.52 |
| agt | S | 0 | 77.66 |
| agc | S | 0 | 70.33 |
| --- | -- | ------ | ------ |
| mPD | | 0 | 1.7 |
| | | nPD: | 0. |
| | | N. weight: | 3. |
| | | Sc. PD: | -0.76 |
| | | Sc. rank: | -597.4 |

,

PA

| Pos. | 710 | obs: | exp: |
|---|---|---|---|
| ttt | F | 0 | 145.10 |
| ttc | F | 353 | 207.90 |
| --- | -- | ------ | ------ |
| mPD | | 0 | 0.48 |
| | | nPD: | 0. |
| | | N. weight: | 0.8 |
| | | Sc. PD: | -0.2 |
| | | Sc. rank: | -160.5 |

,

PA

| Pos. | 711 | obs: | exp: |
|---|---|---|---|
| tta | L | 0 | 30.84 |
| ttg | L | 0 | 67.98 |
| ctt | L | 24 | 65.86 |
| ctc | L | 329 | 57.10 |
| cta | L | 0 | 53.18 |
| ctg | L | 0 | 78.03 |
| --- | -- | ------ | ------ |
| mPD | | 0.13 | 1.1 |
| | | nPD: | 0.11 |
| | | N. weight: | 2.4 |
| | | Sc. PD: | -0.34 |
| | | Sc. rank: | -110.0 |

,

PA

| Pos. | 712 | obs: | exp: |
|---|---|---|---|
| act | T | 0 | 93.26 |
| acc | T | 0 | 75.75 |
| aca | T | 347 | 154.60 |
| acg | T | 6 | 29.35 |
| --- | -- | ------ | ------ |
| mPD | | 0.034 | 0.69 |
| | | nPD: | 0.05 |
| | | N. weight: | 1.2 |
| | | Sc. PD: | -0.24 |
| | | Sc. rank: | -111.5 |

,

PA

| Pos. | 713 | obs: | exp: |
|---|---|---|---|
| cat | H | 353 | 191.30 |
| cac | H | 0 | 161.70 |
| --- | -- | ------ | ------ |
| mPD | | 0 | 0.50 |
| | | nPD: | 0. |
| | | N. weight: | 0.93 |
| | | Sc. PD: | -0.24 |
| | | Sc. rank: | -185.7 |

,

PA

| Pos. | 714 | obs: | exp: |
|---|---|---|---|
| act | T | 0 | 0.26 |
| acc | T | 0 | 0.21 |
| aca | T | 1 | 0.44 |
| acg | T | 0 | 0.08 |
| gct | A | 0 | 88.66 |
| gcc | A | 0 | 72.11 |
| gca | A | 352 | 156.90 |
| gcg | A | 0 | 34.35 |
| --- | -- | ------ | ------ |
| mPD | | 0.0057 | 0.69 |
| | | nPD: | 0.01 |
| | | N. weight: | 1.2 |
| | | Sc. PD: | -0.3 |
| | | Sc. rank: | -191.1 |

,

PA

| Pos. | 715 | obs: | exp: |
|---|---|---|---|
| tta | L | 0 | 30.84 |
| ttg | L | 1 | 67.98 |
| ctt | L | 0 | 65.86 |
| ctc | L | 0 | 57.10 |
| cta | L | 4 | 53.18 |
| ctg | L | 348 | 78.03 |
| --- | -- | ------ | ------ |
| mPD | | 0.028 | 1.1 |
| | | nPD: | 0.03 |
| | | N. weight: | 2.2 |
| | | Sc. PD: | -0.5 |
| | | Sc. rank: | -263.3 |

,

PA

| Pos. | 716 | obs: | exp: |
|---|---|---|---|
| cgt | R | 0 | 1.70 |
| cgc | R | 0 | 2.01 |
| cga | R | 0 | 3.94 |
| cgg | R | 0 | 3.42 |
| act | T | 0 | 0.26 |
| acc | T | 0 | 0.21 |
| aca | T | 1 | 0.44 |
| acg | T | 0 | 0.08 |
| aaa | K | 300 | 173.00 |
| aag | K | 9 | 136.00 |
| aga | R | 41 | 18.44 |
| agg | R | 0 | 11.49 |
| gaa | E | 2 | 1.15 |
| gag | E | 0 | 0.85 |
| --- | -- | ------ | ------ |
| mPD | | 0.27 | 0.79 |
| | | nPD: | 0.35 |
| | | N. weight: | 0.76 |
| | | Sc. PD: | 0.06 |
| | | Sc. rank: | 55.8 |

,

PA

| Pos. | 717 | obs: | exp: |
|---|---|---|---|
| taa | . | 9 | 165.30 |
| tag | . | 344 | 91.35 |
| tga | . | 0 | 96.33 |
| --- | -- | ------ | ------ |
| mPD | | 0.050 | 0.78 |
| | | nPD: | 0.06 |
| | | N. weight: | 1.9 |
| | | Sc. PD: | -0.36 |
| | | Sc. rank: | -146.2 |

}

Amino acid level conservation per codon

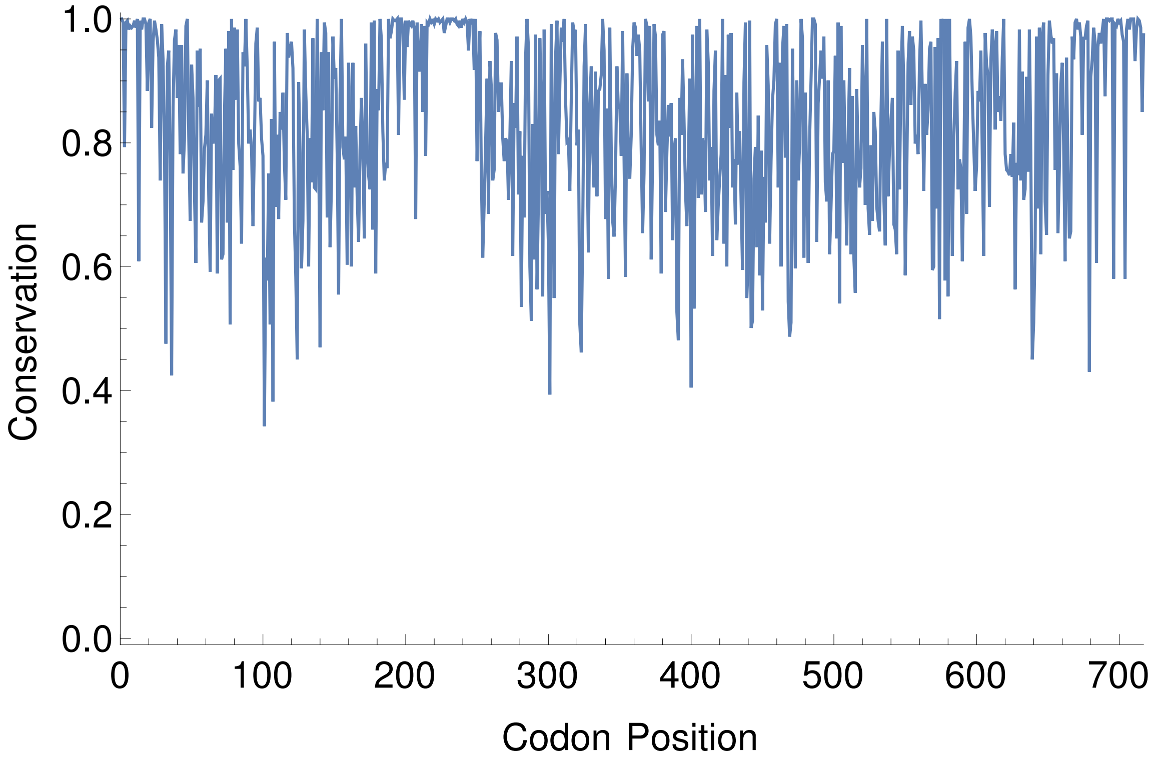

Conservation analysis plots

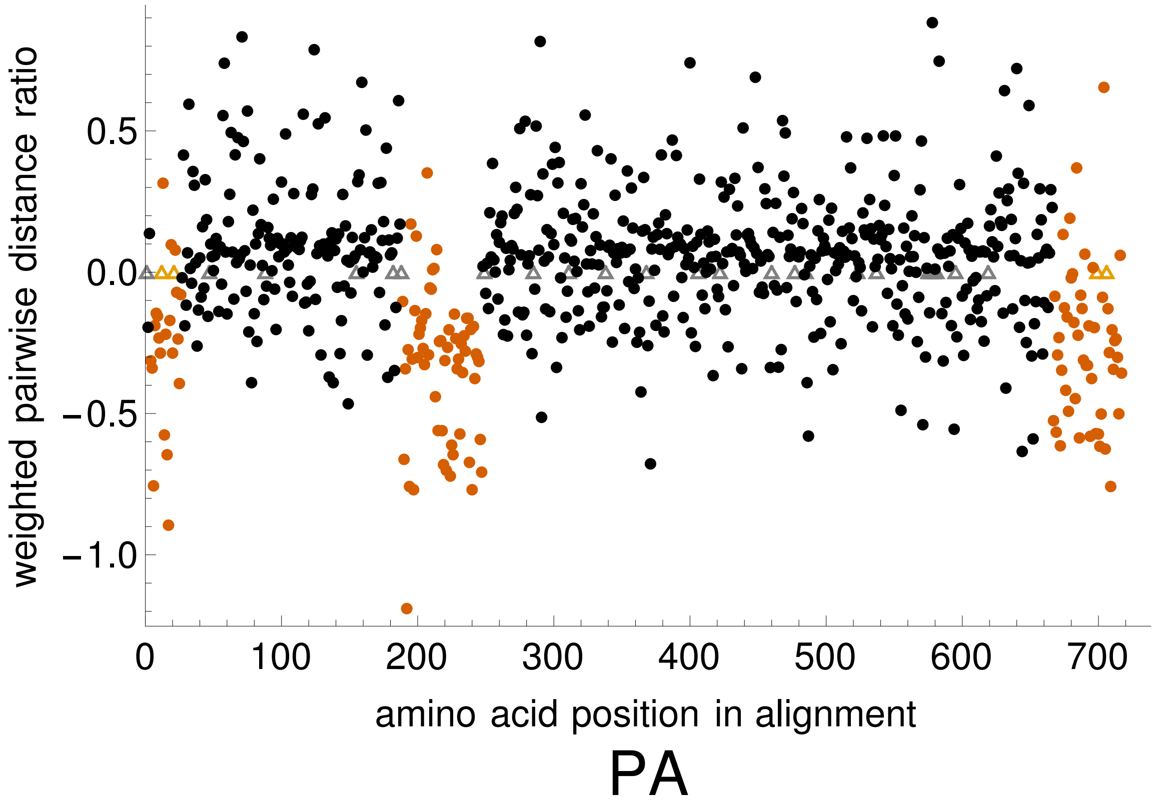


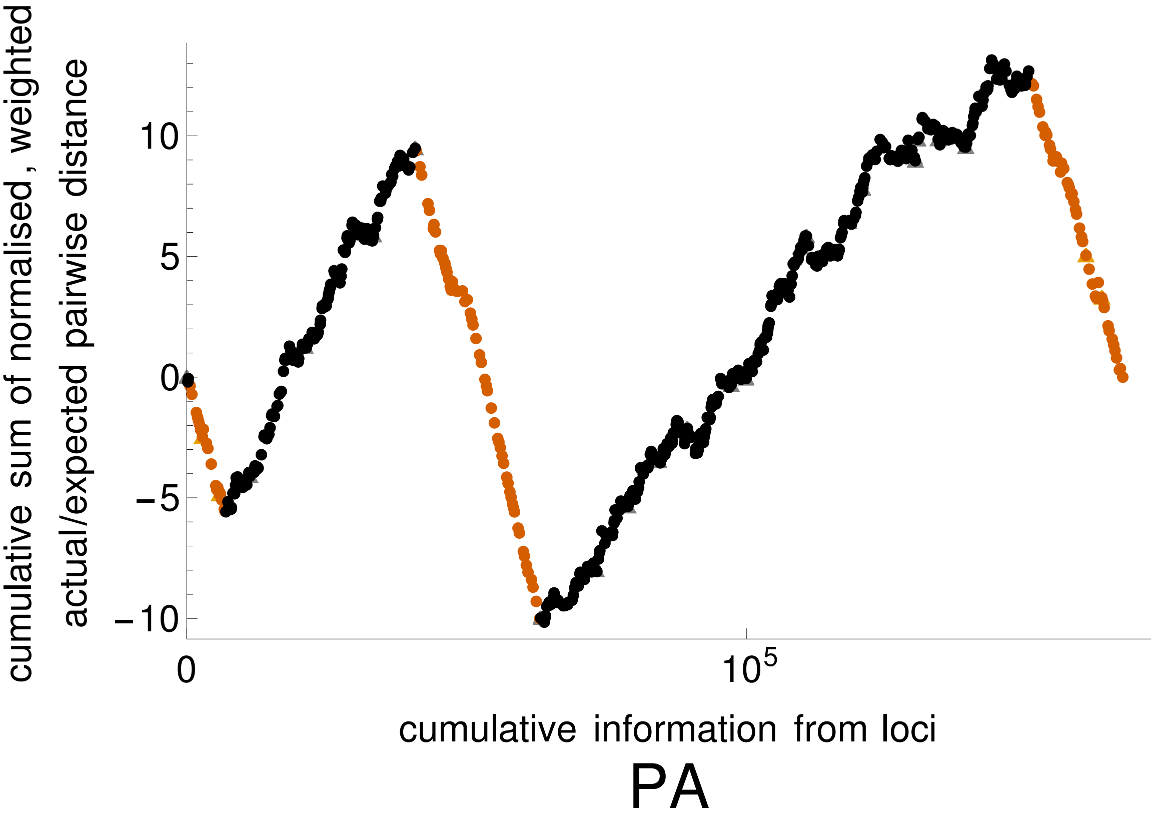

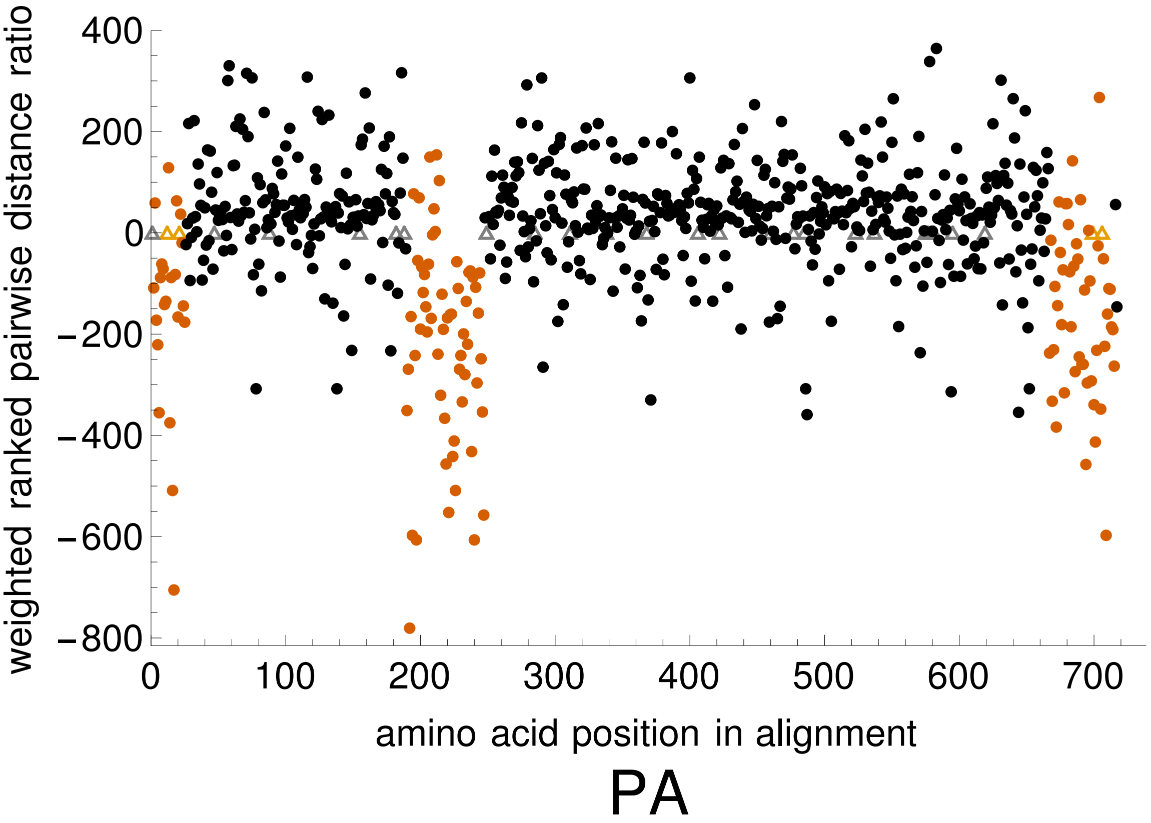
weighted ranked pairwise distance ratio
400
200
0
−200
−400
−600
−800
0
100
200
300
400
500
600
700
amino acid position in alignment
PA

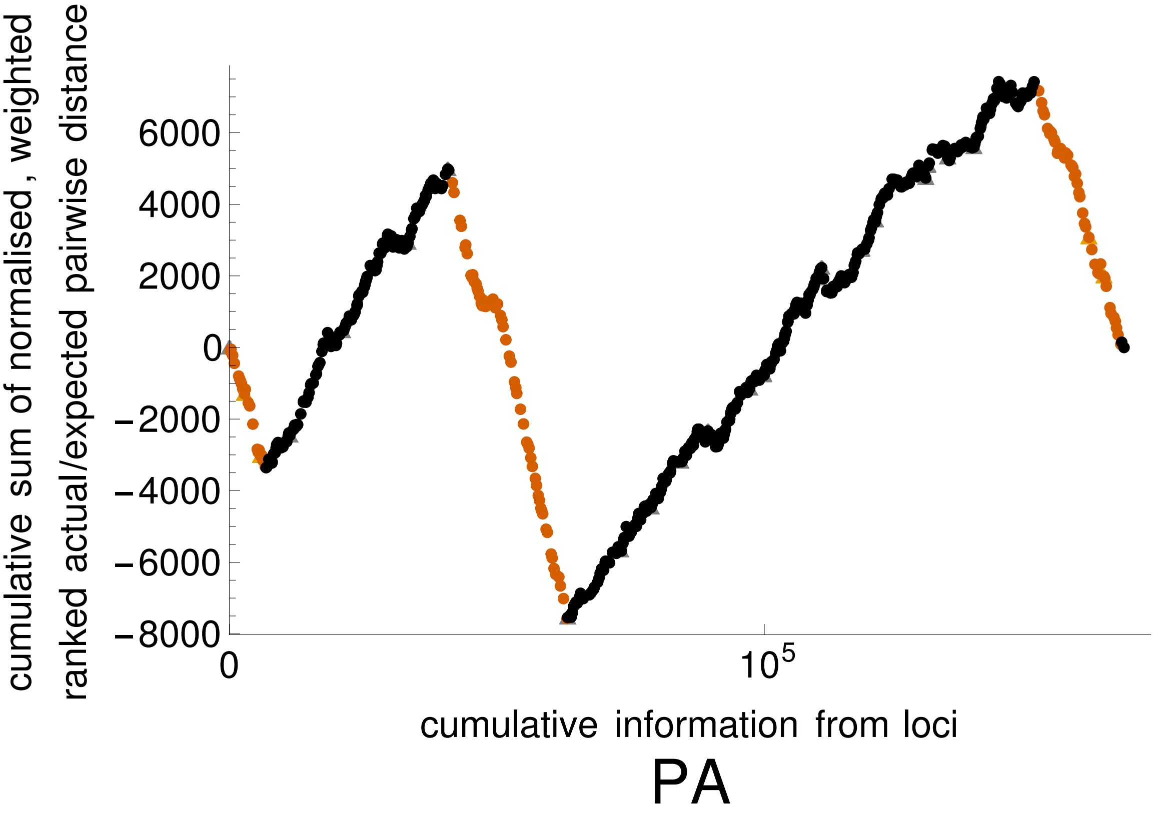
cumulative sum of normalised, weighted ranked actual/expected pairwise distance
6000
4000
2000
0
−2000
−4000
−6000
−8000
0
$10^5$
cumulative information from loci
PA

# PA-X

## Gene length histogram

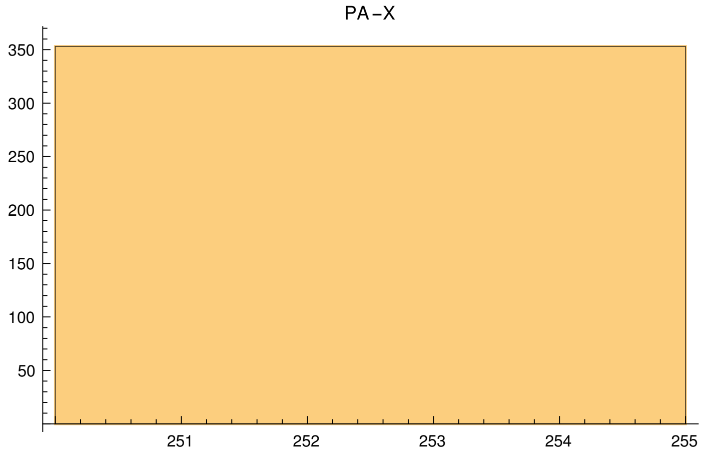

## Information vs. nPD

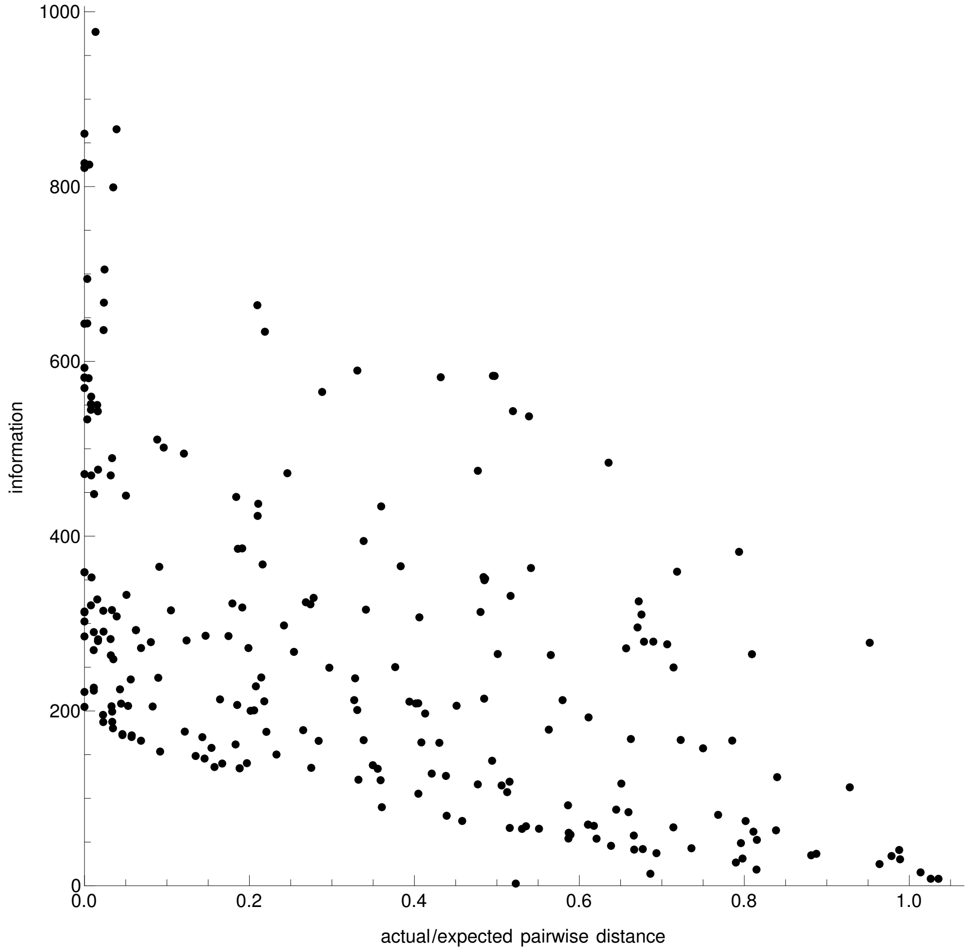


PA–X

## Example sequences highlighted by regions found in analyses to be conserved

Interesting points (by weighted raw PD) highlighted for gene PA-X:

ATGGAAGAT
**TTTGTGCGACAATGCTTCAATCCAATGATTGTCGAGCTTGCGGAAAAGGCAATGA
AAGAATATGGGGAA**GATCCGAAAATCGAAACGAACAAATTTGCCGCAATATGCACACACTTAGAGG
TCTGTTTCATGTATTCAGATTTCCACTTTATTGATGAACGGGGCGAGTCAATAATTGTGGAATCTGGCGA
TCCAAATGCATTATTGAAACACCGATTTGAAATAATTGAAGGGAGAGACCGAACGATGGCTTGGACAGTG
GTGAATAGCATCTGCAACACCACAGGAGTCGAGAAGCCCAAGTTCCTCCCAGATTTGTATGACTACAAAG
AGAATCGATTCATTGAAATTGGAGTAACACGGAGGGAAGTTCATATATATTATCTAGAAAAGGCCAACAA
GATAAAATCAGAGAAAACACACATTCACATATTCTCATTCACTGGAGAGGAAATGGCCACCAAAGCGGAC
TACACTCTTGACGAAGAGAGTAGGGCAAGGATCAAAACCAGGCTGTTCACTATAAGGCAGGAAATGGCCA
GTAGGGGCCTATGG
**GATTCCTTTGCCAGTCCGAGAGAGGCGAAGAGACAGTTGAAGAAAGATTTGAAAT
CACAGGAACCATGCGCAGGCTTGCCGACCAAAGTCTCCCACCGAACTTCTCC
AGCCTTGAAAACTTTAGAGCCTATGTGGATGGATTCGAACCGAACGGCTGCA
TTGAGGGCAAGCTTTCTCAAATGT**CAAAAGAAGTGA

Interesting points (by weighted ranked PD) highlighted for gene PA-X:

ATGGAAGAT
**TTTGTGCGACAATGCTTCAATCCAATGATTGTCGAGCTTGCGGAAAAGGCAATGA
AAGAATATGGG**GAAGATCCGAAAATCGAAACGAACAAATTTGCCGCAATATGCACACACTTAGAGGT
CTGTTTCATGTATTCAGATTTCCACTTTATTGATGAACGGGGCGAGTCAATAATTGTGGAATCTGGCGAT
CCAAATGCATTATTGAAACACCGATTTGAAATAATTGAAGGGAGAGACCGAACGATGGCTTGGACAGTGG
TGAATAGCATCTGCAACACCACAGGAGTCGAGAAGCCCAAGTTCCTCCCAGATTTGTATGACTACAAAGA
GAATCGATTCATTGAAATTGGAGTAACACGGAGGGAAGTTCATATATATTATCTAGAAAAGGCCAACAAG
ATAAAATCAGAGAAAACACACATTCACATATTCTCATTCACTGGAGAGGAAATGGCCACCAAAGCGGACT
ACACTCTTGACGAAGAGAGTAGGGCAAGGATCAAAACCAGGCTGTTCACTATAAGGCAGGAAATGGCCAG
TAGGGGCCTATGGGAT
**TCCTTTGCCAGTCCGAGAGAGGCGAAGAGACAGTTGAAGAAAGATTTGAAATCAC
AGGAACCATGCGCAGGCTTGCCGACCAAAGTCTCCCACCGAACTTCTCCAGC
CTTGAAAACTTTAGAGCCTATGTGGATGGATTCGAACCGAACGGCTGCATTG
AGGGCAAGCTTTCTCAAATGT**CAAAAGAAGTGA

# Per codon conservation report

PA-X

| Pos. | 1 | obs: | exp: |
|---|---|---|---|
| atg | M | 353 | 353.00 |
| --- | -- | ------ | ------ |
| mPD | | 0 | 0 |
| | | nPD: | 1. |
| | | N. weight: | 0. |
| | | Sc. PD: | 0 |
| | | Sc. rank: | 0 |

PA-X

| Pos. | 2 | obs: | exp: |
|---|---|---|---|
| gct | A | 0 | 0.25 |
| gcc | A | 0 | 0.20 |
| gca | A | 1 | 0.45 |
| gcg | A | 0 | 0.10 |
| gaa | E | 351 | 202.00 |
| gag | E | 0 | 149.00 |
| ggt | G | 0 | 0.12 |
| ggc | G | 0 | 0.14 |
| gga | G | 1 | 0.48 |
| ggg | G | 0 | 0.26 |
| --- | -- | ------ | ------ |
| mPD | | 0.011 | 0.50 |
| | | nPD: | 0.02 |
| | | N. weight: | 0.73 |
| | | Sc. PD: | -0.13 |
| | | Sc. rank: | -59.8 |

PA-X

| Pos. | 3 | obs: | exp: |
|---|---|---|---|
| gat | D | 72 | 187.80 |
| gac | D | 280 | 164.20 |
| gaa | E | 0 | 0.58 |
| gag | E | 1 | 0.42 |
| --- | -- | ------ | ------ |
| mPD | | 0.33 | 0.50 |
| | | nPD: | 0.66 |
| | | N. weight: | 0.31 |
| | | Sc. PD: | 0.13 |
| | | Sc. rank: | 63.1 |

PA-X

| Pos. | 4 | obs: | exp: |
|---|---|---|---|
| ttt | F | 351 | 144.30 |
| ttc | F | 0 | 206.70 |
| tta | L | 0 | 0.17 |
| ttg | L | 1 | 0.39 |
| ctt | L | 1 | 0.37 |
| ctc | L | 0 | 0.32 |
| cta | L | 0 | 0.30 |
| ctg | L | 0 | 0.44 |
| --- | -- | ------ | ------ |
| mPD | | 0.011 | 0.50 |
| | | nPD: | 0.02 |
| | | N. weight: | 1.2 |
| | | Sc. PD: | -0.21 |
| | | Sc. rank: | -92.2 |

PA-X

| Pos. | 5 | obs: | exp: |
|---|---|---|---|
| gtt | V | 0 | 72.56 |
| gtc | V | 0 | 74.09 |
| gta | V | 1 | 66.16 |
| gtg | V | 352 | 140.20 |
| --- | -- | ------ | ------ |
| mPD | | 0.0057 | 0.72 |
| | | nPD: | 0.01 |
| | | N. weight: | 1.2 |
| | | Sc. PD: | -0.23 |
| | | Sc. rank: | -154.4 |

PA-X

| Pos. | 6 | obs: | exp: |
|---|---|---|---|
| cgt | R | 0 | 14.65 |
| cgc | R | 0 | 17.28 |
| cga | R | 347 | 33.93 |
| cgg | R | 6 | 29.46 |
| aga | R | 0 | 158.80 |
| agg | R | 0 | 98.92 |
| --- | -- | ------ | ------ |
| mPD | | 0.034 | 0.96 |
| | | nPD: | 0.03 |
| | | N. weight: | 3. |
| | | Sc. PD: | -0.5 |
| | | Sc. rank: | -118.4 |

PA-X

| Pos. | 7 | obs: | exp: |
|---|---|---|---|
| caa | Q | 349 | 187.70 |
| cag | Q | 3 | 164.30 |
| cgt | R | 0 | 0.04 |
| cgc | R | 0 | 0.05 |
| cga | R | 1 | 0.10 |
| cgg | R | 0 | 0.08 |
| aga | R | 0 | 0.45 |
| agg | R | 0 | 0.28 |
| --- | -- | ------ | ------ |
| mPD | | 0.023 | 0.51 |
| | | nPD: | 0.04 |
| | | N. weight: | 0.77 |
| | | Sc. PD: | -0.12 |
| | | Sc. rank: | -21.0 |

PA-X

| Pos. | 8 | obs: | exp: |
|---|---|---|---|
| tgt | C | 4 | 145.10 |
| tgc | C | 348 | 206.90 |
| ggt | G | 0 | 0.12 |
| ggc | G | 1 | 0.14 |
| gga | G | 0 | 0.48 |
| ggg | G | 0 | 0.26 |
| --- | -- | ------ | ------ |
| mPD | | 0.028 | 0.49 |
| | | nPD: | 0.06 |
| | | N. weight: | 0.63 |
| | | Sc. PD: | -0.093 |
| | | Sc. rank: | -9.6 |

PA-X

| Pos. | 9 | obs: | exp: |
|---|---|---|---|
| ttt | F | 3 | 144.70 |
| ttc | F | 349 | 207.30 |
| tat | Y | 0 | 0.52 |
| tac | Y | 1 | 0.48 |
| --- | -- | ------ | ------ |
| mPD | | 0.023 | 0.49 |
| | | nPD: | 0.05 |
| | | N. weight: | 0.64 |
| | | Sc. PD: | -0.1 |
| | | Sc. rank: | -15.9 |

PA-X

| Pos. | 10 | obs: | exp: |
|---|---|---|---|
| aat | N | 352 | 184.50 |
| aac | N | 1 | 168.50 |
| --- | -- | ------ | ------ |
| mPD | | 0.0057 | 0.50 |
| | | nPD: | 0.01 |
| | | N. weight: | 0.83 |
| | | Sc. PD: | -0.16 |
| | | Sc. rank: | -91.4 |

PA-X

| Pos. | 11 | obs: | exp: |
|---|---|---|---|
| cct | P | 0 | 92.95 |
| ccc | P | 0 | 63.67 |
| cca | P | 348 | 139.30 |
| ccg | P | 5 | 57.08 |
| --- | -- | ------ | ------ |
| mPD | | 0.028 | 0.72 |
| | | nPD: | 0.04 |
| | | N. weight: | 1.1 |
| | | Sc. PD: | -0.19 |
| | | Sc. rank: | -34.4 |

PA-X

| Pos. | 12 | obs: | exp: |
|---|---|---|---|
| atg | M | 353 | 353.00 |
| --- | -- | ------ | ------ |
| mPD | | 0 | 0 |
| | | nPD: | 1. |
| | | N. weight: | 0. |
| | | Sc. PD: | 0 |
| | | Sc. rank: | 0 |

PA-X

| Pos. | 13 | obs: | exp: |
|---|---|---|---|
| att | I | 215 | 131.20 |
| atc | I | 134 | 91.47 |
| ata | I | 0 | 126.30 |
| gtt | V | 4 | 0.82 |
| gtc | V | 0 | 0.84 |
| gta | V | 0 | 0.75 |
| gtg | V | 0 | 1.59 |
| --- | -- | ------ | ------ |
| mPD | | 0.49 | 0.68 |
| | | nPD: | 0.72 |
| | | N. weight: | 0.62 |
| | | Sc. PD: | 0.3 |
| | | Sc. rank: | 137.5 |

PA-X

| Pos. | 14 | obs: | exp: |
|---|---|---|---|
| gtt | V | 0 | 72.56 |
| gtc | V | 352 | 74.09 |
| gta | V | 0 | 66.16 |
| gtg | V | 1 | 140.20 |
| --- | -- | ------ | ------ |
| mPD | | 0.0057 | 0.72 |
| | | nPD: | 0.01 |
| | | N. weight: | 2. |
| | | Sc. PD: | -0.39 |
| | | Sc. rank: | -262.1 |

PA-X

| Pos. | 15 | obs: | exp: |
|---|---|---|---|
| gaa | E | 6 | 203.10 |
| gag | E | 347 | 149.90 |
| --- | -- | ------ | ------ |
| mPD | | 0.034 | 0.49 |
| | | nPD: | 0.07 |
| | | N. weight: | 1. |
| | | Sc. PD: | -0.14 |
| | | Sc. rank: | -7.1 |

PA-X

| Pos. | 16 | obs: | exp: |
|---|---|---|---|
| tta | L | 0 | 30.84 |
| ttg | L | 0 | 67.98 |
| ctt | L | 353 | 65.86 |
| ctc | L | 0 | 57.10 |
| cta | L | 0 | 53.18 |
| ctg | L | 0 | 78.03 |
| --- | -- | ------ | ------ |
| mPD | | 0 | 1.1 |
| | | nPD: | 0. |
| | | N. weight: | 2.2 |
| | | Sc. PD: | -0.44 |
| | | Sc. rank: | -497.7 |

PA-X

| Pos. | 17 | obs: | exp: |
|---|---|---|---|
| gct | A | 0 | 88.91 |
| gcc | A | 0 | 72.31 |
| gca | A | 0 | 157.30 |
| gcg | A | 353 | 34.45 |
| --- | -- | ------ | ------ |
| mPD | | 0 | 0.69 |
| | | nPD: | 0. |
| | | N. weight: | 3. |
| | | Sc. PD: | -0.62 |
| | | Sc. rank: | -689.9 |

PA-X

| Pos. | 18 | obs: | exp: |
|---|---|---|---|
| gaa | E | 350 | 203.10 |
| gag | E | 3 | 149.90 |
| --- | -- | ------ | ------ |
| mPD | | 0.017 | 0.49 |
| | | nPD: | 0.03 |
| | | N. weight: | 0.67 |
| | | Sc. PD: | -0.11 |
| | | Sc. rank: | -30.4 |

PA-X

| Pos. | 19 | obs: | exp: |
|---|---|---|---|
| cgt | R | 0 | 0.08 |
| cgc | R | 0 | 0.10 |
| cga | R | 0 | 0.19 |
| cgg | R | 0 | 0.17 |
| aaa | K | 38 | 196.00 |
| aag | K | 312 | 154.00 |
| aga | R | 0 | 0.90 |
| agg | R | 2 | 0.56 |
| gaa | E | 0 | 0.58 |
| gag | E | 1 | 0.42 |
| --- | -- | ------ | ------ |
| mPD | | 0.21 | 0.51 |
| | | nPD: | 0.41 |
| | | N. weight: | 0.61 |
| | | Sc. PD: | 0.12 |
| | | Sc. rank: | 80.5 |

PA-X

| Pos. | 20 | obs: | exp: |
|---|---|---|---|
| act | T | 0 | 0.26 |
| acc | T | 0 | 0.21 |
| aca | T | 1 | 0.44 |
| acg | T | 0 | 0.08 |
| gct | A | 1 | 88.66 |
| gcc | A | 0 | 72.11 |
| gca | A | 351 | 156.90 |
| gcg | A | 0 | 34.35 |
| --- | -- | ------ | ------ |
| mPD | | 0.011 | 0.69 |
| | | nPD: | 0.02 |
| | | N. weight: | 1. |
| | | Sc. PD: | -0.19 |
| | | Sc. rank: | -92.4 |

PA-X

| Pos. | 21 | obs: | exp: |
|---|---|---|---|
| atg | M | 353 | 353.00 |
| --- | -- | ------ | ------ |
| mPD | | 0 | 0 |
| | | nPD: | 1. |
| | | N. weight: | 0. |
| | | Sc. PD: | 0 |
| | | Sc. rank: | 0 |

PA-X

| Pos. | 22 | obs: | exp: |
|---|---|---|---|
| aaa | K | 291 | 197.70 |
| aag | K | 62 | 155.30 |
| --- | -- | ------ | ------ |
| mPD | | 0.29 | 0.49 |
| | | nPD: | 0.59 |
| | | N. weight: | 0.22 |
| | | Sc. PD: | 0.079 |
| | | Sc. rank: | 41.5 |

PA-X

| Pos. | 23 | obs: | exp: |
|---|---|---|---|
| gaa | E | 340 | 203.10 |
| gag | E | 13 | 149.90 |
| --- | -- | ------ | ------ |
| mPD | | 0.071 | 0.49 |
| | | nPD: | 0.15 |
| | | N. weight: | 0.54 |
| | | Sc. PD: | -0.034 |
| | | Sc. rank: | 10.9 |

PA-X

| Pos. | 24 | obs: | exp: |
|---|---|---|---|
| tat | Y | 352 | 182.70 |
| tac | Y | 1 | 170.30 |
| --- | -- | ------ | ------ |
| mPD | | 0.0057 | 0.50 |
| | | nPD: | 0.01 |
| | | N. weight: | 0.84 |
| | | Sc. PD: | -0.16 |
| | | Sc. rank: | -94.1 |

PA-X

| Pos. | 25 | obs: | exp: |
|---|---|---|---|
| ggt | G | 0 | 43.56 |
| ggc | G | 0 | 47.74 |
| gga | G | 6 | 170.80 |
| ggg | G | 347 | 90.95 |
| --- | -- | ------ | ------ |
| mPD | | 0.034 | 0.67 |
| | | nPD: | 0.05 |
| | | N. weight: | 1.7 |
| | | Sc. PD: | -0.26 |
| | | Sc. rank: | -36.0 |

| | | | |
|---|---|---|---|
| PA-X | | | |
| Pos. 26 | | obs: | exp: |
| gaa | E | 341 | 203.10 |
| gag | E | 12 | 149.90 |
| --- | -- | ------ | ------ |
| mPD | | 0.066 | 0.49 |
| | | nPD: | 0.13 |
| | | N. weight: | 0.55 |
| | | Sc. PD: | -0.04 |
| | | Sc. rank: | 10.0 |

| | | | |
|---|---|---|---|
| PA-X | | | |
| Pos. 27 | | obs: | exp: |
| aat | N | 2 | 1.05 |
| aac | N | 0 | 0.95 |
| gat | D | 331 | 187.30 |
| gac | D | 20 | 163.70 |
| --- | -- | ------ | ------ |
| mPD | | 0.12 | 0.51 |
| | | nPD: | 0.23 |
| | | N. weight: | 0.56 |
| | | Sc. PD: | 0.012 |
| | | Sc. rank: | 42.0 |

| | | | |
|---|---|---|---|
| PA-X | | | |
| Pos. 28 | | obs: | exp: |
| tct | S | 0 | 0.16 |
| tcc | S | 0 | 0.14 |
| tca | S | 1 | 0.22 |
| tcg | S | 0 | 0.06 |
| cct | P | 0 | 92.69 |
| ccc | P | 0 | 63.49 |
| cca | P | 91 | 138.90 |
| ccg | P | 261 | 56.91 |
| agt | S | 0 | 0.22 |
| agc | S | 0 | 0.20 |
| --- | -- | ------ | ------ |
| mPD | | 0.39 | 0.72 |
| | | nPD: | 0.54 |
| | | N. weight: | 1.3 |
| | | Sc. PD: | 0.43 |
| | | Sc. rank: | 248.9 |

| | | | |
|---|---|---|---|
| PA-X | | | |
| Pos. 29 | | obs: | exp: |
| cgt | R | 0 | 0.04 |
| cgc | R | 0 | 0.05 |
| cga | R | 0 | 0.10 |
| cgg | R | 0 | 0.08 |
| aaa | K | 350 | 196.50 |
| aag | K | 1 | 154.50 |
| aga | R | 1 | 0.45 |
| agg | R | 0 | 0.28 |
| gaa | E | 1 | 0.58 |
| gag | E | 0 | 0.42 |
| --- | -- | ------ | ------ |
| mPD | | 0.017 | 0.51 |
| | | nPD: | 0.03 |
| | | N. weight: | 0.74 |
| | | Sc. PD: | -0.13 |
| | | Sc. rank: | -35.5 |

| | | | |
|---|---|---|---|
| PA-X | | | |
| Pos. 30 | | obs: | exp: |
| tta | L | 0 | 0.17 |
| ttg | L | 0 | 0.39 |
| ctt | L | 0 | 0.37 |
| ctc | L | 2 | 0.32 |
| cta | L | 0 | 0.30 |
| ctg | L | 0 | 0.44 |
| att | I | 14 | 131.20 |
| atc | I | 330 | 91.47 |
| ata | I | 5 | 126.30 |
| gtt | V | 1 | 0.41 |
| gtc | V | 1 | 0.42 |
| gta | V | 0 | 0.37 |
| gtg | V | 0 | 0.79 |
| --- | -- | ------ | ------ |
| mPD | | 0.13 | 0.68 |
| | | nPD: | 0.19 |
| | | N. weight: | 1.4 |
| | | Sc. PD: | -0.027 |
| | | Sc. rank: | 62.3 |

| | | | |
|---|---|---|---|
| PA-X | | | |
| Pos. 31 | | obs: | exp: |
| gaa | E | 281 | 203.10 |
| gag | E | 72 | 149.90 |
| --- | -- | ------ | ------ |
| mPD | | 0.33 | 0.49 |
| | | nPD: | 0.67 |
| | | N. weight: | 0.15 |
| | | Sc. PD: | 0.067 |
| | | Sc. rank: | 31.2 |

| | | | |
|---|---|---|---|
| PA-X | | | |
| Pos. 32 | | obs: | exp: |
| act | T | 15 | 93.26 |
| acc | T | 5 | 75.75 |
| aca | T | 168 | 154.60 |
| acg | T | 165 | 29.35 |
| --- | -- | ------ | ------ |
| mPD | | 0.55 | 0.69 |
| | | nPD: | 0.81 |
| | | N. weight: | 0.98 |
| | | Sc. PD: | 0.56 |
| | | Sc. rank: | 227.0 |

| | | | |
|---|---|---|---|
| PA-X | | | |
| Pos. 33 | | obs: | exp: |
| aat | N | 27 | 184.50 |
| aac | N | 326 | 168.50 |
| --- | -- | ------ | ------ |
| mPD | | 0.14 | 0.50 |
| | | nPD: | 0.28 |
| | | N. weight: | 0.62 |
| | | Sc. PD: | 0.043 |
| | | Sc. rank: | 56.9 |

| | | | |
|---|---|---|---|
| PA-X | | | |
| Pos. 34 | | obs: | exp: |
| aaa | K | 335 | 197.70 |
| aag | K | 18 | 155.30 |
| --- | -- | ------ | ------ |
| mPD | | 0.097 | 0.49 |
| | | nPD: | 0.2 |
| | | N. weight: | 0.52 |
| | | Sc. PD: | -0.0071 |
| | | Sc. rank: | 24.3 |

| | | | |
|---|---|---|---|
| PA-X | | | |
| Pos. 35 | | obs: | exp: |
| ttt | F | 270 | 134.80 |
| ttc | F | 58 | 193.20 |
| tta | L | 0 | 2.18 |
| ttg | L | 0 | 4.81 |
| ctt | L | 0 | 4.66 |
| ctc | L | 25 | 4.04 |
| cta | L | 0 | 3.77 |
| ctg | L | 0 | 5.53 |
| --- | -- | ------ | ------ |
| mPD | | 0.49 | 0.63 |
| | | nPD: | 0.79 |
| | | N. weight: | 0.62 |
| | | Sc. PD: | 0.34 |
| | | Sc. rank: | 138.8 |

| | | | |
|---|---|---|---|
| PA-X | | | |
| Pos. 36 | | obs: | exp: |
| act | T | 1 | 0.26 |
| acc | T | 0 | 0.21 |
| aca | T | 0 | 0.44 |
| acg | T | 0 | 0.08 |
| gct | A | 150 | 88.66 |
| gcc | A | 136 | 72.11 |
| gca | A | 65 | 156.90 |
| gcg | A | 1 | 34.35 |
| --- | -- | ------ | ------ |
| mPD | | 0.64 | 0.69 |
| | | nPD: | 0.93 |
| | | N. weight: | 0.42 |
| | | Sc. PD: | 0.29 |
| | | Sc. rank: | 98.0 |

| | | | |
|---|---|---|---|
| PA-X | | | |
| Pos. 37 | | obs: | exp: |
| tct | S | 0 | 4.13 |
| tcc | S | 0 | 3.62 |
| tca | S | 26 | 5.76 |
| tcg | S | 0 | 1.58 |
| agt | S | 0 | 5.72 |
| agc | S | 0 | 5.18 |
| gct | A | 0 | 82.36 |
| gcc | A | 2 | 66.99 |
| gca | A | 303 | 145.70 |
| gcg | A | 22 | 31.91 |
| --- | -- | ------ | ------ |
| mPD | | 0.26 | 0.89 |
| | | nPD: | 0.3 |
| | | N. weight: | 0.93 |
| | | Sc. PD: | 0.076 |
| | | Sc. rank: | 90.8 |

| | | | |
|---|---|---|---|
| PA-X | | | |
| Pos. 38 | | obs: | exp: |
| att | I | 0 | 128.20 |
| atc | I | 0 | 89.37 |
| ata | I | 341 | 123.40 |
| atg | M | 1 | 1.00 |
| gtt | V | 0 | 2.26 |
| gtc | V | 0 | 2.31 |
| gta | V | 11 | 2.06 |
| gtg | V | 0 | 4.37 |
| --- | -- | ------ | ------ |
| mPD | | 0.066 | 0.73 |
| | | nPD: | 0.09 |
| | | N. weight: | 1.4 |
| | | Sc. PD: | -0.16 |
| | | Sc. rank: | 5.3 |

| | | | |
|---|---|---|---|
| PA-X | | | |
| Pos. 39 | | obs: | exp: |
| tct | S | 0 | 0.16 |
| tcc | S | 0 | 0.14 |
| tca | S | 0 | 0.22 |
| tcg | S | 0 | 0.06 |
| tgt | C | 5 | 145.10 |
| tgc | C | 347 | 206.90 |
| agt | S | 0 | 0.22 |
| agc | S | 1 | 0.20 |
| --- | -- | ------ | ------ |
| mPD | | 0.034 | 0.49 |
| | | nPD: | 0.07 |
| | | N. weight: | 0.62 |
| | | Sc. PD: | -0.084 |
| | | Sc. rank: | -5.5 |

| | | | |
|---|---|---|---|
| PA-X | | | |
| Pos. 40 | | obs: | exp: |
| act | T | 25 | 93.26 |
| acc | T | 3 | 75.75 |
| aca | T | 308 | 154.60 |
| acg | T | 17 | 29.35 |
| --- | -- | ------ | ------ |
| mPD | | 0.23 | 0.69 |
| | | nPD: | 0.34 |
| | | N. weight: | 0.62 |
| | | Sc. PD: | 0.075 |
| | | Sc. rank: | 68.8 |

| | | | |
|---|---|---|---|
| PA-X | | | |
| Pos. 41 | | obs: | exp: |
| cat | H | 15 | 191.30 |
| cac | H | 338 | 161.70 |
| --- | -- | ------ | ------ |
| mPD | | 0.082 | 0.50 |
| | | nPD: | 0.16 |
| | | N. weight: | 0.79 |
| | | Sc. PD: | -0.035 |
| | | Sc. rank: | 20.3 |

| | | | |
|---|---|---|---|
| PA-X | | | |
| Pos. 42 | | obs: | exp: |
| tta | L | 276 | 30.84 |
| ttg | L | 68 | 67.98 |
| ctt | L | 0 | 65.86 |
| ctc | L | 0 | 57.10 |
| cta | L | 7 | 53.18 |
| ctg | L | 2 | 78.03 |
| --- | -- | ------ | ------ |
| mPD | | 0.37 | 1.1 |
| | | nPD: | 0.33 |
| | | N. weight: | 2.2 |
| | | Sc. PD: | 0.25 |
| | | Sc. rank: | 233.2 |

| | | | |
|---|---|---|---|
| PA-X | | | |
| Pos. 43 | | obs: | exp: |
| gaa | E | 338 | 203.10 |
| gag | E | 15 | 149.90 |
| --- | -- | ------ | ------ |
| mPD | | 0.082 | 0.49 |
| | | nPD: | 0.17 |
| | | N. weight: | 0.52 |
| | | Sc. PD: | -0.022 |
| | | Sc. rank: | 13.8 |

| | | | |
|---|---|---|---|
| PA-X | | | |
| Pos. 44 | | obs: | exp: |
| att | I | 0 | 1.50 |
| atc | I | 3 | 1.05 |
| ata | I | 1 | 1.45 |
| gtt | V | 8 | 71.53 |
| gtc | V | 265 | 73.04 |
| gta | V | 33 | 65.22 |
| gtg | V | 42 | 138.20 |
| gct | A | 0 | 0.25 |
| gcc | A | 1 | 0.20 |
| gca | A | 0 | 0.45 |
| gcg | A | 0 | 0.10 |
| --- | -- | ------ | ------ |
| mPD | | 0.42 | 0.75 |
| | | nPD: | 0.57 |
| | | N. weight: | 0.98 |
| | | Sc. PD: | 0.33 |
| | | Sc. rank: | 184.1 |

| | | | |
|---|---|---|---|
| PA-X | | | |
| Pos. 45 | | obs: | exp: |
| tgt | C | 284 | 145.50 |
| tgc | C | 69 | 207.50 |
| --- | -- | ------ | ------ |
| mPD | | 0.32 | 0.48 |
| | | nPD: | 0.65 |
| | | N. weight: | 0.43 |
| | | Sc. PD: | 0.18 |
| | | Sc. rank: | 86.5 |

| | | | |
|---|---|---|---|
| PA-X | | | |
| Pos. 46 | | obs: | exp: |
| ttt | F | 3 | 144.70 |
| ttc | F | 349 | 207.30 |
| tta | L | 0 | 0.09 |
| ttg | L | 0 | 0.19 |
| ctt | L | 0 | 0.19 |
| ctc | L | 1 | 0.16 |
| cta | L | 0 | 0.15 |
| ctg | L | 0 | 0.22 |
| --- | -- | ------ | ------ |
| mPD | | 0.023 | 0.49 |
| | | nPD: | 0.05 |
| | | N. weight: | 0.64 |
| | | Sc. PD: | -0.1 |
| | | Sc. rank: | -16.7 |

| | | | |
|---|---|---|---|
| PA-X | | | |
| Pos. 47 | | obs: | exp: |
| atg | M | 353 | 353.00 |
| --- | -- | ------ | ------ |
| mPD | | 0 | 0 |
| | | nPD: | 1. |
| | | N. weight: | 0. |
| | | Sc. PD: | 0 |
| | | Sc. rank: | 0 |

| | | | |
|---|---|---|---|
| PA-X | | | |
| Pos. 48 | | obs: | exp: |
| tat | Y | 286 | 182.70 |
| tac | Y | 67 | 170.30 |
| --- | -- | ------ | ------ |
| mPD | | 0.31 | 0.50 |
| | | nPD: | 0.62 |
| | | N. weight: | 0.25 |
| | | Sc. PD: | 0.099 |
| | | Sc. rank: | 49.3 |

| | | | |
|---|---|---|---|
| PA-X | | | |
| Pos. 49 | | obs: | exp: |
| tct | S | 26 | 56.12 |
| tcc | S | 1 | 49.17 |
| tca | S | 88 | 78.20 |
| tcg | S | 238 | 21.52 |
| agt | S | 0 | 77.66 |
| agc | S | 0 | 70.33 |
| --- | -- | ------ | ------ |
| mPD | | 0.48 | 1.7 |
| | | nPD: | 0.29 |
| | | N. weight: | 2.1 |
| | | Sc. PD: | 0.15 |
| | | Sc. rank: | 202.2 |

| | | | |
|---|---|---|---|
| PA-X | | | |
| Pos. 50 | | obs: | exp: |
| gat | D | 327 | 188.30 |
| gac | D | 26 | 164.70 |
| --- | -- | ------ | ------ |
| mPD | | 0.14 | 0.50 |
| | | nPD: | 0.27 |
| | | N. weight: | 0.5 |
| | | Sc. PD: | 0.031 |
| | | Sc. rank: | 44.6 |

| PA-X | | | |
|---|---|---|---|
| Pos. | 51 | obs: | exp: |
| ttt | F | 44 | 144.70 |
| ttc | F | 308 | 207.30 |
| tta | L | 0 | 0.09 |
| ttg | L | 0 | 0.19 |
| ctt | L | 0 | 0.19 |
| ctc | L | 1 | 0.16 |
| cta | L | 0 | 0.15 |
| ctg | L | 0 | 0.22 |
| --- | -- | ------ | ------ |
| mPD | | 0.22 | 0.49 |
| | | nPD: | 0.46 |
| | | N. weight: | 0.28 |
| | | Sc. PD: | 0.065 |
| | | Sc. rank: | 39.3 |

,

| PA-X | | | |
|---|---|---|---|
| Pos. | 52 | obs: | exp: |
| cat | H | 99 | 191.30 |
| cac | H | 254 | 161.70 |
| --- | -- | ------ | ------ |
| mPD | | 0.40 | 0.50 |
| | | nPD: | 0.82 |
| | | N. weight: | 0.19 |
| | | Sc. PD: | 0.11 |
| | | Sc. rank: | 45.2 |

,

| PA-X | | | |
|---|---|---|---|
| Pos. | 53 | obs: | exp: |
| ttt | F | 214 | 145.10 |
| ttc | F | 139 | 207.90 |
| --- | -- | ------ | ------ |
| mPD | | 0.48 | 0.48 |
| | | nPD: | 0.99 |
| | | N. weight: | 0.11 |
| | | Sc. PD: | 0.084 |
| | | Sc. rank: | 26.7 |

,

| PA-X | | | |
|---|---|---|---|
| Pos. | 54 | obs: | exp: |
| att | I | 335 | 132.30 |
| atc | I | 17 | 92.25 |
| ata | I | 0 | 127.40 |
| act | T | 1 | 0.26 |
| acc | T | 0 | 0.21 |
| aca | T | 0 | 0.44 |
| acg | T | 0 | 0.08 |
| --- | -- | ------ | ------ |
| mPD | | 0.098 | 0.66 |
| | | nPD: | 0.15 |
| | | N. weight: | 1.1 |
| | | Sc. PD: | -0.065 |
| | | Sc. rank: | 23.6 |

,

| PA-X | | | |
|---|---|---|---|
| Pos. | 55 | obs: | exp: |
| aat | N | 19 | 9.93 |
| aac | N | 0 | 9.07 |
| gat | D | 303 | 178.20 |
| gac | D | 31 | 155.80 |
| --- | -- | ------ | ------ |
| mPD | | 0.26 | 0.60 |
| | | nPD: | 0.44 |
| | | N. weight: | 0.47 |
| | | Sc. PD: | 0.1 |
| | | Sc. rank: | 65.5 |

,

| PA-X | | | |
|---|---|---|---|
| Pos. | 56 | obs: | exp: |
| gaa | E | 336 | 203.10 |
| gag | E | 17 | 149.90 |
| --- | -- | ------ | ------ |
| mPD | | 0.092 | 0.49 |
| | | nPD: | 0.19 |
| | | N. weight: | 0.5 |
| | | Sc. PD: | -0.011 |
| | | Sc. rank: | 19.9 |

,

| PA-X | | | |
|---|---|---|---|
| Pos. | 57 | obs: | exp: |
| caa | Q | 2 | 1.07 |
| cag | Q | 0 | 0.93 |
| cgt | R | 0 | 14.57 |
| cgc | R | 0 | 17.19 |
| cga | R | 237 | 33.73 |
| cgg | R | 107 | 29.29 |
| aga | R | 3 | 157.90 |
| agg | R | 4 | 98.36 |
| --- | -- | ------ | ------ |
| mPD | | 0.48 | 0.97 |
| | | nPD: | 0.5 |
| | | N. weight: | 2.2 |
| | | Sc. PD: | 0.59 |
| | | Sc. rank: | 352.4 |

,

| PA-X | | | |
|---|---|---|---|
| Pos. | 58 | obs: | exp: |
| ggt | G | 100 | 43.56 |
| ggc | G | 249 | 47.74 |
| gga | G | 4 | 170.80 |
| ggg | G | 0 | 90.95 |
| --- | -- | ------ | ------ |
| mPD | | 0.42 | 0.67 |
| | | nPD: | 0.64 |
| | | N. weight: | 1.8 |
| | | Sc. PD: | 0.73 |
| | | Sc. rank: | 355.0 |

,

| PA-X | | | |
|---|---|---|---|
| Pos. | 59 | obs: | exp: |
| gat | D | 2 | 1.07 |
| gac | D | 0 | 0.93 |
| gaa | E | 279 | 202.00 |
| gag | E | 72 | 149.00 |
| --- | -- | ------ | ------ |
| mPD | | 0.33 | 0.49 |
| | | nPD: | 0.68 |
| | | N. weight: | 0.16 |
| | | Sc. PD: | 0.069 |
| | | Sc. rank: | 32.7 |

,

| PA-X | | | |
|---|---|---|---|
| Pos. | 60 | obs: | exp: |
| tct | S | 1 | 56.12 |
| tcc | S | 0 | 49.17 |
| tca | S | 287 | 78.20 |
| tcg | S | 65 | 21.52 |
| agt | S | 0 | 77.66 |
| agc | S | 0 | 70.33 |
| --- | -- | ------ | ------ |
| mPD | | 0.31 | 1.7 |
| | | nPD: | 0.18 |
| | | N. weight: | 1.7 |
| | | Sc. PD: | -0.043 |
| | | Sc. rank: | 57.7 |

,

| PA-X | | | |
|---|---|---|---|
| Pos. | 61 | obs: | exp: |
| att | I | 0 | 119.50 |
| atc | I | 0 | 83.34 |
| ata | I | 318 | 115.10 |
| atg | M | 3 | 3.00 |
| act | T | 1 | 7.13 |
| acc | T | 25 | 5.79 |
| aca | T | 1 | 11.83 |
| acg | T | 0 | 2.24 |
| gtt | V | 0 | 1.03 |
| gtc | V | 0 | 1.05 |
| gta | V | 5 | 0.94 |
| gtg | V | 0 | 1.99 |
| --- | -- | ------ | ------ |
| mPD | | 0.32 | 0.84 |
| | | nPD: | 0.38 |
| | | N. weight: | 1.4 |
| | | Sc. PD: | 0.22 |
| | | Sc. rank: | 168.2 |

,

| PA-X | | | |
|---|---|---|---|
| Pos. | 62 | obs: | exp: |
| att | I | 253 | 123.70 |
| atc | I | 76 | 86.23 |
| ata | I | 0 | 119.10 |
| gtt | V | 24 | 4.93 |
| gtc | V | 0 | 5.04 |
| gta | V | 0 | 4.50 |
| gtg | V | 0 | 9.53 |
| --- | -- | ------ | ------ |
| mPD | | 0.47 | 0.80 |
| | | nPD: | 0.58 |
| | | N. weight: | 0.79 |
| | | Sc. PD: | 0.28 |
| | | Sc. rank: | 149.3 |

,

| PA-X | | | |
|---|---|---|---|
| Pos. | 63 | obs: | exp: |
| tta | L | 8 | 0.96 |
| ttg | L | 2 | 2.12 |
| ctt | L | 0 | 2.05 |
| ctc | L | 0 | 1.78 |
| cta | L | 1 | 1.66 |
| ctg | L | 0 | 2.43 |
| att | I | 0 | 10.15 |
| atc | I | 0 | 7.08 |
| ata | I | 27 | 9.77 |
| gtt | V | 0 | 63.92 |
| gtc | V | 1 | 65.27 |
| gta | V | 209 | 58.29 |
| gtg | V | 101 | 123.50 |
| gct | A | 0 | 1.01 |
| gcc | A | 0 | 0.82 |
| gca | A | 4 | 1.78 |
| gcg | A | 0 | 0.39 |
| --- | -- | ------ | ------ |
| mPD | | 0.64 | 0.95 |
| | | nPD: | 0.67 |
| | | N. weight: | 1.1 |
| | | Sc. PD: | 0.48 |
| | | Sc. rank: | 225.6 |

,

| PA-X | | | |
|---|---|---|---|
| Pos. | 64 | obs: | exp: |
| gat | D | 0 | 0.53 |
| gac | D | 1 | 0.47 |
| gaa | E | 299 | 202.60 |
| gag | E | 53 | 149.40 |
| --- | -- | ------ | ------ |
| mPD | | 0.26 | 0.49 |
| | | nPD: | 0.53 |
| | | N. weight: | 0.24 |
| | | Sc. PD: | 0.074 |
| | | Sc. rank: | 43.0 |

,

| PA-X | | | |
|---|---|---|---|
| Pos. | 65 | obs: | exp: |
| tct | S | 282 | 56.12 |
| tcc | S | 22 | 49.17 |
| tca | S | 22 | 78.20 |
| tcg | S | 27 | 21.52 |
| agt | S | 0 | 77.66 |
| agc | S | 0 | 70.33 |
| --- | -- | ------ | ------ |
| mPD | | 0.35 | 1.7 |
| | | nPD: | 0.21 |
| | | N. weight: | 1.6 |
| | | Sc. PD: | -0.0017 |
| | | Sc. rank: | 94.7 |

,

| PA-X | | | |
|---|---|---|---|
| Pos. | 66 | obs: | exp: |
| tct | S | 0 | 0.79 |
| tcc | S | 0 | 0.70 |
| tca | S | 0 | 1.11 |
| tcg | S | 0 | 0.30 |
| agt | S | 3 | 1.10 |
| agc | S | 2 | 1.00 |
| gat | D | 0 | 0.53 |
| gac | D | 1 | 0.47 |
| ggt | G | 38 | 42.82 |
| ggc | G | 287 | 46.93 |
| gga | G | 17 | 167.90 |
| ggg | G | 5 | 89.40 |
| --- | -- | ------ | ------ |
| mPD | | 0.34 | 0.72 |
| | | nPD: | 0.48 |
| | | N. weight: | 1.8 |
| | | Sc. PD: | 0.45 |
| | | Sc. rank: | 258.9 |

,

| PA-X | | | |
|---|---|---|---|
| Pos. | 67 | obs: | exp: |
| gat | D | 321 | 188.30 |
| gac | D | 32 | 164.70 |
| --- | -- | ------ | ------ |
| mPD | | 0.17 | 0.50 |
| | | nPD: | 0.33 |
| | | N. weight: | 0.45 |
| | | Sc. PD: | 0.052 |
| | | Sc. rank: | 48.4 |

,

| PA-X | | | |
|---|---|---|---|
| Pos. | 68 | obs: | exp: |
| tct | S | 0 | 0.32 |
| tcc | S | 0 | 0.28 |
| tca | S | 0 | 0.44 |
| tcg | S | 2 | 0.12 |
| cct | P | 0 | 92.42 |
| ccc | P | 1 | 63.31 |
| cca | P | 142 | 138.50 |
| ccg | P | 208 | 56.75 |
| agt | S | 0 | 0.44 |
| agc | S | 0 | 0.40 |
| --- | -- | ------ | ------ |
| mPD | | 0.50 | 0.73 |
| | | nPD: | 0.68 |
| | | N. weight: | 1. |
| | | Sc. PD: | 0.46 |
| | | Sc. rank: | 219.9 |

,

| PA-X | | | |
|---|---|---|---|
| Pos. | 69 | obs: | exp: |
| aat | N | 318 | 184.50 |
| aac | N | 35 | 168.50 |
| --- | -- | ------ | ------ |
| mPD | | 0.18 | 0.50 |
| | | nPD: | 0.36 |
| | | N. weight: | 0.45 |
| | | Sc. PD: | 0.064 |
| | | Sc. rank: | 52.1 |

,

| PA-X | | | |
|---|---|---|---|
| Pos. | 70 | obs: | exp: |
| gtt | V | 0 | 3.49 |
| gtc | V | 0 | 3.57 |
| gta | V | 2 | 3.19 |
| gtg | V | 15 | 6.75 |
| gct | A | 1 | 84.63 |
| gcc | A | 1 | 68.83 |
| gca | A | 319 | 149.80 |
| gcg | A | 15 | 32.79 |
| --- | -- | ------ | ------ |
| mPD | | 0.26 | 0.79 |
| | | nPD: | 0.33 |
| | | N. weight: | 0.88 |
| | | Sc. PD: | 0.099 |
| | | Sc. rank: | 89.1 |

,

| PA-X | | | |
|---|---|---|---|
| Pos. | 71 | obs: | exp: |
| tta | L | 216 | 30.84 |
| ttg | L | 40 | 67.98 |
| ctt | L | 2 | 65.86 |
| ctc | L | 66 | 57.10 |
| cta | L | 25 | 53.18 |
| ctg | L | 4 | 78.03 |
| --- | -- | ------ | ------ |
| mPD | | 0.88 | 1.1 |
| | | nPD: | 0.79 |
| | | N. weight: | 1.4 |
| | | Sc. PD: | 0.79 |
| | | Sc. rank: | 323.3 |

,

| PA-X | | | |
|---|---|---|---|
| Pos. | 72 | obs: | exp: |
| tta | L | 10 | 30.84 |
| ttg | L | 219 | 67.98 |
| ctt | L | 0 | 65.86 |
| ctc | L | 0 | 57.10 |
| cta | L | 66 | 53.18 |
| ctg | L | 58 | 78.03 |
| --- | -- | ------ | ------ |
| mPD | | 0.80 | 1.1 |
| | | nPD: | 0.71 |
| | | N. weight: | 0.93 |
| | | Sc. PD: | 0.45 |
| | | Sc. rank: | 202.6 |

,

| PA-X | | | |
|---|---|---|---|
| Pos. | 73 | obs: | exp: |
| aaa | K | 298 | 197.70 |
| aag | K | 55 | 155.30 |
| --- | -- | ------ | ------ |
| mPD | | 0.26 | 0.49 |
| | | nPD: | 0.54 |
| | | N. weight: | 0.25 |
| | | Sc. PD: | 0.078 |
| | | Sc. rank: | 45.0 |

,

| PA-X | | | |
|---|---|---|---|
| Pos. | 74 | obs: | exp: |
| cat | H | 17 | 191.30 |
| cac | H | 336 | 161.70 |
| --- | -- | ------ | ------ |
| mPD | | 0.092 | 0.50 |
| | | nPD: | 0.19 |
| | | N. weight: | 0.77 |
| | | Sc. PD: | -0.019 |
| | | Sc. rank: | 27.9 |

,

| PA-X | | | |
|---|---|---|---|
| Pos. | 75 | obs: | exp: |
| cgt | R | 22 | 14.65 |
| cgc | R | 5 | 17.28 |
| cga | R | 237 | 33.93 |
| cgg | R | 84 | 29.46 |
| aga | R | 5 | 158.80 |
| agg | R | 0 | 98.92 |
| --- | -- | ------ | ------ |
| mPD | | 0.50 | 0.96 |
| | | nPD: | 0.52 |
| | | N. weight: | 2. |
| | | Sc. PD: | 0.6 |
| | | Sc. rank: | 357.4 |

,

| PA-X | | | |
|---|---|---|---|
| Pos. | 76 | obs: | exp: |
| ttt | F | 346 | 145.10 |
| ttc | F | 7 | 207.90 |
| --- | -- | ------ | ------ |
| mPD | | 0.039 | 0.48 |
| | | nPD: | 0.08 |
| | | N. weight: | 1. |
| | | Sc. PD: | -0.13 |
| | | Sc. rank: | -5.3 |

| PA-X | | | |
|---|---|---|---|
| Pos. | 77 | obs: | exp: |
| gaa | E | 174 | 203.10 |
| gag | E | 179 | 149.90 |
| --- | -- | ------ | ------ |
| mPD | | 0.50 | 0.49 |
| | | nPD: | 1.03 |
| | | N. weight: | 0.03 |
| | | Sc. PD: | 0.023 |
| | | Sc. rank: | 7.1 |

| PA-X | | | |
|---|---|---|---|
| Pos. | 78 | obs: | exp: |
| att | I | 0 | 132.70 |
| atc | I | 0 | 92.52 |
| ata | I | 353 | 127.80 |
| --- | -- | ------ | ------ |
| mPD | | 0 | 0.66 |
| | | nPD: | 0. |
| | | N. weight: | 1.3 |
| | | Sc. PD: | -0.27 |
| | | Sc. rank: | -301.2 |

| PA-X | | | |
|---|---|---|---|
| Pos. | 79 | obs: | exp: |
| att | I | 267 | 132.70 |
| atc | I | 85 | 92.52 |
| ata | I | 1 | 127.80 |
| --- | -- | ------ | ------ |
| mPD | | 0.37 | 0.66 |
| | | nPD: | 0.56 |
| | | N. weight: | 0.66 |
| | | Sc. PD: | 0.22 |
| | | Sc. rank: | 123.4 |

| PA-X | | | |
|---|---|---|---|
| Pos. | 80 | obs: | exp: |
| gaa | E | 348 | 203.10 |
| gag | E | 5 | 149.90 |
| --- | -- | ------ | ------ |
| mPD | | 0.028 | 0.49 |
| | | nPD: | 0.06 |
| | | N. weight: | 0.64 |
| | | Sc. PD: | -0.094 |
| | | Sc. rank: | -9.0 |

| PA-X | | | |
|---|---|---|---|
| Pos. | 81 | obs: | exp: |
| ggt | G | 0 | 43.56 |
| ggc | G | 0 | 47.74 |
| gga | G | 46 | 170.80 |
| ggg | G | 307 | 90.95 |
| --- | -- | ------ | ------ |
| mPD | | 0.23 | 0.67 |
| | | nPD: | 0.34 |
| | | N. weight: | 1.2 |
| | | Sc. PD: | 0.15 |
| | | Sc. rank: | 133.0 |

| PA-X | | | |
|---|---|---|---|
| Pos. | 82 | obs: | exp: |
| cgt | R | 0 | 14.61 |
| cgc | R | 0 | 17.24 |
| cga | R | 1 | 33.83 |
| cgg | R | 0 | 29.37 |
| aaa | K | 1 | 0.56 |
| aag | K | 0 | 0.44 |
| aga | R | 347 | 158.30 |
| agg | R | 4 | 98.64 |
| --- | -- | ------ | ------ |
| mPD | | 0.034 | 0.96 |
| | | nPD: | 0.04 |
| | | N. weight: | 0.96 |
| | | Sc. PD: | -0.16 |
| | | Sc. rank: | -36.7 |

| PA-X | | | |
|---|---|---|---|
| Pos. | 83 | obs: | exp: |
| tat | Y | 0 | 0.52 |
| tac | Y | 1 | 0.48 |
| gat | D | 70 | 187.80 |
| gac | D | 282 | 164.20 |
| --- | -- | ------ | ------ |
| mPD | | 0.32 | 0.50 |
| | | nPD: | 0.64 |
| | | N. weight: | 0.32 |
| | | Sc. PD: | 0.13 |
| | | Sc. rank: | 64.2 |

| PA-X | | | |
|---|---|---|---|
| Pos. | 84 | obs: | exp: |
| cgt | R | 27 | 14.65 |
| cgc | R | 42 | 17.28 |
| cga | R | 269 | 33.93 |
| cgg | R | 1 | 29.46 |
| aga | R | 14 | 158.80 |
| agg | R | 0 | 98.92 |
| --- | -- | ------ | ------ |
| mPD | | 0.41 | 0.96 |
| | | nPD: | 0.43 |
| | | N. weight: | 2.2 |
| | | Sc. PD: | 0.46 |
| | | Sc. rank: | 301.3 |

| PA-X | | | |
|---|---|---|---|
| Pos. | 85 | obs: | exp: |
| cct | P | 0 | 0.26 |
| ccc | P | 0 | 0.18 |
| cca | P | 1 | 0.39 |
| ccg | P | 0 | 0.16 |
| att | I | 0 | 0.38 |
| atc | I | 0 | 0.26 |
| ata | I | 1 | 0.36 |
| act | T | 72 | 91.94 |
| acc | T | 8 | 74.68 |
| aca | T | 225 | 152.50 |
| acg | T | 43 | 28.93 |
| gct | A | 0 | 0.76 |
| gcc | A | 0 | 0.61 |
| gca | A | 3 | 1.34 |
| gcg | A | 0 | 0.29 |
| --- | -- | ------ | ------ |
| mPD | | 0.55 | 0.71 |
| | | nPD: | 0.77 |
| | | N. weight: | 0.3 |
| | | Sc. PD: | 0.16 |
| | | Sc. rank: | 67.5 |

| PA-X | | | |
|---|---|---|---|
| Pos. | 86 | obs: | exp: |
| atg | M | 334 | 334.00 |
| gtt | V | 2 | 3.91 |
| gtc | V | 0 | 3.99 |
| gta | V | 0 | 3.56 |
| gtg | V | 17 | 7.55 |
| --- | -- | ------ | ------ |
| mPD | | 0.11 | 0.17 |
| | | nPD: | 0.69 |
| | | N. weight: | 0.051 |
| | | Sc. PD: | 0.023 |
| | | Sc. rank: | 10.8 |

| PA-X | | | |
|---|---|---|---|
| Pos. | 87 | obs: | exp: |
| gct | A | 14 | 88.91 |
| gcc | A | 326 | 72.31 |
| gca | A | 13 | 157.30 |
| gcg | A | 0 | 34.45 |
| --- | -- | ------ | ------ |
| mPD | | 0.14 | 0.69 |
| | | nPD: | 0.21 |
| | | N. weight: | 1.6 |
| | | Sc. PD: | -0.00072 |
| | | Sc. rank: | 102.6 |

| PA-X | | | |
|---|---|---|---|
| Pos. | 88 | obs: | exp: |
| tgg | W | 353 | 353.00 |
| --- | -- | ------ | ------ |
| mPD | | 0 | 0 |
| | | nPD: | 1. |
| | | N. weight: | 0. |
| | | Sc. PD: | 0 |
| | | Sc. rank: | 0 |

| PA-X | | | |
|---|---|---|---|
| Pos. | 89 | obs: | exp: |
| act | T | 0 | 93.26 |
| acc | T | 1 | 75.75 |
| aca | T | 325 | 154.60 |
| acg | T | 27 | 29.35 |
| --- | -- | ------ | ------ |
| mPD | | 0.15 | 0.69 |
| | | nPD: | 0.21 |
| | | N. weight: | 0.88 |
| | | Sc. PD: | 0.0027 |
| | | Sc. rank: | 57.4 |

| PA-X | | | |
|---|---|---|---|
| Pos. | 90 | obs: | exp: |
| att | I | 0 | 0.38 |
| atc | I | 0 | 0.26 |
| ata | I | 1 | 0.36 |
| gtt | V | 0 | 72.35 |
| gtc | V | 0 | 73.88 |
| gta | V | 70 | 65.97 |
| gtg | V | 282 | 139.80 |
| --- | -- | ------ | ------ |
| mPD | | 0.33 | 0.73 |
| | | nPD: | 0.45 |
| | | N. weight: | 0.76 |
| | | Sc. PD: | 0.18 |
| | | Sc. rank: | 108.8 |

| PA-X | | | |
|---|---|---|---|
| Pos. | 91 | obs: | exp: |
| atg | M | 1 | 1.00 |
| gtt | V | 4 | 72.35 |
| gtc | V | 0 | 73.88 |
| gta | V | 58 | 65.97 |
| gtg | V | 290 | 139.80 |
| --- | -- | ------ | ------ |
| mPD | | 0.30 | 0.73 |
| | | nPD: | 0.41 |
| | | N. weight: | 0.73 |
| | | Sc. PD: | 0.14 |
| | | Sc. rank: | 97.7 |

| PA-X | | | |
|---|---|---|---|
| Pos. | 92 | obs: | exp: |
| aat | N | 279 | 184.50 |
| aac | N | 74 | 168.50 |
| --- | -- | ------ | ------ |
| mPD | | 0.33 | 0.50 |
| | | nPD: | 0.67 |
| | | N. weight: | 0.21 |
| | | Sc. PD: | 0.093 |
| | | Sc. rank: | 43.4 |

| PA-X | | | |
|---|---|---|---|
| Pos. | 93 | obs: | exp: |
| tct | S | 0 | 56.12 |
| tcc | S | 0 | 49.17 |
| tca | S | 0 | 78.20 |
| tcg | S | 0 | 21.52 |
| agt | S | 235 | 77.66 |
| agc | S | 118 | 70.33 |
| --- | -- | ------ | ------ |
| mPD | | 0.45 | 1.7 |
| | | nPD: | 0.27 |
| | | N. weight: | 1.2 |
| | | Sc. PD: | 0.066 |
| | | Sc. rank: | 103.5 |

| PA-X | | | |
|---|---|---|---|
| Pos. | 94 | obs: | exp: |
| att | I | 72 | 132.70 |
| atc | I | 280 | 92.52 |
| ata | I | 1 | 127.80 |
| --- | -- | ------ | ------ |
| mPD | | 0.33 | 0.66 |
| | | nPD: | 0.5 |
| | | N. weight: | 0.98 |
| | | Sc. PD: | 0.27 |
| | | Sc. rank: | 165.9 |

| PA-X | | | |
|---|---|---|---|
| Pos. | 95 | obs: | exp: |
| tgt | C | 14 | 145.50 |
| tgc | C | 339 | 207.50 |
| --- | -- | ------ | ------ |
| mPD | | 0.076 | 0.48 |
| | | nPD: | 0.16 |
| | | N. weight: | 0.5 |
| | | Sc. PD: | -0.026 |
| | | Sc. rank: | 12.2 |

| PA-X | | | |
|---|---|---|---|
| Pos. | 96 | obs: | exp: |
| aat | N | 5 | 184.50 |
| aac | N | 348 | 168.50 |
| --- | -- | ------ | ------ |
| mPD | | 0.028 | 0.50 |
| | | nPD: | 0.06 |
| | | N. weight: | 0.88 |
| | | Sc. PD: | -0.13 |
| | | Sc. rank: | -14.4 |

| PA-X | | | |
|---|---|---|---|
| Pos. | 97 | obs: | exp: |
| act | T | 43 | 92.73 |
| acc | T | 306 | 75.32 |
| aca | T | 2 | 153.80 |
| acg | T | 0 | 29.18 |
| gct | A | 0 | 0.50 |
| gcc | A | 2 | 0.41 |
| gca | A | 0 | 0.89 |
| gcg | A | 0 | 0.20 |
| --- | -- | ------ | ------ |
| mPD | | 0.24 | 0.70 |
| | | nPD: | 0.34 |
| | | N. weight: | 1.5 |
| | | Sc. PD: | 0.18 |
| | | Sc. rank: | 161.2 |

| PA-X | | | |
|---|---|---|---|
| Pos. | 98 | obs: | exp: |
| act | T | 5 | 92.47 |
| acc | T | 4 | 75.10 |
| aca | T | 308 | 153.30 |
| acg | T | 33 | 29.10 |
| gct | A | 0 | 0.76 |
| gcc | A | 0 | 0.61 |
| gca | A | 3 | 1.34 |
| gcg | A | 0 | 0.29 |
| --- | -- | ------ | ------ |
| mPD | | 0.23 | 0.70 |
| | | nPD: | 0.33 |
| | | N. weight: | 0.75 |
| | | Sc. PD: | 0.086 |
| | | Sc. rank: | 76.5 |

| PA-X | | | |
|---|---|---|---|
| Pos. | 99 | obs: | exp: |
| cgt | R | 0 | 0.08 |
| cgc | R | 0 | 0.10 |
| cga | R | 0 | 0.19 |
| cgg | R | 0 | 0.17 |
| aga | R | 2 | 0.90 |
| agg | R | 0 | 0.56 |
| ggt | G | 7 | 43.31 |
| ggc | G | 0 | 47.47 |
| gga | G | 285 | 169.80 |
| ggg | G | 59 | 90.43 |
| --- | -- | ------ | ------ |
| mPD | | 0.32 | 0.68 |
| | | nPD: | 0.48 |
| | | N. weight: | 0.43 |
| | | Sc. PD: | 0.11 |
| | | Sc. rank: | 61.9 |

| PA-X | | | |
|---|---|---|---|
| Pos. | 100 | obs: | exp: |
| att | I | 0 | 1.13 |
| atc | I | 3 | 0.79 |
| ata | I | 0 | 1.09 |
| gtt | V | 64 | 71.12 |
| gtc | V | 275 | 72.62 |
| gta | V | 4 | 64.85 |
| gtg | V | 3 | 137.40 |
| gct | A | 0 | 1.01 |
| gcc | A | 4 | 0.82 |
| gca | A | 0 | 1.78 |
| gcg | A | 0 | 0.39 |
| --- | -- | ------ | ------ |
| mPD | | 0.37 | 0.76 |
| | | nPD: | 0.48 |
| | | N. weight: | 1.3 |
| | | Sc. PD: | 0.34 |
| | | Sc. rank: | 198.6 |

| PA-X | | | |
|---|---|---|---|
| Pos. | 101 | obs: | exp: |
| aat | N | 0 | 1.05 |
| aac | N | 2 | 0.95 |
| gat | D | 99 | 65.09 |
| gac | D | 23 | 56.91 |
| gaa | E | 107 | 131.20 |
| gag | E | 121 | 96.80 |
| ggt | G | 0 | 0.12 |
| ggc | G | 0 | 0.14 |
| gga | G | 0 | 0.48 |
| ggg | G | 1 | 0.26 |
| --- | -- | ------ | ------ |
| mPD | | 0.72 | 0.74 |
| | | nPD: | 0.98 |
| | | N. weight: | 0.13 |
| | | Sc. PD: | 0.093 |
| | | Sc. rank: | 29.9 |

| PA-X | | | |
|---|---|---|---|
| Pos. | 102 | obs: | exp: |
| aaa | K | 136 | 197.70 |
| aag | K | 217 | 155.30 |
| --- | -- | ------ | ------ |
| mPD | | 0.48 | 0.49 |
| | | nPD: | 0.96 |
| | | N. weight: | 0.092 |
| | | Sc. PD: | 0.066 |
| | | Sc. rank: | 21.8 |

| PA-X | | | |
|---|---|---|---|
| Pos. | 103 | obs: | exp: |
| cct | P | 204 | 92.95 |
| ccc | P | 147 | 63.67 |
| cca | P | 2 | 139.30 |
| ccg | P | 0 | 57.08 |
| --- | -- | ------ | ------ |
| mPD | | 0.49 | 0.72 |
| | | nPD: | 0.69 |
| | | N. weight: | 1. |
| | | Sc. PD: | 0.48 |
| | | Sc. rank: | 222.0 |

| PA-X | | | |
|---|---|---|---|
| Pos. | 104 | obs: | exp: |
| cgt | R | 0 | 0.46 |
| cgc | R | 0 | 0.54 |
| cga | R | 0 | 1.06 |
| cgg | R | 0 | 0.92 |
| aaa | K | 265 | 191.50 |
| aag | K | 77 | 150.50 |
| aga | R | 5 | 4.95 |
| agg | R | 6 | 3.08 |
| --- | -- | ------ | ------ |
| mPD | | 0.42 | 0.57 |
| | | nPD: | 0.74 |
| | | N. weight: | 0.16 |
| | | Sc. PD: | 0.08 |
| | | Sc. rank: | 35.4 |

| PA-X | | | |
|---|---|---|---|
| Pos. | 105 | obs: | exp: |
| ttt | F | 174 | 145.10 |
| ttc | F | 179 | 207.90 |
| --- | -- | ------ | ------ |
| mPD | | 0.50 | 0.48 |
| | | nPD: | 1.04 |
| | | N. weight: | 0.03 |
| | | Sc. PD: | 0.023 |
| | | Sc. rank: | 7.0 |

| PA-X | | | |
|---|---|---|---|
| Pos. | 106 | obs: | exp: |
| tta | L | 0 | 30.84 |
| ttg | L | 0 | 67.98 |
| ctt | L | 54 | 65.86 |
| ctc | L | 296 | 57.10 |
| cta | L | 3 | 53.18 |
| ctg | L | 0 | 78.03 |
| --- | -- | ------ | ------ |
| mPD | | 0.27 | 1.1 |
| | | nPD: | 0.25 |
| | | N. weight: | 1.8 |
| | | Sc. PD: | 0.059 |
| | | Sc. rank: | 141.4 |

| PA-X | | | |
|---|---|---|---|
| Pos. | 107 | obs: | exp: |
| cct | P | 58 | 92.95 |
| ccc | P | 46 | 63.67 |
| cca | P | 135 | 139.30 |
| ccg | P | 114 | 57.08 |
| --- | -- | ------ | ------ |
| mPD | | 0.71 | 0.72 |
| | | nPD: | 0.99 |
| | | N. weight: | 0.15 |
| | | Sc. PD: | 0.11 |
| | | Sc. rank: | 36.0 |

| PA-X | | | |
|---|---|---|---|
| Pos. | 108 | obs: | exp: |
| gat | D | 340 | 188.30 |
| gac | D | 13 | 164.70 |
| --- | -- | ------ | ------ |
| mPD | | 0.071 | 0.50 |
| | | nPD: | 0.14 |
| | | N. weight: | 0.63 |
| | | Sc. PD: | -0.041 |
| | | Sc. rank: | 12.1 |

| PA-X | | | |
|---|---|---|---|
| Pos. | 109 | obs: | exp: |
| tta | L | 48 | 30.84 |
| ttg | L | 246 | 67.98 |
| ctt | L | 0 | 65.86 |
| ctc | L | 0 | 57.10 |
| cta | L | 5 | 53.18 |
| ctg | L | 54 | 78.03 |
| --- | -- | ------ | ------ |
| mPD | | 0.54 | 1.1 |
| | | nPD: | 0.48 |
| | | N. weight: | 1.2 |
| | | Sc. PD: | 0.3 |
| | | Sc. rank: | 173.3 |

| PA-X | | | |
|---|---|---|---|
| Pos. | 110 | obs: | exp: |
| tat | Y | 287 | 182.70 |
| tac | Y | 66 | 170.30 |
| --- | -- | ------ | ------ |
| mPD | | 0.30 | 0.50 |
| | | nPD: | 0.61 |
| | | N. weight: | 0.26 |
| | | Sc. PD: | 0.099 |
| | | Sc. rank: | 49.8 |

| PA-X | | | |
|---|---|---|---|
| Pos. | 111 | obs: | exp: |
| gat | D | 114 | 188.30 |
| gac | D | 239 | 164.70 |
| --- | -- | ------ | ------ |
| mPD | | 0.44 | 0.50 |
| | | nPD: | 0.88 |
| | | N. weight: | 0.13 |
| | | Sc. PD: | 0.083 |
| | | Sc. rank: | 30.2 |

| PA-X | | | |
|---|---|---|---|
| Pos. | 112 | obs: | exp: |
| tat | Y | 53 | 182.70 |
| tac | Y | 300 | 170.30 |
| --- | -- | ------ | ------ |
| mPD | | 0.26 | 0.50 |
| | | nPD: | 0.51 |
| | | N. weight: | 0.4 |
| | | Sc. PD: | 0.11 |
| | | Sc. rank: | 67.6 |

| PA-X | | | |
|---|---|---|---|
| Pos. | 113 | obs: | exp: |
| cgt | R | 0 | 0.08 |
| cgc | R | 0 | 0.10 |
| cga | R | 0 | 0.19 |
| cgg | R | 0 | 0.17 |
| aaa | K | 290 | 196.50 |
| aag | K | 61 | 154.50 |
| aga | R | 2 | 0.90 |
| agg | R | 0 | 0.56 |
| --- | -- | ------ | ------ |
| mPD | | 0.30 | 0.51 |
| | | nPD: | 0.59 |
| | | N. weight: | 0.22 |
| | | Sc. PD: | 0.081 |
| | | Sc. rank: | 42.9 |

| PA-X | | | |
|---|---|---|---|
| Pos. | 114 | obs: | exp: |
| gaa | E | 42 | 203.10 |
| gag | E | 311 | 149.90 |
| --- | -- | ------ | ------ |
| mPD | | 0.21 | 0.49 |
| | | nPD: | 0.43 |
| | | N. weight: | 0.61 |
| | | Sc. PD: | 0.13 |
| | | Sc. rank: | 82.3 |

| PA-X | | | |
|---|---|---|---|
| Pos. | 115 | obs: | exp: |
| aat | N | 81 | 184.00 |
| aac | N | 271 | 168.00 |
| gat | D | 0 | 0.53 |
| gac | D | 1 | 0.47 |
| --- | -- | ------ | ------ |
| mPD | | 0.36 | 0.50 |
| | | nPD: | 0.71 |
| | | N. weight: | 0.25 |
| | | Sc. PD: | 0.12 |
| | | Sc. rank: | 53.8 |

| PA-X | | | |
|---|---|---|---|
| Pos. | 116 | obs: | exp: |
| caa | Q | 0 | 1.07 |
| cag | Q | 2 | 0.93 |
| cgt | R | 64 | 14.57 |
| cgc | R | 13 | 17.19 |
| cga | R | 250 | 33.73 |
| cgg | R | 21 | 29.29 |
| aga | R | 1 | 157.90 |
| agg | R | 2 | 98.36 |
| --- | -- | ------ | ------ |
| mPD | | 0.48 | 0.97 |
| | | nPD: | 0.5 |
| | | N. weight: | 2.2 |
| | | Sc. PD: | 0.59 |
| | | Sc. rank: | 361.0 |

| PA-X | | | |
|---|---|---|---|
| Pos. | 117 | obs: | exp: |
| ttt | F | 8 | 145.10 |
| ttc | F | 345 | 207.90 |
| --- | -- | ------ | ------ |
| mPD | | 0.044 | 0.48 |
| | | nPD: | 0.09 |
| | | N. weight: | 0.57 |
| | | Sc. PD: | -0.065 |
| | | Sc. rank: | 2.8 |

| PA-X | | | |
|---|---|---|---|
| Pos. | 118 | obs: | exp: |
| att | I | 331 | 131.20 |
| atc | I | 18 | 91.47 |
| ata | I | 0 | 126.30 |
| gtt | V | 4 | 0.82 |
| gtc | V | 0 | 0.84 |
| gta | V | 0 | 0.75 |
| gtg | V | 0 | 1.59 |
| --- | -- | ------ | ------ |
| mPD | | 0.12 | 0.68 |
| | | nPD: | 0.17 |
| | | N. weight: | 1.1 |
| | | Sc. PD: | -0.037 |
| | | Sc. rank: | 30.3 |

| PA-X | | | |
|---|---|---|---|
| Pos. | 119 | obs: | exp: |
| gaa | E | 336 | 203.10 |
| gag | E | 17 | 149.90 |
| --- | -- | ------ | ------ |
| mPD | | 0.092 | 0.49 |
| | | nPD: | 0.19 |
| | | N. weight: | 0.5 |
| | | Sc. PD: | -0.011 |
| | | Sc. rank: | 19.9 |

| PA-X | | | |
|---|---|---|---|
| Pos. | 120 | obs: | exp: |
| att | I | 340 | 130.80 |
| atc | I | 8 | 91.20 |
| ata | I | 0 | 126.00 |
| gtt | V | 5 | 1.03 |
| gtc | V | 0 | 1.05 |
| gta | V | 0 | 0.94 |
| gtg | V | 0 | 1.99 |
| --- | -- | ------ | ------ |
| mPD | | 0.072 | 0.69 |
| | | nPD: | 0.1 |
| | | N. weight: | 1.2 |
| | | Sc. PD: | -0.12 |
| | | Sc. rank: | 12.3 |

| PA-X | | | |
|---|---|---|---|
| Pos. | 121 | obs: | exp: |
| ggt | G | 1 | 43.56 |
| ggc | G | 0 | 47.74 |
| gga | G | 325 | 170.80 |
| ggg | G | 27 | 90.95 |
| --- | -- | ------ | ------ |
| mPD | | 0.15 | 0.67 |
| | | nPD: | 0.22 |
| | | N. weight: | 0.65 |
| | | Sc. PD: | 0.0059 |
| | | Sc. rank: | 48.6 |

| PA-X | | | |
|---|---|---|---|
| Pos. | 122 | obs: | exp: |
| gtt | V | 0 | 72.56 |
| gtc | V | 0 | 74.09 |
| gta | V | 115 | 66.16 |
| gtg | V | 238 | 140.20 |
| --- | -- | ------ | ------ |
| mPD | | 0.44 | 0.72 |
| | | nPD: | 0.61 |
| | | N. weight: | 0.71 |
| | | Sc. PD: | 0.27 |
| | | Sc. rank: | 138.3 |

| PA-X | | | |
|---|---|---|---|
| Pos. | 123 | obs: | exp: |
| act | T | 1 | 93.26 |
| acc | T | 74 | 75.75 |
| aca | T | 206 | 154.60 |
| acg | T | 72 | 29.35 |
| --- | -- | ------ | ------ |
| mPD | | 0.58 | 0.69 |
| | | nPD: | 0.84 |
| | | N. weight: | 0.46 |
| | | Sc. PD: | 0.28 |
| | | Sc. rank: | 107.5 |

| PA-X | | | |
|---|---|---|---|
| Pos. | 124 | obs: | exp: |
| cgt | R | 0 | 14.65 |
| cgc | R | 0 | 17.28 |
| cga | R | 159 | 33.93 |
| cgg | R | 90 | 29.46 |
| aga | R | 34 | 158.80 |
| agg | R | 70 | 98.92 |
| --- | -- | ------ | ------ |
| mPD | | 0.91 | 0.96 |
| | | nPD: | 0.95 |
| | | N. weight: | 1. |
| | | Sc. PD: | 0.73 |
| | | Sc. rank: | 243.8 |

| PA-X | | | |
|---|---|---|---|
| Pos. | 125 | obs: | exp: |
| cgt | R | 0 | 14.65 |
| cgc | R | 0 | 17.28 |
| cga | R | 0 | 33.93 |
| cgg | R | 0 | 29.46 |
| aga | R | 36 | 158.80 |
| agg | R | 317 | 98.92 |
| --- | -- | ------ | ------ |
| mPD | | 0.18 | 0.96 |
| | | nPD: | 0.19 |
| | | N. weight: | 1.2 |
| | | Sc. PD: | -0.022 |
| | | Sc. rank: | 54.0 |

| PA-X | | | |
|---|---|---|---|
| Pos. | 126 | obs: | exp: |
| gaa | E | 277 | 203.10 |
| gag | E | 76 | 149.90 |
| --- | -- | ------ | ------ |
| mPD | | 0.34 | 0.49 |
| | | nPD: | 0.69 |
| | | N. weight: | 0.14 |
| | | Sc. PD: | 0.064 |
| | | Sc. rank: | 29.7 |

| PA-X | | | |
|---|---|---|---|
| Pos. | 127 | obs: | exp: |
| gtt | V | 211 | 72.56 |
| gtc | V | 140 | 74.09 |
| gta | V | 1 | 66.16 |
| gtg | V | 1 | 140.20 |
| --- | -- | ------ | ------ |
| mPD | | 0.49 | 0.72 |
| | | nPD: | 0.68 |
| | | N. weight: | 1.2 |
| | | Sc. PD: | 0.51 |
| | | Sc. rank: | 241.9 |

| PA-X | | | |
|---|---|---|---|
| Pos. | 128 | obs: | exp: |
| cat | H | 115 | 191.30 |
| cac | H | 238 | 161.70 |
| --- | -- | ------ | ------ |
| mPD | | 0.44 | 0.50 |
| | | nPD: | 0.89 |
| | | N. weight: | 0.14 |
| | | Sc. PD: | 0.088 |
| | | Sc. rank: | 31.7 |

| PA-X | | | |
|---|---|---|---|
| Pos. | 129 | obs: | exp: |
| att | I | 1 | 132.30 |
| atc | I | 4 | 92.25 |
| ata | I | 347 | 127.40 |
| atg | M | 1 | 1.00 |
| --- | -- | ------ | ------ |
| mPD | | 0.034 | 0.66 |
| | | nPD: | 0.05 |
| | | N. weight: | 1.2 |
| | | Sc. PD: | -0.19 |
| | | Sc. rank: | -24.0 |

| PA-X | | | |
|---|---|---|---|
| Pos. | 130 | obs: | exp: |
| tat | Y | 42 | 182.70 |
| tac | Y | 311 | 170.30 |
| --- | -- | ------ | ------ |
| mPD | | 0.21 | 0.50 |
| | | nPD: | 0.42 |
| | | N. weight: | 0.48 |
| | | Sc. PD: | 0.096 |
| | | Sc. rank: | 64.0 |

PA-X

| Pos. | 131 | obs: | exp: |
|---|---|---|---|
| tat | Y | 287 | 182.70 |
| tac | Y | 66 | 170.30 |
| --- | -- | ------ | ------ |
| mPD | | 0.30 | 0.50 |
| | | nPD: | 0.61 |
| | | N. weight: | 0.26 |
| | | Sc. PD: | 0.099 |
| | | Sc. rank: | 49.8 |

,

PA-X

| Pos. | 132 | obs: | exp: |
|---|---|---|---|
| tta | L | 76 | 30.75 |
| ttg | L | 44 | 67.79 |
| ctt | L | 0 | 65.68 |
| ctc | L | 0 | 56.94 |
| cta | L | 212 | 53.03 |
| ctg | L | 20 | 77.81 |
| att | I | 0 | 0.38 |
| atc | I | 0 | 0.26 |
| ata | I | 1 | 0.36 |
| --- | -- | ------ | ------ |
| mPD | | 0.75 | 1.1 |
| | | nPD: | 0.67 |
| | | N. weight: | 1.2 |
| | | Sc. PD: | 0.53 |
| | | Sc. rank: | 251.1 |

,

PA-X

| Pos. | 133 | obs: | exp: |
|---|---|---|---|
| gaa | E | 285 | 203.10 |
| gag | E | 68 | 149.90 |
| --- | -- | ------ | ------ |
| mPD | | 0.31 | 0.49 |
| | | nPD: | 0.64 |
| | | N. weight: | 0.17 |
| | | Sc. PD: | 0.069 |
| | | Sc. rank: | 33.6 |

,

PA-X

| Pos. | 134 | obs: | exp: |
|---|---|---|---|
| aaa | K | 260 | 197.70 |
| aag | K | 93 | 155.30 |
| --- | -- | ------ | ------ |
| mPD | | 0.39 | 0.49 |
| | | nPD: | 0.79 |
| | | N. weight: | 0.099 |
| | | Sc. PD: | 0.055 |
| | | Sc. rank: | 22.2 |

,

PA-X

| Pos. | 135 | obs: | exp: |
|---|---|---|---|
| gct | A | 11 | 88.91 |
| gcc | A | 342 | 72.31 |
| gca | A | 0 | 157.30 |
| gcg | A | 0 | 34.45 |
| --- | -- | ------ | ------ |
| mPD | | 0.061 | 0.69 |
| | | nPD: | 0.09 |
| | | N. weight: | 1.9 |
| | | Sc. PD: | -0.22 |
| | | Sc. rank: | -0.5 |

,

PA-X

| Pos. | 136 | obs: | exp: |
|---|---|---|---|
| aat | N | 96 | 184.50 |
| aac | N | 257 | 168.50 |
| --- | -- | ------ | ------ |
| mPD | | 0.40 | 0.50 |
| | | nPD: | 0.8 |
| | | N. weight: | 0.18 |
| | | Sc. PD: | 0.1 |
| | | Sc. rank: | 41.4 |

,

PA-X

| Pos. | 137 | obs: | exp: |
|---|---|---|---|
| aaa | K | 97 | 197.70 |
| aag | K | 256 | 155.30 |
| --- | -- | ------ | ------ |
| mPD | | 0.40 | 0.49 |
| | | nPD: | 0.81 |
| | | N. weight: | 0.23 |
| | | Sc. PD: | 0.13 |
| | | Sc. rank: | 53.1 |

,

PA-X

| Pos. | 138 | obs: | exp: |
|---|---|---|---|
| att | I | 0 | 132.70 |
| atc | I | 0 | 92.52 |
| ata | I | 353 | 127.80 |
| --- | -- | ------ | ------ |
| mPD | | 0 | 0.66 |
| | | nPD: | 0. |
| | | N. weight: | 1.3 |
| | | Sc. PD: | -0.27 |
| | | Sc. rank: | -301.2 |

,

PA-X

| Pos. | 139 | obs: | exp: |
|---|---|---|---|
| aaa | K | 296 | 197.70 |
| aag | K | 57 | 155.30 |
| --- | -- | ------ | ------ |
| mPD | | 0.27 | 0.49 |
| | | nPD: | 0.55 |
| | | N. weight: | 0.24 |
| | | Sc. PD: | 0.079 |
| | | Sc. rank: | 44.8 |

,

PA-X

| Pos. | 140 | obs: | exp: |
|---|---|---|---|
| tct | S | 98 | 56.12 |
| tcc | S | 60 | 49.17 |
| tca | S | 166 | 78.20 |
| tcg | S | 29 | 21.52 |
| agt | S | 0 | 77.66 |
| agc | S | 0 | 70.33 |
| --- | -- | ------ | ------ |
| mPD | | 0.67 | 1.7 |
| | | nPD: | 0.4 |
| | | N. weight: | 0.77 |
| | | Sc. PD: | 0.14 |
| | | Sc. rank: | 98.2 |

,

PA-X

| Pos. | 141 | obs: | exp: |
|---|---|---|---|
| aaa | K | 0 | 0.56 |
| aag | K | 1 | 0.44 |
| gaa | E | 18 | 202.60 |
| gag | E | 334 | 149.40 |
| --- | -- | ------ | ------ |
| mPD | | 0.10 | 0.49 |
| | | nPD: | 0.21 |
| | | N. weight: | 0.85 |
| | | Sc. PD: | -0.0027 |
| | | Sc. rank: | 44.7 |

,

PA-X

| Pos. | 142 | obs: | exp: |
|---|---|---|---|
| cgt | R | 0 | 0.17 |
| cgc | R | 0 | 0.20 |
| cga | R | 0 | 0.38 |
| cgg | R | 0 | 0.33 |
| aaa | K | 48 | 195.40 |
| aag | K | 301 | 153.60 |
| aga | R | 0 | 1.80 |
| agg | R | 4 | 1.12 |
| --- | -- | ------ | ------ |
| mPD | | 0.26 | 0.52 |
| | | nPD: | 0.49 |
| | | N. weight: | 0.53 |
| | | Sc. PD: | 0.14 |
| | | Sc. rank: | 84.3 |

,

PA-X

| Pos. | 143 | obs: | exp: |
|---|---|---|---|
| act | T | 0 | 93.26 |
| acc | T | 1 | 75.75 |
| aca | T | 351 | 154.60 |
| acg | T | 1 | 29.35 |
| --- | -- | ------ | ------ |
| mPD | | 0.011 | 0.69 |
| | | nPD: | 0.02 |
| | | N. weight: | 1. |
| | | Sc. PD: | -0.2 |
| | | Sc. rank: | -87.6 |

,

PA-X

| Pos. | 144 | obs: | exp: |
|---|---|---|---|
| cat | H | 8 | 191.30 |
| cac | H | 345 | 161.70 |
| --- | -- | ------ | ------ |
| mPD | | 0.044 | 0.50 |
| | | nPD: | 0.09 |
| | | N. weight: | 0.88 |
| | | Sc. PD: | -0.1 |
| | | Sc. rank: | 1.2 |

,

PA-X

| Pos. | 145 | obs: | exp: |
|---|---|---|---|
| att | I | 240 | 132.70 |
| atc | I | 113 | 92.52 |
| ata | I | 0 | 127.80 |
| --- | -- | ------ | ------ |
| mPD | | 0.44 | 0.66 |
| | | nPD: | 0.66 |
| | | N. weight: | 0.62 |
| | | Sc. PD: | 0.27 |
| | | Sc. rank: | 126.4 |

,

PA-X

| Pos. | 146 | obs: | exp: |
|---|---|---|---|
| cat | H | 19 | 191.30 |
| cac | H | 334 | 161.70 |
| --- | -- | ------ | ------ |
| mPD | | 0.10 | 0.50 |
| | | nPD: | 0.21 |
| | | N. weight: | 0.74 |
| | | Sc. PD: | -0.0039 |
| | | Sc. rank: | 38.2 |

,

PA-X

| Pos. | 147 | obs: | exp: |
|---|---|---|---|
| att | I | 45 | 132.70 |
| atc | I | 85 | 92.52 |
| ata | I | 223 | 127.80 |
| --- | -- | ------ | ------ |
| mPD | | 0.53 | 0.66 |
| | | nPD: | 0.8 |
| | | N. weight: | 0.27 |
| | | Sc. PD: | 0.16 |
| | | Sc. rank: | 62.9 |

,

PA-X

| Pos. | 148 | obs: | exp: |
|---|---|---|---|
| ttt | F | 95 | 145.10 |
| ttc | F | 258 | 207.90 |
| --- | -- | ------ | ------ |
| mPD | | 0.39 | 0.48 |
| | | nPD: | 0.81 |
| | | N. weight: | 0.069 |
| | | Sc. PD: | 0.04 |
| | | Sc. rank: | 15.9 |

,

PA-X

| Pos. | 149 | obs: | exp: |
|---|---|---|---|
| tct | S | 2 | 56.12 |
| tcc | S | 3 | 49.17 |
| tca | S | 343 | 78.20 |
| tcg | S | 5 | 21.52 |
| agt | S | 0 | 77.66 |
| agc | S | 0 | 70.33 |
| --- | -- | ------ | ------ |
| mPD | | 0.056 | 1.7 |
| | | nPD: | 0.03 |
| | | N. weight: | 1.8 |
| | | Sc. PD: | -0.31 |
| | | Sc. rank: | -89.5 |

,

PA-X

| Pos. | 150 | obs: | exp: |
|---|---|---|---|
| ttt | F | 34 | 145.10 |
| ttc | F | 319 | 207.90 |
| --- | -- | ------ | ------ |
| mPD | | 0.17 | 0.48 |
| | | nPD: | 0.36 |
| | | N. weight: | 0.33 |
| | | Sc. PD: | 0.048 |
| | | Sc. rank: | 40.0 |

,

PA-X

| Pos. | 151 | obs: | exp: |
|---|---|---|---|
| act | T | 325 | 93.26 |
| acc | T | 24 | 75.75 |
| aca | T | 4 | 154.60 |
| acg | T | 0 | 29.35 |
| --- | -- | ------ | ------ |
| mPD | | 0.15 | 0.69 |
| | | nPD: | 0.22 |
| | | N. weight: | 1.4 |
| | | Sc. PD: | 0.0064 |
| | | Sc. rank: | 92.0 |

,

PA-X

| Pos. | 152 | obs: | exp: |
|---|---|---|---|
| ggt | G | 1 | 43.56 |
| ggc | G | 0 | 47.74 |
| gga | G | 276 | 170.80 |
| ggg | G | 76 | 90.95 |
| --- | -- | ------ | ------ |
| mPD | | 0.34 | 0.67 |
| | | nPD: | 0.52 |
| | | N. weight: | 0.44 |
| | | Sc. PD: | 0.13 |
| | | Sc. rank: | 75.5 |

,

PA-X

| Pos. | 153 | obs: | exp: |
|---|---|---|---|
| gaa | E | 157 | 203.10 |
| gag | E | 196 | 149.90 |
| --- | -- | ------ | ------ |
| mPD | | 0.50 | 0.49 |
| | | nPD: | 1.01 |
| | | N. weight: | 0.057 |
| | | Sc. PD: | 0.044 |
| | | Sc. rank: | 13.5 |

,

PA-X

| Pos. | 154 | obs: | exp: |
|---|---|---|---|
| gaa | E | 310 | 203.10 |
| gag | E | 43 | 149.90 |
| --- | -- | ------ | ------ |
| mPD | | 0.21 | 0.49 |
| | | nPD: | 0.44 |
| | | N. weight: | 0.3 |
| | | Sc. PD: | 0.065 |
| | | Sc. rank: | 41.9 |

,

PA-X

| Pos. | 155 | obs: | exp: |
|---|---|---|---|
| atg | M | 353 | 353.00 |
| --- | -- | ------ | ------ |
| mPD | | 0 | 0 |
| | | nPD: | 1. |
| | | N. weight: | 0. |
| | | Sc. PD: | 0 |
| | | Sc. rank: | 0 |

,

PA-X

| Pos. | 156 | obs: | exp: |
|---|---|---|---|
| gct | A | 69 | 88.91 |
| gcc | A | 280 | 72.31 |
| gca | A | 4 | 157.30 |
| gcg | A | 0 | 34.45 |
| --- | -- | ------ | ------ |
| mPD | | 0.33 | 0.69 |
| | | nPD: | 0.49 |
| | | N. weight: | 1.3 |
| | | Sc. PD: | 0.34 |
| | | Sc. rank: | 205.8 |

,

PA-X

| Pos. | 157 | obs: | exp: |
|---|---|---|---|
| act | T | 78 | 93.26 |
| acc | T | 273 | 75.75 |
| aca | T | 2 | 154.60 |
| acg | T | 0 | 29.35 |
| --- | -- | ------ | ------ |
| mPD | | 0.35 | 0.69 |
| | | nPD: | 0.52 |
| | | N. weight: | 1.2 |
| | | Sc. PD: | 0.36 |
| | | Sc. rank: | 213.7 |

,

PA-X

| Pos. | 158 | obs: | exp: |
|---|---|---|---|
| cgt | R | 0 | 0.04 |
| cgc | R | 0 | 0.05 |
| cga | R | 0 | 0.10 |
| cgg | R | 0 | 0.08 |
| aaa | K | 286 | 197.10 |
| aag | K | 66 | 154.90 |
| aga | R | 1 | 0.45 |
| agg | R | 0 | 0.28 |
| --- | -- | ------ | ------ |
| mPD | | 0.31 | 0.50 |
| | | nPD: | 0.62 |
| | | N. weight: | 0.2 |
| | | Sc. PD: | 0.078 |
| | | Sc. rank: | 38.8 |

,

PA-X

| Pos. | 159 | obs: | exp: |
|---|---|---|---|
| tct | S | 0 | 0.48 |
| tcc | S | 0 | 0.42 |
| tca | S | 2 | 0.66 |
| tcg | S | 1 | 0.18 |
| agt | S | 0 | 0.66 |
| agc | S | 0 | 0.60 |
| gtt | V | 0 | 0.21 |
| gtc | V | 0 | 0.21 |
| gta | V | 0 | 0.19 |
| gtg | V | 1 | 0.40 |
| gct | A | 2 | 87.40 |
| gcc | A | 5 | 71.08 |
| gca | A | 127 | 154.70 |
| gcg | A | 213 | 33.86 |
| gaa | E | 0 | 1.15 |
| gag | E | 2 | 0.85 |
| mPD | | 0.52 | 0.73 |
| | | nPD: | 0.72 |
| | | N. weight: | 1.3 |
| | | Sc. PD: | 0.65 |
| | | Sc. rank: | 294.7 |

PA-X

| Pos. | 160 | obs: | exp: |
|---|---|---|---|
| gat | D | 25 | 188.30 |
| gac | D | 328 | 164.70 |
| mPD | | 0.13 | 0.50 |
| | | nPD: | 0.27 |
| | | N. weight: | 0.66 |
| | | Sc. PD: | 0.034 |
| | | Sc. rank: | 55.4 |

PA-X

| Pos. | 161 | obs: | exp: |
|---|---|---|---|
| tat | Y | 62 | 182.20 |
| tac | Y | 290 | 169.80 |
| cat | H | 0 | 0.54 |
| cac | H | 1 | 0.46 |
| mPD | | 0.30 | 0.51 |
| | | nPD: | 0.59 |
| | | N. weight: | 0.34 |
| | | Sc. PD: | 0.12 |
| | | Sc. rank: | 64.9 |

PA-X

| Pos. | 162 | obs: | exp: |
|---|---|---|---|
| tct | S | 0 | 0.16 |
| tcc | S | 0 | 0.14 |
| tca | S | 0 | 0.22 |
| tcg | S | 0 | 0.06 |
| att | I | 0 | 0.38 |
| atc | I | 1 | 0.26 |
| ata | I | 0 | 0.36 |
| act | T | 138 | 92.73 |
| acc | T | 212 | 75.32 |
| aca | T | 1 | 153.80 |
| acg | T | 0 | 29.18 |
| agt | S | 0 | 0.22 |
| agc | S | 1 | 0.20 |
| mPD | | 0.49 | 0.70 |
| | | nPD: | 0.71 |
| | | N. weight: | 1. |
| | | Sc. PD: | 0.49 |
| | | Sc. rank: | 221.9 |

PA-X

| Pos. | 163 | obs: | exp: |
|---|---|---|---|
| tta | L | 0 | 30.84 |
| ttg | L | 0 | 67.98 |
| ctt | L | 328 | 65.86 |
| ctc | L | 10 | 57.10 |
| cta | L | 15 | 53.18 |
| ctg | L | 0 | 78.03 |
| mPD | | 0.13 | 1.1 |
| | | nPD: | 0.12 |
| | | N. weight: | 1.8 |
| | | Sc. PD: | -0.16 |
| | | Sc. rank: | 25.6 |

PA-X

| Pos. | 164 | obs: | exp: |
|---|---|---|---|
| gat | D | 257 | 188.30 |
| gac | D | 96 | 164.70 |
| mPD | | 0.40 | 0.50 |
| | | nPD: | 0.8 |
| | | N. weight: | 0.12 |
| | | Sc. PD: | 0.065 |
| | | Sc. rank: | 26.4 |

PA-X

| Pos. | 165 | obs: | exp: |
|---|---|---|---|
| gaa | E | 292 | 203.10 |
| gag | E | 61 | 149.90 |
| mPD | | 0.29 | 0.49 |
| | | nPD: | 0.59 |
| | | N. weight: | 0.2 |
| | | Sc. PD: | 0.072 |
| | | Sc. rank: | 38.2 |

PA-X

| Pos. | 166 | obs: | exp: |
|---|---|---|---|
| gaa | E | 101 | 203.10 |
| gag | E | 252 | 149.90 |
| mPD | | 0.41 | 0.49 |
| | | nPD: | 0.84 |
| | | N. weight: | 0.24 |
| | | Sc. PD: | 0.14 |
| | | Sc. rank: | 54.7 |

PA-X

| Pos. | 167 | obs: | exp: |
|---|---|---|---|
| tct | S | 0 | 56.12 |
| tcc | S | 0 | 49.17 |
| tca | S | 0 | 78.20 |
| tcg | S | 0 | 21.52 |
| agt | S | 127 | 77.66 |
| agc | S | 226 | 70.33 |
| mPD | | 0.46 | 1.7 |
| | | nPD: | 0.28 |
| | | N. weight: | 1.2 |
| | | Sc. PD: | 0.078 |
| | | Sc. rank: | 111.7 |

PA-X

| Pos. | 168 | obs: | exp: |
|---|---|---|---|
| cgt | R | 0 | 14.65 |
| cgc | R | 0 | 17.28 |
| cga | R | 0 | 33.93 |
| cgg | R | 0 | 29.46 |
| aga | R | 278 | 158.80 |
| agg | R | 75 | 98.92 |
| mPD | | 0.34 | 0.96 |
| | | nPD: | 0.35 |
| | | N. weight: | 0.51 |
| | | Sc. PD: | 0.068 |
| | | Sc. rank: | 58.6 |

PA-X

| Pos. | 169 | obs: | exp: |
|---|---|---|---|
| gct | A | 1 | 88.91 |
| gcc | A | 1 | 72.31 |
| gca | A | 308 | 157.30 |
| gcg | A | 43 | 34.45 |
| mPD | | 0.22 | 0.69 |
| | | nPD: | 0.33 |
| | | N. weight: | 0.79 |
| | | Sc. PD: | 0.088 |
| | | Sc. rank: | 78.5 |

PA-X

| Pos. | 170 | obs: | exp: |
|---|---|---|---|
| cgt | R | 0 | 14.65 |
| cgc | R | 0 | 17.28 |
| cga | R | 1 | 33.93 |
| cgg | R | 0 | 29.46 |
| aga | R | 277 | 158.80 |
| agg | R | 75 | 98.92 |
| mPD | | 0.34 | 0.96 |
| | | nPD: | 0.36 |
| | | N. weight: | 0.5 |
| | | Sc. PD: | 0.069 |
| | | Sc. rank: | 57.3 |

PA-X

| Pos. | 171 | obs: | exp: |
|---|---|---|---|
| att | I | 19 | 132.00 |
| atc | I | 228 | 91.99 |
| ata | I | 104 | 127.10 |
| gtt | V | 1 | 0.41 |
| gtc | V | 0 | 0.42 |
| gta | V | 1 | 0.37 |
| gtg | V | 0 | 0.79 |
| mPD | | 0.50 | 0.67 |
| | | nPD: | 0.75 |
| | | N. weight: | 0.58 |
| | | Sc. PD: | 0.3 |
| | | Sc. rank: | 130.5 |

PA-X

| Pos. | 172 | obs: | exp: |
|---|---|---|---|
| aat | N | 0 | 1.05 |
| aac | N | 2 | 0.95 |
| aaa | K | 339 | 196.50 |
| aag | K | 12 | 154.50 |
| mPD | | 0.077 | 0.50 |
| | | nPD: | 0.15 |
| | | N. weight: | 0.59 |
| | | Sc. PD: | -0.032 |
| | | Sc. rank: | 13.6 |

PA-X

| Pos. | 173 | obs: | exp: |
|---|---|---|---|
| act | T | 74 | 93.26 |
| acc | T | 279 | 75.75 |
| aca | T | 0 | 154.60 |
| acg | T | 0 | 29.35 |
| mPD | | 0.33 | 0.69 |
| | | nPD: | 0.48 |
| | | N. weight: | 1.3 |
| | | Sc. PD: | 0.34 |
| | | Sc. rank: | 201.6 |

PA-X

| Pos. | 174 | obs: | exp: |
|---|---|---|---|
| cgt | R | 0 | 14.65 |
| cgc | R | 0 | 17.28 |
| cga | R | 0 | 33.93 |
| cgg | R | 0 | 29.46 |
| aga | R | 89 | 158.80 |
| agg | R | 264 | 98.92 |
| mPD | | 0.38 | 0.96 |
| | | nPD: | 0.39 |
| | | N. weight: | 0.78 |
| | | Sc. PD: | 0.14 |
| | | Sc. rank: | 98.0 |

PA-X

| Pos. | 175 | obs: | exp: |
|---|---|---|---|
| tta | L | 5 | 30.84 |
| ttg | L | 6 | 67.98 |
| ctt | L | 6 | 65.86 |
| ctc | L | 0 | 57.10 |
| cta | L | 80 | 53.18 |
| ctg | L | 256 | 78.03 |
| mPD | | 0.45 | 1.1 |
| | | nPD: | 0.41 |
| | | N. weight: | 1.1 |
| | | Sc. PD: | 0.21 |
| | | Sc. rank: | 149.4 |

PA-X

| Pos. | 176 | obs: | exp: |
|---|---|---|---|
| ttt | F | 0 | 144.30 |
| ttc | F | 351 | 206.70 |
| tat | Y | 0 | 1.04 |
| tac | Y | 2 | 0.96 |
| mPD | | 0.011 | 0.50 |
| | | nPD: | 0.02 |
| | | N. weight: | 0.7 |
| | | Sc. PD: | -0.13 |
| | | Sc. rank: | -56.4 |

PA-X

| Pos. | 177 | obs: | exp: |
|---|---|---|---|
| act | T | 233 | 93.26 |
| acc | T | 120 | 75.75 |
| aca | T | 0 | 154.60 |
| acg | T | 0 | 29.35 |
| mPD | | 0.45 | 0.69 |
| | | nPD: | 0.66 |
| | | N. weight: | 1. |
| | | Sc. PD: | 0.43 |
| | | Sc. rank: | 202.9 |

PA-X

| Pos. | 178 | obs: | exp: |
|---|---|---|---|
| att | I | 1 | 132.70 |
| atc | I | 0 | 92.52 |
| ata | I | 352 | 127.80 |
| mPD | | 0.0057 | 0.66 |
| | | nPD: | 0.01 |
| | | N. weight: | 1.3 |
| | | Sc. PD: | -0.25 |
| | | Sc. rank: | -153.5 |

PA-X

| Pos. | 179 | obs: | exp: |
|---|---|---|---|
| cgt | R | 0 | 14.65 |
| cgc | R | 0 | 17.28 |
| cga | R | 0 | 33.93 |
| cgg | R | 0 | 29.46 |
| aga | R | 208 | 158.80 |
| agg | R | 145 | 98.92 |
| mPD | | 0.49 | 0.96 |
| | | nPD: | 0.51 |
| | | N. weight: | 0.43 |
| | | Sc. PD: | 0.12 |
| | | Sc. rank: | 72.1 |

PA-X

| Pos. | 180 | obs: | exp: |
|---|---|---|---|
| caa | Q | 313 | 188.20 |
| cag | Q | 40 | 164.80 |
| mPD | | 0.20 | 0.50 |
| | | nPD: | 0.4 |
| | | N. weight: | 0.39 |
| | | Sc. PD: | 0.073 |
| | | Sc. rank: | 50.4 |

PA-X

| Pos. | 181 | obs: | exp: |
|---|---|---|---|
| gaa | E | 301 | 203.10 |
| gag | E | 52 | 149.90 |
| mPD | | 0.25 | 0.49 |
| | | nPD: | 0.52 |
| | | N. weight: | 0.25 |
| | | Sc. PD: | 0.072 |
| | | Sc. rank: | 42.1 |

PA-X

| Pos. | 182 | obs: | exp: |
|---|---|---|---|
| atg | M | 353 | 353.00 |
| mPD | | 0 | 0 |
| | | nPD: | 1. |
| | | N. weight: | 0. |
| | | Sc. PD: | 0 |
| | | Sc. rank: | 0 |

PA-X

| Pos. | 183 | obs: | exp: |
|---|---|---|---|
| act | T | 0 | 0.26 |
| acc | T | 1 | 0.21 |
| aca | T | 0 | 0.44 |
| acg | T | 0 | 0.08 |
| gct | A | 2 | 88.66 |
| gcc | A | 341 | 72.11 |
| gca | A | 9 | 156.90 |
| gcg | A | 0 | 34.35 |
| mPD | | 0.067 | 0.69 |
| | | nPD: | 0.1 |
| | | N. weight: | 1.9 |
| | | Sc. PD: | -0.21 |
| | | Sc. rank: | 15.6 |

| PA-X | | | |
|---|---|---|---|
| Pos. 184 | | obs: | exp: |
| tct | S | 0 | 55.01 |
| tcc | S | 0 | 48.20 |
| tca | S | 0 | 76.65 |
| tcg | S | 0 | 21.09 |
| act | T | 5 | 1.32 |
| acc | T | 0 | 1.07 |
| aca | T | 0 | 2.19 |
| acg | T | 0 | 0.42 |
| aat | N | 2 | 1.05 |
| aac | N | 0 | 0.95 |
| agt | S | 289 | 76.12 |
| agc | S | 57 | 68.93 |
| --- | -- | ------ | ------ |
| mPD | | 0.31 | 1.7 |
| | | nPD: | 0.19 |
| | | N. weight: | 1.4 |
| | | Sc. PD: | -0.034 |
| | | Sc. rank: | 55.8 |

| PA-X | | | |
|---|---|---|---|
| Pos. 185 | | obs: | exp: |
| cgt | R | 0 | 14.57 |
| cgc | R | 0 | 17.19 |
| cga | R | 0 | 33.73 |
| cgg | R | 0 | 29.29 |
| aaa | K | 0 | 1.12 |
| aag | K | 2 | 0.88 |
| aga | R | 90 | 157.90 |
| agg | R | 261 | 98.36 |
| --- | -- | ------ | ------ |
| mPD | | 0.39 | 0.97 |
| | | nPD: | 0.4 |
| | | N. weight: | 0.77 |
| | | Sc. PD: | 0.14 |
| | | Sc. rank: | 99.4 |

| PA-X | | | |
|---|---|---|---|
| Pos. 186 | | obs: | exp: |
| ggt | G | 271 | 43.56 |
| ggc | G | 81 | 47.74 |
| gga | G | 1 | 170.80 |
| ggg | G | 0 | 90.95 |
| --- | -- | ------ | ------ |
| mPD | | 0.36 | 0.67 |
| | | nPD: | 0.54 |
| | | N. weight: | 2. |
| | | Sc. PD: | 0.63 |
| | | Sc. rank: | 362.7 |

| PA-X | | | |
|---|---|---|---|
| Pos. 187 | | obs: | exp: |
| tta | L | 14 | 30.84 |
| ttg | L | 0 | 67.98 |
| ctt | L | 1 | 65.86 |
| ctc | L | 69 | 57.10 |
| cta | L | 268 | 53.18 |
| ctg | L | 1 | 78.03 |
| --- | -- | ------ | ------ |
| mPD | | 0.40 | 1.1 |
| | | nPD: | 0.36 |
| | | N. weight: | 1.6 |
| | | Sc. PD: | 0.23 |
| | | Sc. rank: | 191.9 |

| PA-X | | | |
|---|---|---|---|
| Pos. 188 | | obs: | exp: |
| tgg | W | 353 | 353.00 |
| --- | -- | ------ | ------ |
| mPD | | 0 | 0 |
| | | nPD: | 1. |
| | | N. weight: | 0. |
| | | Sc. PD: | 0 |
| | | Sc. rank: | 0 |

| PA-X | | | |
|---|---|---|---|
| Pos. 189 | | obs: | exp: |
| gat | D | 342 | 188.30 |
| gac | D | 11 | 164.70 |
| --- | -- | ------ | ------ |
| mPD | | 0.061 | 0.50 |
| | | nPD: | 0.12 |
| | | N. weight: | 0.65 |
| | | Sc. PD: | -0.056 |
| | | Sc. rank: | 9.9 |

| PA-X | | | |
|---|---|---|---|
| Pos. 190 | | obs: | exp: |
| tct | S | 1 | 55.80 |
| tcc | S | 346 | 48.90 |
| tca | S | 4 | 77.76 |
| tcg | S | 0 | 21.39 |
| cct | P | 0 | 0.53 |
| ccc | P | 2 | 0.36 |
| cca | P | 0 | 0.79 |
| ccg | P | 0 | 0.32 |
| agt | S | 0 | 77.22 |
| agc | S | 0 | 69.93 |
| --- | -- | ------ | ------ |
| mPD | | 0.039 | 1.7 |
| | | nPD: | 0.02 |
| | | N. weight: | 2.5 |
| | | Sc. PD: | -0.44 |
| | | Sc. rank: | -168.4 |

| PA-X | | | |
|---|---|---|---|
| Pos. 191 | | obs: | exp: |
| ttt | F | 353 | 145.10 |
| ttc | F | 0 | 207.90 |
| --- | -- | ------ | ------ |
| mPD | | 0 | 0.48 |
| | | nPD: | 0. |
| | | N. weight: | 1.2 |
| | | Sc. PD: | -0.24 |
| | | Sc. rank: | -263.5 |

| PA-X | | | |
|---|---|---|---|
| Pos. 192 | | obs: | exp: |
| gtt | V | 0 | 72.35 |
| gtc | V | 352 | 73.88 |
| gta | V | 0 | 65.97 |
| gtg | V | 0 | 139.80 |
| gct | A | 0 | 0.25 |
| gcc | A | 1 | 0.20 |
| gca | A | 0 | 0.45 |
| gcg | A | 0 | 0.10 |
| --- | -- | ------ | ------ |
| mPD | | 0.0057 | 0.73 |
| | | nPD: | 0.01 |
| | | N. weight: | 2. |
| | | Sc. PD: | -0.4 |
| | | Sc. rank: | -271.3 |

| PA-X | | | |
|---|---|---|---|
| Pos. 193 | | obs: | exp: |
| tct | S | 0 | 55.96 |
| tcc | S | 0 | 49.04 |
| tca | S | 0 | 77.98 |
| tcg | S | 0 | 21.46 |
| aat | N | 1 | 0.52 |
| aac | N | 0 | 0.48 |
| agt | S | 352 | 77.44 |
| agc | S | 0 | 70.13 |
| --- | -- | ------ | ------ |
| mPD | | 0.0057 | 1.7 |
| | | nPD: | 0. |
| | | N. weight: | 2. |
| | | Sc. PD: | -0.39 |
| | | Sc. rank: | -307.4 |

| PA-X | | | |
|---|---|---|---|
| Pos. 194 | | obs: | exp: |
| cct | P | 0 | 92.95 |
| ccc | P | 0 | 63.67 |
| cca | P | 0 | 139.30 |
| ccg | P | 353 | 57.08 |
| --- | -- | ------ | ------ |
| mPD | | 0 | 0.72 |
| | | nPD: | 0. |
| | | N. weight: | 2.4 |
| | | Sc. PD: | -0.48 |
| | | Sc. rank: | -540.2 |

| PA-X | | | |
|---|---|---|---|
| Pos. 195 | | obs: | exp: |
| cgt | R | 0 | 11.91 |
| cgc | R | 0 | 14.05 |
| cga | R | 0 | 27.58 |
| cgg | R | 0 | 23.95 |
| aaa | K | 66 | 36.96 |
| aag | K | 0 | 29.04 |
| aga | R | 287 | 129.10 |
| agg | R | 0 | 80.43 |
| --- | -- | ------ | ------ |
| mPD | | 0.30 | 1.2 |
| | | nPD: | 0.25 |
| | | N. weight: | 0.99 |
| | | Sc. PD: | 0.041 |
| | | Sc. rank: | 82.0 |

| PA-X | | | |
|---|---|---|---|
| Pos. 196 | | obs: | exp: |
| gaa | E | 0 | 203.10 |
| gag | E | 353 | 149.90 |
| --- | -- | ------ | ------ |
| mPD | | 0 | 0.49 |
| | | nPD: | 0. |
| | | N. weight: | 1.1 |
| | | Sc. PD: | -0.23 |
| | | Sc. rank: | -254.0 |

| PA-X | | | |
|---|---|---|---|
| Pos. 197 | | obs: | exp: |
| gct | A | 0 | 88.91 |
| gcc | A | 0 | 72.31 |
| gca | A | 0 | 157.30 |
| gcg | A | 353 | 34.45 |
| --- | -- | ------ | ------ |
| mPD | | 0 | 0.69 |
| | | nPD: | 0. |
| | | N. weight: | 3. |
| | | Sc. PD: | -0.62 |
| | | Sc. rank: | -689.9 |

| PA-X | | | |
|---|---|---|---|
| Pos. 198 | | obs: | exp: |
| cgt | R | 0 | 0.25 |
| cgc | R | 0 | 0.29 |
| cga | R | 0 | 0.58 |
| cgg | R | 0 | 0.50 |
| aaa | K | 0 | 194.30 |
| aag | K | 347 | 152.70 |
| aga | R | 0 | 2.70 |
| agg | R | 6 | 1.68 |
| --- | -- | ------ | ------ |
| mPD | | 0.034 | 0.54 |
| | | nPD: | 0.06 |
| | | N. weight: | 1.1 |
| | | Sc. PD: | -0.15 |
| | | Sc. rank: | -13.1 |

| PA-X | | | |
|---|---|---|---|
| Pos. 199 | | obs: | exp: |
| cgt | R | 0 | 12.75 |
| cgc | R | 0 | 15.03 |
| cga | R | 0 | 29.50 |
| cgg | R | 0 | 25.62 |
| aaa | K | 46 | 25.76 |
| aag | K | 0 | 20.24 |
| aga | R | 307 | 138.10 |
| agg | R | 0 | 86.03 |
| --- | -- | ------ | ------ |
| mPD | | 0.23 | 1.1 |
| | | nPD: | 0.2 |
| | | N. weight: | 1. |
| | | Sc. PD: | -0.012 |
| | | Sc. rank: | 49.0 |

| PA-X | | | |
|---|---|---|---|
| Pos. 200 | | obs: | exp: |
| cct | P | 0 | 0.26 |
| ccc | P | 0 | 0.18 |
| cca | P | 1 | 0.39 |
| ccg | P | 0 | 0.16 |
| caa | Q | 336 | 187.70 |
| cag | Q | 16 | 164.30 |
| --- | -- | ------ | ------ |
| mPD | | 0.092 | 0.50 |
| | | nPD: | 0.18 |
| | | N. weight: | 0.6 |
| | | Sc. PD: | -0.016 |
| | | Sc. rank: | 19.1 |

| PA-X | | | |
|---|---|---|---|
| Pos. 201 | | obs: | exp: |
| tta | L | 0 | 30.84 |
| ttg | L | 353 | 67.98 |
| ctt | L | 0 | 65.86 |
| ctc | L | 0 | 57.10 |
| cta | L | 0 | 53.18 |
| ctg | L | 0 | 78.03 |
| --- | -- | ------ | ------ |
| mPD | | 0 | 1.1 |
| | | nPD: | 0. |
| | | N. weight: | 2.2 |
| | | Sc. PD: | -0.44 |
| | | Sc. rank: | -488.3 |

| PA-X | | | |
|---|---|---|---|
| Pos. 202 | | obs: | exp: |
| cgt | R | 0 | 0.04 |
| cgc | R | 0 | 0.05 |
| cga | R | 0 | 0.10 |
| cgg | R | 0 | 0.08 |
| aaa | K | 0 | 197.10 |
| aag | K | 352 | 154.90 |
| aga | R | 0 | 0.45 |
| agg | R | 1 | 0.28 |
| --- | -- | ------ | ------ |
| mPD | | 0.0057 | 0.50 |
| | | nPD: | 0.01 |
| | | N. weight: | 1.1 |
| | | Sc. PD: | -0.21 |
| | | Sc. rank: | -122.2 |

| PA-X | | | |
|---|---|---|---|
| Pos. 203 | | obs: | exp: |
| cgt | R | 0 | 0.12 |
| cgc | R | 0 | 0.15 |
| cga | R | 0 | 0.29 |
| cgg | R | 0 | 0.25 |
| aaa | K | 350 | 196.00 |
| aag | K | 0 | 154.00 |
| aga | R | 3 | 1.35 |
| agg | R | 0 | 0.84 |
| --- | -- | ------ | ------ |
| mPD | | 0.017 | 0.51 |
| | | nPD: | 0.03 |
| | | N. weight: | 0.76 |
| | | Sc. PD: | -0.13 |
| | | Sc. rank: | -41.8 |

| PA-X | | | |
|---|---|---|---|
| Pos. 204 | | obs: | exp: |
| aat | N | 2 | 1.05 |
| aac | N | 0 | 0.95 |
| gat | D | 349 | 186.20 |
| gac | D | 0 | 162.80 |
| ggt | G | 2 | 0.25 |
| ggc | G | 0 | 0.27 |
| gga | G | 0 | 0.97 |
| ggg | G | 0 | 0.52 |
| --- | -- | ------ | ------ |
| mPD | | 0.023 | 0.52 |
| | | nPD: | 0.04 |
| | | N. weight: | 0.83 |
| | | Sc. PD: | -0.13 |
| | | Sc. rank: | -23.8 |

| PA-X | | | |
|---|---|---|---|
| Pos. 205 | | obs: | exp: |
| tta | L | 0 | 30.75 |
| ttg | L | 352 | 67.79 |
| ctt | L | 0 | 65.68 |
| ctc | L | 0 | 56.94 |
| cta | L | 0 | 53.03 |
| ctg | L | 0 | 77.81 |
| gtt | V | 0 | 0.21 |
| gtc | V | 0 | 0.21 |
| gta | V | 0 | 0.19 |
| gtg | V | 1 | 0.40 |
| --- | -- | ------ | ------ |
| mPD | | 0.0057 | 1.1 |
| | | nPD: | 0.01 |
| | | N. weight: | 2.2 |
| | | Sc. PD: | -0.43 |
| | | Sc. rank: | -306.2 |

| PA-X | | | |
|---|---|---|---|
| Pos. 206 | | obs: | exp: |
| cgt | R | 0 | 0.21 |
| cgc | R | 0 | 0.24 |
| cga | R | 0 | 0.48 |
| cgg | R | 0 | 0.42 |
| aaa | K | 348 | 194.90 |
| aag | K | 0 | 153.10 |
| aga | R | 5 | 2.25 |
| agg | R | 0 | 1.40 |
| --- | -- | ------ | ------ |
| mPD | | 0.028 | 0.53 |
| | | nPD: | 0.05 |
| | | N. weight: | 0.76 |
| | | Sc. PD: | -0.12 |
| | | Sc. rank: | -13.8 |

| PA-X | | | |
|---|---|---|---|
| Pos. 207 | | obs: | exp: |
| tta | L | 114 | 9.96 |
| ttg | L | 0 | 21.96 |
| tct | S | 0 | 38.00 |
| tcc | S | 0 | 33.29 |
| tca | S | 238 | 52.95 |
| tcg | S | 1 | 14.57 |
| ctt | L | 0 | 21.27 |
| ctc | L | 0 | 18.44 |
| cta | L | 0 | 17.17 |
| ctg | L | 0 | 25.20 |
| agt | S | 0 | 52.58 |
| agc | S | 0 | 47.62 |
| --- | -- | ------ | ------ |
| mPD | | 0.44 | 2.0 |
| | | nPD: | 0.22 |
| | | N. weight: | 2.4 |
| | | Sc. PD: | 0.017 |
| | | Sc. rank: | 172.3 |

| PA-X | | | |
|---|---|---|---|
| Pos. 208 | | obs: | exp: |
| tta | L | 0 | 0.09 |
| ttg | L | 0 | 0.19 |
| ctt | L | 0 | 0.19 |
| ctc | L | 0 | 0.16 |
| cta | L | 0 | 0.15 |
| ctg | L | 1 | 0.22 |
| caa | Q | 0 | 187.70 |
| cag | Q | 352 | 164.30 |
| --- | -- | ------ | ------ |
| mPD | | 0.0057 | 0.51 |
| | | nPD: | 0.01 |
| | | N. weight: | 1. |
| | | Sc. PD: | -0.19 |
| | | Sc. rank: | -115.5 |

PA-X

| Pos. | 209 | obs: | exp: |
|---|---|---|---|
| gtt | V | 0 | 0.21 |
| gtc | V | 0 | 0.21 |
| gta | V | 1 | 0.19 |
| gtg | V | 0 | 0.40 |
| gaa | E | 329 | 189.30 |
| gag | E | 0 | 139.70 |
| ggt | G | 0 | 2.84 |
| ggc | G | 0 | 3.11 |
| gga | G | 23 | 11.13 |
| ggg | G | 0 | 5.93 |
| mPD | | 0.13 | 0.63 |
| | | nPD: | 0.2 |
| | | N. weight: | 0.74 |
| | | Sc. PD: | -0.0069 |
| | | Sc. rank: | 37.1 |

PA-X

| Pos. | 210 | obs: | exp: |
|---|---|---|---|
| tta | L | 0 | 0.26 |
| ttg | L | 0 | 0.58 |
| ctt | L | 0 | 0.56 |
| ctc | L | 0 | 0.49 |
| cta | L | 3 | 0.45 |
| ctg | L | 0 | 0.66 |
| cct | P | 0 | 85.05 |
| ccc | P | 0 | 58.26 |
| cca | P | 323 | 127.50 |
| ccg | P | 0 | 52.23 |
| caa | Q | 27 | 14.39 |
| cag | Q | 0 | 12.61 |
| mPD | | 0.16 | 0.88 |
| | | nPD: | 0.18 |
| | | N. weight: | 1.2 |
| | | Sc. PD: | -0.037 |
| | | Sc. rank: | 36.9 |

PA-X

| Pos. | 211 | obs: | exp: |
|---|---|---|---|
| ttt | F | 0 | 1.23 |
| ttc | F | 3 | 1.77 |
| tgt | C | 0 | 144.30 |
| tgc | C | 350 | 205.70 |
| mPD | | 0.017 | 0.50 |
| | | nPD: | 0.03 |
| | | N. weight: | 0.7 |
| | | Sc. PD: | -0.12 |
| | | Sc. rank: | -32.5 |

PA-X

| Pos. | 212 | obs: | exp: |
|---|---|---|---|
| act | T | 0 | 0.26 |
| acc | T | 0 | 0.21 |
| aca | T | 1 | 0.44 |
| acg | T | 0 | 0.08 |
| gtt | V | 0 | 10.07 |
| gtc | V | 0 | 10.28 |
| gta | V | 49 | 9.18 |
| gtg | V | 0 | 19.46 |
| gct | A | 0 | 75.56 |
| gcc | A | 1 | 61.45 |
| gca | A | 299 | 133.70 |
| gcg | A | 0 | 29.27 |
| gaa | E | 3 | 1.73 |
| gag | E | 0 | 1.27 |
| mPD | | 0.27 | 0.97 |
| | | nPD: | 0.27 |
| | | N. weight: | 1.2 |
| | | Sc. PD: | 0.072 |
| | | Sc. rank: | 105.4 |

PA-X

| Pos. | 213 | obs: | exp: |
|---|---|---|---|
| tct | S | 0 | 0.48 |
| tcc | S | 0 | 0.42 |
| tca | S | 0 | 0.66 |
| tcg | S | 0 | 0.18 |
| agt | S | 0 | 0.66 |
| agc | S | 3 | 0.60 |
| ggt | G | 0 | 43.19 |
| ggc | G | 350 | 47.33 |
| gga | G | 0 | 169.30 |
| ggg | G | 0 | 90.17 |
| mPD | | 0.017 | 0.69 |
| | | nPD: | 0.02 |
| | | N. weight: | 2.6 |
| | | Sc. PD: | -0.47 |
| | | Sc. rank: | -165.3 |

PA-X

| Pos. | 214 | obs: | exp: |
|---|---|---|---|
| tta | L | 0 | 24.11 |
| ttg | L | 275 | 53.16 |
| tct | S | 0 | 12.24 |
| tcc | S | 0 | 10.73 |
| tca | S | 0 | 17.06 |
| tcg | S | 77 | 4.69 |
| ctt | L | 0 | 51.50 |
| ctc | L | 0 | 44.65 |
| cta | L | 0 | 41.58 |
| ctg | L | 1 | 61.01 |
| agt | S | 0 | 16.94 |
| agc | S | 0 | 15.34 |
| mPD | | 0.35 | 1.7 |
| | | nPD: | 0.21 |
| | | N. weight: | 2.5 |
| | | Sc. PD: | -0.0035 |
| | | Sc. rank: | 141.5 |

PA-X

| Pos. | 215 | obs: | exp: |
|---|---|---|---|
| tta | L | 0 | 0.17 |
| ttg | L | 0 | 0.39 |
| ctt | L | 0 | 0.37 |
| ctc | L | 0 | 0.32 |
| cta | L | 0 | 0.30 |
| ctg | L | 2 | 0.44 |
| cct | P | 0 | 92.42 |
| ccc | P | 0 | 63.31 |
| cca | P | 1 | 138.50 |
| ccg | P | 350 | 56.75 |
| mPD | | 0.017 | 0.73 |
| | | nPD: | 0.02 |
| | | N. weight: | 2.4 |
| | | Sc. PD: | -0.42 |
| | | Sc. rank: | -171.3 |

PA-X

| Pos. | 216 | obs: | exp: |
|---|---|---|---|
| att | I | 0 | 0.75 |
| atc | I | 2 | 0.52 |
| ata | I | 0 | 0.72 |
| act | T | 0 | 92.73 |
| acc | T | 351 | 75.32 |
| aca | T | 0 | 153.80 |
| acg | T | 0 | 29.18 |
| mPD | | 0.011 | 0.70 |
| | | nPD: | 0.02 |
| | | N. weight: | 2. |
| | | Sc. PD: | -0.38 |
| | | Sc. rank: | -183.0 |

PA-X

| Pos. | 217 | obs: | exp: |
|---|---|---|---|
| aaa | K | 353 | 197.70 |
| aag | K | 0 | 155.30 |
| mPD | | 0 | 0.49 |
| | | nPD: | 0. |
| | | N. weight: | 0.76 |
| | | Sc. PD: | -0.15 |
| | | Sc. rank: | -171.9 |

PA-X

| Pos. | 218 | obs: | exp: |
|---|---|---|---|
| ttt | F | 0 | 0.41 |
| ttc | F | 1 | 0.59 |
| gtt | V | 0 | 72.14 |
| gtc | V | 351 | 73.67 |
| gta | V | 0 | 65.78 |
| gtg | V | 0 | 139.40 |
| gct | A | 0 | 0.25 |
| gcc | A | 1 | 0.20 |
| gca | A | 0 | 0.45 |
| gcg | A | 0 | 0.10 |
| mPD | | 0.011 | 0.73 |
| | | nPD: | 0.02 |
| | | N. weight: | 2. |
| | | Sc. PD: | -0.38 |
| | | Sc. rank: | -197.6 |

PA-X

| Pos. | 219 | obs: | exp: |
|---|---|---|---|
| ttt | F | 0 | 0.41 |
| ttc | F | 1 | 0.59 |
| tct | S | 0 | 55.96 |
| tcc | S | 352 | 49.04 |
| tca | S | 0 | 77.98 |
| tcg | S | 0 | 21.46 |
| agt | S | 0 | 77.44 |
| agc | S | 0 | 70.13 |
| mPD | | 0.0057 | 1.7 |
| | | nPD: | 0. |
| | | N. weight: | 2.6 |
| | | Sc. PD: | -0.51 |
| | | Sc. rank: | -387.8 |

PA-X

| Pos. | 220 | obs: | exp: |
|---|---|---|---|
| cat | H | 0 | 189.70 |
| cac | H | 350 | 160.30 |
| cgt | R | 0 | 0.12 |
| cgc | R | 3 | 0.15 |
| cga | R | 0 | 0.29 |
| cgg | R | 0 | 0.25 |
| aga | R | 0 | 1.35 |
| agg | R | 0 | 0.84 |
| mPD | | 0.017 | 0.53 |
| | | nPD: | 0.03 |
| | | N. weight: | 1. |
| | | Sc. PD: | -0.18 |
| | | Sc. rank: | -64.2 |

PA-X

| Pos. | 221 | obs: | exp: |
|---|---|---|---|
| cgt | R | 0 | 14.65 |
| cgc | R | 0 | 17.28 |
| cga | R | 353 | 33.93 |
| cgg | R | 0 | 29.46 |
| aga | R | 0 | 158.80 |
| agg | R | 0 | 98.92 |
| mPD | | 0 | 0.96 |
| | | nPD: | 0. |
| | | N. weight: | 3.1 |
| | | Sc. PD: | -0.62 |
| | | Sc. rank: | -694.4 |

PA-X

| Pos. | 222 | obs: | exp: |
|---|---|---|---|
| att | I | 1 | 0.38 |
| atc | I | 0 | 0.26 |
| ata | I | 0 | 0.36 |
| act | T | 352 | 93.00 |
| acc | T | 0 | 75.53 |
| aca | T | 0 | 154.20 |
| acg | T | 0 | 29.26 |
| mPD | | 0.0057 | 0.69 |
| | | nPD: | 0.01 |
| | | N. weight: | 1.7 |
| | | Sc. PD: | -0.34 |
| | | Sc. rank: | -208.5 |

PA-X

| Pos. | 223 | obs: | exp: |
|---|---|---|---|
| tct | S | 352 | 56.12 |
| tcc | S | 1 | 49.17 |
| tca | S | 0 | 78.20 |
| tcg | S | 0 | 21.52 |
| agt | S | 0 | 77.66 |
| agc | S | 0 | 70.33 |
| mPD | | 0.0057 | 1.7 |
| | | nPD: | 0. |
| | | N. weight: | 2.4 |
| | | Sc. PD: | -0.48 |
| | | Sc. rank: | -348.8 |

PA-X

| Pos. | 224 | obs: | exp: |
|---|---|---|---|
| cct | P | 0 | 92.42 |
| ccc | P | 0 | 63.31 |
| cca | P | 351 | 138.50 |
| ccg | P | 0 | 56.75 |
| caa | Q | 2 | 1.07 |
| cag | Q | 0 | 0.93 |
| mPD | | 0.011 | 0.73 |
| | | nPD: | 0.02 |
| | | N. weight: | 1.2 |
| | | Sc. PD: | -0.23 |
| | | Sc. rank: | -115.0 |

PA-X

| Pos. | 225 | obs: | exp: |
|---|---|---|---|
| act | T | 0 | 0.26 |
| acc | T | 1 | 0.21 |
| aca | T | 0 | 0.44 |
| acg | T | 0 | 0.08 |
| gct | A | 0 | 88.66 |
| gcc | A | 352 | 72.11 |
| gca | A | 0 | 156.90 |
| gcg | A | 0 | 34.35 |
| mPD | | 0.0057 | 0.69 |
| | | nPD: | 0.01 |
| | | N. weight: | 2.1 |
| | | Sc. PD: | -0.4 |
| | | Sc. rank: | -255.2 |

PA-X

| Pos. | 226 | obs: | exp: |
|---|---|---|---|
| tta | L | 0 | 30.84 |
| ttg | L | 353 | 67.98 |
| ctt | L | 0 | 65.86 |
| ctc | L | 0 | 57.10 |
| cta | L | 0 | 53.18 |
| ctg | L | 0 | 78.03 |
| mPD | | 0 | 1.1 |
| | | nPD: | 0. |
| | | N. weight: | 2.2 |
| | | Sc. PD: | -0.44 |
| | | Sc. rank: | -488.3 |

PA-X

| Pos. | 227 | obs: | exp: |
|---|---|---|---|
| caa | Q | 8 | 4.26 |
| cag | Q | 0 | 3.74 |
| aaa | K | 345 | 193.20 |
| aag | K | 0 | 151.80 |
| mPD | | 0.044 | 0.54 |
| | | nPD: | 0.08 |
| | | N. weight: | 0.76 |
| | | Sc. PD: | -0.094 |
| | | Sc. rank: | -2.8 |

PA-X

| Pos. | 228 | obs: | exp: |
|---|---|---|---|
| cct | P | 3 | 0.79 |
| ccc | P | 0 | 0.54 |
| cca | P | 0 | 1.18 |
| ccg | P | 0 | 0.49 |
| att | I | 1 | 0.38 |
| atc | I | 0 | 0.26 |
| ata | I | 0 | 0.36 |
| act | T | 349 | 92.20 |
| acc | T | 0 | 74.89 |
| aca | T | 0 | 152.90 |
| acg | T | 0 | 29.01 |
| mPD | | 0.023 | 0.71 |
| | | nPD: | 0.03 |
| | | N. weight: | 1.7 |
| | | Sc. PD: | -0.3 |
| | | Sc. rank: | -101.1 |

PA-X

| Pos. | 229 | obs: | exp: |
|---|---|---|---|
| tta | L | 353 | 30.84 |
| ttg | L | 0 | 67.98 |
| ctt | L | 0 | 65.86 |
| ctc | L | 0 | 57.10 |
| cta | L | 0 | 53.18 |
| ctg | L | 0 | 78.03 |
| mPD | | 0 | 1.1 |
| | | nPD: | 0. |
| | | N. weight: | 3.2 |
| | | Sc. PD: | -0.65 |
| | | Sc. rank: | -722.7 |

PA-X

| Pos. | 230 | obs: | exp: |
|---|---|---|---|
| gaa | E | 2 | 203.10 |
| gag | E | 351 | 149.90 |
| mPD | | 0.011 | 0.49 |
| | | nPD: | 0.02 |
| | | N. weight: | 1.1 |
| | | Sc. PD: | -0.19 |
| | | Sc. rank: | -83.0 |

PA-X

| Pos. | 231 | obs: | exp: |
|---|---|---|---|
| cct | P | 353 | 92.95 |
| ccc | P | 0 | 63.67 |
| cca | P | 0 | 139.30 |
| ccg | P | 0 | 57.08 |
| mPD | | 0 | 0.72 |
| | | nPD: | 0. |
| | | N. weight: | 1.7 |
| | | Sc. PD: | -0.35 |
| | | Sc. rank: | -395.6 |

PA-X

| Pos. | 232 | obs: | exp: |
|---|---|---|---|
| atg | M | 353 | 353.00 |
| mPD | | 0 | 0 |
| | | nPD: | 1. |
| | | N. weight: | 0. |
| | | Sc. PD: | 0 |
| | | Sc. rank: | 0 |

PA-X

| Pos. | 233 | obs: | exp: |
|---|---|---|---|
| tgg | W | 353 | 353.00 |
| mPD | | 0 | 0 |
| | | nPD: | 1. |
| | | N. weight: | 0. |
| | | Sc. PD: | 0 |
| | | Sc. rank: | 0 |

PA-X

| Pos. | 234 | obs: | exp: |
|---|---|---|---|
| atg | M | 352 | 352.00 |
| act | T | 0 | 0.26 |
| acc | T | 0 | 0.21 |
| aca | T | 0 | 0.44 |
| acg | T | 1 | 0.08 |
| --- | -- | ------ | ------ |
| mPD | | 0.0057 | 0.011 |
| | | nPD: | 0.52 |
| | | N. weight: | 0.0092 |
| | | Sc. PD: | 0.0028 |
| | | Sc. rank: | 1.6 |

PA-X

| Pos. | 235 | obs: | exp: |
|---|---|---|---|
| gat | D | 353 | 188.30 |
| gac | D | 0 | 164.70 |
| --- | -- | ------ | ------ |
| mPD | | 0 | 0.50 |
| | | nPD: | 0. |
| | | N. weight: | 0.82 |
| | | Sc. PD: | -0.17 |
| | | Sc. rank: | -186.2 |

PA-X

| Pos. | 236 | obs: | exp: |
|---|---|---|---|
| tta | L | 0 | 0.26 |
| ttg | L | 3 | 0.58 |
| tct | S | 0 | 55.64 |
| tcc | S | 0 | 48.76 |
| tca | S | 1 | 77.54 |
| tcg | S | 349 | 21.33 |
| ctt | L | 0 | 0.56 |
| ctc | L | 0 | 0.49 |
| cta | L | 0 | 0.45 |
| ctg | L | 0 | 0.66 |
| agt | S | 0 | 77.00 |
| agc | S | 0 | 69.73 |
| --- | -- | ------ | ------ |
| mPD | | 0.023 | 1.7 |
| | | nPD: | 0.01 |
| | | N. weight: | 3.6 |
| | | Sc. PD: | -0.69 |
| | | Sc. rank: | -364.6 |

PA-X

| Pos. | 237 | obs: | exp: |
|---|---|---|---|
| tct | S | 0 | 0.48 |
| tcc | S | 0 | 0.42 |
| tca | S | 0 | 0.66 |
| tcg | S | 0 | 0.18 |
| aat | N | 0 | 182.90 |
| aac | N | 350 | 167.10 |
| agt | S | 0 | 0.66 |
| agc | S | 3 | 0.60 |
| --- | -- | ------ | ------ |
| mPD | | 0.017 | 0.53 |
| | | nPD: | 0.03 |
| | | N. weight: | 0.98 |
| | | Sc. PD: | -0.17 |
| | | Sc. rank: | -55.0 |

PA-X

| Pos. | 238 | obs: | exp: |
|---|---|---|---|
| caa | Q | 1 | 0.53 |
| cag | Q | 0 | 0.47 |
| cgt | R | 0 | 14.61 |
| cgc | R | 0 | 17.24 |
| cga | R | 352 | 33.83 |
| cgg | R | 0 | 29.37 |
| aga | R | 0 | 158.30 |
| agg | R | 0 | 98.64 |
| --- | -- | ------ | ------ |
| mPD | | 0.0057 | 0.97 |
| | | nPD: | 0.01 |
| | | N. weight: | 3.1 |
| | | Sc. PD: | -0.6 |
| | | Sc. rank: | -417.8 |

PA-X

| Pos. | 239 | obs: | exp: |
|---|---|---|---|
| atg | M | 5 | 5.00 |
| act | T | 0 | 91.94 |
| acc | T | 0 | 74.68 |
| aca | T | 0 | 152.50 |
| acg | T | 348 | 28.93 |
| --- | -- | ------ | ------ |
| mPD | | 0.028 | 0.72 |
| | | nPD: | 0.04 |
| | | N. weight: | 3.2 |
| | | Sc. PD: | -0.53 |
| | | Sc. rank: | -103.5 |

PA-X

| Pos. | 240 | obs: | exp: |
|---|---|---|---|
| gct | A | 351 | 88.91 |
| gcc | A | 2 | 72.31 |
| gca | A | 0 | 157.30 |
| gcg | A | 0 | 34.45 |
| --- | -- | ------ | ------ |
| mPD | | 0.011 | 0.69 |
| | | nPD: | 0.02 |
| | | N. weight: | 1.8 |
| | | Sc. PD: | -0.33 |
| | | Sc. rank: | -151.3 |

PA-X

| Pos. | 241 | obs: | exp: |
|---|---|---|---|
| gct | A | 0 | 88.91 |
| gcc | A | 0 | 72.31 |
| gca | A | 353 | 157.30 |
| gcg | A | 0 | 34.45 |
| --- | -- | ------ | ------ |
| mPD | | 0 | 0.69 |
| | | nPD: | 0. |
| | | N. weight: | 1.1 |
| | | Sc. PD: | -0.21 |
| | | Sc. rank: | -239.6 |

PA-X

| Pos. | 242 | obs: | exp: |
|---|---|---|---|
| tta | L | 0 | 30.84 |
| ttg | L | 353 | 67.98 |
| ctt | L | 0 | 65.86 |
| ctc | L | 0 | 57.10 |
| cta | L | 0 | 53.18 |
| ctg | L | 0 | 78.03 |
| --- | -- | ------ | ------ |
| mPD | | 0 | 1.1 |
| | | nPD: | 0. |
| | | N. weight: | 2.2 |
| | | Sc. PD: | -0.44 |
| | | Sc. rank: | -488.3 |

PA-X

| Pos. | 243 | obs: | exp: |
|---|---|---|---|
| cgt | R | 0 | 14.57 |
| cgc | R | 0 | 17.19 |
| cga | R | 0 | 33.73 |
| cgg | R | 0 | 29.29 |
| aaa | K | 0 | 1.12 |
| aag | K | 2 | 0.88 |
| aga | R | 0 | 157.90 |
| agg | R | 351 | 98.36 |
| --- | -- | ------ | ------ |
| mPD | | 0.011 | 0.97 |
| | | nPD: | 0.01 |
| | | N. weight: | 1.7 |
| | | Sc. PD: | -0.32 |
| | | Sc. rank: | -178.4 |

PA-X

| Pos. | 244 | obs: | exp: |
|---|---|---|---|
| gct | A | 0 | 84.38 |
| gcc | A | 0 | 68.62 |
| gca | A | 335 | 149.30 |
| gcg | A | 0 | 32.69 |
| gaa | E | 18 | 10.36 |
| gag | E | 0 | 7.64 |
| --- | -- | ------ | ------ |
| mPD | | 0.097 | 0.78 |
| | | nPD: | 0.12 |
| | | N. weight: | 1. |
| | | Sc. PD: | -0.087 |
| | | Sc. rank: | 17.8 |

PA-X

| Pos. | 245 | obs: | exp: |
|---|---|---|---|
| tct | S | 0 | 56.12 |
| tcc | S | 0 | 49.17 |
| tca | S | 0 | 78.20 |
| tcg | S | 0 | 21.52 |
| agt | S | 0 | 77.66 |
| agc | S | 353 | 70.33 |
| --- | -- | ------ | ------ |
| mPD | | 0 | 1.7 |
| | | nPD: | 0. |
| | | N. weight: | 2.1 |
| | | Sc. PD: | -0.43 |
| | | Sc. rank: | -478.3 |

PA-X

| Pos. | 246 | obs: | exp: |
|---|---|---|---|
| ttt | F | 350 | 143.90 |
| ttc | F | 0 | 206.10 |
| tct | S | 2 | 0.32 |
| tcc | S | 0 | 0.28 |
| tca | S | 0 | 0.44 |
| tcg | S | 0 | 0.12 |
| tat | Y | 1 | 0.52 |
| tac | Y | 0 | 0.48 |
| agt | S | 0 | 0.44 |
| agc | S | 0 | 0.40 |
| --- | -- | ------ | ------ |
| mPD | | 0.017 | 0.51 |
| | | nPD: | 0.03 |
| | | N. weight: | 1.2 |
| | | Sc. PD: | -0.2 |
| | | Sc. rank: | -61.6 |

PA-X

| Pos. | 247 | obs: | exp: |
|---|---|---|---|
| tta | L | 0 | 30.84 |
| ttg | L | 0 | 67.98 |
| ctt | L | 0 | 65.86 |
| ctc | L | 353 | 57.10 |
| cta | L | 0 | 53.18 |
| ctg | L | 0 | 78.03 |
| --- | -- | ------ | ------ |
| mPD | | 0 | 1.1 |
| | | nPD: | 0. |
| | | N. weight: | 2.4 |
| | | Sc. PD: | -0.48 |
| | | Sc. rank: | -540.0 |

PA-X

| Pos. | 248 | obs: | exp: |
|---|---|---|---|
| cgt | R | 0 | 1.20 |
| cgc | R | 0 | 1.42 |
| cga | R | 0 | 2.79 |
| cgg | R | 0 | 2.42 |
| aaa | K | 324 | 181.40 |
| aag | K | 0 | 142.60 |
| aga | R | 29 | 13.04 |
| agg | R | 0 | 8.13 |
| --- | -- | ------ | ------ |
| mPD | | 0.15 | 0.69 |
| | | nPD: | 0.22 |
| | | N. weight: | 0.78 |
| | | Sc. PD: | 0.0052 |
| | | Sc. rank: | 53.9 |

PA-X

| Pos. | 249 | obs: | exp: |
|---|---|---|---|
| tgt | C | 353 | 145.50 |
| tgc | C | 0 | 207.50 |
| --- | -- | ------ | ------ |
| mPD | | 0 | 0.48 |
| | | nPD: | 0. |
| | | N. weight: | 1.2 |
| | | Sc. PD: | -0.23 |
| | | Sc. rank: | -262.7 |

PA-X

| Pos. | 250 | obs: | exp: |
|---|---|---|---|
| tta | L | 0 | 0.09 |
| ttg | L | 0 | 0.19 |
| ctt | L | 0 | 0.19 |
| ctc | L | 0 | 0.16 |
| cta | L | 1 | 0.15 |
| ctg | L | 0 | 0.22 |
| cct | P | 0 | 20.54 |
| ccc | P | 0 | 14.07 |
| cca | P | 78 | 30.78 |
| ccg | P | 0 | 12.61 |
| caa | Q | 272 | 145.00 |
| cag | Q | 0 | 127.00 |
| cgt | R | 0 | 0.08 |
| cgc | R | 0 | 0.10 |
| cga | R | 2 | 0.19 |
| cgg | R | 0 | 0.17 |
| aga | R | 0 | 0.90 |
| agg | R | 0 | 0.56 |
| --- | -- | ------ | ------ |
| mPD | | 0.36 | 0.95 |
| | | nPD: | 0.38 |
| | | N. weight: | 0.93 |
| | | Sc. PD: | 0.15 |
| | | Sc. rank: | 112.8 |

PA-X

| Pos. | 251 | obs: | exp: |
|---|---|---|---|
| cgt | R | 0 | 1.25 |
| cgc | R | 0 | 1.47 |
| cga | R | 0 | 2.88 |
| cgg | R | 0 | 2.50 |
| aaa | K | 1 | 180.30 |
| aag | K | 321 | 141.70 |
| aga | R | 0 | 13.49 |
| agg | R | 30 | 8.41 |
| ggt | G | 0 | 0.12 |
| ggc | G | 0 | 0.14 |
| gga | G | 0 | 0.48 |
| ggg | G | 1 | 0.26 |
| --- | -- | ------ | ------ |
| mPD | | 0.17 | 0.71 |
| | | nPD: | 0.24 |
| | | N. weight: | 1.1 |
| | | Sc. PD: | 0.033 |
| | | Sc. rank: | 85.7 |

PA-X

| Pos. | 252 | obs: | exp: |
|---|---|---|---|
| cgt | R | 0 | 0.25 |
| cgc | R | 0 | 0.29 |
| cga | R | 0 | 0.58 |
| cgg | R | 0 | 0.50 |
| aaa | K | 0 | 194.30 |
| aag | K | 347 | 152.70 |
| aga | R | 0 | 2.70 |
| agg | R | 6 | 1.68 |
| --- | -- | ------ | ------ |
| mPD | | 0.034 | 0.54 |
| | | nPD: | 0.06 |
| | | N. weight: | 1.1 |
| | | Sc. PD: | -0.15 |
| | | Sc. rank: | -13.1 |

PA-X

| Pos. | 253 | obs: | exp: |
|---|---|---|---|
| taa | . | 89 | 165.30 |
| tag | . | 0 | 91.35 |
| tga | . | 264 | 96.33 |
| --- | -- | ------ | ------ |
| mPD | | 0.38 | 0.78 |
| | | nPD: | 0.48 |
| | | N. weight: | 0.79 |
| | | Sc. PD: | 0.21 |
| | | Sc. rank: | 121.5 |

Amino acid level conservation per codon

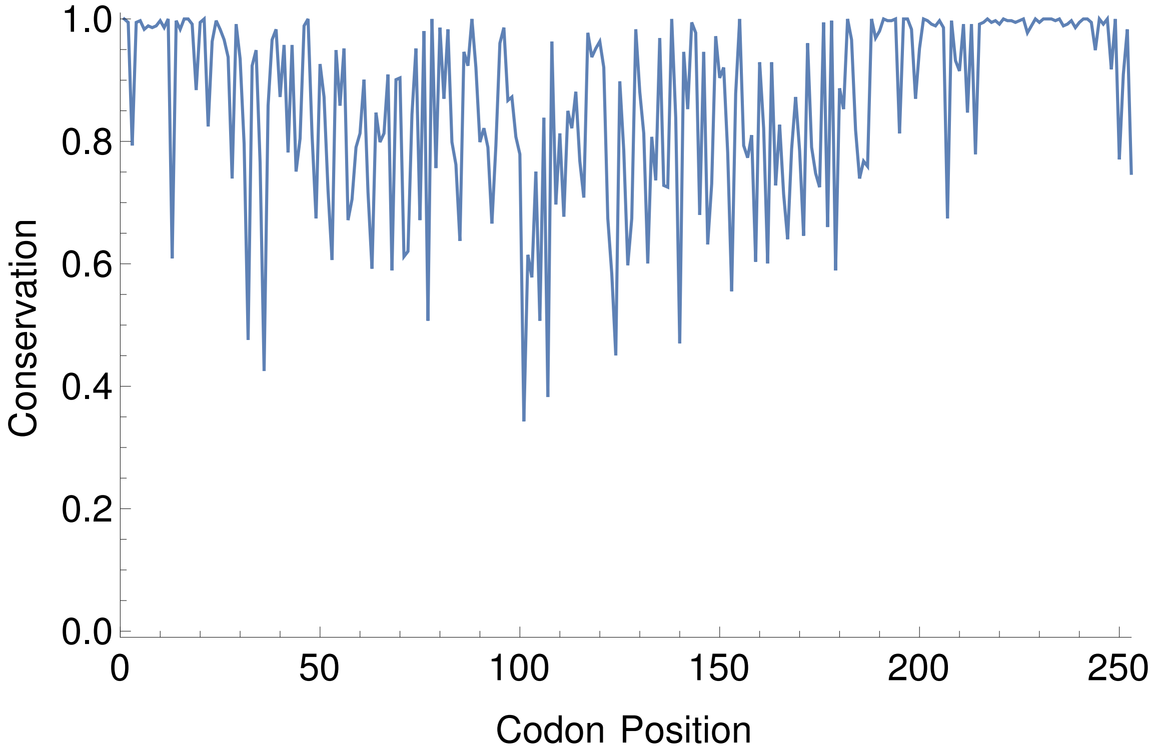

## Conservation analysis plots

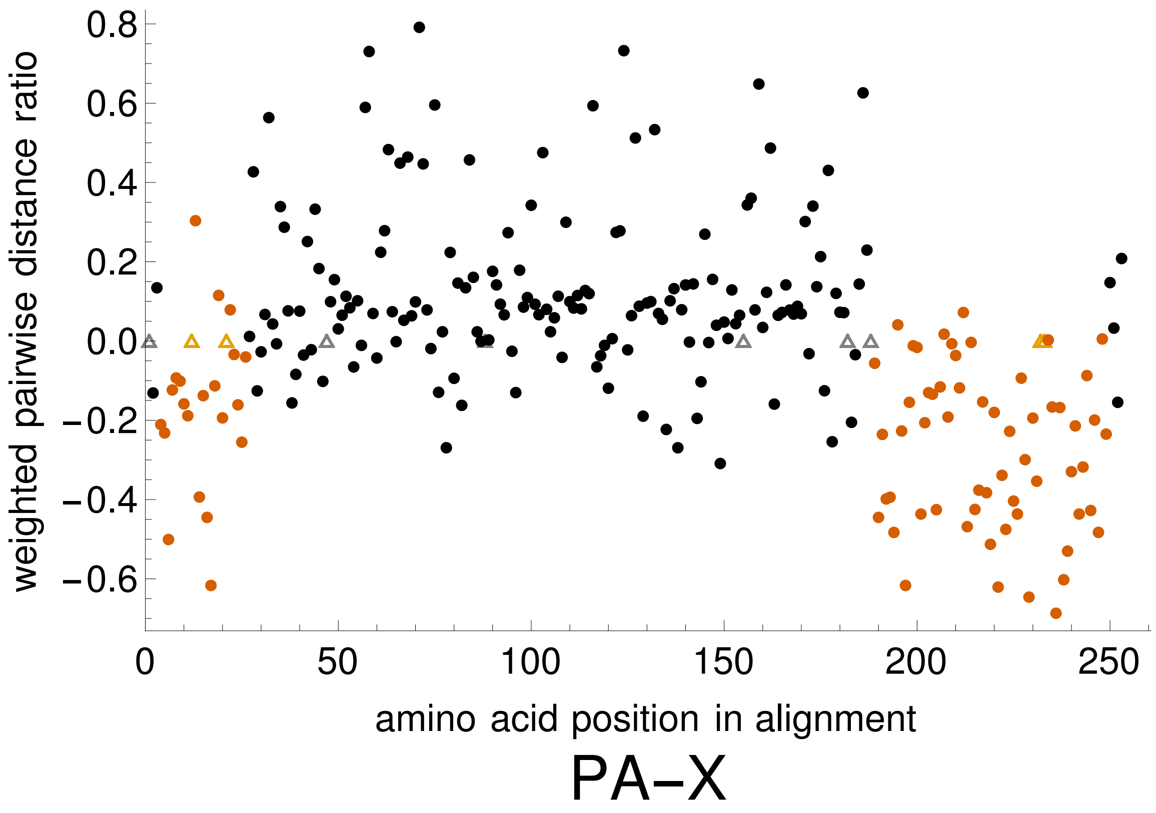


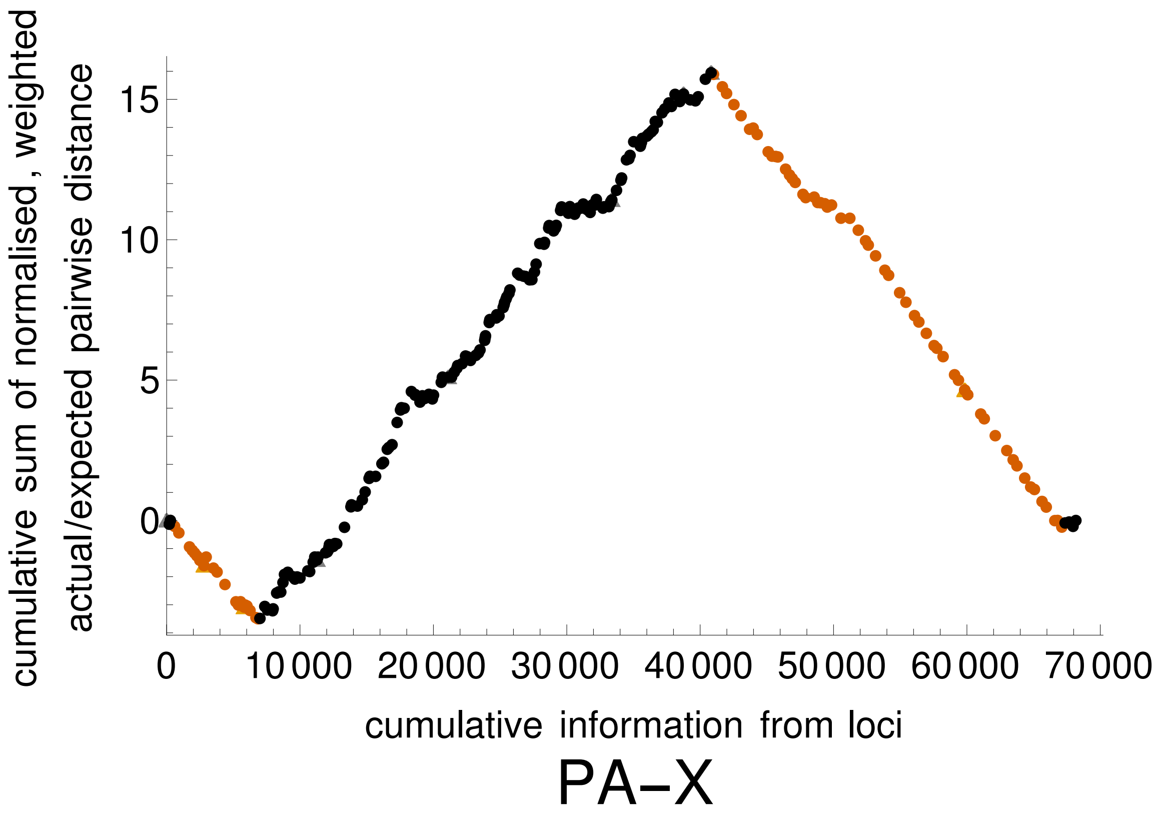

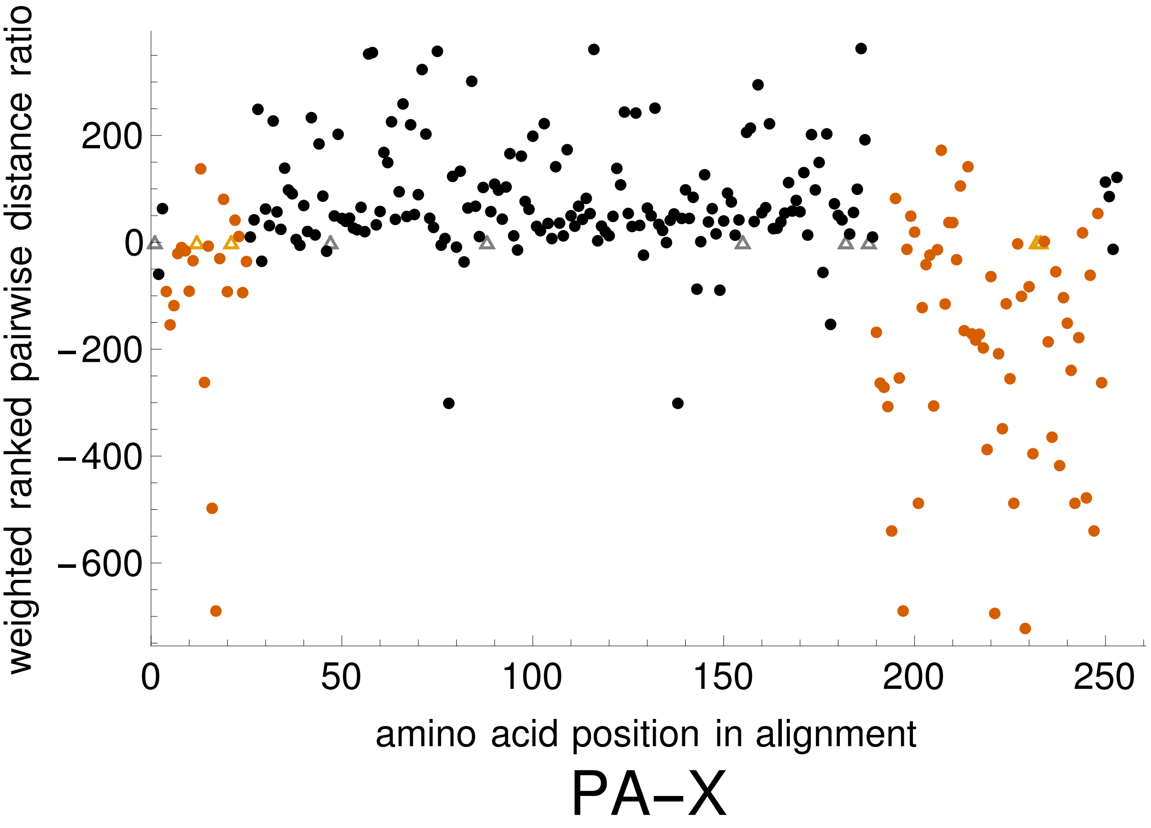
weighted ranked pairwise distance ratio
200
0
−200
−400
−600
0
50
100
150
200
250
amino acid position in alignment
PA–X

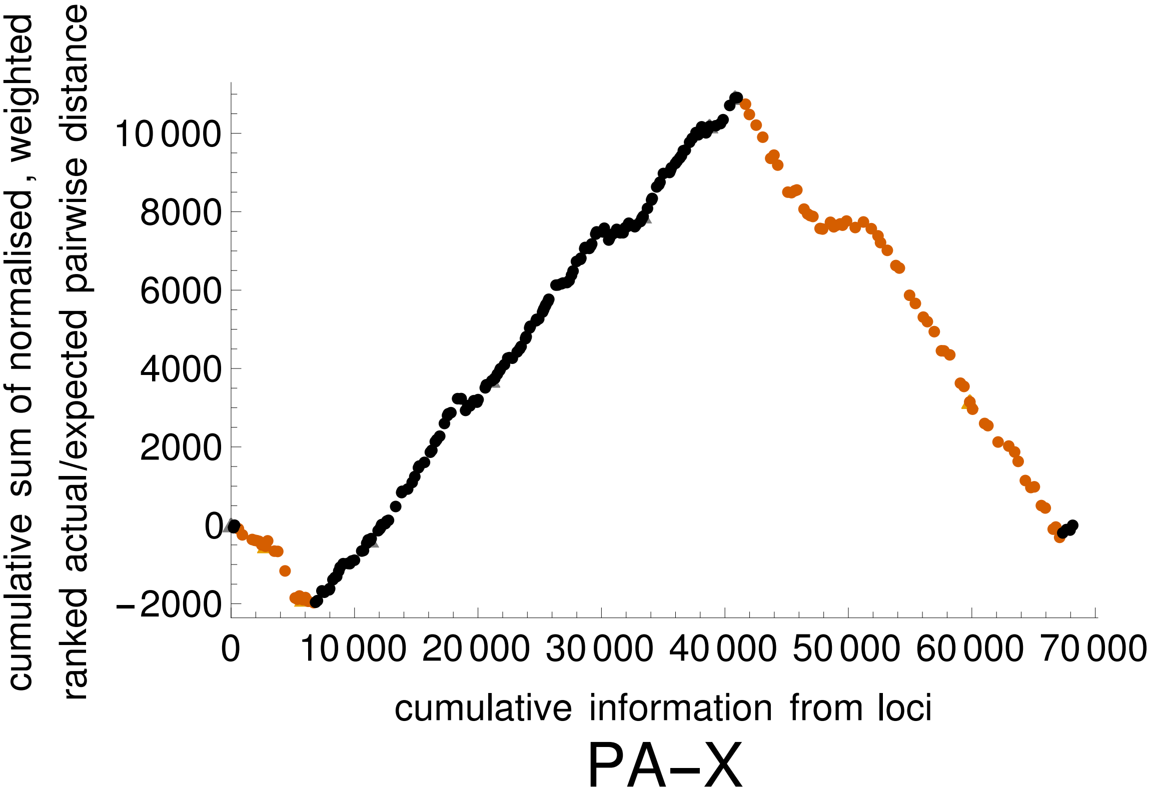
cumulative sum of normalised, weighted
ranked actual/expected pairwise distance
10 000
8000
6000
4000
2000
0
−2000
0
10 000
20 000
30 000
40 000
50 000
60 000
70 000
cumulative information from loci
PA–X

# HA

## Gene length histogram

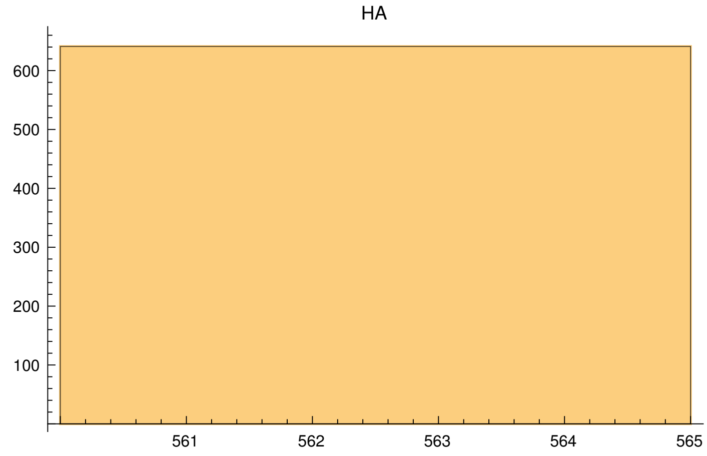

## Information vs. nPD

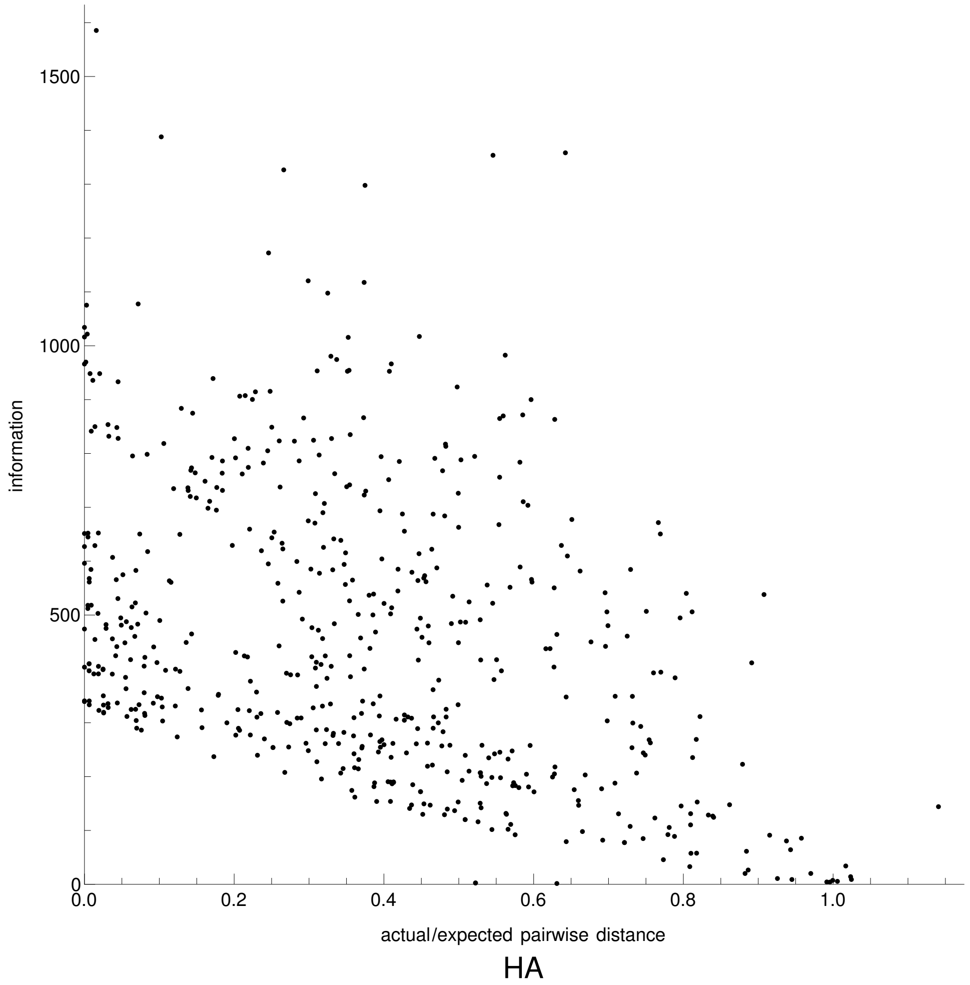

## Example sequences highlighted by regions found in analyses to be conserved

Interesting points (by weighted raw PD) highlighted for gene HA:

ATGAACACTCAAATCCTGGTATTCGCTCTGGTGGCGAGCATTCCGACAAATGCAGACAAGATCTGCCTTGGGCATC⋱
ATGCCGTGTCAAACGGGACTAAAGTAAACACATTAACTGAGAGAGGAGTGGAAGTCGTTAATGCAACTGAAACGG⋱
TGGAACGAACAAACGTTCCCAGGATCTGCTCAAAAGGGAAAAGGACAGTTGACCTCGGTCAATGTGGACTTCTGG⋱
GAACAATCACTGGGCCACCCCAATGTGACCAATTCCTAGAATTTTCGGCCGACTTAATTATTGAGAGGCGAGAAG⋱
GAAGTGATGTCTGTTATCCTGGGAAATTCGTGAATGAAGAAGCTCTGAGGCAAATTCTCAGAGAGTCAGGCGGAA⋱
TTGACAAGGAGACAATGGGATTCACCTACAGCGGAATAAGAACTAATGGAACAACCAGTGCATGTAGGAGATCAG⋱
GATCTTCATTCTATGCAGAGATGAAATGGCTCCTGTCAAACACAGACAATGCTGCTTTCCCGCAAATGACTAAGT⋱
CATACAAGAACACAAGGAAAGCCCCAGCTCTGATAATATGGGGGATCCACCATTCCGGATCAACTACAGAACAGA⋱
CCAAGCTATATGGGAGTGGAAACAAACTGATAACAGTTGGGAGTTCTAATTACCAACAGTCCTTTGTACCGAGTC⋱
CAGGAGCGAGACCACAAGTGAATGGCCAATCTGGAAGAATTGACTTTCATTGGCTGATACTAAACCCTAATGACA⋱
CGGTCACTTTCAGTTTCAATGGGGCCTTCATAGCTCCAGACCGTGCAAGCTTTCTGAGAGGGAAGTCCATGGGAA⋱
TTCAGAGTGAAGTACAGGTTGATGCCAATTGTGAAGGAGATTGCTATCATAGTGGAGGGACAATAATAAGTAATT⋱
TGCCCTTTCAGAACATAAATAGCAGGGCAGTAGGAAAATGTCCGAGATATGTTAAGCAAGAGAGTCTGCTGTTGG⋱
CAACAGGAATGAAGAATGTTCCCGAAATCCCA ------AAG------AGGAGG---AGGAGAGGCCTATTTGGTGCT⋱
ATAGCGGGTTTCATTGAAAATGGATGGGAAGGTTTGATTGATGGGTGGTATGGCTTCAGGCATCAAAATGCACAA⋱
GGGGAGGGAACTGCTGCAGATTACAAAAGCACCCAATCAGCAATTGATCAAATAACAGGGAAATTAAATCGGCTT⋱
ATAGAAAAAACTAACCAACAGTTTGAGTTAATAGACAACGAATTCACTGAGGTTGAAAAGCAAATTGGCAATGTG⋱
ATAAACTGGACCAGAGATTCCATGACAGAAGTGTGGTCCTATAACGCTGAACTCTTAGTAGCAATGGAGAATCAG⋱
CACACAATTGATCTGGCCGACTCAGAAATGAACAAACTGTACGAACGAGTGAAGAGACAACTGAGAGAGAATGCC⋱
GAAGAAGATGGCACTGGTTGCTTCGAAATATTTCACAAGTGTGATGACGACTGCATGGCCAGTATTAGAAACAAC⋱
ACCTATGATCACAGCAAGTACAGGGAAGAAGCAATACAAAATAGAATACAGATTGACCCAGTCAAACTAAGCAGC⋱
GGCTACAAAGATGTGATACTTTGG
**TTTAGCTTCGGGGCATCATGTTTCATACTTCTGGCCATTGCAATGGGCCTTGTCT**
**TCATATGTGTGAAGAATGGAAACATGCGGTGCACTATTTGTATA**TAA

Interesting points (by weighted ranked PD) highlighted for gene HA:

ATGAACACTCAAATCCTGGTATTCGCTCTGGTGGCGAGCATTCCGACAAATGCAGACAAGATCTGCCTTGGGCATCAT⋱
GCCGTGTCAAACGGGACTAAAGTAAACACATTAACTGAGAGAGGAGTGGAAGTCGTTAATGCAACTGAAACGGTGGA⋱
ACGAACAAACGTTCCCAGGATCTGCTCAAAAGGGAAAAGGACAGTTGACCTCGGTCAATGTGGACTTCTGGGAACAA⋱
TCACTGGGCCACCCCAATGTGACCAATTCCTAGAATTTTCGGCCGACTTAATTATTGAGAGGCGAGAAGGAAGTGAT⋱
GTCTGTTATCCTGGGAAATTCGTGAATGAAGAAGCTCTGAGGCAAATTCTCAGAGAGTCAGGCGGAATTGACAAGGA⋱
GACAATGGGATTCACCTACAGCGGAATAAGAACTAATGGAACAACCAGTGCATGTAGGAGATCAGGATCTTCATTCT⋱
ATGCAGAGATGAAATGGCTCCTGTCAAACACAGACAATGCTGCTTTCCCGCAAATGACTAAGTCATACAAGAACACA⋱
AGGAAAGCCCCAGCTCTGATAATATGGGGGATCCACCATTCCGGATCAACTACAGAACAGACCAAGCTATATGGGAG⋱
TGGAAACAAACTGATAACAGTTGGGAGTTCTAATTACCAACAGTCCTTTGTACCGAGTCCAGGAGCGAGACCACAAG⋱
TGAATGGCCAATCTGGAAGAATTGACTTTCATTGGCTGATACTAAACCCTAATGACACGGTCACTTTCAGTTTCAAT⋱
GGGGCCTTCATAGCTCCAGACCGTGCAAGCTTTCTGAGAGGGAAGTCCATGGGAATTCAGAGTGAAGTACAGGTTGA⋱
TGCCAATTGTGAAGGAGATTGCTATCATAGTGGAGGGACAATAATAAGTAATTTGCCCTTTCAGAACATAAATAGCA⋱
GGGCAGTAGGAAAATGTCCGAGATATGTTAAGCAAGAGAGTCTGCTGTTGGCAACAGGAATGAAGAATGTTCCCGAA⋱
ATCCCA------AAG------AGGAGG---AGGAGAGGCCTATTTGGTGCTATAGCGGGTTTCATTGAAAATGGATGGG⋱
AAGGTTTGATTGATGGGTGGTATGGCTTCAGGCATCAAAATGCACAAGGGGAGGGAACTGCTGCAGATTACAAAA⋱
GCACCCAATCAGCAATTGATCAAATAACAGGGAAATTAAATCGGCTTATAGAAAAAACTAACCAACAGTTTGAGT⋱
TAATAGACAACGAATTCACTGAGGTTGAAAAGCAAATTGGCAATGTGATAAACTGGACCAGAGATTCCATGACAG⋱

AAGTGTGGTCCTATAACGCTGAACTCTTAGTAGCAATGGAGAATCAGCACACAATTGATCTGGCCGACTCAGAAA∵.

TGAACAAACTGTACGAACGAGTGAAGAGACAACTGAGAGAGAATGCCGAAGAAGATGGCACTGGTTGCTTCGAAA∵.

TATTTCACAAGTGTGATGACGACTGCATGGCCAGTATTAGAAACAACACCTATGATCACAGCAAGTACAGGGAAG∵.

AAGCAATACAAAATAGAATACAGATTGACCCAGTCAAACTAAGCAGCGGCTACAAAGATGTGATACTTTGG

**TTTAGCTTCGGGGCATCATGTTTCATACTTCTGGCCATTGCAATGGGCCTTGTCT**

**TCATATGTGTGAAGAATGGAAACATGCGGTGCACTATTTGTATA**TAA

# Per codon conservation report

HA

| Pos. | 1 | obs: | exp: |
|---|---|---|---|
| atg | M | 641 | 641.00 |
| --- | -- | ------ | ------ |
| mPD | | 0 | 0 |
| | | nPD: | 1. |
| | | N. weight: | 0. |
| | | Sc. PD: | 0 |
| | | Sc. rank: | 0 |

HA

| Pos. | 2 | obs: | exp: |
|---|---|---|---|
| act | T | 0 | 0.53 |
| acc | T | 1 | 0.43 |
| aca | T | 1 | 0.88 |
| acg | T | 0 | 0.17 |
| aat | N | 13 | 334.00 |
| aac | N | 626 | 305.00 |
| --- | -- | ------ | ------ |
| mPD | | 0.049 | 0.51 |
| | | nPD: | 0.1 |
| | | N. weight: | 0.94 |
| | | Sc. PD: | -0.2 |
| | | Sc. rank: | -201.6 |

HA

| Pos. | 3 | obs: | exp: |
|---|---|---|---|
| att | I | 8 | 3.38 |
| atc | I | 1 | 2.36 |
| ata | I | 0 | 3.26 |
| act | T | 632 | 167.00 |
| acc | T | 0 | 135.60 |
| aca | T | 0 | 276.90 |
| acg | T | 0 | 52.54 |
| --- | -- | ------ | ------ |
| mPD | | 0.031 | 0.71 |
| | | nPD: | 0.04 |
| | | N. weight: | 1.9 |
| | | Sc. PD: | -0.51 |
| | | Sc. rank: | -527.2 |

HA

| Pos. | 4 | obs: | exp: |
|---|---|---|---|
| caa | Q | 641 | 341.70 |
| cag | Q | 0 | 299.30 |
| --- | -- | ------ | ------ |
| mPD | | 0 | 0.50 |
| | | nPD: | 0. |
| | | N. weight: | 0.92 |
| | | Sc. PD: | -0.28 |
| | | Sc. rank: | -348.0 |

HA

| Pos. | 5 | obs: | exp: |
|---|---|---|---|
| att | I | 79 | 239.10 |
| atc | I | 557 | 166.70 |
| ata | I | 0 | 230.20 |
| act | T | 1 | 0.79 |
| acc | T | 2 | 0.64 |
| aca | T | 0 | 1.31 |
| acg | T | 0 | 0.25 |
| gtt | V | 0 | 0.41 |
| gtc | V | 2 | 0.42 |
| gta | V | 0 | 0.37 |
| gtg | V | 0 | 0.79 |
| --- | -- | ------ | ------ |
| mPD | | 0.23 | 0.68 |
| | | nPD: | 0.35 |
| | | N. weight: | 1.4 |
| | | Sc. PD: | 0.041 |
| | | Sc. rank: | 63.8 |

HA

| Pos. | 6 | obs: | exp: |
|---|---|---|---|
| tta | L | 1 | 55.82 |
| ttg | L | 81 | 123.10 |
| ctt | L | 1 | 119.20 |
| ctc | L | 0 | 103.40 |
| cta | L | 47 | 96.27 |
| ctg | L | 509 | 141.30 |
| atg | M | 2 | 2.00 |
| --- | -- | ------ | ------ |
| mPD | | 0.37 | 1.1 |
| | | nPD: | 0.33 |
| | | N. weight: | 1.3 |
| | | Sc. PD: | 0.021 |
| | | Sc. rank: | 39.9 |

HA

| Pos. | 7 | obs: | exp: |
|---|---|---|---|
| tta | L | 5 | 0.61 |
| ttg | L | 0 | 1.35 |
| ctt | L | 0 | 1.31 |
| ctc | L | 0 | 1.13 |
| cta | L | 2 | 1.05 |
| ctg | L | 0 | 1.55 |
| att | I | 0 | 6.77 |
| atc | I | 0 | 4.72 |
| ata | I | 18 | 6.52 |
| atg | M | 29 | 29.00 |
| gtt | V | 0 | 106.10 |
| gtc | V | 0 | 108.30 |
| gta | V | 516 | 96.71 |
| gtg | V | 0 | 204.90 |
| gct | A | 0 | 17.88 |
| gcc | A | 0 | 14.54 |
| gca | A | 71 | 31.64 |
| gcg | A | 0 | 6.93 |
| --- | -- | ------ | ------ |
| mPD | | 0.44 | 1.1 |
| | | nPD: | 0.41 |
| | | N. weight: | 2.2 |
| | | Sc. PD: | 0.19 |
| | | Sc. rank: | 284.5 |

HA

| Pos. | 8 | obs: | exp: |
|---|---|---|---|
| ttt | F | 16 | 204.70 |
| ttc | F | 482 | 293.30 |
| tta | L | 0 | 12.41 |
| ttg | L | 0 | 27.35 |
| ctt | L | 0 | 26.50 |
| ctc | L | 139 | 22.97 |
| cta | L | 3 | 21.39 |
| ctg | L | 0 | 31.39 |
| gct | A | 0 | 0.25 |
| gcc | A | 1 | 0.20 |
| gca | A | 0 | 0.45 |
| gcg | A | 0 | 0.10 |
| --- | -- | ------ | ------ |
| mPD | | 0.41 | 0.89 |
| | | nPD: | 0.46 |
| | | N. weight: | 1. |
| | | Sc. PD: | 0.14 |
| | | Sc. rank: | 189.5 |

HA

| Pos. | 9 | obs: | exp: |
|---|---|---|---|
| att | I | 71 | 27.07 |
| atc | I | 1 | 18.87 |
| ata | I | 0 | 26.06 |
| act | T | 1 | 0.26 |
| acc | T | 0 | 0.21 |
| aca | T | 0 | 0.44 |
| acg | T | 0 | 0.08 |
| gct | A | 528 | 143.10 |
| gcc | A | 27 | 116.40 |
| gca | A | 13 | 253.20 |
| gcg | A | 0 | 55.43 |
| --- | -- | ------ | ------ |
| mPD | | 0.52 | 1.1 |
| | | nPD: | 0.48 |
| | | N. weight: | 1.6 |
| | | Sc. PD: | 0.25 |
| | | Sc. rank: | 321.5 |

HA

| Pos. | 10 | obs: | exp: |
|---|---|---|---|
| tta | L | 0 | 49.36 |
| ttg | L | 16 | 108.80 |
| ctt | L | 13 | 105.40 |
| ctc | L | 0 | 91.39 |
| cta | L | 1 | 85.12 |
| ctg | L | 535 | 124.90 |
| att | I | 0 | 1.50 |
| atc | I | 0 | 1.05 |
| ata | I | 4 | 1.45 |
| gct | A | 70 | 18.13 |
| gcc | A | 2 | 14.75 |
| gca | A | 0 | 32.09 |
| gcg | A | 0 | 7.03 |
| --- | -- | ------ | ------ |
| mPD | | 0.70 | 1.4 |
| | | nPD: | 0.48 |
| | | N. weight: | 1.9 |
| | | Sc. PD: | 0.3 |
| | | Sc. rank: | 388.9 |

HA

| Pos. | 11 | obs: | exp: |
|---|---|---|---|
| tat | Y | 1 | 0.52 |
| tac | Y | 0 | 0.48 |
| tgt | C | 67 | 29.27 |
| tgc | C | 4 | 41.73 |
| att | I | 54 | 20.68 |
| atc | I | 0 | 14.41 |
| ata | I | 1 | 19.91 |
| atg | M | 7 | 7.00 |
| act | T | 0 | 0.79 |
| acc | T | 0 | 0.64 |
| aca | T | 2 | 1.31 |
| acg | T | 1 | 0.25 |
| gtt | V | 40 | 103.40 |
| gtc | V | 0 | 105.60 |
| gta | V | 19 | 94.27 |
| gtg | V | 444 | 199.80 |
| gct | A | 1 | 0.25 |
| gcc | A | 0 | 0.20 |
| gca | A | 0 | 0.45 |
| gcg | A | 0 | 0.10 |
| --- | -- | ------ | ------ |
| mPD | | 1.0 | 1.3 |
| | | nPD: | 0.77 |
| | | N. weight: | 0.9 |
| | | Sc. PD: | 0.39 |
| | | Sc. rank: | 323.6 |

HA

| Pos. | 12 | obs: | exp: |
|---|---|---|---|
| att | I | 0 | 0.38 |
| atc | I | 1 | 0.26 |
| ata | I | 0 | 0.36 |
| atg | M | 68 | 68.00 |
| act | T | 0 | 1.06 |
| acc | T | 0 | 0.86 |
| aca | T | 0 | 1.75 |
| acg | T | 4 | 0.33 |
| gtt | V | 0 | 1.03 |
| gtc | V | 0 | 1.05 |
| gta | V | 0 | 0.94 |
| gtg | V | 5 | 1.99 |
| gct | A | 0 | 141.80 |
| gcc | A | 0 | 115.30 |
| gca | A | 4 | 250.90 |
| gcg | A | 559 | 54.94 |
| --- | -- | ------ | ------ |
| mPD | | 0.42 | 1.1 |
| | | nPD: | 0.37 |
| | | N. weight: | 3. |
| | | Sc. PD: | 0.17 |
| | | Sc. rank: | 289.9 |

HA

| Pos. | 13 | obs: | exp: |
|---|---|---|---|
| tta | L | 0 | 6.29 |
| ttg | L | 0 | 13.87 |
| tct | S | 0 | 1.75 |
| tcc | S | 0 | 1.53 |
| tca | S | 0 | 2.44 |
| tcg | S | 0 | 0.67 |
| ctt | L | 0 | 13.43 |
| ctc | L | 1 | 11.65 |
| cta | L | 0 | 10.85 |
| ctg | L | 71 | 15.92 |
| att | I | 6 | 207.90 |
| atc | I | 486 | 144.90 |
| ata | I | 61 | 200.20 |
| act | T | 0 | 0.53 |
| acc | T | 2 | 0.43 |
| aca | T | 0 | 0.88 |
| acg | T | 0 | 0.17 |
| agt | S | 0 | 2.42 |
| agc | S | 11 | 2.19 |
| gtt | V | 0 | 0.62 |
| gtc | V | 3 | 0.63 |
| gta | V | 0 | 0.56 |
| gtg | V | 0 | 1.19 |
| --- | -- | ------ | ------ |
| mPD | | 0.61 | 0.96 |
| | | nPD: | 0.64 |
| | | N. weight: | 1.4 |
| | | Sc. PD: | 0.44 |
| | | Sc. rank: | 452.8 |

HA

| Pos. | 14 | obs: | exp: |
|---|---|---|---|
| att | I | 615 | 237.60 |
| atc | I | 17 | 165.60 |
| ata | I | 0 | 228.80 |
| act | T | 8 | 2.11 |
| acc | T | 0 | 1.72 |
| aca | T | 0 | 3.50 |
| acg | T | 0 | 0.67 |
| gtt | V | 1 | 0.21 |
| gtc | V | 0 | 0.21 |
| gta | V | 0 | 0.19 |
| gtg | V | 0 | 0.40 |
| --- | -- | ------ | ------ |
| mPD | | 0.080 | 0.69 |
| | | nPD: | 0.12 |
| | | N. weight: | 1.3 |
| | | Sc. PD: | -0.25 |
| | | Sc. rank: | -253.0 |

HA

| Pos. | 15 | obs: | exp: |
|---|---|---|---|
| cct | P | 3 | 148.80 |
| ccc | P | 0 | 101.90 |
| cca | P | 204 | 223.00 |
| ccg | P | 358 | 91.35 |
| cat | H | 1 | 1.63 |
| cac | H | 2 | 1.37 |
| caa | Q | 0 | 0.53 |
| cag | Q | 1 | 0.47 |
| cgt | R | 0 | 0.04 |
| cgc | R | 0 | 0.05 |
| cga | R | 0 | 0.10 |
| cgg | R | 0 | 0.08 |
| aga | R | 1 | 0.45 |
| agg | R | 0 | 0.28 |
| gaa | E | 2 | 1.15 |
| gag | E | 0 | 0.85 |
| ggt | G | 0 | 8.51 |
| ggc | G | 1 | 9.33 |
| gga | G | 65 | 33.38 |
| ggg | G | 3 | 17.78 |
| --- | -- | ------ | ------ |
| mPD | | 0.91 | 1.1 |
| | | nPD: | 0.81 |
| | | N. weight: | 1.2 |
| | | Sc. PD: | 0.55 |
| | | Sc. rank: | 425.2 |

| HA | | | |
|---|---|---|---|
| Pos. | 16 | obs: | exp: |
| att | I | 0 | 18.04 |
| atc | I | 0 | 12.58 |
| ata | I | 48 | 17.38 |
| act | T | 3 | 137.90 |
| acc | T | 2 | 112.00 |
| aca | T | 512 | 228.70 |
| acg | T | 5 | 43.39 |
| gtt | V | 4 | 0.82 |
| gtc | V | 0 | 0.84 |
| gta | V | 0 | 0.75 |
| gtg | V | 0 | 1.59 |
| gct | A | 64 | 16.88 |
| gcc | A | 0 | 13.72 |
| gca | A | 3 | 29.86 |
| gcg | A | 0 | 6.54 |
| --- | -- | ------ | ------ |
| mPD | | 0.56 | 1.0 |
| | | nPD: | 0.55 |
| | | N. weight: | 1.2 |
| | | Sc. PD: | 0.26 |
| | | Sc. rank: | 300.6 |

| HA | | | |
|---|---|---|---|
| Pos. | 17 | obs: | exp: |
| tct | S | 0 | 9.86 |
| tcc | S | 0 | 8.64 |
| tca | S | 0 | 13.74 |
| tcg | S | 0 | 3.78 |
| cgt | R | 0 | 1.25 |
| cgc | R | 0 | 1.47 |
| cga | R | 0 | 2.88 |
| cgg | R | 0 | 2.50 |
| aat | N | 505 | 264.50 |
| aac | N | 1 | 241.50 |
| aaa | K | 41 | 24.08 |
| aag | K | 2 | 18.92 |
| agt | S | 62 | 13.64 |
| agc | S | 0 | 12.35 |
| aga | R | 30 | 13.49 |
| agg | R | 0 | 8.41 |
| --- | -- | ------ | ------ |
| mPD | | 0.45 | 1.0 |
| | | nPD: | 0.45 |
| | | N. weight: | 1. |
| | | Sc. PD: | 0.14 |
| | | Sc. rank: | 183.0 |

| HA | | | |
|---|---|---|---|
| Pos. | 18 | obs: | exp: |
| tct | S | 0 | 2.54 |
| tcc | S | 0 | 2.23 |
| tca | S | 16 | 3.54 |
| tcg | S | 0 | 0.98 |
| agt | S | 0 | 3.52 |
| agc | S | 0 | 3.19 |
| gct | A | 1 | 139.50 |
| gcc | A | 0 | 113.50 |
| gca | A | 537 | 246.90 |
| gcg | A | 16 | 54.06 |
| ggt | G | 0 | 8.76 |
| ggc | G | 0 | 9.60 |
| gga | G | 71 | 34.35 |
| ggg | G | 0 | 18.29 |
| --- | -- | ------ | ------ |
| mPD | | 0.30 | 0.95 |
| | | nPD: | 0.31 |
| | | N. weight: | 1.1 |
| | | Sc. PD: | -0.0032 |
| | | Sc. rank: | -1.8 |

| HA | | | |
|---|---|---|---|
| Pos. | 19 | obs: | exp: |
| gat | D | 20 | 342.00 |
| gac | D | 621 | 299.00 |
| --- | -- | ------ | ------ |
| mPD | | 0.061 | 0.50 |
| | | nPD: | 0.12 |
| | | N. weight: | 0.91 |
| | | Sc. PD: | -0.17 |
| | | Sc. rank: | -176.1 |

| HA | | | |
|---|---|---|---|
| Pos. | 20 | obs: | exp: |
| aaa | K | 176 | 358.90 |
| aag | K | 465 | 282.10 |
| --- | -- | ------ | ------ |
| mPD | | 0.40 | 0.49 |
| | | nPD: | 0.81 |
| | | N. weight: | 0.25 |
| | | Sc. PD: | 0.12 |
| | | Sc. rank: | 92.2 |

| HA | | | |
|---|---|---|---|
| Pos. | 21 | obs: | exp: |
| att | I | 11 | 241.00 |
| atc | I | 558 | 168.00 |
| ata | I | 72 | 232.00 |
| --- | -- | ------ | ------ |
| mPD | | 0.23 | 0.66 |
| | | nPD: | 0.35 |
| | | N. weight: | 1.3 |
| | | Sc. PD: | 0.04 |
| | | Sc. rank: | 61.9 |

| HA | | | |
|---|---|---|---|
| Pos. | 22 | obs: | exp: |
| tgt | C | 100 | 264.20 |
| tgc | C | 541 | 376.80 |
| --- | -- | ------ | ------ |
| mPD | | 0.26 | 0.48 |
| | | nPD: | 0.54 |
| | | N. weight: | 0.23 |
| | | Sc. PD: | 0.051 |
| | | Sc. rank: | 58.2 |

| HA | | | |
|---|---|---|---|
| Pos. | 23 | obs: | exp: |
| tta | L | 0 | 56.00 |
| ttg | L | 0 | 123.50 |
| ctt | L | 537 | 119.60 |
| ctc | L | 103 | 103.70 |
| cta | L | 0 | 96.57 |
| ctg | L | 1 | 141.70 |
| --- | -- | ------ | ------ |
| mPD | | 0.27 | 1.1 |
| | | nPD: | 0.24 |
| | | N. weight: | 1.8 |
| | | Sc. PD: | -0.12 |
| | | Sc. rank: | -158.6 |

| HA | | | |
|---|---|---|---|
| Pos. | 24 | obs: | exp: |
| ggt | G | 0 | 79.09 |
| ggc | G | 0 | 86.68 |
| gga | G | 117 | 310.10 |
| ggg | G | 524 | 165.10 |
| --- | -- | ------ | ------ |
| mPD | | 0.30 | 0.67 |
| | | nPD: | 0.45 |
| | | N. weight: | 1.1 |
| | | Sc. PD: | 0.14 |
| | | Sc. rank: | 194.4 |

| HA | | | |
|---|---|---|---|
| Pos. | 25 | obs: | exp: |
| cat | H | 432 | 347.30 |
| cac | H | 209 | 293.70 |
| --- | -- | ------ | ------ |
| mPD | | 0.44 | 0.50 |
| | | nPD: | 0.89 |
| | | N. weight: | 0.061 |
| | | Sc. PD: | 0.033 |
| | | Sc. rank: | 22.7 |

| HA | | | |
|---|---|---|---|
| Pos. | 26 | obs: | exp: |
| cat | H | 615 | 347.30 |
| cac | H | 26 | 293.70 |
| --- | -- | ------ | ------ |
| mPD | | 0.078 | 0.50 |
| | | nPD: | 0.16 |
| | | N. weight: | 0.66 |
| | | Sc. PD: | -0.1 |
| | | Sc. rank: | -107.6 |

| HA | | | |
|---|---|---|---|
| Pos. | 27 | obs: | exp: |
| gct | A | 170 | 161.50 |
| gcc | A | 467 | 131.30 |
| gca | A | 4 | 285.70 |
| gcg | A | 0 | 62.55 |
| --- | -- | ------ | ------ |
| mPD | | 0.40 | 0.69 |
| | | nPD: | 0.58 |
| | | N. weight: | 1.3 |
| | | Sc. PD: | 0.34 |
| | | Sc. rank: | 384.9 |

| HA | | | |
|---|---|---|---|
| Pos. | 28 | obs: | exp: |
| att | I | 2 | 0.75 |
| atc | I | 0 | 0.52 |
| ata | I | 0 | 0.72 |
| atg | M | 1 | 1.00 |
| gtt | V | 0 | 131.10 |
| gtc | V | 1 | 133.90 |
| gta | V | 4 | 119.60 |
| gtg | V | 633 | 253.40 |
| --- | -- | ------ | ------ |
| mPD | | 0.031 | 0.73 |
| | | nPD: | 0.04 |
| | | N. weight: | 1.3 |
| | | Sc. PD: | -0.34 |
| | | Sc. rank: | -356.6 |

| HA | | | |
|---|---|---|---|
| Pos. | 29 | obs: | exp: |
| tct | S | 5 | 89.98 |
| tcc | S | 0 | 78.85 |
| tca | S | 560 | 125.40 |
| tcg | S | 1 | 34.50 |
| cct | P | 0 | 0.79 |
| ccc | P | 0 | 0.54 |
| cca | P | 3 | 1.18 |
| ccg | P | 0 | 0.49 |
| agt | S | 0 | 124.50 |
| agc | S | 0 | 112.80 |
| gct | A | 0 | 18.13 |
| gcc | A | 0 | 14.75 |
| gca | A | 72 | 32.09 |
| gcg | A | 0 | 7.03 |
| --- | -- | ------ | ------ |
| mPD | | 0.23 | 1.8 |
| | | nPD: | 0.13 |
| | | N. weight: | 2. |
| | | Sc. PD: | -0.36 |
| | | Sc. rank: | -376.1 |

| HA | | | |
|---|---|---|---|
| Pos. | 30 | obs: | exp: |
| aat | N | 169 | 335.00 |
| aac | N | 472 | 306.00 |
| --- | -- | ------ | ------ |
| mPD | | 0.39 | 0.50 |
| | | nPD: | 0.78 |
| | | N. weight: | 0.21 |
| | | Sc. PD: | 0.094 |
| | | Sc. rank: | 75.9 |

| HA | | | |
|---|---|---|---|
| Pos. | 31 | obs: | exp: |
| ggt | G | 1 | 79.09 |
| ggc | G | 2 | 86.68 |
| gga | G | 278 | 310.10 |
| ggg | G | 360 | 165.10 |
| --- | -- | ------ | ------ |
| mPD | | 0.50 | 0.67 |
| | | nPD: | 0.75 |
| | | N. weight: | 0.56 |
| | | Sc. PD: | 0.23 |
| | | Sc. rank: | 194.5 |

| HA | | | |
|---|---|---|---|
| Pos. | 32 | obs: | exp: |
| att | I | 1 | 0.75 |
| atc | I | 1 | 0.52 |
| ata | I | 0 | 0.72 |
| act | T | 423 | 168.30 |
| acc | T | 142 | 136.70 |
| aca | T | 66 | 279.10 |
| acg | T | 6 | 52.95 |
| gct | A | 2 | 0.50 |
| gcc | A | 0 | 0.41 |
| gca | A | 0 | 0.89 |
| gcg | A | 0 | 0.20 |
| --- | -- | ------ | ------ |
| mPD | | 0.51 | 0.70 |
| | | nPD: | 0.73 |
| | | N. weight: | 0.68 |
| | | Sc. PD: | 0.27 |
| | | Sc. rank: | 237.0 |

| HA | | | |
|---|---|---|---|
| Pos. | 33 | obs: | exp: |
| cgt | R | 0 | 0.17 |
| cgc | R | 0 | 0.20 |
| cga | R | 0 | 0.38 |
| cgg | R | 0 | 0.33 |
| aaa | K | 629 | 356.70 |
| aag | K | 8 | 280.30 |
| aga | R | 4 | 1.80 |
| agg | R | 0 | 1.12 |
| --- | -- | ------ | ------ |
| mPD | | 0.037 | 0.51 |
| | | nPD: | 0.07 |
| | | N. weight: | 0.76 |
| | | Sc. PD: | -0.18 |
| | | Sc. rank: | -176.9 |

| HA | | | |
|---|---|---|---|
| Pos. | 34 | obs: | exp: |
| att | I | 0 | 1.13 |
| atc | I | 0 | 0.79 |
| ata | I | 3 | 1.09 |
| gtt | V | 0 | 131.10 |
| gtc | V | 1 | 133.90 |
| gta | V | 546 | 119.60 |
| gtg | V | 91 | 253.40 |
| --- | -- | ------ | ------ |
| mPD | | 0.26 | 0.73 |
| | | nPD: | 0.35 |
| | | N. weight: | 1.7 |
| | | Sc. PD: | 0.056 |
| | | Sc. rank: | 89.1 |

| HA | | | |
|---|---|---|---|
| Pos. | 35 | obs: | exp: |
| aat | N | 6 | 334.50 |
| aac | N | 634 | 305.50 |
| gat | D | 0 | 0.53 |
| gac | D | 1 | 0.47 |
| --- | -- | ------ | ------ |
| mPD | | 0.022 | 0.50 |
| | | nPD: | 0.04 |
| | | N. weight: | 1. |
| | | Sc. PD: | -0.26 |
| | | Sc. rank: | -276.9 |

| HA | | | |
|---|---|---|---|
| Pos. | 36 | obs: | exp: |
| act | T | 0 | 169.30 |
| acc | T | 0 | 137.50 |
| aca | T | 625 | 280.80 |
| acg | T | 16 | 53.29 |
| --- | -- | ------ | ------ |
| mPD | | 0.049 | 0.69 |
| | | nPD: | 0.07 |
| | | N. weight: | 1.1 |
| | | Sc. PD: | -0.26 |
| | | Sc. rank: | -261.0 |

,

| HA | | | |
|---|---|---|---|
| Pos. | 37 | obs: | exp: |
| tta | L | 607 | 56.00 |
| ttg | L | 5 | 123.50 |
| ctt | L | 0 | 119.60 |
| ctc | L | 3 | 103.70 |
| cta | L | 25 | 96.57 |
| ctg | L | 1 | 141.70 |
| --- | -- | ------ | ------ |
| mPD | | 0.11 | 1.1 |
| | | nPD: | 0.1 |
| | | N. weight: | 3.2 |
| | | Sc. PD: | -0.65 |
| | | Sc. rank: | -658.2 |

,

| HA | | | |
|---|---|---|---|
| Pos. | 38 | obs: | exp: |
| act | T | 553 | 169.30 |
| acc | T | 14 | 137.50 |
| aca | T | 73 | 280.80 |
| acg | T | 1 | 53.29 |
| --- | -- | ------ | ------ |
| mPD | | 0.24 | 0.69 |
| | | nPD: | 0.35 |
| | | N. weight: | 1.2 |
| | | Sc. PD: | 0.044 |
| | | Sc. rank: | 76.2 |

,

| HA | | | |
|---|---|---|---|
| Pos. | 39 | obs: | exp: |
| gaa | E | 312 | 368.30 |
| gag | E | 328 | 271.70 |
| ggt | G | 0 | 0.12 |
| ggc | G | 0 | 0.14 |
| gga | G | 1 | 0.48 |
| ggg | G | 0 | 0.26 |
| --- | -- | ------ | ------ |
| mPD | | 0.50 | 0.49 |
| | | nPD: | 1.02 |
| | | N. weight: | 0.032 |
| | | Sc. PD: | 0.022 |
| | | Sc. rank: | 12.2 |

,

| HA | | | |
|---|---|---|---|
| Pos. | 40 | obs: | exp: |
| cgt | R | 0 | 26.36 |
| cgc | R | 0 | 31.09 |
| cga | R | 0 | 61.03 |
| cgg | R | 0 | 52.99 |
| aaa | K | 2 | 3.36 |
| aag | K | 4 | 2.64 |
| aga | R | 515 | 285.60 |
| agg | R | 120 | 178.00 |
| --- | -- | ------ | ------ |
| mPD | | 0.33 | 0.98 |
| | | nPD: | 0.34 |
| | | N. weight: | 0.6 |
| | | Sc. PD: | 0.014 |
| | | Sc. rank: | 24.5 |

,

| HA | | | |
|---|---|---|---|
| Pos. | 41 | obs: | exp: |
| gaa | E | 6 | 3.45 |
| gag | E | 0 | 2.55 |
| ggt | G | 1 | 78.35 |
| ggc | G | 0 | 85.87 |
| gga | G | 600 | 307.20 |
| ggg | G | 34 | 163.60 |
| --- | -- | ------ | ------ |
| mPD | | 0.12 | 0.68 |
| | | nPD: | 0.18 |
| | | N. weight: | 0.8 |
| | | Sc. PD: | -0.11 |
| | | Sc. rank: | -115.4 |

,

| HA | | | |
|---|---|---|---|
| Pos. | 42 | obs: | exp: |
| att | I | 16 | 28.20 |
| atc | I | 55 | 19.66 |
| ata | I | 4 | 27.15 |
| gtt | V | 1 | 116.30 |
| gtc | V | 0 | 118.80 |
| gta | V | 22 | 106.10 |
| gtg | V | 543 | 224.80 |
| --- | -- | ------ | ------ |
| mPD | | 0.48 | 0.94 |
| | | nPD: | 0.51 |
| | | N. weight: | 1.1 |
| | | Sc. PD: | 0.21 |
| | | Sc. rank: | 261.6 |

,

| HA | | | |
|---|---|---|---|
| Pos. | 43 | obs: | exp: |
| gaa | E | 548 | 368.80 |
| gag | E | 93 | 272.20 |
| --- | -- | ------ | ------ |
| mPD | | 0.25 | 0.49 |
| | | nPD: | 0.51 |
| | | N. weight: | 0.27 |
| | | Sc. PD: | 0.051 |
| | | Sc. rank: | 63.9 |

,

| HA | | | |
|---|---|---|---|
| Pos. | 44 | obs: | exp: |
| gtt | V | 52 | 131.80 |
| gtc | V | 514 | 134.50 |
| gta | V | 69 | 120.10 |
| gtg | V | 6 | 254.60 |
| --- | -- | ------ | ------ |
| mPD | | 0.34 | 0.72 |
| | | nPD: | 0.47 |
| | | N. weight: | 1.3 |
| | | Sc. PD: | 0.2 |
| | | Sc. rank: | 264.7 |

,

| HA | | | |
|---|---|---|---|
| Pos. | 45 | obs: | exp: |
| gtt | V | 491 | 131.80 |
| gtc | V | 78 | 134.50 |
| gta | V | 68 | 120.10 |
| gtg | V | 4 | 254.60 |
| --- | -- | ------ | ------ |
| mPD | | 0.39 | 0.72 |
| | | nPD: | 0.54 |
| | | N. weight: | 1.3 |
| | | Sc. PD: | 0.27 |
| | | Sc. rank: | 316.1 |

,

| HA | | | |
|---|---|---|---|
| Pos. | 46 | obs: | exp: |
| tct | S | 0 | 0.16 |
| tcc | S | 0 | 0.14 |
| tca | S | 0 | 0.22 |
| tcg | S | 0 | 0.06 |
| aat | N | 606 | 334.50 |
| aac | N | 34 | 305.50 |
| agt | S | 1 | 0.22 |
| agc | S | 0 | 0.20 |
| --- | -- | ------ | ------ |
| mPD | | 0.10 | 0.50 |
| | | nPD: | 0.21 |
| | | N. weight: | 0.66 |
| | | Sc. PD: | -0.07 |
| | | Sc. rank: | -82.2 |

,

| HA | | | |
|---|---|---|---|
| Pos. | 47 | obs: | exp: |
| gct | A | 2 | 161.50 |
| gcc | A | 79 | 131.30 |
| gca | A | 528 | 285.70 |
| gcg | A | 32 | 62.55 |
| --- | -- | ------ | ------ |
| mPD | | 0.30 | 0.69 |
| | | nPD: | 0.44 |
| | | N. weight: | 0.6 |
| | | Sc. PD: | 0.073 |
| | | Sc. rank: | 95.6 |

,

| HA | | | |
|---|---|---|---|
| Pos. | 48 | obs: | exp: |
| act | T | 565 | 169.30 |
| acc | T | 0 | 137.50 |
| aca | T | 54 | 280.80 |
| acg | T | 22 | 53.29 |
| --- | -- | ------ | ------ |
| mPD | | 0.22 | 0.69 |
| | | nPD: | 0.31 |
| | | N. weight: | 1.3 |
| | | Sc. PD: | -0.0021 |
| | | Sc. rank: | 3.3 |

,

| HA | | | |
|---|---|---|---|
| Pos. | 49 | obs: | exp: |
| gaa | E | 596 | 368.80 |
| gag | E | 45 | 272.20 |
| --- | -- | ------ | ------ |
| mPD | | 0.13 | 0.49 |
| | | nPD: | 0.27 |
| | | N. weight: | 0.47 |
| | | Sc. PD: | -0.022 |
| | | Sc. rank: | -24.3 |

,

| HA | | | |
|---|---|---|---|
| Pos. | 50 | obs: | exp: |
| atg | M | 1 | 1.00 |
| act | T | 0 | 169.10 |
| acc | T | 1 | 137.30 |
| aca | T | 198 | 280.40 |
| acg | T | 441 | 53.20 |
| --- | -- | ------ | ------ |
| mPD | | 0.43 | 0.69 |
| | | nPD: | 0.63 |
| | | N. weight: | 2. |
| | | Sc. PD: | 0.59 |
| | | Sc. rank: | 613.3 |

,

| HA | | | |
|---|---|---|---|
| Pos. | 51 | obs: | exp: |
| gtt | V | 5 | 131.80 |
| gtc | V | 0 | 134.50 |
| gta | V | 11 | 120.10 |
| gtg | V | 625 | 254.60 |
| --- | -- | ------ | ------ |
| mPD | | 0.049 | 0.72 |
| | | nPD: | 0.07 |
| | | N. weight: | 1.2 |
| | | Sc. PD: | -0.28 |
| | | Sc. rank: | -289.5 |

,

| HA | | | |
|---|---|---|---|
| Pos. | 52 | obs: | exp: |
| caa | Q | 0 | 0.53 |
| cag | Q | 1 | 0.47 |
| gaa | E | 564 | 368.30 |
| gag | E | 76 | 271.70 |
| --- | -- | ------ | ------ |
| mPD | | 0.21 | 0.49 |
| | | nPD: | 0.44 |
| | | N. weight: | 0.34 |
| | | Sc. PD: | 0.039 |
| | | Sc. rank: | 52.6 |

,

| HA | | | |
|---|---|---|---|
| Pos. | 53 | obs: | exp: |
| cgt | R | 0 | 23.62 |
| cgc | R | 2 | 27.86 |
| cga | R | 556 | 54.68 |
| cgg | R | 8 | 47.48 |
| act | T | 69 | 18.49 |
| acc | T | 1 | 15.02 |
| aca | T | 0 | 30.67 |
| acg | T | 0 | 5.82 |
| aga | R | 3 | 255.90 |
| agg | R | 0 | 159.50 |
| gct | A | 0 | 0.50 |
| gcc | A | 2 | 0.41 |
| gca | A | 0 | 0.89 |
| gcg | A | 0 | 0.20 |
| --- | -- | ------ | ------ |
| mPD | | 0.64 | 1.2 |
| | | nPD: | 0.55 |
| | | N. weight: | 3.1 |
| | | Sc. PD: | 0.68 |
| | | Sc. rank: | 792.6 |

,

| HA | | | |
|---|---|---|---|
| Pos. | 54 | obs: | exp: |
| tct | S | 0 | 0.16 |
| tcc | S | 0 | 0.14 |
| tca | S | 1 | 0.22 |
| tcg | S | 0 | 0.06 |
| att | I | 0 | 1.88 |
| atc | I | 0 | 1.31 |
| ata | I | 5 | 1.81 |
| atg | M | 1 | 1.00 |
| act | T | 0 | 148.70 |
| acc | T | 0 | 120.80 |
| aca | T | 563 | 246.60 |
| acg | T | 0 | 46.80 |
| agt | S | 0 | 0.22 |
| agc | S | 0 | 0.20 |
| gtt | V | 0 | 0.82 |
| gtc | V | 0 | 0.84 |
| gta | V | 4 | 0.75 |
| gtg | V | 0 | 1.59 |
| gct | A | 0 | 16.88 |
| gcc | A | 0 | 13.72 |
| gca | A | 64 | 29.86 |
| gcg | A | 3 | 6.54 |
| --- | -- | ------ | ------ |
| mPD | | 0.24 | 0.92 |
| | | nPD: | 0.26 |
| | | N. weight: | 1.2 |
| | | Sc. PD: | -0.058 |
| | | Sc. rank: | -69.3 |

,

| HA | | | |
|---|---|---|---|
| Pos. | 55 | obs: | exp: |
| tct | S | 0 | 14.47 |
| tcc | S | 0 | 12.68 |
| tca | S | 0 | 20.16 |
| tcg | S | 0 | 5.55 |
| aat | N | 232 | 287.50 |
| aac | N | 318 | 262.50 |
| agt | S | 14 | 20.02 |
| agc | S | 77 | 18.13 |
| --- | -- | ------ | ------ |
| mPD | | 0.72 | 0.94 |
| | | nPD: | 0.76 |
| | | N. weight: | 0.28 |
| | | Sc. PD: | 0.12 |
| | | Sc. rank: | 99.6 |

,

| HA | | | |
|---|---|---|---|
| Pos. | 56 | obs: | exp: |
| tta | L | 0 | 0.09 |
| ttg | L | 0 | 0.19 |
| ctt | L | 1 | 0.19 |
| ctc | L | 0 | 0.16 |
| cta | L | 0 | 0.15 |
| ctg | L | 0 | 0.22 |
| att | I | 68 | 66.92 |
| atc | I | 107 | 46.65 |
| ata | I | 3 | 64.43 |
| atg | M | 3 | 3.00 |
| act | T | 1 | 0.26 |
| acc | T | 0 | 0.21 |
| aca | T | 0 | 0.44 |
| acg | T | 0 | 0.08 |
| gtt | V | 242 | 94.14 |
| gtc | V | 216 | 96.13 |
| gta | V | 0 | 85.84 |
| gtg | V | 0 | 181.90 |
| --- | -- | ------ | ------ |
| mPD | | 0.92 | 1.2 |
| | | nPD: | 0.8 |
| | | N. weight: | 1.1 |
| | | Sc. PD: | 0.52 |
| | | Sc. rank: | 410.5 |

,

| HA | | | |
|---|---|---|---|
| Pos. | 57 | obs: | exp: |
| cct | P | 0 | 149.80 |
| ccc | P | 560 | 102.60 |
| cca | P | 9 | 224.50 |
| ccg | P | 0 | 92.00 |
| aaa | K | 0 | 38.64 |
| aag | K | 69 | 30.36 |
| gaa | E | 0 | 1.73 |
| gag | E | 3 | 1.27 |
| --- | -- | ------ | ------ |
| mPD | | 0.62 | 1.1 |
| | | nPD: | 0.56 |
| | | N. weight: | 2.2 |
| | | Sc. PD: | 0.53 |
| | | Sc. rank: | 616.4 |

,

| HA | | | |
|---|---|---|---|
| Pos. | 58 | obs: | exp: |
| cgt | R | 0 | 23.21 |
| cgc | R | 0 | 27.37 |
| cga | R | 0 | 53.72 |
| cgg | R | 0 | 46.65 |
| aaa | K | 74 | 45.92 |
| aag | K | 8 | 36.08 |
| aga | R | 41 | 251.40 |
| agg | R | 518 | 156.70 |
| --- | -- | ------ | ------ |
| mPD | | 0.52 | 1.1 |
| | | nPD: | 0.45 |
| | | N. weight: | 1.3 |
| | | Sc. PD: | 0.17 |
| | | Sc. rank: | 234.3 |

,

| HA | | | |
|---|---|---|---|
| Pos. | 59 | obs: | exp: |
| att | I | 12 | 240.60 |
| atc | I | 549 | 167.70 |
| ata | I | 79 | 231.70 |
| act | T | 0 | 0.26 |
| acc | T | 1 | 0.21 |
| aca | T | 0 | 0.44 |
| acg | T | 0 | 0.08 |
| --- | -- | ------ | ------ |
| mPD | | 0.25 | 0.66 |
| | | nPD: | 0.38 |
| | | N. weight: | 1.2 |
| | | Sc. PD: | 0.076 |
| | | Sc. rank: | 124.6 |

,

| HA | | | |
|---|---|---|---|
| Pos. | 60 | obs: | exp: |
| tgt | C | 4 | 264.20 |
| tgc | C | 637 | 376.80 |
| --- | -- | ------ | ------ |
| mPD | | 0.012 | 0.48 |
| | | nPD: | 0.03 |
| | | N. weight: | 0.73 |
| | | Sc. PD: | -0.2 |
| | | Sc. rank: | -214.9 |

,

| HA | | | |
|---|---|---|---|
| Pos. | 61 | obs: | exp: |
| tct | S | 0 | 90.30 |
| tcc | S | 0 | 79.13 |
| tca | S | 565 | 125.80 |
| tcg | S | 3 | 34.62 |
| act | T | 65 | 19.29 |
| acc | T | 7 | 15.66 |
| aca | T | 1 | 31.98 |
| acg | T | 0 | 6.07 |
| agt | S | 0 | 125.00 |
| agc | S | 0 | 113.20 |
| --- | -- | ------ | ------ |
| mPD | | 0.41 | 1.7 |
| | | nPD: | 0.25 |
| | | N. weight: | 2.1 |
| | | Sc. PD: | -0.14 |
| | | Sc. rank: | -163.3 |

,

| HA | | | |
|---|---|---|---|
| Pos. | 62 | obs: | exp: |
| caa | Q | 18 | 25.06 |
| cag | Q | 29 | 21.94 |
| cgt | R | 0 | 0.33 |
| cgc | R | 0 | 0.39 |
| cga | R | 0 | 0.77 |
| cgg | R | 0 | 0.67 |
| aaa | K | 562 | 328.10 |
| aag | K | 24 | 257.90 |
| aga | R | 7 | 3.60 |
| agg | R | 1 | 2.24 |
| --- | -- | ------ | ------ |
| mPD | | 0.32 | 0.66 |
| | | nPD: | 0.48 |
| | | N. weight: | 0.59 |
| | | Sc. PD: | 0.091 |
| | | Sc. rank: | 117.5 |

,

| HA | | | |
|---|---|---|---|
| Pos. | 63 | obs: | exp: |
| ggt | G | 0 | 79.09 |
| ggc | G | 0 | 86.68 |
| gga | G | 14 | 310.10 |
| ggg | G | 627 | 165.10 |
| --- | -- | ------ | ------ |
| mPD | | 0.043 | 0.67 |
| | | nPD: | 0.06 |
| | | N. weight: | 1.8 |
| | | Sc. PD: | -0.44 |
| | | Sc. rank: | -446.1 |

,

| HA | | | |
|---|---|---|---|
| Pos. | 64 | obs: | exp: |
| aaa | K | 637 | 358.90 |
| aag | K | 4 | 282.10 |
| --- | -- | ------ | ------ |
| mPD | | 0.012 | 0.49 |
| | | nPD: | 0.03 |
| | | N. weight: | 0.8 |
| | | Sc. PD: | -0.22 |
| | | Sc. rank: | -237.1 |

,

| HA | | | |
|---|---|---|---|
| Pos. | 65 | obs: | exp: |
| cgt | R | 0 | 25.20 |
| cgc | R | 0 | 29.72 |
| cga | R | 1 | 58.34 |
| cgg | R | 2 | 50.65 |
| att | I | 0 | 0.75 |
| atc | I | 0 | 0.52 |
| ata | I | 2 | 0.72 |
| act | T | 0 | 0.53 |
| acc | T | 0 | 0.43 |
| aca | T | 0 | 0.88 |
| acg | T | 2 | 0.17 |
| aaa | K | 2 | 16.80 |
| aag | K | 28 | 13.20 |
| aga | R | 263 | 273.00 |
| agg | R | 341 | 170.10 |
| --- | -- | ------ | ------ |
| mPD | | 0.60 | 1.0 |
| | | nPD: | 0.57 |
| | | N. weight: | 0.57 |
| | | Sc. PD: | 0.14 |
| | | Sc. rank: | 158.0 |

,

| HA | | | |
|---|---|---|---|
| Pos. | 66 | obs: | exp: |
| tct | S | 0 | 0.48 |
| tcc | S | 0 | 0.42 |
| tca | S | 3 | 0.66 |
| tcg | S | 0 | 0.18 |
| cct | P | 0 | 18.17 |
| ccc | P | 0 | 12.45 |
| cca | P | 69 | 27.23 |
| ccg | P | 0 | 11.16 |
| act | T | 1 | 138.40 |
| acc | T | 2 | 112.40 |
| aca | T | 500 | 229.60 |
| acg | T | 21 | 43.56 |
| agt | S | 0 | 0.66 |
| agc | S | 0 | 0.60 |
| gct | A | 0 | 11.33 |
| gcc | A | 0 | 9.22 |
| gca | A | 44 | 20.06 |
| gcg | A | 1 | 4.39 |
| --- | -- | ------ | ------ |
| mPD | | 0.39 | 1.0 |
| | | nPD: | 0.39 |
| | | N. weight: | 1.1 |
| | | Sc. PD: | 0.075 |
| | | Sc. rank: | 118.3 |

,

| HA | | | |
|---|---|---|---|
| Pos. | 67 | obs: | exp: |
| att | I | 5 | 4.89 |
| atc | I | 6 | 3.41 |
| ata | I | 2 | 4.71 |
| act | T | 0 | 18.49 |
| acc | T | 2 | 15.02 |
| aca | T | 68 | 30.67 |
| acg | T | 0 | 5.82 |
| gtt | V | 542 | 114.30 |
| gtc | V | 13 | 116.70 |
| gta | V | 0 | 104.20 |
| gtg | V | 1 | 220.80 |
| gct | A | 2 | 0.50 |
| gcc | A | 0 | 0.41 |
| gca | A | 0 | 0.89 |
| gcg | A | 0 | 0.20 |
| --- | -- | ------ | ------ |
| mPD | | 0.68 | 1.2 |
| | | nPD: | 0.59 |
| | | N. weight: | 2. |
| | | Sc. PD: | 0.52 |
| | | Sc. rank: | 574.6 |

,

| HA | | | |
|---|---|---|---|
| Pos. | 68 | obs: | exp: |
| gat | D | 78 | 342.00 |
| gac | D | 563 | 299.00 |
| --- | -- | ------ | ------ |
| mPD | | 0.21 | 0.50 |
| | | nPD: | 0.43 |
| | | N. weight: | 0.56 |
| | | Sc. PD: | 0.061 |
| | | Sc. rank: | 86.2 |

,

| HA | | | |
|---|---|---|---|
| Pos. | 69 | obs: | exp: |
| tta | L | 0 | 56.00 |
| ttg | L | 1 | 123.50 |
| ctt | L | 62 | 119.60 |
| ctc | L | 507 | 103.70 |
| cta | L | 3 | 96.57 |
| ctg | L | 68 | 141.70 |
| --- | -- | ------ | ------ |
| mPD | | 0.36 | 1.1 |
| | | nPD: | 0.32 |
| | | N. weight: | 1.6 |
| | | Sc. PD: | 0.0076 |
| | | Sc. rank: | 20.8 |

,

| HA | | | |
|---|---|---|---|
| Pos. | 70 | obs: | exp: |
| tct | S | 0 | 0.32 |
| tcc | S | 0 | 0.28 |
| tca | S | 0 | 0.44 |
| tcg | S | 0 | 0.12 |
| agt | S | 2 | 0.44 |
| agc | S | 0 | 0.40 |
| gat | D | 1 | 0.53 |
| gac | D | 0 | 0.47 |
| ggt | G | 559 | 78.72 |
| ggc | G | 8 | 86.28 |
| gga | G | 69 | 308.60 |
| ggg | G | 2 | 164.40 |
| --- | -- | ------ | ------ |
| mPD | | 0.23 | 0.68 |
| | | nPD: | 0.34 |
| | | N. weight: | 2.2 |
| | | Sc. PD: | 0.046 |
| | | Sc. rank: | 89.7 |

,

| HA | | | |
|---|---|---|---|
| Pos. | 71 | obs: | exp: |
| caa | Q | 632 | 341.20 |
| cag | Q | 8 | 298.80 |
| aaa | K | 1 | 0.56 |
| aag | K | 0 | 0.44 |
| --- | -- | ------ | ------ |
| mPD | | 0.028 | 0.50 |
| | | nPD: | 0.06 |
| | | N. weight: | 0.83 |
| | | Sc. PD: | -0.21 |
| | | Sc. rank: | -212.1 |

,

| HA | | | |
|---|---|---|---|
| Pos. | 72 | obs: | exp: |
| tgt | C | 596 | 264.20 |
| tgc | C | 45 | 376.80 |
| --- | -- | ------ | ------ |
| mPD | | 0.13 | 0.48 |
| | | nPD: | 0.27 |
| | | N. weight: | 0.89 |
| | | Sc. PD: | -0.039 |
| | | Sc. rank: | -44.7 |

,

| HA | | | |
|---|---|---|---|
| Pos. | 73 | obs: | exp: |
| ggt | G | 2 | 79.09 |
| ggc | G | 0 | 86.68 |
| gga | G | 549 | 310.10 |
| ggg | G | 90 | 165.10 |
| --- | -- | ------ | ------ |
| mPD | | 0.25 | 0.67 |
| | | nPD: | 0.37 |
| | | N. weight: | 0.58 |
| | | Sc. PD: | 0.031 |
| | | Sc. rank: | 48.7 |

,

| HA | | | |
|---|---|---|---|
| Pos. | 74 | obs: | exp: |
| tta | L | 0 | 55.91 |
| ttg | L | 4 | 123.30 |
| ctt | L | 511 | 119.40 |
| ctc | L | 117 | 103.50 |
| cta | L | 3 | 96.42 |
| ctg | L | 5 | 141.50 |
| att | I | 1 | 0.38 |
| atc | I | 0 | 0.26 |
| ata | I | 0 | 0.36 |
| --- | -- | ------ | ------ |
| mPD | | 0.34 | 1.1 |
| | | nPD: | 0.31 |
| | | N. weight: | 1.7 |
| | | Sc. PD: | -0.011 |
| | | Sc. rank: | -16.6 |

,

| HA | | | |
|---|---|---|---|
| Pos. | 75 | obs: | exp: |
| tta | L | 2 | 56.00 |
| ttg | L | 2 | 123.50 |
| ctt | L | 1 | 119.60 |
| ctc | L | 0 | 103.70 |
| cta | L | 125 | 96.57 |
| ctg | L | 511 | 141.70 |
| --- | -- | ------ | ------ |
| mPD | | 0.33 | 1.1 |
| | | nPD: | 0.3 |
| | | N. weight: | 1.5 |
| | | Sc. PD: | -0.025 |
| | | Sc. rank: | -37.4 |

,

| | | | |
|---|---|---|---|
| HA | | | |
| Pos. | 76 | obs: | exp: |
| ggt | G | 4 | 79.09 |
| ggc | G | 0 | 86.68 |
| gga | G | 486 | 310.10 |
| ggg | G | 151 | 165.10 |
| --- | -- | ------ | ------ |
| mPD | | 0.37 | 0.67 |
| | | nPD: | 0.56 |
| | | N. weight: | 0.45 |
| | | Sc. PD: | 0.1 |
| | | Sc. rank: | 121.1 |

,

| | | | |
|---|---|---|---|
| HA | | | |
| Pos. | 77 | obs: | exp: |
| att | I | 1 | 0.75 |
| atc | I | 0 | 0.52 |
| ata | I | 1 | 0.72 |
| act | T | 5 | 168.60 |
| acc | T | 71 | 136.90 |
| aca | T | 496 | 279.50 |
| acg | T | 66 | 53.04 |
| gct | A | 0 | 0.25 |
| gcc | A | 1 | 0.20 |
| gca | A | 0 | 0.45 |
| gcg | A | 0 | 0.10 |
| --- | -- | ------ | ------ |
| mPD | | 0.39 | 0.69 |
| | | nPD: | 0.55 |
| | | N. weight: | 0.56 |
| | | Sc. PD: | 0.13 |
| | | Sc. rank: | 149.7 |

,

| | | | |
|---|---|---|---|
| HA | | | |
| Pos. | 78 | obs: | exp: |
| tta | L | 1 | 6.29 |
| ttg | L | 1 | 13.87 |
| ctt | L | 0 | 13.43 |
| ctc | L | 0 | 11.65 |
| cta | L | 66 | 10.85 |
| ctg | L | 4 | 15.92 |
| att | I | 4 | 213.50 |
| atc | I | 557 | 148.90 |
| ata | I | 7 | 205.60 |
| gtt | V | 0 | 0.21 |
| gtc | V | 1 | 0.21 |
| gta | V | 0 | 0.19 |
| gtg | V | 0 | 0.40 |
| --- | -- | ------ | ------ |
| mPD | | 0.43 | 0.90 |
| | | nPD: | 0.48 |
| | | N. weight: | 1.9 |
| | | Sc. PD: | 0.3 |
| | | Sc. rank: | 391.5 |

,

| | | | |
|---|---|---|---|
| HA | | | |
| Pos. | 79 | obs: | exp: |
| att | I | 7 | 29.32 |
| atc | I | 0 | 20.44 |
| ata | I | 71 | 28.23 |
| act | T | 556 | 148.70 |
| acc | T | 5 | 120.80 |
| aca | T | 2 | 246.60 |
| acg | T | 0 | 46.80 |
| --- | -- | ------ | ------ |
| mPD | | 0.43 | 0.90 |
| | | nPD: | 0.48 |
| | | N. weight: | 1.8 |
| | | Sc. PD: | 0.27 |
| | | Sc. rank: | 355.1 |

,

| | | | |
|---|---|---|---|
| HA | | | |
| Pos. | 80 | obs: | exp: |
| ggt | G | 1 | 79.09 |
| ggc | G | 0 | 86.68 |
| gga | G | 197 | 310.10 |
| ggg | G | 443 | 165.10 |
| --- | -- | ------ | ------ |
| mPD | | 0.43 | 0.67 |
| | | nPD: | 0.64 |
| | | N. weight: | 0.79 |
| | | Sc. PD: | 0.25 |
| | | Sc. rank: | 254.4 |

,

| | | | |
|---|---|---|---|
| HA | | | |
| Pos. | 81 | obs: | exp: |
| cct | P | 71 | 168.80 |
| ccc | P | 9 | 115.60 |
| cca | P | 561 | 253.00 |
| ccg | P | 0 | 103.60 |
| --- | -- | ------ | ------ |
| mPD | | 0.22 | 0.72 |
| | | nPD: | 0.31 |
| | | N. weight: | 0.84 |
| | | Sc. PD: | -0.0046 |
| | | Sc. rank: | -5.7 |

,

| | | | |
|---|---|---|---|
| HA | | | |
| Pos. | 82 | obs: | exp: |
| cct | P | 79 | 168.80 |
| ccc | P | 561 | 115.60 |
| cca | P | 1 | 253.00 |
| ccg | P | 0 | 103.60 |
| --- | -- | ------ | ------ |
| mPD | | 0.22 | 0.72 |
| | | nPD: | 0.31 |
| | | N. weight: | 1.9 |
| | | Sc. PD: | -0.017 |
| | | Sc. rank: | -29.2 |

,

| | | | |
|---|---|---|---|
| HA | | | |
| Pos. | 83 | obs: | exp: |
| caa | Q | 624 | 341.70 |
| cag | Q | 17 | 299.30 |
| --- | -- | ------ | ------ |
| mPD | | 0.052 | 0.50 |
| | | nPD: | 0.1 |
| | | N. weight: | 0.75 |
| | | Sc. PD: | -0.15 |
| | | Sc. rank: | -154.6 |

,

| | | | |
|---|---|---|---|
| HA | | | |
| Pos. | 84 | obs: | exp: |
| tgt | C | 577 | 264.20 |
| tgc | C | 64 | 376.80 |
| --- | -- | ------ | ------ |
| mPD | | 0.18 | 0.48 |
| | | nPD: | 0.37 |
| | | N. weight: | 0.78 |
| | | Sc. PD: | 0.042 |
| | | Sc. rank: | 65.6 |

,

| | | | |
|---|---|---|---|
| HA | | | |
| Pos. | 85 | obs: | exp: |
| gat | D | 110 | 341.50 |
| gac | D | 530 | 298.50 |
| gaa | E | 1 | 0.58 |
| gag | E | 0 | 0.42 |
| --- | -- | ------ | ------ |
| mPD | | 0.29 | 0.50 |
| | | nPD: | 0.58 |
| | | N. weight: | 0.42 |
| | | Sc. PD: | 0.1 |
| | | Sc. rank: | 117.8 |

,

| | | | |
|---|---|---|---|
| HA | | | |
| Pos. | 86 | obs: | exp: |
| tta | L | 0 | 0.52 |
| ttg | L | 0 | 1.16 |
| ctt | L | 0 | 1.12 |
| ctc | L | 0 | 0.97 |
| cta | L | 6 | 0.90 |
| ctg | L | 0 | 1.33 |
| caa | Q | 614 | 338.50 |
| cag | Q | 21 | 296.50 |
| --- | -- | ------ | ------ |
| mPD | | 0.082 | 0.52 |
| | | nPD: | 0.16 |
| | | N. weight: | 0.74 |
| | | Sc. PD: | -0.11 |
| | | Sc. rank: | -120.5 |

,

| | | | |
|---|---|---|---|
| HA | | | |
| Pos. | 87 | obs: | exp: |
| ttt | F | 75 | 262.70 |
| ttc | F | 564 | 376.30 |
| tta | L | 0 | 0.17 |
| ttg | L | 0 | 0.39 |
| ctt | L | 0 | 0.37 |
| ctc | L | 2 | 0.32 |
| cta | L | 0 | 0.30 |
| ctg | L | 0 | 0.44 |
| --- | -- | ------ | ------ |
| mPD | | 0.21 | 0.49 |
| | | nPD: | 0.43 |
| | | N. weight: | 0.32 |
| | | Sc. PD: | 0.037 |
| | | Sc. rank: | 50.2 |

,

| | | | |
|---|---|---|---|
| HA | | | |
| Pos. | 88 | obs: | exp: |
| tta | L | 12 | 56.00 |
| ttg | L | 0 | 123.50 |
| ctt | L | 1 | 119.60 |
| ctc | L | 0 | 103.70 |
| cta | L | 556 | 96.57 |
| ctg | L | 72 | 141.70 |
| --- | -- | ------ | ------ |
| mPD | | 0.24 | 1.1 |
| | | nPD: | 0.21 |
| | | N. weight: | 2.1 |
| | | Sc. PD: | -0.2 |
| | | Sc. rank: | -236.6 |

,

| | | | |
|---|---|---|---|
| HA | | | |
| Pos. | 89 | obs: | exp: |
| gat | D | 1 | 0.53 |
| gac | D | 0 | 0.47 |
| gaa | E | 544 | 368.30 |
| gag | E | 96 | 271.70 |
| --- | -- | ------ | ------ |
| mPD | | 0.26 | 0.49 |
| | | nPD: | 0.53 |
| | | N. weight: | 0.27 |
| | | Sc. PD: | 0.054 |
| | | Sc. rank: | 63.8 |

,

| | | | |
|---|---|---|---|
| HA | | | |
| Pos. | 90 | obs: | exp: |
| ttt | F | 608 | 263.50 |
| ttc | F | 33 | 377.50 |
| --- | -- | ------ | ------ |
| mPD | | 0.098 | 0.48 |
| | | nPD: | 0.2 |
| | | N. weight: | 0.98 |
| | | Sc. PD: | -0.11 |
| | | Sc. rank: | -124.8 |

,

| | | | |
|---|---|---|---|
| HA | | | |
| Pos. | 91 | obs: | exp: |
| tct | S | 25 | 90.30 |
| tcc | S | 0 | 79.13 |
| tca | S | 297 | 125.80 |
| tcg | S | 246 | 34.62 |
| act | T | 0 | 0.26 |
| acc | T | 0 | 0.21 |
| aca | T | 0 | 0.44 |
| acg | T | 1 | 0.08 |
| agt | S | 0 | 125.00 |
| agc | S | 0 | 113.20 |
| gat | D | 3 | 36.81 |
| gac | D | 66 | 32.19 |
| gaa | E | 2 | 1.73 |
| gag | E | 1 | 1.27 |
| --- | -- | ------ | ------ |
| mPD | | 1.0 | 1.8 |
| | | nPD: | 0.55 |
| | | N. weight: | 1.7 |
| | | Sc. PD: | 0.4 |
| | | Sc. rank: | 455.6 |

,

| | | | |
|---|---|---|---|
| HA | | | |
| Pos. | 92 | obs: | exp: |
| tct | S | 1 | 0.64 |
| tcc | S | 3 | 0.56 |
| tca | S | 0 | 0.89 |
| tcg | S | 0 | 0.24 |
| agt | S | 0 | 0.88 |
| agc | S | 0 | 0.80 |
| gct | A | 105 | 160.40 |
| gcc | A | 528 | 130.50 |
| gca | A | 4 | 283.90 |
| gcg | A | 0 | 62.16 |
| --- | -- | ------ | ------ |
| mPD | | 0.30 | 0.70 |
| | | nPD: | 0.42 |
| | | N. weight: | 1.6 |
| | | Sc. PD: | 0.16 |
| | | Sc. rank: | 235.5 |

,

| | | | |
|---|---|---|---|
| HA | | | |
| Pos. | 93 | obs: | exp: |
| gat | D | 359 | 342.00 |
| gac | D | 282 | 299.00 |
| --- | -- | ------ | ------ |
| mPD | | 0.49 | 0.50 |
| | | nPD: | 0.99 |
| | | N. weight: | 0.01 |
| | | Sc. PD: | 0.0065 |
| | | Sc. rank: | 3.8 |

,

| | | | |
|---|---|---|---|
| HA | | | |
| Pos. | 94 | obs: | exp: |
| tta | L | 525 | 56.00 |
| ttg | L | 20 | 123.50 |
| ctt | L | 0 | 119.60 |
| ctc | L | 0 | 103.70 |
| cta | L | 90 | 96.57 |
| ctg | L | 6 | 141.70 |
| --- | -- | ------ | ------ |
| mPD | | 0.33 | 1.1 |
| | | nPD: | 0.3 |
| | | N. weight: | 2.6 |
| | | Sc. PD: | -0.041 |
| | | Sc. rank: | -67.4 |

,

| | | | |
|---|---|---|---|
| HA | | | |
| Pos. | 95 | obs: | exp: |
| att | I | 529 | 239.80 |
| atc | I | 35 | 167.20 |
| ata | I | 74 | 230.90 |
| gtt | V | 0 | 0.62 |
| gtc | V | 3 | 0.63 |
| gta | V | 0 | 0.56 |
| gtg | V | 0 | 1.19 |
| --- | -- | ------ | ------ |
| mPD | | 0.31 | 0.67 |
| | | nPD: | 0.47 |
| | | N. weight: | 0.66 |
| | | Sc. PD: | 0.096 |
| | | Sc. rank: | 127.2 |

,

| | | | |
|---|---|---|---|
| HA | | | |
| Pos. | 96 | obs: | exp: |
| att | I | 570 | 220.30 |
| atc | I | 16 | 153.60 |
| ata | I | 0 | 212.10 |
| gtt | V | 55 | 11.30 |
| gtc | V | 0 | 11.54 |
| gta | V | 0 | 10.31 |
| gtg | V | 0 | 21.84 |
| --- | -- | ------ | ------ |
| mPD | | 0.21 | 0.84 |
| | | nPD: | 0.25 |
| | | N. weight: | 1.4 |
| | | Sc. PD: | -0.091 |
| | | Sc. rank: | -114.8 |

,

| | | | |
|---|---|---|---|
| HA | | | |
| Pos. | 97 | obs: | exp: |
| gaa | E | 230 | 368.80 |
| gag | E | 411 | 272.20 |
| --- | -- | ------ | ------ |
| mPD | | 0.46 | 0.49 |
| | | nPD: | 0.94 |
| | | N. weight: | 0.15 |
| | | Sc. PD: | 0.088 |
| | | Sc. rank: | 55.3 |

,

| | | | |
|---|---|---|---|
| HA | | | |
| Pos. | 98 | obs: | exp: |
| cgt | R | 0 | 26.61 |
| cgc | R | 0 | 31.39 |
| cga | R | 54 | 61.60 |
| cgg | R | 18 | 53.49 |
| aga | R | 19 | 288.30 |
| agg | R | 550 | 179.60 |
| --- | -- | ------ | ------ |
| mPD | | 0.40 | 0.96 |
| | | nPD: | 0.42 |
| | | N. weight: | 1.2 |
| | | Sc. PD: | 0.12 |
| | | Sc. rank: | 178.7 |

,

| | | | |
|---|---|---|---|
| HA | | | |
| Pos. | 99 | obs: | exp: |
| caa | Q | 0 | 0.53 |
| cag | Q | 1 | 0.47 |
| cgt | R | 0 | 26.57 |
| cgc | R | 0 | 31.34 |
| cga | R | 500 | 61.51 |
| cgg | R | 65 | 53.41 |
| aga | R | 75 | 287.80 |
| agg | R | 0 | 179.40 |
| --- | -- | ------ | ------ |
| mPD | | 0.40 | 0.96 |
| | | nPD: | 0.41 |
| | | N. weight: | 2.2 |
| | | Sc. PD: | 0.2 |
| | | Sc. rank: | 302.4 |

,

| | | | |
|---|---|---|---|
| HA | | | |
| Pos. | 100 | obs: | exp: |
| gat | D | 0 | 2.67 |
| gac | D | 5 | 2.33 |
| gaa | E | 630 | 366.00 |
| gag | E | 6 | 270.00 |
| --- | -- | ------ | ------ |
| mPD | | 0.034 | 0.50 |
| | | nPD: | 0.07 |
| | | N. weight: | 0.74 |
| | | Sc. PD: | -0.18 |
| | | Sc. rank: | -179.3 |

,

| HA | | | |
|---|---|---|---|
| Pos. | 101 | obs: | exp: |
| ggt | G | 0 | 79.09 |
| ggc | G | 0 | 86.68 |
| gga | G | 610 | 310.10 |
| ggg | G | 31 | 165.10 |
| --- | -- | ------ | ------ |
| mPD | | 0.092 | 0.67 |
| | | nPD: | 0.14 |
| | | N. weight: | 0.83 |
| | | Sc. PD: | -0.14 |
| | | Sc. rank: | -150.8 |

,

| HA | | | |
|---|---|---|---|
| Pos. | 102 | obs: | exp: |
| tct | S | 0 | 87.75 |
| tcc | S | 0 | 76.90 |
| tca | S | 0 | 122.30 |
| tcg | S | 0 | 33.65 |
| act | T | 0 | 17.70 |
| acc | T | 53 | 14.38 |
| aca | T | 0 | 29.35 |
| acg | T | 14 | 5.57 |
| aat | N | 20 | 11.50 |
| aac | N | 2 | 10.50 |
| agt | S | 514 | 121.40 |
| agc | S | 38 | 110.00 |
| --- | -- | ------ | ------ |
| mPD | | 0.53 | 1.7 |
| | | nPD: | 0.31 |
| | | N. weight: | 1.8 |
| | | Sc. PD: | -0.0036 |
| | | Sc. rank: | 1.3 |

,

| HA | | | |
|---|---|---|---|
| Pos. | 103 | obs: | exp: |
| gat | D | 640 | 342.00 |
| gac | D | 1 | 299.00 |
| --- | -- | ------ | ------ |
| mPD | | 0.0031 | 0.50 |
| | | nPD: | 0.01 |
| | | N. weight: | 0.9 |
| | | Sc. PD: | -0.27 |
| | | Sc. rank: | -306.8 |

,

| HA | | | |
|---|---|---|---|
| Pos. | 104 | obs: | exp: |
| att | I | 1 | 1.88 |
| atc | I | 4 | 1.31 |
| ata | I | 0 | 1.81 |
| gtt | V | 46 | 130.70 |
| gtc | V | 518 | 133.50 |
| gta | V | 5 | 119.20 |
| gtg | V | 67 | 252.60 |
| --- | -- | ------ | ------ |
| mPD | | 0.34 | 0.74 |
| | | nPD: | 0.46 |
| | | N. weight: | 1.3 |
| | | Sc. PD: | 0.17 |
| | | Sc. rank: | 231.9 |

,

| HA | | | |
|---|---|---|---|
| Pos. | 105 | obs: | exp: |
| tgt | C | 595 | 264.20 |
| tgc | C | 46 | 376.80 |
| --- | -- | ------ | ------ |
| mPD | | 0.13 | 0.48 |
| | | nPD: | 0.28 |
| | | N. weight: | 0.89 |
| | | Sc. PD: | -0.034 |
| | | Sc. rank: | -41.0 |

,

| HA | | | |
|---|---|---|---|
| Pos. | 106 | obs: | exp: |
| tat | Y | 589 | 331.80 |
| tac | Y | 52 | 309.20 |
| --- | -- | ------ | ------ |
| mPD | | 0.15 | 0.50 |
| | | nPD: | 0.3 |
| | | N. weight: | 0.57 |
| | | Sc. PD: | -0.009 |
| | | Sc. rank: | -13.3 |

,

| HA | | | |
|---|---|---|---|
| Pos. | 107 | obs: | exp: |
| cct | P | 577 | 168.80 |
| ccc | P | 59 | 115.60 |
| cca | P | 3 | 253.00 |
| ccg | P | 2 | 103.60 |
| --- | -- | ------ | ------ |
| mPD | | 0.18 | 0.72 |
| | | nPD: | 0.25 |
| | | N. weight: | 1.5 |
| | | Sc. PD: | -0.089 |
| | | Sc. rank: | -104.6 |

,

| HA | | | |
|---|---|---|---|
| Pos. | 108 | obs: | exp: |
| ggt | G | 0 | 79.09 |
| ggc | G | 1 | 86.68 |
| gga | G | 39 | 310.10 |
| ggg | G | 601 | 165.10 |
| --- | -- | ------ | ------ |
| mPD | | 0.12 | 0.67 |
| | | nPD: | 0.18 |
| | | N. weight: | 1.6 |
| | | Sc. PD: | -0.21 |
| | | Sc. rank: | -233.8 |

,

| HA | | | |
|---|---|---|---|
| Pos. | 109 | obs: | exp: |
| aaa | K | 552 | 358.40 |
| aag | K | 88 | 281.60 |
| gaa | E | 1 | 0.58 |
| gag | E | 0 | 0.42 |
| --- | -- | ------ | ------ |
| mPD | | 0.24 | 0.50 |
| | | nPD: | 0.48 |
| | | N. weight: | 0.32 |
| | | Sc. PD: | 0.052 |
| | | Sc. rank: | 68.2 |

,

| HA | | | |
|---|---|---|---|
| Pos. | 110 | obs: | exp: |
| ttt | F | 80 | 263.50 |
| ttc | F | 561 | 377.50 |
| --- | -- | ------ | ------ |
| mPD | | 0.22 | 0.48 |
| | | nPD: | 0.45 |
| | | N. weight: | 0.3 |
| | | Sc. PD: | 0.039 |
| | | Sc. rank: | 52.0 |

,

| HA | | | |
|---|---|---|---|
| Pos. | 111 | obs: | exp: |
| tct | S | 0 | 0.32 |
| tcc | S | 0 | 0.28 |
| tca | S | 1 | 0.44 |
| tcg | S | 1 | 0.12 |
| att | I | 1 | 0.38 |
| atc | I | 0 | 0.26 |
| ata | I | 0 | 0.36 |
| atg | M | 2 | 2.00 |
| act | T | 0 | 18.76 |
| acc | T | 0 | 15.24 |
| aca | T | 65 | 31.10 |
| acg | T | 6 | 5.90 |
| agt | S | 0 | 0.44 |
| agc | S | 0 | 0.40 |
| gtt | V | 0 | 113.90 |
| gtc | V | 0 | 116.30 |
| gta | V | 40 | 103.80 |
| gtg | V | 514 | 220.00 |
| gct | A | 0 | 2.77 |
| gcc | A | 0 | 2.25 |
| gca | A | 11 | 4.90 |
| gcg | A | 0 | 1.07 |
| --- | -- | ------ | ------ |
| mPD | | 0.74 | 1.2 |
| | | nPD: | 0.63 |
| | | N. weight: | 1.1 |
| | | Sc. PD: | 0.32 |
| | | Sc. rank: | 331.8 |

,

| HA | | | |
|---|---|---|---|
| Pos. | 112 | obs: | exp: |
| aat | N | 606 | 335.00 |
| aac | N | 35 | 306.00 |
| --- | -- | ------ | ------ |
| mPD | | 0.10 | 0.50 |
| | | nPD: | 0.21 |
| | | N. weight: | 0.65 |
| | | Sc. PD: | -0.068 |
| | | Sc. rank: | -80.5 |

,

| HA | | | |
|---|---|---|---|
| Pos. | 113 | obs: | exp: |
| gaa | E | 586 | 366.50 |
| gag | E | 51 | 270.50 |
| ggt | G | 0 | 0.49 |
| ggc | G | 0 | 0.54 |
| gga | G | 4 | 1.93 |
| ggg | G | 0 | 1.03 |
| --- | -- | ------ | ------ |
| mPD | | 0.16 | 0.50 |
| | | nPD: | 0.32 |
| | | N. weight: | 0.45 |
| | | Sc. PD: | 0.00045 |
| | | Sc. rank: | 1.9 |

,

| HA | | | |
|---|---|---|---|
| Pos. | 114 | obs: | exp: |
| gaa | E | 637 | 368.80 |
| gag | E | 4 | 272.20 |
| --- | -- | ------ | ------ |
| mPD | | 0.012 | 0.49 |
| | | nPD: | 0.03 |
| | | N. weight: | 0.76 |
| | | Sc. PD: | -0.21 |
| | | Sc. rank: | -224.6 |

,

| HA | | | |
|---|---|---|---|
| Pos. | 115 | obs: | exp: |
| tct | S | 4 | 12.08 |
| tcc | S | 0 | 10.59 |
| tca | S | 72 | 16.84 |
| tcg | S | 0 | 4.63 |
| act | T | 4 | 1.06 |
| acc | T | 0 | 0.86 |
| aca | T | 0 | 1.75 |
| acg | T | 0 | 0.33 |
| agt | S | 0 | 16.72 |
| agc | S | 0 | 15.14 |
| gct | A | 540 | 141.30 |
| gcc | A | 19 | 114.90 |
| gca | A | 2 | 250.00 |
| gcg | A | 0 | 54.74 |
| --- | -- | ------ | ------ |
| mPD | | 0.48 | 1.0 |
| | | nPD: | 0.47 |
| | | N. weight: | 1.8 |
| | | Sc. PD: | 0.26 |
| | | Sc. rank: | 353.2 |

,

| HA | | | |
|---|---|---|---|
| Pos. | 116 | obs: | exp: |
| tta | L | 5 | 56.00 |
| ttg | L | 17 | 123.50 |
| ctt | L | 0 | 119.60 |
| ctc | L | 0 | 103.70 |
| cta | L | 12 | 96.57 |
| ctg | L | 607 | 141.70 |
| --- | -- | ------ | ------ |
| mPD | | 0.12 | 1.1 |
| | | nPD: | 0.11 |
| | | N. weight: | 1.9 |
| | | Sc. PD: | -0.38 |
| | | Sc. rank: | -377.9 |

,

| HA | | | |
|---|---|---|---|
| Pos. | 117 | obs: | exp: |
| cgt | R | 0 | 26.57 |
| cgc | R | 1 | 31.34 |
| cga | R | 0 | 61.51 |
| cgg | R | 6 | 53.41 |
| att | I | 0 | 0.38 |
| atc | I | 0 | 0.26 |
| ata | I | 1 | 0.36 |
| aga | R | 31 | 287.80 |
| agg | R | 602 | 179.40 |
| --- | -- | ------ | ------ |
| mPD | | 0.12 | 0.96 |
| | | nPD: | 0.13 |
| | | N. weight: | 1.5 |
| | | Sc. PD: | -0.27 |
| | | Sc. rank: | -282.3 |

,

| HA | | | |
|---|---|---|---|
| Pos. | 118 | obs: | exp: |
| cat | H | 1 | 0.54 |
| cac | H | 0 | 0.46 |
| caa | Q | 533 | 341.20 |
| cag | Q | 107 | 298.80 |
| --- | -- | ------ | ------ |
| mPD | | 0.28 | 0.50 |
| | | nPD: | 0.56 |
| | | N. weight: | 0.3 |
| | | Sc. PD: | 0.071 |
| | | Sc. rank: | 82.7 |

,

| HA | | | |
|---|---|---|---|
| Pos. | 119 | obs: | exp: |
| att | I | 549 | 241.00 |
| atc | I | 92 | 168.00 |
| ata | I | 0 | 232.00 |
| --- | -- | ------ | ------ |
| mPD | | 0.25 | 0.66 |
| | | nPD: | 0.37 |
| | | N. weight: | 0.91 |
| | | Sc. PD: | 0.051 |
| | | Sc. rank: | 83.7 |

,

| HA | | | |
|---|---|---|---|
| Pos. | 120 | obs: | exp: |
| tta | L | 0 | 55.82 |
| ttg | L | 0 | 123.10 |
| ctt | L | 62 | 119.20 |
| ctc | L | 574 | 103.40 |
| cta | L | 3 | 96.27 |
| ctg | L | 0 | 141.30 |
| att | I | 1 | 0.75 |
| atc | I | 1 | 0.52 |
| ata | I | 0 | 0.72 |
| --- | -- | ------ | ------ |
| mPD | | 0.19 | 1.1 |
| | | nPD: | 0.17 |
| | | N. weight: | 2.1 |
| | | Sc. PD: | -0.3 |
| | | Sc. rank: | -322.1 |

,

| HA | | | |
|---|---|---|---|
| Pos. | 121 | obs: | exp: |
| cgt | R | 0 | 26.61 |
| cgc | R | 0 | 31.39 |
| cga | R | 72 | 61.60 |
| cgg | R | 23 | 53.49 |
| aga | R | 336 | 288.30 |
| agg | R | 210 | 179.60 |
| --- | -- | ------ | ------ |
| mPD | | 0.72 | 0.96 |
| | | nPD: | 0.75 |
| | | N. weight: | 0.19 |
| | | Sc. PD: | 0.08 |
| | | Sc. rank: | 67.5 |

,

| HA | | | |
|---|---|---|---|
| Pos. | 122 | obs: | exp: |
| cgt | R | 0 | 0.17 |
| cgc | R | 0 | 0.20 |
| cga | R | 0 | 0.38 |
| cgg | R | 0 | 0.33 |
| aaa | K | 3 | 1.68 |
| aag | K | 0 | 1.32 |
| aga | R | 0 | 1.80 |
| agg | R | 4 | 1.12 |
| gaa | E | 110 | 293.50 |
| gag | E | 400 | 216.50 |
| ggt | G | 0 | 15.30 |
| ggc | G | 0 | 16.77 |
| gga | G | 15 | 59.98 |
| ggg | G | 109 | 31.95 |
| --- | -- | ------ | ------ |
| mPD | | 0.66 | 0.88 |
| | | nPD: | 0.76 |
| | | N. weight: | 0.6 |
| | | Sc. PD: | 0.25 |
| | | Sc. rank: | 211.7 |

,

| HA | | | |
|---|---|---|---|
| Pos. | 123 | obs: | exp: |
| tct | S | 23 | 101.90 |
| tcc | S | 0 | 89.29 |
| tca | S | 543 | 142.00 |
| tcg | S | 75 | 39.07 |
| agt | S | 0 | 141.00 |
| agc | S | 0 | 127.70 |
| --- | -- | ------ | ------ |
| mPD | | 0.27 | 1.7 |
| | | nPD: | 0.16 |
| | | N. weight: | 1.7 |
| | | Sc. PD: | -0.25 |
| | | Sc. rank: | -273.1 |

,

| HA | | | |
|---|---|---|---|
| Pos. | 124 | obs: | exp: |
| ggt | G | 48 | 79.09 |
| ggc | G | 515 | 86.68 |
| gga | G | 77 | 310.10 |
| ggg | G | 1 | 165.10 |
| --- | -- | ------ | ------ |
| mPD | | 0.33 | 0.67 |
| | | nPD: | 0.5 |
| | | N. weight: | 1.8 |
| | | Sc. PD: | 0.32 |
| | | Sc. rank: | 417.4 |

,

| HA | | | |
|---|---|---|---|
| Pos. | 125 | obs: | exp: |
| act | T | 0 | 0.26 |
| acc | T | 0 | 0.21 |
| aca | T | 1 | 0.44 |
| acg | T | 0 | 0.08 |
| ggt | G | 29 | 78.97 |
| ggc | G | 0 | 86.55 |
| gga | G | 556 | 309.60 |
| ggg | G | 55 | 164.90 |
| --- | -- | ------ | ------ |
| mPD | | 0.24 | 0.67 |
| | | nPD: | 0.36 |
| | | N. weight: | 0.55 |
| | | Sc. PD: | 0.024 |
| | | Sc. rank: | 41.7 |

,

| HA | | | |
|---|---|---|---|
| Pos. | 126 | obs: | exp: |
| att | I | 521 | 240.60 |
| atc | I | 118 | 167.70 |
| ata | I | 1 | 231.70 |
| gtt | V | 0 | 0.21 |
| gtc | V | 1 | 0.21 |
| gta | V | 0 | 0.19 |
| gtg | V | 0 | 0.40 |
| --- | -- | ------ | ------ |
| mPD | | 0.31 | 0.66 |
| | | nPD: | 0.47 |
| | | N. weight: | 0.83 |
| | | Sc. PD: | 0.12 |
| | | Sc. rank: | 156.1 |

,

| HA | | | |
|---|---|---|---|
| Pos. | 127 | obs: | exp: |
| tat | Y | 0 | 24.85 |
| tac | Y | 48 | 23.15 |
| aat | N | 1 | 7.32 |
| aac | N | 13 | 6.68 |
| gtt | V | 0 | 1.85 |
| gtc | V | 9 | 1.89 |
| gta | V | 0 | 1.69 |
| gtg | V | 0 | 3.57 |
| gat | D | 77 | 303.10 |
| gac | D | 491 | 264.90 |
| gaa | E | 1 | 1.15 |
| gag | E | 1 | 0.85 |
| --- | -- | ------ | ------ |
| mPD | | 0.43 | 0.71 |
| | | nPD: | 0.6 |
| | | N. weight: | 0.59 |
| | | Sc. PD: | 0.16 |
| | | Sc. rank: | 173.7 |

,

| HA | | | |
|---|---|---|---|
| Pos. | 128 | obs: | exp: |
| cat | H | 1 | 0.54 |
| cac | H | 0 | 0.46 |
| aaa | K | 7 | 358.40 |
| aag | K | 633 | 281.60 |
| --- | -- | ------ | ------ |
| mPD | | 0.028 | 0.50 |
| | | nPD: | 0.06 |
| | | N. weight: | 1.1 |
| | | Sc. PD: | -0.28 |
| | | Sc. rank: | -283.2 |

,

| HA | | | |
|---|---|---|---|
| Pos. | 129 | obs: | exp: |
| aaa | K | 0 | 3.36 |
| aag | K | 6 | 2.64 |
| gaa | E | 171 | 364.80 |
| gag | E | 463 | 269.20 |
| ggt | G | 0 | 0.12 |
| ggc | G | 0 | 0.14 |
| gga | G | 0 | 0.48 |
| ggg | G | 1 | 0.26 |
| --- | -- | ------ | ------ |
| mPD | | 0.41 | 0.51 |
| | | nPD: | 0.81 |
| | | N. weight: | 0.3 |
| | | Sc. PD: | 0.14 |
| | | Sc. rank: | 109.7 |

,

| HA | | | |
|---|---|---|---|
| Pos. | 130 | obs: | exp: |
| tct | S | 0 | 11.29 |
| tcc | S | 0 | 9.89 |
| tca | S | 71 | 15.73 |
| tcg | S | 0 | 4.33 |
| cct | P | 0 | 0.79 |
| ccc | P | 0 | 0.54 |
| cca | P | 1 | 1.18 |
| ccg | P | 2 | 0.49 |
| cgt | R | 0 | 0.12 |
| cgc | R | 0 | 0.15 |
| cga | R | 0 | 0.29 |
| cgg | R | 0 | 0.25 |
| act | T | 1 | 135.30 |
| acc | T | 0 | 109.90 |
| aca | T | 484 | 224.30 |
| acg | T | 27 | 42.56 |
| agt | S | 0 | 15.62 |
| agc | S | 0 | 14.15 |
| aga | R | 3 | 1.35 |
| agg | R | 0 | 0.84 |
| gct | A | 0 | 13.10 |
| gcc | A | 0 | 10.65 |
| gca | A | 52 | 23.18 |
| gcg | A | 0 | 5.07 |
| --- | -- | ------ | ------ |
| mPD | | 0.43 | 1.1 |
| | | nPD: | 0.41 |
| | | N. weight: | 1.2 |
| | | Sc. PD: | 0.11 |
| | | Sc. rank: | 162.5 |

,

| HA | | | |
|---|---|---|---|
| Pos. | 131 | obs: | exp: |
| atg | M | 640 | 640.00 |
| act | T | 0 | 0.26 |
| acc | T | 0 | 0.21 |
| aca | T | 0 | 0.44 |
| acg | T | 1 | 0.08 |
| --- | -- | ------ | ------ |
| mPD | | 0.0031 | 0.0060 |
| | | nPD: | 0.52 |
| | | N. weight: | 0.0057 |
| | | Sc. PD: | 0.0011 |
| | | Sc. rank: | 1.4 |

,

| HA | | | |
|---|---|---|---|
| Pos. | 132 | obs: | exp: |
| cgt | R | 0 | 0.21 |
| cgc | R | 0 | 0.24 |
| cga | R | 0 | 0.48 |
| cgg | R | 0 | 0.42 |
| aga | R | 5 | 2.25 |
| agg | R | 0 | 1.40 |
| ggt | G | 71 | 78.47 |
| ggc | G | 2 | 86.01 |
| gga | G | 525 | 307.70 |
| ggg | G | 38 | 163.90 |
| --- | -- | ------ | ------ |
| mPD | | 0.32 | 0.68 |
| | | nPD: | 0.46 |
| | | N. weight: | 0.51 |
| | | Sc. PD: | 0.072 |
| | | Sc. rank: | 95.1 |

,

| HA | | | |
|---|---|---|---|
| Pos. | 133 | obs: | exp: |
| ttt | F | 1 | 262.30 |
| ttc | F | 637 | 375.70 |
| tta | L | 0 | 0.17 |
| ttg | L | 0 | 0.39 |
| ctt | L | 0 | 0.37 |
| ctc | L | 2 | 0.32 |
| cta | L | 0 | 0.30 |
| ctg | L | 0 | 0.44 |
| atg | M | 1 | 1.00 |
| --- | -- | ------ | ------ |
| mPD | | 0.016 | 0.50 |
| | | nPD: | 0.03 |
| | | N. weight: | 0.77 |
| | | Sc. PD: | -0.21 |
| | | Sc. rank: | -221.9 |

,

| HA | | | |
|---|---|---|---|
| Pos. | 134 | obs: | exp: |
| tct | S | 0 | 0.16 |
| tcc | S | 0 | 0.14 |
| tca | S | 1 | 0.22 |
| tcg | S | 0 | 0.06 |
| act | T | 4 | 169.10 |
| acc | T | 316 | 137.30 |
| aca | T | 319 | 280.40 |
| acg | T | 1 | 53.20 |
| agt | S | 0 | 0.22 |
| agc | S | 0 | 0.20 |
| --- | -- | ------ | ------ |
| mPD | | 0.51 | 0.69 |
| | | nPD: | 0.74 |
| | | N. weight: | 0.67 |
| | | Sc. PD: | 0.27 |
| | | Sc. rank: | 233.0 |

,

| HA | | | |
|---|---|---|---|
| Pos. | 135 | obs: | exp: |
| tat | Y | 74 | 331.80 |
| tac | Y | 567 | 309.20 |
| --- | -- | ------ | ------ |
| mPD | | 0.20 | 0.50 |
| | | nPD: | 0.41 |
| | | N. weight: | 0.54 |
| | | Sc. PD: | 0.049 |
| | | Sc. rank: | 72.2 |

,

| HA | | | |
|---|---|---|---|
| Pos. | 136 | obs: | exp: |
| tct | S | 0 | 101.30 |
| tcc | S | 0 | 88.74 |
| tca | S | 0 | 141.10 |
| tcg | S | 0 | 38.83 |
| att | I | 0 | 0.38 |
| atc | I | 1 | 0.26 |
| ata | I | 0 | 0.36 |
| aat | N | 3 | 1.57 |
| aac | N | 0 | 1.43 |
| agt | S | 106 | 140.10 |
| agc | S | 531 | 126.90 |
| --- | -- | ------ | ------ |
| mPD | | 0.30 | 1.7 |
| | | nPD: | 0.18 |
| | | N. weight: | 1.7 |
| | | Sc. PD: | -0.22 |
| | | Sc. rank: | -244.2 |

,

| HA | | | |
|---|---|---|---|
| Pos. | 137 | obs: | exp: |
| ggt | G | 0 | 79.09 |
| ggc | G | 0 | 86.68 |
| gga | G | 560 | 310.10 |
| ggg | G | 81 | 165.10 |
| --- | -- | ------ | ------ |
| mPD | | 0.22 | 0.67 |
| | | nPD: | 0.33 |
| | | N. weight: | 0.63 |
| | | Sc. PD: | 0.01 |
| | | Sc. rank: | 19.4 |

,

| HA | | | |
|---|---|---|---|
| Pos. | 138 | obs: | exp: |
| att | I | 0 | 239.50 |
| atc | I | 0 | 166.90 |
| ata | I | 637 | 230.60 |
| atg | M | 1 | 1.00 |
| gtt | V | 0 | 0.62 |
| gtc | V | 0 | 0.63 |
| gta | V | 3 | 0.56 |
| gtg | V | 0 | 1.19 |
| --- | -- | ------ | ------ |
| mPD | | 0.012 | 0.67 |
| | | nPD: | 0.02 |
| | | N. weight: | 1.5 |
| | | Sc. PD: | -0.42 |
| | | Sc. rank: | -456.4 |

,

| HA | | | |
|---|---|---|---|
| Pos. | 139 | obs: | exp: |
| cgt | R | 0 | 26.53 |
| cgc | R | 0 | 31.29 |
| cga | R | 1 | 61.41 |
| cgg | R | 0 | 53.32 |
| aaa | K | 2 | 1.12 |
| aag | K | 0 | 0.88 |
| aga | R | 624 | 287.40 |
| agg | R | 14 | 179.10 |
| --- | -- | ------ | ------ |
| mPD | | 0.052 | 0.97 |
| | | nPD: | 0.05 |
| | | N. weight: | 1. |
| | | Sc. PD: | -0.26 |
| | | Sc. rank: | -264.3 |

,

| HA | | | |
|---|---|---|---|
| Pos. | 140 | obs: | exp: |
| tgt | C | 1 | 0.41 |
| tgc | C | 0 | 0.59 |
| act | T | 553 | 168.80 |
| acc | T | 85 | 137.10 |
| aca | T | 1 | 279.90 |
| acg | T | 0 | 53.12 |
| aat | N | 1 | 0.52 |
| aac | N | 0 | 0.48 |
| --- | -- | ------ | ------ |
| mPD | | 0.24 | 0.70 |
| | | nPD: | 0.35 |
| | | N. weight: | 1.4 |
| | | Sc. PD: | 0.045 |
| | | Sc. rank: | 71.1 |

,

| HA | | | |
|---|---|---|---|
| Pos. | 141 | obs: | exp: |
| aat | N | 632 | 333.40 |
| aac | N | 6 | 304.60 |
| gat | D | 3 | 1.60 |
| gac | D | 0 | 1.40 |
| --- | -- | ------ | ------ |
| mPD | | 0.028 | 0.51 |
| | | nPD: | 0.05 |
| | | N. weight: | 0.88 |
| | | Sc. PD: | -0.22 |
| | | Sc. rank: | -225.4 |

| HA | | | |
|---|---|---|---|
| Pos. | 142 | obs: | exp: |
| ggt | G | 0 | 79.09 |
| ggc | G | 0 | 86.68 |
| gga | G | 577 | 310.10 |
| ggg | G | 64 | 165.10 |
| --- | -- | ------ | ------ |
| mPD | | 0.18 | 0.67 |
| | | nPD: | 0.27 |
| | | N. weight: | 0.69 |
| | | Sc. PD: | -0.03 |
| | | Sc. rank: | -33.6 |

| HA | | | |
|---|---|---|---|
| Pos. | 143 | obs: | exp: |
| act | T | 0 | 10.83 |
| acc | T | 0 | 8.80 |
| aca | T | 41 | 17.96 |
| acg | T | 0 | 3.41 |
| gtt | V | 0 | 4.11 |
| gtc | V | 0 | 4.20 |
| gta | V | 18 | 3.75 |
| gtg | V | 2 | 7.94 |
| gct | A | 0 | 145.30 |
| gcc | A | 0 | 118.20 |
| gca | A | 528 | 257.20 |
| gcg | A | 49 | 56.30 |
| gaa | E | 3 | 1.73 |
| gag | E | 0 | 1.27 |
| --- | -- | ------ | ------ |
| mPD | | 0.34 | 0.88 |
| | | nPD: | 0.38 |
| | | N. weight: | 1. |
| | | Sc. PD: | 0.063 |
| | | Sc. rank: | 103.1 |

| HA | | | |
|---|---|---|---|
| Pos. | 144 | obs: | exp: |
| act | T | 5 | 169.10 |
| acc | T | 549 | 137.30 |
| aca | T | 17 | 280.40 |
| acg | T | 69 | 53.20 |
| gct | A | 0 | 0.25 |
| gcc | A | 0 | 0.20 |
| gca | A | 0 | 0.45 |
| gcg | A | 1 | 0.10 |
| --- | -- | ------ | ------ |
| mPD | | 0.26 | 0.69 |
| | | nPD: | 0.37 |
| | | N. weight: | 1.7 |
| | | Sc. PD: | 0.092 |
| | | Sc. rank: | 154.9 |

| HA | | | |
|---|---|---|---|
| Pos. | 145 | obs: | exp: |
| tct | S | 0 | 98.57 |
| tcc | S | 0 | 86.37 |
| tca | S | 0 | 137.40 |
| tcg | S | 0 | 37.79 |
| aat | N | 19 | 9.93 |
| aac | N | 0 | 9.07 |
| agt | S | 616 | 136.40 |
| agc | S | 4 | 123.50 |
| ggt | G | 2 | 0.25 |
| ggc | G | 0 | 0.27 |
| gga | G | 0 | 0.97 |
| ggg | G | 0 | 0.52 |
| --- | -- | ------ | ------ |
| mPD | | 0.076 | 1.7 |
| | | nPD: | 0.04 |
| | | N. weight: | 2.1 |
| | | Sc. PD: | -0.55 |
| | | Sc. rank: | -568.3 |

| HA | | | |
|---|---|---|---|
| Pos. | 146 | obs: | exp: |
| act | T | 0 | 0.53 |
| acc | T | 0 | 0.43 |
| aca | T | 2 | 0.88 |
| acg | T | 0 | 0.17 |
| gct | A | 58 | 160.70 |
| gcc | A | 18 | 130.70 |
| gca | A | 526 | 284.40 |
| gcg | A | 36 | 62.26 |
| gat | D | 1 | 0.53 |
| gac | D | 0 | 0.47 |
| --- | -- | ------ | ------ |
| mPD | | 0.32 | 0.70 |
| | | nPD: | 0.46 |
| | | N. weight: | 0.5 |
| | | Sc. PD: | 0.069 |
| | | Sc. rank: | 91.2 |

| HA | | | |
|---|---|---|---|
| Pos. | 147 | obs: | exp: |
| tgt | C | 565 | 264.20 |
| tgc | C | 76 | 376.80 |
| --- | -- | ------ | ------ |
| mPD | | 0.21 | 0.48 |
| | | nPD: | 0.43 |
| | | N. weight: | 0.71 |
| | | Sc. PD: | 0.079 |
| | | Sc. rank: | 110.4 |

| HA | | | |
|---|---|---|---|
| Pos. | 148 | obs: | exp: |
| tgg | W | 1 | 1.00 |
| cgt | R | 0 | 25.57 |
| cgc | R | 0 | 30.16 |
| cga | R | 0 | 59.20 |
| cgg | R | 0 | 51.40 |
| atg | M | 1 | 1.00 |
| aaa | K | 7 | 12.88 |
| aag | K | 16 | 10.12 |
| aga | R | 94 | 277.00 |
| agg | R | 522 | 172.60 |
| --- | -- | ------ | ------ |
| mPD | | 0.34 | 1.0 |
| | | nPD: | 0.33 |
| | | N. weight: | 1.1 |
| | | Sc. PD: | 0.019 |
| | | Sc. rank: | 38.7 |

| HA | | | |
|---|---|---|---|
| Pos. | 149 | obs: | exp: |
| cgt | R | 0 | 26.57 |
| cgc | R | 0 | 31.34 |
| cga | R | 0 | 61.51 |
| cgg | R | 0 | 53.41 |
| att | I | 0 | 0.38 |
| atc | I | 0 | 0.26 |
| ata | I | 1 | 0.36 |
| aga | R | 632 | 287.80 |
| agg | R | 8 | 179.40 |
| --- | -- | ------ | ------ |
| mPD | | 0.028 | 0.96 |
| | | nPD: | 0.03 |
| | | N. weight: | 1.1 |
| | | Sc. PD: | -0.3 |
| | | Sc. rank: | -315.5 |

| HA | | | |
|---|---|---|---|
| Pos. | 150 | obs: | exp: |
| tta | L | 4 | 0.35 |
| ttg | L | 0 | 0.77 |
| tct | S | 1 | 100.80 |
| tcc | S | 0 | 88.32 |
| tca | S | 630 | 140.50 |
| tcg | S | 3 | 38.65 |
| ctt | L | 0 | 0.75 |
| ctc | L | 0 | 0.65 |
| cta | L | 0 | 0.60 |
| ctg | L | 0 | 0.88 |
| cct | P | 0 | 0.79 |
| ccc | P | 0 | 0.54 |
| cca | P | 3 | 1.18 |
| ccg | P | 0 | 0.49 |
| agt | S | 0 | 139.50 |
| agc | S | 0 | 126.30 |
| --- | -- | ------ | ------ |
| mPD | | 0.034 | 1.7 |
| | | nPD: | 0.02 |
| | | N. weight: | 2.2 |
| | | Sc. PD: | -0.61 |
| | | Sc. rank: | -649.8 |

| HA | | | |
|---|---|---|---|
| Pos. | 151 | obs: | exp: |
| tta | L | 1 | 0.09 |
| ttg | L | 0 | 0.19 |
| ctt | L | 0 | 0.19 |
| ctc | L | 0 | 0.16 |
| cta | L | 0 | 0.15 |
| ctg | L | 0 | 0.22 |
| cgt | R | 0 | 0.04 |
| cgc | R | 0 | 0.05 |
| cga | R | 0 | 0.10 |
| cgg | R | 0 | 0.08 |
| aga | R | 1 | 0.45 |
| agg | R | 0 | 0.28 |
| gaa | E | 1 | 0.58 |
| gag | E | 0 | 0.42 |
| ggt | G | 68 | 78.72 |
| ggc | G | 4 | 86.28 |
| gga | G | 515 | 308.60 |
| ggg | G | 51 | 164.40 |
| --- | -- | ------ | ------ |
| mPD | | 0.34 | 0.68 |
| | | nPD: | 0.5 |
| | | N. weight: | 0.44 |
| | | Sc. PD: | 0.08 |
| | | Sc. rank: | 102.5 |

| HA | | | |
|---|---|---|---|
| Pos. | 152 | obs: | exp: |
| tct | S | 601 | 99.04 |
| tcc | S | 21 | 86.79 |
| tca | S | 0 | 138.00 |
| tcg | S | 1 | 37.97 |
| cct | P | 18 | 4.74 |
| ccc | P | 0 | 3.25 |
| cca | P | 0 | 7.10 |
| ccg | P | 0 | 2.91 |
| agt | S | 0 | 137.10 |
| agc | S | 0 | 124.10 |
| --- | -- | ------ | ------ |
| mPD | | 0.12 | 1.7 |
| | | nPD: | 0.07 |
| | | N. weight: | 2.5 |
| | | Sc. PD: | -0.58 |
| | | Sc. rank: | -574.2 |

| HA | | | |
|---|---|---|---|
| Pos. | 153 | obs: | exp: |
| tct | S | 12 | 101.90 |
| tcc | S | 72 | 89.29 |
| tca | S | 556 | 142.00 |
| tcg | S | 1 | 39.07 |
| agt | S | 0 | 141.00 |
| agc | S | 0 | 127.70 |
| --- | -- | ------ | ------ |
| mPD | | 0.24 | 1.7 |
| | | nPD: | 0.14 |
| | | N. weight: | 1.6 |
| | | Sc. PD: | -0.28 |
| | | Sc. rank: | -291.5 |

| HA | | | |
|---|---|---|---|
| Pos. | 154 | obs: | exp: |
| ttt | F | 3 | 263.50 |
| ttc | F | 638 | 377.50 |
| --- | -- | ------ | ------ |
| mPD | | 0.0093 | 0.48 |
| | | nPD: | 0.02 |
| | | N. weight: | 0.74 |
| | | Sc. PD: | -0.21 |
| | | Sc. rank: | -223.4 |

| HA | | | |
|---|---|---|---|
| Pos. | 155 | obs: | exp: |
| tat | Y | 635 | 331.80 |
| tac | Y | 6 | 309.20 |
| --- | -- | ------ | ------ |
| mPD | | 0.019 | 0.50 |
| | | nPD: | 0.04 |
| | | N. weight: | 0.89 |
| | | Sc. PD: | -0.24 |
| | | Sc. rank: | -251.7 |

| HA | | | |
|---|---|---|---|
| Pos. | 156 | obs: | exp: |
| act | T | 0 | 1.32 |
| acc | T | 0 | 1.07 |
| aca | T | 5 | 2.19 |
| acg | T | 0 | 0.42 |
| gct | A | 3 | 160.20 |
| gcc | A | 0 | 130.30 |
| gca | A | 550 | 283.50 |
| gcg | A | 83 | 62.06 |
| --- | -- | ------ | ------ |
| mPD | | 0.25 | 0.70 |
| | | nPD: | 0.36 |
| | | N. weight: | 0.88 |
| | | Sc. PD: | 0.034 |
| | | Sc. rank: | 62.2 |

| HA | | | |
|---|---|---|---|
| Pos. | 157 | obs: | exp: |
| gaa | E | 106 | 368.80 |
| gag | E | 535 | 272.20 |
| --- | -- | ------ | ------ |
| mPD | | 0.28 | 0.49 |
| | | nPD: | 0.57 |
| | | N. weight: | 0.53 |
| | | Sc. PD: | 0.13 |
| | | Sc. rank: | 146.6 |

| HA | | | |
|---|---|---|---|
| Pos. | 158 | obs: | exp: |
| atg | M | 641 | 641.00 |
| --- | -- | ------ | ------ |
| mPD | | 0 | 0 |
| | | nPD: | 1. |
| | | N. weight: | 0. |
| | | Sc. PD: | 0 |
| | | Sc. rank: | 0 |

| HA | | | |
|---|---|---|---|
| Pos. | 159 | obs: | exp: |
| aaa | K | 559 | 358.90 |
| aag | K | 82 | 282.10 |
| --- | -- | ------ | ------ |
| mPD | | 0.22 | 0.49 |
| | | nPD: | 0.45 |
| | | N. weight: | 0.34 |
| | | Sc. PD: | 0.045 |
| | | Sc. rank: | 60.5 |

| HA | | | |
|---|---|---|---|
| Pos. | 160 | obs: | exp: |
| tgg | W | 641 | 641.00 |
| --- | -- | ------ | ------ |
| mPD | | 0 | 0 |
| | | nPD: | 1. |
| | | N. weight: | 0. |
| | | Sc. PD: | 0 |
| | | Sc. rank: | 0 |

| | | | |
|---|---|---|---|
| HA | | | |
| Pos. | 161 | obs: | exp: |
| tta | L | 68 | 56.00 |
| ttg | L | 5 | 123.50 |
| ctt | L | 39 | 119.60 |
| ctc | L | 506 | 103.70 |
| cta | L | 21 | 96.57 |
| ctg | L | 2 | 141.70 |
| --- | -- | ------ | ------ |
| mPD | | 0.56 | 1.1 |
| | | nPD: | 0.5 |
| | | N. weight: | 1.7 |
| | | Sc. PD: | 0.29 |
| | | Sc. rank: | 372.5 |

| | | | |
|---|---|---|---|
| HA | | | |
| Pos. | 162 | obs: | exp: |
| tta | L | 1 | 56.00 |
| ttg | L | 16 | 123.50 |
| ctt | L | 97 | 119.60 |
| ctc | L | 0 | 103.70 |
| cta | L | 49 | 96.57 |
| ctg | L | 478 | 141.70 |
| --- | -- | ------ | ------ |
| mPD | | 0.43 | 1.1 |
| | | nPD: | 0.39 |
| | | N. weight: | 1.1 |
| | | Sc. PD: | 0.077 |
| | | Sc. rank: | 120.4 |

| | | | |
|---|---|---|---|
| HA | | | |
| Pos. | 163 | obs: | exp: |
| tct | S | 0 | 101.90 |
| tcc | S | 14 | 89.29 |
| tca | S | 524 | 142.00 |
| tcg | S | 103 | 39.07 |
| agt | S | 0 | 141.00 |
| agc | S | 0 | 127.70 |
| --- | -- | ------ | ------ |
| mPD | | 0.31 | 1.7 |
| | | nPD: | 0.18 |
| | | N. weight: | 1.7 |
| | | Sc. PD: | -0.22 |
| | | Sc. rank: | -245.2 |

| | | | |
|---|---|---|---|
| HA | | | |
| Pos. | 164 | obs: | exp: |
| tct | S | 0 | 0.48 |
| tcc | S | 0 | 0.42 |
| tca | S | 0 | 0.66 |
| tcg | S | 0 | 0.18 |
| tat | Y | 1 | 0.52 |
| tac | Y | 0 | 0.48 |
| aat | N | 92 | 330.80 |
| aac | N | 541 | 302.20 |
| aaa | K | 2 | 1.12 |
| aag | K | 0 | 0.88 |
| agt | S | 1 | 0.66 |
| agc | S | 2 | 0.60 |
| gat | D | 2 | 1.07 |
| gac | D | 0 | 0.93 |
| --- | -- | ------ | ------ |
| mPD | | 0.28 | 0.53 |
| | | nPD: | 0.53 |
| | | N. weight: | 0.47 |
| | | Sc. PD: | 0.097 |
| | | Sc. rank: | 115.3 |

| | | | |
|---|---|---|---|
| HA | | | |
| Pos. | 165 | obs: | exp: |
| tct | S | 0 | 11.76 |
| tcc | S | 0 | 10.31 |
| tca | S | 72 | 16.39 |
| tcg | S | 2 | 4.51 |
| caa | Q | 1 | 0.53 |
| cag | Q | 0 | 0.47 |
| act | T | 0 | 148.20 |
| acc | T | 1 | 120.40 |
| aca | T | 558 | 245.80 |
| acg | T | 2 | 46.64 |
| aaa | K | 3 | 1.68 |
| aag | K | 0 | 1.32 |
| agt | S | 0 | 16.28 |
| agc | S | 0 | 14.74 |
| gct | A | 0 | 0.50 |
| gcc | A | 0 | 0.41 |
| gca | A | 2 | 0.89 |
| gcg | A | 0 | 0.20 |
| --- | -- | ------ | ------ |
| mPD | | 0.24 | 0.93 |
| | | nPD: | 0.26 |
| | | N. weight: | 1.3 |
| | | Sc. PD: | -0.07 |
| | | Sc. rank: | -85.9 |

| | | | |
|---|---|---|---|
| HA | | | |
| Pos. | 166 | obs: | exp: |
| aat | N | 0 | 0.52 |
| aac | N | 1 | 0.48 |
| gat | D | 71 | 341.50 |
| gac | D | 569 | 298.50 |
| --- | -- | ------ | ------ |
| mPD | | 0.20 | 0.50 |
| | | nPD: | 0.4 |
| | | N. weight: | 0.59 |
| | | Sc. PD: | 0.048 |
| | | Sc. rank: | 73.1 |

| | | | |
|---|---|---|---|
| HA | | | |
| Pos. | 167 | obs: | exp: |
| aat | N | 637 | 334.50 |
| aac | N | 3 | 305.50 |
| gat | D | 1 | 0.53 |
| gac | D | 0 | 0.47 |
| --- | -- | ------ | ------ |
| mPD | | 0.012 | 0.50 |
| | | nPD: | 0.02 |
| | | N. weight: | 0.91 |
| | | Sc. PD: | -0.25 |
| | | Sc. rank: | -272.0 |

| | | | |
|---|---|---|---|
| HA | | | |
| Pos. | 168 | obs: | exp: |
| gtt | V | 1 | 0.21 |
| gtc | V | 0 | 0.21 |
| gta | V | 0 | 0.19 |
| gtg | V | 0 | 0.40 |
| gct | A | 563 | 160.90 |
| gcc | A | 5 | 130.90 |
| gca | A | 3 | 284.80 |
| gcg | A | 68 | 62.35 |
| ggt | G | 0 | 0.12 |
| ggc | G | 0 | 0.14 |
| gga | G | 0 | 0.48 |
| ggg | G | 1 | 0.26 |
| --- | -- | ------ | ------ |
| mPD | | 0.22 | 0.69 |
| | | nPD: | 0.32 |
| | | N. weight: | 1.6 |
| | | Sc. PD: | 0.0045 |
| | | Sc. rank: | 13.9 |

| | | | |
|---|---|---|---|
| HA | | | |
| Pos. | 169 | obs: | exp: |
| gtt | V | 3 | 0.62 |
| gtc | V | 0 | 0.63 |
| gta | V | 0 | 0.56 |
| gtg | V | 0 | 1.19 |
| gct | A | 622 | 160.70 |
| gcc | A | 4 | 130.70 |
| gca | A | 12 | 284.40 |
| gcg | A | 0 | 62.26 |
| --- | -- | ------ | ------ |
| mPD | | 0.058 | 0.70 |
| | | nPD: | 0.08 |
| | | N. weight: | 1.8 |
| | | Sc. PD: | -0.41 |
| | | Sc. rank: | -401.0 |

| | | | |
|---|---|---|---|
| HA | | | |
| Pos. | 170 | obs: | exp: |
| ttt | F | 107 | 263.50 |
| ttc | F | 534 | 377.50 |
| --- | -- | ------ | ------ |
| mPD | | 0.28 | 0.48 |
| | | nPD: | 0.58 |
| | | N. weight: | 0.21 |
| | | Sc. PD: | 0.052 |
| | | Sc. rank: | 59.0 |

| | | | |
|---|---|---|---|
| HA | | | |
| Pos. | 171 | obs: | exp: |
| tct | S | 0 | 0.16 |
| tcc | S | 0 | 0.14 |
| tca | S | 0 | 0.22 |
| tcg | S | 1 | 0.06 |
| cct | P | 19 | 168.50 |
| ccc | P | 61 | 115.40 |
| cca | P | 25 | 252.60 |
| ccg | P | 535 | 103.50 |
| agt | S | 0 | 0.22 |
| agc | S | 0 | 0.20 |
| --- | -- | ------ | ------ |
| mPD | | 0.29 | 0.72 |
| | | nPD: | 0.41 |
| | | N. weight: | 1.7 |
| | | Sc. PD: | 0.15 |
| | | Sc. rank: | 219.4 |

| | | | |
|---|---|---|---|
| HA | | | |
| Pos. | 172 | obs: | exp: |
| tct | S | 0 | 0.32 |
| tcc | S | 0 | 0.28 |
| tca | S | 2 | 0.44 |
| tcg | S | 0 | 0.12 |
| cct | P | 0 | 11.32 |
| ccc | P | 0 | 7.76 |
| cca | P | 0 | 16.97 |
| ccg | P | 43 | 6.95 |
| caa | Q | 333 | 317.70 |
| cag | Q | 263 | 278.30 |
| agt | S | 0 | 0.44 |
| agc | S | 0 | 0.40 |
| --- | -- | ------ | ------ |
| mPD | | 0.64 | 0.66 |
| | | nPD: | 0.96 |
| | | N. weight: | 0.2 |
| | | Sc. PD: | 0.12 |
| | | Sc. rank: | 73.8 |

| | | | |
|---|---|---|---|
| HA | | | |
| Pos. | 173 | obs: | exp: |
| tta | L | 0 | 0.09 |
| ttg | L | 1 | 0.19 |
| ctt | L | 0 | 0.19 |
| ctc | L | 0 | 0.16 |
| cta | L | 0 | 0.15 |
| ctg | L | 0 | 0.22 |
| atg | M | 586 | 586.00 |
| act | T | 0 | 14.00 |
| acc | T | 0 | 11.37 |
| aca | T | 5 | 23.22 |
| acg | T | 48 | 4.41 |
| gtt | V | 0 | 0.21 |
| gtc | V | 0 | 0.21 |
| gta | V | 0 | 0.19 |
| gtg | V | 1 | 0.40 |
| --- | -- | ------ | ------ |
| mPD | | 0.17 | 0.31 |
| | | nPD: | 0.57 |
| | | N. weight: | 0.25 |
| | | Sc. PD: | 0.062 |
| | | Sc. rank: | 70.8 |

| | | | |
|---|---|---|---|
| HA | | | |
| Pos. | 174 | obs: | exp: |
| tct | S | 0 | 0.16 |
| tcc | S | 0 | 0.14 |
| tca | S | 0 | 0.22 |
| tcg | S | 0 | 0.06 |
| act | T | 614 | 169.10 |
| acc | T | 15 | 137.30 |
| aca | T | 4 | 280.40 |
| acg | T | 7 | 53.20 |
| agt | S | 1 | 0.22 |
| agc | S | 0 | 0.20 |
| --- | -- | ------ | ------ |
| mPD | | 0.082 | 0.69 |
| | | nPD: | 0.12 |
| | | N. weight: | 1.7 |
| | | Sc. PD: | -0.32 |
| | | Sc. rank: | -327.7 |

| | | | |
|---|---|---|---|
| HA | | | |
| Pos. | 175 | obs: | exp: |
| aaa | K | 54 | 358.40 |
| aag | K | 586 | 281.60 |
| gaa | E | 0 | 0.58 |
| gag | E | 1 | 0.42 |
| --- | -- | ------ | ------ |
| mPD | | 0.16 | 0.50 |
| | | nPD: | 0.32 |
| | | N. weight: | 0.76 |
| | | Sc. PD: | 0.0017 |
| | | Sc. rank: | 4.0 |

| | | | |
|---|---|---|---|
| HA | | | |
| Pos. | 176 | obs: | exp: |
| tct | S | 3 | 101.90 |
| tcc | S | 2 | 89.29 |
| tca | S | 533 | 142.00 |
| tcg | S | 103 | 39.07 |
| agt | S | 0 | 141.00 |
| agc | S | 0 | 127.70 |
| --- | -- | ------ | ------ |
| mPD | | 0.28 | 1.7 |
| | | nPD: | 0.17 |
| | | N. weight: | 1.8 |
| | | Sc. PD: | -0.25 |
| | | Sc. rank: | -277.0 |

| | | | |
|---|---|---|---|
| HA | | | |
| Pos. | 177 | obs: | exp: |
| tct | S | 0 | 0.16 |
| tcc | S | 1 | 0.14 |
| tca | S | 0 | 0.22 |
| tcg | S | 0 | 0.06 |
| tat | Y | 70 | 331.30 |
| tac | Y | 570 | 308.70 |
| agt | S | 0 | 0.22 |
| agc | S | 0 | 0.20 |
| --- | -- | ------ | ------ |
| mPD | | 0.20 | 0.50 |
| | | nPD: | 0.39 |
| | | N. weight: | 0.56 |
| | | Sc. PD: | 0.042 |
| | | Sc. rank: | 62.7 |

| | | | |
|---|---|---|---|
| HA | | | |
| Pos. | 178 | obs: | exp: |
| caa | Q | 4 | 2.13 |
| cag | Q | 0 | 1.87 |
| cgt | R | 0 | 3.07 |
| cgc | R | 0 | 3.62 |
| cga | R | 0 | 7.11 |
| cgg | R | 0 | 6.18 |
| aaa | K | 321 | 315.30 |
| aag | K | 242 | 247.70 |
| aga | R | 73 | 33.28 |
| agg | R | 1 | 20.74 |
| --- | -- | ------ | ------ |
| mPD | | 0.69 | 0.78 |
| | | nPD: | 0.88 |
| | | N. weight: | 0.14 |
| | | Sc. PD: | 0.076 |
| | | Sc. rank: | 52.3 |

| | | | |
|---|---|---|---|
| HA | | | |
| Pos. | 179 | obs: | exp: |
| aat | N | 172 | 335.00 |
| aac | N | 469 | 306.00 |
| --- | -- | ------ | ------ |
| mPD | | 0.39 | 0.50 |
| | | nPD: | 0.79 |
| | | N. weight: | 0.2 |
| | | Sc. PD: | 0.092 |
| | | Sc. rank: | 73.3 |

| | | | |
|---|---|---|---|
| HA | | | |
| Pos. | 180 | obs: | exp: |
| cct | P | 1 | 19.22 |
| ccc | P | 48 | 13.17 |
| cca | P | 24 | 28.81 |
| ccg | P | 0 | 11.80 |
| att | I | 0 | 0.75 |
| atc | I | 0 | 0.52 |
| ata | I | 2 | 0.72 |
| act | T | 0 | 148.50 |
| acc | T | 1 | 120.60 |
| aca | T | 561 | 246.20 |
| acg | T | 0 | 46.72 |
| gct | A | 0 | 1.01 |
| gcc | A | 0 | 0.82 |
| gca | A | 4 | 1.78 |
| gcg | A | 0 | 0.39 |
| --- | -- | ------ | ------ |
| mPD | | 0.36 | 0.91 |
| | | nPD: | 0.4 |
| | | N. weight: | 1.2 |
| | | Sc. PD: | 0.097 |
| | | Sc. rank: | 148.9 |

HA

| Pos. | 181 | obs: | exp: |
|---|---|---|---|
| tct | S | 0 | 0.16 |
| tcc | S | 0 | 0.14 |
| tca | S | 0 | 0.22 |
| tcg | S | 0 | 0.06 |
| cgt | R | 0 | 26.32 |
| cgc | R | 0 | 31.04 |
| cga | R | 0 | 60.93 |
| cgg | R | 0 | 52.91 |
| aaa | K | 0 | 3.36 |
| aag | K | 6 | 2.64 |
| agt | S | 1 | 0.22 |
| agc | S | 0 | 0.20 |
| aga | R | 100 | 285.10 |
| agg | R | 534 | 177.70 |
| --- | -- | ------ | ------ |
| mPD | | 0.28 | 0.98 |
| | | nPD: | 0.29 |
| | | N. weight: | 1.1 |
| | | Sc. PD: | -0.026 |
| | | Sc. rank: | -37.3 |

,

HA

| Pos. | 182 | obs: | exp: |
|---|---|---|---|
| tct | S | 0 | 2.07 |
| tcc | S | 0 | 1.81 |
| tca | S | 0 | 2.88 |
| tcg | S | 0 | 0.79 |
| cgt | R | 0 | 0.87 |
| cgc | R | 0 | 1.03 |
| cga | R | 0 | 2.02 |
| cgg | R | 0 | 1.75 |
| aat | N | 3 | 31.36 |
| aac | N | 57 | 28.64 |
| aaa | K | 482 | 303.50 |
| aag | K | 60 | 238.50 |
| agt | S | 0 | 2.86 |
| agc | S | 13 | 2.59 |
| aga | R | 21 | 9.44 |
| agg | R | 0 | 5.89 |
| gaa | E | 1 | 0.58 |
| gag | E | 0 | 0.42 |
| ggt | G | 0 | 0.49 |
| ggc | G | 0 | 0.54 |
| gga | G | 4 | 1.93 |
| ggg | G | 0 | 1.03 |
| --- | -- | ------ | ------ |
| mPD | | 0.48 | 0.76 |
| | | nPD: | 0.63 |
| | | N. weight: | 0.5 |
| | | Sc. PD: | 0.15 |
| | | Sc. rank: | 155.2 |

,

HA

| Pos. | 183 | obs: | exp: |
|---|---|---|---|
| aat | N | 0 | 0.52 |
| aac | N | 1 | 0.48 |
| aaa | K | 69 | 40.32 |
| aag | K | 3 | 31.68 |
| gct | A | 0 | 13.10 |
| gcc | A | 52 | 10.65 |
| gca | A | 0 | 23.18 |
| gcg | A | 0 | 5.07 |
| gat | D | 17 | 270.00 |
| gac | D | 489 | 236.00 |
| gaa | E | 4 | 2.88 |
| gag | E | 1 | 2.12 |
| ggt | G | 0 | 0.62 |
| ggc | G | 5 | 0.68 |
| gga | G | 0 | 2.42 |
| ggg | G | 0 | 1.29 |
| --- | -- | ------ | ------ |
| mPD | | 0.62 | 1.0 |
| | | nPD: | 0.62 |
| | | N. weight: | 1. |
| | | Sc. PD: | 0.29 |
| | | Sc. rank: | 304.2 |

,

HA

| Pos. | 184 | obs: | exp: |
|---|---|---|---|
| cct | P | 2 | 168.80 |
| ccc | P | 0 | 115.60 |
| cca | P | 639 | 253.00 |
| ccg | P | 0 | 103.60 |
| --- | -- | ------ | ------ |
| mPD | | 0.0062 | 0.72 |
| | | nPD: | 0.01 |
| | | N. weight: | 1.3 |
| | | Sc. PD: | -0.39 |
| | | Sc. rank: | -439.0 |

,

HA

| Pos. | 185 | obs: | exp: |
|---|---|---|---|
| act | T | 2 | 0.53 |
| acc | T | 0 | 0.43 |
| aca | T | 0 | 0.88 |
| acg | T | 0 | 0.17 |
| gct | A | 608 | 160.90 |
| gcc | A | 31 | 130.90 |
| gca | A | 0 | 284.80 |
| gcg | A | 0 | 62.35 |
| --- | -- | ------ | ------ |
| mPD | | 0.098 | 0.69 |
| | | nPD: | 0.14 |
| | | N. weight: | 1.8 |
| | | Sc. PD: | -0.29 |
| | | Sc. rank: | -307.0 |

,

HA

| Pos. | 186 | obs: | exp: |
|---|---|---|---|
| tta | L | 45 | 55.30 |
| ttg | L | 65 | 121.90 |
| ctt | L | 0 | 118.10 |
| ctc | L | 0 | 102.40 |
| cta | L | 61 | 95.36 |
| ctg | L | 462 | 139.90 |
| att | I | 0 | 3.01 |
| atc | I | 0 | 2.10 |
| ata | I | 8 | 2.90 |
| --- | -- | ------ | ------ |
| mPD | | 0.60 | 1.1 |
| | | nPD: | 0.53 |
| | | N. weight: | 1.1 |
| | | Sc. PD: | 0.23 |
| | | Sc. rank: | 272.7 |

,

HA

| Pos. | 187 | obs: | exp: |
|---|---|---|---|
| att | I | 1 | 235.00 |
| atc | I | 0 | 163.80 |
| ata | I | 624 | 226.20 |
| atg | M | 3 | 3.00 |
| gtt | V | 0 | 2.67 |
| gtc | V | 0 | 2.73 |
| gta | V | 13 | 2.44 |
| gtg | V | 0 | 5.16 |
| --- | -- | ------ | ------ |
| mPD | | 0.052 | 0.71 |
| | | nPD: | 0.07 |
| | | N. weight: | 1.5 |
| | | Sc. PD: | -0.34 |
| | | Sc. rank: | -342.1 |

,

HA

| Pos. | 188 | obs: | exp: |
|---|---|---|---|
| tta | L | 1 | 0.09 |
| ttg | L | 0 | 0.19 |
| ctt | L | 0 | 0.19 |
| ctc | L | 0 | 0.16 |
| cta | L | 0 | 0.15 |
| ctg | L | 0 | 0.22 |
| att | I | 69 | 206.80 |
| atc | I | 4 | 144.10 |
| ata | I | 477 | 199.10 |
| atg | M | 1 | 1.00 |
| act | T | 0 | 0.26 |
| acc | T | 0 | 0.21 |
| aca | T | 1 | 0.44 |
| acg | T | 0 | 0.08 |
| gtt | V | 5 | 18.09 |
| gtc | V | 3 | 18.47 |
| gta | V | 70 | 16.49 |
| gtg | V | 10 | 34.95 |
| --- | -- | ------ | ------ |
| mPD | | 0.50 | 0.94 |
| | | nPD: | 0.53 |
| | | N. weight: | 0.95 |
| | | Sc. PD: | 0.2 |
| | | Sc. rank: | 233.0 |

,

HA

| Pos. | 189 | obs: | exp: |
|---|---|---|---|
| tgg | W | 641 | 641.00 |
| --- | -- | ------ | ------ |
| mPD | | 0 | 0 |
| | | nPD: | 1. |
| | | N. weight: | 0. |
| | | Sc. PD: | 0 |
| | | Sc. rank: | 0 |

,

HA

| Pos. | 190 | obs: | exp: |
|---|---|---|---|
| ggt | G | 0 | 79.09 |
| ggc | G | 0 | 86.68 |
| gga | G | 235 | 310.10 |
| ggg | G | 406 | 165.10 |
| --- | -- | ------ | ------ |
| mPD | | 0.47 | 0.67 |
| | | nPD: | 0.7 |
| | | N. weight: | 0.69 |
| | | Sc. PD: | 0.25 |
| | | Sc. rank: | 233.0 |

,

HA

| Pos. | 191 | obs: | exp: |
|---|---|---|---|
| att | I | 37 | 213.90 |
| atc | I | 532 | 149.10 |
| ata | I | 0 | 206.00 |
| gtt | V | 2 | 14.80 |
| gtc | V | 2 | 15.11 |
| gta | V | 0 | 13.49 |
| gtg | V | 68 | 28.60 |
| --- | -- | ------ | ------ |
| mPD | | 0.49 | 0.89 |
| | | nPD: | 0.55 |
| | | N. weight: | 1.5 |
| | | Sc. PD: | 0.35 |
| | | Sc. rank: | 399.0 |

,

HA

| Pos. | 192 | obs: | exp: |
|---|---|---|---|
| cat | H | 132 | 347.30 |
| cac | H | 509 | 293.70 |
| --- | -- | ------ | ------ |
| mPD | | 0.33 | 0.50 |
| | | nPD: | 0.66 |
| | | N. weight: | 0.36 |
| | | Sc. PD: | 0.12 |
| | | Sc. rank: | 115.6 |

,

HA

| Pos. | 193 | obs: | exp: |
|---|---|---|---|
| cat | H | 572 | 347.30 |
| cac | H | 69 | 293.70 |
| --- | -- | ------ | ------ |
| mPD | | 0.19 | 0.50 |
| | | nPD: | 0.39 |
| | | N. weight: | 0.43 |
| | | Sc. PD: | 0.03 |
| | | Sc. rank: | 47.0 |

,

HA

| Pos. | 194 | obs: | exp: |
|---|---|---|---|
| tct | S | 126 | 100.90 |
| tcc | S | 505 | 88.46 |
| tca | S | 4 | 140.70 |
| tcg | S | 0 | 38.71 |
| cct | P | 1 | 1.58 |
| ccc | P | 5 | 1.08 |
| cca | P | 0 | 2.37 |
| ccg | P | 0 | 0.97 |
| agt | S | 0 | 139.70 |
| agc | S | 0 | 126.50 |
| --- | -- | ------ | ------ |
| mPD | | 0.35 | 1.7 |
| | | nPD: | 0.21 |
| | | N. weight: | 2.1 |
| | | Sc. PD: | -0.22 |
| | | Sc. rank: | -249.6 |

,

HA

| Pos. | 195 | obs: | exp: |
|---|---|---|---|
| gtt | V | 0 | 1.23 |
| gtc | V | 0 | 1.26 |
| gta | V | 6 | 1.12 |
| gtg | V | 0 | 2.38 |
| ggt | G | 0 | 78.35 |
| ggc | G | 0 | 85.87 |
| gga | G | 623 | 307.20 |
| ggg | G | 12 | 163.60 |
| --- | -- | ------ | ------ |
| mPD | | 0.055 | 0.69 |
| | | nPD: | 0.08 |
| | | N. weight: | 0.96 |
| | | Sc. PD: | -0.22 |
| | | Sc. rank: | -216.4 |

,

HA

| Pos. | 196 | obs: | exp: |
|---|---|---|---|
| tct | S | 2 | 101.90 |
| tcc | S | 2 | 89.29 |
| tca | S | 506 | 142.00 |
| tcg | S | 131 | 39.07 |
| agt | S | 0 | 141.00 |
| agc | S | 0 | 127.70 |
| --- | -- | ------ | ------ |
| mPD | | 0.34 | 1.7 |
| | | nPD: | 0.2 |
| | | N. weight: | 1.8 |
| | | Sc. PD: | -0.2 |
| | | Sc. rank: | -231.9 |

HA

| Pos. | 197 | obs: | exp: |
|---|---|---|---|
| att | I | 0 | 1.50 |
| atc | I | 3 | 1.05 |
| ata | I | 1 | 1.45 |
| act | T | 375 | 140.30 |
| acc | T | 153 | 113.90 |
| aca | T | 3 | 232.60 |
| acg | T | 0 | 44.14 |
| gtt | V | 0 | 2.88 |
| gtc | V | 14 | 2.94 |
| gta | V | 0 | 2.62 |
| gtg | V | 0 | 5.56 |
| gct | A | 66 | 23.17 |
| gcc | A | 25 | 18.85 |
| gca | A | 1 | 41.00 |
| gcg | A | 0 | 8.98 |
| --- | -- | ------ | ------ |
| mPD | | 0.77 | 1.0 |
| | | nPD: | 0.75 |
| | | N. weight: | 1.2 |
| | | Sc. PD: | 0.48 |
| | | Sc. rank: | 406.6 |

HA

| Pos. | 198 | obs: | exp: |
|---|---|---|---|
| act | T | 30 | 148.70 |
| acc | T | 35 | 120.80 |
| aca | T | 498 | 246.60 |
| acg | T | 0 | 46.80 |
| aat | N | 1 | 0.52 |
| aac | N | 0 | 0.48 |
| aaa | K | 1 | 0.56 |
| aag | K | 0 | 0.44 |
| gct | A | 4 | 18.13 |
| gcc | A | 1 | 14.75 |
| gca | A | 67 | 32.09 |
| gcg | A | 0 | 7.03 |
| gat | D | 4 | 2.13 |
| gac | D | 0 | 1.87 |
| --- | -- | ------ | ------ |
| mPD | | 0.44 | 0.91 |
| | | nPD: | 0.48 |
| | | N. weight: | 0.71 |
| | | Sc. PD: | 0.11 |
| | | Sc. rank: | 151.0 |

HA

| Pos. | 199 | obs: | exp: |
|---|---|---|---|
| gaa | E | 516 | 368.80 |
| gag | E | 125 | 272.20 |
| --- | -- | ------ | ------ |
| mPD | | 0.31 | 0.49 |
| | | nPD: | 0.64 |
| | | N. weight: | 0.18 |
| | | Sc. PD: | 0.057 |
| | | Sc. rank: | 58.0 |

HA

| Pos. | 200 | obs: | exp: |
|---|---|---|---|
| caa | Q | 110 | 341.70 |
| cag | Q | 531 | 299.30 |
| --- | -- | ------ | ------ |
| mPD | | 0.28 | 0.50 |
| | | nPD: | 0.57 |
| | | N. weight: | 0.42 |
| | | Sc. PD: | 0.1 |
| | | Sc. rank: | 116.9 |

HA

| Pos. | 201 | obs: | exp: |
|---|---|---|---|
| att | I | 0 | 0.75 |
| atc | I | 2 | 0.52 |
| ata | I | 0 | 0.72 |
| act | T | 24 | 167.00 |
| acc | T | 607 | 135.60 |
| aca | T | 1 | 276.90 |
| acg | T | 0 | 52.54 |
| gct | A | 0 | 1.76 |
| gcc | A | 7 | 1.43 |
| gca | A | 0 | 3.12 |
| gcg | A | 0 | 0.68 |
| --- | -- | ------ | ------ |
| mPD | | 0.10 | 0.71 |
| | | nPD: | 0.14 |
| | | N. weight: | 2. |
| | | Sc. PD: | -0.33 |
| | | Sc. rank: | -336.5 |

HA

| Pos. | 202 | obs: | exp: |
|---|---|---|---|
| aaa | K | 83 | 358.90 |
| aag | K | 558 | 282.10 |
| --- | -- | ------ | ------ |
| mPD | | 0.23 | 0.49 |
| | | nPD: | 0.46 |
| | | N. weight: | 0.6 |
| | | Sc. PD: | 0.082 |
| | | Sc. rank: | 108.7 |

HA

| Pos. | 203 | obs: | exp: |
|---|---|---|---|
| tta | L | 10 | 56.00 |
| ttg | L | 0 | 123.50 |
| ctt | L | 1 | 119.60 |
| ctc | L | 71 | 103.70 |
| cta | L | 552 | 96.57 |
| ctg | L | 7 | 141.70 |
| --- | -- | ------ | ------ |
| mPD | | 0.25 | 1.1 |
| | | nPD: | 0.22 |
| | | N. weight: | 2.1 |
| | | Sc. PD: | -0.18 |
| | | Sc. rank: | -206.3 |

HA

| Pos. | 204 | obs: | exp: |
|---|---|---|---|
| tat | Y | 461 | 331.80 |
| tac | Y | 180 | 309.20 |
| --- | -- | ------ | ------ |
| mPD | | 0.40 | 0.50 |
| | | nPD: | 0.81 |
| | | N. weight: | 0.13 |
| | | Sc. PD: | 0.062 |
| | | Sc. rank: | 48.2 |

HA

| Pos. | 205 | obs: | exp: |
|---|---|---|---|
| ggt | G | 1 | 79.09 |
| ggc | G | 0 | 86.68 |
| gga | G | 50 | 310.10 |
| ggg | G | 590 | 165.10 |
| --- | -- | ------ | ------ |
| mPD | | 0.15 | 0.67 |
| | | nPD: | 0.22 |
| | | N. weight: | 1.5 |
| | | Sc. PD: | -0.14 |
| | | Sc. rank: | -158.3 |

HA

| Pos. | 206 | obs: | exp: |
|---|---|---|---|
| tct | S | 0 | 101.70 |
| tcc | S | 0 | 89.16 |
| tca | S | 0 | 141.80 |
| tcg | S | 0 | 39.01 |
| aat | N | 1 | 0.52 |
| aac | N | 0 | 0.48 |
| agt | S | 640 | 140.80 |
| agc | S | 0 | 127.50 |
| --- | -- | ------ | ------ |
| mPD | | 0.0031 | 1.7 |
| | | nPD: | 0. |
| | | N. weight: | 2.2 |
| | | Sc. PD: | -0.67 |
| | | Sc. rank: | -788.7 |

HA

| Pos. | 207 | obs: | exp: |
|---|---|---|---|
| gaa | E | 1 | 0.58 |
| gag | E | 0 | 0.42 |
| ggt | G | 0 | 78.97 |
| ggc | G | 0 | 86.55 |
| gga | G | 586 | 309.60 |
| ggg | G | 54 | 164.90 |
| --- | -- | ------ | ------ |
| mPD | | 0.16 | 0.67 |
| | | nPD: | 0.24 |
| | | N. weight: | 0.72 |
| | | Sc. PD: | -0.056 |
| | | Sc. rank: | -67.9 |

HA

| Pos. | 208 | obs: | exp: |
|---|---|---|---|
| tct | S | 0 | 15.10 |
| tcc | S | 0 | 13.23 |
| tca | S | 0 | 21.05 |
| tcg | S | 0 | 5.79 |
| aat | N | 2 | 283.80 |
| aac | N | 541 | 259.20 |
| agt | S | 1 | 20.90 |
| agc | S | 94 | 18.93 |
| gct | A | 1 | 0.25 |
| gcc | A | 0 | 0.20 |
| gca | A | 0 | 0.45 |
| gcg | A | 0 | 0.10 |
| gat | D | 0 | 0.53 |
| gac | D | 1 | 0.47 |
| ggt | G | 0 | 0.12 |
| ggc | G | 1 | 0.14 |
| gga | G | 0 | 0.48 |
| ggg | G | 0 | 0.26 |
| --- | -- | ------ | ------ |
| mPD | | 0.28 | 0.97 |
| | | nPD: | 0.29 |
| | | N. weight: | 1.2 |
| | | Sc. PD: | -0.034 |
| | | Sc. rank: | -44.1 |

HA

| Pos. | 209 | obs: | exp: |
|---|---|---|---|
| aat | N | 0 | 0.52 |
| aac | N | 1 | 0.48 |
| aaa | K | 550 | 358.40 |
| aag | K | 90 | 281.60 |
| --- | -- | ------ | ------ |
| mPD | | 0.24 | 0.49 |
| | | nPD: | 0.49 |
| | | N. weight: | 0.31 |
| | | Sc. PD: | 0.054 |
| | | Sc. rank: | 68.1 |

HA

| Pos. | 210 | obs: | exp: |
|---|---|---|---|
| tta | L | 14 | 55.65 |
| ttg | L | 240 | 122.70 |
| tgg | W | 1 | 1.00 |
| ctt | L | 0 | 118.90 |
| ctc | L | 0 | 103.00 |
| cta | L | 11 | 95.96 |
| ctg | L | 372 | 140.80 |
| atg | M | 3 | 3.00 |
| --- | -- | ------ | ------ |
| mPD | | 0.56 | 1.1 |
| | | nPD: | 0.5 |
| | | N. weight: | 1.1 |
| | | Sc. PD: | 0.2 |
| | | Sc. rank: | 255.3 |

HA

| Pos. | 211 | obs: | exp: |
|---|---|---|---|
| att | I | 0 | 222.90 |
| atc | I | 0 | 155.40 |
| ata | I | 593 | 214.70 |
| gtt | V | 0 | 9.87 |
| gtc | V | 0 | 10.07 |
| gta | V | 2 | 9.00 |
| gtg | V | 46 | 19.06 |
| --- | -- | ------ | ------ |
| mPD | | 0.27 | 0.82 |
| | | nPD: | 0.33 |
| | | N. weight: | 1.5 |
| | | Sc. PD: | 0.025 |
| | | Sc. rank: | 49.1 |

HA

| Pos. | 212 | obs: | exp: |
|---|---|---|---|
| act | T | 0 | 169.30 |
| acc | T | 2 | 137.50 |
| aca | T | 639 | 280.80 |
| acg | T | 0 | 53.29 |
| --- | -- | ------ | ------ |
| mPD | | 0.0062 | 0.69 |
| | | nPD: | 0.01 |
| | | N. weight: | 1.2 |
| | | Sc. PD: | -0.35 |
| | | Sc. rank: | -386.7 |

HA

| Pos. | 213 | obs: | exp: |
|---|---|---|---|
| att | I | 1 | 0.38 |
| atc | I | 0 | 0.26 |
| ata | I | 0 | 0.36 |
| gtt | V | 526 | 131.50 |
| gtc | V | 35 | 134.30 |
| gta | V | 79 | 119.90 |
| gtg | V | 0 | 254.20 |
| --- | -- | ------ | ------ |
| mPD | | 0.31 | 0.72 |
| | | nPD: | 0.43 |
| | | N. weight: | 1.5 |
| | | Sc. PD: | 0.16 |
| | | Sc. rank: | 228.9 |

HA

| Pos. | 214 | obs: | exp: |
|---|---|---|---|
| cgt | R | 0 | 1.79 |
| cgc | R | 0 | 2.11 |
| cga | R | 0 | 4.13 |
| cgg | R | 0 | 3.59 |
| aga | R | 0 | 19.34 |
| agg | R | 43 | 12.05 |
| gaa | E | 0 | 0.58 |
| gag | E | 1 | 0.42 |
| ggt | G | 0 | 73.66 |
| ggc | G | 0 | 80.73 |
| gga | G | 150 | 288.80 |
| ggg | G | 447 | 153.80 |
| --- | -- | ------ | ------ |
| mPD | | 0.49 | 0.79 |
| | | nPD: | 0.62 |
| | | N. weight: | 1. |
| | | Sc. PD: | 0.29 |
| | | Sc. rank: | 302.7 |

HA

| Pos. | 215 | obs: | exp: |
|---|---|---|---|
| tct | S | 0 | 101.90 |
| tcc | S | 0 | 89.29 |
| tca | S | 0 | 142.00 |
| tcg | S | 0 | 39.07 |
| agt | S | 548 | 141.00 |
| agc | S | 93 | 127.70 |
| --- | -- | ------ | ------ |
| mPD | | 0.25 | 1.7 |
| | | nPD: | 0.15 |
| | | N. weight: | 1.6 |
| | | Sc. PD: | -0.26 |
| | | Sc. rank: | -268.6 |

| HA | | | |
|---|---|---|---|
| Pos. | 216 | obs: | exp: |
| tct | S | 511 | 101.90 |
| tcc | S | 58 | 89.29 |
| tca | S | 31 | 142.00 |
| tcg | S | 41 | 39.07 |
| agt | S | 0 | 141.00 |
| agc | S | 0 | 127.70 |
| --- | -- | ------ | ------ |
| mPD | | 0.35 | 1.7 |
| | | nPD: | 0.21 |
| | | N. weight: | 1.7 |
| | | Sc. PD: | -0.18 |
| | | Sc. rank: | -205.7 |

| HA | | | |
|---|---|---|---|
| Pos. | 217 | obs: | exp: |
| cgt | R | 0 | 0.08 |
| cgc | R | 0 | 0.10 |
| cga | R | 0 | 0.19 |
| cgg | R | 0 | 0.17 |
| aat | N | 474 | 292.70 |
| aac | N | 86 | 267.30 |
| aaa | K | 77 | 43.68 |
| aag | K | 1 | 34.32 |
| aga | R | 2 | 0.90 |
| agg | R | 0 | 0.56 |
| gat | D | 1 | 0.53 |
| gac | D | 0 | 0.47 |
| --- | -- | ------ | ------ |
| mPD | | 0.43 | 0.62 |
| | | nPD: | 0.69 |
| | | N. weight: | 0.41 |
| | | Sc. PD: | 0.15 |
| | | Sc. rank: | 133.9 |

| HA | | | |
|---|---|---|---|
| Pos. | 218 | obs: | exp: |
| tat | Y | 153 | 331.80 |
| tac | Y | 488 | 309.20 |
| --- | -- | ------ | ------ |
| mPD | | 0.36 | 0.50 |
| | | nPD: | 0.73 |
| | | N. weight: | 0.25 |
| | | Sc. PD: | 0.098 |
| | | Sc. rank: | 84.0 |

| HA | | | |
|---|---|---|---|
| Pos. | 219 | obs: | exp: |
| caa | Q | 559 | 341.70 |
| cag | Q | 82 | 299.30 |
| --- | -- | ------ | ------ |
| mPD | | 0.22 | 0.50 |
| | | nPD: | 0.45 |
| | | N. weight: | 0.39 |
| | | Sc. PD: | 0.05 |
| | | Sc. rank: | 67.9 |

| HA | | | |
|---|---|---|---|
| Pos. | 220 | obs: | exp: |
| caa | Q | 112 | 341.70 |
| cag | Q | 529 | 299.30 |
| --- | -- | ------ | ------ |
| mPD | | 0.29 | 0.50 |
| | | nPD: | 0.58 |
| | | N. weight: | 0.41 |
| | | Sc. PD: | 0.1 |
| | | Sc. rank: | 115.6 |

| HA | | | |
|---|---|---|---|
| Pos. | 221 | obs: | exp: |
| tct | S | 152 | 101.90 |
| tcc | S | 404 | 89.29 |
| tca | S | 82 | 142.00 |
| tcg | S | 3 | 39.07 |
| agt | S | 0 | 141.00 |
| agc | S | 0 | 127.70 |
| --- | -- | ------ | ------ |
| mPD | | 0.53 | 1.7 |
| | | nPD: | 0.32 |
| | | N. weight: | 1.4 |
| | | Sc. PD: | 0.0052 |
| | | Sc. rank: | 15.3 |

| HA | | | |
|---|---|---|---|
| Pos. | 222 | obs: | exp: |
| ttt | F | 566 | 263.50 |
| ttc | F | 75 | 377.50 |
| --- | -- | ------ | ------ |
| mPD | | 0.21 | 0.48 |
| | | nPD: | 0.43 |
| | | N. weight: | 0.72 |
| | | Sc. PD: | 0.077 |
| | | Sc. rank: | 110.5 |

| HA | | | |
|---|---|---|---|
| Pos. | 223 | obs: | exp: |
| att | I | 0 | 0.75 |
| atc | I | 0 | 0.52 |
| ata | I | 2 | 0.72 |
| atg | M | 1 | 1.00 |
| act | T | 8 | 19.02 |
| acc | T | 64 | 15.45 |
| aca | T | 0 | 31.54 |
| acg | T | 0 | 5.99 |
| gtt | V | 10 | 116.30 |
| gtc | V | 28 | 118.80 |
| gta | V | 425 | 106.10 |
| gtg | V | 103 | 224.80 |
| --- | -- | ------ | ------ |
| mPD | | 0.92 | 1.1 |
| | | nPD: | 0.8 |
| | | N. weight: | 1.2 |
| | | Sc. PD: | 0.58 |
| | | Sc. rank: | 450.9 |

| HA | | | |
|---|---|---|---|
| Pos. | 224 | obs: | exp: |
| cct | P | 1 | 168.80 |
| ccc | P | 0 | 115.60 |
| cca | P | 75 | 253.00 |
| ccg | P | 565 | 103.60 |
| --- | -- | ------ | ------ |
| mPD | | 0.21 | 0.72 |
| | | nPD: | 0.29 |
| | | N. weight: | 2. |
| | | Sc. PD: | -0.043 |
| | | Sc. rank: | -60.4 |

| HA | | | |
|---|---|---|---|
| Pos. | 225 | obs: | exp: |
| tct | S | 0 | 101.70 |
| tcc | S | 0 | 89.16 |
| tca | S | 0 | 141.80 |
| tcg | S | 0 | 39.01 |
| aat | N | 1 | 0.52 |
| aac | N | 0 | 0.48 |
| agt | S | 470 | 140.80 |
| agc | S | 170 | 127.50 |
| --- | -- | ------ | ------ |
| mPD | | 0.39 | 1.7 |
| | | nPD: | 0.24 |
| | | N. weight: | 1.4 |
| | | Sc. PD: | -0.11 |
| | | Sc. rank: | -130.0 |

| HA | | | |
|---|---|---|---|
| Pos. | 226 | obs: | exp: |
| cct | P | 0 | 168.80 |
| ccc | P | 0 | 115.60 |
| cca | P | 556 | 253.00 |
| ccg | P | 85 | 103.60 |
| --- | -- | ------ | ------ |
| mPD | | 0.23 | 0.72 |
| | | nPD: | 0.32 |
| | | N. weight: | 0.97 |
| | | Sc. PD: | 0.0057 |
| | | Sc. rank: | 14.5 |

| HA | | | |
|---|---|---|---|
| Pos. | 227 | obs: | exp: |
| gaa | E | 4 | 2.30 |
| gag | E | 0 | 1.70 |
| ggt | G | 1 | 78.60 |
| ggc | G | 0 | 86.14 |
| gga | G | 541 | 308.10 |
| ggg | G | 95 | 164.10 |
| --- | -- | ------ | ------ |
| mPD | | 0.27 | 0.68 |
| | | nPD: | 0.4 |
| | | N. weight: | 0.58 |
| | | Sc. PD: | 0.045 |
| | | Sc. rank: | 68.3 |

| HA | | | |
|---|---|---|---|
| Pos. | 228 | obs: | exp: |
| act | T | 0 | 1.06 |
| acc | T | 0 | 0.86 |
| aca | T | 3 | 1.75 |
| acg | T | 1 | 0.33 |
| gtt | V | 0 | 0.21 |
| gtc | V | 0 | 0.21 |
| gta | V | 0 | 0.19 |
| gtg | V | 1 | 0.40 |
| gct | A | 0 | 159.90 |
| gcc | A | 0 | 130.10 |
| gca | A | 113 | 283.00 |
| gcg | A | 522 | 61.96 |
| gaa | E | 0 | 0.58 |
| gag | E | 1 | 0.42 |
| --- | -- | ------ | ------ |
| mPD | | 0.32 | 0.71 |
| | | nPD: | 0.45 |
| | | N. weight: | 2.3 |
| | | Sc. PD: | 0.29 |
| | | Sc. rank: | 396.7 |

| HA | | | |
|---|---|---|---|
| Pos. | 229 | obs: | exp: |
| cgt | R | 0 | 24.83 |
| cgc | R | 0 | 29.28 |
| cga | R | 72 | 57.47 |
| cgg | R | 5 | 49.90 |
| aga | R | 371 | 268.90 |
| agg | R | 150 | 167.60 |
| ggt | G | 0 | 5.31 |
| ggc | G | 0 | 5.81 |
| gga | G | 43 | 20.80 |
| ggg | G | 0 | 11.08 |
| --- | -- | ------ | ------ |
| mPD | | 0.69 | 1.0 |
| | | nPD: | 0.66 |
| | | N. weight: | 0.33 |
| | | Sc. PD: | 0.11 |
| | | Sc. rank: | 109.2 |

| HA | | | |
|---|---|---|---|
| Pos. | 230 | obs: | exp: |
| cct | P | 0 | 168.80 |
| ccc | P | 0 | 115.60 |
| cca | P | 641 | 253.00 |
| ccg | P | 0 | 103.60 |
| --- | -- | ------ | ------ |
| mPD | | 0 | 0.72 |
| | | nPD: | 0. |
| | | N. weight: | 1.4 |
| | | Sc. PD: | -0.41 |
| | | Sc. rank: | -514.4 |

| HA | | | |
|---|---|---|---|
| Pos. | 231 | obs: | exp: |
| cct | P | 0 | 0.26 |
| ccc | P | 0 | 0.18 |
| cca | P | 1 | 0.39 |
| ccg | P | 0 | 0.16 |
| caa | Q | 566 | 340.70 |
| cag | Q | 73 | 298.30 |
| aaa | K | 1 | 0.56 |
| aag | K | 0 | 0.44 |
| --- | -- | ------ | ------ |
| mPD | | 0.21 | 0.50 |
| | | nPD: | 0.41 |
| | | N. weight: | 0.43 |
| | | Sc. PD: | 0.04 |
| | | Sc. rank: | 60.9 |

| HA | | | |
|---|---|---|---|
| Pos. | 232 | obs: | exp: |
| gtt | V | 100 | 131.80 |
| gtc | V | 0 | 134.50 |
| gta | V | 8 | 120.10 |
| gtg | V | 533 | 254.60 |
| --- | -- | ------ | ------ |
| mPD | | 0.28 | 0.72 |
| | | nPD: | 0.39 |
| | | N. weight: | 0.8 |
| | | Sc. PD: | 0.061 |
| | | Sc. rank: | 93.2 |

| HA | | | |
|---|---|---|---|
| Pos. | 233 | obs: | exp: |
| aat | N | 625 | 334.50 |
| aac | N | 15 | 305.50 |
| aaa | K | 1 | 0.56 |
| aag | K | 0 | 0.44 |
| --- | -- | ------ | ------ |
| mPD | | 0.049 | 0.50 |
| | | nPD: | 0.1 |
| | | N. weight: | 0.8 |
| | | Sc. PD: | -0.17 |
| | | Sc. rank: | -169.7 |

| HA | | | |
|---|---|---|---|
| Pos. | 234 | obs: | exp: |
| ggt | G | 65 | 79.09 |
| ggc | G | 493 | 86.68 |
| gga | G | 44 | 310.10 |
| ggg | G | 39 | 165.10 |
| --- | -- | ------ | ------ |
| mPD | | 0.39 | 0.67 |
| | | nPD: | 0.59 |
| | | N. weight: | 1.6 |
| | | Sc. PD: | 0.42 |
| | | Sc. rank: | 471.9 |

| HA | | | |
|---|---|---|---|
| Pos. | 235 | obs: | exp: |
| caa | Q | 626 | 341.70 |
| cag | Q | 15 | 299.30 |
| --- | -- | ------ | ------ |
| mPD | | 0.046 | 0.50 |
| | | nPD: | 0.09 |
| | | N. weight: | 0.77 |
| | | Sc. PD: | -0.16 |
| | | Sc. rank: | -166.7 |

| | | | |
|---|---|---|---|
| HA | | | |
| Pos. | 236 | obs: | exp: |
| tct | S | 529 | 101.60 |
| tcc | S | 34 | 89.02 |
| tca | S | 65 | 141.60 |
| tcg | S | 11 | 38.95 |
| cct | P | 2 | 0.53 |
| ccc | P | 0 | 0.36 |
| cca | P | 0 | 0.79 |
| ccg | P | 0 | 0.32 |
| agt | S | 0 | 140.60 |
| agc | S | 0 | 127.30 |
| --- | -- | ------ | ------ |
| mPD | | 0.31 | 1.7 |
| | | nPD: | 0.18 |
| | | N. weight: | 1.8 |
| | | Sc. PD: | -0.23 |
| | | Sc. rank: | -248.1 |

,

| | | | |
|---|---|---|---|
| HA | | | |
| Pos. | 237 | obs: | exp: |
| ggt | G | 0 | 79.09 |
| ggc | G | 0 | 86.68 |
| gga | G | 590 | 310.10 |
| ggg | G | 51 | 165.10 |
| --- | -- | ------ | ------ |
| mPD | | 0.15 | 0.67 |
| | | nPD: | 0.22 |
| | | N. weight: | 0.74 |
| | | Sc. PD: | -0.067 |
| | | Sc. rank: | -78.9 |

,

| | | | |
|---|---|---|---|
| HA | | | |
| Pos. | 238 | obs: | exp: |
| cgt | R | 0 | 26.61 |
| cgc | R | 0 | 31.39 |
| cga | R | 2 | 61.60 |
| cgg | R | 5 | 53.49 |
| aga | R | 316 | 288.30 |
| agg | R | 318 | 179.60 |
| --- | -- | ------ | ------ |
| mPD | | 0.52 | 0.96 |
| | | nPD: | 0.54 |
| | | N. weight: | 0.45 |
| | | Sc. PD: | 0.1 |
| | | Sc. rank: | 113.5 |

,

| | | | |
|---|---|---|---|
| HA | | | |
| Pos. | 239 | obs: | exp: |
| att | I | 630 | 241.00 |
| atc | I | 10 | 168.00 |
| ata | I | 1 | 232.00 |
| --- | -- | ------ | ------ |
| mPD | | 0.034 | 0.66 |
| | | nPD: | 0.05 |
| | | N. weight: | 1.3 |
| | | Sc. PD: | -0.33 |
| | | Sc. rank: | -340.6 |

,

| | | | |
|---|---|---|---|
| HA | | | |
| Pos. | 240 | obs: | exp: |
| aat | N | 0 | 2.09 |
| aac | N | 4 | 1.91 |
| gat | D | 97 | 339.90 |
| gac | D | 540 | 297.10 |
| --- | -- | ------ | ------ |
| mPD | | 0.27 | 0.51 |
| | | nPD: | 0.53 |
| | | N. weight: | 0.47 |
| | | Sc. PD: | 0.097 |
| | | Sc. rank: | 114.2 |

,

| | | | |
|---|---|---|---|
| HA | | | |
| Pos. | 241 | obs: | exp: |
| ttt | F | 618 | 263.50 |
| ttc | F | 23 | 377.50 |
| --- | -- | ------ | ------ |
| mPD | | 0.069 | 0.48 |
| | | nPD: | 0.14 |
| | | N. weight: | 1.1 |
| | | Sc. PD: | -0.18 |
| | | Sc. rank: | -181.6 |

,

| | | | |
|---|---|---|---|
| HA | | | |
| Pos. | 242 | obs: | exp: |
| cat | H | 581 | 347.30 |
| cac | H | 60 | 293.70 |
| --- | -- | ------ | ------ |
| mPD | | 0.17 | 0.50 |
| | | nPD: | 0.34 |
| | | N. weight: | 0.47 |
| | | Sc. PD: | 0.012 |
| | | Sc. rank: | 19.7 |

,

| | | | |
|---|---|---|---|
| HA | | | |
| Pos. | 243 | obs: | exp: |
| tgg | W | 641 | 641.00 |
| --- | -- | ------ | ------ |
| mPD | | 0 | 0 |
| | | nPD: | 1. |
| | | N. weight: | 0. |
| | | Sc. PD: | 0 |
| | | Sc. rank: | 0 |

,

| | | | |
|---|---|---|---|
| HA | | | |
| Pos. | 244 | obs: | exp: |
| tta | L | 30 | 53.29 |
| ttg | L | 11 | 117.50 |
| ctt | L | 0 | 113.80 |
| ctc | L | 0 | 98.67 |
| cta | L | 122 | 91.90 |
| ctg | L | 447 | 134.80 |
| atg | M | 31 | 31.00 |
| --- | -- | ------ | ------ |
| mPD | | 0.57 | 1.2 |
| | | nPD: | 0.49 |
| | | N. weight: | 1.2 |
| | | Sc. PD: | 0.21 |
| | | Sc. rank: | 265.9 |

,

| | | | |
|---|---|---|---|
| HA | | | |
| Pos. | 245 | obs: | exp: |
| tta | L | 0 | 6.20 |
| ttg | L | 0 | 13.67 |
| ctt | L | 4 | 13.25 |
| ctc | L | 66 | 11.48 |
| cta | L | 1 | 10.70 |
| ctg | L | 0 | 15.69 |
| att | I | 0 | 112.80 |
| atc | I | 1 | 78.62 |
| ata | I | 299 | 108.60 |
| atg | M | 269 | 269.00 |
| gtt | V | 0 | 0.21 |
| gtc | V | 0 | 0.21 |
| gta | V | 0 | 0.19 |
| gtg | V | 1 | 0.40 |
| --- | -- | ------ | ------ |
| mPD | | 0.79 | 0.89 |
| | | nPD: | 0.89 |
| | | N. weight: | 0.94 |
| | | Sc. PD: | 0.52 |
| | | Sc. rank: | 352.2 |

,

| | | | |
|---|---|---|---|
| HA | | | |
| Pos. | 246 | obs: | exp: |
| tta | L | 10 | 56.00 |
| ttg | L | 0 | 123.50 |
| ctt | L | 72 | 119.60 |
| ctc | L | 0 | 103.70 |
| cta | L | 541 | 96.57 |
| ctg | L | 18 | 141.70 |
| --- | -- | ------ | ------ |
| mPD | | 0.28 | 1.1 |
| | | nPD: | 0.25 |
| | | N. weight: | 1.9 |
| | | Sc. PD: | -0.12 |
| | | Sc. rank: | -146.4 |

,

| | | | |
|---|---|---|---|
| HA | | | |
| Pos. | 247 | obs: | exp: |
| aat | N | 108 | 285.90 |
| aac | N | 439 | 261.10 |
| gat | D | 51 | 50.15 |
| gac | D | 43 | 43.85 |
| --- | -- | ------ | ------ |
| mPD | | 0.62 | 0.75 |
| | | nPD: | 0.83 |
| | | N. weight: | 0.29 |
| | | Sc. PD: | 0.15 |
| | | Sc. rank: | 109.1 |

,

| | | | |
|---|---|---|---|
| HA | | | |
| Pos. | 248 | obs: | exp: |
| tct | S | 0 | 0.95 |
| tcc | S | 6 | 0.84 |
| tca | S | 0 | 1.33 |
| tcg | S | 0 | 0.37 |
| cct | P | 243 | 167.20 |
| ccc | P | 391 | 114.50 |
| cca | P | 1 | 250.60 |
| ccg | P | 0 | 102.70 |
| agt | S | 0 | 1.32 |
| agc | S | 0 | 1.20 |
| --- | -- | ------ | ------ |
| mPD | | 0.49 | 0.74 |
| | | nPD: | 0.66 |
| | | N. weight: | 1.3 |
| | | Sc. PD: | 0.44 |
| | | Sc. rank: | 435.3 |

,

| | | | |
|---|---|---|---|
| HA | | | |
| Pos. | 249 | obs: | exp: |
| aat | N | 594 | 335.00 |
| aac | N | 47 | 306.00 |
| --- | -- | ------ | ------ |
| mPD | | 0.14 | 0.50 |
| | | nPD: | 0.27 |
| | | N. weight: | 0.58 |
| | | Sc. PD: | -0.024 |
| | | Sc. rank: | -28.1 |

,

| | | | |
|---|---|---|---|
| HA | | | |
| Pos. | 250 | obs: | exp: |
| gat | D | 61 | 342.00 |
| gac | D | 580 | 299.00 |
| --- | -- | ------ | ------ |
| mPD | | 0.17 | 0.50 |
| | | nPD: | 0.35 |
| | | N. weight: | 0.64 |
| | | Sc. PD: | 0.019 |
| | | Sc. rank: | 29.1 |

,

| | | | |
|---|---|---|---|
| HA | | | |
| Pos. | 251 | obs: | exp: |
| act | T | 0 | 169.30 |
| acc | T | 2 | 137.50 |
| aca | T | 392 | 280.80 |
| acg | T | 247 | 53.29 |
| --- | -- | ------ | ------ |
| mPD | | 0.48 | 0.69 |
| | | nPD: | 0.7 |
| | | N. weight: | 1.2 |
| | | Sc. PD: | 0.42 |
| | | Sc. rank: | 387.4 |

,

| | | | |
|---|---|---|---|
| HA | | | |
| Pos. | 252 | obs: | exp: |
| att | I | 26 | 10.15 |
| atc | I | 1 | 7.08 |
| ata | I | 0 | 9.77 |
| gtt | V | 170 | 126.20 |
| gtc | V | 372 | 128.90 |
| gta | V | 1 | 115.10 |
| gtg | V | 71 | 243.90 |
| --- | -- | ------ | ------ |
| mPD | | 0.64 | 0.81 |
| | | nPD: | 0.79 |
| | | N. weight: | 0.88 |
| | | Sc. PD: | 0.4 |
| | | Sc. rank: | 316.9 |

,

| | | | |
|---|---|---|---|
| HA | | | |
| Pos. | 253 | obs: | exp: |
| act | T | 518 | 169.30 |
| acc | T | 123 | 137.50 |
| aca | T | 0 | 280.80 |
| acg | T | 0 | 53.29 |
| --- | -- | ------ | ------ |
| mPD | | 0.31 | 0.69 |
| | | nPD: | 0.45 |
| | | N. weight: | 1.3 |
| | | Sc. PD: | 0.17 |
| | | Sc. rank: | 229.6 |

,

| | | | |
|---|---|---|---|
| HA | | | |
| Pos. | 254 | obs: | exp: |
| ttt | F | 12 | 263.50 |
| ttc | F | 629 | 377.50 |
| --- | -- | ------ | ------ |
| mPD | | 0.037 | 0.48 |
| | | nPD: | 0.08 |
| | | N. weight: | 0.65 |
| | | Sc. PD: | -0.15 |
| | | Sc. rank: | -150.0 |

,

| | | | |
|---|---|---|---|
| HA | | | |
| Pos. | 255 | obs: | exp: |
| tct | S | 0 | 88.39 |
| tcc | S | 0 | 77.45 |
| tca | S | 0 | 123.20 |
| tcg | S | 0 | 33.89 |
| act | T | 67 | 18.76 |
| acc | T | 4 | 15.24 |
| aca | T | 0 | 31.10 |
| acg | T | 0 | 5.90 |
| aat | N | 1 | 0.52 |
| aac | N | 0 | 0.48 |
| agt | S | 502 | 122.30 |
| agc | S | 54 | 110.80 |
| ggt | G | 13 | 1.60 |
| ggc | G | 0 | 1.76 |
| gga | G | 0 | 6.29 |
| ggg | G | 0 | 3.35 |
| --- | -- | ------ | ------ |
| mPD | | 0.40 | 1.7 |
| | | nPD: | 0.24 |
| | | N. weight: | 1.8 |
| | | Sc. PD: | -0.13 |
| | | Sc. rank: | -160.0 |

,

| | | | |
|---|---|---|---|
| HA | | | |
| Pos. | 256 | obs: | exp: |
| ttt | F | 4 | 263.50 |
| ttc | F | 637 | 377.50 |
| --- | -- | ------ | ------ |
| mPD | | 0.012 | 0.48 |
| | | nPD: | 0.03 |
| | | N. weight: | 0.73 |
| | | Sc. PD: | -0.2 |
| | | Sc. rank: | -213.4 |

,

| | | | |
|---|---|---|---|
| HA | | | |
| Pos. | 257 | obs: | exp: |
| aat | N | 640 | 335.00 |
| aac | N | 1 | 306.00 |
| --- | -- | ------ | ------ |
| mPD | | 0.0031 | 0.50 |
| | | nPD: | 0.01 |
| | | N. weight: | 0.94 |
| | | Sc. PD: | -0.28 |
| | | Sc. rank: | -319.3 |

,

| | | | |
|---|---|---|---|
| HA | | | |
| Pos. | 258 | obs: | exp: |
| ggt | G | 5 | 79.09 |
| ggc | G | 0 | 86.68 |
| gga | G | 2 | 310.10 |
| ggg | G | 634 | 165.10 |
| --- | -- | ------ | ------ |
| mPD | | 0.022 | 0.67 |
| | | nPD: | 0.03 |
| | | N. weight: | 1.9 |
| | | Sc. PD: | -0.52 |
| | | Sc. rank: | -538.7 |

,

| | | | |
|---|---|---|---|
| HA | | | |
| Pos. | 259 | obs: | exp: |
| gct | A | 60 | 161.50 |
| gcc | A | 508 | 131.30 |
| gca | A | 67 | 285.70 |
| gcg | A | 6 | 62.55 |
| --- | -- | ------ | ------ |
| mPD | | 0.35 | 0.69 |
| | | nPD: | 0.51 |
| | | N. weight: | 1.2 |
| | | Sc. PD: | 0.23 |
| | | Sc. rank: | 284.6 |

,

| | | | |
|---|---|---|---|
| HA | | | |
| Pos. | 260 | obs: | exp: |
| ttt | F | 11 | 263.50 |
| ttc | F | 630 | 377.50 |
| --- | -- | ------ | ------ |
| mPD | | 0.034 | 0.48 |
| | | nPD: | 0.07 |
| | | N. weight: | 0.66 |
| | | Sc. PD: | -0.16 |
| | | Sc. rank: | -157.6 |

,

| HA | | | |
|---|---|---|---|
| Pos. | 261 | obs: | exp: |
| att | I | 0 | 240.20 |
| atc | I | 23 | 167.50 |
| ata | I | 616 | 231.30 |
| gtt | V | 0 | 0.41 |
| gtc | V | 0 | 0.42 |
| gta | V | 2 | 0.37 |
| gtg | V | 0 | 0.79 |
| --- | -- | ------ | ------ |
| mPD | | 0.076 | 0.67 |
| | | nPD: | 0.11 |
| | | N. weight: | 1.3 |
| | | Sc. PD: | -0.25 |
| | | Sc. rank: | -256.4 |

| HA | | | |
|---|---|---|---|
| Pos. | 262 | obs: | exp: |
| gct | A | 631 | 161.50 |
| gcc | A | 6 | 131.30 |
| gca | A | 4 | 285.70 |
| gcg | A | 0 | 62.55 |
| --- | -- | ------ | ------ |
| mPD | | 0.031 | 0.69 |
| | | nPD: | 0.04 |
| | | N. weight: | 1.9 |
| | | Sc. PD: | -0.49 |
| | | Sc. rank: | -499.5 |

| HA | | | |
|---|---|---|---|
| Pos. | 263 | obs: | exp: |
| cct | P | 68 | 168.80 |
| ccc | P | 3 | 115.60 |
| cca | P | 509 | 253.00 |
| ccg | P | 61 | 103.60 |
| --- | -- | ------ | ------ |
| mPD | | 0.35 | 0.72 |
| | | nPD: | 0.49 |
| | | N. weight: | 0.59 |
| | | Sc. PD: | 0.098 |
| | | Sc. rank: | 126.5 |

| HA | | | |
|---|---|---|---|
| Pos. | 264 | obs: | exp: |
| aat | N | 2 | 1.05 |
| aac | N | 0 | 0.95 |
| gat | D | 107 | 340.90 |
| gac | D | 532 | 298.10 |
| --- | -- | ------ | ------ |
| mPD | | 0.29 | 0.50 |
| | | nPD: | 0.57 |
| | | N. weight: | 0.43 |
| | | Sc. PD: | 0.11 |
| | | Sc. rank: | 120.8 |

| HA | | | |
|---|---|---|---|
| Pos. | 265 | obs: | exp: |
| cgt | R | 477 | 26.61 |
| cgc | R | 92 | 31.39 |
| cga | R | 0 | 61.60 |
| cgg | R | 0 | 53.49 |
| aga | R | 61 | 288.30 |
| agg | R | 11 | 179.60 |
| --- | -- | ------ | ------ |
| mPD | | 0.62 | 0.96 |
| | | nPD: | 0.64 |
| | | N. weight: | 3.1 |
| | | Sc. PD: | 0.97 |
| | | Sc. rank: | 990.3 |

| HA | | | |
|---|---|---|---|
| Pos. | 266 | obs: | exp: |
| gtt | V | 0 | 0.21 |
| gtc | V | 0 | 0.21 |
| gta | V | 1 | 0.19 |
| gtg | V | 0 | 0.40 |
| gct | A | 3 | 161.20 |
| gcc | A | 0 | 131.10 |
| gca | A | 637 | 285.20 |
| gcg | A | 0 | 62.45 |
| --- | -- | ------ | ------ |
| mPD | | 0.012 | 0.69 |
| | | nPD: | 0.02 |
| | | N. weight: | 1.1 |
| | | Sc. PD: | -0.33 |
| | | Sc. rank: | -354.2 |

| HA | | | |
|---|---|---|---|
| Pos. | 267 | obs: | exp: |
| tct | S | 0 | 100.60 |
| tcc | S | 0 | 88.18 |
| tca | S | 0 | 140.20 |
| tcg | S | 0 | 38.58 |
| tgt | C | 0 | 1.65 |
| tgc | C | 4 | 2.35 |
| act | T | 1 | 0.26 |
| acc | T | 0 | 0.21 |
| aca | T | 0 | 0.44 |
| acg | T | 0 | 0.08 |
| aat | N | 0 | 1.05 |
| aac | N | 2 | 0.95 |
| agt | S | 108 | 139.30 |
| agc | S | 525 | 126.10 |
| ggt | G | 0 | 0.12 |
| ggc | G | 1 | 0.14 |
| gga | G | 0 | 0.48 |
| ggg | G | 0 | 0.26 |
| --- | -- | ------ | ------ |
| mPD | | 0.31 | 1.7 |
| | | nPD: | 0.18 |
| | | N. weight: | 1.7 |
| | | Sc. PD: | -0.21 |
| | | Sc. rank: | -227.2 |

| HA | | | |
|---|---|---|---|
| Pos. | 268 | obs: | exp: |
| ttt | F | 459 | 263.10 |
| ttc | F | 181 | 376.90 |
| tat | Y | 1 | 0.52 |
| tac | Y | 0 | 0.48 |
| --- | -- | ------ | ------ |
| mPD | | 0.41 | 0.49 |
| | | nPD: | 0.84 |
| | | N. weight: | 0.29 |
| | | Sc. PD: | 0.15 |
| | | Sc. rank: | 107.6 |

| HA | | | |
|---|---|---|---|
| Pos. | 269 | obs: | exp: |
| ttt | F | 71 | 29.19 |
| ttc | F | 0 | 41.81 |
| tta | L | 1 | 49.45 |
| ttg | L | 94 | 109.00 |
| ctt | L | 1 | 105.60 |
| ctc | L | 0 | 91.56 |
| cta | L | 1 | 85.27 |
| ctg | L | 469 | 125.10 |
| atg | M | 3 | 3.00 |
| gtt | V | 0 | 0.21 |
| gtc | V | 0 | 0.21 |
| gta | V | 0 | 0.19 |
| gtg | V | 1 | 0.40 |
| --- | -- | ------ | ------ |
| mPD | | 0.60 | 1.2 |
| | | nPD: | 0.5 |
| | | N. weight: | 1.5 |
| | | Sc. PD: | 0.27 |
| | | Sc. rank: | 345.2 |

| HA | | | |
|---|---|---|---|
| Pos. | 270 | obs: | exp: |
| cgt | R | 0 | 26.15 |
| cgc | R | 0 | 30.85 |
| cga | R | 0 | 60.55 |
| cgg | R | 0 | 52.57 |
| aaa | K | 8 | 6.16 |
| aag | K | 3 | 4.84 |
| aga | R | 614 | 283.30 |
| agg | R | 16 | 176.60 |
| --- | -- | ------ | ------ |
| mPD | | 0.091 | 0.99 |
| | | nPD: | 0.09 |
| | | N. weight: | 1. |
| | | Sc. PD: | -0.22 |
| | | Sc. rank: | -217.1 |

| HA | | | |
|---|---|---|---|
| Pos. | 271 | obs: | exp: |
| ggt | G | 0 | 79.09 |
| ggc | G | 0 | 86.68 |
| gga | G | 97 | 310.10 |
| ggg | G | 544 | 165.10 |
| --- | -- | ------ | ------ |
| mPD | | 0.26 | 0.67 |
| | | nPD: | 0.39 |
| | | N. weight: | 1.2 |
| | | Sc. PD: | 0.084 |
| | | Sc. rank: | 133.0 |

| HA | | | |
|---|---|---|---|
| Pos. | 272 | obs: | exp: |
| cgt | R | 0 | 0.08 |
| cgc | R | 0 | 0.10 |
| cga | R | 0 | 0.19 |
| cgg | R | 0 | 0.17 |
| aat | N | 2 | 1.05 |
| aac | N | 0 | 0.95 |
| aaa | K | 144 | 316.90 |
| aag | K | 422 | 249.10 |
| aga | R | 2 | 0.90 |
| agg | R | 0 | 0.56 |
| gat | D | 0 | 0.53 |
| gac | D | 1 | 0.47 |
| gaa | E | 7 | 40.28 |
| gag | E | 63 | 29.72 |
| --- | -- | ------ | ------ |
| mPD | | 0.57 | 0.70 |
| | | nPD: | 0.82 |
| | | N. weight: | 0.35 |
| | | Sc. PD: | 0.17 |
| | | Sc. rank: | 129.2 |

| HA | | | |
|---|---|---|---|
| Pos. | 273 | obs: | exp: |
| tct | S | 307 | 101.90 |
| tcc | S | 263 | 89.29 |
| tca | S | 54 | 142.00 |
| tcg | S | 17 | 39.07 |
| agt | S | 0 | 141.00 |
| agc | S | 0 | 127.70 |
| --- | -- | ------ | ------ |
| mPD | | 0.60 | 1.7 |
| | | nPD: | 0.36 |
| | | N. weight: | 1.3 |
| | | Sc. PD: | 0.053 |
| | | Sc. rank: | 94.0 |

| HA | | | |
|---|---|---|---|
| Pos. | 274 | obs: | exp: |
| tta | L | 0 | 6.29 |
| ttg | L | 0 | 13.87 |
| ctt | L | 0 | 13.43 |
| ctc | L | 1 | 11.65 |
| cta | L | 69 | 10.85 |
| ctg | L | 2 | 15.92 |
| att | I | 0 | 6.77 |
| atc | I | 0 | 4.72 |
| ata | I | 18 | 6.52 |
| atg | M | 550 | 550.00 |
| act | T | 0 | 0.26 |
| acc | T | 0 | 0.21 |
| aca | T | 0 | 0.44 |
| acg | T | 1 | 0.08 |
| --- | -- | ------ | ------ |
| mPD | | 0.44 | 0.39 |
| | | nPD: | 1.14 |
| | | N. weight: | 0.33 |
| | | Sc. PD: | 0.26 |
| | | Sc. rank: | 124.6 |

| HA | | | |
|---|---|---|---|
| Pos. | 275 | obs: | exp: |
| ggt | G | 1 | 79.09 |
| ggc | G | 0 | 86.68 |
| gga | G | 632 | 310.10 |
| ggg | G | 8 | 165.10 |
| --- | -- | ------ | ------ |
| mPD | | 0.028 | 0.67 |
| | | nPD: | 0.04 |
| | | N. weight: | 0.97 |
| | | Sc. PD: | -0.25 |
| | | Sc. rank: | -269.3 |

| HA | | | |
|---|---|---|---|
| Pos. | 276 | obs: | exp: |
| att | I | 504 | 212.00 |
| atc | I | 57 | 147.80 |
| ata | I | 3 | 204.20 |
| gtt | V | 70 | 15.83 |
| gtc | V | 7 | 16.16 |
| gta | V | 0 | 14.43 |
| gtg | V | 0 | 30.58 |
| --- | -- | ------ | ------ |
| mPD | | 0.40 | 0.90 |
| | | nPD: | 0.44 |
| | | N. weight: | 1.1 |
| | | Sc. PD: | 0.13 |
| | | Sc. rank: | 175.2 |

| HA | | | |
|---|---|---|---|
| Pos. | 277 | obs: | exp: |
| caa | Q | 21 | 341.70 |
| cag | Q | 620 | 299.30 |
| --- | -- | ------ | ------ |
| mPD | | 0.063 | 0.50 |
| | | nPD: | 0.13 |
| | | N. weight: | 0.9 |
| | | Sc. PD: | -0.16 |
| | | Sc. rank: | -170.7 |

| HA | | | |
|---|---|---|---|
| Pos. | 278 | obs: | exp: |
| tct | S | 0 | 101.90 |
| tcc | S | 0 | 89.29 |
| tca | S | 0 | 142.00 |
| tcg | S | 0 | 39.07 |
| agt | S | 637 | 141.00 |
| agc | S | 4 | 127.70 |
| --- | -- | ------ | ------ |
| mPD | | 0.012 | 1.7 |
| | | nPD: | 0.01 |
| | | N. weight: | 2.2 |
| | | Sc. PD: | -0.64 |
| | | Sc. rank: | -715.9 |

| HA | | | |
|---|---|---|---|
| Pos. | 279 | obs: | exp: |
| aat | N | 3 | 1.57 |
| aac | N | 0 | 1.43 |
| aaa | K | 1 | 0.56 |
| aag | K | 0 | 0.44 |
| gat | D | 66 | 42.15 |
| gac | D | 13 | 36.85 |
| gaa | E | 262 | 153.60 |
| gag | E | 5 | 113.40 |
| ggt | G | 4 | 35.91 |
| ggc | G | 0 | 39.35 |
| gga | G | 245 | 140.80 |
| ggg | G | 42 | 74.97 |
| --- | -- | ------ | ------ |
| mPD | | 0.86 | 1.2 |
| | | nPD: | 0.73 |
| | | N. weight: | 0.58 |
| | | Sc. PD: | 0.23 |
| | | Sc. rank: | 199.6 |

| HA | | | |
|---|---|---|---|
| Pos. | 280 | obs: | exp: |
| atg | M | 1 | 1.00 |
| gtt | V | 68 | 131.50 |
| gtc | V | 1 | 134.30 |
| gta | V | 555 | 119.90 |
| gtg | V | 16 | 254.20 |
| --- | -- | ------ | ------ |
| mPD | | 0.24 | 0.72 |
| | | nPD: | 0.33 |
| | | N. weight: | 1.7 |
| | | Sc. PD: | 0.031 |
| | | Sc. rank: | 65.0 |

| HA | | | |
|---|---|---|---|
| Pos. | 281 | obs: | exp: |
| cct | P | 72 | 19.75 |
| ccc | P | 0 | 13.53 |
| cca | P | 1 | 29.60 |
| ccg | P | 2 | 12.13 |
| caa | Q | 49 | 301.70 |
| cag | Q | 517 | 264.30 |
| --- | -- | ------ | ------ |
| mPD | | 0.53 | 0.75 |
| | | nPD: | 0.71 |
| | | N. weight: | 0.8 |
| | | Sc. PD: | 0.3 |
| | | Sc. rank: | 270.6 |

,

| HA | | | |
|---|---|---|---|
| Pos. | 282 | obs: | exp: |
| tta | L | 2 | 6.20 |
| ttg | L | 69 | 13.67 |
| ctt | L | 0 | 13.25 |
| ctc | L | 0 | 11.48 |
| cta | L | 0 | 10.70 |
| ctg | L | 0 | 15.69 |
| att | I | 13 | 5.26 |
| atc | I | 1 | 3.67 |
| ata | I | 0 | 5.07 |
| atg | M | 4 | 4.00 |
| gtt | V | 518 | 113.50 |
| gtc | V | 34 | 115.90 |
| gta | V | 0 | 103.50 |
| gtg | V | 0 | 219.20 |
| --- | -- | ------ | ------ |
| mPD | | 0.54 | 0.97 |
| | | nPD: | 0.56 |
| | | N. weight: | 2. |
| | | Sc. PD: | 0.47 |
| | | Sc. rank: | 539.7 |

,

| HA | | | |
|---|---|---|---|
| Pos. | 283 | obs: | exp: |
| aat | N | 4 | 2.09 |
| aac | N | 0 | 1.91 |
| gat | D | 603 | 339.90 |
| gac | D | 34 | 297.10 |
| --- | -- | ------ | ------ |
| mPD | | 0.11 | 0.51 |
| | | nPD: | 0.22 |
| | | N. weight: | 0.63 |
| | | Sc. PD: | -0.057 |
| | | Sc. rank: | -66.0 |

,

| HA | | | |
|---|---|---|---|
| Pos. | 284 | obs: | exp: |
| tct | S | 70 | 11.29 |
| tcc | S | 1 | 9.89 |
| tca | S | 0 | 15.73 |
| tcg | S | 0 | 4.33 |
| cct | P | 1 | 0.26 |
| ccc | P | 0 | 0.18 |
| cca | P | 0 | 0.39 |
| ccg | P | 0 | 0.16 |
| act | T | 0 | 2.64 |
| acc | T | 0 | 2.15 |
| aca | T | 10 | 4.38 |
| acg | T | 0 | 0.83 |
| agt | S | 0 | 15.62 |
| agc | S | 0 | 14.15 |
| gtt | V | 0 | 0.82 |
| gtc | V | 4 | 0.84 |
| gta | V | 0 | 0.75 |
| gtg | V | 0 | 1.59 |
| gct | A | 1 | 139.80 |
| gcc | A | 413 | 113.70 |
| gca | A | 137 | 247.40 |
| gcg | A | 4 | 54.16 |
| --- | -- | ------ | ------ |
| mPD | | 0.75 | 1.0 |
| | | nPD: | 0.73 |
| | | N. weight: | 1.3 |
| | | Sc. PD: | 0.53 |
| | | Sc. rank: | 458.9 |

,

| HA | | | |
|---|---|---|---|
| Pos. | 285 | obs: | exp: |
| tct | S | 0 | 18.76 |
| tcc | S | 0 | 16.44 |
| tca | S | 0 | 26.14 |
| tcg | S | 0 | 7.19 |
| aat | N | 460 | 240.90 |
| aac | N | 1 | 220.10 |
| agt | S | 117 | 25.96 |
| agc | S | 1 | 23.51 |
| gat | D | 8 | 4.27 |
| gac | D | 0 | 3.73 |
| ggt | G | 54 | 6.66 |
| ggc | G | 0 | 7.30 |
| gga | G | 0 | 26.12 |
| ggg | G | 0 | 13.91 |
| --- | -- | ------ | ------ |
| mPD | | 0.57 | 1.4 |
| | | nPD: | 0.42 |
| | | N. weight: | 1.3 |
| | | Sc. PD: | 0.13 |
| | | Sc. rank: | 194.4 |

,

| HA | | | |
|---|---|---|---|
| Pos. | 286 | obs: | exp: |
| tgt | C | 625 | 264.20 |
| tgc | C | 16 | 376.80 |
| --- | -- | ------ | ------ |
| mPD | | 0.049 | 0.48 |
| | | nPD: | 0.1 |
| | | N. weight: | 1.1 |
| | | Sc. PD: | -0.23 |
| | | Sc. rank: | -237.0 |

,

| HA | | | |
|---|---|---|---|
| Pos. | 287 | obs: | exp: |
| gtt | V | 0 | 0.21 |
| gtc | V | 0 | 0.21 |
| gta | V | 0 | 0.19 |
| gtg | V | 1 | 0.40 |
| gct | A | 0 | 0.25 |
| gcc | A | 0 | 0.20 |
| gca | A | 1 | 0.45 |
| gcg | A | 0 | 0.10 |
| gat | D | 0 | 0.53 |
| gac | D | 1 | 0.47 |
| gaa | E | 558 | 365.40 |
| gag | E | 77 | 269.60 |
| ggt | G | 0 | 0.37 |
| ggc | G | 0 | 0.41 |
| gga | G | 2 | 1.45 |
| ggg | G | 1 | 0.77 |
| --- | -- | ------ | ------ |
| mPD | | 0.23 | 0.51 |
| | | nPD: | 0.46 |
| | | N. weight: | 0.34 |
| | | Sc. PD: | 0.047 |
| | | Sc. rank: | 62.3 |

,

| HA | | | |
|---|---|---|---|
| Pos. | 288 | obs: | exp: |
| ggt | G | 0 | 79.09 |
| ggc | G | 0 | 86.68 |
| gga | G | 469 | 310.10 |
| ggg | G | 172 | 165.10 |
| --- | -- | ------ | ------ |
| mPD | | 0.39 | 0.67 |
| | | nPD: | 0.59 |
| | | N. weight: | 0.47 |
| | | Sc. PD: | 0.12 |
| | | Sc. rank: | 136.1 |

,

| HA | | | |
|---|---|---|---|
| Pos. | 289 | obs: | exp: |
| aat | N | 8 | 4.18 |
| aac | N | 0 | 3.82 |
| gat | D | 573 | 334.50 |
| gac | D | 54 | 292.50 |
| gaa | E | 4 | 2.30 |
| gag | E | 0 | 1.70 |
| ggt | G | 2 | 0.25 |
| ggc | G | 0 | 0.27 |
| gga | G | 0 | 0.97 |
| ggg | G | 0 | 0.52 |
| --- | -- | ------ | ------ |
| mPD | | 0.20 | 0.54 |
| | | nPD: | 0.37 |
| | | N. weight: | 0.53 |
| | | Sc. PD: | 0.026 |
| | | Sc. rank: | 42.0 |

,

| HA | | | |
|---|---|---|---|
| Pos. | 290 | obs: | exp: |
| tgt | C | 62 | 264.20 |
| tgc | C | 579 | 376.80 |
| --- | -- | ------ | ------ |
| mPD | | 0.18 | 0.48 |
| | | nPD: | 0.36 |
| | | N. weight: | 0.37 |
| | | Sc. PD: | 0.016 |
| | | Sc. rank: | 28.3 |

,

| HA | | | |
|---|---|---|---|
| Pos. | 291 | obs: | exp: |
| ttt | F | 29 | 29.60 |
| ttc | F | 43 | 42.40 |
| tat | Y | 560 | 294.50 |
| tac | Y | 9 | 274.50 |
| --- | -- | ------ | ------ |
| mPD | | 0.35 | 0.70 |
| | | nPD: | 0.5 |
| | | N. weight: | 0.76 |
| | | Sc. PD: | 0.13 |
| | | Sc. rank: | 169.0 |

,

| HA | | | |
|---|---|---|---|
| Pos. | 292 | obs: | exp: |
| tat | Y | 8 | 4.14 |
| tac | Y | 0 | 3.86 |
| cat | H | 504 | 343.00 |
| cac | H | 129 | 290.00 |
| --- | -- | ------ | ------ |
| mPD | | 0.35 | 0.52 |
| | | nPD: | 0.67 |
| | | N. weight: | 0.22 |
| | | Sc. PD: | 0.075 |
| | | Sc. rank: | 73.4 |

,

| HA | | | |
|---|---|---|---|
| Pos. | 293 | obs: | exp: |
| tct | S | 0 | 75.99 |
| tcc | S | 0 | 66.59 |
| tca | S | 0 | 105.90 |
| tcg | S | 0 | 29.14 |
| aat | N | 161 | 84.67 |
| aac | N | 1 | 77.33 |
| agt | S | 475 | 105.20 |
| agc | S | 3 | 95.23 |
| ggt | G | 1 | 0.12 |
| ggc | G | 0 | 0.14 |
| gga | G | 0 | 0.48 |
| ggg | G | 0 | 0.26 |
| --- | -- | ------ | ------ |
| mPD | | 0.39 | 1.8 |
| | | nPD: | 0.22 |
| | | N. weight: | 1.8 |
| | | Sc. PD: | -0.17 |
| | | Sc. rank: | -204.2 |

,

| HA | | | |
|---|---|---|---|
| Pos. | 294 | obs: | exp: |
| cgt | R | 0 | 0.33 |
| cgc | R | 0 | 0.39 |
| cga | R | 0 | 0.77 |
| cgg | R | 0 | 0.67 |
| aga | R | 8 | 3.60 |
| agg | R | 0 | 2.24 |
| gaa | E | 2 | 1.15 |
| gag | E | 0 | 0.85 |
| ggt | G | 1 | 77.86 |
| ggc | G | 0 | 85.33 |
| gga | G | 551 | 305.20 |
| ggg | G | 79 | 162.60 |
| --- | -- | ------ | ------ |
| mPD | | 0.25 | 0.70 |
| | | nPD: | 0.36 |
| | | N. weight: | 0.63 |
| | | Sc. PD: | 0.027 |
| | | Sc. rank: | 46.4 |

,

| HA | | | |
|---|---|---|---|
| Pos. | 295 | obs: | exp: |
| ggt | G | 66 | 79.09 |
| ggc | G | 9 | 86.68 |
| gga | G | 46 | 310.10 |
| ggg | G | 520 | 165.10 |
| --- | -- | ------ | ------ |
| mPD | | 0.33 | 0.67 |
| | | nPD: | 0.49 |
| | | N. weight: | 1.1 |
| | | Sc. PD: | 0.19 |
| | | Sc. rank: | 238.9 |

,

HA

| Pos. | 296 | obs: | exp: |
|---|---|---|---|
| tct | S | 0 | 0.16 |
| tcc | S | 0 | 0.14 |
| tca | S | 1 | 0.22 |
| tcg | S | 0 | 0.06 |
| act | T | 0 | 168.80 |
| acc | T | 3 | 137.10 |
| aca | T | 564 | 279.90 |
| acg | T | 72 | 53.12 |
| agt | S | 0 | 0.22 |
| agc | S | 0 | 0.20 |
| gct | A | 0 | 0.25 |
| gcc | A | 0 | 0.20 |
| gca | A | 1 | 0.45 |
| gcg | A | 0 | 0.10 |
| --- | -- | ------ | ------ |
| mPD | | 0.21 | 0.69 |
| | | nPD: | 0.31 |
| | | N. weight: | 0.94 |
| | | Sc. PD: | -0.0053 |
| | | Sc. rank: | -7.4 |

,

HA

| Pos. | 297 | obs: | exp: |
|---|---|---|---|
| att | I | 1 | 241.00 |
| atc | I | 0 | 168.00 |
| ata | I | 640 | 232.00 |
| --- | -- | ------ | ------ |
| mPD | | 0.0031 | 0.66 |
| | | nPD: | 0. |
| | | N. weight: | 1.5 |
| | | Sc. PD: | -0.44 |
| | | Sc. rank: | -504.6 |

,

HA

| Pos. | 298 | obs: | exp: |
|---|---|---|---|
| tta | L | 0 | 0.17 |
| ttg | L | 0 | 0.39 |
| ctt | L | 0 | 0.37 |
| ctc | L | 0 | 0.32 |
| cta | L | 2 | 0.30 |
| ctg | L | 0 | 0.44 |
| att | I | 0 | 209.40 |
| atc | I | 0 | 146.00 |
| ata | I | 557 | 201.60 |
| act | T | 0 | 1.32 |
| acc | T | 0 | 1.07 |
| aca | T | 5 | 2.19 |
| acg | T | 0 | 0.42 |
| gtt | V | 0 | 15.83 |
| gtc | V | 2 | 16.16 |
| gta | V | 7 | 14.43 |
| gtg | V | 68 | 30.58 |
| --- | -- | ------ | ------ |
| mPD | | 0.43 | 0.92 |
| | | nPD: | 0.46 |
| | | N. weight: | 1.4 |
| | | Sc. PD: | 0.2 |
| | | Sc. rank: | 266.3 |

,

HA

| Pos. | 299 | obs: | exp: |
|---|---|---|---|
| tct | S | 0 | 101.90 |
| tcc | S | 0 | 89.29 |
| tca | S | 0 | 142.00 |
| tcg | S | 0 | 39.07 |
| agt | S | 536 | 141.00 |
| agc | S | 105 | 127.70 |
| --- | -- | ------ | ------ |
| mPD | | 0.27 | 1.7 |
| | | nPD: | 0.17 |
| | | N. weight: | 1.6 |
| | | Sc. PD: | -0.23 |
| | | Sc. rank: | -251.4 |

,

HA

| Pos. | 300 | obs: | exp: |
|---|---|---|---|
| tct | S | 25 | 12.24 |
| tcc | S | 46 | 10.73 |
| tca | S | 0 | 17.06 |
| tcg | S | 0 | 4.69 |
| cct | P | 0 | 0.26 |
| ccc | P | 0 | 0.18 |
| cca | P | 0 | 0.39 |
| ccg | P | 1 | 0.16 |
| aat | N | 519 | 284.80 |
| aac | N | 26 | 260.20 |
| aaa | K | 18 | 10.08 |
| aag | K | 0 | 7.92 |
| agt | S | 6 | 16.94 |
| agc | S | 0 | 15.34 |
| --- | -- | ------ | ------ |
| mPD | | 0.67 | 0.91 |
| | | nPD: | 0.73 |
| | | N. weight: | 0.8 |
| | | Sc. PD: | 0.32 |
| | | Sc. rank: | 275.4 |

,

HA

| Pos. | 301 | obs: | exp: |
|---|---|---|---|
| ttt | F | 1 | 0.41 |
| ttc | F | 0 | 0.59 |
| tta | L | 6 | 55.91 |
| ttg | L | 555 | 123.30 |
| ctt | L | 0 | 119.40 |
| ctc | L | 0 | 103.50 |
| cta | L | 2 | 96.42 |
| ctg | L | 77 | 141.50 |
| --- | -- | ------ | ------ |
| mPD | | 0.24 | 1.1 |
| | | nPD: | 0.22 |
| | | N. weight: | 1.8 |
| | | Sc. PD: | -0.16 |
| | | Sc. rank: | -191.1 |

,

HA

| Pos. | 302 | obs: | exp: |
|---|---|---|---|
| cct | P | 8 | 168.80 |
| ccc | P | 465 | 115.60 |
| cca | P | 166 | 253.00 |
| ccg | P | 2 | 103.60 |
| --- | -- | ------ | ------ |
| mPD | | 0.41 | 0.72 |
| | | nPD: | 0.57 |
| | | N. weight: | 1.3 |
| | | Sc. PD: | 0.31 |
| | | Sc. rank: | 350.5 |

,

HA

| Pos. | 303 | obs: | exp: |
|---|---|---|---|
| ttt | F | 511 | 263.50 |
| ttc | F | 130 | 377.50 |
| --- | -- | ------ | ------ |
| mPD | | 0.32 | 0.48 |
| | | nPD: | 0.67 |
| | | N. weight: | 0.46 |
| | | Sc. PD: | 0.16 |
| | | Sc. rank: | 152.4 |

,

HA

| Pos. | 304 | obs: | exp: |
|---|---|---|---|
| caa | Q | 50 | 341.70 |
| cag | Q | 591 | 299.30 |
| --- | -- | ------ | ------ |
| mPD | | 0.14 | 0.50 |
| | | nPD: | 0.29 |
| | | N. weight: | 0.71 |
| | | Sc. PD: | -0.018 |
| | | Sc. rank: | -24.5 |

,

HA

| Pos. | 305 | obs: | exp: |
|---|---|---|---|
| aat | N | 54 | 335.00 |
| aac | N | 587 | 306.00 |
| --- | -- | ------ | ------ |
| mPD | | 0.15 | 0.50 |
| | | nPD: | 0.31 |
| | | N. weight: | 0.65 |
| | | Sc. PD: | -0.0037 |
| | | Sc. rank: | -6.0 |

,

HA

| Pos. | 306 | obs: | exp: |
|---|---|---|---|
| att | I | 2 | 240.60 |
| atc | I | 82 | 167.70 |
| ata | I | 556 | 231.70 |
| gtt | V | 1 | 0.21 |
| gtc | V | 0 | 0.21 |
| gta | V | 0 | 0.19 |
| gtg | V | 0 | 0.40 |
| --- | -- | ------ | ------ |
| mPD | | 0.23 | 0.66 |
| | | nPD: | 0.35 |
| | | N. weight: | 0.97 |
| | | Sc. PD: | 0.036 |
| | | Sc. rank: | 64.9 |

,

HA

| Pos. | 307 | obs: | exp: |
|---|---|---|---|
| aat | N | 513 | 308.40 |
| aac | N | 77 | 281.60 |
| gat | D | 5 | 27.21 |
| gac | D | 46 | 23.79 |
| --- | -- | ------ | ------ |
| mPD | | 0.46 | 0.65 |
| | | nPD: | 0.71 |
| | | N. weight: | 0.43 |
| | | Sc. PD: | 0.16 |
| | | Sc. rank: | 145.1 |

,

HA

| Pos. | 308 | obs: | exp: |
|---|---|---|---|
| tct | S | 0 | 89.34 |
| tcc | S | 0 | 78.29 |
| tca | S | 0 | 124.50 |
| tcg | S | 0 | 34.26 |
| cct | P | 64 | 18.96 |
| ccc | P | 6 | 12.99 |
| cca | P | 0 | 28.41 |
| ccg | P | 2 | 11.64 |
| aat | N | 0 | 3.66 |
| aac | N | 7 | 3.34 |
| agt | S | 6 | 123.60 |
| agc | S | 556 | 112.00 |
| --- | -- | ------ | ------ |
| mPD | | 0.62 | 1.8 |
| | | nPD: | 0.35 |
| | | N. weight: | 2.2 |
| | | Sc. PD: | 0.075 |
| | | Sc. rank: | 121.3 |

,

HA

| Pos. | 309 | obs: | exp: |
|---|---|---|---|
| cgt | R | 0 | 26.61 |
| cgc | R | 0 | 31.39 |
| cga | R | 1 | 61.60 |
| cgg | R | 0 | 53.49 |
| aga | R | 218 | 288.30 |
| agg | R | 422 | 179.60 |
| --- | -- | ------ | ------ |
| mPD | | 0.45 | 0.96 |
| | | nPD: | 0.47 |
| | | N. weight: | 0.68 |
| | | Sc. PD: | 0.1 |
| | | Sc. rank: | 135.7 |

,

HA

| Pos. | 310 | obs: | exp: |
|---|---|---|---|
| act | T | 1 | 20.08 |
| acc | T | 0 | 16.31 |
| aca | T | 73 | 33.30 |
| acg | T | 2 | 6.32 |
| gtt | V | 0 | 8.84 |
| gtc | V | 0 | 9.02 |
| gta | V | 43 | 8.06 |
| gtg | V | 0 | 17.08 |
| gct | A | 9 | 131.50 |
| gcc | A | 0 | 106.90 |
| gca | A | 511 | 232.70 |
| gcg | A | 2 | 50.94 |
| --- | -- | ------ | ------ |
| mPD | | 0.38 | 1.0 |
| | | nPD: | 0.37 |
| | | N. weight: | 1.1 |
| | | Sc. PD: | 0.055 |
| | | Sc. rank: | 89.4 |

HA

| Pos. | 311 | obs: | exp: |
|---|---|---|---|
| att | I | 1 | 1.88 |
| atc | I | 0 | 1.31 |
| ata | I | 4 | 1.81 |
| gtt | V | 5 | 130.70 |
| gtc | V | 1 | 133.50 |
| gta | V | 533 | 119.20 |
| gtg | V | 97 | 252.60 |
| --- | -- | ------ | ------ |
| mPD | | 0.29 | 0.74 |
| | | nPD: | 0.39 |
| | | N. weight: | 1.6 |
| | | Sc. PD: | 0.12 |
| | | Sc. rank: | 183.2 |

,

HA

| Pos. | 312 | obs: | exp: |
|---|---|---|---|
| ggt | G | 0 | 79.09 |
| ggc | G | 3 | 86.68 |
| gga | G | 502 | 310.10 |
| ggg | G | 136 | 165.10 |
| --- | -- | ------ | ------ |
| mPD | | 0.34 | 0.67 |
| | | nPD: | 0.51 |
| | | N. weight: | 0.48 |
| | | Sc. PD: | 0.091 |
| | | Sc. rank: | 113.3 |

,

HA

| Pos. | 313 | obs: | exp: |
|---|---|---|---|
| cgt | R | 0 | 0.17 |
| cgc | R | 0 | 0.20 |
| cga | R | 0 | 0.38 |
| cgg | R | 0 | 0.33 |
| aaa | K | 571 | 356.10 |
| aag | K | 65 | 279.90 |
| aga | R | 4 | 1.80 |
| agg | R | 0 | 1.12 |
| gaa | E | 1 | 0.58 |
| gag | E | 0 | 0.42 |
| --- | -- | ------ | ------ |
| mPD | | 0.20 | 0.51 |
| | | nPD: | 0.39 |
| | | N. weight: | 0.41 |
| | | Sc. PD: | 0.028 |
| | | Sc. rank: | 45.0 |

,

HA

| Pos. | 314 | obs: | exp: |
|---|---|---|---|
| tgt | C | 459 | 264.20 |
| tgc | C | 182 | 376.80 |
| --- | -- | ------ | ------ |
| mPD | | 0.41 | 0.48 |
| | | nPD: | 0.84 |
| | | N. weight: | 0.28 |
| | | Sc. PD: | 0.14 |
| | | Sc. rank: | 105.8 |

,

HA

| Pos. | 315 | obs: | exp: |
|---|---|---|---|
| cct | P | 68 | 168.80 |
| ccc | P | 8 | 115.60 |
| cca | P | 9 | 253.00 |
| ccg | P | 556 | 103.60 |
| --- | -- | ------ | ------ |
| mPD | | 0.24 | 0.72 |
| | | nPD: | 0.33 |
| | | N. weight: | 1.9 |
| | | Sc. PD: | 0.026 |
| | | Sc. rank: | 53.2 |

,

| HA | | | |
|---|---|---|---|
| Pos. | 316 | obs: | exp: |
| cgt | R | 0 | 26.61 |
| cgc | R | 0 | 31.39 |
| cga | R | 66 | 61.60 |
| cgg | R | 4 | 53.49 |
| aga | R | 549 | 288.30 |
| agg | R | 22 | 179.60 |
| --- | -- | ------ | ------ |
| mPD | | 0.27 | 0.96 |
| | | nPD: | 0.28 |
| | | N. weight: | 0.7 |
| | | Sc. PD: | -0.021 |
| | | Sc. rank: | -28.8 |

,

| HA | | | |
|---|---|---|---|
| Pos. | 317 | obs: | exp: |
| tat | Y | 637 | 331.80 |
| tac | Y | 4 | 309.20 |
| --- | -- | ------ | ------ |
| mPD | | 0.012 | 0.50 |
| | | nPD: | 0.02 |
| | | N. weight: | 0.91 |
| | | Sc. PD: | -0.25 |
| | | Sc. rank: | -271.7 |

,

| HA | | | |
|---|---|---|---|
| Pos. | 318 | obs: | exp: |
| gtt | V | 450 | 131.80 |
| gtc | V | 178 | 134.50 |
| gta | V | 3 | 120.10 |
| gtg | V | 10 | 254.60 |
| --- | -- | ------ | ------ |
| mPD | | 0.43 | 0.72 |
| | | nPD: | 0.6 |
| | | N. weight: | 1.3 |
| | | Sc. PD: | 0.35 |
| | | Sc. rank: | 387.1 |

,

| HA | | | |
|---|---|---|---|
| Pos. | 319 | obs: | exp: |
| cgt | R | 0 | 0.21 |
| cgc | R | 0 | 0.24 |
| cga | R | 0 | 0.48 |
| cgg | R | 0 | 0.42 |
| aaa | K | 96 | 356.10 |
| aag | K | 540 | 279.90 |
| aga | R | 3 | 2.25 |
| agg | R | 2 | 1.40 |
| --- | -- | ------ | ------ |
| mPD | | 0.28 | 0.51 |
| | | nPD: | 0.54 |
| | | N. weight: | 0.54 |
| | | Sc. PD: | 0.12 |
| | | Sc. rank: | 133.9 |

,

| HA | | | |
|---|---|---|---|
| Pos. | 320 | obs: | exp: |
| caa | Q | 491 | 341.70 |
| cag | Q | 150 | 299.30 |
| --- | -- | ------ | ------ |
| mPD | | 0.36 | 0.50 |
| | | nPD: | 0.72 |
| | | N. weight: | 0.18 |
| | | Sc. PD: | 0.069 |
| | | Sc. rank: | 60.3 |

,

| HA | | | |
|---|---|---|---|
| Pos. | 321 | obs: | exp: |
| tct | S | 0 | 0.32 |
| tcc | S | 0 | 0.28 |
| tca | S | 0 | 0.44 |
| tcg | S | 2 | 0.12 |
| cct | P | 0 | 0.26 |
| ccc | P | 0 | 0.18 |
| cca | P | 0 | 0.39 |
| ccg | P | 1 | 0.16 |
| cgt | R | 0 | 0.21 |
| cgc | R | 0 | 0.24 |
| cga | R | 0 | 0.48 |
| cgg | R | 0 | 0.42 |
| act | T | 0 | 18.23 |
| acc | T | 0 | 14.81 |
| aca | T | 69 | 30.23 |
| acg | T | 0 | 5.74 |
| aaa | K | 1 | 28.00 |
| aag | K | 49 | 22.00 |
| agt | S | 0 | 0.44 |
| agc | S | 0 | 0.40 |
| aga | R | 0 | 2.25 |
| agg | R | 5 | 1.40 |
| gaa | E | 20 | 294.60 |
| gag | E | 492 | 217.40 |
| ggt | G | 0 | 0.25 |
| ggc | G | 0 | 0.27 |
| gga | G | 0 | 0.97 |
| ggg | G | 2 | 0.52 |
| --- | -- | ------ | ------ |
| mPD | | 0.78 | 1.1 |
| | | nPD: | 0.72 |
| | | N. weight: | 1.1 |
| | | Sc. PD: | 0.41 |
| | | Sc. rank: | 359.5 |

,

| HA | | | |
|---|---|---|---|
| Pos. | 322 | obs: | exp: |
| tct | S | 0 | 101.90 |
| tcc | S | 0 | 89.29 |
| tca | S | 0 | 142.00 |
| tcg | S | 0 | 39.07 |
| agt | S | 556 | 141.00 |
| agc | S | 85 | 127.70 |
| --- | -- | ------ | ------ |
| mPD | | 0.23 | 1.7 |
| | | nPD: | 0.14 |
| | | N. weight: | 1.7 |
| | | Sc. PD: | -0.28 |
| | | Sc. rank: | -299.6 |

,

| HA | | | |
|---|---|---|---|
| Pos. | 323 | obs: | exp: |
| tta | L | 3 | 56.00 |
| ttg | L | 3 | 123.50 |
| ctt | L | 6 | 119.60 |
| ctc | L | 66 | 103.70 |
| cta | L | 19 | 96.57 |
| ctg | L | 544 | 141.70 |
| --- | -- | ------ | ------ |
| mPD | | 0.28 | 1.1 |
| | | nPD: | 0.25 |
| | | N. weight: | 1.5 |
| | | Sc. PD: | -0.092 |
| | | Sc. rank: | -108.1 |

,

| HA | | | |
|---|---|---|---|
| Pos. | 324 | obs: | exp: |
| tta | L | 3 | 39.58 |
| ttg | L | 4 | 87.24 |
| ctt | L | 57 | 84.52 |
| ctc | L | 14 | 73.28 |
| cta | L | 12 | 68.24 |
| ctg | L | 363 | 100.10 |
| cgt | R | 0 | 0.17 |
| cgc | R | 0 | 0.20 |
| cga | R | 0 | 0.38 |
| cgg | R | 0 | 0.33 |
| atg | M | 184 | 184.00 |
| aga | R | 0 | 1.80 |
| agg | R | 4 | 1.12 |
| --- | -- | ------ | ------ |
| mPD | | 0.68 | 1.2 |
| | | nPD: | 0.56 |
| | | N. weight: | 0.91 |
| | | Sc. PD: | 0.21 |
| | | Sc. rank: | 243.6 |

,

| HA | | | |
|---|---|---|---|
| Pos. | 325 | obs: | exp: |
| tta | L | 2 | 56.00 |
| ttg | L | 372 | 123.50 |
| ctt | L | 0 | 119.60 |
| ctc | L | 0 | 103.70 |
| cta | L | 110 | 96.57 |
| ctg | L | 157 | 141.70 |
| --- | -- | ------ | ------ |
| mPD | | 0.78 | 1.1 |
| | | nPD: | 0.7 |
| | | N. weight: | 1. |
| | | Sc. PD: | 0.37 |
| | | Sc. rank: | 336.8 |

,

| HA | | | |
|---|---|---|---|
| Pos. | 326 | obs: | exp: |
| gct | A | 62 | 161.50 |
| gcc | A | 9 | 131.30 |
| gca | A | 562 | 285.70 |
| gcg | A | 8 | 62.55 |
| --- | -- | ------ | ------ |
| mPD | | 0.22 | 0.69 |
| | | nPD: | 0.32 |
| | | N. weight: | 0.66 |
| | | Sc. PD: | 0.0049 |
| | | Sc. rank: | 10.4 |

,

| HA | | | |
|---|---|---|---|
| Pos. | 327 | obs: | exp: |
| atg | M | 1 | 1.00 |
| act | T | 0 | 169.10 |
| acc | T | 9 | 137.30 |
| aca | T | 628 | 280.40 |
| acg | T | 3 | 53.20 |
| --- | -- | ------ | ------ |
| mPD | | 0.043 | 0.69 |
| | | nPD: | 0.06 |
| | | N. weight: | 1.1 |
| | | Sc. PD: | -0.26 |
| | | Sc. rank: | -271.8 |

,

| HA | | | |
|---|---|---|---|
| Pos. | 328 | obs: | exp: |
| ggt | G | 0 | 79.09 |
| ggc | G | 0 | 86.68 |
| gga | G | 452 | 310.10 |
| ggg | G | 189 | 165.10 |
| --- | -- | ------ | ------ |
| mPD | | 0.42 | 0.67 |
| | | nPD: | 0.63 |
| | | N. weight: | 0.45 |
| | | Sc. PD: | 0.14 |
| | | Sc. rank: | 138.7 |

,

| HA | | | |
|---|---|---|---|
| Pos. | 329 | obs: | exp: |
| atg | M | 641 | 641.00 |
| --- | -- | ------ | ------ |
| mPD | | 0 | 0 |
| | | nPD: | 1. |
| | | N. weight: | 0. |
| | | Sc. PD: | 0 |
| | | Sc. rank: | 0 |

,

| HA | | | |
|---|---|---|---|
| Pos. | 330 | obs: | exp: |
| cgt | R | 0 | 2.99 |
| cgc | R | 0 | 3.53 |
| cga | R | 0 | 6.92 |
| cgg | R | 0 | 6.01 |
| aaa | K | 155 | 318.60 |
| aag | K | 414 | 250.40 |
| aga | R | 69 | 32.38 |
| agg | R | 3 | 20.18 |
| --- | -- | ------ | ------ |
| mPD | | 0.66 | 0.76 |
| | | nPD: | 0.86 |
| | | N. weight: | 0.34 |
| | | Sc. PD: | 0.18 |
| | | Sc. rank: | 125.8 |

,

| | | | |
|---|---|---|---|
| HA | | | |
| Pos. | 331 | obs: | exp: |
| aat | N | 561 | 335.00 |
| aac | N | 80 | 306.00 |
| --- | -- | ------ | ------ |
| mPD | | 0.22 | 0.50 |
| | | nPD: | 0.44 |
| | | N. weight: | 0.42 |
| | | Sc. PD: | 0.05 |
| | | Sc. rank: | 67.4 |

,

| | | | |
|---|---|---|---|
| HA | | | |
| Pos. | 332 | obs: | exp: |
| gtt | V | 566 | 131.50 |
| gtc | V | 74 | 134.30 |
| gta | V | 0 | 119.90 |
| gtg | V | 0 | 254.20 |
| gct | A | 1 | 0.25 |
| gcc | A | 0 | 0.20 |
| gca | A | 0 | 0.45 |
| gcg | A | 0 | 0.10 |
| --- | -- | ------ | ------ |
| mPD | | 0.21 | 0.72 |
| | | nPD: | 0.29 |
| | | N. weight: | 1.8 |
| | | Sc. PD: | -0.05 |
| | | Sc. rank: | -66.9 |

,

| | | | |
|---|---|---|---|
| HA | | | |
| Pos. | 333 | obs: | exp: |
| cct | P | 183 | 168.80 |
| ccc | P | 386 | 115.60 |
| cca | P | 71 | 253.00 |
| ccg | P | 1 | 103.60 |
| --- | -- | ------ | ------ |
| mPD | | 0.54 | 0.72 |
| | | nPD: | 0.76 |
| | | N. weight: | 0.9 |
| | | Sc. PD: | 0.38 |
| | | Sc. rank: | 317.5 |

,

| | | | |
|---|---|---|---|
| HA | | | |
| Pos. | 334 | obs: | exp: |
| gaa | E | 410 | 368.80 |
| gag | E | 231 | 272.20 |
| --- | -- | ------ | ------ |
| mPD | | 0.46 | 0.49 |
| | | nPD: | 0.94 |
| | | N. weight: | 0.02 |
| | | Sc. PD: | 0.012 |
| | | Sc. rank: | 7.7 |

,

| | | | |
|---|---|---|---|
| HA | | | |
| Pos. | 335 | obs: | exp: |
| tta | L | 0 | 7.34 |
| ttg | L | 0 | 16.18 |
| tct | S | 0 | 0.48 |
| tcc | S | 1 | 0.42 |
| tca | S | 0 | 0.66 |
| tcg | S | 0 | 0.18 |
| ctt | L | 3 | 15.67 |
| ctc | L | 81 | 13.59 |
| cta | L | 0 | 12.65 |
| ctg | L | 0 | 18.57 |
| cct | P | 2 | 2.11 |
| ccc | P | 6 | 1.44 |
| cca | P | 0 | 3.16 |
| ccg | P | 0 | 1.29 |
| att | I | 95 | 167.30 |
| atc | I | 350 | 116.60 |
| ata | I | 0 | 161.10 |
| act | T | 15 | 7.13 |
| acc | T | 12 | 5.79 |
| aca | T | 0 | 11.83 |
| acg | T | 0 | 2.24 |
| aat | N | 9 | 36.06 |
| aac | N | 60 | 32.94 |
| aaa | K | 2 | 1.12 |
| aag | K | 0 | 0.88 |
| agt | S | 1 | 0.66 |
| agc | S | 1 | 0.60 |
| ggt | G | 1 | 0.37 |
| ggc | G | 2 | 0.41 |
| gga | G | 0 | 1.45 |
| ggg | G | 0 | 0.77 |
| --- | -- | ------ | ------ |
| mPD | | 0.88 | 1.3 |
| | | nPD: | 0.7 |
| | | N. weight: | 1.2 |
| | | Sc. PD: | 0.45 |
| | | Sc. rank: | 411.0 |

,

| | | | |
|---|---|---|---|
| HA | | | |
| Pos. | 336 | obs: | exp: |
| cct | P | 0 | 168.80 |
| ccc | P | 74 | 115.60 |
| cca | P | 561 | 253.00 |
| ccg | P | 6 | 103.60 |
| --- | -- | ------ | ------ |
| mPD | | 0.22 | 0.72 |
| | | nPD: | 0.31 |
| | | N. weight: | 0.92 |
| | | Sc. PD: | -0.0062 |
| | | Sc. rank: | -11.2 |

,

| | | | |
|---|---|---|---|
| HA | | | |
| Pos. | 337 | obs: | exp: |
| aaa | K | 0 | 2.80 |
| aag | K | 5 | 2.20 |
| --- | - | 636 | 636.00 |
| --- | -- | ------ | ------ |
| mPD | | 0 | 0.000030 |
| | | nPD: | 0. |
| | | N. weight: | 0. |
| | | Sc. PD: | 0 |
| | | Sc. rank: | 0 |

,

| | | | |
|---|---|---|---|
| HA | | | |
| Pos. | 338 | obs: | exp: |
| aaa | K | 6 | 3.92 |
| aag | K | 1 | 3.08 |
| --- | - | 634 | 634.00 |
| --- | -- | ------ | ------ |
| mPD | | 0.000029 | 0.000059 |
| | | nPD: | 0.5 |
| | | N. weight: | 0. |
| | | Sc. PD: | 0 |
| | | Sc. rank: | 0 |

,

| | | | |
|---|---|---|---|
| HA | | | |
| Pos. | 339 | obs: | exp: |
| cgt | R | 0 | 0.08 |
| cgc | R | 0 | 0.10 |
| cga | R | 0 | 0.19 |
| cgg | R | 0 | 0.17 |
| aaa | K | 27 | 319.20 |
| aag | K | 543 | 250.80 |
| aga | R | 1 | 0.90 |
| agg | R | 1 | 0.56 |
| ggt | G | 0 | 0.12 |
| ggc | G | 0 | 0.14 |
| gga | G | 0 | 0.48 |
| ggg | G | 1 | 0.26 |
| --- | - | 68 | 68.00 |
| --- | -- | ------ | ------ |
| mPD | | 0.086 | 0.41 |
| | | nPD: | 0.21 |
| | | N. weight: | 0. |
| | | Sc. PD: | 0 |
| | | Sc. rank: | 0 |

,

| | | | |
|---|---|---|---|
| HA | | | |
| Pos. | 340 | obs: | exp: |
| aaa | K | 0 | 0.56 |
| aag | K | 1 | 0.44 |
| --- | - | 640 | 640.00 |
| --- | -- | ------ | ------ |
| mPD | | 0 | $1.2 \times 10^{-6}$ |
| | | nPD: | 0. |
| | | N. weight: | 0. |
| | | Sc. PD: | 0 |
| | | Sc. rank: | 0 |

,

| | | | |
|---|---|---|---|
| HA | | | |
| Pos. | 341 | obs: | exp: |
| cat | H | 1 | 0.54 |
| cac | H | 0 | 0.46 |
| aaa | K | 2 | 1.12 |
| aag | K | 0 | 0.88 |
| --- | - | 638 | 638.00 |
| --- | -- | ------ | ------ |
| mPD | | 0.000020 | 0.000025 |
| | | nPD: | 0.77 |
| | | N. weight: | 0. |
| | | Sc. PD: | 0 |
| | | Sc. rank: | 0 |

,

| | | | |
|---|---|---|---|
| HA | | | |
| Pos. | 342 | obs: | exp: |
| cgt | R | 1 | 10.67 |
| cgc | R | 0 | 12.58 |
| cga | R | 0 | 24.70 |
| cgg | R | 0 | 21.45 |
| aaa | K | 10 | 6.16 |
| aag | K | 1 | 4.84 |
| aga | R | 17 | 115.60 |
| agg | R | 239 | 72.02 |
| --- | - | 373 | 373.00 |
| --- | -- | ------ | ------ |
| mPD | | 0.048 | 0.18 |
| | | nPD: | 0.27 |
| | | N. weight: | 0. |
| | | Sc. PD: | 0 |
| | | Sc. rank: | 0 |

,

| | | | |
|---|---|---|---|
| HA | | | |
| Pos. | 343 | obs: | exp: |
| cgt | R | 0 | 10.42 |
| cgc | R | 0 | 12.29 |
| cga | R | 0 | 24.12 |
| cgg | R | 0 | 20.95 |
| aaa | K | 2 | 47.60 |
| aag | K | 83 | 37.40 |
| aga | R | 12 | 112.90 |
| agg | R | 239 | 70.34 |
| --- | - | 305 | 305.00 |
| --- | -- | ------ | ------ |
| mPD | | 0.13 | 0.34 |
| | | nPD: | 0.37 |
| | | N. weight: | 0. |
| | | Sc. PD: | 0 |
| | | Sc. rank: | 0 |

,

HA

| Pos. | 344 | obs: | exp: |
|---|---|---|---|
| gaa | E | 0 | 5.18 |
| gag | E | 9 | 3.82 |
| --- | - | 632 | 632.00 |
| --- | -- | ------ | ------ |
| mPD | | 0 | 0.000096 |
| | | nPD: | 0. |
| | | N. weight: | 0. |
| | | Sc. PD: | 0 |
| | | Sc. rank: | 0 |

,

HA

| Pos. | 345 | obs: | exp: |
|---|---|---|---|
| cgt | R | 0 | 10.34 |
| cgc | R | 0 | 12.19 |
| cga | R | 0 | 23.93 |
| cgg | R | 0 | 20.78 |
| act | T | 2 | 18.49 |
| acc | T | 67 | 15.02 |
| aca | T | 1 | 30.67 |
| acg | T | 0 | 5.82 |
| aaa | K | 16 | 11.76 |
| aag | K | 5 | 9.24 |
| aga | R | 12 | 112.00 |
| agg | R | 237 | 69.78 |
| gct | A | 0 | 0.50 |
| gcc | A | 2 | 0.41 |
| gca | A | 0 | 0.89 |
| gcg | A | 0 | 0.20 |
| gaa | E | 4 | 2.30 |
| gag | E | 0 | 1.70 |
| ggt | G | 1 | 36.40 |
| ggc | G | 0 | 39.89 |
| gga | G | 282 | 142.70 |
| ggg | G | 12 | 76.00 |
| --- | -- | ------ | ------ |
| mPD | | 1.4 | 1.5 |
| | | nPD: | 0.91 |
| | | N. weight: | 1.2 |
| | | Sc. PD: | 0.7 |
| | | Sc. rank: | 462.8 |

,

HA

| Pos. | 346 | obs: | exp: |
|---|---|---|---|
| cgt | R | 0 | 26.49 |
| cgc | R | 0 | 31.24 |
| cga | R | 0 | 61.32 |
| cgg | R | 0 | 53.24 |
| aaa | K | 3 | 1.68 |
| aag | K | 0 | 1.32 |
| aga | R | 632 | 286.90 |
| agg | R | 6 | 178.80 |
| --- | -- | ------ | ------ |
| mPD | | 0.028 | 0.97 |
| | | nPD: | 0.03 |
| | | N. weight: | 1.1 |
| | | Sc. PD: | -0.3 |
| | | Sc. rank: | -321.8 |

,

HA

| Pos. | 347 | obs: | exp: |
|---|---|---|---|
| ggt | G | 81 | 79.09 |
| ggc | G | 555 | 86.68 |
| gga | G | 4 | 310.10 |
| ggg | G | 1 | 165.10 |
| --- | -- | ------ | ------ |
| mPD | | 0.23 | 0.67 |
| | | nPD: | 0.35 |
| | | N. weight: | 2.3 |
| | | Sc. PD: | 0.082 |
| | | Sc. rank: | 136.5 |

,

HA

| Pos. | 348 | obs: | exp: |
|---|---|---|---|
| tta | L | 5 | 56.00 |
| ttg | L | 0 | 123.50 |
| ctt | L | 69 | 119.60 |
| ctc | L | 2 | 103.70 |
| cta | L | 536 | 96.57 |
| ctg | L | 29 | 141.70 |
| --- | -- | ------ | ------ |
| mPD | | 0.29 | 1.1 |
| | | nPD: | 0.26 |
| | | N. weight: | 1.9 |
| | | Sc. PD: | -0.1 |
| | | Sc. rank: | -119.3 |

,

HA

| Pos. | 349 | obs: | exp: |
|---|---|---|---|
| ttt | F | 628 | 263.50 |
| ttc | F | 13 | 377.50 |
| --- | -- | ------ | ------ |
| mPD | | 0.040 | 0.48 |
| | | nPD: | 0.08 |
| | | N. weight: | 1.2 |
| | | Sc. PD: | -0.26 |
| | | Sc. rank: | -255.8 |

,

HA

| Pos. | 350 | obs: | exp: |
|---|---|---|---|
| ggt | G | 562 | 79.09 |
| ggc | G | 7 | 86.68 |
| gga | G | 71 | 310.10 |
| ggg | G | 1 | 165.10 |
| --- | -- | ------ | ------ |
| mPD | | 0.22 | 0.67 |
| | | nPD: | 0.33 |
| | | N. weight: | 2.2 |
| | | Sc. PD: | 0.029 |
| | | Sc. rank: | 54.6 |

,

HA

| Pos. | 351 | obs: | exp: |
|---|---|---|---|
| gct | A | 557 | 161.50 |
| gcc | A | 13 | 131.30 |
| gca | A | 8 | 285.70 |
| gcg | A | 63 | 62.55 |
| --- | -- | ------ | ------ |
| mPD | | 0.24 | 0.69 |
| | | nPD: | 0.34 |
| | | N. weight: | 1.5 |
| | | Sc. PD: | 0.038 |
| | | Sc. rank: | 63.7 |

,

HA

| Pos. | 352 | obs: | exp: |
|---|---|---|---|
| att | I | 71 | 241.00 |
| atc | I | 1 | 168.00 |
| ata | I | 569 | 232.00 |
| --- | -- | ------ | ------ |
| mPD | | 0.20 | 0.66 |
| | | nPD: | 0.3 |
| | | N. weight: | 0.96 |
| | | Sc. PD: | -0.011 |
| | | Sc. rank: | -19.7 |

,

HA

| Pos. | 353 | obs: | exp: |
|---|---|---|---|
| gct | A | 72 | 161.50 |
| gcc | A | 1 | 131.30 |
| gca | A | 74 | 285.70 |
| gcg | A | 494 | 62.55 |
| --- | -- | ------ | ------ |
| mPD | | 0.38 | 0.69 |
| | | nPD: | 0.55 |
| | | N. weight: | 2. |
| | | Sc. PD: | 0.45 |
| | | Sc. rank: | 526.5 |

,

HA

| Pos. | 354 | obs: | exp: |
|---|---|---|---|
| ggt | G | 555 | 79.09 |
| ggc | G | 2 | 86.68 |
| gga | G | 79 | 310.10 |
| ggg | G | 5 | 165.10 |
| --- | -- | ------ | ------ |
| mPD | | 0.24 | 0.67 |
| | | nPD: | 0.35 |
| | | N. weight: | 2.2 |
| | | Sc. PD: | 0.079 |
| | | Sc. rank: | 134.6 |

,

HA

| Pos. | 355 | obs: | exp: |
|---|---|---|---|
| ttt | F | 28 | 263.50 |
| ttc | F | 613 | 377.50 |
| --- | -- | ------ | ------ |
| mPD | | 0.084 | 0.48 |
| | | nPD: | 0.17 |
| | | N. weight: | 0.54 |
| | | Sc. PD: | -0.074 |
| | | Sc. rank: | -80.9 |

,

HA

| Pos. | 356 | obs: | exp: |
|---|---|---|---|
| att | I | 533 | 241.00 |
| atc | I | 36 | 168.00 |
| ata | I | 72 | 232.00 |
| --- | -- | ------ | ------ |
| mPD | | 0.29 | 0.66 |
| | | nPD: | 0.45 |
| | | N. weight: | 0.66 |
| | | Sc. PD: | 0.082 |
| | | Sc. rank: | 107.5 |

,

HA

| Pos. | 357 | obs: | exp: |
|---|---|---|---|
| gaa | E | 569 | 368.80 |
| gag | E | 72 | 272.20 |
| --- | -- | ------ | ------ |
| mPD | | 0.20 | 0.49 |
| | | nPD: | 0.41 |
| | | N. weight: | 0.35 |
| | | Sc. PD: | 0.031 |
| | | Sc. rank: | 46.1 |

,

HA

| Pos. | 358 | obs: | exp: |
|---|---|---|---|
| aat | N | 600 | 335.00 |
| aac | N | 41 | 306.00 |
| --- | -- | ------ | ------ |
| mPD | | 0.12 | 0.50 |
| | | nPD: | 0.24 |
| | | N. weight: | 0.62 |
| | | Sc. PD: | -0.045 |
| | | Sc. rank: | -54.7 |

,

HA

| Pos. | 359 | obs: | exp: |
|---|---|---|---|
| ggt | G | 2 | 79.09 |
| ggc | G | 0 | 86.68 |
| gga | G | 594 | 310.10 |
| ggg | G | 45 | 165.10 |
| --- | -- | ------ | ------ |
| mPD | | 0.14 | 0.67 |
| | | nPD: | 0.2 |
| | | N. weight: | 0.74 |
| | | Sc. PD: | -0.079 |
| | | Sc. rank: | -92.7 |

,

HA

| Pos. | 360 | obs: | exp: |
|---|---|---|---|
| tgg | W | 641 | 641.00 |
| --- | -- | ------ | ------ |
| mPD | | 0 | 0 |
| | | nPD: | 1. |
| | | N. weight: | 0. |
| | | Sc. PD: | 0 |
| | | Sc. rank: | 0 |

,

HA

| Pos. | 361 | obs: | exp: |
|---|---|---|---|
| gaa | E | 636 | 368.80 |
| gag | E | 5 | 272.20 |
| --- | -- | ------ | ------ |
| mPD | | 0.016 | 0.49 |
| | | nPD: | 0.03 |
| | | N. weight: | 0.75 |
| | | Sc. PD: | -0.2 |
| | | Sc. rank: | -214.4 |

,

HA

| Pos. | 362 | obs: | exp: |
|---|---|---|---|
| ggt | G | 562 | 79.09 |
| ggc | G | 79 | 86.68 |
| gga | G | 0 | 310.10 |
| ggg | G | 0 | 165.10 |
| --- | -- | ------ | ------ |
| mPD | | 0.22 | 0.67 |
| | | nPD: | 0.32 |
| | | N. weight: | 2.5 |
| | | Sc. PD: | 0.023 |
| | | Sc. rank: | 51.0 |

,

HA

| Pos. | 363 | obs: | exp: |
|---|---|---|---|
| tta | L | 5 | 56.00 |
| ttg | L | 240 | 123.50 |
| ctt | L | 1 | 119.60 |
| ctc | L | 71 | 103.70 |
| cta | L | 97 | 96.57 |
| ctg | L | 227 | 141.70 |
| --- | -- | ------ | ------ |
| mPD | | 0.91 | 1.1 |
| | | nPD: | 0.81 |
| | | N. weight: | 0.54 |
| | | Sc. PD: | 0.26 |
| | | Sc. rank: | 198.2 |

,

HA

| Pos. | 364 | obs: | exp: |
|---|---|---|---|
| att | I | 587 | 222.20 |
| atc | I | 4 | 154.90 |
| ata | I | 0 | 213.90 |
| gtt | V | 48 | 10.28 |
| gtc | V | 2 | 10.49 |
| gta | V | 0 | 9.37 |
| gtg | V | 0 | 19.86 |
| --- | -- | ------ | ------ |
| mPD | | 0.16 | 0.82 |
| | | nPD: | 0.2 |
| | | N. weight: | 1.4 |
| | | Sc. PD: | -0.16 |
| | | Sc. rank: | -191.4 |

,

HA

| Pos. | 365 | obs: | exp: |
|---|---|---|---|
| gat | D | 548 | 342.00 |
| gac | D | 93 | 299.00 |
| --- | -- | ------ | ------ |
| mPD | | 0.25 | 0.50 |
| | | nPD: | 0.5 |
| | | N. weight: | 0.35 |
| | | Sc. PD: | 0.062 |
| | | Sc. rank: | 77.6 |

,

HA

| Pos. | 366 | obs: | exp: |
|---|---|---|---|
| ggt | G | 33 | 79.09 |
| ggc | G | 0 | 86.68 |
| gga | G | 81 | 310.10 |
| ggg | G | 527 | 165.10 |
| --- | -- | ------ | ------ |
| mPD | | 0.31 | 0.67 |
| | | nPD: | 0.46 |
| | | N. weight: | 1.1 |
| | | Sc. PD: | 0.15 |
| | | Sc. rank: | 201.2 |

,

HA

| Pos. | 367 | obs: | exp: |
|---|---|---|---|
| tgg | W | 641 | 641.00 |
| --- | -- | ------ | ------ |
| mPD | | 0 | 0 |
| | | nPD: | 1. |
| | | N. weight: | 0. |
| | | Sc. PD: | 0 |
| | | Sc. rank: | 0 |

,

HA

| Pos. | 368 | obs: | exp: |
|---|---|---|---|
| tat | Y | 609 | 331.80 |
| tac | Y | 32 | 309.20 |
| --- | -- | ------ | ------ |
| mPD | | 0.095 | 0.50 |
| | | nPD: | 0.19 |
| | | N. weight: | 0.68 |
| | | Sc. PD: | -0.082 |
| | | Sc. rank: | -92.5 |

,

| | | | |
|---|---|---|---|
| HA | | | |
| Pos. | 369 | obs: | exp: |
| ggt | G | 167 | 79.09 |
| ggc | G | 469 | 86.68 |
| gga | G | 3 | 310.10 |
| ggg | G | 2 | 165.10 |
| --- | -- | ------ | ------ |
| mPD | | 0.40 | 0.67 |
| | | nPD: | 0.6 |
| | | N. weight: | 2.1 |
| | | Sc. PD: | 0.55 |
| | | Sc. rank: | 611.9 |

,

| | | | |
|---|---|---|---|
| HA | | | |
| Pos. | 370 | obs: | exp: |
| ttt | F | 1 | 263.50 |
| ttc | F | 640 | 377.50 |
| --- | -- | ------ | ------ |
| mPD | | 0.0031 | 0.48 |
| | | nPD: | 0.01 |
| | | N. weight: | 0.76 |
| | | Sc. PD: | -0.23 |
| | | Sc. rank: | -253.8 |

,

| | | | |
|---|---|---|---|
| HA | | | |
| Pos. | 371 | obs: | exp: |
| cgt | R | 0 | 26.49 |
| cgc | R | 0 | 31.24 |
| cga | R | 0 | 61.32 |
| cgg | R | 2 | 53.24 |
| aaa | K | 0 | 1.68 |
| aag | K | 3 | 1.32 |
| aga | R | 205 | 286.90 |
| agg | R | 431 | 178.80 |
| --- | -- | ------ | ------ |
| mPD | | 0.45 | 0.97 |
| | | nPD: | 0.47 |
| | | N. weight: | 0.71 |
| | | Sc. PD: | 0.1 |
| | | Sc. rank: | 137.1 |

,

| | | | |
|---|---|---|---|
| HA | | | |
| Pos. | 372 | obs: | exp: |
| cat | H | 500 | 347.30 |
| cac | H | 141 | 293.70 |
| --- | -- | ------ | ------ |
| mPD | | 0.34 | 0.50 |
| | | nPD: | 0.69 |
| | | N. weight: | 0.19 |
| | | Sc. PD: | 0.068 |
| | | Sc. rank: | 61.9 |

,

| | | | |
|---|---|---|---|
| HA | | | |
| Pos. | 373 | obs: | exp: |
| caa | Q | 580 | 341.20 |
| cag | Q | 60 | 298.80 |
| aaa | K | 1 | 0.56 |
| aag | K | 0 | 0.44 |
| --- | -- | ------ | ------ |
| mPD | | 0.17 | 0.50 |
| | | nPD: | 0.35 |
| | | N. weight: | 0.49 |
| | | Sc. PD: | 0.014 |
| | | Sc. rank: | 21.8 |

,

| | | | |
|---|---|---|---|
| HA | | | |
| Pos. | 374 | obs: | exp: |
| aat | N | 640 | 335.00 |
| aac | N | 1 | 306.00 |
| --- | -- | ------ | ------ |
| mPD | | 0.0031 | 0.50 |
| | | nPD: | 0.01 |
| | | N. weight: | 0.94 |
| | | Sc. PD: | -0.28 |
| | | Sc. rank: | -319.3 |

,

| | | | |
|---|---|---|---|
| HA | | | |
| Pos. | 375 | obs: | exp: |
| tct | S | 0 | 0.16 |
| tcc | S | 0 | 0.14 |
| tca | S | 1 | 0.22 |
| tcg | S | 0 | 0.06 |
| agt | S | 0 | 0.22 |
| agc | S | 0 | 0.20 |
| gct | A | 0 | 160.20 |
| gcc | A | 0 | 130.30 |
| gca | A | 630 | 283.50 |
| gcg | A | 6 | 62.06 |
| gaa | E | 4 | 2.30 |
| gag | E | 0 | 1.70 |
| --- | -- | ------ | ------ |
| mPD | | 0.034 | 0.70 |
| | | nPD: | 0.05 |
| | | N. weight: | 1.1 |
| | | Sc. PD: | -0.29 |
| | | Sc. rank: | -296.7 |

,

| | | | |
|---|---|---|---|
| HA | | | |
| Pos. | 376 | obs: | exp: |
| caa | Q | 533 | 341.70 |
| cag | Q | 108 | 299.30 |
| --- | -- | ------ | ------ |
| mPD | | 0.28 | 0.50 |
| | | nPD: | 0.56 |
| | | N. weight: | 0.3 |
| | | Sc. PD: | 0.071 |
| | | Sc. rank: | 81.8 |

,

| | | | |
|---|---|---|---|
| HA | | | |
| Pos. | 377 | obs: | exp: |
| ggt | G | 0 | 79.09 |
| ggc | G | 0 | 86.68 |
| gga | G | 301 | 310.10 |
| ggg | G | 340 | 165.10 |
| --- | -- | ------ | ------ |
| mPD | | 0.50 | 0.67 |
| | | nPD: | 0.75 |
| | | N. weight: | 0.55 |
| | | Sc. PD: | 0.23 |
| | | Sc. rank: | 191.7 |

,

| | | | |
|---|---|---|---|
| HA | | | |
| Pos. | 378 | obs: | exp: |
| aaa | K | 3 | 1.68 |
| aag | K | 0 | 1.32 |
| gaa | E | 93 | 367.10 |
| gag | E | 545 | 270.90 |
| --- | -- | ------ | ------ |
| mPD | | 0.26 | 0.50 |
| | | nPD: | 0.53 |
| | | N. weight: | 0.59 |
| | | Sc. PD: | 0.12 |
| | | Sc. rank: | 145.5 |

,

| | | | |
|---|---|---|---|
| HA | | | |
| Pos. | 379 | obs: | exp: |
| ggt | G | 15 | 79.09 |
| ggc | G | 3 | 86.68 |
| gga | G | 572 | 310.10 |
| ggg | G | 51 | 165.10 |
| --- | -- | ------ | ------ |
| mPD | | 0.20 | 0.67 |
| | | nPD: | 0.3 |
| | | N. weight: | 0.6 |
| | | Sc. PD: | -0.011 |
| | | Sc. rank: | -17.8 |

,

| | | | |
|---|---|---|---|
| HA | | | |
| Pos. | 380 | obs: | exp: |
| att | I | 4 | 1.50 |
| atc | I | 0 | 1.05 |
| ata | I | 0 | 1.45 |
| act | T | 566 | 168.30 |
| acc | T | 4 | 136.70 |
| aca | T | 67 | 279.10 |
| acg | T | 0 | 52.95 |
| --- | -- | ------ | ------ |
| mPD | | 0.21 | 0.70 |
| | | nPD: | 0.3 |
| | | N. weight: | 1.3 |
| | | Sc. PD: | -0.017 |
| | | Sc. rank: | -29.0 |

,

| | | | |
|---|---|---|---|
| HA | | | |
| Pos. | 381 | obs: | exp: |
| gct | A | 538 | 161.50 |
| gcc | A | 22 | 131.30 |
| gca | A | 76 | 285.70 |
| gcg | A | 5 | 62.55 |
| --- | -- | ------ | ------ |
| mPD | | 0.28 | 0.69 |
| | | nPD: | 0.41 |
| | | N. weight: | 1.1 |
| | | Sc. PD: | 0.1 |
| | | Sc. rank: | 152.9 |

,

| | | | |
|---|---|---|---|
| HA | | | |
| Pos. | 382 | obs: | exp: |
| gct | A | 75 | 161.50 |
| gcc | A | 0 | 131.30 |
| gca | A | 565 | 285.70 |
| gcg | A | 1 | 62.55 |
| --- | -- | ------ | ------ |
| mPD | | 0.21 | 0.69 |
| | | nPD: | 0.31 |
| | | N. weight: | 0.75 |
| | | Sc. PD: | -0.0072 |
| | | Sc. rank: | -13.5 |

,

| | | | |
|---|---|---|---|
| HA | | | |
| Pos. | 383 | obs: | exp: |
| gat | D | 607 | 342.00 |
| gac | D | 34 | 299.00 |
| --- | -- | ------ | ------ |
| mPD | | 0.10 | 0.50 |
| | | nPD: | 0.2 |
| | | N. weight: | 0.63 |
| | | Sc. PD: | -0.069 |
| | | Sc. rank: | -79.7 |

,

| | | | |
|---|---|---|---|
| HA | | | |
| Pos. | 384 | obs: | exp: |
| ttt | F | 0 | 0.41 |
| ttc | F | 1 | 0.59 |
| tat | Y | 15 | 330.20 |
| tac | Y | 623 | 307.80 |
| cat | H | 0 | 1.08 |
| cac | H | 2 | 0.92 |
| --- | -- | ------ | ------ |
| mPD | | 0.055 | 0.51 |
| | | nPD: | 0.11 |
| | | N. weight: | 0.91 |
| | | Sc. PD: | -0.18 |
| | | Sc. rank: | -182.4 |

,

| | | | |
|---|---|---|---|
| HA | | | |
| Pos. | 385 | obs: | exp: |
| cgt | R | 0 | 0.04 |
| cgc | R | 0 | 0.05 |
| cga | R | 0 | 0.10 |
| cgg | R | 0 | 0.08 |
| aaa | K | 624 | 358.40 |
| aag | K | 16 | 281.60 |
| aga | R | 1 | 0.45 |
| agg | R | 0 | 0.28 |
| --- | -- | ------ | ------ |
| mPD | | 0.052 | 0.50 |
| | | nPD: | 0.1 |
| | | N. weight: | 0.69 |
| | | Sc. PD: | -0.14 |
| | | Sc. rank: | -141.7 |

,

| | | | |
|---|---|---|---|
| HA | | | |
| Pos. | 386 | obs: | exp: |
| tct | S | 0 | 101.90 |
| tcc | S | 0 | 89.29 |
| tca | S | 0 | 142.00 |
| tcg | S | 0 | 39.07 |
| agt | S | 2 | 141.00 |
| agc | S | 639 | 127.70 |
| --- | -- | ------ | ------ |
| mPD | | 0.0062 | 1.7 |
| | | nPD: | 0. |
| | | N. weight: | 2.3 |
| | | Sc. PD: | -0.7 |
| | | Sc. rank: | -816.1 |

,

| | | | |
|---|---|---|---|
| HA | | | |
| Pos. | 387 | obs: | exp: |
| act | T | 200 | 169.30 |
| acc | T | 441 | 137.50 |
| aca | T | 0 | 280.80 |
| acg | T | 0 | 53.29 |
| --- | -- | ------ | ------ |
| mPD | | 0.43 | 0.69 |
| | | nPD: | 0.63 |
| | | N. weight: | 1.3 |
| | | Sc. PD: | 0.38 |
| | | Sc. rank: | 387.0 |

,

| | | | |
|---|---|---|---|
| HA | | | |
| Pos. | 388 | obs: | exp: |
| caa | Q | 598 | 341.70 |
| cag | Q | 43 | 299.30 |
| --- | -- | ------ | ------ |
| mPD | | 0.13 | 0.50 |
| | | nPD: | 0.25 |
| | | N. weight: | 0.58 |
| | | Sc. PD: | -0.036 |
| | | Sc. rank: | -42.2 |

,

| | | | |
|---|---|---|---|
| HA | | | |
| Pos. | 389 | obs: | exp: |
| tct | S | 94 | 101.70 |
| tcc | S | 1 | 89.16 |
| tca | S | 446 | 141.80 |
| tcg | S | 99 | 39.01 |
| agt | S | 0 | 140.80 |
| agc | S | 0 | 127.50 |
| gct | A | 0 | 0.25 |
| gcc | A | 0 | 0.20 |
| gca | A | 1 | 0.45 |
| gcg | A | 0 | 0.10 |
| --- | -- | ------ | ------ |
| mPD | | 0.47 | 1.7 |
| | | nPD: | 0.28 |
| | | N. weight: | 1.4 |
| | | Sc. PD: | -0.042 |
| | | Sc. rank: | -57.2 |

,

| | | | |
|---|---|---|---|
| HA | | | |
| Pos. | 390 | obs: | exp: |
| gct | A | 1 | 161.50 |
| gcc | A | 0 | 131.30 |
| gca | A | 640 | 285.70 |
| gcg | A | 0 | 62.55 |
| --- | -- | ------ | ------ |
| mPD | | 0.0031 | 0.69 |
| | | nPD: | 0. |
| | | N. weight: | 1.2 |
| | | Sc. PD: | -0.35 |
| | | Sc. rank: | -405.4 |

,

| | | | |
|---|---|---|---|
| HA | | | |
| Pos. | 391 | obs: | exp: |
| att | I | 526 | 239.50 |
| atc | I | 41 | 166.90 |
| ata | I | 70 | 230.60 |
| gtt | V | 0 | 0.82 |
| gtc | V | 2 | 0.84 |
| gta | V | 2 | 0.75 |
| gtg | V | 0 | 1.59 |
| --- | -- | ------ | ------ |
| mPD | | 0.32 | 0.67 |
| | | nPD: | 0.48 |
| | | N. weight: | 0.65 |
| | | Sc. PD: | 0.1 |
| | | Sc. rank: | 131.6 |

,

| | | | |
|---|---|---|---|
| HA | | | |
| Pos. | 392 | obs: | exp: |
| aat | N | 45 | 23.52 |
| aac | N | 0 | 21.48 |
| gat | D | 556 | 318.00 |
| gac | D | 40 | 278.00 |
| --- | -- | ------ | ------ |
| mPD | | 0.25 | 0.63 |
| | | nPD: | 0.39 |
| | | N. weight: | 0.61 |
| | | Sc. PD: | 0.046 |
| | | Sc. rank: | 68.7 |

,

| | | | |
|---|---|---|---|
| HA | | | |
| Pos. | 393 | obs: | exp: |
| caa | Q | 559 | 341.70 |
| cag | Q | 82 | 299.30 |
| --- | -- | ------ | ------ |
| mPD | | 0.22 | 0.50 |
| | | nPD: | 0.45 |
| | | N. weight: | 0.39 |
| | | Sc. PD: | 0.05 |
| | | Sc. rank: | 67.9 |

,

| HA | | | |
|---|---|---|---|
| Pos. | 394 | obs: | exp: |
| att | I | 0 | 224.40 |
| atc | I | 71 | 156.50 |
| ata | I | 526 | 216.10 |
| atg | M | 43 | 43.00 |
| gtt | V | 0 | 0.21 |
| gtc | V | 1 | 0.21 |
| gta | V | 0 | 0.19 |
| gtg | V | 0 | 0.40 |
| --- | -- | ------ | ------ |
| mPD | | 0.31 | 0.70 |
| | | nPD: | 0.45 |
| | | N. weight: | 0.95 |
| | | Sc. PD: | 0.12 |
| | | Sc. rank: | 157.6 |

| HA | | | |
|---|---|---|---|
| Pos. | 395 | obs: | exp: |
| act | T | 6 | 169.30 |
| acc | T | 3 | 137.50 |
| aca | T | 572 | 280.80 |
| acg | T | 60 | 53.29 |
| --- | -- | ------ | ------ |
| mPD | | 0.20 | 0.69 |
| | | nPD: | 0.28 |
| | | N. weight: | 0.89 |
| | | Sc. PD: | -0.026 |
| | | Sc. rank: | -35.2 |

| HA | | | |
|---|---|---|---|
| Pos. | 396 | obs: | exp: |
| gat | D | 0 | 0.53 |
| gac | D | 1 | 0.47 |
| ggt | G | 4 | 78.97 |
| ggc | G | 67 | 86.55 |
| gga | G | 146 | 309.60 |
| ggg | G | 423 | 164.90 |
| --- | -- | ------ | ------ |
| mPD | | 0.51 | 0.67 |
| | | nPD: | 0.75 |
| | | N. weight: | 0.61 |
| | | Sc. PD: | 0.26 |
| | | Sc. rank: | 215.7 |

| HA | | | |
|---|---|---|---|
| Pos. | 397 | obs: | exp: |
| aaa | K | 634 | 358.90 |
| aag | K | 7 | 282.10 |
| --- | -- | ------ | ------ |
| mPD | | 0.022 | 0.49 |
| | | nPD: | 0.04 |
| | | N. weight: | 0.77 |
| | | Sc. PD: | -0.2 |
| | | Sc. rank: | -208.5 |

| HA | | | |
|---|---|---|---|
| Pos. | 398 | obs: | exp: |
| tta | L | 545 | 56.00 |
| ttg | L | 77 | 123.50 |
| ctt | L | 0 | 119.60 |
| ctc | L | 0 | 103.70 |
| cta | L | 17 | 96.57 |
| ctg | L | 2 | 141.70 |
| --- | -- | ------ | ------ |
| mPD | | 0.27 | 1.1 |
| | | nPD: | 0.25 |
| | | N. weight: | 2.7 |
| | | Sc. PD: | -0.18 |
| | | Sc. rank: | -216.6 |

| HA | | | |
|---|---|---|---|
| Pos. | 399 | obs: | exp: |
| aat | N | 349 | 335.00 |
| aac | N | 292 | 306.00 |
| --- | -- | ------ | ------ |
| mPD | | 0.50 | 0.50 |
| | | nPD: | 1. |
| | | N. weight: | 0.0093 |
| | | Sc. PD: | 0.0061 |
| | | Sc. rank: | 3.5 |

| HA | | | |
|---|---|---|---|
| Pos. | 400 | obs: | exp: |
| cgt | R | 73 | 26.61 |
| cgc | R | 1 | 31.39 |
| cga | R | 10 | 61.60 |
| cgg | R | 552 | 53.49 |
| aga | R | 3 | 288.30 |
| agg | R | 2 | 179.60 |
| --- | -- | ------ | ------ |
| mPD | | 0.26 | 0.96 |
| | | nPD: | 0.27 |
| | | N. weight: | 3. |
| | | Sc. PD: | -0.14 |
| | | Sc. rank: | -156.8 |

| HA | | | |
|---|---|---|---|
| Pos. | 401 | obs: | exp: |
| tta | L | 3 | 56.00 |
| ttg | L | 1 | 123.50 |
| ctt | L | 518 | 119.60 |
| ctc | L | 44 | 103.70 |
| cta | L | 67 | 96.57 |
| ctg | L | 8 | 141.70 |
| --- | -- | ------ | ------ |
| mPD | | 0.34 | 1.1 |
| | | nPD: | 0.31 |
| | | N. weight: | 1.5 |
| | | Sc. PD: | -0.011 |
| | | Sc. rank: | -20.6 |

| HA | | | |
|---|---|---|---|
| Pos. | 402 | obs: | exp: |
| att | I | 69 | 240.60 |
| atc | I | 5 | 167.70 |
| ata | I | 566 | 231.70 |
| gtt | V | 0 | 0.21 |
| gtc | V | 0 | 0.21 |
| gta | V | 1 | 0.19 |
| gtg | V | 0 | 0.40 |
| --- | -- | ------ | ------ |
| mPD | | 0.21 | 0.66 |
| | | nPD: | 0.32 |
| | | N. weight: | 0.93 |
| | | Sc. PD: | 0.00041 |
| | | Sc. rank: | 3.5 |

| HA | | | |
|---|---|---|---|
| Pos. | 403 | obs: | exp: |
| gat | D | 1 | 38.42 |
| gac | D | 71 | 33.58 |
| gaa | E | 557 | 327.40 |
| gag | E | 12 | 241.60 |
| --- | -- | ------ | ------ |
| mPD | | 0.23 | 0.59 |
| | | nPD: | 0.39 |
| | | N. weight: | 0.71 |
| | | Sc. PD: | 0.054 |
| | | Sc. rank: | 80.5 |

| HA | | | |
|---|---|---|---|
| Pos. | 404 | obs: | exp: |
| cgt | R | 0 | 0.04 |
| cgc | R | 0 | 0.05 |
| cga | R | 0 | 0.10 |
| cgg | R | 0 | 0.08 |
| aaa | K | 628 | 358.40 |
| aag | K | 12 | 281.60 |
| aga | R | 1 | 0.45 |
| agg | R | 0 | 0.28 |
| --- | -- | ------ | ------ |
| mPD | | 0.040 | 0.50 |
| | | nPD: | 0.08 |
| | | N. weight: | 0.72 |
| | | Sc. PD: | -0.16 |
| | | Sc. rank: | -163.9 |

| HA | | | |
|---|---|---|---|
| Pos. | 405 | obs: | exp: |
| act | T | 368 | 168.80 |
| acc | T | 198 | 137.10 |
| aca | T | 73 | 279.90 |
| acg | T | 0 | 53.12 |
| aat | N | 2 | 1.05 |
| aac | N | 0 | 0.95 |
| --- | -- | ------ | ------ |
| mPD | | 0.57 | 0.69 |
| | | nPD: | 0.82 |
| | | N. weight: | 0.61 |
| | | Sc. PD: | 0.3 |
| | | Sc. rank: | 227.1 |

| HA | | | |
|---|---|---|---|
| Pos. | 406 | obs: | exp: |
| aat | N | 102 | 335.00 |
| aac | N | 539 | 306.00 |
| --- | -- | ------ | ------ |
| mPD | | 0.27 | 0.50 |
| | | nPD: | 0.54 |
| | | N. weight: | 0.43 |
| | | Sc. PD: | 0.091 |
| | | Sc. rank: | 105.6 |

| HA | | | |
|---|---|---|---|
| Pos. | 407 | obs: | exp: |
| tta | L | 0 | 0.09 |
| ttg | L | 0 | 0.19 |
| ctt | L | 0 | 0.19 |
| ctc | L | 0 | 0.16 |
| cta | L | 1 | 0.15 |
| ctg | L | 0 | 0.22 |
| cat | H | 1 | 0.54 |
| cac | H | 0 | 0.46 |
| caa | Q | 566 | 340.70 |
| cag | Q | 73 | 298.30 |
| --- | -- | ------ | ------ |
| mPD | | 0.21 | 0.50 |
| | | nPD: | 0.41 |
| | | N. weight: | 0.43 |
| | | Sc. PD: | 0.041 |
| | | Sc. rank: | 61.0 |

| HA | | | |
|---|---|---|---|
| Pos. | 408 | obs: | exp: |
| caa | Q | 316 | 341.70 |
| cag | Q | 325 | 299.30 |
| --- | -- | ------ | ------ |
| mPD | | 0.50 | 0.50 |
| | | nPD: | 1.01 |
| | | N. weight: | 0.013 |
| | | Sc. PD: | 0.0084 |
| | | Sc. rank: | 4.8 |

| HA | | | |
|---|---|---|---|
| Pos. | 409 | obs: | exp: |
| ttt | F | 606 | 263.50 |
| ttc | F | 35 | 377.50 |
| --- | -- | ------ | ------ |
| mPD | | 0.10 | 0.48 |
| | | nPD: | 0.21 |
| | | N. weight: | 0.97 |
| | | Sc. PD: | -0.095 |
| | | Sc. rank: | -113.3 |

| HA | | | |
|---|---|---|---|
| Pos. | 410 | obs: | exp: |
| aaa | K | 0 | 0.56 |
| aag | K | 1 | 0.44 |
| gaa | E | 73 | 366.50 |
| gag | E | 564 | 270.50 |
| ggt | G | 0 | 0.37 |
| ggc | G | 0 | 0.41 |
| gga | G | 0 | 1.45 |
| ggg | G | 3 | 0.77 |
| --- | -- | ------ | ------ |
| mPD | | 0.21 | 0.50 |
| | | nPD: | 0.43 |
| | | N. weight: | 0.7 |
| | | Sc. PD: | 0.075 |
| | | Sc. rank: | 105.0 |

| HA | | | |
|---|---|---|---|
| Pos. | 411 | obs: | exp: |
| tta | L | 346 | 55.48 |
| ttg | L | 207 | 122.30 |
| tct | S | 0 | 0.32 |
| tcc | S | 0 | 0.28 |
| tca | S | 0 | 0.44 |
| tcg | S | 2 | 0.12 |
| ctt | L | 1 | 118.50 |
| ctc | L | 0 | 102.70 |
| cta | L | 13 | 95.66 |
| ctg | L | 68 | 140.40 |
| atg | M | 4 | 4.00 |
| agt | S | 0 | 0.44 |
| agc | S | 0 | 0.40 |
| --- | -- | ------ | ------ |
| mPD | | 0.74 | 1.1 |
| | | nPD: | 0.65 |
| | | N. weight: | 1.5 |
| | | Sc. PD: | 0.5 |
| | | Sc. rank: | 502.0 |

| HA | | | |
|---|---|---|---|
| Pos. | 412 | obs: | exp: |
| att | I | 5 | 241.00 |
| atc | I | 3 | 168.00 |
| ata | I | 633 | 232.00 |
| --- | -- | ------ | ------ |
| mPD | | 0.025 | 0.66 |
| | | nPD: | 0.04 |
| | | N. weight: | 1.4 |
| | | Sc. PD: | -0.37 |
| | | Sc. rank: | -386.9 |

| HA | | | |
|---|---|---|---|
| Pos. | 413 | obs: | exp: |
| gat | D | 6 | 342.00 |
| gac | D | 635 | 299.00 |
| --- | -- | ------ | ------ |
| mPD | | 0.019 | 0.50 |
| | | nPD: | 0.04 |
| | | N. weight: | 1. |
| | | Sc. PD: | -0.28 |
| | | Sc. rank: | -292.5 |

| HA | | | |
|---|---|---|---|
| Pos. | 414 | obs: | exp: |
| aat | N | 576 | 335.00 |
| aac | N | 65 | 306.00 |
| --- | -- | ------ | ------ |
| mPD | | 0.18 | 0.50 |
| | | nPD: | 0.37 |
| | | N. weight: | 0.49 |
| | | Sc. PD: | 0.024 |
| | | Sc. rank: | 38.6 |

,

| HA | | | |
|---|---|---|---|
| Pos. | 415 | obs: | exp: |
| gaa | E | 548 | 368.80 |
| gag | E | 93 | 272.20 |
| --- | -- | ------ | ------ |
| mPD | | 0.25 | 0.49 |
| | | nPD: | 0.51 |
| | | N. weight: | 0.27 |
| | | Sc. PD: | 0.051 |
| | | Sc. rank: | 63.9 |

,

| HA | | | |
|---|---|---|---|
| Pos. | 416 | obs: | exp: |
| ttt | F | 0 | 263.50 |
| ttc | F | 641 | 377.50 |
| --- | -- | ------ | ------ |
| mPD | | 0 | 0.48 |
| | | nPD: | 0. |
| | | N. weight: | 0.77 |
| | | Sc. PD: | -0.23 |
| | | Sc. rank: | -292.9 |

,

| HA | | | |
|---|---|---|---|
| Pos. | 417 | obs: | exp: |
| tct | S | 0 | 10.33 |
| tcc | S | 0 | 9.05 |
| tca | S | 0 | 14.40 |
| tcg | S | 0 | 3.96 |
| att | I | 4 | 1.50 |
| atc | I | 0 | 1.05 |
| ata | I | 0 | 1.45 |
| act | T | 553 | 147.40 |
| acc | T | 4 | 119.70 |
| aca | T | 0 | 244.50 |
| acg | T | 1 | 46.39 |
| aat | N | 8 | 6.27 |
| aac | N | 4 | 5.73 |
| agt | S | 65 | 14.30 |
| agc | S | 0 | 12.95 |
| gct | A | 1 | 0.50 |
| gcc | A | 1 | 0.41 |
| gca | A | 0 | 0.89 |
| gcg | A | 0 | 0.20 |
| --- | -- | ------ | ------ |
| mPD | | 0.26 | 0.94 |
| | | nPD: | 0.28 |
| | | N. weight: | 1.9 |
| | | Sc. PD: | -0.063 |
| | | Sc. rank: | -82.0 |

,

| HA | | | |
|---|---|---|---|
| Pos. | 418 | obs: | exp: |
| gat | D | 1 | 0.53 |
| gac | D | 0 | 0.47 |
| gaa | E | 101 | 368.30 |
| gag | E | 539 | 271.70 |
| --- | -- | ------ | ------ |
| mPD | | 0.27 | 0.49 |
| | | nPD: | 0.55 |
| | | N. weight: | 0.55 |
| | | Sc. PD: | 0.12 |
| | | Sc. rank: | 142.9 |

,

| HA | | | |
|---|---|---|---|
| Pos. | 419 | obs: | exp: |
| att | I | 8 | 28.95 |
| atc | I | 0 | 20.18 |
| ata | I | 69 | 27.87 |
| gtt | V | 494 | 115.90 |
| gtc | V | 15 | 118.40 |
| gta | V | 54 | 105.70 |
| gtg | V | 1 | 224.00 |
| --- | -- | ------ | ------ |
| mPD | | 0.56 | 0.95 |
| | | nPD: | 0.59 |
| | | N. weight: | 1.6 |
| | | Sc. PD: | 0.43 |
| | | Sc. rank: | 471.9 |

,

| HA | | | |
|---|---|---|---|
| Pos. | 420 | obs: | exp: |
| gaa | E | 554 | 368.80 |
| gag | E | 87 | 272.20 |
| --- | -- | ------ | ------ |
| mPD | | 0.23 | 0.49 |
| | | nPD: | 0.48 |
| | | N. weight: | 0.3 |
| | | Sc. PD: | 0.047 |
| | | Sc. rank: | 60.1 |

,

| HA | | | |
|---|---|---|---|
| Pos. | 421 | obs: | exp: |
| cat | H | 0 | 0.54 |
| cac | H | 1 | 0.46 |
| caa | Q | 67 | 37.85 |
| cag | Q | 4 | 33.15 |
| cgt | R | 0 | 2.82 |
| cgc | R | 0 | 3.33 |
| cga | R | 0 | 6.54 |
| cgg | R | 0 | 5.67 |
| act | T | 0 | 0.26 |
| acc | T | 0 | 0.21 |
| aca | T | 0 | 0.44 |
| acg | T | 1 | 0.08 |
| aaa | K | 10 | 278.90 |
| aag | K | 488 | 219.10 |
| aga | R | 11 | 30.58 |
| agg | R | 57 | 19.06 |
| ggt | G | 0 | 0.25 |
| ggc | G | 0 | 0.27 |
| gga | G | 0 | 0.97 |
| ggg | G | 2 | 0.52 |
| --- | -- | ------ | ------ |
| mPD | | 0.64 | 0.95 |
| | | nPD: | 0.68 |
| | | N. weight: | 1. |
| | | Sc. PD: | 0.36 |
| | | Sc. rank: | 339.2 |

,

| HA | | | |
|---|---|---|---|
| Pos. | 422 | obs: | exp: |
| caa | Q | 598 | 341.70 |
| cag | Q | 43 | 299.30 |
| --- | -- | ------ | ------ |
| mPD | | 0.13 | 0.50 |
| | | nPD: | 0.25 |
| | | N. weight: | 0.58 |
| | | Sc. PD: | -0.036 |
| | | Sc. rank: | -42.2 |

,

| HA | | | |
|---|---|---|---|
| Pos. | 423 | obs: | exp: |
| tta | L | 0 | 0.26 |
| ttg | L | 0 | 0.58 |
| ctt | L | 3 | 0.56 |
| ctc | L | 0 | 0.49 |
| cta | L | 0 | 0.45 |
| ctg | L | 0 | 0.66 |
| att | I | 626 | 239.10 |
| atc | I | 10 | 166.70 |
| ata | I | 0 | 230.20 |
| atg | M | 1 | 1.00 |
| act | T | 1 | 0.26 |
| acc | T | 0 | 0.21 |
| aca | T | 0 | 0.44 |
| acg | T | 0 | 0.08 |
| --- | -- | ------ | ------ |
| mPD | | 0.046 | 0.67 |
| | | nPD: | 0.07 |
| | | N. weight: | 1.3 |
| | | Sc. PD: | -0.32 |
| | | Sc. rank: | -319.3 |

,

| HA | | | |
|---|---|---|---|
| Pos. | 424 | obs: | exp: |
| ggt | G | 78 | 79.09 |
| ggc | G | 491 | 86.68 |
| gga | G | 3 | 310.10 |
| ggg | G | 69 | 165.10 |
| --- | -- | ------ | ------ |
| mPD | | 0.39 | 0.67 |
| | | nPD: | 0.58 |
| | | N. weight: | 1.8 |
| | | Sc. PD: | 0.46 |
| | | Sc. rank: | 508.8 |

,

| HA | | | |
|---|---|---|---|
| Pos. | 425 | obs: | exp: |
| aat | N | 628 | 335.00 |
| aac | N | 13 | 306.00 |
| --- | -- | ------ | ------ |
| mPD | | 0.040 | 0.50 |
| | | nPD: | 0.08 |
| | | N. weight: | 0.81 |
| | | Sc. PD: | -0.18 |
| | | Sc. rank: | -184.5 |

,

| HA | | | |
|---|---|---|---|
| Pos. | 426 | obs: | exp: |
| atg | M | 1 | 1.00 |
| gtt | V | 0 | 131.50 |
| gtc | V | 72 | 134.30 |
| gta | V | 38 | 119.90 |
| gtg | V | 530 | 254.20 |
| --- | -- | ------ | ------ |
| mPD | | 0.30 | 0.72 |
| | | nPD: | 0.42 |
| | | N. weight: | 0.7 |
| | | Sc. PD: | 0.068 |
| | | Sc. rank: | 99.4 |

,

| HA | | | |
|---|---|---|---|
| Pos. | 427 | obs: | exp: |
| att | I | 77 | 241.00 |
| atc | I | 2 | 168.00 |
| ata | I | 562 | 232.00 |
| --- | -- | ------ | ------ |
| mPD | | 0.22 | 0.66 |
| | | nPD: | 0.33 |
| | | N. weight: | 0.92 |
| | | Sc. PD: | 0.012 |
| | | Sc. rank: | 23.7 |

,

| HA | | | |
|---|---|---|---|
| Pos. | 428 | obs: | exp: |
| aat | N | 262 | 335.00 |
| aac | N | 379 | 306.00 |
| --- | -- | ------ | ------ |
| mPD | | 0.48 | 0.50 |
| | | nPD: | 0.97 |
| | | N. weight: | 0.046 |
| | | Sc. PD: | 0.029 |
| | | Sc. rank: | 17.4 |

,

| HA | | | |
|---|---|---|---|
| Pos. | 429 | obs: | exp: |
| tgg | W | 641 | 641.00 |
| --- | -- | ------ | ------ |
| mPD | | 0 | 0 |
| | | nPD: | 1. |
| | | N. weight: | 0. |
| | | Sc. PD: | 0 |
| | | Sc. rank: | 0 |

,

| HA | | | |
|---|---|---|---|
| Pos. | 430 | obs: | exp: |
| act | T | 14 | 169.30 |
| acc | T | 540 | 137.50 |
| aca | T | 86 | 280.80 |
| acg | T | 1 | 53.29 |
| --- | -- | ------ | ------ |
| mPD | | 0.27 | 0.69 |
| | | nPD: | 0.4 |
| | | N. weight: | 1.4 |
| | | Sc. PD: | 0.11 |
| | | Sc. rank: | 168.0 |

,

| HA | | | |
|---|---|---|---|
| Pos. | 431 | obs: | exp: |
| cgt | R | 0 | 26.61 |
| cgc | R | 0 | 31.39 |
| cga | R | 71 | 61.60 |
| cgg | R | 0 | 53.49 |
| aga | R | 565 | 288.30 |
| agg | R | 5 | 179.60 |
| --- | -- | ------ | ------ |
| mPD | | 0.21 | 0.96 |
| | | nPD: | 0.22 |
| | | N. weight: | 0.86 |
| | | Sc. PD: | -0.078 |
| | | Sc. rank: | -88.8 |

,

| HA | | | |
|---|---|---|---|
| Pos. | 432 | obs: | exp: |
| gat | D | 491 | 342.00 |
| gac | D | 150 | 299.00 |
| --- | -- | ------ | ------ |
| mPD | | 0.36 | 0.50 |
| | | nPD: | 0.72 |
| | | N. weight: | 0.18 |
| | | Sc. PD: | 0.069 |
| | | Sc. rank: | 60.1 |

,

| HA | | | |
|---|---|---|---|
| Pos. | 433 | obs: | exp: |
| tct | S | 53 | 101.90 |
| tcc | S | 516 | 89.29 |
| tca | S | 72 | 142.00 |
| tcg | S | 0 | 39.07 |
| agt | S | 0 | 141.00 |
| agc | S | 0 | 127.70 |
| --- | -- | ------ | ------ |
| mPD | | 0.33 | 1.7 |
| | | nPD: | 0.2 |
| | | N. weight: | 1.9 |
| | | Sc. PD: | -0.21 |
| | | Sc. rank: | -246.9 |

,

| | | | |
|---|---|---|---|
| HA | | | |
| Pos. | 434 | obs: | exp: |
| tta | L | 0 | 0.26 |
| ttg | L | 2 | 0.58 |
| ctt | L | 0 | 0.56 |
| ctc | L | 0 | 0.49 |
| cta | L | 0 | 0.45 |
| ctg | L | 1 | 0.66 |
| att | I | 0 | 30.83 |
| atc | I | 2 | 21.49 |
| ata | I | 80 | 29.68 |
| atg | M | 555 | 555.00 |
| gtt | V | 0 | 0.21 |
| gtc | V | 0 | 0.21 |
| gta | V | 0 | 0.19 |
| gtg | V | 1 | 0.40 |
| --- | -- | ------ | ------ |
| mPD | | 0.24 | 0.25 |
| | | nPD: | 0.94 |
| | | N. weight: | 0.18 |
| | | Sc. PD: | 0.11 |
| | | Sc. rank: | 69.3 |

,

| | | | |
|---|---|---|---|
| HA | | | |
| Pos. | 435 | obs: | exp: |
| act | T | 71 | 166.70 |
| acc | T | 1 | 135.40 |
| aca | T | 550 | 276.40 |
| acg | T | 9 | 52.46 |
| gct | A | 1 | 2.52 |
| gcc | A | 0 | 2.05 |
| gca | A | 9 | 4.46 |
| gcg | A | 0 | 0.98 |
| --- | -- | ------ | ------ |
| mPD | | 0.26 | 0.72 |
| | | nPD: | 0.36 |
| | | N. weight: | 0.71 |
| | | Sc. PD: | 0.03 |
| | | Sc. rank: | 52.7 |

,

| | | | |
|---|---|---|---|
| HA | | | |
| Pos. | 436 | obs: | exp: |
| gaa | E | 440 | 368.80 |
| gag | E | 201 | 272.20 |
| --- | -- | ------ | ------ |
| mPD | | 0.43 | 0.49 |
| | | nPD: | 0.88 |
| | | N. weight: | 0.046 |
| | | Sc. PD: | 0.025 |
| | | Sc. rank: | 17.1 |

,

| | | | |
|---|---|---|---|
| HA | | | |
| Pos. | 437 | obs: | exp: |
| tta | L | 0 | 0.09 |
| ttg | L | 1 | 0.19 |
| ctt | L | 0 | 0.19 |
| ctc | L | 0 | 0.16 |
| cta | L | 0 | 0.15 |
| ctg | L | 0 | 0.22 |
| att | I | 0 | 0.38 |
| atc | I | 0 | 0.26 |
| ata | I | 1 | 0.36 |
| atg | M | 12 | 12.00 |
| gtt | V | 0 | 128.90 |
| gtc | V | 0 | 131.60 |
| gta | V | 53 | 117.50 |
| gtg | V | 574 | 249.00 |
| --- | -- | ------ | ------ |
| mPD | | 0.20 | 0.76 |
| | | nPD: | 0.26 |
| | | N. weight: | 1. |
| | | Sc. PD: | -0.054 |
| | | Sc. rank: | -66.7 |

,

| | | | |
|---|---|---|---|
| HA | | | |
| Pos. | 438 | obs: | exp: |
| tgg | W | 641 | 641.00 |
| --- | -- | ------ | ------ |
| mPD | | 0 | 0 |
| | | nPD: | 1. |
| | | N. weight: | 0. |
| | | Sc. PD: | 0 |
| | | Sc. rank: | 0 |

,

| | | | |
|---|---|---|---|
| HA | | | |
| Pos. | 439 | obs: | exp: |
| tct | S | 42 | 101.90 |
| tcc | S | 472 | 89.29 |
| tca | S | 63 | 142.00 |
| tcg | S | 64 | 39.07 |
| agt | S | 0 | 141.00 |
| agc | S | 0 | 127.70 |
| --- | -- | ------ | ------ |
| mPD | | 0.43 | 1.7 |
| | | nPD: | 0.26 |
| | | N. weight: | 1.7 |
| | | Sc. PD: | -0.088 |
| | | Sc. rank: | -103.1 |

,

| | | | |
|---|---|---|---|
| HA | | | |
| Pos. | 440 | obs: | exp: |
| tct | S | 0 | 0.16 |
| tcc | S | 1 | 0.14 |
| tca | S | 0 | 0.22 |
| tcg | S | 0 | 0.06 |
| tat | Y | 457 | 330.80 |
| tac | Y | 182 | 308.20 |
| aat | N | 1 | 0.52 |
| aac | N | 0 | 0.48 |
| agt | S | 0 | 0.22 |
| agc | S | 0 | 0.20 |
| --- | -- | ------ | ------ |
| mPD | | 0.41 | 0.51 |
| | | nPD: | 0.82 |
| | | N. weight: | 0.13 |
| | | Sc. PD: | 0.064 |
| | | Sc. rank: | 48.8 |

,

| | | | |
|---|---|---|---|
| HA | | | |
| Pos. | 441 | obs: | exp: |
| aat | N | 300 | 335.00 |
| aac | N | 341 | 306.00 |
| --- | -- | ------ | ------ |
| mPD | | 0.50 | 0.50 |
| | | nPD: | 1. |
| | | N. weight: | 0.017 |
| | | Sc. PD: | 0.011 |
| | | Sc. rank: | 6.3 |

,

| | | | |
|---|---|---|---|
| HA | | | |
| Pos. | 442 | obs: | exp: |
| gct | A | 634 | 161.20 |
| gcc | A | 6 | 131.10 |
| gca | A | 0 | 285.20 |
| gcg | A | 0 | 62.45 |
| ggt | G | 1 | 0.12 |
| ggc | G | 0 | 0.14 |
| gga | G | 0 | 0.48 |
| ggg | G | 0 | 0.26 |
| --- | -- | ------ | ------ |
| mPD | | 0.022 | 0.69 |
| | | nPD: | 0.03 |
| | | N. weight: | 1.9 |
| | | Sc. PD: | -0.53 |
| | | Sc. rank: | -559.6 |

,

| | | | |
|---|---|---|---|
| HA | | | |
| Pos. | 443 | obs: | exp: |
| gaa | E | 621 | 368.80 |
| gag | E | 20 | 272.20 |
| --- | -- | ------ | ------ |
| mPD | | 0.061 | 0.49 |
| | | nPD: | 0.12 |
| | | N. weight: | 0.62 |
| | | Sc. PD: | -0.12 |
| | | Sc. rank: | -120.2 |

,

| | | | |
|---|---|---|---|
| HA | | | |
| Pos. | 444 | obs: | exp: |
| tta | L | 0 | 56.00 |
| ttg | L | 61 | 123.50 |
| ctt | L | 3 | 119.60 |
| ctc | L | 543 | 103.70 |
| cta | L | 24 | 96.57 |
| ctg | L | 10 | 141.70 |
| --- | -- | ------ | ------ |
| mPD | | 0.44 | 1.1 |
| | | nPD: | 0.4 |
| | | N. weight: | 1.8 |
| | | Sc. PD: | 0.14 |
| | | Sc. rank: | 217.5 |

,

| | | | |
|---|---|---|---|
| HA | | | |
| Pos. | 445 | obs: | exp: |
| tta | L | 276 | 56.00 |
| ttg | L | 284 | 123.50 |
| ctt | L | 0 | 119.60 |
| ctc | L | 2 | 103.70 |
| cta | L | 8 | 96.57 |
| ctg | L | 71 | 141.70 |
| --- | -- | ------ | ------ |
| mPD | | 0.72 | 1.1 |
| | | nPD: | 0.65 |
| | | N. weight: | 1.4 |
| | | Sc. PD: | 0.44 |
| | | Sc. rank: | 448.9 |

,

| | | | |
|---|---|---|---|
| HA | | | |
| Pos. | 446 | obs: | exp: |
| att | I | 0 | 8.27 |
| atc | I | 0 | 5.77 |
| ata | I | 22 | 7.96 |
| gtt | V | 32 | 127.20 |
| gtc | V | 3 | 129.90 |
| gta | V | 544 | 116.00 |
| gtg | V | 40 | 245.80 |
| --- | -- | ------ | ------ |
| mPD | | 0.28 | 0.79 |
| | | nPD: | 0.35 |
| | | N. weight: | 1.7 |
| | | Sc. PD: | 0.063 |
| | | Sc. rank: | 111.1 |

,

| | | | |
|---|---|---|---|
| HA | | | |
| Pos. | 447 | obs: | exp: |
| act | T | 0 | 0.53 |
| acc | T | 0 | 0.43 |
| aca | T | 2 | 0.88 |
| acg | T | 0 | 0.17 |
| gct | A | 52 | 158.40 |
| gcc | A | 0 | 128.80 |
| gca | A | 557 | 280.30 |
| gcg | A | 20 | 61.38 |
| gat | D | 1 | 0.53 |
| gac | D | 0 | 0.47 |
| gaa | E | 0 | 5.18 |
| gag | E | 9 | 3.82 |
| --- | -- | ------ | ------ |
| mPD | | 0.27 | 0.72 |
| | | nPD: | 0.37 |
| | | N. weight: | 0.72 |
| | | Sc. PD: | 0.038 |
| | | Sc. rank: | 59.3 |

,

| | | | |
|---|---|---|---|
| HA | | | |
| Pos. | 448 | obs: | exp: |
| atg | M | 640 | 640.00 |
| act | T | 0 | 0.26 |
| acc | T | 0 | 0.21 |
| aca | T | 0 | 0.44 |
| acg | T | 1 | 0.08 |
| --- | -- | ------ | ------ |
| mPD | | 0.0031 | 0.0060 |
| | | nPD: | 0.52 |
| | | N. weight: | 0.0057 |
| | | Sc. PD: | 0.0011 |
| | | Sc. rank: | 1.4 |

,

| | | | |
|---|---|---|---|
| HA | | | |
| Pos. | 449 | obs: | exp: |
| gaa | E | 121 | 368.80 |
| gag | E | 520 | 272.20 |
| --- | -- | ------ | ------ |
| mPD | | 0.31 | 0.49 |
| | | nPD: | 0.63 |
| | | N. weight: | 0.47 |
| | | Sc. PD: | 0.14 |
| | | Sc. rank: | 144.4 |

,

| | | | |
|---|---|---|---|
| HA | | | |
| Pos. | 450 | obs: | exp: |
| aat | N | 541 | 335.00 |
| aac | N | 100 | 306.00 |
| --- | -- | ------ | ------ |
| mPD | | 0.26 | 0.50 |
| | | nPD: | 0.53 |
| | | N. weight: | 0.34 |
| | | Sc. PD: | 0.07 |
| | | Sc. rank: | 82.8 |

,

| | | | |
|---|---|---|---|
| HA | | | |
| Pos. | 451 | obs: | exp: |
| caa | Q | 100 | 341.70 |
| cag | Q | 541 | 299.30 |
| --- | -- | ------ | ------ |
| mPD | | 0.26 | 0.50 |
| | | nPD: | 0.53 |
| | | N. weight: | 0.46 |
| | | Sc. PD: | 0.094 |
| | | Sc. rank: | 112.4 |

,

| | | | |
|---|---|---|---|
| HA | | | |
| Pos. | 452 | obs: | exp: |
| cat | H | 67 | 346.80 |
| cac | H | 573 | 293.20 |
| caa | Q | 1 | 0.53 |
| cag | Q | 0 | 0.47 |
| --- | -- | ------ | ------ |
| mPD | | 0.19 | 0.50 |
| | | nPD: | 0.38 |
| | | N. weight: | 0.63 |
| | | Sc. PD: | 0.04 |
| | | Sc. rank: | 65.8 |

,

| | | | |
|---|---|---|---|
| HA | | | |
| Pos. | 453 | obs: | exp: |
| act | T | 9 | 169.30 |
| acc | T | 3 | 137.50 |
| aca | T | 626 | 280.80 |
| acg | T | 3 | 53.29 |
| --- | -- | ------ | ------ |
| mPD | | 0.046 | 0.69 |
| | | nPD: | 0.07 |
| | | N. weight: | 1.1 |
| | | Sc. PD: | -0.25 |
| | | Sc. rank: | -256.7 |

,

| | | | |
|---|---|---|---|
| HA | | | |
| Pos. | 454 | obs: | exp: |
| tta | L | 0 | 0.09 |
| ttg | L | 0 | 0.19 |
| ctt | L | 1 | 0.19 |
| ctc | L | 0 | 0.16 |
| cta | L | 0 | 0.15 |
| ctg | L | 0 | 0.22 |
| att | I | 548 | 240.20 |
| atc | I | 19 | 167.50 |
| ata | I | 72 | 231.30 |
| gtt | V | 1 | 0.21 |
| gtc | V | 0 | 0.21 |
| gta | V | 0 | 0.19 |
| gtg | V | 0 | 0.40 |
| --- | -- | ------ | ------ |
| mPD | | 0.26 | 0.67 |
| | | nPD: | 0.39 |
| | | N. weight: | 0.77 |
| | | Sc. PD: | 0.052 |
| | | Sc. rank: | 81.5 |

,

| | | | |
|---|---|---|---|
| HA | | | |
| Pos. | 455 | obs: | exp: |
| gat | D | 587 | 342.00 |
| gac | D | 54 | 299.00 |
| --- | -- | ------ | ------ |
| mPD | | 0.15 | 0.50 |
| | | nPD: | 0.31 |
| | | N. weight: | 0.52 |
| | | Sc. PD: | -0.0025 |
| | | Sc. rank: | -3.1 |

,

| | | | |
|---|---|---|---|
| HA | | | |
| Pos. | 456 | obs: | exp: |
| tta | L | 0 | 55.91 |
| ttg | L | 27 | 123.30 |
| ctt | L | 72 | 119.40 |
| ctc | L | 0 | 103.50 |
| cta | L | 4 | 96.42 |
| ctg | L | 537 | 141.50 |
| atg | M | 1 | 1.00 |
| --- | -- | ------ | ------ |
| mPD | | 0.29 | 1.1 |
| | | nPD: | 0.26 |
| | | N. weight: | 1.4 |
| | | Sc. PD: | -0.072 |
| | | Sc. rank: | -85.8 |

,

| | | | |
|---|---|---|---|
| HA | | | |
| Pos. | 457 | obs: | exp: |
| act | T | 1 | 0.79 |
| acc | T | 2 | 0.64 |
| aca | T | 0 | 1.31 |
| acg | T | 0 | 0.25 |
| gct | A | 130 | 160.70 |
| gcc | A | 345 | 130.70 |
| gca | A | 162 | 284.40 |
| gcg | A | 1 | 62.26 |
| --- | -- | ------ | ------ |
| mPD | | 0.61 | 0.70 |
| | | nPD: | 0.88 |
| | | N. weight: | 0.51 |
| | | Sc. PD: | 0.28 |
| | | Sc. rank: | 190.1 |

,

| | | | |
|---|---|---|---|
| HA | | | |
| Pos. | 458 | obs: | exp: |
| gat | D | 117 | 342.00 |
| gac | D | 524 | 299.00 |
| --- | -- | ------ | ------ |
| mPD | | 0.30 | 0.50 |
| | | nPD: | 0.6 |
| | | N. weight: | 0.39 |
| | | Sc. PD: | 0.11 |
| | | Sc. rank: | 118.3 |

,

| HA | | | |
|---|---|---|---|
| Pos. | 459 | obs: | exp: |
| tct | S | 3 | 101.90 |
| tcc | S | 0 | 89.29 |
| tca | S | 635 | 142.00 |
| tcg | S | 3 | 39.07 |
| agt | S | 0 | 141.00 |
| agc | S | 0 | 127.70 |
| --- | -- | ------ | ------ |
| mPD | | 0.019 | 1.7 |
| | | nPD: | 0.01 |
| | | N. weight: | 2.1 |
| | | Sc. PD: | -0.62 |
| | | Sc. rank: | -687.7 |

,

| HA | | | |
|---|---|---|---|
| Pos. | 460 | obs: | exp: |
| gaa | E | 632 | 368.80 |
| gag | E | 9 | 272.20 |
| --- | -- | ------ | ------ |
| mPD | | 0.028 | 0.49 |
| | | nPD: | 0.06 |
| | | N. weight: | 0.71 |
| | | Sc. PD: | -0.18 |
| | | Sc. rank: | -180.3 |

,

| HA | | | |
|---|---|---|---|
| Pos. | 461 | obs: | exp: |
| tta | L | 0 | 0.09 |
| ttg | L | 0 | 0.19 |
| ctt | L | 0 | 0.19 |
| ctc | L | 0 | 0.16 |
| cta | L | 0 | 0.15 |
| ctg | L | 1 | 0.22 |
| atg | M | 640 | 640.00 |
| --- | -- | ------ | ------ |
| mPD | | 0.0031 | 0.0049 |
| | | nPD: | 0.63 |
| | | N. weight: | 0.0034 |
| | | Sc. PD: | 0.001 |
| | | Sc. rank: | 1.1 |

,

| HA | | | |
|---|---|---|---|
| Pos. | 462 | obs: | exp: |
| tct | S | 0 | 0.16 |
| tcc | S | 0 | 0.14 |
| tca | S | 0 | 0.22 |
| tcg | S | 0 | 0.06 |
| aat | N | 57 | 334.00 |
| aac | N | 582 | 305.00 |
| aaa | K | 1 | 0.56 |
| aag | K | 0 | 0.44 |
| agt | S | 0 | 0.22 |
| agc | S | 1 | 0.20 |
| --- | -- | ------ | ------ |
| mPD | | 0.17 | 0.51 |
| | | nPD: | 0.33 |
| | | N. weight: | 0.64 |
| | | Sc. PD: | 0.01 |
| | | Sc. rank: | 20.2 |

,

| HA | | | |
|---|---|---|---|
| Pos. | 463 | obs: | exp: |
| aaa | K | 628 | 358.90 |
| aag | K | 13 | 282.10 |
| --- | -- | ------ | ------ |
| mPD | | 0.040 | 0.49 |
| | | nPD: | 0.08 |
| | | N. weight: | 0.72 |
| | | Sc. PD: | -0.16 |
| | | Sc. rank: | -160.3 |

,

| HA | | | |
|---|---|---|---|
| Pos. | 464 | obs: | exp: |
| tta | L | 3 | 56.00 |
| ttg | L | 7 | 123.50 |
| ctt | L | 71 | 119.60 |
| ctc | L | 1 | 103.70 |
| cta | L | 232 | 96.57 |
| ctg | L | 327 | 141.70 |
| --- | -- | ------ | ------ |
| mPD | | 0.61 | 1.1 |
| | | nPD: | 0.55 |
| | | N. weight: | 0.95 |
| | | Sc. PD: | 0.21 |
| | | Sc. rank: | 247.2 |

,

| HA | | | |
|---|---|---|---|
| Pos. | 465 | obs: | exp: |
| tat | Y | 66 | 331.80 |
| tac | Y | 575 | 309.20 |
| --- | -- | ------ | ------ |
| mPD | | 0.19 | 0.50 |
| | | nPD: | 0.37 |
| | | N. weight: | 0.58 |
| | | Sc. PD: | 0.031 |
| | | Sc. rank: | 47.7 |

,

| HA | | | |
|---|---|---|---|
| Pos. | 466 | obs: | exp: |
| gat | D | 0 | 0.53 |
| gac | D | 1 | 0.47 |
| gaa | E | 479 | 368.30 |
| gag | E | 161 | 271.70 |
| --- | -- | ------ | ------ |
| mPD | | 0.38 | 0.49 |
| | | nPD: | 0.77 |
| | | N. weight: | 0.1 |
| | | Sc. PD: | 0.046 |
| | | Sc. rank: | 37.6 |

,

| HA | | | |
|---|---|---|---|
| Pos. | 467 | obs: | exp: |
| cgt | R | 71 | 26.61 |
| cgc | R | 0 | 31.39 |
| cga | R | 508 | 61.60 |
| cgg | R | 53 | 53.49 |
| aga | R | 9 | 288.30 |
| agg | R | 0 | 179.60 |
| --- | -- | ------ | ------ |
| mPD | | 0.36 | 0.96 |
| | | nPD: | 0.37 |
| | | N. weight: | 2.6 |
| | | Sc. PD: | 0.14 |
| | | Sc. rank: | 230.8 |

,

| HA | | | |
|---|---|---|---|
| Pos. | 468 | obs: | exp: |
| gtt | V | 0 | 131.80 |
| gtc | V | 12 | 134.50 |
| gta | V | 42 | 120.10 |
| gtg | V | 587 | 254.60 |
| --- | -- | ------ | ------ |
| mPD | | 0.16 | 0.72 |
| | | nPD: | 0.22 |
| | | N. weight: | 0.96 |
| | | Sc. PD: | -0.091 |
| | | Sc. rank: | -108.8 |

,

| HA | | | |
|---|---|---|---|
| Pos. | 469 | obs: | exp: |
| cgt | R | 0 | 7.47 |
| cgc | R | 0 | 8.81 |
| cga | R | 0 | 17.30 |
| cgg | R | 0 | 15.02 |
| aaa | K | 125 | 258.10 |
| aag | K | 336 | 202.90 |
| aga | R | 36 | 80.95 |
| agg | R | 144 | 50.44 |
| --- | -- | ------ | ------ |
| mPD | | 0.78 | 1.1 |
| | | nPD: | 0.74 |
| | | N. weight: | 0.47 |
| | | Sc. PD: | 0.19 |
| | | Sc. rank: | 163.7 |

,

| HA | | | |
|---|---|---|---|
| Pos. | 470 | obs: | exp: |
| cgt | R | 0 | 23.37 |
| cgc | R | 0 | 27.57 |
| cga | R | 11 | 54.11 |
| cgg | R | 1 | 46.98 |
| aaa | K | 74 | 43.68 |
| aag | K | 4 | 34.32 |
| aga | R | 363 | 253.20 |
| agg | R | 188 | 157.80 |
| --- | -- | ------ | ------ |
| mPD | | 0.67 | 1.1 |
| | | nPD: | 0.59 |
| | | N. weight: | 0.41 |
| | | Sc. PD: | 0.11 |
| | | Sc. rank: | 121.5 |

,

| HA | | | |
|---|---|---|---|
| Pos. | 471 | obs: | exp: |
| caa | Q | 541 | 341.70 |
| cag | Q | 100 | 299.30 |
| --- | -- | ------ | ------ |
| mPD | | 0.26 | 0.50 |
| | | nPD: | 0.53 |
| | | N. weight: | 0.32 |
| | | Sc. PD: | 0.067 |
| | | Sc. rank: | 79.6 |

,

| HA | | | |
|---|---|---|---|
| Pos. | 472 | obs: | exp: |
| tta | L | 0 | 56.00 |
| ttg | L | 44 | 123.50 |
| ctt | L | 0 | 119.60 |
| ctc | L | 0 | 103.70 |
| cta | L | 12 | 96.57 |
| ctg | L | 585 | 141.70 |
| --- | -- | ------ | ------ |
| mPD | | 0.16 | 1.1 |
| | | nPD: | 0.15 |
| | | N. weight: | 1.7 |
| | | Sc. PD: | -0.28 |
| | | Sc. rank: | -289.8 |

,

| HA | | | |
|---|---|---|---|
| Pos. | 473 | obs: | exp: |
| cgt | R | 0 | 26.61 |
| cgc | R | 0 | 31.39 |
| cga | R | 0 | 61.60 |
| cgg | R | 0 | 53.49 |
| aga | R | 560 | 288.30 |
| agg | R | 81 | 179.60 |
| --- | -- | ------ | ------ |
| mPD | | 0.22 | 0.96 |
| | | nPD: | 0.23 |
| | | N. weight: | 0.71 |
| | | Sc. PD: | -0.058 |
| | | Sc. rank: | -67.7 |

,

| HA | | | |
|---|---|---|---|
| Pos. | 474 | obs: | exp: |
| gaa | E | 22 | 368.80 |
| gag | E | 619 | 272.20 |
| --- | -- | ------ | ------ |
| mPD | | 0.066 | 0.49 |
| | | nPD: | 0.14 |
| | | N. weight: | 1. |
| | | Sc. PD: | -0.18 |
| | | Sc. rank: | -189.7 |

,

| HA | | | |
|---|---|---|---|
| Pos. | 475 | obs: | exp: |
| aat | N | 621 | 335.00 |
| aac | N | 20 | 306.00 |
| --- | -- | ------ | ------ |
| mPD | | 0.061 | 0.50 |
| | | nPD: | 0.12 |
| | | N. weight: | 0.76 |
| | | Sc. PD: | -0.14 |
| | | Sc. rank: | -146.9 |

,

| HA | | | |
|---|---|---|---|
| Pos. | 476 | obs: | exp: |
| gct | A | 396 | 161.50 |
| gcc | A | 239 | 131.30 |
| gca | A | 5 | 285.70 |
| gcg | A | 1 | 62.55 |
| --- | -- | ------ | ------ |
| mPD | | 0.48 | 0.69 |
| | | nPD: | 0.7 |
| | | N. weight: | 1.1 |
| | | Sc. PD: | 0.4 |
| | | Sc. rank: | 370.4 |

,

| HA | | | |
|---|---|---|---|
| Pos. | 477 | obs: | exp: |
| gaa | E | 630 | 368.80 |
| gag | E | 11 | 272.20 |
| --- | -- | ------ | ------ |
| mPD | | 0.034 | 0.49 |
| | | nPD: | 0.07 |
| | | N. weight: | 0.69 |
| | | Sc. PD: | -0.16 |
| | | Sc. rank: | -165.9 |

,

| HA | | | |
|---|---|---|---|
| Pos. | 478 | obs: | exp: |
| aaa | K | 1 | 0.56 |
| aag | K | 0 | 0.44 |
| gaa | E | 579 | 368.30 |
| gag | E | 61 | 271.70 |
| --- | -- | ------ | ------ |
| mPD | | 0.18 | 0.49 |
| | | nPD: | 0.36 |
| | | N. weight: | 0.4 |
| | | Sc. PD: | 0.016 |
| | | Sc. rank: | 28.3 |

,

| HA | | | |
|---|---|---|---|
| Pos. | 479 | obs: | exp: |
| gat | D | 639 | 342.00 |
| gac | D | 2 | 299.00 |
| --- | -- | ------ | ------ |
| mPD | | 0.0062 | 0.50 |
| | | nPD: | 0.01 |
| | | N. weight: | 0.89 |
| | | Sc. PD: | -0.26 |
| | | Sc. rank: | -286.1 |

,

| HA | | | |
|---|---|---|---|
| Pos. | 480 | obs: | exp: |
| ggt | G | 14 | 79.09 |
| ggc | G | 506 | 86.68 |
| gga | G | 11 | 310.10 |
| ggg | G | 110 | 165.10 |
| --- | -- | ------ | ------ |
| mPD | | 0.35 | 0.67 |
| | | nPD: | 0.52 |
| | | N. weight: | 1.8 |
| | | Sc. PD: | 0.36 |
| | | Sc. rank: | 435.7 |

,

| HA | | | |
|---|---|---|---|
| Pos. | 481 | obs: | exp: |
| tct | S | 1 | 0.32 |
| tcc | S | 0 | 0.28 |
| tca | S | 0 | 0.44 |
| tcg | S | 0 | 0.12 |
| act | T | 602 | 168.80 |
| acc | T | 34 | 137.10 |
| aca | T | 3 | 279.90 |
| acg | T | 0 | 53.12 |
| agt | S | 1 | 0.44 |
| agc | S | 0 | 0.40 |
| --- | -- | ------ | ------ |
| mPD | | 0.12 | 0.69 |
| | | nPD: | 0.17 |
| | | N. weight: | 1.6 |
| | | Sc. PD: | -0.23 |
| | | Sc. rank: | -252.5 |

,

| HA | | | |
|---|---|---|---|
| Pos. | 482 | obs: | exp: |
| ggt | G | 416 | 79.09 |
| ggc | G | 154 | 86.68 |
| gga | G | 68 | 310.10 |
| ggg | G | 3 | 165.10 |
| --- | -- | ------ | ------ |
| mPD | | 0.51 | 0.67 |
| | | nPD: | 0.77 |
| | | N. weight: | 1.5 |
| | | Sc. PD: | 0.66 |
| | | Sc. rank: | 546.8 |

,

| HA | | | |
|---|---|---|---|
| Pos. | 483 | obs: | exp: |
| tgt | C | 171 | 264.20 |
| tgc | C | 470 | 376.80 |
| --- | -- | ------ | ------ |
| mPD | | 0.39 | 0.48 |
| | | nPD: | 0.81 |
| | | N. weight: | 0.075 |
| | | Sc. PD: | 0.036 |
| | | Sc. rank: | 27.4 |

,

HA

| Pos. | 484 | obs: | exp: |
|---|---|---|---|
| ttt | F | 362 | 263.50 |
| ttc | F | 279 | 377.50 |
| mPD | | 0.49 | 0.48 |
| | | nPD: | 1.02 |
| | | N. weight: | 0.078 |
| | | Sc. PD: | 0.052 |
| | | Sc. rank: | 29.4 |

,

HA

| Pos. | 485 | obs: | exp: |
|---|---|---|---|
| gaa | E | 535 | 368.80 |
| gag | E | 106 | 272.20 |
| mPD | | 0.28 | 0.49 |
| | | nPD: | 0.57 |
| | | N. weight: | 0.23 |
| | | Sc. PD: | 0.056 |
| | | Sc. rank: | 64.4 |

,

HA

| Pos. | 486 | obs: | exp: |
|---|---|---|---|
| tta | L | 10 | 0.87 |
| ttg | L | 0 | 1.93 |
| ctt | L | 0 | 1.87 |
| ctc | L | 0 | 1.62 |
| cta | L | 0 | 1.51 |
| ctg | L | 0 | 2.21 |
| att | I | 3 | 237.20 |
| atc | I | 6 | 165.40 |
| ata | I | 622 | 228.40 |
| mPD | | 0.059 | 0.69 |
| | | nPD: | 0.08 |
| | | N. weight: | 1.4 |
| | | Sc. PD: | -0.31 |
| | | Sc. rank: | -307.6 |

,

HA

| Pos. | 487 | obs: | exp: |
|---|---|---|---|
| ttt | F | 474 | 263.50 |
| ttc | F | 167 | 377.50 |
| mPD | | 0.39 | 0.48 |
| | | nPD: | 0.8 |
| | | N. weight: | 0.33 |
| | | Sc. PD: | 0.15 |
| | | Sc. rank: | 120.8 |

,

HA

| Pos. | 488 | obs: | exp: |
|---|---|---|---|
| cat | H | 74 | 347.30 |
| cac | H | 567 | 293.70 |
| mPD | | 0.20 | 0.50 |
| | | nPD: | 0.41 |
| | | N. weight: | 0.6 |
| | | Sc. PD: | 0.055 |
| | | Sc. rank: | 83.6 |

,

HA

| Pos. | 489 | obs: | exp: |
|---|---|---|---|
| aaa | K | 94 | 358.90 |
| aag | K | 547 | 282.10 |
| mPD | | 0.25 | 0.49 |
| | | nPD: | 0.51 |
| | | N. weight: | 0.55 |
| | | Sc. PD: | 0.1 |
| | | Sc. rank: | 127.9 |

,

HA

| Pos. | 490 | obs: | exp: |
|---|---|---|---|
| tgt | C | 640 | 264.20 |
| tgc | C | 1 | 376.80 |
| mPD | | 0.0031 | 0.48 |
| | | nPD: | 0.01 |
| | | N. weight: | 1.3 |
| | | Sc. PD: | -0.38 |
| | | Sc. rank: | -428.7 |

,

HA

| Pos. | 491 | obs: | exp: |
|---|---|---|---|
| aat | N | 1 | 0.52 |
| aac | N | 0 | 0.48 |
| gat | D | 638 | 341.50 |
| gac | D | 2 | 298.50 |
| mPD | | 0.0094 | 0.50 |
| | | nPD: | 0.02 |
| | | N. weight: | 0.89 |
| | | Sc. PD: | -0.25 |
| | | Sc. rank: | -272.3 |

,

HA

| Pos. | 492 | obs: | exp: |
|---|---|---|---|
| aat | N | 1 | 1.57 |
| aac | N | 2 | 1.43 |
| gat | D | 145 | 339.90 |
| gac | D | 492 | 297.10 |
| ggt | G | 0 | 0.12 |
| ggc | G | 1 | 0.14 |
| gga | G | 0 | 0.48 |
| ggg | G | 0 | 0.26 |
| mPD | | 0.36 | 0.51 |
| | | nPD: | 0.71 |
| | | N. weight: | 0.3 |
| | | Sc. PD: | 0.11 |
| | | Sc. rank: | 101.6 |

,

HA

| Pos. | 493 | obs: | exp: |
|---|---|---|---|
| caa | Q | 12 | 38.38 |
| cag | Q | 60 | 33.62 |
| aat | N | 18 | 9.93 |
| aac | N | 1 | 9.07 |
| gat | D | 10 | 291.80 |
| gac | D | 537 | 255.20 |
| gaa | E | 2 | 1.15 |
| gag | E | 0 | 0.85 |
| ggt | G | 0 | 0.12 |
| ggc | G | 1 | 0.14 |
| gga | G | 0 | 0.48 |
| ggg | G | 0 | 0.26 |
| mPD | | 0.54 | 0.85 |
| | | nPD: | 0.63 |
| | | N. weight: | 0.92 |
| | | Sc. PD: | 0.28 |
| | | Sc. rank: | 282.1 |

,

HA

| Pos. | 494 | obs: | exp: |
|---|---|---|---|
| tgt | C | 217 | 264.20 |
| tgc | C | 424 | 376.80 |
| mPD | | 0.45 | 0.48 |
| | | nPD: | 0.93 |
| | | N. weight: | 0.025 |
| | | Sc. PD: | 0.014 |
| | | Sc. rank: | 9.2 |

,

HA

| Pos. | 495 | obs: | exp: |
|---|---|---|---|
| atg | M | 641 | 641.00 |
| mPD | | 0 | 0 |
| | | nPD: | 1. |
| | | N. weight: | 0. |
| | | Sc. PD: | 0 |
| | | Sc. rank: | 0 |

,

HA

| Pos. | 496 | obs: | exp: |
|---|---|---|---|
| act | T | 0 | 1.32 |
| acc | T | 5 | 1.07 |
| aca | T | 0 | 2.19 |
| acg | T | 0 | 0.42 |
| gct | A | 8 | 141.30 |
| gcc | A | 553 | 114.90 |
| gca | A | 0 | 250.00 |
| gcg | A | 0 | 54.74 |
| gaa | E | 0 | 41.43 |
| gag | E | 72 | 30.57 |
| ggt | G | 3 | 0.37 |
| ggc | G | 0 | 0.41 |
| gga | G | 0 | 1.45 |
| ggg | G | 0 | 0.77 |
| mPD | | 0.45 | 0.91 |
| | | nPD: | 0.5 |
| | | N. weight: | 2.1 |
| | | Sc. PD: | 0.37 |
| | | Sc. rank: | 465.9 |

,

HA

| Pos. | 497 | obs: | exp: |
|---|---|---|---|
| tct | S | 0 | 101.40 |
| tcc | S | 0 | 88.88 |
| tca | S | 0 | 141.30 |
| tcg | S | 0 | 38.89 |
| aat | N | 3 | 1.57 |
| aac | N | 0 | 1.43 |
| agt | S | 557 | 140.40 |
| agc | S | 81 | 127.10 |
| mPD | | 0.23 | 1.7 |
| | | nPD: | 0.14 |
| | | N. weight: | 1.7 |
| | | Sc. PD: | -0.29 |
| | | Sc. rank: | -307.2 |

,

HA

| Pos. | 498 | obs: | exp: |
|---|---|---|---|
| att | I | 468 | 241.00 |
| atc | I | 91 | 168.00 |
| ata | I | 82 | 232.00 |
| mPD | | 0.43 | 0.66 |
| | | nPD: | 0.65 |
| | | N. weight: | 0.4 |
| | | Sc. PD: | 0.13 |
| | | Sc. rank: | 130.4 |

,

HA

| Pos. | 499 | obs: | exp: |
|---|---|---|---|
| cgt | R | 0 | 26.61 |
| cgc | R | 0 | 31.39 |
| cga | R | 0 | 61.60 |
| cgg | R | 0 | 53.49 |
| aga | R | 519 | 288.30 |
| agg | R | 122 | 179.60 |
| mPD | | 0.31 | 0.96 |
| | | nPD: | 0.32 |
| | | N. weight: | 0.6 |
| | | Sc. PD: | 0.0035 |
| | | Sc. rank: | 8.2 |

,

HA

| Pos. | 500 | obs: | exp: |
|---|---|---|---|
| aat | N | 90 | 335.00 |
| aac | N | 551 | 306.00 |
| mPD | | 0.24 | 0.50 |
| | | nPD: | 0.48 |
| | | N. weight: | 0.48 |
| | | Sc. PD: | 0.077 |
| | | Sc. rank: | 101.8 |

,

HA

| Pos. | 501 | obs: | exp: |
|---|---|---|---|
| aat | N | 30 | 335.00 |
| aac | N | 611 | 306.00 |
| mPD | | 0.089 | 0.50 |
| | | nPD: | 0.18 |
| | | N. weight: | 0.81 |
| | | Sc. PD: | -0.11 |
| | | Sc. rank: | -115.2 |

,

HA

| Pos. | 502 | obs: | exp: |
|---|---|---|---|
| tct | S | 0 | 0.16 |
| tcc | S | 1 | 0.14 |
| tca | S | 0 | 0.22 |
| tcg | S | 0 | 0.06 |
| act | T | 178 | 169.10 |
| acc | T | 459 | 137.30 |
| aca | T | 0 | 280.40 |
| acg | T | 3 | 53.20 |
| agt | S | 0 | 0.22 |
| agc | S | 0 | 0.20 |
| mPD | | 0.41 | 0.69 |
| | | nPD: | 0.6 |
| | | N. weight: | 1.3 |
| | | Sc. PD: | 0.35 |
| | | Sc. rank: | 385.9 |

,

HA

| Pos. | 503 | obs: | exp: |
|---|---|---|---|
| tat | Y | 638 | 331.80 |
| tac | Y | 3 | 309.20 |
| mPD | | 0.0093 | 0.50 |
| | | nPD: | 0.02 |
| | | N. weight: | 0.92 |
| | | Sc. PD: | -0.26 |
| | | Sc. rank: | -280.9 |

,

| | | | |
|---|---|---|---|
| HA | | | |
| Pos. | 504 | obs: | exp: |
| gat | D | 567 | 341.50 |
| gac | D | 73 | 298.50 |
| ggt | G | 1 | 0.12 |
| ggc | G | 0 | 0.14 |
| gga | G | 0 | 0.48 |
| ggg | G | 0 | 0.26 |
| --- | -- | ------ | ------ |
| mPD | | 0.21 | 0.50 |
| | | nPD: | 0.41 |
| | | N. weight: | 0.43 |
| | | Sc. PD: | 0.039 |
| | | Sc. rank: | 57.1 |

| | | | |
|---|---|---|---|
| HA | | | |
| Pos. | 505 | obs: | exp: |
| cat | H | 71 | 347.30 |
| cac | H | 570 | 293.70 |
| --- | -- | ------ | ------ |
| mPD | | 0.20 | 0.50 |
| | | nPD: | 0.4 |
| | | N. weight: | 0.61 |
| | | Sc. PD: | 0.048 |
| | | Sc. rank: | 75.1 |

| | | | |
|---|---|---|---|
| HA | | | |
| Pos. | 506 | obs: | exp: |
| tct | S | 0 | 90.30 |
| tcc | S | 0 | 79.13 |
| tca | S | 0 | 125.80 |
| tcg | S | 0 | 34.62 |
| cgt | R | 0 | 0.04 |
| cgc | R | 0 | 0.05 |
| cga | R | 0 | 0.10 |
| cgg | R | 0 | 0.08 |
| act | T | 50 | 19.02 |
| acc | T | 22 | 15.45 |
| aca | T | 0 | 31.54 |
| acg | T | 0 | 5.99 |
| agt | S | 13 | 125.00 |
| agc | S | 555 | 113.20 |
| aga | R | 1 | 0.45 |
| agg | R | 0 | 0.28 |
| --- | -- | ------ | ------ |
| mPD | | 0.38 | 1.7 |
| | | nPD: | 0.23 |
| | | N. weight: | 2.1 |
| | | Sc. PD: | -0.17 |
| | | Sc. rank: | -203.7 |

| | | | |
|---|---|---|---|
| HA | | | |
| Pos. | 507 | obs: | exp: |
| caa | Q | 38 | 38.38 |
| cag | Q | 34 | 33.62 |
| cgt | R | 0 | 0.08 |
| cgc | R | 0 | 0.10 |
| cga | R | 0 | 0.19 |
| cgg | R | 0 | 0.17 |
| act | T | 0 | 0.26 |
| acc | T | 0 | 0.21 |
| aca | T | 1 | 0.44 |
| acg | T | 0 | 0.08 |
| aaa | K | 165 | 316.90 |
| aag | K | 401 | 249.10 |
| aga | R | 1 | 0.90 |
| agg | R | 1 | 0.56 |
| --- | -- | ------ | ------ |
| mPD | | 0.64 | 0.70 |
| | | nPD: | 0.92 |
| | | N. weight: | 0.21 |
| | | Sc. PD: | 0.12 |
| | | Sc. rank: | 78.5 |

| | | | |
|---|---|---|---|
| HA | | | |
| Pos. | 508 | obs: | exp: |
| tat | Y | 10 | 331.80 |
| tac | Y | 631 | 309.20 |
| --- | -- | ------ | ------ |
| mPD | | 0.031 | 0.50 |
| | | nPD: | 0.06 |
| | | N. weight: | 0.95 |
| | | Sc. PD: | -0.23 |
| | | Sc. rank: | -240.1 |

| | | | |
|---|---|---|---|
| HA | | | |
| Pos. | 509 | obs: | exp: |
| cgt | R | 0 | 26.49 |
| cgc | R | 0 | 31.24 |
| cga | R | 0 | 61.32 |
| cgg | R | 0 | 53.24 |
| att | I | 0 | 0.38 |
| atc | I | 0 | 0.26 |
| ata | I | 1 | 0.36 |
| aaa | K | 1 | 0.56 |
| aag | K | 0 | 0.44 |
| aga | R | 108 | 286.90 |
| agg | R | 530 | 178.80 |
| ggt | G | 0 | 0.12 |
| ggc | G | 0 | 0.14 |
| gga | G | 0 | 0.48 |
| ggg | G | 1 | 0.26 |
| --- | -- | ------ | ------ |
| mPD | | 0.29 | 0.97 |
| | | nPD: | 0.3 |
| | | N. weight: | 1.1 |
| | | Sc. PD: | -0.012 |
| | | Sc. rank: | -20.7 |

| | | | |
|---|---|---|---|
| HA | | | |
| Pos. | 510 | obs: | exp: |
| act | T | 0 | 13.47 |
| acc | T | 0 | 10.94 |
| aca | T | 51 | 22.34 |
| acg | T | 0 | 4.24 |
| aaa | K | 0 | 0.56 |
| aag | K | 1 | 0.44 |
| gct | A | 0 | 5.29 |
| gcc | A | 1 | 4.30 |
| gca | A | 16 | 9.36 |
| gcg | A | 4 | 2.05 |
| gaa | E | 435 | 326.30 |
| gag | E | 132 | 240.70 |
| ggt | G | 0 | 0.12 |
| ggc | G | 0 | 0.14 |
| gga | G | 0 | 0.48 |
| ggg | G | 1 | 0.26 |
| --- | -- | ------ | ------ |
| mPD | | 0.69 | 0.89 |
| | | nPD: | 0.78 |
| | | N. weight: | 0.24 |
| | | Sc. PD: | 0.11 |
| | | Sc. rank: | 86.9 |

| | | | |
|---|---|---|---|
| HA | | | |
| Pos. | 511 | obs: | exp: |
| gaa | E | 327 | 368.80 |
| gag | E | 314 | 272.20 |
| --- | -- | ------ | ------ |
| mPD | | 0.50 | 0.49 |
| | | nPD: | 1.02 |
| | | N. weight: | 0.021 |
| | | Sc. PD: | 0.014 |
| | | Sc. rank: | 7.8 |

| | | | |
|---|---|---|---|
| HA | | | |
| Pos. | 512 | obs: | exp: |
| tct | S | 2 | 11.45 |
| tcc | S | 0 | 10.03 |
| tca | S | 68 | 15.95 |
| tcg | S | 2 | 4.39 |
| agt | S | 0 | 15.84 |
| agc | S | 0 | 14.34 |
| gct | A | 1 | 143.30 |
| gcc | A | 0 | 116.60 |
| gca | A | 515 | 253.60 |
| gcg | A | 53 | 55.52 |
| --- | -- | ------ | ------ |
| mPD | | 0.37 | 0.99 |
| | | nPD: | 0.37 |
| | | N. weight: | 1. |
| | | Sc. PD: | 0.053 |
| | | Sc. rank: | 84.4 |

| | | | |
|---|---|---|---|
| HA | | | |
| Pos. | 513 | obs: | exp: |
| tta | L | 6 | 6.29 |
| ttg | L | 46 | 13.87 |
| ctt | L | 1 | 13.43 |
| ctc | L | 0 | 11.65 |
| cta | L | 0 | 10.85 |
| ctg | L | 19 | 15.92 |
| att | I | 0 | 92.10 |
| atc | I | 0 | 64.21 |
| ata | I | 245 | 88.69 |
| atg | M | 322 | 322.00 |
| gtt | V | 0 | 0.41 |
| gtc | V | 0 | 0.42 |
| gta | V | 0 | 0.37 |
| gtg | V | 2 | 0.79 |
| --- | -- | ------ | ------ |
| mPD | | 0.69 | 0.84 |
| | | nPD: | 0.82 |
| | | N. weight: | 0.71 |
| | | Sc. PD: | 0.35 |
| | | Sc. rank: | 264.0 |

| | | | |
|---|---|---|---|
| HA | | | |
| Pos. | 514 | obs: | exp: |
| caa | Q | 567 | 341.70 |
| cag | Q | 74 | 299.30 |
| --- | -- | ------ | ------ |
| mPD | | 0.20 | 0.50 |
| | | nPD: | 0.41 |
| | | N. weight: | 0.43 |
| | | Sc. PD: | 0.039 |
| | | Sc. rank: | 59.3 |

| | | | |
|---|---|---|---|
| HA | | | |
| Pos. | 515 | obs: | exp: |
| aat | N | 577 | 335.00 |
| aac | N | 64 | 306.00 |
| --- | -- | ------ | ------ |
| mPD | | 0.18 | 0.50 |
| | | nPD: | 0.36 |
| | | N. weight: | 0.49 |
| | | Sc. PD: | 0.021 |
| | | Sc. rank: | 37.6 |

| | | | |
|---|---|---|---|
| HA | | | |
| Pos. | 516 | obs: | exp: |
| cgt | R | 0 | 26.61 |
| cgc | R | 0 | 31.39 |
| cga | R | 61 | 61.60 |
| cgg | R | 0 | 53.49 |
| aga | R | 555 | 288.30 |
| agg | R | 25 | 179.60 |
| --- | -- | ------ | ------ |
| mPD | | 0.25 | 0.96 |
| | | nPD: | 0.26 |
| | | N. weight: | 0.73 |
| | | Sc. PD: | -0.04 |
| | | Sc. rank: | -50.3 |

| | | | |
|---|---|---|---|
| HA | | | |
| Pos. | 517 | obs: | exp: |
| att | I | 0 | 240.60 |
| atc | I | 0 | 167.70 |
| ata | I | 640 | 231.70 |
| gtt | V | 0 | 0.21 |
| gtc | V | 0 | 0.21 |
| gta | V | 1 | 0.19 |
| gtg | V | 0 | 0.40 |
| --- | -- | ------ | ------ |
| mPD | | 0.0031 | 0.66 |
| | | nPD: | 0. |
| | | N. weight: | 1.5 |
| | | Sc. PD: | -0.44 |
| | | Sc. rank: | -513.2 |

| | | | |
|---|---|---|---|
| HA | | | |
| Pos. | 518 | obs: | exp: |
| caa | Q | 33 | 338.50 |
| cag | Q | 602 | 296.50 |
| cgt | R | 0 | 0.04 |
| cgc | R | 0 | 0.05 |
| cga | R | 0 | 0.10 |
| cgg | R | 1 | 0.08 |
| aaa | K | 1 | 2.80 |
| aag | K | 4 | 2.20 |
| aga | R | 0 | 0.45 |
| agg | R | 0 | 0.28 |
| --- | -- | ------ | ------ |
| mPD | | 0.12 | 0.52 |
| | | nPD: | 0.23 |
| | | N. weight: | 0.81 |
| | | Sc. PD: | -0.067 |
| | | Sc. rank: | -78.6 |

| | | | |
|---|---|---|---|
| HA | | | |
| Pos. | 519 | obs: | exp: |
| att | I | 564 | 241.00 |
| atc | I | 5 | 168.00 |
| ata | I | 72 | 232.00 |
| --- | -- | ------ | ------ |
| mPD | | 0.21 | 0.66 |
| | | nPD: | 0.32 |
| | | N. weight: | 0.87 |
| | | Sc. PD: | 0.0071 |
| | | Sc. rank: | 14.9 |

| | | | |
|---|---|---|---|
| HA | | | |
| Pos. | 520 | obs: | exp: |
| cat | H | 0 | 0.54 |
| cac | H | 1 | 0.46 |
| aat | N | 1 | 39.72 |
| aac | N | 75 | 36.28 |
| gat | D | 44 | 300.90 |
| gac | D | 520 | 263.10 |
| --- | -- | ------ | ------ |
| mPD | | 0.34 | 0.71 |
| | | nPD: | 0.48 |
| | | N. weight: | 0.74 |
| | | Sc. PD: | 0.12 |
| | | Sc. rank: | 157.2 |

| | | | |
|---|---|---|---|
| HA | | | |
| Pos. | 521 | obs: | exp: |
| cct | P | 0 | 168.80 |
| ccc | P | 0 | 115.60 |
| cca | P | 470 | 253.00 |
| ccg | P | 171 | 103.60 |
| --- | -- | ------ | ------ |
| mPD | | 0.39 | 0.72 |
| | | nPD: | 0.55 |
| | | N. weight: | 0.87 |
| | | Sc. PD: | 0.19 |
| | | Sc. rank: | 223.6 |

| | | | |
|---|---|---|---|
| HA | | | |
| Pos. | 522 | obs: | exp: |
| att | I | 0 | 8.65 |
| atc | I | 23 | 6.03 |
| ata | I | 0 | 8.33 |
| gtt | V | 3 | 127.00 |
| gtc | V | 514 | 129.70 |
| gta | V | 52 | 115.80 |
| gtg | V | 49 | 245.40 |
| --- | -- | ------ | ------ |
| mPD | | 0.36 | 0.80 |
| | | nPD: | 0.45 |
| | | N. weight: | 1.4 |
| | | Sc. PD: | 0.18 |
| | | Sc. rank: | 235.1 |

| | | | |
|---|---|---|---|
| HA | | | |
| Pos. | 523 | obs: | exp: |
| aaa | K | 631 | 358.90 |
| aag | K | 10 | 282.10 |
| --- | -- | ------ | ------ |
| mPD | | 0.031 | 0.49 |
| | | nPD: | 0.06 |
| | | N. weight: | 0.74 |
| | | Sc. PD: | -0.18 |
| | | Sc. rank: | -186.2 |

| HA | | | |
|---|---|---|---|
| Pos. | 524 | obs: | exp: |
| tta | L | 9 | 55.82 |
| ttg | L | 60 | 123.10 |
| ctt | L | 1 | 119.20 |
| ctc | L | 0 | 103.40 |
| cta | L | 550 | 96.27 |
| ctg | L | 19 | 141.30 |
| att | I | 0 | 0.75 |
| atc | I | 0 | 0.52 |
| ata | I | 2 | 0.72 |
| --- | -- | ------ | ------ |
| mPD | | 0.42 | 1.1 |
| | | nPD: | 0.37 |
| | | N. weight: | 2. |
| | | Sc. PD: | 0.11 |
| | | Sc. rank: | 172.2 |

,

| HA | | | |
|---|---|---|---|
| Pos. | 525 | obs: | exp: |
| tct | S | 0 | 101.70 |
| tcc | S | 0 | 89.16 |
| tca | S | 0 | 141.80 |
| tcg | S | 0 | 39.01 |
| aat | N | 0 | 0.52 |
| aac | N | 1 | 0.48 |
| agt | S | 87 | 140.80 |
| agc | S | 553 | 127.50 |
| --- | -- | ------ | ------ |
| mPD | | 0.24 | 1.7 |
| | | nPD: | 0.14 |
| | | N. weight: | 1.8 |
| | | Sc. PD: | -0.29 |
| | | Sc. rank: | -304.6 |

,

| HA | | | |
|---|---|---|---|
| Pos. | 526 | obs: | exp: |
| tct | S | 0 | 101.30 |
| tcc | S | 0 | 88.74 |
| tca | S | 0 | 141.10 |
| tcg | S | 0 | 38.83 |
| cgt | R | 0 | 0.04 |
| cgc | R | 0 | 0.05 |
| cga | R | 0 | 0.10 |
| cgg | R | 0 | 0.08 |
| aat | N | 1 | 0.52 |
| aac | N | 0 | 0.48 |
| agt | S | 198 | 140.10 |
| agc | S | 439 | 126.90 |
| aga | R | 0 | 0.45 |
| agg | R | 1 | 0.28 |
| ggt | G | 2 | 0.25 |
| ggc | G | 0 | 0.27 |
| gga | G | 0 | 0.97 |
| ggg | G | 0 | 0.52 |
| --- | -- | ------ | ------ |
| mPD | | 0.44 | 1.7 |
| | | nPD: | 0.27 |
| | | N. weight: | 1.4 |
| | | Sc. PD: | -0.069 |
| | | Sc. rank: | -79.3 |

,

| HA | | | |
|---|---|---|---|
| Pos. | 527 | obs: | exp: |
| ggt | G | 157 | 79.09 |
| ggc | G | 414 | 86.68 |
| gga | G | 68 | 310.10 |
| ggg | G | 2 | 165.10 |
| --- | -- | ------ | ------ |
| mPD | | 0.51 | 0.67 |
| | | nPD: | 0.77 |
| | | N. weight: | 1.5 |
| | | Sc. PD: | 0.65 |
| | | Sc. rank: | 532.6 |

,

| HA | | | |
|---|---|---|---|
| Pos. | 528 | obs: | exp: |
| tat | Y | 13 | 331.80 |
| tac | Y | 628 | 309.20 |
| --- | -- | ------ | ------ |
| mPD | | 0.040 | 0.50 |
| | | nPD: | 0.08 |
| | | N. weight: | 0.92 |
| | | Sc. PD: | -0.21 |
| | | Sc. rank: | -211.1 |

,

| HA | | | |
|---|---|---|---|
| Pos. | 529 | obs: | exp: |
| cgt | R | 0 | 0.04 |
| cgc | R | 0 | 0.05 |
| cga | R | 0 | 0.10 |
| cgg | R | 0 | 0.08 |
| aaa | K | 602 | 358.40 |
| aag | K | 38 | 281.60 |
| aga | R | 1 | 0.45 |
| agg | R | 0 | 0.28 |
| --- | -- | ------ | ------ |
| mPD | | 0.11 | 0.50 |
| | | nPD: | 0.23 |
| | | N. weight: | 0.55 |
| | | Sc. PD: | -0.044 |
| | | Sc. rank: | -51.9 |

,

| HA | | | |
|---|---|---|---|
| Pos. | 530 | obs: | exp: |
| gat | D | 568 | 341.50 |
| gac | D | 72 | 298.50 |
| gaa | E | 0 | 0.58 |
| gag | E | 1 | 0.42 |
| --- | -- | ------ | ------ |
| mPD | | 0.20 | 0.50 |
| | | nPD: | 0.41 |
| | | N. weight: | 0.43 |
| | | Sc. PD: | 0.038 |
| | | Sc. rank: | 54.6 |

,

| HA | | | |
|---|---|---|---|
| Pos. | 531 | obs: | exp: |
| att | I | 0 | 27.07 |
| atc | I | 0 | 18.87 |
| ata | I | 72 | 26.06 |
| gtt | V | 0 | 117.00 |
| gtc | V | 0 | 119.40 |
| gta | V | 4 | 106.60 |
| gtg | V | 565 | 226.00 |
| --- | -- | ------ | ------ |
| mPD | | 0.41 | 0.94 |
| | | nPD: | 0.44 |
| | | N. weight: | 1.3 |
| | | Sc. PD: | 0.15 |
| | | Sc. rank: | 210.6 |

,

| HA | | | |
|---|---|---|---|
| Pos. | 532 | obs: | exp: |
| att | I | 0 | 239.10 |
| atc | I | 72 | 166.70 |
| ata | I | 564 | 230.20 |
| gtt | V | 0 | 1.03 |
| gtc | V | 0 | 1.05 |
| gta | V | 5 | 0.94 |
| gtg | V | 0 | 1.99 |
| --- | -- | ------ | ------ |
| mPD | | 0.22 | 0.68 |
| | | nPD: | 0.32 |
| | | N. weight: | 1. |
| | | Sc. PD: | 0.0025 |
| | | Sc. rank: | 7.0 |

,

| HA | | | |
|---|---|---|---|
| Pos. | 533 | obs: | exp: |
| tta | L | 71 | 55.91 |
| ttg | L | 1 | 123.30 |
| ctt | L | 541 | 119.40 |
| ctc | L | 12 | 103.50 |
| cta | L | 15 | 96.42 |
| ctg | L | 0 | 141.50 |
| att | I | 1 | 0.38 |
| atc | I | 0 | 0.26 |
| ata | I | 0 | 0.36 |
| --- | -- | ------ | ------ |
| mPD | | 0.47 | 1.1 |
| | | nPD: | 0.42 |
| | | N. weight: | 1.8 |
| | | Sc. PD: | 0.18 |
| | | Sc. rank: | 265.1 |

,

| HA | | | |
|---|---|---|---|
| Pos. | 534 | obs: | exp: |
| tgg | W | 641 | 641.00 |
| --- | -- | ------ | ------ |
| mPD | | 0 | 0 |
| | | nPD: | 1. |
| | | N. weight: | 0. |
| | | Sc. PD: | 0 |
| | | Sc. rank: | 0 |

,

| HA | | | |
|---|---|---|---|
| Pos. | 535 | obs: | exp: |
| ttt | F | 634 | 263.50 |
| ttc | F | 7 | 377.50 |
| --- | -- | ------ | ------ |
| mPD | | 0.022 | 0.48 |
| | | nPD: | 0.04 |
| | | N. weight: | 1.2 |
| | | Sc. PD: | -0.32 |
| | | Sc. rank: | -326.6 |

,

| HA | | | |
|---|---|---|---|
| Pos. | 536 | obs: | exp: |
| tct | S | 0 | 101.90 |
| tcc | S | 0 | 89.29 |
| tca | S | 0 | 142.00 |
| tcg | S | 0 | 39.07 |
| agt | S | 0 | 141.00 |
| agc | S | 641 | 127.70 |
| --- | -- | ------ | ------ |
| mPD | | 0 | 1.7 |
| | | nPD: | 0. |
| | | N. weight: | 2.4 |
| | | Sc. PD: | -0.72 |
| | | Sc. rank: | -892.5 |

,

| HA | | | |
|---|---|---|---|
| Pos. | 537 | obs: | exp: |
| ttt | F | 0 | 263.50 |
| ttc | F | 641 | 377.50 |
| --- | -- | ------ | ------ |
| mPD | | 0 | 0.48 |
| | | nPD: | 0. |
| | | N. weight: | 0.77 |
| | | Sc. PD: | -0.23 |
| | | Sc. rank: | -292.9 |

,

| HA | | | |
|---|---|---|---|
| Pos. | 538 | obs: | exp: |
| ggt | G | 0 | 79.09 |
| ggc | G | 0 | 86.68 |
| gga | G | 3 | 310.10 |
| ggg | G | 638 | 165.10 |
| --- | -- | ------ | ------ |
| mPD | | 0.0093 | 0.67 |
| | | nPD: | 0.01 |
| | | N. weight: | 1.9 |
| | | Sc. PD: | -0.56 |
| | | Sc. rank: | -610.8 |

,

| HA | | | |
|---|---|---|---|
| Pos. | 539 | obs: | exp: |
| gct | A | 0 | 161.50 |
| gcc | A | 0 | 131.30 |
| gca | A | 630 | 285.70 |
| gcg | A | 11 | 62.55 |
| --- | -- | ------ | ------ |
| mPD | | 0.034 | 0.69 |
| | | nPD: | 0.05 |
| | | N. weight: | 1.1 |
| | | Sc. PD: | -0.28 |
| | | Sc. rank: | -287.0 |

,

| HA | | | |
|---|---|---|---|
| Pos. | 540 | obs: | exp: |
| tct | S | 0 | 101.90 |
| tcc | S | 641 | 89.29 |
| tca | S | 0 | 142.00 |
| tcg | S | 0 | 39.07 |
| agt | S | 0 | 141.00 |
| agc | S | 0 | 127.70 |
| --- | -- | ------ | ------ |
| mPD | | 0 | 1.7 |
| | | nPD: | 0. |
| | | N. weight: | 2.2 |
| | | Sc. PD: | -0.67 |
| | | Sc. rank: | -833.8 |

,

| HA | | | |
|---|---|---|---|
| Pos. | 541 | obs: | exp: |
| tgt | C | 631 | 264.20 |
| tgc | C | 10 | 376.80 |
| --- | -- | ------ | ------ |
| mPD | | 0.031 | 0.48 |
| | | nPD: | 0.06 |
| | | N. weight: | 1.2 |
| | | Sc. PD: | -0.28 |
| | | Sc. rank: | -291.8 |

,

| HA | | | |
|---|---|---|---|
| Pos. | 542 | obs: | exp: |
| ttt | F | 67 | 263.10 |
| ttc | F | 573 | 376.90 |
| tta | L | 1 | 0.09 |
| ttg | L | 0 | 0.19 |
| ctt | L | 0 | 0.19 |
| ctc | L | 0 | 0.16 |
| cta | L | 0 | 0.15 |
| ctg | L | 0 | 0.22 |
| --- | -- | ------ | ------ |
| mPD | | 0.19 | 0.49 |
| | | nPD: | 0.39 |
| | | N. weight: | 0.35 |
| | | Sc. PD: | 0.025 |
| | | Sc. rank: | 39.0 |

,

| HA | | | |
|---|---|---|---|
| Pos. | 543 | obs: | exp: |
| tta | L | 3 | 6.64 |
| ttg | L | 0 | 14.64 |
| ctt | L | 72 | 14.18 |
| ctc | L | 0 | 12.29 |
| cta | L | 1 | 11.45 |
| ctg | L | 0 | 16.80 |
| cgt | R | 0 | 0.04 |
| cgc | R | 0 | 0.05 |
| cga | R | 0 | 0.10 |
| cgg | R | 0 | 0.08 |
| att | I | 0 | 209.80 |
| atc | I | 0 | 146.20 |
| ata | I | 558 | 202.00 |
| act | T | 0 | 1.59 |
| acc | T | 0 | 1.29 |
| aca | T | 6 | 2.63 |
| acg | T | 0 | 0.50 |
| aga | R | 1 | 0.45 |
| agg | R | 0 | 0.28 |
| --- | -- | ------ | ------ |
| mPD | | 0.43 | 0.93 |
| | | nPD: | 0.47 |
| | | N. weight: | 1.6 |
| | | Sc. PD: | 0.23 |
| | | Sc. rank: | 300.3 |

HA

| Pos. | 544 | obs: | exp: |
|---|---|---|---|
| ttt | F | 1 | 0.41 |
| ttc | F | 0 | 0.59 |
| tta | L | 0 | 55.91 |
| ttg | L | 0 | 123.30 |
| ctt | L | 640 | 119.40 |
| ctc | L | 0 | 103.50 |
| cta | L | 0 | 96.42 |
| ctg | L | 0 | 141.50 |
| mPD | | 0.0031 | 1.1 |
| | | nPD: | 0. |
| | | N. weight: | 2.5 |
| | | Sc. PD: | -0.74 |
| | | Sc. rank: | -866.6 |

HA

| Pos. | 545 | obs: | exp: |
|---|---|---|---|
| tta | L | 0 | 55.65 |
| ttg | L | 0 | 122.70 |
| ctt | L | 116 | 118.90 |
| ctc | L | 0 | 103.00 |
| cta | L | 127 | 95.96 |
| ctg | L | 394 | 140.80 |
| att | I | 0 | 1.50 |
| atc | I | 0 | 1.05 |
| ata | I | 4 | 1.45 |
| mPD | | 0.56 | 1.1 |
| | | nPD: | 0.5 |
| | | N. weight: | 1. |
| | | Sc. PD: | 0.18 |
| | | Sc. rank: | 231.5 |

HA

| Pos. | 546 | obs: | exp: |
|---|---|---|---|
| gct | A | 0 | 161.50 |
| gcc | A | 641 | 131.30 |
| gca | A | 0 | 285.70 |
| gcg | A | 0 | 62.55 |
| mPD | | 0 | 0.69 |
| | | nPD: | 0. |
| | | N. weight: | 2.3 |
| | | Sc. PD: | -0.7 |
| | | Sc. rank: | -877.1 |

HA

| Pos. | 547 | obs: | exp: |
|---|---|---|---|
| att | I | 638 | 239.80 |
| atc | I | 0 | 167.20 |
| ata | I | 0 | 230.90 |
| gtt | V | 3 | 0.62 |
| gtc | V | 0 | 0.63 |
| gta | V | 0 | 0.56 |
| gtg | V | 0 | 1.19 |
| mPD | | 0.0093 | 0.67 |
| | | nPD: | 0.01 |
| | | N. weight: | 1.4 |
| | | Sc. PD: | -0.42 |
| | | Sc. rank: | -457.7 |

HA

| Pos. | 548 | obs: | exp: |
|---|---|---|---|
| act | T | 0 | 0.26 |
| acc | T | 0 | 0.21 |
| aca | T | 1 | 0.44 |
| acg | T | 0 | 0.08 |
| gct | A | 0 | 161.20 |
| gcc | A | 0 | 131.10 |
| gca | A | 640 | 285.20 |
| gcg | A | 0 | 62.45 |
| mPD | | 0.0031 | 0.69 |
| | | nPD: | 0. |
| | | N. weight: | 1.2 |
| | | Sc. PD: | -0.35 |
| | | Sc. rank: | -411.9 |

HA

| Pos. | 549 | obs: | exp: |
|---|---|---|---|
| atg | M | 641 | 641.00 |
| mPD | | 0 | 0 |
| | | nPD: | 1. |
| | | N. weight: | 0. |
| | | Sc. PD: | 0 |
| | | Sc. rank: | 0 |

HA

| Pos. | 550 | obs: | exp: |
|---|---|---|---|
| tgt | C | 0 | 0.82 |
| tgc | C | 2 | 1.18 |
| ggt | G | 0 | 78.84 |
| ggc | G | 566 | 86.41 |
| gga | G | 70 | 309.10 |
| ggg | G | 3 | 164.60 |
| mPD | | 0.21 | 0.67 |
| | | nPD: | 0.31 |
| | | N. weight: | 2.2 |
| | | Sc. PD: | -0.0098 |
| | | Sc. rank: | -6.9 |

HA

| Pos. | 551 | obs: | exp: |
|---|---|---|---|
| tta | L | 3 | 56.00 |
| ttg | L | 67 | 123.50 |
| ctt | L | 569 | 119.60 |
| ctc | L | 0 | 103.70 |
| cta | L | 0 | 96.57 |
| ctg | L | 2 | 141.70 |
| mPD | | 0.40 | 1.1 |
| | | nPD: | 0.35 |
| | | N. weight: | 1.9 |
| | | Sc. PD: | 0.072 |
| | | Sc. rank: | 132.4 |

HA

| Pos. | 552 | obs: | exp: |
|---|---|---|---|
| att | I | 2 | 1.88 |
| atc | I | 3 | 1.31 |
| ata | I | 0 | 1.81 |
| gtt | V | 97 | 130.70 |
| gtc | V | 539 | 133.50 |
| gta | V | 0 | 119.20 |
| gtg | V | 0 | 252.60 |
| mPD | | 0.28 | 0.74 |
| | | nPD: | 0.38 |
| | | N. weight: | 1.7 |
| | | Sc. PD: | 0.096 |
| | | Sc. rank: | 166.8 |

HA

| Pos. | 553 | obs: | exp: |
|---|---|---|---|
| ttt | F | 0 | 263.10 |
| ttc | F | 640 | 376.90 |
| gtt | V | 0 | 0.21 |
| gtc | V | 1 | 0.21 |
| gta | V | 0 | 0.19 |
| gtg | V | 0 | 0.40 |
| mPD | | 0.0031 | 0.49 |
| | | nPD: | 0.01 |
| | | N. weight: | 0.78 |
| | | Sc. PD: | -0.23 |
| | | Sc. rank: | -262.7 |

HA

| Pos. | 554 | obs: | exp: |
|---|---|---|---|
| cgt | R | 0 | 0.04 |
| cgc | R | 0 | 0.05 |
| cga | R | 0 | 0.10 |
| cgg | R | 0 | 0.08 |
| att | I | 72 | 220.70 |
| atc | I | 0 | 153.80 |
| ata | I | 515 | 212.50 |
| atg | M | 53 | 53.00 |
| aga | R | 1 | 0.45 |
| agg | R | 0 | 0.28 |
| mPD | | 0.34 | 0.71 |
| | | nPD: | 0.47 |
| | | N. weight: | 0.87 |
| | | Sc. PD: | 0.13 |
| | | Sc. rank: | 172.6 |

HA

| Pos. | 555 | obs: | exp: |
|---|---|---|---|
| tgt | C | 564 | 264.20 |
| tgc | C | 77 | 376.80 |
| mPD | | 0.21 | 0.48 |
| | | nPD: | 0.44 |
| | | N. weight: | 0.7 |
| | | Sc. PD: | 0.082 |
| | | Sc. rank: | 110.8 |

HA

| Pos. | 556 | obs: | exp: |
|---|---|---|---|
| att | I | 0 | 27.07 |
| atc | I | 1 | 18.87 |
| ata | I | 71 | 26.06 |
| gtt | V | 0 | 117.00 |
| gtc | V | 0 | 119.40 |
| gta | V | 7 | 106.60 |
| gtg | V | 562 | 226.00 |
| mPD | | 0.42 | 0.94 |
| | | nPD: | 0.45 |
| | | N. weight: | 1.3 |
| | | Sc. PD: | 0.16 |
| | | Sc. rank: | 211.9 |

HA

| Pos. | 557 | obs: | exp: |
|---|---|---|---|
| cgt | R | 0 | 0.12 |
| cgc | R | 0 | 0.15 |
| cga | R | 0 | 0.29 |
| cgg | R | 0 | 0.25 |
| aaa | K | 53 | 356.70 |
| aag | K | 584 | 280.30 |
| aga | R | 0 | 1.35 |
| agg | R | 3 | 0.84 |
| gaa | E | 1 | 0.58 |
| gag | E | 0 | 0.42 |
| mPD | | 0.17 | 0.51 |
| | | nPD: | 0.33 |
| | | N. weight: | 0.76 |
| | | Sc. PD: | 0.0096 |
| | | Sc. rank: | 16.3 |

HA

| Pos. | 558 | obs: | exp: |
|---|---|---|---|
| tct | S | 0 | 0.16 |
| tcc | S | 0 | 0.14 |
| tca | S | 0 | 0.22 |
| tcg | S | 0 | 0.06 |
| aat | N | 624 | 334.50 |
| aac | N | 16 | 305.50 |
| agt | S | 1 | 0.22 |
| agc | S | 0 | 0.20 |
| mPD | | 0.052 | 0.50 |
| | | nPD: | 0.1 |
| | | N. weight: | 0.79 |
| | | Sc. PD: | -0.16 |
| | | Sc. rank: | -163.1 |

HA

| Pos. | 559 | obs: | exp: |
|---|---|---|---|
| cgt | R | 0 | 0.04 |
| cgc | R | 0 | 0.05 |
| cga | R | 0 | 0.10 |
| cgg | R | 0 | 0.08 |
| aga | R | 1 | 0.45 |
| agg | R | 0 | 0.28 |
| ggt | G | 0 | 78.97 |
| ggc | G | 0 | 86.55 |
| gga | G | 638 | 309.60 |
| ggg | G | 2 | 164.90 |
| mPD | | 0.0094 | 0.67 |
| | | nPD: | 0.01 |
| | | N. weight: | 1. |
| | | Sc. PD: | -0.3 |
| | | Sc. rank: | -329.4 |

HA

| Pos. | 560 | obs: | exp: |
|---|---|---|---|
| aat | N | 0 | 335.00 |
| aac | N | 641 | 306.00 |
| mPD | | 0 | 0.50 |
| | | nPD: | 0. |
| | | N. weight: | 1.1 |
| | | Sc. PD: | -0.33 |
| | | Sc. rank: | -409.1 |

HA

| Pos. | 561 | obs: | exp: |
|---|---|---|---|
| atg | M | 641 | 641.00 |
| mPD | | 0 | 0 |
| | | nPD: | 1. |
| | | N. weight: | 0. |
| | | Sc. PD: | 0 |
| | | Sc. rank: | 0 |

HA

| Pos. | 562 | obs: | exp: |
|---|---|---|---|
| cct | P | 0 | 0.53 |
| ccc | P | 0 | 0.36 |
| cca | P | 0 | 0.79 |
| ccg | P | 2 | 0.32 |
| caa | Q | 0 | 1.60 |
| cag | Q | 3 | 1.40 |
| cgt | R | 0 | 26.40 |
| cgc | R | 0 | 31.14 |
| cga | R | 0 | 61.12 |
| cgg | R | 636 | 53.07 |
| aga | R | 0 | 286.00 |
| agg | R | 0 | 178.20 |
| mPD | | 0.016 | 0.98 |
| | | nPD: | 0.02 |
| | | N. weight: | 3.6 |
| | | Sc. PD: | -1. |
| | | Sc. rank: | -1122.0 |

HA

| Pos. | 563 | obs: | exp: |
|---|---|---|---|
| tgt | C | 0 | 264.20 |
| tgc | C | 641 | 376.80 |
| mPD | | 0 | 0.48 |
| | | nPD: | 0. |
| | | N. weight: | 0.78 |
| | | Sc. PD: | -0.24 |
| | | Sc. rank: | -294.0 |

HA

| Pos. | 564 | obs: | exp: |
|---|---|---|---|
| act | T | 639 | 169.30 |
| acc | T | 2 | 137.50 |
| aca | T | 0 | 280.80 |
| acg | T | 0 | 53.29 |
| mPD | | 0.0062 | 0.69 |
| | | nPD: | 0.01 |
| | | N. weight: | 1.9 |
| | | Sc. PD: | -0.57 |
| | | Sc. rank: | -627.8 |

HA

| Pos. | 565 | obs: | exp: |
|---|---|---|---|
| att | I | 641 | 241.00 |
| atc | I | 0 | 168.00 |
| ata | I | 0 | 232.00 |
| mPD | | 0 | 0.66 |
| | | nPD: | 0. |
| | | N. weight: | 1.4 |
| | | Sc. PD: | -0.43 |
| | | Sc. rank: | -541.2 |

HA

| Pos. | 566 | obs: | exp: |
|---|---|---|---|
| tat | Y | 1 | 0.52 |
| tac | Y | 0 | 0.48 |
| tgt | C | 640 | 263.80 |
| tgc | C | 0 | 376.20 |
| mPD | | 0.0031 | 0.49 |
| | | nPD: | 0.01 |
| | | N. weight: | 1.3 |
| | | Sc. PD: | -0.39 |
| | | Sc. rank: | -436.0 |

HA

| Pos. | 567 | obs: | exp: |
|---|---|---|---|
| att | I | 0 | 241.00 |
| atc | I | 0 | 168.00 |
| ata | I | 641 | 232.00 |
| mPD | | 0 | 0.66 |
| | | nPD: | 0. |
| | | N. weight: | 1.5 |
| | | Sc. PD: | -0.45 |
| | | Sc. rank: | -562.2 |

HA

| Pos. | 568 | obs: | exp: |
|---|---|---|---|
| taa | . | 563 | 300.20 |
| tag | . | 78 | 165.90 |
| tga | . | 0 | 174.90 |
| mPD | | 0.21 | 0.78 |
| | | nPD: | 0.27 |
| | | N. weight: | 0.68 |
| | | Sc. PD: | -0.027 |
| | | Sc. rank: | -32.2 |

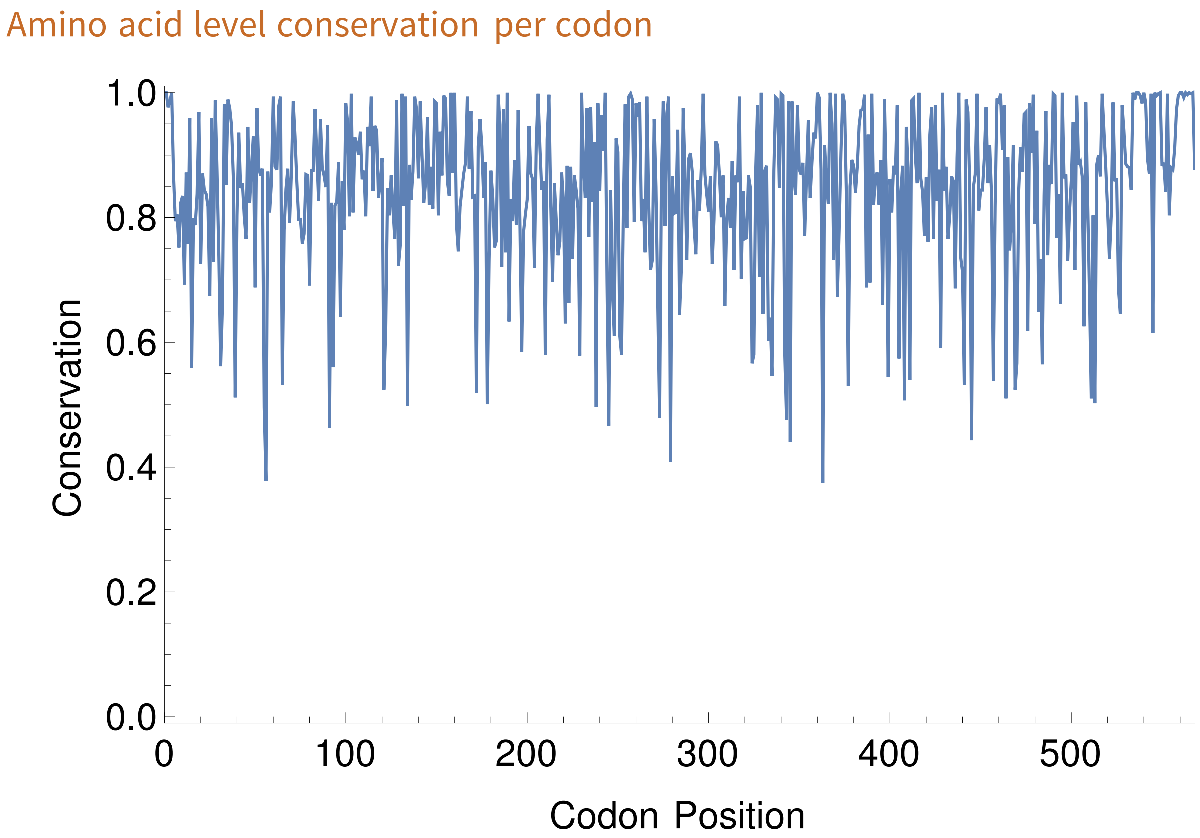
Amino acid level conservation per codon
Conservation
1.0
0.8
0.6
0.4
0.2
0.0
0
100
200
300
400
500
Codon Position

## Conservation analysis plots

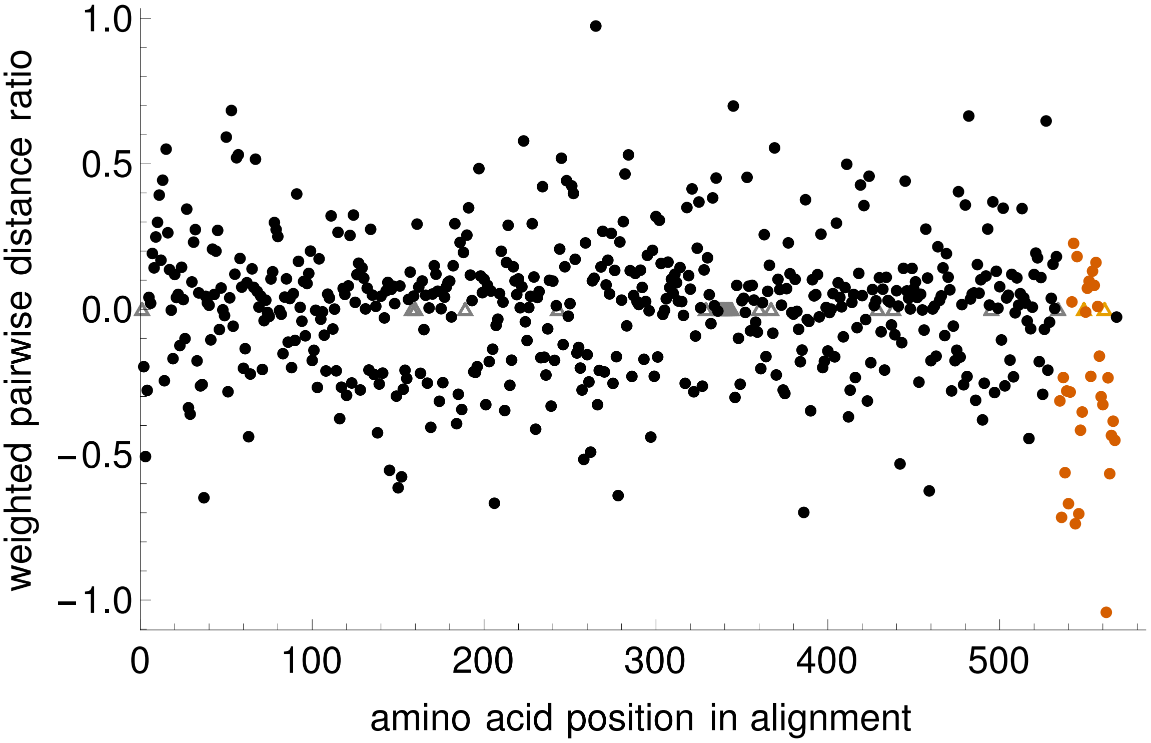


HA

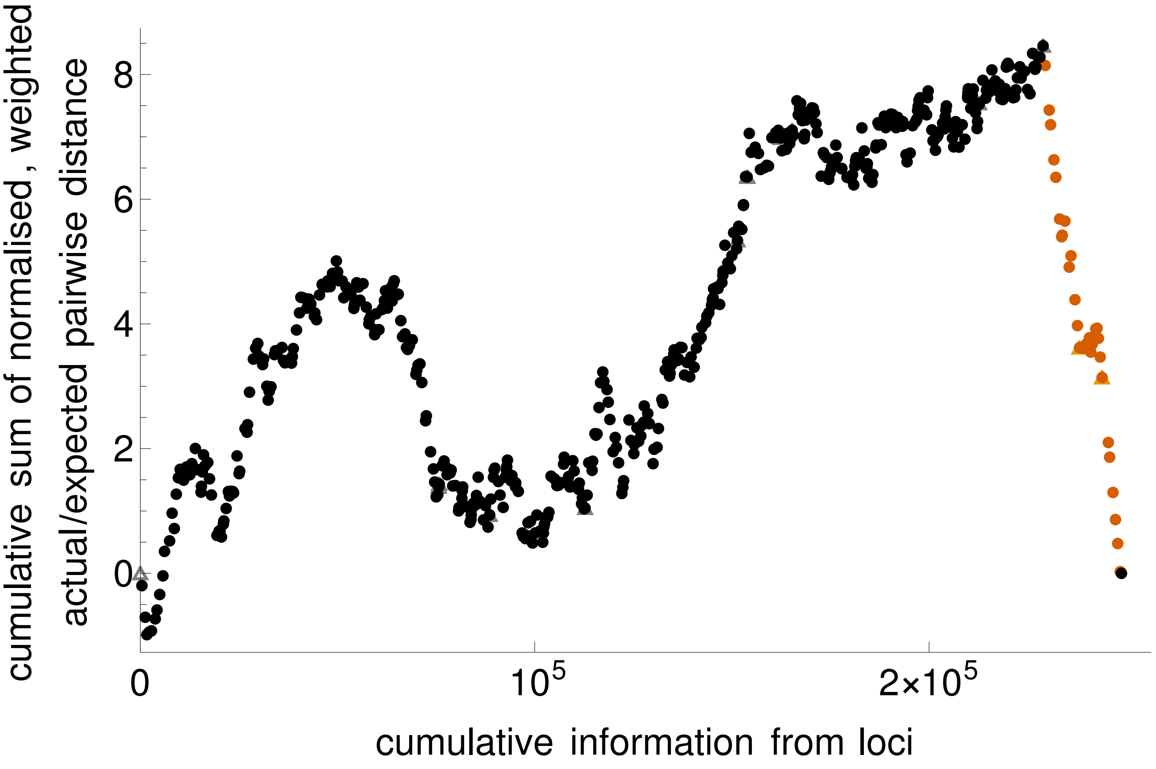


HA

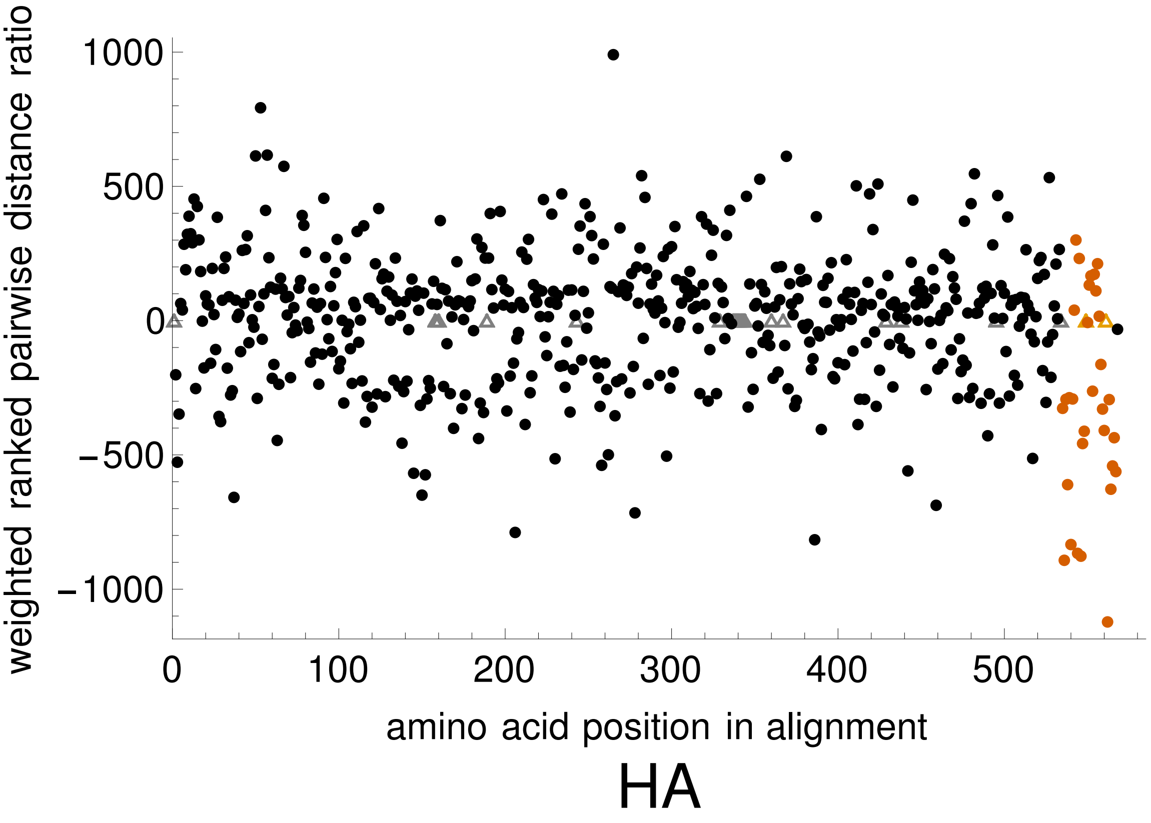
weighted ranked pairwise distance ratio
1000
500
0
−500
−1000
0
100
200
300
400
500
amino acid position in alignment
HA

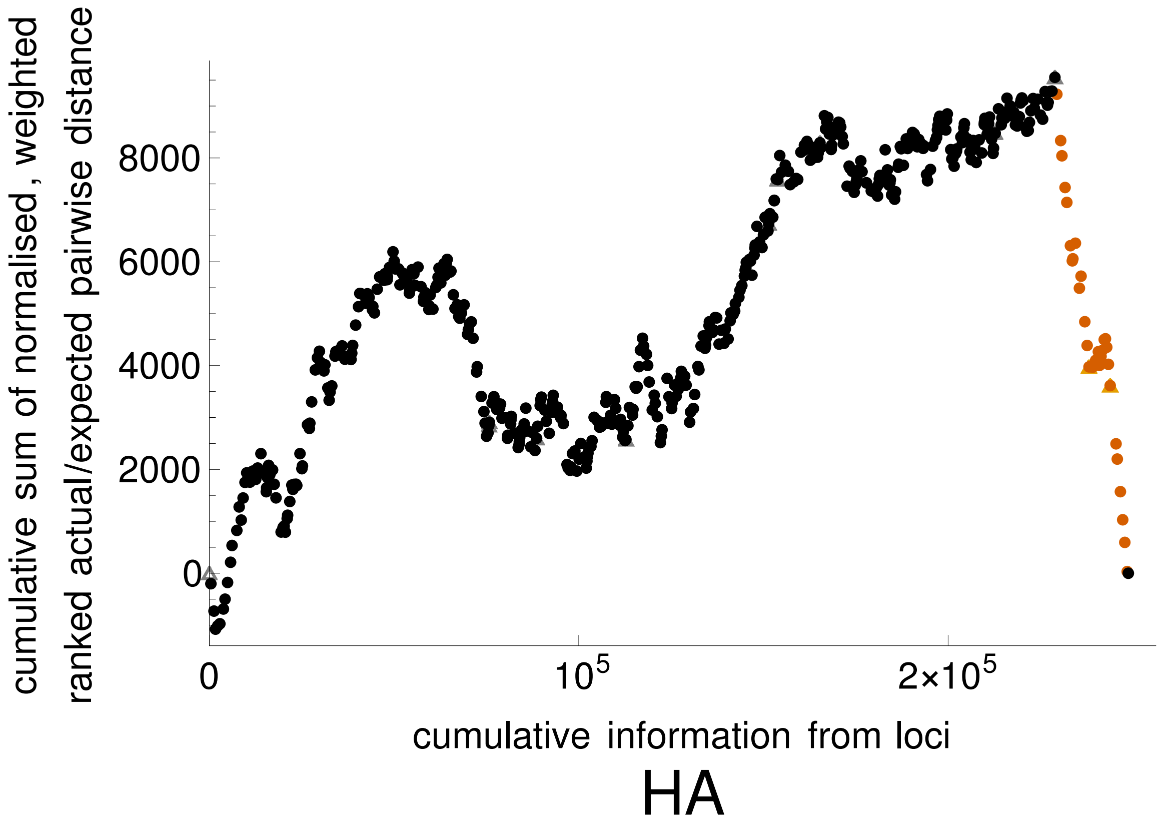
cumulative sum of normalised, weighted ranked actual/expected pairwise distance
8000
6000
4000
2000
0
0
10^5
2×10^5
cumulative information from loci
HA

# NP

## Gene length histogram

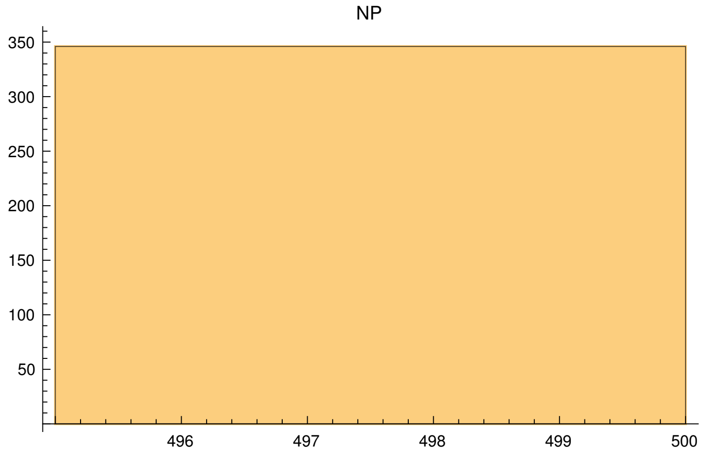

## Information vs. nPD

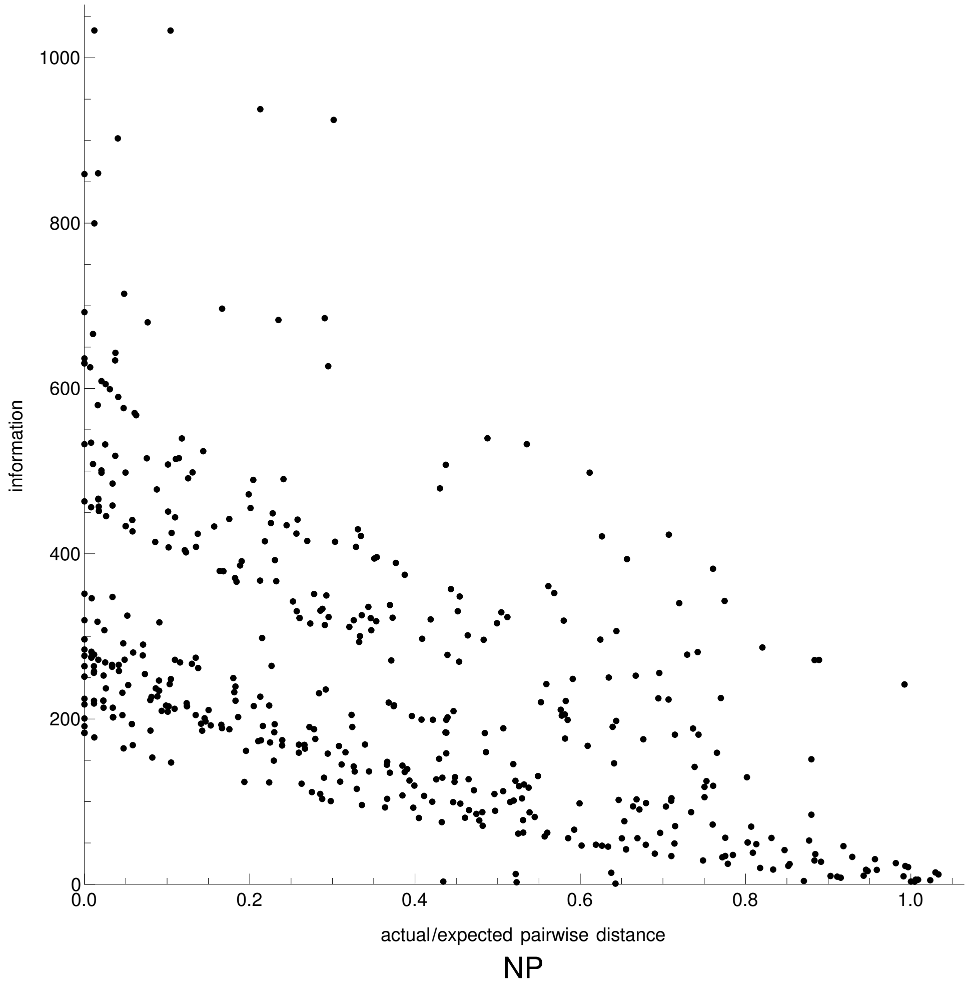

## Example sequences highlighted by regions found in analyses to be conserved

Interesting points (by weighted raw PD) highlighted for gene NP:

ATG
**GCGTCTCAAGGCACCAAACGATCTTATGAACAGATGGAAACTGGTGGAGAACGCC**
**AGAATGCCACCGAAATCAGAGCATCT**GTTGGAAGAATGGTTGGTGGAATTGGAAGATTTTA⋱
CATACAGATGTGCACTGAGCTCAAACTCAGCGATTATGAAGGGAGGCTGATCCAGAACAGCATAACGATA⋱
GAGAGAATGGTCCTCTCTGCATTCGATGAAAGAAGGAACAAATACCTGGAAGAACATCCCAGTGCGGGGA⋱
AAGATCCGAAGAAAACTGGAGGTCCAATTTATCGAAGGAGGGACGGGAAATGGATGAGAGAGCTGATTCT⋱
GTATGACAAAGAGGAGATCAGGAGAATCTGGCGTCAGGCGAACAATGGAGAAGACGCAACTGCTGGTCTC⋱
ACTCACCTGATGATCTGGCATTCCAATCTAAATGATGCCACATACCAGAGAACTAGAGCTCTCGTGCGTA⋱
CTGGGATGGACCCCAGAATGTGCTCTCTGATGCAAGGATCAACCCTCCCGAGGAGGTCTGGAGCTGCTGG⋱
CGCAGCAGTAAAGGGAGTCGGGACAATGGTGATGGAACTGATTCGGATGATAAAACGAGGAATTAATGAT⋱
CGGAATTTCTGGAGAGGCGAAAATGGGCGGAGAACAAGGATTGCATATGAGAGGATGTGCAACATCCTCA⋱
AAGGGAAGTTCCAAACAGCAGCACAAAGAGCAATGATGGACCAAGTGCGAGAAAGCAGAAATCCCGGGAA⋱
TGCTGAAATTGAAGATCTCATTTTCCTGGCAAGGTCTGCGCTCATTCTGAGAGGATCAGTGGCCCATAAG⋱
TCCTGCCTTCCTGCTTGTGTGTACGGGCTTGCTGTGGCCAGTGGATATGACTTTGAAAGAGAAGGGTACT⋱
CTCTAGTTGGAATAGATCCTTTCCGTCTGCTTCAAAACAGCCAGGTCTTCAGCCTCATTAGACCAAATGA⋱
GAACCCAGCACACAAGAGTCAATTGGTGTGGATGGCATGCCATTCTGCAGCATTTGAGGACCTGAGAGTC⋱
TCAAGTTTCATCAGAGGGACAAGAGTAGTCCCAAGAGGGCAACTATCCACCAGAGGAGTTCAAATTGCTT⋱
CAAATGAGAACATGGAAACAATGGACTCCAGCACTCTTGAACTGAGGAGCAGGTATTGGGCTATAAGGAC⋱
CAGGAGTGGAGGAAACACCAACCAACAGAGAGCATCTGCAGGACAAATCAGTGTACAGCCTACTTTCTCG⋱
GTACAGAGAAATCTTCCCTTCGAAAGAGCGACCATTATGGCTGCGTTCACAGGAAATACTGAAGGCAGAA⋱
CATCTGACATGAGGACTGAAATTATAAGAATGATGGAAAGTGCCAGACCAGAA
**GATGTGTCTTTCCAGGGGCGGGGAGTCTTCGAGCTCTCGGACGAAAAGGCAACGA**
**ACCCGATCGTGCCTTCCTTTGACATGAGTAATGAAGGATCTTATTTCTTCGG**
**AGACAATGCAGAGGAGTATGACAAT**TAA

Interesting points (by weighted ranked PD) highlighted for gene NP:

ATGGCG
**TCTCAAGGCACCAAACGATCTTATGAACAGATGGAAACTGGTGGAGAACGCCAGA**
**AT**GCCACCGAAATCAGAGCATCTGTTGGAAGAATGGTTGGTGGAATTGGAAGATTTTACATACAGATGTGC⋱
ACTGAGCTCAAACTCAGCGATTATGAAGGGAGGCTGATCCAGAACAGCATAACGATAGAGAGAATGGTCC⋱
TCTCTGCATTCGATGAAAGAAGGAACAAATACCTGGAAGAACATCCCAGTGCGGGGAAAGATCCGAAGAA⋱
AACTGGAGGTCCAATTTATCGAAGGAGGGACGGGAAATGGATGAGAGAGCTGATTCTGTATGACAAAGAG⋱
GAGATCAGGAGAATCTGGCGTCAGGCGAACAATGGAGAAGACGCAACTGCTGGTCTCACTCACCTGATGA⋱
TCTGGCATTCCAATCTAAATGATGCCACATACCAGAGAACTAGAGCTCTCGTGCGTACTGGGATGGACCC⋱
CAGAATGTGCTCTCTGATGCAAGGATCAACCCTCCCGAGGAGGTCTGGAGCTGCTGGCGCAGCAGTAAAG⋱
GGAGTCGGGACAATGGTGATGGAACTGATTCGGATGATAAAACGAGGAATTAATGATCGGAATTTCTGGA⋱
GAGGCGAAAATGGGCGGAGAACAAGGATTGCATATGAGAGGATGTGCAACATCCTCAAAGGGAAGTTCCA⋱
AACAGCAGCACAAAGAGCAATGATGGACCAAGTGCGAGAAAGCAGAAATCCCGGGAATGCTGAAATTGAA⋱
GATCTCATTTTCCTGGCAAGGTCTGCGCTCATTCTGAGAGGATCAGTGGCCCATAAGTCCTGCCTTCCTG⋱
CTTGTGTGTACGGGCTTGCTGTGGCCAGTGGATATGACTTTGAAAGAGAAGGGTACTCTCTAGTTGGAAT⋱
AGATCCTTTCCGTCTGCTTCAAAACAGCCAGGTCTTCAGCCTCATTAGACCAAATGAGAACCCAGCACAC⋱
AAGAGTCAATTGGTGTGGATGGCATGCCATTCTGCAGCATTTGAGGACCTGAGAGTCTCAAGTTTCATCA⋱
GAGGGACAAGAGTAGTCCCAAGAGGGCAACTATCCACCAGAGGAGTTCAAATTGCTTCAAATGAGAACAT⋱

GGAAACAATGGACTCCAGCACTCTTGAACTGAGGAGCAGGTATTGGGCTATAAGGACCAGGAGTGGAGGA∵.
AACACCAACCAACAGAGAGCATCTGCAGGACAAATCAGTGTACAGCCTACTTTCTCGGTACAGAGAAATC∵.
TTCCCTTCGAAAGAGCGACCATTATGGCTGCGTTCACAGGAAATACTGAAGGCAGAACATCTGACATGAG∵.
GACTGAAATTATAAGAATGATGGAAAGTGCCAGACCAGAAGATGTGTCT
**TTCCAGGGGCGGGGAGTCTTCGAGCTCTCGGACGAAAAGGCAACGAACCCGATCG**
**TGCCTTCCTTTGACATGAGTAATGAAGGATCTTATTTCTTCGGAGACAATGC**
**AGAG**GAGTATGACAATTAA

# Per codon conservation report

NP

| Pos. | 1 | obs: | exp: |
|---|---|---|---|
| atg | M | 346 | 346.00 |
| --- | -- | ------ | ------ |
| mPD | | 0 | 0 |
| | | nPD: | 1. |
| | | N. weight: | 0. |
| | | Sc. PD: | 0 |
| | | Sc. rank: | 0 |

NP

| Pos. | 2 | obs: | exp: |
|---|---|---|---|
| gct | A | 0 | 87.15 |
| gcc | A | 0 | 70.88 |
| gca | A | 21 | 154.20 |
| gcg | A | 325 | 33.76 |
| --- | -- | ------ | ------ |
| mPD | | 0.11 | 0.69 |
| | | nPD: | 0.17 |
| | | N. weight: | 2.9 |
| | | Sc. PD: | -0.19 |
| | | Sc. rank: | -4.8 |

NP

| Pos. | 3 | obs: | exp: |
|---|---|---|---|
| tct | S | 344 | 55.01 |
| tcc | S | 2 | 48.20 |
| tca | S | 0 | 76.65 |
| tcg | S | 0 | 21.09 |
| agt | S | 0 | 76.12 |
| agc | S | 0 | 68.93 |
| --- | -- | ------ | ------ |
| mPD | | 0.012 | 1.7 |
| | | nPD: | 0.01 |
| | | N. weight: | 2.6 |
| | | Sc. PD: | -0.57 |
| | | Sc. rank: | -456.8 |

NP

| Pos. | 4 | obs: | exp: |
|---|---|---|---|
| caa | Q | 346 | 184.50 |
| cag | Q | 0 | 161.50 |
| --- | -- | ------ | ------ |
| mPD | | 0 | 0.50 |
| | | nPD: | 0. |
| | | N. weight: | 0.9 |
| | | Sc. PD: | -0.2 |
| | | Sc. rank: | -186.9 |

NP

| Pos. | 5 | obs: | exp: |
|---|---|---|---|
| ggt | G | 0 | 42.69 |
| ggc | G | 346 | 46.79 |
| gga | G | 0 | 167.40 |
| ggg | G | 0 | 89.14 |
| --- | -- | ------ | ------ |
| mPD | | 0 | 0.67 |
| | | nPD: | 0. |
| | | N. weight: | 2.9 |
| | | Sc. PD: | -0.65 |
| | | Sc. rank: | -594.5 |

NP

| Pos. | 6 | obs: | exp: |
|---|---|---|---|
| act | T | 0 | 91.41 |
| acc | T | 346 | 74.25 |
| aca | T | 0 | 151.60 |
| acg | T | 0 | 28.76 |
| --- | -- | ------ | ------ |
| mPD | | 0 | 0.69 |
| | | nPD: | 0. |
| | | N. weight: | 2.2 |
| | | Sc. PD: | -0.5 |
| | | Sc. rank: | -457.3 |

NP

| Pos. | 7 | obs: | exp: |
|---|---|---|---|
| aaa | K | 346 | 193.70 |
| aag | K | 0 | 152.30 |
| --- | -- | ------ | ------ |
| mPD | | 0 | 0.49 |
| | | nPD: | 0. |
| | | N. weight: | 0.83 |
| | | Sc. PD: | -0.19 |
| | | Sc. rank: | -172.3 |

NP

| Pos. | 8 | obs: | exp: |
|---|---|---|---|
| cgt | R | 0 | 14.36 |
| cgc | R | 0 | 16.94 |
| cga | R | 344 | 33.25 |
| cgg | R | 2 | 28.87 |
| aga | R | 0 | 155.60 |
| agg | R | 0 | 96.96 |
| --- | -- | ------ | ------ |
| mPD | | 0.012 | 0.96 |
| | | nPD: | 0.01 |
| | | N. weight: | 3.3 |
| | | Sc. PD: | -0.71 |
| | | Sc. rank: | -512.6 |

NP

| Pos. | 9 | obs: | exp: |
|---|---|---|---|
| tct | S | 274 | 55.01 |
| tcc | S | 72 | 48.20 |
| tca | S | 0 | 76.65 |
| tcg | S | 0 | 21.09 |
| agt | S | 0 | 76.12 |
| agc | S | 0 | 68.93 |
| --- | -- | ------ | ------ |
| mPD | | 0.33 | 1.7 |
| | | nPD: | 0.2 |
| | | N. weight: | 2. |
| | | Sc. PD: | -0.072 |
| | | Sc. rank: | 26.8 |

NP

| Pos. | 10 | obs: | exp: |
|---|---|---|---|
| tat | Y | 345 | 179.10 |
| tac | Y | 1 | 166.90 |
| --- | -- | ------ | ------ |
| mPD | | 0.0058 | 0.50 |
| | | nPD: | 0.01 |
| | | N. weight: | 0.92 |
| | | Sc. PD: | -0.2 |
| | | Sc. rank: | -148.2 |

NP

| Pos. | 11 | obs: | exp: |
|---|---|---|---|
| gaa | E | 269 | 199.10 |
| gag | E | 77 | 146.90 |
| --- | -- | ------ | ------ |
| mPD | | 0.35 | 0.49 |
| | | nPD: | 0.71 |
| | | N. weight: | 0.14 |
| | | Sc. PD: | 0.064 |
| | | Sc. rank: | 26.6 |

NP

| Pos. | 12 | obs: | exp: |
|---|---|---|---|
| caa | Q | 35 | 184.50 |
| cag | Q | 311 | 161.50 |
| --- | -- | ------ | ------ |
| mPD | | 0.18 | 0.50 |
| | | nPD: | 0.37 |
| | | N. weight: | 0.61 |
| | | Sc. PD: | 0.076 |
| | | Sc. rank: | 64.0 |

NP

| Pos. | 13 | obs: | exp: |
|---|---|---|---|
| atg | M | 346 | 346.00 |
| --- | -- | ------ | ------ |
| mPD | | 0 | 0 |
| | | nPD: | 1. |
| | | N. weight: | 0. |
| | | Sc. PD: | 0 |
| | | Sc. rank: | 0 |

NP

| Pos. | 14 | obs: | exp: |
|---|---|---|---|
| gaa | E | 280 | 199.10 |
| gag | E | 66 | 146.90 |
| --- | -- | ------ | ------ |
| mPD | | 0.31 | 0.49 |
| | | nPD: | 0.63 |
| | | N. weight: | 0.19 |
| | | Sc. PD: | 0.072 |
| | | Sc. rank: | 32.8 |

NP

| Pos. | 15 | obs: | exp: |
|---|---|---|---|
| act | T | 340 | 91.41 |
| acc | T | 6 | 74.25 |
| aca | T | 0 | 151.60 |
| acg | T | 0 | 28.76 |
| --- | -- | ------ | ------ |
| mPD | | 0.034 | 0.69 |
| | | nPD: | 0.05 |
| | | N. weight: | 1.8 |
| | | Sc. PD: | -0.32 |
| | | Sc. rank: | -152.3 |

NP

| Pos. | 16 | obs: | exp: |
|---|---|---|---|
| tct | S | 0 | 0.79 |
| tcc | S | 0 | 0.70 |
| tca | S | 0 | 1.11 |
| tcg | S | 0 | 0.30 |
| agt | S | 5 | 1.10 |
| agc | S | 0 | 1.00 |
| ggt | G | 340 | 42.07 |
| ggc | G | 0 | 46.11 |
| gga | G | 0 | 165.00 |
| ggg | G | 1 | 87.86 |
| --- | -- | ------ | ------ |
| mPD | | 0.034 | 0.71 |
| | | nPD: | 0.05 |
| | | N. weight: | 3. |
| | | Sc. PD: | -0.53 |
| | | Sc. rank: | -263.5 |

NP

| Pos. | 17 | obs: | exp: |
|---|---|---|---|
| ggt | G | 0 | 42.69 |
| ggc | G | 0 | 46.79 |
| gga | G | 268 | 167.40 |
| ggg | G | 78 | 89.14 |
| --- | -- | ------ | ------ |
| mPD | | 0.35 | 0.67 |
| | | nPD: | 0.53 |
| | | N. weight: | 0.49 |
| | | Sc. PD: | 0.14 |
| | | Sc. rank: | 74.9 |

NP

| Pos. | 18 | obs: | exp: |
|---|---|---|---|
| gaa | E | 277 | 199.10 |
| gag | E | 69 | 146.90 |
| --- | -- | ------ | ------ |
| mPD | | 0.32 | 0.49 |
| | | nPD: | 0.66 |
| | | N. weight: | 0.18 |
| | | Sc. PD: | 0.07 |
| | | Sc. rank: | 31.2 |

NP

| Pos. | 19 | obs: | exp: |
|---|---|---|---|
| cgt | R | 2 | 14.36 |
| cgc | R | 344 | 16.94 |
| cga | R | 0 | 33.25 |
| cgg | R | 0 | 28.87 |
| aga | R | 0 | 155.60 |
| agg | R | 0 | 96.96 |
| --- | -- | ------ | ------ |
| mPD | | 0.012 | 0.96 |
| | | nPD: | 0.01 |
| | | N. weight: | 4.3 |
| | | Sc. PD: | -0.92 |
| | | Sc. rank: | -662.1 |

NP

| Pos. | 20 | obs: | exp: |
|---|---|---|---|
| caa | Q | 1 | 184.50 |
| cag | Q | 345 | 161.50 |
| --- | -- | ------ | ------ |
| mPD | | 0.0058 | 0.50 |
| | | nPD: | 0.01 |
| | | N. weight: | 1.1 |
| | | Sc. PD: | -0.23 |
| | | Sc. rank: | -170.2 |

NP

| Pos. | 21 | obs: | exp: |
|---|---|---|---|
| aat | N | 343 | 180.30 |
| aac | N | 2 | 164.70 |
| gat | D | 1 | 0.53 |
| gac | D | 0 | 0.47 |
| --- | -- | ------ | ------ |
| mPD | | 0.017 | 0.50 |
| | | nPD: | 0.03 |
| | | N. weight: | 0.89 |
| | | Sc. PD: | -0.17 |
| | | Sc. rank: | -99.1 |

NP

| Pos. | 22 | obs: | exp: |
|---|---|---|---|
| gtt | V | 0 | 0.21 |
| gtc | V | 1 | 0.21 |
| gta | V | 0 | 0.19 |
| gtg | V | 0 | 0.40 |
| gct | A | 89 | 86.90 |
| gcc | A | 253 | 70.67 |
| gca | A | 3 | 153.80 |
| gcg | A | 0 | 33.67 |
| --- | -- | ------ | ------ |
| mPD | | 0.40 | 0.69 |
| | | nPD: | 0.58 |
| | | N. weight: | 1.3 |
| | | Sc. PD: | 0.43 |
| | | Sc. rank: | 216.5 |

NP

| Pos. | 23 | obs: | exp: |
|---|---|---|---|
| act | T | 340 | 91.41 |
| acc | T | 6 | 74.25 |
| aca | T | 0 | 151.60 |
| acg | T | 0 | 28.76 |
| --- | -- | ------ | ------ |
| mPD | | 0.034 | 0.69 |
| | | nPD: | 0.05 |
| | | N. weight: | 1.8 |
| | | Sc. PD: | -0.32 |
| | | Sc. rank: | -152.3 |

NP

| Pos. | 24 | obs: | exp: |
|---|---|---|---|
| gaa | E | 137 | 199.10 |
| gag | E | 209 | 146.90 |
| --- | -- | ------ | ------ |
| mPD | | 0.48 | 0.49 |
| | | nPD: | 0.98 |
| | | N. weight: | 0.11 |
| | | Sc. PD: | 0.075 |
| | | Sc. rank: | 22.1 |

NP

| Pos. | 25 | obs: | exp: |
|---|---|---|---|
| att | I | 2 | 130.10 |
| atc | I | 343 | 90.68 |
| ata | I | 1 | 125.20 |
| --- | -- | ------ | ------ |
| mPD | | 0.017 | 0.66 |
| | | nPD: | 0.03 |
| | | N. weight: | 1.8 |
| | | Sc. PD: | -0.37 |
| | | Sc. rank: | -223.4 |

| | | | |
|---|---|---|---|
| NP | | | |
| Pos. | 26 | obs: | exp: |
| cgt | R | 0 | 14.32 |
| cgc | R | 0 | 16.89 |
| cga | R | 0 | 33.16 |
| cgg | R | 0 | 28.79 |
| aaa | K | 1 | 0.56 |
| aag | K | 0 | 0.44 |
| aga | R | 316 | 155.20 |
| agg | R | 29 | 96.68 |
| mPD | | 0.16 | 0.96 |
| | | nPD: | 0.17 |
| | | N. weight: | 0.8 |
| | | Sc. PD: | -0.055 |
| | | Sc. rank: | -3.8 |

| | | | |
|---|---|---|---|
| NP | | | |
| Pos. | 27 | obs: | exp: |
| gct | A | 1 | 87.15 |
| gcc | A | 1 | 70.88 |
| gca | A | 341 | 154.20 |
| gcg | A | 3 | 33.76 |
| mPD | | 0.029 | 0.69 |
| | | nPD: | 0.04 |
| | | N. weight: | 1.1 |
| | | Sc. PD: | -0.2 |
| | | Sc. rank: | -103.3 |

| | | | |
|---|---|---|---|
| NP | | | |
| Pos. | 28 | obs: | exp: |
| tct | S | 332 | 55.01 |
| tcc | S | 7 | 48.20 |
| tca | S | 1 | 76.65 |
| tcg | S | 6 | 21.09 |
| agt | S | 0 | 76.12 |
| agc | S | 0 | 68.93 |
| mPD | | 0.079 | 1.7 |
| | | nPD: | 0.05 |
| | | N. weight: | 2.4 |
| | | Sc. PD: | -0.43 |
| | | Sc. rank: | -218.4 |

| | | | |
|---|---|---|---|
| NP | | | |
| Pos. | 29 | obs: | exp: |
| gtt | V | 309 | 71.12 |
| gtc | V | 32 | 72.62 |
| gta | V | 3 | 64.85 |
| gtg | V | 2 | 137.40 |
| mPD | | 0.19 | 0.72 |
| | | nPD: | 0.27 |
| | | N. weight: | 1.7 |
| | | Sc. PD: | 0.053 |
| | | Sc. rank: | 96.6 |

| | | | |
|---|---|---|---|
| NP | | | |
| Pos. | 30 | obs: | exp: |
| ggt | G | 0 | 42.69 |
| ggc | G | 0 | 46.79 |
| gga | G | 283 | 167.40 |
| ggg | G | 63 | 89.14 |
| mPD | | 0.30 | 0.67 |
| | | nPD: | 0.45 |
| | | N. weight: | 0.54 |
| | | Sc. PD: | 0.11 |
| | | Sc. rank: | 71.7 |

| | | | |
|---|---|---|---|
| NP | | | |
| Pos. | 31 | obs: | exp: |
| cgt | R | 0 | 14.36 |
| cgc | R | 0 | 16.94 |
| cga | R | 2 | 33.25 |
| cgg | R | 0 | 28.87 |
| aga | R | 280 | 155.60 |
| agg | R | 64 | 96.96 |
| mPD | | 0.31 | 0.96 |
| | | nPD: | 0.33 |
| | | N. weight: | 0.57 |
| | | Sc. PD: | 0.049 |
| | | Sc. rank: | 48.6 |

| | | | |
|---|---|---|---|
| NP | | | |
| Pos. | 32 | obs: | exp: |
| atg | M | 345 | 345.00 |
| aaa | K | 0 | 0.56 |
| aag | K | 1 | 0.44 |
| mPD | | 0.0058 | 0.0090 |
| | | nPD: | 0.64 |
| | | N. weight: | 0.0034 |
| | | Sc. PD: | 0.0013 |
| | | Sc. rank: | 0.6 |

| | | | |
|---|---|---|---|
| NP | | | |
| Pos. | 33 | obs: | exp: |
| att | I | 2 | 0.75 |
| atc | I | 0 | 0.52 |
| ata | I | 0 | 0.72 |
| gtt | V | 303 | 70.71 |
| gtc | V | 39 | 72.20 |
| gta | V | 1 | 64.47 |
| gtg | V | 1 | 136.60 |
| mPD | | 0.22 | 0.73 |
| | | nPD: | 0.3 |
| | | N. weight: | 1.7 |
| | | Sc. PD: | 0.11 |
| | | Sc. rank: | 136.3 |

| | | | |
|---|---|---|---|
| NP | | | |
| Pos. | 34 | obs: | exp: |
| tct | S | 0 | 5.56 |
| tcc | S | 0 | 4.88 |
| tca | S | 0 | 7.75 |
| tcg | S | 0 | 2.13 |
| agt | S | 33 | 7.70 |
| agc | S | 2 | 6.97 |
| gat | D | 1 | 0.53 |
| gac | D | 0 | 0.47 |
| ggt | G | 293 | 38.25 |
| ggc | G | 16 | 41.92 |
| gga | G | 1 | 150.00 |
| ggg | G | 0 | 79.87 |
| mPD | | 0.29 | 0.99 |
| | | nPD: | 0.29 |
| | | N. weight: | 2.6 |
| | | Sc. PD: | 0.14 |
| | | Sc. rank: | 190.2 |

| | | | |
|---|---|---|---|
| NP | | | |
| Pos. | 35 | obs: | exp: |
| cgt | R | 0 | 0.04 |
| cgc | R | 0 | 0.05 |
| cga | R | 0 | 0.10 |
| cgg | R | 0 | 0.08 |
| aga | R | 1 | 0.45 |
| agg | R | 0 | 0.28 |
| ggt | G | 0 | 42.57 |
| ggc | G | 38 | 46.66 |
| gga | G | 279 | 166.90 |
| ggg | G | 28 | 88.89 |
| mPD | | 0.33 | 0.67 |
| | | nPD: | 0.5 |
| | | N. weight: | 0.45 |
| | | Sc. PD: | 0.11 |
| | | Sc. rank: | 65.7 |

| | | | |
|---|---|---|---|
| NP | | | |
| Pos. | 36 | obs: | exp: |
| att | I | 286 | 130.10 |
| atc | I | 59 | 90.68 |
| ata | I | 1 | 125.20 |
| mPD | | 0.29 | 0.66 |
| | | nPD: | 0.44 |
| | | N. weight: | 0.82 |
| | | Sc. PD: | 0.16 |
| | | Sc. rank: | 105.5 |

| | | | |
|---|---|---|---|
| NP | | | |
| Pos. | 37 | obs: | exp: |
| ggt | G | 24 | 42.69 |
| ggc | G | 0 | 46.79 |
| gga | G | 36 | 167.40 |
| ggg | G | 286 | 89.14 |
| mPD | | 0.30 | 0.67 |
| | | nPD: | 0.45 |
| | | N. weight: | 1.1 |
| | | Sc. PD: | 0.23 |
| | | Sc. rank: | 151.3 |

| | | | |
|---|---|---|---|
| NP | | | |
| Pos. | 38 | obs: | exp: |
| cgt | R | 0 | 14.28 |
| cgc | R | 1 | 16.84 |
| cga | R | 0 | 33.06 |
| cgg | R | 0 | 28.71 |
| aaa | K | 2 | 1.12 |
| aag | K | 0 | 0.88 |
| aga | R | 176 | 154.70 |
| agg | R | 167 | 96.40 |
| mPD | | 0.52 | 0.97 |
| | | nPD: | 0.54 |
| | | N. weight: | 0.48 |
| | | Sc. PD: | 0.14 |
| | | Sc. rank: | 75.4 |

| | | | |
|---|---|---|---|
| NP | | | |
| Pos. | 39 | obs: | exp: |
| ttt | F | 186 | 142.20 |
| ttc | F | 160 | 203.80 |
| mPD | | 0.50 | 0.48 |
| | | nPD: | 1.03 |
| | | N. weight: | 0.059 |
| | | Sc. PD: | 0.045 |
| | | Sc. rank: | 12.4 |

| | | | |
|---|---|---|---|
| NP | | | |
| Pos. | 40 | obs: | exp: |
| tat | Y | 4 | 179.10 |
| tac | Y | 342 | 166.90 |
| mPD | | 0.023 | 0.50 |
| | | nPD: | 0.05 |
| | | N. weight: | 0.96 |
| | | Sc. PD: | -0.18 |
| | | Sc. rank: | -92.0 |

| | | | |
|---|---|---|---|
| NP | | | |
| Pos. | 41 | obs: | exp: |
| att | I | 1 | 128.90 |
| atc | I | 0 | 89.89 |
| ata | I | 342 | 124.20 |
| gtt | V | 0 | 0.62 |
| gtc | V | 0 | 0.63 |
| gta | V | 3 | 0.56 |
| gtg | V | 0 | 1.19 |
| mPD | | 0.023 | 0.68 |
| | | nPD: | 0.03 |
| | | N. weight: | 1.4 |
| | | Sc. PD: | -0.28 |
| | | Sc. rank: | -167.0 |

| | | | |
|---|---|---|---|
| NP | | | |
| Pos. | 42 | obs: | exp: |
| caa | Q | 37 | 184.50 |
| cag | Q | 309 | 161.50 |
| mPD | | 0.19 | 0.50 |
| | | nPD: | 0.38 |
| | | N. weight: | 0.59 |
| | | Sc. PD: | 0.084 |
| | | Sc. rank: | 66.6 |

| | | | |
|---|---|---|---|
| NP | | | |
| Pos. | 43 | obs: | exp: |
| atg | M | 345 | 345.00 |
| act | T | 0 | 0.26 |
| acc | T | 0 | 0.21 |
| aca | T | 0 | 0.44 |
| acg | T | 1 | 0.08 |
| mPD | | 0.0058 | 0.011 |
| | | nPD: | 0.52 |
| | | N. weight: | 0.01 |
| | | Sc. PD: | 0.0028 |
| | | Sc. rank: | 1.6 |

| | | | |
|---|---|---|---|
| NP | | | |
| Pos. | 44 | obs: | exp: |
| tgt | C | 41 | 142.60 |
| tgc | C | 305 | 203.40 |
| mPD | | 0.21 | 0.48 |
| | | nPD: | 0.43 |
| | | N. weight: | 0.31 |
| | | Sc. PD: | 0.058 |
| | | Sc. rank: | 38.6 |

| | | | |
|---|---|---|---|
| NP | | | |
| Pos. | 45 | obs: | exp: |
| act | T | 308 | 91.41 |
| acc | T | 6 | 74.25 |
| aca | T | 32 | 151.60 |
| acg | T | 0 | 28.76 |
| mPD | | 0.20 | 0.69 |
| | | nPD: | 0.29 |
| | | N. weight: | 1.3 |
| | | Sc. PD: | 0.067 |
| | | Sc. rank: | 89.0 |

| | | | |
|---|---|---|---|
| NP | | | |
| Pos. | 46 | obs: | exp: |
| gaa | E | 251 | 199.10 |
| gag | E | 95 | 146.90 |
| mPD | | 0.40 | 0.49 |
| | | nPD: | 0.82 |
| | | N. weight: | 0.082 |
| | | Sc. PD: | 0.045 |
| | | Sc. rank: | 16.6 |

| | | | |
|---|---|---|---|
| NP | | | |
| Pos. | 47 | obs: | exp: |
| tta | L | 0 | 30.23 |
| ttg | L | 0 | 66.64 |
| ctt | L | 3 | 64.56 |
| ctc | L | 316 | 55.97 |
| cta | L | 27 | 52.12 |
| ctg | L | 0 | 76.48 |
| mPD | | 0.16 | 1.1 |
| | | nPD: | 0.14 |
| | | N. weight: | 2.2 |
| | | Sc. PD: | -0.19 |
| | | Sc. rank: | -23.9 |

| | | | |
|---|---|---|---|
| NP | | | |
| Pos. | 48 | obs: | exp: |
| cgt | R | 0 | 0.12 |
| cgc | R | 0 | 0.15 |
| cga | R | 0 | 0.29 |
| cgg | R | 0 | 0.25 |
| aaa | K | 302 | 192.10 |
| aag | K | 41 | 150.90 |
| aga | R | 0 | 1.35 |
| agg | R | 3 | 0.84 |
| mPD | | 0.24 | 0.52 |
| | | nPD: | 0.47 |
| | | N. weight: | 0.37 |
| | | Sc. PD: | 0.081 |
| | | Sc. rank: | 51.8 |

| | | | |
|---|---|---|---|
| NP | | | |
| Pos. | 49 | obs: | exp: |
| tta | L | 0 | 30.23 |
| ttg | L | 0 | 66.64 |
| ctt | L | 0 | 64.56 |
| ctc | L | 346 | 55.97 |
| cta | L | 0 | 52.12 |
| ctg | L | 0 | 76.48 |
| mPD | | 0 | 1.1 |
| | | nPD: | 0. |
| | | N. weight: | 2.6 |
| | | Sc. PD: | -0.59 |
| | | Sc. rank: | -541.3 |

| | | | |
|---|---|---|---|
| NP | | | |
| Pos. | 50 | obs: | exp: |
| tct | S | 0 | 53.89 |
| tcc | S | 0 | 47.22 |
| tca | S | 0 | 75.10 |
| tcg | S | 0 | 20.66 |
| aat | N | 6 | 3.66 |
| aac | N | 1 | 3.34 |
| agt | S | 116 | 74.58 |
| agc | S | 223 | 67.54 |
| mPD | | 0.50 | 1.7 |
| | | nPD: | 0.3 |
| | | N. weight: | 1.3 |
| | | Sc. PD: | 0.075 |
| | | Sc. rank: | 99.6 |

NP

| Pos. | 51 | obs: | exp: |
|---|---|---|---|
| gat | D | 11 | 184.60 |
| gac | D | 335 | 161.40 |
| --- | -- | ------ | ------ |
| mPD | | 0.062 | 0.50 |
| | | nPD: | 0.12 |
| | | N. weight: | 0.89 |
| | | Sc. PD: | -0.097 |
| | | Sc. rank: | -21.4 |

NP

| Pos. | 52 | obs: | exp: |
|---|---|---|---|
| tat | Y | 280 | 156.30 |
| tac | Y | 22 | 145.70 |
| cat | H | 9 | 7.59 |
| cac | H | 5 | 6.41 |
| caa | Q | 0 | 0.53 |
| cag | Q | 1 | 0.47 |
| aat | N | 29 | 15.16 |
| aac | N | 0 | 13.84 |
| --- | -- | ------ | ------ |
| mPD | | 0.38 | 0.73 |
| | | nPD: | 0.52 |
| | | N. weight: | 0.6 |
| | | Sc. PD: | 0.16 |
| | | Sc. rank: | 90.8 |

NP

| Pos. | 53 | obs: | exp: |
|---|---|---|---|
| gaa | E | 320 | 199.10 |
| gag | E | 26 | 146.90 |
| --- | -- | ------ | ------ |
| mPD | | 0.14 | 0.49 |
| | | nPD: | 0.29 |
| | | N. weight: | 0.45 |
| | | Sc. PD: | 0.021 |
| | | Sc. rank: | 28.0 |

NP

| Pos. | 54 | obs: | exp: |
|---|---|---|---|
| ggt | G | 0 | 42.69 |
| ggc | G | 0 | 46.79 |
| gga | G | 171 | 167.40 |
| ggg | G | 175 | 89.14 |
| --- | -- | ------ | ------ |
| mPD | | 0.50 | 0.67 |
| | | nPD: | 0.75 |
| | | N. weight: | 0.52 |
| | | Sc. PD: | 0.25 |
| | | Sc. rank: | 101.4 |

NP

| Pos. | 55 | obs: | exp: |
|---|---|---|---|
| cgt | R | 0 | 14.36 |
| cgc | R | 0 | 16.94 |
| cga | R | 0 | 33.25 |
| cgg | R | 0 | 28.87 |
| aga | R | 92 | 155.60 |
| agg | R | 254 | 96.96 |
| --- | -- | ------ | ------ |
| mPD | | 0.39 | 0.96 |
| | | nPD: | 0.41 |
| | | N. weight: | 0.83 |
| | | Sc. PD: | 0.13 |
| | | Sc. rank: | 97.0 |

NP

| Pos. | 56 | obs: | exp: |
|---|---|---|---|
| tta | L | 0 | 30.23 |
| ttg | L | 4 | 66.64 |
| ctt | L | 0 | 64.56 |
| ctc | L | 1 | 55.97 |
| cta | L | 23 | 52.12 |
| ctg | L | 318 | 76.48 |
| --- | -- | ------ | ------ |
| mPD | | 0.15 | 1.1 |
| | | nPD: | 0.14 |
| | | N. weight: | 1.8 |
| | | Sc. PD: | -0.17 |
| | | Sc. rank: | -26.4 |

NP

| Pos. | 57 | obs: | exp: |
|---|---|---|---|
| att | I | 34 | 130.10 |
| atc | I | 310 | 90.68 |
| ata | I | 2 | 125.20 |
| --- | -- | ------ | ------ |
| mPD | | 0.19 | 0.66 |
| | | nPD: | 0.29 |
| | | N. weight: | 1.4 |
| | | Sc. PD: | 0.063 |
| | | Sc. rank: | 86.5 |

NP

| Pos. | 58 | obs: | exp: |
|---|---|---|---|
| caa | Q | 95 | 184.50 |
| cag | Q | 251 | 161.50 |
| --- | -- | ------ | ------ |
| mPD | | 0.40 | 0.50 |
| | | nPD: | 0.8 |
| | | N. weight: | 0.21 |
| | | Sc. PD: | 0.11 |
| | | Sc. rank: | 42.3 |

NP

| Pos. | 59 | obs: | exp: |
|---|---|---|---|
| aat | N | 24 | 180.80 |
| aac | N | 322 | 165.20 |
| --- | -- | ------ | ------ |
| mPD | | 0.13 | 0.50 |
| | | nPD: | 0.26 |
| | | N. weight: | 0.7 |
| | | Sc. PD: | 0.015 |
| | | Sc. rank: | 35.6 |

NP

| Pos. | 60 | obs: | exp: |
|---|---|---|---|
| tct | S | 0 | 55.01 |
| tcc | S | 0 | 48.20 |
| tca | S | 0 | 76.65 |
| tcg | S | 0 | 21.09 |
| agt | S | 32 | 76.12 |
| agc | S | 314 | 68.93 |
| --- | -- | ------ | ------ |
| mPD | | 0.17 | 1.7 |
| | | nPD: | 0.1 |
| | | N. weight: | 1.9 |
| | | Sc. PD: | -0.24 |
| | | Sc. rank: | -91.1 |

NP

| Pos. | 61 | obs: | exp: |
|---|---|---|---|
| att | I | 1 | 129.70 |
| atc | I | 26 | 90.42 |
| ata | I | 318 | 124.90 |
| act | T | 0 | 0.26 |
| acc | T | 0 | 0.21 |
| aca | T | 1 | 0.44 |
| acg | T | 0 | 0.08 |
| --- | -- | ------ | ------ |
| mPD | | 0.15 | 0.66 |
| | | nPD: | 0.23 |
| | | N. weight: | 1.1 |
| | | Sc. PD: | -0.011 |
| | | Sc. rank: | 34.6 |

NP

| Pos. | 62 | obs: | exp: |
|---|---|---|---|
| act | T | 0 | 91.41 |
| acc | T | 25 | 74.25 |
| aca | T | 311 | 151.60 |
| acg | T | 10 | 28.76 |
| --- | -- | ------ | ------ |
| mPD | | 0.19 | 0.69 |
| | | nPD: | 0.27 |
| | | N. weight: | 0.79 |
| | | Sc. PD: | 0.026 |
| | | Sc. rank: | 44.7 |

NP

| Pos. | 63 | obs: | exp: |
|---|---|---|---|
| att | I | 0 | 130.10 |
| atc | I | 6 | 90.68 |
| ata | I | 340 | 125.20 |
| --- | -- | ------ | ------ |
| mPD | | 0.034 | 0.66 |
| | | nPD: | 0.05 |
| | | N. weight: | 1.3 |
| | | Sc. PD: | -0.24 |
| | | Sc. rank: | -110.7 |

NP

| Pos. | 64 | obs: | exp: |
|---|---|---|---|
| gaa | E | 42 | 199.10 |
| gag | E | 304 | 146.90 |
| --- | -- | ------ | ------ |
| mPD | | 0.21 | 0.49 |
| | | nPD: | 0.44 |
| | | N. weight: | 0.66 |
| | | Sc. PD: | 0.13 |
| | | Sc. rank: | 83.6 |

NP

| Pos. | 65 | obs: | exp: |
|---|---|---|---|
| cgt | R | 0 | 14.32 |
| cgc | R | 0 | 16.89 |
| cga | R | 0 | 33.16 |
| cgg | R | 0 | 28.79 |
| aaa | K | 1 | 0.56 |
| aag | K | 0 | 0.44 |
| aga | R | 320 | 155.20 |
| agg | R | 25 | 96.68 |
| --- | -- | ------ | ------ |
| mPD | | 0.14 | 0.96 |
| | | nPD: | 0.15 |
| | | N. weight: | 0.83 |
| | | Sc. PD: | -0.073 |
| | | Sc. rank: | -8.6 |

NP

| Pos. | 66 | obs: | exp: |
|---|---|---|---|
| atg | M | 346 | 346.00 |
| --- | -- | ------ | ------ |
| mPD | | 0 | 0 |
| | | nPD: | 1. |
| | | N. weight: | 0. |
| | | Sc. PD: | 0 |
| | | Sc. rank: | 0 |

NP

| Pos. | 67 | obs: | exp: |
|---|---|---|---|
| tta | L | 0 | 0.79 |
| ttg | L | 0 | 1.73 |
| ctt | L | 9 | 1.68 |
| ctc | L | 0 | 1.46 |
| cta | L | 0 | 1.36 |
| ctg | L | 0 | 1.99 |
| gtt | V | 278 | 69.27 |
| gtc | V | 26 | 70.73 |
| gta | V | 30 | 63.16 |
| gtg | V | 3 | 133.80 |
| --- | -- | ------ | ------ |
| mPD | | 0.35 | 0.77 |
| | | nPD: | 0.45 |
| | | N. weight: | 1.4 |
| | | Sc. PD: | 0.3 |
| | | Sc. rank: | 197.4 |

NP

| Pos. | 68 | obs: | exp: |
|---|---|---|---|
| tta | L | 0 | 30.23 |
| ttg | L | 0 | 66.64 |
| ctt | L | 4 | 64.56 |
| ctc | L | 338 | 55.97 |
| cta | L | 4 | 52.12 |
| ctg | L | 0 | 76.48 |
| --- | -- | ------ | ------ |
| mPD | | 0.046 | 1.1 |
| | | nPD: | 0.04 |
| | | N. weight: | 2.4 |
| | | Sc. PD: | -0.46 |
| | | Sc. rank: | -240.4 |

NP

| Pos. | 69 | obs: | exp: |
|---|---|---|---|
| tct | S | 242 | 55.01 |
| tcc | S | 31 | 48.20 |
| tca | S | 50 | 76.65 |
| tcg | S | 23 | 21.09 |
| agt | S | 0 | 76.12 |
| agc | S | 0 | 68.93 |
| --- | -- | ------ | ------ |
| mPD | | 0.48 | 1.7 |
| | | nPD: | 0.29 |
| | | N. weight: | 1.4 |
| | | Sc. PD: | 0.067 |
| | | Sc. rank: | 89.2 |

NP

| Pos. | 70 | obs: | exp: |
|---|---|---|---|
| gct | A | 1 | 87.15 |
| gcc | A | 0 | 70.88 |
| gca | A | 342 | 154.20 |
| gcg | A | 3 | 33.76 |
| --- | -- | ------ | ------ |
| mPD | | 0.023 | 0.69 |
| | | nPD: | 0.03 |
| | | N. weight: | 1.1 |
| | | Sc. PD: | -0.21 |
| | | Sc. rank: | -127.7 |

NP

| Pos. | 71 | obs: | exp: |
|---|---|---|---|
| ttt | F | 330 | 142.20 |
| ttc | F | 16 | 203.80 |
| --- | -- | ------ | ------ |
| mPD | | 0.088 | 0.48 |
| | | nPD: | 0.18 |
| | | N. weight: | 0.99 |
| | | Sc. PD: | -0.052 |
| | | Sc. rank: | 5.6 |

NP

| Pos. | 72 | obs: | exp: |
|---|---|---|---|
| gat | D | 343 | 184.60 |
| gac | D | 3 | 161.40 |
| --- | -- | ------ | ------ |
| mPD | | 0.017 | 0.50 |
| | | nPD: | 0.03 |
| | | N. weight: | 0.84 |
| | | Sc. PD: | -0.16 |
| | | Sc. rank: | -92.5 |

NP

| Pos. | 73 | obs: | exp: |
|---|---|---|---|
| gaa | E | 248 | 199.10 |
| gag | E | 98 | 146.90 |
| --- | -- | ------ | ------ |
| mPD | | 0.41 | 0.49 |
| | | nPD: | 0.83 |
| | | N. weight: | 0.074 |
| | | Sc. PD: | 0.042 |
| | | Sc. rank: | 15.1 |

NP

| Pos. | 74 | obs: | exp: |
|---|---|---|---|
| cgt | R | 0 | 14.36 |
| cgc | R | 0 | 16.94 |
| cga | R | 0 | 33.25 |
| cgg | R | 1 | 28.87 |
| aga | R | 79 | 155.60 |
| agg | R | 266 | 96.96 |
| --- | -- | ------ | ------ |
| mPD | | 0.36 | 0.96 |
| | | nPD: | 0.37 |
| | | N. weight: | 0.89 |
| | | Sc. PD: | 0.12 |
| | | Sc. rank: | 97.7 |

NP

| Pos. | 75 | obs: | exp: |
|---|---|---|---|
| cgt | R | 0 | 14.36 |
| cgc | R | 0 | 16.94 |
| cga | R | 0 | 33.25 |
| cgg | R | 0 | 28.87 |
| aga | R | 81 | 155.60 |
| agg | R | 265 | 96.96 |
| --- | -- | ------ | ------ |
| mPD | | 0.36 | 0.96 |
| | | nPD: | 0.37 |
| | | N. weight: | 0.9 |
| | | Sc. PD: | 0.12 |
| | | Sc. rank: | 98.8 |

| NP | | | |
|---|---|---|---|
| Pos. | 76 | obs: | exp: |
| aat | N | 12 | 180.80 |
| aac | N | 334 | 165.20 |
| --- | -- | ------ | ------ |
| mPD | | 0.067 | 0.50 |
| | | nPD: | 0.13 |
| | | N. weight: | 0.85 |
| | | Sc. PD: | -0.083 |
| | | Sc. rank: | -16.0 |

| NP | | | |
|---|---|---|---|
| Pos. | 77 | obs: | exp: |
| cgt | R | 0 | 2.28 |
| cgc | R | 0 | 2.69 |
| cga | R | 0 | 5.29 |
| cgg | R | 0 | 4.59 |
| aaa | K | 273 | 162.90 |
| aag | K | 18 | 128.10 |
| aga | R | 55 | 24.73 |
| agg | R | 0 | 15.41 |
| --- | -- | ------ | ------ |
| mPD | | 0.37 | 0.86 |
| | | nPD: | 0.43 |
| | | N. weight: | 0.63 |
| | | Sc. PD: | 0.12 |
| | | Sc. rank: | 76.7 |

| NP | | | |
|---|---|---|---|
| Pos. | 78 | obs: | exp: |
| tat | Y | 115 | 179.10 |
| tac | Y | 231 | 166.90 |
| --- | -- | ------ | ------ |
| mPD | | 0.45 | 0.50 |
| | | nPD: | 0.89 |
| | | N. weight: | 0.11 |
| | | Sc. PD: | 0.07 |
| | | Sc. rank: | 23.4 |

| NP | | | |
|---|---|---|---|
| Pos. | 79 | obs: | exp: |
| tta | L | 0 | 30.23 |
| ttg | L | 27 | 66.64 |
| ctt | L | 0 | 64.56 |
| ctc | L | 0 | 55.97 |
| cta | L | 21 | 52.12 |
| ctg | L | 298 | 76.48 |
| --- | -- | ------ | ------ |
| mPD | | 0.26 | 1.1 |
| | | nPD: | 0.23 |
| | | N. weight: | 1.5 |
| | | Sc. PD: | -0.0073 |
| | | Sc. rank: | 57.1 |

| NP | | | |
|---|---|---|---|
| Pos. | 80 | obs: | exp: |
| gaa | E | 277 | 199.10 |
| gag | E | 69 | 146.90 |
| --- | -- | ------ | ------ |
| mPD | | 0.32 | 0.49 |
| | | nPD: | 0.66 |
| | | N. weight: | 0.18 |
| | | Sc. PD: | 0.07 |
| | | Sc. rank: | 31.2 |

| NP | | | |
|---|---|---|---|
| Pos. | 81 | obs: | exp: |
| gaa | E | 272 | 199.10 |
| gag | E | 74 | 146.90 |
| --- | -- | ------ | ------ |
| mPD | | 0.34 | 0.49 |
| | | nPD: | 0.69 |
| | | N. weight: | 0.15 |
| | | Sc. PD: | 0.066 |
| | | Sc. rank: | 28.2 |

| NP | | | |
|---|---|---|---|
| Pos. | 82 | obs: | exp: |
| cat | H | 272 | 187.50 |
| cac | H | 74 | 158.50 |
| --- | -- | ------ | ------ |
| mPD | | 0.34 | 0.50 |
| | | nPD: | 0.68 |
| | | N. weight: | 0.2 |
| | | Sc. PD: | 0.084 |
| | | Sc. rank: | 36.2 |

| NP | | | |
|---|---|---|---|
| Pos. | 83 | obs: | exp: |
| cct | P | 1 | 91.11 |
| ccc | P | 344 | 62.41 |
| cca | P | 1 | 136.50 |
| ccg | P | 0 | 55.94 |
| --- | -- | ------ | ------ |
| mPD | | 0.012 | 0.72 |
| | | nPD: | 0.02 |
| | | N. weight: | 2.4 |
| | | Sc. PD: | -0.51 |
| | | Sc. rank: | -356.5 |

| NP | | | |
|---|---|---|---|
| Pos. | 84 | obs: | exp: |
| tct | S | 0 | 53.10 |
| tcc | S | 0 | 46.53 |
| tca | S | 0 | 73.99 |
| tcg | S | 0 | 20.36 |
| aat | N | 12 | 6.27 |
| aac | N | 0 | 5.73 |
| agt | S | 331 | 73.48 |
| agc | S | 3 | 66.54 |
| --- | -- | ------ | ------ |
| mPD | | 0.084 | 1.7 |
| | | nPD: | 0.05 |
| | | N. weight: | 2.1 |
| | | Sc. PD: | -0.37 |
| | | Sc. rank: | -178.2 |

| NP | | | |
|---|---|---|---|
| Pos. | 85 | obs: | exp: |
| act | T | 1 | 0.26 |
| acc | T | 0 | 0.21 |
| aca | T | 0 | 0.44 |
| acg | T | 0 | 0.08 |
| gtt | V | 1 | 1.03 |
| gtc | V | 0 | 1.05 |
| gta | V | 0 | 0.94 |
| gtg | V | 4 | 1.99 |
| gct | A | 124 | 85.64 |
| gcc | A | 0 | 69.65 |
| gca | A | 49 | 151.50 |
| gcg | A | 167 | 33.18 |
| --- | -- | ------ | ------ |
| mPD | | 0.64 | 0.72 |
| | | nPD: | 0.88 |
| | | N. weight: | 1.1 |
| | | Sc. PD: | 0.69 |
| | | Sc. rank: | 231.9 |

| NP | | | |
|---|---|---|---|
| Pos. | 86 | obs: | exp: |
| cgt | R | 0 | 0.12 |
| cgc | R | 0 | 0.15 |
| cga | R | 0 | 0.29 |
| cgg | R | 0 | 0.25 |
| aaa | K | 0 | 1.12 |
| aag | K | 2 | 0.88 |
| aga | R | 0 | 1.35 |
| agg | R | 3 | 0.84 |
| ggt | G | 0 | 42.07 |
| ggc | G | 0 | 46.11 |
| gga | G | 50 | 165.00 |
| ggg | G | 291 | 87.86 |
| --- | -- | ------ | ------ |
| mPD | | 0.29 | 0.70 |
| | | nPD: | 0.41 |
| | | N. weight: | 1.2 |
| | | Sc. PD: | 0.2 |
| | | Sc. rank: | 145.9 |

| NP | | | |
|---|---|---|---|
| Pos. | 87 | obs: | exp: |
| aaa | K | 99 | 193.70 |
| aag | K | 247 | 152.30 |
| --- | -- | ------ | ------ |
| mPD | | 0.41 | 0.49 |
| | | nPD: | 0.83 |
| | | N. weight: | 0.23 |
| | | Sc. PD: | 0.13 |
| | | Sc. rank: | 47.4 |

| NP | | | |
|---|---|---|---|
| Pos. | 88 | obs: | exp: |
| gat | D | 135 | 184.60 |
| gac | D | 211 | 161.40 |
| --- | -- | ------ | ------ |
| mPD | | 0.48 | 0.50 |
| | | nPD: | 0.96 |
| | | N. weight: | 0.072 |
| | | Sc. PD: | 0.05 |
| | | Sc. rank: | 15.0 |

| NP | | | |
|---|---|---|---|
| Pos. | 89 | obs: | exp: |
| cct | P | 65 | 91.11 |
| ccc | P | 41 | 62.41 |
| cca | P | 50 | 136.50 |
| ccg | P | 190 | 55.94 |
| --- | -- | ------ | ------ |
| mPD | | 0.63 | 0.72 |
| | | nPD: | 0.88 |
| | | N. weight: | 0.63 |
| | | Sc. PD: | 0.38 |
| | | Sc. rank: | 128.4 |

| NP | | | |
|---|---|---|---|
| Pos. | 90 | obs: | exp: |
| aaa | K | 0 | 193.70 |
| aag | K | 346 | 152.30 |
| --- | -- | ------ | ------ |
| mPD | | 0 | 0.49 |
| | | nPD: | 0. |
| | | N. weight: | 1.2 |
| | | Sc. PD: | -0.27 |
| | | Sc. rank: | -243.9 |

| NP | | | |
|---|---|---|---|
| Pos. | 91 | obs: | exp: |
| aaa | K | 299 | 193.70 |
| aag | K | 47 | 152.30 |
| --- | -- | ------ | ------ |
| mPD | | 0.24 | 0.49 |
| | | nPD: | 0.48 |
| | | N. weight: | 0.32 |
| | | Sc. PD: | 0.074 |
| | | Sc. rank: | 44.9 |

| NP | | | |
|---|---|---|---|
| Pos. | 92 | obs: | exp: |
| act | T | 331 | 91.41 |
| acc | T | 15 | 74.25 |
| aca | T | 0 | 151.60 |
| acg | T | 0 | 28.76 |
| --- | -- | ------ | ------ |
| mPD | | 0.083 | 0.69 |
| | | nPD: | 0.12 |
| | | N. weight: | 1.7 |
| | | Sc. PD: | -0.19 |
| | | Sc. rank: | -47.5 |

| NP | | | |
|---|---|---|---|
| Pos. | 93 | obs: | exp: |
| ggt | G | 1 | 42.69 |
| ggc | G | 0 | 46.79 |
| gga | G | 343 | 167.40 |
| ggg | G | 2 | 89.14 |
| --- | -- | ------ | ------ |
| mPD | | 0.017 | 0.67 |
| | | nPD: | 0.03 |
| | | N. weight: | 0.98 |
| | | Sc. PD: | -0.2 |
| | | Sc. rank: | -120.4 |

| NP | | | |
|---|---|---|---|
| Pos. | 94 | obs: | exp: |
| ggt | G | 266 | 42.69 |
| ggc | G | 80 | 46.79 |
| gga | G | 0 | 167.40 |
| ggg | G | 0 | 89.14 |
| --- | -- | ------ | ------ |
| mPD | | 0.36 | 0.67 |
| | | nPD: | 0.54 |
| | | N. weight: | 2.2 |
| | | Sc. PD: | 0.63 |
| | | Sc. rank: | 342.7 |

| NP | | | |
|---|---|---|---|
| Pos. | 95 | obs: | exp: |
| cct | P | 0 | 91.11 |
| ccc | P | 0 | 62.41 |
| cca | P | 337 | 136.50 |
| ccg | P | 9 | 55.94 |
| --- | -- | ------ | ------ |
| mPD | | 0.051 | 0.72 |
| | | nPD: | 0.07 |
| | | N. weight: | 1.2 |
| | | Sc. PD: | -0.19 |
| | | Sc. rank: | -83.6 |

| NP | | | |
|---|---|---|---|
| Pos. | 96 | obs: | exp: |
| att | I | 237 | 129.70 |
| atc | I | 84 | 90.42 |
| ata | I | 24 | 124.90 |
| gtt | V | 0 | 0.21 |
| gtc | V | 1 | 0.21 |
| gta | V | 0 | 0.19 |
| gtg | V | 0 | 0.40 |
| --- | -- | ------ | ------ |
| mPD | | 0.47 | 0.67 |
| | | nPD: | 0.71 |
| | | N. weight: | 0.43 |
| | | Sc. PD: | 0.2 |
| | | Sc. rank: | 81.3 |

| NP | | | |
|---|---|---|---|
| Pos. | 97 | obs: | exp: |
| tat | Y | 184 | 179.10 |
| tac | Y | 162 | 166.90 |
| --- | -- | ------ | ------ |
| mPD | | 0.50 | 0.50 |
| | | nPD: | 1. |
| | | N. weight: | 0.014 |
| | | Sc. PD: | 0.0099 |
| | | Sc. rank: | 2.8 |

| NP | | | |
|---|---|---|---|
| Pos. | 98 | obs: | exp: |
| cgt | R | 1 | 14.28 |
| cgc | R | 0 | 16.84 |
| cga | R | 175 | 33.06 |
| cgg | R | 26 | 28.71 |
| aaa | K | 2 | 1.12 |
| aag | K | 0 | 0.88 |
| aga | R | 45 | 154.70 |
| agg | R | 97 | 96.40 |
| --- | -- | ------ | ------ |
| mPD | | 0.96 | 0.97 |
| | | nPD: | 0.99 |
| | | N. weight: | 1. |
| | | Sc. PD: | 0.72 |
| | | Sc. rank: | 209.5 |

| NP | | | |
|---|---|---|---|
| Pos. | 99 | obs: | exp: |
| cgt | R | 0 | 14.36 |
| cgc | R | 0 | 16.94 |
| cga | R | 1 | 33.25 |
| cgg | R | 0 | 28.87 |
| aga | R | 100 | 155.60 |
| agg | R | 245 | 96.96 |
| --- | -- | ------ | ------ |
| mPD | | 0.42 | 0.96 |
| | | nPD: | 0.44 |
| | | N. weight: | 0.76 |
| | | Sc. PD: | 0.15 |
| | | Sc. rank: | 97.8 |

| NP | | | |
|---|---|---|---|
| Pos. | 100 | obs: | exp: |
| cgt | R | 0 | 14.07 |
| cgc | R | 0 | 16.60 |
| cga | R | 1 | 32.58 |
| cgg | R | 0 | 28.29 |
| aaa | K | 0 | 3.92 |
| aag | K | 7 | 3.08 |
| aga | R | 290 | 152.50 |
| agg | R | 48 | 95.00 |
| --- | -- | ------ | ------ |
| mPD | | 0.31 | 0.99 |
| | | nPD: | 0.32 |
| | | N. weight: | 0.66 |
| | | Sc. PD: | 0.05 |
| | | Sc. rank: | 53.9 |

| NP | | | |
|---|---|---|---|
| Pos. | 101 | obs: | exp: |
| gat | D | 182 | 184.60 |
| gac | D | 164 | 161.40 |
| --- | -- | ------ | ------ |
| mPD | | 0.50 | 0.50 |
| | | nPD: | 1. |
| | | N. weight: | 0.013 |
| | | Sc. PD: | 0.0097 |
| | | Sc. rank: | 2.8 |

| NP | | | |
|---|---|---|---|
| Pos. | 102 | obs: | exp: |
| ggt | G | 5 | 42.69 |
| ggc | G | 15 | 46.79 |
| gga | G | 62 | 167.40 |
| ggg | G | 264 | 89.14 |
| --- | -- | ------ | ------ |
| mPD | | 0.38 | 0.67 |
| | | nPD: | 0.58 |
| | | N. weight: | 0.85 |
| | | Sc. PD: | 0.28 |
| | | Sc. rank: | 137.6 |

| NP | | | |
|---|---|---|---|
| Pos. | 103 | obs: | exp: |
| aaa | K | 301 | 193.70 |
| aag | K | 45 | 152.30 |
| --- | -- | ------ | ------ |
| mPD | | 0.23 | 0.49 |
| | | nPD: | 0.46 |
| | | N. weight: | 0.33 |
| | | Sc. PD: | 0.071 |
| | | Sc. rank: | 45.8 |

| NP | | | |
|---|---|---|---|
| Pos. | 104 | obs: | exp: |
| tta | L | 0 | 0.17 |
| ttg | L | 2 | 0.39 |
| tgg | W | 344 | 344.00 |
| ctt | L | 0 | 0.37 |
| ctc | L | 0 | 0.32 |
| cta | L | 0 | 0.30 |
| ctg | L | 0 | 0.44 |
| --- | -- | ------ | ------ |
| mPD | | 0.012 | 0.027 |
| | | nPD: | 0.43 |
| | | N. weight: | 0.014 |
| | | Sc. PD: | 0.0026 |
| | | Sc. rank: | 1.7 |

| NP | | | |
|---|---|---|---|
| Pos. | 105 | obs: | exp: |
| att | I | 0 | 6.01 |
| atc | I | 0 | 4.19 |
| ata | I | 16 | 5.79 |
| atg | M | 162 | 162.00 |
| act | T | 1 | 1.59 |
| acc | T | 0 | 1.29 |
| aca | T | 0 | 2.63 |
| acg | T | 5 | 0.50 |
| gtt | V | 0 | 32.89 |
| gtc | V | 0 | 33.58 |
| gta | V | 3 | 29.99 |
| gtg | V | 157 | 63.55 |
| ggt | G | 0 | 0.25 |
| ggc | G | 0 | 0.27 |
| gga | G | 0 | 0.97 |
| ggg | G | 2 | 0.52 |
| --- | -- | ------ | ------ |
| mPD | | 0.65 | 1.1 |
| | | nPD: | 0.61 |
| | | N. weight: | 0.69 |
| | | Sc. PD: | 0.25 |
| | | Sc. rank: | 117.3 |

| NP | | | |
|---|---|---|---|
| Pos. | 106 | obs: | exp: |
| cgt | R | 0 | 14.36 |
| cgc | R | 0 | 16.94 |
| cga | R | 2 | 33.25 |
| cgg | R | 0 | 28.87 |
| aga | R | 329 | 155.60 |
| agg | R | 15 | 96.96 |
| --- | -- | ------ | ------ |
| mPD | | 0.095 | 0.96 |
| | | nPD: | 0.1 |
| | | N. weight: | 0.9 |
| | | Sc. PD: | -0.12 |
| | | Sc. rank: | -47.3 |

| NP | | | |
|---|---|---|---|
| Pos. | 107 | obs: | exp: |
| gaa | E | 115 | 198.50 |
| gag | E | 230 | 146.50 |
| ggt | G | 1 | 0.12 |
| ggc | G | 0 | 0.14 |
| gga | G | 0 | 0.48 |
| ggg | G | 0 | 0.26 |
| --- | -- | ------ | ------ |
| mPD | | 0.45 | 0.50 |
| | | nPD: | 0.92 |
| | | N. weight: | 0.19 |
| | | Sc. PD: | 0.12 |
| | | Sc. rank: | 39.7 |

| NP | | | |
|---|---|---|---|
| Pos. | 108 | obs: | exp: |
| tta | L | 24 | 30.23 |
| ttg | L | 80 | 66.64 |
| ctt | L | 0 | 64.56 |
| ctc | L | 1 | 55.97 |
| cta | L | 14 | 52.12 |
| ctg | L | 227 | 76.48 |
| --- | -- | ------ | ------ |
| mPD | | 0.62 | 1.1 |
| | | nPD: | 0.56 |
| | | N. weight: | 1. |
| | | Sc. PD: | 0.31 |
| | | Sc. rank: | 159.2 |

| NP | | | |
|---|---|---|---|
| Pos. | 109 | obs: | exp: |
| att | I | 144 | 129.30 |
| atc | I | 200 | 90.16 |
| ata | I | 0 | 124.50 |
| act | T | 0 | 0.26 |
| acc | T | 1 | 0.21 |
| aca | T | 0 | 0.44 |
| acg | T | 0 | 0.08 |
| gtt | V | 0 | 0.21 |
| gtc | V | 1 | 0.21 |
| gta | V | 0 | 0.19 |
| gtg | V | 0 | 0.40 |
| --- | -- | ------ | ------ |
| mPD | | 0.50 | 0.67 |
| | | nPD: | 0.74 |
| | | N. weight: | 0.75 |
| | | Sc. PD: | 0.36 |
| | | Sc. rank: | 146.1 |

| NP | | | |
|---|---|---|---|
| Pos. | 110 | obs: | exp: |
| tta | L | 1 | 30.23 |
| ttg | L | 13 | 66.64 |
| ctt | L | 1 | 64.56 |
| ctc | L | 1 | 55.97 |
| cta | L | 62 | 52.12 |
| ctg | L | 268 | 76.48 |
| --- | -- | ------ | ------ |
| mPD | | 0.39 | 1.1 |
| | | nPD: | 0.35 |
| | | N. weight: | 1.3 |
| | | Sc. PD: | 0.14 |
| | | Sc. rank: | 130.2 |

| NP | | | |
|---|---|---|---|
| Pos. | 111 | obs: | exp: |
| ttt | F | 2 | 0.82 |
| ttc | F | 0 | 1.18 |
| tat | Y | 333 | 177.50 |
| tac | Y | 10 | 165.50 |
| aat | N | 1 | 0.52 |
| aac | N | 0 | 0.48 |
| --- | -- | ------ | ------ |
| mPD | | 0.074 | 0.52 |
| | | nPD: | 0.14 |
| | | N. weight: | 0.77 |
| | | Sc. PD: | -0.07 |
| | | Sc. rank: | -9.9 |

| NP | | | |
|---|---|---|---|
| Pos. | 112 | obs: | exp: |
| gat | D | 10 | 184.10 |
| gac | D | 335 | 160.90 |
| gaa | E | 1 | 0.58 |
| gag | E | 0 | 0.42 |
| --- | -- | ------ | ------ |
| mPD | | 0.062 | 0.50 |
| | | nPD: | 0.12 |
| | | N. weight: | 0.91 |
| | | Sc. PD: | -0.098 |
| | | Sc. rank: | -23.8 |

| NP | | | |
|---|---|---|---|
| Pos. | 113 | obs: | exp: |
| aaa | K | 308 | 193.70 |
| aag | K | 38 | 152.30 |
| --- | -- | ------ | ------ |
| mPD | | 0.20 | 0.49 |
| | | nPD: | 0.4 |
| | | N. weight: | 0.38 |
| | | Sc. PD: | 0.059 |
| | | Sc. rank: | 44.6 |

| NP | | | |
|---|---|---|---|
| Pos. | 114 | obs: | exp: |
| gaa | E | 93 | 199.10 |
| gag | E | 253 | 146.90 |
| --- | -- | ------ | ------ |
| mPD | | 0.39 | 0.49 |
| | | nPD: | 0.81 |
| | | N. weight: | 0.29 |
| | | Sc. PD: | 0.16 |
| | | Sc. rank: | 58.5 |

| NP | | | |
|---|---|---|---|
| Pos. | 115 | obs: | exp: |
| gaa | E | 107 | 199.10 |
| gag | E | 239 | 146.90 |
| --- | -- | ------ | ------ |
| mPD | | 0.43 | 0.49 |
| | | nPD: | 0.88 |
| | | N. weight: | 0.22 |
| | | Sc. PD: | 0.13 |
| | | Sc. rank: | 44.8 |

| NP | | | |
|---|---|---|---|
| Pos. | 116 | obs: | exp: |
| tta | L | 0 | 0.17 |
| ttg | L | 0 | 0.39 |
| ctt | L | 0 | 0.37 |
| ctc | L | 2 | 0.32 |
| cta | L | 0 | 0.30 |
| ctg | L | 0 | 0.44 |
| att | I | 1 | 128.90 |
| atc | I | 342 | 89.89 |
| ata | I | 0 | 124.20 |
| gtt | V | 0 | 0.21 |
| gtc | V | 1 | 0.21 |
| gta | V | 0 | 0.19 |
| gtg | V | 0 | 0.40 |
| --- | -- | ------ | ------ |
| mPD | | 0.023 | 0.68 |
| | | nPD: | 0.03 |
| | | N. weight: | 1.9 |
| | | Sc. PD: | -0.37 |
| | | Sc. rank: | -217.1 |

| NP | | | |
|---|---|---|---|
| Pos. | 117 | obs: | exp: |
| cgt | R | 0 | 14.36 |
| cgc | R | 0 | 16.94 |
| cga | R | 0 | 33.25 |
| cgg | R | 0 | 28.87 |
| aga | R | 103 | 155.60 |
| agg | R | 243 | 96.96 |
| --- | -- | ------ | ------ |
| mPD | | 0.42 | 0.96 |
| | | nPD: | 0.44 |
| | | N. weight: | 0.76 |
| | | Sc. PD: | 0.15 |
| | | Sc. rank: | 95.2 |

| NP | | | |
|---|---|---|---|
| Pos. | 118 | obs: | exp: |
| cgt | R | 0 | 14.24 |
| cgc | R | 0 | 16.79 |
| cga | R | 0 | 32.96 |
| cgg | R | 0 | 28.62 |
| aaa | K | 3 | 1.68 |
| aag | K | 0 | 1.32 |
| aga | R | 175 | 154.30 |
| agg | R | 168 | 96.12 |
| --- | -- | ------ | ------ |
| mPD | | 0.52 | 0.97 |
| | | nPD: | 0.53 |
| | | N. weight: | 0.5 |
| | | Sc. PD: | 0.14 |
| | | Sc. rank: | 76.8 |

| NP | | | |
|---|---|---|---|
| Pos. | 119 | obs: | exp: |
| att | I | 134 | 127.80 |
| atc | I | 206 | 89.11 |
| ata | I | 0 | 123.10 |
| gtt | V | 3 | 1.23 |
| gtc | V | 3 | 1.26 |
| gta | V | 0 | 1.12 |
| gtg | V | 0 | 2.38 |
| --- | -- | ------ | ------ |
| mPD | | 0.51 | 0.70 |
| | | nPD: | 0.74 |
| | | N. weight: | 0.78 |
| | | Sc. PD: | 0.37 |
| | | Sc. rank: | 150.5 |

| NP | | | |
|---|---|---|---|
| Pos. | 120 | obs: | exp: |
| tgg | W | 346 | 346.00 |
| --- | -- | ------ | ------ |
| mPD | | 0 | 0 |
| | | nPD: | 1. |
| | | N. weight: | 0. |
| | | Sc. PD: | 0 |
| | | Sc. rank: | 0 |

| NP | | | |
|---|---|---|---|
| Pos. | 121 | obs: | exp: |
| cat | H | 0 | 0.54 |
| cac | H | 1 | 0.46 |
| cgt | R | 308 | 14.32 |
| cgc | R | 7 | 16.89 |
| cga | R | 29 | 33.16 |
| cgg | R | 1 | 28.79 |
| aga | R | 0 | 155.20 |
| agg | R | 0 | 96.68 |
| --- | -- | ------ | ------ |
| mPD | | 0.21 | 0.97 |
| | | nPD: | 0.21 |
| | | N. weight: | 3.9 |
| | | Sc. PD: | -0.09 |
| | | Sc. rank: | 91.8 |

| NP | | | |
|---|---|---|---|
| Pos. | 122 | obs: | exp: |
| caa | Q | 305 | 184.50 |
| cag | Q | 41 | 161.50 |
| --- | -- | ------ | ------ |
| mPD | | 0.21 | 0.50 |
| | | nPD: | 0.42 |
| | | N. weight: | 0.41 |
| | | Sc. PD: | 0.073 |
| | | Sc. rank: | 49.8 |

| NP | | | |
|---|---|---|---|
| Pos. | 123 | obs: | exp: |
| gct | A | 17 | 87.15 |
| gcc | A | 5 | 70.88 |
| gca | A | 81 | 154.20 |
| gcg | A | 243 | 33.76 |
| --- | -- | ------ | ------ |
| mPD | | 0.45 | 0.69 |
| | | nPD: | 0.66 |
| | | N. weight: | 1.6 |
| | | Sc. PD: | 0.65 |
| | | Sc. rank: | 293.4 |

| NP | | | |
|---|---|---|---|
| Pos. | 124 | obs: | exp: |
| aat | N | 131 | 180.80 |
| aac | N | 215 | 165.20 |
| --- | -- | ------ | ------ |
| mPD | | 0.47 | 0.50 |
| | | nPD: | 0.95 |
| | | N. weight: | 0.073 |
| | | Sc. PD: | 0.049 |
| | | Sc. rank: | 15.1 |

| NP | | | |
|---|---|---|---|
| Pos. | 125 | obs: | exp: |
| aat | N | 296 | 180.80 |
| aac | N | 50 | 165.20 |
| --- | -- | ------ | ------ |
| mPD | | 0.25 | 0.50 |
| | | nPD: | 0.5 |
| | | N. weight: | 0.37 |
| | | Sc. PD: | 0.091 |
| | | Sc. rank: | 53.6 |

| | | | |
|---|---|---|---|
| NP | | | |
| Pos. | 126 | obs: | exp: |
| ggt | G | 2 | 42.69 |
| ggc | G | 1 | 46.79 |
| gga | G | 297 | 167.40 |
| ggg | G | 46 | 89.14 |
| --- | -- | ------ | ------ |
| mPD | | 0.25 | 0.67 |
| | | nPD: | 0.37 |
| | | N. weight: | 0.56 |
| | | Sc. PD: | 0.071 |
| | | Sc. rank: | 59.0 |

| | | | |
|---|---|---|---|
| NP | | | |
| Pos. | 127 | obs: | exp: |
| gaa | E | 263 | 199.10 |
| gag | E | 83 | 146.90 |
| --- | -- | ------ | ------ |
| mPD | | 0.37 | 0.49 |
| | | nPD: | 0.75 |
| | | N. weight: | 0.12 |
| | | Sc. PD: | 0.058 |
| | | Sc. rank: | 23.3 |

| | | | |
|---|---|---|---|
| NP | | | |
| Pos. | 128 | obs: | exp: |
| gat | D | 79 | 184.10 |
| gac | D | 266 | 160.90 |
| gaa | E | 1 | 0.58 |
| gag | E | 0 | 0.42 |
| --- | -- | ------ | ------ |
| mPD | | 0.36 | 0.50 |
| | | nPD: | 0.71 |
| | | N. weight: | 0.29 |
| | | Sc. PD: | 0.13 |
| | | Sc. rank: | 55.3 |

| | | | |
|---|---|---|---|
| NP | | | |
| Pos. | 129 | obs: | exp: |
| gct | A | 0 | 87.15 |
| gcc | A | 1 | 70.88 |
| gca | A | 335 | 154.20 |
| gcg | A | 10 | 33.76 |
| --- | -- | ------ | ------ |
| mPD | | 0.062 | 0.69 |
| | | nPD: | 0.09 |
| | | N. weight: | 1. |
| | | Sc. PD: | -0.14 |
| | | Sc. rank: | -57.4 |

| | | | |
|---|---|---|---|
| NP | | | |
| Pos. | 130 | obs: | exp: |
| act | T | 325 | 91.41 |
| acc | T | 18 | 74.25 |
| aca | T | 3 | 151.60 |
| acg | T | 0 | 28.76 |
| --- | -- | ------ | ------ |
| mPD | | 0.12 | 0.69 |
| | | nPD: | 0.17 |
| | | N. weight: | 1.6 |
| | | Sc. PD: | -0.1 |
| | | Sc. rank: | -0.5 |

| | | | |
|---|---|---|---|
| NP | | | |
| Pos. | 131 | obs: | exp: |
| gct | A | 344 | 87.15 |
| gcc | A | 2 | 70.88 |
| gca | A | 0 | 154.20 |
| gcg | A | 0 | 33.76 |
| --- | -- | ------ | ------ |
| mPD | | 0.012 | 0.69 |
| | | nPD: | 0.02 |
| | | N. weight: | 1.9 |
| | | Sc. PD: | -0.41 |
| | | Sc. rank: | -278.6 |

| | | | |
|---|---|---|---|
| NP | | | |
| Pos. | 132 | obs: | exp: |
| ggt | G | 337 | 42.69 |
| ggc | G | 8 | 46.79 |
| gga | G | 1 | 167.40 |
| ggg | G | 0 | 89.14 |
| --- | -- | ------ | ------ |
| mPD | | 0.051 | 0.67 |
| | | nPD: | 0.08 |
| | | N. weight: | 2.8 |
| | | Sc. PD: | -0.43 |
| | | Sc. rank: | -181.8 |

| | | | |
|---|---|---|---|
| NP | | | |
| Pos. | 133 | obs: | exp: |
| tta | L | 0 | 30.23 |
| ttg | L | 0 | 66.64 |
| ctt | L | 40 | 64.56 |
| ctc | L | 289 | 55.97 |
| cta | L | 17 | 52.12 |
| ctg | L | 0 | 76.48 |
| --- | -- | ------ | ------ |
| mPD | | 0.29 | 1.1 |
| | | nPD: | 0.26 |
| | | N. weight: | 1.8 |
| | | Sc. PD: | 0.036 |
| | | Sc. rank: | 90.9 |

| | | | |
|---|---|---|---|
| NP | | | |
| Pos. | 134 | obs: | exp: |
| act | T | 278 | 91.41 |
| acc | T | 67 | 74.25 |
| aca | T | 1 | 151.60 |
| acg | T | 0 | 28.76 |
| --- | -- | ------ | ------ |
| mPD | | 0.32 | 0.69 |
| | | nPD: | 0.46 |
| | | N. weight: | 1.2 |
| | | Sc. PD: | 0.27 |
| | | Sc. rank: | 172.8 |

| | | | |
|---|---|---|---|
| NP | | | |
| Pos. | 135 | obs: | exp: |
| cat | H | 167 | 187.50 |
| cac | H | 179 | 158.50 |
| --- | -- | ------ | ------ |
| mPD | | 0.50 | 0.50 |
| | | nPD: | 1.01 |
| | | N. weight: | 0.023 |
| | | Sc. PD: | 0.017 |
| | | Sc. rank: | 4.8 |

| | | | |
|---|---|---|---|
| NP | | | |
| Pos. | 136 | obs: | exp: |
| tta | L | 1 | 28.31 |
| ttg | L | 71 | 62.40 |
| ctt | L | 0 | 60.45 |
| ctc | L | 0 | 52.41 |
| cta | L | 1 | 48.81 |
| ctg | L | 251 | 71.62 |
| atg | M | 21 | 21.00 |
| gtt | V | 0 | 0.21 |
| gtc | V | 0 | 0.21 |
| gta | V | 0 | 0.19 |
| gtg | V | 1 | 0.40 |
| --- | -- | ------ | ------ |
| mPD | | 0.44 | 1.2 |
| | | nPD: | 0.37 |
| | | N. weight: | 1.3 |
| | | Sc. PD: | 0.17 |
| | | Sc. rank: | 145.2 |

| | | | |
|---|---|---|---|
| NP | | | |
| Pos. | 137 | obs: | exp: |
| atg | M | 346 | 346.00 |
| --- | -- | ------ | ------ |
| mPD | | 0 | 0 |
| | | nPD: | 1. |
| | | N. weight: | 0. |
| | | Sc. PD: | 0 |
| | | Sc. rank: | 0 |

| | | | |
|---|---|---|---|
| NP | | | |
| Pos. | 138 | obs: | exp: |
| att | I | 51 | 130.10 |
| atc | I | 265 | 90.68 |
| ata | I | 30 | 125.20 |
| --- | -- | ------ | ------ |
| mPD | | 0.39 | 0.66 |
| | | nPD: | 0.58 |
| | | N. weight: | 0.82 |
| | | Sc. PD: | 0.27 |
| | | Sc. rank: | 137.3 |

| | | | |
|---|---|---|---|
| NP | | | |
| Pos. | 139 | obs: | exp: |
| tgg | W | 346 | 346.00 |
| --- | -- | ------ | ------ |
| mPD | | 0 | 0 |
| | | nPD: | 1. |
| | | N. weight: | 0. |
| | | Sc. PD: | 0 |
| | | Sc. rank: | 0 |

| | | | |
|---|---|---|---|
| NP | | | |
| Pos. | 140 | obs: | exp: |
| cat | H | 315 | 187.50 |
| cac | H | 31 | 158.50 |
| --- | -- | ------ | ------ |
| mPD | | 0.16 | 0.50 |
| | | nPD: | 0.33 |
| | | N. weight: | 0.48 |
| | | Sc. PD: | 0.042 |
| | | Sc. rank: | 42.0 |

| | | | |
|---|---|---|---|
| NP | | | |
| Pos. | 141 | obs: | exp: |
| tct | S | 3 | 55.01 |
| tcc | S | 343 | 48.20 |
| tca | S | 0 | 76.65 |
| tcg | S | 0 | 21.09 |
| agt | S | 0 | 76.12 |
| agc | S | 0 | 68.93 |
| --- | -- | ------ | ------ |
| mPD | | 0.017 | 1.7 |
| | | nPD: | 0.01 |
| | | N. weight: | 2.8 |
| | | Sc. PD: | -0.6 |
| | | Sc. rank: | -457.2 |

| | | | |
|---|---|---|---|
| NP | | | |
| Pos. | 142 | obs: | exp: |
| aat | N | 216 | 180.80 |
| aac | N | 130 | 165.20 |
| --- | -- | ------ | ------ |
| mPD | | 0.47 | 0.50 |
| | | nPD: | 0.94 |
| | | N. weight: | 0.043 |
| | | Sc. PD: | 0.029 |
| | | Sc. rank: | 8.9 |

| | | | |
|---|---|---|---|
| NP | | | |
| Pos. | 143 | obs: | exp: |
| tta | L | 6 | 29.70 |
| ttg | L | 28 | 65.48 |
| ctt | L | 2 | 63.44 |
| ctc | L | 2 | 55.00 |
| cta | L | 168 | 51.22 |
| ctg | L | 134 | 75.16 |
| gtt | V | 0 | 1.23 |
| gtc | V | 0 | 1.26 |
| gta | V | 6 | 1.12 |
| gtg | V | 0 | 2.38 |
| --- | -- | ------ | ------ |
| mPD | | 0.72 | 1.1 |
| | | nPD: | 0.63 |
| | | N. weight: | 1. |
| | | Sc. PD: | 0.39 |
| | | Sc. rank: | 180.9 |

| | | | |
|---|---|---|---|
| NP | | | |
| Pos. | 144 | obs: | exp: |
| aat | N | 342 | 180.80 |
| aac | N | 4 | 165.20 |
| --- | -- | ------ | ------ |
| mPD | | 0.023 | 0.50 |
| | | nPD: | 0.05 |
| | | N. weight: | 0.85 |
| | | Sc. PD: | -0.15 |
| | | Sc. rank: | -80.6 |

| | | | |
|---|---|---|---|
| NP | | | |
| Pos. | 145 | obs: | exp: |
| gat | D | 343 | 184.60 |
| gac | D | 3 | 161.40 |
| --- | -- | ------ | ------ |
| mPD | | 0.017 | 0.50 |
| | | nPD: | 0.03 |
| | | N. weight: | 0.84 |
| | | Sc. PD: | -0.16 |
| | | Sc. rank: | -92.5 |

| | | | |
|---|---|---|---|
| NP | | | |
| Pos. | 146 | obs: | exp: |
| act | T | 0 | 0.79 |
| acc | T | 3 | 0.64 |
| aca | T | 0 | 1.31 |
| acg | T | 0 | 0.25 |
| gtt | V | 0 | 0.41 |
| gtc | V | 2 | 0.42 |
| gta | V | 0 | 0.37 |
| gtg | V | 0 | 0.79 |
| gct | A | 2 | 85.89 |
| gcc | A | 312 | 69.85 |
| gca | A | 27 | 152.00 |
| gcg | A | 0 | 33.28 |
| --- | -- | ------ | ------ |
| mPD | | 0.18 | 0.72 |
| | | nPD: | 0.26 |
| | | N. weight: | 1.8 |
| | | Sc. PD: | 0.033 |
| | | Sc. rank: | 82.7 |

| | | | |
|---|---|---|---|
| NP | | | |
| Pos. | 147 | obs: | exp: |
| act | T | 22 | 91.41 |
| acc | T | 37 | 74.25 |
| aca | T | 285 | 151.60 |
| acg | T | 2 | 28.76 |
| --- | -- | ------ | ------ |
| mPD | | 0.31 | 0.69 |
| | | nPD: | 0.45 |
| | | N. weight: | 0.51 |
| | | Sc. PD: | 0.1 |
| | | Sc. rank: | 68.3 |

| | | | |
|---|---|---|---|
| NP | | | |
| Pos. | 148 | obs: | exp: |
| tat | Y | 113 | 179.10 |
| tac | Y | 233 | 166.90 |
| --- | -- | ------ | ------ |
| mPD | | 0.44 | 0.50 |
| | | nPD: | 0.88 |
| | | N. weight: | 0.12 |
| | | Sc. PD: | 0.074 |
| | | Sc. rank: | 24.5 |

| | | | |
|---|---|---|---|
| NP | | | |
| Pos. | 149 | obs: | exp: |
| caa | Q | 11 | 184.50 |
| cag | Q | 335 | 161.50 |
| --- | -- | ------ | ------ |
| mPD | | 0.062 | 0.50 |
| | | nPD: | 0.12 |
| | | N. weight: | 0.89 |
| | | Sc. PD: | -0.096 |
| | | Sc. rank: | -22.7 |

| | | | |
|---|---|---|---|
| NP | | | |
| Pos. | 150 | obs: | exp: |
| cgt | R | 0 | 14.36 |
| cgc | R | 0 | 16.94 |
| cga | R | 2 | 33.25 |
| cgg | R | 0 | 28.87 |
| aga | R | 259 | 155.60 |
| agg | R | 85 | 96.96 |
| --- | -- | ------ | ------ |
| mPD | | 0.38 | 0.96 |
| | | nPD: | 0.4 |
| | | N. weight: | 0.49 |
| | | Sc. PD: | 0.077 |
| | | Sc. rank: | 57.7 |

| NP | | | |
|---|---|---|---|
| Pos. | 151 | obs: | exp: |
| act | T | 15 | 91.41 |
| acc | T | 45 | 74.25 |
| aca | T | 247 | 151.60 |
| acg | T | 39 | 28.76 |
| mPD | | 0.46 | 0.69 |
| | | nPD: | 0.67 |
| | | N. weight: | 0.37 |
| | | Sc. PD: | 0.16 |
| | | Sc. rank: | 68.3 |

| NP | | | |
|---|---|---|---|
| Pos. | 152 | obs: | exp: |
| cgt | R | 0 | 14.36 |
| cgc | R | 0 | 16.94 |
| cga | R | 0 | 33.25 |
| cgg | R | 0 | 28.87 |
| aga | R | 259 | 155.60 |
| agg | R | 87 | 96.96 |
| mPD | | 0.38 | 0.96 |
| | | nPD: | 0.39 |
| | | N. weight: | 0.52 |
| | | Sc. PD: | 0.078 |
| | | Sc. rank: | 59.8 |

| NP | | | |
|---|---|---|---|
| Pos. | 153 | obs: | exp: |
| gct | A | 262 | 87.15 |
| gcc | A | 5 | 70.88 |
| gca | A | 76 | 154.20 |
| gcg | A | 3 | 33.76 |
| mPD | | 0.38 | 0.69 |
| | | nPD: | 0.55 |
| | | N. weight: | 0.91 |
| | | Sc. PD: | 0.27 |
| | | Sc. rank: | 143.8 |

| NP | | | |
|---|---|---|---|
| Pos. | 154 | obs: | exp: |
| tta | L | 0 | 30.23 |
| ttg | L | 0 | 66.64 |
| ctt | L | 82 | 64.56 |
| ctc | L | 227 | 55.97 |
| cta | L | 37 | 52.12 |
| ctg | L | 0 | 76.48 |
| mPD | | 0.50 | 1.1 |
| | | nPD: | 0.45 |
| | | N. weight: | 1.4 |
| | | Sc. PD: | 0.28 |
| | | Sc. rank: | 184.3 |

| NP | | | |
|---|---|---|---|
| Pos. | 155 | obs: | exp: |
| gtt | V | 1 | 71.12 |
| gtc | V | 0 | 72.62 |
| gta | V | 14 | 64.85 |
| gtg | V | 331 | 137.40 |
| mPD | | 0.083 | 0.72 |
| | | nPD: | 0.12 |
| | | N. weight: | 1.1 |
| | | Sc. PD: | -0.13 |
| | | Sc. rank: | -35.2 |

| NP | | | |
|---|---|---|---|
| Pos. | 156 | obs: | exp: |
| cgt | R | 286 | 14.36 |
| cgc | R | 58 | 16.94 |
| cga | R | 1 | 33.25 |
| cgg | R | 1 | 28.87 |
| aga | R | 0 | 155.60 |
| agg | R | 0 | 96.96 |
| mPD | | 0.29 | 0.96 |
| | | nPD: | 0.3 |
| | | N. weight: | 3.8 |
| | | Sc. PD: | 0.24 |
| | | Sc. rank: | 298.5 |

| NP | | | |
|---|---|---|---|
| Pos. | 157 | obs: | exp: |
| act | T | 307 | 91.41 |
| acc | T | 39 | 74.25 |
| aca | T | 0 | 151.60 |
| acg | T | 0 | 28.76 |
| mPD | | 0.20 | 0.69 |
| | | nPD: | 0.29 |
| | | N. weight: | 1.4 |
| | | Sc. PD: | 0.077 |
| | | Sc. rank: | 102.1 |

| NP | | | |
|---|---|---|---|
| Pos. | 158 | obs: | exp: |
| ggt | G | 7 | 42.69 |
| ggc | G | 0 | 46.79 |
| gga | G | 79 | 167.40 |
| ggg | G | 260 | 89.14 |
| mPD | | 0.38 | 0.67 |
| | | nPD: | 0.58 |
| | | N. weight: | 0.87 |
| | | Sc. PD: | 0.28 |
| | | Sc. rank: | 141.8 |

| NP | | | |
|---|---|---|---|
| Pos. | 159 | obs: | exp: |
| atg | M | 346 | 346.00 |
| mPD | | 0 | 0 |
| | | nPD: | 1. |
| | | N. weight: | 0. |
| | | Sc. PD: | 0 |
| | | Sc. rank: | 0 |

| NP | | | |
|---|---|---|---|
| Pos. | 160 | obs: | exp: |
| gat | D | 97 | 184.60 |
| gac | D | 249 | 161.40 |
| mPD | | 0.40 | 0.50 |
| | | nPD: | 0.81 |
| | | N. weight: | 0.2 |
| | | Sc. PD: | 0.11 |
| | | Sc. rank: | 40.8 |

| NP | | | |
|---|---|---|---|
| Pos. | 161 | obs: | exp: |
| cct | P | 41 | 91.11 |
| ccc | P | 299 | 62.41 |
| cca | P | 6 | 136.50 |
| ccg | P | 0 | 55.94 |
| mPD | | 0.24 | 0.72 |
| | | nPD: | 0.33 |
| | | N. weight: | 1.7 |
| | | Sc. PD: | 0.16 |
| | | Sc. rank: | 162.0 |

| NP | | | |
|---|---|---|---|
| Pos. | 162 | obs: | exp: |
| cgt | R | 0 | 14.36 |
| cgc | R | 0 | 16.94 |
| cga | R | 6 | 33.25 |
| cgg | R | 29 | 28.87 |
| aga | R | 197 | 155.60 |
| agg | R | 114 | 96.96 |
| mPD | | 0.67 | 0.96 |
| | | nPD: | 0.7 |
| | | N. weight: | 0.26 |
| | | Sc. PD: | 0.11 |
| | | Sc. rank: | 47.7 |

| NP | | | |
|---|---|---|---|
| Pos. | 163 | obs: | exp: |
| atg | M | 346 | 346.00 |
| mPD | | 0 | 0 |
| | | nPD: | 1. |
| | | N. weight: | 0. |
| | | Sc. PD: | 0 |
| | | Sc. rank: | 0 |

| NP | | | |
|---|---|---|---|
| Pos. | 164 | obs: | exp: |
| tgt | C | 38 | 142.60 |
| tgc | C | 308 | 203.40 |
| mPD | | 0.20 | 0.48 |
| | | nPD: | 0.4 |
| | | N. weight: | 0.33 |
| | | Sc. PD: | 0.053 |
| | | Sc. rank: | 38.8 |

| NP | | | |
|---|---|---|---|
| Pos. | 165 | obs: | exp: |
| tct | S | 327 | 55.01 |
| tcc | S | 19 | 48.20 |
| tca | S | 0 | 76.65 |
| tcg | S | 0 | 21.09 |
| agt | S | 0 | 76.12 |
| agc | S | 0 | 68.93 |
| mPD | | 0.10 | 1.7 |
| | | nPD: | 0.06 |
| | | N. weight: | 2.4 |
| | | Sc. PD: | -0.39 |
| | | Sc. rank: | -168.2 |

| NP | | | |
|---|---|---|---|
| Pos. | 166 | obs: | exp: |
| tta | L | 0 | 30.23 |
| ttg | L | 0 | 66.64 |
| ctt | L | 50 | 64.56 |
| ctc | L | 22 | 55.97 |
| cta | L | 5 | 52.12 |
| ctg | L | 269 | 76.48 |
| mPD | | 0.37 | 1.1 |
| | | nPD: | 0.33 |
| | | N. weight: | 1.2 |
| | | Sc. PD: | 0.11 |
| | | Sc. rank: | 113.6 |

| NP | | | |
|---|---|---|---|
| Pos. | 167 | obs: | exp: |
| atg | M | 346 | 346.00 |
| mPD | | 0 | 0 |
| | | nPD: | 1. |
| | | N. weight: | 0. |
| | | Sc. PD: | 0 |
| | | Sc. rank: | 0 |

| NP | | | |
|---|---|---|---|
| Pos. | 168 | obs: | exp: |
| caa | Q | 341 | 184.50 |
| cag | Q | 5 | 161.50 |
| mPD | | 0.029 | 0.50 |
| | | nPD: | 0.06 |
| | | N. weight: | 0.8 |
| | | Sc. PD: | -0.14 |
| | | Sc. rank: | -64.8 |

| NP | | | |
|---|---|---|---|
| Pos. | 169 | obs: | exp: |
| ggt | G | 3 | 42.69 |
| ggc | G | 53 | 46.79 |
| gga | G | 252 | 167.40 |
| ggg | G | 38 | 89.14 |
| mPD | | 0.44 | 0.67 |
| | | nPD: | 0.65 |
| | | N. weight: | 0.32 |
| | | Sc. PD: | 0.13 |
| | | Sc. rank: | 56.4 |

| NP | | | |
|---|---|---|---|
| Pos. | 170 | obs: | exp: |
| tct | S | 1 | 55.01 |
| tcc | S | 0 | 48.20 |
| tca | S | 340 | 76.65 |
| tcg | S | 5 | 21.09 |
| agt | S | 0 | 76.12 |
| agc | S | 0 | 68.93 |
| mPD | | 0.034 | 1.7 |
| | | nPD: | 0.02 |
| | | N. weight: | 2.1 |
| | | Sc. PD: | -0.43 |
| | | Sc. rank: | -277.8 |

| NP | | | |
|---|---|---|---|
| Pos. | 171 | obs: | exp: |
| act | T | 299 | 91.41 |
| acc | T | 47 | 74.25 |
| aca | T | 0 | 151.60 |
| acg | T | 0 | 28.76 |
| mPD | | 0.24 | 0.69 |
| | | nPD: | 0.34 |
| | | N. weight: | 1.4 |
| | | Sc. PD: | 0.14 |
| | | Sc. rank: | 133.5 |

| NP | | | |
|---|---|---|---|
| Pos. | 172 | obs: | exp: |
| tta | L | 0 | 30.23 |
| ttg | L | 0 | 66.64 |
| ctt | L | 84 | 64.56 |
| ctc | L | 262 | 55.97 |
| cta | L | 0 | 52.12 |
| ctg | L | 0 | 76.48 |
| mPD | | 0.37 | 1.1 |
| | | nPD: | 0.33 |
| | | N. weight: | 1.8 |
| | | Sc. PD: | 0.16 |
| | | Sc. rank: | 158.8 |

| NP | | | |
|---|---|---|---|
| Pos. | 173 | obs: | exp: |
| cct | P | 6 | 91.11 |
| ccc | P | 1 | 62.41 |
| cca | P | 103 | 136.50 |
| ccg | P | 236 | 55.94 |
| mPD | | 0.45 | 0.72 |
| | | nPD: | 0.62 |
| | | N. weight: | 1.2 |
| | | Sc. PD: | 0.45 |
| | | Sc. rank: | 210.9 |

| NP | | | |
|---|---|---|---|
| Pos. | 174 | obs: | exp: |
| cgt | R | 0 | 14.36 |
| cgc | R | 0 | 16.94 |
| cga | R | 0 | 33.25 |
| cgg | R | 1 | 28.87 |
| aga | R | 39 | 155.60 |
| agg | R | 306 | 96.96 |
| mPD | | 0.21 | 0.96 |
| | | nPD: | 0.22 |
| | | N. weight: | 1.2 |
| | | Sc. PD: | -0.026 |
| | | Sc. rank: | 31.2 |

| NP | | | |
|---|---|---|---|
| Pos. | 175 | obs: | exp: |
| cgt | R | 0 | 14.32 |
| cgc | R | 0 | 16.89 |
| cga | R | 0 | 33.16 |
| cgg | R | 0 | 28.79 |
| aaa | K | 1 | 0.56 |
| aag | K | 0 | 0.44 |
| aga | R | 228 | 155.20 |
| agg | R | 117 | 96.68 |
| mPD | | 0.45 | 0.96 |
| | | nPD: | 0.47 |
| | | N. weight: | 0.47 |
| | | Sc. PD: | 0.11 |
| | | Sc. rank: | 65.8 |

| NP | | | |
|---|---|---|---|
| Pos. | 176 | obs: | exp: |
| tct | S | 304 | 55.01 |
| tcc | S | 36 | 48.20 |
| tca | S | 6 | 76.65 |
| tcg | S | 0 | 21.09 |
| agt | S | 0 | 76.12 |
| agc | S | 0 | 68.93 |
| mPD | | 0.22 | 1.7 |
| | | nPD: | 0.13 |
| | | N. weight: | 2.1 |
| | | Sc. PD: | -0.21 |
| | | Sc. rank: | -40.4 |

| NP | | | |
|---|---|---|---|
| Pos. | 177 | obs: | exp: |
| ggt | G | 0 | 42.69 |
| ggc | G | 0 | 46.79 |
| gga | G | 280 | 167.40 |
| ggg | G | 66 | 89.14 |
| mPD | | 0.31 | 0.67 |
| | | nPD: | 0.46 |
| | | N. weight: | 0.53 |
| | | Sc. PD: | 0.11 |
| | | Sc. rank: | 73.2 |

| NP | | | |
|---|---|---|---|
| Pos. | 178 | obs: | exp: |
| gct | A | 339 | 87.15 |
| gcc | A | 0 | 70.88 |
| gca | A | 7 | 154.20 |
| gcg | A | 0 | 33.76 |
| mPD | | 0.040 | 0.69 |
| | | nPD: | 0.06 |
| | | N. weight: | 1.8 |
| | | Sc. PD: | -0.31 |
| | | Sc. rank: | -143.3 |

| NP | | | |
|---|---|---|---|
| Pos. | 179 | obs: | exp: |
| gct | A | 304 | 87.15 |
| gcc | A | 8 | 70.88 |
| gca | A | 31 | 154.20 |
| gcg | A | 3 | 33.76 |
| mPD | | 0.22 | 0.69 |
| | | nPD: | 0.32 |
| | | N. weight: | 1.3 |
| | | Sc. PD: | 0.1 |
| | | Sc. rank: | 106.4 |

| NP | | | |
|---|---|---|---|
| Pos. | 180 | obs: | exp: |
| ggt | G | 194 | 42.69 |
| ggc | G | 75 | 46.79 |
| gga | G | 76 | 167.40 |
| ggg | G | 1 | 89.14 |
| mPD | | 0.59 | 0.67 |
| | | nPD: | 0.89 |
| | | N. weight: | 1.1 |
| | | Sc. PD: | 0.7 |
| | | Sc. rank: | 233.2 |

| NP | | | |
|---|---|---|---|
| Pos. | 181 | obs: | exp: |
| gct | A | 3 | 87.15 |
| gcc | A | 0 | 70.88 |
| gca | A | 323 | 154.20 |
| gcg | A | 20 | 33.76 |
| --- | -- | ------ | ------ |
| mPD | | 0.13 | 0.69 |
| | | nPD: | 0.18 |
| | | N. weight: | 0.92 |
| | | Sc. PD: | -0.048 |
| | | Sc. rank: | 5.9 |

| NP | | | |
|---|---|---|---|
| Pos. | 182 | obs: | exp: |
| gct | A | 0 | 87.15 |
| gcc | A | 0 | 70.88 |
| gca | A | 342 | 154.20 |
| gcg | A | 4 | 33.76 |
| --- | -- | ------ | ------ |
| mPD | | 0.023 | 0.69 |
| | | nPD: | 0.03 |
| | | N. weight: | 1.1 |
| | | Sc. PD: | -0.21 |
| | | Sc. rank: | -129.8 |

| NP | | | |
|---|---|---|---|
| Pos. | 183 | obs: | exp: |
| att | I | 0 | 0.38 |
| atc | I | 0 | 0.26 |
| ata | I | 1 | 0.36 |
| gtt | V | 1 | 70.91 |
| gtc | V | 0 | 72.41 |
| gta | V | 264 | 64.66 |
| gtg | V | 80 | 137.00 |
| --- | -- | ------ | ------ |
| mPD | | 0.37 | 0.73 |
| | | nPD: | 0.5 |
| | | N. weight: | 1.4 |
| | | Sc. PD: | 0.35 |
| | | Sc. rank: | 201.4 |

| NP | | | |
|---|---|---|---|
| Pos. | 184 | obs: | exp: |
| aaa | K | 86 | 193.70 |
| aag | K | 260 | 152.30 |
| --- | -- | ------ | ------ |
| mPD | | 0.37 | 0.49 |
| | | nPD: | 0.76 |
| | | N. weight: | 0.3 |
| | | Sc. PD: | 0.15 |
| | | Sc. rank: | 58.8 |

| NP | | | |
|---|---|---|---|
| Pos. | 185 | obs: | exp: |
| ggt | G | 1 | 42.69 |
| ggc | G | 0 | 46.79 |
| gga | G | 318 | 167.40 |
| ggg | G | 27 | 89.14 |
| --- | -- | ------ | ------ |
| mPD | | 0.15 | 0.67 |
| | | nPD: | 0.22 |
| | | N. weight: | 0.71 |
| | | Sc. PD: | -0.0085 |
| | | Sc. rank: | 20.7 |

| NP | | | |
|---|---|---|---|
| Pos. | 186 | obs: | exp: |
| att | I | 0 | 10.90 |
| atc | I | 0 | 7.60 |
| ata | I | 29 | 10.50 |
| gtt | V | 119 | 65.16 |
| gtc | V | 189 | 66.53 |
| gta | V | 9 | 59.41 |
| gtg | V | 0 | 125.90 |
| --- | -- | ------ | ------ |
| mPD | | 0.73 | 0.89 |
| | | nPD: | 0.82 |
| | | N. weight: | 1.2 |
| | | Sc. PD: | 0.66 |
| | | Sc. rank: | 241.8 |

| NP | | | |
|---|---|---|---|
| Pos. | 187 | obs: | exp: |
| ggt | G | 2 | 42.69 |
| ggc | G | 0 | 46.79 |
| gga | G | 193 | 167.40 |
| ggg | G | 151 | 89.14 |
| --- | -- | ------ | ------ |
| mPD | | 0.50 | 0.67 |
| | | nPD: | 0.75 |
| | | N. weight: | 0.44 |
| | | Sc. PD: | 0.21 |
| | | Sc. rank: | 85.4 |

| NP | | | |
|---|---|---|---|
| Pos. | 188 | obs: | exp: |
| act | T | 0 | 91.41 |
| acc | T | 19 | 74.25 |
| aca | T | 128 | 151.60 |
| acg | T | 199 | 28.76 |
| --- | -- | ------ | ------ |
| mPD | | 0.53 | 0.69 |
| | | nPD: | 0.77 |
| | | N. weight: | 1.4 |
| | | Sc. PD: | 0.73 |
| | | Sc. rank: | 285.1 |

| NP | | | |
|---|---|---|---|
| Pos. | 189 | obs: | exp: |
| atg | M | 346 | 346.00 |
| --- | -- | ------ | ------ |
| mPD | | 0 | 0 |
| | | nPD: | 1. |
| | | N. weight: | 0. |
| | | Sc. PD: | 0 |
| | | Sc. rank: | 0 |

| NP | | | |
|---|---|---|---|
| Pos. | 190 | obs: | exp: |
| gtt | V | 0 | 70.91 |
| gtc | V | 0 | 72.41 |
| gta | V | 39 | 64.66 |
| gtg | V | 306 | 137.00 |
| gct | A | 0 | 0.25 |
| gcc | A | 0 | 0.20 |
| gca | A | 0 | 0.45 |
| gcg | A | 1 | 0.10 |
| --- | -- | ------ | ------ |
| mPD | | 0.21 | 0.73 |
| | | nPD: | 0.28 |
| | | N. weight: | 0.96 |
| | | Sc. PD: | 0.043 |
| | | Sc. rank: | 58.9 |

| NP | | | |
|---|---|---|---|
| Pos. | 191 | obs: | exp: |
| atg | M | 346 | 346.00 |
| --- | -- | ------ | ------ |
| mPD | | 0 | 0 |
| | | nPD: | 1. |
| | | N. weight: | 0. |
| | | Sc. PD: | 0 |
| | | Sc. rank: | 0 |

| NP | | | |
|---|---|---|---|
| Pos. | 192 | obs: | exp: |
| gaa | E | 290 | 199.10 |
| gag | E | 56 | 146.90 |
| --- | -- | ------ | ------ |
| mPD | | 0.27 | 0.49 |
| | | nPD: | 0.56 |
| | | N. weight: | 0.24 |
| | | Sc. PD: | 0.073 |
| | | Sc. rank: | 37.8 |

| NP | | | |
|---|---|---|---|
| Pos. | 193 | obs: | exp: |
| tta | L | 0 | 30.23 |
| ttg | L | 56 | 66.64 |
| ctt | L | 0 | 64.56 |
| ctc | L | 1 | 55.97 |
| cta | L | 181 | 52.12 |
| ctg | L | 108 | 76.48 |
| --- | -- | ------ | ------ |
| mPD | | 0.78 | 1.1 |
| | | nPD: | 0.7 |
| | | N. weight: | 1.1 |
| | | Sc. PD: | 0.46 |
| | | Sc. rank: | 195.9 |

| NP | | | |
|---|---|---|---|
| Pos. | 194 | obs: | exp: |
| att | I | 283 | 128.20 |
| atc | I | 58 | 89.37 |
| ata | I | 0 | 123.40 |
| act | T | 1 | 0.26 |
| acc | T | 0 | 0.21 |
| aca | T | 0 | 0.44 |
| acg | T | 0 | 0.08 |
| gtt | V | 4 | 0.82 |
| gtc | V | 0 | 0.84 |
| gta | V | 0 | 0.75 |
| gtg | V | 0 | 1.59 |
| --- | -- | ------ | ------ |
| mPD | | 0.31 | 0.69 |
| | | nPD: | 0.45 |
| | | N. weight: | 0.87 |
| | | Sc. PD: | 0.17 |
| | | Sc. rank: | 115.1 |

| NP | | | |
|---|---|---|---|
| Pos. | 195 | obs: | exp: |
| cgt | R | 0 | 14.36 |
| cgc | R | 0 | 16.94 |
| cga | R | 50 | 33.25 |
| cgg | R | 292 | 28.87 |
| aga | R | 2 | 155.60 |
| agg | R | 2 | 96.96 |
| --- | -- | ------ | ------ |
| mPD | | 0.28 | 0.96 |
| | | nPD: | 0.29 |
| | | N. weight: | 2.8 |
| | | Sc. PD: | 0.15 |
| | | Sc. rank: | 191.2 |

| NP | | | |
|---|---|---|---|
| Pos. | 196 | obs: | exp: |
| atg | M | 346 | 346.00 |
| --- | -- | ------ | ------ |
| mPD | | 0 | 0 |
| | | nPD: | 1. |
| | | N. weight: | 0. |
| | | Sc. PD: | 0 |
| | | Sc. rank: | 0 |

| NP | | | |
|---|---|---|---|
| Pos. | 197 | obs: | exp: |
| att | I | 3 | 130.10 |
| atc | I | 69 | 90.68 |
| ata | I | 274 | 125.20 |
| --- | -- | ------ | ------ |
| mPD | | 0.33 | 0.66 |
| | | nPD: | 0.51 |
| | | N. weight: | 0.78 |
| | | Sc. PD: | 0.2 |
| | | Sc. rank: | 116.3 |

| NP | | | |
|---|---|---|---|
| Pos. | 198 | obs: | exp: |
| aaa | K | 122 | 193.70 |
| aag | K | 224 | 152.30 |
| --- | -- | ------ | ------ |
| mPD | | 0.46 | 0.49 |
| | | nPD: | 0.93 |
| | | N. weight: | 0.14 |
| | | Sc. PD: | 0.091 |
| | | Sc. rank: | 28.6 |

| NP | | | |
|---|---|---|---|
| Pos. | 199 | obs: | exp: |
| cgt | R | 1 | 14.36 |
| cgc | R | 0 | 16.94 |
| cga | R | 265 | 33.25 |
| cgg | R | 39 | 28.87 |
| aga | R | 40 | 155.60 |
| agg | R | 1 | 96.96 |
| --- | -- | ------ | ------ |
| mPD | | 0.42 | 0.96 |
| | | nPD: | 0.44 |
| | | N. weight: | 2.1 |
| | | Sc. PD: | 0.4 |
| | | Sc. rank: | 266.6 |

| NP | | | |
|---|---|---|---|
| Pos. | 200 | obs: | exp: |
| ggt | G | 1 | 42.69 |
| ggc | G | 0 | 46.79 |
| gga | G | 104 | 167.40 |
| ggg | G | 241 | 89.14 |
| --- | -- | ------ | ------ |
| mPD | | 0.43 | 0.67 |
| | | nPD: | 0.64 |
| | | N. weight: | 0.79 |
| | | Sc. PD: | 0.3 |
| | | Sc. rank: | 138.3 |

| NP | | | |
|---|---|---|---|
| Pos. | 201 | obs: | exp: |
| att | I | 172 | 130.10 |
| atc | I | 173 | 90.68 |
| ata | I | 1 | 125.20 |
| --- | -- | ------ | ------ |
| mPD | | 0.50 | 0.66 |
| | | nPD: | 0.77 |
| | | N. weight: | 0.66 |
| | | Sc. PD: | 0.33 |
| | | Sc. rank: | 131.0 |

| NP | | | |
|---|---|---|---|
| Pos. | 202 | obs: | exp: |
| aat | N | 302 | 180.80 |
| aac | N | 44 | 165.20 |
| --- | -- | ------ | ------ |
| mPD | | 0.22 | 0.50 |
| | | nPD: | 0.45 |
| | | N. weight: | 0.41 |
| | | Sc. PD: | 0.082 |
| | | Sc. rank: | 54.3 |

| NP | | | |
|---|---|---|---|
| Pos. | 203 | obs: | exp: |
| gat | D | 298 | 184.60 |
| gac | D | 48 | 161.40 |
| --- | -- | ------ | ------ |
| mPD | | 0.24 | 0.50 |
| | | nPD: | 0.48 |
| | | N. weight: | 0.36 |
| | | Sc. PD: | 0.084 |
| | | Sc. rank: | 50.8 |

| NP | | | |
|---|---|---|---|
| Pos. | 204 | obs: | exp: |
| cgt | R | 13 | 14.36 |
| cgc | R | 0 | 16.94 |
| cga | R | 6 | 33.25 |
| cgg | R | 307 | 28.87 |
| aga | R | 2 | 155.60 |
| agg | R | 18 | 96.96 |
| --- | -- | ------ | ------ |
| mPD | | 0.23 | 0.96 |
| | | nPD: | 0.23 |
| | | N. weight: | 2.8 |
| | | Sc. PD: | -0.0064 |
| | | Sc. rank: | 113.1 |

| NP | | | |
|---|---|---|---|
| Pos. | 205 | obs: | exp: |
| aat | N | 301 | 180.80 |
| aac | N | 45 | 165.20 |
| --- | -- | ------ | ------ |
| mPD | | 0.23 | 0.50 |
| | | nPD: | 0.45 |
| | | N. weight: | 0.4 |
| | | Sc. PD: | 0.084 |
| | | Sc. rank: | 55.5 |

| NP | | | |
|---|---|---|---|
| Pos. | 206 | obs: | exp: |
| ttt | F | 7 | 142.20 |
| ttc | F | 339 | 203.80 |
| --- | -- | ------ | ------ |
| mPD | | 0.040 | 0.48 |
| | | nPD: | 0.08 |
| | | N. weight: | 0.64 |
| | | Sc. PD: | -0.094 |
| | | Sc. rank: | -39.3 |

| NP | | | |
|---|---|---|---|
| Pos. | 207 | obs: | exp: |
| tgg | W | 346 | 346.00 |
| --- | -- | ------ | ------ |
| mPD | | 0 | 0 |
| | | nPD: | 1. |
| | | N. weight: | 0. |
| | | Sc. PD: | 0 |
| | | Sc. rank: | 0 |

| NP | | | |
|---|---|---|---|
| Pos. | 208 | obs: | exp: |
| cgt | R | 0 | 14.36 |
| cgc | R | 0 | 16.94 |
| cga | R | 0 | 33.25 |
| cgg | R | 0 | 28.87 |
| aga | R | 346 | 155.60 |
| agg | R | 0 | 96.96 |
| --- | -- | ------ | ------ |
| mPD | | 0 | 0.96 |
| | | nPD: | 0. |
| | | N. weight: | 1.1 |
| | | Sc. PD: | -0.26 |
| | | Sc. rank: | -237.4 |

| NP | | | |
|---|---|---|---|
| Pos. | 209 | obs: | exp: |
| ggt | G | 98 | 42.69 |
| ggc | G | 248 | 46.79 |
| gga | G | 0 | 167.40 |
| ggg | G | 0 | 89.14 |
| --- | -- | ------ | ------ |
| mPD | | 0.41 | 0.67 |
| | | nPD: | 0.61 |
| | | N. weight: | 2.1 |
| | | Sc. PD: | 0.74 |
| | | Sc. rank: | 352.2 |

| NP | | | |
|---|---|---|---|
| Pos. | 210 | obs: | exp: |
| gaa | E | 281 | 199.10 |
| gag | E | 65 | 146.90 |
| --- | -- | ------ | ------ |
| mPD | | 0.31 | 0.49 |
| | | nPD: | 0.63 |
| | | N. weight: | 0.19 |
| | | Sc. PD: | 0.072 |
| | | Sc. rank: | 33.6 |

| NP | | | |
|---|---|---|---|
| Pos. | 211 | obs: | exp: |
| aat | N | 306 | 180.80 |
| aac | N | 40 | 165.20 |
| --- | -- | ------ | ------ |
| mPD | | 0.21 | 0.50 |
| | | nPD: | 0.41 |
| | | N. weight: | 0.44 |
| | | Sc. PD: | 0.074 |
| | | Sc. rank: | 52.7 |

| NP | | | |
|---|---|---|---|
| Pos. | 212 | obs: | exp: |
| ggt | G | 5 | 42.69 |
| ggc | G | 0 | 46.79 |
| gga | G | 304 | 167.40 |
| ggg | G | 37 | 89.14 |
| --- | -- | ------ | ------ |
| mPD | | 0.22 | 0.67 |
| | | nPD: | 0.33 |
| | | N. weight: | 0.59 |
| | | Sc. PD: | 0.05 |
| | | Sc. rank: | 49.8 |

| NP | | | |
|---|---|---|---|
| Pos. | 213 | obs: | exp: |
| cgt | R | 0 | 14.36 |
| cgc | R | 0 | 16.94 |
| cga | R | 184 | 33.25 |
| cgg | R | 118 | 28.87 |
| aga | R | 41 | 155.60 |
| agg | R | 3 | 96.96 |
| --- | -- | ------ | ------ |
| mPD | | 0.68 | 0.96 |
| | | nPD: | 0.71 |
| | | N. weight: | 1.8 |
| | | Sc. PD: | 0.79 |
| | | Sc. rank: | 329.1 |

| NP | | | |
|---|---|---|---|
| Pos. | 214 | obs: | exp: |
| cgt | R | 0 | 14.36 |
| cgc | R | 0 | 16.94 |
| cga | R | 0 | 33.25 |
| cgg | R | 0 | 28.87 |
| aga | R | 166 | 155.60 |
| agg | R | 180 | 96.96 |
| --- | -- | ------ | ------ |
| mPD | | 0.50 | 0.96 |
| | | nPD: | 0.52 |
| | | N. weight: | 0.52 |
| | | Sc. PD: | 0.14 |
| | | Sc. rank: | 78.6 |

| NP | | | |
|---|---|---|---|
| Pos. | 215 | obs: | exp: |
| act | T | 14 | 91.41 |
| acc | T | 27 | 74.25 |
| aca | T | 298 | 151.60 |
| acg | T | 7 | 28.76 |
| --- | -- | ------ | ------ |
| mPD | | 0.25 | 0.69 |
| | | nPD: | 0.37 |
| | | N. weight: | 0.6 |
| | | Sc. PD: | 0.074 |
| | | Sc. rank: | 61.9 |

| NP | | | |
|---|---|---|---|
| Pos. | 216 | obs: | exp: |
| cgt | R | 0 | 14.36 |
| cgc | R | 0 | 16.94 |
| cga | R | 0 | 33.25 |
| cgg | R | 0 | 28.87 |
| aga | R | 79 | 155.60 |
| agg | R | 267 | 96.96 |
| --- | -- | ------ | ------ |
| mPD | | 0.35 | 0.96 |
| | | nPD: | 0.37 |
| | | N. weight: | 0.91 |
| | | Sc. PD: | 0.11 |
| | | Sc. rank: | 95.6 |

| NP | | | |
|---|---|---|---|
| Pos. | 217 | obs: | exp: |
| att | I | 335 | 127.80 |
| atc | I | 4 | 89.11 |
| ata | I | 1 | 123.10 |
| act | T | 3 | 0.79 |
| acc | T | 0 | 0.64 |
| aca | T | 0 | 1.31 |
| acg | T | 0 | 0.25 |
| gtt | V | 3 | 0.62 |
| gtc | V | 0 | 0.63 |
| gta | V | 0 | 0.56 |
| gtg | V | 0 | 1.19 |
| --- | -- | ------ | ------ |
| mPD | | 0.063 | 0.70 |
| | | nPD: | 0.09 |
| | | N. weight: | 1.3 |
| | | Sc. PD: | -0.18 |
| | | Sc. rank: | -71.3 |

| NP | | | |
|---|---|---|---|
| Pos. | 218 | obs: | exp: |
| gct | A | 40 | 87.15 |
| gcc | A | 17 | 70.88 |
| gca | A | 288 | 154.20 |
| gcg | A | 1 | 33.76 |
| --- | -- | ------ | ------ |
| mPD | | 0.29 | 0.69 |
| | | nPD: | 0.43 |
| | | N. weight: | 0.53 |
| | | Sc. PD: | 0.095 |
| | | Sc. rank: | 63.9 |

| NP | | | |
|---|---|---|---|
| Pos. | 219 | obs: | exp: |
| tat | Y | 290 | 179.10 |
| tac | Y | 56 | 166.90 |
| --- | -- | ------ | ------ |
| mPD | | 0.27 | 0.50 |
| | | nPD: | 0.54 |
| | | N. weight: | 0.34 |
| | | Sc. PD: | 0.099 |
| | | Sc. rank: | 52.7 |

| NP | | | |
|---|---|---|---|
| Pos. | 220 | obs: | exp: |
| gaa | E | 55 | 199.10 |
| gag | E | 291 | 146.90 |
| --- | -- | ------ | ------ |
| mPD | | 0.27 | 0.49 |
| | | nPD: | 0.55 |
| | | N. weight: | 0.54 |
| | | Sc. PD: | 0.16 |
| | | Sc. rank: | 85.1 |

| NP | | | |
|---|---|---|---|
| Pos. | 221 | obs: | exp: |
| cgt | R | 0 | 14.36 |
| cgc | R | 0 | 16.94 |
| cga | R | 0 | 33.25 |
| cgg | R | 0 | 28.87 |
| aga | R | 274 | 155.60 |
| agg | R | 72 | 96.96 |
| --- | -- | ------ | ------ |
| mPD | | 0.33 | 0.96 |
| | | nPD: | 0.34 |
| | | N. weight: | 0.57 |
| | | Sc. PD: | 0.058 |
| | | Sc. rank: | 54.6 |

| NP | | | |
|---|---|---|---|
| Pos. | 222 | obs: | exp: |
| atg | M | 346 | 346.00 |
| --- | -- | ------ | ------ |
| mPD | | 0 | 0 |
| | | nPD: | 1. |
| | | N. weight: | 0. |
| | | Sc. PD: | 0 |
| | | Sc. rank: | 0 |

| NP | | | |
|---|---|---|---|
| Pos. | 223 | obs: | exp: |
| tgt | C | 9 | 142.60 |
| tgc | C | 337 | 203.40 |
| --- | -- | ------ | ------ |
| mPD | | 0.051 | 0.48 |
| | | nPD: | 0.1 |
| | | N. weight: | 0.61 |
| | | Sc. PD: | -0.077 |
| | | Sc. rank: | -24.9 |

| NP | | | |
|---|---|---|---|
| Pos. | 224 | obs: | exp: |
| aat | N | 7 | 180.80 |
| aac | N | 339 | 165.20 |
| --- | -- | ------ | ------ |
| mPD | | 0.040 | 0.50 |
| | | nPD: | 0.08 |
| | | N. weight: | 0.92 |
| | | Sc. PD: | -0.14 |
| | | Sc. rank: | -58.9 |

| NP | | | |
|---|---|---|---|
| Pos. | 225 | obs: | exp: |
| att | I | 20 | 130.10 |
| atc | I | 324 | 90.68 |
| ata | I | 2 | 125.20 |
| --- | -- | ------ | ------ |
| mPD | | 0.12 | 0.66 |
| | | nPD: | 0.18 |
| | | N. weight: | 1.5 |
| | | Sc. PD: | -0.08 |
| | | Sc. rank: | 7.4 |

| NP | | | |
|---|---|---|---|
| Pos. | 226 | obs: | exp: |
| tta | L | 0 | 30.23 |
| ttg | L | 0 | 66.64 |
| ctt | L | 5 | 64.56 |
| ctc | L | 341 | 55.97 |
| cta | L | 0 | 52.12 |
| ctg | L | 0 | 76.48 |
| --- | -- | ------ | ------ |
| mPD | | 0.029 | 1.1 |
| | | nPD: | 0.03 |
| | | N. weight: | 2.5 |
| | | Sc. PD: | -0.51 |
| | | Sc. rank: | -309.4 |

| NP | | | |
|---|---|---|---|
| Pos. | 227 | obs: | exp: |
| aaa | K | 289 | 193.70 |
| aag | K | 57 | 152.30 |
| --- | -- | ------ | ------ |
| mPD | | 0.28 | 0.49 |
| | | nPD: | 0.56 |
| | | N. weight: | 0.26 |
| | | Sc. PD: | 0.08 |
| | | Sc. rank: | 41.1 |

| NP | | | |
|---|---|---|---|
| Pos. | 228 | obs: | exp: |
| ggt | G | 0 | 42.69 |
| ggc | G | 0 | 46.79 |
| gga | G | 46 | 167.40 |
| ggg | G | 300 | 89.14 |
| --- | -- | ------ | ------ |
| mPD | | 0.23 | 0.67 |
| | | nPD: | 0.35 |
| | | N. weight: | 1.3 |
| | | Sc. PD: | 0.13 |
| | | Sc. rank: | 125.6 |

| NP | | | |
|---|---|---|---|
| Pos. | 229 | obs: | exp: |
| aaa | K | 322 | 193.70 |
| aag | K | 24 | 152.30 |
| --- | -- | ------ | ------ |
| mPD | | 0.13 | 0.49 |
| | | nPD: | 0.26 |
| | | N. weight: | 0.5 |
| | | Sc. PD: | 0.012 |
| | | Sc. rank: | 27.0 |

| NP | | | |
|---|---|---|---|
| Pos. | 230 | obs: | exp: |
| ttt | F | 30 | 141.80 |
| ttc | F | 315 | 203.20 |
| tta | L | 0 | 0.09 |
| ttg | L | 0 | 0.19 |
| ctt | L | 0 | 0.19 |
| ctc | L | 1 | 0.16 |
| cta | L | 0 | 0.15 |
| ctg | L | 0 | 0.22 |
| --- | -- | ------ | ------ |
| mPD | | 0.16 | 0.49 |
| | | nPD: | 0.34 |
| | | N. weight: | 0.4 |
| | | Sc. PD: | 0.037 |
| | | Sc. rank: | 37.5 |

| NP | | | |
|---|---|---|---|
| Pos. | 231 | obs: | exp: |
| caa | Q | 343 | 184.50 |
| cag | Q | 3 | 161.50 |
| --- | -- | ------ | ------ |
| mPD | | 0.017 | 0.50 |
| | | nPD: | 0.03 |
| | | N. weight: | 0.84 |
| | | Sc. PD: | -0.16 |
| | | Sc. rank: | -93.2 |

| NP | | | |
|---|---|---|---|
| Pos. | 232 | obs: | exp: |
| act | T | 0 | 91.41 |
| acc | T | 0 | 74.25 |
| aca | T | 345 | 151.60 |
| acg | T | 1 | 28.76 |
| --- | -- | ------ | ------ |
| mPD | | 0.0058 | 0.69 |
| | | nPD: | 0.01 |
| | | N. weight: | 1.2 |
| | | Sc. PD: | -0.25 |
| | | Sc. rank: | -197.0 |

| NP | | | |
|---|---|---|---|
| Pos. | 233 | obs: | exp: |
| gct | A | 0 | 87.15 |
| gcc | A | 0 | 70.88 |
| gca | A | 343 | 154.20 |
| gcg | A | 3 | 33.76 |
| --- | -- | ------ | ------ |
| mPD | | 0.017 | 0.69 |
| | | nPD: | 0.03 |
| | | N. weight: | 1.1 |
| | | Sc. PD: | -0.23 |
| | | Sc. rank: | -141.1 |

| NP | | | |
|---|---|---|---|
| Pos. | 234 | obs: | exp: |
| gct | A | 0 | 87.15 |
| gcc | A | 0 | 70.88 |
| gca | A | 345 | 154.20 |
| gcg | A | 1 | 33.76 |
| --- | -- | ------ | ------ |
| mPD | | 0.0058 | 0.69 |
| | | nPD: | 0.01 |
| | | N. weight: | 1.1 |
| | | Sc. PD: | -0.25 |
| | | Sc. rank: | -196.0 |

| NP | | | |
|---|---|---|---|
| Pos. | 235 | obs: | exp: |
| caa | Q | 341 | 184.50 |
| cag | Q | 5 | 161.50 |
| --- | -- | ------ | ------ |
| mPD | | 0.029 | 0.50 |
| | | nPD: | 0.06 |
| | | N. weight: | 0.8 |
| | | Sc. PD: | -0.14 |
| | | Sc. rank: | -64.8 |

| NP | | | |
|---|---|---|---|
| Pos. | 236 | obs: | exp: |
| cgt | R | 0 | 14.36 |
| cgc | R | 0 | 16.94 |
| cga | R | 107 | 33.25 |
| cgg | R | 2 | 28.87 |
| aga | R | 204 | 155.60 |
| agg | R | 33 | 96.96 |
| --- | -- | ------ | ------ |
| mPD | | 0.62 | 0.96 |
| | | nPD: | 0.64 |
| | | N. weight: | 0.61 |
| | | Sc. PD: | 0.23 |
| | | Sc. rank: | 106.6 |

| NP | | | |
|---|---|---|---|
| Pos. | 237 | obs: | exp: |
| gct | A | 1 | 87.15 |
| gcc | A | 0 | 70.88 |
| gca | A | 345 | 154.20 |
| gcg | A | 0 | 33.76 |
| --- | -- | ------ | ------ |
| mPD | | 0.0058 | 0.69 |
| | | nPD: | 0.01 |
| | | N. weight: | 1.1 |
| | | Sc. PD: | -0.25 |
| | | Sc. rank: | -195.3 |

| NP | | | |
|---|---|---|---|
| Pos. | 238 | obs: | exp: |
| atg | M | 346 | 346.00 |
| --- | -- | ------ | ------ |
| mPD | | 0 | 0 |
| | | nPD: | 1. |
| | | N. weight: | 0. |
| | | Sc. PD: | 0 |
| | | Sc. rank: | 0 |

| NP | | | |
|---|---|---|---|
| Pos. | 239 | obs: | exp: |
| att | I | 0 | 0.38 |
| atc | I | 0 | 0.26 |
| ata | I | 1 | 0.36 |
| atg | M | 331 | 331.00 |
| gtt | V | 0 | 2.88 |
| gtc | V | 0 | 2.94 |
| gta | V | 0 | 2.62 |
| gtg | V | 14 | 5.56 |
| --- | -- | ------ | ------ |
| mPD | | 0.084 | 0.13 |
| | | nPD: | 0.64 |
| | | N. weight: | 0.058 |
| | | Sc. PD: | 0.022 |
| | | Sc. rank: | 10.1 |

| NP | | | |
|---|---|---|---|
| Pos. | 240 | obs: | exp: |
| gat | D | 104 | 184.60 |
| gac | D | 242 | 161.40 |
| --- | -- | ------ | ------ |
| mPD | | 0.42 | 0.50 |
| | | nPD: | 0.85 |
| | | N. weight: | 0.17 |
| | | Sc. PD: | 0.1 |
| | | Sc. rank: | 35.0 |

| NP | | | |
|---|---|---|---|
| Pos. | 241 | obs: | exp: |
| cat | H | 1 | 0.54 |
| cac | H | 0 | 0.46 |
| caa | Q | 166 | 183.90 |
| cag | Q | 179 | 161.10 |
| --- | -- | ------ | ------ |
| mPD | | 0.50 | 0.50 |
| | | nPD: | 1.01 |
| | | N. weight: | 0.023 |
| | | Sc. PD: | 0.017 |
| | | Sc. rank: | 4.9 |

,

| NP | | | |
|---|---|---|---|
| Pos. | 242 | obs: | exp: |
| gtt | V | 1 | 71.12 |
| gtc | V | 0 | 72.62 |
| gta | V | 17 | 64.85 |
| gtg | V | 328 | 137.40 |
| --- | -- | ------ | ------ |
| mPD | | 0.099 | 0.72 |
| | | nPD: | 0.14 |
| | | N. weight: | 1.1 |
| | | Sc. PD: | -0.1 |
| | | Sc. rank: | -15.3 |

,

| NP | | | |
|---|---|---|---|
| Pos. | 243 | obs: | exp: |
| cgt | R | 24 | 14.36 |
| cgc | R | 0 | 16.94 |
| cga | R | 232 | 33.25 |
| cgg | R | 18 | 28.87 |
| aga | R | 28 | 155.60 |
| agg | R | 44 | 96.96 |
| --- | -- | ------ | ------ |
| mPD | | 0.73 | 0.96 |
| | | nPD: | 0.76 |
| | | N. weight: | 1.6 |
| | | Sc. PD: | 0.79 |
| | | Sc. rank: | 312.7 |

,

| NP | | | |
|---|---|---|---|
| Pos. | 244 | obs: | exp: |
| gaa | E | 299 | 199.10 |
| gag | E | 47 | 146.90 |
| --- | -- | ------ | ------ |
| mPD | | 0.24 | 0.49 |
| | | nPD: | 0.48 |
| | | N. weight: | 0.29 |
| | | Sc. PD: | 0.069 |
| | | Sc. rank: | 41.3 |

,

| NP | | | |
|---|---|---|---|
| Pos. | 245 | obs: | exp: |
| tct | S | 0 | 54.69 |
| tcc | S | 0 | 47.92 |
| tca | S | 0 | 76.21 |
| tcg | S | 0 | 20.97 |
| agt | S | 9 | 75.68 |
| agc | S | 335 | 68.54 |
| ggt | G | 0 | 0.25 |
| ggc | G | 2 | 0.27 |
| gga | G | 0 | 0.97 |
| ggg | G | 0 | 0.52 |
| --- | -- | ------ | ------ |
| mPD | | 0.062 | 1.7 |
| | | nPD: | 0.04 |
| | | N. weight: | 2.1 |
| | | Sc. PD: | -0.41 |
| | | Sc. rank: | -227.3 |

,

| NP | | | |
|---|---|---|---|
| Pos. | 246 | obs: | exp: |
| cgt | R | 1 | 14.36 |
| cgc | R | 1 | 16.94 |
| cga | R | 1 | 33.25 |
| cgg | R | 71 | 28.87 |
| aga | R | 173 | 155.60 |
| agg | R | 99 | 96.96 |
| --- | -- | ------ | ------ |
| mPD | | 0.84 | 0.96 |
| | | nPD: | 0.88 |
| | | N. weight: | 0.35 |
| | | Sc. PD: | 0.21 |
| | | Sc. rank: | 71.6 |

,

| NP | | | |
|---|---|---|---|
| Pos. | 247 | obs: | exp: |
| aat | N | 325 | 180.80 |
| aac | N | 21 | 165.20 |
| --- | -- | ------ | ------ |
| mPD | | 0.11 | 0.50 |
| | | nPD: | 0.23 |
| | | N. weight: | 0.62 |
| | | Sc. PD: | -0.0047 |
| | | Sc. rank: | 20.9 |

,

| NP | | | |
|---|---|---|---|
| Pos. | 248 | obs: | exp: |
| tct | S | 2 | 0.32 |
| tcc | S | 0 | 0.28 |
| tca | S | 0 | 0.44 |
| tcg | S | 0 | 0.12 |
| cct | P | 330 | 90.58 |
| ccc | P | 10 | 62.05 |
| cca | P | 4 | 135.70 |
| ccg | P | 0 | 55.62 |
| agt | S | 0 | 0.44 |
| agc | S | 0 | 0.40 |
| --- | -- | ------ | ------ |
| mPD | | 0.090 | 0.73 |
| | | nPD: | 0.12 |
| | | N. weight: | 1.7 |
| | | Sc. PD: | -0.18 |
| | | Sc. rank: | -44.8 |

,

| NP | | | |
|---|---|---|---|
| Pos. | 249 | obs: | exp: |
| ggt | G | 0 | 42.69 |
| ggc | G | 48 | 46.79 |
| gga | G | 59 | 167.40 |
| ggg | G | 239 | 89.14 |
| --- | -- | ------ | ------ |
| mPD | | 0.48 | 0.67 |
| | | nPD: | 0.71 |
| | | N. weight: | 0.75 |
| | | Sc. PD: | 0.34 |
| | | Sc. rank: | 142.0 |

,

| NP | | | |
|---|---|---|---|
| Pos. | 250 | obs: | exp: |
| aat | N | 345 | 180.80 |
| aac | N | 1 | 165.20 |
| --- | -- | ------ | ------ |
| mPD | | 0.0058 | 0.50 |
| | | nPD: | 0.01 |
| | | N. weight: | 0.91 |
| | | Sc. PD: | -0.2 |
| | | Sc. rank: | -145.3 |

,

| NP | | | |
|---|---|---|---|
| Pos. | 251 | obs: | exp: |
| gct | A | 344 | 87.15 |
| gcc | A | 0 | 70.88 |
| gca | A | 1 | 154.20 |
| gcg | A | 1 | 33.76 |
| --- | -- | ------ | ------ |
| mPD | | 0.012 | 0.69 |
| | | nPD: | 0.02 |
| | | N. weight: | 1.9 |
| | | Sc. PD: | -0.41 |
| | | Sc. rank: | -272.6 |

,

| NP | | | |
|---|---|---|---|
| Pos. | 252 | obs: | exp: |
| gaa | E | 312 | 199.10 |
| gag | E | 34 | 146.90 |
| --- | -- | ------ | ------ |
| mPD | | 0.18 | 0.49 |
| | | nPD: | 0.36 |
| | | N. weight: | 0.39 |
| | | Sc. PD: | 0.047 |
| | | Sc. rank: | 39.6 |

,

| NP | | | |
|---|---|---|---|
| Pos. | 253 | obs: | exp: |
| att | I | 305 | 129.30 |
| atc | I | 10 | 90.16 |
| ata | I | 29 | 124.50 |
| act | T | 1 | 0.26 |
| acc | T | 0 | 0.21 |
| aca | T | 0 | 0.44 |
| acg | T | 0 | 0.08 |
| gtt | V | 1 | 0.21 |
| gtc | V | 0 | 0.21 |
| gta | V | 0 | 0.19 |
| gtg | V | 0 | 0.40 |
| --- | -- | ------ | ------ |
| mPD | | 0.22 | 0.67 |
| | | nPD: | 0.32 |
| | | N. weight: | 0.85 |
| | | Sc. PD: | 0.07 |
| | | Sc. rank: | 70.7 |

,

| NP | | | |
|---|---|---|---|
| Pos. | 254 | obs: | exp: |
| gaa | E | 326 | 199.10 |
| gag | E | 20 | 146.90 |
| --- | -- | ------ | ------ |
| mPD | | 0.11 | 0.49 |
| | | nPD: | 0.22 |
| | | N. weight: | 0.51 |
| | | Sc. PD: | -0.0066 |
| | | Sc. rank: | 14.3 |

,

| NP | | | |
|---|---|---|---|
| Pos. | 255 | obs: | exp: |
| gat | D | 292 | 184.60 |
| gac | D | 54 | 161.40 |
| --- | -- | ------ | ------ |
| mPD | | 0.26 | 0.50 |
| | | nPD: | 0.53 |
| | | N. weight: | 0.32 |
| | | Sc. PD: | 0.09 |
| | | Sc. rank: | 49.1 |

,

| NP | | | |
|---|---|---|---|
| Pos. | 256 | obs: | exp: |
| tta | L | 0 | 30.23 |
| ttg | L | 0 | 66.64 |
| ctt | L | 44 | 64.56 |
| ctc | L | 301 | 55.97 |
| cta | L | 1 | 52.12 |
| ctg | L | 0 | 76.48 |
| --- | -- | ------ | ------ |
| mPD | | 0.23 | 1.1 |
| | | nPD: | 0.2 |
| | | N. weight: | 2. |
| | | Sc. PD: | -0.063 |
| | | Sc. rank: | 34.4 |

,

| NP | | | |
|---|---|---|---|
| Pos. | 257 | obs: | exp: |
| tta | L | 0 | 0.09 |
| ttg | L | 0 | 0.19 |
| ctt | L | 0 | 0.19 |
| ctc | L | 1 | 0.16 |
| cta | L | 0 | 0.15 |
| ctg | L | 0 | 0.22 |
| att | I | 40 | 128.20 |
| atc | I | 299 | 89.37 |
| ata | I | 2 | 123.40 |
| act | T | 0 | 0.26 |
| acc | T | 1 | 0.21 |
| aca | T | 0 | 0.44 |
| acg | T | 0 | 0.08 |
| gtt | V | 0 | 0.62 |
| gtc | V | 3 | 0.63 |
| gta | V | 0 | 0.56 |
| gtg | V | 0 | 1.19 |
| --- | -- | ------ | ------ |
| mPD | | 0.24 | 0.69 |
| | | nPD: | 0.35 |
| | | N. weight: | 1.3 |
| | | Sc. PD: | 0.15 |
| | | Sc. rank: | 133.4 |

,

| NP | | | |
|---|---|---|---|
| Pos. | 258 | obs: | exp: |
| ttt | F | 181 | 142.20 |
| ttc | F | 165 | 203.80 |
| --- | -- | ------ | ------ |
| mPD | | 0.50 | 0.48 |
| | | nPD: | 1.03 |
| | | N. weight: | 0.049 |
| | | Sc. PD: | 0.038 |
| | | Sc. rank: | 10.4 |

,

| NP | | | |
|---|---|---|---|
| Pos. | 259 | obs: | exp: |
| tta | L | 0 | 30.14 |
| ttg | L | 8 | 66.44 |
| ctt | L | 49 | 64.37 |
| ctc | L | 18 | 55.81 |
| cta | L | 7 | 51.97 |
| ctg | L | 263 | 76.26 |
| att | I | 0 | 0.38 |
| atc | I | 0 | 0.26 |
| ata | I | 1 | 0.36 |
| --- | -- | ------ | ------ |
| mPD | | 0.42 | 1.1 |
| | | nPD: | 0.37 |
| | | N. weight: | 1.1 |
| | | Sc. PD: | 0.14 |
| | | Sc. rank: | 120.7 |

,

NP

| Pos. | 260 | obs: | exp: |
|---|---|---|---|
| act | T | 0 | 1.06 |
| acc | T | 0 | 0.86 |
| aca | T | 4 | 1.75 |
| acg | T | 0 | 0.33 |
| gct | A | 0 | 86.14 |
| gcc | A | 0 | 70.06 |
| gca | A | 340 | 152.40 |
| gcg | A | 2 | 33.37 |
| --- | -- | ------ | ------ |
| mPD | | 0.034 | 0.71 |
| | | nPD: | 0.05 |
| | | N. weight: | 1.1 |
| | | Sc. PD: | -0.2 |
| | | Sc. rank: | -99.2 |

NP

| Pos. | 261 | obs: | exp: |
|---|---|---|---|
| cgt | R | 2 | 14.36 |
| cgc | R | 1 | 16.94 |
| cga | R | 56 | 33.25 |
| cgg | R | 253 | 28.87 |
| aga | R | 1 | 155.60 |
| agg | R | 33 | 96.96 |
| --- | -- | ------ | ------ |
| mPD | | 0.47 | 0.96 |
| | | nPD: | 0.49 |
| | | N. weight: | 2.2 |
| | | Sc. PD: | 0.54 |
| | | Sc. rank: | 323.8 |

NP

| Pos. | 262 | obs: | exp: |
|---|---|---|---|
| tct | S | 315 | 55.01 |
| tcc | S | 10 | 48.20 |
| tca | S | 18 | 76.65 |
| tcg | S | 3 | 21.09 |
| agt | S | 0 | 76.12 |
| agc | S | 0 | 68.93 |
| --- | -- | ------ | ------ |
| mPD | | 0.17 | 1.7 |
| | | nPD: | 0.1 |
| | | N. weight: | 2.1 |
| | | Sc. PD: | -0.27 |
| | | Sc. rank: | -105.9 |

NP

| Pos. | 263 | obs: | exp: |
|---|---|---|---|
| gct | A | 70 | 87.15 |
| gcc | A | 0 | 70.88 |
| gca | A | 247 | 154.20 |
| gcg | A | 29 | 33.76 |
| --- | -- | ------ | ------ |
| mPD | | 0.44 | 0.69 |
| | | nPD: | 0.65 |
| | | N. weight: | 0.42 |
| | | Sc. PD: | 0.17 |
| | | Sc. rank: | 75.2 |

NP

| Pos. | 264 | obs: | exp: |
|---|---|---|---|
| tta | L | 0 | 30.23 |
| ttg | L | 0 | 66.64 |
| ctt | L | 1 | 64.56 |
| ctc | L | 342 | 55.97 |
| cta | L | 3 | 52.12 |
| ctg | L | 0 | 76.48 |
| --- | -- | ------ | ------ |
| mPD | | 0.023 | 1.1 |
| | | nPD: | 0.02 |
| | | N. weight: | 2.5 |
| | | Sc. PD: | -0.52 |
| | | Sc. rank: | -334.2 |

NP

| Pos. | 265 | obs: | exp: |
|---|---|---|---|
| att | I | 58 | 130.10 |
| atc | I | 286 | 90.68 |
| ata | I | 2 | 125.20 |
| --- | -- | ------ | ------ |
| mPD | | 0.29 | 0.66 |
| | | nPD: | 0.44 |
| | | N. weight: | 1.1 |
| | | Sc. PD: | 0.22 |
| | | Sc. rank: | 149.0 |

NP

| Pos. | 266 | obs: | exp: |
|---|---|---|---|
| tta | L | 0 | 30.23 |
| ttg | L | 34 | 66.64 |
| ctt | L | 1 | 64.56 |
| ctc | L | 0 | 55.97 |
| cta | L | 5 | 52.12 |
| ctg | L | 306 | 76.48 |
| --- | -- | ------ | ------ |
| mPD | | 0.21 | 1.1 |
| | | nPD: | 0.19 |
| | | N. weight: | 1.6 |
| | | Sc. PD: | -0.072 |
| | | Sc. rank: | 17.9 |

NP

| Pos. | 267 | obs: | exp: |
|---|---|---|---|
| cgt | R | 0 | 14.36 |
| cgc | R | 0 | 16.94 |
| cga | R | 0 | 33.25 |
| cgg | R | 0 | 28.87 |
| aga | R | 283 | 155.60 |
| agg | R | 63 | 96.96 |
| --- | -- | ------ | ------ |
| mPD | | 0.30 | 0.96 |
| | | nPD: | 0.31 |
| | | N. weight: | 0.6 |
| | | Sc. PD: | 0.043 |
| | | Sc. rank: | 48.6 |

NP

| Pos. | 268 | obs: | exp: |
|---|---|---|---|
| ggt | G | 1 | 42.69 |
| ggc | G | 0 | 46.79 |
| gga | G | 286 | 167.40 |
| ggg | G | 59 | 89.14 |
| --- | -- | ------ | ------ |
| mPD | | 0.29 | 0.67 |
| | | nPD: | 0.43 |
| | | N. weight: | 0.53 |
| | | Sc. PD: | 0.1 |
| | | Sc. rank: | 66.5 |

NP

| Pos. | 269 | obs: | exp: |
|---|---|---|---|
| tct | S | 0 | 55.01 |
| tcc | S | 0 | 48.20 |
| tca | S | 340 | 76.65 |
| tcg | S | 6 | 21.09 |
| agt | S | 0 | 76.12 |
| agc | S | 0 | 68.93 |
| --- | -- | ------ | ------ |
| mPD | | 0.034 | 1.7 |
| | | nPD: | 0.02 |
| | | N. weight: | 2.1 |
| | | Sc. PD: | -0.43 |
| | | Sc. rank: | -282.9 |

NP

| Pos. | 270 | obs: | exp: |
|---|---|---|---|
| gtt | V | 0 | 71.12 |
| gtc | V | 0 | 72.62 |
| gta | V | 17 | 64.85 |
| gtg | V | 329 | 137.40 |
| --- | -- | ------ | ------ |
| mPD | | 0.094 | 0.72 |
| | | nPD: | 0.13 |
| | | N. weight: | 1.1 |
| | | Sc. PD: | -0.11 |
| | | Sc. rank: | -23.6 |

NP

| Pos. | 271 | obs: | exp: |
|---|---|---|---|
| gct | A | 166 | 87.15 |
| gcc | A | 180 | 70.88 |
| gca | A | 0 | 154.20 |
| gcg | A | 0 | 33.76 |
| --- | -- | ------ | ------ |
| mPD | | 0.50 | 0.69 |
| | | nPD: | 0.73 |
| | | N. weight: | 1.2 |
| | | Sc. PD: | 0.54 |
| | | Sc. rank: | 220.8 |

NP

| Pos. | 272 | obs: | exp: |
|---|---|---|---|
| cat | H | 299 | 187.50 |
| cac | H | 47 | 158.50 |
| --- | -- | ------ | ------ |
| mPD | | 0.24 | 0.50 |
| | | nPD: | 0.47 |
| | | N. weight: | 0.35 |
| | | Sc. PD: | 0.08 |
| | | Sc. rank: | 49.4 |

NP

| Pos. | 273 | obs: | exp: |
|---|---|---|---|
| aaa | K | 9 | 193.70 |
| aag | K | 337 | 152.30 |
| --- | -- | ------ | ------ |
| mPD | | 0.051 | 0.49 |
| | | nPD: | 0.1 |
| | | N. weight: | 1. |
| | | Sc. PD: | -0.13 |
| | | Sc. rank: | -45.9 |

NP

| Pos. | 274 | obs: | exp: |
|---|---|---|---|
| tct | S | 28 | 55.01 |
| tcc | S | 309 | 48.20 |
| tca | S | 7 | 76.65 |
| tcg | S | 2 | 21.09 |
| agt | S | 0 | 76.12 |
| agc | S | 0 | 68.93 |
| --- | -- | ------ | ------ |
| mPD | | 0.20 | 1.7 |
| | | nPD: | 0.12 |
| | | N. weight: | 2.2 |
| | | Sc. PD: | -0.25 |
| | | Sc. rank: | -66.5 |

NP

| Pos. | 275 | obs: | exp: |
|---|---|---|---|
| tgt | C | 26 | 142.60 |
| tgc | C | 320 | 203.40 |
| --- | -- | ------ | ------ |
| mPD | | 0.14 | 0.48 |
| | | nPD: | 0.29 |
| | | N. weight: | 0.43 |
| | | Sc. PD: | 0.021 |
| | | Sc. rank: | 27.1 |

NP

| Pos. | 276 | obs: | exp: |
|---|---|---|---|
| tta | L | 5 | 30.23 |
| ttg | L | 209 | 66.64 |
| ctt | L | 56 | 64.56 |
| ctc | L | 0 | 55.97 |
| cta | L | 1 | 52.12 |
| ctg | L | 75 | 76.48 |
| --- | -- | ------ | ------ |
| mPD | | 0.77 | 1.1 |
| | | nPD: | 0.69 |
| | | N. weight: | 0.93 |
| | | Sc. PD: | 0.41 |
| | | Sc. rank: | 171.6 |

NP

| Pos. | 277 | obs: | exp: |
|---|---|---|---|
| cct | P | 311 | 91.11 |
| ccc | P | 3 | 62.41 |
| cca | P | 32 | 136.50 |
| ccg | P | 0 | 55.94 |
| --- | -- | ------ | ------ |
| mPD | | 0.18 | 0.72 |
| | | nPD: | 0.26 |
| | | N. weight: | 1.4 |
| | | Sc. PD: | 0.026 |
| | | Sc. rank: | 65.9 |

NP

| Pos. | 278 | obs: | exp: |
|---|---|---|---|
| gct | A | 314 | 87.15 |
| gcc | A | 7 | 70.88 |
| gca | A | 19 | 154.20 |
| gcg | A | 6 | 33.76 |
| --- | -- | ------ | ------ |
| mPD | | 0.17 | 0.69 |
| | | nPD: | 0.25 |
| | | N. weight: | 1.4 |
| | | Sc. PD: | 0.021 |
| | | Sc. rank: | 64.6 |

NP

| Pos. | 279 | obs: | exp: |
|---|---|---|---|
| tgt | C | 341 | 142.60 |
| tgc | C | 5 | 203.40 |
| --- | -- | ------ | ------ |
| mPD | | 0.029 | 0.48 |
| | | nPD: | 0.06 |
| | | N. weight: | 1.2 |
| | | Sc. PD: | -0.2 |
| | | Sc. rank: | -87.7 |

NP

| Pos. | 280 | obs: | exp: |
|---|---|---|---|
| gtt | V | 0 | 71.12 |
| gtc | V | 0 | 72.62 |
| gta | V | 68 | 64.85 |
| gtg | V | 278 | 137.40 |
| --- | -- | ------ | ------ |
| mPD | | 0.32 | 0.72 |
| | | nPD: | 0.44 |
| | | N. weight: | 0.84 |
| | | Sc. PD: | 0.16 |
| | | Sc. rank: | 109.1 |

NP

| Pos. | 281 | obs: | exp: |
|---|---|---|---|
| tat | Y | 132 | 179.10 |
| tac | Y | 214 | 166.90 |
| --- | -- | ------ | ------ |
| mPD | | 0.47 | 0.50 |
| | | nPD: | 0.95 |
| | | N. weight: | 0.066 |
| | | Sc. PD: | 0.045 |
| | | Sc. rank: | 13.8 |

NP

| Pos. | 282 | obs: | exp: |
|---|---|---|---|
| ggt | G | 0 | 42.69 |
| ggc | G | 1 | 46.79 |
| gga | G | 232 | 167.40 |
| ggg | G | 113 | 89.14 |
| --- | -- | ------ | ------ |
| mPD | | 0.45 | 0.67 |
| | | nPD: | 0.67 |
| | | N. weight: | 0.43 |
| | | Sc. PD: | 0.18 |
| | | Sc. rank: | 77.3 |

NP

| Pos. | 283 | obs: | exp: |
|---|---|---|---|
| tta | L | 0 | 30.23 |
| ttg | L | 0 | 66.64 |
| ctt | L | 320 | 64.56 |
| ctc | L | 25 | 55.97 |
| cta | L | 0 | 52.12 |
| ctg | L | 1 | 76.48 |
| --- | -- | ------ | ------ |
| mPD | | 0.14 | 1.1 |
| | | nPD: | 0.13 |
| | | N. weight: | 2. |
| | | Sc. PD: | -0.22 |
| | | Sc. rank: | -45.3 |

NP

| Pos. | 284 | obs: | exp: |
|---|---|---|---|
| gct | A | 250 | 87.15 |
| gcc | A | 57 | 70.88 |
| gca | A | 35 | 154.20 |
| gcg | A | 4 | 33.76 |
| --- | -- | ------ | ------ |
| mPD | | 0.44 | 0.69 |
| | | nPD: | 0.64 |
| | | N. weight: | 0.82 |
| | | Sc. PD: | 0.32 |
| | | Sc. rank: | 144.5 |

NP

| Pos. | 285 | obs: | exp: |
|---|---|---|---|
| gtt | V | 1 | 71.12 |
| gtc | V | 0 | 72.62 |
| gta | V | 23 | 64.85 |
| gtg | V | 322 | 137.40 |
| --- | -- | ------ | ------ |
| mPD | | 0.13 | 0.72 |
| | | nPD: | 0.18 |
| | | N. weight: | 1. |
| | | Sc. PD: | -0.056 |
| | | Sc. rank: | 2.8 |

NP

| Pos. | 286 | obs: | exp: |
|---|---|---|---|
| gtt | V | 0 | 0.62 |
| gtc | V | 3 | 0.63 |
| gta | V | 0 | 0.56 |
| gtg | V | 0 | 1.19 |
| gct | A | 1 | 85.64 |
| gcc | A | 334 | 69.65 |
| gca | A | 5 | 151.50 |
| gcg | A | 0 | 33.18 |
| ggt | G | 2 | 0.37 |
| ggc | G | 1 | 0.41 |
| gga | G | 0 | 1.45 |
| ggg | G | 0 | 0.77 |
| --- | -- | ------ | ------ |
| mPD | | 0.080 | 0.72 |
| | | nPD: | 0.11 |
| | | N. weight: | 2.1 |
| | | Sc. PD: | -0.26 |
| | | Sc. rank: | -73.2 |

NP

| Pos. | 287 | obs: | exp: |
|---|---|---|---|
| tct | S | 0 | 55.01 |
| tcc | S | 0 | 48.20 |
| tca | S | 0 | 76.65 |
| tcg | S | 0 | 21.09 |
| agt | S | 343 | 76.12 |
| agc | S | 3 | 68.93 |
| --- | -- | ------ | ------ |
| mPD | | 0.017 | 1.7 |
| | | nPD: | 0.01 |
| | | N. weight: | 2.1 |
| | | Sc. PD: | -0.46 |
| | | Sc. rank: | -349.1 |

NP

| Pos. | 288 | obs: | exp: |
|---|---|---|---|
| ggt | G | 0 | 42.69 |
| ggc | G | 0 | 46.79 |
| gga | G | 312 | 167.40 |
| ggg | G | 34 | 89.14 |
| --- | -- | ------ | ------ |
| mPD | | 0.18 | 0.67 |
| | | nPD: | 0.27 |
| | | N. weight: | 0.68 |
| | | Sc. PD: | 0.019 |
| | | Sc. rank: | 37.2 |

NP

| Pos. | 289 | obs: | exp: |
|---|---|---|---|
| tat | Y | 172 | 179.10 |
| tac | Y | 174 | 166.90 |
| --- | -- | ------ | ------ |
| mPD | | 0.50 | 0.50 |
| | | nPD: | 1. |
| | | N. weight: | 0.014 |
| | | Sc. PD: | 0.01 |
| | | Sc. rank: | 3.0 |

| NP | | | |
|---|---|---|---|
| Pos. | 290 | obs: | exp: |
| aat | N | 0 | 1.05 |
| aac | N | 2 | 0.95 |
| gat | D | 15 | 183.50 |
| gac | D | 329 | 160.50 |
| --- | -- | ------ | ------ |
| mPD | | 0.095 | 0.51 |
| | | nPD: | 0.19 |
| | | N. weight: | 0.84 |
| | | Sc. PD: | -0.041 |
| | | Sc. rank: | 7.0 |

| NP | | | |
|---|---|---|---|
| Pos. | 291 | obs: | exp: |
| ttt | F | 340 | 142.20 |
| ttc | F | 6 | 203.80 |
| --- | -- | ------ | ------ |
| mPD | | 0.034 | 0.48 |
| | | nPD: | 0.07 |
| | | N. weight: | 1.1 |
| | | Sc. PD: | -0.18 |
| | | Sc. rank: | -80.9 |

| NP | | | |
|---|---|---|---|
| Pos. | 292 | obs: | exp: |
| gtt | V | 0 | 0.21 |
| gtc | V | 0 | 0.21 |
| gta | V | 0 | 0.19 |
| gtg | V | 1 | 0.40 |
| gaa | E | 145 | 198.50 |
| gag | E | 200 | 146.50 |
| --- | -- | ------ | ------ |
| mPD | | 0.49 | 0.50 |
| | | nPD: | 1. |
| | | N. weight: | 0.086 |
| | | Sc. PD: | 0.062 |
| | | Sc. rank: | 18.0 |

| NP | | | |
|---|---|---|---|
| Pos. | 293 | obs: | exp: |
| cgt | R | 0 | 14.36 |
| cgc | R | 0 | 16.94 |
| cga | R | 1 | 33.25 |
| cgg | R | 0 | 28.87 |
| aga | R | 316 | 155.60 |
| agg | R | 29 | 96.96 |
| --- | -- | ------ | ------ |
| mPD | | 0.16 | 0.96 |
| | | nPD: | 0.17 |
| | | N. weight: | 0.78 |
| | | Sc. PD: | -0.053 |
| | | Sc. rank: | -3.2 |

| NP | | | |
|---|---|---|---|
| Pos. | 294 | obs: | exp: |
| gaa | E | 231 | 199.10 |
| gag | E | 115 | 146.90 |
| --- | -- | ------ | ------ |
| mPD | | 0.45 | 0.49 |
| | | nPD: | 0.91 |
| | | N. weight: | 0.038 |
| | | Sc. PD: | 0.025 |
| | | Sc. rank: | 8.0 |

| NP | | | |
|---|---|---|---|
| Pos. | 295 | obs: | exp: |
| ggt | G | 0 | 42.69 |
| ggc | G | 0 | 46.79 |
| gga | G | 149 | 167.40 |
| ggg | G | 197 | 89.14 |
| --- | -- | ------ | ------ |
| mPD | | 0.49 | 0.67 |
| | | nPD: | 0.74 |
| | | N. weight: | 0.59 |
| | | Sc. PD: | 0.28 |
| | | Sc. rank: | 113.7 |

| NP | | | |
|---|---|---|---|
| Pos. | 296 | obs: | exp: |
| tat | Y | 23 | 178.10 |
| tac | Y | 321 | 165.90 |
| cat | H | 0 | 1.08 |
| cac | H | 2 | 0.92 |
| --- | -- | ------ | ------ |
| mPD | | 0.14 | 0.51 |
| | | nPD: | 0.27 |
| | | N. weight: | 0.7 |
| | | Sc. PD: | 0.019 |
| | | Sc. rank: | 37.8 |

| NP | | | |
|---|---|---|---|
| Pos. | 297 | obs: | exp: |
| tct | S | 250 | 55.01 |
| tcc | S | 93 | 48.20 |
| tca | S | 3 | 76.65 |
| tcg | S | 0 | 21.09 |
| agt | S | 0 | 76.12 |
| agc | S | 0 | 68.93 |
| --- | -- | ------ | ------ |
| mPD | | 0.41 | 1.7 |
| | | nPD: | 0.24 |
| | | N. weight: | 1.8 |
| | | Sc. PD: | 0.013 |
| | | Sc. rank: | 79.9 |

| NP | | | |
|---|---|---|---|
| Pos. | 298 | obs: | exp: |
| tta | L | 1 | 30.23 |
| ttg | L | 7 | 66.64 |
| ctt | L | 2 | 64.56 |
| ctc | L | 0 | 55.97 |
| cta | L | 230 | 52.12 |
| ctg | L | 106 | 76.48 |
| --- | -- | ------ | ------ |
| mPD | | 0.49 | 1.1 |
| | | nPD: | 0.44 |
| | | N. weight: | 1.5 |
| | | Sc. PD: | 0.29 |
| | | Sc. rank: | 194.7 |

| NP | | | |
|---|---|---|---|
| Pos. | 299 | obs: | exp: |
| gtt | V | 225 | 71.12 |
| gtc | V | 117 | 72.62 |
| gta | V | 3 | 64.85 |
| gtg | V | 1 | 137.40 |
| --- | -- | ------ | ------ |
| mPD | | 0.46 | 0.72 |
| | | nPD: | 0.64 |
| | | N. weight: | 1.3 |
| | | Sc. PD: | 0.49 |
| | | Sc. rank: | 225.3 |

| NP | | | |
|---|---|---|---|
| Pos. | 300 | obs: | exp: |
| ggt | G | 0 | 42.69 |
| ggc | G | 1 | 46.79 |
| gga | G | 329 | 167.40 |
| ggg | G | 16 | 89.14 |
| --- | -- | ------ | ------ |
| mPD | | 0.094 | 0.67 |
| | | nPD: | 0.14 |
| | | N. weight: | 0.8 |
| | | Sc. PD: | -0.074 |
| | | Sc. rank: | -10.8 |

| NP | | | |
|---|---|---|---|
| Pos. | 301 | obs: | exp: |
| att | I | 61 | 127.80 |
| atc | I | 45 | 89.11 |
| ata | I | 234 | 123.10 |
| gtt | V | 2 | 1.23 |
| gtc | V | 0 | 1.26 |
| gta | V | 4 | 1.12 |
| gtg | V | 0 | 2.38 |
| --- | -- | ------ | ------ |
| mPD | | 0.51 | 0.70 |
| | | nPD: | 0.73 |
| | | N. weight: | 0.36 |
| | | Sc. PD: | 0.17 |
| | | Sc. rank: | 69.4 |

| NP | | | |
|---|---|---|---|
| Pos. | 302 | obs: | exp: |
| gat | D | 317 | 184.60 |
| gac | D | 29 | 161.40 |
| --- | -- | ------ | ------ |
| mPD | | 0.15 | 0.50 |
| | | nPD: | 0.31 |
| | | N. weight: | 0.51 |
| | | Sc. PD: | 0.036 |
| | | Sc. rank: | 41.3 |

| NP | | | |
|---|---|---|---|
| Pos. | 303 | obs: | exp: |
| cct | P | 333 | 91.11 |
| ccc | P | 11 | 62.41 |
| cca | P | 1 | 136.50 |
| ccg | P | 1 | 55.94 |
| --- | -- | ------ | ------ |
| mPD | | 0.073 | 0.72 |
| | | nPD: | 0.1 |
| | | N. weight: | 1.7 |
| | | Sc. PD: | -0.22 |
| | | Sc. rank: | -80.0 |

| NP | | | |
|---|---|---|---|
| Pos. | 304 | obs: | exp: |
| ttt | F | 4 | 142.20 |
| ttc | F | 342 | 203.80 |
| --- | -- | ------ | ------ |
| mPD | | 0.023 | 0.48 |
| | | nPD: | 0.05 |
| | | N. weight: | 0.68 |
| | | Sc. PD: | -0.12 |
| | | Sc. rank: | -63.7 |

| NP | | | |
|---|---|---|---|
| Pos. | 305 | obs: | exp: |
| cgt | R | 330 | 14.32 |
| cgc | R | 14 | 16.89 |
| cga | R | 1 | 33.16 |
| cgg | R | 0 | 28.79 |
| aaa | K | 1 | 0.56 |
| aag | K | 0 | 0.44 |
| aga | R | 0 | 155.20 |
| agg | R | 0 | 96.68 |
| --- | -- | ------ | ------ |
| mPD | | 0.10 | 0.96 |
| | | nPD: | 0.1 |
| | | N. weight: | 4.3 |
| | | Sc. PD: | -0.54 |
| | | Sc. rank: | -180.6 |

| NP | | | |
|---|---|---|---|
| Pos. | 306 | obs: | exp: |
| tta | L | 0 | 30.23 |
| ttg | L | 10 | 66.64 |
| ctt | L | 9 | 64.56 |
| ctc | L | 0 | 55.97 |
| cta | L | 54 | 52.12 |
| ctg | L | 273 | 76.48 |
| --- | -- | ------ | ------ |
| mPD | | 0.36 | 1.1 |
| | | nPD: | 0.33 |
| | | N. weight: | 1.3 |
| | | Sc. PD: | 0.11 |
| | | Sc. rank: | 113.1 |

| NP | | | |
|---|---|---|---|
| Pos. | 307 | obs: | exp: |
| tta | L | 4 | 30.23 |
| ttg | L | 0 | 66.64 |
| ctt | L | 264 | 64.56 |
| ctc | L | 78 | 55.97 |
| cta | L | 0 | 52.12 |
| ctg | L | 0 | 76.48 |
| --- | -- | ------ | ------ |
| mPD | | 0.39 | 1.1 |
| | | nPD: | 0.35 |
| | | N. weight: | 1.6 |
| | | Sc. PD: | 0.18 |
| | | Sc. rank: | 163.3 |

| NP | | | |
|---|---|---|---|
| Pos. | 308 | obs: | exp: |
| caa | Q | 319 | 184.50 |
| cag | Q | 27 | 161.50 |
| --- | -- | ------ | ------ |
| mPD | | 0.14 | 0.50 |
| | | nPD: | 0.29 |
| | | N. weight: | 0.53 |
| | | Sc. PD: | 0.027 |
| | | Sc. rank: | 34.7 |

| NP | | | |
|---|---|---|---|
| Pos. | 309 | obs: | exp: |
| aat | N | 9 | 180.80 |
| aac | N | 337 | 165.20 |
| --- | -- | ------ | ------ |
| mPD | | 0.051 | 0.50 |
| | | nPD: | 0.1 |
| | | N. weight: | 0.89 |
| | | Sc. PD: | -0.12 |
| | | Sc. rank: | -41.6 |

| NP | | | |
|---|---|---|---|
| Pos. | 310 | obs: | exp: |
| tct | S | 0 | 55.01 |
| tcc | S | 0 | 48.20 |
| tca | S | 0 | 76.65 |
| tcg | S | 0 | 21.09 |
| agt | S | 79 | 76.12 |
| agc | S | 267 | 68.93 |
| --- | -- | ------ | ------ |
| mPD | | 0.35 | 1.7 |
| | | nPD: | 0.21 |
| | | N. weight: | 1.5 |
| | | Sc. PD: | -0.036 |
| | | Sc. rank: | 29.8 |

| NP | | | |
|---|---|---|---|
| Pos. | 311 | obs: | exp: |
| caa | Q | 90 | 184.50 |
| cag | Q | 256 | 161.50 |
| --- | -- | ------ | ------ |
| mPD | | 0.39 | 0.50 |
| | | nPD: | 0.78 |
| | | N. weight: | 0.23 |
| | | Sc. PD: | 0.12 |
| | | Sc. rank: | 46.9 |

| NP | | | |
|---|---|---|---|
| Pos. | 312 | obs: | exp: |
| gtt | V | 13 | 71.12 |
| gtc | V | 287 | 72.62 |
| gta | V | 29 | 64.85 |
| gtg | V | 17 | 137.40 |
| --- | -- | ------ | ------ |
| mPD | | 0.30 | 0.72 |
| | | nPD: | 0.42 |
| | | N. weight: | 1.3 |
| | | Sc. PD: | 0.23 |
| | | Sc. rank: | 159.3 |

| NP | | | |
|---|---|---|---|
| Pos. | 313 | obs: | exp: |
| ttt | F | 51 | 141.40 |
| ttc | F | 293 | 202.60 |
| tta | L | 0 | 0.17 |
| ttg | L | 0 | 0.39 |
| ctt | L | 0 | 0.37 |
| ctc | L | 2 | 0.32 |
| cta | L | 0 | 0.30 |
| ctg | L | 0 | 0.44 |
| --- | -- | ------ | ------ |
| mPD | | 0.26 | 0.50 |
| | | nPD: | 0.53 |
| | | N. weight: | 0.26 |
| | | Sc. PD: | 0.073 |
| | | Sc. rank: | 39.7 |

| NP | | | |
|---|---|---|---|
| Pos. | 314 | obs: | exp: |
| tct | S | 0 | 55.01 |
| tcc | S | 0 | 48.20 |
| tca | S | 0 | 76.65 |
| tcg | S | 0 | 21.09 |
| agt | S | 290 | 76.12 |
| agc | S | 56 | 68.93 |
| --- | -- | ------ | ------ |
| mPD | | 0.27 | 1.7 |
| | | nPD: | 0.16 |
| | | N. weight: | 1.6 |
| | | Sc. PD: | -0.11 |
| | | Sc. rank: | -8.5 |

| NP | | | |
|---|---|---|---|
| Pos. | 315 | obs: | exp: |
| tta | L | 0 | 30.23 |
| ttg | L | 0 | 66.64 |
| ctt | L | 23 | 64.56 |
| ctc | L | 297 | 55.97 |
| cta | L | 0 | 52.12 |
| ctg | L | 26 | 76.48 |
| --- | -- | ------ | ------ |
| mPD | | 0.25 | 1.1 |
| | | nPD: | 0.23 |
| | | N. weight: | 1.9 |
| | | Sc. PD: | -0.017 |
| | | Sc. rank: | 61.6 |

| NP | | | |
|---|---|---|---|
| Pos. | 316 | obs: | exp: |
| att | I | 257 | 130.10 |
| atc | I | 89 | 90.68 |
| ata | I | 0 | 125.20 |
| --- | -- | ------ | ------ |
| mPD | | 0.38 | 0.66 |
| | | nPD: | 0.58 |
| | | N. weight: | 0.73 |
| | | Sc. PD: | 0.24 |
| | | Sc. rank: | 120.7 |

| NP | | | |
|---|---|---|---|
| Pos. | 317 | obs: | exp: |
| cgt | R | 0 | 14.36 |
| cgc | R | 0 | 16.94 |
| cga | R | 0 | 33.25 |
| cgg | R | 0 | 28.87 |
| aga | R | 337 | 155.60 |
| agg | R | 9 | 96.96 |
| --- | -- | ------ | ------ |
| mPD | | 0.051 | 0.96 |
| | | nPD: | 0.05 |
| | | N. weight: | 1. |
| | | Sc. PD: | -0.18 |
| | | Sc. rank: | -81.3 |

| NP | | | |
|---|---|---|---|
| Pos. | 318 | obs: | exp: |
| cct | P | 0 | 91.11 |
| ccc | P | 3 | 62.41 |
| cca | P | 332 | 136.50 |
| ccg | P | 11 | 55.94 |
| --- | -- | ------ | ------ |
| mPD | | 0.078 | 0.72 |
| | | nPD: | 0.11 |
| | | N. weight: | 1.1 |
| | | Sc. PD: | -0.14 |
| | | Sc. rank: | -42.4 |

| NP | | | |
|---|---|---|---|
| Pos. | 319 | obs: | exp: |
| aat | N | 290 | 178.20 |
| aac | N | 51 | 162.80 |
| aaa | K | 1 | 2.80 |
| aag | K | 4 | 2.20 |
| --- | -- | ------ | ------ |
| mPD | | 0.28 | 0.51 |
| | | nPD: | 0.54 |
| | | N. weight: | 0.36 |
| | | Sc. PD: | 0.1 |
| | | Sc. rank: | 56.3 |

| NP | | | |
|---|---|---|---|
| Pos. | 320 | obs: | exp: |
| gaa | E | 76 | 199.10 |
| gag | E | 270 | 146.90 |
| mPD | | 0.34 | 0.49 |
| | | nPD: | 0.7 |
| | | N. weight: | 0.39 |
| | | Sc. PD: | 0.17 |
| | | Sc. rank: | 72.3 |

| NP | | | |
|---|---|---|---|
| Pos. | 321 | obs: | exp: |
| aat | N | 254 | 180.80 |
| aac | N | 92 | 165.20 |
| mPD | | 0.39 | 0.50 |
| | | nPD: | 0.78 |
| | | N. weight: | 0.15 |
| | | Sc. PD: | 0.077 |
| | | Sc. rank: | 29.6 |

| NP | | | |
|---|---|---|---|
| Pos. | 322 | obs: | exp: |
| cct | P | 16 | 91.11 |
| ccc | P | 0 | 62.41 |
| cca | P | 318 | 136.50 |
| ccg | P | 12 | 55.94 |
| mPD | | 0.15 | 0.72 |
| | | nPD: | 0.21 |
| | | N. weight: | 0.94 |
| | | Sc. PD: | -0.022 |
| | | Sc. rank: | 19.2 |

| NP | | | |
|---|---|---|---|
| Pos. | 323 | obs: | exp: |
| gtt | V | 0 | 0.21 |
| gtc | V | 0 | 0.21 |
| gta | V | 1 | 0.19 |
| gtg | V | 0 | 0.40 |
| gct | A | 5 | 86.90 |
| gcc | A | 0 | 70.67 |
| gca | A | 320 | 153.80 |
| gcg | A | 20 | 33.67 |
| mPD | | 0.14 | 0.69 |
| | | nPD: | 0.2 |
| | | N. weight: | 0.89 |
| | | Sc. PD: | -0.028 |
| | | Sc. rank: | 15.8 |

| NP | | | |
|---|---|---|---|
| Pos. | 324 | obs: | exp: |
| cat | H | 112 | 187.50 |
| cac | H | 234 | 158.50 |
| mPD | | 0.44 | 0.50 |
| | | nPD: | 0.88 |
| | | N. weight: | 0.15 |
| | | Sc. PD: | 0.094 |
| | | Sc. rank: | 31.2 |

| NP | | | |
|---|---|---|---|
| Pos. | 325 | obs: | exp: |
| aaa | K | 71 | 193.70 |
| aag | K | 275 | 152.30 |
| mPD | | 0.33 | 0.49 |
| | | nPD: | 0.66 |
| | | N. weight: | 0.39 |
| | | Sc. PD: | 0.16 |
| | | Sc. rank: | 70.4 |

| NP | | | |
|---|---|---|---|
| Pos. | 326 | obs: | exp: |
| tct | S | 0 | 55.01 |
| tcc | S | 0 | 48.20 |
| tca | S | 0 | 76.65 |
| tcg | S | 0 | 21.09 |
| agt | S | 281 | 76.12 |
| agc | S | 65 | 68.93 |
| mPD | | 0.31 | 1.7 |
| | | nPD: | 0.18 |
| | | N. weight: | 1.5 |
| | | Sc. PD: | -0.077 |
| | | Sc. rank: | 11.6 |

| NP | | | |
|---|---|---|---|
| Pos. | 327 | obs: | exp: |
| caa | Q | 241 | 184.50 |
| cag | Q | 105 | 161.50 |
| mPD | | 0.42 | 0.50 |
| | | nPD: | 0.85 |
| | | N. weight: | 0.092 |
| | | Sc. PD: | 0.054 |
| | | Sc. rank: | 18.8 |

| NP | | | |
|---|---|---|---|
| Pos. | 328 | obs: | exp: |
| tta | L | 34 | 30.23 |
| ttg | L | 190 | 66.64 |
| ctt | L | 0 | 64.56 |
| ctc | L | 0 | 55.97 |
| cta | L | 5 | 52.12 |
| ctg | L | 117 | 76.48 |
| mPD | | 0.66 | 1.1 |
| | | nPD: | 0.59 |
| | | N. weight: | 1. |
| | | Sc. PD: | 0.35 |
| | | Sc. rank: | 172.6 |

| NP | | | |
|---|---|---|---|
| Pos. | 329 | obs: | exp: |
| att | I | 0 | 7.89 |
| atc | I | 0 | 5.50 |
| ata | I | 21 | 7.60 |
| gtt | V | 3 | 66.80 |
| gtc | V | 3 | 68.21 |
| gta | V | 88 | 60.91 |
| gtg | V | 231 | 129.10 |
| mPD | | 0.57 | 0.84 |
| | | nPD: | 0.68 |
| | | N. weight: | 0.73 |
| | | Sc. PD: | 0.3 |
| | | Sc. rank: | 132.8 |

| NP | | | |
|---|---|---|---|
| Pos. | 330 | obs: | exp: |
| tgg | W | 346 | 346.00 |
| mPD | | 0 | 0 |
| | | nPD: | 1. |
| | | N. weight: | 0. |
| | | Sc. PD: | 0 |
| | | Sc. rank: | 0 |

| NP | | | |
|---|---|---|---|
| Pos. | 331 | obs: | exp: |
| atg | M | 346 | 346.00 |
| mPD | | 0 | 0 |
| | | nPD: | 1. |
| | | N. weight: | 0. |
| | | Sc. PD: | 0 |
| | | Sc. rank: | 0 |

| NP | | | |
|---|---|---|---|
| Pos. | 332 | obs: | exp: |
| act | T | 0 | 0.26 |
| acc | T | 0 | 0.21 |
| aca | T | 1 | 0.44 |
| acg | T | 0 | 0.08 |
| gct | A | 0 | 86.90 |
| gcc | A | 0 | 70.67 |
| gca | A | 341 | 153.80 |
| gcg | A | 4 | 33.67 |
| mPD | | 0.029 | 0.69 |
| | | nPD: | 0.04 |
| | | N. weight: | 1.1 |
| | | Sc. PD: | -0.21 |
| | | Sc. rank: | -107.2 |

| NP | | | |
|---|---|---|---|
| Pos. | 333 | obs: | exp: |
| tct | S | 0 | 0.16 |
| tcc | S | 0 | 0.14 |
| tca | S | 0 | 0.22 |
| tcg | S | 0 | 0.06 |
| tgt | C | 51 | 142.20 |
| tgc | C | 294 | 202.80 |
| agt | S | 0 | 0.22 |
| agc | S | 1 | 0.20 |
| mPD | | 0.26 | 0.49 |
| | | nPD: | 0.52 |
| | | N. weight: | 0.25 |
| | | Sc. PD: | 0.07 |
| | | Sc. rank: | 38.6 |

| NP | | | |
|---|---|---|---|
| Pos. | 334 | obs: | exp: |
| cat | H | 257 | 187.50 |
| cac | H | 89 | 158.50 |
| mPD | | 0.38 | 0.50 |
| | | nPD: | 0.77 |
| | | N. weight: | 0.14 |
| | | Sc. PD: | 0.069 |
| | | Sc. rank: | 27.0 |

| NP | | | |
|---|---|---|---|
| Pos. | 335 | obs: | exp: |
| tct | S | 328 | 54.37 |
| tcc | S | 9 | 47.64 |
| tca | S | 5 | 75.77 |
| tcg | S | 0 | 20.85 |
| agt | S | 0 | 75.24 |
| agc | S | 0 | 68.14 |
| gct | A | 4 | 1.01 |
| gcc | A | 0 | 0.82 |
| gca | A | 0 | 1.78 |
| gcg | A | 0 | 0.39 |
| mPD | | 0.10 | 1.7 |
| | | nPD: | 0.06 |
| | | N. weight: | 2.4 |
| | | Sc. PD: | -0.4 |
| | | Sc. rank: | -173.6 |

| NP | | | |
|---|---|---|---|
| Pos. | 336 | obs: | exp: |
| gct | A | 7 | 87.15 |
| gcc | A | 7 | 70.88 |
| gca | A | 319 | 154.20 |
| gcg | A | 13 | 33.76 |
| mPD | | 0.15 | 0.69 |
| | | nPD: | 0.22 |
| | | N. weight: | 0.79 |
| | | Sc. PD: | -0.016 |
| | | Sc. rank: | 20.6 |

| NP | | | |
|---|---|---|---|
| Pos. | 337 | obs: | exp: |
| gct | A | 26 | 87.15 |
| gcc | A | 1 | 70.88 |
| gca | A | 306 | 154.20 |
| gcg | A | 13 | 33.76 |
| mPD | | 0.21 | 0.69 |
| | | nPD: | 0.31 |
| | | N. weight: | 0.69 |
| | | Sc. PD: | 0.047 |
| | | Sc. rank: | 55.4 |

| NP | | | |
|---|---|---|---|
| Pos. | 338 | obs: | exp: |
| ttt | F | 270 | 142.20 |
| ttc | F | 76 | 203.80 |
| mPD | | 0.34 | 0.48 |
| | | nPD: | 0.71 |
| | | N. weight: | 0.42 |
| | | Sc. PD: | 0.19 |
| | | Sc. rank: | 78.7 |

| NP | | | |
|---|---|---|---|
| Pos. | 339 | obs: | exp: |
| gaa | E | 142 | 199.10 |
| gag | E | 204 | 146.90 |
| mPD | | 0.49 | 0.49 |
| | | nPD: | 0.99 |
| | | N. weight: | 0.092 |
| | | Sc. PD: | 0.066 |
| | | Sc. rank: | 19.2 |

| NP | | | |
|---|---|---|---|
| Pos. | 340 | obs: | exp: |
| gat | D | 151 | 184.60 |
| gac | D | 195 | 161.40 |
| mPD | | 0.49 | 0.50 |
| | | nPD: | 0.99 |
| | | N. weight: | 0.04 |
| | | Sc. PD: | 0.029 |
| | | Sc. rank: | 8.4 |

| NP | | | |
|---|---|---|---|
| Pos. | 341 | obs: | exp: |
| tta | L | 0 | 30.23 |
| ttg | L | 8 | 66.64 |
| ctt | L | 29 | 64.56 |
| ctc | L | 0 | 55.97 |
| cta | L | 2 | 52.12 |
| ctg | L | 307 | 76.48 |
| mPD | | 0.21 | 1.1 |
| | | nPD: | 0.19 |
| | | N. weight: | 1.6 |
| | | Sc. PD: | -0.074 |
| | | Sc. rank: | 15.5 |

| NP | | | |
|---|---|---|---|
| Pos. | 342 | obs: | exp: |
| cgt | R | 0 | 14.36 |
| cgc | R | 0 | 16.94 |
| cga | R | 1 | 33.25 |
| cgg | R | 0 | 28.87 |
| aga | R | 307 | 155.60 |
| agg | R | 38 | 96.96 |
| mPD | | 0.20 | 0.96 |
| | | nPD: | 0.21 |
| | | N. weight: | 0.72 |
| | | Sc. PD: | -0.018 |
| | | Sc. rank: | 13.1 |

| NP | | | |
|---|---|---|---|
| Pos. | 343 | obs: | exp: |
| gtt | V | 23 | 71.12 |
| gtc | V | 203 | 72.62 |
| gta | V | 30 | 64.85 |
| gtg | V | 90 | 137.40 |
| mPD | | 0.58 | 0.72 |
| | | nPD: | 0.8 |
| | | N. weight: | 0.54 |
| | | Sc. PD: | 0.29 |
| | | Sc. rank: | 108.2 |

| NP | | | |
|---|---|---|---|
| Pos. | 344 | obs: | exp: |
| tta | L | 6 | 0.52 |
| ttg | L | 0 | 1.16 |
| tct | S | 0 | 54.05 |
| tcc | S | 0 | 47.36 |
| tca | S | 319 | 75.32 |
| tcg | S | 21 | 20.72 |
| ctt | L | 0 | 1.12 |
| ctc | L | 0 | 0.97 |
| cta | L | 0 | 0.90 |
| ctg | L | 0 | 1.33 |
| agt | S | 0 | 74.80 |
| agc | S | 0 | 67.74 |
| mPD | | 0.15 | 1.7 |
| | | nPD: | 0.09 |
| | | N. weight: | 2. |
| | | Sc. PD: | -0.28 |
| | | Sc. rank: | -114.5 |

| NP | | | |
|---|---|---|---|
| Pos. | 345 | obs: | exp: |
| tct | S | 0 | 55.01 |
| tcc | S | 0 | 48.20 |
| tca | S | 0 | 76.65 |
| tcg | S | 0 | 21.09 |
| agt | S | 226 | 76.12 |
| agc | S | 120 | 68.93 |
| --- | -- | ------ | ------ |
| mPD | | 0.45 | 1.7 |
| | | nPD: | 0.27 |
| | | N. weight: | 1.3 |
| | | Sc. PD: | 0.045 |
| | | Sc. rank: | 75.6 |

,

| NP | | | |
|---|---|---|---|
| Pos. | 346 | obs: | exp: |
| ttt | F | 17 | 142.20 |
| ttc | F | 329 | 203.80 |
| --- | -- | ------ | ------ |
| mPD | | 0.094 | 0.48 |
| | | nPD: | 0.19 |
| | | N. weight: | 0.51 |
| | | Sc. PD: | -0.021 |
| | | Sc. rank: | 5.9 |

,

| NP | | | |
|---|---|---|---|
| Pos. | 347 | obs: | exp: |
| att | I | 2 | 130.10 |
| atc | I | 344 | 90.68 |
| ata | I | 0 | 125.20 |
| --- | -- | ------ | ------ |
| mPD | | 0.012 | 0.66 |
| | | nPD: | 0.02 |
| | | N. weight: | 1.9 |
| | | Sc. PD: | -0.39 |
| | | Sc. rank: | -258.4 |

,

| NP | | | |
|---|---|---|---|
| Pos. | 348 | obs: | exp: |
| cgt | R | 0 | 14.36 |
| cgc | R | 0 | 16.94 |
| cga | R | 0 | 33.25 |
| cgg | R | 0 | 28.87 |
| aga | R | 332 | 155.60 |
| agg | R | 14 | 96.96 |
| --- | -- | ------ | ------ |
| mPD | | 0.078 | 0.96 |
| | | nPD: | 0.08 |
| | | N. weight: | 0.94 |
| | | Sc. PD: | -0.14 |
| | | Sc. rank: | -58.5 |

,

| NP | | | |
|---|---|---|---|
| Pos. | 349 | obs: | exp: |
| ggt | G | 0 | 42.69 |
| ggc | G | 0 | 46.79 |
| gga | G | 183 | 167.40 |
| ggg | G | 163 | 89.14 |
| --- | -- | ------ | ------ |
| mPD | | 0.50 | 0.67 |
| | | nPD: | 0.75 |
| | | N. weight: | 0.49 |
| | | Sc. PD: | 0.24 |
| | | Sc. rank: | 95.4 |

,

| NP | | | |
|---|---|---|---|
| Pos. | 350 | obs: | exp: |
| act | T | 23 | 89.56 |
| acc | T | 5 | 72.74 |
| aca | T | 309 | 148.50 |
| acg | T | 2 | 28.18 |
| gct | A | 0 | 1.76 |
| gcc | A | 0 | 1.43 |
| gca | A | 7 | 3.12 |
| gcg | A | 0 | 0.68 |
| --- | -- | ------ | ------ |
| mPD | | 0.20 | 0.72 |
| | | nPD: | 0.28 |
| | | N. weight: | 0.78 |
| | | Sc. PD: | 0.03 |
| | | Sc. rank: | 45.8 |

,

| NP | | | |
|---|---|---|---|
| Pos. | 351 | obs: | exp: |
| cgt | R | 0 | 14.16 |
| cgc | R | 0 | 16.70 |
| cga | R | 0 | 32.77 |
| cgg | R | 0 | 28.46 |
| aaa | K | 5 | 2.80 |
| aag | K | 0 | 2.20 |
| aga | R | 331 | 153.40 |
| agg | R | 10 | 95.56 |
| --- | -- | ------ | ------ |
| mPD | | 0.085 | 0.98 |
| | | nPD: | 0.09 |
| | | N. weight: | 0.98 |
| | | Sc. PD: | -0.14 |
| | | Sc. rank: | -58.5 |

,

| NP | | | |
|---|---|---|---|
| Pos. | 352 | obs: | exp: |
| att | I | 0 | 0.75 |
| atc | I | 0 | 0.52 |
| ata | I | 2 | 0.72 |
| atg | M | 31 | 31.00 |
| gtt | V | 1 | 64.33 |
| gtc | V | 0 | 65.69 |
| gta | V | 192 | 58.66 |
| gtg | V | 120 | 124.30 |
| --- | -- | ------ | ------ |
| mPD | | 0.67 | 0.87 |
| | | nPD: | 0.77 |
| | | N. weight: | 0.93 |
| | | Sc. PD: | 0.48 |
| | | Sc. rank: | 186.2 |

,

| NP | | | |
|---|---|---|---|
| Pos. | 353 | obs: | exp: |
| tta | L | 0 | 0.09 |
| ttg | L | 0 | 0.19 |
| ctt | L | 1 | 0.19 |
| ctc | L | 0 | 0.16 |
| cta | L | 0 | 0.15 |
| ctg | L | 0 | 0.22 |
| att | I | 5 | 18.80 |
| atc | I | 41 | 13.10 |
| ata | I | 4 | 18.10 |
| gtt | V | 52 | 60.63 |
| gtc | V | 242 | 61.91 |
| gta | V | 1 | 55.29 |
| gtg | V | 0 | 117.20 |
| --- | -- | ------ | ------ |
| mPD | | 0.56 | 0.99 |
| | | nPD: | 0.56 |
| | | N. weight: | 1.5 |
| | | Sc. PD: | 0.46 |
| | | Sc. rank: | 239.2 |

,

| NP | | | |
|---|---|---|---|
| Pos. | 354 | obs: | exp: |
| cct | P | 0 | 91.11 |
| ccc | P | 1 | 62.41 |
| cca | P | 343 | 136.50 |
| ccg | P | 2 | 55.94 |
| --- | -- | ------ | ------ |
| mPD | | 0.017 | 0.72 |
| | | nPD: | 0.02 |
| | | N. weight: | 1.3 |
| | | Sc. PD: | -0.26 |
| | | Sc. rank: | -163.4 |

,

| NP | | | |
|---|---|---|---|
| Pos. | 355 | obs: | exp: |
| cgt | R | 0 | 14.36 |
| cgc | R | 0 | 16.94 |
| cga | R | 1 | 33.25 |
| cgg | R | 0 | 28.87 |
| aga | R | 327 | 155.60 |
| agg | R | 18 | 96.96 |
| --- | -- | ------ | ------ |
| mPD | | 0.10 | 0.96 |
| | | nPD: | 0.11 |
| | | N. weight: | 0.88 |
| | | Sc. PD: | -0.11 |
| | | Sc. rank: | -34.0 |

,

| NP | | | |
|---|---|---|---|
| Pos. | 356 | obs: | exp: |
| ggt | G | 0 | 42.69 |
| ggc | G | 1 | 46.79 |
| gga | G | 316 | 167.40 |
| ggg | G | 29 | 89.14 |
| --- | -- | ------ | ------ |
| mPD | | 0.16 | 0.67 |
| | | nPD: | 0.24 |
| | | N. weight: | 0.7 |
| | | Sc. PD: | 0.0014 |
| | | Sc. rank: | 28.2 |

,

| NP | | | |
|---|---|---|---|
| Pos. | 357 | obs: | exp: |
| caa | Q | 309 | 184.50 |
| cag | Q | 37 | 161.50 |
| --- | -- | ------ | ------ |
| mPD | | 0.19 | 0.50 |
| | | nPD: | 0.38 |
| | | N. weight: | 0.45 |
| | | Sc. PD: | 0.063 |
| | | Sc. rank: | 49.9 |

,

| NP | | | |
|---|---|---|---|
| Pos. | 358 | obs: | exp: |
| tta | L | 16 | 30.23 |
| ttg | L | 37 | 66.64 |
| ctt | L | 0 | 64.56 |
| ctc | L | 0 | 55.97 |
| cta | L | 190 | 52.12 |
| ctg | L | 103 | 76.48 |
| --- | -- | ------ | ------ |
| mPD | | 0.74 | 1.1 |
| | | nPD: | 0.67 |
| | | N. weight: | 1. |
| | | Sc. PD: | 0.43 |
| | | Sc. rank: | 189.5 |

,

| NP | | | |
|---|---|---|---|
| Pos. | 359 | obs: | exp: |
| tct | S | 35 | 50.40 |
| tcc | S | 267 | 44.16 |
| tca | S | 15 | 70.23 |
| tcg | S | 0 | 19.32 |
| tgt | C | 0 | 0.41 |
| tgc | C | 1 | 0.59 |
| agt | S | 0 | 69.74 |
| agc | S | 0 | 63.16 |
| gct | A | 0 | 7.05 |
| gcc | A | 27 | 5.74 |
| gca | A | 1 | 12.48 |
| gcg | A | 0 | 2.73 |
| --- | -- | ------ | ------ |
| mPD | | 0.42 | 1.7 |
| | | nPD: | 0.24 |
| | | N. weight: | 2. |
| | | Sc. PD: | 0.0072 |
| | | Sc. rank: | 87.1 |

,

| NP | | | |
|---|---|---|---|
| Pos. | 360 | obs: | exp: |
| act | T | 41 | 91.41 |
| acc | T | 279 | 74.25 |
| aca | T | 26 | 151.60 |
| acg | T | 0 | 28.76 |
| --- | -- | ------ | ------ |
| mPD | | 0.33 | 0.69 |
| | | nPD: | 0.48 |
| | | N. weight: | 1.2 |
| | | Sc. PD: | 0.29 |
| | | Sc. rank: | 173.7 |

,

| NP | | | |
|---|---|---|---|
| Pos. | 361 | obs: | exp: |
| cgt | R | 0 | 14.36 |
| cgc | R | 0 | 16.94 |
| cga | R | 3 | 33.25 |
| cgg | R | 0 | 28.87 |
| aga | R | 319 | 155.60 |
| agg | R | 24 | 96.96 |
| --- | -- | ------ | ------ |
| mPD | | 0.15 | 0.96 |
| | | nPD: | 0.15 |
| | | N. weight: | 0.8 |
| | | Sc. PD: | -0.064 |
| | | Sc. rank: | -6.5 |

,

| NP | | | |
|---|---|---|---|
| Pos. | 362 | obs: | exp: |
| ggt | G | 0 | 42.69 |
| ggc | G | 0 | 46.79 |
| gga | G | 308 | 167.40 |
| ggg | G | 38 | 89.14 |
| --- | -- | ------ | ------ |
| mPD | | 0.20 | 0.67 |
| | | nPD: | 0.29 |
| | | N. weight: | 0.65 |
| | | Sc. PD: | 0.036 |
| | | Sc. rank: | 46.6 |

,

| NP | | | |
|---|---|---|---|
| Pos. | 363 | obs: | exp: |
| att | I | 10 | 3.76 |
| atc | I | 0 | 2.62 |
| ata | I | 0 | 3.62 |
| gtt | V | 300 | 68.65 |
| gtc | V | 13 | 70.10 |
| gta | V | 19 | 62.60 |
| gtg | V | 2 | 132.70 |
| gct | A | 0 | 0.50 |
| gcc | A | 2 | 0.41 |
| gca | A | 0 | 0.89 |
| gcg | A | 0 | 0.20 |
| --- | -- | ------ | ------ |
| mPD | | 0.26 | 0.79 |
| | | nPD: | 0.33 |
| | | N. weight: | 1.7 |
| | | Sc. PD: | 0.15 |
| | | Sc. rank: | 147.8 |

,

| NP | | | |
|---|---|---|---|
| Pos. | 364 | obs: | exp: |
| caa | Q | 276 | 184.50 |
| cag | Q | 70 | 161.50 |
| --- | -- | ------ | ------ |
| mPD | | 0.32 | 0.50 |
| | | nPD: | 0.65 |
| | | N. weight: | 0.23 |
| | | Sc. PD: | 0.091 |
| | | Sc. rank: | 41.0 |

,

| NP | | | |
|---|---|---|---|
| Pos. | 365 | obs: | exp: |
| att | I | 330 | 130.10 |
| atc | I | 1 | 90.68 |
| ata | I | 15 | 125.20 |
| --- | -- | ------ | ------ |
| mPD | | 0.089 | 0.66 |
| | | nPD: | 0.13 |
| | | N. weight: | 1.1 |
| | | Sc. PD: | -0.11 |
| | | Sc. rank: | -20.4 |

,

| NP | | | |
|---|---|---|---|
| Pos. | 366 | obs: | exp: |
| tct | S | 1 | 0.16 |
| tcc | S | 0 | 0.14 |
| tca | S | 0 | 0.22 |
| tcg | S | 0 | 0.06 |
| agt | S | 0 | 0.22 |
| agc | S | 0 | 0.20 |
| gct | A | 333 | 86.90 |
| gcc | A | 10 | 70.67 |
| gca | A | 1 | 153.80 |
| gcg | A | 1 | 33.67 |
| --- | -- | ------ | ------ |
| mPD | | 0.073 | 0.69 |
| | | nPD: | 0.11 |
| | | N. weight: | 1.8 |
| | | Sc. PD: | -0.22 |
| | | Sc. rank: | -69.3 |

,

| NP | | | |
|---|---|---|---|
| Pos. | 367 | obs: | exp: |
| tct | S | 1 | 55.01 |
| tcc | S | 1 | 48.20 |
| tca | S | 336 | 76.65 |
| tcg | S | 8 | 21.09 |
| agt | S | 0 | 76.12 |
| agc | S | 0 | 68.93 |
| --- | -- | ------ | ------ |
| mPD | | 0.057 | 1.7 |
| | | nPD: | 0.03 |
| | | N. weight: | 2. |
| | | Sc. PD: | -0.39 |
| | | Sc. rank: | -226.3 |

,

| NP | | | |
|---|---|---|---|
| Pos. | 368 | obs: | exp: |
| tct | S | 0 | 0.16 |
| tcc | S | 0 | 0.14 |
| tca | S | 0 | 0.22 |
| tcg | S | 0 | 0.06 |
| aat | N | 328 | 180.30 |
| aac | N | 17 | 164.70 |
| agt | S | 1 | 0.22 |
| agc | S | 0 | 0.20 |
| --- | -- | ------ | ------ |
| mPD | | 0.099 | 0.51 |
| | | nPD: | 0.2 |
| | | N. weight: | 0.67 |
| | | Sc. PD: | -0.027 |
| | | Sc. rank: | 8.1 |

,

| NP | | | |
|---|---|---|---|
| Pos. | 369 | obs: | exp: |
| gaa | E | 20 | 199.10 |
| gag | E | 326 | 146.90 |
| --- | -- | ------ | ------ |
| mPD | | 0.11 | 0.49 |
| | | nPD: | 0.22 |
| | | N. weight: | 0.9 |
| | | Sc. PD: | -0.012 |
| | | Sc. rank: | 25.1 |

,

| NP | | | |
|---|---|---|---|
| Pos. | 370 | obs: | exp: |
| aat | N | 22 | 180.80 |
| aac | N | 324 | 165.20 |
| --- | -- | ------ | ------ |
| mPD | | 0.12 | 0.50 |
| | | nPD: | 0.24 |
| | | N. weight: | 0.72 |
| | | Sc. PD: | 0.0016 |
| | | Sc. rank: | 29.7 |

| NP | | | |
|---|---|---|---|
| Pos. | 371 | obs: | exp: |
| att | I | 0 | 10.90 |
| atc | I | 0 | 7.60 |
| ata | I | 29 | 10.50 |
| atg | M | 316 | 316.00 |
| gtt | V | 0 | 0.21 |
| gtc | V | 0 | 0.21 |
| gta | V | 0 | 0.19 |
| gtg | V | 1 | 0.40 |
| --- | -- | ------ | ------ |
| mPD | | 0.16 | 0.17 |
| | | nPD: | 0.96 |
| | | N. weight: | 0.13 |
| | | Sc. PD: | 0.087 |
| | | Sc. rank: | 26.2 |

| NP | | | |
|---|---|---|---|
| Pos. | 372 | obs: | exp: |
| gat | D | 2 | 1.07 |
| gac | D | 0 | 0.93 |
| gaa | E | 286 | 197.90 |
| gag | E | 58 | 146.10 |
| --- | -- | ------ | ------ |
| mPD | | 0.29 | 0.49 |
| | | nPD: | 0.59 |
| | | N. weight: | 0.23 |
| | | Sc. PD: | 0.077 |
| | | Sc. rank: | 38.6 |

| NP | | | |
|---|---|---|---|
| Pos. | 373 | obs: | exp: |
| act | T | 1 | 83.22 |
| acc | T | 5 | 67.59 |
| aca | T | 300 | 138.00 |
| acg | T | 9 | 26.19 |
| gct | A | 0 | 7.81 |
| gcc | A | 0 | 6.35 |
| gca | A | 31 | 13.82 |
| gcg | A | 0 | 3.03 |
| --- | -- | ------ | ------ |
| mPD | | 0.25 | 0.85 |
| | | nPD: | 0.29 |
| | | N. weight: | 0.98 |
| | | Sc. PD: | 0.051 |
| | | Sc. rank: | 67.7 |

| NP | | | |
|---|---|---|---|
| Pos. | 374 | obs: | exp: |
| att | I | 0 | 1.13 |
| atc | I | 0 | 0.79 |
| ata | I | 3 | 1.09 |
| atg | M | 342 | 342.00 |
| gtt | V | 0 | 0.21 |
| gtc | V | 0 | 0.21 |
| gta | V | 0 | 0.19 |
| gtg | V | 1 | 0.40 |
| --- | -- | ------ | ------ |
| mPD | | 0.023 | 0.026 |
| | | nPD: | 0.87 |
| | | N. weight: | 0.016 |
| | | Sc. PD: | 0.01 |
| | | Sc. rank: | 3.4 |

| NP | | | |
|---|---|---|---|
| Pos. | 375 | obs: | exp: |
| gat | D | 25 | 180.30 |
| gac | D | 313 | 157.70 |
| gaa | E | 4 | 4.60 |
| gag | E | 4 | 3.40 |
| --- | -- | ------ | ------ |
| mPD | | 0.18 | 0.52 |
| | | nPD: | 0.34 |
| | | N. weight: | 0.7 |
| | | Sc. PD: | 0.069 |
| | | Sc. rank: | 66.4 |

| NP | | | |
|---|---|---|---|
| Pos. | 376 | obs: | exp: |
| tct | S | 11 | 55.01 |
| tcc | S | 335 | 48.20 |
| tca | S | 0 | 76.65 |
| tcg | S | 0 | 21.09 |
| agt | S | 0 | 76.12 |
| agc | S | 0 | 68.93 |
| --- | -- | ------ | ------ |
| mPD | | 0.062 | 1.7 |
| | | nPD: | 0.04 |
| | | N. weight: | 2.6 |
| | | Sc. PD: | -0.5 |
| | | Sc. rank: | -282.6 |

| NP | | | |
|---|---|---|---|
| Pos. | 377 | obs: | exp: |
| tct | S | 0 | 49.12 |
| tcc | S | 0 | 43.05 |
| tca | S | 0 | 68.46 |
| tcg | S | 0 | 18.83 |
| tgt | C | 0 | 0.82 |
| tgc | C | 2 | 1.18 |
| aat | N | 2 | 18.29 |
| aac | N | 33 | 16.71 |
| agt | S | 91 | 67.98 |
| agc | S | 218 | 61.56 |
| --- | -- | ------ | ------ |
| mPD | | 0.59 | 1.8 |
| | | nPD: | 0.34 |
| | | N. weight: | 1.3 |
| | | Sc. PD: | 0.13 |
| | | Sc. rank: | 126.7 |

| NP | | | |
|---|---|---|---|
| Pos. | 378 | obs: | exp: |
| act | T | 339 | 91.41 |
| acc | T | 6 | 74.25 |
| aca | T | 1 | 151.60 |
| acg | T | 0 | 28.76 |
| --- | -- | ------ | ------ |
| mPD | | 0.040 | 0.69 |
| | | nPD: | 0.06 |
| | | N. weight: | 1.8 |
| | | Sc. PD: | -0.3 |
| | | Sc. rank: | -136.3 |

| NP | | | |
|---|---|---|---|
| Pos. | 379 | obs: | exp: |
| tta | L | 0 | 30.23 |
| ttg | L | 0 | 66.64 |
| ctt | L | 331 | 64.56 |
| ctc | L | 9 | 55.97 |
| cta | L | 6 | 52.12 |
| ctg | L | 0 | 76.48 |
| --- | -- | ------ | ------ |
| mPD | | 0.084 | 1.1 |
| | | nPD: | 0.08 |
| | | N. weight: | 2.1 |
| | | Sc. PD: | -0.33 |
| | | Sc. rank: | -142.8 |

| NP | | | |
|---|---|---|---|
| Pos. | 380 | obs: | exp: |
| gaa | E | 341 | 199.10 |
| gag | E | 5 | 146.90 |
| --- | -- | ------ | ------ |
| mPD | | 0.029 | 0.49 |
| | | nPD: | 0.06 |
| | | N. weight: | 0.7 |
| | | Sc. PD: | -0.12 |
| | | Sc. rank: | -53.3 |

| NP | | | |
|---|---|---|---|
| Pos. | 381 | obs: | exp: |
| tta | L | 4 | 30.23 |
| ttg | L | 46 | 66.64 |
| ctt | L | 0 | 64.56 |
| ctc | L | 0 | 55.97 |
| cta | L | 7 | 52.12 |
| ctg | L | 289 | 76.48 |
| --- | -- | ------ | ------ |
| mPD | | 0.31 | 1.1 |
| | | nPD: | 0.28 |
| | | N. weight: | 1.5 |
| | | Sc. PD: | 0.057 |
| | | Sc. rank: | 87.5 |

| NP | | | |
|---|---|---|---|
| Pos. | 382 | obs: | exp: |
| cgt | R | 0 | 14.36 |
| cgc | R | 0 | 16.94 |
| cga | R | 0 | 33.25 |
| cgg | R | 0 | 28.87 |
| aga | R | 203 | 155.60 |
| agg | R | 143 | 96.96 |
| --- | -- | ------ | ------ |
| mPD | | 0.49 | 0.96 |
| | | nPD: | 0.51 |
| | | N. weight: | 0.47 |
| | | Sc. PD: | 0.12 |
| | | Sc. rank: | 69.1 |

| NP | | | |
|---|---|---|---|
| Pos. | 383 | obs: | exp: |
| tct | S | 0 | 55.01 |
| tcc | S | 0 | 48.20 |
| tca | S | 0 | 76.65 |
| tcg | S | 0 | 21.09 |
| agt | S | 35 | 76.12 |
| agc | S | 311 | 68.93 |
| --- | -- | ------ | ------ |
| mPD | | 0.18 | 1.7 |
| | | nPD: | 0.11 |
| | | N. weight: | 1.8 |
| | | Sc. PD: | -0.22 |
| | | Sc. rank: | -66.5 |

| NP | | | |
|---|---|---|---|
| Pos. | 384 | obs: | exp: |
| caa | Q | 4 | 2.13 |
| cag | Q | 0 | 1.87 |
| cgt | R | 0 | 13.95 |
| cgc | R | 0 | 16.45 |
| cga | R | 0 | 32.29 |
| cgg | R | 0 | 28.04 |
| aaa | K | 3 | 3.36 |
| aag | K | 3 | 2.64 |
| aga | R | 299 | 151.10 |
| agg | R | 37 | 94.16 |
| --- | -- | ------ | ------ |
| mPD | | 0.28 | 1.0 |
| | | nPD: | 0.28 |
| | | N. weight: | 0.73 |
| | | Sc. PD: | 0.029 |
| | | Sc. rank: | 44.2 |

| NP | | | |
|---|---|---|---|
| Pos. | 385 | obs: | exp: |
| tat | Y | 266 | 179.10 |
| tac | Y | 80 | 166.90 |
| --- | -- | ------ | ------ |
| mPD | | 0.36 | 0.50 |
| | | nPD: | 0.71 |
| | | N. weight: | 0.2 |
| | | Sc. PD: | 0.093 |
| | | Sc. rank: | 38.6 |

| NP | | | |
|---|---|---|---|
| Pos. | 386 | obs: | exp: |
| tgg | W | 346 | 346.00 |
| --- | -- | ------ | ------ |
| mPD | | 0 | 0 |
| | | nPD: | 1. |
| | | N. weight: | 0. |
| | | Sc. PD: | 0 |
| | | Sc. rank: | 0 |

| NP | | | |
|---|---|---|---|
| Pos. | 387 | obs: | exp: |
| gct | A | 295 | 87.15 |
| gcc | A | 49 | 70.88 |
| gca | A | 1 | 154.20 |
| gcg | A | 1 | 33.76 |
| --- | -- | ------ | ------ |
| mPD | | 0.25 | 0.69 |
| | | nPD: | 0.37 |
| | | N. weight: | 1.4 |
| | | Sc. PD: | 0.18 |
| | | Sc. rank: | 149.2 |

| NP | | | |
|---|---|---|---|
| Pos. | 388 | obs: | exp: |
| att | I | 0 | 130.10 |
| atc | I | 0 | 90.68 |
| ata | I | 346 | 125.20 |
| --- | -- | ------ | ------ |
| mPD | | 0 | 0.66 |
| | | nPD: | 0. |
| | | N. weight: | 1.5 |
| | | Sc. PD: | -0.33 |
| | | Sc. rank: | -301.9 |

| NP | | | |
|---|---|---|---|
| Pos. | 389 | obs: | exp: |
| cgt | R | 0 | 14.16 |
| cgc | R | 0 | 16.70 |
| cga | R | 4 | 32.77 |
| cgg | R | 0 | 28.46 |
| aaa | K | 1 | 2.80 |
| aag | K | 4 | 2.20 |
| aga | R | 218 | 153.40 |
| agg | R | 119 | 95.56 |
| --- | -- | ------ | ------ |
| mPD | | 0.51 | 0.98 |
| | | nPD: | 0.52 |
| | | N. weight: | 0.42 |
| | | Sc. PD: | 0.11 |
| | | Sc. rank: | 63.4 |

| NP | | | |
|---|---|---|---|
| Pos. | 390 | obs: | exp: |
| act | T | 1 | 91.41 |
| acc | T | 318 | 74.25 |
| aca | T | 27 | 151.60 |
| acg | T | 0 | 28.76 |
| --- | -- | ------ | ------ |
| mPD | | 0.15 | 0.69 |
| | | nPD: | 0.22 |
| | | N. weight: | 1.7 |
| | | Sc. PD: | -0.031 |
| | | Sc. rank: | 47.1 |

| NP | | | |
|---|---|---|---|
| Pos. | 391 | obs: | exp: |
| cgt | R | 0 | 14.28 |
| cgc | R | 0 | 16.84 |
| cga | R | 0 | 33.06 |
| cgg | R | 0 | 28.71 |
| aaa | K | 0 | 1.12 |
| aag | K | 2 | 0.88 |
| aga | R | 123 | 154.70 |
| agg | R | 221 | 96.40 |
| --- | -- | ------ | ------ |
| mPD | | 0.47 | 0.97 |
| | | nPD: | 0.49 |
| | | N. weight: | 0.66 |
| | | Sc. PD: | 0.16 |
| | | Sc. rank: | 94.7 |

| NP | | | |
|---|---|---|---|
| Pos. | 392 | obs: | exp: |
| tct | S | 0 | 55.01 |
| tcc | S | 0 | 48.20 |
| tca | S | 0 | 76.65 |
| tcg | S | 0 | 21.09 |
| agt | S | 237 | 76.12 |
| agc | S | 109 | 68.93 |
| --- | -- | ------ | ------ |
| mPD | | 0.43 | 1.7 |
| | | nPD: | 0.26 |
| | | N. weight: | 1.3 |
| | | Sc. PD: | 0.03 |
| | | Sc. rank: | 69.4 |

| NP | | | |
|---|---|---|---|
| Pos. | 393 | obs: | exp: |
| ggt | G | 6 | 42.69 |
| ggc | G | 0 | 46.79 |
| gga | G | 335 | 167.40 |
| ggg | G | 5 | 89.14 |
| --- | -- | ------ | ------ |
| mPD | | 0.062 | 0.67 |
| | | nPD: | 0.09 |
| | | N. weight: | 0.87 |
| | | Sc. PD: | -0.12 |
| | | Sc. rank: | -46.6 |

| NP | | | |
|---|---|---|---|
| Pos. | 394 | obs: | exp: |
| ggt | G | 27 | 42.69 |
| ggc | G | 0 | 46.79 |
| gga | G | 276 | 167.40 |
| ggg | G | 43 | 89.14 |
| --- | -- | ------ | ------ |
| mPD | | 0.34 | 0.67 |
| | | nPD: | 0.52 |
| | | N. weight: | 0.41 |
| | | Sc. PD: | 0.11 |
| | | Sc. rank: | 62.0 |

| NP | | | |
|---|---|---|---|
| Pos. | 395 | obs: | exp: |
| tct | S | 0 | 0.32 |
| tcc | S | 0 | 0.28 |
| tca | S | 0 | 0.44 |
| tcg | S | 0 | 0.12 |
| act | T | 0 | 0.26 |
| acc | T | 1 | 0.21 |
| aca | T | 0 | 0.44 |
| acg | T | 0 | 0.08 |
| aat | N | 11 | 179.30 |
| aac | N | 332 | 163.70 |
| agt | S | 0 | 0.44 |
| agc | S | 2 | 0.40 |
| --- | -- | ------ | ------ |
| mPD | | 0.079 | 0.53 |
| | | nPD: | 0.15 |
| | | N. weight: | 0.87 |
| | | Sc. PD: | -0.072 |
| | | Sc. rank: | -7.8 |

| NP | | | |
|---|---|---|---|
| Pos. | 396 | obs: | exp: |
| act | T | 74 | 91.41 |
| acc | T | 269 | 74.25 |
| aca | T | 3 | 151.60 |
| acg | T | 0 | 28.76 |
| --- | -- | ------ | ------ |
| mPD | | 0.35 | 0.69 |
| | | nPD: | 0.51 |
| | | N. weight: | 1.3 |
| | | Sc. PD: | 0.35 |
| | | Sc. rank: | 200.8 |

| NP | | | |
|---|---|---|---|
| Pos. | 397 | obs: | exp: |
| aat | N | 54 | 180.80 |
| aac | N | 292 | 165.20 |
| --- | -- | ------ | ------ |
| mPD | | 0.26 | 0.50 |
| | | nPD: | 0.53 |
| | | N. weight: | 0.43 |
| | | Sc. PD: | 0.12 |
| | | Sc. rank: | 65.7 |

| NP | | | |
|---|---|---|---|
| Pos. | 398 | obs: | exp: |
| caa | Q | 284 | 184.50 |
| cag | Q | 62 | 161.50 |
| --- | -- | ------ | ------ |
| mPD | | 0.30 | 0.50 |
| | | nPD: | 0.59 |
| | | N. weight: | 0.27 |
| | | Sc. PD: | 0.093 |
| | | Sc. rank: | 46.0 |

| NP | | | |
|---|---|---|---|
| Pos. | 399 | obs: | exp: |
| caa | Q | 61 | 182.90 |
| cag | Q | 282 | 160.10 |
| aaa | K | 0 | 1.68 |
| aag | K | 3 | 1.32 |
| --- | -- | ------ | ------ |
| mPD | | 0.31 | 0.51 |
| | | nPD: | 0.6 |
| | | N. weight: | 0.41 |
| | | Sc. PD: | 0.14 |
| | | Sc. rank: | 68.3 |

| NP | | | |
|---|---|---|---|
| Pos. | 400 | obs: | exp: |
| cgt | R | 0 | 14.24 |
| cgc | R | 0 | 16.79 |
| cga | R | 2 | 32.96 |
| cgg | R | 1 | 28.62 |
| aaa | K | 3 | 1.68 |
| aag | K | 0 | 1.32 |
| aga | R | 298 | 154.30 |
| agg | R | 42 | 96.12 |
| --- | -- | ------ | ------ |
| mPD | | 0.25 | 0.97 |
| | | nPD: | 0.26 |
| | | N. weight: | 0.66 |
| | | Sc. PD: | 0.014 |
| | | Sc. rank: | 33.2 |

| NP | | | |
|---|---|---|---|
| Pos. | 401 | obs: | exp: |
| tct | S | 0 | 0.32 |
| tcc | S | 0 | 0.28 |
| tca | S | 2 | 0.44 |
| tcg | S | 0 | 0.12 |
| agt | S | 0 | 0.44 |
| agc | S | 0 | 0.40 |
| gct | A | 2 | 86.64 |
| gcc | A | 0 | 70.47 |
| gca | A | 337 | 153.30 |
| gcg | A | 5 | 33.57 |
| --- | -- | ------ | ------ |
| mPD | | 0.051 | 0.70 |
| | | nPD: | 0.07 |
| | | N. weight: | 1.1 |
| | | Sc. PD: | -0.17 |
| | | Sc. rank: | -72.4 |

| NP | | | |
|---|---|---|---|
| Pos. | 402 | obs: | exp: |
| tct | S | 310 | 55.01 |
| tcc | S | 30 | 48.20 |
| tca | S | 1 | 76.65 |
| tcg | S | 5 | 21.09 |
| agt | S | 0 | 76.12 |
| agc | S | 0 | 68.93 |
| --- | -- | ------ | ------ |
| mPD | | 0.19 | 1.7 |
| | | nPD: | 0.11 |
| | | N. weight: | 2.1 |
| | | Sc. PD: | -0.25 |
| | | Sc. rank: | -69.5 |

| NP | | | |
|---|---|---|---|
| Pos. | 403 | obs: | exp: |
| gct | A | 0 | 87.15 |
| gcc | A | 0 | 70.88 |
| gca | A | 344 | 154.20 |
| gcg | A | 2 | 33.76 |
| --- | -- | ------ | ------ |
| mPD | | 0.012 | 0.69 |
| | | nPD: | 0.02 |
| | | N. weight: | 1.1 |
| | | Sc. PD: | -0.24 |
| | | Sc. rank: | -162.2 |

| NP | | | |
|---|---|---|---|
| Pos. | 404 | obs: | exp: |
| ggt | G | 0 | 42.69 |
| ggc | G | 0 | 46.79 |
| gga | G | 293 | 167.40 |
| ggg | G | 53 | 89.14 |
| --- | -- | ------ | ------ |
| mPD | | 0.26 | 0.67 |
| | | nPD: | 0.39 |
| | | N. weight: | 0.58 |
| | | Sc. PD: | 0.085 |
| | | Sc. rank: | 66.2 |

| NP | | | |
|---|---|---|---|
| Pos. | 405 | obs: | exp: |
| caa | Q | 256 | 184.50 |
| cag | Q | 90 | 161.50 |
| --- | -- | ------ | ------ |
| mPD | | 0.39 | 0.50 |
| | | nPD: | 0.78 |
| | | N. weight: | 0.14 |
| | | Sc. PD: | 0.073 |
| | | Sc. rank: | 28.5 |

| NP | | | |
|---|---|---|---|
| Pos. | 406 | obs: | exp: |
| att | I | 4 | 119.20 |
| atc | I | 311 | 83.08 |
| ata | I | 2 | 114.70 |
| gtt | V | 0 | 5.96 |
| gtc | V | 29 | 6.09 |
| gta | V | 0 | 5.44 |
| gtg | V | 0 | 11.52 |
| --- | -- | ------ | ------ |
| mPD | | 0.19 | 0.83 |
| | | nPD: | 0.23 |
| | | N. weight: | 1.8 |
| | | Sc. PD: | -0.02 |
| | | Sc. rank: | 55.5 |

| NP | | | |
|---|---|---|---|
| Pos. | 407 | obs: | exp: |
| tct | S | 0 | 55.01 |
| tcc | S | 0 | 48.20 |
| tca | S | 0 | 76.65 |
| tcg | S | 0 | 21.09 |
| agt | S | 237 | 76.12 |
| agc | S | 109 | 68.93 |
| --- | -- | ------ | ------ |
| mPD | | 0.43 | 1.7 |
| | | nPD: | 0.26 |
| | | N. weight: | 1.3 |
| | | Sc. PD: | 0.03 |
| | | Sc. rank: | 69.4 |

| NP | | | |
|---|---|---|---|
| Pos. | 408 | obs: | exp: |
| gtt | V | 29 | 71.12 |
| gtc | V | 1 | 72.62 |
| gta | V | 272 | 64.85 |
| gtg | V | 44 | 137.40 |
| --- | -- | ------ | ------ |
| mPD | | 0.36 | 0.72 |
| | | nPD: | 0.5 |
| | | N. weight: | 1.3 |
| | | Sc. PD: | 0.33 |
| | | Sc. rank: | 191.8 |

| NP | | | |
|---|---|---|---|
| Pos. | 409 | obs: | exp: |
| caa | Q | 95 | 184.50 |
| cag | Q | 251 | 161.50 |
| --- | -- | ------ | ------ |
| mPD | | 0.40 | 0.50 |
| | | nPD: | 0.8 |
| | | N. weight: | 0.21 |
| | | Sc. PD: | 0.11 |
| | | Sc. rank: | 42.3 |

| NP | | | |
|---|---|---|---|
| Pos. | 410 | obs: | exp: |
| cct | P | 98 | 91.11 |
| ccc | P | 248 | 62.41 |
| cca | P | 0 | 136.50 |
| ccg | P | 0 | 55.94 |
| --- | -- | ------ | ------ |
| mPD | | 0.41 | 0.72 |
| | | nPD: | 0.57 |
| | | N. weight: | 1.5 |
| | | Sc. PD: | 0.46 |
| | | Sc. rank: | 235.4 |

| NP | | | |
|---|---|---|---|
| Pos. | 411 | obs: | exp: |
| act | T | 264 | 91.41 |
| acc | T | 18 | 74.25 |
| aca | T | 39 | 151.60 |
| acg | T | 25 | 28.76 |
| --- | -- | ------ | ------ |
| mPD | | 0.40 | 0.69 |
| | | nPD: | 0.58 |
| | | N. weight: | 0.85 |
| | | Sc. PD: | 0.28 |
| | | Sc. rank: | 140.1 |

| NP | | | |
|---|---|---|---|
| Pos. | 412 | obs: | exp: |
| ttt | F | 27 | 142.20 |
| ttc | F | 319 | 203.80 |
| --- | -- | ------ | ------ |
| mPD | | 0.14 | 0.48 |
| | | nPD: | 0.3 |
| | | N. weight: | 0.42 |
| | | Sc. PD: | 0.024 |
| | | Sc. rank: | 31.2 |

| NP | | | |
|---|---|---|---|
| Pos. | 413 | obs: | exp: |
| tct | S | 70 | 55.01 |
| tcc | S | 3 | 48.20 |
| tca | S | 119 | 76.65 |
| tcg | S | 154 | 21.09 |
| agt | S | 0 | 76.12 |
| agc | S | 0 | 68.93 |
| --- | -- | ------ | ------ |
| mPD | | 0.64 | 1.7 |
| | | nPD: | 0.39 |
| | | N. weight: | 1.6 |
| | | Sc. PD: | 0.22 |
| | | Sc. rank: | 177.1 |

| NP | | | |
|---|---|---|---|
| Pos. | 414 | obs: | exp: |
| att | I | 0 | 0.38 |
| atc | I | 0 | 0.26 |
| ata | I | 1 | 0.36 |
| gtt | V | 0 | 70.91 |
| gtc | V | 0 | 72.41 |
| gta | V | 290 | 64.66 |
| gtg | V | 55 | 137.00 |
| --- | -- | ------ | ------ |
| mPD | | 0.27 | 0.73 |
| | | nPD: | 0.38 |
| | | N. weight: | 1.6 |
| | | Sc. PD: | 0.21 |
| | | Sc. rank: | 179.6 |

| NP | | | |
|---|---|---|---|
| Pos. | 415 | obs: | exp: |
| caa | Q | 21 | 184.50 |
| cag | Q | 325 | 161.50 |
| --- | -- | ------ | ------ |
| mPD | | 0.11 | 0.50 |
| | | nPD: | 0.23 |
| | | N. weight: | 0.76 |
| | | Sc. PD: | -0.0054 |
| | | Sc. rank: | 26.1 |

| NP | | | |
|---|---|---|---|
| Pos. | 416 | obs: | exp: |
| cgt | R | 0 | 14.36 |
| cgc | R | 0 | 16.94 |
| cga | R | 0 | 33.25 |
| cgg | R | 0 | 28.87 |
| aga | R | 314 | 155.60 |
| agg | R | 32 | 96.96 |
| --- | -- | ------ | ------ |
| mPD | | 0.17 | 0.96 |
| | | nPD: | 0.18 |
| | | N. weight: | 0.78 |
| | | Sc. PD: | -0.046 |
| | | Sc. rank: | 1.4 |

| NP | | | |
|---|---|---|---|
| Pos. | 417 | obs: | exp: |
| tct | S | 0 | 1.43 |
| tcc | S | 0 | 1.25 |
| tca | S | 0 | 1.99 |
| tcg | S | 0 | 0.55 |
| aat | N | 239 | 176.10 |
| aac | N | 98 | 160.90 |
| agt | S | 2 | 1.98 |
| agc | S | 7 | 1.79 |
| --- | -- | ------ | ------ |
| mPD | | 0.47 | 0.59 |
| | | nPD: | 0.81 |
| | | N. weight: | 0.16 |
| | | Sc. PD: | 0.086 |
| | | Sc. rank: | 32.0 |

| NP | | | |
|---|---|---|---|
| Pos. | 418 | obs: | exp: |
| tta | L | 0 | 30.23 |
| ttg | L | 0 | 66.64 |
| ctt | L | 253 | 64.56 |
| ctc | L | 93 | 55.97 |
| cta | L | 0 | 52.12 |
| ctg | L | 0 | 76.48 |
| --- | -- | ------ | ------ |
| mPD | | 0.39 | 1.1 |
| | | nPD: | 0.35 |
| | | N. weight: | 1.6 |
| | | Sc. PD: | 0.18 |
| | | Sc. rank: | 168.1 |

| NP | | | |
|---|---|---|---|
| Pos. | 419 | obs: | exp: |
| cct | P | 46 | 91.11 |
| ccc | P | 228 | 62.41 |
| cca | P | 72 | 136.50 |
| ccg | P | 0 | 55.94 |
| --- | -- | ------ | ------ |
| mPD | | 0.51 | 0.72 |
| | | nPD: | 0.71 |
| | | N. weight: | 0.93 |
| | | Sc. PD: | 0.42 |
| | | Sc. rank: | 172.5 |

| | | | |
|---|---|---|---|
| NP | | | |
| Pos. | 420 | obs: | exp: |
| ttt | F | 61 | 142.20 |
| ttc | F | 285 | 203.80 |
| --- | -- | ------ | ------ |
| mPD | | 0.29 | 0.48 |
| | | nPD: | 0.6 |
| | | N. weight: | 0.19 |
| | | Sc. PD: | 0.068 |
| | | Sc. rank: | 32.7 |

,

| | | | |
|---|---|---|---|
| NP | | | |
| Pos. | 421 | obs: | exp: |
| gaa | E | 233 | 199.10 |
| gag | E | 113 | 146.90 |
| --- | -- | ------ | ------ |
| mPD | | 0.44 | 0.49 |
| | | nPD: | 0.9 |
| | | N. weight: | 0.042 |
| | | Sc. PD: | 0.027 |
| | | Sc. rank: | 8.7 |

,

| | | | |
|---|---|---|---|
| NP | | | |
| Pos. | 422 | obs: | exp: |
| cgt | R | 0 | 14.32 |
| cgc | R | 0 | 16.89 |
| cga | R | 8 | 33.16 |
| cgg | R | 0 | 28.79 |
| act | T | 0 | 0.26 |
| acc | T | 0 | 0.21 |
| aca | T | 1 | 0.44 |
| acg | T | 0 | 0.08 |
| aga | R | 331 | 155.20 |
| agg | R | 6 | 96.68 |
| --- | -- | ------ | ------ |
| mPD | | 0.085 | 0.97 |
| | | nPD: | 0.09 |
| | | N. weight: | 0.94 |
| | | Sc. PD: | -0.13 |
| | | Sc. rank: | -53.8 |

,

| | | | |
|---|---|---|---|
| NP | | | |
| Pos. | 423 | obs: | exp: |
| gct | A | 0 | 87.15 |
| gcc | A | 0 | 70.88 |
| gca | A | 152 | 154.20 |
| gcg | A | 194 | 33.76 |
| --- | -- | ------ | ------ |
| mPD | | 0.49 | 0.69 |
| | | nPD: | 0.72 |
| | | N. weight: | 1.4 |
| | | Sc. PD: | 0.65 |
| | | Sc. rank: | 268.9 |

,

| | | | |
|---|---|---|---|
| NP | | | |
| Pos. | 424 | obs: | exp: |
| act | T | 20 | 91.41 |
| acc | T | 324 | 74.25 |
| aca | T | 2 | 151.60 |
| acg | T | 0 | 28.76 |
| --- | -- | ------ | ------ |
| mPD | | 0.12 | 0.69 |
| | | nPD: | 0.18 |
| | | N. weight: | 1.8 |
| | | Sc. PD: | -0.11 |
| | | Sc. rank: | 2.2 |

,

| | | | |
|---|---|---|---|
| NP | | | |
| Pos. | 425 | obs: | exp: |
| att | I | 289 | 130.10 |
| atc | I | 55 | 90.68 |
| ata | I | 2 | 125.20 |
| --- | -- | ------ | ------ |
| mPD | | 0.28 | 0.66 |
| | | nPD: | 0.42 |
| | | N. weight: | 0.82 |
| | | Sc. PD: | 0.15 |
| | | Sc. rank: | 99.8 |

,

| | | | |
|---|---|---|---|
| NP | | | |
| Pos. | 426 | obs: | exp: |
| atg | M | 346 | 346.00 |
| --- | -- | ------ | ------ |
| mPD | | 0 | 0 |
| | | nPD: | 1. |
| | | N. weight: | 0. |
| | | Sc. PD: | 0 |
| | | Sc. rank: | 0 |

,

| | | | |
|---|---|---|---|
| NP | | | |
| Pos. | 427 | obs: | exp: |
| gct | A | 10 | 87.15 |
| gcc | A | 0 | 70.88 |
| gca | A | 91 | 154.20 |
| gcg | A | 245 | 33.76 |
| --- | -- | ------ | ------ |
| mPD | | 0.43 | 0.69 |
| | | nPD: | 0.63 |
| | | N. weight: | 1.7 |
| | | Sc. PD: | 0.65 |
| | | Sc. rank: | 302.3 |

,

| | | | |
|---|---|---|---|
| NP | | | |
| Pos. | 428 | obs: | exp: |
| gct | A | 2 | 87.15 |
| gcc | A | 1 | 70.88 |
| gca | A | 178 | 154.20 |
| gcg | A | 165 | 33.76 |
| --- | -- | ------ | ------ |
| mPD | | 0.51 | 0.69 |
| | | nPD: | 0.74 |
| | | N. weight: | 1.2 |
| | | Sc. PD: | 0.56 |
| | | Sc. rank: | 226.0 |

,

| | | | |
|---|---|---|---|
| NP | | | |
| Pos. | 429 | obs: | exp: |
| ttt | F | 114 | 142.20 |
| ttc | F | 232 | 203.80 |
| --- | -- | ------ | ------ |
| mPD | | 0.44 | 0.48 |
| | | nPD: | 0.92 |
| | | N. weight: | 0.033 |
| | | Sc. PD: | 0.021 |
| | | Sc. rank: | 6.9 |

,

| | | | |
|---|---|---|---|
| NP | | | |
| Pos. | 430 | obs: | exp: |
| act | T | 3 | 91.41 |
| acc | T | 18 | 74.25 |
| aca | T | 317 | 151.60 |
| acg | T | 8 | 28.76 |
| --- | -- | ------ | ------ |
| mPD | | 0.16 | 0.69 |
| | | nPD: | 0.23 |
| | | N. weight: | 0.8 |
| | | Sc. PD: | -0.0053 |
| | | Sc. rank: | 28.1 |

,

| | | | |
|---|---|---|---|
| NP | | | |
| Pos. | 431 | obs: | exp: |
| ggt | G | 0 | 42.69 |
| ggc | G | 2 | 46.79 |
| gga | G | 167 | 167.40 |
| ggg | G | 177 | 89.14 |
| --- | -- | ------ | ------ |
| mPD | | 0.51 | 0.67 |
| | | nPD: | 0.76 |
| | | N. weight: | 0.49 |
| | | Sc. PD: | 0.25 |
| | | Sc. rank: | 97.8 |

,

| | | | |
|---|---|---|---|
| NP | | | |
| Pos. | 432 | obs: | exp: |
| tct | S | 0 | 0.16 |
| tcc | S | 0 | 0.14 |
| tca | S | 0 | 0.22 |
| tcg | S | 0 | 0.06 |
| aat | N | 238 | 180.30 |
| aac | N | 107 | 164.70 |
| agt | S | 1 | 0.22 |
| agc | S | 0 | 0.20 |
| --- | -- | ------ | ------ |
| mPD | | 0.43 | 0.51 |
| | | nPD: | 0.85 |
| | | N. weight: | 0.1 |
| | | Sc. PD: | 0.06 |
| | | Sc. rank: | 20.7 |

,

| | | | |
|---|---|---|---|
| NP | | | |
| Pos. | 433 | obs: | exp: |
| act | T | 329 | 90.09 |
| acc | T | 7 | 73.17 |
| aca | T | 1 | 149.40 |
| acg | T | 4 | 28.35 |
| gct | A | 5 | 1.26 |
| gcc | A | 0 | 1.02 |
| gca | A | 0 | 2.23 |
| gcg | A | 0 | 0.49 |
| --- | -- | ------ | ------ |
| mPD | | 0.096 | 0.71 |
| | | nPD: | 0.14 |
| | | N. weight: | 1.7 |
| | | Sc. PD: | -0.16 |
| | | Sc. rank: | -27.9 |

,

| | | | |
|---|---|---|---|
| NP | | | |
| Pos. | 434 | obs: | exp: |
| gaa | E | 182 | 199.10 |
| gag | E | 164 | 146.90 |
| --- | -- | ------ | ------ |
| mPD | | 0.50 | 0.49 |
| | | nPD: | 1.02 |
| | | N. weight: | 0.02 |
| | | Sc. PD: | 0.015 |
| | | Sc. rank: | 4.2 |

,

| | | | |
|---|---|---|---|
| NP | | | |
| Pos. | 435 | obs: | exp: |
| tct | S | 0 | 0.16 |
| tcc | S | 0 | 0.14 |
| tca | S | 0 | 0.22 |
| tcg | S | 0 | 0.06 |
| agt | S | 0 | 0.22 |
| agc | S | 1 | 0.20 |
| ggt | G | 34 | 42.57 |
| ggc | G | 289 | 46.66 |
| gga | G | 21 | 166.90 |
| ggg | G | 1 | 88.89 |
| --- | -- | ------ | ------ |
| mPD | | 0.29 | 0.68 |
| | | nPD: | 0.43 |
| | | N. weight: | 2. |
| | | Sc. PD: | 0.37 |
| | | Sc. rank: | 245.4 |

,

| | | | |
|---|---|---|---|
| NP | | | |
| Pos. | 436 | obs: | exp: |
| cgt | R | 0 | 14.36 |
| cgc | R | 0 | 16.94 |
| cga | R | 0 | 33.25 |
| cgg | R | 0 | 28.87 |
| aga | R | 306 | 155.60 |
| agg | R | 40 | 96.96 |
| --- | -- | ------ | ------ |
| mPD | | 0.21 | 0.96 |
| | | nPD: | 0.21 |
| | | N. weight: | 0.72 |
| | | Sc. PD: | -0.016 |
| | | Sc. rank: | 17.5 |

,

| | | | |
|---|---|---|---|
| NP | | | |
| Pos. | 437 | obs: | exp: |
| att | I | 0 | 0.75 |
| atc | I | 0 | 0.52 |
| ata | I | 2 | 0.72 |
| act | T | 71 | 90.88 |
| acc | T | 2 | 73.82 |
| aca | T | 240 | 150.70 |
| acg | T | 31 | 28.60 |
| --- | -- | ------ | ------ |
| mPD | | 0.47 | 0.70 |
| | | nPD: | 0.68 |
| | | N. weight: | 0.41 |
| | | Sc. PD: | 0.17 |
| | | Sc. rank: | 74.6 |

,

| | | | |
|---|---|---|---|
| NP | | | |
| Pos. | 438 | obs: | exp: |
| tct | S | 210 | 55.01 |
| tcc | S | 49 | 48.20 |
| tca | S | 85 | 76.65 |
| tcg | S | 2 | 21.09 |
| agt | S | 0 | 76.12 |
| agc | S | 0 | 68.93 |
| --- | -- | ------ | ------ |
| mPD | | 0.55 | 1.7 |
| | | nPD: | 0.33 |
| | | N. weight: | 1.2 |
| | | Sc. PD: | 0.11 |
| | | Sc. rank: | 109.7 |

,

| | | | |
|---|---|---|---|
| NP | | | |
| Pos. | 439 | obs: | exp: |
| gat | D | 2 | 184.60 |
| gac | D | 344 | 161.40 |
| --- | -- | ------ | ------ |
| mPD | | 0.012 | 0.50 |
| | | nPD: | 0.02 |
| | | N. weight: | 1. |
| | | Sc. PD: | -0.21 |
| | | Sc. rank: | -135.4 |

,

| | | | |
|---|---|---|---|
| NP | | | |
| Pos. | 440 | obs: | exp: |
| atg | M | 346 | 346.00 |
| --- | -- | ------ | ------ |
| mPD | | 0 | 0 |
| | | nPD: | 1. |
| | | N. weight: | 0. |
| | | Sc. PD: | 0 |
| | | Sc. rank: | 0 |

,

| | | | |
|---|---|---|---|
| NP | | | |
| Pos. | 441 | obs: | exp: |
| cgt | R | 0 | 14.36 |
| cgc | R | 0 | 16.94 |
| cga | R | 0 | 33.25 |
| cgg | R | 2 | 28.87 |
| aga | R | 84 | 155.60 |
| agg | R | 260 | 96.96 |
| --- | -- | ------ | ------ |
| mPD | | 0.38 | 0.96 |
| | | nPD: | 0.4 |
| | | N. weight: | 0.84 |
| | | Sc. PD: | 0.13 |
| | | Sc. rank: | 97.7 |

,

| | | | |
|---|---|---|---|
| NP | | | |
| Pos. | 442 | obs: | exp: |
| tct | S | 0 | 0.16 |
| tcc | S | 0 | 0.14 |
| tca | S | 0 | 0.22 |
| tcg | S | 0 | 0.06 |
| act | T | 260 | 91.15 |
| acc | T | 60 | 74.03 |
| aca | T | 25 | 151.10 |
| acg | T | 0 | 28.68 |
| agt | S | 1 | 0.22 |
| agc | S | 0 | 0.20 |
| --- | -- | ------ | ------ |
| mPD | | 0.40 | 0.69 |
| | | nPD: | 0.58 |
| | | N. weight: | 0.92 |
| | | Sc. PD: | 0.3 |
| | | Sc. rank: | 152.5 |

,

| | | | |
|---|---|---|---|
| NP | | | |
| Pos. | 443 | obs: | exp: |
| gaa | E | 282 | 199.10 |
| gag | E | 64 | 146.90 |
| --- | -- | ------ | ------ |
| mPD | | 0.30 | 0.49 |
| | | nPD: | 0.62 |
| | | N. weight: | 0.2 |
| | | Sc. PD: | 0.072 |
| | | Sc. rank: | 33.9 |

,

| | | | |
|---|---|---|---|
| NP | | | |
| Pos. | 444 | obs: | exp: |
| att | I | 9 | 130.10 |
| atc | I | 336 | 90.68 |
| ata | I | 1 | 125.20 |
| --- | -- | ------ | ------ |
| mPD | | 0.056 | 0.66 |
| | | nPD: | 0.09 |
| | | N. weight: | 1.7 |
| | | Sc. PD: | -0.25 |
| | | Sc. rank: | -103.6 |

,

| NP | | | |
|---|---|---|---|
| Pos. | 445 | obs: | exp: |
| att | I | 0 | 130.10 |
| atc | I | 1 | 90.68 |
| ata | I | 345 | 125.20 |
| mPD | | 0.0058 | 0.66 |
| | | nPD: | 0.01 |
| | | N. weight: | 1.4 |
| | | Sc. PD: | -0.31 |
| | | Sc. rank: | -240.5 |

| NP | | | |
|---|---|---|---|
| Pos. | 446 | obs: | exp: |
| cgt | R | 0 | 13.95 |
| cgc | R | 0 | 16.45 |
| cga | R | 0 | 32.29 |
| cgg | R | 0 | 28.04 |
| aaa | K | 10 | 5.60 |
| aag | K | 0 | 4.40 |
| aga | R | 263 | 151.10 |
| agg | R | 73 | 94.16 |
| mPD | | 0.39 | 1.0 |
| | | nPD: | 0.39 |
| | | N. weight: | 0.56 |
| | | Sc. PD: | 0.081 |
| | | Sc. rank: | 63.5 |

| NP | | | |
|---|---|---|---|
| Pos. | 447 | obs: | exp: |
| atg | M | 341 | 341.00 |
| act | T | 0 | 1.32 |
| acc | T | 0 | 1.07 |
| aca | T | 0 | 2.19 |
| acg | T | 5 | 0.42 |
| mPD | | 0.029 | 0.055 |
| | | nPD: | 0.52 |
| | | N. weight: | 0.052 |
| | | Sc. PD: | 0.014 |
| | | Sc. rank: | 7.8 |

| NP | | | |
|---|---|---|---|
| Pos. | 448 | obs: | exp: |
| atg | M | 346 | 346.00 |
| mPD | | 0 | 0 |
| | | nPD: | 1. |
| | | N. weight: | 0. |
| | | Sc. PD: | 0 |
| | | Sc. rank: | 0 |

| NP | | | |
|---|---|---|---|
| Pos. | 449 | obs: | exp: |
| gaa | E | 321 | 199.10 |
| gag | E | 25 | 146.90 |
| mPD | | 0.13 | 0.49 |
| | | nPD: | 0.28 |
| | | N. weight: | 0.46 |
| | | Sc. PD: | 0.017 |
| | | Sc. rank: | 26.9 |

| NP | | | |
|---|---|---|---|
| Pos. | 450 | obs: | exp: |
| tct | S | 0 | 42.13 |
| tcc | S | 0 | 36.92 |
| tca | S | 0 | 58.71 |
| tcg | S | 0 | 16.15 |
| aat | N | 80 | 41.81 |
| aac | N | 0 | 38.19 |
| agt | S | 265 | 58.30 |
| agc | S | 0 | 52.80 |
| ggt | G | 1 | 0.12 |
| ggc | G | 0 | 0.14 |
| gga | G | 0 | 0.48 |
| ggg | G | 0 | 0.26 |
| mPD | | 0.36 | 1.8 |
| | | nPD: | 0.2 |
| | | N. weight: | 1.9 |
| | | Sc. PD: | -0.065 |
| | | Sc. rank: | 28.8 |

| NP | | | |
|---|---|---|---|
| Pos. | 451 | obs: | exp: |
| gct | A | 3 | 87.15 |
| gcc | A | 343 | 70.88 |
| gca | A | 0 | 154.20 |
| gcg | A | 0 | 33.76 |
| mPD | | 0.017 | 0.69 |
| | | nPD: | 0.03 |
| | | N. weight: | 2.2 |
| | | Sc. PD: | -0.45 |
| | | Sc. rank: | -279.8 |

| NP | | | |
|---|---|---|---|
| Pos. | 452 | obs: | exp: |
| cgt | R | 0 | 13.82 |
| cgc | R | 0 | 16.31 |
| cga | R | 0 | 32.00 |
| cgg | R | 0 | 27.79 |
| aaa | K | 13 | 7.28 |
| aag | K | 0 | 5.72 |
| aga | R | 327 | 149.80 |
| agg | R | 6 | 93.32 |
| mPD | | 0.11 | 1.0 |
| | | nPD: | 0.1 |
| | | N. weight: | 1. |
| | | Sc. PD: | -0.13 |
| | | Sc. rank: | -42.5 |

| NP | | | |
|---|---|---|---|
| Pos. | 453 | obs: | exp: |
| tct | S | 0 | 1.27 |
| tcc | S | 0 | 1.11 |
| tca | S | 8 | 1.77 |
| tcg | S | 0 | 0.49 |
| cct | P | 67 | 88.21 |
| ccc | P | 1 | 60.43 |
| cca | P | 267 | 132.20 |
| ccg | P | 0 | 54.17 |
| cat | H | 2 | 1.08 |
| cac | H | 0 | 0.92 |
| caa | Q | 1 | 0.53 |
| cag | Q | 0 | 0.47 |
| agt | S | 0 | 1.76 |
| agc | S | 0 | 1.59 |
| mPD | | 0.39 | 0.80 |
| | | nPD: | 0.48 |
| | | N. weight: | 0.76 |
| | | Sc. PD: | 0.18 |
| | | Sc. rank: | 107.9 |

| NP | | | |
|---|---|---|---|
| Pos. | 454 | obs: | exp: |
| gaa | E | 311 | 195.60 |
| gag | E | 29 | 144.40 |
| ggt | G | 0 | 0.74 |
| ggc | G | 0 | 0.81 |
| gga | G | 5 | 2.90 |
| ggg | G | 1 | 1.55 |
| mPD | | 0.19 | 0.53 |
| | | nPD: | 0.37 |
| | | N. weight: | 0.43 |
| | | Sc. PD: | 0.053 |
| | | Sc. rank: | 44.4 |

| NP | | | |
|---|---|---|---|
| Pos. | 455 | obs: | exp: |
| gat | D | 339 | 184.60 |
| gac | D | 7 | 161.40 |
| mPD | | 0.040 | 0.50 |
| | | nPD: | 0.08 |
| | | N. weight: | 0.77 |
| | | Sc. PD: | -0.12 |
| | | Sc. rank: | -48.6 |

| NP | | | |
|---|---|---|---|
| Pos. | 456 | obs: | exp: |
| atg | M | 2 | 2.00 |
| gtt | V | 0 | 70.71 |
| gtc | V | 0 | 72.20 |
| gta | V | 0 | 64.47 |
| gtg | V | 344 | 136.60 |
| mPD | | 0.012 | 0.73 |
| | | nPD: | 0.02 |
| | | N. weight: | 1.3 |
| | | Sc. PD: | -0.28 |
| | | Sc. rank: | -198.0 |

| NP | | | |
|---|---|---|---|
| Pos. | 457 | obs: | exp: |
| tct | S | 266 | 55.01 |
| tcc | S | 42 | 48.20 |
| tca | S | 38 | 76.65 |
| tcg | S | 0 | 21.09 |
| agt | S | 0 | 76.12 |
| agc | S | 0 | 68.93 |
| mPD | | 0.38 | 1.7 |
| | | nPD: | 0.23 |
| | | N. weight: | 1.6 |
| | | Sc. PD: | -0.01 |
| | | Sc. rank: | 59.0 |

| NP | | | |
|---|---|---|---|
| Pos. | 458 | obs: | exp: |
| ttt | F | 0 | 142.20 |
| ttc | F | 346 | 203.80 |
| mPD | | 0 | 0.48 |
| | | nPD: | 0. |
| | | N. weight: | 0.76 |
| | | Sc. PD: | -0.17 |
| | | Sc. rank: | -157.3 |

| NP | | | |
|---|---|---|---|
| Pos. | 459 | obs: | exp: |
| caa | Q | 11 | 184.50 |
| cag | Q | 335 | 161.50 |
| mPD | | 0.062 | 0.50 |
| | | nPD: | 0.12 |
| | | N. weight: | 0.89 |
| | | Sc. PD: | -0.096 |
| | | Sc. rank: | -22.7 |

| NP | | | |
|---|---|---|---|
| Pos. | 460 | obs: | exp: |
| ggt | G | 0 | 42.69 |
| ggc | G | 0 | 46.79 |
| gga | G | 2 | 167.40 |
| ggg | G | 344 | 89.14 |
| mPD | | 0.012 | 0.67 |
| | | nPD: | 0.02 |
| | | N. weight: | 1.9 |
| | | Sc. PD: | -0.4 |
| | | Sc. rank: | -264.4 |

| NP | | | |
|---|---|---|---|
| Pos. | 461 | obs: | exp: |
| cgt | R | 0 | 14.36 |
| cgc | R | 0 | 16.94 |
| cga | R | 0 | 33.25 |
| cgg | R | 346 | 28.87 |
| aga | R | 0 | 155.60 |
| agg | R | 0 | 96.96 |
| mPD | | 0 | 0.96 |
| | | nPD: | 0. |
| | | N. weight: | 3.6 |
| | | Sc. PD: | -0.81 |
| | | Sc. rank: | -737.9 |

| NP | | | |
|---|---|---|---|
| Pos. | 462 | obs: | exp: |
| ggt | G | 0 | 42.69 |
| ggc | G | 0 | 46.79 |
| gga | G | 346 | 167.40 |
| ggg | G | 0 | 89.14 |
| mPD | | 0 | 0.67 |
| | | nPD: | 0. |
| | | N. weight: | 1. |
| | | Sc. PD: | -0.24 |
| | | Sc. rank: | -215.8 |

| NP | | | |
|---|---|---|---|
| Pos. | 463 | obs: | exp: |
| gtt | V | 1 | 71.12 |
| gtc | V | 345 | 72.62 |
| gta | V | 0 | 64.85 |
| gtg | V | 0 | 137.40 |
| mPD | | 0.0058 | 0.72 |
| | | nPD: | 0.01 |
| | | N. weight: | 2.2 |
| | | Sc. PD: | -0.48 |
| | | Sc. rank: | -386.1 |

| NP | | | |
|---|---|---|---|
| Pos. | 464 | obs: | exp: |
| ttt | F | 0 | 142.20 |
| ttc | F | 346 | 203.80 |
| mPD | | 0 | 0.48 |
| | | nPD: | 0. |
| | | N. weight: | 0.76 |
| | | Sc. PD: | -0.17 |
| | | Sc. rank: | -157.3 |

| NP | | | |
|---|---|---|---|
| Pos. | 465 | obs: | exp: |
| gaa | E | 0 | 199.10 |
| gag | E | 346 | 146.90 |
| mPD | | 0 | 0.49 |
| | | nPD: | 0. |
| | | N. weight: | 1.2 |
| | | Sc. PD: | -0.28 |
| | | Sc. rank: | -254.5 |

| NP | | | |
|---|---|---|---|
| Pos. | 466 | obs: | exp: |
| tta | L | 0 | 30.23 |
| ttg | L | 0 | 66.64 |
| ctt | L | 5 | 64.56 |
| ctc | L | 340 | 55.97 |
| cta | L | 0 | 52.12 |
| ctg | L | 1 | 76.48 |
| mPD | | 0.034 | 1.1 |
| | | nPD: | 0.03 |
| | | N. weight: | 2.5 |
| | | Sc. PD: | -0.49 |
| | | Sc. rank: | -295.3 |

| NP | | | |
|---|---|---|---|
| Pos. | 467 | obs: | exp: |
| tct | S | 0 | 55.01 |
| tcc | S | 0 | 48.20 |
| tca | S | 12 | 76.65 |
| tcg | S | 334 | 21.09 |
| agt | S | 0 | 76.12 |
| agc | S | 0 | 68.93 |
| mPD | | 0.067 | 1.7 |
| | | nPD: | 0.04 |
| | | N. weight: | 3.7 |
| | | Sc. PD: | -0.7 |
| | | Sc. rank: | -375.6 |

| NP | | | |
|---|---|---|---|
| Pos. | 468 | obs: | exp: |
| gat | D | 0 | 184.60 |
| gac | D | 346 | 161.40 |
| mPD | | 0 | 0.50 |
| | | nPD: | 0. |
| | | N. weight: | 1.1 |
| | | Sc. PD: | -0.25 |
| | | Sc. rank: | -226.6 |

| NP | | | |
|---|---|---|---|
| Pos. | 469 | obs: | exp: |
| gaa | E | 346 | 199.10 |
| gag | E | 0 | 146.90 |
| mPD | | 0 | 0.49 |
| | | nPD: | 0. |
| | | N. weight: | 0.79 |
| | | Sc. PD: | -0.18 |
| | | Sc. rank: | -164.2 |

| | | | |
|---|---|---|---|
| NP | | | |
| Pos. | 470 | obs: | exp: |
| aaa | K | 1 | 193.70 |
| aag | K | 345 | 152.30 |
| --- | -- | ------ | ------ |
| mPD | | 0.0058 | 0.49 |
| | | nPD: | 0.01 |
| | | N. weight: | 1.2 |
| | | Sc. PD: | -0.25 |
| | | Sc. rank: | -182.5 |

| | | | |
|---|---|---|---|
| NP | | | |
| Pos. | 471 | obs: | exp: |
| gct | A | 0 | 87.15 |
| gcc | A | 0 | 70.88 |
| gca | A | 323 | 154.20 |
| gcg | A | 23 | 33.76 |
| --- | -- | ------ | ------ |
| mPD | | 0.12 | 0.69 |
| | | nPD: | 0.18 |
| | | N. weight: | 0.96 |
| | | Sc. PD: | -0.051 |
| | | Sc. rank: | 3.4 |

| | | | |
|---|---|---|---|
| NP | | | |
| Pos. | 472 | obs: | exp: |
| act | T | 0 | 90.88 |
| acc | T | 0 | 73.82 |
| aca | T | 0 | 150.70 |
| acg | T | 344 | 28.60 |
| gct | A | 0 | 0.50 |
| gcc | A | 0 | 0.41 |
| gca | A | 0 | 0.89 |
| gcg | A | 2 | 0.20 |
| --- | -- | ------ | ------ |
| mPD | | 0.012 | 0.70 |
| | | nPD: | 0.02 |
| | | N. weight: | 3.6 |
| | | Sc. PD: | -0.75 |
| | | Sc. rank: | -518.3 |

| | | | |
|---|---|---|---|
| NP | | | |
| Pos. | 473 | obs: | exp: |
| aat | N | 0 | 180.30 |
| aac | N | 345 | 164.70 |
| gat | D | 0 | 0.53 |
| gac | D | 1 | 0.47 |
| --- | -- | ------ | ------ |
| mPD | | 0.0058 | 0.50 |
| | | nPD: | 0.01 |
| | | N. weight: | 1.1 |
| | | Sc. PD: | -0.23 |
| | | Sc. rank: | -172.6 |

| | | | |
|---|---|---|---|
| NP | | | |
| Pos. | 474 | obs: | exp: |
| cct | P | 0 | 91.11 |
| ccc | P | 0 | 62.41 |
| cca | P | 0 | 136.50 |
| ccg | P | 346 | 55.94 |
| --- | -- | ------ | ------ |
| mPD | | 0 | 0.72 |
| | | nPD: | 0. |
| | | N. weight: | 2.6 |
| | | Sc. PD: | -0.59 |
| | | Sc. rank: | -541.4 |

| | | | |
|---|---|---|---|
| NP | | | |
| Pos. | 475 | obs: | exp: |
| att | I | 0 | 130.10 |
| atc | I | 346 | 90.68 |
| ata | I | 0 | 125.20 |
| --- | -- | ------ | ------ |
| mPD | | 0 | 0.66 |
| | | nPD: | 0. |
| | | N. weight: | 1.9 |
| | | Sc. PD: | -0.43 |
| | | Sc. rank: | -397.9 |

| | | | |
|---|---|---|---|
| NP | | | |
| Pos. | 476 | obs: | exp: |
| gtt | V | 0 | 71.12 |
| gtc | V | 0 | 72.62 |
| gta | V | 0 | 64.85 |
| gtg | V | 346 | 137.40 |
| --- | -- | ------ | ------ |
| mPD | | 0 | 0.72 |
| | | nPD: | 0. |
| | | N. weight: | 1.3 |
| | | Sc. PD: | -0.3 |
| | | Sc. rank: | -274.4 |

| | | | |
|---|---|---|---|
| NP | | | |
| Pos. | 477 | obs: | exp: |
| cct | P | 345 | 91.11 |
| ccc | P | 1 | 62.41 |
| cca | P | 0 | 136.50 |
| ccg | P | 0 | 55.94 |
| --- | -- | ------ | ------ |
| mPD | | 0.0058 | 0.72 |
| | | nPD: | 0.01 |
| | | N. weight: | 1.9 |
| | | Sc. PD: | -0.41 |
| | | Sc. rank: | -326.6 |

| | | | |
|---|---|---|---|
| NP | | | |
| Pos. | 478 | obs: | exp: |
| ttt | F | 0 | 1.64 |
| ttc | F | 4 | 2.36 |
| tct | S | 7 | 54.37 |
| tcc | S | 335 | 47.64 |
| tca | S | 0 | 75.77 |
| tcg | S | 0 | 20.85 |
| agt | S | 0 | 75.24 |
| agc | S | 0 | 68.14 |
| --- | -- | ------ | ------ |
| mPD | | 0.063 | 1.7 |
| | | nPD: | 0.04 |
| | | N. weight: | 2.7 |
| | | Sc. PD: | -0.51 |
| | | Sc. rank: | -276.0 |

| | | | |
|---|---|---|---|
| NP | | | |
| Pos. | 479 | obs: | exp: |
| ttt | F | 342 | 141.80 |
| ttc | F | 3 | 203.20 |
| tta | L | 0 | 0.09 |
| ttg | L | 0 | 0.19 |
| ctt | L | 1 | 0.19 |
| ctc | L | 0 | 0.16 |
| cta | L | 0 | 0.15 |
| ctg | L | 0 | 0.22 |
| --- | -- | ------ | ------ |
| mPD | | 0.023 | 0.49 |
| | | nPD: | 0.05 |
| | | N. weight: | 1.2 |
| | | Sc. PD: | -0.22 |
| | | Sc. rank: | -113.6 |

| | | | |
|---|---|---|---|
| NP | | | |
| Pos. | 480 | obs: | exp: |
| aat | N | 0 | 0.52 |
| aac | N | 1 | 0.48 |
| gat | D | 0 | 184.10 |
| gac | D | 345 | 160.90 |
| --- | -- | ------ | ------ |
| mPD | | 0.0058 | 0.50 |
| | | nPD: | 0.01 |
| | | N. weight: | 1.1 |
| | | Sc. PD: | -0.24 |
| | | Sc. rank: | -177.0 |

| | | | |
|---|---|---|---|
| NP | | | |
| Pos. | 481 | obs: | exp: |
| atg | M | 346 | 346.00 |
| --- | -- | ------ | ------ |
| mPD | | 0 | 0 |
| | | nPD: | 1. |
| | | N. weight: | 0. |
| | | Sc. PD: | 0 |
| | | Sc. rank: | 0 |

| | | | |
|---|---|---|---|
| NP | | | |
| Pos. | 482 | obs: | exp: |
| tct | S | 0 | 49.28 |
| tcc | S | 0 | 43.18 |
| tca | S | 0 | 68.68 |
| tcg | S | 0 | 18.90 |
| aat | N | 35 | 18.82 |
| aac | N | 1 | 17.18 |
| agt | S | 295 | 68.20 |
| agc | S | 15 | 61.76 |
| --- | -- | ------ | ------ |
| mPD | | 0.28 | 1.8 |
| | | nPD: | 0.16 |
| | | N. weight: | 1.8 |
| | | Sc. PD: | -0.14 |
| | | Sc. rank: | -12.0 |

| | | | |
|---|---|---|---|
| NP | | | |
| Pos. | 483 | obs: | exp: |
| aat | N | 273 | 180.80 |
| aac | N | 73 | 165.20 |
| --- | -- | ------ | ------ |
| mPD | | 0.33 | 0.50 |
| | | nPD: | 0.67 |
| | | N. weight: | 0.23 |
| | | Sc. PD: | 0.095 |
| | | Sc. rank: | 42.0 |

| | | | |
|---|---|---|---|
| NP | | | |
| Pos. | 484 | obs: | exp: |
| gaa | E | 258 | 199.10 |
| gag | E | 88 | 146.90 |
| --- | -- | ------ | ------ |
| mPD | | 0.38 | 0.49 |
| | | nPD: | 0.78 |
| | | N. weight: | 0.1 |
| | | Sc. PD: | 0.053 |
| | | Sc. rank: | 20.7 |

| | | | |
|---|---|---|---|
| NP | | | |
| Pos. | 485 | obs: | exp: |
| ggt | G | 0 | 42.69 |
| ggc | G | 0 | 46.79 |
| gga | G | 334 | 167.40 |
| ggg | G | 12 | 89.14 |
| --- | -- | ------ | ------ |
| mPD | | 0.067 | 0.67 |
| | | nPD: | 0.1 |
| | | N. weight: | 0.87 |
| | | Sc. PD: | -0.11 |
| | | Sc. rank: | -45.1 |

| | | | |
|---|---|---|---|
| NP | | | |
| Pos. | 486 | obs: | exp: |
| tct | S | 346 | 55.01 |
| tcc | S | 0 | 48.20 |
| tca | S | 0 | 76.65 |
| tcg | S | 0 | 21.09 |
| agt | S | 0 | 76.12 |
| agc | S | 0 | 68.93 |
| --- | -- | ------ | ------ |
| mPD | | 0 | 1.7 |
| | | nPD: | 0. |
| | | N. weight: | 2.6 |
| | | Sc. PD: | -0.6 |
| | | Sc. rank: | -546.4 |

| | | | |
|---|---|---|---|
| NP | | | |
| Pos. | 487 | obs: | exp: |
| tat | Y | 344 | 178.60 |
| tac | Y | 1 | 166.40 |
| aat | N | 1 | 0.52 |
| aac | N | 0 | 0.48 |
| --- | -- | ------ | ------ |
| mPD | | 0.012 | 0.51 |
| | | nPD: | 0.02 |
| | | N. weight: | 0.92 |
| | | Sc. PD: | -0.19 |
| | | Sc. rank: | -120.5 |

| | | | |
|---|---|---|---|
| NP | | | |
| Pos. | 488 | obs: | exp: |
| ttt | F | 0 | 142.20 |
| ttc | F | 346 | 203.80 |
| --- | -- | ------ | ------ |
| mPD | | 0 | 0.48 |
| | | nPD: | 0. |
| | | N. weight: | 0.76 |
| | | Sc. PD: | -0.17 |
| | | Sc. rank: | -157.3 |

| | | | |
|---|---|---|---|
| NP | | | |
| Pos. | 489 | obs: | exp: |
| ttt | F | 1 | 142.20 |
| ttc | F | 345 | 203.80 |
| --- | -- | ------ | ------ |
| mPD | | 0.0058 | 0.48 |
| | | nPD: | 0.01 |
| | | N. weight: | 0.74 |
| | | Sc. PD: | -0.16 |
| | | Sc. rank: | -116.3 |

| | | | |
|---|---|---|---|
| NP | | | |
| Pos. | 490 | obs: | exp: |
| ggt | G | 0 | 42.69 |
| ggc | G | 0 | 46.79 |
| gga | G | 346 | 167.40 |
| ggg | G | 0 | 89.14 |
| --- | -- | ------ | ------ |
| mPD | | 0 | 0.67 |
| | | nPD: | 0. |
| | | N. weight: | 1. |
| | | Sc. PD: | -0.24 |
| | | Sc. rank: | -215.8 |

| | | | |
|---|---|---|---|
| NP | | | |
| Pos. | 491 | obs: | exp: |
| gat | D | 0 | 184.60 |
| gac | D | 346 | 161.40 |
| --- | -- | ------ | ------ |
| mPD | | 0 | 0.50 |
| | | nPD: | 0. |
| | | N. weight: | 1.1 |
| | | Sc. PD: | -0.25 |
| | | Sc. rank: | -226.6 |

| | | | |
|---|---|---|---|
| NP | | | |
| Pos. | 492 | obs: | exp: |
| aat | N | 346 | 180.80 |
| aac | N | 0 | 165.20 |
| --- | -- | ------ | ------ |
| mPD | | 0 | 0.50 |
| | | nPD: | 0. |
| | | N. weight: | 0.93 |
| | | Sc. PD: | -0.21 |
| | | Sc. rank: | -192.8 |

| | | | |
|---|---|---|---|
| NP | | | |
| Pos. | 493 | obs: | exp: |
| gct | A | 1 | 87.15 |
| gcc | A | 0 | 70.88 |
| gca | A | 345 | 154.20 |
| gcg | A | 0 | 33.76 |
| --- | -- | ------ | ------ |
| mPD | | 0.0058 | 0.69 |
| | | nPD: | 0.01 |
| | | N. weight: | 1.1 |
| | | Sc. PD: | -0.25 |
| | | Sc. rank: | -195.3 |

| | | | |
|---|---|---|---|
| NP | | | |
| Pos. | 494 | obs: | exp: |
| gaa | E | 0 | 199.10 |
| gag | E | 346 | 146.90 |
| --- | -- | ------ | ------ |
| mPD | | 0 | 0.49 |
| | | nPD: | 0. |
| | | N. weight: | 1.2 |
| | | Sc. PD: | -0.28 |
| | | Sc. rank: | -254.5 |

| | | | |
|---|---|---|---|
| NP | | | |
| Pos. | 495 | obs: | exp: |
| gat | D | 1 | 0.53 |
| gac | D | 0 | 0.47 |
| gaa | E | 29 | 198.50 |
| gag | E | 316 | 146.50 |
| --- | -- | ------ | ------ |
| mPD | | 0.16 | 0.49 |
| | | nPD: | 0.32 |
| | | N. weight: | 0.79 |
| | | Sc. PD: | 0.066 |
| | | Sc. rank: | 66.1 |

| | | | |
|---|---|---|---|
| NP | | | |
| Pos. | 496 | obs: | exp: |
| ttt | F | 1 | 0.41 |
| ttc | F | 0 | 0.59 |
| tat | Y | 344 | 178.60 |
| tac | Y | 1 | 166.40 |
| --- | -- | ------ | ------ |
| mPD | | 0.012 | 0.51 |
| | | nPD: | 0.02 |
| | | N. weight: | 0.92 |
| | | Sc. PD: | -0.19 |
| | | Sc. rank: | -121.3 |

| | | | |
|---|---|---|---|
| NP | | | |
| Pos. | 497 | obs: | exp: |
| gat | D | 6 | 183.50 |
| gac | D | 338 | 160.50 |
| gaa | E | 2 | 1.15 |
| gag | E | 0 | 0.85 |
| --- | -- | ------ | ------ |
| mPD | | 0.046 | 0.50 |
| | | nPD: | 0.09 |
| | | N. weight: | 0.97 |
| | | Sc. PD: | -0.14 |
| | | Sc. rank: | -53.8 |

| | | | |
|---|---|---|---|
| NP | | | |
| Pos. | 498 | obs: | exp: |
| aat | N | 344 | 180.80 |
| aac | N | 2 | 165.20 |
| --- | -- | ------ | ------ |
| mPD | | 0.012 | 0.50 |
| | | nPD: | 0.02 |
| | | N. weight: | 0.89 |
| | | Sc. PD: | -0.18 |
| | | Sc. rank: | -115.3 |

| | | | |
|---|---|---|---|
| NP | | | |
| Pos. | 499 | obs: | exp: |
| taa | . | 325 | 162.00 |
| tag | . | 0 | 89.54 |
| tga | . | 21 | 94.42 |
| --- | -- | ------ | ------ |
| mPD | | 0.11 | 0.78 |
| | | nPD: | 0.15 |
| | | N. weight: | 0.82 |
| | | Sc. PD: | -0.071 |
| | | Sc. rank: | -7.8 |

Amino acid level conservation per codon

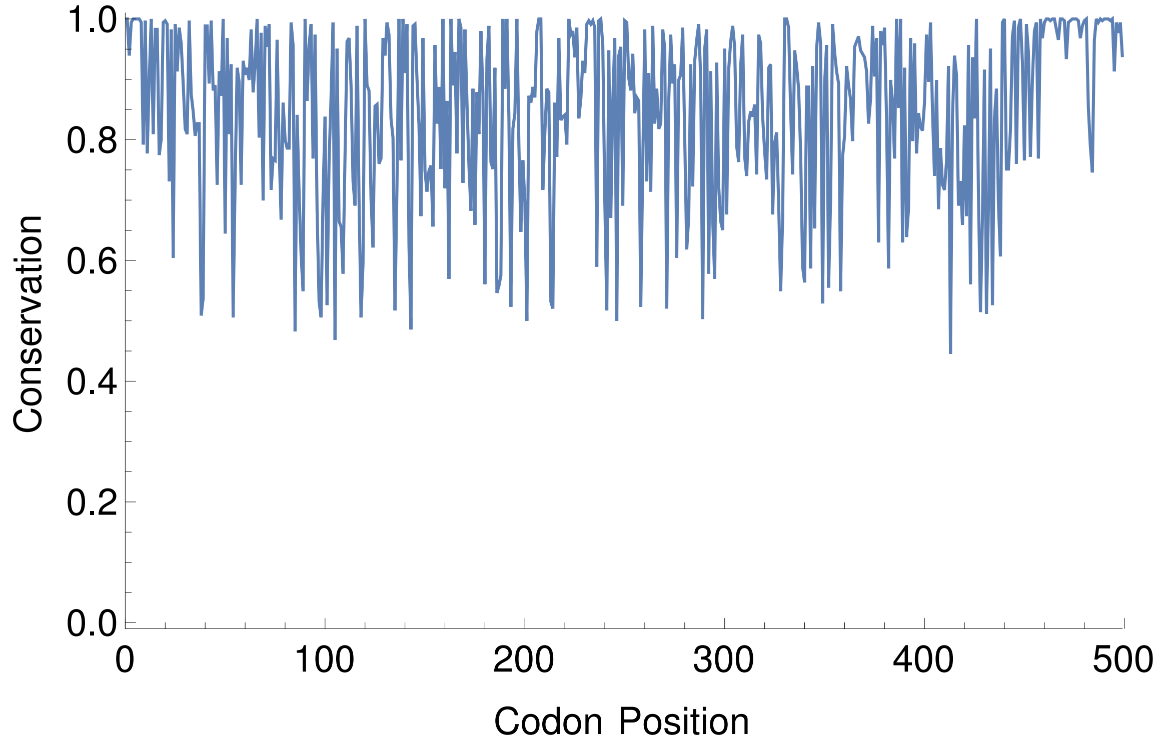

## Conservation analysis plots

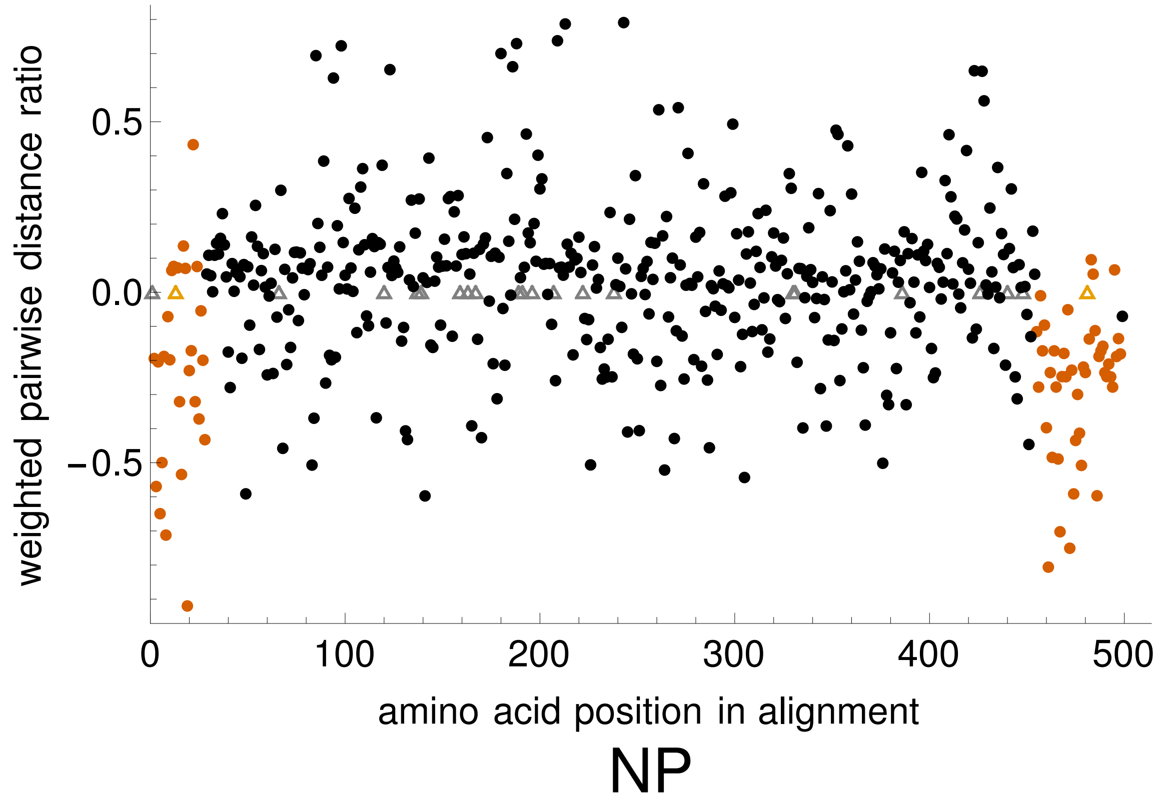


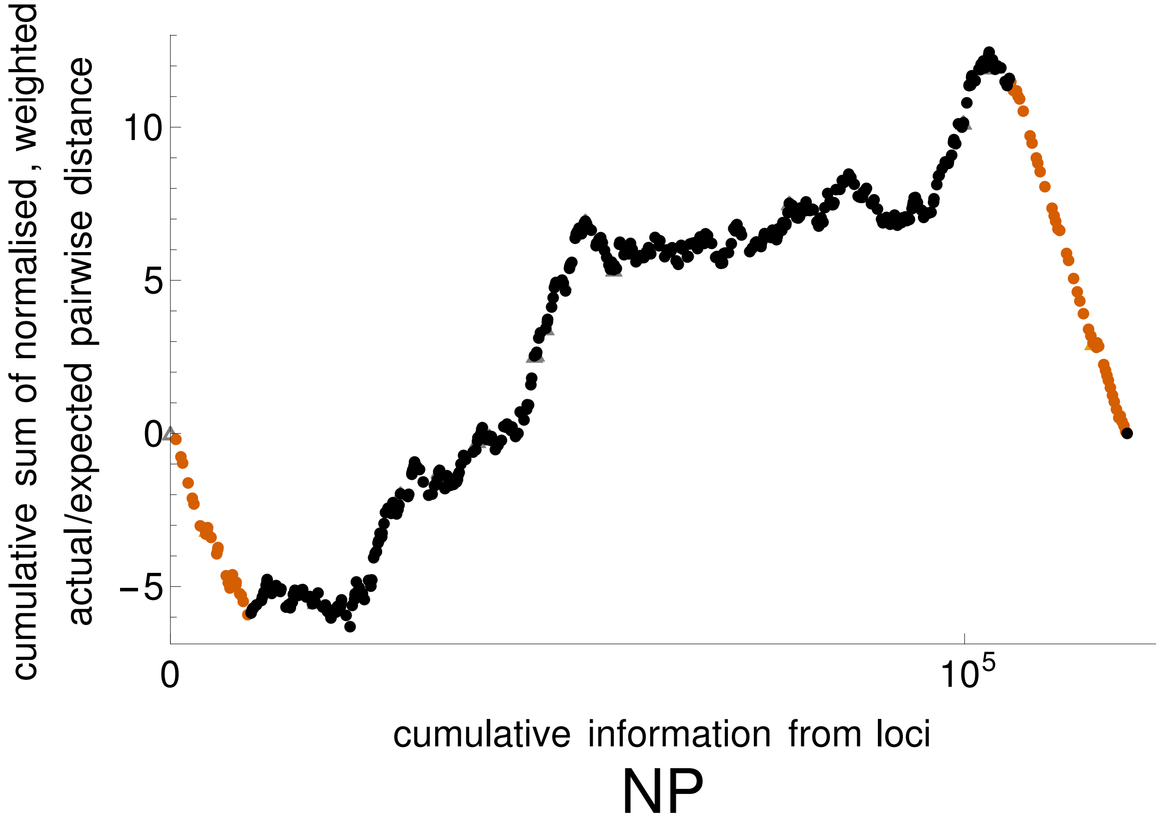

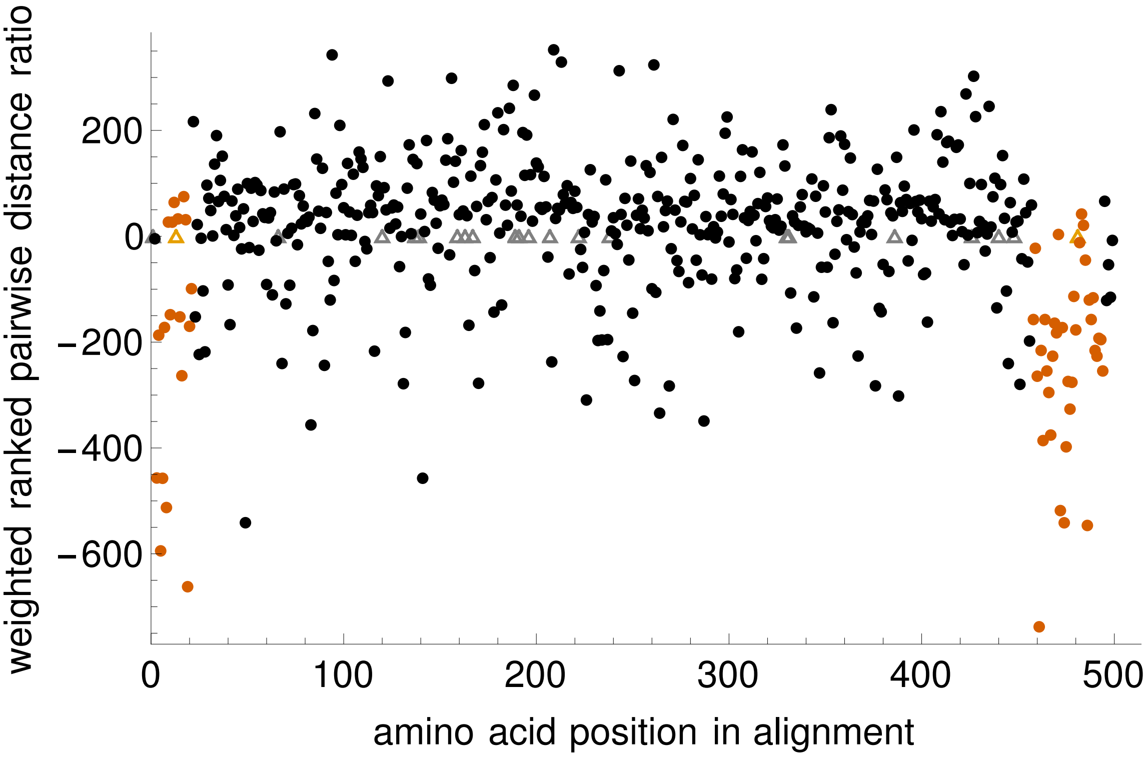
weighted ranked pairwise distance ratio
200
0
−200
−400
−600
0
100
200
300
400
500
amino acid position in alignment

NP

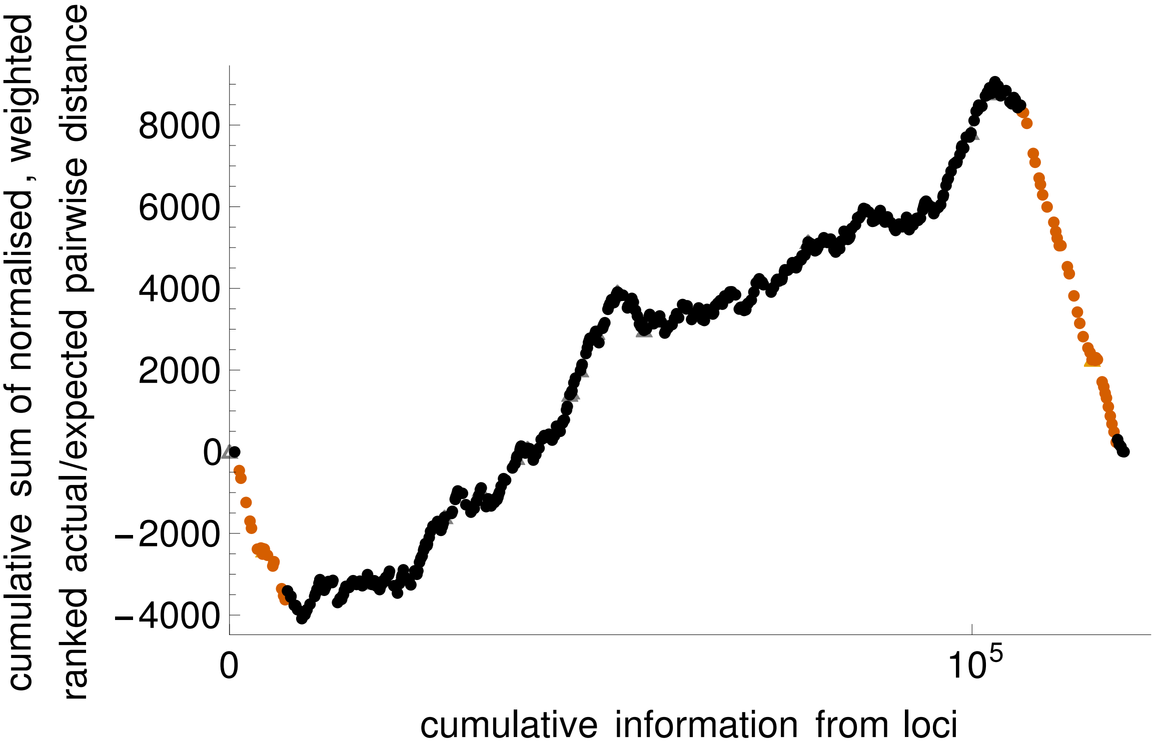
cumulative sum of normalised, weighted ranked actual/expected pairwise distance
8000
6000
4000
2000
0
−2000
−4000
0
$10^5$
cumulative information from loci

NP

# NA

## Gene length histogram

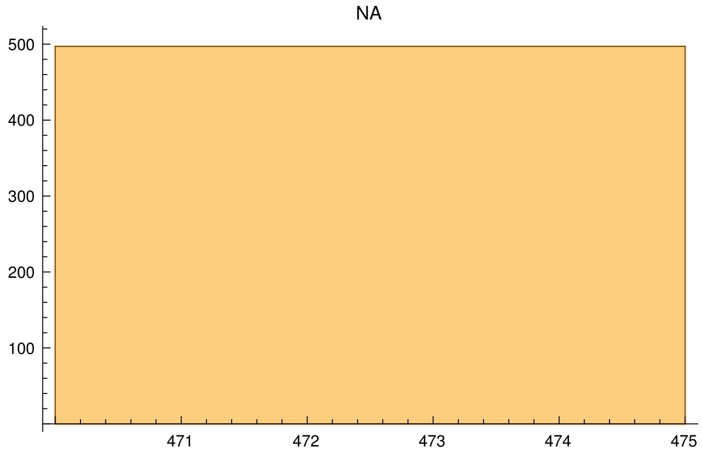

## Information vs. nPD

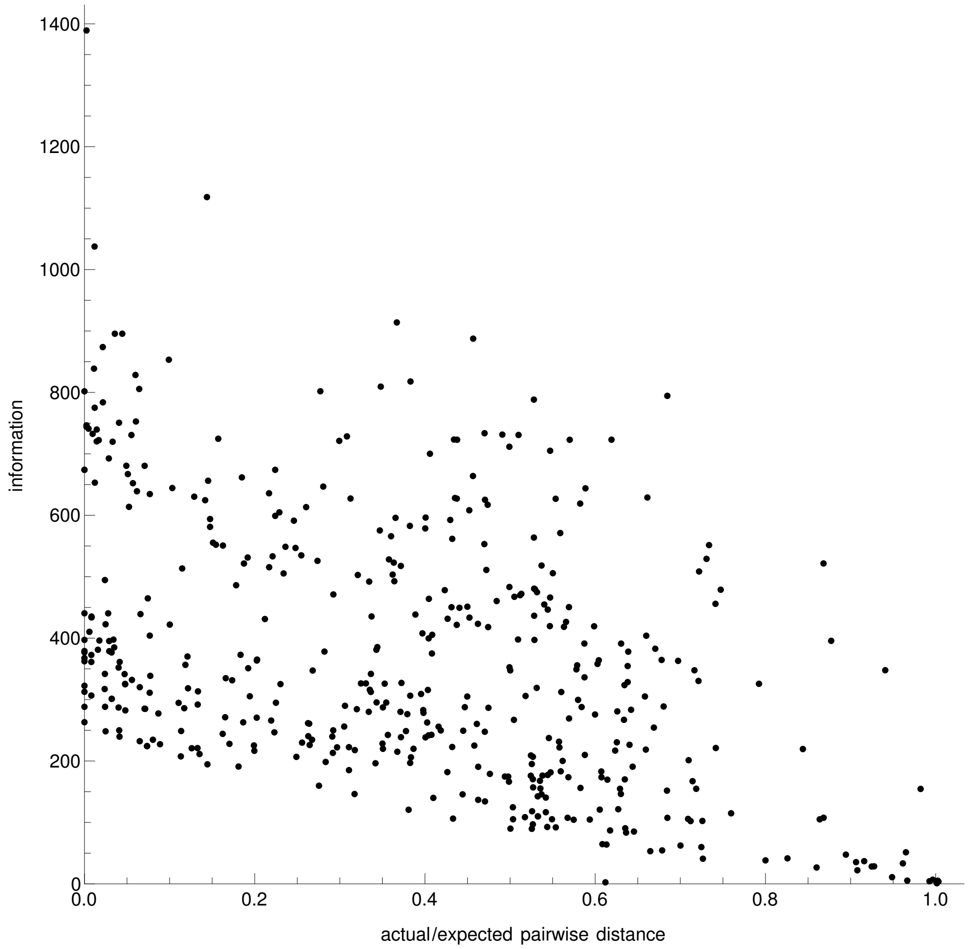


NA

## Example sequences highlighted by regions found in analyses to be conserved

Interesting points (by weighted raw PD) highlighted for gene NA:

ATGAATCCAAATCAGAAACTATTTGCATTATCTGGAGCAGCAATAGCACTCAGTGTGCTGAACCTATTGATAGGAA⋱
TCTCAAATGTCGGATTGAATGTATCTCTACACCTAAAGGAAGGAGGACCCAAACGAGAAGAGAATCTAACATGCA⋱
CGACCATTAATCAAAACAACACTACTGTTGTGGAAAACACATACGTAAATAATACAACAATAATTACCAAGGAAA⋱
CTGAATCGAAAACATCAAGCTATCTGCTGTTGAACAAGAGCCTATGCAATGTTGAAGGATGGGTCGTGATAGCGA⋱
AAGACAATGCAGTAAGATTTGGGGAAAGTGAACAAATCATTGTTACAAGGGAGCCATATGTGTCATGCGACCCAA⋱
CAGGATGTAAAATGTATGCCCTGCACCAAGGGACTACCATTAGGAACAAGCATTCAAATGGAACAATTCACGACA⋱
GAACAGCTTTCAGAGGTCTCATCTCCACTCCATTGGGCACTCCACCAACCGTAAGTAACAGTGACTTTATATGTG⋱
TTGGATGGTCAAGCACAACTTGCCATGATGGGATCGGTAGAATGACTATTTGTATACAAGGAAACAATGACAACG⋱
CTACAGCAACGGTTTATTATAACAGAAGGCTGACAACTACCATTAAGACCTGGGCCAGGAACATCCTGAGGACCC⋱
AAGAGTCAGAATGTGTGTGCCACAATGGTACATGTGCTGTTGTAATGACTGACGGATCGGCCAGTAGTCAAGCCT⋱
ATACAAAGGTGATGTATTTCCACAAGGGGTTGGTAGTAAAGGAAGAGGCATTAAAGGGATCAGCCAGACACATTG⋱
AGGAGTGCTCCTGTTACGGACACAATCAAAAAGTGACCTGTGTGTGCAGAGATAACTGGCAGGGAGCAAATAGGC⋱
CTATTATAGAAATTGATATGAACACATTGGAGCACACAAGCAGGTACGTGTGCACCGGAATTCTCACAGACACCA⋱
GCAGACCTGGGGACAAATCTAGTGGTGATTGTTCCAATCCAATCACTGGAAGTCCTGGCGCCCCAGGAGTGAAGG⋱
GATTCGGGTTTCTAAATGGGGATAACACATGGCTTGGTAGGACCATCAGCCCCAGATCGAGAAGTGGATTCGAAA⋱
TGTTGAAAATACCTAATGCGGGTACTGATCCCAATTCTAGAATAGCAGAACGACAAGAAATTGTCGACAATAACA⋱
ATTGGTCAGGCTATTCCGGAAGCTTCATTGACTATTGGAATGATAACAGTGAATGCTACAATCCGTGCTTCTACG⋱
TAGAGCTAATTAGAGGAAGACCCGAAGAGGCCAAATATGTATGGTGGACAAGTAACAGCCTAATTGCC
**CTATGTGGGAGCCCATTCCCAGTTGGGTCCGGTTCCTTCCCCGATGGGGCACAGA**
**TCCAATACTTTTCGTAA**

Interesting points (by weighted ranked PD) highlighted for gene NA:

ATGAATCCAAATCAGAAACTATTTGCATTATCTGGAGCAGCAATAGCACTCAGTGTGCTGAACCTATTGATAGGAATC⋱
TCAAATGTCGGATTGAATGTATCTCTACACCTAAAGGAAGGAGGACCCAAACGAGAAGAGAATCTAACATGCACGAC⋱
CATTAATCAAAACAACACTACTGTTGTGGAAAACACATACGTAAATAATACAACAATAATTACCAAGGAAACTGAAT⋱
CGAAAACATCAAGCTATCTGCTGTTGAACAAGAGCCTATGCAATGTTGAAGGATGGGTCGTGATAGCGAAAGACAAT⋱
GCAGTAAGATTTGGGGAAAGTGAACAAATCATTGTTACAAGGGAGCCATATGTGTCATGCGACCCAACAGGATGTAA⋱
AATGTATGCCCTGCACCAAGGGACTACCATTAGGAACAAGCATTCAAATGGAACAATTCACGACAGAACAGCTTTCA⋱
GAGGTCTCATCTCCACTCCATTGGGCACTCCACCAACCGTAAGTAACAGTGACTTTATATGTGTTGGATGGTCAAGC⋱
ACAACTTGCCATGATGGGATCGGTAGAATGACTATTTGTATACAAGGAAACAATGACAACGCTACAGCAACGGTTTA⋱
TTATAACAGAAGGCTGACAACTACCATTAAGACCTGGGCCAGGAACATCCTGAGGACCCAAGAGTCAGAATGTGTGT⋱
GCCACAATGGTACATGTGCTGTTGTAATGACTGACGGATCGGCCAGTAGTCAAGCCTATACAAAGGTGATGTATTTC⋱
CACAAGGGGTTGGTAGTAAAGGAAGAGGCATTAAAGGGATCAGCCAGACACATTGAGGAGTGCTCCTGTTACGGACA⋱
CAATCAAAAAGTGACCTGTGTGTGCAGAGATAACTGGCAGGGAGCAAATAGGCCTATTATAGAAATTGATATGAACA⋱
CATTGGAGCACACAAGCAGGTACGTGTGCACCGGAATTCTCACAGACACCAGCAGACCTGGGGACAAATCTAGTGGT⋱
GATTGTTCCAATCCAATCACTGGAAGTCCTGGCGCCCCAGGAGTGAAGGGATTCGGGTTTCTAAATGGGGATAACAC⋱
ATGGCTTGGTAGGACCATCAGCCCCAGATCGAGAAGTGGATTCGAAATGTTGAAAATACCTAATGCGGGTACTGATC⋱
CCAATTCTAGAATAGCAGAACGACAAGAAATTGTCGACAATAACAATTGGTCAGGCTATTCCGGAAGCTTCATTGAC⋱
TATTGGAATGATAACAGTGAATGCTACAATCCGTGCTTCTACGTAGAGCTAATTAGAGGAAGACCCGAAGAGGCCAA⋱
ATATGTATGGTGGACAAGTAACAGCCTAATTGCC
**CTATGTGGGAGCCCATTCCCAGTTGGGTCCGGTTCCTTCCCCGATGGGGCACAGA**
**TCCAATACTTTTCGTAA**

# Per codon conservation report

| | | | |
|---|---|---|---|
| NA | | | |
| Pos. | 1 | obs : | exp : |
| atg | M | 497 | 497.00 |
| --- | -- | ------ | ------ |
| mPD | | 0 | 0 |
| | | nPD : | 1. |
| | | N. weight : | 0. |
| | | Sc. PD : | 0 |
| | | Sc. rank : | 0 |

| | | | |
|---|---|---|---|
| NA | | | |
| Pos. | 2 | obs : | exp : |
| aat | N | 497 | 259.80 |
| aac | N | 0 | 237.20 |
| --- | -- | ------ | ------ |
| mPD | | 0 | 0.50 |
| | | nPD : | 0. |
| | | N. weight : | 0.91 |
| | | Sc. PD : | -0.28 |
| | | Sc. rank : | -278.3 |

| | | | |
|---|---|---|---|
| NA | | | |
| Pos. | 3 | obs : | exp : |
| cct | P | 73 | 130.90 |
| ccc | P | 0 | 89.65 |
| cca | P | 424 | 196.10 |
| ccg | P | 0 | 80.36 |
| --- | -- | ------ | ------ |
| mPD | | 0.25 | 0.72 |
| | | nPD : | 0.35 |
| | | N. weight : | 0.81 |
| | | Sc. PD : | 0.029 |
| | | Sc. rank : | 14.4 |

| | | | |
|---|---|---|---|
| NA | | | |
| Pos. | 4 | obs : | exp : |
| aat | N | 493 | 259.80 |
| aac | N | 4 | 237.20 |
| --- | -- | ------ | ------ |
| mPD | | 0.016 | 0.50 |
| | | nPD : | 0.03 |
| | | N. weight : | 0.85 |
| | | Sc. PD : | -0.23 |
| | | Sc. rank : | -182.4 |

| | | | |
|---|---|---|---|
| NA | | | |
| Pos. | 5 | obs : | exp : |
| caa | Q | 72 | 265.00 |
| cag | Q | 425 | 232.00 |
| --- | -- | ------ | ------ |
| mPD | | 0.25 | 0.50 |
| | | nPD : | 0.5 |
| | | N. weight : | 0.47 |
| | | Sc. PD : | 0.084 |
| | | Sc. rank : | 68.1 |

| | | | |
|---|---|---|---|
| NA | | | |
| Pos. | 6 | obs : | exp : |
| aaa | K | 497 | 278.30 |
| aag | K | 0 | 218.70 |
| --- | -- | ------ | ------ |
| mPD | | 0 | 0.49 |
| | | nPD : | 0. |
| | | N. weight : | 0.81 |
| | | Sc. PD : | -0.25 |
| | | Sc. rank : | -248.7 |

| | | | |
|---|---|---|---|
| NA | | | |
| Pos. | 7 | obs : | exp : |
| tta | L | 103 | 43.33 |
| ttg | L | 0 | 95.53 |
| ctt | L | 0 | 92.55 |
| ctc | L | 0 | 80.23 |
| cta | L | 390 | 74.72 |
| ctg | L | 3 | 109.60 |
| att | I | 0 | 0.38 |
| atc | I | 0 | 0.26 |
| ata | I | 1 | 0.36 |
| --- | -- | ------ | ------ |
| mPD | | 0.34 | 1.1 |
| | | nPD : | 0.31 |
| | | N. weight : | 2. |
| | | Sc. PD : | -0.011 |
| | | Sc. rank : | -24.9 |

| | | | |
|---|---|---|---|
| NA | | | |
| Pos. | 8 | obs : | exp : |
| ttt | F | 414 | 203.10 |
| ttc | F | 80 | 290.90 |
| tct | S | 3 | 0.48 |
| tcc | S | 0 | 0.42 |
| tca | S | 0 | 0.66 |
| tcg | S | 0 | 0.18 |
| agt | S | 0 | 0.66 |
| agc | S | 0 | 0.60 |
| --- | -- | ------ | ------ |
| mPD | | 0.28 | 0.50 |
| | | nPD : | 0.56 |
| | | N. weight : | 0.56 |
| | | Sc. PD : | 0.14 |
| | | Sc. rank : | 120.8 |

| | | | |
|---|---|---|---|
| NA | | | |
| Pos. | 9 | obs : | exp : |
| cgt | R | 0 | 0.04 |
| cgc | R | 0 | 0.05 |
| cga | R | 1 | 0.10 |
| cgg | R | 0 | 0.08 |
| act | T | 0 | 0.79 |
| acc | T | 0 | 0.64 |
| aca | T | 3 | 1.31 |
| acg | T | 0 | 0.25 |
| aga | R | 0 | 0.45 |
| agg | R | 0 | 0.28 |
| gct | A | 1 | 124.20 |
| gcc | A | 0 | 101.00 |
| gca | A | 492 | 219.70 |
| gcg | A | 0 | 48.11 |
| --- | -- | ------ | ------ |
| mPD | | 0.024 | 0.71 |
| | | nPD : | 0.03 |
| | | N. weight : | 1.1 |
| | | Sc. PD : | -0.3 |
| | | Sc. rank : | -234.9 |

| | | | |
|---|---|---|---|
| NA | | | |
| Pos. | 10 | obs : | exp : |
| tta | L | 312 | 43.42 |
| ttg | L | 1 | 95.72 |
| ctt | L | 0 | 92.73 |
| ctc | L | 70 | 80.39 |
| cta | L | 107 | 74.87 |
| ctg | L | 7 | 109.90 |
| --- | -- | ------ | ------ |
| mPD | | 0.74 | 1.1 |
| | | nPD : | 0.66 |
| | | N. weight : | 1.8 |
| | | Sc. PD : | 0.6 |
| | | Sc. rank : | 479.0 |

| | | | |
|---|---|---|---|
| NA | | | |
| Pos. | 11 | obs : | exp : |
| tct | S | 469 | 79.01 |
| tcc | S | 26 | 69.23 |
| tca | S | 2 | 110.10 |
| tcg | S | 0 | 30.29 |
| agt | S | 0 | 109.30 |
| agc | S | 0 | 99.02 |
| --- | -- | ------ | ------ |
| mPD | | 0.11 | 1.7 |
| | | nPD : | 0.06 |
| | | N. weight : | 2.3 |
| | | Sc. PD : | -0.55 |
| | | Sc. rank : | -372.9 |

| | | | |
|---|---|---|---|
| NA | | | |
| Pos. | 12 | obs : | exp : |
| ggt | G | 1 | 61.32 |
| ggc | G | 0 | 67.21 |
| gga | G | 442 | 240.40 |
| ggg | G | 54 | 128.00 |
| --- | -- | ------ | ------ |
| mPD | | 0.20 | 0.67 |
| | | nPD : | 0.3 |
| | | N. weight : | 0.63 |
| | | Sc. PD : | -0.01 |
| | | Sc. rank : | -12.2 |

| | | | |
|---|---|---|---|
| NA | | | |
| Pos. | 13 | obs : | exp : |
| atg | M | 4 | 4.00 |
| gtt | V | 0 | 100.50 |
| gtc | V | 0 | 102.60 |
| gta | V | 8 | 91.65 |
| gtg | V | 481 | 194.20 |
| gct | A | 0 | 1.01 |
| gcc | A | 0 | 0.82 |
| gca | A | 3 | 1.78 |
| gcg | A | 1 | 0.39 |
| --- | -- | ------ | ------ |
| mPD | | 0.075 | 0.75 |
| | | nPD : | 0.1 |
| | | N. weight : | 1.2 |
| | | Sc. PD : | -0.25 |
| | | Sc. rank : | -164.2 |

| | | | |
|---|---|---|---|
| NA | | | |
| Pos. | 14 | obs : | exp : |
| act | T | 0 | 1.32 |
| acc | T | 2 | 1.07 |
| aca | T | 3 | 2.19 |
| acg | T | 0 | 0.42 |
| gct | A | 0 | 123.90 |
| gcc | A | 68 | 100.80 |
| gca | A | 418 | 219.30 |
| gcg | A | 6 | 48.01 |
| --- | -- | ------ | ------ |
| mPD | | 0.28 | 0.71 |
| | | nPD : | 0.4 |
| | | N. weight : | 0.67 |
| | | Sc. PD : | 0.056 |
| | | Sc. rank : | 42.2 |

| | | | |
|---|---|---|---|
| NA | | | |
| Pos. | 15 | obs : | exp : |
| att | I | 2 | 186.10 |
| atc | I | 0 | 129.70 |
| ata | I | 493 | 179.20 |
| gtt | V | 0 | 0.41 |
| gtc | V | 0 | 0.42 |
| gta | V | 2 | 0.37 |
| gtg | V | 0 | 0.79 |
| --- | -- | ------ | ------ |
| mPD | | 0.016 | 0.67 |
| | | nPD : | 0.02 |
| | | N. weight : | 1.4 |
| | | Sc. PD : | -0.39 |
| | | Sc. rank : | -317.5 |

| | | | |
|---|---|---|---|
| NA | | | |
| Pos. | 16 | obs : | exp : |
| gtt | V | 0 | 0.21 |
| gtc | V | 0 | 0.21 |
| gta | V | 1 | 0.19 |
| gtg | V | 0 | 0.40 |
| gct | A | 0 | 124.90 |
| gcc | A | 0 | 101.60 |
| gca | A | 454 | 221.10 |
| gcg | A | 42 | 48.40 |
| --- | -- | ------ | ------ |
| mPD | | 0.16 | 0.69 |
| | | nPD : | 0.23 |
| | | N. weight : | 0.91 |
| | | Sc. PD : | -0.074 |
| | | Sc. rank : | -49.6 |

| | | | |
|---|---|---|---|
| NA | | | |
| Pos. | 17 | obs : | exp : |
| ttt | F | 3 | 1.23 |
| ttc | F | 0 | 1.77 |
| tta | L | 0 | 43.16 |
| ttg | L | 0 | 95.14 |
| ctt | L | 345 | 92.17 |
| ctc | L | 78 | 79.91 |
| cta | L | 0 | 74.42 |
| ctg | L | 71 | 109.20 |
| --- | -- | ------ | ------ |
| mPD | | 0.48 | 1.1 |
| | | nPD : | 0.43 |
| | | N. weight : | 1.2 |
| | | Sc. PD : | 0.13 |
| | | Sc. rank : | 102.0 |

| | | | |
|---|---|---|---|
| NA | | | |
| Pos. | 18 | obs : | exp : |
| tct | S | 0 | 78.38 |
| tcc | S | 0 | 68.68 |
| tca | S | 0 | 109.20 |
| tcg | S | 0 | 30.05 |
| aat | N | 3 | 1.57 |
| aac | N | 0 | 1.43 |
| agt | S | 476 | 108.50 |
| agc | S | 17 | 98.22 |
| ggt | G | 1 | 0.12 |
| ggc | G | 0 | 0.14 |
| gga | G | 0 | 0.48 |
| ggg | G | 0 | 0.26 |
| --- | -- | ------ | ------ |
| mPD | | 0.082 | 1.7 |
| | | nPD : | 0.05 |
| | | N. weight : | 1.9 |
| | | Sc. PD : | -0.49 |
| | | Sc. rank : | -357.3 |

| | | | |
|---|---|---|---|
| NA | | | |
| Pos. | 19 | obs : | exp : |
| att | I | 1 | 42.48 |
| atc | I | 69 | 29.62 |
| ata | I | 43 | 40.90 |
| atg | M | 2 | 2.00 |
| gtt | V | 0 | 78.52 |
| gtc | V | 1 | 80.17 |
| gta | V | 308 | 71.59 |
| gtg | V | 73 | 151.70 |
| --- | -- | ------ | ------ |
| mPD | | 0.82 | 1.1 |
| | | nPD : | 0.74 |
| | | N. weight : | 1.3 |
| | | Sc. PD : | 0.53 |
| | | Sc. rank : | 378.0 |

| | | | |
|---|---|---|---|
| NA | | | |
| Pos. | 20 | obs : | exp : |
| tta | L | 2 | 43.24 |
| ttg | L | 2 | 95.33 |
| ctt | L | 1 | 92.36 |
| ctc | L | 69 | 80.07 |
| cta | L | 0 | 74.57 |
| ctg | L | 421 | 109.40 |
| atg | M | 1 | 1.00 |
| gtt | V | 0 | 0.21 |
| gtc | V | 0 | 0.21 |
| gta | V | 1 | 0.19 |
| gtg | V | 0 | 0.40 |
| --- | -- | ------ | ------ |
| mPD | | 0.28 | 1.1 |
| | | nPD : | 0.25 |
| | | N. weight : | 1.5 |
| | | Sc. PD : | -0.098 |
| | | Sc. rank : | -71.0 |

| | | | |
|---|---|---|---|
| NA | | | |
| Pos. | 21 | obs : | exp : |
| aat | N | 7 | 259.80 |
| aac | N | 490 | 237.20 |
| --- | -- | ------ | ------ |
| mPD | | 0.028 | 0.50 |
| | | nPD : | 0.06 |
| | | N. weight : | 0.93 |
| | | Sc. PD : | -0.23 |
| | | Sc. rank : | -166.3 |

| | | | |
|---|---|---|---|
| NA | | | |
| Pos. | 22 | obs : | exp : |
| tta | L | 350 | 43.42 |
| ttg | L | 3 | 95.72 |
| ctt | L | 0 | 92.73 |
| ctc | L | 0 | 80.39 |
| cta | L | 111 | 74.87 |
| ctg | L | 33 | 109.90 |
| --- | -- | ------ | ------ |
| mPD | | 0.55 | 1.1 |
| | | nPD : | 0.49 |
| | | N. weight : | 2.1 |
| | | Sc. PD : | 0.35 |
| | | Sc. rank : | 295.6 |

| | | | |
|---|---|---|---|
| NA | | | |
| Pos. | 23 | obs : | exp : |
| tta | L | 45 | 43.42 |
| ttg | L | 377 | 95.72 |
| ctt | L | 0 | 92.73 |
| ctc | L | 0 | 80.39 |
| cta | L | 70 | 74.87 |
| ctg | L | 5 | 109.90 |
| --- | -- | ------ | ------ |
| mPD | | 0.61 | 1.1 |
| | | nPD : | 0.55 |
| | | N. weight : | 1.4 |
| | | Sc. PD : | 0.33 |
| | | Sc. rank : | 292.5 |

| | | | |
|---|---|---|---|
| NA | | | |
| Pos. | 24 | obs : | exp : |
| att | I | 23 | 186.80 |
| atc | I | 12 | 130.30 |
| ata | I | 462 | 179.90 |
| --- | -- | ------ | ------ |
| mPD | | 0.13 | 0.66 |
| | | nPD : | 0.2 |
| | | N. weight : | 1. |
| | | Sc. PD : | -0.11 |
| | | Sc. rank : | -76.0 |

| | | | |
|---|---|---|---|
| NA | | | |
| Pos. | 25 | obs : | exp : |
| ggt | G | 0 | 61.32 |
| ggc | G | 0 | 67.21 |
| gga | G | 478 | 240.40 |
| ggg | G | 19 | 128.00 |
| --- | -- | ------ | ------ |
| mPD | | 0.074 | 0.67 |
| | | nPD : | 0.11 |
| | | N. weight : | 0.83 |
| | | Sc. PD : | -0.16 |
| | | Sc. rank : | -111.8 |

| | | | |
|---|---|---|---|
| NA | | | |
| Pos. | 26 | obs: | exp: |
| tta | L | 0 | 0.70 |
| ttg | L | 0 | 1.54 |
| ctt | L | 0 | 1.49 |
| ctc | L | 8 | 1.29 |
| cta | L | 0 | 1.21 |
| ctg | L | 0 | 1.77 |
| att | I | 5 | 182.00 |
| atc | I | 408 | 126.80 |
| ata | I | 71 | 175.20 |
| gtt | V | 0 | 1.03 |
| gtc | V | 5 | 1.05 |
| gta | V | 0 | 0.94 |
| gtg | V | 0 | 1.99 |
| --- | -- | ------ | ------ |
| mPD | | 0.31 | 0.72 |
| | | nPD: | 0.44 |
| | | N. weight: | 1.2 |
| | | Sc. PD: | 0.14 |
| | | Sc. rank: | 115.7 |

,

| | | | |
|---|---|---|---|
| NA | | | |
| Pos. | 27 | obs: | exp: |
| tct | S | 0 | 79.01 |
| tcc | S | 71 | 69.23 |
| tca | S | 426 | 110.10 |
| tcg | S | 0 | 30.29 |
| agt | S | 0 | 109.30 |
| agc | S | 0 | 99.02 |
| --- | -- | ------ | ------ |
| mPD | | 0.25 | 1.7 |
| | | nPD: | 0.15 |
| | | N. weight: | 1.6 |
| | | Sc. PD: | -0.26 |
| | | Sc. rank: | -176.3 |

,

| | | | |
|---|---|---|---|
| NA | | | |
| Pos. | 28 | obs: | exp: |
| aat | N | 275 | 259.80 |
| aac | N | 222 | 237.20 |
| --- | -- | ------ | ------ |
| mPD | | 0.50 | 0.50 |
| | | nPD: | 0.99 |
| | | N. weight: | 0.012 |
| | | Sc. PD: | 0.0079 |
| | | Sc. rank: | 3.7 |

,

| | | | |
|---|---|---|---|
| NA | | | |
| Pos. | 29 | obs: | exp: |
| att | I | 1 | 3.38 |
| atc | I | 8 | 2.36 |
| ata | I | 0 | 3.26 |
| gtt | V | 11 | 100.30 |
| gtc | V | 406 | 102.40 |
| gta | V | 0 | 91.46 |
| gtg | V | 71 | 193.80 |
| --- | -- | ------ | ------ |
| mPD | | 0.32 | 0.76 |
| | | nPD: | 0.42 |
| | | N. weight: | 1.3 |
| | | Sc. PD: | 0.14 |
| | | Sc. rank: | 109.9 |

,

| | | | |
|---|---|---|---|
| NA | | | |
| Pos. | 30 | obs: | exp: |
| ggt | G | 0 | 61.32 |
| ggc | G | 0 | 67.21 |
| gga | G | 468 | 240.40 |
| ggg | G | 29 | 128.00 |
| --- | -- | ------ | ------ |
| mPD | | 0.11 | 0.67 |
| | | nPD: | 0.17 |
| | | N. weight: | 0.76 |
| | | Sc. PD: | -0.11 |
| | | Sc. rank: | -72.5 |

,

| | | | |
|---|---|---|---|
| NA | | | |
| Pos. | 31 | obs: | exp: |
| tta | L | 2 | 43.42 |
| ttg | L | 428 | 95.72 |
| ctt | L | 0 | 92.73 |
| ctc | L | 0 | 80.39 |
| cta | L | 2 | 74.87 |
| ctg | L | 65 | 109.90 |
| --- | -- | ------ | ------ |
| mPD | | 0.25 | 1.1 |
| | | nPD: | 0.22 |
| | | N. weight: | 1.7 |
| | | Sc. PD: | -0.15 |
| | | Sc. rank: | -98.9 |

,

| | | | |
|---|---|---|---|
| NA | | | |
| Pos. | 32 | obs: | exp: |
| tct | S | 0 | 0.16 |
| tcc | S | 0 | 0.14 |
| tca | S | 0 | 0.22 |
| tcg | S | 0 | 0.06 |
| aat | N | 238 | 259.20 |
| aac | N | 258 | 236.80 |
| agt | S | 0 | 0.22 |
| agc | S | 1 | 0.20 |
| --- | -- | ------ | ------ |
| mPD | | 0.50 | 0.51 |
| | | nPD: | 1. |
| | | N. weight: | 0.019 |
| | | Sc. PD: | 0.013 |
| | | Sc. rank: | 5.8 |

,

| | | | |
|---|---|---|---|
| NA | | | |
| Pos. | 33 | obs: | exp: |
| att | I | 11 | 4.89 |
| atc | I | 2 | 3.41 |
| ata | I | 0 | 4.71 |
| atg | M | 1 | 1.00 |
| gtt | V | 34 | 99.28 |
| gtc | V | 69 | 101.40 |
| gta | V | 336 | 90.52 |
| gtg | V | 44 | 191.80 |
| --- | -- | ------ | ------ |
| mPD | | 0.56 | 0.78 |
| | | nPD: | 0.72 |
| | | N. weight: | 0.93 |
| | | Sc. PD: | 0.37 |
| | | Sc. rank: | 266.2 |

,

| | | | |
|---|---|---|---|
| NA | | | |
| Pos. | 34 | obs: | exp: |
| tct | S | 378 | 79.01 |
| tcc | S | 48 | 69.23 |
| tca | S | 71 | 110.10 |
| tcg | S | 0 | 30.29 |
| agt | S | 0 | 109.30 |
| agc | S | 0 | 99.02 |
| --- | -- | ------ | ------ |
| mPD | | 0.39 | 1.7 |
| | | nPD: | 0.24 |
| | | N. weight: | 1.5 |
| | | Sc. PD: | -0.12 |
| | | Sc. rank: | -77.7 |

,

| | | | |
|---|---|---|---|
| NA | | | |
| Pos. | 35 | obs: | exp: |
| tta | L | 24 | 43.16 |
| ttg | L | 1 | 95.14 |
| ctt | L | 0 | 92.17 |
| ctc | L | 0 | 79.91 |
| cta | L | 469 | 74.42 |
| ctg | L | 0 | 109.20 |
| gtt | V | 0 | 0.62 |
| gtc | V | 0 | 0.63 |
| gta | V | 3 | 0.56 |
| gtg | V | 0 | 1.19 |
| --- | -- | ------ | ------ |
| mPD | | 0.11 | 1.1 |
| | | nPD: | 0.1 |
| | | N. weight: | 2.4 |
| | | Sc. PD: | -0.5 |
| | | Sc. rank: | -335.8 |

,

| | | | |
|---|---|---|---|
| NA | | | |
| Pos. | 36 | obs: | exp: |
| cat | H | 344 | 269.30 |
| cac | H | 153 | 227.70 |
| --- | -- | ------ | ------ |
| mPD | | 0.43 | 0.50 |
| | | nPD: | 0.86 |
| | | N. weight: | 0.075 |
| | | Sc. PD: | 0.04 |
| | | Sc. rank: | 22.5 |

,

| | | | |
|---|---|---|---|
| NA | | | |
| Pos. | 37 | obs: | exp: |
| tta | L | 63 | 43.33 |
| ttg | L | 1 | 95.53 |
| ctt | L | 0 | 92.55 |
| ctc | L | 0 | 80.23 |
| cta | L | 360 | 74.72 |
| ctg | L | 72 | 109.60 |
| atg | M | 1 | 1.00 |
| --- | -- | ------ | ------ |
| mPD | | 0.48 | 1.1 |
| | | nPD: | 0.43 |
| | | N. weight: | 1.6 |
| | | Sc. PD: | 0.18 |
| | | Sc. rank: | 143.3 |

,

| | | | |
|---|---|---|---|
| NA | | | |
| Pos. | 38 | obs: | exp: |
| cgt | R | 0 | 0.95 |
| cgc | R | 0 | 1.13 |
| cga | R | 0 | 2.21 |
| cgg | R | 0 | 1.92 |
| aaa | K | 31 | 265.40 |
| aag | K | 443 | 208.60 |
| aga | R | 1 | 10.34 |
| agg | R | 22 | 6.45 |
| --- | -- | ------ | ------ |
| mPD | | 0.21 | 0.61 |
| | | nPD: | 0.34 |
| | | N. weight: | 0.83 |
| | | Sc. PD: | 0.024 |
| | | Sc. rank: | 7.8 |

,

| | | | |
|---|---|---|---|
| NA | | | |
| Pos. | 39 | obs: | exp: |
| cgt | R | 0 | 0.12 |
| cgc | R | 0 | 0.15 |
| cga | R | 0 | 0.29 |
| cgg | R | 0 | 0.25 |
| aat | N | 0 | 1.05 |
| aac | N | 2 | 0.95 |
| aga | R | 3 | 1.35 |
| agg | R | 0 | 0.84 |
| gct | A | 0 | 0.25 |
| gcc | A | 1 | 0.20 |
| gca | A | 0 | 0.45 |
| gcg | A | 0 | 0.10 |
| gaa | E | 337 | 194.50 |
| gag | E | 1 | 143.50 |
| ggt | G | 0 | 18.88 |
| ggc | G | 2 | 20.69 |
| gga | G | 150 | 74.01 |
| ggg | G | 1 | 39.42 |
| --- | -- | ------ | ------ |
| mPD | | 0.48 | 1.0 |
| | | nPD: | 0.47 |
| | | N. weight: | 0.81 |
| | | Sc. PD: | 0.13 |
| | | Sc. rank: | 111.8 |

,

| | | | |
|---|---|---|---|
| NA | | | |
| Pos. | 40 | obs: | exp: |
| cgt | R | 0 | 0.12 |
| cgc | R | 0 | 0.15 |
| cga | R | 0 | 0.29 |
| cgg | R | 0 | 0.25 |
| act | T | 0 | 2.11 |
| acc | T | 3 | 1.72 |
| aca | T | 5 | 3.50 |
| acg | T | 0 | 0.67 |
| aaa | K | 296 | 189.30 |
| aag | K | 42 | 148.70 |
| aga | R | 1 | 1.35 |
| agg | R | 2 | 0.84 |
| gct | A | 0 | 0.25 |
| gcc | A | 0 | 0.20 |
| gca | A | 1 | 0.45 |
| gcg | A | 0 | 0.10 |
| gaa | E | 58 | 34.53 |
| gag | E | 2 | 25.47 |
| ggt | G | 0 | 1.97 |
| ggc | G | 0 | 2.16 |
| gga | G | 16 | 7.74 |
| ggg | G | 0 | 4.12 |
| --- | - | 71 | 71.00 |
| --- | -- | ------ | ------ |
| mPD | | 0.46 | 0.69 |
| | | nPD: | 0.67 |
| | | N. weight: | 0. |
| | | Sc. PD: | 0 |
| | | Sc. rank: | 0 |

,

| | | | |
|---|---|---|---|
| NA | | | |
| Pos. | 41 | obs: | exp: |
| tct | S | 0 | 7.47 |
| tcc | S | 0 | 6.55 |
| tca | S | 0 | 10.41 |
| tcg | S | 0 | 2.86 |
| cgt | R | 0 | 0.50 |
| cgc | R | 0 | 0.59 |
| cga | R | 0 | 1.15 |
| cgg | R | 0 | 1.00 |
| aat | N | 1 | 0.52 |
| aac | N | 0 | 0.48 |
| agt | S | 2 | 10.34 |
| agc | S | 45 | 9.36 |
| aga | R | 12 | 5.40 |
| agg | R | 0 | 3.36 |
| gaa | E | 5 | 3.45 |
| gag | E | 1 | 2.55 |
| ggt | G | 0 | 53.18 |
| ggc | G | 23 | 58.29 |
| gga | G | 357 | 208.50 |
| ggg | G | 51 | 111.00 |
| --- | -- | ------ | ------ |
| mPD | | 0.65 | 1.0 |
| | | nPD: | 0.62 |
| | | N. weight: | 0.61 |
| | | Sc. PD: | 0.18 |
| | | Sc. rank: | 152.5 |

,

| | | | |
|---|---|---|---|
| NA | | | |
| Pos. | 42 | obs: | exp: |
| tct | S | 0 | 7.79 |
| tcc | S | 2 | 6.83 |
| tca | S | 0 | 10.86 |
| tcg | S | 0 | 2.99 |
| cct | P | 0 | 100.30 |
| ccc | P | 381 | 68.72 |
| cca | P | 0 | 150.30 |
| ccg | P | 0 | 61.60 |
| act | T | 0 | 10.57 |
| acc | T | 38 | 8.58 |
| aca | T | 2 | 17.52 |
| acg | T | 0 | 3.33 |
| aat | N | 23 | 12.02 |
| aac | N | 0 | 10.98 |
| agt | S | 47 | 10.78 |
| agc | S | 0 | 9.76 |
| gtt | V | 0 | 0.41 |
| gtc | V | 2 | 0.42 |
| gta | V | 0 | 0.37 |
| gtg | V | 0 | 0.79 |
| gct | A | 0 | 0.25 |
| gcc | A | 1 | 0.20 |
| gca | A | 0 | 0.45 |
| gcg | A | 0 | 0.10 |
| gat | D | 0 | 0.53 |
| gac | D | 1 | 0.47 |
| --- | -- | ------ | ------ |
| mPD | | 0.87 | 1.3 |
| | | nPD: | 0.68 |
| | | N. weight: | 2.2 |
| | | Sc. PD: | 0.8 |
| | | Sc. rank: | 624.3 |

,

| | | | |
|---|---|---|---|
| NA | | | |
| Pos. | 43 | obs: | exp: |
| aat | N | 0 | 2.61 |
| aac | N | 5 | 2.39 |
| aaa | K | 408 | 238.00 |
| aag | K | 17 | 187.00 |
| gat | D | 26 | 32.55 |
| gac | D | 35 | 28.45 |
| gaa | E | 2 | 3.45 |
| gag | E | 4 | 2.55 |
| --- | -- | ------ | ------ |
| mPD | | 0.54 | 0.84 |
| | | nPD: | 0.64 |
| | | N. weight: | 0.54 |
| | | Sc. PD: | 0.17 |
| | | Sc. rank: | 142.0 |

,

| | | | |
|---|---|---|---|
| NA | | | |
| Pos. | 44 | obs: | exp: |
| caa | Q | 168 | 263.40 |
| cag | Q | 326 | 230.60 |
| cgt | R | 0 | 0.12 |
| cgc | R | 0 | 0.15 |
| cga | R | 3 | 0.29 |
| cgg | R | 0 | 0.25 |
| aga | R | 0 | 1.35 |
| agg | R | 0 | 0.84 |
| --- | -- | ------ | ------ |
| mPD | | 0.46 | 0.52 |
| | | nPD: | 0.89 |
| | | N. weight: | 0.13 |
| | | Sc. PD: | 0.075 |
| | | Sc. rank: | 40.7 |

,

| | | | |
|---|---|---|---|
| NA | | | |
| Pos. | 45 | obs: | exp: |
| aaa | K | 0 | 1.12 |
| aag | K | 2 | 0.88 |
| gat | D | 37 | 37.35 |
| gac | D | 33 | 32.65 |
| gaa | E | 104 | 242.80 |
| gag | E | 318 | 179.20 |
| ggt | G | 1 | 0.37 |
| ggc | G | 0 | 0.41 |
| gga | G | 0 | 1.45 |
| ggg | G | 2 | 0.77 |
| --- | -- | ------ | ------ |
| mPD | | 0.55 | 0.63 |
| | | nPD: | 0.86 |
| | | N. weight: | 0.3 |
| | | Sc. PD: | 0.16 |
| | | Sc. rank: | 88.8 |

,

| | | | |
|---|---|---|---|
| NA | | | |
| Pos. | 46 | obs: | exp: |
| aat | N | 0 | 1.05 |
| aac | N | 2 | 0.95 |
| aaa | K | 39 | 39.76 |
| aag | K | 32 | 31.24 |
| gat | D | 6 | 3.20 |
| gac | D | 0 | 2.80 |
| gaa | E | 2 | 238.20 |
| gag | E | 412 | 175.80 |
| ggt | G | 0 | 0.49 |
| ggc | G | 0 | 0.54 |
| gga | G | 0 | 1.93 |
| ggg | G | 4 | 1.03 |
| --- | -- | ------ | ------ |
| mPD | | 0.45 | 0.77 |
| | | nPD: | 0.58 |
| | | N. weight: | 1. |
| | | Sc. PD: | 0.26 |
| | | Sc. rank: | 227.1 |

,

| | | | |
|---|---|---|---|
| NA | | | |
| Pos. | 47 | obs: | exp: |
| tct | S | 0 | 0.16 |
| tcc | S | 0 | 0.14 |
| tca | S | 0 | 0.22 |
| tcg | S | 0 | 0.06 |
| aat | N | 488 | 259.20 |
| aac | N | 8 | 236.80 |
| agt | S | 1 | 0.22 |
| agc | S | 0 | 0.20 |
| --- | -- | ------ | ------ |
| mPD | | 0.036 | 0.51 |
| | | nPD: | 0.07 |
| | | N. weight: | 0.8 |
| | | Sc. PD: | -0.19 |
| | | Sc. rank: | -128.2 |

,

| | | | |
|---|---|---|---|
| NA | | | |
| Pos. | 48 | obs: | exp: |
| tta | L | 373 | 34.94 |
| ttg | L | 6 | 77.04 |
| tct | S | 0 | 4.45 |
| tcc | S | 0 | 3.90 |
| tca | S | 20 | 6.20 |
| tcg | S | 8 | 1.71 |
| tgg | W | 69 | 69.00 |
| ctt | L | 0 | 74.63 |
| ctc | L | 0 | 64.70 |
| cta | L | 21 | 60.26 |
| ctg | L | 0 | 88.42 |
| agt | S | 0 | 6.16 |
| agc | S | 0 | 5.58 |
| --- | -- | ------ | ------ |
| mPD | | 0.69 | 1.5 |
| | | nPD: | 0.46 |
| | | N. weight: | 2.5 |
| | | Sc. PD: | 0.35 |
| | | Sc. rank: | 297.1 |

,

| | | | |
|---|---|---|---|
| NA | | | |
| Pos. | 49 | obs: | exp: |
| act | T | 0 | 131.30 |
| acc | T | 1 | 106.60 |
| aca | T | 476 | 217.70 |
| acg | T | 20 | 41.32 |
| --- | -- | ------ | ------ |
| mPD | | 0.081 | 0.69 |
| | | nPD: | 0.12 |
| | | N. weight: | 1. |
| | | Sc. PD: | -0.19 |
| | | Sc. rank: | -129.4 |

,

| | | | |
|---|---|---|---|
| NA | | | |
| Pos. | 50 | obs: | exp: |
| tat | Y | 2 | 1.04 |
| tac | Y | 0 | 0.96 |
| tgt | C | 32 | 204.00 |
| tgc | C | 463 | 291.00 |
| --- | -- | ------ | ------ |
| mPD | | 0.14 | 0.49 |
| | | nPD: | 0.28 |
| | | N. weight: | 0.45 |
| | | Sc. PD: | -0.017 |
| | | Sc. rank: | -14.4 |

,

| | | | |
|---|---|---|---|
| NA | | | |
| Pos. | 51 | obs: | exp: |
| atg | M | 18 | 18.00 |
| act | T | 0 | 126.50 |
| acc | T | 0 | 102.80 |
| aca | T | 60 | 209.80 |
| acg | T | 419 | 39.82 |
| --- | -- | ------ | ------ |
| mPD | | 0.28 | 0.77 |
| | | nPD: | 0.37 |
| | | N. weight: | 2.6 |
| | | Sc. PD: | 0.13 |
| | | Sc. rank: | 94.9 |

,

| | | | |
|---|---|---|---|
| NA | | | |
| Pos. | 52 | obs: | exp: |
| tct | S | 0 | 11.29 |
| tcc | S | 0 | 9.89 |
| tca | S | 0 | 15.73 |
| tcg | S | 0 | 4.33 |
| act | T | 0 | 112.50 |
| acc | T | 426 | 91.41 |
| aca | T | 0 | 186.60 |
| acg | T | 0 | 35.41 |
| agt | S | 48 | 15.62 |
| agc | S | 23 | 14.15 |
| --- | -- | ------ | ------ |
| mPD | | 0.42 | 0.96 |
| | | nPD: | 0.44 |
| | | N. weight: | 2. |
| | | Sc. PD: | 0.24 |
| | | Sc. rank: | 208.5 |

,

| | | | |
|---|---|---|---|
| NA | | | |
| Pos. | 53 | obs: | exp: |
| att | I | 419 | 161.30 |
| atc | I | 7 | 112.40 |
| ata | I | 3 | 155.30 |
| gtt | V | 0 | 13.98 |
| gtc | V | 0 | 14.27 |
| gta | V | 63 | 12.74 |
| gtg | V | 5 | 27.01 |
| --- | -- | ------ | ------ |
| mPD | | 0.51 | 0.93 |
| | | nPD: | 0.55 |
| | | N. weight: | 1.3 |
| | | Sc. PD: | 0.3 |
| | | Sc. rank: | 265.8 |

,

| | | | |
|---|---|---|---|
| NA | | | |
| Pos. | 54 | obs: | exp: |
| tct | S | 0 | 0.95 |
| tcc | S | 0 | 0.84 |
| tca | S | 0 | 1.33 |
| tcg | S | 0 | 0.37 |
| act | T | 23 | 25.36 |
| acc | T | 2 | 20.60 |
| aca | T | 71 | 42.06 |
| acg | T | 0 | 7.98 |
| aat | N | 389 | 204.90 |
| aac | N | 3 | 187.10 |
| agt | S | 6 | 1.32 |
| agc | S | 0 | 1.20 |
| gat | D | 3 | 1.60 |
| gac | D | 0 | 1.40 |
| --- | -- | ------ | ------ |
| mPD | | 0.61 | 0.94 |
| | | nPD: | 0.64 |
| | | N. weight: | 0.8 |
| | | Sc. PD: | 0.25 |
| | | Sc. rank: | 210.5 |

,

| | | | |
|---|---|---|---|
| NA | | | |
| Pos. | 55 | obs: | exp: |
| caa | Q | 475 | 265.00 |
| cag | Q | 22 | 232.00 |
| --- | -- | ------ | ------ |
| mPD | | 0.085 | 0.50 |
| | | nPD: | 0.17 |
| | | N. weight: | 0.64 |
| | | Sc. PD: | -0.089 |
| | | Sc. rank: | -59.6 |

,

| NA | | | |
|---|---|---|---|
| Pos. | 56 | obs: | exp: |
| aat | N | 1 | 259.80 |
| aac | N | 496 | 237.20 |
| --- | -- | ------ | ------ |
| mPD | | 0.0040 | 0.50 |
| | | nPD: | 0.01 |
| | | N. weight: | 1. |
| | | Sc. PD: | -0.3 |
| | | Sc. rank: | -275.9 |

,

| NA | | | |
|---|---|---|---|
| Pos. | 57 | obs: | exp: |
| tct | S | 0 | 0.32 |
| tcc | S | 0 | 0.28 |
| tca | S | 0 | 0.44 |
| tcg | S | 0 | 0.12 |
| aat | N | 25 | 258.70 |
| aac | N | 470 | 236.30 |
| agt | S | 0 | 0.44 |
| agc | S | 2 | 0.40 |
| --- | -- | ------ | ------ |
| mPD | | 0.10 | 0.51 |
| | | nPD: | 0.2 |
| | | N. weight: | 0.76 |
| | | Sc. PD: | -0.082 |
| | | Sc. rank: | -57.6 |

,

| NA | | | |
|---|---|---|---|
| Pos. | 58 | obs: | exp: |
| act | T | 398 | 131.30 |
| acc | T | 15 | 106.60 |
| aca | T | 3 | 217.70 |
| acg | T | 81 | 41.32 |
| --- | -- | ------ | ------ |
| mPD | | 0.33 | 0.69 |
| | | nPD: | 0.48 |
| | | N. weight: | 1.3 |
| | | Sc. PD: | 0.21 |
| | | Sc. rank: | 182.6 |

,

| NA | | | |
|---|---|---|---|
| Pos. | 59 | obs: | exp: |
| act | T | 488 | 131.30 |
| acc | T | 3 | 106.60 |
| aca | T | 3 | 217.70 |
| acg | T | 3 | 41.32 |
| --- | -- | ------ | ------ |
| mPD | | 0.036 | 0.69 |
| | | nPD: | 0.05 |
| | | N. weight: | 1.7 |
| | | Sc. PD: | -0.44 |
| | | Sc. rank: | -314.1 |

,

| NA | | | |
|---|---|---|---|
| Pos. | 60 | obs: | exp: |
| tta | L | 70 | 6.12 |
| ttg | L | 0 | 13.48 |
| tct | S | 0 | 0.16 |
| tcc | S | 0 | 0.14 |
| tca | S | 1 | 0.22 |
| tcg | S | 0 | 0.06 |
| ctt | L | 0 | 13.06 |
| ctc | L | 0 | 11.32 |
| cta | L | 0 | 10.55 |
| ctg | L | 0 | 15.47 |
| att | I | 6 | 3.01 |
| atc | I | 0 | 2.10 |
| ata | I | 2 | 2.90 |
| agt | S | 0 | 0.22 |
| agc | S | 0 | 0.20 |
| gtt | V | 89 | 85.92 |
| gtc | V | 2 | 87.73 |
| gta | V | 327 | 78.34 |
| gtg | V | 0 | 166.00 |
| --- | -- | ------ | ------ |
| mPD | | 0.59 | 1.0 |
| | | nPD: | 0.59 |
| | | N. weight: | 1.8 |
| | | Sc. PD: | 0.48 |
| | | Sc. rank: | 430.7 |

,

| NA | | | |
|---|---|---|---|
| Pos. | 61 | obs: | exp: |
| att | I | 26 | 26.69 |
| atc | I | 45 | 18.61 |
| ata | I | 0 | 25.70 |
| atg | M | 1 | 1.00 |
| gtt | V | 0 | 87.35 |
| gtc | V | 0 | 89.20 |
| gta | V | 353 | 79.65 |
| gtg | V | 72 | 168.80 |
| --- | -- | ------ | ------ |
| mPD | | 0.71 | 0.99 |
| | | nPD: | 0.72 |
| | | N. weight: | 1.4 |
| | | Sc. PD: | 0.57 |
| | | Sc. rank: | 412.5 |

,

| NA | | | |
|---|---|---|---|
| Pos. | 62 | obs: | exp: |
| aaa | K | 3 | 1.68 |
| aag | K | 0 | 1.32 |
| gaa | E | 480 | 283.70 |
| gag | E | 13 | 209.30 |
| ggt | G | 0 | 0.12 |
| ggc | G | 0 | 0.14 |
| gga | G | 1 | 0.48 |
| ggg | G | 0 | 0.26 |
| --- | -- | ------ | ------ |
| mPD | | 0.067 | 0.51 |
| | | nPD: | 0.13 |
| | | N. weight: | 0.62 |
| | | Sc. PD: | -0.11 |
| | | Sc. rank: | -76.2 |

,

| NA | | | |
|---|---|---|---|
| Pos. | 63 | obs: | exp: |
| act | T | 0 | 0.79 |
| acc | T | 3 | 0.64 |
| aca | T | 0 | 1.31 |
| acg | T | 0 | 0.25 |
| aat | N | 6 | 257.70 |
| aac | N | 487 | 235.30 |
| aaa | K | 1 | 0.56 |
| aag | K | 0 | 0.44 |
| --- | -- | ------ | ------ |
| mPD | | 0.040 | 0.52 |
| | | nPD: | 0.08 |
| | | N. weight: | 0.95 |
| | | Sc. PD: | -0.22 |
| | | Sc. rank: | -138.8 |

,

| NA | | | |
|---|---|---|---|
| Pos. | 64 | obs: | exp: |
| att | I | 0 | 7.52 |
| atc | I | 0 | 5.24 |
| ata | I | 20 | 7.24 |
| act | T | 0 | 126.00 |
| acc | T | 0 | 102.40 |
| aca | T | 461 | 209.00 |
| acg | T | 16 | 39.65 |
| --- | -- | ------ | ------ |
| mPD | | 0.14 | 0.76 |
| | | nPD: | 0.18 |
| | | N. weight: | 1. |
| | | Sc. PD: | -0.13 |
| | | Sc. rank: | -92.2 |

,

| NA | | | |
|---|---|---|---|
| Pos. | 65 | obs: | exp: |
| tat | Y | 179 | 257.30 |
| tac | Y | 318 | 239.70 |
| --- | -- | ------ | ------ |
| mPD | | 0.46 | 0.50 |
| | | nPD: | 0.92 |
| | | N. weight: | 0.079 |
| | | Sc. PD: | 0.047 |
| | | Sc. rank: | 24.2 |

,

| NA | | | |
|---|---|---|---|
| Pos. | 66 | obs: | exp: |
| tta | L | 1 | 0.09 |
| ttg | L | 0 | 0.19 |
| ctt | L | 0 | 0.19 |
| ctc | L | 0 | 0.16 |
| cta | L | 0 | 0.15 |
| ctg | L | 0 | 0.22 |
| att | I | 0 | 2.26 |
| atc | I | 0 | 1.57 |
| ata | I | 6 | 2.17 |
| gtt | V | 4 | 100.70 |
| gtc | V | 71 | 102.80 |
| gta | V | 370 | 91.84 |
| gtg | V | 45 | 194.60 |
| --- | -- | ------ | ------ |
| mPD | | 0.42 | 0.75 |
| | | nPD: | 0.57 |
| | | N. weight: | 1.2 |
| | | Sc. PD: | 0.29 |
| | | Sc. rank: | 261.1 |

,

| NA | | | |
|---|---|---|---|
| Pos. | 67 | obs: | exp: |
| aat | N | 397 | 259.80 |
| aac | N | 100 | 237.20 |
| --- | -- | ------ | ------ |
| mPD | | 0.32 | 0.50 |
| | | nPD: | 0.65 |
| | | N. weight: | 0.24 |
| | | Sc. PD: | 0.077 |
| | | Sc. rank: | 63.5 |

,

| NA | | | |
|---|---|---|---|
| Pos. | 68 | obs: | exp: |
| aat | N | 464 | 259.80 |
| aac | N | 33 | 237.20 |
| --- | -- | ------ | ------ |
| mPD | | 0.12 | 0.50 |
| | | nPD: | 0.25 |
| | | N. weight: | 0.58 |
| | | Sc. PD: | -0.037 |
| | | Sc. rank: | -26.4 |

,

| NA | | | |
|---|---|---|---|
| Pos. | 69 | obs: | exp: |
| act | T | 57 | 131.30 |
| acc | T | 14 | 106.60 |
| aca | T | 426 | 217.70 |
| acg | T | 0 | 41.32 |
| --- | -- | ------ | ------ |
| mPD | | 0.25 | 0.69 |
| | | nPD: | 0.37 |
| | | N. weight: | 0.6 |
| | | Sc. PD: | 0.031 |
| | | Sc. rank: | 22.8 |

,

| NA | | | |
|---|---|---|---|
| Pos. | 70 | obs: | exp: |
| att | I | 1 | 0.38 |
| atc | I | 0 | 0.26 |
| ata | I | 0 | 0.36 |
| act | T | 70 | 131.00 |
| acc | T | 0 | 106.40 |
| aca | T | 424 | 217.30 |
| acg | T | 2 | 41.23 |
| --- | -- | ------ | ------ |
| mPD | | 0.26 | 0.69 |
| | | nPD: | 0.37 |
| | | N. weight: | 0.67 |
| | | Sc. PD: | 0.038 |
| | | Sc. rank: | 27.9 |

,

| NA | | | |
|---|---|---|---|
| Pos. | 71 | obs: | exp: |
| att | I | 0 | 155.30 |
| atc | I | 0 | 108.20 |
| ata | I | 413 | 149.50 |
| gtt | V | 0 | 17.27 |
| gtc | V | 71 | 17.63 |
| gta | V | 13 | 15.74 |
| gtg | V | 0 | 33.36 |
| --- | -- | ------ | ------ |
| mPD | | 0.53 | 0.98 |
| | | nPD: | 0.54 |
| | | N. weight: | 1.5 |
| | | Sc. PD: | 0.32 |
| | | Sc. rank: | 277.2 |

,

| NA | | | |
|---|---|---|---|
| Pos. | 72 | obs: | exp: |
| att | I | 420 | 185.70 |
| atc | I | 74 | 129.50 |
| ata | I | 0 | 178.80 |
| gtt | V | 3 | 0.62 |
| gtc | V | 0 | 0.63 |
| gta | V | 0 | 0.56 |
| gtg | V | 0 | 1.19 |
| --- | -- | ------ | ------ |
| mPD | | 0.27 | 0.67 |
| | | nPD: | 0.4 |
| | | N. weight: | 0.87 |
| | | Sc. PD: | 0.069 |
| | | Sc. rank: | 49.0 |

,

| NA | | | |
|---|---|---|---|
| Pos. | 73 | obs: | exp: |
| tct | S | 0 | 2.70 |
| tcc | S | 17 | 2.37 |
| tca | S | 0 | 3.77 |
| tcg | S | 0 | 1.04 |
| act | T | 13 | 107.80 |
| acc | T | 393 | 87.55 |
| aca | T | 2 | 178.70 |
| acg | T | 0 | 33.92 |
| aat | N | 66 | 37.63 |
| aac | N | 6 | 34.37 |
| agt | S | 0 | 3.74 |
| agc | S | 0 | 3.39 |
| --- | -- | ------ | ------ |
| mPD | | 0.59 | 1.0 |
| | | nPD: | 0.58 |
| | | N. weight: | 1.7 |
| | | Sc. PD: | 0.45 |
| | | Sc. rank: | 401.1 |

,

| NA | | | |
|---|---|---|---|
| Pos. | 74 | obs: | exp: |
| caa | Q | 2 | 1.07 |
| cag | Q | 0 | 0.93 |
| cgt | R | 0 | 0.04 |
| cgc | R | 0 | 0.05 |
| cga | R | 0 | 0.10 |
| cgg | R | 0 | 0.08 |
| aaa | K | 56 | 276.60 |
| aag | K | 438 | 217.40 |
| aga | R | 0 | 0.45 |
| agg | R | 1 | 0.28 |
| --- | -- | ------ | ------ |
| mPD | | 0.22 | 0.51 |
| | | nPD: | 0.43 |
| | | N. weight: | 0.63 |
| | | Sc. PD: | 0.072 |
| | | Sc. rank: | 55.6 |

,

| NA | | | |
|---|---|---|---|
| Pos. | 75 | obs: | exp: |
| aaa | K | 2 | 1.12 |
| aag | K | 0 | 0.88 |
| gaa | E | 246 | 155.90 |
| gag | E | 25 | 115.10 |
| ggt | G | 0 | 27.64 |
| ggc | G | 0 | 30.29 |
| gga | G | 222 | 108.40 |
| ggg | G | 2 | 57.71 |
| --- | -- | ------ | ------ |
| mPD | | 0.61 | 1.1 |
| | | nPD: | 0.56 |
| | | N. weight: | 0.65 |
| | | Sc. PD: | 0.15 |
| | | Sc. rank: | 136.1 |

,

| NA | | | |
|---|---|---|---|
| Pos. | 76 | obs: | exp: |
| cct | P | 0 | 5.27 |
| ccc | P | 0 | 3.61 |
| cca | P | 20 | 7.89 |
| ccg | P | 0 | 3.23 |
| act | T | 344 | 110.70 |
| acc | T | 0 | 89.91 |
| aca | T | 51 | 183.60 |
| acg | T | 24 | 34.83 |
| aat | N | 34 | 17.77 |
| aac | N | 0 | 16.23 |
| gct | A | 22 | 6.04 |
| gcc | A | 0 | 4.92 |
| gca | A | 0 | 10.70 |
| gcg | A | 2 | 2.34 |
| --- | -- | ------ | ------ |
| mPD | | 0.62 | 0.99 |
| | | nPD: | 0.63 |
| | | N. weight: | 1.1 |
| | | Sc. PD: | 0.34 |
| | | Sc. rank: | 280.4 |

,

| NA | | | |
|---|---|---|---|
| Pos. | 77 | obs: | exp: |
| aat | N | 4 | 2.09 |
| aac | N | 0 | 1.91 |
| aaa | K | 1 | 0.56 |
| aag | K | 0 | 0.44 |
| gat | D | 294 | 168.60 |
| gac | D | 22 | 147.40 |
| gaa | E | 106 | 61.57 |
| gag | E | 1 | 45.43 |
| ggt | G | 0 | 8.51 |
| ggc | G | 0 | 9.33 |
| gga | G | 23 | 33.38 |
| ggg | G | 46 | 17.78 |
| --- | -- | ------ | ------ |
| mPD | | 0.82 | 0.97 |
| | | nPD: | 0.84 |
| | | N. weight: | 0.62 |
| | | Sc. PD: | 0.32 |
| | | Sc. rank: | 185.2 |

,

| NA | | | |
|---|---|---|---|
| Pos. | 78 | obs: | exp: |
| tta | L | 5 | 31.01 |
| ttg | L | 346 | 68.37 |
| tct | S | 0 | 3.97 |
| tcc | S | 0 | 3.48 |
| tca | S | 0 | 5.54 |
| tcg | S | 25 | 1.52 |
| tgg | W | 46 | 46.00 |
| ctt | L | 1 | 66.24 |
| ctc | L | 0 | 57.42 |
| cta | L | 0 | 53.48 |
| ctg | L | 3 | 78.47 |
| att | I | 3 | 1.13 |
| atc | I | 0 | 0.79 |
| ata | I | 0 | 1.09 |
| act | T | 67 | 17.70 |
| acc | T | 0 | 14.38 |
| aca | T | 0 | 29.35 |
| acg | T | 0 | 5.57 |
| agt | S | 0 | 5.50 |
| agc | S | 0 | 4.98 |
| gct | A | 0 | 0.25 |
| gcc | A | 0 | 0.20 |
| gca | A | 1 | 0.45 |
| gcg | A | 0 | 0.10 |
| --- | -- | ------ | ------ |
| mPD | | 0.96 | 1.8 |
| | | nPD: | 0.55 |
| | | N. weight: | 2. |
| | | Sc. PD: | 0.45 |
| | | Sc. rank: | 398.6 |

,

| NA | | | |
|---|---|---|---|
| Pos. | 79 | obs: | exp: |
| cgt | R | 0 | 0.04 |
| cgc | R | 0 | 0.05 |
| cga | R | 0 | 0.10 |
| cgg | R | 0 | 0.08 |
| act | T | 0 | 8.19 |
| acc | T | 0 | 6.65 |
| aca | T | 31 | 13.58 |
| acg | T | 0 | 2.58 |
| aat | N | 21 | 10.98 |
| aac | N | 0 | 10.02 |
| aaa | K | 361 | 206.10 |
| aag | K | 7 | 161.90 |
| aga | R | 1 | 0.45 |
| agg | R | 0 | 0.28 |
| gct | A | 0 | 9.82 |
| gcc | A | 2 | 7.99 |
| gca | A | 37 | 17.38 |
| gcg | A | 0 | 3.81 |
| gaa | E | 37 | 21.29 |
| gag | E | 0 | 15.71 |
| --- | -- | ------ | ------ |
| mPD | | 0.62 | 1.1 |
| | | nPD: | 0.57 |
| | | N. weight: | 0.76 |
| | | Sc. PD: | 0.19 |
| | | Sc. rank: | 167.6 |

,

| NA | | | |
|---|---|---|---|
| Pos. | 80 | obs: | exp: |
| cct | P | 0 | 2.63 |
| ccc | P | 0 | 1.80 |
| cca | P | 10 | 3.95 |
| ccg | P | 0 | 1.62 |
| att | I | 0 | 1.50 |
| atc | I | 0 | 1.05 |
| ata | I | 4 | 1.45 |
| act | T | 3 | 107.00 |
| acc | T | 0 | 86.91 |
| aca | T | 401 | 177.40 |
| acg | T | 1 | 33.67 |
| aaa | K | 0 | 39.20 |
| aag | K | 70 | 30.80 |
| gct | A | 3 | 1.76 |
| gcc | A | 0 | 1.43 |
| gca | A | 4 | 3.12 |
| gcg | A | 0 | 0.68 |
| ggt | G | 0 | 0.12 |
| ggc | G | 0 | 0.14 |
| gga | G | 0 | 0.48 |
| ggg | G | 1 | 0.26 |
| --- | -- | ------ | ------ |
| mPD | | 0.60 | 1.0 |
| | | nPD: | 0.59 |
| | | N. weight: | 1.1 |
| | | Sc. PD: | 0.29 |
| | | Sc. rank: | 256.9 |

,

| NA | | | |
|---|---|---|---|
| Pos. | 81 | obs: | exp: |
| tta | L | 0 | 0.09 |
| ttg | L | 0 | 0.19 |
| tct | S | 0 | 11.45 |
| tcc | S | 0 | 10.03 |
| tca | S | 72 | 15.95 |
| tcg | S | 0 | 4.39 |
| ctt | L | 0 | 0.19 |
| ctc | L | 0 | 0.16 |
| cta | L | 1 | 0.15 |
| ctg | L | 0 | 0.22 |
| cct | P | 0 | 97.69 |
| ccc | P | 1 | 66.92 |
| cca | P | 369 | 146.40 |
| ccg | P | 1 | 59.99 |
| caa | Q | 19 | 27.72 |
| cag | Q | 33 | 24.28 |
| agt | S | 0 | 15.84 |
| agc | S | 0 | 14.34 |
| gct | A | 0 | 0.25 |
| gcc | A | 0 | 0.20 |
| gca | A | 1 | 0.45 |
| gcg | A | 0 | 0.10 |
| --- | -- | ------ | ------ |
| mPD | | 0.57 | 1.3 |
| | | nPD: | 0.45 |
| | | N. weight: | 1.3 |
| | | Sc. PD: | 0.17 |
| | | Sc. rank: | 138.9 |

,

| NA | | | |
|---|---|---|---|
| Pos. | 82 | obs: | exp: |
| tct | S | 0 | 66.77 |
| tcc | S | 0 | 58.51 |
| tca | S | 0 | 93.05 |
| tcg | S | 0 | 25.60 |
| cat | H | 1 | 0.54 |
| cac | H | 0 | 0.46 |
| caa | Q | 1 | 0.53 |
| cag | Q | 0 | 0.47 |
| aat | N | 61 | 38.68 |
| aac | N | 13 | 35.32 |
| agt | S | 57 | 92.40 |
| agc | S | 363 | 83.68 |
| ggt | G | 0 | 0.12 |
| ggc | G | 1 | 0.14 |
| gga | G | 0 | 0.48 |
| ggg | G | 0 | 0.26 |
| --- | -- | ------ | ------ |
| mPD | | 0.64 | 1.8 |
| | | nPD: | 0.36 |
| | | N. weight: | 1.5 |
| | | Sc. PD: | 0.063 |
| | | Sc. rank: | 35.3 |

,

| NA | | | |
|---|---|---|---|
| Pos. | 83 | obs: | exp: |
| tat | Y | 476 | 257.30 |
| tac | Y | 21 | 239.70 |
| --- | -- | ------ | ------ |
| mPD | | 0.081 | 0.50 |
| | | nPD: | 0.16 |
| | | N. weight: | 0.69 |
| | | Sc. PD: | -0.1 |
| | | Sc. rank: | -67.4 |

,

| NA | | | |
|---|---|---|---|
| Pos. | 84 | obs: | exp: |
| tta | L | 0 | 43.42 |
| ttg | L | 9 | 95.72 |
| ctt | L | 0 | 92.73 |
| ctc | L | 0 | 80.39 |
| cta | L | 72 | 74.87 |
| ctg | L | 416 | 109.90 |
| --- | -- | ------ | ------ |
| mPD | | 0.28 | 1.1 |
| | | nPD: | 0.25 |
| | | N. weight: | 1.5 |
| | | Sc. PD: | -0.086 |
| | | Sc. rank: | -65.3 |

,

| NA | | | |
|---|---|---|---|
| Pos. | 85 | obs: | exp: |
| tta | L | 0 | 37.22 |
| ttg | L | 39 | 82.04 |
| ctt | L | 1 | 79.49 |
| ctc | L | 0 | 68.91 |
| cta | L | 3 | 64.18 |
| ctg | L | 383 | 94.17 |
| att | I | 0 | 0.38 |
| atc | I | 0 | 0.26 |
| ata | I | 1 | 0.36 |
| atg | M | 68 | 68.00 |
| gtt | V | 0 | 0.41 |
| gtc | V | 0 | 0.42 |
| gta | V | 0 | 0.37 |
| gtg | V | 2 | 0.79 |
| --- | -- | ------ | ------ |
| mPD | | 0.39 | 1.2 |
| | | nPD: | 0.32 |
| | | N. weight: | 1.4 |
| | | Sc. PD: | 0.01 |
| | | Sc. rank: | -5.9 |

,

| NA | | | |
|---|---|---|---|
| Pos. | 86 | obs: | exp: |
| tta | L | 28 | 43.42 |
| ttg | L | 381 | 95.72 |
| ctt | L | 0 | 92.73 |
| ctc | L | 0 | 80.39 |
| cta | L | 2 | 74.87 |
| ctg | L | 86 | 109.90 |
| --- | -- | ------ | ------ |
| mPD | | 0.41 | 1.1 |
| | | nPD: | 0.36 |
| | | N. weight: | 1.4 |
| | | Sc. PD: | 0.067 |
| | | Sc. rank: | 43.5 |

,

| NA | | | |
|---|---|---|---|
| Pos. | 87 | obs: | exp: |
| aat | N | 35 | 259.80 |
| aac | N | 462 | 237.20 |
| --- | -- | ------ | ------ |
| mPD | | 0.13 | 0.50 |
| | | nPD: | 0.26 |
| | | N. weight: | 0.68 |
| | | Sc. PD: | -0.033 |
| | | Sc. rank: | -26.0 |

,

| NA | | | |
|---|---|---|---|
| Pos. | 88 | obs: | exp: |
| aaa | K | 25 | 278.30 |
| aag | K | 472 | 218.70 |
| --- | -- | ------ | ------ |
| mPD | | 0.096 | 0.49 |
| | | nPD: | 0.19 |
| | | N. weight: | 0.86 |
| | | Sc. PD: | -0.1 |
| | | Sc. rank: | -67.2 |

,

| NA | | | |
|---|---|---|---|
| Pos. | 89 | obs: | exp: |
| tct | S | 0 | 78.53 |
| tcc | S | 0 | 68.82 |
| tca | S | 0 | 109.40 |
| tcg | S | 0 | 30.11 |
| agt | S | 54 | 108.70 |
| agc | S | 440 | 98.42 |
| ggt | G | 3 | 0.37 |
| ggc | G | 0 | 0.41 |
| gga | G | 0 | 1.45 |
| ggg | G | 0 | 0.77 |
| --- | -- | ------ | ------ |
| mPD | | 0.22 | 1.7 |
| | | nPD: | 0.13 |
| | | N. weight: | 1.8 |
| | | Sc. PD: | -0.32 |
| | | Sc. rank: | -218.7 |

,

| NA | | | |
|---|---|---|---|
| Pos. | 90 | obs: | exp: |
| tta | L | 70 | 43.42 |
| ttg | L | 0 | 95.72 |
| ctt | L | 0 | 92.73 |
| ctc | L | 0 | 80.39 |
| cta | L | 210 | 74.87 |
| ctg | L | 217 | 109.90 |
| --- | -- | ------ | ------ |
| mPD | | 0.74 | 1.1 |
| | | nPD: | 0.66 |
| | | N. weight: | 1.1 |
| | | Sc. PD: | 0.38 |
| | | Sc. rank: | 305.0 |

,

| | | | |
|---|---|---|---|
| NA | | | |
| Pos. | 91 | obs: | exp: |
| tgt | C | 9 | 204.90 |
| tgc | C | 488 | 292.10 |
| --- | -- | ------ | ------ |
| mPD | | 0.036 | 0.48 |
| | | nPD: | 0.07 |
| | | N. weight: | 0.63 |
| | | Sc. PD: | -0.15 |
| | | Sc. rank: | -97.4 |

,

| | | | |
|---|---|---|---|
| NA | | | |
| Pos. | 92 | obs: | exp: |
| tct | S | 0 | 0.32 |
| tcc | S | 0 | 0.28 |
| tca | S | 0 | 0.44 |
| tcg | S | 0 | 0.12 |
| att | I | 1 | 0.38 |
| atc | I | 0 | 0.26 |
| ata | I | 0 | 0.36 |
| aat | N | 423 | 221.10 |
| aac | N | 0 | 201.90 |
| aaa | K | 70 | 39.76 |
| aag | K | 1 | 31.24 |
| agt | S | 2 | 0.44 |
| agc | S | 0 | 0.40 |
| --- | -- | ------ | ------ |
| mPD | | 0.26 | 0.64 |
| | | nPD: | 0.4 |
| | | N. weight: | 0.89 |
| | | Sc. PD: | 0.077 |
| | | Sc. rank: | 59.7 |

,

| | | | |
|---|---|---|---|
| NA | | | |
| Pos. | 93 | obs: | exp: |
| gtt | V | 487 | 102.20 |
| gtc | V | 3 | 104.30 |
| gta | V | 5 | 93.15 |
| gtg | V | 2 | 197.40 |
| --- | -- | ------ | ------ |
| mPD | | 0.040 | 0.72 |
| | | nPD: | 0.06 |
| | | N. weight: | 2.1 |
| | | Sc. PD: | -0.52 |
| | | Sc. rank: | -368.5 |

,

| | | | |
|---|---|---|---|
| NA | | | |
| Pos. | 94 | obs: | exp: |
| aaa | K | 3 | 1.68 |
| aag | K | 0 | 1.32 |
| gaa | E | 481 | 284.30 |
| gag | E | 13 | 209.70 |
| --- | -- | ------ | ------ |
| mPD | | 0.063 | 0.50 |
| | | nPD: | 0.13 |
| | | N. weight: | 0.62 |
| | | Sc. PD: | -0.11 |
| | | Sc. rank: | -78.0 |

,

| | | | |
|---|---|---|---|
| NA | | | |
| Pos. | 95 | obs: | exp: |
| ggt | G | 1 | 61.32 |
| ggc | G | 44 | 67.21 |
| gga | G | 213 | 240.40 |
| ggg | G | 239 | 128.00 |
| --- | -- | ------ | ------ |
| mPD | | 0.58 | 0.67 |
| | | nPD: | 0.87 |
| | | N. weight: | 0.3 |
| | | Sc. PD: | 0.16 |
| | | Sc. rank: | 91.7 |

,

| | | | |
|---|---|---|---|
| NA | | | |
| Pos. | 96 | obs: | exp: |
| tgg | W | 497 | 497.00 |
| --- | -- | ------ | ------ |
| mPD | | 0 | 0 |
| | | nPD: | 1. |
| | | N. weight: | 0. |
| | | Sc. PD: | 0 |
| | | Sc. rank: | 0 |

,

| | | | |
|---|---|---|---|
| NA | | | |
| Pos. | 97 | obs: | exp: |
| gtt | V | 23 | 102.20 |
| gtc | V | 381 | 104.30 |
| gta | V | 86 | 93.15 |
| gtg | V | 7 | 197.40 |
| --- | -- | ------ | ------ |
| mPD | | 0.38 | 0.72 |
| | | nPD: | 0.53 |
| | | N. weight: | 1.2 |
| | | Sc. PD: | 0.26 |
| | | Sc. rank: | 219.8 |

,

| | | | |
|---|---|---|---|
| NA | | | |
| Pos. | 98 | obs: | exp: |
| gtt | V | 2 | 102.20 |
| gtc | V | 0 | 104.30 |
| gta | V | 35 | 93.15 |
| gtg | V | 460 | 197.40 |
| --- | -- | ------ | ------ |
| mPD | | 0.14 | 0.72 |
| | | nPD: | 0.19 |
| | | N. weight: | 0.99 |
| | | Sc. PD: | -0.12 |
| | | Sc. rank: | -78.4 |

,

| | | | |
|---|---|---|---|
| NA | | | |
| Pos. | 99 | obs: | exp: |
| att | I | 0 | 158.60 |
| atc | I | 0 | 110.60 |
| ata | I | 422 | 152.80 |
| gtt | V | 2 | 15.42 |
| gtc | V | 0 | 15.74 |
| gta | V | 4 | 14.06 |
| gtg | V | 69 | 29.79 |
| --- | -- | ------ | ------ |
| mPD | | 0.50 | 0.95 |
| | | nPD: | 0.53 |
| | | N. weight: | 1.4 |
| | | Sc. PD: | 0.28 |
| | | Sc. rank: | 239.9 |

,

| | | | |
|---|---|---|---|
| NA | | | |
| Pos. | 100 | obs: | exp: |
| gct | A | 2 | 125.20 |
| gcc | A | 73 | 101.80 |
| gca | A | 385 | 221.50 |
| gcg | A | 37 | 48.50 |
| --- | -- | ------ | ------ |
| mPD | | 0.37 | 0.69 |
| | | nPD: | 0.54 |
| | | N. weight: | 0.5 |
| | | Sc. PD: | 0.11 |
| | | Sc. rank: | 96.9 |

,

| | | | |
|---|---|---|---|
| NA | | | |
| Pos. | 101 | obs: | exp: |
| aaa | K | 418 | 278.30 |
| aag | K | 79 | 218.70 |
| --- | -- | ------ | ------ |
| mPD | | 0.27 | 0.49 |
| | | nPD: | 0.54 |
| | | N. weight: | 0.26 |
| | | Sc. PD: | 0.058 |
| | | Sc. rank: | 50.5 |

,

| | | | |
|---|---|---|---|
| NA | | | |
| Pos. | 102 | obs: | exp: |
| gat | D | 0 | 265.20 |
| gac | D | 497 | 231.80 |
| --- | -- | ------ | ------ |
| mPD | | 0 | 0.50 |
| | | nPD: | 0. |
| | | N. weight: | 1.1 |
| | | Sc. PD: | -0.32 |
| | | Sc. rank: | -327.1 |

,

| | | | |
|---|---|---|---|
| NA | | | |
| Pos. | 103 | obs: | exp: |
| aat | N | 424 | 259.80 |
| aac | N | 73 | 237.20 |
| --- | -- | ------ | ------ |
| mPD | | 0.25 | 0.50 |
| | | nPD: | 0.5 |
| | | N. weight: | 0.35 |
| | | Sc. PD: | 0.064 |
| | | Sc. rank: | 53.8 |

,

| | | | |
|---|---|---|---|
| NA | | | |
| Pos. | 104 | obs: | exp: |
| gct | A | 9 | 125.20 |
| gcc | A | 46 | 101.80 |
| gca | A | 442 | 221.50 |
| gcg | A | 0 | 48.50 |
| --- | -- | ------ | ------ |
| mPD | | 0.20 | 0.69 |
| | | nPD: | 0.29 |
| | | N. weight: | 0.7 |
| | | Sc. PD: | -0.015 |
| | | Sc. rank: | -15.5 |

,

| | | | |
|---|---|---|---|
| NA | | | |
| Pos. | 105 | obs: | exp: |
| att | I | 0 | 44.36 |
| atc | I | 0 | 30.93 |
| ata | I | 118 | 42.71 |
| gtt | V | 0 | 77.90 |
| gtc | V | 0 | 79.54 |
| gta | V | 342 | 71.03 |
| gtg | V | 37 | 150.50 |
| --- | -- | ------ | ------ |
| mPD | | 0.50 | 1.1 |
| | | nPD: | 0.45 |
| | | N. weight: | 1.7 |
| | | Sc. PD: | 0.23 |
| | | Sc. rank: | 191.1 |

,

| | | | |
|---|---|---|---|
| NA | | | |
| Pos. | 106 | obs: | exp: |
| cgt | R | 0 | 20.63 |
| cgc | R | 0 | 24.34 |
| cga | R | 0 | 47.76 |
| cgg | R | 0 | 41.47 |
| aga | R | 463 | 223.50 |
| agg | R | 34 | 139.30 |
| --- | -- | ------ | ------ |
| mPD | | 0.13 | 0.96 |
| | | nPD: | 0.13 |
| | | N. weight: | 0.82 |
| | | Sc. PD: | -0.14 |
| | | Sc. rank: | -99.7 |

,

| | | | |
|---|---|---|---|
| NA | | | |
| Pos. | 107 | obs: | exp: |
| ttt | F | 403 | 204.30 |
| ttc | F | 94 | 292.70 |
| --- | -- | ------ | ------ |
| mPD | | 0.31 | 0.48 |
| | | nPD: | 0.63 |
| | | N. weight: | 0.48 |
| | | Sc. PD: | 0.15 |
| | | Sc. rank: | 123.2 |

,

| | | | |
|---|---|---|---|
| NA | | | |
| Pos. | 108 | obs: | exp: |
| tct | S | 0 | 0.32 |
| tcc | S | 0 | 0.28 |
| tca | S | 0 | 0.44 |
| tcg | S | 0 | 0.12 |
| agt | S | 2 | 0.44 |
| agc | S | 0 | 0.40 |
| ggt | G | 68 | 61.08 |
| ggc | G | 0 | 66.94 |
| gga | G | 74 | 239.50 |
| ggg | G | 353 | 127.50 |
| --- | -- | ------ | ------ |
| mPD | | 0.46 | 0.68 |
| | | nPD: | 0.68 |
| | | N. weight: | 0.81 |
| | | Sc. PD: | 0.29 |
| | | Sc. rank: | 224.1 |

,

| | | | |
|---|---|---|---|
| NA | | | |
| Pos. | 109 | obs: | exp: |
| gaa | E | 492 | 286.00 |
| gag | E | 5 | 211.00 |
| --- | -- | ------ | ------ |
| mPD | | 0.020 | 0.49 |
| | | nPD: | 0.04 |
| | | N. weight: | 0.7 |
| | | Sc. PD: | -0.19 |
| | | Sc. rank: | -140.1 |

,

| | | | |
|---|---|---|---|
| NA | | | |
| Pos. | 110 | obs: | exp: |
| tct | S | 0 | 78.38 |
| tcc | S | 0 | 68.68 |
| tca | S | 0 | 109.20 |
| tcg | S | 0 | 30.05 |
| act | T | 3 | 0.79 |
| acc | T | 0 | 0.64 |
| aca | T | 0 | 1.31 |
| acg | T | 0 | 0.25 |
| aat | N | 1 | 0.52 |
| aac | N | 0 | 0.48 |
| agt | S | 491 | 108.50 |
| agc | S | 2 | 98.22 |
| --- | -- | ------ | ------ |
| mPD | | 0.024 | 1.7 |
| | | nPD: | 0.01 |
| | | N. weight: | 2.1 |
| | | Sc. PD: | -0.6 |
| | | Sc. rank: | -507.7 |

,

| | | | |
|---|---|---|---|
| NA | | | |
| Pos. | 111 | obs: | exp: |
| gaa | E | 455 | 286.00 |
| gag | E | 42 | 211.00 |
| --- | -- | ------ | ------ |
| mPD | | 0.16 | 0.49 |
| | | nPD: | 0.32 |
| | | N. weight: | 0.41 |
| | | Sc. PD: | 0.0014 |
| | | Sc. rank: | -3.2 |

,

| | | | |
|---|---|---|---|
| NA | | | |
| Pos. | 112 | obs: | exp: |
| caa | Q | 496 | 265.00 |
| cag | Q | 1 | 232.00 |
| --- | -- | ------ | ------ |
| mPD | | 0.0040 | 0.50 |
| | | nPD: | 0.01 |
| | | N. weight: | 0.86 |
| | | Sc. PD: | -0.26 |
| | | Sc. rank: | -233.1 |

,

| | | | |
|---|---|---|---|
| NA | | | |
| Pos. | 113 | obs: | exp: |
| att | I | 1 | 186.10 |
| atc | I | 424 | 129.70 |
| ata | I | 70 | 179.20 |
| gtt | V | 0 | 0.41 |
| gtc | V | 0 | 0.42 |
| gta | V | 2 | 0.37 |
| gtg | V | 0 | 0.79 |
| --- | -- | ------ | ------ |
| mPD | | 0.26 | 0.67 |
| | | nPD: | 0.39 |
| | | N. weight: | 1.2 |
| | | Sc. PD: | 0.09 |
| | | Sc. rank: | 68.1 |

,

| | | | |
|---|---|---|---|
| NA | | | |
| Pos. | 114 | obs: | exp: |
| att | I | 425 | 186.80 |
| atc | I | 3 | 130.30 |
| ata | I | 69 | 179.90 |
| --- | -- | ------ | ------ |
| mPD | | 0.25 | 0.66 |
| | | nPD: | 0.38 |
| | | N. weight: | 0.78 |
| | | Sc. PD: | 0.049 |
| | | Sc. rank: | 34.7 |

,

| | | | |
|---|---|---|---|
| NA | | | |
| Pos. | 115 | obs: | exp: |
| gtt | V | 407 | 102.20 |
| gtc | V | 19 | 104.30 |
| gta | V | 5 | 93.15 |
| gtg | V | 66 | 197.40 |
| --- | -- | ------ | ------ |
| mPD | | 0.31 | 0.72 |
| | | nPD: | 0.43 |
| | | N. weight: | 1.3 |
| | | Sc. PD: | 0.14 |
| | | Sc. rank: | 111.3 |

,

NA

| Pos. | 116 | obs: | exp: |
|---|---|---|---|
| act | T | 17 | 131.30 |
| acc | T | 406 | 106.60 |
| aca | T | 74 | 217.70 |
| acg | T | 0 | 41.32 |
| --- | -- | ------ | ------ |
| mPD | | 0.31 | 0.69 |
| | | nPD: | 0.45 |
| | | N. weight: | 1.2 |
| | | Sc. PD: | 0.16 |
| | | Sc. rank: | 138.1 |

NA

| Pos. | 117 | obs: | exp: |
|---|---|---|---|
| cgt | R | 0 | 20.63 |
| cgc | R | 0 | 24.34 |
| cga | R | 0 | 47.76 |
| cgg | R | 0 | 41.47 |
| aga | R | 107 | 223.50 |
| agg | R | 390 | 139.30 |
| --- | -- | ------ | ------ |
| mPD | | 0.34 | 0.96 |
| | | nPD: | 0.35 |
| | | N. weight: | 0.92 |
| | | Sc. PD: | 0.035 |
| | | Sc. rank: | 18.2 |

NA

| Pos. | 118 | obs: | exp: |
|---|---|---|---|
| gaa | E | 21 | 286.00 |
| gag | E | 476 | 211.00 |
| --- | -- | ------ | ------ |
| mPD | | 0.081 | 0.49 |
| | | nPD: | 0.17 |
| | | N. weight: | 0.94 |
| | | Sc. PD: | -0.14 |
| | | Sc. rank: | -88.4 |

NA

| Pos. | 119 | obs: | exp: |
|---|---|---|---|
| cct | P | 1 | 130.90 |
| ccc | P | 20 | 89.65 |
| cca | P | 374 | 196.10 |
| ccg | P | 102 | 80.36 |
| --- | -- | ------ | ------ |
| mPD | | 0.39 | 0.72 |
| | | nPD: | 0.55 |
| | | N. weight: | 0.67 |
| | | Sc. PD: | 0.15 |
| | | Sc. rank: | 131.5 |

NA

| Pos. | 120 | obs: | exp: |
|---|---|---|---|
| tat | Y | 459 | 257.30 |
| tac | Y | 38 | 239.70 |
| --- | -- | ------ | ------ |
| mPD | | 0.14 | 0.50 |
| | | nPD: | 0.28 |
| | | N. weight: | 0.56 |
| | | Sc. PD: | -0.017 |
| | | Sc. rank: | -13.7 |

NA

| Pos. | 121 | obs: | exp: |
|---|---|---|---|
| gtt | V | 0 | 101.90 |
| gtc | V | 0 | 104.10 |
| gta | V | 376 | 92.96 |
| gtg | V | 120 | 197.00 |
| gct | A | 0 | 0.25 |
| gcc | A | 0 | 0.20 |
| gca | A | 1 | 0.45 |
| gcg | A | 0 | 0.10 |
| --- | -- | ------ | ------ |
| mPD | | 0.37 | 0.73 |
| | | nPD: | 0.51 |
| | | N. weight: | 1.3 |
| | | Sc. PD: | 0.25 |
| | | Sc. rank: | 214.5 |

NA

| Pos. | 122 | obs: | exp: |
|---|---|---|---|
| tct | S | 0 | 79.01 |
| tcc | S | 0 | 69.23 |
| tca | S | 496 | 110.10 |
| tcg | S | 1 | 30.29 |
| agt | S | 0 | 109.30 |
| agc | S | 0 | 99.02 |
| --- | -- | ------ | ------ |
| mPD | | 0.0040 | 1.7 |
| | | nPD: | 0. |
| | | N. weight: | 2.1 |
| | | Sc. PD: | -0.63 |
| | | Sc. rank: | -585.6 |

NA

| Pos. | 123 | obs: | exp: |
|---|---|---|---|
| tgt | C | 113 | 204.90 |
| tgc | C | 384 | 292.10 |
| --- | -- | ------ | ------ |
| mPD | | 0.35 | 0.48 |
| | | nPD: | 0.73 |
| | | N. weight: | 0.12 |
| | | Sc. PD: | 0.046 |
| | | Sc. rank: | 33.3 |

NA

| Pos. | 124 | obs: | exp: |
|---|---|---|---|
| gat | D | 114 | 264.10 |
| gac | D | 381 | 230.90 |
| ggt | G | 0 | 0.25 |
| ggc | G | 2 | 0.27 |
| gga | G | 0 | 0.97 |
| ggg | G | 0 | 0.52 |
| --- | -- | ------ | ------ |
| mPD | | 0.36 | 0.51 |
| | | nPD: | 0.71 |
| | | N. weight: | 0.29 |
| | | Sc. PD: | 0.11 |
| | | Sc. rank: | 81.3 |

NA

| Pos. | 125 | obs: | exp: |
|---|---|---|---|
| cct | P | 13 | 130.90 |
| ccc | P | 0 | 89.65 |
| cca | P | 483 | 196.10 |
| ccg | P | 1 | 80.36 |
| --- | -- | ------ | ------ |
| mPD | | 0.055 | 0.72 |
| | | nPD: | 0.08 |
| | | N. weight: | 1.1 |
| | | Sc. PD: | -0.26 |
| | | Sc. rank: | -169.7 |

NA

| Pos. | 126 | obs: | exp: |
|---|---|---|---|
| tta | L | 68 | 6.03 |
| ttg | L | 1 | 13.29 |
| tct | S | 0 | 0.95 |
| tcc | S | 0 | 0.84 |
| tca | S | 4 | 1.33 |
| tcg | S | 0 | 0.37 |
| ctt | L | 0 | 12.87 |
| ctc | L | 0 | 11.16 |
| cta | L | 0 | 10.39 |
| ctg | L | 0 | 15.25 |
| att | I | 0 | 1.13 |
| atc | I | 0 | 0.79 |
| ata | I | 3 | 1.09 |
| act | T | 1 | 107.50 |
| acc | T | 0 | 87.34 |
| aca | T | 406 | 178.30 |
| acg | T | 0 | 33.83 |
| aat | N | 9 | 4.70 |
| aac | N | 0 | 4.30 |
| aaa | K | 1 | 0.56 |
| aag | K | 0 | 0.44 |
| agt | S | 2 | 1.32 |
| agc | S | 0 | 1.20 |
| gct | A | 0 | 0.50 |
| gcc | A | 0 | 0.41 |
| gca | A | 2 | 0.89 |
| gcg | A | 0 | 0.20 |
| --- | -- | ------ | ------ |
| mPD | | 0.60 | 1.3 |
| | | nPD: | 0.47 |
| | | N. weight: | 1.4 |
| | | Sc. PD: | 0.22 |
| | | Sc. rank: | 192.3 |

NA

| Pos. | 127 | obs: | exp: |
|---|---|---|---|
| gaa | E | 1 | 0.58 |
| gag | E | 0 | 0.42 |
| ggt | G | 0 | 61.20 |
| ggc | G | 0 | 67.08 |
| gga | G | 486 | 239.90 |
| ggg | G | 10 | 127.80 |
| --- | -- | ------ | ------ |
| mPD | | 0.044 | 0.67 |
| | | nPD: | 0.06 |
| | | N. weight: | 0.9 |
| | | Sc. PD: | -0.22 |
| | | Sc. rank: | -146.4 |

NA

| Pos. | 128 | obs: | exp: |
|---|---|---|---|
| tgt | C | 90 | 204.90 |
| tgc | C | 407 | 292.10 |
| --- | -- | ------ | ------ |
| mPD | | 0.30 | 0.48 |
| | | nPD: | 0.61 |
| | | N. weight: | 0.18 |
| | | Sc. PD: | 0.052 |
| | | Sc. rank: | 44.2 |

NA

| Pos. | 129 | obs: | exp: |
|---|---|---|---|
| cgt | R | 0 | 0.46 |
| cgc | R | 0 | 0.54 |
| cga | R | 0 | 1.06 |
| cgg | R | 0 | 0.92 |
| aaa | K | 355 | 272.10 |
| aag | K | 131 | 213.90 |
| aga | R | 7 | 4.95 |
| agg | R | 4 | 3.08 |
| --- | -- | ------ | ------ |
| mPD | | 0.44 | 0.55 |
| | | nPD: | 0.8 |
| | | N. weight: | 0.11 |
| | | Sc. PD: | 0.051 |
| | | Sc. rank: | 32.2 |

NA

| Pos. | 130 | obs: | exp: |
|---|---|---|---|
| atg | M | 497 | 497.00 |
| --- | -- | ------ | ------ |
| mPD | | 0 | 0 |
| | | nPD: | 1. |
| | | N. weight: | 0. |
| | | Sc. PD: | 0 |
| | | Sc. rank: | 0 |

NA

| Pos. | 131 | obs: | exp: |
|---|---|---|---|
| tat | Y | 434 | 257.30 |
| tac | Y | 63 | 239.70 |
| --- | -- | ------ | ------ |
| mPD | | 0.22 | 0.50 |
| | | nPD: | 0.44 |
| | | N. weight: | 0.41 |
| | | Sc. PD: | 0.052 |
| | | Sc. rank: | 42.9 |

NA

| Pos. | 132 | obs: | exp: |
|---|---|---|---|
| gct | A | 1 | 125.20 |
| gcc | A | 425 | 101.80 |
| gca | A | 70 | 221.50 |
| gcg | A | 1 | 48.50 |
| --- | -- | ------ | ------ |
| mPD | | 0.25 | 0.69 |
| | | nPD: | 0.36 |
| | | N. weight: | 1.5 |
| | | Sc. PD: | 0.071 |
| | | Sc. rank: | 43.5 |

NA

| Pos. | 133 | obs: | exp: |
|---|---|---|---|
| tta | L | 27 | 43.42 |
| ttg | L | 338 | 95.72 |
| ctt | L | 0 | 92.73 |
| ctc | L | 0 | 80.39 |
| cta | L | 6 | 74.87 |
| ctg | L | 126 | 109.90 |
| --- | -- | ------ | ------ |
| mPD | | 0.52 | 1.1 |
| | | nPD: | 0.46 |
| | | N. weight: | 1.2 |
| | | Sc. PD: | 0.17 |
| | | Sc. rank: | 145.6 |

NA

| Pos. | 134 | obs: | exp: |
|---|---|---|---|
| cat | H | 113 | 269.30 |
| cac | H | 384 | 227.70 |
| --- | -- | ------ | ------ |
| mPD | | 0.35 | 0.50 |
| | | nPD: | 0.71 |
| | | N. weight: | 0.3 |
| | | Sc. PD: | 0.11 |
| | | Sc. rank: | 83.8 |

NA

| Pos. | 135 | obs: | exp: |
|---|---|---|---|
| caa | Q | 471 | 265.00 |
| cag | Q | 26 | 232.00 |
| --- | -- | ------ | ------ |
| mPD | | 0.099 | 0.50 |
| | | nPD: | 0.2 |
| | | N. weight: | 0.61 |
| | | Sc. PD: | -0.068 |
| | | Sc. rank: | -46.7 |

NA

| Pos. | 136 | obs: | exp: |
|---|---|---|---|
| ggt | G | 0 | 61.32 |
| ggc | G | 0 | 67.21 |
| gga | G | 34 | 240.40 |
| ggg | G | 463 | 128.00 |
| --- | -- | ------ | ------ |
| mPD | | 0.13 | 0.67 |
| | | nPD: | 0.19 |
| | | N. weight: | 1.5 |
| | | Sc. PD: | -0.18 |
| | | Sc. rank: | -120.6 |

NA

| Pos. | 137 | obs: | exp: |
|---|---|---|---|
| act | T | 371 | 131.30 |
| acc | T | 56 | 106.60 |
| aca | T | 70 | 217.70 |
| acg | T | 0 | 41.32 |
| --- | -- | ------ | ------ |
| mPD | | 0.41 | 0.69 |
| | | nPD: | 0.6 |
| | | N. weight: | 0.78 |
| | | Sc. PD: | 0.22 |
| | | Sc. rank: | 186.7 |

NA

| Pos. | 138 | obs: | exp: |
|---|---|---|---|
| act | T | 25 | 131.30 |
| acc | T | 471 | 106.60 |
| aca | T | 1 | 217.70 |
| acg | T | 0 | 41.32 |
| --- | -- | ------ | ------ |
| mPD | | 0.10 | 0.69 |
| | | nPD: | 0.15 |
| | | N. weight: | 1.8 |
| | | Sc. PD: | -0.3 |
| | | Sc. rank: | -203.1 |

NA

| Pos. | 139 | obs: | exp: |
|---|---|---|---|
| att | I | 486 | 186.50 |
| atc | I | 8 | 130.00 |
| ata | I | 2 | 179.50 |
| gtt | V | 1 | 0.21 |
| gtc | V | 0 | 0.21 |
| gta | V | 0 | 0.19 |
| gtg | V | 0 | 0.40 |
| --- | -- | ------ | ------ |
| mPD | | 0.044 | 0.66 |
| | | nPD: | 0.07 |
| | | N. weight: | 1.2 |
| | | Sc. PD: | -0.3 |
| | | Sc. rank: | -198.8 |

NA

| Pos. | 140 | obs: | exp: |
|---|---|---|---|
| cgt | R | 0 | 20.55 |
| cgc | R | 0 | 24.24 |
| cga | R | 0 | 47.57 |
| cgg | R | 0 | 41.31 |
| aaa | K | 0 | 1.12 |
| aag | K | 2 | 0.88 |
| aga | R | 98 | 222.60 |
| agg | R | 397 | 138.70 |
| --- | -- | ------ | ------ |
| mPD | | 0.33 | 0.97 |
| | | nPD: | 0.34 |
| | | N. weight: | 0.96 |
| | | Sc. PD: | 0.021 |
| | | Sc. rank: | 4.5 |

NA

| Pos. | 141 | obs: | exp: |
|---|---|---|---|
| aat | N | 0 | 259.80 |
| aac | N | 497 | 237.20 |
| --- | -- | ------ | ------ |
| mPD | | 0 | 0.50 |
| | | nPD: | 0. |
| | | N. weight: | 1. |
| | | Sc. PD: | -0.31 |
| | | Sc. rank: | -317.1 |

NA

| Pos. | 142 | obs: | exp: |
|---|---|---|---|
| cgt | R | 0 | 0.08 |
| cgc | R | 0 | 0.10 |
| cga | R | 0 | 0.19 |
| cgg | R | 0 | 0.17 |
| aaa | K | 415 | 277.20 |
| aag | K | 80 | 217.80 |
| aga | R | 2 | 0.90 |
| agg | R | 0 | 0.56 |
| --- | -- | ------ | ------ |
| mPD | | 0.28 | 0.50 |
| | | nPD: | 0.55 |
| | | N. weight: | 0.26 |
| | | Sc. PD: | 0.06 |
| | | Sc. rank: | 53.9 |

NA

| Pos. | 143 | obs: | exp: |
|---|---|---|---|
| cat | H | 440 | 269.30 |
| cac | H | 57 | 227.70 |
| --- | -- | ------ | ------ |
| mPD | | 0.20 | 0.50 |
| | | nPD: | 0.41 |
| | | N. weight: | 0.39 |
| | | Sc. PD: | 0.037 |
| | | Sc. rank: | 30.8 |

NA

| Pos. | 144 | obs: | exp: |
|---|---|---|---|
| tct | S | 0 | 79.01 |
| tcc | S | 0 | 69.23 |
| tca | S | 493 | 110.10 |
| tcg | S | 4 | 30.29 |
| agt | S | 0 | 109.30 |
| agc | S | 0 | 99.02 |
| --- | -- | ------ | ------ |
| mPD | | 0.016 | 1.7 |
| | | nPD: | 0.01 |
| | | N. weight: | 2.1 |
| | | Sc. PD: | -0.61 |
| | | Sc. rank: | -538.9 |

NA

| Pos. | 145 | obs: | exp: |
|---|---|---|---|
| aat | N | 431 | 259.80 |
| aac | N | 66 | 237.20 |
| --- | -- | ------ | ------ |
| mPD | | 0.23 | 0.50 |
| | | nPD: | 0.46 |
| | | N. weight: | 0.38 |
| | | Sc. PD: | 0.055 |
| | | Sc. rank: | 47.2 |

NA

| Pos. | 146 | obs: | exp: |
|---|---|---|---|
| ggt | G | 0 | 61.32 |
| ggc | G | 1 | 67.21 |
| gga | G | 489 | 240.40 |
| ggg | G | 7 | 128.00 |
| --- | -- | ------ | ------ |
| mPD | | 0.032 | 0.67 |
| | | nPD: | 0.05 |
| | | N. weight: | 0.92 |
| | | Sc. PD: | -0.24 |
| | | Sc. rank: | -175.2 |

NA

| Pos. | 147 | obs: | exp: |
|---|---|---|---|
| act | T | 2 | 131.30 |
| acc | T | 8 | 106.60 |
| aca | T | 194 | 217.70 |
| acg | T | 293 | 41.32 |
| --- | -- | ------ | ------ |
| mPD | | 0.50 | 0.69 |
| | | nPD: | 0.73 |
| | | N. weight: | 1.5 |
| | | Sc. PD: | 0.6 |
| | | Sc. rank: | 433.1 |

NA

| Pos. | 148 | obs: | exp: |
|---|---|---|---|
| att | I | 421 | 185.30 |
| atc | I | 2 | 129.20 |
| ata | I | 70 | 178.50 |
| atg | M | 2 | 2.00 |
| gtt | V | 1 | 0.41 |
| gtc | V | 0 | 0.42 |
| gta | V | 1 | 0.37 |
| gtg | V | 0 | 0.79 |
| --- | -- | ------ | ------ |
| mPD | | 0.27 | 0.67 |
| | | nPD: | 0.4 |
| | | N. weight: | 0.78 |
| | | Sc. PD: | 0.064 |
| | | Sc. rank: | 46.9 |

NA

| Pos. | 149 | obs: | exp: |
|---|---|---|---|
| cat | H | 386 | 269.30 |
| cac | H | 111 | 227.70 |
| --- | -- | ------ | ------ |
| mPD | | 0.35 | 0.50 |
| | | nPD: | 0.7 |
| | | N. weight: | 0.18 |
| | | Sc. PD: | 0.066 |
| | | Sc. rank: | 49.3 |

NA

| Pos. | 150 | obs: | exp: |
|---|---|---|---|
| gat | D | 39 | 265.20 |
| gac | D | 458 | 231.80 |
| --- | -- | ------ | ------ |
| mPD | | 0.14 | 0.50 |
| | | nPD: | 0.29 |
| | | N. weight: | 0.67 |
| | | Sc. PD: | -0.015 |
| | | Sc. rank: | -16.0 |

NA

| Pos. | 151 | obs: | exp: |
|---|---|---|---|
| cgt | R | 0 | 20.63 |
| cgc | R | 0 | 24.34 |
| cga | R | 0 | 47.76 |
| cgg | R | 0 | 41.47 |
| aga | R | 426 | 223.50 |
| agg | R | 71 | 139.30 |
| --- | -- | ------ | ------ |
| mPD | | 0.25 | 0.96 |
| | | nPD: | 0.26 |
| | | N. weight: | 0.65 |
| | | Sc. PD: | -0.037 |
| | | Sc. rank: | -27.5 |

NA

| Pos. | 152 | obs: | exp: |
|---|---|---|---|
| act | T | 74 | 131.30 |
| acc | T | 32 | 106.60 |
| aca | T | 388 | 217.70 |
| acg | T | 3 | 41.32 |
| --- | -- | ------ | ------ |
| mPD | | 0.36 | 0.69 |
| | | nPD: | 0.53 |
| | | N. weight: | 0.4 |
| | | Sc. PD: | 0.085 |
| | | Sc. rank: | 74.4 |

NA

| Pos. | 153 | obs: | exp: |
|---|---|---|---|
| tct | S | 1 | 0.16 |
| tcc | S | 0 | 0.14 |
| tca | S | 0 | 0.22 |
| tcg | S | 0 | 0.06 |
| act | T | 2 | 0.53 |
| acc | T | 0 | 0.43 |
| aca | T | 0 | 0.88 |
| acg | T | 0 | 0.17 |
| agt | S | 0 | 0.22 |
| agc | S | 0 | 0.20 |
| gct | A | 487 | 124.40 |
| gcc | A | 7 | 101.20 |
| gca | A | 0 | 220.20 |
| gcg | A | 0 | 48.21 |
| --- | -- | ------ | ------ |
| mPD | | 0.040 | 0.70 |
| | | nPD: | 0.06 |
| | | N. weight: | 1.8 |
| | | Sc. PD: | -0.46 |
| | | Sc. rank: | -322.3 |

NA

| Pos. | 154 | obs: | exp: |
|---|---|---|---|
| ttt | F | 7 | 203.90 |
| ttc | F | 489 | 292.10 |
| tct | S | 0 | 0.16 |
| tcc | S | 1 | 0.14 |
| tca | S | 0 | 0.22 |
| tcg | S | 0 | 0.06 |
| agt | S | 0 | 0.22 |
| agc | S | 0 | 0.20 |
| --- | -- | ------ | ------ |
| mPD | | 0.032 | 0.49 |
| | | nPD: | 0.06 |
| | | N. weight: | 0.65 |
| | | Sc. PD: | -0.16 |
| | | Sc. rank: | -106.9 |

NA

| Pos. | 155 | obs: | exp: |
|---|---|---|---|
| cgt | R | 2 | 20.63 |
| cgc | R | 0 | 24.34 |
| cga | R | 39 | 47.76 |
| cgg | R | 0 | 41.47 |
| aga | R | 443 | 223.50 |
| agg | R | 13 | 139.30 |
| --- | -- | ------ | ------ |
| mPD | | 0.21 | 0.96 |
| | | nPD: | 0.22 |
| | | N. weight: | 0.75 |
| | | Sc. PD: | -0.069 |
| | | Sc. rank: | -49.5 |

NA

| Pos. | 156 | obs: | exp: |
|---|---|---|---|
| ggt | G | 415 | 61.32 |
| ggc | G | 30 | 67.21 |
| gga | G | 1 | 240.40 |
| ggg | G | 51 | 128.00 |
| --- | -- | ------ | ------ |
| mPD | | 0.29 | 0.67 |
| | | nPD: | 0.43 |
| | | N. weight: | 2. |
| | | Sc. PD: | 0.24 |
| | | Sc. rank: | 190.7 |

NA

| Pos. | 157 | obs: | exp: |
|---|---|---|---|
| tta | L | 5 | 43.42 |
| ttg | L | 65 | 95.72 |
| ctt | L | 8 | 92.73 |
| ctc | L | 416 | 80.39 |
| cta | L | 2 | 74.87 |
| ctg | L | 1 | 109.90 |
| --- | -- | ------ | ------ |
| mPD | | 0.52 | 1.1 |
| | | nPD: | 0.47 |
| | | N. weight: | 1.8 |
| | | Sc. PD: | 0.27 |
| | | Sc. rank: | 232.0 |

NA

| Pos. | 158 | obs: | exp: |
|---|---|---|---|
| tta | L | 1 | 0.09 |
| ttg | L | 0 | 0.19 |
| ctt | L | 0 | 0.19 |
| ctc | L | 0 | 0.16 |
| cta | L | 0 | 0.15 |
| ctg | L | 0 | 0.22 |
| att | I | 31 | 186.10 |
| atc | I | 353 | 129.70 |
| ata | I | 111 | 179.20 |
| gtt | V | 0 | 0.21 |
| gtc | V | 1 | 0.21 |
| gta | V | 0 | 0.19 |
| gtg | V | 0 | 0.40 |
| --- | -- | ------ | ------ |
| mPD | | 0.45 | 0.67 |
| | | nPD: | 0.67 |
| | | N. weight: | 0.72 |
| | | Sc. PD: | 0.25 |
| | | Sc. rank: | 194.6 |

NA

| Pos. | 159 | obs: | exp: |
|---|---|---|---|
| tct | S | 3 | 79.01 |
| tcc | S | 421 | 69.23 |
| tca | S | 73 | 110.10 |
| tcg | S | 0 | 30.29 |
| agt | S | 0 | 109.30 |
| agc | S | 0 | 99.02 |
| --- | -- | ------ | ------ |
| mPD | | 0.26 | 1.7 |
| | | nPD: | 0.16 |
| | | N. weight: | 2. |
| | | Sc. PD: | -0.31 |
| | | Sc. rank: | -201.8 |

NA

| Pos. | 160 | obs: | exp: |
|---|---|---|---|
| att | I | 1 | 0.38 |
| atc | I | 0 | 0.26 |
| ata | I | 0 | 0.36 |
| act | T | 456 | 131.00 |
| acc | T | 40 | 106.40 |
| aca | T | 0 | 217.30 |
| acg | T | 0 | 41.23 |
| --- | -- | ------ | ------ |
| mPD | | 0.15 | 0.69 |
| | | nPD: | 0.22 |
| | | N. weight: | 1.5 |
| | | Sc. PD: | -0.14 |
| | | Sc. rank: | -96.4 |

| | | | |
|---|---|---|---|
| NA | | | |
| Pos. | 161 | obs: | exp: |
| tct | S | 0 | 1.11 |
| tcc | S | 0 | 0.98 |
| tca | S | 7 | 1.55 |
| tcg | S | 0 | 0.43 |
| cct | P | 68 | 128.80 |
| ccc | P | 3 | 88.20 |
| cca | P | 417 | 193.00 |
| ccg | P | 1 | 79.07 |
| caa | Q | 1 | 0.53 |
| cag | Q | 0 | 0.47 |
| agt | S | 0 | 1.54 |
| agc | S | 0 | 1.39 |
| --- | -- | ------ | ------ |
| mPD | | 0.28 | 0.76 |
| | | nPD: | 0.37 |
| | | N. weight: | 0.79 |
| | | Sc. PD: | 0.044 |
| | | Sc. rank: | 30.5 |

| | | | |
|---|---|---|---|
| NA | | | |
| Pos. | 162 | obs: | exp: |
| tta | L | 4 | 43.42 |
| ttg | L | 484 | 95.72 |
| ctt | L | 0 | 92.73 |
| ctc | L | 0 | 80.39 |
| cta | L | 4 | 74.87 |
| ctg | L | 5 | 109.90 |
| --- | -- | ------ | ------ |
| mPD | | 0.067 | 1.1 |
| | | nPD: | 0.06 |
| | | N. weight: | 2.1 |
| | | Sc. PD: | -0.52 |
| | | Sc. rank: | -359.6 |

| | | | |
|---|---|---|---|
| NA | | | |
| Pos. | 163 | obs: | exp: |
| ggt | G | 4 | 61.32 |
| ggc | G | 419 | 67.21 |
| gga | G | 5 | 240.40 |
| ggg | G | 69 | 128.00 |
| --- | -- | ------ | ------ |
| mPD | | 0.27 | 0.67 |
| | | nPD: | 0.41 |
| | | N. weight: | 2. |
| | | Sc. PD: | 0.18 |
| | | Sc. rank: | 145.5 |

| | | | |
|---|---|---|---|
| NA | | | |
| Pos. | 164 | obs: | exp: |
| tct | S | 0 | 11.29 |
| tcc | S | 0 | 9.89 |
| tca | S | 0 | 15.73 |
| tcg | S | 0 | 4.33 |
| act | T | 410 | 111.50 |
| acc | T | 12 | 90.56 |
| aca | T | 0 | 184.90 |
| acg | T | 0 | 35.08 |
| aat | N | 0 | 0.52 |
| aac | N | 1 | 0.48 |
| agt | S | 1 | 15.62 |
| agc | S | 70 | 14.15 |
| gct | A | 3 | 0.76 |
| gcc | A | 0 | 0.61 |
| gca | A | 0 | 1.34 |
| gcg | A | 0 | 0.29 |
| --- | -- | ------ | ------ |
| mPD | | 0.54 | 0.97 |
| | | nPD: | 0.55 |
| | | N. weight: | 1.8 |
| | | Sc. PD: | 0.41 |
| | | Sc. rank: | 366.7 |

| | | | |
|---|---|---|---|
| NA | | | |
| Pos. | 165 | obs: | exp: |
| cct | P | 3 | 130.90 |
| ccc | P | 68 | 89.65 |
| cca | P | 424 | 196.10 |
| ccg | P | 2 | 80.36 |
| --- | -- | ------ | ------ |
| mPD | | 0.25 | 0.72 |
| | | nPD: | 0.35 |
| | | N. weight: | 0.83 |
| | | Sc. PD: | 0.033 |
| | | Sc. rank: | 17.4 |

| | | | |
|---|---|---|---|
| NA | | | |
| Pos. | 166 | obs: | exp: |
| cct | P | 70 | 130.90 |
| ccc | P | 1 | 89.65 |
| cca | P | 413 | 196.10 |
| ccg | P | 13 | 80.36 |
| --- | -- | ------ | ------ |
| mPD | | 0.29 | 0.72 |
| | | nPD: | 0.4 |
| | | N. weight: | 0.68 |
| | | Sc. PD: | 0.06 |
| | | Sc. rank: | 46.3 |

| | | | |
|---|---|---|---|
| NA | | | |
| Pos. | 167 | obs: | exp: |
| tct | S | 0 | 0.16 |
| tcc | S | 0 | 0.14 |
| tca | S | 0 | 0.22 |
| tcg | S | 0 | 0.06 |
| att | I | 0 | 6.39 |
| atc | I | 0 | 4.46 |
| ata | I | 17 | 6.15 |
| act | T | 4 | 112.30 |
| acc | T | 418 | 91.20 |
| aca | T | 3 | 186.20 |
| acg | T | 0 | 35.33 |
| agt | S | 0 | 0.22 |
| agc | S | 1 | 0.20 |
| gtt | V | 0 | 11.10 |
| gtc | V | 0 | 11.33 |
| gta | V | 54 | 10.12 |
| gtg | V | 0 | 21.45 |
| --- | -- | ------ | ------ |
| mPD | | 0.71 | 1.1 |
| | | nPD: | 0.62 |
| | | N. weight: | 2. |
| | | Sc. PD: | 0.6 |
| | | Sc. rank: | 506.8 |

| | | | |
|---|---|---|---|
| NA | | | |
| Pos. | 168 | obs: | exp: |
| att | I | 0 | 10.15 |
| atc | I | 23 | 7.08 |
| ata | I | 4 | 9.77 |
| gtt | V | 2 | 96.40 |
| gtc | V | 46 | 98.43 |
| gta | V | 418 | 87.90 |
| gtg | V | 3 | 186.30 |
| gct | A | 0 | 0.25 |
| gcc | A | 0 | 0.20 |
| gca | A | 1 | 0.45 |
| gcg | A | 0 | 0.10 |
| --- | -- | ------ | ------ |
| mPD | | 0.36 | 0.84 |
| | | nPD: | 0.44 |
| | | N. weight: | 1.8 |
| | | Sc. PD: | 0.21 |
| | | Sc. rank: | 169.7 |

| | | | |
|---|---|---|---|
| NA | | | |
| Pos. | 169 | obs: | exp: |
| tct | S | 0 | 79.01 |
| tcc | S | 0 | 69.23 |
| tca | S | 0 | 110.10 |
| tcg | S | 0 | 30.29 |
| agt | S | 424 | 109.30 |
| agc | S | 73 | 99.02 |
| --- | -- | ------ | ------ |
| mPD | | 0.25 | 1.7 |
| | | nPD: | 0.15 |
| | | N. weight: | 1.6 |
| | | Sc. PD: | -0.25 |
| | | Sc. rank: | -161.9 |

| | | | |
|---|---|---|---|
| NA | | | |
| Pos. | 170 | obs: | exp: |
| aat | N | 79 | 259.80 |
| aac | N | 418 | 237.20 |
| --- | -- | ------ | ------ |
| mPD | | 0.27 | 0.50 |
| | | nPD: | 0.54 |
| | | N. weight: | 0.41 |
| | | Sc. PD: | 0.089 |
| | | Sc. rank: | 77.2 |

| | | | |
|---|---|---|---|
| NA | | | |
| Pos. | 171 | obs: | exp: |
| tct | S | 0 | 79.01 |
| tcc | S | 0 | 69.23 |
| tca | S | 0 | 110.10 |
| tcg | S | 0 | 30.29 |
| agt | S | 496 | 109.30 |
| agc | S | 1 | 99.02 |
| --- | -- | ------ | ------ |
| mPD | | 0.0040 | 1.7 |
| | | nPD: | 0. |
| | | N. weight: | 2.1 |
| | | Sc. PD: | -0.63 |
| | | Sc. rank: | -587.4 |

| | | | |
|---|---|---|---|
| NA | | | |
| Pos. | 172 | obs: | exp: |
| gat | D | 47 | 264.60 |
| gac | D | 449 | 231.40 |
| gaa | E | 1 | 0.58 |
| gag | E | 0 | 0.42 |
| --- | -- | ------ | ------ |
| mPD | | 0.18 | 0.50 |
| | | nPD: | 0.35 |
| | | N. weight: | 0.62 |
| | | Sc. PD: | 0.022 |
| | | Sc. rank: | 11.5 |

| | | | |
|---|---|---|---|
| NA | | | |
| Pos. | 173 | obs: | exp: |
| ttt | F | 453 | 204.30 |
| ttc | F | 44 | 292.70 |
| --- | -- | ------ | ------ |
| mPD | | 0.16 | 0.48 |
| | | nPD: | 0.33 |
| | | N. weight: | 0.79 |
| | | Sc. PD: | 0.015 |
| | | Sc. rank: | -0.6 |

| | | | |
|---|---|---|---|
| NA | | | |
| Pos. | 174 | obs: | exp: |
| tta | L | 0 | 6.20 |
| ttg | L | 0 | 13.67 |
| ctt | L | 48 | 13.25 |
| ctc | L | 0 | 11.48 |
| cta | L | 0 | 10.70 |
| ctg | L | 23 | 15.69 |
| att | I | 0 | 27.82 |
| atc | I | 0 | 19.39 |
| ata | I | 74 | 26.79 |
| atg | M | 345 | 345.00 |
| gtt | V | 0 | 1.44 |
| gtc | V | 0 | 1.47 |
| gta | V | 0 | 1.31 |
| gtg | V | 7 | 2.78 |
| --- | -- | ------ | ------ |
| mPD | | 0.67 | 0.68 |
| | | nPD: | 0.98 |
| | | N. weight: | 0.43 |
| | | Sc. PD: | 0.28 |
| | | Sc. rank: | 133.5 |

| | | | |
|---|---|---|---|
| NA | | | |
| Pos. | 175 | obs: | exp: |
| tgt | C | 496 | 204.90 |
| tgc | C | 1 | 292.10 |
| --- | -- | ------ | ------ |
| mPD | | 0.0040 | 0.48 |
| | | nPD: | 0.01 |
| | | N. weight: | 1.2 |
| | | Sc. PD: | -0.36 |
| | | Sc. rank: | -326.4 |

| | | | |
|---|---|---|---|
| NA | | | |
| Pos. | 176 | obs: | exp: |
| att | I | 1 | 0.38 |
| atc | I | 0 | 0.26 |
| ata | I | 0 | 0.36 |
| gtt | V | 425 | 101.90 |
| gtc | V | 3 | 104.10 |
| gta | V | 67 | 92.96 |
| gtg | V | 1 | 197.00 |
| --- | -- | ------ | ------ |
| mPD | | 0.25 | 0.73 |
| | | nPD: | 0.35 |
| | | N. weight: | 1.6 |
| | | Sc. PD: | 0.052 |
| | | Sc. rank: | 21.0 |

| | | | |
|---|---|---|---|
| NA | | | |
| Pos. | 177 | obs: | exp: |
| ggt | G | 0 | 61.32 |
| ggc | G | 0 | 67.21 |
| gga | G | 393 | 240.40 |
| ggg | G | 104 | 128.00 |
| --- | -- | ------ | ------ |
| mPD | | 0.33 | 0.67 |
| | | nPD: | 0.5 |
| | | N. weight: | 0.49 |
| | | Sc. PD: | 0.088 |
| | | Sc. rank: | 71.2 |

| | | | |
|---|---|---|---|
| NA | | | |
| Pos. | 178 | obs: | exp: |
| tgg | W | 497 | 497.00 |
| --- | -- | ------ | ------ |
| mPD | | 0 | 0 |
| | | nPD: | 1. |
| | | N. weight: | 0. |
| | | Sc. PD: | 0 |
| | | Sc. rank: | 0 |

| | | | |
|---|---|---|---|
| NA | | | |
| Pos. | 179 | obs: | exp: |
| tct | S | 4 | 79.01 |
| tcc | S | 4 | 69.23 |
| tca | S | 485 | 110.10 |
| tcg | S | 4 | 30.29 |
| agt | S | 0 | 109.30 |
| agc | S | 0 | 99.02 |
| --- | -- | ------ | ------ |
| mPD | | 0.048 | 1.7 |
| | | nPD: | 0.03 |
| | | N. weight: | 1.9 |
| | | Sc. PD: | -0.54 |
| | | Sc. rank: | -429.7 |

| | | | |
|---|---|---|---|
| NA | | | |
| Pos. | 180 | obs: | exp: |
| tct | S | 0 | 79.01 |
| tcc | S | 0 | 69.23 |
| tca | S | 0 | 110.10 |
| tcg | S | 0 | 30.29 |
| agt | S | 5 | 109.30 |
| agc | S | 492 | 99.02 |
| --- | -- | ------ | ------ |
| mPD | | 0.020 | 1.7 |
| | | nPD: | 0.01 |
| | | N. weight: | 2.2 |
| | | Sc. PD: | -0.64 |
| | | Sc. rank: | -549.0 |

| | | | |
|---|---|---|---|
| NA | | | |
| Pos. | 181 | obs: | exp: |
| act | T | 5 | 131.30 |
| acc | T | 65 | 106.60 |
| aca | T | 370 | 217.70 |
| acg | T | 57 | 41.32 |
| --- | -- | ------ | ------ |
| mPD | | 0.42 | 0.69 |
| | | nPD: | 0.61 |
| | | N. weight: | 0.49 |
| | | Sc. PD: | 0.14 |
| | | Sc. rank: | 119.7 |

,

| | | | |
|---|---|---|---|
| NA | | | |
| Pos. | 182 | obs: | exp: |
| tct | S | 0 | 11.29 |
| tcc | S | 0 | 9.89 |
| tca | S | 0 | 15.73 |
| tcg | S | 0 | 4.33 |
| act | T | 422 | 112.50 |
| acc | T | 4 | 91.41 |
| aca | T | 0 | 186.60 |
| acg | T | 0 | 35.41 |
| agt | S | 69 | 15.62 |
| agc | S | 2 | 14.15 |
| --- | -- | ------ | ------ |
| mPD | | 0.27 | 0.96 |
| | | nPD: | 0.28 |
| | | N. weight: | 1.8 |
| | | Sc. PD: | -0.059 |
| | | Sc. rank: | -48.6 |

,

| | | | |
|---|---|---|---|
| NA | | | |
| Pos. | 183 | obs: | exp: |
| tgt | C | 5 | 204.90 |
| tgc | C | 492 | 292.10 |
| --- | -- | ------ | ------ |
| mPD | | 0.020 | 0.48 |
| | | nPD: | 0.04 |
| | | N. weight: | 0.67 |
| | | Sc. PD: | -0.18 |
| | | Sc. rank: | -133.8 |

,

| | | | |
|---|---|---|---|
| NA | | | |
| Pos. | 184 | obs: | exp: |
| cat | H | 494 | 269.30 |
| cac | H | 3 | 227.70 |
| --- | -- | ------ | ------ |
| mPD | | 0.012 | 0.50 |
| | | nPD: | 0.02 |
| | | N. weight: | 0.81 |
| | | Sc. PD: | -0.23 |
| | | Sc. rank: | -184.2 |

,

| | | | |
|---|---|---|---|
| NA | | | |
| Pos. | 185 | obs: | exp: |
| gat | D | 416 | 265.20 |
| gac | D | 81 | 231.80 |
| --- | -- | ------ | ------ |
| mPD | | 0.27 | 0.50 |
| | | nPD: | 0.55 |
| | | N. weight: | 0.3 |
| | | Sc. PD: | 0.068 |
| | | Sc. rank: | 60.3 |

,

| | | | |
|---|---|---|---|
| NA | | | |
| Pos. | 186 | obs: | exp: |
| ggt | G | 31 | 61.32 |
| ggc | G | 71 | 67.21 |
| gga | G | 26 | 240.40 |
| ggg | G | 369 | 128.00 |
| --- | -- | ------ | ------ |
| mPD | | 0.42 | 0.67 |
| | | nPD: | 0.63 |
| | | N. weight: | 0.91 |
| | | Sc. PD: | 0.28 |
| | | Sc. rank: | 233.9 |

,

| | | | |
|---|---|---|---|
| NA | | | |
| Pos. | 187 | obs: | exp: |
| att | I | 339 | 174.40 |
| atc | I | 122 | 121.60 |
| ata | I | 3 | 168.00 |
| gtt | V | 32 | 6.78 |
| gtc | V | 0 | 6.93 |
| gta | V | 1 | 6.18 |
| gtg | V | 0 | 13.11 |
| --- | -- | ------ | ------ |
| mPD | | 0.51 | 0.80 |
| | | nPD: | 0.63 |
| | | N. weight: | 0.75 |
| | | Sc. PD: | 0.23 |
| | | Sc. rank: | 192.2 |

,

| | | | |
|---|---|---|---|
| NA | | | |
| Pos. | 188 | obs: | exp: |
| tct | S | 0 | 0.32 |
| tcc | S | 0 | 0.28 |
| tca | S | 0 | 0.44 |
| tcg | S | 0 | 0.12 |
| agt | S | 0 | 0.44 |
| agc | S | 2 | 0.40 |
| gct | A | 217 | 54.66 |
| gcc | A | 0 | 44.45 |
| gca | A | 0 | 96.72 |
| gcg | A | 0 | 21.18 |
| ggt | G | 96 | 34.30 |
| ggc | G | 120 | 37.59 |
| gga | G | 4 | 134.50 |
| ggg | G | 58 | 71.62 |
| --- | -- | ------ | ------ |
| mPD | | 1.0 | 1.2 |
| | | nPD: | 0.87 |
| | | N. weight: | 1.5 |
| | | Sc. PD: | 0.79 |
| | | Sc. rank: | 443.7 |

,

| | | | |
|---|---|---|---|
| NA | | | |
| Pos. | 189 | obs: | exp: |
| cgt | R | 0 | 20.63 |
| cgc | R | 0 | 24.34 |
| cga | R | 1 | 47.76 |
| cgg | R | 70 | 41.47 |
| aga | R | 164 | 223.50 |
| agg | R | 262 | 139.30 |
| --- | -- | ------ | ------ |
| mPD | | 0.69 | 0.96 |
| | | nPD: | 0.72 |
| | | N. weight: | 0.44 |
| | | Sc. PD: | 0.17 |
| | | Sc. rank: | 124.2 |

,

| | | | |
|---|---|---|---|
| NA | | | |
| Pos. | 190 | obs: | exp: |
| atg | M | 497 | 497.00 |
| --- | -- | ------ | ------ |
| mPD | | 0 | 0 |
| | | nPD: | 1. |
| | | N. weight: | 0. |
| | | Sc. PD: | 0 |
| | | Sc. rank: | 0 |

,

| | | | |
|---|---|---|---|
| NA | | | |
| Pos. | 191 | obs: | exp: |
| att | I | 0 | 0.38 |
| atc | I | 1 | 0.26 |
| ata | I | 0 | 0.36 |
| act | T | 415 | 129.70 |
| acc | T | 75 | 105.40 |
| aca | T | 1 | 215.10 |
| acg | T | 0 | 40.82 |
| gct | A | 5 | 1.26 |
| gcc | A | 0 | 1.02 |
| gca | A | 0 | 2.23 |
| gcg | A | 0 | 0.49 |
| --- | -- | ------ | ------ |
| mPD | | 0.29 | 0.71 |
| | | nPD: | 0.4 |
| | | N. weight: | 1.3 |
| | | Sc. PD: | 0.11 |
| | | Sc. rank: | 93.0 |

,

| | | | |
|---|---|---|---|
| NA | | | |
| Pos. | 192 | obs: | exp: |
| att | I | 130 | 186.80 |
| atc | I | 367 | 130.30 |
| ata | I | 0 | 179.90 |
| --- | -- | ------ | ------ |
| mPD | | 0.39 | 0.66 |
| | | nPD: | 0.59 |
| | | N. weight: | 0.95 |
| | | Sc. PD: | 0.25 |
| | | Sc. rank: | 221.9 |

,

| | | | |
|---|---|---|---|
| NA | | | |
| Pos. | 193 | obs: | exp: |
| tgt | C | 455 | 204.90 |
| tgc | C | 42 | 292.10 |
| --- | -- | ------ | ------ |
| mPD | | 0.16 | 0.48 |
| | | nPD: | 0.32 |
| | | N. weight: | 0.8 |
| | | Sc. PD: | 0.0047 |
| | | Sc. rank: | -4.8 |

,

| | | | |
|---|---|---|---|
| NA | | | |
| Pos. | 194 | obs: | exp: |
| att | I | 2 | 153.40 |
| atc | I | 0 | 106.90 |
| ata | I | 406 | 147.70 |
| gtt | V | 0 | 18.29 |
| gtc | V | 0 | 18.68 |
| gta | V | 21 | 16.68 |
| gtg | V | 68 | 35.35 |
| --- | -- | ------ | ------ |
| mPD | | 0.54 | 1.0 |
| | | nPD: | 0.54 |
| | | N. weight: | 1.3 |
| | | Sc. PD: | 0.28 |
| | | Sc. rank: | 246.2 |

,

| | | | |
|---|---|---|---|
| NA | | | |
| Pos. | 195 | obs: | exp: |
| caa | Q | 419 | 265.00 |
| cag | Q | 78 | 232.00 |
| --- | -- | ------ | ------ |
| mPD | | 0.27 | 0.50 |
| | | nPD: | 0.53 |
| | | N. weight: | 0.31 |
| | | Sc. PD: | 0.066 |
| | | Sc. rank: | 57.6 |

,

| | | | |
|---|---|---|---|
| NA | | | |
| Pos. | 196 | obs: | exp: |
| ggt | G | 0 | 61.32 |
| ggc | G | 0 | 67.21 |
| gga | G | 448 | 240.40 |
| ggg | G | 49 | 128.00 |
| --- | -- | ------ | ------ |
| mPD | | 0.18 | 0.67 |
| | | nPD: | 0.27 |
| | | N. weight: | 0.66 |
| | | Sc. PD: | -0.03 |
| | | Sc. rank: | -23.6 |

,

| | | | |
|---|---|---|---|
| NA | | | |
| Pos. | 197 | obs: | exp: |
| aat | N | 412 | 259.20 |
| aac | N | 84 | 236.80 |
| gat | D | 1 | 0.53 |
| gac | D | 0 | 0.47 |
| --- | -- | ------ | ------ |
| mPD | | 0.29 | 0.50 |
| | | nPD: | 0.57 |
| | | N. weight: | 0.3 |
| | | Sc. PD: | 0.074 |
| | | Sc. rank: | 65.9 |

,

| | | | |
|---|---|---|---|
| NA | | | |
| Pos. | 198 | obs: | exp: |
| aat | N | 493 | 259.80 |
| aac | N | 4 | 237.20 |
| --- | -- | ------ | ------ |
| mPD | | 0.016 | 0.50 |
| | | nPD: | 0.03 |
| | | N. weight: | 0.85 |
| | | Sc. PD: | -0.23 |
| | | Sc. rank: | -182.4 |

,

| | | | |
|---|---|---|---|
| NA | | | |
| Pos. | 199 | obs: | exp: |
| aat | N | 0 | 20.38 |
| aac | N | 39 | 18.62 |
| gat | D | 6 | 243.80 |
| gac | D | 451 | 213.20 |
| gaa | E | 0 | 0.58 |
| gag | E | 1 | 0.42 |
| --- | -- | ------ | ------ |
| mPD | | 0.17 | 0.64 |
| | | nPD: | 0.27 |
| | | N. weight: | 0.98 |
| | | Sc. PD: | -0.043 |
| | | Sc. rank: | -33.8 |

,

| | | | |
|---|---|---|---|
| NA | | | |
| Pos. | 200 | obs: | exp: |
| cat | H | 2 | 1.08 |
| cac | H | 0 | 0.92 |
| aat | N | 324 | 258.70 |
| aac | N | 171 | 236.30 |
| --- | -- | ------ | ------ |
| mPD | | 0.46 | 0.51 |
| | | nPD: | 0.91 |
| | | N. weight: | 0.062 |
| | | Sc. PD: | 0.036 |
| | | Sc. rank: | 19.0 |

,

| | | | |
|---|---|---|---|
| NA | | | |
| Pos. | 201 | obs: | exp: |
| gct | A | 381 | 125.20 |
| gcc | A | 45 | 101.80 |
| gca | A | 71 | 221.50 |
| gcg | A | 0 | 48.50 |
| --- | -- | ------ | ------ |
| mPD | | 0.38 | 0.69 |
| | | nPD: | 0.56 |
| | | N. weight: | 0.88 |
| | | Sc. PD: | 0.21 |
| | | Sc. rank: | 187.9 |

,

| | | | |
|---|---|---|---|
| NA | | | |
| Pos. | 202 | obs: | exp: |
| act | T | 1 | 131.30 |
| acc | T | 0 | 106.60 |
| aca | T | 494 | 217.70 |
| acg | T | 2 | 41.32 |
| --- | -- | ------ | ------ |
| mPD | | 0.012 | 0.69 |
| | | nPD: | 0.02 |
| | | N. weight: | 1.1 |
| | | Sc. PD: | -0.32 |
| | | Sc. rank: | -265.8 |

,

| | | | |
|---|---|---|---|
| NA | | | |
| Pos. | 203 | obs: | exp: |
| gct | A | 72 | 125.20 |
| gcc | A | 1 | 101.80 |
| gca | A | 420 | 221.50 |
| gcg | A | 4 | 48.50 |
| --- | -- | ------ | ------ |
| mPD | | 0.27 | 0.69 |
| | | nPD: | 0.39 |
| | | N. weight: | 0.62 |
| | | Sc. PD: | 0.044 |
| | | Sc. rank: | 33.2 |

,

| | | | |
|---|---|---|---|
| NA | | | |
| Pos. | 204 | obs: | exp: |
| att | I | 0 | 0.38 |
| atc | I | 0 | 0.26 |
| ata | I | 1 | 0.36 |
| act | T | 0 | 130.20 |
| acc | T | 0 | 105.80 |
| aca | T | 110 | 216.00 |
| acg | T | 383 | 40.98 |
| gct | A | 0 | 0.76 |
| gcc | A | 0 | 0.61 |
| gca | A | 3 | 1.34 |
| gcg | A | 0 | 0.29 |
| --- | -- | ------ | ------ |
| mPD | | 0.37 | 0.70 |
| | | nPD: | 0.53 |
| | | N. weight: | 2.2 |
| | | Sc. PD: | 0.46 |
| | | Sc. rank: | 385.0 |

,

| | | | |
|---|---|---|---|
| NA | | | |
| Pos. | 205 | obs: | exp: |
| gtt | V | 423 | 102.20 |
| gtc | V | 2 | 104.30 |
| gta | V | 12 | 93.15 |
| gtg | V | 60 | 197.40 |
| --- | -- | ------ | ------ |
| mPD | | 0.26 | 0.72 |
| | | nPD: | 0.36 |
| | | N. weight: | 1.4 |
| | | Sc. PD: | 0.066 |
| | | Sc. rank: | 39.2 |

,

| | | | |
|---|---|---|---|
| NA | | | |
| Pos. | 206 | obs: | exp: |
| tat | Y | 399 | 257.30 |
| tac | Y | 98 | 239.70 |
| --- | -- | ------ | ------ |
| mPD | | 0.32 | 0.50 |
| | | nPD: | 0.64 |
| | | N. weight: | 0.25 |
| | | Sc. PD: | 0.079 |
| | | Sc. rank: | 65.7 |

,

| | | | |
|---|---|---|---|
| NA | | | |
| Pos. | 207 | obs: | exp: |
| tat | Y | 247 | 257.30 |
| tac | Y | 250 | 239.70 |
| --- | -- | ------ | ------ |
| mPD | | 0.50 | 0.50 |
| | | nPD: | 1. |
| | | N. weight: | 0.011 |
| | | Sc. PD: | 0.0071 |
| | | Sc. rank: | 3.2 |

,

| | | | |
|---|---|---|---|
| NA | | | |
| Pos. | 208 | obs: | exp: |
| aat | N | 51 | 222.60 |
| aac | N | 375 | 203.40 |
| gat | D | 0 | 36.28 |
| gac | D | 68 | 31.72 |
| gaa | E | 2 | 1.73 |
| gag | E | 1 | 1.27 |
| --- | -- | ------ | ------ |
| mPD | | 0.44 | 0.75 |
| | | nPD: | 0.59 |
| | | N. weight: | 0.59 |
| | | Sc. PD: | 0.16 |
| | | Sc. rank: | 139.0 |

,

| | | | |
|---|---|---|---|
| NA | | | |
| Pos. | 209 | obs: | exp: |
| caa | Q | 1 | 0.53 |
| cag | Q | 0 | 0.47 |
| cgt | R | 0 | 17.60 |
| cgc | R | 0 | 20.76 |
| cga | R | 66 | 40.75 |
| cgg | R | 1 | 35.38 |
| aaa | K | 27 | 15.12 |
| aag | K | 0 | 11.88 |
| aga | R | 345 | 190.70 |
| agg | R | 12 | 118.80 |
| ggt | G | 0 | 5.55 |
| ggc | G | 0 | 6.09 |
| gga | G | 45 | 21.77 |
| ggg | G | 0 | 11.59 |
| --- | -- | ------ | ------ |
| mPD | | 0.53 | 1.2 |
| | | nPD: | 0.46 |
| | | N. weight: | 0.73 |
| | | Sc. PD: | 0.1 |
| | | Sc. rank: | 88.4 |

,

| | | | |
|---|---|---|---|
| NA | | | |
| Pos. | 210 | obs: | exp: |
| cgt | R | 0 | 20.59 |
| cgc | R | 0 | 24.29 |
| cga | R | 0 | 47.67 |
| cgg | R | 0 | 41.39 |
| aaa | K | 0 | 0.56 |
| aag | K | 1 | 0.44 |
| aga | R | 28 | 223.10 |
| agg | R | 468 | 139.00 |
| --- | -- | ------ | ------ |
| mPD | | 0.11 | 0.96 |
| | | nPD: | 0.11 |
| | | N. weight: | 1.4 |
| | | Sc. PD: | -0.28 |
| | | Sc. rank: | -189.7 |

,

| | | | |
|---|---|---|---|
| NA | | | |
| Pos. | 211 | obs: | exp: |
| tta | L | 1 | 43.42 |
| ttg | L | 25 | 95.72 |
| ctt | L | 4 | 92.73 |
| ctc | L | 70 | 80.39 |
| cta | L | 5 | 74.87 |
| ctg | L | 392 | 109.90 |
| --- | -- | ------ | ------ |
| mPD | | 0.38 | 1.1 |
| | | nPD: | 0.34 |
| | | N. weight: | 1.2 |
| | | Sc. PD: | 0.028 |
| | | Sc. rank: | 7.6 |

,

| | | | |
|---|---|---|---|
| NA | | | |
| Pos. | 212 | obs: | exp: |
| att | I | 2 | 0.75 |
| atc | I | 0 | 0.52 |
| ata | I | 0 | 0.72 |
| act | T | 99 | 130.20 |
| acc | T | 377 | 105.80 |
| aca | T | 17 | 216.00 |
| acg | T | 0 | 40.98 |
| gct | A | 1 | 0.50 |
| gcc | A | 1 | 0.41 |
| gca | A | 0 | 0.89 |
| gcg | A | 0 | 0.20 |
| --- | -- | ------ | ------ |
| mPD | | 0.40 | 0.70 |
| | | nPD: | 0.56 |
| | | N. weight: | 1.2 |
| | | Sc. PD: | 0.28 |
| | | Sc. rank: | 254.3 |

,

| | | | |
|---|---|---|---|
| NA | | | |
| Pos. | 213 | obs: | exp: |
| act | T | 357 | 131.30 |
| acc | T | 132 | 106.60 |
| aca | T | 8 | 217.70 |
| acg | T | 0 | 41.32 |
| --- | -- | ------ | ------ |
| mPD | | 0.41 | 0.69 |
| | | nPD: | 0.6 |
| | | N. weight: | 1. |
| | | Sc. PD: | 0.29 |
| | | Sc. rank: | 249.2 |

,

| | | | |
|---|---|---|---|
| NA | | | |
| Pos. | 214 | obs: | exp: |
| act | T | 7 | 131.30 |
| acc | T | 407 | 106.60 |
| aca | T | 83 | 217.70 |
| acg | T | 0 | 41.32 |
| --- | -- | ------ | ------ |
| mPD | | 0.30 | 0.69 |
| | | nPD: | 0.44 |
| | | N. weight: | 1.3 |
| | | Sc. PD: | 0.16 |
| | | Sc. rank: | 131.7 |

,

| | | | |
|---|---|---|---|
| NA | | | |
| Pos. | 215 | obs: | exp: |
| tta | L | 3 | 0.26 |
| ttg | L | 0 | 0.58 |
| ctt | L | 0 | 0.56 |
| ctc | L | 0 | 0.49 |
| cta | L | 0 | 0.45 |
| ctg | L | 0 | 0.66 |
| att | I | 423 | 185.70 |
| atc | I | 3 | 129.50 |
| ata | I | 68 | 178.80 |
| --- | -- | ------ | ------ |
| mPD | | 0.27 | 0.67 |
| | | nPD: | 0.4 |
| | | N. weight: | 0.8 |
| | | Sc. PD: | 0.065 |
| | | Sc. rank: | 46.9 |

,

| | | | |
|---|---|---|---|
| NA | | | |
| Pos. | 216 | obs: | exp: |
| cgt | R | 0 | 0.21 |
| cgc | R | 0 | 0.24 |
| cga | R | 0 | 0.48 |
| cgg | R | 0 | 0.42 |
| aaa | K | 77 | 274.90 |
| aag | K | 414 | 216.10 |
| aga | R | 0 | 2.25 |
| agg | R | 5 | 1.40 |
| gaa | E | 0 | 0.58 |
| gag | E | 1 | 0.42 |
| --- | -- | ------ | ------ |
| mPD | | 0.29 | 0.52 |
| | | nPD: | 0.55 |
| | | N. weight: | 0.51 |
| | | Sc. PD: | 0.12 |
| | | Sc. rank: | 103.8 |

,

| | | | |
|---|---|---|---|
| NA | | | |
| Pos. | 217 | obs: | exp: |
| act | T | 40 | 131.30 |
| acc | T | 376 | 106.60 |
| aca | T | 80 | 217.70 |
| acg | T | 1 | 41.32 |
| --- | -- | ------ | ------ |
| mPD | | 0.40 | 0.69 |
| | | nPD: | 0.58 |
| | | N. weight: | 0.98 |
| | | Sc. PD: | 0.25 |
| | | Sc. rank: | 221.6 |

,

| | | | |
|---|---|---|---|
| NA | | | |
| Pos. | 218 | obs: | exp: |
| tgg | W | 497 | 497.00 |
| --- | -- | ------ | ------ |
| mPD | | 0 | 0 |
| | | nPD: | 1. |
| | | N. weight: | 0. |
| | | Sc. PD: | 0 |
| | | Sc. rank: | 0 |

,

| | | | |
|---|---|---|---|
| NA | | | |
| Pos. | 219 | obs: | exp: |
| gct | A | 27 | 125.20 |
| gcc | A | 353 | 101.80 |
| gca | A | 117 | 221.50 |
| gcg | A | 0 | 48.50 |
| --- | -- | ------ | ------ |
| mPD | | 0.44 | 0.69 |
| | | nPD: | 0.64 |
| | | N. weight: | 0.92 |
| | | Sc. PD: | 0.29 |
| | | Sc. rank: | 241.0 |

,

| | | | |
|---|---|---|---|
| NA | | | |
| Pos. | 220 | obs: | exp: |
| cgt | R | 0 | 17.31 |
| cgc | R | 0 | 20.42 |
| cga | R | 0 | 40.08 |
| cgg | R | 0 | 34.80 |
| aaa | K | 3 | 5.04 |
| aag | K | 6 | 3.96 |
| aga | R | 325 | 187.50 |
| agg | R | 92 | 116.90 |
| gaa | E | 1 | 0.58 |
| gag | E | 0 | 0.42 |
| ggt | G | 0 | 8.64 |
| ggc | G | 0 | 9.47 |
| gga | G | 68 | 33.86 |
| ggg | G | 2 | 18.03 |
| --- | -- | ------ | ------ |
| mPD | | 0.61 | 1.2 |
| | | nPD: | 0.53 |
| | | N. weight: | 0.58 |
| | | Sc. PD: | 0.12 |
| | | Sc. rank: | 99.0 |

,

| | | | |
|---|---|---|---|
| NA | | | |
| Pos. | 221 | obs: | exp: |
| aat | N | 60 | 259.80 |
| aac | N | 437 | 237.20 |
| --- | -- | ------ | ------ |
| mPD | | 0.21 | 0.50 |
| | | nPD: | 0.43 |
| | | N. weight: | 0.51 |
| | | Sc. PD: | 0.056 |
| | | Sc. rank: | 42.2 |

,

| | | | |
|---|---|---|---|
| NA | | | |
| Pos. | 222 | obs: | exp: |
| att | I | 393 | 186.80 |
| atc | I | 103 | 130.30 |
| ata | I | 1 | 179.90 |
| --- | -- | ------ | ------ |
| mPD | | 0.33 | 0.66 |
| | | nPD: | 0.5 |
| | | N. weight: | 0.75 |
| | | Sc. PD: | 0.14 |
| | | Sc. rank: | 116.2 |

,

| | | | |
|---|---|---|---|
| NA | | | |
| Pos. | 223 | obs: | exp: |
| tta | L | 0 | 43.42 |
| ttg | L | 17 | 95.72 |
| ctt | L | 70 | 92.73 |
| ctc | L | 1 | 80.39 |
| cta | L | 4 | 74.87 |
| ctg | L | 405 | 109.90 |
| --- | -- | ------ | ------ |
| mPD | | 0.33 | 1.1 |
| | | nPD: | 0.29 |
| | | N. weight: | 1.3 |
| | | Sc. PD: | -0.028 |
| | | Sc. rank: | -26.9 |

,

| | | | |
|---|---|---|---|
| NA | | | |
| Pos. | 224 | obs: | exp: |
| cgt | R | 0 | 20.63 |
| cgc | R | 0 | 24.34 |
| cga | R | 0 | 47.76 |
| cgg | R | 0 | 41.47 |
| aga | R | 57 | 223.50 |
| agg | R | 440 | 139.30 |
| --- | -- | ------ | ------ |
| mPD | | 0.20 | 0.96 |
| | | nPD: | 0.21 |
| | | N. weight: | 1.2 |
| | | Sc. PD: | -0.12 |
| | | Sc. rank: | -86.6 |

,

| | | | |
|---|---|---|---|
| NA | | | |
| Pos. | 225 | obs: | exp: |
| act | T | 380 | 131.30 |
| acc | T | 46 | 106.60 |
| aca | T | 38 | 217.70 |
| acg | T | 33 | 41.32 |
| --- | -- | ------ | ------ |
| mPD | | 0.40 | 0.69 |
| | | nPD: | 0.58 |
| | | N. weight: | 0.84 |
| | | Sc. PD: | 0.22 |
| | | Sc. rank: | 192.1 |

,

| | | | |
|---|---|---|---|
| NA | | | |
| Pos. | 226 | obs: | exp: |
| caa | Q | 492 | 265.00 |
| cag | Q | 5 | 232.00 |
| --- | -- | ------ | ------ |
| mPD | | 0.020 | 0.50 |
| | | nPD: | 0.04 |
| | | N. weight: | 0.81 |
| | | Sc. PD: | -0.21 |
| | | Sc. rank: | -164.9 |

,

| | | | |
|---|---|---|---|
| NA | | | |
| Pos. | 227 | obs: | exp: |
| gaa | E | 308 | 286.00 |
| gag | E | 189 | 211.00 |
| --- | -- | ------ | ------ |
| mPD | | 0.47 | 0.49 |
| | | nPD: | 0.97 |
| | | N. weight: | 0.015 |
| | | Sc. PD: | 0.0095 |
| | | Sc. rank: | 4.6 |

,

| | | | |
|---|---|---|---|
| NA | | | |
| Pos. | 228 | obs: | exp: |
| tct | S | 4 | 79.01 |
| tcc | S | 0 | 69.23 |
| tca | S | 430 | 110.10 |
| tcg | S | 63 | 30.29 |
| agt | S | 0 | 109.30 |
| agc | S | 0 | 99.02 |
| --- | -- | ------ | ------ |
| mPD | | 0.24 | 1.7 |
| | | nPD: | 0.14 |
| | | N. weight: | 1.8 |
| | | Sc. PD: | -0.29 |
| | | Sc. rank: | -206.0 |

,

| | | | |
|---|---|---|---|
| NA | | | |
| Pos. | 229 | obs: | exp: |
| gaa | E | 474 | 286.00 |
| gag | E | 23 | 211.00 |
| --- | -- | ------ | ------ |
| mPD | | 0.088 | 0.49 |
| | | nPD: | 0.18 |
| | | N. weight: | 0.54 |
| | | Sc. PD: | -0.069 |
| | | Sc. rank: | -48.0 |

,

| | | | |
|---|---|---|---|
| NA | | | |
| Pos. | 230 | obs: | exp: |
| tgt | C | 482 | 204.90 |
| tgc | C | 15 | 292.10 |
| --- | -- | ------ | ------ |
| mPD | | 0.059 | 0.48 |
| | | nPD: | 0.12 |
| | | N. weight: | 1. |
| | | Sc. PD: | -0.19 |
| | | Sc. rank: | -132.9 |

,

| NA | | | |
|---|---|---|---|
| Pos. | 231 | obs: | exp: |
| gtt | V | 0 | 102.20 |
| gtc | V | 0 | 104.30 |
| gta | V | 70 | 93.15 |
| gtg | V | 427 | 197.40 |
| --- | -- | ------ | ------ |
| mPD | | 0.24 | 0.72 |
| | | nPD: | 0.34 |
| | | N. weight: | 0.88 |
| | | Sc. PD: | 0.019 |
| | | Sc. rank: | 3.0 |

,

| NA | | | |
|---|---|---|---|
| Pos. | 232 | obs: | exp: |
| tgt | C | 3 | 204.90 |
| tgc | C | 494 | 292.10 |
| --- | -- | ------ | ------ |
| mPD | | 0.012 | 0.48 |
| | | nPD: | 0.02 |
| | | N. weight: | 0.7 |
| | | Sc. PD: | -0.2 |
| | | Sc. rank: | -157.8 |

,

| NA | | | |
|---|---|---|---|
| Pos. | 233 | obs: | exp: |
| cat | H | 17 | 269.30 |
| cac | H | 480 | 227.70 |
| --- | -- | ------ | ------ |
| mPD | | 0.066 | 0.50 |
| | | nPD: | 0.13 |
| | | N. weight: | 0.88 |
| | | Sc. PD: | -0.15 |
| | | Sc. rank: | -106.0 |

,

| NA | | | |
|---|---|---|---|
| Pos. | 234 | obs: | exp: |
| aat | N | 455 | 259.80 |
| aac | N | 42 | 237.20 |
| --- | -- | ------ | ------ |
| mPD | | 0.16 | 0.50 |
| | | nPD: | 0.31 |
| | | N. weight: | 0.52 |
| | | Sc. PD: | -0.0016 |
| | | Sc. rank: | -5.9 |

,

| NA | | | |
|---|---|---|---|
| Pos. | 235 | obs: | exp: |
| ggt | G | 21 | 61.32 |
| ggc | G | 405 | 67.21 |
| gga | G | 69 | 240.40 |
| ggg | G | 2 | 128.00 |
| --- | -- | ------ | ------ |
| mPD | | 0.32 | 0.67 |
| | | nPD: | 0.47 |
| | | N. weight: | 1.7 |
| | | Sc. PD: | 0.27 |
| | | Sc. rank: | 236.2 |

,

| NA | | | |
|---|---|---|---|
| Pos. | 236 | obs: | exp: |
| att | I | 0 | 1.88 |
| atc | I | 0 | 1.31 |
| ata | I | 5 | 1.81 |
| act | T | 0 | 130.00 |
| acc | T | 1 | 105.60 |
| aca | T | 432 | 215.50 |
| acg | T | 59 | 40.90 |
| --- | -- | ------ | ------ |
| mPD | | 0.23 | 0.71 |
| | | nPD: | 0.33 |
| | | N. weight: | 0.92 |
| | | Sc. PD: | 0.015 |
| | | Sc. rank: | -1.6 |

,

| NA | | | |
|---|---|---|---|
| Pos. | 237 | obs: | exp: |
| tgt | C | 457 | 204.90 |
| tgc | C | 40 | 292.10 |
| --- | -- | ------ | ------ |
| mPD | | 0.15 | 0.48 |
| | | nPD: | 0.31 |
| | | N. weight: | 0.82 |
| | | Sc. PD: | -0.0062 |
| | | Sc. rank: | -12.1 |

,

| NA | | | |
|---|---|---|---|
| Pos. | 238 | obs: | exp: |
| act | T | 0 | 0.53 |
| acc | T | 0 | 0.43 |
| aca | T | 2 | 0.88 |
| acg | T | 0 | 0.17 |
| gtt | V | 0 | 14.59 |
| gtc | V | 0 | 14.90 |
| gta | V | 70 | 13.31 |
| gtg | V | 1 | 28.20 |
| gct | A | 14 | 106.80 |
| gcc | A | 2 | 86.86 |
| gca | A | 405 | 189.00 |
| gcg | A | 3 | 41.37 |
| --- | -- | ------ | ------ |
| mPD | | 0.33 | 0.96 |
| | | nPD: | 0.34 |
| | | N. weight: | 1.1 |
| | | Sc. PD: | 0.032 |
| | | Sc. rank: | 11.8 |

,

| NA | | | |
|---|---|---|---|
| Pos. | 239 | obs: | exp: |
| gtt | V | 422 | 102.20 |
| gtc | V | 4 | 104.30 |
| gta | V | 70 | 93.15 |
| gtg | V | 1 | 197.40 |
| --- | -- | ------ | ------ |
| mPD | | 0.26 | 0.72 |
| | | nPD: | 0.36 |
| | | N. weight: | 1.6 |
| | | Sc. PD: | 0.072 |
| | | Sc. rank: | 41.1 |

,

| NA | | | |
|---|---|---|---|
| Pos. | 240 | obs: | exp: |
| att | I | 0 | 25.56 |
| atc | I | 0 | 17.82 |
| ata | I | 68 | 24.61 |
| gtt | V | 0 | 88.18 |
| gtc | V | 2 | 90.04 |
| gta | V | 379 | 80.40 |
| gtg | V | 48 | 170.40 |
| --- | -- | ------ | ------ |
| mPD | | 0.42 | 0.97 |
| | | nPD: | 0.43 |
| | | N. weight: | 1.7 |
| | | Sc. PD: | 0.19 |
| | | Sc. rank: | 143.6 |

,

| NA | | | |
|---|---|---|---|
| Pos. | 241 | obs: | exp: |
| atg | M | 497 | 497.00 |
| --- | -- | ------ | ------ |
| mPD | | 0 | 0 |
| | | nPD: | 1. |
| | | N. weight: | 0. |
| | | Sc. PD: | 0 |
| | | Sc. rank: | 0 |

,

| NA | | | |
|---|---|---|---|
| Pos. | 242 | obs: | exp: |
| act | T | 130 | 131.30 |
| acc | T | 366 | 106.60 |
| aca | T | 0 | 217.70 |
| acg | T | 1 | 41.32 |
| --- | -- | ------ | ------ |
| mPD | | 0.39 | 0.69 |
| | | nPD: | 0.57 |
| | | N. weight: | 1.3 |
| | | Sc. PD: | 0.31 |
| | | Sc. rank: | 279.2 |

,

| NA | | | |
|---|---|---|---|
| Pos. | 243 | obs: | exp: |
| gat | D | 108 | 265.20 |
| gac | D | 389 | 231.80 |
| --- | -- | ------ | ------ |
| mPD | | 0.34 | 0.50 |
| | | nPD: | 0.68 |
| | | N. weight: | 0.3 |
| | | Sc. PD: | 0.11 |
| | | Sc. rank: | 84.6 |

,

| NA | | | |
|---|---|---|---|
| Pos. | 244 | obs: | exp: |
| ggt | G | 0 | 61.32 |
| ggc | G | 1 | 67.21 |
| gga | G | 457 | 240.40 |
| ggg | G | 39 | 128.00 |
| --- | -- | ------ | ------ |
| mPD | | 0.15 | 0.67 |
| | | nPD: | 0.22 |
| | | N. weight: | 0.69 |
| | | Sc. PD: | -0.061 |
| | | Sc. rank: | -44.0 |

,

| NA | | | |
|---|---|---|---|
| Pos. | 245 | obs: | exp: |
| tct | S | 0 | 78.69 |
| tcc | S | 0 | 68.96 |
| tca | S | 64 | 109.70 |
| tcg | S | 431 | 30.17 |
| cct | P | 0 | 0.53 |
| ccc | P | 2 | 0.36 |
| cca | P | 0 | 0.79 |
| ccg | P | 0 | 0.32 |
| agt | S | 0 | 108.90 |
| agc | S | 0 | 98.62 |
| --- | -- | ------ | ------ |
| mPD | | 0.24 | 1.7 |
| | | nPD: | 0.14 |
| | | N. weight: | 3.1 |
| | | Sc. PD: | -0.52 |
| | | Sc. rank: | -355.8 |

,

| NA | | | |
|---|---|---|---|
| Pos. | 246 | obs: | exp: |
| gct | A | 418 | 125.20 |
| gcc | A | 8 | 101.80 |
| gca | A | 71 | 221.50 |
| gcg | A | 0 | 48.50 |
| --- | -- | ------ | ------ |
| mPD | | 0.27 | 0.69 |
| | | nPD: | 0.4 |
| | | N. weight: | 1.1 |
| | | Sc. PD: | 0.093 |
| | | Sc. rank: | 66.4 |

,

| NA | | | |
|---|---|---|---|
| Pos. | 247 | obs: | exp: |
| tct | S | 0 | 76.31 |
| tcc | S | 0 | 66.87 |
| tca | S | 0 | 106.30 |
| tcg | S | 0 | 29.26 |
| aat | N | 17 | 8.88 |
| aac | N | 0 | 8.12 |
| agt | S | 388 | 105.60 |
| agc | S | 92 | 95.63 |
| --- | -- | ------ | ------ |
| mPD | | 0.37 | 1.7 |
| | | nPD: | 0.22 |
| | | N. weight: | 1.5 |
| | | Sc. PD: | -0.14 |
| | | Sc. rank: | -97.4 |

,

| NA | | | |
|---|---|---|---|
| Pos. | 248 | obs: | exp: |
| tct | S | 0 | 78.53 |
| tcc | S | 0 | 68.82 |
| tca | S | 0 | 109.40 |
| tcg | S | 0 | 30.11 |
| aat | N | 1 | 1.57 |
| aac | N | 2 | 1.43 |
| agt | S | 420 | 108.70 |
| agc | S | 74 | 98.42 |
| --- | -- | ------ | ------ |
| mPD | | 0.27 | 1.7 |
| | | nPD: | 0.16 |
| | | N. weight: | 1.5 |
| | | Sc. PD: | -0.23 |
| | | Sc. rank: | -148.8 |

,

| NA | | | |
|---|---|---|---|
| Pos. | 249 | obs: | exp: |
| cat | H | 1 | 0.54 |
| cac | H | 0 | 0.46 |
| caa | Q | 399 | 264.40 |
| cag | Q | 97 | 231.60 |
| --- | -- | ------ | ------ |
| mPD | | 0.32 | 0.50 |
| | | nPD: | 0.64 |
| | | N. weight: | 0.23 |
| | | Sc. PD: | 0.073 |
| | | Sc. rank: | 60.6 |

,

| NA | | | |
|---|---|---|---|
| Pos. | 250 | obs: | exp: |
| gct | A | 2 | 125.20 |
| gcc | A | 423 | 101.80 |
| gca | A | 72 | 221.50 |
| gcg | A | 0 | 48.50 |
| --- | -- | ------ | ------ |
| mPD | | 0.26 | 0.69 |
| | | nPD: | 0.37 |
| | | N. weight: | 1.5 |
| | | Sc. PD: | 0.082 |
| | | Sc. rank: | 59.2 |

,

| NA | | | |
|---|---|---|---|
| Pos. | 251 | obs: | exp: |
| tat | Y | 410 | 231.90 |
| tac | Y | 38 | 216.10 |
| cat | H | 29 | 26.01 |
| cac | H | 19 | 21.99 |
| gat | D | 0 | 0.53 |
| gac | D | 1 | 0.47 |
| --- | -- | ------ | ------ |
| mPD | | 0.39 | 0.68 |
| | | nPD: | 0.57 |
| | | N. weight: | 0.49 |
| | | Sc. PD: | 0.12 |
| | | Sc. rank: | 106.8 |

,

| NA | | | |
|---|---|---|---|
| Pos. | 252 | obs: | exp: |
| act | T | 0 | 131.30 |
| acc | T | 2 | 106.60 |
| aca | T | 491 | 217.70 |
| acg | T | 4 | 41.32 |
| --- | -- | ------ | ------ |
| mPD | | 0.024 | 0.69 |
| | | nPD: | 0.03 |
| | | N. weight: | 1.1 |
| | | Sc. PD: | -0.29 |
| | | Sc. rank: | -225.9 |

,

| NA | | | |
|---|---|---|---|
| Pos. | 253 | obs: | exp: |
| cgt | R | 0 | 0.12 |
| cgc | R | 0 | 0.15 |
| cga | R | 0 | 0.29 |
| cgg | R | 0 | 0.25 |
| aaa | K | 424 | 276.60 |
| aag | K | 70 | 217.40 |
| aga | R | 0 | 1.35 |
| agg | R | 3 | 0.84 |
| --- | -- | ------ | ------ |
| mPD | | 0.26 | 0.51 |
| | | nPD: | 0.52 |
| | | N. weight: | 0.31 |
| | | Sc. PD: | 0.06 |
| | | Sc. rank: | 50.2 |

,

| NA | | | |
|---|---|---|---|
| Pos. | 254 | obs: | exp: |
| att | I | 7 | 13.53 |
| atc | I | 0 | 9.43 |
| ata | I | 29 | 13.03 |
| gtt | V | 64 | 94.75 |
| gtc | V | 0 | 96.76 |
| gta | V | 325 | 86.40 |
| gtg | V | 72 | 183.10 |
| --- | -- | ------ | ------ |
| mPD | | 0.59 | 0.87 |
| | | nPD: | 0.68 |
| | | N. weight: | 1. |
| | | Sc. PD: | 0.36 |
| | | Sc. rank: | 281.6 |

,

| NA | | | |
|---|---|---|---|
| Pos. | 255 | obs: | exp: |
| tta | L | 0 | 6.12 |
| ttg | L | 3 | 13.48 |
| ctt | L | 0 | 13.06 |
| ctc | L | 0 | 11.32 |
| cta | L | 7 | 10.55 |
| ctg | L | 60 | 15.47 |
| att | I | 0 | 5.26 |
| atc | I | 0 | 3.67 |
| ata | I | 14 | 5.07 |
| atg | M | 396 | 396.00 |
| gtt | V | 0 | 3.49 |
| gtc | V | 0 | 3.57 |
| gta | V | 4 | 3.19 |
| gtg | V | 13 | 6.75 |
| --- | -- | ------ | ------ |
| mPD | | 0.40 | 0.55 |
| | | nPD: | 0.73 |
| | | N. weight: | 0.29 |
| | | Sc. PD: | 0.12 |
| | | Sc. rank: | 83.2 |

,

| NA | | | |
|---|---|---|---|
| Pos. | 256 | obs: | exp: |
| tat | Y | 486 | 257.30 |
| tac | Y | 11 | 239.70 |
| --- | -- | ------ | ------ |
| mPD | | 0.043 | 0.50 |
| | | nPD: | 0.09 |
| | | N. weight: | 0.78 |
| | | Sc. PD: | -0.17 |
| | | Sc. rank: | -112.2 |

,

| NA | | | |
|---|---|---|---|
| Pos. | 257 | obs: | exp: |
| ttt | F | 18 | 204.30 |
| ttc | F | 479 | 292.70 |
| --- | -- | ------ | ------ |
| mPD | | 0.070 | 0.48 |
| | | nPD: | 0.14 |
| | | N. weight: | 0.55 |
| | | Sc. PD: | -0.09 |
| | | Sc. rank: | -61.5 |

,

| NA | | | |
|---|---|---|---|
| Pos. | 258 | obs: | exp: |
| cct | P | 0 | 0.26 |
| ccc | P | 1 | 0.18 |
| cca | P | 0 | 0.39 |
| ccg | P | 0 | 0.16 |
| cat | H | 37 | 267.10 |
| cac | H | 456 | 225.90 |
| caa | Q | 2 | 1.07 |
| cag | Q | 0 | 0.93 |
| cgt | R | 1 | 0.04 |
| cgc | R | 0 | 0.05 |
| cga | R | 0 | 0.10 |
| cgg | R | 0 | 0.08 |
| aga | R | 0 | 0.45 |
| agg | R | 0 | 0.28 |
| --- | -- | ------ | ------ |
| mPD | | 0.16 | 0.51 |
| | | nPD: | 0.31 |
| | | N. weight: | 0.72 |
| | | Sc. PD: | -0.0061 |
| | | Sc. rank: | -11.5 |

,

| NA | | | |
|---|---|---|---|
| Pos. | 259 | obs: | exp: |
| cgt | R | 0 | 0.75 |
| cgc | R | 0 | 0.88 |
| cga | R | 0 | 1.73 |
| cgg | R | 0 | 1.50 |
| aaa | K | 86 | 268.20 |
| aag | K | 393 | 210.80 |
| aga | R | 1 | 8.10 |
| agg | R | 17 | 5.04 |
| --- | -- | ------ | ------ |
| mPD | | 0.36 | 0.58 |
| | | nPD: | 0.61 |
| | | N. weight: | 0.48 |
| | | Sc. PD: | 0.14 |
| | | Sc. rank: | 117.5 |

,

| NA | | | |
|---|---|---|---|
| Pos. | 260 | obs: | exp: |
| ggt | G | 0 | 61.32 |
| ggc | G | 1 | 67.21 |
| gga | G | 367 | 240.40 |
| ggg | G | 129 | 128.00 |
| --- | -- | ------ | ------ |
| mPD | | 0.39 | 0.67 |
| | | nPD: | 0.58 |
| | | N. weight: | 0.44 |
| | | Sc. PD: | 0.11 |
| | | Sc. rank: | 101.4 |

,

| NA | | | |
|---|---|---|---|
| Pos. | 261 | obs: | exp: |
| ttt | F | 1 | 0.41 |
| ttc | F | 0 | 0.59 |
| tta | L | 361 | 43.07 |
| ttg | L | 24 | 94.95 |
| tct | S | 0 | 0.32 |
| tcc | S | 0 | 0.28 |
| tca | S | 2 | 0.44 |
| tcg | S | 0 | 0.12 |
| ctt | L | 31 | 91.99 |
| ctc | L | 0 | 79.75 |
| cta | L | 76 | 74.27 |
| ctg | L | 1 | 109.00 |
| cgt | R | 0 | 0.04 |
| cgc | R | 0 | 0.05 |
| cga | R | 0 | 0.10 |
| cgg | R | 0 | 0.08 |
| agt | S | 0 | 0.44 |
| agc | S | 0 | 0.40 |
| aga | R | 1 | 0.45 |
| agg | R | 0 | 0.28 |
| --- | -- | ------ | ------ |
| mPD | | 0.57 | 1.1 |
| | | nPD: | 0.5 |
| | | N. weight: | 2. |
| | | Sc. PD: | 0.36 |
| | | Sc. rank: | 296.6 |

,

| NA | | | |
|---|---|---|---|
| Pos. | 262 | obs: | exp: |
| att | I | 0 | 7.89 |
| atc | I | 0 | 5.50 |
| ata | I | 21 | 7.60 |
| gtt | V | 0 | 97.84 |
| gtc | V | 0 | 99.90 |
| gta | V | 411 | 89.21 |
| gtg | V | 65 | 189.00 |
| --- | -- | ------ | ------ |
| mPD | | 0.31 | 0.81 |
| | | nPD: | 0.38 |
| | | N. weight: | 1.6 |
| | | Sc. PD: | 0.11 |
| | | Sc. rank: | 77.4 |

,

| NA | | | |
|---|---|---|---|
| Pos. | 263 | obs: | exp: |
| tta | L | 0 | 0.09 |
| ttg | L | 0 | 0.19 |
| ctt | L | 1 | 0.19 |
| ctc | L | 0 | 0.16 |
| cta | L | 0 | 0.15 |
| ctg | L | 0 | 0.22 |
| att | I | 52 | 59.02 |
| atc | I | 0 | 41.15 |
| ata | I | 105 | 56.83 |
| atg | M | 19 | 19.00 |
| gtt | V | 297 | 65.77 |
| gtc | V | 3 | 67.16 |
| gta | V | 19 | 59.97 |
| gtg | V | 1 | 127.10 |
| --- | -- | ------ | ------ |
| mPD | | 0.90 | 1.2 |
| | | nPD: | 0.75 |
| | | N. weight: | 1.3 |
| | | Sc. PD: | 0.57 |
| | | Sc. rank: | 400.4 |

,

| NA | | | |
|---|---|---|---|
| Pos. | 264 | obs: | exp: |
| cgt | R | 0 | 0.04 |
| cgc | R | 0 | 0.05 |
| cga | R | 0 | 0.10 |
| cgg | R | 0 | 0.08 |
| aaa | K | 95 | 277.70 |
| aag | K | 401 | 218.30 |
| aga | R | 0 | 0.45 |
| agg | R | 1 | 0.28 |
| --- | -- | ------ | ------ |
| mPD | | 0.31 | 0.50 |
| | | nPD: | 0.63 |
| | | N. weight: | 0.41 |
| | | Sc. PD: | 0.13 |
| | | Sc. rank: | 104.3 |

,

| NA | | | |
|---|---|---|---|
| Pos. | 265 | obs: | exp: |
| gaa | E | 122 | 286.00 |
| gag | E | 375 | 211.00 |
| --- | -- | ------ | ------ |
| mPD | | 0.37 | 0.49 |
| | | nPD: | 0.76 |
| | | N. weight: | 0.32 |
| | | Sc. PD: | 0.14 |
| | | Sc. rank: | 96.2 |

,

| NA | | | |
|---|---|---|---|
| Pos. | 266 | obs: | exp: |
| gaa | E | 81 | 286.00 |
| gag | E | 416 | 211.00 |
| --- | -- | ------ | ------ |
| mPD | | 0.27 | 0.49 |
| | | nPD: | 0.56 |
| | | N. weight: | 0.52 |
| | | Sc. PD: | 0.12 |
| | | Sc. rank: | 109.6 |

,

| NA | | | |
|---|---|---|---|
| Pos. | 267 | obs: | exp: |
| tct | S | 0 | 3.02 |
| tcc | S | 19 | 2.65 |
| tca | S | 0 | 4.21 |
| tcg | S | 0 | 1.16 |
| cct | P | 0 | 0.79 |
| ccc | P | 0 | 0.54 |
| cca | P | 2 | 1.18 |
| ccg | P | 1 | 0.49 |
| att | I | 0 | 0.38 |
| atc | I | 1 | 0.26 |
| ata | I | 0 | 0.36 |
| act | T | 0 | 0.79 |
| acc | T | 1 | 0.64 |
| aca | T | 2 | 1.31 |
| acg | T | 0 | 0.25 |
| aat | N | 0 | 0.52 |
| aac | N | 1 | 0.48 |
| agt | S | 0 | 4.18 |
| agc | S | 0 | 3.79 |
| gtt | V | 0 | 4.32 |
| gtc | V | 21 | 4.41 |
| gta | V | 0 | 3.94 |
| gtg | V | 0 | 8.34 |
| gct | A | 0 | 54.91 |
| gcc | A | 28 | 44.66 |
| gca | A | 166 | 97.16 |
| gcg | A | 24 | 21.27 |
| gaa | E | 2 | 132.90 |
| gag | E | 229 | 98.08 |
| --- | -- | ------ | ------ |
| mPD | | 1.2 | 1.3 |
| | | nPD: | 0.94 |
| | | N. weight: | 0.98 |
| | | Sc. PD: | 0.59 |
| | | Sc. rank: | 299.6 |

,

| NA | | | |
|---|---|---|---|
| Pos. | 268 | obs: | exp: |
| tta | L | 382 | 43.42 |
| ttg | L | 23 | 95.72 |
| ctt | L | 26 | 92.73 |
| ctc | L | 46 | 80.39 |
| cta | L | 20 | 74.87 |
| ctg | L | 0 | 109.90 |
| --- | -- | ------ | ------ |
| mPD | | 0.64 | 1.1 |
| | | nPD: | 0.57 |
| | | N. weight: | 2. |
| | | Sc. PD: | 0.51 |
| | | Sc. rank: | 455.5 |

,

| NA | | | |
|---|---|---|---|
| Pos. | 269 | obs: | exp: |
| cgt | R | 0 | 9.80 |
| cgc | R | 0 | 11.56 |
| cga | R | 0 | 22.68 |
| cgg | R | 0 | 19.69 |
| aaa | K | 36 | 146.10 |
| aag | K | 225 | 114.90 |
| aga | R | 1 | 106.10 |
| agg | R | 235 | 66.14 |
| --- | -- | ------ | ------ |
| mPD | | 0.64 | 1.3 |
| | | nPD: | 0.51 |
| | | N. weight: | 1.1 |
| | | Sc. PD: | 0.21 |
| | | Sc. rank: | 176.6 |

,

| NA | | | |
|---|---|---|---|
| Pos. | 270 | obs: | exp: |
| ggt | G | 4 | 61.32 |
| ggc | G | 1 | 67.21 |
| gga | G | 489 | 240.40 |
| ggg | G | 3 | 128.00 |
| --- | -- | ------ | ------ |
| mPD | | 0.032 | 0.67 |
| | | nPD: | 0.05 |
| | | N. weight: | 0.91 |
| | | Sc. PD: | -0.24 |
| | | Sc. rank: | -173.8 |

,

| NA | | | |
|---|---|---|---|
| Pos. | 271 | obs: | exp: |
| tta | L | 2 | 0.17 |
| ttg | L | 0 | 0.39 |
| tct | S | 0 | 78.69 |
| tcc | S | 0 | 68.96 |
| tca | S | 466 | 109.70 |
| tcg | S | 29 | 30.17 |
| ctt | L | 0 | 0.37 |
| ctc | L | 0 | 0.32 |
| cta | L | 0 | 0.30 |
| ctg | L | 0 | 0.44 |
| agt | S | 0 | 108.90 |
| agc | S | 0 | 98.62 |
| --- | -- | ------ | ------ |
| mPD | | 0.12 | 1.7 |
| | | nPD: | 0.07 |
| | | N. weight: | 1.9 |
| | | Sc. PD: | -0.45 |
| | | Sc. rank: | -299.2 |

,

| NA | | | |
|---|---|---|---|
| Pos. | 272 | obs: | exp: |
| gct | A | 6 | 125.20 |
| gcc | A | 490 | 101.80 |
| gca | A | 0 | 221.50 |
| gcg | A | 1 | 48.50 |
| --- | -- | ------ | ------ |
| mPD | | 0.028 | 0.69 |
| | | nPD: | 0.04 |
| | | N. weight: | 2.1 |
| | | Sc. PD: | -0.56 |
| | | Sc. rank: | -422.8 |

,

| NA | | | |
|---|---|---|---|
| Pos. | 273 | obs: | exp: |
| cgt | R | 0 | 20.26 |
| cgc | R | 0 | 23.89 |
| cga | R | 0 | 46.90 |
| cgg | R | 0 | 40.72 |
| aaa | K | 9 | 5.04 |
| aag | K | 0 | 3.96 |
| aga | R | 466 | 219.50 |
| agg | R | 22 | 136.80 |
| --- | -- | ------ | ------ |
| mPD | | 0.12 | 0.99 |
| | | nPD: | 0.12 |
| | | N. weight: | 0.9 |
| | | Sc. PD: | -0.17 |
| | | Sc. rank: | -113.2 |

,

| NA | | | |
|---|---|---|---|
| Pos. | 274 | obs: | exp: |
| cct | P | 0 | 0.26 |
| ccc | P | 1 | 0.18 |
| cca | P | 0 | 0.39 |
| ccg | P | 0 | 0.16 |
| cat | H | 265 | 268.80 |
| cac | H | 231 | 227.20 |
| --- | -- | ------ | ------ |
| mPD | | 0.50 | 0.50 |
| | | nPD: | 1. |
| | | N. weight: | 0.014 |
| | | Sc. PD: | 0.0096 |
| | | Sc. rank: | 4.4 |

,

| NA | | | |
|---|---|---|---|
| Pos. | 275 | obs: | exp: |
| att | I | 420 | 186.80 |
| atc | I | 5 | 130.30 |
| ata | I | 72 | 179.90 |
| --- | -- | ------ | ------ |
| mPD | | 0.27 | 0.66 |
| | | nPD: | 0.4 |
| | | N. weight: | 0.74 |
| | | Sc. PD: | 0.064 |
| | | Sc. rank: | 48.8 |

,

| NA | | | |
|---|---|---|---|
| Pos. | 276 | obs: | exp: |
| gaa | E | 22 | 286.00 |
| gag | E | 475 | 211.00 |
| --- | -- | ------ | ------ |
| mPD | | 0.085 | 0.49 |
| | | nPD: | 0.17 |
| | | N. weight: | 0.93 |
| | | Sc. PD: | -0.13 |
| | | Sc. rank: | -85.6 |

,

| NA | | | |
|---|---|---|---|
| Pos. | 277 | obs: | exp: |
| gaa | E | 393 | 286.00 |
| gag | E | 104 | 211.00 |
| --- | -- | ------ | ------ |
| mPD | | 0.33 | 0.49 |
| | | nPD: | 0.68 |
| | | N. weight: | 0.15 |
| | | Sc. PD: | 0.054 |
| | | Sc. rank: | 42.1 |

,

| NA | | | |
|---|---|---|---|
| Pos. | 278 | obs: | exp: |
| tgt | C | 14 | 204.90 |
| tgc | C | 483 | 292.10 |
| --- | -- | ------ | ------ |
| mPD | | 0.055 | 0.48 |
| | | nPD: | 0.11 |
| | | N. weight: | 0.58 |
| | | Sc. PD: | -0.11 |
| | | Sc. rank: | -78.3 |

,

| NA | | | |
|---|---|---|---|
| Pos. | 279 | obs: | exp: |
| tct | S | 19 | 79.01 |
| tcc | S | 408 | 69.23 |
| tca | S | 66 | 110.10 |
| tcg | S | 4 | 30.29 |
| agt | S | 0 | 109.30 |
| agc | S | 0 | 99.02 |
| --- | -- | ------ | ------ |
| mPD | | 0.31 | 1.7 |
| | | nPD: | 0.18 |
| | | N. weight: | 1.9 |
| | | Sc. PD: | -0.23 |
| | | Sc. rank: | -159.2 |

,

| NA | | | |
|---|---|---|---|
| Pos. | 280 | obs: | exp: |
| tgt | C | 393 | 204.90 |
| tgc | C | 104 | 292.10 |
| --- | -- | ------ | ------ |
| mPD | | 0.33 | 0.48 |
| | | nPD: | 0.68 |
| | | N. weight: | 0.43 |
| | | Sc. PD: | 0.15 |
| | | Sc. rank: | 118.0 |

,

| NA | | | |
|---|---|---|---|
| Pos. | 281 | obs: | exp: |
| tat | Y | 353 | 257.30 |
| tac | Y | 144 | 239.70 |
| --- | -- | ------ | ------ |
| mPD | | 0.41 | 0.50 |
| | | nPD: | 0.83 |
| | | N. weight: | 0.12 |
| | | Sc. PD: | 0.058 |
| | | Sc. rank: | 34.9 |

,

| NA | | | |
|---|---|---|---|
| Pos. | 282 | obs: | exp: |
| ggt | G | 0 | 61.32 |
| ggc | G | 0 | 67.21 |
| gga | G | 349 | 240.40 |
| ggg | G | 148 | 128.00 |
| --- | -- | ------ | ------ |
| mPD | | 0.42 | 0.67 |
| | | nPD: | 0.63 |
| | | N. weight: | 0.44 |
| | | Sc. PD: | 0.13 |
| | | Sc. rank: | 110.1 |

,

| NA | | | |
|---|---|---|---|
| Pos. | 283 | obs: | exp: |
| cct | P | 0 | 0.26 |
| ccc | P | 1 | 0.18 |
| cca | P | 0 | 0.39 |
| ccg | P | 0 | 0.16 |
| cat | H | 2 | 268.20 |
| cac | H | 493 | 226.80 |
| caa | Q | 1 | 0.53 |
| cag | Q | 0 | 0.47 |
| --- | -- | ------ | ------ |
| mPD | | 0.016 | 0.50 |
| | | nPD: | 0.03 |
| | | N. weight: | 1.1 |
| | | Sc. PD: | -0.29 |
| | | Sc. rank: | -229.2 |

,

| NA | | | |
|---|---|---|---|
| Pos. | 284 | obs: | exp: |
| tct | S | 0 | 27.18 |
| tcc | S | 0 | 23.82 |
| tca | S | 0 | 37.88 |
| tcg | S | 0 | 10.42 |
| aat | N | 310 | 163.60 |
| aac | N | 3 | 149.40 |
| agt | S | 152 | 37.62 |
| agc | S | 19 | 34.07 |
| gat | D | 13 | 6.94 |
| gac | D | 0 | 6.06 |
| --- | -- | ------ | ------ |
| mPD | | 0.59 | 1.5 |
| | | nPD: | 0.4 |
| | | N. weight: | 1.1 |
| | | Sc. PD: | 0.099 |
| | | Sc. rank: | 78.2 |

,

| NA | | | |
|---|---|---|---|
| Pos. | 285 | obs: | exp: |
| tct | S | 0 | 11.13 |
| tcc | S | 0 | 9.75 |
| tca | S | 55 | 15.51 |
| tcg | S | 15 | 4.27 |
| caa | Q | 417 | 227.10 |
| cag | Q | 9 | 198.90 |
| agt | S | 0 | 15.40 |
| agc | S | 0 | 13.95 |
| gct | A | 0 | 0.25 |
| gcc | A | 0 | 0.20 |
| gca | A | 1 | 0.45 |
| gcg | A | 0 | 0.10 |
| --- | -- | ------ | ------ |
| mPD | | 0.58 | 1.1 |
| | | nPD: | 0.53 |
| | | N. weight: | 0.9 |
| | | Sc. PD: | 0.19 |
| | | Sc. rank: | 164.6 |

,

| NA | | | |
|---|---|---|---|
| Pos. | 286 | obs: | exp: |
| cgt | R | 0 | 0.71 |
| cgc | R | 0 | 0.83 |
| cga | R | 0 | 1.63 |
| cgg | R | 0 | 1.42 |
| aaa | K | 44 | 268.80 |
| aag | K | 436 | 211.20 |
| aga | R | 14 | 7.65 |
| agg | R | 3 | 4.76 |
| --- | -- | ------ | ------ |
| mPD | | 0.27 | 0.58 |
| | | nPD: | 0.47 |
| | | N. weight: | 0.7 |
| | | Sc. PD: | 0.11 |
| | | Sc. rank: | 90.0 |

,

| NA | | | |
|---|---|---|---|
| Pos. | 287 | obs: | exp: |
| att | I | 0 | 0.75 |
| atc | I | 0 | 0.52 |
| ata | I | 2 | 0.72 |
| gtt | V | 0 | 95.78 |
| gtc | V | 1 | 97.80 |
| gta | V | 43 | 87.34 |
| gtg | V | 422 | 185.10 |
| gct | A | 0 | 7.30 |
| gcc | A | 0 | 5.94 |
| gca | A | 1 | 12.93 |
| gcg | A | 28 | 2.83 |
| --- | -- | ------ | ------ |
| mPD | | 0.29 | 0.85 |
| | | nPD: | 0.34 |
| | | N. weight: | 1.1 |
| | | Sc. PD: | 0.03 |
| | | Sc. rank: | 9.0 |

,

| NA | | | |
|---|---|---|---|
| Pos. | 288 | obs: | exp: |
| tct | S | 0 | 0.32 |
| tcc | S | 0 | 0.28 |
| tca | S | 0 | 0.44 |
| tcg | S | 0 | 0.12 |
| act | T | 105 | 130.80 |
| acc | T | 387 | 106.20 |
| aca | T | 3 | 216.90 |
| acg | T | 0 | 41.15 |
| agt | S | 2 | 0.44 |
| agc | S | 0 | 0.40 |
| --- | -- | ------ | ------ |
| mPD | | 0.36 | 0.69 |
| | | nPD: | 0.51 |
| | | N. weight: | 1.3 |
| | | Sc. PD: | 0.26 |
| | | Sc. rank: | 217.8 |

,

| NA | | | |
|---|---|---|---|
| Pos. | 289 | obs: | exp: |
| tgt | C | 496 | 204.90 |
| tgc | C | 1 | 292.10 |
| --- | -- | ------ | ------ |
| mPD | | 0.0040 | 0.48 |
| | | nPD: | 0.01 |
| | | N. weight: | 1.2 |
| | | Sc. PD: | -0.36 |
| | | Sc. rank: | -326.4 |

,

| NA | | | |
|---|---|---|---|
| Pos. | 290 | obs: | exp: |
| gtt | V | 0 | 102.20 |
| gtc | V | 0 | 104.30 |
| gta | V | 100 | 93.15 |
| gtg | V | 397 | 197.40 |
| --- | -- | ------ | ------ |
| mPD | | 0.32 | 0.72 |
| | | nPD: | 0.45 |
| | | N. weight: | 0.81 |
| | | Sc. PD: | 0.1 |
| | | Sc. rank: | 86.3 |

,

| NA | | | |
|---|---|---|---|
| Pos. | 291 | obs: | exp: |
| tgt | C | 51 | 204.90 |
| tgc | C | 446 | 292.10 |
| --- | -- | ------ | ------ |
| mPD | | 0.18 | 0.48 |
| | | nPD: | 0.38 |
| | | N. weight: | 0.34 |
| | | Sc. PD: | 0.022 |
| | | Sc. rank: | 15.3 |

,

| NA | | | |
|---|---|---|---|
| Pos. | 292 | obs: | exp: |
| cgt | R | 0 | 20.63 |
| cgc | R | 0 | 24.34 |
| cga | R | 0 | 47.76 |
| cgg | R | 1 | 41.47 |
| aga | R | 415 | 223.50 |
| agg | R | 81 | 139.30 |
| --- | -- | ------ | ------ |
| mPD | | 0.28 | 0.96 |
| | | nPD: | 0.29 |
| | | N. weight: | 0.6 |
| | | Sc. PD: | -0.013 |
| | | Sc. rank: | -13.8 |

,

| NA | | | |
|---|---|---|---|
| Pos. | 293 | obs: | exp: |
| gat | D | 419 | 265.20 |
| gac | D | 78 | 231.80 |
| --- | -- | ------ | ------ |
| mPD | | 0.27 | 0.50 |
| | | nPD: | 0.53 |
| | | N. weight: | 0.31 |
| | | Sc. PD: | 0.066 |
| | | Sc. rank: | 57.6 |

,

| NA | | | |
|---|---|---|---|
| Pos. | 294 | obs: | exp: |
| aat | N | 42 | 259.80 |
| aac | N | 455 | 237.20 |
| --- | -- | ------ | ------ |
| mPD | | 0.16 | 0.50 |
| | | nPD: | 0.31 |
| | | N. weight: | 0.63 |
| | | Sc. PD: | -0.0019 |
| | | Sc. rank: | -7.1 |

,

| NA | | | |
|---|---|---|---|
| Pos. | 295 | obs: | exp: |
| tgg | W | 497 | 497.00 |
| --- | -- | ------ | ------ |
| mPD | | 0 | 0 |
| | | nPD: | 1. |
| | | N. weight: | 0. |
| | | Sc. PD: | 0 |
| | | Sc. rank: | 0 |

,

| NA | | | |
|---|---|---|---|
| Pos. | 296 | obs: | exp: |
| caa | Q | 77 | 265.00 |
| cag | Q | 420 | 232.00 |
| --- | -- | ------ | ------ |
| mPD | | 0.26 | 0.50 |
| | | nPD: | 0.53 |
| | | N. weight: | 0.44 |
| | | Sc. PD: | 0.091 |
| | | Sc. rank: | 75.4 |

,

| NA | | | |
|---|---|---|---|
| Pos. | 297 | obs: | exp: |
| ggt | G | 3 | 61.32 |
| ggc | G | 3 | 67.21 |
| gga | G | 433 | 240.40 |
| ggg | G | 58 | 128.00 |
| --- | -- | ------ | ------ |
| mPD | | 0.23 | 0.67 |
| | | nPD: | 0.34 |
| | | N. weight: | 0.55 |
| | | Sc. PD: | 0.015 |
| | | Sc. rank: | 3.8 |

,

| NA | | | |
|---|---|---|---|
| Pos. | 298 | obs: | exp: |
| gct | A | 4 | 125.20 |
| gcc | A | 66 | 101.80 |
| gca | A | 427 | 221.50 |
| gcg | A | 0 | 48.50 |
| --- | -- | ------ | ------ |
| mPD | | 0.24 | 0.69 |
| | | nPD: | 0.36 |
| | | N. weight: | 0.68 |
| | | Sc. PD: | 0.028 |
| | | Sc. rank: | 14.9 |

,

| NA | | | |
|---|---|---|---|
| Pos. | 299 | obs: | exp: |
| aat | N | 171 | 259.80 |
| aac | N | 326 | 237.20 |
| --- | -- | ------ | ------ |
| mPD | | 0.45 | 0.50 |
| | | nPD: | 0.91 |
| | | N. weight: | 0.1 |
| | | Sc. PD: | 0.057 |
| | | Sc. rank: | 30.3 |

,

| NA | | | |
|---|---|---|---|
| Pos. | 300 | obs: | exp: |
| cgt | R | 0 | 20.63 |
| cgc | R | 0 | 24.34 |
| cga | R | 0 | 47.76 |
| cgg | R | 0 | 41.47 |
| aga | R | 80 | 223.50 |
| agg | R | 417 | 139.30 |
| --- | -- | ------ | ------ |
| mPD | | 0.27 | 0.96 |
| | | nPD: | 0.28 |
| | | N. weight: | 1.1 |
| | | Sc. PD: | -0.033 |
| | | Sc. rank: | -27.0 |

,

| NA | | | |
|---|---|---|---|
| Pos. | 301 | obs: | exp: |
| cct | P | 368 | 130.90 |
| ccc | P | 56 | 89.65 |
| cca | P | 73 | 196.10 |
| ccg | P | 0 | 80.36 |
| --- | -- | ------ | ------ |
| mPD | | 0.42 | 0.72 |
| | | nPD: | 0.58 |
| | | N. weight: | 0.81 |
| | | Sc. PD: | 0.21 |
| | | Sc. rank: | 187.8 |

,

| NA | | | |
|---|---|---|---|
| Pos. | 302 | obs: | exp: |
| tta | L | 0 | 0.09 |
| ttg | L | 0 | 0.19 |
| ctt | L | 1 | 0.19 |
| ctc | L | 0 | 0.16 |
| cta | L | 0 | 0.15 |
| ctg | L | 0 | 0.22 |
| att | I | 419 | 159.40 |
| atc | I | 3 | 111.10 |
| ata | I | 2 | 153.50 |
| gtt | V | 1 | 14.80 |
| gtc | V | 0 | 15.11 |
| gta | V | 2 | 13.49 |
| gtg | V | 69 | 28.60 |
| --- | -- | ------ | ------ |
| mPD | | 0.52 | 0.95 |
| | | nPD: | 0.54 |
| | | N. weight: | 1.3 |
| | | Sc. PD: | 0.28 |
| | | Sc. rank: | 246.2 |

,

| NA | | | |
|---|---|---|---|
| Pos. | 303 | obs: | exp: |
| att | I | 69 | 185.70 |
| atc | I | 0 | 129.50 |
| ata | I | 425 | 178.80 |
| gtt | V | 2 | 0.62 |
| gtc | V | 0 | 0.63 |
| gta | V | 1 | 0.56 |
| gtg | V | 0 | 1.19 |
| --- | -- | ------ | ------ |
| mPD | | 0.26 | 0.67 |
| | | nPD: | 0.38 |
| | | N. weight: | 0.86 |
| | | Sc. PD: | 0.058 |
| | | Sc. rank: | 42.3 |

,

| NA | | | |
|---|---|---|---|
| Pos. | 304 | obs: | exp: |
| gat | D | 0 | 0.53 |
| gac | D | 1 | 0.47 |
| gaa | E | 480 | 285.40 |
| gag | E | 16 | 210.60 |
| --- | -- | ------ | ------ |
| mPD | | 0.066 | 0.49 |
| | | nPD: | 0.14 |
| | | N. weight: | 0.59 |
| | | Sc. PD: | -0.1 |
| | | Sc. rank: | -71.1 |

,

| NA | | | |
|---|---|---|---|
| Pos. | 305 | obs: | exp: |
| att | I | 409 | 186.80 |
| atc | I | 17 | 130.30 |
| ata | I | 71 | 179.90 |
| --- | -- | ------ | ------ |
| mPD | | 0.30 | 0.66 |
| | | nPD: | 0.46 |
| | | N. weight: | 0.63 |
| | | Sc. PD: | 0.089 |
| | | Sc. rank: | 75.9 |

,

| NA | | | |
|---|---|---|---|
| Pos. | 306 | obs: | exp: |
| aat | N | 1 | 1.57 |
| aac | N | 2 | 1.43 |
| gat | D | 431 | 263.60 |
| gac | D | 63 | 230.40 |
| --- | -- | ------ | ------ |
| mPD | | 0.24 | 0.51 |
| | | nPD: | 0.47 |
| | | N. weight: | 0.38 |
| | | Sc. PD: | 0.057 |
| | | Sc. rank: | 48.9 |

,

| NA | | | |
|---|---|---|---|
| Pos. | 307 | obs: | exp: |
| atg | M | 497 | 497.00 |
| --- | -- | ------ | ------ |
| mPD | | 0 | 0 |
| | | nPD: | 1. |
| | | N. weight: | 0. |
| | | Sc. PD: | 0 |
| | | Sc. rank: | 0 |

,

| NA | | | |
|---|---|---|---|
| Pos. | 308 | obs: | exp: |
| tct | S | 0 | 1.91 |
| tcc | S | 0 | 1.67 |
| tca | S | 0 | 2.66 |
| tcg | S | 0 | 0.73 |
| act | T | 0 | 0.26 |
| acc | T | 1 | 0.21 |
| aca | T | 0 | 0.44 |
| acg | T | 0 | 0.08 |
| aat | N | 73 | 251.90 |
| aac | N | 409 | 230.10 |
| agt | S | 0 | 2.64 |
| agc | S | 12 | 2.39 |
| gat | D | 0 | 0.53 |
| gac | D | 1 | 0.47 |
| gaa | E | 0 | 0.58 |
| gag | E | 1 | 0.42 |
| --- | -- | ------ | ------ |
| mPD | | 0.31 | 0.60 |
| | | nPD: | 0.53 |
| | | N. weight: | 0.48 |
| | | Sc. PD: | 0.099 |
| | | Sc. rank: | 81.2 |

,

| NA | | | |
|---|---|---|---|
| Pos. | 309 | obs: | exp: |
| tct | S | 0 | 0.16 |
| tcc | S | 1 | 0.14 |
| tca | S | 0 | 0.22 |
| tcg | S | 0 | 0.06 |
| cct | P | 0 | 0.26 |
| ccc | P | 0 | 0.18 |
| cca | P | 1 | 0.39 |
| ccg | P | 0 | 0.16 |
| act | T | 3 | 111.80 |
| acc | T | 0 | 90.77 |
| aca | T | 420 | 185.30 |
| acg | T | 0 | 35.16 |
| agt | S | 0 | 0.22 |
| agc | S | 0 | 0.20 |
| gct | A | 7 | 18.13 |
| gcc | A | 63 | 14.75 |
| gca | A | 2 | 32.09 |
| gcg | A | 0 | 7.03 |
| --- | -- | ------ | ------ |
| mPD | | 0.51 | 0.94 |
| | | nPD: | 0.55 |
| | | N. weight: | 1.2 |
| | | Sc. PD: | 0.27 |
| | | Sc. rank: | 234.2 |

,

| NA | | | |
|---|---|---|---|
| Pos. | 310 | obs: | exp: |
| tta | L | 0 | 37.22 |
| ttg | L | 421 | 82.04 |
| ctt | L | 0 | 79.49 |
| ctc | L | 0 | 68.91 |
| cta | L | 2 | 64.18 |
| ctg | L | 3 | 94.17 |
| atg | M | 71 | 71.00 |
| --- | -- | ------ | ------ |
| mPD | | 0.27 | 1.2 |
| | | nPD: | 0.22 |
| | | N. weight: | 1.9 |
| | | Sc. PD: | -0.17 |
| | | Sc. rank: | -115.4 |

,

| NA | | | |
|---|---|---|---|
| Pos. | 311 | obs: | exp: |
| gat | D | 1 | 1.60 |
| gac | D | 2 | 1.40 |
| gaa | E | 53 | 284.30 |
| gag | E | 441 | 209.70 |
| --- | -- | ------ | ------ |
| mPD | | 0.20 | 0.49 |
| | | nPD: | 0.41 |
| | | N. weight: | 0.68 |
| | | Sc. PD: | 0.062 |
| | | Sc. rank: | 51.0 |

,

| NA | | | |
|---|---|---|---|
| Pos. | 312 | obs: | exp: |
| cct | P | 0 | 0.26 |
| ccc | P | 1 | 0.18 |
| cca | P | 0 | 0.39 |
| ccg | P | 0 | 0.16 |
| cat | H | 47 | 268.80 |
| cac | H | 449 | 227.20 |
| --- | -- | ------ | ------ |
| mPD | | 0.18 | 0.50 |
| | | nPD: | 0.35 |
| | | N. weight: | 0.64 |
| | | Sc. PD: | 0.023 |
| | | Sc. rank: | 10.8 |

,

| NA | | | |
|---|---|---|---|
| Pos. | 313 | obs: | exp: |
| cct | P | 0 | 0.26 |
| ccc | P | 0 | 0.18 |
| cca | P | 1 | 0.39 |
| ccg | P | 0 | 0.16 |
| act | T | 0 | 131.00 |
| acc | T | 0 | 106.40 |
| aca | T | 496 | 217.30 |
| acg | T | 0 | 41.23 |
| --- | -- | ------ | ------ |
| mPD | | 0.0040 | 0.69 |
| | | nPD: | 0.01 |
| | | N. weight: | 1.2 |
| | | Sc. PD: | -0.34 |
| | | Sc. rank: | -314.9 |

,

| NA | | | |
|---|---|---|---|
| Pos. | 314 | obs: | exp: |
| tct | S | 0 | 79.01 |
| tcc | S | 0 | 69.23 |
| tca | S | 0 | 110.10 |
| tcg | S | 0 | 30.29 |
| agt | S | 463 | 109.30 |
| agc | S | 34 | 99.02 |
| --- | -- | ------ | ------ |
| mPD | | 0.13 | 1.7 |
| | | nPD: | 0.08 |
| | | N. weight: | 1.8 |
| | | Sc. PD: | -0.41 |
| | | Sc. rank: | -262.3 |

,

| NA | | | |
|---|---|---|---|
| Pos. | 315 | obs: | exp: |
| tct | S | 0 | 0.16 |
| tcc | S | 0 | 0.14 |
| tca | S | 0 | 0.22 |
| tcg | S | 0 | 0.06 |
| caa | Q | 20 | 37.32 |
| cag | Q | 50 | 32.68 |
| cgt | R | 0 | 17.64 |
| cgc | R | 0 | 20.81 |
| cga | R | 3 | 40.84 |
| cgg | R | 0 | 35.47 |
| aaa | K | 1 | 0.56 |
| aag | K | 0 | 0.44 |
| agt | S | 0 | 0.22 |
| agc | S | 1 | 0.20 |
| aga | R | 382 | 191.10 |
| agg | R | 40 | 119.10 |
| --- | -- | ------ | ------ |
| mPD | | 0.80 | 1.3 |
| | | nPD: | 0.63 |
| | | N. weight: | 0.65 |
| | | Sc. PD: | 0.2 |
| | | Sc. rank: | 162.6 |

,

| NA | | | |
|---|---|---|---|
| Pos. | 316 | obs: | exp: |
| tat | Y | 92 | 257.30 |
| tac | Y | 405 | 239.70 |
| --- | -- | ------ | ------ |
| mPD | | 0.30 | 0.50 |
| | | nPD: | 0.61 |
| | | N. weight: | 0.34 |
| | | Sc. PD: | 0.096 |
| | | Sc. rank: | 82.9 |

,

| NA | | | |
|---|---|---|---|
| Pos. | 317 | obs: | exp: |
| tta | L | 1 | 6.29 |
| ttg | L | 0 | 13.87 |
| ctt | L | 0 | 13.43 |
| ctc | L | 0 | 11.65 |
| cta | L | 70 | 10.85 |
| ctg | L | 1 | 15.92 |
| atg | M | 1 | 1.00 |
| gtt | V | 2 | 87.15 |
| gtc | V | 1 | 88.99 |
| gta | V | 79 | 79.47 |
| gtg | V | 342 | 168.40 |
| --- | -- | ------ | ------ |
| mPD | | 0.68 | 0.98 |
| | | nPD: | 0.7 |
| | | N. weight: | 1. |
| | | Sc. PD: | 0.38 |
| | | Sc. rank: | 287.0 |

,

| NA | | | |
|---|---|---|---|
| Pos. | 318 | obs: | exp: |
| tgt | C | 70 | 204.90 |
| tgc | C | 427 | 292.10 |
| --- | -- | ------ | ------ |
| mPD | | 0.24 | 0.48 |
| | | nPD: | 0.5 |
| | | N. weight: | 0.25 |
| | | Sc. PD: | 0.046 |
| | | Sc. rank: | 38.6 |

,

| NA | | | |
|---|---|---|---|
| Pos. | 319 | obs: | exp: |
| cct | P | 1 | 0.26 |
| ccc | P | 0 | 0.18 |
| cca | P | 0 | 0.39 |
| ccg | P | 0 | 0.16 |
| act | T | 326 | 131.00 |
| acc | T | 102 | 106.40 |
| aca | T | 68 | 217.30 |
| acg | T | 0 | 41.23 |
| --- | -- | ------ | ------ |
| mPD | | 0.51 | 0.69 |
| | | nPD: | 0.74 |
| | | N. weight: | 0.62 |
| | | Sc. PD: | 0.26 |
| | | Sc. rank: | 183.9 |

,

| NA | | | |
|---|---|---|---|
| Pos. | 320 | obs: | exp: |
| ggt | G | 0 | 61.32 |
| ggc | G | 0 | 67.21 |
| gga | G | 493 | 240.40 |
| ggg | G | 4 | 128.00 |
| --- | -- | ------ | ------ |
| mPD | | 0.016 | 0.67 |
| | | nPD: | 0.02 |
| | | N. weight: | 0.96 |
| | | Sc. PD: | -0.27 |
| | | Sc. rank: | -221.2 |

,

| NA | | | |
|---|---|---|---|
| Pos. | 321 | obs: | exp: |
| att | I | 309 | 159.00 |
| atc | I | 114 | 110.90 |
| ata | I | 0 | 153.10 |
| gtt | V | 66 | 15.21 |
| gtc | V | 8 | 15.53 |
| gta | V | 0 | 13.87 |
| gtg | V | 0 | 29.39 |
| --- | -- | ------ | ------ |
| mPD | | 0.63 | 0.95 |
| | | nPD: | 0.66 |
| | | N. weight: | 0.86 |
| | | Sc. PD: | 0.29 |
| | | Sc. rank: | 228.4 |

,

| NA | | | |
|---|---|---|---|
| Pos. | 322 | obs: | exp: |
| tta | L | 1 | 43.42 |
| ttg | L | 0 | 95.72 |
| ctt | L | 13 | 92.73 |
| ctc | L | 481 | 80.39 |
| cta | L | 2 | 74.87 |
| ctg | L | 0 | 109.90 |
| --- | -- | ------ | ------ |
| mPD | | 0.067 | 1.1 |
| | | nPD: | 0.06 |
| | | N. weight: | 2.3 |
| | | Sc. PD: | -0.57 |
| | | Sc. rank: | -402.2 |

,

| NA | | | |
|---|---|---|---|
| Pos. | 323 | obs: | exp: |
| cct | P | 0 | 0.26 |
| ccc | P | 0 | 0.18 |
| cca | P | 1 | 0.39 |
| ccg | P | 0 | 0.16 |
| act | T | 103 | 131.00 |
| acc | T | 7 | 106.40 |
| aca | T | 384 | 217.30 |
| acg | T | 2 | 41.23 |
| --- | -- | ------ | ------ |
| mPD | | 0.36 | 0.69 |
| | | nPD: | 0.52 |
| | | N. weight: | 0.5 |
| | | Sc. PD: | 0.1 |
| | | Sc. rank: | 82.3 |

,

| NA | | | |
|---|---|---|---|
| Pos. | 324 | obs: | exp: |
| gat | D | 53 | 265.20 |
| gac | D | 444 | 231.80 |
| --- | -- | ------ | ------ |
| mPD | | 0.19 | 0.50 |
| | | nPD: | 0.38 |
| | | N. weight: | 0.58 |
| | | Sc. PD: | 0.039 |
| | | Sc. rank: | 30.6 |

,

| NA | | | |
|---|---|---|---|
| Pos. | 325 | obs: | exp: |
| cct | P | 0 | 0.26 |
| ccc | P | 1 | 0.18 |
| cca | P | 0 | 0.39 |
| ccg | P | 0 | 0.16 |
| act | T | 36 | 131.00 |
| acc | T | 389 | 106.40 |
| aca | T | 6 | 217.30 |
| acg | T | 65 | 41.23 |
| --- | -- | ------ | ------ |
| mPD | | 0.37 | 0.69 |
| | | nPD: | 0.53 |
| | | N. weight: | 1.3 |
| | | Sc. PD: | 0.28 |
| | | Sc. rank: | 247.3 |

,

| NA | | | |
|---|---|---|---|
| Pos. | 326 | obs: | exp: |
| tct | S | 0 | 79.01 |
| tcc | S | 0 | 69.23 |
| tca | S | 0 | 110.10 |
| tcg | S | 0 | 30.29 |
| agt | S | 47 | 109.30 |
| agc | S | 450 | 99.02 |
| --- | -- | ------ | ------ |
| mPD | | 0.17 | 1.7 |
| | | nPD: | 0.1 |
| | | N. weight: | 1.8 |
| | | Sc. PD: | -0.37 |
| | | Sc. rank: | -246.5 |

,

| NA | | | |
|---|---|---|---|
| Pos. | 327 | obs: | exp: |
| tct | S | 0 | 0.16 |
| tcc | S | 0 | 0.14 |
| tca | S | 0 | 0.22 |
| tcg | S | 0 | 0.06 |
| cgt | R | 0 | 20.59 |
| cgc | R | 0 | 24.29 |
| cga | R | 6 | 47.67 |
| cgg | R | 0 | 41.39 |
| agt | S | 0 | 0.22 |
| agc | S | 1 | 0.20 |
| aga | R | 487 | 223.10 |
| agg | R | 3 | 139.00 |
| --- | -- | ------ | ------ |
| mPD | | 0.040 | 0.96 |
| | | nPD: | 0.04 |
| | | N. weight: | 1. |
| | | Sc. PD: | -0.27 |
| | | Sc. rank: | -200.3 |

,

| NA | | | |
|---|---|---|---|
| Pos. | 328 | obs: | exp: |
| cct | P | 413 | 130.90 |
| ccc | P | 20 | 89.65 |
| cca | P | 63 | 196.10 |
| ccg | P | 1 | 80.36 |
| --- | -- | ------ | ------ |
| mPD | | 0.29 | 0.72 |
| | | nPD: | 0.41 |
| | | N. weight: | 1.1 |
| | | Sc. PD: | 0.096 |
| | | Sc. rank: | 80.3 |

,

| NA | | | |
|---|---|---|---|
| Pos. | 329 | obs: | exp: |
| tct | S | 0 | 12.08 |
| tcc | S | 0 | 10.59 |
| tca | S | 70 | 16.84 |
| tcg | S | 1 | 4.63 |
| cgt | R | 0 | 0.04 |
| cgc | R | 0 | 0.05 |
| cga | R | 0 | 0.10 |
| cgg | R | 0 | 0.08 |
| agt | S | 5 | 16.72 |
| agc | S | 0 | 15.14 |
| aga | R | 0 | 0.45 |
| agg | R | 1 | 0.28 |
| ggt | G | 39 | 51.82 |
| ggc | G | 0 | 56.80 |
| gga | G | 48 | 203.20 |
| ggg | G | 333 | 108.20 |
| --- | -- | ------ | ------ |
| mPD | | 0.99 | 1.1 |
| | | nPD: | 0.88 |
| | | N. weight: | 1.1 |
| | | Sc. PD: | 0.61 |
| | | Sc. rank: | 338.6 |

,

| NA | | | |
|---|---|---|---|
| Pos. | 330 | obs: | exp: |
| gat | D | 1 | 265.20 |
| gac | D | 496 | 231.80 |
| --- | -- | ------ | ------ |
| mPD | | 0.0040 | 0.50 |
| | | nPD: | 0.01 |
| | | N. weight: | 1. |
| | | Sc. PD: | -0.31 |
| | | Sc. rank: | -282.0 |

,

| NA | | | |
|---|---|---|---|
| Pos. | 331 | obs: | exp: |
| cgt | R | 0 | 0.25 |
| cgc | R | 0 | 0.29 |
| cga | R | 0 | 0.58 |
| cgg | R | 0 | 0.50 |
| aaa | K | 482 | 274.90 |
| aag | K | 9 | 216.10 |
| aga | R | 6 | 2.70 |
| agg | R | 0 | 1.68 |
| --- | -- | ------ | ------ |
| mPD | | 0.060 | 0.52 |
| | | nPD: | 0.11 |
| | | N. weight: | 0.7 |
| | | Sc. PD: | -0.14 |
| | | Sc. rank: | -93.2 |

,

| NA | | | |
|---|---|---|---|
| Pos. | 332 | obs: | exp: |
| ttt | F | 1 | 0.41 |
| ttc | F | 0 | 0.59 |
| tta | L | 0 | 0.26 |
| ttg | L | 0 | 0.58 |
| tct | S | 401 | 76.31 |
| tcc | S | 13 | 66.87 |
| tca | S | 66 | 106.30 |
| tcg | S | 0 | 29.26 |
| ctt | L | 2 | 0.56 |
| ctc | L | 0 | 0.49 |
| cta | L | 1 | 0.45 |
| ctg | L | 0 | 0.66 |
| cct | P | 9 | 2.63 |
| ccc | P | 0 | 1.80 |
| cca | P | 1 | 3.95 |
| ccg | P | 0 | 1.62 |
| act | T | 0 | 0.79 |
| acc | T | 0 | 0.64 |
| aca | T | 3 | 1.31 |
| acg | T | 0 | 0.25 |
| agt | S | 0 | 105.60 |
| agc | S | 0 | 95.63 |
| --- | -- | ------ | ------ |
| mPD | | 0.37 | 1.7 |
| | | nPD: | 0.22 |
| | | N. weight: | 1.8 |
| | | Sc. PD: | -0.17 |
| | | Sc. rank: | -123.5 |

,

| NA | | | |
|---|---|---|---|
| Pos. | 333 | obs: | exp: |
| tct | S | 0 | 57.39 |
| tcc | S | 0 | 50.29 |
| tca | S | 0 | 79.98 |
| tcg | S | 0 | 22.00 |
| att | I | 0 | 1.50 |
| atc | I | 0 | 1.05 |
| ata | I | 4 | 1.45 |
| atg | M | 56 | 56.00 |
| act | T | 11 | 3.70 |
| acc | T | 1 | 3.00 |
| aca | T | 0 | 6.13 |
| acg | T | 2 | 1.16 |
| aat | N | 41 | 21.43 |
| aac | N | 0 | 19.57 |
| agt | S | 359 | 79.42 |
| agc | S | 2 | 71.92 |
| gtt | V | 13 | 4.32 |
| gtc | V | 0 | 4.41 |
| gta | V | 0 | 3.94 |
| gtg | V | 8 | 8.34 |
| --- | -- | ------ | ------ |
| mPD | | 0.78 | 1.9 |
| | | nPD: | 0.4 |
| | | N. weight: | 1.7 |
| | | Sc. PD: | 0.14 |
| | | Sc. rank: | 109.2 |

,

| | | | |
|---|---|---|---|
| NA | | | |
| Pos. | 334 | obs: | exp: |
| ggt | G | 447 | 61.32 |
| ggc | G | 10 | 67.21 |
| gga | G | 40 | 240.40 |
| ggg | G | 0 | 128.00 |
| --- | -- | ------ | ------ |
| mPD | | 0.18 | 0.67 |
| | | nPD: | 0.28 |
| | | N. weight: | 2.3 |
| | | Sc. PD: | -0.08 |
| | | Sc. rank: | -65.6 |

,

| | | | |
|---|---|---|---|
| NA | | | |
| Pos. | 335 | obs: | exp: |
| gat | D | 380 | 265.20 |
| gac | D | 117 | 231.80 |
| --- | -- | ------ | ------ |
| mPD | | 0.36 | 0.50 |
| | | nPD: | 0.72 |
| | | N. weight: | 0.17 |
| | | Sc. PD: | 0.067 |
| | | Sc. rank: | 48.6 |

,

| | | | |
|---|---|---|---|
| NA | | | |
| Pos. | 336 | obs: | exp: |
| tgt | C | 494 | 204.90 |
| tgc | C | 3 | 292.10 |
| --- | -- | ------ | ------ |
| mPD | | 0.012 | 0.48 |
| | | nPD: | 0.02 |
| | | N. weight: | 1.2 |
| | | Sc. PD: | -0.33 |
| | | Sc. rank: | -268.6 |

,

| | | | |
|---|---|---|---|
| NA | | | |
| Pos. | 337 | obs: | exp: |
| ttt | F | 0 | 0.41 |
| ttc | F | 1 | 0.59 |
| tct | S | 38 | 67.56 |
| tcc | S | 385 | 59.20 |
| tca | S | 2 | 94.15 |
| tcg | S | 0 | 25.91 |
| cgt | R | 0 | 0.04 |
| cgc | R | 1 | 0.05 |
| cga | R | 0 | 0.10 |
| cgg | R | 0 | 0.08 |
| aat | N | 67 | 36.58 |
| aac | N | 3 | 33.42 |
| agt | S | 0 | 93.50 |
| agc | S | 0 | 84.67 |
| aga | R | 0 | 0.45 |
| agg | R | 0 | 0.28 |
| --- | -- | ------ | ------ |
| mPD | | 0.84 | 1.8 |
| | | nPD: | 0.47 |
| | | N. weight: | 2.1 |
| | | Sc. PD: | 0.31 |
| | | Sc. rank: | 264.6 |

,

| | | | |
|---|---|---|---|
| NA | | | |
| Pos. | 338 | obs: | exp: |
| aat | N | 471 | 259.80 |
| aac | N | 26 | 237.20 |
| --- | -- | ------ | ------ |
| mPD | | 0.099 | 0.50 |
| | | nPD: | 0.2 |
| | | N. weight: | 0.63 |
| | | Sc. PD: | -0.071 |
| | | Sc. rank: | -49.1 |

,

| | | | |
|---|---|---|---|
| NA | | | |
| Pos. | 339 | obs: | exp: |
| cct | P | 0 | 130.90 |
| ccc | P | 0 | 89.65 |
| cca | P | 397 | 196.10 |
| ccg | P | 100 | 80.36 |
| --- | -- | ------ | ------ |
| mPD | | 0.32 | 0.72 |
| | | nPD: | 0.45 |
| | | N. weight: | 0.86 |
| | | Sc. PD: | 0.11 |
| | | Sc. rank: | 92.5 |

,

| | | | |
|---|---|---|---|
| NA | | | |
| Pos. | 340 | obs: | exp: |
| att | I | 7 | 186.80 |
| atc | I | 138 | 130.30 |
| ata | I | 352 | 179.90 |
| --- | -- | ------ | ------ |
| mPD | | 0.42 | 0.66 |
| | | nPD: | 0.64 |
| | | N. weight: | 0.64 |
| | | Sc. PD: | 0.2 |
| | | Sc. rank: | 167.5 |

,

| | | | |
|---|---|---|---|
| NA | | | |
| Pos. | 341 | obs: | exp: |
| att | I | 5 | 1.88 |
| atc | I | 0 | 1.31 |
| ata | I | 0 | 1.81 |
| act | T | 486 | 129.50 |
| acc | T | 4 | 105.10 |
| aca | T | 0 | 214.70 |
| acg | T | 0 | 40.73 |
| gct | A | 2 | 0.50 |
| gcc | A | 0 | 0.41 |
| gca | A | 0 | 0.89 |
| gcg | A | 0 | 0.20 |
| --- | -- | ------ | ------ |
| mPD | | 0.044 | 0.71 |
| | | nPD: | 0.06 |
| | | N. weight: | 1.8 |
| | | Sc. PD: | -0.44 |
| | | Sc. rank: | -301.1 |

,

| | | | |
|---|---|---|---|
| NA | | | |
| Pos. | 342 | obs: | exp: |
| ggt | G | 1 | 61.32 |
| ggc | G | 0 | 67.21 |
| gga | G | 145 | 240.40 |
| ggg | G | 351 | 128.00 |
| --- | -- | ------ | ------ |
| mPD | | 0.42 | 0.67 |
| | | nPD: | 0.63 |
| | | N. weight: | 0.79 |
| | | Sc. PD: | 0.24 |
| | | Sc. rank: | 198.9 |

,

| | | | |
|---|---|---|---|
| NA | | | |
| Pos. | 343 | obs: | exp: |
| tct | S | 0 | 78.85 |
| tcc | S | 0 | 69.10 |
| tca | S | 0 | 109.90 |
| tcg | S | 0 | 30.23 |
| act | T | 1 | 0.26 |
| acc | T | 0 | 0.21 |
| aca | T | 0 | 0.44 |
| acg | T | 0 | 0.08 |
| agt | S | 490 | 109.10 |
| agc | S | 6 | 98.82 |
| --- | -- | ------ | ------ |
| mPD | | 0.028 | 1.7 |
| | | nPD: | 0.02 |
| | | N. weight: | 2. |
| | | Sc. PD: | -0.59 |
| | | Sc. rank: | -487.6 |

,

| | | | |
|---|---|---|---|
| NA | | | |
| Pos. | 344 | obs: | exp: |
| cct | P | 136 | 130.90 |
| ccc | P | 290 | 89.65 |
| cca | P | 4 | 196.10 |
| ccg | P | 67 | 80.36 |
| --- | -- | ------ | ------ |
| mPD | | 0.57 | 0.72 |
| | | nPD: | 0.79 |
| | | N. weight: | 0.92 |
| | | Sc. PD: | 0.43 |
| | | Sc. rank: | 273.9 |

,

| | | | |
|---|---|---|---|
| NA | | | |
| Pos. | 345 | obs: | exp: |
| tgt | C | 0 | 0.41 |
| tgc | C | 1 | 0.59 |
| ggt | G | 39 | 61.20 |
| ggc | G | 385 | 67.08 |
| gga | G | 71 | 239.90 |
| ggg | G | 1 | 127.80 |
| --- | -- | ------ | ------ |
| mPD | | 0.38 | 0.67 |
| | | nPD: | 0.56 |
| | | N. weight: | 1.6 |
| | | Sc. PD: | 0.38 |
| | | Sc. rank: | 340.6 |

,

| | | | |
|---|---|---|---|
| NA | | | |
| Pos. | 346 | obs: | exp: |
| tct | S | 1 | 0.16 |
| tcc | S | 0 | 0.14 |
| tca | S | 0 | 0.22 |
| tcg | S | 0 | 0.06 |
| agt | S | 0 | 0.22 |
| agc | S | 0 | 0.20 |
| gtt | V | 41 | 8.43 |
| gtc | V | 0 | 8.61 |
| gta | V | 0 | 7.68 |
| gtg | V | 0 | 16.28 |
| gct | A | 365 | 114.60 |
| gcc | A | 88 | 93.21 |
| gca | A | 2 | 202.80 |
| gcg | A | 0 | 44.40 |
| --- | -- | ------ | ------ |
| mPD | | 0.45 | 0.86 |
| | | nPD: | 0.53 |
| | | N. weight: | 1.3 |
| | | Sc. PD: | 0.28 |
| | | Sc. rank: | 245.9 |

,

| | | | |
|---|---|---|---|
| NA | | | |
| Pos. | 347 | obs: | exp: |
| cct | P | 70 | 130.90 |
| ccc | P | 0 | 89.65 |
| cca | P | 103 | 196.10 |
| ccg | P | 324 | 80.36 |
| --- | -- | ------ | ------ |
| mPD | | 0.51 | 0.72 |
| | | nPD: | 0.72 |
| | | N. weight: | 0.98 |
| | | Sc. PD: | 0.38 |
| | | Sc. rank: | 278.4 |

,

| | | | |
|---|---|---|---|
| NA | | | |
| Pos. | 348 | obs: | exp: |
| ggt | G | 0 | 61.32 |
| ggc | G | 0 | 67.21 |
| gga | G | 382 | 240.40 |
| ggg | G | 115 | 128.00 |
| --- | -- | ------ | ------ |
| mPD | | 0.36 | 0.67 |
| | | nPD: | 0.54 |
| | | N. weight: | 0.47 |
| | | Sc. PD: | 0.1 |
| | | Sc. rank: | 88.3 |

,

| | | | |
|---|---|---|---|
| NA | | | |
| Pos. | 349 | obs: | exp: |
| gtt | V | 29 | 102.20 |
| gtc | V | 44 | 104.30 |
| gta | V | 40 | 93.15 |
| gtg | V | 384 | 197.40 |
| --- | -- | ------ | ------ |
| mPD | | 0.39 | 0.72 |
| | | nPD: | 0.54 |
| | | N. weight: | 0.44 |
| | | Sc. PD: | 0.094 |
| | | Sc. rank: | 82.0 |

,

| | | | |
|---|---|---|---|
| NA | | | |
| Pos. | 350 | obs: | exp: |
| aaa | K | 78 | 278.30 |
| aag | K | 419 | 218.70 |
| --- | -- | ------ | ------ |
| mPD | | 0.27 | 0.49 |
| | | nPD: | 0.54 |
| | | N. weight: | 0.5 |
| | | Sc. PD: | 0.11 |
| | | Sc. rank: | 94.6 |

,

| | | | |
|---|---|---|---|
| NA | | | |
| Pos. | 351 | obs: | exp: |
| ggt | G | 6 | 61.32 |
| ggc | G | 0 | 67.21 |
| gga | G | 477 | 240.40 |
| ggg | G | 14 | 128.00 |
| --- | -- | ------ | ------ |
| mPD | | 0.078 | 0.67 |
| | | nPD: | 0.12 |
| | | N. weight: | 0.8 |
| | | Sc. PD: | -0.15 |
| | | Sc. rank: | -104.8 |

,

| | | | |
|---|---|---|---|
| NA | | | |
| Pos. | 352 | obs: | exp: |
| ttt | F | 59 | 204.30 |
| ttc | F | 438 | 292.70 |
| --- | -- | ------ | ------ |
| mPD | | 0.21 | 0.48 |
| | | nPD: | 0.43 |
| | | N. weight: | 0.3 |
| | | Sc. PD: | 0.035 |
| | | Sc. rank: | 27.2 |

,

| | | | |
|---|---|---|---|
| NA | | | |
| Pos. | 353 | obs: | exp: |
| ggt | G | 20 | 61.32 |
| ggc | G | 32 | 67.21 |
| gga | G | 43 | 240.40 |
| ggg | G | 402 | 128.00 |
| --- | -- | ------ | ------ |
| mPD | | 0.33 | 0.67 |
| | | nPD: | 0.5 |
| | | N. weight: | 0.98 |
| | | Sc. PD: | 0.18 |
| | | Sc. rank: | 149.1 |

,

| NA | | | |
|---|---|---|---|
| Pos. | 354 | obs: | exp: |
| ttt | F | 407 | 203.10 |
| ttc | F | 87 | 290.90 |
| tat | Y | 3 | 1.55 |
| tac | Y | 0 | 1.45 |
| mPD | | 0.30 | 0.50 |
| | | nPD: | 0.61 |
| | | N. weight: | 0.51 |
| | | Sc. PD: | 0.15 |
| | | Sc. rank: | 125.8 |

,

| NA | | | |
|---|---|---|---|
| Pos. | 355 | obs: | exp: |
| tta | L | 26 | 43.42 |
| ttg | L | 1 | 95.72 |
| ctt | L | 0 | 92.73 |
| ctc | L | 0 | 80.39 |
| cta | L | 400 | 74.87 |
| ctg | L | 70 | 109.90 |
| mPD | | 0.35 | 1.1 |
| | | nPD: | 0.31 |
| | | N. weight: | 1.8 |
| | | Sc. PD: | -0.0022 |
| | | Sc. rank: | -14.8 |

,

| NA | | | |
|---|---|---|---|
| Pos. | 356 | obs: | exp: |
| tct | S | 0 | 0.16 |
| tcc | S | 0 | 0.14 |
| tca | S | 0 | 0.22 |
| tcg | S | 0 | 0.06 |
| aat | N | 425 | 222.60 |
| aac | N | 1 | 203.40 |
| agt | S | 1 | 0.22 |
| agc | S | 0 | 0.20 |
| gat | D | 70 | 37.35 |
| gac | D | 0 | 32.65 |
| mPD | | 0.25 | 0.75 |
| | | nPD: | 0.34 |
| | | N. weight: | 0.89 |
| | | Sc. PD: | 0.018 |
| | | Sc. rank: | 2.0 |

,

| NA | | | |
|---|---|---|---|
| Pos. | 357 | obs: | exp: |
| tct | S | 0 | 11.13 |
| tcc | S | 0 | 9.75 |
| tca | S | 0 | 15.51 |
| tcg | S | 0 | 4.27 |
| agt | S | 70 | 15.40 |
| agc | S | 0 | 13.95 |
| gaa | E | 0 | 2.30 |
| gag | E | 4 | 1.70 |
| ggt | G | 5 | 52.19 |
| ggc | G | 0 | 57.20 |
| gga | G | 22 | 204.60 |
| ggg | G | 396 | 109.00 |
| mPD | | 0.59 | 1.1 |
| | | nPD: | 0.53 |
| | | N. weight: | 1.6 |
| | | Sc. PD: | 0.33 |
| | | Sc. rank: | 278.6 |

,

| NA | | | |
|---|---|---|---|
| Pos. | 358 | obs: | exp: |
| tct | S | 0 | 3.82 |
| tcc | S | 0 | 3.34 |
| tca | S | 0 | 5.32 |
| tcg | S | 0 | 1.46 |
| aat | N | 2 | 1.05 |
| aac | N | 0 | 0.95 |
| agt | S | 0 | 5.28 |
| agc | S | 24 | 4.78 |
| gat | D | 414 | 243.80 |
| gac | D | 43 | 213.20 |
| ggt | G | 1 | 1.73 |
| ggc | G | 13 | 1.89 |
| gga | G | 0 | 6.77 |
| ggg | G | 0 | 3.61 |
| mPD | | 0.51 | 0.78 |
| | | nPD: | 0.66 |
| | | N. weight: | 0.61 |
| | | Sc. PD: | 0.21 |
| | | Sc. rank: | 164.1 |

,

| NA | | | |
|---|---|---|---|
| Pos. | 359 | obs: | exp: |
| tct | S | 0 | 0.48 |
| tcc | S | 0 | 0.42 |
| tca | S | 0 | 0.66 |
| tcg | S | 0 | 0.18 |
| tat | Y | 0 | 0.52 |
| tac | Y | 1 | 0.48 |
| aat | N | 96 | 257.70 |
| aac | N | 397 | 235.30 |
| agt | S | 0 | 0.66 |
| agc | S | 3 | 0.60 |
| mPD | | 0.33 | 0.52 |
| | | nPD: | 0.63 |
| | | N. weight: | 0.34 |
| | | Sc. PD: | 0.1 |
| | | Sc. rank: | 86.2 |

,

| NA | | | |
|---|---|---|---|
| Pos. | 360 | obs: | exp: |
| cct | P | 0 | 0.26 |
| ccc | P | 0 | 0.18 |
| cca | P | 1 | 0.39 |
| ccg | P | 0 | 0.16 |
| act | T | 0 | 131.00 |
| acc | T | 0 | 106.40 |
| aca | T | 492 | 217.30 |
| acg | T | 4 | 41.23 |
| mPD | | 0.020 | 0.69 |
| | | nPD: | 0.03 |
| | | N. weight: | 1.1 |
| | | Sc. PD: | -0.31 |
| | | Sc. rank: | -243.6 |

,

| NA | | | |
|---|---|---|---|
| Pos. | 361 | obs: | exp: |
| tgg | W | 497 | 497.00 |
| mPD | | 0 | 0 |
| | | nPD: | 1. |
| | | N. weight: | 0. |
| | | Sc. PD: | 0 |
| | | Sc. rank: | 0 |

,

| NA | | | |
|---|---|---|---|
| Pos. | 362 | obs: | exp: |
| tta | L | 1 | 43.42 |
| ttg | L | 66 | 95.72 |
| ctt | L | 413 | 92.73 |
| ctc | L | 9 | 80.39 |
| cta | L | 4 | 74.87 |
| ctg | L | 4 | 109.90 |
| mPD | | 0.52 | 1.1 |
| | | nPD: | 0.47 |
| | | N. weight: | 1.6 |
| | | Sc. PD: | 0.24 |
| | | Sc. rank: | 195.3 |

,

| NA | | | |
|---|---|---|---|
| Pos. | 363 | obs: | exp: |
| ggt | G | 423 | 61.32 |
| ggc | G | 72 | 67.21 |
| gga | G | 0 | 240.40 |
| ggg | G | 2 | 128.00 |
| mPD | | 0.26 | 0.67 |
| | | nPD: | 0.38 |
| | | N. weight: | 2.3 |
| | | Sc. PD: | 0.15 |
| | | Sc. rank: | 119.8 |

,

| NA | | | |
|---|---|---|---|
| Pos. | 364 | obs: | exp: |
| cgt | R | 22 | 20.63 |
| cgc | R | 49 | 24.34 |
| cga | R | 0 | 47.76 |
| cgg | R | 0 | 41.47 |
| aga | R | 23 | 223.50 |
| agg | R | 403 | 139.30 |
| mPD | | 0.57 | 0.96 |
| | | nPD: | 0.6 |
| | | N. weight: | 1.2 |
| | | Sc. PD: | 0.33 |
| | | Sc. rank: | 282.7 |

,

| NA | | | |
|---|---|---|---|
| Pos. | 365 | obs: | exp: |
| act | T | 20 | 131.30 |
| acc | T | 404 | 106.60 |
| aca | T | 54 | 217.70 |
| acg | T | 19 | 41.32 |
| mPD | | 0.32 | 0.69 |
| | | nPD: | 0.47 |
| | | N. weight: | 1.2 |
| | | Sc. PD: | 0.18 |
| | | Sc. rank: | 161.8 |

,

| NA | | | |
|---|---|---|---|
| Pos. | 366 | obs: | exp: |
| att | I | 7 | 186.80 |
| atc | I | 419 | 130.30 |
| ata | I | 71 | 179.90 |
| mPD | | 0.27 | 0.66 |
| | | nPD: | 0.41 |
| | | N. weight: | 1.1 |
| | | Sc. PD: | 0.1 |
| | | Sc. rank: | 88.5 |

,

| NA | | | |
|---|---|---|---|
| Pos. | 367 | obs: | exp: |
| tct | S | 0 | 79.01 |
| tcc | S | 0 | 69.23 |
| tca | S | 0 | 110.10 |
| tcg | S | 0 | 30.29 |
| agt | S | 131 | 109.30 |
| agc | S | 366 | 99.02 |
| mPD | | 0.39 | 1.7 |
| | | nPD: | 0.23 |
| | | N. weight: | 1.4 |
| | | Sc. PD: | -0.11 |
| | | Sc. rank: | -74.4 |

,

| NA | | | |
|---|---|---|---|
| Pos. | 368 | obs: | exp: |
| tta | L | 0 | 0.09 |
| ttg | L | 0 | 0.19 |
| ctt | L | 0 | 0.19 |
| ctc | L | 1 | 0.16 |
| cta | L | 0 | 0.15 |
| ctg | L | 0 | 0.22 |
| cct | P | 70 | 130.60 |
| ccc | P | 421 | 89.47 |
| cca | P | 4 | 195.70 |
| ccg | P | 1 | 80.20 |
| mPD | | 0.26 | 0.72 |
| | | nPD: | 0.37 |
| | | N. weight: | 1.7 |
| | | Sc. PD: | 0.084 |
| | | Sc. rank: | 56.3 |

,

| NA | | | |
|---|---|---|---|
| Pos. | 369 | obs: | exp: |
| cgt | R | 70 | 20.55 |
| cgc | R | 0 | 24.24 |
| cga | R | 12 | 47.57 |
| cgg | R | 0 | 41.31 |
| att | I | 0 | 0.75 |
| atc | I | 0 | 0.52 |
| ata | I | 2 | 0.72 |
| aga | R | 360 | 222.60 |
| agg | R | 53 | 138.70 |
| mPD | | 0.69 | 0.97 |
| | | nPD: | 0.71 |
| | | N. weight: | 0.57 |
| | | Sc. PD: | 0.22 |
| | | Sc. rank: | 159.9 |

,

| NA | | | |
|---|---|---|---|
| Pos. | 370 | obs: | exp: |
| tct | S | 2 | 79.01 |
| tcc | S | 71 | 69.23 |
| tca | S | 406 | 110.10 |
| tcg | S | 18 | 30.29 |
| agt | S | 0 | 109.30 |
| agc | S | 0 | 99.02 |
| mPD | | 0.31 | 1.7 |
| | | nPD: | 0.19 |
| | | N. weight: | 1.5 |
| | | Sc. PD: | -0.18 |
| | | Sc. rank: | -121.2 |

,

| NA | | | |
|---|---|---|---|
| Pos. | 371 | obs: | exp: |
| cgt | R | 0 | 20.63 |
| cgc | R | 0 | 24.34 |
| cga | R | 0 | 47.76 |
| cgg | R | 0 | 41.47 |
| aga | R | 423 | 223.50 |
| agg | R | 74 | 139.30 |
| mPD | | 0.25 | 0.96 |
| | | nPD: | 0.26 |
| | | N. weight: | 0.64 |
| | | Sc. PD: | -0.03 |
| | | Sc. rank: | -23.3 |

,

| NA | | | |
|---|---|---|---|
| Pos. | 372 | obs: | exp: |
| tct | S | 0 | 79.01 |
| tcc | S | 0 | 69.23 |
| tca | S | 0 | 110.10 |
| tcg | S | 0 | 30.29 |
| agt | S | 495 | 109.30 |
| agc | S | 2 | 99.02 |
| mPD | | 0.0080 | 1.7 |
| | | nPD: | 0. |
| | | N. weight: | 2.1 |
| | | Sc. PD: | -0.62 |
| | | Sc. rank: | -571.8 |

,

| NA | | | |
|---|---|---|---|
| Pos. | 373 | obs: | exp: |
| ggt | G | 68 | 61.32 |
| ggc | G | 21 | 67.21 |
| gga | G | 401 | 240.40 |
| ggg | G | 7 | 128.00 |
| mPD | | 0.33 | 0.67 |
| | | nPD: | 0.49 |
| | | N. weight: | 0.49 |
| | | Sc. PD: | 0.086 |
| | | Sc. rank: | 70.9 |

,

| NA | | | |
|---|---|---|---|
| Pos. | 374 | obs: | exp: |
| ttt | F | 89 | 204.30 |
| ttc | F | 408 | 292.70 |
| mPD | | 0.29 | 0.48 |
| | | nPD: | 0.61 |
| | | N. weight: | 0.18 |
| | | Sc. PD: | 0.052 |
| | | Sc. rank: | 44.6 |

,

| NA | | | |
|---|---|---|---|
| Pos. | 375 | obs: | exp: |
| gaa | E | 422 | 286.00 |
| gag | E | 75 | 211.00 |
| mPD | | 0.26 | 0.49 |
| | | nPD: | 0.53 |
| | | N. weight: | 0.25 |
| | | Sc. PD: | 0.052 |
| | | Sc. rank: | 42.2 |

,

| NA | | | |
|---|---|---|---|
| Pos. | 376 | obs: | exp: |
| att | I | 0 | 0.38 |
| atc | I | 0 | 0.26 |
| ata | I | 1 | 0.36 |
| atg | M | 496 | 496.00 |
| mPD | | 0.0040 | 0.0040 |
| | | nPD: | 1. |
| | | N. weight: | 0.0029 |
| | | Sc. PD: | 0.0019 |
| | | Sc. rank: | 0.9 |

,

| NA | | | |
|---|---|---|---|
| Pos. | 377 | obs: | exp: |
| tta | L | 38 | 43.42 |
| ttg | L | 429 | 95.72 |
| ctt | L | 0 | 92.73 |
| ctc | L | 0 | 80.39 |
| cta | L | 0 | 74.87 |
| ctg | L | 30 | 109.90 |
| mPD | | 0.26 | 1.1 |
| | | nPD: | 0.23 |
| | | N. weight: | 1.7 |
| | | Sc. PD: | -0.14 |
| | | Sc. rank: | -94.2 |

,

| | | | |
|---|---|---|---|
| NA | | | |
| Pos. | 378 | obs: | exp: |
| cgt | R | 0 | 0.08 |
| cgc | R | 0 | 0.10 |
| cga | R | 0 | 0.19 |
| cgg | R | 0 | 0.17 |
| aaa | K | 424 | 277.20 |
| aag | K | 71 | 217.80 |
| aga | R | 2 | 0.90 |
| agg | R | 0 | 0.56 |
| --- | -- | ------ | ------ |
| mPD | | 0.25 | 0.50 |
| | | nPD: | 0.5 |
| | | N. weight: | 0.3 |
| | | Sc. PD: | 0.054 |
| | | Sc. rank: | 45.5 |

| | | | |
|---|---|---|---|
| NA | | | |
| Pos. | 379 | obs: | exp: |
| att | I | 15 | 186.80 |
| atc | I | 20 | 130.30 |
| ata | I | 462 | 179.90 |
| --- | -- | ------ | ------ |
| mPD | | 0.13 | 0.66 |
| | | nPD: | 0.2 |
| | | N. weight: | 1. |
| | | Sc. PD: | -0.11 |
| | | Sc. rank: | -75.0 |

| | | | |
|---|---|---|---|
| NA | | | |
| Pos. | 380 | obs: | exp: |
| cct | P | 428 | 130.90 |
| ccc | P | 69 | 89.65 |
| cca | P | 0 | 196.10 |
| ccg | P | 0 | 80.36 |
| --- | -- | ------ | ------ |
| mPD | | 0.24 | 0.72 |
| | | nPD: | 0.33 |
| | | N. weight: | 1.4 |
| | | Sc. PD: | 0.028 |
| | | Sc. rank: | 1.5 |

| | | | |
|---|---|---|---|
| NA | | | |
| Pos. | 381 | obs: | exp: |
| tct | S | 0 | 0.16 |
| tcc | S | 0 | 0.14 |
| tca | S | 0 | 0.22 |
| tcg | S | 0 | 0.06 |
| aat | N | 494 | 258.70 |
| aac | N | 1 | 236.30 |
| aaa | K | 1 | 0.56 |
| aag | K | 0 | 0.44 |
| agt | S | 1 | 0.22 |
| agc | S | 0 | 0.20 |
| --- | -- | ------ | ------ |
| mPD | | 0.012 | 0.51 |
| | | nPD: | 0.02 |
| | | N. weight: | 0.89 |
| | | Sc. PD: | -0.25 |
| | | Sc. rank: | -206.4 |

| | | | |
|---|---|---|---|
| NA | | | |
| Pos. | 382 | obs: | exp: |
| gct | A | 71 | 125.20 |
| gcc | A | 0 | 101.80 |
| gca | A | 404 | 221.50 |
| gcg | A | 22 | 48.50 |
| --- | -- | ------ | ------ |
| mPD | | 0.32 | 0.69 |
| | | nPD: | 0.46 |
| | | N. weight: | 0.54 |
| | | Sc. PD: | 0.077 |
| | | Sc. rank: | 66.2 |

| | | | |
|---|---|---|---|
| NA | | | |
| Pos. | 383 | obs: | exp: |
| tct | S | 0 | 0.32 |
| tcc | S | 0 | 0.28 |
| tca | S | 0 | 0.44 |
| tcg | S | 0 | 0.12 |
| aaa | K | 1 | 0.56 |
| aag | K | 0 | 0.44 |
| agt | S | 2 | 0.44 |
| agc | S | 0 | 0.40 |
| gaa | E | 3 | 13.23 |
| gag | E | 20 | 9.77 |
| ggt | G | 413 | 58.12 |
| ggc | G | 1 | 63.69 |
| gga | G | 23 | 227.80 |
| ggg | G | 34 | 121.30 |
| --- | -- | ------ | ------ |
| mPD | | 0.39 | 0.77 |
| | | nPD: | 0.51 |
| | | N. weight: | 2.1 |
| | | Sc. PD: | 0.39 |
| | | Sc. rank: | 330.0 |

| | | | |
|---|---|---|---|
| NA | | | |
| Pos. | 384 | obs: | exp: |
| att | I | 11 | 4.14 |
| atc | I | 0 | 2.88 |
| ata | I | 0 | 3.98 |
| act | T | 388 | 127.90 |
| acc | T | 5 | 103.90 |
| aca | T | 90 | 212.00 |
| acg | T | 1 | 40.24 |
| aat | N | 2 | 1.05 |
| aac | N | 0 | 0.95 |
| --- | -- | ------ | ------ |
| mPD | | 0.37 | 0.74 |
| | | nPD: | 0.5 |
| | | N. weight: | 0.99 |
| | | Sc. PD: | 0.18 |
| | | Sc. rank: | 150.0 |

| | | | |
|---|---|---|---|
| NA | | | |
| Pos. | 385 | obs: | exp: |
| gat | D | 403 | 265.20 |
| gac | D | 94 | 231.80 |
| --- | -- | ------ | ------ |
| mPD | | 0.31 | 0.50 |
| | | nPD: | 0.62 |
| | | N. weight: | 0.24 |
| | | Sc. PD: | 0.072 |
| | | Sc. rank: | 60.3 |

| | | | |
|---|---|---|---|
| NA | | | |
| Pos. | 386 | obs: | exp: |
| tct | S | 0 | 0.16 |
| tcc | S | 1 | 0.14 |
| tca | S | 0 | 0.22 |
| tcg | S | 0 | 0.06 |
| cct | P | 5 | 130.60 |
| ccc | P | 383 | 89.47 |
| cca | P | 108 | 195.70 |
| ccg | P | 0 | 80.20 |
| agt | S | 0 | 0.22 |
| agc | S | 0 | 0.20 |
| --- | -- | ------ | ------ |
| mPD | | 0.36 | 0.72 |
| | | nPD: | 0.5 |
| | | N. weight: | 1.4 |
| | | Sc. PD: | 0.24 |
| | | Sc. rank: | 203.9 |

| | | | |
|---|---|---|---|
| NA | | | |
| Pos. | 387 | obs: | exp: |
| tct | S | 0 | 3.66 |
| tcc | S | 0 | 3.20 |
| tca | S | 0 | 5.10 |
| tcg | S | 0 | 1.40 |
| aat | N | 452 | 247.20 |
| aac | N | 21 | 225.80 |
| agt | S | 23 | 5.06 |
| agc | S | 0 | 4.58 |
| gat | D | 1 | 0.53 |
| gac | D | 0 | 0.47 |
| --- | -- | ------ | ------ |
| mPD | | 0.17 | 0.66 |
| | | nPD: | 0.26 |
| | | N. weight: | 0.73 |
| | | Sc. PD: | -0.035 |
| | | Sc. rank: | -27.5 |

| | | | |
|---|---|---|---|
| NA | | | |
| Pos. | 388 | obs: | exp: |
| ttt | F | 1 | 0.41 |
| ttc | F | 0 | 0.59 |
| tct | S | 488 | 78.85 |
| tcc | S | 7 | 69.10 |
| tca | S | 0 | 109.90 |
| tcg | S | 1 | 30.23 |
| agt | S | 0 | 109.10 |
| agc | S | 0 | 98.82 |
| --- | -- | ------ | ------ |
| mPD | | 0.036 | 1.7 |
| | | nPD: | 0.02 |
| | | N. weight: | 2.5 |
| | | Sc. PD: | -0.7 |
| | | Sc. rank: | -578.5 |

| | | | |
|---|---|---|---|
| NA | | | |
| Pos. | 389 | obs: | exp: |
| cgt | R | 0 | 19.51 |
| cgc | R | 0 | 23.01 |
| cga | R | 0 | 45.17 |
| cgg | R | 0 | 39.22 |
| aaa | K | 25 | 15.12 |
| aag | K | 2 | 11.88 |
| aga | R | 425 | 211.40 |
| agg | R | 45 | 131.70 |
| --- | -- | ------ | ------ |
| mPD | | 0.27 | 1.0 |
| | | nPD: | 0.26 |
| | | N. weight: | 0.74 |
| | | Sc. PD: | -0.037 |
| | | Sc. rank: | -29.0 |

| | | | |
|---|---|---|---|
| NA | | | |
| Pos. | 390 | obs: | exp: |
| att | I | 0 | 186.80 |
| atc | I | 71 | 130.30 |
| ata | I | 426 | 179.90 |
| --- | -- | ------ | ------ |
| mPD | | 0.25 | 0.66 |
| | | nPD: | 0.37 |
| | | N. weight: | 0.92 |
| | | Sc. PD: | 0.052 |
| | | Sc. rank: | 39.3 |

| | | | |
|---|---|---|---|
| NA | | | |
| Pos. | 391 | obs: | exp: |
| att | I | 0 | 0.75 |
| atc | I | 0 | 0.52 |
| ata | I | 2 | 0.72 |
| act | T | 46 | 19.55 |
| acc | T | 28 | 15.88 |
| aca | T | 0 | 32.42 |
| acg | T | 0 | 6.15 |
| gtt | V | 1 | 0.21 |
| gtc | V | 0 | 0.21 |
| gta | V | 0 | 0.19 |
| gtg | V | 0 | 0.40 |
| gct | A | 8 | 104.50 |
| gcc | A | 0 | 85.01 |
| gca | A | 405 | 185.00 |
| gcg | A | 2 | 40.50 |
| gaa | E | 5 | 2.88 |
| gag | E | 0 | 2.12 |
| --- | -- | ------ | ------ |
| mPD | | 0.59 | 0.98 |
| | | nPD: | 0.6 |
| | | N. weight: | 1. |
| | | Sc. PD: | 0.28 |
| | | Sc. rank: | 243.8 |

| | | | |
|---|---|---|---|
| NA | | | |
| Pos. | 392 | obs: | exp: |
| aaa | K | 0 | 3.36 |
| aag | K | 6 | 2.64 |
| gaa | E | 408 | 273.30 |
| gag | E | 67 | 201.70 |
| ggt | G | 0 | 1.97 |
| ggc | G | 0 | 2.16 |
| gga | G | 13 | 7.74 |
| ggg | G | 3 | 4.12 |
| --- | -- | ------ | ------ |
| mPD | | 0.35 | 0.58 |
| | | nPD: | 0.59 |
| | | N. weight: | 0.29 |
| | | Sc. PD: | 0.08 |
| | | Sc. rank: | 70.1 |

| | | | |
|---|---|---|---|
| NA | | | |
| Pos. | 393 | obs: | exp: |
| caa | Q | 0 | 0.53 |
| cag | Q | 1 | 0.47 |
| cgt | R | 0 | 20.59 |
| cgc | R | 1 | 24.29 |
| cga | R | 342 | 47.67 |
| cgg | R | 33 | 41.39 |
| aga | R | 49 | 223.10 |
| agg | R | 71 | 139.00 |
| --- | -- | ------ | ------ |
| mPD | | 0.71 | 0.97 |
| | | nPD: | 0.73 |
| | | N. weight: | 1.6 |
| | | Sc. PD: | 0.63 |
| | | Sc. rank: | 454.6 |

| | | | |
|---|---|---|---|
| NA | | | |
| Pos. | 394 | obs: | exp: |
| caa | Q | 174 | 265.00 |
| cag | Q | 323 | 232.00 |
| --- | -- | ------ | ------ |
| mPD | | 0.46 | 0.50 |
| | | nPD: | 0.92 |
| | | N. weight: | 0.1 |
| | | Sc. PD: | 0.061 |
| | | Sc. rank: | 31.7 |

| | | | |
|---|---|---|---|
| NA | | | |
| Pos. | 395 | obs: | exp: |
| gaa | E | 487 | 285.40 |
| gag | E | 9 | 210.60 |
| ggt | G | 0 | 0.12 |
| ggc | G | 0 | 0.14 |
| gga | G | 1 | 0.48 |
| ggg | G | 0 | 0.26 |
| --- | -- | ------ | ------ |
| mPD | | 0.040 | 0.49 |
| | | nPD: | 0.08 |
| | | N. weight: | 0.66 |
| | | Sc. PD: | -0.15 |
| | | Sc. rank: | -95.6 |

| | | | |
|---|---|---|---|
| NA | | | |
| Pos. | 396 | obs: | exp: |
| att | I | 417 | 186.50 |
| atc | I | 7 | 130.00 |
| ata | I | 72 | 179.50 |
| gtt | V | 1 | 0.21 |
| gtc | V | 0 | 0.21 |
| gta | V | 0 | 0.19 |
| gtg | V | 0 | 0.40 |
| --- | -- | ------ | ------ |
| mPD | | 0.28 | 0.66 |
| | | nPD: | 0.42 |
| | | N. weight: | 0.72 |
| | | Sc. PD: | 0.071 |
| | | Sc. rank: | 56.9 |

| | | | |
|---|---|---|---|
| NA | | | |
| Pos. | 397 | obs: | exp: |
| gtt | V | 52 | 102.20 |
| gtc | V | 445 | 104.30 |
| gta | V | 0 | 93.15 |
| gtg | V | 0 | 197.40 |
| --- | -- | ------ | ------ |
| mPD | | 0.19 | 0.72 |
| | | nPD: | 0.26 |
| | | N. weight: | 1.7 |
| | | Sc. PD: | -0.089 |
| | | Sc. rank: | -69.6 |

NA

| Pos. | 398 | obs: | exp: |
|---|---|---|---|
| gct | A | 0 | 0.25 |
| gcc | A | 1 | 0.20 |
| gca | A | 0 | 0.45 |
| gcg | A | 0 | 0.10 |
| gat | D | 0 | 264.10 |
| gac | D | 495 | 230.90 |
| ggt | G | 0 | 0.12 |
| ggc | G | 1 | 0.14 |
| gga | G | 0 | 0.48 |
| ggg | G | 0 | 0.26 |
| --- | -- | ------ | ------ |
| mPD | | 0.0080 | 0.51 |
| | | nPD: | 0.02 |
| | | N. weight: | 1.1 |
| | | Sc. PD: | -0.31 |
| | | Sc. rank: | -260.1 |

,

NA

| Pos. | 399 | obs: | exp: |
|---|---|---|---|
| aat | N | 420 | 259.80 |
| aac | N | 77 | 237.20 |
| --- | -- | ------ | ------ |
| mPD | | 0.26 | 0.50 |
| | | nPD: | 0.53 |
| | | N. weight: | 0.33 |
| | | Sc. PD: | 0.068 |
| | | Sc. rank: | 56.0 |

,

NA

| Pos. | 400 | obs: | exp: |
|---|---|---|---|
| tct | S | 0 | 2.07 |
| tcc | S | 0 | 1.81 |
| tca | S | 0 | 2.88 |
| tcg | S | 0 | 0.79 |
| aat | N | 19 | 253.00 |
| aac | N | 465 | 231.00 |
| agt | S | 2 | 2.86 |
| agc | S | 11 | 2.59 |
| --- | -- | ------ | ------ |
| mPD | | 0.13 | 0.59 |
| | | nPD: | 0.22 |
| | | N. weight: | 0.83 |
| | | Sc. PD: | -0.072 |
| | | Sc. rank: | -47.8 |

,

NA

| Pos. | 401 | obs: | exp: |
|---|---|---|---|
| tct | S | 0 | 0.16 |
| tcc | S | 0 | 0.14 |
| tca | S | 0 | 0.22 |
| tcg | S | 0 | 0.06 |
| aat | N | 488 | 259.20 |
| aac | N | 8 | 236.80 |
| agt | S | 1 | 0.22 |
| agc | S | 0 | 0.20 |
| --- | -- | ------ | ------ |
| mPD | | 0.036 | 0.51 |
| | | nPD: | 0.07 |
| | | N. weight: | 0.8 |
| | | Sc. PD: | -0.19 |
| | | Sc. rank: | -128.2 |

,

NA

| Pos. | 402 | obs: | exp: |
|---|---|---|---|
| tgg | W | 496 | 496.00 |
| cgt | R | 0 | 0.04 |
| cgc | R | 0 | 0.05 |
| cga | R | 0 | 0.10 |
| cgg | R | 1 | 0.08 |
| aga | R | 0 | 0.45 |
| agg | R | 0 | 0.28 |
| --- | -- | ------ | ------ |
| mPD | | 0.0040 | 0.0066 |
| | | nPD: | 0.61 |
| | | N. weight: | 0.007 |
| | | Sc. PD: | 0.002 |
| | | Sc. rank: | 1.7 |

,

NA

| Pos. | 403 | obs: | exp: |
|---|---|---|---|
| tct | S | 3 | 79.01 |
| tcc | S | 0 | 69.23 |
| tca | S | 491 | 110.10 |
| tcg | S | 3 | 30.29 |
| agt | S | 0 | 109.30 |
| agc | S | 0 | 99.02 |
| --- | -- | ------ | ------ |
| mPD | | 0.024 | 1.7 |
| | | nPD: | 0.01 |
| | | N. weight: | 2. |
| | | Sc. PD: | -0.59 |
| | | Sc. rank: | -500.0 |

,

NA

| Pos. | 404 | obs: | exp: |
|---|---|---|---|
| ggt | G | 11 | 61.32 |
| ggc | G | 415 | 67.21 |
| gga | G | 48 | 240.40 |
| ggg | G | 23 | 128.00 |
| --- | -- | ------ | ------ |
| mPD | | 0.29 | 0.67 |
| | | nPD: | 0.44 |
| | | N. weight: | 1.8 |
| | | Sc. PD: | 0.21 |
| | | Sc. rank: | 176.2 |

,

NA

| Pos. | 405 | obs: | exp: |
|---|---|---|---|
| tat | Y | 417 | 257.30 |
| tac | Y | 80 | 239.70 |
| --- | -- | ------ | ------ |
| mPD | | 0.27 | 0.50 |
| | | nPD: | 0.54 |
| | | N. weight: | 0.33 |
| | | Sc. PD: | 0.073 |
| | | Sc. rank: | 63.3 |

,

NA

| Pos. | 406 | obs: | exp: |
|---|---|---|---|
| tct | S | 15 | 79.01 |
| tcc | S | 363 | 69.23 |
| tca | S | 119 | 110.10 |
| tcg | S | 0 | 30.29 |
| agt | S | 0 | 109.30 |
| agc | S | 0 | 99.02 |
| --- | -- | ------ | ------ |
| mPD | | 0.41 | 1.7 |
| | | nPD: | 0.25 |
| | | N. weight: | 1.7 |
| | | Sc. PD: | -0.11 |
| | | Sc. rank: | -80.1 |

,

NA

| Pos. | 407 | obs: | exp: |
|---|---|---|---|
| ggt | G | 0 | 61.32 |
| ggc | G | 0 | 67.21 |
| gga | G | 464 | 240.40 |
| ggg | G | 33 | 128.00 |
| --- | -- | ------ | ------ |
| mPD | | 0.12 | 0.67 |
| | | nPD: | 0.19 |
| | | N. weight: | 0.74 |
| | | Sc. PD: | -0.091 |
| | | Sc. rank: | -62.5 |

,

NA

| Pos. | 408 | obs: | exp: |
|---|---|---|---|
| tct | S | 0 | 79.01 |
| tcc | S | 0 | 69.23 |
| tca | S | 0 | 110.10 |
| tcg | S | 0 | 30.29 |
| agt | S | 71 | 109.30 |
| agc | S | 426 | 99.02 |
| --- | -- | ------ | ------ |
| mPD | | 0.25 | 1.7 |
| | | nPD: | 0.15 |
| | | N. weight: | 1.7 |
| | | Sc. PD: | -0.27 |
| | | Sc. rank: | -180.2 |

,

NA

| Pos. | 409 | obs: | exp: |
|---|---|---|---|
| ttt | F | 313 | 204.30 |
| ttc | F | 184 | 292.70 |
| --- | -- | ------ | ------ |
| mPD | | 0.47 | 0.48 |
| | | nPD: | 0.97 |
| | | N. weight: | 0.14 |
| | | Sc. PD: | 0.091 |
| | | Sc. rank: | 44.3 |

,

NA

| Pos. | 410 | obs: | exp: |
|---|---|---|---|
| att | I | 484 | 183.80 |
| atc | I | 4 | 128.20 |
| ata | I | 1 | 177.00 |
| gtt | V | 8 | 1.64 |
| gtc | V | 0 | 1.68 |
| gta | V | 0 | 1.50 |
| gtg | V | 0 | 3.18 |
| --- | -- | ------ | ------ |
| mPD | | 0.052 | 0.70 |
| | | nPD: | 0.07 |
| | | N. weight: | 1.3 |
| | | Sc. PD: | -0.3 |
| | | Sc. rank: | -198.6 |

,

NA

| Pos. | 411 | obs: | exp: |
|---|---|---|---|
| gat | D | 5 | 265.20 |
| gac | D | 492 | 231.80 |
| --- | -- | ------ | ------ |
| mPD | | 0.020 | 0.50 |
| | | nPD: | 0.04 |
| | | N. weight: | 0.99 |
| | | Sc. PD: | -0.26 |
| | | Sc. rank: | -201.0 |

,

NA

| Pos. | 412 | obs: | exp: |
|---|---|---|---|
| tat | Y | 488 | 257.30 |
| tac | Y | 9 | 239.70 |
| --- | -- | ------ | ------ |
| mPD | | 0.036 | 0.50 |
| | | nPD: | 0.07 |
| | | N. weight: | 0.8 |
| | | Sc. PD: | -0.19 |
| | | Sc. rank: | -124.4 |

,

NA

| Pos. | 413 | obs: | exp: |
|---|---|---|---|
| tgg | W | 497 | 497.00 |
| --- | -- | ------ | ------ |
| mPD | | 0 | 0 |
| | | nPD: | 1. |
| | | N. weight: | 0. |
| | | Sc. PD: | 0 |
| | | Sc. rank: | 0 |

,

NA

| Pos. | 414 | obs: | exp: |
|---|---|---|---|
| aat | N | 402 | 217.40 |
| aac | N | 14 | 198.60 |
| gat | D | 77 | 43.22 |
| gac | D | 4 | 37.78 |
| --- | -- | ------ | ------ |
| mPD | | 0.34 | 0.77 |
| | | nPD: | 0.44 |
| | | N. weight: | 0.7 |
| | | Sc. PD: | 0.089 |
| | | Sc. rank: | 74.1 |

,

NA

| Pos. | 415 | obs: | exp: |
|---|---|---|---|
| aat | N | 23 | 12.02 |
| aac | N | 0 | 10.98 |
| gat | D | 388 | 215.00 |
| gac | D | 15 | 188.00 |
| gaa | E | 56 | 39.13 |
| gag | E | 12 | 28.87 |
| ggt | G | 0 | 0.37 |
| ggc | G | 0 | 0.41 |
| gga | G | 3 | 1.45 |
| ggg | G | 0 | 0.77 |
| --- | -- | ------ | ------ |
| mPD | | 0.40 | 0.72 |
| | | nPD: | 0.56 |
| | | N. weight: | 0.62 |
| | | Sc. PD: | 0.15 |
| | | Sc. rank: | 131.1 |

,

NA

| Pos. | 416 | obs: | exp: |
|---|---|---|---|
| tct | S | 0 | 9.06 |
| tcc | S | 0 | 7.94 |
| tca | S | 0 | 12.63 |
| tcg | S | 0 | 3.47 |
| act | T | 0 | 0.26 |
| acc | T | 1 | 0.21 |
| aca | T | 0 | 0.44 |
| acg | T | 0 | 0.08 |
| aat | N | 38 | 210.10 |
| aac | N | 364 | 191.90 |
| agt | S | 1 | 12.54 |
| agc | S | 56 | 11.36 |
| gct | A | 2 | 0.50 |
| gcc | A | 0 | 0.41 |
| gca | A | 0 | 0.89 |
| gcg | A | 0 | 0.20 |
| gat | D | 0 | 9.60 |
| gac | D | 18 | 8.40 |
| ggt | G | 2 | 2.10 |
| ggc | G | 15 | 2.30 |
| gga | G | 0 | 8.22 |
| ggg | G | 0 | 4.38 |
| --- | -- | ------ | ------ |
| mPD | | 0.56 | 1.1 |
| | | nPD: | 0.52 |
| | | N. weight: | 0.86 |
| | | Sc. PD: | 0.17 |
| | | Sc. rank: | 142.4 |

,

NA

| Pos. | 417 | obs: | exp: |
|---|---|---|---|
| tct | S | 0 | 77.58 |
| tcc | S | 0 | 67.98 |
| tca | S | 0 | 108.10 |
| tcg | S | 0 | 29.75 |
| cgt | R | 0 | 0.04 |
| cgc | R | 0 | 0.05 |
| cga | R | 0 | 0.10 |
| cgg | R | 0 | 0.08 |
| aat | N | 8 | 4.18 |
| aac | N | 0 | 3.82 |
| agt | S | 483 | 107.40 |
| agc | S | 5 | 97.22 |
| aga | R | 0 | 0.45 |
| agg | R | 1 | 0.28 |
| --- | -- | ------ | ------ |
| mPD | | 0.056 | 1.7 |
| | | nPD: | 0.03 |
| | | N. weight: | 2. |
| | | Sc. PD: | -0.55 |
| | | Sc. rank: | -428.2 |

,

| NA | | | |
|---|---|---|---|
| Pos. | 418 | obs: | exp: |
| gtt | V | 0 | 6.37 |
| gtc | V | 0 | 6.51 |
| gta | V | 0 | 5.81 |
| gtg | V | 31 | 12.31 |
| gat | D | 1 | 0.53 |
| gac | D | 0 | 0.47 |
| gaa | E | 281 | 267.60 |
| gag | E | 184 | 197.40 |
| --- | -- | ------ | ------ |
| mPD | | 0.61 | 0.64 |
| | | nPD: | 0.96 |
| | | N. weight: | 0.094 |
| | | Sc. PD: | 0.059 |
| | | Sc. rank: | 28.7 |

,

| NA | | | |
|---|---|---|---|
| Pos. | 419 | obs: | exp: |
| tgt | C | 9 | 204.90 |
| tgc | C | 488 | 292.10 |
| --- | -- | ------ | ------ |
| mPD | | 0.036 | 0.48 |
| | | nPD: | 0.07 |
| | | N. weight: | 0.63 |
| | | Sc. PD: | -0.15 |
| | | Sc. rank: | -97.4 |

,

| NA | | | |
|---|---|---|---|
| Pos. | 420 | obs: | exp: |
| ttt | F | 0 | 0.41 |
| ttc | F | 1 | 0.59 |
| tct | S | 0 | 0.16 |
| tcc | S | 1 | 0.14 |
| tca | S | 0 | 0.22 |
| tcg | S | 0 | 0.06 |
| tat | Y | 4 | 256.20 |
| tac | Y | 491 | 238.80 |
| agt | S | 0 | 0.22 |
| agc | S | 0 | 0.20 |
| --- | -- | ------ | ------ |
| mPD | | 0.024 | 0.51 |
| | | nPD: | 0.05 |
| | | N. weight: | 0.96 |
| | | Sc. PD: | -0.25 |
| | | Sc. rank: | -185.0 |

,

| NA | | | |
|---|---|---|---|
| Pos. | 421 | obs: | exp: |
| aat | N | 411 | 259.80 |
| aac | N | 86 | 237.20 |
| --- | -- | ------ | ------ |
| mPD | | 0.29 | 0.50 |
| | | nPD: | 0.57 |
| | | N. weight: | 0.29 |
| | | Sc. PD: | 0.074 |
| | | Sc. rank: | 65.9 |

,

| NA | | | |
|---|---|---|---|
| Pos. | 422 | obs: | exp: |
| cct | P | 2 | 130.90 |
| ccc | P | 72 | 89.65 |
| cca | P | 326 | 196.10 |
| ccg | P | 97 | 80.36 |
| --- | -- | ------ | ------ |
| mPD | | 0.51 | 0.72 |
| | | nPD: | 0.71 |
| | | N. weight: | 0.47 |
| | | Sc. PD: | 0.18 |
| | | Sc. rank: | 133.2 |

,

| NA | | | |
|---|---|---|---|
| Pos. | 423 | obs: | exp: |
| tgt | C | 100 | 204.90 |
| tgc | C | 397 | 292.10 |
| --- | -- | ------ | ------ |
| mPD | | 0.32 | 0.48 |
| | | nPD: | 0.66 |
| | | N. weight: | 0.15 |
| | | Sc. PD: | 0.051 |
| | | Sc. rank: | 40.5 |

,

| NA | | | |
|---|---|---|---|
| Pos. | 424 | obs: | exp: |
| ttt | F | 423 | 204.30 |
| ttc | F | 74 | 292.70 |
| --- | -- | ------ | ------ |
| mPD | | 0.25 | 0.48 |
| | | nPD: | 0.52 |
| | | N. weight: | 0.59 |
| | | Sc. PD: | 0.12 |
| | | Sc. rank: | 98.2 |

,

| NA | | | |
|---|---|---|---|
| Pos. | 425 | obs: | exp: |
| tat | Y | 80 | 257.30 |
| tac | Y | 417 | 239.70 |
| --- | -- | ------ | ------ |
| mPD | | 0.27 | 0.50 |
| | | nPD: | 0.54 |
| | | N. weight: | 0.39 |
| | | Sc. PD: | 0.087 |
| | | Sc. rank: | 76.2 |

,

| NA | | | |
|---|---|---|---|
| Pos. | 426 | obs: | exp: |
| att | I | 2 | 1.13 |
| atc | I | 0 | 0.79 |
| ata | I | 1 | 1.09 |
| gtt | V | 71 | 101.50 |
| gtc | V | 0 | 103.70 |
| gta | V | 351 | 92.59 |
| gtg | V | 72 | 196.20 |
| --- | -- | ------ | ------ |
| mPD | | 0.47 | 0.73 |
| | | nPD: | 0.64 |
| | | N. weight: | 1.1 |
| | | Sc. PD: | 0.34 |
| | | Sc. rank: | 278.8 |

,

| NA | | | |
|---|---|---|---|
| Pos. | 427 | obs: | exp: |
| gaa | E | 51 | 286.00 |
| gag | E | 446 | 211.00 |
| --- | -- | ------ | ------ |
| mPD | | 0.18 | 0.49 |
| | | nPD: | 0.38 |
| | | N. weight: | 0.7 |
| | | Sc. PD: | 0.043 |
| | | Sc. rank: | 30.5 |

,

| NA | | | |
|---|---|---|---|
| Pos. | 428 | obs: | exp: |
| tta | L | 389 | 43.42 |
| ttg | L | 58 | 95.72 |
| ctt | L | 0 | 92.73 |
| ctc | L | 0 | 80.39 |
| cta | L | 50 | 74.87 |
| ctg | L | 0 | 109.90 |
| --- | -- | ------ | ------ |
| mPD | | 0.39 | 1.1 |
| | | nPD: | 0.35 |
| | | N. weight: | 2.3 |
| | | Sc. PD: | 0.076 |
| | | Sc. rank: | 36.3 |

,

| NA | | | |
|---|---|---|---|
| Pos. | 429 | obs: | exp: |
| att | I | 417 | 186.80 |
| atc | I | 9 | 130.30 |
| ata | I | 71 | 179.90 |
| --- | -- | ------ | ------ |
| mPD | | 0.28 | 0.66 |
| | | nPD: | 0.42 |
| | | N. weight: | 0.7 |
| | | Sc. PD: | 0.071 |
| | | Sc. rank: | 56.2 |

,

| NA | | | |
|---|---|---|---|
| Pos. | 430 | obs: | exp: |
| cgt | R | 0 | 20.63 |
| cgc | R | 0 | 24.34 |
| cga | R | 0 | 47.76 |
| cgg | R | 0 | 41.47 |
| aga | R | 497 | 223.50 |
| agg | R | 0 | 139.30 |
| --- | -- | ------ | ------ |
| mPD | | 0 | 0.96 |
| | | nPD: | 0. |
| | | N. weight: | 1.1 |
| | | Sc. PD: | -0.34 |
| | | Sc. rank: | -342.7 |

,

| NA | | | |
|---|---|---|---|
| Pos. | 431 | obs: | exp: |
| ggt | G | 0 | 61.32 |
| ggc | G | 0 | 67.21 |
| gga | G | 484 | 240.40 |
| ggg | G | 13 | 128.00 |
| --- | -- | ------ | ------ |
| mPD | | 0.051 | 0.67 |
| | | nPD: | 0.08 |
| | | N. weight: | 0.88 |
| | | Sc. PD: | -0.2 |
| | | Sc. rank: | -132.0 |

,

| NA | | | |
|---|---|---|---|
| Pos. | 432 | obs: | exp: |
| cgt | R | 0 | 20.26 |
| cgc | R | 0 | 23.89 |
| cga | R | 0 | 46.90 |
| cgg | R | 0 | 40.72 |
| att | I | 0 | 3.38 |
| atc | I | 0 | 2.36 |
| ata | I | 9 | 3.26 |
| aga | R | 404 | 219.50 |
| agg | R | 84 | 136.80 |
| --- | -- | ------ | ------ |
| mPD | | 0.32 | 1.0 |
| | | nPD: | 0.32 |
| | | N. weight: | 0.61 |
| | | Sc. PD: | 0.0022 |
| | | Sc. rank: | -4.3 |

,

| NA | | | |
|---|---|---|---|
| Pos. | 433 | obs: | exp: |
| cct | P | 81 | 130.90 |
| ccc | P | 412 | 89.65 |
| cca | P | 4 | 196.10 |
| ccg | P | 0 | 80.36 |
| --- | -- | ------ | ------ |
| mPD | | 0.29 | 0.72 |
| | | nPD: | 0.4 |
| | | N. weight: | 1.6 |
| | | Sc. PD: | 0.14 |
| | | Sc. rank: | 101.0 |

,

| NA | | | |
|---|---|---|---|
| Pos. | 434 | obs: | exp: |
| gaa | E | 486 | 286.00 |
| gag | E | 11 | 211.00 |
| --- | -- | ------ | ------ |
| mPD | | 0.043 | 0.49 |
| | | nPD: | 0.09 |
| | | N. weight: | 0.64 |
| | | Sc. PD: | -0.14 |
| | | Sc. rank: | -91.5 |

,

| NA | | | |
|---|---|---|---|
| Pos. | 435 | obs: | exp: |
| gaa | E | 75 | 286.00 |
| gag | E | 422 | 211.00 |
| --- | -- | ------ | ------ |
| mPD | | 0.26 | 0.49 |
| | | nPD: | 0.53 |
| | | N. weight: | 0.55 |
| | | Sc. PD: | 0.11 |
| | | Sc. rank: | 91.9 |

,

| NA | | | |
|---|---|---|---|
| Pos. | 436 | obs: | exp: |
| gct | A | 324 | 125.20 |
| gcc | A | 169 | 101.80 |
| gca | A | 3 | 221.50 |
| gcg | A | 1 | 48.50 |
| --- | -- | ------ | ------ |
| mPD | | 0.46 | 0.69 |
| | | nPD: | 0.67 |
| | | N. weight: | 1.1 |
| | | Sc. PD: | 0.37 |
| | | Sc. rank: | 294.2 |

,

| NA | | | |
|---|---|---|---|
| Pos. | 437 | obs: | exp: |
| aaa | K | 421 | 278.30 |
| aag | K | 76 | 218.70 |
| --- | -- | ------ | ------ |
| mPD | | 0.26 | 0.49 |
| | | nPD: | 0.53 |
| | | N. weight: | 0.27 |
| | | Sc. PD: | 0.056 |
| | | Sc. rank: | 46.0 |

,

| NA | | | |
|---|---|---|---|
| Pos. | 438 | obs: | exp: |
| tat | Y | 179 | 256.70 |
| tac | Y | 317 | 239.30 |
| cat | H | 1 | 0.54 |
| cac | H | 0 | 0.46 |
| --- | -- | ------ | ------ |
| mPD | | 0.47 | 0.50 |
| | | nPD: | 0.93 |
| | | N. weight: | 0.08 |
| | | Sc. PD: | 0.048 |
| | | Sc. rank: | 24.5 |

,

| NA | | | |
|---|---|---|---|
| Pos. | 439 | obs: | exp: |
| gtt | V | 71 | 101.70 |
| gtc | V | 4 | 103.90 |
| gta | V | 388 | 92.77 |
| gtg | V | 32 | 196.60 |
| gct | A | 0 | 0.50 |
| gcc | A | 0 | 0.41 |
| gca | A | 2 | 0.89 |
| gcg | A | 0 | 0.20 |
| --- | -- | ------ | ------ |
| mPD | | 0.37 | 0.73 |
| | | nPD: | 0.51 |
| | | N. weight: | 1.3 |
| | | Sc. PD: | 0.24 |
| | | Sc. rank: | 205.6 |

,

| NA | | | |
|---|---|---|---|
| Pos. | 440 | obs: | exp: |
| tgg | W | 497 | 497.00 |
| --- | -- | ------ | ------ |
| mPD | | 0 | 0 |
| | | nPD: | 1. |
| | | N. weight: | 0. |
| | | Sc. PD: | 0 |
| | | Sc. rank: | 0 |

,

| NA | | | |
|---|---|---|---|
| Pos. | 441 | obs: | exp: |
| tgg | W | 497 | 497.00 |
| --- | -- | ------ | ------ |
| mPD | | 0 | 0 |
| | | nPD: | 1. |
| | | N. weight: | 0. |
| | | Sc. PD: | 0 |
| | | Sc. rank: | 0 |

,

| NA | | | |
|---|---|---|---|
| Pos. | 442 | obs: | exp: |
| act | T | 0 | 100.70 |
| acc | T | 0 | 81.76 |
| aca | T | 287 | 166.90 |
| acg | T | 94 | 31.67 |
| gct | A | 0 | 29.22 |
| gcc | A | 0 | 23.76 |
| gca | A | 116 | 51.70 |
| gcg | A | 0 | 11.32 |
| --- | -- | ------ | ------ |
| mPD | | 0.67 | 1.0 |
| | | nPD: | 0.64 |
| | | N. weight: | 1. |
| | | Sc. PD: | 0.31 |
| | | Sc. rank: | 258.9 |

,

| | | | |
|---|---|---|---|
| NA | | | |
| Pos. | 443 | obs: | exp: |
| tct | S | 0 | 79.01 |
| tcc | S | 0 | 69.23 |
| tca | S | 0 | 110.10 |
| tcg | S | 0 | 30.29 |
| agt | S | 422 | 109.30 |
| agc | S | 75 | 99.02 |
| --- | -- | ------ | ------ |
| mPD | | 0.26 | 1.7 |
| | | nPD: | 0.15 |
| | | N. weight: | 1.6 |
| | | Sc. PD: | -0.24 |
| | | Sc. rank: | -157.8 |

| | | | |
|---|---|---|---|
| NA | | | |
| Pos. | 444 | obs: | exp: |
| aat | N | 53 | 259.80 |
| aac | N | 444 | 237.20 |
| --- | -- | ------ | ------ |
| mPD | | 0.19 | 0.50 |
| | | nPD: | 0.38 |
| | | N. weight: | 0.55 |
| | | Sc. PD: | 0.037 |
| | | Sc. rank: | 26.6 |

| | | | |
|---|---|---|---|
| NA | | | |
| Pos. | 445 | obs: | exp: |
| tct | S | 0 | 79.01 |
| tcc | S | 0 | 69.23 |
| tca | S | 0 | 110.10 |
| tcg | S | 0 | 30.29 |
| agt | S | 475 | 109.30 |
| agc | S | 22 | 99.02 |
| --- | -- | ------ | ------ |
| mPD | | 0.085 | 1.7 |
| | | nPD: | 0.05 |
| | | N. weight: | 1.9 |
| | | Sc. PD: | -0.48 |
| | | Sc. rank: | -345.6 |

| | | | |
|---|---|---|---|
| NA | | | |
| Pos. | 446 | obs: | exp: |
| tta | L | 90 | 43.16 |
| ttg | L | 2 | 95.14 |
| ctt | L | 0 | 92.17 |
| ctc | L | 3 | 79.91 |
| cta | L | 398 | 74.42 |
| ctg | L | 1 | 109.20 |
| att | I | 0 | 1.13 |
| atc | I | 0 | 0.79 |
| ata | I | 3 | 1.09 |
| --- | -- | ------ | ------ |
| mPD | | 0.34 | 1.1 |
| | | nPD: | 0.3 |
| | | N. weight: | 2. |
| | | Sc. PD: | -0.029 |
| | | Sc. rank: | -34.2 |

| | | | |
|---|---|---|---|
| NA | | | |
| Pos. | 447 | obs: | exp: |
| att | I | 421 | 158.60 |
| atc | I | 1 | 110.60 |
| ata | I | 0 | 152.80 |
| gtt | V | 75 | 15.42 |
| gtc | V | 0 | 15.74 |
| gta | V | 0 | 14.06 |
| gtg | V | 0 | 29.79 |
| --- | -- | ------ | ------ |
| mPD | | 0.26 | 0.95 |
| | | nPD: | 0.27 |
| | | N. weight: | 1.5 |
| | | Sc. PD: | -0.058 |
| | | Sc. rank: | -48.3 |

| | | | |
|---|---|---|---|
| NA | | | |
| Pos. | 448 | obs: | exp: |
| act | T | 0 | 0.26 |
| acc | T | 0 | 0.21 |
| aca | T | 1 | 0.44 |
| acg | T | 0 | 0.08 |
| gct | A | 35 | 124.90 |
| gcc | A | 391 | 101.60 |
| gca | A | 61 | 221.10 |
| gcg | A | 9 | 48.40 |
| --- | -- | ------ | ------ |
| mPD | | 0.36 | 0.69 |
| | | nPD: | 0.53 |
| | | N. weight: | 1.1 |
| | | Sc. PD: | 0.23 |
| | | Sc. rank: | 201.6 |

| | | | |
|---|---|---|---|
| NA | | | |
| Pos. | 449 | obs: | exp: |
| tta | L | 0 | 43.42 |
| ttg | L | 2 | 95.72 |
| ctt | L | 0 | 92.73 |
| ctc | L | 0 | 80.39 |
| cta | L | 489 | 74.87 |
| ctg | L | 6 | 109.90 |
| --- | -- | ------ | ------ |
| mPD | | 0.040 | 1.1 |
| | | nPD: | 0.04 |
| | | N. weight: | 2.5 |
| | | Sc. PD: | -0.68 |
| | | Sc. rank: | -517.3 |

| | | | |
|---|---|---|---|
| NA | | | |
| Pos. | 450 | obs: | exp: |
| tgt | C | 497 | 204.90 |
| tgc | C | 0 | 292.10 |
| --- | -- | ------ | ------ |
| mPD | | 0 | 0.48 |
| | | nPD: | 0. |
| | | N. weight: | 1.2 |
| | | Sc. PD: | -0.38 |
| | | Sc. rank: | -380.1 |

| | | | |
|---|---|---|---|
| NA | | | |
| Pos. | 451 | obs: | exp: |
| ggt | G | 0 | 61.32 |
| ggc | G | 0 | 67.21 |
| gga | G | 399 | 240.40 |
| ggg | G | 98 | 128.00 |
| --- | -- | ------ | ------ |
| mPD | | 0.32 | 0.67 |
| | | nPD: | 0.48 |
| | | N. weight: | 0.5 |
| | | Sc. PD: | 0.079 |
| | | Sc. rank: | 70.2 |

| | | | |
|---|---|---|---|
| NA | | | |
| Pos. | 452 | obs: | exp: |
| tct | S | 0 | 79.01 |
| tcc | S | 0 | 69.23 |
| tca | S | 0 | 110.10 |
| tcg | S | 0 | 30.29 |
| agt | S | 0 | 109.30 |
| agc | S | 497 | 99.02 |
| --- | -- | ------ | ------ |
| mPD | | 0 | 1.7 |
| | | nPD: | 0. |
| | | N. weight: | 2.3 |
| | | Sc. PD: | -0.69 |
| | | Sc. rank: | -691.9 |

| | | | |
|---|---|---|---|
| NA | | | |
| Pos. | 453 | obs: | exp: |
| cct | P | 0 | 130.90 |
| ccc | P | 0 | 89.65 |
| cca | P | 492 | 196.10 |
| ccg | P | 5 | 80.36 |
| --- | -- | ------ | ------ |
| mPD | | 0.020 | 0.72 |
| | | nPD: | 0.03 |
| | | N. weight: | 1.2 |
| | | Sc. PD: | -0.34 |
| | | Sc. rank: | -276.3 |

| | | | |
|---|---|---|---|
| NA | | | |
| Pos. | 454 | obs: | exp: |
| ttt | F | 0 | 173.50 |
| ttc | F | 422 | 248.50 |
| tct | S | 0 | 0.16 |
| tcc | S | 1 | 0.14 |
| tca | S | 0 | 0.22 |
| tcg | S | 0 | 0.06 |
| att | I | 0 | 24.81 |
| atc | I | 66 | 17.30 |
| ata | I | 0 | 23.89 |
| agt | S | 0 | 0.22 |
| agc | S | 0 | 0.20 |
| gtt | V | 0 | 1.64 |
| gtc | V | 8 | 1.68 |
| gta | V | 0 | 1.50 |
| gtg | V | 0 | 3.18 |
| --- | -- | ------ | ------ |
| mPD | | 0.26 | 0.81 |
| | | nPD: | 0.32 |
| | | N. weight: | 0.92 |
| | | Sc. PD: | 0.0098 |
| | | Sc. rank: | -2.7 |

| | | | |
|---|---|---|---|
| NA | | | |
| Pos. | 455 | obs: | exp: |
| tct | S | 0 | 7.31 |
| tcc | S | 0 | 6.41 |
| tca | S | 45 | 10.19 |
| tcg | S | 1 | 2.80 |
| cct | P | 0 | 118.80 |
| ccc | P | 0 | 81.35 |
| cca | P | 451 | 178.00 |
| ccg | P | 0 | 72.92 |
| agt | S | 0 | 10.12 |
| agc | S | 0 | 9.16 |
| --- | -- | ------ | ------ |
| mPD | | 0.17 | 0.97 |
| | | nPD: | 0.18 |
| | | N. weight: | 1.4 |
| | | Sc. PD: | -0.18 |
| | | Sc. rank: | -123.1 |

| | | | |
|---|---|---|---|
| NA | | | |
| Pos. | 456 | obs: | exp: |
| att | I | 4 | 1.50 |
| atc | I | 0 | 1.05 |
| ata | I | 0 | 1.45 |
| gtt | V | 493 | 101.30 |
| gtc | V | 0 | 103.50 |
| gta | V | 0 | 92.40 |
| gtg | V | 0 | 195.80 |
| --- | -- | ------ | ------ |
| mPD | | 0.016 | 0.74 |
| | | nPD: | 0.02 |
| | | N. weight: | 2.2 |
| | | Sc. PD: | -0.62 |
| | | Sc. rank: | -512.6 |

| | | | |
|---|---|---|---|
| NA | | | |
| Pos. | 457 | obs: | exp: |
| ggt | G | 0 | 61.32 |
| ggc | G | 0 | 67.21 |
| gga | G | 0 | 240.40 |
| ggg | G | 497 | 128.00 |
| --- | -- | ------ | ------ |
| mPD | | 0 | 0.67 |
| | | nPD: | 0. |
| | | N. weight: | 1.9 |
| | | Sc. PD: | -0.58 |
| | | Sc. rank: | -581.6 |

| | | | |
|---|---|---|---|
| NA | | | |
| Pos. | 458 | obs: | exp: |
| tct | S | 116 | 63.11 |
| tcc | S | 281 | 55.30 |
| tca | S | 0 | 87.95 |
| tcg | S | 0 | 24.20 |
| cct | P | 100 | 26.33 |
| ccc | P | 0 | 18.04 |
| cca | P | 0 | 39.46 |
| ccg | P | 0 | 16.17 |
| agt | S | 0 | 87.34 |
| agc | S | 0 | 79.09 |
| --- | -- | ------ | ------ |
| mPD | | 0.81 | 1.8 |
| | | nPD: | 0.46 |
| | | N. weight: | 1.9 |
| | | Sc. PD: | 0.26 |
| | | Sc. rank: | 216.1 |

| | | | |
|---|---|---|---|
| NA | | | |
| Pos. | 459 | obs: | exp: |
| tgt | C | 2 | 0.82 |
| tgc | C | 0 | 1.18 |
| ggt | G | 495 | 61.08 |
| ggc | G | 0 | 66.94 |
| gga | G | 0 | 239.50 |
| ggg | G | 0 | 127.50 |
| --- | -- | ------ | ------ |
| mPD | | 0.0080 | 0.68 |
| | | nPD: | 0.01 |
| | | N. weight: | 2.9 |
| | | Sc. PD: | -0.85 |
| | | Sc. rank: | -743.2 |

| | | | |
|---|---|---|---|
| NA | | | |
| Pos. | 460 | obs: | exp: |
| tct | S | 18 | 79.01 |
| tcc | S | 478 | 69.23 |
| tca | S | 1 | 110.10 |
| tcg | S | 0 | 30.29 |
| agt | S | 0 | 109.30 |
| agc | S | 0 | 99.02 |
| --- | -- | ------ | ------ |
| mPD | | 0.074 | 1.7 |
| | | nPD: | 0.04 |
| | | N. weight: | 2.5 |
| | | Sc. PD: | -0.66 |
| | | Sc. rank: | -488.3 |

| | | | |
|---|---|---|---|
| NA | | | |
| Pos. | 461 | obs: | exp: |
| ttt | F | 0 | 204.30 |
| ttc | F | 497 | 292.70 |
| --- | -- | ------ | ------ |
| mPD | | 0 | 0.48 |
| | | nPD: | 0. |
| | | N. weight: | 0.74 |
| | | Sc. PD: | -0.23 |
| | | Sc. rank: | -227.1 |

| | | | |
|---|---|---|---|
| NA | | | |
| Pos. | 462 | obs: | exp: |
| cct | P | 2 | 130.90 |
| ccc | P | 495 | 89.65 |
| cca | P | 0 | 196.10 |
| ccg | P | 0 | 80.36 |
| --- | -- | ------ | ------ |
| mPD | | 0.0080 | 0.72 |
| | | nPD: | 0.01 |
| | | N. weight: | 2.4 |
| | | Sc. PD: | -0.69 |
| | | Sc. rank: | -609.8 |

| | | | |
|---|---|---|---|
| NA | | | |
| Pos. | 463 | obs: | exp: |
| gat | D | 491 | 265.20 |
| gac | D | 6 | 231.80 |
| --- | -- | ------ | ------ |
| mPD | | 0.024 | 0.50 |
| | | nPD: | 0.05 |
| | | N. weight: | 0.79 |
| | | Sc. PD: | -0.2 |
| | | Sc. rank: | -150.2 |

| | | | |
|---|---|---|---|
| NA | | | |
| Pos. | 464 | obs: | exp: |
| ggt | G | 0 | 61.32 |
| ggc | G | 0 | 67.21 |
| gga | G | 0 | 240.40 |
| ggg | G | 497 | 128.00 |
| --- | -- | ------ | ------ |
| mPD | | 0 | 0.67 |
| | | nPD: | 0. |
| | | N. weight: | 1.9 |
| | | Sc. PD: | -0.58 |
| | | Sc. rank: | -581.6 |

| | | | |
|---|---|---|---|
| NA | | | |
| Pos. | 465 | obs: | exp: |
| gct | A | 2 | 125.20 |
| gcc | A | 0 | 101.80 |
| gca | A | 492 | 221.50 |
| gcg | A | 3 | 48.50 |
| --- | -- | ------ | ------ |
| mPD | | 0.020 | 0.69 |
| | | nPD: | 0.03 |
| | | N. weight: | 1.1 |
| | | Sc. PD: | -0.29 |
| | | Sc. rank: | -232.0 |

| | | | |
|---|---|---|---|
| NA | | | |
| Pos. | 466 | obs: | exp: |
| caa | Q | 308 | 265.00 |
| cag | Q | 189 | 232.00 |
| --- | -- | ------ | ------ |
| mPD | | 0.47 | 0.50 |
| | | nPD: | 0.95 |
| | | N. weight: | 0.031 |
| | | Sc. PD: | 0.019 |
| | | Sc. rank: | 9.4 |

| | | | |
|---|---|---|---|
| NA | | | |
| Pos. | 467 | obs: | exp: |
| att | I | 2 | 186.80 |
| atc | I | 495 | 130.30 |
| ata | I | 0 | 179.90 |
| --- | -- | ------ | ------ |
| mPD | | 0.0080 | 0.66 |
| | | nPD: | 0.01 |
| | | N. weight: | 1.8 |
| | | Sc. PD: | -0.54 |
| | | Sc. rank: | -458.2 |

| NA | | | |
|---|---|---|---|
| Pos. | 468 | obs: | exp: |
| caa | Q | 497 | 265.00 |
| cag | Q | 0 | 232.00 |
| --- | -- | ------ | ------ |
| mPD | | 0 | 0.50 |
| | | nPD: | 0. |
| | | N. weight: | 0.88 |
| | | Sc. PD: | -0.27 |
| | | Sc. rank: | -269.8 |

| NA | | | |
|---|---|---|---|
| Pos. | 469 | obs: | exp: |
| tat | Y | 0 | 257.30 |
| tac | Y | 497 | 239.70 |
| --- | -- | ------ | ------ |
| mPD | | 0 | 0.50 |
| | | nPD: | 0. |
| | | N. weight: | 1. |
| | | Sc. PD: | -0.31 |
| | | Sc. rank: | -312.7 |

| NA | | | |
|---|---|---|---|
| Pos. | 470 | obs: | exp: |
| ttt | F | 496 | 204.30 |
| ttc | F | 1 | 292.70 |
| --- | -- | ------ | ------ |
| mPD | | 0.0040 | 0.48 |
| | | nPD: | 0.01 |
| | | N. weight: | 1.2 |
| | | Sc. PD: | -0.36 |
| | | Sc. rank: | -323.4 |

| NA | | | |
|---|---|---|---|
| Pos. | 471 | obs: | exp: |
| tta | L | 0 | 0.09 |
| ttg | L | 1 | 0.19 |
| tct | S | 0 | 78.85 |
| tcc | S | 0 | 69.10 |
| tca | S | 0 | 109.90 |
| tcg | S | 496 | 30.23 |
| ctt | L | 0 | 0.19 |
| ctc | L | 0 | 0.16 |
| cta | L | 0 | 0.15 |
| ctg | L | 0 | 0.22 |
| agt | S | 0 | 109.10 |
| agc | S | 0 | 98.82 |
| --- | -- | ------ | ------ |
| mPD | | 0.0040 | 1.7 |
| | | nPD: | 0. |
| | | N. weight: | 3.9 |
| | | Sc. PD: | -1.2 |
| | | Sc. rank: | -1104.0 |

| NA | | | |
|---|---|---|---|
| Pos. | 472 | obs: | exp: |
| taa | . | 497 | 232.80 |
| tag | . | 0 | 128.60 |
| tga | . | 0 | 135.60 |
| --- | -- | ------ | ------ |
| mPD | | 0 | 0.78 |
| | | nPD: | 0. |
| | | N. weight: | 1.1 |
| | | Sc. PD: | -0.32 |
| | | Sc. rank: | -325.3 |

}

Amino acid level conservation per codon

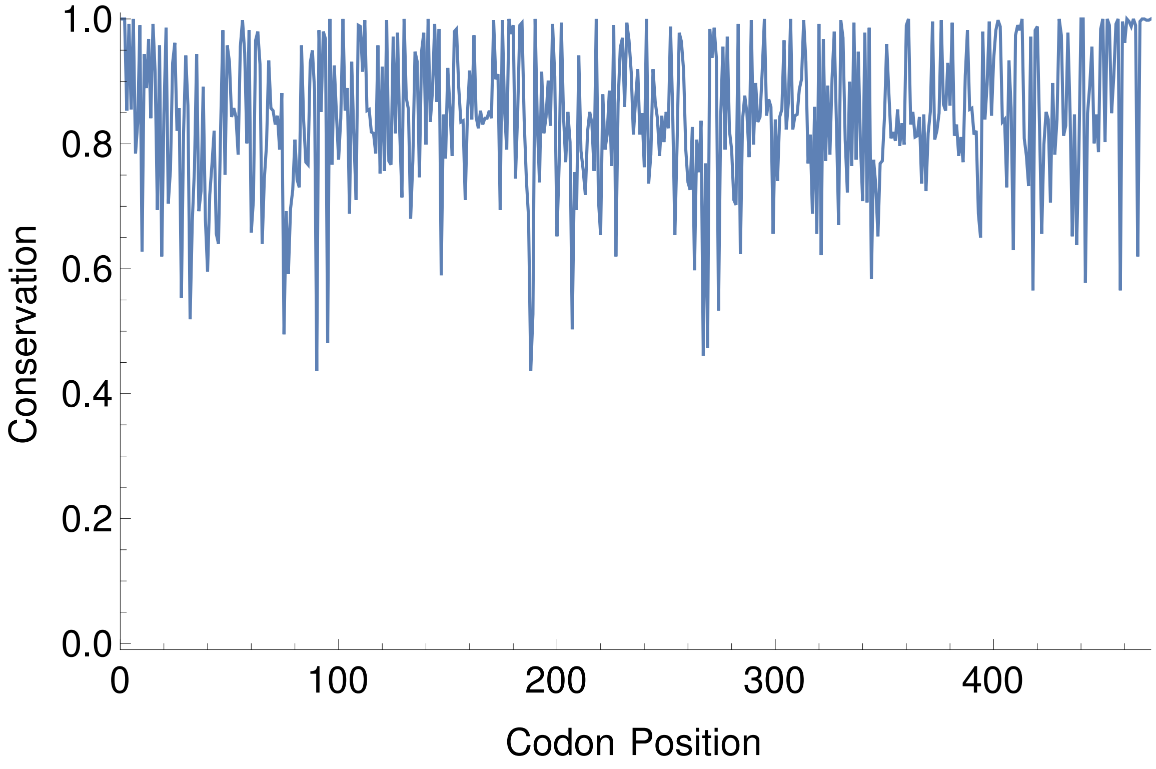

## Conservation analysis plots

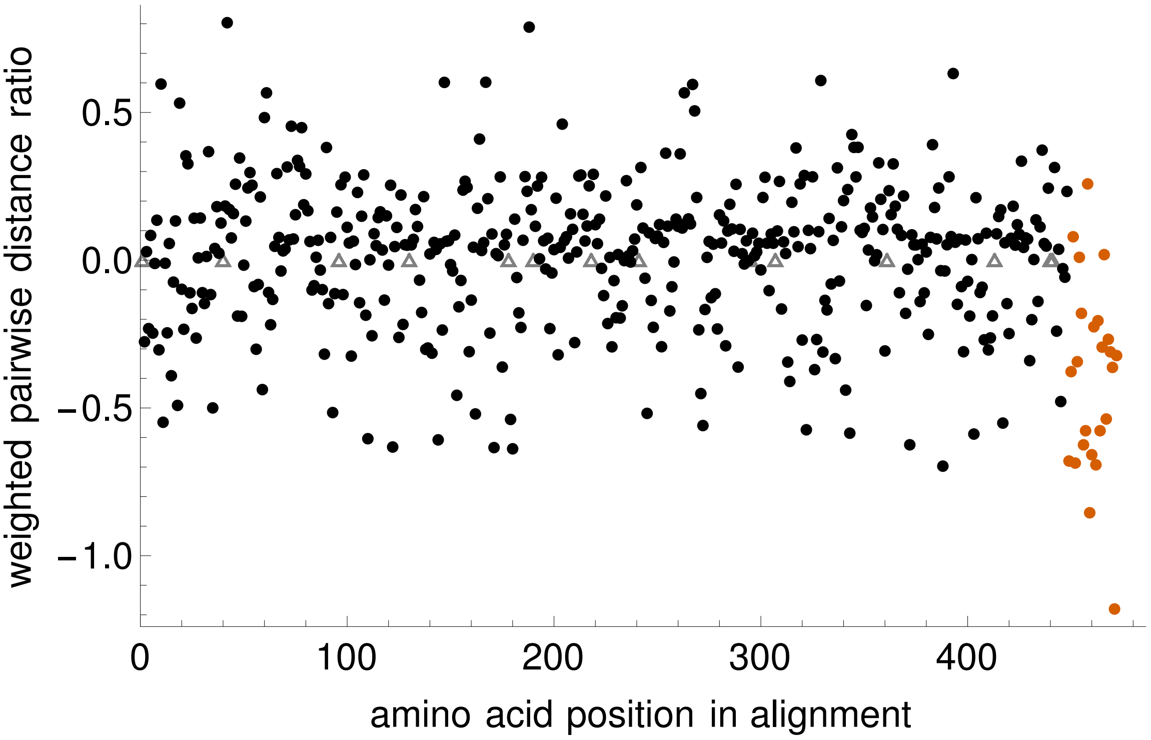


NA

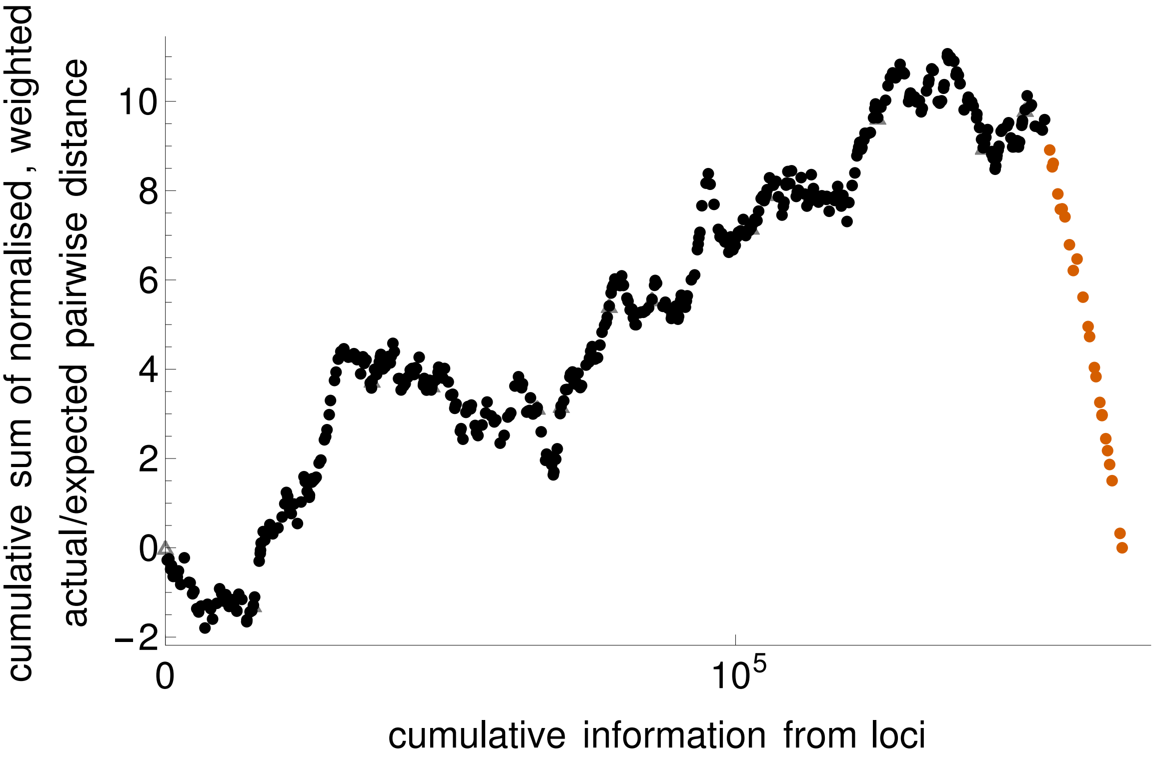


NA

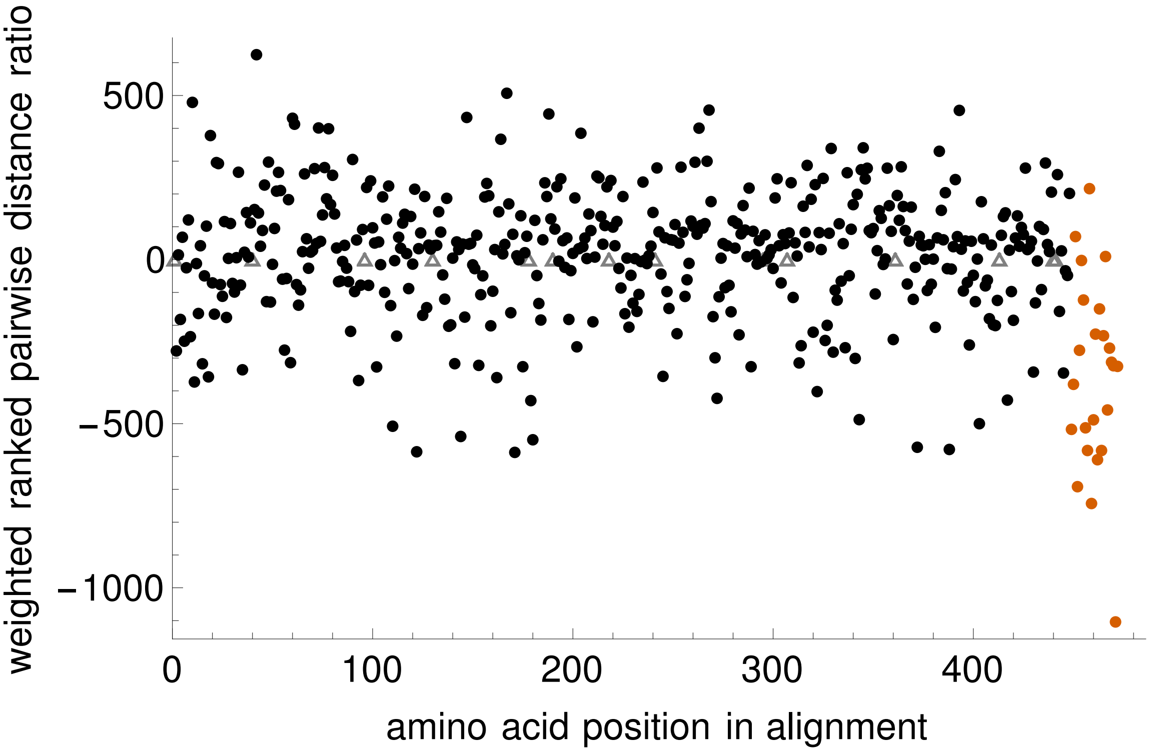
weighted ranked pairwise distance ratio
500
0
−500
−1000
0
100
200
300
400
amino acid position in alignment

NA

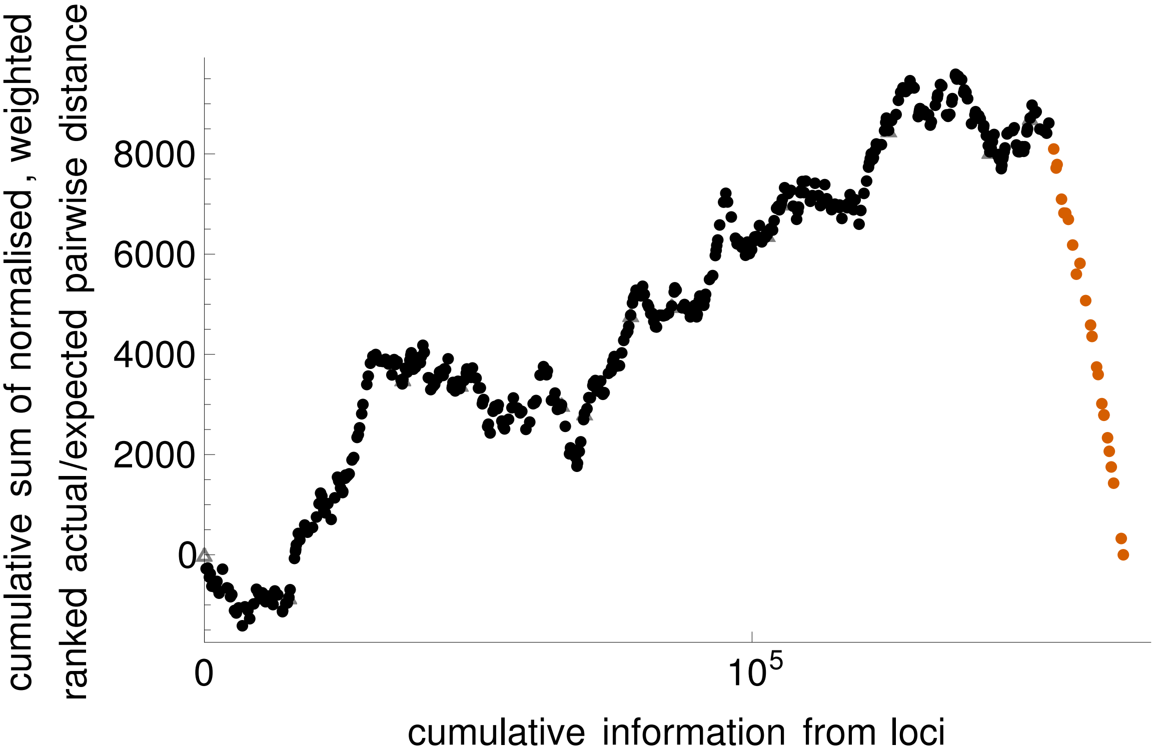
cumulative sum of normalised, weighted ranked actual/expected pairwise distance
8000
6000
4000
2000
0
0
$10^5$
cumulative information from loci

NA

# M1

## Gene length histogram

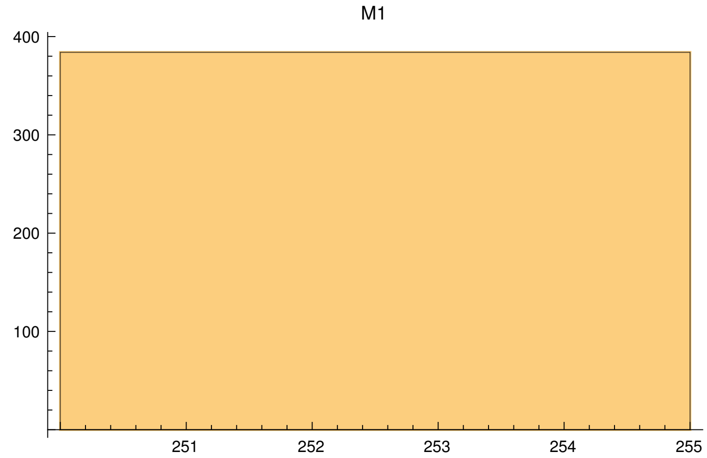

## Information vs. nPD

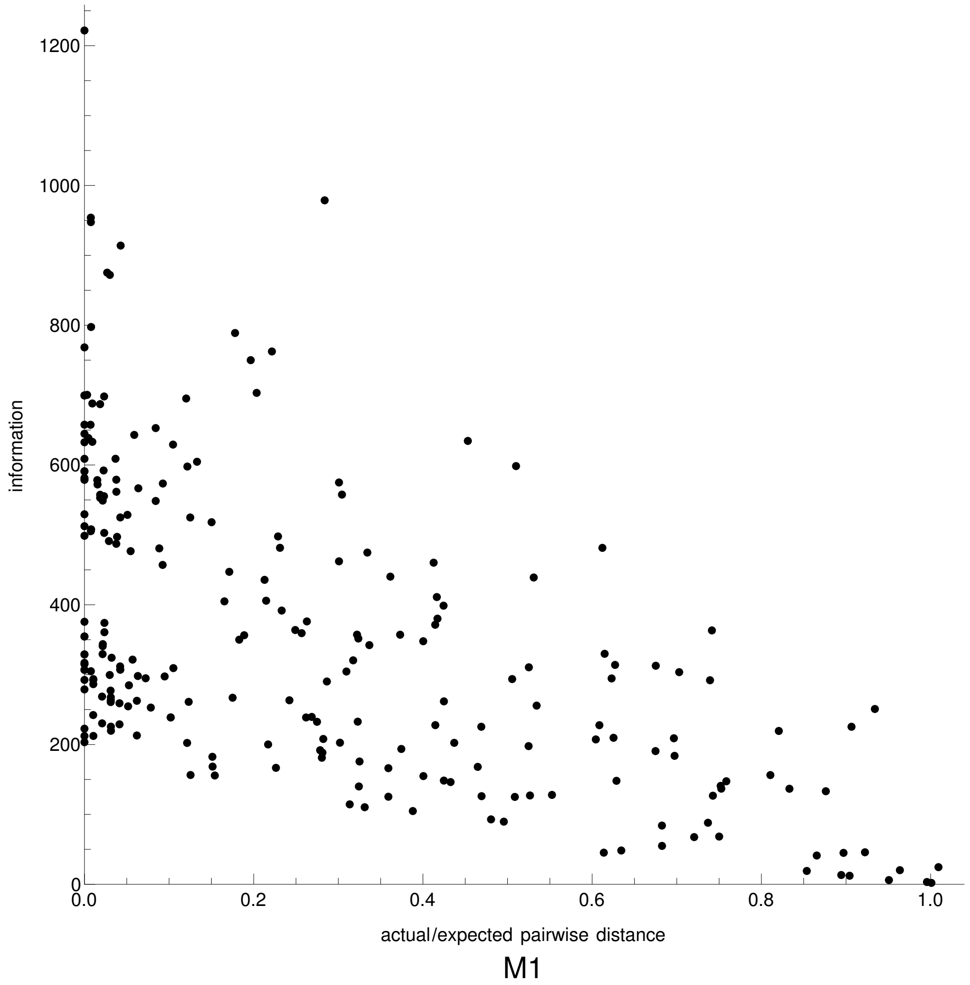

## Example sequences highlighted by regions found in analyses to be conserved

Interesting points (by weighted raw PD) highlighted for gene M1:

ATG
**AGTCTTCTA------ACCGAGGTCGAAACGTACGTTCTCTCTATAATCCCGTCA**
**GGCCCCCTCAAAGCCGAGATCGCGCAGAGACTTGAAGATGTCTTTGCGGGGA**
**AGAACACCGATCTCGAGGCTCTCATGGAATGGCTAAAGACAAGACCAATCCT**
**GTCACCTCTGACTAAGGGGATTTTAGGGTTTGTGTTCACGCTCACCGTGCCC**
**AGTGAGCGAGGACTGCAGCGTAGA**CGCTTTGTCCAAAATGCCCTAAATGGGAATGGAGACCC⋱
AAACAACATGGACAGGGCAGTCAAACTGTACAGGAAGTTGAAAAGAGAGATAACATTCCATGGGGCTAAA⋱
GAAGTTGCACTCAGTTACTCGACCGGTGCGCTTGCCAGTTGTATGGGTCTCATATACAACAGGATGGGAA⋱
CGGTGACCACAGAAGTGGCTTTTGGCCTAGTGTGTGCCACCTGTGAGCAGATTGCTGATTCACAGCATCG⋱
GTCCCACAGACAGATGGTAACTACCACCAACCCACTAATCAGGCATGAAAACAGAATGGTGCTGGCCAGC⋱
ACTACGGCTAAGGCTATGGAGCAGATGGCTGGGTCAAGTGAGCAGGCAGCGGAAGCCATGGAGGTTGCTA⋱
GTCAGGCTAGGCAGATGGTACAGGCGATGAGGACAATTGGA
**ACTCACCCTAGCTCCAGTGCCGGTCTGAAAGATGATCTTCTTGAAAATTTGCAGG**
**CCTACCAGAAACGGATGGGAGTGCAAATGCAGCGATTCAAGTGA**

Interesting points (by weighted ranked PD) highlighted for gene M1:

ATGAGT
**CTTCTA------ACCGAGGTCGAAACGTACGTTCTCTCTATAATCCCGTCAGGC**
**CCCCTCAAAGCCGAGATCGCGCAGAGACTTGAAGATGTCTTTGCGGGGAAGA**
**ACACCGATCTCGAGGCTCTCATGGAATGGCTAAAGACAAGACCAATCCTGTC**
**ACCTCTGACTAAGGGGATTTTAGGGTTTGTGTTCACGCTCACCGTGCCCAGT**
**GAGCGAGGACTGCAGCGT**AGACGCTTTGTCCAAAATGCCCTAAATGGGAATGGAGACCCAAACA⋱
ACATGGACAGGGCAGTCAAACTGTACAGGAAGTTGAAAAGAGAGATAACATTCCATGGGGCTAAAGAAGT⋱
TGCACTCAGTTACTCGACCGGTGCGCTTGCCAGTTGTATGGGTCTCATATACAACAGGATGGGAACGGTG⋱
ACCACAGAAGTGGCTTTTGGCCTAGTGTGTGCCACCTGTGAGCAGATTGCTGATTCACAGCATCGGTCCC⋱
ACAGACAGATGGTAACTACCACCAACCCACTAATCAGGCATGAAAACAGAATGGTGCTGGCCAGCACTAC⋱
GGCTAAGGCTATGGAGCAGATGGCTGGGTCAAGTGAGCAGGCAGCGGAAGCCATGGAGGTTGCTAGTCAG⋱
GCTAGGCAGATGGTACAGGCGATGAGGACAATTGGAACTCACCCTAGCTCCAGTGCCGGTCTGAAAGATG⋱
ATCTTCTTGAAAATTTGCAGGCCTACCAGAAACGGATGGGAGTGCAAATGCAGCGATTCAAGTGA

# Per codon conservation report

| | | | |
|---|---|---|---|
| M1 | | | |
| Pos. | 1 | obs: | exp: |
| atg | M | 384 | 384.00 |
| --- | -- | ------ | ------ |
| mPD | | 0 | 0 |
| | | nPD: | 1. |
| | | N. weight: | 0. |
| | | Sc. PD: | 0 |
| | | Sc. rank: | 0 |

| | | | |
|---|---|---|---|
| M1 | | | |
| Pos. | 2 | obs: | exp: |
| tct | S | 0 | 61.05 |
| tcc | S | 0 | 53.49 |
| tca | S | 0 | 85.07 |
| tcg | S | 0 | 23.41 |
| agt | S | 370 | 84.48 |
| agc | S | 14 | 76.50 |
| --- | -- | ------ | ------ |
| mPD | | 0.070 | 1.7 |
| | | nPD: | 0.04 |
| | | N. weight: | 1.6 |
| | | Sc. PD: | -0.18 |
| | | Sc. rank: | -12.8 |

| | | | |
|---|---|---|---|
| M1 | | | |
| Pos. | 3 | obs: | exp: |
| tta | L | 0 | 33.55 |
| ttg | L | 0 | 73.96 |
| ctt | L | 384 | 71.65 |
| ctc | L | 0 | 62.12 |
| cta | L | 0 | 57.85 |
| ctg | L | 0 | 84.88 |
| --- | -- | ------ | ------ |
| mPD | | 0 | 1.1 |
| | | nPD: | 0. |
| | | N. weight: | 1.9 |
| | | Sc. PD: | -0.3 |
| | | Sc. rank: | -539.9 |

| | | | |
|---|---|---|---|
| M1 | | | |
| Pos. | 4 | obs: | exp: |
| tta | L | 1 | 33.55 |
| ttg | L | 0 | 73.96 |
| ctt | L | 1 | 71.65 |
| ctc | L | 0 | 62.12 |
| cta | L | 379 | 57.85 |
| ctg | L | 3 | 84.88 |
| --- | -- | ------ | ------ |
| mPD | | 0.026 | 1.1 |
| | | nPD: | 0.02 |
| | | N. weight: | 2.1 |
| | | Sc. PD: | -0.28 |
| | | Sc. rank: | -162.1 |

| | | | |
|---|---|---|---|
| M1 | | | |
| Pos. | 5 | obs: | exp: |
| tct | S | 1 | 0.16 |
| tcc | S | 0 | 0.14 |
| tca | S | 0 | 0.22 |
| tcg | S | 0 | 0.06 |
| agt | S | 0 | 0.22 |
| agc | S | 0 | 0.20 |
| --- | - | 383 | 383.00 |
| --- | -- | ------ | ------ |
| mPD | | 0 | 0.000011 |
| | | nPD: | 0. |
| | | N. weight: | 0. |
| | | Sc. PD: | 0 |
| | | Sc. rank: | 0 |

| | | | |
|---|---|---|---|
| M1 | | | |
| Pos. | 6 | obs: | exp: |
| tct | S | 1 | 0.16 |
| tcc | S | 0 | 0.14 |
| tca | S | 0 | 0.22 |
| tcg | S | 0 | 0.06 |
| agt | S | 0 | 0.22 |
| agc | S | 0 | 0.20 |
| --- | - | 383 | 383.00 |
| --- | -- | ------ | ------ |
| mPD | | 0 | 0.000011 |
| | | nPD: | 0. |
| | | N. weight: | 0. |
| | | Sc. PD: | 0 |
| | | Sc. rank: | 0 |

| | | | |
|---|---|---|---|
| M1 | | | |
| Pos. | 7 | obs: | exp: |
| act | T | 0 | 101.40 |
| acc | T | 384 | 82.40 |
| aca | T | 0 | 168.20 |
| acg | T | 0 | 31.92 |
| --- | -- | ------ | ------ |
| mPD | | 0 | 0.69 |
| | | nPD: | 0. |
| | | N. weight: | 1.8 |
| | | Sc. PD: | -0.28 |
| | | Sc. rank: | -494.9 |

| | | | |
|---|---|---|---|
| M1 | | | |
| Pos. | 8 | obs: | exp: |
| gaa | E | 0 | 221.00 |
| gag | E | 384 | 163.00 |
| --- | -- | ------ | ------ |
| mPD | | 0 | 0.49 |
| | | nPD: | 0. |
| | | N. weight: | 0.98 |
| | | Sc. PD: | -0.15 |
| | | Sc. rank: | -275.5 |

| | | | |
|---|---|---|---|
| M1 | | | |
| Pos. | 9 | obs: | exp: |
| gtt | V | 12 | 78.93 |
| gtc | V | 372 | 80.59 |
| gta | V | 0 | 71.97 |
| gtg | V | 0 | 152.50 |
| --- | -- | ------ | ------ |
| mPD | | 0.061 | 0.72 |
| | | nPD: | 0.08 |
| | | N. weight: | 1.6 |
| | | Sc. PD: | -0.13 |
| | | Sc. rank: | 58.9 |

| | | | |
|---|---|---|---|
| M1 | | | |
| Pos. | 10 | obs: | exp: |
| gaa | E | 384 | 221.00 |
| gag | E | 0 | 163.00 |
| --- | -- | ------ | ------ |
| mPD | | 0 | 0.49 |
| | | nPD: | 0. |
| | | N. weight: | 0.63 |
| | | Sc. PD: | -0.099 |
| | | Sc. rank: | -177.7 |

| | | | |
|---|---|---|---|
| M1 | | | |
| Pos. | 11 | obs: | exp: |
| cgt | R | 0 | 0.04 |
| cgc | R | 0 | 0.05 |
| cga | R | 0 | 0.10 |
| cgg | R | 0 | 0.08 |
| act | T | 0 | 101.20 |
| acc | T | 0 | 82.19 |
| aca | T | 0 | 167.80 |
| acg | T | 383 | 31.84 |
| aga | R | 0 | 0.45 |
| agg | R | 1 | 0.28 |
| --- | -- | ------ | ------ |
| mPD | | 0.0052 | 0.69 |
| | | nPD: | 0.01 |
| | | N. weight: | 2.8 |
| | | Sc. PD: | -0.43 |
| | | Sc. rank: | -463.8 |

| | | | |
|---|---|---|---|
| M1 | | | |
| Pos. | 12 | obs: | exp: |
| tat | Y | 3 | 198.80 |
| tac | Y | 381 | 185.20 |
| --- | -- | ------ | ------ |
| mPD | | 0.016 | 0.50 |
| | | nPD: | 0.03 |
| | | N. weight: | 0.78 |
| | | Sc. PD: | -0.1 |
| | | Sc. rank: | -36.8 |

| | | | |
|---|---|---|---|
| M1 | | | |
| Pos. | 13 | obs: | exp: |
| gtt | V | 375 | 78.93 |
| gtc | V | 9 | 80.59 |
| gta | V | 0 | 71.97 |
| gtg | V | 0 | 152.50 |
| --- | -- | ------ | ------ |
| mPD | | 0.046 | 0.72 |
| | | nPD: | 0.06 |
| | | N. weight: | 1.7 |
| | | Sc. PD: | -0.16 |
| | | Sc. rank: | 40.8 |

| | | | |
|---|---|---|---|
| M1 | | | |
| Pos. | 14 | obs: | exp: |
| tta | L | 0 | 33.55 |
| ttg | L | 0 | 73.96 |
| ctt | L | 2 | 71.65 |
| ctc | L | 382 | 62.12 |
| cta | L | 0 | 57.85 |
| ctg | L | 0 | 84.88 |
| --- | -- | ------ | ------ |
| mPD | | 0.010 | 1.1 |
| | | nPD: | 0.01 |
| | | N. weight: | 2. |
| | | Sc. PD: | -0.3 |
| | | Sc. rank: | -282.7 |

| | | | |
|---|---|---|---|
| M1 | | | |
| Pos. | 15 | obs: | exp: |
| tct | S | 383 | 61.05 |
| tcc | S | 1 | 53.49 |
| tca | S | 0 | 85.07 |
| tcg | S | 0 | 23.41 |
| agt | S | 0 | 84.48 |
| agc | S | 0 | 76.50 |
| --- | -- | ------ | ------ |
| mPD | | 0.0052 | 1.7 |
| | | nPD: | 0. |
| | | N. weight: | 2.1 |
| | | Sc. PD: | -0.32 |
| | | Sc. rank: | -372.4 |

| | | | |
|---|---|---|---|
| M1 | | | |
| Pos. | 16 | obs: | exp: |
| att | I | 7 | 144.00 |
| atc | I | 372 | 100.40 |
| ata | I | 4 | 138.60 |
| act | T | 0 | 0.26 |
| acc | T | 1 | 0.21 |
| aca | T | 0 | 0.44 |
| acg | T | 0 | 0.08 |
| --- | -- | ------ | ------ |
| mPD | | 0.061 | 0.66 |
| | | nPD: | 0.09 |
| | | N. weight: | 1.4 |
| | | Sc. PD: | -0.096 |
| | | Sc. rank: | 57.7 |

| | | | |
|---|---|---|---|
| M1 | | | |
| Pos. | 17 | obs: | exp: |
| att | I | 1 | 13.91 |
| atc | I | 8 | 9.70 |
| ata | I | 28 | 13.39 |
| gtt | V | 38 | 71.32 |
| gtc | V | 308 | 72.83 |
| gta | V | 1 | 65.03 |
| gtg | V | 0 | 137.80 |
| --- | -- | ------ | ------ |
| mPD | | 0.48 | 0.91 |
| | | nPD: | 0.53 |
| | | N. weight: | 1.3 |
| | | Sc. PD: | 0.44 |
| | | Sc. rank: | 329.8 |

| | | | |
|---|---|---|---|
| M1 | | | |
| Pos. | 18 | obs: | exp: |
| cct | P | 0 | 101.10 |
| ccc | P | 0 | 69.26 |
| cca | P | 69 | 151.50 |
| ccg | P | 315 | 62.09 |
| --- | -- | ------ | ------ |
| mPD | | 0.30 | 0.72 |
| | | nPD: | 0.41 |
| | | N. weight: | 1.4 |
| | | Sc. PD: | 0.31 |
| | | Sc. rank: | 290.2 |

| | | | |
|---|---|---|---|
| M1 | | | |
| Pos. | 19 | obs: | exp: |
| tct | S | 0 | 61.05 |
| tcc | S | 0 | 53.49 |
| tca | S | 378 | 85.07 |
| tcg | S | 6 | 23.41 |
| agt | S | 0 | 84.48 |
| agc | S | 0 | 76.50 |
| --- | -- | ------ | ------ |
| mPD | | 0.031 | 1.7 |
| | | nPD: | 0.02 |
| | | N. weight: | 1.7 |
| | | Sc. PD: | -0.23 |
| | | Sc. rank: | -179.7 |

| | | | |
|---|---|---|---|
| M1 | | | |
| Pos. | 20 | obs: | exp: |
| ggt | G | 0 | 47.38 |
| ggc | G | 384 | 51.93 |
| gga | G | 0 | 185.80 |
| ggg | G | 0 | 98.93 |
| --- | -- | ------ | ------ |
| mPD | | 0 | 0.67 |
| | | nPD: | 0. |
| | | N. weight: | 2.3 |
| | | Sc. PD: | -0.36 |
| | | Sc. rank: | -643.4 |

| | | | |
|---|---|---|---|
| M1 | | | |
| Pos. | 21 | obs: | exp: |
| cct | P | 0 | 101.10 |
| ccc | P | 384 | 69.26 |
| cca | P | 0 | 151.50 |
| ccg | P | 0 | 62.09 |
| --- | -- | ------ | ------ |
| mPD | | 0 | 0.72 |
| | | nPD: | 0. |
| | | N. weight: | 2. |
| | | Sc. PD: | -0.31 |
| | | Sc. rank: | -550.8 |

| | | | |
|---|---|---|---|
| M1 | | | |
| Pos. | 22 | obs: | exp: |
| tta | L | 0 | 33.55 |
| ttg | L | 0 | 73.96 |
| ctt | L | 0 | 71.65 |
| ctc | L | 384 | 62.12 |
| cta | L | 0 | 57.85 |
| ctg | L | 0 | 84.88 |
| --- | -- | ------ | ------ |
| mPD | | 0 | 1.1 |
| | | nPD: | 0. |
| | | N. weight: | 2.1 |
| | | Sc. PD: | -0.33 |
| | | Sc. rank: | -585.8 |

| | | | |
|---|---|---|---|
| M1 | | | |
| Pos. | 23 | obs: | exp: |
| aaa | K | 384 | 215.00 |
| aag | K | 0 | 169.00 |
| --- | -- | ------ | ------ |
| mPD | | 0 | 0.49 |
| | | nPD: | 0. |
| | | N. weight: | 0.66 |
| | | Sc. PD: | -0.1 |
| | | Sc. rank: | -186.5 |

| | | | |
|---|---|---|---|
| M1 | | | |
| Pos. | 24 | obs: | exp: |
| gct | A | 0 | 96.72 |
| gcc | A | 384 | 78.66 |
| gca | A | 0 | 171.10 |
| gcg | A | 0 | 37.47 |
| --- | -- | ------ | ------ |
| mPD | | 0 | 0.69 |
| | | nPD: | 0. |
| | | N. weight: | 1.8 |
| | | Sc. PD: | -0.28 |
| | | Sc. rank: | -509.9 |

| | | | |
|---|---|---|---|
| M1 | | | |
| Pos. | 25 | obs: | exp: |
| gaa | E | 4 | 221.00 |
| gag | E | 380 | 163.00 |
| --- | -- | ------ | ------ |
| mPD | | 0.021 | 0.49 |
| | | nPD: | 0.04 |
| | | N. weight: | 0.91 |
| | | Sc. PD: | -0.11 |
| | | Sc. rank: | -12.6 |

| M1 | | | |
|---|---|---|---|
| Pos. | 26 | obs: | exp: |
| att | I | 6 | 144.40 |
| atc | I | 377 | 100.60 |
| ata | I | 1 | 139.00 |
| --- | -- | ------ | ------ |
| mPD | | 0.036 | 0.66 |
| | | nPD: | 0.05 |
| | | N. weight: | 1.4 |
| | | Sc. PD: | -0.15 |
| | | Sc. rank: | 12.1 |

| M1 | | | |
|---|---|---|---|
| Pos. | 27 | obs: | exp: |
| gct | A | 0 | 96.47 |
| gcc | A | 0 | 78.46 |
| gca | A | 3 | 170.70 |
| gcg | A | 380 | 37.37 |
| ggt | G | 0 | 0.12 |
| ggc | G | 0 | 0.14 |
| gga | G | 0 | 0.48 |
| ggg | G | 1 | 0.26 |
| --- | -- | ------ | ------ |
| mPD | | 0.021 | 0.69 |
| | | nPD: | 0.03 |
| | | N. weight: | 2.6 |
| | | Sc. PD: | -0.33 |
| | | Sc. rank: | -136.0 |

| M1 | | | |
|---|---|---|---|
| Pos. | 28 | obs: | exp: |
| cat | H | 1 | 0.54 |
| cac | H | 0 | 0.46 |
| caa | Q | 0 | 204.20 |
| cag | Q | 383 | 178.80 |
| --- | -- | ------ | ------ |
| mPD | | 0.0052 | 0.50 |
| | | nPD: | 0.01 |
| | | N. weight: | 0.87 |
| | | Sc. PD: | -0.13 |
| | | Sc. rank: | -113.1 |

| M1 | | | |
|---|---|---|---|
| Pos. | 29 | obs: | exp: |
| cgt | R | 0 | 15.94 |
| cgc | R | 0 | 18.80 |
| cga | R | 0 | 36.90 |
| cgg | R | 0 | 32.04 |
| aga | R | 384 | 172.70 |
| agg | R | 0 | 107.60 |
| --- | -- | ------ | ------ |
| mPD | | 0 | 0.96 |
| | | nPD: | 0. |
| | | N. weight: | 0.91 |
| | | Sc. PD: | -0.14 |
| | | Sc. rank: | -257.0 |

| M1 | | | |
|---|---|---|---|
| Pos. | 30 | obs: | exp: |
| tta | L | 0 | 33.55 |
| ttg | L | 0 | 73.96 |
| ctt | L | 383 | 71.65 |
| ctc | L | 0 | 62.12 |
| cta | L | 0 | 57.85 |
| ctg | L | 1 | 84.88 |
| --- | -- | ------ | ------ |
| mPD | | 0.0052 | 1.1 |
| | | nPD: | 0. |
| | | N. weight: | 1.9 |
| | | Sc. PD: | -0.29 |
| | | Sc. rank: | -331.3 |

| M1 | | | |
|---|---|---|---|
| Pos. | 31 | obs: | exp: |
| gaa | E | 350 | 221.00 |
| gag | E | 34 | 163.00 |
| --- | -- | ------ | ------ |
| mPD | | 0.16 | 0.49 |
| | | nPD: | 0.33 |
| | | N. weight: | 0.33 |
| | | Sc. PD: | 0.05 |
| | | Sc. rank: | 62.5 |

| M1 | | | |
|---|---|---|---|
| Pos. | 32 | obs: | exp: |
| gtt | V | 1 | 0.21 |
| gtc | V | 0 | 0.21 |
| gta | V | 0 | 0.19 |
| gtg | V | 0 | 0.40 |
| gat | D | 383 | 204.30 |
| gac | D | 0 | 178.70 |
| --- | -- | ------ | ------ |
| mPD | | 0.0052 | 0.50 |
| | | nPD: | 0.01 |
| | | N. weight: | 0.72 |
| | | Sc. PD: | -0.11 |
| | | Sc. rank: | -95.1 |

| M1 | | | |
|---|---|---|---|
| Pos. | 33 | obs: | exp: |
| tta | L | 0 | 0.09 |
| ttg | L | 0 | 0.19 |
| ctt | L | 0 | 0.19 |
| ctc | L | 1 | 0.16 |
| cta | L | 0 | 0.15 |
| ctg | L | 0 | 0.22 |
| gtt | V | 43 | 78.72 |
| gtc | V | 257 | 80.38 |
| gta | V | 10 | 71.78 |
| gtg | V | 73 | 152.10 |
| --- | -- | ------ | ------ |
| mPD | | 0.51 | 0.73 |
| | | nPD: | 0.7 |
| | | N. weight: | 0.62 |
| | | Sc. PD: | 0.31 |
| | | Sc. rank: | 172.0 |

| M1 | | | |
|---|---|---|---|
| Pos. | 34 | obs: | exp: |
| ttt | F | 382 | 157.90 |
| ttc | F | 2 | 226.10 |
| --- | -- | ------ | ------ |
| mPD | | 0.010 | 0.48 |
| | | nPD: | 0.02 |
| | | N. weight: | 0.98 |
| | | Sc. PD: | -0.13 |
| | | Sc. rank: | -97.0 |

| M1 | | | |
|---|---|---|---|
| Pos. | 35 | obs: | exp: |
| tct | S | 1 | 0.16 |
| tcc | S | 0 | 0.14 |
| tca | S | 0 | 0.22 |
| tcg | S | 0 | 0.06 |
| act | T | 0 | 1.06 |
| acc | T | 0 | 0.86 |
| aca | T | 4 | 1.75 |
| acg | T | 0 | 0.33 |
| agt | S | 0 | 0.22 |
| agc | S | 0 | 0.20 |
| gct | A | 0 | 95.46 |
| gcc | A | 1 | 77.64 |
| gca | A | 375 | 168.90 |
| gcg | A | 3 | 36.98 |
| --- | -- | ------ | ------ |
| mPD | | 0.052 | 0.71 |
| | | nPD: | 0.07 |
| | | N. weight: | 0.88 |
| | | Sc. PD: | -0.078 |
| | | Sc. rank: | 23.0 |

| M1 | | | |
|---|---|---|---|
| Pos. | 36 | obs: | exp: |
| gaa | E | 0 | 0.58 |
| gag | E | 1 | 0.42 |
| ggt | G | 0 | 47.26 |
| ggc | G | 0 | 51.79 |
| gga | G | 4 | 185.30 |
| ggg | G | 379 | 98.68 |
| --- | -- | ------ | ------ |
| mPD | | 0.026 | 0.67 |
| | | nPD: | 0.04 |
| | | N. weight: | 1.5 |
| | | Sc. PD: | -0.18 |
| | | Sc. rank: | -31.6 |

| M1 | | | |
|---|---|---|---|
| Pos. | 37 | obs: | exp: |
| cgt | R | 0 | 0.12 |
| cgc | R | 0 | 0.15 |
| cga | R | 0 | 0.29 |
| cgg | R | 0 | 0.25 |
| aaa | K | 90 | 213.30 |
| aag | K | 291 | 167.70 |
| aga | R | 1 | 1.35 |
| agg | R | 2 | 0.84 |
| --- | -- | ------ | ------ |
| mPD | | 0.38 | 0.51 |
| | | nPD: | 0.74 |
| | | N. weight: | 0.26 |
| | | Sc. PD: | 0.14 |
| | | Sc. rank: | 73.6 |

| M1 | | | |
|---|---|---|---|
| Pos. | 38 | obs: | exp: |
| aat | N | 1 | 199.60 |
| aac | N | 381 | 182.40 |
| aaa | K | 1 | 1.12 |
| aag | K | 1 | 0.88 |
| --- | -- | ------ | ------ |
| mPD | | 0.016 | 0.50 |
| | | nPD: | 0.03 |
| | | N. weight: | 0.82 |
| | | Sc. PD: | -0.11 |
| | | Sc. rank: | -41.7 |

| M1 | | | |
|---|---|---|---|
| Pos. | 39 | obs: | exp: |
| cat | H | 0 | 0.54 |
| cac | H | 1 | 0.46 |
| att | I | 0 | 0.38 |
| atc | I | 1 | 0.26 |
| ata | I | 0 | 0.36 |
| act | T | 26 | 93.26 |
| acc | T | 282 | 75.75 |
| aca | T | 45 | 154.60 |
| acg | T | 0 | 29.35 |
| gct | A | 0 | 7.30 |
| gcc | A | 0 | 5.94 |
| gca | A | 29 | 12.93 |
| gcg | A | 0 | 2.83 |
| --- | -- | ------ | ------ |
| mPD | | 0.57 | 0.84 |
| | | nPD: | 0.68 |
| | | N. weight: | 0.93 |
| | | Sc. PD: | 0.44 |
| | | Sc. rank: | 255.2 |

| M1 | | | |
|---|---|---|---|
| Pos. | 40 | obs: | exp: |
| gat | D | 378 | 204.90 |
| gac | D | 6 | 179.10 |
| --- | -- | ------ | ------ |
| mPD | | 0.031 | 0.50 |
| | | nPD: | 0.06 |
| | | N. weight: | 0.63 |
| | | Sc. PD: | -0.063 |
| | | Sc. rank: | 11.6 |

| M1 | | | |
|---|---|---|---|
| Pos. | 41 | obs: | exp: |
| tta | L | 0 | 33.55 |
| ttg | L | 0 | 73.96 |
| ctt | L | 123 | 71.65 |
| ctc | L | 253 | 62.12 |
| cta | L | 8 | 57.85 |
| ctg | L | 0 | 84.88 |
| --- | -- | ------ | ------ |
| mPD | | 0.46 | 1.1 |
| | | nPD: | 0.42 |
| | | N. weight: | 1.2 |
| | | Sc. PD: | 0.29 |
| | | Sc. rank: | 267.6 |

| M1 | | | |
|---|---|---|---|
| Pos. | 42 | obs: | exp: |
| gat | D | 1 | 0.53 |
| gac | D | 0 | 0.47 |
| gaa | E | 3 | 220.40 |
| gag | E | 380 | 162.60 |
| --- | -- | ------ | ------ |
| mPD | | 0.021 | 0.49 |
| | | nPD: | 0.04 |
| | | N. weight: | 0.93 |
| | | Sc. PD: | -0.11 |
| | | Sc. rank: | -14.8 |

| M1 | | | |
|---|---|---|---|
| Pos. | 43 | obs: | exp: |
| gtt | V | 2 | 0.41 |
| gtc | V | 0 | 0.42 |
| gta | V | 0 | 0.37 |
| gtg | V | 0 | 0.79 |
| gct | A | 280 | 96.22 |
| gcc | A | 17 | 78.25 |
| gca | A | 14 | 170.30 |
| gcg | A | 71 | 37.28 |
| --- | -- | ------ | ------ |
| mPD | | 0.43 | 0.70 |
| | | nPD: | 0.62 |
| | | N. weight: | 0.87 |
| | | Sc. PD: | 0.37 |
| | | Sc. rank: | 233.0 |

| M1 | | | |
|---|---|---|---|
| Pos. | 44 | obs: | exp: |
| tta | L | 0 | 33.37 |
| ttg | L | 0 | 73.57 |
| ctt | L | 2 | 71.28 |
| ctc | L | 380 | 61.79 |
| cta | L | 0 | 57.55 |
| ctg | L | 0 | 84.44 |
| att | I | 0 | 0.75 |
| atc | I | 2 | 0.52 |
| ata | I | 0 | 0.72 |
| --- | -- | ------ | ------ |
| mPD | | 0.021 | 1.1 |
| | | nPD: | 0.02 |
| | | N. weight: | 2. |
| | | Sc. PD: | -0.29 |
| | | Sc. rank: | -229.2 |

| M1 | | | |
|---|---|---|---|
| Pos. | 45 | obs: | exp: |
| atg | M | 384 | 384.00 |
| --- | -- | ------ | ------ |
| mPD | | 0 | 0 |
| | | nPD: | 1. |
| | | N. weight: | 0. |
| | | Sc. PD: | 0 |
| | | Sc. rank: | 0 |

| M1 | | | |
|---|---|---|---|
| Pos. | 46 | obs: | exp: |
| gaa | E | 352 | 221.00 |
| gag | E | 32 | 163.00 |
| --- | -- | ------ | ------ |
| mPD | | 0.15 | 0.49 |
| | | nPD: | 0.31 |
| | | N. weight: | 0.34 |
| | | Sc. PD: | 0.047 |
| | | Sc. rank: | 60.9 |

| M1 | | | |
|---|---|---|---|
| Pos. | 47 | obs: | exp: |
| tgg | W | 384 | 384.00 |
| --- | -- | ------ | ------ |
| mPD | | 0 | 0 |
| | | nPD: | 1. |
| | | N. weight: | 0. |
| | | Sc. PD: | 0 |
| | | Sc. rank: | 0 |

| M1 | | | |
|---|---|---|---|
| Pos. | 48 | obs: | exp: |
| tta | L | 0 | 31.01 |
| ttg | L | 0 | 68.37 |
| ctt | L | 0 | 66.24 |
| ctc | L | 0 | 57.42 |
| cta | L | 354 | 53.48 |
| ctg | L | 1 | 78.47 |
| att | I | 0 | 10.90 |
| atc | I | 0 | 7.60 |
| ata | I | 29 | 10.50 |
| --- | -- | ------ | ------ |
| mPD | | 0.15 | 1.2 |
| | | nPD: | 0.12 |
| | | N. weight: | 2.1 |
| | | Sc. PD: | -0.092 |
| | | Sc. rank: | 129.7 |

| M1 | | | |
|---|---|---|---|
| Pos. | 49 | obs: | exp: |
| aaa | K | 0 | 215.00 |
| aag | K | 384 | 169.00 |
| --- | -- | ------ | ------ |
| mPD | | 0 | 0.49 |
| | | nPD: | 0. |
| | | N. weight: | 0.94 |
| | | Sc. PD: | -0.15 |
| | | Sc. rank: | -264.0 |

| M1 | | | |
|---|---|---|---|
| Pos. | 50 | obs: | exp: |
| act | T | 0 | 101.40 |
| acc | T | 0 | 82.40 |
| aca | T | 384 | 168.20 |
| acg | T | 0 | 31.92 |
| --- | -- | ------ | ------ |
| mPD | | 0 | 0.69 |
| | | nPD: | 0. |
| | | N. weight: | 0.94 |
| | | Sc. PD: | -0.15 |
| | | Sc. rank: | -265.4 |

| | | | |
|---|---|---|---|
| M1 | | | |
| Pos. | 51 | obs: | exp: |
| cgt | R | 0 | 15.94 |
| cgc | R | 0 | 18.80 |
| cga | R | 0 | 36.90 |
| cgg | R | 0 | 32.04 |
| aga | R | 384 | 172.70 |
| agg | R | 0 | 107.60 |
| --- | -- | ------ | ------ |
| mPD | | 0 | 0.96 |
| | | nPD: | 0. |
| | | N. weight: | 0.91 |
| | | Sc. PD: | -0.14 |
| | | Sc. rank: | -257.0 |

| | | | |
|---|---|---|---|
| M1 | | | |
| Pos. | 52 | obs: | exp: |
| cct | P | 0 | 101.10 |
| ccc | P | 0 | 69.26 |
| cca | P | 381 | 151.50 |
| ccg | P | 3 | 62.09 |
| --- | -- | ------ | ------ |
| mPD | | 0.016 | 0.72 |
| | | nPD: | 0.02 |
| | | N. weight: | 1. |
| | | Sc. PD: | -0.14 |
| | | Sc. rank: | -92.6 |

| | | | |
|---|---|---|---|
| M1 | | | |
| Pos. | 53 | obs: | exp: |
| att | I | 0 | 144.40 |
| atc | I | 383 | 100.60 |
| ata | I | 1 | 139.00 |
| --- | -- | ------ | ------ |
| mPD | | 0.0052 | 0.66 |
| | | nPD: | 0.01 |
| | | N. weight: | 1.5 |
| | | Sc. PD: | -0.23 |
| | | Sc. rank: | -215.5 |

| | | | |
|---|---|---|---|
| M1 | | | |
| Pos. | 54 | obs: | exp: |
| tta | L | 0 | 33.55 |
| ttg | L | 5 | 73.96 |
| ctt | L | 0 | 71.65 |
| ctc | L | 0 | 62.12 |
| cta | L | 0 | 57.85 |
| ctg | L | 379 | 84.88 |
| --- | -- | ------ | ------ |
| mPD | | 0.026 | 1.1 |
| | | nPD: | 0.02 |
| | | N. weight: | 1.6 |
| | | Sc. PD: | -0.22 |
| | | Sc. rank: | -136.8 |

| | | | |
|---|---|---|---|
| M1 | | | |
| Pos. | 55 | obs: | exp: |
| tct | S | 0 | 61.05 |
| tcc | S | 0 | 53.49 |
| tca | S | 384 | 85.07 |
| tcg | S | 0 | 23.41 |
| agt | S | 0 | 84.48 |
| agc | S | 0 | 76.50 |
| --- | -- | ------ | ------ |
| mPD | | 0 | 1.7 |
| | | nPD: | 0. |
| | | N. weight: | 1.7 |
| | | Sc. PD: | -0.27 |
| | | Sc. rank: | -484.7 |

| | | | |
|---|---|---|---|
| M1 | | | |
| Pos. | 56 | obs: | exp: |
| cct | P | 384 | 101.10 |
| ccc | P | 0 | 69.26 |
| cca | P | 0 | 151.50 |
| ccg | P | 0 | 62.09 |
| --- | -- | ------ | ------ |
| mPD | | 0 | 0.72 |
| | | nPD: | 0. |
| | | N. weight: | 1.5 |
| | | Sc. PD: | -0.24 |
| | | Sc. rank: | -429.1 |

| | | | |
|---|---|---|---|
| M1 | | | |
| Pos. | 57 | obs: | exp: |
| tta | L | 0 | 33.55 |
| ttg | L | 5 | 73.96 |
| ctt | L | 0 | 71.65 |
| ctc | L | 0 | 62.12 |
| cta | L | 6 | 57.85 |
| ctg | L | 373 | 84.88 |
| --- | -- | ------ | ------ |
| mPD | | 0.057 | 1.1 |
| | | nPD: | 0.05 |
| | | N. weight: | 1.6 |
| | | Sc. PD: | -0.17 |
| | | Sc. rank: | 2.6 |

| | | | |
|---|---|---|---|
| M1 | | | |
| Pos. | 58 | obs: | exp: |
| act | T | 383 | 101.40 |
| acc | T | 1 | 82.40 |
| aca | T | 0 | 168.20 |
| acg | T | 0 | 31.92 |
| --- | -- | ------ | ------ |
| mPD | | 0.0052 | 0.69 |
| | | nPD: | 0.01 |
| | | N. weight: | 1.5 |
| | | Sc. PD: | -0.23 |
| | | Sc. rank: | -235.2 |

| | | | |
|---|---|---|---|
| M1 | | | |
| Pos. | 59 | obs: | exp: |
| aaa | K | 12 | 215.00 |
| aag | K | 372 | 169.00 |
| --- | -- | ------ | ------ |
| mPD | | 0.061 | 0.49 |
| | | nPD: | 0.12 |
| | | N. weight: | 0.78 |
| | | Sc. PD: | -0.032 |
| | | Sc. rank: | 54.3 |

| | | | |
|---|---|---|---|
| M1 | | | |
| Pos. | 60 | obs: | exp: |
| ggt | G | 0 | 47.38 |
| ggc | G | 0 | 51.93 |
| gga | G | 3 | 185.80 |
| ggg | G | 381 | 98.93 |
| --- | -- | ------ | ------ |
| mPD | | 0.016 | 0.67 |
| | | nPD: | 0.02 |
| | | N. weight: | 1.5 |
| | | Sc. PD: | -0.2 |
| | | Sc. rank: | -111.7 |

| | | | |
|---|---|---|---|
| M1 | | | |
| Pos. | 61 | obs: | exp: |
| att | I | 384 | 144.40 |
| atc | I | 0 | 100.60 |
| ata | I | 0 | 139.00 |
| --- | -- | ------ | ------ |
| mPD | | 0 | 0.66 |
| | | nPD: | 0. |
| | | N. weight: | 1.1 |
| | | Sc. PD: | -0.18 |
| | | Sc. rank: | -314.6 |

| | | | |
|---|---|---|---|
| M1 | | | |
| Pos. | 62 | obs: | exp: |
| tta | L | 337 | 33.55 |
| ttg | L | 44 | 73.96 |
| ctt | L | 0 | 71.65 |
| ctc | L | 0 | 62.12 |
| cta | L | 3 | 57.85 |
| ctg | L | 0 | 84.88 |
| --- | -- | ------ | ------ |
| mPD | | 0.22 | 1.1 |
| | | nPD: | 0.2 |
| | | N. weight: | 2.2 |
| | | Sc. PD: | 0.061 |
| | | Sc. rank: | 242.0 |

| | | | |
|---|---|---|---|
| M1 | | | |
| Pos. | 63 | obs: | exp: |
| ggt | G | 17 | 47.38 |
| ggc | G | 0 | 51.93 |
| gga | G | 131 | 185.80 |
| ggg | G | 236 | 98.93 |
| --- | -- | ------ | ------ |
| mPD | | 0.51 | 0.67 |
| | | nPD: | 0.76 |
| | | N. weight: | 0.44 |
| | | Sc. PD: | 0.24 |
| | | Sc. rank: | 127.1 |

| | | | |
|---|---|---|---|
| M1 | | | |
| Pos. | 64 | obs: | exp: |
| ttt | F | 381 | 157.90 |
| ttc | F | 3 | 226.10 |
| --- | -- | ------ | ------ |
| mPD | | 0.016 | 0.48 |
| | | nPD: | 0.03 |
| | | N. weight: | 0.96 |
| | | Sc. PD: | -0.12 |
| | | Sc. rank: | -38.5 |

| | | | |
|---|---|---|---|
| M1 | | | |
| Pos. | 65 | obs: | exp: |
| gtt | V | 0 | 78.93 |
| gtc | V | 1 | 80.59 |
| gta | V | 7 | 71.97 |
| gtg | V | 376 | 152.50 |
| --- | -- | ------ | ------ |
| mPD | | 0.041 | 0.72 |
| | | nPD: | 0.06 |
| | | N. weight: | 0.95 |
| | | Sc. PD: | -0.099 |
| | | Sc. rank: | 10.3 |

| | | | |
|---|---|---|---|
| M1 | | | |
| Pos. | 66 | obs: | exp: |
| ttt | F | 12 | 157.90 |
| ttc | F | 372 | 226.10 |
| --- | -- | ------ | ------ |
| mPD | | 0.061 | 0.48 |
| | | nPD: | 0.13 |
| | | N. weight: | 0.46 |
| | | Sc. PD: | -0.018 |
| | | Sc. rank: | 34.7 |

| | | | |
|---|---|---|---|
| M1 | | | |
| Pos. | 67 | obs: | exp: |
| act | T | 0 | 101.40 |
| acc | T | 0 | 82.40 |
| aca | T | 1 | 168.20 |
| acg | T | 383 | 31.92 |
| --- | -- | ------ | ------ |
| mPD | | 0.0052 | 0.69 |
| | | nPD: | 0.01 |
| | | N. weight: | 2.8 |
| | | Sc. PD: | -0.42 |
| | | Sc. rank: | -441.0 |

| | | | |
|---|---|---|---|
| M1 | | | |
| Pos. | 68 | obs: | exp: |
| tta | L | 0 | 33.55 |
| ttg | L | 0 | 73.96 |
| ctt | L | 0 | 71.65 |
| ctc | L | 384 | 62.12 |
| cta | L | 0 | 57.85 |
| ctg | L | 0 | 84.88 |
| --- | -- | ------ | ------ |
| mPD | | 0 | 1.1 |
| | | nPD: | 0. |
| | | N. weight: | 2.1 |
| | | Sc. PD: | -0.33 |
| | | Sc. rank: | -585.8 |

| | | | |
|---|---|---|---|
| M1 | | | |
| Pos. | 69 | obs: | exp: |
| act | T | 0 | 101.40 |
| acc | T | 384 | 82.40 |
| aca | T | 0 | 168.20 |
| acg | T | 0 | 31.92 |
| --- | -- | ------ | ------ |
| mPD | | 0 | 0.69 |
| | | nPD: | 0. |
| | | N. weight: | 1.8 |
| | | Sc. PD: | -0.28 |
| | | Sc. rank: | -494.9 |

| | | | |
|---|---|---|---|
| M1 | | | |
| Pos. | 70 | obs: | exp: |
| gtt | V | 0 | 78.93 |
| gtc | V | 0 | 80.59 |
| gta | V | 0 | 71.97 |
| gtg | V | 384 | 152.50 |
| --- | -- | ------ | ------ |
| mPD | | 0 | 0.72 |
| | | nPD: | 0. |
| | | N. weight: | 1.1 |
| | | Sc. PD: | -0.17 |
| | | Sc. rank: | -297.0 |

| | | | |
|---|---|---|---|
| M1 | | | |
| Pos. | 71 | obs: | exp: |
| cct | P | 0 | 100.90 |
| ccc | P | 383 | 69.08 |
| cca | P | 0 | 151.10 |
| ccg | P | 0 | 61.93 |
| gct | A | 0 | 0.25 |
| gcc | A | 1 | 0.20 |
| gca | A | 0 | 0.45 |
| gcg | A | 0 | 0.10 |
| --- | -- | ------ | ------ |
| mPD | | 0.0052 | 0.72 |
| | | nPD: | 0.01 |
| | | N. weight: | 2. |
| | | Sc. PD: | -0.29 |
| | | Sc. rank: | -332.4 |

| | | | |
|---|---|---|---|
| M1 | | | |
| Pos. | 72 | obs: | exp: |
| tct | S | 0 | 60.41 |
| tcc | S | 0 | 52.94 |
| tca | S | 0 | 84.19 |
| tcg | S | 0 | 23.16 |
| aat | N | 4 | 2.09 |
| aac | N | 0 | 1.91 |
| agt | S | 379 | 83.60 |
| agc | S | 1 | 75.71 |
| --- | -- | ------ | ------ |
| mPD | | 0.026 | 1.7 |
| | | nPD: | 0.02 |
| | | N. weight: | 1.7 |
| | | Sc. PD: | -0.24 |
| | | Sc. rank: | -198.8 |

| | | | |
|---|---|---|---|
| M1 | | | |
| Pos. | 73 | obs: | exp: |
| gaa | E | 0 | 221.00 |
| gag | E | 384 | 163.00 |
| --- | -- | ------ | ------ |
| mPD | | 0 | 0.49 |
| | | nPD: | 0. |
| | | N. weight: | 0.98 |
| | | Sc. PD: | -0.15 |
| | | Sc. rank: | -275.5 |

| | | | |
|---|---|---|---|
| M1 | | | |
| Pos. | 74 | obs: | exp: |
| cgt | R | 0 | 15.94 |
| cgc | R | 0 | 18.80 |
| cga | R | 379 | 36.90 |
| cgg | R | 5 | 32.04 |
| aga | R | 0 | 172.70 |
| agg | R | 0 | 107.60 |
| --- | -- | ------ | ------ |
| mPD | | 0.026 | 0.96 |
| | | nPD: | 0.03 |
| | | N. weight: | 2.6 |
| | | Sc. PD: | -0.34 |
| | | Sc. rank: | -165.9 |

| | | | |
|---|---|---|---|
| M1 | | | |
| Pos. | 75 | obs: | exp: |
| ggt | G | 0 | 47.38 |
| ggc | G | 0 | 51.93 |
| gga | G | 384 | 185.80 |
| ggg | G | 0 | 98.93 |
| --- | -- | ------ | ------ |
| mPD | | 0 | 0.67 |
| | | nPD: | 0. |
| | | N. weight: | 0.83 |
| | | Sc. PD: | -0.13 |
| | | Sc. rank: | -233.5 |

| | | | |
|---|---|---|---|
| M1 | | | |
| Pos. | 76 | obs: | exp: |
| tta | L | 0 | 33.55 |
| ttg | L | 0 | 73.96 |
| ctt | L | 0 | 71.65 |
| ctc | L | 0 | 62.12 |
| cta | L | 0 | 57.85 |
| ctg | L | 384 | 84.88 |
| --- | -- | ------ | ------ |
| mPD | | 0 | 1.1 |
| | | nPD: | 0. |
| | | N. weight: | 1.7 |
| | | Sc. PD: | -0.27 |
| | | Sc. rank: | -485.4 |

| | | | |
|---|---|---|---|
| M1 | | | |
| Pos. | 77 | obs: | exp: |
| tta | L | 0 | 0.09 |
| ttg | L | 0 | 0.19 |
| ctt | L | 0 | 0.19 |
| ctc | L | 0 | 0.16 |
| cta | L | 0 | 0.15 |
| ctg | L | 1 | 0.22 |
| caa | Q | 28 | 204.20 |
| cag | Q | 355 | 178.80 |
| --- | -- | ------ | ------ |
| mPD | | 0.14 | 0.51 |
| | | nPD: | 0.28 |
| | | N. weight: | 0.57 |
| | | Sc. PD: | 0.059 |
| | | Sc. rank: | 86.4 |

| | | | |
|---|---|---|---|
| M1 | | | |
| Pos. | 78 | obs: | exp: |
| cgt | R | 384 | 15.94 |
| cgc | R | 0 | 18.80 |
| cga | R | 0 | 36.90 |
| cgg | R | 0 | 32.04 |
| aga | R | 0 | 172.70 |
| agg | R | 0 | 107.60 |
| --- | -- | ------ | ------ |
| mPD | | 0 | 0.96 |
| | | nPD: | 0. |
| | | N. weight: | 3.6 |
| | | Sc. PD: | -0.57 |
| | | Sc. rank: | -1023.2 |

| | | | |
|---|---|---|---|
| M1 | | | |
| Pos. | 79 | obs: | exp: |
| cgt | R | 0 | 15.94 |
| cgc | R | 0 | 18.80 |
| cga | R | 0 | 36.90 |
| cgg | R | 0 | 32.04 |
| aga | R | 369 | 172.70 |
| agg | R | 15 | 107.60 |
| --- | -- | ------ | ------ |
| mPD | | 0.075 | 0.96 |
| | | nPD: | 0.08 |
| | | N. weight: | 0.75 |
| | | Sc. PD: | -0.063 |
| | | Sc. rank: | 21.0 |

| | | | |
|---|---|---|---|
| M1 | | | |
| Pos. | 80 | obs: | exp: |
| cgt | R | 55 | 15.94 |
| cgc | R | 323 | 18.80 |
| cga | R | 5 | 36.90 |
| cgg | R | 1 | 32.04 |
| aga | R | 0 | 172.70 |
| agg | R | 0 | 107.60 |
| --- | -- | ------ | ------ |
| mPD | | 0.27 | 0.96 |
| | | nPD: | 0.28 |
| | | N. weight: | 2.9 |
| | | Sc. PD: | 0.32 |
| | | Sc. rank: | 471.5 |

| M1 | | | |
|---|---|---|---|
| Pos. | 81 | obs: | exp: |
| ttt | F | 375 | 157.90 |
| ttc | F | 9 | 226.10 |
| --- | -- | ------ | ------ |
| mPD | | 0.046 | 0.48 |
| | | nPD: | 0.09 |
| | | N. weight: | 0.88 |
| | | Sc. PD: | -0.06 |
| | | Sc. rank: | 42.8 |

| M1 | | | |
|---|---|---|---|
| Pos. | 82 | obs: | exp: |
| gtt | V | 22 | 78.93 |
| gtc | V | 362 | 80.59 |
| gta | V | 0 | 71.97 |
| gtg | V | 0 | 152.50 |
| --- | -- | ------ | ------ |
| mPD | | 0.11 | 0.72 |
| | | nPD: | 0.15 |
| | | N. weight: | 1.5 |
| | | Sc. PD: | -0.025 |
| | | Sc. rank: | 126.7 |

| M1 | | | |
|---|---|---|---|
| Pos. | 83 | obs: | exp: |
| caa | Q | 369 | 204.70 |
| cag | Q | 15 | 179.30 |
| --- | -- | ------ | ------ |
| mPD | | 0.075 | 0.50 |
| | | nPD: | 0.15 |
| | | N. weight: | 0.54 |
| | | Sc. PD: | -0.0083 |
| | | Sc. rank: | 45.3 |

| M1 | | | |
|---|---|---|---|
| Pos. | 84 | obs: | exp: |
| aat | N | 350 | 200.70 |
| aac | N | 34 | 183.30 |
| --- | -- | ------ | ------ |
| mPD | | 0.16 | 0.50 |
| | | nPD: | 0.32 |
| | | N. weight: | 0.42 |
| | | Sc. PD: | 0.061 |
| | | Sc. rank: | 78.5 |

| M1 | | | |
|---|---|---|---|
| Pos. | 85 | obs: | exp: |
| gct | A | 32 | 96.72 |
| gcc | A | 322 | 78.66 |
| gca | A | 30 | 171.10 |
| gcg | A | 0 | 37.47 |
| --- | -- | ------ | ------ |
| mPD | | 0.28 | 0.69 |
| | | nPD: | 0.41 |
| | | N. weight: | 1.1 |
| | | Sc. PD: | 0.26 |
| | | Sc. rank: | 237.0 |

| M1 | | | |
|---|---|---|---|
| Pos. | 86 | obs: | exp: |
| tta | L | 3 | 33.55 |
| ttg | L | 0 | 73.96 |
| ctt | L | 83 | 71.65 |
| ctc | L | 4 | 62.12 |
| cta | L | 286 | 57.85 |
| ctg | L | 8 | 84.88 |
| --- | -- | ------ | ------ |
| mPD | | 0.40 | 1.1 |
| | | nPD: | 0.36 |
| | | N. weight: | 1.3 |
| | | Sc. PD: | 0.24 |
| | | Sc. rank: | 263.2 |

| M1 | | | |
|---|---|---|---|
| Pos. | 87 | obs: | exp: |
| aat | N | 380 | 200.70 |
| aac | N | 4 | 183.30 |
| --- | -- | ------ | ------ |
| mPD | | 0.021 | 0.50 |
| | | nPD: | 0.04 |
| | | N. weight: | 0.68 |
| | | Sc. PD: | -0.08 |
| | | Sc. rank: | -12.3 |

| M1 | | | |
|---|---|---|---|
| Pos. | 88 | obs: | exp: |
| ggt | G | 0 | 47.38 |
| ggc | G | 0 | 51.93 |
| gga | G | 140 | 185.80 |
| ggg | G | 244 | 98.93 |
| --- | -- | ------ | ------ |
| mPD | | 0.46 | 0.67 |
| | | nPD: | 0.7 |
| | | N. weight: | 0.55 |
| | | Sc. PD: | 0.27 |
| | | Sc. rank: | 152.0 |

| M1 | | | |
|---|---|---|---|
| Pos. | 89 | obs: | exp: |
| aat | N | 361 | 200.70 |
| aac | N | 23 | 183.30 |
| --- | -- | ------ | ------ |
| mPD | | 0.11 | 0.50 |
| | | nPD: | 0.23 |
| | | N. weight: | 0.49 |
| | | Sc. PD: | 0.027 |
| | | Sc. rank: | 62.8 |

| M1 | | | |
|---|---|---|---|
| Pos. | 90 | obs: | exp: |
| ggt | G | 0 | 47.38 |
| ggc | G | 0 | 51.93 |
| gga | G | 291 | 185.80 |
| ggg | G | 93 | 98.93 |
| --- | -- | ------ | ------ |
| mPD | | 0.37 | 0.67 |
| | | nPD: | 0.55 |
| | | N. weight: | 0.38 |
| | | Sc. PD: | 0.14 |
| | | Sc. rank: | 97.1 |

| M1 | | | |
|---|---|---|---|
| Pos. | 91 | obs: | exp: |
| gat | D | 95 | 204.90 |
| gac | D | 289 | 179.10 |
| --- | -- | ------ | ------ |
| mPD | | 0.37 | 0.50 |
| | | nPD: | 0.75 |
| | | N. weight: | 0.2 |
| | | Sc. PD: | 0.11 |
| | | Sc. rank: | 58.3 |

| M1 | | | |
|---|---|---|---|
| Pos. | 92 | obs: | exp: |
| cct | P | 0 | 101.10 |
| ccc | P | 0 | 69.26 |
| cca | P | 369 | 151.50 |
| ccg | P | 15 | 62.09 |
| --- | -- | ------ | ------ |
| mPD | | 0.075 | 0.72 |
| | | nPD: | 0.11 |
| | | N. weight: | 0.92 |
| | | Sc. PD: | -0.054 |
| | | Sc. rank: | 53.4 |

| M1 | | | |
|---|---|---|---|
| Pos. | 93 | obs: | exp: |
| aat | N | 10 | 200.70 |
| aac | N | 374 | 183.30 |
| --- | -- | ------ | ------ |
| mPD | | 0.051 | 0.50 |
| | | nPD: | 0.1 |
| | | N. weight: | 0.71 |
| | | Sc. PD: | -0.044 |
| | | Sc. rank: | 35.5 |

| M1 | | | |
|---|---|---|---|
| Pos. | 94 | obs: | exp: |
| aat | N | 10 | 200.70 |
| aac | N | 374 | 183.30 |
| --- | -- | ------ | ------ |
| mPD | | 0.051 | 0.50 |
| | | nPD: | 0.1 |
| | | N. weight: | 0.71 |
| | | Sc. PD: | -0.044 |
| | | Sc. rank: | 35.5 |

| M1 | | | |
|---|---|---|---|
| Pos. | 95 | obs: | exp: |
| att | I | 0 | 0.75 |
| atc | I | 0 | 0.52 |
| ata | I | 2 | 0.72 |
| atg | M | 382 | 382.00 |
| --- | -- | ------ | ------ |
| mPD | | 0.010 | 0.010 |
| | | nPD: | 1. |
| | | N. weight: | 0.006 |
| | | Sc. PD: | 0.0047 |
| | | Sc. rank: | 1.8 |

| M1 | | | |
|---|---|---|---|
| Pos. | 96 | obs: | exp: |
| gat | D | 22 | 194.70 |
| gac | D | 343 | 170.30 |
| ggt | G | 0 | 2.34 |
| ggc | G | 19 | 2.57 |
| gga | G | 0 | 9.19 |
| ggg | G | 0 | 4.90 |
| --- | -- | ------ | ------ |
| mPD | | 0.20 | 0.63 |
| | | nPD: | 0.32 |
| | | N. weight: | 0.69 |
| | | Sc. PD: | 0.1 |
| | | Sc. rank: | 128.2 |

| M1 | | | |
|---|---|---|---|
| Pos. | 97 | obs: | exp: |
| cgt | R | 0 | 14.70 |
| cgc | R | 0 | 17.33 |
| cga | R | 0 | 34.02 |
| cgg | R | 5 | 29.54 |
| aaa | K | 0 | 16.80 |
| aag | K | 30 | 13.20 |
| aga | R | 104 | 159.20 |
| agg | R | 245 | 99.21 |
| --- | -- | ------ | ------ |
| mPD | | 0.57 | 1.1 |
| | | nPD: | 0.52 |
| | | N. weight: | 0.59 |
| | | Sc. PD: | 0.2 |
| | | Sc. rank: | 145.1 |

| M1 | | | |
|---|---|---|---|
| Pos. | 98 | obs: | exp: |
| gct | A | 0 | 96.72 |
| gcc | A | 2 | 78.66 |
| gca | A | 346 | 171.10 |
| gcg | A | 36 | 37.47 |
| --- | -- | ------ | ------ |
| mPD | | 0.18 | 0.69 |
| | | nPD: | 0.26 |
| | | N. weight: | 0.71 |
| | | Sc. PD: | 0.063 |
| | | Sc. rank: | 102.5 |

| M1 | | | |
|---|---|---|---|
| Pos. | 99 | obs: | exp: |
| gtt | V | 34 | 78.93 |
| gtc | V | 349 | 80.59 |
| gta | V | 0 | 71.97 |
| gtg | V | 1 | 152.50 |
| --- | -- | ------ | ------ |
| mPD | | 0.17 | 0.72 |
| | | nPD: | 0.23 |
| | | N. weight: | 1.4 |
| | | Sc. PD: | 0.085 |
| | | Sc. rank: | 190.9 |

| M1 | | | |
|---|---|---|---|
| Pos. | 100 | obs: | exp: |
| cgt | R | 0 | 0.04 |
| cgc | R | 0 | 0.05 |
| cga | R | 0 | 0.10 |
| cgg | R | 0 | 0.08 |
| aaa | K | 369 | 214.50 |
| aag | K | 14 | 168.50 |
| aga | R | 1 | 0.45 |
| agg | R | 0 | 0.28 |
| --- | -- | ------ | ------ |
| mPD | | 0.076 | 0.50 |
| | | nPD: | 0.15 |
| | | N. weight: | 0.5 |
| | | Sc. PD: | -0.0075 |
| | | Sc. rank: | 42.4 |

| M1 | | | |
|---|---|---|---|
| Pos. | 101 | obs: | exp: |
| tta | L | 29 | 33.55 |
| ttg | L | 7 | 73.96 |
| ctt | L | 7 | 71.65 |
| ctc | L | 5 | 62.12 |
| cta | L | 56 | 57.85 |
| ctg | L | 280 | 84.88 |
| --- | -- | ------ | ------ |
| mPD | | 0.56 | 1.1 |
| | | nPD: | 0.51 |
| | | N. weight: | 0.87 |
| | | Sc. PD: | 0.28 |
| | | Sc. rank: | 210.0 |

| M1 | | | |
|---|---|---|---|
| Pos. | 102 | obs: | exp: |
| tat | Y | 5 | 198.80 |
| tac | Y | 379 | 185.20 |
| --- | -- | ------ | ------ |
| mPD | | 0.026 | 0.50 |
| | | nPD: | 0.05 |
| | | N. weight: | 0.76 |
| | | Sc. PD: | -0.082 |
| | | Sc. rank: | 2.5 |

| M1 | | | |
|---|---|---|---|
| Pos. | 103 | obs: | exp: |
| cgt | R | 0 | 13.95 |
| cgc | R | 0 | 16.45 |
| cga | R | 0 | 32.29 |
| cgg | R | 0 | 28.04 |
| aaa | K | 7 | 26.88 |
| aag | K | 41 | 21.12 |
| aga | R | 24 | 151.10 |
| agg | R | 312 | 94.16 |
| --- | -- | ------ | ------ |
| mPD | | 0.37 | 1.1 |
| | | nPD: | 0.32 |
| | | N. weight: | 1. |
| | | Sc. PD: | 0.15 |
| | | Sc. rank: | 196.2 |

| M1 | | | |
|---|---|---|---|
| Pos. | 104 | obs: | exp: |
| aaa | K | 126 | 215.00 |
| aag | K | 258 | 169.00 |
| --- | -- | ------ | ------ |
| mPD | | 0.44 | 0.49 |
| | | nPD: | 0.9 |
| | | N. weight: | 0.13 |
| | | Sc. PD: | 0.091 |
| | | Sc. rank: | 39.5 |

| M1 | | | |
|---|---|---|---|
| Pos. | 105 | obs: | exp: |
| tta | L | 10 | 33.55 |
| ttg | L | 190 | 73.96 |
| ctt | L | 32 | 71.65 |
| ctc | L | 50 | 62.12 |
| cta | L | 8 | 57.85 |
| ctg | L | 94 | 84.88 |
| --- | -- | ------ | ------ |
| mPD | | 0.93 | 1.1 |
| | | nPD: | 0.83 |
| | | N. weight: | 0.41 |
| | | Sc. PD: | 0.25 |
| | | Sc. rank: | 119.2 |

| M1 | | | |
|---|---|---|---|
| Pos. | 106 | obs: | exp: |
| aaa | K | 256 | 215.00 |
| aag | K | 128 | 169.00 |
| --- | -- | ------ | ------ |
| mPD | | 0.45 | 0.49 |
| | | nPD: | 0.9 |
| | | N. weight: | 0.036 |
| | | Sc. PD: | 0.025 |
| | | Sc. rank: | 10.7 |

| M1 | | | |
|---|---|---|---|
| Pos. | 107 | obs: | exp: |
| cgt | R | 0 | 15.90 |
| cgc | R | 0 | 18.75 |
| cga | R | 0 | 36.81 |
| cgg | R | 0 | 31.96 |
| aaa | K | 1 | 0.56 |
| aag | K | 0 | 0.44 |
| aga | R | 254 | 172.20 |
| agg | R | 129 | 107.30 |
| --- | -- | ------ | ------ |
| mPD | | 0.45 | 0.96 |
| | | nPD: | 0.47 |
| | | N. weight: | 0.37 |
| | | Sc. PD: | 0.11 |
| | | Sc. rank: | 88.9 |

| M1 | | | |
|---|---|---|---|
| Pos. | 108 | obs: | exp: |
| gaa | E | 131 | 221.00 |
| gag | E | 253 | 163.00 |
| --- | -- | ------ | ------ |
| mPD | | 0.45 | 0.49 |
| | | nPD: | 0.92 |
| | | N. weight: | 0.14 |
| | | Sc. PD: | 0.096 |
| | | Sc. rank: | 40.5 |

| M1 | | | |
|---|---|---|---|
| Pos. | 109 | obs: | exp: |
| tta | L | 0 | 0.09 |
| ttg | L | 0 | 0.19 |
| ctt | L | 0 | 0.19 |
| ctc | L | 0 | 0.16 |
| cta | L | 0 | 0.15 |
| ctg | L | 1 | 0.22 |
| att | I | 0 | 133.50 |
| atc | I | 16 | 93.04 |
| ata | I | 339 | 128.50 |
| atg | M | 28 | 28.00 |
| --- | -- | ------ | ------ |
| mPD | | 0.22 | 0.71 |
| | | nPD: | 0.31 |
| | | N. weight: | 0.9 |
| | | Sc. PD: | 0.12 |
| | | Sc. rank: | 161.4 |

| M1 | | | |
|---|---|---|---|
| Pos. | 110 | obs: | exp: |
| act | T | 5 | 101.40 |
| acc | T | 2 | 82.40 |
| aca | T | 377 | 168.20 |
| acg | T | 0 | 31.92 |
| --- | -- | ------ | ------ |
| mPD | | 0.036 | 0.69 |
| | | nPD: | 0.05 |
| | | N. weight: | 0.85 |
| | | Sc. PD: | -0.091 |
| | | Sc. rank: | 4.5 |

| | | | |
|---|---|---|---|
| M1 | | | |
| Pos. | 111 | obs: | exp: |
| ttt | F | 72 | 157.50 |
| ttc | F | 311 | 225.50 |
| tta | L | 0 | 0.09 |
| ttg | L | 0 | 0.19 |
| ctt | L | 0 | 0.19 |
| ctc | L | 1 | 0.16 |
| cta | L | 0 | 0.15 |
| ctg | L | 0 | 0.22 |
| --- | -- | ------ | ------ |
| mPD | | 0.31 | 0.49 |
| | | nPD: | 0.63 |
| | | N. weight: | 0.14 |
| | | Sc. PD: | 0.063 |
| | | Sc. rank: | 39.0 |

| | | | |
|---|---|---|---|
| M1 | | | |
| Pos. | 112 | obs: | exp: |
| cat | H | 381 | 208.10 |
| cac | H | 3 | 175.90 |
| --- | -- | ------ | ------ |
| mPD | | 0.016 | 0.50 |
| | | nPD: | 0.03 |
| | | N. weight: | 0.65 |
| | | Sc. PD: | -0.083 |
| | | Sc. rank: | -27.5 |

| | | | |
|---|---|---|---|
| M1 | | | |
| Pos. | 113 | obs: | exp: |
| ggt | G | 1 | 47.38 |
| ggc | G | 0 | 51.93 |
| gga | G | 36 | 185.80 |
| ggg | G | 347 | 98.93 |
| --- | -- | ------ | ------ |
| mPD | | 0.18 | 0.67 |
| | | nPD: | 0.26 |
| | | N. weight: | 1.1 |
| | | Sc. PD: | 0.1 |
| | | Sc. rank: | 164.3 |

| | | | |
|---|---|---|---|
| M1 | | | |
| Pos. | 114 | obs: | exp: |
| tct | S | 0 | 0.48 |
| tcc | S | 0 | 0.42 |
| tca | S | 3 | 0.66 |
| tcg | S | 0 | 0.18 |
| agt | S | 0 | 0.66 |
| agc | S | 0 | 0.60 |
| gct | A | 257 | 95.96 |
| gcc | A | 8 | 78.05 |
| gca | A | 112 | 169.80 |
| gcg | A | 4 | 37.18 |
| --- | -- | ------ | ------ |
| mPD | | 0.48 | 0.71 |
| | | nPD: | 0.67 |
| | | N. weight: | 0.57 |
| | | Sc. PD: | 0.27 |
| | | Sc. rank: | 154.3 |

| | | | |
|---|---|---|---|
| M1 | | | |
| Pos. | 115 | obs: | exp: |
| aaa | K | 269 | 215.00 |
| aag | K | 115 | 169.00 |
| --- | -- | ------ | ------ |
| mPD | | 0.42 | 0.49 |
| | | nPD: | 0.85 |
| | | N. weight: | 0.057 |
| | | Sc. PD: | 0.037 |
| | | Sc. rank: | 16.7 |

| | | | |
|---|---|---|---|
| M1 | | | |
| Pos. | 116 | obs: | exp: |
| gaa | E | 244 | 221.00 |
| gag | E | 140 | 163.00 |
| --- | -- | ------ | ------ |
| mPD | | 0.46 | 0.49 |
| | | nPD: | 0.95 |
| | | N. weight: | 0.018 |
| | | Sc. PD: | 0.013 |
| | | Sc. rank: | 5.4 |

| | | | |
|---|---|---|---|
| M1 | | | |
| Pos. | 117 | obs: | exp: |
| gtt | V | 291 | 78.93 |
| gtc | V | 5 | 80.59 |
| gta | V | 4 | 71.97 |
| gtg | V | 84 | 152.50 |
| --- | -- | ------ | ------ |
| mPD | | 0.38 | 0.72 |
| | | nPD: | 0.53 |
| | | N. weight: | 0.92 |
| | | Sc. PD: | 0.31 |
| | | Sc. rank: | 229.7 |

| | | | |
|---|---|---|---|
| M1 | | | |
| Pos. | 118 | obs: | exp: |
| tct | S | 0 | 0.16 |
| tcc | S | 0 | 0.14 |
| tca | S | 1 | 0.22 |
| tcg | S | 0 | 0.06 |
| agt | S | 0 | 0.22 |
| agc | S | 0 | 0.20 |
| gct | A | 0 | 96.47 |
| gcc | A | 0 | 78.46 |
| gca | A | 380 | 170.70 |
| gcg | A | 3 | 37.37 |
| --- | -- | ------ | ------ |
| mPD | | 0.021 | 0.69 |
| | | nPD: | 0.03 |
| | | N. weight: | 0.89 |
| | | Sc. PD: | -0.11 |
| | | Sc. rank: | -52.0 |

| | | | |
|---|---|---|---|
| M1 | | | |
| Pos. | 119 | obs: | exp: |
| tta | L | 0 | 33.55 |
| ttg | L | 0 | 73.96 |
| ctt | L | 28 | 71.65 |
| ctc | L | 356 | 62.12 |
| cta | L | 0 | 57.85 |
| ctg | L | 0 | 84.88 |
| --- | -- | ------ | ------ |
| mPD | | 0.14 | 1.1 |
| | | nPD: | 0.12 |
| | | N. weight: | 1.8 |
| | | Sc. PD: | -0.076 |
| | | Sc. rank: | 121.2 |

| | | | |
|---|---|---|---|
| M1 | | | |
| Pos. | 120 | obs: | exp: |
| tct | S | 0 | 61.05 |
| tcc | S | 0 | 53.49 |
| tca | S | 0 | 85.07 |
| tcg | S | 0 | 23.41 |
| agt | S | 266 | 84.48 |
| agc | S | 118 | 76.50 |
| --- | -- | ------ | ------ |
| mPD | | 0.43 | 1.7 |
| | | nPD: | 0.26 |
| | | N. weight: | 1.1 |
| | | Sc. PD: | 0.089 |
| | | Sc. rank: | 152.5 |

| | | | |
|---|---|---|---|
| M1 | | | |
| Pos. | 121 | obs: | exp: |
| tat | Y | 90 | 198.80 |
| tac | Y | 294 | 185.20 |
| --- | -- | ------ | ------ |
| mPD | | 0.36 | 0.50 |
| | | nPD: | 0.72 |
| | | N. weight: | 0.2 |
| | | Sc. PD: | 0.1 |
| | | Sc. rank: | 56.3 |

| | | | |
|---|---|---|---|
| M1 | | | |
| Pos. | 122 | obs: | exp: |
| tct | S | 9 | 61.05 |
| tcc | S | 30 | 53.49 |
| tca | S | 325 | 85.07 |
| tcg | S | 20 | 23.41 |
| agt | S | 0 | 84.48 |
| agc | S | 0 | 76.50 |
| --- | -- | ------ | ------ |
| mPD | | 0.28 | 1.7 |
| | | nPD: | 0.17 |
| | | N. weight: | 1.2 |
| | | Sc. PD: | -0.0024 |
| | | Sc. rank: | 106.4 |

| | | | |
|---|---|---|---|
| M1 | | | |
| Pos. | 123 | obs: | exp: |
| act | T | 154 | 101.40 |
| acc | T | 230 | 82.40 |
| aca | T | 0 | 168.20 |
| acg | T | 0 | 31.92 |
| --- | -- | ------ | ------ |
| mPD | | 0.48 | 0.69 |
| | | nPD: | 0.7 |
| | | N. weight: | 0.9 |
| | | Sc. PD: | 0.45 |
| | | Sc. rank: | 252.8 |

| | | | |
|---|---|---|---|
| M1 | | | |
| Pos. | 124 | obs: | exp: |
| ggt | G | 383 | 47.38 |
| ggc | G | 1 | 51.93 |
| gga | G | 0 | 185.80 |
| ggg | G | 0 | 98.93 |
| --- | -- | ------ | ------ |
| mPD | | 0.0052 | 0.67 |
| | | nPD: | 0.01 |
| | | N. weight: | 2.4 |
| | | Sc. PD: | -0.35 |
| | | Sc. rank: | -346.6 |

| | | | |
|---|---|---|---|
| M1 | | | |
| Pos. | 125 | obs: | exp: |
| gct | A | 2 | 96.72 |
| gcc | A | 1 | 78.66 |
| gca | A | 344 | 171.10 |
| gcg | A | 37 | 37.47 |
| --- | -- | ------ | ------ |
| mPD | | 0.19 | 0.69 |
| | | nPD: | 0.27 |
| | | N. weight: | 0.69 |
| | | Sc. PD: | 0.07 |
| | | Sc. rank: | 103.8 |

| | | | |
|---|---|---|---|
| M1 | | | |
| Pos. | 126 | obs: | exp: |
| tta | L | 0 | 33.55 |
| ttg | L | 0 | 73.96 |
| ctt | L | 382 | 71.65 |
| ctc | L | 0 | 62.12 |
| cta | L | 0 | 57.85 |
| ctg | L | 2 | 84.88 |
| --- | -- | ------ | ------ |
| mPD | | 0.010 | 1.1 |
| | | nPD: | 0.01 |
| | | N. weight: | 1.9 |
| | | Sc. PD: | -0.28 |
| | | Sc. rank: | -260.2 |

| | | | |
|---|---|---|---|
| M1 | | | |
| Pos. | 127 | obs: | exp: |
| gct | A | 1 | 96.72 |
| gcc | A | 379 | 78.66 |
| gca | A | 4 | 171.10 |
| gcg | A | 0 | 37.47 |
| --- | -- | ------ | ------ |
| mPD | | 0.026 | 0.69 |
| | | nPD: | 0.04 |
| | | N. weight: | 1.7 |
| | | Sc. PD: | -0.21 |
| | | Sc. rank: | -49.1 |

| | | | |
|---|---|---|---|
| M1 | | | |
| Pos. | 128 | obs: | exp: |
| tct | S | 0 | 61.05 |
| tcc | S | 0 | 53.49 |
| tca | S | 0 | 85.07 |
| tcg | S | 0 | 23.41 |
| agt | S | 272 | 84.48 |
| agc | S | 112 | 76.50 |
| --- | -- | ------ | ------ |
| mPD | | 0.41 | 1.7 |
| | | nPD: | 0.25 |
| | | N. weight: | 1.1 |
| | | Sc. PD: | 0.083 |
| | | Sc. rank: | 151.8 |

| | | | |
|---|---|---|---|
| M1 | | | |
| Pos. | 129 | obs: | exp: |
| tgt | C | 222 | 158.30 |
| tgc | C | 162 | 225.70 |
| --- | -- | ------ | ------ |
| mPD | | 0.49 | 0.48 |
| | | nPD: | 1.01 |
| | | N. weight: | 0.073 |
| | | Sc. PD: | 0.058 |
| | | Sc. rank: | 21.8 |

| | | | |
|---|---|---|---|
| M1 | | | |
| Pos. | 130 | obs: | exp: |
| atg | M | 384 | 384.00 |
| --- | -- | ------ | ------ |
| mPD | | 0 | 0 |
| | | nPD: | 1. |
| | | N. weight: | 0. |
| | | Sc. PD: | 0 |
| | | Sc. rank: | 0 |

| | | | |
|---|---|---|---|
| M1 | | | |
| Pos. | 131 | obs: | exp: |
| ggt | G | 301 | 47.38 |
| ggc | G | 83 | 51.93 |
| gga | G | 0 | 185.80 |
| ggg | G | 0 | 98.93 |
| --- | -- | ------ | ------ |
| mPD | | 0.34 | 0.67 |
| | | nPD: | 0.51 |
| | | N. weight: | 1.8 |
| | | Sc. PD: | 0.57 |
| | | Sc. rank: | 436.6 |

| | | | |
|---|---|---|---|
| M1 | | | |
| Pos. | 132 | obs: | exp: |
| tta | L | 0 | 33.55 |
| ttg | L | 0 | 73.96 |
| ctt | L | 13 | 71.65 |
| ctc | L | 371 | 62.12 |
| cta | L | 0 | 57.85 |
| ctg | L | 0 | 84.88 |
| --- | -- | ------ | ------ |
| mPD | | 0.066 | 1.1 |
| | | nPD: | 0.06 |
| | | N. weight: | 1.9 |
| | | Sc. PD: | -0.19 |
| | | Sc. rank: | 28.9 |

| | | | |
|---|---|---|---|
| M1 | | | |
| Pos. | 133 | obs: | exp: |
| att | I | 3 | 144.40 |
| atc | I | 0 | 100.60 |
| ata | I | 381 | 139.00 |
| --- | -- | ------ | ------ |
| mPD | | 0.016 | 0.66 |
| | | nPD: | 0.02 |
| | | N. weight: | 1.1 |
| | | Sc. PD: | -0.15 |
| | | Sc. rank: | -80.3 |

| | | | |
|---|---|---|---|
| M1 | | | |
| Pos. | 134 | obs: | exp: |
| tat | Y | 4 | 198.80 |
| tac | Y | 380 | 185.20 |
| --- | -- | ------ | ------ |
| mPD | | 0.021 | 0.50 |
| | | nPD: | 0.04 |
| | | N. weight: | 0.77 |
| | | Sc. PD: | -0.091 |
| | | Sc. rank: | -15.1 |

| | | | |
|---|---|---|---|
| M1 | | | |
| Pos. | 135 | obs: | exp: |
| aat | N | 3 | 200.70 |
| aac | N | 381 | 183.30 |
| --- | -- | ------ | ------ |
| mPD | | 0.016 | 0.50 |
| | | nPD: | 0.03 |
| | | N. weight: | 0.79 |
| | | Sc. PD: | -0.1 |
| | | Sc. rank: | -35.9 |

| | | | |
|---|---|---|---|
| M1 | | | |
| Pos. | 136 | obs: | exp: |
| cgt | R | 0 | 15.94 |
| cgc | R | 0 | 18.80 |
| cga | R | 0 | 36.90 |
| cgg | R | 1 | 32.04 |
| aga | R | 112 | 172.70 |
| agg | R | 271 | 107.60 |
| --- | -- | ------ | ------ |
| mPD | | 0.42 | 0.96 |
| | | nPD: | 0.44 |
| | | N. weight: | 0.6 |
| | | Sc. PD: | 0.15 |
| | | Sc. rank: | 138.1 |

| | | | |
|---|---|---|---|
| M1 | | | |
| Pos. | 137 | obs: | exp: |
| atg | M | 384 | 384.00 |
| --- | -- | ------ | ------ |
| mPD | | 0 | 0 |
| | | nPD: | 1. |
| | | N. weight: | 0. |
| | | Sc. PD: | 0 |
| | | Sc. rank: | 0 |

| | | | |
|---|---|---|---|
| M1 | | | |
| Pos. | 138 | obs: | exp: |
| ggt | G | 0 | 47.38 |
| ggc | G | 0 | 51.93 |
| gga | G | 186 | 185.80 |
| ggg | G | 198 | 98.93 |
| --- | -- | ------ | ------ |
| mPD | | 0.50 | 0.67 |
| | | nPD: | 0.75 |
| | | N. weight: | 0.42 |
| | | Sc. PD: | 0.23 |
| | | Sc. rank: | 120.5 |

| | | | |
|---|---|---|---|
| M1 | | | |
| Pos. | 139 | obs: | exp: |
| act | T | 84 | 101.40 |
| acc | T | 0 | 82.40 |
| aca | T | 125 | 168.20 |
| acg | T | 175 | 31.92 |
| --- | -- | ------ | ------ |
| mPD | | 0.64 | 0.69 |
| | | nPD: | 0.93 |
| | | N. weight: | 0.74 |
| | | Sc. PD: | 0.54 |
| | | Sc. rank: | 222.7 |

| | | | |
|---|---|---|---|
| M1 | | | |
| Pos. | 140 | obs: | exp: |
| gtt | V | 0 | 78.93 |
| gtc | V | 0 | 80.59 |
| gta | V | 70 | 71.97 |
| gtg | V | 314 | 152.50 |
| --- | -- | ------ | ------ |
| mPD | | 0.30 | 0.72 |
| | | nPD: | 0.41 |
| | | N. weight: | 0.68 |
| | | Sc. PD: | 0.16 |
| | | Sc. rank: | 146.4 |

M1

| Pos. | 141 | obs: | exp: |
|---|---|---|---|
| act | T | 3 | 101.40 |
| acc | T | 379 | 82.40 |
| aca | T | 2 | 168.20 |
| acg | T | 0 | 31.92 |
| mPD | | 0.026 | 0.69 |
| | | nPD: | 0.04 |
| | | N. weight: | 1.7 |
| | | Sc. PD: | -0.2 |
| | | Sc. rank: | -41.3 |

M1

| Pos. | 142 | obs: | exp: |
|---|---|---|---|
| act | T | 91 | 93.52 |
| acc | T | 3 | 75.96 |
| aca | T | 260 | 155.10 |
| acg | T | 0 | 29.43 |
| gct | A | 1 | 7.56 |
| gcc | A | 0 | 6.15 |
| gca | A | 29 | 13.37 |
| gcg | A | 0 | 2.93 |
| mPD | | 0.52 | 0.83 |
| | | nPD: | 0.63 |
| | | N. weight: | 0.44 |
| | | Sc. PD: | 0.19 |
| | | Sc. rank: | 119.2 |

M1

| Pos. | 143 | obs: | exp: |
|---|---|---|---|
| gaa | E | 369 | 221.00 |
| gag | E | 15 | 163.00 |
| mPD | | 0.075 | 0.49 |
| | | nPD: | 0.15 |
| | | N. weight: | 0.46 |
| | | Sc. PD: | -0.0058 |
| | | Sc. rank: | 39.7 |

M1

| Pos. | 144 | obs: | exp: |
|---|---|---|---|
| gtt | V | 0 | 72.56 |
| gtc | V | 0 | 74.09 |
| gta | V | 3 | 66.16 |
| gtg | V | 350 | 140.20 |
| gct | A | 0 | 0.50 |
| gcc | A | 0 | 0.41 |
| gca | A | 0 | 0.89 |
| gcg | A | 2 | 0.20 |
| ggt | G | 0 | 3.58 |
| ggc | G | 0 | 3.92 |
| gga | G | 0 | 14.03 |
| ggg | G | 29 | 7.47 |
| mPD | | 0.17 | 0.87 |
| | | nPD: | 0.19 |
| | | N. weight: | 1.1 |
| | | Sc. PD: | 0.021 |
| | | Sc. rank: | 109.6 |

M1

| Pos. | 145 | obs: | exp: |
|---|---|---|---|
| act | T | 0 | 0.26 |
| acc | T | 0 | 0.21 |
| aca | T | 1 | 0.44 |
| acg | T | 0 | 0.08 |
| gct | A | 228 | 96.47 |
| gcc | A | 2 | 78.46 |
| gca | A | 107 | 170.70 |
| gcg | A | 46 | 37.37 |
| mPD | | 0.56 | 0.69 |
| | | nPD: | 0.81 |
| | | N. weight: | 0.46 |
| | | Sc. PD: | 0.28 |
| | | Sc. rank: | 135.2 |

M1

| Pos. | 146 | obs: | exp: |
|---|---|---|---|
| ttt | F | 327 | 144.70 |
| ttc | F | 25 | 207.30 |
| tta | L | 2 | 2.80 |
| ttg | L | 1 | 6.16 |
| ctt | L | 29 | 5.97 |
| ctc | L | 0 | 5.18 |
| cta | L | 0 | 4.82 |
| ctg | L | 0 | 7.07 |
| mPD | | 0.28 | 0.65 |
| | | nPD: | 0.42 |
| | | N. weight: | 0.78 |
| | | Sc. PD: | 0.19 |
| | | Sc. rank: | 176.7 |

M1

| Pos. | 147 | obs: | exp: |
|---|---|---|---|
| ggt | G | 11 | 47.38 |
| ggc | G | 342 | 51.93 |
| gga | G | 30 | 185.80 |
| ggg | G | 1 | 98.93 |
| mPD | | 0.20 | 0.67 |
| | | nPD: | 0.3 |
| | | N. weight: | 1.7 |
| | | Sc. PD: | 0.21 |
| | | Sc. rank: | 292.4 |

M1

| Pos. | 148 | obs: | exp: |
|---|---|---|---|
| tta | L | 1 | 33.55 |
| ttg | L | 2 | 73.96 |
| ctt | L | 0 | 71.65 |
| ctc | L | 0 | 62.12 |
| cta | L | 294 | 57.85 |
| ctg | L | 87 | 84.88 |
| mPD | | 0.37 | 1.1 |
| | | nPD: | 0.33 |
| | | N. weight: | 1.4 |
| | | Sc. PD: | 0.22 |
| | | Sc. rank: | 273.5 |

M1

| Pos. | 149 | obs: | exp: |
|---|---|---|---|
| gtt | V | 0 | 78.93 |
| gtc | V | 0 | 80.59 |
| gta | V | 37 | 71.97 |
| gtg | V | 347 | 152.50 |
| mPD | | 0.17 | 0.72 |
| | | nPD: | 0.24 |
| | | N. weight: | 0.78 |
| | | Sc. PD: | 0.055 |
| | | Sc. rank: | 107.9 |

M1

| Pos. | 150 | obs: | exp: |
|---|---|---|---|
| tct | S | 1 | 0.16 |
| tcc | S | 0 | 0.14 |
| tca | S | 0 | 0.22 |
| tcg | S | 0 | 0.06 |
| tgt | C | 357 | 157.90 |
| tgc | C | 26 | 225.10 |
| agt | S | 0 | 0.22 |
| agc | S | 0 | 0.20 |
| mPD | | 0.13 | 0.49 |
| | | nPD: | 0.27 |
| | | N. weight: | 0.71 |
| | | Sc. PD: | 0.068 |
| | | Sc. rank: | 105.8 |

M1

| Pos. | 151 | obs: | exp: |
|---|---|---|---|
| gct | A | 3 | 96.72 |
| gcc | A | 381 | 78.66 |
| gca | A | 0 | 171.10 |
| gcg | A | 0 | 37.47 |
| mPD | | 0.016 | 0.69 |
| | | nPD: | 0.02 |
| | | N. weight: | 1.8 |
| | | Sc. PD: | -0.24 |
| | | Sc. rank: | -152.5 |

M1

| Pos. | 152 | obs: | exp: |
|---|---|---|---|
| act | T | 100 | 101.40 |
| acc | T | 201 | 82.40 |
| aca | T | 8 | 168.20 |
| acg | T | 75 | 31.92 |
| mPD | | 0.62 | 0.69 |
| | | nPD: | 0.91 |
| | | N. weight: | 0.67 |
| | | Sc. PD: | 0.46 |
| | | Sc. rank: | 198.8 |

M1

| Pos. | 153 | obs: | exp: |
|---|---|---|---|
| tgt | C | 367 | 158.30 |
| tgc | C | 17 | 225.70 |
| mPD | | 0.085 | 0.48 |
| | | nPD: | 0.18 |
| | | N. weight: | 0.79 |
| | | Sc. PD: | 0.0057 |
| | | Sc. rank: | 74.0 |

M1

| Pos. | 154 | obs: | exp: |
|---|---|---|---|
| gaa | E | 6 | 221.00 |
| gag | E | 378 | 163.00 |
| mPD | | 0.031 | 0.49 |
| | | nPD: | 0.06 |
| | | N. weight: | 0.88 |
| | | Sc. PD: | -0.087 |
| | | Sc. rank: | 18.0 |

M1

| Pos. | 155 | obs: | exp: |
|---|---|---|---|
| caa | Q | 34 | 204.70 |
| cag | Q | 350 | 179.30 |
| mPD | | 0.16 | 0.50 |
| | | nPD: | 0.33 |
| | | N. weight: | 0.52 |
| | | Sc. PD: | 0.077 |
| | | Sc. rank: | 99.2 |

M1

| Pos. | 156 | obs: | exp: |
|---|---|---|---|
| att | I | 381 | 144.40 |
| atc | I | 3 | 100.60 |
| ata | I | 0 | 139.00 |
| mPD | | 0.016 | 0.66 |
| | | nPD: | 0.02 |
| | | N. weight: | 1.1 |
| | | Sc. PD: | -0.14 |
| | | Sc. rank: | -77.4 |

M1

| Pos. | 157 | obs: | exp: |
|---|---|---|---|
| gct | A | 384 | 96.72 |
| gcc | A | 0 | 78.66 |
| gca | A | 0 | 171.10 |
| gcg | A | 0 | 37.47 |
| mPD | | 0 | 0.69 |
| | | nPD: | 0. |
| | | N. weight: | 1.6 |
| | | Sc. PD: | -0.25 |
| | | Sc. rank: | -443.4 |

M1

| Pos. | 158 | obs: | exp: |
|---|---|---|---|
| gat | D | 301 | 204.90 |
| gac | D | 83 | 179.10 |
| mPD | | 0.34 | 0.50 |
| | | nPD: | 0.68 |
| | | N. weight: | 0.16 |
| | | Sc. PD: | 0.079 |
| | | Sc. rank: | 45.0 |

M1

| Pos. | 159 | obs: | exp: |
|---|---|---|---|
| tct | S | 2 | 56.44 |
| tcc | S | 84 | 49.45 |
| tca | S | 252 | 78.65 |
| tcg | S | 17 | 21.64 |
| agt | S | 0 | 78.10 |
| agc | S | 0 | 70.73 |
| gct | A | 0 | 7.30 |
| gcc | A | 0 | 5.94 |
| gca | A | 29 | 12.93 |
| gcg | A | 0 | 2.83 |
| mPD | | 0.56 | 1.7 |
| | | nPD: | 0.32 |
| | | N. weight: | 1.1 |
| | | Sc. PD: | 0.15 |
| | | Sc. rank: | 195.1 |

M1

| Pos. | 160 | obs: | exp: |
|---|---|---|---|
| caa | Q | 47 | 204.70 |
| cag | Q | 337 | 179.30 |
| mPD | | 0.22 | 0.50 |
| | | nPD: | 0.43 |
| | | N. weight: | 0.43 |
| | | Sc. PD: | 0.11 |
| | | Sc. rank: | 99.2 |

M1

| Pos. | 161 | obs: | exp: |
|---|---|---|---|
| cat | H | 331 | 208.10 |
| cac | H | 53 | 175.90 |
| mPD | | 0.24 | 0.50 |
| | | nPD: | 0.48 |
| | | N. weight: | 0.28 |
| | | Sc. PD: | 0.081 |
| | | Sc. rank: | 65.7 |

M1

| Pos. | 162 | obs: | exp: |
|---|---|---|---|
| cgt | R | 0 | 15.94 |
| cgc | R | 0 | 18.80 |
| cga | R | 6 | 36.90 |
| cgg | R | 349 | 32.04 |
| aga | R | 0 | 172.70 |
| agg | R | 29 | 107.60 |
| mPD | | 0.17 | 0.96 |
| | | nPD: | 0.18 |
| | | N. weight: | 2.3 |
| | | Sc. PD: | 0.023 |
| | | Sc. rank: | 231.4 |

M1

| Pos. | 163 | obs: | exp: |
|---|---|---|---|
| tct | S | 208 | 61.05 |
| tcc | S | 175 | 53.49 |
| tca | S | 1 | 85.07 |
| tcg | S | 0 | 23.41 |
| agt | S | 0 | 84.48 |
| agc | S | 0 | 76.50 |
| mPD | | 0.50 | 1.7 |
| | | nPD: | 0.3 |
| | | N. weight: | 1.4 |
| | | Sc. PD: | 0.17 |
| | | Sc. rank: | 229.7 |

M1

| Pos. | 164 | obs: | exp: |
|---|---|---|---|
| cat | H | 1 | 208.10 |
| cac | H | 383 | 175.90 |
| mPD | | 0.0052 | 0.50 |
| | | nPD: | 0.01 |
| | | N. weight: | 0.87 |
| | | Sc. PD: | -0.13 |
| | | Sc. rank: | -110.1 |

M1

| Pos. | 165 | obs: | exp: |
|---|---|---|---|
| cgt | R | 0 | 15.94 |
| cgc | R | 0 | 18.80 |
| cga | R | 1 | 36.90 |
| cgg | R | 0 | 32.04 |
| aga | R | 200 | 172.70 |
| agg | R | 183 | 107.60 |
| mPD | | 0.51 | 0.96 |
| | | nPD: | 0.53 |
| | | N. weight: | 0.38 |
| | | Sc. PD: | 0.13 |
| | | Sc. rank: | 94.4 |

| | | | |
|---|---|---|---|
| M1 | | | |
| Pos. | 166 | obs: | exp: |
| caa | Q | 120 | 204.70 |
| cag | Q | 264 | 179.30 |
| --- | -- | ------ | ------ |
| mPD | | 0.43 | 0.50 |
| | | nPD: | 0.87 |
| | | N. weight: | 0.12 |
| | | Sc. PD: | 0.08 |
| | | Sc. rank: | 36.0 |

,

| | | | |
|---|---|---|---|
| M1 | | | |
| Pos. | 167 | obs: | exp: |
| atg | M | 384 | 384.00 |
| --- | -- | ------ | ------ |
| mPD | | 0 | 0 |
| | | nPD: | 1. |
| | | N. weight: | 0. |
| | | Sc. PD: | 0 |
| | | Sc. rank: | 0 |

,

| | | | |
|---|---|---|---|
| M1 | | | |
| Pos. | 168 | obs: | exp: |
| gtt | V | 0 | 72.97 |
| gtc | V | 1 | 74.51 |
| gta | V | 200 | 66.53 |
| gtg | V | 154 | 141.00 |
| gct | A | 0 | 7.30 |
| gcc | A | 0 | 5.94 |
| gca | A | 2 | 12.93 |
| gcg | A | 27 | 2.83 |
| --- | -- | ------ | ------ |
| mPD | | 0.64 | 0.87 |
| | | nPD: | 0.74 |
| | | N. weight: | 0.87 |
| | | Sc. PD: | 0.47 |
| | | Sc. rank: | 245.8 |

,

| | | | |
|---|---|---|---|
| M1 | | | |
| Pos. | 169 | obs: | exp: |
| act | T | 280 | 101.40 |
| acc | T | 15 | 82.40 |
| aca | T | 89 | 168.20 |
| acg | T | 0 | 31.92 |
| --- | -- | ------ | ------ |
| mPD | | 0.41 | 0.69 |
| | | nPD: | 0.6 |
| | | N. weight: | 0.62 |
| | | Sc. PD: | 0.25 |
| | | Sc. rank: | 158.2 |

,

| | | | |
|---|---|---|---|
| M1 | | | |
| Pos. | 170 | obs: | exp: |
| att | I | 0 | 0.38 |
| atc | I | 1 | 0.26 |
| ata | I | 0 | 0.36 |
| act | T | 89 | 101.20 |
| acc | T | 209 | 82.19 |
| aca | T | 85 | 167.80 |
| acg | T | 0 | 31.84 |
| --- | -- | ------ | ------ |
| mPD | | 0.60 | 0.69 |
| | | nPD: | 0.88 |
| | | N. weight: | 0.4 |
| | | Sc. PD: | 0.26 |
| | | Sc. rank: | 116.7 |

,

| | | | |
|---|---|---|---|
| M1 | | | |
| Pos. | 171 | obs: | exp: |
| act | T | 0 | 101.40 |
| acc | T | 382 | 82.40 |
| aca | T | 2 | 168.20 |
| acg | T | 0 | 31.92 |
| --- | -- | ------ | ------ |
| mPD | | 0.010 | 0.69 |
| | | nPD: | 0.02 |
| | | N. weight: | 1.7 |
| | | Sc. PD: | -0.25 |
| | | Sc. rank: | -207.7 |

,

| | | | |
|---|---|---|---|
| M1 | | | |
| Pos. | 172 | obs: | exp: |
| aat | N | 22 | 200.70 |
| aac | N | 362 | 183.30 |
| --- | -- | ------ | ------ |
| mPD | | 0.11 | 0.50 |
| | | nPD: | 0.22 |
| | | N. weight: | 0.59 |
| | | Sc. PD: | 0.028 |
| | | Sc. rank: | 71.6 |

,

| | | | |
|---|---|---|---|
| M1 | | | |
| Pos. | 173 | obs: | exp: |
| cct | P | 2 | 101.10 |
| ccc | P | 0 | 69.26 |
| cca | P | 303 | 151.50 |
| ccg | P | 79 | 62.09 |
| --- | -- | ------ | ------ |
| mPD | | 0.34 | 0.72 |
| | | nPD: | 0.47 |
| | | N. weight: | 0.67 |
| | | Sc. PD: | 0.19 |
| | | Sc. rank: | 158.4 |

,

| | | | |
|---|---|---|---|
| M1 | | | |
| Pos. | 174 | obs: | exp: |
| tta | L | 12 | 33.55 |
| ttg | L | 1 | 73.96 |
| ctt | L | 0 | 71.65 |
| ctc | L | 2 | 62.12 |
| cta | L | 362 | 57.85 |
| ctg | L | 7 | 84.88 |
| --- | -- | ------ | ------ |
| mPD | | 0.12 | 1.1 |
| | | nPD: | 0.1 |
| | | N. weight: | 1.9 |
| | | Sc. PD: | -0.11 |
| | | Sc. rank: | 104.6 |

,

| | | | |
|---|---|---|---|
| M1 | | | |
| Pos. | 175 | obs: | exp: |
| att | I | 31 | 144.40 |
| atc | I | 352 | 100.60 |
| ata | I | 1 | 139.00 |
| --- | -- | ------ | ------ |
| mPD | | 0.15 | 0.66 |
| | | nPD: | 0.23 |
| | | N. weight: | 1.2 |
| | | Sc. PD: | 0.072 |
| | | Sc. rank: | 158.4 |

,

| | | | |
|---|---|---|---|
| M1 | | | |
| Pos. | 176 | obs: | exp: |
| cgt | R | 0 | 14.86 |
| cgc | R | 0 | 17.53 |
| cga | R | 0 | 34.41 |
| cgg | R | 0 | 29.87 |
| aaa | K | 0 | 14.56 |
| aag | K | 26 | 11.44 |
| aga | R | 46 | 161.00 |
| agg | R | 312 | 100.30 |
| --- | -- | ------ | ------ |
| mPD | | 0.34 | 1.1 |
| | | nPD: | 0.32 |
| | | N. weight: | 0.95 |
| | | Sc. PD: | 0.13 |
| | | Sc. rank: | 172.7 |

,

| | | | |
|---|---|---|---|
| M1 | | | |
| Pos. | 177 | obs: | exp: |
| cat | H | 329 | 208.10 |
| cac | H | 55 | 175.90 |
| --- | -- | ------ | ------ |
| mPD | | 0.25 | 0.50 |
| | | nPD: | 0.5 |
| | | N. weight: | 0.27 |
| | | Sc. PD: | 0.082 |
| | | Sc. rank: | 63.5 |

,

| | | | |
|---|---|---|---|
| M1 | | | |
| Pos. | 178 | obs: | exp: |
| gaa | E | 225 | 221.00 |
| gag | E | 159 | 163.00 |
| --- | -- | ------ | ------ |
| mPD | | 0.49 | 0.49 |
| | | nPD: | 1. |
| | | N. weight: | 0.0097 |
| | | Sc. PD: | 0.0076 |
| | | Sc. rank: | 2.9 |

,

| | | | |
|---|---|---|---|
| M1 | | | |
| Pos. | 179 | obs: | exp: |
| aat | N | 29 | 200.70 |
| aac | N | 355 | 183.30 |
| --- | -- | ------ | ------ |
| mPD | | 0.14 | 0.50 |
| | | nPD: | 0.28 |
| | | N. weight: | 0.54 |
| | | Sc. PD: | 0.057 |
| | | Sc. rank: | 82.2 |

,

| | | | |
|---|---|---|---|
| M1 | | | |
| Pos. | 180 | obs: | exp: |
| cgt | R | 0 | 15.94 |
| cgc | R | 0 | 18.80 |
| cga | R | 0 | 36.90 |
| cgg | R | 0 | 32.04 |
| aga | R | 223 | 172.70 |
| agg | R | 161 | 107.60 |
| --- | -- | ------ | ------ |
| mPD | | 0.49 | 0.96 |
| | | nPD: | 0.51 |
| | | N. weight: | 0.37 |
| | | Sc. PD: | 0.12 |
| | | Sc. rank: | 89.7 |

,

| | | | |
|---|---|---|---|
| M1 | | | |
| Pos. | 181 | obs: | exp: |
| atg | M | 384 | 384.00 |
| --- | -- | ------ | ------ |
| mPD | | 0 | 0 |
| | | nPD: | 1. |
| | | N. weight: | 0. |
| | | Sc. PD: | 0 |
| | | Sc. rank: | 0 |

,

| | | | |
|---|---|---|---|
| M1 | | | |
| Pos. | 182 | obs: | exp: |
| gtt | V | 0 | 78.93 |
| gtc | V | 0 | 80.59 |
| gta | V | 131 | 71.97 |
| gtg | V | 253 | 152.50 |
| --- | -- | ------ | ------ |
| mPD | | 0.45 | 0.72 |
| | | nPD: | 0.63 |
| | | N. weight: | 0.62 |
| | | Sc. PD: | 0.27 |
| | | Sc. rank: | 166.8 |

,

| | | | |
|---|---|---|---|
| M1 | | | |
| Pos. | 183 | obs: | exp: |
| tta | L | 0 | 31.10 |
| ttg | L | 5 | 68.56 |
| ctt | L | 3 | 66.42 |
| ctc | L | 0 | 57.59 |
| cta | L | 55 | 53.63 |
| ctg | L | 293 | 78.69 |
| att | I | 0 | 10.53 |
| atc | I | 0 | 7.34 |
| ata | I | 28 | 10.14 |
| --- | -- | ------ | ------ |
| mPD | | 0.51 | 1.2 |
| | | nPD: | 0.42 |
| | | N. weight: | 1.2 |
| | | Sc. PD: | 0.29 |
| | | Sc. rank: | 265.8 |

,

| | | | |
|---|---|---|---|
| M1 | | | |
| Pos. | 184 | obs: | exp: |
| gct | A | 11 | 96.72 |
| gcc | A | 367 | 78.66 |
| gca | A | 5 | 171.10 |
| gcg | A | 1 | 37.47 |
| --- | -- | ------ | ------ |
| mPD | | 0.086 | 0.69 |
| | | nPD: | 0.13 |
| | | N. weight: | 1.6 |
| | | Sc. PD: | -0.062 |
| | | Sc. rank: | 114.8 |

,

| | | | |
|---|---|---|---|
| M1 | | | |
| Pos. | 185 | obs: | exp: |
| tct | S | 0 | 61.05 |
| tcc | S | 0 | 53.49 |
| tca | S | 0 | 85.07 |
| tcg | S | 0 | 23.41 |
| agt | S | 89 | 84.48 |
| agc | S | 295 | 76.50 |
| --- | -- | ------ | ------ |
| mPD | | 0.36 | 1.7 |
| | | nPD: | 0.21 |
| | | N. weight: | 1.2 |
| | | Sc. PD: | 0.053 |
| | | Sc. rank: | 143.6 |

,

| | | | |
|---|---|---|---|
| M1 | | | |
| Pos. | 186 | obs: | exp: |
| act | T | 327 | 101.40 |
| acc | T | 54 | 82.40 |
| aca | T | 0 | 168.20 |
| acg | T | 3 | 31.92 |
| --- | -- | ------ | ------ |
| mPD | | 0.26 | 0.69 |
| | | nPD: | 0.37 |
| | | N. weight: | 1.1 |
| | | Sc. PD: | 0.2 |
| | | Sc. rank: | 216.1 |

,

| | | | |
|---|---|---|---|
| M1 | | | |
| Pos. | 187 | obs: | exp: |
| act | T | 10 | 101.40 |
| acc | T | 0 | 82.40 |
| aca | T | 61 | 168.20 |
| acg | T | 313 | 31.92 |
| --- | -- | ------ | ------ |
| mPD | | 0.31 | 0.69 |
| | | nPD: | 0.45 |
| | | N. weight: | 1.9 |
| | | Sc. PD: | 0.51 |
| | | Sc. rank: | 440.8 |

,

| | | | |
|---|---|---|---|
| M1 | | | |
| Pos. | 188 | obs: | exp: |
| gct | A | 372 | 96.72 |
| gcc | A | 12 | 78.66 |
| gca | A | 0 | 171.10 |
| gcg | A | 0 | 37.47 |
| --- | -- | ------ | ------ |
| mPD | | 0.061 | 0.69 |
| | | nPD: | 0.09 |
| | | N. weight: | 1.4 |
| | | Sc. PD: | -0.11 |
| | | Sc. rank: | 56.3 |

,

| | | | |
|---|---|---|---|
| M1 | | | |
| Pos. | 189 | obs: | exp: |
| aaa | K | 31 | 215.00 |
| aag | K | 353 | 169.00 |
| --- | -- | ------ | ------ |
| mPD | | 0.15 | 0.49 |
| | | nPD: | 0.3 |
| | | N. weight: | 0.6 |
| | | Sc. PD: | 0.076 |
| | | Sc. rank: | 103.9 |

,

| | | | |
|---|---|---|---|
| M1 | | | |
| Pos. | 190 | obs: | exp: |
| gct | A | 265 | 96.72 |
| gcc | A | 118 | 78.66 |
| gca | A | 1 | 171.10 |
| gcg | A | 0 | 37.47 |
| --- | -- | ------ | ------ |
| mPD | | 0.43 | 0.69 |
| | | nPD: | 0.63 |
| | | N. weight: | 0.93 |
| | | Sc. PD: | 0.4 |
| | | Sc. rank: | 251.7 |

,

| | | | |
|---|---|---|---|
| M1 | | | |
| Pos. | 191 | obs: | exp: |
| atg | M | 384 | 384.00 |
| --- | -- | ------ | ------ |
| mPD | | 0 | 0 |
| | | nPD: | 1. |
| | | N. weight: | 0. |
| | | Sc. PD: | 0 |
| | | Sc. rank: | 0 |

,

| | | | |
|---|---|---|---|
| M1 | | | |
| Pos. | 192 | obs: | exp: |
| gaa | E | 4 | 221.00 |
| gag | E | 380 | 163.00 |
| --- | -- | ------ | ------ |
| mPD | | 0.021 | 0.49 |
| | | nPD: | 0.04 |
| | | N. weight: | 0.91 |
| | | Sc. PD: | -0.11 |
| | | Sc. rank: | -12.6 |

,

| | | | |
|---|---|---|---|
| M1 | | | |
| Pos. | 193 | obs: | exp: |
| caa | Q | 83 | 204.70 |
| cag | Q | 301 | 179.30 |
| --- | -- | ------ | ------ |
| mPD | | 0.34 | 0.50 |
| | | nPD: | 0.68 |
| | | N. weight: | 0.25 |
| | | Sc. PD: | 0.12 |
| | | Sc. rank: | 68.7 |

,

| | | | |
|---|---|---|---|
| M1 | | | |
| Pos. | 194 | obs: | exp: |
| atg | M | 384 | 384.00 |
| --- | -- | ------ | ------ |
| mPD | | 0 | 0 |
| | | nPD: | 1. |
| | | N. weight: | 0. |
| | | Sc. PD: | 0 |
| | | Sc. rank: | 0 |

,

| | | | |
|---|---|---|---|
| M1 | | | |
| Pos. | 195 | obs: | exp: |
| gct | A | 294 | 96.72 |
| gcc | A | 3 | 78.66 |
| gca | A | 84 | 171.10 |
| gcg | A | 3 | 37.47 |
| --- | -- | ------ | ------ |
| mPD | | 0.37 | 0.69 |
| | | nPD: | 0.53 |
| | | N. weight: | 0.76 |
| | | Sc. PD: | 0.26 |
| | | Sc. rank: | 193.4 |

,

| M1 | | | |
|---|---|---|---|
| Pos. | 196 | obs: | exp: |
| ggt | G | 0 | 47.38 |
| ggc | G | 0 | 51.93 |
| gga | G | 108 | 185.80 |
| ggg | G | 276 | 98.93 |
| --- | -- | ------ | ------ |
| mPD | | 0.41 | 0.67 |
| | | nPD: | 0.61 |
| | | N. weight: | 0.68 |
| | | Sc. PD: | 0.28 |
| | | Sc. rank: | 174.8 |

| M1 | | | |
|---|---|---|---|
| Pos. | 197 | obs: | exp: |
| tct | S | 0 | 60.89 |
| tcc | S | 0 | 53.35 |
| tca | S | 197 | 84.85 |
| tcg | S | 186 | 23.35 |
| act | T | 0 | 0.26 |
| acc | T | 0 | 0.21 |
| aca | T | 0 | 0.44 |
| acg | T | 1 | 0.08 |
| agt | S | 0 | 84.26 |
| agc | S | 0 | 76.31 |
| --- | -- | ------ | ------ |
| mPD | | 0.51 | 1.7 |
| | | nPD: | 0.3 |
| | | N. weight: | 1.7 |
| | | Sc. PD: | 0.21 |
| | | Sc. rank: | 292.2 |

| M1 | | | |
|---|---|---|---|
| Pos. | 198 | obs: | exp: |
| tct | S | 0 | 61.05 |
| tcc | S | 0 | 53.49 |
| tca | S | 0 | 85.07 |
| tcg | S | 0 | 23.41 |
| agt | S | 377 | 84.48 |
| agc | S | 7 | 76.50 |
| --- | -- | ------ | ------ |
| mPD | | 0.036 | 1.7 |
| | | nPD: | 0.02 |
| | | N. weight: | 1.6 |
| | | Sc. PD: | -0.22 |
| | | Sc. rank: | -151.8 |

| M1 | | | |
|---|---|---|---|
| Pos. | 199 | obs: | exp: |
| gaa | E | 39 | 221.00 |
| gag | E | 345 | 163.00 |
| --- | -- | ------ | ------ |
| mPD | | 0.18 | 0.49 |
| | | nPD: | 0.37 |
| | | N. weight: | 0.57 |
| | | Sc. PD: | 0.11 |
| | | Sc. rank: | 117.9 |

| M1 | | | |
|---|---|---|---|
| Pos. | 200 | obs: | exp: |
| caa | Q | 46 | 204.70 |
| cag | Q | 338 | 179.30 |
| --- | -- | ------ | ------ |
| mPD | | 0.21 | 0.50 |
| | | nPD: | 0.42 |
| | | N. weight: | 0.44 |
| | | Sc. PD: | 0.11 |
| | | Sc. rank: | 99.4 |

| M1 | | | |
|---|---|---|---|
| Pos. | 201 | obs: | exp: |
| gct | A | 1 | 96.72 |
| gcc | A | 0 | 78.66 |
| gca | A | 383 | 171.10 |
| gcg | A | 0 | 37.47 |
| --- | -- | ------ | ------ |
| mPD | | 0.0052 | 0.69 |
| | | nPD: | 0.01 |
| | | N. weight: | 0.91 |
| | | Sc. PD: | -0.14 |
| | | Sc. rank: | -146.4 |

| M1 | | | |
|---|---|---|---|
| Pos. | 202 | obs: | exp: |
| gct | A | 4 | 96.72 |
| gcc | A | 0 | 78.66 |
| gca | A | 108 | 171.10 |
| gcg | A | 272 | 37.47 |
| --- | -- | ------ | ------ |
| mPD | | 0.42 | 0.69 |
| | | nPD: | 0.61 |
| | | N. weight: | 1.4 |
| | | Sc. PD: | 0.6 |
| | | Sc. rank: | 374.3 |

| M1 | | | |
|---|---|---|---|
| Pos. | 203 | obs: | exp: |
| gat | D | 2 | 1.07 |
| gac | D | 0 | 0.93 |
| gaa | E | 260 | 219.80 |
| gag | E | 122 | 162.20 |
| --- | -- | ------ | ------ |
| mPD | | 0.44 | 0.49 |
| | | nPD: | 0.89 |
| | | N. weight: | 0.039 |
| | | Sc. PD: | 0.027 |
| | | Sc. rank: | 11.7 |

| M1 | | | |
|---|---|---|---|
| Pos. | 204 | obs: | exp: |
| gct | A | 104 | 96.72 |
| gcc | A | 273 | 78.66 |
| gca | A | 7 | 171.10 |
| gcg | A | 0 | 37.47 |
| --- | -- | ------ | ------ |
| mPD | | 0.42 | 0.69 |
| | | nPD: | 0.61 |
| | | N. weight: | 0.98 |
| | | Sc. PD: | 0.41 |
| | | Sc. rank: | 259.0 |

| M1 | | | |
|---|---|---|---|
| Pos. | 205 | obs: | exp: |
| atg | M | 384 | 384.00 |
| --- | -- | ------ | ------ |
| mPD | | 0 | 0 |
| | | nPD: | 1. |
| | | N. weight: | 0. |
| | | Sc. PD: | 0 |
| | | Sc. rank: | 0 |

| M1 | | | |
|---|---|---|---|
| Pos. | 206 | obs: | exp: |
| gaa | E | 50 | 221.00 |
| gag | E | 334 | 163.00 |
| --- | -- | ------ | ------ |
| mPD | | 0.23 | 0.49 |
| | | nPD: | 0.46 |
| | | N. weight: | 0.5 |
| | | Sc. PD: | 0.14 |
| | | Sc. rank: | 117.3 |

| M1 | | | |
|---|---|---|---|
| Pos. | 207 | obs: | exp: |
| att | I | 3 | 1.13 |
| atc | I | 0 | 0.79 |
| ata | I | 0 | 1.09 |
| gtt | V | 349 | 78.31 |
| gtc | V | 32 | 79.96 |
| gta | V | 0 | 71.41 |
| gtg | V | 0 | 151.30 |
| --- | -- | ------ | ------ |
| mPD | | 0.17 | 0.74 |
| | | nPD: | 0.23 |
| | | N. weight: | 1.5 |
| | | Sc. PD: | 0.085 |
| | | Sc. rank: | 192.6 |

| M1 | | | |
|---|---|---|---|
| Pos. | 208 | obs: | exp: |
| gct | A | 335 | 96.72 |
| gcc | A | 20 | 78.66 |
| gca | A | 29 | 171.10 |
| gcg | A | 0 | 37.47 |
| --- | -- | ------ | ------ |
| mPD | | 0.23 | 0.69 |
| | | nPD: | 0.34 |
| | | N. weight: | 1. |
| | | Sc. PD: | 0.16 |
| | | Sc. rank: | 199.5 |

| M1 | | | |
|---|---|---|---|
| Pos. | 209 | obs: | exp: |
| tct | S | 0 | 61.05 |
| tcc | S | 0 | 53.49 |
| tca | S | 0 | 85.07 |
| tcg | S | 0 | 23.41 |
| agt | S | 378 | 84.48 |
| agc | S | 6 | 76.50 |
| --- | -- | ------ | ------ |
| mPD | | 0.031 | 1.7 |
| | | nPD: | 0.02 |
| | | N. weight: | 1.6 |
| | | Sc. PD: | -0.23 |
| | | Sc. rank: | -178.3 |

| M1 | | | |
|---|---|---|---|
| Pos. | 210 | obs: | exp: |
| caa | Q | 1 | 204.70 |
| cag | Q | 383 | 179.30 |
| --- | -- | ------ | ------ |
| mPD | | 0.0052 | 0.50 |
| | | nPD: | 0.01 |
| | | N. weight: | 0.85 |
| | | Sc. PD: | -0.13 |
| | | Sc. rank: | -109.1 |

| M1 | | | |
|---|---|---|---|
| Pos. | 211 | obs: | exp: |
| gct | A | 360 | 96.72 |
| gcc | A | 24 | 78.66 |
| gca | A | 0 | 171.10 |
| gcg | A | 0 | 37.47 |
| --- | -- | ------ | ------ |
| mPD | | 0.12 | 0.69 |
| | | nPD: | 0.17 |
| | | N. weight: | 1.3 |
| | | Sc. PD: | 0.0046 |
| | | Sc. rank: | 121.6 |

| M1 | | | |
|---|---|---|---|
| Pos. | 212 | obs: | exp: |
| cgt | R | 0 | 15.94 |
| cgc | R | 0 | 18.80 |
| cga | R | 0 | 36.90 |
| cgg | R | 0 | 32.04 |
| aga | R | 63 | 172.70 |
| agg | R | 321 | 107.60 |
| --- | -- | ------ | ------ |
| mPD | | 0.28 | 0.96 |
| | | nPD: | 0.29 |
| | | N. weight: | 0.86 |
| | | Sc. PD: | 0.096 |
| | | Sc. rank: | 141.5 |

| M1 | | | |
|---|---|---|---|
| Pos. | 213 | obs: | exp: |
| caa | Q | 38 | 204.70 |
| cag | Q | 346 | 179.30 |
| --- | -- | ------ | ------ |
| mPD | | 0.18 | 0.50 |
| | | nPD: | 0.36 |
| | | N. weight: | 0.49 |
| | | Sc. PD: | 0.089 |
| | | Sc. rank: | 97.4 |

| M1 | | | |
|---|---|---|---|
| Pos. | 214 | obs: | exp: |
| atg | M | 384 | 384.00 |
| --- | -- | ------ | ------ |
| mPD | | 0 | 0 |
| | | nPD: | 1. |
| | | N. weight: | 0. |
| | | Sc. PD: | 0 |
| | | Sc. rank: | 0 |

| M1 | | | |
|---|---|---|---|
| Pos. | 215 | obs: | exp: |
| gtt | V | 0 | 78.93 |
| gtc | V | 0 | 80.59 |
| gta | V | 3 | 71.97 |
| gtg | V | 381 | 152.50 |
| --- | -- | ------ | ------ |
| mPD | | 0.016 | 0.72 |
| | | nPD: | 0.02 |
| | | N. weight: | 1. |
| | | Sc. PD: | -0.14 |
| | | Sc. rank: | -98.0 |

| M1 | | | |
|---|---|---|---|
| Pos. | 216 | obs: | exp: |
| caa | Q | 6 | 204.70 |
| cag | Q | 378 | 179.30 |
| --- | -- | ------ | ------ |
| mPD | | 0.031 | 0.50 |
| | | nPD: | 0.06 |
| | | N. weight: | 0.78 |
| | | Sc. PD: | -0.077 |
| | | Sc. rank: | 13.2 |

| M1 | | | |
|---|---|---|---|
| Pos. | 217 | obs: | exp: |
| gct | A | 30 | 96.72 |
| gcc | A | 0 | 78.66 |
| gca | A | 200 | 171.10 |
| gcg | A | 154 | 37.47 |
| --- | -- | ------ | ------ |
| mPD | | 0.56 | 0.69 |
| | | nPD: | 0.82 |
| | | N. weight: | 0.65 |
| | | Sc. PD: | 0.4 |
| | | Sc. rank: | 190.7 |

| M1 | | | |
|---|---|---|---|
| Pos. | 218 | obs: | exp: |
| atg | M | 384 | 384.00 |
| --- | -- | ------ | ------ |
| mPD | | 0 | 0 |
| | | nPD: | 1. |
| | | N. weight: | 0. |
| | | Sc. PD: | 0 |
| | | Sc. rank: | 0 |

| M1 | | | |
|---|---|---|---|
| Pos. | 219 | obs: | exp: |
| cgt | R | 0 | 15.94 |
| cgc | R | 0 | 18.80 |
| cga | R | 0 | 36.90 |
| cgg | R | 1 | 32.04 |
| aga | R | 36 | 172.70 |
| agg | R | 347 | 107.60 |
| --- | -- | ------ | ------ |
| mPD | | 0.18 | 0.96 |
| | | nPD: | 0.18 |
| | | N. weight: | 1. |
| | | Sc. PD: | 0.015 |
| | | Sc. rank: | 105.1 |

| M1 | | | |
|---|---|---|---|
| Pos. | 220 | obs: | exp: |
| act | T | 13 | 101.40 |
| acc | T | 143 | 82.40 |
| aca | T | 228 | 168.20 |
| acg | T | 0 | 31.92 |
| --- | -- | ------ | ------ |
| mPD | | 0.51 | 0.69 |
| | | nPD: | 0.74 |
| | | N. weight: | 0.38 |
| | | Sc. PD: | 0.2 |
| | | Sc. rank: | 108.0 |

| M1 | | | |
|---|---|---|---|
| Pos. | 221 | obs: | exp: |
| att | I | 343 | 132.70 |
| atc | I | 10 | 92.52 |
| ata | I | 0 | 127.80 |
| gtt | V | 2 | 6.37 |
| gtc | V | 29 | 6.51 |
| gta | V | 0 | 5.81 |
| gtg | V | 0 | 12.31 |
| --- | -- | ------ | ------ |
| mPD | | 0.33 | 0.83 |
| | | nPD: | 0.4 |
| | | N. weight: | 1. |
| | | Sc. PD: | 0.23 |
| | | Sc. rank: | 215.0 |

| M1 | | | |
|---|---|---|---|
| Pos. | 222 | obs: | exp: |
| ggt | G | 0 | 47.38 |
| ggc | G | 0 | 51.93 |
| gga | G | 192 | 185.80 |
| ggg | G | 192 | 98.93 |
| --- | -- | ------ | ------ |
| mPD | | 0.50 | 0.67 |
| | | nPD: | 0.75 |
| | | N. weight: | 0.41 |
| | | Sc. PD: | 0.22 |
| | | Sc. rank: | 117.5 |

| M1 | | | |
|---|---|---|---|
| Pos. | 223 | obs: | exp: |
| act | T | 379 | 101.40 |
| acc | T | 5 | 82.40 |
| aca | T | 0 | 168.20 |
| acg | T | 0 | 31.92 |
| --- | -- | ------ | ------ |
| mPD | | 0.026 | 0.69 |
| | | nPD: | 0.04 |
| | | N. weight: | 1.4 |
| | | Sc. PD: | -0.18 |
| | | Sc. rank: | -47.1 |

| M1 | | | |
|---|---|---|---|
| Pos. | 224 | obs: | exp: |
| cat | H | 151 | 208.10 |
| cac | H | 233 | 175.90 |
| --- | -- | ------ | ------ |
| mPD | | 0.48 | 0.50 |
| | | nPD: | 0.96 |
| | | N. weight: | 0.06 |
| | | Sc. PD: | 0.045 |
| | | Sc. rank: | 18.0 |

| M1 | | | |
|---|---|---|---|
| Pos. | 225 | obs: | exp: |
| cct | P | 380 | 101.10 |
| ccc | P | 3 | 69.26 |
| cca | P | 1 | 151.50 |
| ccg | P | 0 | 62.09 |
| --- | -- | ------ | ------ |
| mPD | | 0.021 | 0.72 |
| | | nPD: | 0.03 |
| | | N. weight: | 1.5 |
| | | Sc. PD: | -0.19 |
| | | Sc. rank: | -88.2 |

| M1 | | | |
|---|---|---|---|
| Pos. | 226 | obs: | exp: |
| tct | S | 0 | 56.44 |
| tcc | S | 0 | 49.45 |
| tca | S | 0 | 78.65 |
| tcg | S | 0 | 21.64 |
| aat | N | 0 | 15.16 |
| aac | N | 29 | 13.84 |
| agt | S | 4 | 78.10 |
| agc | S | 351 | 70.73 |
| --- | -- | ------ | ------ |
| mPD | | 0.16 | 1.7 |
| | | nPD: | 0.09 |
| | | N. weight: | 1.7 |
| | | Sc. PD: | -0.12 |
| | | Sc. rank: | 79.1 |

| M1 | | | |
|---|---|---|---|
| Pos. | 227 | obs: | exp: |
| tct | S | 29 | 61.05 |
| tcc | S | 355 | 53.49 |
| tca | S | 0 | 85.07 |
| tcg | S | 0 | 23.41 |
| agt | S | 0 | 84.48 |
| agc | S | 0 | 76.50 |
| --- | -- | ------ | ------ |
| mPD | | 0.14 | 1.7 |
| | | nPD: | 0.08 |
| | | N. weight: | 1.9 |
| | | Sc. PD: | -0.15 |
| | | Sc. rank: | 62.8 |

| M1 | | | |
|---|---|---|---|
| Pos. | 228 | obs: | exp: |
| tct | S | 0 | 61.05 |
| tcc | S | 0 | 53.49 |
| tca | S | 0 | 85.07 |
| tcg | S | 0 | 23.41 |
| agt | S | 384 | 84.48 |
| agc | S | 0 | 76.50 |
| --- | -- | ------ | ------ |
| mPD | | 0 | 1.7 |
| | | nPD: | 0. |
| | | N. weight: | 1.7 |
| | | Sc. PD: | -0.27 |
| | | Sc. rank: | -486.9 |

| M1 | | | |
|---|---|---|---|
| Pos. | 229 | obs: | exp: |
| act | T | 0 | 7.93 |
| acc | T | 1 | 6.44 |
| aca | T | 29 | 13.14 |
| acg | T | 0 | 2.49 |
| gct | A | 93 | 89.16 |
| gcc | A | 261 | 72.52 |
| gca | A | 0 | 157.80 |
| gcg | A | 0 | 34.54 |
| --- | -- | ------ | ------ |
| mPD | | 0.62 | 0.83 |
| | | nPD: | 0.74 |
| | | N. weight: | 1.1 |
| | | Sc. PD: | 0.58 |
| | | Sc. rank: | 308.5 |

| M1 | | | |
|---|---|---|---|
| Pos. | 230 | obs: | exp: |
| ggt | G | 356 | 47.38 |
| ggc | G | 28 | 51.93 |
| gga | G | 0 | 185.80 |
| ggg | G | 0 | 98.93 |
| --- | -- | ------ | ------ |
| mPD | | 0.14 | 0.67 |
| | | nPD: | 0.2 |
| | | N. weight: | 2.1 |
| | | Sc. PD: | 0.071 |
| | | Sc. rank: | 236.9 |

| M1 | | | |
|---|---|---|---|
| Pos. | 231 | obs: | exp: |
| tta | L | 0 | 33.55 |
| ttg | L | 5 | 73.96 |
| ctt | L | 0 | 71.65 |
| ctc | L | 0 | 62.12 |
| cta | L | 46 | 57.85 |
| ctg | L | 333 | 84.88 |
| --- | -- | ------ | ------ |
| mPD | | 0.24 | 1.1 |
| | | nPD: | 0.21 |
| | | N. weight: | 1.3 |
| | | Sc. PD: | 0.055 |
| | | Sc. rank: | 150.6 |

| M1 | | | |
|---|---|---|---|
| Pos. | 232 | obs: | exp: |
| aaa | K | 343 | 215.00 |
| aag | K | 41 | 169.00 |
| --- | -- | ------ | ------ |
| mPD | | 0.19 | 0.49 |
| | | nPD: | 0.39 |
| | | N. weight: | 0.31 |
| | | Sc. PD: | 0.064 |
| | | Sc. rank: | 64.1 |

| M1 | | | |
|---|---|---|---|
| Pos. | 233 | obs: | exp: |
| gat | D | 382 | 204.90 |
| gac | D | 2 | 179.10 |
| --- | -- | ------ | ------ |
| mPD | | 0.010 | 0.50 |
| | | nPD: | 0.02 |
| | | N. weight: | 0.68 |
| | | Sc. PD: | -0.094 |
| | | Sc. rank: | -69.3 |

| M1 | | | |
|---|---|---|---|
| Pos. | 234 | obs: | exp: |
| gat | D | 381 | 204.90 |
| gac | D | 3 | 179.10 |
| --- | -- | ------ | ------ |
| mPD | | 0.016 | 0.50 |
| | | nPD: | 0.03 |
| | | N. weight: | 0.67 |
| | | Sc. PD: | -0.086 |
| | | Sc. rank: | -29.3 |

| M1 | | | |
|---|---|---|---|
| Pos. | 235 | obs: | exp: |
| tta | L | 0 | 33.55 |
| ttg | L | 0 | 73.96 |
| ctt | L | 376 | 71.65 |
| ctc | L | 8 | 62.12 |
| cta | L | 0 | 57.85 |
| ctg | L | 0 | 84.88 |
| --- | -- | ------ | ------ |
| mPD | | 0.041 | 1.1 |
| | | nPD: | 0.04 |
| | | N. weight: | 1.8 |
| | | Sc. PD: | -0.22 |
| | | Sc. rank: | -64.8 |

| M1 | | | |
|---|---|---|---|
| Pos. | 236 | obs: | exp: |
| tta | L | 0 | 31.01 |
| ttg | L | 0 | 68.37 |
| ctt | L | 351 | 66.24 |
| ctc | L | 4 | 57.42 |
| cta | L | 0 | 53.48 |
| ctg | L | 0 | 78.47 |
| att | I | 29 | 10.90 |
| atc | I | 0 | 7.60 |
| ata | I | 0 | 10.50 |
| --- | -- | ------ | ------ |
| mPD | | 0.16 | 1.2 |
| | | nPD: | 0.13 |
| | | N. weight: | 1.8 |
| | | Sc. PD: | -0.058 |
| | | Sc. rank: | 141.5 |

| M1 | | | |
|---|---|---|---|
| Pos. | 237 | obs: | exp: |
| caa | Q | 1 | 0.53 |
| cag | Q | 0 | 0.47 |
| gaa | E | 383 | 220.40 |
| gag | E | 0 | 162.60 |
| --- | -- | ------ | ------ |
| mPD | | 0.0052 | 0.49 |
| | | nPD: | 0.01 |
| | | N. weight: | 0.63 |
| | | Sc. PD: | -0.093 |
| | | Sc. rank: | -78.7 |

| M1 | | | |
|---|---|---|---|
| Pos. | 238 | obs: | exp: |
| aat | N | 372 | 200.20 |
| aac | N | 11 | 182.80 |
| aaa | K | 1 | 0.56 |
| aag | K | 0 | 0.44 |
| --- | -- | ------ | ------ |
| mPD | | 0.061 | 0.50 |
| | | nPD: | 0.12 |
| | | N. weight: | 0.6 |
| | | Sc. PD: | -0.026 |
| | | Sc. rank: | 38.6 |

| M1 | | | |
|---|---|---|---|
| Pos. | 239 | obs: | exp: |
| tta | L | 0 | 33.55 |
| ttg | L | 384 | 73.96 |
| ctt | L | 0 | 71.65 |
| ctc | L | 0 | 62.12 |
| cta | L | 0 | 57.85 |
| ctg | L | 0 | 84.88 |
| --- | -- | ------ | ------ |
| mPD | | 0 | 1.1 |
| | | nPD: | 0. |
| | | N. weight: | 1.9 |
| | | Sc. PD: | -0.3 |
| | | Sc. rank: | -529.7 |

| M1 | | | |
|---|---|---|---|
| Pos. | 240 | obs: | exp: |
| caa | Q | 0 | 204.70 |
| cag | Q | 384 | 179.30 |
| --- | -- | ------ | ------ |
| mPD | | 0 | 0.50 |
| | | nPD: | 0. |
| | | N. weight: | 0.87 |
| | | Sc. PD: | -0.14 |
| | | Sc. rank: | -244.9 |

| M1 | | | |
|---|---|---|---|
| Pos. | 241 | obs: | exp: |
| gct | A | 44 | 96.72 |
| gcc | A | 321 | 78.66 |
| gca | A | 19 | 171.10 |
| gcg | A | 0 | 37.47 |
| --- | -- | ------ | ------ |
| mPD | | 0.29 | 0.69 |
| | | nPD: | 0.42 |
| | | N. weight: | 1.1 |
| | | Sc. PD: | 0.26 |
| | | Sc. rank: | 250.4 |

| M1 | | | |
|---|---|---|---|
| Pos. | 242 | obs: | exp: |
| tat | Y | 2 | 198.80 |
| tac | Y | 382 | 185.20 |
| --- | -- | ------ | ------ |
| mPD | | 0.010 | 0.50 |
| | | nPD: | 0.02 |
| | | N. weight: | 0.8 |
| | | Sc. PD: | -0.11 |
| | | Sc. rank: | -82.1 |

| M1 | | | |
|---|---|---|---|
| Pos. | 243 | obs: | exp: |
| caa | Q | 29 | 204.70 |
| cag | Q | 355 | 179.30 |
| --- | -- | ------ | ------ |
| mPD | | 0.14 | 0.50 |
| | | nPD: | 0.28 |
| | | N. weight: | 0.56 |
| | | Sc. PD: | 0.06 |
| | | Sc. rank: | 86.2 |

| M1 | | | |
|---|---|---|---|
| Pos. | 244 | obs: | exp: |
| aat | N | 0 | 15.16 |
| aac | N | 29 | 13.84 |
| aaa | K | 351 | 198.80 |
| aag | K | 4 | 156.20 |
| --- | -- | ------ | ------ |
| mPD | | 0.16 | 0.56 |
| | | nPD: | 0.28 |
| | | N. weight: | 0.62 |
| | | Sc. PD: | 0.067 |
| | | Sc. rank: | 96.1 |

| M1 | | | |
|---|---|---|---|
| Pos. | 245 | obs: | exp: |
| cgt | R | 2 | 15.94 |
| cgc | R | 0 | 18.80 |
| cga | R | 6 | 36.90 |
| cgg | R | 376 | 32.04 |
| aga | R | 0 | 172.70 |
| agg | R | 0 | 107.60 |
| --- | -- | ------ | ------ |
| mPD | | 0.041 | 0.96 |
| | | nPD: | 0.04 |
| | | N. weight: | 2.7 |
| | | Sc. PD: | -0.32 |
| | | Sc. rank: | -5.3 |

| M1 | | | |
|---|---|---|---|
| Pos. | 246 | obs: | exp: |
| atg | M | 384 | 384.00 |
| --- | -- | ------ | ------ |
| mPD | | 0 | 0 |
| | | nPD: | 1. |
| | | N. weight: | 0. |
| | | Sc. PD: | 0 |
| | | Sc. rank: | 0 |

| M1 | | | |
|---|---|---|---|
| Pos. | 247 | obs: | exp: |
| ggt | G | 0 | 47.38 |
| ggc | G | 0 | 51.93 |
| gga | G | 380 | 185.80 |
| ggg | G | 4 | 98.93 |
| --- | -- | ------ | ------ |
| mPD | | 0.021 | 0.67 |
| | | nPD: | 0.03 |
| | | N. weight: | 0.77 |
| | | Sc. PD: | -0.099 |
| | | Sc. rank: | -37.8 |

| M1 | | | |
|---|---|---|---|
| Pos. | 248 | obs: | exp: |
| gtt | V | 0 | 78.93 |
| gtc | V | 0 | 80.59 |
| gta | V | 0 | 71.97 |
| gtg | V | 384 | 152.50 |
| --- | -- | ------ | ------ |
| mPD | | 0 | 0.72 |
| | | nPD: | 0. |
| | | N. weight: | 1.1 |
| | | Sc. PD: | -0.17 |
| | | Sc. rank: | -297.0 |

| M1 | | | |
|---|---|---|---|
| Pos. | 249 | obs: | exp: |
| caa | Q | 346 | 204.70 |
| cag | Q | 38 | 179.30 |
| --- | -- | ------ | ------ |
| mPD | | 0.18 | 0.50 |
| | | nPD: | 0.36 |
| | | N. weight: | 0.37 |
| | | Sc. PD: | 0.067 |
| | | Sc. rank: | 73.4 |

| M1 | | | |
|---|---|---|---|
| Pos. | 250 | obs: | exp: |
| tta | L | 0 | 2.62 |
| ttg | L | 0 | 5.78 |
| ctt | L | 0 | 5.60 |
| ctc | L | 0 | 4.85 |
| cta | L | 0 | 4.52 |
| ctg | L | 30 | 6.63 |
| atg | M | 354 | 354.00 |
| --- | -- | ------ | ------ |
| mPD | | 0.14 | 0.24 |
| | | nPD: | 0.61 |
| | | N. weight: | 0.13 |
| | | Sc. PD: | 0.056 |
| | | Sc. rank: | 35.3 |

| M1 | | | |
|---|---|---|---|
| Pos. | 251 | obs: | exp: |
| caa | Q | 43 | 204.70 |
| cag | Q | 341 | 179.30 |
| --- | -- | ------ | ------ |
| mPD | | 0.20 | 0.50 |
| | | nPD: | 0.4 |
| | | N. weight: | 0.46 |
| | | Sc. PD: | 0.1 |
| | | Sc. rank: | 96.2 |

| M1 | | | |
|---|---|---|---|
| Pos. | 252 | obs: | exp: |
| cgt | R | 0 | 15.94 |
| cgc | R | 0 | 18.80 |
| cga | R | 338 | 36.90 |
| cgg | R | 45 | 32.04 |
| aga | R | 1 | 172.70 |
| agg | R | 0 | 107.60 |
| --- | -- | ------ | ------ |
| mPD | | 0.21 | 0.96 |
| | | nPD: | 0.22 |
| | | N. weight: | 2.3 |
| | | Sc. PD: | 0.11 |
| | | Sc. rank: | 284.7 |

| M1 | | | |
|---|---|---|---|
| Pos. | 253 | obs: | exp: |
| ttt | F | 0 | 157.90 |
| ttc | F | 384 | 226.10 |
| --- | -- | ------ | ------ |
| mPD | | 0 | 0.48 |
| | | nPD: | 0. |
| | | N. weight: | 0.6 |
| | | Sc. PD: | -0.095 |
| | | Sc. rank: | -170.3 |

| M1 | | | |
|---|---|---|---|
| Pos. | 254 | obs: | exp: |
| aaa | K | 0 | 215.00 |
| aag | K | 384 | 169.00 |
| --- | -- | ------ | ------ |
| mPD | | 0 | 0.49 |
| | | nPD: | 0. |
| | | N. weight: | 0.94 |
| | | Sc. PD: | -0.15 |
| | | Sc. rank: | -264.0 |

| M1 | | | |
|---|---|---|---|
| Pos. | 255 | obs: | exp: |
| taa | . | 0 | 179.80 |
| tag | . | 0 | 99.37 |
| tga | . | 384 | 104.80 |
| --- | -- | ------ | ------ |
| mPD | | 0 | 0.78 |
| | | nPD: | 0. |
| | | N. weight: | 1.5 |
| | | Sc. PD: | -0.23 |
| | | Sc. rank: | -417.7 |

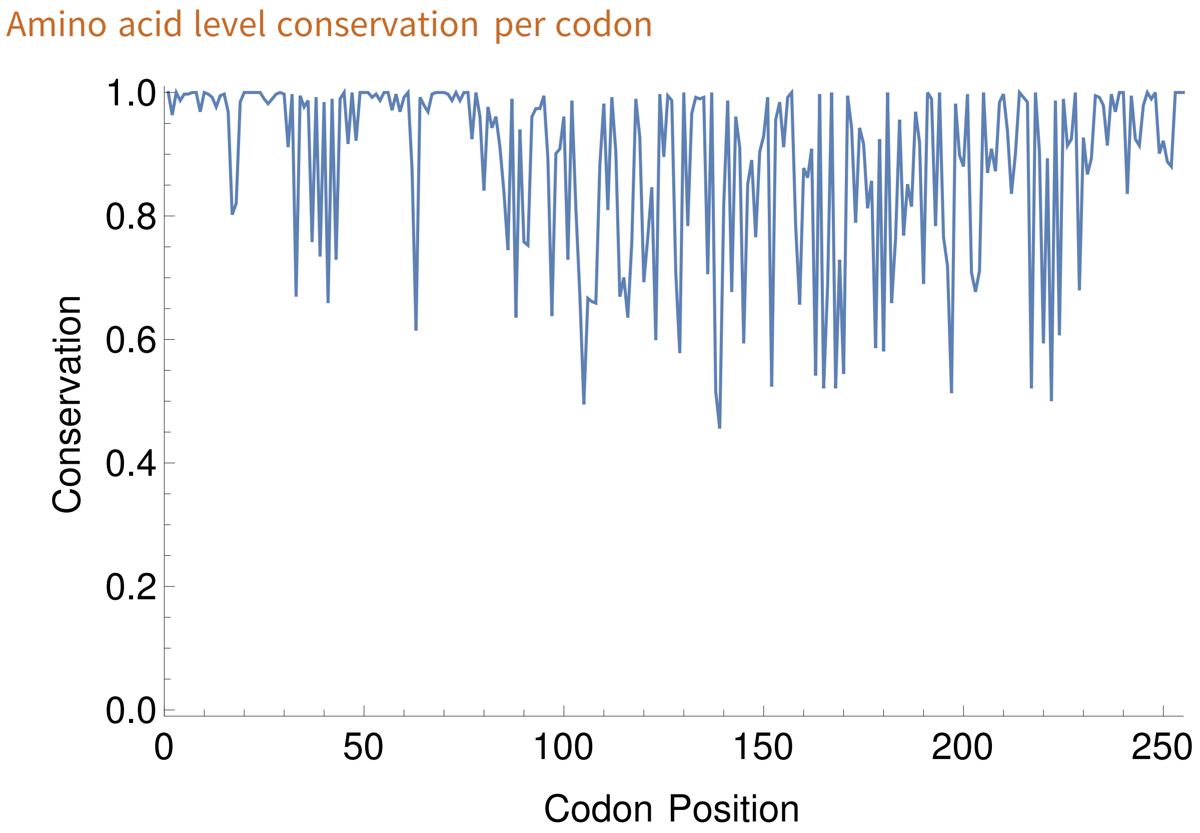
Amino acid level conservation per codon
Conservation
1.0
0.8
0.6
0.4
0.2
0.0
0
50
100
150
200
250
Codon Position

## Conservation analysis plots

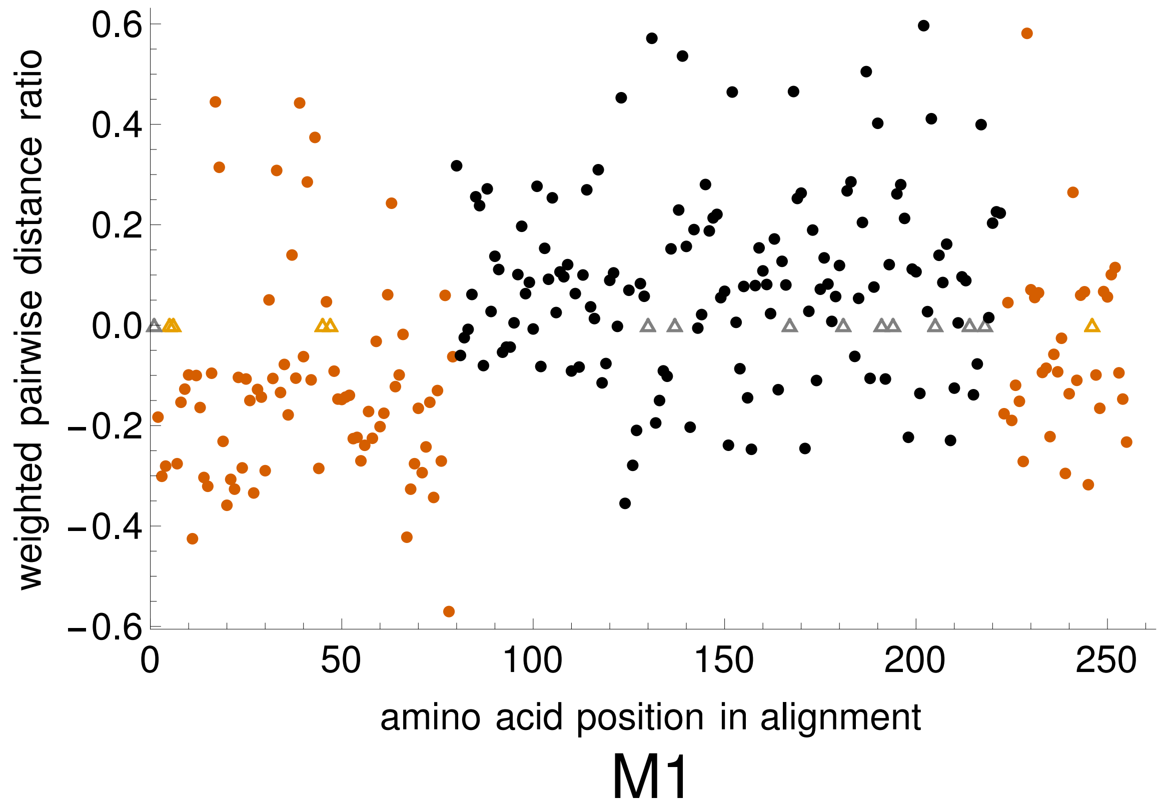


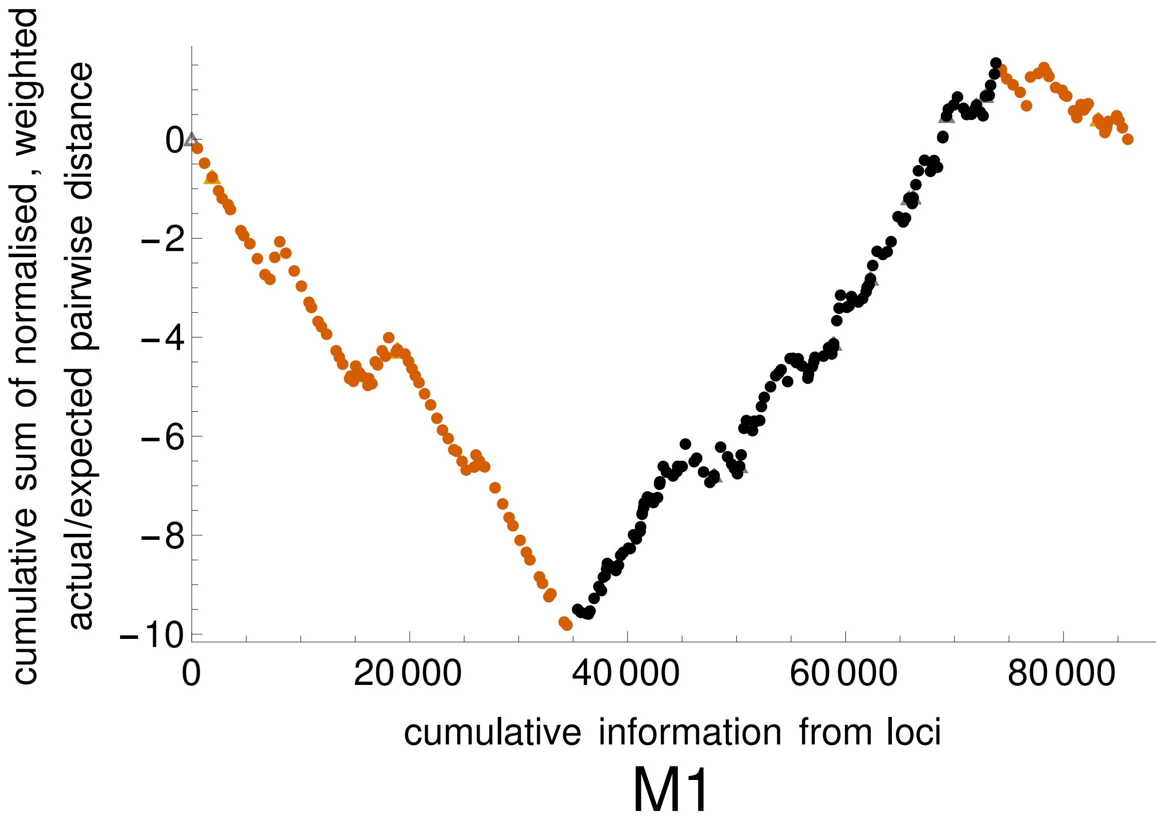

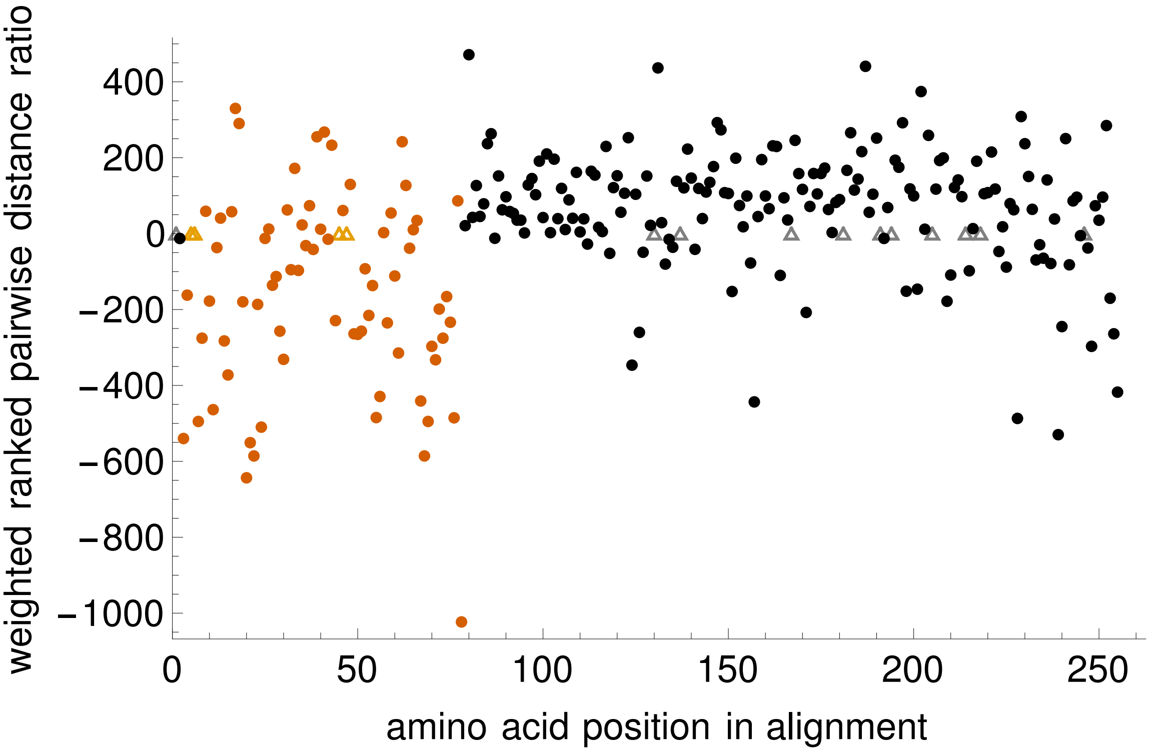
weighted ranked pairwise distance ratio
400
200
0
−200
−400
−600
−800
−1000
0
50
100
150
200
250
amino acid position in alignment

M1

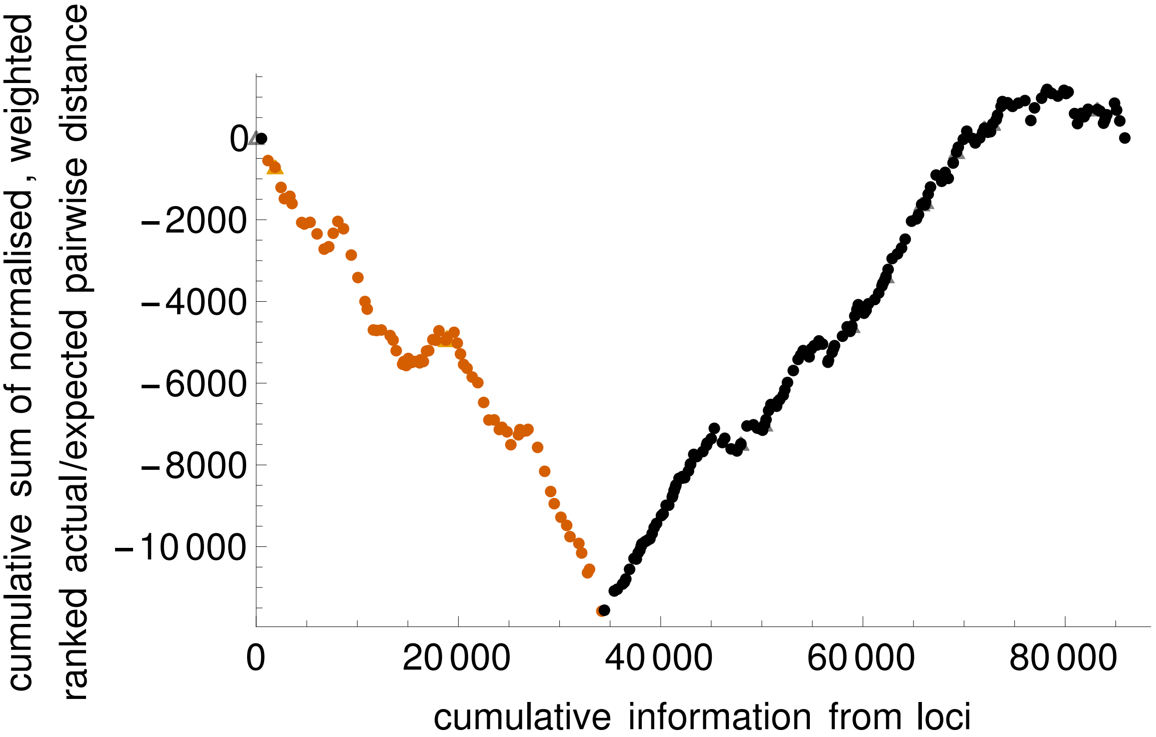
cumulative sum of normalised, weighted ranked actual/expected pairwise distance
0
−2000
−4000
−6000
−8000
−10 000
0
20 000
40 000
60 000
80 000
cumulative information from loci

M1

# M2

## Gene length histogram

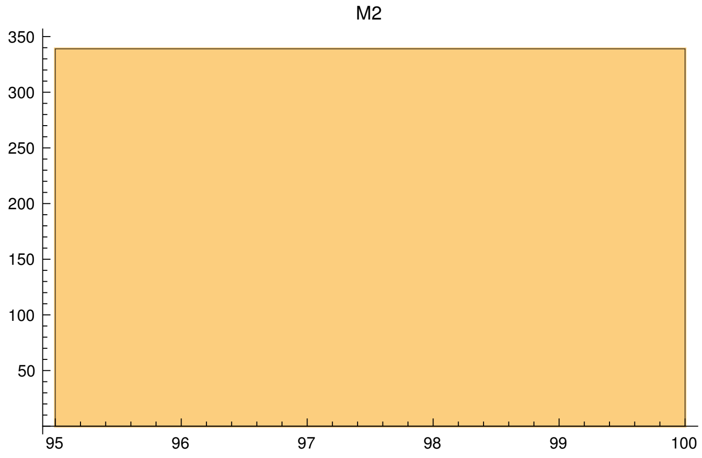

## Information vs. nPD

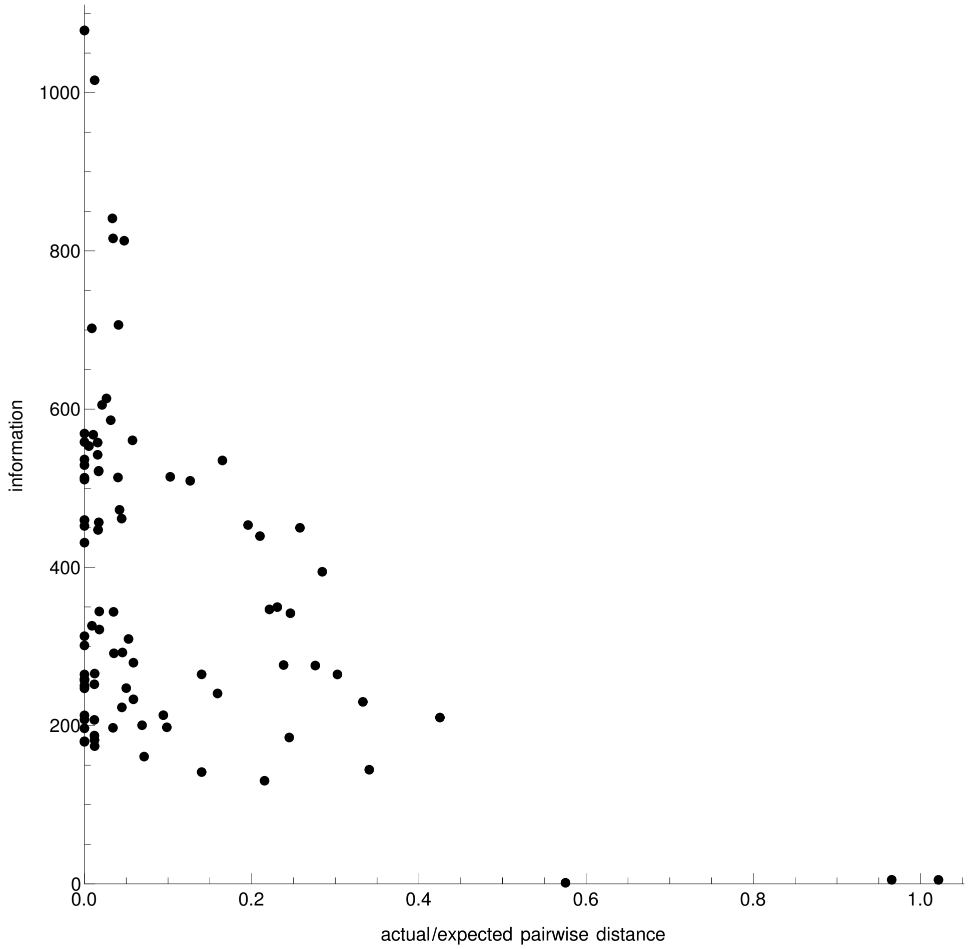


M2

## Example sequences highlighted by regions found in analyses to be conserved

```
Interesting points (by weighted raw PD) highlighted for gene M2:
ATGAGTCTTCTAACCGAGGTCGAAACGCCTACCAGAAACGGATGGGAGTGCAAATGCAGCGATTCAAGTGATCCTC⋱
    TCGTTATTGCCGCAAGTATCATTGGGATCTTGCACTTGATATTGTGGATTCTTGATCGTCTTTTCTTCAAATGCA⋱
    TTTATCGTCGCCTTAAATACGGTTTGAAAAGAGGGCCTTCTACGGAAGGGGTGCCTGAGTCTATGAGGGAAGAGT⋱
    ATCGGCAGGAACAGCAGAGTGCTGTGGATGTTGACGATGGTCATTTTGTCAACATAGAGCTGGAGTAA

Interesting points (by weighted ranked PD) highlighted for gene M2:
ATGAGTCTTCTAACCGAGGTCGAAACGCCTACCAGAAACGGATGGGAGTGCAAATGCAGCGATTCAAGTGATCCTCTC⋱
    GTTATTGCCGCAAGTATCATTGGGATCTTGCACTTGATATTGTGGATTCTTGATCGTCTTTTCTTCAAATGCATTTA⋱
    TCGTCGCCTTAAATACGGTTTGAAAAGAGGGCCTTCTACGGAAGGGGTGCCTGAGTCTATGAGGGAAGAGTATCGGC⋱
    AGGAACAGCAGAGTGCTGTGGATGTTGACGATGGTCATTTTGTCAACATAGAGCTGGAGTAA
```

## Per codon conservation report

| M2 | | | |
|---|---|---|---|
| Pos. | 1 | obs: | exp: |
| atg | M | 339 | 339.00 |
| --- | -- | ------ | ------ |
| mPD | | 0 | 0 |
| | | nPD: | 1. |
| | | N. weight: | 0. |
| | | Sc. PD: | 0 |
| | | Sc. rank: | 0 |

| M2 | | | |
|---|---|---|---|
| Pos. | 2 | obs: | exp: |
| tct | S | 0 | 53.89 |
| tcc | S | 0 | 47.22 |
| tca | S | 0 | 75.10 |
| tcg | S | 0 | 20.66 |
| agt | S | 326 | 74.58 |
| agc | S | 13 | 67.54 |
| --- | -- | ------ | ------ |
| mPD | | 0.074 | 1.7 |
| | | nPD: | 0.04 |
| | | N. weight: | 1.2 |
| | | Sc. PD: | -0.013 |
| | | Sc. rank: | 200.2 |

| M2 | | | |
|---|---|---|---|
| Pos. | 3 | obs: | exp: |
| tta | L | 0 | 29.62 |
| ttg | L | 0 | 65.29 |
| ctt | L | 339 | 63.25 |
| ctc | L | 0 | 54.84 |
| cta | L | 0 | 51.07 |
| ctg | L | 0 | 74.94 |
| --- | -- | ------ | ------ |
| mPD | | 0 | 1.1 |
| | | nPD: | 0. |
| | | N. weight: | 1.5 |
| | | Sc. PD: | -0.082 |
| | | Sc. rank: | -454.0 |

| M2 | | | |
|---|---|---|---|
| Pos. | 4 | obs: | exp: |
| tta | L | 1 | 29.62 |
| ttg | L | 0 | 65.29 |
| ctt | L | 1 | 63.25 |
| ctc | L | 0 | 54.84 |
| cta | L | 334 | 51.07 |
| ctg | L | 3 | 74.94 |
| --- | -- | ------ | ------ |
| mPD | | 0.029 | 1.1 |
| | | nPD: | 0.03 |
| | | N. weight: | 1.6 |
| | | Sc. PD: | -0.046 |
| | | Sc. rank: | 112.2 |

| M2 | | | |
|---|---|---|---|
| Pos. | 5 | obs: | exp: |
| tct | S | 1 | 0.16 |
| tcc | S | 0 | 0.14 |
| tca | S | 0 | 0.22 |
| tcg | S | 0 | 0.06 |
| act | T | 0 | 89.30 |
| acc | T | 338 | 72.53 |
| aca | T | 0 | 148.10 |
| acg | T | 0 | 28.10 |
| agt | S | 0 | 0.22 |
| agc | S | 0 | 0.20 |
| --- | -- | ------ | ------ |
| mPD | | 0.012 | 0.69 |
| | | nPD: | 0.02 |
| | | N. weight: | 1.4 |
| | | Sc. PD: | -0.052 |
| | | Sc. rank: | 36.9 |

| M2 | | | |
|---|---|---|---|
| Pos. | 6 | obs: | exp: |
| tct | S | 1 | 0.16 |
| tcc | S | 0 | 0.14 |
| tca | S | 0 | 0.22 |
| tcg | S | 0 | 0.06 |
| agt | S | 0 | 0.22 |
| agc | S | 0 | 0.20 |
| gaa | E | 0 | 194.50 |
| gag | E | 338 | 143.50 |
| --- | -- | ------ | ------ |
| mPD | | 0.018 | 0.50 |
| | | nPD: | 0.04 |
| | | N. weight: | 0.78 |
| | | Sc. PD: | -0.015 |
| | | Sc. rank: | 96.3 |

| M2 | | | |
|---|---|---|---|
| Pos. | 7 | obs: | exp: |
| act | T | 0 | 0.26 |
| acc | T | 1 | 0.21 |
| aca | T | 0 | 0.44 |
| acg | T | 0 | 0.08 |
| gtt | V | 3 | 69.47 |
| gtc | V | 335 | 70.94 |
| gta | V | 0 | 63.35 |
| gtg | V | 0 | 134.20 |
| --- | -- | ------ | ------ |
| mPD | | 0.029 | 0.73 |
| | | nPD: | 0.04 |
| | | N. weight: | 1.4 |
| | | Sc. PD: | -0.021 |
| | | Sc. rank: | 182.3 |

| M2 | | | |
|---|---|---|---|
| Pos. | 8 | obs: | exp: |
| gaa | E | 338 | 195.10 |
| gag | E | 1 | 143.90 |
| --- | -- | ------ | ------ |
| mPD | | 0.0059 | 0.49 |
| | | nPD: | 0.01 |
| | | N. weight: | 0.49 |
| | | Sc. PD: | -0.02 |
| | | Sc. rank: | -26.3 |

| M2 | | | |
|---|---|---|---|
| Pos. | 9 | obs: | exp: |
| cgt | R | 0 | 0.04 |
| cgc | R | 0 | 0.05 |
| cga | R | 0 | 0.10 |
| cgg | R | 0 | 0.08 |
| act | T | 0 | 89.03 |
| acc | T | 0 | 72.32 |
| aca | T | 0 | 147.60 |
| acg | T | 337 | 28.02 |
| aga | R | 0 | 0.45 |
| agg | R | 1 | 0.28 |
| gtt | V | 0 | 0.21 |
| gtc | V | 1 | 0.21 |
| gta | V | 0 | 0.19 |
| gtg | V | 0 | 0.40 |
| --- | -- | ------ | ------ |
| mPD | | 0.024 | 0.71 |
| | | nPD: | 0.03 |
| | | N. weight: | 2.3 |
| | | Sc. PD: | -0.048 |
| | | Sc. rank: | 211.4 |

| M2 | | | |
|---|---|---|---|
| Pos. | 10 | obs: | exp: |
| tta | L | 0 | 2.88 |
| ttg | L | 0 | 6.36 |
| ctt | L | 33 | 6.16 |
| ctc | L | 0 | 5.34 |
| cta | L | 0 | 4.97 |
| ctg | L | 0 | 7.29 |
| cct | P | 287 | 75.57 |
| ccc | P | 0 | 51.77 |
| cca | P | 0 | 113.30 |
| ccg | P | 0 | 46.40 |
| cat | H | 19 | 10.30 |
| cac | H | 0 | 8.70 |
| --- | -- | ------ | ------ |
| mPD | | 0.27 | 1.1 |
| | | nPD: | 0.26 |
| | | N. weight: | 1.2 |
| | | Sc. PD: | 0.23 |
| | | Sc. rank: | 387.5 |

| M2 | | | |
|---|---|---|---|
| Pos. | 11 | obs: | exp: |
| att | I | 0 | 0.75 |
| atc | I | 2 | 0.52 |
| ata | I | 0 | 0.72 |
| act | T | 0 | 89.03 |
| acc | T | 337 | 72.32 |
| aca | T | 0 | 147.60 |
| acg | T | 0 | 28.02 |
| --- | -- | ------ | ------ |
| mPD | | 0.012 | 0.70 |
| | | nPD: | 0.02 |
| | | N. weight: | 1.4 |
| | | Sc. PD: | -0.052 |
| | | Sc. rank: | 23.8 |

| M2 | | | |
|---|---|---|---|
| Pos. | 12 | obs: | exp: |
| cgt | R | 0 | 12.91 |
| cgc | R | 0 | 15.23 |
| cga | R | 0 | 29.89 |
| cgg | R | 0 | 25.95 |
| aaa | K | 28 | 15.68 |
| aag | K | 0 | 12.32 |
| aga | R | 311 | 139.90 |
| agg | R | 0 | 87.15 |
| --- | -- | ------ | ------ |
| mPD | | 0.15 | 1.1 |
| | | nPD: | 0.14 |
| | | N. weight: | 0.71 |
| | | Sc. PD: | 0.058 |
| | | Sc. rank: | 178.6 |

| M2 | | | |
|---|---|---|---|
| Pos. | 13 | obs: | exp: |
| tct | S | 0 | 0.64 |
| tcc | S | 0 | 0.56 |
| tca | S | 0 | 0.89 |
| tcg | S | 0 | 0.24 |
| act | T | 0 | 7.40 |
| acc | T | 28 | 6.01 |
| aca | T | 0 | 12.27 |
| acg | T | 0 | 2.33 |
| aat | N | 0 | 160.50 |
| aac | N | 307 | 146.50 |
| agt | S | 0 | 0.88 |
| agc | S | 4 | 0.80 |
| --- | -- | ------ | ------ |
| mPD | | 0.17 | 0.73 |
| | | nPD: | 0.24 |
| | | N. weight: | 0.74 |
| | | Sc. PD: | 0.13 |
| | | Sc. rank: | 225.2 |

| M2 | | | |
|---|---|---|---|
| Pos. | 14 | obs: | exp: |
| gtt | V | 0 | 0.41 |
| gtc | V | 0 | 0.42 |
| gta | V | 2 | 0.37 |
| gtg | V | 0 | 0.79 |
| gaa | E | 4 | 2.30 |
| gag | E | 0 | 1.70 |
| ggt | G | 0 | 41.09 |
| ggc | G | 0 | 45.03 |
| gga | G | 333 | 161.10 |
| ggg | G | 0 | 85.79 |
| --- | -- | ------ | ------ |
| mPD | | 0.035 | 0.70 |
| | | nPD: | 0.05 |
| | | N. weight: | 0.66 |
| | | Sc. PD: | -0.0036 |
| | | Sc. rank: | 126.0 |

| M2 | | | |
|---|---|---|---|
| Pos. | 15 | obs: | exp: |
| tgg | W | 339 | 339.00 |
| --- | -- | ------ | ------ |
| mPD | | 0 | 0 |
| | | nPD: | 1. |
| | | N. weight: | 0. |
| | | Sc. PD: | 0 |
| | | Sc. rank: | 0 |

| M2 | | | |
|---|---|---|---|
| Pos. | 16 | obs: | exp: |
| gaa | E | 0 | 192.80 |
| gag | E | 335 | 142.20 |
| ggt | G | 0 | 0.49 |
| ggc | G | 0 | 0.54 |
| gga | G | 0 | 1.93 |
| ggg | G | 4 | 1.03 |
| --- | -- | ------ | ------ |
| mPD | | 0.023 | 0.51 |
| | | nPD: | 0.05 |
| | | N. weight: | 0.78 |
| | | Sc. PD: | -0.0077 |
| | | Sc. rank: | 134.0 |

| M2 | | | |
|---|---|---|---|
| Pos. | 17 | obs: | exp: |
| tgt | C | 0 | 139.70 |
| tgc | C | 339 | 199.30 |
| --- | -- | ------ | ------ |
| mPD | | 0 | 0.48 |
| | | nPD: | 0. |
| | | N. weight: | 0.48 |
| | | Sc. PD: | -0.026 |
| | | Sc. rank: | -143.7 |

| M2 | | | |
|---|---|---|---|
| Pos. | 18 | obs: | exp: |
| tct | S | 0 | 0.16 |
| tcc | S | 0 | 0.14 |
| tca | S | 0 | 0.22 |
| tcg | S | 0 | 0.06 |
| cgt | R | 0 | 1.54 |
| cgc | R | 0 | 1.81 |
| cga | R | 0 | 3.56 |
| cgg | R | 0 | 3.09 |
| aat | N | 0 | 14.63 |
| aac | N | 28 | 13.37 |
| aaa | K | 273 | 152.90 |
| aag | K | 0 | 120.10 |
| agt | S | 0 | 0.22 |
| agc | S | 1 | 0.20 |
| aga | R | 37 | 16.64 |
| agg | R | 0 | 10.37 |
| --- | -- | ------ | ------ |
| mPD | | 0.36 | 0.84 |
| | | nPD: | 0.43 |
| | | N. weight: | 0.56 |
| | | Sc. PD: | 0.2 |
| | | Sc. rank: | 196.3 |

| | | | |
|---|---|---|---|
| M2 | | | |
| Pos. | 19 | obs: | exp: |
| tgt | C | 0 | 139.70 |
| tgc | C | 339 | 199.30 |
| --- | -- | ------ | ------ |
| mPD | | 0 | 0.48 |
| | | nPD: | 0. |
| | | N. weight: | 0.48 |
| | | Sc. PD: | -0.026 |
| | | Sc. rank: | -143.7 |

,

| | | | |
|---|---|---|---|
| M2 | | | |
| Pos. | 20 | obs: | exp: |
| tct | S | 0 | 47.06 |
| tcc | S | 0 | 41.23 |
| tca | S | 0 | 65.58 |
| tcg | S | 0 | 18.04 |
| cgt | R | 0 | 0.04 |
| cgc | R | 0 | 0.05 |
| cga | R | 0 | 0.10 |
| cgg | R | 0 | 0.08 |
| aat | N | 0 | 21.95 |
| aac | N | 42 | 20.05 |
| agt | S | 0 | 65.12 |
| agc | S | 296 | 58.97 |
| aga | R | 1 | 0.45 |
| agg | R | 0 | 0.28 |
| --- | -- | ------ | ------ |
| mPD | | 0.22 | 1.8 |
| | | nPD: | 0.13 |
| | | N. weight: | 1.4 |
| | | Sc. PD: | 0.093 |
| | | Sc. rank: | 337.1 |

,

| | | | |
|---|---|---|---|
| M2 | | | |
| Pos. | 21 | obs: | exp: |
| gat | D | 296 | 157.90 |
| gac | D | 0 | 138.10 |
| ggt | G | 43 | 5.31 |
| ggc | G | 0 | 5.81 |
| gga | G | 0 | 20.80 |
| ggg | G | 0 | 11.08 |
| --- | -- | ------ | ------ |
| mPD | | 0.22 | 0.80 |
| | | nPD: | 0.28 |
| | | N. weight: | 0.74 |
| | | Sc. PD: | 0.16 |
| | | Sc. rank: | 241.2 |

,

| | | | |
|---|---|---|---|
| M2 | | | |
| Pos. | 22 | obs: | exp: |
| tct | S | 0 | 53.89 |
| tcc | S | 0 | 47.22 |
| tca | S | 339 | 75.10 |
| tcg | S | 0 | 20.66 |
| agt | S | 0 | 74.58 |
| agc | S | 0 | 67.54 |
| --- | -- | ------ | ------ |
| mPD | | 0 | 1.7 |
| | | nPD: | 0. |
| | | N. weight: | 1.4 |
| | | Sc. PD: | -0.073 |
| | | Sc. rank: | -407.6 |

,

| | | | |
|---|---|---|---|
| M2 | | | |
| Pos. | 23 | obs: | exp: |
| tct | S | 0 | 53.89 |
| tcc | S | 0 | 47.22 |
| tca | S | 0 | 75.10 |
| tcg | S | 0 | 20.66 |
| agt | S | 339 | 74.58 |
| agc | S | 0 | 67.54 |
| --- | -- | ------ | ------ |
| mPD | | 0 | 1.7 |
| | | nPD: | 0. |
| | | N. weight: | 1.4 |
| | | Sc. PD: | -0.074 |
| | | Sc. rank: | -409.5 |

,

| | | | |
|---|---|---|---|
| M2 | | | |
| Pos. | 24 | obs: | exp: |
| gat | D | 338 | 180.90 |
| gac | D | 1 | 158.10 |
| --- | -- | ------ | ------ |
| mPD | | 0.0059 | 0.50 |
| | | nPD: | 0.01 |
| | | N. weight: | 0.56 |
| | | Sc. PD: | -0.023 |
| | | Sc. rank: | -33.7 |

,

| | | | |
|---|---|---|---|
| M2 | | | |
| Pos. | 25 | obs: | exp: |
| cct | P | 339 | 89.27 |
| ccc | P | 0 | 61.15 |
| cca | P | 0 | 133.80 |
| ccg | P | 0 | 54.81 |
| --- | -- | ------ | ------ |
| mPD | | 0 | 0.72 |
| | | nPD: | 0. |
| | | N. weight: | 1.2 |
| | | Sc. PD: | -0.065 |
| | | Sc. rank: | -360.9 |

,

| | | | |
|---|---|---|---|
| M2 | | | |
| Pos. | 26 | obs: | exp: |
| tta | L | 0 | 29.62 |
| ttg | L | 0 | 65.29 |
| ctt | L | 3 | 63.25 |
| ctc | L | 333 | 54.84 |
| cta | L | 3 | 51.07 |
| ctg | L | 0 | 74.94 |
| --- | -- | ------ | ------ |
| mPD | | 0.035 | 1.1 |
| | | nPD: | 0.03 |
| | | N. weight: | 1.6 |
| | | Sc. PD: | -0.037 |
| | | Sc. rank: | 123.6 |

,

| | | | |
|---|---|---|---|
| M2 | | | |
| Pos. | 27 | obs: | exp: |
| att | I | 10 | 3.76 |
| atc | I | 0 | 2.62 |
| ata | I | 0 | 3.62 |
| gtt | V | 325 | 67.42 |
| gtc | V | 3 | 68.84 |
| gta | V | 0 | 61.47 |
| gtg | V | 0 | 130.30 |
| gct | A | 1 | 0.25 |
| gcc | A | 0 | 0.20 |
| gca | A | 0 | 0.45 |
| gcg | A | 0 | 0.10 |
| --- | -- | ------ | ------ |
| mPD | | 0.081 | 0.79 |
| | | nPD: | 0.1 |
| | | N. weight: | 1.4 |
| | | Sc. PD: | 0.062 |
| | | Sc. rank: | 327.9 |

,

| | | | |
|---|---|---|---|
| M2 | | | |
| Pos. | 28 | obs: | exp: |
| att | I | 303 | 114.30 |
| atc | I | 0 | 79.67 |
| ata | I | 1 | 110.00 |
| act | T | 1 | 0.26 |
| acc | T | 0 | 0.21 |
| aca | T | 0 | 0.44 |
| acg | T | 0 | 0.08 |
| gtt | V | 34 | 6.99 |
| gtc | V | 0 | 7.14 |
| gta | V | 0 | 6.37 |
| gtg | V | 0 | 13.50 |
| --- | -- | ------ | ------ |
| mPD | | 0.19 | 0.87 |
| | | nPD: | 0.22 |
| | | N. weight: | 0.93 |
| | | Sc. PD: | 0.15 |
| | | Sc. rank: | 272.0 |

,

| | | | |
|---|---|---|---|
| M2 | | | |
| Pos. | 29 | obs: | exp: |
| act | T | 0 | 0.26 |
| acc | T | 1 | 0.21 |
| aca | T | 0 | 0.44 |
| acg | T | 0 | 0.08 |
| gct | A | 1 | 85.13 |
| gcc | A | 302 | 69.24 |
| gca | A | 35 | 150.60 |
| gcg | A | 0 | 32.98 |
| --- | -- | ------ | ------ |
| mPD | | 0.20 | 0.69 |
| | | nPD: | 0.28 |
| | | N. weight: | 1.1 |
| | | Sc. PD: | 0.23 |
| | | Sc. rank: | 352.3 |

,

| | | | |
|---|---|---|---|
| M2 | | | |
| Pos. | 30 | obs: | exp: |
| gct | A | 28 | 85.38 |
| gcc | A | 0 | 69.44 |
| gca | A | 308 | 151.10 |
| gcg | A | 3 | 33.08 |
| --- | -- | ------ | ------ |
| mPD | | 0.17 | 0.69 |
| | | nPD: | 0.24 |
| | | N. weight: | 0.5 |
| | | Sc. PD: | 0.09 |
| | | Sc. rank: | 152.2 |

,

| | | | |
|---|---|---|---|
| M2 | | | |
| Pos. | 31 | obs: | exp: |
| tct | S | 0 | 49.12 |
| tcc | S | 0 | 43.05 |
| tca | S | 0 | 68.46 |
| tcg | S | 0 | 18.83 |
| aat | N | 2 | 15.68 |
| aac | N | 28 | 14.32 |
| agt | S | 298 | 67.98 |
| agc | S | 11 | 61.56 |
| --- | -- | ------ | ------ |
| mPD | | 0.37 | 1.7 |
| | | nPD: | 0.21 |
| | | N. weight: | 1.2 |
| | | Sc. PD: | 0.18 |
| | | Sc. rank: | 334.6 |

,

| | | | |
|---|---|---|---|
| M2 | | | |
| Pos. | 32 | obs: | exp: |
| att | I | 28 | 127.40 |
| atc | I | 311 | 88.85 |
| ata | I | 0 | 122.70 |
| --- | -- | ------ | ------ |
| mPD | | 0.15 | 0.66 |
| | | nPD: | 0.23 |
| | | N. weight: | 0.94 |
| | | Sc. PD: | 0.16 |
| | | Sc. rank: | 280.1 |

,

| | | | |
|---|---|---|---|
| M2 | | | |
| Pos. | 33 | obs: | exp: |
| att | I | 337 | 127.40 |
| atc | I | 2 | 88.85 |
| ata | I | 0 | 122.70 |
| --- | -- | ------ | ------ |
| mPD | | 0.012 | 0.66 |
| | | nPD: | 0.02 |
| | | N. weight: | 0.86 |
| | | Sc. PD: | -0.031 |
| | | Sc. rank: | 40.0 |

,

| | | | |
|---|---|---|---|
| M2 | | | |
| Pos. | 34 | obs: | exp: |
| ggt | G | 0 | 41.83 |
| ggc | G | 0 | 45.84 |
| gga | G | 0 | 164.00 |
| ggg | G | 339 | 87.34 |
| --- | -- | ------ | ------ |
| mPD | | 0 | 0.67 |
| | | nPD: | 0. |
| | | N. weight: | 1.2 |
| | | Sc. PD: | -0.066 |
| | | Sc. rank: | -366.8 |

,

| | | | |
|---|---|---|---|
| M2 | | | |
| Pos. | 35 | obs: | exp: |
| att | I | 1 | 127.40 |
| atc | I | 309 | 88.85 |
| ata | I | 29 | 122.70 |
| --- | -- | ------ | ------ |
| mPD | | 0.16 | 0.66 |
| | | nPD: | 0.25 |
| | | N. weight: | 0.92 |
| | | Sc. PD: | 0.17 |
| | | Sc. rank: | 287.1 |

,

| | | | |
|---|---|---|---|
| M2 | | | |
| Pos. | 36 | obs: | exp: |
| tta | L | 0 | 29.62 |
| ttg | L | 339 | 65.29 |
| ctt | L | 0 | 63.25 |
| ctc | L | 0 | 54.84 |
| cta | L | 0 | 51.07 |
| ctg | L | 0 | 74.94 |
| --- | -- | ------ | ------ |
| mPD | | 0 | 1.1 |
| | | nPD: | 0. |
| | | N. weight: | 1.5 |
| | | Sc. PD: | -0.08 |
| | | Sc. rank: | -445.4 |

,

| | | | |
|---|---|---|---|
| M2 | | | |
| Pos. | 37 | obs: | exp: |
| cat | H | 0 | 183.70 |
| cac | H | 339 | 155.30 |
| --- | -- | ------ | ------ |
| mPD | | 0 | 0.50 |
| | | nPD: | 0. |
| | | N. weight: | 0.71 |
| | | Sc. PD: | -0.038 |
| | | Sc. rank: | -211.1 |

,

| | | | |
|---|---|---|---|
| M2 | | | |
| Pos. | 38 | obs: | exp: |
| tta | L | 0 | 29.62 |
| ttg | L | 336 | 65.29 |
| ctt | L | 0 | 63.25 |
| ctc | L | 0 | 54.84 |
| cta | L | 0 | 51.07 |
| ctg | L | 3 | 74.94 |
| --- | -- | ------ | ------ |
| mPD | | 0.018 | 1.1 |
| | | nPD: | 0.02 |
| | | N. weight: | 1.5 |
| | | Sc. PD: | -0.056 |
| | | Sc. rank: | -26.6 |

,

| | | | |
|---|---|---|---|
| M2 | | | |
| Pos. | 39 | obs: | exp: |
| att | I | 0 | 125.90 |
| atc | I | 0 | 87.80 |
| ata | I | 335 | 121.30 |
| atg | M | 2 | 2.00 |
| gtt | V | 0 | 0.41 |
| gtc | V | 0 | 0.42 |
| gta | V | 2 | 0.37 |
| gtg | V | 0 | 0.79 |
| --- | -- | ------ | ------ |
| mPD | | 0.024 | 0.68 |
| | | nPD: | 0.03 |
| | | N. weight: | 0.92 |
| | | Sc. PD: | -0.018 |
| | | Sc. rank: | 108.8 |

,

| | | | |
|---|---|---|---|
| M2 | | | |
| Pos. | 40 | obs: | exp: |
| tta | L | 1 | 29.62 |
| ttg | L | 338 | 65.29 |
| ctt | L | 0 | 63.25 |
| ctc | L | 0 | 54.84 |
| cta | L | 0 | 51.07 |
| ctg | L | 0 | 74.94 |
| --- | -- | ------ | ------ |
| mPD | | 0.0059 | 1.1 |
| | | nPD: | 0.01 |
| | | N. weight: | 1.5 |
| | | Sc. PD: | -0.072 |
| | | Sc. rank: | -159.2 |

,

| | | | |
|---|---|---|---|
| M2 | | | |
| Pos. | 41 | obs: | exp: |
| tgg | W | 338 | 338.00 |
| ggt | G | 0 | 0.12 |
| ggc | G | 0 | 0.14 |
| gga | G | 0 | 0.48 |
| ggg | G | 1 | 0.26 |
| --- | -- | ------ | ------ |
| mPD | | 0.0059 | 0.010 |
| | | nPD: | 0.58 |
| | | N. weight: | 0.0036 |
| | | Sc. PD: | 0.0018 |
| | | Sc. rank: | 1.3 |

,

| | | | |
|---|---|---|---|
| M2 | | | |
| Pos. | 42 | obs: | exp: |
| att | I | 333 | 127.10 |
| atc | I | 5 | 88.58 |
| ata | I | 0 | 122.30 |
| act | T | 1 | 0.26 |
| acc | T | 0 | 0.21 |
| aca | T | 0 | 0.44 |
| acg | T | 0 | 0.08 |
| --- | -- | ------ | ------ |
| mPD | | 0.035 | 0.66 |
| | | nPD: | 0.05 |
| | | N. weight: | 0.83 |
| | | Sc. PD: | -0.0023 |
| | | Sc. rank: | 162.2 |

,

| | | | |
|---|---|---|---|
| M2 | | | |
| Pos. | 43 | obs: | exp: |
| ttt | F | 1 | 0.41 |
| ttc | F | 0 | 0.59 |
| tta | L | 0 | 29.44 |
| ttg | L | 0 | 64.90 |
| ctt | L | 337 | 62.88 |
| ctc | L | 0 | 54.51 |
| cta | L | 0 | 50.77 |
| ctg | L | 0 | 74.49 |
| att | I | 1 | 0.38 |
| atc | I | 0 | 0.26 |
| ata | I | 0 | 0.36 |
| --- | -- | ------ | ------ |
| mPD | | 0.012 | 1.1 |
| | | nPD: | 0.01 |
| | | N. weight: | 1.5 |
| | | Sc. PD: | -0.066 |
| | | Sc. rank: | -104.8 |

,

M2

| Pos. | 44 | obs: | exp: |
|---|---|---|---|
| aat | N | 9 | 4.70 |
| aac | N | 0 | 4.30 |
| gat | D | 330 | 176.10 |
| gac | D | 0 | 153.90 |
| --- | -- | ------ | ------ |
| mPD | | 0.052 | 0.55 |
| | | nPD: | 0.09 |
| | | N. weight: | 0.57 |
| | | Sc. PD: | 0.021 |
| | | Sc. rank: | 128.6 |

,

M2

| Pos. | 45 | obs: | exp: |
|---|---|---|---|
| cgt | R | 339 | 14.07 |
| cgc | R | 0 | 16.60 |
| cga | R | 0 | 32.58 |
| cgg | R | 0 | 28.29 |
| aga | R | 0 | 152.50 |
| agg | R | 0 | 95.00 |
| --- | -- | ------ | ------ |
| mPD | | 0 | 0.96 |
| | | nPD: | 0. |
| | | N. weight: | 2.9 |
| | | Sc. PD: | -0.15 |
| | | Sc. rank: | -860.4 |

,

M2

| Pos. | 46 | obs: | exp: |
|---|---|---|---|
| tta | L | 0 | 29.53 |
| ttg | L | 0 | 65.10 |
| ctt | L | 336 | 63.07 |
| ctc | L | 2 | 54.67 |
| cta | L | 0 | 50.92 |
| ctg | L | 0 | 74.72 |
| att | I | 1 | 0.38 |
| atc | I | 0 | 0.26 |
| ata | I | 0 | 0.36 |
| --- | -- | ------ | ------ |
| mPD | | 0.018 | 1.1 |
| | | nPD: | 0.02 |
| | | N. weight: | 1.5 |
| | | Sc. PD: | -0.057 |
| | | Sc. rank: | -12.5 |

,

M2

| Pos. | 47 | obs: | exp: |
|---|---|---|---|
| ttt | F | 1 | 139.40 |
| ttc | F | 338 | 199.60 |
| --- | -- | ------ | ------ |
| mPD | | 0.0059 | 0.48 |
| | | nPD: | 0.01 |
| | | N. weight: | 0.47 |
| | | Sc. PD: | -0.02 |
| | | Sc. rank: | -15.3 |

,

M2

| Pos. | 48 | obs: | exp: |
|---|---|---|---|
| ttt | F | 0 | 139.40 |
| ttc | F | 339 | 199.60 |
| --- | -- | ------ | ------ |
| mPD | | 0 | 0.48 |
| | | nPD: | 0. |
| | | N. weight: | 0.48 |
| | | Sc. PD: | -0.026 |
| | | Sc. rank: | -143.2 |

,

M2

| Pos. | 49 | obs: | exp: |
|---|---|---|---|
| aaa | K | 320 | 189.80 |
| aag | K | 19 | 149.20 |
| --- | -- | ------ | ------ |
| mPD | | 0.11 | 0.49 |
| | | nPD: | 0.22 |
| | | N. weight: | 0.35 |
| | | Sc. PD: | 0.054 |
| | | Sc. rank: | 100.0 |

,

M2

| Pos. | 50 | obs: | exp: |
|---|---|---|---|
| tat | Y | 1 | 0.52 |
| tac | Y | 0 | 0.48 |
| tgt | C | 124 | 138.90 |
| tgc | C | 213 | 198.10 |
| tgg | W | 1 | 1.00 |
| --- | -- | ------ | ------ |
| mPD | | 0.48 | 0.49 |
| | | nPD: | 0.97 |
| | | N. weight: | 0.014 |
| | | Sc. PD: | 0.012 |
| | | Sc. rank: | 4.8 |

,

M2

| Pos. | 51 | obs: | exp: |
|---|---|---|---|
| att | I | 338 | 127.40 |
| atc | I | 1 | 88.85 |
| ata | I | 0 | 122.70 |
| --- | -- | ------ | ------ |
| mPD | | 0.0059 | 0.66 |
| | | nPD: | 0.01 |
| | | N. weight: | 0.88 |
| | | Sc. PD: | -0.039 |
| | | Sc. rank: | -69.1 |

,

M2

| Pos. | 52 | obs: | exp: |
|---|---|---|---|
| tat | Y | 335 | 173.40 |
| tac | Y | 0 | 161.60 |
| cat | H | 4 | 2.17 |
| cac | H | 0 | 1.83 |
| --- | -- | ------ | ------ |
| mPD | | 0.023 | 0.52 |
| | | nPD: | 0.04 |
| | | N. weight: | 0.6 |
| | | Sc. PD: | -0.0063 |
| | | Sc. rank: | 99.1 |

,

M2

| Pos. | 53 | obs: | exp: |
|---|---|---|---|
| cgt | R | 339 | 14.07 |
| cgc | R | 0 | 16.60 |
| cga | R | 0 | 32.58 |
| cgg | R | 0 | 28.29 |
| aga | R | 0 | 152.50 |
| agg | R | 0 | 95.00 |
| --- | -- | ------ | ------ |
| mPD | | 0 | 0.96 |
| | | nPD: | 0. |
| | | N. weight: | 2.9 |
| | | Sc. PD: | -0.15 |
| | | Sc. rank: | -860.4 |

,

M2

| Pos. | 54 | obs: | exp: |
|---|---|---|---|
| cct | P | 0 | 0.26 |
| ccc | P | 1 | 0.18 |
| cca | P | 0 | 0.39 |
| ccg | P | 0 | 0.16 |
| cgt | R | 1 | 14.03 |
| cgc | R | 337 | 16.55 |
| cga | R | 0 | 32.48 |
| cgg | R | 0 | 28.21 |
| aga | R | 0 | 152.00 |
| agg | R | 0 | 94.72 |
| --- | -- | ------ | ------ |
| mPD | | 0.012 | 0.97 |
| | | nPD: | 0.01 |
| | | N. weight: | 2.7 |
| | | Sc. PD: | -0.11 |
| | | Sc. rank: | -97.7 |

,

M2

| Pos. | 55 | obs: | exp: |
|---|---|---|---|
| ttt | F | 36 | 14.80 |
| ttc | F | 0 | 21.20 |
| tta | L | 0 | 26.47 |
| ttg | L | 0 | 58.36 |
| ctt | L | 302 | 56.54 |
| ctc | L | 1 | 49.01 |
| cta | L | 0 | 45.65 |
| ctg | L | 0 | 66.98 |
| --- | -- | ------ | ------ |
| mPD | | 0.20 | 1.2 |
| | | nPD: | 0.17 |
| | | N. weight: | 1.4 |
| | | Sc. PD: | 0.15 |
| | | Sc. rank: | 384.4 |

,

M2

| Pos. | 56 | obs: | exp: |
|---|---|---|---|
| cgt | R | 0 | 0.12 |
| cgc | R | 0 | 0.15 |
| cga | R | 0 | 0.29 |
| cgg | R | 0 | 0.25 |
| aaa | K | 336 | 188.10 |
| aag | K | 0 | 147.90 |
| aga | R | 3 | 1.35 |
| agg | R | 0 | 0.84 |
| --- | -- | ------ | ------ |
| mPD | | 0.018 | 0.52 |
| | | nPD: | 0.03 |
| | | N. weight: | 0.53 |
| | | Sc. PD: | -0.011 |
| | | Sc. rank: | 51.4 |

,

M2

| Pos. | 57 | obs: | exp: |
|---|---|---|---|
| tat | Y | 0 | 175.50 |
| tac | Y | 339 | 163.50 |
| --- | -- | ------ | ------ |
| mPD | | 0 | 0.50 |
| | | nPD: | 0. |
| | | N. weight: | 0.66 |
| | | Sc. PD: | -0.036 |
| | | Sc. rank: | -197.2 |

,

M2

| Pos. | 58 | obs: | exp: |
|---|---|---|---|
| ggt | G | 338 | 41.83 |
| ggc | G | 0 | 45.84 |
| gga | G | 1 | 164.00 |
| ggg | G | 0 | 87.34 |
| --- | -- | ------ | ------ |
| mPD | | 0.0059 | 0.67 |
| | | nPD: | 0.01 |
| | | N. weight: | 1.9 |
| | | Sc. PD: | -0.085 |
| | | Sc. rank: | -159.7 |

,

M2

| Pos. | 59 | obs: | exp: |
|---|---|---|---|
| tta | L | 1 | 29.62 |
| ttg | L | 338 | 65.29 |
| ctt | L | 0 | 63.25 |
| ctc | L | 0 | 54.84 |
| cta | L | 0 | 51.07 |
| ctg | L | 0 | 74.94 |
| --- | -- | ------ | ------ |
| mPD | | 0.0059 | 1.1 |
| | | nPD: | 0.01 |
| | | N. weight: | 1.5 |
| | | Sc. PD: | -0.072 |
| | | Sc. rank: | -159.2 |

,

M2

| Pos. | 60 | obs: | exp: |
|---|---|---|---|
| aaa | K | 339 | 189.80 |
| aag | K | 0 | 149.20 |
| --- | -- | ------ | ------ |
| mPD | | 0 | 0.49 |
| | | nPD: | 0. |
| | | N. weight: | 0.53 |
| | | Sc. PD: | -0.028 |
| | | Sc. rank: | -156.8 |

,

M2

| Pos. | 61 | obs: | exp: |
|---|---|---|---|
| cgt | R | 0 | 14.03 |
| cgc | R | 0 | 16.55 |
| cga | R | 0 | 32.48 |
| cgg | R | 0 | 28.21 |
| att | I | 0 | 0.38 |
| atc | I | 0 | 0.26 |
| ata | I | 1 | 0.36 |
| aga | R | 337 | 152.00 |
| agg | R | 1 | 94.72 |
| --- | -- | ------ | ------ |
| mPD | | 0.012 | 0.97 |
| | | nPD: | 0.01 |
| | | N. weight: | 0.71 |
| | | Sc. PD: | -0.03 |
| | | Sc. rank: | -20.0 |

,

M2

| Pos. | 62 | obs: | exp: |
|---|---|---|---|
| ggt | G | 0 | 41.83 |
| ggc | G | 0 | 45.84 |
| gga | G | 0 | 164.00 |
| ggg | G | 339 | 87.34 |
| --- | -- | ------ | ------ |
| mPD | | 0 | 0.67 |
| | | nPD: | 0. |
| | | N. weight: | 1.2 |
| | | Sc. PD: | -0.066 |
| | | Sc. rank: | -366.8 |

,

M2

| Pos. | 63 | obs: | exp: |
|---|---|---|---|
| tct | S | 1 | 0.16 |
| tcc | S | 0 | 0.14 |
| tca | S | 0 | 0.22 |
| tcg | S | 0 | 0.06 |
| cct | P | 337 | 89.00 |
| ccc | P | 1 | 60.97 |
| cca | P | 0 | 133.40 |
| ccg | P | 0 | 54.65 |
| agt | S | 0 | 0.22 |
| agc | S | 0 | 0.20 |
| --- | -- | ------ | ------ |
| mPD | | 0.012 | 0.72 |
| | | nPD: | 0.02 |
| | | N. weight: | 1.2 |
| | | Sc. PD: | -0.045 |
| | | Sc. rank: | -0.4 |

,

M2

| Pos. | 64 | obs: | exp: |
|---|---|---|---|
| tct | S | 322 | 53.89 |
| tcc | S | 17 | 47.22 |
| tca | S | 0 | 75.10 |
| tcg | S | 0 | 20.66 |
| agt | S | 0 | 74.58 |
| agc | S | 0 | 67.54 |
| --- | -- | ------ | ------ |
| mPD | | 0.096 | 1.7 |
| | | nPD: | 0.06 |
| | | N. weight: | 1.5 |
| | | Sc. PD: | 0.0027 |
| | | Sc. rank: | 308.8 |

,

M2

| Pos. | 65 | obs: | exp: |
|---|---|---|---|
| act | T | 0 | 89.56 |
| acc | T | 1 | 72.74 |
| aca | T | 3 | 148.50 |
| acg | T | 335 | 28.18 |
| --- | -- | ------ | ------ |
| mPD | | 0.023 | 0.69 |
| | | nPD: | 0.03 |
| | | N. weight: | 2.2 |
| | | Sc. PD: | -0.045 |
| | | Sc. rank: | 244.7 |

,

M2

| Pos. | 66 | obs: | exp: |
|---|---|---|---|
| aaa | K | 1 | 0.56 |
| aag | K | 0 | 0.44 |
| gaa | E | 338 | 194.50 |
| gag | E | 0 | 143.50 |
| --- | -- | ------ | ------ |
| mPD | | 0.0059 | 0.49 |
| | | nPD: | 0.01 |
| | | N. weight: | 0.5 |
| | | Sc. PD: | -0.021 |
| | | Sc. rank: | -28.8 |

,

M2

| Pos. | 67 | obs: | exp: |
|---|---|---|---|
| cgt | R | 0 | 0.04 |
| cgc | R | 0 | 0.05 |
| cga | R | 0 | 0.10 |
| cgg | R | 0 | 0.08 |
| aga | R | 1 | 0.45 |
| agg | R | 0 | 0.28 |
| ggt | G | 1 | 41.70 |
| ggc | G | 0 | 45.71 |
| gga | G | 295 | 163.50 |
| ggg | G | 42 | 87.08 |
| --- | -- | ------ | ------ |
| mPD | | 0.23 | 0.67 |
| | | nPD: | 0.34 |
| | | N. weight: | 0.39 |
| | | Sc. PD: | 0.11 |
| | | Sc. rank: | 133.3 |

,

M2

| Pos. | 68 | obs: | exp: |
|---|---|---|---|
| tta | L | 0 | 0.26 |
| ttg | L | 3 | 0.58 |
| ctt | L | 0 | 0.56 |
| ctc | L | 0 | 0.49 |
| cta | L | 0 | 0.45 |
| ctg | L | 0 | 0.66 |
| atg | M | 29 | 29.00 |
| gtt | V | 0 | 63.10 |
| gtc | V | 0 | 64.43 |
| gta | V | 21 | 57.54 |
| gtg | V | 286 | 121.90 |
| --- | -- | ------ | ------ |
| mPD | | 0.29 | 0.87 |
| | | nPD: | 0.33 |
| | | N. weight: | 0.62 |
| | | Sc. PD: | 0.16 |
| | | Sc. rank: | 210.9 |

,

| | | | |
|---|---|---|---|
| M2 | | | |
| Pos. | 69 | obs: | exp: |
| tta | L | 0 | 0.09 |
| ttg | L | 0 | 0.19 |
| ctt | L | 1 | 0.19 |
| ctc | L | 0 | 0.16 |
| cta | L | 0 | 0.15 |
| ctg | L | 0 | 0.22 |
| cct | P | 337 | 89.00 |
| ccc | P | 0 | 60.97 |
| cca | P | 0 | 133.40 |
| ccg | P | 1 | 54.65 |
| --- | -- | ------ | ------ |
| mPD | | 0.012 | 0.72 |
| | | nPD: | 0.02 |
| | | N. weight: | 1.2 |
| | | Sc. PD: | -0.045 |
| | | Sc. rank: | 9.2 |

,

| | | | |
|---|---|---|---|
| M2 | | | |
| Pos. | 70 | obs: | exp: |
| aaa | K | 0 | 1.68 |
| aag | K | 3 | 1.32 |
| gaa | E | 11 | 193.30 |
| gag | E | 325 | 142.70 |
| --- | -- | ------ | ------ |
| mPD | | 0.081 | 0.51 |
| | | nPD: | 0.16 |
| | | N. weight: | 0.65 |
| | | Sc. PD: | 0.064 |
| | | Sc. rank: | 166.7 |

,

| | | | |
|---|---|---|---|
| M2 | | | |
| Pos. | 71 | obs: | exp: |
| ttt | F | 3 | 1.23 |
| ttc | F | 0 | 1.77 |
| tct | S | 333 | 53.42 |
| tcc | S | 3 | 46.81 |
| tca | S | 0 | 74.44 |
| tcg | S | 0 | 20.48 |
| agt | S | 0 | 73.92 |
| agc | S | 0 | 66.94 |
| --- | -- | ------ | ------ |
| mPD | | 0.035 | 1.7 |
| | | nPD: | 0.02 |
| | | N. weight: | 1.6 |
| | | Sc. PD: | -0.054 |
| | | Sc. rank: | 92.9 |

,

| | | | |
|---|---|---|---|
| M2 | | | |
| Pos. | 72 | obs: | exp: |
| atg | M | 339 | 339.00 |
| --- | -- | ------ | ------ |
| mPD | | 0 | 0 |
| | | nPD: | 1. |
| | | N. weight: | 0. |
| | | Sc. PD: | 0 |
| | | Sc. rank: | 0 |

,

| | | | |
|---|---|---|---|
| M2 | | | |
| Pos. | 73 | obs: | exp: |
| cgt | R | 0 | 14.07 |
| cgc | R | 0 | 16.60 |
| cga | R | 0 | 32.58 |
| cgg | R | 0 | 28.29 |
| aga | R | 0 | 152.50 |
| agg | R | 339 | 95.00 |
| --- | -- | ------ | ------ |
| mPD | | 0 | 0.96 |
| | | nPD: | 0. |
| | | N. weight: | 1.2 |
| | | Sc. PD: | -0.062 |
| | | Sc. rank: | -344.0 |

,

| | | | |
|---|---|---|---|
| M2 | | | |
| Pos. | 74 | obs: | exp: |
| gaa | E | 333 | 195.10 |
| gag | E | 6 | 143.90 |
| --- | -- | ------ | ------ |
| mPD | | 0.035 | 0.49 |
| | | nPD: | 0.07 |
| | | N. weight: | 0.43 |
| | | Sc. PD: | 0.0066 |
| | | Sc. rank: | 95.4 |

,

| | | | |
|---|---|---|---|
| M2 | | | |
| Pos. | 75 | obs: | exp: |
| aaa | K | 0 | 0.56 |
| aag | K | 1 | 0.44 |
| gaa | E | 181 | 194.50 |
| gag | E | 157 | 143.50 |
| --- | -- | ------ | ------ |
| mPD | | 0.51 | 0.49 |
| | | nPD: | 1.02 |
| | | N. weight: | 0.014 |
| | | Sc. PD: | 0.013 |
| | | Sc. rank: | 4.7 |

,

| | | | |
|---|---|---|---|
| M2 | | | |
| Pos. | 76 | obs: | exp: |
| tct | S | 1 | 0.16 |
| tcc | S | 0 | 0.14 |
| tca | S | 0 | 0.22 |
| tcg | S | 0 | 0.06 |
| tat | Y | 333 | 175.00 |
| tac | Y | 5 | 163.00 |
| agt | S | 0 | 0.22 |
| agc | S | 0 | 0.20 |
| --- | -- | ------ | ------ |
| mPD | | 0.035 | 0.51 |
| | | nPD: | 0.07 |
| | | N. weight: | 0.54 |
| | | Sc. PD: | 0.0069 |
| | | Sc. rank: | 117.3 |

,

| | | | |
|---|---|---|---|
| M2 | | | |
| Pos. | 77 | obs: | exp: |
| cct | P | 0 | 0.26 |
| ccc | P | 0 | 0.18 |
| cca | P | 0 | 0.39 |
| ccg | P | 1 | 0.16 |
| caa | Q | 0 | 1.07 |
| cag | Q | 2 | 0.93 |
| cgt | R | 0 | 13.95 |
| cgc | R | 0 | 16.45 |
| cga | R | 5 | 32.29 |
| cgg | R | 331 | 28.04 |
| aga | R | 0 | 151.10 |
| agg | R | 0 | 94.16 |
| --- | -- | ------ | ------ |
| mPD | | 0.047 | 0.98 |
| | | nPD: | 0.05 |
| | | N. weight: | 2.2 |
| | | Sc. PD: | -0.017 |
| | | Sc. rank: | 404.3 |

,

| | | | |
|---|---|---|---|
| M2 | | | |
| Pos. | 78 | obs: | exp: |
| caa | Q | 0 | 180.70 |
| cag | Q | 339 | 158.30 |
| --- | -- | ------ | ------ |
| mPD | | 0 | 0.50 |
| | | nPD: | 0. |
| | | N. weight: | 0.69 |
| | | Sc. PD: | -0.037 |
| | | Sc. rank: | -206.0 |

,

| | | | |
|---|---|---|---|
| M2 | | | |
| Pos. | 79 | obs: | exp: |
| gaa | E | 327 | 195.10 |
| gag | E | 12 | 143.90 |
| --- | -- | ------ | ------ |
| mPD | | 0.068 | 0.49 |
| | | nPD: | 0.14 |
| | | N. weight: | 0.38 |
| | | Sc. PD: | 0.031 |
| | | Sc. rank: | 96.2 |

,

| | | | |
|---|---|---|---|
| M2 | | | |
| Pos. | 80 | obs: | exp: |
| caa | Q | 5 | 180.70 |
| cag | Q | 334 | 158.30 |
| --- | -- | ------ | ------ |
| mPD | | 0.029 | 0.50 |
| | | nPD: | 0.06 |
| | | N. weight: | 0.63 |
| | | Sc. PD: | 0.0018 |
| | | Sc. rank: | 131.1 |

,

| | | | |
|---|---|---|---|
| M2 | | | |
| Pos. | 81 | obs: | exp: |
| caa | Q | 1 | 180.70 |
| cag | Q | 338 | 158.30 |
| --- | -- | ------ | ------ |
| mPD | | 0.0059 | 0.50 |
| | | nPD: | 0.01 |
| | | N. weight: | 0.68 |
| | | Sc. PD: | -0.029 |
| | | Sc. rank: | -43.5 |

,

| | | | |
|---|---|---|---|
| M2 | | | |
| Pos. | 82 | obs: | exp: |
| tct | S | 0 | 42.92 |
| tcc | S | 0 | 37.61 |
| tca | S | 0 | 59.82 |
| tcg | S | 0 | 16.46 |
| aat | N | 64 | 33.45 |
| aac | N | 0 | 30.55 |
| agt | S | 270 | 59.40 |
| agc | S | 0 | 53.79 |
| gat | D | 5 | 2.67 |
| gac | D | 0 | 2.33 |
| --- | -- | ------ | ------ |
| mPD | | 0.35 | 1.8 |
| | | nPD: | 0.2 |
| | | N. weight: | 1.2 |
| | | Sc. PD: | 0.16 |
| | | Sc. rank: | 335.6 |

,

| | | | |
|---|---|---|---|
| M2 | | | |
| Pos. | 83 | obs: | exp: |
| gct | A | 337 | 85.38 |
| gcc | A | 2 | 69.44 |
| gca | A | 0 | 151.10 |
| gcg | A | 0 | 33.08 |
| --- | -- | ------ | ------ |
| mPD | | 0.012 | 0.69 |
| | | nPD: | 0.02 |
| | | N. weight: | 1.2 |
| | | Sc. PD: | -0.045 |
| | | Sc. rank: | 42.3 |

,

| | | | |
|---|---|---|---|
| M2 | | | |
| Pos. | 84 | obs: | exp: |
| gtt | V | 0 | 69.68 |
| gtc | V | 0 | 71.15 |
| gta | V | 0 | 63.54 |
| gtg | V | 339 | 134.60 |
| --- | -- | ------ | ------ |
| mPD | | 0 | 0.72 |
| | | nPD: | 0. |
| | | N. weight: | 0.84 |
| | | Sc. PD: | -0.045 |
| | | Sc. rank: | -249.7 |

,

| | | | |
|---|---|---|---|
| M2 | | | |
| Pos. | 85 | obs: | exp: |
| gat | D | 339 | 180.90 |
| gac | D | 0 | 158.10 |
| --- | -- | ------ | ------ |
| mPD | | 0 | 0.50 |
| | | nPD: | 0. |
| | | N. weight: | 0.57 |
| | | Sc. PD: | -0.031 |
| | | Sc. rank: | -169.9 |

,

| | | | |
|---|---|---|---|
| M2 | | | |
| Pos. | 86 | obs: | exp: |
| gtt | V | 339 | 69.68 |
| gtc | V | 0 | 71.15 |
| gta | V | 0 | 63.54 |
| gtg | V | 0 | 134.60 |
| --- | -- | ------ | ------ |
| mPD | | 0 | 0.72 |
| | | nPD: | 0. |
| | | N. weight: | 1.4 |
| | | Sc. PD: | -0.077 |
| | | Sc. rank: | -427.9 |

,

| | | | |
|---|---|---|---|
| M2 | | | |
| Pos. | 87 | obs: | exp: |
| gat | D | 0 | 180.90 |
| gac | D | 339 | 158.10 |
| --- | -- | ------ | ------ |
| mPD | | 0 | 0.50 |
| | | nPD: | 0. |
| | | N. weight: | 0.69 |
| | | Sc. PD: | -0.037 |
| | | Sc. rank: | -206.2 |

,

| | | | |
|---|---|---|---|
| M2 | | | |
| Pos. | 88 | obs: | exp: |
| aat | N | 6 | 3.14 |
| aac | N | 0 | 2.86 |
| gat | D | 330 | 177.70 |
| gac | D | 3 | 155.30 |
| --- | -- | ------ | ------ |
| mPD | | 0.052 | 0.53 |
| | | nPD: | 0.1 |
| | | N. weight: | 0.53 |
| | | Sc. PD: | 0.022 |
| | | Sc. rank: | 121.2 |

,

| | | | |
|---|---|---|---|
| M2 | | | |
| Pos. | 89 | obs: | exp: |
| tct | S | 0 | 0.79 |
| tcc | S | 0 | 0.70 |
| tca | S | 0 | 1.11 |
| tcg | S | 0 | 0.30 |
| agt | S | 5 | 1.10 |
| agc | S | 0 | 1.00 |
| ggt | G | 334 | 41.21 |
| ggc | G | 0 | 45.17 |
| gga | G | 0 | 161.60 |
| ggg | G | 0 | 86.05 |
| --- | -- | ------ | ------ |
| mPD | | 0.029 | 0.72 |
| | | nPD: | 0.04 |
| | | N. weight: | 1.9 |
| | | Sc. PD: | -0.027 |
| | | Sc. rank: | 274.7 |

,

| | | | |
|---|---|---|---|
| M2 | | | |
| Pos. | 90 | obs: | exp: |
| cat | H | 339 | 183.70 |
| cac | H | 0 | 155.30 |
| --- | -- | ------ | ------ |
| mPD | | 0 | 0.50 |
| | | nPD: | 0. |
| | | N. weight: | 0.56 |
| | | Sc. PD: | -0.03 |
| | | Sc. rank: | -165.7 |

,

| | | | |
|---|---|---|---|
| M2 | | | |
| Pos. | 91 | obs: | exp: |
| ttt | F | 339 | 139.40 |
| ttc | F | 0 | 199.60 |
| --- | -- | ------ | ------ |
| mPD | | 0 | 0.48 |
| | | nPD: | 0. |
| | | N. weight: | 0.81 |
| | | Sc. PD: | -0.043 |
| | | Sc. rank: | -240.4 |

,

| | | | |
|---|---|---|---|
| M2 | | | |
| Pos. | 92 | obs: | exp: |
| gtt | V | 0 | 69.68 |
| gtc | V | 339 | 71.15 |
| gta | V | 0 | 63.54 |
| gtg | V | 0 | 134.60 |
| --- | -- | ------ | ------ |
| mPD | | 0 | 0.72 |
| | | nPD: | 0. |
| | | N. weight: | 1.4 |
| | | Sc. PD: | -0.076 |
| | | Sc. rank: | -422.2 |

,

| | | | |
|---|---|---|---|
| M2 | | | |
| Pos. | 93 | obs: | exp: |
| aat | N | 0 | 177.20 |
| aac | N | 339 | 161.80 |
| --- | -- | ------ | ------ |
| mPD | | 0 | 0.50 |
| | | nPD: | 0. |
| | | N. weight: | 0.67 |
| | | Sc. PD: | -0.036 |
| | | Sc. rank: | -200.0 |

,

| M2 | | | |
|---|---|---|---|
| Pos. | 94 | obs: | exp: |
| att | I | 0 | 127.10 |
| atc | I | 0 | 88.58 |
| ata | I | 338 | 122.30 |
| aaa | K | 0 | 0.56 |
| aag | K | 1 | 0.44 |
| --- | -- | ------ | ------ |
| mPD | | 0.012 | 0.67 |
| | | nPD: | 0.02 |
| | | N. weight: | 0.92 |
| | | Sc. PD: | -0.034 |
| | | Sc. rank: | 37.5 |

,

| M2 | | | |
|---|---|---|---|
| Pos. | 95 | obs: | exp: |
| tct | S | 0 | 0.16 |
| tcc | S | 0 | 0.14 |
| tca | S | 0 | 0.22 |
| tcg | S | 0 | 0.06 |
| agt | S | 1 | 0.22 |
| agc | S | 0 | 0.20 |
| gaa | E | 2 | 194.50 |
| gag | E | 336 | 143.50 |
| --- | -- | ------ | ------ |
| mPD | | 0.029 | 0.50 |
| | | nPD: | 0.06 |
| | | N. weight: | 0.75 |
| | | Sc. PD: | 0.0021 |
| | | Sc. rank: | 160.9 |

,

| M2 | | | |
|---|---|---|---|
| Pos. | 96 | obs: | exp: |
| tta | L | 0 | 29.62 |
| ttg | L | 4 | 65.29 |
| ctt | L | 4 | 63.25 |
| ctc | L | 0 | 54.84 |
| cta | L | 0 | 51.07 |
| ctg | L | 331 | 74.94 |
| --- | -- | ------ | ------ |
| mPD | | 0.047 | 1.1 |
| | | nPD: | 0.04 |
| | | N. weight: | 1.3 |
| | | Sc. PD: | -0.017 |
| | | Sc. rank: | 194.6 |

,

| M2 | | | |
|---|---|---|---|
| Pos. | 97 | obs: | exp: |
| tct | S | 0 | 0.16 |
| tcc | S | 0 | 0.14 |
| tca | S | 0 | 0.22 |
| tcg | S | 0 | 0.06 |
| aaa | K | 0 | 15.68 |
| aag | K | 28 | 12.32 |
| agt | S | 1 | 0.22 |
| agc | S | 0 | 0.20 |
| gaa | E | 5 | 178.40 |
| gag | E | 305 | 131.60 |
| --- | -- | ------ | ------ |
| mPD | | 0.20 | 0.65 |
| | | nPD: | 0.3 |
| | | N. weight: | 0.71 |
| | | Sc. PD: | 0.17 |
| | | Sc. rank: | 239.8 |

,

| M2 | | | |
|---|---|---|---|
| Pos. | 98 | obs: | exp: |
| taa | . | 339 | 158.80 |
| tag | . | 0 | 87.72 |
| tga | . | 0 | 92.51 |
| --- | -- | ------ | ------ |
| mPD | | 0 | 0.78 |
| | | nPD: | 0. |
| | | N. weight: | 0.69 |
| | | Sc. PD: | -0.037 |
| | | Sc. rank: | -205.1 |

}

Amino acid level conservation per codon

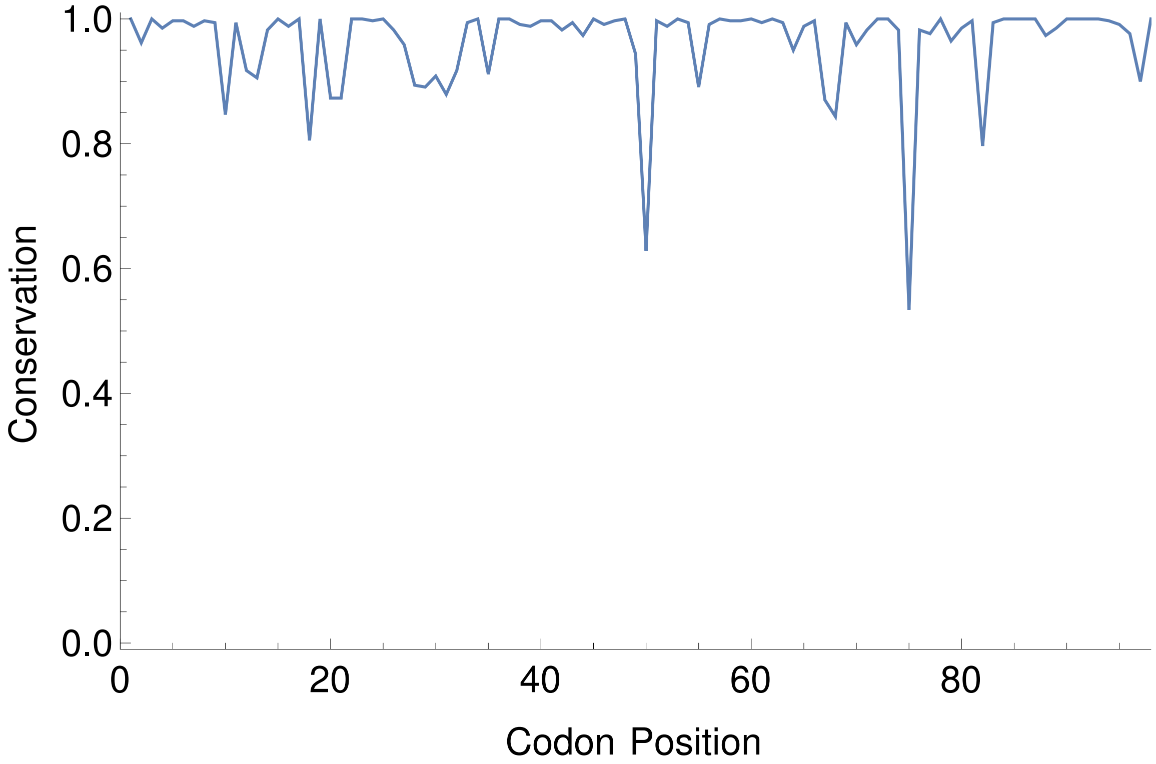

Conservation analysis plots

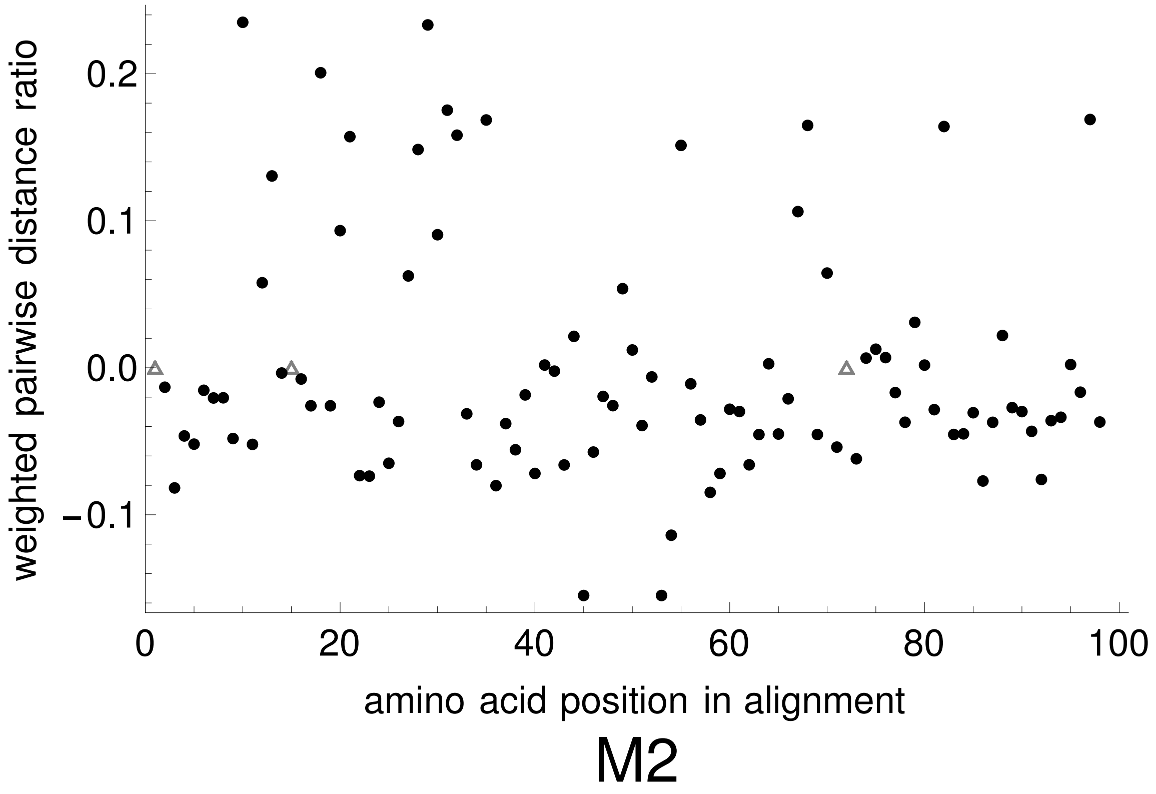


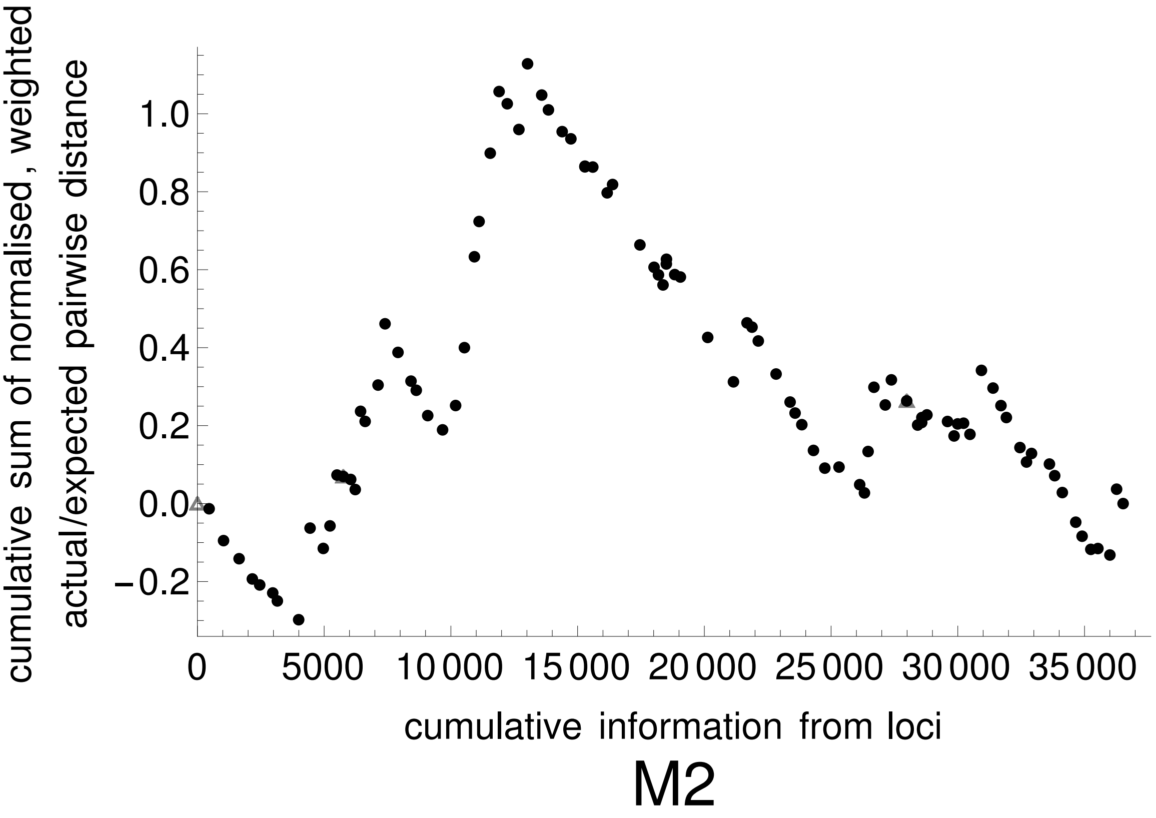

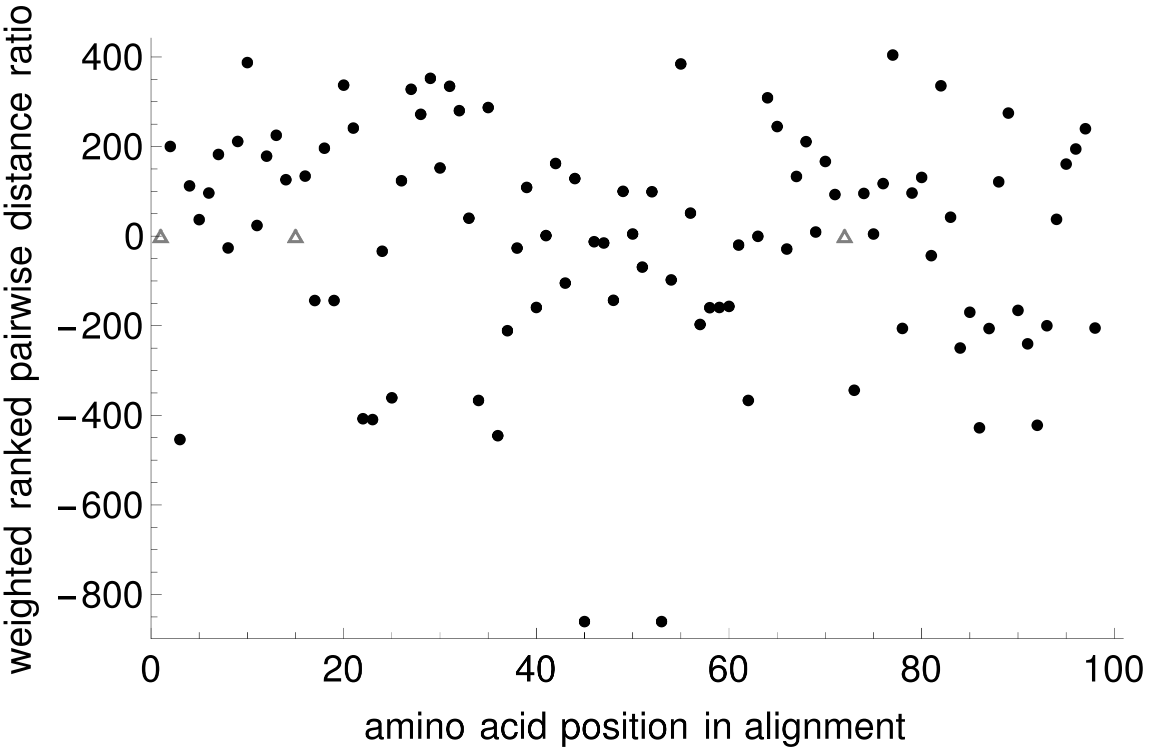
weighted ranked pairwise distance ratio
400
200
0
−200
−400
−600
−800
0
20
40
60
80
100
amino acid position in alignment

M2

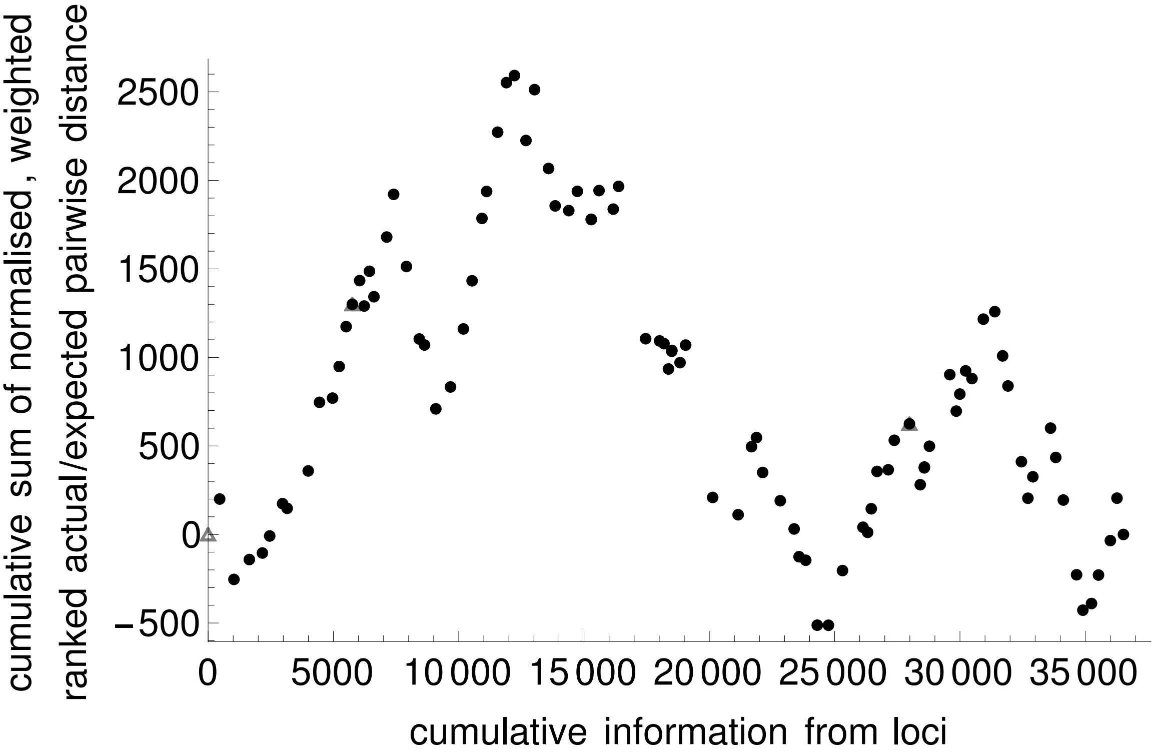
cumulative sum of normalised, weighted ranked actual/expected pairwise distance
2500
2000
1500
1000
500
0
−500
0
5000
10 000
15 000
20 000
25 000
30 000
35 000
cumulative information from loci

M2

# NS1

## Gene length histogram

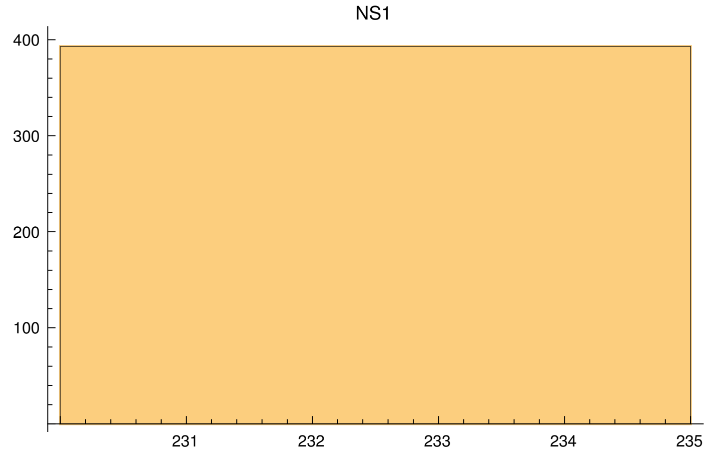

## Information vs. nPD

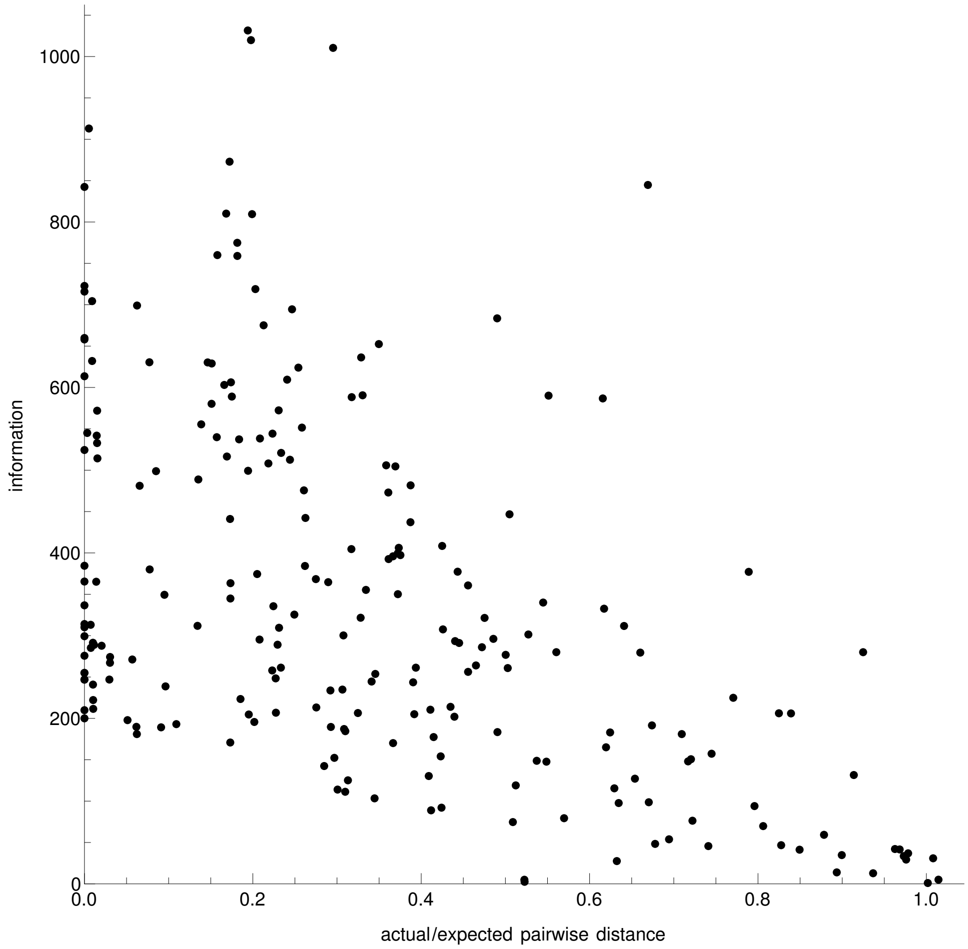


NS1

## Example sequences highlighted by regions found in analyses to be conserved

Interesting points (by weighted raw PD) highlighted for gene NS1:

ATGGAT
**TCCAACACTGTGTCAAGCTTTCAGGTAGACTGCTTTCTTTGGCATGTCCGCAAAC**
**GATTTGCAGACCAAGAACTGGGTGATGCCCCATTCCTTGACCGGCTTCGCCG**
**AGATCAGAAGTCCCTGAGAGGAAGGTGCAGCACTCTTGGTCTG**GACATCGAAACA
GCTACTCGTGCGGGAAAGCAGATAGTGGAGCGGATTCTGGAAGAAGAATCTGATGAGGCACTTAAAATGA
CTATTGCCTCAGTGCCAGCTTCACGCTACCTAACTGACATGACTCTTGAGGAGATGTCAAGGGACTGGTT
CATGCTCATGCCCAAACAAAAAGTGGCAGGTTCCCTTTGCATCAGAATGGACCAAGCAATAATGGATAAG
AACATCATATTGAAAGCGAACTTCAGTGTGATTTTTGACCGGCTGGAAACCCTAATACTACTTAGAGCTT
TCACAGAAGAAGGAGCAATTGTGGGAGAAATCTCACCATTA
**CCTTCTCTTCCAGGACATATTGATGAGGATGTCAAAAATGCAATTGGGGTCCTCA**
**TCGGAGGACTTGAATGGAATGATAACACAGTTCGAGTCTCTGAAACTCTACA**
**GAGATTCGCTTGGAGAAGCAGTAATGAGGATGGGAGACCTCCACTCCCTCCA**
**AAGCAGAAACGGAAAATGGCGAGAACAATTGAGTCAGAAGTT**TGA

Interesting points (by weighted ranked PD) highlighted for gene NS1:

ATGGATTCCAACACTGTGTCAAGCTTT
**CAGGTA**GACTGCTTTCTTTGGCATGTCCGCAAACGATTTGCAGACCAAGAACTG
**GGTGATGCCCCATTCCTTGACCGGCTTCGCCGA**GATCAGAAGTCCCTGAGA
**GGAAGGTGCAGCACTCTTGGTCTG**GACATCGAAACAGCTACTCGTGCGGGAAAGCAGATAGTGGAG
CGGATTCTGGAAGAAGAATCTGATGAGGCACTTAAAATGACTATTGCCTCAGTGCCAGCTTCACGCTACC
TAACTGACATGACTCTTGAGGAGATGTCAAGGGACTGGTTCATGCTCATGCCCAAACAAAAAGTGGCAGG
TTCCCTTTGCATCAGAATGGACCAAGCAATAATGGATAAGAACATCATATTGAAAGCGAACTTCAGTGTG
ATTTTTGACCGGCTGGAAACCCTAATACTACTTAGAGCTTTCACAGAAGAAGGAGCAATTGTGGGAGAAA
TCTCACCATTACCTTCTCTT
**CCAGGACATATTGATGAGGATGTCAAAAATGCAATTGGGGTCCTCATCGGAGGAC**
**TTGAATGGAATGATAACACAGTTCGAGTCTCTGAAACTCTACAGAGATTCGC**
**TTGGAGAAGCAGTAATGAGGATGGGAGACCTCCACTCCCTCCAAAGCAGAAA**
**CGGAAAATGGCGAGAACAATTGAGTCAGAAGTT**TGA

# Per codon conservation report

| | | | |
|---|---|---|---|
| NS1 | | | |
| Pos. | 1 | obs: | exp: |
| atg | M | 393 | 393.00 |
| --- | -- | ------ | ------ |
| mPD | | 0 | 0 |
| | | nPD: | 1. |
| | | N. weight: | 0. |
| | | Sc. PD: | 0 |
| | | Sc. rank: | 0 |

| | | | |
|---|---|---|---|
| NS1 | | | |
| Pos. | 2 | obs: | exp: |
| aaa | K | 2 | 1.12 |
| aag | K | 0 | 0.88 |
| gat | D | 363 | 208.60 |
| gac | D | 28 | 182.40 |
| --- | -- | ------ | ------ |
| mPD | | 0.15 | 0.51 |
| | | nPD: | 0.3 |
| | | N. weight: | 0.46 |
| | | Sc. PD: | 0.021 |
| | | Sc. rank: | 43.2 |

| | | | |
|---|---|---|---|
| NS1 | | | |
| Pos. | 3 | obs: | exp: |
| tct | S | 9 | 61.52 |
| tcc | S | 372 | 53.91 |
| tca | S | 6 | 85.74 |
| tcg | S | 0 | 23.59 |
| cct | P | 0 | 1.05 |
| ccc | P | 4 | 0.72 |
| cca | P | 0 | 1.58 |
| ccg | P | 0 | 0.65 |
| act | T | 0 | 0.53 |
| acc | T | 2 | 0.43 |
| aca | T | 0 | 0.88 |
| acg | T | 0 | 0.17 |
| agt | S | 0 | 85.14 |
| agc | S | 0 | 77.10 |
| --- | -- | ------ | ------ |
| mPD | | 0.10 | 1.7 |
| | | nPD: | 0.06 |
| | | N. weight: | 2.1 |
| | | Sc. PD: | -0.38 |
| | | Sc. rank: | -316.2 |

| | | | |
|---|---|---|---|
| NS1 | | | |
| Pos. | 4 | obs: | exp: |
| aat | N | 3 | 205.40 |
| aac | N | 390 | 187.60 |
| --- | -- | ------ | ------ |
| mPD | | 0.015 | 0.50 |
| | | nPD: | 0.03 |
| | | N. weight: | 0.82 |
| | | Sc. PD: | -0.17 |
| | | Sc. rank: | -133.5 |

| | | | |
|---|---|---|---|
| NS1 | | | |
| Pos. | 5 | obs: | exp: |
| act | T | 337 | 103.80 |
| acc | T | 0 | 84.33 |
| aca | T | 26 | 172.20 |
| acg | T | 30 | 32.67 |
| --- | -- | ------ | ------ |
| mPD | | 0.26 | 0.69 |
| | | nPD: | 0.37 |
| | | N. weight: | 1. |
| | | Sc. PD: | 0.13 |
| | | Sc. rank: | 168.4 |

| | | | |
|---|---|---|---|
| NS1 | | | |
| Pos. | 6 | obs: | exp: |
| att | I | 0 | 11.28 |
| atc | I | 0 | 7.86 |
| ata | I | 30 | 10.86 |
| gtt | V | 1 | 74.61 |
| gtc | V | 0 | 76.19 |
| gta | V | 1 | 68.03 |
| gtg | V | 361 | 144.20 |
| --- | -- | ------ | ------ |
| mPD | | 0.29 | 0.87 |
| | | nPD: | 0.33 |
| | | N. weight: | 1.1 |
| | | Sc. PD: | 0.089 |
| | | Sc. rank: | 135.5 |

| | | | |
|---|---|---|---|
| NS1 | | | |
| Pos. | 7 | obs: | exp: |
| tct | S | 0 | 57.55 |
| tcc | S | 0 | 50.43 |
| tca | S | 362 | 80.20 |
| tcg | S | 0 | 22.07 |
| att | I | 0 | 0.38 |
| atc | I | 1 | 0.26 |
| ata | I | 0 | 0.36 |
| act | T | 0 | 7.93 |
| acc | T | 29 | 6.44 |
| aca | T | 1 | 13.14 |
| acg | T | 0 | 2.49 |
| agt | S | 0 | 79.64 |
| agc | S | 0 | 72.12 |
| --- | -- | ------ | ------ |
| mPD | | 0.29 | 1.7 |
| | | nPD: | 0.18 |
| | | N. weight: | 1.8 |
| | | Sc. PD: | -0.13 |
| | | Sc. rank: | -97.5 |

| | | | |
|---|---|---|---|
| NS1 | | | |
| Pos. | 8 | obs: | exp: |
| tct | S | 0 | 62.48 |
| tcc | S | 0 | 54.75 |
| tca | S | 2 | 87.07 |
| tcg | S | 28 | 23.96 |
| agt | S | 1 | 86.46 |
| agc | S | 362 | 78.30 |
| --- | -- | ------ | ------ |
| mPD | | 0.43 | 1.7 |
| | | nPD: | 0.26 |
| | | N. weight: | 1.7 |
| | | Sc. PD: | 0.016 |
| | | Sc. rank: | 107.1 |

| | | | |
|---|---|---|---|
| NS1 | | | |
| Pos. | 9 | obs: | exp: |
| ttt | F | 361 | 160.70 |
| ttc | F | 30 | 230.30 |
| tat | Y | 2 | 1.04 |
| tac | Y | 0 | 0.96 |
| --- | -- | ------ | ------ |
| mPD | | 0.15 | 0.49 |
| | | nPD: | 0.31 |
| | | N. weight: | 0.7 |
| | | Sc. PD: | 0.04 |
| | | Sc. rank: | 68.4 |

| | | | |
|---|---|---|---|
| NS1 | | | |
| Pos. | 10 | obs: | exp: |
| caa | Q | 0 | 209.50 |
| cag | Q | 393 | 183.50 |
| --- | -- | ------ | ------ |
| mPD | | 0 | 0.50 |
| | | nPD: | 0. |
| | | N. weight: | 0.9 |
| | | Sc. PD: | -0.22 |
| | | Sc. rank: | -255.0 |

| | | | |
|---|---|---|---|
| NS1 | | | |
| Pos. | 11 | obs: | exp: |
| gtt | V | 0 | 80.78 |
| gtc | V | 0 | 82.48 |
| gta | V | 393 | 73.66 |
| gtg | V | 0 | 156.10 |
| --- | -- | ------ | ------ |
| mPD | | 0 | 0.72 |
| | | nPD: | 0. |
| | | N. weight: | 2. |
| | | Sc. PD: | -0.48 |
| | | Sc. rank: | -560.6 |

| | | | |
|---|---|---|---|
| NS1 | | | |
| Pos. | 12 | obs: | exp: |
| gat | D | 33 | 209.70 |
| gac | D | 360 | 183.30 |
| --- | -- | ------ | ------ |
| mPD | | 0.15 | 0.50 |
| | | nPD: | 0.31 |
| | | N. weight: | 0.55 |
| | | Sc. PD: | 0.033 |
| | | Sc. rank: | 57.0 |

| | | | |
|---|---|---|---|
| NS1 | | | |
| Pos. | 13 | obs: | exp: |
| tgt | C | 31 | 162.00 |
| tgc | C | 362 | 231.00 |
| --- | -- | ------ | ------ |
| mPD | | 0.15 | 0.48 |
| | | nPD: | 0.3 |
| | | N. weight: | 0.34 |
| | | Sc. PD: | 0.017 |
| | | Sc. rank: | 32.6 |

| | | | |
|---|---|---|---|
| NS1 | | | |
| Pos. | 14 | obs: | exp: |
| ttt | F | 360 | 149.20 |
| ttc | F | 3 | 213.80 |
| tat | Y | 30 | 15.53 |
| tac | Y | 0 | 14.47 |
| --- | -- | ------ | ------ |
| mPD | | 0.16 | 0.63 |
| | | nPD: | 0.25 |
| | | N. weight: | 0.97 |
| | | Sc. PD: | 0.00077 |
| | | Sc. rank: | 54.6 |

| | | | |
|---|---|---|---|
| NS1 | | | |
| Pos. | 15 | obs: | exp: |
| tta | L | 0 | 34.33 |
| ttg | L | 1 | 75.69 |
| ctt | L | 354 | 73.33 |
| ctc | L | 9 | 63.57 |
| cta | L | 28 | 59.21 |
| ctg | L | 1 | 86.87 |
| --- | -- | ------ | ------ |
| mPD | | 0.19 | 1.1 |
| | | nPD: | 0.17 |
| | | N. weight: | 1.5 |
| | | Sc. PD: | -0.12 |
| | | Sc. rank: | -124.8 |

| | | | |
|---|---|---|---|
| NS1 | | | |
| Pos. | 16 | obs: | exp: |
| tgg | W | 393 | 393.00 |
| --- | -- | ------ | ------ |
| mPD | | 0 | 0 |
| | | nPD: | 1. |
| | | N. weight: | 0. |
| | | Sc. PD: | 0 |
| | | Sc. rank: | 0 |

| | | | |
|---|---|---|---|
| NS1 | | | |
| Pos. | 17 | obs: | exp: |
| cat | H | 363 | 213.00 |
| cac | H | 30 | 180.00 |
| --- | -- | ------ | ------ |
| mPD | | 0.14 | 0.50 |
| | | nPD: | 0.28 |
| | | N. weight: | 0.43 |
| | | Sc. PD: | 0.015 |
| | | Sc. rank: | 34.1 |

| | | | |
|---|---|---|---|
| NS1 | | | |
| Pos. | 18 | obs: | exp: |
| att | I | 0 | 12.03 |
| atc | I | 2 | 8.39 |
| ata | I | 30 | 11.58 |
| gtt | V | 0 | 74.20 |
| gtc | V | 361 | 75.77 |
| gta | V | 0 | 67.66 |
| gtg | V | 0 | 143.40 |
| --- | -- | ------ | ------ |
| mPD | | 0.29 | 0.88 |
| | | nPD: | 0.33 |
| | | N. weight: | 1.8 |
| | | Sc. PD: | 0.14 |
| | | Sc. rank: | 220.4 |

| | | | |
|---|---|---|---|
| NS1 | | | |
| Pos. | 19 | obs: | exp: |
| cgt | R | 0 | 16.32 |
| cgc | R | 363 | 19.24 |
| cga | R | 0 | 37.77 |
| cgg | R | 0 | 32.80 |
| aga | R | 28 | 176.70 |
| agg | R | 2 | 110.10 |
| --- | -- | ------ | ------ |
| mPD | | 0.28 | 0.96 |
| | | nPD: | 0.3 |
| | | N. weight: | 3. |
| | | Sc. PD: | 0.14 |
| | | Sc. rank: | 282.9 |

| | | | |
|---|---|---|---|
| NS1 | | | |
| Pos. | 20 | obs: | exp: |
| aaa | K | 360 | 220.10 |
| aag | K | 33 | 172.90 |
| --- | -- | ------ | ------ |
| mPD | | 0.15 | 0.49 |
| | | nPD: | 0.31 |
| | | N. weight: | 0.37 |
| | | Sc. PD: | 0.023 |
| | | Sc. rank: | 39.0 |

| NS1 | | | |
|---|---|---|---|
| Pos. | 21 | obs: | exp: |
| tta | L | 0 | 2.62 |
| ttg | L | 0 | 5.78 |
| ctt | L | 0 | 5.60 |
| ctc | L | 0 | 4.85 |
| cta | L | 21 | 4.52 |
| ctg | L | 9 | 6.63 |
| caa | Q | 2 | 1.07 |
| cag | Q | 0 | 0.93 |
| cgt | R | 0 | 14.99 |
| cgc | R | 0 | 17.68 |
| cga | R | 359 | 34.69 |
| cgg | R | 2 | 30.12 |
| aga | R | 0 | 162.40 |
| agg | R | 0 | 101.20 |
| --- | -- | ------ | ------ |
| mPD | | 0.21 | 1.2 |
| | | nPD: | 0.17 |
| | | N. weight: | 2.6 |
| | | Sc. PD: | -0.19 |
| | | Sc. rank: | -193.8 |

| NS1 | | | |
|---|---|---|---|
| Pos. | 22 | obs: | exp: |
| ttt | F | 363 | 149.20 |
| ttc | F | 0 | 213.80 |
| tta | L | 0 | 2.62 |
| ttg | L | 0 | 5.78 |
| ctt | L | 0 | 5.60 |
| ctc | L | 30 | 4.85 |
| cta | L | 0 | 4.52 |
| ctg | L | 0 | 6.63 |
| --- | -- | ------ | ------ |
| mPD | | 0.28 | 0.64 |
| | | nPD: | 0.44 |
| | | N. weight: | 1.1 |
| | | Sc. PD: | 0.21 |
| | | Sc. rank: | 224.6 |

| NS1 | | | |
|---|---|---|---|
| Pos. | 23 | obs: | exp: |
| tct | S | 0 | 4.77 |
| tcc | S | 0 | 4.18 |
| tca | S | 0 | 6.65 |
| tcg | S | 0 | 1.83 |
| agt | S | 17 | 6.60 |
| agc | S | 13 | 5.98 |
| gct | A | 0 | 91.43 |
| gcc | A | 0 | 74.36 |
| gca | A | 363 | 161.80 |
| gcg | A | 0 | 35.42 |
| --- | -- | ------ | ------ |
| mPD | | 0.43 | 0.90 |
| | | nPD: | 0.48 |
| | | N. weight: | 0.96 |
| | | Sc. PD: | 0.21 |
| | | Sc. rank: | 204.2 |

| NS1 | | | |
|---|---|---|---|
| Pos. | 24 | obs: | exp: |
| atg | M | 30 | 30.00 |
| gat | D | 0 | 193.70 |
| gac | D | 363 | 169.30 |
| --- | -- | ------ | ------ |
| mPD | | 0.42 | 0.85 |
| | | nPD: | 0.5 |
| | | N. weight: | 0.83 |
| | | Sc. PD: | 0.2 |
| | | Sc. rank: | 184.8 |

| NS1 | | | |
|---|---|---|---|
| Pos. | 25 | obs: | exp: |
| caa | Q | 362 | 193.50 |
| cag | Q | 1 | 169.50 |
| cgt | R | 0 | 1.25 |
| cgc | R | 0 | 1.47 |
| cga | R | 0 | 2.88 |
| cgg | R | 0 | 2.50 |
| aga | R | 29 | 13.49 |
| agg | R | 1 | 8.41 |
| --- | -- | ------ | ------ |
| mPD | | 0.29 | 0.75 |
| | | nPD: | 0.39 |
| | | N. weight: | 0.73 |
| | | Sc. PD: | 0.1 |
| | | Sc. rank: | 127.9 |

| NS1 | | | |
|---|---|---|---|
| Pos. | 26 | obs: | exp: |
| gat | D | 0 | 16.54 |
| gac | D | 31 | 14.46 |
| gaa | E | 360 | 208.30 |
| gag | E | 2 | 153.70 |
| --- | -- | ------ | ------ |
| mPD | | 0.16 | 0.56 |
| | | nPD: | 0.28 |
| | | N. weight: | 0.64 |
| | | Sc. PD: | 0.017 |
| | | Sc. rank: | 50.4 |

| NS1 | | | |
|---|---|---|---|
| Pos. | 27 | obs: | exp: |
| tta | L | 0 | 27.96 |
| ttg | L | 12 | 61.63 |
| ctt | L | 1 | 59.71 |
| ctc | L | 0 | 51.76 |
| cta | L | 6 | 48.21 |
| ctg | L | 301 | 70.74 |
| atg | M | 73 | 73.00 |
| --- | -- | ------ | ------ |
| mPD | | 0.39 | 1.2 |
| | | nPD: | 0.32 |
| | | N. weight: | 1.2 |
| | | Sc. PD: | 0.081 |
| | | Sc. rank: | 129.8 |

| NS1 | | | |
|---|---|---|---|
| Pos. | 28 | obs: | exp: |
| tgt | C | 30 | 12.37 |
| tgc | C | 0 | 17.63 |
| ggt | G | 361 | 44.79 |
| ggc | G | 2 | 49.09 |
| gga | G | 0 | 175.60 |
| ggg | G | 0 | 93.52 |
| --- | -- | ------ | ------ |
| mPD | | 0.15 | 0.83 |
| | | nPD: | 0.18 |
| | | N. weight: | 2.3 |
| | | Sc. PD: | -0.15 |
| | | Sc. rank: | -114.9 |

| NS1 | | | |
|---|---|---|---|
| Pos. | 29 | obs: | exp: |
| gat | D | 393 | 209.70 |
| gac | D | 0 | 183.30 |
| --- | -- | ------ | ------ |
| mPD | | 0 | 0.50 |
| | | nPD: | 0. |
| | | N. weight: | 0.74 |
| | | Sc. PD: | -0.18 |
| | | Sc. rank: | -210.3 |

| NS1 | | | |
|---|---|---|---|
| Pos. | 30 | obs: | exp: |
| gct | A | 32 | 98.99 |
| gcc | A | 361 | 80.51 |
| gca | A | 0 | 175.20 |
| gcg | A | 0 | 38.35 |
| --- | -- | ------ | ------ |
| mPD | | 0.15 | 0.69 |
| | | nPD: | 0.22 |
| | | N. weight: | 1.5 |
| | | Sc. PD: | -0.044 |
| | | Sc. rank: | 20.4 |

| NS1 | | | |
|---|---|---|---|
| Pos. | 31 | obs: | exp: |
| cct | P | 2 | 103.50 |
| ccc | P | 31 | 70.89 |
| cca | P | 348 | 155.10 |
| ccg | P | 12 | 63.54 |
| --- | -- | ------ | ------ |
| mPD | | 0.21 | 0.72 |
| | | nPD: | 0.29 |
| | | N. weight: | 0.7 |
| | | Sc. PD: | 0.03 |
| | | Sc. rank: | 59.2 |

| NS1 | | | |
|---|---|---|---|
| Pos. | 32 | obs: | exp: |
| ttt | F | 32 | 161.60 |
| ttc | F | 361 | 231.40 |
| --- | -- | ------ | ------ |
| mPD | | 0.15 | 0.48 |
| | | nPD: | 0.31 |
| | | N. weight: | 0.33 |
| | | Sc. PD: | 0.02 |
| | | Sc. rank: | 33.9 |

| NS1 | | | |
|---|---|---|---|
| Pos. | 33 | obs: | exp: |
| tta | L | 0 | 31.71 |
| ttg | L | 0 | 69.91 |
| ctt | L | 320 | 67.73 |
| ctc | L | 10 | 58.72 |
| cta | L | 30 | 54.69 |
| ctg | L | 3 | 80.24 |
| gat | D | 29 | 16.01 |
| gac | D | 1 | 13.99 |
| --- | -- | ------ | ------ |
| mPD | | 0.49 | 1.4 |
| | | nPD: | 0.36 |
| | | N. weight: | 1.4 |
| | | Sc. PD: | 0.15 |
| | | Sc. rank: | 204.1 |

| NS1 | | | |
|---|---|---|---|
| Pos. | 34 | obs: | exp: |
| gat | D | 31 | 209.70 |
| gac | D | 362 | 183.30 |
| --- | -- | ------ | ------ |
| mPD | | 0.15 | 0.50 |
| | | nPD: | 0.29 |
| | | N. weight: | 0.57 |
| | | Sc. PD: | 0.024 |
| | | Sc. rank: | 48.8 |

| NS1 | | | |
|---|---|---|---|
| Pos. | 35 | obs: | exp: |
| cgt | R | 0 | 16.32 |
| cgc | R | 0 | 19.24 |
| cga | R | 1 | 37.77 |
| cgg | R | 362 | 32.80 |
| aga | R | 9 | 176.70 |
| agg | R | 21 | 110.10 |
| --- | -- | ------ | ------ |
| mPD | | 0.19 | 0.96 |
| | | nPD: | 0.2 |
| | | N. weight: | 2.4 |
| | | Sc. PD: | -0.12 |
| | | Sc. rank: | -27.6 |

| NS1 | | | |
|---|---|---|---|
| Pos. | 36 | obs: | exp: |
| tta | L | 0 | 34.25 |
| ttg | L | 0 | 75.50 |
| ctt | L | 360 | 73.14 |
| ctc | L | 32 | 63.41 |
| cta | L | 0 | 59.05 |
| ctg | L | 0 | 86.65 |
| att | I | 1 | 0.38 |
| atc | I | 0 | 0.26 |
| ata | I | 0 | 0.36 |
| --- | -- | ------ | ------ |
| mPD | | 0.16 | 1.1 |
| | | nPD: | 0.14 |
| | | N. weight: | 1.7 |
| | | Sc. PD: | -0.18 |
| | | Sc. rank: | -197.4 |

| NS1 | | | |
|---|---|---|---|
| Pos. | 37 | obs: | exp: |
| cgt | R | 0 | 16.32 |
| cgc | R | 363 | 19.24 |
| cga | R | 21 | 37.77 |
| cgg | R | 0 | 32.80 |
| aga | R | 9 | 176.70 |
| agg | R | 0 | 110.10 |
| --- | -- | ------ | ------ |
| mPD | | 0.19 | 0.96 |
| | | nPD: | 0.19 |
| | | N. weight: | 3.1 |
| | | Sc. PD: | -0.16 |
| | | Sc. rank: | -93.8 |

| NS1 | | | |
|---|---|---|---|
| Pos. | 38 | obs: | exp: |
| cgt | R | 1 | 16.32 |
| cgc | R | 0 | 19.24 |
| cga | R | 361 | 37.77 |
| cgg | R | 1 | 32.80 |
| aga | R | 30 | 176.70 |
| agg | R | 0 | 110.10 |
| --- | -- | ------ | ------ |
| mPD | | 0.15 | 0.96 |
| | | nPD: | 0.16 |
| | | N. weight: | 2.3 |
| | | Sc. PD: | -0.2 |
| | | Sc. rank: | -216.6 |

| NS1 | | | |
|---|---|---|---|
| Pos. | 39 | obs: | exp: |
| gat | D | 306 | 209.70 |
| gac | D | 87 | 183.30 |
| --- | -- | ------ | ------ |
| mPD | | 0.35 | 0.50 |
| | | nPD: | 0.69 |
| | | N. weight: | 0.16 |
| | | Sc. PD: | 0.07 |
| | | Sc. rank: | 43.5 |

| NS1 | | | |
|---|---|---|---|
| Pos. | 40 | obs: | exp: |
| cat | H | 1 | 0.54 |
| cac | H | 0 | 0.46 |
| caa | Q | 46 | 209.00 |
| cag | Q | 346 | 183.00 |
| --- | -- | ------ | ------ |
| mPD | | 0.21 | 0.50 |
| | | nPD: | 0.42 |
| | | N. weight: | 0.46 |
| | | Sc. PD: | 0.078 |
| | | Sc. rank: | 85.2 |

| NS1 | | | |
|---|---|---|---|
| Pos. | 41 | obs: | exp: |
| cgt | R | 0 | 0.25 |
| cgc | R | 0 | 0.29 |
| cga | R | 0 | 0.58 |
| cgg | R | 0 | 0.50 |
| aaa | K | 59 | 216.70 |
| aag | K | 328 | 170.30 |
| aga | R | 0 | 2.70 |
| agg | R | 6 | 1.68 |
| --- | -- | ------ | ------ |
| mPD | | 0.29 | 0.53 |
| | | nPD: | 0.54 |
| | | N. weight: | 0.45 |
| | | Sc. PD: | 0.12 |
| | | Sc. rank: | 103.8 |

| NS1 | | | |
|---|---|---|---|
| Pos. | 42 | obs: | exp: |
| tct | S | 105 | 57.71 |
| tcc | S | 257 | 50.57 |
| tca | S | 1 | 80.42 |
| tcg | S | 0 | 22.13 |
| agt | S | 0 | 79.86 |
| agc | S | 0 | 72.32 |
| gct | A | 0 | 7.56 |
| gcc | A | 0 | 6.15 |
| gca | A | 30 | 13.37 |
| gcg | A | 0 | 2.93 |
| --- | -- | ------ | ------ |
| mPD | | 0.64 | 1.7 |
| | | nPD: | 0.37 |
| | | N. weight: | 1.5 |
| | | Sc. PD: | 0.18 |
| | | Sc. rank: | 234.2 |

| NS1 | | | |
|---|---|---|---|
| Pos. | 43 | obs: | exp: |
| tta | L | 40 | 34.33 |
| ttg | L | 32 | 75.69 |
| ctt | L | 0 | 73.33 |
| ctc | L | 0 | 63.57 |
| cta | L | 250 | 59.21 |
| ctg | L | 71 | 86.87 |
| --- | -- | ------ | ------ |
| mPD | | 0.69 | 1.1 |
| | | nPD: | 0.62 |
| | | N. weight: | 1. |
| | | Sc. PD: | 0.36 |
| | | Sc. rank: | 249.7 |

| NS1 | | | |
|---|---|---|---|
| Pos. | 44 | obs: | exp: |
| cgt | R | 0 | 14.99 |
| cgc | R | 0 | 17.68 |
| cga | R | 0 | 34.69 |
| cgg | R | 0 | 30.12 |
| aaa | K | 0 | 16.80 |
| aag | K | 30 | 13.20 |
| aga | R | 321 | 162.40 |
| agg | R | 40 | 101.20 |
| ggt | G | 0 | 0.25 |
| ggc | G | 0 | 0.27 |
| gga | G | 2 | 0.97 |
| ggg | G | 0 | 0.52 |
| --- | -- | ------ | ------ |
| mPD | | 0.45 | 1.1 |
| | | nPD: | 0.41 |
| | | N. weight: | 0.63 |
| | | Sc. PD: | 0.099 |
| | | Sc. rank: | 114.4 |

| NS1 | | | |
|---|---|---|---|
| Pos. | 45 | obs: | exp: |
| ggt | G | 0 | 48.49 |
| ggc | G | 0 | 53.15 |
| gga | G | 380 | 190.10 |
| ggg | G | 13 | 101.30 |
| --- | -- | ------ | ------ |
| mPD | | 0.064 | 0.67 |
| | | nPD: | 0.1 |
| | | N. weight: | 0.71 |
| | | Sc. PD: | -0.11 |
| | | Sc. rank: | -93.1 |

| NS1 | | | |
|---|---|---|---|
| Pos. | 46 | obs: | exp: |
| cgt | R | 0 | 16.32 |
| cgc | R | 0 | 19.24 |
| cga | R | 0 | 37.77 |
| cgg | R | 0 | 32.80 |
| aga | R | 382 | 176.70 |
| agg | R | 11 | 110.10 |
| --- | -- | ------ | ------ |
| mPD | | 0.055 | 0.96 |
| | | nPD: | 0.06 |
| | | N. weight: | 0.81 |
| | | Sc. PD: | -0.15 |
| | | Sc. rank: | -129.2 |

,

| NS1 | | | |
|---|---|---|---|
| Pos. | 47 | obs: | exp: |
| tct | S | 0 | 4.93 |
| tcc | S | 0 | 4.32 |
| tca | S | 0 | 6.87 |
| tcg | S | 0 | 1.89 |
| tgt | C | 0 | 1.24 |
| tgc | C | 3 | 1.76 |
| agt | S | 0 | 6.82 |
| agc | S | 31 | 6.18 |
| ggt | G | 2 | 44.30 |
| ggc | G | 357 | 48.55 |
| gga | G | 0 | 173.70 |
| ggg | G | 0 | 92.49 |
| --- | -- | ------ | ------ |
| mPD | | 0.17 | 0.94 |
| | | nPD: | 0.18 |
| | | N. weight: | 2.3 |
| | | Sc. PD: | -0.15 |
| | | Sc. rank: | -99.6 |

,

| NS1 | | | |
|---|---|---|---|
| Pos. | 48 | obs: | exp: |
| tct | S | 0 | 62.00 |
| tcc | S | 0 | 54.33 |
| tca | S | 0 | 86.40 |
| tcg | S | 0 | 23.77 |
| att | I | 0 | 0.38 |
| atc | I | 1 | 0.26 |
| ata | I | 0 | 0.36 |
| aat | N | 0 | 1.05 |
| aac | N | 2 | 0.95 |
| agt | S | 0 | 85.80 |
| agc | S | 390 | 77.70 |
| --- | -- | ------ | ------ |
| mPD | | 0.015 | 1.7 |
| | | nPD: | 0.01 |
| | | N. weight: | 1.9 |
| | | Sc. PD: | -0.44 |
| | | Sc. rank: | -386.6 |

,

| NS1 | | | |
|---|---|---|---|
| Pos. | 49 | obs: | exp: |
| act | T | 352 | 103.80 |
| acc | T | 2 | 84.33 |
| aca | T | 39 | 172.20 |
| acg | T | 0 | 32.67 |
| --- | -- | ------ | ------ |
| mPD | | 0.19 | 0.69 |
| | | nPD: | 0.27 |
| | | N. weight: | 1.1 |
| | | Sc. PD: | 0.028 |
| | | Sc. rank: | 85.3 |

,

| NS1 | | | |
|---|---|---|---|
| Pos. | 50 | obs: | exp: |
| tta | L | 0 | 34.33 |
| ttg | L | 0 | 75.69 |
| ctt | L | 393 | 73.33 |
| ctc | L | 0 | 63.57 |
| cta | L | 0 | 59.21 |
| ctg | L | 0 | 86.87 |
| --- | -- | ------ | ------ |
| mPD | | 0 | 1.1 |
| | | nPD: | 0. |
| | | N. weight: | 2. |
| | | Sc. PD: | -0.48 |
| | | Sc. rank: | -562.1 |

,

| NS1 | | | |
|---|---|---|---|
| Pos. | 51 | obs: | exp: |
| ggt | G | 363 | 48.49 |
| ggc | G | 0 | 53.15 |
| gga | G | 29 | 190.10 |
| ggg | G | 1 | 101.30 |
| --- | -- | ------ | ------ |
| mPD | | 0.14 | 0.67 |
| | | nPD: | 0.21 |
| | | N. weight: | 2. |
| | | Sc. PD: | -0.07 |
| | | Sc. rank: | 19.4 |

,

| NS1 | | | |
|---|---|---|---|
| Pos. | 52 | obs: | exp: |
| tta | L | 0 | 34.33 |
| ttg | L | 0 | 75.69 |
| ctt | L | 0 | 73.33 |
| ctc | L | 30 | 63.57 |
| cta | L | 2 | 59.21 |
| ctg | L | 361 | 86.87 |
| --- | -- | ------ | ------ |
| mPD | | 0.15 | 1.1 |
| | | nPD: | 0.14 |
| | | N. weight: | 1.5 |
| | | Sc. PD: | -0.16 |
| | | Sc. rank: | -179.8 |

,

| NS1 | | | |
|---|---|---|---|
| Pos. | 53 | obs: | exp: |
| aat | N | 0 | 0.52 |
| aac | N | 1 | 0.48 |
| gat | D | 39 | 209.10 |
| gac | D | 353 | 182.90 |
| --- | -- | ------ | ------ |
| mPD | | 0.18 | 0.50 |
| | | nPD: | 0.37 |
| | | N. weight: | 0.51 |
| | | Sc. PD: | 0.058 |
| | | Sc. rank: | 77.1 |

,

| NS1 | | | |
|---|---|---|---|
| Pos. | 54 | obs: | exp: |
| tta | L | 0 | 2.62 |
| ttg | L | 1 | 5.78 |
| ctt | L | 0 | 5.60 |
| ctc | L | 0 | 4.85 |
| cta | L | 18 | 4.52 |
| ctg | L | 11 | 6.63 |
| att | I | 0 | 136.50 |
| atc | I | 361 | 95.14 |
| ata | I | 2 | 131.40 |
| --- | -- | ------ | ------ |
| mPD | | 0.29 | 0.82 |
| | | nPD: | 0.36 |
| | | N. weight: | 1.5 |
| | | Sc. PD: | 0.16 |
| | | Sc. rank: | 212.9 |

,

| NS1 | | | |
|---|---|---|---|
| Pos. | 55 | obs: | exp: |
| cgt | R | 0 | 2.49 |
| cgc | R | 0 | 2.94 |
| cga | R | 2 | 5.77 |
| cgg | R | 3 | 5.01 |
| aga | R | 55 | 26.98 |
| agg | R | 0 | 16.81 |
| gaa | E | 186 | 191.60 |
| gag | E | 147 | 141.40 |
| --- | -- | ------ | ------ |
| mPD | | 1.0 | 1.0 |
| | | nPD: | 0.97 |
| | | N. weight: | 0.12 |
| | | Sc. PD: | 0.087 |
| | | Sc. rank: | 36.1 |

,

| NS1 | | | |
|---|---|---|---|
| Pos. | 56 | obs: | exp: |
| cct | P | 0 | 0.26 |
| ccc | P | 0 | 0.18 |
| cca | P | 1 | 0.39 |
| ccg | P | 0 | 0.16 |
| act | T | 32 | 95.64 |
| acc | T | 1 | 77.68 |
| aca | T | 291 | 158.60 |
| acg | T | 38 | 30.09 |
| gtt | V | 0 | 6.17 |
| gtc | V | 0 | 6.30 |
| gta | V | 0 | 5.62 |
| gtg | V | 30 | 11.91 |
| --- | -- | ------ | ------ |
| mPD | | 0.70 | 0.99 |
| | | nPD: | 0.71 |
| | | N. weight: | 0.54 |
| | | Sc. PD: | 0.24 |
| | | Sc. rank: | 147.0 |

,

| NS1 | | | |
|---|---|---|---|
| Pos. | 57 | obs: | exp: |
| gct | A | 296 | 98.99 |
| gcc | A | 96 | 80.51 |
| gca | A | 1 | 175.20 |
| gcg | A | 0 | 38.35 |
| --- | -- | ------ | ------ |
| mPD | | 0.37 | 0.69 |
| | | nPD: | 0.54 |
| | | N. weight: | 1. |
| | | Sc. PD: | 0.29 |
| | | Sc. rank: | 239.9 |

,

| NS1 | | | |
|---|---|---|---|
| Pos. | 58 | obs: | exp: |
| act | T | 325 | 103.80 |
| acc | T | 38 | 84.33 |
| aca | T | 30 | 172.20 |
| acg | T | 0 | 32.67 |
| --- | -- | ------ | ------ |
| mPD | | 0.30 | 0.69 |
| | | nPD: | 0.44 |
| | | N. weight: | 0.88 |
| | | Sc. PD: | 0.16 |
| | | Sc. rank: | 172.2 |

,

| NS1 | | | |
|---|---|---|---|
| Pos. | 59 | obs: | exp: |
| cat | H | 5 | 3.25 |
| cac | H | 1 | 2.75 |
| cgt | R | 281 | 14.82 |
| cgc | R | 25 | 17.48 |
| cga | R | 45 | 34.31 |
| cgg | R | 4 | 29.79 |
| atg | M | 28 | 28.00 |
| act | T | 0 | 0.26 |
| acc | T | 0 | 0.21 |
| aca | T | 0 | 0.44 |
| acg | T | 1 | 0.08 |
| aga | R | 2 | 160.60 |
| agg | R | 0 | 100.00 |
| gtt | V | 0 | 0.21 |
| gtc | V | 0 | 0.21 |
| gta | V | 0 | 0.19 |
| gtg | V | 1 | 0.40 |
| --- | -- | ------ | ------ |
| mPD | | 0.77 | 1.1 |
| | | nPD: | 0.67 |
| | | N. weight: | 2.5 |
| | | Sc. PD: | 1. |
| | | Sc. rank: | 675.1 |

,

| NS1 | | | |
|---|---|---|---|
| Pos. | 60 | obs: | exp: |
| act | T | 0 | 1.06 |
| acc | T | 0 | 0.86 |
| aca | T | 0 | 1.75 |
| acg | T | 4 | 0.33 |
| gtt | V | 0 | 0.21 |
| gtc | V | 0 | 0.21 |
| gta | V | 0 | 0.19 |
| gtg | V | 1 | 0.40 |
| gct | A | 86 | 82.61 |
| gcc | A | 1 | 67.19 |
| gca | A | 60 | 146.20 |
| gcg | A | 181 | 32.01 |
| gaa | E | 39 | 34.53 |
| gag | E | 21 | 25.47 |
| --- | -- | ------ | ------ |
| mPD | | 0.90 | 0.97 |
| | | nPD: | 0.92 |
| | | N. weight: | 0.84 |
| | | Sc. PD: | 0.55 |
| | | Sc. rank: | 242.2 |

,

| NS1 | | | |
|---|---|---|---|
| Pos. | 61 | obs: | exp: |
| ggt | G | 0 | 48.49 |
| ggc | G | 6 | 53.15 |
| gga | G | 365 | 190.10 |
| ggg | G | 22 | 101.30 |
| --- | -- | ------ | ------ |
| mPD | | 0.13 | 0.67 |
| | | nPD: | 0.2 |
| | | N. weight: | 0.59 |
| | | Sc. PD: | -0.027 |
| | | Sc. rank: | -5.8 |

,

| NS1 | | | |
|---|---|---|---|
| Pos. | 62 | obs: | exp: |
| cgt | R | 0 | 0.08 |
| cgc | R | 0 | 0.10 |
| cga | R | 0 | 0.19 |
| cgg | R | 0 | 0.17 |
| aaa | K | 32 | 218.90 |
| aag | K | 359 | 172.10 |
| aga | R | 1 | 0.90 |
| agg | R | 1 | 0.56 |
| --- | -- | ------ | ------ |
| mPD | | 0.16 | 0.51 |
| | | nPD: | 0.32 |
| | | N. weight: | 0.62 |
| | | Sc. PD: | 0.046 |
| | | Sc. rank: | 69.9 |

,

| NS1 | | | |
|---|---|---|---|
| Pos. | 63 | obs: | exp: |
| cat | H | 28 | 15.17 |
| cac | H | 0 | 12.83 |
| caa | Q | 9 | 178.10 |
| cag | Q | 325 | 155.90 |
| aat | N | 1 | 0.52 |
| aac | N | 0 | 0.48 |
| aaa | K | 0 | 16.80 |
| aag | K | 30 | 13.20 |
| --- | -- | ------ | ------ |
| mPD | | 0.32 | 0.71 |
| | | nPD: | 0.46 |
| | | N. weight: | 0.77 |
| | | Sc. PD: | 0.15 |
| | | Sc. rank: | 157.7 |

,

| NS1 | | | |
|---|---|---|---|
| Pos. | 64 | obs: | exp: |
| tta | L | 0 | 0.17 |
| ttg | L | 0 | 0.39 |
| ctt | L | 0 | 0.37 |
| ctc | L | 0 | 0.32 |
| cta | L | 2 | 0.30 |
| ctg | L | 0 | 0.44 |
| att | I | 8 | 147.00 |
| atc | I | 22 | 102.50 |
| ata | I | 361 | 141.50 |
| --- | -- | ------ | ------ |
| mPD | | 0.15 | 0.67 |
| | | nPD: | 0.23 |
| | | N. weight: | 0.87 |
| | | Sc. PD: | -0.016 |
| | | Sc. rank: | 23.8 |

,

| NS1 | | | |
|---|---|---|---|
| Pos. | 65 | obs: | exp: |
| att | I | 1 | 0.38 |
| atc | I | 0 | 0.26 |
| ata | I | 0 | 0.36 |
| atg | M | 1 | 1.00 |
| gtt | V | 28 | 80.37 |
| gtc | V | 0 | 82.06 |
| gta | V | 5 | 73.28 |
| gtg | V | 358 | 155.30 |
| --- | -- | ------ | ------ |
| mPD | | 0.17 | 0.73 |
| | | nPD: | 0.23 |
| | | N. weight: | 0.78 |
| | | Sc. PD: | -0.012 |
| | | Sc. rank: | 28.2 |

,

NS1

| Pos. | 66 | obs: | exp: |
|---|---|---|---|
| aaa | K | 0 | 1.12 |
| aag | K | 2 | 0.88 |
| gat | D | 4 | 2.13 |
| gac | D | 0 | 1.87 |
| gaa | E | 37 | 222.70 |
| gag | E | 350 | 164.30 |
| --- | -- | ------ | ------ |
| mPD | | 0.20 | 0.51 |
| | | nPD: | 0.39 |
| | | N. weight: | 0.61 |
| | | Sc. PD: | 0.085 |
| | | Sc. rank: | 108.6 |

NS1

| Pos. | 67 | obs: | exp: |
|---|---|---|---|
| tgg | W | 3 | 3.00 |
| cgt | R | 0 | 14.95 |
| cgc | R | 0 | 17.63 |
| cga | R | 3 | 34.60 |
| cgg | R | 267 | 30.04 |
| aga | R | 0 | 161.90 |
| agg | R | 90 | 100.90 |
| gat | D | 2 | 16.01 |
| gac | D | 28 | 13.99 |
| --- | -- | ------ | ------ |
| mPD | | 0.77 | 1.2 |
| | | nPD: | 0.62 |
| | | N. weight: | 1.8 |
| | | Sc. PD: | 0.63 |
| | | Sc. rank: | 436.2 |

NS1

| Pos. | 68 | obs: | exp: |
|---|---|---|---|
| att | I | 364 | 147.40 |
| atc | I | 28 | 102.70 |
| ata | I | 0 | 141.90 |
| atg | M | 1 | 1.00 |
| --- | -- | ------ | ------ |
| mPD | | 0.14 | 0.66 |
| | | nPD: | 0.21 |
| | | N. weight: | 0.88 |
| | | Sc. PD: | -0.035 |
| | | Sc. rank: | 0.4 |

NS1

| Pos. | 69 | obs: | exp: |
|---|---|---|---|
| tta | L | 30 | 34.33 |
| ttg | L | 11 | 75.69 |
| ctt | L | 0 | 73.33 |
| ctc | L | 1 | 63.57 |
| cta | L | 21 | 59.21 |
| ctg | L | 330 | 86.87 |
| --- | -- | ------ | ------ |
| mPD | | 0.42 | 1.1 |
| | | nPD: | 0.38 |
| | | N. weight: | 1.2 |
| | | Sc. PD: | 0.15 |
| | | Sc. rank: | 198.3 |

NS1

| Pos. | 70 | obs: | exp: |
|---|---|---|---|
| aaa | K | 2 | 16.80 |
| aag | K | 28 | 13.20 |
| gat | D | 0 | 0.53 |
| gac | D | 1 | 0.47 |
| gaa | E | 171 | 207.70 |
| gag | E | 190 | 153.30 |
| ggt | G | 0 | 0.12 |
| ggc | G | 0 | 0.14 |
| gga | G | 0 | 0.48 |
| ggg | G | 1 | 0.26 |
| --- | -- | ------ | ------ |
| mPD | | 0.64 | 0.64 |
| | | nPD: | 1.01 |
| | | N. weight: | 0.093 |
| | | Sc. PD: | 0.068 |
| | | Sc. rank: | 26.9 |

NS1

| Pos. | 71 | obs: | exp: |
|---|---|---|---|
| tct | S | 0 | 4.61 |
| tcc | S | 0 | 4.04 |
| tca | S | 0 | 6.42 |
| tcg | S | 0 | 1.77 |
| aat | N | 1 | 0.52 |
| aac | N | 0 | 0.48 |
| aaa | K | 2 | 1.12 |
| aag | K | 0 | 0.88 |
| agt | S | 28 | 6.38 |
| agc | S | 1 | 5.78 |
| gat | D | 4 | 17.61 |
| gac | D | 29 | 15.39 |
| gaa | E | 302 | 188.70 |
| gag | E | 26 | 139.30 |
| --- | -- | ------ | ------ |
| mPD | | 0.67 | 0.90 |
| | | nPD: | 0.74 |
| | | N. weight: | 0.47 |
| | | Sc. PD: | 0.23 |
| | | Sc. rank: | 129.8 |

NS1

| Pos. | 72 | obs: | exp: |
|---|---|---|---|
| gaa | E | 347 | 226.10 |
| gag | E | 46 | 166.90 |
| --- | -- | ------ | ------ |
| mPD | | 0.21 | 0.49 |
| | | nPD: | 0.42 |
| | | N. weight: | 0.28 |
| | | Sc. PD: | 0.047 |
| | | Sc. rank: | 51.1 |

NS1

| Pos. | 73 | obs: | exp: |
|---|---|---|---|
| tct | S | 216 | 55.80 |
| tcc | S | 99 | 48.90 |
| tca | S | 35 | 77.76 |
| tcg | S | 1 | 21.39 |
| tat | Y | 12 | 6.21 |
| tac | Y | 0 | 5.79 |
| act | T | 3 | 7.93 |
| acc | T | 0 | 6.44 |
| aca | T | 24 | 13.14 |
| acg | T | 3 | 2.49 |
| agt | S | 0 | 77.22 |
| agc | S | 0 | 69.93 |
| --- | -- | ------ | ------ |
| mPD | | 0.77 | 1.7 |
| | | nPD: | 0.46 |
| | | N. weight: | 1.1 |
| | | Sc. PD: | 0.22 |
| | | Sc. rank: | 220.0 |

NS1

| Pos. | 74 | obs: | exp: |
|---|---|---|---|
| aat | N | 1 | 0.52 |
| aac | N | 0 | 0.48 |
| gat | D | 297 | 209.10 |
| gac | D | 95 | 182.90 |
| --- | -- | ------ | ------ |
| mPD | | 0.37 | 0.50 |
| | | nPD: | 0.74 |
| | | N. weight: | 0.14 |
| | | Sc. PD: | 0.065 |
| | | Sc. rank: | 37.5 |

NS1

| Pos. | 75 | obs: | exp: |
|---|---|---|---|
| gaa | E | 76 | 223.30 |
| gag | E | 312 | 164.70 |
| ggt | G | 0 | 0.62 |
| ggc | G | 0 | 0.68 |
| gga | G | 0 | 2.42 |
| ggg | G | 5 | 1.29 |
| --- | -- | ------ | ------ |
| mPD | | 0.34 | 0.52 |
| | | nPD: | 0.65 |
| | | N. weight: | 0.38 |
| | | Sc. PD: | 0.15 |
| | | Sc. rank: | 98.4 |

NS1

| Pos. | 76 | obs: | exp: |
|---|---|---|---|
| aat | N | 8 | 15.68 |
| aac | N | 22 | 14.32 |
| gct | A | 0 | 91.43 |
| gcc | A | 0 | 74.36 |
| gca | A | 361 | 161.80 |
| gcg | A | 2 | 35.42 |
| --- | -- | ------ | ------ |
| mPD | | 0.44 | 0.98 |
| | | nPD: | 0.45 |
| | | N. weight: | 0.87 |
| | | Sc. PD: | 0.17 |
| | | Sc. rank: | 175.2 |

NS1

| Pos. | 77 | obs: | exp: |
|---|---|---|---|
| ttt | F | 1 | 0.41 |
| ttc | F | 0 | 0.59 |
| tta | L | 0 | 34.25 |
| ttg | L | 0 | 75.50 |
| ctt | L | 271 | 73.14 |
| ctc | L | 121 | 63.41 |
| cta | L | 0 | 59.05 |
| ctg | L | 0 | 86.65 |
| --- | -- | ------ | ------ |
| mPD | | 0.43 | 1.1 |
| | | nPD: | 0.39 |
| | | N. weight: | 1.3 |
| | | Sc. PD: | 0.18 |
| | | Sc. rank: | 222.5 |

NS1

| Pos. | 78 | obs: | exp: |
|---|---|---|---|
| cgt | R | 0 | 0.12 |
| cgc | R | 0 | 0.15 |
| cga | R | 0 | 0.29 |
| cgg | R | 0 | 0.25 |
| aaa | K | 375 | 218.40 |
| aag | K | 15 | 171.60 |
| aga | R | 3 | 1.35 |
| agg | R | 0 | 0.84 |
| --- | -- | ------ | ------ |
| mPD | | 0.089 | 0.51 |
| | | nPD: | 0.17 |
| | | N. weight: | 0.51 |
| | | Sc. PD: | -0.037 |
| | | Sc. rank: | -35.6 |

NS1

| Pos. | 79 | obs: | exp: |
|---|---|---|---|
| att | I | 6 | 22.18 |
| atc | I | 0 | 15.46 |
| ata | I | 53 | 21.36 |
| atg | M | 334 | 334.00 |
| --- | -- | ------ | ------ |
| mPD | | 0.26 | 0.27 |
| | | nPD: | 0.96 |
| | | N. weight: | 0.13 |
| | | Sc. PD: | 0.087 |
| | | Sc. rank: | 36.5 |

NS1

| Pos. | 80 | obs: | exp: |
|---|---|---|---|
| tct | S | 0 | 4.61 |
| tcc | S | 0 | 4.04 |
| tca | S | 0 | 6.42 |
| tcg | S | 0 | 1.77 |
| act | T | 312 | 85.60 |
| acc | T | 10 | 69.53 |
| aca | T | 2 | 141.90 |
| acg | T | 0 | 26.93 |
| agt | S | 29 | 6.38 |
| agc | S | 0 | 5.78 |
| gct | A | 5 | 8.82 |
| gcc | A | 24 | 7.17 |
| gca | A | 6 | 15.60 |
| gcg | A | 0 | 3.42 |
| --- | - | 5 | 5.00 |
| --- | -- | ------ | ------ |
| mPD | | 0.49 | 0.97 |
| | | nPD: | 0.5 |
| | | N. weight: | 1.3 |
| | | Sc. PD: | 0.33 |
| | | Sc. rank: | 305.3 |

NS1

| Pos. | 81 | obs: | exp: |
|---|---|---|---|
| att | I | 377 | 142.10 |
| atc | I | 1 | 99.07 |
| ata | I | 0 | 136.80 |
| gtt | V | 10 | 2.06 |
| gtc | V | 0 | 2.10 |
| gta | V | 0 | 1.87 |
| gtg | V | 0 | 3.97 |
| --- | - | 5 | 5.00 |
| --- | -- | ------ | ------ |
| mPD | | 0.054 | 0.70 |
| | | nPD: | 0.08 |
| | | N. weight: | 1.1 |
| | | Sc. PD: | -0.19 |
| | | Sc. rank: | -159.2 |

NS1

| Pos. | 82 | obs: | exp: |
|---|---|---|---|
| gct | A | 263 | 97.73 |
| gcc | A | 115 | 79.48 |
| gca | A | 10 | 172.90 |
| gcg | A | 0 | 37.86 |
| --- | - | 5 | 5.00 |
| --- | -- | ------ | ------ |
| mPD | | 0.44 | 0.67 |
| | | nPD: | 0.66 |
| | | N. weight: | 0.84 |
| | | Sc. PD: | 0.33 |
| | | Sc. rank: | 218.1 |

NS1

| Pos. | 83 | obs: | exp: |
|---|---|---|---|
| tct | S | 105 | 61.68 |
| tcc | S | 25 | 54.05 |
| tca | S | 249 | 85.96 |
| tcg | S | 9 | 23.65 |
| agt | S | 0 | 85.36 |
| agc | S | 0 | 77.30 |
| --- | - | 5 | 5.00 |
| --- | -- | ------ | ------ |
| mPD | | 0.50 | 1.6 |
| | | nPD: | 0.31 |
| | | N. weight: | 0.9 |
| | | Sc. PD: | 0.052 |
| | | Sc. rank: | 89.5 |

NS1

| Pos. | 84 | obs: | exp: |
|---|---|---|---|
| tct | S | 0 | 4.45 |
| tcc | S | 0 | 3.90 |
| tca | S | 0 | 6.20 |
| tcg | S | 0 | 1.71 |
| aat | N | 2 | 1.05 |
| aac | N | 0 | 0.95 |
| agt | S | 23 | 6.16 |
| agc | S | 5 | 5.58 |
| gtt | V | 0 | 73.17 |
| gtc | V | 0 | 74.72 |
| gta | V | 21 | 66.72 |
| gtg | V | 335 | 141.40 |
| gct | A | 0 | 0.50 |
| gcc | A | 0 | 0.41 |
| gca | A | 2 | 0.89 |
| gcg | A | 0 | 0.20 |
| --- | - | 5 | 5.00 |
| --- | -- | ------ | ------ |
| mPD | | 0.53 | 1.0 |
| | | nPD: | 0.53 |
| | | N. weight: | 0.9 |
| | | Sc. PD: | 0.24 |
| | | Sc. rank: | 209.4 |

NS1

| Pos. | 85 | obs: | exp: |
|---|---|---|---|
| tta | L | 0 | 0.09 |
| ttg | L | 0 | 0.19 |
| ctt | L | 0 | 0.19 |
| ctc | L | 0 | 0.16 |
| cta | L | 0 | 0.15 |
| ctg | L | 1 | 0.22 |
| cct | P | 118 | 103.20 |
| ccc | P | 4 | 70.71 |
| cca | P | 61 | 154.70 |
| ccg | P | 209 | 63.38 |
| --- | -- | ------ | ------ |
| mPD | | 0.61 | 0.72 |
| | | nPD: | 0.84 |
| | | N. weight: | 0.62 |
| | | Sc. PD: | 0.35 |
| | | Sc. rank: | 175.6 |

NS1

| Pos. | 86 | obs: | exp: |
|---|---|---|---|
| tct | S | 29 | 4.93 |
| tcc | S | 0 | 4.32 |
| tca | S | 0 | 6.87 |
| tcg | S | 0 | 1.89 |
| act | T | 7 | 1.85 |
| acc | T | 0 | 1.50 |
| aca | T | 0 | 3.07 |
| acg | T | 0 | 0.58 |
| agt | S | 2 | 6.82 |
| agc | S | 0 | 6.18 |
| gtt | V | 4 | 0.82 |
| gtc | V | 0 | 0.84 |
| gta | V | 0 | 0.75 |
| gtg | V | 0 | 1.59 |
| gct | A | 350 | 88.41 |
| gcc | A | 1 | 71.90 |
| gca | A | 0 | 156.40 |
| gcg | A | 0 | 34.25 |
| mPD | | 0.21 | 0.96 |
| | | nPD: | 0.22 |
| | | N. weight: | 1.6 |
| | | Sc. PD: | -0.04 |
| | | Sc. rank: | 31.6 |

NS1

| Pos. | 87 | obs: | exp: |
|---|---|---|---|
| tct | S | 0 | 52.78 |
| tcc | S | 0 | 46.25 |
| tca | S | 330 | 73.55 |
| tcg | S | 2 | 20.24 |
| cct | P | 30 | 16.06 |
| ccc | P | 0 | 11.00 |
| cca | P | 31 | 24.07 |
| ccg | P | 0 | 9.86 |
| agt | S | 0 | 73.04 |
| agc | S | 0 | 66.14 |
| mPD | | 0.41 | 1.8 |
| | | nPD: | 0.23 |
| | | N. weight: | 1.6 |
| | | Sc. PD: | -0.023 |
| | | Sc. rank: | 62.3 |

NS1

| Pos. | 88 | obs: | exp: |
|---|---|---|---|
| tgt | C | 0 | 0.82 |
| tgc | C | 2 | 1.18 |
| cat | H | 0 | 2.71 |
| cac | H | 5 | 2.29 |
| cgt | R | 2 | 16.02 |
| cgc | R | 351 | 18.90 |
| cga | R | 4 | 37.10 |
| cgg | R | 29 | 32.21 |
| aga | R | 0 | 173.60 |
| agg | R | 0 | 108.20 |
| mPD | | 0.20 | 1.0 |
| | | nPD: | 0.2 |
| | | N. weight: | 3.1 |
| | | Sc. PD: | -0.15 |
| | | Sc. rank: | -53.3 |

NS1

| Pos. | 89 | obs: | exp: |
|---|---|---|---|
| tat | Y | 59 | 203.40 |
| tac | Y | 334 | 189.60 |
| mPD | | 0.26 | 0.50 |
| | | nPD: | 0.51 |
| | | N. weight: | 0.36 |
| | | Sc. PD: | 0.091 |
| | | Sc. rank: | 81.9 |

NS1

| Pos. | 90 | obs: | exp: |
|---|---|---|---|
| tta | L | 2 | 31.63 |
| ttg | L | 0 | 69.72 |
| ctt | L | 0 | 67.54 |
| ctc | L | 0 | 58.56 |
| cta | L | 360 | 54.54 |
| ctg | L | 0 | 80.02 |
| att | I | 8 | 11.65 |
| atc | I | 22 | 8.12 |
| ata | I | 1 | 11.22 |
| mPD | | 0.30 | 1.2 |
| | | nPD: | 0.25 |
| | | N. weight: | 2.1 |
| | | Sc. PD: | -0.0039 |
| | | Sc. rank: | 111.4 |

NS1

| Pos. | 91 | obs: | exp: |
|---|---|---|---|
| att | I | 1 | 0.38 |
| atc | I | 0 | 0.26 |
| ata | I | 0 | 0.36 |
| act | T | 384 | 103.60 |
| acc | T | 5 | 84.12 |
| aca | T | 3 | 171.70 |
| acg | T | 0 | 32.59 |
| mPD | | 0.045 | 0.69 |
| | | nPD: | 0.07 |
| | | N. weight: | 1.4 |
| | | Sc. PD: | -0.26 |
| | | Sc. rank: | -212.4 |

NS1

| Pos. | 92 | obs: | exp: |
|---|---|---|---|
| aat | N | 0 | 0.52 |
| aac | N | 1 | 0.48 |
| gat | D | 32 | 209.10 |
| gac | D | 360 | 182.90 |
| mPD | | 0.16 | 0.50 |
| | | nPD: | 0.31 |
| | | N. weight: | 0.56 |
| | | Sc. PD: | 0.033 |
| | | Sc. rank: | 56.5 |

NS1

| Pos. | 93 | obs: | exp: |
|---|---|---|---|
| att | I | 0 | 0.38 |
| atc | I | 0 | 0.26 |
| ata | I | 1 | 0.36 |
| atg | M | 392 | 392.00 |
| mPD | | 0.0051 | 0.0051 |
| | | nPD: | 1. |
| | | N. weight: | 0.003 |
| | | Sc. PD: | 0.0022 |
| | | Sc. rank: | 0.9 |

NS1

| Pos. | 94 | obs: | exp: |
|---|---|---|---|
| tct | S | 0 | 4.77 |
| tcc | S | 0 | 4.18 |
| tca | S | 0 | 6.65 |
| tcg | S | 0 | 1.83 |
| act | T | 350 | 95.11 |
| acc | T | 10 | 77.25 |
| aca | T | 0 | 157.70 |
| acg | T | 0 | 29.93 |
| aat | N | 3 | 1.57 |
| aac | N | 0 | 1.43 |
| agt | S | 2 | 6.60 |
| agc | S | 28 | 5.98 |
| mPD | | 0.33 | 0.85 |
| | | nPD: | 0.39 |
| | | N. weight: | 1.4 |
| | | Sc. PD: | 0.19 |
| | | Sc. rank: | 250.3 |

NS1

| Pos. | 95 | obs: | exp: |
|---|---|---|---|
| tta | L | 0 | 31.28 |
| ttg | L | 0 | 68.95 |
| ctt | L | 356 | 66.80 |
| ctc | L | 2 | 57.91 |
| cta | L | 0 | 53.93 |
| ctg | L | 0 | 79.14 |
| cct | P | 5 | 1.32 |
| ccc | P | 0 | 0.90 |
| cca | P | 0 | 1.97 |
| ccg | P | 0 | 0.81 |
| att | I | 1 | 11.28 |
| atc | I | 0 | 7.86 |
| ata | I | 29 | 10.86 |
| mPD | | 0.31 | 1.2 |
| | | nPD: | 0.25 |
| | | N. weight: | 1.9 |
| | | Sc. PD: | 0.01 |
| | | Sc. rank: | 113.4 |

NS1

| Pos. | 96 | obs: | exp: |
|---|---|---|---|
| gaa | E | 336 | 226.10 |
| gag | E | 57 | 166.90 |
| mPD | | 0.25 | 0.49 |
| | | nPD: | 0.51 |
| | | N. weight: | 0.22 |
| | | Sc. PD: | 0.057 |
| | | Sc. rank: | 51.2 |

NS1

| Pos. | 97 | obs: | exp: |
|---|---|---|---|
| gaa | E | 122 | 226.10 |
| gag | E | 271 | 166.90 |
| mPD | | 0.43 | 0.49 |
| | | nPD: | 0.88 |
| | | N. weight: | 0.18 |
| | | Sc. PD: | 0.11 |
| | | Sc. rank: | 50.7 |

NS1

| Pos. | 98 | obs: | exp: |
|---|---|---|---|
| att | I | 0 | 12.41 |
| atc | I | 0 | 8.65 |
| ata | I | 33 | 11.95 |
| atg | M | 360 | 360.00 |
| mPD | | 0.15 | 0.16 |
| | | nPD: | 0.97 |
| | | N. weight: | 0.1 |
| | | Sc. PD: | 0.071 |
| | | Sc. rank: | 29.1 |

NS1

| Pos. | 99 | obs: | exp: |
|---|---|---|---|
| tct | S | 0 | 62.48 |
| tcc | S | 2 | 54.75 |
| tca | S | 361 | 87.07 |
| tcg | S | 0 | 23.96 |
| agt | S | 2 | 86.46 |
| agc | S | 28 | 78.30 |
| mPD | | 0.43 | 1.7 |
| | | nPD: | 0.26 |
| | | N. weight: | 1.4 |
| | | Sc. PD: | 0.017 |
| | | Sc. rank: | 97.4 |

NS1

| Pos. | 100 | obs: | exp: |
|---|---|---|---|
| cgt | R | 0 | 16.32 |
| cgc | R | 0 | 19.24 |
| cga | R | 20 | 37.77 |
| cgg | R | 4 | 32.80 |
| aga | R | 146 | 176.70 |
| agg | R | 223 | 110.10 |
| mPD | | 0.60 | 0.96 |
| | | nPD: | 0.63 |
| | | N. weight: | 0.35 |
| | | Sc. PD: | 0.13 |
| | | Sc. rank: | 87.9 |

NS1

| Pos. | 101 | obs: | exp: |
|---|---|---|---|
| gat | D | 32 | 193.70 |
| gac | D | 331 | 169.30 |
| gaa | E | 30 | 17.26 |
| gag | E | 0 | 12.74 |
| mPD | | 0.28 | 0.57 |
| | | nPD: | 0.49 |
| | | N. weight: | 0.55 |
| | | Sc. PD: | 0.13 |
| | | Sc. rank: | 121.3 |

NS1

| Pos. | 102 | obs: | exp: |
|---|---|---|---|
| tgg | W | 393 | 393.00 |
| mPD | | 0 | 0 |
| | | nPD: | 1. |
| | | N. weight: | 0. |
| | | Sc. PD: | 0 |
| | | Sc. rank: | 0 |

NS1

| Pos. | 103 | obs: | exp: |
|---|---|---|---|
| ttt | F | 1 | 135.70 |
| ttc | F | 329 | 194.30 |
| tta | L | 30 | 2.62 |
| ttg | L | 0 | 5.78 |
| tct | S | 0 | 0.32 |
| tcc | S | 2 | 0.28 |
| tca | S | 0 | 0.44 |
| tcg | S | 0 | 0.12 |
| tat | Y | 3 | 15.53 |
| tac | Y | 27 | 14.47 |
| ctt | L | 0 | 5.60 |
| ctc | L | 0 | 4.85 |
| cta | L | 0 | 4.52 |
| ctg | L | 0 | 6.63 |
| agt | S | 0 | 0.44 |
| agc | S | 0 | 0.40 |
| gtt | V | 0 | 0.21 |
| gtc | V | 1 | 0.21 |
| gta | V | 0 | 0.19 |
| gtg | V | 0 | 0.40 |
| mPD | | 0.32 | 0.80 |
| | | nPD: | 0.39 |
| | | N. weight: | 0.78 |
| | | Sc. PD: | 0.11 |
| | | Sc. rank: | 140.0 |

NS1

| Pos. | 104 | obs: | exp: |
|---|---|---|---|
| atg | M | 391 | 391.00 |
| act | T | 0 | 0.53 |
| acc | T | 0 | 0.43 |
| aca | T | 0 | 0.88 |
| acg | T | 2 | 0.17 |
| mPD | | 0.010 | 0.019 |
| | | nPD: | 0.52 |
| | | N. weight: | 0.015 |
| | | Sc. PD: | 0.004 |
| | | Sc. rank: | 3.4 |

NS1

| Pos. | 105 | obs: | exp: |
|---|---|---|---|
| tta | L | 0 | 34.33 |
| ttg | L | 5 | 75.69 |
| ctt | L | 2 | 73.33 |
| ctc | L | 363 | 63.57 |
| cta | L | 3 | 59.21 |
| ctg | L | 20 | 86.87 |
| mPD | | 0.17 | 1.1 |
| | | nPD: | 0.15 |
| | | N. weight: | 1.7 |
| | | Sc. PD: | -0.16 |
| | | Sc. rank: | -190.5 |

| | | | |
|---|---|---|---|
| NS1 | | | |
| Pos. | 106 | obs: | exp: |
| att | I | 30 | 11.28 |
| atc | I | 0 | 7.86 |
| ata | I | 0 | 10.86 |
| atg | M | 363 | 363.00 |
| --- | -- | ------ | ------ |
| mPD | | 0.14 | 0.14 |
| | | nPD: | 0.98 |
| | | N. weight: | 0.088 |
| | | Sc. PD: | 0.062 |
| | | Sc. rank: | 25.5 |

,

| | | | |
|---|---|---|---|
| NS1 | | | |
| Pos. | 107 | obs: | exp: |
| cct | P | 26 | 103.50 |
| ccc | P | 356 | 70.89 |
| cca | P | 10 | 155.10 |
| ccg | P | 1 | 63.54 |
| --- | -- | ------ | ------ |
| mPD | | 0.17 | 0.72 |
| | | nPD: | 0.24 |
| | | N. weight: | 1.5 |
| | | Sc. PD: | -0.0065 |
| | | Sc. rank: | 74.3 |

,

| | | | |
|---|---|---|---|
| NS1 | | | |
| Pos. | 108 | obs: | exp: |
| cgt | R | 0 | 1.25 |
| cgc | R | 0 | 1.47 |
| cga | R | 0 | 2.88 |
| cgg | R | 0 | 2.50 |
| aaa | K | 240 | 203.30 |
| aag | K | 123 | 159.70 |
| aga | R | 5 | 13.49 |
| agg | R | 25 | 8.41 |
| --- | -- | ------ | ------ |
| mPD | | 0.61 | 0.68 |
| | | nPD: | 0.9 |
| | | N. weight: | 0.1 |
| | | Sc. PD: | 0.066 |
| | | Sc. rank: | 29.8 |

,

| | | | |
|---|---|---|---|
| NS1 | | | |
| Pos. | 109 | obs: | exp: |
| caa | Q | 92 | 209.50 |
| cag | Q | 301 | 183.50 |
| --- | -- | ------ | ------ |
| mPD | | 0.36 | 0.50 |
| | | nPD: | 0.72 |
| | | N. weight: | 0.23 |
| | | Sc. PD: | 0.11 |
| | | Sc. rank: | 62.6 |

,

| | | | |
|---|---|---|---|
| NS1 | | | |
| Pos. | 110 | obs: | exp: |
| aaa | K | 384 | 220.10 |
| aag | K | 9 | 172.90 |
| --- | -- | ------ | ------ |
| mPD | | 0.045 | 0.49 |
| | | nPD: | 0.09 |
| | | N. weight: | 0.57 |
| | | Sc. PD: | -0.087 |
| | | Sc. rank: | -76.3 |

,

| | | | |
|---|---|---|---|
| NS1 | | | |
| Pos. | 111 | obs: | exp: |
| att | I | 0 | 10.15 |
| atc | I | 0 | 7.08 |
| ata | I | 27 | 9.77 |
| atg | M | 1 | 1.00 |
| gtt | V | 1 | 75.02 |
| gtc | V | 0 | 76.61 |
| gta | V | 44 | 68.41 |
| gtg | V | 320 | 145.00 |
| --- | -- | ------ | ------ |
| mPD | | 0.43 | 0.86 |
| | | nPD: | 0.5 |
| | | N. weight: | 0.78 |
| | | Sc. PD: | 0.19 |
| | | Sc. rank: | 175.6 |

,

| | | | |
|---|---|---|---|
| NS1 | | | |
| Pos. | 112 | obs: | exp: |
| act | T | 19 | 21.14 |
| acc | T | 6 | 17.17 |
| aca | T | 55 | 35.05 |
| acg | T | 0 | 6.65 |
| gtt | V | 0 | 0.82 |
| gtc | V | 0 | 0.84 |
| gta | V | 4 | 0.75 |
| gtg | V | 0 | 1.59 |
| gct | A | 1 | 77.83 |
| gcc | A | 0 | 63.30 |
| gca | A | 306 | 137.70 |
| gcg | A | 2 | 30.15 |
| --- | -- | ------ | ------ |
| mPD | | 0.48 | 1.0 |
| | | nPD: | 0.47 |
| | | N. weight: | 0.79 |
| | | Sc. PD: | 0.17 |
| | | Sc. rank: | 164.1 |

,

| | | | |
|---|---|---|---|
| NS1 | | | |
| Pos. | 113 | obs: | exp: |
| gct | A | 4 | 1.01 |
| gcc | A | 0 | 0.82 |
| gca | A | 0 | 1.78 |
| gcg | A | 0 | 0.39 |
| ggt | G | 254 | 48.00 |
| ggc | G | 75 | 52.61 |
| gga | G | 24 | 188.20 |
| ggg | G | 36 | 100.20 |
| --- | -- | ------ | ------ |
| mPD | | 0.54 | 0.69 |
| | | nPD: | 0.79 |
| | | N. weight: | 1.1 |
| | | Sc. PD: | 0.59 |
| | | Sc. rank: | 316.2 |

,

| | | | |
|---|---|---|---|
| NS1 | | | |
| Pos. | 114 | obs: | exp: |
| tct | S | 8 | 57.71 |
| tcc | S | 354 | 50.57 |
| tca | S | 1 | 80.42 |
| tcg | S | 0 | 22.13 |
| agt | S | 0 | 79.86 |
| agc | S | 0 | 72.32 |
| ggt | G | 6 | 3.70 |
| ggc | G | 24 | 4.06 |
| gga | G | 0 | 14.51 |
| ggg | G | 0 | 7.73 |
| --- | -- | ------ | ------ |
| mPD | | 0.36 | 1.8 |
| | | nPD: | 0.2 |
| | | N. weight: | 2.2 |
| | | Sc. PD: | -0.095 |
| | | Sc. rank: | -9.8 |

,

| | | | |
|---|---|---|---|
| NS1 | | | |
| Pos. | 115 | obs: | exp: |
| tta | L | 1 | 34.33 |
| ttg | L | 0 | 75.69 |
| ctt | L | 288 | 73.33 |
| ctc | L | 91 | 63.57 |
| cta | L | 4 | 59.21 |
| ctg | L | 9 | 86.87 |
| --- | -- | ------ | ------ |
| mPD | | 0.41 | 1.1 |
| | | nPD: | 0.37 |
| | | N. weight: | 1.2 |
| | | Sc. PD: | 0.14 |
| | | Sc. rank: | 189.1 |

,

| | | | |
|---|---|---|---|
| NS1 | | | |
| Pos. | 116 | obs: | exp: |
| tgt | C | 109 | 149.60 |
| tgc | C | 254 | 213.40 |
| atg | M | 30 | 30.00 |
| --- | -- | ------ | ------ |
| mPD | | 0.78 | 0.84 |
| | | nPD: | 0.94 |
| | | N. weight: | 0.038 |
| | | Sc. PD: | 0.026 |
| | | Sc. rank: | 11.1 |

,

| | | | |
|---|---|---|---|
| NS1 | | | |
| Pos. | 117 | obs: | exp: |
| att | I | 56 | 131.20 |
| atc | I | 292 | 91.47 |
| ata | I | 1 | 126.30 |
| gtt | V | 0 | 9.04 |
| gtc | V | 14 | 9.23 |
| gta | V | 1 | 8.25 |
| gtg | V | 29 | 17.48 |
| --- | -- | ------ | ------ |
| mPD | | 0.57 | 0.89 |
| | | nPD: | 0.64 |
| | | N. weight: | 0.93 |
| | | Sc. PD: | 0.36 |
| | | Sc. rank: | 240.4 |

,

| | | | |
|---|---|---|---|
| NS1 | | | |
| Pos. | 118 | obs: | exp: |
| cgt | R | 0 | 14.65 |
| cgc | R | 0 | 17.28 |
| cga | R | 0 | 33.93 |
| cgg | R | 0 | 29.46 |
| aaa | K | 40 | 22.40 |
| aag | K | 0 | 17.60 |
| aga | R | 340 | 158.80 |
| agg | R | 13 | 98.92 |
| --- | -- | ------ | ------ |
| mPD | | 0.25 | 1.1 |
| | | nPD: | 0.22 |
| | | N. weight: | 0.77 |
| | | Sc. PD: | -0.019 |
| | | Sc. rank: | 11.8 |

,

| | | | |
|---|---|---|---|
| NS1 | | | |
| Pos. | 119 | obs: | exp: |
| atg | M | 392 | 392.00 |
| act | T | 0 | 0.26 |
| acc | T | 0 | 0.21 |
| aca | T | 0 | 0.44 |
| acg | T | 1 | 0.08 |
| --- | -- | ------ | ------ |
| mPD | | 0.0051 | 0.0097 |
| | | nPD: | 0.52 |
| | | N. weight: | 0.0074 |
| | | Sc. PD: | 0.002 |
| | | Sc. rank: | 1.7 |

,

| | | | |
|---|---|---|---|
| NS1 | | | |
| Pos. | 120 | obs: | exp: |
| gat | D | 19 | 209.70 |
| gac | D | 374 | 183.30 |
| --- | -- | ------ | ------ |
| mPD | | 0.092 | 0.50 |
| | | nPD: | 0.19 |
| | | N. weight: | 0.67 |
| | | Sc. PD: | -0.041 |
| | | Sc. rank: | -25.5 |

,

| | | | |
|---|---|---|---|
| NS1 | | | |
| Pos. | 121 | obs: | exp: |
| caa | Q | 77 | 209.50 |
| cag | Q | 316 | 183.50 |
| --- | -- | ------ | ------ |
| mPD | | 0.32 | 0.50 |
| | | nPD: | 0.63 |
| | | N. weight: | 0.29 |
| | | Sc. PD: | 0.11 |
| | | Sc. rank: | 74.6 |

,

| | | | |
|---|---|---|---|
| NS1 | | | |
| Pos. | 122 | obs: | exp: |
| act | T | 0 | 0.26 |
| acc | T | 1 | 0.21 |
| aca | T | 0 | 0.44 |
| acg | T | 0 | 0.08 |
| gct | A | 1 | 98.73 |
| gcc | A | 30 | 80.30 |
| gca | A | 288 | 174.70 |
| gcg | A | 73 | 38.25 |
| --- | -- | ------ | ------ |
| mPD | | 0.43 | 0.69 |
| | | nPD: | 0.62 |
| | | N. weight: | 0.49 |
| | | Sc. PD: | 0.18 |
| | | Sc. rank: | 124.6 |

,

| | | | |
|---|---|---|---|
| NS1 | | | |
| Pos. | 123 | obs: | exp: |
| att | I | 80 | 136.80 |
| atc | I | 12 | 95.40 |
| ata | I | 272 | 131.80 |
| gtt | V | 0 | 5.96 |
| gtc | V | 0 | 6.09 |
| gta | V | 29 | 5.44 |
| gtg | V | 0 | 11.52 |
| --- | -- | ------ | ------ |
| mPD | | 0.51 | 0.82 |
| | | nPD: | 0.62 |
| | | N. weight: | 0.55 |
| | | Sc. PD: | 0.2 |
| | | Sc. rank: | 138.9 |

,

| | | | |
|---|---|---|---|
| NS1 | | | |
| Pos. | 124 | obs: | exp: |
| atg | M | 362 | 362.00 |
| act | T | 0 | 0.26 |
| acc | T | 0 | 0.21 |
| aca | T | 0 | 0.44 |
| acg | T | 1 | 0.08 |
| gtt | V | 0 | 6.17 |
| gtc | V | 0 | 6.30 |
| gta | V | 1 | 5.62 |
| gtg | V | 29 | 11.91 |
| --- | -- | ------ | ------ |
| mPD | | 0.15 | 0.24 |
| | | nPD: | 0.63 |
| | | N. weight: | 0.082 |
| | | Sc. PD: | 0.031 |
| | | Sc. rank: | 21.0 |

,

NS1

| Pos. | 125 | obs: | exp: |
|---|---|---|---|
| gat | D | 326 | 209.70 |
| gac | D | 67 | 183.30 |
| --- | -- | ------ | ------ |
| mPD | | 0.28 | 0.50 |
| | | nPD: | 0.57 |
| | | N. weight: | 0.24 |
| | | Sc. PD: | 0.074 |
| | | Sc. rank: | 58.0 |

NS1

| Pos. | 126 | obs: | exp: |
|---|---|---|---|
| aaa | K | 265 | 220.10 |
| aag | K | 128 | 172.90 |
| --- | -- | ------ | ------ |
| mPD | | 0.44 | 0.49 |
| | | nPD: | 0.89 |
| | | N. weight: | 0.042 |
| | | Sc. PD: | 0.026 |
| | | Sc. rank: | 11.9 |

NS1

| Pos. | 127 | obs: | exp: |
|---|---|---|---|
| tct | S | 0 | 0.16 |
| tcc | S | 0 | 0.14 |
| tca | S | 0 | 0.22 |
| tcg | S | 0 | 0.06 |
| cgt | R | 0 | 1.25 |
| cgc | R | 0 | 1.47 |
| cga | R | 0 | 2.88 |
| cgg | R | 0 | 2.50 |
| act | T | 0 | 8.45 |
| acc | T | 32 | 6.87 |
| aca | T | 0 | 14.02 |
| acg | T | 0 | 2.66 |
| aat | N | 2 | 170.40 |
| aac | N | 324 | 155.60 |
| aaa | K | 3 | 1.68 |
| aag | K | 0 | 1.32 |
| agt | S | 0 | 0.22 |
| agc | S | 1 | 0.20 |
| aga | R | 23 | 13.49 |
| agg | R | 7 | 8.41 |
| gat | D | 0 | 0.53 |
| gac | D | 1 | 0.47 |
| --- | -- | ------ | ------ |
| mPD | | 0.45 | 0.93 |
| | | nPD: | 0.49 |
| | | N. weight: | 0.89 |
| | | Sc. PD: | 0.2 |
| | | Sc. rank: | 190.1 |

NS1

| Pos. | 128 | obs: | exp: |
|---|---|---|---|
| att | I | 33 | 147.70 |
| atc | I | 352 | 103.00 |
| ata | I | 8 | 142.30 |
| --- | -- | ------ | ------ |
| mPD | | 0.19 | 0.66 |
| | | nPD: | 0.29 |
| | | N. weight: | 1.1 |
| | | Sc. PD: | 0.044 |
| | | Sc. rank: | 90.4 |

NS1

| Pos. | 129 | obs: | exp: |
|---|---|---|---|
| att | I | 1 | 122.60 |
| atc | I | 23 | 85.44 |
| ata | I | 302 | 118.00 |
| atg | M | 2 | 2.00 |
| act | T | 0 | 16.64 |
| acc | T | 0 | 13.52 |
| aca | T | 63 | 27.60 |
| acg | T | 0 | 5.24 |
| gtt | V | 0 | 0.41 |
| gtc | V | 0 | 0.42 |
| gta | V | 2 | 0.37 |
| gtg | V | 0 | 0.79 |
| --- | -- | ------ | ------ |
| mPD | | 0.40 | 0.95 |
| | | nPD: | 0.43 |
| | | N. weight: | 0.92 |
| | | Sc. PD: | 0.16 |
| | | Sc. rank: | 175.6 |

NS1

| Pos. | 130 | obs: | exp: |
|---|---|---|---|
| tta | L | 4 | 34.33 |
| ttg | L | 247 | 75.69 |
| ctt | L | 24 | 73.33 |
| ctc | L | 6 | 63.57 |
| cta | L | 0 | 59.21 |
| ctg | L | 112 | 86.87 |
| --- | -- | ------ | ------ |
| mPD | | 0.62 | 1.1 |
| | | nPD: | 0.56 |
| | | N. weight: | 0.84 |
| | | Sc. PD: | 0.25 |
| | | Sc. rank: | 204.0 |

NS1

| Pos. | 131 | obs: | exp: |
|---|---|---|---|
| aaa | K | 310 | 220.10 |
| aag | K | 83 | 172.90 |
| --- | -- | ------ | ------ |
| mPD | | 0.33 | 0.49 |
| | | nPD: | 0.68 |
| | | N. weight: | 0.14 |
| | | Sc. PD: | 0.06 |
| | | Sc. rank: | 39.0 |

NS1

| Pos. | 132 | obs: | exp: |
|---|---|---|---|
| gct | A | 6 | 98.99 |
| gcc | A | 4 | 80.51 |
| gca | A | 323 | 175.20 |
| gcg | A | 60 | 38.35 |
| --- | -- | ------ | ------ |
| mPD | | 0.30 | 0.69 |
| | | nPD: | 0.44 |
| | | N. weight: | 0.6 |
| | | Sc. PD: | 0.11 |
| | | Sc. rank: | 117.2 |

NS1

| Pos. | 133 | obs: | exp: |
|---|---|---|---|
| aat | N | 114 | 205.40 |
| aac | N | 279 | 187.60 |
| --- | -- | ------ | ------ |
| mPD | | 0.41 | 0.50 |
| | | nPD: | 0.83 |
| | | N. weight: | 0.14 |
| | | Sc. PD: | 0.079 |
| | | Sc. rank: | 39.6 |

NS1

| Pos. | 134 | obs: | exp: |
|---|---|---|---|
| ttt | F | 44 | 161.60 |
| ttc | F | 349 | 231.40 |
| --- | -- | ------ | ------ |
| mPD | | 0.20 | 0.48 |
| | | nPD: | 0.41 |
| | | N. weight: | 0.27 |
| | | Sc. PD: | 0.042 |
| | | Sc. rank: | 48.5 |

NS1

| Pos. | 135 | obs: | exp: |
|---|---|---|---|
| tct | S | 5 | 62.48 |
| tcc | S | 1 | 54.75 |
| tca | S | 19 | 87.07 |
| tcg | S | 5 | 23.96 |
| agt | S | 356 | 86.46 |
| agc | S | 7 | 78.30 |
| --- | -- | ------ | ------ |
| mPD | | 0.44 | 1.7 |
| | | nPD: | 0.26 |
| | | N. weight: | 1.3 |
| | | Sc. PD: | 0.018 |
| | | Sc. rank: | 98.8 |

NS1

| Pos. | 136 | obs: | exp: |
|---|---|---|---|
| att | I | 0 | 0.75 |
| atc | I | 0 | 0.52 |
| ata | I | 2 | 0.72 |
| gtt | V | 15 | 80.37 |
| gtc | V | 15 | 82.06 |
| gta | V | 33 | 73.28 |
| gtg | V | 328 | 155.30 |
| --- | -- | ------ | ------ |
| mPD | | 0.30 | 0.73 |
| | | nPD: | 0.41 |
| | | N. weight: | 0.53 |
| | | Sc. PD: | 0.086 |
| | | Sc. rank: | 97.5 |

NS1

| Pos. | 137 | obs: | exp: |
|---|---|---|---|
| tta | L | 1 | 2.80 |
| ttg | L | 0 | 6.16 |
| ctt | L | 0 | 5.97 |
| ctc | L | 2 | 5.18 |
| cta | L | 27 | 4.82 |
| ctg | L | 2 | 7.07 |
| att | I | 258 | 135.00 |
| atc | I | 101 | 94.09 |
| ata | I | 0 | 130.00 |
| act | T | 1 | 0.26 |
| acc | T | 0 | 0.21 |
| aca | T | 0 | 0.44 |
| acg | T | 0 | 0.08 |
| gtt | V | 1 | 0.21 |
| gtc | V | 0 | 0.21 |
| gta | V | 0 | 0.19 |
| gtg | V | 0 | 0.40 |
| --- | -- | ------ | ------ |
| mPD | | 0.65 | 0.84 |
| | | nPD: | 0.77 |
| | | N. weight: | 0.67 |
| | | Sc. PD: | 0.34 |
| | | Sc. rank: | 186.7 |

NS1

| Pos. | 138 | obs: | exp: |
|---|---|---|---|
| ttt | F | 289 | 159.90 |
| ttc | F | 100 | 229.10 |
| tat | Y | 2 | 1.04 |
| tac | Y | 0 | 0.96 |
| tgt | C | 1 | 0.41 |
| tgc | C | 0 | 0.59 |
| cat | H | 1 | 0.54 |
| cac | H | 0 | 0.46 |
| --- | -- | ------ | ------ |
| mPD | | 0.41 | 0.51 |
| | | nPD: | 0.8 |
| | | N. weight: | 0.28 |
| | | Sc. PD: | 0.15 |
| | | Sc. rank: | 79.0 |

NS1

| Pos. | 139 | obs: | exp: |
|---|---|---|---|
| aat | N | 31 | 19.34 |
| aac | N | 6 | 17.66 |
| gat | D | 133 | 186.20 |
| gac | D | 216 | 162.80 |
| gaa | E | 2 | 1.73 |
| gag | E | 1 | 1.27 |
| ggt | G | 1 | 0.49 |
| ggc | G | 3 | 0.54 |
| gga | G | 0 | 1.93 |
| ggg | G | 0 | 1.03 |
| --- | -- | ------ | ------ |
| mPD | | 0.69 | 0.70 |
| | | nPD: | 0.98 |
| | | N. weight: | 0.11 |
| | | Sc. PD: | 0.078 |
| | | Sc. rank: | 32.1 |

NS1

| Pos. | 140 | obs: | exp: |
|---|---|---|---|
| caa | Q | 28 | 16.53 |
| cag | Q | 3 | 14.47 |
| cgt | R | 0 | 15.03 |
| cgc | R | 1 | 17.73 |
| cga | R | 75 | 34.79 |
| cgg | R | 281 | 30.21 |
| aga | R | 0 | 162.80 |
| agg | R | 5 | 101.40 |
| --- | -- | ------ | ------ |
| mPD | | 0.56 | 1.1 |
| | | nPD: | 0.49 |
| | | N. weight: | 2. |
| | | Sc. PD: | 0.48 |
| | | Sc. rank: | 449.2 |

NS1

| Pos. | 141 | obs: | exp: |
|---|---|---|---|
| tta | L | 1 | 31.71 |
| ttg | L | 19 | 69.91 |
| ctt | L | 0 | 67.73 |
| ctc | L | 0 | 58.72 |
| cta | L | 33 | 54.69 |
| ctg | L | 310 | 80.24 |
| att | I | 29 | 10.90 |
| atc | I | 0 | 7.60 |
| ata | I | 0 | 10.50 |
| atg | M | 1 | 1.00 |
| --- | -- | ------ | ------ |
| mPD | | 0.51 | 1.2 |
| | | nPD: | 0.42 |
| | | N. weight: | 1.2 |
| | | Sc. PD: | 0.21 |
| | | Sc. rank: | 230.3 |

NS1

| Pos. | 142 | obs: | exp: |
|---|---|---|---|
| gaa | E | 216 | 225.60 |
| gag | E | 176 | 166.40 |
| ggt | G | 0 | 0.12 |
| ggc | G | 0 | 0.14 |
| gga | G | 0 | 0.48 |
| ggg | G | 1 | 0.26 |
| --- | -- | ------ | ------ |
| mPD | | 0.50 | 0.49 |
| | | nPD: | 1.01 |
| | | N. weight: | 0.015 |
| | | Sc. PD: | 0.011 |
| | | Sc. rank: | 4.4 |

NS1

| Pos. | 143 | obs: | exp: |
|---|---|---|---|
| act | T | 76 | 95.90 |
| acc | T | 178 | 77.89 |
| aca | T | 104 | 159.00 |
| acg | T | 5 | 30.18 |
| gct | A | 0 | 7.56 |
| gcc | A | 30 | 6.15 |
| gca | A | 0 | 13.37 |
| gcg | A | 0 | 2.93 |
| --- | -- | ------ | ------ |
| mPD | | 0.76 | 0.83 |
| | | nPD: | 0.91 |
| | | N. weight: | 0.39 |
| | | Sc. PD: | 0.25 |
| | | Sc. rank: | 113.0 |

NS1

| Pos. | 144 | obs: | exp: |
|---|---|---|---|
| tta | L | 35 | 34.33 |
| ttg | L | 5 | 75.69 |
| ctt | L | 1 | 73.33 |
| ctc | L | 0 | 63.57 |
| cta | L | 343 | 59.21 |
| ctg | L | 9 | 86.87 |
| --- | -- | ------ | ------ |
| mPD | | 0.26 | 1.1 |
| | | nPD: | 0.23 |
| | | N. weight: | 1.7 |
| | | Sc. PD: | -0.03 |
| | | Sc. rank: | 54.5 |

| | | | |
|---|---|---|---|
| NS1 | | | |
| Pos. | 145 | obs: | exp: |
| att | I | 0 | 135.70 |
| atc | I | 3 | 94.61 |
| ata | I | 358 | 130.70 |
| gtt | V | 0 | 6.58 |
| gtc | V | 30 | 6.72 |
| gta | V | 2 | 6.00 |
| gtg | V | 0 | 12.71 |
| --- | -- | ------ | ------ |
| mPD | | 0.30 | 0.83 |
| | | nPD: | 0.37 |
| | | N. weight: | 1.2 |
| | | Sc. PD: | 0.14 |
| | | Sc. rank: | 177.8 |

,

| | | | |
|---|---|---|---|
| NS1 | | | |
| Pos. | 146 | obs: | exp: |
| tta | L | 5 | 31.71 |
| ttg | L | 0 | 69.91 |
| tct | S | 23 | 4.77 |
| tcc | S | 1 | 4.18 |
| tca | S | 6 | 6.65 |
| tcg | S | 0 | 1.83 |
| ctt | L | 1 | 67.73 |
| ctc | L | 0 | 58.72 |
| cta | L | 348 | 54.69 |
| ctg | L | 9 | 80.24 |
| agt | S | 0 | 6.60 |
| agc | S | 0 | 5.98 |
| --- | -- | ------ | ------ |
| mPD | | 0.47 | 1.3 |
| | | nPD: | 0.35 |
| | | N. weight: | 2. |
| | | Sc. PD: | 0.19 |
| | | Sc. rank: | 267.1 |

,

| | | | |
|---|---|---|---|
| NS1 | | | |
| Pos. | 147 | obs: | exp: |
| tta | L | 0 | 34.33 |
| ttg | L | 0 | 75.69 |
| ctt | L | 259 | 73.33 |
| ctc | L | 51 | 63.57 |
| cta | L | 53 | 59.21 |
| ctg | L | 30 | 86.87 |
| --- | -- | ------ | ------ |
| mPD | | 0.53 | 1.1 |
| | | nPD: | 0.47 |
| | | N. weight: | 0.86 |
| | | Sc. PD: | 0.19 |
| | | Sc. rank: | 179.6 |

,

| | | | |
|---|---|---|---|
| NS1 | | | |
| Pos. | 148 | obs: | exp: |
| cgt | R | 0 | 16.32 |
| cgc | R | 0 | 19.24 |
| cga | R | 0 | 37.77 |
| cgg | R | 0 | 32.80 |
| aga | R | 352 | 176.70 |
| agg | R | 41 | 110.10 |
| --- | -- | ------ | ------ |
| mPD | | 0.19 | 0.96 |
| | | nPD: | 0.2 |
| | | N. weight: | 0.61 |
| | | Sc. PD: | -0.032 |
| | | Sc. rank: | -15.4 |

,

| | | | |
|---|---|---|---|
| NS1 | | | |
| Pos. | 149 | obs: | exp: |
| gct | A | 355 | 98.99 |
| gcc | A | 14 | 80.51 |
| gca | A | 23 | 175.20 |
| gcg | A | 1 | 38.35 |
| --- | -- | ------ | ------ |
| mPD | | 0.18 | 0.69 |
| | | nPD: | 0.26 |
| | | N. weight: | 1.2 |
| | | Sc. PD: | 0.015 |
| | | Sc. rank: | 82.0 |

,

| | | | |
|---|---|---|---|
| NS1 | | | |
| Pos. | 150 | obs: | exp: |
| ttt | F | 36 | 161.60 |
| ttc | F | 357 | 231.40 |
| --- | -- | ------ | ------ |
| mPD | | 0.17 | 0.48 |
| | | nPD: | 0.34 |
| | | N. weight: | 0.31 |
| | | Sc. PD: | 0.029 |
| | | Sc. rank: | 40.2 |

,

| | | | |
|---|---|---|---|
| NS1 | | | |
| Pos. | 151 | obs: | exp: |
| tct | S | 0 | 0.16 |
| tcc | S | 0 | 0.14 |
| tca | S | 1 | 0.22 |
| tcg | S | 0 | 0.06 |
| act | T | 79 | 103.60 |
| acc | T | 7 | 84.12 |
| aca | T | 276 | 171.70 |
| acg | T | 30 | 32.59 |
| agt | S | 0 | 0.22 |
| agc | S | 0 | 0.20 |
| --- | -- | ------ | ------ |
| mPD | | 0.46 | 0.69 |
| | | nPD: | 0.67 |
| | | N. weight: | 0.3 |
| | | Sc. PD: | 0.12 |
| | | Sc. rank: | 79.1 |

,

| | | | |
|---|---|---|---|
| NS1 | | | |
| Pos. | 152 | obs: | exp: |
| gat | D | 1 | 3.20 |
| gac | D | 5 | 2.80 |
| gaa | E | 382 | 222.70 |
| gag | E | 5 | 164.30 |
| --- | -- | ------ | ------ |
| mPD | | 0.055 | 0.50 |
| | | nPD: | 0.11 |
| | | N. weight: | 0.58 |
| | | Sc. PD: | -0.078 |
| | | Sc. rank: | -74.5 |

,

| | | | |
|---|---|---|---|
| NS1 | | | |
| Pos. | 153 | obs: | exp: |
| gat | D | 30 | 16.01 |
| gac | D | 0 | 13.99 |
| gaa | E | 266 | 208.90 |
| gag | E | 97 | 154.10 |
| --- | -- | ------ | ------ |
| mPD | | 0.48 | 0.56 |
| | | nPD: | 0.85 |
| | | N. weight: | 0.12 |
| | | Sc. PD: | 0.072 |
| | | Sc. rank: | 35.2 |

,

| | | | |
|---|---|---|---|
| NS1 | | | |
| Pos. | 154 | obs: | exp: |
| ggt | G | 26 | 48.49 |
| ggc | G | 4 | 53.15 |
| gga | G | 333 | 190.10 |
| ggg | G | 30 | 101.30 |
| --- | -- | ------ | ------ |
| mPD | | 0.27 | 0.67 |
| | | nPD: | 0.41 |
| | | N. weight: | 0.39 |
| | | Sc. PD: | 0.061 |
| | | Sc. rank: | 70.2 |

,

| | | | |
|---|---|---|---|
| NS1 | | | |
| Pos. | 155 | obs: | exp: |
| tct | S | 0 | 4.61 |
| tcc | S | 0 | 4.04 |
| tca | S | 29 | 6.42 |
| tcg | S | 0 | 1.77 |
| agt | S | 0 | 6.38 |
| agc | S | 0 | 5.78 |
| gtt | V | 0 | 1.23 |
| gtc | V | 0 | 1.26 |
| gta | V | 6 | 1.12 |
| gtg | V | 0 | 2.38 |
| gct | A | 24 | 90.17 |
| gcc | A | 7 | 73.34 |
| gca | A | 327 | 159.60 |
| gcg | A | 0 | 34.93 |
| --- | -- | ------ | ------ |
| mPD | | 0.32 | 0.92 |
| | | nPD: | 0.34 |
| | | N. weight: | 0.73 |
| | | Sc. PD: | 0.066 |
| | | Sc. rank: | 94.6 |

,

| | | | |
|---|---|---|---|
| NS1 | | | |
| Pos. | 156 | obs: | exp: |
| att | I | 380 | 145.90 |
| atc | I | 6 | 101.70 |
| ata | I | 2 | 140.40 |
| gtt | V | 5 | 1.03 |
| gtc | V | 0 | 1.05 |
| gta | V | 0 | 0.94 |
| gtg | V | 0 | 1.99 |
| --- | -- | ------ | ------ |
| mPD | | 0.065 | 0.69 |
| | | nPD: | 0.1 |
| | | N. weight: | 1. |
| | | Sc. PD: | -0.16 |
| | | Sc. rank: | -138.2 |

,

| | | | |
|---|---|---|---|
| NS1 | | | |
| Pos. | 157 | obs: | exp: |
| gtt | V | 15 | 80.78 |
| gtc | V | 75 | 82.48 |
| gta | V | 7 | 73.66 |
| gtg | V | 296 | 156.10 |
| --- | -- | ------ | ------ |
| mPD | | 0.40 | 0.72 |
| | | nPD: | 0.55 |
| | | N. weight: | 0.44 |
| | | Sc. PD: | 0.13 |
| | | Sc. rank: | 104.7 |

,

| | | | |
|---|---|---|---|
| NS1 | | | |
| Pos. | 158 | obs: | exp: |
| gct | A | 29 | 7.56 |
| gcc | A | 1 | 6.15 |
| gca | A | 0 | 13.37 |
| gcg | A | 0 | 2.93 |
| ggt | G | 3 | 44.79 |
| ggc | G | 117 | 49.09 |
| gga | G | 241 | 175.60 |
| ggg | G | 2 | 93.52 |
| --- | -- | ------ | ------ |
| mPD | | 0.67 | 0.81 |
| | | nPD: | 0.82 |
| | | N. weight: | 0.62 |
| | | Sc. PD: | 0.35 |
| | | Sc. rank: | 174.6 |

,

| | | | |
|---|---|---|---|
| NS1 | | | |
| Pos. | 159 | obs: | exp: |
| gaa | E | 392 | 226.10 |
| gag | E | 1 | 166.90 |
| --- | -- | ------ | ------ |
| mPD | | 0.0051 | 0.49 |
| | | nPD: | 0.01 |
| | | N. weight: | 0.63 |
| | | Sc. PD: | -0.15 |
| | | Sc. rank: | -121.5 |

,

| | | | |
|---|---|---|---|
| NS1 | | | |
| Pos. | 160 | obs: | exp: |
| att | I | 86 | 147.70 |
| atc | I | 279 | 103.00 |
| ata | I | 28 | 142.30 |
| --- | -- | ------ | ------ |
| mPD | | 0.44 | 0.66 |
| | | nPD: | 0.67 |
| | | N. weight: | 0.57 |
| | | Sc. PD: | 0.24 |
| | | Sc. rank: | 154.3 |

,

| | | | |
|---|---|---|---|
| NS1 | | | |
| Pos. | 161 | obs: | exp: |
| tct | S | 30 | 62.48 |
| tcc | S | 0 | 54.75 |
| tca | S | 363 | 87.07 |
| tcg | S | 0 | 23.96 |
| agt | S | 0 | 86.46 |
| agc | S | 0 | 78.30 |
| --- | -- | ------ | ------ |
| mPD | | 0.14 | 1.7 |
| | | nPD: | 0.09 |
| | | N. weight: | 1.5 |
| | | Sc. PD: | -0.24 |
| | | Sc. rank: | -203.4 |

,

| | | | |
|---|---|---|---|
| NS1 | | | |
| Pos. | 162 | obs: | exp: |
| cct | P | 3 | 103.50 |
| ccc | P | 30 | 70.89 |
| cca | P | 248 | 155.10 |
| ccg | P | 112 | 63.54 |
| --- | -- | ------ | ------ |
| mPD | | 0.52 | 0.72 |
| | | nPD: | 0.72 |
| | | N. weight: | 0.45 |
| | | Sc. PD: | 0.21 |
| | | Sc. rank: | 123.3 |

,

| | | | |
|---|---|---|---|
| NS1 | | | |
| Pos. | 163 | obs: | exp: |
| tta | L | 268 | 31.54 |
| ttg | L | 79 | 69.53 |
| ctt | L | 0 | 67.36 |
| ctc | L | 0 | 58.40 |
| cta | L | 7 | 54.38 |
| ctg | L | 7 | 79.80 |
| att | I | 30 | 11.28 |
| atc | I | 0 | 7.86 |
| ata | I | 0 | 10.86 |
| gtt | V | 0 | 0.41 |
| gtc | V | 0 | 0.42 |
| gta | V | 0 | 0.37 |
| gtg | V | 2 | 0.79 |
| --- | -- | ------ | ------ |
| mPD | | 0.67 | 1.2 |
| | | nPD: | 0.55 |
| | | N. weight: | 1.8 |
| | | Sc. PD: | 0.52 |
| | | Sc. rank: | 426.2 |

,

| | | | |
|---|---|---|---|
| NS1 | | | |
| Pos. | 164 | obs: | exp: |
| cct | P | 367 | 103.50 |
| ccc | P | 26 | 70.89 |
| cca | P | 0 | 155.10 |
| ccg | P | 0 | 63.54 |
| --- | -- | ------ | ------ |
| mPD | | 0.12 | 0.72 |
| | | nPD: | 0.17 |
| | | N. weight: | 1.3 |
| | | Sc. PD: | -0.097 |
| | | Sc. rank: | -93.6 |

,

NS1

| Pos. | 165 | obs: | exp: |
|---|---|---|---|
| tct | S | 366 | 62.48 |
| tcc | S | 27 | 54.75 |
| tca | S | 0 | 87.07 |
| tcg | S | 0 | 23.96 |
| agt | S | 0 | 86.46 |
| agc | S | 0 | 78.30 |
| mPD | | 0.13 | 1.7 |
| | | nPD: | 0.08 |
| | | N. weight: | 1.9 |
| | | Sc. PD: | -0.31 |
| | | Sc. rank: | -269.4 |

NS1

| Pos. | 166 | obs: | exp: |
|---|---|---|---|
| tta | L | 0 | 31.71 |
| ttg | L | 0 | 69.91 |
| ctt | L | 363 | 67.73 |
| ctc | L | 0 | 58.72 |
| cta | L | 0 | 54.69 |
| ctg | L | 0 | 80.24 |
| atg | M | 30 | 30.00 |
| mPD | | 0.28 | 1.2 |
| | | nPD: | 0.24 |
| | | N. weight: | 1.8 |
| | | Sc. PD: | -0.014 |
| | | Sc. rank: | 81.3 |

NS1

| Pos. | 167 | obs: | exp: |
|---|---|---|---|
| cct | P | 0 | 103.50 |
| ccc | P | 0 | 70.89 |
| cca | P | 393 | 155.10 |
| ccg | P | 0 | 63.54 |
| mPD | | 0 | 0.72 |
| | | nPD: | 0. |
| | | N. weight: | 1.1 |
| | | Sc. PD: | -0.26 |
| | | Sc. rank: | -311.3 |

NS1

| Pos. | 168 | obs: | exp: |
|---|---|---|---|
| gaa | E | 1 | 0.58 |
| gag | E | 0 | 0.42 |
| ggt | G | 0 | 48.37 |
| ggc | G | 0 | 53.01 |
| gga | G | 392 | 189.60 |
| ggg | G | 0 | 101.00 |
| mPD | | 0.0051 | 0.67 |
| | | nPD: | 0.01 |
| | | N. weight: | 0.85 |
| | | Sc. PD: | -0.2 |
| | | Sc. rank: | -178.5 |

NS1

| Pos. | 169 | obs: | exp: |
|---|---|---|---|
| tat | Y | 1 | 0.52 |
| tac | Y | 0 | 0.48 |
| cat | H | 392 | 212.40 |
| cac | H | 0 | 179.60 |
| mPD | | 0.0051 | 0.50 |
| | | nPD: | 0.01 |
| | | N. weight: | 0.72 |
| | | Sc. PD: | -0.17 |
| | | Sc. rank: | -140.6 |

NS1

| Pos. | 170 | obs: | exp: |
|---|---|---|---|
| tct | S | 30 | 4.77 |
| tcc | S | 0 | 4.18 |
| tca | S | 0 | 6.65 |
| tcg | S | 0 | 1.83 |
| att | I | 3 | 1.13 |
| atc | I | 0 | 0.79 |
| ata | I | 0 | 1.09 |
| act | T | 360 | 95.11 |
| acc | T | 0 | 77.25 |
| aca | T | 0 | 157.70 |
| acg | T | 0 | 29.93 |
| agt | S | 0 | 6.60 |
| agc | S | 0 | 5.98 |
| mPD | | 0.16 | 0.85 |
| | | nPD: | 0.18 |
| | | N. weight: | 1.6 |
| | | Sc. PD: | -0.1 |
| | | Sc. rank: | -64.0 |

NS1

| Pos. | 171 | obs: | exp: |
|---|---|---|---|
| act | T | 0 | 7.93 |
| acc | T | 0 | 6.44 |
| aca | T | 30 | 13.14 |
| acg | T | 0 | 2.49 |
| aat | N | 1 | 0.52 |
| aac | N | 0 | 0.48 |
| gat | D | 324 | 191.00 |
| gac | D | 34 | 167.00 |
| ggt | G | 0 | 0.49 |
| ggc | G | 0 | 0.54 |
| gga | G | 4 | 1.93 |
| ggg | G | 0 | 1.03 |
| mPD | | 0.61 | 0.85 |
| | | nPD: | 0.72 |
| | | N. weight: | 0.44 |
| | | Sc. PD: | 0.2 |
| | | Sc. rank: | 120.7 |

NS1

| Pos. | 172 | obs: | exp: |
|---|---|---|---|
| aaa | K | 0 | 16.80 |
| aag | K | 30 | 13.20 |
| gaa | E | 0 | 208.90 |
| gag | E | 363 | 154.10 |
| mPD | | 0.14 | 0.63 |
| | | nPD: | 0.22 |
| | | N. weight: | 1. |
| | | Sc. PD: | -0.024 |
| | | Sc. rank: | 22.0 |

NS1

| Pos. | 173 | obs: | exp: |
|---|---|---|---|
| gat | D | 393 | 209.70 |
| gac | D | 0 | 183.30 |
| mPD | | 0 | 0.50 |
| | | nPD: | 0. |
| | | N. weight: | 0.74 |
| | | Sc. PD: | -0.18 |
| | | Sc. rank: | -210.3 |

NS1

| Pos. | 174 | obs: | exp: |
|---|---|---|---|
| gtt | V | 0 | 80.78 |
| gtc | V | 393 | 82.48 |
| gta | V | 0 | 73.66 |
| gtg | V | 0 | 156.10 |
| mPD | | 0 | 0.72 |
| | | nPD: | 0. |
| | | N. weight: | 1.8 |
| | | Sc. PD: | -0.44 |
| | | Sc. rank: | -522.7 |

NS1

| Pos. | 175 | obs: | exp: |
|---|---|---|---|
| aaa | K | 392 | 220.10 |
| aag | K | 1 | 172.90 |
| mPD | | 0.0051 | 0.49 |
| | | nPD: | 0.01 |
| | | N. weight: | 0.67 |
| | | Sc. PD: | -0.15 |
| | | Sc. rank: | -128.6 |

NS1

| Pos. | 176 | obs: | exp: |
|---|---|---|---|
| aat | N | 393 | 205.40 |
| aac | N | 0 | 187.60 |
| mPD | | 0 | 0.50 |
| | | nPD: | 0. |
| | | N. weight: | 0.76 |
| | | Sc. PD: | -0.18 |
| | | Sc. rank: | -217.2 |

NS1

| Pos. | 177 | obs: | exp: |
|---|---|---|---|
| gct | A | 0 | 98.99 |
| gcc | A | 0 | 80.51 |
| gca | A | 392 | 175.20 |
| gcg | A | 1 | 38.35 |
| mPD | | 0.0051 | 0.69 |
| | | nPD: | 0.01 |
| | | N. weight: | 0.94 |
| | | Sc. PD: | -0.22 |
| | | Sc. rank: | -198.0 |

NS1

| Pos. | 178 | obs: | exp: |
|---|---|---|---|
| att | I | 393 | 147.70 |
| atc | I | 0 | 103.00 |
| ata | I | 0 | 142.30 |
| mPD | | 0 | 0.66 |
| | | nPD: | 0. |
| | | N. weight: | 1.2 |
| | | Sc. PD: | -0.28 |
| | | Sc. rank: | -327.5 |

NS1

| Pos. | 179 | obs: | exp: |
|---|---|---|---|
| gaa | E | 0 | 16.69 |
| gag | E | 29 | 12.31 |
| ggt | G | 0 | 44.91 |
| ggc | G | 0 | 49.22 |
| gga | G | 32 | 176.10 |
| ggg | G | 332 | 93.78 |
| mPD | | 0.29 | 0.79 |
| | | nPD: | 0.36 |
| | | N. weight: | 1.2 |
| | | Sc. PD: | 0.13 |
| | | Sc. rank: | 172.8 |

NS1

| Pos. | 180 | obs: | exp: |
|---|---|---|---|
| att | I | 0 | 12.41 |
| atc | I | 33 | 8.65 |
| ata | I | 0 | 11.95 |
| gtt | V | 0 | 73.99 |
| gtc | V | 360 | 75.56 |
| gta | V | 0 | 67.47 |
| gtg | V | 0 | 143.00 |
| mPD | | 0.15 | 0.89 |
| | | nPD: | 0.17 |
| | | N. weight: | 1.8 |
| | | Sc. PD: | -0.13 |
| | | Sc. rank: | -108.4 |

NS1

| Pos. | 181 | obs: | exp: |
|---|---|---|---|
| tta | L | 0 | 34.33 |
| ttg | L | 0 | 75.69 |
| ctt | L | 0 | 73.33 |
| ctc | L | 393 | 63.57 |
| cta | L | 0 | 59.21 |
| ctg | L | 0 | 86.87 |
| mPD | | 0 | 1.1 |
| | | nPD: | 0. |
| | | N. weight: | 2.1 |
| | | Sc. PD: | -0.52 |
| | | Sc. rank: | -609.9 |

NS1

| Pos. | 182 | obs: | exp: |
|---|---|---|---|
| att | I | 2 | 147.70 |
| atc | I | 391 | 103.00 |
| ata | I | 0 | 142.30 |
| mPD | | 0.010 | 0.66 |
| | | nPD: | 0.02 |
| | | N. weight: | 1.5 |
| | | Sc. PD: | -0.35 |
| | | Sc. rank: | -262.8 |

NS1

| Pos. | 183 | obs: | exp: |
|---|---|---|---|
| ggt | G | 28 | 48.49 |
| ggc | G | 2 | 53.15 |
| gga | G | 361 | 190.10 |
| ggg | G | 2 | 101.30 |
| mPD | | 0.15 | 0.67 |
| | | nPD: | 0.23 |
| | | N. weight: | 0.62 |
| | | Sc. PD: | -0.013 |
| | | Sc. rank: | 15.7 |

NS1

| Pos. | 184 | obs: | exp: |
|---|---|---|---|
| ggt | G | 0 | 48.49 |
| ggc | G | 0 | 53.15 |
| gga | G | 389 | 190.10 |
| ggg | G | 4 | 101.30 |
| mPD | | 0.020 | 0.67 |
| | | nPD: | 0.03 |
| | | N. weight: | 0.8 |
| | | Sc. PD: | -0.17 |
| | | Sc. rank: | -131.7 |

NS1

| Pos. | 185 | obs: | exp: |
|---|---|---|---|
| ttt | F | 39 | 16.03 |
| ttc | F | 0 | 22.97 |
| tta | L | 0 | 30.93 |
| ttg | L | 0 | 68.18 |
| ctt | L | 354 | 66.05 |
| ctc | L | 0 | 57.26 |
| cta | L | 0 | 53.33 |
| ctg | L | 0 | 78.25 |
| mPD | | 0.18 | 1.2 |
| | | nPD: | 0.15 |
| | | N. weight: | 1.9 |
| | | Sc. PD: | -0.18 |
| | | Sc. rank: | -197.6 |

NS1

| Pos. | 186 | obs: | exp: |
|---|---|---|---|
| gaa | E | 388 | 225.60 |
| gag | E | 4 | 166.40 |
| ggt | G | 0 | 0.12 |
| ggc | G | 0 | 0.14 |
| gga | G | 1 | 0.48 |
| ggg | G | 0 | 0.26 |
| mPD | | 0.025 | 0.49 |
| | | nPD: | 0.05 |
| | | N. weight: | 0.59 |
| | | Sc. PD: | -0.11 |
| | | Sc. rank: | -95.5 |

NS1

| Pos. | 187 | obs: | exp: |
|---|---|---|---|
| tgg | W | 393 | 393.00 |
| mPD | | 0 | 0 |
| | | nPD: | 1. |
| | | N. weight: | 0. |
| | | Sc. PD: | 0 |
| | | Sc. rank: | 0 |

NS1

| Pos. | 188 | obs: | exp: |
|---|---|---|---|
| aat | N | 393 | 205.40 |
| aac | N | 0 | 187.60 |
| mPD | | 0 | 0.50 |
| | | nPD: | 0. |
| | | N. weight: | 0.76 |
| | | Sc. PD: | -0.18 |
| | | Sc. rank: | -217.2 |

NS1

| Pos. | 189 | obs: | exp: |
|---|---|---|---|
| aat | N | 3 | 1.57 |
| aac | N | 0 | 1.43 |
| gat | D | 390 | 208.10 |
| gac | D | 0 | 181.90 |
| mPD | | 0.015 | 0.51 |
| | | nPD: | 0.03 |
| | | N. weight: | 0.74 |
| | | Sc. PD: | -0.16 |
| | | Sc. rank: | -123.2 |

| NS1 | | | |
|---|---|---|---|
| Pos. | 190 | obs: | exp: |
| act | T | 0 | 0.26 |
| acc | T | 1 | 0.21 |
| aca | T | 0 | 0.44 |
| acg | T | 0 | 0.08 |
| aat | N | 0 | 204.90 |
| aac | N | 392 | 187.10 |
| --- | -- | ------ | ------ |
| mPD | | 0.0051 | 0.51 |
| | | nPD: | 0.01 |
| | | N. weight: | 0.87 |
| | | Sc. PD: | -0.2 |
| | | Sc. rank: | -171.7 |

,

| NS1 | | | |
|---|---|---|---|
| Pos. | 191 | obs: | exp: |
| tct | S | 0 | 4.77 |
| tcc | S | 0 | 4.18 |
| tca | S | 30 | 6.65 |
| tcg | S | 0 | 1.83 |
| cct | P | 0 | 0.26 |
| ccc | P | 0 | 0.18 |
| cca | P | 1 | 0.39 |
| ccg | P | 0 | 0.16 |
| act | T | 0 | 95.64 |
| acc | T | 0 | 77.68 |
| aca | T | 362 | 158.60 |
| acg | T | 0 | 30.09 |
| agt | S | 0 | 6.60 |
| agc | S | 0 | 5.98 |
| --- | -- | ------ | ------ |
| mPD | | 0.15 | 0.84 |
| | | nPD: | 0.17 |
| | | N. weight: | 1. |
| | | Sc. PD: | -0.075 |
| | | Sc. rank: | -69.2 |

,

| NS1 | | | |
|---|---|---|---|
| Pos. | 192 | obs: | exp: |
| att | I | 31 | 11.65 |
| atc | I | 0 | 8.12 |
| ata | I | 0 | 11.22 |
| gtt | V | 362 | 74.41 |
| gtc | V | 0 | 75.98 |
| gta | V | 0 | 67.85 |
| gtg | V | 0 | 143.80 |
| --- | -- | ------ | ------ |
| mPD | | 0.15 | 0.88 |
| | | nPD: | 0.17 |
| | | N. weight: | 1.8 |
| | | Sc. PD: | -0.14 |
| | | Sc. rank: | -163.7 |

,

| NS1 | | | |
|---|---|---|---|
| Pos. | 193 | obs: | exp: |
| cgt | R | 0 | 16.32 |
| cgc | R | 0 | 19.24 |
| cga | R | 392 | 37.77 |
| cgg | R | 0 | 32.80 |
| aga | R | 1 | 176.70 |
| agg | R | 0 | 110.10 |
| --- | -- | ------ | ------ |
| mPD | | 0.0051 | 0.96 |
| | | nPD: | 0.01 |
| | | N. weight: | 2.7 |
| | | Sc. PD: | -0.65 |
| | | Sc. rank: | -583.7 |

,

| NS1 | | | |
|---|---|---|---|
| Pos. | 194 | obs: | exp: |
| att | I | 0 | 0.38 |
| atc | I | 1 | 0.26 |
| ata | I | 0 | 0.36 |
| gtt | V | 0 | 74.41 |
| gtc | V | 362 | 75.98 |
| gta | V | 0 | 67.85 |
| gtg | V | 0 | 143.80 |
| gct | A | 0 | 7.56 |
| gcc | A | 0 | 6.15 |
| gca | A | 0 | 13.37 |
| gcg | A | 30 | 2.93 |
| --- | -- | ------ | ------ |
| mPD | | 0.29 | 0.88 |
| | | nPD: | 0.33 |
| | | N. weight: | 1.9 |
| | | Sc. PD: | 0.15 |
| | | Sc. rank: | 229.1 |

,

| NS1 | | | |
|---|---|---|---|
| Pos. | 195 | obs: | exp: |
| tct | S | 393 | 62.48 |
| tcc | S | 0 | 54.75 |
| tca | S | 0 | 87.07 |
| tcg | S | 0 | 23.96 |
| agt | S | 0 | 86.46 |
| agc | S | 0 | 78.30 |
| --- | -- | ------ | ------ |
| mPD | | 0 | 1.7 |
| | | nPD: | 0. |
| | | N. weight: | 2.2 |
| | | Sc. PD: | -0.52 |
| | | Sc. rank: | -615.7 |

,

| NS1 | | | |
|---|---|---|---|
| Pos. | 196 | obs: | exp: |
| gaa | E | 387 | 226.10 |
| gag | E | 6 | 166.90 |
| --- | -- | ------ | ------ |
| mPD | | 0.030 | 0.49 |
| | | nPD: | 0.06 |
| | | N. weight: | 0.57 |
| | | Sc. PD: | -0.1 |
| | | Sc. rank: | -89.6 |

,

| NS1 | | | |
|---|---|---|---|
| Pos. | 197 | obs: | exp: |
| act | T | 357 | 94.32 |
| acc | T | 0 | 76.61 |
| aca | T | 0 | 156.40 |
| acg | T | 0 | 29.68 |
| aat | N | 35 | 18.29 |
| aac | N | 0 | 16.71 |
| gct | A | 1 | 0.25 |
| gcc | A | 0 | 0.20 |
| gca | A | 0 | 0.45 |
| gcg | A | 0 | 0.10 |
| --- | -- | ------ | ------ |
| mPD | | 0.17 | 0.86 |
| | | nPD: | 0.19 |
| | | N. weight: | 1.5 |
| | | Sc. PD: | -0.079 |
| | | Sc. rank: | -39.8 |

,

| NS1 | | | |
|---|---|---|---|
| Pos. | 198 | obs: | exp: |
| tta | L | 62 | 31.71 |
| ttg | L | 0 | 69.91 |
| ctt | L | 0 | 67.73 |
| ctc | L | 0 | 58.72 |
| cta | L | 301 | 54.69 |
| ctg | L | 0 | 80.24 |
| att | I | 0 | 11.28 |
| atc | I | 0 | 7.86 |
| ata | I | 30 | 10.86 |
| --- | -- | ------ | ------ |
| mPD | | 0.38 | 1.2 |
| | | nPD: | 0.32 |
| | | N. weight: | 1.8 |
| | | Sc. PD: | 0.12 |
| | | Sc. rank: | 196.4 |

,

| NS1 | | | |
|---|---|---|---|
| Pos. | 199 | obs: | exp: |
| caa | Q | 2 | 209.50 |
| cag | Q | 391 | 183.50 |
| --- | -- | ------ | ------ |
| mPD | | 0.010 | 0.50 |
| | | nPD: | 0.02 |
| | | N. weight: | 0.86 |
| | | Sc. PD: | -0.19 |
| | | Sc. rank: | -145.2 |

,

| NS1 | | | |
|---|---|---|---|
| Pos. | 200 | obs: | exp: |
| cgt | R | 0 | 16.32 |
| cgc | R | 0 | 19.24 |
| cga | R | 0 | 37.77 |
| cgg | R | 0 | 32.80 |
| aga | R | 393 | 176.70 |
| agg | R | 0 | 110.10 |
| --- | -- | ------ | ------ |
| mPD | | 0 | 0.96 |
| | | nPD: | 0. |
| | | N. weight: | 0.94 |
| | | Sc. PD: | -0.23 |
| | | Sc. rank: | -267.5 |

,

| NS1 | | | |
|---|---|---|---|
| Pos. | 201 | obs: | exp: |
| ttt | F | 6 | 161.60 |
| ttc | F | 387 | 231.40 |
| --- | -- | ------ | ------ |
| mPD | | 0.030 | 0.48 |
| | | nPD: | 0.06 |
| | | N. weight: | 0.54 |
| | | Sc. PD: | -0.098 |
| | | Sc. rank: | -84.7 |

,

| NS1 | | | |
|---|---|---|---|
| Pos. | 202 | obs: | exp: |
| act | T | 2 | 0.53 |
| acc | T | 0 | 0.43 |
| aca | T | 0 | 0.88 |
| acg | T | 0 | 0.17 |
| gct | A | 391 | 98.48 |
| gcc | A | 0 | 80.10 |
| gca | A | 0 | 174.30 |
| gcg | A | 0 | 38.15 |
| --- | -- | ------ | ------ |
| mPD | | 0.010 | 0.70 |
| | | nPD: | 0.01 |
| | | N. weight: | 1.6 |
| | | Sc. PD: | -0.37 |
| | | Sc. rank: | -296.5 |

,

| NS1 | | | |
|---|---|---|---|
| Pos. | 203 | obs: | exp: |
| tgg | W | 393 | 393.00 |
| --- | -- | ------ | ------ |
| mPD | | 0 | 0 |
| | | nPD: | 1. |
| | | N. weight: | 0. |
| | | Sc. PD: | 0 |
| | | Sc. rank: | 0 |

,

| NS1 | | | |
|---|---|---|---|
| Pos. | 204 | obs: | exp: |
| cgt | R | 0 | 13.82 |
| cgc | R | 0 | 16.31 |
| cga | R | 0 | 32.00 |
| cgg | R | 0 | 27.79 |
| aaa | K | 1 | 0.56 |
| aag | K | 0 | 0.44 |
| aga | R | 333 | 149.80 |
| agg | R | 0 | 93.32 |
| ggt | G | 0 | 7.28 |
| ggc | G | 0 | 7.98 |
| gga | G | 59 | 28.54 |
| ggg | G | 0 | 15.20 |
| --- | -- | ------ | ------ |
| mPD | | 0.26 | 1.1 |
| | | nPD: | 0.23 |
| | | N. weight: | 0.93 |
| | | Sc. PD: | -0.016 |
| | | Sc. rank: | 31.6 |

,

| NS1 | | | |
|---|---|---|---|
| Pos. | 205 | obs: | exp: |
| tct | S | 0 | 55.32 |
| tcc | S | 0 | 48.48 |
| tca | S | 0 | 77.10 |
| tcg | S | 0 | 21.21 |
| att | I | 0 | 7.14 |
| atc | I | 19 | 4.98 |
| ata | I | 0 | 6.88 |
| aat | N | 0 | 1.57 |
| aac | N | 3 | 1.43 |
| agt | S | 0 | 76.56 |
| agc | S | 348 | 69.33 |
| gtt | V | 0 | 2.26 |
| gtc | V | 11 | 2.31 |
| gta | V | 0 | 2.06 |
| gtg | V | 0 | 4.37 |
| ggt | G | 0 | 1.48 |
| ggc | G | 12 | 1.62 |
| gga | G | 0 | 5.80 |
| ggg | G | 0 | 3.09 |
| --- | -- | ------ | ------ |
| mPD | | 0.27 | 1.8 |
| | | nPD: | 0.15 |
| | | N. weight: | 1.9 |
| | | Sc. PD: | -0.19 |
| | | Sc. rank: | -215.1 |

,

| NS1 | | | |
|---|---|---|---|
| Pos. | 206 | obs: | exp: |
| tct | S | 0 | 55.16 |
| tcc | S | 0 | 48.34 |
| tca | S | 0 | 76.87 |
| tcg | S | 0 | 21.15 |
| tgt | C | 11 | 4.53 |
| tgc | C | 0 | 6.47 |
| cgt | R | 29 | 1.20 |
| cgc | R | 0 | 1.42 |
| cga | R | 0 | 2.79 |
| cgg | R | 0 | 2.42 |
| aat | N | 2 | 1.05 |
| aac | N | 0 | 0.95 |
| agt | S | 316 | 76.34 |
| agc | S | 31 | 69.13 |
| aga | R | 0 | 13.04 |
| agg | R | 0 | 8.13 |
| ggt | G | 4 | 0.49 |
| ggc | G | 0 | 0.54 |
| gga | G | 0 | 1.93 |
| ggg | G | 0 | 1.03 |
| --- | -- | ------ | ------ |
| mPD | | 0.36 | 1.7 |
| | | nPD: | 0.21 |
| | | N. weight: | 1.6 |
| | | Sc. PD: | -0.063 |
| | | Sc. rank: | 7.3 |

,

| NS1 | | | |
|---|---|---|---|
| Pos. | 207 | obs: | exp: |
| aat | N | 332 | 173.50 |
| aac | N | 0 | 158.50 |
| gat | D | 61 | 32.55 |
| gac | D | 0 | 28.45 |
| --- | -- | ------ | ------ |
| mPD | | 0.26 | 0.76 |
| | | nPD: | 0.35 |
| | | N. weight: | 0.76 |
| | | Sc. PD: | 0.072 |
| | | Sc. rank: | 100.2 |

,

| NS1 | | | |
|---|---|---|---|
| Pos. | 208 | obs: | exp: |
| gaa | E | 0 | 226.10 |
| gag | E | 393 | 166.90 |
| --- | -- | ------ | ------ |
| mPD | | 0 | 0.49 |
| | | nPD: | 0. |
| | | N. weight: | 1. |
| | | Sc. PD: | -0.24 |
| | | Sc. rank: | -286.8 |

,

| NS1 | | | |
|---|---|---|---|
| Pos. | 209 | obs: | exp: |
| tct | S | 0 | 0.16 |
| tcc | S | 0 | 0.14 |
| tca | S | 0 | 0.22 |
| tcg | S | 0 | 0.06 |
| aat | N | 29 | 15.16 |
| aac | N | 0 | 13.84 |
| agt | S | 1 | 0.22 |
| agc | S | 0 | 0.20 |
| gat | D | 363 | 193.70 |
| gac | D | 0 | 169.30 |
| --- | -- | ------ | ------ |
| mPD | | 0.15 | 0.64 |
| | | nPD: | 0.23 |
| | | N. weight: | 0.74 |
| | | Sc. PD: | -0.015 |
| | | Sc. rank: | 17.7 |

,

| | | | |
|---|---|---|---|
| NS1 | | | |
| Pos. | 210 | obs: | exp: |
| cgt | R | 0 | 0.08 |
| cgc | R | 0 | 0.10 |
| cga | R | 0 | 0.19 |
| cgg | R | 0 | 0.17 |
| aga | R | 0 | 0.90 |
| agg | R | 2 | 0.56 |
| ggt | G | 0 | 48.24 |
| ggc | G | 0 | 52.88 |
| gga | G | 0 | 189.10 |
| ggg | G | 391 | 100.70 |
| --- | -- | ------ | ------ |
| mPD | | 0.010 | 0.68 |
| | | nPD: | 0.02 |
| | | N. weight: | 1.6 |
| | | Sc. PD: | -0.36 |
| | | Sc. rank: | -285.3 |

| | | | |
|---|---|---|---|
| NS1 | | | |
| Pos. | 211 | obs: | exp: |
| cgt | R | 0 | 15.07 |
| cgc | R | 0 | 17.77 |
| cga | R | 0 | 34.89 |
| cgg | R | 0 | 30.29 |
| aga | R | 363 | 163.30 |
| agg | R | 0 | 101.70 |
| ggt | G | 0 | 3.70 |
| ggc | G | 0 | 4.06 |
| gga | G | 30 | 14.51 |
| ggg | G | 0 | 7.73 |
| --- | -- | ------ | ------ |
| mPD | | 0.14 | 1.1 |
| | | nPD: | 0.13 |
| | | N. weight: | 0.93 |
| | | Sc. PD: | -0.1 |
| | | Sc. rank: | -118.2 |

| | | | |
|---|---|---|---|
| NS1 | | | |
| Pos. | 212 | obs: | exp: |
| cct | P | 393 | 103.50 |
| ccc | P | 0 | 70.89 |
| cca | P | 0 | 155.10 |
| ccg | P | 0 | 63.54 |
| --- | -- | ------ | ------ |
| mPD | | 0 | 0.72 |
| | | nPD: | 0. |
| | | N. weight: | 1.6 |
| | | Sc. PD: | -0.38 |
| | | Sc. rank: | -446.7 |

| | | | |
|---|---|---|---|
| NS1 | | | |
| Pos. | 213 | obs: | exp: |
| cct | P | 0 | 103.00 |
| ccc | P | 0 | 70.53 |
| cca | P | 391 | 154.30 |
| ccg | P | 0 | 63.22 |
| gct | A | 0 | 0.50 |
| gcc | A | 0 | 0.41 |
| gca | A | 2 | 0.89 |
| gcg | A | 0 | 0.20 |
| --- | -- | ------ | ------ |
| mPD | | 0.010 | 0.73 |
| | | nPD: | 0.01 |
| | | N. weight: | 1.1 |
| | | Sc. PD: | -0.25 |
| | | Sc. rank: | -204.3 |

| | | | |
|---|---|---|---|
| NS1 | | | |
| Pos. | 214 | obs: | exp: |
| tta | L | 0 | 34.33 |
| ttg | L | 0 | 75.69 |
| ctt | L | 2 | 73.33 |
| ctc | L | 391 | 63.57 |
| cta | L | 0 | 59.21 |
| ctg | L | 0 | 86.87 |
| --- | -- | ------ | ------ |
| mPD | | 0.010 | 1.1 |
| | | nPD: | 0.01 |
| | | N. weight: | 2.1 |
| | | Sc. PD: | -0.49 |
| | | Sc. rank: | -419.7 |

| | | | |
|---|---|---|---|
| NS1 | | | |
| Pos. | 215 | obs: | exp: |
| tta | L | 0 | 0.09 |
| ttg | L | 0 | 0.19 |
| tct | S | 30 | 4.77 |
| tcc | S | 0 | 4.18 |
| tca | S | 0 | 6.65 |
| tcg | S | 0 | 1.83 |
| ctt | L | 1 | 0.19 |
| ctc | L | 0 | 0.16 |
| cta | L | 0 | 0.15 |
| ctg | L | 0 | 0.22 |
| cct | P | 362 | 95.32 |
| ccc | P | 0 | 65.30 |
| cca | P | 0 | 142.90 |
| ccg | P | 0 | 58.53 |
| agt | S | 0 | 6.60 |
| agc | S | 0 | 5.98 |
| --- | -- | ------ | ------ |
| mPD | | 0.15 | 0.93 |
| | | nPD: | 0.16 |
| | | N. weight: | 1.6 |
| | | Sc. PD: | -0.14 |
| | | Sc. rank: | -163.1 |

| | | | |
|---|---|---|---|
| NS1 | | | |
| Pos. | 216 | obs: | exp: |
| tct | S | 0 | 0.32 |
| tcc | S | 0 | 0.28 |
| tca | S | 2 | 0.44 |
| tcg | S | 0 | 0.12 |
| cct | P | 0 | 95.06 |
| ccc | P | 0 | 65.11 |
| cca | P | 361 | 142.50 |
| ccg | P | 0 | 58.37 |
| act | T | 0 | 7.93 |
| acc | T | 0 | 6.44 |
| aca | T | 30 | 13.14 |
| acg | T | 0 | 2.49 |
| agt | S | 0 | 0.44 |
| agc | S | 0 | 0.40 |
| --- | -- | ------ | ------ |
| mPD | | 0.15 | 0.87 |
| | | nPD: | 0.17 |
| | | N. weight: | 1.1 |
| | | Sc. PD: | -0.079 |
| | | Sc. rank: | -69.9 |

| | | | |
|---|---|---|---|
| NS1 | | | |
| Pos. | 217 | obs: | exp: |
| aaa | K | 107 | 220.10 |
| aag | K | 286 | 172.90 |
| --- | -- | ------ | ------ |
| mPD | | 0.40 | 0.49 |
| | | nPD: | 0.81 |
| | | N. weight: | 0.21 |
| | | Sc. PD: | 0.11 |
| | | Sc. rank: | 58.8 |

| | | | |
|---|---|---|---|
| NS1 | | | |
| Pos. | 218 | obs: | exp: |
| caa | Q | 0 | 193.00 |
| cag | Q | 362 | 169.00 |
| --- | - | 31 | 31.00 |
| --- | -- | ------ | ------ |
| mPD | | 0 | 0.42 |
| | | nPD: | 0. |
| | | N. weight: | 0.83 |
| | | Sc. PD: | -0.2 |
| | | Sc. rank: | -234.9 |

| | | | |
|---|---|---|---|
| NS1 | | | |
| Pos. | 219 | obs: | exp: |
| aaa | K | 362 | 202.70 |
| aag | K | 0 | 159.30 |
| --- | - | 31 | 31.00 |
| --- | -- | ------ | ------ |
| mPD | | 0 | 0.42 |
| | | nPD: | 0. |
| | | N. weight: | 0.63 |
| | | Sc. PD: | -0.15 |
| | | Sc. rank: | -178.8 |

| | | | |
|---|---|---|---|
| NS1 | | | |
| Pos. | 220 | obs: | exp: |
| tgg | W | 1 | 1.00 |
| caa | Q | 0 | 0.53 |
| cag | Q | 1 | 0.47 |
| cgt | R | 0 | 14.95 |
| cgc | R | 30 | 17.63 |
| cga | R | 0 | 34.60 |
| cgg | R | 330 | 30.04 |
| aga | R | 0 | 161.90 |
| agg | R | 0 | 100.90 |
| --- | - | 31 | 31.00 |
| --- | -- | ------ | ------ |
| mPD | | 0.14 | 0.82 |
| | | nPD: | 0.17 |
| | | N. weight: | 2.4 |
| | | Sc. PD: | -0.19 |
| | | Sc. rank: | -205.2 |

| | | | |
|---|---|---|---|
| NS1 | | | |
| Pos. | 221 | obs: | exp: |
| ttt | F | 0 | 0.82 |
| ttc | F | 2 | 1.18 |
| tat | Y | 0 | 14.49 |
| tac | Y | 28 | 13.51 |
| aaa | K | 331 | 185.30 |
| aag | K | 0 | 145.70 |
| gaa | E | 1 | 0.58 |
| gag | E | 0 | 0.42 |
| --- | - | 31 | 31.00 |
| --- | -- | ------ | ------ |
| mPD | | 0.27 | 0.63 |
| | | nPD: | 0.43 |
| | | N. weight: | 0.64 |
| | | Sc. PD: | 0.12 |
| | | Sc. rank: | 123.2 |

| | | | |
|---|---|---|---|
| NS1 | | | |
| Pos. | 222 | obs: | exp: |
| atg | M | 362 | 362.00 |
| --- | - | 31 | 31.00 |
| --- | -- | ------ | ------ |
| mPD | | 0 | 0 |
| | | nPD: | 1. |
| | | N. weight: | 0. |
| | | Sc. PD: | 0 |
| | | Sc. rank: | 0 |

| | | | |
|---|---|---|---|
| NS1 | | | |
| Pos. | 223 | obs: | exp: |
| gct | A | 0 | 91.18 |
| gcc | A | 0 | 74.15 |
| gca | A | 0 | 161.30 |
| gcg | A | 362 | 35.32 |
| --- | - | 31 | 31.00 |
| --- | -- | ------ | ------ |
| mPD | | 0 | 0.58 |
| | | nPD: | 0. |
| | | N. weight: | 2.5 |
| | | Sc. PD: | -0.61 |
| | | Sc. rank: | -717.6 |

| | | | |
|---|---|---|---|
| NS1 | | | |
| Pos. | 224 | obs: | exp: |
| cgt | R | 0 | 14.95 |
| cgc | R | 0 | 17.63 |
| cga | R | 0 | 34.60 |
| cgg | R | 0 | 30.04 |
| aaa | K | 2 | 1.12 |
| aag | K | 0 | 0.88 |
| aga | R | 360 | 161.90 |
| agg | R | 0 | 100.90 |
| --- | - | 31 | 31.00 |
| --- | -- | ------ | ------ |
| mPD | | 0.0093 | 0.82 |
| | | nPD: | 0.01 |
| | | N. weight: | 0.86 |
| | | Sc. PD: | -0.2 |
| | | Sc. rank: | -164.0 |

| | | | |
|---|---|---|---|
| NS1 | | | |
| Pos. | 225 | obs: | exp: |
| cgt | R | 0 | 1.25 |
| cgc | R | 0 | 1.47 |
| cga | R | 24 | 2.88 |
| cgg | R | 0 | 2.50 |
| act | T | 0 | 86.65 |
| acc | T | 0 | 70.38 |
| aca | T | 328 | 143.70 |
| acg | T | 0 | 27.27 |
| aga | R | 6 | 13.49 |
| agg | R | 0 | 8.41 |
| gct | A | 0 | 1.01 |
| gcc | A | 0 | 0.82 |
| gca | A | 4 | 1.78 |
| gcg | A | 0 | 0.39 |
| --- | - | 31 | 31.00 |
| --- | -- | ------ | ------ |
| mPD | | 0.25 | 0.77 |
| | | nPD: | 0.33 |
| | | N. weight: | 0.96 |
| | | Sc. PD: | 0.074 |
| | | Sc. rank: | 111.2 |

| | | | |
|---|---|---|---|
| NS1 | | | |
| Pos. | 226 | obs: | exp: |
| att | I | 328 | 123.30 |
| atc | I | 0 | 85.96 |
| ata | I | 0 | 118.70 |
| gtt | V | 33 | 6.78 |
| gtc | V | 0 | 6.93 |
| gta | V | 0 | 6.18 |
| gtg | V | 0 | 13.11 |
| gct | A | 1 | 0.25 |
| gcc | A | 0 | 0.20 |
| gca | A | 0 | 0.45 |
| gcg | A | 0 | 0.10 |
| --- | - | 31 | 31.00 |
| --- | -- | ------ | ------ |
| mPD | | 0.15 | 0.73 |
| | | nPD: | 0.21 |
| | | N. weight: | 1.1 |
| | | Sc. PD: | -0.047 |
| | | Sc. rank: | -1.9 |

| NS1 | | | |
|---|---|---|---|
| Pos. | 227 | obs: | exp: |
| gaa | E | 0 | 208.30 |
| gag | E | 362 | 153.70 |
| --- | - | 31 | 31.00 |
| --- | -- | ------ | ------ |
| mPD | | 0 | 0.41 |
| | | nPD: | 0. |
| | | N. weight: | 0.93 |
| | | Sc. PD: | -0.22 |
| | | Sc. rank: | -264.2 |

,

| NS1 | | | |
|---|---|---|---|
| Pos. | 228 | obs: | exp: |
| tct | S | 0 | 57.39 |
| tcc | S | 0 | 50.29 |
| tca | S | 361 | 79.98 |
| tcg | S | 0 | 22.00 |
| cct | P | 0 | 0.26 |
| ccc | P | 0 | 0.18 |
| cca | P | 1 | 0.39 |
| ccg | P | 0 | 0.16 |
| agt | S | 0 | 79.42 |
| agc | S | 0 | 71.92 |
| --- | - | 31 | 31.00 |
| --- | -- | ------ | ------ |
| mPD | | 0.0047 | 1.4 |
| | | nPD: | 0. |
| | | N. weight: | 1.6 |
| | | Sc. PD: | -0.39 |
| | | Sc. rank: | -359.6 |

,

| NS1 | | | |
|---|---|---|---|
| Pos. | 229 | obs: | exp: |
| gaa | E | 362 | 208.30 |
| gag | E | 0 | 153.70 |
| --- | - | 31 | 31.00 |
| --- | -- | ------ | ------ |
| mPD | | 0 | 0.41 |
| | | nPD: | 0. |
| | | N. weight: | 0.6 |
| | | Sc. PD: | -0.14 |
| | | Sc. rank: | -170.4 |

,

| NS1 | | | |
|---|---|---|---|
| Pos. | 230 | obs: | exp: |
| att | I | 1 | 0.38 |
| atc | I | 0 | 0.26 |
| ata | I | 0 | 0.36 |
| gtt | V | 360 | 73.99 |
| gtc | V | 0 | 75.56 |
| gta | V | 0 | 67.47 |
| gtg | V | 0 | 143.00 |
| gct | A | 1 | 0.25 |
| gcc | A | 0 | 0.20 |
| gca | A | 0 | 0.45 |
| gcg | A | 0 | 0.10 |
| --- | - | 31 | 31.00 |
| --- | -- | ------ | ------ |
| mPD | | 0.0094 | 0.62 |
| | | nPD: | 0.02 |
| | | N. weight: | 1.7 |
| | | Sc. PD: | -0.39 |
| | | Sc. rank: | -298.9 |

,

| NS1 | | | |
|---|---|---|---|
| Pos. | 231 | obs: | exp: |
| taa | . | 0 | 184.10 |
| tag | . | 31 | 101.70 |
| tga | . | 362 | 107.20 |
| --- | -- | ------ | ------ |
| mPD | | 0.29 | 0.78 |
| | | nPD: | 0.37 |
| | | N. weight: | 1.2 |
| | | Sc. PD: | 0.15 |
| | | Sc. rank: | 199.1 |

}

Amino acid level conservation per codon

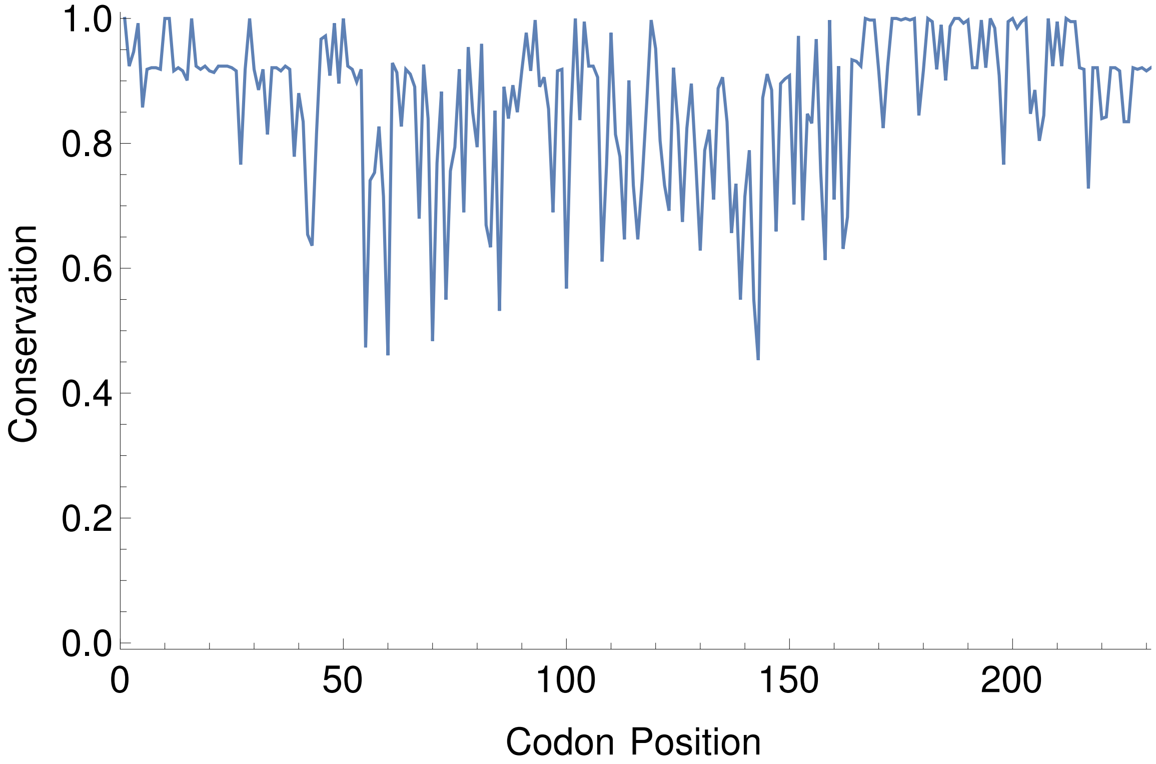

Conservation analysis plots

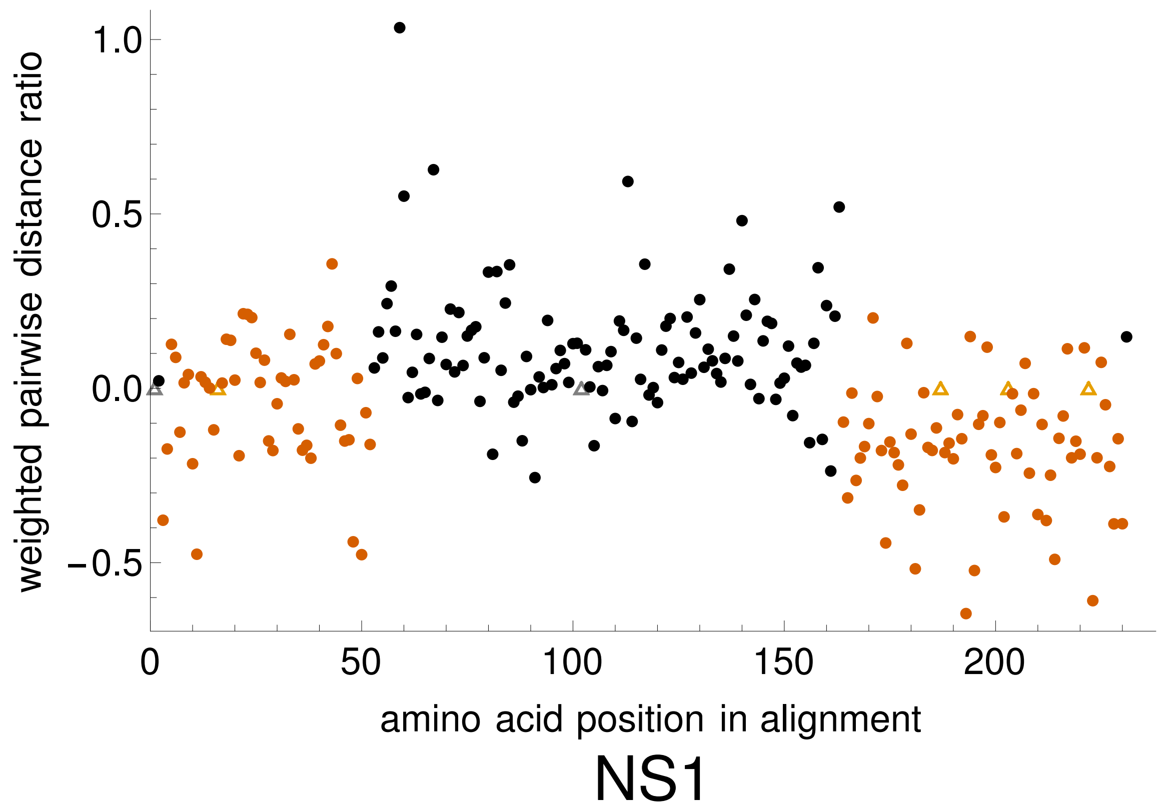


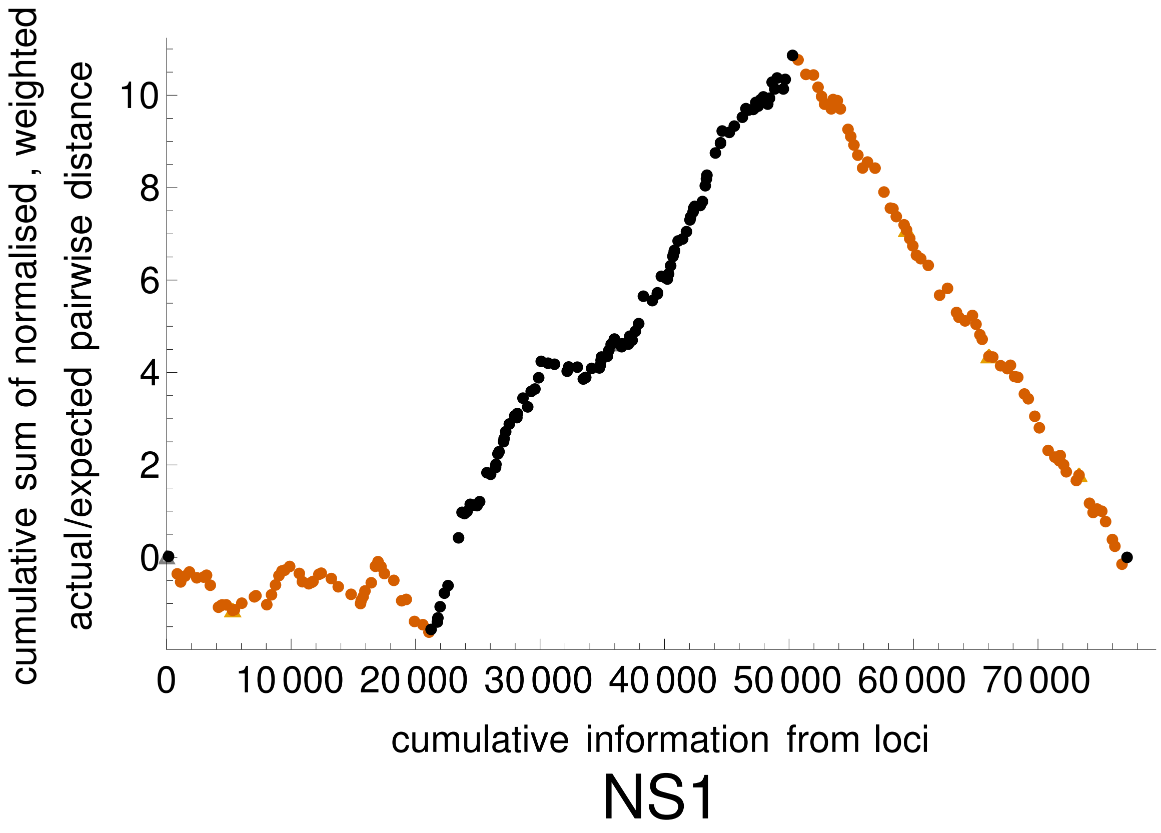

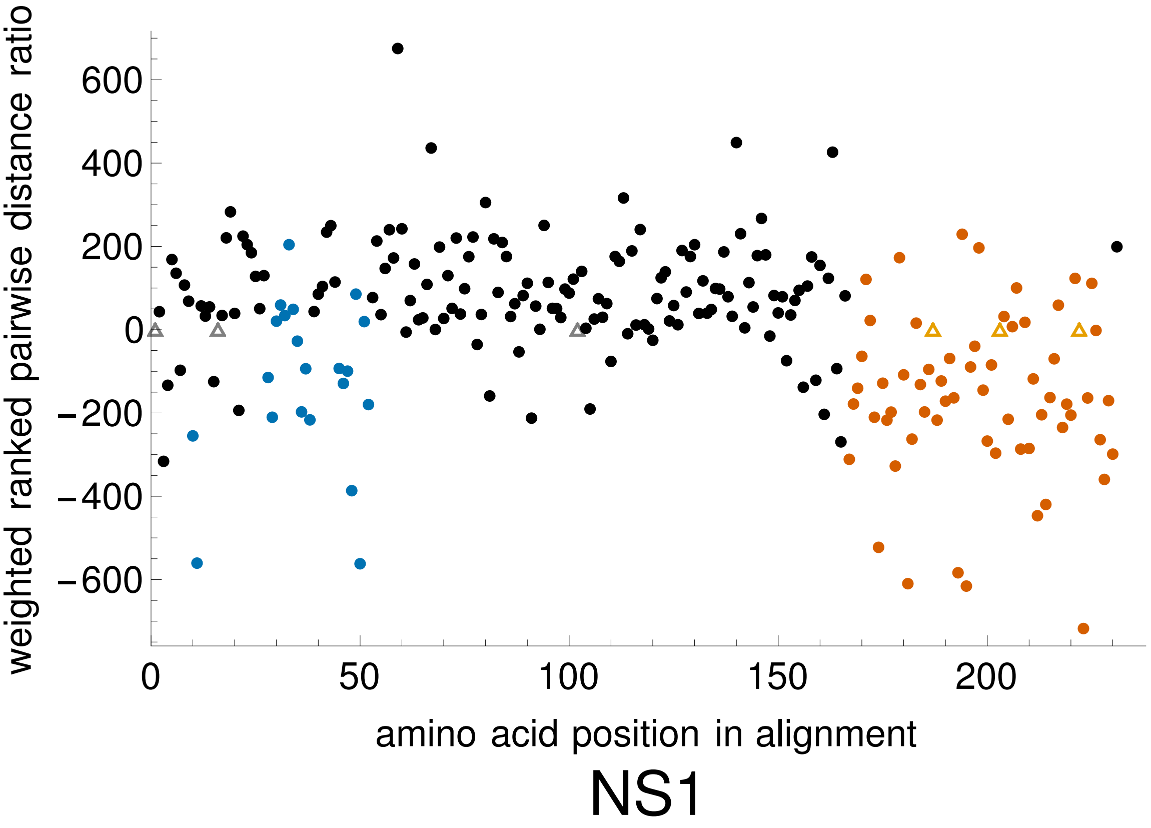

weighted ranked pairwise distance ratio
600
400
200
0
−200
−400
−600
0
50
100
150
200
amino acid position in alignment
NS1


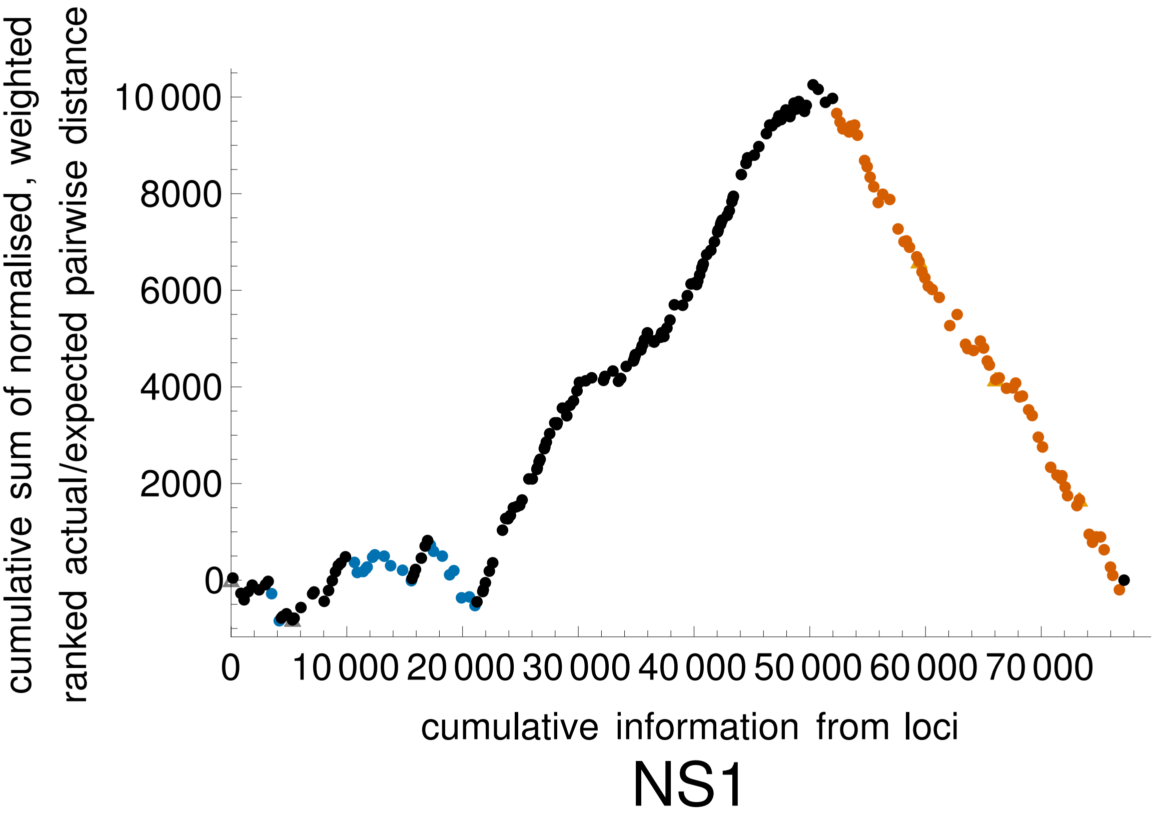

cumulative sum of normalised, weighted ranked actual/expected pairwise distance
10 000
8000
6000
4000
2000
0
10 000
20 000
30 000
40 000
50 000
60 000
70 000
cumulative information from loci
NS1

# NS2

## Gene length histogram

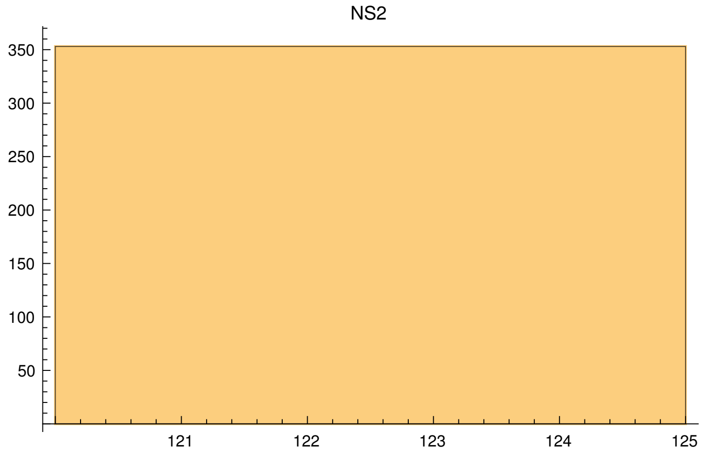

## Information vs. nPD

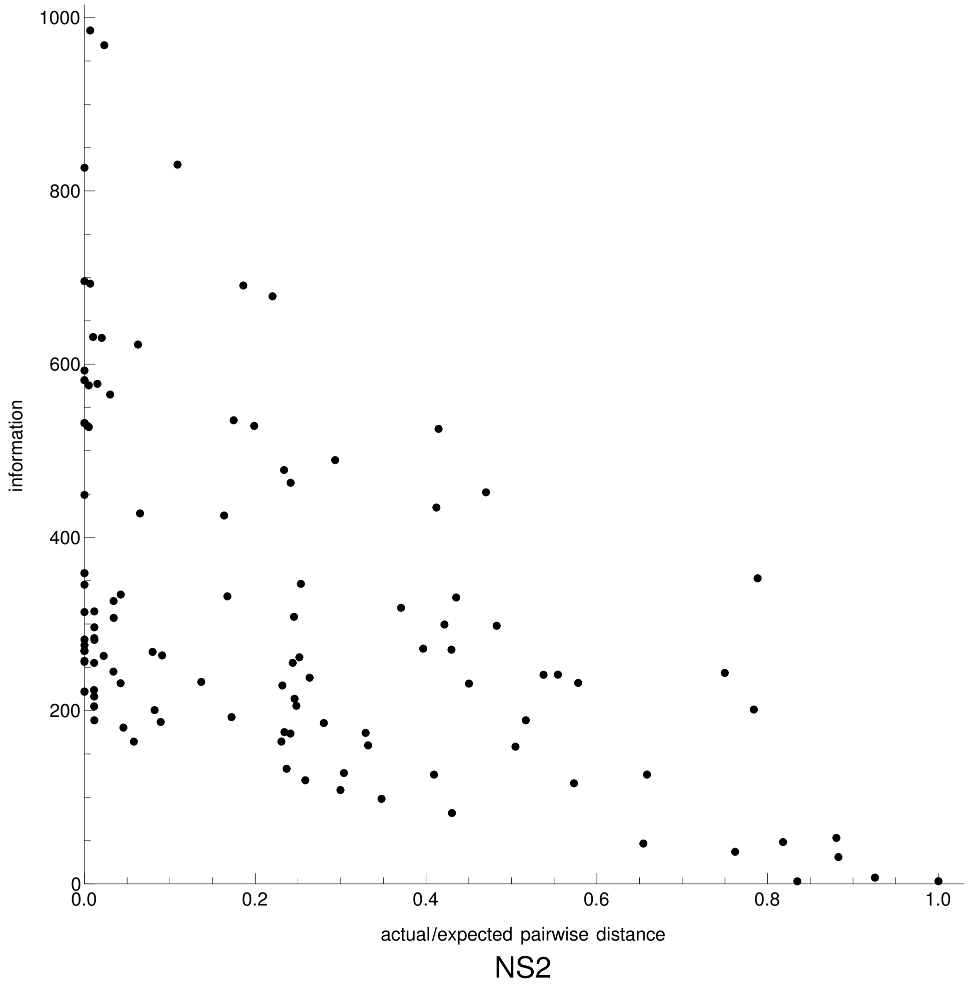

## Example sequences highlighted by regions found in analyses to be conserved

Interesting points (by weighted raw PD) highlighted for gene NS2:

ATGGATTCCAACACTGTGTCAAGC
**TTTCAGGACATATTGATGAGGATGTCAAAAATGCAATTGGGGTCCTCATCGGAGG
ACTTGAATGGAATGATAACACAGTTCGAGTCTCTGAAACTCTACAGAGATTC
GCTTGGAGAAGCAGTAATGAGGATGGGAGACCTCCACTCCCTCCAAAGCAGA
AACGGAAAATGGCGAGAACAATTGAGTCAGAAGTTT**GAAGAAATAAGATGGTTGATT∵
GAAGACGTGCGACACAGACTGAAGATTACAGAGAACAGCTTCGAACAGATAACGTTTATGCAAGCCTTAC∵
AACTATTGCTTGAAGTGGAGCAA
**GAGATAAGAACTTTCTCGTTTCAGCTTATT**TAA

Interesting points (by weighted ranked PD) highlighted for gene NS2:

ATGGATTCCAACACTGTGTCAAGCTTTCAGGACATATTGATG
**AGGATGTCAAAAATGCAATTGGGGTCCTCATCGGAGGACTTGAATGGAATGATA**
ACACAGTTCGAGTCTCTGAAACTC
**TACAGAGATTCGCTT**GGAGAAGCAGTAATGAGGATGGGA
**GACCTCCACTCCCTCCAAAGCAGAAACGGAAAATGGCGAGAACAATTGAGTCAGA
AGTTT**GAAGAAATAAGATGGTTGATTGAAGACGTGCGACACAGACTGAAGATTACAGAGAACAGCTTCG∵
AACAGATAACGTTTATGCAAGCCTTACAACTATTGCTTGAAGTGGAGCAAGAGATAAGAACTTTCTCG
**TTTCAGCTTATT**TAA

# Per codon conservation report

| | | | |
|---|---|---|---|
| NS2 | | | |
| Pos. | 1 | obs: | exp: |
| atg | M | 353 | 353.00 |
| --- | -- | ------ | ------ |
| mPD | | 0 | 0 |
| | | nPD: | 1. |
| | | N. weight: | 0. |
| | | Sc. PD: | 0 |
| | | Sc. rank: | 0 |

| | | | |
|---|---|---|---|
| NS2 | | | |
| Pos. | 2 | obs: | exp: |
| gat | D | 324 | 188.30 |
| gac | D | 29 | 164.70 |
| --- | -- | ------ | ------ |
| mPD | | 0.15 | 0.50 |
| | | nPD: | 0.3 |
| | | N. weight: | 0.42 |
| | | Sc. PD: | 0.062 |
| | | Sc. rank: | 77.8 |

| | | | |
|---|---|---|---|
| NS2 | | | |
| Pos. | 3 | obs: | exp: |
| tct | S | 9 | 55.48 |
| tcc | S | 334 | 48.62 |
| tca | S | 6 | 77.32 |
| tcg | S | 0 | 21.27 |
| cct | P | 0 | 1.05 |
| ccc | P | 4 | 0.72 |
| cca | P | 0 | 1.58 |
| ccg | P | 0 | 0.65 |
| agt | S | 0 | 76.78 |
| agc | S | 0 | 69.53 |
| --- | -- | ------ | ------ |
| mPD | | 0.10 | 1.7 |
| | | nPD: | 0.06 |
| | | N. weight: | 2. |
| | | Sc. PD: | -0.16 |
| | | Sc. rank: | 73.7 |

| | | | |
|---|---|---|---|
| NS2 | | | |
| Pos. | 4 | obs: | exp: |
| aat | N | 3 | 184.50 |
| aac | N | 350 | 168.50 |
| --- | -- | ------ | ------ |
| mPD | | 0.017 | 0.50 |
| | | nPD: | 0.03 |
| | | N. weight: | 0.8 |
| | | Sc. PD: | -0.086 |
| | | Sc. rank: | 4.2 |

| | | | |
|---|---|---|---|
| NS2 | | | |
| Pos. | 5 | obs: | exp: |
| act | T | 295 | 93.26 |
| acc | T | 0 | 75.75 |
| aca | T | 27 | 154.60 |
| acg | T | 31 | 29.35 |
| --- | -- | ------ | ------ |
| mPD | | 0.29 | 0.69 |
| | | nPD: | 0.42 |
| | | N. weight: | 0.98 |
| | | Sc. PD: | 0.25 |
| | | Sc. rank: | 215.7 |

| | | | |
|---|---|---|---|
| NS2 | | | |
| Pos. | 6 | obs: | exp: |
| att | I | 0 | 11.65 |
| atc | I | 0 | 8.12 |
| ata | I | 31 | 11.22 |
| gtt | V | 1 | 66.18 |
| gtc | V | 0 | 67.58 |
| gta | V | 1 | 60.35 |
| gtg | V | 320 | 127.90 |
| --- | -- | ------ | ------ |
| mPD | | 0.33 | 0.89 |
| | | nPD: | 0.37 |
| | | N. weight: | 1. |
| | | Sc. PD: | 0.22 |
| | | Sc. rank: | 204.9 |

| | | | |
|---|---|---|---|
| NS2 | | | |
| Pos. | 7 | obs: | exp: |
| tct | S | 0 | 51.03 |
| tcc | S | 0 | 44.72 |
| tca | S | 321 | 71.11 |
| tcg | S | 0 | 19.57 |
| att | I | 0 | 0.38 |
| atc | I | 1 | 0.26 |
| ata | I | 0 | 0.36 |
| act | T | 0 | 8.19 |
| acc | T | 30 | 6.65 |
| aca | T | 1 | 13.58 |
| acg | T | 0 | 2.58 |
| agt | S | 0 | 70.62 |
| agc | S | 0 | 63.95 |
| --- | -- | ------ | ------ |
| mPD | | 0.33 | 1.7 |
| | | nPD: | 0.2 |
| | | N. weight: | 1.7 |
| | | Sc. PD: | 0.084 |
| | | Sc. rank: | 189.0 |

| | | | |
|---|---|---|---|
| NS2 | | | |
| Pos. | 8 | obs: | exp: |
| tct | S | 0 | 56.12 |
| tcc | S | 0 | 49.17 |
| tca | S | 2 | 78.20 |
| tcg | S | 29 | 21.52 |
| agt | S | 1 | 77.66 |
| agc | S | 321 | 70.33 |
| --- | -- | ------ | ------ |
| mPD | | 0.49 | 1.7 |
| | | nPD: | 0.29 |
| | | N. weight: | 1.6 |
| | | Sc. PD: | 0.22 |
| | | Sc. rank: | 292.0 |

| | | | |
|---|---|---|---|
| NS2 | | | |
| Pos. | 9 | obs: | exp: |
| ttt | F | 350 | 144.30 |
| ttc | F | 1 | 206.70 |
| tat | Y | 2 | 1.04 |
| tac | Y | 0 | 0.96 |
| --- | -- | ------ | ------ |
| mPD | | 0.017 | 0.50 |
| | | nPD: | 0.03 |
| | | N. weight: | 1. |
| | | Sc. PD: | -0.11 |
| | | Sc. rank: | 14.4 |

| | | | |
|---|---|---|---|
| NS2 | | | |
| Pos. | 10 | obs: | exp: |
| caa | Q | 0 | 188.20 |
| cag | Q | 353 | 164.80 |
| --- | -- | ------ | ------ |
| mPD | | 0 | 0.50 |
| | | nPD: | 0. |
| | | N. weight: | 0.88 |
| | | Sc. PD: | -0.12 |
| | | Sc. rank: | -219.3 |

| | | | |
|---|---|---|---|
| NS2 | | | |
| Pos. | 11 | obs: | exp: |
| aat | N | 0 | 0.52 |
| aac | N | 1 | 0.48 |
| gat | D | 1 | 187.80 |
| gac | D | 351 | 164.20 |
| --- | -- | ------ | ------ |
| mPD | | 0.011 | 0.50 |
| | | nPD: | 0.02 |
| | | N. weight: | 0.86 |
| | | Sc. PD: | -0.1 |
| | | Sc. rank: | -17.4 |

| | | | |
|---|---|---|---|
| NS2 | | | |
| Pos. | 12 | obs: | exp: |
| att | I | 31 | 132.70 |
| atc | I | 0 | 92.52 |
| ata | I | 322 | 127.80 |
| --- | -- | ------ | ------ |
| mPD | | 0.16 | 0.66 |
| | | nPD: | 0.24 |
| | | N. weight: | 0.84 |
| | | Sc. PD: | 0.076 |
| | | Sc. rank: | 124.0 |

| | | | |
|---|---|---|---|
| NS2 | | | |
| Pos. | 13 | obs: | exp: |
| tta | L | 0 | 30.84 |
| ttg | L | 3 | 67.98 |
| ctt | L | 0 | 65.86 |
| ctc | L | 0 | 57.10 |
| cta | L | 32 | 53.18 |
| ctg | L | 318 | 78.03 |
| --- | -- | ------ | ------ |
| mPD | | 0.18 | 1.1 |
| | | nPD: | 0.16 |
| | | N. weight: | 1.4 |
| | | Sc. PD: | 0.021 |
| | | Sc. rank: | 106.7 |

| | | | |
|---|---|---|---|
| NS2 | | | |
| Pos. | 14 | obs: | exp: |
| caa | Q | 0 | 16.53 |
| cag | Q | 31 | 14.47 |
| atg | M | 314 | 314.00 |
| act | T | 0 | 1.06 |
| acc | T | 0 | 0.86 |
| aca | T | 0 | 1.75 |
| acg | T | 4 | 0.33 |
| gaa | E | 0 | 2.30 |
| gag | E | 4 | 1.70 |
| --- | -- | ------ | ------ |
| mPD | | 0.38 | 0.50 |
| | | nPD: | 0.76 |
| | | N. weight: | 0.12 |
| | | Sc. PD: | 0.07 |
| | | Sc. rank: | 32.3 |

| | | | |
|---|---|---|---|
| NS2 | | | |
| Pos. | 15 | obs: | exp: |
| cgt | R | 0 | 14.65 |
| cgc | R | 0 | 17.28 |
| cga | R | 0 | 33.93 |
| cgg | R | 0 | 29.46 |
| aga | R | 0 | 158.80 |
| agg | R | 353 | 98.92 |
| --- | -- | ------ | ------ |
| mPD | | 0 | 0.96 |
| | | nPD: | 0. |
| | | N. weight: | 1.5 |
| | | Sc. PD: | -0.21 |
| | | Sc. rank: | -366.3 |

| | | | |
|---|---|---|---|
| NS2 | | | |
| Pos. | 16 | obs: | exp: |
| atg | M | 353 | 353.00 |
| --- | -- | ------ | ------ |
| mPD | | 0 | 0 |
| | | nPD: | 1. |
| | | N. weight: | 0. |
| | | Sc. PD: | 0 |
| | | Sc. rank: | 0 |

| | | | |
|---|---|---|---|
| NS2 | | | |
| Pos. | 17 | obs: | exp: |
| tct | S | 0 | 56.12 |
| tcc | S | 0 | 49.17 |
| tca | S | 353 | 78.20 |
| tcg | S | 0 | 21.52 |
| agt | S | 0 | 77.66 |
| agc | S | 0 | 70.33 |
| --- | -- | ------ | ------ |
| mPD | | 0 | 1.7 |
| | | nPD: | 0. |
| | | N. weight: | 1.7 |
| | | Sc. PD: | -0.24 |
| | | Sc. rank: | -434.0 |

| | | | |
|---|---|---|---|
| NS2 | | | |
| Pos. | 18 | obs: | exp: |
| cgt | R | 0 | 0.04 |
| cgc | R | 0 | 0.05 |
| cga | R | 0 | 0.10 |
| cgg | R | 0 | 0.08 |
| aaa | K | 352 | 197.10 |
| aag | K | 0 | 154.90 |
| aga | R | 1 | 0.45 |
| agg | R | 0 | 0.28 |
| --- | -- | ------ | ------ |
| mPD | | 0.0057 | 0.50 |
| | | nPD: | 0.01 |
| | | N. weight: | 0.67 |
| | | Sc. PD: | -0.087 |
| | | Sc. rank: | -45.2 |

| | | | |
|---|---|---|---|
| NS2 | | | |
| Pos. | 19 | obs: | exp: |
| atg | M | 353 | 353.00 |
| --- | -- | ------ | ------ |
| mPD | | 0 | 0 |
| | | nPD: | 1. |
| | | N. weight: | 0. |
| | | Sc. PD: | 0 |
| | | Sc. rank: | 0 |

| | | | |
|---|---|---|---|
| NS2 | | | |
| Pos. | 20 | obs: | exp: |
| caa | Q | 352 | 187.70 |
| cag | Q | 0 | 164.30 |
| cgt | R | 0 | 0.04 |
| cgc | R | 0 | 0.05 |
| cga | R | 1 | 0.10 |
| cgg | R | 0 | 0.08 |
| aga | R | 0 | 0.45 |
| agg | R | 0 | 0.28 |
| --- | -- | ------ | ------ |
| mPD | | 0.0057 | 0.51 |
| | | nPD: | 0.01 |
| | | N. weight: | 0.73 |
| | | Sc. PD: | -0.095 |
| | | Sc. rank: | -51.5 |

| | | | |
|---|---|---|---|
| NS2 | | | |
| Pos. | 21 | obs: | exp: |
| tta | L | 0 | 30.84 |
| ttg | L | 353 | 67.98 |
| ctt | L | 0 | 65.86 |
| ctc | L | 0 | 57.10 |
| cta | L | 0 | 53.18 |
| ctg | L | 0 | 78.03 |
| --- | -- | ------ | ------ |
| mPD | | 0 | 1.1 |
| | | nPD: | 0. |
| | | N. weight: | 1.9 |
| | | Sc. PD: | -0.27 |
| | | Sc. rank: | -474.3 |

,

| | | | |
|---|---|---|---|
| NS2 | | | |
| Pos. | 22 | obs: | exp: |
| gaa | E | 31 | 18.99 |
| gag | E | 2 | 14.01 |
| ggt | G | 0 | 39.48 |
| ggc | G | 0 | 43.27 |
| gga | G | 2 | 154.80 |
| ggg | G | 318 | 82.45 |
| --- | -- | ------ | ------ |
| mPD | | 0.34 | 0.82 |
| | | nPD: | 0.41 |
| | | N. weight: | 1.4 |
| | | Sc. PD: | 0.36 |
| | | Sc. rank: | 296.2 |

,

| | | | |
|---|---|---|---|
| NS2 | | | |
| Pos. | 23 | obs: | exp: |
| tct | S | 0 | 56.12 |
| tcc | S | 353 | 49.17 |
| tca | S | 0 | 78.20 |
| tcg | S | 0 | 21.52 |
| agt | S | 0 | 77.66 |
| agc | S | 0 | 70.33 |
| --- | -- | ------ | ------ |
| mPD | | 0 | 1.7 |
| | | nPD: | 0. |
| | | N. weight: | 2.3 |
| | | Sc. PD: | -0.32 |
| | | Sc. rank: | -567.6 |

,

| | | | |
|---|---|---|---|
| NS2 | | | |
| Pos. | 24 | obs: | exp: |
| tct | S | 0 | 56.12 |
| tcc | S | 0 | 49.17 |
| tca | S | 353 | 78.20 |
| tcg | S | 0 | 21.52 |
| agt | S | 0 | 77.66 |
| agc | S | 0 | 70.33 |
| --- | -- | ------ | ------ |
| mPD | | 0 | 1.7 |
| | | nPD: | 0. |
| | | N. weight: | 1.7 |
| | | Sc. PD: | -0.24 |
| | | Sc. rank: | -434.0 |

,

| | | | |
|---|---|---|---|
| NS2 | | | |
| Pos. | 25 | obs: | exp: |
| tta | L | 0 | 0.17 |
| ttg | L | 2 | 0.39 |
| tct | S | 0 | 55.80 |
| tcc | S | 0 | 48.90 |
| tca | S | 0 | 77.76 |
| tcg | S | 351 | 21.39 |
| ctt | L | 0 | 0.37 |
| ctc | L | 0 | 0.32 |
| cta | L | 0 | 0.30 |
| ctg | L | 0 | 0.44 |
| agt | S | 0 | 77.22 |
| agc | S | 0 | 69.93 |
| --- | -- | ------ | ------ |
| mPD | | 0.011 | 1.7 |
| | | nPD: | 0.01 |
| | | N. weight: | 3.2 |
| | | Sc. PD: | -0.43 |
| | | Sc. rank: | -298.1 |

,

| | | | |
|---|---|---|---|
| NS2 | | | |
| Pos. | 26 | obs: | exp: |
| gtt | V | 0 | 5.96 |
| gtc | V | 0 | 6.09 |
| gta | V | 0 | 5.44 |
| gtg | V | 29 | 11.52 |
| gct | A | 0 | 0.50 |
| gcc | A | 0 | 0.41 |
| gca | A | 0 | 0.89 |
| gcg | A | 2 | 0.20 |
| gaa | E | 0 | 184.10 |
| gag | E | 320 | 135.90 |
| ggt | G | 0 | 0.25 |
| ggc | G | 0 | 0.27 |
| gga | G | 0 | 0.97 |
| ggg | G | 2 | 0.52 |
| --- | -- | ------ | ------ |
| mPD | | 0.17 | 0.70 |
| | | nPD: | 0.25 |
| | | N. weight: | 1. |
| | | Sc. PD: | 0.093 |
| | | Sc. rank: | 154.3 |

,

| | | | |
|---|---|---|---|
| NS2 | | | |
| Pos. | 27 | obs: | exp: |
| gat | D | 9 | 186.20 |
| gac | D | 340 | 162.80 |
| ggt | G | 0 | 0.49 |
| ggc | G | 4 | 0.54 |
| gga | G | 0 | 1.93 |
| ggg | G | 0 | 1.03 |
| --- | -- | ------ | ------ |
| mPD | | 0.072 | 0.53 |
| | | nPD: | 0.14 |
| | | N. weight: | 0.76 |
| | | Sc. PD: | -0.0079 |
| | | Sc. rank: | 53.9 |

,

| | | | |
|---|---|---|---|
| NS2 | | | |
| Pos. | 28 | obs: | exp: |
| tta | L | 0 | 30.84 |
| ttg | L | 353 | 67.98 |
| ctt | L | 0 | 65.86 |
| ctc | L | 0 | 57.10 |
| cta | L | 0 | 53.18 |
| ctg | L | 0 | 78.03 |
| --- | -- | ------ | ------ |
| mPD | | 0 | 1.1 |
| | | nPD: | 0. |
| | | N. weight: | 1.9 |
| | | Sc. PD: | -0.27 |
| | | Sc. rank: | -474.3 |

,

| | | | |
|---|---|---|---|
| NS2 | | | |
| Pos. | 29 | obs: | exp: |
| tct | S | 0 | 0.48 |
| tcc | S | 0 | 0.42 |
| tca | S | 0 | 0.66 |
| tcg | S | 0 | 0.18 |
| aat | N | 349 | 182.40 |
| aac | N | 0 | 166.60 |
| agt | S | 3 | 0.66 |
| agc | S | 0 | 0.60 |
| gat | D | 1 | 0.53 |
| gac | D | 0 | 0.47 |
| --- | -- | ------ | ------ |
| mPD | | 0.023 | 0.53 |
| | | nPD: | 0.04 |
| | | N. weight: | 0.76 |
| | | Sc. PD: | -0.076 |
| | | Sc. rank: | 13.3 |

,

| | | | |
|---|---|---|---|
| NS2 | | | |
| Pos. | 30 | obs: | exp: |
| ggt | G | 0 | 43.56 |
| ggc | G | 0 | 47.74 |
| gga | G | 353 | 170.80 |
| ggg | G | 0 | 90.95 |
| --- | -- | ------ | ------ |
| mPD | | 0 | 0.67 |
| | | nPD: | 0. |
| | | N. weight: | 0.84 |
| | | Sc. PD: | -0.12 |
| | | Sc. rank: | -209.1 |

,

| | | | |
|---|---|---|---|
| NS2 | | | |
| Pos. | 31 | obs: | exp: |
| att | I | 0 | 1.13 |
| atc | I | 0 | 0.79 |
| ata | I | 3 | 1.09 |
| atg | M | 350 | 350.00 |
| --- | -- | ------ | ------ |
| mPD | | 0.017 | 0.017 |
| | | nPD: | 1. |
| | | N. weight: | 0.01 |
| | | Sc. PD: | 0.008 |
| | | Sc. rank: | 2.8 |

,

| | | | |
|---|---|---|---|
| NS2 | | | |
| Pos. | 32 | obs: | exp: |
| att | I | 0 | 132.70 |
| atc | I | 0 | 92.52 |
| ata | I | 353 | 127.80 |
| --- | -- | ------ | ------ |
| mPD | | 0 | 0.66 |
| | | nPD: | 0. |
| | | N. weight: | 1.2 |
| | | Sc. PD: | -0.16 |
| | | Sc. rank: | -292.6 |

,

| | | | |
|---|---|---|---|
| NS2 | | | |
| Pos. | 33 | obs: | exp: |
| cct | P | 0 | 0.26 |
| ccc | P | 1 | 0.18 |
| cca | P | 0 | 0.39 |
| ccg | P | 0 | 0.16 |
| act | T | 31 | 93.00 |
| acc | T | 0 | 75.53 |
| aca | T | 321 | 154.20 |
| acg | T | 0 | 29.26 |
| --- | -- | ------ | ------ |
| mPD | | 0.17 | 0.69 |
| | | nPD: | 0.25 |
| | | N. weight: | 0.67 |
| | | Sc. PD: | 0.064 |
| | | Sc. rank: | 106.9 |

,

| | | | |
|---|---|---|---|
| NS2 | | | |
| Pos. | 34 | obs: | exp: |
| caa | Q | 32 | 188.20 |
| cag | Q | 321 | 164.80 |
| --- | -- | ------ | ------ |
| mPD | | 0.17 | 0.50 |
| | | nPD: | 0.33 |
| | | N. weight: | 0.52 |
| | | Sc. PD: | 0.091 |
| | | Sc. rank: | 99.7 |

,

| | | | |
|---|---|---|---|
| NS2 | | | |
| Pos. | 35 | obs: | exp: |
| ttt | F | 0 | 144.70 |
| ttc | F | 352 | 207.30 |
| tta | L | 1 | 0.09 |
| ttg | L | 0 | 0.19 |
| ctt | L | 0 | 0.19 |
| ctc | L | 0 | 0.16 |
| cta | L | 0 | 0.15 |
| ctg | L | 0 | 0.22 |
| --- | -- | ------ | ------ |
| mPD | | 0.0057 | 0.49 |
| | | nPD: | 0.01 |
| | | N. weight: | 0.62 |
| | | Sc. PD: | -0.08 |
| | | Sc. rank: | -32.8 |

,

| | | | |
|---|---|---|---|
| NS2 | | | |
| Pos. | 36 | obs: | exp: |
| gaa | E | 1 | 203.10 |
| gag | E | 352 | 149.90 |
| --- | -- | ------ | ------ |
| mPD | | 0.0057 | 0.49 |
| | | nPD: | 0.01 |
| | | N. weight: | 0.97 |
| | | Sc. PD: | -0.12 |
| | | Sc. rank: | -43.8 |

,

| | | | |
|---|---|---|---|
| NS2 | | | |
| Pos. | 37 | obs: | exp: |
| tct | S | 322 | 51.19 |
| tcc | S | 0 | 44.86 |
| tca | S | 0 | 71.34 |
| tcg | S | 0 | 19.63 |
| cgt | R | 31 | 1.29 |
| cgc | R | 0 | 1.52 |
| cga | R | 0 | 2.98 |
| cgg | R | 0 | 2.59 |
| agt | S | 0 | 70.84 |
| agc | S | 0 | 64.15 |
| aga | R | 0 | 13.94 |
| agg | R | 0 | 8.69 |
| --- | -- | ------ | ------ |
| mPD | | 0.32 | 1.7 |
| | | nPD: | 0.19 |
| | | N. weight: | 2.3 |
| | | Sc. PD: | 0.082 |
| | | Sc. rank: | 229.9 |

,

| | | | |
|---|---|---|---|
| NS2 | | | |
| Pos. | 38 | obs: | exp: |
| tta | L | 0 | 30.84 |
| ttg | L | 0 | 67.98 |
| ctt | L | 0 | 65.86 |
| ctc | L | 0 | 57.10 |
| cta | L | 1 | 53.18 |
| ctg | L | 352 | 78.03 |
| --- | -- | ------ | ------ |
| mPD | | 0.0057 | 1.1 |
| | | nPD: | 0.01 |
| | | N. weight: | 1.7 |
| | | Sc. PD: | -0.23 |
| | | Sc. rank: | -197.4 |

,

| | | | |
|---|---|---|---|
| NS2 | | | |
| Pos. | 39 | obs: | exp: |
| cgt | R | 0 | 0.29 |
| cgc | R | 0 | 0.34 |
| cga | R | 0 | 0.67 |
| cgg | R | 0 | 0.58 |
| aaa | K | 345 | 193.70 |
| aag | K | 1 | 152.30 |
| aga | R | 7 | 3.15 |
| agg | R | 0 | 1.96 |
| --- | -- | ------ | ------ |
| mPD | | 0.045 | 0.54 |
| | | nPD: | 0.08 |
| | | N. weight: | 0.66 |
| | | Sc. PD: | -0.041 |
| | | Sc. rank: | 32.2 |

,

| | | | |
|---|---|---|---|
| NS2 | | | |
| Pos. | 40 | obs: | exp: |
| tta | L | 0 | 27.78 |
| ttg | L | 0 | 61.24 |
| ctt | L | 55 | 59.33 |
| ctc | L | 263 | 51.44 |
| cta | L | 0 | 47.91 |
| ctg | L | 0 | 70.29 |
| att | I | 1 | 13.16 |
| atc | I | 3 | 9.17 |
| ata | I | 31 | 12.67 |
| --- | -- | ------ | ------ |
| mPD | | 0.58 | 1.2 |
| | | nPD: | 0.47 |
| | | N. weight: | 1.5 |
| | | Sc. PD: | 0.45 |
| | | Sc. rank: | 354.5 |

,

| | | | |
|---|---|---|---|
| NS2 | | | |
| Pos. | 41 | obs: | exp: |
| tat | Y | 0 | 182.70 |
| tac | Y | 353 | 170.30 |
| --- | -- | ------ | ------ |
| mPD | | 0 | 0.50 |
| | | nPD: | 0. |
| | | N. weight: | 0.84 |
| | | Sc. PD: | -0.12 |
| | | Sc. rank: | -209.9 |

,

| | | | |
|---|---|---|---|
| NS2 | | | |
| Pos. | 42 | obs: | exp: |
| cgt | R | 0 | 14.57 |
| cgc | R | 0 | 17.19 |
| cga | R | 0 | 33.73 |
| cgg | R | 0 | 29.29 |
| aaa | K | 2 | 1.12 |
| aag | K | 0 | 0.88 |
| aga | R | 351 | 157.90 |
| agg | R | 0 | 98.36 |
| --- | -- | ------ | ------ |
| mPD | | 0.011 | 0.97 |
| | | nPD: | 0.01 |
| | | N. weight: | 0.92 |
| | | Sc. PD: | -0.12 |
| | | Sc. rank: | -37.9 |

,

| | | | |
|---|---|---|---|
| NS2 | | | |
| Pos. | 43 | obs: | exp: |
| gat | D | 353 | 188.30 |
| gac | D | 0 | 164.70 |
| --- | -- | ------ | ------ |
| mPD | | 0 | 0.50 |
| | | nPD: | 0. |
| | | N. weight: | 0.73 |
| | | Sc. PD: | -0.1 |
| | | Sc. rank: | -180.9 |

,

| | | | |
|---|---|---|---|
| NS2 | | | |
| Pos. | 44 | obs: | exp: |
| tta | L | 0 | 0.44 |
| ttg | L | 5 | 0.96 |
| tct | S | 0 | 55.32 |
| tcc | S | 0 | 48.48 |
| tca | S | 2 | 77.10 |
| tcg | S | 346 | 21.21 |
| ctt | L | 0 | 0.93 |
| ctc | L | 0 | 0.81 |
| cta | L | 0 | 0.75 |
| ctg | L | 0 | 1.11 |
| agt | S | 0 | 76.56 |
| agc | S | 0 | 69.33 |
| --- | -- | ------ | ------ |
| mPD | | 0.039 | 1.7 |
| | | nPD: | 0.02 |
| | | N. weight: | 3.2 |
| | | Sc. PD: | -0.37 |
| | | Sc. rank: | -20.1 |

,

| | | | |
|---|---|---|---|
| NS2 | | | |
| Pos. | 45 | obs: | exp: |
| tta | L | 0 | 30.84 |
| ttg | L | 0 | 67.98 |
| ctt | L | 353 | 65.86 |
| ctc | L | 0 | 57.10 |
| cta | L | 0 | 53.18 |
| ctg | L | 0 | 78.03 |
| --- | -- | ------ | ------ |
| mPD | | 0 | 1.1 |
| | | nPD: | 0. |
| | | N. weight: | 1.9 |
| | | Sc. PD: | -0.27 |
| | | Sc. rank: | -483.5 |

,

| | | | |
|---|---|---|---|
| NS2 | | | |
| Pos. | 46 | obs: | exp: |
| ggt | G | 0 | 43.56 |
| ggc | G | 0 | 47.74 |
| gga | G | 322 | 170.80 |
| ggg | G | 31 | 90.95 |
| --- | -- | ------ | ------ |
| mPD | | 0.16 | 0.67 |
| | | nPD: | 0.24 |
| | | N. weight: | 0.57 |
| | | Sc. PD: | 0.05 |
| | | Sc. rank: | 78.5 |

,

| | | | |
|---|---|---|---|
| NS2 | | | |
| Pos. | 47 | obs: | exp: |
| aaa | K | 1 | 0.56 |
| aag | K | 0 | 0.44 |
| gaa | E | 329 | 202.60 |
| gag | E | 23 | 149.40 |
| --- | -- | ------ | ------ |
| mPD | | 0.13 | 0.49 |
| | | nPD: | 0.26 |
| | | N. weight: | 0.39 |
| | | Sc. PD: | 0.041 |
| | | Sc. rank: | 66.3 |

,

| | | | |
|---|---|---|---|
| NS2 | | | |
| Pos. | 48 | obs: | exp: |
| tct | S | 1 | 4.93 |
| tcc | S | 30 | 4.32 |
| tca | S | 0 | 6.87 |
| tcg | S | 0 | 1.89 |
| act | T | 0 | 1.06 |
| acc | T | 0 | 0.86 |
| aca | T | 4 | 1.75 |
| acg | T | 0 | 0.33 |
| agt | S | 0 | 6.82 |
| agc | S | 0 | 6.18 |
| gct | A | 9 | 80.10 |
| gcc | A | 0 | 65.14 |
| gca | A | 305 | 141.70 |
| gcg | A | 4 | 31.03 |
| --- | -- | ------ | ------ |
| mPD | | 0.41 | 0.95 |
| | | nPD: | 0.43 |
| | | N. weight: | 0.89 |
| | | Sc. PD: | 0.24 |
| | | Sc. rank: | 198.2 |

,

| | | | |
|---|---|---|---|
| NS2 | | | |
| Pos. | 49 | obs: | exp: |
| atg | M | 1 | 1.00 |
| gtt | V | 0 | 72.14 |
| gtc | V | 0 | 73.67 |
| gta | V | 320 | 65.78 |
| gtg | V | 31 | 139.40 |
| gct | A | 0 | 0.25 |
| gcc | A | 0 | 0.20 |
| gca | A | 1 | 0.45 |
| gcg | A | 0 | 0.10 |
| --- | -- | ------ | ------ |
| mPD | | 0.18 | 0.73 |
| | | nPD: | 0.24 |
| | | N. weight: | 1.5 |
| | | Sc. PD: | 0.13 |
| | | Sc. rank: | 219.5 |

,

| | | | |
|---|---|---|---|
| NS2 | | | |
| Pos. | 50 | obs: | exp: |
| atg | M | 353 | 353.00 |
| --- | -- | ------ | ------ |
| mPD | | 0 | 0 |
| | | nPD: | 1. |
| | | N. weight: | 0. |
| | | Sc. PD: | 0 |
| | | Sc. rank: | 0 |

,

| | | | |
|---|---|---|---|
| NS2 | | | |
| Pos. | 51 | obs: | exp: |
| cgt | R | 0 | 14.65 |
| cgc | R | 0 | 17.28 |
| cga | R | 0 | 33.93 |
| cgg | R | 0 | 29.46 |
| aga | R | 31 | 158.80 |
| agg | R | 322 | 98.92 |
| --- | -- | ------ | ------ |
| mPD | | 0.16 | 0.96 |
| | | nPD: | 0.17 |
| | | N. weight: | 1.1 |
| | | Sc. PD: | 0.02 |
| | | Sc. rank: | 88.5 |

,

| | | | |
|---|---|---|---|
| NS2 | | | |
| Pos. | 52 | obs: | exp: |
| att | I | 0 | 0.75 |
| atc | I | 0 | 0.52 |
| ata | I | 2 | 0.72 |
| atg | M | 350 | 350.00 |
| gtt | V | 0 | 0.21 |
| gtc | V | 0 | 0.21 |
| gta | V | 0 | 0.19 |
| gtg | V | 1 | 0.40 |
| --- | -- | ------ | ------ |
| mPD | | 0.017 | 0.020 |
| | | nPD: | 0.83 |
| | | N. weight: | 0.0097 |
| | | Sc. PD: | 0.0063 |
| | | Sc. rank: | 2.7 |

,

| | | | |
|---|---|---|---|
| NS2 | | | |
| Pos. | 53 | obs: | exp: |
| ggt | G | 0 | 43.56 |
| ggc | G | 0 | 47.74 |
| gga | G | 323 | 170.80 |
| ggg | G | 30 | 90.95 |
| --- | -- | ------ | ------ |
| mPD | | 0.16 | 0.67 |
| | | nPD: | 0.23 |
| | | N. weight: | 0.57 |
| | | Sc. PD: | 0.047 |
| | | Sc. rank: | 76.8 |

,

| | | | |
|---|---|---|---|
| NS2 | | | |
| Pos. | 54 | obs: | exp: |
| gat | D | 0 | 188.30 |
| gac | D | 353 | 164.70 |
| --- | -- | ------ | ------ |
| mPD | | 0 | 0.50 |
| | | nPD: | 0. |
| | | N. weight: | 0.88 |
| | | Sc. PD: | -0.12 |
| | | Sc. rank: | -219.6 |

,

| | | | |
|---|---|---|---|
| NS2 | | | |
| Pos. | 55 | obs: | exp: |
| tta | L | 0 | 30.84 |
| ttg | L | 0 | 67.98 |
| ctt | L | 0 | 65.86 |
| ctc | L | 351 | 57.10 |
| cta | L | 0 | 53.18 |
| ctg | L | 2 | 78.03 |
| --- | -- | ------ | ------ |
| mPD | | 0.011 | 1.1 |
| | | nPD: | 0.01 |
| | | N. weight: | 2.1 |
| | | Sc. PD: | -0.27 |
| | | Sc. rank: | -151.9 |

,

| | | | |
|---|---|---|---|
| NS2 | | | |
| Pos. | 56 | obs: | exp: |
| cat | H | 0 | 191.30 |
| cac | H | 353 | 161.70 |
| --- | -- | ------ | ------ |
| mPD | | 0 | 0.50 |
| | | nPD: | 0. |
| | | N. weight: | 0.9 |
| | | Sc. PD: | -0.13 |
| | | Sc. rank: | -224.8 |

,

| | | | |
|---|---|---|---|
| NS2 | | | |
| Pos. | 57 | obs: | exp: |
| ttt | F | 0 | 0.82 |
| ttc | F | 2 | 1.18 |
| tct | S | 0 | 55.80 |
| tcc | S | 351 | 48.90 |
| tca | S | 0 | 77.76 |
| tcg | S | 0 | 21.39 |
| agt | S | 0 | 77.22 |
| agc | S | 0 | 69.93 |
| --- | -- | ------ | ------ |
| mPD | | 0.011 | 1.7 |
| | | nPD: | 0.01 |
| | | N. weight: | 2.3 |
| | | Sc. PD: | -0.3 |
| | | Sc. rank: | -187.2 |

,

| | | | |
|---|---|---|---|
| NS2 | | | |
| Pos. | 58 | obs: | exp: |
| ttt | F | 1 | 0.41 |
| ttc | F | 0 | 0.59 |
| tta | L | 0 | 30.75 |
| ttg | L | 0 | 67.79 |
| ctt | L | 1 | 65.68 |
| ctc | L | 350 | 56.94 |
| cta | L | 1 | 53.03 |
| ctg | L | 0 | 77.81 |
| --- | -- | ------ | ------ |
| mPD | | 0.023 | 1.1 |
| | | nPD: | 0.02 |
| | | N. weight: | 2.1 |
| | | Sc. PD: | -0.25 |
| | | Sc. rank: | -49.3 |

,

| | | | |
|---|---|---|---|
| NS2 | | | |
| Pos. | 59 | obs: | exp: |
| caa | Q | 353 | 188.20 |
| cag | Q | 0 | 164.80 |
| --- | -- | ------ | ------ |
| mPD | | 0 | 0.50 |
| | | nPD: | 0. |
| | | N. weight: | 0.73 |
| | | Sc. PD: | -0.1 |
| | | Sc. rank: | -181.1 |

,

| | | | |
|---|---|---|---|
| NS2 | | | |
| Pos. | 60 | obs: | exp: |
| tct | S | 0 | 39.43 |
| tcc | S | 0 | 34.55 |
| tca | S | 0 | 54.94 |
| tcg | S | 0 | 15.12 |
| aat | N | 0 | 54.88 |
| aac | N | 105 | 50.12 |
| agt | S | 0 | 54.56 |
| agc | S | 248 | 49.41 |
| --- | -- | ------ | ------ |
| mPD | | 0.42 | 1.8 |
| | | nPD: | 0.23 |
| | | N. weight: | 1.6 |
| | | Sc. PD: | 0.13 |
| | | Sc. rank: | 205.4 |

,

NS2

| Pos. | 61 | obs: | exp: |
|---|---|---|---|
| cgt | R | 0 | 14.65 |
| cgc | R | 0 | 17.28 |
| cga | R | 0 | 33.93 |
| cgg | R | 0 | 29.46 |
| aga | R | 353 | 158.80 |
| agg | R | 0 | 98.92 |
| mPD | | 0 | 0.96 |
| | | nPD: | 0. |
| | | N. weight: | 0.92 |
| | | Sc. PD: | -0.13 |
| | | Sc. rank: | -230.1 |

NS2

| Pos. | 62 | obs: | exp: |
|---|---|---|---|
| aat | N | 1 | 184.50 |
| aac | N | 352 | 168.50 |
| mPD | | 0.0057 | 0.50 |
| | | nPD: | 0.01 |
| | | N. weight: | 0.84 |
| | | Sc. PD: | -0.11 |
| | | Sc. rank: | -53.2 |

NS2

| Pos. | 63 | obs: | exp: |
|---|---|---|---|
| cgt | R | 0 | 0.04 |
| cgc | R | 0 | 0.05 |
| cga | R | 0 | 0.10 |
| cgg | R | 0 | 0.08 |
| aga | R | 1 | 0.45 |
| agg | R | 0 | 0.28 |
| gct | A | 31 | 7.81 |
| gcc | A | 0 | 6.35 |
| gca | A | 0 | 13.82 |
| gcg | A | 0 | 3.03 |
| ggt | G | 0 | 39.61 |
| ggc | G | 0 | 43.41 |
| gga | G | 320 | 155.30 |
| ggg | G | 1 | 82.70 |
| mPD | | 0.33 | 0.84 |
| | | nPD: | 0.4 |
| | | N. weight: | 0.89 |
| | | Sc. PD: | 0.21 |
| | | Sc. rank: | 178.0 |

NS2

| Pos. | 64 | obs: | exp: |
|---|---|---|---|
| tct | S | 0 | 0.32 |
| tcc | S | 0 | 0.28 |
| tca | S | 2 | 0.44 |
| tcg | S | 0 | 0.12 |
| act | T | 0 | 7.66 |
| acc | T | 0 | 6.22 |
| aca | T | 29 | 12.70 |
| acg | T | 0 | 2.41 |
| aaa | K | 322 | 180.30 |
| aag | K | 0 | 141.70 |
| agt | S | 0 | 0.44 |
| agc | S | 0 | 0.40 |
| mPD | | 0.17 | 0.70 |
| | | nPD: | 0.25 |
| | | N. weight: | 0.7 |
| | | Sc. PD: | 0.065 |
| | | Sc. rank: | 109.1 |

NS2

| Pos. | 65 | obs: | exp: |
|---|---|---|---|
| tgg | W | 353 | 353.00 |
| mPD | | 0 | 0 |
| | | nPD: | 1. |
| | | N. weight: | 0. |
| | | Sc. PD: | 0 |
| | | Sc. rank: | 0 |

NS2

| Pos. | 66 | obs: | exp: |
|---|---|---|---|
| cgt | R | 0 | 14.65 |
| cgc | R | 0 | 17.28 |
| cga | R | 353 | 33.93 |
| cgg | R | 0 | 29.46 |
| aga | R | 0 | 158.80 |
| agg | R | 0 | 98.92 |
| mPD | | 0 | 0.96 |
| | | nPD: | 0. |
| | | N. weight: | 2.7 |
| | | Sc. PD: | -0.38 |
| | | Sc. rank: | -674.5 |

NS2

| Pos. | 67 | obs: | exp: |
|---|---|---|---|
| aat | N | 0 | 0.52 |
| aac | N | 1 | 0.48 |
| aaa | K | 1 | 0.56 |
| aag | K | 0 | 0.44 |
| gat | D | 0 | 12.81 |
| gac | D | 24 | 11.19 |
| gaa | E | 324 | 188.20 |
| gag | E | 3 | 138.80 |
| mPD | | 0.16 | 0.57 |
| | | nPD: | 0.28 |
| | | N. weight: | 0.61 |
| | | Sc. PD: | 0.076 |
| | | Sc. rank: | 106.6 |

NS2

| Pos. | 68 | obs: | exp: |
|---|---|---|---|
| caa | Q | 315 | 171.70 |
| cag | Q | 7 | 150.30 |
| gaa | E | 3 | 17.84 |
| gag | E | 28 | 13.16 |
| mPD | | 0.34 | 0.66 |
| | | nPD: | 0.52 |
| | | N. weight: | 0.62 |
| | | Sc. PD: | 0.22 |
| | | Sc. rank: | 153.8 |

NS2

| Pos. | 69 | obs: | exp: |
|---|---|---|---|
| tta | L | 0 | 30.84 |
| ttg | L | 352 | 67.98 |
| ctt | L | 0 | 65.86 |
| ctc | L | 0 | 57.10 |
| cta | L | 0 | 53.18 |
| ctg | L | 1 | 78.03 |
| mPD | | 0.0057 | 1.1 |
| | | nPD: | 0.01 |
| | | N. weight: | 1.9 |
| | | Sc. PD: | -0.25 |
| | | Sc. rank: | -215.4 |

NS2

| Pos. | 70 | obs: | exp: |
|---|---|---|---|
| tct | S | 0 | 56.12 |
| tcc | S | 0 | 49.17 |
| tca | S | 0 | 78.20 |
| tcg | S | 0 | 21.52 |
| agt | S | 352 | 77.66 |
| agc | S | 1 | 70.33 |
| mPD | | 0.0057 | 1.7 |
| | | nPD: | 0. |
| | | N. weight: | 1.7 |
| | | Sc. PD: | -0.24 |
| | | Sc. rank: | -211.6 |

NS2

| Pos. | 71 | obs: | exp: |
|---|---|---|---|
| caa | Q | 0 | 188.20 |
| cag | Q | 353 | 164.80 |
| mPD | | 0 | 0.50 |
| | | nPD: | 0. |
| | | N. weight: | 0.88 |
| | | Sc. PD: | -0.12 |
| | | Sc. rank: | -219.3 |

NS2

| Pos. | 72 | obs: | exp: |
|---|---|---|---|
| aaa | K | 1 | 197.70 |
| aag | K | 352 | 155.30 |
| mPD | | 0.0057 | 0.49 |
| | | nPD: | 0.01 |
| | | N. weight: | 0.93 |
| | | Sc. PD: | -0.12 |
| | | Sc. rank: | -52.6 |

NS2

| Pos. | 73 | obs: | exp: |
|---|---|---|---|
| ttt | F | 352 | 144.70 |
| ttc | F | 0 | 207.30 |
| tta | L | 0 | 0.09 |
| ttg | L | 0 | 0.19 |
| ctt | L | 1 | 0.19 |
| ctc | L | 0 | 0.16 |
| cta | L | 0 | 0.15 |
| ctg | L | 0 | 0.22 |
| mPD | | 0.0057 | 0.49 |
| | | nPD: | 0.01 |
| | | N. weight: | 1. |
| | | Sc. PD: | -0.13 |
| | | Sc. rank: | -54.6 |

NS2

| Pos. | 74 | obs: | exp: |
|---|---|---|---|
| gaa | E | 320 | 203.10 |
| gag | E | 33 | 149.90 |
| mPD | | 0.17 | 0.49 |
| | | nPD: | 0.35 |
| | | N. weight: | 0.32 |
| | | Sc. PD: | 0.061 |
| | | Sc. rank: | 61.6 |

NS2

| Pos. | 75 | obs: | exp: |
|---|---|---|---|
| gaa | E | 311 | 203.10 |
| gag | E | 42 | 149.90 |
| mPD | | 0.21 | 0.49 |
| | | nPD: | 0.43 |
| | | N. weight: | 0.27 |
| | | Sc. PD: | 0.072 |
| | | Sc. rank: | 60.3 |

NS2

| Pos. | 76 | obs: | exp: |
|---|---|---|---|
| att | I | 1 | 132.30 |
| atc | I | 30 | 92.25 |
| ata | I | 321 | 127.40 |
| atg | M | 1 | 1.00 |
| mPD | | 0.17 | 0.66 |
| | | nPD: | 0.25 |
| | | N. weight: | 0.86 |
| | | Sc. PD: | 0.084 |
| | | Sc. rank: | 139.3 |

NS2

| Pos. | 77 | obs: | exp: |
|---|---|---|---|
| cgt | R | 0 | 14.49 |
| cgc | R | 0 | 17.09 |
| cga | R | 0 | 33.54 |
| cgg | R | 1 | 29.12 |
| aaa | K | 4 | 2.24 |
| aag | K | 0 | 1.76 |
| aga | R | 262 | 157.00 |
| agg | R | 86 | 97.80 |
| mPD | | 0.40 | 0.98 |
| | | nPD: | 0.41 |
| | | N. weight: | 0.41 |
| | | Sc. PD: | 0.1 |
| | | Sc. rank: | 83.4 |

NS2

| Pos. | 78 | obs: | exp: |
|---|---|---|---|
| tgg | W | 353 | 353.00 |
| mPD | | 0 | 0 |
| | | nPD: | 1. |
| | | N. weight: | 0. |
| | | Sc. PD: | 0 |
| | | Sc. rank: | 0 |

NS2

| Pos. | 79 | obs: | exp: |
|---|---|---|---|
| tta | L | 3 | 30.84 |
| ttg | L | 40 | 67.98 |
| ctt | L | 7 | 65.86 |
| ctc | L | 25 | 57.10 |
| cta | L | 22 | 53.18 |
| ctg | L | 256 | 78.03 |
| mPD | | 0.50 | 1.1 |
| | | nPD: | 0.45 |
| | | N. weight: | 0.76 |
| | | Sc. PD: | 0.22 |
| | | Sc. rank: | 176.4 |

NS2

| Pos. | 80 | obs: | exp: |
|---|---|---|---|
| att | I | 349 | 132.70 |
| atc | I | 4 | 92.52 |
| ata | I | 0 | 127.80 |
| mPD | | 0.022 | 0.66 |
| | | nPD: | 0.03 |
| | | N. weight: | 1.1 |
| | | Sc. PD: | -0.11 |
| | | Sc. rank: | 10.6 |

NS2

| Pos. | 81 | obs: | exp: |
|---|---|---|---|
| gct | A | 20 | 7.81 |
| gcc | A | 6 | 6.35 |
| gca | A | 5 | 13.82 |
| gcg | A | 0 | 3.03 |
| gaa | E | 313 | 185.30 |
| gag | E | 9 | 136.70 |
| mPD | | 0.35 | 0.68 |
| | | nPD: | 0.5 |
| | | N. weight: | 0.52 |
| | | Sc. PD: | 0.18 |
| | | Sc. rank: | 127.6 |

NS2

| Pos. | 82 | obs: | exp: |
|---|---|---|---|
| gat | D | 0 | 2.13 |
| gac | D | 4 | 1.87 |
| gaa | E | 331 | 200.80 |
| gag | E | 18 | 148.20 |
| mPD | | 0.12 | 0.50 |
| | | nPD: | 0.24 |
| | | N. weight: | 0.44 |
| | | Sc. PD: | 0.037 |
| | | Sc. rank: | 59.0 |

NS2

| Pos. | 83 | obs: | exp: |
|---|---|---|---|
| tgt | C | 11 | 12.78 |
| tgc | C | 20 | 18.22 |
| atg | M | 1 | 1.00 |
| gtt | V | 0 | 65.98 |
| gtc | V | 0 | 67.37 |
| gta | V | 25 | 60.16 |
| gtg | V | 296 | 127.50 |
| mPD | | 0.61 | 1.1 |
| | | nPD: | 0.58 |
| | | N. weight: | 0.76 |
| | | Sc. PD: | 0.31 |
| | | Sc. rank: | 198.0 |

NS2

| Pos. | 84 | obs: | exp: |
|---|---|---|---|
| cgt | R | 0 | 14.65 |
| cgc | R | 0 | 17.28 |
| cga | R | 278 | 33.93 |
| cgg | R | 31 | 29.46 |
| aga | R | 40 | 158.80 |
| agg | R | 4 | 98.92 |
| mPD | | 0.40 | 0.96 |
| | | nPD: | 0.41 |
| | | N. weight: | 1.7 |
| | | Sc. PD: | 0.43 |
| | | Sc. rank: | 371.1 |

NS2

| Pos. | 85 | obs: | exp: |
|---|---|---|---|
| cat | H | 293 | 172.90 |
| cac | H | 26 | 146.10 |
| caa | Q | 3 | 1.60 |
| cag | Q | 0 | 1.40 |
| aat | N | 16 | 16.20 |
| aac | N | 15 | 14.80 |
| mPD | | 0.38 | 0.67 |
| | | nPD: | 0.57 |
| | | N. weight: | 0.38 |
| | | Sc. PD: | 0.15 |
| | | Sc. rank: | 97.8 |

| NS2 | | | |
|---|---|---|---|
| Pos. | 86 | obs: | exp: |
| cgt | R | 0 | 12.83 |
| cgc | R | 0 | 15.13 |
| cga | R | 4 | 29.70 |
| cgg | R | 0 | 25.79 |
| att | I | 0 | 10.90 |
| atc | I | 0 | 7.60 |
| ata | I | 29 | 10.50 |
| aaa | K | 13 | 7.28 |
| aag | K | 0 | 5.72 |
| aga | R | 296 | 139.00 |
| agg | R | 9 | 86.59 |
| gtt | V | 0 | 0.41 |
| gtc | V | 0 | 0.42 |
| gta | V | 1 | 0.37 |
| gtg | V | 1 | 0.79 |
| --- | -- | ------ | ------ |
| mPD | | 0.31 | 1.2 |
| | | nPD: | 0.26 |
| | | N. weight: | 0.78 |
| | | Sc. PD: | 0.085 |
| | | Sc. rank: | 134.5 |

,

| NS2 | | | |
|---|---|---|---|
| Pos. | 87 | obs: | exp: |
| tta | L | 18 | 30.84 |
| ttg | L | 214 | 67.98 |
| ctt | L | 0 | 65.86 |
| ctc | L | 0 | 57.10 |
| cta | L | 73 | 53.18 |
| ctg | L | 48 | 78.03 |
| --- | -- | ------ | ------ |
| mPD | | 0.84 | 1.1 |
| | | nPD: | 0.75 |
| | | N. weight: | 0.8 |
| | | Sc. PD: | 0.45 |
| | | Sc. rank: | 212.6 |

,

| NS2 | | | |
|---|---|---|---|
| Pos. | 88 | obs: | exp: |
| cgt | R | 0 | 0.17 |
| cgc | R | 0 | 0.20 |
| cga | R | 0 | 0.38 |
| cgg | R | 0 | 0.33 |
| act | T | 0 | 8.19 |
| acc | T | 6 | 6.65 |
| aca | T | 25 | 13.58 |
| acg | T | 0 | 2.58 |
| aaa | K | 9 | 178.10 |
| aag | K | 309 | 139.90 |
| aga | R | 0 | 1.80 |
| agg | R | 4 | 1.12 |
| --- | -- | ------ | ------ |
| mPD | | 0.39 | 0.72 |
| | | nPD: | 0.54 |
| | | N. weight: | 0.79 |
| | | Sc. PD: | 0.29 |
| | | Sc. rank: | 199.3 |

,

| NS2 | | | |
|---|---|---|---|
| Pos. | 89 | obs: | exp: |
| ttt | F | 3 | 1.23 |
| ttc | F | 0 | 1.77 |
| cgt | R | 0 | 0.04 |
| cgc | R | 0 | 0.05 |
| cga | R | 0 | 0.10 |
| cgg | R | 0 | 0.08 |
| att | I | 209 | 108.60 |
| atc | I | 77 | 75.74 |
| ata | I | 3 | 104.60 |
| aaa | K | 2 | 16.80 |
| aag | K | 28 | 13.20 |
| aga | R | 0 | 0.45 |
| agg | R | 1 | 0.28 |
| gtt | V | 30 | 6.17 |
| gtc | V | 0 | 6.30 |
| gta | V | 0 | 5.62 |
| gtg | V | 0 | 11.91 |
| --- | -- | ------ | ------ |
| mPD | | 0.81 | 1.0 |
| | | nPD: | 0.78 |
| | | N. weight: | 0.66 |
| | | Sc. PD: | 0.4 |
| | | Sc. rank: | 177.9 |

,

| NS2 | | | |
|---|---|---|---|
| Pos. | 90 | obs: | exp: |
| act | T | 30 | 93.26 |
| acc | T | 0 | 75.75 |
| aca | T | 309 | 154.60 |
| acg | T | 14 | 29.35 |
| --- | -- | ------ | ------ |
| mPD | | 0.23 | 0.69 |
| | | nPD: | 0.33 |
| | | N. weight: | 0.57 |
| | | Sc. PD: | 0.098 |
| | | Sc. rank: | 107.4 |

,

| NS2 | | | |
|---|---|---|---|
| Pos. | 91 | obs: | exp: |
| gaa | E | 110 | 203.10 |
| gag | E | 243 | 149.90 |
| --- | -- | ------ | ------ |
| mPD | | 0.43 | 0.49 |
| | | nPD: | 0.88 |
| | | N. weight: | 0.17 |
| | | Sc. PD: | 0.12 |
| | | Sc. rank: | 48.0 |

,

| NS2 | | | |
|---|---|---|---|
| Pos. | 92 | obs: | exp: |
| aat | N | 115 | 184.50 |
| aac | N | 238 | 168.50 |
| --- | -- | ------ | ------ |
| mPD | | 0.44 | 0.50 |
| | | nPD: | 0.88 |
| | | N. weight: | 0.1 |
| | | Sc. PD: | 0.07 |
| | | Sc. rank: | 28.0 |

,

| NS2 | | | |
|---|---|---|---|
| Pos. | 93 | obs: | exp: |
| tct | S | 0 | 56.12 |
| tcc | S | 0 | 49.17 |
| tca | S | 0 | 78.20 |
| tcg | S | 0 | 21.52 |
| agt | S | 106 | 77.66 |
| agc | S | 247 | 70.33 |
| --- | -- | ------ | ------ |
| mPD | | 0.42 | 1.7 |
| | | nPD: | 0.25 |
| | | N. weight: | 1.1 |
| | | Sc. PD: | 0.11 |
| | | Sc. rank: | 190.0 |

,

| NS2 | | | |
|---|---|---|---|
| Pos. | 94 | obs: | exp: |
| ttt | F | 119 | 145.10 |
| ttc | F | 234 | 207.90 |
| --- | -- | ------ | ------ |
| mPD | | 0.45 | 0.48 |
| | | nPD: | 0.93 |
| | | N. weight: | 0.024 |
| | | Sc. PD: | 0.017 |
| | | Sc. rank: | 6.5 |

,

| NS2 | | | |
|---|---|---|---|
| Pos. | 95 | obs: | exp: |
| gaa | E | 325 | 203.10 |
| gag | E | 28 | 149.90 |
| --- | -- | ------ | ------ |
| mPD | | 0.15 | 0.49 |
| | | nPD: | 0.3 |
| | | N. weight: | 0.35 |
| | | Sc. PD: | 0.051 |
| | | Sc. rank: | 65.2 |

,

| NS2 | | | |
|---|---|---|---|
| Pos. | 96 | obs: | exp: |
| caa | Q | 100 | 188.20 |
| cag | Q | 253 | 164.80 |
| --- | -- | ------ | ------ |
| mPD | | 0.41 | 0.50 |
| | | nPD: | 0.82 |
| | | N. weight: | 0.16 |
| | | Sc. PD: | 0.1 |
| | | Sc. rank: | 43.6 |

,

| NS2 | | | |
|---|---|---|---|
| Pos. | 97 | obs: | exp: |
| att | I | 5 | 132.70 |
| atc | I | 0 | 92.52 |
| ata | I | 348 | 127.80 |
| --- | -- | ------ | ------ |
| mPD | | 0.028 | 0.66 |
| | | nPD: | 0.04 |
| | | N. weight: | 1.1 |
| | | Sc. PD: | -0.11 |
| | | Sc. rank: | 24.5 |

,

| NS2 | | | |
|---|---|---|---|
| Pos. | 98 | obs: | exp: |
| act | T | 11 | 93.26 |
| acc | T | 36 | 75.75 |
| aca | T | 85 | 154.60 |
| acg | T | 221 | 29.35 |
| --- | -- | ------ | ------ |
| mPD | | 0.54 | 0.69 |
| | | nPD: | 0.79 |
| | | N. weight: | 1.2 |
| | | Sc. PD: | 0.7 |
| | | Sc. rank: | 317.6 |

,

| NS2 | | | |
|---|---|---|---|
| Pos. | 99 | obs: | exp: |
| ttt | F | 332 | 145.10 |
| ttc | F | 21 | 207.90 |
| --- | -- | ------ | ------ |
| mPD | | 0.11 | 0.48 |
| | | nPD: | 0.23 |
| | | N. weight: | 0.75 |
| | | Sc. PD: | 0.06 |
| | | Sc. rank: | 93.4 |

,

| NS2 | | | |
|---|---|---|---|
| Pos. | 100 | obs: | exp: |
| tta | L | 2 | 2.71 |
| ttg | L | 29 | 5.97 |
| ctt | L | 0 | 5.78 |
| ctc | L | 0 | 5.01 |
| cta | L | 0 | 4.67 |
| ctg | L | 0 | 6.85 |
| atg | M | 322 | 322.00 |
| --- | -- | ------ | ------ |
| mPD | | 0.17 | 0.26 |
| | | nPD: | 0.65 |
| | | N. weight: | 0.15 |
| | | Sc. PD: | 0.073 |
| | | Sc. rank: | 39.8 |

,

| NS2 | | | |
|---|---|---|---|
| Pos. | 101 | obs: | exp: |
| caa | Q | 345 | 188.20 |
| cag | Q | 8 | 164.80 |
| --- | -- | ------ | ------ |
| mPD | | 0.044 | 0.50 |
| | | nPD: | 0.09 |
| | | N. weight: | 0.61 |
| | | Sc. PD: | -0.034 |
| | | Sc. rank: | 31.6 |

,

| NS2 | | | |
|---|---|---|---|
| Pos. | 102 | obs: | exp: |
| gct | A | 10 | 88.91 |
| gcc | A | 291 | 72.31 |
| gca | A | 52 | 157.30 |
| gcg | A | 0 | 34.45 |
| --- | -- | ------ | ------ |
| mPD | | 0.30 | 0.69 |
| | | nPD: | 0.44 |
| | | N. weight: | 1.1 |
| | | Sc. PD: | 0.29 |
| | | Sc. rank: | 248.7 |

,

| NS2 | | | |
|---|---|---|---|
| Pos. | 103 | obs: | exp: |
| tta | L | 304 | 30.84 |
| ttg | L | 48 | 67.98 |
| ctt | L | 0 | 65.86 |
| ctc | L | 0 | 57.10 |
| cta | L | 0 | 53.18 |
| ctg | L | 1 | 78.03 |
| --- | -- | ------ | ------ |
| mPD | | 0.25 | 1.1 |
| | | nPD: | 0.22 |
| | | N. weight: | 2.2 |
| | | Sc. PD: | 0.15 |
| | | Sc. rank: | 264.1 |

,

| NS2 | | | |
|---|---|---|---|
| Pos. | 104 | obs: | exp: |
| caa | Q | 352 | 188.20 |
| cag | Q | 1 | 164.80 |
| --- | -- | ------ | ------ |
| mPD | | 0.0057 | 0.50 |
| | | nPD: | 0.01 |
| | | N. weight: | 0.71 |
| | | Sc. PD: | -0.091 |
| | | Sc. rank: | -42.9 |

,

| NS2 | | | |
|---|---|---|---|
| Pos. | 105 | obs: | exp: |
| tta | L | 6 | 30.84 |
| ttg | L | 0 | 67.98 |
| ctt | L | 1 | 65.86 |
| ctc | L | 30 | 57.10 |
| cta | L | 316 | 53.18 |
| ctg | L | 0 | 78.03 |
| --- | -- | ------ | ------ |
| mPD | | 0.19 | 1.1 |
| | | nPD: | 0.17 |
| | | N. weight: | 1.8 |
| | | Sc. PD: | 0.045 |
| | | Sc. rank: | 160.9 |

,

| NS2 | | | |
|---|---|---|---|
| Pos. | 106 | obs: | exp: |
| tta | L | 30 | 30.84 |
| ttg | L | 245 | 67.98 |
| ctt | L | 0 | 65.86 |
| ctc | L | 0 | 57.10 |
| cta | L | 8 | 53.18 |
| ctg | L | 70 | 78.03 |
| --- | -- | ------ | ------ |
| mPD | | 0.54 | 1.1 |
| | | nPD: | 0.48 |
| | | N. weight: | 0.98 |
| | | Sc. PD: | 0.31 |
| | | Sc. rank: | 237.8 |

,

| NS2 | | | |
|---|---|---|---|
| Pos. | 107 | obs: | exp: |
| tta | L | 0 | 30.84 |
| ttg | L | 0 | 67.98 |
| ctt | L | 347 | 65.86 |
| ctc | L | 6 | 57.10 |
| cta | L | 0 | 53.18 |
| ctg | L | 0 | 78.03 |
| --- | -- | ------ | ------ |
| mPD | | 0.034 | 1.1 |
| | | nPD: | 0.03 |
| | | N. weight: | 1.9 |
| | | Sc. PD: | -0.21 |
| | | Sc. rank: | 3.2 |

,

| NS2 | | | |
|---|---|---|---|
| Pos. | 108 | obs: | exp: |
| gaa | E | 349 | 202.60 |
| gag | E | 3 | 149.40 |
| ggt | G | 0 | 0.12 |
| ggc | G | 0 | 0.14 |
| gga | G | 1 | 0.48 |
| ggg | G | 0 | 0.26 |
| --- | -- | ------ | ------ |
| mPD | | 0.023 | 0.49 |
| | | nPD: | 0.05 |
| | | N. weight: | 0.59 |
| | | Sc. PD: | -0.057 |
| | | Sc. rank: | 14.7 |

,

| NS2 | | | |
|---|---|---|---|
| Pos. | 109 | obs: | exp: |
| gtt | V | 31 | 72.56 |
| gtc | V | 0 | 74.09 |
| gta | V | 81 | 66.16 |
| gtg | V | 241 | 140.20 |
| --- | -- | ------ | ------ |
| mPD | | 0.47 | 0.72 |
| | | nPD: | 0.66 |
| | | N. weight: | 0.41 |
| | | Sc. PD: | 0.2 |
| | | Sc. rank: | 108.7 |

,

| | | | |
|---|---|---|---|
| NS2 | | | |
| Pos. | 110 | obs: | exp: |
| gaa | E | 8 | 203.10 |
| gag | E | 345 | 149.90 |
| --- | -- | ------ | ------ |
| mPD | | 0.044 | 0.49 |
| | | nPD: | 0.09 |
| | | N. weight: | 0.86 |
| | | Sc. PD: | -0.046 |
| | | Sc. rank: | 47.8 |

| | | | |
|---|---|---|---|
| NS2 | | | |
| Pos. | 111 | obs: | exp: |
| tct | S | 0 | 4.77 |
| tcc | S | 0 | 4.18 |
| tca | S | 0 | 6.65 |
| tcg | S | 0 | 1.83 |
| caa | Q | 320 | 171.10 |
| cag | Q | 1 | 149.90 |
| cgt | R | 0 | 0.04 |
| cgc | R | 0 | 0.05 |
| cga | R | 0 | 0.10 |
| cgg | R | 1 | 0.08 |
| aat | N | 1 | 0.52 |
| aac | N | 0 | 0.48 |
| agt | S | 29 | 6.60 |
| agc | S | 1 | 5.98 |
| aga | R | 0 | 0.45 |
| agg | R | 0 | 0.28 |
| --- | -- | ------ | ------ |
| mPD | | 0.49 | 0.89 |
| | | nPD: | 0.55 |
| | | N. weight: | 0.79 |
| | | Sc. PD: | 0.3 |
| | | Sc. rank: | 202.2 |

| | | | |
|---|---|---|---|
| NS2 | | | |
| Pos. | 112 | obs: | exp: |
| gaa | E | 7 | 203.10 |
| gag | E | 346 | 149.90 |
| --- | -- | ------ | ------ |
| mPD | | 0.039 | 0.49 |
| | | nPD: | 0.08 |
| | | N. weight: | 0.88 |
| | | Sc. PD: | -0.056 |
| | | Sc. rank: | 40.4 |

| | | | |
|---|---|---|---|
| NS2 | | | |
| Pos. | 113 | obs: | exp: |
| att | I | 0 | 132.70 |
| atc | I | 0 | 92.52 |
| ata | I | 353 | 127.80 |
| --- | -- | ------ | ------ |
| mPD | | 0 | 0.66 |
| | | nPD: | 0. |
| | | N. weight: | 1.2 |
| | | Sc. PD: | -0.16 |
| | | Sc. rank: | -292.6 |

| | | | |
|---|---|---|---|
| NS2 | | | |
| Pos. | 114 | obs: | exp: |
| cgt | R | 0 | 14.65 |
| cgc | R | 0 | 17.28 |
| cga | R | 0 | 33.93 |
| cgg | R | 0 | 29.46 |
| aga | R | 321 | 158.80 |
| agg | R | 32 | 98.92 |
| --- | -- | ------ | ------ |
| mPD | | 0.17 | 0.96 |
| | | nPD: | 0.17 |
| | | N. weight: | 0.63 |
| | | Sc. PD: | 0.015 |
| | | Sc. rank: | 53.0 |

| | | | |
|---|---|---|---|
| NS2 | | | |
| Pos. | 115 | obs: | exp: |
| act | T | 345 | 93.26 |
| acc | T | 2 | 75.75 |
| aca | T | 6 | 154.60 |
| acg | T | 0 | 29.35 |
| --- | -- | ------ | ------ |
| mPD | | 0.045 | 0.69 |
| | | nPD: | 0.07 |
| | | N. weight: | 1.4 |
| | | Sc. PD: | -0.11 |
| | | Sc. rank: | 59.2 |

| | | | |
|---|---|---|---|
| NS2 | | | |
| Pos. | 116 | obs: | exp: |
| ttt | F | 5 | 145.10 |
| ttc | F | 348 | 207.90 |
| --- | -- | ------ | ------ |
| mPD | | 0.028 | 0.48 |
| | | nPD: | 0.06 |
| | | N. weight: | 0.54 |
| | | Sc. PD: | -0.046 |
| | | Sc. rank: | 14.7 |

| | | | |
|---|---|---|---|
| NS2 | | | |
| Pos. | 117 | obs: | exp: |
| tct | S | 31 | 56.12 |
| tcc | S | 0 | 49.17 |
| tca | S | 4 | 78.20 |
| tcg | S | 318 | 21.52 |
| agt | S | 0 | 77.66 |
| agc | S | 0 | 70.33 |
| --- | -- | ------ | ------ |
| mPD | | 0.18 | 1.7 |
| | | nPD: | 0.11 |
| | | N. weight: | 2.7 |
| | | Sc. PD: | -0.1 |
| | | Sc. rank: | 182.9 |

| | | | |
|---|---|---|---|
| NS2 | | | |
| Pos. | 118 | obs: | exp: |
| ttt | F | 353 | 145.10 |
| ttc | F | 0 | 207.90 |
| --- | -- | ------ | ------ |
| mPD | | 0 | 0.48 |
| | | nPD: | 0. |
| | | N. weight: | 1. |
| | | Sc. PD: | -0.14 |
| | | Sc. rank: | -256.0 |

| | | | |
|---|---|---|---|
| NS2 | | | |
| Pos. | 119 | obs: | exp: |
| caa | Q | 0 | 188.20 |
| cag | Q | 353 | 164.80 |
| --- | -- | ------ | ------ |
| mPD | | 0 | 0.50 |
| | | nPD: | 0. |
| | | N. weight: | 0.88 |
| | | Sc. PD: | -0.12 |
| | | Sc. rank: | -219.3 |

| | | | |
|---|---|---|---|
| NS2 | | | |
| Pos. | 120 | obs: | exp: |
| tta | L | 0 | 30.84 |
| ttg | L | 0 | 67.98 |
| ctt | L | 350 | 65.86 |
| ctc | L | 3 | 57.10 |
| cta | L | 0 | 53.18 |
| ctg | L | 0 | 78.03 |
| --- | -- | ------ | ------ |
| mPD | | 0.017 | 1.1 |
| | | nPD: | 0.02 |
| | | N. weight: | 1.9 |
| | | Sc. PD: | -0.24 |
| | | Sc. rank: | -62.2 |

| | | | |
|---|---|---|---|
| NS2 | | | |
| Pos. | 121 | obs: | exp: |
| att | I | 353 | 132.70 |
| atc | I | 0 | 92.52 |
| ata | I | 0 | 127.80 |
| --- | -- | ------ | ------ |
| mPD | | 0 | 0.66 |
| | | nPD: | 0. |
| | | N. weight: | 1.1 |
| | | Sc. PD: | -0.16 |
| | | Sc. rank: | -281.7 |

| | | | |
|---|---|---|---|
| NS2 | | | |
| Pos. | 122 | obs: | exp: |
| taa | . | 319 | 165.30 |
| tag | . | 28 | 91.35 |
| tga | . | 6 | 96.33 |
| --- | -- | ------ | ------ |
| mPD | | 0.18 | 0.78 |
| | | nPD: | 0.23 |
| | | N. weight: | 0.54 |
| | | Sc. PD: | 0.042 |
| | | Sc. rank: | 65.2 |

}

Amino acid level conservation per codon

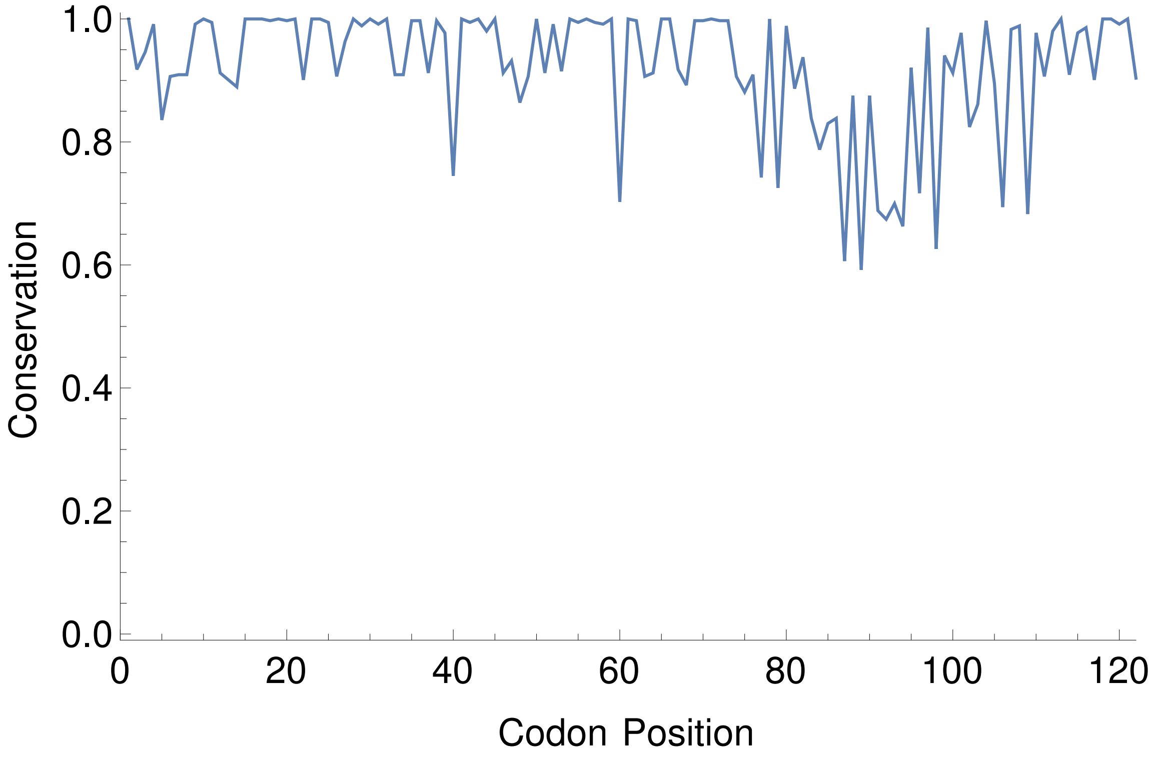

Conservation analysis plots

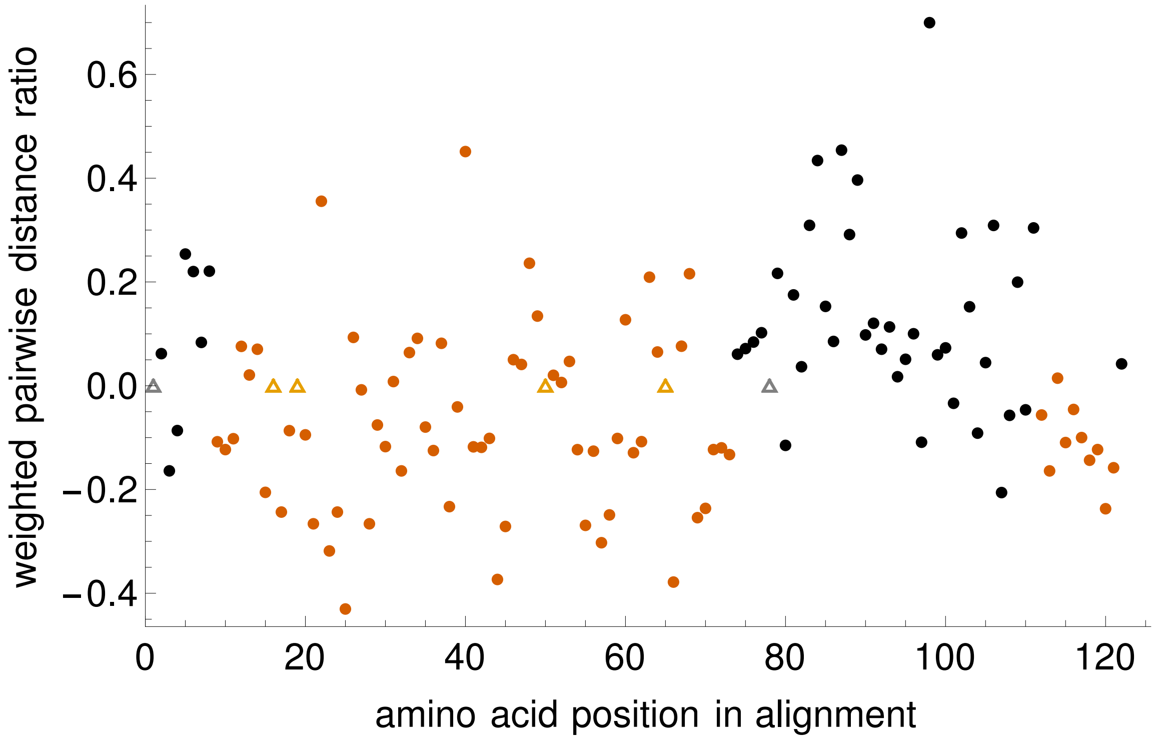


NS2

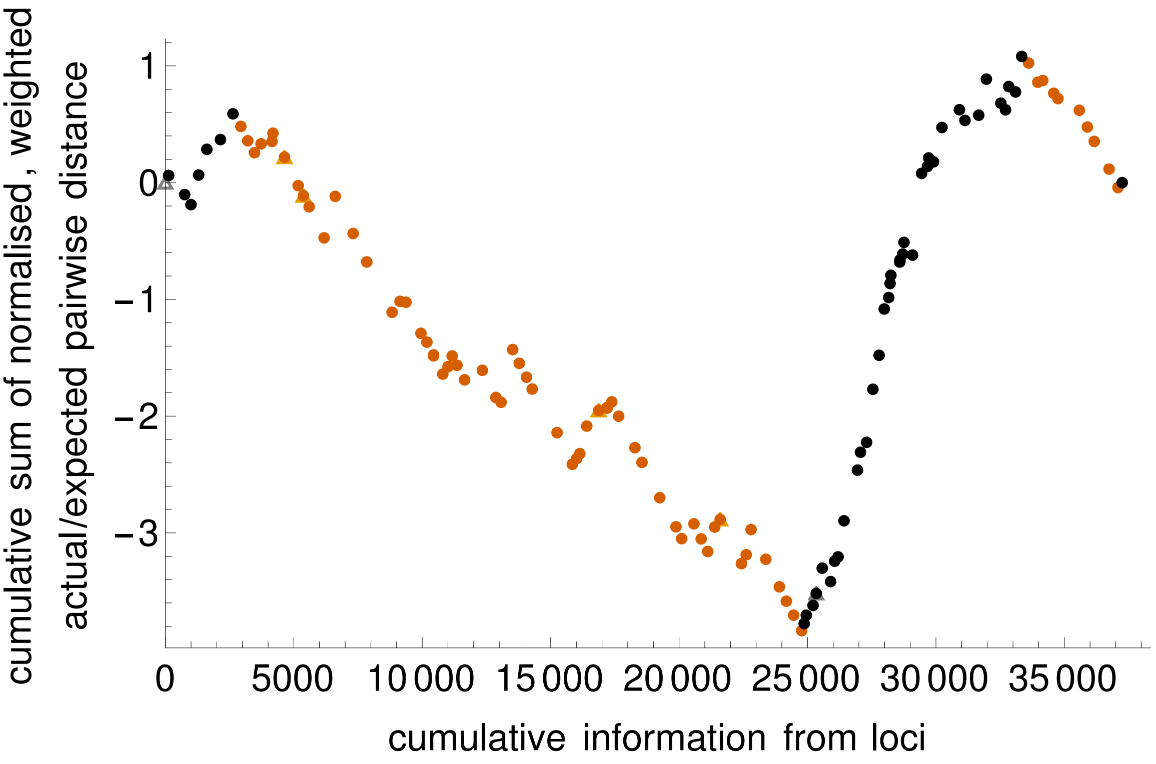


NS2

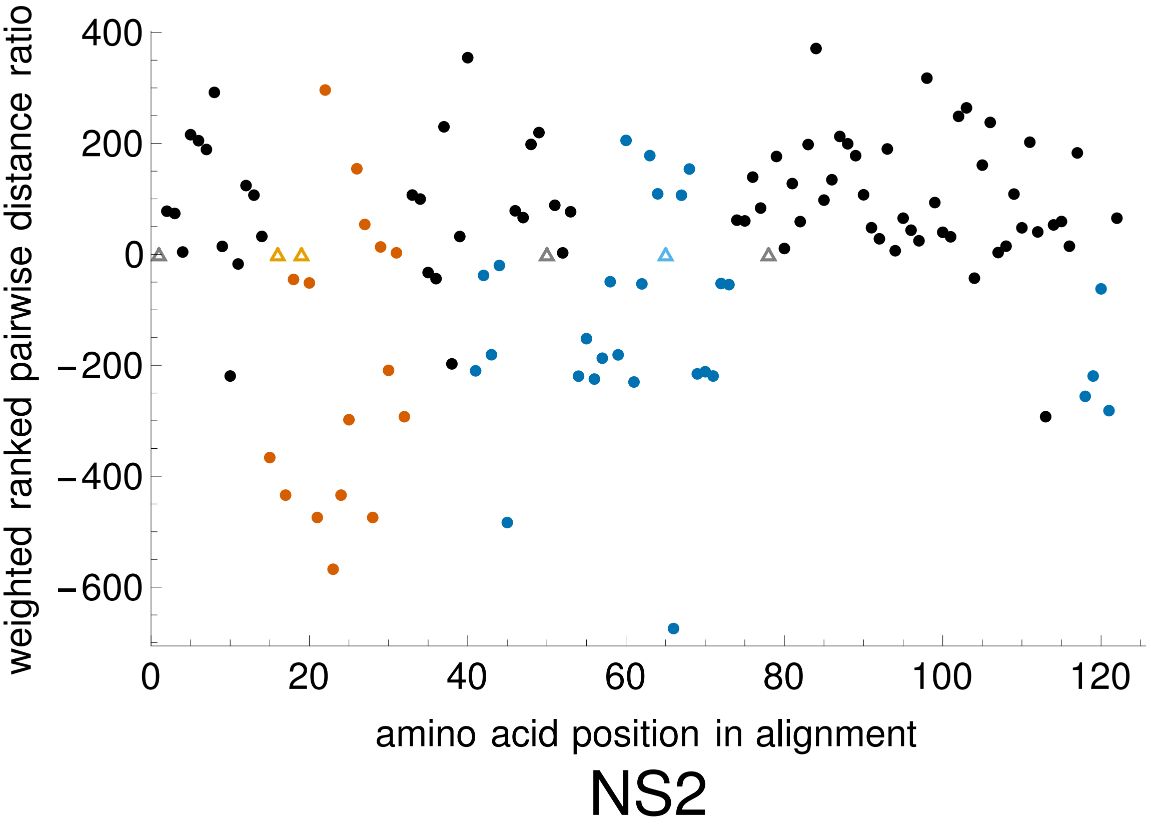
weighted ranked pairwise distance ratio
400
200
0
−200
−400
−600
0
20
40
60
80
100
120
amino acid position in alignment
NS2

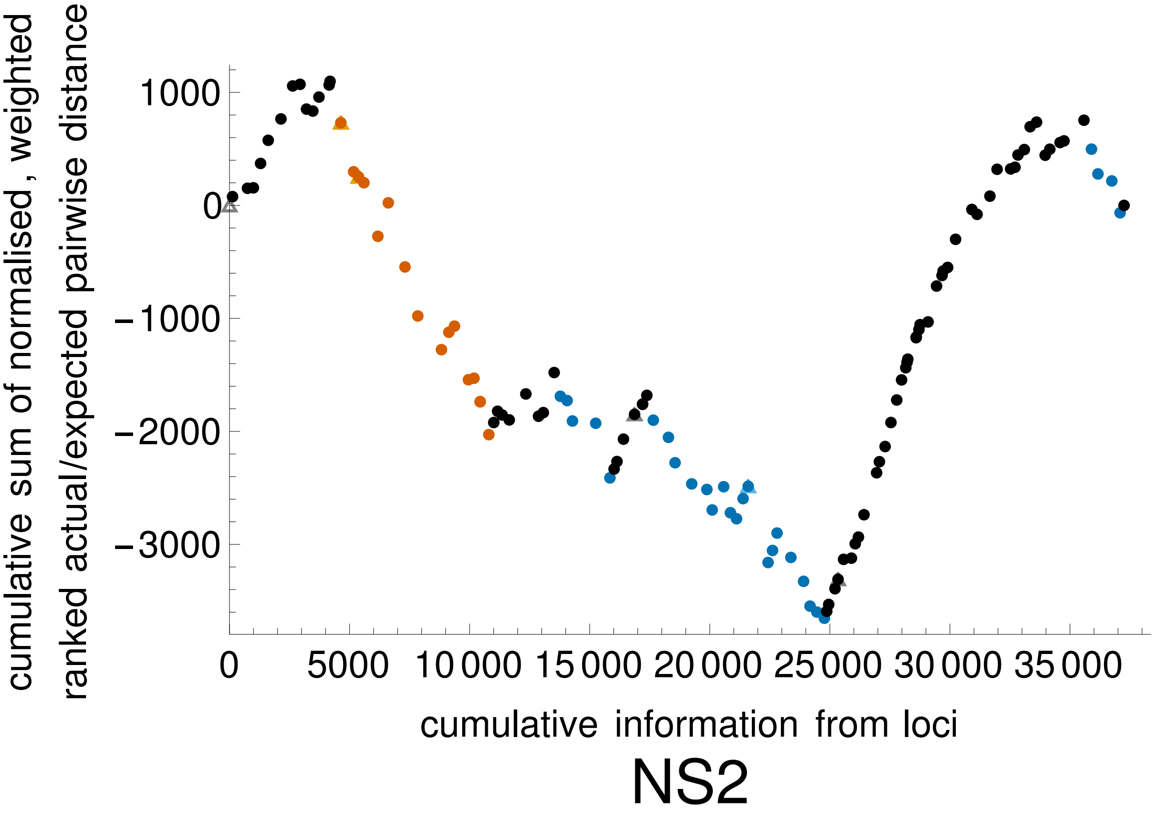
cumulative sum of normalised, weighted ranked actual/expected pairwise distance
1000
0
−1000
−2000
−3000
0
5000
10 000
15 000
20 000
25 000
30 000
35 000
cumulative information from loci
NS2
