# Supplementary material for: Locations and structures of influenza A virus packaging-associated signals and other functional elements via an in silico pipeline for predicting constrained features in RNA viruses
Source: PLoS Comput Biol. 2024 Apr 22;20(4):e1012009. doi: 10.1371/journal.pcbi.1012009 (PMC11034665; doi:10.1371/journal.pcbi.1012009)
Supplement: S7 Code — The content of the notebook follows the same pattern as that in S1 Code. (ZIP) [file pcbi.1012009.s120.zip › S7_Code.pdf]

H7N9 human hosts

PB2

Gene length histogram

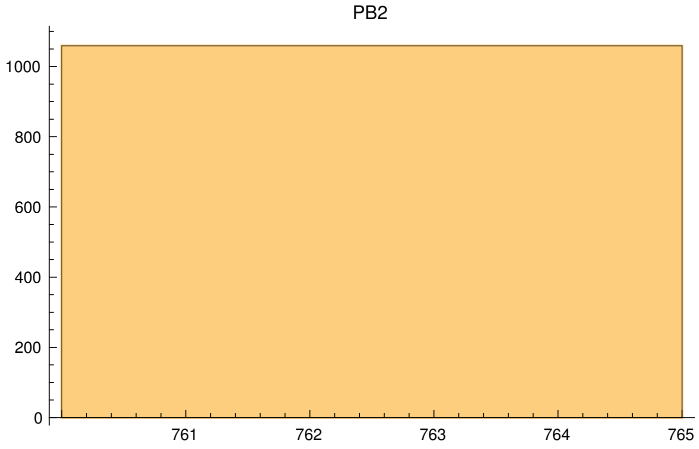

Information vs. nPD

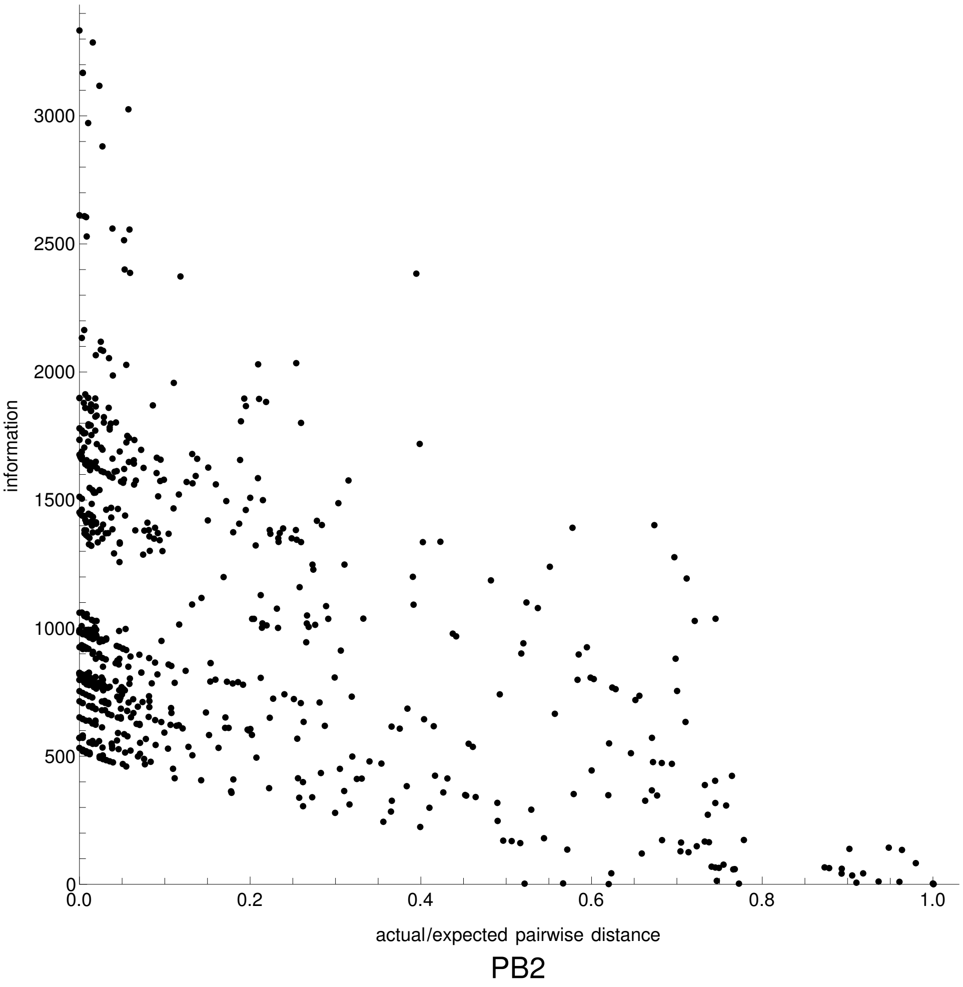

Example sequences highlighted by regions found in analyses to be conserved

Interesting points (by weighted raw PD) highlighted for gene PB2:

ATGGAAAGAATAAAAGAACTAAGAGATTTGATGTCACAGTCTCGCACTCGCGAGATACTGACAAAAACAACTGTGG⋱
ACCATATGGCCATAATCAAGAAATACACATCGGGAAGACAGGAGAAGAATCCTGCCCTTAGGATGAAGTGGATGA⋱
TGGCGATGAAGTATCCAATTACAGCAGACAAAAGGATAATGGAGATGATCCCAGAAAGGAATGAGCAAGGTCAGA⋱
CCCTTTGGAGCAAGACAAATGATGCTGGATCAGACAGAGTAATGGTGTCACCTCTGGCTGTGACGTGGTGGAACA⋱
GAAATGGACCAACGACAAGCACAGTCCATTATCCAAAAGTCTATAAAACCTATTTTGAAAAGGTCGAAAGACTAA⋱
AACATGGGACCTTCGGCCCCGTTCACTTCCGAAACCAGGTTAAAATACGACGCAGGGTCGACATAAACCCAGGCC⋱
ATGCAGATCTTAGTGCTAAGGAAGCACAAGATGTCATCATGGAAGTCGTATTCCCAAACGAAGTTGGAGCCAGAA⋱
TATTGACATCAGAGTCACAGTTAACGATAACCAAGGAAAAGAAGGAGGAGCTTCAAGACTGCAAAATTGCCCCTT⋱
TAATGGTGGCTTACATGTTGGAGAGGGAACTGGTTCGCAAAACACGATTTCTACCAGTAGCTGGAGGAACAAGCA⋱
GCGTGTATATCGAAGTATTACATTTGACCCAAGGGACCTGCTGGGAGCAAATGTACACACCAGGAGGGGAAGTGA⋱
GAAATGATGATGTTGATCAGAGTTTAATTATTGCTGCTAGAAATATTGTTAGAAGGGCAACAGTGTCAGCAGACC⋱
CGTTGGCTTCACTTTTGGAGATGTGCCATAGTACACAGATTGGCGGGGTTAGGATGGTTGACATCCTTAGACAAA⋱
ACCCAACAGAAGAACAGGCTGTGGATATATGTAAGGCAGCAATGGGACTAAGGATCAGTTCATCCTTCAGCTTTG⋱
GAGGTTTCACTTTCAAAAGGACAAGTGGGTCATCCGTCAAAAAGGAAGAAGAAGTGCTCACAGGCAACCTCCAAA⋱
CATTGAAAATAAGAGTACATGAGGGATATGAGGAATTCACAATGGTTGGGCGGAGAGCAACAGCAATTCTAAGGA⋱
AAGCAACCAGAAGGCTGATCCAATTGATAGTGAGTGGGAGAGACGAGCAATCAATTGCCGAGGCAATCATAGTGG⋱
CAATGGTATTCTCACAAGAAGATTGTATGATAAAGGCAGTGAGAGGTGATTTGAACTTTGTCAATAGAGCAAATC⋱
AGAGGCTAAATCCCATGCATCAACTTCTGAGGCATTTCCAAAAGGATGCAAAAGTCCTGTTTCAAAACTGGGGAA⋱
TTGAACCCATTGACAATGTAATGGGGATGATCGGAATATTGCCTGACATGACCCCCAGCACAGAGATGTCATTGA⋱
GAGGAGTGAGAGTTAGTAAAATGGGAGTAGATGAGTATTCCAGTACTGAGAGAGTGGTCGTGAGTATTGATCGTT⋱
TCTTAAGGGTCCGAGACCAGAGGGGAAACGTACTCCTGTCTCCTGAAGAGGTTAGTGAAACACAGGGAACAGAAA⋱
AGCTGACTATAACATATTCATCGTCCATGATGTGGGAGATCAATGGTCCGGAATCAGTGCTAGTTAACACATATC⋱
AATGGATCATTAGAAACTGGGAAACTGTAAAGATTCAATGGTCACAAGATCCTACAATGCTATACAACAAGATGG⋱
AATTTGAACCCTTTCAATCCCTAGTGCCTAAAGCTGTCAGAGGCCAATATAGTGGGTTCGTAAGGGTTCTATTCC⋱
AACAGATGCGTGACGTACTGGGGACATTTGACACTGTCCAAATAATAAAGCTATTACCATTTGCAGCAGCCCCGC⋱
CGAAGCAGAGTAGGATGCAGTTCTCTTCTCTAACTGTGAATGTGAGGGGTTCAGGCATGAGAATAGTTGTGAGAG⋱
GCAATTCTCCTGTGTTTAACTACAACAAGGCAACCAAAAGGCTTACGGTGCTTGGGAAGGATGCAGGTGCATTGG⋱
TGGAAGACCCAGATGAGGGAACAGCAGGAGTGGAATCTGCAGTATTGAGGGGATTTCTGATTCTGGGCAAGGAAG⋱
ACAAGAGGTATGGGCCAGCATTGAGTATCAATGAATTGAGCAATCTTGCGAAAGGAGAGAAGGCTAATGTGTTGA⋱
TAGGGCAAGGAGACGTGGTGTTGGTAATG
AAACGGAAACGGGACTCTAGCATACTTACTGACAGTCAGACAGCGACCAAAAGGA
TTCGGATGGCCATCAATTAA

Interesting points (by weighted ranked PD) highlighted for gene PB2:

ATG
GAAAGAATAAAAGAACTAAGAGATTTGATGTCACAGTCTCGCACTCGCGAGATAC
TGACAAAAACAACTGTGGACCATATGGCCATAATCAAGAAATACACATCGGGAAGACAGGAGAAGAATC⋱
CTGCCCTTAGGATGAAGTGGATGATGGCGATGAAGTATCCAATTACAGCAGACAAAAGGATAATGGAGAT⋱
GATCCCAGAAAGGAATGAGCAAGGTCAGACCCTTTGGAGCAAGACAAATGATGCTGGATCAGACAGAGTA⋱
ATGGTGTCACCTCTGGCTGTGACGTGGTGGAACAGAAATGGACCAACGACAAGCACAGTCCATTATCCAA⋱
AAGTCTATAAAACCTATTTTGAAAAGGTCGAAAGACTAAAACATGGGACCTTCGGCCCCGTTCACTTCCG⋱
AAACCAGGTTAAAATACGACGCAGGGTCGACATAAACCCAGGCCATGCAGATCTTAGTGCTAAGGAAGCA⋱
CAAGATGTCATCATGGAAGTCGTATTCCCAAACGAAGTTGGAGCCAGAATATTGACATCAGAGTCACAGT⋱

TAACGATAACCAAGGAAAAGAAGGAGGAGCTTCAAGACTGCAAAATTGCCCCTTTAATGGTGGCTTACAT⋱
GTTGGAGAGGGAACTGGTTCGCAAAACACGATTTCTACCAGTAGCTGGAGGAACAAGCAGCGTGTATATC⋱
GAAGTATTACATTTGACCCAAGGGACCTGCTGGGAGCAAATGTACACACCAGGAGGGGAAGTGAGAAATG⋱
ATGATGTTGATCAGAGTTTAATTATTGCTGCTAGAAATATTGTTAGAAGGGCAACAGTGTCAGCAGACCC⋱
GTTGGCTTCACTTTTGGAGATGTGCCATAGTACACAGATTGGCGGGGTTAGGATGGTTGACATCCTTAGA⋱
CAAAACCCAACAGAAGAACAGGCTGTGGATATATGTAAGGCAGCAATGGGACTAAGGATCAGTTCATCCT⋱
TCAGCTTTGGAGGTTTCACTTTCAAAAGGACAAGTGGGTCATCCGTCAAAAAGGAAGAAGAAGTGCTCAC⋱
AGGCAACCTCCAAACATTGAAAATAAGAGTACATGAGGGATATGAGGAATTCACAATGGTTGGGCGGAGA⋱
GCAACAGCAATTCTAAGGAAAGCAACCAGAAGGCTGATCCAATTGATAGTGAGTGGGAGAGACGAGCAAT⋱
CAATTGCCGAGGCAATCATAGTGGCAATGGTATTCTCACAAGAAGATTGTATGATAAAGGCAGTGAGAGG⋱
TGATTTGAACTTTGTCAATAGAGCAAATCAGAGGCTAAATCCCATGCATCAACTTCTGAGGCATTTCCAA⋱
AAGGATGCAAAAGTCCTGTTTCAAAACTGGGGAATTGAACCCATTGACAATGTAATGGGGATGATCGGAA⋱
TATTGCCTGACATGACCCCCAGCACAGAGATGTCATTGAGAGGAGTGAGAGTTAGTAAAATGGGAGTAGA⋱
TGAGTATTCCAGTACTGAGAGAGTGGTCGTGAGTATTGATCGTTTCTTAAGGGTCCGAGACCAGAGGGGA⋱
AACGTACTCCTGTCTCCTGAAGAGGTTAGTGAAACACAGGGAACAGAAAAGCTGACTATAACATATTCAT⋱
CGTCCATGATGTGGGAGATCAATGGTCCGGAATCAGTGCTAGTTAACACATATCAATGGATCATTAGAAA⋱
CTGGGAAACTGTAAAGATTCAATGGTCACAAGATCCTACAATGCTATACAACAAGATGGAATTTGAACCC⋱
TTTCAATCCCTAGTGCCTAAAGCTGTCAGAGGCCAATATAGTGGGTTCGTAAGGGTTCTATTCCAACAGA⋱
TGCGTGACGTACTGGGGACATTTGACACTGTCCAAATAATAAAGCTATTACCATTTGCAGCAGCCCCGCC⋱
GAAGCAGAGTAGGATGCAGTTCTCTTCTCTAACTGTGAATGTGAGGGGTTCAGGCATGAGAATAGTTGTG⋱
AGAGGCAATTCTCCTGTGTTTAACTACAACAAGGCAACCAAAAGGCTTACGGTGCTTGGGAAGGATGCAG⋱
GTGCATTGGTGGAAGACCCAGATGAGGGAACAGCAGGAGTGGAATCTGCAGTATTGAGGGGATTTCTGAT⋱
TCTGGGCAAGGAAGACAAGAGGTATGGGCCAGCATTGAGTATCAATGAATTGAGCAATCTTGCGAAAGGA⋱
GAGAAGGCTAATGTG
TTGATAGGGCAAGGAGACGTGGTGTTGGTAATGAAACGGAAACGGGACTCTAGCA
TACTTACTGACAGTCAGACAGCGACCAAAAGGATTCGGATGGCCATCAATTAA

Per codon conservation report

PB2

Pos.	1	obs:	exp:
atg	M	1059	1059.00
---	--	------	------
mPD		0	0
		nPD:	1.
		N. weight:	0.
		Sc. PD:	0
		Sc. rank:	0

PB2

Pos.	2	obs:	exp:
gaa	E	1057	644.40
gag	E	2	414.60
---	--	------	------
mPD		0.0038	0.48
		nPD:	0.01
		N. weight:	0.53
		Sc. PD:	-0.047
		Sc. rank:	-268.3

PB2

Pos.	3	obs:	exp:
cgt	R	0	45.48
cgc	R	0	52.45
cga	R	0	103.60
cgg	R	0	88.36
aga	R	1057	484.20
agg	R	2	284.80
---	--	------	------
mPD		0.0038	0.96
		nPD:	0.
		N. weight:	0.85
		Sc. PD:	-0.078
		Sc. rank:	-541.6

PB2

Pos.	4	obs:	exp:
att	I	0	389.10
atc	I	0	253.80
ata	I	1059	416.10
---	--	------	------
mPD		0	0.65
		nPD:	0.
		N. weight:	1.
		Sc. PD:	-0.098
		Sc. rank:	-852.8

PB2

Pos.	5	obs:	exp:
cgt	R	0	0.26
cgc	R	0	0.30
cga	R	0	0.59
cgg	R	0	0.50
aaa	K	1040	613.70
aag	K	13	439.30
aga	R	6	2.74
agg	R	0	1.61
---	--	------	------
mPD		0.036	0.50
		nPD:	0.07
		N. weight:	0.53
		Sc. PD:	-0.015
		Sc. rank:	158.5

PB2

Pos.	6	obs:	exp:
gaa	E	1052	644.40
gag	E	7	414.60
---	--	------	------
mPD		0.013	0.48
		nPD:	0.03
		N. weight:	0.51
		Sc. PD:	-0.035
		Sc. rank:	-15.4

PB2

Pos.	7	obs:	exp:
tta	L	0	93.98
ttg	L	0	197.20
ctt	L	0	176.30
ctc	L	0	176.70
cta	L	1055	178.50
ctg	L	4	236.30
---	--	------	------
mPD		0.0075	1.1
		nPD:	0.01
		N. weight:	1.9
		Sc. PD:	-0.17
		Sc. rank:	-1063.3

PB2

Pos.	8	obs:	exp:
cgt	R	0	45.44
cgc	R	0	52.40
cga	R	0	103.50
cgg	R	0	88.28
aaa	K	1	0.58
aag	K	0	0.42
aga	R	1053	483.80
agg	R	5	284.60
---	--	------	------
mPD		0.011	0.96
		nPD:	0.01
		N. weight:	0.83
		Sc. PD:	-0.07
		Sc. rank:	-311.2

PB2

Pos.	9	obs:	exp:
tat	Y	3	1.62
tac	Y	0	1.38
aat	N	12	6.11
aac	N	0	5.89
gat	D	1042	563.10
gac	D	0	478.90
gaa	E	1	1.22
gag	E	1	0.78
---	--	------	------
mPD		0.032	0.53
		nPD:	0.06
		N. weight:	0.68
		Sc. PD:	-0.025
		Sc. rank:	179.0

PB2

Pos.	10	obs:	exp:
tta	L	7	93.98
ttg	L	1048	197.20
ctt	L	0	176.30
ctc	L	0	176.70
cta	L	1	178.50
ctg	L	3	236.30
---	--	------	------
mPD		0.023	1.1
		nPD:	0.02
		N. weight:	1.8
		Sc. PD:	-0.14
		Sc. rank:	-238.8

PB2

Pos.	11	obs:	exp:
atg	M	1059	1059.00
---	--	------	------
mPD		0	0
		nPD:	1.
		N. weight:	0.
		Sc. PD:	0
		Sc. rank:	0

PB2

Pos.	12	obs:	exp:
tta	L	0	0.09
ttg	L	0	0.19
tct	S	0	168.30
tcc	S	0	138.20
tca	S	1055	268.50
tcg	S	3	60.03
ctt	L	0	0.17
ctc	L	0	0.17
cta	L	1	0.17
ctg	L	0	0.22
agt	S	0	217.50
agc	S	0	205.50
---	--	------	------
mPD		0.0094	1.7
		nPD:	0.01
		N. weight:	1.5
		Sc. PD:	-0.13
		Sc. rank:	-863.4

PB2

Pos.	13	obs:	exp:
caa	Q	17	539.40
cag	Q	1042	519.60
---	--	------	------
mPD		0.032	0.50
		nPD:	0.06
		N. weight:	0.69
		Sc. PD:	-0.024
		Sc. rank:	185.8

PB2

Pos.	14	obs:	exp:
tct	S	968	168.40
tcc	S	91	138.30
tca	S	0	268.80
tcg	S	0	60.09
agt	S	0	217.70
agc	S	0	205.70
---	--	------	------
mPD		0.16	1.7
		nPD:	0.1
		N. weight:	1.7
		Sc. PD:	-0.0075
		Sc. rank:	668.6

PB2

Pos.	15	obs:	exp:
cgt	R	1	45.48
cgc	R	1057	52.45
cga	R	1	103.60
cgg	R	0	88.36
aga	R	0	484.20
agg	R	0	284.80
---	--	------	------
mPD		0.0038	0.96
		nPD:	0.
		N. weight:	3.3
		Sc. PD:	-0.3
		Sc. rank:	-2039.1

PB2

Pos.	16	obs:	exp:
act	T	1023	269.90
acc	T	36	217.30
aca	T	0	485.70
acg	T	0	86.02
---	--	------	------
mPD		0.066	0.68
		nPD:	0.1
		N. weight:	1.3
		Sc. PD:	-0.0031
		Sc. rank:	533.5

PB2

Pos.	17	obs:	exp:
cgt	R	0	45.48
cgc	R	1057	52.45
cga	R	2	103.60
cgg	R	0	88.36
aga	R	0	484.20
agg	R	0	284.80
---	--	------	------
mPD		0.0038	0.96
		nPD:	0.
		N. weight:	3.3
		Sc. PD:	-0.3
		Sc. rank:	-2101.3

PB2

Pos.	18	obs:	exp:
gaa	E	0	644.40
gag	E	1059	414.60
---	--	------	------
mPD		0	0.48
		nPD:	0.
		N. weight:	1.
		Sc. PD:	-0.098
		Sc. rank:	-856.2

PB2

Pos.	19	obs:	exp:
att	I	0	389.10
atc	I	0	253.80
ata	I	1059	416.10
---	--	------	------
mPD		0	0.65
		nPD:	0.
		N. weight:	1.
		Sc. PD:	-0.098
		Sc. rank:	-852.8

PB2

Pos.	20	obs:	exp:
tta	L	0	93.98
ttg	L	0	197.20
ctt	L	1	176.30
ctc	L	0	176.70
cta	L	8	178.50
ctg	L	1050	236.30
---	--	------	------
mPD		0.017	1.1
		nPD:	0.02
		N. weight:	1.6
		Sc. PD:	-0.13
		Sc. rank:	-436.5

PB2

Pos.	21	obs:	exp:
act	T	0	269.90
acc	T	1	217.30
aca	T	1058	485.70
acg	T	0	86.02
---	--	------	------
mPD		0.0019	0.68
		nPD:	0.
		N. weight:	0.85
		Sc. PD:	-0.079
		Sc. rank:	-594.0

PB2

Pos.	22	obs:	exp:
cgt	R	0	0.30
cgc	R	0	0.35
cga	R	0	0.69
cgg	R	0	0.58
aaa	K	968	613.10
aag	K	84	438.90
aga	R	7	3.20
agg	R	0	1.88
---	--	------	------
mPD		0.16	0.50
		nPD:	0.32
		N. weight:	0.32
		Sc. PD:	0.067
		Sc. rank:	226.4

PB2

Pos.	23	obs:	exp:
act	T	0	269.90
acc	T	1	217.30
aca	T	1056	485.70
acg	T	2	86.02
---	--	------	------
mPD		0.0057	0.68
		nPD:	0.01
		N. weight:	0.84
		Sc. PD:	-0.074
		Sc. rank:	-410.0

PB2

Pos.	24	obs:	exp:
act	T	802	269.90
acc	T	5	217.30
aca	T	237	485.70
acg	T	15	86.02
---	--	------	------
mPD		0.38	0.68
		nPD:	0.56
		N. weight:	0.69
		Sc. PD:	0.3
		Sc. rank:	534.9

PB2

Pos.	25	obs:	exp:
gtt	V	1	219.40
gtc	V	1	213.70
gta	V	63	216.80
gtg	V	994	409.00
---	--	------	------
mPD		0.12	0.73
		nPD:	0.16
		N. weight:	0.83
		Sc. PD:	0.047
		Sc. rank:	404.0

PB2			
Pos.	26	obs:	exp:
gat	D	3	572.30
gac	D	1056	486.70
---	--	------	------
mPD		0.0057	0.50
		nPD:	0.01
		N. weight:	0.83
		Sc. PD:	-0.071
		Sc. rank:	-318.1

,

PB2			
Pos.	27	obs:	exp:
cat	H	955	607.50
cac	H	103	450.50
aat	N	0	0.51
aac	N	1	0.49
---	--	------	------
mPD		0.18	0.49
		nPD:	0.37
		N. weight:	0.29
		Sc. PD:	0.075
		Sc. rank:	209.2

,

PB2			
Pos.	28	obs:	exp:
att	I	0	1.10
atc	I	0	0.72
ata	I	3	1.18
atg	M	1056	1056.00
---	--	------	------
mPD		0.0057	0.0057
		nPD:	1.
		N. weight:	0.0029
		Sc. PD:	0.0025
		Sc. rank:	2.4

,

PB2			
Pos.	29	obs:	exp:
gct	A	17	273.50
gcc	A	1042	197.20
gca	A	0	498.40
gcg	A	0	89.88
---	--	------	------
mPD		0.032	0.67
		nPD:	0.05
		N. weight:	1.8
		Sc. PD:	-0.088
		Sc. rank:	260.6

,

PB2			
Pos.	30	obs:	exp:
att	I	0	389.10
atc	I	1	253.80
ata	I	1058	416.10
---	--	------	------
mPD		0.0019	0.65
		nPD:	0.
		N. weight:	1.
		Sc. PD:	-0.095
		Sc. rank:	-686.2

,

PB2			
Pos.	31	obs:	exp:
att	I	8	388.70
atc	I	1028	253.50
ata	I	22	415.70
gtt	V	0	0.21
gtc	V	1	0.20
gta	V	0	0.20
gtg	V	0	0.39
---	--	------	------
mPD		0.057	0.66
		nPD:	0.09
		N. weight:	1.4
		Sc. PD:	-0.016
		Sc. rank:	496.4

,

PB2			
Pos.	32	obs:	exp:
cgt	R	0	0.04
cgc	R	0	0.05
cga	R	0	0.10
cgg	R	0	0.08
aaa	K	1	616.60
aag	K	1057	441.40
aga	R	0	0.46
agg	R	1	0.27
---	--	------	------
mPD		0.0038	0.49
		nPD:	0.01
		N. weight:	0.95
		Sc. PD:	-0.084
		Sc. rank:	-504.0

,

PB2			
Pos.	33	obs:	exp:
aaa	K	1052	617.20
aag	K	7	441.80
---	--	------	------
mPD		0.013	0.49
		nPD:	0.03
		N. weight:	0.55
		Sc. PD:	-0.039
		Sc. rank:	-25.9

,

PB2			
Pos.	34	obs:	exp:
tat	Y	698	572.00
tac	Y	361	487.00
---	--	------	------
mPD		0.45	0.50
		nPD:	0.91
		N. weight:	0.036
		Sc. PD:	0.028
		Sc. rank:	29.9

,

PB2			
Pos.	35	obs:	exp:
act	T	1	269.90
acc	T	0	217.30
aca	T	1057	485.70
acg	T	1	86.02
---	--	------	------
mPD		0.0038	0.68
		nPD:	0.01
		N. weight:	0.84
		Sc. PD:	-0.076
		Sc. rank:	-503.6

,

PB2			
Pos.	36	obs:	exp:
tct	S	0	168.30
tcc	S	0	138.20
tca	S	547	268.50
tcg	S	511	60.03
act	T	0	0.25
acc	T	0	0.21
aca	T	1	0.46
acg	T	0	0.08
agt	S	0	217.50
agc	S	0	205.50
---	--	------	------
mPD		0.50	1.7
		nPD:	0.3
		N. weight:	1.5
		Sc. PD:	0.3
		Sc. rank:	1063.6

,

PB2			
Pos.	37	obs:	exp:
ggt	G	0	140.40
ggc	G	0	135.30
gga	G	1058	488.50
ggg	G	1	294.80
---	--	------	------
mPD		0.0019	0.68
		nPD:	0.
		N. weight:	0.84
		Sc. PD:	-0.078
		Sc. rank:	-577.9

,

PB2			
Pos.	38	obs:	exp:
cgt	R	0	45.48
cgc	R	0	52.45
cga	R	0	103.60
cgg	R	0	88.36
aga	R	1036	484.20
agg	R	23	284.80
---	--	------	------
mPD		0.043	0.96
		nPD:	0.04
		N. weight:	0.76
		Sc. PD:	-0.04
		Sc. rank:	90.4

,

PB2			
Pos.	39	obs:	exp:
caa	Q	2	539.40
cag	Q	1057	519.60
---	--	------	------
mPD		0.0038	0.50
		nPD:	0.01
		N. weight:	0.77
		Sc. PD:	-0.068
		Sc. rank:	-410.4

,

PB2			
Pos.	40	obs:	exp:
gaa	E	13	644.40
gag	E	1046	414.60
---	--	------	------
mPD		0.024	0.48
		nPD:	0.05
		N. weight:	0.95
		Sc. PD:	-0.045
		Sc. rank:	163.2

,

PB2			
Pos.	41	obs:	exp:
aaa	K	7	617.20
aag	K	1052	441.80
---	--	------	------
mPD		0.013	0.49
		nPD:	0.03
		N. weight:	0.92
		Sc. PD:	-0.064
		Sc. rank:	-42.9

,

PB2			
Pos.	42	obs:	exp:
aat	N	1001	539.40
aac	N	58	519.60
---	--	------	------
mPD		0.10	0.50
		nPD:	0.21
		N. weight:	0.51
		Sc. PD:	0.053
		Sc. rank:	285.4

,

PB2			
Pos.	43	obs:	exp:
cct	P	1055	264.20
ccc	P	3	190.30
cca	P	1	435.90
ccg	P	0	168.60
---	--	------	------
mPD		0.0075	0.71
		nPD:	0.01
		N. weight:	1.5
		Sc. PD:	-0.13
		Sc. rank:	-618.3

,

PB2			
Pos.	44	obs:	exp:
gtt	V	0	0.21
gtc	V	1	0.20
gta	V	0	0.20
gtg	V	0	0.39
gct	A	4	273.20
gcc	A	1054	197.00
gca	A	0	498.00
gcg	A	0	89.79
---	--	------	------
mPD		0.0094	0.67
		nPD:	0.01
		N. weight:	1.8
		Sc. PD:	-0.15
		Sc. rank:	-540.2

,

PB2			
Pos.	45	obs:	exp:
tta	L	0	93.98
ttg	L	0	197.20
ctt	L	955	176.30
ctc	L	104	176.70
cta	L	0	178.50
ctg	L	0	236.30
---	--	------	------
mPD		0.18	1.1
		nPD:	0.16
		N. weight:	1.6
		Sc. PD:	0.094
		Sc. rank:	795.2

,

PB2			
Pos.	46	obs:	exp:
cgt	R	0	45.48
cgc	R	0	52.45
cga	R	0	103.60
cgg	R	0	88.36
aga	R	4	484.20
agg	R	1055	284.80
---	--	------	------
mPD		0.0075	0.96
		nPD:	0.01
		N. weight:	1.4
		Sc. PD:	-0.12
		Sc. rank:	-738.1

,

PB2			
Pos.	47	obs:	exp:
atg	M	1059	1059.00
---	--	------	------
mPD		0	0
		nPD:	1.
		N. weight:	0.
		Sc. PD:	0
		Sc. rank:	0

,

PB2			
Pos.	48	obs:	exp:
aaa	K	4	617.20
aag	K	1055	441.80
---	--	------	------
mPD		0.0075	0.49
		nPD:	0.02
		N. weight:	0.93
		Sc. PD:	-0.075
		Sc. rank:	-252.4

,

PB2			
Pos.	49	obs:	exp:
tgg	W	1059	1059.00
---	--	------	------
mPD		0	0
		nPD:	1.
		N. weight:	0.
		Sc. PD:	0
		Sc. rank:	0

,

PB2			
Pos.	50	obs:	exp:
atg	M	1057	1057.00
act	T	0	0.25
acc	T	0	0.21
aca	T	0	0.46
acg	T	1	0.08
gtt	V	0	0.21
gtc	V	0	0.20
gta	V	0	0.20
gtg	V	1	0.39
---	--	------	------
mPD		0.0038	0.0067
		nPD:	0.57
		N. weight:	0.0036
		Sc. PD:	0.0016
		Sc. rank:	2.8

,

PB2

Pos.	51	obs:	exp:
atg	M	1059	1059.00
---	--	------	------
mPD		0	0
		nPD:	1.
		N. weight:	0.
		Sc. PD:	0
		Sc. rank:	0

,

PB2

Pos.	52	obs:	exp:
gct	A	2	273.50
gcc	A	0	197.20
gca	A	487	498.40
gcg	A	570	89.88
---	--	------	------
mPD		0.50	0.67
		nPD:	0.75
		N. weight:	1.1
		Sc. PD:	0.67
		Sc. rank:	894.3

,

PB2

Pos.	53	obs:	exp:
atg	M	1059	1059.00
---	--	------	------
mPD		0	0
		nPD:	1.
		N. weight:	0.
		Sc. PD:	0
		Sc. rank:	0

,

PB2

Pos.	54	obs:	exp:
cgt	R	0	0.13
cgc	R	0	0.15
cga	R	0	0.29
cgg	R	0	0.25
aaa	K	800	615.50
aag	K	256	440.50
aga	R	3	1.37
agg	R	0	0.81
---	--	------	------
mPD		0.37	0.49
		nPD:	0.75
		N. weight:	0.08
		Sc. PD:	0.05
		Sc. rank:	66.2

,

PB2

Pos.	55	obs:	exp:
tat	Y	970	572.00
tac	Y	89	487.00
---	--	------	------
mPD		0.15	0.50
		nPD:	0.31
		N. weight:	0.38
		Sc. PD:	0.076
		Sc. rank:	261.8

,

PB2

Pos.	56	obs:	exp:
cct	P	0	264.20
ccc	P	2	190.30
cca	P	1053	435.90
ccg	P	4	168.60
---	--	------	------
mPD		0.011	0.71
		nPD:	0.02
		N. weight:	0.94
		Sc. PD:	-0.076
		Sc. rank:	-224.4

,

PB2

Pos.	57	obs:	exp:
att	I	934	389.10
atc	I	125	253.80
ata	I	0	416.10
---	--	------	------
mPD		0.21	0.65
		nPD:	0.32
		N. weight:	0.76
		Sc. PD:	0.16
		Sc. rank:	533.3

,

PB2

Pos.	58	obs:	exp:
act	T	0	269.90
acc	T	4	217.30
aca	T	712	485.70
acg	T	343	86.02
---	--	------	------
mPD		0.44	0.68
		nPD:	0.66
		N. weight:	0.76
		Sc. PD:	0.41
		Sc. rank:	610.6

,

PB2

Pos.	59	obs:	exp:
tct	S	0	0.16
tcc	S	0	0.13
tca	S	1	0.25
tcg	S	0	0.06
agt	S	0	0.21
agc	S	0	0.19
gct	A	0	273.20
gcc	A	9	197.00
gca	A	1049	498.00
gcg	A	0	89.79
---	--	------	------
mPD		0.019	0.67
		nPD:	0.03
		N. weight:	0.79
		Sc. PD:	-0.054
		Sc. rank:	-11.1

,

PB2

Pos.	60	obs:	exp:
aat	N	0	3.57
aac	N	7	3.43
gat	D	66	568.50
gac	D	986	483.50
---	--	------	------
mPD		0.13	0.51
		nPD:	0.26
		N. weight:	0.59
		Sc. PD:	0.088
		Sc. rank:	373.8

,

PB2

Pos.	61	obs:	exp:
cgt	R	0	0.69
cgc	R	0	0.79
cga	R	0	1.57
cgg	R	0	1.34
aaa	K	1039	607.90
aag	K	4	435.10
aga	R	16	7.32
agg	R	0	4.30
---	--	------	------
mPD		0.037	0.53
		nPD:	0.07
		N. weight:	0.57
		Sc. PD:	-0.016
		Sc. rank:	172.5

,

PB2

Pos.	62	obs:	exp:
cgt	R	0	45.23
cgc	R	0	52.15
cga	R	0	103.00
cgg	R	0	87.86
aaa	K	0	3.50
aag	K	6	2.50
aga	R	2	481.50
agg	R	1051	283.20
---	--	------	------
mPD		0.015	0.97
		nPD:	0.02
		N. weight:	1.4
		Sc. PD:	-0.12
		Sc. rank:	-375.8

,

PB2

Pos.	63	obs:	exp:
att	I	0	386.50
atc	I	0	252.10
ata	I	1052	413.40
gtt	V	0	1.45
gtc	V	0	1.41
gta	V	7	1.43
gtg	V	0	2.70
---	--	------	------
mPD		0.013	0.67
		nPD:	0.02
		N. weight:	1.
		Sc. PD:	-0.079
		Sc. rank:	-151.1

,

PB2

Pos.	64	obs:	exp:
att	I	0	0.37
atc	I	0	0.24
ata	I	1	0.39
atg	M	1058	1058.00
---	--	------	------
mPD		0.0019	0.0019
		nPD:	1.
		N. weight:	0.00097
		Sc. PD:	0.00084
		Sc. rank:	0.8

,

PB2

Pos.	65	obs:	exp:
aaa	K	0	0.58
aag	K	1	0.42
gaa	E	3	643.20
gag	E	1054	413.80
ggt	G	0	0.13
ggc	G	0	0.13
gga	G	0	0.46
ggg	G	1	0.28
---	--	------	------
mPD		0.0094	0.48
		nPD:	0.02
		N. weight:	1.
		Sc. PD:	-0.077
		Sc. rank:	-152.0

,

PB2

Pos.	66	obs:	exp:
tta	L	0	0.18
ttg	L	1	0.37
ctt	L	1	0.33
ctc	L	0	0.33
cta	L	0	0.34
ctg	L	0	0.45
att	I	3	1.47
atc	I	0	0.96
ata	I	1	1.57
atg	M	1046	1046.00
act	T	0	0.25
acc	T	0	0.21
aca	T	0	0.46
acg	T	1	0.08
gtt	V	0	1.24
gtc	V	0	1.21
gta	V	0	1.23
gtg	V	6	2.32
---	--	------	------
mPD		0.026	0.035
		nPD:	0.75
		N. weight:	0.014
		Sc. PD:	0.0087
		Sc. rank:	11.7

,

PB2

Pos.	67	obs:	exp:
att	I	28	385.80
atc	I	1021	251.60
ata	I	1	412.60
gtt	V	0	1.86
gtc	V	9	1.82
gta	V	0	1.84
gtg	V	0	3.48
---	--	------	------
mPD		0.070	0.67
		nPD:	0.1
		N. weight:	1.4
		Sc. PD:	0.0066
		Sc. rank:	577.2

,

PB2

Pos.	68	obs:	exp:
cct	P	3	264.20
ccc	P	0	190.30
cca	P	267	435.90
ccg	P	789	168.60
---	--	------	------
mPD		0.38	0.71
		nPD:	0.54
		N. weight:	1.1
		Sc. PD:	0.47
		Sc. rank:	861.3

,

PB2

Pos.	69	obs:	exp:
gaa	E	1052	644.40
gag	E	7	414.60
---	--	------	------
mPD		0.013	0.48
		nPD:	0.03
		N. weight:	0.51
		Sc. PD:	-0.035
		Sc. rank:	-15.4

,

PB2

Pos.	70	obs:	exp:
cgt	R	0	45.48
cgc	R	0	52.45
cga	R	1	103.60
cgg	R	0	88.36
aga	R	706	484.20
agg	R	352	284.80
---	--	------	------
mPD		0.45	0.96
		nPD:	0.46
		N. weight:	0.35
		Sc. PD:	0.12
		Sc. rank:	266.2

,

PB2

Pos.	71	obs:	exp:
aat	N	1056	539.40
aac	N	3	519.60
---	--	------	------
mPD		0.0057	0.50
		nPD:	0.01
		N. weight:	0.72
		Sc. PD:	-0.061
		Sc. rank:	-285.0

,

PB2

Pos.	72	obs:	exp:
gaa	E	105	644.40
gag	E	954	414.60
---	--	------	------
mPD		0.18	0.48
		nPD:	0.38
		N. weight:	0.63
		Sc. PD:	0.17
		Sc. rank:	450.2

,

PB2

Pos.	73	obs:	exp:
caa	Q	1040	538.90
cag	Q	18	519.10
aaa	K	1	0.58
aag	K	0	0.42
---	--	------	------
mPD		0.035	0.50
		nPD:	0.07
		N. weight:	0.65
		Sc. PD:	-0.018
		Sc. rank:	193.1

,

PB2

Pos.	74	obs:	exp:
ggt	G	1050	140.40
ggc	G	8	135.30
gga	G	1	488.50
ggg	G	0	294.80
---	--	------	------
mPD		0.017	0.68
		nPD:	0.02
		N. weight:	2.2
		Sc. PD:	-0.16
		Sc. rank:	-189.7

,

PB2

Pos.	75	obs:	exp:
cat	H	2	1.15
cac	H	0	0.85
caa	Q	136	538.40
cag	Q	921	518.60
---	--	------	------
mPD		0.23	0.50
		nPD:	0.45
		N. weight:	0.36
		Sc. PD:	0.12
		Sc. rank:	269.4

,

PB2

Pos. 76		obs:	exp:
att	I	0	2.94
atc	I	8	1.92
ata	I	0	3.14
act	T	32	267.10
acc	T	1015	215.00
aca	T	0	480.70
acg	T	1	85.12
aat	N	0	0.51
aac	N	1	0.49
gct	A	0	0.52
gcc	A	2	0.37
gca	A	0	0.94
gcg	A	0	0.17
---	--	------	------
mPD		0.081	0.70
		nPD:	0.12
		N. weight:	1.6
		Sc. PD:	0.026
		Sc. rank:	680.3

,

PB2

Pos. 77		obs:	exp:
tta	L	0	93.98
ttg	L	0	197.20
ctt	L	975	176.30
ctc	L	6	176.70
cta	L	0	178.50
ctg	L	78	236.30
---	--	------	------
mPD		0.15	1.1
		nPD:	0.13
		N. weight:	1.6
		Sc. PD:	0.051
		Sc. rank:	745.7

,

PB2

Pos. 78		obs:	exp:
tgg	W	1059	1059.00
---	--	------	------
mPD		0	0
		nPD:	1.
		N. weight:	0.
		Sc. PD:	0
		Sc. rank:	0

,

PB2

Pos. 79		obs:	exp:
tct	S	0	168.40
tcc	S	0	138.30
tca	S	0	268.80
tcg	S	0	60.09
agt	S	32	217.70
agc	S	1027	205.70
---	--	------	------
mPD		0.059	1.7
		nPD:	0.04
		N. weight:	1.7
		Sc. PD:	-0.1
		Sc. rank:	95.4

,

PB2

Pos. 80		obs:	exp:
cgt	R	0	0.17
cgc	R	0	0.20
cga	R	0	0.39
cgg	R	0	0.33
aat	N	6	3.06
aac	N	0	2.94
aaa	K	6	611.40
aag	K	1043	437.60
aga	R	0	1.83
agg	R	4	1.08
---	--	------	------
mPD		0.030	0.50
		nPD:	0.06
		N. weight:	0.92
		Sc. PD:	-0.035
		Sc. rank:	241.7

,

PB2

Pos. 81		obs:	exp:
att	I	0	1.47
atc	I	0	0.96
ata	I	4	1.57
act	T	0	268.90
acc	T	0	216.50
aca	T	1055	483.90
acg	T	0	85.69
---	--	------	------
mPD		0.0075	0.68
		nPD:	0.01
		N. weight:	0.86
		Sc. PD:	-0.073
		Sc. rank:	-348.8

,

PB2

Pos. 82		obs:	exp:
tct	S	0	16.06
tcc	S	0	13.19
tca	S	0	25.63
tcg	S	0	5.73
aat	N	958	488.00
aac	N	0	470.00
agt	S	101	20.76
agc	S	0	19.62
---	--	------	------
mPD		0.17	0.81
		nPD:	0.21
		N. weight:	0.84
		Sc. PD:	0.09
		Sc. rank:	477.2

,

PB2

Pos. 83		obs:	exp:
gat	D	1059	572.30
gac	D	0	486.70
---	--	------	------
mPD		0	0.50
		nPD:	0.
		N. weight:	0.68
		Sc. PD:	-0.065
		Sc. rank:	-561.9

,

PB2

Pos. 84		obs:	exp:
act	T	1	0.25
acc	T	0	0.21
aca	T	0	0.46
acg	T	0	0.08
gct	A	822	273.20
gcc	A	234	197.00
gca	A	2	498.00
gcg	A	0	89.79
---	--	------	------
mPD		0.35	0.67
		nPD:	0.52
		N. weight:	0.98
		Sc. PD:	0.39
		Sc. rank:	745.9

,

PB2

Pos. 85		obs:	exp:
ggt	G	0	140.40
ggc	G	0	135.30
gga	G	1036	488.50
ggg	G	23	294.80
---	--	------	------
mPD		0.043	0.68
		nPD:	0.06
		N. weight:	0.75
		Sc. PD:	-0.026
		Sc. rank:	199.6

,

PB2

Pos. 86		obs:	exp:
tct	S	2	168.30
tcc	S	0	138.20
tca	S	1056	268.50
tcg	S	0	60.03
agt	S	0	217.50
agc	S	0	205.50
gct	A	0	0.26
gcc	A	0	0.19
gca	A	1	0.47
gcg	A	0	0.08
---	--	------	------
mPD		0.0057	1.7
		nPD:	0.
		N. weight:	1.5
		Sc. PD:	-0.14
		Sc. rank:	-982.5

,

PB2

Pos. 87		obs:	exp:
gat	D	17	569.60
gac	D	1037	484.40
gaa	E	5	3.04
gag	E	0	1.96
---	--	------	------
mPD		0.041	0.50
		nPD:	0.08
		N. weight:	0.76
		Sc. PD:	-0.013
		Sc. rank:	253.8

,

PB2

Pos. 88		obs:	exp:
cgt	R	0	45.40
cgc	R	0	52.35
cga	R	0	103.40
cgg	R	0	88.20
aaa	K	2	1.17
aag	K	0	0.83
aga	R	802	483.30
agg	R	255	284.30
---	--	------	------
mPD		0.37	0.96
		nPD:	0.38
		N. weight:	0.4
		Sc. PD:	0.11
		Sc. rank:	284.1

,

PB2

Pos. 89		obs:	exp:
atg	M	1	1.00
gtt	V	2	219.20
gtc	V	0	213.50
gta	V	40	216.60
gtg	V	1016	408.60
---	--	------	------
mPD		0.078	0.73
		nPD:	0.11
		N. weight:	0.88
		Sc. PD:	0.0068
		Sc. rank:	363.9

,

PB2

Pos. 90		obs:	exp:
atg	M	1059	1059.00
---	--	------	------
mPD		0	0
		nPD:	1.
		N. weight:	0.
		Sc. PD:	0
		Sc. rank:	0

,

PB2

Pos. 91		obs:	exp:
gtt	V	2	219.40
gtc	V	0	213.70
gta	V	105	216.80
gtg	V	952	409.00
---	--	------	------
mPD		0.18	0.73
		nPD:	0.25
		N. weight:	0.75
		Sc. PD:	0.11
		Sc. rank:	466.4

,

PB2

Pos. 92		obs:	exp:
tct	S	1	168.40
tcc	S	0	138.30
tca	S	1053	268.80
tcg	S	5	60.09
agt	S	0	217.70
agc	S	0	205.70
---	--	------	------
mPD		0.011	1.7
		nPD:	0.01
		N. weight:	1.5
		Sc. PD:	-0.13
		Sc. rank:	-790.9

,

PB2

Pos. 93		obs:	exp:
cct	P	1054	264.20
ccc	P	5	190.30
cca	P	0	435.90
ccg	P	0	168.60
---	--	------	------
mPD		0.0094	0.71
		nPD:	0.01
		N. weight:	1.5
		Sc. PD:	-0.12
		Sc. rank:	-494.7

,

PB2

Pos. 94		obs:	exp:
tta	L	0	93.89
ttg	L	6	197.00
ctt	L	0	176.20
ctc	L	0	176.60
cta	L	4	178.30
ctg	L	1048	236.10
caa	Q	0	0.51
cag	Q	1	0.49
---	--	------	------
mPD		0.021	1.1
		nPD:	0.02
		N. weight:	1.6
		Sc. PD:	-0.12
		Sc. rank:	-303.3

,

PB2

Pos. 95		obs:	exp:
gct	A	1050	273.50
gcc	A	9	197.20
gca	A	0	498.40
gcg	A	0	89.88
---	--	------	------
mPD		0.017	0.67
		nPD:	0.03
		N. weight:	1.4
		Sc. PD:	-0.1
		Sc. rank:	-109.9

,

PB2

Pos. 96		obs:	exp:
gtt	V	2	219.40
gtc	V	0	213.70
gta	V	8	216.80
gtg	V	1049	409.00
---	--	------	------
mPD		0.019	0.73
		nPD:	0.03
		N. weight:	0.99
		Sc. PD:	-0.07
		Sc. rank:	-66.0

,

PB2

Pos. 97		obs:	exp:
act	T	1	269.90
acc	T	0	217.30
aca	T	13	485.70
acg	T	1045	86.02
---	--	------	------
mPD		0.026	0.68
		nPD:	0.04
		N. weight:	2.7
		Sc. PD:	-0.16
		Sc. rank:	211.2

,

PB2

Pos. 98		obs:	exp:
tgg	W	1059	1059.00
---	--	------	------
mPD		0	0
		nPD:	1.
		N. weight:	0.
		Sc. PD:	0
		Sc. rank:	0

,

PB2

Pos. 99		obs:	exp:
tgg	W	1059	1059.00
---	--	------	------
mPD		0	0
		nPD:	1.
		N. weight:	0.
		Sc. PD:	0
		Sc. rank:	0

,

PB2

Pos. 100		obs:	exp:
aat	N	151	539.40
aac	N	908	519.60
---	--	------	------
mPD		0.24	0.50
		nPD:	0.49
		N. weight:	0.33
		Sc. PD:	0.12
		Sc. rank:	249.6

,

PB2			
Pos.	101	obs:	exp:
cgt	R	0	45.48
cgc	R	0	52.45
cga	R	0	103.60
cgg	R	0	88.36
aga	R	1056	484.20
agg	R	3	284.80
---	--	------	------
mPD		0.0057	0.96
		nPD:	0.01
		N. weight:	0.84
		Sc. PD:	-0.076
		Sc. rank:	-474.4

PB2			
Pos.	102	obs:	exp:
aat	N	1051	538.40
aac	N	6	518.60
aaa	K	0	1.17
aag	K	2	0.83
---	--	------	------
mPD		0.015	0.50
		nPD:	0.03
		N. weight:	0.71
		Sc. PD:	-0.047
		Sc. rank:	0.7

PB2			
Pos.	103	obs:	exp:
ggt	G	1	140.40
ggc	G	0	135.30
gga	G	1051	488.50
ggg	G	7	294.80
---	--	------	------
mPD		0.015	0.68
		nPD:	0.02
		N. weight:	0.81
		Sc. PD:	-0.06
		Sc. rank:	-94.3

PB2			
Pos.	104	obs:	exp:
tta	L	0	0.27
ttg	L	0	0.56
ctt	L	0	0.50
ctc	L	0	0.50
cta	L	3	0.51
ctg	L	0	0.67
cct	P	0	263.50
ccc	P	0	189.70
cca	P	1041	434.70
ccg	P	15	168.10
---	--	------	------
mPD		0.034	0.72
		nPD:	0.05
		N. weight:	0.91
		Sc. PD:	-0.046
		Sc. rank:	128.2

PB2			
Pos.	105	obs:	exp:
att	I	0	1.47
atc	I	0	0.96
ata	I	4	1.57
atg	M	2	2.00
act	T	3	268.20
acc	T	0	215.90
aca	T	383	482.50
acg	T	666	85.45
gct	A	0	0.26
gcc	A	0	0.19
gca	A	0	0.47
gcg	A	1	0.08
---	--	------	------
mPD		0.48	0.69
		nPD:	0.7
		N. weight:	1.3
		Sc. PD:	0.76
		Sc. rank:	1077.5

PB2			
Pos.	106	obs:	exp:
att	I	0	0.37
atc	I	0	0.24
ata	I	1	0.39
act	T	0	260.80
acc	T	0	209.90
aca	T	1022	469.20
acg	T	1	83.09
gct	A	0	9.04
gcc	A	0	6.52
gca	A	35	16.47
gcg	A	0	2.97
---	--	------	------
mPD		0.068	0.74
		nPD:	0.09
		N. weight:	0.85
		Sc. PD:	-0.0067
		Sc. rank:	316.1

PB2			
Pos.	107	obs:	exp:
tct	S	0	163.70
tcc	S	0	134.40
tca	S	0	261.20
tcg	S	0	58.39
aat	N	1	15.28
aac	N	29	14.72
agt	S	339	211.50
agc	S	690	199.90
---	--	------	------
mPD		0.49	1.7
		nPD:	0.29
		N. weight:	1.1
		Sc. PD:	0.2
		Sc. rank:	734.5

PB2			
Pos.	108	obs:	exp:
act	T	1	269.90
acc	T	0	217.30
aca	T	1058	485.70
acg	T	0	86.02
---	--	------	------
mPD		0.0019	0.68
		nPD:	0.
		N. weight:	0.85
		Sc. PD:	-0.079
		Sc. rank:	-593.8

PB2			
Pos.	109	obs:	exp:
att	I	0	30.50
atc	I	83	19.89
ata	I	0	32.61
act	T	0	0.25
acc	T	1	0.21
aca	T	0	0.46
acg	T	0	0.08
gtt	V	4	200.60
gtc	V	964	195.40
gta	V	0	198.20
gtg	V	0	373.90
gct	A	0	1.81
gcc	A	7	1.30
gca	A	0	3.29
gcg	A	0	0.59
---	--	------	------
mPD		0.17	0.90
		nPD:	0.19
		N. weight:	1.7
		Sc. PD:	0.15
		Sc. rank:	894.8

PB2			
Pos.	110	obs:	exp:
cat	H	998	608.10
cac	H	61	450.90
---	--	------	------
mPD		0.11	0.49
		nPD:	0.22
		N. weight:	0.39
		Sc. PD:	0.046
		Sc. rank:	229.2

PB2			
Pos.	111	obs:	exp:
tat	Y	1054	572.00
tac	Y	5	487.00
---	--	------	------
mPD		0.0094	0.50
		nPD:	0.02
		N. weight:	0.65
		Sc. PD:	-0.05
		Sc. rank:	-113.3

PB2			
Pos.	112	obs:	exp:
cct	P	0	264.20
ccc	P	0	190.30
cca	P	1055	435.90
ccg	P	4	168.60
---	--	------	------
mPD		0.0075	0.71
		nPD:	0.01
		N. weight:	0.95
		Sc. PD:	-0.081
		Sc. rank:	-399.5

PB2			
Pos.	113	obs:	exp:
aaa	K	19	617.20
aag	K	1040	441.80
---	--	------	------
mPD		0.035	0.49
		nPD:	0.07
		N. weight:	0.86
		Sc. PD:	-0.022
		Sc. rank:	263.1

PB2			
Pos.	114	obs:	exp:
att	I	0	1.10
atc	I	3	0.72
ata	I	0	1.18
gtt	V	19	218.80
gtc	V	1033	213.10
gta	V	4	216.20
gtg	V	0	407.80
---	--	------	------
mPD		0.048	0.73
		nPD:	0.07
		N. weight:	1.6
		Sc. PD:	-0.053
		Sc. rank:	473.6

PB2			
Pos.	115	obs:	exp:
tat	Y	820	572.00
tac	Y	239	487.00
---	--	------	------
mPD		0.35	0.50
		nPD:	0.7
		N. weight:	0.13
		Sc. PD:	0.078
		Sc. rank:	109.2

PB2			
Pos.	116	obs:	exp:
cgt	R	0	0.09
cgc	R	0	0.10
cga	R	0	0.20
cgg	R	0	0.17
aaa	K	1055	616.00
aag	K	2	441.00
aga	R	2	0.91
agg	R	0	0.54
---	--	------	------
mPD		0.0075	0.49
		nPD:	0.02
		N. weight:	0.58
		Sc. PD:	-0.047
		Sc. rank:	-158.0

PB2			
Pos.	117	obs:	exp:
cct	P	0	2.99
ccc	P	12	2.16
cca	P	0	4.94
ccg	P	0	1.91
att	I	0	0.37
atc	I	1	0.24
ata	I	0	0.39
act	T	14	265.40
acc	T	1023	213.60
aca	T	4	477.50
acg	T	0	84.56
aat	N	0	1.02
aac	N	2	0.98
gct	A	0	0.77
gcc	A	3	0.56
gca	A	0	1.41
gcg	A	0	0.25
---	--	------	------
mPD		0.067	0.71
		nPD:	0.09
		N. weight:	1.6
		Sc. PD:	-0.0078
		Sc. rank:	628.8

PB2			
Pos.	118	obs:	exp:
tat	Y	1058	572.00
tac	Y	1	487.00
---	--	------	------
mPD		0.0019	0.50
		nPD:	0.
		N. weight:	0.67
		Sc. PD:	-0.062
		Sc. rank:	-436.4

PB2			
Pos.	119	obs:	exp:
ttt	F	1055	418.20
ttc	F	4	640.80
---	--	------	------
mPD		0.0075	0.48
		nPD:	0.02
		N. weight:	0.99
		Sc. PD:	-0.08
		Sc. rank:	-242.0

PB2			
Pos.	120	obs:	exp:
aaa	K	1	0.58
aag	K	0	0.42
gat	D	0	0.54
gac	D	1	0.46
gaa	E	1012	643.20
gag	E	45	413.80
---	--	------	------
mPD		0.085	0.48
		nPD:	0.18
		N. weight:	0.38
		Sc. PD:	0.028
		Sc. rank:	190.2

PB2			
Pos.	121	obs:	exp:
cgt	R	0	0.04
cgc	R	0	0.05
cga	R	0	0.10
cgg	R	0	0.08
aaa	K	4	616.60
aag	K	1054	441.40
aga	R	1	0.46
agg	R	0	0.27
---	--	------	------
mPD		0.011	0.49
		nPD:	0.02
		N. weight:	0.93
		Sc. PD:	-0.069
		Sc. rank:	-104.4

PB2			
Pos.	122	obs:	exp:
ttt	F	1	0.39
ttc	F	0	0.61
att	I	0	0.73
atc	I	2	0.48
ata	I	0	0.79
gtt	V	72	201.00
gtc	V	897	195.80
gta	V	1	198.60
gtg	V	0	374.60
gct	A	2	22.21
gcc	A	43	16.02
gca	A	41	40.48
gcg	A	0	7.30
---	--	------	------
mPD		0.36	0.89
		nPD:	0.4
		N. weight:	1.4
		Sc. PD:	0.4
		Sc. rank:	1018.0

PB2			
Pos.	123	obs:	exp:
aaa	K	3	1.75
aag	K	0	1.25
gaa	E	1052	642.60
gag	E	4	413.40
---	--	------	------
mPD		0.013	0.48
		nPD:	0.03
		N. weight:	0.52
		Sc. PD:	-0.036
		Sc. rank:	-20.1

PB2			
Pos.	124	obs:	exp:
cgt	R	0	45.48
cgc	R	0	52.45
cga	R	0	103.60
cgg	R	0	88.36
aga	R	350	484.20
agg	R	709	284.80
---	--	------	------
mPD		0.44	0.96
		nPD:	0.46
		N. weight:	0.56
		Sc. PD:	0.19
		Sc. rank:	418.9

PB2			
Pos.	125	obs:	exp:
tta	L	2	93.98
ttg	L	1	197.20
ctt	L	0	176.30
ctc	L	2	176.70
cta	L	981	178.50
ctg	L	73	236.30
---	--	------	------
mPD		0.14	1.1
		nPD:	0.13
		N. weight:	1.6
		Sc. PD:	0.041
		Sc. rank:	728.2

PB2			
Pos.	126	obs:	exp:
aaa	K	1056	617.20
aag	K	3	441.80
---	--	------	------
mPD		0.0057	0.49
		nPD:	0.01
		N. weight:	0.57
		Sc. PD:	-0.048
		Sc. rank:	-216.1

PB2			
Pos.	127	obs:	exp:
cat	H	1053	607.50
cac	H	5	450.50
aat	N	1	0.51
aac	N	0	0.49
---	--	------	------
mPD		0.011	0.49
		nPD:	0.02
		N. weight:	0.58
		Sc. PD:	-0.043
		Sc. rank:	-66.1

PB2			
Pos.	128	obs:	exp:
ggt	G	112	140.40
ggc	G	0	135.30
gga	G	662	488.50
ggg	G	285	294.80
---	--	------	------
mPD		0.53	0.68
		nPD:	0.78
		N. weight:	0.18
		Sc. PD:	0.12
		Sc. rank:	149.7

PB2			
Pos.	129	obs:	exp:
att	I	0	2.20
atc	I	6	1.44
ata	I	0	2.36
act	T	7	268.40
acc	T	1043	216.10
aca	T	3	483.00
acg	T	0	85.53
---	--	------	------
mPD		0.030	0.69
		nPD:	0.04
		N. weight:	1.7
		Sc. PD:	-0.09
		Sc. rank:	194.1

PB2			
Pos.	130	obs:	exp:
ttt	F	256	418.20
ttc	F	803	640.80
---	--	------	------
mPD		0.37	0.48
		nPD:	0.77
		N. weight:	0.061
		Sc. PD:	0.039
		Sc. rank:	51.1

PB2			
Pos.	131	obs:	exp:
ggt	G	5	140.40
ggc	G	1050	135.30
gga	G	3	488.50
ggg	G	1	294.80
---	--	------	------
mPD		0.017	0.68
		nPD:	0.03
		N. weight:	2.2
		Sc. PD:	-0.16
		Sc. rank:	-174.7

PB2			
Pos.	132	obs:	exp:
cct	P	5	264.20
ccc	P	1052	190.30
cca	P	2	435.90
ccg	P	0	168.60
---	--	------	------
mPD		0.013	0.71
		nPD:	0.02
		N. weight:	1.8
		Sc. PD:	-0.14
		Sc. rank:	-373.2

PB2			
Pos.	133	obs:	exp:
att	I	1	0.37
atc	I	0	0.24
ata	I	0	0.39
gtt	V	998	219.20
gtc	V	55	213.50
gta	V	1	216.60
gtg	V	4	408.60
---	--	------	------
mPD		0.11	0.73
		nPD:	0.15
		N. weight:	1.5
		Sc. PD:	0.072
		Sc. rank:	699.8

PB2			
Pos.	134	obs:	exp:
cat	H	4	607.50
cac	H	1054	450.50
cgt	R	0	0.04
cgc	R	1	0.05
cga	R	0	0.10
cgg	R	0	0.08
aga	R	0	0.46
agg	R	0	0.27
---	--	------	------
mPD		0.0094	0.49
		nPD:	0.02
		N. weight:	0.91
		Sc. PD:	-0.071
		Sc. rank:	-158.2

PB2			
Pos.	135	obs:	exp:
ttt	F	6	418.20
ttc	F	1053	640.80
---	--	------	------
mPD		0.011	0.48
		nPD:	0.02
		N. weight:	0.52
		Sc. PD:	-0.038
		Sc. rank:	-50.5

PB2			
Pos.	136	obs:	exp:
cgt	R	0	45.48
cgc	R	0	52.45
cga	R	686	103.60
cgg	R	5	88.36
aga	R	368	484.20
agg	R	0	284.80
---	--	------	------
mPD		0.46	0.96
		nPD:	0.48
		N. weight:	1.2
		Sc. PD:	0.45
		Sc. rank:	930.5

PB2			
Pos.	137	obs:	exp:
aat	N	33	539.40
aac	N	1026	519.60
---	--	------	------
mPD		0.060	0.50
		nPD:	0.12
		N. weight:	0.63
		Sc. PD:	0.013
		Sc. rank:	278.7

PB2			
Pos.	138	obs:	exp:
caa	Q	88	539.40
cag	Q	971	519.60
---	--	------	------
mPD		0.15	0.50
		nPD:	0.31
		N. weight:	0.47
		Sc. PD:	0.092
		Sc. rank:	322.6

PB2			
Pos.	139	obs:	exp:
att	I	9	29.03
atc	I	70	18.93
ata	I	0	31.04
gtt	V	972	203.10
gtc	V	6	197.80
gta	V	1	200.70
gtg	V	1	378.50
---	--	------	------
mPD		0.28	0.87
		nPD:	0.32
		N. weight:	1.6
		Sc. PD:	0.34
		Sc. rank:	1143.9

PB2			
Pos.	140	obs:	exp:
cgt	R	0	0.04
cgc	R	0	0.05
cga	R	0	0.10
cgg	R	0	0.08
aaa	K	1054	616.60
aag	K	4	441.40
aga	R	1	0.46
agg	R	0	0.27
---	--	------	------
mPD		0.0094	0.49
		nPD:	0.02
		N. weight:	0.57
		Sc. PD:	-0.044
		Sc. rank:	-90.6

PB2			
Pos.	141	obs:	exp:
att	I	0	388.70
atc	I	5	253.50
ata	I	1053	415.70
atg	M	1	1.00
---	--	------	------
mPD		0.011	0.65
		nPD:	0.02
		N. weight:	1.
		Sc. PD:	-0.079
		Sc. rank:	-218.7

PB2			
Pos.	142	obs:	exp:
cgt	R	4	45.48
cgc	R	796	52.45
cga	R	257	103.60
cgg	R	0	88.36
aga	R	2	484.20
agg	R	0	284.80
---	--	------	------
mPD		0.38	0.96
		nPD:	0.39
		N. weight:	2.5
		Sc. PD:	0.7
		Sc. rank:	1798.4

PB2			
Pos.	143	obs:	exp:
cgt	R	11	45.48
cgc	R	1047	52.45
cga	R	1	103.60
cgg	R	0	88.36
aga	R	0	484.20
agg	R	0	284.80
---	--	------	------
mPD		0.022	0.96
		nPD:	0.02
		N. weight:	3.2
		Sc. PD:	-0.24
		Sc. rank:	-332.2

PB2			
Pos.	144	obs:	exp:
cgt	R	0	45.44
cgc	R	0	52.40
cga	R	1	103.50
cgg	R	0	88.28
aaa	K	0	0.58
aag	K	1	0.42
aga	R	8	483.80
agg	R	1049	284.60
---	--	------	------
mPD		0.021	0.96
		nPD:	0.02
		N. weight:	1.4
		Sc. PD:	-0.1
		Sc. rank:	-171.8

PB2			
Pos.	145	obs:	exp:
gtt	V	92	219.40
gtc	V	965	213.70
gta	V	0	216.80
gtg	V	2	409.00
---	--	------	------
mPD		0.16	0.73
		nPD:	0.22
		N. weight:	1.4
		Sc. PD:	0.17
		Sc. rank:	846.7

PB2			
Pos.	146	obs:	exp:
gat	D	6	572.30
gac	D	1053	486.70
---	--	------	------
mPD		0.011	0.50
		nPD:	0.02
		N. weight:	0.82
		Sc. PD:	-0.06
		Sc. rank:	-94.0

PB2			
Pos.	147	obs:	exp:
att	I	0	382.90
atc	I	0	249.70
ata	I	1042	409.40
atg	M	1	1.00
act	T	0	3.06
acc	T	0	2.46
aca	T	12	5.50
acg	T	0	0.97
gtt	V	0	0.83
gtc	V	0	0.81
gta	V	4	0.82
gtg	V	0	1.54
---	--	------	------
mPD		0.032	0.69
		nPD:	0.05
		N. weight:	1.
		Sc. PD:	-0.052
		Sc. rank:	138.0

PB2			
Pos.	148	obs:	exp:
aat	N	8	539.40
aac	N	1051	519.60
---	--	------	------
mPD		0.015	0.50
		nPD:	0.03
		N. weight:	0.74
		Sc. PD:	-0.049
		Sc. rank:	1.9

PB2			
Pos.	149	obs:	exp:
cct	P	76	264.20
ccc	P	0	190.30
cca	P	632	435.90
ccg	P	351	168.60
---	--	------	------
mPD		0.53	0.71
		nPD:	0.74
		N. weight:	0.42
		Sc. PD:	0.26
		Sc. rank:	347.2

PB2			
Pos.	150	obs:	exp:
ggt	G	7	140.40
ggc	G	987	135.30
gga	G	63	488.50
ggg	G	2	294.80
---	--	------	------
mPD		0.13	0.68
		nPD:	0.19
		N. weight:	1.9
		Sc. PD:	0.16
		Sc. rank:	984.1

PB2			
Pos.	151	obs:	exp:
cat	H	1010	607.50
cac	H	48	450.50
aat	N	1	0.51
aac	N	0	0.49
---	--	------	------
mPD		0.089	0.49
		nPD:	0.18
		N. weight:	0.42
		Sc. PD:	0.033
		Sc. rank:	217.4

PB2			
Pos.	152	obs:	exp:
tct	S	0	1.91
tcc	S	0	1.57
tca	S	12	3.05
tcg	S	0	0.68
act	T	0	0.51
acc	T	0	0.41
aca	T	2	0.92
acg	T	0	0.16
agt	S	0	2.47
agc	S	0	2.33
gct	A	1	269.90
gcc	A	1	194.60
gca	A	1043	491.80
gcg	A	0	88.69
---	--	------	------
mPD		0.030	0.71
		nPD:	0.04
		N. weight:	0.82
		Sc. PD:	-0.045
		Sc. rank:	87.3

PB2			
Pos.	153	obs:	exp:
aat	N	1	0.51
aac	N	0	0.49
gat	D	1045	571.70
gac	D	13	486.30
---	--	------	------
mPD		0.026	0.50
		nPD:	0.05
		N. weight:	0.61
		Sc. PD:	-0.028
		Sc. rank:	117.3

PB2			
Pos.	154	obs:	exp:
tta	L	0	93.98
ttg	L	0	197.20
ctt	L	1025	176.30
ctc	L	34	176.70
cta	L	0	178.50
ctg	L	0	236.30
---	--	------	------
mPD		0.062	1.1
		nPD:	0.06
		N. weight:	1.8
		Sc. PD:	-0.076
		Sc. rank:	409.7

PB2			
Pos.	155	obs:	exp:
tct	S	0	168.10
tcc	S	0	138.00
tca	S	0	268.30
tcg	S	0	59.98
aat	N	2	1.02
aac	N	0	0.98
agt	S	1054	217.30
agc	S	3	205.30
---	--	------	------
mPD		0.0094	1.7
		nPD:	0.01
		N. weight:	1.7
		Sc. PD:	-0.15
		Sc. rank:	-999.1

PB2			
Pos.	156	obs:	exp:
act	T	3	0.76
acc	T	0	0.62
aca	T	0	1.38
acg	T	0	0.24
gtt	V	2	0.41
gtc	V	0	0.40
gta	V	0	0.41
gtg	V	0	0.77
gct	A	1045	272.20
gcc	A	8	196.30
gca	A	0	496.10
gcg	A	1	89.46
---	--	------	------
mPD		0.026	0.68
		nPD:	0.04
		N. weight:	1.4
		Sc. PD:	-0.084
		Sc. rank:	124.3

PB2			
Pos.	157	obs:	exp:
cgt	R	0	0.04
cgc	R	0	0.05
cga	R	0	0.10
cgg	R	0	0.08
aaa	K	665	616.60
aag	K	393	441.40
aga	R	0	0.46
agg	R	1	0.27
---	--	------	------
mPD		0.47	0.49
		nPD:	0.96
		N. weight:	0.0099
		Sc. PD:	0.0082
		Sc. rank:	8.3

PB2			
Pos.	158	obs:	exp:
gaa	E	1056	644.40
gag	E	3	414.60
---	--	------	------
mPD		0.0057	0.48
		nPD:	0.01
		N. weight:	0.53
		Sc. PD:	-0.044
		Sc. rank:	-187.6

PB2			
Pos.	159	obs:	exp:
gct	A	0	273.50
gcc	A	0	197.20
gca	A	1057	498.40
gcg	A	2	89.88
---	--	------	------
mPD		0.0038	0.67
		nPD:	0.01
		N. weight:	0.82
		Sc. PD:	-0.074
		Sc. rank:	-482.3

PB2			
Pos.	160	obs:	exp:
caa	Q	715	539.40
cag	Q	344	519.60
---	--	------	------
mPD		0.44	0.50
		nPD:	0.88
		N. weight:	0.066
		Sc. PD:	0.049
		Sc. rank:	54.8

PB2			
Pos.	161	obs:	exp:
gat	D	1052	572.30
gac	D	7	486.70
---	--	------	------
mPD		0.013	0.50
		nPD:	0.03
		N. weight:	0.64
		Sc. PD:	-0.045
		Sc. rank:	-36.9

PB2			
Pos.	162	obs:	exp:
gtt	V	1	219.40
gtc	V	1058	213.70
gta	V	0	216.80
gtg	V	0	409.00
---	--	------	------
mPD		0.0019	0.73
		nPD:	0.
		N. weight:	1.8
		Sc. PD:	-0.16
		Sc. rank:	-1257.7

PB2			
Pos.	163	obs:	exp:
att	I	24	389.10
atc	I	1030	253.80
ata	I	5	416.10
---	--	------	------
mPD		0.054	0.65
		nPD:	0.08
		N. weight:	1.4
		Sc. PD:	-0.024
		Sc. rank:	478.8

PB2			
Pos.	164	obs:	exp:
atg	M	1059	1059.00
---	--	------	------
mPD		0	0
		nPD:	1.
		N. weight:	0.
		Sc. PD:	0
		Sc. rank:	0

PB2			
Pos.	165	obs:	exp:
gaa	E	27	644.40
gag	E	1032	414.60
---	--	------	------
mPD		0.050	0.48
		nPD:	0.1
		N. weight:	0.89
		Sc. PD:	0.0041
		Sc. rank:	359.1

PB2			
Pos.	166	obs:	exp:
att	I	0	0.37
atc	I	1	0.24
ata	I	0	0.39
gtt	V	4	219.20
gtc	V	1043	213.50
gta	V	11	216.60
gtg	V	0	408.60
---	--	------	------
mPD		0.030	0.73
		nPD:	0.04
		N. weight:	1.7
		Sc. PD:	-0.094
		Sc. rank:	170.9

PB2			
Pos.	167	obs:	exp:
gtt	V	5	219.40
gtc	V	2	213.70
gta	V	1048	216.80
gtg	V	4	409.00
---	--	------	------
mPD		0.021	0.73
		nPD:	0.03
		N. weight:	1.7
		Sc. PD:	-0.11
		Sc. rank:	-17.5

PB2			
Pos.	168	obs:	exp:
ttt	F	3	418.20
ttc	F	1056	640.80
---	--	------	------
mPD		0.0057	0.48
		nPD:	0.01
		N. weight:	0.53
		Sc. PD:	-0.045
		Sc. rank:	-196.2

PB2			
Pos.	169	obs:	exp:
cct	P	0	264.20
ccc	P	0	190.30
cca	P	1056	435.90
ccg	P	3	168.60
---	--	------	------
mPD		0.0057	0.71
		nPD:	0.01
		N. weight:	0.96
		Sc. PD:	-0.084
		Sc. rank:	-477.5

PB2			
Pos.	170	obs:	exp:
aat	N	26	539.40
aac	N	1033	519.60
---	--	------	------
mPD		0.048	0.50
		nPD:	0.1
		N. weight:	0.66
		Sc. PD:	-0.0023
		Sc. rank:	256.5

PB2

Pos.	171	obs:	exp:
gaa	E	1058	644.40
gag	E	1	414.60
---	--	------	------
mPD		0.0019	0.48
		nPD:	0.
		N. weight:	0.54
		Sc. PD:	-0.049
		Sc. rank:	-330.7

PB2

Pos.	172	obs:	exp:
gtt	V	1058	219.40
gtc	V	0	213.70
gta	V	1	216.80
gtg	V	0	409.00
---	--	------	------
mPD		0.0019	0.73
		nPD:	0.
		N. weight:	1.7
		Sc. PD:	-0.16
		Sc. rank:	-1237.0

PB2

Pos.	173	obs:	exp:
ggt	G	0	140.40
ggc	G	0	135.30
gga	G	1051	488.50
ggg	G	8	294.80
---	--	------	------
mPD		0.015	0.68
		nPD:	0.02
		N. weight:	0.81
		Sc. PD:	-0.06
		Sc. rank:	-95.9

PB2

Pos.	174	obs:	exp:
gct	A	17	273.50
gcc	A	1023	197.20
gca	A	19	498.40
gcg	A	0	89.88
---	--	------	------
mPD		0.066	0.67
		nPD:	0.1
		N. weight:	1.6
		Sc. PD:	-0.00099
		Sc. rank:	653.7

PB2

Pos.	175	obs:	exp:
cgt	R	0	45.27
cgc	R	0	52.20
cga	R	88	103.10
cgg	R	0	87.95
aaa	K	5	2.91
aag	K	0	2.09
aga	R	964	481.90
agg	R	2	283.50
---	--	------	------
mPD		0.17	0.97
		nPD:	0.17
		N. weight:	0.68
		Sc. PD:	0.046
		Sc. rank:	336.5

PB2

Pos.	176	obs:	exp:
att	I	0	389.10
atc	I	3	253.80
ata	I	1056	416.10
---	--	------	------
mPD		0.0057	0.65
		nPD:	0.01
		N. weight:	1.
		Sc. PD:	-0.088
		Sc. rank:	-463.8

PB2

Pos.	177	obs:	exp:
tta	L	76	93.98
ttg	L	742	197.20
ctt	L	0	176.30
ctc	L	0	176.70
cta	L	0	178.50
ctg	L	241	236.30
---	--	------	------
mPD		0.49	1.1
		nPD:	0.44
		N. weight:	1.
		Sc. PD:	0.33
		Sc. rank:	757.5

PB2

Pos.	178	obs:	exp:
act	T	0	269.70
acc	T	1	217.10
aca	T	1055	485.30
acg	T	2	85.94
gct	A	0	0.26
gcc	A	0	0.19
gca	A	1	0.47
gcg	A	0	0.08
---	--	------	------
mPD		0.0075	0.68
		nPD:	0.01
		N. weight:	0.84
		Sc. PD:	-0.071
		Sc. rank:	-340.2

PB2

Pos.	179	obs:	exp:
tct	S	4	168.40
tcc	S	1	138.30
tca	S	1052	268.80
tcg	S	2	60.09
agt	S	0	217.70
agc	S	0	205.70
---	--	------	------
mPD		0.013	1.7
		nPD:	0.01
		N. weight:	1.5
		Sc. PD:	-0.13
		Sc. rank:	-722.5

PB2

Pos.	180	obs:	exp:
gaa	E	21	644.40
gag	E	1038	414.60
---	--	------	------
mPD		0.039	0.48
		nPD:	0.08
		N. weight:	0.92
		Sc. PD:	-0.016
		Sc. rank:	307.0

PB2

Pos.	181	obs:	exp:
tct	S	0	167.80
tcc	S	2	137.80
tca	S	1048	267.80
tcg	S	5	59.86
cct	P	0	1.00
ccc	P	0	0.72
cca	P	1	1.65
ccg	P	3	0.64
agt	S	0	216.90
agc	S	0	204.90
---	--	------	------
mPD		0.026	1.7
		nPD:	0.02
		N. weight:	1.5
		Sc. PD:	-0.12
		Sc. rank:	-353.4

PB2

Pos.	182	obs:	exp:
caa	Q	24	539.40
cag	Q	1035	519.60
---	--	------	------
mPD		0.044	0.50
		nPD:	0.09
		N. weight:	0.66
		Sc. PD:	-0.007
		Sc. rank:	240.2

PB2

Pos.	183	obs:	exp:
tta	L	1027	93.89
ttg	L	30	197.00
tct	S	0	0.16
tcc	S	0	0.13
tca	S	1	0.25
tcg	S	0	0.06
ctt	L	0	176.20
ctc	L	0	176.60
cta	L	1	178.30
ctg	L	0	236.10
agt	S	0	0.21
agc	S	0	0.19
---	--	------	------
mPD		0.059	1.1
		nPD:	0.05
		N. weight:	2.5
		Sc. PD:	-0.11
		Sc. rank:	494.0

PB2

Pos.	184	obs:	exp:
atg	M	11	11.00
act	T	4	266.90
acc	T	0	214.80
aca	T	6	480.20
acg	T	1037	85.04
gct	A	0	0.26
gcc	A	0	0.19
gca	A	0	0.47
gcg	A	1	0.08
---	--	------	------
mPD		0.041	0.70
		nPD:	0.06
		N. weight:	2.7
		Sc. PD:	-0.1
		Sc. rank:	670.1

PB2

Pos.	185	obs:	exp:
att	I	348	388.70
atc	I	0	253.50
ata	I	710	415.70
gtt	V	0	0.21
gtc	V	0	0.20
gta	V	1	0.20
gtg	V	0	0.39
---	--	------	------
mPD		0.44	0.66
		nPD:	0.68
		N. weight:	0.36
		Sc. PD:	0.2
		Sc. rank:	290.6

PB2

Pos.	186	obs:	exp:
act	T	32	269.90
acc	T	1025	217.30
aca	T	2	485.70
acg	T	0	86.02
---	--	------	------
mPD		0.062	0.68
		nPD:	0.09
		N. weight:	1.6
		Sc. PD:	-0.011
		Sc. rank:	594.5

PB2

Pos.	187	obs:	exp:
aaa	K	12	617.20
aag	K	1047	441.80
---	--	------	------
mPD		0.022	0.49
		nPD:	0.05
		N. weight:	0.89
		Sc. PD:	-0.046
		Sc. rank:	117.7

PB2

Pos.	188	obs:	exp:
aaa	K	7	4.08
aag	K	0	2.92
gaa	E	1021	639.50
gag	E	30	411.50
ggt	G	0	0.13
ggc	G	0	0.13
gga	G	1	0.46
ggg	G	0	0.28
---	--	------	------
mPD		0.070	0.49
		nPD:	0.14
		N. weight:	0.42
		Sc. PD:	0.017
		Sc. rank:	197.0

PB2

Pos.	189	obs:	exp:
aaa	K	1	617.20
aag	K	1058	441.80
---	--	------	------
mPD		0.0019	0.49
		nPD:	0.
		N. weight:	0.95
		Sc. PD:	-0.088
		Sc. rank:	-612.1

PB2

Pos.	190	obs:	exp:
cgt	R	0	0.04
cgc	R	0	0.05
cga	R	0	0.10
cgg	R	0	0.08
aaa	K	14	616.60
aag	K	1044	441.40
aga	R	0	0.46
agg	R	1	0.27
---	--	------	------
mPD		0.028	0.49
		nPD:	0.06
		N. weight:	0.88
		Sc. PD:	-0.036
		Sc. rank:	201.7

PB2

Pos.	191	obs:	exp:
aaa	K	1	212.10
aag	K	363	151.90
gat	D	5	3.24
gac	D	1	2.76
gaa	E	11	419.30
gag	E	678	269.70
---	--	------	------
mPD		0.49	0.94
		nPD:	0.52
		N. weight:	0.93
		Sc. PD:	0.38
		Sc. rank:	712.3

PB2

Pos.	192	obs:	exp:
aaa	K	0	1.17
aag	K	2	0.83
gaa	E	0	643.20
gag	E	1057	413.80
---	--	------	------
mPD		0.0038	0.48
		nPD:	0.01
		N. weight:	1.
		Sc. PD:	-0.091
		Sc. rank:	-521.4

PB2

Pos.	193	obs:	exp:
ttt	F	5	1.97
ttc	F	0	3.03
tta	L	0	93.53
ttg	L	0	196.20
ctt	L	1042	175.50
ctc	L	7	175.90
cta	L	5	177.60
ctg	L	0	235.20
---	--	------	------
mPD		0.032	1.1
		nPD:	0.03
		N. weight:	1.9
		Sc. PD:	-0.13
		Sc. rank:	-4.2

PB2

Pos.	194	obs:	exp:
cat	H	3	1.72
cac	H	0	1.28
caa	Q	255	537.40
cag	Q	800	517.60
aaa	K	0	0.58
aag	K	1	0.42
---	--	------	------
mPD		0.37	0.50
		nPD:	0.74
		N. weight:	0.17
		Sc. PD:	0.1
		Sc. rank:	141.0

PB2

Pos.	195	obs:	exp:
aat	N	0	3.57
aac	N	7	3.43
gat	D	7	567.40
gac	D	1043	482.60
gaa	E	1	0.61
gag	E	0	0.39
ggt	G	0	0.13
ggc	G	1	0.13
gga	G	0	0.46
ggg	G	0	0.28
---	--	------	------
mPD		0.030	0.51
		nPD:	0.06
		N. weight:	0.81
		Sc. PD:	-0.032
		Sc. rank:	200.4

PB2			
Pos.	196	obs:	exp:
tgt	C	14	447.90
tgc	C	1045	611.10
---	--	------	------
mPD		0.026	0.49
		nPD:	0.05
		N. weight:	0.53
		Sc. PD:	-0.024
		Sc. rank:	109.2

PB2			
Pos.	197	obs:	exp:
cgt	R	0	0.26
cgc	R	0	0.30
cga	R	0	0.59
cgg	R	0	0.50
aaa	K	1043	613.70
aag	K	10	439.30
aga	R	6	2.74
agg	R	0	1.61
---	--	------	------
mPD		0.030	0.50
		nPD:	0.06
		N. weight:	0.54
		Sc. PD:	-0.021
		Sc. rank:	142.5

PB2			
Pos.	198	obs:	exp:
att	I	1003	389.10
atc	I	55	253.80
ata	I	1	416.10
---	--	------	------
mPD		0.10	0.65
		nPD:	0.15
		N. weight:	0.9
		Sc. PD:	0.046
		Sc. rank:	433.2

PB2			
Pos.	199	obs:	exp:
tct	S	0	0.80
tcc	S	5	0.65
tca	S	0	1.27
tcg	S	0	0.28
act	T	0	1.27
acc	T	5	1.03
aca	T	0	2.29
acg	T	0	0.41
agt	S	0	1.03
agc	S	0	0.97
gtt	V	0	0.21
gtc	V	1	0.20
gta	V	0	0.20
gtg	V	0	0.39
gct	A	101	270.60
gcc	A	947	195.20
gca	A	0	493.30
gcg	A	0	88.95
---	--	------	------
mPD		0.19	0.70
		nPD:	0.28
		N. weight:	1.5
		Sc. PD:	0.25
		Sc. rank:	987.9

PB2			
Pos.	200	obs:	exp:
cct	P	1055	264.20
ccc	P	4	190.30
cca	P	0	435.90
ccg	P	0	168.60
---	--	------	------
mPD		0.0075	0.71
		nPD:	0.01
		N. weight:	1.5
		Sc. PD:	-0.13
		Sc. rank:	-628.8

PB2			
Pos.	201	obs:	exp:
tta	L	1054	93.98
ttg	L	2	197.20
ctt	L	0	176.30
ctc	L	0	176.70
cta	L	3	178.50
ctg	L	0	236.30
---	--	------	------
mPD		0.0094	1.1
		nPD:	0.01
		N. weight:	2.6
		Sc. PD:	-0.23
		Sc. rank:	-1231.5

PB2			
Pos.	202	obs:	exp:
atg	M	1059	1059.00
---	--	------	------
mPD		0	0
		nPD:	1.
		N. weight:	0.
		Sc. PD:	0
		Sc. rank:	0

PB2			
Pos.	203	obs:	exp:
atg	M	2	2.00
gtt	V	2	219.00
gtc	V	0	213.30
gta	V	0	216.40
gtg	V	1055	408.20
---	--	------	------
mPD		0.0075	0.73
		nPD:	0.01
		N. weight:	1.
		Sc. PD:	-0.088
		Sc. rank:	-438.8

PB2			
Pos.	204	obs:	exp:
gct	A	697	273.50
gcc	A	20	197.20
gca	A	338	498.40
gcg	A	4	89.88
---	--	------	------
mPD		0.47	0.67
		nPD:	0.69
		N. weight:	0.49
		Sc. PD:	0.28
		Sc. rank:	395.4

PB2			
Pos.	205	obs:	exp:
tat	Y	103	572.00
tac	Y	956	487.00
---	--	------	------
mPD		0.18	0.50
		nPD:	0.35
		N. weight:	0.49
		Sc. PD:	0.12
		Sc. rank:	346.8

PB2			
Pos.	206	obs:	exp:
att	I	1	0.37
atc	I	0	0.24
ata	I	0	0.39
atg	M	1058	1058.00
---	--	------	------
mPD		0.0019	0.0019
		nPD:	1.
		N. weight:	0.001
		Sc. PD:	0.0009
		Sc. rank:	0.9

PB2			
Pos.	207	obs:	exp:
tta	L	2	93.98
ttg	L	1056	197.20
ctt	L	1	176.30
ctc	L	0	176.70
cta	L	0	178.50
ctg	L	0	236.30
---	--	------	------
mPD		0.0075	1.1
		nPD:	0.01
		N. weight:	1.8
		Sc. PD:	-0.16
		Sc. rank:	-999.8

PB2			
Pos.	208	obs:	exp:
gaa	E	18	644.40
gag	E	1041	414.60
---	--	------	------
mPD		0.033	0.48
		nPD:	0.07
		N. weight:	0.93
		Sc. PD:	-0.026
		Sc. rank:	275.1

PB2			
Pos.	209	obs:	exp:
cgt	R	0	45.48
cgc	R	0	52.45
cga	R	0	103.60
cgg	R	0	88.36
aga	R	722	484.20
agg	R	337	284.80
---	--	------	------
mPD		0.43	0.96
		nPD:	0.45
		N. weight:	0.36
		Sc. PD:	0.12
		Sc. rank:	271.0

PB2			
Pos.	210	obs:	exp:
gaa	E	1049	644.40
gag	E	10	414.60
---	--	------	------
mPD		0.019	0.48
		nPD:	0.04
		N. weight:	0.49
		Sc. PD:	-0.029
		Sc. rank:	47.2

PB2			
Pos.	211	obs:	exp:
tta	L	0	93.98
ttg	L	42	197.20
ctt	L	11	176.30
ctc	L	0	176.70
cta	L	4	178.50
ctg	L	1002	236.30
---	--	------	------
mPD		0.10	1.1
		nPD:	0.09
		N. weight:	1.4
		Sc. PD:	-0.0075
		Sc. rank:	531.5

PB2			
Pos.	212	obs:	exp:
gtt	V	1049	219.40
gtc	V	10	213.70
gta	V	0	216.80
gtg	V	0	409.00
---	--	------	------
mPD		0.019	0.73
		nPD:	0.03
		N. weight:	1.7
		Sc. PD:	-0.12
		Sc. rank:	-115.1

PB2			
Pos.	213	obs:	exp:
cgt	R	1	45.48
cgc	R	1029	52.45
cga	R	29	103.60
cgg	R	0	88.36
aga	R	0	484.20
agg	R	0	284.80
---	--	------	------
mPD		0.055	0.96
		nPD:	0.06
		N. weight:	3.1
		Sc. PD:	-0.13
		Sc. rank:	740.0

PB2			
Pos.	214	obs:	exp:
cgt	R	0	0.09
cgc	R	0	0.10
cga	R	0	0.20
cgg	R	0	0.17
aaa	K	1039	616.00
aag	K	18	441.00
aga	R	2	0.91
agg	R	0	0.54
---	--	------	------
mPD		0.037	0.49
		nPD:	0.08
		N. weight:	0.51
		Sc. PD:	-0.012
		Sc. rank:	161.5

PB2			
Pos.	215	obs:	exp:
act	T	2	269.90
acc	T	0	217.30
aca	T	1054	485.70
acg	T	3	86.02
---	--	------	------
mPD		0.0094	0.68
		nPD:	0.01
		N. weight:	0.83
		Sc. PD:	-0.068
		Sc. rank:	-252.6

PB2			
Pos.	216	obs:	exp:
cgt	R	0	45.48
cgc	R	0	52.45
cga	R	3	103.60
cgg	R	0	88.36
aga	R	856	484.20
agg	R	200	284.80
---	--	------	------
mPD		0.31	0.96
		nPD:	0.32
		N. weight:	0.43
		Sc. PD:	0.092
		Sc. rank:	300.3

PB2			
Pos.	217	obs:	exp:
ttt	F	679	418.20
ttc	F	380	640.80
---	--	------	------
mPD		0.46	0.48
		nPD:	0.96
		N. weight:	0.14
		Sc. PD:	0.12
		Sc. rank:	116.4

PB2			
Pos.	218	obs:	exp:
tta	L	12	93.98
ttg	L	2	197.20
ctt	L	0	176.30
ctc	L	3	176.70
cta	L	1040	178.50
ctg	L	2	236.30
---	--	------	------
mPD		0.039	1.1
		nPD:	0.04
		N. weight:	1.9
		Sc. PD:	-0.11
		Sc. rank:	89.4

PB2			
Pos.	219	obs:	exp:
cct	P	0	264.20
ccc	P	0	190.30
cca	P	1056	435.90
ccg	P	3	168.60
---	--	------	------
mPD		0.0057	0.71
		nPD:	0.01
		N. weight:	0.96
		Sc. PD:	-0.084
		Sc. rank:	-477.5

PB2			
Pos.	220	obs:	exp:
att	I	0	0.37
atc	I	0	0.24
ata	I	1	0.39
gtt	V	4	219.00
gtc	V	0	213.30
gta	V	941	216.40
gtg	V	112	408.20
gct	A	0	0.26
gcc	A	0	0.19
gca	A	1	0.47
gcg	A	0	0.08
---	--	------	------
mPD		0.20	0.73
		nPD:	0.27
		N. weight:	1.3
		Sc. PD:	0.21
		Sc. rank:	848.3

PB2			
Pos.	221	obs:	exp:
tct	S	1	0.16
tcc	S	0	0.13
tca	S	0	0.25
tcg	S	0	0.06
act	T	3	0.76
acc	T	0	0.62
aca	T	0	1.38
acg	T	0	0.24
agt	S	0	0.21
agc	S	0	0.19
gct	A	1052	272.40
gcc	A	1	196.50
gca	A	2	496.60
gcg	A	0	89.54
---	--	------	------
mPD		0.013	0.68
		nPD:	0.02
		N. weight:	1.5
		Sc. PD:	-0.11
		Sc. rank:	-224.0

,

PB2			
Pos.	222	obs:	exp:
ggt	G	0	140.40
ggc	G	4	135.30
gga	G	1049	488.50
ggg	G	6	294.80
---	--	------	------
mPD		0.019	0.68
		nPD:	0.03
		N. weight:	0.8
		Sc. PD:	-0.055
		Sc. rank:	-12.6

,

PB2			
Pos.	223	obs:	exp:
ggt	G	0	140.40
ggc	G	0	135.30
gga	G	124	488.50
ggg	G	935	294.80
---	--	------	------
mPD		0.21	0.68
		nPD:	0.31
		N. weight:	0.95
		Sc. PD:	0.19
		Sc. rank:	655.1

,

PB2			
Pos.	224	obs:	exp:
tct	S	0	0.32
tcc	S	0	0.26
tca	S	2	0.51
tcg	S	0	0.11
act	T	1	269.40
acc	T	0	216.90
aca	T	1041	484.80
acg	T	15	85.86
agt	S	0	0.41
agc	S	0	0.39
---	--	------	------
mPD		0.034	0.68
		nPD:	0.05
		N. weight:	0.8
		Sc. PD:	-0.038
		Sc. rank:	132.8

,

PB2			
Pos.	225	obs:	exp:
tct	S	0	168.40
tcc	S	0	138.30
tca	S	0	268.80
tcg	S	0	60.09
agt	S	0	217.70
agc	S	1059	205.70
---	--	------	------
mPD		0	1.7
		nPD:	0.
		N. weight:	1.8
		Sc. PD:	-0.17
		Sc. rank:	-1496.2

,

PB2			
Pos.	226	obs:	exp:
tct	S	0	168.40
tcc	S	0	138.30
tca	S	0	268.80
tcg	S	0	60.09
agt	S	347	217.70
agc	S	712	205.70
---	--	------	------
mPD		0.44	1.7
		nPD:	0.27
		N. weight:	1.1
		Sc. PD:	0.17
		Sc. rank:	715.2

,

PB2			
Pos.	227	obs:	exp:
atg	M	1	1.00
gtt	V	49	219.00
gtc	V	0	213.30
gta	V	11	216.40
gtg	V	997	408.20
gct	A	0	0.26
gcc	A	0	0.19
gca	A	0	0.47
gcg	A	1	0.08
---	--	------	------
mPD		0.11	0.73
		nPD:	0.15
		N. weight:	0.82
		Sc. PD:	0.042
		Sc. rank:	395.5

,

PB2			
Pos.	228	obs:	exp:
tat	Y	985	572.00
tac	Y	74	487.00
---	--	------	------
mPD		0.13	0.50
		nPD:	0.26
		N. weight:	0.41
		Sc. PD:	0.064
		Sc. rank:	268.2

,

PB2			
Pos.	229	obs:	exp:
att	I	8	389.10
atc	I	1046	253.80
ata	I	5	416.10
---	--	------	------
mPD		0.024	0.65
		nPD:	0.04
		N. weight:	1.5
		Sc. PD:	-0.089
		Sc. rank:	101.7

,

PB2			
Pos.	230	obs:	exp:
gaa	E	704	644.40
gag	E	355	414.60
---	--	------	------
mPD		0.45	0.48
		nPD:	0.94
		N. weight:	0.011
		Sc. PD:	0.009
		Sc. rank:	9.4

,

PB2			
Pos.	231	obs:	exp:
gtt	V	0	219.40
gtc	V	0	213.70
gta	V	441	216.80
gtg	V	618	409.00
---	--	------	------
mPD		0.49	0.73
		nPD:	0.67
		N. weight:	0.59
		Sc. PD:	0.33
		Sc. rank:	476.3

,

PB2			
Pos.	232	obs:	exp:
tta	L	373	93.98
ttg	L	680	197.20
ctt	L	0	176.30
ctc	L	0	176.70
cta	L	2	178.50
ctg	L	4	236.30
---	--	------	------
mPD		0.47	1.1
		nPD:	0.42
		N. weight:	1.4
		Sc. PD:	0.43
		Sc. rank:	1029.7

,

PB2			
Pos.	233	obs:	exp:
cat	H	983	608.10
cac	H	76	450.90
---	--	------	------
mPD		0.13	0.49
		nPD:	0.27
		N. weight:	0.35
		Sc. PD:	0.059
		Sc. rank:	232.7

,

PB2			
Pos.	234	obs:	exp:
tta	L	7	93.98
ttg	L	1036	197.20
ctt	L	0	176.30
ctc	L	0	176.70
cta	L	0	178.50
ctg	L	16	236.30
---	--	------	------
mPD		0.043	1.1
		nPD:	0.04
		N. weight:	1.7
		Sc. PD:	-0.1
		Sc. rank:	143.6

,

PB2			
Pos.	235	obs:	exp:
act	T	2	269.90
acc	T	1056	217.30
aca	T	1	485.70
acg	T	0	86.02
---	--	------	------
mPD		0.0057	0.68
		nPD:	0.01
		N. weight:	1.7
		Sc. PD:	-0.15
		Sc. rank:	-838.7

,

PB2			
Pos.	236	obs:	exp:
caa	Q	1049	539.40
cag	Q	10	519.60
---	--	------	------
mPD		0.019	0.50
		nPD:	0.04
		N. weight:	0.68
		Sc. PD:	-0.041
		Sc. rank:	47.9

,

PB2			
Pos.	237	obs:	exp:
ggt	G	0	140.40
ggc	G	0	135.30
gga	G	91	488.50
ggg	G	968	294.80
---	--	------	------
mPD		0.16	0.68
		nPD:	0.23
		N. weight:	1.
		Sc. PD:	0.13
		Sc. rank:	626.0

,

PB2			
Pos.	238	obs:	exp:
act	T	88	269.90
acc	T	968	217.30
aca	T	3	485.70
acg	T	0	86.02
---	--	------	------
mPD		0.16	0.68
		nPD:	0.23
		N. weight:	1.4
		Sc. PD:	0.18
		Sc. rank:	844.5

,

PB2			
Pos.	239	obs:	exp:
tgt	C	10	447.90
tgc	C	1049	611.10
---	--	------	------
mPD		0.019	0.49
		nPD:	0.04
		N. weight:	0.55
		Sc. PD:	-0.032
		Sc. rank:	40.6

,

PB2			
Pos.	240	obs:	exp:
tgg	W	1059	1059.00
---	--	------	------
mPD		0	0
		nPD:	1.
		N. weight:	0.
		Sc. PD:	0
		Sc. rank:	0

,

PB2			
Pos.	241	obs:	exp:
gaa	E	7	644.40
gag	E	1052	414.60
---	--	------	------
mPD		0.013	0.48
		nPD:	0.03
		N. weight:	0.99
		Sc. PD:	-0.068
		Sc. rank:	-29.8

,

PB2			
Pos.	242	obs:	exp:
caa	Q	1055	539.40
cag	Q	4	519.60
---	--	------	------
mPD		0.0075	0.50
		nPD:	0.02
		N. weight:	0.72
		Sc. PD:	-0.058
		Sc. rank:	-200.4

,

PB2			
Pos.	243	obs:	exp:
atg	M	1058	1058.00
act	T	0	0.25
acc	T	0	0.21
aca	T	0	0.46
acg	T	1	0.08
---	--	------	------
mPD		0.0019	0.0036
		nPD:	0.52
		N. weight:	0.0026
		Sc. PD:	0.0011
		Sc. rank:	2.0

,

PB2			
Pos.	244	obs:	exp:
tat	Y	55	572.00
tac	Y	1004	487.00
---	--	------	------
mPD		0.099	0.50
		nPD:	0.2
		N. weight:	0.62
		Sc. PD:	0.059
		Sc. rank:	337.1

,

PB2			
Pos.	245	obs:	exp:
act	T	3	269.90
acc	T	0	217.30
aca	T	1054	485.70
acg	T	2	86.02
---	--	------	------
mPD		0.0094	0.68
		nPD:	0.01
		N. weight:	0.83
		Sc. PD:	-0.068
		Sc. rank:	-252.2

,

PB2			
Pos.	246	obs:	exp:
cct	P	11	264.20
ccc	P	1	190.30
cca	P	435	435.90
ccg	P	612	168.60
---	--	------	------
mPD		0.50	0.71
		nPD:	0.7
		N. weight:	0.78
		Sc. PD:	0.45
		Sc. rank:	639.8

PB2			
Pos.	247	obs:	exp:
ggt	G	2	140.40
ggc	G	0	135.30
gga	G	1057	488.50
ggg	G	0	294.80
---	--	------	------
mPD		0.0038	0.68
		nPD:	0.01
		N. weight:	0.84
		Sc. PD:	-0.076
		Sc. rank:	-501.9

PB2			
Pos.	248	obs:	exp:
ggt	G	2	140.40
ggc	G	0	135.30
gga	G	81	488.50
ggg	G	976	294.80
---	--	------	------
mPD		0.14	0.68
		nPD:	0.21
		N. weight:	1.1
		Sc. PD:	0.12
		Sc. rank:	610.7

PB2			
Pos.	249	obs:	exp:
aaa	K	1	0.58
aag	K	0	0.42
gaa	E	1020	620.70
gag	E	0	399.30
ggt	G	0	5.04
ggc	G	0	4.85
gga	G	38	17.53
ggg	G	0	10.58
---	--	------	------
mPD		0.071	0.56
		nPD:	0.13
		N. weight:	0.56
		Sc. PD:	0.015
		Sc. rank:	249.5

PB2			
Pos.	250	obs:	exp:
gtt	V	1	219.40
gtc	V	0	213.70
gta	V	5	216.80
gtg	V	1053	409.00
---	--	------	------
mPD		0.011	0.73
		nPD:	0.02
		N. weight:	1.
		Sc. PD:	-0.082
		Sc. rank:	-256.0

PB2			
Pos.	251	obs:	exp:
cgt	R	0	45.23
cgc	R	0	52.15
cga	R	0	103.00
cgg	R	0	87.86
aaa	K	6	3.50
aag	K	0	2.50
aga	R	1033	481.50
agg	R	20	283.20
---	--	------	------
mPD		0.048	0.97
		nPD:	0.05
		N. weight:	0.77
		Sc. PD:	-0.037
		Sc. rank:	129.2

PB2			
Pos.	252	obs:	exp:
aat	N	1054	539.40
aac	N	5	519.60
---	--	------	------
mPD		0.0094	0.50
		nPD:	0.02
		N. weight:	0.71
		Sc. PD:	-0.055
		Sc. rank:	-128.8

PB2			
Pos.	253	obs:	exp:
aat	N	3	1.53
aac	N	0	1.47
gat	D	1054	570.60
gac	D	2	485.40
---	--	------	------
mPD		0.0094	0.50
		nPD:	0.02
		N. weight:	0.66
		Sc. PD:	-0.051
		Sc. rank:	-123.3

PB2			
Pos.	254	obs:	exp:
tat	Y	1	0.54
tac	Y	0	0.46
gat	D	1056	571.70
gac	D	2	486.30
---	--	------	------
mPD		0.0057	0.50
		nPD:	0.01
		N. weight:	0.66
		Sc. PD:	-0.056
		Sc. rank:	-256.6

PB2			
Pos.	255	obs:	exp:
att	I	2	0.73
atc	I	0	0.48
ata	I	0	0.79
gtt	V	1054	219.00
gtc	V	3	213.30
gta	V	0	216.40
gtg	V	0	408.20
---	--	------	------
mPD		0.0094	0.73
		nPD:	0.01
		N. weight:	1.7
		Sc. PD:	-0.14
		Sc. rank:	-587.9

PB2			
Pos.	256	obs:	exp:
gat	D	1054	571.20
gac	D	3	485.80
ggt	G	2	0.27
ggc	G	0	0.26
gga	G	0	0.92
ggg	G	0	0.56
---	--	------	------
mPD		0.0094	0.50
		nPD:	0.02
		N. weight:	0.66
		Sc. PD:	-0.051
		Sc. rank:	-120.7

PB2			
Pos.	257	obs:	exp:
caa	Q	4	539.40
cag	Q	1055	519.60
---	--	------	------
mPD		0.0075	0.50
		nPD:	0.02
		N. weight:	0.76
		Sc. PD:	-0.061
		Sc. rank:	-211.8

PB2			
Pos.	258	obs:	exp:
tct	S	0	168.40
tcc	S	0	138.30
tca	S	0	268.80
tcg	S	0	60.09
agt	S	984	217.70
agc	S	75	205.70
---	--	------	------
mPD		0.13	1.7
		nPD:	0.08
		N. weight:	1.5
		Sc. PD:	-0.028
		Sc. rank:	478.6

PB2			
Pos.	259	obs:	exp:
tta	L	1023	93.89
ttg	L	35	197.00
ctt	L	0	176.20
ctc	L	0	176.60
cta	L	0	178.30
ctg	L	0	236.10
gtt	V	0	0.21
gtc	V	0	0.20
gta	V	1	0.20
gtg	V	0	0.39
---	--	------	------
mPD		0.066	1.1
		nPD:	0.06
		N. weight:	2.5
		Sc. PD:	-0.096
		Sc. rank:	643.8

PB2			
Pos.	260	obs:	exp:
att	I	1052	388.70
atc	I	6	253.50
ata	I	0	415.70
gtt	V	1	0.21
gtc	V	0	0.20
gta	V	0	0.20
gtg	V	0	0.39
---	--	------	------
mPD		0.013	0.66
		nPD:	0.02
		N. weight:	1.1
		Sc. PD:	-0.081
		Sc. rank:	-151.5

PB2			
Pos.	261	obs:	exp:
att	I	1056	388.40
atc	I	1	253.30
ata	I	0	415.30
gtt	V	2	0.41
gtc	V	0	0.40
gta	V	0	0.41
gtg	V	0	0.77
---	--	------	------
mPD		0.0057	0.66
		nPD:	0.01
		N. weight:	1.1
		Sc. PD:	-0.096
		Sc. rank:	-506.1

PB2			
Pos.	262	obs:	exp:
gct	A	1054	273.50
gcc	A	5	197.20
gca	A	0	498.40
gcg	A	0	89.88
---	--	------	------
mPD		0.0094	0.67
		nPD:	0.01
		N. weight:	1.5
		Sc. PD:	-0.12
		Sc. rank:	-428.2

PB2			
Pos.	263	obs:	exp:
gct	A	1032	273.50
gcc	A	7	197.20
gca	A	20	498.40
gcg	A	0	89.88
---	--	------	------
mPD		0.050	0.67
		nPD:	0.07
		N. weight:	1.3
		Sc. PD:	-0.032
		Sc. rank:	418.3

PB2			
Pos.	264	obs:	exp:
cgt	R	0	45.48
cgc	R	0	52.45
cga	R	0	103.60
cgg	R	0	88.36
aga	R	1057	484.20
agg	R	2	284.80
---	--	------	------
mPD		0.0038	0.96
		nPD:	0.
		N. weight:	0.85
		Sc. PD:	-0.078
		Sc. rank:	-541.6

PB2			
Pos.	265	obs:	exp:
aat	N	1055	538.90
aac	N	3	519.10
aaa	K	1	0.58
aag	K	0	0.42
---	--	------	------
mPD		0.0075	0.50
		nPD:	0.02
		N. weight:	0.72
		Sc. PD:	-0.059
		Sc. rank:	-203.1

PB2			
Pos.	266	obs:	exp:
att	I	1058	388.70
atc	I	0	253.50
ata	I	0	415.70
gtt	V	1	0.21
gtc	V	0	0.20
gta	V	0	0.20
gtg	V	0	0.39
---	--	------	------
mPD		0.0019	0.66
		nPD:	0.
		N. weight:	1.1
		Sc. PD:	-0.1
		Sc. rank:	-743.5

PB2			
Pos.	267	obs:	exp:
att	I	0	0.37
atc	I	1	0.24
ata	I	0	0.39
gtt	V	1047	219.20
gtc	V	9	213.50
gta	V	1	216.60
gtg	V	1	408.60
---	--	------	------
mPD		0.024	0.73
		nPD:	0.03
		N. weight:	1.7
		Sc. PD:	-0.11
		Sc. rank:	43.9

PB2			
Pos.	268	obs:	exp:
cgt	R	0	45.48
cgc	R	0	52.45
cga	R	0	103.60
cgg	R	0	88.36
aga	R	1023	484.20
agg	R	36	284.80
---	--	------	------
mPD		0.066	0.96
		nPD:	0.07
		N. weight:	0.72
		Sc. PD:	-0.022
		Sc. rank:	210.5

PB2			
Pos.	269	obs:	exp:
cgt	R	0	45.48
cgc	R	0	52.45
cga	R	0	103.60
cgg	R	0	88.36
aga	R	755	484.20
agg	R	304	284.80
---	--	------	------
mPD		0.41	0.96
		nPD:	0.43
		N. weight:	0.37
		Sc. PD:	0.12
		Sc. rank:	276.5

PB2			
Pos.	270	obs:	exp:
gct	A	1	273.50
gcc	A	0	197.20
gca	A	1055	498.40
gcg	A	3	89.88
---	--	------	------
mPD		0.0075	0.67
		nPD:	0.01
		N. weight:	0.81
		Sc. PD:	-0.068
		Sc. rank:	-320.0

PB2			
Pos.	271	obs:	exp:
act	T	74	269.40
acc	T	0	216.90
aca	T	983	484.80
acg	T	0	85.86
gct	A	0	0.52
gcc	A	0	0.37
gca	A	2	0.94
gcg	A	0	0.17
mPD		0.13	0.68
		nPD:	0.2
		N. weight:	0.63
		Sc. PD:	0.059
		Sc. rank:	338.1

PB2			
Pos.	272	obs:	exp:
gtt	V	0	219.40
gtc	V	0	213.70
gta	V	718	216.80
gtg	V	341	409.00
mPD		0.44	0.73
		nPD:	0.6
		N. weight:	0.83
		Sc. PD:	0.4
		Sc. rank:	656.3

PB2			
Pos.	273	obs:	exp:
ttt	F	0	0.39
ttc	F	1	0.61
tct	S	0	168.30
tcc	S	15	138.20
tca	S	1041	268.50
tcg	S	2	60.03
agt	S	0	217.50
agc	S	0	205.50
mPD		0.035	1.7
		nPD:	0.02
		N. weight:	1.4
		Sc. PD:	-0.11
		Sc. rank:	-181.3

PB2			
Pos.	274	obs:	exp:
tct	S	0	0.16
tcc	S	0	0.13
tca	S	1	0.25
tcg	S	0	0.06
act	T	0	0.51
acc	T	0	0.41
aca	T	2	0.92
acg	T	0	0.16
agt	S	0	0.21
agc	S	0	0.19
gct	A	27	272.70
gcc	A	0	196.70
gca	A	1029	497.00
gcg	A	0	89.62
mPD		0.055	0.68
		nPD:	0.08
		N. weight:	0.72
		Sc. PD:	-0.012
		Sc. rank:	241.7

PB2			
Pos.	275	obs:	exp:
gat	D	29	572.30
gac	D	1030	486.70
mPD		0.053	0.50
		nPD:	0.11
		N. weight:	0.71
		Sc. PD:	0.0053
		Sc. rank:	292.4

PB2			
Pos.	276	obs:	exp:
cct	P	18	264.20
ccc	P	0	190.30
cca	P	36	435.90
ccg	P	1005	168.60
mPD		0.098	0.71
		nPD:	0.14
		N. weight:	1.7
		Sc. PD:	0.063
		Sc. rank:	804.1

PB2			
Pos.	277	obs:	exp:
tta	L	8	93.98
ttg	L	942	197.20
ctt	L	0	176.30
ctc	L	0	176.70
cta	L	0	178.50
ctg	L	109	236.30
mPD		0.20	1.1
		nPD:	0.18
		N. weight:	1.4
		Sc. PD:	0.11
		Sc. rank:	728.5

PB2			
Pos.	278	obs:	exp:
gtt	V	1	0.21
gtc	V	0	0.20
gta	V	0	0.20
gtg	V	0	0.39
gct	A	1047	273.20
gcc	A	5	197.00
gca	A	2	498.00
gcg	A	4	89.79
mPD		0.023	0.67
		nPD:	0.03
		N. weight:	1.4
		Sc. PD:	-0.09
		Sc. rank:	42.0

PB2			
Pos.	279	obs:	exp:
tct	S	7	168.40
tcc	S	0	138.30
tca	S	17	268.80
tcg	S	1035	60.09
agt	S	0	217.70
agc	S	0	205.70
mPD		0.045	1.7
		nPD:	0.03
		N. weight:	3.
		Sc. PD:	-0.21
		Sc. rank:	-144.6

PB2			
Pos.	280	obs:	exp:
tta	L	0	93.98
ttg	L	0	197.20
ctt	L	1051	176.30
ctc	L	6	176.70
cta	L	1	178.50
ctg	L	1	236.30
mPD		0.015	1.1
		nPD:	0.01
		N. weight:	1.9
		Sc. PD:	-0.16
		Sc. rank:	-626.4

PB2			
Pos.	281	obs:	exp:
tta	L	106	93.80
ttg	L	948	196.80
ctt	L	0	176.00
ctc	L	0	176.40
cta	L	1	178.10
ctg	L	2	235.90
gtt	V	0	0.41
gtc	V	0	0.40
gta	V	0	0.41
gtg	V	2	0.77
mPD		0.19	1.1
		nPD:	0.17
		N. weight:	1.6
		Sc. PD:	0.11
		Sc. rank:	777.9

PB2			
Pos.	282	obs:	exp:
gaa	E	70	644.40
gag	E	989	414.60
mPD		0.12	0.48
		nPD:	0.26
		N. weight:	0.73
		Sc. PD:	0.11
		Sc. rank:	469.5

PB2			
Pos.	283	obs:	exp:
att	I	0	1.10
atc	I	0	0.72
ata	I	3	1.18
atg	M	1056	1056.00
mPD		0.0057	0.0057
		nPD:	1.
		N. weight:	0.0029
		Sc. PD:	0.0025
		Sc. rank:	2.4

PB2			
Pos.	284	obs:	exp:
tgt	C	18	447.90
tgc	C	1041	611.10
mPD		0.033	0.49
		nPD:	0.07
		N. weight:	0.52
		Sc. PD:	-0.015
		Sc. rank:	152.1

PB2			
Pos.	285	obs:	exp:
tat	Y	1	0.54
tac	Y	0	0.46
cat	H	1049	607.50
cac	H	9	450.50
mPD		0.019	0.49
		nPD:	0.04
		N. weight:	0.56
		Sc. PD:	-0.033
		Sc. rank:	40.6

PB2			
Pos.	286	obs:	exp:
tct	S	0	163.70
tcc	S	0	134.40
tca	S	0	261.20
tcg	S	0	58.39
agt	S	1000	211.50
agc	S	29	199.90
ggt	G	30	3.98
ggc	G	0	3.83
gga	G	0	13.84
ggg	G	0	8.35
mPD		0.11	1.7
		nPD:	0.06
		N. weight:	1.6
		Sc. PD:	-0.055
		Sc. rank:	451.6

PB2			
Pos.	287	obs:	exp:
act	T	0	269.90
acc	T	0	217.30
aca	T	1057	485.70
acg	T	2	86.02
mPD		0.0038	0.68
		nPD:	0.01
		N. weight:	0.85
		Sc. PD:	-0.076
		Sc. rank:	-507.8

PB2			
Pos.	288	obs:	exp:
caa	Q	8	538.90
cag	Q	1050	519.10
aaa	K	0	0.58
aag	K	1	0.42
mPD		0.017	0.50
		nPD:	0.03
		N. weight:	0.74
		Sc. PD:	-0.047
		Sc. rank:	22.9

PB2			
Pos.	289	obs:	exp:
att	I	1054	389.10
atc	I	5	253.80
ata	I	0	416.10
mPD		0.0094	0.65
		nPD:	0.01
		N. weight:	1.1
		Sc. PD:	-0.088
		Sc. rank:	-308.4

PB2			
Pos.	290	obs:	exp:
ggt	G	8	140.40
ggc	G	801	135.30
gga	G	250	488.50
ggg	G	0	294.80
mPD		0.37	0.68
		nPD:	0.55
		N. weight:	1.3
		Sc. PD:	0.56
		Sc. rank:	994.2

PB2			
Pos.	291	obs:	exp:
ggt	G	0	140.40
ggc	G	0	135.30
gga	G	5	488.50
ggg	G	1054	294.80
mPD		0.0094	0.68
		nPD:	0.01
		N. weight:	1.4
		Sc. PD:	-0.11
		Sc. rank:	-427.0

PB2			
Pos.	292	obs:	exp:
att	I	110	40.78
atc	I	0	26.60
ata	I	1	43.62
gtt	V	946	196.40
gtc	V	2	191.30
gta	V	0	194.10
gtg	V	0	366.10
mPD		0.19	0.93
		nPD:	0.21
		N. weight:	1.6
		Sc. PD:	0.17
		Sc. rank:	920.9

PB2			
Pos.	293	obs:	exp:
cgt	R	0	45.35
cgc	R	0	52.30
cga	R	0	103.30
cgg	R	0	88.11
aaa	K	0	0.58
aag	K	1	0.42
aga	R	18	482.90
agg	R	1038	284.00
ggt	G	0	0.27
ggc	G	0	0.26
gga	G	0	0.92
ggg	G	2	0.56
mPD		0.039	0.97
		nPD:	0.04
		N. weight:	1.3
		Sc. PD:	-0.076
		Sc. rank:	132.2

PB2			
Pos.	294	obs:	exp:
atg	M	1059	1059.00
mPD		0	0
		nPD:	1.
		N. weight:	0.
		Sc. PD:	0
		Sc. rank:	0

PB2			
Pos.	295	obs:	exp:
att	I	1	0.37
atc	I	0	0.24
ata	I	0	0.39
gtt	V	1051	219.20
gtc	V	6	213.50
gta	V	1	216.60
gtg	V	0	408.60
mPD		0.015	0.73
		nPD:	0.02
		N. weight:	1.7
		Sc. PD:	-0.13
		Sc. rank:	-219.5

PB2			
Pos.	296	obs:	exp:
gat	D	2	571.70
gac	D	1056	486.30
gaa	E	1	0.61
gag	E	0	0.39
---	--	------	------
mPD		0.0057	0.50
		nPD:	0.01
		N. weight:	0.84
		Sc. PD:	-0.071
		Sc. rank:	-323.5

PB2			
Pos.	297	obs:	exp:
att	I	16	389.10
atc	I	1036	253.80
ata	I	7	416.10
---	--	------	------
mPD		0.043	0.65
		nPD:	0.07
		N. weight:	1.4
		Sc. PD:	-0.047
		Sc. rank:	410.0

PB2			
Pos.	298	obs:	exp:
tta	L	0	93.98
ttg	L	0	197.20
ctt	L	1051	176.30
ctc	L	4	176.70
cta	L	0	178.50
ctg	L	4	236.30
---	--	------	------
mPD		0.015	1.1
		nPD:	0.01
		N. weight:	1.9
		Sc. PD:	-0.16
		Sc. rank:	-617.9

PB2			
Pos.	299	obs:	exp:
cgt	R	0	44.92
cgc	R	0	51.81
cga	R	0	102.40
cgg	R	0	87.28
aaa	K	13	7.58
aag	K	0	5.42
aga	R	1031	478.30
agg	R	15	281.30
---	--	------	------
mPD		0.052	0.98
		nPD:	0.05
		N. weight:	0.79
		Sc. PD:	-0.035
		Sc. rank:	157.2

PB2			
Pos.	300	obs:	exp:
cat	H	1	0.57
cac	H	0	0.43
caa	Q	1052	538.90
cag	Q	6	519.10
---	--	------	------
mPD		0.013	0.50
		nPD:	0.03
		N. weight:	0.7
		Sc. PD:	-0.05
		Sc. rank:	-43.0

PB2			
Pos.	301	obs:	exp:
tct	S	0	0.16
tcc	S	0	0.13
tca	S	0	0.25
tcg	S	0	0.06
aat	N	41	538.90
aac	N	1017	519.10
agt	S	0	0.21
agc	S	1	0.19
---	--	------	------
mPD		0.076	0.50
		nPD:	0.15
		N. weight:	0.6
		Sc. PD:	0.03
		Sc. rank:	290.1

PB2			
Pos.	302	obs:	exp:
tta	L	0	0.09
ttg	L	0	0.19
ctt	L	0	0.17
ctc	L	0	0.17
cta	L	1	0.17
ctg	L	0	0.22
cct	P	0	264.00
ccc	P	16	190.10
cca	P	1042	435.50
ccg	P	0	168.40
---	--	------	------
mPD		0.032	0.71
		nPD:	0.04
		N. weight:	0.91
		Sc. PD:	-0.048
		Sc. rank:	109.6

PB2			
Pos.	303	obs:	exp:
act	T	1	269.90
acc	T	1	217.30
aca	T	1053	485.70
acg	T	4	86.02
---	--	------	------
mPD		0.011	0.68
		nPD:	0.02
		N. weight:	0.82
		Sc. PD:	-0.066
		Sc. rank:	-190.9

PB2			
Pos.	304	obs:	exp:
gaa	E	815	644.40
gag	E	244	414.60
---	--	------	------
mPD		0.35	0.48
		nPD:	0.75
		N. weight:	0.068
		Sc. PD:	0.042
		Sc. rank:	56.4

PB2			
Pos.	305	obs:	exp:
gaa	E	1051	644.40
gag	E	8	414.60
---	--	------	------
mPD		0.015	0.48
		nPD:	0.03
		N. weight:	0.5
		Sc. PD:	-0.033
		Sc. rank:	10.0

PB2			
Pos.	306	obs:	exp:
caa	Q	9	539.40
cag	Q	1050	519.60
---	--	------	------
mPD		0.017	0.50
		nPD:	0.03
		N. weight:	0.73
		Sc. PD:	-0.046
		Sc. rank:	23.9

PB2			
Pos.	307	obs:	exp:
gct	A	1056	273.50
gcc	A	2	197.20
gca	A	0	498.40
gcg	A	1	89.88
---	--	------	------
mPD		0.0057	0.67
		nPD:	0.01
		N. weight:	1.5
		Sc. PD:	-0.13
		Sc. rank:	-707.0

PB2			
Pos.	308	obs:	exp:
gtt	V	6	219.40
gtc	V	0	213.70
gta	V	4	216.80
gtg	V	1049	409.00
---	--	------	------
mPD		0.019	0.73
		nPD:	0.03
		N. weight:	0.99
		Sc. PD:	-0.07
		Sc. rank:	-63.8

PB2			
Pos.	309	obs:	exp:
gat	D	1055	572.30
gac	D	4	486.70
---	--	------	------
mPD		0.0075	0.50
		nPD:	0.02
		N. weight:	0.65
		Sc. PD:	-0.053
		Sc. rank:	-180.4

PB2			
Pos.	310	obs:	exp:
att	I	1	389.10
atc	I	0	253.80
ata	I	1058	416.10
---	--	------	------
mPD		0.0019	0.65
		nPD:	0.
		N. weight:	1.
		Sc. PD:	-0.095
		Sc. rank:	-685.9

PB2			
Pos.	311	obs:	exp:
tgt	C	713	447.90
tgc	C	346	611.10
---	--	------	------
mPD		0.44	0.49
		nPD:	0.9
		N. weight:	0.14
		Sc. PD:	0.11
		Sc. rank:	119.8

PB2			
Pos.	312	obs:	exp:
aaa	K	247	617.20
aag	K	812	441.80
---	--	------	------
mPD		0.36	0.49
		nPD:	0.74
		N. weight:	0.28
		Sc. PD:	0.17
		Sc. rank:	232.9

PB2			
Pos.	313	obs:	exp:
gct	A	0	273.50
gcc	A	0	197.20
gca	A	1059	498.40
gcg	A	0	89.88
---	--	------	------
mPD		0	0.67
		nPD:	0.
		N. weight:	0.83
		Sc. PD:	-0.079
		Sc. rank:	-688.0

PB2			
Pos.	314	obs:	exp:
gct	A	2	273.50
gcc	A	0	197.20
gca	A	1056	498.40
gcg	A	1	89.88
---	--	------	------
mPD		0.0057	0.67
		nPD:	0.01
		N. weight:	0.81
		Sc. PD:	-0.071
		Sc. rank:	-390.0

PB2			
Pos.	315	obs:	exp:
tta	L	0	0.09
ttg	L	1	0.19
ctt	L	0	0.17
ctc	L	0	0.17
cta	L	0	0.17
ctg	L	0	0.22
att	I	0	1.84
atc	I	0	1.20
ata	I	5	1.96
atg	M	1053	1053.00
---	--	------	------
mPD		0.011	0.012
		nPD:	0.91
		N. weight:	0.0066
		Sc. PD:	0.0051
		Sc. rank:	5.5

PB2			
Pos.	316	obs:	exp:
ggt	G	704	140.40
ggc	G	340	135.30
gga	G	15	488.50
ggg	G	0	294.80
---	--	------	------
mPD		0.46	0.68
		nPD:	0.67
		N. weight:	1.5
		Sc. PD:	0.8
		Sc. rank:	1174.1

PB2			
Pos.	317	obs:	exp:
tta	L	43	93.98
ttg	L	1	197.20
ctt	L	0	176.30
ctc	L	108	176.70
cta	L	907	178.50
ctg	L	0	236.30
---	--	------	------
mPD		0.26	1.1
		nPD:	0.24
		N. weight:	1.4
		Sc. PD:	0.19
		Sc. rank:	886.8

PB2			
Pos.	318	obs:	exp:
cgt	R	0	45.35
cgc	R	0	52.30
cga	R	0	103.30
cgg	R	0	88.11
aaa	K	0	1.75
aag	K	3	1.25
aga	R	1	482.90
agg	R	1055	284.00
---	--	------	------
mPD		0.0075	0.97
		nPD:	0.01
		N. weight:	1.4
		Sc. PD:	-0.13
		Sc. rank:	-753.3

PB2			
Pos.	319	obs:	exp:
att	I	26	388.00
atc	I	1030	253.10
ata	I	0	414.90
gtt	V	0	0.62
gtc	V	3	0.61
gta	V	0	0.61
gtg	V	0	1.16
---	--	------	------
mPD		0.054	0.66
		nPD:	0.08
		N. weight:	1.4
		Sc. PD:	-0.025
		Sc. rank:	475.5

PB2			
Pos.	320	obs:	exp:
tct	S	0	168.30
tcc	S	0	138.20
tca	S	0	268.50
tcg	S	0	60.03
aat	N	1	0.51
aac	N	0	0.49
agt	S	1051	217.50
agc	S	7	205.50
---	--	------	------
mPD		0.015	1.7
		nPD:	0.01
		N. weight:	1.7
		Sc. PD:	-0.15
		Sc. rank:	-770.1

PB2			
Pos.	321	obs:	exp:
tct	S	0	168.40
tcc	S	1	138.30
tca	S	1046	268.80
tcg	S	12	60.09
agt	S	0	217.70
agc	S	0	205.70
---	--	------	------
mPD		0.024	1.7
		nPD:	0.01
		N. weight:	1.5
		Sc. PD:	-0.12
		Sc. rank:	-413.7

,

PB2			
Pos.	322	obs:	exp:
tct	S	16	168.40
tcc	S	1042	138.30
tca	S	0	268.80
tcg	S	1	60.09
agt	S	0	217.70
agc	S	0	205.70
---	--	------	------
mPD		0.032	1.7
		nPD:	0.02
		N. weight:	2.1
		Sc. PD:	-0.17
		Sc. rank:	-343.9

,

PB2			
Pos.	323	obs:	exp:
ttt	F	6	417.80
ttc	F	1052	640.20
tta	L	1	0.09
ttg	L	0	0.19
ctt	L	0	0.17
ctc	L	0	0.17
cta	L	0	0.17
ctg	L	0	0.22
---	--	------	------
mPD		0.013	0.48
		nPD:	0.03
		N. weight:	0.52
		Sc. PD:	-0.036
		Sc. rank:	-19.4

,

PB2			
Pos.	324	obs:	exp:
tct	S	0	168.40
tcc	S	0	138.30
tca	S	0	268.80
tcg	S	0	60.09
agt	S	5	217.70
agc	S	1054	205.70
---	--	------	------
mPD		0.0094	1.7
		nPD:	0.01
		N. weight:	1.8
		Sc. PD:	-0.16
		Sc. rank:	-1036.3

,

PB2			
Pos.	325	obs:	exp:
ttt	F	1059	418.20
ttc	F	0	640.80
---	--	------	------
mPD		0	0.48
		nPD:	0.
		N. weight:	1.
		Sc. PD:	-0.098
		Sc. rank:	-848.3

,

PB2			
Pos.	326	obs:	exp:
ggt	G	3	140.40
ggc	G	0	135.30
gga	G	1054	488.50
ggg	G	2	294.80
---	--	------	------
mPD		0.0094	0.68
		nPD:	0.01
		N. weight:	0.82
		Sc. PD:	-0.068
		Sc. rank:	-247.2

,

PB2			
Pos.	327	obs:	exp:
tct	S	0	0.16
tcc	S	0	0.13
tca	S	0	0.25
tcg	S	0	0.06
agt	S	1	0.21
agc	S	0	0.19
gat	D	1	0.54
gac	D	0	0.46
ggt	G	1039	140.10
ggc	G	6	135.00
gga	G	1	487.60
ggg	G	11	294.30
---	--	------	------
mPD		0.037	0.68
		nPD:	0.05
		N. weight:	2.1
		Sc. PD:	-0.09
		Sc. rank:	451.5

,

PB2			
Pos.	328	obs:	exp:
ttt	F	1	418.20
ttc	F	1058	640.80
---	--	------	------
mPD		0.0019	0.48
		nPD:	0.
		N. weight:	0.55
		Sc. PD:	-0.05
		Sc. rank:	-337.2

,

PB2			
Pos.	329	obs:	exp:
att	I	1	0.37
atc	I	0	0.24
ata	I	0	0.39
act	T	1052	269.70
acc	T	1	217.10
aca	T	5	485.30
acg	T	0	85.94
---	--	------	------
mPD		0.013	0.68
		nPD:	0.02
		N. weight:	1.5
		Sc. PD:	-0.11
		Sc. rank:	-227.8

,

PB2			
Pos.	330	obs:	exp:
ttt	F	243	418.20
ttc	F	816	640.80
---	--	------	------
mPD		0.35	0.48
		nPD:	0.74
		N. weight:	0.071
		Sc. PD:	0.044
		Sc. rank:	59.1

,

PB2			
Pos.	331	obs:	exp:
aaa	K	988	617.20
aag	K	71	441.80
---	--	------	------
mPD		0.13	0.49
		nPD:	0.26
		N. weight:	0.35
		Sc. PD:	0.053
		Sc. rank:	222.9

,

PB2			
Pos.	332	obs:	exp:
cgt	R	0	45.48
cgc	R	0	52.45
cga	R	0	103.60
cgg	R	0	88.36
aga	R	6	484.20
agg	R	1053	284.80
---	--	------	------
mPD		0.011	0.96
		nPD:	0.01
		N. weight:	1.4
		Sc. PD:	-0.12
		Sc. rank:	-521.0

,

PB2			
Pos.	333	obs:	exp:
act	T	0	269.90
acc	T	0	217.30
aca	T	1059	485.70
acg	T	0	86.02
---	--	------	------
mPD		0	0.68
		nPD:	0.
		N. weight:	0.86
		Sc. PD:	-0.082
		Sc. rank:	-711.6

,

PB2			
Pos.	334	obs:	exp:
tct	S	0	168.40
tcc	S	0	138.30
tca	S	0	268.80
tcg	S	0	60.09
agt	S	1048	217.70
agc	S	11	205.70
---	--	------	------
mPD		0.021	1.7
		nPD:	0.01
		N. weight:	1.7
		Sc. PD:	-0.14
		Sc. rank:	-583.5

,

PB2			
Pos.	335	obs:	exp:
ggt	G	4	140.40
ggc	G	0	135.30
gga	G	13	488.50
ggg	G	1042	294.80
---	--	------	------
mPD		0.032	0.68
		nPD:	0.05
		N. weight:	1.3
		Sc. PD:	-0.066
		Sc. rank:	186.9

,

PB2			
Pos.	336	obs:	exp:
tct	S	20	168.30
tcc	S	0	138.20
tca	S	1035	268.50
tcg	S	3	60.03
act	T	0	0.25
acc	T	0	0.21
aca	T	1	0.46
acg	T	0	0.08
agt	S	0	217.50
agc	S	0	205.50
---	--	------	------
mPD		0.045	1.7
		nPD:	0.03
		N. weight:	1.4
		Sc. PD:	-0.098
		Sc. rank:	-76.9

,

PB2			
Pos.	337	obs:	exp:
ttt	F	1	0.39
ttc	F	0	0.61
tct	S	846	168.30
tcc	S	212	138.20
tca	S	0	268.50
tcg	S	0	60.03
agt	S	0	217.50
agc	S	0	205.50
---	--	------	------
mPD		0.32	1.7
		nPD:	0.19
		N. weight:	1.5
		Sc. PD:	0.14
		Sc. rank:	809.9

,

PB2			
Pos.	338	obs:	exp:
att	I	5	21.31
atc	I	51	13.90
ata	I	2	22.79
gtt	V	3	207.40
gtc	V	996	202.00
gta	V	2	205.00
gtg	V	0	386.60
---	--	------	------
mPD		0.13	0.84
		nPD:	0.15
		N. weight:	1.7
		Sc. PD:	0.083
		Sc. rank:	807.7

,

PB2			
Pos.	339	obs:	exp:
cgt	R	0	0.17
cgc	R	0	0.20
cga	R	0	0.39
cgg	R	0	0.33
aat	N	0	0.51
aac	N	1	0.49
aaa	K	1049	613.70
aag	K	4	439.30
aga	R	4	1.83
agg	R	0	1.08
gaa	E	1	0.61
gag	E	0	0.39
---	--	------	------
mPD		0.019	0.50
		nPD:	0.04
		N. weight:	0.57
		Sc. PD:	-0.034
		Sc. rank:	40.6

,

PB2			
Pos.	340	obs:	exp:
cgt	R	0	30.15
cgc	R	0	34.77
cga	R	0	68.70
cgg	R	0	58.58
aaa	K	16	208.10
aag	K	341	148.90
aga	R	14	321.00
agg	R	688	188.80
---	--	------	------
mPD		0.50	1.3
		nPD:	0.39
		N. weight:	1.1
		Sc. PD:	0.32
		Sc. rank:	817.4

,

PB2			
Pos.	341	obs:	exp:
aaa	K	1	0.58
aag	K	0	0.42
gaa	E	1056	643.80
gag	E	2	414.20
---	--	------	------
mPD		0.0057	0.48
		nPD:	0.01
		N. weight:	0.53
		Sc. PD:	-0.045
		Sc. rank:	-194.6

,

PB2			
Pos.	342	obs:	exp:
aaa	K	1	0.58
aag	K	0	0.42
gaa	E	977	643.80
gag	E	81	414.20
---	--	------	------
mPD		0.14	0.48
		nPD:	0.3
		N. weight:	0.29
		Sc. PD:	0.056
		Sc. rank:	198.4

,

PB2			
Pos.	343	obs:	exp:
gaa	E	907	644.40
gag	E	152	414.60
---	--	------	------
mPD		0.25	0.48
		nPD:	0.52
		N. weight:	0.17
		Sc. PD:	0.067
		Sc. rank:	126.8

,

PB2			
Pos.	344	obs:	exp:
tta	L	0	1.51
ttg	L	17	3.17
ctt	L	0	2.83
ctc	L	0	2.84
cta	L	0	2.86
ctg	L	0	3.79
atg	M	1	1.00
gtt	V	0	215.70
gtc	V	0	210.10
gta	V	4	213.20
gtg	V	1037	402.00
---	--	------	------
mPD		0.041	0.76
		nPD:	0.05
		N. weight:	1.
		Sc. PD:	-0.045
		Sc. rank:	213.9

,

PB2			
Pos.	345	obs:	exp:
tta	L	0	93.98
ttg	L	0	197.20
ctt	L	5	176.30
ctc	L	1042	176.70
cta	L	12	178.50
ctg	L	0	236.30
---	--	------	------
mPD		0.032	1.1
		nPD:	0.03
		N. weight:	1.9
		Sc. PD:	-0.13
		Sc. rank:	-11.9

,

PB2			
Pos.	346	obs:	exp:
act	T	8	269.90
acc	T	0	217.30
aca	T	1048	485.70
acg	T	3	86.02
---	--	------	------
mPD		0.021	0.68
		nPD:	0.03
		N. weight:	0.8
		Sc. PD:	-0.053
		Sc. rank:	4.8

PB2			
Pos.	347	obs:	exp:
ggt	G	71	140.40
ggc	G	985	135.30
gga	G	3	488.50
ggg	G	0	294.80
---	--	------	------
mPD		0.13	0.68
		nPD:	0.19
		N. weight:	2.
		Sc. PD:	0.18
		Sc. rank:	1044.4

PB2			
Pos.	348	obs:	exp:
tct	S	0	0.16
tcc	S	0	0.13
tca	S	0	0.25
tcg	S	0	0.06
aat	N	21	538.90
aac	N	1037	519.10
agt	S	0	0.21
agc	S	1	0.19
---	--	------	------
mPD		0.041	0.50
		nPD:	0.08
		N. weight:	0.68
		Sc. PD:	-0.012
		Sc. rank:	222.6

PB2			
Pos.	349	obs:	exp:
tta	L	0	93.98
ttg	L	0	197.20
ctt	L	86	176.30
ctc	L	972	176.70
cta	L	1	178.50
ctg	L	0	236.30
---	--	------	------
mPD		0.15	1.1
		nPD:	0.14
		N. weight:	1.7
		Sc. PD:	0.058
		Sc. rank:	765.4

PB2			
Pos.	350	obs:	exp:
caa	Q	1041	539.40
cag	Q	18	519.60
---	--	------	------
mPD		0.033	0.50
		nPD:	0.07
		N. weight:	0.65
		Sc. PD:	-0.02
		Sc. rank:	189.1

PB2			
Pos.	351	obs:	exp:
act	T	0	269.90
acc	T	0	217.30
aca	T	1059	485.70
acg	T	0	86.02
---	--	------	------
mPD		0	0.68
		nPD:	0.
		N. weight:	0.86
		Sc. PD:	-0.082
		Sc. rank:	-711.6

PB2			
Pos.	352	obs:	exp:
tta	L	56	93.98
ttg	L	1003	197.20
ctt	L	0	176.30
ctc	L	0	176.70
cta	L	0	178.50
ctg	L	0	236.30
---	--	------	------
mPD		0.10	1.1
		nPD:	0.09
		N. weight:	1.7
		Sc. PD:	-0.015
		Sc. rank:	609.8

PB2			
Pos.	353	obs:	exp:
cgt	R	0	0.04
cgc	R	0	0.05
cga	R	0	0.10
cgg	R	0	0.08
aaa	K	1058	616.60
aag	K	0	441.40
aga	R	1	0.46
agg	R	0	0.27
---	--	------	------
mPD		0.0019	0.49
		nPD:	0.
		N. weight:	0.59
		Sc. PD:	-0.055
		Sc. rank:	-382.4

PB2			
Pos.	354	obs:	exp:
att	I	0	388.00
atc	I	0	253.10
ata	I	1056	414.90
gtt	V	0	0.62
gtc	V	0	0.61
gta	V	3	0.61
gtg	V	0	1.16
---	--	------	------
mPD		0.0057	0.66
		nPD:	0.01
		N. weight:	1.
		Sc. PD:	-0.09
		Sc. rank:	-480.3

PB2			
Pos.	355	obs:	exp:
cgt	R	0	44.32
cgc	R	0	51.11
cga	R	0	101.00
cgg	R	0	86.11
aaa	K	23	15.15
aag	K	3	10.85
aga	R	1030	471.90
agg	R	2	277.60
ggt	G	0	0.13
ggc	G	0	0.13
gga	G	1	0.46
ggg	G	0	0.28
---	--	------	------
mPD		0.059	1.0
		nPD:	0.06
		N. weight:	0.83
		Sc. PD:	-0.032
		Sc. rank:	212.1

PB2			
Pos.	356	obs:	exp:
att	I	0	1.47
atc	I	0	0.96
ata	I	4	1.57
gtt	V	9	218.60
gtc	V	1	212.90
gta	V	1015	216.00
gtg	V	30	407.50
---	--	------	------
mPD		0.081	0.73
		nPD:	0.11
		N. weight:	1.5
		Sc. PD:	0.016
		Sc. rank:	635.5

PB2			
Pos.	357	obs:	exp:
cat	H	1058	608.10
cac	H	1	450.90
---	--	------	------
mPD		0.0019	0.49
		nPD:	0.
		N. weight:	0.6
		Sc. PD:	-0.055
		Sc. rank:	-389.9

PB2			
Pos.	358	obs:	exp:
gaa	E	914	644.40
gag	E	145	414.60
---	--	------	------
mPD		0.24	0.48
		nPD:	0.5
		N. weight:	0.18
		Sc. PD:	0.067
		Sc. rank:	134.3

PB2			
Pos.	359	obs:	exp:
ggt	G	1	140.40
ggc	G	0	135.30
gga	G	1019	488.50
ggg	G	39	294.80
---	--	------	------
mPD		0.073	0.68
		nPD:	0.11
		N. weight:	0.69
		Sc. PD:	0.0054
		Sc. rank:	286.7

PB2			
Pos.	360	obs:	exp:
ttt	F	3	1.18
ttc	F	0	1.82
tat	Y	1038	570.30
tac	Y	18	485.70
---	--	------	------
mPD		0.039	0.50
		nPD:	0.08
		N. weight:	0.59
		Sc. PD:	-0.012
		Sc. rank:	190.5

PB2			
Pos.	361	obs:	exp:
gaa	E	14	644.40
gag	E	1045	414.60
---	--	------	------
mPD		0.026	0.48
		nPD:	0.05
		N. weight:	0.95
		Sc. PD:	-0.041
		Sc. rank:	197.8

PB2			
Pos.	362	obs:	exp:
gaa	E	1056	644.40
gag	E	3	414.60
---	--	------	------
mPD		0.0057	0.48
		nPD:	0.01
		N. weight:	0.53
		Sc. PD:	-0.044
		Sc. rank:	-187.6

PB2			
Pos.	363	obs:	exp:
ttt	F	0	418.20
ttc	F	1059	640.80
---	--	------	------
mPD		0	0.48
		nPD:	0.
		N. weight:	0.55
		Sc. PD:	-0.053
		Sc. rank:	-458.6

PB2			
Pos.	364	obs:	exp:
act	T	0	269.90
acc	T	0	217.30
aca	T	1054	485.70
acg	T	5	86.02
---	--	------	------
mPD		0.0094	0.68
		nPD:	0.01
		N. weight:	0.83
		Sc. PD:	-0.069
		Sc. rank:	-262.3

PB2			
Pos.	365	obs:	exp:
atg	M	1059	1059.00
---	--	------	------
mPD		0	0
		nPD:	1.
		N. weight:	0.
		Sc. PD:	0
		Sc. rank:	0

PB2			
Pos.	366	obs:	exp:
gtt	V	598	219.20
gtc	V	442	213.50
gta	V	17	216.60
gtg	V	1	408.60
gct	A	1	0.26
gcc	A	0	0.19
gca	A	0	0.47
gcg	A	0	0.08
---	--	------	------
mPD		0.51	0.73
		nPD:	0.7
		N. weight:	0.91
		Sc. PD:	0.53
		Sc. rank:	745.0

PB2			
Pos.	367	obs:	exp:
ggt	G	0	140.40
ggc	G	3	135.30
gga	G	82	488.50
ggg	G	974	294.80
---	--	------	------
mPD		0.15	0.68
		nPD:	0.22
		N. weight:	1.
		Sc. PD:	0.12
		Sc. rank:	616.4

PB2			
Pos.	368	obs:	exp:
caa	Q	1	0.51
cag	Q	0	0.49
cgt	R	0	45.44
cgc	R	0	52.40
cga	R	919	103.50
cgg	R	28	88.28
aga	R	106	483.80
agg	R	5	284.60
---	--	------	------
mPD		0.25	0.96
		nPD:	0.26
		N. weight:	1.9
		Sc. PD:	0.29
		Sc. rank:	1209.0

PB2			
Pos.	369	obs:	exp:
cgt	R	0	45.44
cgc	R	0	52.40
cga	R	2	103.50
cgg	R	0	88.28
aga	R	1055	483.80
agg	R	1	284.60
ggt	G	0	0.13
ggc	G	0	0.13
gga	G	1	0.46
ggg	G	0	0.28
---	--	------	------
mPD		0.0075	0.96
		nPD:	0.01
		N. weight:	0.84
		Sc. PD:	-0.074
		Sc. rank:	-428.3

PB2			
Pos.	370	obs:	exp:
gct	A	0	273.50
gcc	A	0	197.20
gca	A	1056	498.40
gcg	A	3	89.88
---	--	------	------
mPD		0.0057	0.67
		nPD:	0.01
		N. weight:	0.81
		Sc. PD:	-0.071
		Sc. rank:	-393.4

PB2

Pos.	371	obs :	exp :
act	T	0	269.90
acc	T	0	217.30
aca	T	1058	485.70
acg	T	1	86.02
---	--	------	------
mPD		0.0019	0.68
		nPD :	0.
		N. weight :	0.85
		Sc. PD :	-0.079
		Sc. rank :	-594.7

,

PB2

Pos.	372	obs :	exp :
gct	A	10	273.50
gcc	A	1036	197.20
gca	A	13	498.40
gcg	A	0	89.88
---	--	------	------
mPD		0.043	0.67
		nPD :	0.06
		N. weight :	1.7
		Sc. PD :	-0.059
		Sc. rank :	469.1

,

PB2

Pos.	373	obs :	exp :
att	I	1056	389.10
atc	I	3	253.80
ata	I	0	416.10
---	--	------	------
mPD		0.0057	0.65
		nPD :	0.01
		N. weight :	1.1
		Sc. PD :	-0.094
		Sc. rank :	-497.6

,

PB2

Pos.	374	obs :	exp :
tta	L	31	93.98
ttg	L	0	197.20
ctt	L	0	176.30
ctc	L	0	176.70
cta	L	1024	178.50
ctg	L	4	236.30
---	--	------	------
mPD		0.064	1.1
		nPD :	0.06
		N. weight :	1.8
		Sc. PD :	-0.072
		Sc. rank :	443.1

,

PB2

Pos.	375	obs :	exp :
cgt	R	0	45.48
cgc	R	0	52.45
cga	R	0	103.60
cgg	R	0	88.36
aga	R	4	484.20
agg	R	1055	284.80
---	--	------	------
mPD		0.0075	0.96
		nPD :	0.01
		N. weight :	1.4
		Sc. PD :	-0.12
		Sc. rank :	-738.1

,

PB2

Pos.	376	obs :	exp :
aaa	K	1030	617.20
aag	K	29	441.80
---	--	------	------
mPD		0.053	0.49
		nPD :	0.11
		N. weight :	0.47
		Sc. PD :	0.0045
		Sc. rank :	193.8

,

PB2

Pos.	377	obs :	exp :
gct	A	0	273.50
gcc	A	1	197.20
gca	A	1056	498.40
gcg	A	2	89.88
---	--	------	------
mPD		0.0057	0.67
		nPD :	0.01
		N. weight :	0.81
		Sc. PD :	-0.071
		Sc. rank :	-390.8

,

PB2

Pos.	378	obs :	exp :
act	T	19	269.90
acc	T	1040	217.30
aca	T	0	485.70
acg	T	0	86.02
---	--	------	------
mPD		0.035	0.68
		nPD :	0.05
		N. weight :	1.6
		Sc. PD :	-0.075
		Sc. rank :	298.6

,

PB2

Pos.	379	obs :	exp :
cgt	R	0	45.48
cgc	R	0	52.45
cga	R	4	103.60
cgg	R	0	88.36
aga	R	1052	484.20
agg	R	3	284.80
---	--	------	------
mPD		0.013	0.96
		nPD :	0.01
		N. weight :	0.82
		Sc. PD :	-0.068
		Sc. rank :	-260.1

,

PB2

Pos.	380	obs :	exp :
cgt	R	0	45.44
cgc	R	0	52.40
cga	R	2	103.50
cgg	R	0	88.28
aaa	K	1	0.58
aag	K	0	0.42
aga	R	1029	483.80
agg	R	27	284.60
---	--	------	------
mPD		0.055	0.96
		nPD :	0.06
		N. weight :	0.74
		Sc. PD :	-0.03
		Sc. rank :	174.7

,

PB2

Pos.	381	obs :	exp :
tta	L	0	93.98
ttg	L	11	197.20
ctt	L	7	176.30
ctc	L	0	176.70
cta	L	4	178.50
ctg	L	1037	236.30
---	--	------	------
mPD		0.041	1.1
		nPD :	0.04
		N. weight :	1.5
		Sc. PD :	-0.091
		Sc. rank :	99.4

,

PB2

Pos.	382	obs :	exp :
att	I	271	389.10
atc	I	788	253.80
ata	I	0	416.10
---	--	------	------
mPD		0.38	0.65
		nPD :	0.58
		N. weight :	0.83
		Sc. PD :	0.38
		Sc. rank :	646.6

,

PB2

Pos.	383	obs :	exp :
caa	Q	1050	539.40
cag	Q	9	519.60
---	--	------	------
mPD		0.017	0.50
		nPD :	0.03
		N. weight :	0.69
		Sc. PD :	-0.044
		Sc. rank :	22.5

,

PB2

Pos.	384	obs :	exp :
ttt	F	1	0.39
ttc	F	0	0.61
tta	L	27	93.71
ttg	L	130	196.60
ctt	L	0	175.80
ctc	L	0	176.20
cta	L	7	178.00
ctg	L	892	235.60
atg	M	2	2.00
---	--	------	------
mPD		0.32	1.1
		nPD :	0.29
		N. weight :	1.1
		Sc. PD :	0.2
		Sc. rank :	767.0

,

PB2

Pos.	385	obs :	exp :
att	I	1	389.10
atc	I	0	253.80
ata	I	1058	416.10
---	--	------	------
mPD		0.0019	0.65
		nPD :	0.
		N. weight :	1.
		Sc. PD :	-0.095
		Sc. rank :	-685.9

,

PB2

Pos.	386	obs :	exp :
gtt	V	1	219.40
gtc	V	0	213.70
gta	V	6	216.80
gtg	V	1052	409.00
---	--	------	------
mPD		0.013	0.73
		nPD :	0.02
		N. weight :	1.
		Sc. PD :	-0.079
		Sc. rank :	-211.4

,

PB2

Pos.	387	obs :	exp :
tct	S	0	168.40
tcc	S	0	138.30
tca	S	0	268.80
tcg	S	0	60.09
agt	S	1057	217.70
agc	S	2	205.70
---	--	------	------
mPD		0.0038	1.7
		nPD :	0.
		N. weight :	1.7
		Sc. PD :	-0.16
		Sc. rank :	-1262.3

,

PB2

Pos.	388	obs :	exp :
ggt	G	0	140.40
ggc	G	0	135.30
gga	G	79	488.50
ggg	G	980	294.80
---	--	------	------
mPD		0.14	0.68
		nPD :	0.2
		N. weight :	1.1
		Sc. PD :	0.11
		Sc. rank :	593.6

,

PB2

Pos.	389	obs :	exp :
cgt	R	0	0.43
cgc	R	0	0.50
cga	R	0	0.98
cgg	R	0	0.83
aaa	K	888	611.40
aag	K	161	437.60
aga	R	9	4.57
agg	R	1	2.69
---	--	------	------
mPD		0.28	0.51
		nPD :	0.54
		N. weight :	0.19
		Sc. PD :	0.08
		Sc. rank :	143.7

,

PB2

Pos.	390	obs :	exp :
gat	D	92	572.30
gac	D	967	486.70
---	--	------	------
mPD		0.16	0.50
		nPD :	0.32
		N. weight :	0.52
		Sc. PD :	0.11
		Sc. rank :	363.7

,

PB2

Pos.	391	obs :	exp :
gat	D	2	1.08
gac	D	0	0.92
gaa	E	48	642.60
gag	E	1008	413.40
ggt	G	0	0.13
ggc	G	0	0.13
gga	G	0	0.46
ggg	G	1	0.28
---	--	------	------
mPD		0.092	0.48
		nPD :	0.19
		N. weight :	0.81
		Sc. PD :	0.071
		Sc. rank :	425.7

,

PB2

Pos.	392	obs :	exp :
caa	Q	1047	539.40
cag	Q	12	519.60
---	--	------	------
mPD		0.022	0.50
		nPD :	0.04
		N. weight :	0.68
		Sc. PD :	-0.036
		Sc. rank :	82.7

,

PB2

Pos.	393	obs :	exp :
tct	S	1	168.40
tcc	S	1	138.30
tca	S	1016	268.80
tcg	S	41	60.09
agt	S	0	217.70
agc	S	0	205.70
---	--	------	------
mPD		0.078	1.7
		nPD :	0.05
		N. weight :	1.4
		Sc. PD :	-0.069
		Sc. rank :	213.5

,

PB2

Pos.	394	obs :	exp :
att	I	361	388.00
atc	I	686	253.10
ata	I	9	414.90
act	T	1	0.25
acc	T	0	0.21
aca	T	0	0.46
acg	T	0	0.08
gtt	V	2	0.41
gtc	V	0	0.40
gta	V	0	0.41
gtg	V	0	0.77
---	--	------	------
mPD		0.47	0.66
		nPD :	0.71
		N. weight :	0.66
		Sc. PD :	0.39
		Sc. rank :	538.6

,

PB2

Pos.	395	obs :	exp :
tct	S	0	2.70
tcc	S	17	2.22
tca	S	0	4.31
tcg	S	0	0.96
agt	S	0	3.49
agc	S	0	3.30
gct	A	66	269.10
gcc	A	973	194.10
gca	A	3	490.40
gcg	A	0	88.44
---	--	------	------
mPD		0.15	0.72
		nPD :	0.21
		N. weight :	1.6
		Sc. PD :	0.17
		Sc. rank :	904.3

,

PB2			
Pos.	396	obs:	exp:
aaa	K	0	0.58
aag	K	1	0.42
gaa	E	6	643.20
gag	E	1051	413.80
ggt	G	0	0.13
ggc	G	0	0.13
gga	G	0	0.46
ggg	G	1	0.28
---	--	------	------
mPD		0.015	0.48
		nPD:	0.03
		N. weight:	0.99
		Sc. PD:	-0.065
		Sc. rank:	13.1

PB2			
Pos.	397	obs:	exp:
gct	A	1	273.50
gcc	A	0	197.20
gca	A	956	498.40
gcg	A	102	89.88
---	--	------	------
mPD		0.18	0.67
		nPD:	0.26
		N. weight:	0.66
		Sc. PD:	0.1
		Sc. rank:	427.6

PB2			
Pos.	398	obs:	exp:
att	I	0	388.70
atc	I	1032	253.50
ata	I	26	415.70
gtt	V	0	0.21
gtc	V	1	0.20
gta	V	0	0.20
gtg	V	0	0.39
---	--	------	------
mPD		0.050	0.66
		nPD:	0.08
		N. weight:	1.4
		Sc. PD:	-0.032
		Sc. rank:	460.1

PB2			
Pos.	399	obs:	exp:
att	I	2	389.10
atc	I	0	253.80
ata	I	1057	416.10
---	--	------	------
mPD		0.0038	0.65
		nPD:	0.01
		N. weight:	1.
		Sc. PD:	-0.091
		Sc. rank:	-581.3

PB2			
Pos.	400	obs:	exp:
gtt	V	0	218.40
gtc	V	0	212.70
gta	V	2	215.80
gtg	V	1052	407.10
gct	A	0	1.29
gcc	A	0	0.93
gca	A	0	2.35
gcg	A	5	0.42
---	--	------	------
mPD		0.013	0.74
		nPD:	0.02
		N. weight:	1.
		Sc. PD:	-0.082
		Sc. rank:	-221.7

PB2			
Pos.	401	obs:	exp:
gct	A	0	273.50
gcc	A	3	197.20
gca	A	1052	498.40
gcg	A	4	89.88
---	--	------	------
mPD		0.013	0.67
		nPD:	0.02
		N. weight:	0.79
		Sc. PD:	-0.061
		Sc. rank:	-118.0

PB2			
Pos.	402	obs:	exp:
att	I	0	0.37
atc	I	0	0.24
ata	I	1	0.39
atg	M	1058	1058.00
---	--	------	------
mPD		0.0019	0.0019
		nPD:	1.
		N. weight:	0.00097
		Sc. PD:	0.00084
		Sc. rank:	0.8

PB2			
Pos.	403	obs:	exp:
gtt	V	0	219.40
gtc	V	0	213.70
gta	V	722	216.80
gtg	V	337	409.00
---	--	------	------
mPD		0.43	0.73
		nPD:	0.6
		N. weight:	0.84
		Sc. PD:	0.4
		Sc. rank:	658.5

PB2			
Pos.	404	obs:	exp:
ttt	F	0	418.20
ttc	F	1059	640.80
---	--	------	------
mPD		0	0.48
		nPD:	0.
		N. weight:	0.55
		Sc. PD:	-0.053
		Sc. rank:	-458.6

PB2			
Pos.	405	obs:	exp:
tct	S	0	168.40
tcc	S	0	138.30
tca	S	1059	268.80
tcg	S	0	60.09
agt	S	0	217.70
agc	S	0	205.70
---	--	------	------
mPD		0	1.7
		nPD:	0.
		N. weight:	1.5
		Sc. PD:	-0.14
		Sc. rank:	-1251.9

PB2			
Pos.	406	obs:	exp:
caa	Q	1056	539.40
cag	Q	3	519.60
---	--	------	------
mPD		0.0057	0.50
		nPD:	0.01
		N. weight:	0.72
		Sc. PD:	-0.061
		Sc. rank:	-282.6

PB2			
Pos.	407	obs:	exp:
gaa	E	102	644.40
gag	E	957	414.60
---	--	------	------
mPD		0.17	0.48
		nPD:	0.37
		N. weight:	0.64
		Sc. PD:	0.16
		Sc. rank:	454.7

PB2			
Pos.	408	obs:	exp:
gat	D	1031	572.30
gac	D	28	486.70
---	--	------	------
mPD		0.052	0.50
		nPD:	0.1
		N. weight:	0.55
		Sc. PD:	0.0022
		Sc. rank:	220.6

PB2			
Pos.	409	obs:	exp:
tgt	C	1029	446.70
tgc	C	27	609.30
cgt	R	3	0.13
cgc	R	0	0.15
cga	R	0	0.29
cgg	R	0	0.25
aga	R	0	1.37
agg	R	0	0.81
---	--	------	------
mPD		0.055	0.50
		nPD:	0.11
		N. weight:	0.82
		Sc. PD:	0.0094
		Sc. rank:	346.5

PB2			
Pos.	410	obs:	exp:
atg	M	1058	1058.00
gtt	V	0	0.21
gtc	V	0	0.20
gta	V	0	0.20
gtg	V	1	0.39
---	--	------	------
mPD		0.0019	0.0030
		nPD:	0.62
		N. weight:	0.00099
		Sc. PD:	0.00049
		Sc. rank:	0.8

PB2			
Pos.	411	obs:	exp:
att	I	0	371.50
atc	I	0	242.30
ata	I	1011	397.30
atg	M	4	4.00
gtt	V	0	9.12
gtc	V	0	8.88
gta	V	44	9.01
gtg	V	0	16.99
---	--	------	------
mPD		0.087	0.75
		nPD:	0.12
		N. weight:	1.1
		Sc. PD:	0.017
		Sc. rank:	457.3

PB2			
Pos.	412	obs:	exp:
aaa	K	19	617.20
aag	K	1040	441.80
---	--	------	------
mPD		0.035	0.49
		nPD:	0.07
		N. weight:	0.86
		Sc. PD:	-0.022
		Sc. rank:	263.1

PB2			
Pos.	413	obs:	exp:
gct	A	0	273.50
gcc	A	0	197.20
gca	A	1059	498.40
gcg	A	0	89.88
---	--	------	------
mPD		0	0.67
		nPD:	0.
		N. weight:	0.83
		Sc. PD:	-0.079
		Sc. rank:	-688.0

PB2			
Pos.	414	obs:	exp:
gtt	V	0	219.40
gtc	V	1	213.70
gta	V	16	216.80
gtg	V	1042	409.00
---	--	------	------
mPD		0.032	0.73
		nPD:	0.04
		N. weight:	0.97
		Sc. PD:	-0.052
		Sc. rank:	108.4

PB2			
Pos.	415	obs:	exp:
cgt	R	0	45.48
cgc	R	0	52.45
cga	R	1	103.60
cgg	R	0	88.36
aga	R	1045	484.20
agg	R	13	284.80
---	--	------	------
mPD		0.026	0.96
		nPD:	0.03
		N. weight:	0.79
		Sc. PD:	-0.055
		Sc. rank:	-31.3

PB2			
Pos.	416	obs:	exp:
tgt	C	1	0.42
tgc	C	0	0.58
ggt	G	984	140.20
ggc	G	72	135.20
gga	G	0	488.10
ggg	G	2	294.50
---	--	------	------
mPD		0.13	0.68
		nPD:	0.2
		N. weight:	1.9
		Sc. PD:	0.18
		Sc. rank:	1043.0

PB2			
Pos.	417	obs:	exp:
gat	D	1057	572.30
gac	D	2	486.70
---	--	------	------
mPD		0.0038	0.50
		nPD:	0.01
		N. weight:	0.66
		Sc. PD:	-0.059
		Sc. rank:	-351.8

PB2			
Pos.	418	obs:	exp:
tta	L	0	93.98
ttg	L	1057	197.20
ctt	L	0	176.30
ctc	L	0	176.70
cta	L	0	178.50
ctg	L	2	236.30
---	--	------	------
mPD		0.0038	1.1
		nPD:	0.
		N. weight:	1.8
		Sc. PD:	-0.17
		Sc. rank:	-1212.1

PB2			
Pos.	419	obs:	exp:
aat	N	3	539.40
aac	N	1056	519.60
---	--	------	------
mPD		0.0057	0.50
		nPD:	0.01
		N. weight:	0.76
		Sc. PD:	-0.065
		Sc. rank:	-301.1

PB2			
Pos.	420	obs:	exp:
ttt	F	1058	418.20
ttc	F	1	640.80
---	--	------	------
mPD		0.0019	0.48
		nPD:	0.
		N. weight:	1.
		Sc. PD:	-0.093
		Sc. rank:	-626.7

PB2			
Pos.	421	obs:	exp:
gtt	V	0	219.40
gtc	V	1058	213.70
gta	V	1	216.80
gtg	V	0	409.00
---	--	------	------
mPD		0.0019	0.73
		nPD:	0.
		N. weight:	1.8
		Sc. PD:	-0.16
		Sc. rank:	-1257.7

PB2			
Pos.	422	obs:	exp:
aat	N	29	539.40
aac	N	1030	519.60
---	--	------	------
mPD		0.053	0.50
		nPD:	0.11
		N. weight:	0.65
		Sc. PD:	0.0044
		Sc. rank:	263.5

PB2			
Pos.	423	obs:	exp:
cgt	R	0	45.48
cgc	R	0	52.45
cga	R	4	103.60
cgg	R	0	88.36
aga	R	1053	484.20
agg	R	2	284.80
---	--	------	------
mPD		0.011	0.96
		nPD:	0.01
		N. weight:	0.83
		Sc. PD:	-0.07
		Sc. rank:	-306.0

PB2			
Pos.	424	obs:	exp:
gct	A	0	273.50
gcc	A	0	197.20
gca	A	719	498.40
gcg	A	340	89.88
---	--	------	------
mPD		0.44	0.67
		nPD:	0.65
		N. weight:	0.75
		Sc. PD:	0.4
		Sc. rank:	595.6

PB2			
Pos.	425	obs:	exp:
aat	N	166	539.40
aac	N	893	519.60
---	--	------	------
mPD		0.26	0.50
		nPD:	0.53
		N. weight:	0.3
		Sc. PD:	0.12
		Sc. rank:	232.0

PB2

Pos.	426	obs:	exp:
caa	Q	3	539.40
cag	Q	1056	519.60
mPD		0.0057	0.50
		nPD:	0.01
		N. weight:	0.76
		Sc. PD:	-0.065
		Sc. rank:	-298.7

PB2

Pos.	427	obs:	exp:
caa	Q	0	0.51
cag	Q	1	0.49
cgt	R	0	45.44
cgc	R	0	52.40
cga	R	104	103.50
cgg	R	695	88.28
aga	R	5	483.80
agg	R	254	284.60
mPD		0.56	0.96
		nPD:	0.58
		N. weight:	1.4
		Sc. PD:	0.66
		Sc. rank:	1123.6

PB2

Pos.	428	obs:	exp:
tta	L	10	93.98
ttg	L	0	197.20
ctt	L	0	176.30
ctc	L	0	176.70
cta	L	1038	178.50
ctg	L	11	236.30
mPD		0.039	1.1
		nPD:	0.04
		N. weight:	1.8
		Sc. PD:	-0.11
		Sc. rank:	96.5

PB2

Pos.	429	obs:	exp:
aat	N	1046	539.40
aac	N	13	519.60
mPD		0.024	0.50
		nPD:	0.05
		N. weight:	0.67
		Sc. PD:	-0.033
		Sc. rank:	107.0

PB2

Pos.	430	obs:	exp:
cct	P	4	264.00
ccc	P	1054	190.10
cca	P	0	435.50
ccg	P	0	168.40
act	T	0	0.25
acc	T	1	0.21
aca	T	0	0.46
acg	T	0	0.08
mPD		0.0094	0.71
		nPD:	0.01
		N. weight:	1.9
		Sc. PD:	-0.15
		Sc. rank:	-620.9

PB2

Pos.	431	obs:	exp:
atg	M	1059	1059.00
mPD		0	0
		nPD:	1.
		N. weight:	0.
		Sc. PD:	0
		Sc. rank:	0

PB2

Pos.	432	obs:	exp:
cat	H	1047	608.10
cac	H	12	450.90
mPD		0.022	0.49
		nPD:	0.05
		N. weight:	0.55
		Sc. PD:	-0.028
		Sc. rank:	71.3

PB2

Pos.	433	obs:	exp:
caa	Q	1054	539.40
cag	Q	5	519.60
mPD		0.0094	0.50
		nPD:	0.02
		N. weight:	0.71
		Sc. PD:	-0.055
		Sc. rank:	-126.6

PB2

Pos.	434	obs:	exp:
tta	L	0	93.98
ttg	L	0	197.20
ctt	L	37	176.30
ctc	L	1015	176.70
cta	L	6	178.50
ctg	L	1	236.30
mPD		0.080	1.1
		nPD:	0.07
		N. weight:	1.8
		Sc. PD:	-0.046
		Sc. rank:	536.6

PB2

Pos.	435	obs:	exp:
tta	L	0	93.98
ttg	L	8	197.20
ctt	L	0	176.30
ctc	L	0	176.70
cta	L	24	178.50
ctg	L	1027	236.30
mPD		0.059	1.1
		nPD:	0.05
		N. weight:	1.5
		Sc. PD:	-0.066
		Sc. rank:	303.8

PB2

Pos.	436	obs:	exp:
cgt	R	0	45.48
cgc	R	0	52.45
cga	R	0	103.60
cgg	R	0	88.36
aga	R	72	484.20
agg	R	987	284.80
mPD		0.13	0.96
		nPD:	0.13
		N. weight:	1.1
		Sc. PD:	0.035
		Sc. rank:	510.7

PB2

Pos.	437	obs:	exp:
cat	H	1058	608.10
cac	H	1	450.90
mPD		0.0019	0.49
		nPD:	0.
		N. weight:	0.6
		Sc. PD:	-0.055
		Sc. rank:	-389.9

PB2

Pos.	438	obs:	exp:
ttt	F	113	418.20
ttc	F	946	640.80
mPD		0.19	0.48
		nPD:	0.4
		N. weight:	0.23
		Sc. PD:	0.067
		Sc. rank:	169.9

PB2

Pos.	439	obs:	exp:
caa	Q	1054	539.40
cag	Q	5	519.60
mPD		0.0094	0.50
		nPD:	0.02
		N. weight:	0.71
		Sc. PD:	-0.055
		Sc. rank:	-126.6

PB2

Pos.	440	obs:	exp:
aaa	K	8	617.20
aag	K	1051	441.80
mPD		0.015	0.49
		nPD:	0.03
		N. weight:	0.91
		Sc. PD:	-0.06
		Sc. rank:	10.1

PB2

Pos.	441	obs:	exp:
aat	N	2	1.02
aac	N	0	0.98
gat	D	986	570.10
gac	D	69	484.90
ggt	G	2	0.27
ggc	G	0	0.26
gga	G	0	0.92
ggg	G	0	0.56
mPD		0.13	0.51
		nPD:	0.26
		N. weight:	0.43
		Sc. PD:	0.064
		Sc. rank:	272.6

PB2

Pos.	442	obs:	exp:
gct	A	1	273.50
gcc	A	65	197.20
gca	A	993	498.40
gcg	A	0	89.88
mPD		0.12	0.67
		nPD:	0.17
		N. weight:	0.63
		Sc. PD:	0.046
		Sc. rank:	319.5

PB2

Pos.	443	obs:	exp:
cgt	R	0	1.03
cgc	R	0	1.19
cga	R	0	2.35
cgg	R	0	2.00
aaa	K	31	603.20
aag	K	1004	431.80
aga	R	0	10.97
agg	R	24	6.46
mPD		0.10	0.54
		nPD:	0.19
		N. weight:	0.82
		Sc. PD:	0.068
		Sc. rank:	420.6

PB2

Pos.	444	obs:	exp:
att	I	0	1.10
atc	I	3	0.72
ata	I	0	1.18
gtt	V	105	218.80
gtc	V	950	213.10
gta	V	0	216.20
gtg	V	1	407.80
mPD		0.19	0.73
		nPD:	0.25
		N. weight:	1.4
		Sc. PD:	0.21
		Sc. rank:	882.7

PB2

Pos.	445	obs:	exp:
tta	L	0	93.98
ttg	L	3	197.20
ctt	L	2	176.30
ctc	L	0	176.70
cta	L	5	178.50
ctg	L	1049	236.30
mPD		0.019	1.1
		nPD:	0.02
		N. weight:	1.6
		Sc. PD:	-0.13
		Sc. rank:	-355.1

PB2

Pos.	446	obs:	exp:
ttt	F	1047	417.40
ttc	F	10	639.60
tta	L	0	0.18
ttg	L	0	0.37
ctt	L	2	0.33
ctc	L	0	0.33
cta	L	0	0.34
ctg	L	0	0.45
mPD		0.022	0.48
		nPD:	0.05
		N. weight:	0.96
		Sc. PD:	-0.049
		Sc. rank:	133.0

PB2

Pos.	447	obs:	exp:
cat	H	0	4.59
cac	H	8	3.41
caa	Q	1051	535.30
cag	Q	0	515.70
mPD		0.015	0.51
		nPD:	0.03
		N. weight:	0.74
		Sc. PD:	-0.05
		Sc. rank:	-0.4

PB2

Pos.	448	obs:	exp:
tct	S	0	0.95
tcc	S	0	0.78
tca	S	0	1.52
tcg	S	0	0.34
aat	N	7	536.40
aac	N	1046	516.60
agt	S	0	1.23
agc	S	6	1.17
mPD		0.024	0.52
		nPD:	0.05
		N. weight:	0.75
		Sc. PD:	-0.038
		Sc. rank:	108.1

PB2

Pos.	449	obs:	exp:
tgg	W	1059	1059.00
mPD		0	0
		nPD:	1.
		N. weight:	0.
		Sc. PD:	0
		Sc. rank:	0

PB2

Pos.	450	obs:	exp:
gaa	E	2	1.22
gag	E	0	0.78
ggt	G	0	140.10
ggc	G	0	135.00
gga	G	1053	487.60
ggg	G	4	294.30
mPD		0.011	0.68
		nPD:	0.02
		N. weight:	0.83
		Sc. PD:	-0.066
		Sc. rank:	-192.6

PB2			
Pos.	451	obs:	exp:
tta	L	0	0.09
ttg	L	0	0.19
ctt	L	1	0.17
ctc	L	0	0.17
cta	L	0	0.17
ctg	L	0	0.22
att	I	948	349.40
atc	I	3	227.90
ata	I	0	373.70
act	T	98	25.49
acc	T	2	20.52
aca	T	0	45.87
acg	T	0	8.12
gtt	V	7	1.45
gtc	V	0	1.41
gta	V	0	1.43
gtg	V	0	2.70
---	--	------	------
mPD		0.20	0.85
		nPD:	0.23
		N. weight:	1.1
		Sc. PD:	0.14
		Sc. rank:	670.4

,

PB2			
Pos.	452	obs:	exp:
gaa	E	805	644.40
gag	E	254	414.60
---	--	------	------
mPD		0.36	0.48
		nPD:	0.77
		N. weight:	0.06
		Sc. PD:	0.039
		Sc. rank:	50.4

,

PB2			
Pos.	453	obs:	exp:
tta	L	0	0.18
ttg	L	0	0.37
tct	S	0	0.48
tcc	S	3	0.39
tca	S	0	0.76
tcg	S	0	0.17
ctt	L	0	0.33
ctc	L	2	0.33
cta	L	0	0.34
ctg	L	0	0.45
cct	P	1	260.20
ccc	P	1042	187.40
cca	P	0	429.30
ccg	P	0	166.00
cat	H	0	5.74
cac	H	10	4.26
act	T	0	0.25
acc	T	1	0.21
aca	T	0	0.46
acg	T	0	0.08
agt	S	0	0.62
agc	S	0	0.58
---	--	------	------
mPD		0.032	0.75
		nPD:	0.04
		N. weight:	1.9
		Sc. PD:	-0.1
		Sc. rank:	206.1

,

PB2			
Pos.	454	obs:	exp:
att	I	1057	389.10
atc	I	2	253.80
ata	I	0	416.10
---	--	------	------
mPD		0.0038	0.65
		nPD:	0.01
		N. weight:	1.1
		Sc. PD:	-0.098
		Sc. rank:	-624.1

,

PB2			
Pos.	455	obs:	exp:
gat	D	3	572.30
gac	D	1056	486.70
---	--	------	------
mPD		0.0057	0.50
		nPD:	0.01
		N. weight:	0.83
		Sc. PD:	-0.071
		Sc. rank:	-318.1

,

PB2			
Pos.	456	obs:	exp:
aat	N	1055	538.90
aac	N	3	519.10
gat	D	1	0.54
gac	D	0	0.46
---	--	------	------
mPD		0.0075	0.50
		nPD:	0.02
		N. weight:	0.72
		Sc. PD:	-0.059
		Sc. rank:	-204.3

,

PB2			
Pos.	457	obs:	exp:
gtt	V	0	219.40
gtc	V	1	213.70
gta	V	1039	216.80
gtg	V	19	409.00
---	--	------	------
mPD		0.037	0.73
		nPD:	0.05
		N. weight:	1.6
		Sc. PD:	-0.076
		Sc. rank:	282.9

,

PB2			
Pos.	458	obs:	exp:
atg	M	1059	1059.00
---	--	------	------
mPD		0	0
		nPD:	1.
		N. weight:	0.
		Sc. PD:	0
		Sc. rank:	0

,

PB2			
Pos.	459	obs:	exp:
ggt	G	1	140.40
ggc	G	0	135.30
gga	G	77	488.50
ggg	G	981	294.80
---	--	------	------
mPD		0.14	0.68
		nPD:	0.2
		N. weight:	1.1
		Sc. PD:	0.11
		Sc. rank:	591.0

,

PB2			
Pos.	460	obs:	exp:
atg	M	1059	1059.00
---	--	------	------
mPD		0	0
		nPD:	1.
		N. weight:	0.
		Sc. PD:	0
		Sc. rank:	0

,

PB2			
Pos.	461	obs:	exp:
att	I	24	386.90
atc	I	1026	252.30
ata	I	3	413.80
gtt	V	0	1.24
gtc	V	6	1.21
gta	V	0	1.23
gtg	V	0	2.32
---	--	------	------
mPD		0.061	0.67
		nPD:	0.09
		N. weight:	1.4
		Sc. PD:	-0.011
		Sc. rank:	533.3

,

PB2			
Pos.	462	obs:	exp:
ggt	G	0	140.40
ggc	G	0	135.30
gga	G	919	488.50
ggg	G	140	294.80
---	--	------	------
mPD		0.23	0.68
		nPD:	0.34
		N. weight:	0.5
		Sc. PD:	0.11
		Sc. rank:	352.5

,

PB2			
Pos.	463	obs:	exp:
att	I	0	388.00
atc	I	4	253.10
ata	I	1052	414.90
gtt	V	0	0.62
gtc	V	0	0.61
gta	V	3	0.61
gtg	V	0	1.16
---	--	------	------
mPD		0.013	0.66
		nPD:	0.02
		N. weight:	1.
		Sc. PD:	-0.077
		Sc. rank:	-145.1

,

PB2			
Pos.	464	obs:	exp:
ttt	F	1	0.39
ttc	F	0	0.61
tta	L	32	93.89
ttg	L	1024	197.00
ctt	L	0	176.20
ctc	L	0	176.60
cta	L	0	178.30
ctg	L	2	236.10
---	--	------	------
mPD		0.064	1.1
		nPD:	0.06
		N. weight:	1.7
		Sc. PD:	-0.069
		Sc. rank:	412.4

,

PB2			
Pos.	465	obs:	exp:
cct	P	1058	264.20
ccc	P	0	190.30
cca	P	1	435.90
ccg	P	0	168.60
---	--	------	------
mPD		0.0019	0.71
		nPD:	0.
		N. weight:	1.5
		Sc. PD:	-0.14
		Sc. rank:	-1068.5

,

PB2			
Pos.	466	obs:	exp:
gat	D	124	572.30
gac	D	935	486.70
---	--	------	------
mPD		0.21	0.50
		nPD:	0.42
		N. weight:	0.44
		Sc. PD:	0.13
		Sc. rank:	325.1

,

PB2			
Pos.	467	obs:	exp:
tta	L	0	0.09
ttg	L	1	0.19
ctt	L	0	0.17
ctc	L	0	0.17
cta	L	0	0.17
ctg	L	0	0.22
att	I	0	0.37
atc	I	0	0.24
ata	I	1	0.39
atg	M	1057	1057.00
---	--	------	------
mPD		0.0038	0.0049
		nPD:	0.77
		N. weight:	0.0027
		Sc. PD:	0.0018
		Sc. rank:	2.3

,

PB2			
Pos.	468	obs:	exp:
act	T	1	269.90
acc	T	1058	217.30
aca	T	0	485.70
acg	T	0	86.02
---	--	------	------
mPD		0.0019	0.68
		nPD:	0.
		N. weight:	1.7
		Sc. PD:	-0.16
		Sc. rank:	-1210.8

,

PB2			
Pos.	469	obs:	exp:
cct	P	3	264.20
ccc	P	1055	190.30
cca	P	1	435.90
ccg	P	0	168.60
---	--	------	------
mPD		0.0075	0.71
		nPD:	0.01
		N. weight:	1.9
		Sc. PD:	-0.16
		Sc. rank:	-766.1

,

PB2			
Pos.	470	obs:	exp:
tct	S	0	168.10
tcc	S	0	138.00
tca	S	0	268.30
tcg	S	0	59.98
aat	N	0	1.02
aac	N	2	0.98
agt	S	33	217.30
agc	S	1024	205.30
---	--	------	------
mPD		0.064	1.7
		nPD:	0.04
		N. weight:	1.6
		Sc. PD:	-0.096
		Sc. rank:	148.3

,

PB2

Pos. 471		obs:	exp:
cct	P	0	0.25
ccc	P	0	0.18
cca	P	1	0.41
ccg	P	0	0.16
act	T	5	269.70
acc	T	1	217.10
aca	T	1049	485.30
acg	T	3	85.94
---	--	------	------
mPD		0.019	0.68
		nPD:	0.03
		N. weight:	0.81
		Sc. PD:	-0.056
		Sc. rank:	-18.1

PB2

Pos. 472		obs:	exp:
gaa	E	2	644.40
gag	E	1057	414.60
---	--	------	------
mPD		0.0038	0.48
		nPD:	0.01
		N. weight:	1.
		Sc. PD:	-0.089
		Sc. rank:	-511.3

PB2

Pos. 473		obs:	exp:
att	I	0	0.73
atc	I	0	0.48
ata	I	2	0.79
atg	M	1017	1017.00
act	T	0	0.51
acc	T	0	0.41
aca	T	0	0.92
acg	T	2	0.16
aaa	K	0	0.58
aag	K	1	0.42
gtt	V	0	7.67
gtc	V	0	7.47
gta	V	0	7.58
gtg	V	37	14.29
---	--	------	------
mPD		0.077	0.12
		nPD:	0.62
		N. weight:	0.045
		Sc. PD:	0.022
		Sc. rank:	35.3

PB2

Pos. 474		obs:	exp:
tct	S	12	168.40
tcc	S	226	138.30
tca	S	821	268.80
tcg	S	0	60.09
agt	S	0	217.70
agc	S	0	205.70
---	--	------	------
mPD		0.35	1.7
		nPD:	0.21
		N. weight:	1.
		Sc. PD:	0.11
		Sc. rank:	598.0

PB2

Pos. 475		obs:	exp:
tta	L	19	93.89
ttg	L	925	197.00
ctt	L	0	176.20
ctc	L	0	176.60
cta	L	0	178.30
ctg	L	114	236.10
atg	M	1	1.00
---	--	------	------
mPD		0.23	1.1
		nPD:	0.21
		N. weight:	1.4
		Sc. PD:	0.14
		Sc. rank:	761.9

PB2

Pos. 476		obs:	exp:
cgt	R	0	45.48
cgc	R	0	52.45
cga	R	0	103.60
cgg	R	0	88.36
aga	R	1054	484.20
agg	R	5	284.80
---	--	------	------
mPD		0.0094	0.96
		nPD:	0.01
		N. weight:	0.83
		Sc. PD:	-0.072
		Sc. rank:	-375.6

PB2

Pos. 477		obs:	exp:
cgt	R	0	0.04
cgc	R	0	0.05
cga	R	0	0.10
cgg	R	0	0.08
aga	R	1	0.46
agg	R	0	0.27
ggt	G	0	140.20
ggc	G	0	135.20
gga	G	1051	488.10
ggg	G	7	294.50
---	--	------	------
mPD		0.015	0.68
		nPD:	0.02
		N. weight:	0.81
		Sc. PD:	-0.06
		Sc. rank:	-97.9

PB2

Pos. 478		obs:	exp:
atg	M	1	1.00
gtt	V	0	219.20
gtc	V	0	213.50
gta	V	5	216.60
gtg	V	1053	408.60
---	--	------	------
mPD		0.011	0.73
		nPD:	0.02
		N. weight:	1.
		Sc. PD:	-0.082
		Sc. rank:	-265.7

PB2

Pos. 479		obs:	exp:
cgt	R	0	45.48
cgc	R	0	52.45
cga	R	0	103.60
cgg	R	0	88.36
aga	R	1057	484.20
agg	R	2	284.80
---	--	------	------
mPD		0.0038	0.96
		nPD:	0.
		N. weight:	0.85
		Sc. PD:	-0.078
		Sc. rank:	-541.6

PB2

Pos. 480		obs:	exp:
gtt	V	1053	219.40
gtc	V	6	213.70
gta	V	0	216.80
gtg	V	0	409.00
---	--	------	------
mPD		0.011	0.73
		nPD:	0.02
		N. weight:	1.7
		Sc. PD:	-0.14
		Sc. rank:	-437.6

PB2

Pos. 481		obs:	exp:
tct	S	0	168.40
tcc	S	0	138.30
tca	S	0	268.80
tcg	S	0	60.09
agt	S	1053	217.70
agc	S	6	205.70
---	--	------	------
mPD		0.011	1.7
		nPD:	0.01
		N. weight:	1.7
		Sc. PD:	-0.15
		Sc. rank:	-924.2

PB2

Pos. 482		obs:	exp:
cgt	R	0	0.43
cgc	R	0	0.50
cga	R	0	0.98
cgg	R	0	0.83
aaa	K	1047	611.40
aag	K	2	437.60
aga	R	10	4.57
agg	R	0	2.69
---	--	------	------
mPD		0.022	0.51
		nPD:	0.04
		N. weight:	0.58
		Sc. PD:	-0.031
		Sc. rank:	68.3

PB2

Pos. 483		obs:	exp:
atg	M	1059	1059.00
---	--	------	------
mPD		0	0
		nPD:	1.
		N. weight:	0.
		Sc. PD:	0
		Sc. rank:	0

PB2

Pos. 484		obs:	exp:
ggt	G	0	140.40
ggc	G	0	135.30
gga	G	1055	488.50
ggg	G	4	294.80
---	--	------	------
mPD		0.0075	0.68
		nPD:	0.01
		N. weight:	0.83
		Sc. PD:	-0.07
		Sc. rank:	-332.8

PB2

Pos. 485		obs:	exp:
gtt	V	2	219.40
gtc	V	0	213.70
gta	V	1054	216.80
gtg	V	3	409.00
---	--	------	------
mPD		0.0094	0.73
		nPD:	0.01
		N. weight:	1.7
		Sc. PD:	-0.14
		Sc. rank:	-577.1

PB2

Pos. 486		obs:	exp:
gat	D	1058	572.30
gac	D	1	486.70
---	--	------	------
mPD		0.0019	0.50
		nPD:	0.
		N. weight:	0.67
		Sc. PD:	-0.062
		Sc. rank:	-435.0

PB2

Pos. 487		obs:	exp:
gaa	E	1012	644.40
gag	E	47	414.60
---	--	------	------
mPD		0.085	0.48
		nPD:	0.18
		N. weight:	0.37
		Sc. PD:	0.028
		Sc. rank:	187.6

PB2

Pos. 488		obs:	exp:
tat	Y	937	571.40
tac	Y	121	486.60
cat	H	1	0.57
cac	H	0	0.43
---	--	------	------
mPD		0.20	0.50
		nPD:	0.41
		N. weight:	0.31
		Sc. PD:	0.092
		Sc. rank:	228.4

PB2

Pos. 489		obs:	exp:
tct	S	75	168.00
tcc	S	981	137.90
tca	S	0	268.00
tcg	S	0	59.92
tgt	C	3	1.27
tgc	C	0	1.73
agt	S	0	217.10
agc	S	0	205.10
---	--	------	------
mPD		0.14	1.7
		nPD:	0.09
		N. weight:	1.9
		Sc. PD:	-0.025
		Sc. rank:	681.9

PB2

Pos. 490		obs:	exp:
tct	S	0	168.40
tcc	S	0	138.30
tca	S	0	268.80
tcg	S	0	60.09
agt	S	686	217.70
agc	S	373	205.70
---	--	------	------
mPD		0.46	1.7
		nPD:	0.28
		N. weight:	1.1
		Sc. PD:	0.18
		Sc. rank:	701.9

PB2

Pos. 491		obs:	exp:
act	T	955	269.90
acc	T	18	217.30
aca	T	86	485.70
acg	T	0	86.02
---	--	------	------
mPD		0.18	0.68
		nPD:	0.27
		N. weight:	1.1
		Sc. PD:	0.17
		Sc. rank:	691.4

PB2

Pos. 492		obs:	exp:
gaa	E	6	644.40
gag	E	1053	414.60
---	--	------	------
mPD		0.011	0.48
		nPD:	0.02
		N. weight:	0.99
		Sc. PD:	-0.072
		Sc. rank:	-93.4

PB2

Pos. 493		obs:	exp:
cgt	R	0	45.48
cgc	R	0	52.45
cga	R	0	103.60
cgg	R	0	88.36
aga	R	1050	484.20
agg	R	9	284.80
---	--	------	------
mPD		0.017	0.96
		nPD:	0.02
		N. weight:	0.81
		Sc. PD:	-0.064
		Sc. rank:	-175.1

PB2

Pos. 494		obs:	exp:
gtt	V	2	219.40
gtc	V	0	213.70
gta	V	48	216.80
gtg	V	1009	409.00
---	--	------	------
mPD		0.090	0.73
		nPD:	0.12
		N. weight:	0.86
		Sc. PD:	0.021
		Sc. rank:	383.3

PB2

Pos. 495		obs:	exp:
att	I	0	1.47
atc	I	4	0.96
ata	I	0	1.57
gtt	V	9	218.60
gtc	V	961	212.90
gta	V	85	216.00
gtg	V	0	407.50
---	--	------	------
mPD		0.17	0.73
		nPD:	0.23
		N. weight:	1.4
		Sc. PD:	0.18
		Sc. rank:	849.3

PB2			
Pos.	496	obs:	exp:
gtt	V	30	219.40
gtc	V	0	213.70
gta	V	5	216.80
gtg	V	1024	409.00
---	--	------	------
mPD		0.064	0.73
		nPD:	0.09
		N. weight:	0.9
		Sc. PD:	-0.0095
		Sc. rank:	323.0

PB2			
Pos.	497	obs:	exp:
tct	S	0	168.30
tcc	S	0	138.20
tca	S	0	268.50
tcg	S	0	60.03
act	T	1	0.25
acc	T	0	0.21
aca	T	0	0.46
acg	T	0	0.08
agt	S	975	217.50
agc	S	83	205.50
---	--	------	------
mPD		0.15	1.7
		nPD:	0.09
		N. weight:	1.4
		Sc. PD:	-0.015
		Sc. rank:	516.7

PB2			
Pos.	498	obs:	exp:
att	I	1059	389.10
atc	I	0	253.80
ata	I	0	416.10
---	--	------	------
mPD		0	0.65
		nPD:	0.
		N. weight:	1.1
		Sc. PD:	-0.11
		Sc. rank:	-914.1

PB2			
Pos.	499	obs:	exp:
gat	D	1044	572.30
gac	D	15	486.70
---	--	------	------
mPD		0.028	0.50
		nPD:	0.06
		N. weight:	0.6
		Sc. PD:	-0.025
		Sc. rank:	136.0

PB2			
Pos.	500	obs:	exp:
tct	S	0	0.16
tcc	S	0	0.13
tca	S	0	0.25
tcg	S	0	0.06
cgt	R	1051	45.44
cgc	R	5	52.40
cga	R	0	103.50
cgg	R	2	88.28
agt	S	1	0.21
agc	S	0	0.19
aga	R	0	483.80
agg	R	0	284.60
---	--	------	------
mPD		0.015	0.96
		nPD:	0.02
		N. weight:	3.4
		Sc. PD:	-0.27
		Sc. rank:	-838.1

PB2			
Pos.	501	obs:	exp:
ttt	F	29	418.20
ttc	F	1030	640.80
---	--	------	------
mPD		0.053	0.48
		nPD:	0.11
		N. weight:	0.43
		Sc. PD:	0.0049
		Sc. rank:	181.6

PB2			
Pos.	502	obs:	exp:
tta	L	204	93.98
ttg	L	853	197.20
ctt	L	0	176.30
ctc	L	0	176.70
cta	L	0	178.50
ctg	L	2	236.30
---	--	------	------
mPD		0.32	1.1
		nPD:	0.28
		N. weight:	1.5
		Sc. PD:	0.26
		Sc. rank:	985.0

PB2			
Pos.	503	obs:	exp:
cgt	R	0	45.48
cgc	R	0	52.45
cga	R	0	103.60
cgg	R	0	88.36
aga	R	5	484.20
agg	R	1054	284.80
---	--	------	------
mPD		0.0094	0.96
		nPD:	0.01
		N. weight:	1.4
		Sc. PD:	-0.12
		Sc. rank:	-636.6

PB2			
Pos.	504	obs:	exp:
gtt	V	106	219.40
gtc	V	953	213.70
gta	V	0	216.80
gtg	V	0	409.00
---	--	------	------
mPD		0.18	0.73
		nPD:	0.25
		N. weight:	1.4
		Sc. PD:	0.2
		Sc. rank:	868.8

PB2			
Pos.	505	obs:	exp:
cgt	R	0	45.48
cgc	R	0	52.45
cga	R	910	103.60
cgg	R	145	88.36
aga	R	4	484.20
agg	R	0	284.80
---	--	------	------
mPD		0.24	0.96
		nPD:	0.25
		N. weight:	2.1
		Sc. PD:	0.31
		Sc. rank:	1328.6

PB2			
Pos.	506	obs:	exp:
gat	D	22	572.30
gac	D	1037	486.70
---	--	------	------
mPD		0.041	0.50
		nPD:	0.08
		N. weight:	0.74
		Sc. PD:	-0.013
		Sc. rank:	253.3

PB2			
Pos.	507	obs:	exp:
cat	H	1	0.57
cac	H	0	0.43
caa	Q	30	538.90
cag	Q	1028	519.10
---	--	------	------
mPD		0.057	0.50
		nPD:	0.11
		N. weight:	0.64
		Sc. PD:	0.0087
		Sc. rank:	273.5

PB2			
Pos.	508	obs:	exp:
cgt	R	0	45.10
cgc	R	0	52.01
cga	R	1	102.80
cgg	R	0	87.61
aaa	K	0	5.25
aag	K	9	3.75
aga	R	244	480.10
agg	R	805	282.40
---	--	------	------
mPD		0.37	0.98
		nPD:	0.38
		N. weight:	0.71
		Sc. PD:	0.19
		Sc. rank:	509.8

PB2			
Pos.	509	obs:	exp:
ggt	G	1	140.40
ggc	G	0	135.30
gga	G	1050	488.50
ggg	G	8	294.80
---	--	------	------
mPD		0.017	0.68
		nPD:	0.02
		N. weight:	0.8
		Sc. PD:	-0.057
		Sc. rank:	-70.2

PB2			
Pos.	510	obs:	exp:
aat	N	7	539.40
aac	N	1052	519.60
---	--	------	------
mPD		0.013	0.50
		nPD:	0.03
		N. weight:	0.74
		Sc. PD:	-0.052
		Sc. rank:	-44.0

PB2			
Pos.	511	obs:	exp:
att	I	0	88.55
atc	I	0	57.75
ata	I	241	94.70
gtt	V	0	169.50
gtc	V	11	165.10
gta	V	626	167.50
gtg	V	181	315.90
---	--	------	------
mPD		0.65	1.1
		nPD:	0.59
		N. weight:	0.96
		Sc. PD:	0.46
		Sc. rank:	753.5

PB2			
Pos.	512	obs:	exp:
tta	L	0	93.98
ttg	L	0	197.20
ctt	L	11	176.30
ctc	L	1047	176.70
cta	L	1	178.50
ctg	L	0	236.30
---	--	------	------
mPD		0.022	1.1
		nPD:	0.02
		N. weight:	1.9
		Sc. PD:	-0.14
		Sc. rank:	-261.8

PB2			
Pos.	513	obs:	exp:
tta	L	1	93.98
ttg	L	23	197.20
ctt	L	0	176.30
ctc	L	0	176.70
cta	L	2	178.50
ctg	L	1033	236.30
---	--	------	------
mPD		0.050	1.1
		nPD:	0.05
		N. weight:	1.5
		Sc. PD:	-0.08
		Sc. rank:	191.2

PB2			
Pos.	514	obs:	exp:
tct	S	1050	168.40
tcc	S	9	138.30
tca	S	0	268.80
tcg	S	0	60.09
agt	S	0	217.70
agc	S	0	205.70
---	--	------	------
mPD		0.017	1.7
		nPD:	0.01
		N. weight:	2.
		Sc. PD:	-0.17
		Sc. rank:	-872.8

PB2			
Pos.	515	obs:	exp:
cct	P	992	264.20
ccc	P	52	190.30
cca	P	15	435.90
ccg	P	0	168.60
---	--	------	------
mPD		0.12	0.71
		nPD:	0.17
		N. weight:	1.2
		Sc. PD:	0.083
		Sc. rank:	615.8

PB2			
Pos.	516	obs:	exp:
gaa	E	887	644.40
gag	E	172	414.60
---	--	------	------
mPD		0.27	0.48
		nPD:	0.57
		N. weight:	0.14
		Sc. PD:	0.064
		Sc. rank:	108.9

PB2			
Pos.	517	obs:	exp:
gaa	E	3	644.40
gag	E	1056	414.60
---	--	------	------
mPD		0.0057	0.48
		nPD:	0.01
		N. weight:	1.
		Sc. PD:	-0.085
		Sc. rank:	-359.1

PB2			
Pos.	518	obs:	exp:
gtt	V	1058	219.40
gtc	V	1	213.70
gta	V	0	216.80
gtg	V	0	409.00
---	--	------	------
mPD		0.0019	0.73
		nPD:	0.
		N. weight:	1.7
		Sc. PD:	-0.16
		Sc. rank:	-1237.0

PB2			
Pos.	519	obs:	exp:
tct	S	0	168.40
tcc	S	0	138.30
tca	S	0	268.80
tcg	S	0	60.09
agt	S	1057	217.70
agc	S	2	205.70
---	--	------	------
mPD		0.0038	1.7
		nPD:	0.
		N. weight:	1.7
		Sc. PD:	-0.16
		Sc. rank:	-1262.3

PB2			
Pos.	520	obs:	exp:
gaa	E	1053	644.40
gag	E	6	414.60
---	--	------	------
mPD		0.011	0.48
		nPD:	0.02
		N. weight:	0.51
		Sc. PD:	-0.037
		Sc. rank:	-48.3

PB2			
Pos.	521	obs:	exp:
att	I	0	1.47
atc	I	0	0.96
ata	I	4	1.57
act	T	1	268.90
acc	T	0	216.50
aca	T	1049	483.90
acg	T	5	85.69
mPD		0.019	0.68
		nPD:	0.03
		N. weight:	0.83
		Sc. PD:	-0.057
		Sc. rank:	-29.4

PB2			
Pos.	522	obs:	exp:
tta	L	0	0.18
ttg	L	0	0.37
ctt	L	0	0.33
ctc	L	0	0.33
cta	L	0	0.34
ctg	L	2	0.45
caa	Q	30	538.40
cag	Q	1027	518.60
mPD		0.059	0.51
		nPD:	0.12
		N. weight:	0.64
		Sc. PD:	0.01
		Sc. rank:	278.4

PB2			
Pos.	523	obs:	exp:
ggt	G	0	140.40
ggc	G	0	135.30
gga	G	1050	488.50
ggg	G	9	294.80
mPD		0.017	0.68
		nPD:	0.02
		N. weight:	0.8
		Sc. PD:	-0.058
		Sc. rank:	-73.0

PB2			
Pos.	524	obs:	exp:
att	I	0	0.73
atc	I	0	0.48
ata	I	2	0.79
act	T	0	269.40
acc	T	70	216.90
aca	T	982	484.80
acg	T	5	85.86
mPD		0.14	0.68
		nPD:	0.2
		N. weight:	0.63
		Sc. PD:	0.061
		Sc. rank:	343.5

PB2			
Pos.	525	obs:	exp:
gaa	E	1058	644.40
gag	E	1	414.60
mPD		0.0019	0.48
		nPD:	0.
		N. weight:	0.54
		Sc. PD:	-0.049
		Sc. rank:	-330.7

PB2			
Pos.	526	obs:	exp:
cgt	R	0	4.64
cgc	R	0	5.35
cga	R	0	10.57
cgg	R	0	9.01
aaa	K	1	554.30
aag	K	950	396.70
aga	R	5	49.38
agg	R	103	29.05
mPD		0.19	0.73
		nPD:	0.27
		N. weight:	0.98
		Sc. PD:	0.16
		Sc. rank:	639.2

PB2			
Pos.	527	obs:	exp:
tta	L	0	93.89
ttg	L	6	197.00
ctt	L	0	176.20
ctc	L	0	176.60
cta	L	4	178.30
ctg	L	1048	236.10
atg	M	1	1.00
mPD		0.021	1.1
		nPD:	0.02
		N. weight:	1.6
		Sc. PD:	-0.12
		Sc. rank:	-297.7

PB2			
Pos.	528	obs:	exp:
act	T	1054	269.90
acc	T	4	217.30
aca	T	1	485.70
acg	T	0	86.02
mPD		0.0094	0.68
		nPD:	0.01
		N. weight:	1.5
		Sc. PD:	-0.12
		Sc. rank:	-453.8

PB2			
Pos.	529	obs:	exp:
att	I	3	389.10
atc	I	1	253.80
ata	I	1055	416.10
mPD		0.0075	0.65
		nPD:	0.01
		N. weight:	1.
		Sc. PD:	-0.085
		Sc. rank:	-377.5

PB2			
Pos.	530	obs:	exp:
act	T	1	269.90
acc	T	0	217.30
aca	T	1054	485.70
acg	T	4	86.02
mPD		0.0094	0.68
		nPD:	0.01
		N. weight:	0.83
		Sc. PD:	-0.068
		Sc. rank:	-256.8

PB2			
Pos.	531	obs:	exp:
tat	Y	952	572.00
tac	Y	107	487.00
mPD		0.18	0.50
		nPD:	0.37
		N. weight:	0.34
		Sc. PD:	0.086
		Sc. rank:	241.1

PB2			
Pos.	532	obs:	exp:
tct	S	0	168.40
tcc	S	1	138.30
tca	S	1016	268.80
tcg	S	42	60.09
agt	S	0	217.70
agc	S	0	205.70
mPD		0.078	1.7
		nPD:	0.05
		N. weight:	1.4
		Sc. PD:	-0.07
		Sc. rank:	210.1

PB2			
Pos.	533	obs:	exp:
tct	S	1	168.40
tcc	S	1	138.30
tca	S	7	268.80
tcg	S	1050	60.09
agt	S	0	217.70
agc	S	0	205.70
mPD		0.017	1.7
		nPD:	0.01
		N. weight:	3.1
		Sc. PD:	-0.26
		Sc. rank:	-1331.7

PB2			
Pos.	534	obs:	exp:
ttt	F	0	7.50
ttc	F	19	11.50
tct	S	9	165.40
tcc	S	1028	135.80
tca	S	3	264.00
tcg	S	0	59.01
agt	S	0	213.80
agc	S	0	202.00
mPD		0.058	1.7
		nPD:	0.03
		N. weight:	2.1
		Sc. PD:	-0.13
		Sc. rank:	92.0

PB2			
Pos.	535	obs:	exp:
tta	L	0	20.41
ttg	L	222	42.82
ctt	L	0	38.30
ctc	L	0	38.38
cta	L	0	38.76
ctg	L	8	51.32
atg	M	829	829.00
mPD		0.34	0.59
		nPD:	0.58
		N. weight:	0.37
		Sc. PD:	0.17
		Sc. rank:	284.8

PB2			
Pos.	536	obs:	exp:
atg	M	1059	1059.00
mPD		0	0
		nPD:	1.
		N. weight:	0.
		Sc. PD:	0
		Sc. rank:	0

PB2			
Pos.	537	obs:	exp:
tgg	W	1059	1059.00
mPD		0	0
		nPD:	1.
		N. weight:	0.
		Sc. PD:	0
		Sc. rank:	0

PB2			
Pos.	538	obs:	exp:
gaa	E	244	644.40
gag	E	815	414.60
mPD		0.35	0.48
		nPD:	0.75
		N. weight:	0.33
		Sc. PD:	0.2
		Sc. rank:	272.9

PB2			
Pos.	539	obs:	exp:
att	I	1	388.00
atc	I	1048	253.10
ata	I	7	414.90
gtt	V	0	0.62
gtc	V	3	0.61
gta	V	0	0.61
gtg	V	0	1.16
mPD		0.021	0.66
		nPD:	0.03
		N. weight:	1.5
		Sc. PD:	-0.099
		Sc. rank:	25.2

PB2			
Pos.	540	obs:	exp:
aat	N	1058	539.40
aac	N	1	519.60
mPD		0.0019	0.50
		nPD:	0.
		N. weight:	0.73
		Sc. PD:	-0.068
		Sc. rank:	-481.8

PB2			
Pos.	541	obs:	exp:
ggt	G	1018	140.40
ggc	G	38	135.30
gga	G	1	488.50
ggg	G	2	294.80
mPD		0.075	0.68
		nPD:	0.11
		N. weight:	2.
		Sc. PD:	0.021
		Sc. rank:	856.8

PB2			
Pos.	542	obs:	exp:
cct	P	5	264.20
ccc	P	0	190.30
cca	P	47	435.90
ccg	P	1007	168.60
mPD		0.094	0.71
		nPD:	0.13
		N. weight:	1.7
		Sc. PD:	0.054
		Sc. rank:	792.0

PB2			
Pos.	543	obs:	exp:
gaa	E	1051	644.40
gag	E	8	414.60
mPD		0.015	0.48
		nPD:	0.03
		N. weight:	0.5
		Sc. PD:	-0.033
		Sc. rank:	10.0

PB2			
Pos.	544	obs:	exp:
tct	S	1	168.40
tcc	S	0	138.30
tca	S	1058	268.80
tcg	S	0	60.09
agt	S	0	217.70
agc	S	0	205.70
mPD		0.0019	1.7
		nPD:	0.
		N. weight:	1.5
		Sc. PD:	-0.14
		Sc. rank:	-1114.3

PB2			
Pos.	545	obs:	exp:
gtt	V	0	219.40
gtc	V	0	213.70
gta	V	122	216.80
gtg	V	937	409.00
mPD		0.20	0.73
		nPD:	0.28
		N. weight:	0.74
		Sc. PD:	0.13
		Sc. rank:	495.4

PB2			
Pos.	546	obs:	exp:
tta	L	81	93.98
ttg	L	0	197.20
ctt	L	1	176.30
ctc	L	2	176.70
cta	L	894	178.50
ctg	L	81	236.30
mPD		0.29	1.1
		nPD:	0.26
		N. weight:	1.4
		Sc. PD:	0.21
		Sc. rank:	891.0

PB2			
Pos.	547	obs:	exp:
att	I	7	2.57
atc	I	0	1.68
ata	I	0	2.75
gtt	V	981	218.00
gtc	V	71	212.30
gta	V	0	215.40
gtg	V	0	406.30
mPD		0.14	0.74
		nPD:	0.19
		N. weight:	1.5
		Sc. PD:	0.12
		Sc. rank:	754.9

PB2			
Pos.	548	obs:	exp:
aat	N	15	539.40
aac	N	1044	519.60
mPD		0.028	0.50
		nPD:	0.06
		N. weight:	0.7
		Sc. PD:	-0.029
		Sc. rank:	154.6

PB2			
Pos.	549	obs:	exp:
act	T	2	269.90
acc	T	10	217.30
aca	T	1047	485.70
acg	T	0	86.02
mPD		0.022	0.68
		nPD:	0.03
		N. weight:	0.8
		Sc. PD:	-0.051
		Sc. rank:	18.1

PB2			
Pos.	550	obs:	exp:
tat	Y	1035	572.00
tac	Y	24	487.00
mPD		0.044	0.50
		nPD:	0.09
		N. weight:	0.56
		Sc. PD:	-0.0056
		Sc. rank:	204.6

PB2			
Pos.	551	obs:	exp:
caa	Q	1032	539.40
cag	Q	27	519.60
mPD		0.050	0.50
		nPD:	0.1
		N. weight:	0.61
		Sc. PD:	-0.000066
		Sc. rank:	245.9

PB2			
Pos.	552	obs:	exp:
tgg	W	1059	1059.00
mPD		0	0
		nPD:	1.
		N. weight:	0.
		Sc. PD:	0
		Sc. rank:	0

PB2			
Pos.	553	obs:	exp:
att	I	3	388.70
atc	I	1054	253.50
ata	I	1	415.70
gtt	V	0	0.21
gtc	V	1	0.20
gta	V	0	0.20
gtg	V	0	0.39
mPD		0.0094	0.66
		nPD:	0.01
		N. weight:	1.5
		Sc. PD:	-0.13
		Sc. rank:	-447.3

PB2			
Pos.	554	obs:	exp:
att	I	1003	389.10
atc	I	55	253.80
ata	I	1	416.10
mPD		0.10	0.65
		nPD:	0.15
		N. weight:	0.9
		Sc. PD:	0.046
		Sc. rank:	433.2

PB2			
Pos.	555	obs:	exp:
cgt	R	0	45.44
cgc	R	0	52.40
cga	R	0	103.50
cgg	R	0	88.28
aaa	K	1	0.58
aag	K	0	0.42
aga	R	1034	483.80
agg	R	24	284.60
mPD		0.046	0.96
		nPD:	0.05
		N. weight:	0.76
		Sc. PD:	-0.037
		Sc. rank:	118.3

PB2			
Pos.	556	obs:	exp:
tct	S	0	0.16
tcc	S	0	0.13
tca	S	0	0.25
tcg	S	0	0.06
aat	N	807	538.90
aac	N	251	519.10
agt	S	1	0.21
agc	S	0	0.19
mPD		0.36	0.50
		nPD:	0.72
		N. weight:	0.15
		Sc. PD:	0.092
		Sc. rank:	127.2

PB2			
Pos.	557	obs:	exp:
tgg	W	1059	1059.00
mPD		0	0
		nPD:	1.
		N. weight:	0.
		Sc. PD:	0
		Sc. rank:	0

PB2			
Pos.	558	obs:	exp:
gaa	E	1050	644.40
gag	E	9	414.60
mPD		0.017	0.48
		nPD:	0.04
		N. weight:	0.5
		Sc. PD:	-0.031
		Sc. rank:	22.0

PB2			
Pos.	559	obs:	exp:
act	T	708	180.70
acc	T	0	145.50
aca	T	0	325.20
acg	T	1	57.59
aat	N	349	177.80
aac	N	0	171.20
gct	A	1	0.26
gcc	A	0	0.19
gca	A	0	0.47
gcg	A	0	0.08
mPD		0.45	1.1
		nPD:	0.39
		N. weight:	1.2
		Sc. PD:	0.35
		Sc. rank:	895.7

PB2			
Pos.	560	obs:	exp:
att	I	0	9.19
atc	I	0	5.99
ata	I	25	9.82
gtt	V	72	214.20
gtc	V	0	208.70
gta	V	957	211.70
gtg	V	5	399.30
mPD		0.18	0.77
		nPD:	0.23
		N. weight:	1.4
		Sc. PD:	0.18
		Sc. rank:	870.8

PB2			
Pos.	561	obs:	exp:
aaa	K	80	617.20
aag	K	979	441.80
mPD		0.14	0.49
		nPD:	0.29
		N. weight:	0.64
		Sc. PD:	0.12
		Sc. rank:	435.3

PB2			
Pos.	562	obs:	exp:
att	I	1057	389.10
atc	I	2	253.80
ata	I	0	416.10
mPD		0.0038	0.65
		nPD:	0.01
		N. weight:	1.1
		Sc. PD:	-0.098
		Sc. rank:	-624.1

PB2			
Pos.	563	obs:	exp:
tta	L	0	0.09
ttg	L	0	0.19
ctt	L	0	0.17
ctc	L	0	0.17
cta	L	1	0.17
ctg	L	0	0.22
caa	Q	1046	538.90
cag	Q	12	519.10
mPD		0.024	0.50
		nPD:	0.05
		N. weight:	0.68
		Sc. PD:	-0.033
		Sc. rank:	106.9

PB2			
Pos.	564	obs:	exp:
tgg	W	1059	1059.00
mPD		0	0
		nPD:	1.
		N. weight:	0.
		Sc. PD:	0
		Sc. rank:	0

PB2			
Pos.	565	obs:	exp:
tct	S	195	168.40
tcc	S	844	138.30
tca	S	20	268.80
tcg	S	0	60.09
agt	S	0	217.70
agc	S	0	205.70
mPD		0.33	1.7
		nPD:	0.2
		N. weight:	1.6
		Sc. PD:	0.15
		Sc. rank:	852.6

PB2			
Pos.	566	obs:	exp:
cat	H	1	0.57
cac	H	0	0.43
caa	Q	828	538.90
cag	Q	230	519.10
mPD		0.34	0.50
		nPD:	0.68
		N. weight:	0.18
		Sc. PD:	0.1
		Sc. rank:	144.8

PB2			
Pos.	567	obs:	exp:
aat	N	0	0.51
aac	N	1	0.49
gat	D	708	571.20
gac	D	349	485.80
gaa	E	0	0.61
gag	E	1	0.39
mPD		0.45	0.50
		nPD:	0.89
		N. weight:	0.043
		Sc. PD:	0.033
		Sc. rank:	36.3

PB2			
Pos.	568	obs:	exp:
cct	P	1053	264.20
ccc	P	3	190.30
cca	P	0	435.90
ccg	P	3	168.60
mPD		0.011	0.71
		nPD:	0.02
		N. weight:	1.5
		Sc. PD:	-0.12
		Sc. rank:	-349.7

PB2			
Pos.	569	obs:	exp:
act	T	8	263.60
acc	T	0	212.20
aca	T	1026	474.30
acg	T	0	83.99
gct	A	0	6.46
gcc	A	0	4.66
gca	A	25	11.77
gcg	A	0	2.12
mPD		0.061	0.72
		nPD:	0.08
		N. weight:	0.81
		Sc. PD:	-0.012
		Sc. rank:	282.7

PB2			
Pos.	570	obs:	exp:
tta	L	0	0.09
ttg	L	0	0.19
ctt	L	1	0.17
ctc	L	0	0.17
cta	L	0	0.17
ctg	L	0	0.22
att	I	238	114.60
atc	I	0	74.77
ata	I	74	122.60
atg	M	744	744.00
gtt	V	0	0.41
gtc	V	0	0.40
gta	V	1	0.41
gtg	V	1	0.77
mPD		0.46	0.48
		nPD:	0.95
		N. weight:	0.15
		Sc. PD:	0.12
		Sc. rank:	124.0

PB2			
Pos.	571	obs:	exp:
tta	L	8	93.98
ttg	L	0	197.20
ctt	L	0	176.30
ctc	L	0	176.70
cta	L	831	178.50
ctg	L	220	236.30
mPD		0.34	1.1
		nPD:	0.31
		N. weight:	1.3
		Sc. PD:	0.26
		Sc. rank:	901.0

PB2			
Pos.	572	obs:	exp:
tat	Y	3	572.00
tac	Y	1056	487.00
mPD		0.0057	0.50
		nPD:	0.01
		N. weight:	0.83
		Sc. PD:	-0.071
		Sc. rank:	-319.4

PB2			
Pos.	573	obs:	exp:
aat	N	1040	539.40
aac	N	19	519.60
mPD		0.035	0.50
		nPD:	0.07
		N. weight:	0.65
		Sc. PD:	-0.018
		Sc. rank:	192.8

PB2			
Pos.	574	obs:	exp:
aaa	K	11	617.20
aag	K	1048	441.80
mPD		0.021	0.49
		nPD:	0.04
		N. weight:	0.9
		Sc. PD:	-0.049
		Sc. rank:	93.4

PB2			
Pos.	575	obs:	exp:
att	I	0	0.37
atc	I	0	0.24
ata	I	1	0.39
atg	M	1058	1058.00
mPD		0.0019	0.0019
		nPD:	1.
		N. weight:	0.00097
		Sc. PD:	0.00084
		Sc. rank:	0.8

PB2			
Pos.	576	obs :	exp :
gaa	E	960	644.40
gag	E	99	414.60
---	--	------	------
mPD		0.17	0.48
		nPD :	0.36
		N. weight :	0.25
		Sc. PD :	0.062
		Sc. rank :	179.7

,

PB2			
Pos.	577	obs :	exp :
ttt	F	1056	418.20
ttc	F	3	640.80
---	--	------	------
mPD		0.0057	0.48
		nPD :	0.01
		N. weight :	1.
		Sc. PD :	-0.084
		Sc. rank :	-367.7

,

PB2			
Pos.	578	obs :	exp :
gaa	E	1057	644.40
gag	E	2	414.60
---	--	------	------
mPD		0.0038	0.48
		nPD :	0.01
		N. weight :	0.53
		Sc. PD :	-0.047
		Sc. rank :	-268.3

,

PB2			
Pos.	579	obs :	exp :
tct	S	0	0.16
tcc	S	1	0.13
tca	S	0	0.25
tcg	S	0	0.06
cct	P	1	264.00
ccc	P	1055	190.10
cca	P	2	435.50
ccg	P	0	168.40
agt	S	0	0.21
agc	S	0	0.19
---	--	------	------
mPD		0.0075	0.71
		nPD :	0.01
		N. weight :	1.9
		Sc. PD :	-0.16
		Sc. rank :	-785.6

,

PB2			
Pos.	580	obs :	exp :
ttt	F	1058	418.20
ttc	F	1	640.80
---	--	------	------
mPD		0.0019	0.48
		nPD :	0.
		N. weight :	1.
		Sc. PD :	-0.093
		Sc. rank :	-626.7

,

PB2			
Pos.	581	obs :	exp :
caa	Q	1014	539.40
cag	Q	45	519.60
---	--	------	------
mPD		0.081	0.50
		nPD :	0.16
		N. weight :	0.55
		Sc. PD :	0.034
		Sc. rank :	272.0

,

PB2			
Pos.	582	obs :	exp :
tct	S	14	168.10
tcc	S	1024	138.00
tca	S	5	268.30
tcg	S	14	59.98
agt	S	0	217.30
agc	S	0	205.30
gct	A	0	0.52
gcc	A	2	0.37
gca	A	0	0.94
gcg	A	0	0.17
---	--	------	------
mPD		0.065	1.7
		nPD :	0.04
		N. weight :	2.1
		Sc. PD :	-0.12
		Sc. rank :	194.9

,

PB2			
Pos.	583	obs :	exp :
tta	L	5	93.98
ttg	L	1	197.20
ctt	L	0	176.30
ctc	L	0	176.70
cta	L	1027	178.50
ctg	L	26	236.30
---	--	------	------
mPD		0.061	1.1
		nPD :	0.06
		N. weight :	1.8
		Sc. PD :	-0.077
		Sc. rank :	391.1

,

PB2			
Pos.	584	obs :	exp :
att	I	0	5.14
atc	I	0	3.35
ata	I	14	5.50
gtt	V	3	216.50
gtc	V	0	210.90
gta	V	8	214.00
gtg	V	1034	403.60
---	--	------	------
mPD		0.072	0.75
		nPD :	0.1
		N. weight :	0.99
		Sc. PD :	-0.0033
		Sc. rank :	386.8

,

PB2			
Pos.	585	obs :	exp :
cct	P	951	264.20
ccc	P	108	190.30
cca	P	0	435.90
ccg	P	0	168.60
---	--	------	------
mPD		0.18	0.71
		nPD :	0.26
		N. weight :	1.2
		Sc. PD :	0.18
		Sc. rank :	767.9

,

PB2			
Pos.	586	obs :	exp :
cgt	R	0	0.04
cgc	R	0	0.05
cga	R	0	0.10
cgg	R	0	0.08
aaa	K	1037	616.60
aag	K	21	441.40
aga	R	1	0.46
agg	R	0	0.27
---	--	------	------
mPD		0.041	0.49
		nPD :	0.08
		N. weight :	0.5
		Sc. PD :	-0.0077
		Sc. rank :	171.4

,

PB2			
Pos.	587	obs :	exp :
gct	A	1048	273.50
gcc	A	6	197.20
gca	A	2	498.40
gcg	A	3	89.88
---	--	------	------
mPD		0.021	0.67
		nPD :	0.03
		N. weight :	1.4
		Sc. PD :	-0.094
		Sc. rank :	12.9

,

PB2			
Pos.	588	obs :	exp :
tta	L	0	0.09
ttg	L	0	0.19
ctt	L	0	0.17
ctc	L	1	0.17
cta	L	0	0.17
ctg	L	0	0.22
att	I	0	1.10
atc	I	3	0.72
ata	I	0	1.18
act	T	0	0.76
acc	T	2	0.62
aca	T	1	1.38
acg	T	0	0.24
gtt	V	3	73.56
gtc	V	351	71.65
gta	V	1	72.69
gtg	V	0	137.10
gct	A	0	180.00
gcc	A	696	129.80
gca	A	1	328.10
gcg	A	0	59.16
---	--	------	------
mPD		0.47	1.2
		nPD :	0.4
		N. weight :	1.8
		Sc. PD :	0.51
		Sc. rank :	1303.9

,

PB2			
Pos.	589	obs :	exp :
cgt	R	0	45.44
cgc	R	0	52.40
cga	R	3	103.50
cgg	R	0	88.28
aaa	K	1	0.58
aag	K	0	0.42
aga	R	1048	483.80
agg	R	7	284.60
---	--	------	------
mPD		0.021	0.96
		nPD :	0.02
		N. weight :	0.81
		Sc. PD :	-0.06
		Sc. rank :	-98.6

,

PB2			
Pos.	590	obs :	exp :
tct	S	0	0.32
tcc	S	0	0.26
tca	S	0	0.51
tcg	S	0	0.11
agt	S	0	0.41
agc	S	2	0.39
ggt	G	80	140.10
ggc	G	977	135.00
gga	G	0	487.60
ggg	G	0	294.30
---	--	------	------
mPD		0.14	0.68
		nPD :	0.21
		N. weight :	2.
		Sc. PD :	0.21
		Sc. rank :	1118.2

,

PB2			
Pos.	591	obs :	exp :
tta	L	0	0.44
ttg	L	0	0.93
ctt	L	0	0.83
ctc	L	0	0.83
cta	L	5	0.84
ctg	L	0	1.12
caa	Q	1014	521.10
cag	Q	9	501.90
cgt	R	0	0.17
cgc	R	0	0.20
cga	R	4	0.39
cgg	R	0	0.33
aaa	K	26	15.74
aag	K	1	11.26
aga	R	0	1.83
agg	R	0	1.08
---	--	------	------
mPD		0.085	0.58
		nPD :	0.15
		N. weight :	0.7
		Sc. PD :	0.032
		Sc. rank :	328.1

,

PB2			
Pos.	592	obs :	exp :
tat	Y	1023	572.00
tac	Y	36	487.00
---	--	------	------
mPD		0.066	0.50
		nPD :	0.13
		N. weight :	0.52
		Sc. PD :	0.016
		Sc. rank :	238.1

,

PB2			
Pos.	593	obs :	exp :
tct	S	0	168.40
tcc	S	0	138.30
tca	S	0	268.90
tcg	S	0	60.09
agt	S	1057	217.70
agc	S	2	205.70
---	--	------	------
mPD		0.0038	1.7
		nPD :	0.
		N. weight :	1.7
		Sc. PD :	-0.16
		Sc. rank :	-1262.3

,

PB2			
Pos.	594	obs :	exp :
ggt	G	4	140.40
ggc	G	0	135.30
gga	G	360	488.50
ggg	G	695	294.80
---	--	------	------
mPD		0.45	0.68
		nPD :	0.67
		N. weight :	0.49
		Sc. PD :	0.27
		Sc. rank :	397.9

,

PB2			
Pos.	595	obs :	exp :
ttt	F	13	418.20
ttc	F	1046	640.80
---	--	------	------
mPD		0.024	0.48
		nPD :	0.05
		N. weight :	0.49
		Sc. PD :	-0.023
		Sc. rank :	82.4

,

PB2

Pos. 596		obs:	exp:
gtt	V	0	219.40
gtc	V	0	213.70
gta	V	688	216.80
gtg	V	371	409.00
mPD		0.46	0.73
		nPD:	0.63
		N. weight:	0.79
		Sc. PD:	0.4
		Sc. rank:	628.5

PB2

Pos. 597		obs:	exp:
cgt	R	0	45.48
cgc	R	0	52.45
cga	R	0	103.60
cgg	R	0	88.36
aga	R	4	484.20
agg	R	1055	284.80
mPD		0.0075	0.96
		nPD:	0.01
		N. weight:	1.4
		Sc. PD:	-0.12
		Sc. rank:	-738.1

PB2

Pos. 598		obs:	exp:
att	I	31	12.49
atc	I	3	8.15
ata	I	0	13.36
act	T	0	0.25
acc	T	0	0.21
aca	T	1	0.46
acg	T	0	0.08
gtt	V	789	211.60
gtc	V	232	206.10
gta	V	0	209.10
gtg	V	0	394.30
gct	A	2	0.77
gcc	A	1	0.56
gca	A	0	1.41
gcg	A	0	0.25
mPD		0.42	0.80
		nPD:	0.52
		N. weight:	1.1
		Sc. PD:	0.46
		Sc. rank:	875.2

PB2

Pos. 599		obs:	exp:
tta	L	8	93.98
ttg	L	0	197.20
ctt	L	0	176.30
ctc	L	0	176.70
cta	L	1048	178.50
ctg	L	3	236.30
mPD		0.021	1.1
		nPD:	0.02
		N. weight:	1.9
		Sc. PD:	-0.15
		Sc. rank:	-368.6

PB2

Pos. 600		obs:	exp:
ttt	F	2	418.20
ttc	F	1057	640.80
mPD		0.0038	0.48
		nPD:	0.01
		N. weight:	0.54
		Sc. PD:	-0.047
		Sc. rank:	-272.2

PB2

Pos. 601		obs:	exp:
caa	Q	682	539.40
cag	Q	377	519.60
mPD		0.46	0.50
		nPD:	0.92
		N. weight:	0.044
		Sc. PD:	0.035
		Sc. rank:	37.0

PB2

Pos. 602		obs:	exp:
caa	Q	1	539.40
cag	Q	1058	519.60
mPD		0.0019	0.50
		nPD:	0.
		N. weight:	0.77
		Sc. PD:	-0.071
		Sc. rank:	-507.5

PB2

Pos. 603		obs:	exp:
atg	M	1059	1059.00
mPD		0	0
		nPD:	1.
		N. weight:	0.
		Sc. PD:	0
		Sc. rank:	0

PB2

Pos. 604		obs:	exp:
cgt	R	1059	45.48
cgc	R	0	52.45
cga	R	0	103.60
cgg	R	0	88.36
aga	R	0	484.20
agg	R	0	284.80
mPD		0	0.96
		nPD:	0.
		N. weight:	3.5
		Sc. PD:	-0.33
		Sc. rank:	-2873.9

PB2

Pos. 605		obs:	exp:
gat	D	129	572.30
gac	D	930	486.70
mPD		0.21	0.50
		nPD:	0.43
		N. weight:	0.43
		Sc. PD:	0.14
		Sc. rank:	318.9

PB2

Pos. 606		obs:	exp:
gtt	V	0	219.40
gtc	V	1	213.70
gta	V	1040	216.80
gtg	V	18	409.00
mPD		0.035	0.73
		nPD:	0.05
		N. weight:	1.6
		Sc. PD:	-0.08
		Sc. rank:	268.4

PB2

Pos. 607		obs:	exp:
tta	L	0	93.44
ttg	L	8	196.00
ctt	L	0	175.30
ctc	L	0	175.70
cta	L	0	177.50
ctg	L	1045	235.00
caa	Q	0	0.51
cag	Q	1	0.49
atg	M	2	2.00
gtt	V	0	0.62
gtc	V	0	0.61
gta	V	0	0.61
gtg	V	3	1.16
mPD		0.026	1.1
		nPD:	0.02
		N. weight:	1.6
		Sc. PD:	-0.12
		Sc. rank:	-158.5

PB2

Pos. 608		obs:	exp:
ggt	G	0	140.40
ggc	G	0	135.30
gga	G	381	488.50
ggg	G	678	294.80
mPD		0.46	0.68
		nPD:	0.68
		N. weight:	0.49
		Sc. PD:	0.27
		Sc. rank:	397.5

PB2

Pos. 609		obs:	exp:
act	T	1	269.90
acc	T	1	217.30
aca	T	887	485.70
acg	T	170	86.02
mPD		0.27	0.68
		nPD:	0.4
		N. weight:	0.67
		Sc. PD:	0.2
		Sc. rank:	492.1

PB2

Pos. 610		obs:	exp:
ttt	F	847	418.20
ttc	F	212	640.80
mPD		0.32	0.48
		nPD:	0.67
		N. weight:	0.38
		Sc. PD:	0.21
		Sc. rank:	305.0

PB2

Pos. 611		obs:	exp:
aat	N	0	1.53
aac	N	3	1.47
gat	D	2	570.60
gac	D	1054	485.40
mPD		0.0094	0.50
		nPD:	0.02
		N. weight:	0.84
		Sc. PD:	-0.065
		Sc. rank:	-156.2

PB2

Pos. 612		obs:	exp:
tct	S	0	0.16
tcc	S	0	0.13
tca	S	0	0.25
tcg	S	0	0.06
act	T	869	269.70
acc	T	181	217.10
aca	T	1	485.30
acg	T	7	85.94
agt	S	0	0.21
agc	S	1	0.19
mPD		0.30	0.68
		nPD:	0.44
		N. weight:	1.
		Sc. PD:	0.33
		Sc. rank:	751.6

PB2

Pos. 613		obs:	exp:
att	I	0	0.37
atc	I	1	0.24
ata	I	0	0.39
gtt	V	92	219.00
gtc	V	965	213.30
gta	V	0	216.40
gtg	V	0	408.20
gct	A	0	0.26
gcc	A	1	0.19
gca	A	0	0.47
gcg	A	0	0.08
mPD		0.16	0.73
		nPD:	0.22
		N. weight:	1.4
		Sc. PD:	0.17
		Sc. rank:	849.1

PB2

Pos. 614		obs:	exp:
caa	Q	1056	539.40
cag	Q	3	519.60
mPD		0.0057	0.50
		nPD:	0.01
		N. weight:	0.72
		Sc. PD:	-0.061
		Sc. rank:	-282.6

PB2

Pos. 615		obs:	exp:
att	I	0	386.90
atc	I	0	252.30
ata	I	1053	413.80
act	T	0	0.25
acc	T	0	0.21
aca	T	1	0.46
acg	T	0	0.08
gtt	V	0	1.04
gtc	V	0	1.01
gta	V	5	1.02
gtg	V	0	1.93
mPD		0.011	0.67
		nPD:	0.02
		N. weight:	1.
		Sc. PD:	-0.082
		Sc. rank:	-228.2

PB2

Pos. 616		obs:	exp:
att	I	0	386.90
atc	I	0	252.30
ata	I	1053	413.80
gtt	V	0	1.24
gtc	V	0	1.21
gta	V	6	1.23
gtg	V	0	2.32
mPD		0.011	0.67
		nPD:	0.02
		N. weight:	1.
		Sc. PD:	-0.082
		Sc. rank:	-234.3

PB2

Pos. 617		obs:	exp:
aaa	K	0	617.20
aag	K	1059	441.80
mPD		0	0.49
		nPD:	0.
		N. weight:	0.96
		Sc. PD:	-0.092
		Sc. rank:	-798.2

PB2

Pos. 618		obs:	exp:
tta	L	42	93.98
ttg	L	0	197.20
ctt	L	0	176.30
ctc	L	1	176.70
cta	L	1004	178.50
ctg	L	12	236.30
mPD		0.10	1.1
		nPD:	0.09
		N. weight:	1.7
		Sc. PD:	-0.015
		Sc. rank:	639.1

PB2

Pos. 619		obs:	exp:
tta	L	921	93.98
ttg	L	12	197.20
ctt	L	0	176.30
ctc	L	0	176.70
cta	L	126	178.50
ctg	L	0	236.30
mPD		0.23	1.1
		nPD:	0.21
		N. weight:	2.1
		Sc. PD:	0.22
		Sc. rank:	1188.8

PB2

Pos. 620		obs:	exp:
cct	P	0	264.20
ccc	P	0	190.30
cca	P	1057	435.90
ccg	P	2	168.60
mPD		0.0038	0.71
		nPD:	0.01
		N. weight:	0.96
		Sc. PD:	-0.087
		Sc. rank:	-580.0

PB2			
Pos.	621	obs:	exp:
ttt	F	1059	418.20
ttc	F	0	640.80
---	--	------	------
mPD		0	0.48
		nPD:	0.
		N. weight:	1.
		Sc. PD:	-0.098
		Sc. rank:	-848.3

PB2			
Pos.	622	obs:	exp:
gct	A	0	273.50
gcc	A	0	197.20
gca	A	973	498.40
gcg	A	86	89.88
---	--	------	------
mPD		0.15	0.67
		nPD:	0.22
		N. weight:	0.67
		Sc. PD:	0.08
		Sc. rank:	400.2

PB2			
Pos.	623	obs:	exp:
gct	A	1	273.50
gcc	A	0	197.20
gca	A	1053	498.40
gcg	A	5	89.88
---	--	------	------
mPD		0.011	0.67
		nPD:	0.02
		N. weight:	0.8
		Sc. PD:	-0.063
		Sc. rank:	-183.5

PB2			
Pos.	624	obs:	exp:
tct	S	0	0.16
tcc	S	1	0.13
tca	S	0	0.25
tcg	S	0	0.06
agt	S	0	0.21
agc	S	0	0.19
gct	A	15	273.20
gcc	A	1036	197.00
gca	A	3	498.00
gcg	A	4	89.79
---	--	------	------
mPD		0.043	0.67
		nPD:	0.06
		N. weight:	1.7
		Sc. PD:	-0.059
		Sc. rank:	466.5

PB2			
Pos.	625	obs:	exp:
cct	P	6	264.20
ccc	P	1	190.30
cca	P	6	435.90
ccg	P	1046	168.60
---	--	------	------
mPD		0.024	0.71
		nPD:	0.03
		N. weight:	1.9
		Sc. PD:	-0.12
		Sc. rank:	74.3

PB2			
Pos.	626	obs:	exp:
cct	P	1	264.20
ccc	P	2	190.30
cca	P	4	435.90
ccg	P	1052	168.60
---	--	------	------
mPD		0.013	0.71
		nPD:	0.02
		N. weight:	2.
		Sc. PD:	-0.15
		Sc. rank:	-391.1

PB2			
Pos.	627	obs:	exp:
caa	Q	1	0.51
cag	Q	0	0.49
aaa	K	127	449.40
aag	K	644	321.60
gtt	V	0	5.80
gtc	V	0	5.65
gta	V	0	5.73
gtg	V	28	10.81
gaa	E	45	157.60
gag	E	214	101.40
---	--	------	------
mPD		0.72	0.94
		nPD:	0.76
		N. weight:	0.44
		Sc. PD:	0.28
		Sc. rank:	366.1

PB2			
Pos.	628	obs:	exp:
caa	Q	8	539.40
cag	Q	1051	519.60
---	--	------	------
mPD		0.015	0.50
		nPD:	0.03
		N. weight:	0.74
		Sc. PD:	-0.049
		Sc. rank:	3.1

PB2			
Pos.	629	obs:	exp:
tct	S	0	168.40
tcc	S	0	138.30
tca	S	0	268.80
tcg	S	0	60.09
agt	S	1058	217.70
agc	S	1	205.70
---	--	------	------
mPD		0.0019	1.7
		nPD:	0.
		N. weight:	1.7
		Sc. PD:	-0.16
		Sc. rank:	-1286.1

PB2			
Pos.	630	obs:	exp:
cgt	R	0	45.48
cgc	R	0	52.45
cga	R	0	103.60
cgg	R	0	88.36
aga	R	3	484.20
agg	R	1056	284.80
---	--	------	------
mPD		0.0057	0.96
		nPD:	0.01
		N. weight:	1.4
		Sc. PD:	-0.13
		Sc. rank:	-801.2

PB2			
Pos.	631	obs:	exp:
atg	M	1059	1059.00
---	--	------	------
mPD		0	0
		nPD:	1.
		N. weight:	0.
		Sc. PD:	0
		Sc. rank:	0

PB2			
Pos.	632	obs:	exp:
caa	Q	13	539.40
cag	Q	1046	519.60
---	--	------	------
mPD		0.024	0.50
		nPD:	0.05
		N. weight:	0.71
		Sc. PD:	-0.035
		Sc. rank:	114.5

PB2			
Pos.	633	obs:	exp:
ttt	F	149	418.20
ttc	F	910	640.80
---	--	------	------
mPD		0.24	0.48
		nPD:	0.51
		N. weight:	0.18
		Sc. PD:	0.068
		Sc. rank:	133.0

PB2			
Pos.	634	obs:	exp:
tct	S	1026	168.40
tcc	S	30	138.30
tca	S	3	268.80
tcg	S	0	60.09
agt	S	0	217.70
agc	S	0	205.70
---	--	------	------
mPD		0.061	1.7
		nPD:	0.04
		N. weight:	1.9
		Sc. PD:	-0.11
		Sc. rank:	115.4

PB2			
Pos.	635	obs:	exp:
tct	S	1050	168.40
tcc	S	9	138.30
tca	S	0	268.80
tcg	S	0	60.09
agt	S	0	217.70
agc	S	0	205.70
---	--	------	------
mPD		0.017	1.7
		nPD:	0.01
		N. weight:	2.
		Sc. PD:	-0.17
		Sc. rank:	-872.8

PB2			
Pos.	636	obs:	exp:
tta	L	36	93.98
ttg	L	0	197.20
ctt	L	0	176.30
ctc	L	0	176.70
cta	L	1020	178.50
ctg	L	3	236.30
---	--	------	------
mPD		0.071	1.1
		nPD:	0.06
		N. weight:	1.8
		Sc. PD:	-0.061
		Sc. rank:	509.0

PB2			
Pos.	637	obs:	exp:
att	I	0	0.37
atc	I	1	0.24
ata	I	0	0.39
act	T	1050	269.70
acc	T	8	217.10
aca	T	0	485.30
acg	T	0	85.94
---	--	------	------
mPD		0.019	0.68
		nPD:	0.03
		N. weight:	1.5
		Sc. PD:	-0.1
		Sc. rank:	-35.2

PB2			
Pos.	638	obs:	exp:
gtt	V	6	219.40
gtc	V	0	213.70
gta	V	0	216.80
gtg	V	1053	409.00
---	--	------	------
mPD		0.011	0.73
		nPD:	0.02
		N. weight:	1.
		Sc. PD:	-0.082
		Sc. rank:	-261.7

PB2			
Pos.	639	obs:	exp:
aat	N	719	539.40
aac	N	340	519.60
---	--	------	------
mPD		0.44	0.50
		nPD:	0.87
		N. weight:	0.069
		Sc. PD:	0.051
		Sc. rank:	57.2

PB2			
Pos.	640	obs:	exp:
att	I	0	31.60
atc	I	0	20.61
ata	I	86	33.79
gtt	V	4	201.60
gtc	V	0	196.40
gta	V	92	199.20
gtg	V	877	375.80
---	--	------	------
mPD		0.44	0.88
		nPD:	0.49
		N. weight:	0.77
		Sc. PD:	0.29
		Sc. rank:	583.8

PB2			
Pos.	641	obs:	exp:
cgt	R	0	45.48
cgc	R	0	52.45
cga	R	0	103.60
cgg	R	0	88.36
aga	R	291	484.20
agg	R	768	284.80
---	--	------	------
mPD		0.40	0.96
		nPD:	0.42
		N. weight:	0.64
		Sc. PD:	0.19
		Sc. rank:	472.6

PB2			
Pos.	642	obs:	exp:
ggt	G	1049	140.40
ggc	G	9	135.30
gga	G	0	488.50
ggg	G	1	294.80
---	--	------	------
mPD		0.019	0.68
		nPD:	0.03
		N. weight:	2.2
		Sc. PD:	-0.15
		Sc. rank:	-38.1

PB2			
Pos.	643	obs:	exp:
tct	S	2	168.40
tcc	S	348	138.30
tca	S	709	268.80
tcg	S	0	60.09
agt	S	0	217.70
agc	S	0	205.70
---	--	------	------
mPD		0.44	1.7
		nPD:	0.27
		N. weight:	1.
		Sc. PD:	0.17
		Sc. rank:	687.1

PB2			
Pos.	644	obs:	exp:
ggt	G	0	140.40
ggc	G	20	135.30
gga	G	1032	488.50
ggg	G	7	294.80
---	--	------	------
mPD		0.050	0.68
		nPD:	0.07
		N. weight:	0.74
		Sc. PD:	-0.018
		Sc. rank:	229.1

PB2			
Pos.	645	obs:	exp:
atg	M	1059	1059.00
---	--	------	------
mPD		0	0
		nPD:	1.
		N. weight:	0.
		Sc. PD:	0
		Sc. rank:	0

PB2			
Pos.	646	obs:	exp:
cgt	R	0	45.48
cgc	R	0	52.45
cga	R	0	103.60
cgg	R	0	88.36
aga	R	1054	484.20
agg	R	5	284.80
---	--	------	------
mPD		0.0094	0.96
		nPD:	0.01
		N. weight:	0.83
		Sc. PD:	-0.072
		Sc. rank:	-375.6

PB2			
Pos.	647	obs:	exp:
att	I	0	302.00
atc	I	0	197.00
ata	I	822	323.00
gtt	V	0	49.11
gtc	V	0	47.83
gta	V	68	48.53
gtg	V	169	91.53
---	--	------	------
mPD		0.62	1.1
		nPD:	0.58
		N. weight:	0.93
		Sc. PD:	0.43
		Sc. rank:	728.6

PB2			
Pos.	648	obs:	exp:
tta	L	0	0.18
ttg	L	0	0.37
ctt	L	1	0.33
ctc	L	1	0.33
cta	L	0	0.34
ctg	L	0	0.45
att	I	7	2.57
atc	I	0	1.68
ata	I	0	2.75
gtt	V	1039	216.50
gtc	V	1	210.90
gta	V	0	214.00
gtg	V	5	403.60
gct	A	5	1.29
gcc	A	0	0.93
gca	A	0	2.35
gcg	A	0	0.42
---	--	------	------
mPD		0.039	0.75
		nPD:	0.05
		N. weight:	1.7
		Sc. PD:	-0.076
		Sc. rank:	322.3

PB2			
Pos.	649	obs:	exp:
tta	L	0	0.09
ttg	L	1	0.19
ctt	L	0	0.17
ctc	L	0	0.17
cta	L	0	0.17
ctg	L	0	0.22
gtt	V	0	219.20
gtc	V	0	213.50
gta	V	0	216.60
gtg	V	1058	408.60
---	--	------	------
mPD		0.0019	0.73
		nPD:	0.
		N. weight:	1.
		Sc. PD:	-0.097
		Sc. rank:	-755.3

PB2			
Pos.	650	obs:	exp:
cgt	R	0	45.48
cgc	R	0	52.45
cga	R	0	103.60
cgg	R	0	88.36
aga	R	1057	484.20
agg	R	2	284.80
---	--	------	------
mPD		0.0038	0.96
		nPD:	0.
		N. weight:	0.85
		Sc. PD:	-0.078
		Sc. rank:	-541.6

PB2			
Pos.	651	obs:	exp:
ggt	G	85	140.40
ggc	G	974	135.30
gga	G	0	488.50
ggg	G	0	294.80
---	--	------	------
mPD		0.15	0.68
		nPD:	0.22
		N. weight:	2.
		Sc. PD:	0.22
		Sc. rank:	1143.9

PB2			
Pos.	652	obs:	exp:
aat	N	978	539.40
aac	N	81	519.60
---	--	------	------
mPD		0.14	0.50
		nPD:	0.28
		N. weight:	0.45
		Sc. PD:	0.079
		Sc. rank:	303.7

PB2			
Pos.	653	obs:	exp:
tct	S	1042	168.40
tcc	S	17	138.30
tca	S	0	268.80
tcg	S	0	60.09
agt	S	0	217.70
agc	S	0	205.70
---	--	------	------
mPD		0.032	1.7
		nPD:	0.02
		N. weight:	1.9
		Sc. PD:	-0.15
		Sc. rank:	-327.5

PB2			
Pos.	654	obs:	exp:
cct	P	972	264.20
ccc	P	0	190.30
cca	P	87	435.90
ccg	P	0	168.60
---	--	------	------
mPD		0.15	0.71
		nPD:	0.21
		N. weight:	1.2
		Sc. PD:	0.13
		Sc. rank:	671.3

PB2			
Pos.	655	obs:	exp:
gtt	V	1	219.40
gtc	V	0	213.70
gta	V	70	216.80
gtg	V	988	409.00
---	--	------	------
mPD		0.13	0.73
		nPD:	0.17
		N. weight:	0.82
		Sc. PD:	0.058
		Sc. rank:	412.7

PB2			
Pos.	656	obs:	exp:
ttt	F	71	418.20
ttc	F	988	640.80
---	--	------	------
mPD		0.13	0.48
		nPD:	0.26
		N. weight:	0.32
		Sc. PD:	0.049
		Sc. rank:	205.1

PB2			
Pos.	657	obs:	exp:
aat	N	15	539.40
aac	N	1044	519.60
---	--	------	------
mPD		0.028	0.50
		nPD:	0.06
		N. weight:	0.7
		Sc. PD:	-0.029
		Sc. rank:	154.6

PB2			
Pos.	658	obs:	exp:
tat	Y	2	572.00
tac	Y	1057	487.00
---	--	------	------
mPD		0.0038	0.50
		nPD:	0.01
		N. weight:	0.84
		Sc. PD:	-0.074
		Sc. rank:	-446.7

PB2			
Pos.	659	obs:	exp:
tct	S	0	0.32
tcc	S	0	0.26
tca	S	0	0.51
tcg	S	0	0.11
aat	N	30	538.40
aac	N	1027	518.60
agt	S	0	0.41
agc	S	2	0.39
---	--	------	------
mPD		0.059	0.51
		nPD:	0.12
		N. weight:	0.64
		Sc. PD:	0.01
		Sc. rank:	275.3

PB2			
Pos.	660	obs:	exp:
cgt	R	0	0.13
cgc	R	0	0.15
cga	R	0	0.29
cgg	R	0	0.25
aaa	K	9	615.50
aag	K	1047	440.50
aga	R	0	1.37
agg	R	3	0.81
---	--	------	------
mPD		0.023	0.49
		nPD:	0.05
		N. weight:	0.91
		Sc. PD:	-0.047
		Sc. rank:	117.1

PB2			
Pos.	661	obs:	exp:
act	T	0	1.53
acc	T	0	1.23
aca	T	6	2.75
acg	T	0	0.49
gtt	V	0	0.21
gtc	V	0	0.20
gta	V	1	0.20
gtg	V	0	0.39
gct	A	2	271.70
gcc	A	0	195.90
gca	A	1049	495.10
gcg	A	1	89.29
---	--	------	------
mPD		0.019	0.68
		nPD:	0.03
		N. weight:	0.81
		Sc. PD:	-0.056
		Sc. rank:	-27.3

PB2			
Pos.	662	obs:	exp:
tct	S	0	0.16
tcc	S	0	0.13
tca	S	0	0.25
tcg	S	0	0.06
act	T	0	269.40
acc	T	950	216.90
aca	T	107	484.80
acg	T	0	85.86
agt	S	0	0.21
agc	S	1	0.19
gct	A	0	0.26
gcc	A	1	0.19
gca	A	0	0.47
gcg	A	0	0.08
---	--	------	------
mPD		0.19	0.68
		nPD:	0.27
		N. weight:	1.3
		Sc. PD:	0.22
		Sc. rank:	858.1

PB2			
Pos.	663	obs:	exp:
cgt	R	0	0.04
cgc	R	0	0.05
cga	R	0	0.10
cgg	R	0	0.08
aaa	K	415	616.60
aag	K	643	441.40
aga	R	0	0.46
agg	R	1	0.27
---	--	------	------
mPD		0.48	0.49
		nPD:	0.98
		N. weight:	0.086
		Sc. PD:	0.072
		Sc. rank:	71.6

PB2			
Pos.	664	obs:	exp:
cgt	R	0	45.40
cgc	R	0	52.35
cga	R	0	103.40
cgg	R	0	88.20
aaa	K	0	1.17
aag	K	2	0.83
aga	R	1	483.30
agg	R	1056	284.30
---	--	------	------
mPD		0.0057	0.96
		nPD:	0.01
		N. weight:	1.4
		Sc. PD:	-0.13
		Sc. rank:	-812.0

PB2			
Pos.	665	obs:	exp:
tta	L	0	93.98
ttg	L	0	197.20
ctt	L	1059	176.30
ctc	L	0	176.70
cta	L	0	178.50
ctg	L	0	236.30
---	--	------	------
mPD		0	1.1
		nPD:	0.
		N. weight:	2.
		Sc. PD:	-0.19
		Sc. rank:	-1636.7

PB2			
Pos.	666	obs:	exp:
tct	S	0	0.16
tcc	S	0	0.13
tca	S	1	0.25
tcg	S	0	0.06
atg	M	167	167.00
act	T	0	227.10
acc	T	0	182.80
aca	T	355	408.70
acg	T	536	72.37
agt	S	0	0.21
agc	S	0	0.19
mPD		0.71	0.99
		nPD:	0.72
		N. weight:	1.1
		Sc. PD:	0.64
		Sc. rank:	879.5

PB2			
Pos.	667	obs:	exp:
gtt	V	0	219.40
gtc	V	1	213.70
gta	V	19	216.80
gtg	V	1039	409.00
mPD		0.037	0.73
		nPD:	0.05
		N. weight:	0.95
		Sc. PD:	-0.044
		Sc. rank:	166.1

PB2			
Pos.	668	obs:	exp:
tta	L	0	93.98
ttg	L	0	197.20
ctt	L	1056	176.30
ctc	L	3	176.70
cta	L	0	178.50
ctg	L	0	236.30
mPD		0.0057	1.1
		nPD:	0.01
		N. weight:	1.9
		Sc. PD:	-0.18
		Sc. rank:	-1185.7

PB2			
Pos.	669	obs:	exp:
ggt	G	1	140.40
ggc	G	0	135.30
gga	G	470	488.50
ggg	G	588	294.80
mPD		0.50	0.68
		nPD:	0.73
		N. weight:	0.4
		Sc. PD:	0.24
		Sc. rank:	332.1

PB2			
Pos.	670	obs:	exp:
aaa	K	4	617.20
aag	K	1055	441.80
mPD		0.0075	0.49
		nPD:	0.02
		N. weight:	0.93
		Sc. PD:	-0.075
		Sc. rank:	-252.4

PB2			
Pos.	671	obs:	exp:
gat	D	909	572.30
gac	D	150	486.70
mPD		0.24	0.50
		nPD:	0.49
		N. weight:	0.26
		Sc. PD:	0.096
		Sc. rank:	194.4

PB2			
Pos.	672	obs:	exp:
gct	A	2	273.50
gcc	A	0	197.20
gca	A	1056	498.40
gcg	A	1	89.88
mPD		0.0057	0.67
		nPD:	0.01
		N. weight:	0.81
		Sc. PD:	-0.071
		Sc. rank:	-390.0

PB2			
Pos.	673	obs:	exp:
ggt	G	1058	140.40
ggc	G	1	135.30
gga	G	0	488.50
ggg	G	0	294.80
mPD		0.0019	0.68
		nPD:	0.
		N. weight:	2.2
		Sc. PD:	-0.21
		Sc. rank:	-1516.7

PB2			
Pos.	674	obs:	exp:
act	T	0	4.59
acc	T	3	3.69
aca	T	7	8.26
acg	T	8	1.46
gtt	V	0	1.45
gtc	V	1	1.41
gta	V	0	1.43
gtg	V	6	2.70
gct	A	1	266.50
gcc	A	73	192.20
gca	A	802	485.70
gcg	A	156	87.59
gaa	E	2	1.22
gag	E	0	0.78
mPD		0.43	0.72
		nPD:	0.6
		N. weight:	0.46
		Sc. PD:	0.22
		Sc. rank:	363.3

PB2			
Pos.	675	obs:	exp:
tta	L	159	93.98
ttg	L	887	197.20
ctt	L	1	176.30
ctc	L	0	176.70
cta	L	0	178.50
ctg	L	12	236.30
mPD		0.28	1.1
		nPD:	0.25
		N. weight:	1.4
		Sc. PD:	0.21
		Sc. rank:	896.5

PB2			
Pos.	676	obs:	exp:
att	I	0	15.43
atc	I	0	10.06
ata	I	42	16.50
atg	M	687	687.00
aaa	K	0	0.58
aag	K	1	0.42
gtt	V	1	68.17
gtc	V	0	66.40
gta	V	0	67.37
gtg	V	328	127.10
mPD		0.51	0.82
		nPD:	0.62
		N. weight:	0.36
		Sc. PD:	0.18
		Sc. rank:	285.3

PB2			
Pos.	677	obs:	exp:
gaa	E	1042	641.40
gag	E	12	412.60
ggt	G	0	0.66
ggc	G	0	0.64
gga	G	2	2.31
ggg	G	3	1.39
mPD		0.037	0.49
		nPD:	0.08
		N. weight:	0.49
		Sc. PD:	-0.011
		Sc. rank:	156.6

PB2			
Pos.	678	obs:	exp:
aat	N	0	0.51
aac	N	1	0.49
gat	D	3	570.10
gac	D	1052	484.90
ggt	G	0	0.40
ggc	G	3	0.38
gga	G	0	1.38
ggg	G	0	0.84
mPD		0.013	0.51
		nPD:	0.03
		N. weight:	0.84
		Sc. PD:	-0.059
		Sc. rank:	-52.4

PB2			
Pos.	679	obs:	exp:
cct	P	0	263.70
ccc	P	348	189.90
cca	P	696	435.10
ccg	P	13	168.30
caa	Q	1	0.51
cag	Q	0	0.49
act	T	0	0.25
acc	T	0	0.21
aca	T	1	0.46
acg	T	0	0.08
mPD		0.46	0.71
		nPD:	0.65
		N. weight:	0.53
		Sc. PD:	0.28
		Sc. rank:	422.8

PB2			
Pos.	680	obs:	exp:
gat	D	815	571.20
gac	D	242	485.80
gaa	E	1	1.22
gag	E	1	0.78
mPD		0.36	0.50
		nPD:	0.71
		N. weight:	0.13
		Sc. PD:	0.077
		Sc. rank:	106.9

PB2			
Pos.	681	obs:	exp:
gat	D	3	1.62
gac	D	0	1.38
gaa	E	5	642.60
gag	E	1051	413.40
mPD		0.015	0.48
		nPD:	0.03
		N. weight:	1.
		Sc. PD:	-0.065
		Sc. rank:	18.7

PB2			
Pos.	682	obs:	exp:
ggt	G	0	140.40
ggc	G	0	135.30
gga	G	981	488.50
ggg	G	78	294.80
mPD		0.14	0.68
		nPD:	0.2
		N. weight:	0.61
		Sc. PD:	0.06
		Sc. rank:	331.2

PB2			
Pos.	683	obs:	exp:
att	I	0	0.37
atc	I	0	0.24
ata	I	1	0.39
act	T	0	269.40
acc	T	0	216.90
aca	T	1056	484.80
acg	T	1	85.86
gct	A	0	0.26
gcc	A	0	0.19
gca	A	1	0.47
gcg	A	0	0.08
mPD		0.0057	0.68
		nPD:	0.01
		N. weight:	0.85
		Sc. PD:	-0.075
		Sc. rank:	-417.9

PB2			
Pos.	684	obs:	exp:
tct	S	0	0.32
tcc	S	0	0.26
tca	S	2	0.51
tcg	S	0	0.11
act	T	0	0.25
acc	T	0	0.21
aca	T	1	0.46
acg	T	0	0.08
agt	S	0	0.41
agc	S	0	0.39
gtt	V	0	1.45
gtc	V	0	1.41
gta	V	7	1.43
gtg	V	0	2.70
gct	A	2	270.90
gcc	A	1	195.40
gca	A	1040	493.70
gcg	A	6	89.03
mPD		0.036	0.69
		nPD:	0.05
		N. weight:	0.79
		Sc. PD:	-0.036
		Sc. rank:	141.2

PB2			
Pos.	685	obs:	exp:
cgt	R	0	0.13
cgc	R	0	0.15
cga	R	0	0.29
cgg	R	0	0.25
aga	R	3	1.37
agg	R	0	0.81
gaa	E	1	0.61
gag	E	0	0.39
ggt	G	0	139.80
ggc	G	0	134.80
gga	G	1042	486.70
ggg	G	13	293.70
mPD		0.032	0.68
		nPD:	0.05
		N. weight:	0.79
		Sc. PD:	-0.04
		Sc. rank:	107.1

PB2

Pos.	686	obs:	exp:
att	I	0	0.73
atc	I	0	0.48
ata	I	2	0.79
gtt	V	1	219.00
gtc	V	0	213.30
gta	V	97	216.40
gtg	V	959	408.20
---	--	------	------
mPD		0.18	0.73
		nPD:	0.24
		N. weight:	0.77
		Sc. PD:	0.1
		Sc. rank:	474.8

PB2

Pos.	687	obs:	exp:
gaa	E	1045	644.40
gag	E	14	414.60
---	--	------	------
mPD		0.026	0.48
		nPD:	0.05
		N. weight:	0.48
		Sc. PD:	-0.021
		Sc. rank:	99.5

PB2

Pos.	688	obs:	exp:
tct	S	1047	168.40
tcc	S	3	138.30
tca	S	8	268.80
tcg	S	1	60.09
agt	S	0	217.70
agc	S	0	205.70
---	--	------	------
mPD		0.022	1.7
		nPD:	0.01
		N. weight:	1.9
		Sc. PD:	-0.16
		Sc. rank:	-618.3

PB2

Pos.	689	obs:	exp:
gct	A	8	273.50
gcc	A	0	197.20
gca	A	393	498.40
gcg	A	658	89.88
---	--	------	------
mPD		0.48	0.67
		nPD:	0.71
		N. weight:	1.2
		Sc. PD:	0.73
		Sc. rank:	1018.0

PB2

Pos.	690	obs:	exp:
gtt	V	0	219.40
gtc	V	1	213.70
gta	V	1052	216.80
gtg	V	6	409.00
---	--	------	------
mPD		0.013	0.73
		nPD:	0.02
		N. weight:	1.7
		Sc. PD:	-0.13
		Sc. rank:	-356.0

PB2

Pos.	691	obs:	exp:
tta	L	44	93.98
ttg	L	1014	197.20
ctt	L	0	176.30
ctc	L	0	176.70
cta	L	1	178.50
ctg	L	0	236.30
---	--	------	------
mPD		0.083	1.1
		nPD:	0.08
		N. weight:	1.7
		Sc. PD:	-0.04
		Sc. rank:	534.6

PB2

Pos.	692	obs:	exp:
cgt	R	0	45.44
cgc	R	0	52.40
cga	R	0	103.50
cgg	R	0	88.28
aaa	K	1	0.58
aag	K	0	0.42
aga	R	340	483.80
agg	R	718	284.60
---	--	------	------
mPD		0.44	0.96
		nPD:	0.46
		N. weight:	0.57
		Sc. PD:	0.19
		Sc. rank:	427.7

PB2

Pos.	693	obs:	exp:
ggt	G	0	140.40
ggc	G	0	135.30
gga	G	994	488.50
ggg	G	65	294.80
---	--	------	------
mPD		0.12	0.68
		nPD:	0.17
		N. weight:	0.63
		Sc. PD:	0.043
		Sc. rank:	314.5

PB2

Pos.	694	obs:	exp:
ttt	F	1053	418.20
ttc	F	6	640.80
---	--	------	------
mPD		0.011	0.48
		nPD:	0.02
		N. weight:	0.98
		Sc. PD:	-0.072
		Sc. rank:	-95.8

PB2

Pos.	695	obs:	exp:
tta	L	0	93.98
ttg	L	6	197.20
ctt	L	0	176.30
ctc	L	0	176.70
cta	L	1	178.50
ctg	L	1052	236.30
---	--	------	------
mPD		0.013	1.1
		nPD:	0.01
		N. weight:	1.6
		Sc. PD:	-0.14
		Sc. rank:	-573.6

PB2

Pos.	696	obs:	exp:
att	I	1057	388.70
atc	I	1	253.50
ata	I	0	415.70
gtt	V	1	0.21
gtc	V	0	0.20
gta	V	0	0.20
gtg	V	0	0.39
---	--	------	------
mPD		0.0038	0.66
		nPD:	0.01
		N. weight:	1.1
		Sc. PD:	-0.099
		Sc. rank:	-630.5

PB2

Pos.	697	obs:	exp:
tta	L	1	93.98
ttg	L	9	197.20
ctt	L	1	176.30
ctc	L	0	176.70
cta	L	237	178.50
ctg	L	811	236.30
---	--	------	------
mPD		0.37	1.1
		nPD:	0.33
		N. weight:	1.1
		Sc. PD:	0.24
		Sc. rank:	761.0

PB2

Pos.	698	obs:	exp:
ggt	G	1	140.40
ggc	G	1057	135.30
gga	G	1	488.50
ggg	G	0	294.80
---	--	------	------
mPD		0.0038	0.68
		nPD:	0.01
		N. weight:	2.2
		Sc. PD:	-0.2
		Sc. rank:	-1327.9

PB2

Pos.	699	obs:	exp:
cgt	R	0	0.17
cgc	R	0	0.20
cga	R	0	0.39
cgg	R	0	0.33
aaa	K	846	614.90
aag	K	209	440.10
aga	R	2	1.83
agg	R	2	1.08
---	--	------	------
mPD		0.33	0.50
		nPD:	0.66
		N. weight:	0.12
		Sc. PD:	0.067
		Sc. rank:	99.7

PB2

Pos.	700	obs:	exp:
gaa	E	1056	644.40
gag	E	3	414.60
---	--	------	------
mPD		0.0057	0.48
		nPD:	0.01
		N. weight:	0.53
		Sc. PD:	-0.044
		Sc. rank:	-187.6

PB2

Pos.	701	obs:	exp:
aat	N	0	28.52
aac	N	56	27.48
gat	D	19	542.00
gac	D	984	461.00
---	--	------	------
mPD		0.14	0.60
		nPD:	0.23
		N. weight:	0.75
		Sc. PD:	0.092
		Sc. rank:	449.6

PB2

Pos.	702	obs:	exp:
cgt	R	0	1.68
cgc	R	0	1.93
cga	R	0	3.82
cgg	R	0	3.25
aaa	K	697	594.50
aag	K	323	425.50
aga	R	5	17.83
agg	R	34	10.49
---	--	------	------
mPD		0.52	0.58
		nPD:	0.89
		N. weight:	0.063
		Sc. PD:	0.048
		Sc. rank:	52.6

PB2

Pos.	703	obs:	exp:
cgt	R	0	45.48
cgc	R	0	52.45
cga	R	1	103.60
cgg	R	0	88.36
aga	R	850	484.20
agg	R	208	284.80
---	--	------	------
mPD		0.32	0.96
		nPD:	0.33
		N. weight:	0.43
		Sc. PD:	0.095
		Sc. rank:	301.8

PB2

Pos.	704	obs:	exp:
tat	Y	1047	572.00
tac	Y	12	487.00
---	--	------	------
mPD		0.022	0.50
		nPD:	0.05
		N. weight:	0.61
		Sc. PD:	-0.032
		Sc. rank:	77.9

PB2

Pos.	705	obs:	exp:
ggt	G	29	140.40
ggc	G	7	135.30
gga	G	17	488.50
ggg	G	1006	294.80
---	--	------	------
mPD		0.097	0.68
		nPD:	0.14
		N. weight:	1.2
		Sc. PD:	0.048
		Sc. rank:	545.2

PB2

Pos.	706	obs:	exp:
cct	P	1	264.20
ccc	P	0	190.30
cca	P	1058	435.90
ccg	P	0	168.60
---	--	------	------
mPD		0.0019	0.71
		nPD:	0.
		N. weight:	0.97
		Sc. PD:	-0.09
		Sc. rank:	-682.0

PB2

Pos.	707	obs:	exp:
tct	S	0	1.11
tcc	S	0	0.91
tca	S	7	1.78
tcg	S	0	0.40
agt	S	0	1.44
agc	S	0	1.36
gct	A	3	271.70
gcc	A	0	195.90
gca	A	1046	495.10
gcg	A	3	89.29
---	--	------	------
mPD		0.024	0.69
		nPD:	0.04
		N. weight:	0.8
		Sc. PD:	-0.049
		Sc. rank:	43.3

PB2

Pos.	708	obs:	exp:
tta	L	9	93.98
ttg	L	1045	197.20
ctt	L	0	176.30
ctc	L	0	176.70
cta	L	1	178.50
ctg	L	4	236.30
---	--	------	------
mPD		0.028	1.1
		nPD:	0.03
		N. weight:	1.8
		Sc. PD:	-0.13
		Sc. rank:	-128.2

PB2

Pos.	709	obs:	exp:
tct	S	0	168.40
tcc	S	0	138.30
tca	S	0	268.80
tcg	S	0	60.09
agt	S	17	217.70
agc	S	1042	205.70
---	--	------	------
mPD		0.032	1.7
		nPD:	0.02
		N. weight:	1.7
		Sc. PD:	-0.13
		Sc. rank:	-289.6

PB2

Pos.	710	obs:	exp:
att	I	0	389.10
atc	I	1058	253.80
ata	I	1	416.10
---	--	------	------
mPD		0.0019	0.65
		nPD:	0.
		N. weight:	1.6
		Sc. PD:	-0.14
		Sc. rank:	-1051.1

PB2			
Pos.	711	obs:	exp:
tct	S	0	0.95
tcc	S	0	0.78
tca	S	0	1.52
tcg	S	0	0.34
aat	N	257	535.80
aac	N	795	516.20
agt	S	1	1.23
agc	S	5	1.17
gat	D	0	0.54
gac	D	1	0.46
---	--	------	------
mPD		0.38	0.52
		nPD:	0.73
		N. weight:	0.17
		Sc. PD:	0.11
		Sc. rank:	142.9

PB2			
Pos.	712	obs:	exp:
gaa	E	813	644.40
gag	E	246	414.60
---	--	------	------
mPD		0.36	0.48
		nPD:	0.75
		N. weight:	0.066
		Sc. PD:	0.041
		Sc. rank:	55.3

PB2			
Pos.	713	obs:	exp:
tta	L	10	93.98
ttg	L	1043	197.20
ctt	L	0	176.30
ctc	L	0	176.70
cta	L	0	178.50
ctg	L	6	236.30
---	--	------	------
mPD		0.030	1.1
		nPD:	0.03
		N. weight:	1.8
		Sc. PD:	-0.12
		Sc. rank:	-72.7

PB2			
Pos.	714	obs:	exp:
tct	S	0	167.20
tcc	S	0	137.30
tca	S	0	266.80
tcg	S	0	59.64
tat	Y	0	0.54
tac	Y	1	0.46
tgt	C	0	2.11
tgc	C	5	2.89
aat	N	0	0.51
aac	N	1	0.49
agt	S	0	216.10
agc	S	1051	204.10
ggt	G	0	0.13
ggc	G	1	0.13
gga	G	0	0.46
ggg	G	0	0.28
---	--	------	------
mPD		0.017	1.7
		nPD:	0.01
		N. weight:	1.8
		Sc. PD:	-0.15
		Sc. rank:	-767.5

PB2			
Pos.	715	obs:	exp:
tct	S	0	0.32
tcc	S	0	0.26
tca	S	0	0.51
tcg	S	0	0.11
cat	H	2	1.15
cac	H	0	0.85
act	T	1	0.25
acc	T	0	0.21
aca	T	0	0.46
acg	T	0	0.08
aat	N	814	536.90
aac	N	240	517.10
agt	S	1	0.41
agc	S	1	0.39
---	--	------	------
mPD		0.36	0.51
		nPD:	0.7
		N. weight:	0.17
		Sc. PD:	0.098
		Sc. rank:	138.4

PB2			
Pos.	716	obs:	exp:
tta	L	0	93.98
ttg	L	0	197.20
ctt	L	1052	176.30
ctc	L	7	176.70
cta	L	0	178.50
ctg	L	0	236.30
---	--	------	------
mPD		0.013	1.1
		nPD:	0.01
		N. weight:	1.9
		Sc. PD:	-0.16
		Sc. rank:	-695.5

PB2			
Pos.	717	obs:	exp:
tct	S	0	0.16
tcc	S	0	0.13
tca	S	0	0.25
tcg	S	1	0.06
act	T	0	1.02
acc	T	0	0.82
aca	T	0	1.83
acg	T	4	0.32
agt	S	0	0.21
agc	S	0	0.19
gct	A	1	272.20
gcc	A	0	196.30
gca	A	13	496.10
gcg	A	1040	89.46
---	--	------	------
mPD		0.036	0.68
		nPD:	0.05
		N. weight:	2.6
		Sc. PD:	-0.12
		Sc. rank:	490.1

PB2			
Pos.	718	obs:	exp:
aaa	K	1055	617.20
aag	K	4	441.80
---	--	------	------
mPD		0.0075	0.49
		nPD:	0.02
		N. weight:	0.57
		Sc. PD:	-0.046
		Sc. rank:	-153.8

PB2			
Pos.	719	obs:	exp:
ggt	G	0	140.40
ggc	G	0	135.30
gga	G	701	488.50
ggg	G	358	294.80
---	--	------	------
mPD		0.45	0.68
		nPD:	0.66
		N. weight:	0.34
		Sc. PD:	0.18
		Sc. rank:	271.0

PB2			
Pos.	720	obs:	exp:
gaa	E	250	644.40
gag	E	809	414.60
---	--	------	------
mPD		0.36	0.48
		nPD:	0.76
		N. weight:	0.32
		Sc. PD:	0.2
		Sc. rank:	265.7

PB2			
Pos.	721	obs:	exp:
aaa	K	6	617.20
aag	K	1053	441.80
---	--	------	------
mPD		0.011	0.49
		nPD:	0.02
		N. weight:	0.92
		Sc. PD:	-0.068
		Sc. rank:	-101.3

PB2			
Pos.	722	obs:	exp:
gct	A	1029	273.50
gcc	A	28	197.20
gca	A	2	498.40
gcg	A	0	89.88
---	--	------	------
mPD		0.055	0.67
		nPD:	0.08
		N. weight:	1.4
		Sc. PD:	-0.022
		Sc. rank:	465.1

PB2			
Pos.	723	obs:	exp:
aat	N	1054	539.40
aac	N	5	519.60
---	--	------	------
mPD		0.0094	0.50
		nPD:	0.02
		N. weight:	0.71
		Sc. PD:	-0.055
		Sc. rank:	-128.8

PB2			
Pos.	724	obs:	exp:
gtt	V	73	219.40
gtc	V	0	213.70
gta	V	1	216.80
gtg	V	985	409.00
---	--	------	------
mPD		0.13	0.73
		nPD:	0.18
		N. weight:	0.81
		Sc. PD:	0.062
		Sc. rank:	413.1

PB2			
Pos.	725	obs:	exp:
tta	L	1	93.98
ttg	L	1056	197.20
ctt	L	0	176.30
ctc	L	0	176.70
cta	L	0	178.50
ctg	L	2	236.30
---	--	------	------
mPD		0.0057	1.1
		nPD:	0.01
		N. weight:	1.8
		Sc. PD:	-0.17
		Sc. rank:	-1103.3

PB2			
Pos.	726	obs:	exp:
att	I	0	388.00
atc	I	0	253.10
ata	I	1056	414.90
gtt	V	0	0.62
gtc	V	0	0.61
gta	V	3	0.61
gtg	V	0	1.16
---	--	------	------
mPD		0.0057	0.66
		nPD:	0.01
		N. weight:	1.
		Sc. PD:	-0.09
		Sc. rank:	-480.3

PB2			
Pos.	727	obs:	exp:
ggt	G	0	140.40
ggc	G	0	135.30
gga	G	4	488.50
ggg	G	1055	294.80
---	--	------	------
mPD		0.0075	0.68
		nPD:	0.01
		N. weight:	1.4
		Sc. PD:	-0.12
		Sc. rank:	-554.6

PB2			
Pos.	728	obs:	exp:
caa	Q	1058	539.40
cag	Q	1	519.60
---	--	------	------
mPD		0.0019	0.50
		nPD:	0.
		N. weight:	0.73
		Sc. PD:	-0.068
		Sc. rank:	-480.6

PB2			
Pos.	729	obs:	exp:
ggt	G	0	140.40
ggc	G	0	135.30
gga	G	1059	488.50
ggg	G	0	294.80
---	--	------	------
mPD		0	0.68
		nPD:	0.
		N. weight:	0.85
		Sc. PD:	-0.081
		Sc. rank:	-706.4

PB2			
Pos.	730	obs:	exp:
gat	D	0	572.30
gac	D	1059	486.70
---	--	------	------
mPD		0	0.50
		nPD:	0.
		N. weight:	0.85
		Sc. PD:	-0.082
		Sc. rank:	-709.7

PB2			
Pos.	731	obs:	exp:
atg	M	1	1.00
gtt	V	358	219.20
gtc	V	0	213.50
gta	V	1	216.60
gtg	V	699	408.60
---	--	------	------
mPD		0.45	0.73
		nPD:	0.62
		N. weight:	0.57
		Sc. PD:	0.29
		Sc. rank:	451.8

PB2			
Pos.	732	obs:	exp:
gtt	V	3	219.00
gtc	V	0	213.30
gta	V	0	216.40
gtg	V	1054	408.20
gct	A	0	0.52
gcc	A	0	0.37
gca	A	0	0.94
gcg	A	2	0.17
---	--	------	------
mPD		0.0094	0.73
		nPD:	0.01
		N. weight:	1.
		Sc. PD:	-0.086
		Sc. rank:	-352.3

PB2			
Pos.	733	obs:	exp:
tta	L	0	93.98
ttg	L	1059	197.20
ctt	L	0	176.30
ctc	L	0	176.70
cta	L	0	178.50
ctg	L	0	236.30
---	--	------	------
mPD		0	1.1
		nPD:	0.
		N. weight:	1.8
		Sc. PD:	-0.18
		Sc. rank:	-1534.8

PB2			
Pos.	734	obs:	exp:
gtt	V	0	219.40
gtc	V	0	213.70
gta	V	693	216.80
gtg	V	366	409.00
---	--	------	------
mPD		0.45	0.73
		nPD:	0.62
		N. weight:	0.8
		Sc. PD:	0.4
		Sc. rank:	632.3

PB2			
Pos.	735	obs:	exp:
atg	M	1059	1059.00
---	--	------	------
mPD		0	0
		nPD:	1.
		N. weight:	0.
		Sc. PD:	0
		Sc. rank:	0

PB2			
Pos.	736	obs:	exp:
aaa	K	1059	617.20
aag	K	0	441.80
---	--	------	------
mPD		0	0.49
		nPD:	0.
		N. weight:	0.59
		Sc. PD:	-0.057
		Sc. rank:	-492.9

,

PB2			
Pos.	737	obs:	exp:
cgt	R	0	45.48
cgc	R	0	52.45
cga	R	2	103.60
cgg	R	1056	88.36
aga	R	0	484.20
agg	R	1	284.80
---	--	------	------
mPD		0.0057	0.96
		nPD:	0.01
		N. weight:	2.7
		Sc. PD:	-0.24
		Sc. rank:	-1502.8

,

PB2			
Pos.	738	obs:	exp:
aaa	K	1059	617.20
aag	K	0	441.80
---	--	------	------
mPD		0	0.49
		nPD:	0.
		N. weight:	0.59
		Sc. PD:	-0.057
		Sc. rank:	-492.9

,

PB2			
Pos.	739	obs:	exp:
cgt	R	36	45.48
cgc	R	0	52.45
cga	R	27	103.60
cgg	R	996	88.36
aga	R	0	484.20
agg	R	0	284.80
---	--	------	------
mPD		0.11	0.96
		nPD:	0.12
		N. weight:	2.5
		Sc. PD:	0.044
		Sc. rank:	1083.3

,

PB2			
Pos.	740	obs:	exp:
gct	A	0	0.77
gcc	A	3	0.56
gca	A	0	1.41
gcg	A	0	0.25
gat	D	0	570.60
gac	D	1056	485.40
---	--	------	------
mPD		0.0057	0.50
		nPD:	0.01
		N. weight:	0.86
		Sc. PD:	-0.073
		Sc. rank:	-341.4

,

PB2			
Pos.	741	obs:	exp:
tct	S	1053	168.40
tcc	S	6	138.30
tca	S	0	268.80
tcg	S	0	60.09
agt	S	0	217.70
agc	S	0	205.70
---	--	------	------
mPD		0.011	1.7
		nPD:	0.01
		N. weight:	2.
		Sc. PD:	-0.18
		Sc. rank:	-1077.7

,

PB2			
Pos.	742	obs:	exp:
tct	S	0	168.40
tcc	S	0	138.30
tca	S	0	268.80
tcg	S	0	60.09
agt	S	0	217.70
agc	S	1059	205.70
---	--	------	------
mPD		0	1.7
		nPD:	0.
		N. weight:	1.8
		Sc. PD:	-0.17
		Sc. rank:	-1496.2

,

PB2			
Pos.	743	obs:	exp:
att	I	0	389.10
atc	I	0	253.80
ata	I	1059	416.10
---	--	------	------
mPD		0	0.65
		nPD:	0.
		N. weight:	1.
		Sc. PD:	-0.098
		Sc. rank:	-852.8

,

PB2			
Pos.	744	obs:	exp:
tta	L	0	93.98
ttg	L	0	197.20
ctt	L	1059	176.30
ctc	L	0	176.70
cta	L	0	178.50
ctg	L	0	236.30
---	--	------	------
mPD		0	1.1
		nPD:	0.
		N. weight:	2.
		Sc. PD:	-0.19
		Sc. rank:	-1636.7

,

PB2			
Pos.	745	obs:	exp:
act	T	1058	269.90
acc	T	1	217.30
aca	T	0	485.70
acg	T	0	86.02
---	--	------	------
mPD		0.0019	0.68
		nPD:	0.
		N. weight:	1.5
		Sc. PD:	-0.14
		Sc. rank:	-1044.6

,

PB2			
Pos.	746	obs:	exp:
gat	D	1	572.30
gac	D	1058	486.70
---	--	------	------
mPD		0.0019	0.50
		nPD:	0.
		N. weight:	0.85
		Sc. PD:	-0.078
		Sc. rank:	-550.3

,

PB2			
Pos.	747	obs:	exp:
tct	S	0	168.40
tcc	S	0	138.30
tca	S	0	268.80
tcg	S	0	60.09
agt	S	1058	217.70
agc	S	1	205.70
---	--	------	------
mPD		0.0019	1.7
		nPD:	0.
		N. weight:	1.7
		Sc. PD:	-0.16
		Sc. rank:	-1286.1

,

PB2			
Pos.	748	obs:	exp:
caa	Q	0	539.40
cag	Q	1059	519.60
---	--	------	------
mPD		0	0.50
		nPD:	0.
		N. weight:	0.78
		Sc. PD:	-0.075
		Sc. rank:	-650.1

,

PB2			
Pos.	749	obs:	exp:
act	T	0	269.90
acc	T	0	217.30
aca	T	1059	485.70
acg	T	0	86.02
---	--	------	------
mPD		0	0.68
		nPD:	0.
		N. weight:	0.86
		Sc. PD:	-0.082
		Sc. rank:	-711.6

,

PB2			
Pos.	750	obs:	exp:
gct	A	0	273.50
gcc	A	0	197.20
gca	A	0	498.40
gcg	A	1059	89.88
---	--	------	------
mPD		0	0.67
		nPD:	0.
		N. weight:	2.7
		Sc. PD:	-0.26
		Sc. rank:	-2252.0

,

PB2			
Pos.	751	obs:	exp:
act	T	0	269.90
acc	T	1059	217.30
aca	T	0	485.70
acg	T	0	86.02
---	--	------	------
mPD		0	0.68
		nPD:	0.
		N. weight:	1.7
		Sc. PD:	-0.17
		Sc. rank:	-1446.0

,

PB2			
Pos.	752	obs:	exp:
aaa	K	1059	617.20
aag	K	0	441.80
---	--	------	------
mPD		0	0.49
		nPD:	0.
		N. weight:	0.59
		Sc. PD:	-0.057
		Sc. rank:	-492.9

,

PB2			
Pos.	753	obs:	exp:
cgt	R	0	45.48
cgc	R	0	52.45
cga	R	0	103.60
cgg	R	0	88.36
aga	R	184	484.20
agg	R	875	284.80
---	--	------	------
mPD		0.29	0.96
		nPD:	0.3
		N. weight:	0.84
		Sc. PD:	0.16
		Sc. rank:	573.8

,

PB2			
Pos.	754	obs:	exp:
att	I	1053	389.10
atc	I	6	253.80
ata	I	0	416.10
---	--	------	------
mPD		0.011	0.65
		nPD:	0.02
		N. weight:	1.1
		Sc. PD:	-0.084
		Sc. rank:	-231.5

,

PB2			
Pos.	755	obs:	exp:
cgt	R	0	45.48
cgc	R	0	52.45
cga	R	4	103.60
cgg	R	1055	88.36
aga	R	0	484.20
agg	R	0	284.80
---	--	------	------
mPD		0.0075	0.96
		nPD:	0.01
		N. weight:	2.7
		Sc. PD:	-0.24
		Sc. rank:	-1409.8

,

PB2			
Pos.	756	obs:	exp:
atg	M	1059	1059.00
---	--	------	------
mPD		0	0
		nPD:	1.
		N. weight:	0.
		Sc. PD:	0
		Sc. rank:	0

,

PB2			
Pos.	757	obs:	exp:
gct	A	0	273.50
gcc	A	1058	197.20
gca	A	1	498.40
gcg	A	0	89.88
---	--	------	------
mPD		0.0019	0.67
		nPD:	0.
		N. weight:	1.8
		Sc. PD:	-0.17
		Sc. rank:	-1246.4

,

PB2			
Pos.	758	obs:	exp:
att	I	0	389.10
atc	I	1059	253.80
ata	I	0	416.10
---	--	------	------
mPD		0	0.65
		nPD:	0.
		N. weight:	1.6
		Sc. PD:	-0.15
		Sc. rank:	-1304.3

,

PB2			
Pos.	759	obs:	exp:
aat	N	1059	539.40
aac	N	0	519.60
---	--	------	------
mPD		0	0.50
		nPD:	0.
		N. weight:	0.74
		Sc. PD:	-0.071
		Sc. rank:	-615.9

,

PB2			
Pos.	760	obs:	exp:
taa	.	1047	449.90
tag	.	12	265.10
tga	.	0	344.00
---	--	------	------
mPD		0.022	0.81
		nPD:	0.03
		N. weight:	0.88
		Sc. PD:	-0.061
		Sc. rank:	-28.0

}

Amino acid level conservation per codon

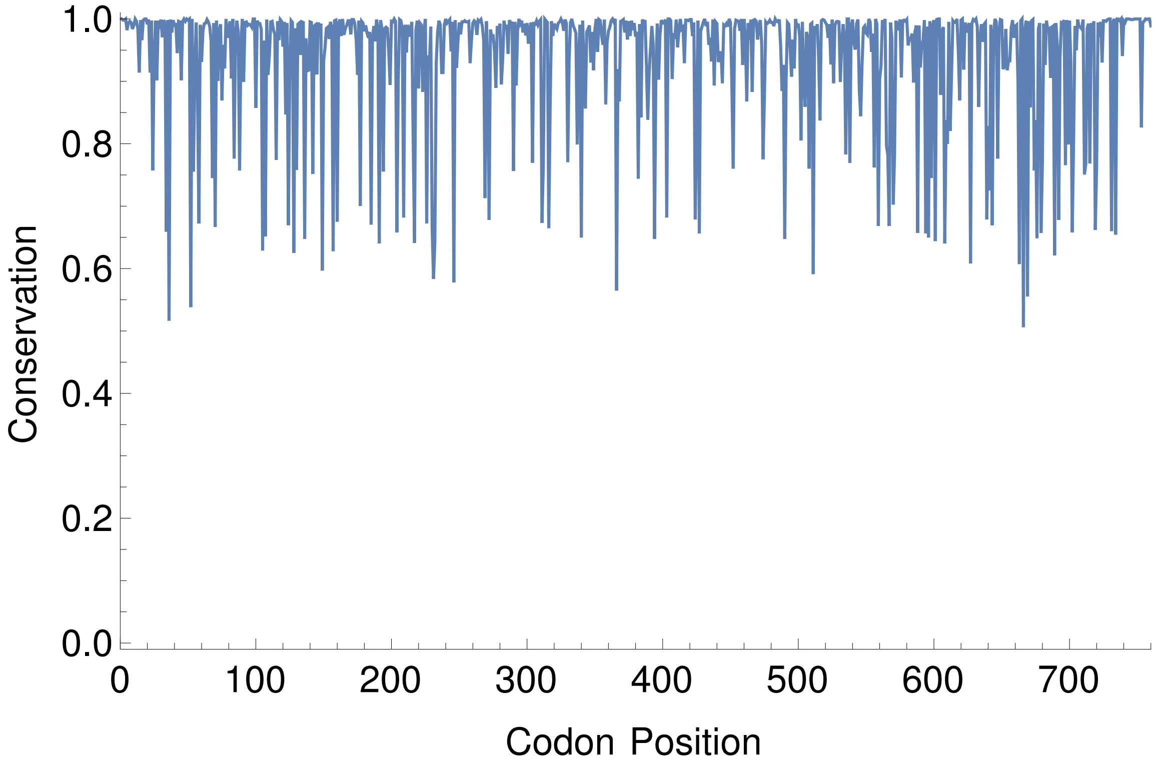

Conservation analysis plots

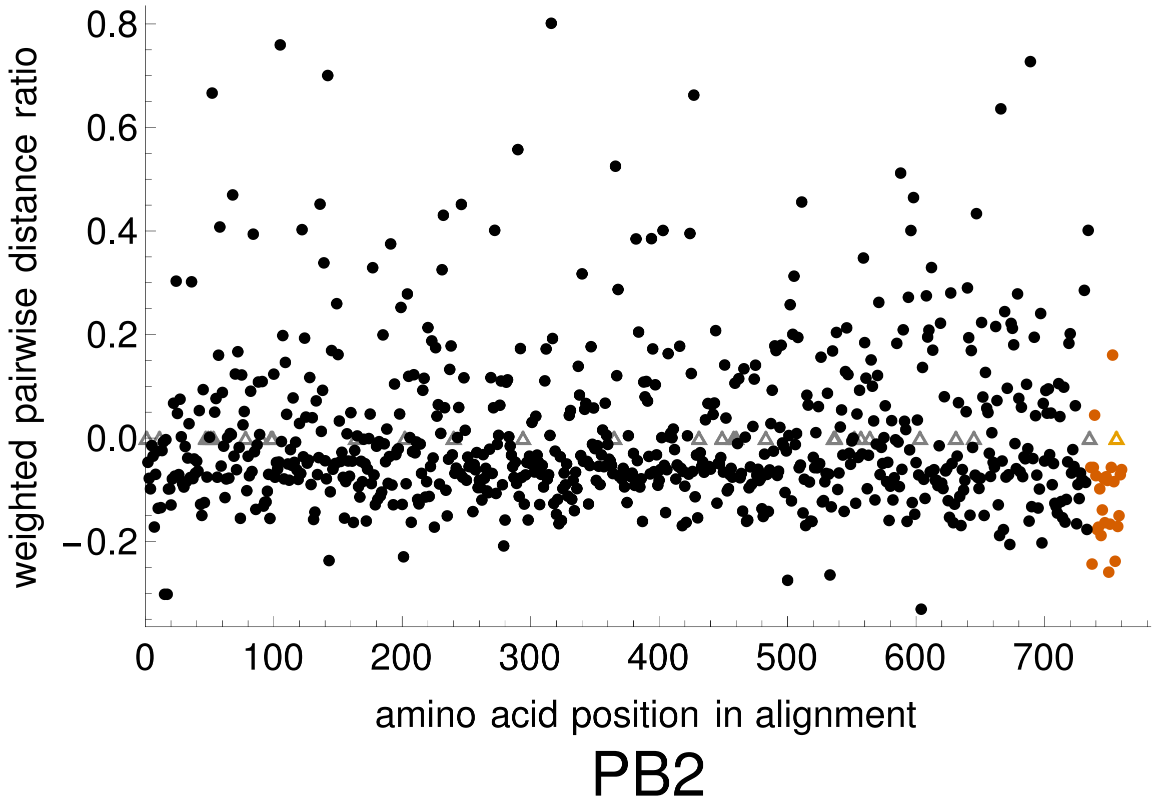

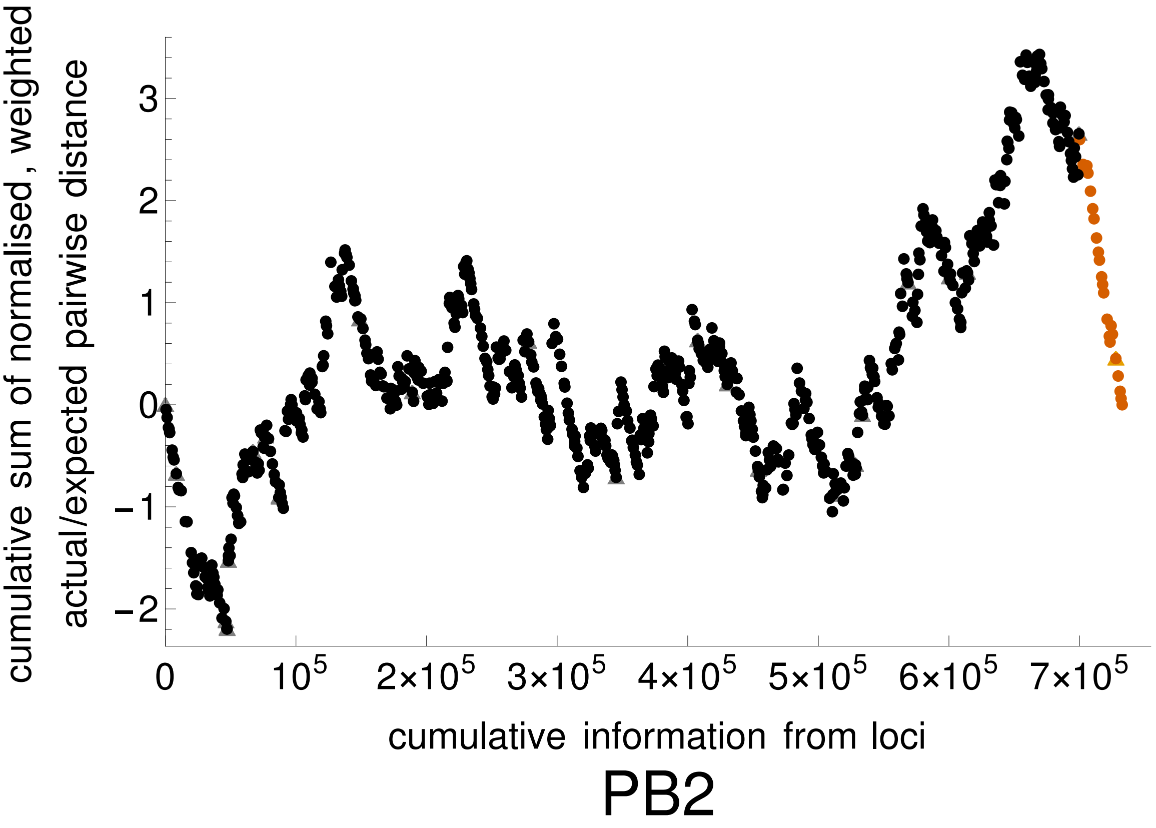

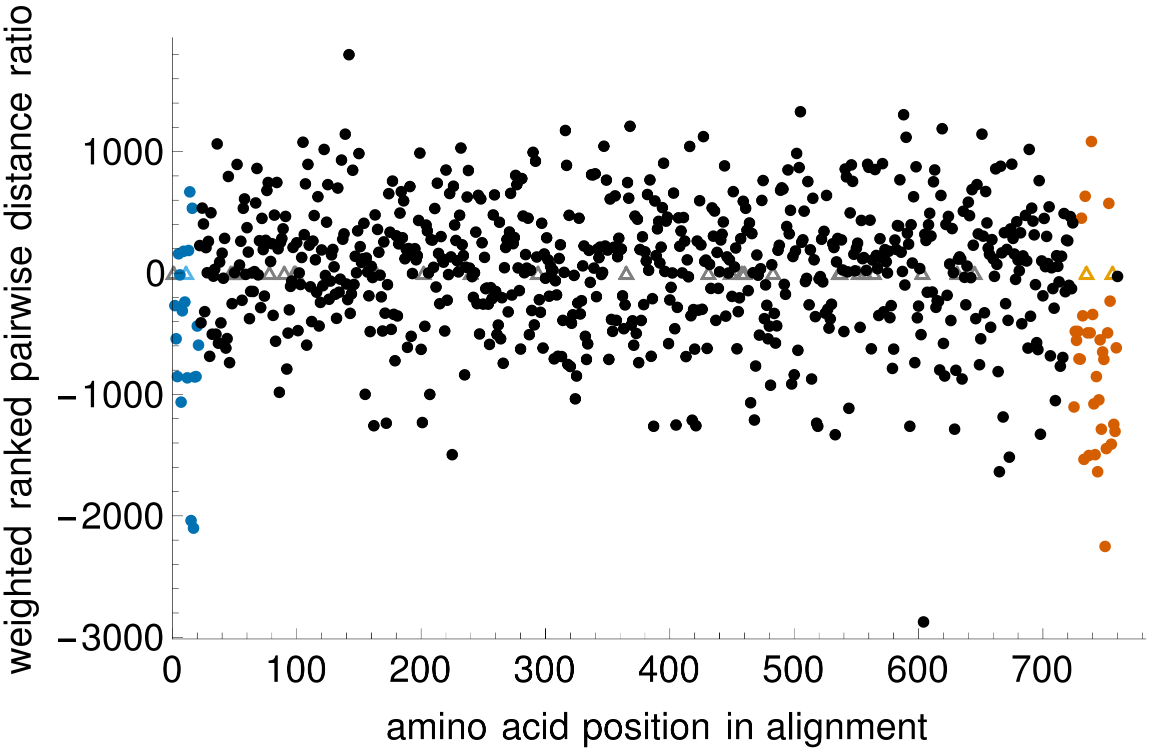

weighted ranked pairwise distance ratio
1000
0
−1000
−2000
−3000
0
100
200
300
400
500
600
700
amino acid position in alignment

PB2

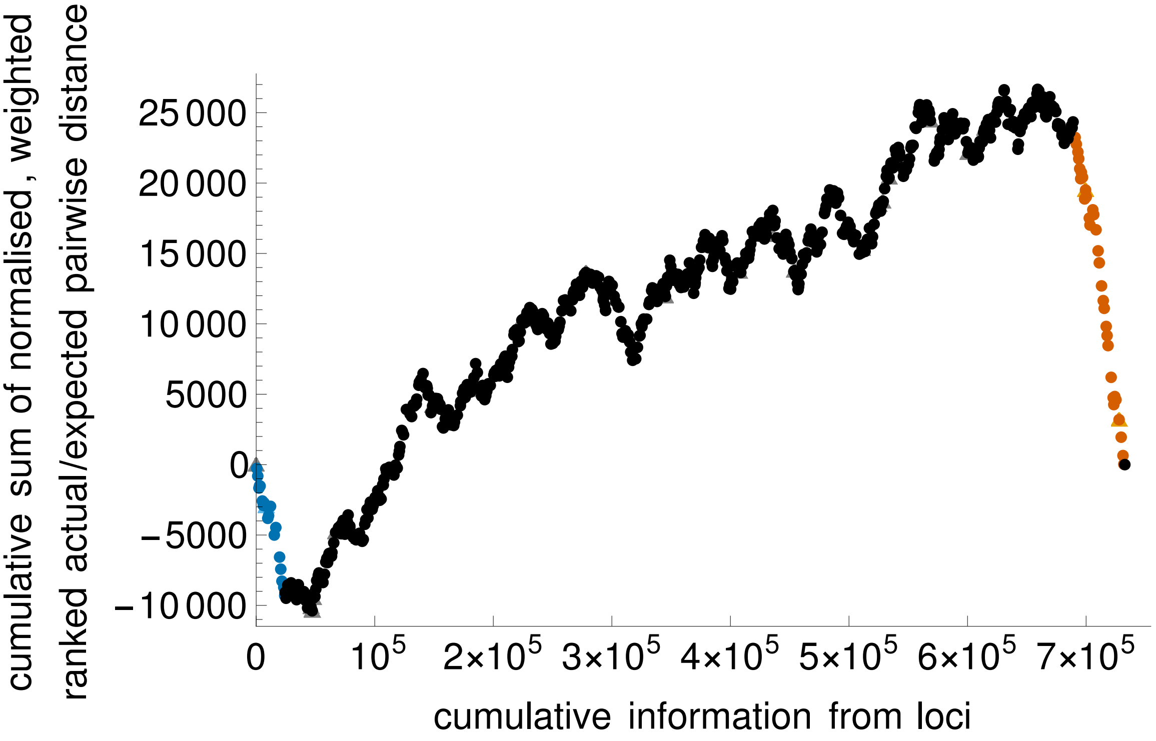

cumulative sum of normalised, weighted ranked actual/expected pairwise distance
25 000
20 000
15 000
10 000
5000
0
−5000
−10 000
0
10^5
2×10^5
3×10^5
4×10^5
5×10^5
6×10^5
7×10^5
cumulative information from loci

PB2

PB1

Gene length histogram

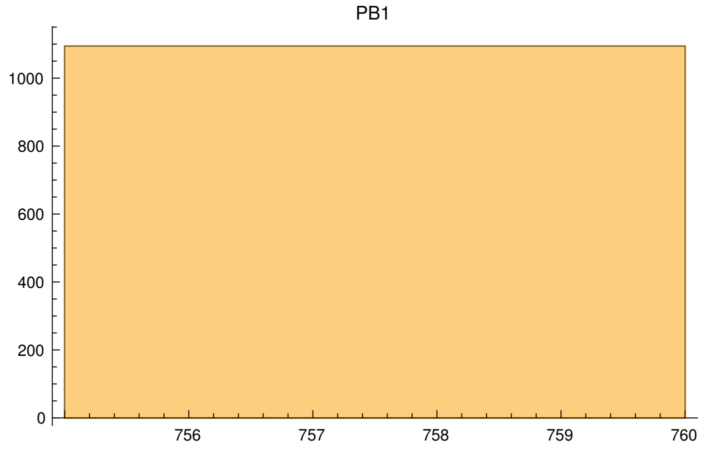

Information vs. nPD

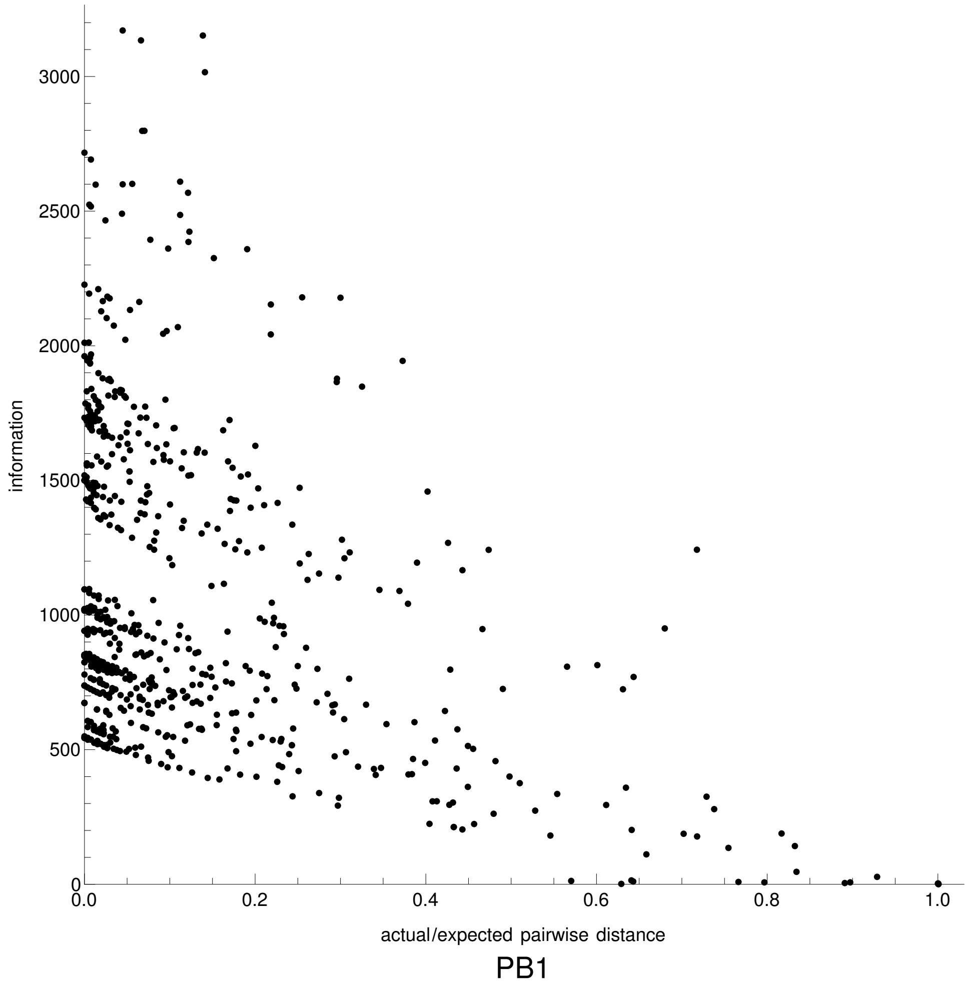

Example sequences highlighted by regions found in analyses to be conserved

Interesting points (by weighted raw PD) highlighted for gene PB1:

ATGGATGTCAATCCGACTTTACTTTTCTTGAAAGTGCCAGTGCAAAATGCTATAAGTACCACTTTCCCTTATACTG⋱
GAGACCCTCCATACAGCCATGGAACAGGAACAGGATACACCATGGACACAGTCAACAGAACACATAAATACTCAG⋱
AAAAAGGAAAGTGGACAACGAACACAGAAACTGGAGCACCCCAACTCAATCCAATTGATGGACCATTACCTGAGG⋱
ACAATGAGCCGAGTGGGTATGCACAAACGGATTGTGTATTGGAAGCAATGGCTTTCCTTGAAGAATCTCACCCAG⋱
GGATCTTTGAAAACTCGTGTCTCGAAACGATGGAAATTGTTCAGCAAACAAGAGTGGACAAACTGACCCAAGGCC⋱
GCCAGACCTATGACTGGACGTTGAATAGAAATCAGCCGGCTGCTACCGCATTGGCCAACACTATAGAGGTATTTA⋱
GATCGAATGGCCTGACAGCTAATGAATCAGGAAGGTTGATCGATTTCCTCAAGGACGTGATGGATTCAATGGACA⋱
AGGAAGAAATGGAGATTACAACACATTTCCAGAGGAAGAGGAGAGTGAGGGACAACATGACCAAGAAAATGGTTA⋱
CACAGAGAACAATAGGGAAGAAAAAACAAAGACTGAACAAAAGGAGCTACCTGATAAGAGCACTGACATTGAACA⋱
CAATGACAAAGGATGCTGAAAGAGGCAAGCTGAAAAGGAGGGCAATCGCAACACCCGGGATGCAAATCAGAGGAT⋱
TCGTGTATTTTGTAGAAGCACTAGCGAGGAGCATCTGTGAGAAACTTGAGCAATCTGGCCTCCCTGTCGGAGGGA⋱
ATGAGAAGAAAGCTAAATTGGCAAATGTTGTGAGGAAGATGATGACCAATTCACAAGATACAGAGCTCTCCTTCA⋱
CAATTACTGGGGACAACACCAAATGGAATGAGAATCAAAACCCCCGGATGTTTCTAGCAATGATAACATACATCA⋱
CAAGAAACCAGCCAGAATGGTTTAGAAATGTCTTAAGCATTGCTCCTATAATGTTCTCAAACAAGATGGCGAGAT⋱
TAGGAAAAGGGTACATGTTCGAAAGTAAGAGTATGAAGTTACGGACACAAGTACCAGCGGAAATGCTCGCAAATA⋱
TTGACCTGAAATACTTCAACAAATCAACAAGAGAGAAAATCGAGAAAATAAGACCTCTACTGATAGATGGCACAG⋱
CCTCATTGAGTCCTGGGATGATGATGGGCATGTTCAACATGTTGAGTACAGTCTTAGGAGTTTCAATTCTGAATC⋱
TTGGGCAGAAGAAGTACACCAAAACCACATATTGGTGGGACGGACTCCAATCCTCAGATGACTTCGCCCTCATAG⋱
TGAATGCACCGAATCATGAGGGAATACAGGCAGGAGTAGATAGGTTCTATAGAACCTGCAAATTAGTTGGGATAA⋱
ACATGAGCAAGAAGAAATCCTACATAAATCGGACAGGAACATTCGAATTCACAAGCTTTTTCTACCGCTATGGAT⋱
TCGTAGCTAACTTCAGTATGGAGTTGCCCAGTTTTGGAGTGTCCGGGATTAATGAGTCAGCTGACATGAGCGTTG⋱
GTGTTACAGTAATAAAGAACAATATGATAAACAACGATCTTGGACCAGCAACAGCCCAAATGGCCCTTCAGCTAT⋱
TTATCAAAGACTACAGATACACATACCGATGTCACAGGGGTGATACGCAAATTCAAACGAGGAGAGCATTCGAGC⋱
TGAAGAAGCTGTGGGAGCAGACCCGTTCGAAGGCTGGACTGTTGGTTTCAGATGGAGGGCCAAACCTGTACAATA⋱
TCCGGAACCTCCACATTCCAGAGGTCTGCTTGAAGTGGGAGTTGATGGATGAAGACTACCAAGGCAGGTTGTGTA⋱
ATCCTATGAACCCGTTTGTCAGCCATAAGGAAATTGATTCAGTCAACAATGCTGTGGTGATGCCAGCTCATGGCC⋱
CCGCCAAAAGCATGGAGTATGATGCCGTTGCAACCACACATTCATGGATTCCTAAGAGGAATCGCTCCATTCTCA⋱
ACACCAGCCAAAGGGGGATTCTTGAGGACGAACAGATGTACCAGAAGTGCTGCAACCTATTCGAAAAGTTCTTCC⋱
CCAGCAGTTCGTACAGG
AGGCCAGTTGGAATTTCCAGCATGGTGGAGGCCATGGTGTCTAGGGCCCGAATTG
ACGCACGAATTGATTTCGAATCTGGAAGGATTAAGAAAGAAGAGTTTGCTGA
GATCATGAAGATCTGTTCCACCATTGAAGAGCTCAGACGGCAAAAA---TAG

Interesting points (by weighted ranked PD) highlighted for gene PB1:

ATGGATGTCAATCCGACTTTACTTTTCTTGAAAGTGCCAGTGCAAAATGCTATAAGTACCACTTTCCCTTATACTGGA⋱
GACCCTCCATACAGCCATGGAACAGGAACAGGATACACCATGGACACAGTCAACAGAACACATAAATACTCAGAAAA⋱
AGGAAAGTGGACAACGAACACAGAAACTGGAGCACCCCAACTCAATCCAATTGATGGACCATTACCTGAGGACAATG⋱
AGCCGAGTGGGTATGCACAAACGGATTGTGTATTGGAAGCAATGGCTTTCCTTGAAGAATCTCACCCAGGGATCTTT⋱
GAAAACTCGTGTCTCGAAACGATGGAAATTGTTCAGCAAACAAGAGTGGACAAACTGACCCAAGGCCGCCAGACCTA⋱
TGACTGGACGTTGAATAGAAATCAGCCGGCTGCTACCGCATTGGCCAACACTATAGAGGTATTTAGATCGAATGGCC⋱
TGACAGCTAATGAATCAGGAAGGTTGATCGATTTCCTCAAGGACGTGATGGATTCAATGGACAAGGAAGAAATGGAG⋱
ATTACAACACATTTCCAGAGGAAGAGGAGAGTGAGGGACAACATGACCAAGAAAATGGTTACACAGAGAACAATAGG⋱
GAAGAAAAAACAAAGACTGAACAAAAGGAGCTACCTGATAAGAGCACTGACATTGAACACAATGACAAAGGATGCTG⋱

AAAGAGGCAAGCTGAAAAGGAGGGCAATCGCAACACCCGGGATGCAAATCAGAGGATTCGTGTATTTTGTAGAAGCA⋱
CTAGCGAGGAGCATCTGTGAGAAACTTGAGCAATCTGGCCTCCCTGTCGGAGGGAATGAGAAGAAAGCTAAATTGGC⋱
AAATGTTGTGAGGAAGATGATGACCAATTCACAAGATACAGAGCTCTCCTTCACAATTACTGGGGACAACACCAAAT⋱
GGAATGAGAATCAAAACCCCCGGATGTTTCTAGCAATGATAACATACATCACAAGAAACCAGCCAGAATGGTTTAGA⋱
AATGTCTTAAGCATTGCTCCTATAATGTTCTCAAACAAGATGGCGAGATTAGGAAAAGGGTACATGTTCGAAAGTAA⋱
GAGTATGAAGTTACGGACACAAGTACCAGCGGAAATGCTCGCAAATATTGACCTGAAATACTTCAACAAATCAACAA⋱
GAGAGAAAATCGAGAAAATAAGACCTCTACTGATAGATGGCACAGCCTCATTGAGTCCTGGGATGATGATGGGCATG⋱
TTCAACATGTTGAGTACAGTCTTAGGAGTTTCAATTCTGAATCTTGGGCAGAAGAAGTACACCAAAACCACATATTG⋱
GTGGGACGGACTCCAATCCTCAGATGACTTCGCCCTCATAGTGAATGCACCGAATCATGAGGGAATACAGGCAGGAG⋱
TAGATAGGTTCTATAGAACCTGCAAATTAGTTGGGATAAACATGAGCAAGAAGAAATCCTACATAAATCGGACAGGA⋱
ACATTCGAATTCACAAGCTTTTTCTACCGCTATGGATTCGTAGCTAACTTCAGTATGGAGTTGCCCAGTTTTGGAGT⋱
GTCCGGGATTAATGAGTCAGCTGACATGAGCGTTGGTGTTACAGTAATAAAGAACAATATGATAAACAACGATCTTG⋱
GACCAGCAACAGCCCAAATGGCCCTTCAGCTATTTATCAAAGACTACAGATACACATACCGATGTCACAGGGGTGAT⋱
ACGCAAATTCAAACGAGGAGAGCATTCGAGCTGAAGAAGCTGTGGGAGCAGACCCGTTCGAAGGCTGGACTGTTGGT⋱
TTCAGATGGAGGGCCAAACCTGTACAATATCCGGAACCTCCACATTCCAGAGGTCTGCTTGAAGTGGGAGTTGATGG⋱
ATGAAGACTACCAAGGCAGGTTGTGTAATCCTATGAACCCGTTTGTCAGCCATAAGGAAATTGATTCAGTCAACAAT⋱
GCTGTGGTGATGCCAGCTCATGGCCCCGCCAAAAGCATGGAGTATGATGCCGTTGCAACCACACATTCATGGATTCC⋱
TAAGAGGAATCGCTCCATTCTCAACACCAGCCAAAGGGGGATTCTTGAGGACGAACAGATGTACCAGAAGTGCTGCA⋱
ACCTATTCGAAAAGTTCTTCCCCAGCAGTTCGTACAGGAGGCCAGTTGGAATTTCCAGCATGGTGGAGGCCATGGTG⋱
TCTAGG
GCCCGAATTGACGCACGAATTGATTTCGAATCTGGAAGGATTAAGAAAGAAGAGT
TTGCTGAGATCATGAAGATCTGTTCCACCATTGAAGAGCTCAGACGGCAAAAA
---TAG

Per codon conservation report

PB1			
Pos.	1	obs:	exp:
atg	M	1094	1094.00
---	--	------	------
mPD		0	0
		nPD:	1.
		N. weight:	0.
		Sc. PD:	0
		Sc. rank:	0

PB1			
Pos.	2	obs:	exp:
gat	D	1094	591.20
gac	D	0	502.80
---	--	------	------
mPD		0	0.50
		nPD:	0.
		N. weight:	0.68
		Sc. PD:	-0.055
		Sc. rank:	-580.7

PB1			
Pos.	3	obs:	exp:
gtt	V	2	226.70
gtc	V	1092	220.80
gta	V	0	224.00
gtg	V	0	422.50
---	--	------	------
mPD		0.0037	0.73
		nPD:	0.01
		N. weight:	1.7
		Sc. PD:	-0.13
		Sc. rank:	-1147.4

PB1			
Pos.	4	obs:	exp:
act	T	0	0.25
acc	T	0	0.21
aca	T	1	0.46
acg	T	0	0.08
aat	N	1060	556.70
aac	N	33	536.30
---	--	------	------
mPD		0.062	0.50
		nPD:	0.12
		N. weight:	0.6
		Sc. PD:	0.022
		Sc. rank:	285.8

PB1			
Pos.	5	obs:	exp:
tct	S	0	0.16
tcc	S	0	0.13
tca	S	0	0.25
tcg	S	1	0.06
cct	P	0	272.50
ccc	P	0	196.20
cca	P	0	449.50
ccg	P	1092	173.80
cgt	R	0	0.04
cgc	R	0	0.05
cga	R	0	0.10
cgg	R	1	0.08
agt	S	0	0.21
agc	S	0	0.19
aga	R	0	0.46
agg	R	0	0.27
---	--	------	------
mPD		0.0037	0.72
		nPD:	0.01
		N. weight:	2.
		Sc. PD:	-0.16
		Sc. rank:	-1319.5

PB1			
Pos.	6	obs:	exp:
act	T	1090	278.90
acc	T	3	224.50
aca	T	0	501.80
acg	T	1	88.86
---	--	------	------
mPD		0.0073	0.68
		nPD:	0.01
		N. weight:	1.5
		Sc. PD:	-0.11
		Sc. rank:	-699.8

PB1			
Pos.	7	obs:	exp:
tta	L	1046	97.08
ttg	L	33	203.70
ctt	L	0	182.20
ctc	L	0	182.60
cta	L	15	184.40
ctg	L	0	244.10
---	--	------	------
mPD		0.086	1.1
		nPD:	0.08
		N. weight:	2.4
		Sc. PD:	-0.019
		Sc. rank:	697.9

PB1			
Pos.	8	obs:	exp:
tta	L	0	96.99
ttg	L	0	203.50
ctt	L	978	182.00
ctc	L	99	182.40
cta	L	0	184.20
ctg	L	16	243.90
att	I	1	0.37
atc	I	0	0.24
ata	I	0	0.39
---	--	------	------
mPD		0.19	1.1
		nPD:	0.17
		N. weight:	1.6
		Sc. PD:	0.13
		Sc. rank:	923.8

PB1			
Pos.	9	obs:	exp:
ttt	F	6	431.60
ttc	F	1087	661.40
tta	L	0	0.09
ttg	L	0	0.19
ctt	L	0	0.17
ctc	L	1	0.17
cta	L	0	0.17
ctg	L	0	0.22
---	--	------	------
mPD		0.013	0.48
		nPD:	0.03
		N. weight:	0.52
		Sc. PD:	-0.029
		Sc. rank:	-71.6

PB1			
Pos.	10	obs:	exp:
tta	L	29	97.08
ttg	L	1062	203.70
ctt	L	0	182.20
ctc	L	0	182.60
cta	L	0	184.40
ctg	L	3	244.10
---	--	------	------
mPD		0.057	1.1
		nPD:	0.05
		N. weight:	1.7
		Sc. PD:	-0.056
		Sc. rank:	193.4

PB1			
Pos.	11	obs:	exp:
aaa	K	1091	637.60
aag	K	3	456.40
---	--	------	------
mPD		0.0055	0.49
		nPD:	0.01
		N. weight:	0.57
		Sc. PD:	-0.041
		Sc. rank:	-254.9

PB1			
Pos.	12	obs:	exp:
gtt	V	7	226.70
gtc	V	0	220.80
gta	V	15	224.00
gtg	V	1072	422.50
---	--	------	------
mPD		0.040	0.73
		nPD:	0.05
		N. weight:	0.94
		Sc. PD:	-0.028
		Sc. rank:	134.5

PB1			
Pos.	13	obs:	exp:
cct	P	0	272.70
ccc	P	0	196.40
cca	P	1080	449.90
ccg	P	13	174.00
gct	A	0	0.26
gcc	A	0	0.19
gca	A	1	0.47
gcg	A	0	0.08
---	--	------	------
mPD		0.025	0.71
		nPD:	0.04
		N. weight:	0.92
		Sc. PD:	-0.044
		Sc. rank:	-15.0

PB1			
Pos.	14	obs:	exp:
tta	L	0	0.09
ttg	L	1	0.19
ctt	L	0	0.17
ctc	L	0	0.17
cta	L	0	0.17
ctg	L	0	0.22
atg	M	2	2.00
gtt	V	0	212.80
gtc	V	0	207.30
gta	V	40	210.30
gtg	V	987	396.60
gct	A	0	16.53
gcc	A	0	11.92
gca	A	1	30.12
gcg	A	63	5.43
---	--	------	------
mPD		0.19	0.85
		nPD:	0.22
		N. weight:	0.99
		Sc. PD:	0.13
		Sc. rank:	678.9

PB1			
Pos.	15	obs:	exp:
caa	Q	1090	557.20
cag	Q	4	536.80
---	--	------	------
mPD		0.0073	0.50
		nPD:	0.01
		N. weight:	0.72
		Sc. PD:	-0.049
		Sc. rank:	-277.7

PB1			
Pos.	16	obs:	exp:
aat	N	1091	557.20
aac	N	3	536.80
---	--	------	------
mPD		0.0055	0.50
		nPD:	0.01
		N. weight:	0.72
		Sc. PD:	-0.051
		Sc. rank:	-332.2

PB1			
Pos.	17	obs:	exp:
cct	P	1	0.25
ccc	P	0	0.18
cca	P	0	0.41
ccg	P	0	0.16
gct	A	1019	282.20
gcc	A	73	203.50
gca	A	1	514.40
gcg	A	0	92.77
---	--	------	------
mPD		0.13	0.67
		nPD:	0.19
		N. weight:	1.2
		Sc. PD:	0.13
		Sc. rank:	777.0

PB1			
Pos.	18	obs:	exp:
att	I	0	401.20
atc	I	0	261.70
ata	I	1092	429.10
gtt	V	0	0.41
gtc	V	0	0.40
gta	V	2	0.41
gtg	V	0	0.77
---	--	------	------
mPD		0.0037	0.66
		nPD:	0.01
		N. weight:	1.
		Sc. PD:	-0.078
		Sc. rank:	-648.5

PB1			
Pos.	19	obs:	exp:
tct	S	0	174.00
tcc	S	0	142.90
tca	S	0	277.70
tcg	S	0	62.08
agt	S	1086	224.90
agc	S	8	212.50
---	--	------	------
mPD		0.015	1.7
		nPD:	0.01
		N. weight:	1.7
		Sc. PD:	-0.12
		Sc. rank:	-829.7

PB1			
Pos.	20	obs:	exp:
act	T	1	278.10
acc	T	1025	223.90
aca	T	63	500.40
acg	T	2	88.62
gct	A	0	0.77
gcc	A	3	0.56
gca	A	0	1.41
gcg	A	0	0.25
---	--	------	------
mPD		0.12	0.68
		nPD:	0.18
		N. weight:	1.4
		Sc. PD:	0.12
		Sc. rank:	858.0

PB1			
Pos.	21	obs:	exp:
act	T	842	278.90
acc	T	5	224.50
aca	T	112	501.80
acg	T	135	88.86
mPD		0.38	0.68
		nPD:	0.57
		N. weight:	0.81
		Sc. PD:	0.37
		Sc. rank:	687.8

,

PB1			
Pos.	22	obs:	exp:
ttt	F	9	432.00
ttc	F	1085	662.00
mPD		0.016	0.48
		nPD:	0.03
		N. weight:	0.5
		Sc. PD:	-0.025
		Sc. rank:	-17.0

,

PB1			
Pos.	23	obs:	exp:
cct	P	1089	273.00
ccc	P	5	196.60
cca	P	0	450.30
ccg	P	0	174.20
mPD		0.0091	0.71
		nPD:	0.01
		N. weight:	1.5
		Sc. PD:	-0.1
		Sc. rank:	-630.0

,

PB1			
Pos.	24	obs:	exp:
tat	Y	1065	590.90
tac	Y	29	503.10
mPD		0.052	0.50
		nPD:	0.1
		N. weight:	0.55
		Sc. PD:	0.0098
		Sc. rank:	219.0

,

PB1			
Pos.	25	obs:	exp:
act	T	970	278.90
acc	T	124	224.50
aca	T	0	501.80
acg	T	0	88.86
mPD		0.20	0.68
		nPD:	0.3
		N. weight:	1.1
		Sc. PD:	0.23
		Sc. rank:	869.7

,

PB1			
Pos.	26	obs:	exp:
ggt	G	0	145.00
ggc	G	6	139.80
gga	G	1087	504.70
ggg	G	1	304.60
mPD		0.013	0.68
		nPD:	0.02
		N. weight:	0.81
		Sc. PD:	-0.052
		Sc. rank:	-216.6

,

PB1			
Pos.	27	obs:	exp:
gat	D	2	590.10
gac	D	1090	501.90
gaa	E	2	1.22
gag	E	0	0.78
mPD		0.0073	0.50
		nPD:	0.01
		N. weight:	0.84
		Sc. PD:	-0.057
		Sc. rank:	-322.6

,

PB1			
Pos.	28	obs:	exp:
cct	P	1093	273.00
ccc	P	1	196.60
cca	P	0	450.30
ccg	P	0	174.20
mPD		0.0018	0.71
		nPD:	0.
		N. weight:	1.5
		Sc. PD:	-0.12
		Sc. rank:	-1138.2

,

PB1			
Pos.	29	obs:	exp:
cct	P	0	273.00
ccc	P	3	196.60
cca	P	1089	450.30
ccg	P	2	174.20
mPD		0.0091	0.71
		nPD:	0.01
		N. weight:	0.95
		Sc. PD:	-0.066
		Sc. rank:	-396.6

,

PB1			
Pos.	30	obs:	exp:
tct	S	0	0.16
tcc	S	1	0.13
tca	S	0	0.25
tcg	S	0	0.06
tat	Y	1	590.30
tac	Y	1092	502.70
agt	S	0	0.21
agc	S	0	0.19
mPD		0.0037	0.50
		nPD:	0.01
		N. weight:	0.85
		Sc. PD:	-0.063
		Sc. rank:	-488.3

,

PB1			
Pos.	31	obs:	exp:
tct	S	0	174.00
tcc	S	0	142.90
tca	S	0	277.70
tcg	S	0	62.08
agt	S	1	224.90
agc	S	1093	212.50
mPD		0.0018	1.7
		nPD:	0.
		N. weight:	1.8
		Sc. PD:	-0.14
		Sc. rank:	-1372.9

,

PB1			
Pos.	32	obs:	exp:
cat	H	1089	628.20
cac	H	5	465.80
mPD		0.0091	0.49
		nPD:	0.02
		N. weight:	0.58
		Sc. PD:	-0.037
		Sc. rank:	-158.9

,

PB1			
Pos.	33	obs:	exp:
ggt	G	0	145.00
ggc	G	0	139.80
gga	G	1092	504.70
ggg	G	2	304.60
mPD		0.0037	0.68
		nPD:	0.01
		N. weight:	0.84
		Sc. PD:	-0.064
		Sc. rank:	-539.3

,

PB1			
Pos.	34	obs:	exp:
act	T	0	278.90
acc	T	0	224.50
aca	T	1091	501.80
acg	T	3	88.86
mPD		0.0055	0.68
		nPD:	0.01
		N. weight:	0.84
		Sc. PD:	-0.062
		Sc. rank:	-439.4

,

PB1			
Pos.	35	obs:	exp:
ggt	G	0	145.00
ggc	G	0	139.80
gga	G	1094	504.70
ggg	G	0	304.60
mPD		0	0.68
		nPD:	0.
		N. weight:	0.85
		Sc. PD:	-0.069
		Sc. rank:	-729.9

,

PB1			
Pos.	36	obs:	exp:
act	T	23	278.90
acc	T	1	224.50
aca	T	1068	501.80
acg	T	2	88.86
mPD		0.047	0.68
		nPD:	0.07
		N. weight:	0.74
		Sc. PD:	-0.012
		Sc. rank:	170.2

,

PB1			
Pos.	37	obs:	exp:
ggt	G	0	145.00
ggc	G	0	139.80
gga	G	1083	504.70
ggg	G	11	304.60
mPD		0.020	0.68
		nPD:	0.03
		N. weight:	0.8
		Sc. PD:	-0.043
		Sc. rank:	-68.7

,

PB1			
Pos.	38	obs:	exp:
tat	Y	1	589.80
tac	Y	1091	502.20
cat	H	0	1.15
cac	H	2	0.85
mPD		0.0055	0.50
		nPD:	0.01
		N. weight:	0.85
		Sc. PD:	-0.06
		Sc. rank:	-382.0

,

PB1			
Pos.	39	obs:	exp:
act	T	81	278.90
acc	T	1010	224.50
aca	T	3	501.80
acg	T	0	88.86
mPD		0.14	0.68
		nPD:	0.21
		N. weight:	1.4
		Sc. PD:	0.17
		Sc. rank:	932.8

,

PB1			
Pos.	40	obs:	exp:
atg	M	1094	1094.00
mPD		0	0
		nPD:	1.
		N. weight:	0.
		Sc. PD:	0
		Sc. rank:	0

,

PB1			
Pos.	41	obs:	exp:
gat	D	4	591.20
gac	D	1090	502.80
mPD		0.0073	0.50
		nPD:	0.01
		N. weight:	0.83
		Sc. PD:	-0.056
		Sc. rank:	-314.1

,

PB1			
Pos.	42	obs:	exp:
act	T	11	278.90
acc	T	0	224.50
aca	T	1082	501.80
acg	T	1	88.86
mPD		0.022	0.68
		nPD:	0.03
		N. weight:	0.8
		Sc. PD:	-0.041
		Sc. rank:	-39.9

,

PB1			
Pos.	43	obs:	exp:
att	I	0	5.51
atc	I	15	3.59
ata	I	0	5.89
gtt	V	1	223.60
gtc	V	1073	217.80
gta	V	5	220.90
gtg	V	0	416.70
mPD		0.038	0.75
		nPD:	0.05
		N. weight:	1.7
		Sc. PD:	-0.057
		Sc. rank:	180.5

,

PB1			
Pos.	44	obs:	exp:
aat	N	6	557.20
aac	N	1088	536.80
mPD		0.011	0.50
		nPD:	0.02
		N. weight:	0.75
		Sc. PD:	-0.045
		Sc. rank:	-162.2

,

PB1			
Pos.	45	obs:	exp:
cgt	R	0	46.99
cgc	R	0	54.19
cga	R	0	107.10
cgg	R	0	91.28
aga	R	1091	500.20
agg	R	3	294.20
mPD		0.0055	0.96
		nPD:	0.01
		N. weight:	0.84
		Sc. PD:	-0.064
		Sc. rank:	-520.5

,

PB1			
Pos.	46	obs:	exp:
act	T	0	278.90
acc	T	2	224.50
aca	T	1090	501.80
acg	T	2	88.86
mPD		0.0073	0.68
		nPD:	0.01
		N. weight:	0.83
		Sc. PD:	-0.06
		Sc. rank:	-391.6

,

PB1			
Pos.	47	obs:	exp:
cat	H	1067	628.20
cac	H	27	465.80
mPD		0.048	0.49
		nPD:	0.1
		N. weight:	0.49
		Sc. PD:	0.0062
		Sc. rank:	186.0

,

PB1			
Pos.	48	obs:	exp:
caa	Q	1	0.51
cag	Q	0	0.49
aaa	K	1091	637.00
aag	K	2	456.00
mPD		0.0055	0.49
		nPD:	0.01
		N. weight:	0.58
		Sc. PD:	-0.041
		Sc. rank:	-258.3

,

PB1			
Pos.	49	obs:	exp:
tat	Y	4	590.90
tac	Y	1090	503.10
mPD		0.0073	0.50
		nPD:	0.01
		N. weight:	0.83
		Sc. PD:	-0.056
		Sc. rank:	-316.6

,

PB1			
Pos.	50	obs:	exp:
tct	S	0	174.00
tcc	S	2	142.90
tca	S	1081	277.70
tcg	S	11	62.08
agt	S	0	224.90
agc	S	0	212.50
mPD		0.024	1.7
		nPD:	0.01
		N. weight:	1.4
		Sc. PD:	-0.099
		Sc. rank:	-567.3

PB1			
Pos.	51	obs:	exp:
gaa	E	1094	665.70
gag	E	0	428.30
---	--	------	------
mPD		0	0.48
		nPD:	0.
		N. weight:	0.55
		Sc. PD:	-0.045
		Sc. rank:	-468.7

PB1			
Pos.	52	obs:	exp:
cat	H	1	0.57
cac	H	0	0.43
cgt	R	0	0.13
cgc	R	0	0.15
cga	R	0	0.29
cgg	R	0	0.25
aat	N	54	27.51
aac	N	0	26.49
aaa	K	993	603.80
aag	K	43	432.20
aga	R	3	1.37
agg	R	0	0.81
---	--	------	------
mPD		0.17	0.54
		nPD:	0.32
		N. weight:	0.44
		Sc. PD:	0.099
		Sc. rank:	342.2

PB1			
Pos.	53	obs:	exp:
ggt	G	0	145.00
ggc	G	0	139.80
gga	G	1094	504.70
ggg	G	0	304.60
---	--	------	------
mPD		0	0.68
		nPD:	0.
		N. weight:	0.85
		Sc. PD:	-0.069
		Sc. rank:	-729.9

PB1			
Pos.	54	obs:	exp:
cgt	R	0	0.86
cgc	R	0	0.99
cga	R	0	1.96
cgg	R	0	1.67
atg	M	7	7.00
aaa	K	98	621.90
aag	K	969	445.10
aga	R	0	9.15
agg	R	20	5.38
---	--	------	------
mPD		0.21	0.55
		nPD:	0.39
		N. weight:	0.6
		Sc. PD:	0.17
		Sc. rank:	487.4

PB1			
Pos.	55	obs:	exp:
tgg	W	1094	1094.00
---	--	------	------
mPD		0	0
		nPD:	1.
		N. weight:	0.
		Sc. PD:	0
		Sc. rank:	0

PB1			
Pos.	56	obs:	exp:
act	T	0	272.80
acc	T	0	219.60
aca	T	1070	490.80
acg	T	0	86.91
gct	A	0	6.20
gcc	A	0	4.47
gca	A	24	11.30
gcg	A	0	2.04
---	--	------	------
mPD		0.043	0.72
		nPD:	0.06
		N. weight:	0.85
		Sc. PD:	-0.021
		Sc. rank:	145.0

PB1			
Pos.	57	obs:	exp:
att	I	0	1.47
atc	I	0	0.96
ata	I	4	1.57
atg	M	23	23.00
act	T	0	271.50
acc	T	0	218.50
aca	T	17	488.50
acg	T	1048	86.51
gct	A	0	0.52
gcc	A	0	0.37
gca	A	0	0.94
gcg	A	2	0.17
---	--	------	------
mPD		0.090	0.74
		nPD:	0.12
		N. weight:	2.6
		Sc. PD:	0.089
		Sc. rank:	1184.5

PB1			
Pos.	58	obs:	exp:
aat	N	6	557.20
aac	N	1088	536.80
---	--	------	------
mPD		0.011	0.50
		nPD:	0.02
		N. weight:	0.75
		Sc. PD:	-0.045
		Sc. rank:	-162.2

PB1			
Pos.	59	obs:	exp:
att	I	0	0.37
atc	I	0	0.24
ata	I	1	0.39
act	T	4	278.60
acc	T	0	224.30
aca	T	1086	501.30
acg	T	3	88.78
---	--	------	------
mPD		0.015	0.68
		nPD:	0.02
		N. weight:	0.82
		Sc. PD:	-0.05
		Sc. rank:	-186.7

PB1			
Pos.	60	obs:	exp:
gaa	E	129	665.70
gag	E	965	428.30
---	--	------	------
mPD		0.21	0.48
		nPD:	0.44
		N. weight:	0.58
		Sc. PD:	0.19
		Sc. rank:	477.9

PB1			
Pos.	61	obs:	exp:
att	I	1	0.37
atc	I	0	0.24
ata	I	0	0.39
act	T	1093	278.60
acc	T	0	224.30
aca	T	0	501.30
acg	T	0	88.78
---	--	------	------
mPD		0.0018	0.68
		nPD:	0.
		N. weight:	1.5
		Sc. PD:	-0.12
		Sc. rank:	-1120.3

PB1			
Pos.	62	obs:	exp:
gaa	E	3	1.83
gag	E	0	1.17
ggt	G	0	144.60
ggc	G	0	139.40
gga	G	1041	503.30
ggg	G	50	303.70
---	--	------	------
mPD		0.093	0.68
		nPD:	0.14
		N. weight:	0.67
		Sc. PD:	0.033
		Sc. rank:	340.8

PB1			
Pos.	63	obs:	exp:
tct	S	0	0.16
tcc	S	0	0.13
tca	S	1	0.25
tcg	S	0	0.06
agt	S	0	0.21
agc	S	0	0.19
gct	A	0	282.20
gcc	A	0	203.50
gca	A	1082	514.40
gcg	A	11	92.77
---	--	------	------
mPD		0.022	0.67
		nPD:	0.03
		N. weight:	0.79
		Sc. PD:	-0.04
		Sc. rank:	-35.1

PB1			
Pos.	64	obs:	exp:
tta	L	0	0.09
ttg	L	0	0.19
ctt	L	0	0.17
ctc	L	1	0.17
cta	L	0	0.17
ctg	L	0	0.22
cct	P	9	272.50
ccc	P	1081	196.20
cca	P	2	449.50
ccg	P	0	173.80
att	I	0	0.37
atc	I	1	0.24
ata	I	0	0.39
---	--	------	------
mPD		0.025	0.72
		nPD:	0.04
		N. weight:	1.8
		Sc. PD:	-0.087
		Sc. rank:	-33.4

PB1			
Pos.	65	obs:	exp:
caa	Q	1079	557.20
cag	Q	15	536.80
---	--	------	------
mPD		0.027	0.50
		nPD:	0.05
		N. weight:	0.66
		Sc. PD:	-0.02
		Sc. rank:	92.6

PB1			
Pos.	66	obs:	exp:
tta	L	1	97.08
ttg	L	0	203.70
ctt	L	3	182.20
ctc	L	1027	182.60
cta	L	63	184.40
ctg	L	0	244.10
---	--	------	------
mPD		0.12	1.1
		nPD:	0.11
		N. weight:	1.7
		Sc. PD:	0.033
		Sc. rank:	696.4

PB1			
Pos.	67	obs:	exp:
aat	N	1089	557.20
aac	N	5	536.80
---	--	------	------
mPD		0.0091	0.50
		nPD:	0.02
		N. weight:	0.71
		Sc. PD:	-0.046
		Sc. rank:	-200.7

PB1			
Pos.	68	obs:	exp:
cct	P	0	273.00
ccc	P	0	196.60
cca	P	893	450.30
ccg	P	201	174.20
---	--	------	------
mPD		0.30	0.71
		nPD:	0.42
		N. weight:	0.65
		Sc. PD:	0.21
		Sc. rank:	528.5

PB1			
Pos.	69	obs:	exp:
att	I	1092	401.20
atc	I	0	261.70
ata	I	0	429.10
gtt	V	2	0.41
gtc	V	0	0.40
gta	V	0	0.41
gtg	V	0	0.77
---	--	------	------
mPD		0.0037	0.66
		nPD:	0.01
		N. weight:	1.1
		Sc. PD:	-0.084
		Sc. rank:	-695.0

PB1			
Pos.	70	obs:	exp:
gat	D	1061	591.20
gac	D	33	502.80
---	--	------	------
mPD		0.059	0.50
		nPD:	0.12
		N. weight:	0.53
		Sc. PD:	0.017
		Sc. rank:	240.4

PB1			
Pos.	71	obs:	exp:
ggt	G	0	145.00
ggc	G	0	139.80
gga	G	1074	504.70
ggg	G	20	304.60
---	--	------	------
mPD		0.036	0.68
		nPD:	0.05
		N. weight:	0.76
		Sc. PD:	-0.023
		Sc. rank:	97.4

PB1			
Pos.	72	obs:	exp:
cct	P	0	272.70
ccc	P	0	196.40
cca	P	987	449.90
ccg	P	106	174.00
caa	Q	1	0.51
cag	Q	0	0.49
---	--	------	------
mPD		0.18	0.71
		nPD:	0.25
		N. weight:	0.73
		Sc. PD:	0.11
		Sc. rank:	520.7

PB1			
Pos.	73	obs:	exp:
tta	L	841	97.08
ttg	L	247	203.70
ctt	L	0	182.20
ctc	L	0	182.60
cta	L	6	184.40
ctg	L	0	244.10
---	--	------	------
mPD		0.36	1.1
		nPD:	0.33
		N. weight:	1.9
		Sc. PD:	0.43
		Sc. rank:	1455.4

PB1			
Pos.	74	obs:	exp:
cct	P	1038	273.00
ccc	P	52	196.60
cca	P	4	450.30
ccg	P	0	174.20
---	--	------	------
mPD		0.098	0.71
		nPD:	0.14
		N. weight:	1.3
		Sc. PD:	0.065
		Sc. rank:	667.6

PB1			
Pos.	75	obs:	exp:
gat	D	2	1.08
gac	D	0	0.92
gaa	E	5	664.50
gag	E	1087	427.50
---	--	------	------
mPD		0.013	0.48
		nPD:	0.03
		N. weight:	1.
		Sc. PD:	-0.056
		Sc. rank:	-131.6

PB1

Pos.	76	obs:	exp:
aat	N	0	4.07
aac	N	8	3.93
gat	D	1	586.30
gac	D	1084	498.70
ggt	G	0	0.13
ggc	G	1	0.13
gga	G	0	0.46
ggg	G	0	0.28
---	--	------	------
mPD		0.018	0.51
		nPD:	0.04
		N. weight:	0.85
		Sc. PD:	-0.041
		Sc. rank:	-19.1

PB1

Pos.	77	obs:	exp:
aat	N	256	557.20
aac	N	838	536.80
---	--	------	------
mPD		0.36	0.50
		nPD:	0.72
		N. weight:	0.18
		Sc. PD:	0.11
		Sc. rank:	153.7

PB1

Pos.	78	obs:	exp:
gaa	E	4	665.70
gag	E	1090	428.30
---	--	------	------
mPD		0.0073	0.48
		nPD:	0.02
		N. weight:	1.
		Sc. PD:	-0.067
		Sc. rank:	-359.6

PB1

Pos.	79	obs:	exp:
cct	P	0	273.00
ccc	P	3	196.60
cca	P	81	450.30
ccg	P	1010	174.20
---	--	------	------
mPD		0.14	0.71
		nPD:	0.2
		N. weight:	1.6
		Sc. PD:	0.18
		Sc. rank:	1050.8

PB1

Pos.	80	obs:	exp:
tct	S	0	173.70
tcc	S	0	142.60
tca	S	0	277.20
tcg	S	0	61.96
aat	N	2	1.02
aac	N	0	0.98
agt	S	1023	224.50
agc	S	69	212.10
---	--	------	------
mPD		0.12	1.7
		nPD:	0.07
		N. weight:	1.5
		Sc. PD:	-0.017
		Sc. rank:	385.0

PB1

Pos.	81	obs:	exp:
ggt	G	0	145.00
ggc	G	0	139.80
gga	G	29	504.70
ggg	G	1065	304.60
---	--	------	------
mPD		0.052	0.68
		nPD:	0.08
		N. weight:	1.3
		Sc. PD:	-0.011
		Sc. rank:	356.3

PB1

Pos.	82	obs:	exp:
tat	Y	1079	590.90
tac	Y	15	503.10
---	--	------	------
mPD		0.027	0.50
		nPD:	0.05
		N. weight:	0.6
		Sc. PD:	-0.018
		Sc. rank:	84.7

PB1

Pos.	83	obs:	exp:
gct	A	0	282.50
gcc	A	1	203.70
gca	A	1091	514.90
gcg	A	2	92.85
---	--	------	------
mPD		0.0055	0.67
		nPD:	0.01
		N. weight:	0.81
		Sc. PD:	-0.06
		Sc. rank:	-403.9

PB1

Pos.	84	obs:	exp:
caa	Q	1073	557.20
cag	Q	21	536.80
---	--	------	------
mPD		0.038	0.50
		nPD:	0.08
		N. weight:	0.64
		Sc. PD:	-0.0061
		Sc. rank:	174.2

PB1

Pos.	85	obs:	exp:
act	T	7	278.90
acc	T	0	224.50
aca	T	14	501.80
acg	T	1073	88.86
---	--	------	------
mPD		0.038	0.68
		nPD:	0.06
		N. weight:	2.6
		Sc. PD:	-0.073
		Sc. rank:	402.4

PB1

Pos.	86	obs:	exp:
gat	D	1090	591.20
gac	D	4	502.80
---	--	------	------
mPD		0.0073	0.50
		nPD:	0.01
		N. weight:	0.65
		Sc. PD:	-0.044
		Sc. rank:	-247.1

PB1

Pos.	87	obs:	exp:
tgt	C	1031	462.70
tgc	C	63	631.30
---	--	------	------
mPD		0.11	0.49
		nPD:	0.22
		N. weight:	0.69
		Sc. PD:	0.09
		Sc. rank:	470.0

PB1

Pos.	88	obs:	exp:
gtt	V	0	226.70
gtc	V	0	220.80
gta	V	1061	224.00
gtg	V	33	422.50
---	--	------	------
mPD		0.059	0.73
		nPD:	0.08
		N. weight:	1.6
		Sc. PD:	-0.0069
		Sc. rank:	480.3

PB1

Pos.	89	obs:	exp:
tta	L	24	97.08
ttg	L	1048	203.70
ctt	L	0	182.20
ctc	L	0	182.60
cta	L	0	184.40
ctg	L	22	244.10
---	--	------	------
mPD		0.082	1.1
		nPD:	0.07
		N. weight:	1.6
		Sc. PD:	-0.017
		Sc. rank:	438.6

PB1

Pos.	90	obs:	exp:
gaa	E	976	665.70
gag	E	118	428.30
---	--	------	------
mPD		0.19	0.48
		nPD:	0.4
		N. weight:	0.23
		Sc. PD:	0.069
		Sc. rank:	183.5

PB1

Pos.	91	obs:	exp:
gct	A	0	282.50
gcc	A	0	203.70
gca	A	1091	514.90
gcg	A	3	92.85
---	--	------	------
mPD		0.0055	0.67
		nPD:	0.01
		N. weight:	0.81
		Sc. PD:	-0.06
		Sc. rank:	-407.9

PB1

Pos.	92	obs:	exp:
att	I	0	0.37
atc	I	0	0.24
ata	I	1	0.39
atg	M	1093	1093.00
---	--	------	------
mPD		0.0018	0.0018
		nPD:	1.
		N. weight:	0.00094
		Sc. PD:	0.00082
		Sc. rank:	0.8

PB1

Pos.	93	obs:	exp:
tct	S	1	0.16
tcc	S	0	0.13
tca	S	0	0.25
tcg	S	0	0.06
agt	S	0	0.21
agc	S	0	0.19
gct	A	1030	282.20
gcc	A	63	203.50
gca	A	0	514.40
gcg	A	0	92.77
---	--	------	------
mPD		0.11	0.67
		nPD:	0.16
		N. weight:	1.3
		Sc. PD:	0.096
		Sc. rank:	720.4

PB1

Pos.	94	obs:	exp:
ttt	F	10	430.40
ttc	F	1080	659.60
tct	S	0	0.32
tcc	S	2	0.26
tca	S	0	0.51
tcg	S	0	0.11
tat	Y	0	1.08
tac	Y	2	0.92
agt	S	0	0.41
agc	S	0	0.39
---	--	------	------
mPD		0.025	0.49
		nPD:	0.05
		N. weight:	0.5
		Sc. PD:	-0.016
		Sc. rank:	57.5

PB1

Pos.	95	obs:	exp:
tta	L	0	97.08
ttg	L	0	203.70
ctt	L	1094	182.20
ctc	L	0	182.60
cta	L	0	184.40
ctg	L	0	244.10
---	--	------	------
mPD		0	1.1
		nPD:	0.
		N. weight:	2.
		Sc. PD:	-0.16
		Sc. rank:	-1691.3

PB1

Pos.	96	obs:	exp:
gaa	E	1068	665.70
gag	E	26	428.30
---	--	------	------
mPD		0.046	0.48
		nPD:	0.1
		N. weight:	0.44
		Sc. PD:	0.0051
		Sc. rank:	161.8

PB1

Pos.	97	obs:	exp:
gat	D	9	49.17
gac	D	82	41.83
gaa	E	993	610.30
gag	E	10	392.70
---	--	------	------
mPD		0.17	0.56
		nPD:	0.31
		N. weight:	0.49
		Sc. PD:	0.1
		Sc. rank:	381.5

PB1

Pos.	98	obs:	exp:
tct	S	1087	174.00
tcc	S	5	142.90
tca	S	2	277.70
tcg	S	0	62.08
agt	S	0	224.90
agc	S	0	212.50
---	--	------	------
mPD		0.013	1.7
		nPD:	0.01
		N. weight:	2.
		Sc. PD:	-0.15
		Sc. rank:	-1047.0

PB1

Pos.	99	obs:	exp:
cat	H	149	628.20
cac	H	945	465.80
---	--	------	------
mPD		0.24	0.49
		nPD:	0.48
		N. weight:	0.46
		Sc. PD:	0.17
		Sc. rank:	386.1

PB1

Pos.	100	obs:	exp:
tct	S	0	0.16
tcc	S	0	0.13
tca	S	1	0.25
tcg	S	0	0.06
cct	P	0	272.70
ccc	P	2	196.40
cca	P	1063	449.90
ccg	P	28	174.00
agt	S	0	0.21
agc	S	0	0.19
---	--	------	------
mPD		0.055	0.71
		nPD:	0.08
		N. weight:	0.86
		Sc. PD:	-0.0064
		Sc. rank:	252.1

PB1			
Pos.	101	obs:	exp:
ggt	G	0	145.00
ggc	G	0	139.80
gga	G	21	504.70
ggg	G	1073	304.60
---	--	------	------
mPD		0.038	0.68
		nPD:	0.06
		N. weight:	1.3
		Sc. PD:	-0.037
		Sc. rank:	191.4

PB1			
Pos.	102	obs:	exp:
att	I	157	402.00
atc	I	936	262.20
ata	I	1	429.90
---	--	------	------
mPD		0.25	0.65
		nPD:	0.38
		N. weight:	1.
		Sc. PD:	0.29
		Sc. rank:	838.8

PB1			
Pos.	103	obs:	exp:
ttt	F	1094	432.00
ttc	F	0	662.00
---	--	------	------
mPD		0	0.48
		nPD:	0.
		N. weight:	1.
		Sc. PD:	-0.083
		Sc. rank:	-876.6

PB1			
Pos.	104	obs:	exp:
gaa	E	1091	665.70
gag	E	3	428.30
---	--	------	------
mPD		0.0055	0.48
		nPD:	0.01
		N. weight:	0.53
		Sc. PD:	-0.037
		Sc. rank:	-228.8

PB1			
Pos.	105	obs:	exp:
tct	S	0	1.75
tcc	S	0	1.44
tca	S	0	2.79
tcg	S	0	0.62
cat	H	0	2.30
cac	H	4	1.70
act	T	0	0.51
acc	T	2	0.41
aca	T	0	0.92
acg	T	0	0.16
aat	N	36	548.60
aac	N	1041	528.40
agt	S	0	2.26
agc	S	11	2.14
---	--	------	------
mPD		0.095	0.55
		nPD:	0.17
		N. weight:	0.64
		Sc. PD:	0.054
		Sc. rank:	377.0

PB1			
Pos.	106	obs:	exp:
tct	S	0	174.00
tcc	S	0	142.90
tca	S	65	277.70
tcg	S	1029	62.08
agt	S	0	224.90
agc	S	0	212.50
---	--	------	------
mPD		0.11	1.7
		nPD:	0.07
		N. weight:	2.8
		Sc. PD:	-0.048
		Sc. rank:	629.6

PB1			
Pos.	107	obs:	exp:
tgt	C	1094	462.70
tgc	C	0	631.30
---	--	------	------
mPD		0	0.49
		nPD:	0.
		N. weight:	0.94
		Sc. PD:	-0.077
		Sc. rank:	-811.8

PB1			
Pos.	108	obs:	exp:
tta	L	0	96.55
ttg	L	0	202.60
ctt	L	52	181.20
ctc	L	1003	181.60
cta	L	32	183.40
ctg	L	1	242.80
att	I	0	2.20
atc	I	6	1.44
ata	I	0	2.36
---	--	------	------
mPD		0.16	1.1
		nPD:	0.14
		N. weight:	1.6
		Sc. PD:	0.086
		Sc. rank:	843.8

PB1			
Pos.	109	obs:	exp:
gaa	E	963	665.70
gag	E	131	428.30
---	--	------	------
mPD		0.21	0.48
		nPD:	0.44
		N. weight:	0.2
		Sc. PD:	0.07
		Sc. rank:	169.2

PB1			
Pos.	110	obs:	exp:
act	T	63	278.60
acc	T	0	224.30
aca	T	11	501.30
acg	T	1019	88.78
gct	A	0	0.26
gcc	A	0	0.19
gca	A	0	0.47
gcg	A	1	0.08
---	--	------	------
mPD		0.13	0.68
		nPD:	0.19
		N. weight:	2.4
		Sc. PD:	0.24
		Sc. rank:	1480.3

PB1			
Pos.	111	obs:	exp:
att	I	4	2.20
atc	I	0	1.44
ata	I	2	2.36
atg	M	1088	1088.00
---	--	------	------
mPD		0.011	0.011
		nPD:	1.
		N. weight:	0.0032
		Sc. PD:	0.0028
		Sc. rank:	2.7

PB1			
Pos.	112	obs:	exp:
aaa	K	1	0.58
aag	K	0	0.42
gaa	E	1093	665.10
gag	E	0	427.90
---	--	------	------
mPD		0.0018	0.48
		nPD:	0.
		N. weight:	0.55
		Sc. PD:	-0.043
		Sc. rank:	-377.3

PB1			
Pos.	113	obs:	exp:
att	I	1090	402.00
atc	I	4	262.20
ata	I	0	429.90
---	--	------	------
mPD		0.0073	0.65
		nPD:	0.01
		N. weight:	1.1
		Sc. PD:	-0.076
		Sc. rank:	-483.4

PB1			
Pos.	114	obs:	exp:
att	I	1	0.37
atc	I	0	0.24
ata	I	0	0.39
gtt	V	1042	226.50
gtc	V	51	220.60
gta	V	0	223.80
gtg	V	0	422.10
---	--	------	------
mPD		0.091	0.73
		nPD:	0.12
		N. weight:	1.5
		Sc. PD:	0.058
		Sc. rank:	736.5

PB1			
Pos.	115	obs:	exp:
caa	Q	3	557.20
cag	Q	1091	536.80
---	--	------	------
mPD		0.0055	0.50
		nPD:	0.01
		N. weight:	0.76
		Sc. PD:	-0.054
		Sc. rank:	-348.4

PB1			
Pos.	116	obs:	exp:
caa	Q	1024	557.20
cag	Q	70	536.80
---	--	------	------
mPD		0.12	0.50
		nPD:	0.24
		N. weight:	0.49
		Sc. PD:	0.072
		Sc. rank:	341.2

PB1			
Pos.	117	obs:	exp:
act	T	51	278.90
acc	T	0	224.50
aca	T	1043	501.80
acg	T	0	88.86
---	--	------	------
mPD		0.089	0.68
		nPD:	0.13
		N. weight:	0.68
		Sc. PD:	0.03
		Sc. rank:	338.2

PB1			
Pos.	118	obs:	exp:
cgt	R	0	46.99
cgc	R	0	54.19
cga	R	0	107.10
cgg	R	0	91.28
aga	R	1083	500.20
agg	R	11	294.20
---	--	------	------
mPD		0.020	0.96
		nPD:	0.02
		N. weight:	0.81
		Sc. PD:	-0.05
		Sc. rank:	-195.4

PB1			
Pos.	119	obs:	exp:
atg	M	2	2.00
gtt	V	7	226.30
gtc	V	0	220.40
gta	V	10	223.60
gtg	V	1075	421.70
---	--	------	------
mPD		0.034	0.73
		nPD:	0.05
		N. weight:	0.96
		Sc. PD:	-0.035
		Sc. rank:	76.9

PB1			
Pos.	120	obs:	exp:
gat	D	943	591.20
gac	D	151	502.80
---	--	------	------
mPD		0.24	0.50
		nPD:	0.48
		N. weight:	0.26
		Sc. PD:	0.099
		Sc. rank:	220.9

PB1			
Pos.	121	obs:	exp:
cgt	R	0	0.04
cgc	R	0	0.05
cga	R	0	0.10
cgg	R	0	0.08
aaa	K	1088	637.00
aag	K	5	456.00
aga	R	1	0.46
agg	R	0	0.27
---	--	------	------
mPD		0.011	0.49
		nPD:	0.02
		N. weight:	0.56
		Sc. PD:	-0.034
		Sc. rank:	-117.4

PB1			
Pos.	122	obs:	exp:
tta	L	0	97.08
ttg	L	0	203.70
ctt	L	2	182.20
ctc	L	0	182.60
cta	L	46	184.40
ctg	L	1046	244.10
---	--	------	------
mPD		0.084	1.1
		nPD:	0.08
		N. weight:	1.5
		Sc. PD:	-0.013
		Sc. rank:	406.7

PB1			
Pos.	123	obs:	exp:
act	T	7	278.60
acc	T	1085	224.30
aca	T	1	501.30
acg	T	0	88.78
gct	A	0	0.26
gcc	A	1	0.19
gca	A	0	0.47
gcg	A	0	0.08
---	--	------	------
mPD		0.016	0.68
		nPD:	0.02
		N. weight:	1.7
		Sc. PD:	-0.099
		Sc. rank:	-291.6

PB1			
Pos.	124	obs:	exp:
caa	Q	1087	556.70
cag	Q	6	536.30
gaa	E	1	0.61
gag	E	0	0.39
---	--	------	------
mPD		0.013	0.50
		nPD:	0.03
		N. weight:	0.7
		Sc. PD:	-0.04
		Sc. rank:	-109.7

PB1			
Pos.	125	obs:	exp:
ggt	G	2	145.00
ggc	G	1083	139.80
gga	G	9	504.70
ggg	G	0	304.60
---	--	------	------
mPD		0.020	0.68
		nPD:	0.03
		N. weight:	2.2
		Sc. PD:	-0.12
		Sc. rank:	-168.2

PB1			
Pos.	126	obs:	exp:
cat	H	0	0.57
cac	H	1	0.43
cgt	R	34	46.94
cgc	R	1058	54.14
cga	R	0	107.00
cgg	R	1	91.20
aga	R	0	499.80
agg	R	0	294.00
---	--	------	------
mPD		0.064	0.96
		nPD:	0.07
		N. weight:	3.1
		Sc. PD:	-0.057
		Sc. rank:	675.3

PB1			
Pos.	127	obs:	exp:
caa	Q	3	557.20
cag	Q	1091	536.80
---	--	------	------
mPD		0.0055	0.50
		nPD:	0.01
		N. weight:	0.76
		Sc. PD:	-0.054
		Sc. rank:	-348.4

PB1			
Pos.	128	obs:	exp:
act	T	7	278.90
acc	T	1086	224.50
aca	T	1	501.80
acg	T	0	88.86
---	--	------	------
mPD		0.015	0.68
		nPD:	0.02
		N. weight:	1.7
		Sc. PD:	-0.1
		Sc. rank:	-378.3

PB1			
Pos.	129	obs:	exp:
tat	Y	1094	590.90
tac	Y	0	503.10
---	--	------	------
mPD		0	0.50
		nPD:	0.
		N. weight:	0.68
		Sc. PD:	-0.055
		Sc. rank:	-581.2

PB1			
Pos.	130	obs:	exp:
aat	N	0	0.51
aac	N	1	0.49
gat	D	6	590.60
gac	D	1087	502.40
---	--	------	------
mPD		0.013	0.50
		nPD:	0.03
		N. weight:	0.82
		Sc. PD:	-0.047
		Sc. rank:	-121.8

PB1			
Pos.	131	obs:	exp:
tgg	W	1094	1094.00
---	--	------	------
mPD		0	0
		nPD:	1.
		N. weight:	0.
		Sc. PD:	0
		Sc. rank:	0

PB1			
Pos.	132	obs:	exp:
cgt	R	0	0.04
cgc	R	0	0.05
cga	R	0	0.10
cgg	R	0	0.08
act	T	29	278.60
acc	T	0	224.30
aca	T	376	501.30
acg	T	688	88.78
aga	R	0	0.46
agg	R	1	0.27
---	--	------	------
mPD		0.49	0.68
		nPD:	0.72
		N. weight:	1.2
		Sc. PD:	0.76
		Sc. rank:	1073.7

PB1			
Pos.	133	obs:	exp:
tta	L	3	97.08
ttg	L	1035	203.70
ctt	L	0	182.20
ctc	L	0	182.60
cta	L	0	184.40
ctg	L	56	244.10
---	--	------	------
mPD		0.10	1.1
		nPD:	0.09
		N. weight:	1.6
		Sc. PD:	0.011
		Sc. rank:	553.6

PB1			
Pos.	134	obs:	exp:
aat	N	1089	557.20
aac	N	5	536.80
---	--	------	------
mPD		0.0091	0.50
		nPD:	0.02
		N. weight:	0.71
		Sc. PD:	-0.046
		Sc. rank:	-200.7

PB1			
Pos.	135	obs:	exp:
cgt	R	0	46.99
cgc	R	0	54.19
cga	R	0	107.10
cgg	R	0	91.28
aga	R	1092	500.20
agg	R	2	294.20
---	--	------	------
mPD		0.0037	0.96
		nPD:	0.
		N. weight:	0.85
		Sc. PD:	-0.066
		Sc. rank:	-589.7

PB1			
Pos.	136	obs:	exp:
aat	N	991	556.70
aac	N	102	536.30
aaa	K	0	0.58
aag	K	1	0.42
---	--	------	------
mPD		0.17	0.50
		nPD:	0.34
		N. weight:	0.41
		Sc. PD:	0.1
		Sc. rank:	321.3

PB1			
Pos.	137	obs:	exp:
caa	Q	71	557.20
cag	Q	1023	536.80
---	--	------	------
mPD		0.12	0.50
		nPD:	0.24
		N. weight:	0.52
		Sc. PD:	0.078
		Sc. rank:	365.1

PB1			
Pos.	138	obs:	exp:
tta	L	0	0.09
ttg	L	0	0.19
ctt	L	0	0.17
ctc	L	0	0.17
cta	L	0	0.17
ctg	L	1	0.22
cct	P	6	272.70
ccc	P	2	196.40
cca	P	29	449.90
ccg	P	1056	174.00
---	--	------	------
mPD		0.068	0.71
		nPD:	0.09
		N. weight:	1.8
		Sc. PD:	0.017
		Sc. rank:	645.5

PB1			
Pos.	139	obs:	exp:
tct	S	1	0.16
tcc	S	0	0.13
tca	S	0	0.25
tcg	S	0	0.06
agt	S	0	0.21
agc	S	0	0.19
gct	A	948	282.20
gcc	A	143	203.50
gca	A	2	514.40
gcg	A	0	92.77
---	--	------	------
mPD		0.23	0.67
		nPD:	0.35
		N. weight:	1.1
		Sc. PD:	0.27
		Sc. rank:	867.5

PB1			
Pos.	140	obs:	exp:
gct	A	1025	282.50
gcc	A	68	203.70
gca	A	1	514.90
gcg	A	0	92.85
---	--	------	------
mPD		0.12	0.67
		nPD:	0.18
		N. weight:	1.2
		Sc. PD:	0.11
		Sc. rank:	752.1

PB1			
Pos.	141	obs:	exp:
act	T	199	278.90
acc	T	894	224.50
aca	T	1	501.80
acg	T	0	88.86
---	--	------	------
mPD		0.30	0.68
		nPD:	0.44
		N. weight:	1.2
		Sc. PD:	0.4
		Sc. rank:	972.4

PB1			
Pos.	142	obs:	exp:
gct	A	5	282.50
gcc	A	4	203.70
gca	A	1056	514.90
gcg	A	29	92.85
---	--	------	------
mPD		0.068	0.67
		nPD:	0.1
		N. weight:	0.7
		Sc. PD:	0.01
		Sc. rank:	272.2

PB1			
Pos.	143	obs:	exp:
tta	L	17	97.08
ttg	L	1031	203.70
ctt	L	0	182.20
ctc	L	0	182.60
cta	L	0	184.40
ctg	L	46	244.10
---	--	------	------
mPD		0.11	1.1
		nPD:	0.1
		N. weight:	1.6
		Sc. PD:	0.023
		Sc. rank:	612.9

PB1			
Pos.	144	obs:	exp:
act	T	0	0.25
acc	T	1	0.21
aca	T	0	0.46
acg	T	0	0.08
gct	A	19	282.20
gcc	A	1050	203.50
gca	A	24	514.40
gcg	A	0	92.77
---	--	------	------
mPD		0.078	0.67
		nPD:	0.12
		N. weight:	1.6
		Sc. PD:	0.048
		Sc. rank:	721.3

PB1			
Pos.	145	obs:	exp:
aat	N	64	557.20
aac	N	1030	536.80
---	--	------	------
mPD		0.11	0.50
		nPD:	0.22
		N. weight:	0.54
		Sc. PD:	0.07
		Sc. rank:	365.1

PB1			
Pos.	146	obs:	exp:
act	T	1050	278.90
acc	T	44	224.50
aca	T	0	501.80
acg	T	0	88.86
---	--	------	------
mPD		0.077	0.68
		nPD:	0.11
		N. weight:	1.3
		Sc. PD:	0.037
		Sc. rank:	583.8

PB1			
Pos.	147	obs:	exp:
att	I	0	402.00
atc	I	0	262.20
ata	I	1094	429.90
---	--	------	------
mPD		0	0.65
		nPD:	0.
		N. weight:	1.
		Sc. PD:	-0.084
		Sc. rank:	-881.3

PB1			
Pos.	148	obs:	exp:
gaa	E	80	665.70
gag	E	1014	428.30
---	--	------	------
mPD		0.14	0.48
		nPD:	0.28
		N. weight:	0.71
		Sc. PD:	0.14
		Sc. rank:	528.0

PB1			
Pos.	149	obs:	exp:
att	I	0	3.31
atc	I	0	2.16
ata	I	9	3.54
gtt	V	2	224.80
gtc	V	2	219.00
gta	V	1010	222.20
gtg	V	71	419.00
---	--	------	------
mPD		0.14	0.74
		nPD:	0.19
		N. weight:	1.4
		Sc. PD:	0.15
		Sc. rank:	895.3

PB1			
Pos.	150	obs:	exp:
ttt	F	128	432.00
ttc	F	966	662.00
---	--	------	------
mPD		0.21	0.48
		nPD:	0.43
		N. weight:	0.21
		Sc. PD:	0.071
		Sc. rank:	176.0

PB1			
Pos.	151	obs:	exp:
cgt	R	0	46.99
cgc	R	0	54.19
cga	R	0	107.10
cgg	R	0	91.28
aga	R	1087	500.20
agg	R	7	294.20
---	--	------	------
mPD		0.013	0.96
		nPD:	0.01
		N. weight:	0.82
		Sc. PD:	-0.057
		Sc. rank:	-332.8

PB1			
Pos.	152	obs:	exp:
tta	L	1	0.62
ttg	L	6	1.30
tct	S	7	172.90
tcc	S	0	142.00
tca	S	52	275.90
tcg	S	1028	61.68
ctt	L	0	1.17
ctc	L	0	1.17
cta	L	0	1.18
ctg	L	0	1.56
agt	S	0	223.50
agc	S	0	211.10
---	--	------	------
mPD		0.12	1.7
		nPD:	0.07
		N. weight:	2.8
		Sc. PD:	-0.04
		Sc. rank:	665.8

PB1			
Pos.	153	obs:	exp:
aat	N	1086	557.20
aac	N	8	536.80
---	--	------	------
mPD		0.015	0.50
		nPD:	0.03
		N. weight:	0.7
		Sc. PD:	-0.037
		Sc. rank:	-65.6

PB1			
Pos.	154	obs:	exp:
tct	S	0	0.32
tcc	S	0	0.26
tca	S	0	0.51
tcg	S	0	0.11
tgt	C	0	3.38
tgc	C	8	4.62
agt	S	0	0.41
agc	S	2	0.39
gat	D	0	1.62
gac	D	3	1.38
gaa	E	1	0.61
gag	E	0	0.39
ggt	G	1	143.20
ggc	G	1070	138.00
gga	G	3	498.20
ggg	G	6	300.70
---	--	------	------
mPD		0.045	0.71
		nPD:	0.06
		N. weight:	2.2
		Sc. PD:	-0.044
		Sc. rank:	417.9

PB1			
Pos.	155	obs:	exp:
tta	L	0	97.08
ttg	L	3	203.70
ctt	L	0	182.20
ctc	L	0	182.60
cta	L	92	184.40
ctg	L	999	244.10
---	--	------	------
mPD		0.16	1.1
		nPD:	0.14
		N. weight:	1.3
		Sc. PD:	0.075
		Sc. rank:	718.8

PB1			
Pos.	156	obs:	exp:
cgt	R	0	0.13
cgc	R	0	0.15
cga	R	0	0.29
cgg	R	0	0.25
att	I	0	0.37
atc	I	0	0.24
ata	I	1	0.39
atg	M	1	1.00
act	T	0	277.10
acc	T	50	223.00
aca	T	1037	498.60
acg	T	0	88.29
aaa	K	1	0.58
aag	K	0	0.42
aga	R	3	1.37
agg	R	0	0.81
gct	A	0	0.26
gcc	A	0	0.19
gca	A	1	0.47
gcg	A	0	0.08
---	--	------	------
mPD		0.10	0.69
		nPD:	0.15
		N. weight:	0.69
		Sc. PD:	0.041
		Sc. rank:	374.2

PB1			
Pos.	157	obs:	exp:
act	T	1	0.25
acc	T	0	0.21
aca	T	0	0.46
acg	T	0	0.08
gct	A	182	282.00
gcc	A	907	203.40
gca	A	3	514.00
gcg	A	0	92.68
gat	D	0	0.54
gac	D	1	0.46
---	--	------	------
mPD		0.29	0.67
		nPD:	0.43
		N. weight:	1.3
		Sc. PD:	0.42
		Sc. rank:	1044.9

PB1			
Pos.	158	obs:	exp:
tct	S	0	8.27
tcc	S	0	6.79
tca	S	0	13.20
tcg	S	0	2.95
cat	H	1	0.57
cac	H	0	0.43
aat	N	1040	530.20
aac	N	1	510.80
agt	S	52	10.69
agc	S	0	10.10
---	--	------	------
mPD		0.094	0.66
		nPD:	0.14
		N. weight:	0.78
		Sc. PD:	0.043
		Sc. rank:	416.4

PB1			
Pos.	159	obs:	exp:
gct	A	0	0.26
gcc	A	0	0.19
gca	A	1	0.47
gcg	A	0	0.08
gat	D	4	2.16
gac	D	0	1.84
gaa	E	1086	662.70
gag	E	3	426.30
---	--	------	------
mPD		0.015	0.48
		nPD:	0.03
		N. weight:	0.53
		Sc. PD:	-0.028
		Sc. rank:	-34.5

PB1			
Pos.	160	obs:	exp:
tct	S	0	174.00
tcc	S	0	142.90
tca	S	1094	277.70
tcg	S	0	62.08
agt	S	0	224.90
agc	S	0	212.50
---	--	------	------
mPD		0	1.7
		nPD:	0.
		N. weight:	1.5
		Sc. PD:	-0.12
		Sc. rank:	-1293.7

PB1			
Pos.	161	obs:	exp:
ggt	G	0	145.00
ggc	G	0	139.80
gga	G	1045	504.70
ggg	G	49	304.60
---	--	------	------
mPD		0.086	0.68
		nPD:	0.13
		N. weight:	0.68
		Sc. PD:	0.027
		Sc. rank:	330.7

PB1			
Pos.	162	obs:	exp:
cgt	R	0	46.99
cgc	R	0	54.19
cga	R	0	107.10
cgg	R	0	91.28
aga	R	23	500.20
agg	R	1071	294.20
---	--	------	------
mPD		0.041	0.96
		nPD:	0.04
		N. weight:	1.3
		Sc. PD:	-0.054
		Sc. rank:	53.8

PB1			
Pos.	163	obs:	exp:
tta	L	2	96.99
ttg	L	1087	203.50
tgg	W	1	1.00
ctt	L	0	182.00
ctc	L	0	182.40
cta	L	1	184.20
ctg	L	3	243.90
---	--	------	------
mPD		0.015	1.1
		nPD:	0.01
		N. weight:	1.8
		Sc. PD:	-0.12
		Sc. rank:	-738.5

PB1			
Pos.	164	obs:	exp:
att	I	200	400.90
atc	I	889	261.40
ata	I	2	428.70
gtt	V	0	0.62
gtc	V	3	0.61
gta	V	0	0.61
gtg	V	0	1.16
---	--	------	------
mPD		0.31	0.66
		nPD:	0.47
		N. weight:	0.95
		Sc. PD:	0.35
		Sc. rank:	795.6

PB1			
Pos.	165	obs:	exp:
gat	D	1094	591.20
gac	D	0	502.80
---	--	------	------
mPD		0	0.50
		nPD:	0.
		N. weight:	0.68
		Sc. PD:	-0.055
		Sc. rank:	-580.7

PB1			
Pos.	166	obs:	exp:
ttt	F	15	431.60
ttc	F	1078	661.40
tta	L	0	0.09
ttg	L	0	0.19
ctt	L	0	0.17
ctc	L	1	0.17
cta	L	0	0.17
ctg	L	0	0.22
---	--	------	------
mPD		0.029	0.48
		nPD:	0.06
		N. weight:	0.48
		Sc. PD:	-0.012
		Sc. rank:	82.3

PB1			
Pos.	167	obs:	exp:
tta	L	0	97.08
ttg	L	0	203.70
ctt	L	45	182.20
ctc	L	1049	182.60
cta	L	0	184.40
ctg	L	0	244.10
---	--	------	------
mPD		0.079	1.1
		nPD:	0.07
		N. weight:	1.8
		Sc. PD:	-0.024
		Sc. rank:	434.9

PB1			
Pos.	168	obs:	exp:
cgt	R	0	0.39
cgc	R	0	0.45
cga	R	0	0.88
cgg	R	0	0.75
aaa	K	33	632.40
aag	K	1052	452.60
aga	R	0	4.12
agg	R	9	2.42
---	--	------	------
mPD		0.075	0.51
		nPD:	0.15
		N. weight:	0.81
		Sc. PD:	0.048
		Sc. rank:	436.3

PB1			
Pos.	169	obs:	exp:
gat	D	319	591.20
gac	D	775	502.80
---	--	------	------
mPD		0.41	0.50
		nPD:	0.83
		N. weight:	0.14
		Sc. PD:	0.1
		Sc. rank:	123.2

PB1			
Pos.	170	obs:	exp:
gtt	V	12	226.70
gtc	V	0	220.80
gta	V	7	224.00
gtg	V	1075	422.50
---	--	------	------
mPD		0.034	0.73
		nPD:	0.05
		N. weight:	0.95
		Sc. PD:	-0.035
		Sc. rank:	78.4

PB1			
Pos.	171	obs:	exp:
att	I	0	6.25
atc	I	0	4.07
ata	I	17	6.68
atg	M	969	969.00
gtt	V	2	22.38
gtc	V	0	21.80
gta	V	0	22.11
gtg	V	106	41.71
---	--	------	------
mPD		0.21	0.32
		nPD:	0.66
		N. weight:	0.11
		Sc. PD:	0.061
		Sc. rank:	95.5

PB1			
Pos.	172	obs:	exp:
aat	N	2	1.02
aac	N	0	0.98
gat	D	1081	587.40
gac	D	6	499.60
gaa	E	1	0.61
gag	E	0	0.39
ggt	G	4	0.53
ggc	G	0	0.51
gga	G	0	1.85
ggg	G	0	1.11
---	--	------	------
mPD		0.024	0.51
		nPD:	0.05
		N. weight:	0.65
		Sc. PD:	-0.024
		Sc. rank:	47.8

PB1			
Pos.	173	obs:	exp:
tct	S	0	174.00
tcc	S	0	142.90
tca	S	1090	277.70
tcg	S	4	62.08
agt	S	0	224.90
agc	S	0	212.50
---	--	------	------
mPD		0.0073	1.7
		nPD:	0.
		N. weight:	1.5
		Sc. PD:	-0.12
		Sc. rank:	-1007.9

PB1			
Pos.	174	obs:	exp:
att	I	0	1.47
atc	I	0	0.96
ata	I	4	1.57
atg	M	1089	1089.00
gtt	V	0	0.21
gtc	V	0	0.20
gta	V	0	0.20
gtg	V	1	0.39
---	--	------	------
mPD		0.0091	0.010
		nPD:	0.89
		N. weight:	0.0047
		Sc. PD:	0.0036
		Sc. rank:	4.1

PB1			
Pos.	175	obs:	exp:
aat	N	3	1.53
aac	N	0	1.47
gat	D	959	587.90
gac	D	129	500.10
gaa	E	0	1.22
gag	E	2	0.78
ggt	G	1	0.13
ggc	G	0	0.13
gga	G	0	0.46
ggg	G	0	0.28
---	--	------	------
mPD		0.22	0.51
		nPD:	0.43
		N. weight:	0.3
		Sc. PD:	0.1
		Sc. rank:	251.2

PB1			
Pos.	176	obs:	exp:
cgt	R	0	0.09
cgc	R	0	0.10
cga	R	0	0.20
cgg	R	0	0.17
aaa	K	33	636.40
aag	K	1059	455.60
aga	R	0	0.91
agg	R	2	0.54
---	--	------	------
mPD		0.062	0.49
		nPD:	0.13
		N. weight:	0.8
		Sc. PD:	0.032
		Sc. rank:	390.4

PB1			
Pos.	177	obs:	exp:
aaa	K	2	1.17
aag	K	0	0.83
gat	D	17	9.19
gac	D	0	7.81
gaa	E	1067	649.30
gag	E	0	417.70
ggt	G	0	1.06
ggc	G	0	1.02
gga	G	8	3.69
ggg	G	0	2.23
---	--	------	------
mPD		0.049	0.51
		nPD:	0.1
		N. weight:	0.55
		Sc. PD:	0.0052
		Sc. rank:	197.1

PB1			
Pos.	178	obs:	exp:
gat	D	3	1.62
gac	D	0	1.38
gaa	E	1046	654.10
gag	E	29	420.90
ggt	G	0	2.12
ggc	G	0	2.04
gga	G	16	7.38
ggg	G	0	4.45
---	--	------	------
mPD		0.086	0.51
		nPD:	0.17
		N. weight:	0.43
		Sc. PD:	0.034
		Sc. rank:	247.1

PB1			
Pos.	179	obs:	exp:
att	I	28	11.02
atc	I	0	7.19
ata	I	2	11.79
atg	M	1060	1060.00
gtt	V	0	0.83
gtc	V	0	0.81
gta	V	0	0.82
gtg	V	4	1.54
---	--	------	------
mPD		0.061	0.065
		nPD:	0.93
		N. weight:	0.028
		Sc. PD:	0.022
		Sc. rank:	24.0

PB1			
Pos.	180	obs:	exp:
aaa	K	0	1.75
aag	K	3	1.25
gtt	V	0	0.21
gtc	V	0	0.20
gta	V	0	0.20
gtg	V	1	0.39
gaa	E	11	662.10
gag	E	1077	425.90
ggt	G	0	0.27
ggc	G	0	0.26
gga	G	0	0.92
ggg	G	2	0.56
---	--	------	------
mPD		0.031	0.49
		nPD:	0.06
		N. weight:	0.97
		Sc. PD:	-0.02
		Sc. rank:	175.4

PB1			
Pos.	181	obs:	exp:
att	I	1028	394.20
atc	I	44	257.10
ata	I	1	421.60
gtt	V	21	4.35
gtc	V	0	4.24
gta	V	0	4.30
gtg	V	0	8.11
---	--	------	------
mPD		0.12	0.70
		nPD:	0.17
		N. weight:	0.94
		Sc. PD:	0.074
		Sc. rank:	541.0

PB1			
Pos.	182	obs:	exp:
act	T	0	278.90
acc	T	2	224.50
aca	T	1087	501.80
acg	T	5	88.86
---	--	------	------
mPD		0.013	0.68
		nPD:	0.02
		N. weight:	0.82
		Sc. PD:	-0.052
		Sc. rank:	-217.0

PB1			
Pos.	183	obs:	exp:
act	T	0	278.60
acc	T	0	224.30
aca	T	1035	501.30
acg	T	58	88.78
gct	A	0	0.26
gcc	A	0	0.19
gca	A	0	0.47
gcg	A	1	0.08
---	--	------	------
mPD		0.10	0.68
		nPD:	0.15
		N. weight:	0.73
		Sc. PD:	0.048
		Sc. rank:	404.8

PB1			
Pos.	184	obs:	exp:
cat	H	1088	628.20
cac	H	6	465.80
---	--	------	------
mPD		0.011	0.49
		nPD:	0.02
		N. weight:	0.58
		Sc. PD:	-0.035
		Sc. rank:	-120.4

PB1			
Pos.	185	obs:	exp:
ttt	F	1	432.00
ttc	F	1093	662.00
---	--	------	------
mPD		0.0018	0.48
		nPD:	0.
		N. weight:	0.54
		Sc. PD:	-0.043
		Sc. rank:	-374.2

PB1			
Pos.	186	obs:	exp:
cat	H	2	1.15
cac	H	0	0.85
caa	Q	16	556.20
cag	Q	1076	535.80
---	--	------	------
mPD		0.032	0.50
		nPD:	0.06
		N. weight:	0.7
		Sc. PD:	-0.014
		Sc. rank:	135.8

PB1			
Pos.	187	obs:	exp:
cgt	R	0	46.99
cgc	R	0	54.19
cga	R	0	107.10
cgg	R	0	91.28
aga	R	57	500.20
agg	R	1037	294.20
---	--	------	------
mPD		0.099	0.96
		nPD:	0.1
		N. weight:	1.2
		Sc. PD:	0.02
		Sc. rank:	472.6

PB1			
Pos.	188	obs:	exp:
aaa	K	48	637.60
aag	K	1046	456.40
---	--	------	------
mPD		0.084	0.49
		nPD:	0.17
		N. weight:	0.75
		Sc. PD:	0.063
		Sc. rank:	441.9

PB1			
Pos.	189	obs:	exp:
tct	S	0	0.16
tcc	S	0	0.13
tca	S	0	0.25
tcg	S	0	0.06
cgt	R	0	46.94
cgc	R	0	54.14
cga	R	0	107.00
cgg	R	0	91.20
agt	S	0	0.21
agc	S	1	0.19
aga	R	3	499.80
agg	R	1090	294.00
---	--	------	------
mPD		0.0073	0.96
		nPD:	0.01
		N. weight:	1.4
		Sc. PD:	-0.11
		Sc. rank:	-787.9

PB1			
Pos.	190	obs:	exp:
cgt	R	0	46.94
cgc	R	0	54.14
cga	R	0	107.00
cgg	R	0	91.20
aaa	K	1	0.58
aag	K	0	0.42
aga	R	1093	499.80
agg	R	0	294.00
---	--	------	------
mPD		0.0018	0.96
		nPD:	0.
		N. weight:	0.86
		Sc. PD:	-0.069
		Sc. rank:	-656.3

PB1			
Pos.	191	obs:	exp:
atg	M	1	1.00
gtt	V	0	226.50
gtc	V	0	220.60
gta	V	22	223.80
gtg	V	1071	422.10
---	--	------	------
mPD		0.041	0.73
		nPD:	0.06
		N. weight:	0.95
		Sc. PD:	-0.026
		Sc. rank:	148.8

PB1			
Pos.	192	obs:	exp:
cgt	R	0	46.99
cgc	R	0	54.19
cga	R	0	107.10
cgg	R	0	91.28
aga	R	7	500.20
agg	R	1087	294.20
---	--	------	------
mPD		0.013	0.96
		nPD:	0.01
		N. weight:	1.4
		Sc. PD:	-0.096
		Sc. rank:	-565.5

PB1			
Pos.	193	obs:	exp:
gat	D	7	591.20
gac	D	1087	502.80
---	--	------	------
mPD		0.013	0.50
		nPD:	0.03
		N. weight:	0.81
		Sc. PD:	-0.046
		Sc. rank:	-119.6

PB1			
Pos.	194	obs:	exp:
tct	S	0	0.32
tcc	S	0	0.26
tca	S	0	0.51
tcg	S	0	0.11
tat	Y	0	0.54
tac	Y	1	0.46
aat	N	63	555.20
aac	N	1027	534.80
aaa	K	1	0.58
aag	K	0	0.42
agt	S	1	0.41
agc	S	1	0.39
---	--	------	------
mPD		0.12	0.51
		nPD:	0.23
		N. weight:	0.54
		Sc. PD:	0.075
		Sc. rank:	377.5

PB1			
Pos.	195	obs:	exp:
tta	L	0	0.27
ttg	L	3	0.56
ctt	L	0	0.50
ctc	L	0	0.50
cta	L	0	0.51
ctg	L	0	0.67
atg	M	1088	1088.00
act	T	0	0.76
acc	T	0	0.62
aca	T	0	1.38
acg	T	3	0.24
---	--	------	------
mPD		0.011	0.019
		nPD:	0.57
		N. weight:	0.013
		Sc. PD:	0.0059
		Sc. rank:	10.7

PB1			
Pos.	196	obs:	exp:
act	T	3	278.90
acc	T	1091	224.50
aca	T	0	501.80
acg	T	0	88.86
---	--	------	------
mPD		0.0055	0.68
		nPD:	0.01
		N. weight:	1.7
		Sc. PD:	-0.13
		Sc. rank:	-897.4

PB1			
Pos.	197	obs:	exp:
cgt	R	0	3.31
cgc	R	0	3.81
cga	R	0	7.54
cgg	R	0	6.42
atg	M	1	1.00
aaa	K	22	592.20
aag	K	994	423.80
aga	R	0	35.21
agg	R	77	20.71
---	--	------	------
mPD		0.17	0.66
		nPD:	0.26
		N. weight:	0.88
		Sc. PD:	0.15
		Sc. rank:	643.4

PB1			
Pos.	198	obs:	exp:
cgt	R	0	0.04
cgc	R	0	0.05
cga	R	0	0.10
cgg	R	0	0.08
aaa	K	1015	637.00
aag	K	78	456.00
aga	R	1	0.46
agg	R	0	0.27
---	--	------	------
mPD		0.13	0.49
		nPD:	0.27
		N. weight:	0.34
		Sc. PD:	0.062
		Sc. rank:	252.4

PB1			
Pos.	199	obs:	exp:
att	I	0	1.47
atc	I	0	0.96
ata	I	4	1.57
atg	M	1088	1088.00
act	T	0	0.51
acc	T	0	0.41
aca	T	0	0.92
acg	T	2	0.16
---	--	------	------
mPD		0.011	0.014
		nPD:	0.77
		N. weight:	0.0088
		Sc. PD:	0.0057
		Sc. rank:	7.6

PB1			
Pos.	200	obs:	exp:
att	I	0	2.94
atc	I	7	1.92
ata	I	1	3.14
gtt	V	24	225.00
gtc	V	1060	219.20
gta	V	2	222.40
gtg	V	0	419.40
---	--	------	------
mPD		0.063	0.74
		nPD:	0.08
		N. weight:	1.6
		Sc. PD:	-0.00075
		Sc. rank:	528.7

PB1			
Pos.	201	obs:	exp:
act	T	0	278.90
acc	T	0	224.50
aca	T	1094	501.80
acg	T	0	88.86
---	--	------	------
mPD		0	0.68
		nPD:	0.
		N. weight:	0.86
		Sc. PD:	-0.07
		Sc. rank:	-735.4

PB1			
Pos.	202	obs:	exp:
cat	H	1	0.57
cac	H	0	0.43
caa	Q	14	556.70
cag	Q	1079	536.30
---	--	------	------
mPD		0.027	0.50
		nPD:	0.05
		N. weight:	0.71
		Sc. PD:	-0.021
		Sc. rank:	97.7

PB1			
Pos.	203	obs:	exp:
cgt	R	0	46.94
cgc	R	0	54.14
cga	R	0	107.00
cgg	R	0	91.20
aaa	K	1	0.58
aag	K	0	0.42
aga	R	1087	499.80
agg	R	6	294.00
---	--	------	------
mPD		0.013	0.96
		nPD:	0.01
		N. weight:	0.83
		Sc. PD:	-0.057
		Sc. rank:	-336.6

PB1			
Pos.	204	obs:	exp:
act	T	0	278.90
acc	T	0	224.50
aca	T	1080	501.80
acg	T	14	88.86
---	--	------	------
mPD		0.025	0.68
		nPD:	0.04
		N. weight:	0.81
		Sc. PD:	-0.037
		Sc. rank:	-0.3

PB1			
Pos.	205	obs:	exp:
att	I	0	401.20
atc	I	7	261.70
ata	I	1085	429.10
gtt	V	0	0.41
gtc	V	0	0.40
gta	V	2	0.41
gtg	V	0	0.77
---	--	------	------
mPD		0.016	0.66
		nPD:	0.02
		N. weight:	0.99
		Sc. PD:	-0.057
		Sc. rank:	-157.7

PB1			
Pos.	206	obs:	exp:
ggt	G	3	145.00
ggc	G	0	139.80
gga	G	625	504.70
ggg	G	466	304.60
---	--	------	------
mPD		0.49	0.68
		nPD:	0.73
		N. weight:	0.33
		Sc. PD:	0.2
		Sc. rank:	281.6

PB1			
Pos.	207	obs:	exp:
aaa	K	1	637.60
aag	K	1093	456.40
---	--	------	------
mPD		0.0018	0.49
		nPD:	0.
		N. weight:	0.95
		Sc. PD:	-0.074
		Sc. rank:	-667.4

PB1			
Pos.	208	obs:	exp:
cgt	R	0	0.04
cgc	R	0	0.05
cga	R	0	0.10
cgg	R	0	0.08
aaa	K	1076	637.00
aag	K	17	456.00
aga	R	1	0.46
agg	R	0	0.27
---	--	------	------
mPD		0.032	0.49
		nPD:	0.07
		N. weight:	0.51
		Sc. PD:	-0.0093
		Sc. rank:	110.7

PB1			
Pos.	209	obs:	exp:
cgt	R	0	0.09
cgc	R	0	0.10
cga	R	0	0.20
cgg	R	0	0.17
aat	N	0	2.55
aac	N	5	2.45
aaa	K	1085	633.50
aag	K	2	453.50
aga	R	2	0.91
agg	R	0	0.54
---	--	------	------
mPD		0.016	0.50
		nPD:	0.03
		N. weight:	0.58
		Sc. PD:	-0.029
		Sc. rank:	-20.9

PB1			
Pos.	210	obs:	exp:
caa	Q	1090	557.20
cag	Q	4	536.80
---	--	------	------
mPD		0.0073	0.50
		nPD:	0.01
		N. weight:	0.72
		Sc. PD:	-0.049
		Sc. rank:	-277.7

PB1			
Pos.	211	obs:	exp:
cgt	R	0	45.40
cgc	R	0	52.35
cga	R	0	103.40
cgg	R	0	88.20
aaa	K	30	21.56
aag	K	7	15.44
aga	R	997	483.30
agg	R	60	284.30
---	--	------	------
mPD		0.18	1.0
		nPD:	0.18
		N. weight:	0.64
		Sc. PD:	0.057
		Sc. rank:	388.0

PB1			
Pos.	212	obs:	exp:
tta	L	0	97.08
ttg	L	9	203.70
ctt	L	2	182.20
ctc	L	0	182.60
cta	L	5	184.40
ctg	L	1078	244.10
---	--	------	------
mPD		0.029	1.1
		nPD:	0.03
		N. weight:	1.6
		Sc. PD:	-0.088
		Sc. rank:	-216.3

PB1			
Pos.	213	obs:	exp:
tct	S	0	0.64
tcc	S	0	0.52
tca	S	0	1.02
tcg	S	0	0.23
att	I	0	0.37
atc	I	1	0.24
ata	I	0	0.39
act	T	0	4.08
acc	T	16	3.28
aca	T	0	7.34
acg	T	0	1.30
aat	N	5	539.40
aac	N	1054	519.60
aaa	K	5	2.91
aag	K	0	2.09
agt	S	4	0.82
agc	S	0	0.78
gct	A	0	0.77
gcc	A	3	0.56
gca	A	0	1.41
gcg	A	0	0.25
gat	D	0	2.70
gac	D	5	2.30
ggt	G	0	0.13
ggc	G	1	0.13
gga	G	0	0.46
ggg	G	0	0.28
---	--	------	------
mPD		0.087	0.58
		nPD:	0.15
		N. weight:	0.77
		Sc. PD:	0.047
		Sc. rank:	421.3

PB1			
Pos.	214	obs:	exp:
aat	N	0	0.51
aac	N	1	0.49
aaa	K	1066	637.00
aag	K	27	456.00
---	--	------	------
mPD		0.050	0.49
		nPD:	0.1
		N. weight:	0.48
		Sc. PD:	0.0079
		Sc. rank:	188.4

PB1			
Pos.	215	obs:	exp:
cgt	R	0	39.04
cgc	R	0	45.02
cga	R	0	88.95
cgg	R	0	75.85
aat	N	1	0.51
aac	N	0	0.49
aaa	K	1	107.20
aag	K	183	76.76
aga	R	2	415.60
agg	R	907	244.50
---	--	------	------
mPD		0.29	1.2
		nPD:	0.24
		N. weight:	1.3
		Sc. PD:	0.2
		Sc. rank:	949.8

PB1

Pos.	216	obs:	exp:
tct	S	0	170.50
tcc	S	0	140.00
tca	S	0	272.10
tcg	S	0	60.83
tgt	C	0	1.69
tgc	C	4	2.31
cgt	R	0	0.26
cgc	R	4	0.30
cga	R	0	0.59
cgg	R	0	0.50
aat	N	0	1.02
aac	N	2	0.98
agt	S	24	220.40
agc	S	1048	208.20
aga	R	2	2.74
agg	R	0	1.61
ggt	G	0	1.33
ggc	G	10	1.28
gga	G	0	4.61
ggg	G	0	2.78
---	--	------	------
mPD		0.083	1.7
		nPD:	0.05
		N. weight:	1.7
		Sc. PD:	-0.058
		Sc. rank:	167.8

,

PB1

Pos.	217	obs:	exp:
tat	Y	0	590.90
tac	Y	1094	503.10
---	--	------	------
mPD		0	0.50
		nPD:	0.
		N. weight:	0.85
		Sc. PD:	-0.07
		Sc. rank:	-732.8

,

PB1

Pos.	218	obs:	exp:
tta	L	16	97.08
ttg	L	0	203.70
ctt	L	0	182.20
ctc	L	2	182.60
cta	L	1049	184.40
ctg	L	27	244.10
---	--	------	------
mPD		0.081	1.1
		nPD:	0.07
		N. weight:	1.7
		Sc. PD:	-0.021
		Sc. rank:	439.6

,

PB1

Pos.	219	obs:	exp:
att	I	0	401.60
atc	I	0	261.90
ata	I	1093	429.50
gtt	V	0	0.21
gtc	V	0	0.20
gta	V	1	0.20
gtg	V	0	0.39
---	--	------	------
mPD		0.0018	0.66
		nPD:	0.
		N. weight:	1.
		Sc. PD:	-0.081
		Sc. rank:	-753.6

,

PB1

Pos.	220	obs:	exp:
cgt	R	0	46.99
cgc	R	0	54.19
cga	R	2	107.10
cgg	R	0	91.28
aga	R	1086	500.20
agg	R	6	294.20
---	--	------	------
mPD		0.015	0.96
		nPD:	0.02
		N. weight:	0.82
		Sc. PD:	-0.055
		Sc. rank:	-299.0

,

PB1

Pos.	221	obs:	exp:
gct	A	0	282.50
gcc	A	0	203.70
gca	A	1084	514.90
gcg	A	10	92.85
---	--	------	------
mPD		0.018	0.67
		nPD:	0.03
		N. weight:	0.79
		Sc. PD:	-0.044
		Sc. rank:	-91.4

,

PB1

Pos.	222	obs:	exp:
tta	L	13	97.08
ttg	L	146	203.70
ctt	L	4	182.20
ctc	L	0	182.60
cta	L	6	184.40
ctg	L	925	244.10
---	--	------	------
mPD		0.29	1.1
		nPD:	0.26
		N. weight:	1.1
		Sc. PD:	0.19
		Sc. rank:	831.1

,

PB1

Pos.	223	obs:	exp:
act	T	0	278.90
acc	T	0	224.50
aca	T	1045	501.80
acg	T	49	88.86
---	--	------	------
mPD		0.086	0.68
		nPD:	0.13
		N. weight:	0.74
		Sc. PD:	0.029
		Sc. rank:	362.1

,

PB1

Pos.	224	obs:	exp:
tta	L	3	97.08
ttg	L	1084	203.70
ctt	L	0	182.20
ctc	L	0	182.60
cta	L	0	184.40
ctg	L	7	244.10
---	--	------	------
mPD		0.018	1.1
		nPD:	0.02
		N. weight:	1.8
		Sc. PD:	-0.12
		Sc. rank:	-550.3

,

PB1

Pos.	225	obs:	exp:
aat	N	2	557.20
aac	N	1092	536.80
---	--	------	------
mPD		0.0037	0.50
		nPD:	0.01
		N. weight:	0.77
		Sc. PD:	-0.057
		Sc. rank:	-447.0

,

PB1

Pos.	226	obs:	exp:
act	T	0	278.90
acc	T	0	224.50
aca	T	1094	501.80
acg	T	0	88.86
---	--	------	------
mPD		0	0.68
		nPD:	0.
		N. weight:	0.86
		Sc. PD:	-0.07
		Sc. rank:	-735.4

,

PB1

Pos.	227	obs:	exp:
atg	M	1094	1094.00
---	--	------	------
mPD		0	0
		nPD:	1.
		N. weight:	0.
		Sc. PD:	0
		Sc. rank:	0

,

PB1

Pos.	228	obs:	exp:
act	T	0	278.90
acc	T	0	224.50
aca	T	1093	501.80
acg	T	1	88.86
---	--	------	------
mPD		0.0018	0.68
		nPD:	0.
		N. weight:	0.85
		Sc. PD:	-0.067
		Sc. rank:	-632.5

,

PB1

Pos.	229	obs:	exp:
aaa	K	11	637.60
aag	K	1083	456.40
---	--	------	------
mPD		0.020	0.49
		nPD:	0.04
		N. weight:	0.9
		Sc. PD:	-0.038
		Sc. rank:	17.3

,

PB1

Pos.	230	obs:	exp:
gat	D	1074	591.20
gac	D	20	502.80
---	--	------	------
mPD		0.036	0.50
		nPD:	0.07
		N. weight:	0.58
		Sc. PD:	-0.0072
		Sc. rank:	144.6

,

PB1

Pos.	231	obs:	exp:
gct	A	1090	282.50
gcc	A	2	203.70
gca	A	2	514.90
gcg	A	0	92.85
---	--	------	------
mPD		0.0073	0.67
		nPD:	0.01
		N. weight:	1.5
		Sc. PD:	-0.1
		Sc. rank:	-674.3

,

PB1

Pos.	232	obs:	exp:
gaa	E	1087	665.70
gag	E	7	428.30
---	--	------	------
mPD		0.013	0.48
		nPD:	0.03
		N. weight:	0.51
		Sc. PD:	-0.029
		Sc. rank:	-66.5

,

PB1

Pos.	233	obs:	exp:
cgt	R	0	46.99
cgc	R	0	54.19
cga	R	3	107.10
cgg	R	0	91.28
aga	R	1006	500.20
agg	R	85	294.20
---	--	------	------
mPD		0.15	0.96
		nPD:	0.15
		N. weight:	0.59
		Sc. PD:	0.04
		Sc. rank:	328.2

,

PB1

Pos.	234	obs:	exp:
ggt	G	1	145.00
ggc	G	1084	139.80
gga	G	9	504.70
ggg	G	0	304.60
---	--	------	------
mPD		0.018	0.68
		nPD:	0.03
		N. weight:	2.2
		Sc. PD:	-0.12
		Sc. rank:	-265.3

,

PB1

Pos.	235	obs:	exp:
aaa	K	20	637.60
aag	K	1074	456.40
---	--	------	------
mPD		0.036	0.49
		nPD:	0.07
		N. weight:	0.85
		Sc. PD:	-0.0094
		Sc. rank:	225.4

,

PB1

Pos.	236	obs:	exp:
tta	L	112	97.08
ttg	L	324	203.70
ctt	L	0	182.20
ctc	L	0	182.60
cta	L	2	184.40
ctg	L	656	244.10
---	--	------	------
mPD		0.67	1.1
		nPD:	0.6
		N. weight:	0.82
		Sc. PD:	0.4
		Sc. rank:	694.2

,

PB1

Pos.	237	obs:	exp:
cgt	R	0	0.04
cgc	R	0	0.05
cga	R	0	0.10
cgg	R	0	0.08
aaa	K	1088	637.00
aag	K	5	456.00
aga	R	1	0.46
agg	R	0	0.27
---	--	------	------
mPD		0.011	0.49
		nPD:	0.02
		N. weight:	0.56
		Sc. PD:	-0.034
		Sc. rank:	-117.4

,

PB1

Pos.	238	obs:	exp:
cgt	R	0	46.99
cgc	R	0	54.19
cga	R	0	107.10
cgg	R	0	91.28
aga	R	21	500.20
agg	R	1073	294.20
---	--	------	------
mPD		0.038	0.96
		nPD:	0.04
		N. weight:	1.3
		Sc. PD:	-0.059
		Sc. rank:	10.5

,

PB1

Pos.	239	obs:	exp:
cgt	R	0	46.99
cgc	R	0	54.19
cga	R	0	107.10
cgg	R	5	91.28
aga	R	115	500.20
agg	R	974	294.20
---	--	------	------
mPD		0.20	0.96
		nPD:	0.21
		N. weight:	0.99
		Sc. PD:	0.11
		Sc. rank:	645.1

,

PB1

Pos.	240	obs:	exp:
gct	A	0	282.50
gcc	A	0	203.70
gca	A	1088	514.90
gcg	A	6	92.85
---	--	------	------
mPD		0.011	0.67
		nPD:	0.02
		N. weight:	0.8
		Sc. PD:	-0.053
		Sc. rank:	-263.1

,

PB1

Pos.	241	obs:	exp:
att	I	37	402.00
atc	I	1051	262.20
ata	I	6	429.90
mPD		0.076	0.65
		nPD:	0.12
		N. weight:	1.4
		Sc. PD:	0.04
		Sc. rank:	602.0

PB1

Pos.	242	obs:	exp:
gct	A	0	282.50
gcc	A	0	203.70
gca	A	1091	514.90
gcg	A	3	92.85
mPD		0.0055	0.67
		nPD:	0.01
		N. weight:	0.81
		Sc. PD:	-0.06
		Sc. rank:	-407.9

PB1

Pos.	243	obs:	exp:
act	T	0	278.90
acc	T	27	224.50
aca	T	1049	501.80
acg	T	18	88.86
mPD		0.080	0.68
		nPD:	0.12
		N. weight:	0.69
		Sc. PD:	0.022
		Sc. rank:	313.3

PB1

Pos.	244	obs:	exp:
cct	P	37	273.00
ccc	P	1019	196.60
cca	P	38	450.30
ccg	P	0	174.20
mPD		0.13	0.71
		nPD:	0.18
		N. weight:	1.5
		Sc. PD:	0.14
		Sc. rank:	939.5

PB1

Pos.	245	obs:	exp:
ggt	G	0	145.00
ggc	G	0	139.80
gga	G	11	504.70
ggg	G	1083	304.60
mPD		0.020	0.68
		nPD:	0.03
		N. weight:	1.3
		Sc. PD:	-0.072
		Sc. rank:	-115.7

PB1

Pos.	246	obs:	exp:
atg	M	1094	1094.00
mPD		0	0
		nPD:	1.
		N. weight:	0.
		Sc. PD:	0
		Sc. rank:	0

PB1

Pos.	247	obs:	exp:
caa	Q	1092	557.20
cag	Q	2	536.80
mPD		0.0037	0.50
		nPD:	0.01
		N. weight:	0.73
		Sc. PD:	-0.054
		Sc. rank:	-420.8

PB1

Pos.	248	obs:	exp:
att	I	0	402.00
atc	I	1093	262.20
ata	I	1	429.90
mPD		0.0018	0.65
		nPD:	0.
		N. weight:	1.6
		Sc. PD:	-0.12
		Sc. rank:	-1127.5

PB1

Pos.	249	obs:	exp:
cgt	R	0	46.99
cgc	R	0	54.19
cga	R	3	107.10
cgg	R	0	91.28
aga	R	1085	500.20
agg	R	6	294.20
mPD		0.016	0.96
		nPD:	0.02
		N. weight:	0.81
		Sc. PD:	-0.053
		Sc. rank:	-243.2

PB1

Pos.	250	obs:	exp:
cgt	R	0	0.04
cgc	R	0	0.05
cga	R	1	0.10
cgg	R	0	0.08
aga	R	0	0.46
agg	R	0	0.27
ggt	G	5	144.90
ggc	G	0	139.60
gga	G	1055	504.20
ggg	G	33	304.30
mPD		0.069	0.68
		nPD:	0.1
		N. weight:	0.7
		Sc. PD:	0.011
		Sc. rank:	273.6

PB1

Pos.	251	obs:	exp:
ttt	F	5	432.00
ttc	F	1089	662.00
mPD		0.0091	0.48
		nPD:	0.02
		N. weight:	0.52
		Sc. PD:	-0.033
		Sc. rank:	-136.1

PB1

Pos.	252	obs:	exp:
gtt	V	2	226.70
gtc	V	0	220.80
gta	V	2	224.00
gtg	V	1090	422.50
mPD		0.0073	0.73
		nPD:	0.01
		N. weight:	1.
		Sc. PD:	-0.074
		Sc. rank:	-494.0

PB1

Pos.	253	obs:	exp:
tat	Y	1027	590.90
tac	Y	67	503.10
mPD		0.12	0.50
		nPD:	0.23
		N. weight:	0.44
		Sc. PD:	0.061
		Sc. rank:	305.1

PB1

Pos.	254	obs:	exp:
ttt	F	1065	432.00
ttc	F	29	662.00
mPD		0.052	0.48
		nPD:	0.11
		N. weight:	0.88
		Sc. PD:	0.019
		Sc. rank:	360.4

PB1

Pos.	255	obs:	exp:
gtt	V	2	226.70
gtc	V	0	220.80
gta	V	1083	224.00
gtg	V	9	422.50
mPD		0.020	0.73
		nPD:	0.03
		N. weight:	1.7
		Sc. PD:	-0.093
		Sc. rank:	-187.1

PB1

Pos.	256	obs:	exp:
gaa	E	1091	665.70
gag	E	3	428.30
mPD		0.0055	0.48
		nPD:	0.01
		N. weight:	0.53
		Sc. PD:	-0.037
		Sc. rank:	-228.8

PB1

Pos.	257	obs:	exp:
act	T	0	1.53
acc	T	0	1.23
aca	T	6	2.75
acg	T	0	0.49
gtt	V	0	0.21
gtc	V	0	0.20
gta	V	1	0.20
gtg	V	0	0.39
gct	A	0	280.70
gcc	A	0	202.40
gca	A	1074	511.60
gcg	A	13	92.26
mPD		0.036	0.68
		nPD:	0.05
		N. weight:	0.78
		Sc. PD:	-0.024
		Sc. rank:	98.6

PB1

Pos.	258	obs:	exp:
tta	L	12	97.08
ttg	L	0	203.70
ctt	L	0	182.20
ctc	L	6	182.60
cta	L	1072	184.40
ctg	L	4	244.10
mPD		0.040	1.1
		nPD:	0.04
		N. weight:	1.8
		Sc. PD:	-0.087
		Sc. rank:	-11.8

PB1

Pos.	259	obs:	exp:
gct	A	1	282.50
gcc	A	0	203.70
gca	A	46	514.90
gcg	A	1047	92.85
mPD		0.082	0.67
		nPD:	0.12
		N. weight:	2.4
		Sc. PD:	0.088
		Sc. rank:	1162.9

PB1

Pos.	260	obs:	exp:
cgt	R	0	46.99
cgc	R	0	54.19
cga	R	0	107.10
cgg	R	0	91.28
aga	R	1	500.20
agg	R	1093	294.20
mPD		0.0018	0.96
		nPD:	0.
		N. weight:	1.4
		Sc. PD:	-0.11
		Sc. rank:	-1091.2

PB1

Pos.	261	obs:	exp:
tct	S	0	173.80
tcc	S	0	142.70
tca	S	0	277.40
tcg	S	0	62.02
tgt	C	1	0.42
tgc	C	0	0.58
agt	S	13	224.70
agc	S	1080	212.30
mPD		0.027	1.7
		nPD:	0.02
		N. weight:	1.7
		Sc. PD:	-0.11
		Sc. rank:	-539.5

PB1

Pos.	262	obs:	exp:
att	I	0	402.00
atc	I	1025	262.20
ata	I	69	429.90
mPD		0.12	0.65
		nPD:	0.18
		N. weight:	1.3
		Sc. PD:	0.12
		Sc. rank:	784.9

PB1

Pos.	263	obs:	exp:
tgt	C	1094	462.70
tgc	C	0	631.30
mPD		0	0.49
		nPD:	0.
		N. weight:	0.94
		Sc. PD:	-0.077
		Sc. rank:	-811.8

PB1

Pos.	264	obs:	exp:
gaa	E	34	665.10
gag	E	1059	427.90
ggt	G	0	0.13
ggc	G	0	0.13
gga	G	1	0.46
ggg	G	0	0.28
mPD		0.064	0.48
		nPD:	0.13
		N. weight:	0.86
		Sc. PD:	0.04
		Sc. rank:	434.0

PB1

Pos.	265	obs:	exp:
aat	N	0	0.51
aac	N	1	0.49
aaa	K	1090	637.00
aag	K	3	456.00
mPD		0.0073	0.49
		nPD:	0.01
		N. weight:	0.57
		Sc. PD:	-0.039
		Sc. rank:	-212.3

PB1			
Pos.	266	obs:	exp:
tta	L	0	97.08
ttg	L	1	203.70
ctt	L	1076	182.20
ctc	L	17	182.60
cta	L	0	184.40
ctg	L	0	244.10
---	--	------	------
mPD		0.034	1.1
		nPD:	0.03
		N. weight:	1.9
		Sc. PD:	-0.098
		Sc. rank:	-106.2

,

PB1			
Pos.	267	obs:	exp:
tct	S	0	0.16
tcc	S	0	0.13
tca	S	0	0.25
tcg	S	0	0.06
agt	S	0	0.21
agc	S	1	0.19
gaa	E	0	665.10
gag	E	1093	427.90
---	--	------	------
mPD		0.0055	0.48
		nPD:	0.01
		N. weight:	1.
		Sc. PD:	-0.073
		Sc. rank:	-448.1

,

PB1			
Pos.	268	obs:	exp:
caa	Q	818	556.70
cag	Q	275	536.30
aaa	K	1	0.58
aag	K	0	0.42
---	--	------	------
mPD		0.38	0.50
		nPD:	0.75
		N. weight:	0.14
		Sc. PD:	0.087
		Sc. rank:	117.1

,

PB1			
Pos.	269	obs:	exp:
tct	S	1053	174.00
tcc	S	37	142.90
tca	S	2	277.70
tcg	S	2	62.08
agt	S	0	224.90
agc	S	0	212.50
---	--	------	------
mPD		0.072	1.7
		nPD:	0.04
		N. weight:	1.8
		Sc. PD:	-0.073
		Sc. rank:	92.1

,

PB1			
Pos.	270	obs:	exp:
ggt	G	5	145.00
ggc	G	1059	139.80
gga	G	29	504.70
ggg	G	1	304.60
---	--	------	------
mPD		0.062	0.68
		nPD:	0.09
		N. weight:	2.1
		Sc. PD:	0.013
		Sc. rank:	702.6

,

PB1			
Pos.	271	obs:	exp:
tta	L	0	97.08
ttg	L	0	203.70
ctt	L	9	182.20
ctc	L	1064	182.60
cta	L	21	184.40
ctg	L	0	244.10
---	--	------	------
mPD		0.054	1.1
		nPD:	0.05
		N. weight:	1.8
		Sc. PD:	-0.064
		Sc. rank:	171.7

,

PB1			
Pos.	272	obs:	exp:
cct	P	1088	273.00
ccc	P	0	196.60
cca	P	5	450.30
ccg	P	1	174.20
---	--	------	------
mPD		0.011	0.71
		nPD:	0.02
		N. weight:	1.5
		Sc. PD:	-0.099
		Sc. rank:	-515.7

,

PB1			
Pos.	273	obs:	exp:
gtt	V	21	226.70
gtc	V	1044	220.80
gta	V	29	224.00
gtg	V	0	422.50
---	--	------	------
mPD		0.088	0.73
		nPD:	0.12
		N. weight:	1.5
		Sc. PD:	0.053
		Sc. rank:	708.5

,

PB1			
Pos.	274	obs:	exp:
ggt	G	0	145.00
ggc	G	0	139.80
gga	G	1083	504.70
ggg	G	11	304.60
---	--	------	------
mPD		0.020	0.68
		nPD:	0.03
		N. weight:	0.8
		Sc. PD:	-0.043
		Sc. rank:	-68.7

,

PB1			
Pos.	275	obs:	exp:
ggt	G	0	145.00
ggc	G	0	139.80
gga	G	6	504.70
ggg	G	1088	304.60
---	--	------	------
mPD		0.011	0.68
		nPD:	0.02
		N. weight:	1.4
		Sc. PD:	-0.09
		Sc. rank:	-465.7

,

PB1			
Pos.	276	obs:	exp:
aat	N	1076	557.20
aac	N	18	536.80
---	--	------	------
mPD		0.032	0.50
		nPD:	0.06
		N. weight:	0.65
		Sc. PD:	-0.013
		Sc. rank:	127.5

,

PB1			
Pos.	277	obs:	exp:
gaa	E	4	665.70
gag	E	1090	428.30
---	--	------	------
mPD		0.0073	0.48
		nPD:	0.02
		N. weight:	1.
		Sc. PD:	-0.067
		Sc. rank:	-359.6

,

PB1			
Pos.	278	obs:	exp:
aaa	K	83	637.00
aag	K	1010	456.00
gaa	E	0	0.61
gag	E	1	0.39
---	--	------	------
mPD		0.14	0.49
		nPD:	0.29
		N. weight:	0.64
		Sc. PD:	0.13
		Sc. rank:	478.1

,

PB1			
Pos.	279	obs:	exp:
aaa	K	785	637.60
aag	K	309	456.40
---	--	------	------
mPD		0.41	0.49
		nPD:	0.83
		N. weight:	0.046
		Sc. PD:	0.033
		Sc. rank:	40.2

,

PB1			
Pos.	280	obs:	exp:
gct	A	1086	282.50
gcc	A	8	203.70
gca	A	0	514.90
gcg	A	0	92.85
---	--	------	------
mPD		0.015	0.67
		nPD:	0.02
		N. weight:	1.4
		Sc. PD:	-0.088
		Sc. rank:	-318.6

,

PB1			
Pos.	281	obs:	exp:
aaa	K	1091	637.60
aag	K	3	456.40
---	--	------	------
mPD		0.0055	0.49
		nPD:	0.01
		N. weight:	0.57
		Sc. PD:	-0.041
		Sc. rank:	-254.9

,

PB1			
Pos.	282	obs:	exp:
ttt	F	1	0.39
ttc	F	0	0.61
tta	L	6	96.99
ttg	L	983	203.50
ctt	L	6	182.00
ctc	L	0	182.40
cta	L	1	184.20
ctg	L	97	243.90
---	--	------	------
mPD		0.20	1.1
		nPD:	0.18
		N. weight:	1.4
		Sc. PD:	0.13
		Sc. rank:	871.8

,

PB1			
Pos.	283	obs:	exp:
gct	A	0	282.50
gcc	A	0	203.70
gca	A	1094	514.90
gcg	A	0	92.85
---	--	------	------
mPD		0	0.67
		nPD:	0.
		N. weight:	0.83
		Sc. PD:	-0.068
		Sc. rank:	-711.0

,

PB1			
Pos.	284	obs:	exp:
aat	N	1094	557.20
aac	N	0	536.80
---	--	------	------
mPD		0	0.50
		nPD:	0.
		N. weight:	0.74
		Sc. PD:	-0.061
		Sc. rank:	-636.5

,

PB1			
Pos.	285	obs:	exp:
gtt	V	1091	226.70
gtc	V	1	220.80
gta	V	1	224.00
gtg	V	1	422.50
---	--	------	------
mPD		0.0055	0.73
		nPD:	0.01
		N. weight:	1.7
		Sc. PD:	-0.13
		Sc. rank:	-957.2

,

PB1			
Pos.	286	obs:	exp:
gtt	V	0	226.70
gtc	V	0	220.80
gta	V	14	224.00
gtg	V	1080	422.50
---	--	------	------
mPD		0.025	0.73
		nPD:	0.03
		N. weight:	0.98
		Sc. PD:	-0.047
		Sc. rank:	-23.9

,

PB1			
Pos.	287	obs:	exp:
cgt	R	0	46.94
cgc	R	0	54.14
cga	R	0	107.00
cgg	R	0	91.20
aaa	K	0	0.58
aag	K	1	0.42
aga	R	131	499.80
agg	R	962	294.00
---	--	------	------
mPD		0.21	0.96
		nPD:	0.22
		N. weight:	0.97
		Sc. PD:	0.13
		Sc. rank:	662.6

,

PB1			
Pos.	288	obs:	exp:
aaa	K	24	637.60
aag	K	1070	456.40
---	--	------	------
mPD		0.043	0.49
		nPD:	0.09
		N. weight:	0.84
		Sc. PD:	0.0024
		Sc. rank:	282.3

,

PB1			
Pos.	289	obs:	exp:
atg	M	1094	1094.00
---	--	------	------
mPD		0	0
		nPD:	1.
		N. weight:	0.
		Sc. PD:	0
		Sc. rank:	0

,

PB1			
Pos.	290	obs:	exp:
atg	M	1094	1094.00
---	--	------	------
mPD		0	0
		nPD:	1.
		N. weight:	0.
		Sc. PD:	0
		Sc. rank:	0

,

PB1			
Pos.	291	obs:	exp:
act	T	1061	278.10
acc	T	30	223.90
aca	T	0	500.40
acg	T	0	88.62
gct	A	3	0.77
gcc	A	0	0.56
gca	A	0	1.41
gcg	A	0	0.25
---	--	------	------
mPD		0.059	0.68
		nPD:	0.09
		N. weight:	1.4
		Sc. PD:	0.0014
		Sc. rank:	454.5

,

PB1			
Pos.	292	obs:	exp:
tct	S	0	0.80
tcc	S	0	0.65
tca	S	0	1.27
tcg	S	0	0.28
aat	N	1082	554.70
aac	N	7	534.30
agt	S	5	1.03
agc	S	0	0.97
---	--	------	------
mPD		0.022	0.52
		nPD:	0.04
		N. weight:	0.7
		Sc. PD:	-0.029
		Sc. rank:	25.6

,

PB1			
Pos.	293	obs:	exp:
tct	S	0	174.00
tcc	S	0	142.90
tca	S	1094	277.70
tcg	S	0	62.08
agt	S	0	224.90
agc	S	0	212.50
---	--	------	------
mPD		0	1.7
		nPD:	0.
		N. weight:	1.5
		Sc. PD:	-0.12
		Sc. rank:	-1293.7

,

PB1			
Pos.	294	obs:	exp:
caa	Q	1091	557.20
cag	Q	3	536.80
---	--	------	------
mPD		0.0055	0.50
		nPD:	0.01
		N. weight:	0.72
		Sc. PD:	-0.051
		Sc. rank:	-329.7

,

PB1			
Pos.	295	obs:	exp:
gat	D	962	591.20
gac	D	132	502.80
---	--	------	------
mPD		0.21	0.50
		nPD:	0.43
		N. weight:	0.3
		Sc. PD:	0.097
		Sc. rank:	243.4

,

PB1			
Pos.	296	obs:	exp:
act	T	6	278.90
acc	T	0	224.50
aca	T	1088	501.80
acg	T	0	88.86
---	--	------	------
mPD		0.011	0.68
		nPD:	0.02
		N. weight:	0.82
		Sc. PD:	-0.055
		Sc. rank:	-283.0

PB1			
Pos.	297	obs:	exp:
gaa	E	53	665.70
gag	E	1041	428.30
---	--	------	------
mPD		0.092	0.48
		nPD:	0.19
		N. weight:	0.8
		Sc. PD:	0.083
		Sc. rank:	504.3

PB1			
Pos.	298	obs:	exp:
tta	L	0	96.91
ttg	L	0	203.30
ctt	L	16	181.80
ctc	L	1076	182.20
cta	L	0	184.00
ctg	L	0	243.70
att	I	0	0.73
atc	I	2	0.48
ata	I	0	0.79
---	--	------	------
mPD		0.033	1.1
		nPD:	0.03
		N. weight:	1.9
		Sc. PD:	-0.1
		Sc. rank:	-169.7

PB1			
Pos.	299	obs:	exp:
tct	S	31	174.00
tcc	S	1062	142.90
tca	S	1	277.70
tcg	S	0	62.08
agt	S	0	224.90
agc	S	0	212.50
---	--	------	------
mPD		0.057	1.7
		nPD:	0.03
		N. weight:	2.1
		Sc. PD:	-0.1
		Sc. rank:	-55.5

PB1			
Pos.	300	obs:	exp:
ttt	F	43	432.00
ttc	F	1051	662.00
---	--	------	------
mPD		0.076	0.48
		nPD:	0.16
		N. weight:	0.39
		Sc. PD:	0.027
		Sc. rank:	218.2

PB1			
Pos.	301	obs:	exp:
act	T	17	278.90
acc	T	1	224.50
aca	T	1054	501.80
acg	T	22	88.86
---	--	------	------
mPD		0.071	0.68
		nPD:	0.11
		N. weight:	0.71
		Sc. PD:	0.014
		Sc. rank:	286.7

PB1			
Pos.	302	obs:	exp:
att	I	969	402.00
atc	I	125	262.20
ata	I	0	429.90
---	--	------	------
mPD		0.20	0.65
		nPD:	0.31
		N. weight:	0.77
		Sc. PD:	0.17
		Sc. rank:	594.9

PB1			
Pos.	303	obs:	exp:
act	T	1092	278.90
acc	T	0	224.50
aca	T	2	501.80
acg	T	0	88.86
---	--	------	------
mPD		0.0037	0.68
		nPD:	0.01
		N. weight:	1.5
		Sc. PD:	-0.11
		Sc. rank:	-965.7

PB1			
Pos.	304	obs:	exp:
ggt	G	0	145.00
ggc	G	0	139.80
gga	G	229	504.70
ggg	G	865	304.60
---	--	------	------
mPD		0.33	0.68
		nPD:	0.49
		N. weight:	0.73
		Sc. PD:	0.28
		Sc. rank:	613.5

PB1			
Pos.	305	obs:	exp:
gat	D	6	591.20
gac	D	1088	502.80
---	--	------	------
mPD		0.011	0.50
		nPD:	0.02
		N. weight:	0.82
		Sc. PD:	-0.05
		Sc. rank:	-173.2

PB1			
Pos.	306	obs:	exp:
aat	N	3	557.20
aac	N	1091	536.80
---	--	------	------
mPD		0.0055	0.50
		nPD:	0.01
		N. weight:	0.76
		Sc. PD:	-0.054
		Sc. rank:	-351.0

PB1			
Pos.	307	obs:	exp:
act	T	7	278.90
acc	T	1074	224.50
aca	T	13	501.80
acg	T	0	88.86
---	--	------	------
mPD		0.036	0.68
		nPD:	0.05
		N. weight:	1.6
		Sc. PD:	-0.049
		Sc. rank:	212.7

PB1			
Pos.	308	obs:	exp:
aaa	K	1088	637.60
aag	K	6	456.40
---	--	------	------
mPD		0.011	0.49
		nPD:	0.02
		N. weight:	0.56
		Sc. PD:	-0.034
		Sc. rank:	-114.2

PB1			
Pos.	309	obs:	exp:
tgg	W	1094	1094.00
---	--	------	------
mPD		0	0
		nPD:	1.
		N. weight:	0.
		Sc. PD:	0
		Sc. rank:	0

PB1			
Pos.	310	obs:	exp:
aat	N	1094	557.20
aac	N	0	536.80
---	--	------	------
mPD		0	0.50
		nPD:	0.
		N. weight:	0.74
		Sc. PD:	-0.061
		Sc. rank:	-636.5

PB1			
Pos.	311	obs:	exp:
gaa	E	25	665.70
gag	E	1069	428.30
---	--	------	------
mPD		0.045	0.48
		nPD:	0.09
		N. weight:	0.9
		Sc. PD:	0.0073
		Sc. rank:	318.4

PB1			
Pos.	312	obs:	exp:
aat	N	1056	557.20
aac	N	38	536.80
---	--	------	------
mPD		0.067	0.50
		nPD:	0.13
		N. weight:	0.58
		Sc. PD:	0.027
		Sc. rank:	291.4

PB1			
Pos.	313	obs:	exp:
caa	Q	1093	557.20
cag	Q	1	536.80
---	--	------	------
mPD		0.0018	0.50
		nPD:	0.
		N. weight:	0.73
		Sc. PD:	-0.057
		Sc. rank:	-517.9

PB1			
Pos.	314	obs:	exp:
tct	S	0	0.32
tcc	S	0	0.26
tca	S	0	0.51
tcg	S	0	0.11
aat	N	3	556.20
aac	N	1089	535.80
agt	S	0	0.41
agc	S	2	0.39
---	--	------	------
mPD		0.0091	0.51
		nPD:	0.02
		N. weight:	0.76
		Sc. PD:	-0.049
		Sc. rank:	-218.5

PB1			
Pos.	315	obs:	exp:
cct	P	11	273.00
ccc	P	1083	196.60
cca	P	0	450.30
ccg	P	0	174.20
---	--	------	------
mPD		0.020	0.71
		nPD:	0.03
		N. weight:	1.8
		Sc. PD:	-0.1
		Sc. rank:	-177.7

PB1			
Pos.	316	obs:	exp:
cgt	R	0	46.99
cgc	R	0	54.19
cga	R	35	107.10
cgg	R	1028	91.28
aga	R	0	500.20
agg	R	31	294.20
---	--	------	------
mPD		0.12	0.96
		nPD:	0.12
		N. weight:	2.4
		Sc. PD:	0.084
		Sc. rank:	1126.6

PB1			
Pos.	317	obs:	exp:
att	I	0	0.73
atc	I	0	0.48
ata	I	2	0.79
atg	M	1082	1082.00
act	T	0	0.51
acc	T	0	0.41
aca	T	0	0.92
acg	T	2	0.16
gtt	V	0	1.66
gtc	V	0	1.61
gta	V	0	1.64
gtg	V	8	3.09
---	--	------	------
mPD		0.022	0.034
		nPD:	0.64
		N. weight:	0.015
		Sc. PD:	0.0077
		Sc. rank:	12.4

PB1			
Pos.	318	obs:	exp:
ttt	F	1083	432.00
ttc	F	11	662.00
---	--	------	------
mPD		0.020	0.48
		nPD:	0.04
		N. weight:	0.96
		Sc. PD:	-0.04
		Sc. rank:	20.0

PB1			
Pos.	319	obs:	exp:
tta	L	0	97.08
ttg	L	2	203.70
ctt	L	0	182.20
ctc	L	6	182.60
cta	L	1083	184.40
ctg	L	3	244.10
---	--	------	------
mPD		0.024	1.1
		nPD:	0.02
		N. weight:	1.9
		Sc. PD:	-0.12
		Sc. rank:	-449.5

PB1			
Pos.	320	obs:	exp:
gct	A	0	282.50
gcc	A	0	203.70
gca	A	1050	514.90
gcg	A	44	92.85
---	--	------	------
mPD		0.077	0.67
		nPD:	0.12
		N. weight:	0.72
		Sc. PD:	0.021
		Sc. rank:	318.0

PB1			
Pos.	321	obs:	exp:
atg	M	1094	1094.00
---	--	------	------
mPD		0	0
		nPD:	1.
		N. weight:	0.
		Sc. PD:	0
		Sc. rank:	0

PB1			
Pos.	322	obs:	exp:
att	I	0	401.60
atc	I	0	261.90
ata	I	1093	429.50
gtt	V	0	0.21
gtc	V	0	0.20
gta	V	1	0.20
gtg	V	0	0.39
---	--	------	------
mPD		0.0018	0.66
		nPD:	0.
		N. weight:	1.
		Sc. PD:	-0.081
		Sc. rank:	-753.6

PB1			
Pos.	323	obs:	exp:
act	T	3	278.90
acc	T	0	224.50
aca	T	1088	501.80
acg	T	3	88.86
---	--	------	------
mPD		0.011	0.68
		nPD:	0.02
		N. weight:	0.82
		Sc. PD:	-0.055
		Sc. rank:	-276.0

PB1			
Pos.	324	obs:	exp:
tat	Y	5	590.90
tac	Y	1089	503.10
---	--	------	------
mPD		0.0091	0.50
		nPD:	0.02
		N. weight:	0.82
		Sc. PD:	-0.053
		Sc. rank:	-227.9

PB1			
Pos.	325	obs:	exp:
att	I	5	402.00
atc	I	1068	262.20
ata	I	21	429.90
---	--	------	------
mPD		0.047	0.65
		nPD:	0.07
		N. weight:	1.4
		Sc. PD:	-0.019
		Sc. rank:	352.5

PB1			
Pos.	326	obs:	exp:
act	T	0	278.90
acc	T	0	224.50
aca	T	1091	501.80
acg	T	3	88.86
---	--	------	------
mPD		0.0055	0.68
		nPD:	0.01
		N. weight:	0.84
		Sc. PD:	-0.062
		Sc. rank:	-439.4

,

PB1			
Pos.	327	obs:	exp:
cgt	R	0	45.74
cgc	R	0	52.75
cga	R	4	104.20
cgg	R	0	88.87
aaa	K	29	16.90
aag	K	0	12.10
aga	R	1026	487.00
agg	R	35	286.40
---	--	------	------
mPD		0.12	1.0
		nPD:	0.12
		N. weight:	0.7
		Sc. PD:	0.024
		Sc. rank:	316.9

,

PB1			
Pos.	328	obs:	exp:
tct	S	0	6.20
tcc	S	0	5.09
tca	S	0	9.90
tcg	S	0	2.21
aat	N	29	535.80
aac	N	1023	516.20
aaa	K	1	1.75
aag	K	2	1.25
agt	S	0	8.02
agc	S	39	7.57
---	--	------	------
mPD		0.13	0.62
		nPD:	0.2
		N. weight:	0.69
		Sc. PD:	0.076
		Sc. rank:	441.8

,

PB1			
Pos.	329	obs:	exp:
caa	Q	10	557.20
cag	Q	1084	536.80
---	--	------	------
mPD		0.018	0.50
		nPD:	0.04
		N. weight:	0.73
		Sc. PD:	-0.034
		Sc. rank:	-3.5

,

PB1			
Pos.	330	obs:	exp:
cct	P	32	273.00
ccc	P	0	196.60
cca	P	963	450.30
ccg	P	99	174.20
---	--	------	------
mPD		0.22	0.71
		nPD:	0.3
		N. weight:	0.62
		Sc. PD:	0.13
		Sc. rank:	474.5

,

PB1			
Pos.	331	obs:	exp:
aaa	K	1	0.58
aag	K	0	0.42
gaa	E	1026	664.50
gag	E	66	427.50
ggt	G	0	0.13
ggc	G	0	0.13
gga	G	1	0.46
ggg	G	0	0.28
---	--	------	------
mPD		0.12	0.48
		nPD:	0.24
		N. weight:	0.33
		Sc. PD:	0.05
		Sc. rank:	232.6

,

PB1			
Pos.	332	obs:	exp:
tgg	W	1094	1094.00
---	--	------	------
mPD		0	0
		nPD:	1.
		N. weight:	0.
		Sc. PD:	0
		Sc. rank:	0

,

PB1			
Pos.	333	obs:	exp:
ttt	F	1078	432.00
ttc	F	16	662.00
---	--	------	------
mPD		0.029	0.48
		nPD:	0.06
		N. weight:	0.93
		Sc. PD:	-0.022
		Sc. rank:	161.0

,

PB1			
Pos.	334	obs:	exp:
cgt	R	0	46.99
cgc	R	0	54.19
cga	R	0	107.10
cgg	R	0	91.28
aga	R	1043	500.20
agg	R	51	294.20
---	--	------	------
mPD		0.089	0.96
		nPD:	0.09
		N. weight:	0.68
		Sc. PD:	0.0048
		Sc. rank:	237.2

,

PB1			
Pos.	335	obs:	exp:
aat	N	1060	557.20
aac	N	34	536.80
---	--	------	------
mPD		0.060	0.50
		nPD:	0.12
		N. weight:	0.59
		Sc. PD:	0.02
		Sc. rank:	268.9

,

PB1			
Pos.	336	obs:	exp:
att	I	0	0.37
atc	I	1	0.24
ata	I	0	0.39
gtt	V	10	226.50
gtc	V	1077	220.60
gta	V	6	223.80
gtg	V	0	422.10
---	--	------	------
mPD		0.031	0.73
		nPD:	0.04
		N. weight:	1.7
		Sc. PD:	-0.069
		Sc. rank:	57.8

,

PB1			
Pos.	337	obs:	exp:
tta	L	1067	97.08
ttg	L	12	203.70
ctt	L	0	182.20
ctc	L	0	182.60
cta	L	15	184.40
ctg	L	0	244.10
---	--	------	------
mPD		0.049	1.1
		nPD:	0.04
		N. weight:	2.5
		Sc. PD:	-0.099
		Sc. rank:	139.2

,

PB1			
Pos.	338	obs:	exp:
tct	S	0	173.70
tcc	S	0	142.60
tca	S	0	277.20
tcg	S	0	61.96
att	I	0	0.73
atc	I	2	0.48
ata	I	0	0.79
agt	S	9	224.50
agc	S	1083	212.10
---	--	------	------
mPD		0.020	1.7
		nPD:	0.01
		N. weight:	1.7
		Sc. PD:	-0.12
		Sc. rank:	-749.1

,

PB1			
Pos.	339	obs:	exp:
att	I	1088	402.00
atc	I	0	262.20
ata	I	6	429.90
---	--	------	------
mPD		0.011	0.65
		nPD:	0.02
		N. weight:	1.1
		Sc. PD:	-0.07
		Sc. rank:	-322.3

,

PB1			
Pos.	340	obs:	exp:
gct	A	1083	282.50
gcc	A	11	203.70
gca	A	0	514.90
gcg	A	0	92.85
---	--	------	------
mPD		0.020	0.67
		nPD:	0.03
		N. weight:	1.4
		Sc. PD:	-0.076
		Sc. rank:	-102.9

,

PB1			
Pos.	341	obs:	exp:
cct	P	1090	273.00
ccc	P	1	196.60
cca	P	3	450.30
ccg	P	0	174.20
---	--	------	------
mPD		0.0073	0.71
		nPD:	0.01
		N. weight:	1.5
		Sc. PD:	-0.11
		Sc. rank:	-716.3

,

PB1			
Pos.	342	obs:	exp:
att	I	0	402.00
atc	I	1	262.20
ata	I	1093	429.90
---	--	------	------
mPD		0.0018	0.65
		nPD:	0.
		N. weight:	1.
		Sc. PD:	-0.081
		Sc. rank:	-736.0

,

PB1			
Pos.	343	obs:	exp:
atg	M	1094	1094.00
---	--	------	------
mPD		0	0
		nPD:	1.
		N. weight:	0.
		Sc. PD:	0
		Sc. rank:	0

,

PB1			
Pos.	344	obs:	exp:
ttt	F	5	432.00
ttc	F	1089	662.00
---	--	------	------
mPD		0.0091	0.48
		nPD:	0.02
		N. weight:	0.52
		Sc. PD:	-0.033
		Sc. rank:	-136.1

,

PB1			
Pos.	345	obs:	exp:
tct	S	0	174.00
tcc	S	0	142.90
tca	S	1088	277.70
tcg	S	6	62.08
agt	S	0	224.90
agc	S	0	212.50
---	--	------	------
mPD		0.011	1.7
		nPD:	0.01
		N. weight:	1.5
		Sc. PD:	-0.11
		Sc. rank:	-884.0

,

PB1			
Pos.	346	obs:	exp:
aat	N	0	557.20
aac	N	1094	536.80
---	--	------	------
mPD		0	0.50
		nPD:	0.
		N. weight:	0.78
		Sc. PD:	-0.064
		Sc. rank:	-671.8

,

PB1			
Pos.	347	obs:	exp:
aaa	K	123	637.60
aag	K	971	456.40
---	--	------	------
mPD		0.20	0.49
		nPD:	0.41
		N. weight:	0.54
		Sc. PD:	0.17
		Sc. rank:	437.3

,

PB1			
Pos.	348	obs:	exp:
tta	L	0	0.09
ttg	L	0	0.19
ctt	L	0	0.17
ctc	L	0	0.17
cta	L	0	0.17
ctg	L	1	0.22
atg	M	1093	1093.00
---	--	------	------
mPD		0.0018	0.0029
		nPD:	0.63
		N. weight:	0.0015
		Sc. PD:	0.00078
		Sc. rank:	1.3

,

PB1			
Pos.	349	obs:	exp:
gct	A	1	282.50
gcc	A	0	203.70
gca	A	102	514.90
gcg	A	991	92.85
---	--	------	------
mPD		0.17	0.67
		nPD:	0.26
		N. weight:	2.2
		Sc. PD:	0.36
		Sc. rank:	1592.2

,

PB1			
Pos.	350	obs:	exp:
cgt	R	0	46.99
cgc	R	0	54.19
cga	R	0	107.10
cgg	R	0	91.28
aga	R	1061	500.20
agg	R	33	294.20
---	--	------	------
mPD		0.059	0.96
		nPD:	0.06
		N. weight:	0.73
		Sc. PD:	-0.017
		Sc. rank:	128.9

,

PB1			
Pos.	351	obs:	exp:
tta	L	1086	97.08
ttg	L	8	203.70
ctt	L	0	182.20
ctc	L	0	182.60
cta	L	0	184.40
ctg	L	0	244.10
---	--	------	------
mPD		0.015	1.1
		nPD:	0.01
		N. weight:	2.6
		Sc. PD:	-0.18
		Sc. rank:	-1077.4

PB1			
Pos.	352	obs:	exp:
ggt	G	0	145.00
ggc	G	0	139.80
gga	G	926	504.70
ggg	G	168	304.60
---	--	------	------
mPD		0.26	0.68
		nPD:	0.39
		N. weight:	0.47
		Sc. PD:	0.13
		Sc. rank:	376.1

PB1			
Pos.	353	obs:	exp:
aaa	K	1089	637.60
aag	K	5	456.40
---	--	------	------
mPD		0.0091	0.49
		nPD:	0.02
		N. weight:	0.56
		Sc. PD:	-0.036
		Sc. rank:	-153.8

PB1			
Pos.	354	obs:	exp:
ggt	G	0	145.00
ggc	G	0	139.80
gga	G	192	504.70
ggg	G	902	304.60
---	--	------	------
mPD		0.29	0.68
		nPD:	0.43
		N. weight:	0.8
		Sc. PD:	0.26
		Sc. rank:	659.0

PB1			
Pos.	355	obs:	exp:
tat	Y	11	590.90
tac	Y	1083	503.10
---	--	------	------
mPD		0.020	0.50
		nPD:	0.04
		N. weight:	0.79
		Sc. PD:	-0.034
		Sc. rank:	10.6

PB1			
Pos.	356	obs:	exp:
atg	M	1094	1094.00
---	--	------	------
mPD		0	0
		nPD:	1.
		N. weight:	0.
		Sc. PD:	0
		Sc. rank:	0

PB1			
Pos.	357	obs:	exp:
ttt	F	29	431.60
ttc	F	1064	661.40
tct	S	0	0.16
tcc	S	1	0.13
tca	S	0	0.25
tcg	S	0	0.06
agt	S	0	0.21
agc	S	0	0.19
---	--	------	------
mPD		0.053	0.48
		nPD:	0.11
		N. weight:	0.43
		Sc. PD:	0.011
		Sc. rank:	181.9

PB1			
Pos.	358	obs:	exp:
gaa	E	1090	665.70
gag	E	4	428.30
---	--	------	------
mPD		0.0073	0.48
		nPD:	0.02
		N. weight:	0.52
		Sc. PD:	-0.035
		Sc. rank:	-187.2

PB1			
Pos.	359	obs:	exp:
tct	S	0	174.00
tcc	S	0	142.90
tca	S	0	277.70
tcg	S	0	62.08
agt	S	1090	224.90
agc	S	4	212.50
---	--	------	------
mPD		0.0073	1.7
		nPD:	0.
		N. weight:	1.7
		Sc. PD:	-0.13
		Sc. rank:	-1161.0

PB1			
Pos.	360	obs:	exp:
aaa	K	38	637.60
aag	K	1056	456.40
---	--	------	------
mPD		0.067	0.49
		nPD:	0.14
		N. weight:	0.78
		Sc. PD:	0.04
		Sc. rank:	402.8

PB1			
Pos.	361	obs:	exp:
tct	S	0	172.40
tcc	S	0	141.60
tca	S	0	275.10
tcg	S	0	61.51
aat	N	10	5.09
aac	N	0	4.91
agt	S	1051	222.80
agc	S	33	210.50
---	--	------	------
mPD		0.077	1.7
		nPD:	0.05
		N. weight:	1.6
		Sc. PD:	-0.06
		Sc. rank:	114.8

PB1			
Pos.	362	obs:	exp:
atg	M	1094	1094.00
---	--	------	------
mPD		0	0
		nPD:	1.
		N. weight:	0.
		Sc. PD:	0
		Sc. rank:	0

PB1			
Pos.	363	obs:	exp:
cgt	R	0	0.90
cgc	R	0	1.04
cga	R	0	2.06
cgg	R	0	1.75
aaa	K	37	625.40
aag	K	1036	447.60
aga	R	5	9.60
agg	R	16	5.65
---	--	------	------
mPD		0.11	0.54
		nPD:	0.21
		N. weight:	0.78
		Sc. PD:	0.092
		Sc. rank:	515.9

PB1			
Pos.	364	obs:	exp:
tta	L	888	96.99
ttg	L	168	203.50
ctt	L	0	182.00
ctc	L	0	182.40
cta	L	36	184.20
ctg	L	1	243.90
att	I	0	0.37
atc	I	0	0.24
ata	I	1	0.39
---	--	------	------
mPD		0.33	1.1
		nPD:	0.3
		N. weight:	1.9
		Sc. PD:	0.38
		Sc. rank:	1428.0

PB1			
Pos.	365	obs:	exp:
cgt	R	0	46.99
cgc	R	0	54.19
cga	R	22	107.10
cgg	R	1071	91.28
aga	R	1	500.20
agg	R	0	294.20
---	--	------	------
mPD		0.043	0.96
		nPD:	0.04
		N. weight:	2.6
		Sc. PD:	-0.1
		Sc. rank:	179.6

PB1			
Pos.	366	obs:	exp:
act	T	0	277.60
acc	T	0	223.50
aca	T	1041	499.50
acg	T	48	88.45
gct	A	0	1.29
gcc	A	0	0.93
gca	A	5	2.35
gcg	A	0	0.42
---	--	------	------
mPD		0.093	0.69
		nPD:	0.14
		N. weight:	0.74
		Sc. PD:	0.036
		Sc. rank:	375.7

PB1			
Pos.	367	obs:	exp:
caa	Q	1038	557.20
cag	Q	56	536.80
---	--	------	------
mPD		0.097	0.50
		nPD:	0.19
		N. weight:	0.52
		Sc. PD:	0.055
		Sc. rank:	332.5

PB1			
Pos.	368	obs:	exp:
tta	L	1	0.09
ttg	L	0	0.19
ctt	L	0	0.17
ctc	L	0	0.17
cta	L	0	0.17
ctg	L	0	0.22
att	I	0	16.53
atc	I	0	10.78
ata	I	45	17.68
gtt	V	0	217.10
gtc	V	7	211.50
gta	V	1033	214.60
gtg	V	8	404.80
---	--	------	------
mPD		0.11	0.81
		nPD:	0.13
		N. weight:	1.6
		Sc. PD:	0.074
		Sc. rank:	810.8

PB1			
Pos.	369	obs:	exp:
tct	S	0	5.88
tcc	S	0	4.83
tca	S	37	9.39
tcg	S	0	2.10
cct	P	0	263.70
ccc	P	1	189.90
cca	P	1039	435.10
ccg	P	17	168.30
agt	S	0	7.61
agc	S	0	7.19
---	--	------	------
mPD		0.098	0.80
		nPD:	0.12
		N. weight:	0.92
		Sc. PD:	0.032
		Sc. rank:	423.6

PB1			
Pos.	370	obs:	exp:
gtt	V	0	0.21
gtc	V	0	0.20
gta	V	0	0.20
gtg	V	1	0.39
gct	A	17	282.20
gcc	A	0	203.50
gca	A	139	514.40
gcg	A	937	92.77
---	--	------	------
mPD		0.25	0.67
		nPD:	0.37
		N. weight:	2.
		Sc. PD:	0.54
		Sc. rank:	1560.3

PB1			
Pos.	371	obs:	exp:
gaa	E	1088	665.70
gag	E	6	428.30
---	--	------	------
mPD		0.011	0.48
		nPD:	0.02
		N. weight:	0.51
		Sc. PD:	-0.031
		Sc. rank:	-93.3

PB1			
Pos.	372	obs:	exp:
tta	L	0	6.30
ttg	L	15	13.22
ctt	L	0	11.82
ctc	L	0	11.85
cta	L	0	11.97
ctg	L	56	15.84
att	I	0	44.46
atc	I	0	29.00
ata	I	121	47.55
atg	M	901	901.00
aaa	K	0	0.58
aag	K	1	0.42
---	--	------	------
mPD		0.32	0.39
		nPD:	0.82
		N. weight:	0.19
		Sc. PD:	0.13
		Sc. rank:	163.5

PB1			
Pos.	373	obs:	exp:
tta	L	0	97.08
ttg	L	0	203.70
ctt	L	22	182.20
ctc	L	1065	182.60
cta	L	7	184.40
ctg	L	0	244.10
---	--	------	------
mPD		0.052	1.1
		nPD:	0.05
		N. weight:	1.8
		Sc. PD:	-0.067
		Sc. rank:	142.0

PB1			
Pos.	374	obs:	exp:
tct	S	0	1.27
tcc	S	0	1.04
tca	S	7	2.03
tcg	S	1	0.45
act	T	0	1.27
acc	T	0	1.03
aca	T	5	2.29
acg	T	0	0.41
agt	S	0	1.64
agc	S	0	1.55
gtt	V	0	0.62
gtc	V	0	0.61
gta	V	1	0.61
gtg	V	2	1.16
gct	A	0	278.40
gcc	A	0	200.80
gca	A	973	507.40
gcg	A	105	91.49
---	--	------	------
mPD		0.21	0.71
		nPD:	0.29
		N. weight:	0.67
		Sc. PD:	0.13
		Sc. rank:	502.2

PB1			
Pos.	375	obs:	exp:
tct	S	0	0.16
tcc	S	0	0.13
tca	S	0	0.25
tcg	S	0	0.06
cat	H	1	0.57
cac	H	0	0.43
aat	N	1073	553.70
aac	N	14	533.30
aaa	K	4	2.33
aag	K	0	1.67
agt	S	1	0.21
agc	S	0	0.19
gat	D	1	0.54
gac	D	0	0.46
---	--	------	------
mPD		0.038	0.51
		nPD:	0.07
		N. weight:	0.67
		Sc. PD:	-0.007
		Sc. rank:	179.7

PB1			
Pos.	376	obs:	exp:
att	I	1081	400.10
atc	I	8	261.00
ata	I	0	427.90
gtt	V	5	1.04
gtc	V	0	1.01
gta	V	0	1.02
gtg	V	0	1.93
mPD		0.024	0.66
		nPD:	0.04
		N. weight:	1.1
		Sc. PD:	-0.05
		Sc. rank:	-14.7

,

PB1			
Pos.	377	obs:	exp:
gat	D	15	587.40
gac	D	1072	499.60
gaa	E	2	4.26
gag	E	5	2.74
mPD		0.040	0.50
		nPD:	0.08
		N. weight:	0.77
		Sc. PD:	-0.0048
		Sc. rank:	226.9

,

PB1			
Pos.	378	obs:	exp:
tta	L	0	97.08
ttg	L	41	203.70
ctt	L	5	182.20
ctc	L	0	182.60
cta	L	0	184.40
ctg	L	1048	244.10
mPD		0.081	1.1
		nPD:	0.07
		N. weight:	1.5
		Sc. PD:	-0.017
		Sc. rank:	372.1

,

PB1			
Pos.	379	obs:	exp:
aaa	K	1086	637.60
aag	K	8	456.40
mPD		0.015	0.49
		nPD:	0.03
		N. weight:	0.55
		Sc. PD:	-0.029
		Sc. rank:	-37.8

,

PB1			
Pos.	380	obs:	exp:
tat	Y	122	590.90
tac	Y	972	503.10
mPD		0.20	0.50
		nPD:	0.4
		N. weight:	0.45
		Sc. PD:	0.14
		Sc. rank:	366.6

,

PB1			
Pos.	381	obs:	exp:
ttt	F	7	432.00
ttc	F	1087	662.00
mPD		0.013	0.48
		nPD:	0.03
		N. weight:	0.51
		Sc. PD:	-0.029
		Sc. rank:	-69.9

,

PB1			
Pos.	382	obs:	exp:
tct	S	0	0.16
tcc	S	0	0.13
tca	S	0	0.25
tcg	S	0	0.06
aat	N	4	556.70
aac	N	1089	536.30
agt	S	0	0.21
agc	S	1	0.19
mPD		0.0091	0.50
		nPD:	0.02
		N. weight:	0.76
		Sc. PD:	-0.049
		Sc. rank:	-215.3

,

PB1			
Pos.	383	obs:	exp:
cgt	R	0	0.04
cgc	R	0	0.05
cga	R	0	0.10
cgg	R	0	0.08
aaa	K	1037	635.30
aag	K	53	454.70
aga	R	1	0.46
agg	R	0	0.27
gaa	E	3	1.83
gag	E	0	1.17
mPD		0.10	0.49
		nPD:	0.2
		N. weight:	0.4
		Sc. PD:	0.045
		Sc. rank:	258.7

,

PB1			
Pos.	384	obs:	exp:
tct	S	0	174.00
tcc	S	0	142.90
tca	S	1089	277.70
tcg	S	5	62.08
agt	S	0	224.90
agc	S	0	212.50
mPD		0.0091	1.7
		nPD:	0.01
		N. weight:	1.5
		Sc. PD:	-0.11
		Sc. rank:	-947.4

,

PB1			
Pos.	385	obs:	exp:
act	T	0	278.90
acc	T	0	224.50
aca	T	1091	501.80
acg	T	3	88.86
mPD		0.0055	0.68
		nPD:	0.01
		N. weight:	0.84
		Sc. PD:	-0.062
		Sc. rank:	-439.4

,

PB1			
Pos.	386	obs:	exp:
cgt	R	0	45.01
cgc	R	0	51.91
cga	R	0	102.60
cgg	R	0	87.45
aaa	K	46	26.81
aag	K	0	19.19
aga	R	1038	479.20
agg	R	10	281.90
mPD		0.099	1.0
		nPD:	0.1
		N. weight:	0.8
		Sc. PD:	0.0082
		Sc. rank:	290.8

,

PB1			
Pos.	387	obs:	exp:
aaa	K	0	0.58
aag	K	1	0.42
gaa	E	1	665.10
gag	E	1092	427.90
mPD		0.0037	0.48
		nPD:	0.01
		N. weight:	1.
		Sc. PD:	-0.076
		Sc. rank:	-546.4

,

PB1			
Pos.	388	obs:	exp:
aaa	K	955	637.60
aag	K	139	456.40
mPD		0.22	0.49
		nPD:	0.46
		N. weight:	0.22
		Sc. PD:	0.08
		Sc. rank:	187.4

,

PB1			
Pos.	389	obs:	exp:
att	I	15	402.00
atc	I	1070	262.20
ata	I	9	429.90
mPD		0.043	0.65
		nPD:	0.07
		N. weight:	1.4
		Sc. PD:	-0.026
		Sc. rank:	296.8

,

PB1			
Pos.	390	obs:	exp:
gaa	E	94	665.70
gag	E	1000	428.30
mPD		0.16	0.48
		nPD:	0.33
		N. weight:	0.67
		Sc. PD:	0.16
		Sc. rank:	526.3

,

PB1			
Pos.	391	obs:	exp:
aat	N	0	0.51
aac	N	1	0.49
aaa	K	1085	637.00
aag	K	8	456.00
mPD		0.016	0.49
		nPD:	0.03
		N. weight:	0.55
		Sc. PD:	-0.027
		Sc. rank:	-19.1

,

PB1			
Pos.	392	obs:	exp:
att	I	1	402.00
atc	I	0	262.20
ata	I	1093	429.90
mPD		0.0018	0.65
		nPD:	0.
		N. weight:	1.
		Sc. PD:	-0.081
		Sc. rank:	-735.7

,

PB1			
Pos.	393	obs:	exp:
cgt	R	0	46.99
cgc	R	0	54.19
cga	R	1	107.10
cgg	R	0	91.28
aga	R	991	500.20
agg	R	102	294.20
mPD		0.17	0.96
		nPD:	0.18
		N. weight:	0.57
		Sc. PD:	0.051
		Sc. rank:	348.8

,

PB1			
Pos.	394	obs:	exp:
tta	L	0	0.09
ttg	L	0	0.19
ctt	L	1	0.17
ctc	L	0	0.17
cta	L	0	0.17
ctg	L	0	0.22
cct	P	1085	272.70
ccc	P	8	196.40
cca	P	0	449.90
ccg	P	0	174.00
mPD		0.016	0.71
		nPD:	0.02
		N. weight:	1.5
		Sc. PD:	-0.089
		Sc. rank:	-264.6

,

PB1			
Pos.	395	obs:	exp:
tta	L	9	96.55
ttg	L	0	202.60
ctt	L	0	181.20
ctc	L	0	181.60
cta	L	1068	183.40
ctg	L	11	242.80
att	I	0	2.20
atc	I	0	1.44
ata	I	6	2.36
mPD		0.047	1.1
		nPD:	0.04
		N. weight:	1.8
		Sc. PD:	-0.076
		Sc. rank:	57.0

,

PB1			
Pos.	396	obs:	exp:
tta	L	25	97.08
ttg	L	7	203.70
ctt	L	0	182.20
ctc	L	0	182.60
cta	L	80	184.40
ctg	L	982	244.10
mPD		0.23	1.1
		nPD:	0.21
		N. weight:	1.3
		Sc. PD:	0.15
		Sc. rank:	821.9

,

PB1			
Pos.	397	obs:	exp:
att	I	0	365.60
atc	I	0	238.40
ata	I	995	391.00
atg	M	99	99.00
mPD		0.16	0.70
		nPD:	0.23
		N. weight:	0.93
		Sc. PD:	0.13
		Sc. rank:	654.8

,

PB1			
Pos.	398	obs:	exp:
aat	N	9	4.58
aac	N	0	4.42
gat	D	1082	586.30
gac	D	3	498.70
mPD		0.022	0.51
		nPD:	0.04
		N. weight:	0.66
		Sc. PD:	-0.027
		Sc. rank:	24.9

,

PB1			
Pos.	399	obs:	exp:
ggt	G	13	145.00
ggc	G	1074	139.80
gga	G	7	504.70
ggg	G	0	304.60
mPD		0.036	0.68
		nPD:	0.05
		N. weight:	2.1
		Sc. PD:	-0.065
		Sc. rank:	291.9

,

PB1			
Pos.	400	obs:	exp:
act	T	9	278.90
acc	T	0	224.50
aca	T	1080	501.80
acg	T	5	88.86
mPD		0.025	0.68
		nPD:	0.04
		N. weight:	0.79
		Sc. PD:	-0.036
		Sc. rank:	1.1

,

PB1			
Pos.	401	obs:	exp:
gct	A	8	282.50
gcc	A	927	203.70
gca	A	159	514.90
gcg	A	0	92.85
mPD		0.26	0.67
		nPD:	0.39
		N. weight:	1.2
		Sc. PD:	0.35
		Sc. rank:	970.1

PB1			
Pos.	402	obs:	exp:
tct	S	0	173.80
tcc	S	0	142.70
tca	S	1092	277.40
tcg	S	1	62.02
cct	P	0	0.25
ccc	P	0	0.18
cca	P	1	0.41
ccg	P	0	0.16
agt	S	0	224.70
agc	S	0	212.30
mPD		0.0037	1.7
		nPD:	0.
		N. weight:	1.5
		Sc. PD:	-0.12
		Sc. rank:	-1135.8

PB1			
Pos.	403	obs:	exp:
tta	L	10	97.08
ttg	L	1084	203.70
ctt	L	0	182.20
ctc	L	0	182.60
cta	L	0	184.40
ctg	L	0	244.10
mPD		0.018	1.1
		nPD:	0.02
		N. weight:	1.8
		Sc. PD:	-0.12
		Sc. rank:	-575.9

PB1			
Pos.	404	obs:	exp:
tct	S	0	174.00
tcc	S	0	142.90
tca	S	0	277.70
tcg	S	0	62.08
agt	S	1044	224.90
agc	S	50	212.50
mPD		0.087	1.7
		nPD:	0.05
		N. weight:	1.5
		Sc. PD:	-0.048
		Sc. rank:	180.6

PB1			
Pos.	405	obs:	exp:
cct	P	1094	273.00
ccc	P	0	196.60
cca	P	0	450.30
ccg	P	0	174.20
mPD		0	0.71
		nPD:	0.
		N. weight:	1.5
		Sc. PD:	-0.12
		Sc. rank:	-1309.8

PB1			
Pos.	406	obs:	exp:
ggt	G	0	145.00
ggc	G	0	139.80
gga	G	860	504.70
ggg	G	234	304.60
mPD		0.34	0.68
		nPD:	0.5
		N. weight:	0.4
		Sc. PD:	0.16
		Sc. rank:	338.8

PB1			
Pos.	407	obs:	exp:
atg	M	1094	1094.00
mPD		0	0
		nPD:	1.
		N. weight:	0.
		Sc. PD:	0
		Sc. rank:	0

PB1			
Pos.	408	obs:	exp:
att	I	0	0.37
atc	I	1	0.24
ata	I	0	0.39
atg	M	1093	1093.00
mPD		0.0018	0.0018
		nPD:	1.
		N. weight:	0.0014
		Sc. PD:	0.0013
		Sc. rank:	1.2

PB1			
Pos.	409	obs:	exp:
atg	M	1094	1094.00
mPD		0	0
		nPD:	1.
		N. weight:	0.
		Sc. PD:	0
		Sc. rank:	0

PB1			
Pos.	410	obs:	exp:
ggt	G	123	145.00
ggc	G	971	139.80
gga	G	0	504.70
ggg	G	0	304.60
mPD		0.20	0.68
		nPD:	0.3
		N. weight:	1.9
		Sc. PD:	0.38
		Sc. rank:	1410.4

PB1			
Pos.	411	obs:	exp:
atg	M	1094	1094.00
mPD		0	0
		nPD:	1.
		N. weight:	0.
		Sc. PD:	0
		Sc. rank:	0

PB1			
Pos.	412	obs:	exp:
ttt	F	2	432.00
ttc	F	1092	662.00
mPD		0.0037	0.48
		nPD:	0.01
		N. weight:	0.54
		Sc. PD:	-0.04
		Sc. rank:	-290.8

PB1			
Pos.	413	obs:	exp:
aat	N	105	557.20
aac	N	989	536.80
mPD		0.17	0.50
		nPD:	0.35
		N. weight:	0.43
		Sc. PD:	0.11
		Sc. rank:	343.5

PB1			
Pos.	414	obs:	exp:
atg	M	1094	1094.00
mPD		0	0
		nPD:	1.
		N. weight:	0.
		Sc. PD:	0
		Sc. rank:	0

PB1			
Pos.	415	obs:	exp:
tta	L	8	97.08
ttg	L	1021	203.70
ctt	L	0	182.20
ctc	L	0	182.60
cta	L	0	184.40
ctg	L	65	244.10
mPD		0.13	1.1
		nPD:	0.11
		N. weight:	1.5
		Sc. PD:	0.043
		Sc. rank:	676.9

PB1			
Pos.	416	obs:	exp:
tct	S	0	174.00
tcc	S	0	142.90
tca	S	0	277.70
tcg	S	0	62.08
agt	S	1044	224.90
agc	S	50	212.50
mPD		0.087	1.7
		nPD:	0.05
		N. weight:	1.5
		Sc. PD:	-0.048
		Sc. rank:	180.6

PB1			
Pos.	417	obs:	exp:
act	T	0	278.90
acc	T	0	224.50
aca	T	1084	501.80
acg	T	10	88.86
mPD		0.018	0.68
		nPD:	0.03
		N. weight:	0.82
		Sc. PD:	-0.046
		Sc. rank:	-104.7

PB1			
Pos.	418	obs:	exp:
gtt	V	29	226.70
gtc	V	961	220.80
gta	V	104	224.00
gtg	V	0	422.50
mPD		0.22	0.73
		nPD:	0.3
		N. weight:	1.3
		Sc. PD:	0.27
		Sc. rank:	988.4

PB1			
Pos.	419	obs:	exp:
tta	L	1031	97.08
ttg	L	62	203.70
ctt	L	0	182.20
ctc	L	0	182.60
cta	L	1	184.40
ctg	L	0	244.10
mPD		0.11	1.1
		nPD:	0.1
		N. weight:	2.4
		Sc. PD:	0.029
		Sc. rank:	892.0

PB1			
Pos.	420	obs:	exp:
ggt	G	0	145.00
ggc	G	0	139.80
gga	G	1092	504.70
ggg	G	2	304.60
mPD		0.0037	0.68
		nPD:	0.01
		N. weight:	0.84
		Sc. PD:	-0.064
		Sc. rank:	-539.3

PB1			
Pos.	421	obs:	exp:
gtt	V	1085	226.70
gtc	V	3	220.80
gta	V	1	224.00
gtg	V	5	422.50
mPD		0.016	0.73
		nPD:	0.02
		N. weight:	1.7
		Sc. PD:	-0.1
		Sc. rank:	-317.8

PB1			
Pos.	422	obs:	exp:
tct	S	0	174.00
tcc	S	0	142.90
tca	S	1094	277.70
tcg	S	0	62.08
agt	S	0	224.90
agc	S	0	212.50
mPD		0	1.7
		nPD:	0.
		N. weight:	1.5
		Sc. PD:	-0.12
		Sc. rank:	-1293.7

PB1			
Pos.	423	obs:	exp:
att	I	1092	402.00
atc	I	1	262.20
ata	I	1	429.90
mPD		0.0037	0.65
		nPD:	0.01
		N. weight:	1.1
		Sc. PD:	-0.083
		Sc. rank:	-677.8

PB1			
Pos.	424	obs:	exp:
tta	L	0	97.08
ttg	L	2	203.70
ctt	L	0	182.20
ctc	L	0	182.60
cta	L	15	184.40
ctg	L	1077	244.10
mPD		0.031	1.1
		nPD:	0.03
		N. weight:	1.6
		Sc. PD:	-0.086
		Sc. rank:	-158.7

PB1			
Pos.	425	obs:	exp:
aat	N	1090	557.20
aac	N	4	536.80
mPD		0.0073	0.50
		nPD:	0.01
		N. weight:	0.72
		Sc. PD:	-0.049
		Sc. rank:	-278.9

PB1			
Pos.	426	obs:	exp:
ttt	F	0	0.39
ttc	F	1	0.61
tta	L	0	96.99
ttg	L	0	203.50
ctt	L	115	182.00
ctc	L	964	182.40
cta	L	14	184.20
ctg	L	0	243.90
mPD		0.21	1.1
		nPD:	0.19
		N. weight:	1.5
		Sc. PD:	0.16
		Sc. rank:	964.8

PB1			
Pos.	427	obs:	exp:
ggt	G	26	145.00
ggc	G	3	139.80
gga	G	34	504.70
ggg	G	1031	304.60
mPD		0.11	0.68
		nPD:	0.16
		N. weight:	1.1
		Sc. PD:	0.084
		Sc. rank:	632.6

PB1			
Pos.	428	obs:	exp:
caa	Q	87	557.20
cag	Q	1007	536.80
mPD		0.15	0.50
		nPD:	0.29
		N. weight:	0.48
		Sc. PD:	0.095
		Sc. rank:	356.5

PB1			
Pos.	429	obs:	exp:
aaa	K	46	637.60
aag	K	1048	456.40
mPD		0.081	0.49
		nPD:	0.17
		N. weight:	0.76
		Sc. PD:	0.058
		Sc. rank:	431.8

PB1			
Pos.	430	obs:	exp:
cgt	R	0	0.13
cgc	R	0	0.15
cga	R	0	0.29
cgg	R	0	0.25
aaa	K	137	635.90
aag	K	954	455.10
aga	R	0	1.37
agg	R	3	0.81
mPD		0.22	0.49
		nPD:	0.46
		N. weight:	0.5
		Sc. PD:	0.18
		Sc. rank:	420.9

PB1			
Pos.	431	obs:	exp:
tat	Y	2	590.90
tac	Y	1092	503.10
mPD		0.0037	0.50
		nPD:	0.01
		N. weight:	0.84
		Sc. PD:	-0.063
		Sc. rank:	-482.2

PB1			
Pos.	432	obs:	exp:
att	I	0	0.73
atc	I	2	0.48
ata	I	0	0.79
act	T	93	278.40
acc	T	967	224.10
aca	T	32	500.90
acg	T	0	88.70
mPD		0.21	0.68
		nPD:	0.31
		N. weight:	1.2
		Sc. PD:	0.27
		Sc. rank:	964.2

PB1			
Pos.	433	obs:	exp:
cgt	R	0	0.04
cgc	R	0	0.05
cga	R	0	0.10
cgg	R	0	0.08
aaa	K	1043	637.00
aag	K	50	456.00
aga	R	1	0.46
agg	R	0	0.27
mPD		0.089	0.49
		nPD:	0.18
		N. weight:	0.41
		Sc. PD:	0.038
		Sc. rank:	251.3

PB1			
Pos.	434	obs:	exp:
act	T	21	268.20
acc	T	1031	215.90
aca	T	0	482.50
acg	T	0	85.45
aat	N	9	21.39
aac	N	33	20.61
mPD		0.13	0.76
		nPD:	0.17
		N. weight:	1.6
		Sc. PD:	0.13
		Sc. rank:	911.2

PB1			
Pos.	435	obs:	exp:
att	I	0	0.37
atc	I	0	0.24
ata	I	1	0.39
act	T	0	278.60
acc	T	0	224.30
aca	T	1087	501.30
acg	T	6	88.78
mPD		0.013	0.68
		nPD:	0.02
		N. weight:	0.83
		Sc. PD:	-0.053
		Sc. rank:	-224.9

PB1			
Pos.	436	obs:	exp:
tat	Y	1087	589.80
tac	Y	5	502.20
cat	H	2	1.15
cac	H	0	0.85
mPD		0.013	0.50
		nPD:	0.03
		N. weight:	0.65
		Sc. PD:	-0.037
		Sc. rank:	-98.6

PB1			
Pos.	437	obs:	exp:
tgg	W	1094	1094.00
mPD		0	0
		nPD:	1.
		N. weight:	0.
		Sc. PD:	0
		Sc. rank:	0

PB1			
Pos.	438	obs:	exp:
tgg	W	1094	1094.00
mPD		0	0
		nPD:	1.
		N. weight:	0.
		Sc. PD:	0
		Sc. rank:	0

PB1			
Pos.	439	obs:	exp:
gat	D	180	591.20
gac	D	914	502.80
mPD		0.28	0.50
		nPD:	0.55
		N. weight:	0.34
		Sc. PD:	0.15
		Sc. rank:	285.0

PB1			
Pos.	440	obs:	exp:
ggt	G	0	145.00
ggc	G	0	139.80
gga	G	1086	504.70
ggg	G	8	304.60
mPD		0.015	0.68
		nPD:	0.02
		N. weight:	0.81
		Sc. PD:	-0.049
		Sc. rank:	-190.2

PB1			
Pos.	441	obs:	exp:
tta	L	0	97.08
ttg	L	0	203.70
ctt	L	17	182.20
ctc	L	1077	182.60
cta	L	0	184.40
ctg	L	0	244.10
mPD		0.031	1.1
		nPD:	0.03
		N. weight:	1.9
		Sc. PD:	-0.1
		Sc. rank:	-202.4

PB1			
Pos.	442	obs:	exp:
caa	Q	1073	557.20
cag	Q	21	536.80
mPD		0.038	0.50
		nPD:	0.08
		N. weight:	0.64
		Sc. PD:	-0.0061
		Sc. rank:	174.2

PB1			
Pos.	443	obs:	exp:
tct	S	22	174.00
tcc	S	1070	142.90
tca	S	2	277.70
tcg	S	0	62.08
agt	S	0	224.90
agc	S	0	212.50
mPD		0.043	1.7
		nPD:	0.03
		N. weight:	2.1
		Sc. PD:	-0.12
		Sc. rank:	-300.7

PB1			
Pos.	444	obs:	exp:
tct	S	1	174.00
tcc	S	0	142.90
tca	S	1088	277.70
tcg	S	5	62.08
agt	S	0	224.90
agc	S	0	212.50
mPD		0.011	1.7
		nPD:	0.01
		N. weight:	1.5
		Sc. PD:	-0.11
		Sc. rank:	-866.3

PB1			
Pos.	445	obs:	exp:
gat	D	1067	591.20
gac	D	27	502.80
mPD		0.048	0.50
		nPD:	0.1
		N. weight:	0.56
		Sc. PD:	0.0062
		Sc. rank:	205.6

PB1			
Pos.	446	obs:	exp:
gat	D	11	591.20
gac	D	1083	502.80
mPD		0.020	0.50
		nPD:	0.04
		N. weight:	0.79
		Sc. PD:	-0.034
		Sc. rank:	12.1

PB1			
Pos.	447	obs:	exp:
ttt	F	11	432.00
ttc	F	1083	662.00
mPD		0.020	0.48
		nPD:	0.04
		N. weight:	0.5
		Sc. PD:	-0.021
		Sc. rank:	10.4

PB1			
Pos.	448	obs:	exp:
gct	A	13	282.50
gcc	A	1058	203.70
gca	A	21	514.90
gcg	A	2	92.85
mPD		0.064	0.67
		nPD:	0.1
		N. weight:	1.6
		Sc. PD:	0.017
		Sc. rank:	594.1

PB1			
Pos.	449	obs:	exp:
tta	L	0	97.08
ttg	L	0	203.70
ctt	L	0	182.20
ctc	L	1090	182.60
cta	L	4	184.40
ctg	L	0	244.10
mPD		0.0073	1.1
		nPD:	0.01
		N. weight:	1.9
		Sc. PD:	-0.15
		Sc. rank:	-1175.9

PB1			
Pos.	450	obs:	exp:
att	I	0	402.00
atc	I	1	262.20
ata	I	1093	429.90
mPD		0.0018	0.65
		nPD:	0.
		N. weight:	1.
		Sc. PD:	-0.081
		Sc. rank:	-736.0

PB1			
Pos.	451	obs:	exp:
gtt	V	7	226.70
gtc	V	0	220.80
gta	V	5	224.00
gtg	V	1082	422.50
mPD		0.022	0.73
		nPD:	0.03
		N. weight:	0.98
		Sc. PD:	-0.052
		Sc. rank:	-65.2

PB1			
Pos.	452	obs:	exp:
aat	N	1094	557.20
aac	N	0	536.80
mPD		0	0.50
		nPD:	0.
		N. weight:	0.74
		Sc. PD:	-0.061
		Sc. rank:	-636.5

PB1			
Pos.	453	obs:	exp:
gct	A	0	282.50
gcc	A	0	203.70
gca	A	1089	514.90
gcg	A	5	92.85
mPD		0.0091	0.67
		nPD:	0.01
		N. weight:	0.81
		Sc. PD:	-0.055
		Sc. rank:	-321.0

PB1			
Pos.	454	obs:	exp:
tta	L	0	0.27
ttg	L	0	0.56
tct	S	0	0.64
tcc	S	0	0.52
tca	S	0	1.02
tcg	S	4	0.23
ctt	L	0	0.50
ctc	L	0	0.50
cta	L	0	0.51
ctg	L	3	0.67
cct	P	0	270.70
ccc	P	0	194.90
cca	P	228	446.60
ccg	P	857	172.70
caa	Q	0	0.51
cag	Q	1	0.49
agt	S	0	0.82
agc	S	0	0.78
gct	A	0	0.26
gcc	A	0	0.19
gca	A	0	0.47
gcg	A	1	0.08
mPD		0.35	0.73
		nPD:	0.47
		N. weight:	1.2
		Sc. PD:	0.46
		Sc. rank:	1046.2

PB1			
Pos.	455	obs:	exp:
tct	S	0	0.48
tcc	S	0	0.39
tca	S	0	0.76
tcg	S	0	0.17
aat	N	1080	555.20
aac	N	10	534.80
agt	S	3	0.62
agc	S	0	0.58
gat	D	1	0.54
gac	D	0	0.46
mPD		0.025	0.51
		nPD:	0.05
		N. weight:	0.69
		Sc. PD:	-0.023
		Sc. rank:	69.7

PB1			
Pos.	456	obs:	exp:
cat	H	1091	628.20
cac	H	3	465.80
---	--	------	------
mPD		0.0055	0.49
		nPD:	0.01
		N. weight:	0.59
		Sc. PD:	-0.042
		Sc. rank:	-264.6

,

PB1			
Pos.	457	obs:	exp:
gat	D	1	0.54
gac	D	0	0.46
gaa	E	8	665.10
gag	E	1085	427.90
---	--	------	------
mPD		0.016	0.48
		nPD:	0.03
		N. weight:	0.98
		Sc. PD:	-0.048
		Sc. rank:	-30.8

,

PB1			
Pos.	458	obs:	exp:
ggt	G	1	145.00
ggc	G	0	139.80
gga	G	1076	504.70
ggg	G	17	304.60
---	--	------	------
mPD		0.032	0.68
		nPD:	0.05
		N. weight:	0.77
		Sc. PD:	-0.027
		Sc. rank:	69.4

,

PB1			
Pos.	459	obs:	exp:
att	I	0	402.00
atc	I	2	262.20
ata	I	1092	429.90
---	--	------	------
mPD		0.0037	0.65
		nPD:	0.01
		N. weight:	1.
		Sc. PD:	-0.077
		Sc. rank:	-635.1

,

PB1			
Pos.	460	obs:	exp:
caa	Q	71	557.20
cag	Q	1023	536.80
---	--	------	------
mPD		0.12	0.50
		nPD:	0.24
		N. weight:	0.52
		Sc. PD:	0.078
		Sc. rank:	365.1

,

PB1			
Pos.	461	obs:	exp:
gct	A	0	282.50
gcc	A	0	203.70
gca	A	1094	514.90
gcg	A	0	92.85
---	--	------	------
mPD		0	0.67
		nPD:	0.
		N. weight:	0.83
		Sc. PD:	-0.068
		Sc. rank:	-711.0

,

PB1			
Pos.	462	obs:	exp:
ggt	G	0	145.00
ggc	G	0	139.80
gga	G	1088	504.70
ggg	G	6	304.60
---	--	------	------
mPD		0.011	0.68
		nPD:	0.02
		N. weight:	0.82
		Sc. PD:	-0.054
		Sc. rank:	-278.6

,

PB1			
Pos.	463	obs:	exp:
gtt	V	69	226.70
gtc	V	0	220.80
gta	V	757	224.00
gtg	V	268	422.50
---	--	------	------
mPD		0.46	0.73
		nPD:	0.63
		N. weight:	0.73
		Sc. PD:	0.38
		Sc. rank:	619.5

,

PB1			
Pos.	464	obs:	exp:
aat	N	0	0.51
aac	N	1	0.49
gat	D	968	590.60
gac	D	125	502.40
---	--	------	------
mPD		0.21	0.50
		nPD:	0.41
		N. weight:	0.31
		Sc. PD:	0.097
		Sc. rank:	252.7

,

PB1			
Pos.	465	obs:	exp:
cgt	R	0	46.99
cgc	R	0	54.19
cga	R	0	107.10
cgg	R	0	91.28
aga	R	2	500.20
agg	R	1092	294.20
---	--	------	------
mPD		0.0037	0.96
		nPD:	0.
		N. weight:	1.4
		Sc. PD:	-0.11
		Sc. rank:	-993.9

,

PB1			
Pos.	466	obs:	exp:
ttt	F	10	432.00
ttc	F	1084	662.00
---	--	------	------
mPD		0.018	0.48
		nPD:	0.04
		N. weight:	0.5
		Sc. PD:	-0.023
		Sc. rank:	1.3

,

PB1			
Pos.	467	obs:	exp:
tat	Y	1021	590.90
tac	Y	73	503.10
---	--	------	------
mPD		0.12	0.50
		nPD:	0.25
		N. weight:	0.42
		Sc. PD:	0.067
		Sc. rank:	302.5

,

PB1			
Pos.	468	obs:	exp:
cgt	R	0	46.99
cgc	R	0	54.19
cga	R	2	107.10
cgg	R	0	91.28
aga	R	1082	500.20
agg	R	10	294.20
---	--	------	------
mPD		0.022	0.96
		nPD:	0.02
		N. weight:	0.8
		Sc. PD:	-0.048
		Sc. rank:	-148.2

,

PB1			
Pos.	469	obs:	exp:
act	T	2	278.90
acc	T	1092	224.50
aca	T	0	501.80
acg	T	0	88.86
---	--	------	------
mPD		0.0037	0.68
		nPD:	0.01
		N. weight:	1.7
		Sc. PD:	-0.13
		Sc. rank:	-1121.0

,

PB1			
Pos.	470	obs:	exp:
tgt	C	3	462.70
tgc	C	1091	631.30
---	--	------	------
mPD		0.0055	0.49
		nPD:	0.01
		N. weight:	0.59
		Sc. PD:	-0.042
		Sc. rank:	-261.4

,

PB1			
Pos.	471	obs:	exp:
aaa	K	1093	637.60
aag	K	1	456.40
---	--	------	------
mPD		0.0018	0.49
		nPD:	0.
		N. weight:	0.59
		Sc. PD:	-0.046
		Sc. rank:	-410.6

,

PB1			
Pos.	472	obs:	exp:
tta	L	956	96.99
ttg	L	53	203.50
ctt	L	0	182.00
ctc	L	0	182.40
cta	L	80	184.20
ctg	L	4	243.90
gtt	V	0	0.21
gtc	V	0	0.20
gta	V	1	0.20
gtg	V	0	0.39
---	--	------	------
mPD		0.24	1.1
		nPD:	0.22
		N. weight:	2.
		Sc. PD:	0.26
		Sc. rank:	1374.1

,

PB1			
Pos.	473	obs:	exp:
att	I	1	0.37
atc	I	0	0.24
ata	I	0	0.39
gtt	V	1078	226.50
gtc	V	8	220.60
gta	V	4	223.80
gtg	V	3	422.10
---	--	------	------
mPD		0.029	0.73
		nPD:	0.04
		N. weight:	1.6
		Sc. PD:	-0.071
		Sc. rank:	19.0

,

PB1			
Pos.	474	obs:	exp:
ggt	G	0	145.00
ggc	G	0	139.80
gga	G	7	504.70
ggg	G	1087	304.60
---	--	------	------
mPD		0.013	0.68
		nPD:	0.02
		N. weight:	1.4
		Sc. PD:	-0.087
		Sc. rank:	-363.9

,

PB1			
Pos.	475	obs:	exp:
att	I	0	402.00
atc	I	1	262.20
ata	I	1093	429.90
---	--	------	------
mPD		0.0018	0.65
		nPD:	0.
		N. weight:	1.
		Sc. PD:	-0.081
		Sc. rank:	-736.0

,

PB1			
Pos.	476	obs:	exp:
aat	N	60	557.20
aac	N	1034	536.80
---	--	------	------
mPD		0.10	0.50
		nPD:	0.21
		N. weight:	0.55
		Sc. PD:	0.064
		Sc. rank:	358.2

,

PB1			
Pos.	477	obs:	exp:
atg	M	1094	1094.00
---	--	------	------
mPD		0	0
		nPD:	1.
		N. weight:	0.
		Sc. PD:	0
		Sc. rank:	0

,

PB1			
Pos.	478	obs:	exp:
tct	S	0	174.00
tcc	S	0	142.90
tca	S	0	277.70
tcg	S	0	62.08
agt	S	4	224.90
agc	S	1090	212.50
---	--	------	------
mPD		0.0073	1.7
		nPD:	0.
		N. weight:	1.8
		Sc. PD:	-0.14
		Sc. rank:	-1202.9

,

PB1			
Pos.	479	obs:	exp:
aaa	K	3	637.60
aag	K	1091	456.40
---	--	------	------
mPD		0.0055	0.49
		nPD:	0.01
		N. weight:	0.94
		Sc. PD:	-0.067
		Sc. rank:	-416.8

,

PB1			
Pos.	480	obs:	exp:
aaa	K	2	637.60
aag	K	1092	456.40
---	--	------	------
mPD		0.0037	0.49
		nPD:	0.01
		N. weight:	0.95
		Sc. PD:	-0.07
		Sc. rank:	-540.2

,

PB1			
Pos.	481	obs:	exp:
aaa	K	1090	637.60
aag	K	4	456.40
---	--	------	------
mPD		0.0073	0.49
		nPD:	0.01
		N. weight:	0.57
		Sc. PD:	-0.038
		Sc. rank:	-209.5

,

PB1			
Pos.	482	obs:	exp:
tct	S	16	174.00
tcc	S	1076	142.90
tca	S	2	277.70
tcg	S	0	62.08
agt	S	0	224.90
agc	S	0	212.50
---	--	------	------
mPD		0.032	1.7
		nPD:	0.02
		N. weight:	2.1
		Sc. PD:	-0.13
		Sc. rank:	-538.3

,

PB1			
Pos.	483	obs:	exp:
tat	Y	22	590.90
tac	Y	1072	503.10
---	--	------	------
mPD		0.039	0.50
		nPD:	0.08
		N. weight:	0.74
		Sc. PD:	-0.0042
		Sc. rank:	222.4

,

PB1			
Pos.	484	obs:	exp:
att	I	0	402.00
atc	I	0	262.20
ata	I	1094	429.90
---	--	------	------
mPD		0	0.65
		nPD:	0.
		N. weight:	1.
		Sc. PD:	-0.084
		Sc. rank:	-881.3

,

PB1			
Pos.	485	obs:	exp:
aat	N	1044	557.20
aac	N	50	536.80
---	--	------	------
mPD		0.087	0.50
		nPD:	0.17
		N. weight:	0.54
		Sc. PD:	0.046
		Sc. rank:	322.9

,

PB1			
Pos.	486	obs:	exp:
caa	Q	0	0.51
cag	Q	1	0.49
cgt	R	0	46.94
cgc	R	0	54.14
cga	R	46	107.00
cgg	R	1013	91.20
aga	R	2	499.80
agg	R	32	294.00
---	--	------	------
mPD		0.15	0.96
		nPD:	0.15
		N. weight:	2.3
		Sc. PD:	0.15
		Sc. rank:	1283.9

,

PB1			
Pos.	487	obs:	exp:
act	T	7	278.90
acc	T	1	224.50
aca	T	1084	501.80
acg	T	2	88.86
---	--	------	------
mPD		0.018	0.68
		nPD:	0.03
		N. weight:	0.8
		Sc. PD:	-0.045
		Sc. rank:	-95.8

,

PB1			
Pos.	488	obs:	exp:
ggt	G	0	145.00
ggc	G	0	139.80
gga	G	1086	504.70
ggg	G	8	304.60
---	--	------	------
mPD		0.015	0.68
		nPD:	0.02
		N. weight:	0.81
		Sc. PD:	-0.049
		Sc. rank:	-190.2

,

PB1			
Pos.	489	obs:	exp:
act	T	4	278.90
acc	T	2	224.50
aca	T	1065	501.80
acg	T	23	88.86
---	--	------	------
mPD		0.052	0.68
		nPD:	0.08
		N. weight:	0.75
		Sc. PD:	-0.0061
		Sc. rank:	214.4

,

PB1			
Pos.	490	obs:	exp:
ttt	F	24	432.00
ttc	F	1070	662.00
---	--	------	------
mPD		0.043	0.48
		nPD:	0.09
		N. weight:	0.45
		Sc. PD:	0.002
		Sc. rank:	151.5

,

PB1			
Pos.	491	obs:	exp:
gaa	E	1093	665.70
gag	E	1	428.30
---	--	------	------
mPD		0.0018	0.48
		nPD:	0.
		N. weight:	0.54
		Sc. PD:	-0.042
		Sc. rank:	-368.3

,

PB1			
Pos.	492	obs:	exp:
ttt	F	2	432.00
ttc	F	1092	662.00
---	--	------	------
mPD		0.0037	0.48
		nPD:	0.01
		N. weight:	0.54
		Sc. PD:	-0.04
		Sc. rank:	-290.8

,

PB1			
Pos.	493	obs:	exp:
act	T	0	278.90
acc	T	1	224.50
aca	T	1090	501.80
acg	T	3	88.86
---	--	------	------
mPD		0.0073	0.68
		nPD:	0.01
		N. weight:	0.84
		Sc. PD:	-0.06
		Sc. rank:	-396.0

,

PB1			
Pos.	494	obs:	exp:
tct	S	0	174.00
tcc	S	0	142.90
tca	S	0	277.70
tcg	S	0	62.08
agt	S	6	224.90
agc	S	1088	212.50
---	--	------	------
mPD		0.011	1.7
		nPD:	0.01
		N. weight:	1.8
		Sc. PD:	-0.13
		Sc. rank:	-1054.0

,

PB1			
Pos.	495	obs:	exp:
ttt	F	1086	432.00
ttc	F	8	662.00
---	--	------	------
mPD		0.015	0.48
		nPD:	0.03
		N. weight:	0.97
		Sc. PD:	-0.051
		Sc. rank:	-59.1

,

PB1			
Pos.	496	obs:	exp:
ttt	F	6	432.00
ttc	F	1088	662.00
---	--	------	------
mPD		0.011	0.48
		nPD:	0.02
		N. weight:	0.52
		Sc. PD:	-0.031
		Sc. rank:	-95.6

,

PB1			
Pos.	497	obs:	exp:
tat	Y	12	590.90
tac	Y	1082	503.10
---	--	------	------
mPD		0.022	0.50
		nPD:	0.04
		N. weight:	0.79
		Sc. PD:	-0.031
		Sc. rank:	36.0

,

PB1			
Pos.	498	obs:	exp:
cgt	R	80	46.99
cgc	R	1014	54.19
cga	R	0	107.10
cgg	R	0	91.28
aga	R	0	500.20
agg	R	0	294.20
---	--	------	------
mPD		0.14	0.96
		nPD:	0.14
		N. weight:	3.
		Sc. PD:	0.16
		Sc. rank:	1608.4

,

PB1			
Pos.	499	obs:	exp:
tat	Y	1075	590.90
tac	Y	19	503.10
---	--	------	------
mPD		0.034	0.50
		nPD:	0.07
		N. weight:	0.59
		Sc. PD:	-0.0093
		Sc. rank:	133.0

,

PB1			
Pos.	500	obs:	exp:
ggt	G	1	145.00
ggc	G	0	139.80
gga	G	898	504.70
ggg	G	195	304.60
---	--	------	------
mPD		0.29	0.68
		nPD:	0.44
		N. weight:	0.43
		Sc. PD:	0.15
		Sc. rank:	356.5

,

PB1			
Pos.	501	obs:	exp:
ttt	F	84	432.00
ttc	F	1010	662.00
---	--	------	------
mPD		0.14	0.48
		nPD:	0.3
		N. weight:	0.29
		Sc. PD:	0.059
		Sc. rank:	222.4

,

PB1			
Pos.	502	obs:	exp:
gtt	V	0	226.70
gtc	V	0	220.80
gta	V	1081	224.00
gtg	V	13	422.50
---	--	------	------
mPD		0.024	0.73
		nPD:	0.03
		N. weight:	1.7
		Sc. PD:	-0.084
		Sc. rank:	-68.0

,

PB1			
Pos.	503	obs:	exp:
gct	A	982	282.50
gcc	A	111	203.70
gca	A	1	514.90
gcg	A	0	92.85
---	--	------	------
mPD		0.18	0.67
		nPD:	0.27
		N. weight:	1.2
		Sc. PD:	0.21
		Sc. rank:	858.4

,

PB1			
Pos.	504	obs:	exp:
aat	N	116	557.20
aac	N	978	536.80
---	--	------	------
mPD		0.19	0.50
		nPD:	0.38
		N. weight:	0.41
		Sc. PD:	0.12
		Sc. rank:	328.8

,

PB1			
Pos.	505	obs:	exp:
ttt	F	2	432.00
ttc	F	1092	662.00
---	--	------	------
mPD		0.0037	0.48
		nPD:	0.01
		N. weight:	0.54
		Sc. PD:	-0.04
		Sc. rank:	-290.8

,

PB1			
Pos.	506	obs:	exp:
tct	S	0	174.00
tcc	S	0	142.90
tca	S	0	277.70
tcg	S	0	62.08
agt	S	1064	224.90
agc	S	30	212.50
---	--	------	------
mPD		0.053	1.7
		nPD:	0.03
		N. weight:	1.6
		Sc. PD:	-0.081
		Sc. rank:	-74.4

,

PB1			
Pos.	507	obs:	exp:
atg	M	1094	1094.00
---	--	------	------
mPD		0	0
		nPD:	1.
		N. weight:	0.
		Sc. PD:	0
		Sc. rank:	0

,

PB1			
Pos.	508	obs:	exp:
gaa	E	35	665.70
gag	E	1059	428.30
---	--	------	------
mPD		0.062	0.48
		nPD:	0.13
		N. weight:	0.86
		Sc. PD:	0.037
		Sc. rank:	422.8

,

PB1			
Pos.	509	obs:	exp:
tta	L	123	97.08
ttg	L	943	203.70
ctt	L	0	182.20
ctc	L	0	182.60
cta	L	1	184.40
ctg	L	27	244.10
---	--	------	------
mPD		0.25	1.1
		nPD:	0.23
		N. weight:	1.4
		Sc. PD:	0.19
		Sc. rank:	982.4

,

PB1			
Pos.	510	obs:	exp:
cct	P	31	273.00
ccc	P	1041	196.60
cca	P	20	450.30
ccg	P	2	174.20
---	--	------	------
mPD		0.093	0.71
		nPD:	0.13
		N. weight:	1.6
		Sc. PD:	0.071
		Sc. rank:	795.6

,

PB1			
Pos.	511	obs:	exp:
tct	S	0	174.00
tcc	S	0	142.90
tca	S	0	277.70
tcg	S	0	62.08
agt	S	1088	224.90
agc	S	6	212.50
---	--	------	------
mPD		0.011	1.7
		nPD:	0.01
		N. weight:	1.7
		Sc. PD:	-0.13
		Sc. rank:	-1017.1

,

PB1			
Pos.	512	obs:	exp:
ttt	F	1090	432.00
ttc	F	4	662.00
---	--	------	------
mPD		0.0073	0.48
		nPD:	0.02
		N. weight:	0.99
		Sc. PD:	-0.067
		Sc. rank:	-358.1

,

PB1			
Pos.	513	obs:	exp:
gaa	E	1	0.61
gag	E	0	0.39
ggt	G	2	144.90
ggc	G	0	139.60
gga	G	1084	504.20
ggg	G	7	304.30
---	--	------	------
mPD		0.018	0.68
		nPD:	0.03
		N. weight:	0.8
		Sc. PD:	-0.045
		Sc. rank:	-101.1

,

PB1			
Pos.	514	obs:	exp:
gtt	V	3	226.70
gtc	V	0	220.80
gta	V	6	224.00
gtg	V	1085	422.50
---	--	------	------
mPD		0.016	0.73
		nPD:	0.02
		N. weight:	1.
		Sc. PD:	-0.06
		Sc. rank:	-193.3

,

PB1			
Pos.	515	obs:	exp:
tct	S	165	171.80
tcc	S	914	141.00
tca	S	1	274.10
tcg	S	0	61.28
cct	P	0	3.49
ccc	P	14	2.52
cca	P	0	5.76
ccg	P	0	2.23
agt	S	0	222.00
agc	S	0	209.80
---	--	------	------
mPD		0.28	1.7
		nPD:	0.17
		N. weight:	1.7
		Sc. PD:	0.14
		Sc. rank:	1007.0

,

PB1

Pos.	516	obs:	exp:
ggt	G	2	145.00
ggc	G	0	139.80
gga	G	86	504.70
ggg	G	1006	304.60
---	--	------	------
mPD		0.15	0.68
		nPD:	0.22
		N. weight:	1.
		Sc. PD:	0.13
		Sc. rank:	711.3

,

PB1

Pos.	517	obs:	exp:
att	I	999	400.50
atc	I	29	261.20
ata	I	62	428.30
gtt	V	4	0.83
gtc	V	0	0.81
gta	V	0	0.82
gtg	V	0	1.54
---	--	------	------
mPD		0.16	0.66
		nPD:	0.25
		N. weight:	0.74
		Sc. PD:	0.11
		Sc. rank:	530.0

,

PB1

Pos.	518	obs:	exp:
aat	N	1072	557.20
aac	N	22	536.80
---	--	------	------
mPD		0.039	0.50
		nPD:	0.08
		N. weight:	0.64
		Sc. PD:	-0.0039
		Sc. rank:	188.0

,

PB1

Pos.	519	obs:	exp:
gaa	E	59	665.70
gag	E	1035	428.30
---	--	------	------
mPD		0.10	0.48
		nPD:	0.21
		N. weight:	0.78
		Sc. PD:	0.096
		Sc. rank:	516.6

,

PB1

Pos.	520	obs:	exp:
tct	S	1	174.00
tcc	S	0	142.90
tca	S	1088	277.70
tcg	S	5	62.08
agt	S	0	224.90
agc	S	0	212.50
---	--	------	------
mPD		0.011	1.7
		nPD:	0.01
		N. weight:	1.5
		Sc. PD:	-0.11
		Sc. rank:	-866.3

,

PB1

Pos.	521	obs:	exp:
gct	A	1071	282.50
gcc	A	3	203.70
gca	A	20	514.90
gcg	A	0	92.85
---	--	------	------
mPD		0.041	0.67
		nPD:	0.06
		N. weight:	1.4
		Sc. PD:	-0.031
		Sc. rank:	243.8

,

PB1

Pos.	522	obs:	exp:
gat	D	4	591.20
gac	D	1090	502.80
---	--	------	------
mPD		0.0073	0.50
		nPD:	0.01
		N. weight:	0.83
		Sc. PD:	-0.056
		Sc. rank:	-314.1

,

PB1

Pos.	523	obs:	exp:
att	I	0	1.10
atc	I	0	0.72
ata	I	3	1.18
atg	M	1091	1091.00
---	--	------	------
mPD		0.0055	0.0055
		nPD:	1.
		N. weight:	0.0028
		Sc. PD:	0.0025
		Sc. rank:	2.4

,

PB1

Pos.	524	obs:	exp:
tct	S	0	174.00
tcc	S	0	142.90
tca	S	0	277.70
tcg	S	0	62.08
agt	S	9	224.90
agc	S	1085	212.50
---	--	------	------
mPD		0.016	1.7
		nPD:	0.01
		N. weight:	1.7
		Sc. PD:	-0.13
		Sc. rank:	-850.5

,

PB1

Pos.	525	obs:	exp:
att	I	270	111.70
atc	I	34	72.85
ata	I	0	119.50
gtt	V	790	163.70
gtc	V	0	159.40
gta	V	0	161.80
gtg	V	0	305.10
---	--	------	------
mPD		0.46	1.1
		nPD:	0.4
		N. weight:	1.5
		Sc. PD:	0.44
		Sc. rank:	1190.9

,

PB1

Pos.	526	obs:	exp:
ggt	G	1086	145.00
ggc	G	8	139.80
gga	G	0	504.70
ggg	G	0	304.60
---	--	------	------
mPD		0.015	0.68
		nPD:	0.02
		N. weight:	2.2
		Sc. PD:	-0.13
		Sc. rank:	-511.8

,

PB1

Pos.	527	obs:	exp:
gtt	V	1087	226.70
gtc	V	7	220.80
gta	V	0	224.00
gtg	V	0	422.50
---	--	------	------
mPD		0.013	0.73
		nPD:	0.02
		N. weight:	1.7
		Sc. PD:	-0.11
		Sc. rank:	-492.2

,

PB1

Pos.	528	obs:	exp:
act	T	0	278.60
acc	T	0	224.30
aca	T	1089	501.30
acg	T	4	88.78
gct	A	0	0.26
gcc	A	0	0.19
gca	A	1	0.47
gcg	A	0	0.08
---	--	------	------
mPD		0.0091	0.68
		nPD:	0.01
		N. weight:	0.84
		Sc. PD:	-0.058
		Sc. rank:	-335.3

,

PB1

Pos.	529	obs:	exp:
gtt	V	0	226.70
gtc	V	0	220.80
gta	V	1085	224.00
gtg	V	9	422.50
---	--	------	------
mPD		0.016	0.73
		nPD:	0.02
		N. weight:	1.7
		Sc. PD:	-0.1
		Sc. rank:	-337.7

,

PB1

Pos.	530	obs:	exp:
att	I	0	402.00
atc	I	0	262.20
ata	I	1094	429.90
---	--	------	------
mPD		0	0.65
		nPD:	0.
		N. weight:	1.
		Sc. PD:	-0.084
		Sc. rank:	-881.3

,

PB1

Pos.	531	obs:	exp:
aaa	K	256	637.60
aag	K	838	456.40
---	--	------	------
mPD		0.36	0.49
		nPD:	0.74
		N. weight:	0.28
		Sc. PD:	0.18
		Sc. rank:	241.7

,

PB1

Pos.	532	obs:	exp:
aat	N	5	557.20
aac	N	1089	536.80
---	--	------	------
mPD		0.0091	0.50
		nPD:	0.02
		N. weight:	0.75
		Sc. PD:	-0.048
		Sc. rank:	-212.2

,

PB1

Pos.	533	obs:	exp:
tct	S	0	0.16
tcc	S	0	0.13
tca	S	0	0.25
tcg	S	0	0.06
aat	N	1055	556.70
aac	N	38	536.30
agt	S	1	0.21
agc	S	0	0.19
---	--	------	------
mPD		0.069	0.50
		nPD:	0.14
		N. weight:	0.58
		Sc. PD:	0.029
		Sc. rank:	294.5

,

PB1

Pos.	534	obs:	exp:
atg	M	1094	1094.00
---	--	------	------
mPD		0	0
		nPD:	1.
		N. weight:	0.
		Sc. PD:	0
		Sc. rank:	0

,

PB1

Pos.	535	obs:	exp:
att	I	1	399.00
atc	I	0	260.20
ata	I	1085	426.70
act	T	0	0.25
acc	T	0	0.21
aca	T	1	0.46
acg	T	0	0.08
gtt	V	0	1.45
gtc	V	0	1.41
gta	V	7	1.43
gtg	V	0	2.70
---	--	------	------
mPD		0.016	0.67
		nPD:	0.02
		N. weight:	1.
		Sc. PD:	-0.06
		Sc. rank:	-171.1

,

PB1

Pos.	536	obs:	exp:
aat	N	24	557.20
aac	N	1070	536.80
---	--	------	------
mPD		0.043	0.50
		nPD:	0.09
		N. weight:	0.67
		Sc. PD:	0.0004
		Sc. rank:	219.1

,

PB1

Pos.	537	obs:	exp:
tct	S	0	0.48
tcc	S	0	0.39
tca	S	0	0.76
tcg	S	0	0.17
aat	N	140	555.70
aac	N	951	535.30
agt	S	0	0.62
agc	S	3	0.58
---	--	------	------
mPD		0.23	0.51
		nPD:	0.45
		N. weight:	0.36
		Sc. PD:	0.13
		Sc. rank:	301.9

,

PB1

Pos.	538	obs:	exp:
gat	D	1087	590.60
gac	D	6	502.40
ggt	G	1	0.13
ggc	G	0	0.13
gga	G	0	0.46
ggg	G	0	0.28
---	--	------	------
mPD		0.013	0.50
		nPD:	0.03
		N. weight:	0.64
		Sc. PD:	-0.037
		Sc. rank:	-97.0

,

PB1

Pos.	539	obs:	exp:
tta	L	0	97.08
ttg	L	0	203.70
ctt	L	1090	182.20
ctc	L	0	182.60
cta	L	4	184.40
ctg	L	0	244.10
---	--	------	------
mPD		0.0073	1.1
		nPD:	0.01
		N. weight:	1.9
		Sc. PD:	-0.15
		Sc. rank:	-1177.4

,

PB1

Pos.	540	obs:	exp:
ggt	G	0	145.00
ggc	G	7	139.80
gga	G	1083	504.70
ggg	G	4	304.60
---	--	------	------
mPD		0.020	0.68
		nPD:	0.03
		N. weight:	0.79
		Sc. PD:	-0.042
		Sc. rank:	-59.8

,

PB1			
Pos.	541	obs:	exp:
cct	P	4	273.00
ccc	P	0	196.60
cca	P	1090	450.30
ccg	P	0	174.20
---	--	------	------
mPD		0.0073	0.71
		nPD:	0.01
		N. weight:	0.95
		Sc. PD:	-0.068
		Sc. rank:	-458.7

,

PB1			
Pos.	542	obs:	exp:
gct	A	0	282.50
gcc	A	0	203.70
gca	A	1088	514.90
gcg	A	6	92.85
---	--	------	------
mPD		0.011	0.67
		nPD:	0.02
		N. weight:	0.8
		Sc. PD:	-0.053
		Sc. rank:	-263.1

,

PB1			
Pos.	543	obs:	exp:
act	T	0	278.90
acc	T	0	224.50
aca	T	1091	501.80
acg	T	3	88.86
---	--	------	------
mPD		0.0055	0.68
		nPD:	0.01
		N. weight:	0.84
		Sc. PD:	-0.062
		Sc. rank:	-439.4

,

PB1			
Pos.	544	obs:	exp:
act	T	0	0.25
acc	T	1	0.21
aca	T	0	0.46
acg	T	0	0.08
gct	A	348	282.20
gcc	A	728	203.50
gca	A	17	514.40
gcg	A	0	92.77
---	--	------	------
mPD		0.46	0.67
		nPD:	0.68
		N. weight:	0.95
		Sc. PD:	0.54
		Sc. rank:	818.0

,

PB1			
Pos.	545	obs:	exp:
caa	Q	875	557.20
cag	Q	219	536.80
---	--	------	------
mPD		0.32	0.50
		nPD:	0.64
		N. weight:	0.2
		Sc. PD:	0.11
		Sc. rank:	173.0

,

PB1			
Pos.	546	obs:	exp:
atg	M	1094	1094.00
---	--	------	------
mPD		0	0
		nPD:	1.
		N. weight:	0.
		Sc. PD:	0
		Sc. rank:	0

,

PB1			
Pos.	547	obs:	exp:
gct	A	4	282.50
gcc	A	1090	203.70
gca	A	0	514.90
gcg	A	0	92.85
---	--	------	------
mPD		0.0073	0.67
		nPD:	0.01
		N. weight:	1.8
		Sc. PD:	-0.13
		Sc. rank:	-846.6

,

PB1			
Pos.	548	obs:	exp:
tta	L	0	97.08
ttg	L	0	203.70
ctt	L	1068	182.20
ctc	L	9	182.60
cta	L	17	184.40
ctg	L	0	244.10
---	--	------	------
mPD		0.047	1.1
		nPD:	0.04
		N. weight:	1.8
		Sc. PD:	-0.076
		Sc. rank:	49.0

,

PB1			
Pos.	549	obs:	exp:
caa	Q	7	557.20
cag	Q	1087	536.80
---	--	------	------
mPD		0.013	0.50
		nPD:	0.03
		N. weight:	0.74
		Sc. PD:	-0.042
		Sc. rank:	-114.0

,

PB1			
Pos.	550	obs:	exp:
tta	L	0	97.08
ttg	L	0	203.70
ctt	L	1	182.20
ctc	L	39	182.60
cta	L	1041	184.40
ctg	L	13	244.10
---	--	------	------
mPD		0.093	1.1
		nPD:	0.08
		N. weight:	1.7
		Sc. PD:	-0.0021
		Sc. rank:	544.7

,

PB1			
Pos.	551	obs:	exp:
ttt	F	1089	432.00
ttc	F	5	662.00
---	--	------	------
mPD		0.0091	0.48
		nPD:	0.02
		N. weight:	0.99
		Sc. PD:	-0.063
		Sc. rank:	-256.8

,

PB1			
Pos.	552	obs:	exp:
att	I	0	401.20
atc	I	1091	261.70
ata	I	1	429.10
gtt	V	0	0.41
gtc	V	2	0.40
gta	V	0	0.41
gtg	V	0	0.77
---	--	------	------
mPD		0.0055	0.66
		nPD:	0.01
		N. weight:	1.6
		Sc. PD:	-0.12
		Sc. rank:	-771.8

,

PB1			
Pos.	553	obs:	exp:
aaa	K	1084	637.60
aag	K	10	456.40
---	--	------	------
mPD		0.018	0.49
		nPD:	0.04
		N. weight:	0.54
		Sc. PD:	-0.025
		Sc. rank:	-1.2

,

PB1			
Pos.	554	obs:	exp:
aat	N	0	1.02
aac	N	2	0.98
gat	D	4	590.10
gac	D	1088	501.90
---	--	------	------
mPD		0.011	0.50
		nPD:	0.02
		N. weight:	0.83
		Sc. PD:	-0.05
		Sc. rank:	-177.0

,

PB1			
Pos.	555	obs:	exp:
ttt	F	0	0.39
ttc	F	1	0.61
tat	Y	15	590.30
tac	Y	1078	502.70
---	--	------	------
mPD		0.029	0.50
		nPD:	0.06
		N. weight:	0.77
		Sc. PD:	-0.02
		Sc. rank:	123.8

,

PB1			
Pos.	556	obs:	exp:
cgt	R	0	46.99
cgc	R	0	54.19
cga	R	0	107.10
cgg	R	0	91.28
aga	R	1085	500.20
agg	R	9	294.20
---	--	------	------
mPD		0.016	0.96
		nPD:	0.02
		N. weight:	0.81
		Sc. PD:	-0.053
		Sc. rank:	-245.1

,

PB1			
Pos.	557	obs:	exp:
tat	Y	0	590.90
tac	Y	1094	503.10
---	--	------	------
mPD		0	0.50
		nPD:	0.
		N. weight:	0.85
		Sc. PD:	-0.07
		Sc. rank:	-732.8

,

PB1			
Pos.	558	obs:	exp:
act	T	0	278.90
acc	T	0	224.50
aca	T	1076	501.80
acg	T	18	88.86
---	--	------	------
mPD		0.032	0.68
		nPD:	0.05
		N. weight:	0.8
		Sc. PD:	-0.029
		Sc. rank:	70.8

,

PB1			
Pos.	559	obs:	exp:
tat	Y	23	590.90
tac	Y	1071	503.10
---	--	------	------
mPD		0.041	0.50
		nPD:	0.08
		N. weight:	0.74
		Sc. PD:	-0.0017
		Sc. rank:	232.7

,

PB1			
Pos.	560	obs:	exp:
cgt	R	0	46.99
cgc	R	0	54.19
cga	R	1081	107.10
cgg	R	10	91.28
aga	R	3	500.20
agg	R	0	294.20
---	--	------	------
mPD		0.024	0.96
		nPD:	0.02
		N. weight:	2.5
		Sc. PD:	-0.14
		Sc. rank:	-399.8

,

PB1			
Pos.	561	obs:	exp:
tgt	C	1086	462.70
tgc	C	8	631.30
---	--	------	------
mPD		0.015	0.49
		nPD:	0.03
		N. weight:	0.9
		Sc. PD:	-0.048
		Sc. rank:	-62.7

,

PB1			
Pos.	562	obs:	exp:
cat	H	11	628.20
cac	H	1083	465.80
---	--	------	------
mPD		0.020	0.49
		nPD:	0.04
		N. weight:	0.87
		Sc. PD:	-0.037
		Sc. rank:	15.1

,

PB1			
Pos.	563	obs:	exp:
cgt	R	0	46.94
cgc	R	0	54.14
cga	R	0	107.00
cgg	R	0	91.20
aaa	K	0	0.58
aag	K	1	0.42
aga	R	12	499.80
agg	R	1081	294.00
---	--	------	------
mPD		0.024	0.96
		nPD:	0.02
		N. weight:	1.4
		Sc. PD:	-0.08
		Sc. rank:	-232.4

,

PB1			
Pos.	564	obs:	exp:
tct	S	0	0.32
tcc	S	0	0.26
tca	S	0	0.51
tcg	S	0	0.11
agt	S	2	0.41
agc	S	0	0.39
ggt	G	1057	144.70
ggc	G	34	139.50
gga	G	0	503.80
ggg	G	1	304.00
---	--	------	------
mPD		0.066	0.68
		nPD:	0.1
		N. weight:	2.1
		Sc. PD:	0.022
		Sc. rank:	760.5

,

PB1			
Pos.	565	obs:	exp:
gat	D	1028	590.60
gac	D	65	502.40
ggt	G	1	0.13
ggc	G	0	0.13
gga	G	0	0.46
ggg	G	0	0.28
---	--	------	------
mPD		0.11	0.50
		nPD:	0.23
		N. weight:	0.44
		Sc. PD:	0.06
		Sc. rank:	306.9

,

PB1			
Pos.	566	obs:	exp:
atg	M	32	32.00
act	T	1	269.40
acc	T	0	216.90
aca	T	94	484.80
acg	T	962	85.86
gct	A	1	1.29
gcc	A	0	0.93
gca	A	0	2.35
gcg	A	4	0.42
---	--	------	------
mPD		0.23	0.76
		nPD:	0.3
		N. weight:	2.2
		Sc. PD:	0.45
		Sc. rank:	1676.2

,

PB1			
Pos.	567	obs:	exp:
cat	H	1	11.48
cac	H	19	8.52
caa	Q	1072	547.10
cag	Q	2	526.90
---	--	------	------
mPD		0.040	0.52
		nPD:	0.08
		N. weight:	0.73
		Sc. PD:	-0.0062
		Sc. rank:	204.1

,

PB1			
Pos.	568	obs:	exp:
att	I	1080	401.60
atc	I	13	261.90
ata	I	0	429.50
gtt	V	1	0.21
gtc	V	0	0.20
gta	V	0	0.20
gtg	V	0	0.39
---	--	------	------
mPD		0.025	0.66
		nPD:	0.04
		N. weight:	1.
		Sc. PD:	-0.046
		Sc. rank:	5.1

,

PB1			
Pos.	569	obs:	exp:
caa	Q	1088	556.20
cag	Q	4	535.80
aaa	K	2	1.17
aag	K	0	0.83
---	--	------	------
mPD		0.011	0.50
		nPD:	0.02
		N. weight:	0.72
		Sc. PD:	-0.044
		Sc. rank:	-156.8

,

PB1			
Pos.	570	obs:	exp:
act	T	3	278.90
acc	T	0	224.50
aca	T	40	501.80
acg	T	1051	88.86
---	--	------	------
mPD		0.076	0.68
		nPD:	0.11
		N. weight:	2.5
		Sc. PD:	0.064
		Sc. rank:	1075.0

,

PB1			
Pos.	571	obs:	exp:
cgt	R	0	46.73
cgc	R	0	53.89
cga	R	0	106.50
cgg	R	0	90.78
aaa	K	0	3.50
aag	K	6	2.50
aga	R	49	497.50
agg	R	1039	292.60
---	--	------	------
mPD		0.097	0.97
		nPD:	0.1
		N. weight:	1.2
		Sc. PD:	0.017
		Sc. rank:	464.2

,

PB1			
Pos.	572	obs:	exp:
cgt	R	0	46.99
cgc	R	0	54.19
cga	R	0	107.10
cgg	R	0	91.28
aga	R	1090	500.20
agg	R	4	294.20
---	--	------	------
mPD		0.0073	0.96
		nPD:	0.01
		N. weight:	0.84
		Sc. PD:	-0.062
		Sc. rank:	-455.5

,

PB1			
Pos.	573	obs:	exp:
tct	S	0	0.32
tcc	S	0	0.26
tca	S	2	0.51
tcg	S	0	0.11
agt	S	0	0.41
agc	S	0	0.39
gct	A	1	282.00
gcc	A	0	203.40
gca	A	1084	514.00
gcg	A	7	92.68
---	--	------	------
mPD		0.018	0.68
		nPD:	0.03
		N. weight:	0.79
		Sc. PD:	-0.044
		Sc. rank:	-93.3

,

PB1			
Pos.	574	obs:	exp:
ttt	F	34	432.00
ttc	F	1060	662.00
---	--	------	------
mPD		0.060	0.48
		nPD:	0.13
		N. weight:	0.42
		Sc. PD:	0.016
		Sc. rank:	201.7

,

PB1			
Pos.	575	obs:	exp:
gaa	E	45	665.70
gag	E	1049	428.30
---	--	------	------
mPD		0.079	0.48
		nPD:	0.17
		N. weight:	0.82
		Sc. PD:	0.063
		Sc. rank:	469.5

,

PB1			
Pos.	576	obs:	exp:
tta	L	0	97.08
ttg	L	31	203.70
ctt	L	1	182.20
ctc	L	0	182.60
cta	L	1	184.40
ctg	L	1061	244.10
---	--	------	------
mPD		0.059	1.1
		nPD:	0.05
		N. weight:	1.5
		Sc. PD:	-0.046
		Sc. rank:	186.5

,

PB1			
Pos.	577	obs:	exp:
cgt	R	0	2.10
cgc	R	0	2.43
cga	R	0	4.80
cgg	R	0	4.09
aaa	K	13	606.70
aag	K	1028	434.30
aga	R	9	22.41
agg	R	40	13.18
gaa	E	0	0.61
gag	E	1	0.39
ggt	G	0	0.40
ggc	G	0	0.38
gga	G	0	1.38
ggg	G	3	0.84
---	--	------	------
mPD		0.14	0.61
		nPD:	0.22
		N. weight:	0.88
		Sc. PD:	0.12
		Sc. rank:	607.4

,

PB1			
Pos.	578	obs:	exp:
cgt	R	0	0.17
cgc	R	0	0.20
cga	R	0	0.39
cgg	R	0	0.33
atg	M	1	1.00
act	T	0	0.25
acc	T	0	0.21
aca	T	0	0.46
acg	T	1	0.08
aaa	K	133	634.10
aag	K	955	453.90
aga	R	0	1.83
agg	R	4	1.08
---	--	------	------
mPD		0.22	0.50
		nPD:	0.45
		N. weight:	0.52
		Sc. PD:	0.18
		Sc. rank:	429.3

,

PB1			
Pos.	579	obs:	exp:
tta	L	0	97.08
ttg	L	6	203.70
ctt	L	3	182.20
ctc	L	0	182.60
cta	L	3	184.40
ctg	L	1082	244.10
---	--	------	------
mPD		0.022	1.1
		nPD:	0.02
		N. weight:	1.6
		Sc. PD:	-0.099
		Sc. rank:	-385.1

,

PB1			
Pos.	580	obs:	exp:
tgg	W	1094	1094.00
---	--	------	------
mPD		0	0
		nPD:	1.
		N. weight:	0.
		Sc. PD:	0
		Sc. rank:	0

,

PB1			
Pos.	581	obs:	exp:
aaa	K	0	0.58
aag	K	1	0.42
gat	D	1	0.54
gac	D	0	0.46
gaa	E	2	664.50
gag	E	1090	427.50
---	--	------	------
mPD		0.0073	0.48
		nPD:	0.02
		N. weight:	1.
		Sc. PD:	-0.068
		Sc. rank:	-368.1

,

PB1			
Pos.	582	obs:	exp:
caa	Q	9	557.20
cag	Q	1085	536.80
---	--	------	------
mPD		0.016	0.50
		nPD:	0.03
		N. weight:	0.73
		Sc. PD:	-0.037
		Sc. rank:	-28.6

,

PB1			
Pos.	583	obs:	exp:
act	T	1	278.90
acc	T	1093	224.50
aca	T	0	501.80
acg	T	0	88.86
---	--	------	------
mPD		0.0018	0.68
		nPD:	0.
		N. weight:	1.7
		Sc. PD:	-0.14
		Sc. rank:	-1287.9

,

PB1			
Pos.	584	obs:	exp:
tta	L	0	0.18
ttg	L	0	0.37
tct	S	0	0.16
tcc	S	0	0.13
tca	S	0	0.25
tcg	S	0	0.06
tgt	C	2	0.85
tgc	C	0	1.15
ctt	L	2	0.33
ctc	L	0	0.33
cta	L	0	0.34
ctg	L	0	0.45
cat	H	10	5.74
cac	H	0	4.26
cgt	R	1014	46.34
cgc	R	64	53.44
cga	R	1	105.60
cgg	R	0	90.03
agt	S	1	0.21
agc	S	0	0.19
aga	R	0	493.40
agg	R	0	290.20
---	--	------	------
mPD		0.14	1.0
		nPD:	0.14
		N. weight:	3.2
		Sc. PD:	0.16
		Sc. rank:	1647.7

,

PB1			
Pos.	585	obs:	exp:
tct	S	0	174.00
tcc	S	0	142.90
tca	S	113	277.70
tcg	S	981	62.08
agt	S	0	224.90
agc	S	0	212.50
---	--	------	------
mPD		0.19	1.7
		nPD:	0.11
		N. weight:	2.6
		Sc. PD:	0.067
		Sc. rank:	1113.5

,

PB1			
Pos.	586	obs:	exp:
cgt	R	0	0.04
cgc	R	0	0.05
cga	R	0	0.10
cgg	R	0	0.08
aaa	K	208	637.00
aag	K	885	456.00
aga	R	0	0.46
agg	R	1	0.27
---	--	------	------
mPD		0.31	0.49
		nPD:	0.63
		N. weight:	0.36
		Sc. PD:	0.19
		Sc. rank:	307.2

PB1			
Pos.	587	obs:	exp:
tct	S	0	0.16
tcc	S	0	0.13
tca	S	1	0.25
tcg	S	0	0.06
act	T	0	0.76
acc	T	0	0.62
aca	T	0	1.38
acg	T	3	0.24
agt	S	0	0.21
agc	S	0	0.19
gtt	V	0	0.21
gtc	V	0	0.20
gta	V	1	0.20
gtg	V	0	0.39
gct	A	124	281.20
gcc	A	1	202.80
gca	A	875	512.60
gcg	A	89	92.43
---	--	------	------
mPD		0.35	0.68
		nPD:	0.51
		N. weight:	0.38
		Sc. PD:	0.15
		Sc. rank:	318.3

PB1			
Pos.	588	obs:	exp:
gat	D	0	1.62
gac	D	3	1.38
ggt	G	0	144.60
ggc	G	0	139.40
gga	G	1059	503.30
ggg	G	32	303.70
---	--	------	------
mPD		0.068	0.68
		nPD:	0.1
		N. weight:	0.72
		Sc. PD:	0.0096
		Sc. rank:	274.3

PB1			
Pos.	589	obs:	exp:
tta	L	0	97.08
ttg	L	3	203.70
ctt	L	0	182.20
ctc	L	0	182.60
cta	L	6	184.40
ctg	L	1085	244.10
---	--	------	------
mPD		0.016	1.1
		nPD:	0.01
		N. weight:	1.6
		Sc. PD:	-0.11
		Sc. rank:	-590.9

PB1			
Pos.	590	obs:	exp:
tta	L	14	97.08
ttg	L	981	203.70
ctt	L	0	182.20
ctc	L	0	182.60
cta	L	0	184.40
ctg	L	99	244.10
---	--	------	------
mPD		0.19	1.1
		nPD:	0.17
		N. weight:	1.4
		Sc. PD:	0.12
		Sc. rank:	845.1

PB1			
Pos.	591	obs:	exp:
att	I	21	7.72
atc	I	0	5.03
ata	I	0	8.25
gtt	V	1067	222.30
gtc	V	6	216.60
gta	V	0	219.70
gtg	V	0	414.40
---	--	------	------
mPD		0.049	0.77
		nPD:	0.06
		N. weight:	1.7
		Sc. PD:	-0.035
		Sc. rank:	311.8

PB1			
Pos.	592	obs:	exp:
tct	S	8	173.20
tcc	S	0	142.20
tca	S	1013	276.40
tcg	S	68	61.79
agt	S	0	223.90
agc	S	0	211.50
gct	A	0	1.29
gcc	A	0	0.93
gca	A	5	2.35
gcg	A	0	0.42
---	--	------	------
mPD		0.14	1.7
		nPD:	0.08
		N. weight:	1.3
		Sc. PD:	-0.0015
		Sc. rank:	421.4

PB1			
Pos.	593	obs:	exp:
gat	D	1070	591.20
gac	D	24	502.80
---	--	------	------
mPD		0.043	0.50
		nPD:	0.09
		N. weight:	0.57
		Sc. PD:	0.00063
		Sc. rank:	188.4

PB1			
Pos.	594	obs:	exp:
ggt	G	0	145.00
ggc	G	1	139.80
gga	G	1088	504.70
ggg	G	5	304.60
---	--	------	------
mPD		0.011	0.68
		nPD:	0.02
		N. weight:	0.82
		Sc. PD:	-0.054
		Sc. rank:	-274.8

PB1			
Pos.	595	obs:	exp:
ggt	G	0	145.00
ggc	G	29	139.80
gga	G	2	504.70
ggg	G	1063	304.60
---	--	------	------
mPD		0.055	0.68
		nPD:	0.08
		N. weight:	1.3
		Sc. PD:	-0.0044
		Sc. rank:	400.7

PB1			
Pos.	596	obs:	exp:
tct	S	0	0.48
tcc	S	0	0.39
tca	S	3	0.76
tcg	S	0	0.17
cct	P	3	272.20
ccc	P	0	196.00
cca	P	1084	449.10
ccg	P	4	173.70
agt	S	0	0.62
agc	S	0	0.58
---	--	------	------
mPD		0.018	0.72
		nPD:	0.03
		N. weight:	0.94
		Sc. PD:	-0.054
		Sc. rank:	-147.3

PB1			
Pos.	597	obs:	exp:
aat	N	19	557.20
aac	N	1075	536.80
---	--	------	------
mPD		0.034	0.50
		nPD:	0.07
		N. weight:	0.69
		Sc. PD:	-0.011
		Sc. rank:	155.2

PB1			
Pos.	598	obs:	exp:
tta	L	41	96.82
ttg	L	20	203.10
ctt	L	2	181.70
ctc	L	0	182.10
cta	L	2	183.90
ctg	L	1026	243.50
cct	P	0	0.25
ccc	P	0	0.18
cca	P	0	0.41
ccg	P	1	0.16
caa	Q	0	0.51
cag	Q	1	0.49
atg	M	1	1.00
---	--	------	------
mPD		0.19	1.1
		nPD:	0.17
		N. weight:	1.4
		Sc. PD:	0.11
		Sc. rank:	814.2

PB1			
Pos.	599	obs:	exp:
tat	Y	71	590.90
tac	Y	1023	503.10
---	--	------	------
mPD		0.12	0.50
		nPD:	0.24
		N. weight:	0.58
		Sc. PD:	0.089
		Sc. rank:	412.6

PB1			
Pos.	600	obs:	exp:
aat	N	1092	557.20
aac	N	2	536.80
---	--	------	------
mPD		0.0037	0.50
		nPD:	0.01
		N. weight:	0.73
		Sc. PD:	-0.054
		Sc. rank:	-423.2

PB1			
Pos.	601	obs:	exp:
att	I	99	402.00
atc	I	995	262.20
ata	I	0	429.90
---	--	------	------
mPD		0.16	0.65
		nPD:	0.25
		N. weight:	1.2
		Sc. PD:	0.19
		Sc. rank:	864.4

PB1			
Pos.	602	obs:	exp:
cgt	R	0	46.99
cgc	R	0	54.19
cga	R	4	107.10
cgg	R	1090	91.28
aga	R	0	500.20
agg	R	0	294.20
---	--	------	------
mPD		0.0073	0.96
		nPD:	0.01
		N. weight:	2.7
		Sc. PD:	-0.2
		Sc. rank:	-1471.3

PB1			
Pos.	603	obs:	exp:
aat	N	18	557.20
aac	N	1076	536.80
---	--	------	------
mPD		0.032	0.50
		nPD:	0.06
		N. weight:	0.69
		Sc. PD:	-0.014
		Sc. rank:	135.3

PB1			
Pos.	604	obs:	exp:
tta	L	0	97.08
ttg	L	0	203.70
ctt	L	5	182.20
ctc	L	1084	182.60
cta	L	5	184.40
ctg	L	0	244.10
---	--	------	------
mPD		0.018	1.1
		nPD:	0.02
		N. weight:	1.9
		Sc. PD:	-0.13
		Sc. rank:	-601.9

PB1			
Pos.	605	obs:	exp:
cat	H	1	628.20
cac	H	1093	465.80
---	--	------	------
mPD		0.0018	0.49
		nPD:	0.
		N. weight:	0.93
		Sc. PD:	-0.073
		Sc. rank:	-653.3

PB1			
Pos.	606	obs:	exp:
att	I	996	402.00
atc	I	98	262.20
ata	I	0	429.90
---	--	------	------
mPD		0.16	0.65
		nPD:	0.25
		N. weight:	0.81
		Sc. PD:	0.13
		Sc. rank:	582.3

PB1			
Pos.	607	obs:	exp:
cct	P	0	273.00
ccc	P	0	196.60
cca	P	1085	450.30
ccg	P	9	174.20
---	--	------	------
mPD		0.016	0.71
		nPD:	0.02
		N. weight:	0.93
		Sc. PD:	-0.056
		Sc. rank:	-164.6

PB1			
Pos.	608	obs:	exp:
gaa	E	1	665.70
gag	E	1093	428.30
---	--	------	------
mPD		0.0018	0.48
		nPD:	0.
		N. weight:	1.
		Sc. PD:	-0.08
		Sc. rank:	-698.7

PB1			
Pos.	609	obs:	exp:
gtt	V	2	226.70
gtc	V	1091	220.80
gta	V	1	224.00
gtg	V	0	422.50
---	--	------	------
mPD		0.0055	0.73
		nPD:	0.01
		N. weight:	1.7
		Sc. PD:	-0.13
		Sc. rank:	-980.8

PB1			
Pos.	610	obs:	exp:
tgt	C	173	462.70
tgc	C	921	631.30
---	--	------	------
mPD		0.27	0.49
		nPD:	0.55
		N. weight:	0.18
		Sc. PD:	0.08
		Sc. rank:	153.6

PB1

Pos. 611		obs:	exp:
tta	L	6	97.08
ttg	L	1082	203.70
ctt	L	0	182.20
ctc	L	0	182.60
cta	L	0	184.40
ctg	L	6	244.10
---	--	------	------
mPD		0.022	1.1
		nPD:	0.02
		N. weight:	1.8
		Sc. PD:	-0.11
		Sc. rank:	-441.0

,

PB1

Pos. 612		obs:	exp:
aaa	K	1008	637.60
aag	K	86	456.40
---	--	------	------
mPD		0.14	0.49
		nPD:	0.3
		N. weight:	0.32
		Sc. PD:	0.066
		Sc. rank:	245.6

,

PB1

Pos. 613		obs:	exp:
tgt	C	0	0.42
tgc	C	1	0.58
tgg	W	1093	1093.00
---	--	------	------
mPD		0.0018	0.0018
		nPD:	1.
		N. weight:	0.00055
		Sc. PD:	0.00048
		Sc. rank:	0.5

,

PB1

Pos. 614		obs:	exp:
gat	D	0	2.16
gac	D	4	1.84
gaa	E	1079	660.80
gag	E	7	425.20
ggt	G	0	0.53
ggc	G	0	0.51
gga	G	3	1.85
ggg	G	1	1.11
---	--	------	------
mPD		0.029	0.49
		nPD:	0.06
		N. weight:	0.51
		Sc. PD:	-0.013
		Sc. rank:	85.4

,

PB1

Pos. 615		obs:	exp:
tta	L	1	97.08
ttg	L	1083	203.70
ctt	L	0	182.20
ctc	L	0	182.60
cta	L	0	184.40
ctg	L	10	244.10
---	--	------	------
mPD		0.020	1.1
		nPD:	0.02
		N. weight:	1.8
		Sc. PD:	-0.11
		Sc. rank:	-512.6

,

PB1

Pos. 616		obs:	exp:
atg	M	1094	1094.00
---	--	------	------
mPD		0	0
		nPD:	1.
		N. weight:	0.
		Sc. PD:	0
		Sc. rank:	0

,

PB1

Pos. 617		obs:	exp:
gat	D	1086	591.20
gac	D	8	502.80
---	--	------	------
mPD		0.015	0.50
		nPD:	0.03
		N. weight:	0.63
		Sc. PD:	-0.034
		Sc. rank:	-56.0

,

PB1

Pos. 618		obs:	exp:
gaa	E	1094	665.70
gag	E	0	428.30
---	--	------	------
mPD		0	0.48
		nPD:	0.
		N. weight:	0.55
		Sc. PD:	-0.045
		Sc. rank:	-468.7

,

PB1

Pos. 619		obs:	exp:
aat	N	0	0.51
aac	N	1	0.49
gat	D	7	590.10
gac	D	1085	501.90
ggt	G	0	0.13
ggc	G	1	0.13
gga	G	0	0.46
ggg	G	0	0.28
---	--	------	------
mPD		0.016	0.50
		nPD:	0.03
		N. weight:	0.81
		Sc. PD:	-0.041
		Sc. rank:	-30.4

,

PB1

Pos. 620		obs:	exp:
tat	Y	29	590.90
tac	Y	1065	503.10
---	--	------	------
mPD		0.052	0.50
		nPD:	0.1
		N. weight:	0.72
		Sc. PD:	0.013
		Sc. rank:	285.6

,

PB1

Pos. 621		obs:	exp:
caa	Q	991	552.70
cag	Q	94	532.30
cgt	R	0	0.13
cgc	R	0	0.15
cga	R	2	0.29
cgg	R	1	0.25
aat	N	0	0.51
aac	N	1	0.49
aaa	K	5	2.91
aag	K	0	2.09
aga	R	0	1.37
agg	R	0	0.81
---	--	------	------
mPD		0.18	0.52
		nPD:	0.34
		N. weight:	0.43
		Sc. PD:	0.1
		Sc. rank:	338.4

,

PB1

Pos. 622		obs:	exp:
ggt	G	4	145.00
ggc	G	1088	139.80
gga	G	2	504.70
ggg	G	0	304.60
---	--	------	------
mPD		0.011	0.68
		nPD:	0.02
		N. weight:	2.2
		Sc. PD:	-0.15
		Sc. rank:	-731.6

,

PB1

Pos. 623		obs:	exp:
tta	L	0	0.09
ttg	L	0	0.19
ctt	L	0	0.17
ctc	L	0	0.17
cta	L	0	0.17
ctg	L	1	0.22
cgt	R	0	46.94
cgc	R	0	54.14
cga	R	0	107.00
cgg	R	0	91.20
aga	R	135	499.80
agg	R	958	294.00
---	--	------	------
mPD		0.22	0.96
		nPD:	0.23
		N. weight:	0.96
		Sc. PD:	0.13
		Sc. rank:	668.8

,

PB1

Pos. 624		obs:	exp:
tta	L	7	96.99
ttg	L	867	203.50
ctt	L	1	182.00
ctc	L	0	182.40
cta	L	1	184.20
ctg	L	217	243.90
gtt	V	0	0.21
gtc	V	0	0.20
gta	V	0	0.20
gtg	V	1	0.39
---	--	------	------
mPD		0.34	1.1
		nPD:	0.3
		N. weight:	1.2
		Sc. PD:	0.26
		Sc. rank:	940.2

,

PB1

Pos. 625		obs:	exp:
ttt	F	1	0.39
ttc	F	0	0.61
tgt	C	1075	462.30
tgc	C	18	630.70
---	--	------	------
mPD		0.034	0.49
		nPD:	0.07
		N. weight:	0.85
		Sc. PD:	-0.013
		Sc. rank:	196.0

,

PB1

Pos. 626		obs:	exp:
aat	N	1055	557.20
aac	N	39	536.80
---	--	------	------
mPD		0.069	0.50
		nPD:	0.14
		N. weight:	0.58
		Sc. PD:	0.029
		Sc. rank:	294.8

,

PB1

Pos. 627		obs:	exp:
cct	P	1068	273.00
ccc	P	11	196.60
cca	P	15	450.30
ccg	P	0	174.20
---	--	------	------
mPD		0.047	0.71
		nPD:	0.07
		N. weight:	1.4
		Sc. PD:	-0.026
		Sc. rank:	282.6

,

PB1

Pos. 628		obs:	exp:
tta	L	0	0.18
ttg	L	1	0.37
ctt	L	0	0.33
ctc	L	0	0.33
cta	L	0	0.34
ctg	L	1	0.45
att	I	0	1.47
atc	I	0	0.96
ata	I	4	1.57
atg	M	1087	1087.00
gtt	V	0	0.21
gtc	V	0	0.20
gta	V	0	0.20
gtg	V	1	0.39
---	--	------	------
mPD		0.013	0.016
		nPD:	0.8
		N. weight:	0.0072
		Sc. PD:	0.0049
		Sc. rank:	6.2

,

PB1

Pos. 629		obs:	exp:
aat	N	248	557.20
aac	N	846	536.80
---	--	------	------
mPD		0.35	0.50
		nPD:	0.7
		N. weight:	0.19
		Sc. PD:	0.11
		Sc. rank:	161.6

,

PB1

Pos. 630		obs:	exp:
cct	P	4	273.00
ccc	P	0	196.60
cca	P	63	450.30
ccg	P	1027	174.20
---	--	------	------
mPD		0.12	0.71
		nPD:	0.16
		N. weight:	1.7
		Sc. PD:	0.13
		Sc. rank:	951.6

,

PB1			
Pos.	631	obs:	exp:
ttt	F	1094	432.00
ttc	F	0	662.00
---	--	------	------
mPD		0	0.48
		nPD:	0.
		N. weight:	1.
		Sc. PD:	-0.083
		Sc. rank:	-876.6

PB1			
Pos.	632	obs:	exp:
gtt	V	6	226.50
gtc	V	1085	220.60
gta	V	2	223.80
gtg	V	0	422.10
gct	A	0	0.26
gcc	A	1	0.19
gca	A	0	0.47
gcg	A	0	0.08
---	--	------	------
mPD		0.016	0.73
		nPD:	0.02
		N. weight:	1.7
		Sc. PD:	-0.1
		Sc. rank:	-335.6

PB1			
Pos.	633	obs:	exp:
tct	S	0	173.70
tcc	S	1	142.60
tca	S	0	277.20
tcg	S	0	61.96
aat	N	2	1.02
aac	N	0	0.98
agt	S	997	224.50
agc	S	94	212.10
---	--	------	------
mPD		0.17	1.7
		nPD:	0.1
		N. weight:	1.4
		Sc. PD:	0.02
		Sc. rank:	545.3

PB1			
Pos.	634	obs:	exp:
tta	L	0	0.09
ttg	L	0	0.19
ctt	L	1	0.17
ctc	L	0	0.17
cta	L	0	0.17
ctg	L	0	0.22
cct	P	1	0.25
ccc	P	0	0.18
cca	P	0	0.41
ccg	P	0	0.16
cat	H	1088	626.50
cac	H	3	464.50
aat	N	1	0.51
aac	N	0	0.49
---	--	------	------
mPD		0.011	0.50
		nPD:	0.02
		N. weight:	0.59
		Sc. PD:	-0.036
		Sc. rank:	-124.9

PB1			
Pos.	635	obs:	exp:
cgt	R	0	0.60
cgc	R	0	0.69
cga	R	0	1.37
cgg	R	0	1.17
aaa	K	0	628.90
aag	K	1079	450.10
aga	R	0	6.40
agg	R	14	3.77
gtt	V	1	0.21
gtc	V	0	0.20
gta	V	0	0.20
gtg	V	0	0.39
---	--	------	------
mPD		0.031	0.52
		nPD:	0.06
		N. weight:	0.97
		Sc. PD:	-0.025
		Sc. rank:	160.9

PB1			
Pos.	636	obs:	exp:
tct	S	0	0.16
tcc	S	0	0.13
tca	S	1	0.25
tcg	S	0	0.06
agt	S	0	0.21
agc	S	0	0.19
gaa	E	1075	663.90
gag	E	16	427.10
ggt	G	0	0.27
ggc	G	0	0.26
gga	G	2	0.92
ggg	G	0	0.56
---	--	------	------
mPD		0.036	0.48
		nPD:	0.07
		N. weight:	0.47
		Sc. PD:	-0.0049
		Sc. rank:	127.5

PB1			
Pos.	637	obs:	exp:
ttt	F	3	1.18
ttc	F	0	1.82
tta	L	0	0.18
ttg	L	0	0.37
ctt	L	2	0.33
ctc	L	0	0.33
cta	L	0	0.34
ctg	L	0	0.45
att	I	1063	394.20
atc	I	10	257.10
ata	I	0	421.60
act	T	1	0.25
acc	T	0	0.21
aca	T	0	0.46
acg	T	0	0.08
gtt	V	15	3.11
gtc	V	0	3.03
gta	V	0	3.07
gtg	V	0	5.79
---	--	------	------
mPD		0.056	0.70
		nPD:	0.08
		N. weight:	1.1
		Sc. PD:	-0.0049
		Sc. rank:	319.3

PB1			
Pos.	638	obs:	exp:
gat	D	1045	573.90
gac	D	17	488.10
gaa	E	0	3.65
gag	E	6	2.35
ggt	G	26	3.45
ggc	G	0	3.32
gga	G	0	11.99
ggg	G	0	7.24
---	--	------	------
mPD		0.088	0.57
		nPD:	0.16
		N. weight:	0.63
		Sc. PD:	0.042
		Sc. rank:	350.5

PB1			
Pos.	639	obs:	exp:
tct	S	1	174.00
tcc	S	107	142.90
tca	S	943	277.70
tcg	S	43	62.08
agt	S	0	224.90
agc	S	0	212.50
---	--	------	------
mPD		0.25	1.7
		nPD:	0.15
		N. weight:	1.1
		Sc. PD:	0.068
		Sc. rank:	603.9

PB1			
Pos.	640	obs:	exp:
att	I	0	0.37
atc	I	1	0.24
ata	I	0	0.39
atg	M	1	1.00
gtt	V	7	226.30
gtc	V	1057	220.40
gta	V	27	223.60
gtg	V	1	421.70
---	--	------	------
mPD		0.068	0.73
		nPD:	0.09
		N. weight:	1.6
		Sc. PD:	0.012
		Sc. rank:	555.6

PB1			
Pos.	641	obs:	exp:
aat	N	66	556.70
aac	N	1027	536.30
gat	D	0	0.54
gac	D	1	0.46
---	--	------	------
mPD		0.12	0.50
		nPD:	0.23
		N. weight:	0.53
		Sc. PD:	0.074
		Sc. rank:	370.2

PB1			
Pos.	642	obs:	exp:
tct	S	0	10.82
tcc	S	0	8.88
tca	S	0	17.26
tcg	S	0	3.86
cct	P	1	0.25
ccc	P	0	0.18
cca	P	0	0.41
ccg	P	0	0.16
act	T	1	0.25
acc	T	0	0.21
aca	T	0	0.46
acg	T	0	0.08
aat	N	1013	521.60
aac	N	11	502.40
agt	S	62	13.98
agc	S	6	13.21
---	--	------	------
mPD		0.15	0.71
		nPD:	0.21
		N. weight:	0.73
		Sc. PD:	0.089
		Sc. rank:	482.9

PB1			
Pos.	643	obs:	exp:
act	T	1	0.25
acc	T	0	0.21
aca	T	0	0.46
acg	T	0	0.08
gct	A	988	282.20
gcc	A	5	203.50
gca	A	1	514.40
gcg	A	99	92.77
---	--	------	------
mPD		0.18	0.67
		nPD:	0.26
		N. weight:	1.2
		Sc. PD:	0.21
		Sc. rank:	905.2

PB1			
Pos.	644	obs:	exp:
gtt	V	1	226.70
gtc	V	0	220.80
gta	V	32	224.00
gtg	V	1061	422.50
---	--	------	------
mPD		0.059	0.73
		nPD:	0.08
		N. weight:	0.92
		Sc. PD:	-0.0039
		Sc. rank:	281.4

PB1			
Pos.	645	obs:	exp:
atg	M	2	2.00
gtt	V	2	226.30
gtc	V	0	220.40
gta	V	26	223.60
gtg	V	1064	421.70
---	--	------	------
mPD		0.054	0.73
		nPD:	0.07
		N. weight:	0.93
		Sc. PD:	-0.01
		Sc. rank:	242.3

PB1

Pos.	646	obs:	exp:
tta	L	0	0.35
ttg	L	0	0.74
ctt	L	0	0.67
ctc	L	0	0.67
cta	L	0	0.67
ctg	L	4	0.89
att	I	1	0.37
atc	I	0	0.24
ata	I	0	0.39
atg	M	1087	1087.00
act	T	0	0.25
acc	T	0	0.21
aca	T	0	0.46
acg	T	1	0.08
gtt	V	0	0.21
gtc	V	0	0.20
gta	V	0	0.20
gtg	V	1	0.39
---	--	------	------
mPD		0.013	0.020
		nPD:	0.64
		N. weight:	0.01
		Sc. PD:	0.0056
		Sc. rank:	9.0

,

PB1

Pos.	647	obs:	exp:
cct	P	12	273.00
ccc	P	4	196.60
cca	P	1070	450.30
ccg	P	8	174.20
---	--	------	------
mPD		0.043	0.71
		nPD:	0.06
		N. weight:	0.86
		Sc. PD:	-0.02
		Sc. rank:	149.8

,

PB1

Pos.	648	obs:	exp:
tct	S	20	3.18
tcc	S	0	2.61
tca	S	0	5.08
tcg	S	0	1.13
tgt	C	1	0.42
tgc	C	0	0.58
act	T	2	0.51
acc	T	0	0.41
aca	T	0	0.92
acg	T	0	0.16
agt	S	0	4.11
agc	S	0	3.88
gct	A	1031	276.60
gcc	A	36	199.50
gca	A	4	504.10
gcg	A	0	90.90
---	--	------	------
mPD		0.11	0.73
		nPD:	0.16
		N. weight:	1.3
		Sc. PD:	0.09
		Sc. rank:	738.8

,

PB1

Pos.	649	obs:	exp:
cat	H	1093	627.60
cac	H	0	465.40
caa	Q	0	0.51
cag	Q	1	0.49
---	--	------	------
mPD		0.0018	0.49
		nPD:	0.
		N. weight:	0.61
		Sc. PD:	-0.048
		Sc. rank:	-429.2

,

PB1

Pos.	650	obs:	exp:
ggt	G	41	145.00
ggc	G	1052	139.80
gga	G	1	504.70
ggg	G	0	304.60
---	--	------	------
mPD		0.074	0.68
		nPD:	0.11
		N. weight:	2.1
		Sc. PD:	0.048
		Sc. rank:	864.2

,

PB1

Pos.	651	obs:	exp:
cct	P	2	273.00
ccc	P	122	196.60
cca	P	967	450.30
ccg	P	3	174.20
---	--	------	------
mPD		0.21	0.71
		nPD:	0.29
		N. weight:	0.67
		Sc. PD:	0.13
		Sc. rank:	497.2

,

PB1

Pos.	652	obs:	exp:
tct	S	0	0.16
tcc	S	1	0.13
tca	S	0	0.25
tcg	S	0	0.06
agt	S	0	0.21
agc	S	0	0.19
gct	A	101	282.20
gcc	A	992	203.50
gca	A	0	514.40
gcg	A	0	92.77
---	--	------	------
mPD		0.17	0.67
		nPD:	0.25
		N. weight:	1.5
		Sc. PD:	0.24
		Sc. rank:	1064.3

,

PB1

Pos.	653	obs:	exp:
aaa	K	1088	637.00
aag	K	5	456.00
gaa	E	1	0.61
gag	E	0	0.39
---	--	------	------
mPD		0.011	0.49
		nPD:	0.02
		N. weight:	0.56
		Sc. PD:	-0.034
		Sc. rank:	-115.9

,

PB1

Pos.	654	obs:	exp:
tta	L	0	0.09
ttg	L	0	0.19
tct	S	0	172.90
tcc	S	0	142.00
tca	S	0	275.90
tcg	S	0	61.68
ctt	L	0	0.17
ctc	L	0	0.17
cta	L	0	0.17
ctg	L	1	0.22
aat	N	0	3.06
aac	N	6	2.94
agt	S	5	223.50
agc	S	1082	211.10
---	--	------	------
mPD		0.025	1.7
		nPD:	0.02
		N. weight:	1.8
		Sc. PD:	-0.12
		Sc. rank:	-618.2

,

PB1

Pos.	655	obs:	exp:
att	I	0	0.37
atc	I	0	0.24
ata	I	1	0.39
atg	M	1093	1093.00
---	--	------	------
mPD		0.0018	0.0018
		nPD:	1.
		N. weight:	0.00094
		Sc. PD:	0.00082
		Sc. rank:	0.8

,

PB1

Pos.	656	obs:	exp:
gat	D	1	0.54
gac	D	0	0.46
gaa	E	16	665.10
gag	E	1077	427.90
---	--	------	------
mPD		0.031	0.48
		nPD:	0.06
		N. weight:	0.94
		Sc. PD:	-0.019
		Sc. rank:	176.5

,

PB1

Pos.	657	obs:	exp:
tat	Y	1090	590.90
tac	Y	4	503.10
---	--	------	------
mPD		0.0073	0.50
		nPD:	0.01
		N. weight:	0.65
		Sc. PD:	-0.044
		Sc. rank:	-249.6

,

PB1

Pos.	658	obs:	exp:
gat	D	1090	591.20
gac	D	4	502.80
---	--	------	------
mPD		0.0073	0.50
		nPD:	0.01
		N. weight:	0.65
		Sc. PD:	-0.044
		Sc. rank:	-247.1

,

PB1

Pos.	659	obs:	exp:
gct	A	54	282.50
gcc	A	1015	203.70
gca	A	25	514.90
gcg	A	0	92.85
---	--	------	------
mPD		0.14	0.67
		nPD:	0.2
		N. weight:	1.5
		Sc. PD:	0.17
		Sc. rank:	957.4

,

PB1

Pos.	660	obs:	exp:
att	I	1	0.37
atc	I	0	0.24
ata	I	0	0.39
gtt	V	1093	226.50
gtc	V	0	220.60
gta	V	0	223.80
gtg	V	0	422.10
---	--	------	------
mPD		0.0018	0.73
		nPD:	0.
		N. weight:	1.7
		Sc. PD:	-0.14
		Sc. rank:	-1301.8

,

PB1

Pos.	661	obs:	exp:
gct	A	0	282.50
gcc	A	4	203.70
gca	A	1088	514.90
gcg	A	2	92.85
---	--	------	------
mPD		0.011	0.67
		nPD:	0.02
		N. weight:	0.8
		Sc. PD:	-0.053
		Sc. rank:	-258.3

,

PB1

Pos.	662	obs:	exp:
act	T	195	278.90
acc	T	792	224.50
aca	T	107	501.80
acg	T	0	88.86
---	--	------	------
mPD		0.43	0.68
		nPD:	0.64
		N. weight:	0.77
		Sc. PD:	0.41
		Sc. rank:	661.0

,

PB1

Pos.	663	obs:	exp:
cct	P	0	0.25
ccc	P	1	0.18
cca	P	0	0.41
ccg	P	0	0.16
act	T	0	278.60
acc	T	2	224.30
aca	T	1091	501.30
acg	T	0	88.78
---	--	------	------
mPD		0.0073	0.68
		nPD:	0.01
		N. weight:	0.84
		Sc. PD:	-0.06
		Sc. rank:	-402.8

,

PB1

Pos.	664	obs:	exp:
tta	L	0	0.09
ttg	L	0	0.19
ctt	L	1	0.17
ctc	L	0	0.17
cta	L	0	0.17
ctg	L	0	0.22
cat	H	1084	627.00
cac	H	8	465.00
cgt	R	1	0.04
cgc	R	0	0.05
cga	R	0	0.10
cgg	R	0	0.08
aga	R	0	0.46
agg	R	0	0.27
---	--	------	------
mPD		0.018	0.50
		nPD:	0.04
		N. weight:	0.57
		Sc. PD:	-0.027
		Sc. rank:	-2.0

,

PB1

Pos.	665	obs:	exp:
tct	S	12	174.00
tcc	S	3	142.90
tca	S	1065	277.70
tcg	S	14	62.08
agt	S	0	224.90
agc	S	0	212.50
---	--	------	------
mPD		0.052	1.7
		nPD:	0.03
		N. weight:	1.4
		Sc. PD:	-0.071
		Sc. rank:	-73.7

,

PB1			
Pos.	666	obs:	exp:
tgg	W	1094	1094.00
---	--	------	------
mPD		0	0
		nPD:	1.
		N. weight:	0.
		Sc. PD:	0
		Sc. rank:	0

,

PB1			
Pos.	667	obs:	exp:
att	I	985	399.00
atc	I	99	260.20
ata	I	2	426.70
act	T	3	0.76
acc	T	0	0.62
aca	T	0	1.38
acg	T	0	0.24
gtt	V	5	1.04
gtc	V	0	1.01
gta	V	0	1.02
gtg	V	0	1.93
---	--	------	------
mPD		0.18	0.67
		nPD:	0.27
		N. weight:	0.8
		Sc. PD:	0.14
		Sc. rank:	593.1

,

PB1			
Pos.	668	obs:	exp:
cct	P	1080	273.00
ccc	P	11	196.60
cca	P	2	450.30
ccg	P	1	174.20
---	--	------	------
mPD		0.025	0.71
		nPD:	0.04
		N. weight:	1.4
		Sc. PD:	-0.069
		Sc. rank:	-15.3

,

PB1			
Pos.	669	obs:	exp:
aaa	K	18	637.60
aag	K	1076	456.40
---	--	------	------
mPD		0.032	0.49
		nPD:	0.07
		N. weight:	0.86
		Sc. PD:	-0.015
		Sc. rank:	188.2

,

PB1			
Pos.	670	obs:	exp:
cgt	R	0	46.99
cgc	R	0	54.19
cga	R	0	107.10
cgg	R	0	91.28
aga	R	6	500.20
agg	R	1088	294.20
---	--	------	------
mPD		0.011	0.96
		nPD:	0.01
		N. weight:	1.4
		Sc. PD:	-0.099
		Sc. rank:	-613.5

,

PB1			
Pos.	671	obs:	exp:
aat	N	923	557.20
aac	N	171	536.80
---	--	------	------
mPD		0.26	0.50
		nPD:	0.53
		N. weight:	0.27
		Sc. PD:	0.12
		Sc. rank:	232.0

,

PB1			
Pos.	672	obs:	exp:
cgt	R	22	46.99
cgc	R	1070	54.19
cga	R	2	107.10
cgg	R	0	91.28
aga	R	0	500.20
agg	R	0	294.20
---	--	------	------
mPD		0.043	0.96
		nPD:	0.04
		N. weight:	3.2
		Sc. PD:	-0.12
		Sc. rank:	200.2

,

PB1			
Pos.	673	obs:	exp:
tct	S	4	174.00
tcc	S	1089	142.90
tca	S	1	277.70
tcg	S	0	62.08
agt	S	0	224.90
agc	S	0	212.50
---	--	------	------
mPD		0.0091	1.7
		nPD:	0.01
		N. weight:	2.2
		Sc. PD:	-0.17
		Sc. rank:	-1395.7

,

PB1			
Pos.	674	obs:	exp:
att	I	1074	402.00
atc	I	20	262.20
ata	I	0	429.90
---	--	------	------
mPD		0.036	0.65
		nPD:	0.06
		N. weight:	1.
		Sc. PD:	-0.029
		Sc. rank:	146.7

,

PB1			
Pos.	675	obs:	exp:
tta	L	0	97.08
ttg	L	0	203.70
ctt	L	2	182.20
ctc	L	1092	182.60
cta	L	0	184.40
ctg	L	0	244.10
---	--	------	------
mPD		0.0037	1.1
		nPD:	0.
		N. weight:	2.
		Sc. PD:	-0.15
		Sc. rank:	-1381.5

,

PB1			
Pos.	676	obs:	exp:
cct	P	0	0.25
ccc	P	1	0.18
cca	P	0	0.41
ccg	P	0	0.16
aat	N	116	556.70
aac	N	977	536.30
---	--	------	------
mPD		0.19	0.50
		nPD:	0.38
		N. weight:	0.41
		Sc. PD:	0.12
		Sc. rank:	330.1

,

PB1			
Pos.	677	obs:	exp:
tct	S	0	0.16
tcc	S	1	0.13
tca	S	0	0.25
tcg	S	0	0.06
act	T	14	278.40
acc	T	1075	224.10
aca	T	3	500.90
acg	T	0	88.70
agt	S	0	0.21
agc	S	0	0.19
gct	A	0	0.26
gcc	A	1	0.19
gca	A	0	0.47
gcg	A	0	0.08
---	--	------	------
mPD		0.034	0.68
		nPD:	0.05
		N. weight:	1.6
		Sc. PD:	-0.055
		Sc. rank:	178.7

,

PB1			
Pos.	678	obs:	exp:
tta	L	0	0.09
ttg	L	0	0.19
tct	S	0	173.70
tcc	S	0	142.60
tca	S	0	277.20
tcg	S	0	61.96
ctt	L	0	0.17
ctc	L	1	0.17
cta	L	0	0.17
ctg	L	0	0.22
cgt	R	0	0.04
cgc	R	1	0.05
cga	R	0	0.10
cgg	R	0	0.08
agt	S	13	224.50
agc	S	1079	212.10
aga	R	0	0.46
agg	R	0	0.27
---	--	------	------
mPD		0.029	1.7
		nPD:	0.02
		N. weight:	1.7
		Sc. PD:	-0.11
		Sc. rank:	-511.6

,

PB1			
Pos.	679	obs:	exp:
caa	Q	1087	556.70
cag	Q	6	536.30
cgt	R	0	0.04
cgc	R	0	0.05
cga	R	0	0.10
cgg	R	1	0.08
aga	R	0	0.46
agg	R	0	0.27
---	--	------	------
mPD		0.015	0.50
		nPD:	0.03
		N. weight:	0.71
		Sc. PD:	-0.038
		Sc. rank:	-67.8

,

PB1			
Pos.	680	obs:	exp:
cct	P	0	0.25
ccc	P	0	0.18
cca	P	0	0.41
ccg	P	1	0.16
cgt	R	0	46.86
cgc	R	0	54.04
cga	R	0	106.80
cgg	R	30	91.03
aaa	K	0	1.17
aag	K	2	0.83
aga	R	99	498.90
agg	R	962	293.40
---	--	------	------
mPD		0.23	0.97
		nPD:	0.23
		N. weight:	0.96
		Sc. PD:	0.14
		Sc. rank:	673.1

,

PB1			
Pos.	681	obs:	exp:
ggt	G	1	145.00
ggc	G	0	139.80
gga	G	30	504.70
ggg	G	1063	304.60
---	--	------	------
mPD		0.055	0.68
		nPD:	0.08
		N. weight:	1.2
		Sc. PD:	-0.0044
		Sc. rank:	386.6

,

PB1			
Pos.	682	obs:	exp:
tct	S	0	0.16
tcc	S	0	0.13
tca	S	0	0.25
tcg	S	0	0.06
att	I	1003	383.20
atc	I	40	249.90
ata	I	0	409.80
agt	S	1	0.21
agc	S	0	0.19
gtt	V	50	10.36
gtc	V	0	10.09
gta	V	0	10.24
gtg	V	0	19.31
---	--	------	------
mPD		0.16	0.76
		nPD:	0.21
		N. weight:	0.98
		Sc. PD:	0.12
		Sc. rank:	648.0

,

PB1			
Pos.	683	obs:	exp:
tta	L	0	96.99
ttg	L	0	203.50
ctt	L	1091	182.00
ctc	L	2	182.40
cta	L	0	184.20
ctg	L	0	243.90
att	I	1	0.37
atc	I	0	0.24
ata	I	0	0.39
---	--	------	------
mPD		0.0055	1.1
		nPD:	0.
		N. weight:	2.
		Sc. PD:	-0.15
		Sc. rank:	-1293.6

,

PB1			
Pos.	684	obs:	exp:
gaa	E	15	665.70
gag	E	1079	428.30
---	--	------	------
mPD		0.027	0.48
		nPD:	0.06
		N. weight:	0.95
		Sc. PD:	-0.026
		Sc. rank:	149.8

,

PB1			
Pos.	685	obs:	exp:
gat	D	204	591.20
gac	D	890	502.80
---	--	------	------
mPD		0.30	0.50
		nPD:	0.61
		N. weight:	0.3
		Sc. PD:	0.15
		Sc. rank:	251.5

,

PB1

Pos.	686	obs:	exp:
caa	Q	1	0.51
cag	Q	0	0.49
gaa	E	1074	665.10
gag	E	19	427.90
---	--	------	------
mPD		0.036	0.48
		nPD:	0.08
		N. weight:	0.46
		Sc. PD:	-0.0044
		Sc. rank:	124.7

PB1

Pos.	687	obs:	exp:
tta	L	0	0.09
ttg	L	0	0.19
ctt	L	1	0.17
ctc	L	0	0.17
cta	L	0	0.17
ctg	L	0	0.22
caa	Q	22	556.70
cag	Q	1071	536.30
---	--	------	------
mPD		0.043	0.50
		nPD:	0.09
		N. weight:	0.68
		Sc. PD:	0.00026
		Sc. rank:	221.0

PB1

Pos.	688	obs:	exp:
atg	M	1094	1094.00
---	--	------	------
mPD		0	0
		nPD:	1.
		N. weight:	0.
		Sc. PD:	0
		Sc. rank:	0

PB1

Pos.	689	obs:	exp:
tta	L	0	0.09
ttg	L	1	0.19
tat	Y	20	590.30
tac	Y	1073	502.70
ctt	L	0	0.17
ctc	L	0	0.17
cta	L	0	0.17
ctg	L	0	0.22
---	--	------	------
mPD		0.040	0.50
		nPD:	0.08
		N. weight:	0.75
		Sc. PD:	-0.0045
		Sc. rank:	223.8

PB1

Pos.	690	obs:	exp:
tta	L	0	0.09
ttg	L	0	0.19
ctt	L	0	0.17
ctc	L	0	0.17
cta	L	0	0.17
ctg	L	1	0.22
caa	Q	27	556.20
cag	Q	1065	535.80
cgt	R	0	0.04
cgc	R	0	0.05
cga	R	0	0.10
cgg	R	1	0.08
aga	R	0	0.46
agg	R	0	0.27
---	--	------	------
mPD		0.052	0.51
		nPD:	0.1
		N. weight:	0.66
		Sc. PD:	0.011
		Sc. rank:	259.2

PB1

Pos.	691	obs:	exp:
cgt	R	0	0.26
cgc	R	0	0.30
cga	R	0	0.59
cgg	R	0	0.50
aaa	K	100	634.10
aag	K	988	453.90
aga	R	0	2.74
agg	R	6	1.61
---	--	------	------
mPD		0.18	0.50
		nPD:	0.35
		N. weight:	0.6
		Sc. PD:	0.15
		Sc. rank:	473.4

PB1

Pos.	692	obs:	exp:
tgt	C	64	462.70
tgc	C	1030	631.30
---	--	------	------
mPD		0.11	0.49
		nPD:	0.23
		N. weight:	0.38
		Sc. PD:	0.051
		Sc. rank:	262.8

PB1

Pos.	693	obs:	exp:
tct	S	0	0.16
tcc	S	0	0.13
tca	S	0	0.25
tcg	S	0	0.06
tat	Y	0	0.54
tac	Y	1	0.46
tgt	C	0	461.90
tgc	C	1092	630.10
agt	S	0	0.21
agc	S	1	0.19
---	--	------	------
mPD		0.0037	0.49
		nPD:	0.01
		N. weight:	0.6
		Sc. PD:	-0.045
		Sc. rank:	-346.8

PB1

Pos.	694	obs:	exp:
tct	S	0	11.61
tcc	S	0	9.53
tca	S	0	18.53
tcg	S	0	4.14
aat	N	41	520.10
aac	N	980	500.90
agt	S	0	15.01
agc	S	73	14.18
---	--	------	------
mPD		0.20	0.72
		nPD:	0.27
		N. weight:	0.68
		Sc. PD:	0.12
		Sc. rank:	499.7

PB1

Pos.	695	obs:	exp:
tta	L	6	97.08
ttg	L	0	203.70
ctt	L	0	182.20
ctc	L	0	182.60
cta	L	1058	184.40
ctg	L	30	244.10
---	--	------	------
mPD		0.064	1.1
		nPD:	0.06
		N. weight:	1.8
		Sc. PD:	-0.047
		Sc. rank:	292.3

PB1

Pos.	696	obs:	exp:
ttt	F	128	432.00
ttc	F	966	662.00
---	--	------	------
mPD		0.21	0.48
		nPD:	0.43
		N. weight:	0.21
		Sc. PD:	0.071
		Sc. rank:	176.0

PB1

Pos.	697	obs:	exp:
gaa	E	1055	665.70
gag	E	39	428.30
---	--	------	------
mPD		0.069	0.48
		nPD:	0.14
		N. weight:	0.4
		Sc. PD:	0.022
		Sc. rank:	213.0

PB1

Pos.	698	obs:	exp:
cgt	R	0	1.16
cgc	R	0	1.34
cga	R	0	2.64
cgg	R	0	2.25
aaa	K	7	621.90
aag	K	1060	445.10
aga	R	0	12.35
agg	R	27	7.26
---	--	------	------
mPD		0.061	0.55
		nPD:	0.11
		N. weight:	0.93
		Sc. PD:	0.023
		Sc. rank:	388.6

PB1

Pos.	699	obs:	exp:
ttt	F	6	432.00
ttc	F	1088	662.00
---	--	------	------
mPD		0.011	0.48
		nPD:	0.02
		N. weight:	0.52
		Sc. PD:	-0.031
		Sc. rank:	-95.6

PB1

Pos.	700	obs:	exp:
ttt	F	3	431.60
ttc	F	1090	661.40
gtt	V	0	0.21
gtc	V	1	0.20
gta	V	0	0.20
gtg	V	0	0.39
---	--	------	------
mPD		0.0073	0.48
		nPD:	0.02
		N. weight:	0.53
		Sc. PD:	-0.036
		Sc. rank:	-195.2

PB1

Pos.	701	obs:	exp:
tta	L	0	0.09
ttg	L	0	0.19
ctt	L	0	0.17
ctc	L	1	0.17
cta	L	0	0.17
ctg	L	0	0.22
cct	P	4	272.70
ccc	P	1068	196.40
cca	P	21	449.90
ccg	P	0	174.00
---	--	------	------
mPD		0.047	0.71
		nPD:	0.07
		N. weight:	1.7
		Sc. PD:	-0.033
		Sc. rank:	349.9

PB1

Pos.	702	obs:	exp:
tct	S	0	173.80
tcc	S	0	142.70
tca	S	0	277.40
tcg	S	0	62.02
tgt	C	1	0.42
tgc	C	0	0.58
agt	S	2	224.70
agc	S	1091	212.30
---	--	------	------
mPD		0.0073	1.7
		nPD:	0.
		N. weight:	1.8
		Sc. PD:	-0.14
		Sc. rank:	-1189.6

PB1

Pos.	703	obs:	exp:
ttt	F	1	0.39
ttc	F	0	0.61
tct	S	0	173.80
tcc	S	0	142.70
tca	S	0	277.40
tcg	S	0	62.02
agt	S	1088	224.70
agc	S	5	212.30
---	--	------	------
mPD		0.013	1.7
		nPD:	0.01
		N. weight:	1.7
		Sc. PD:	-0.13
		Sc. rank:	-897.9

PB1

Pos.	704	obs:	exp:
tct	S	1	174.00
tcc	S	0	142.90
tca	S	257	277.70
tcg	S	836	62.08
agt	S	0	224.90
agc	S	0	212.50
---	--	------	------
mPD		0.36	1.7
		nPD:	0.22
		N. weight:	2.2
		Sc. PD:	0.28
		Sc. rank:	1459.5

PB1

Pos.	705	obs:	exp:
tat	Y	18	590.30
tac	Y	1075	502.70
gct	A	1	0.26
gcc	A	0	0.19
gca	A	0	0.47
gcg	A	0	0.08
---	--	------	------
mPD		0.038	0.50
		nPD:	0.08
		N. weight:	0.76
		Sc. PD:	-0.0072
		Sc. rank:	209.6

PB1

Pos.	706	obs:	exp:
tgg	W	1	1.00
cgt	R	0	46.90
cgc	R	0	54.09
cga	R	9	106.90
cgg	R	217	91.12
aga	R	3	499.30
agg	R	863	293.70
gtt	V	1	0.21
gtc	V	0	0.20
gta	V	0	0.20
gtg	V	0	0.39
---	--	------	------
mPD		0.36	0.97
		nPD:	0.37
		N. weight:	1.1
		Sc. PD:	0.3
		Sc. rank:	869.6

PB1

Pos.	707	obs:	exp:
cgt	R	0	46.94
cgc	R	0	54.14
cga	R	0	107.00
cgg	R	0	91.20
aaa	K	0	0.58
aag	K	1	0.42
aga	R	11	499.80
agg	R	1082	294.00
---	--	------	------
mPD		0.022	0.96
		nPD:	0.02
		N. weight:	1.4
		Sc. PD:	-0.083
		Sc. rank:	-257.7

PB1

Pos.	708	obs:	exp:
tct	S	0	0.64
tcc	S	0	0.52
tca	S	4	1.02
tcg	S	0	0.23
cct	P	0	272.00
ccc	P	0	195.80
cca	P	1082	448.70
ccg	P	8	173.50
agt	S	0	0.82
agc	S	0	0.78
---	--	------	------
mPD		0.022	0.72
		nPD:	0.03
		N. weight:	0.94
		Sc. PD:	-0.049
		Sc. rank:	-59.2

PB1

Pos.	709	obs:	exp:
tta	L	0	0.09
ttg	L	0	0.19
ctt	L	1	0.17
ctc	L	0	0.17
cta	L	0	0.17
ctg	L	0	0.22
att	I	4	1.47
atc	I	0	0.96
ata	I	0	1.57
gtt	V	1089	225.60
gtc	V	0	219.80
gta	V	0	223.00
gtg	V	0	420.60
---	--	------	------
mPD		0.0091	0.73
		nPD:	0.01
		N. weight:	1.7
		Sc. PD:	-0.12
		Sc. rank:	-733.0

PB1			
Pos.	710	obs:	exp:
att	I	0	0.37
atc	I	0	0.24
ata	I	1	0.39
ggt	G	0	144.90
ggc	G	0	139.60
gga	G	1092	504.20
ggg	G	1	304.30
---	--	------	------
mPD		0.0055	0.68
		nPD:	0.01
		N. weight:	0.84
		Sc. PD:	-0.062
		Sc. rank:	-442.1

PB1			
Pos.	711	obs:	exp:
att	I	1062	401.60
atc	I	31	261.90
ata	I	0	429.50
gtt	V	1	0.21
gtc	V	0	0.20
gta	V	0	0.20
gtg	V	0	0.39
---	--	------	------
mPD		0.057	0.66
		nPD:	0.09
		N. weight:	0.97
		Sc. PD:	0.0015
		Sc. rank:	326.2

PB1			
Pos.	712	obs:	exp:
tct	S	42	174.00
tcc	S	1049	142.90
tca	S	3	277.70
tcg	S	0	62.08
agt	S	0	224.90
agc	S	0	212.50
---	--	------	------
mPD		0.079	1.7
		nPD:	0.05
		N. weight:	2.
		Sc. PD:	-0.073
		Sc. rank:	176.6

PB1			
Pos.	713	obs:	exp:
tct	S	0	173.80
tcc	S	0	142.70
tca	S	0	277.40
tcg	S	0	62.02
agt	S	12	224.70
agc	S	1081	212.30
ggt	G	0	0.13
ggc	G	1	0.13
gga	G	0	0.46
ggg	G	0	0.28
---	--	------	------
mPD		0.024	1.7
		nPD:	0.01
		N. weight:	1.7
		Sc. PD:	-0.12
		Sc. rank:	-684.3

PB1			
Pos.	714	obs:	exp:
atg	M	1094	1094.00
---	--	------	------
mPD		0	0
		nPD:	1.
		N. weight:	0.
		Sc. PD:	0
		Sc. rank:	0

PB1			
Pos.	715	obs:	exp:
atg	M	2	2.00
gtt	V	0	226.30
gtc	V	0	220.40
gta	V	1	223.60
gtg	V	1091	421.70
---	--	------	------
mPD		0.0055	0.73
		nPD:	0.01
		N. weight:	1.
		Sc. PD:	-0.077
		Sc. rank:	-589.3

PB1			
Pos.	716	obs:	exp:
aaa	K	0	0.58
aag	K	1	0.42
gaa	E	0	665.10
gag	E	1093	427.90
---	--	------	------
mPD		0.0018	0.48
		nPD:	0.
		N. weight:	1.
		Sc. PD:	-0.08
		Sc. rank:	-712.2

PB1			
Pos.	717	obs:	exp:
gct	A	0	282.50
gcc	A	1093	203.70
gca	A	1	514.90
gcg	A	0	92.85
---	--	------	------
mPD		0.0018	0.67
		nPD:	0.
		N. weight:	1.8
		Sc. PD:	-0.15
		Sc. rank:	-1353.5

PB1			
Pos.	718	obs:	exp:
atg	M	1094	1094.00
---	--	------	------
mPD		0	0
		nPD:	1.
		N. weight:	0.
		Sc. PD:	0
		Sc. rank:	0

PB1			
Pos.	719	obs:	exp:
atg	M	5	5.00
gtt	V	70	225.00
gtc	V	0	219.20
gta	V	3	222.40
gtg	V	1013	419.40
gct	A	0	0.77
gcc	A	0	0.56
gca	A	0	1.41
gcg	A	3	0.25
---	--	------	------
mPD		0.14	0.74
		nPD:	0.19
		N. weight:	0.81
		Sc. PD:	0.081
		Sc. rank:	504.3

PB1			
Pos.	720	obs:	exp:
ttt	F	1	0.39
ttc	F	0	0.61
tct	S	990	173.70
tcc	S	102	142.60
tca	S	0	277.20
tcg	S	0	61.96
tat	Y	1	0.54
tac	Y	0	0.46
agt	S	0	224.50
agc	S	0	212.10
---	--	------	------
mPD		0.17	1.7
		nPD:	0.1
		N. weight:	1.7
		Sc. PD:	0.031
		Sc. rank:	686.6

PB1			
Pos.	721	obs:	exp:
cgt	R	0	46.00
cgc	R	0	53.05
cga	R	0	104.80
cgg	R	1	89.37
aaa	K	0	13.41
aag	K	23	9.59
aga	R	0	489.70
agg	R	1070	288.10
---	--	------	------
mPD		0.043	1.0
		nPD:	0.04
		N. weight:	1.4
		Sc. PD:	-0.057
		Sc. rank:	62.8

PB1			
Pos.	722	obs:	exp:
gct	A	0	281.70
gcc	A	1091	203.20
gca	A	0	513.50
gcg	A	0	92.60
ggt	G	0	0.40
ggc	G	3	0.38
gga	G	0	1.38
ggg	G	0	0.84
---	--	------	------
mPD		0.0055	0.68
		nPD:	0.01
		N. weight:	1.8
		Sc. PD:	-0.14
		Sc. rank:	-939.1

PB1			
Pos.	723	obs:	exp:
caa	Q	1	0.51
cag	Q	0	0.49
cgt	R	0	46.94
cgc	R	0	54.14
cga	R	1090	107.00
cgg	R	0	91.20
aga	R	3	499.80
agg	R	0	294.00
---	--	------	------
mPD		0.0073	0.96
		nPD:	0.01
		N. weight:	2.5
		Sc. PD:	-0.19
		Sc. rank:	-1386.1

PB1			
Pos.	724	obs:	exp:
att	I	1094	402.00
atc	I	0	262.20
ata	I	0	429.90
---	--	------	------
mPD		0	0.65
		nPD:	0.
		N. weight:	1.1
		Sc. PD:	-0.09
		Sc. rank:	-944.7

PB1			
Pos.	725	obs:	exp:
gat	D	969	591.20
gac	D	125	502.80
---	--	------	------
mPD		0.20	0.50
		nPD:	0.41
		N. weight:	0.31
		Sc. PD:	0.096
		Sc. rank:	252.0

PB1			
Pos.	726	obs:	exp:
gtt	V	0	0.21
gtc	V	0	0.20
gta	V	1	0.20
gtg	V	0	0.39
gct	A	0	282.20
gcc	A	0	203.50
gca	A	1091	514.40
gcg	A	2	92.77
---	--	------	------
mPD		0.0055	0.67
		nPD:	0.01
		N. weight:	0.82
		Sc. PD:	-0.06
		Sc. rank:	-411.9

PB1			
Pos.	727	obs:	exp:
cgt	R	0	46.99
cgc	R	0	54.19
cga	R	1091	107.10
cgg	R	3	91.28
aga	R	0	500.20
agg	R	0	294.20
---	--	------	------
mPD		0.0055	0.96
		nPD:	0.01
		N. weight:	2.5
		Sc. PD:	-0.19
		Sc. rank:	-1566.9

PB1			
Pos.	728	obs:	exp:
att	I	1084	400.90
atc	I	4	261.40
ata	I	3	428.70
act	T	2	0.51
acc	T	0	0.41
aca	T	0	0.92
acg	T	0	0.16
gtt	V	1	0.21
gtc	V	0	0.20
gta	V	0	0.20
gtg	V	0	0.39
---	--	------	------
mPD		0.018	0.66
		nPD:	0.03
		N. weight:	1.1
		Sc. PD:	-0.058
		Sc. rank:	-111.3

PB1			
Pos.	729	obs:	exp:
gat	D	54	590.10
gac	D	1038	501.90
gaa	E	2	1.22
gag	E	0	0.78
---	--	------	------
mPD		0.097	0.50
		nPD:	0.2
		N. weight:	0.63
		Sc. PD:	0.067
		Sc. rank:	403.5

PB1			
Pos.	730	obs:	exp:
ttt	F	0	432.00
ttc	F	1094	662.00
---	--	------	------
mPD		0	0.48
		nPD:	0.
		N. weight:	0.55
		Sc. PD:	-0.045
		Sc. rank:	-474.0

PB1			
Pos.	731	obs:	exp:
gaa	E	1087	665.70
gag	E	7	428.30
---	--	------	------
mPD		0.013	0.48
		nPD:	0.03
		N. weight:	0.51
		Sc. PD:	-0.029
		Sc. rank:	-66.5

PB1			
Pos.	732	obs:	exp:
tct	S	1094	174.00
tcc	S	0	142.90
tca	S	0	277.70
tcg	S	0	62.08
agt	S	0	224.90
agc	S	0	212.50
---	--	------	------
mPD		0	1.7
		nPD:	0.
		N. weight:	2.
		Sc. PD:	-0.16
		Sc. rank:	-1734.6

PB1			
Pos.	733	obs:	exp:
cgt	R	0	0.09
cgc	R	0	0.10
cga	R	0	0.20
cgg	R	0	0.17
aga	R	2	0.91
agg	R	0	0.54
ggt	G	0	144.70
ggc	G	0	139.50
gga	G	1088	503.80
ggg	G	4	304.00
---	--	------	------
mPD		0.011	0.68
		nPD:	0.02
		N. weight:	0.83
		Sc. PD:	-0.055
		Sc. rank:	-285.5

PB1			
Pos.	734	obs:	exp:
cgt	R	0	46.81
cgc	R	0	53.99
cga	R	0	106.70
cgg	R	0	90.95
aaa	K	0	0.58
aag	K	1	0.42
aga	R	0	498.40
agg	R	1090	293.20
ggt	G	0	0.40
ggc	G	0	0.38
gga	G	0	1.38
ggg	G	3	0.84
---	--	------	------
mPD		0.0073	0.97
		nPD:	0.01
		N. weight:	1.4
		Sc. PD:	-0.11
		Sc. rank:	-803.7

PB1			
Pos.	735	obs:	exp:
att	I	1088	401.20
atc	I	4	261.70
ata	I	0	429.10
aat	N	1	0.51
aac	N	0	0.49
gtt	V	1	0.21
gtc	V	0	0.20
gta	V	0	0.20
gtg	V	0	0.39
---	--	------	------
mPD		0.011	0.66
		nPD:	0.02
		N. weight:	1.1
		Sc. PD:	-0.071
		Sc. rank:	-328.8

,

PB1			
Pos.	736	obs:	exp:
cgt	R	0	0.94
cgc	R	0	1.09
cga	R	0	2.15
cgg	R	0	1.84
aat	N	1	0.51
aac	N	0	0.49
aaa	K	1	620.10
aag	K	1063	443.90
aga	R	0	10.06
agg	R	22	5.92
gaa	E	0	1.83
gag	E	3	1.17
ggt	G	0	0.53
ggc	G	0	0.51
gga	G	0	1.85
ggg	G	4	1.11
---	--	------	------
mPD		0.063	0.56
		nPD:	0.11
		N. weight:	0.96
		Sc. PD:	0.025
		Sc. rank:	417.4

,

PB1			
Pos.	737	obs:	exp:
cgt	R	0	1.37
cgc	R	0	1.58
cga	R	0	3.13
cgg	R	0	2.67
aaa	K	1038	619.00
aag	K	24	443.00
aga	R	32	14.63
agg	R	0	8.61
---	--	------	------
mPD		0.10	0.56
		nPD:	0.18
		N. weight:	0.5
		Sc. PD:	0.044
		Sc. rank:	300.0

,

PB1			
Pos.	738	obs:	exp:
gat	D	1	1.62
gac	D	2	1.38
gaa	E	1081	663.90
gag	E	10	427.10
---	--	------	------
mPD		0.024	0.48
		nPD:	0.05
		N. weight:	0.49
		Sc. PD:	-0.017
		Sc. rank:	47.4

,

PB1			
Pos.	739	obs:	exp:
gat	D	1	0.54
gac	D	0	0.46
gaa	E	31	664.50
gag	E	1061	427.50
ggt	G	0	0.13
ggc	G	0	0.13
gga	G	0	0.46
ggg	G	1	0.28
---	--	------	------
mPD		0.059	0.48
		nPD:	0.12
		N. weight:	0.88
		Sc. PD:	0.031
		Sc. rank:	414.5

,

PB1			
Pos.	740	obs:	exp:
ttt	F	1087	432.00
ttc	F	7	662.00
---	--	------	------
mPD		0.013	0.48
		nPD:	0.03
		N. weight:	0.98
		Sc. PD:	-0.055
		Sc. rank:	-132.9

,

PB1			
Pos.	741	obs:	exp:
tct	S	1	0.16
tcc	S	0	0.13
tca	S	0	0.25
tcg	S	0	0.06
agt	S	0	0.21
agc	S	0	0.19
gct	A	1091	282.20
gcc	A	2	203.50
gca	A	0	514.40
gcg	A	0	92.77
---	--	------	------
mPD		0.0055	0.67
		nPD:	0.01
		N. weight:	1.5
		Sc. PD:	-0.11
		Sc. rank:	-744.9

,

PB1			
Pos.	742	obs:	exp:
aaa	K	0	0.58
aag	K	1	0.42
gaa	E	0	665.10
gag	E	1093	427.90
---	--	------	------
mPD		0.0018	0.48
		nPD:	0.
		N. weight:	1.
		Sc. PD:	-0.08
		Sc. rank:	-712.2

,

PB1			
Pos.	743	obs:	exp:
att	I	0	401.60
atc	I	1093	261.90
ata	I	0	429.50
act	T	0	0.25
acc	T	1	0.21
aca	T	0	0.46
acg	T	0	0.08
---	--	------	------
mPD		0.0018	0.66
		nPD:	0.
		N. weight:	1.6
		Sc. PD:	-0.12
		Sc. rank:	-1142.6

,

PB1			
Pos.	744	obs:	exp:
att	I	1	2.57
atc	I	0	1.68
ata	I	6	2.75
atg	M	1086	1086.00
act	T	0	0.25
acc	T	0	0.21
aca	T	0	0.46
acg	T	1	0.08
---	--	------	------
mPD		0.015	0.016
		nPD:	0.9
		N. weight:	0.0072
		Sc. PD:	0.0056
		Sc. rank:	6.2

,

PB1			
Pos.	745	obs:	exp:
cgt	R	0	0.13
cgc	R	0	0.15
cga	R	0	0.29
cgg	R	0	0.25
aaa	K	2	635.90
aag	K	1089	455.10
aga	R	0	1.37
agg	R	3	0.81
---	--	------	------
mPD		0.0091	0.49
		nPD:	0.02
		N. weight:	0.95
		Sc. PD:	-0.061
		Sc. rank:	-260.6

,

PB1			
Pos.	746	obs:	exp:
att	I	0	401.60
atc	I	1093	261.90
ata	I	0	429.50
act	T	0	0.25
acc	T	1	0.21
aca	T	0	0.46
acg	T	0	0.08
---	--	------	------
mPD		0.0018	0.66
		nPD:	0.
		N. weight:	1.6
		Sc. PD:	-0.12
		Sc. rank:	-1142.6

,

PB1			
Pos.	747	obs:	exp:
tgt	C	1094	462.70
tgc	C	0	631.30
---	--	------	------
mPD		0	0.49
		nPD:	0.
		N. weight:	0.94
		Sc. PD:	-0.077
		Sc. rank:	-811.8

,

PB1			
Pos.	748	obs:	exp:
tct	S	0	174.00
tcc	S	1094	142.90
tca	S	0	277.70
tcg	S	0	62.08
agt	S	0	224.90
agc	S	0	212.50
---	--	------	------
mPD		0	1.7
		nPD:	0.
		N. weight:	2.2
		Sc. PD:	-0.18
		Sc. rank:	-1920.6

,

PB1			
Pos.	749	obs:	exp:
act	T	0	278.90
acc	T	1094	224.50
aca	T	0	501.80
acg	T	0	88.86
---	--	------	------
mPD		0	0.68
		nPD:	0.
		N. weight:	1.7
		Sc. PD:	-0.14
		Sc. rank:	-1494.3

,

PB1			
Pos.	750	obs:	exp:
att	I	1094	402.00
atc	I	0	262.20
ata	I	0	429.90
---	--	------	------
mPD		0	0.65
		nPD:	0.
		N. weight:	1.1
		Sc. PD:	-0.09
		Sc. rank:	-944.7

,

PB1			
Pos.	751	obs:	exp:
gat	D	1	0.54
gac	D	0	0.46
gaa	E	1093	665.10
gag	E	0	427.90
---	--	------	------
mPD		0.0018	0.48
		nPD:	0.
		N. weight:	0.55
		Sc. PD:	-0.043
		Sc. rank:	-373.9

,

PB1			
Pos.	752	obs:	exp:
aaa	K	0	1.17
aag	K	2	0.83
gaa	E	2	663.90
gag	E	1089	427.10
ggt	G	0	0.13
ggc	G	0	0.13
gga	G	0	0.46
ggg	G	1	0.28
---	--	------	------
mPD		0.0091	0.48
		nPD:	0.02
		N. weight:	1.
		Sc. PD:	-0.065
		Sc. rank:	-265.6

,

PB1			
Pos.	753	obs:	exp:
ttt	F	0	0.79
ttc	F	2	1.21
tta	L	0	96.73
ttg	L	0	202.90
ctt	L	0	181.50
ctc	L	1090	181.90
cta	L	0	183.70
ctg	L	0	243.20
att	I	0	0.73
atc	I	2	0.48
ata	I	0	0.79
---	--	------	------
mPD		0.0073	1.1
		nPD:	0.01
		N. weight:	2.
		Sc. PD:	-0.15
		Sc. rank:	-1197.7

,

PB1			
Pos.	754	obs:	exp:
cgt	R	0	46.86
cgc	R	0	54.04
cga	R	0	106.80
cgg	R	0	91.03
aaa	K	2	1.17
aag	K	0	0.83
aga	R	1091	498.90
agg	R	0	293.40
ggt	G	0	0.13
ggc	G	0	0.13
gga	G	1	0.46
ggg	G	0	0.28
---	--	------	------
mPD		0.0055	0.97
		nPD:	0.01
		N. weight:	0.86
		Sc. PD:	-0.066
		Sc. rank:	-534.4

,

PB1			
Pos.	755	obs:	exp:
cgt	R	0	46.99
cgc	R	0	54.19
cga	R	0	107.10
cgg	R	1094	91.28
aga	R	0	500.20
agg	R	0	294.20
---	--	------	------
mPD		0	0.96
		nPD:	0.
		N. weight:	2.7
		Sc. PD:	-0.22
		Sc. rank:	-2343.2

,

PB1			
Pos.	756	obs:	exp:
tta	L	0	0.09
ttg	L	0	0.19
ctt	L	0	0.17
ctc	L	0	0.17
cta	L	1	0.17
ctg	L	0	0.22
caa	Q	1085	554.70
cag	Q	4	534.30
cgt	R	0	0.04
cgc	R	0	0.05
cga	R	1	0.10
cgg	R	0	0.08
aaa	K	3	1.75
aag	K	0	1.25
aga	R	0	0.46
agg	R	0	0.27
---	--	------	------
mPD		0.016	0.51
		nPD:	0.03
		N. weight:	0.72
		Sc. PD:	-0.037
		Sc. rank:	-37.3

,

PB1			
Pos.	757	obs:	exp:
aat	N	50	25.47
aac	N	0	24.53
aaa	K	1040	608.50
aag	K	4	435.50
---	--	------	------
mPD		0.094	0.53
		nPD:	0.18
		N. weight:	0.58
		Sc. PD:	0.051
		Sc. rank:	347.8

,

PB1			
Pos.	758	obs:	exp:
tta	L	0	0.09
ttg	L	1	0.19
tgg	W	2	2.00
ctt	L	0	0.17
ctc	L	0	0.17
cta	L	0	0.17
ctg	L	0	0.22
---	-	1091	1091.00
---	--	------	------
mPD		3.3×10^{-6}	8.7×10^{-6}
		nPD:	0.39
		N. weight:	0.
		Sc. PD:	0
		Sc. rank:	0

,

PB1			
Pos.	759	obs:	exp:
taa	.	18	464.80
tag	.	1069	273.90
tga	.	7	355.40
---	--	------	------
mPD		0.057	0.81
		nPD:	0.07
		N. weight:	1.4
		Sc. PD:	-0.019
		Sc. rank:	331.3

}

Amino acid level conservation per codon

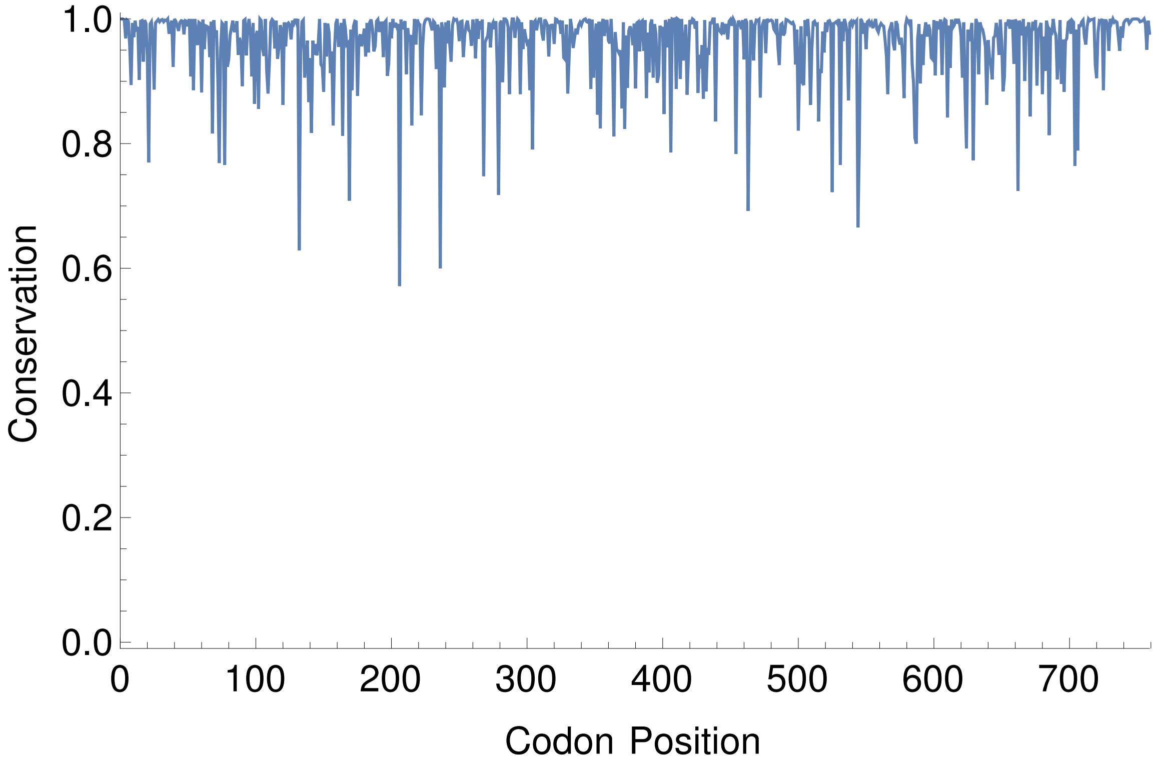

Conservation analysis plots

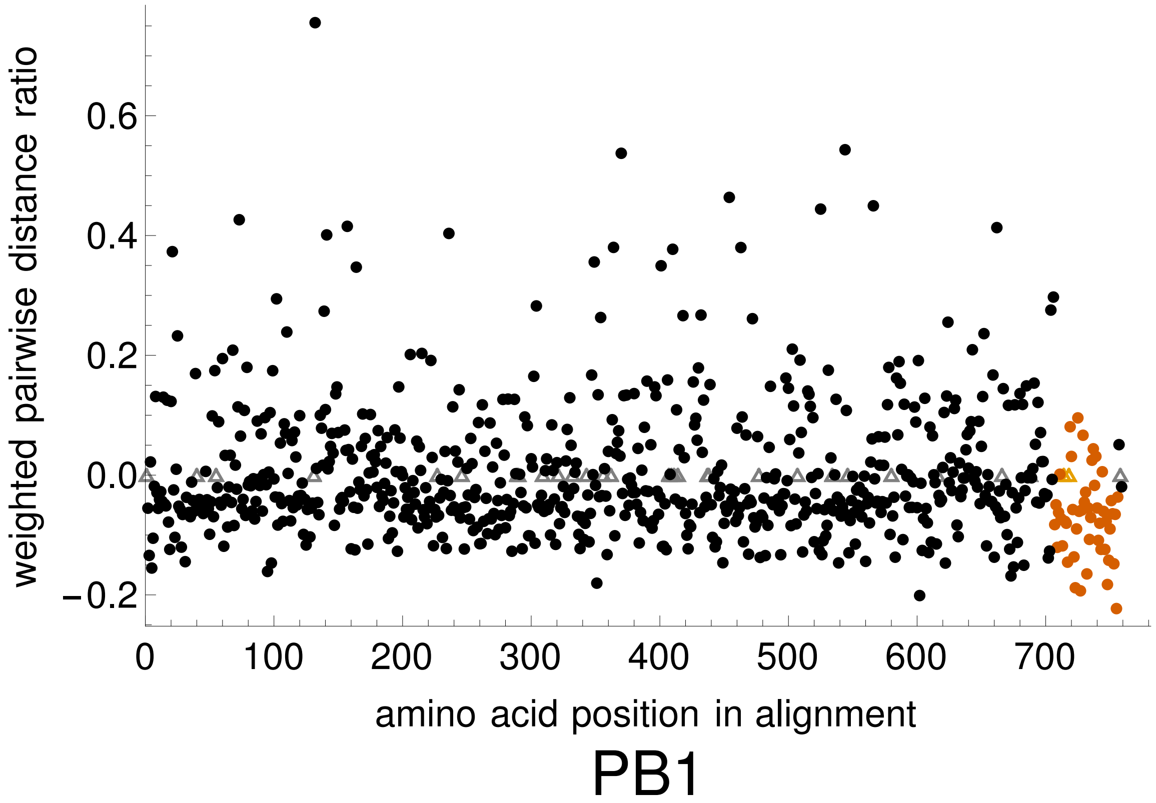

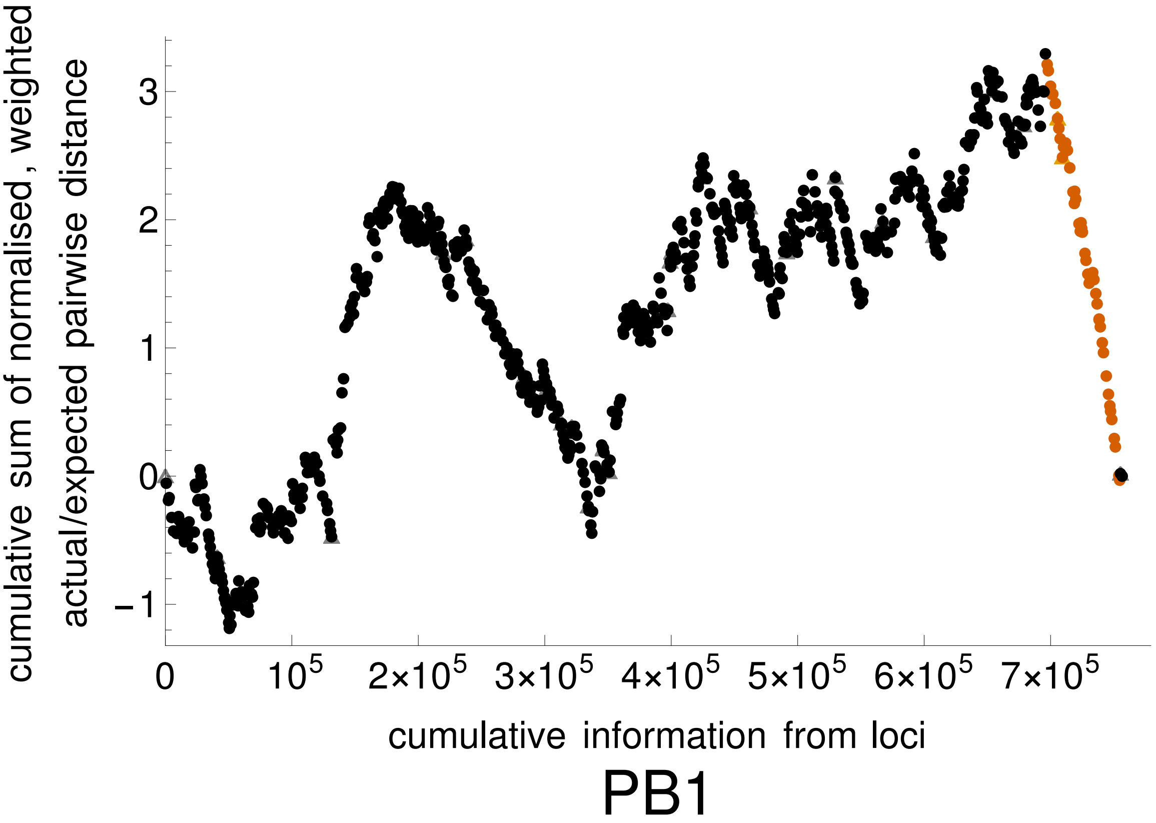

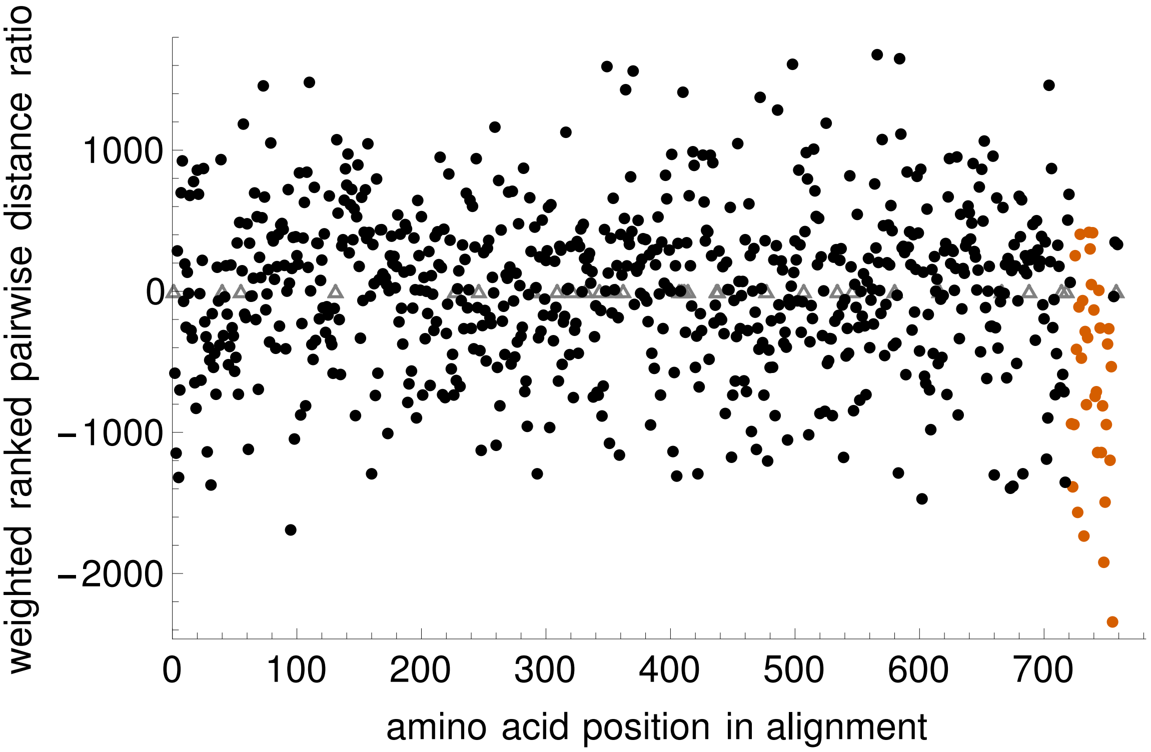
weighted ranked pairwise distance ratio
1000
0
−1000
−2000
0
100
200
300
400
500
600
700
amino acid position in alignment

PB1

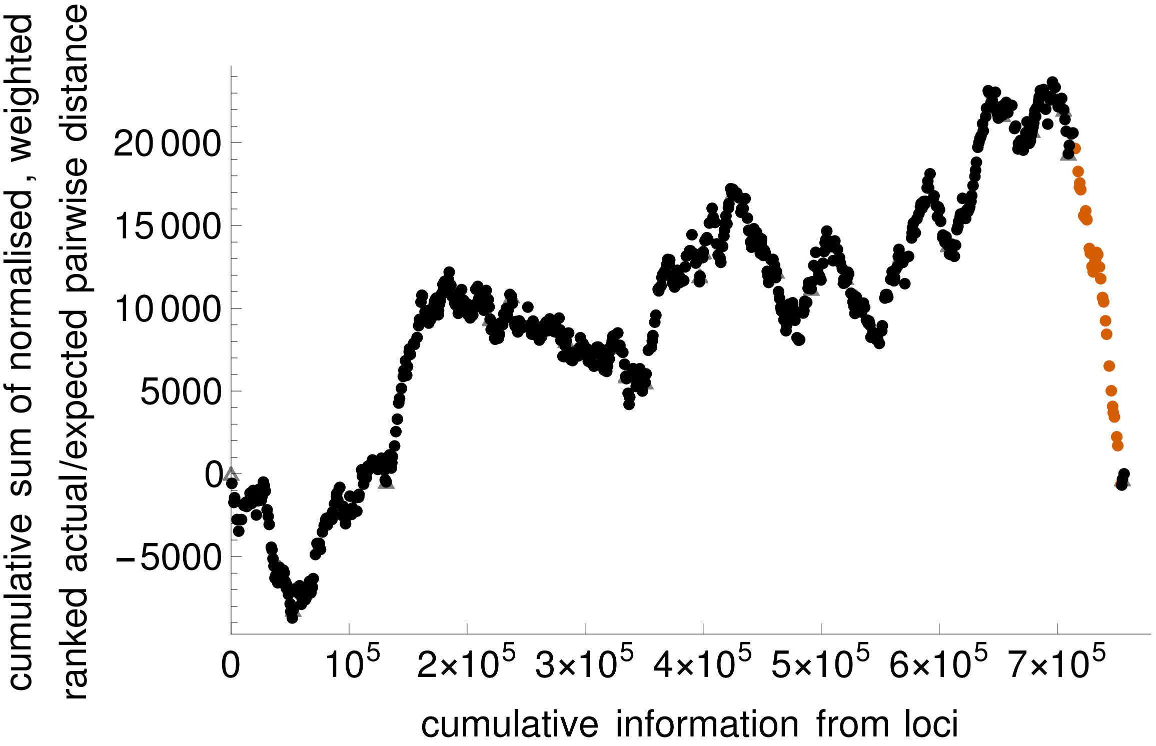
cumulative sum of normalised, weighted ranked actual/expected pairwise distance
20 000
15 000
10 000
5000
0
−5000
0
10^5
2×10^5
3×10^5
4×10^5
5×10^5
6×10^5
7×10^5
cumulative information from loci

PB1

PB1-F2

Gene length histogram

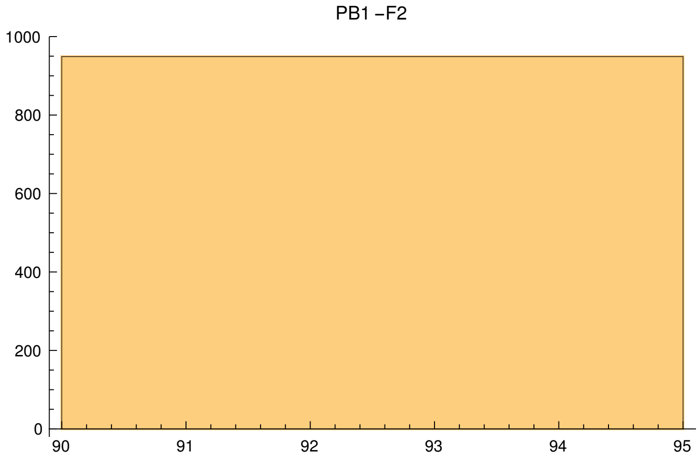

Information vs. nPD

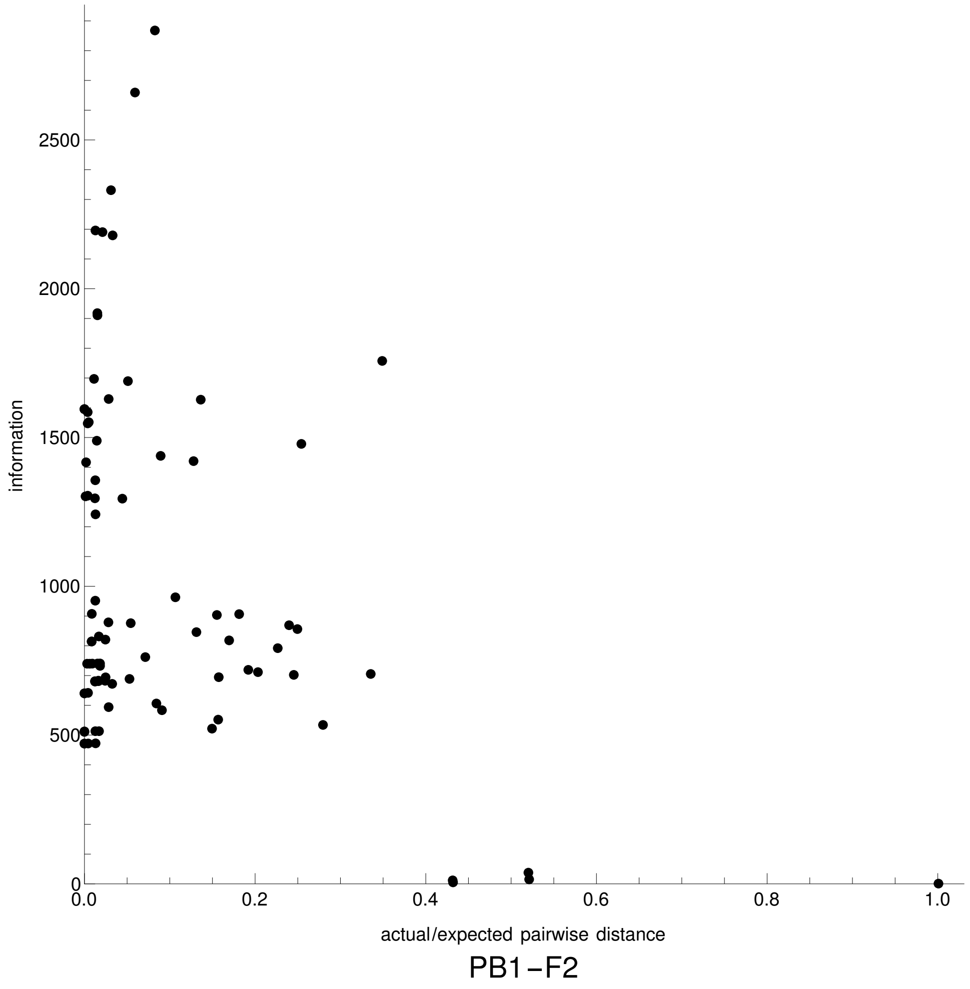

Example sequences highlighted by regions found in analyses to be conserved

```
Interesting points (by weighted raw PD) highlighted for gene PB1-F2:
ATGGAACAGGAACAGGATACACCATGGACACAGTCAACAGAACACATAAATACTCAGAAAAAGGAAAGTGGACAACGA⋱
    ACACAGAAACTGGAGCACCCCAACTCAATCCAATTGATGGACCATTACCTGAGGACAATGAGCCGAGTGGGTATGCA⋱
    CAAACGGATTGTGTATTGGAAGCAATGGCTTTCCTTGAAGAATCTCACCCAGGGATCTTTGAAAACTCGTGTCTCGA⋱
    AACGATGGAAATTGTTCAGCAAACAAGAGTGGACAAACTGA

Interesting points (by weighted ranked PD) highlighted for gene PB1-F2:
ATGGAACAGGAACAGGATACACCATGGACACAGTCAACAGAACACATAAATACTCAGAAAAAGGAAAGTGGACAACGA⋱
    ACACAGAAACTGGAGCACCCCAACTCAATCCAATTGATGGACCATTACCTGAGGACAATGAGCCGAGTGGGTATGCA⋱
    CAAACGGATTGTGTATTGGAAGCAATGGCTTTCCTTGAAGAATCTCACCCAGGGATCTTTGAAAACTCGTGTCTCGA⋱
    AACGATGGAAATTGTTCAGCAAACAAGAGTGGACAAACTGA
```

Per codon conservation report

{

PB1-F2

Pos.	1	obs:	exp:
atg	M	949	949.00
---	--	------	------
mPD		0	0
		nPD:	1.
		N. weight:	0.
		Sc. PD:	0
		Sc. rank:	0

,

PB1-F2

Pos.	2	obs:	exp:
gaa	E	948	576.90
gag	E	0	371.10
ggt	G	0	0.13
ggc	G	0	0.13
gga	G	1	0.46
ggg	G	0	0.28
---	--	------	------
mPD		0.0021	0.48
		nPD:	0.
		N. weight:	0.48
		Sc. PD:	-0.027
		Sc. rank:	-253.7

,

PB1-F2

Pos.	3	obs:	exp:
caa	Q	0	481.90
cag	Q	946	464.10
cgt	R	0	0.13
cgc	R	0	0.15
cga	R	0	0.29
cgg	R	3	0.25
aga	R	0	1.37
agg	R	0	0.81
---	--	------	------
mPD		0.0063	0.51
		nPD:	0.01
		N. weight:	0.7
		Sc. PD:	-0.033
		Sc. rank:	-243.4

,

PB1-F2

Pos.	4	obs:	exp:
gaa	E	949	577.50
gag	E	0	371.50
---	--	------	------
mPD		0	0.48
		nPD:	0.
		N. weight:	0.48
		Sc. PD:	-0.029
		Sc. rank:	-402.3

,

PB1-F2

Pos.	5	obs:	exp:
tta	L	0	0.09
ttg	L	0	0.19
ctt	L	0	0.17
ctc	L	0	0.17
cta	L	0	0.17
ctg	L	1	0.22
cct	P	0	0.25
ccc	P	0	0.18
cca	P	0	0.41
ccg	P	1	0.16
caa	Q	0	481.30
cag	Q	945	463.70
cgt	R	0	0.09
cgc	R	0	0.10
cga	R	0	0.20
cgg	R	2	0.17
aga	R	0	0.91
agg	R	0	0.54
---	--	------	------
mPD		0.0084	0.51
		nPD:	0.02
		N. weight:	0.7
		Sc. PD:	-0.03
		Sc. rank:	-46.0

,

PB1-F2

Pos.	6	obs:	exp:
gat	D	942	509.00
gac	D	0	433.00
ggt	G	7	0.93
ggc	G	0	0.89
gga	G	0	3.23
ggg	G	0	1.95
---	--	------	------
mPD		0.015	0.52
		nPD:	0.03
		N. weight:	0.61
		Sc. PD:	-0.019
		Sc. rank:	60.6

,

PB1-F2

Pos.	7	obs:	exp:
att	I	0	0.37
atc	I	0	0.24
ata	I	1	0.39
act	T	0	241.70
acc	T	0	194.50
aca	T	948	434.80
acg	T	0	77.00
---	--	------	------
mPD		0.0021	0.68
		nPD:	0.
		N. weight:	0.76
		Sc. PD:	-0.043
		Sc. rank:	-478.1

,

PB1-F2

Pos.	8	obs:	exp:
tta	L	0	6.12
ttg	L	0	12.85
ctt	L	0	11.49
ctc	L	0	11.51
cta	L	69	11.63
ctg	L	0	15.40
cct	P	0	219.10
ccc	P	0	157.70
cca	P	878	361.40
ccg	P	0	139.80
caa	Q	2	1.02
cag	Q	0	0.98
---	--	------	------
mPD		0.14	0.89
		nPD:	0.16
		N. weight:	0.93
		Sc. PD:	0.084
		Sc. rank:	560.9

,

PB1-F2

Pos.	9	obs:	exp:
tgg	W	949	949.00
---	--	------	------
mPD		0	0
		nPD:	1.
		N. weight:	0.
		Sc. PD:	0
		Sc. rank:	0

,

PB1-F2

Pos.	10	obs:	exp:
att	I	0	1.10
atc	I	0	0.72
ata	I	3	1.18
act	T	0	241.10
acc	T	0	194.10
aca	T	946	433.90
acg	T	0	76.84
---	--	------	------
mPD		0.0063	0.68
		nPD:	0.01
		N. weight:	0.76
		Sc. PD:	-0.038
		Sc. rank:	-317.6

,

PB1-F2

Pos.	11	obs:	exp:
tta	L	0	0.44
ttg	L	0	0.93
ctt	L	0	0.83
ctc	L	0	0.83
cta	L	0	0.84
ctg	L	5	1.12
caa	Q	15	480.30
cag	Q	928	462.70
cgt	R	0	0.04
cgc	R	0	0.05
cga	R	0	0.10
cgg	R	1	0.08
aga	R	0	0.46
agg	R	0	0.27
---	--	------	------
mPD		0.044	0.52
		nPD:	0.08
		N. weight:	0.62
		Sc. PD:	0.014
		Sc. rank:	278.0

,

PB1-F2

Pos.	12	obs:	exp:
tta	L	1	0.09
ttg	L	0	0.19
tct	S	0	150.80
tcc	S	0	123.80
tca	S	948	240.60
tcg	S	0	53.79
ctt	L	0	0.17
ctc	L	0	0.17
cta	L	0	0.17
ctg	L	0	0.22
agt	S	0	194.90
agc	S	0	184.10
---	--	------	------
mPD		0.0021	1.7
		nPD:	0.
		N. weight:	1.3
		Sc. PD:	-0.077
		Sc. rank:	-896.5

,

PB1-F2

Pos.	13	obs:	exp:
att	I	0	2.20
atc	I	0	1.44
ata	I	6	2.36
act	T	0	240.40
acc	T	0	193.50
aca	T	943	432.50
acg	T	0	76.60
---	--	------	------
mPD		0.013	0.69
		nPD:	0.02
		N. weight:	0.76
		Sc. PD:	-0.032
		Sc. rank:	-19.9

,

PB1-F2

Pos.	14	obs:	exp:
gaa	E	946	575.60
gag	E	0	370.40
ggt	G	0	0.40
ggc	G	0	0.38
gga	G	3	1.38
ggg	G	0	0.84
---	--	------	------
mPD		0.0063	0.48
		nPD:	0.01
		N. weight:	0.49
		Sc. PD:	-0.023
		Sc. rank:	-100.4

,

PB1-F2

Pos.	15	obs:	exp:
cat	H	0	543.80
cac	H	947	403.20
cgt	R	0	0.09
cgc	R	2	0.10
cga	R	0	0.20
cgg	R	0	0.17
aga	R	0	0.91
agg	R	0	0.54
---	--	------	------
mPD		0.0042	0.50
		nPD:	0.01
		N. weight:	0.84
		Sc. PD:	-0.043
		Sc. rank:	-388.7

,

PB1-F2

Pos.	16	obs:	exp:
att	I	0	345.70
atc	I	1	225.50
ata	I	940	369.80
act	T	0	2.04
acc	T	0	1.64
aca	T	8	3.67
acg	T	0	0.65
---	--	------	------
mPD		0.019	0.67
		nPD:	0.03
		N. weight:	0.9
		Sc. PD:	-0.029
		Sc. rank:	79.4

,

PB1-F2

Pos.	17	obs:	exp:
aat	N	949	483.40
aac	N	0	465.60
---	--	------	------
mPD		0	0.50
		nPD:	0.
		N. weight:	0.66
		Sc. PD:	-0.039
		Sc. rank:	-546.3

,

PB1-F2

Pos.	18	obs:	exp:
att	I	4	1.47
atc	I	0	0.96
ata	I	0	1.57
act	T	945	240.90
acc	T	0	193.90
aca	T	0	433.40
acg	T	0	76.76
---	--	------	------
mPD		0.0084	0.68
		nPD:	0.01
		N. weight:	1.3
		Sc. PD:	-0.063
		Sc. rank:	-480.2

,

PB1-F2

Pos.	19	obs:	exp:
caa	Q	0	481.30
cag	Q	945	463.70
cgt	R	0	0.17
cgc	R	0	0.20
cga	R	0	0.39
cgg	R	4	0.33
aga	R	0	1.83
agg	R	0	1.08
---	--	------	------
mPD		0.0084	0.51
		nPD:	0.02
		N. weight:	0.7
		Sc. PD:	-0.03
		Sc. rank:	-55.2

,

PB1-F2

Pos.	20	obs:	exp:
aaa	K	949	553.10
aag	K	0	395.90
---	--	------	------
mPD		0	0.49
		nPD:	0.
		N. weight:	0.53
		Sc. PD:	-0.031
		Sc. rank:	-437.2

,

PB1-F2

Pos.	21	obs:	exp:
cgt	R	0	1.59
cgc	R	0	1.83
cga	R	0	3.62
cgg	R	0	3.09
aaa	K	0	531.00
aag	K	911	380.00
aga	R	0	16.92
agg	R	37	9.95
gaa	E	0	0.61
gag	E	1	0.39
mPD		0.077	0.59
		nPD:	0.13
		N. weight:	0.87
		Sc. PD:	0.058
		Sc. rank:	474.7

PB1-F2

Pos.	22	obs:	exp:
gaa	E	949	577.50
gag	E	0	371.50
mPD		0	0.48
		nPD:	0.
		N. weight:	0.48
		Sc. PD:	-0.029
		Sc. rank:	-402.3

PB1-F2

Pos.	23	obs:	exp:
tct	S	0	132.30
tcc	S	0	108.70
tca	S	0	211.20
tcg	S	0	47.21
tgt	C	7	2.96
tgc	C	0	4.04
aat	N	92	46.86
aac	N	0	45.14
agt	S	832	171.00
agc	S	0	161.60
ggt	G	18	2.39
ggc	G	0	2.30
gga	G	0	8.30
ggg	G	0	5.01
mPD		0.23	1.8
		nPD:	0.13
		N. weight:	1.5
		Sc. PD:	0.093
		Sc. rank:	773.7

PB1-F2

Pos.	24	obs:	exp:
ggt	G	0	125.80
ggc	G	0	121.20
gga	G	941	437.80
ggg	G	8	264.20
mPD		0.017	0.68
		nPD:	0.02
		N. weight:	0.71
		Sc. PD:	-0.025
		Sc. rank:	50.8

PB1-F2

Pos.	25	obs:	exp:
caa	Q	949	483.40
cag	Q	0	465.60
mPD		0	0.50
		nPD:	0.
		N. weight:	0.66
		Sc. PD:	-0.039
		Sc. rank:	-546.3

PB1-F2

Pos.	26	obs:	exp:
caa	Q	16	8.15
cag	Q	0	7.85
cgt	R	0	40.07
cgc	R	0	46.21
cga	R	933	91.30
cgg	R	0	77.85
aga	R	0	426.60
agg	R	0	250.90
mPD		0.033	1.0
		nPD:	0.03
		N. weight:	2.2
		Sc. PD:	-0.061
		Sc. rank:	512.2

PB1-F2

Pos.	27	obs:	exp:
att	I	0	1.84
atc	I	0	1.20
ata	I	5	1.96
act	T	0	240.60
acc	T	0	193.70
aca	T	944	433.00
acg	T	0	76.68
mPD		0.010	0.69
		nPD:	0.02
		N. weight:	0.76
		Sc. PD:	-0.034
		Sc. rank:	-69.8

PB1-F2

Pos.	28	obs:	exp:
tta	L	0	0.35
ttg	L	0	0.74
ctt	L	0	0.67
ctc	L	0	0.67
cta	L	0	0.67
ctg	L	4	0.89
caa	Q	0	480.30
cag	Q	943	462.70
cgt	R	0	0.09
cgc	R	0	0.10
cga	R	0	0.20
cgg	R	2	0.17
aga	R	0	0.91
agg	R	0	0.54
mPD		0.013	0.52
		nPD:	0.02
		N. weight:	0.7
		Sc. PD:	-0.025
		Sc. rank:	29.7

PB1-F2

Pos.	29	obs:	exp:
cgt	R	0	35.26
cgc	R	0	40.66
cga	R	0	80.34
cgg	R	0	68.51
aaa	K	128	74.60
aag	K	0	53.40
aga	R	821	375.40
agg	R	0	220.80
mPD		0.23	1.1
		nPD:	0.2
		N. weight:	0.73
		Sc. PD:	0.1
		Sc. rank:	502.9

PB1-F2

Pos.	30	obs:	exp:
tta	L	0	84.22
ttg	L	1	176.70
ctt	L	0	158.00
ctc	L	0	158.40
cta	L	0	159.90
ctg	L	948	211.80
mPD		0.0021	1.1
		nPD:	0.
		N. weight:	1.5
		Sc. PD:	-0.083
		Sc. rank:	-936.1

PB1-F2

Pos.	31	obs:	exp:
aaa	K	0	1.75
aag	K	3	1.25
gat	D	1	0.54
gac	D	0	0.46
gaa	E	0	544.60
gag	E	895	350.40
ggt	G	0	6.63
ggc	G	0	6.39
gga	G	0	23.07
ggg	G	50	13.92
mPD		0.11	0.60
		nPD:	0.18
		N. weight:	0.93
		Sc. PD:	0.11
		Sc. rank:	615.3

PB1-F2

Pos.	32	obs:	exp:
cat	H	0	541.50
cac	H	943	401.50
caa	Q	1	0.51
cag	Q	0	0.49
cgt	R	0	0.21
cgc	R	5	0.25
cga	R	0	0.49
cgg	R	0	0.42
aga	R	0	2.29
agg	R	0	1.34
mPD		0.013	0.51
		nPD:	0.02
		N. weight:	0.84
		Sc. PD:	-0.03
		Sc. rank:	48.9

PB1-F2

Pos.	33	obs:	exp:
tta	L	0	0.62
ttg	L	0	1.30
tct	S	0	0.32
tcc	S	2	0.26
tca	S	0	0.51
tcg	S	0	0.11
ctt	L	0	1.17
ctc	L	7	1.17
cta	L	0	1.18
ctg	L	0	1.56
cct	P	0	234.30
ccc	P	939	168.70
cca	P	0	386.50
ccg	P	0	149.50
cat	H	0	0.57
cac	H	1	0.43
agt	S	0	0.41
agc	S	0	0.39
mPD		0.021	0.74
		nPD:	0.03
		N. weight:	1.7
		Sc. PD:	-0.053
		Sc. rank:	218.0

PB1-F2

Pos.	34	obs:	exp:
tct	S	0	2.23
tcc	S	0	1.83
tca	S	0	3.55
tcg	S	0	0.79
aat	N	0	476.30
aac	N	935	458.70
agt	S	0	2.88
agc	S	14	2.72
mPD		0.029	0.55
		nPD:	0.05
		N. weight:	0.71
		Sc. PD:	-0.0058
		Sc. rank:	211.3

PB1-F2

Pos.	35	obs:	exp:
tta	L	3	0.27
ttg	L	0	0.56
tct	S	0	150.50
tcc	S	0	123.50
tca	S	946	240.10
tcg	S	0	53.68
ctt	L	0	0.50
ctc	L	0	0.50
cta	L	0	0.51
ctg	L	0	0.67
agt	S	0	194.50
agc	S	0	183.70
mPD		0.0063	1.7
		nPD:	0.
		N. weight:	1.3
		Sc. PD:	-0.074
		Sc. rank:	-769.4

PB1-F2

Pos.	36	obs:	exp:
att	I	0	347.20
atc	I	945	226.50
ata	I	0	371.30
act	T	0	1.02
acc	T	4	0.82
aca	T	0	1.83
acg	T	0	0.32
mPD		0.0084	0.66
		nPD:	0.01
		N. weight:	1.4
		Sc. PD:	-0.065
		Sc. rank:	-436.1

PB1-F2

Pos.	37	obs:	exp:
caa	Q	852	434.00
cag	Q	0	418.00
cgt	R	0	4.17
cgc	R	0	4.80
cga	R	95	9.49
cgg	R	2	8.09
aga	R	0	44.35
agg	R	0	26.09
mPD		0.19	0.83
		nPD:	0.23
		N. weight:	0.81
		Sc. PD:	0.13
		Sc. rank:	572.1

PB1-F2

Pos.	38	obs:	exp:
tta	L	0	84.22
ttg	L	949	176.70
ctt	L	0	158.00
ctc	L	0	158.40
cta	L	0	159.90
ctg	L	0	211.80
mPD		0	1.1
		nPD:	0.
		N. weight:	1.6
		Sc. PD:	-0.097
		Sc. rank:	-1361.3

PB1-F2

Pos.	39	obs:	exp:
atg	M	943	943.00
act	T	0	1.53
acc	T	0	1.23
aca	T	0	2.75
acg	T	6	0.49
mPD		0.013	0.024
		nPD:	0.52
		N. weight:	0.015
		Sc. PD:	0.0068
		Sc. rank:	12.9

PB1-F2

Pos.	40	obs:	exp:
gat	D	0	502.60
gac	D	930	427.40
ggt	G	0	2.52
ggc	G	19	2.43
gga	G	0	8.77
ggg	G	0	5.29
mPD		0.039	0.55
		nPD:	0.07
		N. weight:	0.78
		Sc. PD:	0.0075
		Sc. rank:	297.7

PB1-F2

Pos.	41	obs:	exp:
cat	H	867	499.00
cac	H	2	370.00
cgt	R	79	3.39
cgc	R	0	3.91
cga	R	0	7.73
cgg	R	0	6.59
aat	N	1	0.51
aac	N	0	0.49
aga	R	0	36.12
agg	R	0	21.25
mPD		0.16	0.83
		nPD:	0.19
		N. weight:	0.74
		Sc. PD:	0.093
		Sc. rank:	498.3

PB1-F2

Pos.	42	obs:	exp:
tat	Y	0	436.90
tac	Y	809	372.10
tgt	C	0	59.22
tgc	C	140	80.78
mPD		0.25	0.75
		nPD:	0.34
		N. weight:	0.73
		Sc. PD:	0.19
		Sc. rank:	580.3

PB1-F2

Pos.	43	obs:	exp:
tta	L	0	79.42
ttg	L	0	166.60
ctt	L	0	149.00
ctc	L	0	149.40
cta	L	0	150.80
ctg	L	895	199.70
cct	P	0	12.73
ccc	P	0	9.16
cca	P	0	20.99
ccg	P	51	8.12
caa	Q	0	1.53
cag	Q	3	1.47
mPD		0.11	1.2
		nPD:	0.09
		N. weight:	1.5
		Sc. PD:	0.04
		Sc. rank:	700.0

PB1-F2

Pos.	44	obs:	exp:
cgt	R	0	40.50
cgc	R	0	46.71
cga	R	0	92.28
cgg	R	0	78.69
atg	M	2	2.00
aaa	K	0	2.33
aag	K	4	1.67
aga	R	0	431.20
agg	R	943	253.60
mPD		0.013	0.97
		nPD:	0.01
		N. weight:	1.3
		Sc. PD:	-0.059
		Sc. rank:	-275.4

PB1-F2

Pos.	45	obs:	exp:
att	I	0	0.37
atc	I	0	0.24
ata	I	1	0.39
act	T	0	241.40
acc	T	0	194.30
aca	T	947	434.40
acg	T	0	76.92
gct	A	0	0.26
gcc	A	0	0.19
gca	A	1	0.47
gcg	A	0	0.08
mPD		0.0042	0.68
		nPD:	0.01
		N. weight:	0.76
		Sc. PD:	-0.04
		Sc. rank:	-364.8

PB1-F2

Pos.	46	obs:	exp:
atg	M	249	249.00
act	T	0	178.40
acc	T	0	143.60
aca	T	0	321.10
acg	T	700	56.86
mPD		0.39	1.1
		nPD:	0.35
		N. weight:	1.8
		Sc. PD:	0.5
		Sc. rank:	1506.2

PB1-F2

Pos.	47	obs:	exp:
tct	S	0	150.30
tcc	S	0	123.40
tca	S	0	239.90
tcg	S	0	53.62
aat	N	0	2.04
aac	N	4	1.96
agt	S	0	194.30
agc	S	945	183.50
mPD		0.0084	1.7
		nPD:	0.01
		N. weight:	1.6
		Sc. PD:	-0.086
		Sc. rank:	-787.4

PB1-F2

Pos.	48	obs:	exp:
caa	Q	6	3.06
cag	Q	0	2.94
cgt	R	0	40.50
cgc	R	0	46.71
cga	R	943	92.28
cgg	R	0	78.69
aga	R	0	431.20
agg	R	0	253.60
mPD		0.013	0.98
		nPD:	0.01
		N. weight:	2.3
		Sc. PD:	-0.11
		Sc. rank:	-540.3

PB1-F2

Pos.	49	obs:	exp:
atg	M	2	2.00
gtt	V	0	187.70
gtc	V	0	182.90
gta	V	0	185.50
gtg	V	906	349.90
gct	A	0	10.59
gcc	A	0	7.64
gca	A	0	19.30
gcg	A	41	3.48
mPD		0.087	0.82
		nPD:	0.11
		N. weight:	0.99
		Sc. PD:	0.043
		Sc. rank:	497.8

PB1-F2

Pos.	50	obs:	exp:
gat	D	5	2.70
gac	D	0	2.30
ggt	G	944	125.10
ggc	G	0	120.60
gga	G	0	435.50
ggg	G	0	262.80
mPD		0.010	0.69
		nPD:	0.02
		N. weight:	2.
		Sc. PD:	-0.087
		Sc. rank:	-207.7

PB1-F2

Pos.	51	obs:	exp:
atg	M	934	934.00
act	T	0	3.82
acc	T	0	3.08
aca	T	0	6.88
acg	T	15	1.22
mPD		0.031	0.060
		nPD:	0.52
		N. weight:	0.039
		Sc. PD:	0.017
		Sc. rank:	32.3

PB1-F2

Pos.	52	obs:	exp:
cat	H	0	543.80
cac	H	947	403.20
cgt	R	0	0.09
cgc	R	2	0.10
cga	R	0	0.20
cgg	R	0	0.17
aga	R	0	0.91
agg	R	0	0.54
mPD		0.0042	0.50
		nPD:	0.01
		N. weight:	0.84
		Sc. PD:	-0.043
		Sc. rank:	-388.7

PB1-F2

Pos.	53	obs:	exp:
cgt	R	0	0.13
cgc	R	0	0.15
cga	R	0	0.29
cgg	R	0	0.25
aaa	K	946	551.40
aag	K	0	394.60
aga	R	3	1.37
agg	R	0	0.81
mPD		0.0063	0.49
		nPD:	0.01
		N. weight:	0.53
		Sc. PD:	-0.025
		Sc. rank:	-148.2

PB1-F2

Pos.	54	obs:	exp:
tta	L	0	0.09
ttg	L	0	0.19
ctt	L	0	0.17
ctc	L	0	0.17
cta	L	0	0.17
ctg	L	1	0.22
caa	Q	0	7.13
cag	Q	14	6.87
cgt	R	0	40.11
cgc	R	0	46.26
cga	R	0	91.40
cgg	R	934	77.93
aga	R	0	427.10
agg	R	0	251.20
mPD		0.031	1.0
		nPD:	0.03
		N. weight:	2.4
		Sc. PD:	-0.07
		Sc. rank:	418.0

PB1-F2

Pos.	55	obs:	exp:
att	I	945	347.20
atc	I	0	226.50
ata	I	0	371.30
act	T	4	1.02
acc	T	0	0.82
aca	T	0	1.83
acg	T	0	0.32
mPD		0.0084	0.66
		nPD:	0.01
		N. weight:	0.98
		Sc. PD:	-0.046
		Sc. rank:	-306.0

PB1-F2

Pos.	56	obs:	exp:
gtt	V	0	196.00
gtc	V	0	190.90
gta	V	0	193.70
gtg	V	946	365.40
gct	A	0	0.77
gcc	A	0	0.56
gca	A	0	1.41
gcg	A	3	0.25
mPD		0.0063	0.73
		nPD:	0.01
		N. weight:	0.93
		Sc. PD:	-0.047
		Sc. rank:	-402.5

PB1-F2

Pos.	57	obs:	exp:
tat	Y	925	500.10
tac	Y	1	425.90
tgt	C	23	9.73
tgc	C	0	13.27
mPD		0.049	0.54
		nPD:	0.09
		N. weight:	0.6
		Sc. PD:	0.017
		Sc. rank:	290.6

PB1-F2

Pos.	58	obs:	exp:
tgg	W	949	949.00
mPD		0	0
		nPD:	1.
		N. weight:	0.
		Sc. PD:	0
		Sc. rank:	0

PB1-F2

Pos.	59	obs:	exp:
cgt	R	0	3.87
cgc	R	0	4.46
cga	R	0	8.81
cgg	R	0	7.51
aaa	K	0	500.60
aag	K	859	358.40
aga	R	0	41.15
agg	R	90	24.21
---	--	------	------
mPD		0.17	0.72
		nPD:	0.24
		N. weight:	0.89
		Sc. PD:	0.15
		Sc. rank:	642.5

PB1-F2

Pos.	60	obs:	exp:
caa	Q	948	482.90
cag	Q	0	465.10
cgt	R	0	0.04
cgc	R	0	0.05
cga	R	1	0.10
cgg	R	0	0.08
aga	R	0	0.46
agg	R	0	0.27
---	--	------	------
mPD		0.0021	0.50
		nPD:	0.
		N. weight:	0.66
		Sc. PD:	-0.036
		Sc. rank:	-351.1

PB1-F2

Pos.	61	obs:	exp:
tgt	C	1	0.42
tgc	C	0	0.58
tgg	W	948	948.00
---	--	------	------
mPD		0.0021	0.0021
		nPD:	1.
		N. weight:	0.00088
		Sc. PD:	0.0008
		Sc. rank:	0.7

PB1-F2

Pos.	62	obs:	exp:
tta	L	0	81.64
ttg	L	0	171.30
ctt	L	920	153.20
ctc	L	0	153.50
cta	L	0	155.00
ctg	L	0	205.30
cct	P	29	7.24
ccc	P	0	5.21
cca	P	0	11.94
ccg	P	0	4.62
---	--	------	------
mPD		0.059	1.2
		nPD:	0.05
		N. weight:	1.7
		Sc. PD:	-0.017
		Sc. rank:	495.5

PB1-F2

Pos.	63	obs:	exp:
ttt	F	0	3.16
ttc	F	8	4.84
tct	S	0	149.00
tcc	S	937	122.40
tca	S	0	237.80
tcg	S	0	53.17
cct	P	0	0.50
ccc	P	2	0.36
cca	P	0	0.82
ccg	P	0	0.32
act	T	0	0.51
acc	T	2	0.41
aca	T	0	0.92
acg	T	0	0.16
agt	S	0	192.60
agc	S	0	182.00
---	--	------	------
mPD		0.025	1.7
		nPD:	0.02
		N. weight:	2.
		Sc. PD:	-0.088
		Sc. rank:	-280.0

PB1-F2

Pos.	64	obs:	exp:
tta	L	0	84.22
ttg	L	949	176.70
ctt	L	0	158.00
ctc	L	0	158.40
cta	L	0	159.90
ctg	L	0	211.80
---	--	------	------
mPD		0	1.1
		nPD:	0.
		N. weight:	1.6
		Sc. PD:	-0.097
		Sc. rank:	-1361.3

PB1-F2

Pos.	65	obs:	exp:
cgt	R	0	0.17
cgc	R	0	0.20
cga	R	0	0.39
cgg	R	0	0.33
aaa	K	0	550.80
aag	K	945	394.20
aga	R	0	1.83
agg	R	4	1.08
---	--	------	------
mPD		0.0084	0.50
		nPD:	0.02
		N. weight:	0.85
		Sc. PD:	-0.037
		Sc. rank:	-42.7

PB1-F2

Pos.	66	obs:	exp:
tct	S	0	0.48
tcc	S	0	0.39
tca	S	0	0.76
tcg	S	0	0.17
att	I	8	2.94
atc	I	0	1.92
ata	I	0	3.14
act	T	82	20.90
acc	T	0	16.83
aca	T	0	37.61
acg	T	0	6.66
aat	N	856	436.00
aac	N	0	420.00
agt	S	3	0.62
agc	S	0	0.58
---	--	------	------
mPD		0.18	0.73
		nPD:	0.25
		N. weight:	0.72
		Sc. PD:	0.13
		Sc. rank:	528.8

PB1-F2

Pos.	67	obs:	exp:
tta	L	0	83.68
ttg	L	0	175.60
ctt	L	0	157.00
ctc	L	943	157.40
cta	L	0	158.90
ctg	L	0	210.40
cct	P	0	1.00
ccc	P	4	0.72
cca	P	0	1.65
ccg	P	0	0.64
cat	H	0	1.15
cac	H	2	0.85
---	--	------	------
mPD		0.013	1.1
		nPD:	0.01
		N. weight:	1.7
		Sc. PD:	-0.084
		Sc. rank:	-671.9

PB1-F2

Pos.	68	obs:	exp:
att	I	1	45.56
atc	I	123	29.72
ata	I	0	48.72
act	T	0	210.30
acc	T	825	169.30
aca	T	0	378.40
acg	T	0	67.01
---	--	------	------
mPD		0.23	0.90
		nPD:	0.25
		N. weight:	1.5
		Sc. PD:	0.28
		Sc. rank:	1180.7

PB1-F2

Pos.	69	obs:	exp:
cct	P	0	0.50
ccc	P	0	0.36
cca	P	0	0.82
ccg	P	2	0.32
caa	Q	0	481.90
cag	Q	946	464.10
cgt	R	0	0.04
cgc	R	0	0.05
cga	R	0	0.10
cgg	R	1	0.08
aga	R	0	0.46
agg	R	0	0.27
---	--	------	------
mPD		0.0063	0.51
		nPD:	0.01
		N. weight:	0.7
		Sc. PD:	-0.033
		Sc. rank:	-233.9

PB1-F2

Pos.	70	obs:	exp:
gaa	E	6	3.65
gag	E	0	2.35
ggt	G	0	125.00
ggc	G	0	120.50
gga	G	943	435.00
ggg	G	0	262.50
---	--	------	------
mPD		0.013	0.69
		nPD:	0.02
		N. weight:	0.75
		Sc. PD:	-0.031
		Sc. rank:	-9.2

PB1-F2

Pos.	71	obs:	exp:
ttt	F	128	50.55
ttc	F	0	77.45
tct	S	820	130.40
tcc	S	0	107.10
tca	S	0	208.10
tcg	S	0	46.53
tat	Y	1	0.54
tac	Y	0	0.46
agt	S	0	168.60
agc	S	0	159.30
---	--	------	------
mPD		0.24	1.7
		nPD:	0.14
		N. weight:	1.7
		Sc. PD:	0.12
		Sc. rank:	964.8

PB1-F2

Pos.	72	obs:	exp:
tta	L	0	84.22
ttg	L	949	176.70
ctt	L	0	158.00
ctc	L	0	158.40
cta	L	0	159.90
ctg	L	0	211.80
---	--	------	------
mPD		0	1.1
		nPD:	0.
		N. weight:	1.6
		Sc. PD:	-0.097
		Sc. rank:	-1361.3

PB1-F2

Pos.	73	obs:	exp:
cgt	R	0	0.04
cgc	R	0	0.05
cga	R	0	0.10
cgg	R	0	0.08
aat	N	0	1.53
aac	N	3	1.47
aaa	K	945	550.80
aag	K	0	394.20
aga	R	1	0.46
agg	R	0	0.27
---	--	------	------
mPD		0.0084	0.49
		nPD:	0.02
		N. weight:	0.53
		Sc. PD:	-0.022
		Sc. rank:	-21.2

PB1-F2

Pos.	74	obs:	exp:
cct	P	1	0.25
ccc	P	0	0.18
cca	P	0	0.41
ccg	P	0	0.16
att	I	7	2.57
atc	I	0	1.68
ata	I	0	2.75
act	T	934	238.10
acc	T	0	191.70
aca	T	0	428.40
acg	T	0	75.86
gct	A	7	1.81
gcc	A	0	1.30
gca	A	0	3.29
gcg	A	0	0.59
---	--	------	------
mPD		0.031	0.71
		nPD:	0.04
		N. weight:	1.3
		Sc. PD:	-0.022
		Sc. rank:	337.0

PB1-F2

Pos.	75	obs:	exp:
cat	H	46	26.41
cac	H	0	19.59
cgt	R	903	38.78
cgc	R	0	44.73
cga	R	0	88.37
cgg	R	0	75.35
aga	R	0	412.90
agg	R	0	242.90
---	--	------	------
mPD		0.092	1.1
		nPD:	0.08
		N. weight:	2.9
		Sc. PD:	0.06
		Sc. rank:	1281.2

PB1-F2

Pos.	76	obs:	exp:
gtt	V	0	196.60
gtc	V	944	191.50
gta	V	5	194.30
gtg	V	0	366.50
---	--	------	------
mPD		0.010	0.73
		nPD:	0.01
		N. weight:	1.5
		Sc. PD:	-0.069
		Sc. rank:	-273.2

PB1-F2

Pos.	77	obs:	exp:
tta	L	0	4.53
ttg	L	51	9.50
tct	S	0	142.70
tcc	S	0	117.10
tca	S	0	227.70
tcg	S	897	50.90
tgg	W	1	1.00
ctt	L	0	8.49
ctc	L	0	8.51
cta	L	0	8.59
ctg	L	0	11.38
agt	S	0	184.40
agc	S	0	174.20
---	--	------	------
mPD		0.10	1.8
		nPD:	0.06
		N. weight:	2.7
		Sc. PD:	-0.0056
		Sc. rank:	999.5

PB1-F2

Pos.	78	obs:	exp:
cgt	R	0	4.98
cgc	R	0	5.75
cga	R	0	11.35
cgg	R	0	9.68
aaa	K	832	485.50
aag	K	1	347.50
aga	R	116	53.04
agg	R	0	31.20
---	--	------	------
mPD		0.22	0.78
		nPD:	0.28
		N. weight:	0.55
		Sc. PD:	0.11
		Sc. rank:	432.1

PB1-F2

Pos.	79	obs:	exp:
tta	L	0	0.09
ttg	L	0	0.19
ctt	L	0	0.17
ctc	L	0	0.17
cta	L	1	0.17
ctg	L	0	0.22
caa	Q	9	4.58
cag	Q	0	4.42
cgt	R	0	40.33
cgc	R	0	46.51
cga	R	939	91.89
cgg	R	0	78.35
aga	R	0	429.40
agg	R	0	252.60
---	--	------	------
mPD		0.021	0.99
		nPD:	0.02
		N. weight:	2.3
		Sc. PD:	-0.087
		Sc. rank:	66.3

PB1-F2

Pos.	80	obs:	exp:
tta	L	0	0.27
ttg	L	3	0.56
tgg	W	943	943.00
ctt	L	0	0.50
ctc	L	0	0.50
cta	L	0	0.51
ctg	L	0	0.67
---	-	3	3.00
---	--	------	------
mPD		0.0063	0.015
		nPD:	0.43
		N. weight:	0.0052
		Sc. PD:	0.0018
		Sc. rank:	4.3

PB1-F2

Pos.	81	obs:	exp:
aaa	K	946	551.40
aag	K	0	394.60
---	-	3	3.00
---	--	------	------
mPD		0	0.48
		nPD:	0.
		N. weight:	0.53
		Sc. PD:	-0.031
		Sc. rank:	-435.8

PB1-F2

Pos.	82	obs:	exp:
tta	L	1	83.86
ttg	L	944	175.90
tct	S	0	0.16
tcc	S	0	0.13
tca	S	0	0.25
tcg	S	1	0.06
ctt	L	0	157.40
ctc	L	0	157.70
cta	L	0	159.30
ctg	L	0	210.90
agt	S	0	0.21
agc	S	0	0.19
---	-	3	3.00
---	--	------	------
mPD		0.0042	1.1
		nPD:	0.
		N. weight:	1.6
		Sc. PD:	-0.09
		Sc. rank:	-975.5

PB1-F2

Pos.	83	obs:	exp:
ttt	F	0	353.80
ttc	F	896	542.20
tct	S	0	7.95
tcc	S	50	6.53
tca	S	0	12.69
tcg	S	0	2.84
agt	S	0	10.28
agc	S	0	9.71
---	-	3	3.00
---	--	------	------
mPD		0.10	0.64
		nPD:	0.16
		N. weight:	0.57
		Sc. PD:	0.052
		Sc. rank:	348.6

PB1-F2

Pos.	84	obs:	exp:
tct	S	0	150.00
tcc	S	0	123.20
tca	S	0	239.30
tcg	S	0	53.51
aat	N	0	1.53
aac	N	3	1.47
agt	S	0	193.90
agc	S	943	183.20
---	-	3	3.00
---	--	------	------
mPD		0.0063	1.6
		nPD:	0.
		N. weight:	1.6
		Sc. PD:	-0.088
		Sc. rank:	-866.0

PB1-F2

Pos.	85	obs:	exp:
cgt	R	0	1.93
cgc	R	0	2.23
cga	R	0	4.40
cgg	R	0	3.75
aaa	K	901	525.10
aag	K	0	375.90
aga	R	45	20.58
agg	R	0	12.10
---	-	3	3.00
---	--	------	------
mPD		0.090	0.60
		nPD:	0.15
		N. weight:	0.54
		Sc. PD:	0.045
		Sc. rank:	314.6

PB1-F2

Pos.	86	obs:	exp:
tta	L	0	4.53
ttg	L	0	9.50
ctt	L	0	8.49
ctc	L	0	8.51
cta	L	51	8.59
ctg	L	0	11.38
caa	Q	895	455.90
cag	Q	0	439.10
---	-	3	3.00
---	--	------	------
mPD		0.10	0.64
		nPD:	0.16
		N. weight:	0.71
		Sc. PD:	0.066
		Sc. rank:	448.1

PB1-F2

Pos.	87	obs:	exp:
gaa	E	2	568.30
gag	E	932	365.70
ggt	G	0	1.46
ggc	G	0	1.41
gga	G	0	5.07
ggg	G	11	3.06
---	-	4	4.00
---	--	------	------
mPD		0.027	0.50
		nPD:	0.05
		N. weight:	0.9
		Sc. PD:	-0.0062
		Sc. rank:	283.7

PB1-F2

Pos.	88	obs:	exp:
tta	L	0	0.62
ttg	L	7	1.30
tgg	W	929	929.00
ctt	L	0	1.17
ctc	L	0	1.17
cta	L	0	1.18
ctg	L	0	1.56
---	-	13	13.00
---	--	------	------
mPD		0.014	0.034
		nPD:	0.43
		N. weight:	0.012
		Sc. PD:	0.0043
		Sc. rank:	10.1

PB1-F2

Pos.	89	obs:	exp:
att	I	0	300.90
atc	I	0	196.30
ata	I	819	321.80
act	T	0	29.82
acc	T	0	24.01
aca	T	117	53.66
acg	T	0	9.50
---	-	13	13.00
---	--	------	------
mPD		0.21	0.85
		nPD:	0.25
		N. weight:	0.88
		Sc. PD:	0.16
		Sc. rank:	659.0

PB1-F2

Pos.	90	obs:	exp:
tct	S	0	0.80
tcc	S	0	0.65
tca	S	0	1.27
tcg	S	0	0.28
aat	N	0	473.70
aac	N	930	456.30
agt	S	0	1.03
agc	S	5	0.97
gat	D	0	0.54
gac	D	1	0.46
gaa	E	0	0.61
gag	E	1	0.39
---	-	12	12.00
---	--	------	------
mPD		0.017	0.51
		nPD:	0.03
		N. weight:	0.69
		Sc. PD:	-0.019
		Sc. rank:	129.3

PB1-F2

Pos.	91	obs:	exp:
taa	.	45	403.20
tag	.	12	237.60
tga	.	892	308.30
---	--	------	------
mPD		0.14	0.81
		nPD:	0.17
		N. weight:	0.84
		Sc. PD:	0.087
		Sc. rank:	541.0

Amino acid level conservation per codon

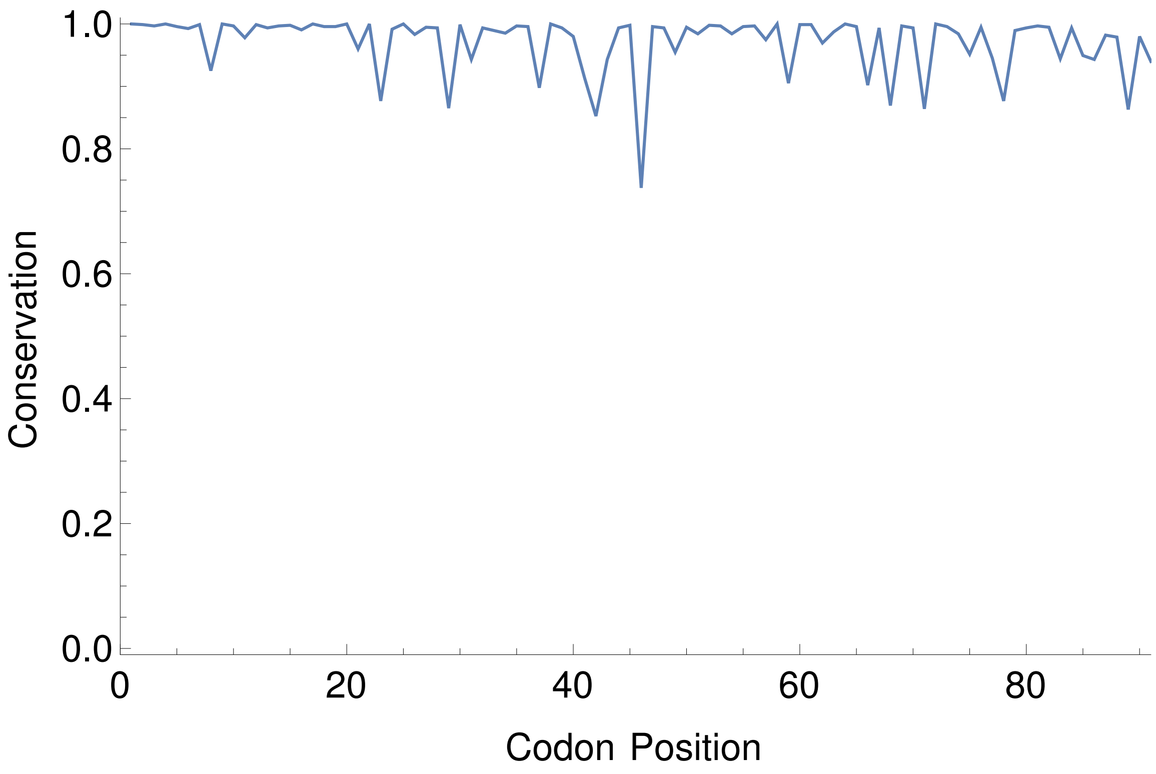

Conservation analysis plots

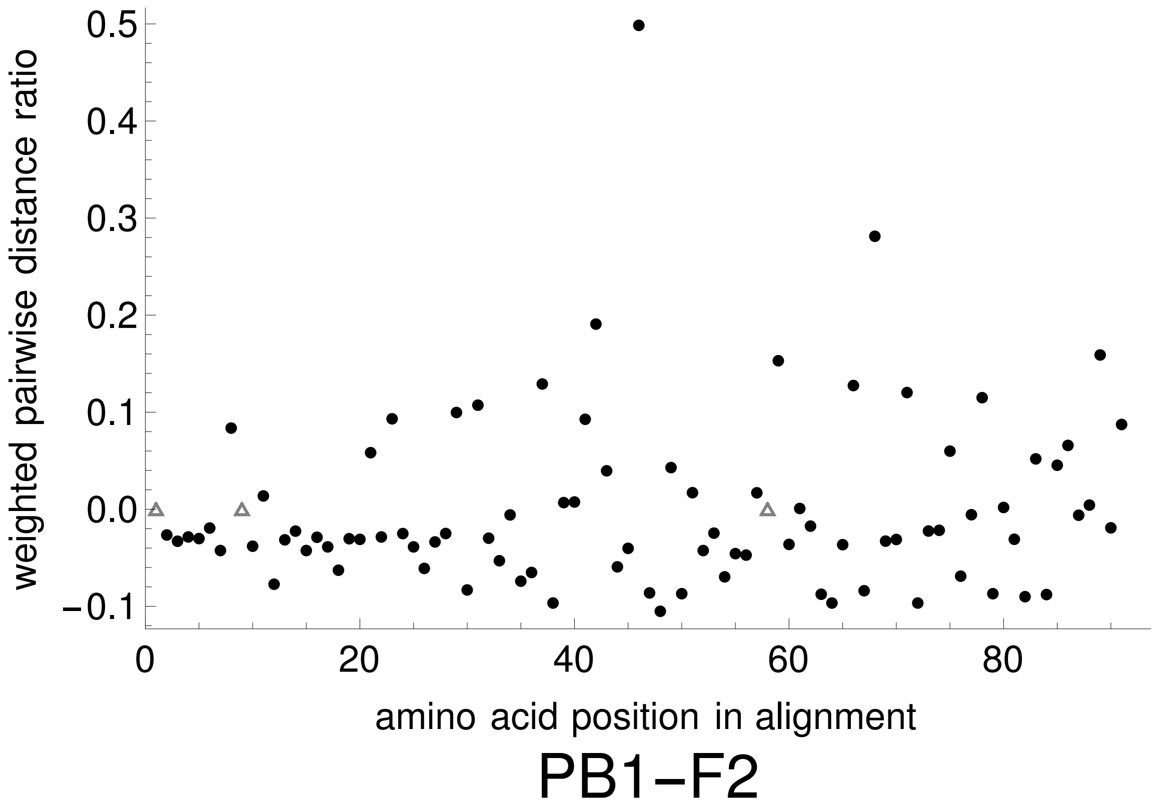

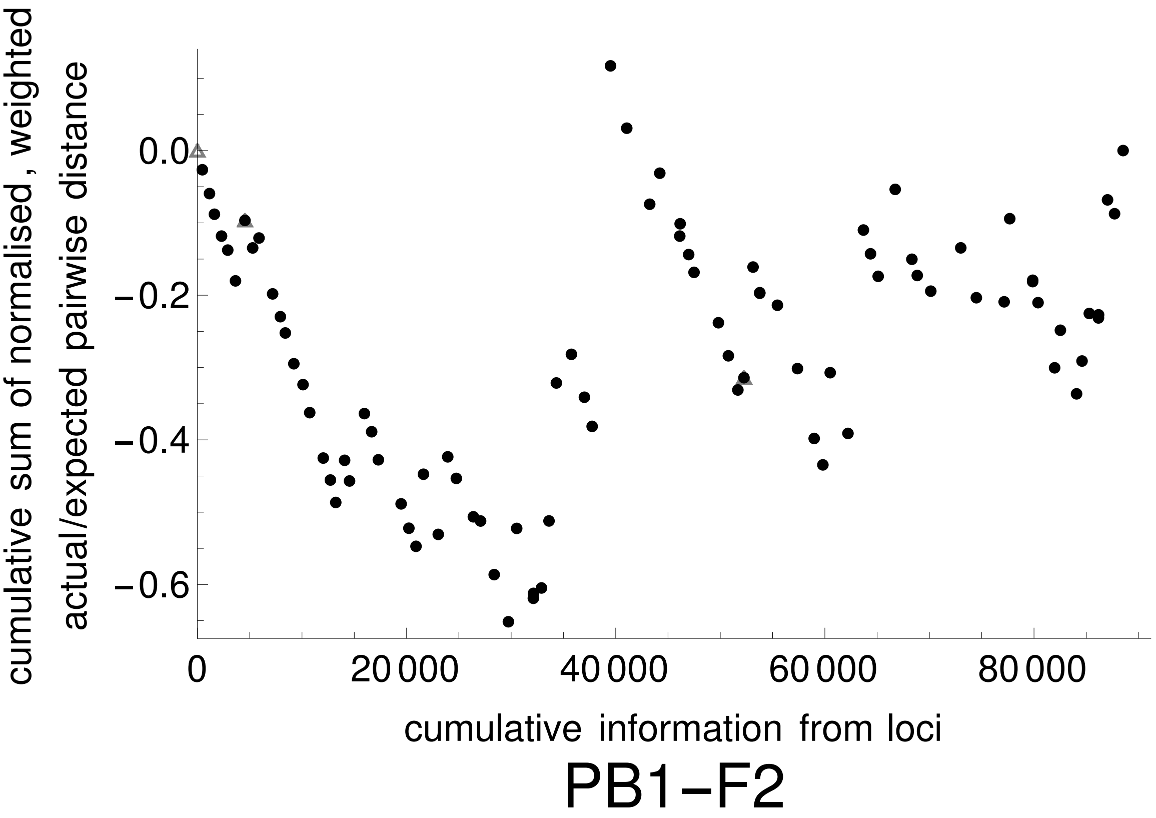

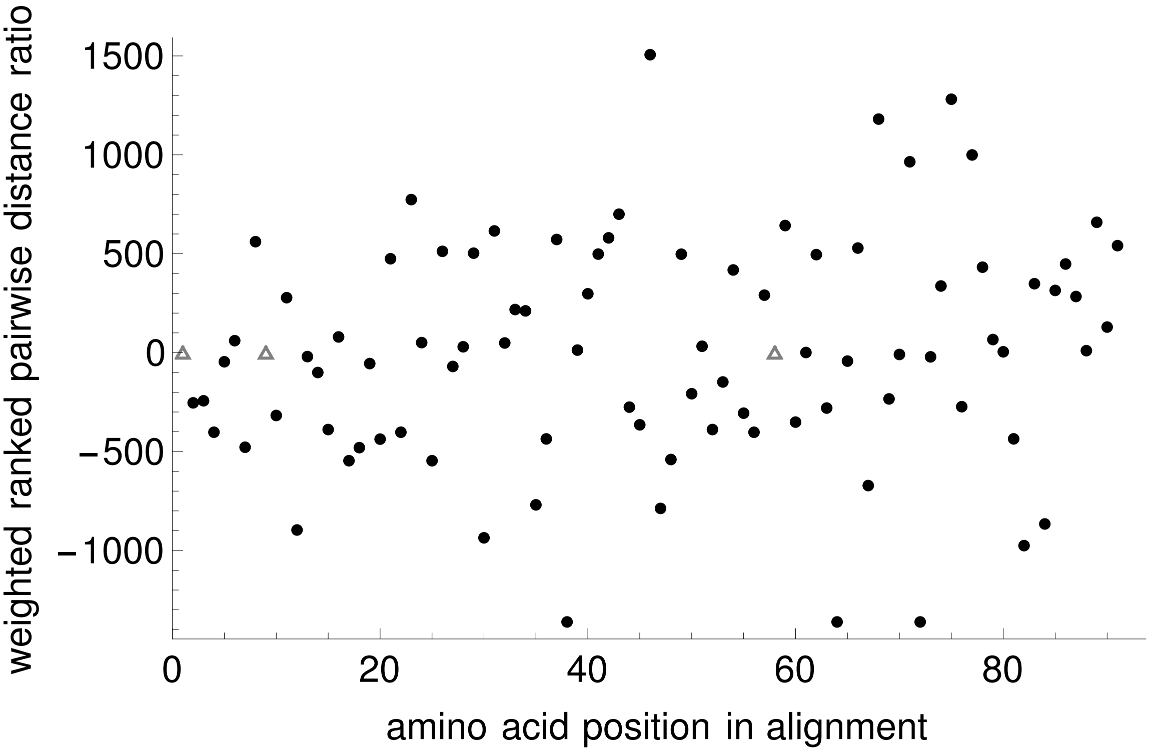
weighted ranked pairwise distance ratio
1500
1000
500
0
−500
−1000
0
20
40
60
80
amino acid position in alignment

PB1−F2

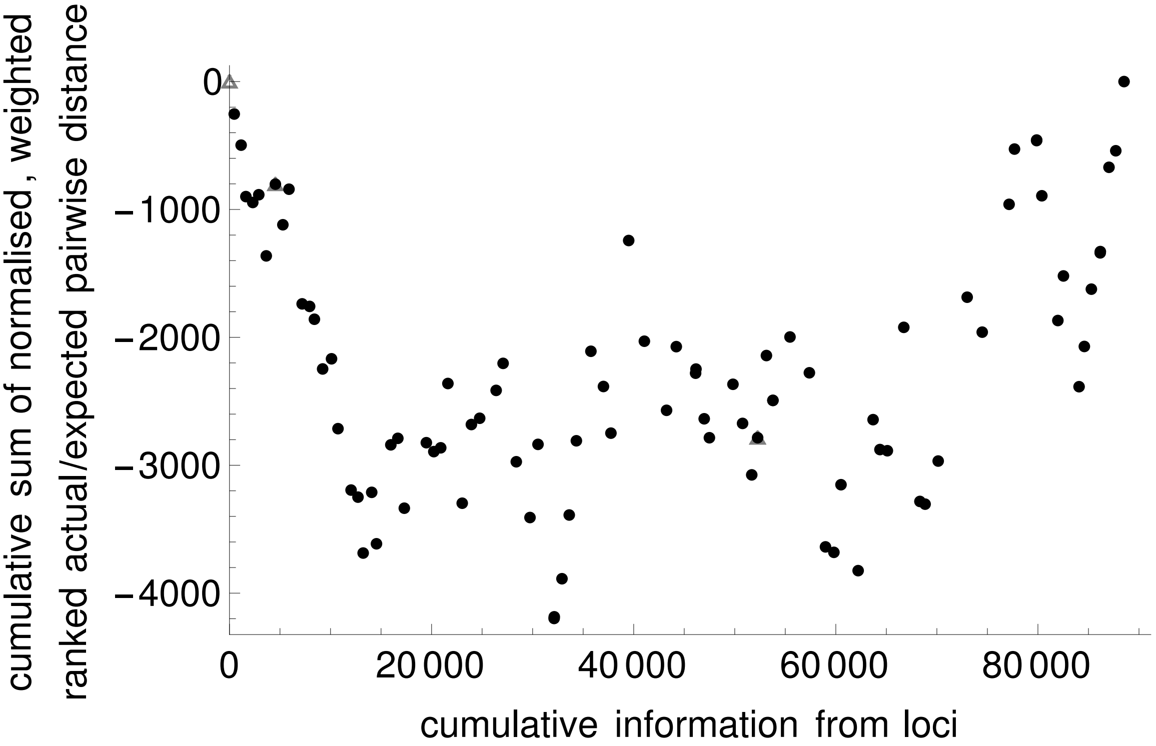
cumulative sum of normalised, weighted ranked actual/expected pairwise distance
0
−1000
−2000
−3000
−4000
0
20 000
40 000
60 000
80 000
cumulative information from loci

PB1−F2

PA

Gene length histogram

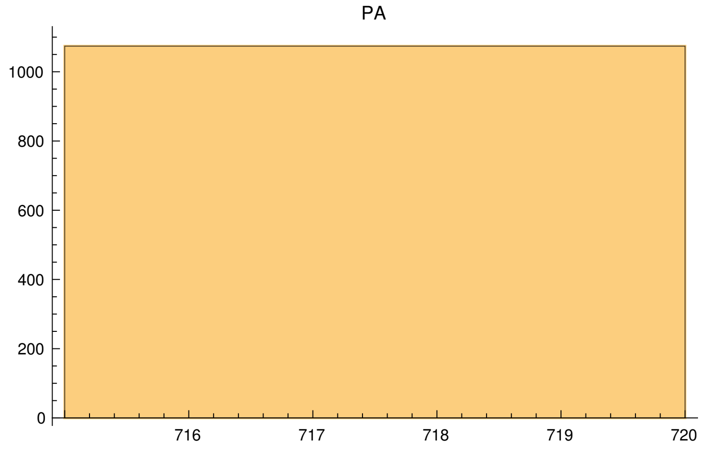

Information vs. nPD

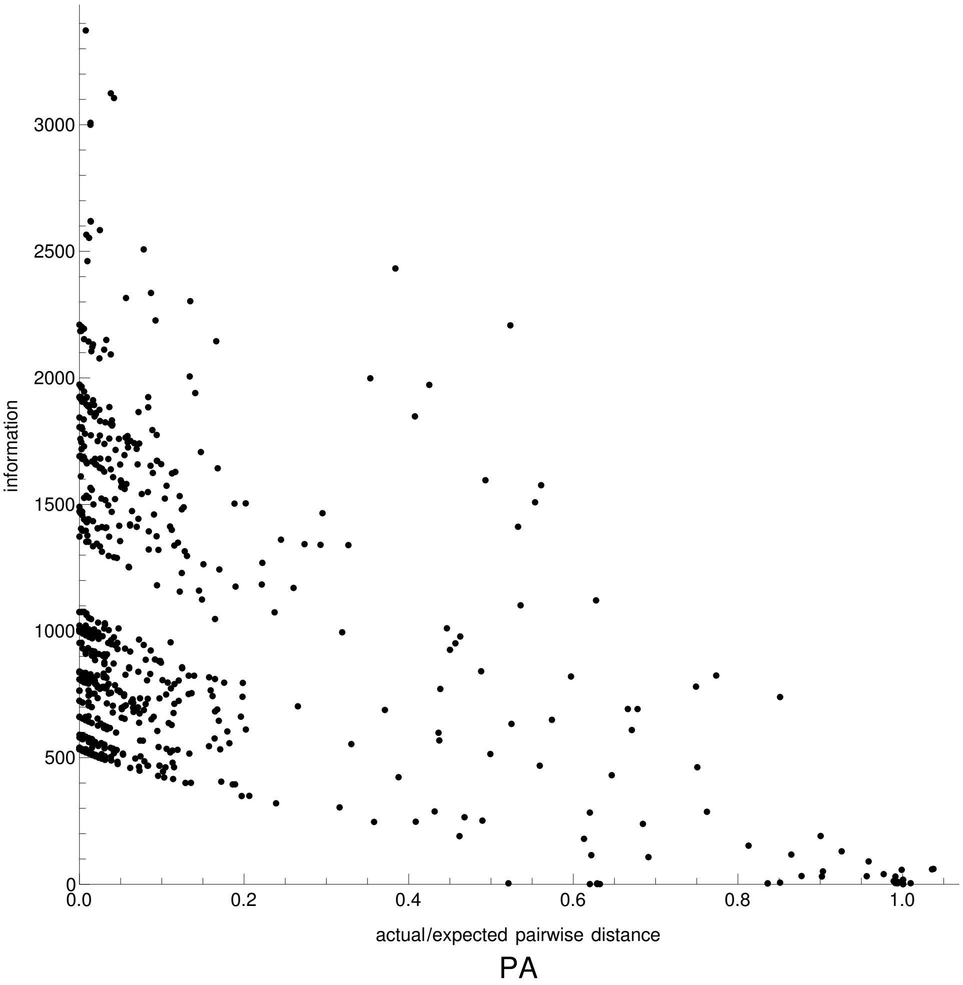

Example sequences highlighted by regions found in analyses to be conserved

Interesting points (by weighted raw PD) highlighted for gene PA:

ATG

GAAGACTTTGTGCGACAATGCTTCAATCCAATGATCGTCGAGCTTGCGGAAAAGG
CAATGAAAGAGTATGGGGAAGATCCGAAAATCGAAACAAACAAATTCGCATC
AATATGCACACACTTAGAAGTCTGCTTCATGTATTCTGATTTCCACTTCATC⋱
GACGAACGGGGCGAATCAACTATTATAGAATCTGGCGATCCAAATGTGTTGTTGAAACATCGATTTGAAA⋱
TAATCGAAGGAAGAGACCGAACAATGGCCTGGACAGTGGTGAATAGTATCTGCAACACCACGGGAGTCGA⋱
AAAACCCAAATTTCTCCCGGATCTGTATGACTACAAGGAAAACCGATTCATTGAAATTGGAGTGACGAGG⋱
AGGGAAGTCCACATATATTACCTAGAGAAAGCCAATAAAATAAAATCCGAGAAGACACACATACACATTT⋱
TCTCATTCACTGGAGAAGAGATGGCCACCAAAGCAGATTACACTCTTGATGAAGAGAGCAGGGCAAGAAT⋱
CAAAACCAGGTTGTTCACCATAAGGCAGGAAATGGCCAGC
AGGGGTCTATGGGATTCCTTTCGTCAGTCCGAAAGAGGCGAAGAAACAATTGAAG
AAAGATTTGAAATCACAGGAACCATGCGCAGGCTTGCCGACCAAAGTCTCCC
ACCGAACTTCTCCAGCCTTGAAAACTTTAGAGCCTATGTGGATGGATTCGAA
CCGAACGGCTGCATTGAGGGCAAGCTTTCTCAAATGTCAAAAGAAGTGAACG
CCAGAATCGAGCCATTTCTAAGGACAACACCACGACCACTCAGATTGCCTAATGGGCCTCCCTGTTCCCAG⋱
CGGTCGAAATTCTTGCTGATGGATGCTCTGAAATTAAGCATTGAGGACCCGAGCCACGAAGGGGAGGGA⋱
TACCGCTATATGATGCGATCAAATGCATGAAAACGTTCTTCGGATGGAAAGAGCCCAACATTATCAAACC⋱
ACATGAGAAAGGCATAAACCCCAACTACCTCCTGACTTGGAAGCAGGTGCTGTCAGAACTTCAGGACATT⋱
GAAAATGAAGAGAAGATTCCAAGGACGAAAAACATGAAGAAAACAAGCCAATTAAAGTGGGCACTCGGTG⋱
AGAACATGGCACCAGAGAAGGTGGACTTTGAGGATTGTAAAGATGTCAACGACCTGAAACAGTATGACAG⋱
TGATGAGCCAGAGCCCAGATCACTAGCATGTTGGATCCAGAATGAATTCAACAAGGCATGTGAATTAACT⋱
GATTCAAGCTGGGTAGAACTTGATGAAATAGGGGAAGATGTTGCCCCAATCGAACACATTGCAAGCATGA⋱
GAAGGAACTATTTTACAGCAGAGGTATCCCACTGCAGGGCTACTGAATACATAATGAAGGGAGTGTACAT⋱
AAATACAGCTTTGCTCAATGCATCTTGTGCAGCCATGGATGACTTTCAACTGATTCCAATGATAAGTAAA⋱
TGCAGAACCAAAGAAGGAAGACGGAAAACAAACCTATAT
GGATTCATTATAAAAGGAAGATCTCATTTGAGGAATGATACCGATGTGGTGAACT
TTGTAAGTATGGAATTTTCCCTTACCGATCCAAGGTTGGAACCACATAAATG
GGAAAAGTATTGTGTTCTCGAAATAGGGGATATGCTCCTGCGAACTGCAGTA
GGCCAAGTGTCAAGACCCATGTTTCTGTACGTGAGGACCAATGGGACCTCTA
AGATCAAGATGAAATGGGGTATGGAAATGAGACGCTGCCTTCTACAATCTCT
CCAACAGATTGAGAGCATGATTGAAGCTGAATCCTCCGTCAAAGAGAAAGAC
CTGACCAAAGAATTCTTTGAAAACAAATCAGAAACATGGCCAATTGGAGAAT
CACCTAAGGGAGTGGAGGAAGGTTCCATTGGGAAGGTGTGCAGAACCTTACT
AGCAAAATCTGTATTCAATAGCCTATATGCATCTCCGCAACTCGAAGGGTTC
TCAGCTGAATCGAGAAAACTGCTACTCATTGTTCAGGCACTTAGGGATAACC
TGGAACCTGGAACCTTTGATCTTGAGGGGCTATATGAAGCAATCGAGGAGTG
CCTGATTAATGATCCCTGGGTTTTGCTTAATGCATCTTGGTTCAACTCCTTC
CTCACACATGCACTAAGATAG

Interesting points (by weighted ranked PD) highlighted for gene PA:

ATGGAAGACTTTGTGCGACAATGCTTCAATCCAATGATCGTCGAGCTTGCGGAAAAGGCAATGAAAGAGTATGGGGAA⋱
GATCCGAAAATCGAAACAAACAAATTCGCATCAATATGCACACACTTAGAAGTCTGCTTCATGTATTCTGATTTCCA⋱
CTTCATCGACGAACGGGGCGAATCAACTATTATAGAATCTGGCGATCCAAATGTGTTGTTGAAACATCGATTTGAAA⋱

TAATCGAAGGAAGAGACCGAACAATGGCCTGGACAGTGGTGAATAGTATCTGCAACACCACGGGAGTCGAAAAACCC⋱
AAATTTCTCCCGGATCTGTATGACTACAAGGAAAACCGATTCATTGAAATTGGAGTGACGAGGAGGGAAGTCCACAT⋱
ATATTACCTAGAGAAAGCCAATAAAATAAAATCCGAGAAGACACACATACACATTTTCTCATTCACTGGAGAAGAGA⋱
TGGCCACCAAAGCAGATTACACTCTTGATGAAGAGAGCAGGGCAAGAATCAAAACCAGGTTGTTCACCATAAGGCAG⋱
GAAATGGCCAGCAGGGGTCTATGGGAT
TCCTTTCGTCAGTCCGAAAGAGGCGAAGAAACAATTGAAGAAAGATTTGAAATCA
CAGGAACCATGCGCAGGCTTGCCGACCAAAGTCTCCCACCGAACTTCTCCAG
CCTTGAAAACTTTAGAGCCTATGTGGATGGATTCGAACCGAACGGCTGCATT
GAGGGCAAGCTTTCTCAAATGTCAAAAGAAGTGAACGCCAGAATCGAGCCATTTCTAAGGACAACAC⋱
CACGACCACTCAGATTGCCTAATGGGCCTCCCTGTTCCCAGCGGTCGAAATTCTTGCTGATGGATGCTCT⋱
GAAATTAAGCATTGAGGACCCGAGCCACGAAGGGGAGGGGATACCGCTATATGATGCGATCAAATGCATG⋱
AAAACGTTCTTCGGATGGAAAGAGCCCAACATTATCAAACCACATGAGAAAGGCATAAACCCCAACTACC⋱
TCCTGACTTGGAAGCAGGTGCTGTCAGAACTTCAGGACATTGAAAATGAAGAGAAGATTCCAAGGACGAA⋱
AAACATGAAGAAAACAAGCCAATTAAAGTGGGCACTCGGTGAGAACATGGCACCAGAGAAGGTGGACTTT⋱
GAGGATTGTAAAGATGTCAACGACCTGAAACAGTATGACAGTGATGAGCCAGAGCCCAGATCACTAGCAT⋱
GTTGGATCCAGAATGAATTCAACAAGGCATGTGAATTAACTGATTCAAGCTGGGTAGAACTTGATGAAAT⋱
AGGGGAAGATGTTGCCCCAATCGAACACATTGCAAGCATGAGAAGGAACTATTTTACAGCAGAGGTATCC⋱
CACTGCAGGGCTACTGAATACATAATGAAGGGAGTGTACATAAATACAGCTTTGCTCAATGCATCTTGTG⋱
CAGCCATGGATGACTTTCAACTGATTCCAATGATAAGTAAATGCAGAACCAAAGAAGGAAGACGGAAAAC⋱
AAACCTATATGGATTCATTATAAAAGGAAGATCTCATTTGAGGAATGATACCGATGTGGTGAACTTTGTA⋱
AGTATGGAATTTTCCCTTACCGATCCAAGGTTGGAACCACATAAATGGGAAAAGTATTGTGTTCTCGAAA⋱
TAGGGGATATGCTCCTGCGAACTGCAGTAGGCCAAGTGTCAAGACCCATGTTTCTGTACGTGAGGACCAA⋱
TGGGACCTCTAAGATCAAGATGAAATGGGGTATGGAAATGAGACGCTGCCTTCTACAATCTCTCCAACAG⋱
ATTGAGAGCATGATTGAAGCTGAATCCTCCGTCAAAGAGAAAGACCTGACCAAAGAATTCTTTGAAAACA⋱
AATCAGAAACATGGCCAATTGGAGAATCACCTAAGGGAGTGGAGGAAGGTTCCATTGGGAAGGTGTGCAG⋱
AACCTTACTAGCAAAATCTGTATTCAATAGCCTATATGCATCTCCGCAACTCGAAGGGTTCTCAGCTGAA⋱
TCGAGAAAACTGCTACTCATTGTTCAGGCACTTAGGGATAACCTGGAACCTGGAACCTTT
GATCTTGAGGGGCTATATGAAGCAATCGAGGAGTGCCTGATTAATGATCCCTGGG
TTTTGCTTAATGCATCTTGGTTCAACTCCTTCCTCACACATGCACTAAGATAG

Per codon conservation report

PA			
Pos.	1	obs:	exp:
atg	M	1074	1074.00
---	--	------	------
mPD		0	0
		nPD:	1.
		N. weight:	0.
		Sc. PD:	0
		Sc. rank:	0

PA			
Pos.	2	obs:	exp:
gat	D	1	0.54
gac	D	0	0.46
gaa	E	1070	652.90
gag	E	3	420.10
---	--	------	------
mPD		0.0074	0.48
		nPD:	0.02
		N. weight:	0.52
		Sc. PD:	-0.029
		Sc. rank:	-98.2

PA			
Pos.	3	obs:	exp:
aat	N	0	0.51
aac	N	1	0.49
gat	D	3	579.30
gac	D	1069	492.70
ggt	G	0	0.13
ggc	G	1	0.13
gga	G	0	0.46
ggg	G	0	0.28
---	--	------	------
mPD		0.0093	0.50
		nPD:	0.02
		N. weight:	0.83
		Sc. PD:	-0.043
		Sc. rank:	-114.2

PA			
Pos.	4	obs:	exp:
ttt	F	1068	422.10
ttc	F	1	646.90
tta	L	0	0.35
ttg	L	0	0.74
ctt	L	4	0.67
ctc	L	0	0.67
cta	L	0	0.67
ctg	L	0	0.89
gtt	V	1	0.21
gtc	V	0	0.20
gta	V	0	0.20
gtg	V	0	0.39
---	--	------	------
mPD		0.011	0.49
		nPD:	0.02
		N. weight:	1.
		Sc. PD:	-0.048
		Sc. rank:	-64.8

PA			
Pos.	5	obs:	exp:
tta	L	0	0.09
ttg	L	1	0.19
ctt	L	0	0.17
ctc	L	0	0.17
cta	L	0	0.17
ctg	L	0	0.22
atg	M	1	1.00
gtt	V	0	221.90
gtc	V	0	216.20
gta	V	0	219.30
gtg	V	1071	413.60
ggt	G	0	0.13
ggc	G	0	0.13
gga	G	0	0.46
ggg	G	1	0.28
---	--	------	------
mPD		0.0056	0.73
		nPD:	0.01
		N. weight:	1.
		Sc. PD:	-0.064
		Sc. rank:	-482.1

PA			
Pos.	6	obs:	exp:
cgt	R	0	46.13
cgc	R	0	53.19
cga	R	1044	105.10
cgg	R	0	89.62
aga	R	30	491.10
agg	R	0	288.90
---	--	------	------
mPD		0.054	0.96
		nPD:	0.06
		N. weight:	2.3
		Sc. PD:	-0.035
		Sc. rank:	754.4

PA			
Pos.	7	obs:	exp:
caa	Q	478	547.10
cag	Q	596	526.90
---	--	------	------
mPD		0.49	0.50
		nPD:	0.99
		N. weight:	0.013
		Sc. PD:	0.011
		Sc. rank:	10.9

PA			
Pos.	8	obs:	exp:
tgt	C	10	454.30
tgc	C	1064	619.70
---	--	------	------
mPD		0.018	0.49
		nPD:	0.04
		N. weight:	0.55
		Sc. PD:	-0.018
		Sc. rank:	83.8

PA			
Pos.	9	obs:	exp:
ttt	F	1	424.10
ttc	F	1073	649.90
---	--	------	------
mPD		0.0019	0.48
		nPD:	0.
		N. weight:	0.54
		Sc. PD:	-0.036
		Sc. rank:	-303.7

PA			
Pos.	10	obs:	exp:
aat	N	1073	547.10
aac	N	1	526.90
---	--	------	------
mPD		0.0019	0.50
		nPD:	0.
		N. weight:	0.73
		Sc. PD:	-0.048
		Sc. rank:	-428.5

PA			
Pos.	11	obs:	exp:
cct	P	0	268.00
ccc	P	0	193.00
cca	P	1074	442.10
ccg	P	0	171.00
---	--	------	------
mPD		0	0.71
		nPD:	0.
		N. weight:	0.97
		Sc. PD:	-0.067
		Sc. rank:	-820.9

PA			
Pos.	12	obs:	exp:
atg	M	1074	1074.00
---	--	------	------
mPD		0	0
		nPD:	1.
		N. weight:	0.
		Sc. PD:	0
		Sc. rank:	0

PA			
Pos.	13	obs:	exp:
tta	L	0	0.09
ttg	L	0	0.19
ctt	L	0	0.17
ctc	L	1	0.17
cta	L	0	0.17
ctg	L	0	0.22
att	I	97	393.50
atc	I	974	256.70
ata	I	0	420.80
gtt	V	1	0.41
gtc	V	1	0.40
gta	V	0	0.41
gtg	V	0	0.77
---	--	------	------
mPD		0.17	0.66
		nPD:	0.26
		N. weight:	1.2
		Sc. PD:	0.22
		Sc. rank:	882.7

PA			
Pos.	14	obs:	exp:
att	I	0	0.37
atc	I	1	0.24
ata	I	0	0.39
gtt	V	0	222.30
gtc	V	1073	216.60
gta	V	0	219.70
gtg	V	0	414.40
---	--	------	------
mPD		0.0019	0.73
		nPD:	0.
		N. weight:	1.7
		Sc. PD:	-0.12
		Sc. rank:	-1152.9

PA			
Pos.	15	obs:	exp:
gaa	E	2	653.50
gag	E	1072	420.50
---	--	------	------
mPD		0.0037	0.48
		nPD:	0.01
		N. weight:	1.
		Sc. PD:	-0.062
		Sc. rank:	-432.0

PA			
Pos.	16	obs:	exp:
tta	L	0	95.31
ttg	L	0	200.00
ctt	L	1072	178.80
ctc	L	2	179.20
cta	L	0	181.00
ctg	L	0	239.70
---	--	------	------
mPD		0.0037	1.1
		nPD:	0.
		N. weight:	1.9
		Sc. PD:	-0.13
		Sc. rank:	-1184.6

PA			
Pos.	17	obs:	exp:
gct	A	0	277.30
gcc	A	0	200.00
gca	A	9	505.50
gcg	A	1065	91.15
---	--	------	------
mPD		0.017	0.67
		nPD:	0.02
		N. weight:	2.6
		Sc. PD:	-0.12
		Sc. rank:	-47.9

PA			
Pos.	18	obs:	exp:
gat	D	0	4.32
gac	D	8	3.68
gaa	E	1063	648.70
gag	E	3	417.30
---	--	------	------
mPD		0.020	0.48
		nPD:	0.04
		N. weight:	0.53
		Sc. PD:	-0.015
		Sc. rank:	111.5

PA			
Pos.	19	obs:	exp:
aat	N	1	0.51
aac	N	0	0.49
aaa	K	15	625.40
aag	K	1058	447.60
---	--	------	------
mPD		0.029	0.49
		nPD:	0.06
		N. weight:	0.87
		Sc. PD:	-0.0097
		Sc. rank:	300.6

PA			
Pos.	20	obs:	exp:
act	T	0	18.86
acc	T	0	15.18
aca	T	74	33.94
acg	T	0	6.01
gct	A	0	257.70
gcc	A	0	185.90
gca	A	998	469.70
gcg	A	0	84.70
gaa	E	2	1.22
gag	E	0	0.78
---	--	------	------
mPD		0.13	0.80
		nPD:	0.16
		N. weight:	0.82
		Sc. PD:	0.074
		Sc. rank:	560.0

PA

Pos.	21	obs:	exp:
atg	M	1073	1073.00
gtt	V	0	0.21
gtc	V	0	0.20
gta	V	0	0.20
gtg	V	1	0.39
mPD		0.0019	0.0030
		nPD:	0.62
		N. weight:	0.00096
		Sc. PD:	0.00051
		Sc. rank:	0.8

PA

Pos.	22	obs:	exp:
cgt	R	0	0.09
cgc	R	0	0.10
cga	R	0	0.20
cgg	R	0	0.17
aaa	K	1066	624.80
aag	K	6	447.20
aga	R	2	0.91
agg	R	0	0.54
mPD		0.015	0.49
		nPD:	0.03
		N. weight:	0.55
		Sc. PD:	-0.022
		Sc. rank:	28.1

PA

Pos.	23	obs:	exp:
gaa	E	939	653.50
gag	E	135	420.50
mPD		0.22	0.48
		nPD:	0.46
		N. weight:	0.19
		Sc. PD:	0.073
		Sc. rank:	154.3

PA

Pos.	24	obs:	exp:
tat	Y	1072	580.10
tac	Y	2	493.90
mPD		0.0037	0.50
		nPD:	0.01
		N. weight:	0.66
		Sc. PD:	-0.041
		Sc. rank:	-312.5

PA

Pos.	25	obs:	exp:
ggt	G	0	142.40
ggc	G	0	137.20
gga	G	3	495.50
ggg	G	1071	299.00
mPD		0.0056	0.68
		nPD:	0.01
		N. weight:	1.4
		Sc. PD:	-0.084
		Sc. rank:	-571.7

PA

Pos.	26	obs:	exp:
gaa	E	1062	653.50
gag	E	12	420.50
mPD		0.022	0.48
		nPD:	0.05
		N. weight:	0.48
		Sc. PD:	-0.012
		Sc. rank:	120.8

PA

Pos.	27	obs:	exp:
gat	D	1072	579.80
gac	D	1	493.20
ggt	G	1	0.13
ggc	G	0	0.13
gga	G	0	0.46
ggg	G	0	0.28
mPD		0.0037	0.50
		nPD:	0.01
		N. weight:	0.66
		Sc. PD:	-0.041
		Sc. rank:	-316.9

PA

Pos.	28	obs:	exp:
tct	S	0	0.32
tcc	S	0	0.26
tca	S	0	0.51
tcg	S	2	0.11
cct	P	1	267.50
ccc	P	1	192.60
cca	P	31	441.30
ccg	P	1039	170.60
agt	S	0	0.41
agc	S	0	0.39
mPD		0.063	0.72
		nPD:	0.09
		N. weight:	1.8
		Sc. PD:	0.03
		Sc. rank:	885.1

PA

Pos.	29	obs:	exp:
aaa	K	1073	626.00
aag	K	1	448.00
mPD		0.0019	0.49
		nPD:	0.
		N. weight:	0.58
		Sc. PD:	-0.038
		Sc. rank:	-333.0

PA

Pos.	30	obs:	exp:
att	I	11	393.50
atc	I	1060	256.70
ata	I	0	420.80
gtt	V	0	0.62
gtc	V	3	0.61
gta	V	0	0.61
gtg	V	0	1.16
mPD		0.026	0.66
		nPD:	0.04
		N. weight:	1.5
		Sc. PD:	-0.047
		Sc. rank:	272.0

PA

Pos.	31	obs:	exp:
gaa	E	1068	653.50
gag	E	6	420.50
mPD		0.011	0.48
		nPD:	0.02
		N. weight:	0.51
		Sc. PD:	-0.024
		Sc. rank:	-28.8

PA

Pos.	32	obs:	exp:
act	T	0	273.80
acc	T	0	220.40
aca	T	1061	492.60
acg	T	13	87.24
mPD		0.024	0.68
		nPD:	0.04
		N. weight:	0.8
		Sc. PD:	-0.028
		Sc. rank:	101.7

PA

Pos.	33	obs:	exp:
aat	N	3	547.10
aac	N	1071	526.90
mPD		0.0056	0.50
		nPD:	0.01
		N. weight:	0.76
		Sc. PD:	-0.044
		Sc. rank:	-250.9

PA

Pos.	34	obs:	exp:
aaa	K	1073	626.00
aag	K	1	448.00
mPD		0.0019	0.49
		nPD:	0.
		N. weight:	0.58
		Sc. PD:	-0.038
		Sc. rank:	-333.0

PA

Pos.	35	obs:	exp:
ttt	F	23	422.50
ttc	F	1047	647.50
tta	L	2	0.35
ttg	L	0	0.74
ctt	L	0	0.67
ctc	L	2	0.67
cta	L	0	0.67
ctg	L	0	0.89
mPD		0.049	0.49
		nPD:	0.1
		N. weight:	0.45
		Sc. PD:	0.013
		Sc. rank:	246.6

PA

Pos.	36	obs:	exp:
gct	A	0	277.30
gcc	A	0	200.00
gca	A	1073	505.50
gcg	A	1	91.15
mPD		0.0019	0.67
		nPD:	0.
		N. weight:	0.82
		Sc. PD:	-0.055
		Sc. rank:	-522.6

PA

Pos.	37	obs:	exp:
tct	S	0	170.70
tcc	S	0	140.10
tca	S	1073	272.30
tcg	S	0	60.89
agt	S	0	220.60
agc	S	0	208.40
gct	A	0	0.26
gcc	A	0	0.19
gca	A	0	0.47
gcg	A	1	0.08
mPD		0.0037	1.7
		nPD:	0.
		N. weight:	1.5
		Sc. PD:	-0.1
		Sc. rank:	-994.9

PA

Pos.	38	obs:	exp:
att	I	0	394.60
atc	I	0	257.40
ata	I	1074	422.00
mPD		0	0.65
		nPD:	0.
		N. weight:	1.
		Sc. PD:	-0.071
		Sc. rank:	-863.8

PA

Pos.	39	obs:	exp:
tgt	C	4	454.30
tgc	C	1070	619.70
mPD		0.0074	0.49
		nPD:	0.02
		N. weight:	0.58
		Sc. PD:	-0.032
		Sc. rank:	-119.8

PA

Pos.	40	obs:	exp:
act	T	0	273.80
acc	T	2	220.40
aca	T	1069	492.60
acg	T	3	87.24
mPD		0.0093	0.68
		nPD:	0.01
		N. weight:	0.82
		Sc. PD:	-0.046
		Sc. rank:	-201.5

PA

Pos.	41	obs:	exp:
cat	H	30	616.70
cac	H	1044	457.30
mPD		0.054	0.49
		nPD:	0.11
		N. weight:	0.78
		Sc. PD:	0.03
		Sc. rank:	448.1

PA

Pos.	42	obs:	exp:
tta	L	1020	95.31
ttg	L	49	200.00
ctt	L	0	178.80
ctc	L	0	179.20
cta	L	5	181.00
ctg	L	0	239.70
mPD		0.096	1.1
		nPD:	0.09
		N. weight:	2.4
		Sc. PD:	0.035
		Sc. rank:	1138.3

PA

Pos.	43	obs:	exp:
gaa	E	1069	653.50
gag	E	5	420.50
mPD		0.0093	0.48
		nPD:	0.02
		N. weight:	0.51
		Sc. PD:	-0.026
		Sc. rank:	-54.9

PA

Pos.	44	obs:	exp:
att	I	0	0.37
atc	I	1	0.24
ata	I	0	0.39
gtt	V	10	222.30
gtc	V	1059	216.60
gta	V	4	219.70
gtg	V	0	414.40
mPD		0.028	0.73
		nPD:	0.04
		N. weight:	1.7
		Sc. PD:	-0.054
		Sc. rank:	261.5

PA

Pos.	45	obs:	exp:
tgt	C	8	454.30
tgc	C	1066	619.70
mPD		0.015	0.49
		nPD:	0.03
		N. weight:	0.56
		Sc. PD:	-0.022
		Sc. rank:	33.4

PA			
Pos.	46	obs:	exp:
ttt	F	2	424.10
ttc	F	1072	649.90
---	--	------	------
mPD		0.0037	0.48
		nPD:	0.01
		N. weight :	0.53
		Sc. PD:	-0.033
		Sc. rank :	-234.6

,

PA			
Pos.	47	obs:	exp:
tta	L	0	0.09
ttg	L	1	0.19
ctt	L	0	0.17
ctc	L	0	0.17
cta	L	0	0.17
ctg	L	0	0.22
atg	M	1073	1073.00
---	--	------	------
mPD		0.0019	0.0030
		nPD:	0.63
		N. weight :	0.0017
		Sc. PD:	0.00092
		Sc. rank :	1.4

,

PA			
Pos.	48	obs:	exp:
tat	Y	335	580.10
tac	Y	739	493.90
---	--	------	------
mPD		0.43	0.50
		nPD:	0.86
		N. weight :	0.12
		Sc. PD:	0.091
		Sc. rank :	101.8

,

PA			
Pos.	49	obs:	exp:
tct	S	1073	170.80
tcc	S	0	140.30
tca	S	0	272.60
tcg	S	1	60.94
agt	S	0	220.80
agc	S	0	208.60
---	--	------	------
mPD		0.0019	1.7
		nPD:	0.
		N. weight :	2.
		Sc. PD:	-0.14
		Sc. rank :	-1430.7

,

PA			
Pos.	50	obs:	exp:
cat	H	1	0.57
cac	H	0	0.43
gat	D	1053	579.80
gac	D	20	493.20
---	--	------	------
mPD		0.038	0.50
		nPD:	0.08
		N. weight :	0.58
		Sc. PD:	0.0029
		Sc. rank :	245.1

,

PA			
Pos.	51	obs:	exp:
ttt	F	0	422.50
ttc	F	1070	647.50
tgt	C	0	1.69
tgc	C	4	2.31
---	--	------	------
mPD		0.0074	0.49
		nPD:	0.02
		N. weight :	0.55
		Sc. PD:	-0.03
		Sc. rank :	-109.3

,

PA			
Pos.	52	obs:	exp:
cat	H	8	616.70
cac	H	1066	457.30
---	--	------	------
mPD		0.015	0.49
		nPD:	0.03
		N. weight :	0.88
		Sc. PD:	-0.036
		Sc. rank :	46.5

,

PA			
Pos.	53	obs:	exp:
ttt	F	1	424.10
ttc	F	1073	649.90
---	--	------	------
mPD		0.0019	0.48
		nPD:	0.
		N. weight :	0.54
		Sc. PD:	-0.036
		Sc. rank :	-303.7

,

PA			
Pos.	54	obs:	exp:
att	I	41	394.60
atc	I	1032	257.40
ata	I	1	422.00
---	--	------	------
mPD		0.075	0.65
		nPD:	0.12
		N. weight :	1.4
		Sc. PD:	0.057
		Sc. rank :	799.7

,

PA			
Pos.	55	obs:	exp:
cat	H	0	0.57
cac	H	1	0.43
aat	N	0	3.06
aac	N	6	2.94
gat	D	20	576.60
gac	D	1047	490.40
---	--	------	------
mPD		0.050	0.51
		nPD:	0.1
		N. weight :	0.74
		Sc. PD:	0.018
		Sc. rank :	396.6

,

PA			
Pos.	56	obs:	exp:
gaa	E	1068	653.50
gag	E	6	420.50
---	--	------	------
mPD		0.011	0.48
		nPD:	0.02
		N. weight :	0.51
		Sc. PD:	-0.024
		Sc. rank :	-28.8

,

PA			
Pos.	57	obs:	exp:
tta	L	0	0.18
ttg	L	0	0.37
ctt	L	0	0.33
ctc	L	0	0.33
cta	L	0	0.34
ctg	L	2	0.45
caa	Q	29	19.87
cag	Q	10	19.13
cgt	R	50	44.37
cgc	R	9	51.16
cga	R	687	101.10
cgg	R	254	86.20
aga	R	31	472.30
agg	R	2	277.80
---	--	------	------
mPD		0.59	1.1
		nPD:	0.55
		N. weight :	1.5
		Sc. PD:	0.71
		Sc. rank :	1265.4

,

PA			
Pos.	58	obs:	exp:
ggt	G	46	142.40
ggc	G	773	137.20
gga	G	255	495.50
ggg	G	0	299.00
---	--	------	------
mPD		0.42	0.68
		nPD:	0.63
		N. weight :	1.1
		Sc. PD:	0.61
		Sc. rank :	954.8

,

PA			
Pos.	59	obs:	exp:
gaa	E	1073	653.50
gag	E	1	420.50
---	--	------	------
mPD		0.0019	0.48
		nPD:	0.
		N. weight :	0.53
		Sc. PD:	-0.035
		Sc. rank :	-296.3

,

PA			
Pos.	60	obs:	exp:
tct	S	0	170.30
tcc	S	0	139.90
tca	S	1071	271.80
tcg	S	0	60.77
cct	P	0	0.75
ccc	P	0	0.54
cca	P	3	1.23
ccg	P	0	0.48
agt	S	0	220.20
agc	S	0	208.00
---	--	------	------
mPD		0.0056	1.7
		nPD:	0.
		N. weight :	1.5
		Sc. PD:	-0.099
		Sc. rank :	-892.4

,

PA			
Pos.	61	obs:	exp:
att	I	6	2.20
atc	I	0	1.44
ata	I	0	2.36
atg	M	1	1.00
act	T	1017	272.00
acc	T	47	218.90
aca	T	3	489.40
acg	T	0	86.67
---	--	------	------
mPD		0.10	0.69
		nPD:	0.15
		N. weight :	1.3
		Sc. PD:	0.098
		Sc. rank :	859.9

,

PA			
Pos.	62	obs:	exp:
att	I	478	394.20
atc	I	3	257.10
ata	I	592	421.60
gtt	V	1	0.21
gtc	V	0	0.20
gta	V	0	0.20
gtg	V	0	0.39
---	--	------	------
mPD		0.50	0.66
		nPD:	0.76
		N. weight :	0.29
		Sc. PD:	0.19
		Sc. rank :	246.9

,

PA			
Pos.	63	obs:	exp:
att	I	0	390.60
atc	I	0	254.70
ata	I	1063	417.70
gtt	V	0	2.28
gtc	V	0	2.22
gta	V	11	2.25
gtg	V	0	4.25
---	--	------	------
mPD		0.020	0.68
		nPD:	0.03
		N. weight :	1.
		Sc. PD:	-0.042
		Sc. rank :	37.1

,

PA			
Pos.	64	obs:	exp:
gaa	E	1072	653.50
gag	E	2	420.50
---	--	------	------
mPD		0.0037	0.48
		nPD:	0.01
		N. weight :	0.53
		Sc. PD:	-0.033
		Sc. rank :	-226.7

,

PA			
Pos.	65	obs:	exp:
tct	S	1058	170.80
tcc	S	10	140.30
tca	S	0	272.60
tcg	S	6	60.94
agt	S	0	220.80
agc	S	0	208.60
---	--	------	------
mPD		0.029	1.7
		nPD:	0.02
		N. weight :	1.9
		Sc. PD:	-0.1
		Sc. rank :	-284.9

,

PA

Pos.	66	obs:	exp:
tct	S	0	0.16
tcc	S	0	0.13
tca	S	0	0.25
tcg	S	0	0.06
agt	S	0	0.21
agc	S	1	0.19
ggt	G	11	142.20
ggc	G	1062	137.10
gga	G	0	495.00
ggg	G	0	298.70
mPD		0.022	0.68
		nPD:	0.03
		N. weight:	2.2
		Sc. PD:	-0.083
		Sc. rank:	206.8

,

PA

Pos.	67	obs:	exp:
tat	Y	1	0.54
tac	Y	0	0.46
aat	N	1	0.51
aac	N	0	0.49
gat	D	1065	579.30
gac	D	7	492.70
mPD		0.017	0.50
		nPD:	0.03
		N. weight:	0.63
		Sc. PD:	-0.024
		Sc. rank:	67.6

,

PA

Pos.	68	obs:	exp:
tta	L	0	0.18
ttg	L	0	0.37
tct	S	0	0.16
tcc	S	0	0.13
tca	S	1	0.25
tcg	S	0	0.06
ctt	L	0	0.33
ctc	L	0	0.33
cta	L	2	0.34
ctg	L	0	0.45
cct	P	3	267.00
ccc	P	0	192.20
cca	P	1061	440.40
ccg	P	6	170.30
act	T	0	0.25
acc	T	0	0.21
aca	T	1	0.46
acg	T	0	0.08
agt	S	0	0.21
agc	S	0	0.19
mPD		0.024	0.72
		nPD:	0.03
		N. weight:	0.92
		Sc. PD:	-0.034
		Sc. rank:	100.8

,

PA

Pos.	69	obs:	exp:
aat	N	1070	547.10
aac	N	4	526.90
mPD		0.0074	0.50
		nPD:	0.01
		N. weight:	0.71
		Sc. PD:	-0.039
		Sc. rank:	-158.3

,

PA

Pos.	70	obs:	exp:
gtt	V	0	55.12
gtc	V	0	53.69
gta	V	0	54.47
gtg	V	266	102.70
gct	A	1	208.70
gcc	A	0	150.50
gca	A	49	380.30
gcg	A	758	68.58
mPD		0.46	1.1
		nPD:	0.43
		N. weight:	2.
		Sc. PD:	0.68
		Sc. rank:	1573.9

,

PA

Pos.	71	obs:	exp:
tta	L	6	95.31
ttg	L	430	200.00
ctt	L	0	178.80
ctc	L	0	179.20
cta	L	3	181.00
ctg	L	635	239.70
mPD		0.50	1.1
		nPD:	0.45
		N. weight:	0.94
		Sc. PD:	0.34
		Sc. rank:	748.4

,

PA

Pos.	72	obs:	exp:
tta	L	11	95.22
ttg	L	435	199.80
tct	S	0	0.16
tcc	S	0	0.13
tca	S	0	0.25
tcg	S	1	0.06
ctt	L	4	178.70
ctc	L	0	179.10
cta	L	15	180.80
ctg	L	608	239.40
agt	S	0	0.21
agc	S	0	0.19
mPD		0.54	1.1
		nPD:	0.49
		N. weight:	0.85
		Sc. PD:	0.34
		Sc. rank:	686.6

,

PA

Pos.	73	obs:	exp:
aaa	K	1056	626.00
aag	K	18	448.00
mPD		0.033	0.49
		nPD:	0.07
		N. weight:	0.5
		Sc. PD:	-0.002
		Sc. rank:	191.4

,

PA

Pos.	74	obs:	exp:
cat	H	402	616.70
cac	H	672	457.30
mPD		0.47	0.49
		nPD:	0.96
		N. weight:	0.092
		Sc. PD:	0.079
		Sc. rank:	78.4

,

PA

Pos.	75	obs:	exp:
cgt	R	0	46.13
cgc	R	0	53.19
cga	R	811	105.10
cgg	R	246	89.62
aga	R	10	491.10
agg	R	7	288.90
mPD		0.39	0.96
		nPD:	0.41
		N. weight:	1.9
		Sc. PD:	0.61
		Sc. rank:	1464.6

,

PA

Pos.	76	obs:	exp:
ttt	F	1073	424.10
ttc	F	1	649.90
mPD		0.0019	0.48
		nPD:	0.
		N. weight:	1.
		Sc. PD:	-0.066
		Sc. rank:	-564.3

,

PA

Pos.	77	obs:	exp:
gaa	E	1068	653.50
gag	E	6	420.50
mPD		0.011	0.48
		nPD:	0.02
		N. weight:	0.51
		Sc. PD:	-0.024
		Sc. rank:	-28.8

,

PA

Pos.	78	obs:	exp:
tta	L	2	0.18
ttg	L	0	0.37
ctt	L	0	0.33
ctc	L	0	0.33
cta	L	0	0.34
ctg	L	0	0.45
att	I	0	393.90
atc	I	0	256.90
ata	I	1072	421.20
mPD		0.0037	0.66
		nPD:	0.01
		N. weight:	1.
		Sc. PD:	-0.065
		Sc. rank:	-511.5

,

PA

Pos.	79	obs:	exp:
att	I	12	393.90
atc	I	1052	256.90
ata	I	8	421.20
gtt	V	0	0.41
gtc	V	2	0.40
gta	V	0	0.41
gtg	V	0	0.77
mPD		0.040	0.66
		nPD:	0.06
		N. weight:	1.4
		Sc. PD:	-0.014
		Sc. rank:	509.7

,

PA

Pos.	80	obs:	exp:
gaa	E	1071	653.50
gag	E	3	420.50
mPD		0.0056	0.48
		nPD:	0.01
		N. weight:	0.52
		Sc. PD:	-0.03
		Sc. rank:	-156.4

,

PA

Pos.	81	obs:	exp:
ggt	G	0	142.40
ggc	G	0	137.20
gga	G	194	495.50
ggg	G	880	299.00
mPD		0.30	0.68
		nPD:	0.44
		N. weight:	0.78
		Sc. PD:	0.28
		Sc. rank:	619.8

,

PA

Pos.	82	obs:	exp:
cgt	R	0	46.13
cgc	R	0	53.19
cga	R	0	105.10
cgg	R	0	89.62
aga	R	1064	491.10
agg	R	10	288.90
mPD		0.018	0.96
		nPD:	0.02
		N. weight:	0.8
		Sc. PD:	-0.041
		Sc. rank:	-89.7

,

PA

Pos.	83	obs:	exp:
gat	D	1	580.40
gac	D	1073	493.60
mPD		0.0019	0.50
		nPD:	0.
		N. weight:	0.84
		Sc. PD:	-0.055
		Sc. rank:	-483.4

,

PA

Pos.	84	obs:	exp:
cgt	R	0	46.13
cgc	R	0	53.19
cga	R	1069	105.10
cgg	R	3	89.62
aga	R	2	491.10
agg	R	0	288.90
mPD		0.0093	0.96
		nPD:	0.01
		N. weight:	2.5
		Sc. PD:	-0.15
		Sc. rank:	-907.9

,

PA

Pos.	85	obs:	exp:
att	I	0	0.73
atc	I	0	0.48
ata	I	2	0.79
act	T	0	271.20
acc	T	0	218.30
aca	T	1063	488.00
acg	T	1	86.42
gct	A	0	2.07
gcc	A	0	1.49
gca	A	8	3.77
gcg	A	0	0.68
mPD		0.020	0.69
		nPD:	0.03
		N. weight:	0.84
		Sc. PD:	-0.035
		Sc. rank:	24.3

,

PA

Pos. 86		obs:	exp:
att	I	0	1.84
atc	I	0	1.20
ata	I	5	1.96
atg	M	1067	1067.00
gtt	V	0	0.41
gtc	V	0	0.40
gta	V	0	0.41
gtg	V	2	0.77
mPD		0.013	0.015
		nPD:	0.85
		N. weight:	0.0067
		Sc. PD:	0.005
		Sc. rank:	5.7

PA

Pos. 87		obs:	exp:
gct	A	2	277.30
gcc	A	1041	200.00
gca	A	31	505.50
gcg	A	0	91.15
mPD		0.060	0.67
		nPD:	0.09
		N. weight:	1.6
		Sc. PD:	0.028
		Sc. rank:	808.0

PA

Pos. 88		obs:	exp:
tgg	W	1074	1074.00
mPD		0	0
		nPD:	1.
		N. weight:	0.
		Sc. PD:	0
		Sc. rank:	0

PA

Pos. 89		obs:	exp:
act	T	2	273.50
acc	T	10	220.20
aca	T	1054	492.20
acg	T	7	87.16
gct	A	0	0.26
gcc	A	0	0.19
gca	A	1	0.47
gcg	A	0	0.08
mPD		0.037	0.68
		nPD:	0.05
		N. weight:	0.76
		Sc. PD:	-0.013
		Sc. rank:	223.9

PA

Pos. 90		obs:	exp:
att	I	0	0.37
atc	I	0	0.24
ata	I	1	0.39
atg	M	3	3.00
gtt	V	1	221.70
gtc	V	0	216.00
gta	V	45	219.10
gtg	V	1024	413.20
mPD		0.091	0.73
		nPD:	0.12
		N. weight:	0.87
		Sc. PD:	0.044
		Sc. rank:	539.3

PA

Pos. 91		obs:	exp:
atg	M	1	1.00
gtt	V	1	222.10
gtc	V	0	216.40
gta	V	48	219.50
gtg	V	1023	414.00
gct	A	0	0.26
gcc	A	0	0.19
gca	A	0	0.47
gcg	A	1	0.08
mPD		0.091	0.73
		nPD:	0.12
		N. weight:	0.87
		Sc. PD:	0.044
		Sc. rank:	538.5

PA

Pos. 92		obs:	exp:
tct	S	0	0.16
tcc	S	0	0.13
tca	S	0	0.25
tcg	S	0	0.06
aat	N	1060	546.50
aac	N	13	526.50
agt	S	1	0.21
agc	S	0	0.19
mPD		0.026	0.50
		nPD:	0.05
		N. weight:	0.67
		Sc. PD:	-0.013
		Sc. rank:	190.0

PA

Pos. 93		obs:	exp:
tct	S	0	170.80
tcc	S	0	140.30
tca	S	0	272.60
tcg	S	0	60.94
agt	S	1014	220.80
agc	S	60	208.60
mPD		0.11	1.7
		nPD:	0.06
		N. weight:	1.5
		Sc. PD:	-0.012
		Sc. rank:	549.5

PA

Pos. 94		obs:	exp:
att	I	65	386.20
atc	I	986	251.90
ata	I	0	413.00
gtt	V	0	4.77
gtc	V	23	4.64
gta	V	0	4.71
gtg	V	0	8.88
mPD		0.16	0.70
		nPD:	0.22
		N. weight:	1.3
		Sc. PD:	0.19
		Sc. rank:	945.2

PA

Pos. 95		obs:	exp:
tat	Y	0	0.54
tac	Y	1	0.46
tgt	C	3	453.80
tgc	C	1070	619.20
mPD		0.0074	0.49
		nPD:	0.02
		N. weight:	0.58
		Sc. PD:	-0.032
		Sc. rank:	-121.8

PA

Pos. 96		obs:	exp:
cat	H	0	16.65
cac	H	29	12.35
aat	N	3	532.30
aac	N	1042	512.70
mPD		0.058	0.55
		nPD:	0.11
		N. weight:	0.76
		Sc. PD:	0.025
		Sc. rank:	420.9

PA

Pos. 97		obs:	exp:
att	I	1	1.84
atc	I	4	1.20
ata	I	0	1.96
act	T	89	272.20
acc	T	977	219.10
aca	T	2	489.90
acg	T	0	86.75
gct	A	0	0.26
gcc	A	1	0.19
gca	A	0	0.47
gcg	A	0	0.08
mPD		0.17	0.69
		nPD:	0.24
		N. weight:	1.4
		Sc. PD:	0.23
		Sc. rank:	1022.5

PA

Pos. 98		obs:	exp:
act	T	23	273.30
acc	T	0	220.00
aca	T	621	491.70
acg	T	428	87.07
gct	A	0	0.52
gcc	A	0	0.37
gca	A	0	0.94
gcg	A	2	0.17
mPD		0.51	0.68
		nPD:	0.75
		N. weight:	0.79
		Sc. PD:	0.52
		Sc. rank:	671.5

PA

Pos. 99		obs:	exp:
cgt	R	0	0.04
cgc	R	0	0.05
cga	R	0	0.10
cgg	R	0	0.08
aga	R	1	0.46
agg	R	0	0.27
ggt	G	57	142.20
ggc	G	0	137.10
gga	G	1009	495.00
ggg	G	7	298.70
mPD		0.11	0.68
		nPD:	0.17
		N. weight:	0.65
		Sc. PD:	0.062
		Sc. rank:	456.9

PA

Pos. 100		obs:	exp:
att	I	0	10.29
atc	I	28	6.71
ata	I	0	11.00
act	T	0	1.02
acc	T	4	0.82
aca	T	0	1.83
acg	T	0	0.32
gtt	V	41	92.00
gtc	V	403	89.61
gta	V	0	90.91
gtg	V	0	171.50
gct	A	4	154.40
gcc	A	593	111.40
gca	A	1	281.50
gcg	A	0	50.75
mPD		0.63	1.3
		nPD:	0.49
		N. weight:	1.6
		Sc. PD:	0.66
		Sc. rank:	1309.6

PA

Pos. 101		obs:	exp:
caa	Q	3	1.53
cag	Q	0	1.47
gaa	E	1018	651.70
gag	E	53	419.30
mPD		0.099	0.48
		nPD:	0.21
		N. weight:	0.35
		Sc. PD:	0.046
		Sc. rank:	258.0

PA

Pos. 102		obs:	exp:
aaa	K	1048	626.00
aag	K	26	448.00
mPD		0.047	0.49
		nPD:	0.1
		N. weight:	0.48
		Sc. PD:	0.012
		Sc. rank:	253.6

PA

Pos. 103		obs:	exp:
cct	P	77	268.00
ccc	P	991	193.00
cca	P	6	442.10
ccg	P	0	171.00
mPD		0.14	0.71
		nPD:	0.2
		N. weight:	1.5
		Sc. PD:	0.19
		Sc. rank:	1107.5

PA

Pos. 104		obs:	exp:
aaa	K	1071	626.00
aag	K	3	448.00
mPD		0.0056	0.49
		nPD:	0.01
		N. weight:	0.57
		Sc. PD:	-0.033
		Sc. rank:	-178.9

PA

Pos. 105		obs:	exp:
ttt	F	1048	422.90
ttc	F	23	648.10
tta	L	0	0.27
ttg	L	0	0.56
ctt	L	3	0.50
ctc	L	0	0.50
cta	L	0	0.51
ctg	L	0	0.67
mPD		0.048	0.48
		nPD:	0.1
		N. weight:	0.89
		Sc. PD:	0.023
		Sc. rank:	480.3

PA

Pos. 106		obs:	exp:
tta	L	0	95.13
ttg	L	0	199.60
ctt	L	42	178.50
ctc	L	1029	178.90
cta	L	1	180.70
ctg	L	0	239.20
att	I	0	0.73
atc	I	2	0.48
ata	I	0	0.79
mPD		0.081	1.1
		nPD:	0.07
		N. weight:	1.8
		Sc. PD:	0.0013
		Sc. rank:	730.9

PA

Pos. 107		obs:	exp:
cct	P	6	268.00
ccc	P	0	193.00
cca	P	62	442.10
ccg	P	1006	171.00
mPD		0.12	0.71
		nPD:	0.17
		N. weight:	1.7
		Sc. PD:	0.16
		Sc. rank:	1159.9

PA

Pos. 108		obs:	exp:
gat	D	1071	580.40
gac	D	3	493.60
mPD		0.0056	0.50
		nPD:	0.01
		N. weight:	0.65
		Sc. PD:	-0.038
		Sc. rank:	-212.9

PA

Pos. 109		obs:	exp:
tta	L	0	95.31
ttg	L	11	200.00
ctt	L	4	178.80
ctc	L	0	179.20
cta	L	1	181.00
ctg	L	1058	239.70
mPD		0.030	1.1
		nPD:	0.03
		N. weight:	1.5
		Sc. PD:	-0.068
		Sc. rank:	2.9

PA

Pos. 110		obs:	exp:
tat	Y	1073	580.10
tac	Y	1	493.90
mPD		0.0019	0.50
		nPD:	0.
		N. weight:	0.66
		Sc. PD:	-0.044
		Sc. rank:	-386.0

PA			
Pos.	111	obs:	exp:
gat	D	9	580.40
gac	D	1065	493.60
mPD		0.017	0.50
		nPD:	0.03
		N. weight:	0.79
		Sc. PD:	-0.03
		Sc. rank:	88.2

PA			
Pos.	112	obs:	exp:
tat	Y	0	579.50
tac	Y	1073	493.50
tgt	C	0	0.42
tgc	C	1	0.58
mPD		0.0019	0.50
		nPD:	0.
		N. weight:	0.85
		Sc. PD:	-0.056
		Sc. rank:	-494.8

PA			
Pos.	113	obs:	exp:
aaa	K	19	626.00
aag	K	1055	448.00
mPD		0.035	0.49
		nPD:	0.07
		N. weight:	0.85
		Sc. PD:	-0.00025
		Sc. rank:	337.8

PA			
Pos.	114	obs:	exp:
gaa	E	1072	653.50
gag	E	2	420.50
mPD		0.0037	0.48
		nPD:	0.01
		N. weight:	0.53
		Sc. PD:	-0.033
		Sc. rank:	-226.7

PA			
Pos.	115	obs:	exp:
tct	S	0	4.61
tcc	S	0	3.79
tca	S	0	7.36
tcg	S	0	1.65
aat	N	23	531.30
aac	N	1020	511.70
agt	S	0	5.96
agc	S	29	5.63
gat	D	0	1.08
gac	D	2	0.92
mPD		0.098	0.60
		nPD:	0.16
		N. weight:	0.69
		Sc. PD:	0.062
		Sc. rank:	472.8

PA			
Pos.	116	obs:	exp:
cgt	R	592	46.13
cgc	R	1	53.19
cga	R	475	105.10
cgg	R	4	89.62
aga	R	2	491.10
agg	R	0	288.90
mPD		0.50	0.96
		nPD:	0.52
		N. weight:	2.2
		Sc. PD:	0.98
		Sc. rank:	1826.2

PA			
Pos.	117	obs:	exp:
ttt	F	8	424.10
ttc	F	1066	649.90
mPD		0.015	0.48
		nPD:	0.03
		N. weight:	0.5
		Sc. PD:	-0.02
		Sc. rank:	34.3

PA			
Pos.	118	obs:	exp:
att	I	1073	394.20
atc	I	0	257.10
ata	I	0	421.60
gtt	V	1	0.21
gtc	V	0	0.20
gta	V	0	0.20
gtg	V	0	0.39
mPD		0.0019	0.66
		nPD:	0.
		N. weight:	1.1
		Sc. PD:	-0.073
		Sc. rank:	-692.2

PA			
Pos.	119	obs:	exp:
gaa	E	1073	653.50
gag	E	1	420.50
mPD		0.0019	0.48
		nPD:	0.
		N. weight:	0.53
		Sc. PD:	-0.035
		Sc. rank:	-296.3

PA			
Pos.	120	obs:	exp:
att	I	1071	393.90
atc	I	1	256.90
ata	I	0	421.20
gtt	V	2	0.41
gtc	V	0	0.40
gta	V	0	0.41
gtg	V	0	0.77
mPD		0.0056	0.66
		nPD:	0.01
		N. weight:	1.1
		Sc. PD:	-0.067
		Sc. rank:	-422.8

PA			
Pos.	121	obs:	exp:
ggt	G	0	142.40
ggc	G	0	137.20
gga	G	1044	495.50
ggg	G	30	299.00
mPD		0.054	0.68
		nPD:	0.08
		N. weight:	0.72
		Sc. PD:	0.006
		Sc. rank:	316.9

PA			
Pos.	122	obs:	exp:
gtt	V	8	222.50
gtc	V	0	216.80
gta	V	46	219.90
gtg	V	1020	414.80
mPD		0.096	0.73
		nPD:	0.13
		N. weight:	0.84
		Sc. PD:	0.049
		Sc. rank:	532.3

PA			
Pos.	123	obs:	exp:
act	T	4	273.80
acc	T	0	220.40
aca	T	25	492.60
acg	T	1045	87.24
mPD		0.053	0.68
		nPD:	0.08
		N. weight:	2.5
		Sc. PD:	0.015
		Sc. rank:	1099.2

PA			
Pos.	124	obs:	exp:
cgt	R	0	46.13
cgc	R	0	53.19
cga	R	0	105.10
cgg	R	1	89.62
aga	R	12	491.10
agg	R	1061	288.90
mPD		0.024	0.96
		nPD:	0.02
		N. weight:	1.4
		Sc. PD:	-0.061
		Sc. rank:	-17.8

PA			
Pos.	125	obs:	exp:
cgt	R	0	46.13
cgc	R	0	53.19
cga	R	0	105.10
cgg	R	3	89.62
aga	R	19	491.10
agg	R	1052	288.90
mPD		0.040	0.96
		nPD:	0.04
		N. weight:	1.3
		Sc. PD:	-0.038
		Sc. rank:	282.2

PA			
Pos.	126	obs:	exp:
gaa	E	1067	651.70
gag	E	4	419.30
ggt	G	0	0.40
ggc	G	0	0.38
gga	G	3	1.38
ggg	G	0	0.84
mPD		0.013	0.48
		nPD:	0.03
		N. weight:	0.52
		Sc. PD:	-0.023
		Sc. rank:	1.6

PA			
Pos.	127	obs:	exp:
att	I	0	0.73
atc	I	2	0.48
ata	I	0	0.79
gtt	V	5	221.70
gtc	V	1064	216.00
gta	V	1	219.10
gtg	V	0	413.20
gct	A	0	0.52
gcc	A	2	0.37
gca	A	0	0.94
gcg	A	0	0.17
mPD		0.019	0.73
		nPD:	0.03
		N. weight:	1.7
		Sc. PD:	-0.077
		Sc. rank:	-8.0

PA			
Pos.	128	obs:	exp:
cat	H	2	615.60
cac	H	1070	456.40
caa	Q	1	0.51
cag	Q	0	0.49
aat	N	0	0.51
aac	N	1	0.49
mPD		0.0074	0.49
		nPD:	0.02
		N. weight:	0.92
		Sc. PD:	-0.05
		Sc. rank:	-195.8

PA			
Pos.	129	obs:	exp:
att	I	0	392.00
atc	I	3	255.70
ata	I	1064	419.30
atg	M	6	6.00
act	T	0	0.25
acc	T	0	0.21
aca	T	1	0.46
acg	T	0	0.08
mPD		0.019	0.66
		nPD:	0.03
		N. weight:	0.99
		Sc. PD:	-0.042
		Sc. rank:	22.6

PA			
Pos.	130	obs:	exp:
tat	Y	922	580.10
tac	Y	152	493.90
mPD		0.24	0.50
		nPD:	0.49
		N. weight:	0.26
		Sc. PD:	0.1
		Sc. rank:	205.4

PA			
Pos.	131	obs:	exp:
tta	L	0	0.09
ttg	L	1	0.19
tat	Y	0	579.50
tac	Y	1073	493.50
ctt	L	0	0.17
ctc	L	0	0.17
cta	L	0	0.17
ctg	L	0	0.22
mPD		0.0037	0.50
		nPD:	0.01
		N. weight:	0.85
		Sc. PD:	-0.053
		Sc. rank:	-405.0

PA			
Pos.	132	obs:	exp:
tta	L	3	95.31
ttg	L	0	200.00
ctt	L	0	178.80
ctc	L	0	179.20
cta	L	1063	181.00
ctg	L	8	239.70
mPD		0.020	1.1
		nPD:	0.02
		N. weight:	1.9
		Sc. PD:	-0.097
		Sc. rank:	-265.6

PA			
Pos.	133	obs:	exp:
gaa	E	47	653.50
gag	E	1027	420.50
mPD		0.084	0.48
		nPD:	0.18
		N. weight:	0.81
		Sc. PD:	0.081
		Sc. rank:	569.2

PA			
Pos.	134	obs:	exp:
aaa	K	1027	626.00
aag	K	47	448.00
mPD		0.084	0.49
		nPD:	0.17
		N. weight:	0.41
		Sc. PD:	0.04
		Sc. rank:	289.0

PA			
Pos.	135	obs:	exp:
gct	A	5	277.30
gcc	A	1069	200.00
gca	A	0	505.50
gcg	A	0	91.15
mPD		0.0093	0.67
		nPD:	0.01
		N. weight:	1.8
		Sc. PD:	-0.1
		Sc. rank:	-421.2

PA

Pos.	136	obs:	exp:
tct	S	0	0.32
tcc	S	0	0.26
tca	S	0	0.51
tcg	S	0	0.11
aat	N	1054	546.00
aac	N	18	526.00
agt	S	2	0.41
agc	S	0	0.39
mPD		0.037	0.51
		nPD:	0.07
		N. weight:	0.65
		Sc. PD:	0.00041
		Sc. rank:	265.1

,

PA

Pos.	137	obs:	exp:
aaa	K	1053	626.00
aag	K	21	448.00
mPD		0.038	0.49
		nPD:	0.08
		N. weight:	0.49
		Sc. PD:	0.0034
		Sc. rank:	215.5

,

PA

Pos.	138	obs:	exp:
tta	L	3	0.27
ttg	L	0	0.56
ctt	L	0	0.50
ctc	L	0	0.50
cta	L	0	0.51
ctg	L	0	0.67
att	I	0	393.10
atc	I	0	256.40
ata	I	1070	420.40
aaa	K	1	0.58
aag	K	0	0.42
mPD		0.0074	0.66
		nPD:	0.01
		N. weight:	1.
		Sc. PD:	-0.06
		Sc. rank:	-331.3

,

PA

Pos.	139	obs:	exp:
aaa	K	1073	626.00
aag	K	1	448.00
mPD		0.0019	0.49
		nPD:	0.
		N. weight:	0.58
		Sc. PD:	-0.038
		Sc. rank:	-333.0

,

PA

Pos.	140	obs:	exp:
tct	S	19	170.80
tcc	S	1007	140.30
tca	S	48	272.60
tcg	S	0	60.94
agt	S	0	220.80
agc	S	0	208.60
mPD		0.12	1.7
		nPD:	0.07
		N. weight:	1.9
		Sc. PD:	-0.00015
		Sc. rank:	765.7

,

PA

Pos.	141	obs:	exp:
aaa	K	0	1.17
aag	K	2	0.83
gaa	E	9	651.70
gag	E	1062	419.30
ggt	G	0	0.13
ggc	G	0	0.13
gga	G	0	0.46
ggg	G	1	0.28
mPD		0.022	0.48
		nPD:	0.05
		N. weight:	0.97
		Sc. PD:	-0.024
		Sc. rank:	235.1

,

PA

Pos.	142	obs:	exp:
cgt	R	0	0.09
cgc	R	0	0.10
cga	R	0	0.20
cgg	R	0	0.17
aaa	K	38	622.50
aag	K	1030	445.50
aga	R	0	0.91
agg	R	2	0.54
gaa	E	0	2.43
gag	E	4	1.57
mPD		0.079	0.50
		nPD:	0.16
		N. weight:	0.78
		Sc. PD:	0.066
		Sc. rank:	525.1

,

PA

Pos.	143	obs:	exp:
act	T	0	272.50
acc	T	11	219.40
aca	T	1058	490.30
acg	T	0	86.83
aaa	K	2	1.17
aag	K	0	0.83
gct	A	0	0.77
gcc	A	0	0.56
gca	A	3	1.41
gcg	A	0	0.25
mPD		0.030	0.69
		nPD:	0.04
		N. weight:	0.8
		Sc. PD:	-0.022
		Sc. rank:	181.1

,

PA

Pos.	144	obs:	exp:
cat	H	5	616.70
cac	H	1069	457.30
mPD		0.0093	0.49
		nPD:	0.02
		N. weight:	0.9
		Sc. PD:	-0.046
		Sc. rank:	-113.6

,

PA

Pos.	145	obs:	exp:
tta	L	0	0.09
ttg	L	0	0.19
ctt	L	0	0.17
ctc	L	0	0.17
cta	L	1	0.17
ctg	L	0	0.22
att	I	2	393.90
atc	I	734	256.90
ata	I	336	421.20
gtt	V	0	0.21
gtc	V	0	0.20
gta	V	1	0.20
gtg	V	0	0.39
mPD		0.44	0.66
		nPD:	0.67
		N. weight:	0.7
		Sc. PD:	0.4
		Sc. rank:	591.4

,

PA

Pos.	146	obs:	exp:
cat	H	599	616.70
cac	H	475	457.30
mPD		0.49	0.49
		nPD:	1.01
		N. weight:	0.0044
		Sc. PD:	0.004
		Sc. rank:	3.7

,

PA

Pos.	147	obs:	exp:
att	I	1063	394.20
atc	I	10	257.10
ata	I	0	421.60
act	T	1	0.25
acc	T	0	0.21
aca	T	0	0.46
acg	T	0	0.08
mPD		0.020	0.66
		nPD:	0.03
		N. weight:	1.
		Sc. PD:	-0.041
		Sc. rank:	74.5

,

PA

Pos.	148	obs:	exp:
ttt	F	592	423.70
ttc	F	481	649.30
tta	L	0	0.09
ttg	L	0	0.19
ctt	L	0	0.17
ctc	L	1	0.17
cta	L	0	0.17
ctg	L	0	0.22
mPD		0.50	0.48
		nPD:	1.04
		N. weight:	0.06
		Sc. PD:	0.056
		Sc. rank:	51.4

,

PA

Pos.	149	obs:	exp:
tct	S	0	170.80
tcc	S	0	140.30
tca	S	1071	272.60
tcg	S	3	60.94
agt	S	0	220.80
agc	S	0	208.60
mPD		0.0056	1.7
		nPD:	0.
		N. weight:	1.5
		Sc. PD:	-0.098
		Sc. rank:	-878.8

,

PA

Pos.	150	obs:	exp:
ttt	F	5	423.30
ttc	F	1067	648.70
tta	L	0	0.18
ttg	L	0	0.37
ctt	L	0	0.33
ctc	L	2	0.33
cta	L	0	0.34
ctg	L	0	0.45
mPD		0.013	0.48
		nPD:	0.03
		N. weight:	0.52
		Sc. PD:	-0.023
		Sc. rank:	2.3

,

PA

Pos.	151	obs:	exp:
act	T	1068	273.50
acc	T	2	220.20
aca	T	2	492.20
acg	T	1	87.16
aat	N	1	0.51
aac	N	0	0.49
mPD		0.011	0.68
		nPD:	0.02
		N. weight:	1.5
		Sc. PD:	-0.078
		Sc. rank:	-250.2

,

PA

Pos.	152	obs:	exp:
ggt	G	0	142.40
ggc	G	0	137.20
gga	G	1050	495.50
ggg	G	24	299.00
mPD		0.044	0.68
		nPD:	0.06
		N. weight:	0.74
		Sc. PD:	-0.0051
		Sc. rank:	273.7

,

PA

Pos.	153	obs:	exp:
gaa	E	1072	652.90
gag	E	1	420.10
ggt	G	0	0.13
ggc	G	0	0.13
gga	G	1	0.46
ggg	G	0	0.28
mPD		0.0037	0.48
		nPD:	0.01
		N. weight:	0.53
		Sc. PD:	-0.033
		Sc. rank:	-235.6

,

PA

Pos.	154	obs:	exp:
gaa	E	16	653.50
gag	E	1058	420.50
mPD		0.029	0.48
		nPD:	0.06
		N. weight:	0.93
		Sc. PD:	-0.0092
		Sc. rank:	332.3

,

PA

Pos.	155	obs:	exp:
atg	M	1074	1074.00
mPD		0	0
		nPD:	1.
		N. weight:	0.
		Sc. PD:	0
		Sc. rank:	0

,

PA

Pos.	156	obs:	exp:
gct	A	1	277.30
gcc	A	1048	200.00
gca	A	25	505.50
gcg	A	0	91.15
mPD		0.047	0.67
		nPD:	0.07
		N. weight:	1.7
		Sc. PD:	-0.0019
		Sc. rank:	661.2

,

PA

Pos.	157	obs:	exp:
act	T	9	273.80
acc	T	1064	220.40
aca	T	1	492.60
acg	T	0	87.24
mPD		0.018	0.68
		nPD:	0.03
		N. weight:	1.7
		Sc. PD:	-0.072
		Sc. rank:	25.1

,

PA

Pos.	158	obs:	exp:
caa	Q	7	3.57
cag	Q	0	3.43
cgt	R	0	0.04
cgc	R	0	0.05
cga	R	0	0.10
cgg	R	0	0.08
aaa	K	1043	621.30
aag	K	23	444.70
aga	R	1	0.46
agg	R	0	0.27
mPD		0.057	0.50
		nPD:	0.11
		N. weight:	0.49
		Sc. PD:	0.019
		Sc. rank:	283.6

,

PA

Pos.	159	obs:	exp:
gct	A	0	277.30
gcc	A	0	200.00
gca	A	1073	505.50
gcg	A	1	91.15
mPD		0.0019	0.67
		nPD:	0.
		N. weight:	0.82
		Sc. PD:	-0.055
		Sc. rank:	-522.6

,

PA

Pos.	160	obs:	exp:
aat	N	2	1.02
aac	N	0	0.98
gat	D	1064	579.30
gac	D	8	492.70
mPD		0.019	0.50
		nPD:	0.04
		N. weight:	0.63
		Sc. PD:	-0.021
		Sc. rank:	92.4

,

PA			
Pos.	161	obs:	exp:
tat	Y	1	580.10
tac	Y	1073	493.90
---	--	------	------
mPD		0.0019	0.50
		nPD:	0.
		N. weight:	0.84
		Sc. PD:	-0.055
		Sc. rank:	-487.6

,

PA			
Pos.	162	obs:	exp:
act	T	981	273.80
acc	T	14	220.40
aca	T	79	492.60
acg	T	0	87.24
---	--	------	------
mPD		0.16	0.68
		nPD:	0.24
		N. weight:	1.1
		Sc. PD:	0.17
		Sc. rank:	802.5

,

PA			
Pos.	163	obs:	exp:
tta	L	0	95.31
ttg	L	0	200.00
ctt	L	1069	178.80
ctc	L	5	179.20
cta	L	0	181.00
ctg	L	0	239.70
---	--	------	------
mPD		0.0093	1.1
		nPD:	0.01
		N. weight:	1.9
		Sc. PD:	-0.12
		Sc. rank:	-772.9

,

PA			
Pos.	164	obs:	exp:
gat	D	301	580.40
gac	D	773	493.60
---	--	------	------
mPD		0.40	0.50
		nPD:	0.81
		N. weight:	0.15
		Sc. PD:	0.11
		Sc. rank:	132.0

,

PA			
Pos.	165	obs:	exp:
gaa	E	1067	653.50
gag	E	7	420.50
---	--	------	------
mPD		0.013	0.48
		nPD:	0.03
		N. weight:	0.5
		Sc. PD:	-0.022
		Sc. rank:	3.4

,

PA			
Pos.	166	obs:	exp:
gaa	E	880	653.50
gag	E	194	420.50
---	--	------	------
mPD		0.30	0.48
		nPD:	0.62
		N. weight:	0.12
		Sc. PD:	0.062
		Sc. rank:	97.8

,

PA			
Pos.	167	obs:	exp:
tct	S	0	170.70
tcc	S	0	140.10
tca	S	0	272.30
tcg	S	0	60.89
tgt	C	0	0.42
tgc	C	1	0.58
agt	S	46	220.60
agc	S	1027	208.40
---	--	------	------
mPD		0.084	1.7
		nPD:	0.05
		N. weight:	1.6
		Sc. PD:	-0.033
		Sc. rank:	444.6

,

PA			
Pos.	168	obs:	exp:
cgt	R	0	46.13
cgc	R	0	53.19
cga	R	0	105.10
cgg	R	0	89.62
aga	R	2	491.10
agg	R	1072	288.90
---	--	------	------
mPD		0.0037	0.96
		nPD:	0.
		N. weight:	1.4
		Sc. PD:	-0.093
		Sc. rank:	-797.5

,

PA			
Pos.	169	obs:	exp:
gct	A	0	277.30
gcc	A	0	200.00
gca	A	1074	505.50
gcg	A	0	91.15
---	--	------	------
mPD		0	0.67
		nPD:	0.
		N. weight:	0.82
		Sc. PD:	-0.057
		Sc. rank:	-696.9

,

PA			
Pos.	170	obs:	exp:
cgt	R	0	46.13
cgc	R	0	53.19
cga	R	0	105.10
cgg	R	0	89.62
aga	R	1071	491.10
agg	R	3	288.90
---	--	------	------
mPD		0.0056	0.96
		nPD:	0.01
		N. weight:	0.83
		Sc. PD:	-0.053
		Sc. rank:	-407.4

,

PA			
Pos.	171	obs:	exp:
att	I	1	394.20
atc	I	1068	257.10
ata	I	4	421.60
gtt	V	0	0.21
gtc	V	1	0.20
gta	V	0	0.20
gtg	V	0	0.39
---	--	------	------
mPD		0.011	0.66
		nPD:	0.02
		N. weight:	1.5
		Sc. PD:	-0.081
		Sc. rank:	-236.5

,

PA			
Pos.	172	obs:	exp:
aat	N	0	0.51
aac	N	1	0.49
aaa	K	1071	625.40
aag	K	2	447.60
---	--	------	------
mPD		0.0056	0.49
		nPD:	0.01
		N. weight:	0.58
		Sc. PD:	-0.034
		Sc. rank:	-181.5

,

PA			
Pos.	173	obs:	exp:
act	T	7	273.80
acc	T	1065	220.40
aca	T	2	492.60
acg	T	0	87.24
---	--	------	------
mPD		0.017	0.68
		nPD:	0.02
		N. weight:	1.7
		Sc. PD:	-0.076
		Sc. rank:	-48.0

,

PA			
Pos.	174	obs:	exp:
cgt	R	0	46.13
cgc	R	0	53.19
cga	R	0	105.10
cgg	R	0	89.62
aga	R	51	491.10
agg	R	1023	288.90
---	--	------	------
mPD		0.091	0.96
		nPD:	0.09
		N. weight:	1.2
		Sc. PD:	0.026
		Sc. rank:	619.9

,

PA			
Pos.	175	obs:	exp:
tta	L	0	95.31
ttg	L	147	200.00
ctt	L	0	178.80
ctc	L	0	179.20
cta	L	5	181.00
ctg	L	922	239.70
---	--	------	------
mPD		0.25	1.1
		nPD:	0.22
		N. weight:	1.2
		Sc. PD:	0.17
		Sc. rank:	877.9

,

PA			
Pos.	176	obs:	exp:
ttt	F	7	424.10
ttc	F	1067	649.90
---	--	------	------
mPD		0.013	0.48
		nPD:	0.03
		N. weight:	0.51
		Sc. PD:	-0.022
		Sc. rank:	2.8

,

PA			
Pos.	177	obs:	exp:
act	T	29	273.80
acc	T	1043	220.40
aca	T	2	492.60
acg	T	0	87.24
---	--	------	------
mPD		0.056	0.68
		nPD:	0.08
		N. weight:	1.6
		Sc. PD:	0.017
		Sc. rank:	706.7

,

PA			
Pos.	178	obs:	exp:
att	I	0	394.60
atc	I	29	257.40
ata	I	1045	422.00
---	--	------	------
mPD		0.053	0.65
		nPD:	0.08
		N. weight:	0.9
		Sc. PD:	0.0076
		Sc. rank:	396.8

,

PA			
Pos.	179	obs:	exp:
cgt	R	0	46.13
cgc	R	0	53.19
cga	R	0	105.10
cgg	R	0	89.62
aga	R	32	491.10
agg	R	1042	288.90
---	--	------	------
mPD		0.058	0.96
		nPD:	0.06
		N. weight:	1.3
		Sc. PD:	-0.014
		Sc. rank:	436.5

,

PA			
Pos.	180	obs:	exp:
cat	H	2	1.15
cac	H	0	0.85
caa	Q	18	546.00
cag	Q	1054	526.00
---	--	------	------
mPD		0.037	0.50
		nPD:	0.07
		N. weight:	0.68
		Sc. PD:	0.0008
		Sc. rank:	285.1

,

PA			
Pos.	181	obs:	exp:
gaa	E	1009	653.50
gag	E	65	420.50
---	--	------	------
mPD		0.11	0.48
		nPD:	0.24
		N. weight:	0.32
		Sc. PD:	0.052
		Sc. rank:	239.2

,

PA			
Pos.	182	obs:	exp:
tta	L	0	0.27
ttg	L	1	0.56
ctt	L	0	0.50
ctc	L	0	0.50
cta	L	0	0.51
ctg	L	2	0.67
atg	M	1071	1071.00
---	--	------	------
mPD		0.0056	0.0089
		nPD:	0.63
		N. weight:	0.0036
		Sc. PD:	0.002
		Sc. rank:	3.1

,

PA			
Pos.	183	obs:	exp:
gct	A	18	277.30
gcc	A	1054	200.00
gca	A	2	505.50
gcg	A	0	91.15
---	--	------	------
mPD		0.037	0.67
		nPD:	0.05
		N. weight:	1.7
		Sc. PD:	-0.029
		Sc. rank:	515.8

,

PA			
Pos.	184	obs:	exp:
tct	S	0	169.70
tcc	S	0	139.40
tca	S	0	270.80
tcg	S	0	60.55
tgt	C	0	0.85
tgc	C	2	1.15
att	I	0	0.37
atc	I	1	0.24
ata	I	0	0.39
aat	N	0	0.51
aac	N	1	0.49
agt	S	40	219.30
agc	S	1027	207.20
ggt	G	0	0.40
ggc	G	3	0.38
gga	G	0	1.38
ggg	G	0	0.84
---	--	------	------
mPD		0.085	1.7
		nPD:	0.05
		N. weight:	1.6
		Sc. PD:	-0.032
		Sc. rank:	456.1

,

PA			
Pos.	185	obs:	exp:
cgt	R	0	46.13
cgc	R	0	53.19
cga	R	0	105.10
cgg	R	0	89.62
aga	R	11	491.10
agg	R	1063	288.90
---	--	------	------
mPD		0.020	0.96
		nPD:	0.02
		N. weight:	1.4
		Sc. PD:	-0.067
		Sc. rank:	-124.8

,

PA

Pos.	186	obs:	exp:
tct	S	0	0.16
tcc	S	0	0.13
tca	S	0	0.25
tcg	S	0	0.06
agt	S	1	0.21
agc	S	0	0.19
ggt	G	1060	142.20
ggc	G	10	137.10
gga	G	3	495.00
ggg	G	0	298.70
---	--	------	------
mPD		0.026	0.68
		nPD:	0.04
		N. weight:	2.1
		Sc. PD:	-0.069
		Sc. rank:	344.7

PA

Pos.	187	obs:	exp:
tta	L	1	95.22
ttg	L	0	199.80
ctt	L	0	178.70
ctc	L	2	179.10
cta	L	1066	180.80
ctg	L	4	239.40
att	I	0	0.37
atc	I	0	0.24
ata	I	1	0.39
---	--	------	------
mPD		0.015	1.1
		nPD:	0.01
		N. weight:	1.9
		Sc. PD:	-0.11
		Sc. rank:	-508.7

PA

Pos.	188	obs:	exp:
tgg	W	1074	1074.00
---	--	------	------
mPD		0	0
		nPD:	1.
		N. weight:	0.
		Sc. PD:	0
		Sc. rank:	0

PA

Pos.	189	obs:	exp:
gat	D	1066	579.30
gac	D	6	492.70
gaa	E	0	1.22
gag	E	2	0.78
---	--	------	------
mPD		0.015	0.50
		nPD:	0.03
		N. weight:	0.64
		Sc. PD:	-0.026
		Sc. rank:	20.6

PA

Pos.	190	obs:	exp:
ttt	F	0	0.39
ttc	F	1	0.61
tct	S	0	170.70
tcc	S	1073	140.10
tca	S	0	272.30
tcg	S	0	60.89
agt	S	0	220.60
agc	S	0	208.40
---	--	------	------
mPD		0.0019	1.7
		nPD:	0.
		N. weight:	2.2
		Sc. PD:	-0.15
		Sc. rank:	-1617.6

PA

Pos.	191	obs:	exp:
ttt	F	1071	423.70
ttc	F	2	649.30
tct	S	1	0.16
tcc	S	0	0.13
tca	S	0	0.25
tcg	S	0	0.06
agt	S	0	0.21
agc	S	0	0.19
---	--	------	------
mPD		0.0056	0.48
		nPD:	0.01
		N. weight:	1.
		Sc. PD:	-0.058
		Sc. rank:	-311.2

PA

Pos.	192	obs:	exp:
tct	S	0	0.16
tcc	S	0	0.13
tca	S	0	0.25
tcg	S	0	0.06
tgt	C	3	1.27
tgc	C	0	1.73
cgt	R	1070	45.96
cgc	R	0	53.00
cga	R	0	104.70
cgg	R	0	89.28
agt	S	1	0.21
agc	S	0	0.19
aga	R	0	489.30
agg	R	0	287.80
---	--	------	------
mPD		0.0074	0.97
		nPD:	0.01
		N. weight:	3.4
		Sc. PD:	-0.21
		Sc. rank:	-1547.9

PA

Pos.	193	obs:	exp:
caa	Q	0	547.10
cag	Q	1074	526.90
---	--	------	------
mPD		0	0.50
		nPD:	0.
		N. weight:	0.78
		Sc. PD:	-0.054
		Sc. rank:	-658.5

PA

Pos.	194	obs:	exp:
tct	S	414	170.80
tcc	S	651	140.30
tca	S	9	272.60
tcg	S	0	60.94
agt	S	0	220.80
agc	S	0	208.60
---	--	------	------
mPD		0.48	1.7
		nPD:	0.29
		N. weight:	1.4
		Sc. PD:	0.29
		Sc. rank:	1022.2

PA

Pos.	195	obs:	exp:
gaa	E	1074	653.50
gag	E	0	420.50
---	--	------	------
mPD		0	0.48
		nPD:	0.
		N. weight:	0.54
		Sc. PD:	-0.038
		Sc. rank:	-459.4

PA

Pos.	196	obs:	exp:
cgt	R	0	46.13
cgc	R	0	53.19
cga	R	0	105.10
cgg	R	0	89.62
aga	R	1074	491.10
agg	R	0	288.90
---	--	------	------
mPD		0	0.96
		nPD:	0.
		N. weight:	0.85
		Sc. PD:	-0.059
		Sc. rank:	-723.7

PA

Pos.	197	obs:	exp:
ggt	G	0	142.40
ggc	G	1074	137.20
gga	G	0	495.50
ggg	G	0	299.00
---	--	------	------
mPD		0	0.68
		nPD:	0.
		N. weight:	2.2
		Sc. PD:	-0.16
		Sc. rank:	-1902.9

PA

Pos.	198	obs:	exp:
gaa	E	1021	653.50
gag	E	53	420.50
---	--	------	------
mPD		0.094	0.48
		nPD:	0.2
		N. weight:	0.35
		Sc. PD:	0.043
		Sc. rank:	254.0

PA

Pos.	199	obs:	exp:
gaa	E	1062	652.30
gag	E	10	419.70
ggt	G	0	0.27
ggc	G	0	0.26
gga	G	2	0.92
ggg	G	0	0.56
---	--	------	------
mPD		0.022	0.48
		nPD:	0.05
		N. weight:	0.49
		Sc. PD:	-0.012
		Sc. rank:	119.9

PA

Pos.	200	obs:	exp:
act	T	0	273.80
acc	T	0	220.40
aca	T	1074	492.60
acg	T	0	87.24
---	--	------	------
mPD		0	0.68
		nPD:	0.
		N. weight:	0.85
		Sc. PD:	-0.059
		Sc. rank:	-720.8

PA

Pos.	201	obs:	exp:
att	I	1072	393.90
atc	I	0	256.90
ata	I	0	421.20
act	T	2	0.51
acc	T	0	0.41
aca	T	0	0.92
acg	T	0	0.16
---	--	------	------
mPD		0.0037	0.66
		nPD:	0.01
		N. weight:	1.1
		Sc. PD:	-0.07
		Sc. rank:	-541.5

PA

Pos.	202	obs:	exp:
gaa	E	1074	653.50
gag	E	0	420.50
---	--	------	------
mPD		0	0.48
		nPD:	0.
		N. weight:	0.54
		Sc. PD:	-0.038
		Sc. rank:	-459.4

PA

Pos.	203	obs:	exp:
gaa	E	1066	653.50
gag	E	8	420.50
---	--	------	------
mPD		0.015	0.48
		nPD:	0.03
		N. weight:	0.5
		Sc. PD:	-0.02
		Sc. rank:	36.3

PA

Pos.	204	obs:	exp:
cgt	R	0	44.84
cgc	R	0	51.71
cga	R	0	102.20
cgg	R	0	87.11
att	I	0	0.73
atc	I	0	0.48
ata	I	2	0.79
aaa	K	28	16.32
aag	K	0	11.68
aga	R	1043	477.40
agg	R	1	280.80
---	--	------	------
mPD		0.056	1.0
		nPD:	0.06
		N. weight:	0.84
		Sc. PD:	-0.013
		Sc. rank:	259.9

PA

Pos.	205	obs:	exp:
ttt	F	1074	424.10
ttc	F	0	649.90
---	--	------	------
mPD		0	0.48
		nPD:	0.
		N. weight:	1.
		Sc. PD:	-0.07
		Sc. rank:	-859.2

PA

Pos.	206	obs:	exp:
gtt	V	0	0.41
gtc	V	0	0.40
gta	V	2	0.41
gtg	V	0	0.77
gat	D	0	0.54
gac	D	1	0.46
gaa	E	1064	651.10
gag	E	6	418.90
ggt	G	0	0.13
ggc	G	0	0.13
gga	G	1	0.46
ggg	G	0	0.28
---	--	------	------
mPD		0.019	0.48
		nPD:	0.04
		N. weight:	0.51
		Sc. PD:	-0.017
		Sc. rank:	87.4

PA

Pos.	207	obs:	exp:
att	I	1	394.20
atc	I	1072	257.10
ata	I	0	421.60
aat	N	0	0.51
aac	N	1	0.49
---	--	------	------
mPD		0.0037	0.66
		nPD:	0.01
		N. weight:	1.5
		Sc. PD:	-0.099
		Sc. rank:	-762.3

PA

Pos.	208	obs:	exp:
act	T	1	273.50
acc	T	0	220.20
aca	T	1072	492.20
acg	T	0	87.16
gct	A	0	0.26
gcc	A	0	0.19
gca	A	1	0.47
gcg	A	0	0.08
---	--	------	------
mPD		0.0037	0.68
		nPD:	0.01
		N. weight:	0.84
		Sc. PD:	-0.054
		Sc. rank:	-451.9

PA

Pos.	209	obs:	exp:
gaa	E	2	1.22
gag	E	0	0.78
ggt	G	0	142.10
ggc	G	28	136.90
gga	G	1043	494.50
ggg	G	1	298.40
---	--	------	------
mPD		0.056	0.68
		nPD:	0.08
		N. weight:	0.74
		Sc. PD:	0.008
		Sc. rank:	331.5

PA

Pos.	210	obs:	exp:
act	T	3	273.80
acc	T	1059	220.40
aca	T	12	492.60
acg	T	0	87.24
---	--	------	------
mPD		0.028	0.68
		nPD:	0.04
		N. weight:	1.6
		Sc. PD:	-0.049
		Sc. rank:	330.6

PA			
Pos.	211	obs:	exp:
atg	M	1074	1074.00
mPD		0	0
		nPD:	1.
		N. weight:	0.
		Sc. PD:	0
		Sc. rank:	0

PA			
Pos.	212	obs:	exp:
tta	L	0	0.18
ttg	L	0	0.37
ctt	L	0	0.33
ctc	L	2	0.33
cta	L	0	0.34
ctg	L	0	0.45
cgt	R	13	46.04
cgc	R	1054	53.10
cga	R	4	104.90
cgg	R	1	89.45
aga	R	0	490.20
agg	R	0	288.30
mPD		0.037	0.97
		nPD:	0.04
		N. weight:	3.2
		Sc. PD:	-0.1
		Sc. rank:	538.4

PA			
Pos.	213	obs:	exp:
cgt	R	0	46.13
cgc	R	0	53.19
cga	R	0	105.10
cgg	R	1	89.62
aga	R	0	491.10
agg	R	1073	288.90
mPD		0.0019	0.96
		nPD:	0.
		N. weight:	1.4
		Sc. PD:	-0.096
		Sc. rank:	-973.5

PA			
Pos.	214	obs:	exp:
tta	L	0	95.31
ttg	L	0	200.00
ctt	L	1067	178.80
ctc	L	7	179.20
cta	L	0	181.00
ctg	L	0	239.70
mPD		0.013	1.1
		nPD:	0.01
		N. weight:	1.9
		Sc. PD:	-0.11
		Sc. rank:	-578.0

PA			
Pos.	215	obs:	exp:
gct	A	15	277.30
gcc	A	1058	200.00
gca	A	1	505.50
gcg	A	0	91.15
mPD		0.029	0.67
		nPD:	0.04
		N. weight:	1.7
		Sc. PD:	-0.047
		Sc. rank:	408.8

PA			
Pos.	216	obs:	exp:
aat	N	0	0.51
aac	N	1	0.49
gat	D	0	579.80
gac	D	1073	493.20
mPD		0.0019	0.50
		nPD:	0.
		N. weight:	0.85
		Sc. PD:	-0.056
		Sc. rank:	-493.6

PA			
Pos.	217	obs:	exp:
caa	Q	1074	547.10
cag	Q	0	526.90
mPD		0	0.50
		nPD:	0.
		N. weight:	0.73
		Sc. PD:	-0.051
		Sc. rank:	-623.9

PA			
Pos.	218	obs:	exp:
tct	S	0	170.80
tcc	S	0	140.30
tca	S	0	272.60
tcg	S	0	60.94
agt	S	1073	220.80
agc	S	1	208.60
mPD		0.0019	1.7
		nPD:	0.
		N. weight:	1.7
		Sc. PD:	-0.12
		Sc. rank:	-1229.7

PA			
Pos.	219	obs:	exp:
tta	L	0	95.31
ttg	L	0	200.00
ctt	L	1	178.80
ctc	L	1073	179.20
cta	L	0	181.00
ctg	L	0	239.70
mPD		0.0019	1.1
		nPD:	0.
		N. weight:	1.9
		Sc. PD:	-0.13
		Sc. rank:	-1343.6

PA			
Pos.	220	obs:	exp:
tct	S	0	0.16
tcc	S	0	0.13
tca	S	1	0.25
tcg	S	0	0.06
cct	P	0	267.70
ccc	P	0	192.80
cca	P	1073	441.70
ccg	P	0	170.80
agt	S	0	0.21
agc	S	0	0.19
mPD		0.0019	0.71
		nPD:	0.
		N. weight:	0.97
		Sc. PD:	-0.065
		Sc. rank:	-637.6

PA			
Pos.	221	obs:	exp:
cct	P	0	268.00
ccc	P	0	193.00
cca	P	0	442.10
ccg	P	1074	171.00
mPD		0	0.71
		nPD:	0.
		N. weight:	2.
		Sc. PD:	-0.14
		Sc. rank:	-1699.4

PA			
Pos.	222	obs:	exp:
aat	N	0	547.10
aac	N	1074	526.90
mPD		0	0.50
		nPD:	0.
		N. weight:	0.78
		Sc. PD:	-0.054
		Sc. rank:	-658.5

PA			
Pos.	223	obs:	exp:
ttt	F	0	424.10
ttc	F	1074	649.90
mPD		0	0.48
		nPD:	0.
		N. weight:	0.55
		Sc. PD:	-0.038
		Sc. rank:	-464.6

PA			
Pos.	224	obs:	exp:
tct	S	0	170.50
tcc	S	1072	140.00
tca	S	0	272.10
tcg	S	0	60.83
cct	P	0	0.50
ccc	P	2	0.36
cca	P	0	0.82
ccg	P	0	0.32
agt	S	0	220.40
agc	S	0	208.20
mPD		0.0037	1.7
		nPD:	0.
		N. weight:	2.2
		Sc. PD:	-0.15
		Sc. rank:	-1503.2

PA			
Pos.	225	obs:	exp:
tct	S	0	170.70
tcc	S	0	140.10
tca	S	0	272.30
tcg	S	0	60.89
tgt	C	0	0.42
tgc	C	1	0.58
agt	S	0	220.60
agc	S	1073	208.40
mPD		0.0019	1.7
		nPD:	0.
		N. weight:	1.8
		Sc. PD:	-0.12
		Sc. rank:	-1285.4

PA			
Pos.	226	obs:	exp:
ttt	F	5	1.97
ttc	F	0	3.03
tta	L	0	94.86
ttg	L	0	199.00
ctt	L	1069	178.00
ctc	L	0	178.40
cta	L	0	180.20
ctg	L	0	238.50
mPD		0.0093	1.1
		nPD:	0.01
		N. weight:	1.9
		Sc. PD:	-0.12
		Sc. rank:	-792.4

PA			
Pos.	227	obs:	exp:
gaa	E	1066	653.50
gag	E	8	420.50
mPD		0.015	0.48
		nPD:	0.03
		N. weight:	0.5
		Sc. PD:	-0.02
		Sc. rank:	36.3

PA			
Pos.	228	obs:	exp:
tct	S	0	0.16
tcc	S	0	0.13
tca	S	0	0.25
tcg	S	0	0.06
aat	N	0	545.50
aac	N	1071	525.50
agt	S	0	0.21
agc	S	1	0.19
gat	D	0	1.08
gac	D	2	0.92
mPD		0.0056	0.51
		nPD:	0.01
		N. weight:	0.78
		Sc. PD:	-0.046
		Sc. rank:	-265.9

PA			
Pos.	229	obs:	exp:
ttt	F	1074	424.10
ttc	F	0	649.90
mPD		0	0.48
		nPD:	0.
		N. weight:	1.
		Sc. PD:	-0.07
		Sc. rank:	-859.2

PA			
Pos.	230	obs:	exp:
cgt	R	0	46.13
cgc	R	0	53.19
cga	R	0	105.10
cgg	R	0	89.62
aga	R	1074	491.10
agg	R	0	288.90
mPD		0	0.96
		nPD:	0.
		N. weight:	0.85
		Sc. PD:	-0.059
		Sc. rank:	-723.7

PA			
Pos.	231	obs:	exp:
act	T	0	0.25
acc	T	1	0.21
aca	T	0	0.46
acg	T	0	0.08
gct	A	0	277.10
gcc	A	1073	199.80
gca	A	0	505.00
gcg	A	0	91.07
mPD		0.0019	0.67
		nPD:	0.
		N. weight:	1.8
		Sc. PD:	-0.12
		Sc. rank:	-1176.6

PA			
Pos.	232	obs:	exp:
tat	Y	1074	580.10
tac	Y	0	493.90
mPD		0	0.50
		nPD:	0.
		N. weight:	0.67
		Sc. PD:	-0.047
		Sc. rank:	-569.7

PA			
Pos.	233	obs:	exp:
gtt	V	0	222.50
gtc	V	0	216.80
gta	V	0	219.90
gtg	V	1074	414.80
mPD		0	0.73
		nPD:	0.
		N. weight:	1.
		Sc. PD:	-0.072
		Sc. rank:	-879.8

PA			
Pos.	234	obs:	exp:
gat	D	1074	580.40
gac	D	0	493.60
mPD		0	0.50
		nPD:	0.
		N. weight:	0.67
		Sc. PD:	-0.047
		Sc. rank:	-569.2

PA			
Pos.	235	obs:	exp:
cgt	R	0	0.09
cgc	R	0	0.10
cga	R	0	0.20
cgg	R	0	0.17
aga	R	2	0.91
agg	R	0	0.54
ggt	G	0	142.10
ggc	G	0	136.90
gga	G	1072	494.50
ggg	G	0	298.40
mPD		0.0037	0.68
		nPD:	0.01
		N. weight:	0.84
		Sc. PD:	-0.054
		Sc. rank:	-453.7

PA

Pos.	236	obs:	exp:
ttt	F	12	424.10
ttc	F	1062	649.90
mPD		0.022	0.48
		nPD:	0.05
		N. weight:	0.49
		Sc. PD:	-0.012
		Sc. rank:	120.9

PA

Pos.	237	obs:	exp:
aaa	K	5	2.91
aag	K	0	2.09
gat	D	0	0.54
gac	D	1	0.46
gaa	E	1052	648.70
gag	E	14	417.30
ggt	G	0	0.27
ggc	G	0	0.26
gga	G	2	0.92
ggg	G	0	0.56
mPD		0.041	0.49
		nPD:	0.08
		N. weight:	0.48
		Sc. PD:	0.005
		Sc. rank:	211.1

PA

Pos.	238	obs:	exp:
cct	P	0	268.00
ccc	P	0	193.00
cca	P	1	442.10
ccg	P	1073	171.00
mPD		0.0019	0.71
		nPD:	0.
		N. weight:	2.
		Sc. PD:	-0.13
		Sc. rank:	-1304.7

PA

Pos.	239	obs:	exp:
tct	S	0	0.16
tcc	S	0	0.13
tca	S	0	0.25
tcg	S	0	0.06
act	T	0	0.25
acc	T	1	0.21
aca	T	0	0.46
acg	T	0	0.08
aat	N	2	545.50
aac	N	1069	525.50
aaa	K	1	0.58
aag	K	0	0.42
agt	S	0	0.21
agc	S	1	0.19
mPD		0.0093	0.51
		nPD:	0.02
		N. weight:	0.76
		Sc. PD:	-0.04
		Sc. rank:	-106.8

PA

Pos.	240	obs:	exp:
ggt	G	0	142.40
ggc	G	1072	137.20
gga	G	2	495.50
ggg	G	0	299.00
mPD		0.0037	0.68
		nPD:	0.01
		N. weight:	2.2
		Sc. PD:	-0.14
		Sc. rank:	-1181.9

PA

Pos.	241	obs:	exp:
tct	S	0	0.16
tcc	S	0	0.13
tca	S	0	0.25
tcg	S	0	0.06
tgt	C	3	453.80
tgc	C	1070	619.20
agt	S	0	0.21
agc	S	1	0.19
mPD		0.0074	0.49
		nPD:	0.02
		N. weight:	0.58
		Sc. PD:	-0.032
		Sc. rank:	-122.8

PA

Pos.	242	obs:	exp:
att	I	1074	394.60
atc	I	0	257.40
ata	I	0	422.00
mPD		0	0.65
		nPD:	0.
		N. weight:	1.1
		Sc. PD:	-0.076
		Sc. rank:	-925.9

PA

Pos.	243	obs:	exp:
gaa	E	4	652.90
gag	E	1069	420.10
ggt	G	0	0.13
ggc	G	0	0.13
gga	G	0	0.46
ggg	G	1	0.28
mPD		0.0093	0.48
		nPD:	0.02
		N. weight:	0.99
		Sc. PD:	-0.051
		Sc. rank:	-107.7

PA

Pos.	244	obs:	exp:
ggt	G	0	142.40
ggc	G	1073	137.20
gga	G	1	495.50
ggg	G	0	299.00
mPD		0.0019	0.68
		nPD:	0.
		N. weight:	2.2
		Sc. PD:	-0.15
		Sc. rank:	-1444.9

PA

Pos.	245	obs:	exp:
aaa	K	2	626.00
aag	K	1072	448.00
mPD		0.0037	0.49
		nPD:	0.01
		N. weight:	0.94
		Sc. PD:	-0.058
		Sc. rank:	-434.3

PA

Pos.	246	obs:	exp:
tta	L	0	95.31
ttg	L	0	200.00
ctt	L	1074	178.80
ctc	L	0	179.20
cta	L	0	181.00
ctg	L	0	239.70
mPD		0	1.1
		nPD:	0.
		N. weight:	2.
		Sc. PD:	-0.14
		Sc. rank:	-1657.8

PA

Pos.	247	obs:	exp:
tct	S	991	157.90
tcc	S	0	129.70
tca	S	2	252.00
tcg	S	0	56.35
cct	P	81	20.21
ccc	P	0	14.55
cca	P	0	33.34
ccg	P	0	12.89
agt	S	0	204.10
agc	S	0	192.90
mPD		0.14	1.7
		nPD:	0.08
		N. weight:	2.
		Sc. PD:	0.022
		Sc. rank:	889.0

PA

Pos.	248	obs:	exp:
caa	Q	493	547.10
cag	Q	581	526.90
mPD		0.50	0.50
		nPD:	0.99
		N. weight:	0.0093
		Sc. PD:	0.0083
		Sc. rank:	7.9

PA

Pos.	249	obs:	exp:
atg	M	1074	1074.00
mPD		0	0
		nPD:	1.
		N. weight:	0.
		Sc. PD:	0
		Sc. rank:	0

PA

Pos.	250	obs:	exp:
tct	S	0	170.80
tcc	S	0	140.30
tca	S	1074	272.60
tcg	S	0	60.94
agt	S	0	220.80
agc	S	0	208.60
mPD		0	1.7
		nPD:	0.
		N. weight:	1.5
		Sc. PD:	-0.1
		Sc. rank:	-1268.0

PA

Pos.	251	obs:	exp:
cgt	R	0	0.04
cgc	R	0	0.05
cga	R	0	0.10
cgg	R	0	0.08
aaa	K	1063	625.40
aag	K	10	447.60
aga	R	1	0.46
agg	R	0	0.27
mPD		0.020	0.49
		nPD:	0.04
		N. weight:	0.54
		Sc. PD:	-0.016
		Sc. rank:	111.2

PA

Pos.	252	obs:	exp:
aaa	K	2	1.17
aag	K	0	0.83
gaa	E	1072	652.30
gag	E	0	419.70
mPD		0.0037	0.48
		nPD:	0.01
		N. weight:	0.54
		Sc. PD:	-0.034
		Sc. rank:	-239.2

PA

Pos.	253	obs:	exp:
gtt	V	0	222.50
gtc	V	0	216.80
gta	V	2	219.90
gtg	V	1072	414.80
mPD		0.0037	0.73
		nPD:	0.01
		N. weight:	1.
		Sc. PD:	-0.066
		Sc. rank:	-558.0

PA

Pos.	254	obs:	exp:
aat	N	28	546.00
aac	N	1044	526.00
aaa	K	0	0.58
aag	K	1	0.42
gat	D	0	0.54
gac	D	1	0.46
mPD		0.055	0.50
		nPD:	0.11
		N. weight:	0.65
		Sc. PD:	0.023
		Sc. rank:	363.1

PA

Pos.	255	obs:	exp:
gct	A	2	277.30
gcc	A	1066	200.00
gca	A	6	505.50
gcg	A	0	91.15
mPD		0.015	0.67
		nPD:	0.02
		N. weight:	1.8
		Sc. PD:	-0.085
		Sc. rank:	-135.3

PA

Pos.	256	obs:	exp:
cgt	R	0	46.00
cgc	R	0	53.05
cga	R	17	104.80
cgg	R	0	89.37
att	I	0	0.37
atc	I	0	0.24
ata	I	1	0.39
aaa	K	2	1.17
aag	K	0	0.83
aga	R	1041	489.70
agg	R	13	288.10
mPD		0.061	0.97
		nPD:	0.06
		N. weight:	0.73
		Sc. PD:	-0.0064
		Sc. rank:	266.0

PA

Pos.	257	obs:	exp:
att	I	650	394.60
atc	I	407	257.40
ata	I	17	422.00
mPD		0.49	0.65
		nPD:	0.75
		N. weight:	0.47
		Sc. PD:	0.31
		Sc. rank:	398.2

PA

Pos.	258	obs:	exp:
gat	D	1	0.54
gac	D	0	0.46
gaa	E	23	652.90
gag	E	1050	420.10
mPD		0.044	0.48
		nPD:	0.09
		N. weight:	0.9
		Sc. PD:	0.017
		Sc. rank:	448.2

PA

Pos.	259	obs:	exp:
cct	P	6	268.00
ccc	P	2	193.00
cca	P	1058	442.10
ccg	P	8	171.00
mPD		0.030	0.71
		nPD:	0.04
		N. weight:	0.88
		Sc. PD:	-0.026
		Sc. rank:	182.3

PA

Pos.	260	obs:	exp:
ttt	F	1070	424.10
ttc	F	4	649.90
mPD		0.0074	0.48
		nPD:	0.02
		N. weight:	0.99
		Sc. PD:	-0.054
		Sc. rank:	-187.4

PA			
Pos.	261	obs:	exp:
tta	L	6	95.22
ttg	L	0	199.80
ctt	L	0	178.70
ctc	L	4	179.10
cta	L	1059	180.80
ctg	L	4	239.40
att	I	0	0.37
atc	I	0	0.24
ata	I	1	0.39
---	--	------	------
mPD		0.028	1.1
		nPD:	0.03
		N. weight:	1.9
		Sc. PD:	-0.084
		Sc. rank:	-16.3

PA			
Pos.	262	obs:	exp:
cgt	R	0	38.95
cgc	R	0	44.92
cga	R	0	88.76
cgg	R	0	75.68
aaa	K	2	97.33
aag	K	165	69.67
aga	R	595	414.70
agg	R	312	244.00
---	--	------	------
mPD		0.76	1.2
		nPD:	0.65
		N. weight:	0.44
		Sc. PD:	0.24
		Sc. rank:	367.3

PA			
Pos.	263	obs:	exp:
act	T	30	266.90
acc	T	0	214.80
aca	T	1013	480.20
acg	T	4	85.04
aaa	K	15	11.66
aag	K	5	8.34
gct	A	0	1.55
gcc	A	0	1.12
gca	A	6	2.82
gcg	A	0	0.51
gaa	E	1	0.61
gag	E	0	0.39
---	--	------	------
mPD		0.12	0.73
		nPD:	0.17
		N. weight:	0.7
		Sc. PD:	0.065
		Sc. rank:	484.6

PA			
Pos.	264	obs:	exp:
act	T	0	273.80
acc	T	0	220.40
aca	T	1074	492.60
acg	T	0	87.24
---	--	------	------
mPD		0	0.68
		nPD:	0.
		N. weight:	0.85
		Sc. PD:	-0.059
		Sc. rank:	-720.8

PA			
Pos.	265	obs:	exp:
cct	P	0	268.00
ccc	P	0	193.00
cca	P	1067	442.10
ccg	P	7	171.00
---	--	------	------
mPD		0.013	0.71
		nPD:	0.02
		N. weight:	0.93
		Sc. PD:	-0.048
		Sc. rank:	-136.3

PA			
Pos.	266	obs:	exp:
cgt	R	4	46.13
cgc	R	817	53.19
cga	R	250	105.10
cgg	R	2	89.62
aga	R	1	491.10
agg	R	0	288.90
---	--	------	------
mPD		0.37	0.96
		nPD:	0.38
		N. weight:	2.5
		Sc. PD:	0.75
		Sc. rank:	1914.1

PA			
Pos.	267	obs:	exp:
cct	P	740	268.00
ccc	P	2	193.00
cca	P	142	442.10
ccg	P	190	171.00
---	--	------	------
mPD		0.48	0.71
		nPD:	0.67
		N. weight:	0.62
		Sc. PD:	0.36
		Sc. rank:	521.4

PA			
Pos.	268	obs:	exp:
ttt	F	0	3.16
ttc	F	8	4.84
tta	L	0	94.42
ttg	L	0	198.10
ctt	L	0	177.20
ctc	L	1064	177.60
cta	L	0	179.30
ctg	L	0	237.40
att	I	0	0.73
atc	I	2	0.48
ata	I	0	0.79
---	--	------	------
mPD		0.018	1.1
		nPD:	0.02
		N. weight:	1.9
		Sc. PD:	-0.1
		Sc. rank:	-308.4

PA			
Pos.	269	obs:	exp:
tta	L	1	0.09
ttg	L	0	0.19
ctt	L	0	0.17
ctc	L	0	0.17
cta	L	0	0.17
ctg	L	0	0.22
cgt	R	0	45.96
cgc	R	0	53.00
cga	R	0	104.70
cgg	R	0	89.28
att	I	0	0.37
atc	I	0	0.24
ata	I	1	0.39
aaa	K	2	1.17
aag	K	0	0.83
aga	R	1068	489.30
agg	R	2	287.80
---	--	------	------
mPD		0.013	0.97
		nPD:	0.01
		N. weight:	0.84
		Sc. PD:	-0.048
		Sc. rank:	-214.5

PA			
Pos.	270	obs:	exp:
ttt	F	4	1.58
ttc	F	0	2.42
tta	L	3	94.51
ttg	L	1061	198.30
tct	S	0	0.16
tcc	S	0	0.13
tca	S	1	0.25
tcg	S	0	0.06
ctt	L	0	177.30
ctc	L	0	177.70
cta	L	0	179.50
ctg	L	1	237.70
atg	M	3	3.00
aaa	K	0	0.58
aag	K	1	0.42
agt	S	0	0.21
agc	S	0	0.19
---	--	------	------
mPD		0.028	1.1
		nPD:	0.02
		N. weight:	1.8
		Sc. PD:	-0.082
		Sc. rank:	-44.1

PA			
Pos.	271	obs:	exp:
tct	S	6	0.95
tcc	S	0	0.78
tca	S	0	1.52
tcg	S	0	0.34
cct	P	1055	266.50
ccc	P	3	191.90
cca	P	10	439.60
ccg	P	0	170.00
agt	S	0	1.23
agc	S	0	1.17
---	--	------	------
mPD		0.035	0.73
		nPD:	0.05
		N. weight:	1.4
		Sc. PD:	-0.033
		Sc. rank:	377.1

PA			
Pos.	272	obs:	exp:
tct	S	0	0.64
tcc	S	0	0.52
tca	S	0	1.02
tcg	S	0	0.23
tat	Y	1	0.54
tac	Y	0	0.46
act	T	2	0.51
acc	T	0	0.41
aca	T	0	0.92
acg	T	0	0.16
aat	N	1037	528.20
aac	N	0	508.80
agt	S	4	0.82
agc	S	0	0.78
gat	D	29	16.21
gac	D	1	13.79
---	--	------	------
mPD		0.069	0.57
		nPD:	0.12
		N. weight:	0.73
		Sc. PD:	0.035
		Sc. rank:	444.6

PA			
Pos.	273	obs:	exp:
ggt	G	0	142.40
ggc	G	43	137.20
gga	G	12	495.50
ggg	G	1019	299.00
---	--	------	------
mPD		0.098	0.68
		nPD:	0.15
		N. weight:	1.2
		Sc. PD:	0.084
		Sc. rank:	777.4

PA			
Pos.	274	obs:	exp:
cct	P	1041	268.00
ccc	P	4	193.00
cca	P	29	442.10
ccg	P	0	171.00
---	--	------	------
mPD		0.060	0.71
		nPD:	0.08
		N. weight:	1.3
		Sc. PD:	0.016
		Sc. rank:	621.4

PA			
Pos.	275	obs:	exp:
tta	L	0	0.98
ttg	L	0	2.05
tct	S	0	0.16
tcc	S	1	0.13
tca	S	0	0.25
tcg	S	0	0.06
ctt	L	0	1.83
ctc	L	11	1.84
cta	L	0	1.85
ctg	L	0	2.45
cct	P	2	265.00
ccc	P	1058	190.80
cca	P	2	437.10
ccg	P	0	169.10
agt	S	0	0.21
agc	S	0	0.19
---	--	------	------
mPD		0.030	0.74
		nPD:	0.04
		N. weight:	1.8
		Sc. PD:	-0.057
		Sc. rank:	360.1

PA			
Pos.	276	obs:	exp:
tgt	C	443	454.30
tgc	C	631	619.70
---	--	------	------
mPD		0.49	0.49
		nPD:	0.99
		N. weight:	0.004
		Sc. PD:	0.0036
		Sc. rank:	3.4

PA			
Pos.	277	obs:	exp:
ttt	F	6	2.37
ttc	F	0	3.63
tct	S	872	169.10
tcc	S	191	138.80
tca	S	0	269.80
tcg	S	0	60.32
tat	Y	5	2.70
tac	Y	0	2.30
agt	S	0	218.50
agc	S	0	206.50
---	--	------	------
mPD		0.31	1.7
		nPD:	0.19
		N. weight:	1.5
		Sc. PD:	0.17
		Sc. rank:	1086.0

PA			
Pos.	278	obs:	exp:
caa	Q	30	546.50
cag	Q	1043	526.50
aaa	K	0	0.58
aag	K	1	0.42
---	--	------	------
mPD		0.056	0.50
		nPD:	0.11
		N. weight:	0.64
		Sc. PD:	0.025
		Sc. rank:	365.6

PA			
Pos.	279	obs:	exp:
cgt	R	0	46.13
cgc	R	0	53.19
cga	R	25	105.10
cgg	R	1003	89.62
aga	R	1	491.10
agg	R	45	288.90
---	--	------	------
mPD		0.13	0.96
		nPD:	0.13
		N. weight:	2.3
		Sc. PD:	0.14
		Sc. rank:	1515.0

PA			
Pos.	280	obs:	exp:
tct	S	3	170.50
tcc	S	0	140.00
tca	S	7	272.10
tcg	S	1062	60.83
agt	S	0	220.40
agc	S	0	208.20
gct	A	0	0.52
gcc	A	0	0.37
gca	A	0	0.94
gcg	A	2	0.17
---	--	------	------
mPD		0.022	1.7
		nPD:	0.01
		N. weight:	3.1
		Sc. PD:	-0.17
		Sc. rank:	-784.0

PA			
Pos.	281	obs:	exp:
aaa	K	954	626.00
aag	K	120	448.00
---	--	------	------
mPD		0.20	0.49
		nPD:	0.41
		N. weight:	0.25
		Sc. PD:	0.082
		Sc. rank:	196.0

,

PA			
Pos.	282	obs:	exp:
ttt	F	0	424.10
ttc	F	1074	649.90
---	--	------	------
mPD		0	0.48
		nPD:	0.
		N. weight:	0.55
		Sc. PD:	-0.038
		Sc. rank:	-464.6

,

PA			
Pos.	283	obs:	exp:
tta	L	56	95.31
ttg	L	1008	200.00
ctt	L	0	178.80
ctc	L	0	179.20
cta	L	0	181.00
ctg	L	10	239.70
---	--	------	------
mPD		0.12	1.1
		nPD:	0.11
		N. weight:	1.6
		Sc. PD:	0.053
		Sc. rank:	889.9

,

PA			
Pos.	284	obs:	exp:
tta	L	0	95.31
ttg	L	10	200.00
ctt	L	8	178.80
ctc	L	0	179.20
cta	L	3	181.00
ctg	L	1053	239.70
---	--	------	------
mPD		0.039	1.1
		nPD:	0.03
		N. weight:	1.5
		Sc. PD:	-0.054
		Sc. rank:	180.2

,

PA			
Pos.	285	obs:	exp:
atg	M	1073	1073.00
aaa	K	0	0.58
aag	K	1	0.42
---	--	------	------
mPD		0.0019	0.0029
		nPD:	0.63
		N. weight:	0.00089
		Sc. PD:	0.00048
		Sc. rank:	0.7

,

PA			
Pos.	286	obs:	exp:
gat	D	1068	580.40
gac	D	6	493.60
---	--	------	------
mPD		0.011	0.50
		nPD:	0.02
		N. weight:	0.64
		Sc. PD:	-0.03
		Sc. rank:	-43.9

,

PA			
Pos.	287	obs:	exp:
gct	A	1066	277.30
gcc	A	6	200.00
gca	A	2	505.50
gcg	A	0	91.15
---	--	------	------
mPD		0.015	0.67
		nPD:	0.02
		N. weight:	1.4
		Sc. PD:	-0.069
		Sc. rank:	-108.7

,

PA			
Pos.	288	obs:	exp:
tta	L	6	95.31
ttg	L	31	200.00
ctt	L	8	178.80
ctc	L	0	179.20
cta	L	69	181.00
ctg	L	960	239.70
---	--	------	------
mPD		0.21	1.1
		nPD:	0.19
		N. weight:	1.2
		Sc. PD:	0.14
		Sc. rank:	853.9

,

PA			
Pos.	289	obs:	exp:
aaa	K	1046	626.00
aag	K	28	448.00
---	--	------	------
mPD		0.051	0.49
		nPD:	0.1
		N. weight:	0.47
		Sc. PD:	0.015
		Sc. rank:	258.6

,

PA			
Pos.	290	obs:	exp:
tta	L	967	95.31
ttg	L	15	200.00
ctt	L	0	178.80
ctc	L	0	179.20
cta	L	92	181.00
ctg	L	0	239.70
---	--	------	------
mPD		0.18	1.1
		nPD:	0.17
		N. weight:	2.2
		Sc. PD:	0.2
		Sc. rank:	1502.0

,

PA			
Pos.	291	obs:	exp:
tct	S	0	170.80
tcc	S	0	140.30
tca	S	0	272.60
tcg	S	0	60.94
agt	S	5	220.80
agc	S	1069	208.60
---	--	------	------
mPD		0.0093	1.7
		nPD:	0.01
		N. weight:	1.8
		Sc. PD:	-0.11
		Sc. rank:	-900.9

,

PA			
Pos.	292	obs:	exp:
att	I	1066	394.60
atc	I	8	257.40
ata	I	0	422.00
---	--	------	------
mPD		0.015	0.65
		nPD:	0.02
		N. weight:	1.
		Sc. PD:	-0.05
		Sc. rank:	-69.8

,

PA			
Pos.	293	obs:	exp:
gat	D	1	0.54
gac	D	0	0.46
gaa	E	169	652.90
gag	E	904	420.10
---	--	------	------
mPD		0.27	0.48
		nPD:	0.56
		N. weight:	0.48
		Sc. PD:	0.22
		Sc. rank:	393.4

,

PA			
Pos.	294	obs:	exp:
gat	D	5	580.40
gac	D	1069	493.60
---	--	------	------
mPD		0.0093	0.50
		nPD:	0.02
		N. weight:	0.82
		Sc. PD:	-0.042
		Sc. rank:	-110.9

,

PA			
Pos.	295	obs:	exp:
cct	P	9	268.00
ccc	P	0	193.00
cca	P	5	442.10
ccg	P	1060	171.00
---	--	------	------
mPD		0.026	0.71
		nPD:	0.04
		N. weight:	1.9
		Sc. PD:	-0.066
		Sc. rank:	279.4

,

PA			
Pos.	296	obs:	exp:
tct	S	0	170.20
tcc	S	0	139.70
tca	S	0	271.60
tcg	S	0	60.72
aat	N	1	2.04
aac	N	3	1.96
agt	S	103	220.00
agc	S	967	207.80
---	--	------	------
mPD		0.18	1.7
		nPD:	0.11
		N. weight:	1.4
		Sc. PD:	0.053
		Sc. rank:	810.7

,

PA			
Pos.	297	obs:	exp:
tat	Y	0	19.98
tac	Y	37	17.02
cat	H	24	595.50
cac	H	1013	441.50
---	--	------	------
mPD		0.11	0.56
		nPD:	0.2
		N. weight:	0.81
		Sc. PD:	0.099
		Sc. rank:	582.5

,

PA			
Pos.	298	obs:	exp:
gat	D	1	0.54
gac	D	0	0.46
gaa	E	1068	652.90
gag	E	5	420.10
---	--	------	------
mPD		0.011	0.48
		nPD:	0.02
		N. weight:	0.51
		Sc. PD:	-0.024
		Sc. rank:	-29.8

,

PA			
Pos.	299	obs:	exp:
cgt	R	0	0.04
cgc	R	0	0.05
cga	R	0	0.10
cgg	R	0	0.08
aga	R	0	0.46
agg	R	1	0.27
ggt	G	0	142.20
ggc	G	0	137.10
gga	G	3	495.00
ggg	G	1070	298.70
---	--	------	------
mPD		0.0074	0.68
		nPD:	0.01
		N. weight:	1.4
		Sc. PD:	-0.081
		Sc. rank:	-482.1

,

PA			
Pos.	300	obs:	exp:
gaa	E	9	653.50
gag	E	1065	420.50
---	--	------	------
mPD		0.017	0.48
		nPD:	0.03
		N. weight:	0.97
		Sc. PD:	-0.035
		Sc. rank:	111.2

,

PA			
Pos.	301	obs:	exp:
ggt	G	4	142.40
ggc	G	0	137.20
gga	G	9	495.50
ggg	G	1061	299.00
---	--	------	------
mPD		0.024	0.68
		nPD:	0.04
		N. weight:	1.3
		Sc. PD:	-0.046
		Sc. rank:	170.8

,

PA			
Pos.	302	obs:	exp:
att	I	0	394.60
atc	I	0	257.40
ata	I	1074	422.00
---	--	------	------
mPD		0	0.65
		nPD:	0.
		N. weight:	1.
		Sc. PD:	-0.071
		Sc. rank:	-863.8

,

PA			
Pos.	303	obs:	exp:
cct	P	2	268.00
ccc	P	0	193.00
cca	P	35	442.10
ccg	P	1037	171.00
---	--	------	------
mPD		0.067	0.71
		nPD:	0.09
		N. weight:	1.8
		Sc. PD:	0.038
		Sc. rank:	919.1

,

PA			
Pos.	304	obs:	exp:
tta	L	4	95.31
ttg	L	1	200.00
ctt	L	0	178.80
ctc	L	0	179.20
cta	L	1004	181.00
ctg	L	65	239.70
---	--	------	------
mPD		0.12	1.1
		nPD:	0.11
		N. weight:	1.6
		Sc. PD:	0.065
		Sc. rank:	954.5

,

PA			
Pos.	305	obs:	exp:
tat	Y	1068	577.40
tac	Y	1	491.60
cat	H	5	2.87
cac	H	0	2.13
---	--	------	------
mPD		0.011	0.51
		nPD:	0.02
		N. weight:	0.66
		Sc. PD:	-0.032
		Sc. rank:	-56.6

,

PA			
Pos.	306	obs:	exp:
gat	D	1074	580.40
gac	D	0	493.60
---	--	------	------
mPD		0	0.50
		nPD:	0.
		N. weight:	0.67
		Sc. PD:	-0.047
		Sc. rank:	-569.2

,

PA			
Pos.	307	obs:	exp:
act	T	0	0.25
acc	T	0	0.21
aca	T	0	0.46
acg	T	1	0.08
gct	A	0	277.10
gcc	A	0	199.80
gca	A	4	505.00
gcg	A	1069	91.07
---	--	------	------
mPD		0.0093	0.67
		nPD:	0.01
		N. weight:	2.7
		Sc. PD:	-0.15
		Sc. rank:	-633.0

,

PA			
Pos.	308	obs:	exp:
ttt	F	0	0.39
ttc	F	1	0.61
att	I	0	389.50
atc	I	1058	254.00
ata	I	2	416.50
gtt	V	0	2.69
gtc	V	13	2.62
gta	V	0	2.66
gtg	V	0	5.02
---	--	------	------
mPD		0.029	0.68
		nPD:	0.04
		N. weight:	1.5
		Sc. PD:	-0.043
		Sc. rank:	356.1

,

PA			
Pos.	309	obs:	exp:
aaa	K	1073	626.00
aag	K	1	448.00
---	--	------	------
mPD		0.0019	0.49
		nPD:	0.
		N. weight:	0.58
		Sc. PD:	-0.038
		Sc. rank:	-333.0

,

PA			
Pos.	310	obs:	exp:
tgt	C	5	454.30
tgc	C	1069	619.70
---	--	------	------
mPD		0.0093	0.49
		nPD:	0.02
		N. weight:	0.57
		Sc. PD:	-0.029
		Sc. rank:	-71.3

,

PA

Pos. 311		obs:	exp:
att	I	1	0.73
atc	I	0	0.48
ata	I	1	0.79
atg	M	1072	1072.00
---	--	------	------
mPD		0.0037	0.0037
		nPD:	1.
		N. weight:	0.0013
		Sc. PD:	0.0011
		Sc. rank:	1.1

,

PA

Pos. 312		obs:	exp:
aaa	K	1068	626.00
aag	K	6	448.00
---	--	------	------
mPD		0.011	0.49
		nPD:	0.02
		N. weight:	0.55
		Sc. PD:	-0.026
		Sc. rank:	-34.9

,

PA

Pos. 313		obs:	exp:
act	T	4	273.80
acc	T	0	220.40
aca	T	144	492.60
acg	T	926	87.24
---	--	------	------
mPD		0.24	0.68
		nPD:	0.35
		N. weight:	2.
		Sc. PD:	0.55
		Sc. rank:	1556.1

,

PA

Pos. 314		obs:	exp:
ttt	F	2	424.10
ttc	F	1072	649.90
---	--	------	------
mPD		0.0037	0.48
		nPD:	0.01
		N. weight:	0.53
		Sc. PD:	-0.033
		Sc. rank:	-234.6

,

PA

Pos. 315		obs:	exp:
ttt	F	10	424.10
ttc	F	1064	649.90
---	--	------	------
mPD		0.018	0.48
		nPD:	0.04
		N. weight:	0.5
		Sc. PD:	-0.016
		Sc. rank:	87.6

,

PA

Pos. 316		obs:	exp:
ggt	G	3	142.40
ggc	G	29	137.20
gga	G	204	495.50
ggg	G	838	299.00
---	--	------	------
mPD		0.35	0.68
		nPD:	0.52
		N. weight:	0.64
		Sc. PD:	0.28
		Sc. rank:	525.1

,

PA

Pos. 317		obs:	exp:
tgg	W	1074	1074.00
---	--	------	------
mPD		0	0
		nPD:	1.
		N. weight:	0.
		Sc. PD:	0
		Sc. rank:	0

,

PA

Pos. 318		obs:	exp:
aaa	K	984	626.00
aag	K	90	448.00
---	--	------	------
mPD		0.15	0.49
		nPD:	0.32
		N. weight:	0.31
		Sc. PD:	0.073
		Sc. rank:	232.9

,

PA

Pos. 319		obs:	exp:
gaa	E	4	653.50
gag	E	1070	420.50
---	--	------	------
mPD		0.0074	0.48
		nPD:	0.02
		N. weight:	0.99
		Sc. PD:	-0.054
		Sc. rank:	-185.1

,

PA

Pos. 320		obs:	exp:
tta	L	0	0.09
ttg	L	0	0.19
ctt	L	0	0.17
ctc	L	1	0.17
cta	L	0	0.17
ctg	L	0	0.22
cct	P	0	267.70
ccc	P	1072	192.80
cca	P	1	441.70
ccg	P	0	170.80
---	--	------	------
mPD		0.0037	0.71
		nPD:	0.01
		N. weight:	1.9
		Sc. PD:	-0.12
		Sc. rank:	-1006.3

,

PA

Pos. 321		obs:	exp:
tct	S	0	0.16
tcc	S	0	0.13
tca	S	0	0.25
tcg	S	0	0.06
tgt	C	0	3.38
tgc	C	8	4.62
aat	N	104	542.50
aac	N	961	522.50
agt	S	0	0.21
agc	S	1	0.19
---	--	------	------
mPD		0.21	0.53
		nPD:	0.39
		N. weight:	0.43
		Sc. PD:	0.13
		Sc. rank:	333.3

,

PA

Pos. 322		obs:	exp:
att	I	1046	394.20
atc	I	19	257.10
ata	I	8	421.60
gtt	V	1	0.21
gtc	V	0	0.20
gta	V	0	0.20
gtg	V	0	0.39
---	--	------	------
mPD		0.051	0.66
		nPD:	0.08
		N. weight:	0.96
		Sc. PD:	0.0058
		Sc. rank:	416.6

,

PA

Pos. 323		obs:	exp:
att	I	1	393.50
atc	I	1070	256.70
ata	I	0	420.80
gtt	V	0	0.62
gtc	V	3	0.61
gta	V	0	0.61
gtg	V	0	1.16
---	--	------	------
mPD		0.0074	0.66
		nPD:	0.01
		N. weight:	1.5
		Sc. PD:	-0.091
		Sc. rank:	-496.7

,

PA

Pos. 324		obs:	exp:
aaa	K	1048	626.00
aag	K	26	448.00
---	--	------	------
mPD		0.047	0.49
		nPD:	0.1
		N. weight:	0.48
		Sc. PD:	0.012
		Sc. rank:	253.6

,

PA

Pos. 325		obs:	exp:
cct	P	0	268.00
ccc	P	1	193.00
cca	P	1065	442.10
ccg	P	8	171.00
---	--	------	------
mPD		0.017	0.71
		nPD:	0.02
		N. weight:	0.92
		Sc. PD:	-0.043
		Sc. rank:	-42.7

,

PA

Pos. 326		obs:	exp:
cat	H	1071	616.70
cac	H	3	457.30
---	--	------	------
mPD		0.0056	0.49
		nPD:	0.01
		N. weight:	0.59
		Sc. PD:	-0.034
		Sc. rank:	-185.5

,

PA

Pos. 327		obs:	exp:
aaa	K	0	0.58
aag	K	1	0.42
gat	D	1	0.54
gac	D	0	0.46
gaa	E	5	649.90
gag	E	1063	418.10
ggt	G	0	0.53
ggc	G	0	0.51
gga	G	0	1.85
ggg	G	4	1.11
---	--	------	------
mPD		0.020	0.49
		nPD:	0.04
		N. weight:	0.99
		Sc. PD:	-0.029
		Sc. rank:	208.9

,

PA

Pos. 328		obs:	exp:
cgt	R	0	0.09
cgc	R	0	0.10
cga	R	0	0.20
cgg	R	0	0.17
aaa	K	845	624.80
aag	K	227	447.20
aga	R	0	0.91
agg	R	2	0.54
---	--	------	------
mPD		0.34	0.49
		nPD:	0.69
		N. weight:	0.11
		Sc. PD:	0.065
		Sc. rank:	92.1

,

PA

Pos. 329		obs:	exp:
ggt	G	32	142.40
ggc	G	1021	137.20
gga	G	21	495.50
ggg	G	0	299.00
---	--	------	------
mPD		0.095	0.68
		nPD:	0.14
		N. weight:	2.
		Sc. PD:	0.13
		Sc. rank:	1294.9

,

PA

Pos. 330		obs:	exp:
att	I	0	394.60
atc	I	0	257.40
ata	I	1074	422.00
---	--	------	------
mPD		0	0.65
		nPD:	0.
		N. weight:	1.
		Sc. PD:	-0.071
		Sc. rank:	-863.8

,

PA

Pos. 331		obs:	exp:
aat	N	24	547.10
aac	N	1050	526.90
---	--	------	------
mPD		0.044	0.50
		nPD:	0.09
		N. weight:	0.66
		Sc. PD:	0.01
		Sc. rank:	318.5

,

PA

Pos. 332		obs:	exp:
tct	S	0	0.16
tcc	S	1	0.13
tca	S	0	0.25
tcg	S	0	0.06
cct	P	5	267.70
ccc	P	1060	192.80
cca	P	8	441.70
ccg	P	0	170.80
agt	S	0	0.21
agc	S	0	0.19
---	--	------	------
mPD		0.026	0.71
		nPD:	0.04
		N. weight:	1.8
		Sc. PD:	-0.062
		Sc. rank:	252.9

,

PA

Pos. 333		obs:	exp:
aat	N	705	547.10
aac	N	369	526.90
---	--	------	------
mPD		0.45	0.50
		nPD:	0.9
		N. weight:	0.052
		Sc. PD:	0.042
		Sc. rank:	44.2

,

PA

Pos. 334		obs:	exp:
tat	Y	930	580.10
tac	Y	144	493.90
---	--	------	------
mPD		0.23	0.50
		nPD:	0.47
		N. weight:	0.27
		Sc. PD:	0.1
		Sc. rank:	215.6

,

PA

Pos. 335		obs:	exp:
tta	L	0	95.31
ttg	L	0	200.00
ctt	L	11	178.80
ctc	L	1062	179.20
cta	L	1	181.00
ctg	L	0	239.70
---	--	------	------
mPD		0.022	1.1
		nPD:	0.02
		N. weight:	1.9
		Sc. PD:	-0.095
		Sc. rank:	-184.6

,

PA			
Pos.	336	obs:	exp:
tta	L	0	92.11
ttg	L	4	193.30
ctt	L	14	172.80
ctc	L	0	173.20
cta	L	1	174.90
ctg	L	1019	231.60
att	I	2	0.73
atc	I	0	0.48
ata	I	0	0.79
atg	M	34	34.00
mPD		0.10	1.1
		nPD:	0.09
		N. weight:	1.5
		Sc. PD:	0.027
		Sc. rank:	734.0

PA			
Pos.	337	obs:	exp:
act	T	1024	272.20
acc	T	44	219.10
aca	T	0	489.90
acg	T	0	86.75
gct	A	6	1.55
gcc	A	0	1.12
gca	A	0	2.82
gcg	A	0	0.51
mPD		0.090	0.69
		nPD:	0.13
		N. weight:	1.3
		Sc. PD:	0.075
		Sc. rank:	832.8

PA			
Pos.	338	obs:	exp:
tgg	W	1074	1074.00
mPD		0	0
		nPD:	1.
		N. weight:	0.
		Sc. PD:	0
		Sc. rank:	0

PA			
Pos.	339	obs:	exp:
cgt	R	0	0.04
cgc	R	0	0.05
cga	R	0	0.10
cgg	R	0	0.08
aaa	K	21	625.40
aag	K	1052	447.60
aga	R	1	0.46
agg	R	0	0.27
mPD		0.042	0.49
		nPD:	0.09
		N. weight:	0.84
		Sc. PD:	0.012
		Sc. rank:	394.9

PA			
Pos.	340	obs:	exp:
caa	Q	11	547.10
cag	Q	1063	526.90
mPD		0.020	0.50
		nPD:	0.04
		N. weight:	0.72
		Sc. PD:	-0.022
		Sc. rank:	142.2

PA			
Pos.	341	obs:	exp:
tta	L	0	0.09
ttg	L	0	0.19
ctt	L	0	0.17
ctc	L	0	0.17
cta	L	0	0.17
ctg	L	1	0.22
atg	M	1	1.00
gtt	V	0	222.10
gtc	V	0	216.40
gta	V	4	219.50
gtg	V	1068	414.00
mPD		0.011	0.73
		nPD:	0.02
		N. weight:	1.
		Sc. PD:	-0.055
		Sc. rank:	-203.7

PA			
Pos.	342	obs:	exp:
tta	L	0	95.31
ttg	L	0	200.00
ctt	L	0	178.80
ctc	L	0	179.20
cta	L	874	181.00
ctg	L	200	239.70
mPD		0.30	1.1
		nPD:	0.27
		N. weight:	1.4
		Sc. PD:	0.27
		Sc. rank:	1019.8

PA			
Pos.	343	obs:	exp:
tct	S	0	54.24
tcc	S	0	44.53
tca	S	341	86.55
tcg	S	0	19.35
act	T	0	0.51
acc	T	0	0.41
aca	T	2	0.92
acg	T	0	0.16
agt	S	0	70.10
agc	S	0	66.23
gtt	V	0	0.21
gtc	V	0	0.20
gta	V	1	0.20
gtg	V	0	0.39
gct	A	1	188.50
gcc	A	4	135.90
gca	A	725	343.60
gcg	A	0	61.96
mPD		0.45	1.4
		nPD:	0.32
		N. weight:	1.
		Sc. PD:	0.24
		Sc. rank:	765.6

PA			
Pos.	344	obs:	exp:
gaa	E	1058	653.50
gag	E	16	420.50
mPD		0.029	0.48
		nPD:	0.06
		N. weight:	0.47
		Sc. PD:	-0.0046
		Sc. rank:	166.2

PA			
Pos.	345	obs:	exp:
tta	L	0	95.04
ttg	L	0	199.40
ctt	L	1066	178.30
ctc	L	5	178.70
cta	L	0	180.50
ctg	L	0	239.00
att	I	3	1.10
atc	I	0	0.72
ata	I	0	1.18
mPD		0.015	1.1
		nPD:	0.01
		N. weight:	1.9
		Sc. PD:	-0.11
		Sc. rank:	-545.8

PA			
Pos.	346	obs:	exp:
caa	Q	17	546.50
cag	Q	1056	526.50
cgt	R	0	0.04
cgc	R	0	0.05
cga	R	0	0.10
cgg	R	1	0.08
aga	R	0	0.46
agg	R	0	0.27
mPD		0.033	0.50
		nPD:	0.07
		N. weight:	0.69
		Sc. PD:	-0.0041
		Sc. rank:	258.5

PA			
Pos.	347	obs:	exp:
gtt	V	0	0.21
gtc	V	1	0.20
gta	V	0	0.20
gtg	V	0	0.39
gat	D	3	577.70
gac	D	1066	491.30
gaa	E	2	1.22
gag	E	0	0.78
ggt	G	0	0.27
ggc	G	2	0.26
gga	G	0	0.92
ggg	G	0	0.56
mPD		0.015	0.51
		nPD:	0.03
		N. weight:	0.83
		Sc. PD:	-0.034
		Sc. rank:	25.5

PA			
Pos.	348	obs:	exp:
tta	L	0	0.09
ttg	L	0	0.19
ctt	L	1	0.17
ctc	L	0	0.17
cta	L	0	0.17
ctg	L	0	0.22
att	I	1071	394.20
atc	I	2	257.10
ata	I	0	421.60
mPD		0.0056	0.66
		nPD:	0.01
		N. weight:	1.1
		Sc. PD:	-0.066
		Sc. rank:	-417.6

PA			
Pos.	349	obs:	exp:
aaa	K	2	1.17
aag	K	0	0.83
gaa	E	1066	651.70
gag	E	5	419.30
ggt	G	0	0.13
ggc	G	0	0.13
gga	G	1	0.46
ggg	G	0	0.28
mPD		0.015	0.48
		nPD:	0.03
		N. weight:	0.51
		Sc. PD:	-0.02
		Sc. rank:	33.0

PA			
Pos.	350	obs:	exp:
tct	S	0	0.64
tcc	S	0	0.52
tca	S	0	1.02
tcg	S	0	0.23
cat	H	8	4.59
cac	H	0	3.41
att	I	1	0.37
atc	I	0	0.24
ata	I	0	0.39
act	T	3	0.76
acc	T	0	0.62
aca	T	0	1.38
acg	T	0	0.24
aat	N	1055	538.40
aac	N	2	518.60
agt	S	4	0.82
agc	S	0	0.78
gat	D	1	0.54
gac	D	0	0.46
mPD		0.035	0.54
		nPD:	0.07
		N. weight:	0.73
		Sc. PD:	-0.0046
		Sc. rank:	270.8

PA			
Pos.	351	obs:	exp:
aaa	K	1	0.58
aag	K	0	0.42
gaa	E	1038	652.30
gag	E	34	419.70
ggt	G	0	0.13
ggc	G	0	0.13
gga	G	1	0.46
ggg	G	0	0.28
mPD		0.065	0.48
		nPD:	0.14
		N. weight:	0.41
		Sc. PD:	0.025
		Sc. rank:	264.0

PA			
Pos.	352	obs:	exp:
gaa	E	2	653.50
gag	E	1072	420.50
mPD		0.0037	0.48
		nPD:	0.01
		N. weight:	1.
		Sc. PD:	-0.062
		Sc. rank:	-432.0

PA			
Pos.	353	obs:	exp:
aaa	K	16	626.00
aag	K	1058	448.00
mPD		0.029	0.49
		nPD:	0.06
		N. weight:	0.86
		Sc. PD:	-0.0096
		Sc. rank:	300.8

PA			
Pos.	354	obs:	exp:
att	I	1033	390.90
atc	I	31	255.00
ata	I	0	418.10
act	T	1	0.25
acc	T	0	0.21
aca	T	0	0.46
acg	T	0	0.08
aat	N	1	0.51
aac	N	0	0.49
gtt	V	8	1.66
gtc	V	0	1.61
gta	V	0	1.64
gtg	V	0	3.09
mPD		0.075	0.67
		nPD:	0.11
		N. weight:	0.97
		Sc. PD:	0.037
		Sc. rank:	551.7

PA			
Pos.	355	obs:	exp:
cct	P	0	268.00
ccc	P	29	193.00
cca	P	1020	442.10
ccg	P	25	171.00
mPD		0.097	0.71
		nPD:	0.14
		N. weight:	0.77
		Sc. PD:	0.048
		Sc. rank:	498.7

PA			
Pos.	356	obs:	exp:
cgt	R	0	44.58
cgc	R	0	51.41
cga	R	0	101.60
cgg	R	0	86.61
aaa	K	0	20.98
aag	K	36	15.02
aga	R	34	474.60
agg	R	1004	279.20
---	--	------	------
mPD		0.13	1.0
		nPD:	0.12
		N. weight:	1.2
		Sc. PD:	0.063
		Sc. rank:	766.3

PA			
Pos.	357	obs:	exp:
tct	S	0	4.14
tcc	S	0	3.40
tca	S	26	6.60
tcg	S	0	1.48
act	T	0	265.10
acc	T	0	213.40
aca	T	910	477.00
acg	T	130	84.47
aaa	K	1	0.58
aag	K	0	0.42
agt	S	0	5.34
agc	S	0	5.05
gct	A	0	1.81
gcc	A	0	1.30
gca	A	7	3.29
gcg	A	0	0.59
---	--	------	------
mPD		0.27	0.74
		nPD:	0.37
		N. weight:	0.7
		Sc. PD:	0.2
		Sc. rank:	537.8

PA			
Pos.	358	obs:	exp:
aaa	K	367	626.00
aag	K	707	448.00
---	--	------	------
mPD		0.45	0.49
		nPD:	0.93
		N. weight:	0.13
		Sc. PD:	0.11
		Sc. rank:	113.0

PA			
Pos.	359	obs:	exp:
act	T	0	0.76
acc	T	3	0.62
aca	T	0	1.38
acg	T	0	0.24
aat	N	22	545.50
aac	N	1049	525.50
---	--	------	------
mPD		0.046	0.51
		nPD:	0.09
		N. weight:	0.67
		Sc. PD:	0.012
		Sc. rank:	330.2

PA			
Pos.	360	obs:	exp:
tta	L	0	0.09
ttg	L	1	0.19
ctt	L	0	0.17
ctc	L	0	0.17
cta	L	0	0.17
ctg	L	0	0.22
atg	M	1073	1073.00
---	--	------	------
mPD		0.0019	0.0030
		nPD:	0.63
		N. weight:	0.0017
		Sc. PD:	0.00092
		Sc. rank:	1.4

PA			
Pos.	361	obs:	exp:
cgt	R	0	0.21
cgc	R	0	0.25
cga	R	0	0.49
cgg	R	0	0.42
aaa	K	28	623.00
aag	K	1041	446.00
aga	R	0	2.29
agg	R	5	1.34
---	--	------	------
mPD		0.060	0.50
		nPD:	0.12
		N. weight:	0.82
		Sc. PD:	0.039
		Sc. rank:	492.4

PA			
Pos.	362	obs:	exp:
cgt	R	0	0.04
cgc	R	0	0.05
cga	R	0	0.10
cgg	R	0	0.08
aaa	K	1022	625.40
aag	K	51	447.60
aga	R	1	0.46
agg	R	0	0.27
---	--	------	------
mPD		0.092	0.49
		nPD:	0.19
		N. weight:	0.4
		Sc. PD:	0.045
		Sc. rank:	285.6

PA			
Pos.	363	obs:	exp:
act	T	0	273.80
acc	T	0	220.40
aca	T	1070	492.60
acg	T	4	87.24
---	--	------	------
mPD		0.0074	0.68
		nPD:	0.01
		N. weight:	0.83
		Sc. PD:	-0.049
		Sc. rank:	-290.3

PA			
Pos.	364	obs:	exp:
tct	S	0	165.40
tcc	S	0	135.80
tca	S	0	264.00
tcg	S	0	59.01
tgt	C	0	0.42
tgc	C	1	0.58
att	I	0	1.84
atc	I	5	1.20
ata	I	0	1.96
aat	N	0	0.51
aac	N	1	0.49
agt	S	2	213.80
agc	S	1038	202.00
gtt	V	0	3.94
gtc	V	19	3.83
gta	V	0	3.89
gtg	V	0	7.34
ggt	G	0	1.06
ggc	G	8	1.02
gga	G	0	3.69
ggg	G	0	2.23
---	--	------	------
mPD		0.10	1.7
		nPD:	0.06
		N. weight:	1.8
		Sc. PD:	-0.023
		Sc. rank:	590.7

PA			
Pos.	365	obs:	exp:
cat	H	2	1.72
cac	H	1	1.28
caa	Q	1063	545.50
cag	Q	8	525.50
---	--	------	------
mPD		0.020	0.50
		nPD:	0.04
		N. weight:	0.69
		Sc. PD:	-0.021
		Sc. rank:	135.7

PA			
Pos.	366	obs:	exp:
tta	L	1067	95.31
ttg	L	5	200.00
ctt	L	0	178.80
ctc	L	0	179.20
cta	L	2	181.00
ctg	L	0	239.70
---	--	------	------
mPD		0.013	1.1
		nPD:	0.01
		N. weight:	2.6
		Sc. PD:	-0.15
		Sc. rank:	-748.0

PA			
Pos.	367	obs:	exp:
cgt	R	0	0.13
cgc	R	0	0.15
cga	R	0	0.29
cgg	R	0	0.25
aaa	K	5	624.20
aag	K	1066	446.80
aga	R	0	1.37
agg	R	3	0.81
---	--	------	------
mPD		0.015	0.49
		nPD:	0.03
		N. weight:	0.92
		Sc. PD:	-0.037
		Sc. rank:	41.8

PA			
Pos.	368	obs:	exp:
tgg	W	1074	1074.00
---	--	------	------
mPD		0	0
		nPD:	1.
		N. weight:	0.
		Sc. PD:	0
		Sc. rank:	0

PA			
Pos.	369	obs:	exp:
gct	A	0	277.30
gcc	A	0	200.00
gca	A	1072	505.50
gcg	A	2	91.15
---	--	------	------
mPD		0.0037	0.67
		nPD:	0.01
		N. weight:	0.81
		Sc. PD:	-0.052
		Sc. rank:	-424.2

PA			
Pos.	370	obs:	exp:
tta	L	0	95.31
ttg	L	0	200.00
ctt	L	49	178.80
ctc	L	1015	179.20
cta	L	10	181.00
ctg	L	0	239.70
---	--	------	------
mPD		0.10	1.1
		nPD:	0.09
		N. weight:	1.7
		Sc. PD:	0.037
		Sc. rank:	884.7

PA			
Pos.	371	obs:	exp:
ggt	G	1070	142.40
ggc	G	3	137.20
gga	G	0	495.50
ggg	G	1	299.00
---	--	------	------
mPD		0.0074	0.68
		nPD:	0.01
		N. weight:	2.2
		Sc. PD:	-0.13
		Sc. rank:	-748.3

PA			
Pos.	372	obs:	exp:
gat	D	1	1.08
gac	D	1	0.92
gaa	E	125	652.30
gag	E	947	419.70
---	--	------	------
mPD		0.21	0.48
		nPD:	0.44
		N. weight:	0.58
		Sc. PD:	0.2
		Sc. rank:	455.3

PA			
Pos.	373	obs:	exp:
tct	S	0	0.16
tcc	S	0	0.13
tca	S	0	0.25
tcg	S	0	0.06
aat	N	5	546.50
aac	N	1068	526.50
agt	S	0	0.21
agc	S	1	0.19
---	--	------	------
mPD		0.011	0.50
		nPD:	0.02
		N. weight:	0.75
		Sc. PD:	-0.036
		Sc. rank:	-52.7

PA			
Pos.	374	obs:	exp:
cgt	R	0	0.04
cgc	R	0	0.05
cga	R	0	0.10
cgg	R	0	0.08
atg	M	1072	1072.00
act	T	0	0.25
acc	T	0	0.21
aca	T	0	0.46
acg	T	1	0.08
aga	R	0	0.46
agg	R	1	0.27
---	--	------	------
mPD		0.0037	0.0071
		nPD:	0.52
		N. weight:	0.0039
		Sc. PD:	0.0017
		Sc. rank:	3.1

PA			
Pos.	375	obs:	exp:
gct	A	0	277.30
gcc	A	0	200.00
gca	A	1066	505.50
gcg	A	8	91.15
---	--	------	------
mPD		0.015	0.67
		nPD:	0.02
		N. weight:	0.79
		Sc. PD:	-0.038
		Sc. rank:	-65.8

PA

Pos.	376	obs:	exp:
cct	P	1	268.00
ccc	P	0	193.00
cca	P	273	442.10
ccg	P	800	171.00
mPD		0.38	0.71
		nPD:	0.54
		N. weight:	1.1
		Sc. PD:	0.5
		Sc. rank:	920.1

PA

Pos.	377	obs:	exp:
gaa	E	6	653.50
gag	E	1068	420.50
mPD		0.011	0.48
		nPD:	0.02
		N. weight:	0.98
		Sc. PD:	-0.046
		Sc. rank:	-55.8

PA

Pos.	378	obs:	exp:
aaa	K	44	626.00
aag	K	1030	448.00
mPD		0.079	0.49
		nPD:	0.16
		N. weight:	0.75
		Sc. PD:	0.066
		Sc. rank:	510.9

PA

Pos.	379	obs:	exp:
gtt	V	0	222.50
gtc	V	0	216.80
gta	V	39	219.90
gtg	V	1035	414.80
mPD		0.070	0.73
		nPD:	0.1
		N. weight:	0.89
		Sc. PD:	0.021
		Sc. rank:	474.5

PA

Pos.	380	obs:	exp:
gat	D	20	580.40
gac	D	1054	493.60
mPD		0.037	0.50
		nPD:	0.07
		N. weight:	0.74
		Sc. PD:	0.0013
		Sc. rank:	312.8

PA

Pos.	381	obs:	exp:
ttt	F	1071	424.10
ttc	F	3	649.90
mPD		0.0056	0.48
		nPD:	0.01
		N. weight:	0.99
		Sc. PD:	-0.058
		Sc. rank:	-306.6

PA

Pos.	382	obs:	exp:
gaa	E	10	653.50
gag	E	1064	420.50
mPD		0.018	0.48
		nPD:	0.04
		N. weight:	0.96
		Sc. PD:	-0.031
		Sc. rank:	171.9

PA

Pos.	383	obs:	exp:
gat	D	1045	580.40
gac	D	29	493.60
mPD		0.053	0.50
		nPD:	0.11
		N. weight:	0.54
		Sc. PD:	0.018
		Sc. rank:	303.2

PA

Pos.	384	obs:	exp:
tgt	C	333	454.30
tgc	C	741	619.70
mPD		0.43	0.49
		nPD:	0.88
		N. weight:	0.033
		Sc. PD:	0.026
		Sc. rank:	28.3

PA

Pos.	385	obs:	exp:
cgt	R	0	0.09
cgc	R	0	0.10
cga	R	0	0.20
cgg	R	0	0.17
aaa	K	1070	624.80
aag	K	2	447.20
aga	R	2	0.91
agg	R	0	0.54
mPD		0.0074	0.49
		nPD:	0.02
		N. weight:	0.58
		Sc. PD:	-0.032
		Sc. rank:	-122.3

PA

Pos.	386	obs:	exp:
aat	N	1	0.51
aac	N	0	0.49
gat	D	1071	578.70
gac	D	0	492.30
ggt	G	2	0.27
ggc	G	0	0.26
gga	G	0	0.92
ggg	G	0	0.56
mPD		0.0056	0.50
		nPD:	0.01
		N. weight:	0.67
		Sc. PD:	-0.04
		Sc. rank:	-224.6

PA

Pos.	387	obs:	exp:
att	I	0	12.49
atc	I	34	8.15
ata	I	0	13.36
gtt	V	7	215.50
gtc	V	1033	209.90
gta	V	0	212.90
gtg	V	0	401.70
mPD		0.074	0.79
		nPD:	0.09
		N. weight:	1.7
		Sc. PD:	0.036
		Sc. rank:	873.1

PA

Pos.	388	obs:	exp:
tct	S	0	1.11
tcc	S	0	0.91
tca	S	0	1.78
tcg	S	0	0.40
aat	N	49	524.60
aac	N	981	505.40
aaa	K	1	0.58
aag	K	0	0.42
agt	S	4	1.44
agc	S	3	1.36
gat	D	9	17.29
gac	D	23	14.71
gaa	E	4	2.43
gag	E	0	1.57
mPD		0.20	0.59
		nPD:	0.33
		N. weight:	0.56
		Sc. PD:	0.14
		Sc. rank:	428.6

PA

Pos.	389	obs:	exp:
gat	D	5	580.40
gac	D	1069	493.60
mPD		0.0093	0.50
		nPD:	0.02
		N. weight:	0.82
		Sc. PD:	-0.042
		Sc. rank:	-110.9

PA

Pos.	390	obs:	exp:
tta	L	0	95.31
ttg	L	590	200.00
ctt	L	5	178.80
ctc	L	0	179.20
cta	L	1	181.00
ctg	L	478	239.70
mPD		0.51	1.1
		nPD:	0.46
		N. weight:	0.97
		Sc. PD:	0.36
		Sc. rank:	771.4

PA

Pos.	391	obs:	exp:
cgt	R	0	0.04
cgc	R	0	0.05
cga	R	0	0.10
cgg	R	0	0.08
aaa	K	1069	625.40
aag	K	4	447.60
aga	R	1	0.46
agg	R	0	0.27
mPD		0.0093	0.49
		nPD:	0.02
		N. weight:	0.56
		Sc. PD:	-0.029
		Sc. rank:	-69.9

PA

Pos.	392	obs:	exp:
caa	Q	9	547.10
cag	Q	1065	526.90
mPD		0.017	0.50
		nPD:	0.03
		N. weight:	0.72
		Sc. PD:	-0.027
		Sc. rank:	76.6

PA

Pos.	393	obs:	exp:
tat	Y	233	580.10
tac	Y	841	493.90
mPD		0.34	0.50
		nPD:	0.68
		N. weight:	0.24
		Sc. PD:	0.14
		Sc. rank:	204.7

PA

Pos.	394	obs:	exp:
aat	N	0	302.00
aac	N	593	291.00
gat	D	42	259.90
gac	D	439	221.10
mPD		0.57	0.99
		nPD:	0.57
		N. weight:	0.66
		Sc. PD:	0.32
		Sc. rank:	549.1

PA

Pos.	395	obs:	exp:
tct	S	0	170.50
tcc	S	0	140.00
tca	S	0	272.10
tcg	S	0	60.83
tgt	C	1	0.42
tgc	C	0	0.58
aat	N	1	0.51
aac	N	0	0.49
agt	S	1066	220.40
agc	S	6	208.20
mPD		0.015	1.7
		nPD:	0.01
		N. weight:	1.7
		Sc. PD:	-0.1
		Sc. rank:	-632.2

PA

Pos.	396	obs:	exp:
gct	A	1	0.26
gcc	A	0	0.19
gca	A	0	0.47
gcg	A	0	0.08
gat	D	1037	579.30
gac	D	35	492.70
gaa	E	0	0.61
gag	E	1	0.39
mPD		0.067	0.50
		nPD:	0.13
		N. weight:	0.52
		Sc. PD:	0.031
		Sc. rank:	334.2

PA

Pos.	397	obs:	exp:
aaa	K	0	1.75
aag	K	3	1.25
gat	D	3	1.62
gac	D	0	1.38
gaa	E	15	648.70
gag	E	1051	417.30
ggt	G	0	0.27
ggc	G	0	0.26
gga	G	0	0.92
ggg	G	2	0.56
mPD		0.042	0.49
		nPD:	0.09
		N. weight:	0.94
		Sc. PD:	0.013
		Sc. rank:	444.8

PA

Pos.	398	obs:	exp:
cct	P	32	267.70
ccc	P	38	192.80
cca	P	995	441.70
ccg	P	8	170.80
act	T	0	0.25
acc	T	0	0.21
aca	T	1	0.46
acg	T	0	0.08
mPD		0.14	0.71
		nPD:	0.2
		N. weight:	0.67
		Sc. PD:	0.081
		Sc. rank:	481.8

PA

Pos.	399	obs:	exp:
aaa	K	0	1.17
aag	K	2	0.83
gaa	E	7	651.70
gag	E	1064	419.30
ggt	G	0	0.13
ggc	G	0	0.13
gga	G	0	0.46
ggg	G	1	0.28
mPD		0.019	0.48
		nPD:	0.04
		N. weight:	0.98
		Sc. PD:	-0.032
		Sc. rank:	171.4

PA

Pos.	400	obs:	exp:
tct	S	0	1.11
tcc	S	7	0.91
tca	S	0	1.78
tcg	S	0	0.40
cct	P	4	265.20
ccc	P	1059	191.00
cca	P	0	437.60
ccg	P	0	169.20
cat	H	0	0.57
cac	H	1	0.43
agt	S	0	1.44
agc	S	0	1.36
gct	A	0	0.77
gcc	A	3	0.56
gca	A	0	1.41
gcg	A	0	0.25
mPD		0.028	0.74
		nPD:	0.04
		N. weight:	1.8
		Sc. PD:	-0.061
		Sc. rank:	280.5

PA			
Pos.	401	obs:	exp:
cgt	R	0	46.13
cgc	R	0	53.19
cga	R	13	105.10
cgg	R	0	89.62
aga	R	1059	491.10
agg	R	2	288.90
mPD		0.028	0.96
		nPD:	0.03
		N. weight:	0.79
		Sc. PD:	-0.033
		Sc. rank:	21.2

PA			
Pos.	402	obs:	exp:
tct	S	0	170.80
tcc	S	0	140.30
tca	S	1054	272.60
tcg	S	20	60.94
agt	S	0	220.80
agc	S	0	208.60
mPD		0.037	1.7
		nPD:	0.02
		N. weight:	1.4
		Sc. PD:	-0.069
		Sc. rank:	-114.1

PA			
Pos.	403	obs:	exp:
tta	L	17	95.04
ttg	L	0	199.40
ctt	L	0	178.30
ctc	L	4	178.70
cta	L	1050	180.50
ctg	L	0	239.00
att	I	0	0.73
atc	I	0	0.48
ata	I	2	0.79
gtt	V	0	0.21
gtc	V	0	0.20
gta	V	1	0.20
gtg	V	0	0.39
mPD		0.044	1.1
		nPD:	0.04
		N. weight:	1.8
		Sc. PD:	-0.057
		Sc. rank:	351.2

PA			
Pos.	404	obs:	exp:
tct	S	0	0.32
tcc	S	0	0.26
tca	S	2	0.51
tcg	S	0	0.11
act	T	0	0.25
acc	T	0	0.21
aca	T	1	0.46
acg	T	0	0.08
agt	S	0	0.41
agc	S	0	0.39
gct	A	0	276.60
gcc	A	0	199.50
gca	A	1067	504.10
gcg	A	4	90.90
mPD		0.013	0.68
		nPD:	0.02
		N. weight:	0.8
		Sc. PD:	-0.041
		Sc. rank:	-91.5

PA			
Pos.	405	obs:	exp:
tct	S	0	0.16
tcc	S	0	0.13
tca	S	0	0.25
tcg	S	0	0.06
tat	Y	1	0.54
tac	Y	0	0.46
tgt	C	1046	453.40
tgc	C	26	618.60
agt	S	0	0.21
agc	S	1	0.19
mPD		0.053	0.49
		nPD:	0.11
		N. weight:	0.81
		Sc. PD:	0.028
		Sc. rank:	453.0

PA			
Pos.	406	obs:	exp:
tgg	W	1074	1074.00
mPD		0	0
		nPD:	1.
		N. weight:	0.
		Sc. PD:	0
		Sc. rank:	0

PA			
Pos.	407	obs:	exp:
att	I	36	392.80
atc	I	1031	256.20
ata	I	2	420.10
gtt	V	1	1.04
gtc	V	4	1.01
gta	V	0	1.02
gtg	V	0	1.93
mPD		0.079	0.66
		nPD:	0.12
		N. weight:	1.4
		Sc. PD:	0.063
		Sc. rank:	823.0

PA			
Pos.	408	obs:	exp:
caa	Q	46	547.10
cag	Q	1028	526.90
mPD		0.082	0.50
		nPD:	0.16
		N. weight:	0.58
		Sc. PD:	0.052
		Sc. rank:	396.7

PA			
Pos.	409	obs:	exp:
tct	S	0	4.93
tcc	S	0	4.05
tca	S	0	7.87
tcg	S	0	1.76
aat	N	1036	531.30
aac	N	7	511.70
agt	S	31	6.37
agc	S	0	6.02
mPD		0.069	0.60
		nPD:	0.12
		N. weight:	0.72
		Sc. PD:	0.03
		Sc. rank:	427.3

PA			
Pos.	410	obs:	exp:
gaa	E	1068	653.50
gag	E	6	420.50
mPD		0.011	0.48
		nPD:	0.02
		N. weight:	0.51
		Sc. PD:	-0.024
		Sc. rank:	-28.8

PA			
Pos.	411	obs:	exp:
ttt	F	5	424.10
ttc	F	1069	649.90
mPD		0.0093	0.48
		nPD:	0.02
		N. weight:	0.52
		Sc. PD:	-0.026
		Sc. rank:	-57.4

PA			
Pos.	412	obs:	exp:
aat	N	3	547.10
aac	N	1071	526.90
mPD		0.0056	0.50
		nPD:	0.01
		N. weight:	0.76
		Sc. PD:	-0.044
		Sc. rank:	-250.9

PA			
Pos.	413	obs:	exp:
aaa	K	31	626.00
aag	K	1043	448.00
mPD		0.056	0.49
		nPD:	0.12
		N. weight:	0.8
		Sc. PD:	0.034
		Sc. rank:	475.5

PA			
Pos.	414	obs:	exp:
gct	A	12	277.30
gcc	A	71	200.00
gca	A	488	505.50
gcg	A	503	91.15
mPD		0.57	0.67
		nPD:	0.85
		N. weight:	0.75
		Sc. PD:	0.57
		Sc. rank:	640.7

PA			
Pos.	415	obs:	exp:
tct	S	1	0.16
tcc	S	0	0.13
tca	S	0	0.25
tcg	S	0	0.06
tgt	C	1072	453.80
tgc	C	1	619.20
agt	S	0	0.21
agc	S	0	0.19
mPD		0.0037	0.49
		nPD:	0.01
		N. weight:	0.93
		Sc. PD:	-0.058
		Sc. rank:	-438.7

PA			
Pos.	416	obs:	exp:
gaa	E	1067	653.50
gag	E	7	420.50
mPD		0.013	0.48
		nPD:	0.03
		N. weight:	0.5
		Sc. PD:	-0.022
		Sc. rank:	3.4

PA			
Pos.	417	obs:	exp:
tta	L	256	95.31
ttg	L	224	200.00
ctt	L	0	178.80
ctc	L	0	179.20
cta	L	0	181.00
ctg	L	594	239.70
mPD		0.86	1.1
		nPD:	0.77
		N. weight:	0.84
		Sc. PD:	0.57
		Sc. rank:	712.4

PA			
Pos.	418	obs:	exp:
act	T	1038	273.80
acc	T	36	220.40
aca	T	0	492.60
acg	T	0	87.24
mPD		0.065	0.68
		nPD:	0.1
		N. weight:	1.3
		Sc. PD:	0.031
		Sc. rank:	706.3

PA			
Pos.	419	obs:	exp:
aat	N	1	0.51
aac	N	0	0.49
gat	D	474	578.20
gac	D	596	491.80
ggt	G	3	0.40
ggc	G	0	0.38
gga	G	0	1.38
ggg	G	0	0.84
mPD		0.50	0.51
		nPD:	0.99
		N. weight:	0.031
		Sc. PD:	0.028
		Sc. rank:	26.7

PA			
Pos.	420	obs:	exp:
tct	S	0	170.80
tcc	S	1	140.30
tca	S	1041	272.60
tcg	S	32	60.94
agt	S	0	220.80
agc	S	0	208.60
mPD		0.060	1.7
		nPD:	0.04
		N. weight:	1.4
		Sc. PD:	-0.048
		Sc. rank:	191.3

PA			
Pos.	421	obs:	exp:
tct	S	0	170.80
tcc	S	0	140.30
tca	S	0	272.60
tcg	S	0	60.94
agt	S	111	220.80
agc	S	963	208.60
mPD		0.19	1.7
		nPD:	0.11
		N. weight:	1.4
		Sc. PD:	0.055
		Sc. rank:	817.7

PA			
Pos.	422	obs:	exp:
tgg	W	1074	1074.00
mPD		0	0
		nPD:	1.
		N. weight:	0.
		Sc. PD:	0
		Sc. rank:	0

PA			
Pos.	423	obs:	exp:
att	I	0	1.47
atc	I	0	0.96
ata	I	4	1.57
gtt	V	0	221.70
gtc	V	0	216.00
gta	V	1023	219.10
gtg	V	47	413.20
mPD		0.091	0.73
		nPD:	0.12
		N. weight:	1.5
		Sc. PD:	0.076
		Sc. rank:	928.4

PA			
Pos.	424	obs:	exp:
gaa	E	1055	653.50
gag	E	19	420.50
mPD		0.035	0.48
		nPD:	0.07
		N. weight:	0.46
		Sc. PD:	0.00052
		Sc. rank:	188.9

PA

Pos. 425		obs:	exp:
ttt	F	1	0.39
ttc	F	0	0.61
tta	L	0	95.22
ttg	L	0	199.80
ctt	L	1071	178.70
ctc	L	2	179.10
cta	L	0	180.80
ctg	L	0	239.40
mPD		0.0056	1.1
		nPD:	0.01
		N. weight:	1.9
		Sc. PD:	-0.13
		Sc. rank:	-1060.8

PA

Pos. 426		obs:	exp:
gat	D	1065	580.40
gac	D	9	493.60
mPD		0.017	0.50
		nPD:	0.03
		N. weight:	0.62
		Sc. PD:	-0.023
		Sc. rank:	69.0

PA

Pos. 427		obs:	exp:
gaa	E	1070	653.50
gag	E	4	420.50
mPD		0.0074	0.48
		nPD:	0.02
		N. weight:	0.52
		Sc. PD:	-0.028
		Sc. rank:	-96.3

PA

Pos. 428		obs:	exp:
att	I	0	394.60
atc	I	1	257.40
ata	I	1073	422.00
mPD		0.0019	0.65
		nPD:	0.
		N. weight:	1.
		Sc. PD:	-0.068
		Sc. rank:	-631.2

PA

Pos. 429		obs:	exp:
ggt	G	0	142.40
ggc	G	0	137.20
gga	G	10	495.50
ggg	G	1064	299.00
mPD		0.018	0.68
		nPD:	0.03
		N. weight:	1.3
		Sc. PD:	-0.057
		Sc. rank:	14.8

PA

Pos. 430		obs:	exp:
gat	D	8	4.32
gac	D	0	3.68
gaa	E	1052	648.70
gag	E	14	417.30
mPD		0.040	0.48
		nPD:	0.08
		N. weight:	0.47
		Sc. PD:	0.0053
		Sc. rank:	214.2

PA

Pos. 431		obs:	exp:
gat	D	1062	580.40
gac	D	12	493.60
mPD		0.022	0.50
		nPD:	0.04
		N. weight:	0.61
		Sc. PD:	-0.016
		Sc. rank:	143.7

PA

Pos. 432		obs:	exp:
att	I	47	17.27
atc	I	0	11.26
ata	I	0	18.47
gtt	V	1021	212.80
gtc	V	0	207.30
gta	V	6	210.30
gtg	V	0	396.60
mPD		0.095	0.81
		nPD:	0.12
		N. weight:	1.7
		Sc. PD:	0.071
		Sc. rank:	985.9

PA

Pos. 433		obs:	exp:
gct	A	10	277.30
gcc	A	1063	200.00
gca	A	1	505.50
gcg	A	0	91.15
mPD		0.020	0.67
		nPD:	0.03
		N. weight:	1.8
		Sc. PD:	-0.071
		Sc. rank:	102.9

PA

Pos. 434		obs:	exp:
cct	P	0	268.00
ccc	P	0	193.00
cca	P	1070	442.10
ccg	P	4	171.00
mPD		0.0074	0.71
		nPD:	0.01
		N. weight:	0.95
		Sc. PD:	-0.056
		Sc. rank:	-337.5

PA

Pos. 435		obs:	exp:
att	I	20	394.20
atc	I	1052	257.10
ata	I	1	421.60
gtt	V	0	0.21
gtc	V	1	0.20
gta	V	0	0.20
gtg	V	0	0.39
mPD		0.040	0.66
		nPD:	0.06
		N. weight:	1.4
		Sc. PD:	-0.014
		Sc. rank:	506.4

PA

Pos. 436		obs:	exp:
gaa	E	1068	653.50
gag	E	6	420.50
mPD		0.011	0.48
		nPD:	0.02
		N. weight:	0.51
		Sc. PD:	-0.024
		Sc. rank:	-28.8

PA

Pos. 437		obs:	exp:
cat	H	36	616.70
cac	H	1038	457.30
mPD		0.065	0.49
		nPD:	0.13
		N. weight:	0.76
		Sc. PD:	0.045
		Sc. rank:	483.8

PA

Pos. 438		obs:	exp:
att	I	1069	394.60
atc	I	5	257.40
ata	I	0	422.00
mPD		0.0093	0.65
		nPD:	0.01
		N. weight:	1.1
		Sc. PD:	-0.059
		Sc. rank:	-246.1

PA

Pos. 439		obs:	exp:
gct	A	0	277.30
gcc	A	1	200.00
gca	A	1073	505.50
gcg	A	0	91.15
mPD		0.0019	0.67
		nPD:	0.
		N. weight:	0.81
		Sc. PD:	-0.054
		Sc. rank:	-522.1

PA

Pos. 440		obs:	exp:
tct	S	0	170.80
tcc	S	0	140.30
tca	S	0	272.60
tcg	S	0	60.94
agt	S	2	220.80
agc	S	1072	208.60
mPD		0.0037	1.7
		nPD:	0.
		N. weight:	1.8
		Sc. PD:	-0.12
		Sc. rank:	-1193.3

PA

Pos. 441		obs:	exp:
att	I	0	0.37
atc	I	0	0.24
ata	I	1	0.39
atg	M	1073	1073.00
mPD		0.0019	0.0019
		nPD:	1.
		N. weight:	0.00095
		Sc. PD:	0.00085
		Sc. rank:	0.8

PA

Pos. 442		obs:	exp:
cgt	R	0	46.13
cgc	R	0	53.19
cga	R	0	105.10
cgg	R	0	89.62
aga	R	1070	491.10
agg	R	4	288.90
mPD		0.0074	0.96
		nPD:	0.01
		N. weight:	0.83
		Sc. PD:	-0.051
		Sc. rank:	-367.6

PA

Pos. 443		obs:	exp:
cgt	R	0	46.13
cgc	R	0	53.19
cga	R	31	105.10
cgg	R	705	89.62
aga	R	14	491.10
agg	R	324	288.90
mPD		0.51	0.96
		nPD:	0.53
		N. weight:	1.4
		Sc. PD:	0.64
		Sc. rank:	1175.3

PA

Pos. 444		obs:	exp:
tct	S	0	0.16
tcc	S	0	0.13
tca	S	0	0.25
tcg	S	0	0.06
aat	N	13	544.50
aac	N	1056	524.50
agt	S	0	0.21
agc	S	1	0.19
gat	D	0	2.16
gac	D	4	1.84
mPD		0.033	0.51
		nPD:	0.07
		N. weight:	0.71
		Sc. PD:	-0.0046
		Sc. rank:	262.4

PA

Pos. 445		obs:	exp:
tat	Y	1066	580.10
tac	Y	8	493.90
mPD		0.015	0.50
		nPD:	0.03
		N. weight:	0.63
		Sc. PD:	-0.026
		Sc. rank:	21.2

PA

Pos. 446		obs:	exp:
ttt	F	1037	424.10
ttc	F	37	649.90
mPD		0.067	0.48
		nPD:	0.14
		N. weight:	0.84
		Sc. PD:	0.055
		Sc. rank:	545.9

PA

Pos. 447		obs:	exp:
act	T	11	273.50
acc	T	1	220.20
aca	T	1058	492.20
acg	T	3	87.16
gct	A	0	0.26
gcc	A	0	0.19
gca	A	0	0.47
gcg	A	1	0.08
mPD		0.031	0.68
		nPD:	0.05
		N. weight:	0.78
		Sc. PD:	-0.019
		Sc. rank:	191.2

PA

Pos. 448		obs:	exp:
tct	S	0	0.16
tcc	S	0	0.13
tca	S	1	0.25
tcg	S	0	0.06
agt	S	0	0.21
agc	S	0	0.19
gct	A	6	276.60
gcc	A	0	199.50
gca	A	1060	504.10
gcg	A	5	90.90
gaa	E	2	1.22
gag	E	0	0.78
mPD		0.026	0.68
		nPD:	0.04
		N. weight:	0.77
		Sc. PD:	-0.025
		Sc. rank:	132.7

PA

Pos. 449		obs:	exp:
caa	Q	0	0.51
cag	Q	1	0.49
gaa	E	41	652.90
gag	E	1032	420.10
mPD		0.075	0.48
		nPD:	0.16
		N. weight:	0.83
		Sc. PD:	0.069
		Sc. rank:	558.9

PA			
Pos.	450	obs:	exp:
att	I	0	0.37
atc	I	0	0.24
ata	I	1	0.39
gtt	V	0	222.30
gtc	V	8	216.60
gta	V	199	219.70
gtg	V	866	414.40
---	--	------	------
mPD		0.32	0.73
		nPD:	0.44
		N. weight:	0.61
		Sc. PD:	0.21
		Sc. rank:	478.9

,

PA			
Pos.	451	obs:	exp:
tct	S	22	170.80
tcc	S	1052	140.30
tca	S	0	272.60
tcg	S	0	60.94
agt	S	0	220.80
agc	S	0	208.60
---	--	------	------
mPD		0.040	1.7
		nPD:	0.02
		N. weight:	2.1
		Sc. PD:	-0.097
		Sc. rank:	-87.5

,

PA			
Pos.	452	obs:	exp:
cat	H	22	616.70
cac	H	1052	457.30
---	--	------	------
mPD		0.040	0.49
		nPD:	0.08
		N. weight:	0.82
		Sc. PD:	0.0081
		Sc. rank:	362.1

,

PA			
Pos.	453	obs:	exp:
tgt	C	12	454.30
tgc	C	1062	619.70
---	--	------	------
mPD		0.022	0.49
		nPD:	0.05
		N. weight:	0.54
		Sc. PD:	-0.014
		Sc. rank:	127.9

,

PA			
Pos.	454	obs:	exp:
cgt	R	0	46.13
cgc	R	0	53.19
cga	R	0	105.10
cgg	R	0	89.62
aga	R	93	491.10
agg	R	981	288.90
---	--	------	------
mPD		0.16	0.96
		nPD:	0.16
		N. weight:	1.1
		Sc. PD:	0.096
		Sc. rank:	728.2

,

PA			
Pos.	455	obs:	exp:
gct	A	1072	277.30
gcc	A	1	200.00
gca	A	0	505.50
gcg	A	1	91.15
---	--	------	------
mPD		0.0037	0.67
		nPD:	0.01
		N. weight:	1.5
		Sc. PD:	-0.094
		Sc. rank:	-760.9

,

PA			
Pos.	456	obs:	exp:
act	T	1064	273.80
acc	T	8	220.40
aca	T	1	492.60
acg	T	1	87.24
---	--	------	------
mPD		0.018	0.68
		nPD:	0.03
		N. weight:	1.4
		Sc. PD:	-0.062
		Sc. rank:	26.5

,

PA			
Pos.	457	obs:	exp:
gaa	E	973	653.50
gag	E	101	420.50
---	--	------	------
mPD		0.17	0.48
		nPD:	0.36
		N. weight:	0.25
		Sc. PD:	0.069
		Sc. rank:	192.0

,

PA			
Pos.	458	obs:	exp:
tat	Y	605	580.10
tac	Y	469	493.90
---	--	------	------
mPD		0.49	0.50
		nPD:	0.99
		N. weight:	0.0049
		Sc. PD:	0.0044
		Sc. rank:	4.2

,

PA			
Pos.	459	obs:	exp:
att	I	0	394.20
atc	I	0	257.10
ata	I	1073	421.60
atg	M	1	1.00
---	--	------	------
mPD		0.0019	0.65
		nPD:	0.
		N. weight:	1.
		Sc. PD:	-0.068
		Sc. rank:	-637.2

,

PA			
Pos.	460	obs:	exp:
tta	L	0	0.09
ttg	L	0	0.19
ctt	L	0	0.17
ctc	L	0	0.17
cta	L	0	0.17
ctg	L	1	0.22
atg	M	1073	1073.00
---	--	------	------
mPD		0.0019	0.0030
		nPD:	0.63
		N. weight:	0.0015
		Sc. PD:	0.00082
		Sc. rank:	1.3

,

PA			
Pos.	461	obs:	exp:
aaa	K	4	626.00
aag	K	1070	448.00
---	--	------	------
mPD		0.0074	0.49
		nPD:	0.02
		N. weight:	0.93
		Sc. PD:	-0.051
		Sc. rank:	-191.4

,

PA			
Pos.	462	obs:	exp:
ggt	G	0	142.40
ggc	G	0	137.20
gga	G	1069	495.50
ggg	G	5	299.00
---	--	------	------
mPD		0.0093	0.68
		nPD:	0.01
		N. weight:	0.81
		Sc. PD:	-0.046
		Sc. rank:	-201.0

,

PA			
Pos.	463	obs:	exp:
gtt	V	0	222.50
gtc	V	0	216.80
gta	V	22	219.90
gtg	V	1052	414.80
---	--	------	------
mPD		0.040	0.73
		nPD:	0.06
		N. weight:	0.94
		Sc. PD:	-0.015
		Sc. rank:	290.4

,

PA			
Pos.	464	obs:	exp:
tat	Y	3	576.80
tac	Y	1065	491.20
cat	H	0	3.45
cac	H	6	2.55
---	--	------	------
mPD		0.017	0.51
		nPD:	0.03
		N. weight:	0.83
		Sc. PD:	-0.031
		Sc. rank:	85.9

,

PA			
Pos.	465	obs:	exp:
tta	L	2	0.18
ttg	L	0	0.37
ctt	L	0	0.33
ctc	L	0	0.33
cta	L	0	0.34
ctg	L	0	0.45
att	I	0	390.20
atc	I	1	254.50
ata	I	1061	417.30
atg	M	1	1.00
act	T	0	0.25
acc	T	0	0.21
aca	T	1	0.46
acg	T	0	0.08
gtt	V	0	1.66
gtc	V	0	1.61
gta	V	8	1.64
gtg	V	0	3.09
---	--	------	------
mPD		0.024	0.68
		nPD:	0.04
		N. weight:	1.
		Sc. PD:	-0.036
		Sc. rank:	134.6

,

PA			
Pos.	466	obs:	exp:
tct	S	0	0.16
tcc	S	0	0.13
tca	S	0	0.25
tcg	S	0	0.06
aat	N	1059	546.50
aac	N	14	526.50
agt	S	1	0.21
agc	S	0	0.19
---	--	------	------
mPD		0.028	0.50
		nPD:	0.05
		N. weight:	0.66
		Sc. PD:	-0.011
		Sc. rank:	199.9

,

PA			
Pos.	467	obs:	exp:
act	T	38	273.80
acc	T	0	220.40
aca	T	1031	492.60
acg	T	5	87.24
---	--	------	------
mPD		0.077	0.68
		nPD:	0.11
		N. weight:	0.69
		Sc. PD:	0.028
		Sc. rank:	401.4

,

PA			
Pos.	468	obs:	exp:
gct	A	1056	277.30
gcc	A	12	200.00
gca	A	6	505.50
gcg	A	0	91.15
---	--	------	------
mPD		0.033	0.67
		nPD:	0.05
		N. weight:	1.4
		Sc. PD:	-0.03
		Sc. rank:	365.5

,

PA			
Pos.	469	obs:	exp:
tta	L	1	95.31
ttg	L	1070	200.00
ctt	L	0	178.80
ctc	L	0	179.20
cta	L	0	181.00
ctg	L	3	239.70
---	--	------	------
mPD		0.0074	1.1
		nPD:	0.01
		N. weight:	1.8
		Sc. PD:	-0.11
		Sc. rank:	-869.6

,

PA			
Pos.	470	obs:	exp:
ttt	F	0	0.39
ttc	F	1	0.61
tta	L	0	95.22
ttg	L	0	199.80
ctt	L	36	178.70
ctc	L	1033	179.10
cta	L	4	180.80
ctg	L	0	239.40
---	--	------	------
mPD		0.074	1.1
		nPD:	0.07
		N. weight:	1.8
		Sc. PD:	-0.009
		Sc. rank:	669.0

,

PA			
Pos.	471	obs:	exp:
aat	N	1030	547.10
aac	N	44	526.90
---	--	------	------
mPD		0.079	0.50
		nPD:	0.16
		N. weight:	0.55
		Sc. PD:	0.046
		Sc. rank:	371.7

,

PA			
Pos.	472	obs:	exp:
tct	S	0	0.16
tcc	S	0	0.13
tca	S	1	0.25
tcg	S	0	0.06
agt	S	0	0.21
agc	S	0	0.19
gct	A	6	277.10
gcc	A	0	199.80
gca	A	1061	505.00
gcg	A	6	91.07
---	--	------	------
mPD		0.024	0.67
		nPD:	0.04
		N. weight:	0.76
		Sc. PD:	-0.027
		Sc. rank:	102.4

,

PA			
Pos.	473	obs:	exp:
tct	S	1066	170.80
tcc	S	4	140.30
tca	S	4	272.60
tcg	S	0	60.94
agt	S	0	220.80
agc	S	0	208.60
---	--	------	------
mPD		0.015	1.7
		nPD:	0.01
		N. weight:	2.
		Sc. PD:	-0.12
		Sc. rank:	-739.5

,

PA			
Pos.	474	obs:	exp:
tgt	C	1066	454.30
tgc	C	8	619.70
---	--	------	------
mPD		0.015	0.49
		nPD:	0.03
		N. weight:	0.89
		Sc. PD:	-0.036
		Sc. rank:	53.4

,

PA			
Pos.	475	obs:	exp:
act	T	0	0.51
acc	T	0	0.41
aca	T	2	0.92
acg	T	0	0.16
gct	A	28	276.80
gcc	A	0	199.60
gca	A	1006	504.60
gcg	A	38	90.98
mPD		0.12	0.67
		nPD:	0.18
		N. weight:	0.61
		Sc. PD:	0.064
		Sc. rank:	432.5

PA			
Pos.	476	obs:	exp:
act	T	0	0.25
acc	T	1	0.21
aca	T	0	0.46
acg	T	0	0.08
gct	A	30	277.10
gcc	A	1042	199.80
gca	A	1	505.00
gcg	A	0	91.07
mPD		0.058	0.67
		nPD:	0.09
		N. weight:	1.7
		Sc. PD:	0.024
		Sc. rank:	792.5

PA			
Pos.	477	obs:	exp:
atg	M	1074	1074.00
mPD		0	0
		nPD:	1.
		N. weight:	0.
		Sc. PD:	0
		Sc. rank:	0

PA			
Pos.	478	obs:	exp:
gat	D	1072	580.40
gac	D	2	493.60
mPD		0.0037	0.50
		nPD:	0.01
		N. weight:	0.66
		Sc. PD:	-0.041
		Sc. rank:	-311.2

PA			
Pos.	479	obs:	exp:
gat	D	55	579.30
gac	D	1017	492.70
gaa	E	2	1.22
gag	E	0	0.78
mPD		0.10	0.50
		nPD:	0.2
		N. weight:	0.62
		Sc. PD:	0.078
		Sc. rank:	451.0

PA			
Pos.	480	obs:	exp:
ttt	F	1001	424.10
ttc	F	73	649.90
mPD		0.13	0.48
		nPD:	0.27
		N. weight:	0.71
		Sc. PD:	0.13
		Sc. rank:	531.4

PA			
Pos.	481	obs:	exp:
caa	Q	1048	547.10
cag	Q	26	526.90
mPD		0.047	0.50
		nPD:	0.09
		N. weight:	0.61
		Sc. PD:	0.014
		Sc. rank:	321.3

PA			
Pos.	482	obs:	exp:
tta	L	0	95.31
ttg	L	7	200.00
ctt	L	2	178.80
ctc	L	0	179.20
cta	L	43	181.00
ctg	L	1022	239.70
mPD		0.093	1.1
		nPD:	0.08
		N. weight:	1.4
		Sc. PD:	0.017
		Sc. rank:	659.7

PA			
Pos.	483	obs:	exp:
att	I	1048	394.60
atc	I	26	257.40
ata	I	0	422.00
mPD		0.047	0.65
		nPD:	0.07
		N. weight:	0.98
		Sc. PD:	0.00053
		Sc. rank:	400.2

PA			
Pos.	484	obs:	exp:
cct	P	0	268.00
ccc	P	1	193.00
cca	P	1062	442.10
ccg	P	11	171.00
mPD		0.022	0.71
		nPD:	0.03
		N. weight:	0.91
		Sc. PD:	-0.036
		Sc. rank:	69.4

PA			
Pos.	485	obs:	exp:
atg	M	1074	1074.00
mPD		0	0
		nPD:	1.
		N. weight:	0.
		Sc. PD:	0
		Sc. rank:	0

PA			
Pos.	486	obs:	exp:
att	I	1	394.20
atc	I	0	257.10
ata	I	1072	421.60
atg	M	1	1.00
mPD		0.0037	0.65
		nPD:	0.01
		N. weight:	1.
		Sc. PD:	-0.065
		Sc. rank:	-494.8

PA			
Pos.	487	obs:	exp:
tct	S	0	170.80
tcc	S	0	140.30
tca	S	0	272.60
tcg	S	0	60.94
agt	S	945	220.80
agc	S	129	208.60
mPD		0.21	1.7
		nPD:	0.13
		N. weight:	1.3
		Sc. PD:	0.072
		Sc. rank:	839.2

PA			
Pos.	488	obs:	exp:
aaa	K	1023	626.00
aag	K	51	448.00
mPD		0.091	0.49
		nPD:	0.19
		N. weight:	0.4
		Sc. PD:	0.044
		Sc. rank:	283.5

PA			
Pos.	489	obs:	exp:
tgt	C	623	454.30
tgc	C	451	619.70
mPD		0.49	0.49
		nPD:	1.
		N. weight:	0.058
		Sc. PD:	0.052
		Sc. rank:	49.6

PA			
Pos.	490	obs:	exp:
cgt	R	0	46.13
cgc	R	0	53.19
cga	R	0	105.10
cgg	R	0	89.62
aga	R	1063	491.10
agg	R	11	288.90
mPD		0.020	0.96
		nPD:	0.02
		N. weight:	0.8
		Sc. PD:	-0.039
		Sc. rank:	-73.0

PA			
Pos.	491	obs:	exp:
act	T	46	273.80
acc	T	1026	220.40
aca	T	2	492.60
acg	T	0	87.24
mPD		0.086	0.68
		nPD:	0.13
		N. weight:	1.5
		Sc. PD:	0.08
		Sc. rank:	945.2

PA			
Pos.	492	obs:	exp:
aaa	K	1074	626.00
aag	K	0	448.00
mPD		0	0.49
		nPD:	0.
		N. weight:	0.59
		Sc. PD:	-0.041
		Sc. rank:	-499.3

PA			
Pos.	493	obs:	exp:
gaa	E	1049	653.50
gag	E	25	420.50
mPD		0.046	0.48
		nPD:	0.1
		N. weight:	0.43
		Sc. PD:	0.01
		Sc. rank:	227.7

PA			
Pos.	494	obs:	exp:
ggt	G	0	142.40
ggc	G	0	137.20
gga	G	1054	495.50
ggg	G	20	299.00
mPD		0.037	0.68
		nPD:	0.05
		N. weight:	0.75
		Sc. PD:	-0.013
		Sc. rank:	220.6

PA			
Pos.	495	obs:	exp:
cgt	R	0	46.08
cgc	R	0	53.15
cga	R	2	105.00
cgg	R	0	89.53
att	I	0	0.37
atc	I	0	0.24
ata	I	1	0.39
aga	R	1066	490.60
agg	R	5	288.60
mPD		0.015	0.96
		nPD:	0.02
		N. weight:	0.81
		Sc. PD:	-0.044
		Sc. rank:	-157.8

PA			
Pos.	496	obs:	exp:
cgt	R	0	46.13
cgc	R	0	53.19
cga	R	4	105.10
cgg	R	1067	89.62
aga	R	0	491.10
agg	R	3	288.90
mPD		0.013	0.96
		nPD:	0.01
		N. weight:	2.7
		Sc. PD:	-0.15
		Sc. rank:	-660.6

PA			
Pos.	497	obs:	exp:
cgt	R	0	0.86
cgc	R	0	0.99
cga	R	0	1.96
cgg	R	0	1.67
aaa	K	1046	614.30
aag	K	8	439.70
aga	R	20	9.15
agg	R	0	5.38
mPD		0.051	0.53
		nPD:	0.1
		N. weight:	0.55
		Sc. PD:	0.013
		Sc. rank:	290.7

PA			
Pos.	498	obs:	exp:
act	T	0	273.80
acc	T	0	220.40
aca	T	1073	492.60
acg	T	1	87.24
mPD		0.0019	0.68
		nPD:	0.
		N. weight:	0.84
		Sc. PD:	-0.056
		Sc. rank:	-550.4

PA			
Pos.	499	obs:	exp:
tct	S	0	0.16
tcc	S	0	0.13
tca	S	0	0.25
tcg	S	0	0.06
att	I	0	0.37
atc	I	1	0.24
ata	I	0	0.39
aat	N	2	546.00
aac	N	1070	526.00
agt	S	0	0.21
agc	S	1	0.19
mPD		0.0074	0.51
		nPD:	0.01
		N. weight:	0.76
		Sc. PD:	-0.042
		Sc. rank:	-171.6

PA			
Pos.	500	obs:	exp:
tta	L	0	95.31
ttg	L	0	200.00
ctt	L	0	178.80
ctc	L	0	179.20
cta	L	482	181.00
ctg	L	592	239.70
mPD		0.50	1.1
		nPD:	0.45
		N. weight:	1.
		Sc. PD:	0.37
		Sc. rank:	814.8

PA			
Pos.	501	obs:	exp:
tat	Y	1054	580.10
tac	Y	20	493.90
mPD		0.037	0.50
		nPD:	0.07
		N. weight:	0.58
		Sc. PD:	0.001
		Sc. rank:	240.7

PA			
Pos.	502	obs:	exp:
ggt	G	1	142.40
ggc	G	0	137.20
gga	G	1063	495.50
ggg	G	10	299.00
mPD		0.020	0.68
		nPD:	0.03
		N. weight:	0.79
		Sc. PD:	-0.032
		Sc. rank:	34.0

PA			
Pos.	503	obs:	exp:
ttt	F	6	424.10
ttc	F	1068	649.90
mPD		0.011	0.48
		nPD:	0.02
		N. weight:	0.51
		Sc. PD:	-0.024
		Sc. rank:	-30.5

PA			
Pos.	504	obs:	exp:
att	I	1070	394.60
atc	I	4	257.40
ata	I	0	422.00
mPD		0.0074	0.65
		nPD:	0.01
		N. weight:	1.1
		Sc. PD:	-0.062
		Sc. rank:	-339.5

PA			
Pos .	505	obs :	exp :
att	I	0	393.90
atc	I	0	256.90
ata	I	1072	421.20
gtt	V	0	0.41
gtc	V	0	0.40
gta	V	2	0.41
gtg	V	0	0.77
---	--	------	------
mPD		0.0037	0.66
		nPD :	0.01
		N. weight :	1.
		Sc. PD :	-0.065
		Sc. rank :	-508.2

PA			
Pos .	506	obs :	exp :
caa	Q	1	0.51
cag	Q	0	0.49
aaa	K	1060	625.40
aag	K	13	447.60
---	--	------	------
mPD		0.026	0.49
		nPD :	0.05
		N. weight :	0.52
		Sc. PD :	-0.0096
		Sc. rank :	151.5

PA			
Pos .	507	obs :	exp :
ggt	G	0	142.40
ggc	G	0	137.20
gga	G	1067	495.50
ggg	G	7	299.00
---	--	------	------
mPD		0.013	0.68
		nPD :	0.02
		N. weight :	0.81
		Sc. PD :	-0.041
		Sc. rank :	-93.2

PA			
Pos .	508	obs :	exp :
cgt	R	0	46.13
cgc	R	0	53.19
cga	R	5	105.10
cgg	R	0	89.62
aga	R	1037	491.10
agg	R	32	288.90
---	--	------	------
mPD		0.067	0.96
		nPD :	0.07
		N. weight :	0.7
		Sc. PD :	-0.0013
		Sc. rank :	273.8

PA			
Pos .	509	obs :	exp :
tct	S	1052	170.80
tcc	S	22	140.30
tca	S	0	272.60
tcg	S	0	60.94
agt	S	0	220.80
agc	S	0	208.60
---	--	------	------
mPD		0.040	1.7
		nPD :	0.02
		N. weight :	1.9
		Sc. PD :	-0.088
		Sc. rank :	-78.9

PA			
Pos .	510	obs :	exp :
cat	H	1067	616.70
cac	H	7	457.30
---	--	------	------
mPD		0.013	0.49
		nPD :	0.03
		N. weight :	0.57
		Sc. PD :	-0.025
		Sc. rank :	-1.0

PA			
Pos .	511	obs :	exp :
tta	L	6	95.31
ttg	L	1044	200.00
ctt	L	0	178.80
ctc	L	0	179.20
cta	L	0	181.00
ctg	L	24	239.70
---	--	------	------
mPD		0.055	1.1
		nPD :	0.05
		N. weight :	1.7
		Sc. PD :	-0.036
		Sc. rank :	453.8

PA			
Pos .	512	obs :	exp :
cgt	R	0	46.13
cgc	R	0	53.19
cga	R	0	105.10
cgg	R	0	89.62
aga	R	5	491.10
agg	R	1069	288.90
---	--	------	------
mPD		0.0093	0.96
		nPD :	0.01
		N. weight :	1.4
		Sc. PD :	-0.084
		Sc. rank :	-520.2

PA			
Pos .	513	obs :	exp :
aat	N	1073	547.10
aac	N	1	526.90
---	--	------	------
mPD		0.0019	0.50
		nPD :	0.
		N. weight :	0.73
		Sc. PD :	-0.048
		Sc. rank :	-428.5

PA			
Pos .	514	obs :	exp :
gat	D	1041	579.30
gac	D	31	492.70
ggt	G	2	0.27
ggc	G	0	0.26
gga	G	0	0.92
ggg	G	0	0.56
---	--	------	------
mPD		0.060	0.50
		nPD :	0.12
		N. weight :	0.54
		Sc. PD :	0.025
		Sc. rank :	322.2

PA			
Pos .	515	obs :	exp :
act	T	15	273.80
acc	T	1053	220.40
aca	T	6	492.60
acg	T	0	87.24
---	--	------	------
mPD		0.039	0.68
		nPD :	0.06
		N. weight :	1.6
		Sc. PD :	-0.023
		Sc. rank :	521.2

PA			
Pos .	516	obs :	exp :
gat	D	493	580.40
gac	D	581	493.60
---	--	------	------
mPD		0.50	0.50
		nPD :	1.
		N. weight :	0.018
		Sc. PD :	0.016
		Sc. rank :	15.6

PA			
Pos .	517	obs :	exp :
gtt	V	1	222.50
gtc	V	0	216.80
gta	V	8	219.90
gtg	V	1065	414.80
---	--	------	------
mPD		0.017	0.73
		nPD :	0.02
		N. weight :	0.99
		Sc. PD :	-0.047
		Sc. rank :	-59.9

PA			
Pos .	518	obs :	exp :
gtt	V	0	222.50
gtc	V	0	216.80
gta	V	734	219.90
gtg	V	340	414.80
---	--	------	------
mPD		0.43	0.73
		nPD :	0.6
		N. weight :	0.83
		Sc. PD :	0.42
		Sc. rank :	695.4

PA			
Pos .	519	obs :	exp :
tct	S	0	0.16
tcc	S	0	0.13
tca	S	0	0.25
tcg	S	0	0.06
act	T	0	0.51
acc	T	2	0.41
aca	T	0	0.92
acg	T	0	0.16
aat	N	12	545.50
aac	N	1059	525.50
agt	S	0	0.21
agc	S	1	0.19
---	--	------	------
mPD		0.028	0.51
		nPD :	0.05
		N. weight :	0.71
		Sc. PD :	-0.012
		Sc. rank :	211.1

PA			
Pos .	520	obs :	exp :
ttt	F	1072	424.10
ttc	F	2	649.90
---	--	------	------
mPD		0.0037	0.48
		nPD :	0.01
		N. weight :	1.
		Sc. PD :	-0.062
		Sc. rank :	-437.9

PA			
Pos .	521	obs :	exp :
tta	L	1	0.09
ttg	L	0	0.19
ctt	L	0	0.17
ctc	L	0	0.17
cta	L	0	0.17
ctg	L	0	0.22
gtt	V	0	222.30
gtc	V	1	216.60
gta	V	1068	219.70
gtg	V	4	414.40
---	--	------	------
mPD		0.011	0.73
		nPD :	0.02
		N. weight :	1.7
		Sc. PD :	-0.093
		Sc. rank :	-331.5

PA			
Pos .	522	obs :	exp :
tct	S	0	170.80
tcc	S	0	140.30
tca	S	0	272.60
tcg	S	0	60.94
agt	S	1071	220.80
agc	S	3	208.60
---	--	------	------
mPD		0.0056	1.7
		nPD :	0.
		N. weight :	1.7
		Sc. PD :	-0.11
		Sc. rank :	-1012.6

PA			
Pos .	523	obs :	exp :
atg	M	1074	1074.00
---	--	------	------
mPD		0	0
		nPD :	1.
		N. weight :	0.
		Sc. PD :	0
		Sc. rank :	0

PA			
Pos .	524	obs :	exp :
gaa	E	1073	653.50
gag	E	1	420.50
---	--	------	------
mPD		0.0019	0.48
		nPD :	0.
		N. weight :	0.53
		Sc. PD :	-0.035
		Sc. rank :	-296.3

PA			
Pos .	525	obs :	exp :
ttt	F	1062	424.10
ttc	F	12	649.90
---	--	------	------
mPD		0.022	0.48
		nPD :	0.05
		N. weight :	0.94
		Sc. PD :	-0.023
		Sc. rank :	233.5

PA			
Pos .	526	obs :	exp :
tct	S	4	170.80
tcc	S	1069	140.30
tca	S	1	272.60
tcg	S	0	60.94
agt	S	0	220.80
agc	S	0	208.60
---	--	------	------
mPD		0.0093	1.7
		nPD :	0.01
		N. weight :	2.2
		Sc. PD :	-0.14
		Sc. rank :	-1110.1

PA			
Pos .	527	obs :	exp :
tta	L	0	95.31
ttg	L	0	200.00
ctt	L	1062	178.80
ctc	L	12	179.20
cta	L	0	181.00
ctg	L	0	239.70
---	--	------	------
mPD		0.022	1.1
		nPD :	0.02
		N. weight :	1.9
		Sc. PD :	-0.095
		Sc. rank :	-193.6

PA			
Pos .	528	obs :	exp :
act	T	13	273.80
acc	T	1046	220.40
aca	T	15	492.60
acg	T	0	87.24
---	--	------	------
mPD		0.051	0.68
		nPD :	0.08
		N. weight :	1.6
		Sc. PD :	0.0058
		Sc. rank :	664.1

PA			
Pos .	529	obs :	exp :
gat	D	204	580.40
gac	D	870	493.60
---	--	------	------
mPD		0.31	0.50
		nPD :	0.62
		N. weight :	0.29
		Sc. PD :	0.15
		Sc. rank :	240.3

PA

Pos.	530	obs:	exp:
cct	P	1	268.00
ccc	P	0	193.00
cca	P	1068	442.10
ccg	P	5	171.00
mPD		0.011	0.71
		nPD:	0.02
		N. weight:	0.93
		Sc. PD:	-0.051
		Sc. rank:	-164.0

PA

Pos.	531	obs:	exp:
cgt	R	0	46.08
cgc	R	0	53.15
cga	R	0	105.00
cgg	R	0	89.53
aaa	K	0	0.58
aag	K	1	0.42
aga	R	23	490.60
agg	R	1050	288.60
mPD		0.044	0.96
		nPD:	0.05
		N. weight:	1.3
		Sc. PD:	-0.033
		Sc. rank:	314.8

PA

Pos.	532	obs:	exp:
tta	L	1	95.22
ttg	L	1072	199.80
ctt	L	0	178.70
ctc	L	0	179.10
cta	L	0	180.80
ctg	L	0	239.40
gtt	V	0	0.21
gtc	V	0	0.20
gta	V	0	0.20
gtg	V	1	0.39
mPD		0.0037	1.1
		nPD:	0.
		N. weight:	1.8
		Sc. PD:	-0.12
		Sc. rank:	-1122.7

PA

Pos.	533	obs:	exp:
gct	A	0	0.26
gcc	A	0	0.19
gca	A	1	0.47
gcg	A	0	0.08
gaa	E	1070	652.90
gag	E	3	420.10
mPD		0.0074	0.48
		nPD:	0.02
		N. weight:	0.52
		Sc. PD:	-0.029
		Sc. rank:	-100.7

PA

Pos.	534	obs:	exp:
cct	P	0	268.00
ccc	P	1	193.00
cca	P	1063	442.10
ccg	P	10	171.00
mPD		0.020	0.71
		nPD:	0.03
		N. weight:	0.91
		Sc. PD:	-0.038
		Sc. rank:	22.8

PA

Pos.	535	obs:	exp:
cat	H	1072	616.70
cac	H	2	457.30
mPD		0.0037	0.49
		nPD:	0.01
		N. weight:	0.59
		Sc. PD:	-0.037
		Sc. rank:	-277.9

PA

Pos.	536	obs:	exp:
cgt	R	0	0.17
cgc	R	0	0.20
cga	R	0	0.39
cgg	R	0	0.33
aaa	K	1054	623.60
aag	K	16	446.40
aga	R	4	1.83
agg	R	0	1.08
mPD		0.037	0.50
		nPD:	0.07
		N. weight:	0.51
		Sc. PD:	0.0012
		Sc. rank:	215.8

PA

Pos.	537	obs:	exp:
tgg	W	1074	1074.00
mPD		0	0
		nPD:	1.
		N. weight:	0.
		Sc. PD:	0
		Sc. rank:	0

PA

Pos.	538	obs:	exp:
gaa	E	1070	652.90
gag	E	3	420.10
ggt	G	0	0.13
ggc	G	0	0.13
gga	G	1	0.46
ggg	G	0	0.28
mPD		0.0074	0.48
		nPD:	0.02
		N. weight:	0.52
		Sc. PD:	-0.029
		Sc. rank:	-98.9

PA

Pos.	539	obs:	exp:
aaa	K	2	626.00
aag	K	1072	448.00
mPD		0.0037	0.49
		nPD:	0.01
		N. weight:	0.94
		Sc. PD:	-0.058
		Sc. rank:	-434.3

PA

Pos.	540	obs:	exp:
tct	S	2	0.32
tcc	S	0	0.26
tca	S	0	0.51
tcg	S	0	0.11
tat	Y	1067	579.00
tac	Y	5	493.00
agt	S	0	0.41
agc	S	0	0.39
mPD		0.013	0.50
		nPD:	0.03
		N. weight:	0.64
		Sc. PD:	-0.029
		Sc. rank:	-2.0

PA

Pos.	541	obs:	exp:
tgt	C	1072	454.30
tgc	C	2	619.70
mPD		0.0037	0.49
		nPD:	0.01
		N. weight:	0.92
		Sc. PD:	-0.057
		Sc. rank:	-431.7

PA

Pos.	542	obs:	exp:
gtt	V	1071	222.50
gtc	V	3	216.80
gta	V	0	219.90
gtg	V	0	414.80
mPD		0.0056	0.73
		nPD:	0.01
		N. weight:	1.7
		Sc. PD:	-0.11
		Sc. rank:	-760.4

PA

Pos.	543	obs:	exp:
tta	L	0	95.31
ttg	L	0	200.00
ctt	L	819	178.80
ctc	L	255	179.20
cta	L	0	181.00
ctg	L	0	239.70
mPD		0.36	1.1
		nPD:	0.33
		N. weight:	1.4
		Sc. PD:	0.34
		Sc. rank:	1034.8

PA

Pos.	544	obs:	exp:
gat	D	1	0.54
gac	D	0	0.46
gaa	E	1071	652.90
gag	E	2	420.10
mPD		0.0056	0.48
		nPD:	0.01
		N. weight:	0.53
		Sc. PD:	-0.031
		Sc. rank:	-159.2

PA

Pos.	545	obs:	exp:
att	I	0	394.20
atc	I	0	257.10
ata	I	1073	421.60
gtt	V	0	0.21
gtc	V	0	0.20
gta	V	1	0.20
gtg	V	0	0.39
mPD		0.0019	0.66
		nPD:	0.
		N. weight:	1.
		Sc. PD:	-0.068
		Sc. rank:	-645.9

PA

Pos.	546	obs:	exp:
ggt	G	0	142.40
ggc	G	0	137.20
gga	G	6	495.50
ggg	G	1068	299.00
mPD		0.011	0.68
		nPD:	0.02
		N. weight:	1.4
		Sc. PD:	-0.073
		Sc. rank:	-228.6

PA

Pos.	547	obs:	exp:
aat	N	1	0.51
aac	N	0	0.49
gat	D	442	579.30
gac	D	630	492.70
gaa	E	1	0.61
gag	E	0	0.39
mPD		0.49	0.50
		nPD:	0.98
		N. weight:	0.041
		Sc. PD:	0.036
		Sc. rank:	34.9

PA

Pos.	548	obs:	exp:
att	I	0	0.73
atc	I	0	0.48
ata	I	2	0.79
atg	M	1072	1072.00
mPD		0.0037	0.0037
		nPD:	1.
		N. weight:	0.0019
		Sc. PD:	0.0017
		Sc. rank:	1.6

PA

Pos.	549	obs:	exp:
ttt	F	0	0.39
ttc	F	1	0.61
tta	L	0	95.22
ttg	L	0	199.80
ctt	L	3	178.70
ctc	L	1068	179.10
cta	L	2	180.80
ctg	L	0	239.40
mPD		0.011	1.1
		nPD:	0.01
		N. weight:	1.9
		Sc. PD:	-0.11
		Sc. rank:	-688.1

PA

Pos.	550	obs:	exp:
tta	L	0	95.31
ttg	L	1	200.00
ctt	L	1	178.80
ctc	L	0	179.20
cta	L	17	181.00
ctg	L	1055	239.70
mPD		0.035	1.1
		nPD:	0.03
		N. weight:	1.5
		Sc. PD:	-0.06
		Sc. rank:	122.9

PA

Pos.	551	obs:	exp:
cgt	R	0	46.13
cgc	R	1	53.19
cga	R	1029	105.10
cgg	R	8	89.62
aga	R	32	491.10
agg	R	4	288.90
mPD		0.089	0.96
		nPD:	0.09
		N. weight:	2.3
		Sc. PD:	0.045
		Sc. rank:	1136.2

PA

Pos.	552	obs:	exp:
act	T	1062	273.30
acc	T	8	220.00
aca	T	2	491.70
acg	T	0	87.07
aat	N	1	0.51
aac	N	0	0.49
gct	A	1	0.26
gcc	A	0	0.19
gca	A	0	0.47
gcg	A	0	0.08
mPD		0.022	0.68
		nPD:	0.03
		N. weight:	1.4
		Sc. PD:	-0.054
		Sc. rank:	140.4

PA

Pos.	553	obs:	exp:
tct	S	0	0.16
tcc	S	0	0.13
tca	S	1	0.25
tcg	S	0	0.06
agt	S	0	0.21
agc	S	0	0.19
gct	A	2	277.10
gcc	A	0	199.80
gca	A	1070	505.00
gcg	A	1	91.07
mPD		0.0074	0.67
		nPD:	0.01
		N. weight:	0.8
		Sc. PD:	-0.047
		Sc. rank:	-270.9

PA

Pos.	554	obs:	exp:
tta	L	2	0.18
ttg	L	0	0.37
ctt	L	0	0.33
ctc	L	0	0.33
cta	L	0	0.34
ctg	L	0	0.45
att	I	0	5.88
atc	I	0	3.83
ata	I	16	6.29
atg	M	1	1.00
gtt	V	0	218.60
gtc	V	1	212.90
gta	V	1024	216.00
gtg	V	30	407.50
mPD		0.093	0.76
		nPD:	0.12
		N. weight:	1.6
		Sc. PD:	0.075
		Sc. rank:	946.7

PA			
Pos.	555	obs:	exp:
ggt	G	51	142.40
ggc	G	1023	137.20
gga	G	0	495.50
ggg	G	0	299.00
---	--	------	------
mPD		0.091	0.68
		nPD:	0.13
		N. weight:	2.
		Sc. PD:	0.12
		Sc. rank:	1308.1

,

PA			
Pos.	556	obs:	exp:
cat	H	1	1.15
cac	H	1	0.85
caa	Q	1058	540.90
cag	Q	4	521.10
cgt	R	0	0.21
cgc	R	0	0.25
cga	R	5	0.49
cgg	R	0	0.42
aaa	K	5	2.91
aag	K	0	2.09
aga	R	0	2.29
agg	R	0	1.34
---	--	------	------
mPD		0.030	0.53
		nPD:	0.06
		N. weight:	0.72
		Sc. PD:	-0.011
		Sc. rank:	226.3

,

PA			
Pos.	557	obs:	exp:
atg	M	1	1.00
gtt	V	2	221.70
gtc	V	0	216.00
gta	V	1	219.10
gtg	V	1067	413.20
gct	A	0	0.77
gcc	A	0	0.56
gca	A	0	1.41
gcg	A	3	0.25
---	--	------	------
mPD		0.013	0.73
		nPD:	0.02
		N. weight:	1.
		Sc. PD:	-0.053
		Sc. rank:	-156.2

,

PA			
Pos.	558	obs:	exp:
tct	S	6	170.80
tcc	S	0	140.30
tca	S	1067	272.60
tcg	S	1	60.94
agt	S	0	220.80
agc	S	0	208.60
---	--	------	------
mPD		0.013	1.7
		nPD:	0.01
		N. weight:	1.5
		Sc. PD:	-0.09
		Sc. rank:	-611.1

,

PA			
Pos.	559	obs:	exp:
cgt	R	0	46.13
cgc	R	0	53.19
cga	R	0	105.10
cgg	R	0	89.62
aga	R	1036	491.10
agg	R	38	288.90
---	--	------	------
mPD		0.068	0.96
		nPD:	0.07
		N. weight:	0.71
		Sc. PD:	-0.00051
		Sc. rank:	279.9

,

PA			
Pos.	560	obs:	exp:
tct	S	0	0.16
tcc	S	1	0.13
tca	S	0	0.25
tcg	S	0	0.06
cct	P	20	267.70
ccc	P	1051	192.80
cca	P	2	441.70
ccg	P	0	170.80
agt	S	0	0.21
agc	S	0	0.19
---	--	------	------
mPD		0.042	0.71
		nPD:	0.06
		N. weight:	1.7
		Sc. PD:	-0.022
		Sc. rank:	590.9

,

PA			
Pos.	561	obs:	exp:
tta	L	0	0.09
ttg	L	1	0.19
ctt	L	0	0.17
ctc	L	0	0.17
cta	L	0	0.17
ctg	L	0	0.22
att	I	0	0.73
atc	I	0	0.48
ata	I	2	0.79
atg	M	1071	1071.00
---	--	------	------
mPD		0.0056	0.0067
		nPD:	0.84
		N. weight:	0.0036
		Sc. PD:	0.0027
		Sc. rank:	3.1

,

PA			
Pos.	562	obs:	exp:
ttt	F	1071	424.10
ttc	F	3	649.90
---	--	------	------
mPD		0.0056	0.48
		nPD:	0.01
		N. weight:	0.99
		Sc. PD:	-0.058
		Sc. rank:	-306.6

,

PA			
Pos.	563	obs:	exp:
tta	L	0	95.31
ttg	L	1	200.00
ctt	L	0	178.80
ctc	L	0	179.20
cta	L	7	181.00
ctg	L	1066	239.70
---	--	------	------
mPD		0.015	1.1
		nPD:	0.01
		N. weight:	1.6
		Sc. PD:	-0.09
		Sc. rank:	-445.5

,

PA			
Pos.	564	obs:	exp:
tat	Y	943	580.10
tac	Y	131	493.90
---	--	------	------
mPD		0.21	0.50
		nPD:	0.43
		N. weight:	0.29
		Sc. PD:	0.1
		Sc. rank:	229.7

,

PA			
Pos.	565	obs:	exp:
att	I	0	0.37
atc	I	0	0.24
ata	I	1	0.39
gtt	V	29	222.30
gtc	V	0	216.60
gta	V	51	219.70
gtg	V	993	414.40
---	--	------	------
mPD		0.14	0.73
		nPD:	0.2
		N. weight:	0.75
		Sc. PD:	0.092
		Sc. rank:	541.0

,

PA			
Pos.	566	obs:	exp:
cgt	R	0	46.13
cgc	R	0	53.19
cga	R	0	105.10
cgg	R	0	89.62
aga	R	970	491.10
agg	R	104	288.90
---	--	------	------
mPD		0.18	0.96
		nPD:	0.18
		N. weight:	0.57
		Sc. PD:	0.06
		Sc. rank:	399.9

,

PA			
Pos.	567	obs:	exp:
act	T	7	273.80
acc	T	1067	220.40
aca	T	0	492.60
acg	T	0	87.24
---	--	------	------
mPD		0.013	0.68
		nPD:	0.02
		N. weight:	1.7
		Sc. PD:	-0.086
		Sc. rank:	-197.9

,

PA			
Pos.	568	obs:	exp:
aat	N	1070	547.10
aac	N	4	526.90
---	--	------	------
mPD		0.0074	0.50
		nPD:	0.01
		N. weight:	0.71
		Sc. PD:	-0.039
		Sc. rank:	-158.3

,

PA			
Pos.	569	obs:	exp:
ggt	G	4	142.40
ggc	G	0	137.20
gga	G	42	495.50
ggg	G	1028	299.00
---	--	------	------
mPD		0.082	0.68
		nPD:	0.12
		N. weight:	1.2
		Sc. PD:	0.057
		Sc. rank:	717.1

,

PA			
Pos.	570	obs:	exp:
act	T	2	273.50
acc	T	1065	220.20
aca	T	6	492.20
acg	T	0	87.16
aat	N	0	0.51
aac	N	1	0.49
---	--	------	------
mPD		0.017	0.68
		nPD:	0.02
		N. weight:	1.7
		Sc. PD:	-0.076
		Sc. rank:	-54.7

,

PA			
Pos.	571	obs:	exp:
tct	S	149	170.80
tcc	S	922	140.30
tca	S	3	272.60
tcg	S	0	60.94
agt	S	0	220.80
agc	S	0	208.60
---	--	------	------
mPD		0.24	1.7
		nPD:	0.15
		N. weight:	1.7
		Sc. PD:	0.13
		Sc. rank:	1151.4

,

PA			
Pos.	572	obs:	exp:
aaa	K	1	626.00
aag	K	1073	448.00
---	--	------	------
mPD		0.0019	0.49
		nPD:	0.
		N. weight:	0.94
		Sc. PD:	-0.062
		Sc. rank:	-541.3

,

PA			
Pos.	573	obs:	exp:
att	I	0	393.50
atc	I	1071	256.70
ata	I	0	420.80
gtt	V	0	0.62
gtc	V	3	0.61
gta	V	0	0.61
gtg	V	0	1.16
---	--	------	------
mPD		0.0056	0.66
		nPD:	0.01
		N. weight:	1.6
		Sc. PD:	-0.096
		Sc. rank:	-610.5

,

PA			
Pos.	574	obs:	exp:
aaa	K	27	626.00
aag	K	1047	448.00
---	--	------	------
mPD		0.049	0.49
		nPD:	0.1
		N. weight:	0.82
		Sc. PD:	0.023
		Sc. rank:	445.7

,

PA			
Pos.	575	obs:	exp:
atg	M	1074	1074.00
---	--	------	------
mPD		0	0
		nPD:	1.
		N. weight:	0.
		Sc. PD:	0
		Sc. rank:	0

,

PA			
Pos.	576	obs:	exp:
aaa	K	1070	626.00
aag	K	4	448.00
---	--	------	------
mPD		0.0074	0.49
		nPD:	0.02
		N. weight:	0.56
		Sc. PD:	-0.031
		Sc. rank:	-116.7

,

PA			
Pos.	577	obs:	exp:
tgg	W	1074	1074.00
---	--	------	------
mPD		0	0
		nPD:	1.
		N. weight:	0.
		Sc. PD:	0
		Sc. rank:	0

,

PA			
Pos.	578	obs:	exp:
ggt	G	1068	142.40
ggc	G	5	137.20
gga	G	1	495.50
ggg	G	0	299.00
---	--	------	------
mPD		0.011	0.68
		nPD:	0.02
		N. weight:	2.2
		Sc. PD:	-0.12
		Sc. rank:	-353.9

,

PA

Pos.	579	obs:	exp:
atg	M	1074	1074.00
---	--	------	------
mPD		0	0
		nPD:	1.
		N. weight:	0.
		Sc. PD:	0
		Sc. rank:	0

PA

Pos.	580	obs:	exp:
gaa	E	1074	653.50
gag	E	0	420.50
---	--	------	------
mPD		0	0.48
		nPD:	0.
		N. weight:	0.54
		Sc. PD:	-0.038
		Sc. rank:	-459.4

PA

Pos.	581	obs:	exp:
tta	L	0	0.09
ttg	L	0	0.19
ctt	L	0	0.17
ctc	L	0	0.17
cta	L	0	0.17
ctg	L	1	0.22
att	I	2	0.73
atc	I	0	0.48
ata	I	0	0.79
atg	M	1071	1071.00
---	--	------	------
mPD		0.0056	0.0067
		nPD:	0.84
		N. weight:	0.0036
		Sc. PD:	0.0026
		Sc. rank:	3.0

PA

Pos.	582	obs:	exp:
cgt	R	0	46.13
cgc	R	0	53.19
cga	R	0	105.10
cgg	R	0	89.62
aga	R	1032	491.10
agg	R	42	288.90
---	--	------	------
mPD		0.075	0.96
		nPD:	0.08
		N. weight:	0.7
		Sc. PD:	0.0043
		Sc. rank:	304.4

PA

Pos.	583	obs:	exp:
cgt	R	13	46.13
cgc	R	1052	53.19
cga	R	9	105.10
cgg	R	0	89.62
aga	R	0	491.10
agg	R	0	288.90
---	--	------	------
mPD		0.040	0.96
		nPD:	0.04
		N. weight:	3.1
		Sc. PD:	-0.091
		Sc. rank:	702.1

PA

Pos.	584	obs:	exp:
tgt	C	7	453.80
tgc	C	1066	619.20
tgg	W	1	1.00
---	--	------	------
mPD		0.015	0.49
		nPD:	0.03
		N. weight:	0.56
		Sc. PD:	-0.023
		Sc. rank:	30.3

PA

Pos.	585	obs:	exp:
tta	L	0	95.31
ttg	L	0	200.00
ctt	L	1072	178.80
ctc	L	2	179.20
cta	L	0	181.00
ctg	L	0	239.70
---	--	------	------
mPD		0.0037	1.1
		nPD:	0.
		N. weight:	1.9
		Sc. PD:	-0.13
		Sc. rank:	-1184.6

PA

Pos.	586	obs:	exp:
tta	L	0	95.31
ttg	L	0	200.00
ctt	L	702	178.80
ctc	L	182	179.20
cta	L	190	181.00
ctg	L	0	239.70
---	--	------	------
mPD		0.51	1.1
		nPD:	0.46
		N. weight:	0.99
		Sc. PD:	0.38
		Sc. rank:	796.1

PA

Pos.	587	obs:	exp:
caa	Q	1026	547.10
cag	Q	48	526.90
---	--	------	------
mPD		0.085	0.50
		nPD:	0.17
		N. weight:	0.54
		Sc. PD:	0.052
		Sc. rank:	379.5

PA

Pos.	588	obs:	exp:
tct	S	1022	170.80
tcc	S	48	140.30
tca	S	4	272.60
tcg	S	0	60.94
agt	S	0	220.80
agc	S	0	208.60
---	--	------	------
mPD		0.093	1.7
		nPD:	0.06
		N. weight:	1.8
		Sc. PD:	-0.028
		Sc. rank:	561.7

PA

Pos.	589	obs:	exp:
tta	L	0	95.22
ttg	L	0	199.80
ctt	L	32	178.70
ctc	L	1036	179.10
cta	L	5	180.80
ctg	L	0	239.40
att	I	0	0.37
atc	I	1	0.24
ata	I	0	0.39
---	--	------	------
mPD		0.069	1.1
		nPD:	0.06
		N. weight:	1.8
		Sc. PD:	-0.017
		Sc. rank:	643.5

PA

Pos.	590	obs:	exp:
caa	Q	1071	547.10
cag	Q	3	526.90
---	--	------	------
mPD		0.0056	0.50
		nPD:	0.01
		N. weight:	0.72
		Sc. PD:	-0.042
		Sc. rank:	-234.9

PA

Pos.	591	obs:	exp:
caa	Q	14	547.10
cag	Q	1060	526.90
---	--	------	------
mPD		0.026	0.50
		nPD:	0.05
		N. weight:	0.7
		Sc. PD:	-0.014
		Sc. rank:	202.2

PA

Pos.	592	obs:	exp:
att	I	1057	393.50
atc	I	14	256.70
ata	I	0	420.80
gtt	V	3	0.62
gtc	V	0	0.61
gta	V	0	0.61
gtg	V	0	1.16
---	--	------	------
mPD		0.031	0.66
		nPD:	0.05
		N. weight:	1.
		Sc. PD:	-0.024
		Sc. rank:	259.4

PA

Pos.	593	obs:	exp:
gaa	E	0	653.50
gag	E	1074	420.50
---	--	------	------
mPD		0	0.48
		nPD:	0.
		N. weight:	1.
		Sc. PD:	-0.071
		Sc. rank:	-867.2

PA

Pos.	594	obs:	exp:
tct	S	0	170.50
tcc	S	0	140.00
tca	S	0	272.10
tcg	S	0	60.83
aat	N	0	1.02
aac	N	2	0.98
agt	S	15	220.40
agc	S	1057	208.20
---	--	------	------
mPD		0.031	1.7
		nPD:	0.02
		N. weight:	1.7
		Sc. PD:	-0.087
		Sc. rank:	-221.6

PA

Pos.	595	obs:	exp:
att	I	0	0.37
atc	I	0	0.24
ata	I	1	0.39
atg	M	1073	1073.00
---	--	------	------
mPD		0.0019	0.0019
		nPD:	1.
		N. weight:	0.00095
		Sc. PD:	0.00085
		Sc. rank:	0.8

PA

Pos.	596	obs:	exp:
att	I	1074	394.60
atc	I	0	257.40
ata	I	0	422.00
---	--	------	------
mPD		0	0.65
		nPD:	0.
		N. weight:	1.1
		Sc. PD:	-0.076
		Sc. rank:	-925.9

PA

Pos.	597	obs:	exp:
gaa	E	1070	653.50
gag	E	4	420.50
---	--	------	------
mPD		0.0074	0.48
		nPD:	0.02
		N. weight:	0.52
		Sc. PD:	-0.028
		Sc. rank:	-96.3

PA

Pos.	598	obs:	exp:
gtt	V	2	0.41
gtc	V	0	0.40
gta	V	0	0.41
gtg	V	0	0.77
gct	A	1070	276.80
gcc	A	2	199.60
gca	A	0	504.60
gcg	A	0	90.98
---	--	------	------
mPD		0.0074	0.67
		nPD:	0.01
		N. weight:	1.5
		Sc. PD:	-0.086
		Sc. rank:	-495.6

PA

Pos.	599	obs:	exp:
gaa	E	1066	652.90
gag	E	7	420.10
ggt	G	0	0.13
ggc	G	0	0.13
gga	G	1	0.46
ggg	G	0	0.28
---	--	------	------
mPD		0.015	0.48
		nPD:	0.03
		N. weight:	0.5
		Sc. PD:	-0.02
		Sc. rank:	34.8

PA

Pos.	600	obs:	exp:
tct	S	3	170.80
tcc	S	1061	140.30
tca	S	10	272.60
tcg	S	0	60.94
agt	S	0	220.80
agc	S	0	208.60
---	--	------	------
mPD		0.024	1.7
		nPD:	0.01
		N. weight:	2.1
		Sc. PD:	-0.12
		Sc. rank:	-483.7

PA

Pos.	601	obs:	exp:
tct	S	79	170.80
tcc	S	994	140.30
tca	S	1	272.60
tcg	S	0	60.94
agt	S	0	220.80
agc	S	0	208.60
---	--	------	------
mPD		0.14	1.7
		nPD:	0.08
		N. weight:	1.9
		Sc. PD:	0.022
		Sc. rank:	879.2

PA

Pos.	602	obs:	exp:
att	I	1	2.57
atc	I	6	1.68
ata	I	0	2.75
gtt	V	6	221.10
gtc	V	1061	215.30
gta	V	0	218.50
gtg	V	0	412.10
---	--	------	------
mPD		0.026	0.74
		nPD:	0.04
		N. weight:	1.7
		Sc. PD:	-0.061
		Sc. rank:	209.1

PA

Pos.	603	obs:	exp:
cgt	R	0	0.82
cgc	R	0	0.94
cga	R	0	1.86
cgg	R	0	1.59
aaa	K	1042	614.90
aag	K	13	440.10
aga	R	19	8.69
agg	R	0	5.11
---	--	------	------
mPD		0.059	0.53
		nPD:	0.11
		N. weight:	0.53
		Sc. PD:	0.02
		Sc. rank:	299.5

PA			
Pos.	604	obs:	exp:
gaa	E	3	653.50
gag	E	1071	420.50
---	--	------	------
mPD		0.0056	0.48
		nPD:	0.01
		N. weight:	1.
		Sc. PD:	-0.058
		Sc. rank:	-299.3

PA			
Pos.	605	obs:	exp:
cgt	R	0	0.09
cgc	R	0	0.10
cga	R	0	0.20
cgg	R	0	0.17
aaa	K	1069	624.80
aag	K	3	447.20
aga	R	2	0.91
agg	R	0	0.54
---	--	------	------
mPD		0.0093	0.49
		nPD:	0.02
		N. weight:	0.57
		Sc. PD:	-0.029
		Sc. rank:	-73.3

PA			
Pos.	606	obs:	exp:
gat	D	13	580.40
gac	D	1061	493.60
---	--	------	------
mPD		0.024	0.50
		nPD:	0.05
		N. weight:	0.78
		Sc. PD:	-0.018
		Sc. rank:	201.0

PA			
Pos.	607	obs:	exp:
tta	L	0	94.69
ttg	L	3	198.70
ctt	L	0	177.70
ctc	L	0	178.10
cta	L	34	179.80
ctg	L	1030	238.10
atg	M	7	7.00
---	--	------	------
mPD		0.080	1.1
		nPD:	0.07
		N. weight:	1.5
		Sc. PD:	-0.00035
		Sc. rank:	586.0

PA			
Pos.	608	obs:	exp:
act	T	5	273.80
acc	T	1063	220.40
aca	T	6	492.60
acg	T	0	87.24
---	--	------	------
mPD		0.020	0.68
		nPD:	0.03
		N. weight:	1.7
		Sc. PD:	-0.067
		Sc. rank:	81.6

PA			
Pos.	609	obs:	exp:
aaa	K	1066	626.00
aag	K	8	448.00
---	--	------	------
mPD		0.015	0.49
		nPD:	0.03
		N. weight:	0.54
		Sc. PD:	-0.022
		Sc. rank:	34.4

PA			
Pos.	610	obs:	exp:
gat	D	0	0.54
gac	D	1	0.46
gaa	E	1072	652.90
gag	E	1	420.10
---	--	------	------
mPD		0.0037	0.48
		nPD:	0.01
		N. weight:	0.53
		Sc. PD:	-0.033
		Sc. rank:	-230.4

PA			
Pos.	611	obs:	exp:
ttt	F	2	424.10
ttc	F	1072	649.90
---	--	------	------
mPD		0.0037	0.48
		nPD:	0.01
		N. weight:	0.53
		Sc. PD:	-0.033
		Sc. rank:	-234.6

PA			
Pos.	612	obs:	exp:
ttt	F	1072	424.10
ttc	F	2	649.90
---	--	------	------
mPD		0.0037	0.48
		nPD:	0.01
		N. weight:	1.
		Sc. PD:	-0.062
		Sc. rank:	-437.9

PA			
Pos.	613	obs:	exp:
gaa	E	1047	653.50
gag	E	27	420.50
---	--	------	------
mPD		0.049	0.48
		nPD:	0.1
		N. weight:	0.43
		Sc. PD:	0.013
		Sc. rank:	234.1

PA			
Pos.	614	obs:	exp:
act	T	0	2.04
acc	T	8	1.64
aca	T	0	3.67
acg	T	0	0.65
aat	N	2	540.90
aac	N	1060	521.10
aaa	K	0	1.17
aag	K	2	0.83
gat	D	0	1.08
gac	D	2	0.92
---	--	------	------
mPD		0.026	0.52
		nPD:	0.05
		N. weight:	0.77
		Sc. PD:	-0.017
		Sc. rank:	209.1

PA			
Pos.	615	obs:	exp:
aaa	K	1060	626.00
aag	K	14	448.00
---	--	------	------
mPD		0.026	0.49
		nPD:	0.05
		N. weight:	0.52
		Sc. PD:	-0.0095
		Sc. rank:	150.9

PA			
Pos.	616	obs:	exp:
tct	S	6	170.80
tcc	S	0	140.30
tca	S	1066	272.60
tcg	S	2	60.94
agt	S	0	220.80
agc	S	0	208.60
---	--	------	------
mPD		0.015	1.7
		nPD:	0.01
		N. weight:	1.5
		Sc. PD:	-0.088
		Sc. rank:	-556.3

PA			
Pos.	617	obs:	exp:
gaa	E	1044	652.90
gag	E	29	420.10
ggt	G	0	0.13
ggc	G	0	0.13
gga	G	1	0.46
ggg	G	0	0.28
---	--	------	------
mPD		0.054	0.48
		nPD:	0.11
		N. weight:	0.42
		Sc. PD:	0.017
		Sc. rank:	246.0

PA			
Pos.	618	obs:	exp:
act	T	0	271.70
acc	T	0	218.70
aca	T	1062	488.90
acg	T	4	86.59
aaa	K	6	3.50
aag	K	0	2.50
gct	A	0	0.52
gcc	A	0	0.37
gca	A	2	0.94
gcg	A	0	0.17
---	--	------	------
mPD		0.022	0.69
		nPD:	0.03
		N. weight:	0.83
		Sc. PD:	-0.032
		Sc. rank:	74.3

PA			
Pos.	619	obs:	exp:
tgg	W	1074	1074.00
---	--	------	------
mPD		0	0
		nPD:	1.
		N. weight:	0.
		Sc. PD:	0
		Sc. rank:	0

PA			
Pos.	620	obs:	exp:
cct	P	0	268.00
ccc	P	0	193.00
cca	P	1070	442.10
ccg	P	4	171.00
---	--	------	------
mPD		0.0074	0.71
		nPD:	0.01
		N. weight:	0.95
		Sc. PD:	-0.056
		Sc. rank:	-337.5

PA			
Pos.	621	obs:	exp:
tct	S	0	0.32
tcc	S	0	0.26
tca	S	0	0.51
tcg	S	0	0.11
att	I	1063	393.50
atc	I	6	256.70
ata	I	2	420.80
agt	S	2	0.41
agc	S	0	0.39
gtt	V	1	0.21
gtc	V	0	0.20
gta	V	0	0.20
gtg	V	0	0.39
---	--	------	------
mPD		0.020	0.66
		nPD:	0.03
		N. weight:	1.
		Sc. PD:	-0.042
		Sc. rank:	69.8

PA			
Pos.	622	obs:	exp:
ggt	G	4	142.40
ggc	G	0	137.20
gga	G	1064	495.50
ggg	G	6	299.00
---	--	------	------
mPD		0.019	0.68
		nPD:	0.03
		N. weight:	0.79
		Sc. PD:	-0.034
		Sc. rank:	16.1

PA			
Pos.	623	obs:	exp:
aaa	K	5	6.41
aag	K	6	4.59
gaa	E	477	646.80
gag	E	586	416.20
---	--	------	------
mPD		0.52	0.50
		nPD:	1.04
		N. weight:	0.062
		Sc. PD:	0.058
		Sc. rank:	52.8

PA			
Pos.	624	obs:	exp:
tct	S	0	170.80
tcc	S	0	140.30
tca	S	1073	272.60
tcg	S	1	60.94
agt	S	0	220.80
agc	S	0	208.60
---	--	------	------
mPD		0.0019	1.7
		nPD:	0.
		N. weight:	1.5
		Sc. PD:	-0.1
		Sc. rank:	-1066.2

PA			
Pos.	625	obs:	exp:
cct	P	1005	268.00
ccc	P	65	193.00
cca	P	4	442.10
ccg	P	0	171.00
---	--	------	------
mPD		0.12	0.71
		nPD:	0.17
		N. weight:	1.3
		Sc. PD:	0.12
		Sc. rank:	883.6

PA			
Pos.	626	obs:	exp:
cgt	R	0	0.21
cgc	R	0	0.25
cga	R	0	0.49
cgg	R	0	0.42
aat	N	16	8.15
aac	N	0	7.85
aaa	K	603	613.70
aag	K	450	439.30
aga	R	4	2.29
agg	R	1	1.34
---	--	------	------
mPD		0.51	0.51
		nPD:	1.
		N. weight:	0.018
		Sc. PD:	0.016
		Sc. rank:	15.2

PA			
Pos.	627	obs:	exp:
ggt	G	0	142.40
ggc	G	0	137.20
gga	G	1062	495.50
ggg	G	12	299.00
---	--	------	------
mPD		0.022	0.68
		nPD:	0.03
		N. weight:	0.78
		Sc. PD:	-0.03
		Sc. rank:	80.0

PA			
Pos.	628	obs:	exp:
gtt	V	1	222.50
gtc	V	0	216.80
gta	V	39	219.90
gtg	V	1034	414.80
---	--	------	------
mPD		0.072	0.73
		nPD:	0.1
		N. weight:	0.89
		Sc. PD:	0.023
		Sc. rank:	478.5

PA			
Pos.	629	obs:	exp:
gaa	E	3	653.50
gag	E	1071	420.50
---	--	------	------
mPD		0.0056	0.48
		nPD:	0.01
		N. weight:	1.
		Sc. PD:	-0.058
		Sc. rank:	-299.3

PA			
Pos.	630	obs:	exp:
gaa	E	1074	653.50
gag	E	0	420.50
---	--	------	------
mPD		0	0.48
		nPD:	0.
		N. weight:	0.54
		Sc. PD:	-0.038
		Sc. rank:	-459.4

PA			
Pos.	631	obs:	exp:
ggt	G	1063	142.40
ggc	G	11	137.20
gga	G	0	495.50
ggg	G	0	299.00
---	--	------	------
mPD		0.020	0.68
		nPD:	0.03
		N. weight:	2.1
		Sc. PD:	-0.087
		Sc. rank:	88.5

PA			
Pos.	632	obs:	exp:
ttt	F	0	0.39
ttc	F	1	0.61
tct	S	0	170.20
tcc	S	1060	139.70
tca	S	10	271.60
tcg	S	0	60.72
cct	P	0	0.75
ccc	P	3	0.54
cca	P	0	1.23
ccg	P	0	0.48
agt	S	0	220.00
agc	S	0	207.80
---	--	------	------
mPD		0.026	1.7
		nPD:	0.02
		N. weight:	2.2
		Sc. PD:	-0.12
		Sc. rank:	-382.6

PA			
Pos.	633	obs:	exp:
att	I	349	394.60
atc	I	722	257.40
ata	I	3	422.00
---	--	------	------
mPD		0.44	0.65
		nPD:	0.68
		N. weight:	0.7
		Sc. PD:	0.41
		Sc. rank:	593.5

PA			
Pos.	634	obs:	exp:
ggt	G	0	142.40
ggc	G	0	137.20
gga	G	57	495.50
ggg	G	1017	299.00
---	--	------	------
mPD		0.10	0.68
		nPD:	0.15
		N. weight:	1.1
		Sc. PD:	0.085
		Sc. rank:	761.6

PA			
Pos.	635	obs:	exp:
aaa	K	5	626.00
aag	K	1069	448.00
---	--	------	------
mPD		0.0093	0.49
		nPD:	0.02
		N. weight:	0.92
		Sc. PD:	-0.047
		Sc. rank:	-111.9

PA			
Pos.	636	obs:	exp:
gtt	V	0	222.50
gtc	V	1	216.80
gta	V	16	219.90
gtg	V	1057	414.80
---	--	------	------
mPD		0.031	0.73
		nPD:	0.04
		N. weight:	0.96
		Sc. PD:	-0.027
		Sc. rank:	215.8

PA			
Pos.	637	obs:	exp:
tgt	C	31	454.30
tgc	C	1043	619.70
---	--	------	------
mPD		0.056	0.49
		nPD:	0.11
		N. weight:	0.47
		Sc. PD:	0.02
		Sc. rank:	274.8

PA			
Pos.	638	obs:	exp:
cgt	R	0	46.13
cgc	R	0	53.19
cga	R	0	105.10
cgg	R	0	89.62
aga	R	1071	491.10
agg	R	3	288.90
---	--	------	------
mPD		0.0056	0.96
		nPD:	0.01
		N. weight:	0.83
		Sc. PD:	-0.053
		Sc. rank:	-407.4

PA			
Pos.	639	obs:	exp:
act	T	1	273.80
acc	T	1072	220.40
aca	T	1	492.60
acg	T	0	87.24
---	--	------	------
mPD		0.0037	0.68
		nPD:	0.01
		N. weight:	1.7
		Sc. PD:	-0.11
		Sc. rank:	-901.2

PA			
Pos.	640	obs:	exp:
tta	L	1069	95.31
ttg	L	2	200.00
ctt	L	0	178.80
ctc	L	0	179.20
cta	L	3	181.00
ctg	L	0	239.70
---	--	------	------
mPD		0.0093	1.1
		nPD:	0.01
		N. weight:	2.6
		Sc. PD:	-0.16
		Sc. rank:	-1030.1

PA			
Pos.	641	obs:	exp:
tta	L	4	95.31
ttg	L	0	200.00
ctt	L	0	178.80
ctc	L	0	179.20
cta	L	1066	181.00
ctg	L	4	239.70
---	--	------	------
mPD		0.015	1.1
		nPD:	0.01
		N. weight:	1.9
		Sc. PD:	-0.11
		Sc. rank:	-499.7

PA			
Pos.	642	obs:	exp:
gct	A	0	277.30
gcc	A	0	200.00
gca	A	1071	505.50
gcg	A	3	91.15
---	--	------	------
mPD		0.0056	0.67
		nPD:	0.01
		N. weight:	0.81
		Sc. PD:	-0.05
		Sc. rank:	-331.8

PA			
Pos.	643	obs:	exp:
cgt	R	0	0.04
cgc	R	0	0.05
cga	R	0	0.10
cgg	R	0	0.08
aaa	K	1063	625.40
aag	K	10	447.60
aga	R	1	0.46
agg	R	0	0.27
---	--	------	------
mPD		0.020	0.49
		nPD:	0.04
		N. weight:	0.54
		Sc. PD:	-0.016
		Sc. rank:	111.2

PA			
Pos.	644	obs:	exp:
tct	S	1038	170.80
tcc	S	6	140.30
tca	S	1	272.60
tcg	S	29	60.94
agt	S	0	220.80
agc	S	0	208.60
---	--	------	------
mPD		0.065	1.7
		nPD:	0.04
		N. weight:	1.9
		Sc. PD:	-0.058
		Sc. rank:	347.1

PA			
Pos.	645	obs:	exp:
gtt	V	0	222.30
gtc	V	0	216.60
gta	V	1066	219.70
gtg	V	7	414.40
gct	A	0	0.26
gcc	A	0	0.19
gca	A	1	0.47
gcg	A	0	0.08
---	--	------	------
mPD		0.015	0.73
		nPD:	0.02
		N. weight:	1.7
		Sc. PD:	-0.084
		Sc. rank:	-158.1

PA			
Pos.	646	obs:	exp:
ttt	F	1	424.10
ttc	F	1073	649.90
---	--	------	------
mPD		0.0019	0.48
		nPD:	0.
		N. weight:	0.54
		Sc. PD:	-0.036
		Sc. rank:	-303.7

PA			
Pos.	647	obs:	exp:
tct	S	0	0.32
tcc	S	0	0.26
tca	S	0	0.51
tcg	S	0	0.11
aat	N	429	546.00
aac	N	643	526.00
agt	S	1	0.41
agc	S	1	0.39
---	--	------	------
mPD		0.48	0.51
		nPD:	0.96
		N. weight:	0.032
		Sc. PD:	0.028
		Sc. rank:	27.7

PA			
Pos.	648	obs:	exp:
tct	S	0	170.30
tcc	S	0	139.90
tca	S	0	271.80
tcg	S	0	60.77
aat	N	0	1.53
aac	N	3	1.47
agt	S	48	220.20
agc	S	1023	208.00
---	--	------	------
mPD		0.091	1.7
		nPD:	0.05
		N. weight:	1.6
		Sc. PD:	-0.026
		Sc. rank:	483.6

PA			
Pos.	649	obs:	exp:
tta	L	17	95.31
ttg	L	0	200.00
ctt	L	0	178.80
ctc	L	0	179.20
cta	L	1055	181.00
ctg	L	2	239.70
---	--	------	------
mPD		0.035	1.1
		nPD:	0.03
		N. weight:	1.9
		Sc. PD:	-0.072
		Sc. rank:	155.9

PA			
Pos.	650	obs:	exp:
tat	Y	873	580.10
tac	Y	201	493.90
---	--	------	------
mPD		0.30	0.50
		nPD:	0.61
		N. weight:	0.18
		Sc. PD:	0.095
		Sc. rank:	152.3

PA			
Pos.	651	obs:	exp:
gct	A	1	277.30
gcc	A	0	200.00
gca	A	1072	505.50
gcg	A	1	91.15
---	--	------	------
mPD		0.0037	0.67
		nPD:	0.01
		N. weight:	0.81
		Sc. PD:	-0.052
		Sc. rank:	-421.1

PA			
Pos.	652	obs:	exp:
ttt	F	1	0.39
ttc	F	0	0.61
tct	S	1069	170.70
tcc	S	1	140.10
tca	S	3	272.30
tcg	S	0	60.89
agt	S	0	220.60
agc	S	0	208.40
---	--	------	------
mPD		0.0093	1.7
		nPD:	0.01
		N. weight:	2.
		Sc. PD:	-0.13
		Sc. rank:	-994.2

PA			
Pos.	653	obs:	exp:
tta	L	0	0.09
ttg	L	0	0.19
ctt	L	0	0.17
ctc	L	0	0.17
cta	L	0	0.17
ctg	L	1	0.22
cct	P	0	267.70
ccc	P	0	192.80
cca	P	127	441.70
ccg	P	946	170.80
---	--	------	------
mPD		0.21	0.71
		nPD:	0.3
		N. weight:	1.5
		Sc. PD:	0.32
		Sc. rank:	1122.7

PA

Pos.	654	obs:	exp:
caa	Q	1074	547.10
cag	Q	0	526.90
mPD		0	0.50
		nPD:	0.
		N. weight:	0.73
		Sc. PD:	-0.051
		Sc. rank:	-623.9

PA

Pos.	655	obs:	exp:
tta	L	0	95.22
ttg	L	0	199.80
ctt	L	46	178.70
ctc	L	1012	179.10
cta	L	15	180.80
ctg	L	0	239.40
att	I	0	0.37
atc	I	1	0.24
ata	I	0	0.39
mPD		0.11	1.1
		nPD:	0.1
		N. weight:	1.7
		Sc. PD:	0.045
		Sc. rank:	913.9

PA

Pos.	656	obs:	exp:
gaa	E	148	653.50
gag	E	926	420.50
mPD		0.24	0.48
		nPD:	0.5
		N. weight:	0.52
		Sc. PD:	0.22
		Sc. rank:	422.9

PA

Pos.	657	obs:	exp:
ggt	G	2	142.40
ggc	G	9	137.20
gga	G	11	495.50
ggg	G	1052	299.00
mPD		0.040	0.68
		nPD:	0.06
		N. weight:	1.3
		Sc. PD:	-0.015
		Sc. rank:	433.3

PA

Pos.	658	obs:	exp:
ttt	F	17	423.30
ttc	F	1055	648.70
tta	L	1	0.18
ttg	L	1	0.37
ctt	L	0	0.33
ctc	L	0	0.33
cta	L	0	0.34
ctg	L	0	0.45
mPD		0.035	0.48
		nPD:	0.07
		N. weight:	0.47
		Sc. PD:	0.00021
		Sc. rank:	191.1

PA

Pos.	659	obs:	exp:
tct	S	0	170.80
tcc	S	1	140.30
tca	S	1073	272.60
tcg	S	0	60.94
agt	S	0	220.80
agc	S	0	208.60
mPD		0.0019	1.7
		nPD:	0.
		N. weight:	1.5
		Sc. PD:	-0.1
		Sc. rank:	-1065.6

PA

Pos.	660	obs:	exp:
tct	S	1	0.16
tcc	S	0	0.13
tca	S	0	0.25
tcg	S	0	0.06
agt	S	0	0.21
agc	S	0	0.19
gct	A	1071	277.10
gcc	A	0	199.80
gca	A	2	505.00
gcg	A	0	91.07
mPD		0.0056	0.67
		nPD:	0.01
		N. weight:	1.5
		Sc. PD:	-0.09
		Sc. rank:	-603.6

PA

Pos.	661	obs:	exp:
gaa	E	1040	653.50
gag	E	34	420.50
mPD		0.061	0.48
		nPD:	0.13
		N. weight:	0.41
		Sc. PD:	0.022
		Sc. rank:	255.8

PA

Pos.	662	obs:	exp:
tct	S	0	170.80
tcc	S	0	140.30
tca	S	12	272.60
tcg	S	1062	60.94
agt	S	0	220.80
agc	S	0	208.60
mPD		0.022	1.7
		nPD:	0.01
		N. weight:	3.
		Sc. PD:	-0.17
		Sc. rank:	-831.4

PA

Pos.	663	obs:	exp:
cgt	R	0	46.13
cgc	R	0	53.19
cga	R	0	105.10
cgg	R	0	89.62
aga	R	1069	491.10
agg	R	5	288.90
mPD		0.0093	0.96
		nPD:	0.01
		N. weight:	0.82
		Sc. PD:	-0.05
		Sc. rank:	-307.0

PA

Pos.	664	obs:	exp:
aaa	K	1070	626.00
aag	K	4	448.00
mPD		0.0074	0.49
		nPD:	0.02
		N. weight:	0.56
		Sc. PD:	-0.031
		Sc. rank:	-116.7

PA

Pos.	665	obs:	exp:
tta	L	0	95.04
ttg	L	45	199.40
ctt	L	3	178.30
ctc	L	0	178.70
cta	L	7	180.50
ctg	L	1016	239.00
atg	M	3	3.00
mPD		0.10	1.1
		nPD:	0.09
		N. weight:	1.4
		Sc. PD:	0.03
		Sc. rank:	705.9

PA

Pos.	666	obs:	exp:
tta	L	6	95.31
ttg	L	0	200.00
ctt	L	3	178.80
ctc	L	0	179.20
cta	L	1045	181.00
ctg	L	20	239.70
mPD		0.053	1.1
		nPD:	0.05
		N. weight:	1.8
		Sc. PD:	-0.041
		Sc. rank:	459.2

PA

Pos.	667	obs:	exp:
tta	L	0	95.31
ttg	L	0	200.00
ctt	L	2	178.80
ctc	L	1072	179.20
cta	L	0	181.00
ctg	L	0	239.70
mPD		0.0037	1.1
		nPD:	0.
		N. weight:	1.9
		Sc. PD:	-0.13
		Sc. rank:	-1183.2

PA

Pos.	668	obs:	exp:
att	I	1073	394.20
atc	I	0	257.10
ata	I	0	421.60
act	T	1	0.25
acc	T	0	0.21
aca	T	0	0.46
acg	T	0	0.08
mPD		0.0019	0.66
		nPD:	0.
		N. weight:	1.1
		Sc. PD:	-0.073
		Sc. rank:	-686.5

PA

Pos.	669	obs:	exp:
att	I	4	1.47
atc	I	0	0.96
ata	I	0	1.57
gtt	V	1054	221.70
gtc	V	1	216.00
gta	V	0	219.10
gtg	V	15	413.20
mPD		0.037	0.73
		nPD:	0.05
		N. weight:	1.6
		Sc. PD:	-0.034
		Sc. rank:	445.9

PA

Pos.	670	obs:	exp:
caa	Q	0	547.10
cag	Q	1074	526.90
mPD		0	0.50
		nPD:	0.
		N. weight:	0.78
		Sc. PD:	-0.054
		Sc. rank:	-658.5

PA

Pos.	671	obs:	exp:
gct	A	4	277.30
gcc	A	0	200.00
gca	A	263	505.50
gcg	A	807	91.15
mPD		0.38	0.67
		nPD:	0.56
		N. weight:	1.6
		Sc. PD:	0.76
		Sc. rank:	1329.9

PA

Pos.	672	obs:	exp:
tta	L	0	95.31
ttg	L	0	200.00
ctt	L	1038	178.80
ctc	L	36	179.20
cta	L	0	181.00
ctg	L	0	239.70
mPD		0.065	1.1
		nPD:	0.06
		N. weight:	1.8
		Sc. PD:	-0.023
		Sc. rank:	591.2

PA

Pos.	673	obs:	exp:
cgt	R	0	46.04
cgc	R	0	53.10
cga	R	0	104.90
cgg	R	1	89.45
aaa	K	0	0.58
aag	K	1	0.42
aga	R	1	490.20
agg	R	1070	288.30
ggt	G	0	0.13
ggc	G	0	0.13
gga	G	0	0.46
ggg	G	1	0.28
mPD		0.0074	0.96
		nPD:	0.01
		N. weight:	1.4
		Sc. PD:	-0.088
		Sc. rank:	-630.5

PA

Pos.	674	obs:	exp:
gat	D	1043	580.40
gac	D	31	493.60
mPD		0.056	0.50
		nPD:	0.11
		N. weight:	0.54
		Sc. PD:	0.021
		Sc. rank:	311.3

PA

Pos.	675	obs:	exp:
tct	S	0	0.16
tcc	S	0	0.13
tca	S	0	0.25
tcg	S	0	0.06
aat	N	13	546.50
aac	N	1060	526.50
agt	S	0	0.21
agc	S	1	0.19
mPD		0.026	0.50
		nPD:	0.05
		N. weight:	0.71
		Sc. PD:	-0.014
		Sc. rank:	201.4

PA

Pos.	676	obs:	exp:
tta	L	0	95.31
ttg	L	1	200.00
ctt	L	2	178.80
ctc	L	0	179.20
cta	L	6	181.00
ctg	L	1065	239.70
mPD		0.017	1.1
		nPD:	0.02
		N. weight:	1.6
		Sc. PD:	-0.087
		Sc. rank:	-341.3

PA

Pos.	677	obs:	exp:
gaa	E	1068	653.50
gag	E	6	420.50
mPD		0.011	0.48
		nPD:	0.02
		N. weight:	0.51
		Sc. PD:	-0.024
		Sc. rank:	-28.8

PA

Pos.	678	obs:	exp:
cct	P	1074	268.00
ccc	P	0	193.00
cca	P	0	442.10
ccg	P	0	171.00
mPD		0	0.71
		nPD:	0.
		N. weight:	1.5
		Sc. PD:	-0.11
		Sc. rank:	-1283.8

PA			
Pos.	679	obs:	exp:
gat	D	0	0.54
gac	D	1	0.46
ggt	G	0	142.20
ggc	G	0	137.10
gga	G	1063	495.00
ggg	G	10	298.70
---	--	------	------
mPD		0.022	0.68
		nPD:	0.03
		N. weight:	0.79
		Sc. PD:	-0.03
		Sc. rank:	79.3

PA			
Pos.	680	obs:	exp:
act	T	38	273.80
acc	T	1035	220.40
aca	T	1	492.60
acg	T	0	87.24
---	--	------	------
mPD		0.070	0.68
		nPD:	0.1
		N. weight:	1.5
		Sc. PD:	0.048
		Sc. rank:	850.9

PA			
Pos.	681	obs:	exp:
ttt	F	738	424.10
ttc	F	336	649.90
---	--	------	------
mPD		0.43	0.48
		nPD:	0.9
		N. weight:	0.19
		Sc. PD:	0.16
		Sc. rank:	165.4

PA			
Pos.	682	obs:	exp:
aat	N	2	1.02
aac	N	0	0.98
gat	D	1072	579.30
gac	D	0	492.70
---	--	------	------
mPD		0.0037	0.50
		nPD:	0.01
		N. weight:	0.67
		Sc. PD:	-0.042
		Sc. rank:	-322.0

PA			
Pos.	683	obs:	exp:
tta	L	0	95.22
ttg	L	0	199.80
ctt	L	1073	178.70
ctc	L	0	179.10
cta	L	0	180.80
ctg	L	0	239.40
att	I	1	0.37
atc	I	0	0.24
ata	I	0	0.39
---	--	------	------
mPD		0.0019	1.1
		nPD:	0.
		N. weight:	2.
		Sc. PD:	-0.13
		Sc. rank:	-1358.6

PA			
Pos.	684	obs:	exp:
aaa	K	0	0.58
aag	K	1	0.42
gct	A	0	0.26
gcc	A	0	0.19
gca	A	1	0.47
gcg	A	0	0.08
gaa	E	712	634.70
gag	E	331	408.30
ggt	G	0	3.84
ggc	G	0	3.70
gga	G	24	13.38
ggg	G	5	8.07
---	--	------	------
mPD		0.49	0.54
		nPD:	0.9
		N. weight:	0.031
		Sc. PD:	0.025
		Sc. rank:	26.8

PA			
Pos.	685	obs:	exp:
ggt	G	0	142.40
ggc	G	0	137.20
gga	G	0	495.50
ggg	G	1074	299.00
---	--	------	------
mPD		0	0.68
		nPD:	0.
		N. weight:	1.4
		Sc. PD:	-0.097
		Sc. rank:	-1182.6

PA			
Pos.	686	obs:	exp:
tta	L	1	95.22
ttg	L	0	199.80
ctt	L	0	178.70
ctc	L	0	179.10
cta	L	1072	180.80
ctg	L	0	239.40
gtt	V	0	0.21
gtc	V	0	0.20
gta	V	1	0.20
gtg	V	0	0.39
---	--	------	------
mPD		0.0037	1.1
		nPD:	0.
		N. weight:	1.9
		Sc. PD:	-0.13
		Sc. rank:	-1198.1

PA			
Pos.	687	obs:	exp:
tat	Y	1073	580.10
tac	Y	1	493.90
---	--	------	------
mPD		0.0019	0.50
		nPD:	0.
		N. weight:	0.66
		Sc. PD:	-0.044
		Sc. rank:	-386.0

PA			
Pos.	688	obs:	exp:
gat	D	1	1.08
gac	D	1	0.92
gaa	E	1063	646.80
gag	E	0	416.20
ggt	G	0	1.19
ggc	G	0	1.15
gga	G	9	4.15
ggg	G	0	2.51
---	--	------	------
mPD		0.020	0.50
		nPD:	0.04
		N. weight:	0.54
		Sc. PD:	-0.016
		Sc. rank:	110.9

PA			
Pos.	689	obs:	exp:
gct	A	0	277.30
gcc	A	0	200.00
gca	A	1074	505.50
gcg	A	0	91.15
---	--	------	------
mPD		0	0.67
		nPD:	0.
		N. weight:	0.82
		Sc. PD:	-0.057
		Sc. rank:	-696.9

PA			
Pos.	690	obs:	exp:
att	I	24	394.20
atc	I	1049	257.10
ata	I	0	421.60
gtt	V	0	0.21
gtc	V	1	0.20
gta	V	0	0.20
gtg	V	0	0.39
---	--	------	------
mPD		0.046	0.66
		nPD:	0.07
		N. weight:	1.4
		Sc. PD:	-0.0031
		Sc. rank:	554.7

PA			
Pos.	691	obs:	exp:
gaa	E	0	653.50
gag	E	1074	420.50
---	--	------	------
mPD		0	0.48
		nPD:	0.
		N. weight:	1.
		Sc. PD:	-0.071
		Sc. rank:	-867.2

PA			
Pos.	692	obs:	exp:
gaa	E	0	653.50
gag	E	1074	420.50
---	--	------	------
mPD		0	0.48
		nPD:	0.
		N. weight:	1.
		Sc. PD:	-0.071
		Sc. rank:	-867.2

PA			
Pos.	693	obs:	exp:
tgt	C	0	454.30
tgc	C	1074	619.70
---	--	------	------
mPD		0	0.49
		nPD:	0.
		N. weight:	0.6
		Sc. PD:	-0.042
		Sc. rank:	-508.5

PA			
Pos.	694	obs:	exp:
tta	L	0	95.22
ttg	L	0	199.80
ctt	L	0	178.70
ctc	L	0	179.10
cta	L	0	180.80
ctg	L	1073	239.40
cct	P	0	0.25
ccc	P	0	0.18
cca	P	0	0.41
ccg	P	1	0.16
---	--	------	------
mPD		0.0019	1.1
		nPD:	0.
		N. weight:	1.6
		Sc. PD:	-0.11
		Sc. rank:	-1145.1

PA			
Pos.	695	obs:	exp:
att	I	1074	394.60
atc	I	0	257.40
ata	I	0	422.00
---	--	------	------
mPD		0	0.65
		nPD:	0.
		N. weight:	1.1
		Sc. PD:	-0.076
		Sc. rank:	-925.9

PA			
Pos.	696	obs:	exp:
aat	N	1074	547.10
aac	N	0	526.90
---	--	------	------
mPD		0	0.50
		nPD:	0.
		N. weight:	0.73
		Sc. PD:	-0.051
		Sc. rank:	-623.9

PA			
Pos.	697	obs:	exp:
gat	D	1070	580.40
gac	D	4	493.60
---	--	------	------
mPD		0.0074	0.50
		nPD:	0.01
		N. weight:	0.65
		Sc. PD:	-0.036
		Sc. rank:	-142.0

PA			
Pos.	698	obs:	exp:
cct	P	0	268.00
ccc	P	1074	193.00
cca	P	0	442.10
ccg	P	0	171.00
---	--	------	------
mPD		0	0.71
		nPD:	0.
		N. weight:	1.9
		Sc. PD:	-0.13
		Sc. rank:	-1587.5

PA			
Pos.	699	obs:	exp:
tgg	W	1074	1074.00
---	--	------	------
mPD		0	0
		nPD:	1.
		N. weight:	0.
		Sc. PD:	0
		Sc. rank:	0

PA			
Pos.	700	obs:	exp:
gtt	V	1074	222.50
gtc	V	0	216.80
gta	V	0	219.90
gtg	V	0	414.80
---	--	------	------
mPD		0	0.73
		nPD:	0.
		N. weight:	1.7
		Sc. PD:	-0.12
		Sc. rank:	-1455.7

PA			
Pos.	701	obs:	exp:
tta	L	0	95.31
ttg	L	1074	200.00
ctt	L	0	178.80
ctc	L	0	179.20
cta	L	0	181.00
ctg	L	0	239.70
---	--	------	------
mPD		0	1.1
		nPD:	0.
		N. weight:	1.8
		Sc. PD:	-0.13
		Sc. rank:	-1554.6

PA			
Pos.	702	obs:	exp:
tta	L	0	95.31
ttg	L	0	200.00
ctt	L	1073	178.80
ctc	L	1	179.20
cta	L	0	181.00
ctg	L	0	239.70
---	--	------	------
mPD		0.0019	1.1
		nPD:	0.
		N. weight:	1.9
		Sc. PD:	-0.13
		Sc. rank:	-1345.3

PA

Pos.	703	obs:	exp:
aat	N	1072	546.50
aac	N	1	526.50
ggt	G	1	0.13
ggc	G	0	0.13
gga	G	0	0.46
ggg	G	0	0.28
---	--	------	------
mPD		0.0056	0.50
		nPD:	0.01
		N. weight:	0.73
		Sc. PD:	-0.043
		Sc. rank:	-244.4

,

PA

Pos.	704	obs:	exp:
gct	A	0	277.30
gcc	A	1	200.00
gca	A	1065	505.50
gcg	A	8	91.15
---	--	------	------
mPD		0.017	0.67
		nPD:	0.02
		N. weight:	0.78
		Sc. PD:	-0.036
		Sc. rank:	-12.8

,

PA

Pos.	705	obs:	exp:
tct	S	1072	170.80
tcc	S	2	140.30
tca	S	0	272.60
tcg	S	0	60.94
agt	S	0	220.80
agc	S	0	208.60
---	--	------	------
mPD		0.0037	1.7
		nPD:	0.
		N. weight:	2.
		Sc. PD:	-0.13
		Sc. rank:	-1340.3

,

PA

Pos.	706	obs:	exp:
tgg	W	1074	1074.00
---	--	------	------
mPD		0	0
		nPD:	1.
		N. weight:	0.
		Sc. PD:	0
		Sc. rank:	0

,

PA

Pos.	707	obs:	exp:
ttt	F	1	424.10
ttc	F	1073	649.90
---	--	------	------
mPD		0.0019	0.48
		nPD:	0.
		N. weight:	0.54
		Sc. PD:	-0.036
		Sc. rank:	-303.7

,

PA

Pos.	708	obs:	exp:
aat	N	0	547.10
aac	N	1074	526.90
---	--	------	------
mPD		0	0.50
		nPD:	0.
		N. weight:	0.78
		Sc. PD:	-0.054
		Sc. rank:	-658.5

,

PA

Pos.	709	obs:	exp:
tct	S	0	170.70
tcc	S	1073	140.10
tca	S	0	272.30
tcg	S	0	60.89
act	T	0	0.25
acc	T	1	0.21
aca	T	0	0.46
acg	T	0	0.08
agt	S	0	220.60
agc	S	0	208.40
---	--	------	------
mPD		0.0019	1.7
		nPD:	0.
		N. weight:	2.2
		Sc. PD:	-0.15
		Sc. rank:	-1606.7

,

PA

Pos.	710	obs:	exp:
ttt	F	0	424.10
ttc	F	1074	649.90
---	--	------	------
mPD		0	0.48
		nPD:	0.
		N. weight:	0.55
		Sc. PD:	-0.038
		Sc. rank:	-464.6

,

PA

Pos.	711	obs:	exp:
tta	L	0	95.31
ttg	L	0	200.00
ctt	L	0	178.80
ctc	L	1074	179.20
cta	L	0	181.00
ctg	L	0	239.70
---	--	------	------
mPD		0	1.1
		nPD:	0.
		N. weight:	2.
		Sc. PD:	-0.14
		Sc. rank:	-1655.8

,

PA

Pos.	712	obs:	exp:
att	I	0	0.37
atc	I	0	0.24
ata	I	1	0.39
act	T	0	272.20
acc	T	2	219.10
aca	T	1066	489.90
acg	T	0	86.75
gct	A	0	1.29
gcc	A	0	0.93
gca	A	5	2.35
gcg	A	0	0.42
---	--	------	------
mPD		0.015	0.69
		nPD:	0.02
		N. weight:	0.84
		Sc. PD:	-0.041
		Sc. rank:	-72.7

,

PA

Pos.	713	obs:	exp:
cat	H	1073	616.70
cac	H	1	457.30
---	--	------	------
mPD		0.0019	0.49
		nPD:	0.
		N. weight:	0.6
		Sc. PD:	-0.039
		Sc. rank:	-343.2

,

PA

Pos.	714	obs:	exp:
gtt	V	0	0.21
gtc	V	0	0.20
gta	V	1	0.20
gtg	V	0	0.39
gct	A	0	276.80
gcc	A	0	199.60
gca	A	1072	504.60
gcg	A	0	90.98
gaa	E	1	0.61
gag	E	0	0.39
---	--	------	------
mPD		0.0037	0.67
		nPD:	0.01
		N. weight:	0.82
		Sc. PD:	-0.053
		Sc. rank:	-431.3

,

PA

Pos.	715	obs:	exp:
tta	L	1	95.31
ttg	L	0	200.00
ctt	L	0	178.80
ctc	L	0	179.20
cta	L	1031	181.00
ctg	L	42	239.70
---	--	------	------
mPD		0.077	1.1
		nPD:	0.07
		N. weight:	1.7
		Sc. PD:	-0.004
		Sc. rank:	669.7

,

PA

Pos.	716	obs:	exp:
tct	S	0	1.43
tcc	S	0	1.18
tca	S	0	2.28
tcg	S	0	0.51
cgt	R	0	45.31
cgc	R	0	52.25
cga	R	0	103.20
cgg	R	0	88.03
att	I	0	0.37
atc	I	0	0.24
ata	I	1	0.39
aaa	K	5	2.91
aag	K	0	2.09
agt	S	0	1.85
agc	S	9	1.75
aga	R	1055	482.40
agg	R	0	283.80
ggt	G	0	0.53
ggc	G	0	0.51
gga	G	4	1.85
ggg	G	0	1.11
---	--	------	------
mPD		0.035	0.99
		nPD:	0.04
		N. weight:	0.86
		Sc. PD:	-0.03
		Sc. rank:	107.2

,

PA

Pos.	717	obs:	exp:
taa	.	14	456.30
tag	.	1060	268.90
tga	.	0	348.90
---	--	------	------
mPD		0.026	0.81
		nPD:	0.03
		N. weight:	1.4
		Sc. PD:	-0.056
		Sc. rank:	125.1

}

Amino acid level conservation per codon

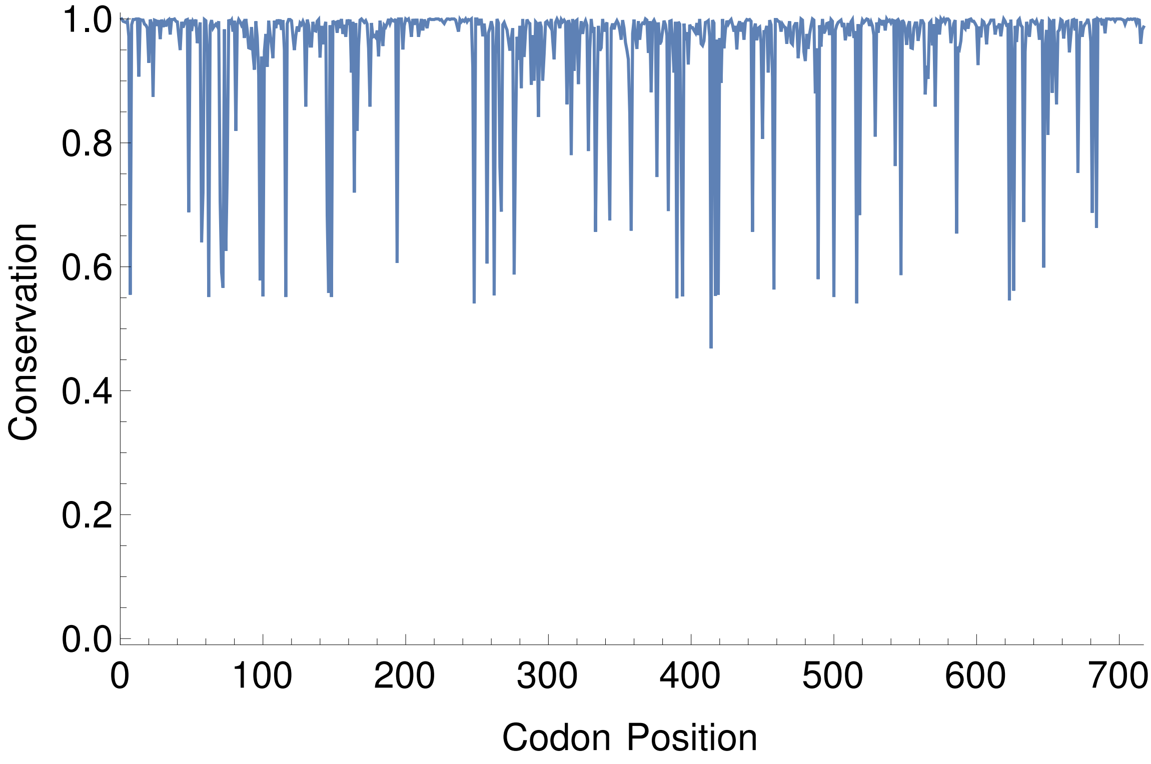

Conservation analysis plots

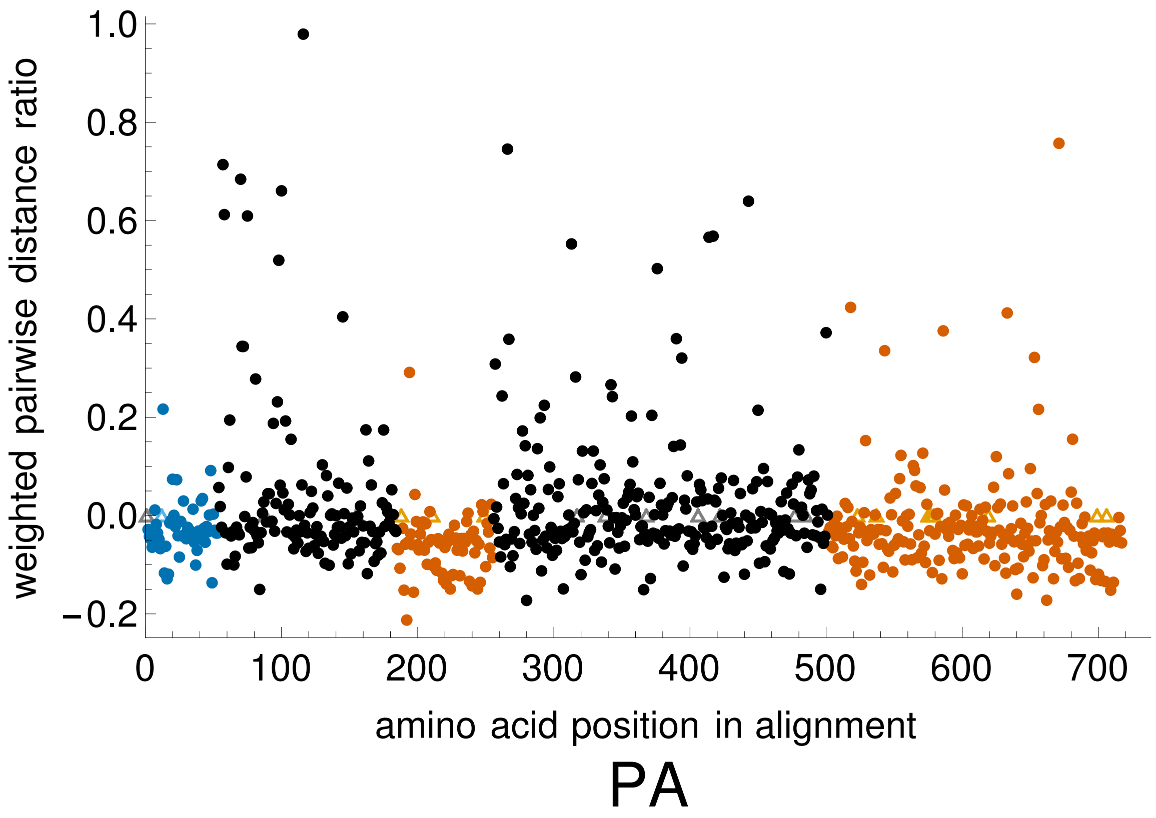

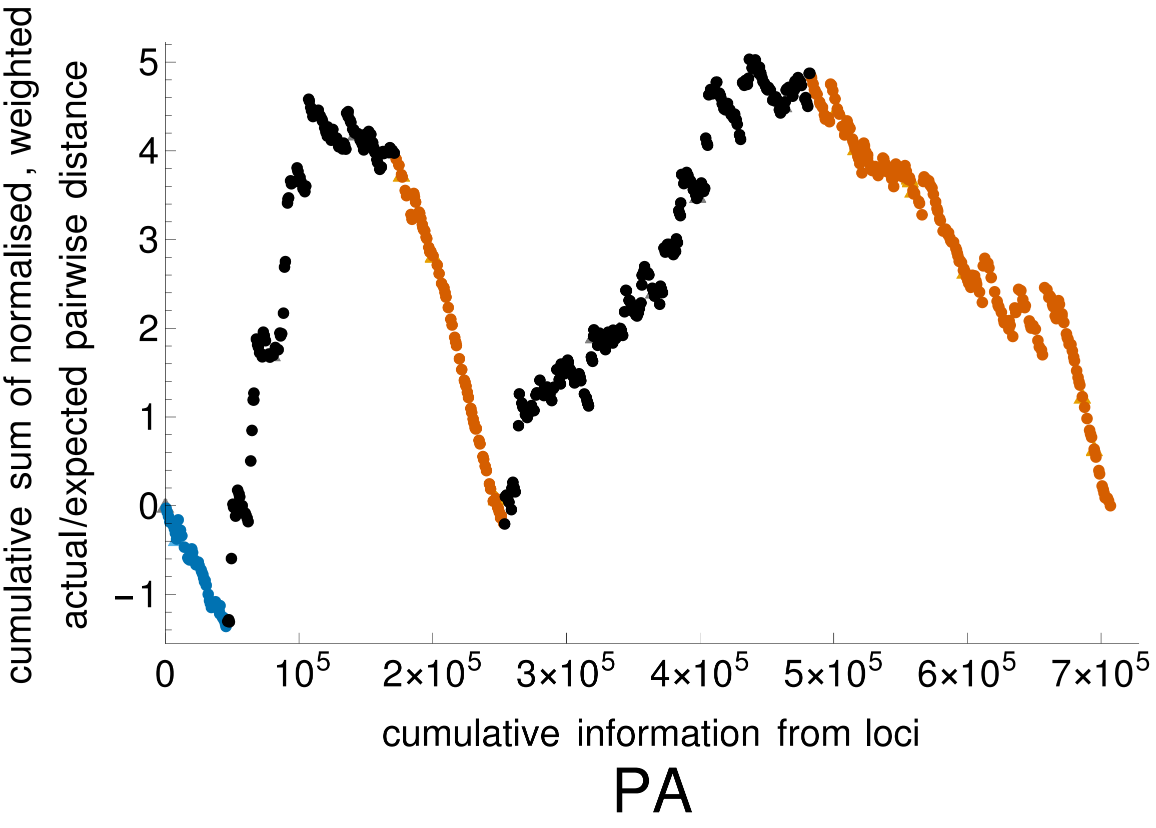

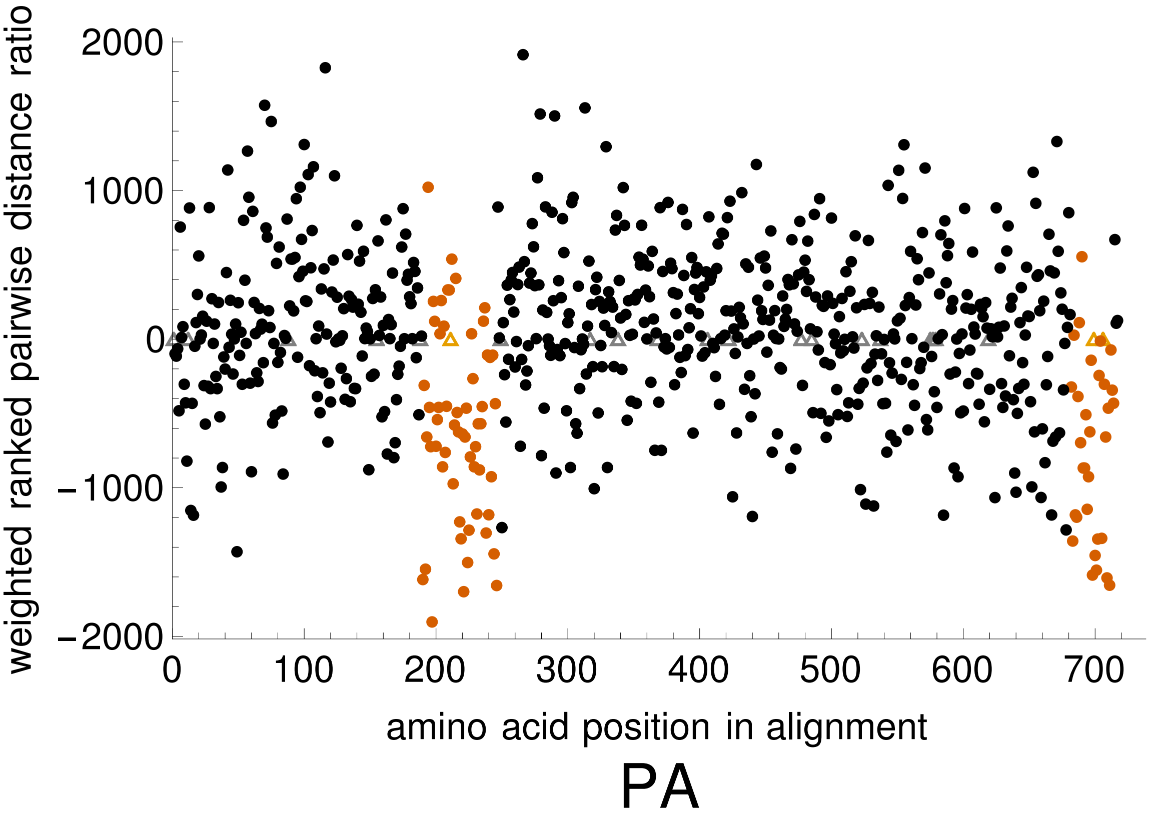

weighted ranked pairwise distance ratio
2000
1000
0
−1000
−2000
0
100
200
300
400
500
600
700
amino acid position in alignment
PA

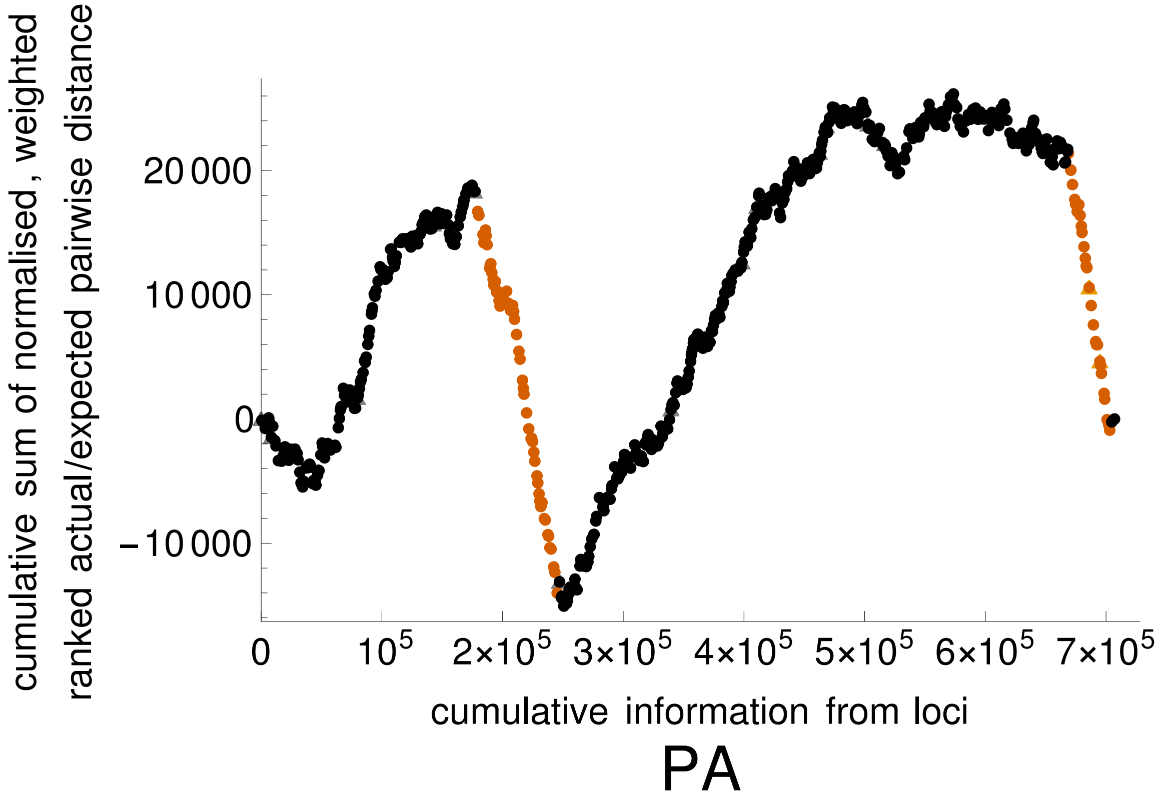

cumulative sum of normalised, weighted ranked actual/expected pairwise distance
20 000
10 000
0
−10 000
0
10^5
2×10^5
3×10^5
4×10^5
5×10^5
6×10^5
7×10^5
cumulative information from loci
PA

PA-X

Gene length histogram

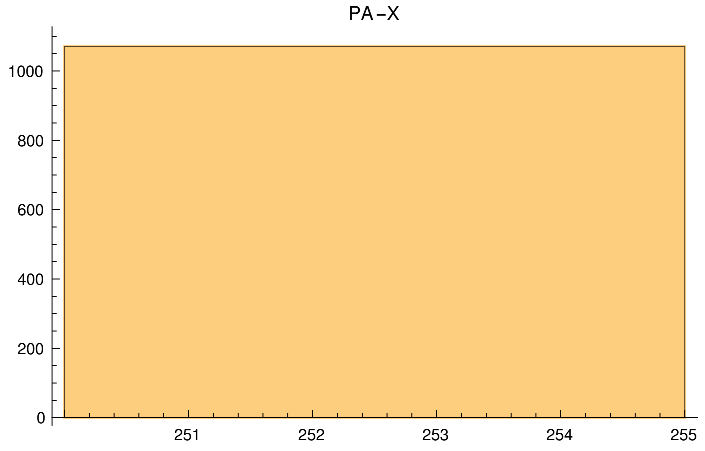

Information vs. nPD

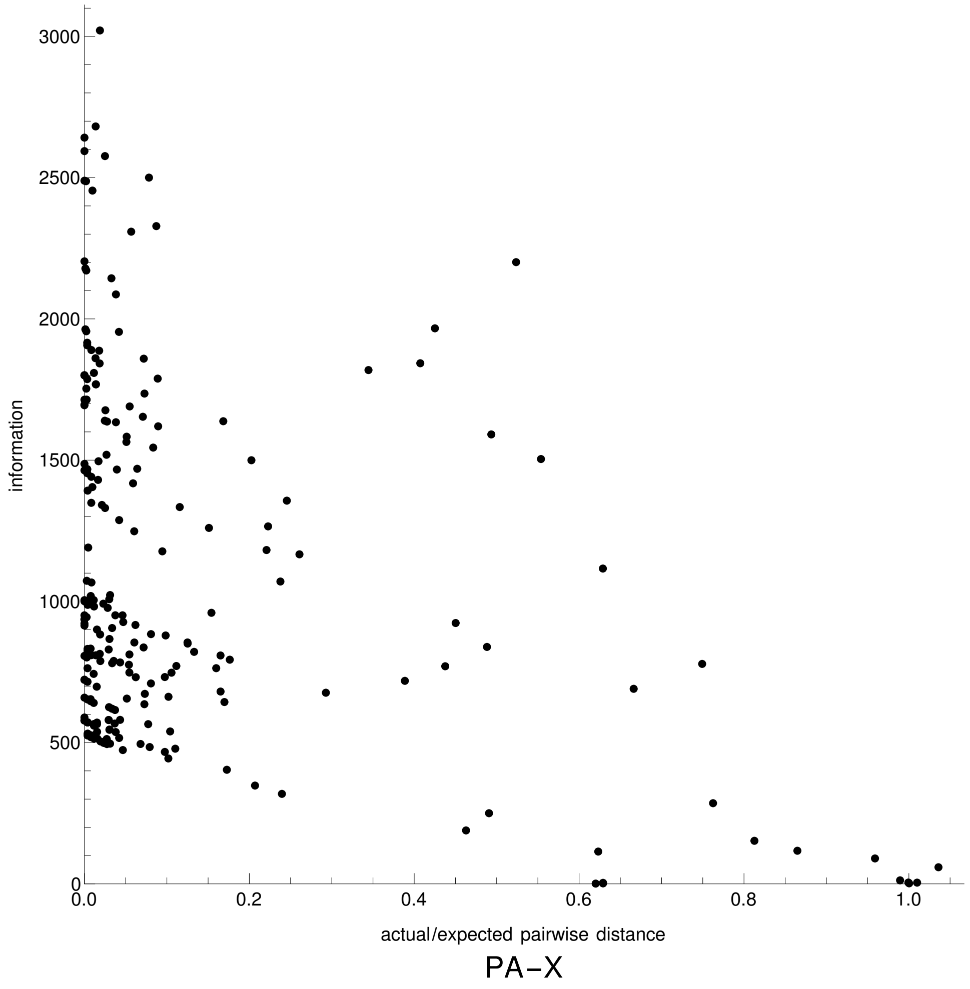

Example sequences highlighted by regions found in analyses to be conserved

Interesting points (by weighted raw PD) highlighted for gene PA-X:

ATG
GAAGACTTTGTGCGACAATGCTTCAATCCAATGATCGTCGAGCTTGCGGAAAAGG
CAATGAAAGAGTATGGGGAAGATCCGAAAATCGAAACAAACAAATTCGCATC
AATATGCACACACTTAGAAGTCTGCTTCATGTATTCTGATTTCCACTTCATC⋱
GACGAACGGGGCGAATCAACTATTATAGAATCTGGCGATCCAAATGTGTTGTTGAAACATCGATTTGAAA⋱
TAATCGAAGGAAGAGACCGAACAATGGCCTGGACAGTGGTGAATAGTATCTGCAACACCACGGGAGTCGA⋱
AAAACCCAAATTTCTCCCGGATCTGTATGACTACAAGGAAAACCGA
TTCATTGAAATTGGAGTGACGAGGAGGGAAGTCCACATATATTACCTAGAGAAAG
CCAATAAAATAAAATCCGAGAAGACACACATACACATTTTCTCATTCACTGG
AGAAGAGATGGCCACCAAAGCAGATTACACTCTTGATGAAGAGAGCAGGGCA
AGAATCAAAACCAGGTTGTTCACCATAAGGCAGGAAATGGCCAGCAGGGGTC
TATGGGATTCCTTTGTCAGTCCG
AAAGAGGCGAAGAAACAATTGAAGAAAGATTTGAAATCACAGGAACCATGCGCAG
GCTTGCCGACCAAAGTCTCCCACCGAACTTCTCCAGCCTTGAAAACTTTAGA
GCCTATGTGGATGGATTCGAACCGAACGGCTGCATTGAGGGCAAGC
TTTCTCAAATGTCAAAAGAAGTGA

Interesting points (by weighted ranked PD) highlighted for gene PA-X:

ATGGAAGACTTTGTGCGACAATGCTTCAATCCAATGATCGTCGAGCTTGCGGAAAAGGCAATGAAAGAGTATGGGGAA⋱
GATCCGAAAATCGAAACAAACAAATTCGCATCAATATGCACACACTTAGAAGTCTGCTTCATGTATTCTGATTTCCA⋱
CTTCATCGACGAACGGGGCGAATCAACTATTATAGAATCTGGCGATCCAAATGTGTTGTTGAAACATCGATTTGAAA⋱
TAATCGAAGGAAGAGACCGAACAATGGCCTGGACAGTGGTGAATAGTATCTGCAACACCACGGGAGTCGAAAAACCC⋱
AAATTTCTCCCGGATCTGTATGACTACAAGGAAAACCGATTCATTGAAATTGGAGTGACGAGGAGGGAAGTCCACAT⋱
ATATTACCTAGAGAAAGCCAATAAAATAAAATCCGAGAAGACACACATACACATTTTCTCATTCACTGGAGAAGAGA⋱
TGGCCACCAAAGCAGATTACACTCTTGATGAAGAGAGCAGGGCAAGAATCAAAACCAGGTTGTTCACCATAAGGCAG⋱
GAAATGGCCAGCAGGGGTCTATGGGAT
TCCTTTGTCAGTCCGAAAGAGGCGAAGAAACAATTGAAGAAAGATTTGAAATCAC
AGGAACCATGCGCAGGCTTGCCGACCAAAGTCTCCCACCGAACTTCTCCAGC
CTTGAAAACTTTAGAGCCTATGTGGATGGATTCGAACCGAACGGCTGCATTG
AGGGCAAGCTTTCTCAAATGTCAAAAGAAGTGA

Per codon conservation report

PA-X

Pos. 1		obs:	exp:
atg	M	1071	1071.00
---	--	------	------
mPD		0	0
		nPD:	1.
		N. weight:	0.
		Sc. PD:	0
		Sc. rank:	0

PA-X

Pos. 2		obs:	exp:
gat	D	1	0.54
gac	D	0	0.46
gaa	E	1067	651.10
gag	E	3	418.90
---	--	------	------
mPD		0.0075	0.48
		nPD:	0.02
		N. weight:	0.5
		Sc. PD:	-0.027
		Sc. rank:	-31.1

PA-X

Pos. 3		obs:	exp:
aat	N	0	0.51
aac	N	1	0.49
gat	D	3	577.70
gac	D	1066	491.30
ggt	G	0	0.13
ggc	G	1	0.13
gga	G	0	0.46
ggg	G	0	0.28
---	--	------	------
mPD		0.0093	0.50
		nPD:	0.02
		N. weight:	0.79
		Sc. PD:	-0.04
		Sc. rank:	-8.6

PA-X

Pos. 4		obs:	exp:
ttt	F	1065	421.00
ttc	F	1	645.00
tta	L	0	0.35
ttg	L	0	0.74
ctt	L	4	0.67
ctc	L	0	0.67
cta	L	0	0.67
ctg	L	0	0.89
gtt	V	1	0.21
gtc	V	0	0.20
gta	V	0	0.20
gtg	V	0	0.39
---	--	------	------
mPD		0.011	0.49
		nPD:	0.02
		N. weight:	0.97
		Sc. PD:	-0.045
		Sc. rank:	39.5

PA-X

Pos. 5		obs:	exp:
tta	L	0	0.09
ttg	L	1	0.19
ctt	L	0	0.17
ctc	L	0	0.17
cta	L	0	0.17
ctg	L	0	0.22
atg	M	1	1.00
gtt	V	0	221.30
gtc	V	0	215.50
gta	V	0	218.70
gtg	V	1068	412.50
ggt	G	0	0.13
ggc	G	0	0.13
gga	G	0	0.46
ggg	G	1	0.28
---	--	------	------
mPD		0.0056	0.73
		nPD:	0.01
		N. weight:	0.99
		Sc. PD:	-0.061
		Sc. rank:	-286.4

PA-X

Pos. 6		obs:	exp:
cgt	R	0	46.00
cgc	R	0	53.05
cga	R	1041	104.80
cgg	R	0	89.37
aga	R	30	489.70
agg	R	0	288.10
---	--	------	------
mPD		0.055	0.96
		nPD:	0.06
		N. weight:	2.3
		Sc. PD:	-0.031
		Sc. rank:	905.3

PA-X

Pos. 7		obs:	exp:
caa	Q	477	545.50
cag	Q	594	525.50
---	--	------	------
mPD		0.49	0.50
		nPD:	0.99
		N. weight:	0.012
		Sc. PD:	0.011
		Sc. rank:	11.0

PA-X

Pos. 8		obs:	exp:
tgt	C	10	453.00
tgc	C	1061	618.00
---	--	------	------
mPD		0.019	0.49
		nPD:	0.04
		N. weight:	0.52
		Sc. PD:	-0.017
		Sc. rank:	128.8

PA-X

Pos. 9		obs:	exp:
ttt	F	1	422.90
ttc	F	1070	648.10
---	--	------	------
mPD		0.0019	0.48
		nPD:	0.
		N. weight:	0.52
		Sc. PD:	-0.034
		Sc. rank:	-183.4

PA-X

Pos. 10		obs:	exp:
aat	N	1070	545.50
aac	N	1	525.50
---	--	------	------
mPD		0.0019	0.50
		nPD:	0.
		N. weight:	0.7
		Sc. PD:	-0.046
		Sc. rank:	-280.1

PA-X

Pos. 11		obs:	exp:
cct	P	0	267.20
ccc	P	0	192.40
cca	P	1071	440.80
ccg	P	0	170.50
---	--	------	------
mPD		0	0.71
		nPD:	0.
		N. weight:	0.93
		Sc. PD:	-0.064
		Sc. rank:	-805.4

PA-X

Pos. 12		obs:	exp:
atg	M	1071	1071.00
---	--	------	------
mPD		0	0
		nPD:	1.
		N. weight:	0.
		Sc. PD:	0
		Sc. rank:	0

PA-X

Pos. 13		obs:	exp:
tta	L	0	0.09
ttg	L	0	0.19
ctt	L	0	0.17
ctc	L	1	0.17
cta	L	0	0.17
ctg	L	0	0.22
att	I	97	392.40
atc	I	971	255.90
ata	I	0	419.70
gtt	V	1	0.41
gtc	V	1	0.40
gta	V	0	0.41
gtg	V	0	0.77
---	--	------	------
mPD		0.17	0.66
		nPD:	0.26
		N. weight:	1.1
		Sc. PD:	0.21
		Sc. rank:	877.7

PA-X

Pos. 14		obs:	exp:
att	I	0	0.37
atc	I	1	0.24
ata	I	0	0.39
gtt	V	0	221.70
gtc	V	1070	216.00
gta	V	0	219.10
gtg	V	0	413.20
---	--	------	------
mPD		0.0019	0.73
		nPD:	0.
		N. weight:	1.7
		Sc. PD:	-0.11
		Sc. rank:	-889.6

PA-X

Pos. 15		obs:	exp:
gaa	E	2	651.70
gag	E	1069	419.30
---	--	------	------
mPD		0.0037	0.48
		nPD:	0.01
		N. weight:	0.97
		Sc. PD:	-0.059
		Sc. rank:	-266.9

PA-X

Pos. 16		obs:	exp:
tta	L	0	95.04
ttg	L	0	199.40
ctt	L	1069	178.30
ctc	L	2	178.70
cta	L	0	180.50
ctg	L	0	239.00
---	--	------	------
mPD		0.0037	1.1
		nPD:	0.
		N. weight:	1.9
		Sc. PD:	-0.12
		Sc. rank:	-844.7

PA-X

Pos. 17		obs:	exp:
gct	A	0	276.60
gcc	A	0	199.50
gca	A	9	504.10
gcg	A	1062	90.90
---	--	------	------
mPD		0.017	0.67
		nPD:	0.02
		N. weight:	2.5
		Sc. PD:	-0.11
		Sc. rank:	201.1

PA-X

Pos. 18		obs:	exp:
gat	D	0	4.32
gac	D	8	3.68
gaa	E	1060	646.80
gag	E	3	416.20
---	--	------	------
mPD		0.020	0.48
		nPD:	0.04
		N. weight:	0.5
		Sc. PD:	-0.014
		Sc. rank:	150.2

PA-X

Pos. 19		obs:	exp:
aat	N	1	0.51
aac	N	0	0.49
aaa	K	15	623.60
aag	K	1055	446.40
---	--	------	------
mPD		0.029	0.49
		nPD:	0.06
		N. weight:	0.83
		Sc. PD:	-0.0085
		Sc. rank:	355.2

PA-X

Pos. 20		obs:	exp:
act	T	0	18.86
acc	T	0	15.18
aca	T	74	33.94
acg	T	0	6.01
gct	A	0	256.90
gcc	A	0	185.30
gca	A	995	468.30
gcg	A	0	84.45
gaa	E	2	1.22
gag	E	0	0.78
---	--	------	------
mPD		0.13	0.80
		nPD:	0.17
		N. weight:	0.79
		Sc. PD:	0.072
		Sc. rank:	541.4

PA-X			
Pos.	21	obs:	exp:
atg	M	1070	1070.00
gtt	V	0	0.21
gtc	V	0	0.20
gta	V	0	0.20
gtg	V	1	0.39
mPD		0.0019	0.0030
		nPD:	0.62
		N. weight:	0.00093
		Sc. PD:	0.00049
		Sc. rank:	0.8

PA-X			
Pos.	22	obs:	exp:
cgt	R	0	0.09
cgc	R	0	0.10
cga	R	0	0.20
cgg	R	0	0.17
aaa	K	1063	623.00
aag	K	6	446.00
aga	R	2	0.91
agg	R	0	0.54
mPD		0.015	0.49
		nPD:	0.03
		N. weight:	0.53
		Sc. PD:	-0.021
		Sc. rank:	91.2

PA-X			
Pos.	23	obs:	exp:
gaa	E	936	651.70
gag	E	135	419.30
mPD		0.22	0.48
		nPD:	0.46
		N. weight:	0.18
		Sc. PD:	0.07
		Sc. rank:	153.6

PA-X			
Pos.	24	obs:	exp:
tat	Y	1069	578.40
tac	Y	2	492.60
mPD		0.0037	0.50
		nPD:	0.01
		N. weight:	0.63
		Sc. PD:	-0.039
		Sc. rank:	-186.2

PA-X			
Pos.	25	obs:	exp:
ggt	G	0	142.00
ggc	G	0	136.80
gga	G	3	494.10
ggg	G	1068	298.20
mPD		0.0056	0.68
		nPD:	0.01
		N. weight:	1.3
		Sc. PD:	-0.08
		Sc. rank:	-331.9

PA-X			
Pos.	26	obs:	exp:
gaa	E	1059	651.70
gag	E	12	419.30
mPD		0.022	0.48
		nPD:	0.05
		N. weight:	0.46
		Sc. PD:	-0.011
		Sc. rank:	150.7

PA-X			
Pos.	27	obs:	exp:
gat	D	1069	578.20
gac	D	1	491.80
ggt	G	1	0.13
ggc	G	0	0.13
gga	G	0	0.46
ggg	G	0	0.28
mPD		0.0037	0.50
		nPD:	0.01
		N. weight:	0.64
		Sc. PD:	-0.039
		Sc. rank:	-191.0

PA-X			
Pos.	28	obs:	exp:
tct	S	0	0.32
tcc	S	0	0.26
tca	S	0	0.51
tcg	S	2	0.11
cct	P	1	266.70
ccc	P	1	192.10
cca	P	31	440.00
ccg	P	1036	170.20
agt	S	0	0.41
agc	S	0	0.39
mPD		0.064	0.72
		nPD:	0.09
		N. weight:	1.7
		Sc. PD:	0.03
		Sc. rank:	1004.3

PA-X			
Pos.	29	obs:	exp:
aaa	K	1070	624.20
aag	K	1	446.80
mPD		0.0019	0.49
		nPD:	0.
		N. weight:	0.56
		Sc. PD:	-0.036
		Sc. rank:	-209.6

PA-X			
Pos.	30	obs:	exp:
att	I	11	392.40
atc	I	1057	255.90
ata	I	0	419.70
gtt	V	0	0.62
gtc	V	3	0.61
gta	V	0	0.61
gtg	V	0	1.16
mPD		0.026	0.66
		nPD:	0.04
		N. weight:	1.4
		Sc. PD:	-0.044
		Sc. rank:	402.4

PA-X			
Pos.	31	obs:	exp:
gaa	E	1065	651.70
gag	E	6	419.30
mPD		0.011	0.48
		nPD:	0.02
		N. weight:	0.49
		Sc. PD:	-0.023
		Sc. rank:	21.6

PA-X			
Pos.	32	obs:	exp:
act	T	0	273.00
acc	T	0	219.80
aca	T	1058	491.20
acg	T	13	86.99
mPD		0.024	0.68
		nPD:	0.04
		N. weight:	0.77
		Sc. PD:	-0.027
		Sc. rank:	175.0

PA-X			
Pos.	33	obs:	exp:
aat	N	3	545.50
aac	N	1068	525.50
mPD		0.0056	0.50
		nPD:	0.01
		N. weight:	0.73
		Sc. PD:	-0.042
		Sc. rank:	-138.2

PA-X			
Pos.	34	obs:	exp:
aaa	K	1070	624.20
aag	K	1	446.80
mPD		0.0019	0.49
		nPD:	0.
		N. weight:	0.56
		Sc. PD:	-0.036
		Sc. rank:	-209.6

PA-X			
Pos.	35	obs:	exp:
ttt	F	23	421.40
ttc	F	1044	645.60
tta	L	2	0.35
ttg	L	0	0.74
ctt	L	0	0.67
ctc	L	2	0.67
cta	L	0	0.67
ctg	L	0	0.89
mPD		0.049	0.49
		nPD:	0.1
		N. weight:	0.43
		Sc. PD:	0.013
		Sc. rank:	265.1

PA-X			
Pos.	36	obs:	exp:
gct	A	0	276.60
gcc	A	0	199.50
gca	A	1070	504.10
gcg	A	1	90.90
mPD		0.0019	0.67
		nPD:	0.
		N. weight:	0.78
		Sc. PD:	-0.052
		Sc. rank:	-385.5

PA-X			
Pos.	37	obs:	exp:
tct	S	0	170.20
tcc	S	0	139.70
tca	S	1070	271.60
tcg	S	0	60.72
agt	S	0	220.00
agc	S	0	207.80
gct	A	0	0.26
gcc	A	0	0.19
gca	A	0	0.47
gcg	A	1	0.08
mPD		0.0037	1.7
		nPD:	0.
		N. weight:	1.4
		Sc. PD:	-0.096
		Sc. rank:	-801.0

PA-X			
Pos.	38	obs:	exp:
att	I	0	393.50
atc	I	0	256.70
ata	I	1071	420.80
mPD		0	0.65
		nPD:	0.
		N. weight:	0.98
		Sc. PD:	-0.067
		Sc. rank:	-847.5

PA-X			
Pos.	39	obs:	exp:
tgt	C	4	453.00
tgc	C	1067	618.00
mPD		0.0074	0.49
		nPD:	0.02
		N. weight:	0.55
		Sc. PD:	-0.03
		Sc. rank:	-38.2

PA-X			
Pos.	40	obs:	exp:
act	T	0	273.00
acc	T	2	219.80
aca	T	1066	491.20
acg	T	3	86.99
mPD		0.0093	0.68
		nPD:	0.01
		N. weight:	0.79
		Sc. PD:	-0.044
		Sc. rank:	-88.6

PA-X			
Pos.	41	obs:	exp:
cat	H	30	615.00
cac	H	1041	456.00
mPD		0.055	0.49
		nPD:	0.11
		N. weight:	0.75
		Sc. PD:	0.03
		Sc. rank:	477.0

PA-X			
Pos.	42	obs:	exp:
tta	L	1017	95.04
ttg	L	49	199.40
ctt	L	0	178.30
ctc	L	0	178.70
cta	L	5	180.50
ctg	L	0	239.00
mPD		0.097	1.1
		nPD:	0.09
		N. weight:	2.3
		Sc. PD:	0.036
		Sc. rank:	1279.6

PA-X			
Pos.	43	obs:	exp:
gaa	E	1066	651.70
gag	E	5	419.30
mPD		0.0093	0.48
		nPD:	0.02
		N. weight:	0.49
		Sc. PD:	-0.025
		Sc. rank:	12.2

PA-X			
Pos.	44	obs:	exp:
att	I	0	0.37
atc	I	1	0.24
ata	I	0	0.39
gtt	V	10	221.70
gtc	V	1056	216.00
gta	V	4	219.10
gtg	V	0	413.20
mPD		0.028	0.73
		nPD:	0.04
		N. weight:	1.6
		Sc. PD:	-0.051
		Sc. rank:	409.5

PA-X			
Pos.	45	obs:	exp:
tgt	C	8	453.00
tgc	C	1063	618.00
mPD		0.015	0.49
		nPD:	0.03
		N. weight:	0.53
		Sc. PD:	-0.021
		Sc. rank:	96.5

PA-X

Pos.	46	obs:	exp:
ttt	F	2	422.90
ttc	F	1069	648.10
mPD		0.0037	0.48
		nPD:	0.01
		N. weight:	0.51
		Sc. PD:	-0.031
		Sc. rank:	-144.0

PA-X

Pos.	47	obs:	exp:
tta	L	0	0.09
ttg	L	1	0.19
ctt	L	0	0.17
ctc	L	0	0.17
cta	L	0	0.17
ctg	L	0	0.22
atg	M	1070	1070.00
mPD		0.0019	0.0030
		nPD:	0.63
		N. weight:	0.0016
		Sc. PD:	0.00089
		Sc. rank:	1.5

PA-X

Pos.	48	obs:	exp:
tat	Y	334	578.40
tac	Y	737	492.60
mPD		0.43	0.50
		nPD:	0.86
		N. weight:	0.11
		Sc. PD:	0.088
		Sc. rank:	102.7

PA-X

Pos.	49	obs:	exp:
tct	S	1070	170.30
tcc	S	0	139.90
tca	S	0	271.80
tcg	S	1	60.77
agt	S	0	220.20
agc	S	0	208.00
mPD		0.0019	1.7
		nPD:	0.
		N. weight:	1.9
		Sc. PD:	-0.13
		Sc. rank:	-1170.6

PA-X

Pos.	50	obs:	exp:
cat	H	1	0.57
cac	H	0	0.43
gat	D	1050	578.20
gac	D	20	491.80
mPD		0.039	0.50
		nPD:	0.08
		N. weight:	0.55
		Sc. PD:	0.0034
		Sc. rank:	278.7

PA-X

Pos.	51	obs:	exp:
ttt	F	0	421.40
ttc	F	1067	645.60
tgt	C	0	1.69
tgc	C	4	2.31
mPD		0.0074	0.49
		nPD:	0.02
		N. weight:	0.53
		Sc. PD:	-0.028
		Sc. rank:	-34.4

PA-X

Pos.	52	obs:	exp:
cat	H	8	615.00
cac	H	1063	456.00
mPD		0.015	0.49
		nPD:	0.03
		N. weight:	0.85
		Sc. PD:	-0.033
		Sc. rank:	149.9

PA-X

Pos.	53	obs:	exp:
ttt	F	1	422.90
ttc	F	1070	648.10
mPD		0.0019	0.48
		nPD:	0.
		N. weight:	0.52
		Sc. PD:	-0.034
		Sc. rank:	-183.4

PA-X

Pos.	54	obs:	exp:
att	I	41	393.50
atc	I	1029	256.70
ata	I	1	420.80
mPD		0.075	0.65
		nPD:	0.12
		N. weight:	1.3
		Sc. PD:	0.056
		Sc. rank:	836.9

PA-X

Pos.	55	obs:	exp:
cat	H	0	0.57
cac	H	1	0.43
aat	N	0	3.06
aac	N	6	2.94
gat	D	20	575.00
gac	D	1044	489.00
mPD		0.050	0.51
		nPD:	0.1
		N. weight:	0.71
		Sc. PD:	0.018
		Sc. rank:	428.4

PA-X

Pos.	56	obs:	exp:
gaa	E	1065	651.70
gag	E	6	419.30
mPD		0.011	0.48
		nPD:	0.02
		N. weight:	0.49
		Sc. PD:	-0.023
		Sc. rank:	21.6

PA-X

Pos.	57	obs:	exp:
tta	L	0	0.18
ttg	L	0	0.37
ctt	L	0	0.33
ctc	L	0	0.33
cta	L	0	0.34
ctg	L	2	0.45
caa	Q	29	19.87
cag	Q	10	19.13
cgt	R	50	44.24
cgc	R	9	51.02
cga	R	685	100.80
cgg	R	253	85.94
aga	R	31	471.00
agg	R	2	277.00
mPD		0.59	1.1
		nPD:	0.55
		N. weight:	1.5
		Sc. PD:	0.69
		Sc. rank:	1285.2

PA-X

Pos.	58	obs:	exp:
ggt	G	46	142.00
ggc	G	770	136.80
gga	G	255	494.10
ggg	G	0	298.20
mPD		0.42	0.68
		nPD:	0.63
		N. weight:	1.1
		Sc. PD:	0.59
		Sc. rank:	963.1

PA-X

Pos.	59	obs:	exp:
gaa	E	1070	651.70
gag	E	1	419.30
mPD		0.0019	0.48
		nPD:	0.
		N. weight:	0.51
		Sc. PD:	-0.033
		Sc. rank:	-174.2

PA-X

Pos.	60	obs:	exp:
tct	S	0	169.90
tcc	S	0	139.50
tca	S	1068	271.10
tcg	S	0	60.60
cct	P	0	0.75
ccc	P	0	0.54
cca	P	3	1.23
ccg	P	0	0.48
agt	S	0	219.50
agc	S	0	207.40
mPD		0.0056	1.7
		nPD:	0.
		N. weight:	1.4
		Sc. PD:	-0.094
		Sc. rank:	-617.5

PA-X

Pos.	61	obs:	exp:
att	I	6	2.20
atc	I	0	1.44
ata	I	0	2.36
atg	M	1	1.00
act	T	1014	271.20
acc	T	47	218.30
aca	T	3	488.00
acg	T	0	86.42
mPD		0.10	0.69
		nPD:	0.15
		N. weight:	1.2
		Sc. PD:	0.095
		Sc. rank:	822.4

PA-X

Pos.	62	obs:	exp:
att	I	477	393.10
atc	I	3	256.40
ata	I	590	420.40
gtt	V	1	0.21
gtc	V	0	0.20
gta	V	0	0.20
gtg	V	0	0.39
mPD		0.50	0.66
		nPD:	0.76
		N. weight:	0.28
		Sc. PD:	0.19
		Sc. rank:	249.7

PA-X

Pos.	63	obs:	exp:
att	I	0	389.50
atc	I	0	254.00
ata	I	1060	416.50
gtt	V	0	2.28
gtc	V	0	2.22
gta	V	11	2.25
gtg	V	0	4.25
mPD		0.020	0.68
		nPD:	0.03
		N. weight:	0.98
		Sc. PD:	-0.039
		Sc. rank:	164.7

PA-X

Pos.	64	obs:	exp:
gaa	E	1069	651.70
gag	E	2	419.30
mPD		0.0037	0.48
		nPD:	0.01
		N. weight:	0.51
		Sc. PD:	-0.031
		Sc. rank:	-140.1

PA-X

Pos.	65	obs:	exp:
tct	S	1055	170.30
tcc	S	10	139.90
tca	S	0	271.80
tcg	S	6	60.77
agt	S	0	220.20
agc	S	0	208.00
mPD		0.030	1.7
		nPD:	0.02
		N. weight:	1.8
		Sc. PD:	-0.095
		Sc. rank:	-53.4

PA-X

Pos.	66	obs:	exp:
tct	S	0	0.16
tcc	S	0	0.13
tca	S	0	0.25
tcg	S	0	0.06
agt	S	0	0.21
agc	S	1	0.19
ggt	G	11	141.80
ggc	G	1059	136.70
gga	G	0	493.60
ggg	G	0	297.90
mPD		0.022	0.68
		nPD:	0.03
		N. weight:	2.1
		Sc. PD:	-0.078
		Sc. rank:	431.1

PA-X

Pos.	67	obs:	exp:
tat	Y	1	0.54
tac	Y	0	0.46
aat	N	1	0.51
aac	N	0	0.49
gat	D	1062	577.70
gac	D	7	491.30
mPD		0.017	0.50
		nPD:	0.03
		N. weight:	0.61
		Sc. PD:	-0.022
		Sc. rank:	127.3

PA-X

Pos.	68	obs:	exp:
tta	L	0	0.18
ttg	L	0	0.37
tct	S	0	0.16
tcc	S	0	0.13
tca	S	1	0.25
tcg	S	0	0.06
ctt	L	0	0.33
ctc	L	0	0.33
cta	L	2	0.34
ctg	L	0	0.45
cct	P	3	266.20
ccc	P	0	191.70
cca	P	1058	439.20
ccg	P	6	169.90
act	T	0	0.25
acc	T	0	0.21
aca	T	1	0.46
acg	T	0	0.08
agt	S	0	0.21
agc	S	0	0.19
mPD		0.024	0.72
		nPD:	0.03
		N. weight:	0.88
		Sc. PD:	-0.032
		Sc. rank:	191.4

PA-X

Pos.	69	obs:	exp:
aat	N	1067	545.50
aac	N	4	525.50
mPD		0.0074	0.50
		nPD:	0.01
		N. weight:	0.68
		Sc. PD:	-0.037
		Sc. rank:	-56.6

PA-X

Pos.	70	obs:	exp:
gtt	V	0	54.91
gtc	V	0	53.48
gta	V	0	54.26
gtg	V	265	102.30
gct	A	1	208.10
gcc	A	0	150.10
gca	A	49	379.40
gcg	A	756	68.41
mPD		0.46	1.1
		nPD:	0.43
		N. weight:	1.9
		Sc. PD:	0.66
		Sc. rank:	1571.9

PA-X

Pos.	71	obs:	exp:
tta	L	6	95.04
ttg	L	429	199.40
ctt	L	0	178.30
ctc	L	0	178.70
cta	L	3	180.50
ctg	L	633	239.00
mPD		0.50	1.1
		nPD:	0.45
		N. weight:	0.9
		Sc. PD:	0.33
		Sc. rank:	748.5

PA-X

Pos.	72	obs:	exp:
tta	L	11	94.95
ttg	L	434	199.20
tct	S	0	0.16
tcc	S	0	0.13
tca	S	0	0.25
tcg	S	1	0.06
ctt	L	4	178.20
ctc	L	0	178.60
cta	L	15	180.30
ctg	L	606	238.80
agt	S	0	0.21
agc	S	0	0.19
mPD		0.54	1.1
		nPD:	0.49
		N. weight:	0.82
		Sc. PD:	0.33
		Sc. rank:	685.7

PA-X

Pos.	73	obs:	exp:
aaa	K	1053	624.20
aag	K	18	446.80
mPD		0.033	0.49
		nPD:	0.07
		N. weight:	0.48
		Sc. PD:	-0.0014
		Sc. rank:	217.8

PA-X

Pos.	74	obs:	exp:
cat	H	401	615.00
cac	H	670	456.00
mPD		0.47	0.49
		nPD:	0.96
		N. weight:	0.088
		Sc. PD:	0.076
		Sc. rank:	78.9

PA-X

Pos.	75	obs:	exp:
cgt	R	0	46.00
cgc	R	0	53.05
cga	R	809	104.80
cgg	R	245	89.37
aga	R	10	489.70
agg	R	7	288.10
mPD		0.39	0.96
		nPD:	0.41
		N. weight:	1.8
		Sc. PD:	0.59
		Sc. rank:	1448.9

PA-X

Pos.	76	obs:	exp:
ttt	F	1070	422.90
ttc	F	1	648.10
mPD		0.0019	0.48
		nPD:	0.
		N. weight:	0.96
		Sc. PD:	-0.063
		Sc. rank:	-340.9

PA-X

Pos.	77	obs:	exp:
gaa	E	1065	651.70
gag	E	6	419.30
mPD		0.011	0.48
		nPD:	0.02
		N. weight:	0.49
		Sc. PD:	-0.023
		Sc. rank:	21.6

PA-X

Pos.	78	obs:	exp:
tta	L	2	0.18
ttg	L	0	0.37
ctt	L	0	0.33
ctc	L	0	0.33
cta	L	0	0.34
ctg	L	0	0.45
att	I	0	392.80
atc	I	0	256.20
ata	I	1069	420.10
mPD		0.0037	0.66
		nPD:	0.01
		N. weight:	0.98
		Sc. PD:	-0.062
		Sc. rank:	-308.7

PA-X

Pos.	79	obs:	exp:
att	I	12	392.80
atc	I	1050	256.20
ata	I	7	420.10
gtt	V	0	0.41
gtc	V	2	0.40
gta	V	0	0.41
gtg	V	0	0.77
mPD		0.039	0.66
		nPD:	0.06
		N. weight:	1.4
		Sc. PD:	-0.016
		Sc. rank:	569.4

PA-X

Pos.	80	obs:	exp:
gaa	E	1068	651.70
gag	E	3	419.30
mPD		0.0056	0.48
		nPD:	0.01
		N. weight:	0.5
		Sc. PD:	-0.029
		Sc. rank:	-74.7

PA-X

Pos.	81	obs:	exp:
ggt	G	0	142.00
ggc	G	0	136.80
gga	G	193	494.10
ggg	G	878	298.20
mPD		0.30	0.68
		nPD:	0.44
		N. weight:	0.75
		Sc. PD:	0.27
		Sc. rank:	619.7

PA-X

Pos.	82	obs:	exp:
cgt	R	0	46.00
cgc	R	0	53.05
cga	R	0	104.80
cgg	R	0	89.37
aga	R	1061	489.70
agg	R	10	288.10
mPD		0.019	0.96
		nPD:	0.02
		N. weight:	0.77
		Sc. PD:	-0.039
		Sc. rank:	16.5

PA-X

Pos.	83	obs:	exp:
gat	D	1	578.70
gac	D	1070	492.30
mPD		0.0019	0.50
		nPD:	0.
		N. weight:	0.81
		Sc. PD:	-0.053
		Sc. rank:	-305.8

PA-X

Pos.	84	obs:	exp:
cgt	R	0	46.00
cgc	R	0	53.05
cga	R	1066	104.80
cgg	R	3	89.37
aga	R	2	489.70
agg	R	0	288.10
mPD		0.0093	0.96
		nPD:	0.01
		N. weight:	2.4
		Sc. PD:	-0.14
		Sc. rank:	-468.3

PA-X

Pos.	85	obs:	exp:
att	I	0	0.73
atc	I	0	0.48
ata	I	2	0.79
act	T	0	270.50
acc	T	0	217.70
aca	T	1060	486.60
acg	T	1	86.18
gct	A	0	2.07
gcc	A	0	1.49
gca	A	8	3.77
gcg	A	0	0.68
mPD		0.020	0.69
		nPD:	0.03
		N. weight:	0.81
		Sc. PD:	-0.033
		Sc. rank:	123.4

PA-X			
Pos.	86	obs:	exp:
att	I	0	1.84
atc	I	0	1.20
ata	I	5	1.96
atg	M	1066	1066.00
---	--	------	------
mPD		0.0093	0.0093
		nPD:	1.
		N. weight:	0.0046
		Sc. PD:	0.0041
		Sc. rank:	4.1

,

PA-X			
Pos.	87	obs:	exp:
gct	A	2	276.60
gcc	A	1038	199.50
gca	A	31	504.10
gcg	A	0	90.90
---	--	------	------
mPD		0.060	0.67
		nPD:	0.09
		N. weight:	1.6
		Sc. PD:	0.028
		Sc. rank:	927.1

,

PA-X			
Pos.	88	obs:	exp:
tgg	W	1071	1071.00
---	--	------	------
mPD		0	0
		nPD:	1.
		N. weight:	0.
		Sc. PD:	0
		Sc. rank:	0

,

PA-X			
Pos.	89	obs:	exp:
act	T	2	272.80
acc	T	10	219.60
aca	T	1051	490.80
acg	T	7	86.91
gct	A	0	0.26
gcc	A	0	0.19
gca	A	1	0.47
gcg	A	0	0.08
---	--	------	------
mPD		0.037	0.68
		nPD:	0.05
		N. weight:	0.73
		Sc. PD:	-0.012
		Sc. rank:	269.2

,

PA-X			
Pos.	90	obs:	exp:
att	I	0	0.37
atc	I	0	0.24
ata	I	1	0.39
atg	M	3	3.00
gtt	V	1	221.10
gtc	V	0	215.30
gta	V	45	218.50
gtg	V	1021	412.10
---	--	------	------
mPD		0.092	0.73
		nPD:	0.13
		N. weight:	0.83
		Sc. PD:	0.044
		Sc. rank:	541.0

,

PA-X			
Pos.	91	obs:	exp:
atg	M	1	1.00
gtt	V	1	221.50
gtc	V	0	215.80
gta	V	48	218.90
gtg	V	1020	412.90
gct	A	0	0.26
gcc	A	0	0.19
gca	A	0	0.47
gcg	A	1	0.08
---	--	------	------
mPD		0.091	0.73
		nPD:	0.13
		N. weight:	0.83
		Sc. PD:	0.044
		Sc. rank:	543.3

,

PA-X			
Pos.	92	obs:	exp:
tct	S	0	0.16
tcc	S	0	0.13
tca	S	0	0.25
tcg	S	0	0.06
aat	N	1057	545.00
aac	N	13	525.00
agt	S	1	0.21
agc	S	0	0.19
---	--	------	------
mPD		0.026	0.50
		nPD:	0.05
		N. weight:	0.64
		Sc. PD:	-0.012
		Sc. rank:	229.4

,

PA-X			
Pos.	93	obs:	exp:
tct	S	0	170.30
tcc	S	0	139.90
tca	S	0	271.80
tcg	S	0	60.77
agt	S	1011	220.20
agc	S	60	208.00
---	--	------	------
mPD		0.11	1.7
		nPD:	0.06
		N. weight:	1.4
		Sc. PD:	-0.0098
		Sc. rank:	641.4

,

PA-X			
Pos.	94	obs:	exp:
att	I	65	385.10
atc	I	983	251.10
ata	I	0	411.80
gtt	V	0	4.77
gtc	V	23	4.64
gta	V	0	4.71
gtg	V	0	8.88
---	--	------	------
mPD		0.16	0.70
		nPD:	0.22
		N. weight:	1.2
		Sc. PD:	0.18
		Sc. rank:	919.0

,

PA-X			
Pos.	95	obs:	exp:
tat	Y	0	0.54
tac	Y	1	0.46
tgt	C	3	452.60
tgc	C	1067	617.40
---	--	------	------
mPD		0.0075	0.49
		nPD:	0.02
		N. weight:	0.56
		Sc. PD:	-0.03
		Sc. rank:	-40.7

,

PA-X			
Pos.	96	obs:	exp:
cat	H	0	16.65
cac	H	29	12.35
aat	N	3	530.80
aac	N	1039	511.20
---	--	------	------
mPD		0.058	0.55
		nPD:	0.11
		N. weight:	0.73
		Sc. PD:	0.024
		Sc. rank:	456.1

,

PA-X			
Pos.	97	obs:	exp:
att	I	1	1.84
atc	I	4	1.20
ata	I	0	1.96
act	T	89	271.50
acc	T	974	218.50
aca	T	2	488.50
acg	T	0	86.51
gct	A	0	0.26
gcc	A	1	0.19
gca	A	0	0.47
gcg	A	0	0.08
---	--	------	------
mPD		0.17	0.69
		nPD:	0.25
		N. weight:	1.3
		Sc. PD:	0.22
		Sc. rank:	1010.0

,

PA-X			
Pos.	98	obs:	exp:
act	T	23	272.50
acc	T	0	219.40
aca	T	619	490.30
acg	T	427	86.83
gct	A	0	0.52
gcc	A	0	0.37
gca	A	0	0.94
gcg	A	2	0.17
---	--	------	------
mPD		0.51	0.68
		nPD:	0.75
		N. weight:	0.76
		Sc. PD:	0.5
		Sc. rank:	679.5

,

PA-X			
Pos.	99	obs:	exp:
cgt	R	0	0.04
cgc	R	0	0.05
cga	R	0	0.10
cgg	R	0	0.08
aga	R	1	0.46
agg	R	0	0.27
ggt	G	57	141.80
ggc	G	0	136.70
gga	G	1006	493.60
ggg	G	7	297.90
---	--	------	------
mPD		0.12	0.68
		nPD:	0.17
		N. weight:	0.63
		Sc. PD:	0.06
		Sc. rank:	443.8

,

PA-X			
Pos.	100	obs:	exp:
att	I	0	10.29
atc	I	28	6.71
ata	I	0	11.00
act	T	0	1.02
acc	T	4	0.82
aca	T	0	1.83
acg	T	0	0.32
gtt	V	41	91.79
gtc	V	402	89.41
gta	V	0	90.71
gtg	V	0	171.10
gct	A	4	153.90
gcc	A	591	111.00
gca	A	1	280.50
gcg	A	0	50.58
---	--	------	------
mPD		0.63	1.3
		nPD:	0.49
		N. weight:	1.6
		Sc. PD:	0.64
		Sc. rank:	1320.3

,

PA-X			
Pos.	101	obs:	exp:
caa	Q	3	1.53
cag	Q	0	1.47
gaa	E	1015	649.90
gag	E	53	418.10
---	--	------	------
mPD		0.10	0.48
		nPD:	0.21
		N. weight:	0.34
		Sc. PD:	0.045
		Sc. rank:	247.0

,

PA-X			
Pos.	102	obs:	exp:
aaa	K	1045	624.20
aag	K	26	446.80
---	--	------	------
mPD		0.047	0.49
		nPD:	0.1
		N. weight:	0.46
		Sc. PD:	0.012
		Sc. rank:	274.7

,

PA-X			
Pos.	103	obs:	exp:
cct	P	77	267.20
ccc	P	988	192.40
cca	P	6	440.80
ccg	P	0	170.50
---	--	------	------
mPD		0.14	0.71
		nPD:	0.2
		N. weight:	1.5
		Sc. PD:	0.19
		Sc. rank:	1061.0

,

PA-X			
Pos.	104	obs:	exp:
aaa	K	1068	624.20
aag	K	3	446.80
---	--	------	------
mPD		0.0056	0.49
		nPD:	0.01
		N. weight:	0.55
		Sc. PD:	-0.032
		Sc. rank:	-93.7

,

PA-X			
Pos.	105	obs:	exp:
ttt	F	1045	421.70
ttc	F	23	646.30
tta	L	0	0.27
ttg	L	0	0.56
ctt	L	3	0.50
ctc	L	0	0.50
cta	L	0	0.51
ctg	L	0	0.67
---	--	------	------
mPD		0.048	0.48
		nPD:	0.1
		N. weight:	0.86
		Sc. PD:	0.023
		Sc. rank:	522.4

PA-X			
Pos.	106	obs:	exp:
tta	L	0	94.86
ttg	L	0	199.00
ctt	L	42	178.00
ctc	L	1026	178.40
cta	L	1	180.20
ctg	L	0	238.50
att	I	0	0.73
atc	I	2	0.48
ata	I	0	0.79
---	--	------	------
mPD		0.081	1.1
		nPD:	0.07
		N. weight:	1.7
		Sc. PD:	0.0029
		Sc. rank:	841.4

,

PA-X			
Pos.	107	obs:	exp:
cct	P	6	267.20
ccc	P	0	192.40
cca	P	62	440.80
ccg	P	1003	170.50
---	--	------	------
mPD		0.12	0.71
		nPD:	0.17
		N. weight:	1.6
		Sc. PD:	0.15
		Sc. rank:	1122.1

,

PA-X			
Pos.	108	obs:	exp:
gat	D	1068	578.70
gac	D	3	492.30
---	--	------	------
mPD		0.0056	0.50
		nPD:	0.01
		N. weight:	0.63
		Sc. PD:	-0.036
		Sc. rank:	-116.3

,

PA-X			
Pos.	109	obs:	exp:
tta	L	0	95.04
ttg	L	11	199.40
ctt	L	4	178.30
ctc	L	0	178.70
cta	L	1	180.50
ctg	L	1055	239.00
---	--	------	------
mPD		0.030	1.1
		nPD:	0.03
		N. weight:	1.5
		Sc. PD:	-0.064
		Sc. rank:	164.5

,

PA-X			
Pos.	110	obs:	exp:
tat	Y	1070	578.40
tac	Y	1	492.60
---	--	------	------
mPD		0.0019	0.50
		nPD:	0.
		N. weight:	0.64
		Sc. PD:	-0.042
		Sc. rank:	-248.7

,

PA-X

Pos.	111	obs:	exp:
gat	D	9	578.70
gac	D	1062	492.30
mPD		0.017	0.50
		nPD:	0.03
		N. weight:	0.76
		Sc. PD:	-0.028
		Sc. rank:	169.1

PA-X

Pos.	112	obs:	exp:
tat	Y	0	577.90
tac	Y	1070	492.10
tgt	C	0	0.42
tgc	C	1	0.58
mPD		0.0019	0.50
		nPD:	0.
		N. weight:	0.81
		Sc. PD:	-0.053
		Sc. rank:	-320.9

PA-X

Pos.	113	obs:	exp:
aaa	K	19	624.20
aag	K	1052	446.80
mPD		0.035	0.49
		nPD:	0.07
		N. weight:	0.82
		Sc. PD:	0.00055
		Sc. rank:	382.0

PA-X

Pos.	114	obs:	exp:
gaa	E	1069	651.70
gag	E	2	419.30
mPD		0.0037	0.48
		nPD:	0.01
		N. weight:	0.51
		Sc. PD:	-0.031
		Sc. rank:	-140.1

PA-X

Pos.	115	obs:	exp:
tct	S	0	4.61
tcc	S	0	3.79
tca	S	0	7.36
tcg	S	0	1.65
aat	N	23	529.70
aac	N	1017	510.30
agt	S	0	5.96
agc	S	29	5.63
gat	D	0	1.08
gac	D	2	0.92
mPD		0.099	0.60
		nPD:	0.17
		N. weight:	0.66
		Sc. PD:	0.061
		Sc. rank:	459.0

PA-X

Pos.	116	obs:	exp:
cgt	R	590	46.00
cgc	R	1	53.05
cga	R	474	104.80
cgg	R	4	89.37
aga	R	2	489.70
agg	R	0	288.10
mPD		0.50	0.96
		nPD:	0.52
		N. weight:	2.1
		Sc. PD:	0.94
		Sc. rank:	1858.7

PA-X

Pos.	117	obs:	exp:
ttt	F	8	422.90
ttc	F	1063	648.10
mPD		0.015	0.48
		nPD:	0.03
		N. weight:	0.48
		Sc. PD:	-0.019
		Sc. rank:	89.3

PA-X

Pos.	118	obs:	exp:
att	I	1070	393.10
atc	I	0	256.40
ata	I	0	420.40
gtt	V	1	0.21
gtc	V	0	0.20
gta	V	0	0.20
gtg	V	0	0.39
mPD		0.0019	0.66
		nPD:	0.
		N. weight:	1.
		Sc. PD:	-0.069
		Sc. rank:	-502.3

PA-X

Pos.	119	obs:	exp:
gaa	E	1070	651.70
gag	E	1	419.30
mPD		0.0019	0.48
		nPD:	0.
		N. weight:	0.51
		Sc. PD:	-0.033
		Sc. rank:	-174.2

PA-X

Pos.	120	obs:	exp:
att	I	1068	392.80
atc	I	1	256.20
ata	I	0	420.10
gtt	V	2	0.41
gtc	V	0	0.40
gta	V	0	0.41
gtg	V	0	0.77
mPD		0.0056	0.66
		nPD:	0.01
		N. weight:	1.
		Sc. PD:	-0.063
		Sc. rank:	-231.1

PA-X

Pos.	121	obs:	exp:
ggt	G	0	142.00
ggc	G	0	136.80
gga	G	1041	494.10
ggg	G	30	298.20
mPD		0.055	0.68
		nPD:	0.08
		N. weight:	0.69
		Sc. PD:	0.0065
		Sc. rank:	367.3

PA-X

Pos.	122	obs:	exp:
gtt	V	8	221.90
gtc	V	0	216.20
gta	V	46	219.30
gtg	V	1017	413.60
mPD		0.096	0.73
		nPD:	0.13
		N. weight:	0.8
		Sc. PD:	0.048
		Sc. rank:	529.1

PA-X

Pos.	123	obs:	exp:
act	T	4	273.00
acc	T	0	219.80
aca	T	25	491.20
acg	T	1042	86.99
mPD		0.053	0.68
		nPD:	0.08
		N. weight:	2.4
		Sc. PD:	0.017
		Sc. rank:	1274.5

PA-X

Pos.	124	obs:	exp:
cgt	R	0	46.00
cgc	R	0	53.05
cga	R	0	104.80
cgg	R	1	89.37
aga	R	12	489.70
agg	R	1058	288.10
mPD		0.024	0.96
		nPD:	0.03
		N. weight:	1.3
		Sc. PD:	-0.058
		Sc. rank:	115.7

PA-X

Pos.	125	obs:	exp:
cgt	R	0	46.00
cgc	R	0	53.05
cga	R	0	104.80
cgg	R	3	89.37
aga	R	19	489.70
agg	R	1049	288.10
mPD		0.040	0.96
		nPD:	0.04
		N. weight:	1.3
		Sc. PD:	-0.035
		Sc. rank:	385.6

PA-X

Pos.	126	obs:	exp:
gaa	E	1064	649.90
gag	E	4	418.10
ggt	G	0	0.40
ggc	G	0	0.38
gga	G	3	1.38
ggg	G	0	0.84
mPD		0.013	0.48
		nPD:	0.03
		N. weight:	0.5
		Sc. PD:	-0.021
		Sc. rank:	57.0

PA-X

Pos.	127	obs:	exp:
att	I	0	0.73
atc	I	2	0.48
ata	I	0	0.79
gtt	V	5	221.10
gtc	V	1061	215.30
gta	V	1	218.50
gtg	V	0	412.10
gct	A	0	0.52
gcc	A	2	0.37
gca	A	0	0.94
gcg	A	0	0.17
mPD		0.019	0.73
		nPD:	0.03
		N. weight:	1.6
		Sc. PD:	-0.072
		Sc. rank:	164.6

PA-X

Pos.	128	obs:	exp:
cat	H	2	613.80
cac	H	1067	455.20
caa	Q	1	0.51
cag	Q	0	0.49
aat	N	0	0.51
aac	N	1	0.49
mPD		0.0075	0.49
		nPD:	0.02
		N. weight:	0.88
		Sc. PD:	-0.048
		Sc. rank:	-67.6

PA-X

Pos.	129	obs:	exp:
att	I	0	390.90
atc	I	3	255.00
ata	I	1061	418.10
atg	M	6	6.00
act	T	0	0.25
acc	T	0	0.21
aca	T	1	0.46
acg	T	0	0.08
mPD		0.019	0.66
		nPD:	0.03
		N. weight:	0.95
		Sc. PD:	-0.04
		Sc. rank:	136.1

PA-X

Pos.	130	obs:	exp:
tat	Y	919	578.40
tac	Y	152	492.60
mPD		0.24	0.50
		nPD:	0.49
		N. weight:	0.24
		Sc. PD:	0.099
		Sc. rank:	204.9

PA-X

Pos.	131	obs:	exp:
tta	L	0	0.09
ttg	L	1	0.19
tat	Y	0	577.90
tac	Y	1070	492.10
ctt	L	0	0.17
ctc	L	0	0.17
cta	L	0	0.17
ctg	L	0	0.22
mPD		0.0037	0.50
		nPD:	0.01
		N. weight:	0.81
		Sc. PD:	-0.05
		Sc. rank:	-247.0

PA-X

Pos.	132	obs:	exp:
tta	L	3	95.04
ttg	L	0	199.40
ctt	L	0	178.30
ctc	L	0	178.70
cta	L	1060	180.50
ctg	L	8	239.00
mPD		0.020	1.1
		nPD:	0.02
		N. weight:	1.8
		Sc. PD:	-0.092
		Sc. rank:	-29.4

PA-X

Pos.	133	obs:	exp:
gaa	E	47	651.70
gag	E	1024	419.30
mPD		0.084	0.48
		nPD:	0.18
		N. weight:	0.77
		Sc. PD:	0.079
		Sc. rank:	553.5

PA-X

Pos.	134	obs:	exp:
aaa	K	1024	624.20
aag	K	47	446.80
mPD		0.084	0.49
		nPD:	0.17
		N. weight:	0.39
		Sc. PD:	0.039
		Sc. rank:	279.5

PA-X

Pos.	135	obs:	exp:
gct	A	5	276.60
gcc	A	1066	199.50
gca	A	0	504.10
gcg	A	0	90.90
mPD		0.0093	0.67
		nPD:	0.01
		N. weight:	1.7
		Sc. PD:	-0.096
		Sc. rank:	-172.6

PA-X

Pos.	136	obs:	exp:
tct	S	0	0.32
tcc	S	0	0.26
tca	S	0	0.51
tcg	S	0	0.11
aat	N	1051	544.50
aac	N	18	524.50
agt	S	2	0.41
agc	S	0	0.39
---	--	------	------
mPD		0.037	0.51
		nPD:	0.07
		N. weight:	0.62
		Sc. PD:	0.001
		Sc. rank:	300.9

PA-X

Pos.	137	obs:	exp:
aaa	K	1050	624.20
aag	K	21	446.80
---	--	------	------
mPD		0.038	0.49
		nPD:	0.08
		N. weight:	0.47
		Sc. PD:	0.0037
		Sc. rank:	248.4

PA-X

Pos.	138	obs:	exp:
tta	L	3	0.27
ttg	L	0	0.56
ctt	L	0	0.50
ctc	L	0	0.50
cta	L	0	0.51
ctg	L	0	0.67
att	I	0	392.00
atc	I	0	255.70
ata	I	1067	419.30
aaa	K	1	0.58
aag	K	0	0.42
---	--	------	------
mPD		0.0075	0.66
		nPD:	0.01
		N. weight:	0.98
		Sc. PD:	-0.057
		Sc. rank:	-175.6

PA-X

Pos.	139	obs:	exp:
aaa	K	1070	624.20
aag	K	1	446.80
---	--	------	------
mPD		0.0019	0.49
		nPD:	0.
		N. weight:	0.56
		Sc. PD:	-0.036
		Sc. rank:	-209.6

PA-X

Pos.	140	obs:	exp:
tct	S	19	170.30
tcc	S	1004	139.90
tca	S	48	271.80
tcg	S	0	60.77
agt	S	0	220.20
agc	S	0	208.00
---	--	------	------
mPD		0.12	1.7
		nPD:	0.07
		N. weight:	1.8
		Sc. PD:	0.0016
		Sc. rank:	871.8

PA-X

Pos.	141	obs:	exp:
aaa	K	0	1.17
aag	K	2	0.83
gaa	E	9	649.90
gag	E	1059	418.10
ggt	G	0	0.13
ggc	G	0	0.13
gga	G	0	0.46
ggg	G	1	0.28
---	--	------	------
mPD		0.022	0.48
		nPD:	0.05
		N. weight:	0.93
		Sc. PD:	-0.022
		Sc. rank:	299.4

PA-X

Pos.	142	obs:	exp:
cgt	R	0	0.09
cgc	R	0	0.10
cga	R	0	0.20
cgg	R	0	0.17
aaa	K	38	620.70
aag	K	1027	444.30
aga	R	0	0.91
agg	R	2	0.54
gaa	E	0	2.43
gag	E	4	1.57
---	--	------	------
mPD		0.080	0.50
		nPD:	0.16
		N. weight:	0.75
		Sc. PD:	0.064
		Sc. rank:	507.1

PA-X

Pos.	143	obs:	exp:
act	T	0	271.70
acc	T	11	218.70
aca	T	1055	488.90
acg	T	0	86.59
aaa	K	2	1.17
aag	K	0	0.83
gct	A	0	0.77
gcc	A	0	0.56
gca	A	3	1.41
gcg	A	0	0.25
---	--	------	------
mPD		0.030	0.69
		nPD:	0.04
		N. weight:	0.77
		Sc. PD:	-0.021
		Sc. rank:	241.9

PA-X

Pos.	144	obs:	exp:
cat	H	5	615.00
cac	H	1066	456.00
---	--	------	------
mPD		0.0093	0.49
		nPD:	0.02
		N. weight:	0.86
		Sc. PD:	-0.043
		Sc. rank:	13.8

PA-X

Pos.	145	obs:	exp:
tta	L	0	0.09
ttg	L	0	0.19
ctt	L	0	0.17
ctc	L	0	0.17
cta	L	1	0.17
ctg	L	0	0.22
att	I	2	392.80
atc	I	732	256.20
ata	I	335	420.10
gtt	V	0	0.21
gtc	V	0	0.20
gta	V	1	0.20
gtg	V	0	0.39
---	--	------	------
mPD		0.44	0.66
		nPD:	0.67
		N. weight:	0.67
		Sc. PD:	0.39
		Sc. rank:	599.0

PA-X

Pos.	146	obs:	exp:
cat	H	597	615.00
cac	H	474	456.00
---	--	------	------
mPD		0.49	0.49
		nPD:	1.01
		N. weight:	0.0042
		Sc. PD:	0.0038
		Sc. rank:	3.8

PA-X

Pos.	147	obs:	exp:
att	I	1060	393.10
atc	I	10	256.40
ata	I	0	420.40
act	T	1	0.25
acc	T	0	0.21
aca	T	0	0.46
acg	T	0	0.08
---	--	------	------
mPD		0.020	0.66
		nPD:	0.03
		N. weight:	1.
		Sc. PD:	-0.039
		Sc. rank:	190.9

PA-X

Pos.	148	obs:	exp:
ttt	F	590	422.50
ttc	F	480	647.50
tta	L	0	0.09
ttg	L	0	0.19
ctt	L	0	0.17
ctc	L	1	0.17
cta	L	0	0.17
ctg	L	0	0.22
---	--	------	------
mPD		0.50	0.48
		nPD:	1.04
		N. weight:	0.057
		Sc. PD:	0.054
		Sc. rank:	51.6

PA-X

Pos.	149	obs:	exp:
tct	S	0	170.30
tcc	S	0	139.90
tca	S	1068	271.80
tcg	S	3	60.77
agt	S	0	220.20
agc	S	0	208.00
---	--	------	------
mPD		0.0056	1.7
		nPD:	0.
		N. weight:	1.4
		Sc. PD:	-0.093
		Sc. rank:	-597.8

PA-X

Pos.	150	obs:	exp:
ttt	F	5	422.10
ttc	F	1064	646.90
tta	L	0	0.18
ttg	L	0	0.37
ctt	L	0	0.33
ctc	L	2	0.33
cta	L	0	0.34
ctg	L	0	0.45
---	--	------	------
mPD		0.013	0.48
		nPD:	0.03
		N. weight:	0.5
		Sc. PD:	-0.021
		Sc. rank:	59.0

PA-X

Pos.	151	obs:	exp:
act	T	1065	272.80
acc	T	2	219.60
aca	T	2	490.80
acg	T	1	86.91
aat	N	1	0.51
aac	N	0	0.49
---	--	------	------
mPD		0.011	0.68
		nPD:	0.02
		N. weight:	1.4
		Sc. PD:	-0.074
		Sc. rank:	-72.8

PA-X

Pos.	152	obs:	exp:
ggt	G	0	142.00
ggc	G	0	136.80
gga	G	1048	494.10
ggg	G	23	298.20
---	--	------	------
mPD		0.042	0.68
		nPD:	0.06
		N. weight:	0.71
		Sc. PD:	-0.0061
		Sc. rank:	312.2

PA-X

Pos.	153	obs:	exp:
gaa	E	1069	651.10
gag	E	1	418.90
ggt	G	0	0.13
ggc	G	0	0.13
gga	G	1	0.46
ggg	G	0	0.28
---	--	------	------
mPD		0.0037	0.48
		nPD:	0.01
		N. weight:	0.51
		Sc. PD:	-0.031
		Sc. rank:	-145.9

PA-X

Pos.	154	obs:	exp:
gaa	E	16	651.70
gag	E	1055	419.30
---	--	------	------
mPD		0.029	0.48
		nPD:	0.06
		N. weight:	0.89
		Sc. PD:	-0.008
		Sc. rank:	386.5

PA-X

Pos.	155	obs:	exp:
atg	M	1071	1071.00
---	--	------	------
mPD		0	0
		nPD:	1.
		N. weight:	0.
		Sc. PD:	0
		Sc. rank:	0

PA-X

Pos.	156	obs:	exp:
gct	A	1	276.60
gcc	A	1045	199.50
gca	A	25	504.10
gcg	A	0	90.90
---	--	------	------
mPD		0.047	0.67
		nPD:	0.07
		N. weight:	1.6
		Sc. PD:	-0.0003
		Sc. rank:	745.5

PA-X

Pos.	157	obs:	exp:
act	T	9	273.00
acc	T	1061	219.80
aca	T	1	491.20
acg	T	0	86.99
---	--	------	------
mPD		0.019	0.68
		nPD:	0.03
		N. weight:	1.6
		Sc. PD:	-0.068
		Sc. rank:	217.2

PA-X

Pos.	158	obs:	exp:
caa	Q	6	3.06
cag	Q	0	2.94
cgt	R	0	0.04
cgc	R	0	0.05
cga	R	0	0.10
cgg	R	0	0.08
aaa	K	1041	620.10
aag	K	23	443.90
aga	R	1	0.46
agg	R	0	0.27
---	--	------	------
mPD		0.055	0.50
		nPD:	0.11
		N. weight:	0.47
		Sc. PD:	0.018
		Sc. rank:	293.5

PA-X

Pos.	159	obs:	exp:
gct	A	0	276.60
gcc	A	0	199.50
gca	A	1070	504.10
gcg	A	1	90.90
---	--	------	------
mPD		0.0019	0.67
		nPD:	0.
		N. weight:	0.78
		Sc. PD:	-0.052
		Sc. rank:	-385.5

PA-X

Pos.	160	obs:	exp:
aat	N	2	1.02
aac	N	0	0.98
gat	D	1061	577.70
gac	D	8	491.30
---	--	------	------
mPD		0.019	0.50
		nPD:	0.04
		N. weight:	0.6
		Sc. PD:	-0.02
		Sc. rank:	141.4

PA-X			
Pos.	161	obs:	exp:
tat	Y	1	578.40
tac	Y	1070	492.60
---	--	------	------
mPD		0.0019	0.50
		nPD:	0.
		N. weight:	0.8
		Sc. PD:	-0.053
		Sc. rank:	-314.2

PA-X			
Pos.	162	obs:	exp:
act	T	978	273.00
acc	T	14	219.80
aca	T	79	491.20
acg	T	0	86.99
---	--	------	------
mPD		0.16	0.68
		nPD:	0.24
		N. weight:	1.
		Sc. PD:	0.17
		Sc. rank:	784.9

PA-X			
Pos.	163	obs:	exp:
tta	L	0	95.04
ttg	L	0	199.40
ctt	L	1066	178.30
ctc	L	5	178.70
cta	L	0	180.50
ctg	L	0	239.00
---	--	------	------
mPD		0.0093	1.1
		nPD:	0.01
		N. weight:	1.8
		Sc. PD:	-0.11
		Sc. rank:	-422.9

PA-X			
Pos.	164	obs:	exp:
gat	D	300	578.70
gac	D	771	492.30
---	--	------	------
mPD		0.40	0.50
		nPD:	0.81
		N. weight:	0.15
		Sc. PD:	0.11
		Sc. rank:	133.5

PA-X			
Pos.	165	obs:	exp:
gaa	E	1064	651.70
gag	E	7	419.30
---	--	------	------
mPD		0.013	0.48
		nPD:	0.03
		N. weight:	0.48
		Sc. PD:	-0.02
		Sc. rank:	60.3

PA-X			
Pos.	166	obs:	exp:
gaa	E	877	651.70
gag	E	194	419.30
---	--	------	------
mPD		0.30	0.48
		nPD:	0.62
		N. weight:	0.11
		Sc. PD:	0.06
		Sc. rank:	97.8

PA-X			
Pos.	167	obs:	exp:
tct	S	0	170.20
tcc	S	0	139.70
tca	S	0	271.60
tcg	S	0	60.72
tgt	C	0	0.42
tgc	C	1	0.58
agt	S	46	220.00
agc	S	1024	207.80
---	--	------	------
mPD		0.084	1.7
		nPD:	0.05
		N. weight:	1.5
		Sc. PD:	-0.03
		Sc. rank:	523.7

PA-X			
Pos.	168	obs:	exp:
cgt	R	0	46.00
cgc	R	0	53.05
cga	R	0	104.80
cgg	R	0	89.37
aga	R	2	489.70
agg	R	1069	288.10
---	--	------	------
mPD		0.0037	0.96
		nPD:	0.
		N. weight:	1.4
		Sc. PD:	-0.088
		Sc. rank:	-486.8

PA-X			
Pos.	169	obs:	exp:
gct	A	0	276.60
gcc	A	0	199.50
gca	A	1071	504.10
gcg	A	0	90.90
---	--	------	------
mPD		0	0.67
		nPD:	0.
		N. weight:	0.79
		Sc. PD:	-0.054
		Sc. rank:	-683.7

PA-X			
Pos.	170	obs:	exp:
cgt	R	0	46.00
cgc	R	0	53.05
cga	R	0	104.80
cgg	R	0	89.37
aga	R	1068	489.70
agg	R	3	288.10
---	--	------	------
mPD		0.0056	0.96
		nPD:	0.01
		N. weight:	0.8
		Sc. PD:	-0.051
		Sc. rank:	-247.9

PA-X			
Pos.	171	obs:	exp:
att	I	1	393.10
atc	I	1065	256.40
ata	I	4	420.40
gtt	V	0	0.21
gtc	V	1	0.20
gta	V	0	0.20
gtg	V	0	0.39
---	--	------	------
mPD		0.011	0.66
		nPD:	0.02
		N. weight:	1.5
		Sc. PD:	-0.076
		Sc. rank:	-61.2

PA-X			
Pos.	172	obs:	exp:
aat	N	0	0.51
aac	N	1	0.49
aaa	K	1068	623.60
aag	K	2	446.40
---	--	------	------
mPD		0.0056	0.49
		nPD:	0.01
		N. weight:	0.55
		Sc. PD:	-0.032
		Sc. rank:	-96.8

PA-X			
Pos.	173	obs:	exp:
act	T	7	273.00
acc	T	1062	219.80
aca	T	2	491.20
acg	T	0	86.99
---	--	------	------
mPD		0.017	0.68
		nPD:	0.02
		N. weight:	1.6
		Sc. PD:	-0.072
		Sc. rank:	99.7

PA-X			
Pos.	174	obs:	exp:
cgt	R	0	46.00
cgc	R	0	53.05
cga	R	0	104.80
cgg	R	0	89.37
aga	R	51	489.70
agg	R	1020	288.10
---	--	------	------
mPD		0.091	0.96
		nPD:	0.09
		N. weight:	1.1
		Sc. PD:	0.026
		Sc. rank:	683.0

PA-X			
Pos.	175	obs:	exp:
tta	L	0	95.04
ttg	L	146	199.40
ctt	L	0	178.30
ctc	L	0	178.70
cta	L	5	180.50
ctg	L	920	239.00
---	--	------	------
mPD		0.24	1.1
		nPD:	0.22
		N. weight:	1.2
		Sc. PD:	0.17
		Sc. rank:	848.1

PA-X			
Pos.	176	obs:	exp:
ttt	F	7	422.90
ttc	F	1064	648.10
---	--	------	------
mPD		0.013	0.48
		nPD:	0.03
		N. weight:	0.49
		Sc. PD:	-0.021
		Sc. rank:	59.3

PA-X			
Pos.	177	obs:	exp:
act	T	29	273.00
acc	T	1040	219.80
aca	T	2	491.20
acg	T	0	86.99
---	--	------	------
mPD		0.056	0.68
		nPD:	0.08
		N. weight:	1.5
		Sc. PD:	0.018
		Sc. rank:	824.7

PA-X			
Pos.	178	obs:	exp:
att	I	0	393.50
atc	I	29	256.70
ata	I	1042	420.80
---	--	------	------
mPD		0.053	0.65
		nPD:	0.08
		N. weight:	0.86
		Sc. PD:	0.0081
		Sc. rank:	462.9

PA-X			
Pos.	179	obs:	exp:
cgt	R	0	46.00
cgc	R	0	53.05
cga	R	0	104.80
cgg	R	0	89.37
aga	R	32	489.70
agg	R	1039	288.10
---	--	------	------
mPD		0.058	0.96
		nPD:	0.06
		N. weight:	1.2
		Sc. PD:	-0.013
		Sc. rank:	511.7

PA-X			
Pos.	180	obs:	exp:
cat	H	2	1.15
cac	H	0	0.85
caa	Q	18	544.50
cag	Q	1051	524.50
---	--	------	------
mPD		0.037	0.50
		nPD:	0.07
		N. weight:	0.66
		Sc. PD:	0.0014
		Sc. rank:	329.1

PA-X			
Pos.	181	obs:	exp:
gaa	E	1006	651.70
gag	E	65	419.30
---	--	------	------
mPD		0.11	0.48
		nPD:	0.24
		N. weight:	0.31
		Sc. PD:	0.051
		Sc. rank:	234.3

PA-X			
Pos.	182	obs:	exp:
tta	L	0	0.27
ttg	L	1	0.56
ctt	L	0	0.50
ctc	L	0	0.50
cta	L	0	0.51
ctg	L	2	0.67
atg	M	1068	1068.00
---	--	------	------
mPD		0.0056	0.0089
		nPD:	0.63
		N. weight:	0.0035
		Sc. PD:	0.0019
		Sc. rank:	3.1

PA-X			
Pos.	183	obs:	exp:
gct	A	18	276.60
gcc	A	1051	199.50
gca	A	2	504.10
gcg	A	0	90.90
---	--	------	------
mPD		0.037	0.67
		nPD:	0.05
		N. weight:	1.6
		Sc. PD:	-0.026
		Sc. rank:	636.6

PA-X			
Pos.	184	obs:	exp:
tct	S	0	169.20
tcc	S	0	139.00
tca	S	0	270.10
tcg	S	0	60.38
tgt	C	0	0.85
tgc	C	2	1.15
att	I	0	0.37
atc	I	1	0.24
ata	I	0	0.39
aat	N	0	0.51
aac	N	1	0.49
agt	S	40	218.70
agc	S	1024	206.70
ggt	G	0	0.40
ggc	G	3	0.38
gga	G	0	1.38
ggg	G	0	0.84
---	--	------	------
mPD		0.085	1.7
		nPD:	0.05
		N. weight:	1.5
		Sc. PD:	-0.03
		Sc. rank:	546.7

PA-X			
Pos.	185	obs:	exp:
cgt	R	0	46.00
cgc	R	0	53.05
cga	R	0	104.80
cgg	R	0	89.37
aga	R	11	489.70
agg	R	1060	288.10
---	--	------	------
mPD		0.020	0.96
		nPD:	0.02
		N. weight:	1.3
		Sc. PD:	-0.063
		Sc. rank:	44.5

PA-X

Pos.	186	obs:	exp:
tct	S	0	0.16
tcc	S	0	0.13
tca	S	0	0.25
tcg	S	0	0.06
agt	S	1	0.21
agc	S	0	0.19
ggt	G	1057	141.80
ggc	G	10	136.70
gga	G	3	493.60
ggg	G	0	297.90
---	--	------	------
mPD		0.026	0.68
		nPD:	0.04
		N. weight:	2.
		Sc. PD:	-0.065
		Sc. rank:	552.0

,

PA-X

Pos.	187	obs:	exp:
tta	L	1	94.95
ttg	L	0	199.20
ctt	L	0	178.20
ctc	L	2	178.60
cta	L	1063	180.30
ctg	L	4	238.80
att	I	0	0.37
atc	I	0	0.24
ata	I	1	0.39
---	--	------	------
mPD		0.015	1.1
		nPD:	0.01
		N. weight:	1.8
		Sc. PD:	-0.1
		Sc. rank:	-247.1

,

PA-X

Pos.	188	obs:	exp:
tgg	W	1071	1071.00
---	--	------	------
mPD		0	0
		nPD:	1.
		N. weight:	0.
		Sc. PD:	0
		Sc. rank:	0

,

PA-X

Pos.	189	obs:	exp:
gat	D	1063	577.70
gac	D	6	491.30
gaa	E	0	1.22
gag	E	2	0.78
---	--	------	------
mPD		0.015	0.50
		nPD:	0.03
		N. weight:	0.61
		Sc. PD:	-0.024
		Sc. rank:	98.1

,

PA-X

Pos.	190	obs:	exp:
ttt	F	0	0.39
ttc	F	1	0.61
tct	S	0	170.20
tcc	S	1070	139.70
tca	S	0	271.60
tcg	S	0	60.72
agt	S	0	220.00
agc	S	0	207.80
---	--	------	------
mPD		0.0019	1.7
		nPD:	0.
		N. weight:	2.1
		Sc. PD:	-0.14
		Sc. rank:	-1327.7

,

PA-X

Pos.	191	obs:	exp:
ttt	F	1068	422.50
ttc	F	2	647.50
tct	S	1	0.16
tcc	S	0	0.13
tca	S	0	0.25
tcg	S	0	0.06
agt	S	0	0.21
agc	S	0	0.19
---	--	------	------
mPD		0.0056	0.48
		nPD:	0.01
		N. weight:	0.96
		Sc. PD:	-0.055
		Sc. rank:	-146.0

,

PA-X

Pos.	192	obs:	exp:
gtt	V	0	221.90
gtc	V	1071	216.20
gta	V	0	219.30
gtg	V	0	413.60
---	--	------	------
mPD		0	0.73
		nPD:	0.
		N. weight:	1.7
		Sc. PD:	-0.12
		Sc. rank:	-1452.0

,

PA-X

Pos.	193	obs:	exp:
tct	S	0	170.30
tcc	S	0	139.90
tca	S	0	271.80
tcg	S	0	60.77
agt	S	1071	220.20
agc	S	0	208.00
---	--	------	------
mPD		0	1.7
		nPD:	0.
		N. weight:	1.7
		Sc. PD:	-0.11
		Sc. rank:	-1435.3

,

PA-X

Pos.	194	obs:	exp:
tta	L	0	36.56
ttg	L	0	76.71
ctt	L	0	68.60
ctc	L	0	68.76
cta	L	0	69.43
ctg	L	412	91.94
cct	P	0	162.20
ccc	P	0	116.80
cca	P	0	267.60
ccg	P	650	103.50
caa	Q	0	4.58
cag	Q	9	4.42
---	--	------	------
mPD		0.48	1.4
		nPD:	0.34
		N. weight:	1.8
		Sc. PD:	0.47
		Sc. rank:	1398.7

,

PA-X

Pos.	195	obs:	exp:
aaa	K	1071	624.20
aag	K	0	446.80
---	--	------	------
mPD		0	0.49
		nPD:	0.
		N. weight:	0.56
		Sc. PD:	-0.039
		Sc. rank:	-489.8

,

PA-X

Pos.	196	obs:	exp:
gaa	E	0	651.70
gag	E	1071	419.30
---	--	------	------
mPD		0	0.48
		nPD:	0.
		N. weight:	0.98
		Sc. PD:	-0.068
		Sc. rank:	-850.8

,

PA-X

Pos.	197	obs:	exp:
gct	A	0	276.60
gcc	A	0	199.50
gca	A	0	504.10
gcg	A	1071	90.90
---	--	------	------
mPD		0	0.67
		nPD:	0.
		N. weight:	2.6
		Sc. PD:	-0.18
		Sc. rank:	-2237.9

,

PA-X

Pos.	198	obs:	exp:
cgt	R	0	2.28
cgc	R	0	2.63
cga	R	0	5.19
cgg	R	0	4.42
aaa	K	0	593.30
aag	K	1018	424.70
aga	R	0	24.23
agg	R	53	14.26
---	--	------	------
mPD		0.094	0.61
		nPD:	0.15
		N. weight:	0.94
		Sc. PD:	0.075
		Sc. rank:	632.5

,

PA-X

Pos.	199	obs:	exp:
cgt	R	0	0.43
cgc	R	0	0.50
cga	R	0	0.98
cgg	R	0	0.83
aaa	K	1059	617.20
aag	K	0	441.80
aga	R	10	4.57
agg	R	0	2.69
gaa	E	2	1.22
gag	E	0	0.78
---	--	------	------
mPD		0.022	0.51
		nPD:	0.04
		N. weight:	0.57
		Sc. PD:	-0.015
		Sc. rank:	176.1

,

PA-X

Pos.	200	obs:	exp:
caa	Q	1071	545.50
cag	Q	0	525.50
---	--	------	------
mPD		0	0.50
		nPD:	0.
		N. weight:	0.71
		Sc. PD:	-0.049
		Sc. rank:	-612.1

,

PA-X

Pos.	201	obs:	exp:
tta	L	0	95.04
ttg	L	1069	199.40
ctt	L	0	178.30
ctc	L	0	178.70
cta	L	0	180.50
ctg	L	2	239.00
---	--	------	------
mPD		0.0037	1.1
		nPD:	0.
		N. weight:	1.7
		Sc. PD:	-0.11
		Sc. rank:	-791.6

,

PA-X

Pos.	202	obs:	exp:
aaa	K	0	624.20
aag	K	1071	446.80
---	--	------	------
mPD		0	0.49
		nPD:	0.
		N. weight:	0.91
		Sc. PD:	-0.063
		Sc. rank:	-793.2

,

PA-X

Pos.	203	obs:	exp:
cgt	R	0	0.34
cgc	R	0	0.40
cga	R	0	0.78
cgg	R	0	0.67
aaa	K	1063	619.50
aag	K	0	443.50
aga	R	8	3.66
agg	R	0	2.15
---	--	------	------
mPD		0.015	0.51
		nPD:	0.03
		N. weight:	0.57
		Sc. PD:	-0.023
		Sc. rank:	83.1

,

PA-X

Pos.	204	obs:	exp:
tat	Y	2	1.08
tac	Y	0	0.92
aat	N	28	14.26
aac	N	0	13.74
gat	D	1040	562.00
gac	D	0	478.00
ggt	G	1	0.13
ggc	G	0	0.13
gga	G	0	0.46
ggg	G	0	0.28
---	--	------	------
mPD		0.056	0.55
		nPD:	0.1
		N. weight:	0.65
		Sc. PD:	0.019
		Sc. rank:	398.3

,

PA-X

Pos.	205	obs:	exp:
tta	L	0	95.04
ttg	L	1071	199.40
ctt	L	0	178.30
ctc	L	0	178.70
cta	L	0	180.50
ctg	L	0	239.00
---	--	------	------
mPD		0	1.1
		nPD:	0.
		N. weight:	1.8
		Sc. PD:	-0.12
		Sc. rank:	-1525.2

,

PA-X			
Pos.	206	obs:	exp:
cgt	R	0	0.26
cgc	R	0	0.30
cga	R	0	0.59
cgg	R	0	0.50
act	T	0	0.25
acc	T	0	0.21
aca	T	1	0.46
acg	T	0	0.08
aaa	K	1063	619.50
aag	K	0	443.50
aga	R	6	2.74
agg	R	0	1.61
gaa	E	1	0.61
gag	E	0	0.39
---	--	------	------
mPD		0.015	0.51
		nPD:	0.03
		N. weight:	0.57
		Sc. PD:	-0.023
		Sc. rank:	88.5

,

PA-X			
Pos.	207	obs:	exp:
tta	L	0	0.09
ttg	L	1	0.19
tct	S	0	170.00
tcc	S	0	139.60
tca	S	1069	271.30
tcg	S	0	60.66
ctt	L	0	0.17
ctc	L	0	0.17
cta	L	0	0.17
ctg	L	0	0.22
act	T	0	0.25
acc	T	0	0.21
aca	T	1	0.46
acg	T	0	0.08
agt	S	0	219.80
agc	S	0	207.60
---	--	------	------
mPD		0.0056	1.7
		nPD:	0.
		N. weight:	1.4
		Sc. PD:	-0.094
		Sc. rank:	-589.3

,

PA-X			
Pos.	208	obs:	exp:
tta	L	0	0.09
ttg	L	0	0.19
ctt	L	0	0.17
ctc	L	0	0.17
cta	L	0	0.17
ctg	L	1	0.22
caa	Q	0	545.00
cag	Q	1070	525.00
---	--	------	------
mPD		0.0019	0.50
		nPD:	0.
		N. weight:	0.74
		Sc. PD:	-0.049
		Sc. rank:	-302.5

,

PA-X			
Pos.	209	obs:	exp:
aaa	K	2	1.17
aag	K	0	0.83
gct	A	0	7.23
gcc	A	0	5.21
gca	A	28	13.18
gcg	A	0	2.38
gaa	E	1040	632.80
gag	E	0	407.20
ggt	G	0	0.13
ggc	G	0	0.13
gga	G	1	0.46
ggg	G	0	0.28
---	--	------	------
mPD		0.057	0.54
		nPD:	0.1
		N. weight:	0.53
		Sc. PD:	0.017
		Sc. rank:	326.5

,

PA-X			
Pos.	210	obs:	exp:
tta	L	0	0.27
ttg	L	0	0.56
ctt	L	0	0.50
ctc	L	0	0.50
cta	L	3	0.51
ctg	L	0	0.67
cct	P	0	263.50
ccc	P	0	189.70
cca	P	1056	434.70
ccg	P	0	168.10
caa	Q	12	6.11
cag	Q	0	5.89
---	--	------	------
mPD		0.028	0.74
		nPD:	0.04
		N. weight:	0.93
		Sc. PD:	-0.03
		Sc. rank:	224.4

,

PA-X			
Pos.	211	obs:	exp:
tgt	C	0	453.00
tgc	C	1071	618.00
---	--	------	------
mPD		0	0.49
		nPD:	0.
		N. weight:	0.57
		Sc. PD:	-0.04
		Sc. rank:	-498.9

,

PA-X			
Pos.	212	obs:	exp:
tct	S	0	0.32
tcc	S	0	0.26
tca	S	2	0.51
tcg	S	0	0.11
agt	S	0	0.41
agc	S	0	0.39
gtt	V	0	2.69
gtc	V	0	2.62
gta	V	13	2.66
gtg	V	0	5.02
gct	A	0	271.40
gcc	A	1	195.70
gca	A	1050	494.70
gcg	A	0	89.20
gaa	E	4	2.43
gag	E	0	1.57
ggt	G	0	0.13
ggc	G	0	0.13
gga	G	1	0.46
ggg	G	0	0.28
---	--	------	------
mPD		0.039	0.71
		nPD:	0.05
		N. weight:	0.79
		Sc. PD:	-0.013
		Sc. rank:	296.7

,

PA-X			
Pos.	213	obs:	exp:
ggt	G	0	142.00
ggc	G	1071	136.80
gga	G	0	494.10
ggg	G	0	298.20
---	--	------	------
mPD		0	0.68
		nPD:	0.
		N. weight:	2.2
		Sc. PD:	-0.15
		Sc. rank:	-1866.9

,

PA-X			
Pos.	214	obs:	exp:
tta	L	0	94.42
ttg	L	1064	198.10
tct	S	0	1.11
tcc	S	0	0.91
tca	S	0	1.78
tcg	S	7	0.40
ctt	L	0	177.20
ctc	L	0	177.60
cta	L	0	179.30
ctg	L	0	237.40
agt	S	0	1.44
agc	S	0	1.36
---	--	------	------
mPD		0.013	1.1
		nPD:	0.01
		N. weight:	1.8
		Sc. PD:	-0.1
		Sc. rank:	-280.8

,

PA-X			
Pos.	215	obs:	exp:
tta	L	0	1.33
ttg	L	0	2.79
ctt	L	0	2.50
ctc	L	0	2.50
cta	L	0	2.53
ctg	L	15	3.35
cct	P	0	263.20
ccc	P	0	189.60
cca	P	1	434.30
ccg	P	1054	167.90
caa	Q	0	0.51
cag	Q	1	0.49
---	--	------	------
mPD		0.031	0.75
		nPD:	0.04
		N. weight:	1.9
		Sc. PD:	-0.054
		Sc. rank:	561.6

,

PA-X			
Pos.	216	obs:	exp:
act	T	0	273.00
acc	T	1071	219.80
aca	T	0	491.20
acg	T	0	86.99
---	--	------	------
mPD		0	0.68
		nPD:	0.
		N. weight:	1.7
		Sc. PD:	-0.11
		Sc. rank:	-1437.0

,

PA-X			
Pos.	217	obs:	exp:
aaa	K	1071	624.20
aag	K	0	446.80
---	--	------	------
mPD		0	0.49
		nPD:	0.
		N. weight:	0.56
		Sc. PD:	-0.039
		Sc. rank:	-489.8

,

PA-X			
Pos.	218	obs:	exp:
gtt	V	0	221.70
gtc	V	1070	216.00
gta	V	0	219.10
gtg	V	0	413.20
gct	A	0	0.26
gcc	A	1	0.19
gca	A	0	0.47
gcg	A	0	0.08
---	--	------	------
mPD		0.0019	0.73
		nPD:	0.
		N. weight:	1.7
		Sc. PD:	-0.11
		Sc. rank:	-870.1

,

PA-X			
Pos.	219	obs:	exp:
ttt	F	0	0.39
ttc	F	1	0.61
tct	S	1	170.20
tcc	S	1069	139.70
tca	S	0	271.60
tcg	S	0	60.72
agt	S	0	220.00
agc	S	0	207.80
---	--	------	------
mPD		0.0037	1.7
		nPD:	0.
		N. weight:	2.1
		Sc. PD:	-0.14
		Sc. rank:	-1152.0

,

PA-X			
Pos.	220	obs:	exp:
cat	H	0	615.00
cac	H	1071	456.00
---	--	------	------
mPD		0	0.49
		nPD:	0.
		N. weight:	0.89
		Sc. PD:	-0.061
		Sc. rank:	-774.7

,

PA-X			
Pos.	221	obs:	exp:
cgt	R	0	46.00
cgc	R	0	53.05
cga	R	1071	104.80
cgg	R	0	89.37
aga	R	0	489.70
agg	R	0	288.10
---	--	------	------
mPD		0	0.96
		nPD:	0.
		N. weight:	2.4
		Sc. PD:	-0.17
		Sc. rank:	-2108.8

,

PA-X			
Pos.	222	obs:	exp:
act	T	1071	273.00
acc	T	0	219.80
aca	T	0	491.20
acg	T	0	86.99
---	--	------	------
mPD		0	0.68
		nPD:	0.
		N. weight:	1.4
		Sc. PD:	-0.098
		Sc. rank:	-1240.1

,

PA-X			
Pos.	223	obs:	exp:
tct	S	1069	170.30
tcc	S	2	139.90
tca	S	0	271.80
tcg	S	0	60.77
agt	S	0	220.20
agc	S	0	208.00
---	--	------	------
mPD		0.0037	1.7
		nPD:	0.
		N. weight:	1.9
		Sc. PD:	-0.13
		Sc. rank:	-1085.4

,

PA-X			
Pos.	224	obs:	exp:
cct	P	1	267.20
ccc	P	0	192.40
cca	P	1070	440.80
ccg	P	0	170.50
---	--	------	------
mPD		0.0019	0.71
		nPD:	0.
		N. weight:	0.92
		Sc. PD:	-0.061
		Sc. rank:	-473.3

,

PA-X

Pos.	225	obs:	exp:
gct	A	5	276.60
gcc	A	1066	199.50
gca	A	0	504.10
gcg	A	0	90.90
---	--	------	------
mPD		0.0093	0.67
		nPD:	0.01
		N. weight:	1.7
		Sc. PD:	-0.096
		Sc. rank:	-172.6

,

PA-X

Pos.	226	obs:	exp:
tta	L	0	95.04
ttg	L	1071	199.40
ctt	L	0	178.30
ctc	L	0	178.70
cta	L	0	180.50
ctg	L	0	239.00
---	--	------	------
mPD		0	1.1
		nPD:	0.
		N. weight:	1.8
		Sc. PD:	-0.12
		Sc. rank:	-1525.2

,

PA-X

Pos.	227	obs:	exp:
cgt	R	0	0.34
cgc	R	0	0.40
cga	R	0	0.78
cgg	R	0	0.67
aaa	K	1061	619.50
aag	K	2	443.50
aga	R	8	3.66
agg	R	0	2.15
---	--	------	------
mPD		0.019	0.51
		nPD:	0.04
		N. weight:	0.55
		Sc. PD:	-0.018
		Sc. rank:	128.0

,

PA-X

Pos.	228	obs:	exp:
act	T	1070	272.80
acc	T	0	219.60
aca	T	0	490.80
acg	T	0	86.91
gct	A	1	0.26
gcc	A	0	0.19
gca	A	0	0.47
gcg	A	0	0.08
---	--	------	------
mPD		0.0019	0.68
		nPD:	0.
		N. weight:	1.4
		Sc. PD:	-0.095
		Sc. rank:	-719.5

,

PA-X

Pos.	229	obs:	exp:
tta	L	1071	95.04
ttg	L	0	199.40
ctt	L	0	178.30
ctc	L	0	178.70
cta	L	0	180.50
ctg	L	0	239.00
---	--	------	------
mPD		0	1.1
		nPD:	0.
		N. weight:	2.5
		Sc. PD:	-0.17
		Sc. rank:	-2197.5

,

PA-X

Pos.	230	obs:	exp:
gaa	E	1	651.70
gag	E	1070	419.30
---	--	------	------
mPD		0.0019	0.48
		nPD:	0.
		N. weight:	0.97
		Sc. PD:	-0.063
		Sc. rank:	-330.4

,

PA-X

Pos.	231	obs:	exp:
cct	P	1071	267.20
ccc	P	0	192.40
cca	P	0	440.80
ccg	P	0	170.50
---	--	------	------
mPD		0	0.71
		nPD:	0.
		N. weight:	1.5
		Sc. PD:	-0.1
		Sc. rank:	-1259.5

,

PA-X

Pos.	232	obs:	exp:
atg	M	1071	1071.00
---	--	------	------
mPD		0	0
		nPD:	1.
		N. weight:	0.
		Sc. PD:	0
		Sc. rank:	0

,

PA-X

Pos.	233	obs:	exp:
tgg	W	1071	1071.00
---	--	------	------
mPD		0	0
		nPD:	1.
		N. weight:	0.
		Sc. PD:	0
		Sc. rank:	0

,

PA-X

Pos.	234	obs:	exp:
att	I	0	0.73
atc	I	0	0.48
ata	I	2	0.79
atg	M	1069	1069.00
---	--	------	------
mPD		0.0037	0.0037
		nPD:	1.
		N. weight:	0.0018
		Sc. PD:	0.0016
		Sc. rank:	1.6

,

PA-X

Pos.	235	obs:	exp:
gat	D	1071	578.70
gac	D	0	492.30
---	--	------	------
mPD		0	0.50
		nPD:	0.
		N. weight:	0.64
		Sc. PD:	-0.044
		Sc. rank:	-558.4

,

PA-X

Pos.	236	obs:	exp:
tta	L	0	1.06
ttg	L	12	2.23
tct	S	0	168.40
tcc	S	0	138.30
tca	S	5	268.80
tcg	S	1054	60.09
ctt	L	0	2.00
ctc	L	0	2.00
cta	L	0	2.02
ctg	L	0	2.68
agt	S	0	217.70
agc	S	0	205.70
---	--	------	------
mPD		0.031	1.7
		nPD:	0.02
		N. weight:	2.9
		Sc. PD:	-0.15
		Sc. rank:	29.2

,

PA-X

Pos.	237	obs:	exp:
tct	S	0	2.07
tcc	S	0	1.70
tca	S	0	3.30
tcg	S	0	0.74
act	T	0	0.25
acc	T	1	0.21
aca	T	0	0.46
acg	T	0	0.08
aat	N	0	537.40
aac	N	1055	517.60
agt	S	0	2.67
agc	S	13	2.52
gat	D	0	1.08
gac	D	2	0.92
---	--	------	------
mPD		0.030	0.55
		nPD:	0.05
		N. weight:	0.76
		Sc. PD:	-0.013
		Sc. rank:	275.3

,

PA-X

Pos.	238	obs:	exp:
caa	Q	1	0.51
cag	Q	0	0.49
cgt	R	0	45.96
cgc	R	0	53.00
cga	R	1070	104.70
cgg	R	0	89.28
aga	R	0	489.30
agg	R	0	287.80
---	--	------	------
mPD		0.0019	0.96
		nPD:	0.
		N. weight:	2.4
		Sc. PD:	-0.16
		Sc. rank:	-1442.0

,

PA-X

Pos.	239	obs:	exp:
cct	P	0	0.25
ccc	P	0	0.18
cca	P	0	0.41
ccg	P	1	0.16
atg	M	2	2.00
act	T	0	271.70
acc	T	0	218.70
aca	T	0	488.90
acg	T	1066	86.59
aaa	K	0	0.58
aag	K	1	0.42
gct	A	0	0.26
gcc	A	0	0.19
gca	A	0	0.47
gcg	A	1	0.08
---	--	------	------
mPD		0.0093	0.69
		nPD:	0.01
		N. weight:	2.6
		Sc. PD:	-0.15
		Sc. rank:	-308.0

,

PA-X

Pos.	240	obs:	exp:
gct	A	1068	276.10
gcc	A	0	199.10
gca	A	1	503.10
gcg	A	0	90.73
gat	D	2	1.08
gac	D	0	0.92
---	--	------	------
mPD		0.0056	0.67
		nPD:	0.01
		N. weight:	1.4
		Sc. PD:	-0.086
		Sc. rank:	-340.7

,

PA-X

Pos.	241	obs:	exp:
gtt	V	0	0.62
gtc	V	0	0.61
gta	V	3	0.61
gtg	V	0	1.16
gct	A	0	275.80
gcc	A	0	198.90
gca	A	1068	502.70
gcg	A	0	90.64
---	--	------	------
mPD		0.0056	0.68
		nPD:	0.01
		N. weight:	0.79
		Sc. PD:	-0.048
		Sc. rank:	-206.5

,

PA-X

Pos.	242	obs:	exp:
tta	L	0	95.04
ttg	L	1071	199.40
ctt	L	0	178.30
ctc	L	0	178.70
cta	L	0	180.50
ctg	L	0	239.00
---	--	------	------
mPD		0	1.1
		nPD:	0.
		N. weight:	1.8
		Sc. PD:	-0.12
		Sc. rank:	-1525.2

,

PA-X

Pos.	243	obs:	exp:
cgt	R	0	45.78
cgc	R	0	52.80
cga	R	0	104.30
cgg	R	0	88.95
aaa	K	0	2.33
aag	K	4	1.67
aga	R	0	487.40
agg	R	1066	286.70
ggt	G	0	0.13
ggc	G	0	0.13
gga	G	0	0.46
ggg	G	1	0.28
---	--	------	------
mPD		0.0093	0.97
		nPD:	0.01
		N. weight:	1.4
		Sc. PD:	-0.082
		Sc. rank:	-291.1

,

PA-X

Pos.	244	obs:	exp:
gct	A	0	276.30
gcc	A	0	199.30
gca	A	1070	503.60
gcg	A	0	90.81
gaa	E	1	0.61
gag	E	0	0.39
---	--	------	------
mPD		0.0019	0.67
		nPD:	0.
		N. weight:	0.79
		Sc. PD:	-0.052
		Sc. rank:	-392.2

,

PA-X

Pos.	245	obs:	exp:
tct	S	0	170.00
tcc	S	0	139.60
tca	S	0	271.30
tcg	S	0	60.66
aat	N	0	1.02
aac	N	2	0.98
agt	S	0	219.80
agc	S	1069	207.60
---	--	------	------
mPD		0.0037	1.7
		nPD:	0.
		N. weight:	1.7
		Sc. PD:	-0.11
		Sc. rank:	-995.7

,

PA-X

Pos.	246	obs:	exp:
ttt	F	990	422.90
ttc	F	81	648.10
---	--	------	------
mPD		0.14	0.48
		nPD:	0.29
		N. weight:	0.66
		Sc. PD:	0.14
		Sc. rank:	512.1

,

PA-X

Pos.	247	obs:	exp:
tta	L	0	94.86
ttg	L	0	199.00
ctt	L	0	178.00
ctc	L	1069	178.40
cta	L	0	180.20
ctg	L	0	238.50
cat	H	0	1.15
cac	H	2	0.85
---	--	------	------
mPD		0.0037	1.1
		nPD:	0.
		N. weight:	1.9
		Sc. PD:	-0.12
		Sc. rank:	-872.4

,

PA-X

Pos.	248	obs:	exp:
cgt	R	0	24.87
cgc	R	0	28.68
cga	R	0	56.66
cgg	R	0	48.31
aaa	K	492	286.80
aag	K	0	205.20
aga	R	579	264.80
agg	R	0	155.70
---	--	------	------
mPD		0.50	1.3
		nPD:	0.39
		N. weight:	0.7
		Sc. PD:	0.22
		Sc. rank:	556.1

PA-X			
Pos.	249	obs:	exp:
tgt	C	1071	453.00
tgc	C	0	618.00
---	--	------	------
mPD		0	0.49
		nPD:	0.
		N. weight:	0.9
		Sc. PD:	-0.062
		Sc. rank:	-780.7

,

PA-X			
Pos.	250	obs:	exp:
caa	Q	1071	545.50
cag	Q	0	525.50
---	--	------	------
mPD		0	0.50
		nPD:	0.
		N. weight:	0.71
		Sc. PD:	-0.049
		Sc. rank:	-612.1

,

PA-X			
Pos.	251	obs:	exp:
cgt	R	0	0.43
cgc	R	0	0.50
cga	R	0	0.98
cgg	R	0	0.83
aaa	K	2	617.80
aag	K	1058	442.20
aga	R	0	4.57
agg	R	10	2.69
gaa	E	0	0.61
gag	E	1	0.39
---	--	------	------
mPD		0.024	0.51
		nPD:	0.05
		N. weight:	0.9
		Sc. PD:	-0.021
		Sc. rank:	300.6

,

PA-X			
Pos.	252	obs:	exp:
aaa	K	0	624.20
aag	K	1071	446.80
---	--	------	------
mPD		0	0.49
		nPD:	0.
		N. weight:	0.91
		Sc. PD:	-0.063
		Sc. rank:	-793.2

,

PA-X			
Pos.	253	obs:	exp:
taa	.	2	455.00
tag	.	0	268.10
tga	.	1069	347.90
---	--	------	------
mPD		0.0037	0.81
		nPD:	0.
		N. weight:	1.2
		Sc. PD:	-0.075
		Sc. rank:	-374.2

}

Amino acid level conservation per codon

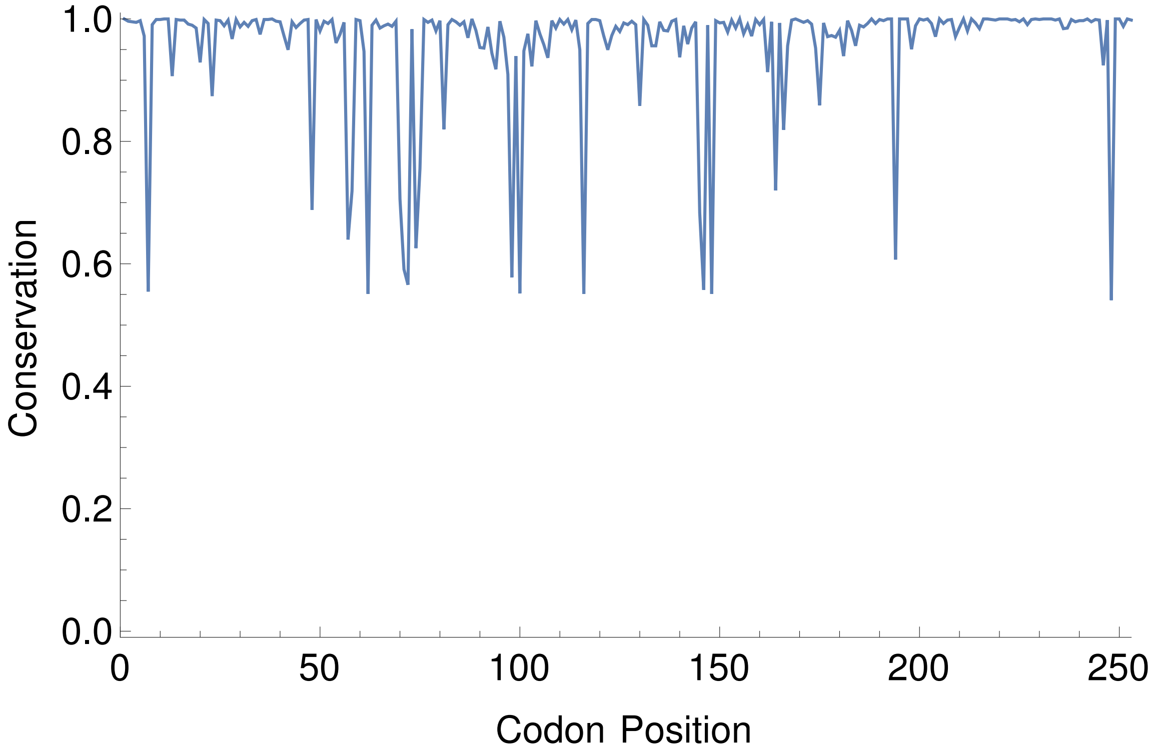

Conservation analysis plots

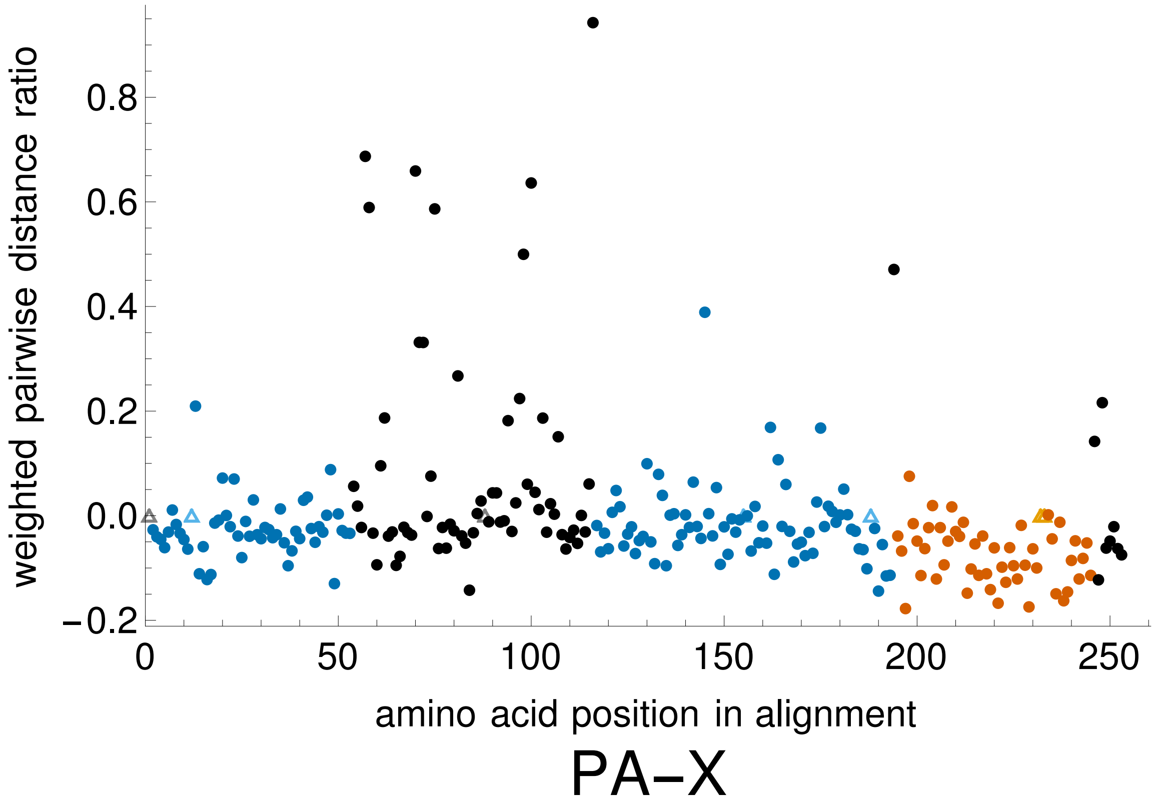

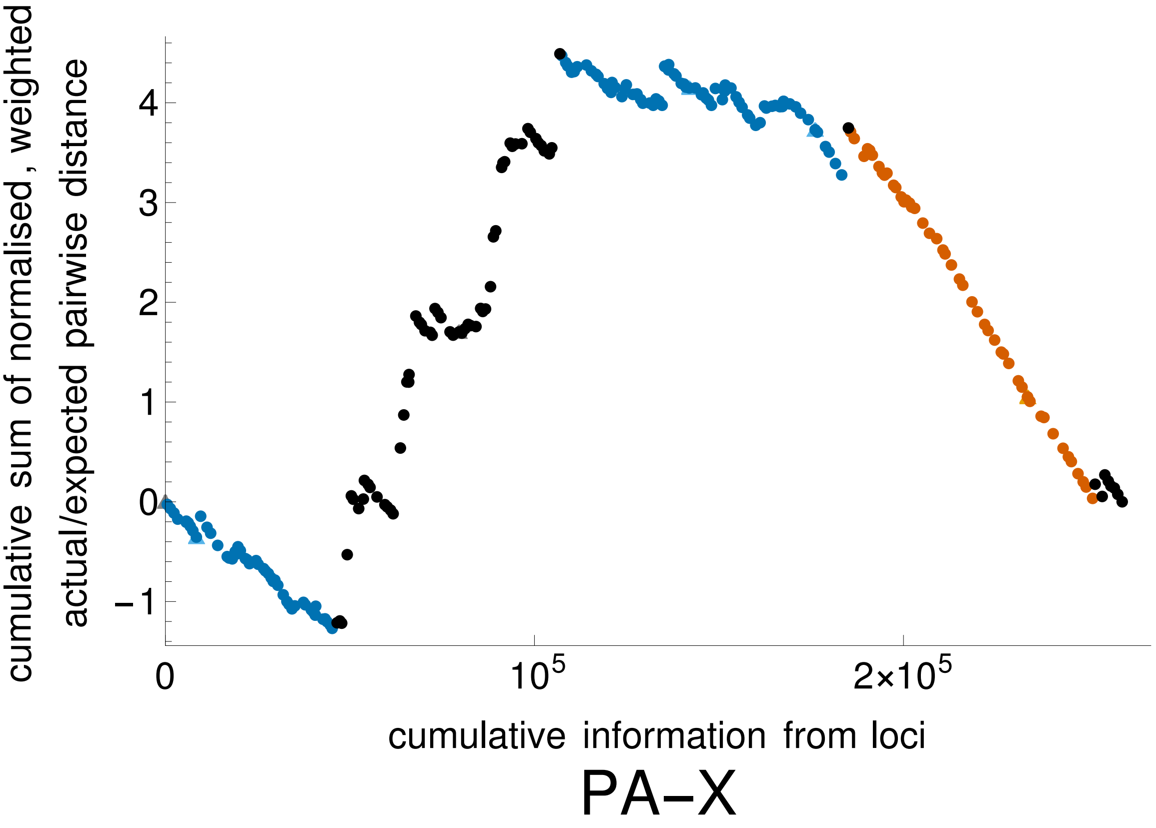

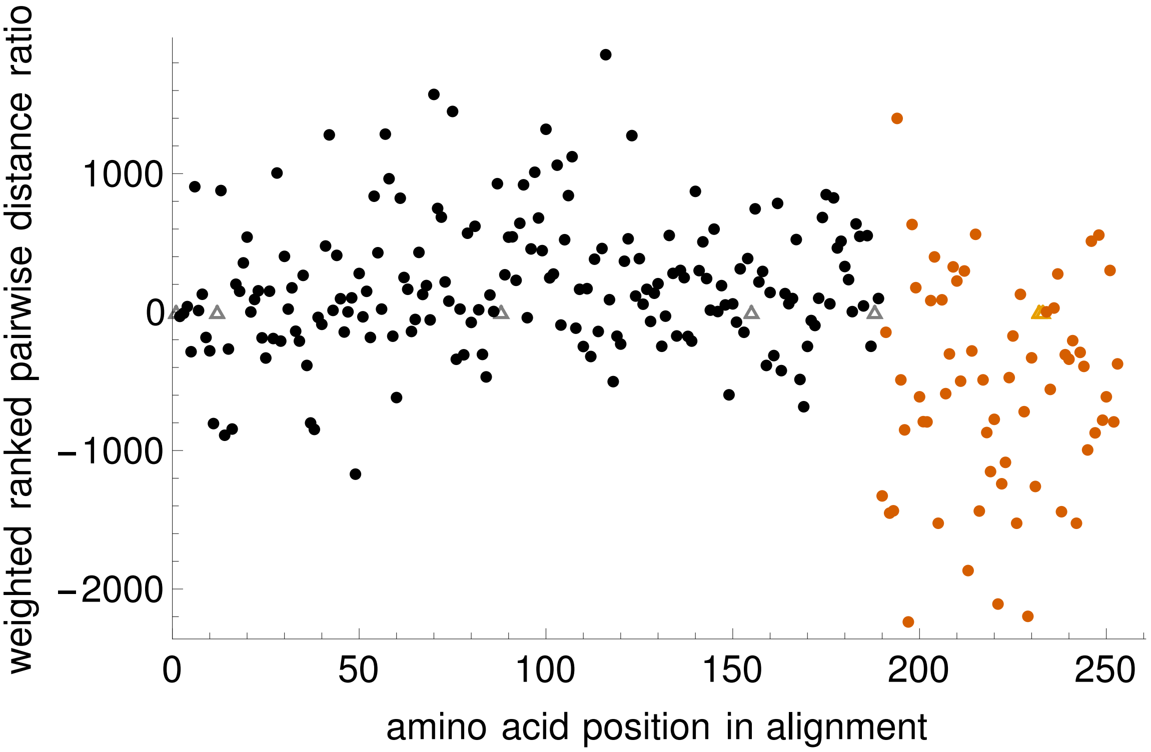
weighted ranked pairwise distance ratio
1000
0
−1000
−2000
0
50
100
150
200
250
amino acid position in alignment

PA–X

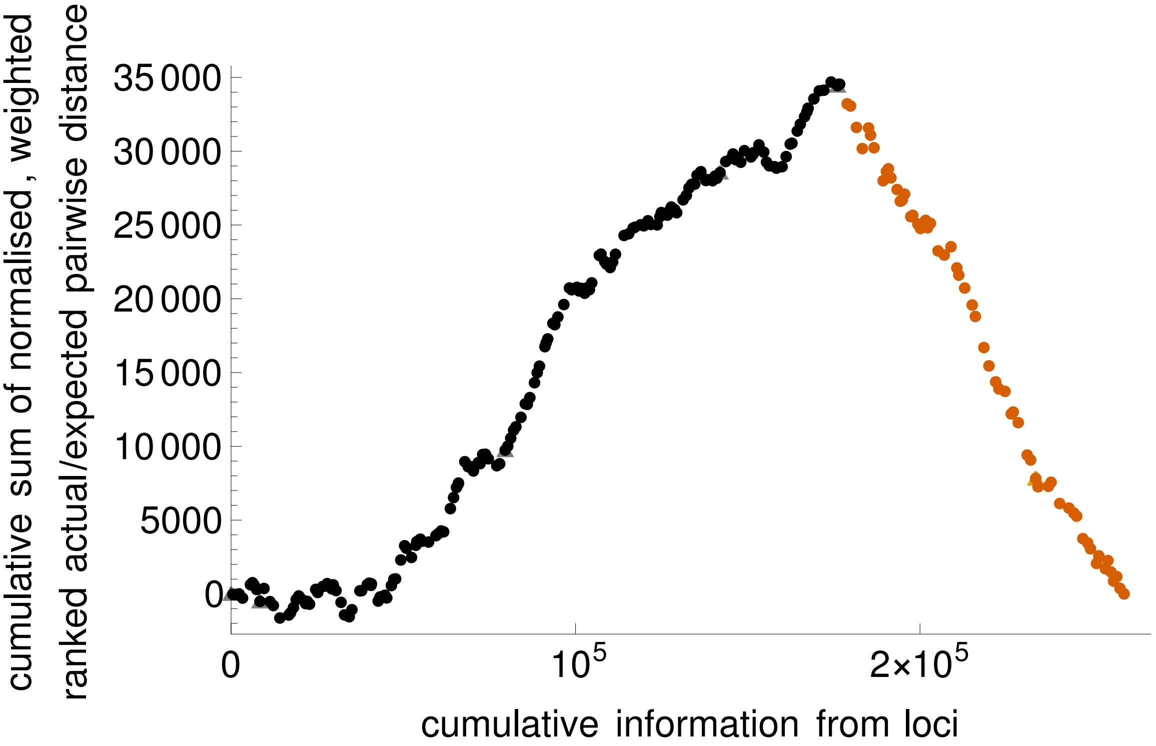
cumulative sum of normalised, weighted ranked actual/expected pairwise distance
35 000
30 000
25 000
20 000
15 000
10 000
5000
0
0
10^5
2×10^5
cumulative information from loci

PA–X

HA

Gene length histogram

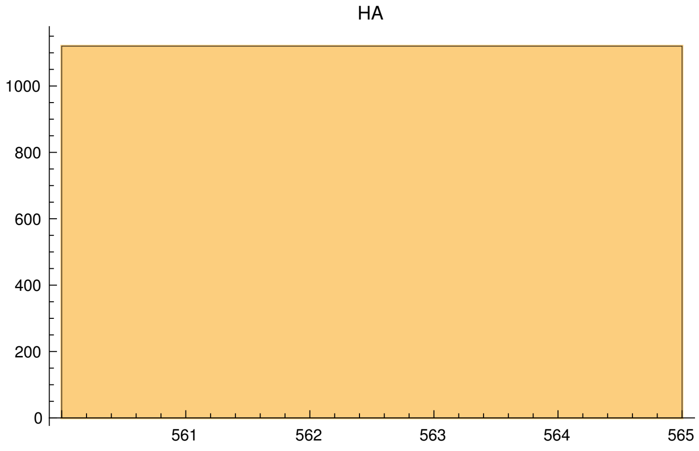

Information vs. nPD

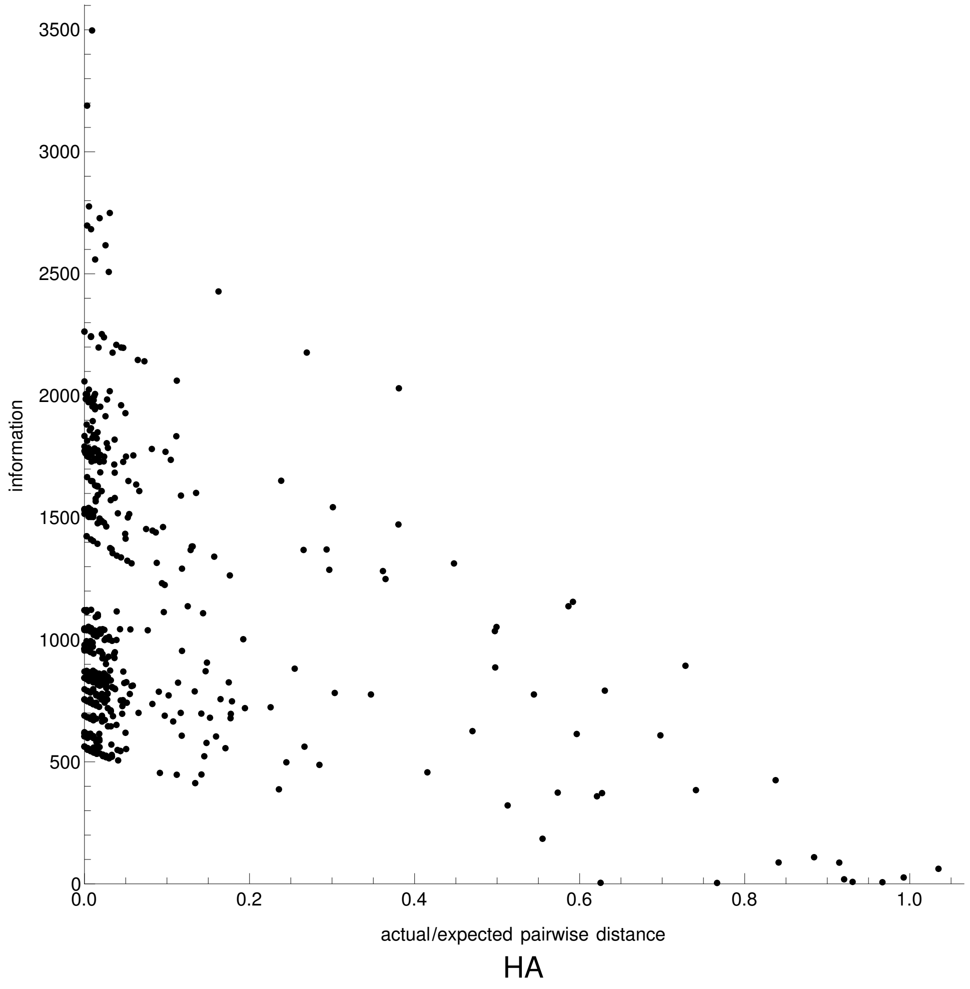

Example sequences highlighted by regions found in analyses to be conserved

```
Interesting points (by weighted raw PD) highlighted for gene HA:
ATGAACACTCAAATCCTGGTATTCGCTCTGATTGCGATCATTCCAACAAATGCAGATAAAATCTGCCTCGGACACC⋱
    ATGCCGTGTCAAACGGGACCAAAGTAAACACATTAACTGAAAGAGGAGTGGAAGTCGTCAATGCAACTGAAACAG⋱
    TGGAACGAACAAACATCCCTAGGATCTGCTCAAAAGGGAAACGGACAGTTGACCTCGGTCAATGTGGACTCCTGG⋱
    GGACAATCACTGGACCACCTCAATGTGACCAATTCCTAGAATTTTCGGCCGATTTAATTATTGAGAGGCGAGAAG⋱
    GAAGTGATGTCTGTTATCCTGGAAAATTCGTGAATGAAGAAGCTCTGAGGCAAATTCTCAGAGAATCAGGCGGAA⋱
    TTGACAAGGAAACAATGGGGTTCACATACAATGGAATAAGAACCAATGGAGTAACCAGTGCATGTAAGAGATCAG⋱
    GATCTTCATTCTATGCAGAAATGAAATGGCTCCTGTCAAACACAGATAATGCTGCATTCCCGCAGATGACTAAAT⋱
    CATATAAAAATACAAGAAAAAGCCCAGCTATAATAGTATGGGGGATCCATCATTCCGTTTCAACTGCAGAACAAA⋱
    CCAAGCTATATGGGAGTGGAAACAAACTGGTGACAGTTGGGAGCTCTAATTATCAACAATCTTTCGTACCAAGTC⋱
    CAGGAGCGAGACCACAAGTTAATGGTCTATCAGGGAGAATTGACTTTCATTGGCTAATACTAAATCCCAATGATA⋱
    CAGTCACTTTCAGTTTCAATGGGGCTTTCATAGCTCCAGACCGTGCAAGCTTCCTGAGAGGAAAATCTATGGGAA⋱
    TCCAGAGTGGAGCACAGGTTGATGCCAATTGTGAAGGGGACTGCTATCATAGTGGTGGGACAATAATAAGTAACT⋱
    TGCCATTTCAGAACATAGATAGCAGGGCAGTTGGAAAATGTCCGAGATATGTTAAGCAAAGGAGTCTGCTGCTTG⋱
    CAACAGGGATGAAGAATGTTCCTGAGATTCCAAAG ------------GGAAGAGGCCTATTTGGTGCTATAGCGGGT⋱
    TTCATTGAAAATGGATGGGAAGGCCTAATTGATGGTTGGTATGGTTTCAGACACCAGAATGCACAGGGAGAGGGA⋱
    ACTGCTGCAGATTACAAAAGCACTCAATCGGCAATTGATCAAATAACAGGGAAATTAAACCGGCTTATAGCAAAA⋱
    ACCAACCAACAATTTGAGTTGATAGACAATGAATTCAATGAGGTAGAGAAGCAAATCGGTAATGTGATAAATTGG⋱
    ACCAGAGATTCTATAACAGAAATGTGGTCATACAATGCTGAACTCTTGATAGCAATGGAGAACCAGCATACAATT⋱
    GATCTGGCTGATTCAGAAATGGACAAACTGTATGAACGGGTGAAAAGACAGCTGAGAGAGAATGCTGAAGAGGAT⋱
    GGCACTGGTTGCTTTGAAATATTTCACAAGTGTGATGATGACTGTATGGCCAGTATTAGAAACAACACCTATGAT⋱
    CACAGAAAATACAGG
```

GAAGAGGCAATGCAAAATAGAATACAGATTGACCCAGTCAAGCTAAGCAGCGGCT
ACAAAGATGTGATACTTTGGTTTAGCTTCGGGGCATCATGTTTCATACTTCT
AGCCATTGTAATGGGCCTTGTCTTCATATGT

```
GTGAAGAATGGAAACATGCGGTGCACTATTTGTATATAA
```

```
Interesting points (by weighted ranked PD) highlighted for gene HA:
ATGAACACTCAAATCCTGGTATTCGCTCTGATTGCGATCATTCCAACAAATGCAGATAAAATCTGCCTCGGACACCAT⋱
    GCCGTGTCAAACGGGACCAAAGTAAACACATTAACTGAAAGAGGAGTGGAAGTCGTCAATGCAACTGAAACAGTGGA⋱
    ACGAACAAACATCCCTAGGATCTGCTCAAAAGGGAAACGGACAGTTGACCTCGGTCAATGTGGACTCCTGGGGACAA⋱
    TCACTGGACCACCTCAATGTGACCAATTCCTAGAATTTTCGGCCGATTTAATTATTGAGAGGCGAGAAGGAAGTGAT⋱
    GTCTGTTATCCTGGAAAATTCGTGAATGAAGAAGCTCTGAGGCAAATTCTCAGAGAATCAGGCGGAATTGACAAGGA⋱
    AACAATGGGGTTCACATACAATGGAATAAGAACCAATGGAGTAACCAGTGCATGTAAGAGATCAGGATCTTCATTCT⋱
    ATGCAGAAATGAAATGGCTCCTGTCAAACACAGATAATGCTGCATTCCCGCAGATGACTAAATCATATAAAAATACA⋱
    AGAAAAAGCCCAGCTATAATAGTATGGGGGATCCATCATTCCGTTTCAACTGCAGAACAAACCAAGCTATATGGGAG⋱
    TGGAAACAAACTGGTGACAGTTGGGAGCTCTAATTATCAACAATCTTTCGTACCAAGTCCAGGAGCGAGACCACAAG⋱
    TTAATGGTCTATCAGGGAGAATTGACTTTCATTGGCTAATACTAAATCCCAATGATACAGTCACTTTCAGTTTCAAT⋱
    GGGGCTTTCATAGCTCCAGACCGTGCAAGCTTCCTGAGAGGAAAATCTATGGGAATCCAGAGTGGAGCACAGGTTGA⋱
    TGCCAATTGTGAAGGGGACTGCTATCATAGTGGTGGGACAATAATAAGTAACTTGCCATTTCAGAACATAGATAGCA⋱
    GGGCAGTTGGAAAATGTCCGAGATATGTTAAGCAAAGGAGTCTGCTGCTTGCAACAGGGATGAAGAATGTTCCTGAG⋱
    ATTCCAAAG ------------GGAAGAGGC
```

CTATTTGGTGCTATAGCGGGTTTCATTGAAAATGGATGGGAAGGCCTAATTGATG
GTTGGTATGGTTTCAGACACCAGAATGCACAGGGAGAGGGAACTGCTGCAGA

TTACAAAAGCACTCAATCGGCAATTGATCAAATAACAGGGAAATTAAACCGG
CTTATAGCAAAAACCAACCAACAATTTGAGTTGATAGACAATGAATTCAATG
AGGTAGAGAAGCAAATCGGTAATGTGATAAATTGGACCAGAGATTCTATAAC
AGAAATGTGGTCATACAATGCTGAACTCTTGATAGCAATGGAGAACCAGCATACAATTGATCTGG∵.
CTGATTCAGAAATGGACAAACTGTATGAACGGGTGAAAAGACAGCTGAGAGAGAATGCTGAAGAGGATGG∵.
CACTGGTTGCTTTGAAATATTTCACAAGTGTGATGATGACTGTATGGCCAGTATTAGAAACAACACCTAT∵.
GATCACAGAAAATACAGGGAAGAGGCAATGCAAAATAGAATACAGATTGACCCAGTCAAGCTAAGCAGCG∵.
GC
TACAAAGATGTGATACTTTGGTTTAGCTTCGGGGCATCATGTTTCATACTTCTAG
CCATTGTAATGGGCCTTGTCTTCATATGTGTGAAGAATGGAAACATGCGGTG
CACTATTTGTATATAA

Per codon conservation report

HA

Pos.	1	obs:	exp:
atg	M	1120	1120.00
---	--	------	------
mPD		0	0
		nPD:	1.
		N. weight:	0.
		Sc. PD:	0
		Sc. rank:	0

HA

Pos.	2	obs:	exp:
tct	S	0	0.16
tcc	S	0	0.13
tca	S	0	0.25
tcg	S	0	0.06
aat	N	0	568.40
aac	N	1116	547.60
agt	S	0	0.21
agc	S	1	0.19
gat	D	0	1.08
gac	D	2	0.92
---	-	1	1.00
---	--	------	------
mPD		0.0053	0.51
		nPD:	0.01
		N. weight:	0.75
		Sc. PD:	-0.028
		Sc. rank:	-154.1

HA

Pos.	3	obs:	exp:
att	I	18	6.61
atc	I	0	4.31
ata	I	0	7.07
act	T	1098	280.10
acc	T	1	225.50
aca	T	0	504.10
acg	T	0	89.27
aat	N	3	1.53
aac	N	0	1.47
---	--	------	------
mPD		0.039	0.71
		nPD:	0.05
		N. weight:	1.4
		Sc. PD:	0.0064
		Sc. rank:	859.7

HA

Pos.	4	obs:	exp:
tct	S	0	0.16
tcc	S	0	0.13
tca	S	1	0.25
tcg	S	0	0.06
caa	Q	1119	570.00
cag	Q	0	549.00
agt	S	0	0.21
agc	S	0	0.19
---	--	------	------
mPD		0.0036	0.50
		nPD:	0.01
		N. weight:	0.71
		Sc. PD:	-0.029
		Sc. rank:	-309.5

HA

Pos.	5	obs:	exp:
ttt	F	0	0.39
ttc	F	1	0.61
att	I	2	394.20
atc	I	1071	257.10
ata	I	0	421.60
act	T	0	11.47
acc	T	45	9.23
aca	T	0	20.64
acg	T	0	3.66
aat	N	1	0.51
aac	N	0	0.49
---	--	------	------
mPD		0.086	0.74
		nPD:	0.12
		N. weight:	1.5
		Sc. PD:	0.097
		Sc. rank:	1100.5

HA

Pos.	6	obs:	exp:
tta	L	0	99.30
ttg	L	0	208.30
ctt	L	0	186.30
ctc	L	0	186.70
cta	L	5	188.60
ctg	L	1114	249.70
gtt	V	0	0.21
gtc	V	0	0.20
gta	V	0	0.20
gtg	V	1	0.39
---	--	------	------
mPD		0.011	1.1
		nPD:	0.01
		N. weight:	1.5
		Sc. PD:	-0.059
		Sc. rank:	-405.4

HA

Pos.	7	obs:	exp:
att	I	0	2.57
atc	I	0	1.68
ata	I	7	2.75
gtt	V	0	226.70
gtc	V	0	220.80
gta	V	1094	224.00
gtg	V	0	422.50
gct	A	0	4.91
gcc	A	0	3.54
gca	A	19	8.94
gcg	A	0	1.61
---	--	------	------
mPD		0.046	0.77
		nPD:	0.06
		N. weight:	1.6
		Sc. PD:	0.015
		Sc. rank:	1028.9

HA

Pos.	8	obs:	exp:
ttt	F	1	440.70
ttc	F	1115	675.30
tta	L	1	0.35
ttg	L	0	0.74
ctt	L	0	0.67
ctc	L	3	0.67
cta	L	0	0.67
ctg	L	0	0.89
---	--	------	------
mPD		0.0089	0.49
		nPD:	0.02
		N. weight:	0.52
		Sc. PD:	-0.016
		Sc. rank:	49.5

HA

Pos.	9	obs:	exp:
act	T	3	0.76
acc	T	0	0.62
aca	T	0	1.38
acg	T	0	0.24
gct	A	1111	288.40
gcc	A	5	208.00
gca	A	1	525.70
gcg	A	0	94.80
---	--	------	------
mPD		0.016	0.68
		nPD:	0.02
		N. weight:	1.4
		Sc. PD:	-0.034
		Sc. rank:	362.2

HA

Pos.	10	obs:	exp:
tta	L	0	99.39
ttg	L	0	208.50
ctt	L	0	186.50
ctc	L	0	186.90
cta	L	5	188.80
ctg	L	1115	249.90
---	--	------	------
mPD		0.0089	1.1
		nPD:	0.01
		N. weight:	1.5
		Sc. PD:	-0.062
		Sc. rank:	-495.3

HA

Pos.	11	obs:	exp:
att	I	1096	403.10
atc	I	1	262.90
ata	I	0	431.10
act	T	8	5.61
acc	T	14	4.51
aca	T	0	10.09
acg	T	0	1.79
gtt	V	1	0.21
gtc	V	0	0.20
gta	V	0	0.20
gtg	V	0	0.39
---	--	------	------
mPD		0.067	0.69
		nPD:	0.1
		N. weight:	1.
		Sc. PD:	0.047
		Sc. rank:	724.5

HA

Pos.	12	obs:	exp:
tct	S	0	0.16
tcc	S	0	0.13
tca	S	0	0.25
tcg	S	1	0.06
act	T	0	0.51
acc	T	0	0.41
aca	T	0	0.92
acg	T	2	0.16
agt	S	0	0.21
agc	S	0	0.19
gtt	V	0	1.86
gtc	V	0	1.82
gta	V	0	1.84
gtg	V	9	3.48
gct	A	0	286.10
gcc	A	0	206.30
gca	A	0	521.50
gcg	A	1108	94.04
---	--	------	------
mPD		0.021	0.69
		nPD:	0.03
		N. weight:	2.6
		Sc. PD:	-0.047
		Sc. rank:	1010.6

HA

Pos.	13	obs:	exp:
att	I	1	409.70
atc	I	1114	267.20
ata	I	0	438.10
gtt	V	0	1.04
gtc	V	5	1.01
gta	V	0	1.02
gtg	V	0	1.93
---	--	------	------
mPD		0.011	0.66
		nPD:	0.02
		N. weight:	1.5
		Sc. PD:	-0.048
		Sc. rank:	57.1

HA

Pos.	14	obs:	exp:
tct	S	0	0.48
tcc	S	0	0.39
tca	S	0	0.76
tcg	S	0	0.17
att	I	1065	392.00
atc	I	2	255.70
ata	I	0	419.30
act	T	4	1.02
acc	T	0	0.82
aca	T	0	1.83
acg	T	0	0.32
aat	N	1	0.51
aac	N	0	0.49
agt	S	3	0.62
agc	S	0	0.58
gtt	V	45	9.32
gtc	V	0	9.08
gta	V	0	9.21
gtg	V	0	17.38
---	--	------	------
mPD		0.095	0.76
		nPD:	0.13
		N. weight:	1.1
		Sc. PD:	0.077
		Sc. rank:	799.8

HA

Pos.	15	obs:	exp:
tta	L	0	0.09
ttg	L	0	0.19
ctt	L	0	0.17
ctc	L	1	0.17
cta	L	0	0.17
ctg	L	0	0.22
cct	P	0	279.00
ccc	P	0	200.90
cca	P	1118	460.20
ccg	P	0	178.00
act	T	0	0.25
acc	T	0	0.21
aca	T	1	0.46
acg	T	0	0.08
---	--	------	------
mPD		0.0054	0.71
		nPD:	0.01
		N. weight:	0.93
		Sc. PD:	-0.038
		Sc. rank:	-357.0

HA

Pos.	16	obs:	exp:
att	I	0	1.10
atc	I	0	0.72
ata	I	3	1.18
act	T	0	284.50
acc	T	0	229.00
aca	T	1116	511.90
acg	T	0	90.65
gct	A	0	0.26
gcc	A	0	0.19
gca	A	1	0.47
gcg	A	0	0.08
---	--	------	------
mPD		0.0071	0.68
		nPD:	0.01
		N. weight:	0.82
		Sc. PD:	-0.031
		Sc. rank:	-185.8

HA

Pos.	17	obs:	exp:
aat	N	1114	567.40
aac	N	0	546.60
gat	D	6	3.24
gac	D	0	2.76
---	--	------	------
mPD		0.011	0.51
		nPD:	0.02
		N. weight:	0.71
		Sc. PD:	-0.019
		Sc. rank:	132.7

HA

Pos.	18	obs:	exp:
gct	A	0	289.20
gcc	A	1	208.60
gca	A	1114	527.10
gcg	A	5	95.06
---	--	------	------
mPD		0.011	0.67
		nPD:	0.02
		N. weight:	0.76
		Sc. PD:	-0.025
		Sc. rank:	10.3

HA

Pos.	19	obs:	exp:
gat	D	364	605.20
gac	D	756	514.80
---	--	------	------
mPD		0.44	0.50
		nPD:	0.88
		N. weight:	0.1
		Sc. PD:	0.082
		Sc. rank:	95.2

HA

Pos.	20	obs:	exp:
cgt	R	0	0.13
cgc	R	0	0.15
cga	R	0	0.29
cgg	R	0	0.25
aaa	K	1117	651.00
aag	K	0	466.00
aga	R	3	1.37
agg	R	0	0.81
---	--	------	------
mPD		0.0053	0.49
		nPD:	0.01
		N. weight:	0.57
		Sc. PD:	-0.021
		Sc. rank:	-103.3

HA			
Pos.	21	obs:	exp:
att	I	45	411.50
atc	I	1071	268.40
ata	I	4	440.10
---	--	------	------
mPD		0.084	0.65
		nPD:	0.13
		N. weight:	1.3
		Sc. PD:	0.098
		Sc. rank:	967.2

HA			
Pos.	22	obs:	exp:
tgt	C	3	473.70
tgc	C	1117	646.30
---	--	------	------
mPD		0.0053	0.49
		nPD:	0.01
		N. weight:	0.56
		Sc. PD:	-0.021
		Sc. rank:	-99.0

HA			
Pos.	23	obs:	exp:
tta	L	0	99.39
ttg	L	0	208.50
ctt	L	1	186.50
ctc	L	1112	186.90
cta	L	7	188.80
ctg	L	0	249.90
---	--	------	------
mPD		0.014	1.1
		nPD:	0.01
		N. weight:	1.8
		Sc. PD:	-0.065
		Sc. rank:	-174.6

HA			
Pos.	24	obs:	exp:
ggt	G	0	148.50
ggc	G	0	143.10
gga	G	1113	516.70
ggg	G	7	311.80
---	--	------	------
mPD		0.012	0.68
		nPD:	0.02
		N. weight:	0.78
		Sc. PD:	-0.023
		Sc. rank:	86.4

HA			
Pos.	25	obs:	exp:
tta	L	0	0.09
ttg	L	0	0.19
ctt	L	1	0.17
ctc	L	0	0.17
cta	L	0	0.17
ctg	L	0	0.22
cat	H	938	642.50
cac	H	181	476.50
---	--	------	------
mPD		0.27	0.49
		nPD:	0.56
		N. weight:	0.17
		Sc. PD:	0.084
		Sc. rank:	157.3

HA			
Pos.	26	obs:	exp:
cat	H	1119	643.10
cac	H	1	476.90
---	--	------	------
mPD		0.0018	0.49
		nPD:	0.
		N. weight:	0.57
		Sc. PD:	-0.025
		Sc. rank:	-328.7

HA			
Pos.	27	obs:	exp:
tct	S	0	0.64
tcc	S	4	0.52
tca	S	0	1.02
tcg	S	0	0.23
agt	S	0	0.82
agc	S	0	0.78
gct	A	8	288.20
gcc	A	1106	207.80
gca	A	2	525.30
gcg	A	0	94.72
---	--	------	------
mPD		0.025	0.68
		nPD:	0.04
		N. weight:	1.7
		Sc. PD:	-0.021
		Sc. rank:	791.8

HA			
Pos.	28	obs:	exp:
gtt	V	0	232.10
gtc	V	0	226.00
gta	V	8	229.30
gtg	V	1112	432.60
---	--	------	------
mPD		0.014	0.73
		nPD:	0.02
		N. weight:	0.96
		Sc. PD:	-0.028
		Sc. rank:	155.7

HA			
Pos.	29	obs:	exp:
tct	S	0	177.50
tcc	S	0	145.70
tca	S	1112	283.30
tcg	S	4	63.33
cct	P	0	1.00
ccc	P	0	0.72
cca	P	4	1.65
ccg	P	0	0.64
agt	S	0	229.40
agc	S	0	216.80
---	--	------	------
mPD		0.014	1.7
		nPD:	0.01
		N. weight:	1.4
		Sc. PD:	-0.056
		Sc. rank:	-403.0

HA			
Pos.	30	obs:	exp:
aat	N	5	570.00
aac	N	1114	549.00
gat	D	0	0.54
gac	D	1	0.46
---	--	------	------
mPD		0.011	0.50
		nPD:	0.02
		N. weight:	0.72
		Sc. PD:	-0.02
		Sc. rank:	156.3

HA			
Pos.	31	obs:	exp:
ggt	G	0	148.50
ggc	G	0	143.10
gga	G	826	516.70
ggg	G	294	311.80
---	--	------	------
mPD		0.39	0.68
		nPD:	0.57
		N. weight:	0.35
		Sc. PD:	0.18
		Sc. rank:	318.5

HA			
Pos.	32	obs:	exp:
act	T	6	285.50
acc	T	1106	229.80
aca	T	8	513.70
acg	T	0	90.97
---	--	------	------
mPD		0.025	0.68
		nPD:	0.04
		N. weight:	1.6
		Sc. PD:	-0.02
		Sc. rank:	744.9

HA			
Pos.	33	obs:	exp:
cgt	R	0	0.04
cgc	R	0	0.05
cga	R	0	0.10
cgg	R	0	0.08
atg	M	1	1.00
aaa	K	1112	651.60
aag	K	6	466.40
aga	R	1	0.46
agg	R	0	0.27
---	--	------	------
mPD		0.016	0.49
		nPD:	0.03
		N. weight:	0.53
		Sc. PD:	-0.0087
		Sc. rank:	224.3

HA			
Pos.	34	obs:	exp:
gtt	V	0	232.10
gtc	V	0	226.00
gta	V	1115	229.30
gtg	V	5	432.60
---	--	------	------
mPD		0.0089	0.73
		nPD:	0.01
		N. weight:	1.6
		Sc. PD:	-0.059
		Sc. rank:	-179.8

HA			
Pos.	35	obs:	exp:
aat	N	3	567.90
aac	N	1112	547.10
gat	D	0	2.70
gac	D	5	2.30
---	--	------	------
mPD		0.014	0.51
		nPD:	0.03
		N. weight:	0.73
		Sc. PD:	-0.015
		Sc. rank:	249.9

HA			
Pos.	36	obs:	exp:
act	T	0	285.50
acc	T	1	229.80
aca	T	1119	513.70
acg	T	0	90.97
---	--	------	------
mPD		0.0018	0.68
		nPD:	0.
		N. weight:	0.81
		Sc. PD:	-0.037
		Sc. rank:	-562.2

HA			
Pos.	37	obs:	exp:
tta	L	1105	99.39
ttg	L	12	208.50
ctt	L	0	186.50
ctc	L	0	186.90
cta	L	2	188.80
ctg	L	1	249.90
---	--	------	------
mPD		0.028	1.1
		nPD:	0.03
		N. weight:	2.4
		Sc. PD:	-0.057
		Sc. rank:	738.8

HA			
Pos.	38	obs:	exp:
tct	S	1	0.16
tcc	S	0	0.13
tca	S	0	0.25
tcg	S	0	0.06
act	T	1113	285.20
acc	T	6	229.60
aca	T	0	513.30
acg	T	0	90.89
agt	S	0	0.21
agc	S	0	0.19
---	--	------	------
mPD		0.012	0.68
		nPD:	0.02
		N. weight:	1.4
		Sc. PD:	-0.042
		Sc. rank:	138.6

HA			
Pos.	39	obs:	exp:
gaa	E	1119	681.50
gag	E	1	438.50
---	--	------	------
mPD		0.0018	0.48
		nPD:	0.
		N. weight:	0.51
		Sc. PD:	-0.023
		Sc. rank:	-284.7

HA			
Pos.	40	obs:	exp:
cgt	R	0	46.94
cgc	R	0	54.14
cga	R	0	107.00
cgg	R	0	91.20
aaa	K	27	15.74
aag	K	0	11.26
aga	R	1093	499.80
agg	R	0	294.00
---	--	------	------
mPD		0.047	1.0
		nPD:	0.05
		N. weight:	0.81
		Sc. PD:	-0.0019
		Sc. rank:	453.3

HA			
Pos.	41	obs:	exp:
gaa	E	13	7.91
gag	E	0	5.09
ggt	G	5	146.70
ggc	G	0	141.40
gga	G	1100	510.70
ggg	G	2	308.20
---	--	------	------
mPD		0.035	0.70
		nPD:	0.05
		N. weight:	0.77
		Sc. PD:	0.00094
		Sc. rank:	452.9

HA			
Pos.	42	obs:	exp:
gtt	V	0	232.10
gtc	V	0	226.00
gta	V	2	229.30
gtg	V	1118	432.60
---	--	------	------
mPD		0.0036	0.73
		nPD:	0.
		N. weight:	0.98
		Sc. PD:	-0.042
		Sc. rank:	-519.8

HA			
Pos.	43	obs:	exp:
gtt	V	0	0.21
gtc	V	0	0.20
gta	V	1	0.20
gtg	V	0	0.39
gaa	E	1095	680.90
gag	E	24	438.10
---	--	------	------
mPD		0.044	0.48
		nPD:	0.09
		N. weight:	0.43
		Sc. PD:	0.017
		Sc. rank:	290.8

HA			
Pos.	44	obs:	exp:
gtt	V	1	232.10
gtc	V	1113	226.00
gta	V	6	229.30
gtg	V	0	432.60
---	--	------	------
mPD		0.012	0.73
		nPD:	0.02
		N. weight:	1.6
		Sc. PD:	-0.051
		Sc. rank:	117.9

HA			
Pos.	45	obs:	exp:
gtt	V	0	232.10
gtc	V	1118	226.00
gta	V	2	229.30
gtg	V	0	432.60
---	--	------	------
mPD		0.0036	0.73
		nPD:	0.
		N. weight:	1.7
		Sc. PD:	-0.072
		Sc. rank:	-877.8

HA			
Pos.	46	obs:	exp:
aat	N	1117	570.50
aac	N	3	549.50
---	--	------	------
mPD		0.0053	0.50
		nPD:	0.01
		N. weight:	0.69
		Sc. PD:	-0.026
		Sc. rank:	-137.3

HA			
Pos.	47	obs:	exp:
gct	A	0	289.20
gcc	A	0	208.60
gca	A	1099	527.10
gcg	A	21	95.06
---	--	------	------
mPD		0.037	0.67
		nPD:	0.05
		N. weight:	0.73
		Sc. PD:	0.0038
		Sc. rank:	443.2

HA			
Pos.	48	obs:	exp:
act	T	1112	285.50
acc	T	8	229.80
aca	T	0	513.70
acg	T	0	90.97
---	--	------	------
mPD		0.014	0.68
		nPD:	0.02
		N. weight:	1.4
		Sc. PD:	-0.038
		Sc. rank:	271.1

HA			
Pos.	49	obs:	exp:
gaa	E	1117	681.50
gag	E	3	438.50
---	--	------	------
mPD		0.0053	0.48
		nPD:	0.01
		N. weight:	0.5
		Sc. PD:	-0.019
		Sc. rank:	-79.0

HA			
Pos.	50	obs:	exp:
act	T	0	285.50
acc	T	0	229.80
aca	T	1080	513.70
acg	T	40	90.97
---	--	------	------
mPD		0.069	0.68
		nPD:	0.1
		N. weight:	0.72
		Sc. PD:	0.037
		Sc. rank:	512.3

HA			
Pos.	51	obs:	exp:
gtt	V	5	232.10
gtc	V	0	226.00
gta	V	0	229.30
gtg	V	1115	432.60
---	--	------	------
mPD		0.0089	0.73
		nPD:	0.01
		N. weight:	0.97
		Sc. PD:	-0.035
		Sc. rank:	-107.1

HA			
Pos.	52	obs:	exp:
gaa	E	1114	681.50
gag	E	6	438.50
---	--	------	------
mPD		0.011	0.48
		nPD:	0.02
		N. weight:	0.49
		Sc. PD:	-0.013
		Sc. rank:	116.0

HA			
Pos.	53	obs:	exp:
caa	Q	1	0.51
cag	Q	0	0.49
cgt	R	0	48.06
cgc	R	0	55.42
cga	R	1113	109.50
cgg	R	3	93.37
aga	R	3	511.70
agg	R	0	301.00
---	--	------	------
mPD		0.012	0.96
		nPD:	0.01
		N. weight:	2.4
		Sc. PD:	-0.084
		Sc. rank:	-154.1

HA			
Pos.	54	obs:	exp:
act	T	2	284.70
acc	T	0	229.20
aca	T	1114	512.30
acg	T	1	90.73
aaa	K	2	1.17
aag	K	0	0.83
gct	A	0	0.26
gcc	A	0	0.19
gca	A	1	0.47
gcg	A	0	0.08
---	--	------	------
mPD		0.011	0.68
		nPD:	0.02
		N. weight:	0.8
		Sc. PD:	-0.026
		Sc. rank:	0.8

HA			
Pos.	55	obs:	exp:
tct	S	0	0.95
tcc	S	0	0.78
tca	S	0	1.52
tcg	S	0	0.34
cat	H	0	1.15
cac	H	2	0.85
act	T	0	0.51
acc	T	2	0.41
aca	T	0	0.92
acg	T	0	0.16
aat	N	1	565.40
aac	N	1109	544.60
agt	S	0	1.23
agc	S	6	1.17
---	--	------	------
mPD		0.020	0.53
		nPD:	0.04
		N. weight:	0.75
		Sc. PD:	-0.0089
		Sc. rank:	360.4

HA			
Pos.	56	obs:	exp:
tta	L	0	0.09
ttg	L	0	0.19
ctt	L	0	0.17
ctc	L	1	0.17
cta	L	0	0.17
ctg	L	0	0.22
att	I	1	389.80
atc	I	1060	254.30
ata	I	0	416.90
act	T	0	14.02
acc	T	55	11.29
aca	T	0	25.23
acg	T	0	4.47
gtt	V	0	0.62
gtc	V	3	0.61
gta	V	0	0.61
gtg	V	0	1.16
---	--	------	------
mPD		0.10	0.76
		nPD:	0.14
		N. weight:	1.5
		Sc. PD:	0.12
		Sc. rank:	1158.2

HA			
Pos.	57	obs:	exp:
cct	P	331	279.50
ccc	P	789	201.20
cca	P	0	461.00
ccg	P	0	178.30
---	--	------	------
mPD		0.42	0.71
		nPD:	0.59
		N. weight:	1.1
		Sc. PD:	0.55
		Sc. rank:	973.0

HA			
Pos.	58	obs:	exp:
cgt	R	0	48.06
cgc	R	0	55.42
cga	R	0	109.50
cgg	R	45	93.37
atg	M	1	1.00
aga	R	3	511.70
agg	R	1071	301.00
---	--	------	------
mPD		0.084	0.96
		nPD:	0.09
		N. weight:	1.2
		Sc. PD:	0.045
		Sc. rank:	836.7

HA			
Pos.	59	obs:	exp:
att	I	13	411.10
atc	I	1105	268.20
ata	I	1	439.70
gtt	V	0	0.21
gtc	V	1	0.20
gta	V	0	0.20
gtg	V	0	0.39
---	--	------	------
mPD		0.027	0.66
		nPD:	0.04
		N. weight:	1.4
		Sc. PD:	-0.012
		Sc. rank:	722.3

HA			
Pos.	60	obs:	exp:
tgt	C	0	473.70
tgc	C	1120	646.30
---	--	------	------
mPD		0	0.49
		nPD:	0.
		N. weight:	0.58
		Sc. PD:	-0.027
		Sc. rank:	-528.2

HA			
Pos.	61	obs:	exp:
tct	S	0	178.10
tcc	S	0	146.30
tca	S	1114	284.30
tcg	S	6	63.55
agt	S	0	230.20
agc	S	0	217.50
---	--	------	------
mPD		0.011	1.7
		nPD:	0.01
		N. weight:	1.4
		Sc. PD:	-0.059
		Sc. rank:	-620.9

HA			
Pos.	62	obs:	exp:
aaa	K	1116	652.80
aag	K	4	467.20
---	--	------	------
mPD		0.0071	0.49
		nPD:	0.01
		N. weight:	0.54
		Sc. PD:	-0.018
		Sc. rank:	-12.8

HA			
Pos.	63	obs:	exp:
ggt	G	0	148.50
ggc	G	0	143.10
gga	G	3	516.70
ggg	G	1117	311.80
---	--	------	------
mPD		0.0053	0.68
		nPD:	0.01
		N. weight:	1.3
		Sc. PD:	-0.053
		Sc. rank:	-480.4

HA			
Pos.	64	obs:	exp:
aaa	K	1116	652.80
aag	K	4	467.20
---	--	------	------
mPD		0.0071	0.49
		nPD:	0.01
		N. weight:	0.54
		Sc. PD:	-0.018
		Sc. rank:	-12.8

HA			
Pos.	65	obs:	exp:
tta	L	0	0.98
ttg	L	0	2.05
ctt	L	0	1.83
ctc	L	0	1.84
cta	L	0	1.85
ctg	L	11	2.45
cgt	R	0	39.38
cgc	R	0	45.42
cga	R	1	89.74
cgg	R	318	76.52
atg	M	13	13.00
aaa	K	0	103.70
aag	K	178	74.26
aga	R	0	419.30
agg	R	598	246.60
gtt	V	0	0.21
gtc	V	0	0.20
gta	V	0	0.20
gtg	V	1	0.39
---	--	------	------
mPD		0.72	1.2
		nPD:	0.59
		N. weight:	1.1
		Sc. PD:	0.56
		Sc. rank:	992.3

HA			
Pos.	66	obs:	exp:
act	T	0	285.20
acc	T	0	229.60
aca	T	1118	513.30
acg	T	1	90.89
gct	A	0	0.26
gcc	A	0	0.19
gca	A	1	0.47
gcg	A	0	0.08
---	--	------	------
mPD		0.0036	0.68
		nPD:	0.01
		N. weight:	0.81
		Sc. PD:	-0.035
		Sc. rank:	-403.4

HA			
Pos.	67	obs:	exp:
tta	L	0	0.09
ttg	L	0	0.19
ctt	L	1	0.17
ctc	L	0	0.17
cta	L	0	0.17
ctg	L	0	0.22
att	I	21	7.72
atc	I	0	5.03
ata	I	0	8.25
gtt	V	1098	227.50
gtc	V	0	221.60
gta	V	0	224.80
gtg	V	0	424.10
---	--	------	------
mPD		0.039	0.77
		nPD:	0.05
		N. weight:	1.6
		Sc. PD:	0.0013
		Sc. rank:	952.7

HA			
Pos.	68	obs:	exp:
gat	D	44	605.20
gac	D	1076	514.80
---	--	------	------
mPD		0.076	0.50
		nPD:	0.15
		N. weight:	0.64
		Sc. PD:	0.063
		Sc. rank:	503.5

HA			
Pos.	69	obs:	exp:
tta	L	0	99.39
ttg	L	0	208.50
ctt	L	49	186.50
ctc	L	1067	186.90
cta	L	4	188.80
ctg	L	0	249.90
---	--	------	------
mPD		0.091	1.1
		nPD:	0.08
		N. weight:	1.7
		Sc. PD:	0.052
		Sc. rank:	1108.3

HA			
Pos.	70	obs:	exp:
ggt	G	1107	148.50
ggc	G	4	143.10
gga	G	9	516.70
ggg	G	0	311.80
---	--	------	------
mPD		0.023	0.68
		nPD:	0.03
		N. weight:	2.
		Sc. PD:	-0.03
		Sc. rank:	897.8

HA			
Pos.	71	obs:	exp:
caa	Q	1119	570.50
cag	Q	1	549.50
---	--	------	------
mPD		0.0018	0.50
		nPD:	0.
		N. weight:	0.7
		Sc. PD:	-0.031
		Sc. rank:	-416.1

HA			
Pos.	72	obs:	exp:
tgt	C	1120	473.70
tgc	C	0	646.30
---	--	------	------
mPD		0	0.49
		nPD:	0.
		N. weight:	0.9
		Sc. PD:	-0.043
		Sc. rank:	-826.6

HA			
Pos.	73	obs:	exp:
ggt	G	0	148.50
ggc	G	0	143.10
gga	G	1074	516.70
ggg	G	46	311.80
---	--	------	------
mPD		0.079	0.68
		nPD:	0.12
		N. weight:	0.66
		Sc. PD:	0.042
		Sc. rank:	481.4

HA			
Pos.	74	obs:	exp:
tta	L	0	99.39
ttg	L	0	208.50
ctt	L	3	186.50
ctc	L	1051	186.90
cta	L	66	188.80
ctg	L	0	249.90
---	--	------	------
mPD		0.12	1.1
		nPD:	0.1
		N. weight:	1.6
		Sc. PD:	0.086
		Sc. rank:	1161.3

HA			
Pos.	75	obs:	exp:
tta	L	0	99.30
ttg	L	3	208.30
ctt	L	0	186.30
ctc	L	0	186.70
cta	L	6	188.60
ctg	L	1110	249.70
cgt	R	0	0.04
cgc	R	0	0.05
cga	R	0	0.10
cgg	R	1	0.08
aga	R	0	0.46
agg	R	0	0.27
---	--	------	------
mPD		0.018	1.1
		nPD:	0.02
		N. weight:	1.5
		Sc. PD:	-0.049
		Sc. rank:	42.6

HA			
Pos.	76	obs:	exp:
ggt	G	4	148.50
ggc	G	0	143.10
gga	G	9	516.70
ggg	G	1107	311.80
---	--	------	------
mPD		0.023	0.68
		nPD:	0.03
		N. weight:	1.3
		Sc. PD:	-0.019
		Sc. rank:	558.9

HA			
Pos.	77	obs:	exp:
att	I	0	1.10
atc	I	0	0.72
ata	I	3	1.18
act	T	0	284.50
acc	T	1	229.00
aca	T	1115	511.90
acg	T	0	90.65
gct	A	0	0.26
gcc	A	0	0.19
gca	A	1	0.47
gcg	A	0	0.08
---	--	------	------
mPD		0.0089	0.68
		nPD:	0.01
		N. weight:	0.81
		Sc. PD:	-0.028
		Sc. rank:	-47.9

HA			
Pos.	78	obs:	exp:
att	I	2	410.80
atc	I	1115	267.90
ata	I	1	439.30
gtt	V	0	0.41
gtc	V	2	0.40
gta	V	0	0.41
gtg	V	0	0.77
---	--	------	------
mPD		0.0089	0.66
		nPD:	0.01
		N. weight:	1.5
		Sc. PD:	-0.051
		Sc. rank:	-76.3

HA			
Pos.	79	obs:	exp:
att	I	3	1.10
atc	I	0	0.72
ata	I	0	1.18
act	T	1101	284.70
acc	T	5	229.20
aca	T	11	512.30
acg	T	0	90.73
---	--	------	------
mPD		0.034	0.68
		nPD:	0.05
		N. weight:	1.3
		Sc. PD:	-0.00023
		Sc. rank:	756.9

HA			
Pos.	80	obs:	exp:
ggt	G	0	148.50
ggc	G	6	143.10
gga	G	1113	516.70
ggg	G	1	311.80
---	--	------	------
mPD		0.012	0.68
		nPD:	0.02
		N. weight:	0.78
		Sc. PD:	-0.023
		Sc. rank:	92.8

HA			
Pos.	81	obs:	exp:
cct	P	0	279.50
ccc	P	0	201.20
cca	P	1118	461.00
ccg	P	2	178.30
---	--	------	------
mPD		0.0036	0.71
		nPD:	0.01
		N. weight:	0.92
		Sc. PD:	-0.039
		Sc. rank:	-467.9

HA			
Pos.	82	obs:	exp:
tct	S	1	0.16
tcc	S	0	0.13
tca	S	0	0.25
tcg	S	0	0.06
cct	P	1115	279.20
ccc	P	2	201.00
cca	P	2	460.60
ccg	P	0	178.10
agt	S	0	0.21
agc	S	0	0.19
---	--	------	------
mPD		0.0089	0.71
		nPD:	0.01
		N. weight:	1.4
		Sc. PD:	-0.051
		Sc. rank:	-144.9

HA			
Pos.	83	obs:	exp:
caa	Q	1120	570.50
cag	Q	0	549.50
---	--	------	------
mPD		0	0.50
		nPD:	0.
		N. weight:	0.71
		Sc. PD:	-0.034
		Sc. rank:	-648.0

HA			
Pos.	84	obs:	exp:
tgt	C	1118	473.70
tgc	C	2	646.30
---	--	------	------
mPD		0.0036	0.49
		nPD:	0.01
		N. weight:	0.89
		Sc. PD:	-0.036
		Sc. rank:	-367.7

HA			
Pos.	85	obs:	exp:
gat	D	2	605.20
gac	D	1118	514.80
---	--	------	------
mPD		0.0036	0.50
		nPD:	0.01
		N. weight:	0.8
		Sc. PD:	-0.033
		Sc. rank:	-339.4

HA			
Pos.	86	obs:	exp:
cat	H	0	0.57
cac	H	1	0.43
caa	Q	1118	570.00
cag	Q	1	549.00
---	--	------	------
mPD		0.0036	0.50
		nPD:	0.01
		N. weight:	0.7
		Sc. PD:	-0.029
		Sc. rank:	-304.8

HA			
Pos.	87	obs:	exp:
ttt	F	2	442.30
ttc	F	1118	677.70
---	--	------	------
mPD		0.0036	0.48
		nPD:	0.01
		N. weight:	0.51
		Sc. PD:	-0.021
		Sc. rank:	-204.9

HA			
Pos.	88	obs:	exp:
tta	L	0	99.39
ttg	L	0	208.50
ctt	L	0	186.50
ctc	L	0	186.90
cta	L	1119	188.80
ctg	L	1	249.90
---	--	------	------
mPD		0.0018	1.1
		nPD:	0.
		N. weight:	1.9
		Sc. PD:	-0.086
		Sc. rank:	-1352.1

HA			
Pos.	89	obs:	exp:
aaa	K	4	2.33
aag	K	0	1.67
gaa	E	1111	679.10
gag	E	5	436.90
---	--	------	------
mPD		0.016	0.48
		nPD:	0.03
		N. weight:	0.49
		Sc. PD:	-0.0078
		Sc. rank:	210.5

HA			
Pos.	90	obs:	exp:
ttt	F	1118	441.90
ttc	F	1	677.10
tta	L	0	0.09
ttg	L	0	0.19
ctt	L	1	0.17
ctc	L	0	0.17
cta	L	0	0.17
ctg	L	0	0.22
---	--	------	------
mPD		0.0036	0.48
		nPD:	0.01
		N. weight:	0.97
		Sc. PD:	-0.039
		Sc. rank:	-386.8

HA			
Pos.	91	obs:	exp:
tct	S	0	178.10
tcc	S	0	146.30
tca	S	601	284.30
tcg	S	519	63.55
agt	S	0	230.20
agc	S	0	217.50
---	--	------	------
mPD		0.50	1.7
		nPD:	0.3
		N. weight:	1.4
		Sc. PD:	0.35
		Sc. rank:	1250.4

,

HA			
Pos.	92	obs:	exp:
act	T	0	0.25
acc	T	1	0.21
aca	T	0	0.46
acg	T	0	0.08
gct	A	1	289.00
gcc	A	1117	208.40
gca	A	1	526.70
gcg	A	0	94.97
---	--	------	------
mPD		0.0054	0.67
		nPD:	0.01
		N. weight:	1.7
		Sc. PD:	-0.07
		Sc. rank:	-569.0

,

HA			
Pos.	93	obs:	exp:
gat	D	1120	605.20
gac	D	0	514.80
---	--	------	------
mPD		0	0.50
		nPD:	0.
		N. weight:	0.64
		Sc. PD:	-0.031
		Sc. rank:	-591.3

,

HA			
Pos.	94	obs:	exp:
tta	L	1118	99.39
ttg	L	2	208.50
ctt	L	0	186.50
ctc	L	0	186.90
cta	L	0	188.80
ctg	L	0	249.90
---	--	------	------
mPD		0.0036	1.1
		nPD:	0.
		N. weight:	2.5
		Sc. PD:	-0.11
		Sc. rank:	-1623.7

,

HA			
Pos.	95	obs:	exp:
att	I	1115	411.50
atc	I	5	268.40
ata	I	0	440.10
---	--	------	------
mPD		0.0089	0.65
		nPD:	0.01
		N. weight:	1.
		Sc. PD:	-0.035
		Sc. rank:	-48.7

,

HA			
Pos.	96	obs:	exp:
att	I	1105	406.70
atc	I	2	265.30
ata	I	0	435.00
gtt	V	13	2.69
gtc	V	0	2.62
gta	V	0	2.66
gtg	V	0	5.02
---	--	------	------
mPD		0.027	0.68
		nPD:	0.04
		N. weight:	1.
		Sc. PD:	-0.011
		Sc. rank:	520.1

,

HA			
Pos.	97	obs:	exp:
gaa	E	2	681.50
gag	E	1118	438.50
---	--	------	------
mPD		0.0036	0.48
		nPD:	0.01
		N. weight:	0.97
		Sc. PD:	-0.039
		Sc. rank:	-377.0

,

HA			
Pos.	98	obs:	exp:
cgt	R	0	48.10
cgc	R	0	55.47
cga	R	0	109.60
cgg	R	0	93.45
aga	R	18	512.10
agg	R	1102	301.20
---	--	------	------
mPD		0.032	0.96
		nPD:	0.03
		N. weight:	1.3
		Sc. PD:	-0.021
		Sc. rank:	544.2

,

HA			
Pos.	99	obs:	exp:
caa	Q	1	0.51
cag	Q	0	0.49
cgt	R	0	48.06
cgc	R	0	55.42
cga	R	1104	109.50
cgg	R	9	93.37
aga	R	6	511.70
agg	R	0	301.00
---	--	------	------
mPD		0.028	0.96
		nPD:	0.03
		N. weight:	2.3
		Sc. PD:	-0.045
		Sc. rank:	869.3

,

HA			
Pos.	100	obs:	exp:
gaa	E	1116	680.30
gag	E	2	437.70
ggt	G	0	0.27
ggc	G	0	0.26
gga	G	2	0.92
ggg	G	0	0.56
---	--	------	------
mPD		0.0071	0.48
		nPD:	0.01
		N. weight:	0.51
		Sc. PD:	-0.017
		Sc. rank:	-7.8

,

HA			
Pos.	101	obs:	exp:
ggt	G	0	148.50
ggc	G	0	143.10
gga	G	1119	516.70
ggg	G	1	311.80
---	--	------	------
mPD		0.0018	0.68
		nPD:	0.
		N. weight:	0.8
		Sc. PD:	-0.036
		Sc. rank:	-551.6

,

HA			
Pos.	102	obs:	exp:
tct	S	0	178.00
tcc	S	0	146.10
tca	S	0	284.00
tcg	S	0	63.50
aat	N	1	0.51
aac	N	0	0.49
agt	S	1112	230.00
agc	S	7	217.30
---	--	------	------
mPD		0.014	1.7
		nPD:	0.01
		N. weight:	1.6
		Sc. PD:	-0.064
		Sc. rank:	-468.0

,

HA			
Pos.	103	obs:	exp:
aat	N	1	0.51
aac	N	0	0.49
gat	D	1114	602.00
gac	D	0	512.00
gaa	E	5	3.04
gag	E	0	1.96
---	--	------	------
mPD		0.011	0.50
		nPD:	0.02
		N. weight:	0.64
		Sc. PD:	-0.018
		Sc. rank:	138.9

,

HA			
Pos.	104	obs:	exp:
ttt	F	0	1.58
ttc	F	4	2.42
att	I	0	2.94
atc	I	8	1.92
ata	I	0	3.14
gtt	V	0	229.60
gtc	V	1108	223.60
gta	V	0	226.90
gtg	V	0	427.90
---	--	------	------
mPD		0.021	0.75
		nPD:	0.03
		N. weight:	1.7
		Sc. PD:	-0.034
		Sc. rank:	590.7

,

HA			
Pos.	105	obs:	exp:
tgt	C	1119	473.70
tgc	C	1	646.30
---	--	------	------
mPD		0.0018	0.49
		nPD:	0.
		N. weight:	0.89
		Sc. PD:	-0.04
		Sc. rank:	-507.2

,

HA			
Pos.	106	obs:	exp:
tat	Y	1111	604.40
tac	Y	8	514.60
cat	H	1	0.57
cac	H	0	0.43
---	--	------	------
mPD		0.016	0.50
		nPD:	0.03
		N. weight:	0.6
		Sc. PD:	-0.01
		Sc. rank:	252.6

,

HA			
Pos.	107	obs:	exp:
cct	P	1118	279.50
ccc	P	0	201.20
cca	P	2	461.00
ccg	P	0	178.30
---	--	------	------
mPD		0.0036	0.71
		nPD:	0.01
		N. weight:	1.4
		Sc. PD:	-0.062
		Sc. rank:	-733.5

,

HA			
Pos.	108	obs:	exp:
ggt	G	0	148.50
ggc	G	0	143.10
gga	G	313	516.70
ggg	G	807	311.80
---	--	------	------
mPD		0.40	0.68
		nPD:	0.6
		N. weight:	0.57
		Sc. PD:	0.3
		Sc. rank:	528.4

,

HA			
Pos.	109	obs:	exp:
aaa	K	1080	652.80
aag	K	40	467.20
---	--	------	------
mPD		0.069	0.49
		nPD:	0.14
		N. weight:	0.42
		Sc. PD:	0.037
		Sc. rank:	325.5

,

HA			
Pos.	110	obs:	exp:
ttt	F	2	442.30
ttc	F	1118	677.70
---	--	------	------
mPD		0.0036	0.48
		nPD:	0.01
		N. weight:	0.51
		Sc. PD:	-0.021
		Sc. rank:	-204.9

,

HA			
Pos.	111	obs:	exp:
atg	M	2	2.00
gtt	V	0	231.70
gtc	V	0	225.60
gta	V	14	228.90
gtg	V	1104	431.80
---	--	------	------
mPD		0.028	0.73
		nPD:	0.04
		N. weight:	0.93
		Sc. PD:	-0.0096
		Sc. rank:	462.4

,

HA			
Pos.	112	obs:	exp:
aat	N	1117	570.50
aac	N	3	549.50
---	--	------	------
mPD		0.0053	0.50
		nPD:	0.01
		N. weight:	0.69
		Sc. PD:	-0.026
		Sc. rank:	-137.3

,

HA			
Pos.	113	obs:	exp:
gaa	E	1083	681.50
gag	E	37	438.50
---	--	------	------
mPD		0.064	0.48
		nPD:	0.13
		N. weight:	0.39
		Sc. PD:	0.031
		Sc. rank:	296.6

,

HA			
Pos.	114	obs:	exp:
gaa	E	1118	681.50
gag	E	2	438.50
---	--	------	------
mPD		0.0036	0.48
		nPD:	0.01
		N. weight:	0.51
		Sc. PD:	-0.021
		Sc. rank:	-197.9

,

HA			
Pos.	115	obs:	exp:
act	T	1	0.25
acc	T	0	0.21
aca	T	0	0.46
acg	T	0	0.08
gct	A	1117	289.00
gcc	A	2	208.40
gca	A	0	526.70
gcg	A	0	94.97
---	--	------	------
mPD		0.0054	0.67
		nPD:	0.01
		N. weight:	1.4
		Sc. PD:	-0.056
		Sc. rank:	-466.3

,

HA			
Pos.	116	obs:	exp:
tta	L	0	99.39
ttg	L	11	208.50
ctt	L	0	186.50
ctc	L	0	186.90
cta	L	2	188.80
ctg	L	1107	249.90
---	--	------	------
mPD		0.023	1.1
		nPD:	0.02
		N. weight:	1.5
		Sc. PD:	-0.042
		Sc. rank:	275.7

,

HA			
Pos.	117	obs:	exp:
cgt	R	0	48.10
cgc	R	0	55.47
cga	R	0	109.60
cgg	R	0	93.45
aga	R	17	512.10
agg	R	1103	301.20
---	--	------	------
mPD		0.030	0.96
		nPD:	0.03
		N. weight:	1.3
		Sc. PD:	-0.023
		Sc. rank:	517.9

,

HA			
Pos.	118	obs:	exp:
caa	Q	1119	570.50
cag	Q	1	549.50
---	--	------	------
mPD		0.0018	0.50
		nPD:	0.
		N. weight:	0.7
		Sc. PD:	-0.031
		Sc. rank:	-416.1

,

HA			
Pos.	119	obs:	exp:
att	I	1104	410.80
atc	I	4	267.90
ata	I	10	439.30
gtt	V	2	0.41
gtc	V	0	0.40
gta	V	0	0.41
gtg	V	0	0.77
---	--	------	------
mPD		0.028	0.66
		nPD:	0.04
		N. weight:	0.98
		Sc. PD:	-0.006
		Sc. rank:	501.1

,

HA			
Pos.	120	obs:	exp:
tta	L	0	99.39
ttg	L	0	208.50
ctt	L	0	186.50
ctc	L	1114	186.90
cta	L	6	188.80
ctg	L	0	249.90
---	--	------	------
mPD		0.011	1.1
		nPD:	0.01
		N. weight:	1.8
		Sc. PD:	-0.071
		Sc. rank:	-493.2

,

HA			
Pos.	121	obs:	exp:
cgt	R	0	48.10
cgc	R	0	55.47
cga	R	0	109.60
cgg	R	0	93.45
aga	R	1116	512.10
agg	R	4	301.20
mPD		0.0071	0.96
		nPD:	0.01
		N. weight:	0.8
		Sc. PD:	-0.032
		Sc. rank:	-323.9

HA			
Pos.	122	obs:	exp:
cgt	R	0	0.13
cgc	R	0	0.15
cga	R	0	0.29
cgg	R	0	0.25
aaa	K	68	39.63
aag	K	0	28.37
aga	R	3	1.37
agg	R	0	0.81
gaa	E	1036	638.30
gag	E	13	410.70
mPD		0.15	0.60
		nPD:	0.24
		N. weight:	0.47
		Sc. PD:	0.088
		Sc. rank:	389.7

HA			
Pos.	123	obs:	exp:
tct	S	0	178.10
tcc	S	0	146.30
tca	S	1120	284.30
tcg	S	0	63.55
agt	S	0	230.20
agc	S	0	217.50
mPD		0	1.7
		nPD:	0.
		N. weight:	1.4
		Sc. PD:	-0.069
		Sc. rank:	-1317.2

HA			
Pos.	124	obs:	exp:
ggt	G	15	148.50
ggc	G	1092	143.10
gga	G	13	516.70
ggg	G	0	311.80
mPD		0.049	0.68
		nPD:	0.07
		N. weight:	2.
		Sc. PD:	0.045
		Sc. rank:	1305.5

HA			
Pos.	125	obs:	exp:
ggt	G	0	148.50
ggc	G	0	143.10
gga	G	1117	516.70
ggg	G	3	311.80
mPD		0.0053	0.68
		nPD:	0.01
		N. weight:	0.79
		Sc. PD:	-0.032
		Sc. rank:	-288.9

HA			
Pos.	126	obs:	exp:
tta	L	0	0.27
ttg	L	0	0.56
ctt	L	3	0.50
ctc	L	0	0.50
cta	L	0	0.51
ctg	L	0	0.67
att	I	1063	410.40
atc	I	45	267.70
ata	I	9	438.90
mPD		0.098	0.66
		nPD:	0.15
		N. weight:	0.85
		Sc. PD:	0.081
		Sc. rank:	668.8

HA			
Pos.	127	obs:	exp:
tat	Y	0	0.54
tac	Y	1	0.46
aat	N	0	1.02
aac	N	2	0.98
gtt	V	0	0.41
gtc	V	2	0.40
gta	V	0	0.41
gtg	V	0	0.77
gat	D	7	590.60
gac	D	1086	502.40
gaa	E	22	13.39
gag	E	0	8.61
mPD		0.060	0.53
		nPD:	0.11
		N. weight:	0.77
		Sc. PD:	0.047
		Sc. rank:	564.7

HA			
Pos.	128	obs:	exp:
aaa	K	6	652.80
aag	K	1114	467.20
mPD		0.011	0.49
		nPD:	0.02
		N. weight:	0.88
		Sc. PD:	-0.023
		Sc. rank:	203.1

HA			
Pos.	129	obs:	exp:
gaa	E	1116	681.50
gag	E	4	438.50
mPD		0.0071	0.48
		nPD:	0.01
		N. weight:	0.5
		Sc. PD:	-0.017
		Sc. rank:	-2.4

HA			
Pos.	130	obs:	exp:
tct	S	0	0.16
tcc	S	0	0.13
tca	S	1	0.25
tcg	S	0	0.06
cct	P	0	12.98
ccc	P	52	9.34
cca	P	0	21.40
ccg	P	0	8.28
act	T	0	84.63
acc	T	0	68.13
aca	T	332	152.30
acg	T	0	26.97
agt	S	0	0.21
agc	S	0	0.19
gct	A	3	189.50
gcc	A	0	136.70
gca	A	731	345.50
gcg	A	0	62.30
gaa	E	1	0.61
gag	E	0	0.39
mPD		0.58	1.2
		nPD:	0.5
		N. weight:	0.83
		Sc. PD:	0.36
		Sc. rank:	748.7

HA			
Pos.	131	obs:	exp:
atg	M	1120	1120.00
mPD		0	0
		nPD:	1.
		N. weight:	0.
		Sc. PD:	0
		Sc. rank:	0

HA			
Pos.	132	obs:	exp:
cgt	R	0	0.17
cgc	R	0	0.20
cga	R	0	0.39
cgg	R	0	0.33
aga	R	4	1.83
agg	R	0	1.08
ggt	G	0	147.90
ggc	G	0	142.60
gga	G	785	514.80
ggg	G	331	310.70
mPD		0.42	0.68
		nPD:	0.62
		N. weight:	0.34
		Sc. PD:	0.18
		Sc. rank:	309.1

HA			
Pos.	133	obs:	exp:
ttt	F	6	442.30
ttc	F	1114	677.70
mPD		0.011	0.48
		nPD:	0.02
		N. weight:	0.5
		Sc. PD:	-0.013
		Sc. rank:	116.6

HA			
Pos.	134	obs:	exp:
act	T	0	284.50
acc	T	0	229.00
aca	T	1109	511.90
acg	T	7	90.65
aaa	K	4	2.33
aag	K	0	1.67
mPD		0.020	0.68
		nPD:	0.03
		N. weight:	0.79
		Sc. PD:	-0.016
		Sc. rank:	284.1

HA			
Pos.	135	obs:	exp:
tat	Y	2	604.90
tac	Y	1118	515.10
mPD		0.0036	0.50
		nPD:	0.01
		N. weight:	0.8
		Sc. PD:	-0.033
		Sc. rank:	-341.0

HA			
Pos.	136	obs:	exp:
tct	S	0	96.22
tcc	S	0	79.01
tca	S	0	153.60
tcg	S	0	34.33
aat	N	512	260.80
aac	N	0	251.20
agt	S	602	124.40
agc	S	3	117.50
gat	D	3	1.62
gac	D	0	1.38
mPD		0.51	1.7
		nPD:	0.3
		N. weight:	1.2
		Sc. PD:	0.29
		Sc. rank:	1036.9

HA			
Pos.	137	obs:	exp:
gaa	E	1	0.61
gag	E	0	0.39
ggt	G	0	148.30
ggc	G	0	142.90
gga	G	1112	516.20
ggg	G	7	311.50
mPD		0.014	0.68
		nPD:	0.02
		N. weight:	0.78
		Sc. PD:	-0.021
		Sc. rank:	147.6

HA			
Pos.	138	obs:	exp:
att	I	0	403.40
atc	I	0	263.10
ata	I	1098	431.40
act	T	0	5.61
acc	T	0	4.51
aca	T	22	10.09
acg	T	0	1.79
mPD		0.039	0.69
		nPD:	0.06
		N. weight:	0.98
		Sc. PD:	0.0058
		Sc. rank:	597.0

HA			
Pos.	139	obs:	exp:
cgt	R	0	48.10
cgc	R	0	55.47
cga	R	0	109.60
cgg	R	0	93.45
aga	R	1119	512.10
agg	R	1	301.20
mPD		0.0018	0.96
		nPD:	0.
		N. weight:	0.81
		Sc. PD:	-0.037
		Sc. rank:	-584.8

HA			
Pos.	140	obs:	exp:
att	I	4	1.47
atc	I	0	0.96
ata	I	0	1.57
act	T	870	273.00
acc	T	200	219.80
aca	T	1	491.20
acg	T	0	86.99
gct	A	45	11.62
gcc	A	0	8.38
gca	A	0	21.18
gcg	A	0	3.82
mPD		0.38	0.76
		nPD:	0.5
		N. weight:	0.98
		Sc. PD:	0.43
		Sc. rank:	891.9

HA

Pos.	141	obs:	exp:
tct	S	0	0.64
tcc	S	0	0.52
tca	S	0	1.02
tcg	S	0	0.23
aat	N	1107	564.40
aac	N	1	543.60
agt	S	4	0.82
agc	S	0	0.78
gat	D	8	4.32
gac	D	0	3.68
---	--	------	------
mPD		0.023	0.53
		nPD:	0.04
		N. weight:	0.7
		Sc. PD:	-0.0038
		Sc. rank:	364.9

HA

Pos.	142	obs:	exp:
ggt	G	0	148.50
ggc	G	0	143.10
gga	G	1008	516.70
ggg	G	112	311.80
---	--	------	------
mPD		0.18	0.68
		nPD:	0.27
		N. weight:	0.53
		Sc. PD:	0.11
		Sc. rank:	444.3

HA

Pos.	143	obs:	exp:
tct	S	0	0.48
tcc	S	0	0.39
tca	S	3	0.76
tcg	S	0	0.17
act	T	0	4.08
acc	T	0	3.28
aca	T	15	7.34
acg	T	1	1.30
agt	S	0	0.62
agc	S	0	0.58
gtt	V	0	104.00
gtc	V	0	101.30
gta	V	453	102.80
gtg	V	49	193.90
gct	A	0	154.70
gcc	A	6	111.60
gca	A	590	281.90
gcg	A	3	50.84
---	--	------	------
mPD		0.63	1.3
		nPD:	0.5
		N. weight:	0.97
		Sc. PD:	0.42
		Sc. rank:	871.9

HA

Pos.	144	obs:	exp:
tct	S	0	0.16
tcc	S	0	0.13
tca	S	0	0.25
tcg	S	0	0.06
act	T	1	285.20
acc	T	1118	229.60
aca	T	0	513.30
acg	T	0	90.89
agt	S	0	0.21
agc	S	1	0.19
---	--	------	------
mPD		0.0036	0.68
		nPD:	0.01
		N. weight:	1.7
		Sc. PD:	-0.07
		Sc. rank:	-825.9

HA

Pos.	145	obs:	exp:
tct	S	0	175.90
tcc	S	0	144.40
tca	S	0	280.70
tcg	S	0	62.76
aat	N	1	0.51
aac	N	0	0.49
agt	S	1104	227.40
agc	S	2	214.80
ggt	G	13	1.72
ggc	G	0	1.66
gga	G	0	6.00
ggg	G	0	3.62
---	--	------	------
mPD		0.028	1.7
		nPD:	0.02
		N. weight:	1.6
		Sc. PD:	-0.052
		Sc. rank:	88.5

HA

Pos.	146	obs:	exp:
tct	S	0	0.16
tcc	S	0	0.13
tca	S	1	0.25
tcg	S	0	0.06
act	T	0	0.25
acc	T	0	0.21
aca	T	1	0.46
acg	T	0	0.08
agt	S	0	0.21
agc	S	0	0.19
gct	A	0	288.70
gcc	A	0	208.20
gca	A	1117	526.20
gcg	A	1	94.89
---	--	------	------
mPD		0.0054	0.67
		nPD:	0.01
		N. weight:	0.78
		Sc. PD:	-0.031
		Sc. rank:	-263.9

HA

Pos.	147	obs:	exp:
tgt	C	1073	473.70
tgc	C	47	646.30
---	--	------	------
mPD		0.080	0.49
		nPD:	0.16
		N. weight:	0.71
		Sc. PD:	0.079
		Sc. rank:	570.9

HA

Pos.	148	obs:	exp:
cgt	R	0	30.92
cgc	R	0	35.66
cga	R	0	70.46
cgg	R	0	60.08
aat	N	1	0.51
aac	N	0	0.49
aaa	K	0	232.00
aag	K	398	166.00
aga	R	2	329.20
agg	R	718	193.70
ggt	G	0	0.13
ggc	G	0	0.13
gga	G	0	0.46
ggg	G	1	0.28
---	--	------	------
mPD		0.47	1.3
		nPD:	0.36
		N. weight:	1.2
		Sc. PD:	0.36
		Sc. rank:	1048.8

HA

Pos.	149	obs:	exp:
cgt	R	0	48.10
cgc	R	0	55.47
cga	R	0	109.60
cgg	R	0	93.45
aga	R	1116	512.10
agg	R	4	301.20
---	--	------	------
mPD		0.0071	0.96
		nPD:	0.01
		N. weight:	0.8
		Sc. PD:	-0.032
		Sc. rank:	-323.9

HA

Pos.	150	obs:	exp:
tct	S	0	178.10
tcc	S	0	146.30
tca	S	1120	284.30
tcg	S	0	63.55
agt	S	0	230.20
agc	S	0	217.50
---	--	------	------
mPD		0	1.7
		nPD:	0.
		N. weight:	1.4
		Sc. PD:	-0.069
		Sc. rank:	-1317.2

HA

Pos.	151	obs:	exp:
gaa	E	4	2.43
gag	E	0	1.57
ggt	G	0	147.90
ggc	G	0	142.60
gga	G	1115	514.80
ggg	G	1	310.70
---	--	------	------
mPD		0.0089	0.68
		nPD:	0.01
		N. weight:	0.8
		Sc. PD:	-0.028
		Sc. rank:	-49.6

HA

Pos.	152	obs:	exp:
ttt	F	13	5.13
ttc	F	0	7.87
tct	S	1091	174.20
tcc	S	1	143.00
tca	S	0	277.90
tcg	S	3	62.13
cct	P	12	2.99
ccc	P	0	2.16
cca	P	0	4.94
ccg	P	0	1.91
agt	S	0	225.10
agc	S	0	212.70
---	--	------	------
mPD		0.051	1.7
		nPD:	0.03
		N. weight:	1.9
		Sc. PD:	-0.034
		Sc. rank:	726.2

HA

Pos.	153	obs:	exp:
tct	S	0	178.10
tcc	S	0	146.30
tca	S	1119	284.30
tcg	S	1	63.55
agt	S	0	230.20
agc	S	0	217.50
---	--	------	------
mPD		0.0018	1.7
		nPD:	0.
		N. weight:	1.4
		Sc. PD:	-0.067
		Sc. rank:	-1066.0

HA

Pos.	154	obs:	exp:
ttt	F	2	442.30
ttc	F	1118	677.70
---	--	------	------
mPD		0.0036	0.48
		nPD:	0.01
		N. weight:	0.51
		Sc. PD:	-0.021
		Sc. rank:	-204.9

HA

Pos.	155	obs:	exp:
tat	Y	1119	604.90
tac	Y	1	515.10
---	--	------	------
mPD		0.0018	0.50
		nPD:	0.
		N. weight:	0.64
		Sc. PD:	-0.028
		Sc. rank:	-373.8

HA

Pos.	156	obs:	exp:
gct	A	0	289.20
gcc	A	0	208.60
gca	A	1120	527.10
gcg	A	0	95.06
---	--	------	------
mPD		0	0.67
		nPD:	0.
		N. weight:	0.79
		Sc. PD:	-0.038
		Sc. rank:	-723.9

HA

Pos.	157	obs:	exp:
gat	D	0	0.54
gac	D	1	0.46
gaa	E	1109	680.90
gag	E	10	438.10
---	--	------	------
mPD		0.019	0.48
		nPD:	0.04
		N. weight:	0.47
		Sc. PD:	-0.004
		Sc. rank:	241.4

HA

Pos.	158	obs:	exp:
atg	M	1120	1120.00
---	--	------	------
mPD		0	0
		nPD:	1.
		N. weight:	0.
		Sc. PD:	0
		Sc. rank:	0

HA

Pos.	159	obs:	exp:
cgt	R	0	0.04
cgc	R	0	0.05
cga	R	0	0.10
cgg	R	0	0.08
aaa	K	1119	652.20
aag	K	0	466.80
aga	R	1	0.46
agg	R	0	0.27
---	--	------	------
mPD		0.0018	0.49
		nPD:	0.
		N. weight:	0.57
		Sc. PD:	-0.025
		Sc. rank:	-322.4

HA

Pos.	160	obs:	exp:
tgg	W	1120	1120.00
---	--	------	------
mPD		0	0
		nPD:	1.
		N. weight:	0.
		Sc. PD:	0
		Sc. rank:	0

HA			
Pos.	161	obs:	exp:
tta	L	0	99.39
ttg	L	0	208.50
ctt	L	3	186.50
ctc	L	1114	186.90
cta	L	3	188.80
ctg	L	0	249.90
mPD		0.011	1.1
		nPD:	0.01
		N. weight:	1.8
		Sc. PD:	-0.071
		Sc. rank:	-472.0

HA			
Pos.	162	obs:	exp:
tta	L	0	99.39
ttg	L	2	208.50
ctt	L	0	186.50
ctc	L	0	186.90
cta	L	6	188.80
ctg	L	1112	249.90
mPD		0.014	1.1
		nPD:	0.01
		N. weight:	1.5
		Sc. PD:	-0.054
		Sc. rank:	-128.1

HA			
Pos.	163	obs:	exp:
tct	S	0	178.10
tcc	S	0	146.30
tca	S	1117	284.30
tcg	S	3	63.55
agt	S	0	230.20
agc	S	0	217.50
mPD		0.0053	1.7
		nPD:	0.
		N. weight:	1.4
		Sc. PD:	-0.063
		Sc. rank:	-898.5

HA			
Pos.	164	obs:	exp:
tct	S	0	0.32
tcc	S	0	0.26
tca	S	0	0.51
tcg	S	0	0.11
aat	N	1	569.50
aac	N	1117	548.50
agt	S	0	0.41
agc	S	2	0.39
mPD		0.0054	0.51
		nPD:	0.01
		N. weight:	0.74
		Sc. PD:	-0.028
		Sc. rank:	-154.8

HA			
Pos.	165	obs:	exp:
act	T	0	285.20
acc	T	0	229.60
aca	T	1119	513.30
acg	T	0	90.89
gct	A	0	0.26
gcc	A	0	0.19
gca	A	1	0.47
gcg	A	0	0.08
mPD		0.0018	0.68
		nPD:	0.
		N. weight:	0.82
		Sc. PD:	-0.037
		Sc. rank:	-569.6

HA			
Pos.	166	obs:	exp:
aat	N	5	2.55
aac	N	0	2.45
gct	A	15	3.87
gcc	A	0	2.79
gca	A	0	7.06
gcg	A	0	1.27
gat	D	1100	594.40
gac	D	0	505.60
mPD		0.035	0.54
		nPD:	0.07
		N. weight:	0.66
		Sc. PD:	0.01
		Sc. rank:	419.6

HA			
Pos.	167	obs:	exp:
aat	N	1112	566.40
aac	N	0	545.60
gat	D	8	4.32
gac	D	0	3.68
mPD		0.014	0.51
		nPD:	0.03
		N. weight:	0.71
		Sc. PD:	-0.015
		Sc. rank:	239.0

HA			
Pos.	168	obs:	exp:
act	T	3	0.76
acc	T	0	0.62
aca	T	0	1.38
acg	T	0	0.24
gct	A	1116	288.40
gcc	A	1	208.00
gca	A	0	525.70
gcg	A	0	94.80
mPD		0.0071	0.68
		nPD:	0.01
		N. weight:	1.4
		Sc. PD:	-0.053
		Sc. rank:	-298.4

HA			
Pos.	169	obs:	exp:
tct	S	0	0.16
tcc	S	0	0.13
tca	S	1	0.25
tcg	S	0	0.06
act	T	0	3.31
acc	T	0	2.67
aca	T	13	5.96
acg	T	0	1.06
agt	S	0	0.21
agc	S	0	0.19
gtt	V	0	0.21
gtc	V	0	0.20
gta	V	1	0.20
gtg	V	0	0.39
gct	A	1	285.30
gcc	A	3	205.80
gca	A	1101	520.10
gcg	A	0	93.78
mPD		0.034	0.70
		nPD:	0.05
		N. weight:	0.77
		Sc. PD:	-0.00096
		Sc. rank:	430.8

HA			
Pos.	170	obs:	exp:
ttt	F	5	442.30
ttc	F	1115	677.70
mPD		0.0089	0.48
		nPD:	0.02
		N. weight:	0.5
		Sc. PD:	-0.015
		Sc. rank:	68.1

HA			
Pos.	171	obs:	exp:
cct	P	42	279.50
ccc	P	0	201.20
cca	P	4	461.00
ccg	P	1074	178.30
mPD		0.079	0.71
		nPD:	0.11
		N. weight:	1.7
		Sc. PD:	0.1
		Sc. rank:	1239.1

HA			
Pos.	172	obs:	exp:
cat	H	0	0.57
cac	H	1	0.43
caa	Q	168	570.00
cag	Q	951	549.00
mPD		0.26	0.50
		nPD:	0.51
		N. weight:	0.3
		Sc. PD:	0.13
		Sc. rank:	272.7

HA			
Pos.	173	obs:	exp:
att	I	1	0.73
atc	I	0	0.48
ata	I	1	0.79
atg	M	1117	1117.00
act	T	0	0.25
acc	T	0	0.21
aca	T	0	0.46
acg	T	1	0.08
mPD		0.0054	0.0070
		nPD:	0.77
		N. weight:	0.0035
		Sc. PD:	0.0024
		Sc. rank:	3.3

HA			
Pos.	174	obs:	exp:
act	T	1090	281.20
acc	T	13	226.30
aca	T	0	505.90
acg	T	0	89.59
aat	N	16	8.15
aac	N	0	7.85
gct	A	1	0.26
gcc	A	0	0.19
gca	A	0	0.47
gcg	A	0	0.08
mPD		0.053	0.71
		nPD:	0.07
		N. weight:	1.4
		Sc. PD:	0.033
		Sc. rank:	892.5

HA			
Pos.	175	obs:	exp:
aaa	K	210	652.80
aag	K	910	467.20
mPD		0.30	0.49
		nPD:	0.63
		N. weight:	0.35
		Sc. PD:	0.19
		Sc. rank:	320.8

HA			
Pos.	176	obs:	exp:
tct	S	0	178.00
tcc	S	0	146.10
tca	S	1115	284.00
tcg	S	4	63.50
act	T	0	0.25
acc	T	0	0.21
aca	T	1	0.46
acg	T	0	0.08
agt	S	0	230.00
agc	S	0	217.30
mPD		0.0089	1.7
		nPD:	0.01
		N. weight:	1.4
		Sc. PD:	-0.06
		Sc. rank:	-658.8

HA			
Pos.	177	obs:	exp:
tat	Y	1106	604.90
tac	Y	14	515.10
mPD		0.025	0.50
		nPD:	0.05
		N. weight:	0.58
		Sc. PD:	0.00012
		Sc. rank:	331.2

HA			
Pos.	178	obs:	exp:
caa	Q	1	0.51
cag	Q	0	0.49
cgt	R	0	0.04
cgc	R	0	0.05
cga	R	0	0.10
cgg	R	0	0.08
aaa	K	1116	651.60
aag	K	2	466.40
aga	R	1	0.46
agg	R	0	0.27
mPD		0.0071	0.49
		nPD:	0.01
		N. weight:	0.55
		Sc. PD:	-0.019
		Sc. rank:	-14.0

HA			
Pos.	179	obs:	exp:
aat	N	1115	570.50
aac	N	5	549.50
mPD		0.0089	0.50
		nPD:	0.02
		N. weight:	0.68
		Sc. PD:	-0.021
		Sc. rank:	54.2

HA			
Pos.	180	obs:	exp:
att	I	0	0.37
atc	I	0	0.24
ata	I	1	0.39
act	T	0	285.00
acc	T	1	229.40
aca	T	1117	512.80
acg	T	0	90.81
gct	A	0	0.26
gcc	A	0	0.19
gca	A	1	0.47
gcg	A	0	0.08
mPD		0.0054	0.68
		nPD:	0.01
		N. weight:	0.81
		Sc. PD:	-0.033
		Sc. rank:	-305.0

HA			
Pos.	181	obs:	exp:
cgt	R	0	47.72
cgc	R	0	55.03
cga	R	0	108.70
cgg	R	0	92.70
aaa	K	9	5.25
aag	K	0	3.75
aga	R	1103	508.00
agg	R	8	298.80
---	--	------	------
mPD		0.030	0.97
		nPD:	0.03
		N. weight:	0.78
		Sc. PD:	-0.014
		Sc. rank:	308.2

HA			
Pos.	182	obs:	exp:
cgt	R	0	0.26
cgc	R	0	0.30
cga	R	0	0.59
cgg	R	0	0.50
aaa	K	1066	621.30
aag	K	0	444.70
aga	R	6	2.74
agg	R	0	1.61
gaa	E	48	29.21
gag	E	0	18.79
---	--	------	------
mPD		0.093	0.58
		nPD:	0.16
		N. weight:	0.56
		Sc. PD:	0.06
		Sc. rank:	450.1

HA			
Pos.	183	obs:	exp:
tct	S	0	176.20
tcc	S	0	144.70
tca	S	0	281.20
tcg	S	0	62.87
aat	N	0	5.09
aac	N	10	4.91
agt	S	0	227.80
agc	S	1108	215.20
gat	D	0	0.54
gac	D	1	0.46
ggt	G	1	0.13
ggc	G	0	0.13
gga	G	0	0.46
ggg	G	0	0.28
---	--	------	------
mPD		0.025	1.7
		nPD:	0.01
		N. weight:	1.7
		Sc. PD:	-0.057
		Sc. rank:	-16.5

HA			
Pos.	184	obs:	exp:
cct	P	0	279.50
ccc	P	1	201.20
cca	P	1116	461.00
ccg	P	3	178.30
---	--	------	------
mPD		0.0071	0.71
		nPD:	0.01
		N. weight:	0.91
		Sc. PD:	-0.035
		Sc. rank:	-209.2

HA			
Pos.	185	obs:	exp:
gct	A	1110	289.20
gcc	A	10	208.60
gca	A	0	527.10
gcg	A	0	95.06
---	--	------	------
mPD		0.018	0.67
		nPD:	0.03
		N. weight:	1.4
		Sc. PD:	-0.03
		Sc. rank:	441.1

HA			
Pos.	186	obs:	exp:
tta	L	3	47.65
ttg	L	0	99.98
ctt	L	0	89.42
ctc	L	0	89.62
cta	L	533	90.50
ctg	L	1	119.80
att	I	1	214.20
atc	I	0	139.70
ata	I	582	229.10
---	--	------	------
mPD		0.51	1.3
		nPD:	0.38
		N. weight:	1.4
		Sc. PD:	0.44
		Sc. rank:	1216.5

HA			
Pos.	187	obs:	exp:
att	I	4	401.60
atc	I	0	261.90
ata	I	1089	429.50
gtt	V	0	5.59
gtc	V	0	5.45
gta	V	27	5.53
gtg	V	0	10.43
---	--	------	------
mPD		0.054	0.71
		nPD:	0.08
		N. weight:	0.97
		Sc. PD:	0.025
		Sc. rank:	641.0

HA			
Pos.	188	obs:	exp:
att	I	0	2.20
atc	I	0	1.44
ata	I	6	2.36
gtt	V	0	230.80
gtc	V	0	224.80
gta	V	1112	228.10
gtg	V	2	430.20
---	--	------	------
mPD		0.014	0.74
		nPD:	0.02
		N. weight:	1.6
		Sc. PD:	-0.048
		Sc. rank:	261.8

HA			
Pos.	189	obs:	exp:
tgg	W	1120	1120.00
---	--	------	------
mPD		0	0
		nPD:	1.
		N. weight:	0.
		Sc. PD:	0
		Sc. rank:	0

HA			
Pos.	190	obs:	exp:
ggt	G	1	148.50
ggc	G	0	143.10
gga	G	21	516.70
ggg	G	1098	311.80
---	--	------	------
mPD		0.039	0.68
		nPD:	0.06
		N. weight:	1.2
		Sc. PD:	0.0089
		Sc. rank:	760.0

HA			
Pos.	191	obs:	exp:
att	I	5	411.50
atc	I	1115	268.40
ata	I	0	440.10
---	--	------	------
mPD		0.0089	0.65
		nPD:	0.01
		N. weight:	1.5
		Sc. PD:	-0.051
		Sc. rank:	-69.8

HA			
Pos.	192	obs:	exp:
cat	H	1106	643.10
cac	H	14	476.90
---	--	------	------
mPD		0.025	0.49
		nPD:	0.05
		N. weight:	0.52
		Sc. PD:	0.0005
		Sc. rank:	301.4

HA			
Pos.	193	obs:	exp:
cat	H	1120	643.10
cac	H	0	476.90
---	--	------	------
mPD		0	0.49
		nPD:	0.
		N. weight:	0.58
		Sc. PD:	-0.028
		Sc. rank:	-532.9

HA			
Pos.	194	obs:	exp:
tct	S	12	177.80
tcc	S	1104	146.00
tca	S	2	283.80
tcg	S	0	63.44
cct	P	0	0.50
ccc	P	2	0.36
cca	P	0	0.82
ccg	P	0	0.32
agt	S	0	229.80
agc	S	0	217.10
---	--	------	------
mPD		0.028	1.7
		nPD:	0.02
		N. weight:	2.1
		Sc. PD:	-0.064
		Sc. rank:	124.1

HA			
Pos.	195	obs:	exp:
att	I	6	2.20
atc	I	0	1.44
ata	I	0	2.36
gtt	V	519	226.90
gtc	V	0	221.00
gta	V	555	224.20
gtg	V	21	422.90
gct	A	1	4.13
gcc	A	0	2.98
gca	A	15	7.53
gcg	A	0	1.36
ggt	G	0	0.40
ggc	G	0	0.38
gga	G	3	1.38
ggg	G	0	0.84
---	--	------	------
mPD		0.56	0.77
		nPD:	0.73
		N. weight:	0.84
		Sc. PD:	0.55
		Sc. rank:	776.7

HA			
Pos.	196	obs:	exp:
tct	S	0	178.10
tcc	S	0	146.30
tca	S	1115	284.30
tcg	S	5	63.55
agt	S	0	230.20
agc	S	0	217.50
---	--	------	------
mPD		0.0089	1.7
		nPD:	0.01
		N. weight:	1.4
		Sc. PD:	-0.06
		Sc. rank:	-672.7

HA			
Pos.	197	obs:	exp:
att	I	5	1.84
atc	I	0	1.20
ata	I	0	1.96
act	T	1099	280.90
acc	T	1	226.10
aca	T	2	505.50
acg	T	0	89.51
aat	N	9	4.58
aac	N	0	4.42
gct	A	4	1.03
gcc	A	0	0.74
gca	A	0	1.88
gcg	A	0	0.34
---	--	------	------
mPD		0.037	0.71
		nPD:	0.05
		N. weight:	1.4
		Sc. PD:	0.0041
		Sc. rank:	838.1

HA			
Pos.	198	obs:	exp:
gtt	V	0	0.21
gtc	V	0	0.20
gta	V	1	0.20
gtg	V	0	0.39
gct	A	0	289.00
gcc	A	0	208.40
gca	A	1112	526.70
gcg	A	7	94.97
---	--	------	------
mPD		0.014	0.67
		nPD:	0.02
		N. weight:	0.76
		Sc. PD:	-0.021
		Sc. rank:	161.1

HA			
Pos.	199	obs:	exp:
gaa	E	8	681.50
gag	E	1112	438.50
---	--	------	------
mPD		0.014	0.48
		nPD:	0.03
		N. weight:	0.94
		Sc. PD:	-0.018
		Sc. rank:	352.1

HA			
Pos.	200	obs:	exp:
caa	Q	1120	570.50
cag	Q	0	549.50
---	--	------	------
mPD		0	0.50
		nPD:	0.
		N. weight:	0.71
		Sc. PD:	-0.034
		Sc. rank:	-648.0

HA			
Pos.	201	obs:	exp:
act	T	7	285.50
acc	T	1113	229.80
aca	T	0	513.70
acg	T	0	90.97
---	--	------	------
mPD		0.012	0.68
		nPD:	0.02
		N. weight:	1.6
		Sc. PD:	-0.049
		Sc. rank:	168.3

,

HA			
Pos.	202	obs:	exp:
cgt	R	0	0.17
cgc	R	0	0.20
cga	R	0	0.39
cgg	R	0	0.33
act	T	0	46.14
acc	T	0	37.14
aca	T	0	83.02
acg	T	181	14.70
aat	N	12	6.11
aac	N	0	5.89
aaa	K	4	537.90
aag	K	919	385.10
aga	R	0	1.83
agg	R	4	1.08
---	--	------	------
mPD		0.31	0.84
		nPD:	0.36
		N. weight:	1.2
		Sc. PD:	0.35
		Sc. rank:	1026.6

,

HA			
Pos.	203	obs:	exp:
tta	L	2	99.39
ttg	L	0	208.50
ctt	L	0	186.50
ctc	L	0	186.90
cta	L	1112	188.80
ctg	L	6	249.90
---	--	------	------
mPD		0.014	1.1
		nPD:	0.01
		N. weight:	1.8
		Sc. PD:	-0.064
		Sc. rank:	-152.6

,

HA			
Pos.	204	obs:	exp:
tat	Y	1119	604.90
tac	Y	1	515.10
---	--	------	------
mPD		0.0018	0.50
		nPD:	0.
		N. weight:	0.64
		Sc. PD:	-0.028
		Sc. rank:	-373.8

,

HA			
Pos.	205	obs:	exp:
ggt	G	1	148.50
ggc	G	0	143.10
gga	G	3	516.70
ggg	G	1116	311.80
---	--	------	------
mPD		0.0071	0.68
		nPD:	0.01
		N. weight:	1.3
		Sc. PD:	-0.049
		Sc. rank:	-289.9

,

HA			
Pos.	206	obs:	exp:
tct	S	0	177.80
tcc	S	0	146.00
tca	S	0	283.80
tcg	S	0	63.44
cgt	R	0	0.04
cgc	R	0	0.05
cga	R	0	0.10
cgg	R	0	0.08
aat	N	1	0.51
aac	N	0	0.49
agt	S	1118	229.80
agc	S	0	217.10
aga	R	0	0.46
agg	R	1	0.27
---	--	------	------
mPD		0.0036	1.7
		nPD:	0.
		N. weight:	1.7
		Sc. PD:	-0.076
		Sc. rank:	-1172.5

,

HA			
Pos.	207	obs:	exp:
ggt	G	0	148.50
ggc	G	0	143.10
gga	G	1106	516.70
ggg	G	14	311.80
---	--	------	------
mPD		0.025	0.68
		nPD:	0.04
		N. weight:	0.75
		Sc. PD:	-0.0094
		Sc. rank:	350.0

,

HA			
Pos.	208	obs:	exp:
tct	S	0	1.59
tcc	S	0	1.31
tca	S	0	2.54
tcg	S	0	0.57
aat	N	0	565.40
aac	N	1110	544.60
agt	S	0	2.06
agc	S	10	1.94
---	--	------	------
mPD		0.018	0.53
		nPD:	0.03
		N. weight:	0.75
		Sc. PD:	-0.012
		Sc. rank:	324.4

,

HA			
Pos.	209	obs:	exp:
aaa	K	1108	652.80
aag	K	12	467.20
---	--	------	------
mPD		0.021	0.49
		nPD:	0.04
		N. weight:	0.51
		Sc. PD:	-0.0029
		Sc. rank:	262.2

,

HA			
Pos.	210	obs:	exp:
tta	L	0	99.21
ttg	L	6	208.10
ctt	L	0	186.20
ctc	L	0	186.60
cta	L	2	188.40
ctg	L	1110	249.50
atg	M	1	1.00
gtt	V	0	0.21
gtc	V	0	0.20
gta	V	0	0.20
gtg	V	1	0.39
---	--	------	------
mPD		0.018	1.1
		nPD:	0.02
		N. weight:	1.5
		Sc. PD:	-0.049
		Sc. rank:	35.0

,

HA			
Pos.	211	obs:	exp:
att	I	0	0.37
atc	I	0	0.24
ata	I	1	0.39
gtt	V	1	231.90
gtc	V	0	225.80
gta	V	4	229.10
gtg	V	1114	432.20
---	--	------	------
mPD		0.012	0.73
		nPD:	0.02
		N. weight:	0.97
		Sc. PD:	-0.03
		Sc. rank:	61.6

,

HA			
Pos.	212	obs:	exp:
att	I	0	0.37
atc	I	0	0.24
ata	I	1	0.39
act	T	0	285.20
acc	T	0	229.60
aca	T	1117	513.30
acg	T	2	90.89
---	--	------	------
mPD		0.0054	0.68
		nPD:	0.01
		N. weight:	0.81
		Sc. PD:	-0.032
		Sc. rank:	-301.6

,

HA			
Pos.	213	obs:	exp:
gtt	V	1098	232.10
gtc	V	20	226.00
gta	V	0	229.30
gtg	V	2	432.60
---	--	------	------
mPD		0.039	0.73
		nPD:	0.05
		N. weight:	1.5
		Sc. PD:	0.0055
		Sc. rank:	929.4

,

HA			
Pos.	214	obs:	exp:
ggt	G	3	148.50
ggc	G	0	143.10
gga	G	12	516.70
ggg	G	1105	311.80
---	--	------	------
mPD		0.027	0.68
		nPD:	0.04
		N. weight:	1.3
		Sc. PD:	-0.012
		Sc. rank:	631.8

,

HA			
Pos.	215	obs:	exp:
tct	S	0	178.10
tcc	S	0	146.30
tca	S	0	284.30
tcg	S	0	63.55
agt	S	922	230.20
agc	S	198	217.50
---	--	------	------
mPD		0.29	1.7
		nPD:	0.18
		N. weight:	1.2
		Sc. PD:	0.14
		Sc. rank:	963.1

,

HA			
Pos.	216	obs:	exp:
tct	S	1115	178.10
tcc	S	1	146.30
tca	S	4	284.30
tcg	S	0	63.55
agt	S	0	230.20
agc	S	0	217.50
---	--	------	------
mPD		0.0089	1.7
		nPD:	0.01
		N. weight:	1.9
		Sc. PD:	-0.081
		Sc. rank:	-888.9

,

HA			
Pos.	217	obs:	exp:
aat	N	1116	569.50
aac	N	2	548.50
gat	D	2	1.08
gac	D	0	0.92
---	--	------	------
mPD		0.0071	0.50
		nPD:	0.01
		N. weight:	0.69
		Sc. PD:	-0.024
		Sc. rank:	-28.6

,

HA			
Pos.	218	obs:	exp:
tat	Y	1112	604.90
tac	Y	8	515.10
---	--	------	------
mPD		0.014	0.50
		nPD:	0.03
		N. weight:	0.6
		Sc. PD:	-0.012
		Sc. rank:	214.7

,

HA			
Pos.	219	obs:	exp:
caa	Q	1086	570.50
cag	Q	34	549.50
---	--	------	------
mPD		0.059	0.50
		nPD:	0.12
		N. weight:	0.57
		Sc. PD:	0.037
		Sc. rank:	421.0

,

HA			
Pos.	220	obs:	exp:
caa	Q	1112	569.50
cag	Q	6	548.50
aaa	K	2	1.17
aag	K	0	0.83
---	--	------	------
mPD		0.014	0.50
		nPD:	0.03
		N. weight:	0.67
		Sc. PD:	-0.014
		Sc. rank:	232.6

,

HA			
Pos.	221	obs:	exp:
ttt	F	2	0.79
ttc	F	0	1.21
tct	S	1108	177.70
tcc	S	9	145.90
tca	S	0	283.50
tcg	S	0	63.38
tat	Y	1	0.54
tac	Y	0	0.46
agt	S	0	229.60
agc	S	0	217.00
---	--	------	------
mPD		0.021	1.7
		nPD:	0.01
		N. weight:	1.9
		Sc. PD:	-0.066
		Sc. rank:	-135.6

,

HA			
Pos.	222	obs:	exp:
ttt	F	621	442.30
ttc	F	499	677.70
---	--	------	------
mPD		0.49	0.48
		nPD:	1.03
		N. weight:	0.058
		Sc. PD:	0.055
		Sc. rank:	53.9

,

HA			
Pos.	223	obs:	exp:
gtt	V	0	230.60
gtc	V	0	224.60
gta	V	1105	227.90
gtg	V	8	429.90
gct	A	0	0.52
gcc	A	0	0.37
gca	A	2	0.94
gcg	A	0	0.17
gaa	E	5	3.04
gag	E	0	1.96
---	--	------	------
mPD		0.027	0.74
		nPD:	0.04
		N. weight:	1.6
		Sc. PD:	-0.021
		Sc. rank:	733.9

,

HA			
Pos.	224	obs:	exp:
tta	L	0	0.18
ttg	L	0	0.37
tct	S	0	0.16
tcc	S	0	0.13
tca	S	0	0.25
tcg	S	1	0.06
ctt	L	0	0.33
ctc	L	0	0.33
cta	L	0	0.34
ctg	L	2	0.45
cct	P	6	278.70
ccc	P	0	200.70
cca	P	9	459.80
ccg	P	1102	177.80
agt	S	0	0.21
agc	S	0	0.19
---	--	------	------
mPD		0.032	0.72
		nPD:	0.04
		N. weight:	1.8
		Sc. PD:	-0.009
		Sc. rank:	970.7

,

HA			
Pos.	225	obs:	exp:
tct	S	0	178.00
tcc	S	0	146.10
tca	S	0	284.00
tcg	S	0	63.50
att	I	1	0.37
atc	I	0	0.24
ata	I	0	0.39
agt	S	1102	230.00
agc	S	17	217.30
---	--	------	------
mPD		0.032	1.7
		nPD:	0.02
		N. weight:	1.6
		Sc. PD:	-0.046
		Sc. rank:	233.2

,

HA			
Pos.	226	obs:	exp:
cct	P	5	277.70
ccc	P	0	200.00
cca	P	1105	458.10
ccg	P	3	177.20
act	T	0	1.78
acc	T	0	1.44
aca	T	7	3.21
acg	T	0	0.57
---	--	------	------
mPD		0.027	0.72
		nPD:	0.04
		N. weight:	0.89
		Sc. PD:	-0.011
		Sc. rank:	426.5

,

HA			
Pos.	227	obs:	exp:
gaa	E	3	1.83
gag	E	0	1.17
ggt	G	0	148.10
ggc	G	0	142.70
gga	G	1113	515.30
ggg	G	4	311.00
---	--	------	------
mPD		0.012	0.68
		nPD:	0.02
		N. weight:	0.79
		Sc. PD:	-0.024
		Sc. rank:	73.0

,

HA			
Pos.	228	obs:	exp:
act	T	0	0.51
acc	T	0	0.41
aca	T	0	0.92
acg	T	2	0.16
gtt	V	0	0.21
gtc	V	0	0.20
gta	V	0	0.20
gtg	V	1	0.39
gct	A	0	287.70
gcc	A	0	207.50
gca	A	59	524.30
gcg	A	1055	94.55
gaa	E	0	1.83
gag	E	3	1.17
---	--	------	------
mPD		0.11	0.68
		nPD:	0.16
		N. weight:	2.3
		Sc. PD:	0.25
		Sc. rank:	1826.0

,

HA			
Pos.	229	obs:	exp:
tct	S	0	0.16
tcc	S	0	0.13
tca	S	0	0.25
tcg	S	0	0.06
cgt	R	0	47.67
cgc	R	0	54.98
cga	R	3	108.60
cgg	R	0	92.62
att	I	0	0.37
atc	I	0	0.24
ata	I	1	0.39
agt	S	0	0.21
agc	S	1	0.19
aga	R	1107	507.60
agg	R	0	298.60
ggt	G	0	1.06
ggc	G	0	1.02
gga	G	8	3.69
ggg	G	0	2.23
---	--	------	------
mPD		0.023	0.97
		nPD:	0.02
		N. weight:	0.81
		Sc. PD:	-0.02
		Sc. rank:	205.3

,

HA			
Pos.	230	obs:	exp:
tct	S	0	0.16
tcc	S	0	0.13
tca	S	1	0.25
tcg	S	0	0.06
cct	P	0	278.70
ccc	P	0	200.70
cca	P	1116	459.80
ccg	P	1	177.80
act	T	0	0.51
acc	T	0	0.41
aca	T	2	0.92
acg	T	0	0.16
agt	S	0	0.21
agc	S	0	0.19
---	--	------	------
mPD		0.0071	0.72
		nPD:	0.01
		N. weight:	0.92
		Sc. PD:	-0.035
		Sc. rank:	-223.7

,

HA			
Pos.	231	obs:	exp:
cct	P	0	0.75
ccc	P	0	0.54
cca	P	3	1.23
ccg	P	0	0.48
caa	Q	1113	567.90
cag	Q	2	547.10
gaa	E	2	1.22
gag	E	0	0.78
---	--	------	------
mPD		0.012	0.51
		nPD:	0.02
		N. weight:	0.69
		Sc. PD:	-0.017
		Sc. rank:	193.1

,

HA			
Pos.	232	obs:	exp:
ttt	F	2	0.79
ttc	F	0	1.21
gtt	V	1115	231.70
gtc	V	3	225.60
gta	V	0	228.90
gtg	V	0	431.80
---	--	------	------
mPD		0.0089	0.73
		nPD:	0.01
		N. weight:	1.6
		Sc. PD:	-0.059
		Sc. rank:	-195.4

,

HA			
Pos.	233	obs:	exp:
aat	N	1117	570.00
aac	N	2	549.00
gat	D	1	0.54
gac	D	0	0.46
---	--	------	------
mPD		0.0054	0.50
		nPD:	0.01
		N. weight:	0.69
		Sc. PD:	-0.026
		Sc. rank:	-140.0

,

HA			
Pos.	234	obs:	exp:
gct	A	1	0.26
gcc	A	0	0.19
gca	A	0	0.47
gcg	A	0	0.08
gat	D	6	3.24
gac	D	0	2.76
ggt	G	1105	147.50
ggc	G	8	142.20
gga	G	0	513.50
ggg	G	0	309.80
---	--	------	------
mPD		0.027	0.69
		nPD:	0.04
		N. weight:	2.1
		Sc. PD:	-0.022
		Sc. rank:	1011.6

,

HA			
Pos.	235	obs:	exp:
tta	L	91	92.47
ttg	L	0	194.00
tct	S	0	0.32
tcc	S	0	0.26
tca	S	2	0.51
tcg	S	0	0.11
ctt	L	0	173.50
ctc	L	0	173.90
cta	L	949	175.60
ctg	L	2	232.50
cct	P	0	0.25
ccc	P	0	0.18
cca	P	1	0.41
ccg	P	0	0.16
cat	H	1	1.15
cac	H	1	0.85
caa	Q	58	29.54
cag	Q	0	28.46
att	I	0	5.51
atc	I	0	3.59
ata	I	15	5.89
agt	S	0	0.41
agc	S	0	0.39
---	--	------	------
mPD		0.29	1.2
		nPD:	0.24
		N. weight:	1.5
		Sc. PD:	0.28
		Sc. rank:	1290.0

,

HA

Pos.	236	obs:	exp:
ttt	F	6	3.95
ttc	F	4	6.05
tta	L	1	0.09
ttg	L	0	0.19
tct	S	789	176.20
tcc	S	302	144.70
tca	S	17	281.20
tcg	S	0	62.87
tat	Y	1	0.54
tac	Y	0	0.46
ctt	L	0	0.17
ctc	L	0	0.17
cta	L	0	0.17
ctg	L	0	0.22
agt	S	0	227.80
agc	S	0	215.20
---	--	------	------
mPD		0.44	1.7
		nPD:	0.27
		N. weight:	1.3
		Sc. PD:	0.27
		Sc. rank:	1079.3

,

HA

Pos.	237	obs:	exp:
ggt	G	0	148.50
ggc	G	0	143.10
gga	G	931	516.70
ggg	G	189	311.80
---	--	------	------
mPD		0.28	0.68
		nPD:	0.42
		N. weight:	0.43
		Sc. PD:	0.15
		Sc. rank:	380.8

,

HA

Pos.	238	obs:	exp:
cgt	R	0	48.10
cgc	R	0	55.47
cga	R	0	109.60
cgg	R	0	93.45
aga	R	1119	512.10
agg	R	1	301.20
---	--	------	------
mPD		0.0018	0.96
		nPD:	0.
		N. weight:	0.81
		Sc. PD:	-0.037
		Sc. rank:	-584.8

,

HA

Pos.	239	obs:	exp:
att	I	1119	411.50
atc	I	1	268.40
ata	I	0	440.10
---	--	------	------
mPD		0.0018	0.65
		nPD:	0.
		N. weight:	1.
		Sc. PD:	-0.047
		Sc. rank:	-691.3

,

HA

Pos.	240	obs:	exp:
aat	N	0	0.51
aac	N	1	0.49
gat	D	5	604.70
gac	D	1114	514.30
---	--	------	------
mPD		0.011	0.50
		nPD:	0.02
		N. weight:	0.78
		Sc. PD:	-0.021
		Sc. rank:	176.6

,

HA

Pos.	241	obs:	exp:
ttt	F	1116	442.30
ttc	F	4	677.70
---	--	------	------
mPD		0.0071	0.48
		nPD:	0.01
		N. weight:	0.95
		Sc. PD:	-0.032
		Sc. rank:	-6.2

,

HA

Pos.	242	obs:	exp:
cat	H	1120	643.10
cac	H	0	476.90
---	--	------	------
mPD		0	0.49
		nPD:	0.
		N. weight:	0.58
		Sc. PD:	-0.028
		Sc. rank:	-532.9

,

HA

Pos.	243	obs:	exp:
tgg	W	1120	1120.00
---	--	------	------
mPD		0	0
		nPD:	1.
		N. weight:	0.
		Sc. PD:	0
		Sc. rank:	0

,

HA

Pos.	244	obs:	exp:
tta	L	0	99.39
ttg	L	0	208.50
ctt	L	0	186.50
ctc	L	0	186.90
cta	L	1117	188.80
ctg	L	3	249.90
---	--	------	------
mPD		0.0053	1.1
		nPD:	0.
		N. weight:	1.8
		Sc. PD:	-0.08
		Sc. rank:	-993.2

,

HA

Pos.	245	obs:	exp:
tta	L	1	3.55
ttg	L	1	7.45
ctt	L	0	6.66
ctc	L	0	6.68
cta	L	0	6.74
ctg	L	38	8.93
att	I	0	144.80
atc	I	0	94.42
ata	I	394	154.80
atg	M	682	682.00
gtt	V	0	0.83
gtc	V	0	0.81
gta	V	1	0.82
gtg	V	3	1.54
---	--	------	------
mPD		0.53	0.64
		nPD:	0.84
		N. weight:	0.4
		Sc. PD:	0.3
		Sc. rank:	370.2

,

HA

Pos.	246	obs:	exp:
tta	L	14	99.39
ttg	L	0	208.50
ctt	L	0	186.50
ctc	L	0	186.90
cta	L	1104	188.80
ctg	L	2	249.90
---	--	------	------
mPD		0.028	1.1
		nPD:	0.03
		N. weight:	1.8
		Sc. PD:	-0.042
		Sc. rank:	526.6

,

HA

Pos.	247	obs:	exp:
aat	N	1111	570.00
aac	N	8	549.00
gat	D	1	0.54
gac	D	0	0.46
---	--	------	------
mPD		0.016	0.50
		nPD:	0.03
		N. weight:	0.66
		Sc. PD:	-0.011
		Sc. rank:	273.4

,

HA

Pos.	248	obs:	exp:
cct	P	4	279.50
ccc	P	1116	201.20
cca	P	0	461.00
ccg	P	0	178.30
---	--	------	------
mPD		0.0071	0.71
		nPD:	0.01
		N. weight:	1.8
		Sc. PD:	-0.068
		Sc. rank:	-421.4

,

HA

Pos.	249	obs:	exp:
aat	N	1120	570.50
aac	N	0	549.50
---	--	------	------
mPD		0	0.50
		nPD:	0.
		N. weight:	0.71
		Sc. PD:	-0.034
		Sc. rank:	-648.0

,

HA

Pos.	250	obs:	exp:
gat	D	1117	605.20
gac	D	3	514.80
---	--	------	------
mPD		0.0053	0.50
		nPD:	0.01
		N. weight:	0.63
		Sc. PD:	-0.023
		Sc. rank:	-115.6

,

HA

Pos.	251	obs:	exp:
act	T	0	285.50
acc	T	2	229.80
aca	T	1118	513.70
acg	T	0	90.97
---	--	------	------
mPD		0.0036	0.68
		nPD:	0.01
		N. weight:	0.81
		Sc. PD:	-0.034
		Sc. rank:	-398.3

,

HA

Pos.	252	obs:	exp:
att	I	0	0.37
atc	I	1	0.24
ata	I	0	0.39
gtt	V	0	231.90
gtc	V	1118	225.80
gta	V	1	229.10
gtg	V	0	432.20
---	--	------	------
mPD		0.0036	0.73
		nPD:	0.
		N. weight:	1.7
		Sc. PD:	-0.072
		Sc. rank:	-889.0

,

HA

Pos.	253	obs:	exp:
act	T	1113	285.50
acc	T	7	229.80
aca	T	0	513.70
acg	T	0	90.97
---	--	------	------
mPD		0.012	0.68
		nPD:	0.02
		N. weight:	1.4
		Sc. PD:	-0.042
		Sc. rank:	145.0

,

HA

Pos.	254	obs:	exp:
ttt	F	7	442.30
ttc	F	1113	677.70
---	--	------	------
mPD		0.012	0.48
		nPD:	0.03
		N. weight:	0.49
		Sc. PD:	-0.011
		Sc. rank:	149.0

,

HA

Pos.	255	obs:	exp:
tct	S	0	177.00
tcc	S	0	145.40
tca	S	0	282.50
tcg	S	0	63.16
aat	N	7	3.57
aac	N	0	3.43
agt	S	1112	228.80
agc	S	1	216.20
---	--	------	------
mPD		0.014	1.7
		nPD:	0.01
		N. weight:	1.6
		Sc. PD:	-0.065
		Sc. rank:	-484.2

,

HA

Pos.	256	obs:	exp:
ttt	F	1	442.30
ttc	F	1119	677.70
---	--	------	------
mPD		0.0018	0.48
		nPD:	0.
		N. weight:	0.52
		Sc. PD:	-0.023
		Sc. rank:	-289.1

,

HA

Pos.	257	obs:	exp:
aat	N	1120	570.50
aac	N	0	549.50
---	--	------	------
mPD		0	0.50
		nPD:	0.
		N. weight:	0.71
		Sc. PD:	-0.034
		Sc. rank:	-648.0

,

HA

Pos.	258	obs:	exp:
ggt	G	0	148.50
ggc	G	0	143.10
gga	G	1	516.70
ggg	G	1119	311.80
---	--	------	------
mPD		0.0018	0.68
		nPD:	0.
		N. weight:	1.3
		Sc. PD:	-0.06
		Sc. rank:	-913.7

,

HA

Pos.	259	obs:	exp:
tct	S	1	0.16
tcc	S	0	0.13
tca	S	0	0.25
tcg	S	0	0.06
agt	S	0	0.21
agc	S	0	0.19
gct	A	1101	289.00
gcc	A	0	208.40
gca	A	15	526.70
gcg	A	3	94.97
---	--	------	------
mPD		0.034	0.67
		nPD:	0.05
		N. weight:	1.3
		Sc. PD:	0.00038
		Sc. rank:	762.5

,

HA

Pos.	260	obs:	exp:
ttt	F	7	442.30
ttc	F	1113	677.70
---	--	------	------
mPD		0.012	0.48
		nPD:	0.03
		N. weight:	0.49
		Sc. PD:	-0.011
		Sc. rank:	149.0

,

HA

Pos.	261	obs:	exp:
att	I	1	411.50
atc	I	0	268.40
ata	I	1119	440.10
---	--	------	------
mPD		0.0018	0.65
		nPD:	0.
		N. weight:	0.97
		Sc. PD:	-0.044
		Sc. rank:	-644.5

HA

Pos.	262	obs:	exp:
gct	A	1120	289.20
gcc	A	0	208.60
gca	A	0	527.10
gcg	A	0	95.06
---	--	------	------
mPD		0	0.67
		nPD:	0.
		N. weight:	1.4
		Sc. PD:	-0.068
		Sc. rank:	-1300.6

HA

Pos.	263	obs:	exp:
cct	P	0	279.50
ccc	P	0	201.20
cca	P	1116	461.00
ccg	P	4	178.30
---	--	------	------
mPD		0.0071	0.71
		nPD:	0.01
		N. weight:	0.91
		Sc. PD:	-0.035
		Sc. rank:	-216.2

HA

Pos.	264	obs:	exp:
gat	D	44	600.90
gac	D	1068	511.10
gaa	E	8	4.87
gag	E	0	3.13
---	--	------	------
mPD		0.089	0.50
		nPD:	0.18
		N. weight:	0.63
		Sc. PD:	0.078
		Sc. rank:	518.5

HA

Pos.	265	obs:	exp:
cat	H	1	0.57
cac	H	0	0.43
cgt	R	1115	48.06
cgc	R	4	55.42
cga	R	0	109.50
cgg	R	0	93.37
aga	R	0	511.70
agg	R	0	301.00
---	--	------	------
mPD		0.0089	0.96
		nPD:	0.01
		N. weight:	3.3
		Sc. PD:	-0.13
		Sc. rank:	-895.4

HA

Pos.	266	obs:	exp:
gct	A	0	289.20
gcc	A	0	208.60
gca	A	1120	527.10
gcg	A	0	95.06
---	--	------	------
mPD		0	0.67
		nPD:	0.
		N. weight:	0.79
		Sc. PD:	-0.038
		Sc. rank:	-723.9

HA

Pos.	267	obs:	exp:
tct	S	0	178.00
tcc	S	0	146.10
tca	S	0	284.00
tcg	S	0	63.50
tgt	C	0	0.42
tgc	C	1	0.58
agt	S	21	230.00
agc	S	1098	217.30
---	--	------	------
mPD		0.039	1.7
		nPD:	0.02
		N. weight:	1.6
		Sc. PD:	-0.041
		Sc. rank:	392.4

HA

Pos.	268	obs:	exp:
ttt	F	8	441.90
ttc	F	1111	677.10
tat	Y	0	0.54
tac	Y	1	0.46
---	--	------	------
mPD		0.016	0.48
		nPD:	0.03
		N. weight:	0.49
		Sc. PD:	-0.0076
		Sc. rank:	208.2

HA

Pos.	269	obs:	exp:
tta	L	0	99.39
ttg	L	2	208.50
ctt	L	0	186.50
ctc	L	6	186.90
cta	L	12	188.80
ctg	L	1100	249.90
---	--	------	------
mPD		0.035	1.1
		nPD:	0.03
		N. weight:	1.5
		Sc. PD:	-0.025
		Sc. rank:	602.1

HA

Pos.	270	obs:	exp:
cgt	R	0	47.72
cgc	R	0	55.03
cga	R	0	108.70
cgg	R	0	92.70
aaa	K	9	5.25
aag	K	0	3.75
aga	R	1107	508.00
agg	R	4	298.80
---	--	------	------
mPD		0.023	0.97
		nPD:	0.02
		N. weight:	0.8
		Sc. PD:	-0.02
		Sc. rank:	204.7

HA

Pos.	271	obs:	exp:
ggt	G	0	148.50
ggc	G	1	143.10
gga	G	1088	516.70
ggg	G	31	311.80
---	--	------	------
mPD		0.056	0.68
		nPD:	0.08
		N. weight:	0.69
		Sc. PD:	0.022
		Sc. rank:	460.1

HA

Pos.	272	obs:	exp:
aaa	K	1119	652.80
aag	K	1	467.20
---	--	------	------
mPD		0.0018	0.49
		nPD:	0.
		N. weight:	0.56
		Sc. PD:	-0.025
		Sc. rank:	-316.1

HA

Pos.	273	obs:	exp:
tct	S	1120	178.10
tcc	S	0	146.30
tca	S	0	284.30
tcg	S	0	63.55
agt	S	0	230.20
agc	S	0	217.50
---	--	------	------
mPD		0	1.7
		nPD:	0.
		N. weight:	1.9
		Sc. PD:	-0.092
		Sc. rank:	-1766.2

HA

Pos.	274	obs:	exp:
att	I	0	10.29
atc	I	0	6.71
ata	I	28	11.00
atg	M	1092	1092.00
---	--	------	------
mPD		0.049	0.049
		nPD:	0.99
		N. weight:	0.024
		Sc. PD:	0.022
		Sc. rank:	22.8

HA

Pos.	275	obs:	exp:
ggt	G	0	148.50
ggc	G	0	143.10
gga	G	1112	516.70
ggg	G	8	311.80
---	--	------	------
mPD		0.014	0.68
		nPD:	0.02
		N. weight:	0.77
		Sc. PD:	-0.021
		Sc. rank:	152.4

HA

Pos.	276	obs:	exp:
att	I	3	407.50
atc	I	1106	265.80
ata	I	0	435.80
gtt	V	0	2.28
gtc	V	11	2.22
gta	V	0	2.25
gtg	V	0	4.25
---	--	------	------
mPD		0.025	0.68
		nPD:	0.04
		N. weight:	1.5
		Sc. PD:	-0.018
		Sc. rank:	706.3

HA

Pos.	277	obs:	exp:
caa	Q	0	570.50
cag	Q	1120	549.50
---	--	------	------
mPD		0	0.50
		nPD:	0.
		N. weight:	0.75
		Sc. PD:	-0.036
		Sc. rank:	-684.0

HA

Pos.	278	obs:	exp:
tct	S	0	178.10
tcc	S	0	146.30
tca	S	0	284.30
tcg	S	0	63.55
agt	S	1119	230.20
agc	S	1	217.50
---	--	------	------
mPD		0.0018	1.7
		nPD:	0.
		N. weight:	1.7
		Sc. PD:	-0.077
		Sc. rank:	-1229.5

HA

Pos.	279	obs:	exp:
cgt	R	0	0.39
cgc	R	0	0.45
cga	R	0	0.88
cgg	R	0	0.75
aga	R	9	4.12
agg	R	0	2.42
gaa	E	3	1.83
gag	E	0	1.17
ggt	G	8	146.90
ggc	G	0	141.50
gga	G	1097	511.10
ggg	G	3	308.50
---	--	------	------
mPD		0.041	0.70
		nPD:	0.06
		N. weight:	0.76
		Sc. PD:	0.0067
		Sc. rank:	472.1

HA

Pos.	280	obs:	exp:
gtt	V	0	229.00
gtc	V	2	223.00
gta	V	1100	226.30
gtg	V	3	426.80
gct	A	0	3.87
gcc	A	0	2.79
gca	A	15	7.06
gcg	A	0	1.27
---	--	------	------
mPD		0.035	0.75
		nPD:	0.05
		N. weight:	1.6
		Sc. PD:	-0.004
		Sc. rank:	886.1

HA

Pos.	281	obs:	exp:
caa	Q	5	570.50
cag	Q	1115	549.50
---	--	------	------
mPD		0.0089	0.50
		nPD:	0.02
		N. weight:	0.72
		Sc. PD:	-0.022
		Sc. rank:	60.7

HA

Pos.	282	obs:	exp:
att	I	9	3.31
atc	I	0	2.16
ata	I	0	3.54
gtt	V	1110	230.20
gtc	V	1	224.20
gta	V	0	227.50
gtg	V	0	429.10
---	--	------	------
mPD		0.018	0.74
		nPD:	0.02
		N. weight:	1.6
		Sc. PD:	-0.04
		Sc. rank:	441.4

HA

Pos.	283	obs:	exp:
tat	Y	1	0.54
tac	Y	0	0.46
gct	A	4	1.03
gcc	A	0	0.74
gca	A	0	1.88
gcg	A	0	0.34
gat	D	1114	602.50
gac	D	1	512.50
---	--	------	------
mPD		0.011	0.51
		nPD:	0.02
		N. weight:	0.64
		Sc. PD:	-0.018
		Sc. rank:	132.4

HA

Pos.	284	obs:	exp:
act	T	0	1.02
acc	T	4	0.82
aca	T	0	1.83
acg	T	0	0.32
gtt	V	0	1.24
gtc	V	6	1.21
gta	V	0	1.23
gtg	V	0	2.32
gct	A	20	285.30
gcc	A	1085	205.80
gca	A	0	520.10
gcg	A	0	93.78
gat	D	4	2.70
gac	D	1	2.30
---	--	------	------
mPD		0.069	0.70
		nPD:	0.1
		N. weight:	1.7
		Sc. PD:	0.078
		Sc. rank:	1170.5

HA

Pos.	285	obs:	exp:
tct	S	0	0.32
tcc	S	0	0.26
tca	S	0	0.51
tcg	S	0	0.11
cat	H	5	2.87
cac	H	0	2.13
aat	N	1057	538.90
aac	N	1	519.10
agt	S	2	0.41
agc	S	0	0.39
gat	D	54	29.18
gac	D	0	24.82
ggt	G	1	0.13
ggc	G	0	0.13
gga	G	0	0.46
ggg	G	0	0.28
---	--	------	------
mPD		0.11	0.61
		nPD:	0.18
		N. weight:	0.7
		Sc. PD:	0.087
		Sc. rank:	574.5

HA

Pos.	286	obs:	exp:
tgt	C	1120	473.70
tgc	C	0	646.30
mPD		0	0.49
		nPD:	0.
		N. weight:	0.9
		Sc. PD:	-0.043
		Sc. rank:	-826.6

HA

Pos.	287	obs:	exp:
aaa	K	1	0.58
aag	K	0	0.42
gtt	V	0	0.41
gtc	V	0	0.40
gta	V	2	0.41
gtg	V	0	0.77
gaa	E	1117	679.70
gag	E	0	437.30
mPD		0.0054	0.48
		nPD:	0.01
		N. weight:	0.52
		Sc. PD:	-0.019
		Sc. rank:	-89.3

HA

Pos.	288	obs:	exp:
ggt	G	0	148.50
ggc	G	0	143.10
gga	G	6	516.70
ggg	G	1114	311.80
mPD		0.011	0.68
		nPD:	0.02
		N. weight:	1.3
		Sc. PD:	-0.042
		Sc. rank:	6.9

HA

Pos.	289	obs:	exp:
tat	Y	0	0.54
tac	Y	1	0.46
aat	N	0	4.58
aac	N	9	4.42
gat	D	39	597.70
gac	D	1067	508.30
gaa	E	4	2.43
gag	E	0	1.57
mPD		0.092	0.52
		nPD:	0.18
		N. weight:	0.65
		Sc. PD:	0.08
		Sc. rank:	533.5

HA

Pos.	290	obs:	exp:
tgt	C	3	473.70
tgc	C	1117	646.30
mPD		0.0053	0.49
		nPD:	0.01
		N. weight:	0.56
		Sc. PD:	-0.021
		Sc. rank:	-99.0

HA

Pos.	291	obs:	exp:
tat	Y	1116	603.80
tac	Y	2	514.20
cat	H	2	1.15
cac	H	0	0.85
mPD		0.0071	0.50
		nPD:	0.01
		N. weight:	0.63
		Sc. PD:	-0.022
		Sc. rank:	-20.4

HA

Pos.	292	obs:	exp:
tat	Y	4	2.16
tac	Y	0	1.84
cat	H	1115	640.80
cac	H	1	475.20
mPD		0.0089	0.50
		nPD:	0.02
		N. weight:	0.57
		Sc. PD:	-0.017
		Sc. rank:	49.7

HA

Pos.	293	obs:	exp:
tct	S	0	178.00
tcc	S	0	146.10
tca	S	0	284.00
tcg	S	0	63.50
aat	N	1	0.51
aac	N	0	0.49
agt	S	1116	230.00
agc	S	3	217.30
mPD		0.0071	1.7
		nPD:	0.
		N. weight:	1.6
		Sc. PD:	-0.071
		Sc. rank:	-891.3

HA

Pos.	294	obs:	exp:
cgt	R	0	0.26
cgc	R	0	0.30
cga	R	0	0.59
cgg	R	0	0.50
aga	R	6	2.74
agg	R	0	1.61
ggt	G	401	147.70
ggc	G	0	142.30
gga	G	706	513.90
ggg	G	7	310.10
mPD		0.48	0.69
		nPD:	0.7
		N. weight:	0.57
		Sc. PD:	0.36
		Sc. rank:	527.3

HA

Pos.	295	obs:	exp:
ggt	G	0	148.50
ggc	G	0	143.10
gga	G	17	516.70
ggg	G	1103	311.80
mPD		0.030	0.68
		nPD:	0.04
		N. weight:	1.3
		Sc. PD:	-0.0063
		Sc. rank:	654.5

HA

Pos.	296	obs:	exp:
act	T	0	285.50
acc	T	0	229.80
aca	T	1110	513.70
acg	T	10	90.97
mPD		0.018	0.68
		nPD:	0.03
		N. weight:	0.78
		Sc. PD:	-0.018
		Sc. rank:	246.1

HA

Pos.	297	obs:	exp:
att	I	0	411.50
atc	I	0	268.40
ata	I	1120	440.10
mPD		0	0.65
		nPD:	0.
		N. weight:	0.98
		Sc. PD:	-0.047
		Sc. rank:	-897.3

HA

Pos.	298	obs:	exp:
cgt	R	0	0.04
cgc	R	0	0.05
cga	R	0	0.10
cgg	R	0	0.08
att	I	0	408.20
atc	I	0	266.20
ata	I	1111	436.60
atg	M	5	5.00
act	T	0	0.76
acc	T	0	0.62
aca	T	3	1.38
acg	T	0	0.24
aga	R	1	0.46
agg	R	0	0.27
mPD		0.016	0.66
		nPD:	0.02
		N. weight:	0.97
		Sc. PD:	-0.024
		Sc. rank:	265.5

HA

Pos.	299	obs:	exp:
tct	S	0	178.00
tcc	S	0	146.10
tca	S	0	284.00
tcg	S	0	63.50
aat	N	1	0.51
aac	N	0	0.49
agt	S	1117	230.00
agc	S	2	217.30
mPD		0.0054	1.7
		nPD:	0.
		N. weight:	1.6
		Sc. PD:	-0.073
		Sc. rank:	-1012.4

HA

Pos.	300	obs:	exp:
tct	S	0	1.91
tcc	S	0	1.57
tca	S	0	3.05
tcg	S	0	0.68
cat	H	0	0.57
cac	H	1	0.43
aat	N	3	557.70
aac	N	1092	537.30
aaa	K	12	6.99
aag	K	0	5.01
agt	S	0	2.47
agc	S	12	2.33
mPD		0.050	0.55
		nPD:	0.09
		N. weight:	0.74
		Sc. PD:	0.029
		Sc. rank:	502.6

HA

Pos.	301	obs:	exp:
tta	L	3	99.39
ttg	L	1116	208.50
ctt	L	0	186.50
ctc	L	0	186.90
cta	L	0	188.80
ctg	L	1	249.90
mPD		0.0071	1.1
		nPD:	0.01
		N. weight:	1.7
		Sc. PD:	-0.072
		Sc. rank:	-772.6

HA

Pos.	302	obs:	exp:
cct	P	2	279.50
ccc	P	3	201.20
cca	P	1113	461.00
ccg	P	2	178.30
mPD		0.012	0.71
		nPD:	0.02
		N. weight:	0.89
		Sc. PD:	-0.027
		Sc. rank:	66.9

HA

Pos.	303	obs:	exp:
ttt	F	1117	442.30
ttc	F	3	677.70
mPD		0.0053	0.48
		nPD:	0.01
		N. weight:	0.95
		Sc. PD:	-0.035
		Sc. rank:	-151.9

HA

Pos.	304	obs:	exp:
caa	Q	1	570.50
cag	Q	1119	549.50
mPD		0.0018	0.50
		nPD:	0.
		N. weight:	0.74
		Sc. PD:	-0.033
		Sc. rank:	-439.4

HA

Pos.	305	obs:	exp:
aat	N	5	570.50
aac	N	1115	549.50
mPD		0.0089	0.50
		nPD:	0.02
		N. weight:	0.72
		Sc. PD:	-0.022
		Sc. rank:	57.3

HA

Pos.	306	obs:	exp:
att	I	0	410.80
atc	I	2	267.90
ata	I	1116	439.30
gtt	V	0	0.41
gtc	V	0	0.40
gta	V	2	0.41
gtg	V	0	0.77
mPD		0.0071	0.66
		nPD:	0.01
		N. weight:	0.97
		Sc. PD:	-0.036
		Sc. rank:	-173.6

HA

Pos.	307	obs:	exp:
aat	N	3	1.53
aac	N	0	1.47
gat	D	1109	603.10
gac	D	7	512.90
ggt	G	1	0.13
ggc	G	0	0.13
gga	G	0	0.46
ggg	G	0	0.28
mPD		0.020	0.50
		nPD:	0.04
		N. weight:	0.61
		Sc. PD:	-0.0063
		Sc. rank:	299.6

HA

Pos.	308	obs:	exp:
tct	S	0	176.70
tcc	S	0	145.10
tca	S	0	282.00
tcg	S	0	63.04
cgt	R	0	0.26
cgc	R	0	0.30
cga	R	0	0.59
cgg	R	0	0.50
aat	N	0	1.53
aac	N	3	1.47
agt	S	0	228.40
agc	S	1111	215.80
aga	R	6	2.74
agg	R	0	1.61
mPD		0.016	1.7
		nPD:	0.01
		N. weight:	1.7
		Sc. PD:	-0.066
		Sc. rank:	-418.7

HA

Pos.	309	obs:	exp:
cgt	R	0	48.10
cgc	R	0	55.47
cga	R	0	109.60
cgg	R	0	93.45
aga	R	55	512.10
agg	R	1065	301.20
mPD		0.093	0.96
		nPD:	0.1
		N. weight:	1.1
		Sc. PD:	0.053
		Sc. rank:	800.3

HA

Pos.	310	obs:	exp:
act	T	0	11.73
acc	T	0	9.44
aca	T	46	21.10
acg	T	0	3.74
gct	A	12	277.30
gcc	A	0	200.00
gca	A	1062	505.50
gcg	A	0	91.15
mPD		0.10	0.75
		nPD:	0.13
		N. weight:	0.74
		Sc. PD:	0.06
		Sc. rank:	565.6

HA			
Pos.	311	obs:	exp:
att	I	1	0.37
atc	I	0	0.24
ata	I	0	0.39
gtt	V	1115	231.90
gtc	V	0	225.80
gta	V	3	229.10
gtg	V	1	432.20
---	--	------	------
mPD		0.0089	0.73
		nPD:	0.01
		N. weight:	1.6
		Sc. PD:	-0.058
		Sc. rank:	-186.0

HA			
Pos.	312	obs:	exp:
ggt	G	0	148.50
ggc	G	0	143.10
gga	G	1110	516.70
ggg	G	10	311.80
---	--	------	------
mPD		0.018	0.68
		nPD:	0.03
		N. weight:	0.76
		Sc. PD:	-0.017
		Sc. rank:	242.5

HA			
Pos.	313	obs:	exp:
cgt	R	0	0.13
cgc	R	0	0.15
cga	R	0	0.29
cgg	R	0	0.25
aaa	K	1115	651.00
aag	K	2	466.00
aga	R	3	1.37
agg	R	0	0.81
---	--	------	------
mPD		0.0089	0.49
		nPD:	0.02
		N. weight:	0.55
		Sc. PD:	-0.017
		Sc. rank:	48.9

HA			
Pos.	314	obs:	exp:
tgt	C	1113	473.70
tgc	C	7	646.30
---	--	------	------
mPD		0.012	0.49
		nPD:	0.03
		N. weight:	0.86
		Sc. PD:	-0.02
		Sc. rank:	248.0

HA			
Pos.	315	obs:	exp:
cct	P	2	279.50
ccc	P	0	201.20
cca	P	9	461.00
ccg	P	1109	178.30
---	--	------	------
mPD		0.019	0.71
		nPD:	0.03
		N. weight:	1.9
		Sc. PD:	-0.04
		Sc. rank:	624.1

HA			
Pos.	316	obs:	exp:
cgt	R	0	48.06
cgc	R	0	55.42
cga	R	0	109.50
cgg	R	0	93.37
aaa	K	1	0.58
aag	K	0	0.42
aga	R	1109	511.70
agg	R	10	301.00
---	--	------	------
mPD		0.019	0.96
		nPD:	0.02
		N. weight:	0.77
		Sc. PD:	-0.022
		Sc. rank:	132.2

HA			
Pos.	317	obs:	exp:
tat	Y	1117	604.90
tac	Y	3	515.10
---	--	------	------
mPD		0.0053	0.50
		nPD:	0.01
		N. weight:	0.63
		Sc. PD:	-0.023
		Sc. rank:	-117.0

HA			
Pos.	318	obs:	exp:
gtt	V	1118	232.10
gtc	V	2	226.00
gta	V	0	229.30
gtg	V	0	432.60
---	--	------	------
mPD		0.0036	0.73
		nPD:	0.
		N. weight:	1.6
		Sc. PD:	-0.07
		Sc. rank:	-863.3

HA			
Pos.	319	obs:	exp:
cgt	R	0	0.17
cgc	R	0	0.20
cga	R	0	0.39
cgg	R	0	0.33
aaa	K	62	650.40
aag	K	1054	465.60
aga	R	0	1.83
agg	R	4	1.08
---	--	------	------
mPD		0.11	0.50
		nPD:	0.23
		N. weight:	0.68
		Sc. PD:	0.11
		Sc. rank:	560.9

HA			
Pos.	320	obs:	exp:
caa	Q	1116	570.50
cag	Q	4	549.50
---	--	------	------
mPD		0.0071	0.50
		nPD:	0.01
		N. weight:	0.68
		Sc. PD:	-0.023
		Sc. rank:	-23.4

HA			
Pos.	321	obs:	exp:
cgt	R	0	44.49
cgc	R	0	51.31
cga	R	0	101.40
cgg	R	0	86.45
aaa	K	0	48.37
aag	K	83	34.63
aga	R	17	473.70
agg	R	1019	278.60
ggt	G	0	0.13
ggc	G	0	0.13
gga	G	0	0.46
ggg	G	1	0.28
---	--	------	------
mPD		0.17	1.1
		nPD:	0.16
		N. weight:	1.3
		Sc. PD:	0.13
		Sc. rank:	996.9

HA			
Pos.	322	obs:	exp:
tct	S	0	178.10
tcc	S	0	146.30
tca	S	0	284.30
tcg	S	0	63.55
agt	S	1117	230.20
agc	S	3	217.50
---	--	------	------
mPD		0.0053	1.7
		nPD:	0.
		N. weight:	1.6
		Sc. PD:	-0.073
		Sc. rank:	-1035.4

HA			
Pos.	323	obs:	exp:
tta	L	0	99.39
ttg	L	0	208.50
ctt	L	81	186.50
ctc	L	0	186.90
cta	L	6	188.80
ctg	L	1033	249.90
---	--	------	------
mPD		0.14	1.1
		nPD:	0.13
		N. weight:	1.3
		Sc. PD:	0.1
		Sc. rank:	982.9

HA			
Pos.	324	obs:	exp:
tta	L	0	99.30
ttg	L	4	208.30
ctt	L	0	186.30
ctc	L	0	186.70
cta	L	4	188.60
ctg	L	1111	249.70
atg	M	1	1.00
---	--	------	------
mPD		0.016	1.1
		nPD:	0.01
		N. weight:	1.5
		Sc. PD:	-0.052
		Sc. rank:	-41.4

HA			
Pos.	325	obs:	exp:
tta	L	1	99.39
ttg	L	2	208.50
ctt	L	180	186.50
ctc	L	0	186.90
cta	L	591	188.80
ctg	L	346	249.90
---	--	------	------
mPD		0.60	1.1
		nPD:	0.54
		N. weight:	0.73
		Sc. PD:	0.35
		Sc. rank:	660.0

HA			
Pos.	326	obs:	exp:
gct	A	1	289.20
gcc	A	0	208.60
gca	A	1110	527.10
gcg	A	9	95.06
---	--	------	------
mPD		0.018	0.67
		nPD:	0.03
		N. weight:	0.75
		Sc. PD:	-0.017
		Sc. rank:	243.6

HA			
Pos.	327	obs:	exp:
act	T	0	285.50
acc	T	0	229.80
aca	T	1118	513.70
acg	T	2	90.97
---	--	------	------
mPD		0.0036	0.68
		nPD:	0.01
		N. weight:	0.81
		Sc. PD:	-0.034
		Sc. rank:	-399.2

HA			
Pos.	328	obs:	exp:
ggt	G	0	148.50
ggc	G	0	143.10
gga	G	20	516.70
ggg	G	1100	311.80
---	--	------	------
mPD		0.035	0.68
		nPD:	0.05
		N. weight:	1.2
		Sc. PD:	0.0029
		Sc. rank:	733.5

HA			
Pos.	329	obs:	exp:
atg	M	1120	1120.00
---	--	------	------
mPD		0	0
		nPD:	1.
		N. weight:	0.
		Sc. PD:	0
		Sc. rank:	0

HA			
Pos.	330	obs:	exp:
aaa	K	0	652.80
aag	K	1120	467.20
---	--	------	------
mPD		0	0.49
		nPD:	0.
		N. weight:	0.92
		Sc. PD:	-0.044
		Sc. rank:	-839.8

HA			
Pos.	331	obs:	exp:
aat	N	1119	570.50
aac	N	1	549.50
---	--	------	------
mPD		0.0018	0.50
		nPD:	0.
		N. weight:	0.7
		Sc. PD:	-0.031
		Sc. rank:	-419.4

HA			
Pos.	332	obs:	exp:
gtt	V	1094	232.10
gtc	V	2	226.00
gta	V	24	229.30
gtg	V	0	432.60
---	--	------	------
mPD		0.045	0.73
		nPD:	0.06
		N. weight:	1.5
		Sc. PD:	0.019
		Sc. rank:	966.6

HA			
Pos.	333	obs:	exp:
cct	P	1117	279.50
ccc	P	0	201.20
cca	P	3	461.00
ccg	P	0	178.30
---	--	------	------
mPD		0.0053	0.71
		nPD:	0.01
		N. weight:	1.4
		Sc. PD:	-0.058
		Sc. rank:	-543.6

HA			
Pos.	334	obs:	exp:
gaa	E	1	681.50
gag	E	1119	438.50
---	--	------	------
mPD		0.0018	0.48
		nPD:	0.
		N. weight:	0.98
		Sc. PD:	-0.043
		Sc. rank:	-540.1

HA			
Pos.	335	obs:	exp:
att	I	1057	391.30
atc	I	7	255.20
ata	I	1	418.50
gtt	V	54	11.19
gtc	V	0	10.90
gta	V	0	11.06
gtg	V	0	20.86
ggt	G	1	0.13
ggc	G	0	0.13
gga	G	0	0.46
ggg	G	0	0.28
---	--	------	------
mPD		0.11	0.76
		nPD:	0.14
		N. weight:	1.
		Sc. PD:	0.094
		Sc. rank:	809.0

HA			
Pos.	336	obs:	exp:
cct	P	0	279.50
ccc	P	2	201.20
cca	P	1112	461.00
ccg	P	6	178.30
---	--	------	------
mPD		0.014	0.71
		nPD:	0.02
		N. weight:	0.89
		Sc. PD:	-0.025
		Sc. rank:	150.1

,

HA			
Pos.	337	obs:	exp:
caa	Q	0	0.51
cag	Q	1	0.49
cgt	R	0	0.13
cgc	R	0	0.15
cga	R	0	0.29
cgg	R	0	0.25
aaa	K	6	650.40
aag	K	1110	465.60
aga	R	0	1.37
agg	R	3	0.81
---	--	------	------
mPD		0.018	0.50
		nPD:	0.04
		N. weight:	0.88
		Sc. PD:	-0.012
		Sc. rank:	398.0

,

HA			
Pos.	338	obs:	exp:
cgt	R	0	2.15
cgc	R	0	2.48
cga	R	0	4.89
cgg	R	0	4.17
aga	R	50	22.86
agg	R	0	13.45
ggt	G	0	0.13
ggc	G	0	0.13
gga	G	1	0.46
ggg	G	0	0.28
---	-	1069	1069.00
---	--	------	------
mPD		0.000080	0.0020
		nPD:	0.04
		N. weight:	0.
		Sc. PD:	0
		Sc. rank:	0

,

HA			
Pos.	339	obs:	exp:
aaa	K	51	29.72
aag	K	0	21.28
---	-	1069	1069.00
---	--	------	------
mPD		0	0.0010
		nPD:	0.
		N. weight:	0.
		Sc. PD:	0
		Sc. rank:	0

,

HA			
Pos.	340	obs:	exp:
cgt	R	0	2.19
cgc	R	0	2.53
cga	R	0	4.99
cgg	R	51	4.26
aga	R	0	23.32
agg	R	0	13.72
---	-	1069	1069.00
---	--	------	------
mPD		0	0.0020
		nPD:	0.
		N. weight:	0.
		Sc. PD:	0
		Sc. rank:	0

,

HA			
Pos.	341	obs:	exp:
att	I	1	0.37
atc	I	0	0.24
ata	I	0	0.39
act	T	49	12.49
acc	T	0	10.05
aca	T	0	22.47
acg	T	0	3.98
gct	A	1	0.26
gcc	A	0	0.19
gca	A	0	0.47
gcg	A	0	0.08
---	-	1069	1069.00
---	--	------	------
mPD		0.00016	0.0016
		nPD:	0.1
		N. weight:	0.
		Sc. PD:	0
		Sc. rank:	0

,

HA			
Pos.	342	obs:	exp:
gct	A	0	13.17
gcc	A	0	9.50
gca	A	1	24.00
gcg	A	50	4.33
ggt	G	0	141.70
ggc	G	0	136.60
gga	G	1055	493.20
ggg	G	14	297.60
---	--	------	------
mPD		0.19	0.76
		nPD:	0.25
		N. weight:	0.82
		Sc. PD:	0.16
		Sc. rank:	692.1

,

HA			
Pos.	343	obs:	exp:
cgt	R	0	48.10
cgc	R	0	55.47
cga	R	0	109.60
cgg	R	0	93.45
aga	R	1119	512.10
agg	R	1	301.20
---	--	------	------
mPD		0.0018	0.96
		nPD:	0.
		N. weight:	0.81
		Sc. PD:	-0.037
		Sc. rank:	-584.8

,

HA			
Pos.	344	obs:	exp:
ggt	G	6	148.50
ggc	G	1112	143.10
gga	G	2	516.70
ggg	G	0	311.80
---	--	------	------
mPD		0.014	0.68
		nPD:	0.02
		N. weight:	2.1
		Sc. PD:	-0.058
		Sc. rank:	430.6

,

HA			
Pos.	345	obs:	exp:
tta	L	0	99.39
ttg	L	0	208.50
ctt	L	0	186.50
ctc	L	0	186.90
cta	L	1119	188.80
ctg	L	1	249.90
---	--	------	------
mPD		0.0018	1.1
		nPD:	0.
		N. weight:	1.9
		Sc. PD:	-0.086
		Sc. rank:	-1352.1

,

HA			
Pos.	346	obs:	exp:
ttt	F	1116	441.50
ttc	F	2	676.50
tta	L	0	0.18
ttg	L	0	0.37
ctt	L	2	0.33
ctc	L	0	0.33
cta	L	0	0.34
ctg	L	0	0.45
---	--	------	------
mPD		0.0071	0.48
		nPD:	0.01
		N. weight:	0.96
		Sc. PD:	-0.032
		Sc. rank:	-16.3

,

HA			
Pos.	347	obs:	exp:
ggt	G	1120	148.50
ggc	G	0	143.10
gga	G	0	516.70
ggg	G	0	311.80
---	--	------	------
mPD		0	0.68
		nPD:	0.
		N. weight:	2.1
		Sc. PD:	-0.1
		Sc. rank:	-1941.3

,

HA			
Pos.	348	obs:	exp:
gct	A	1118	289.20
gcc	A	2	208.60
gca	A	0	527.10
gcg	A	0	95.06
---	--	------	------
mPD		0.0036	0.67
		nPD:	0.01
		N. weight:	1.4
		Sc. PD:	-0.06
		Sc. rank:	-680.2

,

HA			
Pos.	349	obs:	exp:
att	I	0	411.50
atc	I	0	268.40
ata	I	1120	440.10
---	--	------	------
mPD		0	0.65
		nPD:	0.
		N. weight:	0.98
		Sc. PD:	-0.047
		Sc. rank:	-897.3

,

HA			
Pos.	350	obs:	exp:
gct	A	45	289.20
gcc	A	0	208.60
gca	A	64	527.10
gcg	A	1011	95.06
---	--	------	------
mPD		0.18	0.67
		nPD:	0.27
		N. weight:	2.
		Sc. PD:	0.43
		Sc. rank:	1734.5

,

HA			
Pos.	351	obs:	exp:
ggt	G	1117	148.50
ggc	G	0	143.10
gga	G	0	516.70
ggg	G	3	311.80
---	--	------	------
mPD		0.0053	0.68
		nPD:	0.01
		N. weight:	2.1
		Sc. PD:	-0.084
		Sc. rank:	-763.0

,

HA			
Pos.	352	obs:	exp:
ttt	F	2	442.30
ttc	F	1118	677.70
---	--	------	------
mPD		0.0036	0.48
		nPD:	0.01
		N. weight:	0.51
		Sc. PD:	-0.021
		Sc. rank:	-204.9

,

HA			
Pos.	353	obs:	exp:
att	I	1120	411.50
atc	I	0	268.40
ata	I	0	440.10
---	--	------	------
mPD		0	0.65
		nPD:	0.
		N. weight:	1.
		Sc. PD:	-0.05
		Sc. rank:	-961.8

,

HA			
Pos.	354	obs:	exp:
gaa	E	1112	681.50
gag	E	8	438.50
---	--	------	------
mPD		0.014	0.48
		nPD:	0.03
		N. weight:	0.48
		Sc. PD:	-0.0091
		Sc. rank:	180.9

,

HA			
Pos.	355	obs:	exp:
aat	N	1120	570.50
aac	N	0	549.50
---	--	------	------
mPD		0	0.50
		nPD:	0.
		N. weight:	0.71
		Sc. PD:	-0.034
		Sc. rank:	-648.0

,

HA			
Pos.	356	obs:	exp:
ggt	G	0	148.50
ggc	G	1	143.10
gga	G	1115	516.70
ggg	G	4	311.80
---	--	------	------
mPD		0.0089	0.68
		nPD:	0.01
		N. weight:	0.78
		Sc. PD:	-0.027
		Sc. rank:	-44.3

,

HA			
Pos.	357	obs:	exp:
tgg	W	1120	1120.00
---	--	------	------
mPD		0	0
		nPD:	1.
		N. weight:	0.
		Sc. PD:	0
		Sc. rank:	0

,

HA			
Pos.	358	obs:	exp:
gaa	E	1117	681.50
gag	E	3	438.50
---	--	------	------
mPD		0.0053	0.48
		nPD:	0.01
		N. weight:	0.5
		Sc. PD:	-0.019
		Sc. rank:	-79.0

,

HA			
Pos.	359	obs:	exp:
ggt	G	0	148.50
ggc	G	1076	143.10
gga	G	43	516.70
ggg	G	1	311.80
---	--	------	------
mPD		0.076	0.68
		nPD:	0.11
		N. weight:	1.9
		Sc. PD:	0.12
		Sc. rank:	1407.7

,

HA			
Pos.	360	obs:	exp:
tta	L	1	99.39
ttg	L	0	208.50
ctt	L	0	186.50
ctc	L	0	186.90
cta	L	1114	188.80
ctg	L	5	249.90
---	--	------	------
mPD		0.011	1.1
		nPD:	0.01
		N. weight:	1.8
		Sc. PD:	-0.07
		Sc. rank:	-458.5

,

HA			
Pos.	361	obs :	exp :
ttt	F	1	0.39
ttc	F	0	0.61
att	I	1114	410.40
atc	I	3	267.70
ata	I	0	438.90
act	T	2	0.51
acc	T	0	0.41
aca	T	0	0.92
acg	T	0	0.16
---	--	------	------
mPD		0.011	0.66
		nPD :	0.02
		N. weight :	1.
		Sc. PD :	-0.033
		Sc. rank :	43.0

HA			
Pos.	362	obs :	exp :
aat	N	6	3.06
aac	N	0	2.94
gat	D	1110	600.40
gac	D	1	510.60
ggt	G	3	0.40
ggc	G	0	0.38
gga	G	0	1.38
ggg	G	0	0.84
---	--	------	------
mPD		0.018	0.51
		nPD :	0.03
		N. weight :	0.64
		Sc. PD :	-0.0093
		Sc. rank :	288.2

HA			
Pos.	363	obs :	exp :
ggt	G	1120	148.50
ggc	G	0	143.10
gga	G	0	516.70
ggg	G	0	311.80
---	--	------	------
mPD		0	0.68
		nPD :	0.
		N. weight :	2.1
		Sc. PD :	-0.1
		Sc. rank :	-1941.3

HA			
Pos.	364	obs :	exp :
tgg	W	1120	1120.00
---	--	------	------
mPD		0	0
		nPD :	1.
		N. weight :	0.
		Sc. PD :	0
		Sc. rank :	0

HA			
Pos.	365	obs :	exp :
tat	Y	1120	604.90
tac	Y	0	515.10
---	--	------	------
mPD		0	0.50
		nPD :	0.
		N. weight :	0.65
		Sc. PD :	-0.031
		Sc. rank :	-591.8

HA			
Pos.	366	obs :	exp :
ggt	G	1117	148.50
ggc	G	0	143.10
gga	G	0	516.70
ggg	G	3	311.80
---	--	------	------
mPD		0.0053	0.68
		nPD :	0.01
		N. weight :	2.1
		Sc. PD :	-0.084
		Sc. rank :	-763.0

HA			
Pos.	367	obs :	exp :
ttt	F	0	442.30
ttc	F	1120	677.70
---	--	------	------
mPD		0	0.48
		nPD :	0.
		N. weight :	0.53
		Sc. PD :	-0.025
		Sc. rank :	-482.6

HA			
Pos.	368	obs :	exp :
cgt	R	0	47.63
cgc	R	0	54.93
cga	R	0	108.50
cgg	R	0	92.54
aaa	K	11	6.41
aag	K	0	4.59
aga	R	1106	507.10
agg	R	3	298.30
---	--	------	------
mPD		0.025	0.98
		nPD :	0.03
		N. weight :	0.8
		Sc. PD :	-0.019
		Sc. rank :	228.3

HA			
Pos.	369	obs :	exp :
cat	H	56	643.10
cac	H	1064	476.90
---	--	------	------
mPD		0.095	0.49
		nPD :	0.19
		N. weight :	0.67
		Sc. PD :	0.094
		Sc. rank :	556.6

HA			
Pos.	370	obs :	exp :
caa	Q	6	570.50
cag	Q	1114	549.50
---	--	------	------
mPD		0.011	0.50
		nPD :	0.02
		N. weight :	0.71
		Sc. PD :	-0.019
		Sc. rank :	156.9

HA			
Pos.	371	obs :	exp :
aat	N	1077	570.50
aac	N	43	549.50
---	--	------	------
mPD		0.074	0.50
		nPD :	0.15
		N. weight :	0.54
		Sc. PD :	0.051
		Sc. rank :	424.6

HA			
Pos.	372	obs :	exp :
gct	A	0	289.20
gcc	A	0	208.60
gca	A	1116	527.10
gcg	A	4	95.06
---	--	------	------
mPD		0.0071	0.67
		nPD :	0.01
		N. weight :	0.77
		Sc. PD :	-0.029
		Sc. rank :	-157.5

HA			
Pos.	373	obs :	exp :
caa	Q	2	570.50
cag	Q	1118	549.50
---	--	------	------
mPD		0.0036	0.50
		nPD :	0.01
		N. weight :	0.73
		Sc. PD :	-0.03
		Sc. rank :	-317.5

HA			
Pos.	374	obs :	exp :
ggt	G	0	148.50
ggc	G	0	143.10
gga	G	1117	516.70
ggg	G	3	311.80
---	--	------	------
mPD		0.0053	0.68
		nPD :	0.01
		N. weight :	0.79
		Sc. PD :	-0.032
		Sc. rank :	-288.9

HA			
Pos.	375	obs :	exp :
gaa	E	3	681.50
gag	E	1117	438.50
---	--	------	------
mPD		0.0053	0.48
		nPD :	0.01
		N. weight :	0.96
		Sc. PD :	-0.036
		Sc. rank :	-151.1

HA			
Pos.	376	obs :	exp :
ggt	G	0	148.50
ggc	G	0	143.10
gga	G	1107	516.70
ggg	G	13	311.80
---	--	------	------
mPD		0.023	0.68
		nPD :	0.03
		N. weight :	0.75
		Sc. PD :	-0.011
		Sc. rank :	327.6

HA			
Pos.	377	obs :	exp :
act	T	1118	285.50
acc	T	0	229.80
aca	T	1	513.70
acg	T	1	90.97
---	--	------	------
mPD		0.0036	0.68
		nPD :	0.01
		N. weight :	1.4
		Sc. PD :	-0.06
		Sc. rank :	-691.5

HA			
Pos.	378	obs :	exp :
gtt	V	3	0.62
gtc	V	0	0.61
gta	V	0	0.61
gtg	V	0	1.16
gct	A	1117	288.40
gcc	A	0	208.00
gca	A	0	525.70
gcg	A	0	94.80
---	--	------	------
mPD		0.0053	0.68
		nPD :	0.01
		N. weight :	1.4
		Sc. PD :	-0.057
		Sc. rank :	-523.4

HA			
Pos.	379	obs :	exp :
gct	A	2	289.20
gcc	A	0	208.60
gca	A	1118	527.10
gcg	A	0	95.06
---	--	------	------
mPD		0.0036	0.67
		nPD :	0.01
		N. weight :	0.78
		Sc. PD :	-0.033
		Sc. rank :	-376.3

HA			
Pos.	380	obs :	exp :
gat	D	1119	605.20
gac	D	1	514.80
---	--	------	------
mPD		0.0018	0.50
		nPD :	0.
		N. weight :	0.64
		Sc. PD :	-0.028
		Sc. rank :	-369.1

HA			
Pos.	381	obs :	exp :
ttt	F	0	1.58
ttc	F	4	2.42
tat	Y	1	602.70
tac	Y	1115	513.30
---	--	------	------
mPD		0.0089	0.50
		nPD :	0.02
		N. weight :	0.81
		Sc. PD :	-0.025
		Sc. rank :	62.6

HA			
Pos.	382	obs :	exp :
aaa	K	1109	652.80
aag	K	11	467.20
---	--	------	------
mPD		0.019	0.49
		nPD :	0.04
		N. weight :	0.51
		Sc. PD :	-0.0047
		Sc. rank :	258.6

HA			
Pos.	383	obs :	exp :
tct	S	0	178.10
tcc	S	0	146.30
tca	S	0	284.30
tcg	S	0	63.55
agt	S	3	230.20
agc	S	1117	217.50
---	--	------	------
mPD		0.0053	1.7
		nPD :	0.
		N. weight :	1.7
		Sc. PD :	-0.076
		Sc. rank :	-1072.7

HA			
Pos.	384	obs :	exp :
act	T	1116	285.50
acc	T	2	229.80
aca	T	2	513.70
acg	T	0	90.97
---	--	------	------
mPD		0.0071	0.68
		nPD :	0.01
		N. weight :	1.4
		Sc. PD :	-0.053
		Sc. rank :	-303.7

HA			
Pos.	385	obs :	exp :
caa	Q	1106	564.90
cag	Q	3	544.10
cgt	R	0	0.43
cgc	R	0	0.50
cga	R	10	0.98
cgg	R	0	0.83
aaa	K	1	0.58
aag	K	0	0.42
aga	R	0	4.57
agg	R	0	2.69
---	--	------	------
mPD		0.025	0.53
		nPD :	0.05
		N. weight :	0.7
		Sc. PD :	-0.002
		Sc. rank :	382.0

HA

Pos.	386	obs:	exp:
tct	S	1	178.10
tcc	S	0	146.30
tca	S	2	284.30
tcg	S	1117	63.55
agt	S	0	230.20
agc	S	0	217.50
mPD		0.0054	1.7
		nPD:	0.
		N. weight:	3.
		Sc. PD:	-0.13
		Sc. rank:	-1815.3

,

HA

Pos.	387	obs:	exp:
gct	A	0	289.20
gcc	A	0	208.60
gca	A	1120	527.10
gcg	A	0	95.06
mPD		0	0.67
		nPD:	0.
		N. weight:	0.79
		Sc. PD:	-0.038
		Sc. rank:	-723.9

,

HA

Pos.	388	obs:	exp:
att	I	1114	410.80
atc	I	0	267.90
ata	I	4	439.30
gtt	V	2	0.41
gtc	V	0	0.40
gta	V	0	0.41
gtg	V	0	0.77
mPD		0.011	0.66
		nPD:	0.02
		N. weight:	1.
		Sc. PD:	-0.033
		Sc. rank:	49.5

,

HA

Pos.	389	obs:	exp:
aat	N	2	1.02
aac	N	0	0.98
gat	D	1114	604.10
gac	D	4	513.90
mPD		0.011	0.50
		nPD:	0.02
		N. weight:	0.62
		Sc. PD:	-0.017
		Sc. rank:	138.3

,

HA

Pos.	390	obs:	exp:
caa	Q	1118	570.50
cag	Q	2	549.50
mPD		0.0036	0.50
		nPD:	0.01
		N. weight:	0.69
		Sc. PD:	-0.028
		Sc. rank:	-300.5

,

HA

Pos.	391	obs:	exp:
att	I	0	410.40
atc	I	0	267.70
ata	I	1117	438.90
gtt	V	0	0.62
gtc	V	0	0.61
gta	V	3	0.61
gtg	V	0	1.16
mPD		0.0053	0.66
		nPD:	0.01
		N. weight:	0.98
		Sc. PD:	-0.039
		Sc. rank:	-297.2

,

HA

Pos.	392	obs:	exp:
act	T	0	284.20
acc	T	0	228.80
aca	T	1111	511.40
acg	T	4	90.57
gct	A	0	1.29
gcc	A	0	0.93
gca	A	5	2.35
gcg	A	0	0.42
mPD		0.016	0.68
		nPD:	0.02
		N. weight:	0.8
		Sc. PD:	-0.02
		Sc. rank:	195.9

,

HA

Pos.	393	obs:	exp:
ggt	G	2	148.50
ggc	G	0	143.10
gga	G	528	516.70
ggg	G	590	311.80
mPD		0.50	0.68
		nPD:	0.74
		N. weight:	0.36
		Sc. PD:	0.24
		Sc. rank:	334.3

,

HA

Pos.	394	obs:	exp:
aaa	K	1120	652.80
aag	K	0	467.20
mPD		0	0.49
		nPD:	0.
		N. weight:	0.57
		Sc. PD:	-0.027
		Sc. rank:	-518.6

,

HA

Pos.	395	obs:	exp:
tta	L	1115	99.30
ttg	L	1	208.30
ctt	L	0	186.30
ctc	L	0	186.70
cta	L	3	188.60
ctg	L	0	249.70
gtt	V	0	0.21
gtc	V	0	0.20
gta	V	1	0.20
gtg	V	0	0.39
mPD		0.0089	1.1
		nPD:	0.01
		N. weight:	2.5
		Sc. PD:	-0.1
		Sc. rank:	-769.0

,

HA

Pos.	396	obs:	exp:
tct	S	0	0.16
tcc	S	0	0.13
tca	S	0	0.25
tcg	S	0	0.06
aat	N	3	569.00
aac	N	1114	548.00
aaa	K	2	1.17
aag	K	0	0.83
agt	S	0	0.21
agc	S	1	0.19
mPD		0.011	0.50
		nPD:	0.02
		N. weight:	0.73
		Sc. PD:	-0.02
		Sc. rank:	155.5

,

HA

Pos.	397	obs:	exp:
caa	Q	0	0.51
cag	Q	1	0.49
cgt	R	0	48.06
cgc	R	0	55.42
cga	R	9	109.50
cgg	R	1110	93.37
aga	R	0	511.70
agg	R	0	301.00
mPD		0.018	0.96
		nPD:	0.02
		N. weight:	2.6
		Sc. PD:	-0.076
		Sc. rank:	325.6

,

HA

Pos.	398	obs:	exp:
tta	L	0	97.53
ttg	L	0	204.60
ctt	L	1088	183.00
ctc	L	10	183.40
cta	L	0	185.20
ctg	L	1	245.20
att	I	21	7.72
atc	I	0	5.03
ata	I	0	8.25
mPD		0.056	1.1
		nPD:	0.05
		N. weight:	1.8
		Sc. PD:	0.00018
		Sc. rank:	1028.5

,

HA

Pos.	399	obs:	exp:
att	I	0	411.50
atc	I	0	268.40
ata	I	1120	440.10
mPD		0	0.65
		nPD:	0.
		N. weight:	0.98
		Sc. PD:	-0.047
		Sc. rank:	-897.3

,

HA

Pos.	400	obs:	exp:
caa	Q	1	0.51
cag	Q	0	0.49
gct	A	0	136.10
gcc	A	5	98.14
gca	A	518	248.00
gcg	A	4	44.73
gaa	E	586	360.20
gag	E	6	231.80
mPD		0.53	1.1
		nPD:	0.47
		N. weight:	0.59
		Sc. PD:	0.24
		Sc. rank:	525.0

,

HA

Pos.	401	obs:	exp:
aaa	K	1120	652.80
aag	K	0	467.20
mPD		0	0.49
		nPD:	0.
		N. weight:	0.57
		Sc. PD:	-0.027
		Sc. rank:	-518.6

,

HA

Pos.	402	obs:	exp:
act	T	0	285.50
acc	T	1120	229.80
aca	T	0	513.70
acg	T	0	90.97
mPD		0	0.68
		nPD:	0.
		N. weight:	1.7
		Sc. PD:	-0.079
		Sc. rank:	-1521.4

,

HA

Pos.	403	obs:	exp:
aat	N	13	570.50
aac	N	1107	549.50
mPD		0.023	0.50
		nPD:	0.05
		N. weight:	0.68
		Sc. PD:	-0.0024
		Sc. rank:	368.0

,

HA

Pos.	404	obs:	exp:
cat	H	2	1.15
cac	H	0	0.85
caa	Q	1114	567.40
cag	Q	0	546.60
cgt	R	0	0.13
cgc	R	0	0.15
cga	R	3	0.29
cgg	R	0	0.25
aaa	K	1	0.58
aag	K	0	0.42
aga	R	0	1.37
agg	R	0	0.81
mPD		0.011	0.51
		nPD:	0.02
		N. weight:	0.71
		Sc. PD:	-0.02
		Sc. rank:	131.9

,

HA

Pos.	405	obs:	exp:
caa	Q	1119	570.50
cag	Q	1	549.50
mPD		0.0018	0.50
		nPD:	0.
		N. weight:	0.7
		Sc. PD:	-0.031
		Sc. rank:	-416.1

,

HA

Pos.	406	obs:	exp:
ttt	F	1120	442.30
ttc	F	0	677.70
mPD		0	0.48
		nPD:	0.
		N. weight:	0.97
		Sc. PD:	-0.046
		Sc. rank:	-892.6

,

HA

Pos.	407	obs:	exp:
aaa	K	0	26.23
aag	K	45	18.77
gaa	E	8	647.40
gag	E	1056	416.60
ggt	G	0	1.46
ggc	G	0	1.41
gga	G	0	5.07
ggg	G	11	3.06
mPD		0.11	0.58
		nPD:	0.19
		N. weight:	0.94
		Sc. PD:	0.13
		Sc. rank:	772.8

,

HA

Pos.	408	obs:	exp:
tta	L	2	99.39
ttg	L	1113	208.50
ctt	L	0	186.50
ctc	L	0	186.90
cta	L	0	188.80
ctg	L	5	249.90
mPD		0.012	1.1
		nPD:	0.01
		N. weight:	1.7
		Sc. PD:	-0.063
		Sc. rank:	-244.8

,

HA

Pos.	409	obs:	exp:
att	I	4	411.10
atc	I	4	268.20
ata	I	1111	439.70
gtt	V	0	0.21
gtc	V	0	0.20
gta	V	1	0.20
gtg	V	0	0.39
mPD		0.016	0.66
		nPD:	0.02
		N. weight:	0.93
		Sc. PD:	-0.023
		Sc. rank:	257.8

,

HA

Pos.	410	obs:	exp:
gat	D	1	605.20
gac	D	1119	514.80
mPD		0.0018	0.50
		nPD:	0.
		N. weight:	0.81
		Sc. PD:	-0.036
		Sc. rank:	-467.0

,

HA

Pos.	411	obs:	exp:
aat	N	1117	570.50
aac	N	3	549.50
mPD		0.0053	0.50
		nPD:	0.01
		N. weight:	0.69
		Sc. PD:	-0.026
		Sc. rank:	-137.3

HA

Pos.	412	obs:	exp:
gaa	E	1116	681.50
gag	E	4	438.50
mPD		0.0071	0.48
		nPD:	0.01
		N. weight:	0.5
		Sc. PD:	-0.017
		Sc. rank:	-2.4

HA

Pos.	413	obs:	exp:
ttt	F	0	442.30
ttc	F	1120	677.70
mPD		0	0.48
		nPD:	0.
		N. weight:	0.53
		Sc. PD:	-0.025
		Sc. rank:	-482.6

HA

Pos.	414	obs:	exp:
tct	S	0	1.11
tcc	S	0	0.91
tca	S	0	1.78
tcg	S	0	0.40
att	I	0	0.37
atc	I	1	0.24
ata	I	0	0.39
act	T	2	0.51
acc	T	0	0.41
aca	T	0	0.92
acg	T	0	0.16
aat	N	1031	564.90
aac	N	78	544.10
aaa	K	1	0.58
aag	K	0	0.42
agt	S	7	1.44
agc	S	0	1.36
mPD		0.15	0.53
		nPD:	0.28
		N. weight:	0.46
		Sc. PD:	0.1
		Sc. rank:	389.2

HA

Pos.	415	obs:	exp:
gaa	E	7	681.50
gag	E	1113	438.50
mPD		0.012	0.48
		nPD:	0.03
		N. weight:	0.94
		Sc. PD:	-0.021
		Sc. rank:	292.0

HA

Pos.	416	obs:	exp:
gtt	V	0	232.10
gtc	V	0	226.00
gta	V	1118	229.30
gtg	V	2	432.60
mPD		0.0036	0.73
		nPD:	0.
		N. weight:	1.6
		Sc. PD:	-0.071
		Sc. rank:	-869.2

HA

Pos.	417	obs:	exp:
aat	N	0	0.51
aac	N	1	0.49
gaa	E	6	680.90
gag	E	1113	438.10
mPD		0.014	0.48
		nPD:	0.03
		N. weight:	0.95
		Sc. PD:	-0.018
		Sc. rank:	353.7

HA

Pos.	418	obs:	exp:
cgt	R	0	0.13
cgc	R	0	0.15
cga	R	0	0.29
cgg	R	0	0.25
act	T	0	8.92
acc	T	0	7.18
aca	T	0	16.05
acg	T	35	2.84
aat	N	1	0.51
aac	N	0	0.49
aaa	K	62	630.00
aag	K	1019	451.00
aga	R	0	1.37
agg	R	3	0.81
mPD		0.17	0.57
		nPD:	0.3
		N. weight:	0.73
		Sc. PD:	0.18
		Sc. rank:	635.5

HA

Pos.	419	obs:	exp:
caa	Q	1120	570.50
cag	Q	0	549.50
mPD		0	0.50
		nPD:	0.
		N. weight:	0.71
		Sc. PD:	-0.034
		Sc. rank:	-648.0

HA

Pos.	420	obs:	exp:
att	I	31	411.50
atc	I	1089	268.40
ata	I	0	440.10
mPD		0.054	0.65
		nPD:	0.08
		N. weight:	1.4
		Sc. PD:	0.043
		Sc. rank:	909.3

HA

Pos.	421	obs:	exp:
ggt	G	1117	148.50
ggc	G	3	143.10
gga	G	0	516.70
ggg	G	0	311.80
mPD		0.0053	0.68
		nPD:	0.01
		N. weight:	2.1
		Sc. PD:	-0.084
		Sc. rank:	-763.8

HA

Pos.	422	obs:	exp:
tct	S	0	0.16
tcc	S	0	0.13
tca	S	0	0.25
tcg	S	0	0.06
aat	N	1111	570.00
aac	N	8	549.00
agt	S	1	0.21
agc	S	0	0.19
mPD		0.016	0.50
		nPD:	0.03
		N. weight:	0.67
		Sc. PD:	-0.011
		Sc. rank:	269.2

HA

Pos.	423	obs:	exp:
gtt	V	0	232.10
gtc	V	0	226.00
gta	V	3	229.30
gtg	V	1117	432.60
mPD		0.0053	0.73
		nPD:	0.01
		N. weight:	0.98
		Sc. PD:	-0.04
		Sc. rank:	-400.3

HA

Pos.	424	obs:	exp:
att	I	0	411.50
atc	I	0	268.40
ata	I	1120	440.10
mPD		0	0.65
		nPD:	0.
		N. weight:	0.98
		Sc. PD:	-0.047
		Sc. rank:	-897.3

HA

Pos.	425	obs:	exp:
aat	N	1070	570.50
aac	N	50	549.50
mPD		0.085	0.50
		nPD:	0.17
		N. weight:	0.52
		Sc. PD:	0.061
		Sc. rank:	420.3

HA

Pos.	426	obs:	exp:
tgg	W	1120	1120.00
mPD		0	0
		nPD:	1.
		N. weight:	0.
		Sc. PD:	0
		Sc. rank:	0

HA

Pos.	427	obs:	exp:
act	T	0	285.50
acc	T	1119	229.80
aca	T	1	513.70
acg	T	0	90.97
mPD		0.0018	0.68
		nPD:	0.
		N. weight:	1.7
		Sc. PD:	-0.075
		Sc. rank:	-1145.6

HA

Pos.	428	obs:	exp:
cgt	R	0	48.10
cgc	R	0	55.47
cga	R	0	109.60
cgg	R	0	93.45
aga	R	1114	512.10
agg	R	6	301.20
mPD		0.011	0.96
		nPD:	0.01
		N. weight:	0.79
		Sc. PD:	-0.029
		Sc. rank:	-132.9

HA

Pos.	429	obs:	exp:
cat	H	1	0.57
cac	H	0	0.43
gat	D	1118	604.70
gac	D	1	514.30
mPD		0.0036	0.50
		nPD:	0.01
		N. weight:	0.64
		Sc. PD:	-0.026
		Sc. rank:	-273.5

HA

Pos.	430	obs:	exp:
tct	S	1109	178.10
tcc	S	10	146.30
tca	S	1	284.30
tcg	S	0	63.55
agt	S	0	230.20
agc	S	0	217.50
mPD		0.019	1.7
		nPD:	0.01
		N. weight:	1.9
		Sc. PD:	-0.068
		Sc. rank:	-254.5

HA			
Pos.	431	obs:	exp:
tta	L	0	0.18
ttg	L	0	0.37
ctt	L	0	0.33
ctc	L	0	0.33
cta	L	2	0.34
ctg	L	0	0.45
att	I	1	409.30
atc	I	0	267.00
ata	I	1113	437.70
atg	M	4	4.00
---	--	------	------
mPD		0.012	0.66
		nPD:	0.02
		N. weight:	0.97
		Sc. PD:	-0.029
		Sc. rank:	138.4

HA			
Pos.	432	obs:	exp:
att	I	0	0.37
atc	I	0	0.24
ata	I	1	0.39
act	T	1	285.20
acc	T	0	229.60
aca	T	1116	513.30
acg	T	2	90.89
---	--	------	------
mPD		0.0071	0.68
		nPD:	0.01
		N. weight:	0.8
		Sc. PD:	-0.03
		Sc. rank:	-180.2

HA			
Pos.	433	obs:	exp:
caa	Q	1	0.51
cag	Q	0	0.49
gaa	E	1119	680.90
gag	E	0	438.10
---	--	------	------
mPD		0.0018	0.48
		nPD:	0.
		N. weight:	0.52
		Sc. PD:	-0.023
		Sc. rank:	-290.3

HA			
Pos.	434	obs:	exp:
tta	L	0	1.86
ttg	L	16	3.91
ctt	L	0	3.50
ctc	L	0	3.50
cta	L	1	3.54
ctg	L	4	4.69
atg	M	118	118.00
gtt	V	1	203.30
gtc	V	0	198.00
gta	V	55	200.90
gtg	V	925	378.90
---	--	------	------
mPD		0.32	0.92
		nPD:	0.35
		N. weight:	0.73
		Sc. PD:	0.21
		Sc. rank:	632.2

HA			
Pos.	435	obs:	exp:
tgg	W	1120	1120.00
---	--	------	------
mPD		0	0
		nPD:	1.
		N. weight:	0.
		Sc. PD:	0
		Sc. rank:	0

HA			
Pos.	436	obs:	exp:
tct	S	0	178.10
tcc	S	0	146.30
tca	S	1118	284.30
tcg	S	2	63.55
agt	S	0	230.20
agc	S	0	217.50
---	--	------	------
mPD		0.0036	1.7
		nPD:	0.
		N. weight:	1.4
		Sc. PD:	-0.065
		Sc. rank:	-1003.4

HA			
Pos.	437	obs:	exp:
tat	Y	1	604.90
tac	Y	1119	515.10
---	--	------	------
mPD		0.0018	0.50
		nPD:	0.
		N. weight:	0.81
		Sc. PD:	-0.036
		Sc. rank:	-472.1

HA			
Pos.	438	obs:	exp:
cct	P	1	0.25
ccc	P	0	0.18
cca	P	0	0.41
ccg	P	0	0.16
aat	N	1108	570.00
aac	N	11	549.00
---	--	------	------
mPD		0.023	0.50
		nPD:	0.05
		N. weight:	0.65
		Sc. PD:	-0.0024
		Sc. rank:	350.7

HA			
Pos.	439	obs:	exp:
gct	A	1074	289.20
gcc	A	1	208.60
gca	A	41	527.10
gcg	A	4	95.06
---	--	------	------
mPD		0.079	0.67
		nPD:	0.12
		N. weight:	1.2
		Sc. PD:	0.08
		Sc. rank:	900.4

HA			
Pos.	440	obs:	exp:
gaa	E	1119	681.50
gag	E	1	438.50
---	--	------	------
mPD		0.0018	0.48
		nPD:	0.
		N. weight:	0.51
		Sc. PD:	-0.023
		Sc. rank:	-284.7

HA			
Pos.	441	obs:	exp:
ttt	F	0	1.97
ttc	F	5	3.03
tta	L	0	98.95
ttg	L	0	207.60
ctt	L	5	185.70
ctc	L	1108	186.10
cta	L	2	187.90
ctg	L	0	248.80
---	--	------	------
mPD		0.021	1.1
		nPD:	0.02
		N. weight:	1.8
		Sc. PD:	-0.053
		Sc. rank:	281.3

HA			
Pos.	442	obs:	exp:
tta	L	16	99.39
ttg	L	1103	208.50
ctt	L	0	186.50
ctc	L	0	186.90
cta	L	0	188.80
ctg	L	1	249.90
---	--	------	------
mPD		0.030	1.1
		nPD:	0.03
		N. weight:	1.7
		Sc. PD:	-0.037
		Sc. rank:	557.5

HA			
Pos.	443	obs:	exp:
att	I	0	137.80
atc	I	0	89.86
ata	I	375	147.40
gtt	V	0	154.40
gtc	V	0	150.40
gta	V	693	152.50
gtg	V	52	287.70
---	--	------	------
mPD		0.53	1.2
		nPD:	0.45
		N. weight:	1.2
		Sc. PD:	0.47
		Sc. rank:	1098.9

HA			
Pos.	444	obs:	exp:
act	T	0	0.25
acc	T	0	0.21
aca	T	1	0.46
acg	T	0	0.08
gct	A	6	289.00
gcc	A	0	208.40
gca	A	1113	526.70
gcg	A	0	94.97
---	--	------	------
mPD		0.012	0.67
		nPD:	0.02
		N. weight:	0.76
		Sc. PD:	-0.023
		Sc. rank:	99.6

HA			
Pos.	445	obs:	exp:
atg	M	1120	1120.00
---	--	------	------
mPD		0	0
		nPD:	1.
		N. weight:	0.
		Sc. PD:	0
		Sc. rank:	0

HA			
Pos.	446	obs:	exp:
gaa	E	3	681.50
gag	E	1117	438.50
---	--	------	------
mPD		0.0053	0.48
		nPD:	0.01
		N. weight:	0.96
		Sc. PD:	-0.036
		Sc. rank:	-151.1

HA			
Pos.	447	obs:	exp:
aat	N	1	570.50
aac	N	1119	549.50
---	--	------	------
mPD		0.0018	0.50
		nPD:	0.
		N. weight:	0.74
		Sc. PD:	-0.033
		Sc. rank:	-442.8

HA			
Pos.	448	obs:	exp:
cat	H	0	0.57
cac	H	1	0.43
caa	Q	3	570.00
cag	Q	1116	549.00
---	--	------	------
mPD		0.0071	0.50
		nPD:	0.01
		N. weight:	0.73
		Sc. PD:	-0.025
		Sc. rank:	-28.3

HA			
Pos.	449	obs:	exp:
cct	P	1	0.25
ccc	P	0	0.18
cca	P	0	0.41
ccg	P	0	0.16
cat	H	1118	642.50
cac	H	1	476.50
---	--	------	------
mPD		0.0036	0.49
		nPD:	0.01
		N. weight:	0.58
		Sc. PD:	-0.023
		Sc. rank:	-239.6

HA			
Pos.	450	obs:	exp:
cct	P	0	0.25
ccc	P	1	0.18
cca	P	0	0.41
ccg	P	0	0.16
act	T	0	285.20
acc	T	0	229.60
aca	T	1099	513.30
acg	T	20	90.89
---	--	------	------
mPD		0.039	0.68
		nPD:	0.06
		N. weight:	0.76
		Sc. PD:	0.0055
		Sc. rank:	466.0

HA			
Pos.	451	obs:	exp:
att	I	1120	411.50
atc	I	0	268.40
ata	I	0	440.10
---	--	------	------
mPD		0	0.65
		nPD:	0.
		N. weight:	1.
		Sc. PD:	-0.05
		Sc. rank:	-961.8

HA			
Pos.	452	obs:	exp:
gat	D	1118	605.20
gac	D	2	514.80
---	--	------	------
mPD		0.0036	0.50
		nPD:	0.01
		N. weight:	0.63
		Sc. PD:	-0.026
		Sc. rank:	-267.9

HA			
Pos.	453	obs:	exp:
tta	L	0	99.39
ttg	L	1	208.50
ctt	L	1	186.50
ctc	L	0	186.90
cta	L	11	188.80
ctg	L	1107	249.90
---	--	------	------
mPD		0.023	1.1
		nPD:	0.02
		N. weight:	1.5
		Sc. PD:	-0.042
		Sc. rank:	268.4

HA			
Pos.	454	obs:	exp:
act	T	1	0.25
acc	T	0	0.21
aca	T	0	0.46
acg	T	0	0.08
gct	A	1113	289.00
gcc	A	6	208.40
gca	A	0	526.70
gcg	A	0	94.97
---	--	------	------
mPD		0.012	0.67
		nPD:	0.02
		N. weight:	1.4
		Sc. PD:	-0.041
		Sc. rank:	181.9

HA			
Pos.	455	obs:	exp:
gat	D	1119	605.20
gac	D	1	514.80
---	--	------	------
mPD		0.0018	0.50
		nPD:	0.
		N. weight:	0.64
		Sc. PD:	-0.028
		Sc. rank:	-369.1

HA			
Pos.	456	obs :	exp :
tct	S	1	178.10
tcc	S	0	146.30
tca	S	1117	284.30
tcg	S	2	63.55
agt	S	0	230.20
agc	S	0	217.50
---	--	------	------
mPD		0.0054	1.7
		nPD :	0.
		N. weight :	1.4
		Sc. PD :	-0.063
		Sc. rank :	-864.5

,

HA			
Pos.	457	obs :	exp :
caa	Q	1	0.51
cag	Q	0	0.49
gaa	E	1118	680.90
gag	E	1	438.10
---	--	------	------
mPD		0.0036	0.48
		nPD :	0.01
		N. weight :	0.51
		Sc. PD :	-0.021
		Sc. rank :	-201.4

,

HA			
Pos.	458	obs :	exp :
atg	M	1120	1120.00
---	--	------	------
mPD		0	0
		nPD :	1.
		N. weight :	0.
		Sc. PD :	0
		Sc. rank :	0

,

HA			
Pos.	459	obs :	exp :
aat	N	0	3.06
aac	N	6	2.94
gat	D	0	600.40
gac	D	1111	510.60
ggt	G	0	0.40
ggc	G	3	0.38
gga	G	0	1.38
ggg	G	0	0.84
---	--	------	------
mPD		0.016	0.51
		nPD :	0.03
		N. weight :	0.82
		Sc. PD :	-0.015
		Sc. rank :	325.6

,

HA			
Pos.	460	obs :	exp :
aaa	K	1108	652.80
aag	K	12	467.20
---	--	------	------
mPD		0.021	0.49
		nPD :	0.04
		N. weight :	0.51
		Sc. PD :	-0.0029
		Sc. rank :	262.2

,

HA			
Pos.	461	obs :	exp :
tta	L	0	99.39
ttg	L	2	208.50
ctt	L	0	186.50
ctc	L	0	186.90
cta	L	0	188.80
ctg	L	1118	249.90
---	--	------	------
mPD		0.0036	1.1
		nPD :	0.
		N. weight :	1.6
		Sc. PD :	-0.07
		Sc. rank :	-1003.3

,

HA			
Pos.	462	obs :	exp :
tat	Y	390	604.90
tac	Y	730	515.10
---	--	------	------
mPD		0.45	0.50
		nPD :	0.91
		N. weight :	0.081
		Sc. PD :	0.068
		Sc. rank :	75.9

,

HA			
Pos.	463	obs :	exp :
aaa	K	5	2.91
aag	K	0	2.09
gaa	E	1089	678.50
gag	E	26	436.50
---	--	------	------
mPD		0.054	0.49
		nPD :	0.11
		N. weight :	0.42
		Sc. PD :	0.025
		Sc. rank :	302.6

,

HA			
Pos.	464	obs :	exp :
cct	P	0	0.25
ccc	P	0	0.18
cca	P	1	0.41
ccg	P	0	0.16
cgt	R	0	48.06
cgc	R	0	55.42
cga	R	850	109.50
cgg	R	269	93.37
aga	R	0	511.70
agg	R	0	301.00
---	--	------	------
mPD		0.37	0.96
		nPD :	0.38
		N. weight :	1.9
		Sc. PD :	0.61
		Sc. rank :	1689.4

,

HA			
Pos.	465	obs :	exp :
tta	L	0	0.09
ttg	L	0	0.19
ctt	L	0	0.17
ctc	L	0	0.17
cta	L	0	0.17
ctg	L	1	0.22
gtt	V	2	231.90
gtc	V	0	225.80
gta	V	5	229.10
gtg	V	1112	432.20
---	--	------	------
mPD		0.014	0.73
		nPD :	0.02
		N. weight :	0.96
		Sc. PD :	-0.028
		Sc. rank :	159.0

,

HA			
Pos.	466	obs :	exp :
caa	Q	1	0.51
cag	Q	0	0.49
cgt	R	0	0.04
cgc	R	0	0.05
cga	R	0	0.10
cgg	R	0	0.08
aaa	K	1115	651.60
aag	K	3	466.40
aga	R	1	0.46
agg	R	0	0.27
---	--	------	------
mPD		0.0089	0.49
		nPD :	0.02
		N. weight :	0.55
		Sc. PD :	-0.017
		Sc. rank :	49.4

,

HA			
Pos.	467	obs :	exp :
tta	L	0	0.09
ttg	L	0	0.19
ctt	L	0	0.17
ctc	L	0	0.17
cta	L	1	0.17
ctg	L	0	0.22
cgt	R	0	47.89
cgc	R	0	55.23
cga	R	0	109.10
cgg	R	0	93.04
aaa	K	3	1.75
aag	K	0	1.25
aga	R	1108	509.80
agg	R	7	299.90
ggt	G	0	0.13
ggc	G	0	0.13
gga	G	1	0.46
ggg	G	0	0.28
---	--	------	------
mPD		0.023	0.97
		nPD :	0.02
		N. weight :	0.79
		Sc. PD :	-0.019
		Sc. rank :	207.5

,

HA			
Pos.	468	obs :	exp :
tta	L	0	0.09
ttg	L	0	0.19
ctt	L	0	0.17
ctc	L	1	0.17
cta	L	0	0.17
ctg	L	0	0.22
caa	Q	29	570.00
cag	Q	1090	549.00
---	--	------	------
mPD		0.054	0.50
		nPD :	0.11
		N. weight :	0.62
		Sc. PD :	0.035
		Sc. rank :	446.0

,

HA			
Pos.	469	obs :	exp :
tta	L	0	99.39
ttg	L	29	208.50
ctt	L	0	186.50
ctc	L	0	186.90
cta	L	26	188.80
ctg	L	1065	249.90
---	--	------	------
mPD		0.096	1.1
		nPD :	0.09
		N. weight :	1.3
		Sc. PD :	0.048
		Sc. rank :	910.6

,

HA			
Pos.	470	obs :	exp :
cgt	R	0	48.06
cgc	R	0	55.42
cga	R	0	109.50
cgg	R	0	93.37
act	T	0	0.25
acc	T	0	0.21
aca	T	1	0.46
acg	T	0	0.08
aga	R	1118	511.70
agg	R	1	301.00
---	--	------	------
mPD		0.0036	0.96
		nPD :	0.
		N. weight :	0.81
		Sc. PD :	-0.036
		Sc. rank :	-457.7

,

HA			
Pos.	471	obs :	exp :
cat	H	0	0.57
cac	H	1	0.43
gaa	E	1	679.10
gag	E	1115	436.90
ggt	G	0	0.40
ggc	G	0	0.38
gga	G	0	1.38
ggg	G	3	0.84
---	--	------	------
mPD		0.011	0.49
		nPD :	0.02
		N. weight :	0.98
		Sc. PD :	-0.026
		Sc. rank :	228.2

,

HA			
Pos.	472	obs :	exp :
aat	N	1117	570.50
aac	N	3	549.50
---	--	------	------
mPD		0.0053	0.50
		nPD :	0.01
		N. weight :	0.69
		Sc. PD :	-0.026
		Sc. rank :	-137.3

,

HA			
Pos.	473	obs :	exp :
gct	A	1114	289.20
gcc	A	3	208.60
gca	A	2	527.10
gcg	A	1	95.06
---	--	------	------
mPD		0.011	0.67
		nPD :	0.02
		N. weight :	1.4
		Sc. PD :	-0.045
		Sc. rank :	24.8

,

HA			
Pos.	474	obs :	exp :
gaa	E	1113	681.50
gag	E	7	438.50
---	--	------	------
mPD		0.012	0.48
		nPD :	0.03
		N. weight :	0.49
		Sc. PD :	-0.011
		Sc. rank :	150.5

,

HA			
Pos.	475	obs :	exp :
aaa	K	1	0.58
aag	K	0	0.42
gaa	E	717	680.90
gag	E	402	438.10
---	--	------	------
mPD		0.46	0.48
		nPD :	0.97
		N. weight :	0.0063
		Sc. PD :	0.0055
		Sc. rank :	5.8

,

HA			
Pos.	476	obs :	exp :
cat	H	1	0.57
cac	H	0	0.43
gat	D	1078	604.70
gac	D	41	514.30
---	--	------	------
mPD		0.072	0.50
		nPD :	0.15
		N. weight :	0.49
		Sc. PD :	0.045
		Sc. rank :	382.2

,

HA			
Pos.	477	obs :	exp :
ggt	G	0	148.50
ggc	G	1111	143.10
gga	G	5	516.70
ggg	G	4	311.80
---	--	------	------
mPD		0.016	0.68
		nPD :	0.02
		N. weight :	2.1
		Sc. PD :	-0.052
		Sc. rank :	527.6

,

HA			
Pos.	478	obs :	exp :
act	T	1068	285.50
acc	T	0	229.80
aca	T	0	513.70
acg	T	52	90.97
---	--	------	------
mPD		0.089	0.68
		nPD :	0.13
		N. weight :	1.3
		Sc. PD :	0.1
		Sc. rank :	988.6

,

HA			
Pos.	479	obs :	exp :
ggt	G	1095	148.50
ggc	G	25	143.10
gga	G	0	516.70
ggg	G	0	311.80
---	--	------	------
mPD		0.044	0.68
		nPD :	0.06
		N. weight :	2.
		Sc. PD :	0.029
		Sc. rank :	1281.4

,

HA			
Pos.	480	obs :	exp :
tgt	C	2	473.70
tgc	C	1118	646.30
---	--	------	------
mPD		0.0036	0.49
		nPD :	0.01
		N. weight :	0.56
		Sc. PD :	-0.023
		Sc. rank :	-233.5

,

HA			
Pos.	481	obs:	exp:
ttt	F	1120	442.30
ttc	F	0	677.70
---	--	------	------
mPD		0	0.48
		nPD:	0.
		N. weight:	0.97
		Sc. PD:	-0.046
		Sc. rank:	-892.6

HA			
Pos.	482	obs:	exp:
gaa	E	1117	681.50
gag	E	3	438.50
---	--	------	------
mPD		0.0053	0.48
		nPD:	0.01
		N. weight:	0.5
		Sc. PD:	-0.019
		Sc. rank:	-79.0

HA			
Pos.	483	obs:	exp:
tta	L	0	0.18
ttg	L	0	0.37
ctt	L	0	0.33
ctc	L	0	0.33
cta	L	2	0.34
ctg	L	0	0.45
att	I	0	410.80
atc	I	0	267.90
ata	I	1118	439.30
---	--	------	------
mPD		0.0036	0.66
		nPD:	0.01
		N. weight:	0.98
		Sc. PD:	-0.042
		Sc. rank:	-452.1

HA			
Pos.	484	obs:	exp:
ttt	F	1071	441.90
ttc	F	48	677.10
tta	L	0	0.09
ttg	L	0	0.19
ctt	L	1	0.17
ctc	L	0	0.17
cta	L	0	0.17
ctg	L	0	0.22
---	--	------	------
mPD		0.084	0.48
		nPD:	0.17
		N. weight:	0.77
		Sc. PD:	0.093
		Sc. rank:	626.1

HA			
Pos.	485	obs:	exp:
cat	H	5	642.50
cac	H	1114	476.50
caa	Q	1	0.51
cag	Q	0	0.49
---	--	------	------
mPD		0.011	0.49
		nPD:	0.02
		N. weight:	0.86
		Sc. PD:	-0.023
		Sc. rank:	196.9

HA			
Pos.	486	obs:	exp:
aat	N	0	0.51
aac	N	1	0.49
aaa	K	9	652.20
aag	K	1110	466.80
---	--	------	------
mPD		0.018	0.49
		nPD:	0.04
		N. weight:	0.87
		Sc. PD:	-0.011
		Sc. rank:	397.8

HA			
Pos.	487	obs:	exp:
tgt	C	1120	473.70
tgc	C	0	646.30
---	--	------	------
mPD		0	0.49
		nPD:	0.
		N. weight:	0.9
		Sc. PD:	-0.043
		Sc. rank:	-826.6

HA			
Pos.	488	obs:	exp:
aat	N	3	1.53
aac	N	0	1.47
gct	A	1	0.26
gcc	A	0	0.19
gca	A	0	0.47
gcg	A	0	0.08
gat	D	1113	603.10
gac	D	3	512.90
---	--	------	------
mPD		0.012	0.50
		nPD:	0.02
		N. weight:	0.63
		Sc. PD:	-0.015
		Sc. rank:	175.9

HA			
Pos.	489	obs:	exp:
aat	N	5	2.55
aac	N	0	2.45
gat	D	1114	602.50
gac	D	1	512.50
---	--	------	------
mPD		0.011	0.51
		nPD:	0.02
		N. weight:	0.64
		Sc. PD:	-0.017
		Sc. rank:	133.2

HA			
Pos.	490	obs:	exp:
aat	N	0	23.94
aac	N	47	23.06
gat	D	0	578.20
gac	D	1070	491.80
ggt	G	0	0.40
ggc	G	3	0.38
gga	G	0	1.38
ggg	G	0	0.84
---	--	------	------
mPD		0.086	0.58
		nPD:	0.15
		N. weight:	0.81
		Sc. PD:	0.076
		Sc. rank:	639.1

HA			
Pos.	491	obs:	exp:
tgt	C	1120	473.70
tgc	C	0	646.30
---	--	------	------
mPD		0	0.49
		nPD:	0.
		N. weight:	0.9
		Sc. PD:	-0.043
		Sc. rank:	-826.6

HA			
Pos.	492	obs:	exp:
atg	M	1120	1120.00
---	--	------	------
mPD		0	0
		nPD:	1.
		N. weight:	0.
		Sc. PD:	0
		Sc. rank:	0

HA			
Pos.	493	obs:	exp:
cct	P	0	0.25
ccc	P	1	0.18
cca	P	0	0.41
ccg	P	0	0.16
gct	A	5	289.00
gcc	A	1114	208.40
gca	A	0	526.70
gcg	A	0	94.97
---	--	------	------
mPD		0.011	0.67
		nPD:	0.02
		N. weight:	1.7
		Sc. PD:	-0.056
		Sc. rank:	18.9

HA			
Pos.	494	obs:	exp:
tct	S	0	177.80
tcc	S	0	146.00
tca	S	0	283.80
tcg	S	0	63.44
cgt	R	0	0.04
cgc	R	0	0.05
cga	R	0	0.10
cgg	R	1	0.08
aat	N	0	0.51
aac	N	1	0.49
agt	S	1026	229.80
agc	S	92	217.10
aga	R	0	0.46
agg	R	0	0.27
---	--	------	------
mPD		0.16	1.7
		nPD:	0.1
		N. weight:	1.4
		Sc. PD:	0.06
		Sc. rank:	946.6

HA			
Pos.	495	obs:	exp:
att	I	1075	411.50
atc	I	45	268.40
ata	I	0	440.10
---	--	------	------
mPD		0.077	0.65
		nPD:	0.12
		N. weight:	0.89
		Sc. PD:	0.059
		Sc. rank:	668.1

HA			
Pos.	496	obs:	exp:
cgt	R	0	48.10
cgc	R	0	55.47
cga	R	0	109.60
cgg	R	0	93.45
aga	R	1065	512.10
agg	R	55	301.20
---	--	------	------
mPD		0.093	0.96
		nPD:	0.1
		N. weight:	0.64
		Sc. PD:	0.03
		Sc. rank:	450.2

HA			
Pos.	497	obs:	exp:
aat	N	784	570.50
aac	N	336	549.50
---	--	------	------
mPD		0.42	0.50
		nPD:	0.84
		N. weight:	0.082
		Sc. PD:	0.062
		Sc. rank:	76.4

HA			
Pos.	498	obs:	exp:
aat	N	7	570.50
aac	N	1113	549.50
---	--	------	------
mPD		0.012	0.50
		nPD:	0.02
		N. weight:	0.71
		Sc. PD:	-0.017
		Sc. rank:	199.9

HA			
Pos.	499	obs:	exp:
act	T	6	285.50
acc	T	1113	229.80
aca	T	1	513.70
acg	T	0	90.97
---	--	------	------
mPD		0.012	0.68
		nPD:	0.02
		N. weight:	1.6
		Sc. PD:	-0.048
		Sc. rank:	188.7

HA			
Pos.	500	obs:	exp:
tat	Y	1112	604.90
tac	Y	8	515.10
---	--	------	------
mPD		0.014	0.50
		nPD:	0.03
		N. weight:	0.6
		Sc. PD:	-0.012
		Sc. rank:	214.7

HA			
Pos.	501	obs:	exp:
aat	N	2	1.02
aac	N	0	0.98
gat	D	1117	604.10
gac	D	1	513.90
---	--	------	------
mPD		0.0054	0.50
		nPD:	0.01
		N. weight:	0.64
		Sc. PD:	-0.024
		Sc. rank:	-121.6

HA			
Pos.	502	obs:	exp:
cat	H	0	643.10
cac	H	1120	476.90
---	--	------	------
mPD		0	0.49
		nPD:	0.
		N. weight:	0.89
		Sc. PD:	-0.043
		Sc. rank:	-820.2

HA			
Pos.	503	obs:	exp:
tct	S	0	93.84
tcc	S	0	77.05
tca	S	0	149.70
tcg	S	0	33.48
cgt	R	0	22.55
cgc	R	0	26.00
cga	R	0	51.38
cgg	R	0	43.81
aat	N	0	2.04
aac	N	4	1.96
aaa	K	1	0.58
aag	K	0	0.42
agt	S	0	121.30
agc	S	590	114.60
aga	R	523	240.10
agg	R	2	141.20
---	--	------	------
mPD		0.51	1.7
		nPD:	0.29
		N. weight:	1.3
		Sc. PD:	0.3
		Sc. rank:	1098.9

HA			
Pos.	504	obs:	exp:
tta	L	0	0.09
ttg	L	0	0.19
ctt	L	0	0.17
ctc	L	0	0.17
cta	L	1	0.17
ctg	L	0	0.22
caa	Q	1	0.51
cag	Q	0	0.49
aat	N	1	1.02
aac	N	1	0.98
aaa	K	1052	649.90
aag	K	63	465.10
gaa	E	1	0.61
gag	E	0	0.39
---	--	------	------
mPD		0.12	0.50
		nPD:	0.24
		N. weight:	0.36
		Sc. PD:	0.065
		Sc. rank:	300.7

HA			
Pos.	505	obs:	exp:
tct	S	0	0.16
tcc	S	0	0.13
tca	S	0	0.25
tcg	S	0	0.06
tat	Y	6	603.80
tac	Y	1112	514.20
cat	H	0	0.57
cac	H	1	0.43
agt	S	0	0.21
agc	S	1	0.19
---	--	------	------
mPD		0.016	0.50
		nPD:	0.03
		N. weight:	0.78
		Sc. PD:	-0.013
		Sc. rank:	324.9

HA			
Pos.	506	obs:	exp:
cgt	R	0	48.10
cgc	R	0	55.47
cga	R	0	109.60
cgg	R	0	93.45
aga	R	53	512.10
agg	R	1067	301.20
---	--	------	------
mPD		0.090	0.96
		nPD:	0.09
		N. weight:	1.2
		Sc. PD:	0.049
		Sc. rank:	792.4

HA			
Pos.	507	obs:	exp:
gaa	E	1116	681.50
gag	E	4	438.50
---	--	------	------
mPD		0.0071	0.48
		nPD:	0.01
		N. weight:	0.5
		Sc. PD:	-0.017
		Sc. rank:	-2.4

HA			
Pos.	508	obs:	exp:
gaa	E	9	681.50
gag	E	1111	438.50
---	--	------	------
mPD		0.016	0.48
		nPD:	0.03
		N. weight:	0.93
		Sc. PD:	-0.014
		Sc. rank:	403.4

HA			
Pos.	509	obs:	exp:
gct	A	0	289.20
gcc	A	0	208.60
gca	A	1120	527.10
gcg	A	0	95.06
---	--	------	------
mPD		0	0.67
		nPD:	0.
		N. weight:	0.79
		Sc. PD:	-0.038
		Sc. rank:	-723.9

HA			
Pos.	510	obs:	exp:
tta	L	0	0.09
ttg	L	0	0.19
ctt	L	0	0.17
ctc	L	0	0.17
cta	L	0	0.17
ctg	L	1	0.22
att	I	0	2.57
atc	I	0	1.68
ata	I	7	2.75
atg	M	1112	1112.00
---	--	------	------
mPD		0.014	0.015
		nPD:	0.93
		N. weight:	0.0075
		Sc. PD:	0.0064
		Sc. rank:	7.0

HA			
Pos.	511	obs:	exp:
caa	Q	1111	570.50
cag	Q	9	549.50
---	--	------	------
mPD		0.016	0.50
		nPD:	0.03
		N. weight:	0.66
		Sc. PD:	-0.011
		Sc. rank:	273.1

HA			
Pos.	512	obs:	exp:
tct	S	0	0.95
tcc	S	0	0.78
tca	S	0	1.52
tcg	S	0	0.34
aat	N	1111	567.40
aac	N	3	546.60
agt	S	0	1.23
agc	S	6	1.17
---	--	------	------
mPD		0.027	0.52
		nPD:	0.05
		N. weight:	0.69
		Sc. PD:	0.0012
		Sc. rank:	408.3

HA			
Pos.	513	obs:	exp:
cgt	R	0	48.10
cgc	R	0	55.47
cga	R	2	109.60
cgg	R	0	93.45
aga	R	1117	512.10
agg	R	1	301.20
---	--	------	------
mPD		0.0054	0.96
		nPD:	0.01
		N. weight:	0.8
		Sc. PD:	-0.034
		Sc. rank:	-362.0

HA			
Pos.	514	obs:	exp:
att	I	0	411.50
atc	I	0	268.40
ata	I	1120	440.10
---	--	------	------
mPD		0	0.65
		nPD:	0.
		N. weight:	0.98
		Sc. PD:	-0.047
		Sc. rank:	-897.3

HA			
Pos.	515	obs:	exp:
caa	Q	3	567.90
cag	Q	1112	547.10
cgt	R	0	0.04
cgc	R	0	0.05
cga	R	0	0.10
cgg	R	1	0.08
aaa	K	0	2.33
aag	K	4	1.67
aga	R	0	0.46
agg	R	0	0.27
---	--	------	------
mPD		0.014	0.51
		nPD:	0.03
		N. weight:	0.73
		Sc. PD:	-0.015
		Sc. rank:	248.8

HA			
Pos.	516	obs:	exp:
att	I	1119	411.50
atc	I	1	268.40
ata	I	0	440.10
---	--	------	------
mPD		0.0018	0.65
		nPD:	0.
		N. weight:	1.
		Sc. PD:	-0.047
		Sc. rank:	-691.3

HA			
Pos.	517	obs:	exp:
aat	N	0	1.02
aac	N	2	0.98
gat	D	39	604.10
gac	D	1079	513.90
---	--	------	------
mPD		0.071	0.50
		nPD:	0.14
		N. weight:	0.65
		Sc. PD:	0.058
		Sc. rank:	505.8

HA			
Pos.	518	obs:	exp:
tct	S	0	0.16
tcc	S	0	0.13
tca	S	1	0.25
tcg	S	0	0.06
cct	P	0	279.20
ccc	P	0	201.00
cca	P	1119	460.60
ccg	P	0	178.10
agt	S	0	0.21
agc	S	0	0.19
---	--	------	------
mPD		0.0018	0.71
		nPD:	0.
		N. weight:	0.93
		Sc. PD:	-0.042
		Sc. rank:	-651.5

HA			
Pos.	519	obs:	exp:
gtt	V	0	232.10
gtc	V	1120	226.00
gta	V	0	229.30
gtg	V	0	432.60
---	--	------	------
mPD		0	0.73
		nPD:	0.
		N. weight:	1.7
		Sc. PD:	-0.08
		Sc. rank:	-1537.4

HA			
Pos.	520	obs:	exp:
aaa	K	742	652.80
aag	K	378	467.20
---	--	------	------
mPD		0.45	0.49
		nPD:	0.92
		N. weight:	0.017
		Sc. PD:	0.015
		Sc. rank:	16.3

HA			
Pos.	521	obs:	exp:
tta	L	4	99.30
ttg	L	1	208.30
ctt	L	0	186.30
ctc	L	0	186.70
cta	L	1113	188.60
ctg	L	1	249.70
att	I	0	0.37
atc	I	0	0.24
ata	I	1	0.39
---	--	------	------
mPD		0.014	1.1
		nPD:	0.01
		N. weight:	1.8
		Sc. PD:	-0.065
		Sc. rank:	-163.5

HA			
Pos.	522	obs:	exp:
tct	S	0	177.30
tcc	S	0	145.60
tca	S	0	283.00
tcg	S	0	63.27
cgt	R	0	0.09
cgc	R	0	0.10
cga	R	0	0.20
cgg	R	0	0.17
aat	N	0	1.02
aac	N	2	0.98
agt	S	10	229.20
agc	S	1105	216.60
aga	R	2	0.91
agg	R	0	0.54
ggt	G	0	0.13
ggc	G	1	0.13
gga	G	0	0.46
ggg	G	0	0.28
---	--	------	------
mPD		0.027	1.7
		nPD:	0.02
		N. weight:	1.7
		Sc. PD:	-0.054
		Sc. rank:	55.5

HA			
Pos.	523	obs:	exp:
tct	S	0	176.40
tcc	S	0	144.80
tca	S	0	281.50
tcg	S	0	62.93
cgt	R	0	0.43
cgc	R	0	0.50
cga	R	0	0.98
cgg	R	0	0.83
aat	N	0	0.51
aac	N	1	0.49
agt	S	54	228.00
agc	S	1055	215.40
aga	R	10	4.57
agg	R	0	2.69
---	--	------	------
mPD		0.11	1.7
		nPD:	0.07
		N. weight:	1.5
		Sc. PD:	0.025
		Sc. rank:	971.5

HA			
Pos.	524	obs:	exp:
ggt	G	12	148.50
ggc	G	1102	143.10
gga	G	6	516.70
ggg	G	0	311.80
---	--	------	------
mPD		0.032	0.68
		nPD:	0.05
		N. weight:	2.1
		Sc. PD:	-0.005
		Sc. rank:	1139.4

HA			
Pos.	525	obs:	exp:
tat	Y	0	604.90
tac	Y	1120	515.10
---	--	------	------
mPD		0	0.50
		nPD:	0.
		N. weight:	0.81
		Sc. PD:	-0.039
		Sc. rank:	-746.2

HA

Pos.	526	obs:	exp:
aaa	K	1120	652.80
aag	K	0	467.20
---	--	------	------
mPD		0	0.49
		nPD:	0.
		N. weight:	0.57
		Sc. PD:	-0.027
		Sc. rank:	-518.6

HA

Pos.	527	obs:	exp:
gat	D	1117	603.60
gac	D	0	513.40
gaa	E	1	1.83
gag	E	2	1.17
---	--	------	------
mPD		0.0054	0.50
		nPD:	0.01
		N. weight:	0.64
		Sc. PD:	-0.024
		Sc. rank:	-121.4

HA

Pos.	528	obs:	exp:
gtt	V	0	232.10
gtc	V	0	226.00
gta	V	7	229.30
gtg	V	1113	432.60
---	--	------	------
mPD		0.012	0.73
		nPD:	0.02
		N. weight:	0.96
		Sc. PD:	-0.03
		Sc. rank:	64.2

HA

Pos.	529	obs:	exp:
att	I	0	411.50
atc	I	0	268.40
ata	I	1120	440.10
---	--	------	------
mPD		0	0.65
		nPD:	0.
		N. weight:	0.98
		Sc. PD:	-0.047
		Sc. rank:	-897.3

HA

Pos.	530	obs:	exp:
ttt	F	1	0.39
ttc	F	0	0.61
tta	L	0	99.21
ttg	L	0	208.10
ctt	L	1115	186.20
ctc	L	3	186.60
cta	L	0	188.40
ctg	L	0	249.50
att	I	1	0.37
atc	I	0	0.24
ata	I	0	0.39
---	--	------	------
mPD		0.0089	1.1
		nPD:	0.01
		N. weight:	1.9
		Sc. PD:	-0.074
		Sc. rank:	-584.8

HA

Pos.	531	obs:	exp:
tgg	W	1120	1120.00
---	--	------	------
mPD		0	0
		nPD:	1.
		N. weight:	0.
		Sc. PD:	0
		Sc. rank:	0

HA

Pos.	532	obs:	exp:
ttt	F	1119	441.90
ttc	F	0	677.10
tat	Y	1	0.54
tac	Y	0	0.46
---	--	------	------
mPD		0.0018	0.48
		nPD:	0.
		N. weight:	0.97
		Sc. PD:	-0.043
		Sc. rank:	-544.2

HA

Pos.	533	obs:	exp:
tct	S	0	178.10
tcc	S	0	146.30
tca	S	0	284.30
tcg	S	0	63.55
agt	S	0	230.20
agc	S	1120	217.50
---	--	------	------
mPD		0	1.7
		nPD:	0.
		N. weight:	1.7
		Sc. PD:	-0.082
		Sc. rank:	-1574.2

HA

Pos.	534	obs:	exp:
ttt	F	0	442.30
ttc	F	1120	677.70
---	--	------	------
mPD		0	0.48
		nPD:	0.
		N. weight:	0.53
		Sc. PD:	-0.025
		Sc. rank:	-482.6

HA

Pos.	535	obs:	exp:
ggt	G	0	148.50
ggc	G	0	143.10
gga	G	1	516.70
ggg	G	1119	311.80
---	--	------	------
mPD		0.0018	0.68
		nPD:	0.
		N. weight:	1.3
		Sc. PD:	-0.06
		Sc. rank:	-913.7

HA

Pos.	536	obs:	exp:
gct	A	0	289.20
gcc	A	0	208.60
gca	A	1119	527.10
gcg	A	1	95.06
---	--	------	------
mPD		0.0018	0.67
		nPD:	0.
		N. weight:	0.78
		Sc. PD:	-0.035
		Sc. rank:	-525.4

HA

Pos.	537	obs:	exp:
tct	S	0	178.10
tcc	S	0	146.30
tca	S	1120	284.30
tcg	S	0	63.55
agt	S	0	230.20
agc	S	0	217.50
---	--	------	------
mPD		0	1.7
		nPD:	0.
		N. weight:	1.4
		Sc. PD:	-0.069
		Sc. rank:	-1317.2

HA

Pos.	538	obs:	exp:
tgt	C	1117	473.70
tgc	C	3	646.30
---	--	------	------
mPD		0.0053	0.49
		nPD:	0.01
		N. weight:	0.88
		Sc. PD:	-0.033
		Sc. rank:	-156.3

HA

Pos.	539	obs:	exp:
ttt	F	1	441.10
ttc	F	1116	675.90
tta	L	0	0.27
ttg	L	0	0.56
ctt	L	0	0.50
ctc	L	3	0.50
cta	L	0	0.51
ctg	L	0	0.67
---	--	------	------
mPD		0.0071	0.48
		nPD:	0.01
		N. weight:	0.52
		Sc. PD:	-0.018
		Sc. rank:	-10.5

HA

Pos.	540	obs:	exp:
att	I	0	408.90
atc	I	0	266.70
ata	I	1113	437.30
atg	M	3	3.00
act	T	0	1.02
acc	T	0	0.82
aca	T	4	1.83
acg	T	0	0.32
---	--	------	------
mPD		0.012	0.66
		nPD:	0.02
		N. weight:	0.97
		Sc. PD:	-0.029
		Sc. rank:	136.0

HA

Pos.	541	obs:	exp:
tta	L	0	99.30
ttg	L	0	208.30
ctt	L	1119	186.30
ctc	L	0	186.70
cta	L	0	188.60
ctg	L	0	249.70
att	I	1	0.37
atc	I	0	0.24
ata	I	0	0.39
---	--	------	------
mPD		0.0018	1.1
		nPD:	0.
		N. weight:	1.9
		Sc. PD:	-0.087
		Sc. rank:	-1377.1

HA

Pos.	542	obs:	exp:
tta	L	0	98.86
ttg	L	0	207.40
ctt	L	1	185.50
ctc	L	0	185.90
cta	L	1113	187.70
ctg	L	0	248.60
cct	P	0	0.25
ccc	P	0	0.18
cca	P	1	0.41
ccg	P	0	0.16
att	I	0	1.84
atc	I	0	1.20
ata	I	5	1.96
---	--	------	------
mPD		0.012	1.1
		nPD:	0.01
		N. weight:	1.9
		Sc. PD:	-0.069
		Sc. rank:	-300.9

HA

Pos.	543	obs:	exp:
gtt	V	0	0.21
gtc	V	1	0.20
gta	V	0	0.20
gtg	V	0	0.39
gct	A	0	289.00
gcc	A	1119	208.40
gca	A	0	526.70
gcg	A	0	94.97
---	--	------	------
mPD		0.0018	0.67
		nPD:	0.
		N. weight:	1.8
		Sc. PD:	-0.079
		Sc. rank:	-1183.8

HA

Pos.	544	obs:	exp:
att	I	1117	410.40
atc	I	0	267.70
ata	I	0	438.90
gtt	V	3	0.62
gtc	V	0	0.61
gta	V	0	0.61
gtg	V	0	1.16
---	--	------	------
mPD		0.0053	0.66
		nPD:	0.01
		N. weight:	1.1
		Sc. PD:	-0.042
		Sc. rank:	-318.4

HA

Pos.	545	obs:	exp:
gtt	V	0	231.00
gtc	V	0	225.00
gta	V	1115	228.30
gtg	V	0	430.60
gct	A	0	1.29
gcc	A	0	0.93
gca	A	5	2.35
gcg	A	0	0.42
---	--	------	------
mPD		0.0089	0.73
		nPD:	0.01
		N. weight:	1.7
		Sc. PD:	-0.06
		Sc. rank:	-216.7

HA

Pos.	546	obs:	exp:
atg	M	1120	1120.00
---	--	------	------
mPD		0	0
		nPD:	1.
		N. weight:	0.
		Sc. PD:	0
		Sc. rank:	0

HA

Pos.	547	obs:	exp:
ggt	G	10	148.50
ggc	G	1103	143.10
gga	G	6	516.70
ggg	G	1	311.80
---	--	------	------
mPD		0.030	0.68
		nPD:	0.04
		N. weight:	2.1
		Sc. PD:	-0.01
		Sc. rank:	1101.7

HA

Pos.	548	obs:	exp:
tta	L	0	99.21
ttg	L	0	208.10
ctt	L	1118	186.20
ctc	L	0	186.60
cta	L	0	188.40
ctg	L	0	249.50
att	I	2	0.73
atc	I	0	0.48
ata	I	0	0.79
---	--	------	------
mPD		0.0036	1.1
		nPD:	0.
		N. weight:	1.9
		Sc. PD:	-0.084
		Sc. rank:	-1220.0

HA

Pos.	549	obs:	exp:
att	I	0	0.37
atc	I	1	0.24
ata	I	0	0.39
gtt	V	1	231.40
gtc	V	1116	225.40
gta	V	0	228.70
gtg	V	0	431.40
gct	A	1	0.52
gcc	A	1	0.37
gca	A	0	0.94
gcg	A	0	0.17
---	--	------	------
mPD		0.0089	0.73
		nPD:	0.01
		N. weight:	1.7
		Sc. PD:	-0.06
		Sc. rank:	-209.0

HA

Pos.	550	obs:	exp:
ttt	F	0	442.30
ttc	F	1120	677.70
---	--	------	------
mPD		0	0.48
		nPD:	0.
		N. weight:	0.53
		Sc. PD:	-0.025
		Sc. rank:	-482.6

HA

Pos.	551	obs:	exp:
att	I	1	409.70
atc	I	0	267.20
ata	I	1114	438.10
atg	M	4	4.00
act	T	0	0.25
acc	T	0	0.21
aca	T	1	0.46
acg	T	0	0.08
---	--	------	------
mPD		0.011	0.66

nPD: 0.02
N. weight: 0.97
Sc. PD: -0.031
Sc. rank: 43.4

HA

Pos.	552	obs:	exp:
tgt	C	1120	473.70
tgc	C	0	646.30
---	--	------	------
mPD		0	0.49

nPD: 0.
N. weight: 0.9
Sc. PD: -0.043
Sc. rank: -826.6

HA

Pos.	553	obs:	exp:
gtt	V	3	232.10
gtc	V	0	226.00
gta	V	728	229.30
gtg	V	389	432.60
---	--	------	------
mPD		0.46	0.73

nPD: 0.63
N. weight: 0.74
Sc. PD: 0.41
Sc. rank: 684.7

HA

Pos.	554	obs:	exp:
aaa	K	8	652.80
aag	K	1112	467.20
---	--	------	------
mPD		0.014	0.49

nPD: 0.03
N. weight: 0.87
Sc. PD: -0.017
Sc. rank: 316.0

HA

Pos.	555	obs:	exp:
tct	S	0	0.95
tcc	S	0	0.78
tca	S	0	1.52
tcg	S	0	0.34
aat	N	1114	567.40
aac	N	0	546.60
agt	S	6	1.23
agc	S	0	1.17
---	--	------	------
mPD		0.011	0.52

nPD: 0.02
N. weight: 0.71
Sc. PD: -0.02
Sc. rank: 123.4

HA

Pos.	556	obs:	exp:
cgt	R	0	0.34
cgc	R	0	0.40
cga	R	0	0.78
cgg	R	0	0.67
aga	R	8	3.66
agg	R	0	2.15
ggt	G	0	147.40
ggc	G	0	142.10
gga	G	1109	513.00
ggg	G	3	309.60
---	--	------	------
mPD		0.020	0.69

nPD: 0.03
N. weight: 0.79
Sc. PD: -0.016
Sc. rank: 276.3

HA

Pos.	557	obs:	exp:
aat	N	4	570.50
aac	N	1116	549.50
---	--	------	------
mPD		0.0071	0.50

nPD: 0.01
N. weight: 0.72
Sc. PD: -0.025
Sc. rank: -26.4

HA

Pos.	558	obs:	exp:
tta	L	0	0.09
ttg	L	0	0.19
ctt	L	0	0.17
ctc	L	0	0.17
cta	L	0	0.17
ctg	L	1	0.22
atg	M	1116	1116.00
aaa	K	0	0.58
aag	K	1	0.42
gtt	V	0	0.41
gtc	V	0	0.40
gta	V	0	0.41
gtg	V	2	0.77
---	--	------	------
mPD		0.0071	0.011

nPD: 0.63
N. weight: 0.004
Sc. PD: 0.0022
Sc. rank: 3.7

HA

Pos.	559	obs:	exp:
caa	Q	0	1.53
cag	Q	3	1.47
cgt	R	0	47.97
cgc	R	0	55.32
cga	R	0	109.30
cgg	R	1117	93.20
aga	R	0	510.80
agg	R	0	300.40
---	--	------	------
mPD		0.0053	0.97

nPD: 0.01
N. weight: 2.6
Sc. PD: -0.11
Sc. rank: -1175.8

HA

Pos.	560	obs:	exp:
tgt	C	0	473.70
tgc	C	1120	646.30
---	--	------	------
mPD		0	0.49

nPD: 0.
N. weight: 0.58
Sc. PD: -0.027
Sc. rank: -528.2

HA

Pos.	561	obs:	exp:
act	T	1120	285.50
acc	T	0	229.80
aca	T	0	513.70
acg	T	0	90.97
---	--	------	------
mPD		0	0.68

nPD: 0.
N. weight: 1.4
Sc. PD: -0.068
Sc. rank: -1313.0

HA

Pos.	562	obs:	exp:
att	I	1119	411.10
atc	I	0	268.20
ata	I	0	439.70
gtt	V	1	0.21
gtc	V	0	0.20
gta	V	0	0.20
gtg	V	0	0.39
---	--	------	------
mPD		0.0018	0.66

nPD: 0.
N. weight: 1.
Sc. PD: -0.047
Sc. rank: -699.3

HA

Pos.	563	obs:	exp:
tgt	C	1118	472.90
tgc	C	0	645.10
ggt	G	2	0.27
ggc	G	0	0.26
gga	G	0	0.92
ggg	G	0	0.56
---	--	------	------
mPD		0.0036	0.49

nPD: 0.01
N. weight: 0.9
Sc. PD: -0.037
Sc. rank: -377.8

HA

Pos.	564	obs:	exp:
att	I	0	411.50
atc	I	0	268.40
ata	I	1120	440.10
---	--	------	------
mPD		0	0.65

nPD: 0.
N. weight: 0.98
Sc. PD: -0.047
Sc. rank: -897.3

HA

Pos.	565	obs:	exp:
taa	.	1108	475.80
tag	.	12	280.40
tga	.	0	363.80
---	--	------	------
mPD		0.021	0.81

nPD: 0.03
N. weight: 0.84
Sc. PD: -0.019
Sc. rank: 259.4 }

Amino acid level conservation per codon

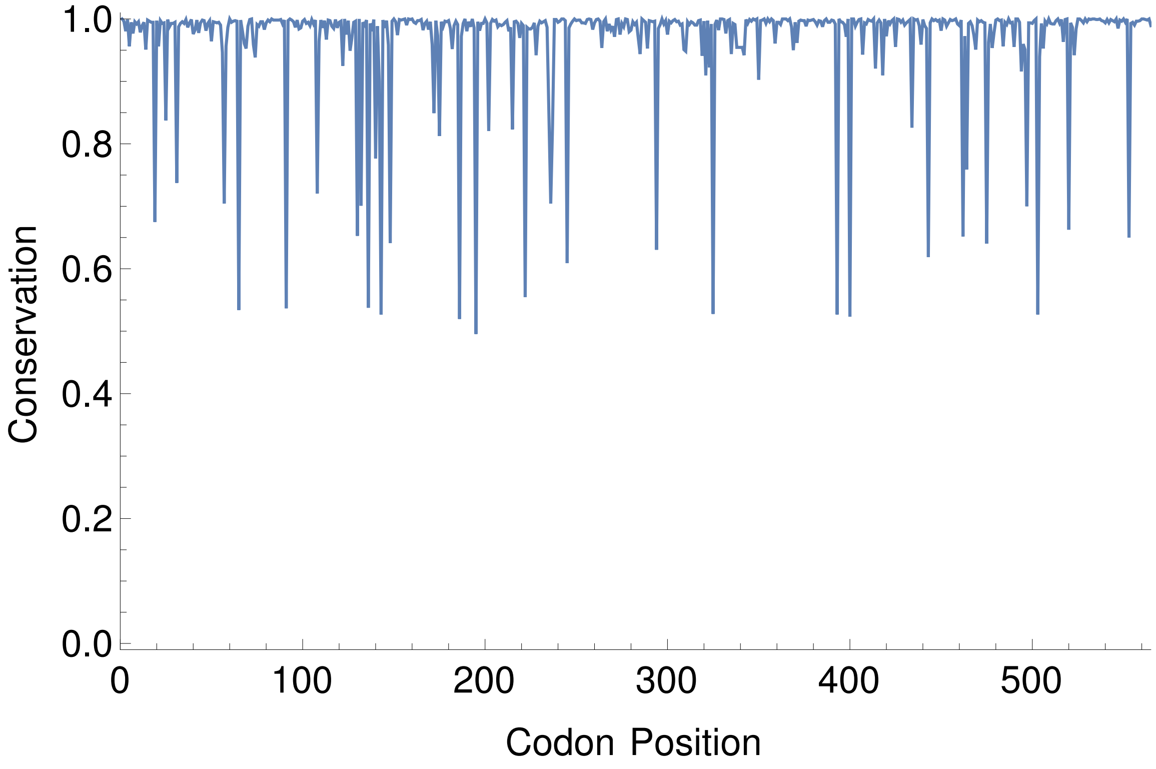

Conservation analysis plots

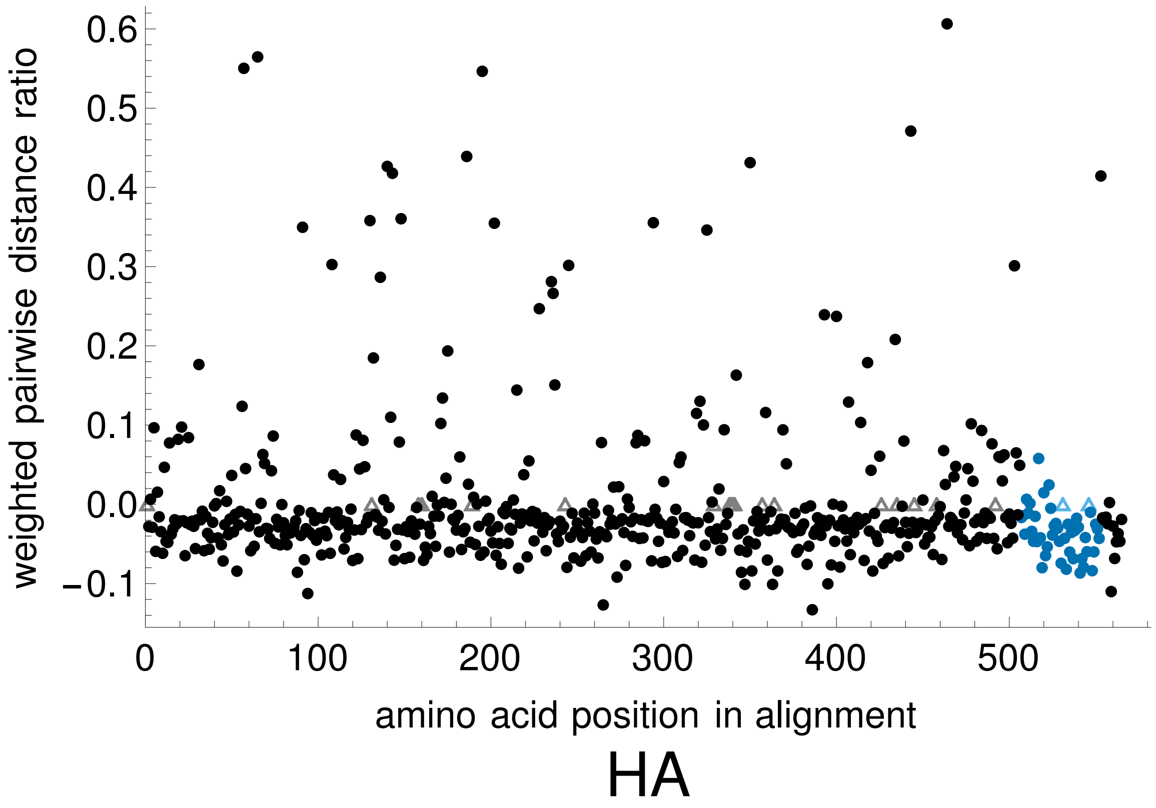

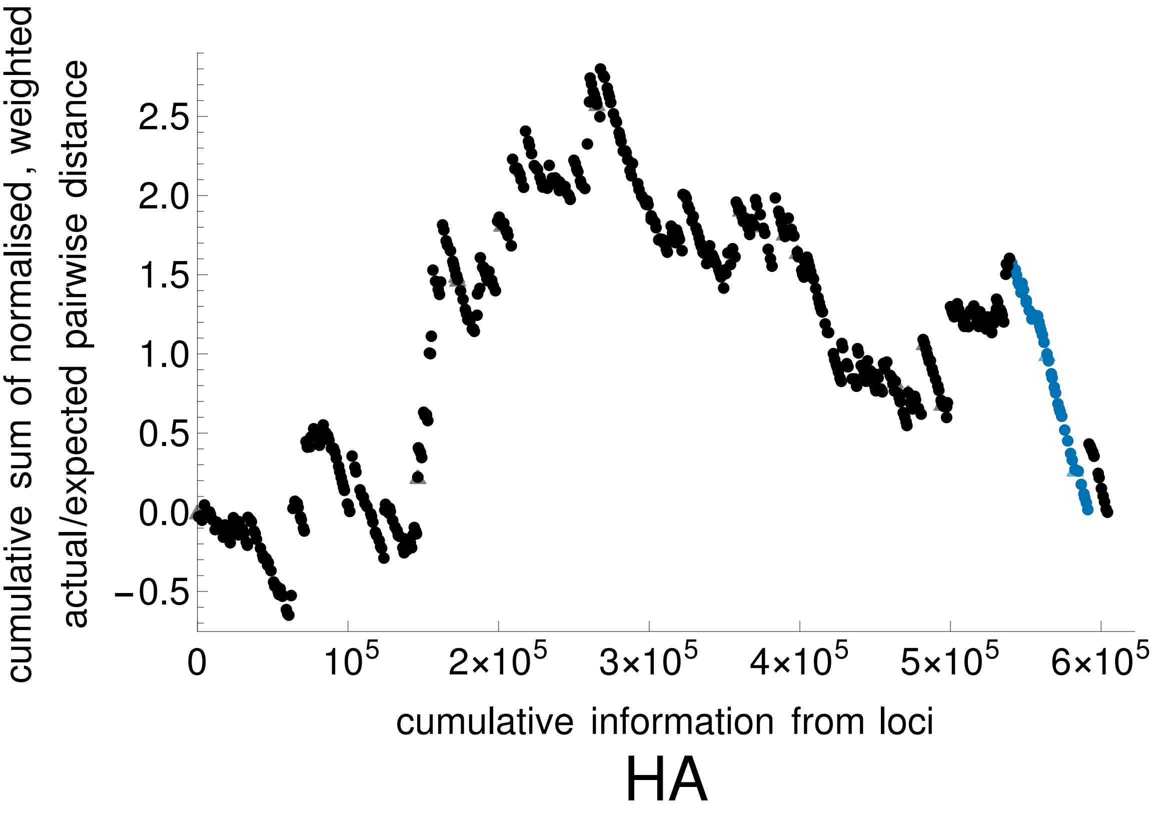

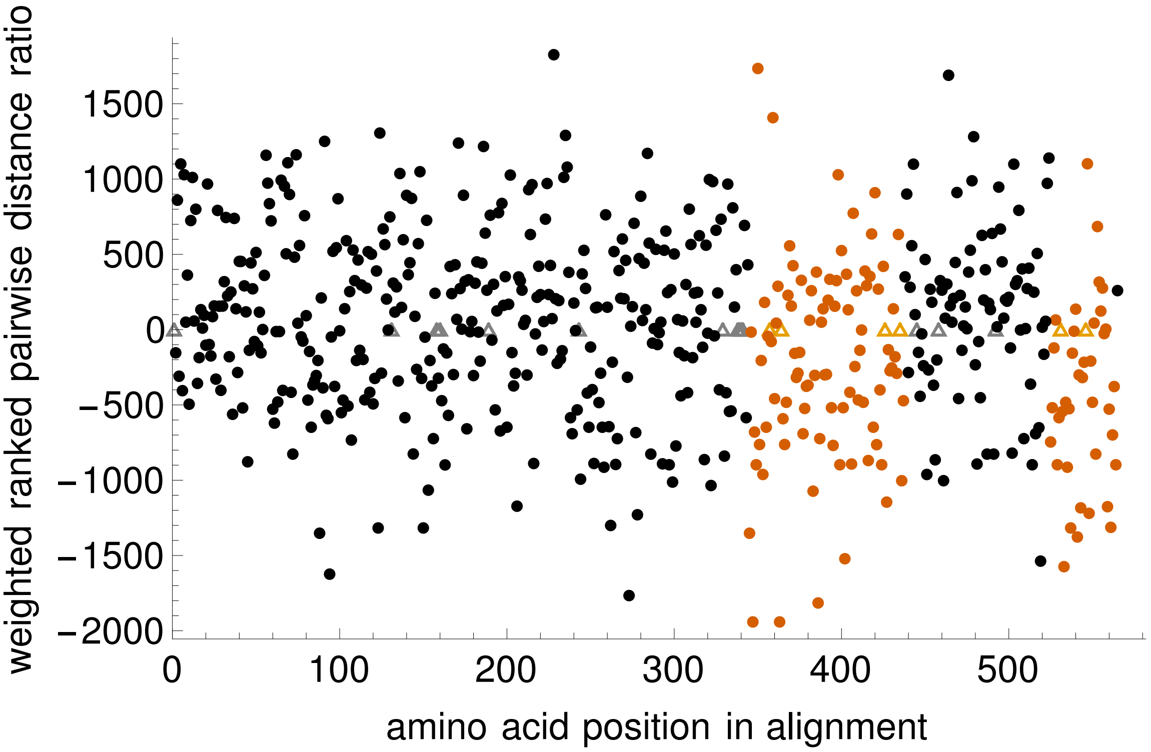
weighted ranked pairwise distance ratio
1500
1000
500
0
−500
−1000
−1500
−2000
0
100
200
300
400
500
amino acid position in alignment

HA

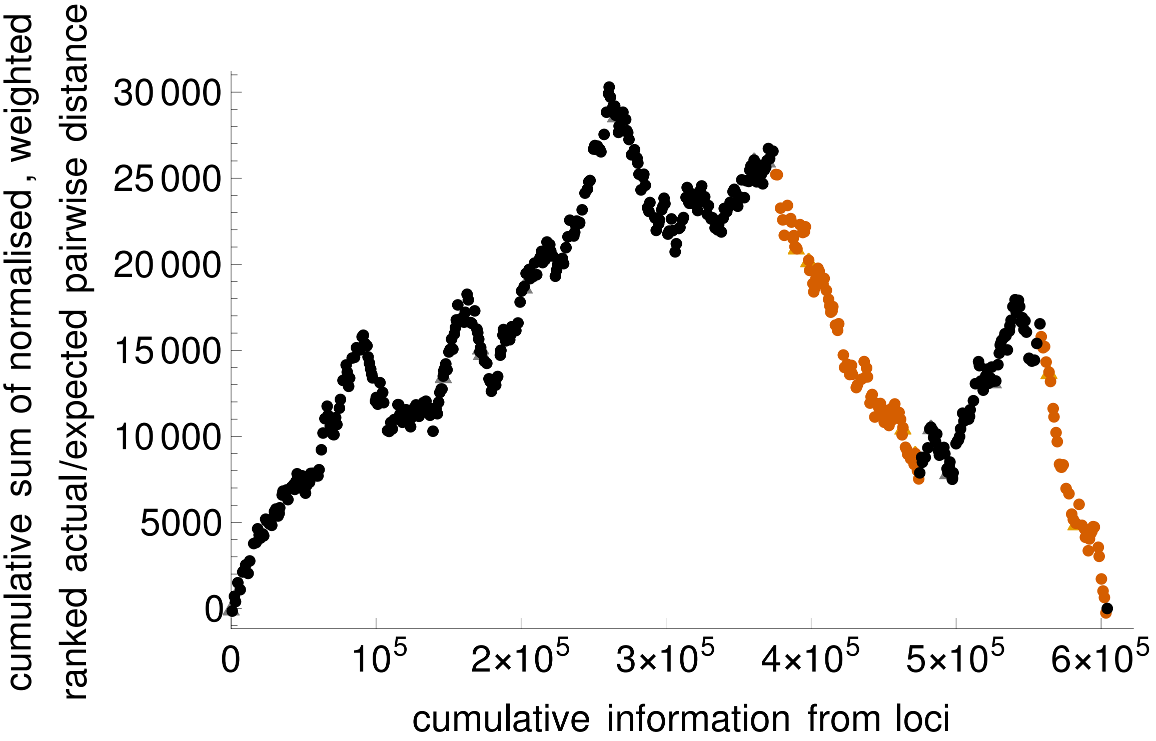
cumulative sum of normalised, weighted ranked actual/expected pairwise distance
30 000
25 000
20 000
15 000
10 000
5000
0
0
10^5
2×10^5
3×10^5
4×10^5
5×10^5
6×10^5
cumulative information from loci

HA

NP

Gene length histogram

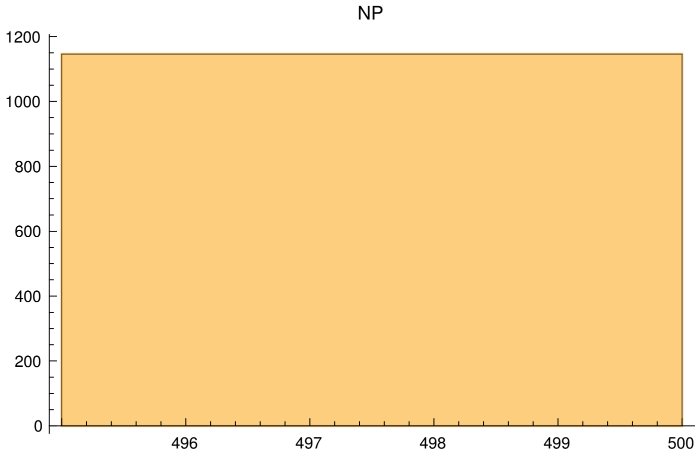

Information vs. nPD

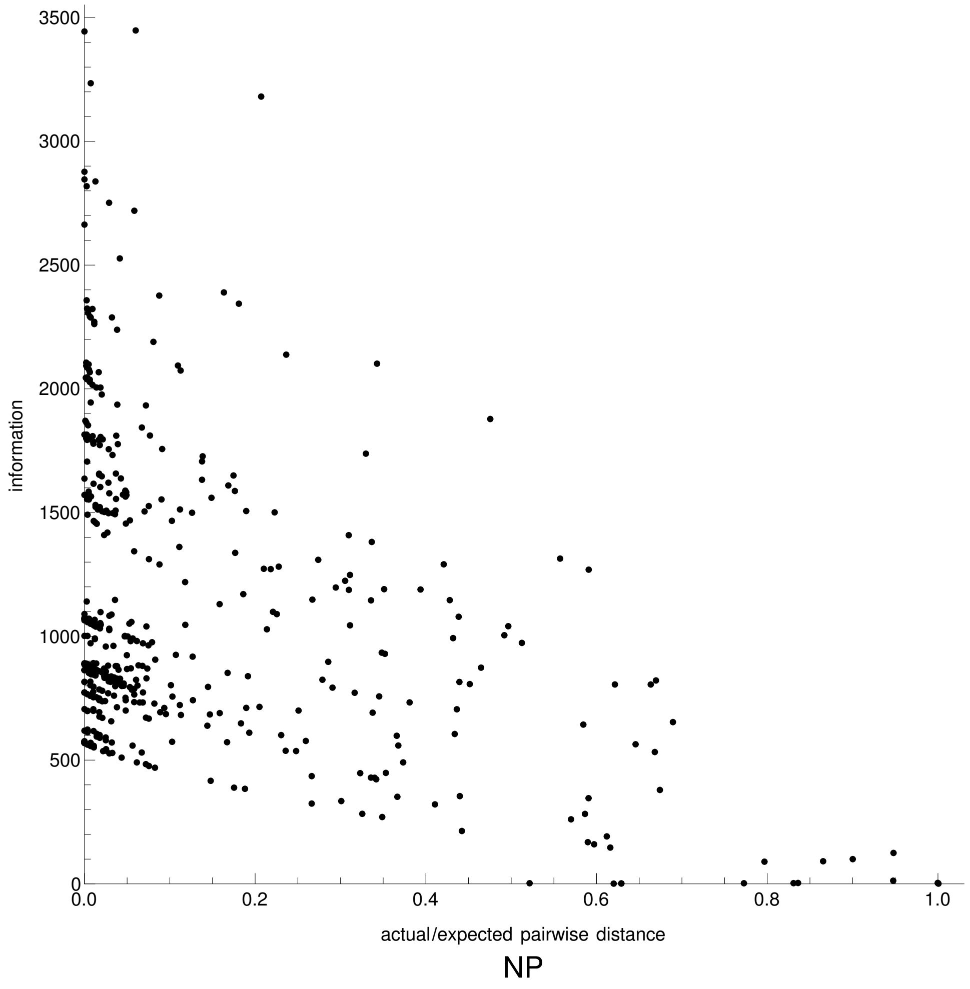

Example sequences highlighted by regions found in analyses to be conserved

Interesting points (by weighted raw PD) highlighted for gene NP:

ATGGCGTCTCAAGGCACCAAACGATCCTATGAACAGATGGAAACTGGTGGGGAACGCCAGAATGCTACTGAGATCA⋱
GGGCATCTGTTGGAAGAATGGTTAGCGGAATTGGGAGATTCTACATACAGATGTGTACAGAACTCAAACTCAATG⋱
ACAATGAAGGGAGGCTGATTCAAAACAGTATAACAATAGAGAGAATGGTACTCTCTGCATTTGATGAAAGAAGGA⋱
ACAGATACTTGGAAGAGCACCCCAGTGCAGGAAAGGATCCCAAGAAAACTGGAGGTCCAATTTACAGGAGAAGAG⋱
ACGGAAAATGGGTGAGAGAGCTGATCCTGTATGACAAAGAGGAAATCAGGAGAATTTGGCGACAAGCGAACAATG⋱
GAGAGGATGCAACTGCTGGTCTTACCCACCTGATGATATGGCATTCCAACCTGAATGATGCTACTTACCAGAGAA⋱
CGAGAGCTCTCGTGCGTACTGGAATGGATCCCCGGATGTGCTCTCTGATGCAAGGGTCAACTCTCCCGAGGAGAT⋱
CTGGAGCTGCAGGAGCAGCAGTAAAGGGAATAGGAACAATGGTGATGGAACTGGTTCGGATGATAAAACGAGGAA⋱
TCAACGACCGGAATTTCTGGAGAGGCGAAAATGGAAGAAGGACAAGAATTGCATATGAGAGAATGTGCAACATCC⋱
TCAAAGGGAAATTCCAAACAGCAGCACAAAGGGCAATGATGGATCAAGTGCGAGAGAGCAGAAATCCTGGGAATG⋱
CTGAAATAGAAGATCTTATTTTTCTGGCAAGGTCTGCACTCATCCTGAGAGGATCAGTGGCTCATAAATCCTGCT⋱
TGCCCGCTTGTGTGTACGGACTTGCAGTGGCTAGTGGATATGACTTTGAGAGAGAAGGGTACTCCTTGGTGGGAA⋱
TAGATCCTTTCCGTCTGCTTCAAAACAGCCAGGTCTTTAGTCTCATTAGACCAAATGAGAACCCAGCACACAAGA⋱
GCCAACTGGTATGGATGGCATGCCACTCTGCAGCGTTTGAGGACCTTAGGGTCTCAAGTTTCATTAGAGGAACAA⋱
AAATGGTCCCAAGAGGACAGCTATCCACTAGAGGAGTTCAAATTGCTTCAAATGAGAACATGGAAGCAATGGACT⋱
CCAATACTCTTGAATTGAGAAGTAGATATTGGGCTATAAGAACCAGAAGCGGAGGGAACACCAACCAACAGAGGG⋱
CATCTGCAGGACAGGTCAGCGTTCAACCCACTTTCTCAGTACAGAGAAACCTTCCTTTCGAAAGAGCAACCATTA⋱
TGGCAGCATTTACAGGAAATACTGAGGGTAGAACGTCCGACATGAGGACTGAAATCATAAGAATGATGGAAAGTG⋱
CCAGACCAGAAGATGTGTCATTCCAGGGGCGGGGAGTCTTCGAGCTATCGGACGAAAAGGCAACGAACCCGATCG⋱
TGCCTTCCTTTGACATGAGTAATGAAGGATCTTATTTCTTCGGAGACAATGCAGAGGAGTATGACAATTGA

Interesting points (by weighted ranked PD) highlighted for gene NP:

ATG
GCGTCTCAAGGCACCAAACGATCCTATGAACAGATGGAAACTGGTGGGGAACGCC
AGAATGCTACTGAGATCAGGGCATCTGTTGGAAGAATGGTTAGCGGAATTGGGAGATTCTACATACAGA⋱
TGTGTACAGAACTCAAACTCAATGACAATGAAGGGAGGCTGATTCAAAACAGTATAACAATAGAGAGAAT⋱
GGTACTCTCTGCATTTGATGAAAGAAGGAACAGATACTTGGAAGAGCACCCCAGTGCAGGAAAGGATCCC⋱
AAGAAAACTGGAGGTCCAATTTACAGGAGAAGAGACGGAAAATGGGTGAGAGAGCTGATCCTGTATGACA⋱
AAGAGGAAATCAGGAGAATTTGGCGACAAGCGAACAATGGAGAGGATGCAACTGCTGGTCTTACCCACCT⋱
GATGATATGGCATTCCAACCTGAATGATGCTACTTACCAGAGAACGAGAGCTCTCGTGCGTACTGGAATG⋱
GATCCCCGGATGTGCTCTCTGATGCAAGGGTCAACTCTCCCGAGGAGATCTGGAGCTGCAGGAGCAGCAG⋱
TAAAGGGAATAGGAACAATGGTGATGGAACTGGTTCGGATGATAAAACGAGGAATCAACGACCGGAATTT⋱
CTGGAGAGGCGAAAATGGAAGAAGGACAAGAATTGCATATGAGAGAATGTGCAACATCCTCAAAGGGAAA⋱
TTCCAAACAGCAGCACAAAGGGCAATGATGGATCAAGTGCGAGAGAGCAGAAATCCTGGGAATGCTGAAA⋱
TAGAAGATCTTATTTTTCTGGCAAGGTCTGCACTCATCCTGAGAGGATCAGTGGCTCATAAATCCTGCTT⋱
GCCCGCTTGTGTGTACGGACTTGCAGTGGCTAGTGGATATGACTTTGAGAGAGAAGGGTACTCCTTGGTG⋱
GGAATAGATCCTTTCCGTCTGCTTCAAAACAGCCAGGTCTTTAGTCTCATTAGACCAAATGAGAACCCAG⋱
CACACAAGAGCCAACTGGTATGGATGGCATGCCACTCTGCAGCGTTTGAGGACCTTAGGGTCTCAAGTTT⋱
CATTAGAGGAACAAAAATGGTCCCAAGAGGACAGCTATCCACTAGAGGAGTTCAAATTGCTTCAAATGAG⋱
AACATGGAAGCAATGGACTCCAATACTCTTGAATTGAGAAGTAGATATTGGGCTATAAGAACCAGAAGCG⋱
GAGGGAACACCAACCAACAGAGGGCATCTGCAGGACAGGTCAGCGTTCAACCCACTTTCTCAGTACAGAG⋱
AAACCTTCCTTTCGAAAGAGCAACCATTATGGCAGCATTTACAGGAAATACTGAGGGTAGAACGTCCGAC⋱
ATGAGGACTGAAATCATAAGAATGATGGAAAGTGCCAGACCAGAAGATGTGTCATTCCAGGGG

CGGGGAGTCTTCGAGCTATCGGACGAAAAGGCAACGAACCCGATCGTGCCTTCCT
TTGACATGAGTAATGAAGGATCTTATTTCTTCGGAGACAATGCA

GAGGAGTATGACAATTGA

Per codon conservation report

NP			
Pos.	1	obs:	exp:
atg	M	1146	1146.00
---	--	------	------
mPD		0	0
		nPD:	1.
		N. weight:	0.
		Sc. PD:	0
		Sc. rank:	0

NP			
Pos.	2	obs:	exp:
gct	A	1	295.90
gcc	A	0	213.40
gca	A	0	539.40
gcg	A	1145	97.26
---	--	------	------
mPD		0.0017	0.67
		nPD:	0.
		N. weight:	2.7
		Sc. PD:	-0.21
		Sc. rank:	-1874.0

NP			
Pos.	3	obs:	exp:
tct	S	1130	180.70
tcc	S	6	148.40
tca	S	0	288.30
tcg	S	0	64.46
tat	Y	1	0.54
tac	Y	0	0.46
cct	P	8	2.00
ccc	P	0	1.44
cca	P	0	3.29
ccg	P	0	1.27
act	T	1	0.25
acc	T	0	0.21
aca	T	0	0.46
acg	T	0	0.08
agt	S	0	233.50
agc	S	0	220.60
---	--	------	------
mPD		0.028	1.7
		nPD:	0.02
		N. weight:	2.
		Sc. PD:	-0.12
		Sc. rank:	-302.9

NP			
Pos.	4	obs:	exp:
caa	Q	1146	583.70
cag	Q	0	562.30
---	--	------	------
mPD		0	0.50
		nPD:	0.
		N. weight:	0.74
		Sc. PD:	-0.058
		Sc. rank:	-664.7

NP			
Pos.	5	obs:	exp:
tct	S	0	0.16
tcc	S	0	0.13
tca	S	0	0.25
tcg	S	0	0.06
agt	S	0	0.21
agc	S	1	0.19
ggt	G	0	151.80
ggc	G	1145	146.30
gga	G	0	528.20
ggg	G	0	318.80
---	--	------	------
mPD		0.0017	0.68
		nPD:	0.
		N. weight:	2.2
		Sc. PD:	-0.17
		Sc. rank:	-1617.5

NP			
Pos.	6	obs:	exp:
act	T	0	292.10
acc	T	1146	235.20
aca	T	0	525.60
acg	T	0	93.08
---	--	------	------
mPD		0	0.68
		nPD:	0.
		N. weight:	1.7
		Sc. PD:	-0.14
		Sc. rank:	-1560.5

NP			
Pos.	7	obs:	exp:
aaa	K	1146	667.90
aag	K	0	478.10
---	--	------	------
mPD		0	0.49
		nPD:	0.
		N. weight:	0.59
		Sc. PD:	-0.047
		Sc. rank:	-531.9

NP			
Pos.	8	obs:	exp:
cgt	R	0	49.22
cgc	R	0	56.76
cga	R	1146	112.10
cgg	R	0	95.62
aga	R	0	524.00
agg	R	0	308.20
---	--	------	------
mPD		0	0.96
		nPD:	0.
		N. weight:	2.5
		Sc. PD:	-0.2
		Sc. rank:	-2290.1

NP			
Pos.	9	obs:	exp:
tct	S	0	182.10
tcc	S	1144	149.50
tca	S	1	290.60
tcg	S	0	64.97
agt	S	0	235.40
agc	S	0	222.40
gct	A	1	0.26
gcc	A	0	0.19
gca	A	0	0.47
gcg	A	0	0.08
---	--	------	------
mPD		0.0052	1.7
		nPD:	0.
		N. weight:	2.2
		Sc. PD:	-0.17
		Sc. rank:	-1370.2

NP			
Pos.	10	obs:	exp:
tat	Y	1145	619.00
tac	Y	1	527.00
---	--	------	------
mPD		0.0017	0.50
		nPD:	0.
		N. weight:	0.67
		Sc. PD:	-0.05
		Sc. rank:	-403.9

NP			
Pos.	11	obs:	exp:
gaa	E	1138	697.30
gag	E	8	448.70
---	--	------	------
mPD		0.014	0.48
		nPD:	0.03
		N. weight:	0.5
		Sc. PD:	-0.026
		Sc. rank:	36.5

NP			
Pos.	12	obs:	exp:
caa	Q	2	583.70
cag	Q	1144	562.30
---	--	------	------
mPD		0.0035	0.50
		nPD:	0.01
		N. weight:	0.76
		Sc. PD:	-0.055
		Sc. rank:	-356.4

NP			
Pos.	13	obs:	exp:
atg	M	1146	1146.00
---	--	------	------
mPD		0	0
		nPD:	1.
		N. weight:	0.
		Sc. PD:	0
		Sc. rank:	0

NP			
Pos.	14	obs:	exp:
gat	D	0	0.54
gac	D	1	0.46
gaa	E	1068	696.70
gag	E	77	448.30
---	--	------	------
mPD		0.13	0.48
		nPD:	0.27
		N. weight:	0.31
		Sc. PD:	0.055
		Sc. rank:	222.6

NP			
Pos.	15	obs:	exp:
att	I	1	0.37
atc	I	0	0.24
ata	I	0	0.39
act	T	1144	291.60
acc	T	0	234.70
aca	T	0	524.70
acg	T	0	92.92
aaa	K	1	0.58
aag	K	0	0.42
---	--	------	------
mPD		0.0052	0.68
		nPD:	0.01
		N. weight:	1.5
		Sc. PD:	-0.11
		Sc. rank:	-609.2

NP			
Pos.	16	obs:	exp:
tct	S	0	0.48
tcc	S	0	0.39
tca	S	0	0.76
tcg	S	0	0.17
agt	S	3	0.62
agc	S	0	0.58
gat	D	8	4.32
gac	D	0	3.68
ggt	G	1133	150.40
ggc	G	1	145.00
gga	G	0	523.60
ggg	G	1	316.00
---	--	------	------
mPD		0.023	0.70
		nPD:	0.03
		N. weight:	2.2
		Sc. PD:	-0.1
		Sc. rank:	226.0

NP			
Pos.	17	obs:	exp:
ggt	G	0	151.90
ggc	G	1	146.40
gga	G	94	528.70
ggg	G	1051	319.00
---	--	------	------
mPD		0.15	0.68
		nPD:	0.23
		N. weight:	1.
		Sc. PD:	0.14
		Sc. rank:	723.7

NP			
Pos.	18	obs:	exp:
gaa	E	1123	697.30
gag	E	23	448.70
---	--	------	------
mPD		0.039	0.48
		nPD:	0.08
		N. weight:	0.45
		Sc. PD:	0.00018
		Sc. rank:	204.4

NP			
Pos.	19	obs:	exp:
cgt	R	0	49.22
cgc	R	1146	56.76
cga	R	0	112.10
cgg	R	0	95.62
aga	R	0	524.00
agg	R	0	308.20
---	--	------	------
mPD		0	0.96
		nPD:	0.
		N. weight:	3.3
		Sc. PD:	-0.26
		Sc. rank:	-2961.0

NP			
Pos.	20	obs:	exp:
caa	Q	0	583.70
cag	Q	1146	562.30
---	--	------	------
mPD		0	0.50
		nPD:	0.
		N. weight:	0.78
		Sc. PD:	-0.061
		Sc. rank:	-701.6

NP			
Pos.	21	obs:	exp:
tct	S	0	0.48
tcc	S	0	0.39
tca	S	0	0.76
tcg	S	0	0.17
aat	N	1143	582.20
aac	N	0	560.80
agt	S	3	0.62
agc	S	0	0.58
---	--	------	------
mPD		0.0052	0.51
		nPD:	0.01
		N. weight:	0.74
		Sc. PD:	-0.051
		Sc. rank:	-266.3

NP			
Pos.	22	obs:	exp:
act	T	41	10.45
acc	T	0	8.41
aca	T	0	18.81
acg	T	0	3.33
gtt	V	1	0.21
gtc	V	0	0.20
gta	V	0	0.20
gtg	V	0	0.39
gct	A	1097	285.10
gcc	A	5	205.60
gca	A	0	519.60
gcg	A	2	93.70
---	--	------	------
mPD		0.083	0.74
		nPD:	0.11
		N. weight:	1.4
		Sc. PD:	0.041
		Sc. rank:	759.9

NP			
Pos.	23	obs:	exp:
act	T	1139	292.10
acc	T	3	235.20
aca	T	2	525.60
acg	T	2	93.08
---	--	------	------
mPD		0.012	0.68
		nPD:	0.02
		N. weight:	1.4
		Sc. PD:	-0.09
		Sc. rank:	-153.6

NP			
Pos.	24	obs:	exp:
gaa	E	17	697.30
gag	E	1129	448.70
---	--	------	------
mPD		0.029	0.48
		nPD:	0.06
		N. weight:	0.93
		Sc. PD:	-0.019
		Sc. rank:	338.7

NP			
Pos.	25	obs:	exp:
att	I	4	420.30
atc	I	1139	274.10
ata	I	1	449.50
gtt	V	0	0.41
gtc	V	2	0.40
gta	V	0	0.41
gtg	V	0	0.77
---	--	------	------
mPD		0.012	0.66
		nPD:	0.02
		N. weight:	1.5
		Sc. PD:	-0.094
		Sc. rank:	-131.2

NP			
Pos.	26	obs:	exp:
cgt	R	0	49.18
cgc	R	0	56.71
cga	R	0	112.00
cgg	R	0	95.54
aaa	K	0	0.58
aag	K	1	0.42
aga	R	132	523.60
agg	R	1013	308.00
---	--	------	------
mPD		0.21	0.96
		nPD:	0.21
		N. weight:	0.98
		Sc. PD:	0.12
		Sc. rank:	666.1

NP			
Pos.	27	obs:	exp:
act	T	0	0.25
acc	T	0	0.21
aca	T	1	0.46
acg	T	0	0.08
gct	A	1	295.70
gcc	A	0	213.20
gca	A	1105	538.90
gcg	A	39	97.18
---	--	------	------
mPD		0.069	0.67
		nPD:	0.1
		N. weight:	0.72
		Sc. PD:	0.014
		Sc. rank:	363.6

NP			
Pos.	28	obs:	exp:
tct	S	1143	182.30
tcc	S	2	149.70
tca	S	0	290.90
tcg	S	1	65.03
agt	S	0	235.60
agc	S	0	222.60
---	--	------	------
mPD		0.0052	1.7
		nPD:	0.
		N. weight:	2.
		Sc. PD:	-0.15
		Sc. rank:	-1275.7

NP			
Pos.	29	obs:	exp:
gtt	V	1061	237.50
gtc	V	85	231.30
gta	V	0	234.70
gtg	V	0	442.60
---	--	------	------
mPD		0.14	0.73
		nPD:	0.19
		N. weight:	1.4
		Sc. PD:	0.15
		Sc. rank:	934.0

NP			
Pos.	30	obs:	exp:
ggt	G	0	151.90
ggc	G	0	146.40
gga	G	1070	528.70
ggg	G	76	319.00
---	--	------	------
mPD		0.12	0.68
		nPD:	0.18
		N. weight:	0.62
		Sc. PD:	0.06
		Sc. rank:	395.5

NP			
Pos.	31	obs:	exp:
cgt	R	0	49.18
cgc	R	0	56.71
cga	R	0	112.00
cgg	R	0	95.54
aga	R	1141	523.60
agg	R	4	308.00
ggt	G	0	0.13
ggc	G	0	0.13
gga	G	1	0.46
ggg	G	0	0.28
---	--	------	------
mPD		0.0087	0.96
		nPD:	0.01
		N. weight:	0.83
		Sc. PD:	-0.059
		Sc. rank:	-319.9

NP			
Pos.	32	obs:	exp:
atg	M	1146	1146.00
---	--	------	------
mPD		0	0
		nPD:	1.
		N. weight:	0.
		Sc. PD:	0
		Sc. rank:	0

NP			
Pos.	33	obs:	exp:
att	I	4	1.47
atc	I	0	0.96
ata	I	0	1.57
gtt	V	1134	236.60
gtc	V	8	230.50
gta	V	0	233.80
gtg	V	0	441.10
---	--	------	------
mPD		0.021	0.73
		nPD:	0.03
		N. weight:	1.7
		Sc. PD:	-0.087
		Sc. rank:	85.9

NP			
Pos.	34	obs:	exp:
tct	S	0	179.90
tcc	S	0	147.70
tca	S	0	287.10
tcg	S	0	64.18
aat	N	0	1.02
aac	N	2	0.98
agt	S	117	232.50
agc	S	1014	219.70
ggt	G	1	1.72
ggc	G	12	1.66
gga	G	0	6.00
ggg	G	0	3.62
---	--	------	------
mPD		0.21	1.7
		nPD:	0.13
		N. weight:	1.4
		Sc. PD:	0.06
		Sc. rank:	785.5

NP			
Pos.	35	obs:	exp:
tct	S	0	0.64
tcc	S	0	0.52
tca	S	0	1.02
tcg	S	0	0.23
agt	S	0	0.82
agc	S	4	0.78
ggt	G	2	151.40
ggc	G	829	145.90
gga	G	309	526.80
ggg	G	2	317.90
---	--	------	------
mPD		0.41	0.69
		nPD:	0.59
		N. weight:	1.2
		Sc. PD:	0.59
		Sc. rank:	1079.9

NP			
Pos.	36	obs:	exp:
att	I	1145	421.10
atc	I	1	274.60
ata	I	0	450.30
---	--	------	------
mPD		0.0017	0.65
		nPD:	0.
		N. weight:	1.1
		Sc. PD:	-0.083
		Sc. rank:	-750.3

NP			
Pos.	37	obs:	exp:
ggt	G	107	151.90
ggc	G	2	146.40
gga	G	5	528.70
ggg	G	1032	319.00
---	--	------	------
mPD		0.18	0.68
		nPD:	0.27
		N. weight:	1.1
		Sc. PD:	0.19
		Sc. rank:	794.1

NP			
Pos.	38	obs:	exp:
cgt	R	0	49.13
cgc	R	0	56.66
cga	R	0	112.00
cgg	R	0	95.46
aaa	K	1	0.58
aag	K	0	0.42
aga	R	1138	523.10
agg	R	6	307.70
ggt	G	0	0.13
ggc	G	0	0.13
gga	G	1	0.46
ggg	G	0	0.28
---	--	------	------
mPD		0.014	0.96
		nPD:	0.01
		N. weight:	0.82
		Sc. PD:	-0.054
		Sc. rank:	-154.7

NP			
Pos.	39	obs:	exp:
ttt	F	2	452.50
ttc	F	1144	693.50
---	--	------	------
mPD		0.0035	0.48
		nPD:	0.01
		N. weight:	0.54
		Sc. PD:	-0.039
		Sc. rank:	-239.6

NP			
Pos.	40	obs:	exp:
tat	Y	0	616.80
tac	Y	1142	525.20
cat	H	0	2.30
cac	H	4	1.70
---	--	------	------
mPD		0.0070	0.50
		nPD:	0.01
		N. weight:	0.85
		Sc. PD:	-0.056
		Sc. rank:	-177.9

NP			
Pos.	41	obs:	exp:
att	I	0	420.30
atc	I	0	274.10
ata	I	1144	449.50
atg	M	1	1.00
gtt	V	0	0.21
gtc	V	0	0.20
gta	V	1	0.20
gtg	V	0	0.39
---	--	------	------
mPD		0.0035	0.66
		nPD:	0.01
		N. weight:	1.
		Sc. PD:	-0.075
		Sc. rank:	-517.6

NP			
Pos.	42	obs:	exp:
caa	Q	6	583.70
cag	Q	1140	562.30
---	--	------	------
mPD		0.010	0.50
		nPD:	0.02
		N. weight:	0.74
		Sc. PD:	-0.044
		Sc. rank:	-33.3

NP			
Pos.	43	obs:	exp:
atg	M	1146	1146.00
---	--	------	------
mPD		0	0
		nPD:	1.
		N. weight:	0.
		Sc. PD:	0
		Sc. rank:	0

NP			
Pos.	44	obs:	exp:
tgt	C	1104	484.70
tgc	C	42	661.30
---	--	------	------
mPD		0.071	0.49
		nPD:	0.14
		N. weight:	0.76
		Sc. PD:	0.046
		Sc. rank:	438.3

NP			
Pos.	45	obs:	exp:
act	T	1	292.10
acc	T	0	235.20
aca	T	1145	525.60
acg	T	0	93.08
---	--	------	------
mPD		0.0017	0.68
		nPD:	0.
		N. weight:	0.84
		Sc. PD:	-0.065
		Sc. rank:	-600.4

NP			
Pos.	46	obs:	exp:
gaa	E	1125	697.30
gag	E	21	448.70
---	--	------	------
mPD		0.036	0.48
		nPD:	0.08
		N. weight:	0.45
		Sc. PD:	-0.0029
		Sc. rank:	197.7

NP			
Pos.	47	obs:	exp:
tta	L	0	101.70
ttg	L	0	213.40
ctt	L	10	190.80
ctc	L	1133	191.20
cta	L	3	193.10
ctg	L	0	255.70
---	--	------	------
mPD		0.022	1.1
		nPD:	0.02
		N. weight:	1.9
		Sc. PD:	-0.11
		Sc. rank:	-110.0

NP			
Pos.	48	obs:	exp:
aaa	K	1142	667.90
aag	K	4	478.10
---	--	------	------
mPD		0.0070	0.49
		nPD:	0.01
		N. weight:	0.57
		Sc. PD:	-0.037
		Sc. rank:	-111.1

NP			
Pos.	49	obs:	exp:
tta	L	0	101.70
ttg	L	0	213.40
ctt	L	0	190.80
ctc	L	1144	191.20
cta	L	2	193.10
ctg	L	0	255.70
---	--	------	------
mPD		0.0035	1.1
		nPD:	0.
		N. weight:	1.9
		Sc. PD:	-0.15
		Sc. rank:	-1320.7

NP			
Pos.	50	obs:	exp:
tct	S	0	149.30
tcc	S	0	122.60
tca	S	0	238.30
tcg	S	0	53.28
cgt	R	1	0.04
cgc	R	0	0.05
cga	R	0	0.10
cgg	R	0	0.08
act	T	1	0.25
acc	T	0	0.21
aca	T	0	0.46
acg	T	0	0.08
aat	N	205	104.40
aac	N	0	100.60
agt	S	936	193.00
agc	S	3	182.40
aga	R	0	0.46
agg	R	0	0.27
---	--	------	------
mPD		0.30	1.8
		nPD:	0.17
		N. weight:	1.5
		Sc. PD:	0.13
		Sc. rank:	939.5

NP			
Pos.	51	obs:	exp:
gat	D	10	619.30
gac	D	1136	526.70
---	--	------	------
mPD		0.017	0.50
		nPD:	0.03
		N. weight:	0.79
		Sc. PD:	-0.036
		Sc. rank:	102.0

NP			
Pos.	52	obs:	exp:
tat	Y	1	0.54
tac	Y	0	0.46
aat	N	1040	583.20
aac	N	105	561.80
---	--	------	------
mPD		0.17	0.50
		nPD:	0.34
		N. weight:	0.41
		Sc. PD:	0.1
		Sc. rank:	318.3

NP			
Pos.	53	obs:	exp:
gaa	E	1129	697.30
gag	E	17	448.70
---	--	------	------
mPD		0.029	0.48
		nPD:	0.06
		N. weight:	0.47
		Sc. PD:	-0.0094
		Sc. rank:	169.4

NP			
Pos.	54	obs:	exp:
ggt	G	1	151.90
ggc	G	0	146.40
gga	G	29	528.70
ggg	G	1116	319.00
---	--	------	------
mPD		0.051	0.68
		nPD:	0.08
		N. weight:	1.2
		Sc. PD:	-0.008
		Sc. rank:	542.6

NP			
Pos.	55	obs:	exp:
cgt	R	0	49.22
cgc	R	0	56.76
cga	R	0	112.10
cgg	R	0	95.62
aga	R	15	524.00
agg	R	1131	308.20
---	--	------	------
mPD		0.026	0.96
		nPD:	0.03
		N. weight:	1.4
		Sc. PD:	-0.072
		Sc. rank:	34.9

NP			
Pos.	56	obs:	exp:
tta	L	0	101.70
ttg	L	0	213.40
ctt	L	0	190.80
ctc	L	0	191.20
cta	L	2	193.10
ctg	L	1144	255.70
---	--	------	------
mPD		0.0035	1.1
		nPD:	0.
		N. weight:	1.6
		Sc. PD:	-0.12
		Sc. rank:	-1105.3

NP			
Pos.	57	obs:	exp:
att	I	1030	421.10
atc	I	116	274.60
ata	I	0	450.30
---	--	------	------
mPD		0.18	0.65
		nPD:	0.28
		N. weight:	0.79
		Sc. PD:	0.15
		Sc. rank:	576.0

NP			
Pos.	58	obs:	exp:
caa	Q	362	583.70
cag	Q	784	562.30
---	--	------	------
mPD		0.43	0.50
		nPD:	0.87
		N. weight:	0.087
		Sc. PD:	0.066
		Sc. rank:	79.3

NP			
Pos.	59	obs:	exp:
aat	N	3	583.70
aac	N	1143	562.30
---	--	------	------
mPD		0.0052	0.50
		nPD:	0.01
		N. weight:	0.76
		Sc. PD:	-0.052
		Sc. rank:	-253.4

NP			
Pos.	60	obs:	exp:
tct	S	0	182.30
tcc	S	0	149.70
tca	S	0	290.90
tcg	S	0	65.03
agt	S	1104	235.60
agc	S	42	222.60
---	--	------	------
mPD		0.071	1.7
		nPD:	0.04
		N. weight:	1.6
		Sc. PD:	-0.059
		Sc. rank:	345.0

NP			
Pos.	61	obs:	exp:
att	I	0	419.20
atc	I	2	273.40
ata	I	1139	448.30
atg	M	5	5.00
---	--	------	------
mPD		0.012	0.66
		nPD:	0.02
		N. weight:	1.
		Sc. PD:	-0.061
		Sc. rank:	-91.8

NP			
Pos.	62	obs:	exp:
act	T	2	292.10
acc	T	0	235.20
aca	T	1144	525.60
acg	T	0	93.08
---	--	------	------
mPD		0.0035	0.68
		nPD:	0.01
		N. weight:	0.84
		Sc. PD:	-0.062
		Sc. rank:	-444.8

NP			
Pos.	63	obs:	exp:
att	I	0	420.70
atc	I	0	274.40
ata	I	1145	449.90
gtt	V	0	0.21
gtc	V	0	0.20
gta	V	1	0.20
gtg	V	0	0.39
---	--	------	------
mPD		0.0017	0.66
		nPD:	0.
		N. weight:	1.
		Sc. PD:	-0.078
		Sc. rank:	-708.4

NP			
Pos.	64	obs:	exp:
gaa	E	5	697.30
gag	E	1141	448.70
---	--	------	------
mPD		0.0087	0.48
		nPD:	0.02
		N. weight:	0.99
		Sc. PD:	-0.061
		Sc. rank:	-98.0

NP			
Pos.	65	obs:	exp:
cgt	R	0	49.18
cgc	R	0	56.71
cga	R	0	112.00
cgg	R	0	95.54
aaa	K	1	0.58
aag	K	0	0.42
aga	R	1141	523.60
agg	R	4	308.00
---	--	------	------
mPD		0.0087	0.96
		nPD:	0.01
		N. weight:	0.83
		Sc. PD:	-0.059
		Sc. rank:	-324.9

NP			
Pos.	66	obs:	exp:
att	I	0	0.37
atc	I	0	0.24
ata	I	1	0.39
atg	M	1145	1145.00
---	--	------	------
mPD		0.0017	0.0017
		nPD:	1.
		N. weight:	0.00089
		Sc. PD:	0.00079
		Sc. rank:	0.8

NP			
Pos.	67	obs:	exp:
att	I	0	1.84
atc	I	0	1.20
ata	I	5	1.96
gtt	V	0	236.00
gtc	V	1	229.90
gta	V	1138	233.20
gtg	V	0	439.90
gct	A	0	0.52
gcc	A	0	0.37
gca	A	2	0.94
gcg	A	0	0.17
---	--	------	------
mPD		0.014	0.74
		nPD:	0.02
		N. weight:	1.7
		Sc. PD:	-0.1
		Sc. rank:	-118.5

NP			
Pos.	68	obs:	exp:
tta	L	0	101.50
ttg	L	0	213.00
ctt	L	1	190.50
ctc	L	1142	190.90
cta	L	1	192.80
ctg	L	0	255.30
att	I	0	0.73
atc	I	2	0.48
ata	I	0	0.79
---	--	------	------
mPD		0.0070	1.1
		nPD:	0.01
		N. weight:	1.9
		Sc. PD:	-0.14
		Sc. rank:	-958.4

NP			
Pos.	69	obs:	exp:
tct	S	1143	182.10
tcc	S	0	149.50
tca	S	1	290.60
tcg	S	1	64.97
tgt	C	1	0.42
tgc	C	0	0.58
agt	S	0	235.40
agc	S	0	222.40
---	--	------	------
mPD		0.0052	1.7
		nPD:	0.
		N. weight:	2.
		Sc. PD:	-0.15
		Sc. rank:	-1219.0

NP			
Pos.	70	obs:	exp:
tct	S	0	0.16
tcc	S	0	0.13
tca	S	1	0.25
tcg	S	0	0.06
agt	S	0	0.21
agc	S	0	0.19
gtt	V	1	0.21
gtc	V	0	0.20
gta	V	0	0.20
gtg	V	0	0.39
gct	A	0	295.40
gcc	A	24	213.00
gca	A	1120	538.40
gcg	A	0	97.09
---	--	------	------
mPD		0.046	0.67
		nPD:	0.07
		N. weight:	0.74
		Sc. PD:	-0.0097
		Sc. rank:	289.7

NP

Pos.	71	obs:	exp:
ttt	F	1141	452.50
ttc	F	5	693.50
mPD		0.0087	0.48
		nPD:	0.02
		N. weight:	0.98
		Sc. PD:	-0.061
		Sc. rank:	-100.7

NP

Pos.	72	obs:	exp:
gat	D	1116	619.30
gac	D	30	526.70
mPD		0.051	0.50
		nPD:	0.1
		N. weight:	0.55
		Sc. PD:	0.011
		Sc. rank:	274.3

NP

Pos.	73	obs:	exp:
gaa	E	1041	696.70
gag	E	104	448.30
ggt	G	0	0.13
ggc	G	0	0.13
gga	G	1	0.46
ggg	G	0	0.28
mPD		0.17	0.48
		nPD:	0.35
		N. weight:	0.26
		Sc. PD:	0.066
		Sc. rank:	207.5

NP

Pos.	74	obs:	exp:
cgt	R	0	49.22
cgc	R	0	56.76
cga	R	1	112.10
cgg	R	0	95.62
aga	R	1113	524.00
agg	R	32	308.20
mPD		0.056	0.96
		nPD:	0.06
		N. weight:	0.73
		Sc. PD:	-0.017
		Sc. rank:	244.5

NP

Pos.	75	obs:	exp:
cgt	R	0	49.22
cgc	R	0	56.76
cga	R	98	112.10
cgg	R	12	95.62
aga	R	5	524.00
agg	R	1031	308.20
mPD		0.34	0.96
		nPD:	0.35
		N. weight:	1.1
		Sc. PD:	0.29
		Sc. rank:	919.5

NP

Pos.	76	obs:	exp:
aat	N	10	583.70
aac	N	1136	562.30
mPD		0.017	0.50
		nPD:	0.03
		N. weight:	0.72
		Sc. PD:	-0.033
		Sc. rank:	90.9

NP

Pos.	77	obs:	exp:
cgt	R	0	49.05
cgc	R	0	56.56
cga	R	0	111.80
cgg	R	0	95.29
aaa	K	4	2.33
aag	K	0	1.67
aga	R	1134	522.20
agg	R	8	307.20
mPD		0.021	0.97
		nPD:	0.02
		N. weight:	0.81
		Sc. PD:	-0.048
		Sc. rank:	-20.1

NP

Pos.	78	obs:	exp:
tat	Y	17	618.40
tac	Y	1128	526.60
cat	H	0	0.57
cac	H	1	0.43
mPD		0.031	0.50
		nPD:	0.06
		N. weight:	0.76
		Sc. PD:	-0.015
		Sc. rank:	280.7

NP

Pos.	79	obs:	exp:
tta	L	2	101.70
ttg	L	405	213.40
ctt	L	0	190.80
ctc	L	0	191.20
cta	L	10	193.10
ctg	L	729	255.70
mPD		0.48	1.1
		nPD:	0.43
		N. weight:	0.95
		Sc. PD:	0.32
		Sc. rank:	796.5

NP

Pos.	80	obs:	exp:
gaa	E	1134	697.30
gag	E	12	448.70
mPD		0.021	0.48
		nPD:	0.04
		N. weight:	0.49
		Sc. PD:	-0.018
		Sc. rank:	109.7

NP

Pos.	81	obs:	exp:
gaa	E	101	697.30
gag	E	1045	448.70
mPD		0.16	0.48
		nPD:	0.34
		N. weight:	0.66
		Sc. PD:	0.16
		Sc. rank:	520.2

NP

Pos.	82	obs:	exp:
cat	H	18	658.10
cac	H	1128	487.90
mPD		0.031	0.49
		nPD:	0.06
		N. weight:	0.84
		Sc. PD:	-0.015
		Sc. rank:	311.8

NP

Pos.	83	obs:	exp:
cct	P	107	286.00
ccc	P	1006	205.90
cca	P	33	471.70
ccg	P	0	182.40
mPD		0.22	0.71
		nPD:	0.31
		N. weight:	1.3
		Sc. PD:	0.29
		Sc. rank:	1011.3

NP

Pos.	84	obs:	exp:
tct	S	0	181.80
tcc	S	0	149.30
tca	S	0	290.10
tcg	S	0	64.86
aat	N	2	1.02
aac	N	0	0.98
agt	S	1143	235.00
agc	S	0	222.00
ggt	G	1	0.13
ggc	G	0	0.13
gga	G	0	0.46
ggg	G	0	0.28
mPD		0.0052	1.7
		nPD:	0.
		N. weight:	1.7
		Sc. PD:	-0.13
		Sc. rank:	-1139.3

NP

Pos.	85	obs:	exp:
tct	S	0	5.73
tcc	S	0	4.70
tca	S	34	9.14
tcg	S	2	2.04
act	T	0	0.51
acc	T	0	0.41
aca	T	2	0.92
acg	T	0	0.16
agt	S	0	7.40
agc	S	0	6.99
gct	A	54	286.10
gcc	A	0	206.30
gca	A	974	521.50
gcg	A	80	94.04
mPD		0.28	0.76
		nPD:	0.37
		N. weight:	0.53
		Sc. PD:	0.15
		Sc. rank:	437.0

NP

Pos.	86	obs:	exp:
gaa	E	1	0.61
gag	E	0	0.39
ggt	G	0	151.80
ggc	G	0	146.30
gga	G	1136	528.20
ggg	G	9	318.80
mPD		0.017	0.68
		nPD:	0.03
		N. weight:	0.8
		Sc. PD:	-0.044
		Sc. rank:	12.6

NP

Pos.	87	obs:	exp:
aaa	K	113	667.90
aag	K	1033	478.10
mPD		0.18	0.49
		nPD:	0.37
		N. weight:	0.57
		Sc. PD:	0.16
		Sc. rank:	466.0

NP

Pos.	88	obs:	exp:
gat	D	386	619.30
gac	D	760	526.70
mPD		0.45	0.50
		nPD:	0.9
		N. weight:	0.095
		Sc. PD:	0.075
		Sc. rank:	86.7

NP

Pos.	89	obs:	exp:
cct	P	783	285.50
ccc	P	272	205.50
cca	P	1	470.90
ccg	P	88	182.10
cat	H	0	1.15
cac	H	2	0.85
mPD		0.47	0.71
		nPD:	0.66
		N. weight:	0.77
		Sc. PD:	0.43
		Sc. rank:	692.4

NP

Pos.	90	obs:	exp:
aat	N	1	0.51
aac	N	0	0.49
aaa	K	0	667.30
aag	K	1145	477.70
mPD		0.0017	0.49
		nPD:	0.
		N. weight:	0.95
		Sc. PD:	-0.072
		Sc. rank:	-568.7

NP

Pos.	91	obs:	exp:
aaa	K	1145	667.90
aag	K	1	478.10
mPD		0.0017	0.49
		nPD:	0.
		N. weight:	0.58
		Sc. PD:	-0.044
		Sc. rank:	-346.2

NP

Pos.	92	obs:	exp:
act	T	1132	292.10
acc	T	12	235.20
aca	T	0	525.60
acg	T	2	93.08
mPD		0.024	0.68
		nPD:	0.04
		N. weight:	1.4
		Sc. PD:	-0.064
		Sc. rank:	190.1

NP

Pos.	93	obs:	exp:
ggt	G	0	151.90
ggc	G	0	146.40
gga	G	1143	528.70
ggg	G	3	319.00
mPD		0.0052	0.68
		nPD:	0.01
		N. weight:	0.83
		Sc. PD:	-0.059
		Sc. rank:	-330.5

NP

Pos.	94	obs:	exp:
ggt	G	1101	151.90
ggc	G	10	146.40
gga	G	2	528.70
ggg	G	33	319.00
mPD		0.076	0.68
		nPD:	0.11
		N. weight:	2.
		Sc. PD:	0.058
		Sc. rank:	1056.1

NP

Pos.	95	obs:	exp:
cct	P	111	286.00
ccc	P	0	205.90
cca	P	1033	471.70
ccg	P	2	182.40
mPD		0.18	0.71
		nPD:	0.25
		N. weight:	0.67
		Sc. PD:	0.11
		Sc. rank:	478.4

NP

Pos. 96		obs:	exp:
att	I	1135	421.10
atc	I	3	274.60
ata	I	8	450.30
mPD		0.019	0.65
		nPD:	0.03
		N. weight:	1.
		Sc. PD:	-0.053
		Sc. rank:	81.6

NP

Pos. 97		obs:	exp:
tat	Y	9	619.00
tac	Y	1137	527.00
mPD		0.016	0.50
		nPD:	0.03
		N. weight:	0.8
		Sc. PD:	-0.039
		Sc. rank:	69.8

NP

Pos. 98		obs:	exp:
cgt	R	0	48.75
cgc	R	0	56.22
cga	R	80	111.10
cgg	R	7	94.71
aaa	K	0	6.41
aag	K	11	4.59
aga	R	6	519.00
agg	R	1042	305.30
mPD		0.30	0.98
		nPD:	0.31
		N. weight:	1.2
		Sc. PD:	0.25
		Sc. rank:	873.3

NP

Pos. 99		obs:	exp:
cgt	R	0	49.13
cgc	R	0	56.66
cga	R	0	112.00
cgg	R	0	95.46
aaa	K	2	1.17
aag	K	0	0.83
aga	R	1140	523.10
agg	R	4	307.70
mPD		0.010	0.96
		nPD:	0.01
		N. weight:	0.83
		Sc. PD:	-0.057
		Sc. rank:	-249.1

NP

Pos. 100		obs:	exp:
cgt	R	0	48.96
cgc	R	0	56.46
cga	R	2	111.60
cgg	R	0	95.12
att	I	0	1.10
atc	I	0	0.72
ata	I	3	1.18
atg	M	1	1.00
aaa	K	1	0.58
aag	K	0	0.42
aga	R	1133	521.30
agg	R	5	306.60
ggt	G	0	0.13
ggc	G	0	0.13
gga	G	1	0.46
ggg	G	0	0.28
mPD		0.024	0.97
		nPD:	0.03
		N. weight:	0.82
		Sc. PD:	-0.045
		Sc. rank:	7.4

NP

Pos. 101		obs:	exp:
gat	D	11	618.70
gac	D	1134	526.30
gaa	E	1	0.61
gag	E	0	0.39
mPD		0.021	0.50
		nPD:	0.04
		N. weight:	0.79
		Sc. PD:	-0.031
		Sc. rank:	170.1

NP

Pos. 102		obs:	exp:
ggt	G	0	151.90
ggc	G	1	146.40
gga	G	1035	528.70
ggg	G	110	319.00
mPD		0.18	0.68
		nPD:	0.26
		N. weight:	0.55
		Sc. PD:	0.094
		Sc. rank:	395.6

NP

Pos. 103		obs:	exp:
aaa	K	1137	667.90
aag	K	9	478.10
mPD		0.016	0.49
		nPD:	0.03
		N. weight:	0.54
		Sc. PD:	-0.026
		Sc. rank:	52.1

NP

Pos. 104		obs:	exp:
tgg	W	1146	1146.00
mPD		0	0
		nPD:	1.
		N. weight:	0.
		Sc. PD:	0
		Sc. rank:	0

NP

Pos. 105		obs:	exp:
tta	L	0	0.44
ttg	L	5	0.93
ctt	L	0	0.83
ctc	L	0	0.83
cta	L	0	0.84
ctg	L	0	1.12
gtt	V	3	236.20
gtc	V	0	230.10
gta	V	25	233.40
gtg	V	1112	440.30
gct	A	0	0.26
gcc	A	0	0.19
gca	A	0	0.47
gcg	A	1	0.08
mPD		0.058	0.74
		nPD:	0.08
		N. weight:	0.93
		Sc. PD:	-0.0027
		Sc. rank:	414.3

NP

Pos. 106		obs:	exp:
cgt	R	0	48.40
cgc	R	0	55.82
cga	R	0	110.30
cgg	R	0	94.04
aaa	K	19	11.07
aag	K	0	7.93
aga	R	1125	515.30
agg	R	2	303.10
mPD		0.036	0.99
		nPD:	0.04
		N. weight:	0.84
		Sc. PD:	-0.037
		Sc. rank:	124.6

NP

Pos. 107		obs:	exp:
gaa	E	4	697.30
gag	E	1142	448.70
mPD		0.0070	0.48
		nPD:	0.01
		N. weight:	1.
		Sc. PD:	-0.065
		Sc. rank:	-184.0

NP

Pos. 108		obs:	exp:
tta	L	0	101.70
ttg	L	1	213.40
ctt	L	1	190.80
ctc	L	0	191.20
cta	L	9	193.10
ctg	L	1135	255.70
mPD		0.019	1.1
		nPD:	0.02
		N. weight:	1.6
		Sc. PD:	-0.099
		Sc. rank:	-233.7

NP

Pos. 109		obs:	exp:
att	I	26	420.70
atc	I	1119	274.40
ata	I	0	449.90
gtt	V	0	0.21
gtc	V	1	0.20
gta	V	0	0.20
gtg	V	0	0.39
mPD		0.046	0.66
		nPD:	0.07
		N. weight:	1.4
		Sc. PD:	-0.016
		Sc. rank:	571.1

NP

Pos. 110		obs:	exp:
tta	L	0	101.70
ttg	L	17	213.40
ctt	L	1	190.80
ctc	L	0	191.20
cta	L	11	193.10
ctg	L	1117	255.70
mPD		0.050	1.1
		nPD:	0.05
		N. weight:	1.5
		Sc. PD:	-0.054
		Sc. rank:	350.4

NP

Pos. 111		obs:	exp:
tat	Y	1143	617.90
tac	Y	1	526.10
cat	H	2	1.15
cac	H	0	0.85
mPD		0.0052	0.50
		nPD:	0.01
		N. weight:	0.67
		Sc. PD:	-0.046
		Sc. rank:	-217.1

NP

Pos. 112		obs:	exp:
gat	D	6	619.30
gac	D	1140	526.70
mPD		0.010	0.50
		nPD:	0.02
		N. weight:	0.81
		Sc. PD:	-0.048
		Sc. rank:	-32.1

NP

Pos. 113		obs:	exp:
aaa	K	1055	667.90
aag	K	91	478.10
mPD		0.15	0.49
		nPD:	0.3
		N. weight:	0.32
		Sc. PD:	0.067
		Sc. rank:	237.2

NP

Pos. 114		obs:	exp:
gat	D	1	0.54
gac	D	0	0.46
gaa	E	13	696.70
gag	E	1132	448.30
mPD		0.024	0.48
		nPD:	0.05
		N. weight:	0.95
		Sc. PD:	-0.029
		Sc. rank:	279.3

NP

Pos. 115		obs:	exp:
gaa	E	1144	697.30
gag	E	2	448.70
mPD		0.0035	0.48
		nPD:	0.01
		N. weight:	0.53
		Sc. PD:	-0.038
		Sc. rank:	-233.3

NP

Pos. 116		obs:	exp:
att	I	0	420.70
atc	I	1142	274.40
ata	I	3	449.90
gtt	V	0	0.21
gtc	V	1	0.20
gta	V	0	0.20
gtg	V	0	0.39
mPD		0.0070	0.66
		nPD:	0.01
		N. weight:	1.5
		Sc. PD:	-0.11
		Sc. rank:	-469.0

NP

Pos. 117		obs:	exp:
cgt	R	0	49.22
cgc	R	0	56.76
cga	R	0	112.10
cgg	R	1	95.62
aga	R	1	524.00
agg	R	1144	308.20
mPD		0.0035	0.96
		nPD:	0.
		N. weight:	1.4
		Sc. PD:	-0.11
		Sc. rank:	-814.5

NP

Pos. 118		obs:	exp:
cgt	R	0	49.22
cgc	R	0	56.76
cga	R	0	112.10
cgg	R	0	95.62
aga	R	1099	524.00
agg	R	47	308.20
mPD		0.079	0.96
		nPD:	0.08
		N. weight:	0.69
		Sc. PD:	-0.00019
		Sc. rank:	316.2

NP

Pos. 119		obs:	exp:
att	I	1132	416.30
atc	I	0	271.50
ata	I	1	445.20
act	T	3	0.76
acc	T	0	0.62
aca	T	0	1.38
acg	T	0	0.24
gtt	V	10	2.07
gtc	V	0	2.02
gta	V	0	2.05
gtg	V	0	3.86
mPD		0.024	0.68
		nPD:	0.04
		N. weight:	1.1
		Sc. PD:	-0.049
		Sc. rank:	150.4

NP

Pos. 120		obs:	exp:
tgg	W	1146	1146.00
mPD		0	0
		nPD:	1.
		N. weight:	0.
		Sc. PD:	0
		Sc. rank:	0

NP			
Pos.	121	obs:	exp:
cgt	R	87	49.22
cgc	R	2	56.76
cga	R	1007	112.10
cgg	R	3	95.62
aga	R	47	524.00
agg	R	0	308.20
---	--	------	------
mPD		0.23	0.96
		nPD:	0.24
		N. weight:	2.
		Sc. PD:	0.3
		Sc. rank:	1452.1

NP			
Pos.	122	obs:	exp:
caa	Q	1132	583.70
cag	Q	14	562.30
---	--	------	------
mPD		0.024	0.50
		nPD:	0.05
		N. weight:	0.67
		Sc. PD:	-0.022
		Sc. rank:	176.8

NP			
Pos.	123	obs:	exp:
gct	A	9	295.90
gcc	A	0	213.40
gca	A	141	539.40
gcg	A	996	97.26
---	--	------	------
mPD		0.23	0.67
		nPD:	0.34
		N. weight:	2.
		Sc. PD:	0.5
		Sc. rank:	1601.4

NP			
Pos.	124	obs:	exp:
aat	N	5	583.70
aac	N	1141	562.30
---	--	------	------
mPD		0.0087	0.50
		nPD:	0.02
		N. weight:	0.75
		Sc. PD:	-0.047
		Sc. rank:	-100.8

NP			
Pos.	125	obs:	exp:
aat	N	1124	583.70
aac	N	22	562.30
---	--	------	------
mPD		0.038	0.50
		nPD:	0.08
		N. weight:	0.64
		Sc. PD:	-0.0042
		Sc. rank:	273.2

NP			
Pos.	126	obs:	exp:
ggt	G	0	151.90
ggc	G	5	146.40
gga	G	1128	528.70
ggg	G	13	319.00
---	--	------	------
mPD		0.031	0.68
		nPD:	0.05
		N. weight:	0.76
		Sc. PD:	-0.027
		Sc. rank:	180.4

NP			
Pos.	127	obs:	exp:
gat	D	1	0.54
gac	D	0	0.46
gaa	E	32	694.90
gag	E	1110	447.10
ggt	G	0	0.40
ggc	G	0	0.38
gga	G	0	1.38
ggg	G	3	0.84
---	--	------	------
mPD		0.061	0.48
		nPD:	0.13
		N. weight:	0.87
		Sc. PD:	0.037
		Sc. rank:	483.7

NP			
Pos.	128	obs:	exp:
aat	N	1	0.51
aac	N	0	0.49
gat	D	1064	617.70
gac	D	79	525.30
gaa	E	1	0.61
gag	E	0	0.39
ggt	G	1	0.13
ggc	G	0	0.13
gga	G	0	0.46
ggg	G	0	0.28
---	--	------	------
mPD		0.13	0.50
		nPD:	0.27
		N. weight:	0.41
		Sc. PD:	0.073
		Sc. rank:	299.3

NP			
Pos.	129	obs:	exp:
gct	A	14	295.90
gcc	A	0	213.40
gca	A	1132	539.40
gcg	A	0	97.26
---	--	------	------
mPD		0.024	0.67
		nPD:	0.04
		N. weight:	0.76
		Sc. PD:	-0.034
		Sc. rank:	106.8

NP			
Pos.	130	obs:	exp:
act	T	1144	292.10
acc	T	0	235.20
aca	T	1	525.60
acg	T	1	93.08
---	--	------	------
mPD		0.0035	0.68
		nPD:	0.01
		N. weight:	1.5
		Sc. PD:	-0.11
		Sc. rank:	-776.0

NP			
Pos.	131	obs:	exp:
gtt	V	2	0.41
gtc	V	0	0.40
gta	V	0	0.41
gtg	V	0	0.77
gct	A	1141	295.40
gcc	A	1	213.00
gca	A	2	538.40
gcg	A	0	97.09
---	--	------	------
mPD		0.0087	0.67
		nPD:	0.01
		N. weight:	1.5
		Sc. PD:	-0.097
		Sc. rank:	-327.6

NP			
Pos.	132	obs:	exp:
ggt	G	1131	151.90
ggc	G	15	146.40
gga	G	0	528.70
ggg	G	0	319.00
---	--	------	------
mPD		0.026	0.68
		nPD:	0.04
		N. weight:	2.1
		Sc. PD:	-0.09
		Sc. rank:	388.5

NP			
Pos.	133	obs:	exp:
ttt	F	1	0.39
ttc	F	0	0.61
tta	L	0	101.60
ttg	L	0	213.20
ctt	L	1144	190.70
ctc	L	1	191.10
cta	L	0	193.00
ctg	L	0	255.50
---	--	------	------
mPD		0.0035	1.1
		nPD:	0.
		N. weight:	1.9
		Sc. PD:	-0.15
		Sc. rank:	-1299.3

NP			
Pos.	134	obs:	exp:
act	T	7	292.10
acc	T	1139	235.20
aca	T	0	525.60
acg	T	0	93.08
---	--	------	------
mPD		0.012	0.68
		nPD:	0.02
		N. weight:	1.7
		Sc. PD:	-0.1
		Sc. rank:	-187.8

NP			
Pos.	135	obs:	exp:
cat	H	946	658.10
cac	H	200	487.90
---	--	------	------
mPD		0.29	0.49
		nPD:	0.59
		N. weight:	0.16
		Sc. PD:	0.078
		Sc. rank:	142.4

NP			
Pos.	136	obs:	exp:
tta	L	0	101.00
ttg	L	15	211.90
ctt	L	0	189.50
ctc	L	0	189.90
cta	L	8	191.80
ctg	L	1115	253.90
atg	M	8	8.00
---	--	------	------
mPD		0.053	1.1
		nPD:	0.05
		N. weight:	1.5
		Sc. PD:	-0.05
		Sc. rank:	381.3

NP			
Pos.	137	obs:	exp:
atg	M	1146	1146.00
---	--	------	------
mPD		0	0
		nPD:	1.
		N. weight:	0.
		Sc. PD:	0
		Sc. rank:	0

NP			
Pos.	138	obs:	exp:
att	I	4	421.10
atc	I	1	274.60
ata	I	1141	450.30
---	--	------	------
mPD		0.0087	0.65
		nPD:	0.01
		N. weight:	0.99
		Sc. PD:	-0.066
		Sc. rank:	-210.7

NP			
Pos.	139	obs:	exp:
tgg	W	1146	1146.00
---	--	------	------
mPD		0	0
		nPD:	1.
		N. weight:	0.
		Sc. PD:	0
		Sc. rank:	0

NP			
Pos.	140	obs:	exp:
cat	H	1143	658.10
cac	H	3	487.90
---	--	------	------
mPD		0.0052	0.49
		nPD:	0.01
		N. weight:	0.59
		Sc. PD:	-0.04
		Sc. rank:	-177.8

NP			
Pos.	141	obs:	exp:
tct	S	4	182.30
tcc	S	1142	149.70
tca	S	0	290.90
tcg	S	0	65.03
agt	S	0	235.60
agc	S	0	222.60
---	--	------	------
mPD		0.0070	1.7
		nPD:	0.
		N. weight:	2.2
		Sc. PD:	-0.16
		Sc. rank:	-1227.4

NP			
Pos.	142	obs:	exp:
aat	N	26	583.70
aac	N	1120	562.30
---	--	------	------
mPD		0.044	0.50
		nPD:	0.09
		N. weight:	0.66
		Sc. PD:	0.0042
		Sc. rank:	314.1

NP			
Pos.	143	obs:	exp:
tta	L	0	101.70
ttg	L	0	213.40
ctt	L	1	190.80
ctc	L	0	191.20
cta	L	49	193.10
ctg	L	1096	255.70
---	--	------	------
mPD		0.084	1.1
		nPD:	0.08
		N. weight:	1.5
		Sc. PD:	-0.0096
		Sc. rank:	621.3

NP			
Pos.	144	obs:	exp:
aat	N	1141	583.70
aac	N	5	562.30
---	--	------	------
mPD		0.0087	0.50
		nPD:	0.02
		N. weight:	0.71
		Sc. PD:	-0.044
		Sc. rank:	-95.3

NP			
Pos.	145	obs:	exp:
gat	D	1145	619.30
gac	D	1	526.70
---	--	------	------
mPD		0.0017	0.50
		nPD:	0.
		N. weight:	0.67
		Sc. PD:	-0.05
		Sc. rank:	-400.3

NP			
Pos.	146	obs:	exp:
act	T	0	0.25
acc	T	1	0.21
aca	T	0	0.46
acg	T	0	0.08
gct	A	912	295.70
gcc	A	223	213.20
gca	A	10	538.90
gcg	A	0	97.18
---	--	------	------
mPD		0.33	0.67
		nPD:	0.49
		N. weight:	0.96
		Sc. PD:	0.38
		Sc. rank:	833.6

NP			
Pos.	147	obs:	exp:
act	T	21	292.10
acc	T	1030	235.20
aca	T	95	525.60
acg	T	0	93.08
---	--	------	------
mPD		0.19	0.68
		nPD:	0.27
		N. weight:	1.2
		Sc. PD:	0.23
		Sc. rank:	910.9

NP			
Pos.	148	obs:	exp:
tat	Y	835	619.00
tac	Y	311	527.00
---	--	------	------
mPD		0.40	0.50
		nPD:	0.8
		N. weight:	0.085
		Sc. PD:	0.059
		Sc. rank:	77.7

NP			
Pos.	149	obs:	exp:
caa	Q	8	583.70
cag	Q	1138	562.30
---	--	------	------
mPD		0.014	0.50
		nPD:	0.03
		N. weight:	0.73
		Sc. PD:	-0.038
		Sc. rank:	23.2

NP			
Pos.	150	obs:	exp:
cgt	R	0	49.22
cgc	R	0	56.76
cga	R	0	112.10
cgg	R	0	95.62
aga	R	1144	524.00
agg	R	2	308.20
---	--	------	------
mPD		0.0035	0.96
		nPD:	0.
		N. weight:	0.84
		Sc. PD:	-0.064
		Sc. rank:	-497.7

NP			
Pos.	151	obs:	exp:
act	T	5	292.10
acc	T	0	235.20
aca	T	18	525.60
acg	T	1123	93.08
---	--	------	------
mPD		0.040	0.68
		nPD:	0.06
		N. weight:	2.6
		Sc. PD:	-0.059
		Sc. rank:	893.8

NP			
Pos.	152	obs:	exp:
cgt	R	0	49.22
cgc	R	0	56.76
cga	R	2	112.10
cgg	R	0	95.62
aga	R	1144	524.00
agg	R	0	308.20
---	--	------	------
mPD		0.0035	0.96
		nPD:	0.
		N. weight:	0.84
		Sc. PD:	-0.064
		Sc. rank:	-498.9

NP			
Pos.	153	obs:	exp:
tct	S	1	0.16
tcc	S	0	0.13
tca	S	0	0.25
tcg	S	0	0.06
act	T	1	0.25
acc	T	0	0.21
aca	T	0	0.46
acg	T	0	0.08
agt	S	0	0.21
agc	S	0	0.19
gct	A	1011	295.40
gcc	A	133	213.00
gca	A	0	538.40
gcg	A	0	97.09
---	--	------	------
mPD		0.21	0.67
		nPD:	0.31
		N. weight:	1.1
		Sc. PD:	0.25
		Sc. rank:	857.3

NP			
Pos.	154	obs:	exp:
tta	L	0	101.60
ttg	L	0	213.20
ctt	L	25	190.70
ctc	L	1102	191.10
cta	L	18	193.00
ctg	L	0	255.50
att	I	0	0.37
atc	I	1	0.24
ata	I	0	0.39
---	--	------	------
mPD		0.075	1.1
		nPD:	0.07
		N. weight:	1.8
		Sc. PD:	-0.025
		Sc. rank:	670.2

NP			
Pos.	155	obs:	exp:
gtt	V	1	237.50
gtc	V	0	231.30
gta	V	19	234.70
gtg	V	1126	442.60
---	--	------	------
mPD		0.034	0.73
		nPD:	0.05
		N. weight:	0.95
		Sc. PD:	-0.032
		Sc. rank:	235.1

NP			
Pos.	156	obs:	exp:
cgt	R	1112	49.22
cgc	R	33	56.76
cga	R	0	112.10
cgg	R	1	95.62
aga	R	0	524.00
agg	R	0	308.20
---	--	------	------
mPD		0.058	0.96
		nPD:	0.06
		N. weight:	3.3
		Sc. PD:	-0.07
		Sc. rank:	1172.5

NP			
Pos.	157	obs:	exp:
act	T	1136	292.10
acc	T	8	235.20
aca	T	2	525.60
acg	T	0	93.08
---	--	------	------
mPD		0.017	0.68
		nPD:	0.03
		N. weight:	1.4
		Sc. PD:	-0.078
		Sc. rank:	30.0

NP			
Pos.	158	obs:	exp:
ggt	G	1	151.90
ggc	G	0	146.40
gga	G	1133	528.70
ggg	G	12	319.00
---	--	------	------
mPD		0.022	0.68
		nPD:	0.03
		N. weight:	0.78
		Sc. PD:	-0.037
		Sc. rank:	89.6

NP			
Pos.	159	obs:	exp:
atg	M	1146	1146.00
---	--	------	------
mPD		0	0
		nPD:	1.
		N. weight:	0.
		Sc. PD:	0
		Sc. rank:	0

NP			
Pos.	160	obs:	exp:
gat	D	932	619.30
gac	D	214	526.70
---	--	------	------
mPD		0.30	0.50
		nPD:	0.61
		N. weight:	0.18
		Sc. PD:	0.093
		Sc. rank:	163.3

NP			
Pos.	161	obs:	exp:
cct	P	1	286.00
ccc	P	1143	205.90
cca	P	2	471.70
ccg	P	0	182.40
---	--	------	------
mPD		0.0052	0.71
		nPD:	0.01
		N. weight:	1.9
		Sc. PD:	-0.13
		Sc. rank:	-767.0

NP			
Pos.	162	obs:	exp:
cgt	R	1	49.22
cgc	R	0	56.76
cga	R	3	112.10
cgg	R	1052	95.62
aga	R	3	524.00
agg	R	87	308.20
---	--	------	------
mPD		0.16	0.96
		nPD:	0.16
		N. weight:	2.3
		Sc. PD:	0.18
		Sc. rank:	1370.6

NP			
Pos.	163	obs:	exp:
atg	M	1146	1146.00
---	--	------	------
mPD		0	0
		nPD:	1.
		N. weight:	0.
		Sc. PD:	0
		Sc. rank:	0

NP			
Pos.	164	obs:	exp:
tgt	C	1	484.70
tgc	C	1145	661.30
---	--	------	------
mPD		0.0017	0.49
		nPD:	0.
		N. weight:	0.59
		Sc. PD:	-0.045
		Sc. rank:	-356.0

NP			
Pos.	165	obs:	exp:
tct	S	1141	182.30
tcc	S	5	149.70
tca	S	0	290.90
tcg	S	0	65.03
agt	S	0	235.60
agc	S	0	222.60
---	--	------	------
mPD		0.0087	1.7
		nPD:	0.01
		N. weight:	2.
		Sc. PD:	-0.15
		Sc. rank:	-1012.2

NP			
Pos.	166	obs:	exp:
tta	L	0	101.70
ttg	L	2	213.40
ctt	L	4	190.80
ctc	L	0	191.20
cta	L	5	193.10
ctg	L	1135	255.70
---	--	------	------
mPD		0.019	1.1
		nPD:	0.02
		N. weight:	1.6
		Sc. PD:	-0.098
		Sc. rank:	-224.1

NP			
Pos.	167	obs:	exp:
atg	M	1146	1146.00
---	--	------	------
mPD		0	0
		nPD:	1.
		N. weight:	0.
		Sc. PD:	0
		Sc. rank:	0

NP			
Pos.	168	obs:	exp:
caa	Q	1144	583.70
cag	Q	2	562.30
---	--	------	------
mPD		0.0035	0.50
		nPD:	0.01
		N. weight:	0.72
		Sc. PD:	-0.052
		Sc. rank:	-337.4

NP			
Pos.	169	obs:	exp:
ggt	G	0	151.90
ggc	G	9	146.40
gga	G	836	528.70
ggg	G	301	319.00
---	--	------	------
mPD		0.40	0.68
		nPD:	0.59
		N. weight:	0.33
		Sc. PD:	0.16
		Sc. rank:	293.0

NP			
Pos.	170	obs:	exp:
tct	S	0	182.30
tcc	S	3	149.70
tca	S	1143	290.90
tcg	S	0	65.03
agt	S	0	235.60
agc	S	0	222.60
---	--	------	------
mPD		0.0052	1.7
		nPD:	0.
		N. weight:	1.5
		Sc. PD:	-0.11
		Sc. rank:	-968.8

NP

Pos.	171	obs:	exp:
act	T	1125	292.10
acc	T	21	235.20
aca	T	0	525.60
acg	T	0	93.08
---	--	------	------
mPD		0.036	0.68
		nPD:	0.05
		N. weight:	1.4
		Sc. PD:	-0.039
		Sc. rank:	422.5

NP

Pos.	172	obs:	exp:
tta	L	0	101.70
ttg	L	0	213.40
ctt	L	121	190.80
ctc	L	1022	191.20
cta	L	3	193.10
ctg	L	0	255.70
---	--	------	------
mPD		0.19	1.1
		nPD:	0.17
		N. weight:	1.6
		Sc. PD:	0.14
		Sc. rank:	972.0

NP

Pos.	173	obs:	exp:
cct	P	4	286.00
ccc	P	0	205.90
cca	P	26	471.70
ccg	P	1116	182.40
---	--	------	------
mPD		0.051	0.71
		nPD:	0.07
		N. weight:	1.8
		Sc. PD:	-0.018
		Sc. rank:	749.1

NP

Pos.	174	obs:	exp:
cgt	R	0	49.22
cgc	R	0	56.76
cga	R	0	112.10
cgg	R	0	95.62
aga	R	7	524.00
agg	R	1139	308.20
---	--	------	------
mPD		0.012	0.96
		nPD:	0.01
		N. weight:	1.4
		Sc. PD:	-0.093
		Sc. rank:	-343.4

NP

Pos.	175	obs:	exp:
cgt	R	0	49.22
cgc	R	0	56.76
cga	R	5	112.10
cgg	R	0	95.62
aga	R	1129	524.00
agg	R	12	308.20
---	--	------	------
mPD		0.029	0.96
		nPD:	0.03
		N. weight:	0.78
		Sc. PD:	-0.039
		Sc. rank:	63.7

NP

Pos.	176	obs:	exp:
tct	S	1140	182.30
tcc	S	3	149.70
tca	S	3	290.90
tcg	S	0	65.03
agt	S	0	235.60
agc	S	0	222.60
---	--	------	------
mPD		0.010	1.7
		nPD:	0.01
		N. weight:	2.
		Sc. PD:	-0.14
		Sc. rank:	-929.0

NP

Pos.	177	obs:	exp:
ggt	G	0	151.90
ggc	G	0	146.40
gga	G	1137	528.70
ggg	G	9	319.00
---	--	------	------
mPD		0.016	0.68
		nPD:	0.02
		N. weight:	0.8
		Sc. PD:	-0.046
		Sc. rank:	-13.5

NP

Pos.	178	obs:	exp:
gct	A	1137	295.90
gcc	A	8	213.40
gca	A	0	539.40
gcg	A	1	97.26
---	--	------	------
mPD		0.016	0.67
		nPD:	0.02
		N. weight:	1.4
		Sc. PD:	-0.081
		Sc. rank:	-9.8

NP

Pos.	179	obs:	exp:
gct	A	0	295.90
gcc	A	0	213.40
gca	A	1142	539.40
gcg	A	4	97.26
---	--	------	------
mPD		0.0070	0.67
		nPD:	0.01
		N. weight:	0.81
		Sc. PD:	-0.056
		Sc. rank:	-275.6

NP

Pos.	180	obs:	exp:
ggt	G	858	151.90
ggc	G	0	146.40
gga	G	288	528.70
ggg	G	0	319.00
---	--	------	------
mPD		0.38	0.68
		nPD:	0.56
		N. weight:	1.3
		Sc. PD:	0.57
		Sc. rank:	1105.0

NP

Pos.	181	obs:	exp:
tct	S	0	0.32
tcc	S	0	0.26
tca	S	2	0.51
tcg	S	0	0.11
agt	S	0	0.41
agc	S	0	0.39
gct	A	1	295.40
gcc	A	0	213.00
gca	A	1143	538.40
gcg	A	0	97.09
---	--	------	------
mPD		0.0052	0.67
		nPD:	0.01
		N. weight:	0.82
		Sc. PD:	-0.059
		Sc. rank:	-324.1

NP

Pos.	182	obs:	exp:
tct	S	0	3.50
tcc	S	0	2.87
tca	S	22	5.58
tcg	S	0	1.25
agt	S	0	4.52
agc	S	0	4.27
gct	A	1	290.30
gcc	A	0	209.30
gca	A	1123	529.00
gcg	A	0	95.40
---	--	------	------
mPD		0.039	0.72
		nPD:	0.05
		N. weight:	0.83
		Sc. PD:	-0.022
		Sc. rank:	258.1

NP

Pos.	183	obs:	exp:
gtt	V	0	237.50
gtc	V	0	231.30
gta	V	557	234.70
gtg	V	589	442.60
---	--	------	------
mPD		0.50	0.73
		nPD:	0.69
		N. weight:	0.62
		Sc. PD:	0.36
		Sc. rank:	566.9

NP

Pos.	184	obs:	exp:
aaa	K	14	667.90
aag	K	1132	478.10
---	--	------	------
mPD		0.024	0.49
		nPD:	0.05
		N. weight:	0.88
		Sc. PD:	-0.028
		Sc. rank:	252.4

NP

Pos.	185	obs:	exp:
ggt	G	0	151.90
ggc	G	0	146.40
gga	G	368	528.70
ggg	G	778	319.00
---	--	------	------
mPD		0.44	0.68
		nPD:	0.65
		N. weight:	0.54
		Sc. PD:	0.29
		Sc. rank:	483.2

NP

Pos.	186	obs:	exp:
att	I	0	420.70
atc	I	0	274.40
ata	I	1145	449.90
gtt	V	0	0.21
gtc	V	1	0.20
gta	V	0	0.20
gtg	V	0	0.39
---	--	------	------
mPD		0.0035	0.66
		nPD:	0.01
		N. weight:	1.
		Sc. PD:	-0.075
		Sc. rank:	-514.3

NP

Pos.	187	obs:	exp:
ggt	G	0	151.90
ggc	G	0	146.40
gga	G	156	528.70
ggg	G	990	319.00
---	--	------	------
mPD		0.24	0.68
		nPD:	0.35
		N. weight:	0.89
		Sc. PD:	0.23
		Sc. rank:	716.9

NP

Pos.	188	obs:	exp:
act	T	100	292.10
acc	T	0	235.20
aca	T	984	525.60
acg	T	62	93.08
---	--	------	------
mPD		0.25	0.68
		nPD:	0.37
		N. weight:	0.47
		Sc. PD:	0.13
		Sc. rank:	384.5

NP

Pos.	189	obs:	exp:
tta	L	0	0.09
ttg	L	0	0.19
ctt	L	0	0.17
ctc	L	0	0.17
cta	L	0	0.17
ctg	L	1	0.22
atg	M	1145	1145.00
---	--	------	------
mPD		0.0017	0.0028
		nPD:	0.63
		N. weight:	0.0014
		Sc. PD:	0.00075
		Sc. rank:	1.3

NP

Pos.	190	obs:	exp:
gtt	V	1	237.50
gtc	V	0	231.30
gta	V	7	234.70
gtg	V	1138	442.60
---	--	------	------
mPD		0.014	0.73
		nPD:	0.02
		N. weight:	1.
		Sc. PD:	-0.061
		Sc. rank:	-65.1

NP

Pos.	191	obs:	exp:
atg	M	1145	1145.00
gtt	V	0	0.21
gtc	V	0	0.20
gta	V	0	0.20
gtg	V	1	0.39
---	--	------	------
mPD		0.0017	0.0028
		nPD:	0.62
		N. weight:	0.00091
		Sc. PD:	0.00047
		Sc. rank:	0.8

NP

Pos.	192	obs:	exp:
gaa	E	1049	697.30
gag	E	97	448.70
---	--	------	------
mPD		0.16	0.48
		nPD:	0.33
		N. weight:	0.27
		Sc. PD:	0.063
		Sc. rank:	207.8

NP

Pos.	193	obs:	exp:
tta	L	0	101.70
ttg	L	4	213.40
ctt	L	0	190.80
ctc	L	0	191.20
cta	L	9	193.10
ctg	L	1133	255.70
---	--	------	------
mPD		0.023	1.1
		nPD:	0.02
		N. weight:	1.6
		Sc. PD:	-0.093
		Sc. rank:	-82.6

NP

Pos.	194	obs:	exp:
att	I	831	314.90
atc	I	26	205.40
ata	I	0	336.70
gtt	V	285	59.88
gtc	V	3	58.33
gta	V	1	59.17
gtg	V	0	111.60
---	--	------	------
mPD		0.43	1.1
		nPD:	0.39
		N. weight:	1.1
		Sc. PD:	0.34
		Sc. rank:	939.4

NP

Pos.	195	obs:	exp:
cgt	R	0	49.22
cgc	R	0	56.76
cga	R	10	112.10
cgg	R	1038	95.62
aga	R	0	524.00
agg	R	98	308.20
---	--	------	------
mPD		0.17	0.96
		nPD:	0.18
		N. weight:	2.2
		Sc. PD:	0.21
		Sc. rank:	1424.7

NP

Pos.	196	obs:	exp:
atg	M	1146	1146.00
---	--	------	------
mPD		0	0
		nPD:	1.
		N. weight:	0.
		Sc. PD:	0
		Sc. rank:	0

NP

Pos.	197	obs:	exp:
att	I	0	421.10
atc	I	0	274.60
ata	I	1146	450.30
---	--	------	------
mPD		0	0.65
		nPD:	0.
		N. weight:	1.
		Sc. PD:	-0.081
		Sc. rank:	-920.4

NP

Pos.	198	obs:	exp:
aaa	K	1127	667.90
aag	K	19	478.10
---	--	------	------
mPD		0.033	0.49
		nPD:	0.07
		N. weight:	0.51
		Sc. PD:	-0.0073
		Sc. rank:	189.7

NP

Pos.	199	obs:	exp:
cgt	R	1	49.22
cgc	R	0	56.76
cga	R	1096	112.10
cgg	R	4	95.62
aga	R	45	524.00
agg	R	0	308.20
---	--	------	------
mPD		0.084	0.96
		nPD:	0.09
		N. weight:	2.3
		Sc. PD:	0.012
		Sc. rank:	1061.1

NP

Pos.	200	obs:	exp:
ggt	G	0	151.90
ggc	G	0	146.40
gga	G	215	528.70
ggg	G	931	319.00
---	--	------	------
mPD		0.31	0.68
		nPD:	0.45
		N. weight:	0.77
		Sc. PD:	0.27
		Sc. rank:	659.8

NP			
Pos.	201	obs:	exp:
att	I	1	419.60
atc	I	1054	273.70
ata	I	87	448.70
gtt	V	0	0.83
gtc	V	3	0.81
gta	V	1	0.82
gtg	V	0	1.54
---	--	------	------
mPD		0.15	0.66
		nPD:	0.23
		N. weight:	1.2
		Sc. PD:	0.17
		Sc. rank:	856.5

NP			
Pos.	202	obs:	exp:
aat	N	76	583.70
aac	N	1070	562.30
---	--	------	------
mPD		0.12	0.50
		nPD:	0.25
		N. weight:	0.51
		Sc. PD:	0.082
		Sc. rank:	365.4

NP			
Pos.	203	obs:	exp:
gat	D	47	619.30
gac	D	1099	526.70
---	--	------	------
mPD		0.079	0.50
		nPD:	0.16
		N. weight:	0.66
		Sc. PD:	0.048
		Sc. rank:	390.3

NP			
Pos.	204	obs:	exp:
cgt	R	9	49.22
cgc	R	0	56.76
cga	R	1	112.10
cgg	R	1130	95.62
aga	R	0	524.00
agg	R	6	308.20
---	--	------	------
mPD		0.028	0.96
		nPD:	0.03
		N. weight:	2.6
		Sc. PD:	-0.13
		Sc. rank:	159.6

NP			
Pos.	205	obs:	exp:
aat	N	1125	583.70
aac	N	21	562.30
---	--	------	------
mPD		0.036	0.50
		nPD:	0.07
		N. weight:	0.64
		Sc. PD:	-0.0063
		Sc. rank:	261.7

NP			
Pos.	206	obs:	exp:
ttt	F	7	452.50
ttc	F	1139	693.50
---	--	------	------
mPD		0.012	0.48
		nPD:	0.03
		N. weight:	0.51
		Sc. PD:	-0.028
		Sc. rank:	6.5

NP			
Pos.	207	obs:	exp:
tgg	W	1146	1146.00
---	--	------	------
mPD		0	0
		nPD:	1.
		N. weight:	0.
		Sc. PD:	0
		Sc. rank:	0

NP			
Pos.	208	obs:	exp:
cgt	R	0	49.22
cgc	R	0	56.76
cga	R	0	112.10
cgg	R	0	95.62
aga	R	1141	524.00
agg	R	5	308.20
---	--	------	------
mPD		0.0087	0.96
		nPD:	0.01
		N. weight:	0.83
		Sc. PD:	-0.058
		Sc. rank:	-320.6

NP			
Pos.	209	obs:	exp:
ggt	G	25	151.90
ggc	G	1114	146.40
gga	G	7	528.70
ggg	G	0	319.00
---	--	------	------
mPD		0.055	0.68
		nPD:	0.08
		N. weight:	2.1
		Sc. PD:	-0.0028
		Sc. rank:	945.2

NP			
Pos.	210	obs:	exp:
gat	D	0	0.54
gac	D	1	0.46
gaa	E	1144	696.70
gag	E	1	448.30
---	--	------	------
mPD		0.0035	0.48
		nPD:	0.01
		N. weight:	0.54
		Sc. PD:	-0.039
		Sc. rank:	-236.9

NP			
Pos.	211	obs:	exp:
tct	S	0	0.16
tcc	S	0	0.13
tca	S	0	0.25
tcg	S	0	0.06
aat	N	1139	583.20
aac	N	6	561.80
agt	S	1	0.21
agc	S	0	0.19
---	--	------	------
mPD		0.012	0.50
		nPD:	0.02
		N. weight:	0.7
		Sc. PD:	-0.039
		Sc. rank:	-1.0

NP			
Pos.	212	obs:	exp:
ggt	G	0	151.90
ggc	G	1	146.40
gga	G	1142	528.70
ggg	G	3	319.00
---	--	------	------
mPD		0.0070	0.68
		nPD:	0.01
		N. weight:	0.82
		Sc. PD:	-0.057
		Sc. rank:	-291.2

NP			
Pos.	213	obs:	exp:
cgt	R	0	49.22
cgc	R	0	56.76
cga	R	0	112.10
cgg	R	1	95.62
aga	R	1141	524.00
agg	R	4	308.20
---	--	------	------
mPD		0.010	0.96
		nPD:	0.01
		N. weight:	0.83
		Sc. PD:	-0.057
		Sc. rank:	-238.5

NP			
Pos.	214	obs:	exp:
cgt	R	0	49.13
cgc	R	0	56.66
cga	R	4	112.00
cgg	R	0	95.46
aaa	K	0	1.17
aag	K	2	0.83
aga	R	262	523.10
agg	R	878	307.70
---	--	------	------
mPD		0.37	0.96
		nPD:	0.38
		N. weight:	0.7
		Sc. PD:	0.2
		Sc. rank:	576.1

NP			
Pos.	215	obs:	exp:
tct	S	0	0.16
tcc	S	0	0.13
tca	S	0	0.25
tcg	S	1	0.06
act	T	0	291.60
acc	T	0	234.70
aca	T	1138	524.70
acg	T	6	92.92
agt	S	0	0.21
agc	S	0	0.19
gct	A	0	0.26
gcc	A	0	0.19
gca	A	1	0.47
gcg	A	0	0.08
---	--	------	------
mPD		0.016	0.68
		nPD:	0.02
		N. weight:	0.83
		Sc. PD:	-0.047
		Sc. rank:	-16.4

NP			
Pos.	216	obs:	exp:
cgt	R	0	49.18
cgc	R	0	56.71
cga	R	0	112.00
cgg	R	0	95.54
aaa	K	1	0.58
aag	K	0	0.42
aga	R	997	523.60
agg	R	148	308.00
---	--	------	------
mPD		0.23	0.96
		nPD:	0.24
		N. weight:	0.51
		Sc. PD:	0.076
		Sc. rank:	361.3

NP			
Pos.	217	obs:	exp:
att	I	1052	388.40
atc	I	5	253.30
ata	I	0	415.30
gtt	V	89	18.44
gtc	V	0	17.96
gta	V	0	18.22
gtg	V	0	34.37
---	--	------	------
mPD		0.15	0.82
		nPD:	0.19
		N. weight:	1.1
		Sc. PD:	0.11
		Sc. rank:	718.5

NP			
Pos.	218	obs:	exp:
gct	A	1	295.90
gcc	A	0	213.40
gca	A	1144	539.40
gcg	A	1	97.26
---	--	------	------
mPD		0.0035	0.67
		nPD:	0.01
		N. weight:	0.81
		Sc. PD:	-0.06
		Sc. rank:	-420.9

NP			
Pos.	219	obs:	exp:
tat	Y	1014	619.00
tac	Y	132	527.00
---	--	------	------
mPD		0.20	0.50
		nPD:	0.41
		N. weight:	0.31
		Sc. PD:	0.097
		Sc. rank:	254.0

NP			
Pos.	220	obs:	exp:
gaa	E	8	697.30
gag	E	1138	448.70
---	--	------	------
mPD		0.014	0.48
		nPD:	0.03
		N. weight:	0.98
		Sc. PD:	-0.05
		Sc. rank:	70.9

NP			
Pos.	221	obs:	exp:
cgt	R	0	49.22
cgc	R	0	56.76
cga	R	5	112.10
cgg	R	0	95.62
aga	R	914	524.00
agg	R	227	308.20
---	--	------	------
mPD		0.33	0.96
		nPD:	0.34
		N. weight:	0.41
		Sc. PD:	0.1
		Sc. rank:	323.1

NP			
Pos.	222	obs:	exp:
atg	M	1146	1146.00
---	--	------	------
mPD		0	0
		nPD:	1.
		N. weight:	0.
		Sc. PD:	0
		Sc. rank:	0

NP			
Pos.	223	obs:	exp:
tgt	C	4	484.70
tgc	C	1142	661.30
---	--	------	------
mPD		0.0070	0.49
		nPD:	0.01
		N. weight:	0.58
		Sc. PD:	-0.038
		Sc. rank:	-114.4

NP			
Pos.	224	obs:	exp:
aat	N	14	583.70
aac	N	1132	562.30
---	--	------	------
mPD		0.024	0.50
		nPD:	0.05
		N. weight:	0.71
		Sc. PD:	-0.023
		Sc. rank:	185.6

NP			
Pos.	225	obs:	exp:
tta	L	0	0.09
ttg	L	0	0.19
ctt	L	0	0.17
ctc	L	1	0.17
cta	L	0	0.17
ctg	L	0	0.22
att	I	10	420.70
atc	I	1135	274.40
ata	I	0	449.90
---	--	------	------
mPD		0.019	0.66
		nPD:	0.03
		N. weight:	1.5
		Sc. PD:	-0.077
		Sc. rank:	105.1

NP			
Pos.	226	obs:	exp:
tta	L	0	101.70
ttg	L	0	213.40
ctt	L	6	190.80
ctc	L	1140	191.20
cta	L	0	193.10
ctg	L	0	255.70
---	--	------	------
mPD		0.010	1.1
		nPD:	0.01
		N. weight:	1.9
		Sc. PD:	-0.13
		Sc. rank:	-724.7

,

NP			
Pos.	227	obs:	exp:
aaa	K	1141	667.90
aag	K	5	478.10
---	--	------	------
mPD		0.0087	0.49
		nPD:	0.02
		N. weight:	0.56
		Sc. PD:	-0.035
		Sc. rank:	-66.0

,

NP			
Pos.	228	obs:	exp:
ggt	G	1	151.90
ggc	G	0	146.40
gga	G	8	528.70
ggg	G	1137	319.00
---	--	------	------
mPD		0.016	0.68
		nPD:	0.02
		N. weight:	1.3
		Sc. PD:	-0.076
		Sc. rank:	-16.2

,

NP			
Pos.	229	obs:	exp:
aaa	K	1139	667.90
aag	K	7	478.10
---	--	------	------
mPD		0.012	0.49
		nPD:	0.02
		N. weight:	0.55
		Sc. PD:	-0.03
		Sc. rank:	1.9

,

NP			
Pos.	230	obs:	exp:
ttt	F	3	452.50
ttc	F	1143	693.50
---	--	------	------
mPD		0.0052	0.48
		nPD:	0.01
		N. weight:	0.53
		Sc. PD:	-0.036
		Sc. rank:	-151.7

,

NP			
Pos.	231	obs:	exp:
caa	Q	1141	583.70
cag	Q	5	562.30
---	--	------	------
mPD		0.0087	0.50
		nPD:	0.02
		N. weight:	0.71
		Sc. PD:	-0.044
		Sc. rank:	-89.8

,

NP			
Pos.	232	obs:	exp:
act	T	3	292.10
acc	T	0	235.20
aca	T	1142	525.60
acg	T	1	93.08
---	--	------	------
mPD		0.0070	0.68
		nPD:	0.01
		N. weight:	0.83
		Sc. PD:	-0.057
		Sc. rank:	-296.2

,

NP			
Pos.	233	obs:	exp:
tct	S	0	0.80
tcc	S	0	0.65
tca	S	5	1.27
tcg	S	0	0.28
agt	S	0	1.03
agc	S	0	0.97
gct	A	5	294.60
gcc	A	0	212.50
gca	A	1129	537.00
gcg	A	7	96.84
---	--	------	------
mPD		0.029	0.68
		nPD:	0.04
		N. weight:	0.77
		Sc. PD:	-0.029
		Sc. rank:	172.9

,

NP			
Pos.	234	obs:	exp:
gct	A	0	295.90
gcc	A	0	213.40
gca	A	1146	539.40
gcg	A	0	97.26
---	--	------	------
mPD		0	0.67
		nPD:	0.
		N. weight:	0.82
		Sc. PD:	-0.065
		Sc. rank:	-742.5

,

NP			
Pos.	235	obs:	exp:
caa	Q	1146	583.70
cag	Q	0	562.30
---	--	------	------
mPD		0	0.50
		nPD:	0.
		N. weight:	0.74
		Sc. PD:	-0.058
		Sc. rank:	-664.7

,

NP			
Pos.	236	obs:	exp:
cgt	R	0	49.22
cgc	R	0	56.76
cga	R	0	112.10
cgg	R	0	95.62
aga	R	188	524.00
agg	R	958	308.20
---	--	------	------
mPD		0.27	0.96
		nPD:	0.29
		N. weight:	0.85
		Sc. PD:	0.17
		Sc. rank:	629.3

,

NP			
Pos.	237	obs:	exp:
act	T	0	0.25
acc	T	0	0.21
aca	T	1	0.46
acg	T	0	0.08
gct	A	0	295.70
gcc	A	1	213.20
gca	A	1095	538.90
gcg	A	49	97.18
---	--	------	------
mPD		0.085	0.67
		nPD:	0.13
		N. weight:	0.71
		Sc. PD:	0.031
		Sc. rank:	392.8

,

NP			
Pos.	238	obs:	exp:
atg	M	1145	1145.00
act	T	0	0.25
acc	T	0	0.21
aca	T	0	0.46
acg	T	1	0.08
---	--	------	------
mPD		0.0017	0.0033
		nPD:	0.52
		N. weight:	0.0024
		Sc. PD:	0.001
		Sc. rank:	2.1

,

NP			
Pos.	239	obs:	exp:
atg	M	978	978.00
gtt	V	0	34.81
gtc	V	0	33.91
gta	V	0	34.40
gtg	V	168	64.88
---	--	------	------
mPD		0.25	0.42
		nPD:	0.6
		N. weight:	0.15
		Sc. PD:	0.075
		Sc. rank:	136.0

,

NP			
Pos.	240	obs:	exp:
gat	D	1141	619.30
gac	D	5	526.70
---	--	------	------
mPD		0.0087	0.50
		nPD:	0.02
		N. weight:	0.64
		Sc. PD:	-0.04
		Sc. rank:	-78.2

,

NP			
Pos.	241	obs:	exp:
caa	Q	1144	583.70
cag	Q	2	562.30
---	--	------	------
mPD		0.0035	0.50
		nPD:	0.01
		N. weight:	0.72
		Sc. PD:	-0.052
		Sc. rank:	-337.4

,

NP			
Pos.	242	obs:	exp:
gtt	V	0	237.50
gtc	V	1	231.30
gta	V	31	234.70
gtg	V	1114	442.60
---	--	------	------
mPD		0.054	0.73
		nPD:	0.07
		N. weight:	0.92
		Sc. PD:	-0.0064
		Sc. rank:	387.4

,

NP			
Pos.	243	obs:	exp:
cgt	R	8	49.22
cgc	R	0	56.76
cga	R	1123	112.10
cgg	R	7	95.62
aga	R	8	524.00
agg	R	0	308.20
---	--	------	------
mPD		0.040	0.96
		nPD:	0.04
		N. weight:	2.4
		Sc. PD:	-0.094
		Sc. rank:	511.6

,

NP			
Pos.	244	obs:	exp:
gaa	E	134	697.30
gag	E	1012	448.70
---	--	------	------
mPD		0.21	0.48
		nPD:	0.43
		N. weight:	0.58
		Sc. PD:	0.2
		Sc. rank:	487.0

,

NP			
Pos.	245	obs:	exp:
tct	S	0	182.30
tcc	S	0	149.70
tca	S	0	290.90
tcg	S	0	65.03
agt	S	1	235.60
agc	S	1145	222.60
---	--	------	------
mPD		0.0017	1.7
		nPD:	0.
		N. weight:	1.8
		Sc. PD:	-0.14
		Sc. rank:	-1364.9

,

NP			
Pos.	246	obs:	exp:
cgt	R	0	48.36
cgc	R	0	55.77
cga	R	0	110.20
cgg	R	0	93.96
aaa	K	20	11.66
aag	K	0	8.34
aga	R	1124	514.90
agg	R	2	302.90
---	--	------	------
mPD		0.038	0.99
		nPD:	0.04
		N. weight:	0.84
		Sc. PD:	-0.035
		Sc. rank:	146.1

,

NP			
Pos.	247	obs:	exp:
tct	S	0	0.48
tcc	S	0	0.39
tca	S	0	0.76
tcg	S	0	0.17
aat	N	1001	582.20
aac	N	142	560.80
agt	S	2	0.62
agc	S	1	0.58
---	--	------	------
mPD		0.22	0.51
		nPD:	0.44
		N. weight:	0.34
		Sc. PD:	0.12
		Sc. rank:	288.6

,

NP			
Pos.	248	obs:	exp:
cct	P	1131	286.00
ccc	P	10	205.90
cca	P	1	471.70
ccg	P	4	182.40
---	--	------	------
mPD		0.026	0.71
		nPD:	0.04
		N. weight:	1.4
		Sc. PD:	-0.063
		Sc. rank:	209.2

,

NP			
Pos.	249	obs:	exp:
ggt	G	0	151.90
ggc	G	0	146.40
gga	G	93	528.70
ggg	G	1053	319.00
---	--	------	------
mPD		0.15	0.68
		nPD:	0.22
		N. weight:	1.
		Sc. PD:	0.14
		Sc. rank:	720.4

,

NP			
Pos.	250	obs:	exp:
aat	N	1146	583.70
aac	N	0	562.30
---	--	------	------
mPD		0	0.50
		nPD:	0.
		N. weight:	0.74
		Sc. PD:	-0.058
		Sc. rank:	-664.7

,

NP			
Pos.	251	obs:	exp:
gct	A	1140	295.90
gcc	A	6	213.40
gca	A	0	539.40
gcg	A	0	97.26
---	--	------	------
mPD		0.010	0.67
		nPD:	0.02
		N. weight:	1.4
		Sc. PD:	-0.093
		Sc. rank:	-254.6

NP			
Pos.	252	obs:	exp:
gaa	E	1143	697.30
gag	E	3	448.70
---	--	------	------
mPD		0.0052	0.48
		nPD:	0.01
		N. weight:	0.52
		Sc. PD:	-0.036
		Sc. rank:	-147.0

NP			
Pos.	253	obs:	exp:
att	I	4	420.70
atc	I	1	274.40
ata	I	1140	449.90
gtt	V	0	0.21
gtc	V	0	0.20
gta	V	1	0.20
gtg	V	0	0.39
---	--	------	------
mPD		0.010	0.66
		nPD:	0.02
		N. weight:	0.99
		Sc. PD:	-0.063
		Sc. rank:	-165.6

NP			
Pos.	254	obs:	exp:
gaa	E	1145	697.30
gag	E	1	448.70
---	--	------	------
mPD		0.0017	0.48
		nPD:	0.
		N. weight:	0.54
		Sc. PD:	-0.041
		Sc. rank:	-304.3

NP			
Pos.	255	obs:	exp:
gat	D	1140	619.30
gac	D	6	526.70
---	--	------	------
mPD		0.010	0.50
		nPD:	0.02
		N. weight:	0.64
		Sc. PD:	-0.038
		Sc. rank:	-25.2

NP			
Pos.	256	obs:	exp:
tta	L	0	101.70
ttg	L	0	213.40
ctt	L	420	190.80
ctc	L	724	191.20
cta	L	2	193.10
ctg	L	0	255.70
---	--	------	------
mPD		0.47	1.1
		nPD:	0.42
		N. weight:	1.2
		Sc. PD:	0.4
		Sc. rank:	1026.6

NP			
Pos.	257	obs:	exp:
att	I	1134	421.10
atc	I	12	274.60
ata	I	0	450.30
---	--	------	------
mPD		0.021	0.65
		nPD:	0.03
		N. weight:	1.
		Sc. PD:	-0.05
		Sc. rank:	97.2

NP			
Pos.	258	obs:	exp:
ttt	F	1131	452.50
ttc	F	15	693.50
---	--	------	------
mPD		0.026	0.48
		nPD:	0.05
		N. weight:	0.93
		Sc. PD:	-0.025
		Sc. rank:	287.9

NP			
Pos.	259	obs:	exp:
tta	L	0	101.70
ttg	L	4	213.40
ctt	L	2	190.80
ctc	L	0	191.20
cta	L	12	193.10
ctg	L	1128	255.70
---	--	------	------
mPD		0.031	1.1
		nPD:	0.03
		N. weight:	1.5
		Sc. PD:	-0.08
		Sc. rank:	57.4

NP			
Pos.	260	obs:	exp:
act	T	0	0.25
acc	T	0	0.21
aca	T	1	0.46
acg	T	0	0.08
gct	A	0	295.70
gcc	A	0	213.20
gca	A	1144	538.90
gcg	A	1	97.18
---	--	------	------
mPD		0.0035	0.67
		nPD:	0.01
		N. weight:	0.82
		Sc. PD:	-0.061
		Sc. rank:	-426.5

NP			
Pos.	261	obs:	exp:
cgt	R	0	49.22
cgc	R	0	56.76
cga	R	0	112.10
cgg	R	0	95.62
aga	R	6	524.00
agg	R	1140	308.20
---	--	------	------
mPD		0.010	0.96
		nPD:	0.01
		N. weight:	1.4
		Sc. PD:	-0.096
		Sc. rank:	-412.4

NP			
Pos.	262	obs:	exp:
tct	S	1143	182.30
tcc	S	2	149.70
tca	S	1	290.90
tcg	S	0	65.03
agt	S	0	235.60
agc	S	0	222.60
---	--	------	------
mPD		0.0052	1.7
		nPD:	0.
		N. weight:	2.
		Sc. PD:	-0.15
		Sc. rank:	-1274.7

NP			
Pos.	263	obs:	exp:
gct	A	0	295.90
gcc	A	0	213.40
gca	A	1141	539.40
gcg	A	5	97.26
---	--	------	------
mPD		0.0087	0.67
		nPD:	0.01
		N. weight:	0.8
		Sc. PD:	-0.053
		Sc. rank:	-177.8

NP			
Pos.	264	obs:	exp:
tta	L	0	101.70
ttg	L	0	213.40
ctt	L	0	190.80
ctc	L	1145	191.20
cta	L	1	193.10
ctg	L	0	255.70
---	--	------	------
mPD		0.0017	1.1
		nPD:	0.
		N. weight:	1.9
		Sc. PD:	-0.15
		Sc. rank:	-1478.0

NP			
Pos.	265	obs:	exp:
att	I	8	417.40
atc	I	1127	272.20
ata	I	1	446.40
gtt	V	0	2.07
gtc	V	10	2.02
gta	V	0	2.05
gtg	V	0	3.86
---	--	------	------
mPD		0.033	0.67
		nPD:	0.05
		N. weight:	1.5
		Sc. PD:	-0.048
		Sc. rank:	419.2

NP			
Pos.	266	obs:	exp:
tta	L	1	101.70
ttg	L	7	213.40
ctt	L	18	190.80
ctc	L	0	191.20
cta	L	4	193.10
ctg	L	1116	255.70
---	--	------	------
mPD		0.053	1.1
		nPD:	0.05
		N. weight:	1.5
		Sc. PD:	-0.049
		Sc. rank:	387.5

NP			
Pos.	267	obs:	exp:
cgt	R	0	49.22
cgc	R	0	56.76
cga	R	0	112.10
cgg	R	0	95.62
aga	R	1092	524.00
agg	R	54	308.20
---	--	------	------
mPD		0.090	0.96
		nPD:	0.09
		N. weight:	0.68
		Sc. PD:	0.0074
		Sc. rank:	331.4

NP			
Pos.	268	obs:	exp:
ggt	G	4	151.90
ggc	G	1	146.40
gga	G	1134	528.70
ggg	G	7	319.00
---	--	------	------
mPD		0.021	0.68
		nPD:	0.03
		N. weight:	0.78
		Sc. PD:	-0.039
		Sc. rank:	66.5

NP			
Pos.	269	obs:	exp:
tct	S	86	182.30
tcc	S	0	149.70
tca	S	1056	290.90
tcg	S	4	65.03
agt	S	0	235.60
agc	S	0	222.60
---	--	------	------
mPD		0.15	1.7
		nPD:	0.09
		N. weight:	1.2
		Sc. PD:	0.0068
		Sc. rank:	581.7

NP			
Pos.	270	obs:	exp:
gtt	V	0	237.50
gtc	V	0	231.30
gta	V	24	234.70
gtg	V	1122	442.60
---	--	------	------
mPD		0.041	0.73
		nPD:	0.06
		N. weight:	0.94
		Sc. PD:	-0.023
		Sc. rank:	305.0

NP			
Pos.	271	obs:	exp:
gct	A	1056	295.70
gcc	A	89	213.20
gca	A	0	538.90
gcg	A	0	97.18
ggt	G	0	0.13
ggc	G	1	0.13
gga	G	0	0.46
ggg	G	0	0.28
---	--	------	------
mPD		0.15	0.67
		nPD:	0.22
		N. weight:	1.2
		Sc. PD:	0.16
		Sc. rank:	829.0

NP			
Pos.	272	obs:	exp:
cat	H	1130	658.10
cac	H	16	487.90
---	--	------	------
mPD		0.028	0.49
		nPD:	0.06
		N. weight:	0.53
		Sc. PD:	-0.013
		Sc. rank:	170.0

NP			
Pos.	273	obs:	exp:
aaa	K	936	667.90
aag	K	210	478.10
---	--	------	------
mPD		0.30	0.49
		nPD:	0.62
		N. weight:	0.14
		Sc. PD:	0.072
		Sc. rank:	124.9

NP			
Pos.	274	obs:	exp:
tct	S	6	182.30
tcc	S	1139	149.70
tca	S	1	290.90
tcg	S	0	65.03
agt	S	0	235.60
agc	S	0	222.60
---	--	------	------
mPD		0.012	1.7
		nPD:	0.01
		N. weight:	2.2
		Sc. PD:	-0.16
		Sc. rank:	-941.6

NP			
Pos.	275	obs:	exp:
tgt	C	7	484.70
tgc	C	1139	661.30
---	--	------	------
mPD		0.012	0.49
		nPD:	0.02
		N. weight:	0.56
		Sc. PD:	-0.031
		Sc. rank:	0.4

NP			
Pos.	276	obs:	exp:
ttt	F	1	0.39
ttc	F	0	0.61
tta	L	3	101.60
ttg	L	1124	213.20
ctt	L	0	190.70
ctc	L	0	191.10
cta	L	2	193.00
ctg	L	16	255.50
---	--	------	------
mPD		0.041	1.1
		nPD:	0.04
		N. weight:	1.7
		Sc. PD:	-0.075
		Sc. rank:	286.5

,

NP			
Pos.	277	obs:	exp:
cct	P	808	286.00
ccc	P	278	205.90
cca	P	60	471.70
ccg	P	0	182.40
---	--	------	------
mPD		0.44	0.71
		nPD:	0.62
		N. weight:	0.77
		Sc. PD:	0.4
		Sc. rank:	688.7

,

NP			
Pos.	278	obs:	exp:
gct	A	1019	295.90
gcc	A	127	213.40
gca	A	0	539.40
gcg	A	0	97.26
---	--	------	------
mPD		0.20	0.67
		nPD:	0.29
		N. weight:	1.1
		Sc. PD:	0.23
		Sc. rank:	847.8

,

NP			
Pos.	279	obs:	exp:
tgt	C	1144	484.70
tgc	C	2	661.30
---	--	------	------
mPD		0.0035	0.49
		nPD:	0.01
		N. weight:	0.93
		Sc. PD:	-0.067
		Sc. rank:	-420.8

,

NP			
Pos.	280	obs:	exp:
atg	M	3	3.00
gtt	V	0	234.30
gtc	V	0	228.30
gta	V	136	231.60
gtg	V	995	436.80
gct	A	0	3.10
gcc	A	0	2.23
gca	A	2	5.65
gcg	A	10	1.02
---	--	------	------
mPD		0.24	0.75
		nPD:	0.32
		N. weight:	0.74
		Sc. PD:	0.17
		Sc. rank:	565.1

,

NP			
Pos.	281	obs:	exp:
tat	Y	3	619.00
tac	Y	1143	527.00
---	--	------	------
mPD		0.0052	0.50
		nPD:	0.01
		N. weight:	0.83
		Sc. PD:	-0.057
		Sc. rank:	-262.7

,

NP			
Pos.	282	obs:	exp:
ggt	G	0	151.90
ggc	G	0	146.40
gga	G	1101	528.70
ggg	G	45	319.00
---	--	------	------
mPD		0.076	0.68
		nPD:	0.11
		N. weight:	0.69
		Sc. PD:	0.02
		Sc. rank:	359.3

,

NP			
Pos.	283	obs:	exp:
ttt	F	2	0.79
ttc	F	0	1.21
tta	L	0	101.50
ttg	L	3	213.00
ctt	L	1137	190.50
ctc	L	1	190.90
cta	L	2	192.80
ctg	L	1	255.30
---	--	------	------
mPD		0.021	1.1
		nPD:	0.02
		N. weight:	1.9
		Sc. PD:	-0.12
		Sc. rank:	-150.8

,

NP			
Pos.	284	obs:	exp:
act	T	0	5.86
acc	T	0	4.72
aca	T	23	10.55
acg	T	0	1.87
gct	A	2	290.00
gcc	A	0	209.10
gca	A	1116	528.60
gcg	A	5	95.31
---	--	------	------
mPD		0.052	0.71
		nPD:	0.07
		N. weight:	0.79
		Sc. PD:	-0.0073
		Sc. rank:	328.8

,

NP			
Pos.	285	obs:	exp:
atg	M	1	1.00
gtt	V	0	237.20
gtc	V	0	231.10
gta	V	4	234.40
gtg	V	1141	442.20
---	--	------	------
mPD		0.0087	0.73
		nPD:	0.01
		N. weight:	1.
		Sc. PD:	-0.069
		Sc. rank:	-261.9

,

NP			
Pos.	286	obs:	exp:
act	T	1	0.25
acc	T	0	0.21
aca	T	0	0.46
acg	T	0	0.08
gct	A	905	295.70
gcc	A	239	213.20
gca	A	0	538.90
gcg	A	1	97.18
---	--	------	------
mPD		0.33	0.67
		nPD:	0.5
		N. weight:	0.99
		Sc. PD:	0.4
		Sc. rank:	867.4

,

NP			
Pos.	287	obs:	exp:
tct	S	0	182.30
tcc	S	0	149.70
tca	S	0	290.90
tcg	S	0	65.03
agt	S	1143	235.60
agc	S	3	222.60
---	--	------	------
mPD		0.0052	1.7
		nPD:	0.
		N. weight:	1.7
		Sc. PD:	-0.13
		Sc. rank:	-1118.2

,

NP			
Pos.	288	obs:	exp:
ggt	G	0	151.90
ggc	G	0	146.40
gga	G	1141	528.70
ggg	G	5	319.00
---	--	------	------
mPD		0.0087	0.68
		nPD:	0.01
		N. weight:	0.82
		Sc. PD:	-0.055
		Sc. rank:	-188.1

,

NP			
Pos.	289	obs:	exp:
tat	Y	1145	619.00
tac	Y	1	527.00
---	--	------	------
mPD		0.0017	0.50
		nPD:	0.
		N. weight:	0.67
		Sc. PD:	-0.05
		Sc. rank:	-403.9

,

NP			
Pos.	290	obs:	exp:
gat	D	6	619.30
gac	D	1140	526.70
---	--	------	------
mPD		0.010	0.50
		nPD:	0.02
		N. weight:	0.81
		Sc. PD:	-0.048
		Sc. rank:	-32.1

,

NP			
Pos.	291	obs:	exp:
ttt	F	1144	452.50
ttc	F	2	693.50
---	--	------	------
mPD		0.0035	0.48
		nPD:	0.01
		N. weight:	1.
		Sc. PD:	-0.072
		Sc. rank:	-446.9

,

NP			
Pos.	292	obs:	exp:
gaa	E	13	697.30
gag	E	1133	448.70
---	--	------	------
mPD		0.022	0.48
		nPD:	0.05
		N. weight:	0.95
		Sc. PD:	-0.032
		Sc. rank:	231.5

,

NP			
Pos.	293	obs:	exp:
cgt	R	0	49.22
cgc	R	0	56.76
cga	R	0	112.10
cgg	R	0	95.62
aga	R	1144	524.00
agg	R	2	308.20
---	--	------	------
mPD		0.0035	0.96
		nPD:	0.
		N. weight:	0.84
		Sc. PD:	-0.064
		Sc. rank:	-497.7

,

NP			
Pos.	294	obs:	exp:
gaa	E	1145	697.30
gag	E	1	448.70
---	--	------	------
mPD		0.0017	0.48
		nPD:	0.
		N. weight:	0.54
		Sc. PD:	-0.041
		Sc. rank:	-304.3

,

NP			
Pos.	295	obs:	exp:
ggt	G	2	151.90
ggc	G	0	146.40
gga	G	205	528.70
ggg	G	939	319.00
---	--	------	------
mPD		0.30	0.68
		nPD:	0.44
		N. weight:	0.78
		Sc. PD:	0.27
		Sc. rank:	663.0

,

NP			
Pos.	296	obs:	exp:
tat	Y	5	619.00
tac	Y	1141	527.00
---	--	------	------
mPD		0.0087	0.50
		nPD:	0.02
		N. weight:	0.82
		Sc. PD:	-0.051
		Sc. rank:	-101.3

,

NP			
Pos.	297	obs:	exp:
tct	S	3	182.30
tcc	S	1135	149.70
tca	S	8	290.90
tcg	S	0	65.03
agt	S	0	235.60
agc	S	0	222.60
---	--	------	------
mPD		0.019	1.7
		nPD:	0.01
		N. weight:	2.2
		Sc. PD:	-0.15
		Sc. rank:	-563.3

,

NP			
Pos.	298	obs:	exp:
tta	L	4	101.70
ttg	L	925	213.40
ctt	L	10	190.80
ctc	L	0	191.20
cta	L	8	193.10
ctg	L	199	255.70
---	--	------	------
mPD		0.35	1.1
		nPD:	0.31
		N. weight:	1.2
		Sc. PD:	0.26
		Sc. rank:	906.0

,

NP			
Pos.	299	obs:	exp:
gtt	V	866	237.50
gtc	V	3	231.30
gta	V	2	234.70
gtg	V	275	442.60
---	--	------	------
mPD		0.37	0.73
		nPD:	0.51
		N. weight:	0.93
		Sc. PD:	0.38
		Sc. rank:	814.3

,

NP			
Pos.	300	obs:	exp:
ggt	G	3	151.90
ggc	G	0	146.40
gga	G	1131	528.70
ggg	G	12	319.00
---	--	------	------
mPD		0.026	0.68
		nPD:	0.04
		N. weight:	0.77
		Sc. PD:	-0.033
		Sc. rank:	145.4

,

NP			
Pos.	301	obs:	exp:
att	I	2	420.30
atc	I	0	274.10
ata	I	1142	449.50
gtt	V	0	0.41
gtc	V	0	0.40
gta	V	2	0.41
gtg	V	0	0.77
---	--	------	------
mPD		0.0070	0.66
		nPD:	0.01
		N. weight:	1.
		Sc. PD:	-0.069
		Sc. rank:	-312.7

,

NP			
Pos.	302	obs:	exp:
gat	D	1030	619.30
gac	D	116	526.70
---	--	------	------
mPD		0.18	0.50
		nPD:	0.37
		N. weight:	0.33
		Sc. PD:	0.092
		Sc. rank:	274.4

,

NP			
Pos.	303	obs:	exp:
tct	S	1	0.16
tcc	S	0	0.13
tca	S	0	0.25
tcg	S	0	0.06
cct	P	1144	285.70
ccc	P	1	205.70
cca	P	0	471.30
ccg	P	0	182.30
agt	S	0	0.21
agc	S	0	0.19
---	--	------	------
mPD		0.0035	0.71
		nPD:	0.
		N. weight:	1.5
		Sc. PD:	-0.11
		Sc. rank:	-822.5

,

NP			
Pos.	304	obs:	exp:
ttt	F	54	452.50
ttc	F	1092	693.50
---	--	------	------
mPD		0.090	0.48
		nPD:	0.19
		N. weight:	0.37
		Sc. PD:	0.037
		Sc. rank:	236.2

,

NP			
Pos.	305	obs:	exp:
cgt	R	1018	49.22
cgc	R	126	56.76
cga	R	2	112.10
cgg	R	0	95.62
aga	R	0	524.00
agg	R	0	308.20
---	--	------	------
mPD		0.20	0.96
		nPD:	0.21
		N. weight:	3.
		Sc. PD:	0.36
		Sc. rank:	2036.0

,

NP			
Pos.	306	obs:	exp:
tta	L	0	101.70
ttg	L	1	213.40
ctt	L	4	190.80
ctc	L	0	191.20
cta	L	27	193.10
ctg	L	1114	255.70
---	--	------	------
mPD		0.055	1.1
		nPD:	0.05
		N. weight:	1.5
		Sc. PD:	-0.048
		Sc. rank:	424.3

,

NP			
Pos.	307	obs:	exp:
tta	L	0	101.60
ttg	L	0	213.20
ctt	L	1137	190.70
ctc	L	7	191.10
cta	L	0	193.00
ctg	L	1	255.50
att	I	1	0.37
atc	I	0	0.24
ata	I	0	0.39
---	--	------	------
mPD		0.016	1.1
		nPD:	0.01
		N. weight:	1.9
		Sc. PD:	-0.13
		Sc. rank:	-382.4

,

NP			
Pos.	308	obs:	exp:
caa	Q	1096	583.70
cag	Q	50	562.30
---	--	------	------
mPD		0.084	0.50
		nPD:	0.17
		N. weight:	0.55
		Sc. PD:	0.045
		Sc. rank:	329.4

,

NP			
Pos.	309	obs:	exp:
aat	N	0	583.70
aac	N	1146	562.30
---	--	------	------
mPD		0	0.50
		nPD:	0.
		N. weight:	0.78
		Sc. PD:	-0.061
		Sc. rank:	-701.6

,

NP			
Pos.	310	obs:	exp:
tct	S	0	182.30
tcc	S	0	149.70
tca	S	0	290.90
tcg	S	0	65.03
agt	S	4	235.60
agc	S	1142	222.60
---	--	------	------
mPD		0.0070	1.7
		nPD:	0.
		N. weight:	1.8
		Sc. PD:	-0.13
		Sc. rank:	-985.7

,

NP			
Pos.	311	obs:	exp:
caa	Q	112	583.70
cag	Q	1034	562.30
---	--	------	------
mPD		0.18	0.50
		nPD:	0.35
		N. weight:	0.43
		Sc. PD:	0.11
		Sc. rank:	348.2

,

NP			
Pos.	312	obs:	exp:
gtt	V	55	237.50
gtc	V	1081	231.30
gta	V	10	234.70
gtg	V	0	442.60
---	--	------	------
mPD		0.11	0.73
		nPD:	0.15
		N. weight:	1.5
		Sc. PD:	0.095
		Sc. rank:	873.0

,

NP			
Pos.	313	obs:	exp:
ttt	F	1098	452.20
ttc	F	47	692.80
tta	L	0	0.09
ttg	L	0	0.19
ctt	L	1	0.17
ctc	L	0	0.17
cta	L	0	0.17
ctg	L	0	0.22
---	--	------	------
mPD		0.080	0.48
		nPD:	0.17
		N. weight:	0.81
		Sc. PD:	0.067
		Sc. rank:	492.9

,

NP			
Pos.	314	obs:	exp:
tct	S	0	182.30
tcc	S	0	149.70
tca	S	0	290.90
tcg	S	0	65.03
agt	S	1144	235.60
agc	S	2	222.60
---	--	------	------
mPD		0.0035	1.7
		nPD:	0.
		N. weight:	1.7
		Sc. PD:	-0.13
		Sc. rank:	-1272.1

,

NP			
Pos.	315	obs:	exp:
tta	L	0	101.70
ttg	L	0	213.40
ctt	L	4	190.80
ctc	L	1051	191.20
cta	L	90	193.10
ctg	L	1	255.70
---	--	------	------
mPD		0.15	1.1
		nPD:	0.14
		N. weight:	1.6
		Sc. PD:	0.087
		Sc. rank:	922.4

,

NP			
Pos.	316	obs:	exp:
tta	L	0	0.18
ttg	L	0	0.37
ctt	L	2	0.33
ctc	L	0	0.33
cta	L	0	0.34
ctg	L	0	0.45
att	I	1118	418.50
atc	I	12	272.90
ata	I	9	447.60
atg	M	1	1.00
gtt	V	4	0.83
gtc	V	0	0.81
gta	V	0	0.82
gtg	V	0	1.54
---	--	------	------
mPD		0.048	0.67
		nPD:	0.07
		N. weight:	0.99
		Sc. PD:	-0.0092
		Sc. rank:	408.9

,

NP			
Pos.	317	obs:	exp:
cgt	R	0	49.22
cgc	R	0	56.76
cga	R	0	112.10
cgg	R	0	95.62
aga	R	1125	524.00
agg	R	21	308.20
---	--	------	------
mPD		0.036	0.96
		nPD:	0.04
		N. weight:	0.77
		Sc. PD:	-0.033
		Sc. rank:	129.6

,

NP			
Pos.	318	obs:	exp:
tta	L	0	0.09
ttg	L	0	0.19
ctt	L	0	0.17
ctc	L	0	0.17
cta	L	1	0.17
ctg	L	0	0.22
cct	P	0	285.70
ccc	P	0	205.70
cca	P	1132	471.30
ccg	P	13	182.30
---	--	------	------
mPD		0.024	0.71
		nPD:	0.03
		N. weight:	0.92
		Sc. PD:	-0.043
		Sc. rank:	112.6

,

NP			
Pos.	319	obs:	exp:
act	T	0	0.51
acc	T	2	0.41
aca	T	0	0.92
acg	T	0	0.16
aat	N	1046	582.70
aac	N	98	561.30
---	--	------	------
mPD		0.16	0.50
		nPD:	0.32
		N. weight:	0.43
		Sc. PD:	0.099
		Sc. rank:	328.0

,

NP			
Pos.	320	obs:	exp:
gaa	E	3	697.30
gag	E	1143	448.70
---	--	------	------
mPD		0.0052	0.48
		nPD:	0.01
		N. weight:	1.
		Sc. PD:	-0.069
		Sc. rank:	-281.1

,

NP

Pos.	321	obs :	exp :
tct	S	0	1.59
tcc	S	0	1.31
tca	S	0	2.54
tcg	S	0	0.57
aat	N	7	578.60
aac	N	1129	557.40
agt	S	0	2.06
agc	S	10	1.94
---	--	------	------
mPD		0.029	0.53
		nPD :	0.06
		N. weight :	0.75
		Sc. PD :	-0.019
		Sc. rank :	237.3

NP

Pos.	322	obs :	exp :
cct	P	0	286.00
ccc	P	0	205.90
cca	P	1141	471.70
ccg	P	5	182.40
---	--	------	------
mPD		0.0087	0.71
		nPD :	0.01
		N. weight :	0.94
		Sc. PD :	-0.064
		Sc. rank :	-240.2

NP

Pos.	323	obs :	exp :
tct	S	0	0.32
tcc	S	0	0.26
tca	S	2	0.51
tcg	S	0	0.11
act	T	1	0.25
acc	T	0	0.21
aca	T	0	0.46
acg	T	0	0.08
agt	S	0	0.41
agc	S	0	0.39
gct	A	2	295.20
gcc	A	0	212.90
gca	A	1129	538.00
gcg	A	12	97.01
---	--	------	------
mPD		0.031	0.68
		nPD :	0.05
		N. weight :	0.77
		Sc. PD :	-0.027
		Sc. rank :	184.7

NP

Pos.	324	obs :	exp :
cat	H	729	658.10
cac	H	417	487.90
---	--	------	------
mPD		0.46	0.49
		nPD :	0.95
		N. weight :	0.012
		Sc. PD :	0.01
		Sc. rank :	11.1

NP

Pos.	325	obs :	exp :
aaa	K	7	667.90
aag	K	1139	478.10
---	--	------	------
mPD		0.012	0.49
		nPD :	0.02
		N. weight :	0.91
		Sc. PD :	-0.05
		Sc. rank :	3.1

NP

Pos.	326	obs :	exp :
tct	S	0	182.30
tcc	S	0	149.70
tca	S	0	290.90
tcg	S	0	65.03
agt	S	93	235.60
agc	S	1053	222.60
---	--	------	------
mPD		0.15	1.7
		nPD :	0.09
		N. weight :	1.5
		Sc. PD :	0.011
		Sc. rank :	711.6

NP

Pos.	327	obs :	exp :
caa	Q	1038	583.70
cag	Q	108	562.30
---	--	------	------
mPD		0.17	0.50
		nPD :	0.34
		N. weight :	0.4
		Sc. PD :	0.1
		Sc. rank :	319.0

NP

Pos.	328	obs :	exp :
tta	L	101	101.70
ttg	L	138	213.40
ctt	L	0	190.80
ctc	L	1	191.20
cta	L	713	193.10
ctg	L	193	255.70
---	--	------	------
mPD		0.74	1.1
		nPD :	0.67
		N. weight :	0.78
		Sc. PD :	0.44
		Sc. rank :	710.0

NP

Pos.	329	obs :	exp :
atg	M	14	14.00
gtt	V	108	234.60
gtc	V	0	228.50
gta	V	260	231.80
gtg	V	764	437.20
---	--	------	------
mPD		0.50	0.75
		nPD :	0.67
		N. weight :	0.36
		Sc. PD :	0.21
		Sc. rank :	328.2

NP

Pos.	330	obs :	exp :
tgg	W	1146	1146.00
---	--	------	------
mPD		0	0
		nPD :	1.
		N. weight :	0.
		Sc. PD :	0
		Sc. rank :	0

NP

Pos.	331	obs :	exp :
att	I	0	1.10
atc	I	0	0.72
ata	I	3	1.18
atg	M	1143	1143.00
---	--	------	------
mPD		0.0052	0.0052
		nPD :	1.
		N. weight :	0.0027
		Sc. PD :	0.0024
		Sc. rank :	2.4

NP

Pos.	332	obs :	exp :
gct	A	0	295.90
gcc	A	2	213.40
gca	A	1143	539.40
gcg	A	1	97.26
---	--	------	------
mPD		0.0052	0.67
		nPD :	0.01
		N. weight :	0.81
		Sc. PD :	-0.058
		Sc. rank :	-317.3

NP

Pos.	333	obs :	exp :
tgt	C	5	484.70
tgc	C	1141	661.30
---	--	------	------
mPD		0.0087	0.49
		nPD :	0.02
		N. weight :	0.57
		Sc. PD :	-0.035
		Sc. rank :	-68.4

NP

Pos.	334	obs :	exp :
cat	H	21	658.10
cac	H	1125	487.90
---	--	------	------
mPD		0.036	0.49
		nPD :	0.07
		N. weight :	0.83
		Sc. PD :	-0.0068
		Sc. rank :	346.9

NP

Pos.	335	obs :	exp :
tct	S	1144	182.00
tcc	S	0	149.40
tca	S	0	290.40
tcg	S	0	64.91
agt	S	0	235.20
agc	S	0	222.20
gct	A	2	0.52
gcc	A	0	0.37
gca	A	0	0.94
gcg	A	0	0.17
---	--	------	------
mPD		0.0035	1.7
		nPD :	0.
		N. weight :	2.
		Sc. PD :	-0.15
		Sc. rank :	-1501.3

NP

Pos.	336	obs :	exp :
act	T	0	0.51
acc	T	0	0.41
aca	T	2	0.92
acg	T	0	0.16
gct	A	5	295.40
gcc	A	0	213.00
gca	A	1135	538.40
gcg	A	4	97.09
---	--	------	------
mPD		0.019	0.67
		nPD :	0.03
		N. weight :	0.78
		Sc. PD :	-0.04
		Sc. rank :	35.3

NP

Pos.	337	obs :	exp :
tct	S	0	0.48
tcc	S	0	0.39
tca	S	2	0.76
tcg	S	1	0.17
agt	S	0	0.62
agc	S	0	0.58
gct	A	0	295.20
gcc	A	0	212.90
gca	A	224	538.00
gcg	A	919	97.01
---	--	------	------
mPD		0.32	0.68
		nPD :	0.48
		N. weight :	1.8
		Sc. PD :	0.68
		Sc. rank :	1552.3

NP

Pos.	338	obs :	exp :
ttt	F	1145	452.50
ttc	F	1	693.50
---	--	------	------
mPD		0.0017	0.48
		nPD :	0.
		N. weight :	1.
		Sc. PD :	-0.076
		Sc. rank :	-573.7

NP

Pos.	339	obs :	exp :
gaa	E	5	697.30
gag	E	1141	448.70
---	--	------	------
mPD		0.0087	0.48
		nPD :	0.02
		N. weight :	0.99
		Sc. PD :	-0.061
		Sc. rank :	-98.0

NP

Pos.	340	obs :	exp :
gat	D	2	619.30
gac	D	1144	526.70
---	--	------	------
mPD		0.0035	0.50
		nPD :	0.01
		N. weight :	0.84
		Sc. PD :	-0.06
		Sc. rank :	-382.5

NP

Pos.	341	obs :	exp :
tta	L	0	101.70
ttg	L	0	213.40
ctt	L	1142	190.80
ctc	L	3	191.20
cta	L	0	193.10
ctg	L	1	255.70
---	--	------	------
mPD		0.0070	1.1
		nPD :	0.01
		N. weight :	1.9
		Sc. PD :	-0.14
		Sc. rank :	-940.4

NP

Pos.	342	obs :	exp :
cgt	R	0	49.22
cgc	R	0	56.76
cga	R	0	112.10
cgg	R	0	95.62
aga	R	95	524.00
agg	R	1051	308.20
---	--	------	------
mPD		0.15	0.96
		nPD :	0.16
		N. weight :	1.1
		Sc. PD :	0.079
		Sc. rank :	636.7

NP

Pos.	343	obs :	exp :
att	I	0	1.10
atc	I	3	0.72
ata	I	0	1.18
gtt	V	1	236.80
gtc	V	1137	230.70
gta	V	5	234.00
gtg	V	0	441.40
---	--	------	------
mPD		0.016	0.73
		nPD :	0.02
		N. weight :	1.7
		Sc. PD :	-0.1
		Sc. rank :	-47.4

NP

Pos.	344	obs :	exp :
tct	S	0	182.30
tcc	S	0	149.70
tca	S	1146	290.90
tcg	S	0	65.03
agt	S	0	235.60
agc	S	0	222.60
---	--	------	------
mPD		0	1.7
		nPD :	0.
		N. weight :	1.5
		Sc. PD :	-0.12
		Sc. rank :	-1351.0

NP

Pos.	345	obs :	exp :
tct	S	0	182.30
tcc	S	0	149.70
tca	S	0	290.90
tcg	S	0	65.03
agt	S	1110	235.60
agc	S	36	222.60
---	--	------	------
mPD		0.061	1.7
		nPD :	0.04
		N. weight :	1.6
		Sc. PD :	-0.069
		Sc. rank :	243.8

NP

Pos.	346	obs:	exp:
ttt	F	6	452.20
ttc	F	1139	692.80
tct	S	0	0.16
tcc	S	1	0.13
tca	S	0	0.25
tcg	S	0	0.06
agt	S	0	0.21
agc	S	0	0.19
---	--	------	------
mPD		0.012	0.48
		nPD:	0.03
		N. weight:	0.52
		Sc. PD:	-0.028
		Sc. rank:	5.6

,

NP

Pos.	347	obs:	exp:
att	I	949	420.70
atc	I	195	274.40
ata	I	1	449.90
gtt	V	1	0.21
gtc	V	0	0.20
gta	V	0	0.20
gtg	V	0	0.39
---	--	------	------
mPD		0.29	0.66
		nPD:	0.44
		N. weight:	0.67
		Sc. PD:	0.23
		Sc. rank:	568.9

,

NP

Pos.	348	obs:	exp:
cgt	R	0	47.50
cgc	R	0	54.78
cga	R	0	108.20
cgg	R	0	92.29
aaa	K	40	23.31
aag	K	0	16.69
aga	R	1105	505.70
agg	R	1	297.50
---	--	------	------
mPD		0.069	1.0
		nPD:	0.07
		N. weight:	0.84
		Sc. PD:	-0.011
		Sc. rank:	322.6

,

NP

Pos.	349	obs:	exp:
ggt	G	0	151.90
ggc	G	0	146.40
gga	G	394	528.70
ggg	G	752	319.00
---	--	------	------
mPD		0.45	0.68
		nPD:	0.67
		N. weight:	0.51
		Sc. PD:	0.29
		Sc. rank:	458.8

,

NP

Pos.	350	obs:	exp:
att	I	0	1.47
atc	I	0	0.96
ata	I	4	1.57
act	T	0	289.80
acc	T	2	233.30
aca	T	1135	521.50
acg	T	0	92.35
aaa	K	1	0.58
aag	K	0	0.42
gct	A	0	1.03
gcc	A	0	0.74
gca	A	4	1.88
gcg	A	0	0.34
---	--	------	------
mPD		0.019	0.69
		nPD:	0.03
		N. weight:	0.84
		Sc. PD:	-0.044
		Sc. rank:	24.3

,

NP

Pos.	351	obs:	exp:
cgt	R	0	38.53
cgc	R	0	44.43
cga	R	0	87.78
cgg	R	0	74.85
aaa	K	249	145.10
aag	K	0	103.90
aga	R	888	410.20
agg	R	9	241.30
---	--	------	------
mPD		0.36	1.2
		nPD:	0.29
		N. weight:	0.75
		Sc. PD:	0.15
		Sc. rank:	558.2

,

NP

Pos.	352	obs:	exp:
att	I	0	0.73
atc	I	0	0.48
ata	I	2	0.79
atg	M	1143	1143.00
gtt	V	0	0.21
gtc	V	0	0.20
gta	V	0	0.20
gtg	V	1	0.39
---	--	------	------
mPD		0.0052	0.0063
		nPD:	0.83
		N. weight:	0.0027
		Sc. PD:	0.0019
		Sc. rank:	2.4

,

NP

Pos.	353	obs:	exp:
tgt	C	2	0.85
tgc	C	0	1.15
att	I	0	4.04
atc	I	11	2.64
ata	I	0	4.32
gtt	V	1	233.10
gtc	V	1122	227.10
gta	V	2	230.40
gtg	V	0	434.50
gct	A	0	0.26
gcc	A	1	0.19
gca	A	0	0.47
gcg	A	0	0.08
ggt	G	6	0.93
ggc	G	1	0.89
gga	G	0	3.23
ggg	G	0	1.95
---	--	------	------
mPD		0.059	0.77
		nPD:	0.08
		N. weight:	1.7
		Sc. PD:	-0.009
		Sc. rank:	762.9

,

NP

Pos.	354	obs:	exp:
cct	P	0	286.00
ccc	P	2	205.90
cca	P	1141	471.70
ccg	P	3	182.40
---	--	------	------
mPD		0.0087	0.71
		nPD:	0.01
		N. weight:	0.94
		Sc. PD:	-0.063
		Sc. rank:	-236.3

,

NP

Pos.	355	obs:	exp:
cgt	R	0	49.22
cgc	R	0	56.76
cga	R	0	112.10
cgg	R	0	95.62
aga	R	1140	524.00
agg	R	6	308.20
---	--	------	------
mPD		0.010	0.96
		nPD:	0.01
		N. weight:	0.82
		Sc. PD:	-0.057
		Sc. rank:	-243.1

,

NP

Pos.	356	obs:	exp:
ggt	G	0	151.90
ggc	G	0	146.40
gga	G	1086	528.70
ggg	G	60	319.00
---	--	------	------
mPD		0.099	0.68
		nPD:	0.15
		N. weight:	0.65
		Sc. PD:	0.041
		Sc. rank:	378.6

,

NP

Pos.	357	obs:	exp:
caa	Q	42	583.20
cag	Q	1103	561.80
cgt	R	0	0.04
cgc	R	0	0.05
cga	R	0	0.10
cgg	R	1	0.08
aga	R	0	0.46
agg	R	0	0.27
---	--	------	------
mPD		0.072	0.50
		nPD:	0.14
		N. weight:	0.61
		Sc. PD:	0.036
		Sc. rank:	350.3

,

NP

Pos.	358	obs:	exp:
tta	L	85	101.70
ttg	L	4	213.40
ctt	L	0	190.80
ctc	L	0	191.20
cta	L	821	193.10
ctg	L	236	255.70
---	--	------	------
mPD		0.47	1.1
		nPD:	0.43
		N. weight:	1.1
		Sc. PD:	0.36
		Sc. rank:	915.8

,

NP

Pos.	359	obs:	exp:
tct	S	1	181.00
tcc	S	1137	148.60
tca	S	0	288.80
tcg	S	0	64.57
agt	S	0	233.90
agc	S	0	221.00
gct	A	0	2.07
gcc	A	8	1.49
gca	A	0	3.77
gcg	A	0	0.68
---	--	------	------
mPD		0.016	1.7
		nPD:	0.01
		N. weight:	2.2
		Sc. PD:	-0.16
		Sc. rank:	-817.0

,

NP

Pos.	360	obs:	exp:
act	T	1058	292.10
acc	T	87	235.20
aca	T	1	525.60
acg	T	0	93.08
---	--	------	------
mPD		0.14	0.68
		nPD:	0.21
		N. weight:	1.2
		Sc. PD:	0.15
		Sc. rank:	820.1

,

NP

Pos.	361	obs:	exp:
cgt	R	0	49.22
cgc	R	0	56.76
cga	R	0	112.10
cgg	R	0	95.62
aga	R	1145	524.00
agg	R	1	308.20
---	--	------	------
mPD		0.0017	0.96
		nPD:	0.
		N. weight:	0.85
		Sc. PD:	-0.066
		Sc. rank:	-640.8

,

NP

Pos.	362	obs:	exp:
ggt	G	0	151.90
ggc	G	0	146.40
gga	G	310	528.70
ggg	G	836	319.00
---	--	------	------
mPD		0.40	0.68
		nPD:	0.58
		N. weight:	0.61
		Sc. PD:	0.3
		Sc. rank:	543.0

,

NP

Pos.	363	obs:	exp:
att	I	14	5.14
atc	I	0	3.35
ata	I	0	5.50
gtt	V	1129	234.60
gtc	V	3	228.50
gta	V	0	231.80
gtg	V	0	437.20
---	--	------	------
mPD		0.029	0.75
		nPD:	0.04
		N. weight:	1.7
		Sc. PD:	-0.07
		Sc. rank:	339.8

,

NP

Pos.	364	obs:	exp:
caa	Q	1135	583.70
cag	Q	11	562.30
---	--	------	------
mPD		0.019	0.50
		nPD:	0.04
		N. weight:	0.68
		Sc. PD:	-0.029
		Sc. rank:	116.5

,

NP

Pos.	365	obs:	exp:
att	I	1126	421.10
atc	I	18	274.60
ata	I	2	450.30
---	--	------	------
mPD		0.034	0.65
		nPD:	0.05
		N. weight:	1.
		Sc. PD:	-0.028
		Sc. rank:	297.4

,

NP			
Pos.	366	obs:	exp:
gct	A	1141	295.90
gcc	A	2	213.40
gca	A	0	539.40
gcg	A	3	97.26
mPD		0.0087	0.67
		nPD:	0.01
		N. weight:	1.5
		Sc. PD:	-0.097
		Sc. rank:	-314.0

NP			
Pos.	367	obs:	exp:
tct	S	1	182.30
tcc	S	0	149.70
tca	S	1131	290.90
tcg	S	14	65.03
agt	S	0	235.60
agc	S	0	222.60
mPD		0.026	1.7
		nPD:	0.02
		N. weight:	1.4
		Sc. PD:	-0.092
		Sc. rank:	-246.1

NP			
Pos.	368	obs:	exp:
tct	S	0	0.16
tcc	S	0	0.13
tca	S	0	0.25
tcg	S	0	0.06
aat	N	1141	583.20
aac	N	4	561.80
agt	S	1	0.21
agc	S	0	0.19
mPD		0.0087	0.50
		nPD:	0.02
		N. weight:	0.71
		Sc. PD:	-0.045
		Sc. rank:	-99.7

NP			
Pos.	369	obs:	exp:
gaa	E	19	697.30
gag	E	1127	448.70
mPD		0.033	0.48
		nPD:	0.07
		N. weight:	0.93
		Sc. PD:	-0.012
		Sc. rank:	361.6

NP			
Pos.	370	obs:	exp:
tct	S	0	0.16
tcc	S	0	0.13
tca	S	0	0.25
tcg	S	0	0.06
aat	N	12	582.70
aac	N	1132	561.30
aaa	K	1	0.58
aag	K	0	0.42
agt	S	0	0.21
agc	S	1	0.19
mPD		0.024	0.50
		nPD:	0.05
		N. weight:	0.72
		Sc. PD:	-0.024
		Sc. rank:	182.3

NP			
Pos.	371	obs:	exp:
att	I	0	47.40
atc	I	0	30.91
ata	I	129	50.69
atg	M	1014	1014.00
act	T	0	0.25
acc	T	0	0.21
aca	T	0	0.46
acg	T	1	0.08
gtt	V	0	0.41
gtc	V	0	0.40
gta	V	0	0.41
gtg	V	2	0.77
mPD		0.21	0.22
		nPD:	0.95
		N. weight:	0.12
		Sc. PD:	0.099
		Sc. rank:	108.5

NP			
Pos.	372	obs:	exp:
gaa	E	1140	697.30
gag	E	6	448.70
mPD		0.010	0.48
		nPD:	0.02
		N. weight:	0.51
		Sc. PD:	-0.03
		Sc. rank:	-11.7

NP			
Pos.	373	obs:	exp:
tct	S	0	0.16
tcc	S	0	0.13
tca	S	1	0.25
tcg	S	0	0.06
act	T	0	0.76
acc	T	0	0.62
aca	T	3	1.38
acg	T	0	0.24
agt	S	0	0.21
agc	S	0	0.19
gtt	V	0	0.21
gtc	V	0	0.20
gta	V	1	0.20
gtg	V	0	0.39
gct	A	0	294.60
gcc	A	0	212.50
gca	A	1140	537.00
gcg	A	1	96.84
mPD		0.010	0.68
		nPD:	0.02
		N. weight:	0.82
		Sc. PD:	-0.053
		Sc. rank:	-148.5

NP			
Pos.	374	obs:	exp:
tta	L	0	0.09
ttg	L	0	0.19
ctt	L	0	0.17
ctc	L	0	0.17
cta	L	0	0.17
ctg	L	1	0.22
att	I	0	0.37
atc	I	0	0.24
ata	I	1	0.39
atg	M	1144	1144.00
mPD		0.0035	0.0045
		nPD:	0.77
		N. weight:	0.0023
		Sc. PD:	0.0015
		Sc. rank:	2.1

NP			
Pos.	375	obs:	exp:
gat	D	21	562.00
gac	D	1019	478.00
gaa	E	106	64.50
gag	E	0	41.50
mPD		0.20	0.58
		nPD:	0.35
		N. weight:	0.72
		Sc. PD:	0.18
		Sc. rank:	579.0

NP			
Pos.	376	obs:	exp:
tct	S	4	182.30
tcc	S	1140	149.70
tca	S	2	290.90
tcg	S	0	65.03
agt	S	0	235.60
agc	S	0	222.60
mPD		0.010	1.7
		nPD:	0.01
		N. weight:	2.2
		Sc. PD:	-0.16
		Sc. rank:	-1045.7

NP			
Pos.	377	obs:	exp:
tct	S	0	0.16
tcc	S	0	0.13
tca	S	0	0.25
tcg	S	0	0.06
aat	N	949	583.20
aac	N	196	561.80
agt	S	0	0.21
agc	S	1	0.19
mPD		0.29	0.50
		nPD:	0.57
		N. weight:	0.25
		Sc. PD:	0.12
		Sc. rank:	219.5

NP			
Pos.	378	obs:	exp:
act	T	1135	292.10
acc	T	2	235.20
aca	T	9	525.60
acg	T	0	93.08
mPD		0.019	0.68
		nPD:	0.03
		N. weight:	1.4
		Sc. PD:	-0.074
		Sc. rank:	60.5

NP			
Pos.	379	obs:	exp:
tta	L	0	101.70
ttg	L	0	213.40
ctt	L	1121	190.80
ctc	L	0	191.20
cta	L	25	193.10
ctg	L	0	255.70
mPD		0.043	1.1
		nPD:	0.04
		N. weight:	1.8
		Sc. PD:	-0.078
		Sc. rank:	358.9

NP			
Pos.	380	obs:	exp:
gaa	E	1009	697.30
gag	E	137	448.70
mPD		0.21	0.48
		nPD:	0.44
		N. weight:	0.2
		Sc. PD:	0.07
		Sc. rank:	174.0

NP			
Pos.	381	obs:	exp:
tta	L	0	101.70
ttg	L	348	213.40
ctt	L	12	190.80
ctc	L	0	191.20
cta	L	43	193.10
ctg	L	743	255.70
mPD		0.52	1.1
		nPD:	0.46
		N. weight:	0.83
		Sc. PD:	0.31
		Sc. rank:	716.5

NP			
Pos.	382	obs:	exp:
cgt	R	0	49.22
cgc	R	0	56.76
cga	R	0	112.10
cgg	R	0	95.62
aga	R	1144	524.00
agg	R	2	308.20
mPD		0.0035	0.96
		nPD:	0.
		N. weight:	0.84
		Sc. PD:	-0.064
		Sc. rank:	-497.7

NP			
Pos.	383	obs:	exp:
tct	S	0	182.30
tcc	S	0	149.70
tca	S	0	290.90
tcg	S	0	65.03
agt	S	1039	235.60
agc	S	107	222.60
mPD		0.17	1.7
		nPD:	0.1
		N. weight:	1.4
		Sc. PD:	0.027
		Sc. rank:	698.2

NP			
Pos.	384	obs:	exp:
cgt	R	0	49.18
cgc	R	0	56.71
cga	R	0	112.00
cgg	R	0	95.54
aaa	K	1	0.58
aag	K	0	0.42
aga	R	1143	523.60
agg	R	2	308.00
mPD		0.0052	0.96
		nPD:	0.01
		N. weight:	0.84
		Sc. PD:	-0.062
		Sc. rank:	-421.9

NP			
Pos.	385	obs:	exp:
tat	Y	1137	619.00
tac	Y	9	527.00
mPD		0.016	0.50
		nPD:	0.03
		N. weight:	0.63
		Sc. PD:	-0.031
		Sc. rank:	54.7

NP			
Pos.	386	obs:	exp:
tgg	W	1146	1146.00
mPD		0	0
		nPD:	1.
		N. weight:	0.
		Sc. PD:	0
		Sc. rank:	0

NP			
Pos.	387	obs:	exp:
gct	A	1138	295.90
gcc	A	7	213.40
gca	A	1	539.40
gcg	A	0	97.26
mPD		0.014	0.67
		nPD:	0.02
		N. weight:	1.4
		Sc. PD:	-0.085
		Sc. rank:	-68.0

NP			
Pos.	388	obs:	exp:
att	I	1	421.10
atc	I	0	274.60
ata	I	1145	450.30
mPD		0.0017	0.65
		nPD:	0.
		N. weight:	1.
		Sc. PD:	-0.078
		Sc. rank:	-699.5

NP			
Pos.	389	obs:	exp:
cgt	R	0	49.22
cgc	R	0	56.76
cga	R	0	112.10
cgg	R	0	95.62
aga	R	1144	524.00
agg	R	2	308.20
mPD		0.0035	0.96
		nPD:	0.
		N. weight:	0.84
		Sc. PD:	-0.064
		Sc. rank:	-497.7

NP			
Pos.	390	obs:	exp:
act	T	2	292.10
acc	T	1142	235.20
aca	T	2	525.60
acg	T	0	93.08
mPD		0.0070	0.68
		nPD:	0.01
		N. weight:	1.7
		Sc. PD:	-0.12
		Sc. rank:	-592.2

NP			
Pos.	391	obs:	exp:
cgt	R	0	49.22
cgc	R	0	56.76
cga	R	0	112.10
cgg	R	0	95.62
aga	R	1080	524.00
agg	R	66	308.20
---	--	------	------
mPD		0.11	0.96
		nPD:	0.11
		N. weight:	0.65
		Sc. PD:	0.019
		Sc. rank:	349.0

NP			
Pos.	392	obs:	exp:
tct	S	0	182.30
tcc	S	0	149.70
tca	S	0	290.90
tcg	S	0	65.03
agt	S	32	235.60
agc	S	1114	222.60
---	--	------	------
mPD		0.054	1.7
		nPD:	0.03
		N. weight:	1.7
		Sc. PD:	-0.078
		Sc. rank:	184.1

NP			
Pos.	393	obs:	exp:
ggt	G	1	151.90
ggc	G	0	146.40
gga	G	1145	528.70
ggg	G	0	319.00
---	--	------	------
mPD		0.0017	0.68
		nPD:	0.
		N. weight:	0.84
		Sc. PD:	-0.064
		Sc. rank:	-593.9

NP			
Pos.	394	obs:	exp:
ggt	G	0	151.90
ggc	G	0	146.40
gga	G	158	528.70
ggg	G	988	319.00
---	--	------	------
mPD		0.24	0.68
		nPD:	0.35
		N. weight:	0.89
		Sc. PD:	0.23
		Sc. rank:	720.6

NP			
Pos.	395	obs:	exp:
tct	S	0	0.16
tcc	S	0	0.13
tca	S	0	0.25
tcg	S	0	0.06
aat	N	16	583.20
aac	N	1129	561.80
agt	S	0	0.21
agc	S	1	0.19
---	--	------	------
mPD		0.029	0.50
		nPD:	0.06
		N. weight:	0.7
		Sc. PD:	-0.016
		Sc. rank:	231.0

NP			
Pos.	396	obs:	exp:
act	T	3	292.10
acc	T	1143	235.20
aca	T	0	525.60
acg	T	0	93.08
---	--	------	------
mPD		0.0052	0.68
		nPD:	0.01
		N. weight:	1.7
		Sc. PD:	-0.12
		Sc. rank:	-687.9

NP			
Pos.	397	obs:	exp:
aat	N	3	583.70
aac	N	1143	562.30
---	--	------	------
mPD		0.0052	0.50
		nPD:	0.01
		N. weight:	0.76
		Sc. PD:	-0.052
		Sc. rank:	-253.4

NP			
Pos.	398	obs:	exp:
cat	H	0	0.57
cac	H	1	0.43
caa	Q	1142	583.20
cag	Q	3	561.80
---	--	------	------
mPD		0.0070	0.50
		nPD:	0.01
		N. weight:	0.72
		Sc. PD:	-0.047
		Sc. rank:	-148.8

NP			
Pos.	399	obs:	exp:
cat	H	4	2.30
cac	H	0	1.70
caa	Q	16	581.70
cag	Q	1126	560.30
---	--	------	------
mPD		0.034	0.50
		nPD:	0.07
		N. weight:	0.7
		Sc. PD:	-0.0093
		Sc. rank:	270.3

NP			
Pos.	400	obs:	exp:
cgt	R	0	49.09
cgc	R	0	56.61
cga	R	0	111.90
cgg	R	0	95.37
aaa	K	0	1.75
aag	K	3	1.25
aga	R	66	522.60
agg	R	1077	307.40
---	--	------	------
mPD		0.11	0.97
		nPD:	0.12
		N. weight:	1.2
		Sc. PD:	0.04
		Sc. rank:	628.5

NP			
Pos.	401	obs:	exp:
act	T	0	0.51
acc	T	0	0.41
aca	T	2	0.92
acg	T	0	0.16
gct	A	0	295.40
gcc	A	0	213.00
gca	A	1142	538.40
gcg	A	2	97.09
---	--	------	------
mPD		0.0070	0.67
		nPD:	0.01
		N. weight:	0.81
		Sc. PD:	-0.056
		Sc. rank:	-280.7

NP			
Pos.	402	obs:	exp:
tct	S	1144	182.30
tcc	S	0	149.70
tca	S	1	290.90
tcg	S	1	65.03
agt	S	0	235.60
agc	S	0	222.60
---	--	------	------
mPD		0.0035	1.7
		nPD:	0.
		N. weight:	2.
		Sc. PD:	-0.15
		Sc. rank:	-1452.5

NP			
Pos.	403	obs:	exp:
act	T	0	0.25
acc	T	0	0.21
aca	T	1	0.46
acg	T	0	0.08
gct	A	1	295.70
gcc	A	0	213.20
gca	A	1068	538.90
gcg	A	76	97.18
---	--	------	------
mPD		0.13	0.67
		nPD:	0.19
		N. weight:	0.68
		Sc. PD:	0.07
		Sc. rank:	442.5

NP			
Pos.	404	obs:	exp:
ggt	G	0	151.90
ggc	G	0	146.40
gga	G	1125	528.70
ggg	G	21	319.00
---	--	------	------
mPD		0.036	0.68
		nPD:	0.05
		N. weight:	0.76
		Sc. PD:	-0.021
		Sc. rank:	230.8

NP			
Pos.	405	obs:	exp:
caa	Q	28	583.70
cag	Q	1118	562.30
---	--	------	------
mPD		0.048	0.50
		nPD:	0.1
		N. weight:	0.65
		Sc. PD:	0.0083
		Sc. rank:	321.4

NP			
Pos.	406	obs:	exp:
att	I	85	34.90
atc	I	10	22.77
ata	I	0	37.33
gtt	V	2	217.80
gtc	V	1049	212.10
gta	V	0	215.20
gtg	V	0	405.90
---	--	------	------
mPD		0.29	0.89
		nPD:	0.33
		N. weight:	1.7
		Sc. PD:	0.39
		Sc. rank:	1286.4

NP			
Pos.	407	obs:	exp:
tct	S	0	182.30
tcc	S	0	149.70
tca	S	0	290.90
tcg	S	0	65.03
agt	S	2	235.60
agc	S	1144	222.60
---	--	------	------
mPD		0.0035	1.7
		nPD:	0.
		N. weight:	1.8
		Sc. PD:	-0.14
		Sc. rank:	-1317.9

NP			
Pos.	408	obs:	exp:
att	I	28	10.29
atc	I	0	6.71
ata	I	0	11.00
gtt	V	1083	231.40
gtc	V	32	225.40
gta	V	2	228.70
gtg	V	0	431.40
gct	A	1	0.26
gcc	A	0	0.19
gca	A	0	0.47
gcg	A	0	0.08
---	--	------	------
mPD		0.11	0.78
		nPD:	0.14
		N. weight:	1.6
		Sc. PD:	0.083
		Sc. rank:	873.1

NP			
Pos.	409	obs:	exp:
caa	Q	1140	583.70
cag	Q	6	562.30
---	--	------	------
mPD		0.010	0.50
		nPD:	0.02
		N. weight:	0.7
		Sc. PD:	-0.041
		Sc. rank:	-31.5

NP			
Pos.	410	obs:	exp:
cct	P	19	286.00
ccc	P	1108	205.90
cca	P	19	471.70
ccg	P	0	182.40
---	--	------	------
mPD		0.065	0.71
		nPD:	0.09
		N. weight:	1.7
		Sc. PD:	0.014
		Sc. rank:	815.1

NP			
Pos.	411	obs:	exp:
act	T	1133	292.10
acc	T	11	235.20
aca	T	0	525.60
acg	T	2	93.08
---	--	------	------
mPD		0.022	0.68
		nPD:	0.03
		N. weight:	1.4
		Sc. PD:	-0.067
		Sc. rank:	170.6

NP			
Pos.	412	obs:	exp:
ttt	F	0	452.50
ttc	F	1146	693.50
---	--	------	------
mPD		0	0.48
		nPD:	0.
		N. weight:	0.55
		Sc. PD:	-0.043
		Sc. rank:	-495.0

NP			
Pos.	413	obs:	exp:
tct	S	1	182.30
tcc	S	0	149.70
tca	S	943	290.90
tcg	S	202	65.03
agt	S	0	235.60
agc	S	0	222.60
---	--	------	------
mPD		0.29	1.7
		nPD:	0.18
		N. weight:	1.3
		Sc. PD:	0.12
		Sc. rank:	802.5

NP			
Pos.	414	obs:	exp:
att	I	0	1.47
atc	I	0	0.96
ata	I	4	1.57
gtt	V	1	236.60
gtc	V	0	230.50
gta	V	1139	233.80
gtg	V	2	441.10
---	--	------	------
mPD		0.012	0.73
		nPD:	0.02
		N. weight:	1.7
		Sc. PD:	-0.11
		Sc. rank:	-275.0

NP			
Pos.	415	obs:	exp:
caa	Q	17	582.70
cag	Q	1127	561.30
cgt	R	0	0.09
cgc	R	0	0.10
cga	R	0	0.20
cgg	R	2	0.17
aga	R	0	0.91
agg	R	0	0.54
---	--	------	------
mPD		0.033	0.51
		nPD:	0.06
		N. weight:	0.7
		Sc. PD:	-0.012
		Sc. rank:	260.6

NP

Pos. 416		obs:	exp:
cgt	R	0	49.22
cgc	R	0	56.76
cga	R	0	112.10
cgg	R	0	95.62
aga	R	1142	524.00
agg	R	4	308.20
---	--	------	------
mPD		0.0070	0.96
		nPD:	0.01
		N. weight:	0.83
		Sc. PD:	-0.06
		Sc. rank:	-375.7

,

NP

Pos. 417		obs:	exp:
tat	Y	1	0.54
tac	Y	0	0.46
aat	N	200	581.20
aac	N	941	559.80
gat	D	0	2.16
gac	D	4	1.84
---	--	------	------
mPD		0.30	0.51
		nPD:	0.59
		N. weight:	0.27
		Sc. PD:	0.13
		Sc. rank:	238.7

,

NP

Pos. 418		obs:	exp:
tta	L	0	101.70
ttg	L	0	213.40
ctt	L	1050	190.80
ctc	L	96	191.20
cta	L	0	193.10
ctg	L	0	255.70
---	--	------	------
mPD		0.15	1.1
		nPD:	0.14
		N. weight:	1.6
		Sc. PD:	0.089
		Sc. rank:	943.4

,

NP

Pos. 419		obs:	exp:
tct	S	2	0.32
tcc	S	0	0.26
tca	S	0	0.51
tcg	S	0	0.11
cct	P	926	285.50
ccc	P	212	205.50
cca	P	6	470.90
ccg	P	0	182.10
agt	S	0	0.41
agc	S	0	0.39
---	--	------	------
mPD		0.31	0.72
		nPD:	0.44
		N. weight:	1.
		Sc. PD:	0.35
		Sc. rank:	874.2

,

NP

Pos. 420		obs:	exp:
ttt	F	42	452.50
ttc	F	1104	693.50
---	--	------	------
mPD		0.071	0.48
		nPD:	0.15
		N. weight:	0.4
		Sc. PD:	0.025
		Sc. rank:	230.8

,

NP

Pos. 421		obs:	exp:
gaa	E	1146	697.30
gag	E	0	448.70
---	--	------	------
mPD		0	0.48
		nPD:	0.
		N. weight:	0.54
		Sc. PD:	-0.043
		Sc. rank:	-489.5

,

NP

Pos. 422		obs:	exp:
cgt	R	0	49.22
cgc	R	0	56.76
cga	R	2	112.10
cgg	R	0	95.62
aga	R	1133	524.00
agg	R	11	308.20
---	--	------	------
mPD		0.023	0.96
		nPD:	0.02
		N. weight:	0.79
		Sc. PD:	-0.045
		Sc. rank:	-3.1

,

NP

Pos. 423		obs:	exp:
tct	S	0	0.32
tcc	S	0	0.26
tca	S	2	0.51
tcg	S	0	0.11
act	T	0	2.29
acc	T	0	1.85
aca	T	9	4.13
acg	T	0	0.73
agt	S	0	0.41
agc	S	0	0.39
gct	A	1	293.10
gcc	A	0	211.40
gca	A	1060	534.20
gcg	A	74	96.33
---	--	------	------
mPD		0.14	0.69
		nPD:	0.21
		N. weight:	0.68
		Sc. PD:	0.081
		Sc. rank:	450.1

,

NP

Pos. 424		obs:	exp:
act	T	0	291.90
acc	T	1145	234.90
aca	T	0	525.20
acg	T	0	93.00
gct	A	0	0.26
gcc	A	1	0.19
gca	A	0	0.47
gcg	A	0	0.08
---	--	------	------
mPD		0.0017	0.68
		nPD:	0.
		N. weight:	1.7
		Sc. PD:	-0.13
		Sc. rank:	-1234.4

,

NP

Pos. 425		obs:	exp:
att	I	1125	420.70
atc	I	20	274.40
ata	I	0	449.90
gtt	V	1	0.21
gtc	V	0	0.20
gta	V	0	0.20
gtg	V	0	0.39
---	--	------	------
mPD		0.036	0.66
		nPD:	0.06
		N. weight:	1.
		Sc. PD:	-0.026
		Sc. rank:	317.4

,

NP

Pos. 426		obs:	exp:
tta	L	0	0.09
ttg	L	1	0.19
ctt	L	0	0.17
ctc	L	0	0.17
cta	L	0	0.17
ctg	L	0	0.22
att	I	0	0.73
atc	I	0	0.48
ata	I	2	0.79
atg	M	1143	1143.00
---	--	------	------
mPD		0.0052	0.0063
		nPD:	0.84
		N. weight:	0.0034
		Sc. PD:	0.0025
		Sc. rank:	3.1

,

NP

Pos. 427		obs:	exp:
gct	A	2	295.90
gcc	A	0	213.40
gca	A	1129	539.40
gcg	A	15	97.26
---	--	------	------
mPD		0.029	0.67
		nPD:	0.04
		N. weight:	0.76
		Sc. PD:	-0.028
		Sc. rank:	174.0

,

NP

Pos. 428		obs:	exp:
cat	H	1	0.57
cac	H	0	0.43
act	T	0	1.02
acc	T	0	0.82
aca	T	4	1.83
acg	T	0	0.32
gtt	V	0	0.21
gtc	V	0	0.20
gta	V	1	0.20
gtg	V	0	0.39
gct	A	87	294.40
gcc	A	1	212.30
gca	A	1051	536.60
gcg	A	1	96.75
---	--	------	------
mPD		0.16	0.68
		nPD:	0.23
		N. weight:	0.57
		Sc. PD:	0.082
		Sc. rank:	402.9

,

NP

Pos. 429		obs:	exp:
ttt	F	1143	452.50
ttc	F	3	693.50
---	--	------	------
mPD		0.0052	0.48
		nPD:	0.01
		N. weight:	0.99
		Sc. PD:	-0.068
		Sc. rank:	-284.0

,

NP

Pos. 430		obs:	exp:
tct	S	0	0.32
tcc	S	0	0.26
tca	S	2	0.51
tcg	S	0	0.11
act	T	2	289.30
acc	T	3	232.90
aca	T	1130	520.60
acg	T	0	92.19
agt	S	0	0.41
agc	S	0	0.39
gct	A	0	2.32
gcc	A	0	1.68
gca	A	9	4.24
gcg	A	0	0.76
---	--	------	------
mPD		0.028	0.70
		nPD:	0.04
		N. weight:	0.82
		Sc. PD:	-0.034
		Sc. rank:	167.9

,

NP

Pos. 431		obs:	exp:
gtt	V	5	1.04
gtc	V	0	1.01
gta	V	0	1.02
gtg	V	0	1.93
ggt	G	0	151.20
ggc	G	0	145.80
gga	G	1127	526.40
ggg	G	14	317.60
---	--	------	------
mPD		0.041	0.69
		nPD:	0.06
		N. weight:	0.79
		Sc. PD:	-0.016
		Sc. rank:	282.6

,

NP

Pos. 432		obs:	exp:
aat	N	1141	583.70
aac	N	5	562.30
---	--	------	------
mPD		0.0087	0.50
		nPD:	0.02
		N. weight:	0.71
		Sc. PD:	-0.044
		Sc. rank:	-95.3

,

NP

Pos. 433		obs:	exp:
tct	S	0	0.16
tcc	S	0	0.13
tca	S	0	0.25
tcg	S	0	0.06
att	I	1	0.37
atc	I	0	0.24
ata	I	0	0.39
act	T	983	275.80
acc	T	0	222.00
aca	T	99	496.30
acg	T	0	87.89
aat	N	50	25.47
aac	N	0	24.53
agt	S	1	0.21
agc	S	0	0.19
gct	A	12	3.10
gcc	A	0	2.23
gca	A	0	5.65
gcg	A	0	1.02
---	--	------	------
mPD		0.27	0.79
		nPD:	0.34
		N. weight:	1.1
		Sc. PD:	0.27
		Sc. rank:	853.7

,

NP

Pos. 434		obs:	exp:
gtt	V	0	0.21
gtc	V	0	0.20
gta	V	0	0.20
gtg	V	1	0.39
gaa	E	7	696.70
gag	E	1138	448.30
---	--	------	------
mPD		0.014	0.48
		nPD:	0.03
		N. weight:	0.98
		Sc. PD:	-0.05
		Sc. rank:	63.3

,

NP

Pos. 435		obs:	exp:
ggt	G	1102	151.90
ggc	G	2	146.40
gga	G	0	528.70
ggg	G	42	319.00
---	--	------	------
mPD		0.074	0.68
		nPD:	0.11
		N. weight:	2.
		Sc. PD:	0.052
		Sc. rank:	1027.1

,

NP			
Pos.	436	obs:	exp:
cgt	R	0	49.22
cgc	R	0	56.76
cga	R	0	112.10
cgg	R	0	95.62
aga	R	1139	524.00
agg	R	7	308.20
---	--	------	------
mPD		0.012	0.96
		nPD:	0.01
		N. weight:	0.82
		Sc. PD:	-0.055
		Sc. rank:	-202.1

NP			
Pos.	437	obs:	exp:
act	T	0	292.10
acc	T	0	235.20
aca	T	5	525.60
acg	T	1141	93.08
---	--	------	------
mPD		0.0087	0.68
		nPD:	0.01
		N. weight:	2.7
		Sc. PD:	-0.18
		Sc. rank:	-629.7

NP			
Pos.	438	obs:	exp:
tct	S	870	182.30
tcc	S	270	149.70
tca	S	5	290.90
tcg	S	1	65.03
agt	S	0	235.60
agc	S	0	222.60
---	--	------	------
mPD		0.37	1.7
		nPD:	0.22
		N. weight:	1.4
		Sc. PD:	0.19
		Sc. rank:	991.3

NP			
Pos.	439	obs:	exp:
gat	D	0	619.30
gac	D	1146	526.70
---	--	------	------
mPD		0	0.50
		nPD:	0.
		N. weight:	0.85
		Sc. PD:	-0.067
		Sc. rank:	-765.9

NP			
Pos.	440	obs:	exp:
atg	M	1146	1146.00
---	--	------	------
mPD		0	0
		nPD:	1.
		N. weight:	0.
		Sc. PD:	0
		Sc. rank:	0

NP			
Pos.	441	obs:	exp:
cgt	R	0	49.22
cgc	R	0	56.76
cga	R	0	112.10
cgg	R	0	95.62
aga	R	8	524.00
agg	R	1138	308.20
---	--	------	------
mPD		0.014	0.96
		nPD:	0.01
		N. weight:	1.4
		Sc. PD:	-0.09
		Sc. rank:	-264.6

NP			
Pos.	442	obs:	exp:
act	T	1127	292.10
acc	T	8	235.20
aca	T	9	525.60
acg	T	2	93.08
---	--	------	------
mPD		0.033	0.68
		nPD:	0.05
		N. weight:	1.4
		Sc. PD:	-0.045
		Sc. rank:	378.0

NP			
Pos.	443	obs:	exp:
gaa	E	1096	697.30
gag	E	50	448.70
---	--	------	------
mPD		0.084	0.48
		nPD:	0.18
		N. weight:	0.37
		Sc. PD:	0.033
		Sc. rank:	229.5

NP			
Pos.	444	obs:	exp:
att	I	13	421.10
atc	I	1132	274.60
ata	I	1	450.30
---	--	------	------
mPD		0.024	0.65
		nPD:	0.04
		N. weight:	1.5
		Sc. PD:	-0.064
		Sc. rank:	236.7

NP			
Pos.	445	obs:	exp:
att	I	0	421.10
atc	I	0	274.60
ata	I	1146	450.30
---	--	------	------
mPD		0	0.65
		nPD:	0.
		N. weight:	1.
		Sc. PD:	-0.081
		Sc. rank:	-920.4

NP			
Pos.	446	obs:	exp:
cgt	R	0	48.58
cgc	R	0	56.02
cga	R	0	110.70
cgg	R	0	94.37
aaa	K	12	7.58
aag	K	1	5.42
aga	R	1031	517.20
agg	R	100	304.20
ggt	G	0	0.27
ggc	G	0	0.26
gga	G	0	0.92
ggg	G	2	0.56
---	--	------	------
mPD		0.19	0.98
		nPD:	0.19
		N. weight:	0.58
		Sc. PD:	0.062
		Sc. rank:	383.0

NP			
Pos.	447	obs:	exp:
atg	M	1146	1146.00
---	--	------	------
mPD		0	0
		nPD:	1.
		N. weight:	0.
		Sc. PD:	0
		Sc. rank:	0

NP			
Pos.	448	obs:	exp:
tta	L	0	0.09
ttg	L	1	0.19
ctt	L	0	0.17
ctc	L	0	0.17
cta	L	0	0.17
ctg	L	0	0.22
atg	M	1145	1145.00
---	--	------	------
mPD		0.0017	0.0028
		nPD:	0.63
		N. weight:	0.0016
		Sc. PD:	0.00084
		Sc. rank:	1.4

NP			
Pos.	449	obs:	exp:
gaa	E	1146	697.30
gag	E	0	448.70
---	--	------	------
mPD		0	0.48
		nPD:	0.
		N. weight:	0.54
		Sc. PD:	-0.043
		Sc. rank:	-489.5

NP			
Pos.	450	obs:	exp:
tct	S	0	181.50
tcc	S	0	149.00
tca	S	0	289.60
tcg	S	0	64.74
aat	N	5	2.55
aac	N	0	2.45
agt	S	1136	234.60
agc	S	5	221.60
---	--	------	------
mPD		0.017	1.7
		nPD:	0.01
		N. weight:	1.7
		Sc. PD:	-0.12
		Sc. rank:	-541.9

NP			
Pos.	451	obs:	exp:
gct	A	1	295.90
gcc	A	1074	213.40
gca	A	70	539.40
gcg	A	1	97.26
---	--	------	------
mPD		0.12	0.67
		nPD:	0.18
		N. weight:	1.5
		Sc. PD:	0.14
		Sc. rank:	945.4

NP			
Pos.	452	obs:	exp:
cgt	R	0	48.15
cgc	R	0	55.52
cga	R	0	109.70
cgg	R	0	93.54
aaa	K	25	14.57
aag	K	0	10.43
aga	R	1117	512.60
agg	R	4	301.50
---	--	------	------
mPD		0.050	1.0
		nPD:	0.05
		N. weight:	0.83
		Sc. PD:	-0.026
		Sc. rank:	239.6

NP			
Pos.	453	obs:	exp:
tct	S	0	0.48
tcc	S	0	0.39
tca	S	3	0.76
tcg	S	0	0.17
cct	P	0	285.20
ccc	P	0	205.40
cca	P	1111	470.50
ccg	P	32	182.00
agt	S	0	0.62
agc	S	0	0.58
---	--	------	------
mPD		0.060	0.72
		nPD:	0.08
		N. weight:	0.86
		Sc. PD:	0.00061
		Sc. rank:	397.3

NP			
Pos.	454	obs:	exp:
gaa	E	1144	696.10
gag	E	0	447.90
ggt	G	0	0.27
ggc	G	0	0.26
gga	G	2	0.92
ggg	G	0	0.56
---	--	------	------
mPD		0.0035	0.48
		nPD:	0.01
		N. weight:	0.54
		Sc. PD:	-0.039
		Sc. rank:	-243.9

NP			
Pos.	455	obs:	exp:
aat	N	3	1.53
aac	N	0	1.47
gat	D	1143	617.70
gac	D	0	525.30
---	--	------	------
mPD		0.0052	0.50
		nPD:	0.01
		N. weight:	0.67
		Sc. PD:	-0.046
		Sc. rank:	-228.3

NP			
Pos.	456	obs:	exp:
tta	L	0	0.09
ttg	L	1	0.19
ctt	L	0	0.17
ctc	L	0	0.17
cta	L	0	0.17
ctg	L	0	0.22
atg	M	1	1.00
gtt	V	0	235.80
gtc	V	0	229.70
gta	V	0	233.00
gtg	V	1138	439.50
gct	A	0	1.29
gcc	A	0	0.93
gca	A	0	2.35
gcg	A	5	0.42
ggt	G	0	0.13
ggc	G	0	0.13
gga	G	0	0.46
ggg	G	1	0.28
---	--	------	------
mPD		0.014	0.74
		nPD:	0.02
		N. weight:	1.
		Sc. PD:	-0.064
		Sc. rank:	-78.6

NP			
Pos.	457	obs:	exp:
tct	S	2	182.30
tcc	S	0	149.70
tca	S	1029	290.90
tcg	S	115	65.03
agt	S	0	235.60
agc	S	0	222.60
---	--	------	------
mPD		0.18	1.7
		nPD:	0.11
		N. weight:	1.3
		Sc. PD:	0.036
		Sc. rank:	673.7

NP			
Pos.	458	obs:	exp:
ttt	F	0	452.50
ttc	F	1146	693.50
---	--	------	------
mPD		0	0.48
		nPD:	0.
		N. weight:	0.55
		Sc. PD:	-0.043
		Sc. rank:	-495.0

NP			
Pos.	459	obs:	exp:
caa	Q	14	583.70
cag	Q	1132	562.30
---	--	------	------
mPD		0.024	0.50
		nPD:	0.05
		N. weight:	0.71
		Sc. PD:	-0.023
		Sc. rank:	187.4

NP			
Pos.	460	obs:	exp:
ggt	G	0	151.90
ggc	G	0	146.40
gga	G	23	528.70
ggg	G	1123	319.00
mPD		0.039	0.68
		nPD:	0.06
		N. weight:	1.3
		Sc. PD:	-0.029
		Sc. rank:	419.4

NP			
Pos.	461	obs:	exp:
cgt	R	0	49.22
cgc	R	0	56.76
cga	R	0	112.10
cgg	R	1146	95.62
aga	R	0	524.00
agg	R	0	308.20
mPD		0	0.96
		nPD:	0.
		N. weight:	2.7
		Sc. PD:	-0.21
		Sc. rank:	-2447.1

NP			
Pos.	462	obs:	exp:
ggt	G	0	151.90
ggc	G	0	146.40
gga	G	1146	528.70
ggg	G	0	319.00
mPD		0	0.68
		nPD:	0.
		N. weight:	0.84
		Sc. PD:	-0.067
		Sc. rank:	-762.3

NP			
Pos.	463	obs:	exp:
gtt	V	4	237.50
gtc	V	1142	231.30
gta	V	0	234.70
gtg	V	0	442.60
mPD		0.0070	0.73
		nPD:	0.01
		N. weight:	1.7
		Sc. PD:	-0.12
		Sc. rank:	-625.5

NP			
Pos.	464	obs:	exp:
ttt	F	0	452.50
ttc	F	1146	693.50
mPD		0	0.48
		nPD:	0.
		N. weight:	0.55
		Sc. PD:	-0.043
		Sc. rank:	-495.0

NP			
Pos.	465	obs:	exp:
gaa	E	0	697.30
gag	E	1146	448.70
mPD		0	0.48
		nPD:	0.
		N. weight:	1.
		Sc. PD:	-0.081
		Sc. rank:	-924.0

NP			
Pos.	466	obs:	exp:
tta	L	0	101.70
ttg	L	0	213.40
ctt	L	6	190.80
ctc	L	865	191.20
cta	L	274	193.10
ctg	L	1	255.70
mPD		0.37	1.1
		nPD:	0.34
		N. weight:	1.3
		Sc. PD:	0.32
		Sc. rank:	1035.8

NP			
Pos.	467	obs:	exp:
tct	S	2	182.30
tcc	S	0	149.70
tca	S	5	290.90
tcg	S	1139	65.03
agt	S	0	235.60
agc	S	0	222.60
mPD		0.012	1.7
		nPD:	0.01
		N. weight:	3.1
		Sc. PD:	-0.22
		Sc. rank:	-1296.4

NP			
Pos.	468	obs:	exp:
gat	D	3	619.30
gac	D	1143	526.70
mPD		0.0052	0.50
		nPD:	0.01
		N. weight:	0.83
		Sc. PD:	-0.057
		Sc. rank:	-260.4

NP			
Pos.	469	obs:	exp:
gaa	E	1146	697.30
gag	E	0	448.70
mPD		0	0.48
		nPD:	0.
		N. weight:	0.54
		Sc. PD:	-0.043
		Sc. rank:	-489.5

NP			
Pos.	470	obs:	exp:
aaa	K	0	667.90
aag	K	1146	478.10
mPD		0	0.49
		nPD:	0.
		N. weight:	0.95
		Sc. PD:	-0.076
		Sc. rank:	-861.4

NP			
Pos.	471	obs:	exp:
gct	A	0	295.90
gcc	A	0	213.40
gca	A	1146	539.40
gcg	A	0	97.26
mPD		0	0.67
		nPD:	0.
		N. weight:	0.82
		Sc. PD:	-0.065
		Sc. rank:	-742.5

NP			
Pos.	472	obs:	exp:
act	T	0	292.10
acc	T	0	235.20
aca	T	0	525.60
acg	T	1146	93.08
mPD		0	0.68
		nPD:	0.
		N. weight:	2.7
		Sc. PD:	-0.22
		Sc. rank:	-2473.6

NP			
Pos.	473	obs:	exp:
act	T	0	0.25
acc	T	1	0.21
aca	T	0	0.46
acg	T	0	0.08
aat	N	0	582.70
aac	N	1144	561.30
gat	D	0	0.54
gac	D	1	0.46
mPD		0.0035	0.50
		nPD:	0.01
		N. weight:	0.78
		Sc. PD:	-0.056
		Sc. rank:	-364.9

NP			
Pos.	474	obs:	exp:
tct	S	0	0.16
tcc	S	0	0.13
tca	S	0	0.25
tcg	S	1	0.06
cct	P	0	285.70
ccc	P	0	205.70
cca	P	1	471.30
ccg	P	1144	182.30
agt	S	0	0.21
agc	S	0	0.19
mPD		0.0035	0.71
		nPD:	0.
		N. weight:	2.
		Sc. PD:	-0.15
		Sc. rank:	-1089.5

NP			
Pos.	475	obs:	exp:
att	I	0	421.10
atc	I	1146	274.60
ata	I	0	450.30
mPD		0	0.65
		nPD:	0.
		N. weight:	1.6
		Sc. PD:	-0.12
		Sc. rank:	-1407.6

NP			
Pos.	476	obs:	exp:
gtt	V	0	237.50
gtc	V	0	231.30
gta	V	0	234.70
gtg	V	1146	442.60
mPD		0	0.73
		nPD:	0.
		N. weight:	1.
		Sc. PD:	-0.082
		Sc. rank:	-937.4

NP			
Pos.	477	obs:	exp:
cct	P	1144	286.00
ccc	P	2	205.90
cca	P	0	471.70
ccg	P	0	182.40
mPD		0.0035	0.71
		nPD:	0.
		N. weight:	1.5
		Sc. PD:	-0.11
		Sc. rank:	-801.3

NP			
Pos.	478	obs:	exp:
tct	S	11	182.30
tcc	S	1135	149.70
tca	S	0	290.90
tcg	S	0	65.03
agt	S	0	235.60
agc	S	0	222.60
mPD		0.019	1.7
		nPD:	0.01
		N. weight:	2.2
		Sc. PD:	-0.15
		Sc. rank:	-582.4

NP			
Pos.	479	obs:	exp:
ttt	F	1146	452.50
ttc	F	0	693.50
mPD		0	0.48
		nPD:	0.
		N. weight:	1.
		Sc. PD:	-0.08
		Sc. rank:	-915.5

NP			
Pos.	480	obs:	exp:
gat	D	11	619.30
gac	D	1135	526.70
mPD		0.019	0.50
		nPD:	0.04
		N. weight:	0.79
		Sc. PD:	-0.033
		Sc. rank:	146.2

NP			
Pos.	481	obs:	exp:
att	I	0	1.47
atc	I	0	0.96
ata	I	4	1.57
atg	M	1142	1142.00
mPD		0.0070	0.0070
		nPD:	1.
		N. weight:	0.0036
		Sc. PD:	0.0031
		Sc. rank:	3.2

NP			
Pos.	482	obs:	exp:
tct	S	0	48.51
tcc	S	0	39.83
tca	S	0	77.41
tcg	S	0	17.31
aat	N	839	427.90
aac	N	1	412.10
agt	S	305	62.70
agc	S	0	59.24
ggt	G	1	0.13
ggc	G	0	0.13
gga	G	0	0.46
ggg	G	0	0.28
mPD		0.40	1.3
		nPD:	0.31
		N. weight:	0.99
		Sc. PD:	0.22
		Sc. rank:	761.8

NP			
Pos.	483	obs:	exp:
aat	N	1143	583.70
aac	N	3	562.30
mPD		0.0052	0.50
		nPD:	0.01
		N. weight:	0.72
		Sc. PD:	-0.05
		Sc. rank:	-239.8

NP			
Pos.	484	obs:	exp:
gat	D	0	0.54
gac	D	1	0.46
gaa	E	1126	696.70
gag	E	19	448.30
mPD		0.034	0.48
		nPD:	0.07
		N. weight:	0.46
		Sc. PD:	-0.0045
		Sc. rank:	184.3

NP

Pos.	485	obs:	exp:
cgt	R	0	0.04
cgc	R	0	0.05
cga	R	0	0.10
cgg	R	0	0.08
aga	R	1	0.46
agg	R	0	0.27
ggt	G	2	151.80
ggc	G	0	146.30
gga	G	1123	528.20
ggg	G	20	318.80
---	--	------	------
mPD		0.039	0.68
		nPD:	0.06
		N. weight:	0.75
		Sc. PD:	-0.017
		Sc. rank:	249.7

,

NP

Pos.	486	obs:	exp:
tct	S	1143	182.30
tcc	S	1	149.70
tca	S	0	290.90
tcg	S	2	65.03
agt	S	0	235.60
agc	S	0	222.60
---	--	------	------
mPD		0.0052	1.7
		nPD:	0.
		N. weight:	2.
		Sc. PD:	-0.15
		Sc. rank:	-1276.2

,

NP

Pos.	487	obs:	exp:
tat	Y	1146	619.00
tac	Y	0	527.00
---	--	------	------
mPD		0	0.50
		nPD:	0.
		N. weight:	0.67
		Sc. PD:	-0.053
		Sc. rank:	-607.0

,

NP

Pos.	488	obs:	exp:
ttt	F	0	452.50
ttc	F	1146	693.50
---	--	------	------
mPD		0	0.48
		nPD:	0.
		N. weight:	0.55
		Sc. PD:	-0.043
		Sc. rank:	-495.0

,

NP

Pos.	489	obs:	exp:
ttt	F	9	452.50
ttc	F	1137	693.50
---	--	------	------
mPD		0.016	0.48
		nPD:	0.03
		N. weight:	0.5
		Sc. PD:	-0.024
		Sc. rank:	53.2

,

NP

Pos.	490	obs:	exp:
ggt	G	0	151.90
ggc	G	0	146.40
gga	G	1146	528.70
ggg	G	0	319.00
---	--	------	------
mPD		0	0.68
		nPD:	0.
		N. weight:	0.84
		Sc. PD:	-0.067
		Sc. rank:	-762.3

,

NP

Pos.	491	obs:	exp:
gat	D	0	619.30
gac	D	1146	526.70
---	--	------	------
mPD		0	0.50
		nPD:	0.
		N. weight:	0.85
		Sc. PD:	-0.067
		Sc. rank:	-765.9

,

NP

Pos.	492	obs:	exp:
aat	N	1145	583.70
aac	N	1	562.30
---	--	------	------
mPD		0.0017	0.50
		nPD:	0.
		N. weight:	0.73
		Sc. PD:	-0.055
		Sc. rank:	-444.3

,

NP

Pos.	493	obs:	exp:
gct	A	0	295.90
gcc	A	0	213.40
gca	A	1146	539.40
gcg	A	0	97.26
---	--	------	------
mPD		0	0.67
		nPD:	0.
		N. weight:	0.82
		Sc. PD:	-0.065
		Sc. rank:	-742.5

,

NP

Pos.	494	obs:	exp:
gaa	E	30	697.30
gag	E	1116	448.70
---	--	------	------
mPD		0.051	0.48
		nPD:	0.11
		N. weight:	0.88
		Sc. PD:	0.021
		Sc. rank:	447.4

,

NP

Pos.	495	obs:	exp:
gat	D	2	1.08
gac	D	0	0.92
gaa	E	53	696.10
gag	E	1091	447.90
---	--	------	------
mPD		0.092	0.48
		nPD:	0.19
		N. weight:	0.8
		Sc. PD:	0.084
		Sc. rank:	524.5

,

NP

Pos.	496	obs:	exp:
ttt	F	2	0.79
ttc	F	0	1.21
tat	Y	1141	617.30
tac	Y	2	525.70
aat	N	1	0.51
aac	N	0	0.49
---	--	------	------
mPD		0.0087	0.50
		nPD:	0.02
		N. weight:	0.66
		Sc. PD:	-0.041
		Sc. rank:	-90.7

,

NP

Pos.	497	obs:	exp:
aat	N	0	0.51
aac	N	1	0.49
gat	D	0	617.70
gac	D	1143	525.30
gaa	E	1	0.61
gag	E	0	0.39
ggt	G	0	0.13
ggc	G	1	0.13
gga	G	0	0.46
ggg	G	0	0.28
---	--	------	------
mPD		0.0052	0.50
		nPD:	0.01
		N. weight:	0.85
		Sc. PD:	-0.059
		Sc. rank:	-286.0

,

NP

Pos.	498	obs:	exp:
tct	S	0	5.25
tcc	S	0	4.31
tca	S	0	8.38
tcg	S	0	1.87
cat	H	3	1.72
cac	H	0	1.28
aat	N	1110	565.40
aac	N	0	544.60
agt	S	33	6.78
agc	S	0	6.41
---	--	------	------
mPD		0.061	0.60
		nPD:	0.1
		N. weight:	0.76
		Sc. PD:	0.014
		Sc. rank:	378.2

,

NP

Pos.	499	obs:	exp:
taa	.	58	486.90
tag	.	0	286.90
tga	.	1088	372.20
---	--	------	------
mPD		0.096	0.81
		nPD:	0.12
		N. weight:	1.
		Sc. PD:	0.034
		Sc. rank:	543.1

}

Amino acid level conservation per codon

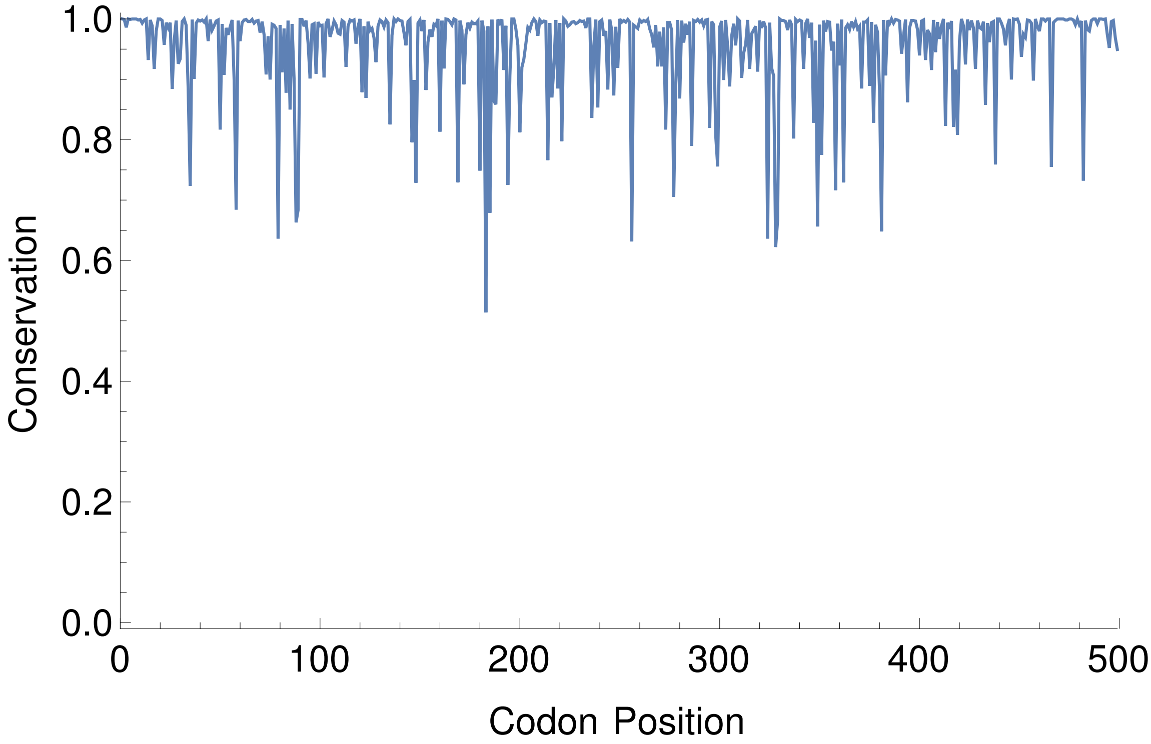

Conservation analysis plots

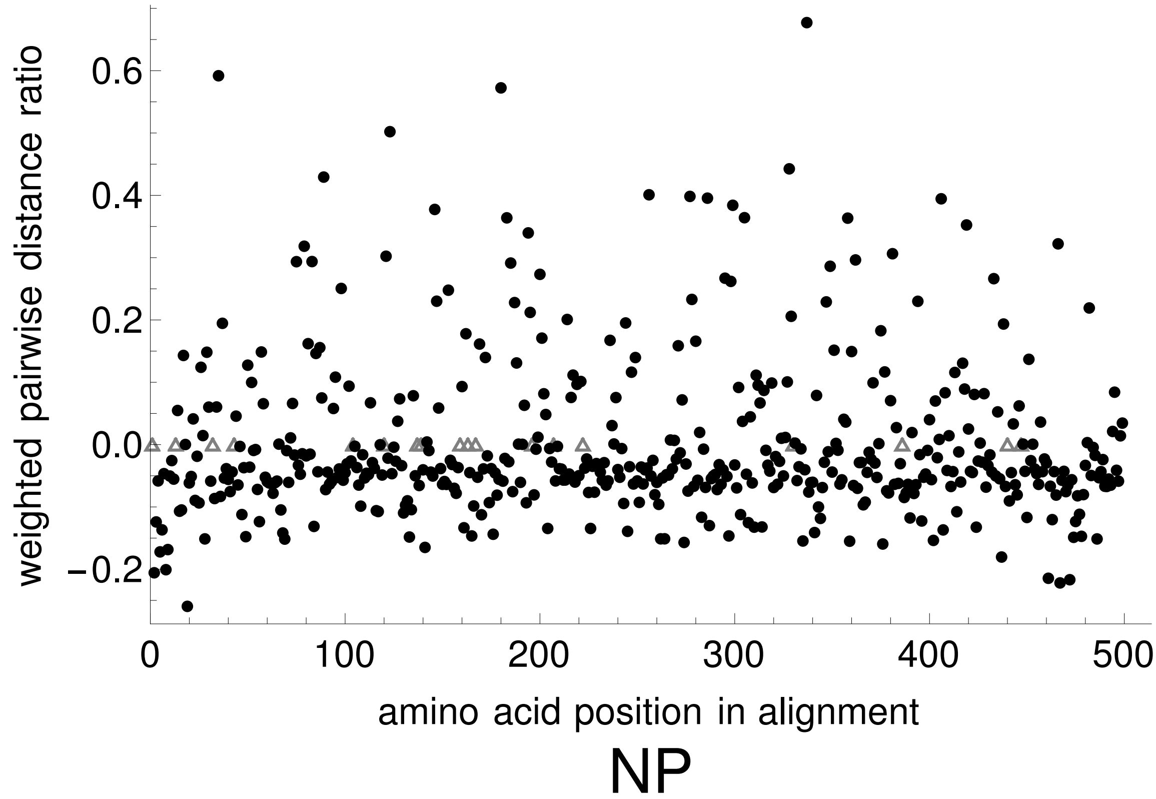

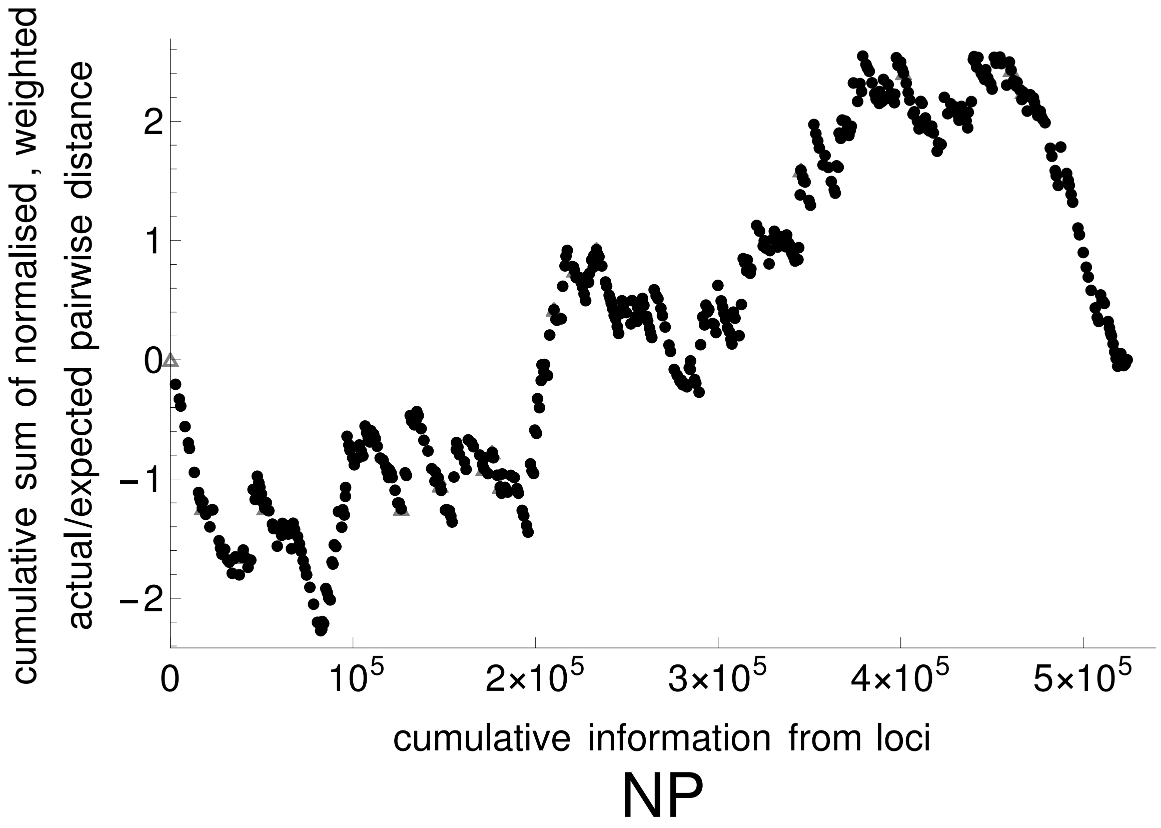

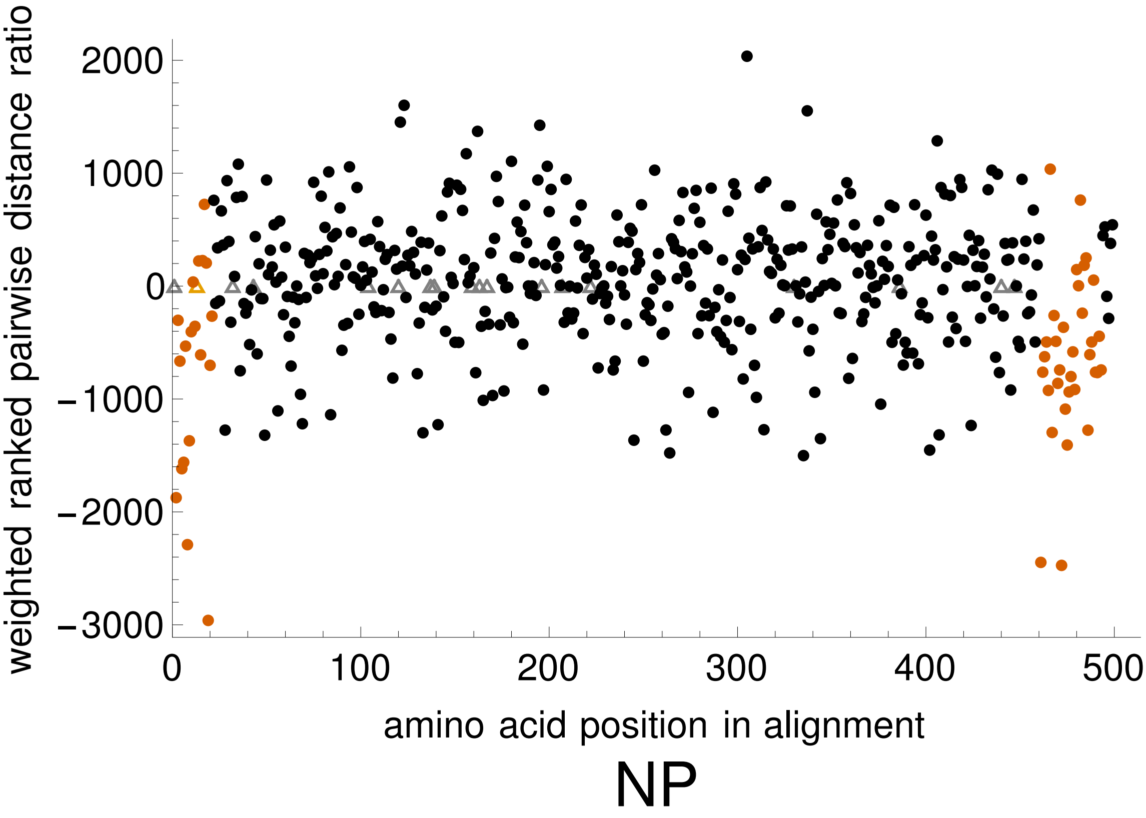
weighted ranked pairwise distance ratio
2000
1000
0
−1000
−2000
−3000
0
100
200
300
400
500
amino acid position in alignment
NP

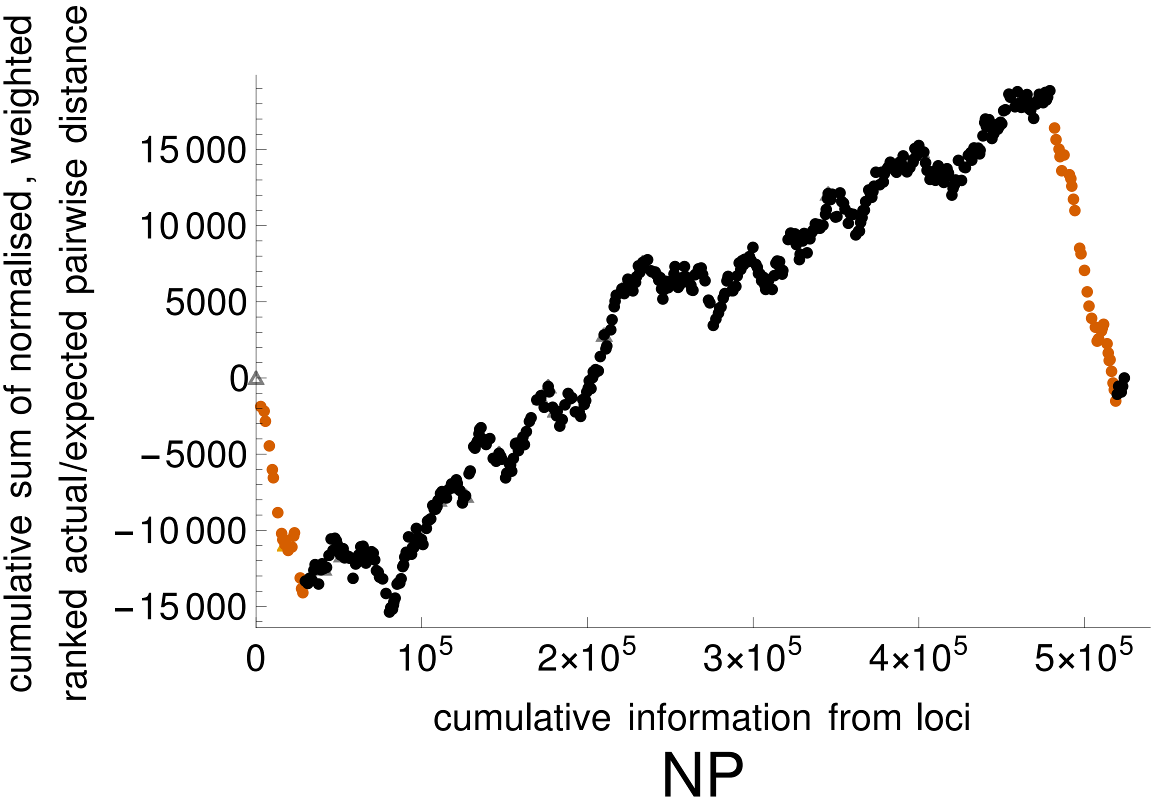
cumulative sum of normalised, weighted ranked actual/expected pairwise distance
15 000
10 000
5000
0
−5000
−10 000
−15 000
0
10^5
2×10^5
3×10^5
4×10^5
5×10^5
cumulative information from loci
NP

NA

Gene length histogram

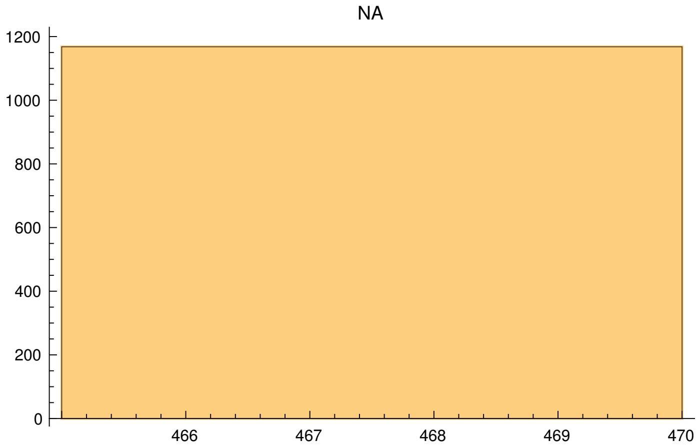

Information vs. nPD

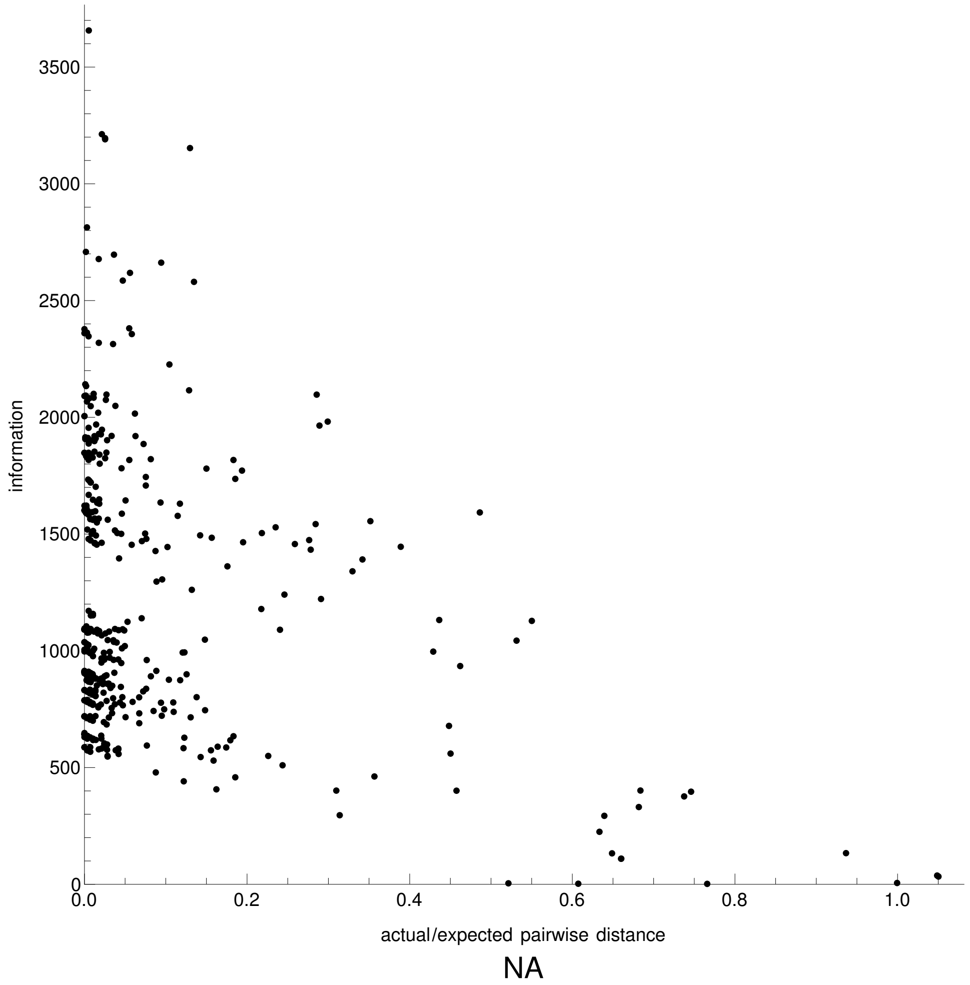

Example sequences highlighted by regions found in analyses to be conserved

```
Interesting points (by weighted raw PD) highlighted for gene NA:
ATGAATCCAAATCAGAAGATTCTATGCACTTCAGCCACTGCCATCACAATAGGCGCAATCGCAGTACTCATTGGAA⋱
    TAGCAAACCTAGGATTGAACATAGGACTGCATCTAAAACCGGGCTGCAACTGCACACACTCACAACCTGAAACAA⋱
    CCAACACAAGCCAAACAATAATAAACAACTATTATAATGAA ---------------ACAAACATC---------------
    CCAACATCCAAATGGAAGAAAGAACAAGCAGGAATTTCAATAACCTAACTAAAGGGCTCTGTACTATAAATTCAT⋱
    GGCACATATATGGGAAAGACAATGCAGTAAGAATTGGAGAAAGCTCGGATGTTTTAGTTACAAGAGAACCCTATG⋱
    TTTCATGCGACCCAGATGAATGCAGGTTCTATGCTCTCAGCCAAGGAACAACAATCAGAGGGAAACACTCAAACG⋱
    GAACAATACACGATAGGTCCCAGTATCGCGCCCTGATAAGCTGGCCACTATCATCACCGCCCACAGTGCACAACA⋱
    GCAGGGTGGAATGCATTGGGTGGTCAAGTGCTAGTTGCCATGATGGCAAGTCCAGGATGTCAATATGTATATCAG⋱
    GACCGAACAACAATGCATCTGCAGTAGTATGGTACAACAGAAGGCCTATAGCAGAAATTAACACATGGGCCCGAA⋱
    ACATACTAAGAACACAGGAATCTGAATGTGTGTGCCACAACGGCGTATGCCCAGTAGTGTTCACCGATGGGCCCG⋱
    CCACTGGACCTGCAGACACAAGAATATACTATTTTAAAGAGGGGAAAATATTAAAATGGGAGTCTCTGACTGGAA⋱
    CTGCTAAGCATATTGAAGAATGCTCATGTTACGGGGAACGAACAGGGATTACCTGCACATGCAGGGACAATTGGC⋱
    AGGGCTCAAATAGACCAGTGATTCAGATTGACCCAGTAGCAATGACACACACTAGTCAATATATATGCAGTCCTG⋱
    TTCTTACAGACAGTCCCCGGCCGAATGACCCAAATATAGGTAAGTGTAATGACCCTTATCCAGGTAATAATAACA⋱
    ATGGAGTCAAGGGATTCTCATACCTGGATGGGGCTAACACCTGGCTAGGGAGGACAATAAGCACAGCATCGAGGT⋱
    CTGGATACGAGATGTTGAAAGTGCCAAATGCATTGACAGATGATAGATCAAAGCCCATTCAAGGTCAGACAATTG⋱
    TATTAAACGCTGATTGGAGTGGTTACAGTGGGTCCTTTATGGACTATTGGGCTGAAGGGGACTGCTATCGAGCGT⋱
    GCTTTTATGTGGAGTTGATACGTGGAAGACCCAAGGAGGATAAAGTGTGGTGGACCAGCAATAGTATAGTATCGA⋱
    TGTGTTCCAGTACAGAGTTCCTGGGACAATGGAACTGGCCTGATGGGGCTAAAATAGAGTACTTCCTC ---TAA
```

```
Interesting points (by weighted ranked PD) highlighted for gene NA:
ATGAATCCAAATCAGAAGATTCTATGCACTTCAGCCACTGCCATCACAATAGGCGCAATCGCAGTACTCATTGGAATA⋱
    GCAAACCTAGGATTGAACATAGGACTGCATCTAAAACCGGGCTGCAACTGCACACACTCACAACCTGAAACAACCAA⋱
    CACAAGCCAAACAATAATAAACAACTATTATAATGAA ---------------ACAAACATC---------------ACCA
    CATCCAAATGGAAGAAAGAACAAGCAGGAATTTCAATAACCTAACTAAAGGGCTCTGTACTATAAATTCATGGCA⋱
    CATATATGGGAAAGACAATGCAGTAAGAATTGGAGAAAGCTCGGATGTTTTAGTTACAAGAGAACCCTATGTTTC⋱
    ATGCGACCCAGATGAATGCAGGTTCTATGCTCTCAGCCAAGGAACAACAATCAGAGGGAAACACTCAAACGGAAC⋱
    AATACACGATAGGTCCCAGTATCGCGCCCTGATAAGCTGGCCACTATCATCACCGCCCACAGTGCACAACAGCAG⋱
    GGTGGAATGCATTGGGTGGTCAAGTGCTAGTTGCCATGATGGCAAGTCCAGGATGTCAATATGTATATCAGGACC⋱
    GAACAACAATGCATCTGCAGTAGTATGGTACAACAGAAGGCCTATAGCAGAAATTAACACATGGGCCCGAAACAT⋱
    ACTAAGAACACAGGAATCTGAATGTGTGTGCCACAACGGCGTATGCCCAGTAGTGTTCACCGATGGGCCCGCCAC⋱
    TGGACCTGCAGACACAAGAATATACTATTTTAAAGAGGGGAAAATATTAAAATGGGAGTCTCTGACTGGAACTGC⋱
    TAAGCATATTGAAGAATGCTCATGTTACGGGGAACGAACAGGGATTACCTGCACATGCAGGGACAATTGGCAGGG⋱
    CTCAAATAGACCAGTGATTCAGATTGACCCAGTAGCAATGACACACACTAGTCAATATATATGCAGTCCTGTTCT⋱
    TACAGACAGTCCCCGGCCGAATGACCCAAATATAGGTAAGTGTAATGACCCTTATCCAGGTAATAATAACAATGG⋱
    AGTCAAGGGATTCTCATACCTGGATGGGGCTAACACCTGGCTAGGGAGGACAATAAGCACAGCATCGAGGTCTGG⋱
    ATACGAGATGTTGAAAGTGCCAAATGCATTGACAGATGATAGATCAAAGCCCATTCAAGGTCAGACAATTGTATT⋱
    AAACGCTGATTGGAGTGGTTACAGTGGGTCCTTTATGGACTATTGGGCTGAAGGGGACTGCTATCGAGCGTGCTT⋱
    TTATGTGGAGTTGATACGTGGAAGACCCAAGGAGGATAAAGTGTGGTGGACCAGCAATAGTATAGTATCGATGTG⋱
    TTCCAGTACAGAGTTCCTGGGACAATGGAACTGGCCTGATGGGGCTAAAATAGAGTACTTCCTC ---TAA
```

Per codon conservation report

NA

Pos.	1	obs:	exp:
atg	M	1168	1168.00
---	--	------	------
mPD		0	0
		nPD:	1.
		N. weight:	0.
		Sc. PD:	0
		Sc. rank:	0

NA

Pos.	2	obs:	exp:
aat	N	1168	594.90
aac	N	0	573.10
---	--	------	------
mPD		0	0.50
		nPD:	0.
		N. weight:	0.71
		Sc. PD:	-0.037
		Sc. rank:	-673.9

NA

Pos.	3	obs:	exp:
cct	P	0	291.40
ccc	P	0	209.90
cca	P	1168	480.80
ccg	P	0	185.90
---	--	------	------
mPD		0	0.71
		nPD:	0.
		N. weight:	0.94
		Sc. PD:	-0.048
		Sc. rank:	-886.7

NA

Pos.	4	obs:	exp:
tct	S	0	0.48
tcc	S	0	0.39
tca	S	0	0.76
tcg	S	0	0.17
aat	N	1114	593.40
aac	N	51	571.60
agt	S	3	0.62
agc	S	0	0.58
---	--	------	------
mPD		0.089	0.51
		nPD:	0.17
		N. weight:	0.53
		Sc. PD:	0.06
		Sc. rank:	419.0

NA

Pos.	5	obs:	exp:
caa	Q	0	594.90
cag	Q	1168	573.10
---	--	------	------
mPD		0	0.50
		nPD:	0.
		N. weight:	0.75
		Sc. PD:	-0.039
		Sc. rank:	-711.3

NA

Pos.	6	obs:	exp:
aaa	K	0	680.20
aag	K	1167	486.80
gaa	E	0	0.61
gag	E	1	0.39
---	--	------	------
mPD		0.0017	0.49
		nPD:	0.
		N. weight:	0.92
		Sc. PD:	-0.045
		Sc. rank:	-489.8

NA

Pos.	7	obs:	exp:
att	I	1149	428.80
atc	I	18	279.70
ata	I	0	458.60
gtt	V	1	0.21
gtc	V	0	0.20
gta	V	0	0.20
gtg	V	0	0.39
---	--	------	------
mPD		0.032	0.66
		nPD:	0.05
		N. weight:	0.98
		Sc. PD:	-0.0054
		Sc. rank:	467.6

NA

Pos.	8	obs:	exp:
tta	L	2	103.60
ttg	L	0	217.50
ctt	L	0	194.50
ctc	L	0	194.90
cta	L	1166	196.80
ctg	L	0	260.60
---	--	------	------
mPD		0.0034	1.1
		nPD:	0.
		N. weight:	1.9
		Sc. PD:	-0.091
		Sc. rank:	-1123.4

NA

Pos.	9	obs:	exp:
tat	Y	0	2.70
tac	Y	5	2.30
tgt	C	1	491.90
tgc	C	1162	671.10
---	--	------	------
mPD		0.010	0.50
		nPD:	0.02
		N. weight:	0.58
		Sc. PD:	-0.019
		Sc. rank:	75.4

NA

Pos.	10	obs:	exp:
tct	S	1	0.16
tcc	S	0	0.13
tca	S	0	0.25
tcg	S	0	0.06
att	I	3	1.10
atc	I	0	0.72
ata	I	0	1.18
act	T	1137	296.50
acc	T	26	238.60
aca	T	0	533.40
acg	T	0	94.47
agt	S	0	0.21
agc	S	0	0.19
gct	A	1	0.26
gcc	A	0	0.19
gca	A	0	0.47
gcg	A	0	0.08
---	--	------	------
mPD		0.052	0.68
		nPD:	0.08
		N. weight:	1.3
		Sc. PD:	0.027
		Sc. rank:	804.8

NA

Pos.	11	obs:	exp:
tct	S	0	185.80
tcc	S	0	152.50
tca	S	1167	296.50
tcg	S	1	66.28
agt	S	0	240.10
agc	S	0	226.90
---	--	------	------
mPD		0.0017	1.7
		nPD:	0.
		N. weight:	1.4
		Sc. PD:	-0.073
		Sc. rank:	-1059.9

NA

Pos.	12	obs:	exp:
gtt	V	0	0.21
gtc	V	1	0.20
gta	V	0	0.20
gtg	V	0	0.39
gct	A	0	301.40
gcc	A	1166	217.30
gca	A	1	549.30
gcg	A	0	99.05
---	--	------	------
mPD		0.0034	0.67
		nPD:	0.01
		N. weight:	1.8
		Sc. PD:	-0.083
		Sc. rank:	-744.6

NA

Pos.	13	obs:	exp:
att	I	3	1.10
atc	I	0	0.72
ata	I	0	1.18
atg	M	2	2.00
act	T	1128	295.90
acc	T	31	238.20
aca	T	2	532.50
acg	T	0	94.30
gct	A	2	0.52
gcc	A	0	0.37
gca	A	0	0.94
gcg	A	0	0.17
---	--	------	------
mPD		0.070	0.69
		nPD:	0.1
		N. weight:	1.3
		Sc. PD:	0.058
		Sc. rank:	873.2

NA

Pos.	14	obs:	exp:
act	T	8	2.04
acc	T	0	1.64
aca	T	0	3.67
acg	T	0	0.65
gct	A	897	299.50
gcc	A	262	216.00
gca	A	1	546.00
gcg	A	0	98.45
---	--	------	------
mPD		0.36	0.68
		nPD:	0.53
		N. weight:	0.94
		Sc. PD:	0.42
		Sc. rank:	899.8

NA

Pos.	15	obs:	exp:
ttt	F	0	0.39
ttc	F	1	0.61
att	I	0	428.40
atc	I	1166	279.40
ata	I	0	458.20
act	T	0	0.25
acc	T	1	0.21
aca	T	0	0.46
acg	T	0	0.08
---	--	------	------
mPD		0.0034	0.66
		nPD:	0.01
		N. weight:	1.5
		Sc. PD:	-0.07
		Sc. rank:	-588.6

NA

Pos.	16	obs:	exp:
att	I	0	202.80
atc	I	0	132.30
ata	I	552	216.90
act	T	0	156.00
acc	T	0	125.60
aca	T	612	280.70
acg	T	0	49.71
gtt	V	0	0.41
gtc	V	0	0.40
gta	V	2	0.41
gtg	V	0	0.77
gct	A	0	0.26
gcc	A	0	0.19
gca	A	1	0.47
gcg	A	0	0.08
---	-	1	1.00
---	--	------	------
mPD		0.50	1.2
		nPD:	0.43
		N. weight:	0.9
		Sc. PD:	0.32
		Sc. rank:	838.5

NA

Pos.	17	obs:	exp:
att	I	1	425.10
atc	I	0	277.30
ata	I	1156	454.60
act	T	0	2.80
acc	T	0	2.26
aca	T	11	5.05
acg	T	0	0.89
---	--	------	------
mPD		0.020	0.67
		nPD:	0.03
		N. weight:	0.98
		Sc. PD:	-0.023
		Sc. rank:	301.5

NA

Pos.	18	obs:	exp:
tct	S	0	2.70
tcc	S	0	2.22
tca	S	0	4.31
tcg	S	0	0.96
cgt	R	0	0.04
cgc	R	0	0.05
cga	R	0	0.10
cgg	R	0	0.08
agt	S	0	3.49
agc	S	17	3.30
aga	R	0	0.46
agg	R	1	0.27
ggt	G	5	152.40
ggc	G	1144	146.90
gga	G	1	530.50
ggg	G	0	320.10
---	--	------	------
mPD		0.042	0.73
		nPD:	0.06
		N. weight:	2.1
		Sc. PD:	0.0068
		Sc. rank:	1130.8

NA

Pos.	19	obs:	exp:
cgt	R	0	0.04
cgc	R	1	0.05
cga	R	0	0.10
cgg	R	0	0.08
act	T	0	1.27
acc	T	0	1.03
aca	T	5	2.29
acg	T	0	0.41
aga	R	0	0.46
agg	R	0	0.27
gtt	V	0	0.21
gtc	V	0	0.20
gta	V	1	0.20
gtg	V	0	0.39
gct	A	0	299.80
gcc	A	0	216.20
gca	A	1161	546.40
gcg	A	0	98.54
---	--	------	------
mPD		0.015	0.68
		nPD:	0.02
		N. weight:	0.8
		Sc. PD:	-0.024
		Sc. rank:	141.0

NA

Pos.	20	obs:	exp:
att	I	3	427.70
atc	I	1161	278.90
ata	I	0	457.40
act	T	0	1.02
acc	T	4	0.82
aca	T	0	1.83
acg	T	0	0.32
---	--	------	------
mPD		0.012	0.66
		nPD:	0.02
		N. weight:	1.5
		Sc. PD:	-0.052
		Sc. rank:	134.4

NA			
Pos.	21	obs:	exp:
act	T	0	11.73
acc	T	0	9.44
aca	T	46	21.10
acg	T	0	3.74
gct	A	0	289.70
gcc	A	0	209.00
gca	A	1121	528.10
gcg	A	1	95.23
---	--	------	------
mPD		0.077	0.75
		nPD:	0.1
		N. weight:	0.79
		Sc. PD:	0.037
		Sc. rank:	532.1

NA			
Pos.	22	obs:	exp:
act	T	0	0.25
acc	T	0	0.21
aca	T	1	0.46
acg	T	0	0.08
gtt	V	0	234.10
gtc	V	0	228.10
gta	V	1130	231.40
gtg	V	0	436.40
gct	A	0	9.55
gcc	A	0	6.89
gca	A	37	17.41
gcg	A	0	3.14
---	--	------	------
mPD		0.065	0.79
		nPD:	0.08
		N. weight:	1.6
		Sc. PD:	0.041
		Sc. rank:	1010.6

NA			
Pos.	23	obs:	exp:
ttt	F	0	0.39
ttc	F	1	0.61
tta	L	0	103.40
ttg	L	0	216.90
ctt	L	1	194.00
ctc	L	1164	194.40
cta	L	0	196.30
ctg	L	0	260.00
att	I	0	0.73
atc	I	2	0.48
ata	I	0	0.79
---	--	------	------
mPD		0.0068	1.1
		nPD:	0.01
		N. weight:	1.9
		Sc. PD:	-0.086
		Sc. rank:	-682.1

NA			
Pos.	24	obs:	exp:
att	I	1165	429.10
atc	I	3	279.90
ata	I	0	459.00
---	--	------	------
mPD		0.0051	0.65
		nPD:	0.01
		N. weight:	1.
		Sc. PD:	-0.046
		Sc. rank:	-226.3

NA			
Pos.	25	obs:	exp:
ggt	G	0	154.80
ggc	G	0	149.20
gga	G	1166	538.80
ggg	G	2	325.20
---	--	------	------
mPD		0.0034	0.68
		nPD:	0.01
		N. weight:	0.81
		Sc. PD:	-0.038
		Sc. rank:	-365.0

NA			
Pos.	26	obs:	exp:
att	I	0	426.20
atc	I	2	278.00
ata	I	1158	455.80
atg	M	4	4.00
act	T	0	1.02
acc	T	0	0.82
aca	T	4	1.83
acg	T	0	0.32
---	--	------	------
mPD		0.017	0.66
		nPD:	0.03
		N. weight:	0.97
		Sc. PD:	-0.027
		Sc. rank:	233.4

NA			
Pos.	27	obs:	exp:
act	T	0	0.76
acc	T	0	0.62
aca	T	3	1.38
acg	T	0	0.24
gct	A	0	300.80
gcc	A	0	217.00
gca	A	1165	548.30
gcg	A	0	98.88
---	--	------	------
mPD		0.0051	0.68
		nPD:	0.01
		N. weight:	0.8
		Sc. PD:	-0.035
		Sc. rank:	-197.9

NA			
Pos.	28	obs:	exp:
aat	N	0	594.90
aac	N	1168	573.10
---	--	------	------
mPD		0	0.50
		nPD:	0.
		N. weight:	0.75
		Sc. PD:	-0.039
		Sc. rank:	-711.3

NA			
Pos.	29	obs:	exp:
tta	L	0	100.30
ttg	L	0	210.40
ctt	L	0	188.20
ctc	L	0	188.60
cta	L	1125	190.40
ctg	L	5	252.20
att	I	0	13.96
atc	I	0	9.11
ata	I	38	14.93
---	--	------	------
mPD		0.072	1.2
		nPD:	0.06
		N. weight:	1.8
		Sc. PD:	0.012
		Sc. rank:	985.8

NA			
Pos.	30	obs:	exp:
ggt	G	0	154.80
ggc	G	0	149.20
gga	G	1166	538.80
ggg	G	2	325.20
---	--	------	------
mPD		0.0034	0.68
		nPD:	0.01
		N. weight:	0.81
		Sc. PD:	-0.038
		Sc. rank:	-365.0

NA			
Pos.	31	obs:	exp:
tta	L	2	103.60
ttg	L	1160	217.30
ctt	L	0	194.30
ctc	L	0	194.80
cta	L	0	196.70
ctg	L	5	260.40
gtt	V	0	0.21
gtc	V	0	0.20
gta	V	0	0.20
gtg	V	1	0.39
---	--	------	------
mPD		0.014	1.1
		nPD:	0.01
		N. weight:	1.7
		Sc. PD:	-0.07
		Sc. rank:	-149.2

NA			
Pos.	32	obs:	exp:
aat	N	20	594.90
aac	N	1148	573.10
---	--	------	------
mPD		0.034	0.50
		nPD:	0.07
		N. weight:	0.66
		Sc. PD:	0.0078
		Sc. rank:	366.3

NA			
Pos.	33	obs:	exp:
att	I	0	429.10
atc	I	0	279.90
ata	I	1168	459.00
---	--	------	------
mPD		0	0.65
		nPD:	0.
		N. weight:	0.99
		Sc. PD:	-0.051
		Sc. rank:	-933.1

NA			
Pos.	34	obs:	exp:
cgt	R	0	0.04
cgc	R	0	0.05
cga	R	0	0.10
cgg	R	0	0.08
aga	R	1	0.46
agg	R	0	0.27
ggt	G	0	154.70
ggc	G	0	149.10
gga	G	1166	538.40
ggg	G	1	324.90
---	--	------	------
mPD		0.0034	0.68
		nPD:	0.01
		N. weight:	0.81
		Sc. PD:	-0.038
		Sc. rank:	-373.9

NA			
Pos.	35	obs:	exp:
tta	L	0	103.60
ttg	L	0	217.50
ctt	L	0	194.50
ctc	L	0	194.90
cta	L	3	196.80
ctg	L	1165	260.60
---	--	------	------
mPD		0.0051	1.1
		nPD:	0.
		N. weight:	1.6
		Sc. PD:	-0.074
		Sc. rank:	-754.9

NA			
Pos.	36	obs:	exp:
cat	H	1168	670.70
cac	H	0	497.30
---	--	------	------
mPD		0	0.49
		nPD:	0.
		N. weight:	0.59
		Sc. PD:	-0.03
		Sc. rank:	-554.1

NA			
Pos.	37	obs:	exp:
tta	L	131	103.60
ttg	L	1	217.50
ctt	L	0	194.50
ctc	L	0	194.90
cta	L	1034	196.80
ctg	L	2	260.60
---	--	------	------
mPD		0.21	1.1
		nPD:	0.19
		N. weight:	1.6
		Sc. PD:	0.19
		Sc. rank:	1276.5

NA			
Pos.	38	obs:	exp:
aaa	K	1167	680.70
aag	K	1	487.30
---	--	------	------
mPD		0.0017	0.49
		nPD:	0.
		N. weight:	0.56
		Sc. PD:	-0.027
		Sc. rank:	-295.9

NA			
Pos.	39	obs:	exp:
tta	L	0	0.35
ttg	L	4	0.74
tct	S	0	7.00
tcc	S	0	5.75
tca	S	0	11.17
tcg	S	44	2.50
ctt	L	0	0.67
ctc	L	0	0.67
cta	L	0	0.67
ctg	L	0	0.89
cct	P	0	279.50
ccc	P	0	201.20
cca	P	12	461.00
ccg	P	1108	178.30
agt	S	0	9.04
agc	S	0	8.55
---	--	------	------
mPD		0.11	0.82
		nPD:	0.13
		N. weight:	1.9
		Sc. PD:	0.13
		Sc. rank:	1385.4

NA			
Pos.	40	obs:	exp:
tct	S	0	2.86
tcc	S	0	2.35
tca	S	0	4.57
tcg	S	0	1.02
agt	S	0	3.70
agc	S	18	3.50
gat	D	0	2.70
gac	D	5	2.30
ggt	G	0	151.80
ggc	G	1144	146.30
gga	G	1	528.20
ggg	G	0	318.80
---	--	------	------
mPD		0.041	0.74
		nPD:	0.06
		N. weight:	2.2
		Sc. PD:	0.00042
		Sc. rank:	1078.1

NA			
Pos.	41	obs:	exp:
tgt	C	0	494.00
tgc	C	1168	674.00
---	--	------	------
mPD		0	0.49
		nPD:	0.
		N. weight:	0.58
		Sc. PD:	-0.03
		Sc. rank:	-549.3

NA			
Pos.	42	obs:	exp:
tct	S	0	0.16
tcc	S	0	0.13
tca	S	0	0.25
tcg	S	0	0.06
aat	N	937	593.40
aac	N	228	571.60
agt	S	0	0.21
agc	S	1	0.19
gat	D	2	1.08
gac	D	0	0.92
---	--	------	------
mPD		0.32	0.51
		nPD:	0.63
		N. weight:	0.2
		Sc. PD:	0.11
		Sc. rank:	194.9

NA			
Pos.	43	obs:	exp:
tgt	C	3	494.00
tgc	C	1165	674.00
---	--	------	------
mPD		0.0051	0.49
		nPD:	0.01
		N. weight:	0.56
		Sc. PD:	-0.024
		Sc. rank:	-75.6

NA			
Pos.	44	obs:	exp:
tct	S	0	149.50
tcc	S	0	122.80
tca	S	940	238.60
tcg	S	0	53.34
cct	P	0	6.49
ccc	P	0	4.67
cca	P	26	10.70
ccg	P	0	4.14
act	T	0	51.49
acc	T	1	41.45
aca	T	201	92.65
acg	T	0	16.41
agt	S	0	193.20
agc	S	0	182.60
---	--	------	------
mPD		0.32	1.7
		nPD:	0.2
		N. weight:	1.3
		Sc. PD:	0.17
		Sc. rank:	1094.8

NA			
Pos.	45	obs:	exp:
tta	L	0	0.09
ttg	L	0	0.19
ctt	L	0	0.17
ctc	L	1	0.17
cta	L	0	0.17
ctg	L	0	0.22
cct	P	0	0.25
ccc	P	1	0.18
cca	P	0	0.41
ccg	P	0	0.16
cat	H	5	606.40
cac	H	1051	449.60
cgt	R	0	4.25
cgc	R	99	4.90
cga	R	0	9.69
cgg	R	0	8.26
aat	N	0	5.60
aac	N	11	5.40
aga	R	0	45.27
agg	R	0	26.63
---	--	------	------
mPD		0.19	0.85
		nPD:	0.22
		N. weight:	1.1
		Sc. PD:	0.16
		Sc. rank:	885.6

NA			
Pos.	46	obs:	exp:
tta	L	5	0.44
ttg	L	0	0.93
tct	S	0	183.90
tcc	S	0	151.00
tca	S	1155	293.40
tcg	S	1	65.60
ctt	L	0	0.83
ctc	L	0	0.83
cta	L	0	0.84
ctg	L	0	1.12
cct	P	0	1.50
ccc	P	0	1.08
cca	P	6	2.47
ccg	P	0	0.96
act	T	0	0.25
acc	T	0	0.21
aca	T	1	0.46
acg	T	0	0.08
agt	S	0	237.60
agc	S	0	224.50
---	--	------	------
mPD		0.022	1.7
		nPD:	0.01
		N. weight:	1.4
		Sc. PD:	-0.057
		Sc. rank:	-74.8

NA			
Pos.	47	obs:	exp:
caa	Q	1165	594.90
cag	Q	3	573.10
---	--	------	------
mPD		0.0051	0.50
		nPD:	0.01
		N. weight:	0.69
		Sc. PD:	-0.029
		Sc. rank:	-115.1

NA			
Pos.	48	obs:	exp:
tta	L	0	0.09
ttg	L	0	0.19
tct	S	9	1.43
tcc	S	0	1.18
tca	S	0	2.28
tcg	S	0	0.51
ctt	L	1	0.17
ctc	L	0	0.17
cta	L	0	0.17
ctg	L	0	0.22
cct	P	1148	288.20
ccc	P	7	207.50
cca	P	0	475.40
ccg	P	0	183.90
act	T	3	0.76
acc	T	0	0.62
aca	T	0	1.38
acg	T	0	0.24
agt	S	0	1.85
agc	S	0	1.75
---	--	------	------
mPD		0.034	0.74
		nPD:	0.05
		N. weight:	1.4
		Sc. PD:	-0.012
		Sc. rank:	644.5

NA			
Pos.	49	obs:	exp:
aaa	K	6	3.50
aag	K	0	2.50
gaa	E	1128	687.00
gag	E	1	442.00
ggt	G	0	4.37
ggc	G	0	4.22
gga	G	33	15.22
ggg	G	0	9.19
---	--	------	------
mPD		0.067	0.55
		nPD:	0.12
		N. weight:	0.53
		Sc. PD:	0.033
		Sc. rank:	372.1

NA			
Pos.	50	obs:	exp:
att	I	0	9.92
atc	I	0	6.47
ata	I	27	10.61
act	T	0	287.80
acc	T	0	231.70
aca	T	1129	517.80
acg	T	0	91.70
aaa	K	1	0.58
aag	K	0	0.42
gct	A	0	2.84
gcc	A	0	2.05
gca	A	11	5.18
gcg	A	0	0.93
---	--	------	------
mPD		0.066	0.74
		nPD:	0.09
		N. weight:	0.83
		Sc. PD:	0.026
		Sc. rank:	520.7

NA			
Pos.	51	obs:	exp:
att	I	0	2.94
atc	I	8	1.92
ata	I	0	3.14
act	T	0	294.90
acc	T	1157	237.40
aca	T	0	530.70
acg	T	0	93.98
gct	A	0	0.77
gcc	A	3	0.56
gca	A	0	1.41
gcg	A	0	0.25
---	--	------	------
mPD		0.019	0.69
		nPD:	0.03
		N. weight:	1.7
		Sc. PD:	-0.044
		Sc. rank:	425.5

NA			
Pos.	52	obs:	exp:
tct	S	0	0.32
tcc	S	0	0.26
tca	S	0	0.51
tcg	S	0	0.11
aat	N	1	593.40
aac	N	1164	571.60
agt	S	0	0.41
agc	S	2	0.39
gat	D	0	0.54
gac	D	1	0.46
---	--	------	------
mPD		0.0068	0.51
		nPD:	0.01
		N. weight:	0.75
		Sc. PD:	-0.029
		Sc. rank:	-34.2

NA			
Pos.	53	obs:	exp:
cct	P	0	2.25
ccc	P	0	1.62
cca	P	9	3.70
ccg	P	0	1.43
att	I	0	0.73
atc	I	0	0.48
ata	I	2	0.79
atg	M	1	1.00
act	T	0	294.70
acc	T	0	237.20
aca	T	1111	530.20
acg	T	45	93.90
---	--	------	------
mPD		0.096	0.70
		nPD:	0.14
		N. weight:	0.72
		Sc. PD:	0.057
		Sc. rank:	547.2

NA			
Pos.	54	obs:	exp:
tct	S	0	185.10
tcc	S	0	152.00
tca	S	0	295.40
tcg	S	0	66.05
att	I	0	0.73
atc	I	2	0.48
ata	I	0	0.79
aat	N	0	1.02
aac	N	2	0.98
agt	S	0	239.30
agc	S	1164	226.10
---	--	------	------
mPD		0.0068	1.7
		nPD:	0.
		N. weight:	1.7
		Sc. PD:	-0.083
		Sc. rank:	-853.5

NA			
Pos.	55	obs:	exp:
caa	Q	1167	594.40
cag	Q	0	572.60
cgt	R	0	0.04
cgc	R	0	0.05
cga	R	1	0.10
cgg	R	0	0.08
aga	R	0	0.46
agg	R	0	0.27
---	--	------	------
mPD		0.0017	0.50
		nPD:	0.
		N. weight:	0.71
		Sc. PD:	-0.035
		Sc. rank:	-404.7

NA			
Pos.	56	obs:	exp:
act	T	0	297.70
acc	T	0	239.70
aca	T	1168	535.70
acg	T	0	94.87
---	--	------	------
mPD		0	0.68
		nPD:	0.
		N. weight:	0.82
		Sc. PD:	-0.043
		Sc. rank:	-778.6

NA			
Pos.	57	obs:	exp:
att	I	0	423.60
atc	I	0	276.30
ata	I	1153	453.10
act	T	0	2.29
acc	T	0	1.85
aca	T	9	4.13
acg	T	0	0.73
gtt	V	0	1.24
gtc	V	0	1.21
gta	V	6	1.23
gtg	V	0	2.32
---	--	------	------
mPD		0.026	0.68
		nPD:	0.04
		N. weight:	0.99
		Sc. PD:	-0.016
		Sc. rank:	380.2

NA			
Pos.	58	obs:	exp:
att	I	0	429.10
atc	I	0	279.90
ata	I	1168	459.00
---	--	------	------
mPD		0	0.65
		nPD:	0.
		N. weight:	0.99
		Sc. PD:	-0.051
		Sc. rank:	-933.1

NA			
Pos.	59	obs:	exp:
aat	N	4	594.90
aac	N	1164	573.10
---	--	------	------
mPD		0.0068	0.50
		nPD:	0.01
		N. weight:	0.73
		Sc. PD:	-0.028
		Sc. rank:	-29.3

NA			
Pos.	60	obs:	exp:
aat	N	2	594.90
aac	N	1166	573.10
---	--	------	------
mPD		0.0034	0.50
		nPD:	0.01
		N. weight:	0.74
		Sc. PD:	-0.033
		Sc. rank:	-256.9

NA			
Pos.	61	obs:	exp:
tat	Y	1167	630.80
tac	Y	1	537.20
---	--	------	------
mPD		0.0017	0.50
		nPD:	0.
		N. weight:	0.64
		Sc. PD:	-0.031
		Sc. rank:	-359.8

,

NA			
Pos.	62	obs:	exp:
tat	Y	1160	629.80
tac	Y	6	536.20
cat	H	2	1.15
cac	H	0	0.85
---	--	------	------
mPD		0.014	0.50
		nPD:	0.03
		N. weight:	0.62
		Sc. PD:	-0.016
		Sc. rank:	167.6

,

NA			
Pos.	63	obs:	exp:
tct	S	0	2.39
tcc	S	0	1.96
tca	S	0	3.81
tcg	S	0	0.85
aat	N	1153	587.30
aac	N	0	565.70
agt	S	15	3.08
agc	S	0	2.91
---	--	------	------
mPD		0.025	0.54
		nPD:	0.05
		N. weight:	0.72
		Sc. PD:	-0.0056
		Sc. rank:	330.2

,

NA			
Pos.	64	obs:	exp:
aaa	K	2	1.17
aag	K	0	0.83
gaa	E	1162	707.10
gag	E	0	454.90
ggt	G	0	0.53
ggc	G	0	0.51
gga	G	4	1.85
ggg	G	0	1.11
---	--	------	------
mPD		0.010	0.49
		nPD:	0.02
		N. weight:	0.53
		Sc. PD:	-0.017
		Sc. rank:	74.8

,

NA			
Pos.	65	obs:	exp:
act	T	0	1.27
acc	T	0	1.03
aca	T	5	2.29
acg	T	0	0.41
---	-	1163	1163.00
---	--	------	------
mPD		0	0.000012
		nPD:	0.
		N. weight:	0.
		Sc. PD:	0
		Sc. rank:	0

,

NA			
Pos.	66	obs:	exp:
aat	N	0	2.55
aac	N	5	2.45
---	-	1163	1163.00
---	--	------	------
mPD		0	9.2×10^{-6}
		nPD:	0.
		N. weight:	0.
		Sc. PD:	0
		Sc. rank:	0

,

NA			
Pos.	67	obs:	exp:
att	I	0	1.84
atc	I	5	1.20
ata	I	0	1.96
---	-	1163	1163.00
---	--	------	------
mPD		0	0.000012
		nPD:	0.
		N. weight:	0.
		Sc. PD:	0
		Sc. rank:	0

,

NA			
Pos.	68	obs:	exp:
act	T	0	1.27
acc	T	5	1.03
aca	T	0	2.29
acg	T	0	0.41
---	-	1163	1163.00
---	--	------	------
mPD		0	0.000012
		nPD:	0.
		N. weight:	0.
		Sc. PD:	0
		Sc. rank:	0

,

NA			
Pos.	69	obs:	exp:
caa	Q	3	1.53
cag	Q	0	1.47
gaa	E	2	1.22
gag	E	0	0.78
---	-	1163	1163.00
---	--	------	------
mPD		8.8×10^{-6}	0.000018
		nPD:	0.49
		N. weight:	0.
		Sc. PD:	0
		Sc. rank:	0

,

NA			
Pos.	70	obs:	exp:
act	T	0	297.20
acc	T	0	239.30
aca	T	1165	534.80
acg	T	1	94.71
gct	A	0	0.52
gcc	A	0	0.37
gca	A	2	0.94
gcg	A	0	0.17
---	--	------	------
mPD		0.0051	0.68
		nPD:	0.01
		N. weight:	0.82
		Sc. PD:	-0.036
		Sc. rank:	-218.6

,

NA			
Pos.	71	obs:	exp:
tct	S	0	1.59
tcc	S	0	1.31
tca	S	0	2.54
tcg	S	0	0.57
aat	N	0	589.80
aac	N	1158	568.20
agt	S	0	2.06
agc	S	10	1.94
---	--	------	------
mPD		0.017	0.53
		nPD:	0.03
		N. weight:	0.76
		Sc. PD:	-0.016
		Sc. rank:	244.4

,

NA			
Pos.	72	obs:	exp:
att	I	0	428.80
atc	I	1161	279.70
ata	I	6	458.60
gtt	V	0	0.21
gtc	V	1	0.20
gta	V	0	0.20
gtg	V	0	0.39
---	--	------	------
mPD		0.012	0.66
		nPD:	0.02
		N. weight:	1.5
		Sc. PD:	-0.051
		Sc. rank:	141.6

,

NA			
Pos.	73	obs:	exp:
act	T	0	0.25
acc	T	1	0.21
aca	T	0	0.46
acg	T	0	0.08
---	-	1167	1167.00
---	--	------	------
mPD		0	5.0×10^{-7}
		nPD:	0.
		N. weight:	0.
		Sc. PD:	0
		Sc. rank:	0

,

NA			
Pos.	74	obs:	exp:
caa	Q	1	0.51
cag	Q	0	0.49
---	-	1167	1167.00
---	--	------	------
mPD		0	3.7×10^{-7}
		nPD:	0.
		N. weight:	0.
		Sc. PD:	0
		Sc. rank:	0

,

NA			
Pos.	75	obs:	exp:
att	I	0	0.37
atc	I	0	0.24
ata	I	1	0.39
---	-	1167	1167.00
---	--	------	------
mPD		0	4.8×10^{-7}
		nPD:	0.
		N. weight:	0.
		Sc. PD:	0
		Sc. rank:	0

,

NA			
Pos.	76	obs:	exp:
tct	S	0	0.16
tcc	S	0	0.13
tca	S	0	0.25
tcg	S	0	0.06
agt	S	1	0.21
agc	S	0	0.19
---	-	1167	1167.00
---	--	------	------
mPD		0	1.2×10^{-6}
		nPD:	0.
		N. weight:	0.
		Sc. PD:	0
		Sc. rank:	0

,

NA			
Pos.	77	obs:	exp:
aat	N	0	0.51
aac	N	1	0.49
---	-	1167	1167.00
---	--	------	------
mPD		0	3.7×10^{-7}
		nPD:	0.
		N. weight:	0.
		Sc. PD:	0
		Sc. rank:	0

,

NA			
Pos.	78	obs:	exp:
act	T	0	297.20
acc	T	1166	239.30
aca	T	0	534.80
acg	T	0	94.71
aat	N	0	1.02
aac	N	2	0.98
---	--	------	------
mPD		0.0034	0.68
		nPD:	0.01
		N. weight:	1.7
		Sc. PD:	-0.078
		Sc. rank:	-781.2

,

NA			
Pos.	79	obs:	exp:
tct	S	0	0.32
tcc	S	0	0.26
tca	S	0	0.51
tcg	S	0	0.11
aat	N	1	593.90
aac	N	1165	572.10
agt	S	0	0.41
agc	S	2	0.39
---	--	------	------
mPD		0.0051	0.51
		nPD:	0.01
		N. weight:	0.75
		Sc. PD:	-0.031
		Sc. rank:	-132.4

,

NA			
Pos.	80	obs:	exp:
att	I	2	428.80
atc	I	1164	279.70
ata	I	1	458.60
gtt	V	0	0.21
gtc	V	1	0.20
gta	V	0	0.20
gtg	V	0	0.39
---	--	------	------
mPD		0.0068	0.66
		nPD:	0.01
		N. weight:	1.5
		Sc. PD:	-0.062
		Sc. rank:	-208.2

,

NA			
Pos.	81	obs:	exp:
caa	Q	1162	593.40
cag	Q	3	571.60
cgt	R	0	0.04
cgc	R	0	0.05
cga	R	1	0.10
cgg	R	0	0.08
aaa	K	2	1.17
aag	K	0	0.83
aga	R	0	0.46
agg	R	0	0.27
---	--	------	------
mPD		0.010	0.51
		nPD:	0.02
		N. weight:	0.7
		Sc. PD:	-0.023
		Sc. rank:	85.1

,

NA			
Pos.	82	obs:	exp:
att	I	0	48.50
atc	I	0	31.63
ata	I	132	51.87
atg	M	1030	1030.00
act	T	0	1.27
acc	T	0	1.03
aca	T	1	2.29
acg	T	4	0.41
gtt	V	0	0.21
gtc	V	0	0.20
gta	V	0	0.20
gtg	V	1	0.39
---	--	------	------
mPD		0.21	0.23
		nPD:	0.94
		N. weight:	0.12
		Sc. PD:	0.1
		Sc. rank:	116.7

,

NA			
Pos.	83	obs:	exp:
cgt	R	0	0.21
cgc	R	0	0.25
cga	R	0	0.49
cgg	R	0	0.42
aaa	K	3	1.75
aag	K	0	1.25
aga	R	5	2.29
agg	R	0	1.34
gat	D	1	0.54
gac	D	0	0.46
gaa	E	1120	683.30
gag	E	3	439.70
ggt	G	0	4.77
ggc	G	0	4.60
gga	G	36	16.61
ggg	G	0	10.02
---	--	------	------
mPD		0.088	0.57
		nPD:	0.16
		N. weight:	0.52
		Sc. PD:	0.049
		Sc. rank:	403.5

,

NA			
Pos.	84	obs:	exp:
aaa	K	5	2.91
aag	K	0	2.09
gaa	E	576	707.10
gag	E	586	454.90
ggt	G	0	0.13
ggc	G	0	0.13
gga	G	1	0.46
ggg	G	0	0.28
---	--	------	------
mPD		0.51	0.49
		nPD:	1.05
		N. weight:	0.034
		Sc. PD:	0.032
		Sc. rank:	32.8

,

NA			
Pos.	85	obs:	exp:
tct	S	0	0.16
tcc	S	0	0.13
tca	S	0	0.25
tcg	S	0	0.06
cgt	R	0	49.91
cgc	R	0	57.55
cga	R	0	113.70
cgg	R	0	96.96
aaa	K	4	2.33
aag	K	0	1.67
agt	S	0	0.21
agc	S	1	0.19
aga	R	1151	531.30
agg	R	11	312.50
ggt	G	0	0.13
ggc	G	0	0.13
gga	G	1	0.46
ggg	G	0	0.28
---	--	------	------
mPD		0.029	0.97
		nPD:	0.03
		N. weight:	0.78
		Sc. PD:	-0.018
		Sc. rank:	234.4

NA			
Pos.	86	obs:	exp:
cgt	R	0	0.04
cgc	R	0	0.05
cga	R	0	0.10
cgg	R	0	0.08
act	T	0	296.20
acc	T	0	238.40
aca	T	1158	533.00
acg	T	4	94.38
aaa	K	5	2.91
aag	K	0	2.09
aga	R	1	0.46
agg	R	0	0.27
---	--	------	------
mPD		0.017	0.69
		nPD:	0.02
		N. weight:	0.81
		Sc. PD:	-0.023
		Sc. rank:	166.4

NA			
Pos.	87	obs:	exp:
tct	S	0	184.00
tcc	S	0	151.10
tca	S	0	293.70
tcg	S	0	65.65
att	I	0	0.73
atc	I	2	0.48
ata	I	0	0.79
aat	N	0	4.58
aac	N	9	4.42
agt	S	1	237.80
agc	S	1156	224.70
---	--	------	------
mPD		0.020	1.7
		nPD:	0.01
		N. weight:	1.7
		Sc. PD:	-0.069
		Sc. rank:	-159.5

NA			
Pos.	88	obs:	exp:
tgg	W	1	1.00
cgt	R	0	45.40
cgc	R	0	52.35
cga	R	0	103.40
cgg	R	0	88.20
aaa	K	0	64.11
aag	K	110	45.89
aga	R	0	483.30
agg	R	1057	284.30
---	--	------	------
mPD		0.17	1.1
		nPD:	0.16
		N. weight:	1.3
		Sc. PD:	0.13
		Sc. rank:	1050.8

NA			
Pos.	89	obs:	exp:
tct	S	0	2.23
tcc	S	0	1.83
tca	S	0	3.55
tcg	S	0	0.79
tat	Y	1	0.54
tac	Y	0	0.46
att	I	2	0.73
atc	I	0	0.48
ata	I	0	0.79
aat	N	1146	583.70
aac	N	0	562.30
aaa	K	1	0.58
aag	K	0	0.42
agt	S	14	2.88
agc	S	0	2.72
gat	D	4	2.16
gac	D	0	1.84
---	--	------	------
mPD		0.037	0.55
		nPD:	0.07
		N. weight:	0.72
		Sc. PD:	0.0085
		Sc. rank:	398.8

NA			
Pos.	90	obs:	exp:
ttt	F	0	461.20
ttc	F	1168	706.80
---	--	------	------
mPD		0	0.48
		nPD:	0.
		N. weight:	0.53
		Sc. PD:	-0.027
		Sc. rank:	-501.8

NA			
Pos.	91	obs:	exp:
aat	N	1163	594.90
aac	N	5	573.10
---	--	------	------
mPD		0.0085	0.50
		nPD:	0.02
		N. weight:	0.68
		Sc. PD:	-0.024
		Sc. rank:	30.2

NA			
Pos.	92	obs:	exp:
tct	S	0	0.32
tcc	S	0	0.26
tca	S	0	0.51
tcg	S	0	0.11
act	T	0	0.25
acc	T	1	0.21
aca	T	0	0.46
acg	T	0	0.08
aat	N	2	592.40
aac	N	1161	570.60
aaa	K	2	1.17
aag	K	0	0.83
agt	S	0	0.41
agc	S	2	0.39
---	--	------	------
mPD		0.012	0.51
		nPD:	0.02
		N. weight:	0.74
		Sc. PD:	-0.022
		Sc. rank:	137.8

NA			
Pos.	93	obs:	exp:
tta	L	939	103.60
ttg	L	2	217.50
ctt	L	0	194.50
ctc	L	0	194.90
cta	L	227	196.80
ctg	L	0	260.60
---	--	------	------
mPD		0.32	1.1
		nPD:	0.29
		N. weight:	1.9
		Sc. PD:	0.41
		Sc. rank:	1674.7

NA			
Pos.	94	obs:	exp:
act	T	1163	297.70
acc	T	3	239.70
aca	T	0	535.70
acg	T	2	94.87
---	--	------	------
mPD		0.0085	0.68
		nPD:	0.01
		N. weight:	1.4
		Sc. PD:	-0.056
		Sc. rank:	-89.1

NA			
Pos.	95	obs:	exp:
aat	N	1	0.51
aac	N	0	0.49
aaa	K	1165	680.20
aag	K	2	486.80
---	--	------	------
mPD		0.0051	0.49
		nPD:	0.01
		N. weight:	0.56
		Sc. PD:	-0.023
		Sc. rank:	-73.6

NA			
Pos.	96	obs:	exp:
cgt	R	0	0.90
cgc	R	0	1.04
cga	R	0	2.06
cgg	R	0	1.75
aga	R	0	9.60
agg	R	21	5.65
gaa	E	1	3.04
gag	E	4	1.96
ggt	G	0	151.40
ggc	G	0	145.90
gga	G	3	526.80
ggg	G	1139	317.90
---	--	------	------
mPD		0.051	0.72
		nPD:	0.07
		N. weight:	1.3
		Sc. PD:	0.02
		Sc. rank:	751.3

NA			
Pos.	97	obs:	exp:
tta	L	0	103.60
ttg	L	0	217.50
ctt	L	0	194.50
ctc	L	1168	194.90
cta	L	0	196.80
ctg	L	0	260.60
---	--	------	------
mPD		0	1.1
		nPD:	0.
		N. weight:	1.9
		Sc. PD:	-0.098
		Sc. rank:	-1788.5

NA			
Pos.	98	obs:	exp:
tgt	C	1161	494.00
tgc	C	7	674.00
---	--	------	------
mPD		0.012	0.49
		nPD:	0.02
		N. weight:	0.87
		Sc. PD:	-0.025
		Sc. rank:	172.5

NA			
Pos.	99	obs:	exp:
act	T	1161	297.20
acc	T	4	239.30
aca	T	0	534.80
acg	T	1	94.71
gct	A	2	0.52
gcc	A	0	0.37
gca	A	0	0.94
gcg	A	0	0.17
---	--	------	------
mPD		0.012	0.68
		nPD:	0.02
		N. weight:	1.4
		Sc. PD:	-0.05
		Sc. rank:	88.8

NA			
Pos.	100	obs:	exp:
att	I	0	429.10
atc	I	0	279.90
ata	I	1168	459.00
---	--	------	------
mPD		0	0.65
		nPD:	0.
		N. weight:	0.99
		Sc. PD:	-0.051
		Sc. rank:	-933.1

NA			
Pos.	101	obs:	exp:
aat	N	1166	594.40
aac	N	1	572.60
gat	D	1	0.54
gac	D	0	0.46
---	--	------	------
mPD		0.0034	0.50
		nPD:	0.01
		N. weight:	0.71
		Sc. PD:	-0.032
		Sc. rank:	-247.2

NA			
Pos.	102	obs:	exp:
tct	S	1	185.80
tcc	S	0	152.50
tca	S	1109	296.50
tcg	S	58	66.28
agt	S	0	240.10
agc	S	0	226.90
---	--	------	------
mPD		0.096	1.7
		nPD:	0.06
		N. weight:	1.3
		Sc. PD:	0.004
		Sc. rank:	686.5

NA			
Pos.	103	obs:	exp:
tgg	W	1168	1168.00
---	--	------	------
mPD		0	0
		nPD:	1.
		N. weight:	0.
		Sc. PD:	0
		Sc. rank:	0

NA			
Pos.	104	obs:	exp:
tta	L	0	0.09
ttg	L	0	0.19
ctt	L	0	0.17
ctc	L	0	0.17
cta	L	1	0.17
ctg	L	0	0.22
cat	H	4	669.50
cac	H	1162	496.50
caa	Q	1	0.51
cag	Q	0	0.49
---	--	------	------
mPD		0.012	0.49
		nPD:	0.02
		N. weight:	0.88
		Sc. PD:	-0.025
		Sc. rank:	171.7

NA			
Pos.	105	obs:	exp:
att	I	0	428.80
atc	I	0	279.70
ata	I	1167	458.60
gtt	V	0	0.21
gtc	V	0	0.20
gta	V	1	0.20
gtg	V	0	0.39
---	--	------	------
mPD		0.0017	0.66
		nPD:	0.
		N. weight:	0.99
		Sc. PD:	-0.049
		Sc. rank:	-600.5

,

NA			
Pos.	106	obs:	exp:
tat	Y	1166	629.80
tac	Y	0	536.20
tgt	C	1	0.42
tgc	C	0	0.58
cat	H	1	0.57
cac	H	0	0.43
---	--	------	------
mPD		0.0034	0.50
		nPD:	0.01
		N. weight:	0.65
		Sc. PD:	-0.029
		Sc. rank:	-216.6

,

NA			
Pos.	107	obs:	exp:
ggt	G	35	154.80
ggc	G	0	149.20
gga	G	19	538.80
ggg	G	1114	325.20
---	--	------	------
mPD		0.089	0.68
		nPD:	0.13
		N. weight:	1.1
		Sc. PD:	0.083
		Sc. rank:	847.2

,

NA			
Pos.	108	obs:	exp:
cgt	R	0	0.04
cgc	R	0	0.05
cga	R	0	0.10
cgg	R	0	0.08
aaa	K	1161	680.20
aag	K	6	486.80
aga	R	1	0.46
agg	R	0	0.27
---	--	------	------
mPD		0.012	0.49
		nPD:	0.02
		N. weight:	0.54
		Sc. PD:	-0.015
		Sc. rank:	109.7

,

NA			
Pos.	109	obs:	exp:
gat	D	0	631.20
gac	D	1168	536.80
---	--	------	------
mPD		0	0.50
		nPD:	0.
		N. weight:	0.82
		Sc. PD:	-0.042
		Sc. rank:	-776.5

,

NA			
Pos.	110	obs:	exp:
aat	N	1131	594.90
aac	N	37	573.10
---	--	------	------
mPD		0.061	0.50
		nPD:	0.12
		N. weight:	0.57
		Sc. PD:	0.036
		Sc. rank:	402.9

,

NA			
Pos.	111	obs:	exp:
gct	A	1	301.60
gcc	A	0	217.50
gca	A	1162	549.70
gcg	A	5	99.13
---	--	------	------
mPD		0.010	0.67
		nPD:	0.02
		N. weight:	0.77
		Sc. PD:	-0.029
		Sc. rank:	10.1

,

NA			
Pos.	112	obs:	exp:
tta	L	3	0.27
ttg	L	0	0.56
ctt	L	0	0.50
ctc	L	0	0.50
cta	L	0	0.51
ctg	L	0	0.67
att	I	0	1.47
atc	I	0	0.96
ata	I	4	1.57
gtt	V	0	240.60
gtc	V	0	234.30
gta	V	1157	237.70
gtg	V	4	448.40
---	--	------	------
mPD		0.019	0.74
		nPD:	0.03
		N. weight:	1.6
		Sc. PD:	-0.046
		Sc. rank:	389.9

,

NA			
Pos.	113	obs:	exp:
cgt	R	0	50.16
cgc	R	0	57.85
cga	R	0	114.30
cgg	R	0	97.46
aga	R	1168	534.10
agg	R	0	314.20
---	--	------	------
mPD		0	0.96
		nPD:	0.
		N. weight:	0.83
		Sc. PD:	-0.043
		Sc. rank:	-781.7

,

NA			
Pos.	114	obs:	exp:
tta	L	0	0.18
ttg	L	0	0.37
ctt	L	2	0.33
ctc	L	0	0.33
cta	L	0	0.34
ctg	L	0	0.45
att	I	1166	428.40
atc	I	0	279.40
ata	I	0	458.20
---	--	------	------
mPD		0.0034	0.66
		nPD:	0.01
		N. weight:	1.1
		Sc. PD:	-0.049
		Sc. rank:	-423.9

,

NA			
Pos.	115	obs:	exp:
ggt	G	0	154.80
ggc	G	0	149.20
gga	G	1168	538.80
ggg	G	0	325.20
---	--	------	------
mPD		0	0.68
		nPD:	0.
		N. weight:	0.82
		Sc. PD:	-0.042
		Sc. rank:	-772.8

,

NA			
Pos.	116	obs:	exp:
gaa	E	581	710.70
gag	E	587	457.30
---	--	------	------
mPD		0.50	0.48
		nPD:	1.05
		N. weight:	0.03
		Sc. PD:	0.028
		Sc. rank:	29.1

,

NA			
Pos.	117	obs:	exp:
tct	S	0	184.80
tcc	S	0	151.80
tca	S	0	294.90
tcg	S	0	65.94
aat	N	0	3.06
aac	N	6	2.94
agt	S	1	238.90
agc	S	1161	225.70
---	--	------	------
mPD		0.012	1.7
		nPD:	0.01
		N. weight:	1.7
		Sc. PD:	-0.077
		Sc. rank:	-484.4

,

NA			
Pos.	118	obs:	exp:
tct	S	2	185.80
tcc	S	0	152.50
tca	S	19	296.50
tcg	S	1147	66.28
agt	S	0	240.10
agc	S	0	226.90
---	--	------	------
mPD		0.035	1.7
		nPD:	0.02
		N. weight:	2.9
		Sc. PD:	-0.091
		Sc. rank:	483.0

,

NA			
Pos.	119	obs:	exp:
aat	N	1	0.51
aac	N	0	0.49
gat	D	1166	630.10
gac	D	0	535.90
gaa	E	1	0.61
gag	E	0	0.39
---	--	------	------
mPD		0.0034	0.50
		nPD:	0.01
		N. weight:	0.65
		Sc. PD:	-0.029
		Sc. rank:	-214.7

,

NA			
Pos.	120	obs:	exp:
att	I	11	4.04
atc	I	0	2.64
ata	I	0	4.32
gtt	V	1148	239.70
gtc	V	9	233.50
gta	V	0	236.90
gtg	V	0	446.90
---	--	------	------
mPD		0.034	0.75
		nPD:	0.05
		N. weight:	1.6
		Sc. PD:	-0.014
		Sc. rank:	714.2

,

NA			
Pos.	121	obs:	exp:
tta	L	1166	103.60
ttg	L	1	217.50
ctt	L	0	194.50
ctc	L	0	194.90
cta	L	1	196.80
ctg	L	0	260.60
---	--	------	------
mPD		0.0034	1.1
		nPD:	0.
		N. weight:	2.5
		Sc. PD:	-0.12
		Sc. rank:	-1502.6

,

NA			
Pos.	122	obs:	exp:
att	I	1	1.47
atc	I	3	0.96
ata	I	0	1.57
gtt	V	150	239.10
gtc	V	999	232.90
gta	V	5	236.30
gtg	V	0	445.70
gct	A	0	2.58
gcc	A	10	1.86
gca	A	0	4.71
gcg	A	0	0.85
---	--	------	------
mPD		0.26	0.75
		nPD:	0.34
		N. weight:	1.3
		Sc. PD:	0.34
		Sc. rank:	1150.3

,

NA			
Pos.	123	obs:	exp:
act	T	2	297.50
acc	T	0	239.50
aca	T	1164	535.30
acg	T	1	94.79
gct	A	0	0.26
gcc	A	0	0.19
gca	A	1	0.47
gcg	A	0	0.08
---	--	------	------
mPD		0.0068	0.68
		nPD:	0.01
		N. weight:	0.81
		Sc. PD:	-0.034
		Sc. rank:	-153.2

,

NA			
Pos.	124	obs:	exp:
cgt	R	0	50.12
cgc	R	0	57.80
cga	R	0	114.20
cgg	R	0	97.38
att	I	0	0.37
atc	I	0	0.24
ata	I	1	0.39
aga	R	1166	533.60
agg	R	1	313.90
---	--	------	------
mPD		0.0034	0.96
		nPD:	0.
		N. weight:	0.82
		Sc. PD:	-0.04
		Sc. rank:	-425.3

,

NA			
Pos.	125	obs:	exp:
gtt	V	0	0.83
gtc	V	0	0.81
gta	V	4	0.82
gtg	V	0	1.54
gaa	E	1163	708.30
gag	E	1	455.70
---	--	------	------
mPD		0.0085	0.48
		nPD:	0.02
		N. weight:	0.52
		Sc. PD:	-0.018
		Sc. rank:	33.9

,

NA			
Pos.	126	obs:	exp:
cct	P	0	291.40
ccc	P	1159	209.90
cca	P	9	480.80
ccg	P	0	185.90
---	--	------	------
mPD		0.015	0.71
		nPD:	0.02
		N. weight:	1.8
		Sc. PD:	-0.055
		Sc. rank:	305.1

,

NA			
Pos.	127	obs:	exp:
tat	Y	1167	630.80
tac	Y	1	537.20
---	--	------	------
mPD		0.0017	0.50
		nPD:	0.
		N. weight:	0.64
		Sc. PD:	-0.031
		Sc. rank:	-359.8

,

NA			
Pos.	128	obs:	exp:
gtt	V	1127	242.00
gtc	V	1	235.70
gta	V	0	239.20
gtg	V	40	451.10
---	--	------	------
mPD		0.068	0.73
		nPD:	0.09
		N. weight:	1.5
		Sc. PD:	0.054
		Sc. rank:	947.2

,

NA			
Pos.	129	obs:	exp:
tct	S	0	185.80
tcc	S	0	152.50
tca	S	1168	296.50
tcg	S	0	66.28
agt	S	0	240.10
agc	S	0	226.90
---	--	------	------
mPD		0	1.7
		nPD:	0.
		N. weight:	1.4
		Sc. PD:	-0.075
		Sc. rank:	-1369.7

,

NA			
Pos.	130	obs:	exp:
tgt	C	2	494.00
tgc	C	1166	674.00
---	--	------	------
mPD		0.0034	0.49
		nPD:	0.01
		N. weight:	0.57
		Sc. PD:	-0.026
		Sc. rank:	-174.7

,

NA			
Pos.	131	obs:	exp:
aat	N	0	20.88
aac	N	41	20.12
gat	D	1	608.50
gac	D	1125	517.50
ggt	G	0	0.13
ggc	G	1	0.13
gga	G	0	0.46
ggg	G	0	0.28
---	--	------	------
mPD		0.071	0.57
		nPD:	0.13
		N. weight:	0.81
		Sc. PD:	0.054
		Sc. rank:	582.7

,

NA			
Pos.	132	obs:	exp:
cct	P	1	291.40
ccc	P	0	209.90
cca	P	1117	480.80
ccg	P	50	185.90
---	--	------	------
mPD		0.084	0.71
		nPD:	0.12
		N. weight:	0.79
		Sc. PD:	0.047
		Sc. rank:	553.4

,

NA			
Pos.	133	obs:	exp:
tat	Y	1	0.54
tac	Y	0	0.46
aat	N	2	1.02
aac	N	0	0.98
gat	D	1164	629.00
gac	D	0	535.00
ggt	G	1	0.13
ggc	G	0	0.13
gga	G	0	0.46
ggg	G	0	0.28
---	--	------	------
mPD		0.0068	0.50
		nPD:	0.01
		N. weight:	0.65
		Sc. PD:	-0.025
		Sc. rank:	-28.1

,

NA			
Pos.	134	obs:	exp:
aaa	K	2	1.17
aag	K	0	0.83
gaa	E	1160	709.50
gag	E	6	456.50
---	--	------	------
mPD		0.014	0.48
		nPD:	0.03
		N. weight:	0.49
		Sc. PD:	-0.012
		Sc. rank:	143.0

,

NA			
Pos.	135	obs:	exp:
tgt	C	1	494.00
tgc	C	1167	674.00
---	--	------	------
mPD		0.0017	0.49
		nPD:	0.
		N. weight:	0.57
		Sc. PD:	-0.028
		Sc. rank:	-313.1

,

NA			
Pos.	136	obs:	exp:
cgt	R	0	48.58
cgc	R	0	56.02
cga	R	0	110.70
cgg	R	0	94.37
aaa	K	0	21.56
aag	K	37	15.44
aga	R	16	517.20
agg	R	1115	304.20
---	--	------	------
mPD		0.088	1.0
		nPD:	0.09
		N. weight:	1.3
		Sc. PD:	0.04
		Sc. rank:	806.6

,

NA			
Pos.	137	obs:	exp:
ttt	F	3	459.30
ttc	F	1160	703.70
tct	S	0	0.80
tcc	S	5	0.65
tca	S	0	1.27
tcg	S	0	0.28
agt	S	0	1.03
agc	S	0	0.97
---	--	------	------
mPD		0.014	0.49
		nPD:	0.03
		N. weight:	0.52
		Sc. PD:	-0.013
		Sc. rank:	142.2

,

NA			
Pos.	138	obs:	exp:
tat	Y	1168	630.80
tac	Y	0	537.20
---	--	------	------
mPD		0	0.50
		nPD:	0.
		N. weight:	0.65
		Sc. PD:	-0.034
		Sc. rank:	-615.3

,

NA			
Pos.	139	obs:	exp:
tct	S	1	0.16
tcc	S	0	0.13
tca	S	0	0.25
tcg	S	0	0.06
agt	S	0	0.21
agc	S	0	0.19
gct	A	1150	301.40
gcc	A	17	217.30
gca	A	0	549.30
gcg	A	0	99.05
---	--	------	------
mPD		0.030	0.67
		nPD:	0.05
		N. weight:	1.4
		Sc. PD:	-0.012
		Sc. rank:	588.2

,

NA			
Pos.	140	obs:	exp:
ttt	F	0	0.39
ttc	F	1	0.61
tta	L	0	103.60
ttg	L	0	217.30
ctt	L	46	194.30
ctc	L	1119	194.80
cta	L	2	196.70
ctg	L	0	260.40
---	--	------	------
mPD		0.081	1.1
		nPD:	0.07
		N. weight:	1.7
		Sc. PD:	0.029
		Sc. rank:	980.7

,

NA			
Pos.	141	obs:	exp:
tct	S	0	185.60
tcc	S	0	152.40
tca	S	0	296.20
tcg	S	0	66.22
att	I	0	0.37
atc	I	1	0.24
ata	I	0	0.39
agt	S	11	239.90
agc	S	1156	226.70
---	--	------	------
mPD		0.020	1.7
		nPD:	0.01
		N. weight:	1.7
		Sc. PD:	-0.067
		Sc. rank:	-132.7

,

NA			
Pos.	142	obs:	exp:
caa	Q	1166	594.90
cag	Q	2	573.10
---	--	------	------
mPD		0.0034	0.50
		nPD:	0.01
		N. weight:	0.7
		Sc. PD:	-0.032
		Sc. rank:	-237.0

,

NA			
Pos.	143	obs:	exp:
ggt	G	0	154.80
ggc	G	0	149.20
gga	G	1164	538.80
ggg	G	4	325.20
---	--	------	------
mPD		0.0068	0.68
		nPD:	0.01
		N. weight:	0.8
		Sc. PD:	-0.034
		Sc. rank:	-148.6

,

NA			
Pos.	144	obs:	exp:
act	T	0	297.70
acc	T	0	239.70
aca	T	1166	535.70
acg	T	2	94.87
---	--	------	------
mPD		0.0034	0.68
		nPD:	0.01
		N. weight:	0.81
		Sc. PD:	-0.038
		Sc. rank:	-372.6

,

NA			
Pos.	145	obs:	exp:
act	T	0	297.70
acc	T	0	239.70
aca	T	1167	535.70
acg	T	1	94.87
---	--	------	------
mPD		0.0017	0.68
		nPD:	0.
		N. weight:	0.82
		Sc. PD:	-0.04
		Sc. rank:	-512.7

,

NA			
Pos.	146	obs:	exp:
tta	L	0	0.09
ttg	L	0	0.19
ctt	L	0	0.17
ctc	L	1	0.17
cta	L	0	0.17
ctg	L	0	0.22
att	I	3	428.80
atc	I	1162	279.70
ata	I	2	458.60
---	--	------	------
mPD		0.010	0.66
		nPD:	0.02
		N. weight:	1.5
		Sc. PD:	-0.055
		Sc. rank:	40.4

,

NA			
Pos.	147	obs:	exp:
cgt	R	0	50.16
cgc	R	0	57.85
cga	R	3	114.30
cgg	R	0	97.46
aga	R	1165	534.10
agg	R	0	314.20
---	--	------	------
mPD		0.0051	0.96
		nPD:	0.01
		N. weight:	0.81
		Sc. PD:	-0.038
		Sc. rank:	-307.5

,

NA			
Pos.	148	obs:	exp:
ggt	G	0	154.80
ggc	G	0	149.20
gga	G	6	538.80
ggg	G	1162	325.20
---	--	------	------
mPD		0.010	0.68
		nPD:	0.02
		N. weight:	1.3
		Sc. PD:	-0.049
		Sc. rank:	11.5

,

NA			
Pos.	149	obs:	exp:
aaa	K	1157	680.70
aag	K	11	487.30
---	--	------	------
mPD		0.019	0.49
		nPD:	0.04
		N. weight:	0.52
		Sc. PD:	-0.008
		Sc. rank:	204.6

,

NA			
Pos.	150	obs:	exp:
cat	H	1	670.10
cac	H	1166	496.90
caa	Q	1	0.51
cag	Q	0	0.49
---	--	------	------
mPD		0.0034	0.49
		nPD:	0.01
		N. weight:	0.89
		Sc. PD:	-0.04
		Sc. rank:	-277.3

,

NA			
Pos.	151	obs:	exp:
tct	S	0	185.60
tcc	S	0	152.40
tca	S	1165	296.20
tcg	S	2	66.22
act	T	0	0.25
acc	T	0	0.21
aca	T	1	0.46
acg	T	0	0.08
agt	S	0	239.90
agc	S	0	226.70
---	--	------	------
mPD		0.0051	1.7
		nPD:	0.
		N. weight:	1.4
		Sc. PD:	-0.07
		Sc. rank:	-819.3

,

NA			
Pos.	152	obs:	exp:
aat	N	3	594.90
aac	N	1165	573.10
---	--	------	------
mPD		0.0051	0.50
		nPD:	0.01
		N. weight:	0.73
		Sc. PD:	-0.031
		Sc. rank:	-123.7

,

NA			
Pos.	153	obs:	exp:
gaa	E	6	3.65
gag	E	0	2.35
ggt	G	55	154.00
ggc	G	0	148.40
gga	G	1106	536.10
ggg	G	1	323.50
---	--	------	------
mPD		0.10	0.69
		nPD:	0.15
		N. weight:	0.67
		Sc. PD:	0.059
		Sc. rank:	518.4

,

NA			
Pos.	154	obs:	exp:
act	T	0	297.70
acc	T	0	239.70
aca	T	1168	535.70
acg	T	0	94.87
---	--	------	------
mPD		0	0.68
		nPD:	0.
		N. weight:	0.82
		Sc. PD:	-0.043
		Sc. rank:	-778.6

,

NA			
Pos.	155	obs:	exp:
cgt	R	0	0.09
cgc	R	0	0.10
cga	R	0	0.20
cgg	R	0	0.17
att	I	0	422.20
atc	I	0	275.30
ata	I	1149	451.50
atg	M	1	1.00
act	T	0	2.55
acc	T	0	2.05
aca	T	10	4.59
acg	T	0	0.81
aga	R	2	0.91
agg	R	0	0.54
gtt	V	0	1.24
gtc	V	0	1.21
gta	V	6	1.23
gtg	V	0	2.32
---	--	------	------
mPD		0.032	0.69
		nPD:	0.05
		N. weight:	0.99
		Sc. PD:	-0.0073
		Sc. rank:	453.9

NA			
Pos.	156	obs:	exp:
tat	Y	0	0.54
tac	Y	1	0.46
cat	H	1	667.20
cac	H	1161	494.80
cgt	R	0	0.04
cgc	R	1	0.05
cga	R	0	0.10
cgg	R	0	0.08
aat	N	0	2.04
aac	N	4	1.96
aga	R	0	0.46
agg	R	0	0.27
---	--	------	------
mPD		0.012	0.50
		nPD:	0.02
		N. weight:	0.9
		Sc. PD:	-0.026
		Sc. rank:	172.0

NA			
Pos.	157	obs:	exp:
gat	D	1168	631.20
gac	D	0	536.80
---	--	------	------
mPD		0	0.50
		nPD:	0.
		N. weight:	0.65
		Sc. PD:	-0.034
		Sc. rank:	-614.8

NA			
Pos.	158	obs:	exp:
cgt	R	0	50.04
cgc	R	0	57.70
cga	R	0	114.00
cgg	R	0	97.21
act	T	0	0.25
acc	T	0	0.21
aca	T	0	0.46
acg	T	1	0.08
aaa	K	0	1.17
aag	K	2	0.83
aga	R	3	532.70
agg	R	1162	313.30
---	--	------	------
mPD		0.010	0.97
		nPD:	0.01
		N. weight:	1.4
		Sc. PD:	-0.057
		Sc. rank:	-167.7

NA			
Pos.	159	obs:	exp:
tct	S	0	185.80
tcc	S	1166	152.50
tca	S	2	296.50
tcg	S	0	66.28
agt	S	0	240.10
agc	S	0	226.90
---	--	------	------
mPD		0.0034	1.7
		nPD:	0.
		N. weight:	2.1
		Sc. PD:	-0.11
		Sc. rank:	-1489.0

NA			
Pos.	160	obs:	exp:
caa	Q	4	594.90
cag	Q	1164	573.10
---	--	------	------
mPD		0.0068	0.50
		nPD:	0.01
		N. weight:	0.73
		Sc. PD:	-0.028
		Sc. rank:	-27.2

NA			
Pos.	161	obs:	exp:
tat	Y	1168	630.80
tac	Y	0	537.20
---	--	------	------
mPD		0	0.50
		nPD:	0.
		N. weight:	0.65
		Sc. PD:	-0.034
		Sc. rank:	-615.3

NA			
Pos.	162	obs:	exp:
cat	H	0	0.57
cac	H	1	0.43
cgt	R	20	50.12
cgc	R	1091	57.80
cga	R	56	114.20
cgg	R	0	97.38
aga	R	0	533.60
agg	R	0	313.90
---	--	------	------
mPD		0.13	0.96
		nPD:	0.13
		N. weight:	2.8
		Sc. PD:	0.2
		Sc. rank:	2097.5

NA			
Pos.	163	obs:	exp:
tct	S	0	0.16
tcc	S	1	0.13
tca	S	0	0.25
tcg	S	0	0.06
act	T	0	0.25
acc	T	1	0.21
aca	T	0	0.46
acg	T	0	0.08
agt	S	0	0.21
agc	S	0	0.19
gct	A	4	301.10
gcc	A	1161	217.10
gca	A	1	548.80
gcg	A	0	98.96
---	--	------	------
mPD		0.012	0.67
		nPD:	0.02
		N. weight:	1.7
		Sc. PD:	-0.061
		Sc. rank:	139.9

NA			
Pos.	164	obs:	exp:
tta	L	0	103.60
ttg	L	3	217.30
ctt	L	0	194.30
ctc	L	0	194.80
cta	L	5	196.70
ctg	L	1159	260.40
atg	M	1	1.00
---	--	------	------
mPD		0.015	1.1
		nPD:	0.01
		N. weight:	1.5
		Sc. PD:	-0.059
		Sc. rank:	-28.2

NA			
Pos.	165	obs:	exp:
att	I	1	426.90
atc	I	0	278.50
ata	I	1161	456.60
atg	M	2	2.00
gtt	V	0	0.83
gtc	V	0	0.81
gta	V	4	0.82
gtg	V	0	1.54
---	--	------	------
mPD		0.012	0.66
		nPD:	0.02
		N. weight:	0.98
		Sc. PD:	-0.034
		Sc. rank:	82.5

NA			
Pos.	166	obs:	exp:
tct	S	0	185.80
tcc	S	0	152.50
tca	S	0	296.50
tcg	S	0	66.28
agt	S	1	240.10
agc	S	1167	226.90
---	--	------	------
mPD		0.0017	1.7
		nPD:	0.
		N. weight:	1.7
		Sc. PD:	-0.087
		Sc. rank:	-1266.4

NA			
Pos.	167	obs:	exp:
tgg	W	1168	1168.00
---	--	------	------
mPD		0	0
		nPD:	1.
		N. weight:	0.
		Sc. PD:	0
		Sc. rank:	0

NA			
Pos.	168	obs:	exp:
cct	P	1	291.40
ccc	P	0	209.90
cca	P	1163	480.80
ccg	P	4	185.90
---	--	------	------
mPD		0.0085	0.71
		nPD:	0.01
		N. weight:	0.91
		Sc. PD:	-0.037
		Sc. rank:	-91.1

NA			
Pos.	169	obs:	exp:
tta	L	40	103.60
ttg	L	0	217.50
ctt	L	1	194.50
ctc	L	0	194.90
cta	L	1126	196.80
ctg	L	1	260.60
---	--	------	------
mPD		0.070	1.1
		nPD:	0.06
		N. weight:	1.7
		Sc. PD:	0.013
		Sc. rank:	950.8

NA			
Pos.	170	obs:	exp:
tct	S	0	185.80
tcc	S	0	152.50
tca	S	1168	296.50
tcg	S	0	66.28
agt	S	0	240.10
agc	S	0	226.90
---	--	------	------
mPD		0	1.7
		nPD:	0.
		N. weight:	1.4
		Sc. PD:	-0.075
		Sc. rank:	-1369.7

NA			
Pos.	171	obs:	exp:
tct	S	0	185.10
tcc	S	0	152.00
tca	S	1164	295.40
tcg	S	0	66.05
act	T	0	1.02
acc	T	0	0.82
aca	T	4	1.83
acg	T	0	0.32
agt	S	0	239.30
agc	S	0	226.10
---	--	------	------
mPD		0.0068	1.7
		nPD:	0.
		N. weight:	1.4
		Sc. PD:	-0.069
		Sc. rank:	-705.5

NA			
Pos.	172	obs:	exp:
cct	P	8	291.40
ccc	P	0	209.90
cca	P	3	480.80
ccg	P	1157	185.90
---	--	------	------
mPD		0.019	0.71
		nPD:	0.03
		N. weight:	1.9
		Sc. PD:	-0.05
		Sc. rank:	464.8

NA			
Pos.	173	obs:	exp:
cct	P	0	291.40
ccc	P	1168	209.90
cca	P	0	480.80
ccg	P	0	185.90
---	--	------	------
mPD		0	0.71
		nPD:	0.
		N. weight:	1.8
		Sc. PD:	-0.094
		Sc. rank:	-1714.7

NA			
Pos.	174	obs:	exp:
act	T	0	297.20
acc	T	0	239.30
aca	T	1164	534.80
acg	T	2	94.71
gct	A	0	0.52
gcc	A	0	0.37
gca	A	2	0.94
gcg	A	0	0.17
---	--	------	------
mPD		0.0068	0.68
		nPD:	0.01
		N. weight:	0.81
		Sc. PD:	-0.034
		Sc. rank:	-162.5

NA			
Pos.	175	obs:	exp:
gtt	V	7	242.00
gtc	V	0	235.70
gta	V	0	239.20
gtg	V	1161	451.10
---	--	------	------
mPD		0.012	0.73
		nPD:	0.02
		N. weight:	0.97
		Sc. PD:	-0.035
		Sc. rank:	30.3

NA			
Pos.	176	obs:	exp:
tat	Y	0	438.00
tac	Y	811	373.00
cat	H	0	205.00
cac	H	357	152.00
---	--	------	------
mPD		0.42	0.92
		nPD:	0.46
		N. weight:	0.84
		Sc. PD:	0.32
		Sc. rank:	797.9

NA			
Pos.	177	obs:	exp:
aat	N	2	594.90
aac	N	1166	573.10
---	--	------	------
mPD		0.0034	0.50
		nPD:	0.01
		N. weight:	0.74
		Sc. PD:	-0.033
		Sc. rank:	-256.9

NA			
Pos.	178	obs:	exp:
tct	S	0	185.60
tcc	S	0	152.40
tca	S	0	296.20
tcg	S	0	66.22
aat	N	0	0.51
aac	N	1	0.49
agt	S	4	239.90
agc	S	1163	226.70
---	--	------	------
mPD		0.0085	1.7
		nPD:	0.01
		N. weight:	1.7
		Sc. PD:	-0.08
		Sc. rank:	-690.6

NA			
Pos.	179	obs:	exp:
cgt	R	0	50.16
cgc	R	0	57.85
cga	R	0	114.30
cgg	R	0	97.46
aga	R	12	534.10
agg	R	1156	314.20
---	--	------	------
mPD		0.020	0.96
		nPD:	0.02
		N. weight:	1.3
		Sc. PD:	-0.042
		Sc. rank:	204.5

NA			
Pos.	180	obs:	exp:
gtt	V	0	242.00
gtc	V	0	235.70
gta	V	17	239.20
gtg	V	1151	451.10
---	--	------	------
mPD		0.029	0.73
		nPD:	0.04
		N. weight:	0.94
		Sc. PD:	-0.013
		Sc. rank:	372.5

NA			
Pos.	181	obs:	exp:
gaa	E	1133	710.70
gag	E	35	457.30
---	--	------	------
mPD		0.058	0.48
		nPD:	0.12
		N. weight:	0.4
		Sc. PD:	0.025
		Sc. rank:	282.1

NA			
Pos.	182	obs:	exp:
tgt	C	0	494.00
tgc	C	1168	674.00
---	--	------	------
mPD		0	0.49
		nPD:	0.
		N. weight:	0.58
		Sc. PD:	-0.03
		Sc. rank:	-549.3

NA			
Pos.	183	obs:	exp:
att	I	1120	429.10
atc	I	48	279.90
ata	I	0	459.00
---	--	------	------
mPD		0.079	0.65
		nPD:	0.12
		N. weight:	0.9
		Sc. PD:	0.056
		Sc. rank:	631.6

NA			
Pos.	184	obs:	exp:
ggt	G	0	154.80
ggc	G	0	149.20
gga	G	3	538.80
ggg	G	1165	325.20
---	--	------	------
mPD		0.0051	0.68
		nPD:	0.01
		N. weight:	1.3
		Sc. PD:	-0.059
		Sc. rank:	-339.7

NA			
Pos.	185	obs:	exp:
tgg	W	1168	1168.00
---	--	------	------
mPD		0	0
		nPD:	1.
		N. weight:	0.
		Sc. PD:	0
		Sc. rank:	0

NA			
Pos.	186	obs:	exp:
tct	S	7	185.80
tcc	S	0	152.50
tca	S	1161	296.50
tcg	S	0	66.28
agt	S	0	240.10
agc	S	0	226.90
---	--	------	------
mPD		0.012	1.7
		nPD:	0.01
		N. weight:	1.4
		Sc. PD:	-0.063
		Sc. rank:	-390.4

NA			
Pos.	187	obs:	exp:
tct	S	0	185.80
tcc	S	0	152.50
tca	S	0	296.50
tcg	S	0	66.28
agt	S	1117	240.10
agc	S	51	226.90
---	--	------	------
mPD		0.084	1.7
		nPD:	0.05
		N. weight:	1.5
		Sc. PD:	-0.006
		Sc. rank:	721.5

NA			
Pos.	188	obs:	exp:
act	T	902	231.20
acc	T	5	186.10
aca	T	0	416.00
acg	T	0	73.67
gct	A	259	67.40
gcc	A	2	48.61
gca	A	0	122.80
gcg	A	0	22.15
---	--	------	------
mPD		0.36	1.0
		nPD:	0.35
		N. weight:	1.4
		Sc. PD:	0.39
		Sc. rank:	1293.7

NA			
Pos.	189	obs:	exp:
tct	S	0	185.80
tcc	S	0	152.50
tca	S	0	296.50
tcg	S	0	66.28
agt	S	1163	240.10
agc	S	5	226.90
---	--	------	------
mPD		0.0085	1.7
		nPD:	0.01
		N. weight:	1.6
		Sc. PD:	-0.077
		Sc. rank:	-676.2

NA			
Pos.	190	obs:	exp:
tgt	C	4	494.00
tgc	C	1164	674.00
---	--	------	------
mPD		0.0068	0.49
		nPD:	0.01
		N. weight:	0.56
		Sc. PD:	-0.022
		Sc. rank:	-5.1

NA			
Pos.	191	obs:	exp:
tat	Y	2	1.08
tac	Y	0	0.92
cat	H	1162	669.50
cac	H	4	496.50
---	--	------	------
mPD		0.010	0.49
		nPD:	0.02
		N. weight:	0.56
		Sc. PD:	-0.018
		Sc. rank:	75.2

NA			
Pos.	192	obs:	exp:
gat	D	1168	631.20
gac	D	0	536.80
---	--	------	------
mPD		0	0.50
		nPD:	0.
		N. weight:	0.65
		Sc. PD:	-0.034
		Sc. rank:	-614.8

NA			
Pos.	193	obs:	exp:
ggt	G	132	154.80
ggc	G	1035	149.20
gga	G	1	538.80
ggg	G	0	325.20
---	--	------	------
mPD		0.20	0.68
		nPD:	0.3
		N. weight:	1.8
		Sc. PD:	0.41
		Sc. rank:	1616.0

NA			
Pos.	194	obs:	exp:
aaa	K	939	680.70
aag	K	229	487.30
---	--	------	------
mPD		0.32	0.49
		nPD:	0.65
		N. weight:	0.12
		Sc. PD:	0.067
		Sc. rank:	115.1

NA			
Pos.	195	obs:	exp:
tct	S	2	185.60
tcc	S	1165	152.40
tca	S	0	296.20
tcg	S	0	66.22
tat	Y	0	0.54
tac	Y	1	0.46
agt	S	0	239.90
agc	S	0	226.70
---	--	------	------
mPD		0.0051	1.7
		nPD:	0.
		N. weight:	2.1
		Sc. PD:	-0.1
		Sc. rank:	-1229.3

NA			
Pos.	196	obs:	exp:
cgt	R	0	50.16
cgc	R	0	57.85
cga	R	0	114.30
cgg	R	0	97.46
aga	R	52	534.10
agg	R	1116	314.20
---	--	------	------
mPD		0.085	0.96
		nPD:	0.09
		N. weight:	1.2
		Sc. PD:	0.037
		Sc. rank:	744.1

NA			
Pos.	197	obs:	exp:
atg	M	1168	1168.00
---	--	------	------
mPD		0	0
		nPD:	1.
		N. weight:	0.
		Sc. PD:	0
		Sc. rank:	0

NA			
Pos.	198	obs:	exp:
tct	S	0	185.80
tcc	S	0	152.50
tca	S	1166	296.50
tcg	S	2	66.28
agt	S	0	240.10
agc	S	0	226.90
---	--	------	------
mPD		0.0034	1.7
		nPD:	0.
		N. weight:	1.4
		Sc. PD:	-0.071
		Sc. rank:	-1002.7

NA			
Pos.	199	obs:	exp:
att	I	0	428.00
atc	I	0	279.20
ata	I	1165	457.80
gtt	V	0	0.62
gtc	V	0	0.61
gta	V	3	0.61
gtg	V	0	1.16
---	--	------	------
mPD		0.0051	0.66
		nPD:	0.01
		N. weight:	0.99
		Sc. PD:	-0.044
		Sc. rank:	-219.0

NA			
Pos.	200	obs:	exp:
tgt	C	1167	494.00
tgc	C	1	674.00
---	--	------	------
mPD		0.0017	0.49
		nPD:	0.
		N. weight:	0.9
		Sc. PD:	-0.044
		Sc. rank:	-491.5

NA			
Pos.	201	obs:	exp:
att	I	0	428.00
atc	I	11	279.20
ata	I	1154	457.80
gtt	V	0	0.62
gtc	V	0	0.61
gta	V	3	0.61
gtg	V	0	1.16
---	--	------	------
mPD		0.024	0.66
		nPD:	0.04
		N. weight:	0.94
		Sc. PD:	-0.017
		Sc. rank:	337.2

NA			
Pos.	202	obs:	exp:
tct	S	0	185.80
tcc	S	0	152.50
tca	S	1166	296.50
tcg	S	2	66.28
agt	S	0	240.10
agc	S	0	226.90
---	--	------	------
mPD		0.0034	1.7
		nPD:	0.
		N. weight:	1.4
		Sc. PD:	-0.071
		Sc. rank:	-1002.7

NA			
Pos.	203	obs:	exp:
ggt	G	0	154.80
ggc	G	0	149.20
gga	G	1159	538.80
ggg	G	9	325.20
---	--	------	------
mPD		0.015	0.68
		nPD:	0.02
		N. weight:	0.77
		Sc. PD:	-0.024
		Sc. rank:	139.3

NA			
Pos.	204	obs:	exp:
cct	P	0	291.40
ccc	P	0	209.90
cca	P	936	480.80
ccg	P	232	185.90
---	--	------	------
mPD		0.32	0.71
		nPD:	0.45
		N. weight:	0.61
		Sc. PD:	0.23
		Sc. rank:	574.9

NA

Pos.	205	obs:	exp:
aat	N	1	593.90
aac	N	1165	572.10
aaa	K	2	1.17
aag	K	0	0.83
---	--	------	------
mPD		0.0051	0.50
		nPD:	0.01
		N. weight:	0.74
		Sc. PD:	-0.031
		Sc. rank:	-129.8

NA

Pos.	206	obs:	exp:
aat	N	1	594.90
aac	N	1167	573.10
---	--	------	------
mPD		0.0017	0.50
		nPD:	0.
		N. weight:	0.75
		Sc. PD:	-0.036
		Sc. rank:	-420.4

NA

Pos.	207	obs:	exp:
aat	N	1168	594.90
aac	N	0	573.10
---	--	------	------
mPD		0	0.50
		nPD:	0.
		N. weight:	0.71
		Sc. PD:	-0.037
		Sc. rank:	-673.9

NA

Pos.	208	obs:	exp:
gct	A	0	301.60
gcc	A	1	217.50
gca	A	1167	549.70
gcg	A	0	99.13
---	--	------	------
mPD		0.0017	0.67
		nPD:	0.
		N. weight:	0.79
		Sc. PD:	-0.039
		Sc. rank:	-484.0

NA

Pos.	209	obs:	exp:
tct	S	1167	185.80
tcc	S	0	152.50
tca	S	0	296.50
tcg	S	1	66.28
agt	S	0	240.10
agc	S	0	226.90
---	--	------	------
mPD		0.0017	1.7
		nPD:	0.
		N. weight:	1.9
		Sc. PD:	-0.098
		Sc. rank:	-1422.1

NA

Pos.	210	obs:	exp:
gct	A	23	301.60
gcc	A	0	217.50
gca	A	1134	549.70
gcg	A	11	99.13
---	--	------	------
mPD		0.057	0.67
		nPD:	0.09
		N. weight:	0.67
		Sc. PD:	0.019
		Sc. rank:	416.0

NA

Pos.	211	obs:	exp:
att	I	0	2.57
atc	I	0	1.68
ata	I	7	2.75
gtt	V	0	240.60
gtc	V	0	234.30
gta	V	1160	237.70
gtg	V	1	448.40
---	--	------	------
mPD		0.014	0.74
		nPD:	0.02
		N. weight:	1.7
		Sc. PD:	-0.057
		Sc. rank:	170.9

NA

Pos.	212	obs:	exp:
att	I	0	19.11
atc	I	0	12.46
ata	I	52	20.43
gtt	V	0	231.20
gtc	V	0	225.20
gta	V	1050	228.50
gtg	V	66	431.00
---	--	------	------
mPD		0.19	0.82
		nPD:	0.24
		N. weight:	1.4
		Sc. PD:	0.23
		Sc. rank:	1166.5

NA

Pos.	213	obs:	exp:
tgg	W	1168	1168.00
---	--	------	------
mPD		0	0
		nPD:	1.
		N. weight:	0.
		Sc. PD:	0
		Sc. rank:	0

NA

Pos.	214	obs:	exp:
tat	Y	2	630.80
tac	Y	1166	537.20
---	--	------	------
mPD		0.0034	0.50
		nPD:	0.01
		N. weight:	0.81
		Sc. PD:	-0.036
		Sc. rank:	-264.5

NA

Pos.	215	obs:	exp:
aat	N	2	594.90
aac	N	1166	573.10
---	--	------	------
mPD		0.0034	0.50
		nPD:	0.01
		N. weight:	0.74
		Sc. PD:	-0.033
		Sc. rank:	-256.9

NA

Pos.	216	obs:	exp:
cgt	R	0	49.95
cgc	R	0	57.60
cga	R	0	113.80
cgg	R	0	97.04
aaa	K	5	2.91
aag	K	0	2.09
aga	R	1152	531.80
agg	R	11	312.80
---	--	------	------
mPD		0.027	0.97
		nPD:	0.03
		N. weight:	0.78
		Sc. PD:	-0.02
		Sc. rank:	221.2

NA

Pos.	217	obs:	exp:
cgt	R	0	50.16
cgc	R	0	57.85
cga	R	0	114.30
cgg	R	0	97.46
aga	R	5	534.10
agg	R	1163	314.20
---	--	------	------
mPD		0.0085	0.96
		nPD:	0.01
		N. weight:	1.4
		Sc. PD:	-0.059
		Sc. rank:	-287.6

NA

Pos.	218	obs:	exp:
cct	P	1167	291.40
ccc	P	0	209.90
cca	P	1	480.80
ccg	P	0	185.90
---	--	------	------
mPD		0.0017	0.71
		nPD:	0.
		N. weight:	1.5
		Sc. PD:	-0.072
		Sc. rank:	-927.9

NA

Pos.	219	obs:	exp:
att	I	191	78.63
atc	I	0	51.28
ata	I	23	84.09
gtt	V	912	197.50
gtc	V	1	192.30
gta	V	1	195.10
gtg	V	39	368.10
gct	A	1	0.26
gcc	A	0	0.19
gca	A	0	0.47
gcg	A	0	0.08
---	--	------	------
mPD		0.41	1.0
		nPD:	0.39
		N. weight:	1.3
		Sc. PD:	0.41
		Sc. rank:	1211.6

NA

Pos.	220	obs:	exp:
act	T	0	0.76
acc	T	0	0.62
aca	T	3	1.38
acg	T	0	0.24
gtt	V	0	0.62
gtc	V	0	0.61
gta	V	3	0.61
gtg	V	0	1.16
gct	A	0	300.10
gcc	A	0	216.40
gca	A	1161	546.90
gcg	A	1	98.62
---	--	------	------
mPD		0.012	0.68
		nPD:	0.02
		N. weight:	0.79
		Sc. PD:	-0.028
		Sc. rank:	45.2

NA

Pos.	221	obs:	exp:
gaa	E	1167	710.70
gag	E	1	457.30
---	--	------	------
mPD		0.0017	0.48
		nPD:	0.
		N. weight:	0.52
		Sc. PD:	-0.025
		Sc. rank:	-262.5

NA

Pos.	222	obs:	exp:
att	I	1164	428.80
atc	I	3	279.70
ata	I	0	458.60
gtt	V	1	0.21
gtc	V	0	0.20
gta	V	0	0.20
gtg	V	0	0.39
---	--	------	------
mPD		0.0068	0.66
		nPD:	0.01
		N. weight:	1.
		Sc. PD:	-0.044
		Sc. rank:	-151.8

NA

Pos.	223	obs:	exp:
tct	S	0	0.16
tcc	S	0	0.13
tca	S	0	0.25
tcg	S	0	0.06
act	T	0	0.25
acc	T	1	0.21
aca	T	0	0.46
acg	T	0	0.08
aat	N	0	593.90
aac	N	1166	572.10
agt	S	0	0.21
agc	S	1	0.19
---	--	------	------
mPD		0.0034	0.50
		nPD:	0.01
		N. weight:	0.75
		Sc. PD:	-0.034
		Sc. rank:	-266.0

NA

Pos.	224	obs:	exp:
act	T	0	297.70
acc	T	0	239.70
aca	T	1160	535.70
acg	T	8	94.87
---	--	------	------
mPD		0.014	0.68
		nPD:	0.02
		N. weight:	0.79
		Sc. PD:	-0.026
		Sc. rank:	89.3

NA

Pos.	225	obs:	exp:
tgg	W	1168	1168.00
---	--	------	------
mPD		0	0
		nPD:	1.
		N. weight:	0.
		Sc. PD:	0
		Sc. rank:	0

NA

Pos.	226	obs:	exp:
gct	A	9	301.40
gcc	A	1157	217.30
gca	A	1	549.30
gcg	A	0	99.05
ggt	G	0	0.13
ggc	G	1	0.13
gga	G	0	0.46
ggg	G	0	0.28
---	--	------	------
mPD		0.019	0.67
		nPD:	0.03
		N. weight:	1.7
		Sc. PD:	-0.044
		Sc. rank:	481.0

NA

Pos.	227	obs:	exp:
caa	Q	27	13.75
cag	Q	0	13.25
cgt	R	0	49.00
cgc	R	0	56.51
cga	R	1134	111.70
cgg	R	0	95.21
aga	R	7	521.70
agg	R	0	306.90
---	--	------	------
mPD		0.057	1.0
		nPD:	0.06
		N. weight:	2.4
		Sc. PD:	0.0026
		Sc. rank:	1223.7

NA

Pos.	228	obs:	exp:
cat	H	0	1.15
cac	H	2	0.85
aat	N	1	583.70
aac	N	1145	562.30
gat	D	0	10.81
gac	D	20	9.19
---	--	------	------
mPD		0.039	0.54
		nPD:	0.07
		N. weight:	0.75
		Sc. PD:	0.012
		Sc. rank:	424.6

NA

Pos.	229	obs:	exp:
cgt	R	0	0.04
cgc	R	0	0.05
cga	R	0	0.10
cgg	R	0	0.08
att	I	0	426.90
atc	I	0	278.50
ata	I	1162	456.60
act	T	0	1.27
acc	T	0	1.03
aca	T	5	2.29
acg	T	0	0.41
aga	R	1	0.46
agg	R	0	0.27
---	--	------	------
mPD		0.010	0.66
		nPD:	0.02
		N. weight:	0.99
		Sc. PD:	-0.037
		Sc. rank:	21.1

NA			
Pos.	230	obs:	exp:
tta	L	0	103.60
ttg	L	0	217.50
ctt	L	0	194.50
ctc	L	0	194.90
cta	L	1163	196.80
ctg	L	5	260.60
---	--	------	------
mPD		0.0085	1.1
		nPD:	0.01
		N. weight:	1.9
		Sc. PD:	-0.082
		Sc. rank:	-440.8

NA			
Pos.	231	obs:	exp:
cgt	R	0	50.16
cgc	R	0	57.85
cga	R	0	114.30
cgg	R	0	97.46
aga	R	1164	534.10
agg	R	4	314.20
---	--	------	------
mPD		0.0068	0.96
		nPD:	0.01
		N. weight:	0.81
		Sc. PD:	-0.036
		Sc. rank:	-234.2

NA			
Pos.	232	obs:	exp:
act	T	0	297.70
acc	T	4	239.70
aca	T	1159	535.70
acg	T	5	94.87
---	--	------	------
mPD		0.015	0.68
		nPD:	0.02
		N. weight:	0.78
		Sc. PD:	-0.024
		Sc. rank:	143.3

NA			
Pos.	233	obs:	exp:
caa	Q	8	594.90
cag	Q	1160	573.10
---	--	------	------
mPD		0.014	0.50
		nPD:	0.03
		N. weight:	0.71
		Sc. PD:	-0.018
		Sc. rank:	190.5

NA			
Pos.	234	obs:	exp:
gaa	E	1073	710.70
gag	E	95	457.30
---	--	------	------
mPD		0.15	0.48
		nPD:	0.31
		N. weight:	0.27
		Sc. PD:	0.065
		Sc. rank:	242.0

NA			
Pos.	235	obs:	exp:
tct	S	1166	185.80
tcc	S	0	152.50
tca	S	1	296.50
tcg	S	1	66.28
agt	S	0	240.10
agc	S	0	226.90
---	--	------	------
mPD		0.0034	1.7
		nPD:	0.
		N. weight:	1.9
		Sc. PD:	-0.096
		Sc. rank:	-1255.3

NA			
Pos.	236	obs:	exp:
gaa	E	1167	710.70
gag	E	1	457.30
---	--	------	------
mPD		0.0017	0.48
		nPD:	0.
		N. weight:	0.52
		Sc. PD:	-0.025
		Sc. rank:	-262.5

NA			
Pos.	237	obs:	exp:
tgt	C	1167	494.00
tgc	C	1	674.00
---	--	------	------
mPD		0.0017	0.49
		nPD:	0.
		N. weight:	0.9
		Sc. PD:	-0.044
		Sc. rank:	-491.5

NA			
Pos.	238	obs:	exp:
gtt	V	0	241.80
gtc	V	0	235.50
gta	V	938	239.00
gtg	V	229	450.70
gct	A	0	0.26
gcc	A	0	0.19
gca	A	1	0.47
gcg	A	0	0.08
---	--	------	------
mPD		0.32	0.73
		nPD:	0.44
		N. weight:	1.
		Sc. PD:	0.37
		Sc. rank:	956.6

NA			
Pos.	239	obs:	exp:
tgt	C	8	494.00
tgc	C	1160	674.00
---	--	------	------
mPD		0.014	0.49
		nPD:	0.03
		N. weight:	0.54
		Sc. PD:	-0.014
		Sc. rank:	152.6

NA			
Pos.	240	obs:	exp:
cat	H	3	670.70
cac	H	1165	497.30
---	--	------	------
mPD		0.0051	0.49
		nPD:	0.01
		N. weight:	0.88
		Sc. PD:	-0.037
		Sc. rank:	-120.3

NA			
Pos.	241	obs:	exp:
aat	N	12	593.90
aac	N	1154	572.10
gat	D	0	1.08
gac	D	2	0.92
---	--	------	------
mPD		0.024	0.50
		nPD:	0.05
		N. weight:	0.69
		Sc. PD:	-0.005
		Sc. rank:	327.0

NA			
Pos.	242	obs:	exp:
tct	S	0	0.32
tcc	S	0	0.26
tca	S	0	0.51
tcg	S	0	0.11
agt	S	0	0.41
agc	S	2	0.39
ggt	G	41	154.50
ggc	G	1125	149.00
gga	G	0	537.90
ggg	G	0	324.60
---	--	------	------
mPD		0.071	0.68
		nPD:	0.1
		N. weight:	2.
		Sc. PD:	0.094
		Sc. rank:	1368.4

NA			
Pos.	243	obs:	exp:
att	I	0	41.52
atc	I	0	27.08
ata	I	113	44.40
gtt	V	0	218.60
gtc	V	1	212.90
gta	V	1054	216.00
gtg	V	0	407.50
---	--	------	------
mPD		0.18	0.91
		nPD:	0.19
		N. weight:	1.6
		Sc. PD:	0.21
		Sc. rank:	1315.6

NA			
Pos.	244	obs:	exp:
tgt	C	1	494.00
tgc	C	1167	674.00
---	--	------	------
mPD		0.0017	0.49
		nPD:	0.
		N. weight:	0.57
		Sc. PD:	-0.028
		Sc. rank:	-313.1

NA			
Pos.	245	obs:	exp:
cct	P	11	291.40
ccc	P	2	209.90
cca	P	1155	480.80
ccg	P	0	185.90
---	--	------	------
mPD		0.022	0.71
		nPD:	0.03
		N. weight:	0.88
		Sc. PD:	-0.02
		Sc. rank:	273.4

NA			
Pos.	246	obs:	exp:
att	I	0	24.98
atc	I	0	16.30
ata	I	68	26.72
gtt	V	0	226.30
gtc	V	0	220.40
gta	V	1089	223.60
gtg	V	3	421.70
gct	A	0	2.07
gcc	A	0	1.49
gca	A	8	3.77
gcg	A	0	0.68
---	--	------	------
mPD		0.13	0.86
		nPD:	0.15
		N. weight:	1.6
		Sc. PD:	0.14
		Sc. rank:	1248.4

NA			
Pos.	247	obs:	exp:
gtt	V	1	242.00
gtc	V	0	235.70
gta	V	2	239.20
gtg	V	1165	451.10
---	--	------	------
mPD		0.0051	0.73
		nPD:	0.01
		N. weight:	0.99
		Sc. PD:	-0.044
		Sc. rank:	-292.5

NA			
Pos.	248	obs:	exp:
ttt	F	0	461.20
ttc	F	1168	706.80
---	--	------	------
mPD		0	0.48
		nPD:	0.
		N. weight:	0.53
		Sc. PD:	-0.027
		Sc. rank:	-501.8

NA			
Pos.	249	obs:	exp:
act	T	113	297.70
acc	T	1055	239.70
aca	T	0	535.70
acg	T	0	94.87
---	--	------	------
mPD		0.17	0.68
		nPD:	0.26
		N. weight:	1.3
		Sc. PD:	0.25
		Sc. rank:	1132.4

NA			
Pos.	250	obs:	exp:
gat	D	1166	631.20
gac	D	2	536.80
---	--	------	------
mPD		0.0034	0.50
		nPD:	0.01
		N. weight:	0.64
		Sc. PD:	-0.029
		Sc. rank:	-201.7

NA			
Pos.	251	obs:	exp:
ggt	G	0	154.80
ggc	G	1	149.20
gga	G	103	538.80
ggg	G	1064	325.20
---	--	------	------
mPD		0.16	0.68
		nPD:	0.24
		N. weight:	0.99
		Sc. PD:	0.17
		Sc. rank:	835.6

NA			
Pos.	252	obs:	exp:
tct	S	606	96.38
tcc	S	0	79.14
tca	S	0	153.80
tcg	S	0	34.39
cct	P	333	139.70
ccc	P	227	100.60
cca	P	0	230.50
ccg	P	0	89.15
cat	H	2	1.15
cac	H	0	0.85
agt	S	0	124.60
agc	S	0	117.70
---	--	------	------
mPD		0.82	1.7
		nPD:	0.49
		N. weight:	1.4
		Sc. PD:	0.58
		Sc. rank:	1367.7

NA			
Pos.	253	obs:	exp:
act	T	0	0.51
acc	T	2	0.41
aca	T	0	0.92
acg	T	0	0.16
gct	A	0	300.80
gcc	A	1160	217.00
gca	A	5	548.30
gcg	A	0	98.88
gat	D	0	0.54
gac	D	1	0.46
---	--	------	------
mPD		0.014	0.68
		nPD:	0.02
		N. weight:	1.7
		Sc. PD:	-0.057
		Sc. rank:	208.1

NA			
Pos.	254	obs:	exp:
act	T	1153	297.20
acc	T	2	239.30
aca	T	11	534.80
acg	T	0	94.71
aat	N	2	1.02
aac	N	0	0.98
---	--	------	------
mPD		0.025	0.68
		nPD:	0.04
		N. weight:	1.4
		Sc. PD:	-0.022
		Sc. rank:	521.5

NA			
Pos.	255	obs:	exp:
ggt	G	0	154.80
ggc	G	1	149.20
gga	G	1164	538.80
ggg	G	3	325.20
---	--	------	------
mPD		0.0068	0.68
		nPD:	0.01
		N. weight:	0.8
		Sc. PD:	-0.034
		Sc. rank:	-145.9

NA			
Pos.	256	obs:	exp:
cct	P	1168	291.40
ccc	P	0	209.90
cca	P	0	480.80
ccg	P	0	185.90
---	--	------	------
mPD		0	0.71
		nPD:	0.
		N. weight:	1.5
		Sc. PD:	-0.076
		Sc. rank:	-1386.7

NA			
Pos.	257	obs:	exp:
gct	A	2	301.60
gcc	A	0	217.50
gca	A	1166	549.70
gcg	A	0	99.13
---	--	------	------
mPD		0.0034	0.67
		nPD:	0.01
		N. weight:	0.78
		Sc. PD:	-0.037
		Sc. rank:	-328.1

NA			
Pos.	258	obs:	exp:
tat	Y	0	0.54
tac	Y	1	0.46
aat	N	0	2.04
aac	N	4	1.96
gat	D	0	625.20
gac	D	1157	531.80
gaa	E	6	3.65
gag	E	0	2.35
---	--	------	------
mPD		0.019	0.51
		nPD:	0.04
		N. weight:	0.82
		Sc. PD:	-0.014
		Sc. rank:	305.0

NA			
Pos.	259	obs:	exp:
act	T	0	297.70
acc	T	0	239.70
aca	T	1168	535.70
acg	T	0	94.87
---	--	------	------
mPD		0	0.68
		nPD:	0.
		N. weight:	0.82
		Sc. PD:	-0.043
		Sc. rank:	-778.6

NA			
Pos.	260	obs:	exp:
cgt	R	0	50.16
cgc	R	0	57.85
cga	R	17	114.30
cgg	R	0	97.46
aga	R	1150	534.10
agg	R	1	314.20
---	--	------	------
mPD		0.030	0.96
		nPD:	0.03
		N. weight:	0.77
		Sc. PD:	-0.017
		Sc. rank:	243.8

NA			
Pos.	261	obs:	exp:
att	I	0	429.10
atc	I	0	279.90
ata	I	1168	459.00
---	--	------	------
mPD		0	0.65
		nPD:	0.
		N. weight:	0.99
		Sc. PD:	-0.051
		Sc. rank:	-933.1

NA			
Pos.	262	obs:	exp:
tat	Y	10	630.80
tac	Y	1158	537.20
---	--	------	------
mPD		0.017	0.50
		nPD:	0.03
		N. weight:	0.77
		Sc. PD:	-0.015
		Sc. rank:	258.9

NA			
Pos.	263	obs:	exp:
tat	Y	1125	630.80
tac	Y	43	537.20
---	--	------	------
mPD		0.071	0.50
		nPD:	0.14
		N. weight:	0.49
		Sc. PD:	0.041
		Sc. rank:	375.8

NA			
Pos.	264	obs:	exp:
ttt	F	1167	461.20
ttc	F	1	706.80
---	--	------	------
mPD		0.0017	0.48
		nPD:	0.
		N. weight:	0.97
		Sc. PD:	-0.047
		Sc. rank:	-495.8

NA			
Pos.	265	obs:	exp:
cgt	R	0	0.04
cgc	R	0	0.05
cga	R	0	0.10
cgg	R	0	0.08
aaa	K	1167	680.20
aag	K	0	486.80
aga	R	1	0.46
agg	R	0	0.27
---	--	------	------
mPD		0.0017	0.49
		nPD:	0.
		N. weight:	0.57
		Sc. PD:	-0.028
		Sc. rank:	-312.4

NA			
Pos.	266	obs:	exp:
aaa	K	0	0.58
aag	K	1	0.42
gaa	E	13	710.10
gag	E	1154	456.90
---	--	------	------
mPD		0.024	0.48
		nPD:	0.05
		N. weight:	0.92
		Sc. PD:	-0.0045
		Sc. rank:	442.3

NA			
Pos.	267	obs:	exp:
ggt	G	0	154.80
ggc	G	0	149.20
gga	G	5	538.80
ggg	G	1163	325.20
---	--	------	------
mPD		0.0085	0.68
		nPD:	0.01
		N. weight:	1.3
		Sc. PD:	-0.053
		Sc. rank:	-97.6

NA			
Pos.	268	obs:	exp:
aaa	K	1167	680.20
aag	K	0	486.80
gaa	E	1	0.61
gag	E	0	0.39
---	--	------	------
mPD		0.0017	0.49
		nPD:	0.
		N. weight:	0.57
		Sc. PD:	-0.028
		Sc. rank:	-302.4

NA			
Pos.	269	obs:	exp:
att	I	0	428.40
atc	I	0	279.40
ata	I	1166	458.20
gtt	V	0	0.41
gtc	V	0	0.40
gta	V	2	0.41
gtg	V	0	0.77
---	--	------	------
mPD		0.0034	0.66
		nPD:	0.01
		N. weight:	0.99
		Sc. PD:	-0.046
		Sc. rank:	-391.5

NA			
Pos.	270	obs:	exp:
tta	L	223	103.60
ttg	L	942	217.50
ctt	L	0	194.50
ctc	L	0	194.90
cta	L	1	196.80
ctg	L	2	260.60
---	--	------	------
mPD		0.32	1.1
		nPD:	0.28
		N. weight:	1.4
		Sc. PD:	0.3
		Sc. rank:	1221.3

NA			
Pos.	271	obs:	exp:
aaa	K	1156	679.60
aag	K	10	486.40
gaa	E	2	1.22
gag	E	0	0.78
---	--	------	------
mPD		0.020	0.49
		nPD:	0.04
		N. weight:	0.52
		Sc. PD:	-0.0065
		Sc. rank:	213.8

NA			
Pos.	272	obs:	exp:
tgg	W	1168	1168.00
---	--	------	------
mPD		0	0
		nPD:	1.
		N. weight:	0.
		Sc. PD:	0
		Sc. rank:	0

NA			
Pos.	273	obs:	exp:
gat	D	2	1.08
gac	D	0	0.92
gaa	E	8	709.50
gag	E	1158	456.50
---	--	------	------
mPD		0.017	0.48
		nPD:	0.04
		N. weight:	0.94
		Sc. PD:	-0.017
		Sc. rank:	332.6

NA			
Pos.	274	obs:	exp:
tct	S	1157	185.30
tcc	S	7	152.10
tca	S	0	295.70
tcg	S	1	66.11
cct	P	3	0.75
ccc	P	0	0.54
cca	P	0	1.23
ccg	P	0	0.48
agt	S	0	239.50
agc	S	0	226.30
---	--	------	------
mPD		0.019	1.7
		nPD:	0.01
		N. weight:	1.9
		Sc. PD:	-0.078
		Sc. rank:	-204.0

NA			
Pos.	275	obs:	exp:
tta	L	0	103.60
ttg	L	1	217.50
ctt	L	0	194.50
ctc	L	0	194.90
cta	L	4	196.80
ctg	L	1163	260.60
---	--	------	------
mPD		0.0085	1.1
		nPD:	0.01
		N. weight:	1.6
		Sc. PD:	-0.069
		Sc. rank:	-360.8

NA			
Pos.	276	obs:	exp:
att	I	45	16.53
atc	I	0	10.78
ata	I	0	17.68
act	T	1115	284.20
acc	T	0	228.80
aca	T	0	511.40
acg	T	0	90.57
aat	N	3	1.53
aac	N	0	1.47
gct	A	5	1.29
gcc	A	0	0.93
gca	A	0	2.35
gcg	A	0	0.42
---	--	------	------
mPD		0.088	0.76
		nPD:	0.11
		N. weight:	1.4
		Sc. PD:	0.08
		Sc. rank:	985.9

NA			
Pos.	277	obs:	exp:
ggt	G	0	154.80
ggc	G	0	149.20
gga	G	1167	538.80
ggg	G	1	325.20
---	--	------	------
mPD		0.0017	0.68
		nPD:	0.
		N. weight:	0.81
		Sc. PD:	-0.04
		Sc. rank:	-501.9

NA			
Pos.	278	obs:	exp:
act	T	1167	297.70
acc	T	0	239.70
aca	T	1	535.70
acg	T	0	94.87
---	--	------	------
mPD		0.0017	0.68
		nPD:	0.
		N. weight:	1.4
		Sc. PD:	-0.071
		Sc. rank:	-900.1

NA			
Pos.	279	obs:	exp:
gct	A	1152	301.60
gcc	A	16	217.50
gca	A	0	549.70
gcg	A	0	99.13
---	--	------	------
mPD		0.027	0.67
		nPD:	0.04
		N. weight:	1.4
		Sc. PD:	-0.019
		Sc. rank:	548.6

NA

Pos.	280	obs:	exp:
aaa	K	3	680.70
aag	K	1165	487.30
---	--	------	------
mPD		0.0051	0.49
		nPD:	0.01
		N. weight:	0.9
		Sc. PD:	-0.038
		Sc. rank:	-115.9

,

NA

Pos.	281	obs:	exp:
tat	Y	2	2.16
tac	Y	2	1.84
cat	H	1114	668.40
cac	H	50	495.60
---	--	------	------
mPD		0.092	0.50
		nPD:	0.19
		N. weight:	0.41
		Sc. PD:	0.051
		Sc. rank:	337.6

,

NA

Pos.	282	obs:	exp:
att	I	1139	421.10
atc	I	1	274.60
ata	I	6	450.30
gtt	V	22	4.56
gtc	V	0	4.44
gta	V	0	4.50
gtg	V	0	8.50
---	--	------	------
mPD		0.049	0.70
		nPD:	0.07
		N. weight:	1.
		Sc. PD:	0.015
		Sc. rank:	576.9

,

NA

Pos.	283	obs:	exp:
gaa	E	1121	710.70
gag	E	47	457.30
---	--	------	------
mPD		0.077	0.48
		nPD:	0.16
		N. weight:	0.37
		Sc. PD:	0.037
		Sc. rank:	289.2

,

NA

Pos.	284	obs:	exp:
gaa	E	1166	710.70
gag	E	2	457.30
---	--	------	------
mPD		0.0034	0.48
		nPD:	0.01
		N. weight:	0.51
		Sc. PD:	-0.023
		Sc. rank:	-148.2

,

NA

Pos.	285	obs:	exp:
tgt	C	7	494.00
tgc	C	1161	674.00
---	--	------	------
mPD		0.012	0.49
		nPD:	0.02
		N. weight:	0.55
		Sc. PD:	-0.016
		Sc. rank:	108.2

,

NA

Pos.	286	obs:	exp:
tct	S	0	185.80
tcc	S	0	152.50
tca	S	1166	296.50
tcg	S	2	66.28
agt	S	0	240.10
agc	S	0	226.90
---	--	------	------
mPD		0.0034	1.7
		nPD:	0.
		N. weight:	1.4
		Sc. PD:	-0.071
		Sc. rank:	-1002.7

,

NA

Pos.	287	obs:	exp:
tgt	C	1162	494.00
tgc	C	6	674.00
---	--	------	------
mPD		0.010	0.49
		nPD:	0.02
		N. weight:	0.87
		Sc. PD:	-0.028
		Sc. rank:	122.6

,

NA

Pos.	288	obs:	exp:
tat	Y	4	630.80
tac	Y	1164	537.20
---	--	------	------
mPD		0.0068	0.50
		nPD:	0.01
		N. weight:	0.8
		Sc. PD:	-0.031
		Sc. rank:	-27.2

,

NA

Pos.	289	obs:	exp:
ggt	G	0	154.80
ggc	G	0	149.20
gga	G	2	538.80
ggg	G	1166	325.20
---	--	------	------
mPD		0.0034	0.68
		nPD:	0.01
		N. weight:	1.3
		Sc. PD:	-0.063
		Sc. rank:	-605.8

,

NA

Pos.	290	obs:	exp:
aaa	K	63	38.47
aag	K	3	27.53
gtt	V	0	0.41
gtc	V	0	0.40
gta	V	2	0.41
gtg	V	0	0.77
gat	D	0	1.62
gac	D	3	1.38
gaa	E	1083	667.50
gag	E	14	429.50
---	--	------	------
mPD		0.14	0.59
		nPD:	0.24
		N. weight:	0.46
		Sc. PD:	0.082
		Sc. rank:	391.6

,

NA

Pos.	291	obs:	exp:
tta	L	0	0.09
ttg	L	0	0.19
ctt	L	0	0.17
ctc	L	0	0.17
cta	L	1	0.17
ctg	L	0	0.22
caa	Q	4	2.04
cag	Q	0	1.96
cgt	R	0	49.95
cgc	R	0	57.60
cga	R	1158	113.80
cgg	R	5	97.04
aga	R	0	531.80
agg	R	0	312.80
---	--	------	------
mPD		0.017	0.97
		nPD:	0.02
		N. weight:	2.4
		Sc. PD:	-0.085
		Sc. rank:	130.3

,

NA

Pos.	292	obs:	exp:
act	T	0	295.90
acc	T	0	238.20
aca	T	1137	532.50
acg	T	24	94.30
gct	A	0	1.81
gcc	A	0	1.30
gca	A	7	3.29
gcg	A	0	0.59
---	--	------	------
mPD		0.052	0.69
		nPD:	0.08
		N. weight:	0.76
		Sc. PD:	0.015
		Sc. rank:	451.4

,

NA

Pos.	293	obs:	exp:
cgt	R	0	0.26
cgc	R	0	0.30
cga	R	0	0.59
cgg	R	0	0.50
aga	R	0	2.74
agg	R	6	1.61
gtt	V	0	0.21
gtc	V	0	0.20
gta	V	1	0.20
gtg	V	0	0.39
gaa	E	1	0.61
gag	E	0	0.39
ggt	G	0	153.80
ggc	G	0	148.20
gga	G	587	535.10
ggg	G	573	322.90
---	--	------	------
mPD		0.51	0.69
		nPD:	0.75
		N. weight:	0.36
		Sc. PD:	0.23
		Sc. rank:	346.6

,

NA

Pos.	294	obs:	exp:
att	I	1164	428.40
atc	I	2	279.40
ata	I	0	458.20
gtt	V	2	0.41
gtc	V	0	0.40
gta	V	0	0.41
gtg	V	0	0.77
---	--	------	------
mPD		0.0068	0.66
		nPD:	0.01
		N. weight:	1.
		Sc. PD:	-0.044
		Sc. rank:	-157.0

,

NA

Pos.	295	obs:	exp:
att	I	0	0.37
atc	I	1	0.24
ata	I	0	0.39
act	T	0	297.50
acc	T	1164	239.50
aca	T	3	535.30
acg	T	0	94.79
---	--	------	------
mPD		0.0068	0.68
		nPD:	0.01
		N. weight:	1.7
		Sc. PD:	-0.07
		Sc. rank:	-319.0

,

NA

Pos.	296	obs:	exp:
tgt	C	12	494.00
tgc	C	1156	674.00
---	--	------	------
mPD		0.020	0.49
		nPD:	0.04
		N. weight:	0.53
		Sc. PD:	-0.0065
		Sc. rank:	216.0

,

NA

Pos.	297	obs:	exp:
act	T	2	297.70
acc	T	0	239.70
aca	T	1164	535.70
acg	T	2	94.87
---	--	------	------
mPD		0.0068	0.68
		nPD:	0.01
		N. weight:	0.8
		Sc. PD:	-0.034
		Sc. rank:	-144.7

,

NA

Pos.	298	obs:	exp:
tgt	C	0	494.00
tgc	C	1168	674.00
---	--	------	------
mPD		0	0.49
		nPD:	0.
		N. weight:	0.58
		Sc. PD:	-0.03
		Sc. rank:	-549.3

,

NA

Pos.	299	obs:	exp:
cgt	R	1	48.32
cgc	R	0	55.72
cga	R	0	110.10
cgg	R	0	93.87
aaa	K	0	25.06
aag	K	43	17.94
aga	R	1	514.40
agg	R	1123	302.60
---	--	------	------
mPD		0.076	1.0
		nPD:	0.07
		N. weight:	1.4
		Sc. PD:	0.025
		Sc. rank:	788.7

,

NA

Pos.	300	obs:	exp:
gat	D	1	631.20
gac	D	1167	536.80
---	--	------	------
mPD		0.0017	0.50
		nPD:	0.
		N. weight:	0.81
		Sc. PD:	-0.039
		Sc. rank:	-447.9

,

NA

Pos.	301	obs:	exp:
tct	S	0	0.16
tcc	S	0	0.13
tca	S	0	0.25
tcg	S	0	0.06
aat	N	1153	594.40
aac	N	14	572.60
agt	S	1	0.21
agc	S	0	0.19
---	--	------	------
mPD		0.025	0.50
		nPD:	0.05
		N. weight:	0.65
		Sc. PD:	-0.0026
		Sc. rank:	315.8

,

NA

Pos.	302	obs:	exp:
tgg	W	1168	1168.00
---	--	------	------
mPD		0	0
		nPD:	1.
		N. weight:	0.
		Sc. PD:	0
		Sc. rank:	0

,

NA

Pos.	303	obs:	exp:
tta	L	0	0.35
ttg	L	0	0.74
ctt	L	0	0.67
ctc	L	0	0.67
cta	L	0	0.67
ctg	L	4	0.89
caa	Q	10	590.90
cag	Q	1150	569.10
cgt	R	0	0.04
cgc	R	0	0.05
cga	R	0	0.10
cgg	R	1	0.08
aaa	K	0	1.75
aag	K	3	1.25
aga	R	0	0.46
agg	R	0	0.27
---	--	------	------
mPD		0.031	0.52
		nPD:	0.06
		N. weight:	0.71
		Sc. PD:	0.0029
		Sc. rank:	376.9

,

NA

Pos.	304	obs:	exp:
ggt	G	8	154.80
ggc	G	1154	149.20
gga	G	6	538.80
ggg	G	0	325.20
---	--	------	------
mPD		0.024	0.68
		nPD:	0.04
		N. weight:	2.1
		Sc. PD:	-0.039
		Sc. rank:	722.3

,

NA			
Pos. 305		obs:	exp:
tct	S	0	185.80
tcc	S	3	152.50
tca	S	1161	296.50
tcg	S	4	66.28
agt	S	0	240.10
agc	S	0	226.90
---	--	------	------
mPD		0.012	1.7
		nPD:	0.01
		N. weight:	1.4
		Sc. PD:	-0.063
		Sc. rank:	-382.6

NA			
Pos. 306		obs:	exp:
aat	N	1168	594.90
aac	N	0	573.10
---	--	------	------
mPD		0	0.50
		nPD:	0.
		N. weight:	0.71
		Sc. PD:	-0.037
		Sc. rank:	-673.9

NA			
Pos. 307		obs:	exp:
cgt	R	0	50.16
cgc	R	0	57.85
cga	R	0	114.30
cgg	R	0	97.46
aga	R	1112	534.10
agg	R	56	314.20
---	--	------	------
mPD		0.091	0.96
		nPD:	0.1
		N. weight:	0.65
		Sc. PD:	0.025
		Sc. rank:	428.0

NA			
Pos. 308		obs:	exp:
cct	P	0	291.40
ccc	P	0	209.90
cca	P	1166	480.80
ccg	P	2	185.90
---	--	------	------
mPD		0.0034	0.71
		nPD:	0.
		N. weight:	0.93
		Sc. PD:	-0.044
		Sc. rank:	-443.0

NA			
Pos. 309		obs:	exp:
gtt	V	1	242.00
gtc	V	0	235.70
gta	V	4	239.20
gtg	V	1163	451.10
---	--	------	------
mPD		0.0085	0.73
		nPD:	0.01
		N. weight:	0.98
		Sc. PD:	-0.04
		Sc. rank:	-101.3

NA			
Pos. 310		obs:	exp:
att	I	1020	375.50
atc	I	2	244.90
ata	I	0	401.60
gtt	V	146	30.25
gtc	V	0	29.47
gta	V	0	29.89
gtg	V	0	56.39
---	--	------	------
mPD		0.22	0.90
		nPD:	0.25
		N. weight:	1.1
		Sc. PD:	0.2
		Sc. rank:	958.2

NA			
Pos. 311		obs:	exp:
caa	Q	11	594.90
cag	Q	1157	573.10
---	--	------	------
mPD		0.019	0.50
		nPD:	0.04
		N. weight:	0.7
		Sc. PD:	-0.011
		Sc. rank:	261.5

NA			
Pos. 312		obs:	exp:
att	I	209	429.10
atc	I	0	279.90
ata	I	959	459.00
---	--	------	------
mPD		0.29	0.65
		nPD:	0.45
		N. weight:	0.51
		Sc. PD:	0.19
		Sc. rank:	475.4

NA			
Pos. 313		obs:	exp:
aat	N	0	2.55
aac	N	5	2.45
gat	D	27	627.90
gac	D	1135	534.10
ggt	G	0	0.13
ggc	G	1	0.13
gga	G	0	0.46
ggg	G	0	0.28
---	--	------	------
mPD		0.055	0.51
		nPD:	0.11
		N. weight:	0.7
		Sc. PD:	0.036
		Sc. rank:	480.7

NA			
Pos. 314		obs:	exp:
cct	P	1	291.40
ccc	P	0	209.90
cca	P	1166	480.80
ccg	P	1	185.90
---	--	------	------
mPD		0.0034	0.71
		nPD:	0.
		N. weight:	0.93
		Sc. PD:	-0.044
		Sc. rank:	-439.1

NA			
Pos. 315		obs:	exp:
att	I	0	7.35
atc	I	0	4.79
ata	I	20	7.86
act	T	0	0.25
acc	T	0	0.21
aca	T	1	0.46
acg	T	0	0.08
gtt	V	0	237.70
gtc	V	0	231.50
gta	V	1144	234.90
gtg	V	3	443.00
---	--	------	------
mPD		0.042	0.76
		nPD:	0.06
		N. weight:	1.6
		Sc. PD:	0.00049
		Sc. rank:	833.6

NA			
Pos. 316		obs:	exp:
act	T	0	1.02
acc	T	0	0.82
aca	T	4	1.83
acg	T	0	0.32
gtt	V	0	0.83
gtc	V	0	0.81
gta	V	4	0.82
gtg	V	0	1.54
gct	A	0	299.50
gcc	A	0	216.00
gca	A	1150	546.00
gcg	A	10	98.45
---	--	------	------
mPD		0.031	0.68
		nPD:	0.04
		N. weight:	0.76
		Sc. PD:	-0.0072
		Sc. rank:	327.1

NA			
Pos. 317		obs:	exp:
atg	M	1168	1168.00
---	--	------	------
mPD		0	0
		nPD:	1.
		N. weight:	0.
		Sc. PD:	0
		Sc. rank:	0

NA			
Pos. 318		obs:	exp:
act	T	0	297.20
acc	T	2	239.30
aca	T	1164	534.80
acg	T	0	94.71
aaa	K	2	1.17
aag	K	0	0.83
---	--	------	------
mPD		0.0068	0.68
		nPD:	0.01
		N. weight:	0.81
		Sc. PD:	-0.034
		Sc. rank:	-164.8

NA			
Pos. 319		obs:	exp:
cat	H	0	670.70
cac	H	1168	497.30
---	--	------	------
mPD		0	0.49
		nPD:	0.
		N. weight:	0.9
		Sc. PD:	-0.047
		Sc. rank:	-852.9

NA			
Pos. 320		obs:	exp:
att	I	1	0.37
atc	I	0	0.24
ata	I	0	0.39
act	T	991	254.90
acc	T	9	205.20
aca	T	0	458.70
acg	T	0	81.23
aat	N	167	85.06
aac	N	0	81.94
---	--	------	------
mPD		0.26	0.94
		nPD:	0.28
		N. weight:	1.3
		Sc. PD:	0.27
		Sc. rank:	1127.3

NA			
Pos. 321		obs:	exp:
tct	S	0	185.80
tcc	S	0	152.50
tca	S	0	296.50
tcg	S	0	66.28
agt	S	1166	240.10
agc	S	2	226.90
---	--	------	------
mPD		0.0034	1.7
		nPD:	0.
		N. weight:	1.7
		Sc. PD:	-0.082
		Sc. rank:	-1156.0

NA			
Pos. 322		obs:	exp:
caa	Q	1148	594.90
cag	Q	20	573.10
---	--	------	------
mPD		0.034	0.50
		nPD:	0.07
		N. weight:	0.62
		Sc. PD:	0.0074
		Sc. rank:	346.8

NA			
Pos. 323		obs:	exp:
tat	Y	1165	630.80
tac	Y	3	537.20
---	--	------	------
mPD		0.0051	0.50
		nPD:	0.01
		N. weight:	0.63
		Sc. PD:	-0.027
		Sc. rank:	-97.7

NA			
Pos. 324		obs:	exp:
att	I	0	429.10
atc	I	0	279.90
ata	I	1168	459.00
---	--	------	------
mPD		0	0.65
		nPD:	0.
		N. weight:	0.99
		Sc. PD:	-0.051
		Sc. rank:	-933.1

NA			
Pos. 325		obs:	exp:
tgt	C	0	494.00
tgc	C	1168	674.00
---	--	------	------
mPD		0	0.49
		nPD:	0.
		N. weight:	0.58
		Sc. PD:	-0.03
		Sc. rank:	-549.3

NA			
Pos. 326		obs:	exp:
tct	S	0	185.80
tcc	S	0	152.50
tca	S	0	296.50
tcg	S	0	66.28
agt	S	1166	240.10
agc	S	2	226.90
---	--	------	------
mPD		0.0034	1.7
		nPD:	0.
		N. weight:	1.7
		Sc. PD:	-0.082
		Sc. rank:	-1156.0

NA			
Pos. 327		obs:	exp:
cct	P	1164	291.40
ccc	P	1	209.90
cca	P	3	480.80
ccg	P	0	185.90
---	--	------	------
mPD		0.0068	0.71
		nPD:	0.01
		N. weight:	1.4
		Sc. PD:	-0.061
		Sc. rank:	-297.1

NA			
Pos. 328		obs:	exp:
gtt	V	1116	242.00
gtc	V	52	235.70
gta	V	0	239.20
gtg	V	0	451.10
---	--	------	------
mPD		0.085	0.73
		nPD:	0.12
		N. weight:	1.5
		Sc. PD:	0.087
		Sc. rank:	1027.2

NA			
Pos.	329	obs:	exp:
tta	L	0	103.60
ttg	L	0	217.30
ctt	L	1167	194.30
ctc	L	0	194.80
cta	L	0	196.70
ctg	L	0	260.40
att	I	1	0.37
atc	I	0	0.24
ata	I	0	0.39
---	--	------	------
mPD		0.0017	1.1
		nPD:	0.
		N. weight:	1.9
		Sc. PD:	-0.095
		Sc. rank:	-1349.8

,

NA			
Pos.	330	obs:	exp:
act	T	0	297.70
acc	T	0	239.70
aca	T	1168	535.70
acg	T	0	94.87
---	--	------	------
mPD		0	0.68
		nPD:	0.
		N. weight:	0.82
		Sc. PD:	-0.043
		Sc. rank:	-778.6

,

NA			
Pos.	331	obs:	exp:
gat	D	4	631.20
gac	D	1164	536.80
---	--	------	------
mPD		0.0068	0.50
		nPD:	0.01
		N. weight:	0.8
		Sc. PD:	-0.031
		Sc. rank:	-22.1

,

NA			
Pos.	332	obs:	exp:
tct	S	0	89.70
tcc	S	0	73.66
tca	S	0	143.10
tcg	S	0	32.00
act	T	68	17.33
acc	T	0	13.95
aca	T	0	31.19
acg	T	0	5.52
aat	N	536	273.00
aac	N	0	263.00
agt	S	563	115.90
agc	S	1	109.50
---	--	------	------
mPD		0.56	1.7
		nPD:	0.33
		N. weight:	1.2
		Sc. PD:	0.31
		Sc. rank:	1102.2

,

NA			
Pos.	333	obs:	exp:
cct	P	0	291.40
ccc	P	1168	209.90
cca	P	0	480.80
ccg	P	0	185.90
---	--	------	------
mPD		0	0.71
		nPD:	0.
		N. weight:	1.8
		Sc. PD:	-0.094
		Sc. rank:	-1714.7

,

NA			
Pos.	334	obs:	exp:
cgt	R	1	50.16
cgc	R	0	57.85
cga	R	1141	114.30
cgg	R	25	97.46
aga	R	1	534.10
agg	R	0	314.20
---	--	------	------
mPD		0.045	0.96
		nPD:	0.05
		N. weight:	2.3
		Sc. PD:	-0.017
		Sc. rank:	1096.4

,

NA			
Pos.	335	obs:	exp:
cct	P	0	291.40
ccc	P	0	209.90
cca	P	16	480.80
ccg	P	1152	185.90
---	--	------	------
mPD		0.027	0.71
		nPD:	0.04
		N. weight:	1.9
		Sc. PD:	-0.029
		Sc. rank:	726.2

,

NA			
Pos.	336	obs:	exp:
tct	S	0	0.48
tcc	S	0	0.39
tca	S	0	0.76
tcg	S	0	0.17
aat	N	1158	592.90
aac	N	6	571.10
agt	S	3	0.62
agc	S	0	0.58
gat	D	1	0.54
gac	D	0	0.46
---	--	------	------
mPD		0.017	0.51
		nPD:	0.03
		N. weight:	0.68
		Sc. PD:	-0.014
		Sc. rank:	226.0

,

NA			
Pos.	337	obs:	exp:
gat	D	4	631.20
gac	D	1164	536.80
---	--	------	------
mPD		0.0068	0.50
		nPD:	0.01
		N. weight:	0.8
		Sc. PD:	-0.031
		Sc. rank:	-22.1

,

NA			
Pos.	338	obs:	exp:
cct	P	0	291.40
ccc	P	0	209.90
cca	P	1168	480.80
ccg	P	0	185.90
---	--	------	------
mPD		0	0.71
		nPD:	0.
		N. weight:	0.94
		Sc. PD:	-0.048
		Sc. rank:	-886.7

,

NA			
Pos.	339	obs:	exp:
tct	S	0	1.11
tcc	S	0	0.91
tca	S	0	1.78
tcg	S	0	0.40
act	T	0	0.25
acc	T	1	0.21
aca	T	0	0.46
acg	T	0	0.08
aat	N	1048	584.20
aac	N	99	562.80
aaa	K	3	1.75
aag	K	0	1.25
agt	S	7	1.44
agc	S	0	1.36
gat	D	9	5.40
gac	D	1	4.60
---	--	------	------
mPD		0.19	0.54
		nPD:	0.36
		N. weight:	0.42
		Sc. PD:	0.12
		Sc. rank:	384.9

,

NA			
Pos.	340	obs:	exp:
tta	L	2	0.18
ttg	L	0	0.37
ctt	L	0	0.33
ctc	L	0	0.33
cta	L	0	0.34
ctg	L	0	0.45
att	I	0	406.70
atc	I	0	265.30
ata	I	1107	435.00
atg	M	50	50.00
act	T	0	0.25
acc	T	0	0.21
aca	T	1	0.46
acg	T	0	0.08
gtt	V	0	1.66
gtc	V	0	1.61
gta	V	6	1.64
gtg	V	2	3.09
---	--	------	------
mPD		0.10	0.70
		nPD:	0.15
		N. weight:	0.95
		Sc. PD:	0.083
		Sc. rank:	726.0

,

NA			
Pos.	341	obs:	exp:
ggt	G	1040	154.80
ggc	G	128	149.20
gga	G	0	538.80
ggg	G	0	325.20
---	--	------	------
mPD		0.20	0.68
		nPD:	0.29
		N. weight:	1.8
		Sc. PD:	0.39
		Sc. rank:	1581.5

,

NA			
Pos.	342	obs:	exp:
cgt	R	0	0.09
cgc	R	0	0.10
cga	R	0	0.20
cgg	R	0	0.17
aat	N	2	1.02
aac	N	0	0.98
aaa	K	4	677.80
aag	K	1159	485.20
aga	R	0	0.91
agg	R	2	0.54
gaa	E	0	0.61
gag	E	1	0.39
---	--	------	------
mPD		0.015	0.49
		nPD:	0.03
		N. weight:	0.9
		Sc. PD:	-0.02
		Sc. rank:	283.7

,

NA			
Pos.	343	obs:	exp:
tgt	C	1166	494.00
tgc	C	2	674.00
---	--	------	------
mPD		0.0034	0.49
		nPD:	0.01
		N. weight:	0.9
		Sc. PD:	-0.04
		Sc. rank:	-275.1

,

NA			
Pos.	344	obs:	exp:
cat	H	1	0.57
cac	H	0	0.43
aat	N	1163	594.40
aac	N	4	572.60
---	--	------	------
mPD		0.0085	0.50
		nPD:	0.02
		N. weight:	0.69
		Sc. PD:	-0.025
		Sc. rank:	28.5

,

NA			
Pos.	345	obs:	exp:
gtt	V	0	0.21
gtc	V	1	0.20
gta	V	0	0.20
gtg	V	0	0.39
gat	D	2	627.90
gac	D	1160	534.10
gaa	E	1	2.43
gag	E	3	1.57
ggt	G	0	0.13
ggc	G	1	0.13
gga	G	0	0.46
ggg	G	0	0.28
---	--	------	------
mPD		0.014	0.50
		nPD:	0.03
		N. weight:	0.81
		Sc. PD:	-0.021
		Sc. rank:	214.8

,

NA			
Pos.	346	obs:	exp:
cct	P	1156	291.20
ccc	P	7	209.70
cca	P	0	480.40
ccg	P	4	185.80
cgt	R	1	0.04
cgc	R	0	0.05
cga	R	0	0.10
cgg	R	0	0.08
aga	R	0	0.46
agg	R	0	0.27
---	--	------	------
mPD		0.020	0.71
		nPD:	0.03
		N. weight:	1.4
		Sc. PD:	-0.035
		Sc. rank:	421.3

,

NA			
Pos.	347	obs:	exp:
tat	Y	1120	630.80
tac	Y	48	537.20
---	--	------	------
mPD		0.079	0.50
		nPD:	0.16
		N. weight:	0.48
		Sc. PD:	0.047
		Sc. rank:	376.1

,

NA			
Pos.	348	obs:	exp:
cct	P	1	291.40
ccc	P	6	209.90
cca	P	1153	480.80
ccg	P	8	185.90
---	--	------	------
mPD		0.025	0.71
		nPD:	0.04
		N. weight:	0.87
		Sc. PD:	-0.016
		Sc. rank:	308.9

,

NA			
Pos.	349	obs:	exp:
ggt	G	1166	154.80
ggc	G	2	149.20
gga	G	0	538.80
ggg	G	0	325.20
---	--	------	------
mPD		0.0034	0.68
		nPD:	0.01
		N. weight:	2.1
		Sc. PD:	-0.099
		Sc. rank:	-961.2

,

NA			
Pos.	350	obs:	exp:
tct	S	0	0.64
tcc	S	0	0.52
tca	S	0	1.02
tcg	S	0	0.23
att	I	32	11.76
atc	I	0	7.67
ata	I	0	12.57
aat	N	1000	576.60
aac	N	132	555.40
agt	S	2	0.82
agc	S	2	0.78
---	--	------	------
mPD		0.26	0.58
		nPD:	0.46
		N. weight:	0.36
		Sc. PD:	0.14
		Sc. rank:	341.0

,

NA			
Pos.	351	obs:	exp:
tct	S	0	0.16
tcc	S	0	0.13
tca	S	0	0.25
tcg	S	0	0.06
tat	Y	1	0.54
tac	Y	0	0.46
att	I	1	0.37
atc	I	0	0.24
ata	I	0	0.39
aat	N	1100	592.40
aac	N	63	570.60
agt	S	0	0.21
agc	S	1	0.19
gat	D	0	1.08
gac	D	2	0.92
---	--	------	------
mPD		0.12	0.51
		nPD:	0.23
		N. weight:	0.5
		Sc. PD:	0.08
		Sc. rank:	416.7

,

NA			
Pos.	352	obs:	exp:
tat	Y	1	0.54
tac	Y	0	0.46
aat	N	53	594.40
aac	N	1114	572.60
---	--	------	------
mPD		0.090	0.50
		nPD:	0.18
		N. weight:	0.56
		Sc. PD:	0.065
		Sc. rank:	445.0

,

NA			
Pos.	353	obs:	exp:
tct	S	0	1.59
tcc	S	0	1.31
tca	S	0	2.54
tcg	S	0	0.57
aat	N	1157	589.30
aac	N	0	567.70
aaa	K	1	0.58
aag	K	0	0.42
agt	S	10	2.06
agc	S	0	1.94
---	--	------	------
mPD		0.019	0.53
		nPD:	0.04
		N. weight:	0.72
		Sc. PD:	-0.013
		Sc. rank:	250.8

,

NA			
Pos.	354	obs:	exp:
ggt	G	0	154.80
ggc	G	0	149.20
gga	G	1166	538.80
ggg	G	2	325.20
---	--	------	------
mPD		0.0034	0.68
		nPD:	0.01
		N. weight:	0.81
		Sc. PD:	-0.038
		Sc. rank:	-365.0

,

NA			
Pos.	355	obs:	exp:
att	I	0	35.64
atc	I	97	23.25
ata	I	0	38.12
gtt	V	0	221.90
gtc	V	1065	216.20
gta	V	6	219.30
gtg	V	0	413.60
---	--	------	------
mPD		0.16	0.89
		nPD:	0.18
		N. weight:	1.6
		Sc. PD:	0.2
		Sc. rank:	1321.8

,

NA			
Pos.	356	obs:	exp:
aaa	K	13	680.70
aag	K	1155	487.30
---	--	------	------
mPD		0.022	0.49
		nPD:	0.05
		N. weight:	0.86
		Sc. PD:	-0.0077
		Sc. rank:	374.3

,

NA			
Pos.	357	obs:	exp:
ggt	G	0	154.80
ggc	G	0	149.20
gga	G	1123	538.80
ggg	G	45	325.20
---	--	------	------
mPD		0.074	0.68
		nPD:	0.11
		N. weight:	0.67
		Sc. PD:	0.034
		Sc. rank:	457.6

,

NA			
Pos.	358	obs:	exp:
ttt	F	7	460.80
ttc	F	1160	706.20
tta	L	0	0.09
ttg	L	1	0.19
ctt	L	0	0.17
ctc	L	0	0.17
cta	L	0	0.17
ctg	L	0	0.22
---	--	------	------
mPD		0.014	0.48
		nPD:	0.03
		N. weight:	0.5
		Sc. PD:	-0.012
		Sc. rank:	142.7

,

NA			
Pos.	359	obs:	exp:
tct	S	0	185.80
tcc	S	0	152.50
tca	S	1166	296.50
tcg	S	2	66.28
agt	S	0	240.10
agc	S	0	226.90
---	--	------	------
mPD		0.0034	1.7
		nPD:	0.
		N. weight:	1.4
		Sc. PD:	-0.071
		Sc. rank:	-1002.7

,

NA			
Pos.	360	obs:	exp:
ttt	F	0	0.79
ttc	F	2	1.21
tat	Y	4	629.80
tac	Y	1162	536.20
---	--	------	------
mPD		0.010	0.50
		nPD:	0.02
		N. weight:	0.8
		Sc. PD:	-0.026
		Sc. rank:	99.9

,

NA			
Pos.	361	obs:	exp:
tta	L	0	103.60
ttg	L	4	217.50
ctt	L	0	194.50
ctc	L	0	194.90
cta	L	1	196.80
ctg	L	1163	260.60
---	--	------	------
mPD		0.0085	1.1
		nPD:	0.01
		N. weight:	1.6
		Sc. PD:	-0.069
		Sc. rank:	-360.7

,

NA			
Pos.	362	obs:	exp:
gat	D	1168	631.20
gac	D	0	536.80
---	--	------	------
mPD		0	0.50
		nPD:	0.
		N. weight:	0.65
		Sc. PD:	-0.034
		Sc. rank:	-614.8

,

NA			
Pos.	363	obs:	exp:
ggt	G	0	154.80
ggc	G	0	149.20
gga	G	39	538.80
ggg	G	1129	325.20
---	--	------	------
mPD		0.065	0.68
		nPD:	0.1
		N. weight:	1.2
		Sc. PD:	0.045
		Sc. rank:	779.8

,

NA			
Pos.	364	obs:	exp:
act	T	44	11.47
acc	T	1	9.23
aca	T	0	20.64
acg	T	0	3.66
gtt	V	7	1.45
gtc	V	0	1.41
gta	V	0	1.43
gtg	V	0	2.70
gct	A	1056	274.50
gcc	A	7	198.00
gca	A	0	500.30
gcg	A	0	90.22
gat	D	52	28.10
gac	D	0	23.90
ggt	G	1	0.13
ggc	G	0	0.13
gga	G	0	0.46
ggg	G	0	0.28
---	--	------	------
mPD		0.19	0.85
		nPD:	0.22
		N. weight:	1.4
		Sc. PD:	0.21
		Sc. rank:	1137.4

,

NA			
Pos.	365	obs:	exp:
aat	N	0	594.90
aac	N	1168	573.10
---	--	------	------
mPD		0	0.50
		nPD:	0.
		N. weight:	0.75
		Sc. PD:	-0.039
		Sc. rank:	-711.3

,

NA			
Pos.	366	obs:	exp:
act	T	1039	297.70
acc	T	129	239.70
aca	T	0	535.70
acg	T	0	94.87
---	--	------	------
mPD		0.20	0.68
		nPD:	0.29
		N. weight:	1.1
		Sc. PD:	0.25
		Sc. rank:	988.6

,

NA			
Pos.	367	obs:	exp:
tgg	W	1168	1168.00
---	--	------	------
mPD		0	0
		nPD:	1.
		N. weight:	0.
		Sc. PD:	0
		Sc. rank:	0

,

NA			
Pos.	368	obs:	exp:
tta	L	4	103.60
ttg	L	0	217.30
ctt	L	0	194.30
ctc	L	0	194.80
cta	L	1157	196.70
ctg	L	6	260.40
gtt	V	0	0.21
gtc	V	0	0.20
gta	V	1	0.20
gtg	V	0	0.39
---	--	------	------
mPD		0.019	1.1
		nPD:	0.02
		N. weight:	1.8
		Sc. PD:	-0.065
		Sc. rank:	70.4

,

NA			
Pos.	369	obs:	exp:
ggt	G	4	154.80
ggc	G	0	149.20
gga	G	13	538.80
ggg	G	1151	325.20
---	--	------	------
mPD		0.029	0.68
		nPD:	0.04
		N. weight:	1.3
		Sc. PD:	-0.015
		Sc. rank:	532.6

,

NA			
Pos.	370	obs:	exp:
cgt	R	0	50.16
cgc	R	0	57.85
cga	R	0	114.30
cgg	R	0	97.46
aga	R	2	534.10
agg	R	1166	314.20
---	--	------	------
mPD		0.0034	0.96
		nPD:	0.
		N. weight:	1.4
		Sc. PD:	-0.066
		Sc. rank:	-704.3

,

NA			
Pos.	371	obs:	exp:
act	T	0	297.70
acc	T	0	239.70
aca	T	1168	535.70
acg	T	0	94.87
---	--	------	------
mPD		0	0.68
		nPD:	0.
		N. weight:	0.82
		Sc. PD:	-0.043
		Sc. rank:	-778.6

,

NA			
Pos.	372	obs:	exp:
att	I	0	422.90
atc	I	0	275.80
ata	I	1151	452.30
act	T	0	4.33
acc	T	0	3.49
aca	T	17	7.80
acg	T	0	1.38
---	--	------	------
mPD		0.029	0.68
		nPD:	0.04
		N. weight:	0.98
		Sc. PD:	-0.012
		Sc. rank:	410.4

,

NA			
Pos.	373	obs:	exp:
tct	S	0	185.80
tcc	S	0	152.50
tca	S	0	296.50
tcg	S	0	66.28
agt	S	206	240.10
agc	S	962	226.90
---	--	------	------
mPD		0.29	1.7
		nPD:	0.18
		N. weight:	1.2
		Sc. PD:	0.14
		Sc. rank:	979.6

,

NA			
Pos.	374	obs:	exp:
act	T	0	297.70
acc	T	1	239.70
aca	T	1165	535.70
acg	T	2	94.87
---	--	------	------
mPD		0.0051	0.68
		nPD:	0.01
		N. weight:	0.81
		Sc. PD:	-0.036
		Sc. rank:	-203.6

,

NA			
Pos.	375	obs:	exp:
tct	S	0	1.11
tcc	S	7	0.91
tca	S	0	1.78
tcg	S	0	0.40
att	I	0	0.37
atc	I	1	0.24
ata	I	0	0.39
act	T	0	12.49
acc	T	49	10.05
aca	T	0	22.47
acg	T	0	3.98
agt	S	0	1.44
agc	S	0	1.36
gct	A	12	286.90
gcc	A	873	206.90
gca	A	226	522.90
gcg	A	0	94.29
---	--	------	------
mPD		0.42	0.77
		nPD:	0.55
		N. weight:	1.
		Sc. PD:	0.48
		Sc. rank:	976.9

,

NA			
Pos.	376	obs:	exp:
tct	S	2	185.80
tcc	S	0	152.50
tca	S	23	296.50
tcg	S	1143	66.28
agt	S	0	240.10
agc	S	0	226.90
---	--	------	------
mPD		0.042	1.7
		nPD:	0.03
		N. weight:	2.9
		Sc. PD:	-0.08
		Sc. rank:	662.8

,

NA			
Pos.	377	obs:	exp:
cgt	R	0	50.08
cgc	R	0	57.75
cga	R	0	114.10
cgg	R	0	97.29
aaa	K	0	1.17
aag	K	2	0.83
aga	R	6	533.20
agg	R	1160	313.60
---	--	------	------
mPD		0.014	0.96
		nPD:	0.01
		N. weight:	1.3
		Sc. PD:	-0.052
		Sc. rank:	-5.0

,

NA			
Pos.	378	obs:	exp:
tct	S	1157	185.80
tcc	S	7	152.50
tca	S	1	296.50
tcg	S	3	66.28
agt	S	0	240.10
agc	S	0	226.90
---	--	------	------
mPD		0.019	1.7
		nPD:	0.01
		N. weight:	1.9
		Sc. PD:	-0.077
		Sc. rank:	-216.8

,

NA			
Pos.	379	obs:	exp:
ggt	G	0	154.80
ggc	G	1	149.20
gga	G	1128	538.80
ggg	G	39	325.20
---	--	------	------
mPD		0.066	0.68
		nPD:	0.1
		N. weight:	0.68
		Sc. PD:	0.028
		Sc. rank:	449.4

,

NA			
Pos.	380	obs:	exp:
tat	Y	2	630.80
tac	Y	1166	537.20
---	--	------	------
mPD		0.0034	0.50
		nPD:	0.01
		N. weight:	0.81
		Sc. PD:	-0.036
		Sc. rank:	-264.5

,

NA			
Pos.	381	obs:	exp:
gaa	E	8	710.70
gag	E	1160	457.30
---	--	------	------
mPD		0.014	0.48
		nPD:	0.03
		N. weight:	0.95
		Sc. PD:	-0.023
		Sc. rank:	276.9

,

NA			
Pos.	382	obs:	exp:
att	I	0	0.37
atc	I	0	0.24
ata	I	1	0.39
atg	M	1166	1166.00
gtt	V	0	0.21
gtc	V	0	0.20
gta	V	0	0.20
gtg	V	1	0.39
---	--	------	------
mPD		0.0034	0.0045
		nPD:	0.77
		N. weight:	0.0017
		Sc. PD:	0.0011
		Sc. rank:	1.6

,

NA			
Pos.	383	obs:	exp:
tta	L	1144	103.60
ttg	L	24	217.50
ctt	L	0	194.50
ctc	L	0	194.90
cta	L	0	196.80
ctg	L	0	260.60
---	--	------	------
mPD		0.040	1.1
		nPD:	0.04
		N. weight:	2.4
		Sc. PD:	-0.043
		Sc. rank:	899.7

,

NA			
Pos.	384	obs:	exp:
aaa	K	1168	680.70
aag	K	0	487.30
---	--	------	------
mPD		0	0.49
		nPD:	0.
		N. weight:	0.57
		Sc. PD:	-0.029
		Sc. rank:	-539.3

,

NA			
Pos.	385	obs:	exp:
gtt	V	0	242.00
gtc	V	0	235.70
gta	V	9	239.20
gtg	V	1159	451.10
---	--	------	------
mPD		0.015	0.73
		nPD:	0.02
		N. weight:	0.96
		Sc. PD:	-0.031
		Sc. rank:	140.9

,

NA			
Pos.	386	obs:	exp:
cct	P	0	291.40
ccc	P	1	209.90
cca	P	1167	480.80
ccg	P	0	185.90
---	--	------	------
mPD		0.0017	0.71
		nPD:	0.
		N. weight:	0.93
		Sc. PD:	-0.046
		Sc. rank:	-592.5

,

NA			
Pos.	387	obs:	exp:
aat	N	1165	594.40
aac	N	2	572.60
gat	D	1	0.54
gac	D	0	0.46
---	--	------	------
mPD		0.0051	0.50
		nPD:	0.01
		N. weight:	0.7
		Sc. PD:	-0.029
		Sc. rank:	-120.0

,

NA			
Pos.	388	obs:	exp:
gct	A	0	301.60
gcc	A	0	217.50
gca	A	1165	549.70
gcg	A	3	99.13
---	--	------	------
mPD		0.0051	0.67
		nPD:	0.01
		N. weight:	0.78
		Sc. PD:	-0.035
		Sc. rank:	-192.2

,

NA			
Pos.	389	obs:	exp:
tta	L	4	103.60
ttg	L	1159	217.50
ctt	L	0	194.50
ctc	L	0	194.90
cta	L	0	196.80
ctg	L	5	260.60
---	--	------	------
mPD		0.015	1.1
		nPD:	0.01
		N. weight:	1.7
		Sc. PD:	-0.067
		Sc. rank:	-19.7

,

NA			
Pos.	390	obs:	exp:
act	T	0	297.70
acc	T	0	239.70
aca	T	1168	535.70
acg	T	0	94.87
---	--	------	------
mPD		0	0.68
		nPD:	0.
		N. weight:	0.82
		Sc. PD:	-0.043
		Sc. rank:	-778.6

,

NA			
Pos.	391	obs:	exp:
gat	D	1166	631.20
gac	D	2	536.80
---	--	------	------
mPD		0.0034	0.50
		nPD:	0.01
		N. weight:	0.64
		Sc. PD:	-0.029
		Sc. rank:	-201.7

,

NA			
Pos.	392	obs:	exp:
aat	N	3	1.53
aac	N	0	1.47
gat	D	1161	629.50
gac	D	4	535.50
---	--	------	------
mPD		0.012	0.50
		nPD:	0.02
		N. weight:	0.63
		Sc. PD:	-0.018
		Sc. rank:	118.3

,

NA			
Pos.	393	obs:	exp:
cgt	R	0	50.04
cgc	R	0	57.70
cga	R	0	114.00
cgg	R	0	97.21
aaa	K	3	1.75
aag	K	0	1.25
aga	R	1156	532.70
agg	R	9	313.30
---	--	------	------
mPD		0.020	0.97
		nPD:	0.02
		N. weight:	0.78
		Sc. PD:	-0.025
		Sc. rank:	117.1

,

NA			
Pos.	394	obs:	exp:
tct	S	0	185.80
tcc	S	0	152.50
tca	S	1158	296.50
tcg	S	10	66.28
agt	S	0	240.10
agc	S	0	226.90
---	--	------	------
mPD		0.017	1.7
		nPD:	0.01
		N. weight:	1.4
		Sc. PD:	-0.059
		Sc. rank:	-225.8

,

NA			
Pos.	395	obs:	exp:
aaa	K	1	680.70
aag	K	1167	487.30
---	--	------	------
mPD		0.0017	0.49
		nPD:	0.
		N. weight:	0.92
		Sc. PD:	-0.044
		Sc. rank:	-480.8

,

NA			
Pos.	396	obs:	exp:
cct	P	5	291.20
ccc	P	1154	209.70
cca	P	8	480.40
ccg	P	0	185.80
gct	A	0	0.26
gcc	A	1	0.19
gca	A	0	0.47
gcg	A	0	0.08
---	--	------	------
mPD		0.024	0.71
		nPD:	0.03
		N. weight:	1.7
		Sc. PD:	-0.035
		Sc. rank:	570.1

,

NA			
Pos.	397	obs:	exp:
att	I	1147	425.10
atc	I	10	277.30
ata	I	0	454.60
act	T	2	0.51
acc	T	0	0.41
aca	T	0	0.92
acg	T	0	0.16
gtt	V	9	1.86
gtc	V	0	1.82
gta	V	0	1.84
gtg	V	0	3.48
---	--	------	------
mPD		0.036	0.67
		nPD:	0.05
		N. weight:	1.
		Sc. PD:	-0.0018
		Sc. rank:	500.2

,

NA			
Pos.	398	obs:	exp:
caa	Q	1167	594.90
cag	Q	1	573.10
---	--	------	------
mPD		0.0017	0.50
		nPD:	0.
		N. weight:	0.71
		Sc. PD:	-0.034
		Sc. rank:	-396.1

,

NA			
Pos.	399	obs:	exp:
ggt	G	1168	154.80
ggc	G	0	149.20
gga	G	0	538.80
ggg	G	0	325.20
---	--	------	------
mPD		0	0.68
		nPD:	0.
		N. weight:	2.1
		Sc. PD:	-0.11
		Sc. rank:	-2018.6

NA			
Pos.	400	obs:	exp:
caa	Q	2	594.90
cag	Q	1166	573.10
---	--	------	------
mPD		0.0034	0.50
		nPD:	0.01
		N. weight:	0.74
		Sc. PD:	-0.033
		Sc. rank:	-250.3

NA			
Pos.	401	obs:	exp:
act	T	0	297.70
acc	T	0	239.70
aca	T	1168	535.70
acg	T	0	94.87
---	--	------	------
mPD		0	0.68
		nPD:	0.
		N. weight:	0.82
		Sc. PD:	-0.043
		Sc. rank:	-778.6

NA			
Pos.	402	obs:	exp:
att	I	1119	428.80
atc	I	48	279.70
ata	I	0	458.60
gtt	V	1	0.21
gtc	V	0	0.20
gta	V	0	0.20
gtg	V	0	0.39
---	--	------	------
mPD		0.081	0.66
		nPD:	0.12
		N. weight:	0.9
		Sc. PD:	0.058
		Sc. rank:	640.6

NA			
Pos.	403	obs:	exp:
tta	L	4	0.35
ttg	L	0	0.74
ctt	L	0	0.67
ctc	L	0	0.67
cta	L	0	0.67
ctg	L	0	0.89
att	I	0	0.37
atc	I	0	0.24
ata	I	1	0.39
gtt	V	0	241.00
gtc	V	0	234.70
gta	V	1135	238.10
gtg	V	28	449.20
---	--	------	------
mPD		0.055	0.73
		nPD:	0.08
		N. weight:	1.5
		Sc. PD:	0.03
		Sc. rank:	906.3

NA			
Pos.	404	obs:	exp:
tta	L	1097	98.95
ttg	L	5	207.60
tct	S	0	8.43
tcc	S	0	6.92
tca	S	53	13.45
tcg	S	0	3.01
ctt	L	0	185.70
ctc	L	0	186.10
cta	L	13	187.90
ctg	L	0	248.80
agt	S	0	10.90
agc	S	0	10.29
---	--	------	------
mPD		0.12	1.2
		nPD:	0.09
		N. weight:	2.4
		Sc. PD:	0.09
		Sc. rank:	1572.4

NA			
Pos.	405	obs:	exp:
act	T	0	0.76
acc	T	3	0.62
aca	T	0	1.38
acg	T	0	0.24
aat	N	10	593.40
aac	N	1155	571.60
---	--	------	------
mPD		0.022	0.51
		nPD:	0.04
		N. weight:	0.7
		Sc. PD:	-0.0074
		Sc. rank:	299.0

NA			
Pos.	406	obs:	exp:
tct	S	6	0.95
tcc	S	0	0.78
tca	S	0	1.52
tcg	S	0	0.34
act	T	17	6.37
acc	T	8	5.13
aca	T	0	11.47
acg	T	0	2.03
agt	S	0	1.23
agc	S	0	1.17
gtt	V	7	1.45
gtc	V	0	1.41
gta	V	0	1.43
gtg	V	0	2.70
gct	A	1113	291.00
gcc	A	11	209.90
gca	A	3	530.40
gcg	A	0	95.65
gat	D	3	1.62
gac	D	0	1.38
---	--	------	------
mPD		0.11	0.75
		nPD:	0.14
		N. weight:	1.4
		Sc. PD:	0.11
		Sc. rank:	1027.8

NA			
Pos.	407	obs:	exp:
gat	D	231	631.20
gac	D	937	536.80
---	--	------	------
mPD		0.32	0.50
		nPD:	0.64
		N. weight:	0.26
		Sc. PD:	0.15
		Sc. rank:	254.4

NA			
Pos.	408	obs:	exp:
tgg	W	1167	1167.00
cgt	R	0	0.04
cgc	R	0	0.05
cga	R	0	0.10
cgg	R	1	0.08
aga	R	0	0.46
agg	R	0	0.27
---	--	------	------
mPD		0.0017	0.0028
		nPD:	0.61
		N. weight:	0.0022
		Sc. PD:	0.0012
		Sc. rank:	2.2

NA			
Pos.	409	obs:	exp:
tct	S	0	185.80
tcc	S	0	152.50
tca	S	0	296.50
tcg	S	0	66.28
agt	S	1165	240.10
agc	S	3	226.90
---	--	------	------
mPD		0.0051	1.7
		nPD:	0.
		N. weight:	1.7
		Sc. PD:	-0.081
		Sc. rank:	-965.6

NA			
Pos.	410	obs:	exp:
gtt	V	1	0.21
gtc	V	0	0.20
gta	V	0	0.20
gtg	V	0	0.39
ggt	G	1161	154.70
ggc	G	4	149.10
gga	G	2	538.40
ggg	G	0	324.90
---	--	------	------
mPD		0.012	0.68
		nPD:	0.02
		N. weight:	2.1
		Sc. PD:	-0.073
		Sc. rank:	153.5

NA			
Pos.	411	obs:	exp:
tat	Y	4	630.80
tac	Y	1164	537.20
---	--	------	------
mPD		0.0068	0.50
		nPD:	0.01
		N. weight:	0.8
		Sc. PD:	-0.031
		Sc. rank:	-27.2

NA			
Pos.	412	obs:	exp:
tct	S	0	185.80
tcc	S	0	152.50
tca	S	0	296.50
tcg	S	0	66.28
agt	S	1168	240.10
agc	S	0	226.90
---	--	------	------
mPD		0	1.7
		nPD:	0.
		N. weight:	1.7
		Sc. PD:	-0.086
		Sc. rank:	-1580.2

NA			
Pos.	413	obs:	exp:
ggt	G	0	154.80
ggc	G	0	149.20
gga	G	621	538.80
ggg	G	547	325.20
---	--	------	------
mPD		0.50	0.68
		nPD:	0.74
		N. weight:	0.34
		Sc. PD:	0.22
		Sc. rank:	328.4

NA			
Pos.	414	obs:	exp:
tct	S	756	185.80
tcc	S	412	152.50
tca	S	0	296.50
tcg	S	0	66.28
agt	S	0	240.10
agc	S	0	226.90
---	--	------	------
mPD		0.46	1.7
		nPD:	0.28
		N. weight:	1.3
		Sc. PD:	0.28
		Sc. rank:	1152.6

NA			
Pos.	415	obs:	exp:
ttt	F	229	461.20
ttc	F	939	706.80
---	--	------	------
mPD		0.32	0.48
		nPD:	0.66
		N. weight:	0.099
		Sc. PD:	0.057
		Sc. rank:	95.5

NA			
Pos.	416	obs:	exp:
att	I	2	3.67
atc	I	0	2.40
ata	I	8	3.93
atg	M	1158	1158.00
---	--	------	------
mPD		0.017	0.017
		nPD:	1.
		N. weight:	0.0051
		Sc. PD:	0.0046
		Sc. rank:	5.0

NA			
Pos.	417	obs:	exp:
gat	D	6	631.20
gac	D	1162	536.80
---	--	------	------
mPD		0.010	0.50
		nPD:	0.02
		N. weight:	0.79
		Sc. PD:	-0.025
		Sc. rank:	101.3

NA			
Pos.	418	obs:	exp:
tat	Y	1166	630.80
tac	Y	2	537.20
---	--	------	------
mPD		0.0034	0.50
		nPD:	0.01
		N. weight:	0.64
		Sc. PD:	-0.029
		Sc. rank:	-209.1

NA			
Pos.	419	obs:	exp:
tgg	W	1168	1168.00
---	--	------	------
mPD		0	0
		nPD:	1.
		N. weight:	0.
		Sc. PD:	0
		Sc. rank:	0

NA			
Pos.	420	obs:	exp:
gct	A	1162	301.60
gcc	A	1	217.50
gca	A	0	549.70
gcg	A	5	99.13
---	--	------	------
mPD		0.010	0.67
		nPD:	0.02
		N. weight:	1.4
		Sc. PD:	-0.052
		Sc. rank:	18.3

NA			
Pos.	421	obs:	exp:
aaa	K	1	0.58
aag	K	0	0.42
gat	D	4	2.16
gac	D	0	1.84
gaa	E	1156	705.90
gag	E	4	454.10
ggt	G	0	0.40
ggc	G	0	0.38
gga	G	3	1.38
ggg	G	0	0.84
---	--	------	------
mPD		0.020	0.49
		nPD:	0.04
		N. weight:	0.5
		Sc. PD:	-0.0061
		Sc. rank:	208.5

NA			
Pos.	422	obs:	exp:
ggt	G	3	154.80
ggc	G	0	149.20
gga	G	2	538.80
ggg	G	1163	325.20
---	--	------	------
mPD		0.0085	0.68
		nPD:	0.01
		N. weight:	1.3
		Sc. PD:	-0.053
		Sc. rank:	-76.2

NA			
Pos.	423	obs:	exp:
aat	N	0	0.51
aac	N	1	0.49
gat	D	27	630.60
gac	D	1140	536.40
---	--	------	------
mPD		0.047	0.50
		nPD:	0.09
		N. weight:	0.7
		Sc. PD:	0.026
		Sc. rank:	452.5

NA			
Pos.	424	obs:	exp:
tgt	C	1	494.00
tgc	C	1167	674.00
---	--	------	------
mPD		0.0017	0.49
		nPD:	0.
		N. weight:	0.57
		Sc. PD:	-0.028
		Sc. rank:	-313.1

NA			
Pos.	425	obs:	exp:
tat	Y	1167	630.80
tac	Y	1	537.20
---	--	------	------
mPD		0.0017	0.50
		nPD:	0.
		N. weight:	0.64
		Sc. PD:	-0.031
		Sc. rank:	-359.8

NA			
Pos.	426	obs:	exp:
cgt	R	1	50.16
cgc	R	0	57.85
cga	R	1167	114.30
cgg	R	0	97.46
aga	R	0	534.10
agg	R	0	314.20
---	--	------	------
mPD		0.0017	0.96
		nPD:	0.
		N. weight:	2.4
		Sc. PD:	-0.12
		Sc. rank:	-1722.5

NA			
Pos.	427	obs:	exp:
gct	A	49	301.60
gcc	A	0	217.50
gca	A	6	549.70
gcg	A	1113	99.13
---	--	------	------
mPD		0.090	0.67
		nPD:	0.13
		N. weight:	2.3
		Sc. PD:	0.18
		Sc. rank:	1755.0

NA			
Pos.	428	obs:	exp:
tgt	C	922	494.00
tgc	C	246	674.00
---	--	------	------
mPD		0.33	0.49
		nPD:	0.68
		N. weight:	0.3
		Sc. PD:	0.18
		Sc. rank:	287.9

NA			
Pos.	429	obs:	exp:
ttt	F	1155	461.20
ttc	F	13	706.80
---	--	------	------
mPD		0.022	0.48
		nPD:	0.05
		N. weight:	0.91
		Sc. PD:	-0.0075
		Sc. rank:	413.6

NA			
Pos.	430	obs:	exp:
tat	Y	1166	630.80
tac	Y	2	537.20
---	--	------	------
mPD		0.0034	0.50
		nPD:	0.01
		N. weight:	0.64
		Sc. PD:	-0.029
		Sc. rank:	-209.1

NA			
Pos.	431	obs:	exp:
gtt	V	0	242.00
gtc	V	0	235.70
gta	V	1	239.20
gtg	V	1167	451.10
---	--	------	------
mPD		0.0017	0.73
		nPD:	0.
		N. weight:	1.
		Sc. PD:	-0.049
		Sc. rank:	-645.9

NA			
Pos.	432	obs:	exp:
gaa	E	1	710.70
gag	E	1167	457.30
---	--	------	------
mPD		0.0017	0.48
		nPD:	0.
		N. weight:	0.98
		Sc. PD:	-0.047
		Sc. rank:	-497.8

NA			
Pos.	433	obs:	exp:
tta	L	1	103.60
ttg	L	1117	217.50
ctt	L	0	194.50
ctc	L	0	194.90
cta	L	0	196.80
ctg	L	50	260.60
---	--	------	------
mPD		0.084	1.1
		nPD:	0.08
		N. weight:	1.6
		Sc. PD:	0.031
		Sc. rank:	935.7

NA			
Pos.	434	obs:	exp:
att	I	29	428.80
atc	I	0	279.70
ata	I	1138	458.60
gtt	V	0	0.21
gtc	V	0	0.20
gta	V	1	0.20
gtg	V	0	0.39
---	--	------	------
mPD		0.050	0.66
		nPD:	0.08
		N. weight:	0.87
		Sc. PD:	0.018
		Sc. rank:	525.5

NA			
Pos.	435	obs:	exp:
cgt	R	1165	50.16
cgc	R	3	57.85
cga	R	0	114.30
cgg	R	0	97.46
aga	R	0	534.10
agg	R	0	314.20
---	--	------	------
mPD		0.0051	0.96
		nPD:	0.01
		N. weight:	3.3
		Sc. PD:	-0.15
		Sc. rank:	-1250.3

NA			
Pos.	436	obs:	exp:
ggt	G	0	154.80
ggc	G	0	149.20
gga	G	1114	538.80
ggg	G	54	325.20
---	--	------	------
mPD		0.088	0.68
		nPD:	0.13
		N. weight:	0.65
		Sc. PD:	0.046
		Sc. rank:	477.4

NA			
Pos.	437	obs:	exp:
tct	S	0	0.16
tcc	S	0	0.13
tca	S	0	0.25
tcg	S	0	0.06
cgt	R	0	48.06
cgc	R	0	55.42
cga	R	0	109.50
cgg	R	0	93.37
aaa	K	48	27.98
aag	K	0	20.02
agt	S	1	0.21
agc	S	0	0.19
aga	R	1117	511.70
agg	R	2	301.00
---	--	------	------
mPD		0.084	1.0
		nPD:	0.08
		N. weight:	0.8
		Sc. PD:	0.02
		Sc. rank:	497.1

NA			
Pos.	438	obs:	exp:
tta	L	0	0.09
ttg	L	0	0.19
ctt	L	0	0.17
ctc	L	1	0.17
cta	L	0	0.17
ctg	L	0	0.22
cct	P	2	291.20
ccc	P	1162	209.70
cca	P	3	480.40
ccg	P	0	185.80
---	--	------	------
mPD		0.010	0.71
		nPD:	0.01
		N. weight:	1.8
		Sc. PD:	-0.068
		Sc. rank:	6.1

NA			
Pos.	439	obs:	exp:
cgt	R	0	0.04
cgc	R	0	0.05
cga	R	0	0.10
cgg	R	0	0.08
aaa	K	10	679.60
aag	K	1156	486.40
aga	R	0	0.46
agg	R	1	0.27
gaa	E	0	0.61
gag	E	1	0.39
---	--	------	------
mPD		0.020	0.49
		nPD:	0.04
		N. weight:	0.87
		Sc. PD:	-0.011
		Sc. rank:	354.1

NA			
Pos.	440	obs:	exp:
gaa	E	239	710.70
gag	E	929	457.30
---	--	------	------
mPD		0.33	0.48
		nPD:	0.68
		N. weight:	0.36
		Sc. PD:	0.22
		Sc. rank:	349.9

NA			
Pos.	441	obs:	exp:
aat	N	1	0.51
aac	N	0	0.49
gat	D	1159	627.40
gac	D	2	533.60
ggt	G	6	0.80
ggc	G	0	0.77
gga	G	0	2.77
ggg	G	0	1.67
---	--	------	------
mPD		0.015	0.51
		nPD:	0.03
		N. weight:	0.65
		Sc. PD:	-0.015
		Sc. rank:	196.4

NA			
Pos.	442	obs:	exp:
cgt	R	0	0.21
cgc	R	0	0.25
cga	R	0	0.49
cgg	R	0	0.42
aat	N	8	4.07
aac	N	0	3.93
aaa	K	1145	671.40
aag	K	7	480.60
aga	R	5	2.29
agg	R	0	1.34
gaa	E	3	1.83
gag	E	0	1.17
---	--	------	------
mPD		0.039	0.51
		nPD:	0.08
		N. weight:	0.54
		Sc. PD:	0.011
		Sc. rank:	326.3

NA			
Pos.	443	obs:	exp:
gtt	V	0	242.00
gtc	V	0	235.70
gta	V	3	239.20
gtg	V	1165	451.10
---	--	------	------
mPD		0.0051	0.73
		nPD:	0.01
		N. weight:	0.99
		Sc. PD:	-0.044
		Sc. rank:	-296.7

NA

Pos.	444	obs:	exp:
tta	L	0	0.09
ttg	L	1	0.19
tgg	W	1165	1165.00
ctt	L	0	0.17
ctc	L	0	0.17
cta	L	0	0.17
ctg	L	0	0.22
ggt	G	0	0.27
ggc	G	0	0.26
gga	G	0	0.92
ggg	G	2	0.56
---	--	------	------
mPD		0.0051	0.0098
		nPD:	0.52
		N. weight:	0.0038
		Sc. PD:	0.0017
		Sc. rank:	3.6

,

NA

Pos.	445	obs:	exp:
tgg	W	1168	1168.00
---	--	------	------
mPD		0	0
		nPD:	1.
		N. weight:	0.
		Sc. PD:	0
		Sc. rank:	0

,

NA

Pos.	446	obs:	exp:
act	T	1	297.50
acc	T	1165	239.50
aca	T	1	535.30
acg	T	0	94.79
gct	A	0	0.26
gcc	A	1	0.19
gca	A	0	0.47
gcg	A	0	0.08
---	--	------	------
mPD		0.0051	0.68
		nPD:	0.01
		N. weight:	1.7
		Sc. PD:	-0.074
		Sc. rank:	-432.1

,

NA

Pos.	447	obs:	exp:
tct	S	0	185.60
tcc	S	0	152.40
tca	S	0	296.20
tcg	S	0	66.22
agt	S	0	239.90
agc	S	1167	226.70
ggt	G	0	0.13
ggc	G	1	0.13
gga	G	0	0.46
ggg	G	0	0.28
---	--	------	------
mPD		0.0017	1.7
		nPD:	0.
		N. weight:	1.7
		Sc. PD:	-0.088
		Sc. rank:	-1283.2

,

NA

Pos.	448	obs:	exp:
aat	N	1118	594.40
aac	N	49	572.60
gat	D	1	0.54
gac	D	0	0.46
---	--	------	------
mPD		0.082	0.50
		nPD:	0.16
		N. weight:	0.53
		Sc. PD:	0.055
		Sc. rank:	420.1

,

NA

Pos.	449	obs:	exp:
tct	S	0	185.80
tcc	S	0	152.50
tca	S	0	296.50
tcg	S	0	66.28
agt	S	1168	240.10
agc	S	0	226.90
---	--	------	------
mPD		0	1.7
		nPD:	0.
		N. weight:	1.7
		Sc. PD:	-0.086
		Sc. rank:	-1580.2

,

NA

Pos.	450	obs:	exp:
att	I	0	429.10
atc	I	2	279.90
ata	I	1166	459.00
---	--	------	------
mPD		0.0034	0.65
		nPD:	0.01
		N. weight:	0.97
		Sc. PD:	-0.046
		Sc. rank:	-376.9

,

NA

Pos.	451	obs:	exp:
gtt	V	0	242.00
gtc	V	0	235.70
gta	V	1160	239.20
gtg	V	8	451.10
---	--	------	------
mPD		0.014	0.73
		nPD:	0.02
		N. weight:	1.6
		Sc. PD:	-0.055
		Sc. rank:	178.0

,

NA

Pos.	452	obs:	exp:
tct	S	0	185.80
tcc	S	0	152.50
tca	S	25	296.50
tcg	S	1143	66.28
agt	S	0	240.10
agc	S	0	226.90
---	--	------	------
mPD		0.042	1.7
		nPD:	0.03
		N. weight:	2.9
		Sc. PD:	-0.08
		Sc. rank:	630.3

,

NA

Pos.	453	obs:	exp:
atg	M	1168	1168.00
---	--	------	------
mPD		0	0
		nPD:	1.
		N. weight:	0.
		Sc. PD:	0
		Sc. rank:	0

,

NA

Pos.	454	obs:	exp:
tgt	C	1168	494.00
tgc	C	0	674.00
---	--	------	------
mPD		0	0.49
		nPD:	0.
		N. weight:	0.91
		Sc. PD:	-0.047
		Sc. rank:	-859.5

,

NA

Pos.	455	obs:	exp:
tct	S	0	185.80
tcc	S	1168	152.50
tca	S	0	296.50
tcg	S	0	66.28
agt	S	0	240.10
agc	S	0	226.90
---	--	------	------
mPD		0	1.7
		nPD:	0.
		N. weight:	2.1
		Sc. PD:	-0.11
		Sc. rank:	-2033.4

,

NA

Pos.	456	obs:	exp:
tct	S	0	185.80
tcc	S	0	152.50
tca	S	0	296.50
tcg	S	0	66.28
agt	S	1168	240.10
agc	S	0	226.90
---	--	------	------
mPD		0	1.7
		nPD:	0.
		N. weight:	1.7
		Sc. PD:	-0.086
		Sc. rank:	-1580.2

,

NA

Pos.	457	obs:	exp:
act	T	0	297.20
acc	T	0	239.30
aca	T	1166	534.80
acg	T	0	94.71
gct	A	0	0.52
gcc	A	0	0.37
gca	A	2	0.94
gcg	A	0	0.17
---	--	------	------
mPD		0.0034	0.68
		nPD:	0.01
		N. weight:	0.82
		Sc. PD:	-0.039
		Sc. rank:	-382.1

,

NA

Pos.	458	obs:	exp:
aaa	K	1	0.58
aag	K	0	0.42
gaa	E	939	709.50
gag	E	227	456.50
ggt	G	0	0.13
ggc	G	0	0.13
gga	G	1	0.46
ggg	G	0	0.28
---	--	------	------
mPD		0.32	0.48
		nPD:	0.66
		N. weight:	0.099
		Sc. PD:	0.056
		Sc. rank:	95.0

,

NA

Pos.	459	obs:	exp:
ttt	F	0	460.40
ttc	F	1166	705.60
tat	Y	0	1.08
tac	Y	2	0.92
---	--	------	------
mPD		0.0034	0.48
		nPD:	0.01
		N. weight:	0.53
		Sc. PD:	-0.024
		Sc. rank:	-156.1

,

NA

Pos.	460	obs:	exp:
tta	L	0	103.60
ttg	L	0	217.50
ctt	L	0	194.50
ctc	L	0	194.90
cta	L	4	196.80
ctg	L	1164	260.60
---	--	------	------
mPD		0.0068	1.1
		nPD:	0.01
		N. weight:	1.6
		Sc. PD:	-0.072
		Sc. rank:	-556.3

,

NA

Pos.	461	obs:	exp:
ggt	G	0	154.80
ggc	G	0	149.20
gga	G	1167	538.80
ggg	G	1	325.20
---	--	------	------
mPD		0.0017	0.68
		nPD:	0.
		N. weight:	0.81
		Sc. PD:	-0.04
		Sc. rank:	-501.9

,

NA

Pos.	462	obs:	exp:
caa	Q	1158	594.90
cag	Q	10	573.10
---	--	------	------
mPD		0.017	0.50
		nPD:	0.03
		N. weight:	0.66
		Sc. PD:	-0.013
		Sc. rank:	221.1

,

NA

Pos.	463	obs:	exp:
tgg	W	1168	1168.00
---	--	------	------
mPD		0	0
		nPD:	1.
		N. weight:	0.
		Sc. PD:	0
		Sc. rank:	0

,

NA

Pos.	464	obs:	exp:
aat	N	0	592.90
aac	N	1164	571.10
gat	D	0	2.16
gac	D	4	1.84
---	--	------	------
mPD		0.0068	0.51
		nPD:	0.01
		N. weight:	0.75
		Sc. PD:	-0.029
		Sc. rank:	-36.7

,

NA

Pos.	465	obs:	exp:
tgg	W	1168	1168.00
---	--	------	------
mPD		0	0
		nPD:	1.
		N. weight:	0.
		Sc. PD:	0
		Sc. rank:	0

,

NA

Pos.	466	obs:	exp:
tct	S	1	0.16
tcc	S	0	0.13
tca	S	0	0.25
tcg	S	0	0.06
cct	P	1167	291.20
ccc	P	0	209.70
cca	P	0	480.40
ccg	P	0	185.80
agt	S	0	0.21
agc	S	0	0.19
---	--	------	------
mPD		0.0017	0.71
		nPD:	0.
		N. weight:	1.5
		Sc. PD:	-0.072
		Sc. rank:	-939.6

,

NA

Pos.	467	obs:	exp:
gat	D	1070	631.20
gac	D	98	536.80
---	--	------	------
mPD		0.15	0.50
		nPD:	0.31
		N. weight:	0.36
		Sc. PD:	0.087
		Sc. rank:	327.8

,

NA

Pos.	468	obs:	exp:
ggt	G	0	154.80
ggc	G	0	149.20
gga	G	5	538.80
ggg	G	1163	325.20
---	--	------	------
mPD		0.0085	0.68
		nPD:	0.01
		N. weight:	1.3
		Sc. PD:	-0.053
		Sc. rank:	-97.6

,

NA

Pos.	469	obs:	exp:
gct	A	1166	301.10
gcc	A	0	217.10
gca	A	0	548.80
gcg	A	0	98.96
ggt	G	2	0.27
ggc	G	0	0.26
gga	G	0	0.92
ggg	G	0	0.56
---	--	------	------
mPD		0.0034	0.67
		nPD:	0.01
		N. weight:	1.4
		Sc. PD:	-0.067
		Sc. rank:	-612.9

,

NA

Pos.	470	obs:	exp:
cgt	R	0	2.75
cgc	R	0	3.17
cga	R	0	6.26
cgg	R	0	5.34
act	T	0	0.25
acc	T	0	0.21
aca	T	1	0.46
acg	T	0	0.08
aat	N	0	1.53
aac	N	3	1.47
aaa	K	1097	640.50
aag	K	2	458.50
aga	R	64	29.26
agg	R	0	17.21
gaa	E	1	0.61
gag	E	0	0.39
---	--	------	------
mPD		0.12	0.63
		nPD:	0.18
		N. weight:	0.57
		Sc. PD:	0.069
		Sc. rank:	462.6

,

NA

Pos.	471	obs:	exp:
att	I	0	429.10
atc	I	0	279.90
ata	I	1168	459.00
---	--	------	------
mPD		0	0.65
		nPD:	0.
		N. weight:	0.99
		Sc. PD:	-0.051
		Sc. rank:	-933.1

,

NA

Pos.	472	obs:	exp:
gaa	E	0	710.70
gag	E	1168	457.30
---	--	------	------
mPD		0	0.48
		nPD:	0.
		N. weight:	0.99
		Sc. PD:	-0.051
		Sc. rank:	-936.7

,

NA

Pos.	473	obs:	exp:
tat	Y	2	630.30
tac	Y	1165	536.70
cat	H	0	0.57
cac	H	1	0.43
---	--	------	------
mPD		0.0051	0.50
		nPD:	0.01
		N. weight:	0.81
		Sc. PD:	-0.034
		Sc. rank:	-126.6

,

NA

Pos.	474	obs:	exp:
ttt	F	25	461.20
ttc	F	1143	706.80
---	--	------	------
mPD		0.042	0.48
		nPD:	0.09
		N. weight:	0.43
		Sc. PD:	0.013
		Sc. rank:	271.5

,

NA

Pos.	475	obs:	exp:
tta	L	0	102.90
ttg	L	0	215.80
ctt	L	0	193.00
ctc	L	1159	193.40
cta	L	0	195.30
ctg	L	0	258.60
cgt	R	0	0.39
cgc	R	0	0.45
cga	R	0	0.88
cgg	R	9	0.75
aga	R	0	4.12
agg	R	0	2.42
---	--	------	------
mPD		0.031	1.1
		nPD:	0.03
		N. weight:	1.9
		Sc. PD:	-0.05
		Sc. rank:	497.1

,

NA

Pos.	476	obs:	exp:
tct	S	0	1.43
tcc	S	0	1.18
tca	S	9	2.28
tcg	S	0	0.51
agt	S	0	1.85
agc	S	0	1.75
---	-	1159	1159.00
---	--	------	------
mPD		0	0.000098
		nPD:	0.
		N. weight:	0.
		Sc. PD:	0
		Sc. rank:	0

,

NA

Pos.	477	obs:	exp:
taa	.	1158	496.20
tag	.	10	292.40
tga	.	0	379.40
---	--	------	------
mPD		0.017	0.81
		nPD:	0.02
		N. weight:	0.86
		Sc. PD:	-0.027
		Sc. rank:	117.3

}

Amino acid level conservation per codon

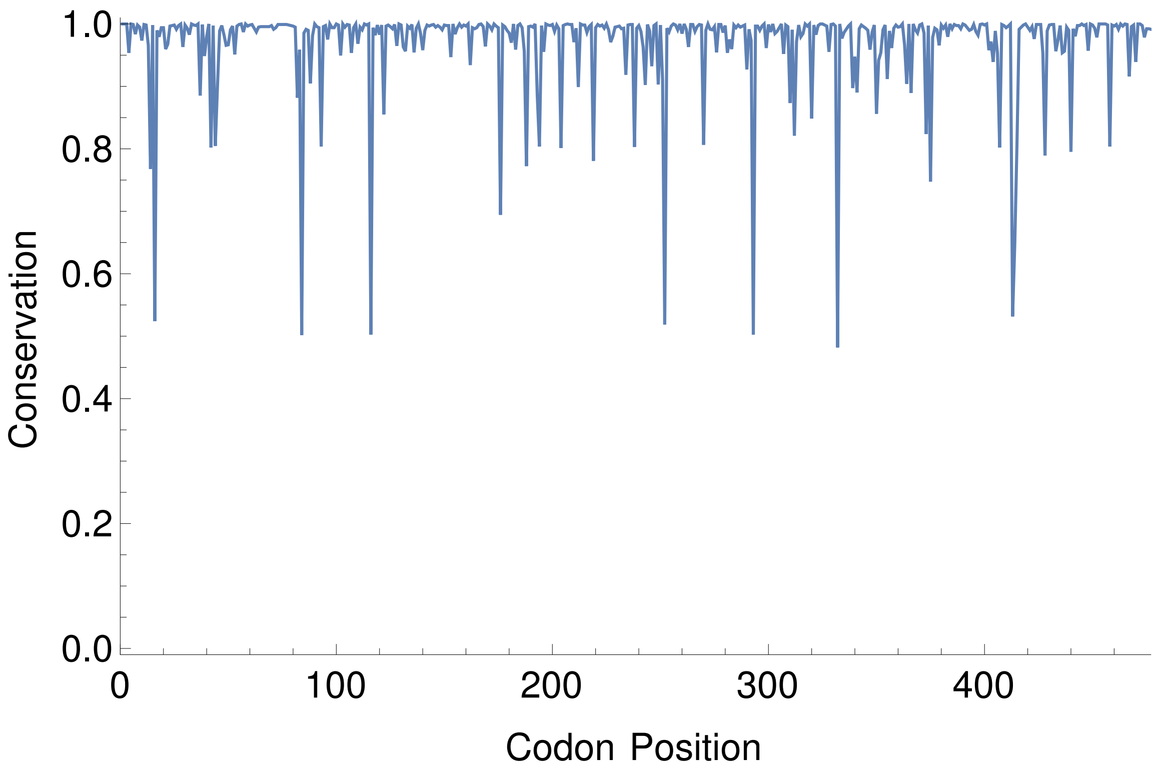

Conservation analysis plots

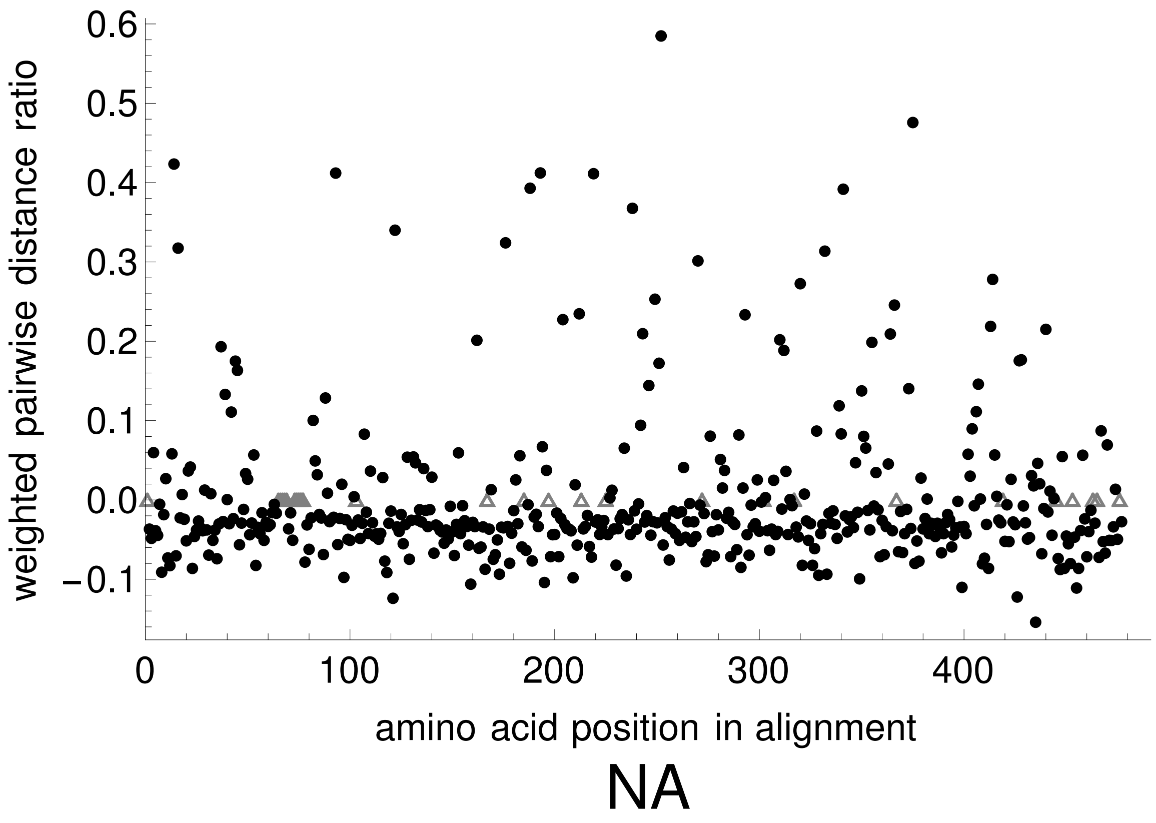

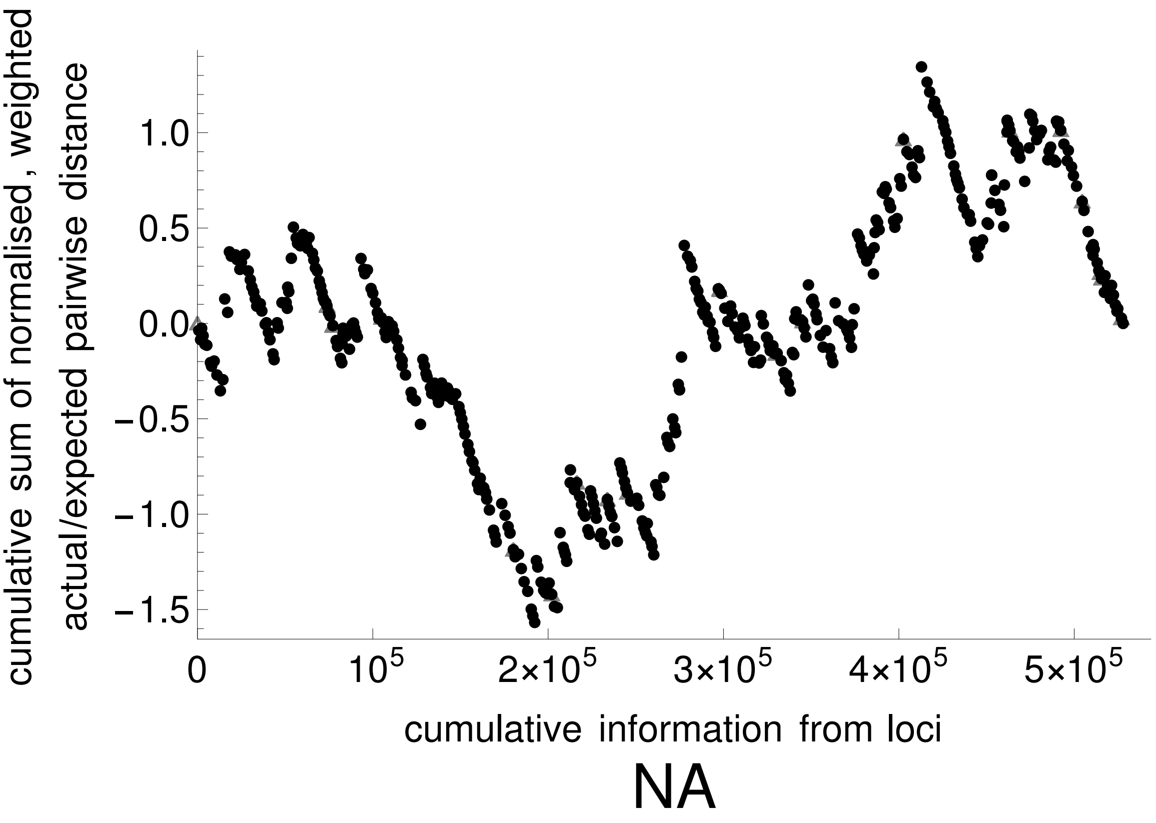

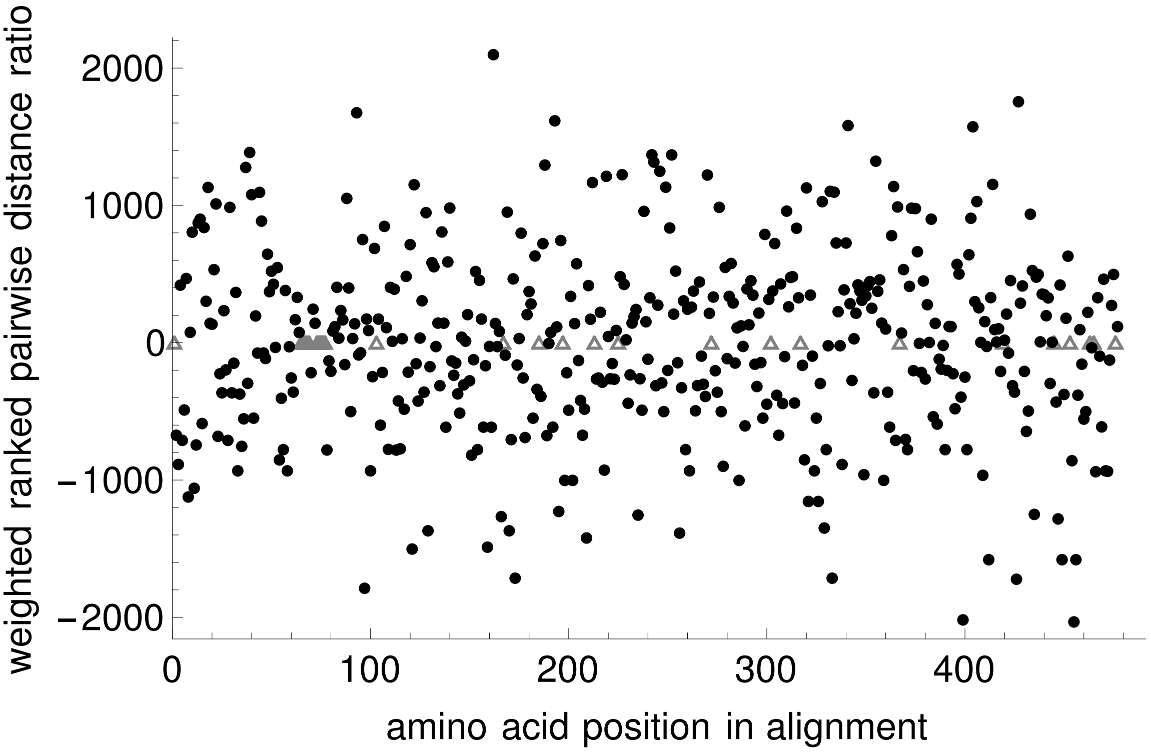
2000
1000
0
−1000
−2000
weighted ranked pairwise distance ratio
0
100
200
300
400
amino acid position in alignment

NA

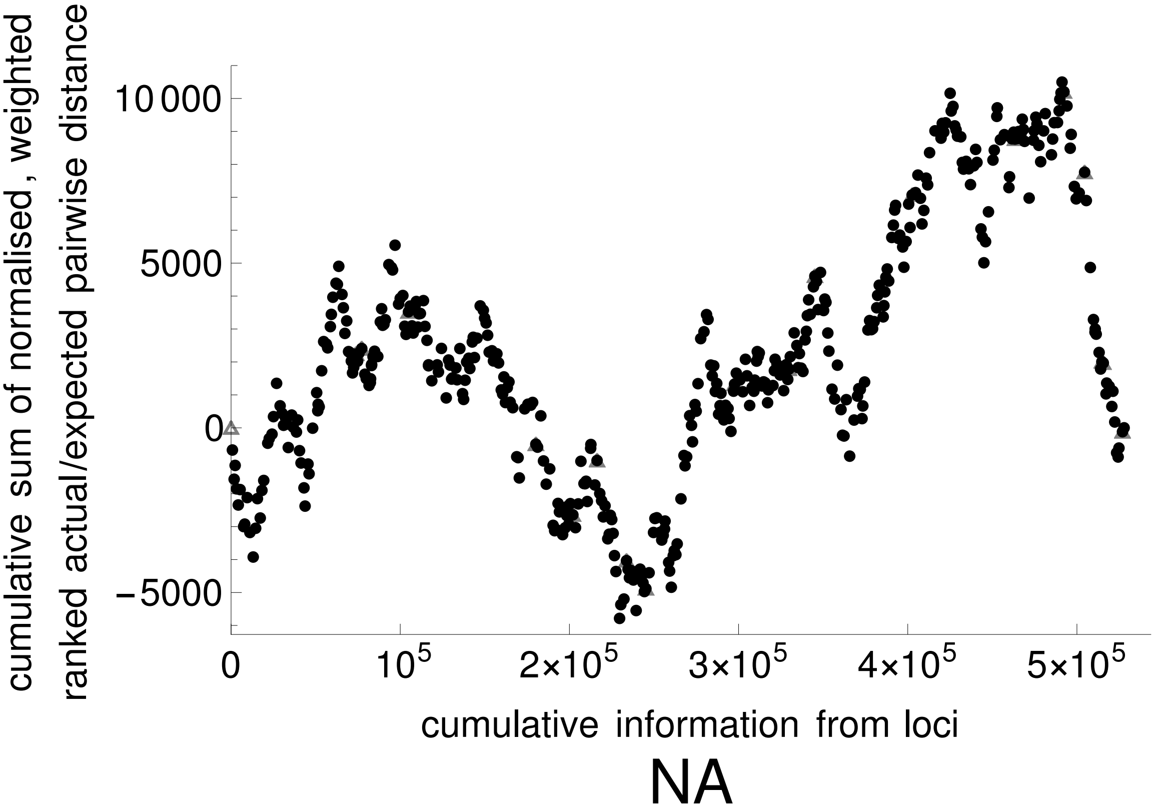
10 000
5000
0
−5000
cumulative sum of normalised, weighted
ranked actual/expected pairwise distance
0
10^5
2×10^5
3×10^5
4×10^5
5×10^5
cumulative information from loci

NA

M1

Gene length histogram

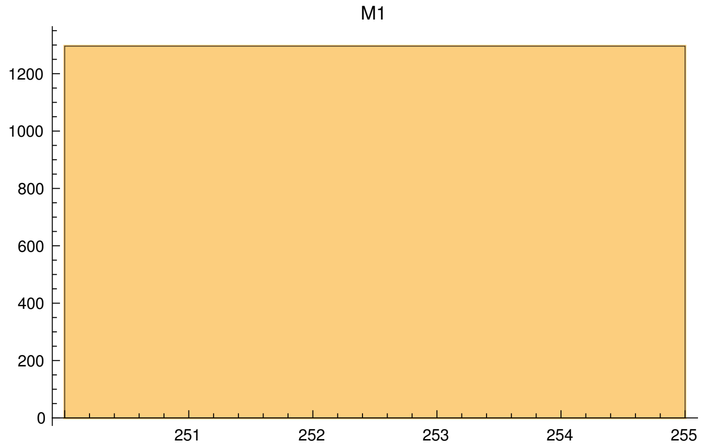

Information vs. nPD

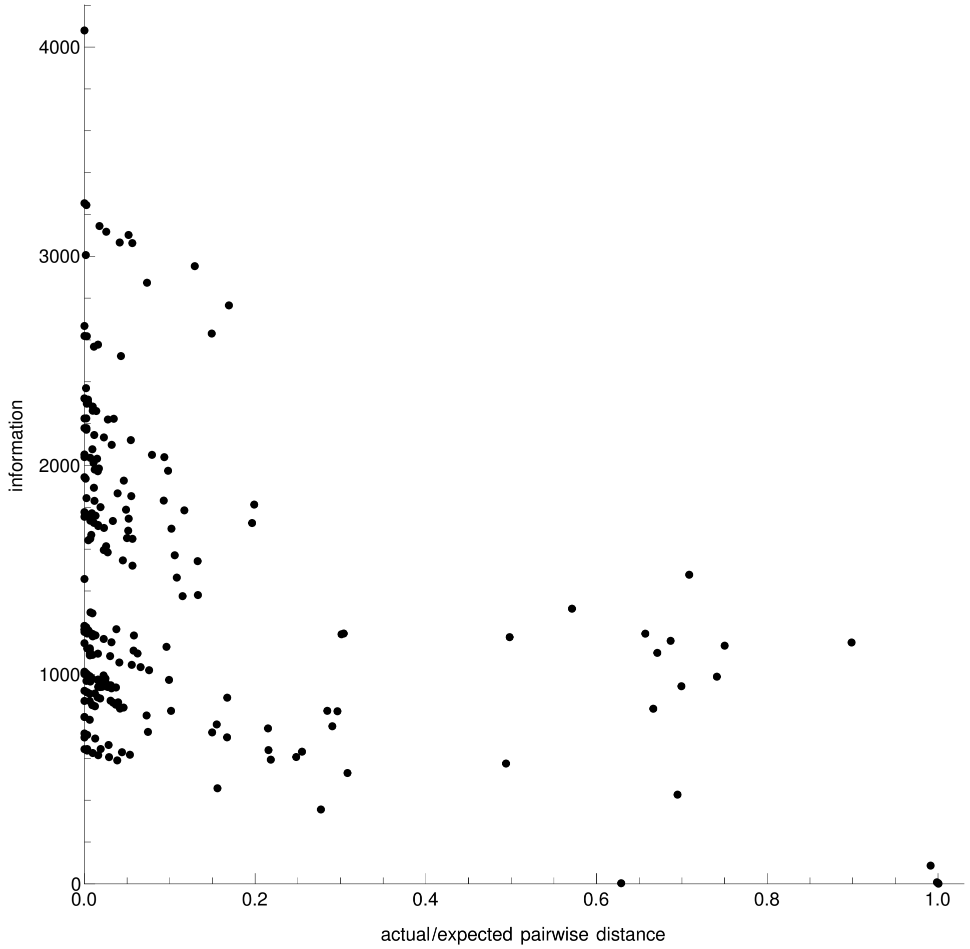

M1

Example sequences highlighted by regions found in analyses to be conserved

Interesting points (by weighted raw PD) highlighted for gene M1:

ATG
AGTCTTCTAACCGAGGTCGAAACGTACGTTCTCTCTATCATTCCATCAGGCCCCC
TCAAAGCCGAGATCGCGCAGAGACTTGAGGATGTTTTTGCAGGGAAGAACGC
AGATCTCGAGGCTCTCATGGAGTGGATAAAGACAAGACCAATCCTGTCACCT
CTGACTAAGGGGATTTTAGGGTTTGTGTTCACGCTCACCGTGCCCAGTGAGC
GAGGACTGCAGCGTAGACGGTTTGTCCAAAACGCCCTAAATGGGAATGGAGACCCAAACAACATG⋱
GACAAGGCAGTTAAATTATACAAGAAACTGAAGAGGGAAATGACATTTCATGGAGCAAAGGAAGTTGCAC⋱
TCAGTTACTCAACTGGTGCGCTTGCCAGCTGCATGGGTCTCATATACAACAGGATGGGGACAGTAACTGC⋱
AGAAGGGGCTCTTGGATTGGTATGTGCCACTTGTGAGCAGATTGCCGACGCACAACATCGGTCCCACAGG⋱
CAGATGGCAACTACTACCAACCCACTAATTAGGCATGAGAATAGAATGGTACTAGCCAGTACTACGGCTA⋱
AGGCTATGGAGCAGATGGCTGGATCAAGTGAACAGGCAGCGGAAGCCATGGAAGTCGCAAGCCAGGCTAG⋱
GCAAATGGTGCAGGCTATGAGGACAGTCGGGACTCACCCTAACTCCAGTACAGGTCTTAAGGATGATCTT⋱
ATTGAAAATTTGCAGGCCTACCAGAACCGGATGGGAGTGCAACTGCAGCGGTTCAAGTGA

Interesting points (by weighted ranked PD) highlighted for gene M1:

ATGAGT
CTTCTAACCGAGGTCGAAACGTACGTTCTCTCTATCATTCCATCAGGCCCCCTCA
AAGCCGAG
ATCGCGCAGAGACTTGAGGATGTTTTTGCAGGGAAGAACGCAGATCTCGAGGCTCTCATGGAGTGG
ATAAAGACAAGACCAATCCTGTCACCTCTGACTAAGGGGATTTTAGGGTTTGTGTTC
ACGCTCACCGTGCCCAGTGAGCGAGGACTGCAGCGTAGACGGTTTGTCCAAAACGCCCTAA⋱
ATGGGAATGGAGACCCAAACAACATGGACAAGGCAGTTAAATTATACAAGAAACTGAAGAGGGAAATGAC⋱
ATTTCATGGAGCAAAGGAAGTTGCACTCAGTTACTCAACTGGTGCGCTTGCCAGCTGCATGGGTCTCATA⋱
TACAACAGGATGGGGACAGTAACTGCAGAAGGGGCTCTTGGATTGGTATGTGCCACTTGTGAGCAGATTG⋱
CCGACGCACAACATCGGTCCCACAGGCAGATGGCAACTACTACCAACCCACTAATTAGGCATGAGAATAG⋱
AATGGTACTAGCCAGTACTACGGCTAAGGCTATGGAGCAGATGGCTGGATCAAGTGAACAGGCAGCGGAA⋱
GCCATGGAAGTCGCAAGCCAGGCTAGGCAAATGGTGCAGGCTATGAGGACAGTCGGGACTCACCCTAACT⋱
CCAGTACAGGTCTTAAGGATGATCTTATTGAAAATTTGCAGGCCTACCAGAACCGGATGGGAGTGCAACT⋱
GCAGCGGTTCAAGTGA

Per codon conservation report

M1			
Pos.	1	obs:	exp:
atg	M	1296	1296.00
---	--	------	------
mPD		0	0
		nPD:	1.
		N. weight:	0.
		Sc. PD:	0
		Sc. rank:	0

M1			
Pos.	2	obs:	exp:
tct	S	0	206.10
tcc	S	0	169.30
tca	S	0	328.90
tcg	S	0	73.54
agt	S	1253	266.40
agc	S	43	251.70
---	--	------	------
mPD		0.064	1.7
		nPD:	0.04
		N. weight:	1.4
		Sc. PD:	-0.031
		Sc. rank:	657.5

M1			
Pos.	3	obs:	exp:
tta	L	0	115.00
ttg	L	0	241.30
ctt	L	1295	215.80
ctc	L	1	216.30
cta	L	0	218.40
ctg	L	0	289.20
---	--	------	------
mPD		0.0015	1.1
		nPD:	0.
		N. weight:	1.7
		Sc. PD:	-0.099
		Sc. rank:	-1304.5

M1			
Pos.	4	obs:	exp:
tta	L	1	115.00
ttg	L	0	241.30
ctt	L	1	215.80
ctc	L	0	216.30
cta	L	1289	218.40
ctg	L	5	289.20
---	--	------	------
mPD		0.011	1.1
		nPD:	0.01
		N. weight:	1.7
		Sc. PD:	-0.084
		Sc. rank:	-218.2

M1			
Pos.	5	obs:	exp:
act	T	0	330.10
acc	T	1293	265.70
aca	T	2	594.00
acg	T	0	105.20
aat	N	0	0.51
aac	N	1	0.49
---	--	------	------
mPD		0.0046	0.68
		nPD:	0.01
		N. weight:	1.5
		Sc. PD:	-0.079
		Sc. rank:	-520.8

M1			
Pos.	6	obs:	exp:
gaa	E	0	788.60
gag	E	1296	507.40
---	--	------	------
mPD		0	0.48
		nPD:	0.
		N. weight:	0.9
		Sc. PD:	-0.053
		Sc. rank:	-1024.9

M1			
Pos.	7	obs:	exp:
gtt	V	7	268.50
gtc	V	1289	261.60
gta	V	0	265.40
gtg	V	0	500.50
---	--	------	------
mPD		0.011	0.73
		nPD:	0.01
		N. weight:	1.5
		Sc. PD:	-0.068
		Sc. rank:	50.4

M1			
Pos.	8	obs:	exp:
gaa	E	1296	788.60
gag	E	0	507.40
---	--	------	------
mPD		0	0.48
		nPD:	0.
		N. weight:	0.48
		Sc. PD:	-0.028
		Sc. rank:	-542.9

M1			
Pos.	9	obs:	exp:
act	T	0	330.40
acc	T	0	265.90
aca	T	0	594.40
acg	T	1296	105.30
---	--	------	------
mPD		0	0.68
		nPD:	0.
		N. weight:	2.4
		Sc. PD:	-0.14
		Sc. rank:	-2743.7

M1			
Pos.	10	obs:	exp:
tat	Y	5	700.00
tac	Y	1291	596.00
---	--	------	------
mPD		0.0077	0.50
		nPD:	0.02
		N. weight:	0.72
		Sc. PD:	-0.032
		Sc. rank:	33.4

M1			
Pos.	11	obs:	exp:
gtt	V	1296	268.50
gtc	V	0	261.60
gta	V	0	265.40
gtg	V	0	500.50
---	--	------	------
mPD		0	0.73
		nPD:	0.
		N. weight:	1.5
		Sc. PD:	-0.089
		Sc. rank:	-1720.3

M1			
Pos.	12	obs:	exp:
tta	L	0	115.00
ttg	L	0	241.30
ctt	L	2	215.80
ctc	L	1225	216.30
cta	L	69	218.40
ctg	L	0	289.20
---	--	------	------
mPD		0.10	1.1
		nPD:	0.09
		N. weight:	1.5
		Sc. PD:	0.046
		Sc. rank:	1246.9

M1			
Pos.	13	obs:	exp:
tct	S	1294	206.10
tcc	S	2	169.30
tca	S	0	328.90
tcg	S	0	73.54
agt	S	0	266.40
agc	S	0	251.70
---	--	------	------
mPD		0.0031	1.7
		nPD:	0.
		N. weight:	1.8
		Sc. PD:	-0.1
		Sc. rank:	-1208.5

M1			
Pos.	14	obs:	exp:
att	I	3	475.40
atc	I	1291	310.10
ata	I	0	508.50
gtt	V	0	0.41
gtc	V	2	0.40
gta	V	0	0.41
gtg	V	0	0.77
---	--	------	------
mPD		0.0077	0.66
		nPD:	0.01
		N. weight:	1.4
		Sc. PD:	-0.065
		Sc. rank:	-63.3

M1			
Pos.	15	obs:	exp:
att	I	1280	476.20
atc	I	15	310.60
ata	I	1	509.30
---	--	------	------
mPD		0.024	0.65
		nPD:	0.04
		N. weight:	0.9
		Sc. PD:	-0.021
		Sc. rank:	413.5

M1			
Pos.	16	obs:	exp:
cct	P	0	323.40
ccc	P	1	232.90
cca	P	1270	533.50
ccg	P	25	206.30
---	--	------	------
mPD		0.039	0.71
		nPD:	0.06
		N. weight:	0.78
		Sc. PD:	-0.0049
		Sc. rank:	522.7

M1			
Pos.	17	obs:	exp:
tct	S	0	206.10
tcc	S	0	169.30
tca	S	1294	328.90
tcg	S	2	73.54
agt	S	0	266.40
agc	S	0	251.70
---	--	------	------
mPD		0.0031	1.7
		nPD:	0.
		N. weight:	1.3
		Sc. PD:	-0.075
		Sc. rank:	-900.9

M1			
Pos.	18	obs:	exp:
ggt	G	0	171.80
ggc	G	1296	165.60
gga	G	0	597.90
ggg	G	0	360.80
---	--	------	------
mPD		0	0.68
		nPD:	0.
		N. weight:	2.
		Sc. PD:	-0.12
		Sc. rank:	-2248.8

M1			
Pos.	19	obs:	exp:
cct	P	0	323.40
ccc	P	1296	232.90
cca	P	0	533.50
ccg	P	0	206.30
---	--	------	------
mPD		0	0.71
		nPD:	0.
		N. weight:	1.6
		Sc. PD:	-0.098
		Sc. rank:	-1876.1

M1			
Pos.	20	obs:	exp:
tta	L	0	115.00
ttg	L	0	241.30
ctt	L	0	215.80
ctc	L	1296	216.30
cta	L	0	218.40
ctg	L	0	289.20
---	--	------	------
mPD		0	1.1
		nPD:	0.
		N. weight:	1.7
		Sc. PD:	-0.1
		Sc. rank:	-1956.8

M1			
Pos.	21	obs:	exp:
aaa	K	1296	755.30
aag	K	0	540.70
---	--	------	------
mPD		0	0.49
		nPD:	0.
		N. weight:	0.52
		Sc. PD:	-0.031
		Sc. rank:	-590.0

M1			
Pos.	22	obs:	exp:
gct	A	0	334.40
gcc	A	1295	241.20
gca	A	0	609.50
gcg	A	0	109.90
ggt	G	0	0.13
ggc	G	1	0.13
gga	G	0	0.46
ggg	G	0	0.28
---	--	------	------
mPD		0.0015	0.67
		nPD:	0.
		N. weight:	1.6
		Sc. PD:	-0.092
		Sc. rank:	-989.9

M1			
Pos.	23	obs:	exp:
gaa	E	0	788.60
gag	E	1296	507.40
---	--	------	------
mPD		0	0.48
		nPD:	0.
		N. weight:	0.9
		Sc. PD:	-0.053
		Sc. rank:	-1024.9

M1			
Pos.	24	obs:	exp:
att	I	0	476.20
atc	I	1288	310.60
ata	I	8	509.30
---	--	------	------
mPD		0.012	0.65
		nPD:	0.02
		N. weight:	1.3
		Sc. PD:	-0.055
		Sc. rank:	257.2

M1			
Pos.	25	obs:	exp:
gct	A	0	334.70
gcc	A	0	241.40
gca	A	501	610.00
gcg	A	795	110.00
---	--	------	------
mPD		0.47	0.67
		nPD:	0.71
		N. weight:	1.1
		Sc. PD:	0.68
		Sc. rank:	1281.0

M1			
Pos.	26	obs:	exp:
caa	Q	6	660.10
cag	Q	1290	635.90
---	--	------	------
mPD		0.0092	0.50
		nPD:	0.02
		N. weight:	0.66
		Sc. PD:	-0.027
		Sc. rank:	114.1

M1			
Pos.	27	obs:	exp:
cgt	R	0	55.66
cgc	R	0	64.19
cga	R	0	126.80
cgg	R	0	108.10
aga	R	1294	592.60
agg	R	2	348.60
---	--	------	------
mPD		0.0031	0.96
		nPD:	0.
		N. weight:	0.74
		Sc. PD:	-0.042
		Sc. rank:	-375.8

M1			
Pos.	28	obs:	exp:
tta	L	0	115.00
ttg	L	0	241.30
ctt	L	1295	215.80
ctc	L	0	216.30
cta	L	0	218.40
ctg	L	1	289.20
---	--	------	------
mPD		0.0015	1.1
		nPD:	0.
		N. weight:	1.7
		Sc. PD:	-0.099
		Sc. rank:	-1304.3

M1			
Pos.	29	obs:	exp:
gaa	E	3	788.60
gag	E	1293	507.40
---	--	------	------
mPD		0.0046	0.48
		nPD:	0.01
		N. weight:	0.89
		Sc. PD:	-0.044
		Sc. rank:	-134.5

M1			
Pos.	30	obs:	exp:
gat	D	1296	700.30
gac	D	0	595.70
---	--	------	------
mPD		0	0.50
		nPD:	0.
		N. weight:	0.59
		Sc. PD:	-0.035
		Sc. rank:	-672.7

M1			
Pos.	31	obs:	exp:
gtt	V	1288	268.50
gtc	V	1	261.60
gta	V	1	265.40
gtg	V	6	500.50
---	--	------	------
mPD		0.012	0.73
		nPD:	0.02
		N. weight:	1.5
		Sc. PD:	-0.063
		Sc. rank:	205.4

,

M1			
Pos.	32	obs:	exp:
ttt	F	1296	511.80
ttc	F	0	784.20
---	--	------	------
mPD		0	0.48
		nPD:	0.
		N. weight:	0.89
		Sc. PD:	-0.053
		Sc. rank:	-1015.4

,

M1			
Pos.	33	obs:	exp:
tct	S	0	0.64
tcc	S	0	0.52
tca	S	4	1.02
tcg	S	0	0.23
agt	S	0	0.82
agc	S	0	0.78
gct	A	0	333.60
gcc	A	0	240.60
gca	A	1292	608.10
gcg	A	0	109.70
---	--	------	------
mPD		0.0062	0.68
		nPD:	0.01
		N. weight:	0.73
		Sc. PD:	-0.037
		Sc. rank:	-177.9

,

M1			
Pos.	34	obs:	exp:
ggt	G	0	171.80
ggc	G	0	165.60
gga	G	10	597.90
ggg	G	1286	360.80
---	--	------	------
mPD		0.015	0.68
		nPD:	0.02
		N. weight:	1.2
		Sc. PD:	-0.044
		Sc. rank:	279.0

,

M1			
Pos.	35	obs:	exp:
aaa	K	55	755.30
aag	K	1241	540.70
---	--	------	------
mPD		0.081	0.49
		nPD:	0.17
		N. weight:	0.66
		Sc. PD:	0.066
		Sc. rank:	656.5

,

M1			
Pos.	36	obs:	exp:
tct	S	0	0.32
tcc	S	0	0.26
tca	S	0	0.51
tcg	S	0	0.11
aat	N	54	659.10
aac	N	1240	634.90
agt	S	1	0.41
agc	S	1	0.39
---	--	------	------
mPD		0.084	0.51
		nPD:	0.17
		N. weight:	0.52
		Sc. PD:	0.052
		Sc. rank:	513.5

,

M1			
Pos.	37	obs:	exp:
act	T	0	1.27
acc	T	0	1.03
aca	T	5	2.29
acg	T	0	0.41
gtt	V	0	1.24
gtc	V	0	1.21
gta	V	6	1.23
gtg	V	0	2.32
gct	A	0	331.80
gcc	A	0	239.30
gca	A	1285	604.80
gcg	A	0	109.10
---	--	------	------
mPD		0.017	0.69
		nPD:	0.02
		N. weight:	0.73
		Sc. PD:	-0.026
		Sc. rank:	195.7

,

M1			
Pos.	38	obs:	exp:
gat	D	1294	700.30
gac	D	2	595.70
---	--	------	------
mPD		0.0031	0.50
		nPD:	0.01
		N. weight:	0.58
		Sc. PD:	-0.031
		Sc. rank:	-231.2

,

M1			
Pos.	39	obs:	exp:
tta	L	0	115.00
ttg	L	0	241.30
ctt	L	16	215.80
ctc	L	1237	216.30
cta	L	43	218.40
ctg	L	0	289.20
---	--	------	------
mPD		0.088	1.1
		nPD:	0.08
		N. weight:	1.5
		Sc. PD:	0.025
		Sc. rank:	1213.2

,

M1			
Pos.	40	obs:	exp:
gaa	E	2	788.60
gag	E	1294	507.40
---	--	------	------
mPD		0.0031	0.48
		nPD:	0.01
		N. weight:	0.89
		Sc. PD:	-0.047
		Sc. rank:	-319.5

,

M1			
Pos.	41	obs:	exp:
tct	S	8	1.27
tcc	S	0	1.04
tca	S	0	2.03
tcg	S	0	0.45
act	T	2	0.51
acc	T	0	0.41
aca	T	0	0.92
acg	T	0	0.16
agt	S	0	1.64
agc	S	0	1.55
gtt	V	1	0.21
gtc	V	0	0.20
gta	V	0	0.20
gtg	V	0	0.39
gct	A	1281	331.80
gcc	A	4	239.30
gca	A	0	604.80
gcg	A	0	109.10
---	--	------	------
mPD		0.023	0.69
		nPD:	0.03
		N. weight:	1.3
		Sc. PD:	-0.035
		Sc. rank:	535.4

,

M1			
Pos.	42	obs:	exp:
tta	L	0	115.00
ttg	L	0	241.30
ctt	L	0	215.80
ctc	L	1293	216.30
cta	L	3	218.40
ctg	L	0	289.20
---	--	------	------
mPD		0.0046	1.1
		nPD:	0.
		N. weight:	1.7
		Sc. PD:	-0.094
		Sc. rank:	-807.2

,

M1			
Pos.	43	obs:	exp:
tta	L	0	0.18
ttg	L	0	0.37
ctt	L	0	0.33
ctc	L	0	0.33
cta	L	0	0.34
ctg	L	2	0.45
atg	M	1294	1294.00
---	--	------	------
mPD		0.0031	0.0049
		nPD:	0.63
		N. weight:	0.0022
		Sc. PD:	0.0012
		Sc. rank:	2.5

,

M1			
Pos.	44	obs:	exp:
gaa	E	99	788.60
gag	E	1197	507.40
---	--	------	------
mPD		0.14	0.48
		nPD:	0.3
		N. weight:	0.61
		Sc. PD:	0.14
		Sc. rank:	660.2

,

M1			
Pos.	45	obs:	exp:
tgg	W	1296	1296.00
---	--	------	------
mPD		0	0
		nPD:	1.
		N. weight:	0.
		Sc. PD:	0
		Sc. rank:	0

,

M1			
Pos.	46	obs:	exp:
tta	L	0	0.09
ttg	L	0	0.19
ctt	L	0	0.17
ctc	L	0	0.17
cta	L	1	0.17
ctg	L	0	0.22
att	I	0	475.40
atc	I	0	310.10
ata	I	1294	508.50
act	T	0	0.25
acc	T	0	0.21
aca	T	1	0.46
acg	T	0	0.08
---	--	------	------
mPD		0.0031	0.66
		nPD:	0.
		N. weight:	0.9
		Sc. PD:	-0.049
		Sc. rank:	-379.0

,

M1			
Pos.	47	obs:	exp:
aaa	K	1	755.30
aag	K	1295	540.70
---	--	------	------
mPD		0.0015	0.49
		nPD:	0.
		N. weight:	0.83
		Sc. PD:	-0.047
		Sc. rank:	-428.0

,

M1			
Pos.	48	obs:	exp:
act	T	0	330.40
acc	T	0	265.90
aca	T	1296	594.40
acg	T	0	105.30
---	--	------	------
mPD		0	0.68
		nPD:	0.
		N. weight:	0.75
		Sc. PD:	-0.044
		Sc. rank:	-851.8

,

M1			
Pos.	49	obs:	exp:
cgt	R	0	55.66
cgc	R	0	64.19
cga	R	0	126.80
cgg	R	0	108.10
aga	R	1295	592.60
agg	R	1	348.60
---	--	------	------
mPD		0.0015	0.96
		nPD:	0.
		N. weight:	0.75
		Sc. PD:	-0.043
		Sc. rank:	-534.7

,

M1			
Pos.	50	obs:	exp:
cct	P	0	323.40
ccc	P	0	232.90
cca	P	1296	533.50
ccg	P	0	206.30
---	--	------	------
mPD		0	0.71
		nPD:	0.
		N. weight:	0.85
		Sc. PD:	-0.05
		Sc. rank:	-970.1

,

M1

Pos. 51		obs:	exp:
att	I	1	476.20
atc	I	1295	310.60
ata	I	0	509.30
mPD		0.0015	0.65
		nPD:	0.
		N. weight:	1.4
		Sc. PD:	-0.078
		Sc. rank:	-791.0

M1

Pos. 52		obs:	exp:
tta	L	0	115.00
ttg	L	36	241.30
ctt	L	0	215.80
ctc	L	0	216.30
cta	L	0	218.40
ctg	L	1260	289.20
mPD		0.054	1.1
		nPD:	0.05
		N. weight:	1.3
		Sc. PD:	-0.017
		Sc. rank:	767.2

M1

Pos. 53		obs:	exp:
tct	S	0	206.10
tcc	S	0	169.30
tca	S	1296	328.90
tcg	S	0	73.54
agt	S	0	266.40
agc	S	0	251.70
mPD		0	1.7
		nPD:	0.
		N. weight:	1.3
		Sc. PD:	-0.078
		Sc. rank:	-1498.5

M1

Pos. 54		obs:	exp:
cct	P	1290	323.40
ccc	P	0	232.90
cca	P	6	533.50
ccg	P	0	206.30
mPD		0.0092	0.71
		nPD:	0.01
		N. weight:	1.3
		Sc. PD:	-0.061
		Sc. rank:	5.2

M1

Pos. 55		obs:	exp:
tta	L	0	115.00
ttg	L	1	241.30
ctt	L	0	215.80
ctc	L	0	216.30
cta	L	0	218.40
ctg	L	1295	289.20
mPD		0.0015	1.1
		nPD:	0.
		N. weight:	1.4
		Sc. PD:	-0.083
		Sc. rank:	-1090.9

M1

Pos. 56		obs:	exp:
act	T	1293	330.40
acc	T	0	265.90
aca	T	0	594.40
acg	T	3	105.30
mPD		0.0046	0.68
		nPD:	0.01
		N. weight:	1.3
		Sc. PD:	-0.068
		Sc. rank:	-418.4

M1

Pos. 57		obs:	exp:
aaa	K	32	755.30
aag	K	1264	540.70
mPD		0.048	0.49
		nPD:	0.1
		N. weight:	0.72
		Sc. PD:	0.026
		Sc. rank:	616.2

M1

Pos. 58		obs:	exp:
ggt	G	0	171.80
ggc	G	0	165.60
gga	G	2	597.90
ggg	G	1294	360.80
mPD		0.0031	0.68
		nPD:	0.
		N. weight:	1.2
		Sc. PD:	-0.067
		Sc. rank:	-524.0

M1

Pos. 59		obs:	exp:
att	I	1293	475.10
atc	I	0	309.90
ata	I	0	508.10
atg	M	1	1.00
gtt	V	2	0.41
gtc	V	0	0.40
gta	V	0	0.41
gtg	V	0	0.77
mPD		0.0046	0.66
		nPD:	0.01
		N. weight:	0.96
		Sc. PD:	-0.05
		Sc. rank:	-271.4

M1

Pos. 60		obs:	exp:
tta	L	1241	115.00
ttg	L	55	241.30
ctt	L	0	215.80
ctc	L	0	216.30
cta	L	0	218.40
ctg	L	0	289.20
mPD		0.081	1.1
		nPD:	0.07
		N. weight:	2.1
		Sc. PD:	0.023
		Sc. rank:	1644.4

M1

Pos. 61		obs:	exp:
ggt	G	1	171.80
ggc	G	0	165.60
gga	G	60	597.90
ggg	G	1235	360.80
mPD		0.090	0.68
		nPD:	0.13
		N. weight:	1.
		Sc. PD:	0.069
		Sc. rank:	975.1

M1

Pos. 62		obs:	exp:
ttt	F	1295	511.80
ttc	F	1	784.20
mPD		0.0015	0.48
		nPD:	0.
		N. weight:	0.89
		Sc. PD:	-0.05
		Sc. rank:	-443.3

M1

Pos. 63		obs:	exp:
gtt	V	1	268.50
gtc	V	0	261.60
gta	V	0	265.40
gtg	V	1295	500.50
mPD		0.0015	0.73
		nPD:	0.
		N. weight:	0.91
		Sc. PD:	-0.052
		Sc. rank:	-604.9

M1

Pos. 64		obs:	exp:
ttt	F	12	511.80
ttc	F	1284	784.20
mPD		0.018	0.48
		nPD:	0.04
		N. weight:	0.44
		Sc. PD:	-0.0098
		Sc. rank:	202.3

M1

Pos. 65		obs:	exp:
act	T	0	330.40
acc	T	0	265.90
aca	T	1	594.40
acg	T	1295	105.30
mPD		0.0015	0.68
		nPD:	0.
		N. weight:	2.4
		Sc. PD:	-0.14
		Sc. rank:	-1510.2

M1

Pos. 66		obs:	exp:
tta	L	0	115.00
ttg	L	0	241.30
ctt	L	0	215.80
ctc	L	1296	216.30
cta	L	0	218.40
ctg	L	0	289.20
mPD		0	1.1
		nPD:	0.
		N. weight:	1.7
		Sc. PD:	-0.1
		Sc. rank:	-1956.8

M1

Pos. 67		obs:	exp:
act	T	0	330.40
acc	T	1296	265.90
aca	T	0	594.40
acg	T	0	105.30
mPD		0	0.68
		nPD:	0.
		N. weight:	1.5
		Sc. PD:	-0.09
		Sc. rank:	-1730.9

M1

Pos. 68		obs:	exp:
gtt	V	0	268.50
gtc	V	0	261.60
gta	V	0	265.40
gtg	V	1296	500.50
mPD		0	0.73
		nPD:	0.
		N. weight:	0.91
		Sc. PD:	-0.054
		Sc. rank:	-1039.7

M1

Pos. 69		obs:	exp:
tct	S	0	0.16
tcc	S	1	0.13
tca	S	0	0.25
tcg	S	0	0.06
cct	P	0	323.10
ccc	P	1295	232.70
cca	P	0	533.10
ccg	P	0	206.10
agt	S	0	0.21
agc	S	0	0.19
mPD		0.0015	0.71
		nPD:	0.
		N. weight:	1.6
		Sc. PD:	-0.094
		Sc. rank:	-1072.4

M1

Pos. 70		obs:	exp:
tct	S	0	206.10
tcc	S	0	169.30
tca	S	0	328.90
tcg	S	0	73.54
agt	S	1296	266.40
agc	S	0	251.70
mPD		0	1.7
		nPD:	0.
		N. weight:	1.5
		Sc. PD:	-0.09
		Sc. rank:	-1728.9

M1

Pos. 71		obs:	exp:
gaa	E	0	788.60
gag	E	1296	507.40
mPD		0	0.48
		nPD:	0.
		N. weight:	0.9
		Sc. PD:	-0.053
		Sc. rank:	-1024.9

M1

Pos. 72		obs:	exp:
cgt	R	1	55.66
cgc	R	0	64.19
cga	R	1295	126.80
cgg	R	0	108.10
aga	R	0	592.60
agg	R	0	348.60
mPD		0.0015	0.96
		nPD:	0.
		N. weight:	2.2
		Sc. PD:	-0.13
		Sc. rank:	-1595.3

M1

Pos. 73		obs:	exp:
ggt	G	0	171.80
ggc	G	0	165.60
gga	G	1296	597.90
ggg	G	0	360.80
mPD		0	0.68
		nPD:	0.
		N. weight:	0.74
		Sc. PD:	-0.044
		Sc. rank:	-845.5

M1

Pos. 74		obs:	exp:
tta	L	0	115.00
ttg	L	0	241.30
ctt	L	0	215.80
ctc	L	0	216.30
cta	L	0	218.40
ctg	L	1296	289.20
mPD		0	1.1
		nPD:	0.
		N. weight:	1.4
		Sc. PD:	-0.085
		Sc. rank:	-1639.2

M1

Pos. 75		obs:	exp:
caa	Q	1	660.10
cag	Q	1295	635.90
mPD		0.0015	0.50
		nPD:	0.
		N. weight:	0.68
		Sc. PD:	-0.038
		Sc. rank:	-361.4

M1

Pos. 76		obs:	exp:
cgt	R	1296	55.66
cgc	R	0	64.19
cga	R	0	126.80
cgg	R	0	108.10
aga	R	0	592.60
agg	R	0	348.60
mPD		0	0.96
		nPD:	0.
		N. weight:	3.
		Sc. PD:	-0.18
		Sc. rank:	-3440.1

M1

Pos. 77		obs:	exp:
cgt	R	0	55.66
cgc	R	0	64.19
cga	R	0	126.80
cgg	R	0	108.10
aga	R	1296	592.60
agg	R	0	348.60
mPD		0	0.96
		nPD:	0.
		N. weight:	0.75
		Sc. PD:	-0.044
		Sc. rank:	-855.2

M1

Pos. 78		obs:	exp:
cgt	R	84	55.66
cgc	R	0	64.19
cga	R	2	126.80
cgg	R	1210	108.10
aga	R	0	592.60
agg	R	0	348.60
mPD		0.12	0.96
		nPD:	0.13
		N. weight:	2.2
		Sc. PD:	0.14
		Sc. rank:	2041.6

M1

Pos. 79		obs:	exp:
ttt	F	1293	511.80
ttc	F	3	784.20
mPD		0.0046	0.48
		nPD:	0.01
		N. weight:	0.88
		Sc. PD:	-0.044
		Sc. rank:	-152.5

M1

Pos. 80		obs:	exp:
att	I	0	0.37
atc	I	1	0.24
ata	I	0	0.39
gtt	V	99	268.30
gtc	V	1196	261.40
gta	V	0	265.20
gtg	V	0	500.10
mPD		0.14	0.73
		nPD:	0.2
		N. weight:	1.3
		Sc. PD:	0.16
		Sc. rank:	1311.7

M1			
Pos.	81	obs:	exp:
caa	Q	1296	660.10
cag	Q	0	635.90
---	--	------	------
mPD		0	0.50
		nPD:	0.
		N. weight:	0.65
		Sc. PD:	-0.038
		Sc. rank:	-737.3

,

M1			
Pos.	82	obs:	exp:
tct	S	0	0.80
tcc	S	0	0.65
tca	S	0	1.27
tcg	S	0	0.28
act	T	0	5.61
acc	T	22	4.51
aca	T	0	10.09
acg	T	0	1.79
aat	N	48	646.40
aac	N	1221	622.60
agt	S	5	1.03
agc	S	0	0.97
---	--	------	------
mPD		0.12	0.56
		nPD:	0.22
		N. weight:	0.55
		Sc. PD:	0.08
		Sc. rank:	574.8

,

M1			
Pos.	83	obs:	exp:
gct	A	1	334.70
gcc	A	1295	241.40
gca	A	0	610.00
gcg	A	0	110.00
---	--	------	------
mPD		0.0015	0.67
		nPD:	0.
		N. weight:	1.6
		Sc. PD:	-0.092
		Sc. rank:	-969.0

,

M1			
Pos.	84	obs:	exp:
tta	L	2	115.00
ttg	L	0	241.30
ctt	L	0	215.80
ctc	L	0	216.30
cta	L	1294	218.40
ctg	L	0	289.20
---	--	------	------
mPD		0.0031	1.1
		nPD:	0.
		N. weight:	1.7
		Sc. PD:	-0.096
		Sc. rank:	-957.9

,

M1			
Pos.	85	obs:	exp:
aat	N	1292	660.10
aac	N	4	635.90
---	--	------	------
mPD		0.0062	0.50
		nPD:	0.01
		N. weight:	0.63
		Sc. PD:	-0.03
		Sc. rank:	-13.2

,

M1			
Pos.	86	obs:	exp:
ggt	G	5	171.80
ggc	G	0	165.60
gga	G	7	597.90
ggg	G	1284	360.80
---	--	------	------
mPD		0.018	0.68
		nPD:	0.03
		N. weight:	1.2
		Sc. PD:	-0.039
		Sc. rank:	389.8

,

M1			
Pos.	87	obs:	exp:
aat	N	1293	660.10
aac	N	3	635.90
---	--	------	------
mPD		0.0046	0.50
		nPD:	0.01
		N. weight:	0.63
		Sc. PD:	-0.032
		Sc. rank:	-147.3

,

M1			
Pos.	88	obs:	exp:
ggt	G	0	171.80
ggc	G	1	165.60
gga	G	1250	597.90
ggg	G	45	360.80
---	--	------	------
mPD		0.069	0.68
		nPD:	0.1
		N. weight:	0.61
		Sc. PD:	0.023
		Sc. rank:	526.1

,

M1			
Pos.	89	obs:	exp:
gat	D	0	700.30
gac	D	1296	595.70
---	--	------	------
mPD		0	0.50
		nPD:	0.
		N. weight:	0.75
		Sc. PD:	-0.044
		Sc. rank:	-849.6

,

M1			
Pos.	90	obs:	exp:
cct	P	0	323.40
ccc	P	0	232.90
cca	P	1265	533.50
ccg	P	31	206.30
---	--	------	------
mPD		0.047	0.71
		nPD:	0.07
		N. weight:	0.77
		Sc. PD:	0.0028
		Sc. rank:	573.6

,

M1			
Pos.	91	obs:	exp:
aat	N	1	660.10
aac	N	1295	635.90
---	--	------	------
mPD		0.0015	0.50
		nPD:	0.
		N. weight:	0.68
		Sc. PD:	-0.038
		Sc. rank:	-365.6

,

M1			
Pos.	92	obs:	exp:
aat	N	15	660.10
aac	N	1281	635.90
---	--	------	------
mPD		0.023	0.50
		nPD:	0.05
		N. weight:	0.62
		Sc. PD:	-0.0097
		Sc. rank:	345.6

,

M1			
Pos.	93	obs:	exp:
atg	M	1296	1296.00
---	--	------	------
mPD		0	0
		nPD:	1.
		N. weight:	0.
		Sc. PD:	0
		Sc. rank:	0

,

M1			
Pos.	94	obs:	exp:
gat	D	10	700.30
gac	D	1286	595.70
---	--	------	------
mPD		0.015	0.50
		nPD:	0.03
		N. weight:	0.7
		Sc. PD:	-0.021
		Sc. rank:	264.4

,

M1			
Pos.	95	obs:	exp:
cgt	R	0	0.64
cgc	R	0	0.74
cga	R	0	1.47
cgg	R	0	1.25
aaa	K	6	746.60
aag	K	1275	534.40
aga	R	0	6.86
agg	R	15	4.03
---	--	------	------
mPD		0.032	0.52
		nPD:	0.06
		N. weight:	0.82
		Sc. PD:	0.00015
		Sc. rank:	603.8

,

M1			
Pos.	96	obs:	exp:
gct	A	1	334.70
gcc	A	0	241.40
gca	A	813	610.00
gcg	A	482	110.00
---	--	------	------
mPD		0.47	0.67
		nPD:	0.7
		N. weight:	0.7
		Sc. PD:	0.43
		Sc. rank:	811.9

,

M1			
Pos.	97	obs:	exp:
gtt	V	1251	268.50
gtc	V	6	261.60
gta	V	39	265.40
gtg	V	0	500.50
---	--	------	------
mPD		0.067	0.73
		nPD:	0.09
		N. weight:	1.4
		Sc. PD:	0.04
		Sc. rank:	1100.9

,

M1			
Pos.	98	obs:	exp:
aaa	K	1279	755.30
aag	K	17	540.70
---	--	------	------
mPD		0.026	0.49
		nPD:	0.05
		N. weight:	0.46
		Sc. PD:	-0.0038
		Sc. rank:	292.4

,

M1			
Pos.	99	obs:	exp:
tta	L	1184	115.00
ttg	L	97	241.30
ctt	L	0	215.80
ctc	L	0	216.30
cta	L	12	218.40
ctg	L	3	289.20
---	--	------	------
mPD		0.17	1.1
		nPD:	0.15
		N. weight:	2.
		Sc. PD:	0.16
		Sc. rank:	1893.2

,

M1			
Pos.	100	obs:	exp:
tat	Y	12	700.00
tac	Y	1284	596.00
---	--	------	------
mPD		0.018	0.50
		nPD:	0.04
		N. weight:	0.7
		Sc. PD:	-0.017
		Sc. rank:	313.1

,

M1			
Pos.	101	obs:	exp:
cgt	R	0	0.04
cgc	R	0	0.05
cga	R	0	0.10
cgg	R	0	0.08
aaa	K	1	754.80
aag	K	1294	540.20
aga	R	0	0.46
agg	R	1	0.27
---	--	------	------
mPD		0.0031	0.49
		nPD:	0.01
		N. weight:	0.83
		Sc. PD:	-0.044
		Sc. rank:	-312.9

,

M1			
Pos.	102	obs:	exp:
aaa	K	1282	755.30
aag	K	14	540.70
---	--	------	------
mPD		0.021	0.49
		nPD:	0.04
		N. weight:	0.47
		Sc. PD:	-0.008
		Sc. rank:	250.4

,

M1			
Pos.	103	obs:	exp:
tta	L	0	115.00
ttg	L	4	241.30
ctt	L	1	215.80
ctc	L	0	216.30
cta	L	3	218.40
ctg	L	1288	289.20
---	--	------	------
mPD		0.012	1.1
		nPD:	0.01
		N. weight:	1.4
		Sc. PD:	-0.068
		Sc. rank:	-145.1

,

M1			
Pos.	104	obs:	exp:
aaa	K	5	755.30
aag	K	1291	540.70
---	--	------	------
mPD		0.0077	0.49
		nPD:	0.02
		N. weight:	0.82
		Sc. PD:	-0.036
		Sc. rank:	54.9

,

M1			
Pos.	105	obs:	exp:
cgt	R	0	55.66
cgc	R	0	64.19
cga	R	0	126.80
cgg	R	0	108.10
aga	R	36	592.60
agg	R	1260	348.60
---	--	------	------
mPD		0.054	0.96
		nPD:	0.06
		N. weight:	1.1
		Sc. PD:	-0.0062
		Sc. rank:	807.6

,

M1			
Pos.	106	obs:	exp:
gaa	E	1291	788.60
gag	E	5	507.40
---	--	------	------
mPD		0.0077	0.48
		nPD:	0.02
		N. weight:	0.46
		Sc. PD:	-0.02
		Sc. rank:	57.4

,

M1			
Pos.	107	obs:	exp:
att	I	0	0.37
atc	I	0	0.24
ata	I	1	0.39
atg	M	1295	1295.00
---	--	------	------
mPD		0.0015	0.0015
		nPD:	1.
		N. weight:	0.00069
		Sc. PD:	0.00062
		Sc. rank:	0.8

,

M1			
Pos.	108	obs:	exp:
act	T	4	330.40
acc	T	55	265.90
aca	T	1225	594.40
acg	T	12	105.30
---	--	------	------
mPD		0.10	0.68
		nPD:	0.15
		N. weight:	0.57
		Sc. PD:	0.05
		Sc. rank:	554.2

,

M1			
Pos.	109	obs:	exp:
ttt	F	1201	511.80
ttc	F	95	784.20
---	--	------	------
mPD		0.14	0.48
		nPD:	0.28
		N. weight:	0.61
		Sc. PD:	0.13
		Sc. rank:	655.1

,

M1			
Pos.	110	obs:	exp:
cat	H	1295	744.20
cac	H	1	551.80
---	--	------	------
mPD		0.0015	0.49
		nPD:	0.
		N. weight:	0.53
		Sc. PD:	-0.03
		Sc. rank:	-274.9

,

M1

Pos.	111	obs:	exp:
ggt	G	0	171.80
ggc	G	0	165.60
gga	G	1285	597.90
ggg	G	11	360.80
mPD		0.017	0.68
		nPD:	0.02
		N. weight:	0.7
		Sc. PD:	-0.025
		Sc. rank:	198.0

M1

Pos.	112	obs:	exp:
gct	A	0	334.70
gcc	A	1	241.40
gca	A	1288	610.00
gcg	A	7	110.00
mPD		0.012	0.67
		nPD:	0.02
		N. weight:	0.7
		Sc. PD:	-0.029
		Sc. rank:	116.9

M1

Pos.	113	obs:	exp:
aaa	K	13	755.30
aag	K	1283	540.70
mPD		0.020	0.49
		nPD:	0.04
		N. weight:	0.78
		Sc. PD:	-0.016
		Sc. rank:	383.1

M1

Pos.	114	obs:	exp:
gaa	E	1246	788.60
gag	E	50	507.40
mPD		0.074	0.48
		nPD:	0.16
		N. weight:	0.34
		Sc. PD:	0.03
		Sc. rank:	333.4

M1

Pos.	115	obs:	exp:
att	I	1	0.37
atc	I	0	0.24
ata	I	0	0.39
gtt	V	1291	268.30
gtc	V	3	261.40
gta	V	1	265.20
gtg	V	0	500.10
mPD		0.0077	0.73
		nPD:	0.01
		N. weight:	1.5
		Sc. PD:	-0.073
		Sc. rank:	-173.6

M1

Pos.	116	obs:	exp:
tct	S	0	0.16
tcc	S	0	0.13
tca	S	1	0.25
tcg	S	0	0.06
agt	S	0	0.21
agc	S	0	0.19
gct	A	0	334.40
gcc	A	0	241.20
gca	A	1293	609.50
gcg	A	2	109.90
mPD		0.0046	0.67
		nPD:	0.01
		N. weight:	0.72
		Sc. PD:	-0.038
		Sc. rank:	-217.2

M1

Pos.	117	obs:	exp:
tta	L	0	115.00
ttg	L	0	241.30
ctt	L	13	215.80
ctc	L	1256	216.30
cta	L	27	218.40
ctg	L	0	289.20
mPD		0.060	1.1
		nPD:	0.05
		N. weight:	1.6
		Sc. PD:	-0.011
		Sc. rank:	1027.8

M1

Pos.	118	obs:	exp:
tct	S	0	205.80
tcc	S	0	169.00
tca	S	0	328.40
tcg	S	0	73.43
agt	S	1279	266.00
agc	S	15	251.30
ggt	G	2	0.27
ggc	G	0	0.26
gga	G	0	0.92
ggg	G	0	0.56
mPD		0.026	1.7
		nPD:	0.02
		N. weight:	1.5
		Sc. PD:	-0.065
		Sc. rank:	87.5

M1

Pos.	119	obs:	exp:
tat	Y	6	700.00
tac	Y	1290	596.00
mPD		0.0092	0.50
		nPD:	0.02
		N. weight:	0.72
		Sc. PD:	-0.03
		Sc. rank:	129.6

M1

Pos.	120	obs:	exp:
tct	S	0	206.10
tcc	S	0	169.30
tca	S	1295	328.90
tcg	S	1	73.54
agt	S	0	266.40
agc	S	0	251.70
mPD		0.0015	1.7
		nPD:	0.
		N. weight:	1.3
		Sc. PD:	-0.077
		Sc. rank:	-1017.4

M1

Pos.	121	obs:	exp:
act	T	1293	330.40
acc	T	3	265.90
aca	T	0	594.40
acg	T	0	105.30
mPD		0.0046	0.68
		nPD:	0.01
		N. weight:	1.3
		Sc. PD:	-0.068
		Sc. rank:	-417.8

M1

Pos.	122	obs:	exp:
ggt	G	1296	171.80
ggc	G	0	165.60
gga	G	0	597.90
ggg	G	0	360.80
mPD		0	0.68
		nPD:	0.
		N. weight:	1.9
		Sc. PD:	-0.11
		Sc. rank:	-2208.5

M1

Pos.	123	obs:	exp:
gct	A	0	334.70
gcc	A	2	241.40
gca	A	76	610.00
gcg	A	1218	110.00
mPD		0.11	0.67
		nPD:	0.17
		N. weight:	2.1
		Sc. PD:	0.21
		Sc. rank:	2078.7

M1

Pos.	124	obs:	exp:
tta	L	0	115.00
ttg	L	0	241.30
ctt	L	1286	215.80
ctc	L	6	216.30
cta	L	0	218.40
ctg	L	4	289.20
mPD		0.015	1.1
		nPD:	0.01
		N. weight:	1.7
		Sc. PD:	-0.077
		Sc. rank:	32.7

M1

Pos.	125	obs:	exp:
act	T	0	0.76
acc	T	3	0.62
aca	T	0	1.38
acg	T	0	0.24
gct	A	7	333.90
gcc	A	1286	240.80
gca	A	0	608.60
gcg	A	0	109.70
mPD		0.015	0.67
		nPD:	0.02
		N. weight:	1.6
		Sc. PD:	-0.059
		Sc. rank:	396.3

M1

Pos.	126	obs:	exp:
tct	S	0	205.80
tcc	S	0	169.00
tca	S	0	328.40
tcg	S	0	73.43
agt	S	8	266.00
agc	S	1286	251.30
ggt	G	0	0.27
ggc	G	2	0.26
gga	G	0	0.92
ggg	G	0	0.56
mPD		0.015	1.7
		nPD:	0.01
		N. weight:	1.5
		Sc. PD:	-0.077
		Sc. rank:	-318.0

M1

Pos.	127	obs:	exp:
tgt	C	9	548.20
tgc	C	1287	747.80
mPD		0.014	0.49
		nPD:	0.03
		N. weight:	0.49
		Sc. PD:	-0.016
		Sc. rank:	173.0

M1

Pos.	128	obs:	exp:
atg	M	1296	1296.00
mPD		0	0
		nPD:	1.
		N. weight:	0.
		Sc. PD:	0
		Sc. rank:	0

M1

Pos.	129	obs:	exp:
ggt	G	1289	171.80
ggc	G	7	165.60
gga	G	0	597.90
ggg	G	0	360.80
mPD		0.011	0.68
		nPD:	0.02
		N. weight:	1.9
		Sc. PD:	-0.084
		Sc. rank:	175.3

M1

Pos.	130	obs:	exp:
tta	L	0	115.00
ttg	L	0	241.30
ctt	L	0	215.80
ctc	L	1293	216.30
cta	L	3	218.40
ctg	L	0	289.20
mPD		0.0046	1.1
		nPD:	0.
		N. weight:	1.7
		Sc. PD:	-0.094
		Sc. rank:	-807.2

M1

Pos.	131	obs:	exp:
att	I	1	476.20
atc	I	3	310.60
ata	I	1292	509.30
mPD		0.0062	0.65
		nPD:	0.01
		N. weight:	0.88
		Sc. PD:	-0.044
		Sc. rank:	-166.5

M1

Pos.	132	obs:	exp:
tat	Y	2	700.00
tac	Y	1294	596.00
mPD		0.0031	0.50
		nPD:	0.01
		N. weight:	0.74
		Sc. PD:	-0.039
		Sc. rank:	-296.6

M1

Pos.	133	obs:	exp:
aat	N	12	660.10
aac	N	1284	635.90
mPD		0.018	0.50
		nPD:	0.04
		N. weight:	0.63
		Sc. PD:	-0.015
		Sc. rank:	281.5

M1

Pos.	134	obs:	exp:
cgt	R	0	55.66
cgc	R	0	64.19
cga	R	0	126.80
cgg	R	0	108.10
aga	R	501	592.60
agg	R	795	348.60
mPD		0.47	0.96
		nPD:	0.49
		N. weight:	0.43
		Sc. PD:	0.18
		Sc. rank:	470.4

M1

Pos.	135	obs:	exp:
atg	M	1296	1296.00
mPD		0	0
		nPD:	1.
		N. weight:	0.
		Sc. PD:	0
		Sc. rank:	0

M1			
Pos.	136	obs:	exp:
ggt	G	0	171.80
ggc	G	0	165.60
gga	G	20	597.90
ggg	G	1276	360.80
mPD		0.030	0.68
		nPD:	0.05
		N. weight:	1.1
		Sc. PD:	-0.019
		Sc. rank:	627.4

M1			
Pos.	137	obs:	exp:
act	T	480	330.40
acc	T	1	265.90
aca	T	813	594.40
acg	T	2	105.30
mPD		0.47	0.68
		nPD:	0.69
		N. weight:	0.32
		Sc. PD:	0.19
		Sc. rank:	364.5

M1			
Pos.	138	obs:	exp:
gtt	V	0	268.50
gtc	V	4	261.60
gta	V	776	265.40
gtg	V	516	500.50
mPD		0.48	0.73
		nPD:	0.67
		N. weight:	0.62
		Sc. PD:	0.36
		Sc. rank:	703.9

M1			
Pos.	139	obs:	exp:
act	T	705	329.90
acc	T	586	265.50
aca	T	3	593.50
acg	T	0	105.10
gct	A	0	0.52
gcc	A	0	0.37
gca	A	2	0.94
gcg	A	0	0.17
mPD		0.50	0.68
		nPD:	0.74
		N. weight:	0.73
		Sc. PD:	0.48
		Sc. rank:	863.5

M1			
Pos.	140	obs:	exp:
act	T	0	0.51
acc	T	0	0.41
aca	T	2	0.92
acg	T	0	0.16
gct	A	0	334.20
gcc	A	7	241.00
gca	A	1287	609.00
gcg	A	0	109.80
mPD		0.014	0.67
		nPD:	0.02
		N. weight:	0.7
		Sc. PD:	-0.028
		Sc. rank:	146.6

M1			
Pos.	141	obs:	exp:
gaa	E	1295	788.60
gag	E	1	507.40
mPD		0.0015	0.48
		nPD:	0.
		N. weight:	0.47
		Sc. PD:	-0.026
		Sc. rank:	-231.0

M1			
Pos.	142	obs:	exp:
gtt	V	0	0.21
gtc	V	0	0.20
gta	V	1	0.20
gtg	V	0	0.39
ggt	G	0	171.60
ggc	G	0	165.40
gga	G	1	597.40
ggg	G	1294	360.50
mPD		0.0046	0.68
		nPD:	0.01
		N. weight:	1.2
		Sc. PD:	-0.064
		Sc. rank:	-408.1

M1			
Pos.	143	obs:	exp:
gct	A	1289	334.70
gcc	A	7	241.40
gca	A	0	610.00
gcg	A	0	110.00
mPD		0.011	0.67
		nPD:	0.02
		N. weight:	1.3
		Sc. PD:	-0.056
		Sc. rank:	131.6

M1			
Pos.	144	obs:	exp:
ttt	F	0	0.39
ttc	F	1	0.61
tta	L	0	114.90
ttg	L	0	241.10
ctt	L	1294	215.60
ctc	L	1	216.10
cta	L	0	218.20
ctg	L	0	289.00
mPD		0.0046	1.1
		nPD:	0.
		N. weight:	1.7
		Sc. PD:	-0.095
		Sc. rank:	-757.8

M1			
Pos.	145	obs:	exp:
ggt	G	0	171.80
ggc	G	1	165.60
gga	G	1284	597.90
ggg	G	11	360.80
mPD		0.018	0.68
		nPD:	0.03
		N. weight:	0.7
		Sc. PD:	-0.023
		Sc. rank:	223.9

M1			
Pos.	146	obs:	exp:
tta	L	65	115.00
ttg	L	639	241.30
ctt	L	0	215.80
ctc	L	0	216.30
cta	L	587	218.40
ctg	L	5	289.20
mPD		1.0	1.1
		nPD:	0.9
		N. weight:	0.86
		Sc. PD:	0.68
		Sc. rank:	1019.5

M1			
Pos.	147	obs:	exp:
gtt	V	1	268.50
gtc	V	1	261.60
gta	V	1274	265.40
gtg	V	20	500.50
mPD		0.033	0.73
		nPD:	0.05
		N. weight:	1.4
		Sc. PD:	-0.022
		Sc. rank:	809.3

M1			
Pos.	148	obs:	exp:
tgt	C	1294	548.20
tgc	C	2	747.80
mPD		0.0031	0.49
		nPD:	0.01
		N. weight:	0.82
		Sc. PD:	-0.043
		Sc. rank:	-312.2

M1			
Pos.	149	obs:	exp:
gct	A	14	334.70
gcc	A	1282	241.40
gca	A	0	610.00
gcg	A	0	110.00
mPD		0.021	0.67
		nPD:	0.03
		N. weight:	1.6
		Sc. PD:	-0.045
		Sc. rank:	629.3

M1			
Pos.	150	obs:	exp:
att	I	4	1.47
atc	I	0	0.96
ata	I	0	1.57
act	T	1235	329.30
acc	T	55	265.10
aca	T	2	592.60
acg	T	0	104.90
mPD		0.090	0.68
		nPD:	0.13
		N. weight:	1.1
		Sc. PD:	0.077
		Sc. rank:	1078.7

M1			
Pos.	151	obs:	exp:
tgt	C	1293	548.20
tgc	C	3	747.80
mPD		0.0046	0.49
		nPD:	0.01
		N. weight:	0.81
		Sc. PD:	-0.041
		Sc. rank:	-147.6

M1			
Pos.	152	obs:	exp:
gaa	E	18	788.60
gag	E	1278	507.40
mPD		0.027	0.48
		nPD:	0.06
		N. weight:	0.83
		Sc. PD:	-0.0035
		Sc. rank:	598.3

M1			
Pos.	153	obs:	exp:
cat	H	2	1.15
cac	H	0	0.85
caa	Q	8	659.10
cag	Q	1286	634.90
mPD		0.015	0.50
		nPD:	0.03
		N. weight:	0.65
		Sc. PD:	-0.019
		Sc. rank:	239.6

M1			
Pos.	154	obs:	exp:
att	I	1271	476.20
atc	I	25	310.60
ata	I	0	509.30
mPD		0.038	0.65
		nPD:	0.06
		N. weight:	0.88
		Sc. PD:	-0.0034
		Sc. rank:	644.4

M1			
Pos.	155	obs:	exp:
gct	A	1022	334.70
gcc	A	274	241.40
gca	A	0	610.00
gcg	A	0	110.00
mPD		0.33	0.67
		nPD:	0.5
		N. weight:	0.87
		Sc. PD:	0.36
		Sc. rank:	971.6

M1			
Pos.	156	obs:	exp:
gat	D	7	699.80
gac	D	1288	595.20
gaa	E	1	0.61
gag	E	0	0.39
mPD		0.012	0.50
		nPD:	0.02
		N. weight:	0.72
		Sc. PD:	-0.025
		Sc. rank:	197.0

M1			
Pos.	157	obs:	exp:
tct	S	0	0.32
tcc	S	0	0.26
tca	S	1	0.51
tcg	S	1	0.11
agt	S	0	0.41
agc	S	0	0.39
gct	A	4	334.20
gcc	A	0	241.00
gca	A	1288	609.00
gcg	A	2	109.80
mPD		0.014	0.67
		nPD:	0.02
		N. weight:	0.7
		Sc. PD:	-0.028
		Sc. rank:	142.7

M1			
Pos.	158	obs:	exp:
caa	Q	1221	660.10
cag	Q	75	635.90
mPD		0.11	0.50
		nPD:	0.22
		N. weight:	0.44
		Sc. PD:	0.066
		Sc. rank:	462.9

M1			
Pos.	159	obs:	exp:
cat	H	1292	744.20
cac	H	4	551.80
mPD		0.0062	0.49
		nPD:	0.01
		N. weight:	0.52
		Sc. PD:	-0.024
		Sc. rank:	-8.3

M1			
Pos.	160	obs:	exp:
cgt	R	0	55.66
cgc	R	0	64.19
cga	R	6	126.80
cgg	R	1285	108.10
aga	R	0	592.60
agg	R	5	348.60
mPD		0.017	0.96
		nPD:	0.02
		N. weight:	2.3
		Sc. PD:	-0.099
		Sc. rank:	375.6

M1			
Pos.	161	obs:	exp:
tct	S	3	206.10
tcc	S	1293	169.30
tca	S	0	328.90
tcg	S	0	73.54
agt	S	0	266.40
agc	S	0	251.70
mPD		0.0046	1.7
		nPD:	0.
		N. weight:	1.9
		Sc. PD:	-0.11
		Sc. rank:	-1057.6

M1			
Pos.	162	obs:	exp:
cat	H	2	744.20
cac	H	1294	551.80
mPD		0.0031	0.49
		nPD:	0.01
		N. weight:	0.81
		Sc. PD:	-0.043
		Sc. rank:	-315.9

M1			
Pos.	163	obs:	exp:
cgt	R	0	55.66
cgc	R	0	64.19
cga	R	0	126.80
cgg	R	0	108.10
aga	R	76	592.60
agg	R	1220	348.60
mPD		0.11	0.96
		nPD:	0.11
		N. weight:	1.
		Sc. PD:	0.052
		Sc. rank:	917.8

M1			
Pos.	164	obs:	exp:
caa	Q	74	660.10
cag	Q	1222	635.90
mPD		0.11	0.50
		nPD:	0.22
		N. weight:	0.47
		Sc. PD:	0.069
		Sc. rank:	496.6

M1

Pos.	165	obs:	exp:
att	I	0	2.94
atc	I	0	1.92
ata	I	8	3.14
atg	M	1288	1288.00
---	--	------	------
mPD		0.012	0.012
		nPD:	1.
		N. weight:	0.0055
		Sc. PD:	0.005
		Sc. rank:	6.6

,

M1

Pos.	166	obs:	exp:
gtt	V	0	0.21
gtc	V	0	0.20
gta	V	0	0.20
gtg	V	1	0.39
gct	A	3	334.40
gcc	A	0	241.20
gca	A	667	609.50
gcg	A	625	109.90
---	--	------	------
mPD		0.50	0.67
		nPD:	0.75
		N. weight:	0.84
		Sc. PD:	0.55
		Sc. rank:	999.4

,

M1

Pos.	167	obs:	exp:
att	I	4	1.47
atc	I	0	0.96
ata	I	0	1.57
act	T	1274	328.60
acc	T	14	264.50
aca	T	1	591.20
acg	T	0	104.70
aat	N	1	0.51
aac	N	0	0.49
gct	A	1	0.52
gcc	A	0	0.37
gca	A	1	0.94
gcg	A	0	0.17
---	--	------	------
mPD		0.035	0.69
		nPD:	0.05
		N. weight:	1.3
		Sc. PD:	-0.013
		Sc. rank:	752.9

,

M1

Pos.	168	obs:	exp:
tct	S	0	3.02
tcc	S	0	2.48
tca	S	0	4.82
tcg	S	0	1.08
act	T	1272	325.50
acc	T	5	262.00
aca	T	0	585.70
acg	T	0	103.70
agt	S	19	3.91
agc	S	0	3.69
---	--	------	------
mPD		0.037	0.71
		nPD:	0.05
		N. weight:	1.3
		Sc. PD:	-0.013
		Sc. rank:	821.5

,

M1

Pos.	169	obs:	exp:
att	I	0	0.37
atc	I	1	0.24
ata	I	0	0.39
act	T	438	329.30
acc	T	850	265.10
aca	T	4	592.60
acg	T	0	104.90
aat	N	0	1.53
aac	N	3	1.47
---	--	------	------
mPD		0.46	0.68
		nPD:	0.67
		N. weight:	0.82
		Sc. PD:	0.48
		Sc. rank:	934.7

,

M1

Pos.	170	obs:	exp:
aat	N	24	660.10
aac	N	1272	635.90
---	--	------	------
mPD		0.036	0.50
		nPD:	0.07
		N. weight:	0.6
		Sc. PD:	0.0061
		Sc. rank:	448.9

,

M1

Pos.	171	obs:	exp:
cct	P	0	323.40
ccc	P	0	232.90
cca	P	1282	533.50
ccg	P	14	206.30
---	--	------	------
mPD		0.021	0.71
		nPD:	0.03
		N. weight:	0.81
		Sc. PD:	-0.025
		Sc. rank:	293.1

,

M1

Pos.	172	obs:	exp:
tta	L	0	114.60
ttg	L	7	240.40
ctt	L	0	215.00
ctc	L	0	215.40
cta	L	1278	217.60
ctg	L	6	288.10
att	I	0	1.84
atc	I	0	1.20
ata	I	5	1.96
---	--	------	------
mPD		0.038	1.1
		nPD:	0.03
		N. weight:	1.6
		Sc. PD:	-0.044
		Sc. rank:	721.2

,

M1

Pos.	173	obs:	exp:
att	I	1254	476.20
atc	I	42	310.60
ata	I	0	509.30
---	--	------	------
mPD		0.063	0.65
		nPD:	0.1
		N. weight:	0.84
		Sc. PD:	0.027
		Sc. rank:	699.1

,

M1

Pos.	174	obs:	exp:
cgt	R	0	55.62
cgc	R	0	64.14
cga	R	0	126.70
cgg	R	0	108.10
aaa	K	0	0.58
aag	K	1	0.42
aga	R	15	592.10
agg	R	1280	348.30
---	--	------	------
mPD		0.024	0.96
		nPD:	0.03
		N. weight:	1.2
		Sc. PD:	-0.042
		Sc. rank:	350.7

,

M1

Pos.	175	obs:	exp:
cat	H	1296	744.20
cac	H	0	551.80
---	--	------	------
mPD		0	0.49
		nPD:	0.
		N. weight:	0.53
		Sc. PD:	-0.032
		Sc. rank:	-606.3

,

M1

Pos.	176	obs:	exp:
gaa	E	4	788.60
gag	E	1292	507.40
---	--	------	------
mPD		0.0062	0.48
		nPD:	0.01
		N. weight:	0.88
		Sc. PD:	-0.041
		Sc. rank:	-7.1

,

M1

Pos.	177	obs:	exp:
aat	N	1293	660.10
aac	N	3	635.90
---	--	------	------
mPD		0.0046	0.50
		nPD:	0.01
		N. weight:	0.63
		Sc. PD:	-0.032
		Sc. rank:	-147.3

,

M1

Pos.	178	obs:	exp:
cgt	R	0	55.66
cgc	R	0	64.19
cga	R	0	126.80
cgg	R	0	108.10
aga	R	1291	592.60
agg	R	5	348.60
---	--	------	------
mPD		0.0077	0.96
		nPD:	0.01
		N. weight:	0.73
		Sc. PD:	-0.038
		Sc. rank:	-199.5

,

M1

Pos.	179	obs:	exp:
atg	M	1296	1296.00
---	--	------	------
mPD		0	0
		nPD:	1.
		N. weight:	0.
		Sc. PD:	0
		Sc. rank:	0

,

M1

Pos.	180	obs:	exp:
gtt	V	43	268.50
gtc	V	0	261.60
gta	V	1239	265.40
gtg	V	14	500.50
---	--	------	------
mPD		0.085	0.73
		nPD:	0.12
		N. weight:	1.3
		Sc. PD:	0.07
		Sc. rank:	1207.7

,

M1

Pos.	181	obs:	exp:
tta	L	0	114.70
ttg	L	0	240.50
ctt	L	0	215.10
ctc	L	0	215.60
cta	L	1277	217.70
ctg	L	15	288.30
att	I	0	1.10
atc	I	0	0.72
ata	I	3	1.18
atg	M	1	1.00
---	--	------	------
mPD		0.031	1.1
		nPD:	0.03
		N. weight:	1.6
		Sc. PD:	-0.054
		Sc. rank:	571.1

,

M1

Pos.	182	obs:	exp:
gct	A	44	334.70
gcc	A	1252	241.40
gca	A	0	610.00
gcg	A	0	110.00
---	--	------	------
mPD		0.066	0.67
		nPD:	0.1
		N. weight:	1.5
		Sc. PD:	0.05
		Sc. rank:	1238.3

,

M1

Pos.	183	obs:	exp:
tct	S	0	206.10
tcc	S	0	169.30
tca	S	0	328.90
tcg	S	0	73.54
agt	S	693	266.40
agc	S	603	251.70
---	--	------	------
mPD		0.50	1.7
		nPD:	0.3
		N. weight:	0.88
		Sc. PD:	0.2
		Sc. rank:	961.8

,

M1

Pos.	184	obs:	exp:
act	T	1271	330.40
acc	T	18	265.90
aca	T	0	594.40
acg	T	7	105.30
---	--	------	------
mPD		0.038	0.68
		nPD:	0.06
		N. weight:	1.2
		Sc. PD:	-0.0067
		Sc. rank:	863.0

,

M1

Pos.	185	obs:	exp:
atg	M	2	2.00
act	T	12	329.90
acc	T	0	265.50
aca	T	9	593.50
acg	T	1273	105.10
---	--	------	------
mPD		0.035	0.68
		nPD:	0.05
		N. weight:	2.3
		Sc. PD:	-0.023
		Sc. rank:	1432.2

,

M1

Pos.	186	obs:	exp:
gct	A	1274	334.70
gcc	A	22	241.40
gca	A	0	610.00
gcg	A	0	110.00
---	--	------	------
mPD		0.033	0.67
		nPD:	0.05
		N. weight:	1.2
		Sc. PD:	-0.014
		Sc. rank:	722.9

,

M1

Pos.	187	obs:	exp:
aaa	K	99	755.30
aag	K	1197	540.70
---	--	------	------
mPD		0.14	0.49
		nPD:	0.29
		N. weight:	0.56
		Sc. PD:	0.12
		Sc. rank:	599.6

,

M1

Pos.	188	obs:	exp:
gct	A	1286	334.70
gcc	A	10	241.40
gca	A	0	610.00
gcg	A	0	110.00
---	--	------	------
mPD		0.015	0.67
		nPD:	0.02
		N. weight:	1.3
		Sc. PD:	-0.047
		Sc. rank:	330.7

,

M1

Pos.	189	obs:	exp:
atg	M	1296	1296.00
---	--	------	------
mPD		0	0
		nPD:	1.
		N. weight:	0.
		Sc. PD:	0
		Sc. rank:	0

,

M1			
Pos.	190	obs:	exp:
gaa	E	7	788.60
gag	E	1289	507.40
---	--	------	------
mPD		0.011	0.48
		nPD:	0.02
		N. weight:	0.87
		Sc. PD:	-0.033
		Sc. rank:	195.2

M1			
Pos.	191	obs:	exp:
caa	Q	2	660.10
cag	Q	1294	635.90
---	--	------	------
mPD		0.0031	0.50
		nPD:	0.01
		N. weight:	0.67
		Sc. PD:	-0.036
		Sc. rank:	-276.2

M1			
Pos.	192	obs:	exp:
atg	M	1296	1296.00
---	--	------	------
mPD		0	0
		nPD:	1.
		N. weight:	0.
		Sc. PD:	0
		Sc. rank:	0

M1			
Pos.	193	obs:	exp:
gct	A	1296	334.70
gcc	A	0	241.40
gca	A	0	610.00
gcg	A	0	110.00
---	--	------	------
mPD		0	0.67
		nPD:	0.
		N. weight:	1.3
		Sc. PD:	-0.077
		Sc. rank:	-1479.6

M1			
Pos.	194	obs:	exp:
ggt	G	0	171.80
ggc	G	0	165.60
gga	G	1282	597.90
ggg	G	14	360.80
---	--	------	------
mPD		0.021	0.68
		nPD:	0.03
		N. weight:	0.69
		Sc. PD:	-0.02
		Sc. rank:	264.8

M1			
Pos.	195	obs:	exp:
tct	S	1	206.10
tcc	S	0	169.30
tca	S	1284	328.90
tcg	S	11	73.54
agt	S	0	266.40
agc	S	0	251.70
---	--	------	------
mPD		0.018	1.7
		nPD:	0.01
		N. weight:	1.3
		Sc. PD:	-0.062
		Sc. rank:	-117.1

M1			
Pos.	196	obs:	exp:
tct	S	0	203.10
tcc	S	0	166.80
tca	S	0	324.10
tcg	S	0	72.46
aat	N	19	9.68
aac	N	0	9.32
agt	S	1235	262.50
agc	S	42	248.00
---	--	------	------
mPD		0.092	1.7
		nPD:	0.05
		N. weight:	1.4
		Sc. PD:	-0.0094
		Sc. rank:	915.5

M1			
Pos.	197	obs:	exp:
gaa	E	1204	788.60
gag	E	92	507.40
---	--	------	------
mPD		0.13	0.48
		nPD:	0.28
		N. weight:	0.26
		Sc. PD:	0.054
		Sc. rank:	280.1

M1			
Pos.	198	obs:	exp:
caa	Q	10	659.60
cag	Q	1285	635.40
aaa	K	0	0.58
aag	K	1	0.42
---	--	------	------
mPD		0.017	0.50
		nPD:	0.03
		N. weight:	0.64
		Sc. PD:	-0.017
		Sc. rank:	271.2

M1			
Pos.	199	obs:	exp:
gct	A	2	334.70
gcc	A	0	241.40
gca	A	1289	610.00
gcg	A	5	110.00
---	--	------	------
mPD		0.011	0.67
		nPD:	0.02
		N. weight:	0.7
		Sc. PD:	-0.031
		Sc. rank:	78.6

M1			
Pos.	200	obs:	exp:
tct	S	0	0.48
tcc	S	0	0.39
tca	S	0	0.76
tcg	S	3	0.17
act	T	0	0.51
acc	T	0	0.41
aca	T	0	0.92
acg	T	2	0.16
agt	S	0	0.62
agc	S	0	0.58
gct	A	0	333.40
gcc	A	0	240.40
gca	A	20	607.60
gcg	A	1271	109.60
---	--	------	------
mPD		0.038	0.68
		nPD:	0.06
		N. weight:	2.3
		Sc. PD:	-0.013
		Sc. rank:	1577.0

M1			
Pos.	201	obs:	exp:
gaa	E	1287	787.40
gag	E	7	506.60
ggt	G	0	0.27
ggc	G	0	0.26
gga	G	2	0.92
ggg	G	0	0.56
---	--	------	------
mPD		0.014	0.48
		nPD:	0.03
		N. weight:	0.45
		Sc. PD:	-0.014
		Sc. rank:	159.8

M1			
Pos.	202	obs:	exp:
gct	A	5	334.70
gcc	A	1291	241.40
gca	A	0	610.00
gcg	A	0	110.00
---	--	------	------
mPD		0.0077	0.67
		nPD:	0.01
		N. weight:	1.6
		Sc. PD:	-0.077
		Sc. rank:	-94.1

M1			
Pos.	203	obs:	exp:
att	I	4	1.84
atc	I	0	1.20
ata	I	1	1.96
atg	M	1291	1291.00
---	--	------	------
mPD		0.0077	0.0077
		nPD:	1.
		N. weight:	0.0025
		Sc. PD:	0.0022
		Sc. rank:	2.9

M1			
Pos.	204	obs:	exp:
gaa	E	1293	788.60
gag	E	3	507.40
---	--	------	------
mPD		0.0046	0.48
		nPD:	0.01
		N. weight:	0.46
		Sc. PD:	-0.023
		Sc. rank:	-70.4

M1			
Pos.	205	obs:	exp:
att	I	0	4.04
atc	I	11	2.64
ata	I	0	4.32
gtt	V	559	266.30
gtc	V	723	259.30
gta	V	2	263.10
gtg	V	1	496.30
---	--	------	------
mPD		0.51	0.74
		nPD:	0.69
		N. weight:	0.86
		Sc. PD:	0.51
		Sc. rank:	990.3

M1			
Pos.	206	obs:	exp:
gct	A	1	334.70
gcc	A	0	241.40
gca	A	1295	610.00
gcg	A	0	110.00
---	--	------	------
mPD		0.0015	0.67
		nPD:	0.
		N. weight:	0.72
		Sc. PD:	-0.041
		Sc. rank:	-433.0

M1			
Pos.	207	obs:	exp:
tct	S	0	205.60
tcc	S	0	168.90
tca	S	0	328.20
tcg	S	0	73.37
aat	N	3	1.53
aac	N	0	1.47
agt	S	606	265.80
agc	S	687	251.10
---	--	------	------
mPD		0.50	1.7
		nPD:	0.3
		N. weight:	0.89
		Sc. PD:	0.2
		Sc. rank:	972.0

M1			
Pos.	208	obs:	exp:
cat	H	1	0.57
cac	H	0	0.43
caa	Q	10	658.60
cag	Q	1283	634.40
cgt	R	0	0.04
cgc	R	0	0.05
cga	R	0	0.10
cgg	R	1	0.08
aaa	K	0	0.58
aag	K	1	0.42
aga	R	0	0.46
agg	R	0	0.27
---	--	------	------
mPD		0.020	0.50
		nPD:	0.04
		N. weight:	0.64
		Sc. PD:	-0.014
		Sc. rank:	309.4

M1			
Pos.	209	obs:	exp:
gct	A	1293	334.70
gcc	A	3	241.40
gca	A	0	610.00
gcg	A	0	110.00
---	--	------	------
mPD		0.0046	0.67
		nPD:	0.01
		N. weight:	1.3
		Sc. PD:	-0.068
		Sc. rank:	-374.5

M1			
Pos.	210	obs:	exp:
cgt	R	0	55.66
cgc	R	0	64.19
cga	R	0	126.80
cgg	R	0	108.10
aga	R	5	592.60
agg	R	1291	348.60
---	--	------	------
mPD		0.0077	0.96
		nPD:	0.01
		N. weight:	1.2
		Sc. PD:	-0.064
		Sc. rank:	-337.6

M1			
Pos.	211	obs:	exp:
caa	Q	1282	657.10
cag	Q	8	632.90
cgt	R	0	0.26
cgc	R	0	0.30
cga	R	6	0.59
cgg	R	0	0.50
aga	R	0	2.74
agg	R	0	1.61
---	--	------	------
mPD		0.022	0.52
		nPD:	0.04
		N. weight:	0.62
		Sc. PD:	-0.012
		Sc. rank:	319.9

M1			
Pos.	212	obs:	exp:
atg	M	1296	1296.00
---	--	------	------
mPD		0	0
		nPD:	1.
		N. weight:	0.
		Sc. PD:	0
		Sc. rank:	0

M1			
Pos.	213	obs:	exp:
gtt	V	5	268.50
gtc	V	0	261.60
gta	V	10	265.40
gtg	V	1281	500.50
---	--	------	------
mPD		0.023	0.73
		nPD:	0.03
		N. weight:	0.86
		Sc. PD:	-0.025
		Sc. rank:	334.0

M1			
Pos.	214	obs:	exp:
caa	Q	5	660.10
cag	Q	1291	635.90
---	--	------	------
mPD		0.0077	0.50
		nPD:	0.02
		N. weight:	0.66
		Sc. PD:	-0.029
		Sc. rank:	26.2

M1			
Pos.	215	obs:	exp:
gct	A	1289	334.70
gcc	A	5	241.40
gca	A	2	610.00
gcg	A	0	110.00
---	--	------	------
mPD		0.011	0.67
		nPD:	0.02
		N. weight:	1.3
		Sc. PD:	-0.056
		Sc. rank:	142.8

M1			
Pos.	216	obs:	exp:
atg	M	1296	1296.00
---	--	------	------
mPD		0	0
		nPD:	1.
		N. weight:	0.
		Sc. PD:	0
		Sc. rank:	0

M1			
Pos.	217	obs:	exp:
cgt	R	0	55.66
cgc	R	0	64.19
cga	R	0	126.80
cgg	R	0	108.10
aga	R	1062	592.60
agg	R	234	348.60
---	--	------	------
mPD		0.30	0.96
		nPD:	0.31
		N. weight:	0.39
		Sc. PD:	0.092
		Sc. rank:	431.8

M1			
Pos.	218	obs:	exp:
act	T	5	330.10
acc	T	0	265.70
aca	T	1289	594.00
acg	T	1	105.20
gct	A	0	0.26
gcc	A	0	0.19
gca	A	1	0.47
gcg	A	0	0.08
---	--	------	------
mPD		0.011	0.68
		nPD:	0.02
		N. weight:	0.72
		Sc. PD:	-0.032
		Sc. rank:	53.6

M1			
Pos.	219	obs:	exp:
att	I	1	2.20
atc	I	5	1.44
ata	I	0	2.36
gtt	V	495	267.30
gtc	V	794	260.40
gta	V	1	264.10
gtg	V	0	498.20
---	--	------	------
mPD		0.48	0.74
		nPD:	0.66
		N. weight:	0.89
		Sc. PD:	0.5
		Sc. rank:	1000.6

M1			
Pos.	220	obs:	exp:
ggt	G	43	171.80
ggc	G	0	165.60
gga	G	6	597.90
ggg	G	1247	360.80
---	--	------	------
mPD		0.073	0.68
		nPD:	0.11
		N. weight:	1.1
		Sc. PD:	0.048
		Sc. rank:	966.7

M1			
Pos.	221	obs:	exp:
act	T	1248	330.40
acc	T	46	265.90
aca	T	2	594.40
acg	T	0	105.30
---	--	------	------
mPD		0.071	0.68
		nPD:	0.11
		N. weight:	1.2
		Sc. PD:	0.049
		Sc. rank:	1025.5

M1			
Pos.	222	obs:	exp:
cat	H	15	738.40
cac	H	1271	547.60
aat	N	0	5.09
aac	N	10	4.91
---	--	------	------
mPD		0.038	0.50
		nPD:	0.08
		N. weight:	0.76
		Sc. PD:	0.0099
		Sc. rank:	593.5

M1			
Pos.	223	obs:	exp:
cct	P	1292	323.40
ccc	P	2	232.90
cca	P	2	533.50
ccg	P	0	206.30
---	--	------	------
mPD		0.0062	0.71
		nPD:	0.01
		N. weight:	1.3
		Sc. PD:	-0.067
		Sc. rank:	-330.6

M1			
Pos.	224	obs:	exp:
tct	S	0	1.91
tcc	S	0	1.57
tca	S	0	3.05
tcg	S	0	0.68
aat	N	81	654.00
aac	N	1203	630.00
agt	S	0	2.47
agc	S	12	2.33
---	--	------	------
mPD		0.14	0.53
		nPD:	0.25
		N. weight:	0.47
		Sc. PD:	0.086
		Sc. rank:	496.6

M1			
Pos.	225	obs:	exp:
tct	S	8	206.00
tcc	S	1284	169.10
tca	S	3	328.70
tcg	S	0	73.48
act	T	0	0.25
acc	T	1	0.21
aca	T	0	0.46
acg	T	0	0.08
agt	S	0	266.20
agc	S	0	251.50
---	--	------	------
mPD		0.018	1.7
		nPD:	0.01
		N. weight:	1.9
		Sc. PD:	-0.092
		Sc. rank:	-140.7

M1			
Pos.	226	obs:	exp:
tct	S	0	206.10
tcc	S	0	169.30
tca	S	0	328.90
tcg	S	0	73.54
agt	S	1283	266.40
agc	S	13	251.70
---	--	------	------
mPD		0.020	1.7
		nPD:	0.01
		N. weight:	1.5
		Sc. PD:	-0.07
		Sc. rank:	-48.5

M1			
Pos.	227	obs:	exp:
act	T	2	328.60
acc	T	0	264.50
aca	T	1287	591.20
acg	T	0	104.70
gct	A	0	1.81
gcc	A	1	1.30
gca	A	6	3.29
gcg	A	0	0.59
---	--	------	------
mPD		0.015	0.69
		nPD:	0.02
		N. weight:	0.74
		Sc. PD:	-0.028
		Sc. rank:	160.2

M1			
Pos.	228	obs:	exp:
ggt	G	1277	171.80
ggc	G	19	165.60
gga	G	0	597.90
ggg	G	0	360.80
---	--	------	------
mPD		0.029	0.68
		nPD:	0.04
		N. weight:	1.9
		Sc. PD:	-0.034
		Sc. rank:	995.8

M1			
Pos.	229	obs:	exp:
tta	L	1	115.00
ttg	L	0	241.30
ctt	L	161	215.80
ctc	L	0	216.30
cta	L	1133	218.40
ctg	L	1	289.20
---	--	------	------
mPD		0.22	1.1
		nPD:	0.2
		N. weight:	1.3
		Sc. PD:	0.18
		Sc. rank:	1395.5

M1			
Pos.	230	obs:	exp:
aaa	K	524	755.30
aag	K	772	540.70
---	--	------	------
mPD		0.48	0.49
		nPD:	0.99
		N. weight:	0.065
		Sc. PD:	0.057
		Sc. rank:	77.1

M1			
Pos.	231	obs:	exp:
gat	D	1296	700.30
gac	D	0	595.70
---	--	------	------
mPD		0	0.50
		nPD:	0.
		N. weight:	0.59
		Sc. PD:	-0.035
		Sc. rank:	-672.7

M1			
Pos.	232	obs:	exp:
aat	N	10	5.09
aac	N	0	4.91
gct	A	1	0.26
gcc	A	0	0.19
gca	A	0	0.47
gcg	A	0	0.08
gat	D	1271	694.40
gac	D	14	590.60
---	--	------	------
mPD		0.038	0.51
		nPD:	0.07
		N. weight:	0.54
		Sc. PD:	0.0064
		Sc. rank:	418.3

M1			
Pos.	233	obs:	exp:
tta	L	0	115.00
ttg	L	0	241.30
ctt	L	1289	215.80
ctc	L	7	216.30
cta	L	0	218.40
ctg	L	0	289.20
---	--	------	------
mPD		0.011	1.1
		nPD:	0.01
		N. weight:	1.7
		Sc. PD:	-0.084
		Sc. rank:	-267.5

M1			
Pos.	234	obs:	exp:
tta	L	0	0.27
ttg	L	0	0.56
ctt	L	3	0.50
ctc	L	0	0.50
cta	L	0	0.51
ctg	L	0	0.67
att	I	1292	475.10
atc	I	1	309.90
ata	I	0	508.10
---	--	------	------
mPD		0.0062	0.66
		nPD:	0.01
		N. weight:	0.96
		Sc. PD:	-0.048
		Sc. rank:	-189.4

M1			
Pos.	235	obs:	exp:
aaa	K	4	2.33
aag	K	0	1.67
gaa	E	1290	785.00
gag	E	0	505.00
ggt	G	0	0.27
ggc	G	0	0.26
gga	G	2	0.92
ggg	G	0	0.56
---	--	------	------
mPD		0.0092	0.49
		nPD:	0.02
		N. weight:	0.48
		Sc. PD:	-0.02
		Sc. rank:	94.2

M1			
Pos.	236	obs:	exp:
cat	H	2	1.15
cac	H	0	0.85
aat	N	1294	659.10
aac	N	0	634.90
---	--	------	------
mPD		0.0031	0.50
		nPD:	0.01
		N. weight:	0.65
		Sc. PD:	-0.035
		Sc. rank:	-269.6

M1			
Pos.	237	obs:	exp:
tta	L	0	115.00
ttg	L	1296	241.30
ctt	L	0	215.80
ctc	L	0	216.30
cta	L	0	218.40
ctg	L	0	289.20
---	--	------	------
mPD		0	1.1
		nPD:	0.
		N. weight:	1.6
		Sc. PD:	-0.096
		Sc. rank:	-1837.2

M1			
Pos.	238	obs:	exp:
caa	Q	0	660.10
cag	Q	1296	635.90
---	--	------	------
mPD		0	0.50
		nPD:	0.
		N. weight:	0.68
		Sc. PD:	-0.04
		Sc. rank:	-778.2

M1			
Pos.	239	obs:	exp:
act	T	0	0.25
acc	T	1	0.21
aca	T	0	0.46
acg	T	0	0.08
gtt	V	0	0.62
gtc	V	3	0.61
gta	V	0	0.61
gtg	V	0	1.16
gct	A	323	333.60
gcc	A	964	240.60
gca	A	5	608.10
gcg	A	0	109.70
---	--	------	------
mPD		0.39	0.68
		nPD:	0.57
		N. weight:	0.98
		Sc. PD:	0.47
		Sc. rank:	1092.4

,

M1			
Pos.	240	obs:	exp:
tat	Y	1	700.00
tac	Y	1295	596.00
---	--	------	------
mPD		0.0015	0.50
		nPD:	0.
		N. weight:	0.74
		Sc. PD:	-0.042
		Sc. rank:	-389.3

,

M1			
Pos.	241	obs:	exp:
caa	Q	86	660.10
cag	Q	1210	635.90
---	--	------	------
mPD		0.12	0.50
		nPD:	0.25
		N. weight:	0.45
		Sc. PD:	0.08
		Sc. rank:	474.7

,

M1			
Pos.	242	obs:	exp:
tct	S	0	0.16
tcc	S	0	0.13
tca	S	0	0.25
tcg	S	0	0.06
aat	N	2	659.10
aac	N	1292	634.90
aaa	K	1	0.58
aag	K	0	0.42
agt	S	0	0.21
agc	S	1	0.19
---	--	------	------
mPD		0.0062	0.50
		nPD:	0.01
		N. weight:	0.67
		Sc. PD:	-0.032
		Sc. rank:	-18.1

,

M1			
Pos.	243	obs:	exp:
cgt	R	0	55.66
cgc	R	0	64.19
cga	R	10	126.80
cgg	R	1280	108.10
aga	R	0	592.60
agg	R	6	348.60
---	--	------	------
mPD		0.025	0.96
		nPD:	0.03
		N. weight:	2.3
		Sc. PD:	-0.08
		Sc. rank:	726.8

,

M1			
Pos.	244	obs:	exp:
atg	M	1296	1296.00
---	--	------	------
mPD		0	0
		nPD:	1.
		N. weight:	0.
		Sc. PD:	0
		Sc. rank:	0

,

M1			
Pos.	245	obs:	exp:
ggt	G	3	171.80
ggc	G	0	165.60
gga	G	1292	597.90
ggg	G	1	360.80
---	--	------	------
mPD		0.0062	0.68
		nPD:	0.01
		N. weight:	0.73
		Sc. PD:	-0.037
		Sc. rank:	-173.1

,

M1			
Pos.	246	obs:	exp:
gtt	V	0	268.50
gtc	V	0	261.60
gta	V	0	265.40
gtg	V	1296	500.50
---	--	------	------
mPD		0	0.73
		nPD:	0.
		N. weight:	0.91
		Sc. PD:	-0.054
		Sc. rank:	-1039.7

,

M1			
Pos.	247	obs:	exp:
caa	Q	1293	660.10
cag	Q	3	635.90
---	--	------	------
mPD		0.0046	0.50
		nPD:	0.01
		N. weight:	0.63
		Sc. PD:	-0.032
		Sc. rank:	-139.8

,

M1			
Pos.	248	obs:	exp:
tta	L	0	112.60
ttg	L	50	236.30
ctt	L	0	211.30
ctc	L	0	211.80
cta	L	1	213.90
ctg	L	1218	283.20
atg	M	27	27.00
---	--	------	------
mPD		0.12	1.1
		nPD:	0.1
		N. weight:	1.3
		Sc. PD:	0.048
		Sc. rank:	1094.9

,

M1			
Pos.	249	obs:	exp:
caa	Q	48	658.60
cag	Q	1245	634.40
cgt	R	0	0.13
cgc	R	0	0.15
cga	R	0	0.29
cgg	R	3	0.25
aga	R	0	1.37
agg	R	0	0.81
---	--	------	------
mPD		0.076	0.51
		nPD:	0.15
		N. weight:	0.54
		Sc. PD:	0.045
		Sc. rank:	523.7

,

M1			
Pos.	250	obs:	exp:
cgt	R	4	55.66
cgc	R	3	64.19
cga	R	15	126.80
cgg	R	1270	108.10
aga	R	0	592.60
agg	R	4	348.60
---	--	------	------
mPD		0.040	0.96
		nPD:	0.04
		N. weight:	2.3
		Sc. PD:	-0.045
		Sc. rank:	1157.7

,

M1			
Pos.	251	obs:	exp:
ttt	F	1	511.80
ttc	F	1295	784.20
---	--	------	------
mPD		0.0015	0.48
		nPD:	0.
		N. weight:	0.48
		Sc. PD:	-0.027
		Sc. rank:	-238.7

,

M1			
Pos.	252	obs:	exp:
aaa	K	2	755.30
aag	K	1294	540.70
---	--	------	------
mPD		0.0031	0.49
		nPD:	0.01
		N. weight:	0.83
		Sc. PD:	-0.044
		Sc. rank:	-304.5

,

M1			
Pos.	253	obs:	exp:
taa	.	0	550.60
tag	.	0	324.50
tga	.	1296	421.00
---	--	------	------
mPD		0	0.81
		nPD:	0.
		N. weight:	1.1
		Sc. PD:	-0.064
		Sc. rank:	-1228.9

}

Amino acid level conservation per codon

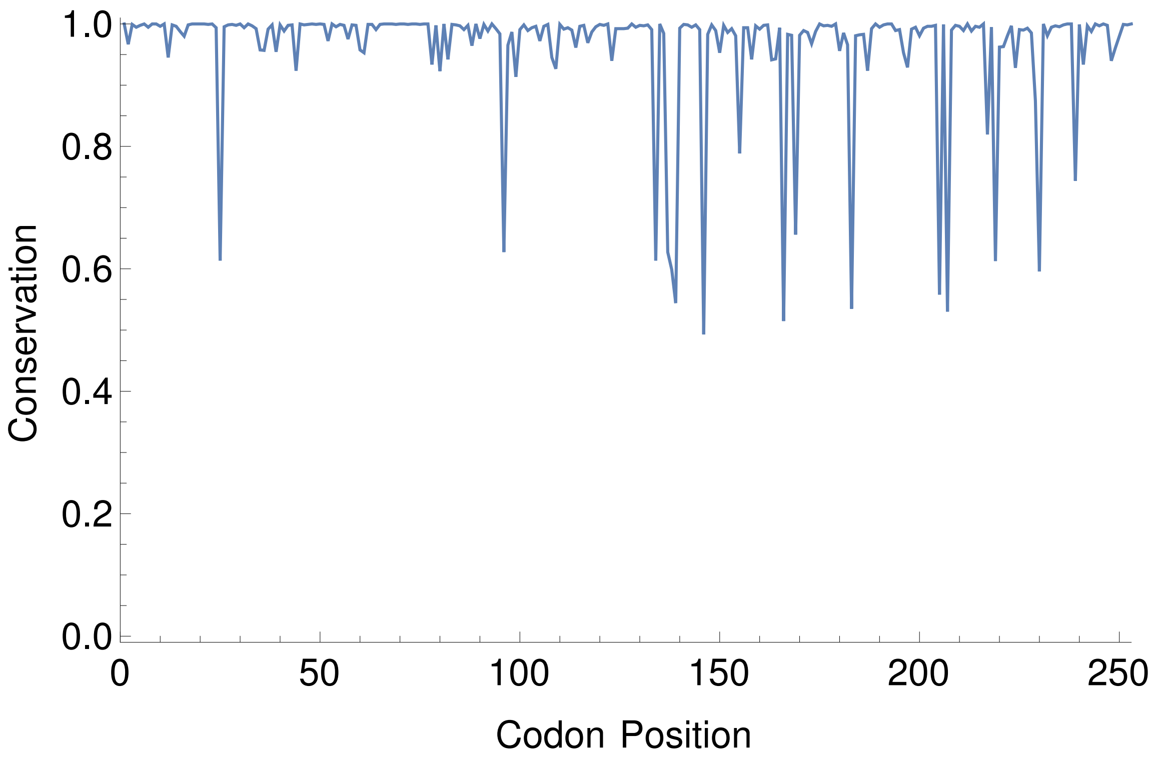

Conservation analysis plots

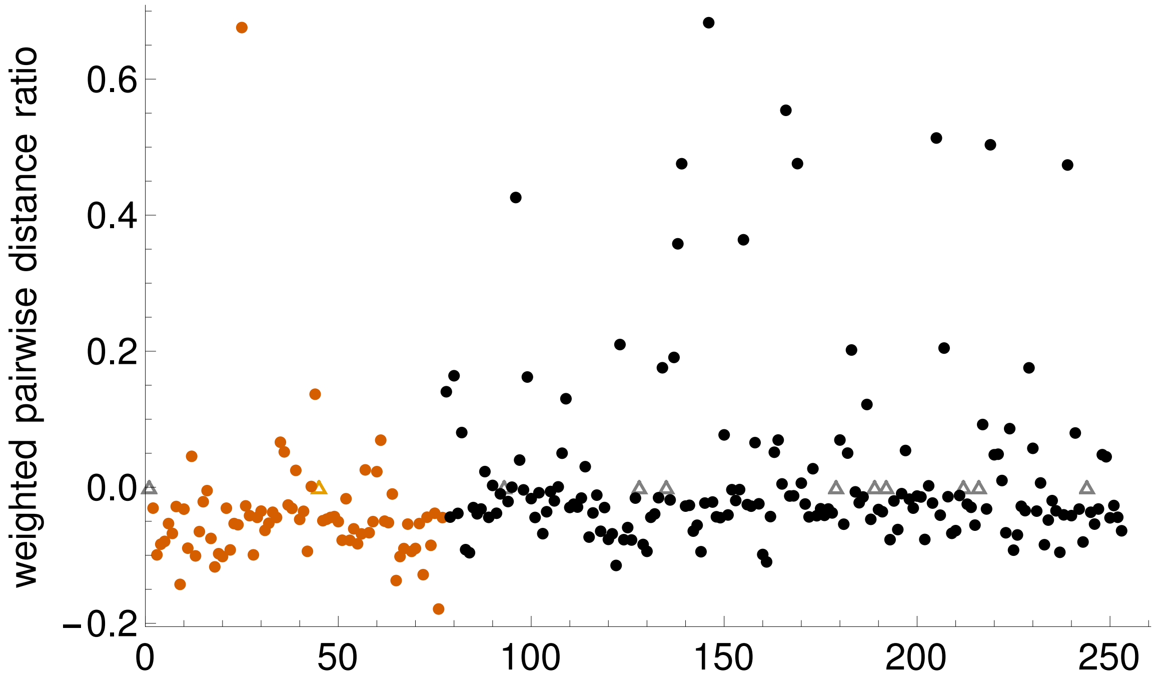

M1

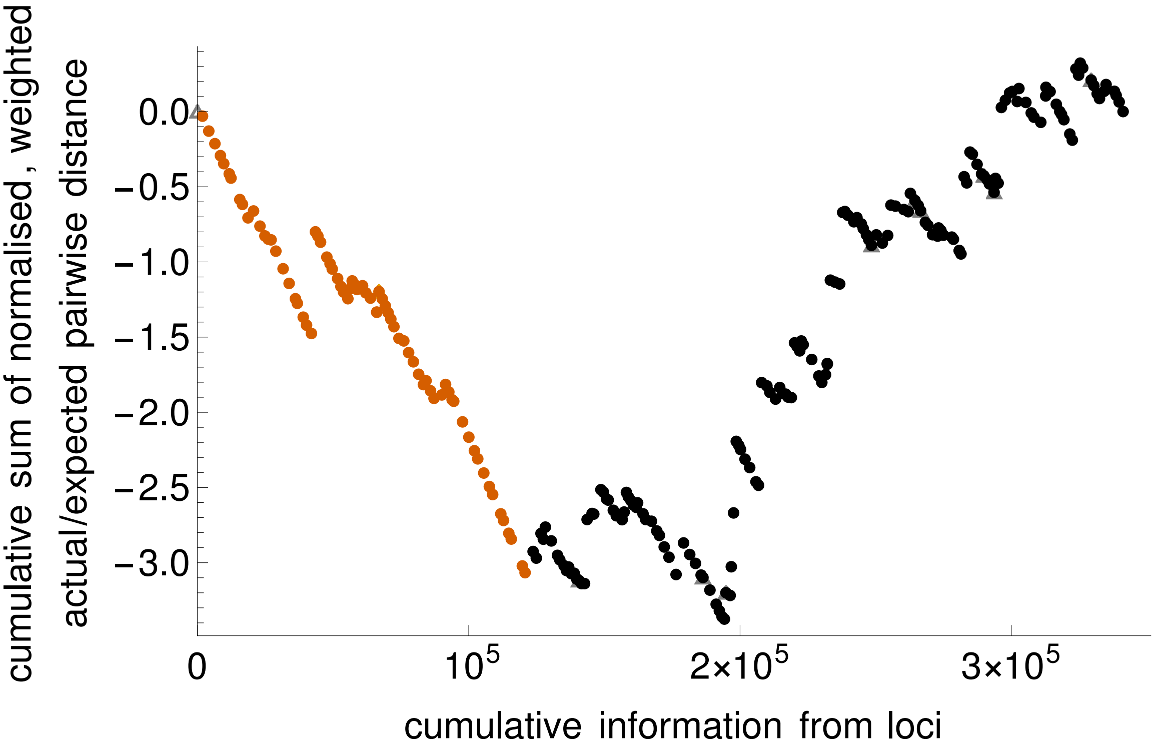

M1

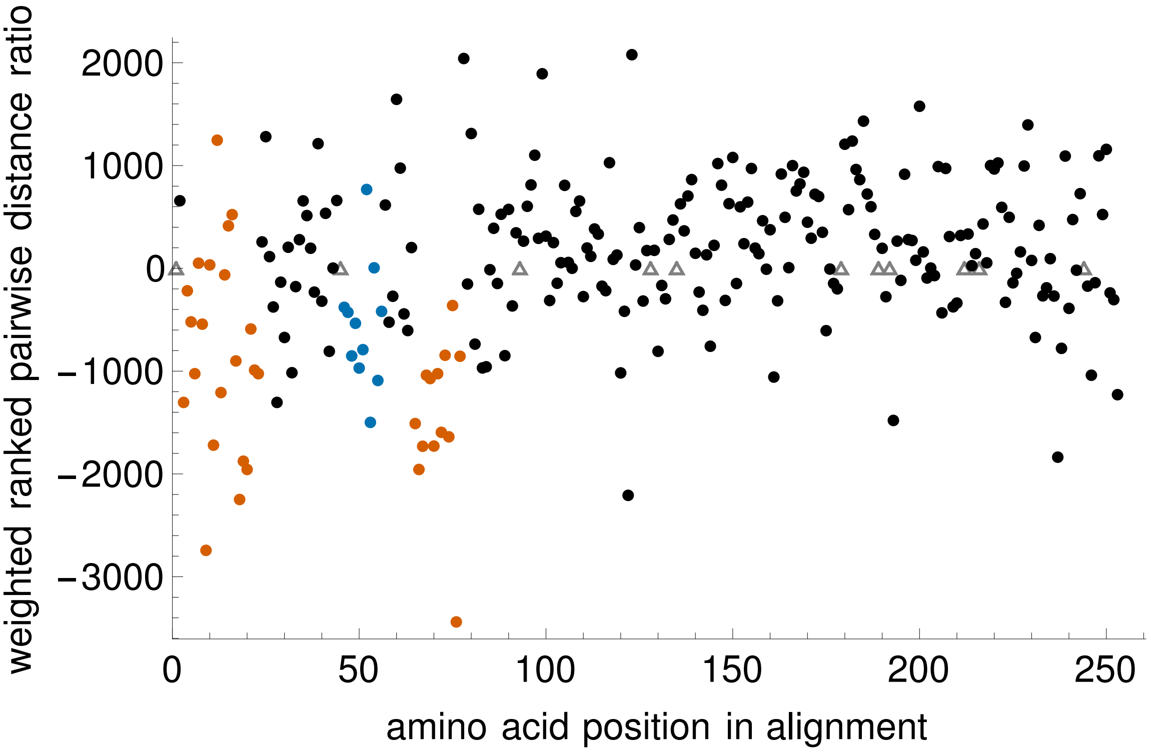
weighted ranked pairwise distance ratio
2000
1000
0
−1000
−2000
−3000
0
50
100
150
200
250
amino acid position in alignment

M1

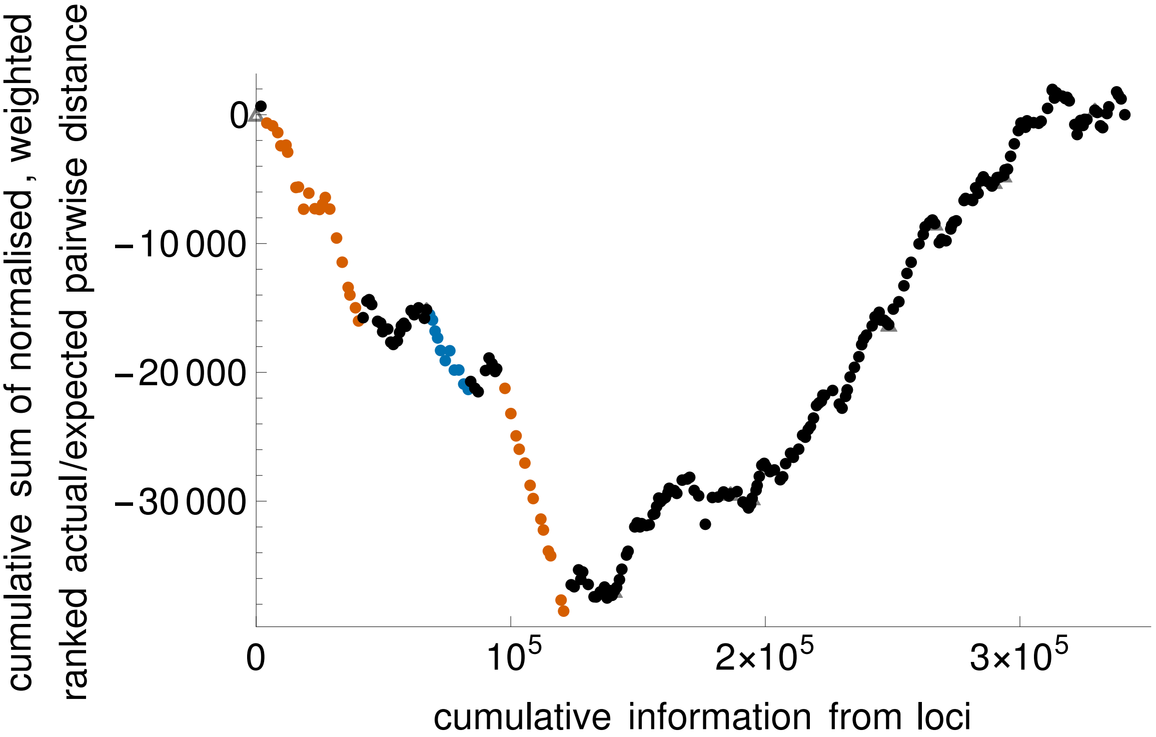
cumulative sum of normalised, weighted ranked actual/expected pairwise distance
0
−10 000
−20 000
−30 000
0
10^5
2×10^5
3×10^5
cumulative information from loci

M1

M2

Gene length histogram

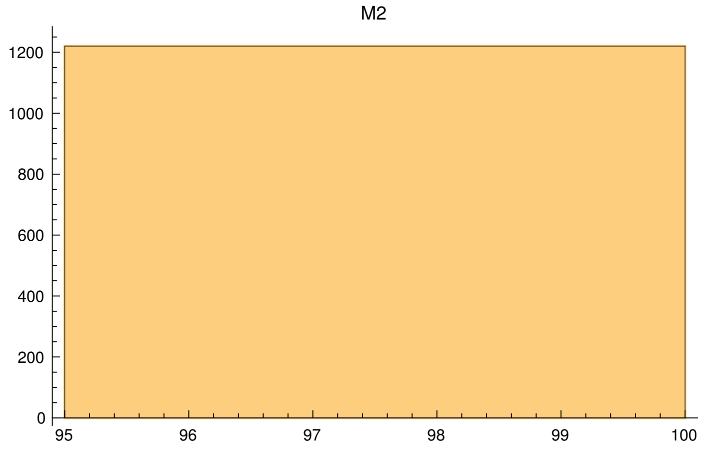

Information vs. nPD

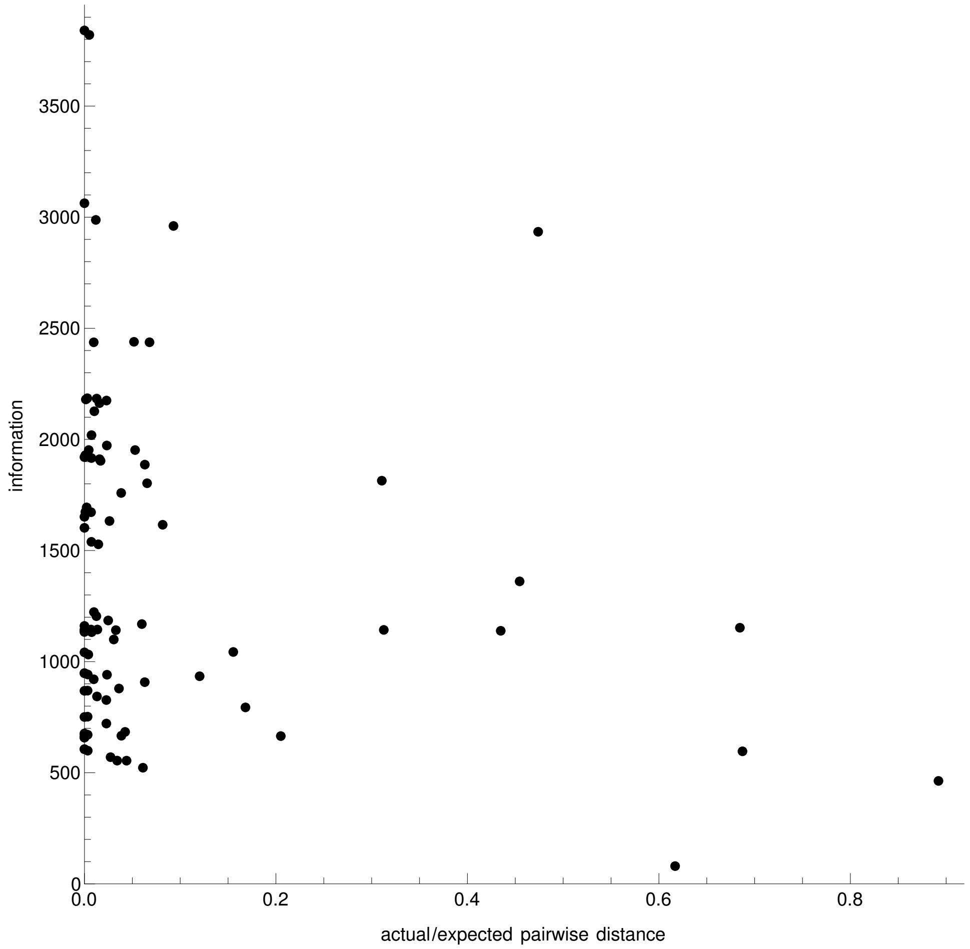

M2

Example sequences highlighted by regions found in analyses to be conserved

```
Interesting points (by weighted raw PD) highlighted for gene M2:
ATGAGTCTTCTAACCGAGGTCGAAACGCCTACCAGAACCGGATGGGAGTGCAACTGCAGCGGTTCAAGTGACCCAC⋱
    TCGTTGTTGCAGCTAACATTATTGGGATATTGCACCTGATATTGTGGATTCTTGATCGTCTTTTCTTCAAATGCA⋱
    TTTATCGTCGCTTTAAATACGGTTTGAAAAGAGGGCCTTCTACGGAAGGAATGCCTGAGTCTATGAGGGAAGAAT⋱
    ATCGGCAGGAACAGCAGAGTGCTGTGGATGTTGACGATGGTCATTTTGTCAACATAGAGCTGAAGTAA

Interesting points (by weighted ranked PD) highlighted for gene M2:
ATGAGTCTTCTAACCGAGGTCGAAACGCCTACCAGAACCGGATGGGAGTGCAACTGCAGCGGTTCAAGTGACCCACTC⋱
    GTTGTTGCAGCTAACATTATTGGGATATTGCACCTGATATTGTGGATTCTTGATCGTCTTTTCTTCAAATGCATTTA⋱
    TCGTCGCTTTAAATACGGTTTGAAAAGAGGGCCTTCTACGGAAGGAATGCCTGAGTCTATGAGGGAAGAATATCGGC⋱
    AGGAACAGCAGAGTGCTGTGGATGTTGACGATGGTCATTTTGTCAACATAGAGCTGAAGTAA
```

Per codon conservation report

M2			
Pos.	1	obs:	exp:
atg	M	1220	1220.00
---	--	------	------
mPD		0	0
		nPD:	1.
		N. weight:	0.
		Sc. PD:	0
		Sc. rank:	0

M2			
Pos.	2	obs:	exp:
tct	S	0	194.00
tcc	S	0	159.30
tca	S	0	309.60
tcg	S	0	69.23
agt	S	1180	250.80
agc	S	40	237.00
---	--	------	------
mPD		0.063	1.7
		nPD:	0.04
		N. weight:	1.3
		Sc. PD:	-0.027
		Sc. rank:	774.1

M2			
Pos.	3	obs:	exp:
tta	L	0	108.30
ttg	L	0	227.10
ctt	L	1219	203.10
ctc	L	1	203.60
cta	L	0	205.60
ctg	L	0	272.20
---	--	------	------
mPD		0.0016	1.1
		nPD:	0.
		N. weight:	1.6
		Sc. PD:	-0.091
		Sc. rank:	-930.8

M2			
Pos.	4	obs:	exp:
tta	L	1	108.30
ttg	L	0	227.10
ctt	L	1	203.10
ctc	L	0	203.60
cta	L	1213	205.60
ctg	L	5	272.20
---	--	------	------
mPD		0.011	1.1
		nPD:	0.01
		N. weight:	1.6
		Sc. PD:	-0.075
		Sc. rank:	47.8

M2			
Pos.	5	obs:	exp:
act	T	0	310.70
acc	T	1217	250.10
aca	T	2	559.10
acg	T	0	99.01
aat	N	0	0.51
aac	N	1	0.49
---	--	------	------
mPD		0.0049	0.68
		nPD:	0.01
		N. weight:	1.4
		Sc. PD:	-0.072
		Sc. rank:	-248.0

M2			
Pos.	6	obs:	exp:
gaa	E	0	742.40
gag	E	1220	477.60
---	--	------	------
mPD		0	0.48
		nPD:	0.
		N. weight:	0.86
		Sc. PD:	-0.049
		Sc. rank:	-950.6

M2			
Pos.	7	obs:	exp:
gtt	V	7	252.80
gtc	V	1213	246.20
gta	V	0	249.80
gtg	V	0	471.20
---	--	------	------
mPD		0.011	0.73
		nPD:	0.02
		N. weight:	1.4
		Sc. PD:	-0.06
		Sc. rank:	398.7

M2			
Pos.	8	obs:	exp:
gaa	E	1220	742.40
gag	E	0	477.60
---	--	------	------
mPD		0	0.48
		nPD:	0.
		N. weight:	0.46
		Sc. PD:	-0.026
		Sc. rank:	-503.5

M2			
Pos.	9	obs:	exp:
act	T	0	311.00
acc	T	0	250.30
aca	T	0	559.60
acg	T	1220	99.10
---	--	------	------
mPD		0	0.68
		nPD:	0.
		N. weight:	2.3
		Sc. PD:	-0.13
		Sc. rank:	-2544.9

M2			
Pos.	10	obs:	exp:
tta	L	0	27.07
ttg	L	0	56.79
tct	S	3	0.48
tcc	S	0	0.39
tca	S	0	0.76
tcg	S	0	0.17
ctt	L	305	50.79
ctc	L	0	50.90
cta	L	0	51.40
ctg	L	0	68.06
cct	P	907	226.30
ccc	P	0	163.00
cca	P	0	373.30
ccg	P	0	144.40
cat	H	5	2.87
cac	H	0	2.13
agt	S	0	0.62
agc	S	0	0.58
---	--	------	------
mPD		0.39	1.2
		nPD:	0.31
		N. weight:	1.4
		Sc. PD:	0.33
		Sc. rank:	1399.2

M2			
Pos.	11	obs:	exp:
att	I	0	0.37
atc	I	1	0.24
ata	I	0	0.39
act	T	0	310.70
acc	T	1219	250.10
aca	T	0	559.10
acg	T	0	99.01
---	--	------	------
mPD		0.0016	0.68
		nPD:	0.
		N. weight:	1.5
		Sc. PD:	-0.079
		Sc. rank:	-731.5

M2			
Pos.	12	obs:	exp:
cgt	R	0	48.79
cgc	R	0	56.27
cga	R	0	111.20
cgg	R	0	94.79
aaa	K	84	48.96
aag	K	0	35.04
aga	R	1136	519.40
agg	R	0	305.50
---	--	------	------
mPD		0.13	1.1
		nPD:	0.12
		N. weight:	0.7
		Sc. PD:	0.041
		Sc. rank:	666.8

M2			
Pos.	13	obs:	exp:
att	I	0	0.73
atc	I	2	0.48
ata	I	0	0.79
act	T	0	310.20
acc	T	1213	249.70
aca	T	4	558.20
acg	T	0	98.85
gct	A	0	0.26
gcc	A	1	0.19
gca	A	0	0.47
gcg	A	0	0.08
---	--	------	------
mPD		0.011	0.68
		nPD:	0.02
		N. weight:	1.4
		Sc. PD:	-0.058
		Sc. rank:	445.4

M2			
Pos.	14	obs:	exp:
gaa	E	10	6.09
gag	E	0	3.91
ggt	G	0	160.40
ggc	G	0	154.60
gga	G	1210	558.20
ggg	G	0	336.80
---	--	------	------
mPD		0.016	0.69
		nPD:	0.02
		N. weight:	0.71
		Sc. PD:	-0.024
		Sc. rank:	303.5

M2			
Pos.	15	obs:	exp:
tgg	W	1220	1220.00
---	--	------	------
mPD		0	0
		nPD:	1.
		N. weight:	0.
		Sc. PD:	0
		Sc. rank:	0

M2			
Pos.	16	obs:	exp:
gtt	V	0	0.62
gtc	V	0	0.61
gta	V	0	0.61
gtg	V	3	1.16
gaa	E	0	739.90
gag	E	1216	476.10
ggt	G	0	0.13
ggc	G	0	0.13
gga	G	0	0.46
ggg	G	1	0.28
---	--	------	------
mPD		0.0065	0.48
		nPD:	0.01
		N. weight:	0.86
		Sc. PD:	-0.038
		Sc. rank:	153.3

M2			
Pos.	17	obs:	exp:
tgt	C	0	516.00
tgc	C	1220	704.00
---	--	------	------
mPD		0	0.49
		nPD:	0.
		N. weight:	0.5
		Sc. PD:	-0.029
		Sc. rank:	-557.4

M2			
Pos.	18	obs:	exp:
tct	S	0	0.48
tcc	S	0	0.39
tca	S	0	0.76
tcg	S	0	0.17
aat	N	49	611.70
aac	N	1152	589.30
aaa	K	16	9.33
aag	K	0	6.67
agt	S	0	0.62
agc	S	3	0.58
---	--	------	------
mPD		0.11	0.52
		nPD:	0.21
		N. weight:	0.5
		Sc. PD:	0.069
		Sc. rank:	496.8

M2			
Pos.	19	obs:	exp:
tat	Y	0	0.54
tac	Y	1	0.46
tgt	C	0	515.60
tgc	C	1219	703.40
---	--	------	------
mPD		0.0016	0.49
		nPD:	0.
		N. weight:	0.5
		Sc. PD:	-0.027
		Sc. rank:	-203.8

M2			
Pos.	20	obs:	exp:
tct	S	0	185.00
tcc	S	0	151.90
tca	S	0	295.20
tcg	S	0	65.99
cgt	R	0	0.17
cgc	R	0	0.20
cga	R	0	0.39
cgg	R	0	0.33
aat	N	0	24.96
aac	N	49	24.04
agt	S	0	239.10
agc	S	1163	225.90
aga	R	4	1.83
agg	R	0	1.08
ggt	G	0	0.53
ggc	G	4	0.51
gga	G	0	1.85
ggg	G	0	1.11
---	--	------	------
mPD		0.090	1.7
		nPD:	0.05
		N. weight:	1.5
		Sc. PD:	-0.0094
		Sc. rank:	1022.8

M2			
Pos.	21	obs:	exp:
gtt	V	4	0.83
gtc	V	0	0.81
gta	V	0	0.82
gtg	V	0	1.54
gct	A	3	0.77
gcc	A	0	0.56
gca	A	0	1.41
gcg	A	0	0.25
gat	D	16	8.65
gac	D	0	7.35
ggt	G	1197	158.70
ggc	G	0	152.90
gga	G	0	552.20
ggg	G	0	333.20
---	--	------	------
mPD		0.037	0.72
		nPD:	0.05
		N. weight:	1.8
		Sc. PD:	-0.014
		Sc. rank:	1214.6

,

M2			
Pos.	22	obs:	exp:
tta	L	1	0.09
ttg	L	0	0.19
tct	S	0	193.90
tcc	S	0	159.20
tca	S	1219	309.40
tcg	S	0	69.17
ctt	L	0	0.17
ctc	L	0	0.17
cta	L	0	0.17
ctg	L	0	0.22
agt	S	0	250.60
agc	S	0	236.80
---	--	------	------
mPD		0.0016	1.7
		nPD:	0.
		N. weight:	1.3
		Sc. PD:	-0.07
		Sc. rank:	-806.4

,

M2			
Pos.	23	obs:	exp:
tct	S	0	193.90
tcc	S	0	159.20
tca	S	0	309.40
tcg	S	0	69.17
aat	N	1	0.51
aac	N	0	0.49
agt	S	1219	250.60
agc	S	0	236.80
---	--	------	------
mPD		0.0016	1.7
		nPD:	0.
		N. weight:	1.5
		Sc. PD:	-0.081
		Sc. rank:	-879.7

,

M2			
Pos.	24	obs:	exp:
gat	D	267	435.50
gac	D	539	370.50
gaa	E	0	251.90
gag	E	414	162.10
---	--	------	------
mPD		0.64	0.72
		nPD:	0.89
		N. weight:	0.35
		Sc. PD:	0.28
		Sc. rank:	412.0

,

M2			
Pos.	25	obs:	exp:
tta	L	0	0.53
ttg	L	0	1.12
tct	S	1	0.16
tcc	S	0	0.13
tca	S	0	0.25
tcg	S	0	0.06
ctt	L	5	1.00
ctc	L	0	1.00
cta	L	1	1.01
ctg	L	0	1.34
cct	P	732	302.20
ccc	P	14	217.60
cca	P	465	498.50
ccg	P	0	192.80
act	T	2	0.51
acc	T	0	0.41
aca	T	0	0.92
acg	T	0	0.16
agt	S	0	0.21
agc	S	0	0.19
---	--	------	------
mPD		0.50	0.73
		nPD:	0.69
		N. weight:	0.45
		Sc. PD:	0.27
		Sc. rank:	526.8

,

M2			
Pos.	26	obs:	exp:
ttt	F	0	15.01
ttc	F	38	22.99
tta	L	0	104.90
ttg	L	0	220.10
ctt	L	0	196.80
ctc	L	754	197.30
cta	L	428	199.20
ctg	L	0	263.80
---	--	------	------
mPD		0.52	1.1
		nPD:	0.45
		N. weight:	1.
		Sc. PD:	0.39
		Sc. rank:	1115.9

,

M2			
Pos.	27	obs:	exp:
att	I	53	22.78
atc	I	9	14.86
ata	I	0	24.36
gtt	V	709	233.10
gtc	V	416	227.10
gta	V	0	230.40
gtg	V	0	434.50
gct	A	1	0.26
gcc	A	0	0.19
gca	A	0	0.47
gcg	A	0	0.08
ggt	G	32	4.24
ggc	G	0	4.09
gga	G	0	14.76
ggg	G	0	8.91
---	--	------	------
mPD		0.60	0.88
		nPD:	0.68
		N. weight:	0.87
		Sc. PD:	0.52
		Sc. rank:	1008.8

,

M2			
Pos.	28	obs:	exp:
att	I	11	4.04
atc	I	0	2.64
ata	I	0	4.32
gtt	V	1190	250.50
gtc	V	12	244.00
gta	V	4	247.60
gtg	V	3	466.90
---	--	------	------
mPD		0.049	0.74
		nPD:	0.07
		N. weight:	1.4
		Sc. PD:	0.0075
		Sc. rank:	1095.7

,

M2			
Pos.	29	obs:	exp:
act	T	0	0.76
acc	T	0	0.62
aca	T	3	1.38
acg	T	0	0.24
gtt	V	0	0.41
gtc	V	0	0.40
gta	V	2	0.41
gtg	V	0	0.77
gct	A	1	313.80
gcc	A	0	226.30
gca	A	1205	571.90
gcg	A	9	103.10
---	--	------	------
mPD		0.024	0.68
		nPD:	0.04
		N. weight:	0.66
		Sc. PD:	-0.015
		Sc. rank:	366.2

,

M2			
Pos.	30	obs:	exp:
tct	S	5	0.80
tcc	S	0	0.65
tca	S	0	1.27
tcg	S	0	0.28
act	T	3	0.76
acc	T	0	0.62
aca	T	0	1.38
acg	T	0	0.24
agt	S	0	1.03
agc	S	0	0.97
gct	A	1209	313.00
gcc	A	1	225.70
gca	A	2	570.40
gcg	A	0	102.90
---	--	------	------
mPD		0.018	0.69
		nPD:	0.03
		N. weight:	1.2
		Sc. PD:	-0.039
		Sc. rank:	587.8

,

M2			
Pos.	31	obs:	exp:
aat	N	7	621.40
aac	N	1213	598.60
---	--	------	------
mPD		0.011	0.50
		nPD:	0.02
		N. weight:	0.62
		Sc. PD:	-0.022
		Sc. rank:	202.7

,

M2			
Pos.	32	obs:	exp:
att	I	1195	443.50
atc	I	12	289.20
ata	I	0	474.30
gtt	V	13	2.69
gtc	V	0	2.62
gta	V	0	2.66
gtg	V	0	5.02
---	--	------	------
mPD		0.041	0.68
		nPD:	0.06
		N. weight:	0.88
		Sc. PD:	0.00028
		Sc. rank:	630.7

,

M2			
Pos.	33	obs:	exp:
tta	L	0	0.09
ttg	L	0	0.19
ctt	L	1	0.17
ctc	L	0	0.17
cta	L	0	0.17
ctg	L	0	0.22
att	I	1215	447.50
atc	I	3	291.90
ata	I	0	478.60
gtt	V	1	0.21
gtc	V	0	0.20
gta	V	0	0.20
gtg	V	0	0.39
---	--	------	------
mPD		0.0082	0.66
		nPD:	0.01
		N. weight:	0.91
		Sc. PD:	-0.041
		Sc. rank:	94.4

,

M2			
Pos.	34	obs:	exp:
ggt	G	0	161.70
ggc	G	0	155.90
gga	G	3	562.80
ggg	G	1217	339.60
---	--	------	------
mPD		0.0049	0.68
		nPD:	0.01
		N. weight:	1.2
		Sc. PD:	-0.058
		Sc. rank:	-167.6

,

M2			
Pos.	35	obs:	exp:
att	I	0	447.50
atc	I	1	291.90
ata	I	1217	478.60
atg	M	1	1.00
gtt	V	0	0.21
gtc	V	0	0.20
gta	V	1	0.20
gtg	V	0	0.39
---	--	------	------
mPD		0.0049	0.66
		nPD:	0.01
		N. weight:	0.85
		Sc. PD:	-0.042
		Sc. rank:	-75.8

,

M2			
Pos.	36	obs:	exp:
tta	L	0	108.30
ttg	L	1215	227.10
ctt	L	0	203.10
ctc	L	0	203.60
cta	L	0	205.60
ctg	L	5	272.20
---	--	------	------
mPD		0.0082	1.1
		nPD:	0.01
		N. weight:	1.5
		Sc. PD:	-0.076
		Sc. rank:	-165.6

,

M2			
Pos.	37	obs:	exp:
cat	H	0	700.50
cac	H	1220	519.50
---	--	------	------
mPD		0	0.49
		nPD:	0.
		N. weight:	0.78
		Sc. PD:	-0.044
		Sc. rank:	-865.5

,

M2			
Pos.	38	obs:	exp:
tta	L	1	108.20
ttg	L	731	227.00
ctt	L	0	203.00
ctc	L	0	203.40
cta	L	0	205.40
ctg	L	487	272.00
atg	M	1	1.00
---	--	------	------
mPD		0.48	1.1
		nPD:	0.43
		N. weight:	0.86
		Sc. PD:	0.31
		Sc. rank:	912.9

,

M2			
Pos.	39	obs:	exp:
att	I	0	447.90
atc	I	0	292.10
ata	I	1219	479.00
act	T	0	0.25
acc	T	0	0.21
aca	T	1	0.46
acg	T	0	0.08
---	--	------	------
mPD		0.0016	0.65
		nPD:	0.
		N. weight:	0.86
		Sc. PD:	-0.047
		Sc. rank:	-414.1

,

M2			
Pos.	40	obs:	exp:
ttt	F	1	0.39
ttc	F	0	0.61
tta	L	0	108.10
ttg	L	1204	226.80
ctt	L	0	202.80
ctc	L	0	203.30
cta	L	0	205.30
ctg	L	14	271.80
gtt	V	0	0.21
gtc	V	0	0.20
gta	V	0	0.20
gtg	V	1	0.39
---	--	------	------
mPD		0.026	1.1
		nPD:	0.02
		N. weight:	1.5
		Sc. PD:	-0.051
		Sc. rank:	611.4

,

M2			
Pos.	41	obs:	exp:
tgg	W	1220	1220.00
---	--	------	------
mPD		0	0
		nPD:	1.
		N. weight:	0.
		Sc. PD:	0
		Sc. rank:	0

,

M2			
Pos.	42	obs:	exp:
att	I	1216	446.80
atc	I	0	291.40
ata	I	0	477.80
act	T	4	1.02
acc	T	0	0.82
aca	T	0	1.83
acg	T	0	0.32
---	--	------	------
mPD		0.0065	0.66
		nPD:	0.01
		N. weight:	0.92
		Sc. PD:	-0.044
		Sc. rank:	-7.2

,

M2			
Pos.	43	obs:	exp:
ttt	F	1	0.39
ttc	F	0	0.61
tta	L	0	106.80
ttg	L	0	224.20
ctt	L	1204	200.50
ctc	L	0	200.90
cta	L	0	202.90
ctg	L	0	268.70
att	I	14	5.14
atc	I	0	3.35
ata	I	0	5.50
gtt	V	1	0.21
gtc	V	0	0.20
gta	V	0	0.20
gtg	V	0	0.39
---	--	------	------
mPD		0.026	1.1
		nPD:	0.02
		N. weight:	1.6
		Sc. PD:	-0.057
		Sc. rank:	616.9

,

M2			
Pos.	44	obs:	exp:
gat	D	1220	659.30
gac	D	0	560.70
---	--	------	------
mPD		0	0.50
		nPD:	0.
		N. weight:	0.56
		Sc. PD:	-0.032
		Sc. rank:	-623.9

,

M2			
Pos.	45	obs:	exp:
cgt	R	1217	52.40
cgc	R	3	60.43
cga	R	0	119.40
cgg	R	0	101.80
aga	R	0	557.90
agg	R	0	328.10
---	--	------	------
mPD		0.0049	0.96
		nPD:	0.01
		N. weight:	2.9
		Sc. PD:	-0.15
		Sc. rank:	-735.5

,

M2			
Pos.	46	obs:	exp:
ttt	F	2	0.79
ttc	F	0	1.21
tta	L	0	108.10
ttg	L	0	226.80
ctt	L	1218	202.80
ctc	L	0	203.30
cta	L	0	205.30
ctg	L	0	271.80
---	--	------	------
mPD		0.0033	1.1
		nPD:	0.
		N. weight:	1.6
		Sc. PD:	-0.089
		Sc. rank:	-730.6

,

M2			
Pos.	47	obs:	exp:
ttt	F	8	481.80
ttc	F	1212	738.20
---	--	------	------
mPD		0.013	0.48
		nPD:	0.03
		N. weight:	0.43
		Sc. PD:	-0.013
		Sc. rank:	209.6

,

M2			
Pos.	48	obs:	exp:
ttt	F	12	481.40
ttc	F	1207	737.60
tct	S	0	0.16
tcc	S	1	0.13
tca	S	0	0.25
tcg	S	0	0.06
agt	S	0	0.21
agc	S	0	0.19
---	--	------	------
mPD		0.021	0.48
		nPD:	0.04
		N. weight:	0.42
		Sc. PD:	-0.0062
		Sc. rank:	258.0

,

M2			
Pos.	49	obs:	exp:
aaa	K	1220	711.00
aag	K	0	509.00
---	--	------	------
mPD		0	0.49
		nPD:	0.
		N. weight:	0.5
		Sc. PD:	-0.028
		Sc. rank:	-547.3

,

M2			
Pos.	50	obs:	exp:
tat	Y	0	5.94
tac	Y	11	5.06
tgt	C	1	511.40
tgc	C	1208	697.60
---	--	------	------
mPD		0.020	0.51
		nPD:	0.04
		N. weight:	0.5
		Sc. PD:	-0.01
		Sc. rank:	299.2

,

M2			
Pos.	51	obs:	exp:
tta	L	0	0.09
ttg	L	0	0.19
ctt	L	1	0.17
ctc	L	0	0.17
cta	L	0	0.17
ctg	L	0	0.22
att	I	1210	447.10
atc	I	7	291.60
ata	I	0	478.20
gtt	V	2	0.41
gtc	V	0	0.40
gta	V	0	0.41
gtg	V	0	0.77
---	--	------	------
mPD		0.016	0.66
		nPD:	0.02
		N. weight:	0.89
		Sc. PD:	-0.03
		Sc. rank:	400.9

,

M2			
Pos.	52	obs:	exp:
tat	Y	1213	657.80
tac	Y	5	560.20
cat	H	2	1.15
cac	H	0	0.85
---	--	------	------
mPD		0.011	0.50
		nPD:	0.02
		N. weight:	0.54
		Sc. PD:	-0.019
		Sc. rank:	183.7

,

M2			
Pos.	53	obs:	exp:
cgt	R	1220	52.40
cgc	R	0	60.43
cga	R	0	119.40
cgg	R	0	101.80
aga	R	0	557.90
agg	R	0	328.10
---	--	------	------
mPD		0	0.96
		nPD:	0.
		N. weight:	2.9
		Sc. PD:	-0.16
		Sc. rank:	-3190.8

,

M2			
Pos.	54	obs:	exp:
tta	L	0	0.09
ttg	L	0	0.19
ctt	L	0	0.17
ctc	L	1	0.17
cta	L	0	0.17
ctg	L	0	0.22
cgt	R	424	52.35
cgc	R	794	60.38
cga	R	1	119.30
cgg	R	0	101.70
aga	R	0	557.40
agg	R	0	327.90
---	--	------	------
mPD		0.46	0.96
		nPD:	0.47
		N. weight:	2.2
		Sc. PD:	0.87
		Sc. rank:	2520.4

,

M2			
Pos.	55	obs:	exp:
ttt	F	1210	477.80
ttc	F	0	732.20
tta	L	0	0.71
ttg	L	0	1.49
ctt	L	8	1.33
ctc	L	0	1.34
cta	L	0	1.35
ctg	L	0	1.79
gtt	V	2	0.41
gtc	V	0	0.40
gta	V	0	0.41
gtg	V	0	0.77
---	--	------	------
mPD		0.016	0.50
		nPD:	0.03
		N. weight:	0.86
		Sc. PD:	-0.022
		Sc. rank:	453.9

,

M2			
Pos.	56	obs:	exp:
aaa	K	1220	711.00
aag	K	0	509.00
---	--	------	------
mPD		0	0.49
		nPD:	0.
		N. weight:	0.5
		Sc. PD:	-0.028
		Sc. rank:	-547.3

,

M2			
Pos.	57	obs:	exp:
tat	Y	0	658.90
tac	Y	1220	561.10
---	--	------	------
mPD		0	0.50
		nPD:	0.
		N. weight:	0.71
		Sc. PD:	-0.04
		Sc. rank:	-787.4

,

M2			
Pos.	58	obs:	exp:
ggt	G	1216	161.70
ggc	G	2	155.90
gga	G	0	562.80
ggg	G	2	339.60
---	--	------	------
mPD		0.0065	0.68
		nPD:	0.01
		N. weight:	1.8
		Sc. PD:	-0.087
		Sc. rank:	-54.0

,

M2			
Pos.	59	obs:	exp:
tta	L	40	108.20
ttg	L	1176	227.00
ctt	L	0	203.00
ctc	L	0	203.40
cta	L	0	205.40
ctg	L	3	272.00
gtt	V	0	0.21
gtc	V	0	0.20
gta	V	0	0.20
gtg	V	1	0.39
---	--	------	------
mPD		0.070	1.1
		nPD:	0.06
		N. weight:	1.4
		Sc. PD:	0.0047
		Sc. rank:	1101.3

,

M2			
Pos.	60	obs:	exp:
aaa	K	1220	711.00
aag	K	0	509.00
---	--	------	------
mPD		0	0.49
		nPD:	0.
		N. weight:	0.5
		Sc. PD:	-0.028
		Sc. rank:	-547.3

,

M2			
Pos.	61	obs:	exp:
cgt	R	0	52.40
cgc	R	0	60.43
cga	R	0	119.40
cgg	R	0	101.80
aga	R	1218	557.90
agg	R	2	328.10
---	--	------	------
mPD		0.0033	0.96
		nPD:	0.
		N. weight:	0.71
		Sc. PD:	-0.038
		Sc. rank:	-274.4

M2			
Pos.	62	obs:	exp:
gaa	E	0	0.61
gag	E	1	0.39
ggt	G	0	161.60
ggc	G	3	155.70
gga	G	2	562.40
ggg	G	1214	339.40
---	--	------	------
mPD		0.0098	0.68
		nPD:	0.01
		N. weight:	1.1
		Sc. PD:	-0.049
		Sc. rank:	235.8

M2			
Pos.	63	obs:	exp:
tct	S	1	0.16
tcc	S	0	0.13
tca	S	0	0.25
tcg	S	0	0.06
cct	P	1219	304.20
ccc	P	0	219.00
cca	P	0	501.80
ccg	P	0	194.00
agt	S	0	0.21
agc	S	0	0.19
---	--	------	------
mPD		0.0016	0.71
		nPD:	0.
		N. weight:	1.3
		Sc. PD:	-0.07
		Sc. rank:	-685.1

M2			
Pos.	64	obs:	exp:
tct	S	1207	193.70
tcc	S	11	159.10
tca	S	0	309.10
tcg	S	0	69.11
cct	P	2	0.50
ccc	P	0	0.36
cca	P	0	0.82
ccg	P	0	0.32
agt	S	0	250.40
agc	S	0	236.60
---	--	------	------
mPD		0.021	1.7
		nPD:	0.01
		N. weight:	1.6
		Sc. PD:	-0.073
		Sc. rank:	234.7

M2			
Pos.	65	obs:	exp:
atg	M	1	1.00
act	T	0	301.30
acc	T	0	242.50
aca	T	5	542.10
acg	T	1177	96.01
aaa	K	0	21.56
aag	K	37	15.44
---	--	------	------
mPD		0.069	0.74
		nPD:	0.09
		N. weight:	2.2
		Sc. PD:	0.071
		Sc. rank:	2076.1

M2			
Pos.	66	obs:	exp:
gaa	E	1220	742.40
gag	E	0	477.60
---	--	------	------
mPD		0	0.48
		nPD:	0.
		N. weight:	0.46
		Sc. PD:	-0.026
		Sc. rank:	-503.5

M2			
Pos.	67	obs:	exp:
ggt	G	0	161.70
ggc	G	0	155.90
gga	G	1216	562.80
ggg	G	4	339.60
---	--	------	------
mPD		0.0065	0.68
		nPD:	0.01
		N. weight:	0.69
		Sc. PD:	-0.033
		Sc. rank:	-50.3

M2			
Pos.	68	obs:	exp:
att	I	0	0.73
atc	I	0	0.48
ata	I	2	0.79
atg	M	1136	1136.00
gtt	V	0	16.99
gtc	V	0	16.55
gta	V	0	16.79
gtg	V	82	31.67
---	--	------	------
mPD		0.13	0.21
		nPD:	0.62
		N. weight:	0.06
		Sc. PD:	0.032
		Sc. rank:	68.7

M2			
Pos.	69	obs:	exp:
cct	P	1217	304.40
ccc	P	1	219.20
cca	P	2	502.20
ccg	P	0	194.20
---	--	------	------
mPD		0.0049	0.71
		nPD:	0.01
		N. weight:	1.3
		Sc. PD:	-0.063
		Sc. rank:	-259.2

M2			
Pos.	70	obs:	exp:
aaa	K	0	1.17
aag	K	2	0.83
gaa	E	7	741.20
gag	E	1211	476.80
---	--	------	------
mPD		0.015	0.48
		nPD:	0.03
		N. weight:	0.83
		Sc. PD:	-0.023
		Sc. rank:	420.4

M2			
Pos.	71	obs:	exp:
tct	S	1204	194.00
tcc	S	16	159.30
tca	S	0	309.60
tcg	S	0	69.23
agt	S	0	250.80
agc	S	0	237.00
---	--	------	------
mPD		0.026	1.7
		nPD:	0.02
		N. weight:	1.6
		Sc. PD:	-0.068
		Sc. rank:	396.2

M2			
Pos.	72	obs:	exp:
atg	M	1220	1220.00
---	--	------	------
mPD		0	0
		nPD:	1.
		N. weight:	0.
		Sc. PD:	0
		Sc. rank:	0

M2			
Pos.	73	obs:	exp:
cgt	R	0	52.40
cgc	R	0	60.43
cga	R	0	119.40
cgg	R	0	101.80
aga	R	0	557.90
agg	R	1220	328.10
---	--	------	------
mPD		0	0.96
		nPD:	0.
		N. weight:	1.2
		Sc. PD:	-0.068
		Sc. rank:	-1331.1

M2			
Pos.	74	obs:	exp:
gaa	E	1219	742.40
gag	E	1	477.60
---	--	------	------
mPD		0.0016	0.48
		nPD:	0.
		N. weight:	0.45
		Sc. PD:	-0.024
		Sc. rank:	-169.7

M2			
Pos.	75	obs:	exp:
gaa	E	1202	742.40
gag	E	18	477.60
---	--	------	------
mPD		0.029	0.48
		nPD:	0.06
		N. weight:	0.39
		Sc. PD:	0.00055
		Sc. rank:	285.6

M2			
Pos.	76	obs:	exp:
tat	Y	1207	658.90
tac	Y	13	561.10
---	--	------	------
mPD		0.021	0.50
		nPD:	0.04
		N. weight:	0.51
		Sc. PD:	-0.0084
		Sc. rank:	313.2

M2			
Pos.	77	obs:	exp:
cgt	R	0	52.40
cgc	R	0	60.43
cga	R	7	119.40
cgg	R	1213	101.80
aga	R	0	557.90
agg	R	0	328.10
---	--	------	------
mPD		0.011	0.96
		nPD:	0.01
		N. weight:	2.2
		Sc. PD:	-0.1
		Sc. rank:	186.2

M2			
Pos.	78	obs:	exp:
caa	Q	0	621.40
cag	Q	1220	598.60
---	--	------	------
mPD		0	0.50
		nPD:	0.
		N. weight:	0.65
		Sc. PD:	-0.037
		Sc. rank:	-721.8

M2			
Pos.	79	obs:	exp:
gaa	E	1210	742.40
gag	E	10	477.60
---	--	------	------
mPD		0.016	0.48
		nPD:	0.03
		N. weight:	0.42
		Sc. PD:	-0.01
		Sc. rank:	224.5

M2			
Pos.	80	obs:	exp:
caa	Q	0	610.20
cag	Q	1198	587.80
cgt	R	0	0.94
cgc	R	0	1.09
cga	R	0	2.15
cgg	R	22	1.84
aga	R	0	10.06
agg	R	0	5.92
---	--	------	------
mPD		0.035	0.56
		nPD:	0.06
		N. weight:	0.68
		Sc. PD:	0.0022
		Sc. rank:	507.0

M2			
Pos.	81	obs:	exp:
caa	Q	4	621.40
cag	Q	1216	598.60
---	--	------	------
mPD		0.0065	0.50
		nPD:	0.01
		N. weight:	0.63
		Sc. PD:	-0.028
		Sc. rank:	100.1

M2			
Pos.	82	obs:	exp:
tct	S	0	55.99
tcc	S	0	45.97
tca	S	0	89.34
tcg	S	0	19.97
att	I	1	0.37
atc	I	0	0.24
ata	I	0	0.39
aat	N	867	441.60
aac	N	0	425.40
agt	S	352	72.36
agc	S	0	68.37
---	--	------	------
mPD		0.41	1.3
		nPD:	0.31
		N. weight:	0.86
		Sc. PD:	0.21
		Sc. rank:	898.8

M2			
Pos.	83	obs:	exp:
gct	A	1220	315.00
gcc	A	0	227.20
gca	A	0	574.20
gcg	A	0	103.50
---	--	------	------
mPD		0	0.67
		nPD:	0.
		N. weight:	1.2
		Sc. PD:	-0.071
		Sc. rank:	-1372.4

M2			
Pos.	84	obs:	exp:
gtt	V	0	252.80
gtc	V	0	246.20
gta	V	0	249.80
gtg	V	1220	471.20
---	--	------	------
mPD		0	0.73
		nPD:	0.
		N. weight:	0.87
		Sc. PD:	-0.05
		Sc. rank:	-964.4

M2			
Pos.	85	obs:	exp:
gat	D	1219	658.70
gac	D	0	560.30
ggt	G	1	0.13
ggc	G	0	0.13
gga	G	0	0.46
ggg	G	0	0.28
---	--	------	------
mPD		0.0016	0.50
		nPD:	0.
		N. weight:	0.57
		Sc. PD:	-0.03
		Sc. rank:	-235.2

M2			
Pos.	86	obs:	exp:
gtt	V	1220	252.80
gtc	V	0	246.20
gta	V	0	249.80
gtg	V	0	471.20
---	--	------	------
mPD		0	0.73
		nPD:	0.
		N. weight:	1.4
		Sc. PD:	-0.082
		Sc. rank:	-1595.6

M2			
Pos.	87	obs:	exp:
gat	D	0	659.30
gac	D	1220	560.70
---	--	------	------
mPD		0	0.50
		nPD:	0.
		N. weight:	0.71
		Sc. PD:	-0.041
		Sc. rank:	-788.0

M2			
Pos.	88	obs:	exp:
tat	Y	2	1.08
tac	Y	0	0.92
aat	N	8	4.07
aac	N	0	3.93
gct	A	9	2.32
gcc	A	0	1.68
gca	A	0	4.24
gcg	A	0	0.76
gat	D	1154	625.80
gac	D	4	532.20
ggt	G	43	5.70
ggc	G	0	5.49
gga	G	0	19.84
ggg	G	0	11.97
---	--	------	------
mPD		0.11	0.62
		nPD:	0.17
		N. weight:	0.6
		Sc. PD:	0.062
		Sc. rank:	586.2

M2			
Pos.	89	obs:	exp:
tct	S	0	4.45
tcc	S	0	3.66
tca	S	0	7.11
tcg	S	0	1.59
tgt	C	1	0.42
tgc	C	0	0.58
agt	S	28	5.76
agc	S	0	5.44
gat	D	1	0.54
gac	D	0	0.46
ggt	G	1188	157.70
ggc	G	2	152.00
gga	G	0	549.00
ggg	G	0	331.30
---	--	------	------
mPD		0.051	0.76
		nPD:	0.07
		N. weight:	1.8
		Sc. PD:	0.015
		Sc. rank:	1560.4

M2			
Pos.	90	obs:	exp:
cat	H	1220	700.50
cac	H	0	519.50
---	--	------	------
mPD		0	0.49
		nPD:	0.
		N. weight:	0.51
		Sc. PD:	-0.029
		Sc. rank:	-562.3

M2			
Pos.	91	obs:	exp:
ttt	F	1220	481.80
ttc	F	0	738.20
---	--	------	------
mPD		0	0.48
		nPD:	0.
		N. weight:	0.85
		Sc. PD:	-0.048
		Sc. rank:	-941.8

M2			
Pos.	92	obs:	exp:
att	I	0	0.73
atc	I	2	0.48
ata	I	0	0.79
gtt	V	0	252.40
gtc	V	1218	245.80
gta	V	0	249.40
gtg	V	0	470.40
---	--	------	------
mPD		0.0033	0.73
		nPD:	0.
		N. weight:	1.5
		Sc. PD:	-0.077
		Sc. rank:	-475.2

M2			
Pos.	93	obs:	exp:
act	T	0	0.25
acc	T	1	0.21
aca	T	0	0.46
acg	T	0	0.08
aat	N	0	620.90
aac	N	1219	598.10
---	--	------	------
mPD		0.0016	0.50
		nPD:	0.
		N. weight:	0.65
		Sc. PD:	-0.035
		Sc. rank:	-280.6

M2			
Pos.	94	obs:	exp:
att	I	0	448.30
atc	I	0	292.40
ata	I	1220	479.40
---	--	------	------
mPD		0	0.65
		nPD:	0.
		N. weight:	0.86
		Sc. PD:	-0.049
		Sc. rank:	-946.9

M2			
Pos.	95	obs:	exp:
aaa	K	0	0.58
aag	K	1	0.42
gaa	E	0	741.20
gag	E	1218	476.80
ggt	G	0	0.13
ggc	G	0	0.13
gga	G	0	0.46
ggg	G	1	0.28
---	--	------	------
mPD		0.0033	0.48
		nPD:	0.01
		N. weight:	0.86
		Sc. PD:	-0.043
		Sc. rank:	-202.9

M2			
Pos.	96	obs:	exp:
tta	L	0	108.30
ttg	L	0	227.10
ctt	L	0	203.10
ctc	L	0	203.60
cta	L	58	205.60
ctg	L	1162	272.20
---	--	------	------
mPD		0.091	1.1
		nPD:	0.08
		N. weight:	1.2
		Sc. PD:	0.026
		Sc. rank:	1069.4

M2			
Pos.	97	obs:	exp:
aaa	K	4	680.70
aag	K	1164	487.30
gaa	E	0	31.64
gag	E	52	20.36
---	--	------	------
mPD		0.088	0.57
		nPD:	0.16
		N. weight:	0.78
		Sc. PD:	0.072
		Sc. rank:	759.3

M2			
Pos.	98	obs:	exp:
taa	.	1218	518.30
tag	.	2	305.40
tga	.	0	396.30
---	--	------	------
mPD		0.0033	0.81
		nPD:	0.
		N. weight:	0.78
		Sc. PD:	-0.041
		Sc. rank:	-278.1

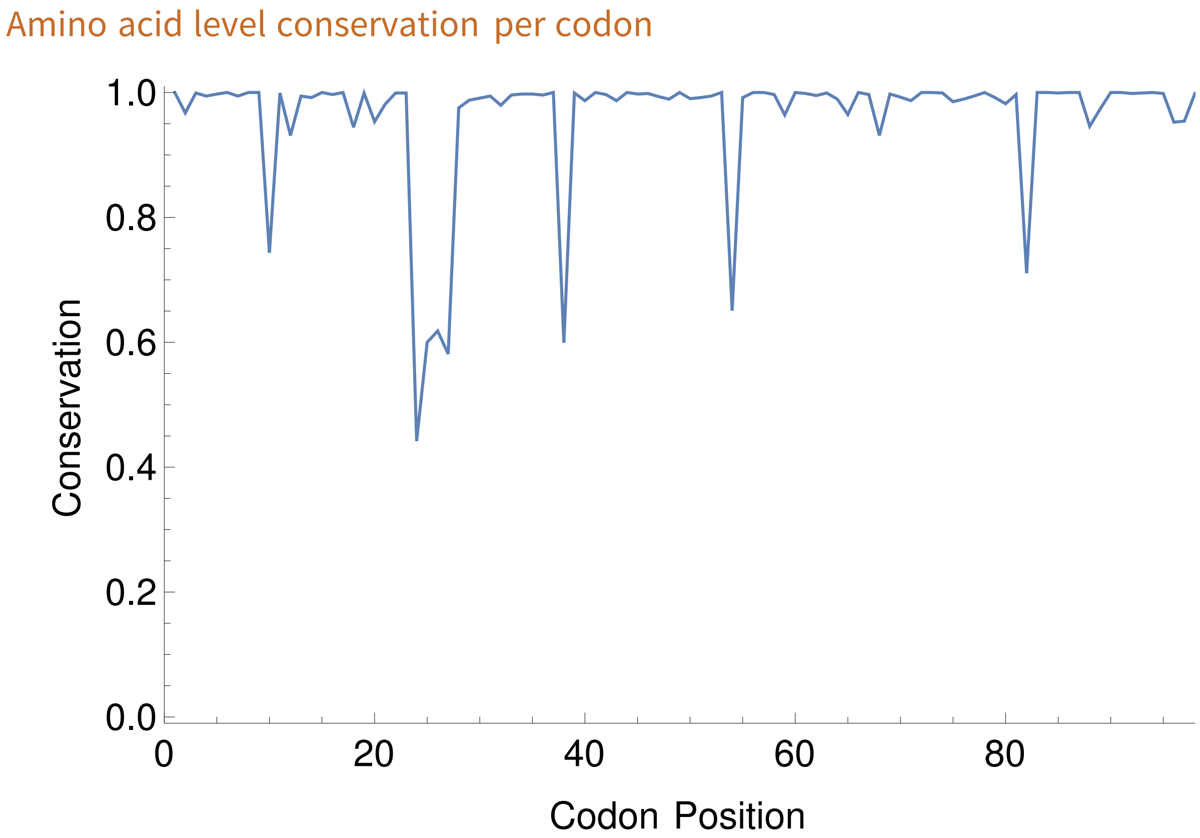
Amino acid level conservation per codon
Conservation
1.0
0.8
0.6
0.4
0.2
0.0
0
20
40
60
80
Codon Position

Conservation analysis plots

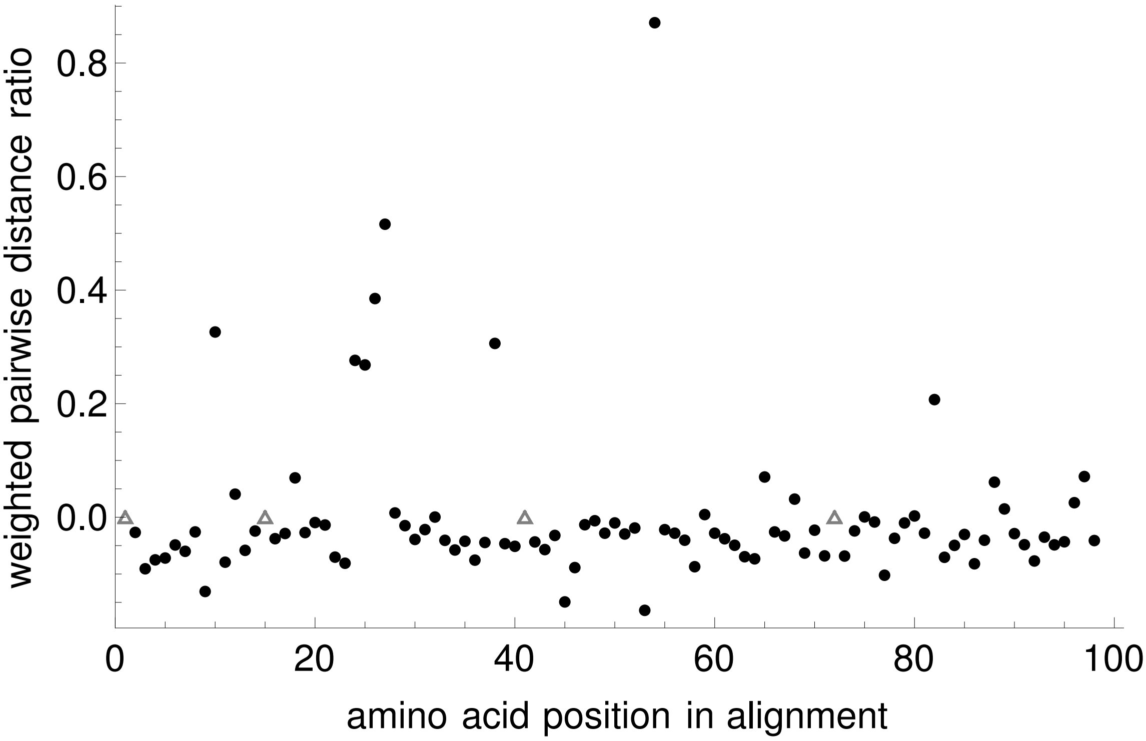

M2

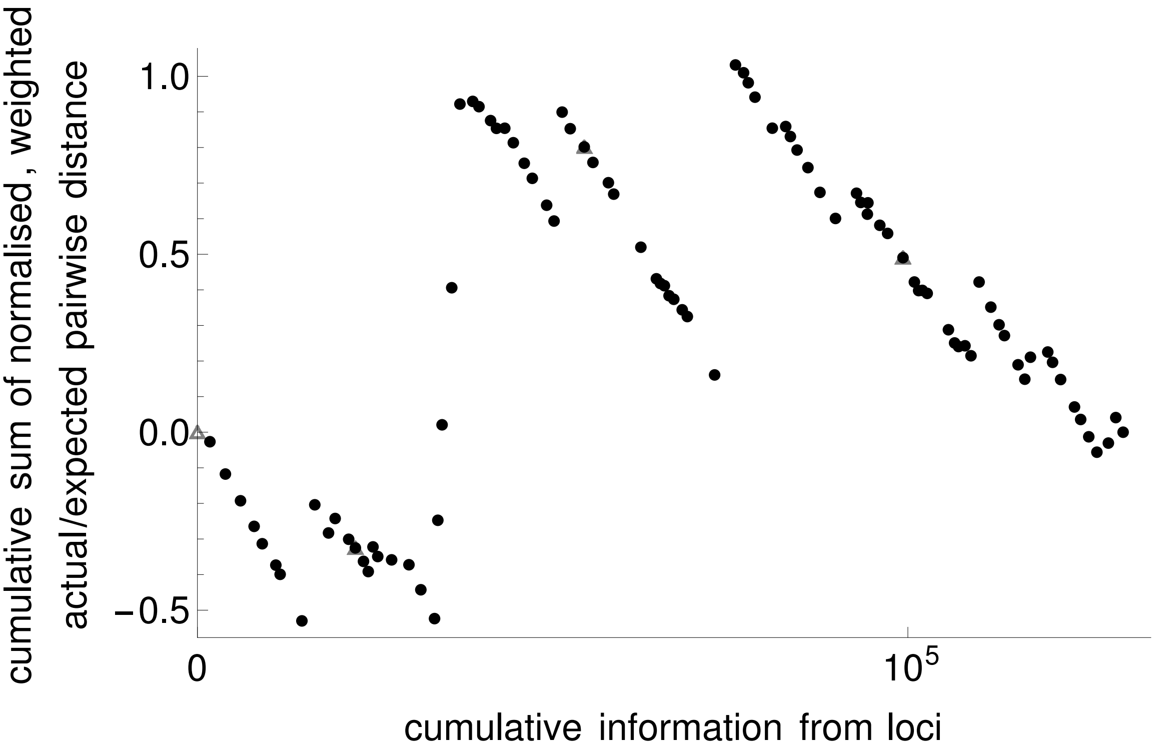

M2

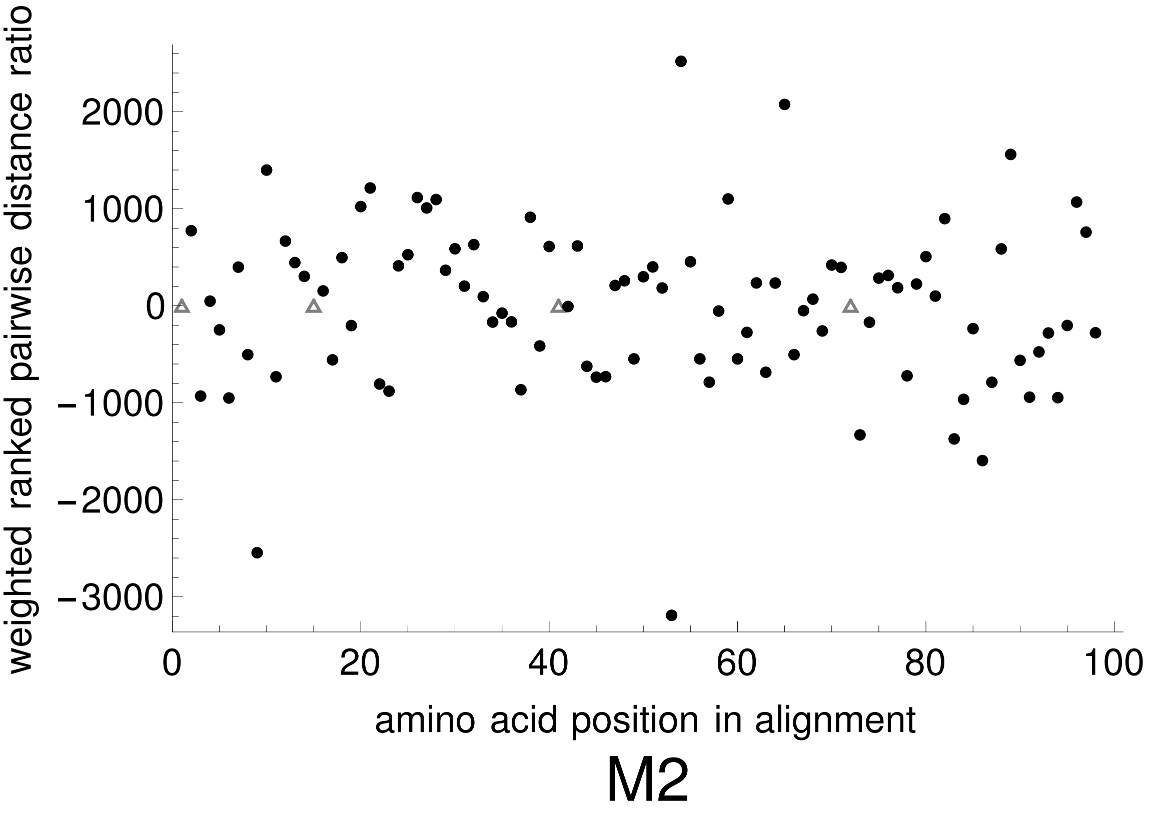
weighted ranked pairwise distance ratio
2000
1000
0
−1000
−2000
−3000
0
20
40
60
80
100
amino acid position in alignment
M2

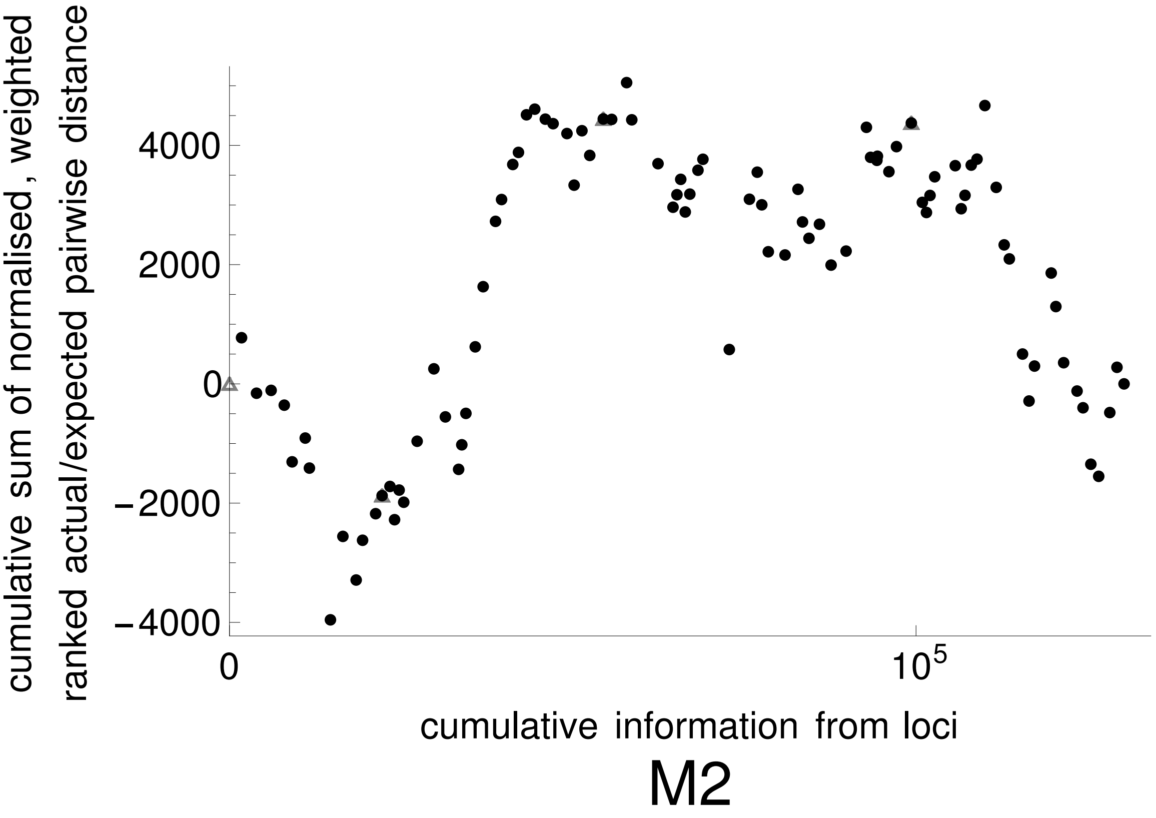
cumulative sum of normalised, weighted ranked actual/expected pairwise distance
4000
2000
0
−2000
−4000
0
10^5
cumulative information from loci
M2

NS1

Gene length histogram

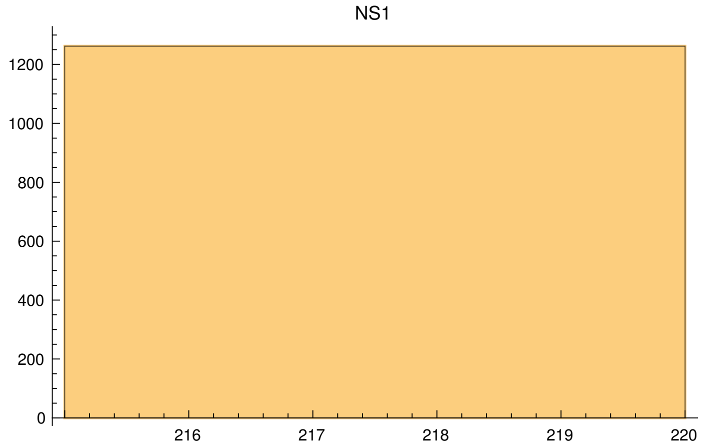

Information vs. nPD

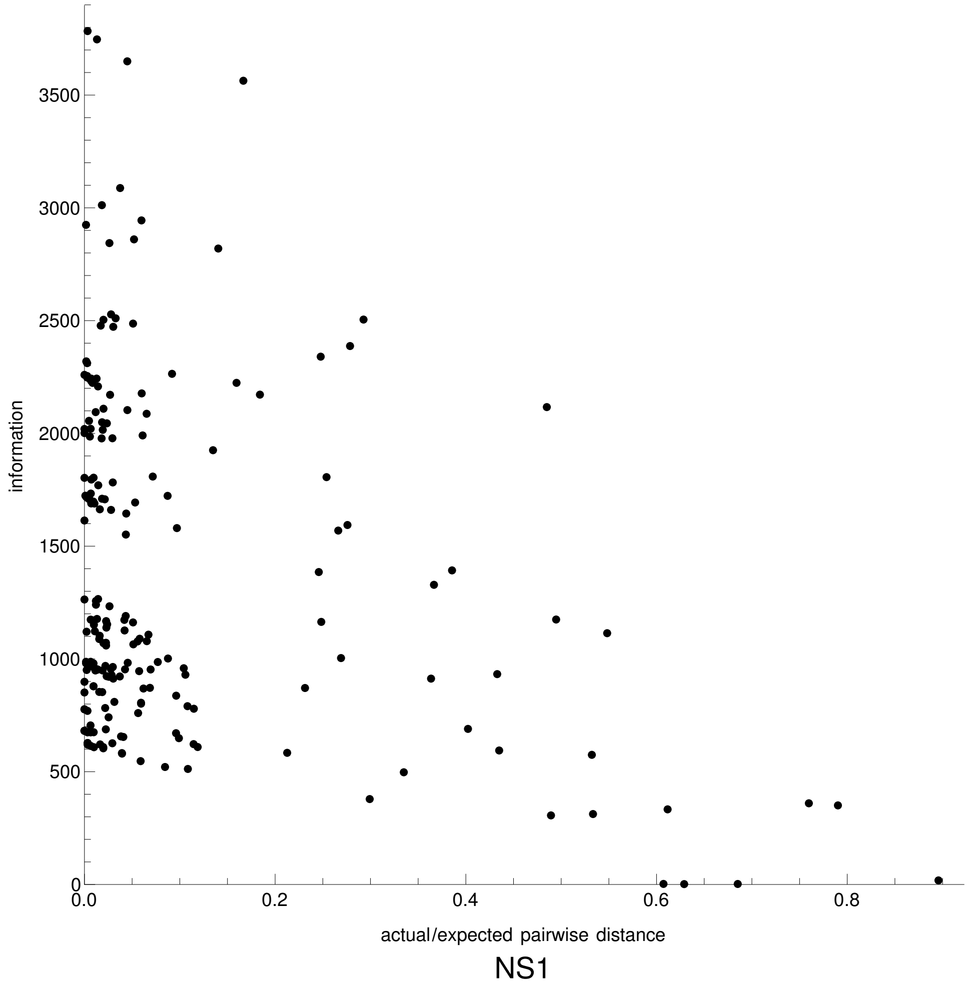

Example sequences highlighted by regions found in analyses to be conserved

Interesting points (by weighted raw PD) highlighted for gene NS1:

ATGGATTCCAAT

**ACTGTGTCAAGCTTCCAGGTAGACTGCTTTCTTTGGCATGTCCGCAAACGATTTG
CAGACCAAGAAATGGGTGATGCCCCATTTCTAGACCGGCTTCGCCGAGATCA
GAAGTCCCTGAGGGGAAGAAGCAGCACTCTTGGT**

CTAGACATCAGAACTGCCACGCGTGAAGGAAAGCATATAGTGGAG ---CGGATTTTAGAAGAAGAGTCAGAT⋱
GAAGCATTTAAAATGAGTATTGCTTCAGTACCAGCTCCACGCTATCTAACTGACATGACTCTTGAAGAAATGTCA⋱
AGAGATTGGTTAATGCTCATCCCCAAACAGAAAATAACAGGGTCCCTATGCATTAGAATGGACCAAGCAATAGTG⋱
GACAAAAACATCACATTGAAAGCAAATTTCAGTGTGATTTTCAATCGGCTTGAAGCCCTGATACTACTTAGAGCT⋱
TTTACGGAAAAAGGAGCAATAGTA

**GGCGAAATCTCACCATTACCTTCTCTTCCAGGACATACTGACAAGGATGTCAAAA
ATGCAATTGAGATCCTCATCGGAGGATTTGAATGGAATGATAACACAGTTCG
AGTCTCTGAAACTCTACAGAGATTCGCTTGGAGAAGC**

AGCGATGAGGATGGGAGATCTCCACTCTCTACAAAG --
--------------------TAG

Interesting points (by weighted ranked PD) highlighted for gene NS1:

ATGGATTCCAATACTGTGTCAAGCTTCCAGGTAGACTGCTTTCTTTGGCATGTCCGCAAACGATTTGCAGACCAAGAA⋱
ATGGGTGATGCCCCATTTCTAGACCGGCTTCGCCGAGATCAGAAGTCCCTGAGGGGAAGAAGCAGCACTCTTGGTCT⋱
AGACATCAGAACTGCCACGCGTGAAGGAAAGCATATAGTGGAG ---CGGATTTTAGAAGAAGAGTCAGATGAAGCATT
TAAAATGAGTATTGCTTCAGTACCAGCTCCACGCTATCTAACTGACATGACTCTTGAAGAAATGTCAAGAGATTG⋱
GTTAATGCTCATCCCCAAACAGAAAATAACAGGGTCCCTATGCATTAGAATGGACCAAGCAATAGTGGACAAAAA⋱
CATCACATTGAAAGCAAATTTCAGTGTGATTTTCAATCGGCTTGAAGCCCTGATACTACTTAGAGCTTTTACGGA⋱
AAAAGGAGCAATAGTAGGCGAA

**ATCTCACCATTACCTTCTCTTCCAGGACATACTGACAAGGATGTCAAAAATGCAA
TTGAGATCCTCATCGGAGGATTTGAATGGAATGATAACACAGTTCGAGTCTC
TGAAACTCTACAGAGATTCGCTTGGAGA**

AGCAGCGATGAGGATGGGAGATCTCCACTCTCTACAAAG ------------------------------------
------------------------TAG

Per codon conservation report

NS1

Pos.	1	obs:	exp:
atg	M	1262	1262.00
---	--	------	------
mPD		0	0
		nPD:	1.
		N. weight:	0.
		Sc. PD:	0
		Sc. rank:	0

NS1

Pos.	2	obs:	exp:
aat	N	2	1.53
aac	N	1	1.47
gat	D	1231	680.30
gac	D	28	578.70
---	--	------	------
mPD		0.050	0.50
		nPD:	0.1
		N. weight:	0.54
		Sc. PD:	0.015
		Sc. rank:	369.8

NS1

Pos.	3	obs:	exp:
ttt	F	0	0.39
ttc	F	1	0.61
tta	L	0	0.09
ttg	L	0	0.19
tct	S	9	199.10
tcc	S	1242	163.50
tca	S	1	317.80
tcg	S	0	71.04
ctt	L	0	0.17
ctc	L	1	0.17
cta	L	0	0.17
ctg	L	0	0.22
cct	P	0	2.00
ccc	P	8	1.44
cca	P	0	3.29
ccg	P	0	1.27
agt	S	0	257.40
agc	S	0	243.20
---	--	------	------
mPD		0.033	1.7
		nPD:	0.02
		N. weight:	2.1
		Sc. PD:	-0.086
		Sc. rank:	-148.3

NS1

Pos.	4	obs:	exp:
cat	H	1	0.57
cac	H	0	0.43
aat	N	1062	642.30
aac	N	199	618.70
---	--	------	------
mPD		0.27	0.50
		nPD:	0.53
		N. weight:	0.26
		Sc. PD:	0.11
		Sc. rank:	265.4

NS1

Pos.	5	obs:	exp:
act	T	1258	321.70
acc	T	0	259.00
aca	T	3	578.80
acg	T	1	102.50
---	--	------	------
mPD		0.0063	0.68
		nPD:	0.01
		N. weight:	1.4
		Sc. PD:	-0.072
		Sc. rank:	-663.5

NS1

Pos.	6	obs:	exp:
att	I	0	0.37
atc	I	0	0.24
ata	I	1	0.39
atg	M	16	16.00
gtt	V	0	258.00
gtc	V	0	251.30
gta	V	2	254.90
gtg	V	1243	480.80
---	--	------	------
mPD		0.031	0.75
		nPD:	0.04
		N. weight:	0.98
		Sc. PD:	-0.022
		Sc. rank:	281.8

NS1

Pos.	7	obs:	exp:
tct	S	0	200.60
tcc	S	0	164.70
tca	S	1258	320.10
tcg	S	3	71.55
act	T	0	0.25
acc	T	1	0.21
aca	T	0	0.46
acg	T	0	0.08
agt	S	0	259.20
agc	S	0	244.90
---	--	------	------
mPD		0.0079	1.7
		nPD:	0.
		N. weight:	1.4
		Sc. PD:	-0.078
		Sc. rank:	-939.4

NS1

Pos.	8	obs:	exp:
tct	S	0	200.70
tcc	S	0	164.80
tca	S	0	320.30
tcg	S	1	71.61
agt	S	16	259.40
agc	S	1245	245.10
---	--	------	------
mPD		0.030	1.7
		nPD:	0.02
		N. weight:	1.6
		Sc. PD:	-0.071
		Sc. rank:	-288.0

NS1

Pos.	9	obs:	exp:
ttt	F	26	498.40
ttc	F	1236	763.60
---	--	------	------
mPD		0.040	0.48
		nPD:	0.08
		N. weight:	0.43
		Sc. PD:	0.0068
		Sc. rank:	269.5

NS1

Pos.	10	obs:	exp:
caa	Q	0	642.80
cag	Q	1262	619.20
---	--	------	------
mPD		0	0.50
		nPD:	0.
		N. weight:	0.75
		Sc. PD:	-0.044
		Sc. rank:	-773.3

NS1

Pos.	11	obs:	exp:
gtt	V	0	261.50
gtc	V	0	254.70
gta	V	1262	258.40
gtg	V	0	487.40
---	--	------	------
mPD		0	0.73
		nPD:	0.
		N. weight:	1.7
		Sc. PD:	-0.098
		Sc. rank:	-1722.5

NS1

Pos.	12	obs:	exp:
gat	D	9	682.00
gac	D	1253	580.00
---	--	------	------
mPD		0.014	0.50
		nPD:	0.03
		N. weight:	0.77
		Sc. PD:	-0.026
		Sc. rank:	112.4

NS1

Pos.	13	obs:	exp:
tgt	C	100	533.80
tgc	C	1162	728.20
---	--	------	------
mPD		0.15	0.49
		nPD:	0.3
		N. weight:	0.32
		Sc. PD:	0.065
		Sc. rank:	297.1

NS1

Pos.	14	obs:	exp:
ttt	F	1255	497.60
ttc	F	5	762.40
tat	Y	2	1.08
tac	Y	0	0.92
---	--	------	------
mPD		0.011	0.48
		nPD:	0.02
		N. weight:	0.95
		Sc. PD:	-0.037
		Sc. rank:	13.4

NS1

Pos.	15	obs:	exp:
ttt	F	1	0.39
ttc	F	0	0.61
tta	L	0	111.90
ttg	L	0	234.80
ctt	L	1258	210.00
ctc	L	0	210.40
cta	L	0	212.50
ctg	L	3	281.40
---	--	------	------
mPD		0.0063	1.1
		nPD:	0.01
		N. weight:	1.9
		Sc. PD:	-0.1
		Sc. rank:	-1196.9

NS1

Pos.	16	obs:	exp:
tgg	W	1262	1262.00
---	--	------	------
mPD		0	0
		nPD:	1.
		N. weight:	0.
		Sc. PD:	0
		Sc. rank:	0

NS1

Pos.	17	obs:	exp:
cat	H	1255	721.80
cac	H	2	535.20
caa	Q	0	2.04
cag	Q	4	1.96
aat	N	1	0.51
aac	N	0	0.49
---	--	------	------
mPD		0.011	0.49
		nPD:	0.02
		N. weight:	0.57
		Sc. PD:	-0.022
		Sc. rank:	-10.3

NS1

Pos.	18	obs:	exp:
att	I	0	2.94
atc	I	7	1.92
ata	I	1	3.14
gtt	V	0	259.80
gtc	V	1254	253.10
gta	V	0	256.80
gtg	V	0	484.30
---	--	------	------
mPD		0.014	0.74
		nPD:	0.02
		N. weight:	1.7
		Sc. PD:	-0.071
		Sc. rank:	-164.7

NS1

Pos.	19	obs:	exp:
cgt	R	0	54.20
cgc	R	1261	62.51
cga	R	0	123.50
cgg	R	0	105.30
aga	R	0	577.10
agg	R	1	339.40
---	--	------	------
mPD		0.0032	0.96
		nPD:	0.
		N. weight:	3.2
		Sc. PD:	-0.18
		Sc. rank:	-2301.7

NS1

Pos.	20	obs:	exp:
cgt	R	0	0.09
cgc	R	0	0.10
cga	R	0	0.20
cgg	R	0	0.17
aaa	K	1259	734.40
aag	K	1	525.60
aga	R	2	0.91
agg	R	0	0.54
---	--	------	------
mPD		0.0048	0.49
		nPD:	0.01
		N. weight:	0.56
		Sc. PD:	-0.028
		Sc. rank:	-237.8

NS1			
Pos.	21	obs:	exp:
tta	L	0	0.35
ttg	L	0	0.74
ctt	L	0	0.67
ctc	L	0	0.67
cta	L	3	0.67
ctg	L	1	0.89
caa	Q	11	5.60
cag	Q	0	5.40
cgt	R	0	53.56
cgc	R	0	61.76
cga	R	1063	122.00
cgg	R	178	104.10
aga	R	6	570.20
agg	R	0	335.40
---	--	------	------
mPD		0.28	0.99
		nPD:	0.28
		N. weight:	2.
		Sc. PD:	0.37
		Sc. rank:	1831.1

NS1			
Pos.	22	obs:	exp:
ttt	F	1256	496.40
ttc	F	1	760.60
tta	L	0	0.44
ttg	L	0	0.93
ctt	L	4	0.83
ctc	L	1	0.83
cta	L	0	0.84
ctg	L	0	1.12
---	--	------	------
mPD		0.011	0.49
		nPD:	0.02
		N. weight:	0.97
		Sc. PD:	-0.038
		Sc. rank:	5.7

NS1			
Pos.	23	obs:	exp:
tct	S	0	0.16
tcc	S	0	0.13
tca	S	0	0.25
tcg	S	0	0.06
act	T	0	0.51
acc	T	0	0.41
aca	T	2	0.92
acg	T	0	0.16
agt	S	0	0.21
agc	S	1	0.19
gtt	V	0	0.21
gtc	V	0	0.20
gta	V	1	0.20
gtg	V	0	0.39
gct	A	0	324.90
gcc	A	0	234.30
gca	A	1258	592.10
gcg	A	0	106.80
---	--	------	------
mPD		0.0095	0.68
		nPD:	0.01
		N. weight:	0.79
		Sc. PD:	-0.037
		Sc. rank:	-225.3

NS1			
Pos.	24	obs:	exp:
atg	M	1	1.00
gat	D	0	681.40
gac	D	1261	579.60
---	--	------	------
mPD		0.0048	0.50
		nPD:	0.01
		N. weight:	0.82
		Sc. PD:	-0.041
		Sc. rank:	-377.3

NS1			
Pos.	25	obs:	exp:
caa	Q	1258	640.80
cag	Q	0	617.20
cgt	R	0	0.13
cgc	R	0	0.15
cga	R	1	0.29
cgg	R	1	0.25
aaa	K	1	0.58
aag	K	0	0.42
aga	R	1	1.37
agg	R	0	0.81
---	--	------	------
mPD		0.0095	0.51
		nPD:	0.02
		N. weight:	0.71
		Sc. PD:	-0.03
		Sc. rank:	-85.0

NS1			
Pos.	26	obs:	exp:
aaa	K	1	0.58
aag	K	0	0.42
gat	D	0	0.54
gac	D	1	0.46
gaa	E	1257	765.50
gag	E	1	492.50
ggt	G	0	0.27
ggc	G	0	0.26
gga	G	2	0.92
ggg	G	0	0.56
---	--	------	------
mPD		0.0079	0.48
		nPD:	0.02
		N. weight:	0.52
		Sc. PD:	-0.023
		Sc. rank:	-107.2

NS1			
Pos.	27	obs:	exp:
tta	L	0	16.06
ttg	L	0	33.70
ctt	L	0	30.14
ctc	L	0	30.21
cta	L	0	30.50
ctg	L	181	40.39
cgt	R	0	0.04
cgc	R	0	0.05
cga	R	0	0.10
cgg	R	0	0.08
atg	M	1011	1011.00
aaa	K	0	40.22
aag	K	69	28.78
aga	R	0	0.46
agg	R	1	0.27
---	--	------	------
mPD		0.35	0.57
		nPD:	0.61
		N. weight:	0.28
		Sc. PD:	0.13
		Sc. rank:	286.0

NS1			
Pos.	28	obs:	exp:
tgt	C	1	0.42
tgc	C	0	0.58
ggt	G	1249	167.10
ggc	G	8	161.10
gga	G	4	581.70
ggg	G	0	351.00
---	--	------	------
mPD		0.020	0.68
		nPD:	0.03
		N. weight:	2.1
		Sc. PD:	-0.067
		Sc. rank:	430.0

NS1			
Pos.	29	obs:	exp:
gat	D	1261	682.00
gac	D	1	580.00
---	--	------	------
mPD		0.0016	0.50
		nPD:	0.
		N. weight:	0.64
		Sc. PD:	-0.036
		Sc. rank:	-493.5

NS1			
Pos.	30	obs:	exp:
act	T	0	0.25
acc	T	1	0.21
aca	T	0	0.46
acg	T	0	0.08
gct	A	4	325.60
gcc	A	1257	234.80
gca	A	0	593.50
gcg	A	0	107.00
---	--	------	------
mPD		0.0079	0.67
		nPD:	0.01
		N. weight:	1.7
		Sc. PD:	-0.085
		Sc. rank:	-641.5

NS1			
Pos.	31	obs:	exp:
cct	P	9	314.90
ccc	P	2	226.70
cca	P	1213	519.50
ccg	P	38	200.90
---	--	------	------
mPD		0.075	0.71
		nPD:	0.11
		N. weight:	0.78
		Sc. PD:	0.027
		Sc. rank:	540.8

NS1			
Pos.	32	obs:	exp:
ttt	F	1245	498.40
ttc	F	17	763.60
---	--	------	------
mPD		0.027	0.48
		nPD:	0.06
		N. weight:	0.9
		Sc. PD:	-0.0088
		Sc. rank:	412.1

NS1			
Pos.	33	obs:	exp:
tta	L	4	111.80
ttg	L	0	234.60
ctt	L	0	209.80
ctc	L	0	210.30
cta	L	1254	212.30
ctg	L	2	281.20
att	I	0	0.37
atc	I	0	0.24
ata	I	1	0.39
gat	D	1	0.54
gac	D	0	0.46
---	--	------	------
mPD		0.016	1.1
		nPD:	0.01
		N. weight:	1.8
		Sc. PD:	-0.086
		Sc. rank:	-492.6

NS1			
Pos.	34	obs:	exp:
aat	N	0	17.32
aac	N	34	16.68
gat	D	3	663.60
gac	D	1225	564.40
---	--	------	------
mPD		0.057	0.55
		nPD:	0.1
		N. weight:	0.8
		Sc. PD:	0.026
		Sc. rank:	552.1

NS1			
Pos.	35	obs:	exp:
cct	P	0	4.74
ccc	P	0	3.41
cca	P	0	7.82
ccg	P	19	3.02
cgt	R	0	53.39
cgc	R	0	61.57
cga	R	4	121.60
cgg	R	1238	103.70
aga	R	0	568.40
agg	R	1	334.30
---	--	------	------
mPD		0.038	1.0
		nPD:	0.04
		N. weight:	2.6
		Sc. PD:	-0.067
		Sc. rank:	673.7

NS1			
Pos.	36	obs:	exp:
tta	L	0	112.00
ttg	L	0	235.00
ctt	L	1256	210.10
ctc	L	5	210.60
cta	L	0	212.70
ctg	L	1	281.60
---	--	------	------
mPD		0.0095	1.1
		nPD:	0.01
		N. weight:	1.9
		Sc. PD:	-0.095
		Sc. rank:	-905.0

NS1			
Pos.	37	obs:	exp:
tgt	C	0	0.42
tgc	C	1	0.58
cgt	R	0	54.16
cgc	R	1255	62.46
cga	R	5	123.40
cgg	R	0	105.20
aga	R	0	576.60
agg	R	1	339.20
---	--	------	------
mPD		0.013	0.96
		nPD:	0.01
		N. weight:	3.1
		Sc. PD:	-0.15
		Sc. rank:	-934.0

NS1			
Pos.	38	obs:	exp:
cgt	R	10	54.20
cgc	R	0	62.51
cga	R	1246	123.50
cgg	R	4	105.30
aga	R	2	577.10
agg	R	0	339.40
---	--	------	------
mPD		0.025	0.96
		nPD:	0.03
		N. weight:	2.4
		Sc. PD:	-0.085
		Sc. rank:	181.7

NS1			
Pos.	39	obs:	exp:
gat	D	1254	680.90
gac	D	6	579.10
gaa	E	2	1.22
gag	E	0	0.78
---	--	------	------
mPD		0.013	0.50
		nPD:	0.03
		N. weight:	0.62
		Sc. PD:	-0.023
		Sc. rank:	30.5

NS1			
Pos.	40	obs:	exp:
caa	Q	3	642.80
cag	Q	1259	619.20
---	--	------	------
mPD		0.0047	0.50
		nPD:	0.01
		N. weight:	0.73
		Sc. PD:	-0.037
		Sc. rank:	-329.0

NS1

Pos.	41	obs:	exp:
aaa	K	7	735.50
aag	K	1255	526.50
mPD		0.011	0.49
		nPD:	0.02
		N. weight:	0.88
		Sc. PD:	-0.034
		Sc. rank:	-2.3

NS1

Pos.	42	obs:	exp:
tct	S	15	200.60
tcc	S	1245	164.70
tca	S	1	320.10
tcg	S	0	71.55
agt	S	0	259.20
agc	S	0	244.90
gct	A	0	0.26
gcc	A	0	0.19
gca	A	1	0.47
gcg	A	0	0.08
mPD		0.028	1.7
		nPD:	0.02
		N. weight:	2.1
		Sc. PD:	-0.091
		Sc. rank:	-390.5

NS1

Pos.	43	obs:	exp:
tta	L	1	112.00
ttg	L	1	235.00
ctt	L	11	210.10
ctc	L	0	210.60
cta	L	7	212.70
ctg	L	1242	281.60
mPD		0.033	1.1
		nPD:	0.03
		N. weight:	1.5
		Sc. PD:	-0.049
		Sc. rank:	263.2

NS1

Pos.	44	obs:	exp:
cgt	R	0	53.94
cgc	R	0	62.21
cga	R	0	122.90
cgg	R	0	104.80
aaa	K	5	3.50
aag	K	1	2.50
aga	R	1010	574.30
agg	R	246	337.80
mPD		0.32	0.97
		nPD:	0.33
		N. weight:	0.41
		Sc. PD:	0.098
		Sc. rank:	391.7

NS1

Pos.	45	obs:	exp:
ggt	G	0	167.30
ggc	G	0	161.20
gga	G	1251	582.20
ggg	G	11	351.30
mPD		0.017	0.68
		nPD:	0.03
		N. weight:	0.77
		Sc. PD:	-0.028
		Sc. rank:	43.0

NS1

Pos.	46	obs:	exp:
cgt	R	0	54.20
cgc	R	0	62.51
cga	R	0	123.50
cgg	R	0	105.30
aga	R	1260	577.10
agg	R	2	339.40
mPD		0.0032	0.96
		nPD:	0.
		N. weight:	0.81
		Sc. PD:	-0.046
		Sc. rank:	-615.4

NS1

Pos.	47	obs:	exp:
tct	S	0	200.20
tcc	S	0	164.40
tca	S	0	319.50
tcg	S	0	71.44
agt	S	2	258.80
agc	S	1257	244.50
ggt	G	0	0.40
ggc	G	3	0.38
gga	G	0	1.38
ggg	G	0	0.84
mPD		0.0079	1.7
		nPD:	0.
		N. weight:	1.7
		Sc. PD:	-0.094
		Sc. rank:	-1147.0

NS1

Pos.	48	obs:	exp:
tct	S	0	191.50
tcc	S	0	157.20
tca	S	0	305.60
tcg	S	0	68.32
att	I	0	16.90
atc	I	46	11.02
ata	I	0	18.08
aat	N	0	6.11
aac	N	12	5.89
agt	S	10	247.50
agc	S	1194	233.90
mPD		0.10	1.7
		nPD:	0.06
		N. weight:	1.7
		Sc. PD:	-0.0084
		Sc. rank:	906.4

NS1

Pos.	49	obs:	exp:
act	T	1250	321.40
acc	T	9	258.80
aca	T	2	578.40
acg	T	0	102.40
gct	A	1	0.26
gcc	A	0	0.19
gca	A	0	0.47
gcg	A	0	0.08
mPD		0.019	0.68
		nPD:	0.03
		N. weight:	1.4
		Sc. PD:	-0.048
		Sc. rank:	166.5

NS1

Pos.	50	obs:	exp:
tta	L	0	111.90
ttg	L	0	234.80
ctt	L	1260	210.00
ctc	L	1	210.40
cta	L	0	212.50
ctg	L	0	281.40
att	I	1	0.37
atc	I	0	0.24
ata	I	0	0.39
mPD		0.0032	1.1
		nPD:	0.
		N. weight:	1.9
		Sc. PD:	-0.11
		Sc. rank:	-1531.3

NS1

Pos.	51	obs:	exp:
tct	S	0	1.91
tcc	S	0	1.57
tca	S	0	3.05
tcg	S	0	0.68
cgt	R	1	0.04
cgc	R	0	0.05
cga	R	0	0.10
cgg	R	0	0.08
agt	S	12	2.47
agc	S	0	2.33
aga	R	0	0.46
agg	R	0	0.27
ggt	G	1239	165.60
ggc	G	9	159.60
gga	G	1	576.20
ggg	G	0	347.70
mPD		0.036	0.71
		nPD:	0.05
		N. weight:	2.1
		Sc. PD:	-0.029
		Sc. rank:	850.4

NS1

Pos.	52	obs:	exp:
tta	L	5	112.00
ttg	L	11	235.00
ctt	L	107	210.10
ctc	L	1	210.60
cta	L	263	212.70
ctg	L	875	281.60
mPD		0.48	1.1
		nPD:	0.43
		N. weight:	0.78
		Sc. PD:	0.25
		Sc. rank:	764.0

NS1

Pos.	53	obs:	exp:
gat	D	0	681.40
gac	D	1261	579.60
gaa	E	1	0.61
gag	E	0	0.39
mPD		0.0016	0.50
		nPD:	0.
		N. weight:	0.82
		Sc. PD:	-0.046
		Sc. rank:	-633.3

NS1

Pos.	54	obs:	exp:
tta	L	0	0.09
ttg	L	0	0.19
ctt	L	0	0.17
ctc	L	0	0.17
cta	L	0	0.17
ctg	L	1	0.22
att	I	1	463.30
atc	I	1260	302.20
ata	I	0	495.50
mPD		0.0048	0.65
		nPD:	0.01
		N. weight:	1.5
		Sc. PD:	-0.079
		Sc. rank:	-754.7

NS1

Pos.	55	obs:	exp:
cgt	R	0	53.77
cgc	R	0	62.01
cga	R	0	122.50
cgg	R	0	104.50
att	I	0	0.37
atc	I	0	0.24
ata	I	1	0.39
aaa	K	9	5.25
aag	K	0	3.75
aga	R	1239	572.50
agg	R	13	336.70
mPD		0.036	0.97
		nPD:	0.04
		N. weight:	0.77
		Sc. PD:	-0.02
		Sc. rank:	184.0

NS1

Pos.	56	obs:	exp:
tct	S	1	0.16
tcc	S	0	0.13
tca	S	0	0.25
tcg	S	0	0.06
act	T	971	294.20
acc	T	160	236.80
aca	T	22	529.30
acg	T	1	93.73
aat	N	1	0.51
aac	N	0	0.49
agt	S	0	0.21
agc	S	0	0.19
gtt	V	0	0.21
gtc	V	0	0.20
gta	V	0	0.20
gtg	V	1	0.39
gct	A	105	27.11
gcc	A	0	19.55
gca	A	0	49.42
gcg	A	0	8.91
mPD		0.41	0.83
		nPD:	0.49
		N. weight:	0.98
		Sc. PD:	0.37
		Sc. rank:	992.1

NS1

Pos.	57	obs:	exp:
gtt	V	0	0.21
gtc	V	1	0.20
gta	V	0	0.20
gtg	V	0	0.39
gct	A	6	325.60
gcc	A	1071	234.80
gca	A	184	593.50
gcg	A	0	107.00
mPD		0.26	0.67
		nPD:	0.39
		N. weight:	1.2
		Sc. PD:	0.33
		Sc. rank:	1127.1

NS1

Pos.	58	obs:	exp:
act	T	202	321.70
acc	T	1	259.00
aca	T	45	578.80
acg	T	1014	102.50
mPD		0.33	0.68
		nPD:	0.48
		N. weight:	1.8
		Sc. PD:	0.65
		Sc. rank:	1768.5

NS1

Pos.	59	obs:	exp:
tta	L	0	0.35
ttg	L	0	0.74
tgt	C	2	0.85
tgc	C	0	1.15
ctt	L	4	0.67
ctc	L	0	0.67
cta	L	0	0.67
ctg	L	0	0.89
cat	H	139	79.82
cac	H	0	59.18
cgt	R	1111	47.93
cgc	R	2	55.28
cga	R	3	109.20
cgg	R	0	93.12
atg	M	1	1.00
aga	R	0	510.30
agg	R	0	300.20
mPD		0.22	1.3
		nPD:	0.17
		N. weight:	3.
		Sc. PD:	0.26
		Sc. rank:	2370.0

NS1

Pos.	60	obs:	exp:
gat	D	0	1.62
gac	D	3	1.38
gaa	E	1250	766.10
gag	E	9	492.90
mPD		0.019	0.48
		nPD:	0.04
		N. weight:	0.48
		Sc. PD:	-0.012
		Sc. rank:	133.0

NS1			
Pos.	61	obs:	exp:
ggt	G	0	167.30
ggc	G	0	161.20
gga	G	1249	582.20
ggg	G	13	351.30
---	--	------	------
mPD		0.020	0.68
		nPD:	0.03
		N. weight:	0.76
		Sc. PD:	-0.025
		Sc. rank:	145.1

,

NS1			
Pos.	62	obs:	exp:
cgt	R	0	0.09
cgc	R	0	0.10
cga	R	0	0.20
cgg	R	0	0.17
aaa	K	5	734.40
aag	K	1255	525.60
aga	R	0	0.91
agg	R	2	0.54
---	--	------	------
mPD		0.011	0.49
		nPD:	0.02
		N. weight:	0.89
		Sc. PD:	-0.035
		Sc. rank:	-9.1

,

NS1			
Pos.	63	obs:	exp:
cat	H	1230	712.60
cac	H	11	528.40
caa	Q	2	2.04
cag	Q	2	1.96
cgt	R	11	0.47
cgc	R	0	0.54
cga	R	0	1.08
cgg	R	0	0.92
aat	N	5	2.55
aac	N	0	2.45
aaa	K	0	0.58
aag	K	1	0.42
aga	R	0	5.03
agg	R	0	2.96
---	--	------	------
mPD		0.052	0.54
		nPD:	0.1
		N. weight:	0.56
		Sc. PD:	0.014
		Sc. rank:	370.0

,

NS1			
Pos.	64	obs:	exp:
att	I	0	462.60
atc	I	1	301.70
ata	I	1258	494.70
atg	M	1	1.00
act	T	0	0.51
acc	T	0	0.41
aca	T	2	0.92
acg	T	0	0.16
---	--	------	------
mPD		0.0063	0.66
		nPD:	0.01
		N. weight:	0.98
		Sc. PD:	-0.049
		Sc. rank:	-417.3

,

NS1			
Pos.	65	obs:	exp:
atg	M	1	1.00
gtt	V	1	261.10
gtc	V	0	254.30
gta	V	8	258.00
gtg	V	1251	486.60
gct	A	0	0.26
gcc	A	0	0.19
gca	A	0	0.47
gcg	A	1	0.08
---	--	------	------
mPD		0.017	0.73
		nPD:	0.02
		N. weight:	0.96
		Sc. PD:	-0.036
		Sc. rank:	42.3

,

NS1			
Pos.	66	obs:	exp:
aaa	K	0	0.58
aag	K	1	0.42
gaa	E	19	767.30
gag	E	1242	493.70
---	--	------	------
mPD		0.031	0.48
		nPD:	0.07
		N. weight:	0.9
		Sc. PD:	-0.0011
		Sc. rank:	517.4

,

NS1			
Pos.	67	obs:	exp:
cgt	R	0	0.04
cgc	R	0	0.05
cga	R	1	0.10
cgg	R	0	0.08
aga	R	0	0.46
agg	R	0	0.27
---	-	1261	1261.00
---	--	------	------
mPD		0	6.0×10^{-7}
		nPD:	0.
		N. weight:	0.
		Sc. PD:	0
		Sc. rank:	0

,

NS1			
Pos.	68	obs:	exp:
tct	S	0	0.16
tcc	S	0	0.13
tca	S	0	0.25
tcg	S	0	0.06
caa	Q	0	6.11
cag	Q	12	5.89
cgt	R	2	53.60
cgc	R	0	61.81
cga	R	186	122.10
cgg	R	1054	104.10
agt	S	1	0.21
agc	S	0	0.19
aga	R	0	570.70
agg	R	6	335.70
gat	D	0	0.54
gac	D	1	0.46
---	--	------	------
mPD		0.29	0.99
		nPD:	0.29
		N. weight:	2.1
		Sc. PD:	0.42
		Sc. rank:	1959.0

,

NS1			
Pos.	69	obs:	exp:
att	I	1257	463.30
atc	I	4	302.20
ata	I	0	495.50
gtt	V	1	0.21
gtc	V	0	0.20
gta	V	0	0.20
gtg	V	0	0.39
---	--	------	------
mPD		0.0079	0.65
		nPD:	0.01
		N. weight:	1.
		Sc. PD:	-0.05
		Sc. rank:	-361.2

,

NS1			
Pos.	70	obs:	exp:
ttt	F	8	3.16
ttc	F	0	4.84
tta	L	1058	111.30
ttg	L	192	233.50
ctt	L	0	208.80
ctc	L	0	209.30
cta	L	3	211.30
ctg	L	1	279.80
---	--	------	------
mPD		0.28	1.1
		nPD:	0.25
		N. weight:	2.
		Sc. PD:	0.31
		Sc. rank:	1660.3

,

NS1			
Pos.	71	obs:	exp:
cgt	R	0	0.04
cgc	R	0	0.05
cga	R	0	0.10
cgg	R	0	0.08
aaa	K	5	6.99
aag	K	7	5.01
aga	R	1	0.46
agg	R	0	0.27
gaa	E	304	758.80
gag	E	943	488.20
ggt	G	0	0.27
ggc	G	0	0.26
gga	G	1	0.92
ggg	G	1	0.56
---	--	------	------
mPD		0.40	0.50
		nPD:	0.79
		N. weight:	0.29
		Sc. PD:	0.19
		Sc. rank:	302.6

,

NS1			
Pos.	72	obs:	exp:
tct	S	0	0.16
tcc	S	0	0.13
tca	S	0	0.25
tcg	S	0	0.06
aaa	K	18	10.49
aag	K	0	7.51
agt	S	1	0.21
agc	S	0	0.19
gat	D	0	3.78
gac	D	7	3.22
gaa	E	1224	747.20
gag	E	4	480.80
ggt	G	0	1.06
ggc	G	0	1.02
gga	G	8	3.69
ggg	G	0	2.23
---	--	------	------
mPD		0.063	0.53
		nPD:	0.12
		N. weight:	0.51
		Sc. PD:	0.023
		Sc. rank:	366.6

,

NS1			
Pos.	73	obs:	exp:
gat	D	1	0.54
gac	D	0	0.46
gaa	E	187	767.30
gag	E	1074	493.70
---	--	------	------
mPD		0.25	0.48
		nPD:	0.53
		N. weight:	0.48
		Sc. PD:	0.2
		Sc. rank:	487.4

,

NS1			
Pos.	74	obs:	exp:
tct	S	2	200.60
tcc	S	0	164.70
tca	S	1245	320.10
tcg	S	14	71.55
act	T	0	0.25
acc	T	0	0.21
aca	T	1	0.46
acg	T	0	0.08
agt	S	0	259.20
agc	S	0	244.90
---	--	------	------
mPD		0.027	1.7
		nPD:	0.02
		N. weight:	1.4
		Sc. PD:	-0.062
		Sc. rank:	-293.3

,

NS1			
Pos.	75	obs:	exp:
tat	Y	1	0.54
tac	Y	0	0.46
aat	N	2	1.02
aac	N	0	0.98
gat	D	1082	678.70
gac	D	174	577.30
ggt	G	2	0.40
ggc	G	1	0.38
gga	G	0	1.38
ggg	G	0	0.84
---	--	------	------
mPD		0.25	0.51
		nPD:	0.49
		N. weight:	0.26
		Sc. PD:	0.095
		Sc. rank:	256.5

,

NS1			
Pos.	76	obs:	exp:
aaa	K	1	0.58
aag	K	0	0.42
gaa	E	1256	766.10
gag	E	3	492.90
ggt	G	0	0.27
ggc	G	0	0.26
gga	G	2	0.92
ggg	G	0	0.56
---	--	------	------
mPD		0.0095	0.48
		nPD:	0.02
		N. weight:	0.51
		Sc. PD:	-0.021
		Sc. rank:	-47.5

,

NS1			
Pos.	77	obs:	exp:
tct	S	0	0.64
tcc	S	0	0.52
tca	S	4	1.02
tcg	S	0	0.23
act	T	0	3.06
acc	T	0	2.46
aca	T	12	5.50
acg	T	0	0.97
aat	N	1	0.51
aac	N	0	0.49
agt	S	0	0.82
agc	S	0	0.78
gct	A	0	321.50
gcc	A	0	231.90
gca	A	1245	586.00
gcg	A	0	105.70
---	--	------	------
mPD		0.030	0.70
		nPD:	0.04
		N. weight:	0.8
		Sc. PD:	-0.017
		Sc. rank:	241.1

,

NS1			
Pos.	78	obs:	exp:
ttt	F	1250	496.80
ttc	F	8	761.20
tta	L	0	0.09
ttg	L	0	0.19
tct	S	3	0.48
tcc	S	0	0.39
tca	S	0	0.76
tcg	S	0	0.17
ctt	L	0	0.17
ctc	L	1	0.17
cta	L	0	0.17
ctg	L	0	0.22
agt	S	0	0.62
agc	S	0	0.58
---	--	------	------
mPD		0.021	0.49
		nPD:	0.04
		N. weight:	0.94
		Sc. PD:	-0.02
		Sc. rank:	278.2

,

NS1			
Pos.	79	obs:	exp:
aaa	K	1262	735.50
aag	K	0	526.50
---	--	------	------
mPD		0	0.49
		nPD:	0.
		N. weight:	0.57
		Sc. PD:	-0.034
		Sc. rank:	-586.3

,

NS1			
Pos.	80	obs:	exp:
cgt	R	0	0.04
cgc	R	0	0.05
cga	R	0	0.10
cgg	R	0	0.08
att	I	0	0.37
atc	I	0	0.24
ata	I	1	0.39
atg	M	1260	1260.00
aga	R	0	0.46
agg	R	1	0.27
---	--	------	------
mPD		0.0032	0.0046
		nPD:	0.69
		N. weight:	0.0019
		Sc. PD:	0.001
		Sc. rank:	1.9

,

NS1

Pos.	81	obs:	exp:
tct	S	0	146.20
tcc	S	0	120.00
tca	S	0	233.30
tcg	S	0	52.15
act	T	180	45.88
acc	T	0	36.94
aca	T	0	82.56
acg	T	0	14.62
aat	N	155	78.95
aac	N	0	76.05
agt	S	917	188.90
agc	S	2	178.50
gct	A	1	0.52
gcc	A	1	0.37
gca	A	0	0.94
gcg	A	0	0.17
ggt	G	6	0.80
ggc	G	0	0.77
gga	G	0	2.77
ggg	G	0	1.67
---	--	------	------
mPD		0.44	1.8
		nPD:	0.25
		N. weight:	1.5
		Sc. PD:	0.25
		Sc. rank:	1313.8

,

NS1

Pos.	82	obs:	exp:
ttt	F	1	0.39
ttc	F	0	0.61
att	I	1256	461.50
atc	I	0	301.00
ata	I	0	493.50
act	T	4	1.02
acc	T	0	0.82
aca	T	0	1.83
acg	T	0	0.32
gtt	V	1	0.21
gtc	V	0	0.20
gta	V	0	0.20
gtg	V	0	0.39
---	--	------	------
mPD		0.0095	0.66
		nPD:	0.01
		N. weight:	1.1
		Sc. PD:	-0.049
		Sc. rank:	-272.6

,

NS1

Pos.	83	obs:	exp:
cct	P	11	2.74
ccc	P	0	1.98
cca	P	0	4.53
ccg	P	0	1.75
act	T	0	0.25
acc	T	1	0.21
aca	T	0	0.46
acg	T	0	0.08
gct	A	1220	322.80
gcc	A	30	232.80
gca	A	0	588.30
gcg	A	0	106.10
---	--	------	------
mPD		0.067	0.69
		nPD:	0.1
		N. weight:	1.3
		Sc. PD:	0.035
		Sc. rank:	894.5

,

NS1

Pos.	84	obs:	exp:
tct	S	1	200.70
tcc	S	0	164.80
tca	S	1260	320.30
tcg	S	1	71.61
agt	S	0	259.40
agc	S	0	245.10
---	--	------	------
mPD		0.0032	1.7
		nPD:	0.
		N. weight:	1.4
		Sc. PD:	-0.082
		Sc. rank:	-1211.9

,

NS1

Pos.	85	obs:	exp:
tct	S	0	0.16
tcc	S	0	0.13
tca	S	0	0.25
tcg	S	0	0.06
agt	S	1	0.21
agc	S	0	0.19
gtt	V	0	261.30
gtc	V	5	254.50
gta	V	19	258.20
gtg	V	1237	487.00
---	--	------	------
mPD		0.042	0.73
		nPD:	0.06
		N. weight:	0.91
		Sc. PD:	-0.0071
		Sc. rank:	435.0

,

NS1

Pos.	86	obs:	exp:
cct	P	1	314.90
ccc	P	4	226.70
cca	P	1253	519.50
ccg	P	4	200.90
---	--	------	------
mPD		0.014	0.71
		nPD:	0.02
		N. weight:	0.89
		Sc. PD:	-0.037
		Sc. rank:	-42.8

,

NS1

Pos.	87	obs:	exp:
tct	S	7	1.11
tcc	S	0	0.91
tca	S	0	1.78
tcg	S	0	0.40
act	T	14	3.57
acc	T	0	2.87
aca	T	0	6.42
acg	T	0	1.14
agt	S	0	1.44
agc	S	0	1.36
gct	A	1238	320.50
gcc	A	3	231.10
gca	A	0	584.10
gcg	A	0	105.30
---	--	------	------
mPD		0.038	0.71
		nPD:	0.05
		N. weight:	1.4
		Sc. PD:	-0.017
		Sc. rank:	636.6

,

NS1

Pos.	88	obs:	exp:
tct	S	0	1.75
tcc	S	0	1.44
tca	S	11	2.79
tcg	S	0	0.62
cct	P	1	312.20
ccc	P	0	224.80
cca	P	1238	514.90
ccg	P	12	199.10
agt	S	0	2.26
agc	S	0	2.14
---	--	------	------
mPD		0.038	0.74
		nPD:	0.05
		N. weight:	0.89
		Sc. PD:	-0.012
		Sc. rank:	371.0

,

NS1

Pos.	89	obs:	exp:
tta	L	0	0.09
ttg	L	0	0.19
tgt	C	0	0.42
tgc	C	1	0.58
ctt	L	0	0.17
ctc	L	1	0.17
cta	L	0	0.17
ctg	L	0	0.22
cat	H	0	2.30
cac	H	4	1.70
cgt	R	1	53.94
cgc	R	1234	62.21
cga	R	19	122.90
cgg	R	2	104.80
aga	R	0	574.30
agg	R	0	337.80
---	--	------	------
mPD		0.044	0.98
		nPD:	0.04
		N. weight:	3.
		Sc. PD:	-0.059
		Sc. rank:	1099.6

,

NS1

Pos.	90	obs:	exp:
tat	Y	1225	681.10
tac	Y	36	579.90
cat	H	1	0.57
cac	H	0	0.43
---	--	------	------
mPD		0.057	0.50
		nPD:	0.11
		N. weight:	0.52
		Sc. PD:	0.022
		Sc. rank:	369.1

,

NS1

Pos.	91	obs:	exp:
tta	L	0	110.30
ttg	L	0	231.40
ctt	L	0	207.00
ctc	L	4	207.40
cta	L	1175	209.50
ctg	L	64	277.40
att	I	0	2.20
atc	I	1	1.44
ata	I	5	2.36
atg	M	13	13.00
---	--	------	------
mPD		0.15	1.1
		nPD:	0.13
		N. weight:	1.6
		Sc. PD:	0.097
		Sc. rank:	1180.5

,

NS1

Pos.	92	obs:	exp:
act	T	1254	320.20
acc	T	1	257.70
aca	T	1	576.10
acg	T	0	102.00
gct	A	6	1.55
gcc	A	0	1.12
gca	A	0	2.82
gcg	A	0	0.51
---	--	------	------
mPD		0.013	0.69
		nPD:	0.02
		N. weight:	1.4
		Sc. PD:	-0.061
		Sc. rank:	-200.3

,

NS1

Pos.	93	obs:	exp:
gat	D	5	681.40
gac	D	1256	579.60
gaa	E	1	0.61
gag	E	0	0.39
---	--	------	------
mPD		0.0095	0.50
		nPD:	0.02
		N. weight:	0.79
		Sc. PD:	-0.033
		Sc. rank:	-89.1

,

NS1

Pos.	94	obs:	exp:
tta	L	0	0.09
ttg	L	1	0.19
ctt	L	0	0.17
ctc	L	0	0.17
cta	L	0	0.17
ctg	L	0	0.22
atg	M	1261	1261.00
---	--	------	------
mPD		0.0016	0.0025
		nPD:	0.63
		N. weight:	0.0014
		Sc. PD:	0.0007
		Sc. rank:	1.4

,

NS1

Pos.	95	obs:	exp:
tct	S	0	0.16
tcc	S	0	0.13
tca	S	0	0.25
tcg	S	0	0.06
act	T	1244	321.20
acc	T	16	258.50
aca	T	0	577.90
acg	T	0	102.30
aat	N	1	0.51
aac	N	0	0.49
agt	S	0	0.21
agc	S	1	0.19
---	--	------	------
mPD		0.030	0.68
		nPD:	0.04
		N. weight:	1.4
		Sc. PD:	-0.028
		Sc. rank:	459.2

,

NS1			
Pos.	96	obs:	exp:
tta	L	0	109.20
ttg	L	0	229.00
ctt	L	1219	204.80
ctc	L	1	205.30
cta	L	0	207.30
ctg	L	10	274.50
cct	P	15	3.74
ccc	P	0	2.70
cca	P	0	6.17
ccg	P	0	2.39
att	I	16	5.88
atc	I	0	3.83
ata	I	0	6.29
gtt	V	0	0.21
gtc	V	0	0.20
gta	V	1	0.20
gtg	V	0	0.39
---	--	------	------
mPD		0.069	1.1
		nPD:	0.06
		N. weight:	1.8
		Sc. PD:	-0.011
		Sc. rank:	965.1

NS1			
Pos.	97	obs:	exp:
gaa	E	1261	767.90
gag	E	1	494.10
---	--	------	------
mPD		0.0016	0.48
		nPD:	0.
		N. weight:	0.52
		Sc. PD:	-0.029
		Sc. rank:	-370.3

NS1			
Pos.	98	obs:	exp:
gaa	E	1260	767.90
gag	E	2	494.10
---	--	------	------
mPD		0.0032	0.48
		nPD:	0.01
		N. weight:	0.51
		Sc. PD:	-0.027
		Sc. rank:	-294.1

NS1			
Pos.	99	obs:	exp:
att	I	0	5.14
atc	I	0	3.35
ata	I	14	5.50
atg	M	1246	1246.00
act	T	0	0.51
acc	T	0	0.41
aca	T	0	0.92
acg	T	2	0.16
---	--	------	------
mPD		0.025	0.028
		nPD:	0.9
		N. weight:	0.015
		Sc. PD:	0.011
		Sc. rank:	15.6

NS1			
Pos.	100	obs:	exp:
tct	S	0	200.70
tcc	S	0	164.80
tca	S	1253	320.30
tcg	S	8	71.61
agt	S	0	259.40
agc	S	1	245.10
---	--	------	------
mPD		0.017	1.7
		nPD:	0.01
		N. weight:	1.4
		Sc. PD:	-0.07
		Sc. rank:	-559.8

NS1			
Pos.	101	obs:	exp:
cgt	R	0	54.20
cgc	R	0	62.51
cga	R	1	123.50
cgg	R	0	105.30
aga	R	1255	577.10
agg	R	6	339.40
---	--	------	------
mPD		0.011	0.96
		nPD:	0.01
		N. weight:	0.79
		Sc. PD:	-0.039
		Sc. rank:	-302.6

NS1			
Pos.	102	obs:	exp:
aat	N	2	1.02
aac	N	0	0.98
gat	D	1255	678.20
gac	D	0	576.80
gaa	E	1	0.61
gag	E	0	0.39
ggt	G	4	0.53
ggc	G	0	0.51
gga	G	0	1.85
ggg	G	0	1.11
---	--	------	------
mPD		0.011	0.51
		nPD:	0.02
		N. weight:	0.65
		Sc. PD:	-0.026
		Sc. rank:	-19.6

NS1			
Pos.	103	obs:	exp:
tgg	W	1262	1262.00
---	--	------	------
mPD		0	0
		nPD:	1.
		N. weight:	0.
		Sc. PD:	0
		Sc. rank:	0

NS1			
Pos.	104	obs:	exp:
tta	L	1227	111.80
ttg	L	21	234.60
tat	Y	1	0.54
tac	Y	0	0.46
ctt	L	0	209.80
ctc	L	0	210.30
cta	L	12	212.30
ctg	L	0	281.20
gtt	V	0	0.21
gtc	V	0	0.20
gta	V	0	0.20
gtg	V	1	0.39
---	--	------	------
mPD		0.058	1.1
		nPD:	0.05
		N. weight:	2.4
		Sc. PD:	-0.031
		Sc. rank:	1045.9

NS1			
Pos.	105	obs:	exp:
atg	M	1262	1262.00
---	--	------	------
mPD		0	0
		nPD:	1.
		N. weight:	0.
		Sc. PD:	0
		Sc. rank:	0

NS1			
Pos.	106	obs:	exp:
tta	L	0	111.90
ttg	L	0	234.80
ctt	L	3	210.00
ctc	L	1257	210.40
cta	L	0	212.50
ctg	L	1	281.40
att	I	0	0.37
atc	I	1	0.24
ata	I	0	0.39
---	--	------	------
mPD		0.0079	1.1
		nPD:	0.01
		N. weight:	1.9
		Sc. PD:	-0.098
		Sc. rank:	-962.5

NS1			
Pos.	107	obs:	exp:
att	I	1067	461.50
atc	I	11	301.00
ata	I	178	493.50
atg	M	1	1.00
gtt	V	5	1.04
gtc	V	0	1.01
gta	V	0	1.02
gtg	V	0	1.93
---	--	------	------
mPD		0.27	0.66
		nPD:	0.4
		N. weight:	0.58
		Sc. PD:	0.17
		Sc. rank:	561.2

NS1			
Pos.	108	obs:	exp:
cct	P	8	314.90
ccc	P	1253	226.70
cca	P	1	519.50
ccg	P	0	200.90
---	--	------	------
mPD		0.014	0.71
		nPD:	0.02
		N. weight:	1.8
		Sc. PD:	-0.073
		Sc. rank:	-98.1

NS1			
Pos.	109	obs:	exp:
cgt	R	0	0.34
cgc	R	0	0.40
cga	R	0	0.78
cgg	R	0	0.67
aaa	K	1249	730.90
aag	K	5	523.10
aga	R	8	3.66
agg	R	0	2.15
---	--	------	------
mPD		0.021	0.50
		nPD:	0.04
		N. weight:	0.55
		Sc. PD:	-0.013
		Sc. rank:	152.5

NS1			
Pos.	110	obs:	exp:
caa	Q	19	642.80
cag	Q	1243	619.20
---	--	------	------
mPD		0.030	0.50
		nPD:	0.06
		N. weight:	0.67
		Sc. PD:	-0.0043
		Sc. rank:	330.5

NS1			
Pos.	111	obs:	exp:
aaa	K	1262	735.50
aag	K	0	526.50
---	--	------	------
mPD		0	0.49
		nPD:	0.
		N. weight:	0.57
		Sc. PD:	-0.034
		Sc. rank:	-586.3

NS1			
Pos.	112	obs:	exp:
att	I	1	386.20
atc	I	0	251.90
ata	I	1050	413.00
atg	M	26	26.00
gtt	V	2	38.33
gtc	V	0	37.34
gta	V	9	37.88
gtg	V	174	71.45
---	--	------	------
mPD		0.52	0.95
		nPD:	0.55
		N. weight:	0.93
		Sc. PD:	0.4
		Sc. rank:	954.4

NS1			
Pos.	113	obs:	exp:
att	I	0	1.47
atc	I	0	0.96
ata	I	4	1.57
act	T	1	320.20
acc	T	0	257.70
aca	T	1250	576.10
acg	T	5	102.00
gct	A	0	0.52
gcc	A	0	0.37
gca	A	2	0.94
gcg	A	0	0.17
---	--	------	------
mPD		0.019	0.69
		nPD:	0.03
		N. weight:	0.8
		Sc. PD:	-0.028
		Sc. rank:	86.2

NS1			
Pos.	114	obs:	exp:
cgt	R	0	0.34
cgc	R	0	0.40
cga	R	0	0.78
cgg	R	0	0.67
aga	R	0	3.66
agg	R	8	2.15
gaa	E	0	0.61
gag	E	1	0.39
ggt	G	1	166.10
ggc	G	0	160.10
gga	G	9	578.00
ggg	G	1243	348.80
---	--	------	------
mPD		0.030	0.69
		nPD:	0.04
		N. weight:	1.3
		Sc. PD:	-0.027
		Sc. rank:	417.8

NS1			
Pos.	115	obs:	exp:
tct	S	8	196.90
tcc	S	1230	161.70
tca	S	0	314.20
tcg	S	0	70.25
cct	P	1	5.74
ccc	P	22	4.13
cca	P	0	9.47
ccg	P	0	3.66
agt	S	0	254.50
agc	S	0	240.50
ggt	G	1	0.13
ggc	G	0	0.13
gga	G	0	0.46
ggg	G	0	0.28
---	--	------	------
mPD		0.055	1.7
		nPD:	0.03
		N. weight:	2.1
		Sc. PD:	-0.063
		Sc. rank:	486.9

NS1

Pos.	116	obs:	exp:
tta	L	15	112.00
ttg	L	0	235.00
ctt	L	185	210.10
ctc	L	3	210.60
cta	L	1046	212.70
ctg	L	13	281.60
---	--	------	------
mPD		0.30	1.1
		nPD:	0.27
		N. weight:	1.3
		Sc. PD:	0.23
		Sc. rank:	1156.3

,

NS1

Pos.	117	obs:	exp:
tct	S	0	0.16
tcc	S	0	0.13
tca	S	0	0.25
tcg	S	1	0.06
tgt	C	7	532.90
tgc	C	1253	727.10
atg	M	1	1.00
agt	S	0	0.21
agc	S	0	0.19
---	--	------	------
mPD		0.019	0.49
		nPD:	0.04
		N. weight:	0.55
		Sc. PD:	-0.014
		Sc. rank:	145.8

,

NS1

Pos.	118	obs:	exp:
att	I	1258	462.60
atc	I	1	301.70
ata	I	0	494.70
aat	N	1	0.51
aac	N	0	0.49
gtt	V	1	0.41
gtc	V	0	0.40
gta	V	1	0.41
gtg	V	0	0.77
---	--	------	------
mPD		0.0079	0.66
		nPD:	0.01
		N. weight:	1.
		Sc. PD:	-0.051
		Sc. rank:	-375.5

,

NS1

Pos.	119	obs:	exp:
cgt	R	0	54.12
cgc	R	0	62.41
cga	R	0	123.30
cgg	R	0	105.10
att	I	0	0.37
atc	I	0	0.24
ata	I	1	0.39
aaa	K	1	0.58
aag	K	0	0.42
aga	R	1258	576.10
agg	R	2	338.90
---	--	------	------
mPD		0.0063	0.96
		nPD:	0.01
		N. weight:	0.81
		Sc. PD:	-0.043
		Sc. rank:	-477.4

,

NS1

Pos.	120	obs:	exp:
atg	M	1262	1262.00
---	--	------	------
mPD		0	0
		nPD:	1.
		N. weight:	0.
		Sc. PD:	0
		Sc. rank:	0

,

NS1

Pos.	121	obs:	exp:
gat	D	4	682.00
gac	D	1258	580.00
---	--	------	------
mPD		0.0063	0.50
		nPD:	0.01
		N. weight:	0.8
		Sc. PD:	-0.038
		Sc. rank:	-272.6

,

NS1

Pos.	122	obs:	exp:
caa	Q	1244	642.80
cag	Q	18	619.20
---	--	------	------
mPD		0.028	0.50
		nPD:	0.06
		N. weight:	0.63
		Sc. PD:	-0.0058
		Sc. rank:	294.1

,

NS1

Pos.	123	obs:	exp:
tct	S	0	4.77
tcc	S	0	3.92
tca	S	30	7.61
tcg	S	0	1.70
agt	S	0	6.17
agc	S	0	5.83
gct	A	0	318.10
gcc	A	1	229.40
gca	A	1229	579.90
gcg	A	2	104.60
---	--	------	------
mPD		0.051	0.74
		nPD:	0.07
		N. weight:	0.79
		Sc. PD:	0.0019
		Sc. rank:	474.1

,

NS1

Pos.	124	obs:	exp:
att	I	0	456.70
atc	I	0	297.90
ata	I	1243	488.40
act	T	0	0.25
acc	T	0	0.21
aca	T	1	0.46
acg	T	0	0.08
gtt	V	0	3.73
gtc	V	0	3.63
gta	V	18	3.69
gtg	V	0	6.95
---	--	------	------
mPD		0.030	0.69
		nPD:	0.04
		N. weight:	0.99
		Sc. PD:	-0.021
		Sc. rank:	309.4

,

NS1

Pos.	125	obs:	exp:
tta	L	0	0.09
ttg	L	1	0.19
ctt	L	0	0.17
ctc	L	0	0.17
cta	L	0	0.17
ctg	L	0	0.22
atg	M	15	15.00
gtt	V	3	258.20
gtc	V	0	251.50
gta	V	13	255.10
gtg	V	1230	481.20
---	--	------	------
mPD		0.050	0.75
		nPD:	0.07
		N. weight:	0.92
		Sc. PD:	0.00031
		Sc. rank:	538.6

,

NS1

Pos.	126	obs:	exp:
aat	N	0	0.51
aac	N	1	0.49
gat	D	2	679.30
gac	D	1255	577.70
gaa	E	2	1.22
gag	E	0	0.78
ggt	G	0	0.27
ggc	G	2	0.26
gga	G	0	0.92
ggg	G	0	0.56
---	--	------	------
mPD		0.011	0.50
		nPD:	0.02
		N. weight:	0.81
		Sc. PD:	-0.032
		Sc. rank:	-18.6

,

NS1

Pos.	127	obs:	exp:
aaa	K	1260	734.90
aag	K	1	526.10
gaa	E	1	0.61
gag	E	0	0.39
---	--	------	------
mPD		0.0032	0.49
		nPD:	0.01
		N. weight:	0.56
		Sc. PD:	-0.03
		Sc. rank:	-346.5

,

NS1

Pos.	128	obs:	exp:
cgt	R	0	0.04
cgc	R	0	0.05
cga	R	0	0.10
cgg	R	0	0.08
act	T	1	45.37
acc	T	177	36.52
aca	T	0	81.64
acg	T	0	14.46
aat	N	30	550.60
aac	N	1051	530.40
aaa	K	1	0.58
aag	K	0	0.42
aga	R	1	0.46
agg	R	0	0.27
gat	D	0	0.54
gac	D	1	0.46
---	--	------	------
mPD		0.30	0.82
		nPD:	0.36
		N. weight:	0.76
		Sc. PD:	0.2
		Sc. rank:	723.7

,

NS1

Pos.	129	obs:	exp:
att	I	0	463.70
atc	I	1262	302.40
ata	I	0	495.90
---	--	------	------
mPD		0	0.65
		nPD:	0.
		N. weight:	1.5
		Sc. PD:	-0.089
		Sc. rank:	-1551.6

,

NS1

Pos.	130	obs:	exp:
att	I	0	0.73
atc	I	1	0.48
ata	I	1	0.79
act	T	3	320.90
acc	T	23	258.30
aca	T	1233	577.50
acg	T	0	102.30
aaa	K	1	0.58
aag	K	0	0.42
---	--	------	------
mPD		0.047	0.68
		nPD:	0.07
		N. weight:	0.73
		Sc. PD:	0.0012
		Sc. rank:	428.6

,

NS1

Pos.	131	obs:	exp:
tta	L	4	112.00
ttg	L	1250	235.00
ctt	L	0	210.10
ctc	L	1	210.60
cta	L	0	212.70
ctg	L	7	281.60
---	--	------	------
mPD		0.021	1.1
		nPD:	0.02
		N. weight:	1.7
		Sc. PD:	-0.073
		Sc. rank:	-214.7

,

NS1

Pos.	132	obs:	exp:
aaa	K	1262	735.50
aag	K	0	526.50
---	--	------	------
mPD		0	0.49
		nPD:	0.
		N. weight:	0.57
		Sc. PD:	-0.034
		Sc. rank:	-586.3

,

NS1

Pos.	133	obs:	exp:
gct	A	0	325.90
gcc	A	1	235.00
gca	A	1220	594.00
gcg	A	41	107.10
---	--	------	------
mPD		0.064	0.67
		nPD:	0.1
		N. weight:	0.7
		Sc. PD:	0.018
		Sc. rank:	465.9

,

NS1

Pos.	134	obs:	exp:
aat	N	1191	642.80
aac	N	71	619.20
---	--	------	------
mPD		0.11	0.50
		nPD:	0.21
		N. weight:	0.49
		Sc. PD:	0.063
		Sc. rank:	397.9

,

NS1

Pos.	135	obs:	exp:
ttt	F	1	498.40
ttc	F	1261	763.60
---	--	------	------
mPD		0.0016	0.48
		nPD:	0.
		N. weight:	0.52
		Sc. PD:	-0.029
		Sc. rank:	-379.2

,

NS1			
Pos.	136	obs:	exp:
tct	S	1	200.10
tcc	S	0	164.30
tca	S	0	319.30
tcg	S	0	71.38
aat	N	4	2.04
aac	N	0	1.96
agt	S	1257	258.60
agc	S	0	244.30
mPD		0.0095	1.7
		nPD:	0.01
		N. weight:	1.7
		Sc. PD:	-0.089
		Sc. rank:	-1036.8

NS1			
Pos.	137	obs:	exp:
gtt	V	5	261.50
gtc	V	0	254.70
gta	V	111	258.40
gtg	V	1146	487.40
mPD		0.17	0.73
		nPD:	0.23
		N. weight:	0.73
		Sc. PD:	0.11
		Sc. rank:	598.5

NS1			
Pos.	138	obs:	exp:
tta	L	0	0.09
ttg	L	0	0.19
ctt	L	0	0.17
ctc	L	0	0.17
cta	L	1	0.17
ctg	L	0	0.22
att	I	1252	461.80
atc	I	1	301.20
ata	I	4	493.90
act	T	4	1.02
acc	T	0	0.82
aca	T	0	1.83
acg	T	0	0.32
mPD		0.017	0.66
		nPD:	0.03
		N. weight:	1.
		Sc. PD:	-0.037
		Sc. rank:	88.0

NS1			
Pos.	139	obs:	exp:
ttt	F	1	498.40
ttc	F	1261	763.60
mPD		0.0016	0.48
		nPD:	0.
		N. weight:	0.52
		Sc. PD:	-0.029
		Sc. rank:	-379.2

NS1			
Pos.	140	obs:	exp:
aat	N	1253	641.80
aac	N	7	618.20
gat	D	1	1.08
gac	D	1	0.92
mPD		0.016	0.50
		nPD:	0.03
		N. weight:	0.67
		Sc. PD:	-0.021
		Sc. rank:	144.7

NS1			
Pos.	141	obs:	exp:
caa	Q	1	0.51
cag	Q	0	0.49
cgt	R	1	54.16
cgc	R	0	62.46
cga	R	27	123.40
cgg	R	1226	105.20
aga	R	0	576.60
agg	R	7	339.20
mPD		0.058	0.96
		nPD:	0.06
		N. weight:	2.5
		Sc. PD:	-0.015
		Sc. rank:	1266.3

NS1			
Pos.	142	obs:	exp:
ttt	F	1	0.39
ttc	F	0	0.61
tta	L	1	110.90
ttg	L	22	232.70
ctt	L	1065	208.10
ctc	L	4	208.60
cta	L	0	210.70
ctg	L	158	278.90
att	I	8	2.94
atc	I	0	1.92
ata	I	0	3.14
atg	M	3	3.00
mPD		0.31	1.1
		nPD:	0.28
		N. weight:	1.3
		Sc. PD:	0.25
		Sc. rank:	1199.5

NS1			
Pos.	143	obs:	exp:
aaa	K	3	1.75
aag	K	0	1.25
gaa	E	1228	763.70
gag	E	27	491.30
ggt	G	0	0.53
ggc	G	0	0.51
gga	G	4	1.85
ggg	G	0	1.11
mPD		0.053	0.49
		nPD:	0.11
		N. weight:	0.43
		Sc. PD:	0.016
		Sc. rank:	301.9

NS1			
Pos.	144	obs:	exp:
tct	S	0	1.43
tcc	S	7	1.18
tca	S	0	2.28
tcg	S	0	0.51
act	T	0	1.27
acc	T	4	1.03
aca	T	0	2.29
acg	T	1	0.41
agt	S	0	1.85
agc	S	2	1.75
gtt	V	0	0.21
gtc	V	1	0.20
gta	V	0	0.20
gtg	V	0	0.39
gct	A	0	322.00
gcc	A	1245	232.20
gca	A	2	586.90
gcg	A	0	105.80
mPD		0.032	0.70
		nPD:	0.05
		N. weight:	1.8
		Sc. PD:	-0.034
		Sc. rank:	660.4

NS1			
Pos.	145	obs:	exp:
tta	L	5	112.00
ttg	L	0	235.00
ctt	L	0	210.10
ctc	L	0	210.60
cta	L	193	212.70
ctg	L	1064	281.60
mPD		0.27	1.1
		nPD:	0.25
		N. weight:	1.2
		Sc. PD:	0.18
		Sc. rank:	963.2

NS1			
Pos.	146	obs:	exp:
att	I	0	408.60
atc	I	0	266.50
ata	I	1112	436.90
atg	M	2	2.00
act	T	0	33.65
acc	T	0	27.09
aca	T	132	60.54
acg	T	0	10.72
gtt	V	0	3.32
gtc	V	1	3.23
gta	V	15	3.28
gtg	V	0	6.18
mPD		0.22	0.87
		nPD:	0.25
		N. weight:	0.97
		Sc. PD:	0.16
		Sc. rank:	834.2

NS1			
Pos.	147	obs:	exp:
tta	L	11	111.80
ttg	L	0	234.60
tct	S	1	0.16
tcc	S	0	0.13
tca	S	0	0.25
tcg	S	0	0.06
ctt	L	0	209.80
ctc	L	0	210.30
cta	L	1246	212.30
ctg	L	3	281.20
atg	M	1	1.00
agt	S	0	0.21
agc	S	0	0.19
mPD		0.030	1.1
		nPD:	0.03
		N. weight:	1.8
		Sc. PD:	-0.064
		Sc. rank:	183.4

NS1			
Pos.	148	obs:	exp:
tta	L	0	112.00
ttg	L	0	235.00
ctt	L	1260	210.10
ctc	L	0	210.60
cta	L	1	212.70
ctg	L	1	281.60
mPD		0.0032	1.1
		nPD:	0.
		N. weight:	1.9
		Sc. PD:	-0.11
		Sc. rank:	-1496.2

NS1			
Pos.	149	obs:	exp:
cgt	R	0	54.20
cgc	R	0	62.51
cga	R	0	123.50
cgg	R	0	105.30
aga	R	1261	577.10
agg	R	1	339.40
mPD		0.0016	0.96
		nPD:	0.
		N. weight:	0.82
		Sc. PD:	-0.047
		Sc. rank:	-727.4

NS1			
Pos.	150	obs:	exp:
gct	A	1259	325.90
gcc	A	0	235.00
gca	A	1	594.00
gcg	A	2	107.10
mPD		0.0047	0.67
		nPD:	0.01
		N. weight:	1.4
		Sc. PD:	-0.074
		Sc. rank:	-751.3

NS1			
Pos.	151	obs:	exp:
ttt	F	1259	498.40
ttc	F	3	763.60
mPD		0.0047	0.48
		nPD:	0.01
		N. weight:	0.96
		Sc. PD:	-0.048
		Sc. rank:	-397.7

NS1			
Pos.	152	obs:	exp:
act	T	0	321.40
acc	T	0	258.80
aca	T	61	578.40
acg	T	1200	102.40
gct	A	0	0.26
gcc	A	0	0.19
gca	A	1	0.47
gcg	A	0	0.08
mPD		0.095	0.68
		nPD:	0.14
		N. weight:	2.4
		Sc. PD:	0.15
		Sc. rank:	1776.7

NS1			
Pos.	153	obs:	exp:
aat	N	1	0.51
aac	N	0	0.49
aaa	K	2	1.17
aag	K	0	0.83
gat	D	180	97.27
gac	D	0	82.73
gaa	E	1068	656.60
gag	E	11	422.40
mPD		0.27	0.61
		nPD:	0.43
		N. weight:	0.5
		Sc. PD:	0.16
		Sc. rank:	488.8

NS1			
Pos.	154	obs:	exp:
aaa	K	3	1.75
aag	K	0	1.25
gat	D	5	3.24
gac	D	1	2.76
gaa	E	1253	762.50
gag	E	0	490.50
mPD		0.014	0.49
		nPD:	0.03
		N. weight:	0.52
		Sc. PD:	-0.017
		Sc. rank:	78.2

NS1			
Pos.	155	obs:	exp:
ggt	G	1	167.30
ggc	G	0	161.20
gga	G	1252	582.20
ggg	G	9	351.30
mPD		0.016	0.68
		nPD:	0.02
		N. weight:	0.77
		Sc. PD:	-0.03
		Sc. rank:	16.1

NS1			
Pos.	156	obs:	exp:
act	T	0	5.10
acc	T	0	4.10
aca	T	20	9.17
acg	T	0	1.62
gtt	V	0	0.41
gtc	V	0	0.40
gta	V	2	0.41
gtg	V	0	0.77
gct	A	1	319.40
gcc	A	0	230.40
gca	A	1236	582.20
gcg	A	0	105.00
gaa	E	3	1.83
gag	E	0	1.17
---	--	------	------
mPD		0.041	0.71
		nPD:	0.06
		N. weight:	0.79
		Sc. PD:	-0.0065
		Sc. rank:	371.5

NS1			
Pos.	157	obs:	exp:
tta	L	0	0.18
ttg	L	0	0.37
ctt	L	2	0.33
ctc	L	0	0.33
cta	L	0	0.34
ctg	L	0	0.45
att	I	695	462.60
atc	I	1	301.70
ata	I	563	494.70
gtt	V	0	0.21
gtc	V	0	0.20
gta	V	1	0.20
gtg	V	0	0.39
---	--	------	------
mPD		0.50	0.66
		nPD:	0.76
		N. weight:	0.3
		Sc. PD:	0.18
		Sc. rank:	309.9

NS1			
Pos.	158	obs:	exp:
gtt	V	0	261.50
gtc	V	0	254.70
gta	V	1063	258.40
gtg	V	199	487.40
---	--	------	------
mPD		0.27	0.73
		nPD:	0.37
		N. weight:	1.1
		Sc. PD:	0.29
		Sc. rank:	1064.2

NS1			
Pos.	159	obs:	exp:
gct	A	1	0.26
gcc	A	0	0.19
gca	A	0	0.47
gcg	A	0	0.08
ggt	G	5	167.10
ggc	G	1251	161.10
gga	G	5	581.70
ggg	G	0	351.00
---	--	------	------
mPD		0.019	0.68
		nPD:	0.03
		N. weight:	2.1
		Sc. PD:	-0.072
		Sc. rank:	292.0

NS1			
Pos.	160	obs:	exp:
gct	A	0	0.26
gcc	A	0	0.19
gca	A	1	0.47
gcg	A	0	0.08
gaa	E	1244	767.30
gag	E	17	493.70
---	--	------	------
mPD		0.028	0.48
		nPD:	0.06
		N. weight:	0.46
		Sc. PD:	-0.0032
		Sc. rank:	220.2

NS1			
Pos.	161	obs:	exp:
att	I	5	463.30
atc	I	1256	302.20
ata	I	0	495.50
gtt	V	0	0.21
gtc	V	1	0.20
gta	V	0	0.20
gtg	V	0	0.39
---	--	------	------
mPD		0.0095	0.65
		nPD:	0.01
		N. weight:	1.5
		Sc. PD:	-0.068
		Sc. rank:	-362.4

NS1			
Pos.	162	obs:	exp:
tct	S	1	200.70
tcc	S	0	164.80
tca	S	1261	320.30
tcg	S	0	71.61
agt	S	0	259.40
agc	S	0	245.10
---	--	------	------
mPD		0.0016	1.7
		nPD:	0.
		N. weight:	1.4
		Sc. PD:	-0.084
		Sc. rank:	-1308.8

NS1			
Pos.	163	obs:	exp:
tct	S	0	0.16
tcc	S	0	0.13
tca	S	1	0.25
tcg	S	0	0.06
cct	P	0	314.60
ccc	P	1	226.60
cca	P	1255	519.10
ccg	P	5	200.70
agt	S	0	0.21
agc	S	0	0.19
---	--	------	------
mPD		0.011	0.71
		nPD:	0.02
		N. weight:	0.91
		Sc. PD:	-0.041
		Sc. rank:	-210.0

NS1			
Pos.	164	obs:	exp:
tta	L	1250	111.40
ttg	L	1	233.70
tct	S	0	0.80
tcc	S	0	0.65
tca	S	5	1.27
tcg	S	0	0.28
ctt	L	0	209.00
ctc	L	0	209.40
cta	L	4	211.50
ctg	L	0	280.10
att	I	1	0.73
atc	I	0	0.48
ata	I	1	0.79
agt	S	0	1.03
agc	S	0	0.97
---	--	------	------
mPD		0.021	1.1
		nPD:	0.02
		N. weight:	2.5
		Sc. PD:	-0.11
		Sc. rank:	-383.9

NS1			
Pos.	165	obs:	exp:
cct	P	1259	314.90
ccc	P	3	226.70
cca	P	0	519.50
ccg	P	0	200.90
---	--	------	------
mPD		0.0047	0.71
		nPD:	0.01
		N. weight:	1.4
		Sc. PD:	-0.077
		Sc. rank:	-811.9

NS1			
Pos.	166	obs:	exp:
ttt	F	2	0.79
ttc	F	0	1.21
tct	S	1259	200.40
tcc	S	1	164.60
tca	S	0	319.80
tcg	S	0	71.50
agt	S	0	259.00
agc	S	0	244.70
---	--	------	------
mPD		0.0048	1.7
		nPD:	0.
		N. weight:	1.9
		Sc. PD:	-0.11
		Sc. rank:	-1506.4

NS1			
Pos.	167	obs:	exp:
tta	L	0	96.73
ttg	L	0	202.90
ctt	L	1087	181.50
ctc	L	3	181.90
cta	L	0	183.70
ctg	L	0	243.20
cct	P	170	42.42
ccc	P	0	30.54
cca	P	0	69.98
ccg	P	0	27.06
att	I	1	0.37
atc	I	0	0.24
ata	I	0	0.39
atg	M	1	1.00
---	--	------	------
mPD		0.24	1.3
		nPD:	0.18
		N. weight:	1.8
		Sc. PD:	0.19
		Sc. rank:	1472.9

NS1			
Pos.	168	obs:	exp:
tct	S	0	0.16
tcc	S	0	0.13
tca	S	1	0.25
tcg	S	0	0.06
cct	P	0	314.60
ccc	P	0	226.60
cca	P	1261	519.10
ccg	P	0	200.70
agt	S	0	0.21
agc	S	0	0.19
---	--	------	------
mPD		0.0016	0.71
		nPD:	0.
		N. weight:	0.93
		Sc. PD:	-0.053
		Sc. rank:	-782.9

NS1			
Pos.	169	obs:	exp:
gaa	E	1	0.61
gag	E	0	0.39
ggt	G	0	167.10
ggc	G	0	161.10
gga	G	1260	581.70
ggg	G	1	351.00
---	--	------	------
mPD		0.0032	0.68
		nPD:	0.
		N. weight:	0.81
		Sc. PD:	-0.044
		Sc. rank:	-563.0

NS1			
Pos.	170	obs:	exp:
cat	H	1260	723.50
cac	H	0	536.50
cgt	R	2	0.09
cgc	R	0	0.10
cga	R	0	0.20
cgg	R	0	0.17
aga	R	0	0.91
agg	R	0	0.54
---	--	------	------
mPD		0.0032	0.50
		nPD:	0.01
		N. weight:	0.59
		Sc. PD:	-0.031
		Sc. rank:	-365.2

NS1			
Pos.	171	obs:	exp:
tct	S	1	0.16
tcc	S	0	0.13
tca	S	0	0.25
tcg	S	0	0.06
att	I	1	0.37
atc	I	0	0.24
ata	I	0	0.39
act	T	1260	321.20
acc	T	0	258.50
aca	T	0	577.90
acg	T	0	102.30
agt	S	0	0.21
agc	S	0	0.19
---	--	------	------
mPD		0.0032	0.68
		nPD:	0.
		N. weight:	1.4
		Sc. PD:	-0.079
		Sc. rank:	-1011.9

NS1			
Pos.	172	obs:	exp:
act	T	0	0.25
acc	T	0	0.21
aca	T	1	0.46
acg	T	0	0.08
aat	N	0	5.09
aac	N	10	4.91
gat	D	0	674.90
gac	D	1249	574.10
ggt	G	0	0.27
ggc	G	2	0.26
gga	G	0	0.92
ggg	G	0	0.56
---	--	------	------
mPD		0.024	0.52
		nPD:	0.05
		N. weight:	0.82
		Sc. PD:	-0.015
		Sc. rank:	314.5

NS1			
Pos.	173	obs:	exp:
caa	Q	0	2.04
cag	Q	4	1.96
aaa	K	0	732.60
aag	K	1257	524.40
gaa	E	0	0.61
gag	E	1	0.39
---	--	------	------
mPD		0.0079	0.49
		nPD:	0.02
		N. weight:	0.92
		Sc. PD:	-0.041
		Sc. rank:	-205.5

NS1			
Pos.	174	obs:	exp:
gat	D	1262	682.00
gac	D	0	580.00
---	--	------	------
mPD		0	0.50
		nPD:	0.
		N. weight:	0.65
		Sc. PD:	-0.038
		Sc. rank:	-668.5

NS1			
Pos.	175	obs:	exp:
gtt	V	0	261.50
gtc	V	1262	254.70
gta	V	0	258.40
gtg	V	0	487.40
---	--	------	------
mPD		0	0.73
		nPD:	0.
		N. weight:	1.7
		Sc. PD:	-0.099
		Sc. rank:	-1738.1

NS1			
Pos.	176	obs:	exp:
aaa	K	1261	735.50
aag	K	1	526.50
---	--	------	------
mPD		0.0016	0.49
		nPD:	0.
		N. weight:	0.56
		Sc. PD:	-0.032
		Sc. rank:	-429.7

,

NS1			
Pos.	177	obs:	exp:
tct	S	0	0.32
tcc	S	0	0.26
tca	S	0	0.51
tcg	S	0	0.11
att	I	2	0.73
atc	I	0	0.48
ata	I	0	0.79
aat	N	1257	640.30
aac	N	0	616.70
agt	S	2	0.41
agc	S	0	0.39
gat	D	1	0.54
gac	D	0	0.46
---	--	------	------
mPD		0.0079	0.51
		nPD:	0.02
		N. weight:	0.71
		Sc. PD:	-0.032
		Sc. rank:	-170.4

,

NS1			
Pos.	178	obs:	exp:
gct	A	0	325.60
gcc	A	0	234.80
gca	A	1261	593.50
gcg	A	0	107.00
ggt	G	0	0.13
ggc	G	0	0.13
gga	G	1	0.46
ggg	G	0	0.28
---	--	------	------
mPD		0.0016	0.67
		nPD:	0.
		N. weight:	0.79
		Sc. PD:	-0.045
		Sc. rank:	-658.9

,

NS1			
Pos.	179	obs:	exp:
att	I	1262	463.70
atc	I	0	302.40
ata	I	0	495.90
---	--	------	------
mPD		0	0.65
		nPD:	0.
		N. weight:	1.1
		Sc. PD:	-0.062
		Sc. rank:	-1087.5

,

NS1			
Pos.	180	obs:	exp:
aaa	K	0	2.33
aag	K	4	1.67
gat	D	1	0.54
gac	D	0	0.46
gaa	E	3	760.60
gag	E	1247	489.40
ggt	G	0	0.93
ggc	G	0	0.89
gga	G	1	3.23
ggg	G	6	1.95
---	--	------	------
mPD		0.025	0.50
		nPD:	0.05
		N. weight:	0.97
		Sc. PD:	-0.014
		Sc. rank:	379.9

,

NS1			
Pos.	181	obs:	exp:
att	I	0	451.60
atc	I	1229	294.50
ata	I	0	482.90
gtt	V	0	6.84
gtc	V	33	6.66
gta	V	0	6.76
gtg	V	0	12.75
---	--	------	------
mPD		0.051	0.71
		nPD:	0.07
		N. weight:	1.5
		Sc. PD:	0.0066
		Sc. rank:	919.2

,

NS1			
Pos.	182	obs:	exp:
tta	L	0	112.00
ttg	L	0	235.00
ctt	L	0	210.10
ctc	L	1262	210.60
cta	L	0	212.70
ctg	L	0	281.60
---	--	------	------
mPD		0	1.1
		nPD:	0.
		N. weight:	1.9
		Sc. PD:	-0.11
		Sc. rank:	-1944.6

,

NS1			
Pos.	183	obs:	exp:
att	I	0	462.20
atc	I	1258	301.50
ata	I	0	494.30
act	T	0	1.02
acc	T	4	0.82
aca	T	0	1.83
acg	T	0	0.32
---	--	------	------
mPD		0.0063	0.66
		nPD:	0.01
		N. weight:	1.5
		Sc. PD:	-0.076
		Sc. rank:	-655.5

,

NS1			
Pos.	184	obs:	exp:
cgt	R	0	0.09
cgc	R	0	0.10
cga	R	0	0.20
cgg	R	0	0.17
aga	R	2	0.91
agg	R	0	0.54
ggt	G	1	167.00
ggc	G	0	161.00
gga	G	1258	581.30
ggg	G	1	350.80
---	--	------	------
mPD		0.0063	0.68
		nPD:	0.01
		N. weight:	0.8
		Sc. PD:	-0.041
		Sc. rank:	-386.6

,

NS1			
Pos.	185	obs:	exp:
cgt	R	0	0.09
cgc	R	0	0.10
cga	R	0	0.20
cgg	R	0	0.17
aga	R	2	0.91
agg	R	0	0.54
ggt	G	0	167.00
ggc	G	0	161.00
gga	G	1235	581.30
ggg	G	25	350.80
---	--	------	------
mPD		0.042	0.68
		nPD:	0.06
		N. weight:	0.72
		Sc. PD:	-0.0031
		Sc. rank:	400.1

,

NS1			
Pos.	186	obs:	exp:
ttt	F	1260	497.60
ttc	F	0	762.40
tta	L	0	0.18
ttg	L	0	0.37
ctt	L	2	0.33
ctc	L	0	0.33
cta	L	0	0.34
ctg	L	0	0.45
---	--	------	------
mPD		0.0032	0.48
		nPD:	0.01
		N. weight:	0.98
		Sc. PD:	-0.052
		Sc. rank:	-566.7

,

NS1			
Pos.	187	obs:	exp:
gaa	E	1256	766.70
gag	E	4	493.30
ggt	G	0	0.27
ggc	G	0	0.26
gga	G	2	0.92
ggg	G	0	0.56
---	--	------	------
mPD		0.0095	0.48
		nPD:	0.02
		N. weight:	0.5
		Sc. PD:	-0.021
		Sc. rank:	-44.9

,

NS1			
Pos.	188	obs:	exp:
tgg	W	1262	1262.00
---	--	------	------
mPD		0	0
		nPD:	1.
		N. weight:	0.
		Sc. PD:	0
		Sc. rank:	0

,

NS1			
Pos.	189	obs:	exp:
aat	N	1262	642.80
aac	N	0	619.20
---	--	------	------
mPD		0	0.50
		nPD:	0.
		N. weight:	0.71
		Sc. PD:	-0.042
		Sc. rank:	-732.7

,

NS1			
Pos.	190	obs:	exp:
gat	D	1262	682.00
gac	D	0	580.00
---	--	------	------
mPD		0	0.50
		nPD:	0.
		N. weight:	0.65
		Sc. PD:	-0.038
		Sc. rank:	-668.5

,

NS1			
Pos.	191	obs:	exp:
aat	N	3	642.80
aac	N	1259	619.20
---	--	------	------
mPD		0.0047	0.50
		nPD:	0.01
		N. weight:	0.73
		Sc. PD:	-0.037
		Sc. rank:	-333.6

,

NS1			
Pos.	192	obs:	exp:
tct	S	0	0.16
tcc	S	0	0.13
tca	S	1	0.25
tcg	S	0	0.06
act	T	0	319.40
acc	T	0	257.10
aca	T	1249	574.70
acg	T	4	101.80
aaa	K	6	3.50
aag	K	0	2.50
agt	S	0	0.21
agc	S	0	0.19
gct	A	0	0.52
gcc	A	0	0.37
gca	A	2	0.94
gcg	A	0	0.17
---	--	------	------
mPD		0.021	0.69
		nPD:	0.03
		N. weight:	0.8
		Sc. PD:	-0.026
		Sc. rank:	148.0

,

NS1			
Pos.	193	obs:	exp:
att	I	14	5.14
atc	I	0	3.35
ata	I	0	5.50
gtt	V	1248	258.60
gtc	V	0	251.90
gta	V	0	255.50
gtg	V	0	482.00
---	--	------	------
mPD		0.022	0.75
		nPD:	0.03
		N. weight:	1.6
		Sc. PD:	-0.055
		Sc. rank:	270.8

,

NS1			
Pos.	194	obs:	exp:
cgt	R	0	54.20
cgc	R	0	62.51
cga	R	1261	123.50
cgg	R	0	105.30
aga	R	1	577.10
agg	R	0	339.40
---	--	------	------
mPD		0.0016	0.96
		nPD:	0.
		N. weight:	2.4
		Sc. PD:	-0.14
		Sc. rank:	-2168.6

,

NS1			
Pos.	195	obs:	exp:
att	I	0	0.37
atc	I	1	0.24
ata	I	0	0.39
gtt	V	0	261.10
gtc	V	1260	254.30
gta	V	0	258.00
gtg	V	0	486.60
gct	A	0	0.26
gcc	A	0	0.19
gca	A	0	0.47
gcg	A	1	0.08
---	--	------	------
mPD		0.0048	0.73
		nPD:	0.01
		N. weight:	1.7
		Sc. PD:	-0.09
		Sc. rank:	-1013.3

NS1			
Pos.	196	obs:	exp:
tct	S	1260	200.40
tcc	S	0	164.60
tca	S	0	319.80
tcg	S	0	71.50
act	T	2	0.51
acc	T	0	0.41
aca	T	0	0.92
acg	T	0	0.16
agt	S	0	259.00
agc	S	0	244.70
---	--	------	------
mPD		0.0032	1.7
		nPD:	0.
		N. weight:	1.9
		Sc. PD:	-0.11
		Sc. rank:	-1660.0

,

NS1			
Pos.	197	obs:	exp:
gaa	E	1259	767.90
gag	E	3	494.10
---	--	------	------
mPD		0.0047	0.48
		nPD:	0.01
		N. weight:	0.51
		Sc. PD:	-0.025
		Sc. rank:	-207.9

,

NS1			
Pos.	198	obs:	exp:
act	T	1220	311.00
acc	T	0	250.30
aca	T	0	559.60
acg	T	0	99.10
aat	N	2	1.02
aac	N	0	0.98
gct	A	40	10.33
gcc	A	0	7.45
gca	A	0	18.83
gcg	A	0	3.39
---	--	------	------
mPD		0.065	0.74
		nPD:	0.09
		N. weight:	1.4
		Sc. PD:	0.026
		Sc. rank:	909.4

,

NS1			
Pos.	199	obs:	exp:
tta	L	0	111.50
ttg	L	0	234.00
ctt	L	0	209.30
ctc	L	0	209.80
cta	L	1257	211.80
ctg	L	0	280.50
att	I	0	1.84
atc	I	0	1.20
ata	I	5	1.96
---	--	------	------
mPD		0.0079	1.1
		nPD:	0.01
		N. weight:	1.9
		Sc. PD:	-0.099
		Sc. rank:	-1020.3

,

NS1			
Pos.	200	obs:	exp:
cat	H	1	0.57
cac	H	0	0.43
caa	Q	18	642.30
cag	Q	1243	618.70
---	--	------	------
mPD		0.030	0.50
		nPD:	0.06
		N. weight:	0.67
		Sc. PD:	-0.0044
		Sc. rank:	328.3

,

NS1			
Pos.	201	obs:	exp:
cgt	R	0	54.16
cgc	R	0	62.46
cga	R	0	123.40
cgg	R	0	105.20
aaa	K	1	0.58
aag	K	0	0.42
aga	R	1261	576.60
agg	R	0	339.20
---	--	------	------
mPD		0.0016	0.96
		nPD:	0.
		N. weight:	0.82
		Sc. PD:	-0.047
		Sc. rank:	-743.8

,

NS1			
Pos.	202	obs:	exp:
ttt	F	10	497.60
ttc	F	1250	762.40
tat	Y	0	1.08
tac	Y	2	0.92
---	--	------	------
mPD		0.019	0.48
		nPD:	0.04
		N. weight:	0.49
		Sc. PD:	-0.012
		Sc. rank:	131.4

,

NS1			
Pos.	203	obs:	exp:
tct	S	1	0.16
tcc	S	0	0.13
tca	S	0	0.25
tcg	S	0	0.06
act	T	1	0.25
acc	T	0	0.21
aca	T	0	0.46
acg	T	0	0.08
agt	S	0	0.21
agc	S	0	0.19
gct	A	1260	325.40
gcc	A	0	234.60
gca	A	0	593.00
gcg	A	0	106.90
---	--	------	------
mPD		0.0032	0.67
		nPD:	0.
		N. weight:	1.4
		Sc. PD:	-0.078
		Sc. rank:	-974.9

,

NS1			
Pos.	204	obs:	exp:
tgg	W	1261	1261.00
cgt	R	0	0.04
cgc	R	0	0.05
cga	R	0	0.10
cgg	R	1	0.08
aga	R	0	0.46
agg	R	0	0.27
---	--	------	------
mPD		0.0016	0.0026
		nPD:	0.61
		N. weight:	0.0021
		Sc. PD:	0.00099
		Sc. rank:	2.1

,

NS1			
Pos.	205	obs:	exp:
cgt	R	0	54.03
cgc	R	0	62.31
cga	R	0	123.10
cgg	R	0	105.00
aaa	K	1	0.58
aag	K	0	0.42
aga	R	1258	575.20
agg	R	0	338.40
ggt	G	0	0.40
ggc	G	0	0.38
gga	G	3	1.38
ggg	G	0	0.84
---	--	------	------
mPD		0.0063	0.97
		nPD:	0.01
		N. weight:	0.82
		Sc. PD:	-0.044
		Sc. rank:	-489.3

,

NS1			
Pos.	206	obs:	exp:
tct	S	0	197.10
tcc	S	0	161.80
tca	S	0	314.50
tcg	S	0	70.31
cgt	R	0	0.43
cgc	R	0	0.50
cga	R	0	0.98
cgg	R	0	0.83
att	I	0	0.73
atc	I	2	0.48
ata	I	0	0.79
aat	N	0	2.04
aac	N	4	1.96
agt	S	2	254.70
agc	S	1237	240.70
aga	R	10	4.57
agg	R	0	2.69
ggt	G	0	0.93
ggc	G	7	0.89
gga	G	0	3.23
ggg	G	0	1.95
---	--	------	------
mPD		0.039	1.7
		nPD:	0.02
		N. weight:	1.7
		Sc. PD:	-0.065
		Sc. rank:	60.8

,

NS1			
Pos.	207	obs:	exp:
tct	S	0	189.10
tcc	S	0	155.30
tca	S	0	301.80
tcg	S	0	67.47
tgt	C	0	2.54
tgc	C	6	3.46
cgt	R	1	0.13
cgc	R	2	0.15
cga	R	0	0.29
cgg	R	0	0.25
att	I	0	1.84
atc	I	5	1.20
ata	I	0	1.96
aat	N	0	0.51
aac	N	1	0.49
agt	S	0	244.40
agc	S	1189	230.90
aga	R	0	1.37
agg	R	0	0.81
ggt	G	0	7.69
ggc	G	58	7.41
gga	G	0	26.76
ggg	G	0	16.15
---	--	------	------
mPD		0.11	1.7
		nPD:	0.07
		N. weight:	1.7
		Sc. PD:	-0.0023
		Sc. rank:	987.4

,

NS1			
Pos.	208	obs:	exp:
aat	N	42	21.39
aac	N	0	20.61
gat	D	1220	659.30
gac	D	0	560.70
---	--	------	------
mPD		0.064	0.56
		nPD:	0.11
		N. weight:	0.65
		Sc. PD:	0.028
		Sc. rank:	465.8

,

NS1			
Pos.	209	obs:	exp:
aaa	K	0	1.17
aag	K	2	0.83
gct	A	0	0.26
gcc	A	0	0.19
gca	A	0	0.47
gcg	A	1	0.08
gaa	E	1	766.10
gag	E	1258	492.90
---	--	------	------
mPD		0.0063	0.48
		nPD:	0.01
		N. weight:	0.98
		Sc. PD:	-0.047
		Sc. rank:	-285.1

,

NS1			
Pos.	210	obs:	exp:
aat	N	26	13.75
aac	N	1	13.25
gat	D	1224	661.40
gac	D	0	562.60
ggt	G	11	1.46
ggc	G	0	1.41
gga	G	0	5.07
ggg	G	0	3.06
---	--	------	------
mPD		0.061	0.56
		nPD:	0.11
		N. weight:	0.66
		Sc. PD:	0.024
		Sc. rank:	463.4

,

NS1			
Pos.	211	obs:	exp:
ggt	G	0	167.30
ggc	G	0	161.20
gga	G	0	582.20
ggg	G	1262	351.30
---	--	------	------
mPD		0	0.68
		nPD:	0.
		N. weight:	1.3
		Sc. PD:	-0.079
		Sc. rank:	-1388.8

NS1			
Pos.	212	obs:	exp:
cgt	R	0	52.01
cgc	R	0	59.98
cga	R	0	118.50
cgg	R	0	101.00
aaa	K	3	1.75
aag	K	0	1.25
aga	R	1211	553.70
agg	R	0	325.70
ggt	G	0	6.36
ggc	G	0	6.13
gga	G	48	22.14
ggg	G	0	13.36
---	--	------	------
mPD		0.078	1.0
		nPD:	0.08
		N. weight:	0.82
		Sc. PD:	0.0074
		Sc. rank:	507.2

NS1			
Pos.	213	obs:	exp:
tta	L	0	0.09
ttg	L	0	0.19
tct	S	1051	167.20
tcc	S	0	137.30
tca	S	0	266.80
tcg	S	0	59.64
tat	Y	2	1.08
tac	Y	0	0.92
ctt	L	1	0.17
ctc	L	0	0.17
cta	L	0	0.17
ctg	L	0	0.22
cct	P	208	51.90
ccc	P	0	37.37
cca	P	0	85.62
ccg	P	0	33.11
agt	S	0	216.10
agc	S	0	204.10
---	--	------	------
mPD		0.28	1.8
		nPD:	0.16
		N. weight:	1.9
		Sc. PD:	0.15
		Sc. rank:	1431.3

NS1			
Pos.	214	obs:	exp:
tct	S	0	0.80
tcc	S	0	0.65
tca	S	5	1.27
tcg	S	0	0.28
cct	P	0	313.60
ccc	P	0	225.80
cca	P	1257	517.40
ccg	P	0	200.10
agt	S	0	1.03
agc	S	0	0.97
---	--	------	------
mPD		0.0079	0.72
		nPD:	0.01
		N. weight:	0.94
		Sc. PD:	-0.046
		Sc. rank:	-364.5

NS1			
Pos.	215	obs:	exp:
ttt	F	0	2.76
ttc	F	7	4.24
tta	L	0	111.30
ttg	L	0	233.50
ctt	L	0	208.80
ctc	L	1253	209.30
cta	L	1	211.30
ctg	L	0	279.80
att	I	0	0.37
atc	I	1	0.24
ata	I	0	0.39
---	--	------	------
mPD		0.014	1.1
		nPD:	0.01
		N. weight:	1.9
		Sc. PD:	-0.089
		Sc. rank:	-609.9

NS1			
Pos.	216	obs:	exp:
tct	S	1153	183.50
tcc	S	0	150.70
tca	S	1	292.90
tcg	S	0	65.48
cct	P	108	26.95
ccc	P	0	19.40
cca	P	0	44.46
ccg	P	0	17.19
agt	S	0	237.20
agc	S	0	224.10
---	--	------	------
mPD		0.16	1.7
		nPD:	0.09
		N. weight:	1.9
		Sc. PD:	0.042
		Sc. rank:	1239.5

NS1			
Pos.	217	obs:	exp:
tct	S	0	0.16
tcc	S	0	0.13
tca	S	1	0.25
tcg	S	0	0.06
cct	P	0	45.41
ccc	P	0	32.70
cca	P	182	74.92
ccg	P	0	28.97
act	T	0	274.50
acc	T	0	221.00
aca	T	1077	494.00
acg	T	0	87.48
agt	S	0	0.21
agc	S	0	0.19
gct	A	0	0.52
gcc	A	0	0.37
gca	A	2	0.94
gcg	A	0	0.17
---	--	------	------
mPD		0.25	0.93
		nPD:	0.27
		N. weight:	0.84
		Sc. PD:	0.15
		Sc. rank:	745.8

NS1			
Pos.	218	obs:	exp:
cgt	R	0	0.04
cgc	R	0	0.05
cga	R	0	0.10
cgg	R	0	0.08
aaa	K	19	730.30
aag	K	1234	522.70
aga	R	0	0.46
agg	R	1	0.27
gaa	E	0	4.87
gag	E	8	3.13
---	--	------	------
mPD		0.044	0.50
		nPD:	0.09
		N. weight:	0.84
		Sc. PD:	0.015
		Sc. rank:	534.8

NS1			
Pos.	219	obs:	exp:
caa	Q	0	1.53
cag	Q	3	1.47
aaa	K	0	1.17
aag	K	2	0.83
---	-	1257	1257.00
---	--	------	------
mPD		7.5×10^{-6}	0.000015
		nPD:	0.49
		N. weight:	0.
		Sc. PD:	0
		Sc. rank:	0

NS1			
Pos.	220	obs:	exp:
aaa	K	3	1.75
aag	K	0	1.25
gaa	E	2	1.22
gag	E	0	0.78
---	-	1257	1257.00
---	--	------	------
mPD		7.5×10^{-6}	0.000015
		nPD:	0.5
		N. weight:	0.
		Sc. PD:	0
		Sc. rank:	0

NS1			
Pos.	221	obs:	exp:
tgg	W	2	2.00
cgt	R	0	0.13
cgc	R	1	0.15
cga	R	0	0.29
cgg	R	2	0.25
aga	R	0	1.37
agg	R	0	0.81
---	-	1257	1257.00
---	--	------	------
mPD		0.000013	0.000018
		nPD:	0.7
		N. weight:	0.
		Sc. PD:	0
		Sc. rank:	0

NS1			
Pos.	222	obs:	exp:
tat	Y	0	0.54
tac	Y	1	0.46
gaa	E	4	2.43
gag	E	0	1.57
---	-	1257	1257.00
---	--	------	------
mPD		0.000010	0.000015
		nPD:	0.66
		N. weight:	0.
		Sc. PD:	0
		Sc. rank:	0

NS1			
Pos.	223	obs:	exp:
atg	M	5	5.00
---	-	1257	1257.00
---	--	------	------
mPD		0	0
		nPD:	1.
		N. weight:	0.
		Sc. PD:	0
		Sc. rank:	0

NS1			
Pos.	224	obs:	exp:
gct	A	0	0.26
gcc	A	0	0.19
gca	A	0	0.47
gcg	A	1	0.08
gaa	E	0	2.43
gag	E	4	1.57
---	-	1257	1257.00
---	--	------	------
mPD		5.0×10^{-6}	0.000014
		nPD:	0.37
		N. weight:	0.
		Sc. PD:	0
		Sc. rank:	0

NS1			
Pos.	225	obs:	exp:
cgt	R	0	0.21
cgc	R	0	0.25
cga	R	0	0.49
cgg	R	0	0.42
aga	R	5	2.29
agg	R	0	1.34
---	-	1257	1257.00
---	--	------	------
mPD		0	0.000015
		nPD:	0.
		N. weight:	0.
		Sc. PD:	0
		Sc. rank:	0

NS1			
Pos.	226	obs:	exp:
cgt	R	0	0.04
cgc	R	0	0.05
cga	R	1	0.10
cgg	R	0	0.08
act	T	0	1.02
acc	T	0	0.82
aca	T	4	1.83
acg	T	0	0.32
aga	R	0	0.46
agg	R	0	0.27
---	-	1257	1257.00
---	--	------	------
mPD		0.000010	0.000017
		nPD:	0.58
		N. weight:	0.
		Sc. PD:	0
		Sc. rank:	0

NS1			
Pos.	227	obs:	exp:
att	I	2	0.73
atc	I	0	0.48
ata	I	0	0.79
gtt	V	3	0.62
gtc	V	0	0.61
gta	V	0	0.61
gtg	V	0	1.16
---	-	1257	1257.00
---	--	------	------
mPD		7.5×10^{-6}	0.000019
		nPD:	0.39
		N. weight:	0.
		Sc. PD:	0
		Sc. rank:	0

NS1			
Pos.	228	obs:	exp:
aaa	K	0	2.33
aag	K	4	1.67
gaa	E	0	0.61
gag	E	1	0.39
---	-	1257	1257.00
---	--	------	------
mPD		5.0×10^{-6}	0.000013
		nPD:	0.4
		N. weight:	0.
		Sc. PD:	0
		Sc. rank:	0

,

NS1			
Pos.	229	obs:	exp:
tct	S	0	0.16
tcc	S	0	0.13
tca	S	1	0.25
tcg	S	0	0.06
cct	P	0	1.00
ccc	P	0	0.72
cca	P	4	1.65
ccg	P	0	0.64
agt	S	0	0.21
agc	S	0	0.19
---	-	1257	1257.00
---	--	------	------
mPD		5.0×10^{-6}	0.000019
		nPD:	0.27
		N. weight:	0.
		Sc. PD:	0
		Sc. rank:	0

,

NS1			
Pos.	230	obs:	exp:
gaa	E	5	3.04
gag	E	0	1.96
---	-	1257	1257.00
---	--	------	------
mPD		0	7.5×10^{-6}
		nPD:	0.
		N. weight:	0.
		Sc. PD:	0
		Sc. rank:	0

,

NS1			
Pos.	231	obs:	exp:
gtt	V	5	1.04
gtc	V	0	1.01
gta	V	0	1.02
gtg	V	0	1.93
---	-	1257	1257.00
---	--	------	------
mPD		0	0.000011
		nPD:	0.
		N. weight:	0.
		Sc. PD:	0
		Sc. rank:	0

,

NS1			
Pos.	232	obs:	exp:
cgt	R	0	0.17
cgc	R	0	0.20
cga	R	4	0.39
cgg	R	0	0.33
aga	R	0	1.83
agg	R	0	1.08
---	-	1258	1258.00
---	--	------	------
mPD		0	9.7×10^{-6}
		nPD:	0.
		N. weight:	0.
		Sc. PD:	0
		Sc. rank:	0

,

NS1			
Pos.	233	obs:	exp:
cgt	R	0	0.17
cgc	R	0	0.20
cga	R	0	0.39
cgg	R	0	0.33
aga	R	4	1.83
agg	R	0	1.08
---	-	1258	1258.00
---	--	------	------
mPD		0	9.7×10^{-6}
		nPD:	0.
		N. weight:	0.
		Sc. PD:	0
		Sc. rank:	0

,

NS1			
Pos.	234	obs:	exp:
aat	N	4	2.04
aac	N	0	1.96
---	-	1258	1258.00
---	--	------	------
mPD		0	5.0×10^{-6}
		nPD:	0.
		N. weight:	0.
		Sc. PD:	0
		Sc. rank:	0

,

NS1			
Pos.	235	obs:	exp:
aaa	K	0	2.33
aag	K	4	1.67
---	-	1258	1258.00
---	--	------	------
mPD		0	4.9×10^{-6}
		nPD:	0.
		N. weight:	0.
		Sc. PD:	0
		Sc. rank:	0

,

NS1			
Pos.	236	obs:	exp:
atg	M	4	4.00
---	-	1258	1258.00
---	--	------	------
mPD		0	0
		nPD:	1.
		N. weight:	0.
		Sc. PD:	0
		Sc. rank:	0

,

NS1			
Pos.	237	obs:	exp:
gtt	V	4	0.83
gtc	V	0	0.81
gta	V	0	0.82
gtg	V	0	1.54
---	-	1258	1258.00
---	--	------	------
mPD		0	7.3×10^{-6}
		nPD:	0.
		N. weight:	0.
		Sc. PD:	0
		Sc. rank:	0

,

NS1			
Pos.	238	obs:	exp:
gat	D	4	2.16
gac	D	0	1.84
---	-	1258	1258.00
---	--	------	------
mPD		0	5.0×10^{-6}
		nPD:	0.
		N. weight:	0.
		Sc. PD:	0
		Sc. rank:	0

,

NS1			
Pos.	239	obs:	exp:
taa	.	1	536.10
tag	.	1256	315.90
tga	.	5	409.90
---	--	------	------
mPD		0.017	0.81
		nPD:	0.02
		N. weight:	1.4
		Sc. PD:	-0.057
		Sc. rank:	-50.8

}

Amino acid level conservation per codon

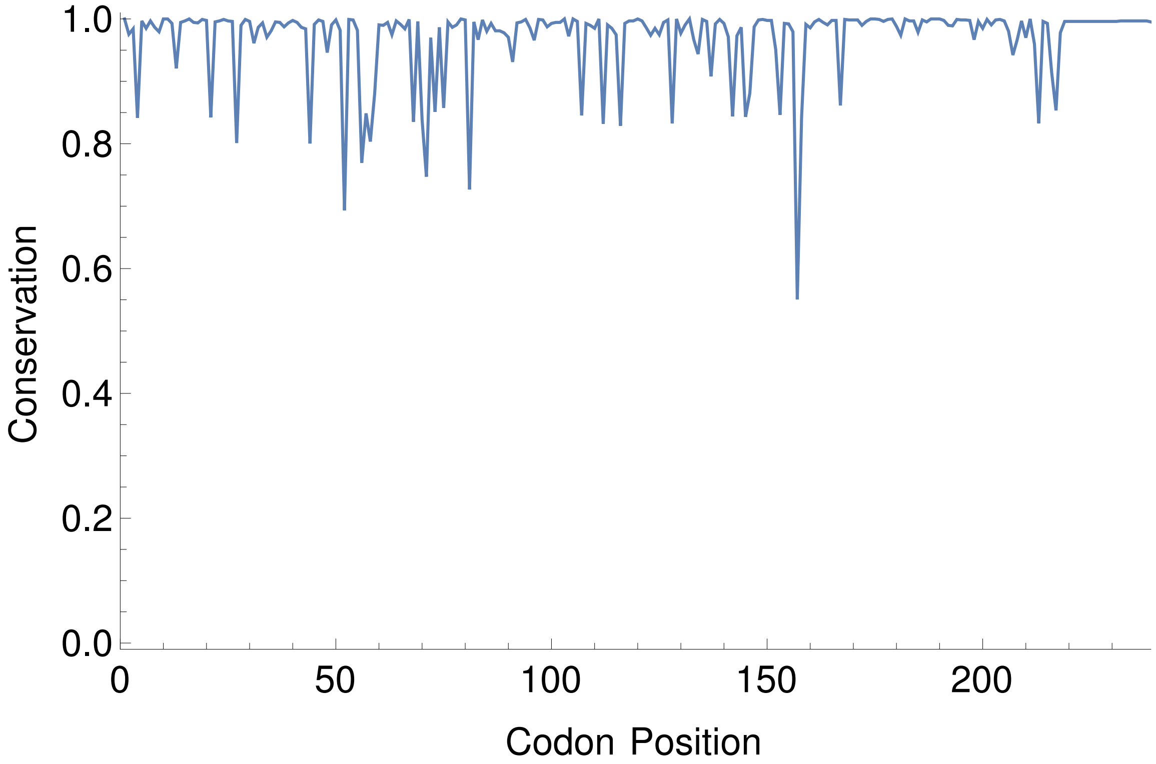

Conservation analysis plots

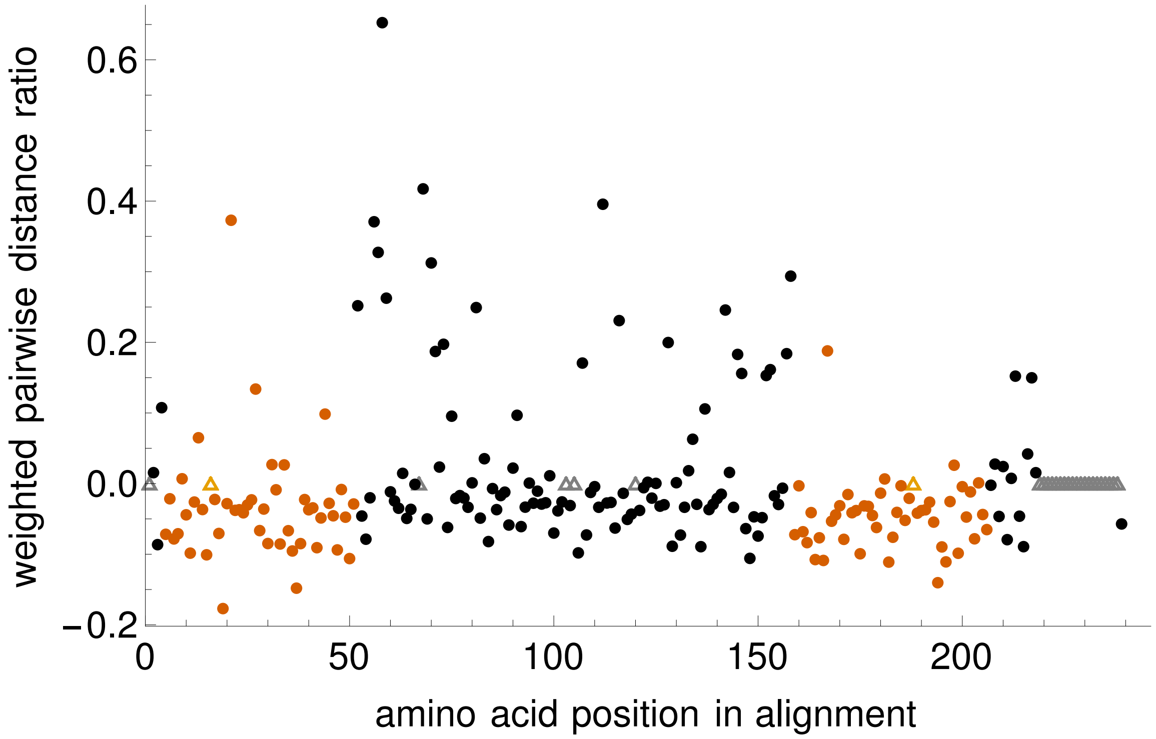

NS1

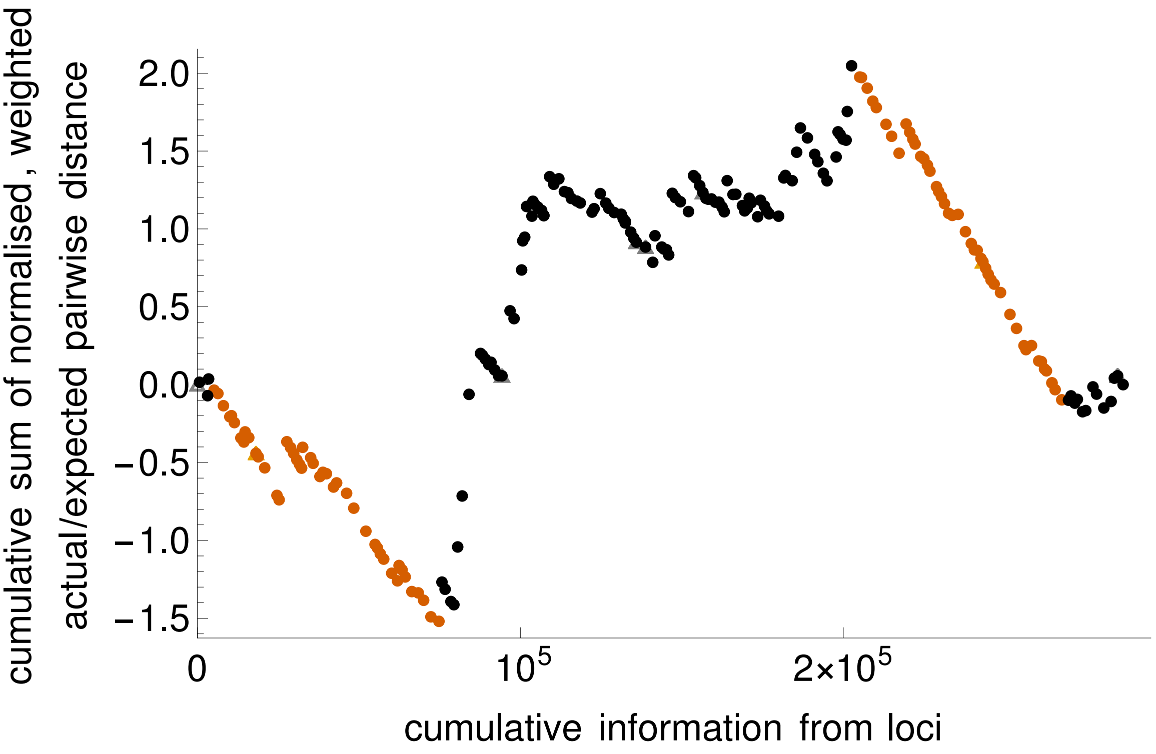

NS1

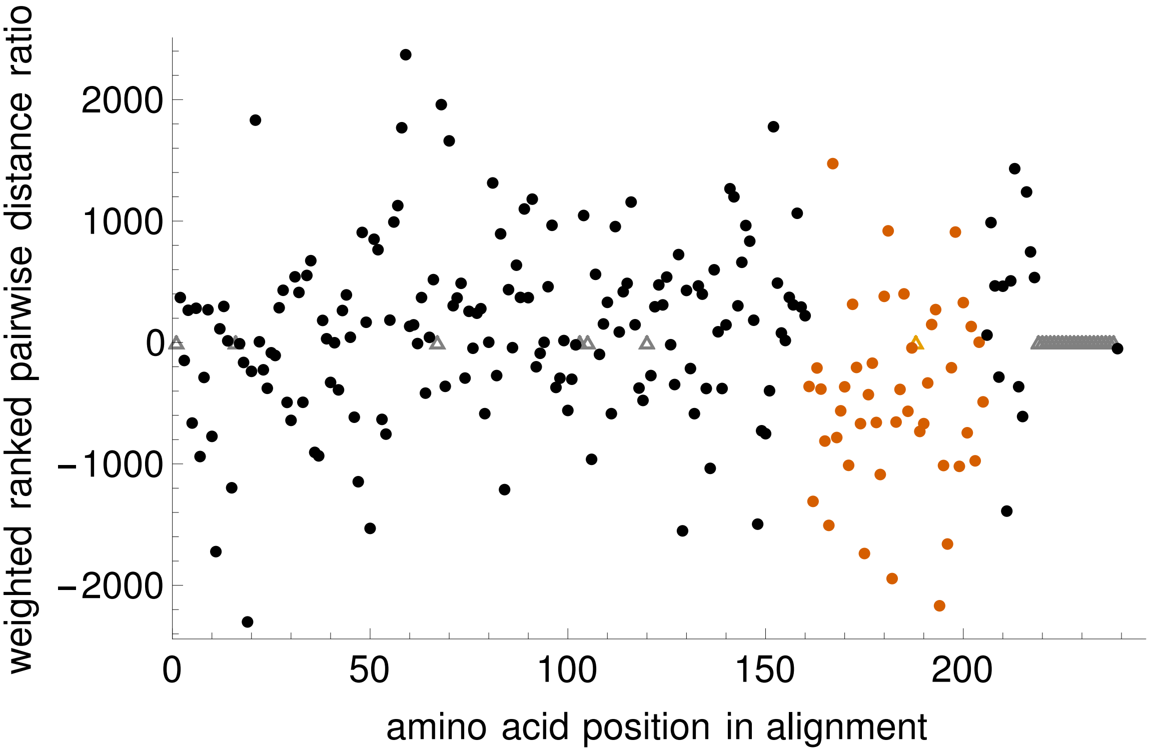
weighted ranked pairwise distance ratio
2000
1000
0
−1000
−2000
0
50
100
150
200
amino acid position in alignment
NS1

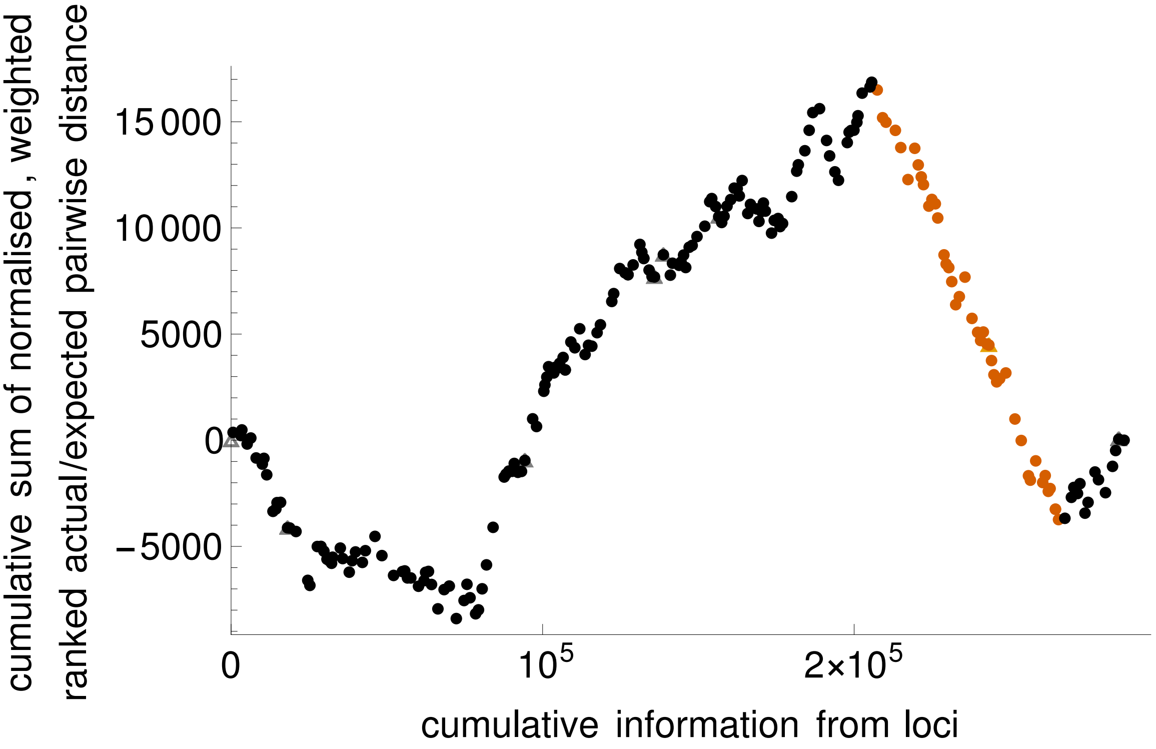
cumulative sum of normalised, weighted ranked actual/expected pairwise distance
15 000
10 000
5000
0
−5000
0
10^5
2×10^5
cumulative information from loci
NS1

NS2

Gene length histogram

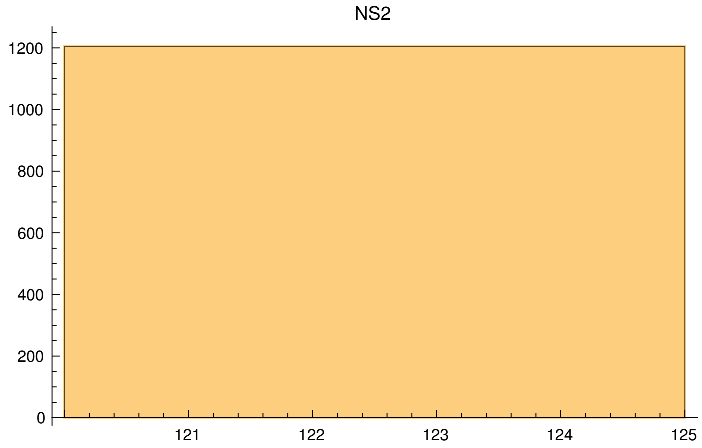

Information vs. nPD

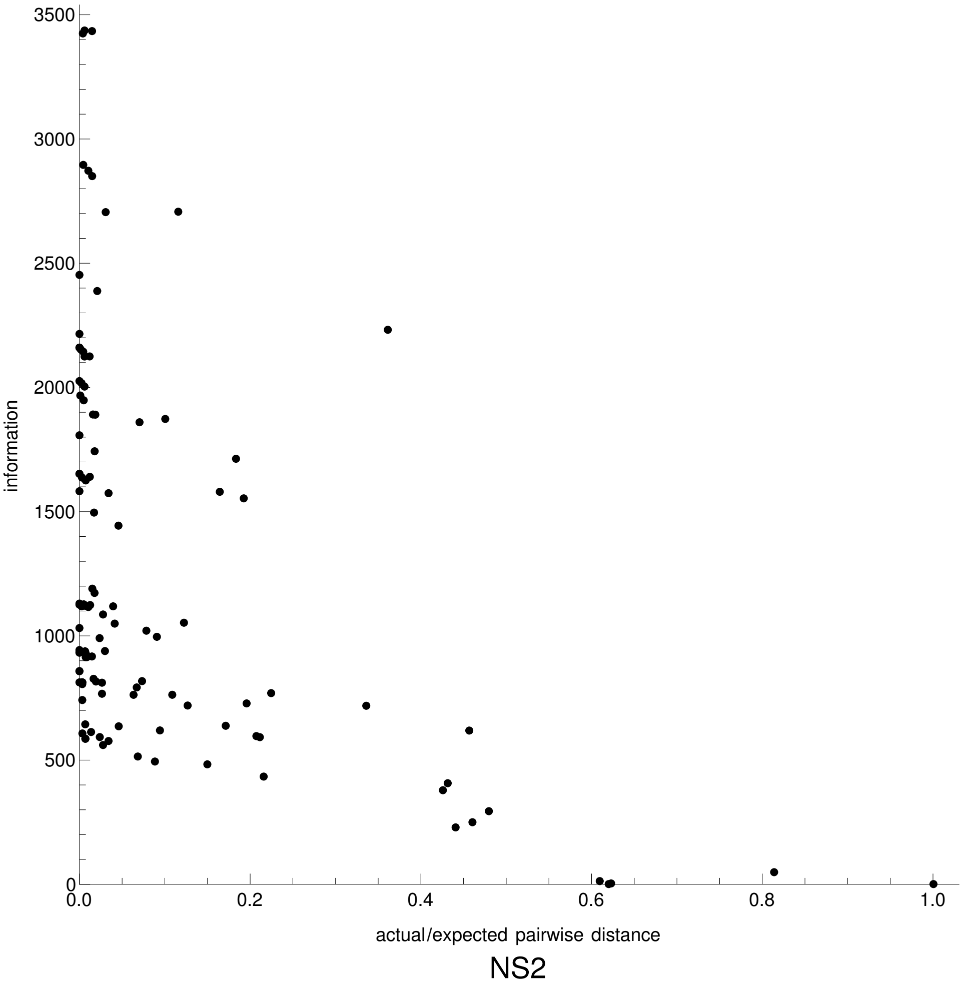

Example sequences highlighted by regions found in analyses to be conserved

Interesting points (by weighted raw PD) highlighted for gene NS2:

ATGGATTCCAAT

**ACTGTGTCAAGCTTCCAGGACATACTGACAAGGATGTCAAAAATGCAATTGAGAT
CCTCATCGGAGGATTTGAATGGAATGATAACACAGTTCGAGTCTCTGAAACT
CTACAGAGATTCGCTTGGAGAA**GCAGCGATGAGGATGGGAGATCTCCACTCTCTACAAAGTAG⋱
AAACGGGAAATGGAGAGAACAGTTAAGCCAGAAGTTCGAAGAGATAAGATGGTTGATTGAAGAAGTACGA⋱
CATAGATTAAAAATTACGGAGAATAGCTTTGAGCAAATAACTTTTATG

**CAAGCCTTACAACTATTGCTTGAAGTGGAGCAAGAGATAAGAACTTTCTCGTTTC
AGCTTATTTAA**

Interesting points (by weighted ranked PD) highlighted for gene NS2:

ATGGATTCCAATACTGTGTCAAGCTTCCAGGACATACTGACA

**AGGATGTCAAAAATGCAATTGAGATCCTCATCGGAGGATTTGAATGGAATGATAA
CACAGTTCGAGTCTCTGAAACTCTAC**AGAGATTCGCTTGGAGAAGCAGCGATGAGGATGGG⋱
AGATCTCCACTCTCTACAAAGTAGAAACGGGAAATGGAGAGAACAGTTAAGCCAGAAGTTCGAAGAGATA⋱
AGATGGTTGATTGAAGAAGTACGACATAGATTAAAAATTACGGAGAATAGCTTTGAGCAAATAACTTTTA⋱
TGCAAGCCTTACAACTATTGCTTGAAGTGGAGCAAGAGATAAGAACTTTCTCGTTTCAGCTTATTTAA

Per codon conservation report

NS2

Pos. 1		obs:	exp:
atg	M	1205	1205.00
---	--	------	------
mPD		0	0
		nPD:	1.
		N. weight:	0.
		Sc. PD:	0
		Sc. rank:	0

NS2

Pos. 2		obs:	exp:
aat	N	2	1.02
aac	N	0	0.98
gat	D	1176	650.10
gac	D	27	552.90
---	--	------	------
mPD		0.047	0.50
		nPD:	0.09
		N. weight:	0.53
		Sc. PD:	0.025
		Sc. rank:	388.1

NS2

Pos. 3		obs:	exp:
ttt	F	0	0.39
ttc	F	1	0.61
tta	L	0	0.09
ttg	L	0	0.19
tct	S	9	190.10
tcc	S	1185	156.10
tca	S	1	303.30
tcg	S	0	67.81
ctt	L	0	0.17
ctc	L	1	0.17
cta	L	0	0.17
ctg	L	0	0.22
cct	P	0	2.00
ccc	P	8	1.44
cca	P	0	3.29
ccg	P	0	1.27
agt	S	0	245.70
agc	S	0	232.10
---	--	------	------
mPD		0.035	1.7
		nPD:	0.02
		N. weight:	2.
		Sc. PD:	-0.045
		Sc. rank:	840.7

NS2

Pos. 4		obs:	exp:
cat	H	1	0.57
cac	H	0	0.43
aat	N	1059	613.30
aac	N	145	590.70
---	--	------	------
mPD		0.21	0.50
		nPD:	0.43
		N. weight:	0.32
		Sc. PD:	0.12
		Sc. rank:	326.6

NS2

Pos. 5		obs:	exp:
act	T	1202	307.20
acc	T	0	247.30
aca	T	3	552.70
acg	T	0	97.88
---	--	------	------
mPD		0.0050	0.68
		nPD:	0.01
		N. weight:	1.4
		Sc. PD:	-0.048
		Sc. rank:	-54.5

NS2

Pos. 6		obs:	exp:
atg	M	16	16.00
gtt	V	0	246.40
gtc	V	0	240.00
gta	V	2	243.50
gtg	V	1187	459.20
---	--	------	------
mPD		0.030	0.75
		nPD:	0.04
		N. weight:	0.95
		Sc. PD:	-0.0042
		Sc. rank:	552.3

NS2

Pos. 7		obs:	exp:
tct	S	0	191.70
tcc	S	0	157.40
tca	S	1202	305.80
tcg	S	3	68.38
agt	S	0	247.70
agc	S	0	234.00
---	--	------	------
mPD		0.0050	1.7
		nPD:	0.
		N. weight:	1.4
		Sc. PD:	-0.055
		Sc. rank:	-694.9

NS2

Pos. 8		obs:	exp:
tct	S	0	191.70
tcc	S	0	157.40
tca	S	0	305.80
tcg	S	0	68.38
agt	S	16	247.70
agc	S	1189	234.00
---	--	------	------
mPD		0.026	1.7
		nPD:	0.02
		N. weight:	1.6
		Sc. PD:	-0.043
		Sc. rank:	429.8

NS2

Pos. 9		obs:	exp:
ttt	F	20	475.80
ttc	F	1185	729.20
---	--	------	------
mPD		0.033	0.48
		nPD:	0.07
		N. weight:	0.44
		Sc. PD:	0.01
		Sc. rank:	286.2

NS2

Pos. 10		obs:	exp:
caa	Q	0	613.80
cag	Q	1205	591.20
---	--	------	------
mPD		0	0.50
		nPD:	0.
		N. weight:	0.73
		Sc. PD:	-0.031
		Sc. rank:	-713.9

NS2

Pos. 11		obs:	exp:
aat	N	0	0.51
aac	N	1	0.49
gat	D	0	650.10
gac	D	1203	552.90
ggt	G	0	0.13
ggc	G	1	0.13
gga	G	0	0.46
ggg	G	0	0.28
---	--	------	------
mPD		0.0033	0.50
		nPD:	0.01
		N. weight:	0.8
		Sc. PD:	-0.028
		Sc. rank:	-93.6

NS2

Pos. 12		obs:	exp:
att	I	0	442.00
atc	I	0	288.30
ata	I	1203	472.70
gtt	V	0	0.41
gtc	V	0	0.40
gta	V	2	0.41
gtg	V	0	0.77
---	--	------	------
mPD		0.0033	0.66
		nPD:	0.01
		N. weight:	0.96
		Sc. PD:	-0.036
		Sc. rank:	-243.5

NS2

Pos. 13		obs:	exp:
tta	L	0	106.90
ttg	L	1	224.30
ctt	L	0	200.70
ctc	L	0	201.10
cta	L	11	203.10
ctg	L	1193	268.90
---	--	------	------
mPD		0.020	1.1
		nPD:	0.02
		N. weight:	1.5
		Sc. PD:	-0.037
		Sc. rank:	506.4

NS2

Pos. 14		obs:	exp:
act	T	0	306.70
acc	T	4	246.90
aca	T	1199	551.80
acg	T	0	97.71
gct	A	0	0.52
gcc	A	0	0.37
gca	A	2	0.94
gcg	A	0	0.17
---	--	------	------
mPD		0.0099	0.68
		nPD:	0.01
		N. weight:	0.78
		Sc. PD:	-0.022
		Sc. rank:	104.7

NS2

Pos. 15		obs:	exp:
cgt	R	0	51.75
cgc	R	0	59.68
cga	R	0	117.90
cgg	R	0	100.50
aga	R	0	551.00
agg	R	1205	324.10
---	--	------	------
mPD		0	0.96
		nPD:	0.
		N. weight:	1.3
		Sc. PD:	-0.057
		Sc. rank:	-1316.7

NS2

Pos. 16		obs:	exp:
atg	M	1205	1205.00
---	--	------	------
mPD		0	0
		nPD:	1.
		N. weight:	0.
		Sc. PD:	0
		Sc. rank:	0

NS2

Pos. 17		obs:	exp:
tct	S	0	191.70
tcc	S	0	157.40
tca	S	1205	305.80
tcg	S	0	68.38
agt	S	0	247.70
agc	S	0	234.00
---	--	------	------
mPD		0	1.7
		nPD:	0.
		N. weight:	1.4
		Sc. PD:	-0.059
		Sc. rank:	-1374.8

NS2

Pos. 18		obs:	exp:
cgt	R	0	0.04
cgc	R	0	0.05
cga	R	0	0.10
cgg	R	0	0.08
aaa	K	1203	701.70
aag	K	1	502.30
aga	R	1	0.46
agg	R	0	0.27
---	--	------	------
mPD		0.0033	0.49
		nPD:	0.01
		N. weight:	0.55
		Sc. PD:	-0.019
		Sc. rank:	-59.3

NS2

Pos. 19		obs:	exp:
tta	L	0	0.09
ttg	L	1	0.19
ctt	L	0	0.17
ctc	L	0	0.17
cta	L	0	0.17
ctg	L	0	0.22
atg	M	1202	1202.00
gtt	V	0	0.41
gtc	V	0	0.40
gta	V	0	0.41
gtg	V	2	0.77
---	--	------	------
mPD		0.0050	0.0080
		nPD:	0.62
		N. weight:	0.003
		Sc. PD:	0.0017
		Sc. rank:	3.2

NS2

Pos. 20		obs:	exp:
caa	Q	1204	613.30
cag	Q	0	590.70
gaa	E	1	0.61
gag	E	0	0.39
---	--	------	------
mPD		0.0017	0.50
		nPD:	0.
		N. weight:	0.69
		Sc. PD:	-0.027
		Sc. rank:	-337.0

NS2

Pos. 21		obs:	exp:
tta	L	4	106.90
ttg	L	1201	224.30
ctt	L	0	200.70
ctc	L	0	201.10
cta	L	0	203.10
ctg	L	0	268.90
mPD		0.0066	1.1
		nPD:	0.01
		N. weight:	1.7
		Sc. PD:	-0.062
		Sc. rank:	-357.2

NS2

Pos. 22		obs:	exp:
cgt	R	0	51.28
cgc	R	0	59.14
cga	R	0	116.80
cgg	R	0	99.63
att	I	0	0.37
atc	I	0	0.24
ata	I	1	0.39
aaa	K	2	1.75
aag	K	1	1.25
aga	R	1164	546.00
agg	R	30	321.10
ggt	G	0	0.93
ggc	G	0	0.89
gga	G	5	3.23
ggg	G	2	1.95
mPD		0.072	0.97
		nPD:	0.07
		N. weight:	0.69
		Sc. PD:	0.019
		Sc. rank:	481.5

NS2

Pos. 23		obs:	exp:
tct	S	0	191.70
tcc	S	1205	157.40
tca	S	0	305.80
tcg	S	0	68.38
agt	S	0	247.70
agc	S	0	234.00
mPD		0	1.7
		nPD:	0.
		N. weight:	2.1
		Sc. PD:	-0.088
		Sc. rank:	-2041.1

NS2

Pos. 24		obs:	exp:
tct	S	0	191.70
tcc	S	0	157.40
tca	S	1205	305.80
tcg	S	0	68.38
agt	S	0	247.70
agc	S	0	234.00
mPD		0	1.7
		nPD:	0.
		N. weight:	1.4
		Sc. PD:	-0.059
		Sc. rank:	-1374.8

NS2

Pos. 25		obs:	exp:
tct	S	0	191.00
tcc	S	0	156.90
tca	S	2	304.80
tcg	S	1199	68.15
cct	P	0	1.00
ccc	P	0	0.72
cca	P	0	1.65
ccg	P	4	0.64
agt	S	0	246.90
agc	S	0	233.30
mPD		0.0099	1.7
		nPD:	0.01
		N. weight:	2.9
		Sc. PD:	-0.11
		Sc. rank:	-382.0

NS2

Pos. 26		obs:	exp:
gaa	E	2	732.60
gag	E	1202	471.40
ggt	G	0	0.13
ggc	G	0	0.13
gga	G	0	0.46
ggg	G	1	0.28
mPD		0.0050	0.48
		nPD:	0.01
		N. weight:	0.95
		Sc. PD:	-0.03
		Sc. rank:	2.3

NS2

Pos. 27		obs:	exp:
gat	D	1184	640.30
gac	D	1	544.70
ggt	G	20	2.65
ggc	G	0	2.55
gga	G	0	9.23
ggg	G	0	5.57
mPD		0.034	0.54
		nPD:	0.06
		N. weight:	0.65
		Sc. PD:	0.012
		Sc. rank:	412.4

NS2

Pos. 28		obs:	exp:
tta	L	0	106.90
ttg	L	1205	224.30
ctt	L	0	200.70
ctc	L	0	201.10
cta	L	0	203.10
ctg	L	0	268.90
mPD		0	1.1
		nPD:	0.
		N. weight:	1.7
		Sc. PD:	-0.072
		Sc. rank:	-1685.5

NS2

Pos. 29		obs:	exp:
tct	S	0	0.64
tcc	S	0	0.52
tca	S	0	1.02
tcg	S	0	0.23
aat	N	1199	610.70
aac	N	0	588.30
agt	S	4	0.82
agc	S	0	0.78
gat	D	2	1.08
gac	D	0	0.92
mPD		0.0099	0.51
		nPD:	0.02
		N. weight:	0.69
		Sc. PD:	-0.016
		Sc. rank:	263.9

NS2

Pos. 30		obs:	exp:
ggt	G	0	159.70
ggc	G	0	153.90
gga	G	1205	555.90
ggg	G	0	335.50
mPD		0	0.68
		nPD:	0.
		N. weight:	0.79
		Sc. PD:	-0.033
		Sc. rank:	-775.7

NS2

Pos. 31		obs:	exp:
atg	M	1205	1205.00
mPD		0	0
		nPD:	1.
		N. weight:	0.
		Sc. PD:	0
		Sc. rank:	0

NS2

Pos. 32		obs:	exp:
att	I	0	442.70
atc	I	0	288.80
ata	I	1205	473.50
mPD		0	0.65
		nPD:	0.
		N. weight:	0.96
		Sc. PD:	-0.04
		Sc. rank:	-936.6

NS2

Pos. 33		obs:	exp:
att	I	0	0.37
atc	I	0	0.24
ata	I	1	0.39
act	T	0	306.90
acc	T	0	247.10
aca	T	1202	552.20
acg	T	2	97.80
mPD		0.0050	0.68
		nPD:	0.01
		N. weight:	0.79
		Sc. PD:	-0.028
		Sc. rank:	-49.4

NS2

Pos. 34		obs:	exp:
caa	Q	13	609.70
cag	Q	1184	587.30
cgt	R	0	0.09
cgc	R	0	0.10
cga	R	0	0.20
cgg	R	2	0.17
aaa	K	0	3.50
aag	K	6	2.50
aga	R	0	0.91
agg	R	0	0.54
mPD		0.035	0.52
		nPD:	0.07
		N. weight:	0.67
		Sc. PD:	0.015
		Sc. rank:	436.0

NS2

Pos. 35		obs:	exp:
ttt	F	0	475.50
ttc	F	1204	728.50
tta	L	1	0.09
ttg	L	0	0.19
ctt	L	0	0.17
ctc	L	0	0.17
cta	L	0	0.17
ctg	L	0	0.22
mPD		0.0017	0.48
		nPD:	0.
		N. weight:	0.52
		Sc. PD:	-0.02
		Sc. rank:	-224.0

NS2

Pos. 36		obs:	exp:
gaa	E	1	733.20
gag	E	1204	471.80
mPD		0.0017	0.48
		nPD:	0.
		N. weight:	0.95
		Sc. PD:	-0.037
		Sc. rank:	-398.9

NS2

Pos. 37		obs:	exp:
tct	S	1205	191.70
tcc	S	0	157.40
tca	S	0	305.80
tcg	S	0	68.38
agt	S	0	247.70
agc	S	0	234.00
mPD		0	1.7
		nPD:	0.
		N. weight:	1.9
		Sc. PD:	-0.079
		Sc. rank:	-1843.5

NS2

Pos. 38		obs:	exp:
tta	L	0	106.90
ttg	L	0	224.30
ctt	L	0	200.70
ctc	L	0	201.10
cta	L	0	203.10
ctg	L	1205	268.90
mPD		0	1.1
		nPD:	0.
		N. weight:	1.5
		Sc. PD:	-0.065
		Sc. rank:	-1503.9

NS2

Pos. 39		obs:	exp:
cgt	R	0	0.13
cgc	R	0	0.15
cga	R	0	0.29
cgg	R	0	0.25
aaa	K	1159	700.60
aag	K	43	501.40
aga	R	3	1.37
agg	R	0	0.81
mPD		0.074	0.49
		nPD:	0.15
		N. weight:	0.41
		Sc. PD:	0.041
		Sc. rank:	346.9

NS2

Pos. 40		obs:	exp:
tta	L	0	106.80
ttg	L	0	224.20
ctt	L	0	200.50
ctc	L	1202	200.90
cta	L	2	202.90
ctg	L	0	268.70
att	I	0	0.37
atc	I	1	0.24
ata	I	0	0.39
mPD		0.0050	1.1
		nPD:	0.
		N. weight:	1.8
		Sc. PD:	-0.069
		Sc. rank:	-617.7

NS2

Pos.	41	obs:	exp:
tat	Y	0	650.80
tac	Y	1205	554.20
mPD		0	0.50
		nPD:	0.
		N. weight:	0.79
		Sc. PD:	-0.033
		Sc. rank:	-778.8

NS2

Pos.	42	obs:	exp:
cgt	R	0	50.98
cgc	R	0	58.79
cga	R	0	116.20
cgg	R	0	99.05
att	I	0	0.37
atc	I	0	0.24
ata	I	1	0.39
aaa	K	17	9.91
aag	K	0	7.09
aga	R	1187	542.80
agg	R	0	319.30
mPD		0.029	0.99
		nPD:	0.03
		N. weight:	0.8
		Sc. PD:	-0.011
		Sc. rank:	395.7

NS2

Pos.	43	obs:	exp:
aat	N	1	0.51
aac	N	0	0.49
gat	D	1204	650.60
gac	D	0	553.40
mPD		0.0017	0.50
		nPD:	0.
		N. weight:	0.63
		Sc. PD:	-0.025
		Sc. rank:	-279.1

NS2

Pos.	44	obs:	exp:
tta	L	0	1.06
ttg	L	12	2.23
tct	S	1	189.40
tcc	S	0	155.50
tca	S	0	302.30
tcg	S	1190	67.58
ctt	L	0	2.00
ctc	L	0	2.00
cta	L	0	2.02
ctg	L	0	2.68
act	T	0	0.51
acc	T	0	0.41
aca	T	0	0.92
acg	T	2	0.16
agt	S	0	244.80
agc	S	0	231.30
mPD		0.025	1.7
		nPD:	0.01
		N. weight:	2.9
		Sc. PD:	-0.082
		Sc. rank:	534.6

NS2

Pos.	45	obs:	exp:
tta	L	0	106.90
ttg	L	0	224.30
ctt	L	1205	200.70
ctc	L	0	201.10
cta	L	0	203.10
ctg	L	0	268.90
mPD		0	1.1
		nPD:	0.
		N. weight:	1.8
		Sc. PD:	-0.077
		Sc. rank:	-1797.5

NS2

Pos.	46	obs:	exp:
ggt	G	0	159.70
ggc	G	0	153.90
gga	G	1202	555.90
ggg	G	3	335.50
mPD		0.0050	0.68
		nPD:	0.01
		N. weight:	0.78
		Sc. PD:	-0.027
		Sc. rank:	-20.6

NS2

Pos.	47	obs:	exp:
aaa	K	1	0.58
aag	K	0	0.42
gaa	E	1197	732.60
gag	E	7	471.40
mPD		0.013	0.48
		nPD:	0.03
		N. weight:	0.48
		Sc. PD:	-0.0075
		Sc. rank:	229.9

NS2

Pos.	48	obs:	exp:
tct	S	0	0.16
tcc	S	0	0.13
tca	S	1	0.25
tcg	S	0	0.06
act	T	0	1.02
acc	T	0	0.82
aca	T	2	1.83
acg	T	2	0.32
agt	S	0	0.21
agc	S	0	0.19
gtt	V	0	0.41
gtc	V	0	0.40
gta	V	2	0.41
gtg	V	0	0.77
gct	A	6	306.80
gcc	A	4	221.20
gca	A	1121	559.20
gcg	A	57	100.80
gaa	E	10	6.09
gag	E	0	3.91
mPD		0.14	0.70
		nPD:	0.2
		N. weight:	0.62
		Sc. PD:	0.089
		Sc. rank:	577.7

NS2

Pos.	49	obs:	exp:
tct	S	0	0.48
tcc	S	0	0.39
tca	S	0	0.76
tcg	S	3	0.17
act	T	0	0.25
acc	T	0	0.21
aca	T	0	0.46
acg	T	1	0.08
agt	S	0	0.62
agc	S	0	0.58
gct	A	0	310.10
gcc	A	0	223.70
gca	A	45	565.30
gcg	A	1156	101.90
mPD		0.079	0.68
		nPD:	0.12
		N. weight:	2.3
		Sc. PD:	0.16
		Sc. rank:	1870.8

NS2

Pos.	50	obs:	exp:
att	I	0	0.37
atc	I	0	0.24
ata	I	1	0.39
atg	M	1204	1204.00
mPD		0.0017	0.0017
		nPD:	1.
		N. weight:	0.00079
		Sc. PD:	0.00072
		Sc. rank:	0.8

NS2

Pos.	51	obs:	exp:
cgt	R	0	51.71
cgc	R	0	59.63
cga	R	0	117.80
cgg	R	1	100.50
aaa	K	0	0.58
aag	K	1	0.42
aga	R	25	550.50
agg	R	1178	323.80
mPD		0.044	0.96
		nPD:	0.05
		N. weight:	1.2
		Sc. PD:	0.0018
		Sc. rank:	756.1

NS2

Pos.	52	obs:	exp:
atg	M	1193	1193.00
act	T	0	0.25
acc	T	0	0.21
aca	T	0	0.46
acg	T	1	0.08
gtt	V	0	2.28
gtc	V	0	2.22
gta	V	0	2.25
gtg	V	11	4.25
mPD		0.020	0.032
		nPD:	0.61
		N. weight:	0.011
		Sc. PD:	0.0059
		Sc. rank:	11.5

NS2

Pos.	53	obs:	exp:
ggt	G	0	159.70
ggc	G	0	153.90
gga	G	1159	555.90
ggg	G	46	335.50
mPD		0.073	0.68
		nPD:	0.11
		N. weight:	0.65
		Sc. PD:	0.04
		Sc. rank:	502.3

NS2

Pos.	54	obs:	exp:
aat	N	0	1.02
aac	N	2	0.98
gat	D	1040	650.10
gac	D	163	552.90
mPD		0.24	0.50
		nPD:	0.48
		N. weight:	0.25
		Sc. PD:	0.1
		Sc. rank:	260.3

NS2

Pos.	55	obs:	exp:
ttt	F	0	0.39
ttc	F	1	0.61
tta	L	0	106.70
ttg	L	0	223.80
ctt	L	5	200.20
ctc	L	1197	200.60
cta	L	0	202.60
ctg	L	0	268.20
att	I	0	0.73
atc	I	2	0.48
ata	I	0	0.79
mPD		0.013	1.1
		nPD:	0.01
		N. weight:	1.8
		Sc. PD:	-0.055
		Sc. rank:	132.5

NS2

Pos.	56	obs:	exp:
cat	H	6	691.40
cac	H	1198	512.60
caa	Q	1	0.51
cag	Q	0	0.49
mPD		0.012	0.49
		nPD:	0.02
		N. weight:	0.84
		Sc. PD:	-0.016
		Sc. rank:	360.7

NS2

Pos.	57	obs:	exp:
tct	S	1095	191.50
tcc	S	109	157.20
tca	S	0	305.60
tcg	S	0	68.32
tat	Y	1	0.54
tac	Y	0	0.46
agt	S	0	247.50
agc	S	0	233.90
mPD		0.17	1.7
		nPD:	0.1
		N. weight:	1.6
		Sc. PD:	0.086
		Sc. rank:	1215.9

NS2

Pos.	58	obs:	exp:
tta	L	0	106.80
ttg	L	0	224.20
ctt	L	0	200.50
ctc	L	135	200.90
cta	L	1067	202.90
ctg	L	2	268.70
caa	Q	1	0.51
cag	Q	0	0.49
mPD		0.20	1.1
		nPD:	0.18
		N. weight:	1.5
		Sc. PD:	0.19
		Sc. rank:	1311.6

NS2

Pos.	59	obs:	exp:
caa	Q	1197	613.80
cag	Q	8	591.20
mPD		0.013	0.50
		nPD:	0.03
		N. weight:	0.65
		Sc. PD:	-0.011
		Sc. rank:	299.3

NS2

Pos.	60	obs:	exp:
tct	S	0	188.60
tcc	S	0	154.90
tca	S	0	301.00
tcg	S	0	67.30
aat	N	18	9.17
aac	N	0	8.83
agt	S	1186	243.80
agc	S	0	230.40
ggt	G	1	0.13
ggc	G	0	0.13
gga	G	0	0.46
ggg	G	0	0.28
mPD		0.031	1.7
		nPD:	0.02
		N. weight:	1.6
		Sc. PD:	-0.039
		Sc. rank:	592.4

NS2			
Pos.	61	obs:	exp:
cgt	R	0	51.71
cgc	R	0	59.63
cga	R	0	117.80
cgg	R	0	100.50
aaa	K	1	0.58
aag	K	0	0.42
aga	R	1070	550.50
agg	R	134	323.80
---	--	------	------
mPD		0.20	0.96
		nPD:	0.21
		N. weight:	0.51
		Sc. PD:	0.079
		Sc. rank:	477.6

NS2			
Pos.	62	obs:	exp:
aat	N	8	613.80
aac	N	1197	591.20
---	--	------	------
mPD		0.013	0.50
		nPD:	0.03
		N. weight:	0.69
		Sc. PD:	-0.012
		Sc. rank:	309.2

NS2			
Pos.	63	obs:	exp:
ggt	G	0	159.70
ggc	G	0	153.90
gga	G	7	555.90
ggg	G	1198	335.50
---	--	------	------
mPD		0.012	0.68
		nPD:	0.02
		N. weight:	1.3
		Sc. PD:	-0.033
		Sc. rank:	382.0

NS2			
Pos.	64	obs:	exp:
cgt	R	0	0.04
cgc	R	0	0.05
cga	R	0	0.10
cgg	R	0	0.08
act	T	0	0.76
acc	T	0	0.62
aca	T	3	1.38
acg	T	0	0.24
aaa	K	1137	698.20
aag	K	61	499.80
aga	R	1	0.46
agg	R	0	0.27
gaa	E	3	1.83
gag	E	0	1.17
---	--	------	------
mPD		0.11	0.50
		nPD:	0.22
		N. weight:	0.37
		Sc. PD:	0.06
		Sc. rank:	352.5

NS2			
Pos.	65	obs:	exp:
tgg	W	1205	1205.00
---	--	------	------
mPD		0	0
		nPD:	1.
		N. weight:	0.
		Sc. PD:	0
		Sc. rank:	0

NS2			
Pos.	66	obs:	exp:
cgt	R	0	51.75
cgc	R	0	59.68
cga	R	0	117.90
cgg	R	0	100.50
aga	R	1205	551.00
agg	R	0	324.10
---	--	------	------
mPD		0	0.96
		nPD:	0.
		N. weight:	0.8
		Sc. PD:	-0.034
		Sc. rank:	-784.6

NS2			
Pos.	67	obs:	exp:
aaa	K	4	2.33
aag	K	0	1.67
gaa	E	1198	729.60
gag	E	1	469.40
ggt	G	0	0.27
ggc	G	0	0.26
gga	G	2	0.92
ggg	G	0	0.56
---	--	------	------
mPD		0.012	0.49
		nPD:	0.02
		N. weight:	0.5
		Sc. PD:	-0.0097
		Sc. rank:	220.0

NS2			
Pos.	68	obs:	exp:
caa	Q	148	613.80
cag	Q	1057	591.20
---	--	------	------
mPD		0.22	0.50
		nPD:	0.43
		N. weight:	0.35
		Sc. PD:	0.13
		Sc. rank:	352.9

NS2			
Pos.	69	obs:	exp:
tta	L	1195	106.90
ttg	L	4	224.30
ctt	L	0	200.70
ctc	L	0	201.10
cta	L	6	203.10
ctg	L	0	268.90
---	--	------	------
mPD		0.017	1.1
		nPD:	0.01
		N. weight:	2.4
		Sc. PD:	-0.067
		Sc. rank:	541.9

NS2			
Pos.	70	obs:	exp:
tct	S	0	191.50
tcc	S	0	157.20
tca	S	0	305.60
tcg	S	0	68.32
aat	N	0	0.51
aac	N	1	0.49
agt	S	4	247.50
agc	S	1200	233.90
---	--	------	------
mPD		0.0083	1.7
		nPD:	0.01
		N. weight:	1.7
		Sc. PD:	-0.062
		Sc. rank:	-447.5

NS2			
Pos.	71	obs:	exp:
caa	Q	5	613.80
cag	Q	1200	591.20
---	--	------	------
mPD		0.0083	0.50
		nPD:	0.02
		N. weight:	0.7
		Sc. PD:	-0.018
		Sc. rank:	196.3

NS2			
Pos.	72	obs:	exp:
cgt	R	0	0.04
cgc	R	0	0.05
cga	R	0	0.10
cgg	R	0	0.08
aaa	K	69	701.70
aag	K	1135	502.30
aga	R	0	0.46
agg	R	1	0.27
---	--	------	------
mPD		0.11	0.49
		nPD:	0.22
		N. weight:	0.65
		Sc. PD:	0.11
		Sc. rank:	632.7

NS2			
Pos.	73	obs:	exp:
ttt	F	144	475.80
ttc	F	1061	729.20
---	--	------	------
mPD		0.21	0.48
		nPD:	0.44
		N. weight:	0.19
		Sc. PD:	0.074
		Sc. rank:	199.4

NS2			
Pos.	74	obs:	exp:
aaa	K	1	0.58
aag	K	0	0.42
gat	D	0	0.54
gac	D	1	0.46
gaa	E	1179	732.00
gag	E	24	471.00
---	--	------	------
mPD		0.042	0.48
		nPD:	0.09
		N. weight:	0.42
		Sc. PD:	0.018
		Sc. rank:	300.0

NS2			
Pos.	75	obs:	exp:
gat	D	0	0.54
gac	D	1	0.46
gaa	E	888	732.60
gag	E	316	471.40
---	--	------	------
mPD		0.39	0.48
		nPD:	0.81
		N. weight:	0.041
		Sc. PD:	0.03
		Sc. rank:	43.2

NS2			
Pos.	76	obs:	exp:
att	I	0	440.90
atc	I	0	287.60
ata	I	1200	471.50
atg	M	1	1.00
act	T	0	1.02
acc	T	0	0.82
aca	T	4	1.83
acg	T	0	0.32
---	--	------	------
mPD		0.0083	0.66
		nPD:	0.01
		N. weight:	0.95
		Sc. PD:	-0.029
		Sc. rank:	107.6

NS2			
Pos.	77	obs:	exp:
cgt	R	0	51.75
cgc	R	0	59.68
cga	R	1	117.90
cgg	R	0	100.50
aga	R	1200	551.00
agg	R	4	324.10
---	--	------	------
mPD		0.0083	0.96
		nPD:	0.01
		N. weight:	0.78
		Sc. PD:	-0.026
		Sc. rank:	-10.5

NS2			
Pos.	78	obs:	exp:
tgg	W	1205	1205.00
---	--	------	------
mPD		0	0
		nPD:	1.
		N. weight:	0.
		Sc. PD:	0
		Sc. rank:	0

NS2			
Pos.	79	obs:	exp:
tta	L	49	106.90
ttg	L	1156	224.30
ctt	L	0	200.70
ctc	L	0	201.10
cta	L	0	203.10
ctg	L	0	268.90
---	--	------	------
mPD		0.078	1.1
		nPD:	0.07
		N. weight:	1.6
		Sc. PD:	0.04
		Sc. rank:	1076.6

NS2			
Pos.	80	obs:	exp:
att	I	1199	441.60
atc	I	3	288.00
ata	I	0	472.30
gtt	V	3	0.62
gtc	V	0	0.61
gta	V	0	0.61
gtg	V	0	1.16
---	--	------	------
mPD		0.0099	0.66
		nPD:	0.02
		N. weight:	1.
		Sc. PD:	-0.028
		Sc. rank:	243.3

NS2			
Pos.	81	obs:	exp:
gaa	E	1203	733.20
gag	E	2	471.80
---	--	------	------
mPD		0.0033	0.48
		nPD:	0.01
		N. weight:	0.5
		Sc. PD:	-0.018
		Sc. rank:	-41.9

NS2			
Pos.	82	obs:	exp:
gat	D	0	1.08
gac	D	2	0.92
gaa	E	1195	729.60
gag	E	4	469.40
ggt	G	0	0.53
ggc	G	0	0.51
gga	G	4	1.85
ggg	G	0	1.11
---	--	------	------
mPD		0.017	0.49
		nPD:	0.03
		N. weight:	0.49
		Sc. PD:	-0.0047
		Sc. rank:	266.0

NS2			
Pos.	83	obs:	exp:
att	I	1	2.57
atc	I	0	1.68
ata	I	6	2.75
gtt	V	38	248.20
gtc	V	1	241.80
gta	V	1130	245.30
gtg	V	29	462.70
---	--	------	------
mPD		0.12	0.74
		nPD:	0.16
		N. weight:	1.3
		Sc. PD:	0.15
		Sc. rank:	1164.9

NS2			
Pos.	84	obs:	exp:
cgt	R	1	51.75
cgc	R	0	59.68
cga	R	1187	117.90
cgg	R	16	100.50
aga	R	1	551.00
agg	R	0	324.10
---	--	------	------
mPD		0.030	0.96
		nPD:	0.03
		N. weight:	2.3
		Sc. PD:	-0.029
		Sc. rank:	1228.7

NS2			
Pos.	85	obs:	exp:
tta	L	0	0.09
ttg	L	0	0.19
tat	Y	5	2.70
tac	Y	0	2.30
ctt	L	1	0.17
ctc	L	0	0.17
cta	L	0	0.17
ctg	L	0	0.22
cat	H	1191	687.30
cac	H	6	509.70
aat	N	1	0.51
aac	N	0	0.49
gat	D	1	0.54
gac	D	0	0.46
---	--	------	------
mPD		0.023	0.50
		nPD:	0.05
		N. weight:	0.54
		Sc. PD:	0.00096
		Sc. rank:	338.0

NS2			
Pos.	86	obs:	exp:
cgt	R	0	51.71
cgc	R	0	59.63
cga	R	0	117.80
cgg	R	0	100.50
aga	R	1067	550.50
agg	R	137	323.80
ggt	G	0	0.13
ggc	G	0	0.13
gga	G	1	0.46
ggg	G	0	0.28
---	--	------	------
mPD		0.20	0.96
		nPD:	0.21
		N. weight:	0.5
		Sc. PD:	0.08
		Sc. rank:	478.3

NS2			
Pos.	87	obs:	exp:
tta	L	1202	106.90
ttg	L	2	224.30
ctt	L	0	200.70
ctc	L	0	201.10
cta	L	1	203.10
ctg	L	0	268.90
---	--	------	------
mPD		0.0050	1.1
		nPD:	0.
		N. weight:	2.5
		Sc. PD:	-0.093
		Sc. rank:	-733.2

NS2			
Pos.	88	obs:	exp:
cgt	R	0	0.21
cgc	R	0	0.25
cga	R	0	0.49
cgg	R	0	0.42
aaa	K	1048	699.40
aag	K	152	500.60
aga	R	5	2.29
agg	R	0	1.34
---	--	------	------
mPD		0.23	0.50
		nPD:	0.46
		N. weight:	0.21
		Sc. PD:	0.084
		Sc. rank:	220.1

NS2			
Pos.	89	obs:	exp:
ttt	F	7	2.76
ttc	F	0	4.24
att	I	1154	438.70
atc	I	40	286.10
ata	I	0	469.20
gtt	V	4	0.83
gtc	V	0	0.81
gta	V	0	0.82
gtg	V	0	1.54
---	--	------	------
mPD		0.082	0.67
		nPD:	0.12
		N. weight:	0.89
		Sc. PD:	0.067
		Sc. rank:	741.0

NS2			
Pos.	90	obs:	exp:
act	T	2	307.20
acc	T	0	247.30
aca	T	169	552.70
acg	T	1034	97.88
---	--	------	------
mPD		0.24	0.68
		nPD:	0.36
		N. weight:	1.9
		Sc. PD:	0.57
		Sc. rank:	1914.6

NS2			
Pos.	91	obs:	exp:
gat	D	3	1.62
gac	D	0	1.38
gaa	E	20	731.40
gag	E	1182	470.60
---	--	------	------
mPD		0.038	0.48
		nPD:	0.08
		N. weight:	0.87
		Sc. PD:	0.028
		Sc. rank:	613.6

NS2			
Pos.	92	obs:	exp:
tct	S	0	2.54
tcc	S	0	2.09
tca	S	0	4.06
tcg	S	0	0.91
att	I	1	0.37
atc	I	0	0.24
ata	I	0	0.39
act	T	3	0.76
acc	T	0	0.62
aca	T	0	1.38
acg	T	0	0.24
aat	N	1162	603.60
aac	N	23	581.40
agt	S	16	3.29
agc	S	0	3.11
---	--	------	------
mPD		0.070	0.55
		nPD:	0.13
		N. weight:	0.61
		Sc. PD:	0.048
		Sc. rank:	512.7

NS2			
Pos.	93	obs:	exp:
tct	S	0	191.70
tcc	S	0	157.40
tca	S	0	305.80
tcg	S	0	68.38
agt	S	1	247.70
agc	S	1204	234.00
---	--	------	------
mPD		0.0017	1.7
		nPD:	0.
		N. weight:	1.7
		Sc. PD:	-0.069
		Sc. rank:	-999.6

NS2			
Pos.	94	obs:	exp:
ttt	F	1193	475.80
ttc	F	12	729.20
---	--	------	------
mPD		0.020	0.48
		nPD:	0.04
		N. weight:	0.89
		Sc. PD:	-0.0024
		Sc. rank:	531.3

NS2			
Pos.	95	obs:	exp:
caa	Q	0	0.51
cag	Q	1	0.49
gat	D	1	0.54
gac	D	0	0.46
gaa	E	148	732.00
gag	E	1055	471.00
---	--	------	------
mPD		0.22	0.48
		nPD:	0.46
		N. weight:	0.53
		Sc. PD:	0.21
		Sc. rank:	543.1

NS2			
Pos.	96	obs:	exp:
caa	Q	1204	613.30
cag	Q	0	590.70
gaa	E	1	0.61
gag	E	0	0.39
---	--	------	------
mPD		0.0017	0.50
		nPD:	0.
		N. weight:	0.69
		Sc. PD:	-0.027
		Sc. rank:	-337.0

NS2			
Pos.	97	obs:	exp:
att	I	0	442.70
atc	I	1	288.80
ata	I	1204	473.50
---	--	------	------
mPD		0.0017	0.65
		nPD:	0.
		N. weight:	0.95
		Sc. PD:	-0.038
		Sc. rank:	-497.0

NS2			
Pos.	98	obs:	exp:
act	T	1191	307.20
acc	T	7	247.30
aca	T	0	552.70
acg	T	7	97.88
---	--	------	------
mPD		0.023	0.68
		nPD:	0.03
		N. weight:	1.3
		Sc. PD:	-0.013
		Sc. rank:	755.8

NS2			
Pos.	99	obs:	exp:
ttt	F	1099	475.80
ttc	F	106	729.20
---	--	------	------
mPD		0.16	0.48
		nPD:	0.34
		N. weight:	0.61
		Sc. PD:	0.17
		Sc. rank:	597.1

NS2			
Pos.	100	obs:	exp:
atg	M	1204	1204.00
gtt	V	0	0.21
gtc	V	0	0.20
gta	V	0	0.20
gtg	V	1	0.39
---	--	------	------
mPD		0.0017	0.0027
		nPD:	0.62
		N. weight:	0.00081
		Sc. PD:	0.00044
		Sc. rank:	0.8

NS2			
Pos.	101	obs:	exp:
caa	Q	1205	613.80
cag	Q	0	591.20
---	--	------	------
mPD		0	0.50
		nPD:	0.
		N. weight:	0.69
		Sc. PD:	-0.029
		Sc. rank:	-676.4

NS2			
Pos.	102	obs:	exp:
gct	A	0	311.20
gcc	A	1204	224.40
gca	A	1	567.20
gcg	A	0	102.30
---	--	------	------
mPD		0.0017	0.67
		nPD:	0.
		N. weight:	1.7
		Sc. PD:	-0.068
		Sc. rank:	-923.3

NS2			
Pos.	103	obs:	exp:
tta	L	1198	106.90
ttg	L	7	224.30
ctt	L	0	200.70
ctc	L	0	201.10
cta	L	0	203.10
ctg	L	0	268.90
---	--	------	------
mPD		0.012	1.1
		nPD:	0.01
		N. weight:	2.4
		Sc. PD:	-0.078
		Sc. rank:	105.4

NS2			
Pos.	104	obs:	exp:
caa	Q	1204	613.80
cag	Q	1	591.20
---	--	------	------
mPD		0.0017	0.50
		nPD:	0.
		N. weight:	0.68
		Sc. PD:	-0.027
		Sc. rank:	-310.4

NS2			
Pos.	105	obs:	exp:
tta	L	0	106.80
ttg	L	0	224.20
ctt	L	0	200.50
ctc	L	0	200.90
cta	L	1201	202.90
ctg	L	3	268.70
att	I	0	0.37
atc	I	0	0.24
ata	I	1	0.39
---	--	------	------
mPD		0.0066	1.1
		nPD:	0.01
		N. weight:	1.8
		Sc. PD:	-0.066
		Sc. rank:	-324.3

NS2			
Pos.	106	obs:	exp:
tta	L	1	106.90
ttg	L	1059	224.30
ctt	L	0	200.70
ctc	L	0	201.10
cta	L	0	203.10
ctg	L	145	268.90
---	--	------	------
mPD		0.21	1.1
		nPD:	0.19
		N. weight:	1.3
		Sc. PD:	0.19
		Sc. rank:	1218.7

NS2			
Pos.	107	obs:	exp:
tta	L	0	106.90
ttg	L	0	224.30
ctt	L	1205	200.70
ctc	L	0	201.10
cta	L	0	203.10
ctg	L	0	268.90
---	--	------	------
mPD		0	1.1
		nPD:	0.
		N. weight:	1.8
		Sc. PD:	-0.077
		Sc. rank:	-1797.5

NS2			
Pos.	108	obs:	exp:
gaa	E	1203	733.20
gag	E	2	471.80
---	--	------	------
mPD		0.0033	0.48
		nPD:	0.01
		N. weight:	0.5
		Sc. PD:	-0.018
		Sc. rank:	-41.9

NS2			
Pos.	109	obs:	exp:
gtt	V	0	249.70
gtc	V	0	243.20
gta	V	41	246.70
gtg	V	1164	465.40
---	--	------	------
mPD		0.066	0.73
		nPD:	0.09
		N. weight:	0.85
		Sc. PD:	0.038
		Sc. rank:	616.7

NS2			
Pos.	110	obs:	exp:
gaa	E	7	732.60
gag	E	1197	471.40
ggt	G	0	0.13
ggc	G	0	0.13
gga	G	0	0.46
ggg	G	1	0.28
---	--	------	------
mPD		0.013	0.48
		nPD:	0.03
		N. weight:	0.92
		Sc. PD:	-0.015
		Sc. rank:	438.1

,

NS2			
Pos.	111	obs:	exp:
cat	H	0	0.57
cac	H	1	0.43
caa	Q	1204	613.30
cag	Q	0	590.70
---	--	------	------
mPD		0.0017	0.50
		nPD:	0.
		N. weight:	0.69
		Sc. PD:	-0.027
		Sc. rank:	-321.1

,

NS2			
Pos.	112	obs:	exp:
gaa	E	0	733.20
gag	E	1205	471.80
---	--	------	------
mPD		0	0.48
		nPD:	0.
		N. weight:	0.96
		Sc. PD:	-0.04
		Sc. rank:	-940.3

,

NS2			
Pos.	113	obs:	exp:
att	I	0	442.00
atc	I	0	288.30
ata	I	1203	472.70
atg	M	1	1.00
gtt	V	0	0.21
gtc	V	0	0.20
gta	V	1	0.20
gtg	V	0	0.39
---	--	------	------
mPD		0.0033	0.66
		nPD:	0.01
		N. weight:	0.96
		Sc. PD:	-0.036
		Sc. rank:	-227.9

,

NS2			
Pos.	114	obs:	exp:
cgt	R	0	51.75
cgc	R	0	59.68
cga	R	0	117.90
cgg	R	0	100.50
aga	R	1096	551.00
agg	R	109	324.10
---	--	------	------
mPD		0.16	0.96
		nPD:	0.17
		N. weight:	0.54
		Sc. PD:	0.066
		Sc. rank:	475.4

,

NS2			
Pos.	115	obs:	exp:
tta	L	0	0.09
ttg	L	0	0.19
ctt	L	1	0.17
ctc	L	0	0.17
cta	L	0	0.17
ctg	L	0	0.22
att	I	1	0.37
atc	I	0	0.24
ata	I	0	0.39
act	T	1201	306.40
acc	T	1	246.60
aca	T	0	551.30
acg	T	0	97.63
gct	A	1	0.26
gcc	A	0	0.19
gca	A	0	0.47
gcg	A	0	0.08
---	--	------	------
mPD		0.0083	0.68
		nPD:	0.01
		N. weight:	1.4
		Sc. PD:	-0.042
		Sc. rank:	134.8

,

NS2			
Pos.	116	obs:	exp:
ttt	F	0	474.30
ttc	F	1201	726.70
tta	L	4	0.35
ttg	L	0	0.74
ctt	L	0	0.67
ctc	L	0	0.67
cta	L	0	0.67
ctg	L	0	0.89
---	--	------	------
mPD		0.0066	0.49
		nPD:	0.01
		N. weight:	0.52
		Sc. PD:	-0.015
		Sc. rank:	63.2

,

NS2			
Pos.	117	obs:	exp:
tct	S	1	191.70
tcc	S	1	157.40
tca	S	2	305.80
tcg	S	1201	68.38
agt	S	0	247.70
agc	S	0	234.00
---	--	------	------
mPD		0.0066	1.7
		nPD:	0.
		N. weight:	2.9
		Sc. PD:	-0.11
		Sc. rank:	-1075.6

,

NS2			
Pos.	118	obs:	exp:
ttt	F	1203	475.10
ttc	F	0	727.90
tta	L	0	0.09
ttg	L	0	0.19
ctt	L	1	0.17
ctc	L	0	0.17
cta	L	0	0.17
ctg	L	0	0.22
gtt	V	1	0.21
gtc	V	0	0.20
gta	V	0	0.20
gtg	V	0	0.39
---	--	------	------
mPD		0.0033	0.48
		nPD:	0.01
		N. weight:	0.95
		Sc. PD:	-0.034
		Sc. rank:	-88.0

,

NS2			
Pos.	119	obs:	exp:
caa	Q	0	613.80
cag	Q	1205	591.20
---	--	------	------
mPD		0	0.50
		nPD:	0.
		N. weight:	0.73
		Sc. PD:	-0.031
		Sc. rank:	-713.9

,

NS2			
Pos.	120	obs:	exp:
tta	L	0	106.90
ttg	L	0	224.30
ctt	L	1204	200.70
ctc	L	1	201.10
cta	L	0	203.10
ctg	L	0	268.90
---	--	------	------
mPD		0.0017	1.1
		nPD:	0.
		N. weight:	1.8
		Sc. PD:	-0.074
		Sc. rank:	-1037.9

,

NS2			
Pos.	121	obs:	exp:
att	I	1198	442.40
atc	I	6	288.50
ata	I	0	473.10
aat	N	1	0.51
aac	N	0	0.49
---	--	------	------
mPD		0.012	0.65
		nPD:	0.02
		N. weight:	1.
		Sc. PD:	-0.025
		Sc. rank:	316.1

,

NS2			
Pos.	122	obs:	exp:
taa	.	1205	511.90
tag	.	0	301.70
tga	.	0	391.40
---	--	------	------
mPD		0	0.81
		nPD:	0.
		N. weight:	0.88
		Sc. PD:	-0.037
		Sc. rank:	-858.4

}

Amino acid level conservation per codon

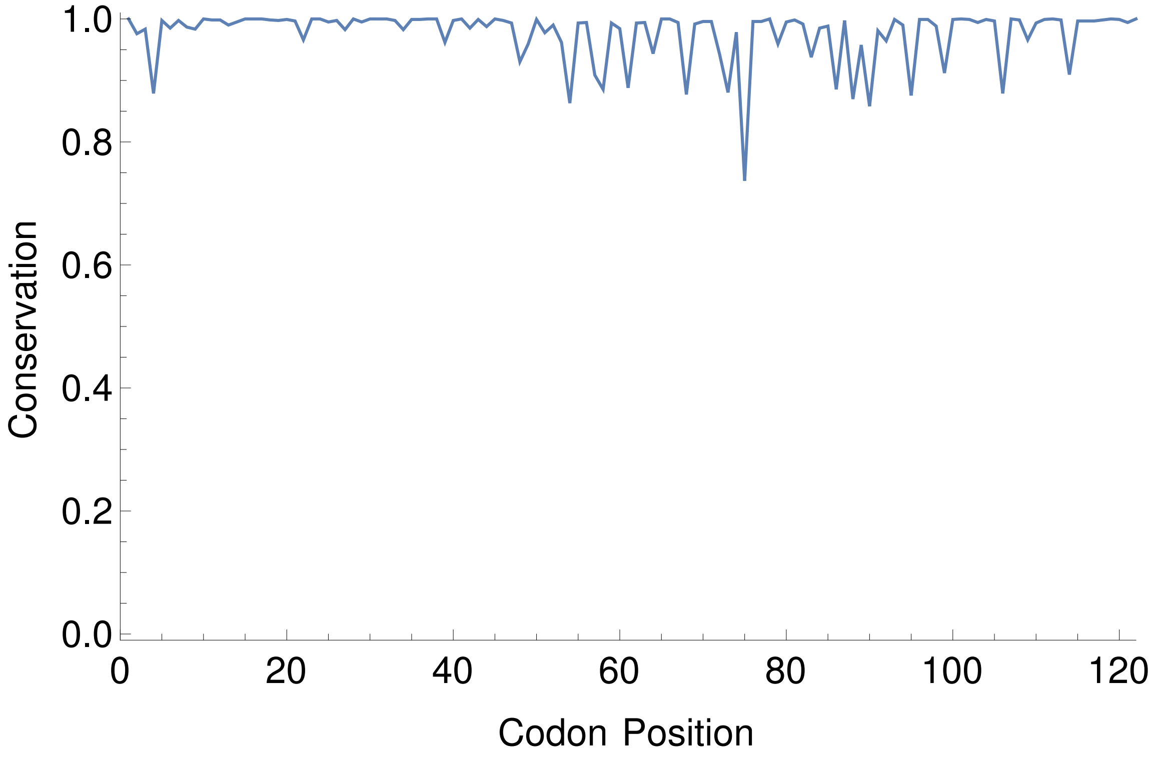

Conservation analysis plots

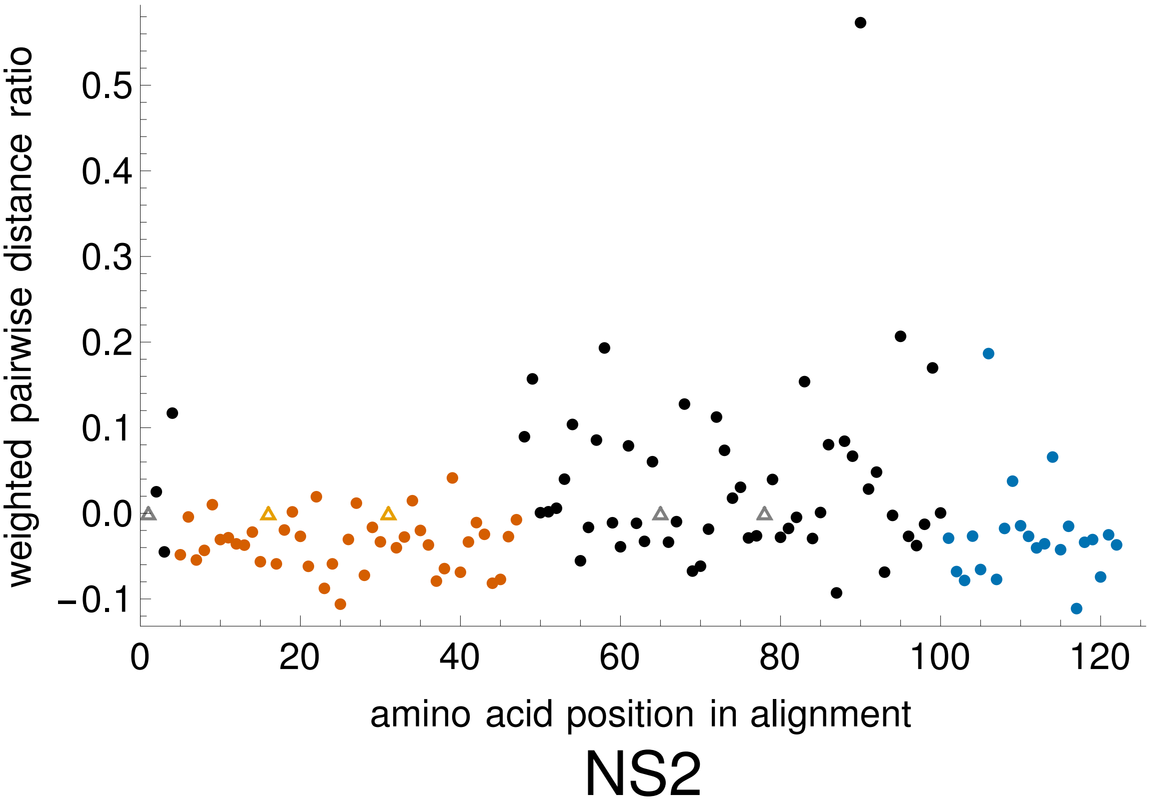

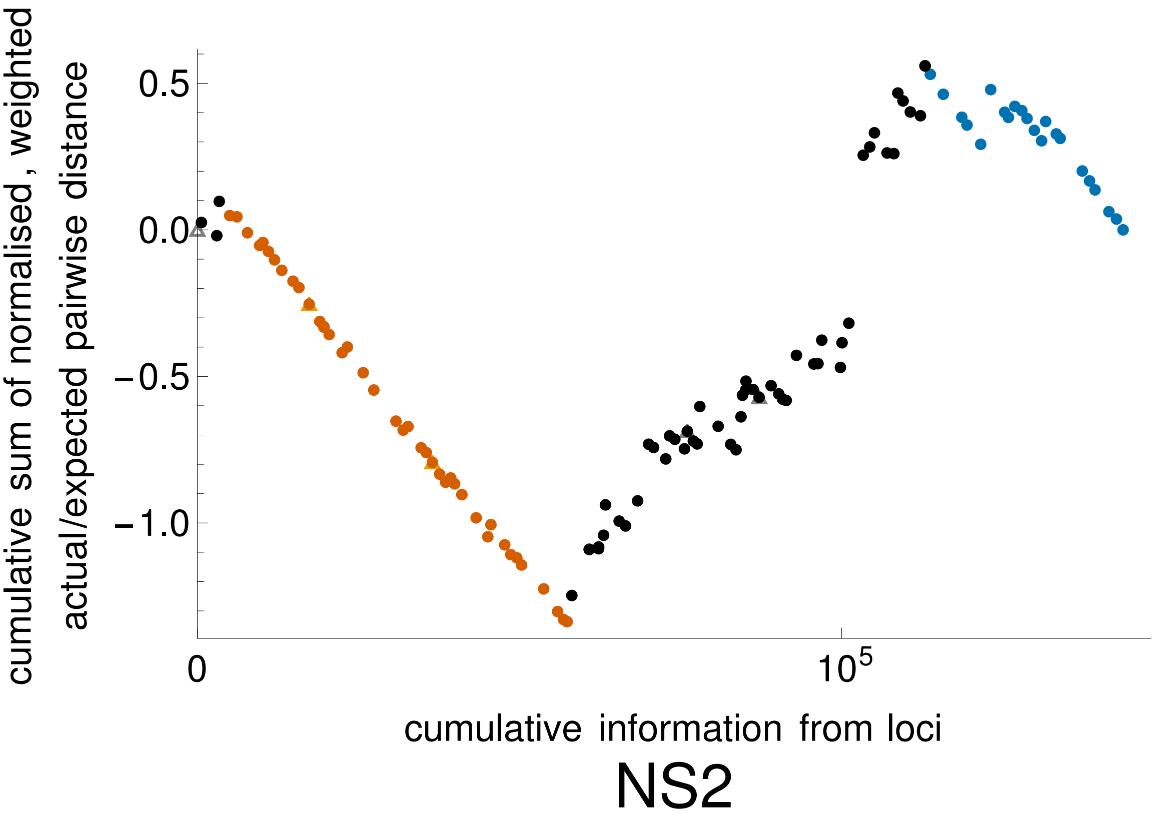

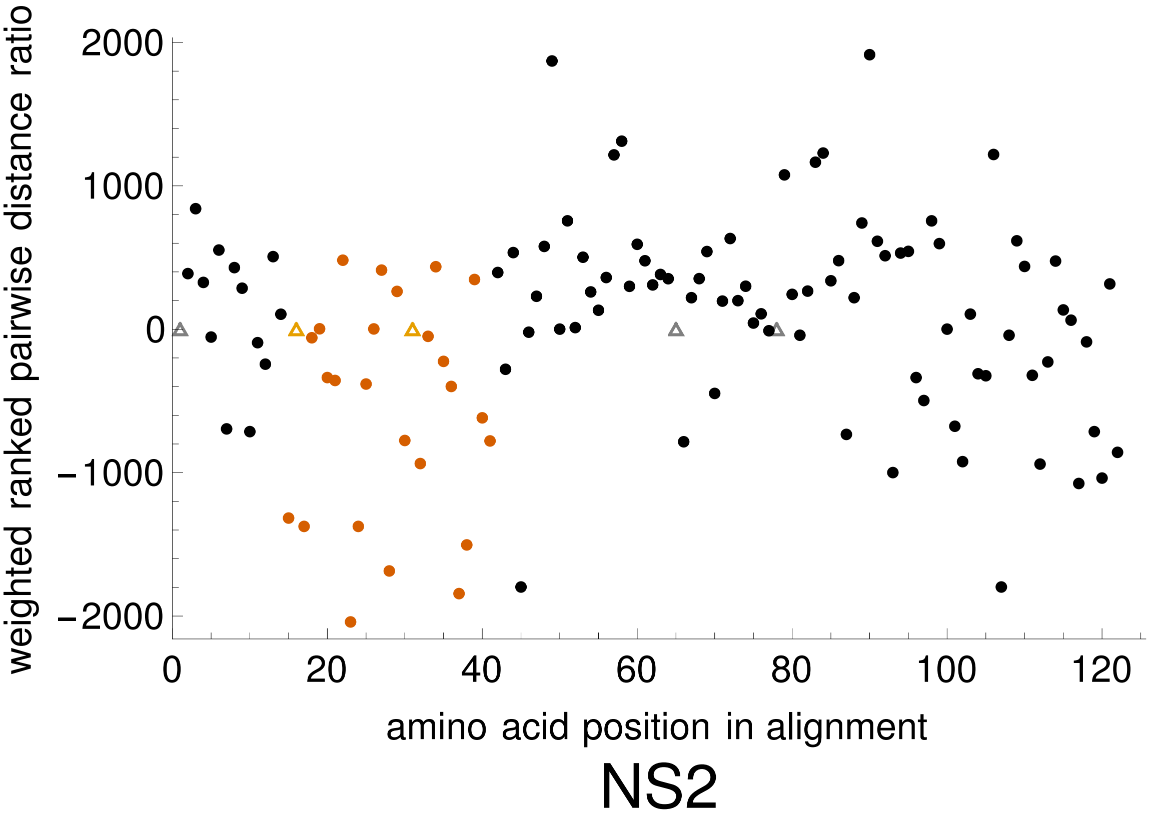
weighted ranked pairwise distance ratio
2000
1000
0
−1000
−2000
0
20
40
60
80
100
120
amino acid position in alignment
NS2

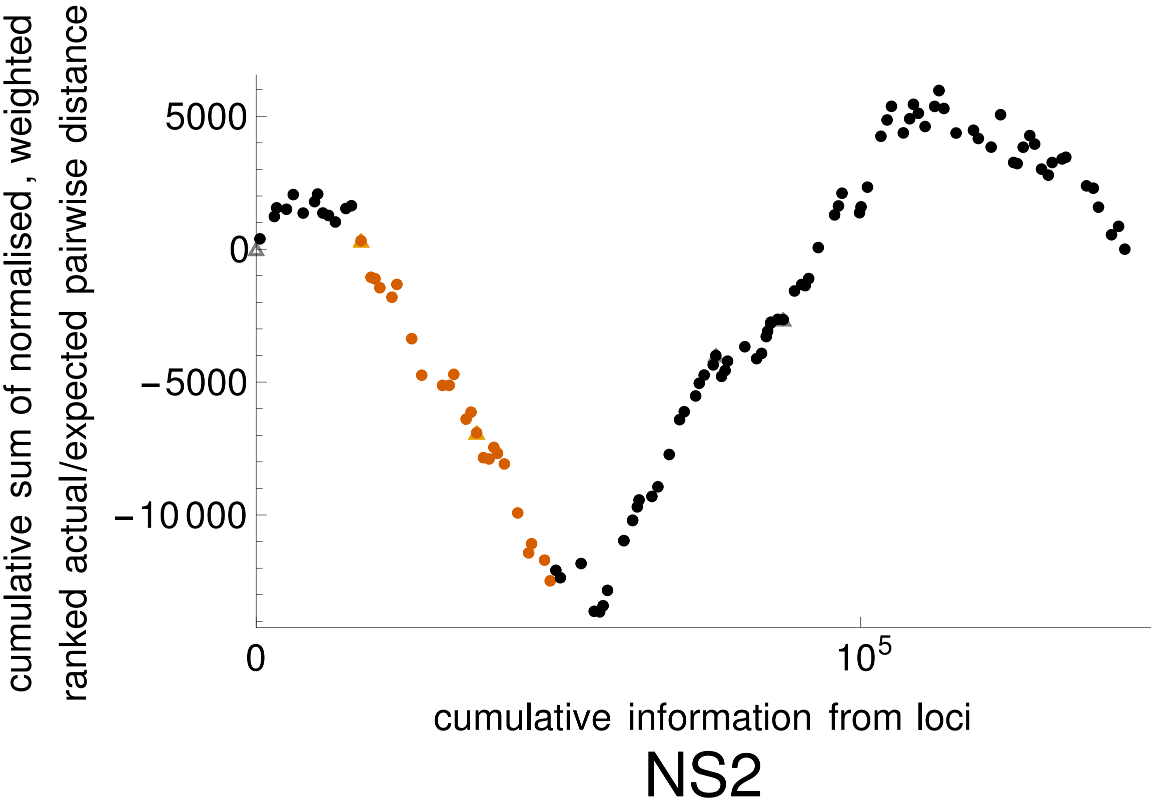
cumulative sum of normalised, weighted ranked actual/expected pairwise distance
5000
0
−5000
−10 000
0
10^5
cumulative information from loci
NS2
