# Supplementary material for: Locations and structures of influenza A virus packaging-associated signals and other functional elements via an in silico pipeline for predicting constrained features in RNA viruses
Source: PLoS Comput Biol. 2024 Apr 22;20(4):e1012009. doi: 10.1371/journal.pcbi.1012009 (PMC11034665; doi:10.1371/journal.pcbi.1012009)
Supplement: S8 Code — The content of the notebook follows the same pattern as that in S1 Code. (ZIP) [file pcbi.1012009.s121.zip › S8_Code.pdf]

H7N9 avian hosts

PB2

Gene length histogram

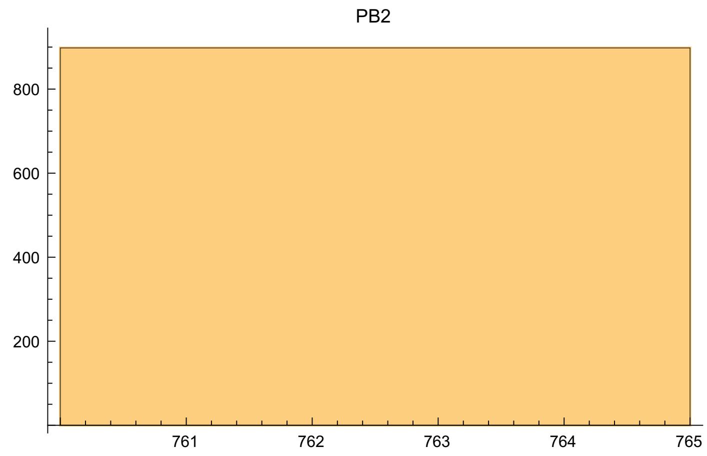

Information vs. nPD

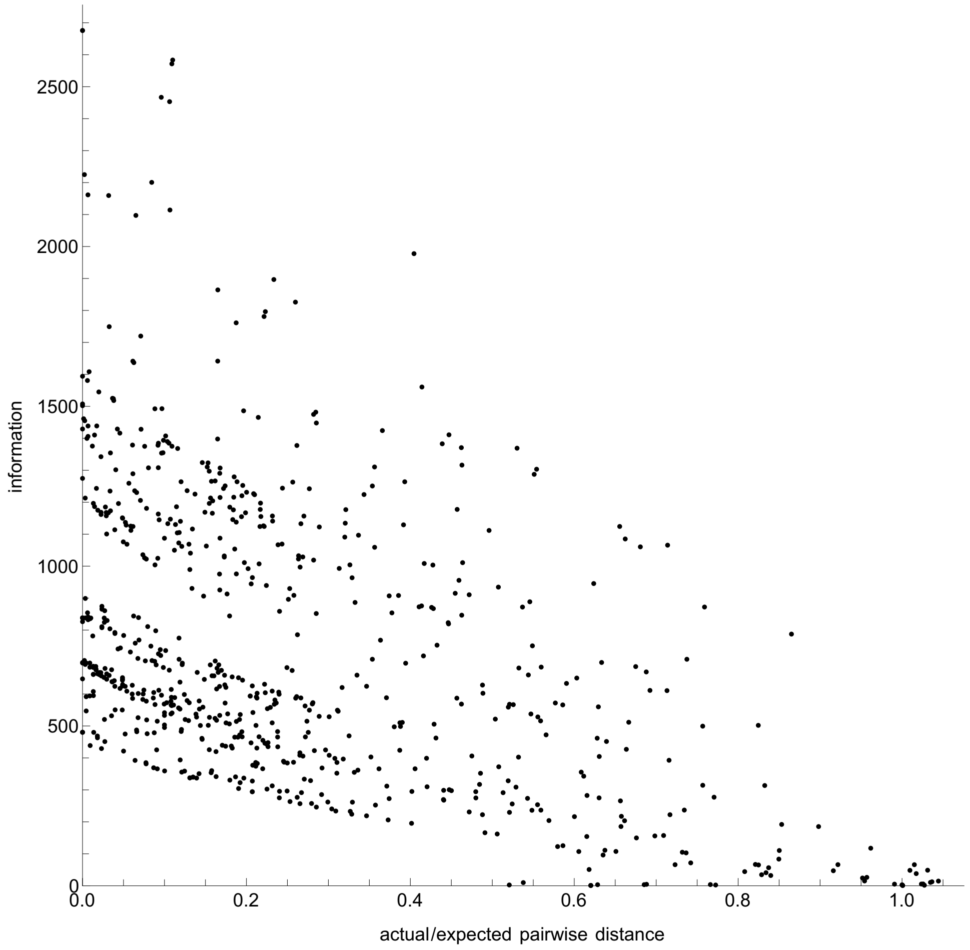

PB2

Example sequences highlighted by regions found in analyses to be conserved

Interesting points (by weighted raw PD) highlighted for gene PB2:

ATGGAG
AGAATAAAAGAACTAAGAGATTTGATGTCGCAGTCTCGCACTCGCGAGATACTGAC⋱
AAAAACCACCGTGGACCATATGGCCATAATCAAGAAATATACATCAGGAAGACAGGAGAAGAATCCTGCA⋱
CTCAGGATGAAATGGATGATGGCAATGAAATATCCGATTACAGCAGATAAAAGGATAATGGAGATGATCC⋱
CTGAAAGAAATGAGCAAGGTCAGACCCTTTGGAGCAAAACAAATGATGCTGGATCAGATAGAGTAATGGT⋱
GTCACCTCTGGCTGTGACGTGGTGGAATAGAAATGGACCAACGACAAGCACAGTCCATTATCCAAAGGTC⋱
TATAAAACCTACTTTGAAAAGGTCGAAAGGTTAAAACATGGAACCTTTGGTCCCGTTCACTTTCGAAATC⋱
AGGTTAAGATACGCCGCAGGGTTGACATAAACCCAGGCCATGCAGATCTCAGTGCTAAAGAAGCACAAGA⋱
TGTCATCATGGAGGTCGTTTTCCCAAACGAAGTTGGAGCCAGGATATTGACATCAGAGTCACAGTTAACA⋱
ATAACAAAGGAAAAGAAGGAAGAGCTTCAGGACTGTAGGATTGCTCCTTTAATGGTGGCATACATGTTGG⋱
AGAGAGAACTGGTTCGTAAAACCAGATTCTTGCCAGTAGCTGGCGGGACAAGCAGCGTGTATATCGAGGT⋱
CTTGCACTTGACTCAAGGGACCTGCTGGGAACAAATGTACACACCAGGAGGAGAGGTGAGAAATGATGAC⋱
GTTGATCAGAGTCTAATTATTGCTGCTAGAAACATCGTTAGGAGAGCAACAGTATCAGCAGACCCGTTGG⋱
CTTCGCTCTTGGAGATGTGCCATAGTACACAAATTGGCGGGGTAAGAATGGTAGACATCCTTAGACAAAA⋱
CCCAACAGAAGAGCAAGCTGTGGATATATGCAAAGCAGCAATGGGTCTAAGGATCAGTTCATCCTTCAGC⋱
TTTGGAGGTTTCACTTTCAAAAGGACAAGTGGGTCATCTATCAAAAGAGAAGAAGAAGTGCTCACCGGCA⋱
ACCTCCAAACATTGAAAATAAGGGTGCACGAAGGGTATGAGGAATTCACAATGGTTGGGCGAAGAGCAAC⋱
AGCCATTCTAAGGAAAGCAACCAGAAGGCTGATTCAATTGATAGTGAGTGGGAGAGACGAGCAGTCAATC⋱
GCCGAAGCAATCATAGTGGCAATGGTGTTCTCCCAAGAGGATTGCATGATAAAAGCAGTACGAGGTGATC⋱
TGAATTTTGTCAACAGAGCGAATCAGCGGTTAAATCCCATGCACCAACTCCTGAGGCATTTCCAAAAAGA⋱
TGCAAAGGTGCTGTTTCAAAACTGGGGGATTGAGCCCATTGACAATGTTATGGGGATGATCGGTATATTG⋱
CCTGACATGACCCCCAGCACAGAGATGTCACTAAGAGGAGTGAGAGTCAGTAAAATGGGAGTGGATGAAT⋱
ATTCCAGTGCTGAGAGAGTGGTCGTGAGTATTGATCGTTTCTTGAGGGTCCGAGATCAGAGGGGAAACGT⋱
GCTCTTGTCTCCTGAAGAGGTTAGTGAAACACAGGGAATAGAGAAGCTGACGATAACATATTCATCGTCC⋱
ATGATGTGGGAAATCAATGGCCCGGAATCAGTGTTAGTTAACACATATCAATGGATCATTAGAAACTGGG⋱
AAACTGTGAAGATTCAGTGGTCCCAAGACCCTACAATGTTATACAACAAGATGGAGTTTGAGCCCTTTCA⋱
ATCCTTGGTGCCTAAGGCTGCCAGAGGCCAGTATAGTGGATTTGTGAGAACGCTATTCCAGCAGATGCGT⋱
GATGTGCTGGGGACCTTTGACACTGTCCAGATAATAAAACTACTTCCATTTGCAGCAGCCCCACCGGAGC⋱
AGAGTAGGATGCAGTTCTCTTCTCTAACTGTAAACGTAAGAGGATCAGGAATGAGAATACTTGTGAGAGG⋱
CAACTCTCCTGTGTTCAACTATAACAAGGCAACCAAGAGGCTCACAGTCCTTGGAAAGGATGCAGGTGCA⋱
TTGACAGAAGACCCAGATGAGGGGACGGCAGGAGTGGAGTCTGCAGTATTAAGAGGATTTCTAATTCTGG⋱
GCAAAGAAGACAAAAGATATGGA
CCAGCATTGAGCATCAACGAATTGAGCAATCTCGCGAAAGGGGAGAAGGCTAATG
TGTTGATAGGGCAAGGAGACGTGGTGTTGGTGATGAAACGGAAACGGGACTC
TAGCATACTTACTGACAGCCAGACAGCGACCAAAAGAATTCGGATGGCCATC
AATTAG

Interesting points (by weighted ranked PD) highlighted for gene PB2:

ATGGAG
AGAATAAAAGAACTAAGAGATTTGATGTCGCAGTCTCGCACTCGCGAGATACTGAC⋱
AAAAACCACCGTGGACCATATGGCCATAATCAAGAAATATACATCAGGAAGACAGGAGAAGAATCCTGCA⋱
CTCAGGATGAAATGGATGATGGCAATGAAATATCCGATTACAGCAGATAAAAGGATAATGGAGATGATCC⋱
CTGAAAGAAATGAGCAAGGTCAGACCCTTTGGAGCAAAACAAATGATGCTGGATCAGATAGAGTAATGGT⋱

GTCACCTCTGGCTGTGACGTGGTGGAATAGAAATGGACCAACGACAAGCACAGTCCATTATCCAAAGGTC⋱
TATAAAACCTACTTTGAAAAGGTCGAAAGGTTAAAACATGGAACCTTTGGTCCCGTTCACTTTCGAAATC⋱
AGGTTAAGATACGCCGCAGGGTTGACATAAACCCAGGCCATGCAGATCTCAGTGCTAAAGAAGCACAAGA⋱
TGTCATCATGGAGGTCGTTTTCCCAAACGAAGTTGGAGCCAGGATATTGACATCAGAGTCACAGTTAACA⋱
ATAACAAAGGAAAAGAAGGAAGAGCTTCAGGACTGTAGGATTGCTCCTTTAATGGTGGCATACATGTTGG⋱
AGAGAGAACTGGTTCGTAAAACCAGATTCTTGCCAGTAGCTGGCGGGACAAGCAGCGTGTATATCGAGGT⋱
CTTGCACTTGACTCAAGGGACCTGCTGGGAACAAATGTACACACCAGGAGGAGAGGTGAGAAATGATGAC⋱
GTTGATCAGAGTCTAATTATTGCTGCTAGAAACATCGTTAGGAGAGCAACAGTATCAGCAGACCCGTTGG⋱
CTTCGCTCTTGGAGATGTGCCATAGTACACAAATTGGCGGGGTAAGAATGGTAGACATCCTTAGACAAAA⋱
CCCAACAGAAGAGCAAGCTGTGGATATATGCAAAGCAGCAATGGGTCTAAGGATCAGTTCATCCTTCAGC⋱
TTTGGAGGTTTCACTTTCAAAAGGACAAGTGGGTCATCTATCAAAAGAGAAGAAGAAGTGCTCACCGGCA⋱
ACCTCCAAACATTGAAAATAAGGGTGCACGAAGGGTATGAGGAATTCACAATGGTTGGGCGAAGAGCAAC⋱
AGCCATTCTAAGGAAAGCAACCAGAAGGCTGATTCAATTGATAGTGAGTGGGAGAGACGAGCAGTCAATC⋱
GCCGAAGCAATCATAGTGGCAATGGTGTTCTCCCAAGAGGATTGCATGATAAAAGCAGTACGAGGTGATC⋱
TGAATTTTGTCAACAGAGCGAATCAGCGGTTAAATCCCATGCACCAACTCCTGAGGCATTTCCAAAAAGA⋱
TGCAAAGGTGCTGTTTCAAAACTGGGGGATTGAGCCCATTGACAATGTTATGGGGATGATCGGTATATTG⋱
CCTGACATGACCCCCAGCACAGAGATGTCACTAAGAGGAGTGAGAGTCAGTAAAATGGGAGTGGATGAAT⋱
ATTCCAGTGCTGAGAGAGTGGTCGTGAGTATTGATCGTTTCTTGAGGGTCCGAGATCAGAGGGGAAACGT⋱
GCTCTTGTCTCCTGAAGAGGTTAGTGAAACACAGGGAATAGAGAAGCTGACGATAACATATTCATCGTCC⋱
ATGATGTGGGAAATCAATGGCCCGGAATCAGTGTTAGTTAACACATATCAATGGATCATTAGAAACTGGG⋱
AAACTGTGAAGATTCAGTGGTCCCAAGACCCTACAATGTTATACAACAAGATGGAGTTTGAGCCCTTTCA⋱
ATCCTTGGTGCCTAAGGCTGCCAGAGGCCAGTATAGTGGATTTGTGAGAACGCTATTCCAGCAGATGCGT⋱
GATGTGCTGGGGACCTTTGACACTGTCCAGATAATAAAACTACTTCCATTTGCAGCAGCCCCACCGGAGC⋱
AGAGTAGGATGCAGTTCTCTTCTCTAACTGTAAACGTAAGAGGATCAGGAATGAGAATACTTGTGAGAGG⋱
CAACTCTCCTGTGTTCAACTATAACAAGGCAACCAAGAGGCTCACAGTCCTTGGAAAGGATGCAGGTGCA⋱
TTGACAGAAGACCCAGATGAGGGGACGGCAGGAGTGGAGTCTGCAGTATTAAGAGGATTTCTAATTCTGG⋱
GCAAAGAAGACAAAAGATATGGA

CCAGCATTGAGCATCAACGAATTGAGCAATCTCGCGAAAGGGGAGAAGGCTAATG
TGTTGATAGGGCAAGGAGACGTGGTGTTGGTGATGAAACGGAAACGGGACTC
TAGCATACTTACTGACAGCCAGACAGCGACCAAAAGAATTCGGATGGCCATC
AATTAG

Per codon conservation report

PB2			
Pos.	1	obs:	exp:
atg	M	898	898.00
---	--	------	------
mPD		0	0
		nPD:	1.
		N. weight:	0.
		Sc. PD:	0
		Sc. rank:	0

PB2			
Pos.	2	obs:	exp:
gaa	E	809	540.20
gag	E	89	357.80
---	--	------	------
mPD		0.18	0.48
		nPD:	0.37
		N. weight:	0.29
		Sc. PD:	0.051
		Sc. rank:	123.6

PB2			
Pos.	3	obs:	exp:
tct	S	0	0.16
tcc	S	0	0.13
tca	S	0	0.25
tcg	S	0	0.06
cgt	R	0	38.32
cgc	R	0	45.57
cga	R	0	89.44
cgg	R	0	74.71
agt	S	1	0.20
agc	S	0	0.20
aga	R	897	409.60
agg	R	0	239.40
---	--	------	------
mPD		0.0022	0.97
		nPD:	0.
		N. weight:	0.99
		Sc. PD:	-0.18
		Sc. rank:	-562.1

PB2			
Pos.	4	obs:	exp:
att	I	0	327.30
atc	I	0	217.00
ata	I	897	352.70
act	T	0	0.26
acc	T	0	0.20
aca	T	1	0.46
acg	T	0	0.08
---	--	------	------
mPD		0.0022	0.66
		nPD:	0.
		N. weight:	1.2
		Sc. PD:	-0.21
		Sc. rank:	-651.1

PB2			
Pos.	5	obs:	exp:
caa	Q	3	1.54
cag	Q	0	1.46
cgt	R	0	0.13
cgc	R	0	0.15
cga	R	0	0.30
cgg	R	0	0.25
aaa	K	886	519.60
aag	K	1	367.40
aga	R	3	1.37
agg	R	0	0.80
gaa	E	5	3.01
gag	E	0	1.99
---	--	------	------
mPD		0.027	0.51
		nPD:	0.05
		N. weight:	0.66
		Sc. PD:	-0.089
		Sc. rank:	-219.2

PB2			
Pos.	6	obs:	exp:
gaa	E	893	540.20
gag	E	5	357.80
---	--	------	------
mPD		0.011	0.48
		nPD:	0.02
		N. weight:	0.6
		Sc. PD:	-0.097
		Sc. rank:	-271.9

PB2			
Pos.	7	obs:	exp:
tta	L	13	78.75
ttg	L	0	167.20
ctt	L	0	151.80
ctc	L	0	150.10
cta	L	879	149.40
ctg	L	4	198.70
caa	Q	2	1.03
cag	Q	0	0.97
---	--	------	------
mPD		0.042	1.1
		nPD:	0.04
		N. weight:	2.1
		Sc. PD:	-0.31
		Sc. rank:	-811.7

PB2			
Pos.	8	obs:	exp:
cgt	R	0	38.19
cgc	R	0	45.42
cga	R	2	89.14
cgg	R	3	74.46
aaa	K	3	1.76
aag	K	0	1.24
aga	R	889	408.20
agg	R	0	238.60
ggt	G	0	0.13
ggc	G	0	0.13
gga	G	1	0.46
ggg	G	0	0.28
---	--	------	------
mPD		0.027	0.97
		nPD:	0.03
		N. weight:	0.95
		Sc. PD:	-0.15
		Sc. rank:	-411.1

PB2			
Pos.	9	obs:	exp:
aat	N	11	5.60
aac	N	0	5.40
gat	D	882	478.80
gac	D	5	408.20
---	--	------	------
mPD		0.035	0.52
		nPD:	0.07
		N. weight:	0.74
		Sc. PD:	-0.087
		Sc. rank:	-198.5

PB2			
Pos.	10	obs:	exp:
tta	L	4	78.92
ttg	L	828	167.60
ctt	L	0	152.20
ctc	L	0	150.40
cta	L	61	149.70
ctg	L	5	199.20
---	--	------	------
mPD		0.27	1.1
		nPD:	0.24
		N. weight:	1.7
		Sc. PD:	0.089
		Sc. rank:	491.2

PB2			
Pos.	11	obs:	exp:
atg	M	898	898.00
---	--	------	------
mPD		0	0
		nPD:	1.
		N. weight:	0.
		Sc. PD:	0
		Sc. rank:	0

PB2			
Pos.	12	obs:	exp:
tct	S	0	143.70
tcc	S	0	116.20
tca	S	852	226.60
tcg	S	46	52.26
agt	S	0	184.00
agc	S	0	175.10
---	--	------	------
mPD		0.097	1.7
		nPD:	0.06
		N. weight:	1.6
		Sc. PD:	-0.2
		Sc. rank:	-491.9

PB2			
Pos.	13	obs:	exp:
tta	L	0	0.09
ttg	L	0	0.19
ctt	L	0	0.17
ctc	L	0	0.17
cta	L	0	0.17
ctg	L	1	0.22
caa	Q	12	460.20
cag	Q	884	435.80
aaa	K	0	0.59
aag	K	1	0.41
---	--	------	------
mPD		0.031	0.51
		nPD:	0.06
		N. weight:	0.82
		Sc. PD:	-0.1
		Sc. rank:	-248.2

PB2			
Pos.	14	obs:	exp:
tct	S	763	143.70
tcc	S	135	116.20
tca	S	0	226.60
tcg	S	0	52.26
agt	S	0	184.00
agc	S	0	175.10
---	--	------	------
mPD		0.26	1.7
		nPD:	0.15
		N. weight:	1.8
		Sc. PD:	-0.063
		Sc. rank:	26.0

PB2			
Pos.	15	obs:	exp:
cgt	R	0	38.36
cgc	R	898	45.62
cga	R	0	89.54
cgg	R	0	74.79
aga	R	0	410.00
agg	R	0	239.70
---	--	------	------
mPD		0	0.96
		nPD:	0.
		N. weight:	3.7
		Sc. PD:	-0.69
		Sc. rank:	-2308.6

PB2			
Pos.	16	obs:	exp:
act	T	879	232.60
acc	T	18	182.90
aca	T	1	412.60
acg	T	0	69.95
---	--	------	------
mPD		0.042	0.67
		nPD:	0.06
		N. weight:	1.6
		Sc. PD:	-0.2
		Sc. rank:	-461.5

PB2			
Pos.	17	obs:	exp:
cgt	R	0	38.36
cgc	R	898	45.62
cga	R	0	89.54
cgg	R	0	74.79
aga	R	0	410.00
agg	R	0	239.70
---	--	------	------
mPD		0	0.96
		nPD:	0.
		N. weight:	3.7
		Sc. PD:	-0.69
		Sc. rank:	-2308.6

PB2			
Pos.	18	obs:	exp:
gaa	E	0	540.20
gag	E	898	357.80
---	--	------	------
mPD		0	0.48
		nPD:	0.
		N. weight:	1.2
		Sc. PD:	-0.21
		Sc. rank:	-712.9

PB2			
Pos.	19	obs:	exp:
att	I	0	326.60
atc	I	0	216.50
ata	I	895	352.00
atg	M	1	1.00
act	T	0	0.26
acc	T	0	0.20
aca	T	1	0.46
acg	T	0	0.08
gtt	V	0	0.21
gtc	V	0	0.20
gta	V	1	0.21
gtg	V	0	0.39
---	--	------	------
mPD		0.0067	0.66
		nPD:	0.01
		N. weight:	1.2
		Sc. PD:	-0.2
		Sc. rank:	-600.3

PB2			
Pos.	20	obs:	exp:
tta	L	0	78.83
ttg	L	0	167.40
ctt	L	0	152.00
ctc	L	16	150.30
cta	L	93	149.50
ctg	L	788	198.90
gtt	V	0	0.21
gtc	V	0	0.20
gta	V	0	0.21
gtg	V	1	0.39
---	--	------	------
mPD		0.22	1.1
		nPD:	0.2
		N. weight:	1.4
		Sc. PD:	0.0092
		Sc. rank:	238.0

PB2

Pos.	21	obs:	exp:
act	T	0	231.60
acc	T	41	182.10
aca	T	851	410.70
acg	T	2	69.64
gct	A	0	1.02
gcc	A	0	0.75
gca	A	4	1.87
gcg	A	0	0.35
---	--	------	------
mPD		0.10	0.68
		nPD:	0.15
		N. weight:	0.78
		Sc. PD:	-0.033
		Sc. rank:	-3.4

PB2

Pos.	22	obs:	exp:
cgt	R	0	0.26
cgc	R	0	0.30
cga	R	0	0.60
cgg	R	0	0.50
aaa	K	722	521.90
aag	K	169	369.10
aga	R	6	2.74
agg	R	0	1.60
gct	A	0	0.26
gcc	A	0	0.19
gca	A	1	0.47
gcg	A	0	0.09
---	--	------	------
mPD		0.32	0.51
		nPD:	0.64
		N. weight:	0.16
		Sc. PD:	0.067
		Sc. rank:	90.2

PB2

Pos.	23	obs:	exp:
att	I	0	0.36
atc	I	0	0.24
ata	I	1	0.39
act	T	0	232.40
acc	T	92	182.70
aca	T	805	412.10
acg	T	0	69.87
---	--	------	------
mPD		0.19	0.68
		nPD:	0.28
		N. weight:	0.67
		Sc. PD:	0.055
		Sc. rank:	226.2

PB2

Pos.	24	obs:	exp:
act	T	542	232.60
acc	T	2	182.90
aca	T	352	412.60
acg	T	2	69.95
---	--	------	------
mPD		0.48	0.67
		nPD:	0.72
		N. weight:	0.55
		Sc. PD:	0.28
		Sc. rank:	331.6

PB2

Pos.	25	obs:	exp:
gtt	V	26	185.40
gtc	V	0	179.90
gta	V	48	185.90
gtg	V	824	346.80
---	--	------	------
mPD		0.15	0.73
		nPD:	0.21
		N. weight:	0.84
		Sc. PD:	0.018
		Sc. rank:	171.2

PB2

Pos.	26	obs:	exp:
gat	D	0	484.80
gac	D	898	413.20
---	--	------	------
mPD		0	0.50
		nPD:	0.
		N. weight:	0.97
		Sc. PD:	-0.18
		Sc. rank:	-601.4

PB2

Pos.	27	obs:	exp:
cat	H	835	515.90
cac	H	63	382.10
---	--	------	------
mPD		0.13	0.49
		nPD:	0.27
		N. weight:	0.41
		Sc. PD:	0.03
		Sc. rank:	132.8

PB2

Pos.	28	obs:	exp:
atg	M	898	898.00
---	--	------	------
mPD		0	0
		nPD:	1.
		N. weight:	0.
		Sc. PD:	0
		Sc. rank:	0

PB2

Pos.	29	obs:	exp:
gct	A	12	230.10
gcc	A	885	168.60
gca	A	1	419.90
gcg	A	0	79.43
---	--	------	------
mPD		0.029	0.67
		nPD:	0.04
		N. weight:	2.
		Sc. PD:	-0.29
		Sc. rank:	-721.2

PB2

Pos.	30	obs:	exp:
att	I	0	327.30
atc	I	6	217.00
ata	I	891	352.70
gtt	V	0	0.21
gtc	V	0	0.20
gta	V	1	0.21
gtg	V	0	0.39
---	--	------	------
mPD		0.016	0.66
		nPD:	0.02
		N. weight:	1.1
		Sc. PD:	-0.18
		Sc. rank:	-505.1

PB2

Pos.	31	obs:	exp:
att	I	18	327.70
atc	I	818	217.20
ata	I	62	353.10
---	--	------	------
mPD		0.17	0.65
		nPD:	0.25
		N. weight:	1.3
		Sc. PD:	0.078
		Sc. rank:	378.9

PB2

Pos.	32	obs:	exp:
cgt	R	0	0.04
cgc	R	0	0.05
cga	R	0	0.10
cgg	R	0	0.08
aaa	K	54	525.50
aag	K	843	371.50
aga	R	0	0.46
agg	R	1	0.27
---	--	------	------
mPD		0.12	0.49
		nPD:	0.24
		N. weight:	0.8
		Sc. PD:	0.035
		Sc. rank:	212.4

PB2

Pos.	33	obs:	exp:
aaa	K	857	526.00
aag	K	41	372.00
---	--	------	------
mPD		0.087	0.49
		nPD:	0.18
		N. weight:	0.46
		Sc. PD:	-0.0049
		Sc. rank:	51.3

PB2

Pos.	34	obs:	exp:
tat	Y	639	476.10
tac	Y	259	421.90
---	--	------	------
mPD		0.41	0.50
		nPD:	0.82
		N. weight:	0.091
		Sc. PD:	0.056
		Sc. rank:	55.9

PB2

Pos.	35	obs:	exp:
act	T	0	232.60
acc	T	0	182.90
aca	T	883	412.60
acg	T	15	69.95
---	--	------	------
mPD		0.033	0.67
		nPD:	0.05
		N. weight:	0.91
		Sc. PD:	-0.12
		Sc. rank:	-311.3

PB2

Pos.	36	obs:	exp:
tct	S	2	143.70
tcc	S	0	116.20
tca	S	327	226.60
tcg	S	569	52.26
agt	S	0	184.00
agc	S	0	175.10
---	--	------	------
mPD		0.47	1.7
		nPD:	0.28
		N. weight:	2.1
		Sc. PD:	0.18
		Sc. rank:	721.2

PB2

Pos.	37	obs:	exp:
ggt	G	0	120.40
ggc	G	1	114.10
gga	G	890	415.20
ggg	G	7	248.30
---	--	------	------
mPD		0.018	0.68
		nPD:	0.03
		N. weight:	0.91
		Sc. PD:	-0.14
		Sc. rank:	-402.7

PB2

Pos.	38	obs:	exp:
cgt	R	0	38.36
cgc	R	0	45.62
cga	R	0	89.54
cgg	R	0	74.79
aga	R	863	410.00
agg	R	35	239.70
---	--	------	------
mPD		0.075	0.96
		nPD:	0.08
		N. weight:	0.81
		Sc. PD:	-0.088
		Sc. rank:	-192.1

PB2

Pos.	39	obs:	exp:
caa	Q	64	461.30
cag	Q	834	436.70
---	--	------	------
mPD		0.13	0.50
		nPD:	0.27
		N. weight:	0.58
		Sc. PD:	0.042
		Sc. rank:	185.2

PB2

Pos.	40	obs:	exp:
gaa	E	21	540.20
gag	E	877	357.80
---	--	------	------
mPD		0.046	0.48
		nPD:	0.1
		N. weight:	1.
		Sc. PD:	-0.093
		Sc. rank:	-176.4

PB2

Pos.	41	obs:	exp:
aaa	K	38	526.00
aag	K	860	372.00
---	--	------	------
mPD		0.081	0.49
		nPD:	0.17
		N. weight:	0.87
		Sc. PD:	-0.02
		Sc. rank:	57.5

PB2

Pos.	42	obs:	exp:
aat	N	760	456.90
aac	N	138	441.10
---	--	------	------
mPD		0.26	0.50
		nPD:	0.52
		N. weight:	0.32
		Sc. PD:	0.1
		Sc. rank:	170.8

PB2

Pos.	43	obs:	exp:
cct	P	859	228.30
ccc	P	35	160.10
cca	P	1	366.90
ccg	P	3	142.70
---	--	------	------
mPD		0.084	0.71
		nPD:	0.12
		N. weight:	1.5
		Sc. PD:	-0.11
		Sc. rank:	-106.8

PB2

Pos.	44	obs:	exp:
tct	S	0	0.32
tcc	S	0	0.26
tca	S	2	0.50
tcg	S	0	0.12
agt	S	0	0.41
agc	S	0	0.39
gct	A	18	229.60
gcc	A	830	168.20
gca	A	44	418.90
gcg	A	4	79.25
---	--	------	------
mPD		0.15	0.68
		nPD:	0.22
		N. weight:	1.6
		Sc. PD:	0.041
		Sc. rank:	355.7

PB2

Pos.	45	obs:	exp:
tta	L	0	78.92
ttg	L	0	167.60
ctt	L	675	152.20
ctc	L	223	150.40
cta	L	0	149.70
ctg	L	0	199.20
---	--	------	------
mPD		0.37	1.1
		nPD:	0.34
		N. weight:	1.5
		Sc. PD:	0.22
		Sc. rank:	620.3

PB2			
Pos.	46	obs:	exp:
cgt	R	0	38.36
cgc	R	0	45.62
cga	R	0	89.54
cgg	R	0	74.79
aga	R	34	410.00
agg	R	864	239.70
mPD		0.073	0.96
		nPD:	0.08
		N. weight:	1.4
		Sc. PD:	-0.16
		Sc. rank:	-353.1

PB2			
Pos.	47	obs:	exp:
atg	M	898	898.00
mPD		0	0
		nPD:	1.
		N. weight:	0.
		Sc. PD:	0
		Sc. rank:	0

PB2			
Pos.	48	obs:	exp:
aaa	K	68	526.00
aag	K	830	372.00
mPD		0.14	0.49
		nPD:	0.29
		N. weight:	0.74
		Sc. PD:	0.07
		Sc. rank:	269.4

PB2			
Pos.	49	obs:	exp:
tgg	W	898	898.00
mPD		0	0
		nPD:	1.
		N. weight:	0.
		Sc. PD:	0
		Sc. rank:	0

PB2			
Pos.	50	obs:	exp:
atg	M	898	898.00
mPD		0	0
		nPD:	1.
		N. weight:	0.
		Sc. PD:	0
		Sc. rank:	0

PB2			
Pos.	51	obs:	exp:
atg	M	898	898.00
mPD		0	0
		nPD:	1.
		N. weight:	0.
		Sc. PD:	0
		Sc. rank:	0

PB2			
Pos.	52	obs:	exp:
gct	A	0	230.10
gcc	A	0	168.60
gca	A	294	419.90
gcg	A	604	79.43
mPD		0.44	0.67
		nPD:	0.66
		N. weight:	1.6
		Sc. PD:	0.71
		Sc. rank:	919.0

PB2			
Pos.	53	obs:	exp:
atg	M	897	897.00
gtt	V	0	0.21
gtc	V	0	0.20
gta	V	0	0.21
gtg	V	1	0.39
mPD		0.0022	0.0036
		nPD:	0.62
		N. weight:	0.0013
		Sc. PD:	0.00055
		Sc. rank:	0.8

PB2			
Pos.	54	obs:	exp:
aaa	K	774	526.00
aag	K	124	372.00
mPD		0.24	0.49
		nPD:	0.49
		N. weight:	0.23
		Sc. PD:	0.067
		Sc. rank:	121.0

PB2			
Pos.	55	obs:	exp:
tat	Y	776	476.10
tac	Y	122	421.90
mPD		0.24	0.50
		nPD:	0.47
		N. weight:	0.32
		Sc. PD:	0.088
		Sc. rank:	165.9

PB2			
Pos.	56	obs:	exp:
cct	P	0	228.30
ccc	P	1	160.10
cca	P	869	366.90
ccg	P	28	142.70
mPD		0.063	0.71
		nPD:	0.09
		N. weight:	0.98
		Sc. PD:	-0.097
		Sc. rank:	-207.8

PB2			
Pos.	57	obs:	exp:
att	I	712	327.70
atc	I	185	217.20
ata	I	1	353.10
mPD		0.33	0.65
		nPD:	0.5
		N. weight:	0.73
		Sc. PD:	0.22
		Sc. rank:	382.8

PB2			
Pos.	58	obs:	exp:
act	T	4	232.40
acc	T	1	182.70
aca	T	824	412.10
acg	T	68	69.87
gct	A	0	0.26
gcc	A	0	0.19
gca	A	1	0.47
gcg	A	0	0.09
mPD		0.15	0.68
		nPD:	0.23
		N. weight:	0.77
		Sc. PD:	0.026
		Sc. rank:	190.2

PB2			
Pos.	59	obs:	exp:
gct	A	3	230.10
gcc	A	11	168.60
gca	A	861	419.90
gcg	A	23	79.43
mPD		0.080	0.67
		nPD:	0.12
		N. weight:	0.77
		Sc. PD:	-0.054
		Sc. rank:	-49.5

PB2			
Pos.	60	obs:	exp:
aat	N	0	1.02
aac	N	2	0.98
gat	D	62	482.60
gac	D	832	411.40
gaa	E	1	0.60
gag	E	0	0.40
ggt	G	0	0.13
ggc	G	1	0.13
gga	G	0	0.46
ggg	G	0	0.28
mPD		0.14	0.51
		nPD:	0.27
		N. weight:	0.65
		Sc. PD:	0.051
		Sc. rank:	218.0

PB2			
Pos.	61	obs:	exp:
cgt	R	0	0.13
cgc	R	0	0.15
cga	R	0	0.30
cgg	R	0	0.25
act	T	0	0.26
acc	T	0	0.20
aca	T	1	0.46
acg	T	0	0.08
aaa	K	839	523.70
aag	K	55	370.30
aga	R	3	1.37
agg	R	0	0.80
mPD		0.12	0.50
		nPD:	0.25
		N. weight:	0.41
		Sc. PD:	0.024
		Sc. rank:	118.7

PB2			
Pos.	62	obs:	exp:
cgt	R	0	38.15
cgc	R	0	45.37
cga	R	26	89.04
cgg	R	1	74.38
aaa	K	0	1.17
aag	K	2	0.83
aga	R	55	407.70
agg	R	811	238.30
ggt	G	0	0.40
ggc	G	0	0.38
gga	G	0	1.39
ggg	G	3	0.83
mPD		0.23	0.97
		nPD:	0.24
		N. weight:	1.2
		Sc. PD:	0.058
		Sc. rank:	332.7

PB2			
Pos.	63	obs:	exp:
att	I	0	327.30
atc	I	1	217.00
ata	I	896	352.70
gtt	V	0	0.21
gtc	V	0	0.20
gta	V	1	0.21
gtg	V	0	0.39
mPD		0.0045	0.66
		nPD:	0.01
		N. weight:	1.2
		Sc. PD:	-0.21
		Sc. rank:	-611.7

PB2			
Pos.	64	obs:	exp:
att	I	0	0.36
atc	I	0	0.24
ata	I	1	0.39
atg	M	896	896.00
act	T	0	0.26
acc	T	0	0.20
aca	T	1	0.46
acg	T	0	0.08
mPD		0.0067	0.0065
		nPD:	1.03
		N. weight:	0.0024
		Sc. PD:	0.0019
		Sc. rank:	1.5

PB2			
Pos.	65	obs:	exp:
aaa	K	0	1.17
aag	K	2	0.83
gaa	E	59	538.40
gag	E	836	356.60
ggt	G	0	0.13
ggc	G	0	0.13
gga	G	0	0.46
ggg	G	1	0.28
mPD		0.13	0.49
		nPD:	0.27
		N. weight:	0.82
		Sc. PD:	0.06
		Sc. rank:	265.1

PB2			
Pos.	66	obs:	exp:
tta	L	0	0.09
ttg	L	1	0.19
ctt	L	0	0.17
ctc	L	0	0.17
cta	L	0	0.17
ctg	L	0	0.22
att	I	1	0.36
atc	I	0	0.24
ata	I	0	0.39
atg	M	894	894.00
gtt	V	0	0.41
gtc	V	0	0.40
gta	V	0	0.41
gtg	V	2	0.77
mPD		0.0089	0.013
		nPD:	0.69
		N. weight:	0.0064
		Sc. PD:	0.0031
		Sc. rank:	3.8

PB2			
Pos.	67	obs:	exp:
ttt	F	0	0.39
ttc	F	1	0.61
att	I	42	324.70
atc	I	848	215.30
ata	I	0	350.00
gtt	V	3	1.45
gtc	V	4	1.40
gta	V	0	1.45
gtg	V	0	2.70
mPD		0.11	0.67
		nPD:	0.17
		N. weight:	1.5
		Sc. PD:	-0.034
		Sc. rank:	113.5

PB2			
Pos.	68	obs:	exp:
cct	P	91	228.30
ccc	P	2	160.10
cca	P	114	366.90
ccg	P	691	142.70
mPD		0.38	0.71
		nPD:	0.54
		N. weight:	1.2
		Sc. PD:	0.41
		Sc. rank:	662.1

PB2			
Pos.	69	obs:	exp:
gaa	E	881	540.20
gag	E	17	357.80
mPD		0.037	0.48
		nPD:	0.08
		N. weight:	0.53
		Sc. PD:	-0.058
		Sc. rank:	-127.9

PB2			
Pos.	70	obs:	exp:
cgt	R	0	38.28
cgc	R	0	45.52
cga	R	10	89.34
cgg	R	0	74.63
aaa	K	2	1.17
aag	K	0	0.83
aga	R	673	409.10
agg	R	213	239.10
mPD		0.39	0.97
		nPD:	0.4
		N. weight:	0.41
		Sc. PD:	0.084
		Sc. rank:	185.4

PB2			
Pos.	71	obs:	exp:
aat	N	887	456.90
aac	N	11	441.10
---	--	------	------
mPD		0.024	0.50
		nPD:	0.05
		N. weight:	0.77
		Sc. PD:	-0.11
		Sc. rank:	-268.4

PB2			
Pos.	72	obs:	exp:
gaa	E	105	540.20
gag	E	793	357.80
---	--	------	------
mPD		0.21	0.48
		nPD:	0.43
		N. weight:	0.65
		Sc. PD:	0.15
		Sc. rank:	307.8

PB2			
Pos.	73	obs:	exp:
caa	Q	864	460.70
cag	Q	33	436.30
aaa	K	1	0.59
aag	K	0	0.41
---	--	------	------
mPD		0.073	0.50
		nPD:	0.15
		N. weight:	0.64
		Sc. PD:	-0.028
		Sc. rank:	-6.3

PB2			
Pos.	74	obs:	exp:
gat	D	1	0.54
gac	D	0	0.46
ggt	G	813	120.30
ggc	G	13	114.00
gga	G	35	414.70
ggg	G	36	248.00
---	--	------	------
mPD		0.18	0.68
		nPD:	0.26
		N. weight:	1.9
		Sc. PD:	0.13
		Sc. rank:	595.8

PB2			
Pos.	75	obs:	exp:
cct	P	0	0.25
ccc	P	0	0.18
cca	P	0	0.41
ccg	P	1	0.16
caa	Q	206	460.70
cag	Q	691	436.30
---	--	------	------
mPD		0.36	0.50
		nPD:	0.71
		N. weight:	0.22
		Sc. PD:	0.11
		Sc. rank:	131.8

PB2			
Pos.	76	obs:	exp:
att	I	0	1.09
atc	I	3	0.73
ata	I	0	1.18
act	T	30	231.10
acc	T	839	181.60
aca	T	12	409.80
acg	T	11	69.48
gct	A	0	0.77
gcc	A	3	0.56
gca	A	0	1.40
gcg	A	0	0.27
---	--	------	------
mPD		0.13	0.69
		nPD:	0.18
		N. weight:	1.6
		Sc. PD:	-0.011
		Sc. rank:	196.9

PB2			
Pos.	77	obs:	exp:
tta	L	0	78.92
ttg	L	0	167.60
ctt	L	698	152.20
ctc	L	45	150.40
cta	L	3	149.70
ctg	L	152	199.20
---	--	------	------
mPD		0.37	1.1
		nPD:	0.33
		N. weight:	1.3
		Sc. PD:	0.18
		Sc. rank:	533.9

PB2			
Pos.	78	obs:	exp:
tgg	W	898	898.00
---	--	------	------
mPD		0	0
		nPD:	1.
		N. weight:	0.
		Sc. PD:	0
		Sc. rank:	0

PB2			
Pos.	79	obs:	exp:
tct	S	0	143.70
tcc	S	0	116.20
tca	S	0	226.60
tcg	S	0	52.26
agt	S	5	184.00
agc	S	893	175.10
---	--	------	------
mPD		0.011	1.7
		nPD:	0.01
		N. weight:	2.
		Sc. PD:	-0.36
		Sc. rank:	-1071.1

PB2			
Pos.	80	obs:	exp:
cgt	R	0	0.13
cgc	R	0	0.15
cga	R	0	0.30
cgg	R	0	0.25
aaa	K	69	524.30
aag	K	826	370.70
aga	R	0	1.37
agg	R	3	0.80
---	--	------	------
mPD		0.15	0.49
		nPD:	0.3
		N. weight:	0.74
		Sc. PD:	0.079
		Sc. rank:	273.7

PB2			
Pos.	81	obs:	exp:
atg	M	1	1.00
act	T	0	232.40
acc	T	1	182.70
aca	T	895	412.10
acg	T	1	69.87
---	--	------	------
mPD		0.0089	0.68
		nPD:	0.01
		N. weight:	0.96
		Sc. PD:	-0.16
		Sc. rank:	-484.5

PB2			
Pos.	82	obs:	exp:
tct	S	0	3.68
tcc	S	0	2.98
tca	S	0	5.80
tcg	S	0	1.34
act	T	1	0.26
acc	T	0	0.20
aca	T	0	0.46
acg	T	0	0.08
aat	N	847	443.60
aac	N	25	428.40
agt	S	23	4.71
agc	S	0	4.49
gtt	V	2	0.41
gtc	V	0	0.40
gta	V	0	0.41
gtg	V	0	0.77
---	--	------	------
mPD		0.12	0.60
		nPD:	0.19
		N. weight:	0.73
		Sc. PD:	0.00056
		Sc. rank:	106.9

PB2			
Pos.	83	obs:	exp:
gat	D	897	484.80
gac	D	1	413.20
---	--	------	------
mPD		0.0022	0.50
		nPD:	0.
		N. weight:	0.76
		Sc. PD:	-0.14
		Sc. rank:	-420.2

PB2			
Pos.	84	obs:	exp:
gct	A	507	230.10
gcc	A	387	168.60
gca	A	4	419.90
gcg	A	0	79.43
---	--	------	------
mPD		0.50	0.67
		nPD:	0.74
		N. weight:	0.99
		Sc. PD:	0.52
		Sc. rank:	601.7

PB2			
Pos.	85	obs:	exp:
ggt	G	0	120.40
ggc	G	15	114.10
gga	G	867	415.20
ggg	G	16	248.30
---	--	------	------
mPD		0.067	0.68
		nPD:	0.1
		N. weight:	0.8
		Sc. PD:	-0.07
		Sc. rank:	-121.3

PB2			
Pos.	86	obs:	exp:
tct	S	3	143.70
tcc	S	1	116.20
tca	S	884	226.60
tcg	S	10	52.26
agt	S	0	184.00
agc	S	0	175.10
---	--	------	------
mPD		0.031	1.7
		nPD:	0.02
		N. weight:	1.6
		Sc. PD:	-0.27
		Sc. rank:	-775.3

PB2			
Pos.	87	obs:	exp:
aat	N	0	0.51
aac	N	1	0.49
gat	D	20	479.90
gac	D	869	409.10
gaa	E	8	4.81
gag	E	0	3.19
---	--	------	------
mPD		0.063	0.51
		nPD:	0.12
		N. weight:	0.84
		Sc. PD:	-0.054
		Sc. rank:	-38.7

PB2			
Pos.	88	obs:	exp:
cgt	R	0	38.32
cgc	R	0	45.57
cga	R	39	89.44
cgg	R	2	74.71
aaa	K	1	0.59
aag	K	0	0.41
aga	R	474	409.60
agg	R	382	239.40
---	--	------	------
mPD		0.58	0.97
		nPD:	0.6
		N. weight:	0.3
		Sc. PD:	0.12
		Sc. rank:	171.9

PB2			
Pos.	89	obs:	exp:
tta	L	0	0.09
ttg	L	1	0.19
ctt	L	0	0.17
ctc	L	0	0.17
cta	L	0	0.17
ctg	L	0	0.22
att	I	0	0.36
atc	I	0	0.24
ata	I	1	0.39
gtt	V	2	185.00
gtc	V	0	179.50
gta	V	18	185.50
gtg	V	876	346.00
---	--	------	------
mPD		0.050	0.73
		nPD:	0.07
		N. weight:	1.1
		Sc. PD:	-0.13
		Sc. rank:	-284.8

PB2			
Pos.	90	obs:	exp:
att	I	0	0.73
atc	I	0	0.48
ata	I	2	0.79
atg	M	894	894.00
gtt	V	0	0.41
gtc	V	0	0.40
gta	V	0	0.41
gtg	V	2	0.77
---	--	------	------
mPD		0.0089	0.012
		nPD:	0.77
		N. weight:	0.0053
		Sc. PD:	0.0029
		Sc. rank:	3.2

PB2			
Pos.	91	obs:	exp:
gtt	V	2	185.20
gtc	V	0	179.70
gta	V	254	185.70
gtg	V	641	346.40
gct	A	0	0.26
gcc	A	0	0.19
gca	A	0	0.47
gcg	A	1	0.09
---	--	------	------
mPD		0.41	0.73
		nPD:	0.57
		N. weight:	0.66
		Sc. PD:	0.24
		Sc. rank:	371.5

,

PB2			
Pos.	92	obs:	exp:
tct	S	22	143.70
tcc	S	0	116.20
tca	S	868	226.60
tcg	S	8	52.26
agt	S	0	184.00
agc	S	0	175.10
---	--	------	------
mPD		0.065	1.7
		nPD:	0.04
		N. weight:	1.6
		Sc. PD:	-0.23
		Sc. rank:	-581.6

,

PB2			
Pos.	93	obs:	exp:
tct	S	1	0.16
tcc	S	0	0.13
tca	S	0	0.25
tcg	S	0	0.06
cct	P	865	228.10
ccc	P	26	159.90
cca	P	6	366.50
ccg	P	0	142.50
agt	S	0	0.20
agc	S	0	0.20
---	--	------	------
mPD		0.071	0.71
		nPD:	0.1
		N. weight:	1.5
		Sc. PD:	-0.13
		Sc. rank:	-228.1

,

PB2			
Pos.	94	obs:	exp:
tta	L	1	78.92
ttg	L	8	167.60
ctt	L	1	152.20
ctc	L	0	150.40
cta	L	29	149.70
ctg	L	859	199.20
---	--	------	------
mPD		0.087	1.1
		nPD:	0.08
		N. weight:	1.7
		Sc. PD:	-0.18
		Sc. rank:	-385.3

,

PB2			
Pos.	95	obs:	exp:
gct	A	883	230.10
gcc	A	14	168.60
gca	A	1	419.90
gcg	A	0	79.43
---	--	------	------
mPD		0.033	0.67
		nPD:	0.05
		N. weight:	1.6
		Sc. PD:	-0.22
		Sc. rank:	-540.9

,

PB2			
Pos.	96	obs:	exp:
gtt	V	0	185.40
gtc	V	0	179.90
gta	V	32	185.90
gtg	V	866	346.80
---	--	------	------
mPD		0.069	0.73
		nPD:	0.09
		N. weight:	1.
		Sc. PD:	-0.096
		Sc. rank:	-182.6

,

PB2			
Pos.	97	obs:	exp:
act	T	22	232.60
acc	T	1	182.90
aca	T	62	412.60
acg	T	813	69.95
---	--	------	------
mPD		0.18	0.67
		nPD:	0.26
		N. weight:	2.6
		Sc. PD:	0.17
		Sc. rank:	775.0

,

PB2			
Pos.	98	obs:	exp:
tgg	W	898	898.00
---	--	------	------
mPD		0	0
		nPD:	1.
		N. weight:	0.
		Sc. PD:	0
		Sc. rank:	0

,

PB2			
Pos.	99	obs:	exp:
tgg	W	898	898.00
---	--	------	------
mPD		0	0
		nPD:	1.
		N. weight:	0.
		Sc. PD:	0
		Sc. rank:	0

,

PB2			
Pos.	100	obs:	exp:
aat	N	202	456.90
aac	N	696	441.10
---	--	------	------
mPD		0.35	0.50
		nPD:	0.7
		N. weight:	0.22
		Sc. PD:	0.11
		Sc. rank:	130.6

,

PB2			
Pos.	101	obs:	exp:
cgt	R	0	38.36
cgc	R	0	45.62
cga	R	1	89.54
cgg	R	0	74.79
aga	R	831	410.00
agg	R	66	239.70
---	--	------	------
mPD		0.14	0.96
		nPD:	0.14
		N. weight:	0.7
		Sc. PD:	-0.032
		Sc. rank:	-7.6

,

PB2			
Pos.	102	obs:	exp:
act	T	1	0.26
acc	T	0	0.20
aca	T	0	0.46
acg	T	0	0.08
aat	N	877	456.30
aac	N	20	440.70
---	--	------	------
mPD		0.046	0.50
		nPD:	0.09
		N. weight:	0.72
		Sc. PD:	-0.069
		Sc. rank:	-143.4

,

PB2			
Pos.	103	obs:	exp:
ggt	G	2	120.40
ggc	G	0	114.10
gga	G	854	415.20
ggg	G	42	248.30
---	--	------	------
mPD		0.094	0.68
		nPD:	0.14
		N. weight:	0.75
		Sc. PD:	-0.038
		Sc. rank:	-14.1

,

PB2			
Pos.	104	obs:	exp:
cct	P	3	228.30
ccc	P	1	160.10
cca	P	848	366.90
ccg	P	46	142.70
---	--	------	------
mPD		0.11	0.71
		nPD:	0.15
		N. weight:	0.9
		Sc. PD:	-0.037
		Sc. rank:	-0.8

,

PB2			
Pos.	105	obs:	exp:
att	I	0	0.73
atc	I	0	0.48
ata	I	2	0.79
atg	M	1	1.00
act	T	2	228.20
acc	T	0	179.40
aca	T	433	404.70
acg	T	446	68.63
gtt	V	0	0.21
gtc	V	0	0.20
gta	V	0	0.21
gtg	V	1	0.39
gct	A	0	3.33
gcc	A	0	2.44
gca	A	13	6.08
gcg	A	0	1.15
---	--	------	------
mPD		0.54	0.71
		nPD:	0.76
		N. weight:	1.2
		Sc. PD:	0.67
		Sc. rank:	745.3

,

PB2			
Pos.	106	obs:	exp:
att	I	0	0.73
atc	I	0	0.48
ata	I	2	0.79
act	T	15	228.50
acc	T	1	179.60
aca	T	863	405.20
acg	T	3	68.71
gct	A	0	3.59
gcc	A	0	2.63
gca	A	14	6.55
gcg	A	0	1.24
---	--	------	------
mPD		0.077	0.71
		nPD:	0.11
		N. weight:	0.86
		Sc. PD:	-0.068
		Sc. rank:	-94.1

,

PB2			
Pos.	107	obs:	exp:
tct	S	0	142.00
tcc	S	0	114.80
tca	S	0	223.90
tcg	S	0	51.62
aat	N	0	5.60
aac	N	11	5.40
agt	S	644	181.80
agc	S	243	173.00
---	--	------	------
mPD		0.43	1.7
		nPD:	0.26
		N. weight:	1.3
		Sc. PD:	0.082
		Sc. rank:	380.3

,

PB2			
Pos.	108	obs:	exp:
act	T	7	231.80
acc	T	0	182.30
aca	T	883	411.20
acg	T	5	69.72
gct	A	0	0.77
gcc	A	0	0.56
gca	A	3	1.40
gcg	A	0	0.27
---	--	------	------
mPD		0.033	0.68
		nPD:	0.05
		N. weight:	0.9
		Sc. PD:	-0.12
		Sc. rank:	-310.1

,

PB2			
Pos.	109	obs:	exp:
tta	L	0	0.09
ttg	L	0	0.19
tct	S	0	1.44
tcc	S	0	1.16
tca	S	0	2.27
tcg	S	0	0.52
ctt	L	0	0.17
ctc	L	1	0.17
cta	L	0	0.17
ctg	L	0	0.22
att	I	0	36.85
atc	I	101	24.43
ata	I	0	39.72
act	T	0	0.78
acc	T	3	0.61
aca	T	0	1.38
acg	T	0	0.23
aat	N	0	0.51
aac	N	1	0.49
agt	S	0	1.84
agc	S	9	1.76
gtt	V	46	161.40
gtc	V	736	156.70
gta	V	0	161.90
gtg	V	0	302.00
gct	A	0	0.26
gcc	A	1	0.19
gca	A	0	0.47
gcg	A	0	0.09
---	--	------	------
mPD		0.35	1.0
		nPD:	0.35
		N. weight:	1.7
		Sc. PD:	0.28
		Sc. rank:	725.1

,

PB2			
Pos.	110	obs:	exp:
cat	H	850	515.90
cac	H	48	382.10
---	--	------	------
mPD		0.10	0.49
		nPD:	0.21
		N. weight:	0.46
		Sc. PD:	0.0073
		Sc. rank:	85.0

,

PB2			
Pos.	111	obs:	exp:
tat	Y	878	476.10
tac	Y	20	421.90
mPD		0.044	0.50
		nPD:	0.09
		N. weight:	0.67
		Sc. PD:	-0.067
		Sc. rank:	-142.8

PB2			
Pos.	112	obs:	exp:
cct	P	0	228.30
ccc	P	0	160.10
cca	P	883	366.90
ccg	P	15	142.70
mPD		0.033	0.71
		nPD:	0.05
		N. weight:	1.
		Sc. PD:	-0.15
		Sc. rank:	-364.3

PB2			
Pos.	113	obs:	exp:
aaa	K	17	526.00
aag	K	881	372.00
mPD		0.037	0.49
		nPD:	0.08
		N. weight:	0.98
		Sc. PD:	-0.11
		Sc. rank:	-237.5

PB2			
Pos.	114	obs:	exp:
gtt	V	30	185.40
gtc	V	823	179.90
gta	V	41	185.90
gtg	V	4	346.80
mPD		0.16	0.73
		nPD:	0.22
		N. weight:	1.6
		Sc. PD:	0.039
		Sc. rank:	335.3

PB2			
Pos.	115	obs:	exp:
tat	Y	502	476.10
tac	Y	396	421.90
mPD		0.49	0.50
		nPD:	0.99
		N. weight:	0.0072
		Sc. PD:	0.0055
		Sc. rank:	4.4

PB2			
Pos.	116	obs:	exp:
cgt	R	0	0.04
cgc	R	0	0.05
cga	R	0	0.10
cgg	R	0	0.08
aaa	K	894	525.50
aag	K	3	371.50
aga	R	1	0.46
agg	R	0	0.27
mPD		0.0089	0.49
		nPD:	0.02
		N. weight:	0.65
		Sc. PD:	-0.11
		Sc. rank:	-308.2

PB2			
Pos.	117	obs:	exp:
cct	P	0	12.46
ccc	P	49	8.74
cca	P	0	20.02
ccg	P	0	7.78
att	I	1	1.09
atc	I	2	0.73
ata	I	0	1.18
act	T	42	218.90
acc	T	779	172.10
aca	T	24	388.20
acg	T	0	65.82
gct	A	0	0.26
gcc	A	1	0.19
gca	A	0	0.47
gcg	A	0	0.09
mPD		0.25	0.79
		nPD:	0.32
		N. weight:	1.6
		Sc. PD:	0.2
		Sc. rank:	613.8

PB2			
Pos.	118	obs:	exp:
tat	Y	862	476.10
tac	Y	36	421.90
mPD		0.077	0.50
		nPD:	0.15
		N. weight:	0.6
		Sc. PD:	-0.021
		Sc. rank:	9.1

PB2			
Pos.	119	obs:	exp:
ttt	F	861	354.40
ttc	F	37	543.60
mPD		0.079	0.48
		nPD:	0.17
		N. weight:	0.93
		Sc. PD:	-0.023
		Sc. rank:	56.7

PB2			
Pos.	120	obs:	exp:
gat	D	1	1.08
gac	D	1	0.92
gaa	E	801	539.00
gag	E	95	357.00
mPD		0.19	0.48
		nPD:	0.4
		N. weight:	0.27
		Sc. PD:	0.056
		Sc. rank:	122.6

PB2			
Pos.	121	obs:	exp:
cgt	R	0	0.17
cgc	R	0	0.20
cga	R	0	0.40
cgg	R	0	0.33
aaa	K	62	523.70
aag	K	832	370.30
aga	R	0	1.83
agg	R	4	1.07
mPD		0.14	0.50
		nPD:	0.28
		N. weight:	0.77
		Sc. PD:	0.064
		Sc. rank:	262.1

PB2			
Pos.	122	obs:	exp:
att	I	0	0.73
atc	I	2	0.48
ata	I	0	0.79
gtt	V	212	161.80
gtc	V	570	157.10
gta	V	2	162.30
gtg	V	0	302.80
gct	A	2	28.70
gcc	A	105	21.03
gca	A	5	52.37
gcg	A	0	9.91
mPD		0.60	0.96
		nPD:	0.62
		N. weight:	1.3
		Sc. PD:	0.55
		Sc. rank:	760.0

PB2			
Pos.	123	obs:	exp:
aaa	K	1	0.59
aag	K	0	0.41
gat	D	0	0.54
gac	D	1	0.46
gaa	E	892	537.20
gag	E	1	355.80
ggt	G	0	0.40
ggc	G	0	0.38
gga	G	3	1.39
ggg	G	0	0.83
mPD		0.013	0.49
		nPD:	0.03
		N. weight:	0.63
		Sc. PD:	-0.1
		Sc. rank:	-273.7

PB2			
Pos.	124	obs:	exp:
cgt	R	0	38.36
cgc	R	0	45.62
cga	R	0	89.54
cgg	R	0	74.79
aga	R	190	410.00
agg	R	708	239.70
mPD		0.33	0.96
		nPD:	0.35
		N. weight:	0.87
		Sc. PD:	0.13
		Sc. rank:	357.1

PB2			
Pos.	125	obs:	exp:
tta	L	26	78.83
ttg	L	65	167.40
ctt	L	0	152.00
ctc	L	0	150.30
cta	L	692	149.50
ctg	L	114	198.90
atg	M	1	1.00
mPD		0.51	1.1
		nPD:	0.45
		N. weight:	1.3
		Sc. PD:	0.33
		Sc. rank:	631.3

PB2			
Pos.	126	obs:	exp:
cgt	R	0	0.38
cgc	R	0	0.46
cga	R	0	0.90
cgg	R	0	0.75
aaa	K	877	520.80
aag	K	12	368.20
aga	R	9	4.11
agg	R	0	2.40
mPD		0.046	0.51
		nPD:	0.09
		N. weight:	0.59
		Sc. PD:	-0.058
		Sc. rank:	-119.1

PB2			
Pos.	127	obs:	exp:
cat	H	872	515.30
cac	H	25	381.70
aat	N	0	0.51
aac	N	1	0.49
mPD		0.059	0.49
		nPD:	0.12
		N. weight:	0.55
		Sc. PD:	-0.038
		Sc. rank:	-34.0

PB2			
Pos.	128	obs:	exp:
ggt	G	28	120.40
ggc	G	0	114.10
gga	G	685	415.20
ggg	G	185	248.30
mPD		0.38	0.68
		nPD:	0.56
		N. weight:	0.35
		Sc. PD:	0.12
		Sc. rank:	197.6

PB2			
Pos.	129	obs:	exp:
att	I	0	5.84
atc	I	16	3.87
ata	I	0	6.29
act	T	12	227.70
acc	T	866	179.00
aca	T	1	403.80
acg	T	0	68.47
gct	A	0	0.77
gcc	A	3	0.56
gca	A	0	1.40
gcg	A	0	0.27
mPD		0.070	0.72
		nPD:	0.1
		N. weight:	1.9
		Sc. PD:	-0.17
		Sc. rank:	-300.3

PB2			
Pos.	130	obs:	exp:
ttt	F	424	354.40
ttc	F	474	543.60
mPD		0.50	0.48
		nPD:	1.04
		N. weight:	0.021
		Sc. PD:	0.017
		Sc. rank:	12.7

PB2			
Pos.	131	obs:	exp:
ggt	G	51	120.40
ggc	G	845	114.10
gga	G	1	415.20
ggg	G	1	248.30
mPD		0.11	0.68
		nPD:	0.16
		N. weight:	2.3
		Sc. PD:	-0.057
		Sc. rank:	126.1

PB2			
Pos.	132	obs:	exp:
cct	P	54	228.30
ccc	P	841	160.10
cca	P	3	366.90
ccg	P	0	142.70
mPD		0.12	0.71
		nPD:	0.17
		N. weight:	1.8
		Sc. PD:	-0.04
		Sc. rank:	147.0

PB2			
Pos.	133	obs:	exp:
att	I	1	0.36
atc	I	0	0.24
ata	I	0	0.39
gtt	V	835	185.20
gtc	V	37	179.70
gta	V	22	185.70
gtg	V	3	346.40
mPD		0.13	0.73
		nPD:	0.18
		N. weight:	1.6
		Sc. PD:	-0.012
		Sc. rank:	187.5

PB2			
Pos.	134	obs:	exp:
cat	H	52	514.80
cac	H	844	381.20
cgt	R	0	0.04
cgc	R	1	0.05
cga	R	0	0.10
cgg	R	0	0.08
aat	N	0	0.51
aac	N	1	0.49
aga	R	0	0.46
agg	R	0	0.27
mPD		0.11	0.50
		nPD:	0.23
		N. weight:	0.78
		Sc. PD:	0.029
		Sc. rank:	194.1

PB2			
Pos.	135	obs:	exp:
ttt	F	29	354.40
ttc	F	869	543.60
mPD		0.063	0.48
		nPD:	0.13
		N. weight:	0.47
		Sc. PD:	-0.027
		Sc. rank:	-15.2

PB2			
Pos.	136	obs:	exp:
cgt	R	0	38.36
cgc	R	0	45.62
cga	R	759	89.54
cgg	R	14	74.79
aga	R	123	410.00
agg	R	2	239.70
---	--	------	------
mPD		0.27	0.96
		nPD:	0.29
		N. weight:	2.
		Sc. PD:	0.18
		Sc. rank:	732.1

PB2			
Pos.	137	obs:	exp:
tct	S	0	1.28
tcc	S	0	1.04
tca	S	0	2.02
tcg	S	0	0.47
aat	N	71	452.80
aac	N	819	437.20
agt	S	0	1.64
agc	S	8	1.56
---	--	------	------
mPD		0.16	0.53
		nPD:	0.31
		N. weight:	0.56
		Sc. PD:	0.063
		Sc. rank:	207.7

PB2			
Pos.	138	obs:	exp:
cat	H	2	1.15
cac	H	0	0.85
caa	Q	184	460.20
cag	Q	712	435.80
---	--	------	------
mPD		0.33	0.50
		nPD:	0.66
		N. weight:	0.26
		Sc. PD:	0.12
		Sc. rank:	151.8

PB2			
Pos.	139	obs:	exp:
att	I	14	58.01
atc	I	145	38.46
ata	I	0	62.53
gtt	V	732	152.60
gtc	V	7	148.00
gta	V	0	153.00
gtg	V	0	285.40
---	--	------	------
mPD		0.57	1.0
		nPD:	0.55
		N. weight:	1.8
		Sc. PD:	0.64
		Sc. rank:	1014.2

PB2			
Pos.	140	obs:	exp:
aaa	K	854	526.00
aag	K	44	372.00
---	--	------	------
mPD		0.093	0.49
		nPD:	0.19
		N. weight:	0.45
		Sc. PD:	0.00065
		Sc. rank:	67.1

PB2			
Pos.	141	obs:	exp:
att	I	0	327.70
atc	I	0	217.20
ata	I	898	353.10
---	--	------	------
mPD		0	0.65
		nPD:	0.
		N. weight:	1.2
		Sc. PD:	-0.22
		Sc. rank:	-723.1

PB2			
Pos.	142	obs:	exp:
cgt	R	1	38.36
cgc	R	732	45.62
cga	R	124	89.54
cgg	R	0	74.79
aga	R	40	410.00
agg	R	1	239.70
---	--	------	------
mPD		0.39	0.96
		nPD:	0.4
		N. weight:	2.8
		Sc. PD:	0.57
		Sc. rank:	1255.0

PB2			
Pos.	143	obs:	exp:
cgt	R	0	38.36
cgc	R	853	45.62
cga	R	3	89.54
cgg	R	39	74.79
aga	R	1	410.00
agg	R	2	239.70
---	--	------	------
mPD		0.10	0.96
		nPD:	0.11
		N. weight:	3.4
		Sc. PD:	-0.28
		Sc. rank:	-419.4

PB2			
Pos.	144	obs:	exp:
cgt	R	0	38.28
cgc	R	0	45.52
cga	R	23	89.34
cgg	R	0	74.63
aaa	K	0	1.17
aag	K	2	0.83
aga	R	11	409.10
agg	R	862	239.10
---	--	------	------
mPD		0.13	0.97
		nPD:	0.13
		N. weight:	1.5
		Sc. PD:	-0.083
		Sc. rank:	-42.2

PB2			
Pos.	145	obs:	exp:
gtt	V	174	185.20
gtc	V	707	179.70
gta	V	5	185.70
gtg	V	11	346.40
gct	A	0	0.26
gcc	A	1	0.19
gca	A	0	0.47
gcg	A	0	0.09
---	--	------	------
mPD		0.34	0.73
		nPD:	0.47
		N. weight:	1.3
		Sc. PD:	0.35
		Sc. rank:	653.5

PB2			
Pos.	146	obs:	exp:
gat	D	33	484.80
gac	D	865	413.20
---	--	------	------
mPD		0.071	0.50
		nPD:	0.14
		N. weight:	0.77
		Sc. PD:	-0.036
		Sc. rank:	-9.2

PB2			
Pos.	147	obs:	exp:
att	I	0	324.70
atc	I	0	215.30
ata	I	890	350.00
atg	M	1	1.00
act	T	0	1.30
acc	T	0	1.02
aca	T	5	2.30
acg	T	0	0.39
gtt	V	0	0.41
gtc	V	0	0.40
gta	V	2	0.41
gtg	V	0	0.77
---	--	------	------
mPD		0.018	0.67
		nPD:	0.03
		N. weight:	1.2
		Sc. PD:	-0.19
		Sc. rank:	-515.4

PB2			
Pos.	148	obs:	exp:
aat	N	23	456.90
aac	N	875	441.10
---	--	------	------
mPD		0.050	0.50
		nPD:	0.1
		N. weight:	0.75
		Sc. PD:	-0.065
		Sc. rank:	-110.9

PB2			
Pos.	149	obs:	exp:
cct	P	172	228.30
ccc	P	2	160.10
cca	P	590	366.90
ccg	P	134	142.70
---	--	------	------
mPD		0.51	0.71
		nPD:	0.72
		N. weight:	0.31
		Sc. PD:	0.16
		Sc. rank:	187.9

PB2			
Pos.	150	obs:	exp:
tct	S	0	0.16
tcc	S	0	0.13
tca	S	0	0.25
tcg	S	0	0.06
agt	S	0	0.20
agc	S	1	0.20
gat	D	0	0.54
gac	D	1	0.46
ggt	G	14	120.20
ggc	G	730	113.80
gga	G	151	414.30
ggg	G	1	247.70
---	--	------	------
mPD		0.31	0.68
		nPD:	0.46
		N. weight:	1.6
		Sc. PD:	0.42
		Sc. rank:	819.0

PB2			
Pos.	151	obs:	exp:
cat	H	855	515.90
cac	H	43	382.10
---	--	------	------
mPD		0.091	0.49
		nPD:	0.19
		N. weight:	0.48
		Sc. PD:	-0.0019
		Sc. rank:	61.8

PB2			
Pos.	152	obs:	exp:
tct	S	0	0.32
tcc	S	0	0.26
tca	S	2	0.50
tcg	S	0	0.12
agt	S	0	0.41
agc	S	0	0.39
gct	A	25	229.60
gcc	A	0	168.20
gca	A	871	418.90
gcg	A	0	79.25
---	--	------	------
mPD		0.059	0.68
		nPD:	0.09
		N. weight:	0.82
		Sc. PD:	-0.083
		Sc. rank:	-177.9

PB2			
Pos.	153	obs:	exp:
gat	D	833	484.80
gac	D	65	413.20
---	--	------	------
mPD		0.13	0.50
		nPD:	0.27
		N. weight:	0.47
		Sc. PD:	0.036
		Sc. rank:	155.1

PB2			
Pos.	154	obs:	exp:
ttt	F	0	0.39
ttc	F	1	0.61
tta	L	0	78.83
ttg	L	0	167.40
ctt	L	795	152.00
ctc	L	102	150.30
cta	L	0	149.50
ctg	L	0	198.90
---	--	------	------
mPD		0.21	1.1
		nPD:	0.19
		N. weight:	1.8
		Sc. PD:	-0.0099
		Sc. rank:	224.4

PB2			
Pos.	155	obs:	exp:
tct	S	0	143.40
tcc	S	0	116.00
tca	S	0	226.10
tcg	S	0	52.14
agt	S	893	183.60
agc	S	3	174.70
ggt	G	2	0.27
ggc	G	0	0.25
gga	G	0	0.92
ggg	G	0	0.55
---	--	------	------
mPD		0.011	1.7
		nPD:	0.01
		N. weight:	2.
		Sc. PD:	-0.35
		Sc. rank:	-1040.5

PB2			
Pos.	156	obs:	exp:
tct	S	0	0.16
tcc	S	1	0.13
tca	S	0	0.25
tcg	S	0	0.06
act	T	2	0.52
acc	T	0	0.41
aca	T	0	0.92
acg	T	0	0.16
agt	S	0	0.20
agc	S	0	0.20
gct	A	828	228.80
gcc	A	64	167.70
gca	A	1	417.50
gcg	A	0	78.98
gat	D	2	1.08
gac	D	0	0.92
---	--	------	------
mPD		0.15	0.69
		nPD:	0.22
		N. weight:	1.4
		Sc. PD:	0.034
		Sc. rank:	296.9

PB2			
Pos.	157	obs:	exp:
cgt	R	0	0.09
cgc	R	0	0.10
cga	R	0	0.20
cgg	R	0	0.17
aaa	K	645	524.90
aag	K	251	371.10
aga	R	0	0.91
agg	R	2	0.53
---	--	------	------
mPD		0.41	0.49
		nPD:	0.83
		N. weight:	0.057
		Sc. PD:	0.036
		Sc. rank:	35.2

PB2			
Pos.	158	obs:	exp:
gaa	E	843	540.20
gag	E	55	357.80
---	--	------	------
mPD		0.12	0.48
		nPD:	0.24
		N. weight:	0.38
		Sc. PD:	0.018
		Sc. rank:	105.6

PB2			
Pos.	159	obs:	exp:
gct	A	0	230.10
gcc	A	2	168.60
gca	A	868	419.90
gcg	A	28	79.43
---	--	------	------
mPD		0.065	0.67
		nPD:	0.1
		N. weight:	0.83
		Sc. PD:	-0.076
		Sc. rank:	-140.1

PB2			
Pos.	160	obs:	exp:
caa	Q	785	461.30
cag	Q	113	436.70
---	--	------	------
mPD		0.22	0.50
		nPD:	0.44
		N. weight:	0.37
		Sc. PD:	0.09
		Sc. rank:	180.9

PB2			
Pos.	161	obs:	exp:
tat	Y	1	0.53
tac	Y	0	0.47
gat	D	848	484.20
gac	D	49	412.80
---	--	------	------
mPD		0.11	0.50
		nPD:	0.21
		N. weight:	0.52
		Sc. PD:	0.01
		Sc. rank:	103.1

,

PB2			
Pos.	162	obs:	exp:
att	I	0	0.73
atc	I	2	0.48
ata	I	0	0.79
gtt	V	15	185.00
gtc	V	836	179.50
gta	V	43	185.50
gtg	V	2	346.00
---	--	------	------
mPD		0.13	0.73
		nPD:	0.18
		N. weight:	1.7
		Sc. PD:	-0.018
		Sc. rank:	182.5

,

PB2			
Pos.	163	obs:	exp:
att	I	8	327.70
atc	I	885	217.20
ata	I	5	353.10
---	--	------	------
mPD		0.029	0.65
		nPD:	0.04
		N. weight:	1.7
		Sc. PD:	-0.24
		Sc. rank:	-596.2

,

PB2			
Pos.	164	obs:	exp:
atg	M	897	897.00
gtt	V	0	0.21
gtc	V	0	0.20
gta	V	0	0.21
gtg	V	1	0.39
---	--	------	------
mPD		0.0022	0.0036
		nPD:	0.62
		N. weight:	0.0013
		Sc. PD:	0.00055
		Sc. rank:	0.8

,

PB2			
Pos.	165	obs:	exp:
gaa	E	47	540.20
gag	E	851	357.80
---	--	------	------
mPD		0.099	0.48
		nPD:	0.21
		N. weight:	0.87
		Sc. PD:	0.014
		Sc. rank:	159.9

,

PB2			
Pos.	166	obs:	exp:
gtt	V	48	185.40
gtc	V	831	179.90
gta	V	13	185.90
gtg	V	6	346.80
---	--	------	------
mPD		0.14	0.73
		nPD:	0.19
		N. weight:	1.6
		Sc. PD:	0.0051
		Sc. rank:	246.4

,

PB2			
Pos.	167	obs:	exp:
gtt	V	63	185.40
gtc	V	29	179.90
gta	V	798	185.90
gtg	V	8	346.80
---	--	------	------
mPD		0.20	0.73
		nPD:	0.28
		N. weight:	1.4
		Sc. PD:	0.13
		Sc. rank:	501.6

,

PB2			
Pos.	168	obs:	exp:
ttt	F	23	352.40
ttc	F	870	540.60
tct	S	0	0.16
tcc	S	1	0.13
tca	S	0	0.25
tcg	S	0	0.06
tat	Y	0	2.12
tac	Y	4	1.88
agt	S	0	0.20
agc	S	0	0.20
---	--	------	------
mPD		0.061	0.49
		nPD:	0.12
		N. weight:	0.5
		Sc. PD:	-0.032
		Sc. rank:	-22.8

,

PB2			
Pos.	169	obs:	exp:
cct	P	0	228.30
ccc	P	3	160.10
cca	P	884	366.90
ccg	P	11	142.70
---	--	------	------
mPD		0.031	0.71
		nPD:	0.04
		N. weight:	1.
		Sc. PD:	-0.15
		Sc. rank:	-372.2

,

PB2			
Pos.	170	obs:	exp:
aat	N	107	456.90
aac	N	791	441.10
---	--	------	------
mPD		0.21	0.50
		nPD:	0.42
		N. weight:	0.43
		Sc. PD:	0.096
		Sc. rank:	202.6

,

PB2			
Pos.	171	obs:	exp:
gaa	E	884	540.20
gag	E	14	357.80
---	--	------	------
mPD		0.031	0.48
		nPD:	0.06
		N. weight:	0.55
		Sc. PD:	-0.067
		Sc. rank:	-155.9

,

PB2			
Pos.	172	obs:	exp:
tta	L	0	0.09
ttg	L	0	0.19
ctt	L	1	0.17
ctc	L	0	0.17
cta	L	0	0.17
ctg	L	0	0.22
att	I	6	2.19
atc	I	0	1.45
ata	I	0	2.36
gtt	V	813	183.90
gtc	V	7	178.50
gta	V	9	184.50
gtg	V	62	344.10
---	--	------	------
mPD		0.18	0.74
		nPD:	0.24
		N. weight:	1.5
		Sc. PD:	0.069
		Sc. rank:	404.4

,

PB2			
Pos.	173	obs:	exp:
gaa	E	1	0.60
gag	E	0	0.40
ggt	G	1	120.30
ggc	G	2	114.00
gga	G	883	414.70
ggg	G	11	248.00
---	--	------	------
mPD		0.033	0.68
		nPD:	0.05
		N. weight:	0.87
		Sc. PD:	-0.12
		Sc. rank:	-300.2

,

PB2			
Pos.	174	obs:	exp:
act	T	0	0.26
acc	T	1	0.20
aca	T	0	0.46
acg	T	0	0.08
gct	A	24	229.80
gcc	A	831	168.40
gca	A	7	419.40
gcg	A	35	79.34
---	--	------	------
mPD		0.14	0.67
		nPD:	0.21
		N. weight:	1.7
		Sc. PD:	0.032
		Sc. rank:	334.2

,

PB2			
Pos.	175	obs:	exp:
cgt	R	0	38.02
cgc	R	0	45.22
cga	R	210	88.74
cgg	R	1	74.13
aaa	K	8	4.69
aag	K	0	3.31
aga	R	639	406.40
agg	R	40	237.50
---	--	------	------
mPD		0.46	0.98
		nPD:	0.48
		N. weight:	0.57
		Sc. PD:	0.16
		Sc. rank:	292.3

,

PB2			
Pos.	176	obs:	exp:
att	I	1	327.70
atc	I	53	217.20
ata	I	844	353.10
---	--	------	------
mPD		0.11	0.65
		nPD:	0.17
		N. weight:	0.92
		Sc. PD:	-0.016
		Sc. rank:	84.7

,

PB2			
Pos.	177	obs:	exp:
tta	L	35	78.92
ttg	L	464	167.60
ctt	L	0	152.20
ctc	L	2	150.40
cta	L	15	149.70
ctg	L	382	199.20
---	--	------	------
mPD		0.60	1.1
		nPD:	0.54
		N. weight:	0.92
		Sc. PD:	0.31
		Sc. rank:	502.8

,

PB2			
Pos.	178	obs:	exp:
tct	S	0	0.16
tcc	S	0	0.13
tca	S	1	0.25
tcg	S	0	0.06
act	T	1	231.80
acc	T	13	182.30
aca	T	857	411.20
acg	T	24	69.72
agt	S	0	0.20
agc	S	0	0.20
gct	A	0	0.51
gcc	A	0	0.38
gca	A	2	0.94
gcg	A	0	0.18
---	--	------	------
mPD		0.089	0.68
		nPD:	0.13
		N. weight:	0.8
		Sc. PD:	-0.047
		Sc. rank:	-28.2

,

PB2			
Pos.	179	obs:	exp:
tct	S	1	143.70
tcc	S	2	116.20
tca	S	852	226.60
tcg	S	43	52.26
agt	S	0	184.00
agc	S	0	175.10
---	--	------	------
mPD		0.098	1.7
		nPD:	0.06
		N. weight:	1.6
		Sc. PD:	-0.2
		Sc. rank:	-482.2

,

PB2			
Pos.	180	obs:	exp:
gaa	E	27	540.20
gag	E	871	357.80
---	--	------	------
mPD		0.058	0.48
		nPD:	0.12
		N. weight:	0.97
		Sc. PD:	-0.065
		Sc. rank:	-46.4

,

PB2			
Pos.	181	obs:	exp:
tta	L	1	0.09
ttg	L	0	0.19
tct	S	0	143.60
tcc	S	0	116.10
tca	S	874	226.40
tcg	S	23	52.20
ctt	L	0	0.17
ctc	L	0	0.17
cta	L	0	0.17
ctg	L	0	0.22
agt	S	0	183.80
agc	S	0	174.90
---	--	------	------
mPD		0.052	1.7
		nPD:	0.03
		N. weight:	1.6
		Sc. PD:	-0.25
		Sc. rank:	-676.0

,

PB2			
Pos.	182	obs:	exp:
caa	Q	139	461.30
cag	Q	759	436.70
---	--	------	------
mPD		0.26	0.50
		nPD:	0.52
		N. weight:	0.36
		Sc. PD:	0.12
		Sc. rank:	191.0

,

PB2			
Pos.	183	obs:	exp:
tta	L	816	78.92
ttg	L	42	167.60
ctt	L	0	152.20
ctc	L	0	150.40
cta	L	3	149.70
ctg	L	37	199.20
---	--	------	------
mPD		0.25	1.1
		nPD:	0.22
		N. weight:	2.5
		Sc. PD:	0.074
		Sc. rank:	572.5

,

PB2			
Pos.	184	obs:	exp:
atg	M	2	2.00
act	T	10	231.10
acc	T	0	181.60
aca	T	60	409.80
acg	T	822	69.48
gct	A	0	1.02
gcc	A	1	0.75
gca	A	0	1.87
gcg	A	3	0.35
---	--	------	------
mPD		0.16	0.69
		nPD:	0.23
		N. weight:	2.7
		Sc. PD:	0.11
		Sc. rank:	693.8

,

PB2			
Pos.	185	obs:	exp:
att	I	67	324.00
atc	I	0	214.80
ata	I	821	349.20
atg	M	10	10.00
---	--	------	------
mPD		0.16	0.66
		nPD:	0.24
		N. weight:	0.84
		Sc. PD:	0.04
		Sc. rank:	229.4

,

PB2			
Pos.	186	obs:	exp:
act	T	66	232.60
acc	T	744	182.90
aca	T	76	412.60
acg	T	12	69.95
---	--	------	------
mPD		0.30	0.67
		nPD:	0.45
		N. weight:	1.1
		Sc. PD:	0.28
		Sc. rank:	557.6

,

PB2			
Pos.	187	obs:	exp:
aaa	K	47	526.00
aag	K	851	372.00
---	--	------	------
mPD		0.099	0.49
		nPD:	0.2
		N. weight:	0.83
		Sc. PD:	0.011
		Sc. rank:	147.3

,

PB2			
Pos.	188	obs:	exp:
aaa	K	19	11.13
aag	K	0	7.87
gaa	E	816	528.10
gag	E	62	349.90
ggt	G	0	0.13
ggc	G	0	0.13
gga	G	1	0.46
ggg	G	0	0.28
---	--	------	------
mPD		0.17	0.52
		nPD:	0.33
		N. weight:	0.37
		Sc. PD:	0.049
		Sc. rank:	145.2

,

PB2			
Pos.	189	obs:	exp:
aaa	K	22	526.00
aag	K	876	372.00
---	--	------	------
mPD		0.048	0.49
		nPD:	0.1
		N. weight:	0.96
		Sc. PD:	-0.085
		Sc. rank:	-149.9

,

PB2			
Pos.	190	obs:	exp:
cgt	R	0	0.13
cgc	R	0	0.15
cga	R	0	0.30
cgg	R	0	0.25
aaa	K	37	524.30
aag	K	858	370.70
aga	R	0	1.37
agg	R	3	0.80
---	--	------	------
mPD		0.086	0.49
		nPD:	0.17
		N. weight:	0.88
		Sc. PD:	-0.015
		Sc. rank:	82.1

,

PB2			
Pos.	191	obs:	exp:
act	T	0	0.26
acc	T	0	0.20
aca	T	0	0.46
acg	T	1	0.08
aaa	K	0	41.01
aag	K	70	28.99
gat	D	2	2.16
gac	D	2	1.84
gaa	E	66	495.10
gag	E	757	327.90
---	--	------	------
mPD		0.29	0.63
		nPD:	0.46
		N. weight:	0.79
		Sc. PD:	0.21
		Sc. rank:	400.6

,

PB2			
Pos.	192	obs:	exp:
gat	D	1	0.54
gac	D	0	0.46
gaa	E	59	539.60
gag	E	838	357.40
---	--	------	------
mPD		0.12	0.48
		nPD:	0.26
		N. weight:	0.82
		Sc. PD:	0.055
		Sc. rank:	250.2

,

PB2			
Pos.	193	obs:	exp:
ttt	F	5	1.97
ttc	F	0	3.03
tta	L	0	78.48
ttg	L	1	166.60
ctt	L	817	151.30
ctc	L	69	149.60
cta	L	6	148.90
ctg	L	0	198.00
---	--	------	------
mPD		0.17	1.1
		nPD:	0.15
		N. weight:	1.8
		Sc. PD:	-0.068
		Sc. rank:	13.2

,

PB2			
Pos.	194	obs:	exp:
cat	H	15	8.62
cac	H	0	6.38
caa	Q	131	452.00
cag	Q	749	428.00
cgt	R	0	0.09
cgc	R	0	0.10
cga	R	1	0.20
cgg	R	1	0.17
aaa	K	0	0.59
aag	K	1	0.41
aga	R	0	0.91
agg	R	0	0.53
---	--	------	------
mPD		0.29	0.53
		nPD:	0.54
		N. weight:	0.38
		Sc. PD:	0.13
		Sc. rank:	207.9

,

PB2			
Pos.	195	obs:	exp:
aat	N	0	3.56
aac	N	7	3.44
gat	D	28	477.20
gac	D	856	406.80
gaa	E	5	3.01
gag	E	0	1.99
ggt	G	0	0.27
ggc	G	2	0.25
gga	G	0	0.92
ggg	G	0	0.55
---	--	------	------
mPD		0.091	0.52
		nPD:	0.17
		N. weight:	0.8
		Sc. PD:	-0.013
		Sc. rank:	75.7

,

PB2			
Pos.	196	obs:	exp:
tgt	C	61	368.70
tgc	C	837	529.30
---	--	------	------
mPD		0.13	0.48
		nPD:	0.26
		N. weight:	0.39
		Sc. PD:	0.027
		Sc. rank:	119.9

,

PB2			
Pos.	197	obs:	exp:
cgt	R	0	0.56
cgc	R	0	0.66
cga	R	0	1.30
cgg	R	0	1.08
aaa	K	809	517.80
aag	K	75	366.20
aga	R	11	5.94
agg	R	2	3.47
gaa	E	1	0.60
gag	E	0	0.40
---	--	------	------
mPD		0.19	0.53
		nPD:	0.36
		N. weight:	0.35
		Sc. PD:	0.057
		Sc. rank:	148.5

,

PB2			
Pos.	198	obs:	exp:
att	I	793	327.30
atc	I	102	217.00
ata	I	2	352.70
act	T	1	0.26
acc	T	0	0.20
aca	T	0	0.46
acg	T	0	0.08
---	--	------	------
mPD		0.21	0.66
		nPD:	0.32
		N. weight:	0.87
		Sc. PD:	0.11
		Sc. rank:	330.4

,

PB2			
Pos.	199	obs:	exp:
tct	S	2	0.64
tcc	S	2	0.52
tca	S	0	1.01
tcg	S	0	0.23
act	T	0	1.04
acc	T	4	0.81
aca	T	0	1.84
acg	T	0	0.31
agt	S	0	0.82
agc	S	0	0.78
gct	A	162	228.10
gcc	A	725	167.10
gca	A	3	416.10
gcg	A	0	78.72
---	--	------	------
mPD		0.32	0.69
		nPD:	0.46
		N. weight:	1.4
		Sc. PD:	0.37
		Sc. rank:	722.7

,

PB2			
Pos.	200	obs:	exp:
cct	P	889	228.10
ccc	P	7	159.90
cca	P	1	366.50
ccg	P	0	142.50
cat	H	1	0.57
cac	H	0	0.43
---	--	------	------
mPD		0.020	0.71
		nPD:	0.03
		N. weight:	1.7
		Sc. PD:	-0.26
		Sc. rank:	-712.0

,

PB2			
Pos.	201	obs:	exp:
tta	L	836	78.92
ttg	L	35	167.60
ctt	L	0	152.20
ctc	L	0	150.40
cta	L	2	149.70
ctg	L	25	199.20
---	--	------	------
mPD		0.18	1.1
		nPD:	0.17
		N. weight:	2.6
		Sc. PD:	-0.065
		Sc. rank:	154.3

,

PB2			
Pos.	202	obs:	exp:
att	I	0	0.36
atc	I	0	0.24
ata	I	1	0.39
atg	M	897	897.00
---	--	------	------
mPD		0.0022	0.0022
		nPD:	1.
		N. weight:	0.0013
		Sc. PD:	0.001
		Sc. rank:	0.8

,

PB2			
Pos.	203	obs:	exp:
atg	M	1	1.00
gtt	V	43	185.20
gtc	V	2	179.70
gta	V	9	185.70
gtg	V	843	346.40
---	--	------	------
mPD		0.12	0.73
		nPD:	0.16
		N. weight:	0.92
		Sc. PD:	-0.027
		Sc. rank:	33.5

,

PB2			
Pos.	204	obs:	exp:
gct	A	254	230.10
gcc	A	1	168.60
gca	A	639	419.90
gcg	A	4	79.43
---	--	------	------
mPD		0.41	0.67
		nPD:	0.62
		N. weight:	0.4
		Sc. PD:	0.16
		Sc. rank:	226.2

,

PB2			
Pos.	205	obs:	exp:
tat	Y	25	476.10
tac	Y	873	421.90
---	--	------	------
mPD		0.054	0.50
		nPD:	0.11
		N. weight:	0.79
		Sc. PD:	-0.062
		Sc. rank:	-84.3

,

PB2			
Pos.	206	obs:	exp:
atg	M	898	898.00
---	--	------	------
mPD		0	0
		nPD:	1.
		N. weight:	0.
		Sc. PD:	0
		Sc. rank:	0

PB2			
Pos.	207	obs:	exp:
tta	L	4	78.92
ttg	L	840	167.60
ctt	L	5	152.20
ctc	L	0	150.40
cta	L	40	149.70
ctg	L	9	199.20
---	--	------	------
mPD		0.22	1.1
		nPD:	0.2
		N. weight:	1.8
		Sc. PD:	0.008
		Sc. rank:	283.5

PB2			
Pos.	208	obs:	exp:
gaa	E	72	539.60
gag	E	825	357.40
ggt	G	0	0.13
ggc	G	0	0.13
gga	G	0	0.46
ggg	G	1	0.28
---	--	------	------
mPD		0.15	0.48
		nPD:	0.31
		N. weight:	0.77
		Sc. PD:	0.089
		Sc. rank:	288.9

PB2			
Pos.	209	obs:	exp:
cgt	R	0	38.36
cgc	R	0	45.62
cga	R	0	89.54
cgg	R	0	74.79
aga	R	719	410.00
agg	R	179	239.70
---	--	------	------
mPD		0.32	0.96
		nPD:	0.33
		N. weight:	0.5
		Sc. PD:	0.067
		Sc. rank:	197.4

PB2			
Pos.	210	obs:	exp:
aaa	K	0	0.59
aag	K	1	0.41
gaa	E	822	539.60
gag	E	75	357.40
---	--	------	------
mPD		0.16	0.48
		nPD:	0.33
		N. weight:	0.33
		Sc. PD:	0.043
		Sc. rank:	128.1

PB2			
Pos.	211	obs:	exp:
tta	L	2	78.92
ttg	L	40	167.60
ctt	L	2	152.20
ctc	L	0	150.40
cta	L	2	149.70
ctg	L	852	199.20
---	--	------	------
mPD		0.10	1.1
		nPD:	0.09
		N. weight:	1.6
		Sc. PD:	-0.15
		Sc. rank:	-309.3

PB2			
Pos.	212	obs:	exp:
gtt	V	828	185.40
gtc	V	68	179.90
gta	V	1	185.90
gtg	V	1	346.80
---	--	------	------
mPD		0.14	0.73
		nPD:	0.2
		N. weight:	1.6
		Sc. PD:	0.013
		Sc. rank:	279.0

PB2			
Pos.	213	obs:	exp:
cgt	R	21	38.36
cgc	R	856	45.62
cga	R	20	89.54
cgg	R	0	74.79
aga	R	1	410.00
agg	R	0	239.70
---	--	------	------
mPD		0.093	0.96
		nPD:	0.1
		N. weight:	3.4
		Sc. PD:	-0.32
		Sc. rank:	-584.5

PB2			
Pos.	214	obs:	exp:
cgt	R	0	0.13
cgc	R	0	0.15
cga	R	0	0.30
cgg	R	0	0.25
aaa	K	862	524.30
aag	K	33	370.70
aga	R	3	1.37
agg	R	0	0.80
---	--	------	------
mPD		0.078	0.49
		nPD:	0.16
		N. weight:	0.49
		Sc. PD:	-0.016
		Sc. rank:	12.0

PB2			
Pos.	215	obs:	exp:
act	T	17	232.60
acc	T	35	182.90
aca	T	806	412.60
acg	T	40	69.95
---	--	------	------
mPD		0.19	0.67
		nPD:	0.28
		N. weight:	0.59
		Sc. PD:	0.053
		Sc. rank:	208.7

PB2			
Pos.	216	obs:	exp:
cgt	R	0	38.36
cgc	R	0	45.62
cga	R	1	89.54
cgg	R	1	74.79
aga	R	513	410.00
agg	R	383	239.70
---	--	------	------
mPD		0.49	0.96
		nPD:	0.51
		N. weight:	0.41
		Sc. PD:	0.13
		Sc. rank:	215.5

PB2			
Pos.	217	obs:	exp:
ttt	F	764	354.40
ttc	F	134	543.60
---	--	------	------
mPD		0.25	0.48
		nPD:	0.53
		N. weight:	0.56
		Sc. PD:	0.19
		Sc. rank:	303.6

PB2			
Pos.	218	obs:	exp:
tta	L	19	78.92
ttg	L	3	167.60
ctt	L	3	152.20
ctc	L	43	150.40
cta	L	819	149.70
ctg	L	11	199.20
---	--	------	------
mPD		0.17	1.1
		nPD:	0.16
		N. weight:	1.8
		Sc. PD:	-0.057
		Sc. rank:	48.2

PB2			
Pos.	219	obs:	exp:
cct	P	5	228.30
ccc	P	0	160.10
cca	P	876	366.90
ccg	P	17	142.70
---	--	------	------
mPD		0.048	0.71
		nPD:	0.07
		N. weight:	0.99
		Sc. PD:	-0.12
		Sc. rank:	-269.6

PB2			
Pos.	220	obs:	exp:
att	I	0	0.73
atc	I	0	0.48
ata	I	2	0.79
gtt	V	4	185.00
gtc	V	0	179.50
gta	V	748	185.50
gtg	V	144	346.00
---	--	------	------
mPD		0.28	0.73
		nPD:	0.39
		N. weight:	1.3
		Sc. PD:	0.24
		Sc. rank:	554.6

PB2			
Pos.	221	obs:	exp:
gtt	V	1	0.21
gtc	V	0	0.20
gta	V	0	0.21
gtg	V	0	0.39
gct	A	863	229.80
gcc	A	7	168.40
gca	A	26	419.40
gcg	A	1	79.34
---	--	------	------
mPD		0.076	0.68
		nPD:	0.11
		N. weight:	1.5
		Sc. PD:	-0.11
		Sc. rank:	-128.0

PB2			
Pos.	222	obs:	exp:
ggt	G	48	120.40
ggc	G	44	114.10
gga	G	793	415.20
ggg	G	13	248.30
---	--	------	------
mPD		0.21	0.68
		nPD:	0.32
		N. weight:	0.55
		Sc. PD:	0.068
		Sc. rank:	211.7

PB2			
Pos.	223	obs:	exp:
ggt	G	0	120.40
ggc	G	0	114.10
gga	G	304	415.20
ggg	G	594	248.30
---	--	------	------
mPD		0.45	0.68
		nPD:	0.66
		N. weight:	0.6
		Sc. PD:	0.27
		Sc. rank:	352.3

PB2			
Pos.	224	obs:	exp:
att	I	0	0.36
atc	I	0	0.24
ata	I	1	0.39
act	T	1	232.40
acc	T	1	182.70
aca	T	883	412.10
acg	T	12	69.87
---	--	------	------
mPD		0.033	0.68
		nPD:	0.05
		N. weight:	0.9
		Sc. PD:	-0.12
		Sc. rank:	-307.7

PB2			
Pos.	225	obs:	exp:
tct	S	0	143.70
tcc	S	0	116.20
tca	S	0	226.60
tcg	S	0	52.26
agt	S	1	184.00
agc	S	897	175.10
---	--	------	------
mPD		0.0022	1.7
		nPD:	0.
		N. weight:	2.
		Sc. PD:	-0.37
		Sc. rank:	-1168.5

PB2			
Pos.	226	obs:	exp:
tct	S	0	143.70
tcc	S	0	116.20
tca	S	0	226.60
tcg	S	0	52.26
agt	S	634	184.00
agc	S	264	175.10
---	--	------	------
mPD		0.42	1.7
		nPD:	0.25
		N. weight:	1.3
		Sc. PD:	0.073
		Sc. rank:	362.5

PB2			
Pos.	227	obs:	exp:
att	I	0	0.36
atc	I	0	0.24
ata	I	1	0.39
atg	M	1	1.00
gtt	V	28	185.00
gtc	V	35	179.50
gta	V	76	185.50
gtg	V	757	346.00
---	--	------	------
mPD		0.28	0.73
		nPD:	0.39
		N. weight:	0.59
		Sc. PD:	0.11
		Sc. rank:	259.5

PB2			
Pos.	228	obs:	exp:
tat	Y	681	476.10
tac	Y	217	421.90
---	--	------	------
mPD		0.37	0.50
		nPD:	0.74
		N. weight:	0.14
		Sc. PD:	0.076
		Sc. rank:	87.1

PB2			
Pos.	229	obs:	exp:
att	I	69	321.10
atc	I	809	212.80
ata	I	2	346.10
gtt	V	0	3.72
gtc	V	18	3.61
gta	V	0	3.73
gtg	V	0	6.95
---	--	------	------
mPD		0.19	0.70
		nPD:	0.27
		N. weight:	1.4
		Sc. PD:	0.1
		Sc. rank:	447.8

PB2			
Pos.	230	obs:	exp:
gaa	E	740	540.20
gag	E	158	357.80
---	--	------	------
mPD		0.29	0.48
		nPD:	0.61
		N. weight:	0.15
		Sc. PD:	0.06
		Sc. rank:	85.4

PB2			
Pos.	231	obs:	exp:
gtt	V	1	185.40
gtc	V	3	179.90
gta	V	318	185.90
gtg	V	576	346.80
---	--	------	------
mPD		0.46	0.73
		nPD:	0.64
		N. weight:	0.63
		Sc. PD:	0.27
		Sc. rank:	367.3

PB2			
Pos.	232	obs:	exp:
tta	L	220	78.92
ttg	L	635	167.60
ctt	L	0	152.20
ctc	L	0	150.40
cta	L	1	149.70
ctg	L	42	199.20
---	--	------	------
mPD		0.46	1.1
		nPD:	0.42
		N. weight:	1.4
		Sc. PD:	0.31
		Sc. rank:	657.2

PB2			
Pos.	233	obs:	exp:
cat	H	720	515.90
cac	H	178	382.10
---	--	------	------
mPD		0.32	0.49
		nPD:	0.65
		N. weight:	0.15
		Sc. PD:	0.067
		Sc. rank:	87.4

PB2			
Pos.	234	obs:	exp:
tta	L	19	78.92
ttg	L	870	167.60
ctt	L	0	152.20
ctc	L	0	150.40
cta	L	3	149.70
ctg	L	6	199.20
---	--	------	------
mPD		0.068	1.1
		nPD:	0.06
		N. weight:	1.9
		Sc. PD:	-0.24
		Sc. rank:	-586.5

PB2			
Pos.	235	obs:	exp:
act	T	54	232.60
acc	T	844	182.90
aca	T	0	412.60
acg	T	0	69.95
---	--	------	------
mPD		0.11	0.67
		nPD:	0.17
		N. weight:	1.7
		Sc. PD:	-0.038
		Sc. rank:	131.5

PB2			
Pos.	236	obs :	exp :
caa	Q	855	461.30
cag	Q	43	436.70
---	--	------	------
mPD		0.091	0.50
		nPD :	0.18
		N. weight :	0.6
		Sc. PD :	-0.0047
		Sc. rank :	68.9

PB2			
Pos.	237	obs :	exp :
ggt	G	0	120.40
ggc	G	0	114.10
gga	G	187	415.20
ggg	G	711	248.30
---	--	------	------
mPD		0.33	0.68
		nPD :	0.49
		N. weight :	0.84
		Sc. PD :	0.24
		Sc. rank :	438.7

PB2			
Pos.	238	obs :	exp :
act	T	121	232.60
acc	T	734	182.90
aca	T	41	412.60
acg	T	2	69.95
---	--	------	------
mPD		0.31	0.67
		nPD :	0.46
		N. weight :	1.2
		Sc. PD :	0.31
		Sc. rank :	598.9

PB2			
Pos.	239	obs :	exp :
tgt	C	11	368.70
tgc	C	887	529.30
---	--	------	------
mPD		0.024	0.48
		nPD :	0.05
		N. weight :	0.59
		Sc. PD :	-0.08
		Sc. rank :	-196.2

PB2			
Pos.	240	obs :	exp :
tgg	W	898	898.00
---	--	------	------
mPD		0	0
		nPD :	1.
		N. weight :	0.
		Sc. PD :	0
		Sc. rank :	0

PB2			
Pos.	241	obs :	exp :
aaa	K	0	0.59
aag	K	1	0.41
gaa	E	52	539.60
gag	E	845	357.40
---	--	------	------
mPD		0.11	0.48
		nPD :	0.23
		N. weight :	0.85
		Sc. PD :	0.033
		Sc. rank :	214.1

PB2			
Pos.	242	obs :	exp :
caa	Q	857	461.30
cag	Q	41	436.70
---	--	------	------
mPD		0.087	0.50
		nPD :	0.17
		N. weight :	0.61
		Sc. PD :	-0.0095
		Sc. rank :	60.2

PB2			
Pos.	243	obs :	exp :
att	I	0	1.09
atc	I	0	0.73
ata	I	3	1.18
atg	M	895	895.00
---	--	------	------
mPD		0.0067	0.0067
		nPD :	1.
		N. weight :	0.0039
		Sc. PD :	0.0031
		Sc. rank :	2.4

PB2			
Pos.	244	obs :	exp :
tat	Y	41	476.10
tac	Y	857	421.90
---	--	------	------
mPD		0.087	0.50
		nPD :	0.18
		N. weight :	0.71
		Sc. PD :	-0.011
		Sc. rank :	72.1

PB2			
Pos.	245	obs :	exp :
act	T	52	232.60
acc	T	15	182.90
aca	T	827	412.60
acg	T	4	69.95
---	--	------	------
mPD		0.15	0.67
		nPD :	0.22
		N. weight :	0.64
		Sc. PD :	0.018
		Sc. rank :	142.1

PB2			
Pos.	246	obs :	exp :
cct	P	13	228.30
ccc	P	0	160.10
cca	P	388	366.90
ccg	P	497	142.70
---	--	------	------
mPD		0.51	0.71
		nPD :	0.71
		N. weight :	0.85
		Sc. PD :	0.43
		Sc. rank :	512.7

PB2			
Pos.	247	obs :	exp :
ggt	G	12	120.40
ggc	G	2	114.10
gga	G	882	415.20
ggg	G	2	248.30
---	--	------	------
mPD		0.035	0.68
		nPD :	0.05
		N. weight :	0.87
		Sc. PD :	-0.12
		Sc. rank :	-286.8

PB2			
Pos.	248	obs :	exp :
ggt	G	0	120.40
ggc	G	1	114.10
gga	G	203	415.20
ggg	G	694	248.30
---	--	------	------
mPD		0.35	0.68
		nPD :	0.52
		N. weight :	0.79
		Sc. PD :	0.25
		Sc. rank :	423.1

PB2			
Pos.	249	obs :	exp :
gat	D	0	0.54
gac	D	1	0.46
gaa	E	779	484.80
gag	E	27	321.20
ggt	G	0	12.20
ggc	G	0	11.56
gga	G	88	42.07
ggg	G	3	25.16
---	--	------	------
mPD		0.25	0.69
		nPD :	0.36
		N. weight :	0.51
		Sc. PD :	0.084
		Sc. rank :	215.6

PB2			
Pos.	250	obs :	exp :
gtt	V	0	185.40
gtc	V	0	179.90
gta	V	11	185.90
gtg	V	887	346.80
---	--	------	------
mPD		0.024	0.73
		nPD :	0.03
		N. weight :	1.1
		Sc. PD :	-0.17
		Sc. rank :	-452.0

PB2			
Pos.	251	obs :	exp :
cgt	R	0	38.19
cgc	R	0	45.42
cga	R	0	89.14
cgg	R	0	74.46
aaa	K	4	2.34
aag	K	0	1.66
aga	R	826	408.20
agg	R	68	238.60
---	--	------	------
mPD		0.15	0.97
		nPD :	0.15
		N. weight :	0.7
		Sc. PD :	-0.025
		Sc. rank :	8.0

PB2			
Pos.	252	obs :	exp :
cat	H	1	0.57
cac	H	0	0.43
aat	N	870	456.30
aac	N	27	440.70
---	--	------	------
mPD		0.061	0.50
		nPD :	0.12
		N. weight :	0.68
		Sc. PD :	-0.046
		Sc. rank :	-36.9

PB2			
Pos.	253	obs :	exp :
gat	D	880	484.20
gac	D	17	412.80
ggt	G	1	0.13
ggc	G	0	0.13
gga	G	0	0.46
ggg	G	0	0.28
---	--	------	------
mPD		0.039	0.50
		nPD :	0.08
		N. weight :	0.67
		Sc. PD :	-0.072
		Sc. rank :	-153.7

PB2			
Pos.	254	obs :	exp :
gat	D	892	484.80
gac	D	6	413.20
---	--	------	------
mPD		0.013	0.50
		nPD :	0.03
		N. weight :	0.73
		Sc. PD :	-0.12
		Sc. rank :	-317.9

PB2			
Pos.	255	obs :	exp :
att	I	5	2.19
atc	I	1	1.45
ata	I	0	2.36
gtt	V	821	184.10
gtc	V	45	178.70
gta	V	26	184.70
gtg	V	0	344.50
---	--	------	------
mPD		0.16	0.74
		nPD :	0.22
		N. weight :	1.6
		Sc. PD :	0.047
		Sc. rank :	365.4

PB2			
Pos.	256	obs :	exp :
gat	D	846	484.80
gac	D	52	413.20
---	--	------	------
mPD		0.11	0.50
		nPD :	0.22
		N. weight :	0.51
		Sc. PD :	0.014
		Sc. rank :	113.8

PB2			
Pos.	257	obs :	exp :
caa	Q	23	461.30
cag	Q	875	436.70
---	--	------	------
mPD		0.050	0.50
		nPD :	0.1
		N. weight :	0.76
		Sc. PD :	-0.066
		Sc. rank :	-110.9

PB2			
Pos.	258	obs :	exp :
tct	S	0	143.70
tcc	S	0	116.20
tca	S	0	226.60
tcg	S	0	52.26
agt	S	701	184.00
agc	S	197	175.10
---	--	------	------
mPD		0.34	1.7
		nPD :	0.21
		N. weight :	1.3
		Sc. PD :	0.021
		Sc. rank :	253.0

PB2			
Pos.	259	obs :	exp :
tta	L	802	78.92
ttg	L	81	167.60
ctt	L	0	152.20
ctc	L	0	150.40
cta	L	9	149.70
ctg	L	6	199.20
---	--	------	------
mPD		0.21	1.1
		nPD :	0.19
		N. weight :	2.5
		Sc. PD :	-0.0078
		Sc. rank :	329.2

PB2			
Pos.	260	obs :	exp :
att	I	817	325.80
atc	I	76	216.00
ata	I	0	351.20
gtt	V	5	1.03
gtc	V	0	1.00
gta	V	0	1.04
gtg	V	0	1.93
---	--	------	------
mPD		0.17	0.67
		nPD :	0.25
		N. weight :	0.95
		Sc. PD :	0.054
		Sc. rank :	272.7

PB2			
Pos.	261	obs :	exp :
att	I	862	327.30
atc	I	35	217.00
ata	I	0	352.70
act	T	1	0.26
acc	T	0	0.20
aca	T	0	0.46
acg	T	0	0.08
---	--	------	------
mPD		0.077	0.66
		nPD :	0.12
		N. weight :	1.1
		Sc. PD :	-0.076
		Sc. rank :	-75.2

PB2			
Pos.	262	obs :	exp :
gct	A	894	230.10
gcc	A	1	168.60
gca	A	2	419.90
gcg	A	1	79.43
---	--	------	------
mPD		0.0089	0.67
		nPD :	0.01
		N. weight :	1.7
		Sc. PD :	-0.29
		Sc. rank :	-832.0

PB2			
Pos.	263	obs :	exp :
gct	A	776	230.10
gcc	A	64	168.60
gca	A	56	419.90
gcg	A	2	79.43
---	--	------	------
mPD		0.24	0.67
		nPD :	0.36
		N. weight :	1.1
		Sc. PD :	0.18
		Sc. rank :	454.6

PB2			
Pos.	264	obs :	exp :
cgt	R	0	38.36
cgc	R	0	45.62
cga	R	2	89.54
cgg	R	1	74.79
aga	R	850	410.00
agg	R	45	239.70
---	--	------	------
mPD		0.10	0.96
		nPD :	0.11
		N. weight :	0.75
		Sc. PD :	-0.06
		Sc. rank :	-85.4

PB2			
Pos.	265	obs :	exp :
tct	S	0	0.32
tcc	S	0	0.26
tca	S	0	0.50
tcg	S	0	0.12
aat	N	826	455.80
aac	N	70	440.20
agt	S	2	0.41
agc	S	0	0.39
---	--	------	------
mPD		0.15	0.51
		nPD :	0.29
		N. weight :	0.52
		Sc. PD :	0.05
		Sc. rank :	189.3

PB2			
Pos.	266	obs:	exp:
att	I	849	327.30
atc	I	7	217.00
ata	I	41	352.70
gtt	V	0	0.21
gtc	V	0	0.20
gta	V	1	0.21
gtg	V	0	0.39
---	--	------	------
mPD		0.11	0.66
		nPD:	0.16
		N. weight:	0.98
		Sc. PD:	-0.027
		Sc. rank:	41.4

PB2			
Pos.	267	obs:	exp:
gtt	V	845	185.40
gtc	V	9	179.90
gta	V	44	185.90
gtg	V	0	346.80
---	--	------	------
mPD		0.11	0.73
		nPD:	0.15
		N. weight:	1.7
		Sc. PD:	-0.058
		Sc. rank:	30.1

PB2			
Pos.	268	obs:	exp:
cgt	R	0	38.36
cgc	R	0	45.62
cga	R	0	89.54
cgg	R	0	74.79
aga	R	848	410.00
agg	R	50	239.70
---	--	------	------
mPD		0.11	0.96
		nPD:	0.11
		N. weight:	0.76
		Sc. PD:	-0.059
		Sc. rank:	-73.1

PB2			
Pos.	269	obs:	exp:
cgt	R	0	38.36
cgc	R	0	45.62
cga	R	0	89.54
cgg	R	0	74.79
aga	R	573	410.00
agg	R	325	239.70
---	--	------	------
mPD		0.46	0.96
		nPD:	0.48
		N. weight:	0.41
		Sc. PD:	0.11
		Sc. rank:	212.2

PB2			
Pos.	270	obs:	exp:
gct	A	0	230.10
gcc	A	3	168.60
gca	A	894	419.90
gcg	A	1	79.43
---	--	------	------
mPD		0.0089	0.67
		nPD:	0.01
		N. weight:	0.93
		Sc. PD:	-0.16
		Sc. rank:	-462.7

PB2			
Pos.	271	obs:	exp:
act	T	143	232.40
acc	T	0	182.70
aca	T	722	412.10
acg	T	32	69.87
gct	A	0	0.26
gcc	A	0	0.19
gca	A	1	0.47
gcg	A	0	0.09
---	--	------	------
mPD		0.33	0.68
		nPD:	0.48
		N. weight:	0.44
		Sc. PD:	0.13
		Sc. rank:	229.2

PB2			
Pos.	272	obs:	exp:
att	I	0	0.36
atc	I	0	0.24
ata	I	1	0.39
gtt	V	0	185.20
gtc	V	1	179.70
gta	V	727	185.70
gtg	V	169	346.40
---	--	------	------
mPD		0.31	0.73
		nPD:	0.43
		N. weight:	1.2
		Sc. PD:	0.28
		Sc. rank:	572.1

PB2			
Pos.	273	obs:	exp:
tct	S	3	143.70
tcc	S	3	116.20
tca	S	881	226.60
tcg	S	11	52.26
agt	S	0	184.00
agc	S	0	175.10
---	--	------	------
mPD		0.037	1.7
		nPD:	0.02
		N. weight:	1.6
		Sc. PD:	-0.26
		Sc. rank:	-739.8

PB2			
Pos.	274	obs:	exp:
act	T	0	0.26
acc	T	0	0.20
aca	T	1	0.46
acg	T	0	0.08
gct	A	2	229.80
gcc	A	0	168.40
gca	A	883	419.40
gcg	A	12	79.34
---	--	------	------
mPD		0.033	0.67
		nPD:	0.05
		N. weight:	0.88
		Sc. PD:	-0.12
		Sc. rank:	-298.4

PB2			
Pos.	275	obs:	exp:
gat	D	49	484.20
gac	D	848	412.80
gaa	E	1	0.60
gag	E	0	0.40
---	--	------	------
mPD		0.11	0.50
		nPD:	0.21
		N. weight:	0.7
		Sc. PD:	0.014
		Sc. rank:	139.0

PB2			
Pos.	276	obs:	exp:
cct	P	1	228.30
ccc	P	1	160.10
cca	P	97	366.90
ccg	P	799	142.70
---	--	------	------
mPD		0.20	0.71
		nPD:	0.28
		N. weight:	1.7
		Sc. PD:	0.14
		Sc. rank:	590.3

PB2			
Pos.	277	obs:	exp:
tta	L	5	78.83
ttg	L	811	167.40
ctt	L	0	152.00
ctc	L	0	150.30
cta	L	37	149.50
ctg	L	44	198.90
cct	P	0	0.25
ccc	P	0	0.18
cca	P	0	0.41
ccg	P	1	0.16
---	--	------	------
mPD		0.26	1.1
		nPD:	0.23
		N. weight:	1.6
		Sc. PD:	0.064
		Sc. rank:	412.0

PB2			
Pos.	278	obs:	exp:
act	T	1	0.26
acc	T	0	0.20
aca	T	0	0.46
acg	T	0	0.08
gct	A	831	229.80
gcc	A	1	168.40
gca	A	64	419.40
gcg	A	1	79.34
---	--	------	------
mPD		0.14	0.67
		nPD:	0.21
		N. weight:	1.3
		Sc. PD:	0.019
		Sc. rank:	240.3

PB2			
Pos.	279	obs:	exp:
tct	S	40	143.60
tcc	S	2	116.10
tca	S	41	226.40
tcg	S	814	52.20
agt	S	0	183.80
agc	S	0	174.90
gct	A	0	0.26
gcc	A	0	0.19
gca	A	1	0.47
gcg	A	0	0.09
---	--	------	------
mPD		0.18	1.7
		nPD:	0.11
		N. weight:	3.
		Sc. PD:	-0.24
		Sc. rank:	-347.2

PB2			
Pos.	280	obs:	exp:
tta	L	2	78.92
ttg	L	0	167.60
ctt	L	807	152.20
ctc	L	24	150.40
cta	L	64	149.70
ctg	L	1	199.20
---	--	------	------
mPD		0.19	1.1
		nPD:	0.17
		N. weight:	1.7
		Sc. PD:	-0.031
		Sc. rank:	149.1

PB2			
Pos.	281	obs:	exp:
tta	L	163	78.92
ttg	L	709	167.60
ctt	L	0	152.20
ctc	L	0	150.40
cta	L	1	149.70
ctg	L	25	199.20
---	--	------	------
mPD		0.36	1.1
		nPD:	0.32
		N. weight:	1.5
		Sc. PD:	0.19
		Sc. rank:	586.3

PB2			
Pos.	282	obs:	exp:
gat	D	10	5.40
gac	D	0	4.60
gaa	E	85	534.20
gag	E	803	353.80
---	--	------	------
mPD		0.19	0.49
		nPD:	0.39
		N. weight:	0.71
		Sc. PD:	0.14
		Sc. rank:	315.3

PB2			
Pos.	283	obs:	exp:
att	I	0	0.73
atc	I	0	0.48
ata	I	2	0.79
atg	M	896	896.00
---	--	------	------
mPD		0.0044	0.0044
		nPD:	1.
		N. weight:	0.0026
		Sc. PD:	0.002
		Sc. rank:	1.6

PB2			
Pos.	284	obs:	exp:
tgt	C	32	368.70
tgc	C	866	529.30
---	--	------	------
mPD		0.069	0.48
		nPD:	0.14
		N. weight:	0.49
		Sc. PD:	-0.023
		Sc. rank:	-7.1

PB2			
Pos.	285	obs:	exp:
tat	Y	4	2.12
tac	Y	0	1.88
cat	H	828	513.60
cac	H	66	380.40
---	--	------	------
mPD		0.15	0.50
		nPD:	0.29
		N. weight:	0.4
		Sc. PD:	0.039
		Sc. rank:	146.3

PB2			
Pos.	286	obs:	exp:
tct	S	0	140.50
tcc	S	0	113.60
tca	S	0	221.60
tcg	S	0	51.09
aat	N	1	0.51
aac	N	0	0.49
agt	S	805	179.90
agc	S	73	171.20
ggt	G	19	2.55
ggc	G	0	2.41
gga	G	0	8.78
ggg	G	0	5.25
---	--	------	------
mPD		0.19	1.7
		nPD:	0.11
		N. weight:	1.7
		Sc. PD:	-0.12
		Sc. rank:	-133.6

PB2			
Pos.	287	obs:	exp:
act	T	0	232.60
acc	T	1	182.90
aca	T	889	412.60
acg	T	8	69.95
---	--	------	------
mPD		0.020	0.67
		nPD:	0.03
		N. weight:	0.93
		Sc. PD:	-0.14
		Sc. rank:	-388.3

PB2			
Pos.	288	obs:	exp:
tta	L	0	0.18
ttg	L	0	0.37
ctt	L	0	0.34
ctc	L	0	0.34
cta	L	0	0.33
ctg	L	2	0.44
caa	Q	69	459.20
cag	Q	825	434.80
cgt	R	0	0.09
cgc	R	0	0.10
cga	R	2	0.20
cgg	R	0	0.17
aga	R	0	0.91
agg	R	0	0.53
---	--	------	------
mPD		0.15	0.51
		nPD:	0.3
		N. weight:	0.57
		Sc. PD:	0.061
		Sc. rank:	211.9

PB2			
Pos.	289	obs:	exp:
att	I	891	327.70
atc	I	5	217.20
ata	I	2	353.10
---	--	------	------
mPD		0.016	0.65
		nPD:	0.02
		N. weight:	1.2
		Sc. PD:	-0.2
		Sc. rank:	-536.4

PB2			
Pos.	290	obs:	exp:
ggt	G	30	120.40
ggc	G	449	114.10
gga	G	393	415.20
ggg	G	26	248.30
---	--	------	------
mPD		0.56	0.68
		nPD:	0.82
		N. weight:	0.7
		Sc. PD:	0.43
		Sc. rank:	430.4

PB2			
Pos.	291	obs:	exp:
ggt	G	0	120.40
ggc	G	0	114.10
gga	G	54	415.20
ggg	G	844	248.30
---	--	------	------
mPD		0.11	0.68
		nPD:	0.17
		N. weight:	1.3
		Sc. PD:	-0.029
		Sc. rank:	93.4

PB2			
Pos.	292	obs:	exp:
att	I	123	76.26
atc	I	0	50.55
ata	I	86	82.19
act	T	0	0.26
acc	T	0	0.20
aca	T	1	0.46
acg	T	0	0.08
gtt	V	681	142.00
gtc	V	2	137.80
gta	V	5	142.40
gtg	V	0	265.70
---	--	------	------
mPD		0.55	1.1
		nPD:	0.5
		N. weight:	1.6
		Sc. PD:	0.46
		Sc. rank:	814.2

PB2			
Pos.	293	obs:	exp:
tgg	W	1	1.00
cgt	R	0	38.28
cgc	R	0	45.52
cga	R	0	89.34
cgg	R	0	74.63
aaa	K	0	0.59
aag	K	1	0.41
aga	R	31	409.10
agg	R	865	239.10
---	--	------	------
mPD		0.071	0.97
		nPD:	0.07
		N. weight:	1.4
		Sc. PD:	-0.16
		Sc. rank:	-363.9

PB2			
Pos.	294	obs:	exp:
atg	M	898	898.00
---	--	------	------
mPD		0	0
		nPD:	1.
		N. weight:	0.
		Sc. PD:	0
		Sc. rank:	0

PB2			
Pos.	295	obs:	exp:
gtt	V	805	185.40
gtc	V	2	179.90
gta	V	56	185.90
gtg	V	35	346.80
---	--	------	------
mPD		0.19	0.73
		nPD:	0.26
		N. weight:	1.4
		Sc. PD:	0.1
		Sc. rank:	456.9

PB2			
Pos.	296	obs:	exp:
gat	D	5	484.80
gac	D	893	413.20
---	--	------	------
mPD		0.011	0.50
		nPD:	0.02
		N. weight:	0.93
		Sc. PD:	-0.15
		Sc. rank:	-432.6

PB2			
Pos.	297	obs:	exp:
att	I	107	327.30
atc	I	787	217.00
ata	I	3	352.70
gtt	V	0	0.21
gtc	V	1	0.20
gta	V	0	0.21
gtg	V	0	0.39
---	--	------	------
mPD		0.22	0.66
		nPD:	0.33
		N. weight:	1.2
		Sc. PD:	0.17
		Sc. rank:	495.4

PB2			
Pos.	298	obs:	exp:
tta	L	0	78.83
ttg	L	0	167.40
ctt	L	888	152.00
ctc	L	9	150.30
cta	L	0	149.50
ctg	L	0	198.90
att	I	1	0.36
atc	I	0	0.24
ata	I	0	0.39
---	--	------	------
mPD		0.022	1.1
		nPD:	0.02
		N. weight:	2.2
		Sc. PD:	-0.36
		Sc. rank:	-1011.9

PB2			
Pos.	299	obs:	exp:
cgt	R	0	38.11
cgc	R	0	45.32
cga	R	1	88.94
cgg	R	33	74.29
aaa	K	6	3.51
aag	K	0	2.49
aga	R	820	407.30
agg	R	38	238.10
---	--	------	------
mPD		0.23	0.97
		nPD:	0.24
		N. weight:	0.67
		Sc. PD:	0.031
		Sc. rank:	179.9

PB2			
Pos.	300	obs:	exp:
caa	Q	858	461.30
cag	Q	40	436.70
---	--	------	------
mPD		0.085	0.50
		nPD:	0.17
		N. weight:	0.61
		Sc. PD:	-0.012
		Sc. rank:	51.0

PB2			
Pos.	301	obs:	exp:
aat	N	125	456.90
aac	N	773	441.10
---	--	------	------
mPD		0.24	0.50
		nPD:	0.48
		N. weight:	0.38
		Sc. PD:	0.11
		Sc. rank:	198.3

PB2			
Pos.	302	obs:	exp:
cct	P	2	228.30
ccc	P	31	160.10
cca	P	863	366.90
ccg	P	2	142.70
---	--	------	------
mPD		0.075	0.71
		nPD:	0.11
		N. weight:	0.94
		Sc. PD:	-0.077
		Sc. rank:	-120.6

PB2			
Pos.	303	obs:	exp:
att	I	0	1.09
atc	I	0	0.73
ata	I	3	1.18
act	T	13	231.80
acc	T	10	182.30
aca	T	866	411.20
acg	T	6	69.72
---	--	------	------
mPD		0.070	0.68
		nPD:	0.1
		N. weight:	0.8
		Sc. PD:	-0.068
		Sc. rank:	-110.7

PB2			
Pos.	304	obs:	exp:
gtt	V	0	0.21
gtc	V	0	0.20
gta	V	1	0.21
gtg	V	0	0.39
gaa	E	483	539.60
gag	E	414	357.40
---	--	------	------
mPD		0.50	0.48
		nPD:	1.04
		N. weight:	0.018
		Sc. PD:	0.014
		Sc. rank:	10.8

PB2			
Pos.	305	obs:	exp:
gaa	E	868	540.20
gag	E	30	357.80
---	--	------	------
mPD		0.065	0.48
		nPD:	0.13
		N. weight:	0.48
		Sc. PD:	-0.026
		Sc. rank:	-11.2

PB2			
Pos.	306	obs:	exp:
caa	Q	76	461.30
cag	Q	822	436.70
---	--	------	------
mPD		0.16	0.50
		nPD:	0.31
		N. weight:	0.54
		Sc. PD:	0.062
		Sc. rank:	202.2

PB2			
Pos.	307	obs:	exp:
gct	A	857	230.10
gcc	A	40	168.60
gca	A	0	419.90
gcg	A	1	79.43
---	--	------	------
mPD		0.087	0.67
		nPD:	0.13
		N. weight:	1.5
		Sc. PD:	-0.088
		Sc. rank:	-54.5

PB2			
Pos.	308	obs:	exp:
gtt	V	7	185.40
gtc	V	0	179.90
gta	V	6	185.90
gtg	V	885	346.80
---	--	------	------
mPD		0.029	0.73
		nPD:	0.04
		N. weight:	1.1
		Sc. PD:	-0.16
		Sc. rank:	-406.6

PB2			
Pos.	309	obs:	exp:
aat	N	2	1.02
aac	N	0	0.98
gat	D	847	482.60
gac	D	47	411.40
ggt	G	2	0.27
ggc	G	0	0.25
gga	G	0	0.92
ggg	G	0	0.55
---	--	------	------
mPD		0.11	0.51
		nPD:	0.21
		N. weight:	0.54
		Sc. PD:	0.012
		Sc. rank:	109.2

PB2			
Pos.	310	obs:	exp:
att	I	0	327.70
atc	I	0	217.20
ata	I	898	353.10
---	--	------	------
mPD		0	0.65
		nPD:	0.
		N. weight:	1.2
		Sc. PD:	-0.22
		Sc. rank:	-723.1

PB2			
Pos.	311	obs:	exp:
tgt	C	239	368.70
tgc	C	659	529.30
---	--	------	------
mPD		0.39	0.48
		nPD:	0.81
		N. weight:	0.062
		Sc. PD:	0.037
		Sc. rank:	37.9

PB2			
Pos.	312	obs:	exp:
cgt	R	0	0.13
cgc	R	0	0.15
cga	R	0	0.30
cgg	R	0	0.25
aaa	K	405	524.30
aag	K	490	370.70
aga	R	1	1.37
agg	R	2	0.80
---	--	------	------
mPD		0.50	0.49
		nPD:	1.02
		N. weight:	0.053
		Sc. PD:	0.042
		Sc. rank:	33.0

PB2			
Pos.	313	obs:	exp:
gct	A	4	230.10
gcc	A	0	168.60
gca	A	894	419.90
gcg	A	0	79.43
---	--	------	------
mPD		0.0089	0.67
		nPD:	0.01
		N. weight:	0.92
		Sc. PD:	-0.16
		Sc. rank:	-463.7

PB2			
Pos.	314	obs:	exp:
gct	A	1	230.10
gcc	A	0	168.60
gca	A	871	419.90
gcg	A	26	79.43
---	--	------	------
mPD		0.058	0.67
		nPD:	0.09
		N. weight:	0.85
		Sc. PD:	-0.085
		Sc. rank:	-181.2

PB2			
Pos.	315	obs:	exp:
tta	L	0	0.09
ttg	L	0	0.19
ctt	L	0	0.17
ctc	L	0	0.17
cta	L	0	0.17
ctg	L	1	0.22
att	I	0	0.36
atc	I	0	0.24
ata	I	1	0.39
atg	M	896	896.00
---	--	------	------
mPD		0.0045	0.0058
		nPD:	0.77
		N. weight:	0.0034
		Sc. PD:	0.0019
		Sc. rank:	2.1

PB2			
Pos.	316	obs:	exp:
ggt	G	679	120.40
ggc	G	213	114.10
gga	G	6	415.20
ggg	G	0	248.30
---	--	------	------
mPD		0.37	0.68
		nPD:	0.55
		N. weight:	1.8
		Sc. PD:	0.63
		Sc. rank:	996.4

PB2			
Pos.	317	obs:	exp:
ttt	F	0	0.39
ttc	F	1	0.61
tta	L	59	78.83
ttg	L	61	167.40
ctt	L	0	152.00
ctc	L	209	150.30
cta	L	556	149.50
ctg	L	12	198.90
---	--	------	------
mPD		0.70	1.1
		nPD:	0.63
		N. weight:	0.98
		Sc. PD:	0.42
		Sc. rank:	566.9

PB2			
Pos.	318	obs:	exp:
cgt	R	0	37.55
cgc	R	0	44.66
cga	R	0	87.65
cgg	R	0	73.21
aaa	K	0	11.13
aag	K	19	7.87
aga	R	42	401.30
agg	R	837	234.60
---	--	------	------
mPD		0.13	1.0
		nPD:	0.13
		N. weight:	1.4
		Sc. PD:	-0.08
		Sc. rank:	-45.6

PB2			
Pos.	319	obs:	exp:
att	I	111	326.20
atc	I	752	216.20
ata	I	31	351.60
gtt	V	2	0.83
gtc	V	2	0.80
gta	V	0	0.83
gtg	V	0	1.54
---	--	------	------
mPD		0.29	0.66
		nPD:	0.43
		N. weight:	1.1
		Sc. PD:	0.25
		Sc. rank:	503.0

PB2			
Pos.	320	obs:	exp:
tct	S	0	143.40
tcc	S	0	116.00
tca	S	0	226.10
tcg	S	0	52.14
aat	N	1	1.02
aac	N	1	0.98
agt	S	855	183.60
agc	S	41	174.70
---	--	------	------
mPD		0.094	1.7
		nPD:	0.06
		N. weight:	1.8
		Sc. PD:	-0.23
		Sc. rank:	-558.0

PB2			
Pos.	321	obs:	exp:
tct	S	2	143.70
tcc	S	1	116.20
tca	S	887	226.60
tcg	S	8	52.26
agt	S	0	184.00
agc	S	0	175.10
---	--	------	------
mPD		0.024	1.7
		nPD:	0.01
		N. weight:	1.7
		Sc. PD:	-0.28
		Sc. rank:	-811.1

PB2			
Pos.	322	obs:	exp:
tct	S	47	143.60
tcc	S	849	116.10
tca	S	1	226.40
tcg	S	0	52.20
cct	P	0	0.25
ccc	P	1	0.18
cca	P	0	0.41
ccg	P	0	0.16
agt	S	0	183.80
agc	S	0	174.90
---	--	------	------
mPD		0.10	1.7
		nPD:	0.06
		N. weight:	2.3
		Sc. PD:	-0.28
		Sc. rank:	-659.0

PB2			
Pos.	323	obs:	exp:
ttt	F	29	354.40
ttc	F	869	543.60
---	--	------	------
mPD		0.063	0.48
		nPD:	0.13
		N. weight:	0.47
		Sc. PD:	-0.027
		Sc. rank:	-15.2

PB2			
Pos.	324	obs:	exp:
tct	S	0	143.60
tcc	S	0	116.10
tca	S	0	226.40
tcg	S	0	52.20
cgt	R	0	0.04
cgc	R	0	0.05
cga	R	0	0.10
cgg	R	0	0.08
agt	S	25	183.80
agc	S	872	174.90
aga	R	1	0.46
agg	R	0	0.27
---	--	------	------
mPD		0.056	1.7
		nPD:	0.03
		N. weight:	1.9
		Sc. PD:	-0.29
		Sc. rank:	-750.3

PB2			
Pos.	325	obs:	exp:
ttt	F	884	354.40
ttc	F	14	543.60
---	--	------	------
mPD		0.031	0.48
		nPD:	0.06
		N. weight:	1.1
		Sc. PD:	-0.13
		Sc. rank:	-299.8

PB2			
Pos.	326	obs:	exp:
ggt	G	35	120.40
ggc	G	8	114.10
gga	G	849	415.20
ggg	G	6	248.30
---	--	------	------
mPD		0.10	0.68
		nPD:	0.15
		N. weight:	0.74
		Sc. PD:	-0.026
		Sc. rank:	14.2

PB2			
Pos.	327	obs:	exp:
ggt	G	829	120.40
ggc	G	26	114.10
gga	G	42	415.20
ggg	G	1	248.30
---	--	------	------
mPD		0.14	0.68
		nPD:	0.21
		N. weight:	2.
		Sc. PD:	0.047
		Sc. rank:	425.1

PB2			
Pos.	328	obs:	exp:
ttt	F	2	354.40
ttc	F	896	543.60
---	--	------	------
mPD		0.0044	0.48
		nPD:	0.01
		N. weight:	0.61
		Sc. PD:	-0.11
		Sc. rank:	-315.6

PB2			
Pos.	329	obs:	exp:
tct	S	0	0.16
tcc	S	0	0.13
tca	S	0	0.25
tcg	S	0	0.06
att	I	0	0.36
atc	I	1	0.24
ata	I	0	0.39
act	T	884	231.80
acc	T	8	182.30
aca	T	2	411.20
acg	T	1	69.72
aat	N	1	0.51
aac	N	0	0.49
agt	S	1	0.20
agc	S	0	0.20
---	--	------	------
mPD		0.033	0.68
		nPD:	0.05
		N. weight:	1.6
		Sc. PD:	-0.22
		Sc. rank:	-557.5

PB2			
Pos.	330	obs:	exp:
ttt	F	384	354.40
ttc	F	514	543.60
---	--	------	------
mPD		0.49	0.48
		nPD:	1.03
		N. weight:	0.0079
		Sc. PD:	0.0064
		Sc. rank:	4.9

PB2			
Pos.	331	obs:	exp:
aaa	K	744	526.00
aag	K	154	372.00
---	--	------	------
mPD		0.28	0.49
		nPD:	0.59
		N. weight:	0.18
		Sc. PD:	0.067
		Sc. rank:	99.3

PB2			
Pos.	332	obs:	exp:
cgt	R	0	38.36
cgc	R	0	45.62
cga	R	0	89.54
cgg	R	0	74.79
aga	R	62	410.00
agg	R	836	239.70
---	--	------	------
mPD		0.13	0.96
		nPD:	0.13
		N. weight:	1.3
		Sc. PD:	-0.072
		Sc. rank:	-35.0

PB2			
Pos.	333	obs:	exp:
act	T	0	232.60
acc	T	0	182.90
aca	T	893	412.60
acg	T	5	69.95
---	--	------	------
mPD		0.011	0.67
		nPD:	0.02
		N. weight:	0.95
		Sc. PD:	-0.16
		Sc. rank:	-460.7

PB2			
Pos.	334	obs:	exp:
tct	S	0	143.70
tcc	S	0	116.20
tca	S	0	226.60
tcg	S	0	52.26
agt	S	881	184.00
agc	S	17	175.10
---	--	------	------
mPD		0.037	1.7
		nPD:	0.02
		N. weight:	1.9
		Sc. PD:	-0.3
		Sc. rank:	-864.9

PB2			
Pos.	335	obs:	exp:
ggt	G	0	120.40
ggc	G	1	114.10
gga	G	56	415.20
ggg	G	841	248.30
---	--	------	------
mPD		0.12	0.68
		nPD:	0.18
		N. weight:	1.3
		Sc. PD:	-0.018
		Sc. rank:	131.8

PB2			
Pos.	336	obs:	exp:
tct	S	2	143.10
tcc	S	0	115.70
tca	S	857	225.60
tcg	S	35	52.03
act	T	0	1.04
acc	T	0	0.81
aca	T	4	1.84
acg	T	0	0.31
agt	S	0	183.20
agc	S	0	174.30
---	--	------	------
mPD		0.088	1.7
		nPD:	0.05
		N. weight:	1.6
		Sc. PD:	-0.21
		Sc. rank:	-510.6

PB2			
Pos.	337	obs:	exp:
tct	S	735	143.70
tcc	S	120	116.20
tca	S	43	226.60
tcg	S	0	52.26
agt	S	0	184.00
agc	S	0	175.10
---	--	------	------
mPD		0.31	1.7
		nPD:	0.19
		N. weight:	1.6
		Sc. PD:	-0.0049
		Sc. rank:	216.8

PB2			
Pos.	338	obs:	exp:
att	I	18	43.78
atc	I	97	29.02
ata	I	5	47.19
gtt	V	14	160.40
gtc	V	753	155.70
gta	V	10	160.90
gtg	V	0	300.10
gat	D	0	0.54
gac	D	1	0.46
---	--	------	------
mPD		0.33	0.97
		nPD:	0.34
		N. weight:	1.7
		Sc. PD:	0.25
		Sc. rank:	697.1

PB2			
Pos.	339	obs:	exp:
cgt	R	0	0.09
cgc	R	0	0.10
cga	R	0	0.20
cgg	R	0	0.17
aat	N	0	0.51
aac	N	1	0.49
aaa	K	852	521.90
aag	K	39	369.10
aga	R	2	0.91
agg	R	0	0.53
gaa	E	4	2.41
gag	E	0	1.59
---	--	------	------
mPD		0.099	0.50
		nPD:	0.2
		N. weight:	0.47
		Sc. PD:	0.0028
		Sc. rank:	78.2

PB2			
Pos.	340	obs:	exp:
cgt	R	0	30.20
cgc	R	0	35.92
cga	R	0	70.50
cgg	R	0	58.89
aaa	K	10	111.90
aag	K	181	79.11
aga	R	51	322.80
agg	R	656	188.70
---	--	------	------
mPD		0.46	1.2
		nPD:	0.38
		N. weight:	1.2
		Sc. PD:	0.22
		Sc. rank:	517.6

PB2			
Pos.	341	obs:	exp:
aaa	K	1	0.59
aag	K	0	0.41
gaa	E	881	539.60
gag	E	16	357.40
---	--	------	------
mPD		0.037	0.48
		nPD:	0.08
		N. weight:	0.54
		Sc. PD:	-0.059
		Sc. rank:	-129.5

,

PB2			
Pos.	342	obs:	exp:
gaa	E	730	540.20
gag	E	168	357.80
---	--	------	------
mPD		0.30	0.48
		nPD:	0.64
		N. weight:	0.13
		Sc. PD:	0.058
		Sc. rank:	78.1

,

PB2			
Pos.	343	obs:	exp:
aaa	K	1	0.59
aag	K	0	0.41
gaa	E	647	539.60
gag	E	250	357.40
---	--	------	------
mPD		0.40	0.48
		nPD:	0.84
		N. weight:	0.045
		Sc. PD:	0.028
		Sc. rank:	27.6

,

PB2			
Pos.	344	obs:	exp:
atg	M	7	7.00
gtt	V	0	183.90
gtc	V	1	178.50
gta	V	2	184.50
gtg	V	888	344.10
---	--	------	------
mPD		0.022	0.74
		nPD:	0.03
		N. weight:	1.2
		Sc. PD:	-0.18
		Sc. rank:	-483.9

,

PB2			
Pos.	345	obs:	exp:
ttt	F	1	0.39
ttc	F	0	0.61
tta	L	0	78.75
ttg	L	0	167.20
ctt	L	69	151.80
ctc	L	820	150.10
cta	L	7	149.40
ctg	L	0	198.70
cct	P	0	0.25
ccc	P	1	0.18
cca	P	0	0.41
ccg	P	0	0.16
---	--	------	------
mPD		0.16	1.1
		nPD:	0.15
		N. weight:	1.9
		Sc. PD:	-0.08
		Sc. rank:	-10.5

,

PB2			
Pos.	346	obs:	exp:
act	T	0	232.60
acc	T	7	182.90
aca	T	853	412.60
acg	T	38	69.95
---	--	------	------
mPD		0.096	0.67
		nPD:	0.14
		N. weight:	0.81
		Sc. PD:	-0.038
		Sc. rank:	-10.7

,

PB2			
Pos.	347	obs:	exp:
ggt	G	151	120.40
ggc	G	738	114.10
gga	G	9	415.20
ggg	G	0	248.30
---	--	------	------
mPD		0.30	0.68
		nPD:	0.44
		N. weight:	1.9
		Sc. PD:	0.46
		Sc. rank:	930.2

,

PB2			
Pos.	348	obs:	exp:
aat	N	64	456.90
aac	N	834	441.10
---	--	------	------
mPD		0.13	0.50
		nPD:	0.27
		N. weight:	0.57
		Sc. PD:	0.041
		Sc. rank:	181.2

,

PB2			
Pos.	349	obs:	exp:
tta	L	0	78.92
ttg	L	0	167.60
ctt	L	83	152.20
ctc	L	814	150.40
cta	L	1	149.70
ctg	L	0	199.20
---	--	------	------
mPD		0.17	1.1
		nPD:	0.15
		N. weight:	1.9
		Sc. PD:	-0.067
		Sc. rank:	18.9

,

PB2			
Pos.	350	obs:	exp:
caa	Q	889	461.30
cag	Q	9	436.70
---	--	------	------
mPD		0.020	0.50
		nPD:	0.04
		N. weight:	0.77
		Sc. PD:	-0.11
		Sc. rank:	-282.6

,

PB2			
Pos.	351	obs:	exp:
act	T	1	232.60
acc	T	0	182.90
aca	T	893	412.60
acg	T	4	69.95
---	--	------	------
mPD		0.011	0.67
		nPD:	0.02
		N. weight:	0.94
		Sc. PD:	-0.16
		Sc. rank:	-457.3

,

PB2			
Pos.	352	obs:	exp:
tta	L	84	78.92
ttg	L	798	167.60
ctt	L	0	152.20
ctc	L	0	150.40
cta	L	0	149.70
ctg	L	16	199.20
---	--	------	------
mPD		0.20	1.1
		nPD:	0.18
		N. weight:	1.7
		Sc. PD:	-0.01
		Sc. rank:	208.2

,

PB2			
Pos.	353	obs:	exp:
cgt	R	0	0.09
cgc	R	0	0.10
cga	R	0	0.20
cgg	R	0	0.17
aaa	K	894	524.90
aag	K	2	371.10
aga	R	2	0.91
agg	R	0	0.53
---	--	------	------
mPD		0.0089	0.49
		nPD:	0.02
		N. weight:	0.66
		Sc. PD:	-0.11
		Sc. rank:	-312.6

,

PB2			
Pos.	354	obs:	exp:
tta	L	2	0.18
ttg	L	0	0.37
ctt	L	0	0.34
ctc	L	0	0.34
cta	L	0	0.33
ctg	L	0	0.44
att	I	0	326.90
atc	I	0	216.70
ata	I	896	352.40
---	--	------	------
mPD		0.0044	0.66
		nPD:	0.01
		N. weight:	1.2
		Sc. PD:	-0.21
		Sc. rank:	-620.3

,

PB2			
Pos.	355	obs:	exp:
cgt	R	0	37.93
cgc	R	0	45.12
cga	R	8	88.54
cgg	R	0	73.96
att	I	0	0.36
atc	I	0	0.24
ata	I	1	0.39
aaa	K	8	4.69
aag	K	0	3.31
aga	R	871	405.40
agg	R	9	237.00
ggt	G	0	0.13
ggc	G	0	0.13
gga	G	1	0.46
ggg	G	0	0.28
---	--	------	------
mPD		0.060	0.98
		nPD:	0.06
		N. weight:	0.88
		Sc. PD:	-0.11
		Sc. rank:	-269.6

,

PB2			
Pos.	356	obs:	exp:
att	I	0	4.74
atc	I	0	3.14
ata	I	13	5.11
gtt	V	2	182.70
gtc	V	0	177.30
gta	V	842	183.20
gtg	V	41	341.80
---	--	------	------
mPD		0.12	0.76
		nPD:	0.16
		N. weight:	1.7
		Sc. PD:	-0.052
		Sc. rank:	58.9

,

PB2			
Pos.	357	obs:	exp:
cat	H	895	515.90
cac	H	3	382.10
---	--	------	------
mPD		0.0067	0.49
		nPD:	0.01
		N. weight:	0.67
		Sc. PD:	-0.11
		Sc. rank:	-332.2

,

PB2			
Pos.	358	obs:	exp:
caa	Q	0	0.51
cag	Q	1	0.49
gtt	V	0	0.21
gtc	V	0	0.20
gta	V	0	0.21
gtg	V	1	0.39
gaa	E	749	538.40
gag	E	146	356.60
ggt	G	0	0.13
ggc	G	0	0.13
gga	G	1	0.46
ggg	G	0	0.28
---	--	------	------
mPD		0.28	0.49
		nPD:	0.58
		N. weight:	0.17
		Sc. PD:	0.064
		Sc. rank:	96.9

,

PB2			
Pos.	359	obs:	exp:
ggt	G	0	120.40
ggc	G	0	114.10
gga	G	846	415.20
ggg	G	52	248.30
---	--	------	------
mPD		0.11	0.68
		nPD:	0.16
		N. weight:	0.73
		Sc. PD:	-0.021
		Sc. rank:	27.6

,

PB2			
Pos.	360	obs:	exp:
tat	Y	890	475.60
tac	Y	7	421.40
tgt	C	1	0.41
tgc	C	0	0.59
---	--	------	------
mPD		0.018	0.50
		nPD:	0.04
		N. weight:	0.74
		Sc. PD:	-0.11
		Sc. rank:	-291.7

,

PB2			
Pos.	361	obs:	exp:
gaa	E	73	540.20
gag	E	825	357.80
---	--	------	------
mPD		0.15	0.48
		nPD:	0.31
		N. weight:	0.76
		Sc. PD:	0.089
		Sc. rank:	288.1

,

PB2			
Pos.	362	obs:	exp:
gaa	E	851	540.20
gag	E	47	357.80
---	--	------	------
mPD		0.099	0.48
		nPD:	0.21
		N. weight:	0.41
		Sc. PD:	0.0065
		Sc. rank:	75.2

,

PB2			
Pos.	363	obs:	exp:
ttt	F	19	354.40
ttc	F	879	543.60
---	--	------	------
mPD		0.041	0.48
		nPD:	0.09
		N. weight:	0.52
		Sc. PD:	-0.052
		Sc. rank:	-111.4

,

PB2			
Pos.	364	obs:	exp:
att	I	0	0.36
atc	I	0	0.24
ata	I	1	0.39
act	T	1	232.40
acc	T	0	182.70
aca	T	894	412.10
acg	T	2	69.87
---	--	------	------
mPD		0.0089	0.68
		nPD:	0.01
		N. weight:	0.96
		Sc. PD:	-0.16
		Sc. rank:	-480.5

,

PB2			
Pos.	365	obs:	exp:
att	I	0	0.36
atc	I	0	0.24
ata	I	1	0.39
atg	M	897	897.00
---	--	------	------
mPD		0.0022	0.0022
		nPD:	1.
		N. weight:	0.0013
		Sc. PD:	0.001
		Sc. rank:	0.8

,

PB2			
Pos.	366	obs:	exp:
att	I	1	0.36
atc	I	0	0.24
ata	I	0	0.39
gtt	V	656	185.20
gtc	V	238	179.70
gta	V	2	185.70
gtg	V	1	346.40
---	--	------	------
mPD		0.40	0.73
		nPD:	0.55
		N. weight:	1.2
		Sc. PD:	0.43
		Sc. rank:	679.8

,

PB2			
Pos.	367	obs:	exp:
ggt	G	1	120.40
ggc	G	2	114.10
gga	G	220	415.20
ggg	G	675	248.30
---	--	------	------
mPD		0.38	0.68
		nPD:	0.56
		N. weight:	0.74
		Sc. PD:	0.26
		Sc. rank:	412.4

,

PB2			
Pos.	368	obs:	exp:
caa	Q	14	7.70
cag	Q	1	7.30
cgt	R	0	37.51
cgc	R	0	44.61
cga	R	687	87.55
cgg	R	13	73.13
aaa	K	5	2.93
aag	K	0	2.07
aga	R	178	400.90
agg	R	0	234.30
---	--	------	------
mPD		0.40	1.0
		nPD:	0.39
		N. weight:	1.8
		Sc. PD:	0.35
		Sc. rank:	789.4

,

PB2			
Pos.	369	obs:	exp:
cgt	R	0	38.19
cgc	R	0	45.42
cga	R	4	89.14
cgg	R	0	74.46
aaa	K	4	2.34
aag	K	0	1.66
aga	R	881	408.20
agg	R	9	238.60
---	--	------	------
mPD		0.038	0.97
		nPD:	0.04
		N. weight:	0.9
		Sc. PD:	-0.13
		Sc. rank:	-337.4

,

PB2			
Pos.	370	obs:	exp:
act	T	0	0.52
acc	T	0	0.41
aca	T	2	0.92
acg	T	0	0.16
gct	A	17	229.60
gcc	A	0	168.20
gca	A	877	418.90
gcg	A	2	79.25
---	--	------	------
mPD		0.046	0.68
		nPD:	0.07
		N. weight:	0.84
		Sc. PD:	-0.1
		Sc. rank:	-225.9

,

PB2			
Pos.	371	obs:	exp:
act	T	2	232.60
acc	T	1	182.90
aca	T	869	412.60
acg	T	26	69.95
---	--	------	------
mPD		0.063	0.67
		nPD:	0.09
		N. weight:	0.86
		Sc. PD:	-0.081
		Sc. rank:	-154.8

,

PB2			
Pos.	372	obs:	exp:
gct	A	27	230.10
gcc	A	868	168.60
gca	A	3	419.90
gcg	A	0	79.43
---	--	------	------
mPD		0.065	0.67
		nPD:	0.1
		N. weight:	1.9
		Sc. PD:	-0.17
		Sc. rank:	-312.3

,

PB2			
Pos.	373	obs:	exp:
att	I	890	327.70
atc	I	6	217.20
ata	I	2	353.10
---	--	------	------
mPD		0.018	0.65
		nPD:	0.03
		N. weight:	1.2
		Sc. PD:	-0.19
		Sc. rank:	-523.4

,

PB2			
Pos.	374	obs:	exp:
tta	L	12	78.66
ttg	L	3	167.00
ctt	L	0	151.70
ctc	L	39	149.90
cta	L	818	149.20
ctg	L	23	198.50
att	I	0	1.09
atc	I	2	0.73
ata	I	1	1.18
---	--	------	------
mPD		0.18	1.1
		nPD:	0.16
		N. weight:	1.8
		Sc. PD:	-0.049
		Sc. rank:	71.8

,

PB2			
Pos.	375	obs:	exp:
cgt	R	0	38.32
cgc	R	0	45.57
cga	R	0	89.44
cgg	R	1	74.71
aaa	K	0	0.59
aag	K	1	0.41
aga	R	20	409.60
agg	R	876	239.40
---	--	------	------
mPD		0.048	0.97
		nPD:	0.05
		N. weight:	1.5
		Sc. PD:	-0.2
		Sc. rank:	-502.5

,

PB2			
Pos.	376	obs:	exp:
aaa	K	846	526.00
aag	K	52	372.00
---	--	------	------
mPD		0.11	0.49
		nPD:	0.23
		N. weight:	0.42
		Sc. PD:	0.014
		Sc. rank:	103.3

,

PB2			
Pos.	377	obs:	exp:
tct	S	0	0.48
tcc	S	0	0.39
tca	S	3	0.76
tcg	S	0	0.17
agt	S	0	0.61
agc	S	0	0.59
gtt	V	0	0.41
gtc	V	0	0.40
gta	V	2	0.41
gtg	V	0	0.77
gct	A	0	228.80
gcc	A	0	167.70
gca	A	893	417.50
gcg	A	0	78.98
---	--	------	------
mPD		0.011	0.69
		nPD:	0.02
		N. weight:	0.96
		Sc. PD:	-0.16
		Sc. rank:	-467.7

,

PB2			
Pos.	378	obs:	exp:
act	T	24	232.60
acc	T	873	182.90
aca	T	1	412.60
acg	T	0	69.95
---	--	------	------
mPD		0.054	0.67
		nPD:	0.08
		N. weight:	1.8
		Sc. PD:	-0.19
		Sc. rank:	-413.7

,

PB2			
Pos.	379	obs:	exp:
cgt	R	0	38.28
cgc	R	0	45.52
cga	R	2	89.34
cgg	R	0	74.63
aaa	K	2	1.17
aag	K	0	0.83
aga	R	889	409.10
agg	R	5	239.10
---	--	------	------
mPD		0.020	0.97
		nPD:	0.02
		N. weight:	0.93
		Sc. PD:	-0.15
		Sc. rank:	-434.1

,

PB2			
Pos.	380	obs:	exp:
cgt	R	0	38.32
cgc	R	0	45.57
cga	R	7	89.44
cgg	R	0	74.71
aaa	K	0	0.59
aag	K	1	0.41
aga	R	797	409.60
agg	R	93	239.40
---	--	------	------
mPD		0.21	0.97
		nPD:	0.21
		N. weight:	0.6
		Sc. PD:	0.013
		Sc. rank:	120.8

,

PB2			
Pos.	381	obs:	exp:
tta	L	0	78.83
ttg	L	33	167.40
ctt	L	5	152.00
ctc	L	0	150.30
cta	L	9	149.50
ctg	L	850	198.90
atg	M	1	1.00
---	--	------	------
mPD		0.10	1.1
		nPD:	0.09
		N. weight:	1.6
		Sc. PD:	-0.15
		Sc. rank:	-285.6

,

PB2			
Pos.	382	obs:	exp:
att	I	397	326.90
atc	I	499	216.70
ata	I	0	352.40
gtt	V	1	0.41
gtc	V	1	0.40
gta	V	0	0.41
gtg	V	0	0.77
---	--	------	------
mPD		0.50	0.66
		nPD:	0.76
		N. weight:	0.7
		Sc. PD:	0.38
		Sc. rank:	424.9

,

PB2			
Pos.	383	obs:	exp:
cct	P	0	0.25
ccc	P	0	0.18
cca	P	1	0.41
ccg	P	0	0.16
caa	Q	839	460.70
cag	Q	58	436.30
---	--	------	------
mPD		0.12	0.50
		nPD:	0.25
		N. weight:	0.54
		Sc. PD:	0.029
		Sc. rank:	154.3

,

PB2			
Pos.	384	obs:	exp:
tta	L	28	78.92
ttg	L	295	167.60
ctt	L	0	152.20
ctc	L	2	150.40
cta	L	59	149.70
ctg	L	514	199.20
---	--	------	------
mPD		0.64	1.1
		nPD:	0.58
		N. weight:	0.8
		Sc. PD:	0.3
		Sc. rank:	451.4

,

PB2			
Pos.	385	obs:	exp:
att	I	5	326.90
atc	I	0	216.70
ata	I	891	352.40
gtt	V	0	0.41
gtc	V	0	0.40
gta	V	2	0.41
gtg	V	0	0.77
---	--	------	------
mPD		0.016	0.66
		nPD:	0.02
		N. weight:	1.1
		Sc. PD:	-0.18
		Sc. rank:	-511.3

,

PB2			
Pos.	386	obs:	exp:
gtt	V	0	185.40
gtc	V	0	179.90
gta	V	57	185.90
gtg	V	841	346.80
---	--	------	------
mPD		0.12	0.73
		nPD:	0.16
		N. weight:	0.95
		Sc. PD:	-0.024
		Sc. rank:	45.7

,

PB2			
Pos.	387	obs:	exp:
tct	S	0	143.70
tcc	S	0	116.20
tca	S	0	226.60
tcg	S	0	52.26
agt	S	894	184.00
agc	S	4	175.10
---	--	------	------
mPD		0.0089	1.7
		nPD:	0.01
		N. weight:	2.
		Sc. PD:	-0.35
		Sc. rank:	-1069.1

,

PB2			
Pos.	388	obs:	exp:
ggt	G	3	120.40
ggc	G	0	114.10
gga	G	215	415.20
ggg	G	680	248.30
---	--	------	------
mPD		0.37	0.68
		nPD:	0.55
		N. weight:	0.75
		Sc. PD:	0.26
		Sc. rank:	412.2

,

PB2			
Pos.	389	obs:	exp:
cgt	R	0	4.06
cgc	R	0	4.83
cga	R	0	9.47
cgg	R	0	7.91
aaa	K	540	470.40
aag	K	263	332.60
aga	R	77	43.38
agg	R	18	25.35
---	--	------	------
mPD		0.62	0.74
		nPD:	0.84
		N. weight:	0.079
		Sc. PD:	0.049
		Sc. rank:	48.6

,

PB2			
Pos.	390	obs:	exp:
aat	N	0	0.51
aac	N	1	0.49
gat	D	183	483.70
gac	D	713	412.30
gaa	E	1	0.60
gag	E	0	0.40
---	--	------	------
mPD		0.33	0.50
		nPD:	0.66
		N. weight:	0.3
		Sc. PD:	0.14
		Sc. rank:	178.1

,

PB2			
Pos.	391	obs:	exp:
gtt	V	0	0.21
gtc	V	0	0.20
gta	V	0	0.21
gtg	V	1	0.39
gaa	E	91	538.40
gag	E	804	356.60
ggt	G	0	0.27
ggc	G	0	0.25
gga	G	0	0.92
ggg	G	2	0.55
---	--	------	------
mPD		0.19	0.49
		nPD:	0.39
		N. weight:	0.7
		Sc. PD:	0.13
		Sc. rank:	306.7

PB2			
Pos.	392	obs:	exp:
cat	H	2	1.15
cac	H	0	0.85
caa	Q	805	460.20
cag	Q	91	435.80
---	--	------	------
mPD		0.19	0.50
		nPD:	0.37
		N. weight:	0.44
		Sc. PD:	0.076
		Sc. rank:	186.8

PB2			
Pos.	393	obs:	exp:
tct	S	0	143.70
tcc	S	0	116.20
tca	S	876	226.60
tcg	S	22	52.26
agt	S	0	184.00
agc	S	0	175.10
---	--	------	------
mPD		0.048	1.7
		nPD:	0.03
		N. weight:	1.6
		Sc. PD:	-0.26
		Sc. rank:	-697.3

PB2			
Pos.	394	obs:	exp:
att	I	214	326.90
atc	I	670	216.70
ata	I	12	352.40
gtt	V	1	0.41
gtc	V	1	0.40
gta	V	0	0.41
gtg	V	0	0.77
---	--	------	------
mPD		0.39	0.66
		nPD:	0.59
		N. weight:	0.89
		Sc. PD:	0.34
		Sc. rank:	502.6

PB2			
Pos.	395	obs:	exp:
tct	S	0	0.32
tcc	S	2	0.26
tca	S	0	0.50
tcg	S	0	0.12
agt	S	0	0.41
agc	S	0	0.39
gct	A	105	229.60
gcc	A	775	168.20
gca	A	16	418.90
gcg	A	0	79.25
---	--	------	------
mPD		0.24	0.68
		nPD:	0.36
		N. weight:	1.5
		Sc. PD:	0.24
		Sc. rank:	621.9

PB2			
Pos.	396	obs:	exp:
gaa	E	91	540.20
gag	E	807	357.80
---	--	------	------
mPD		0.18	0.48
		nPD:	0.38
		N. weight:	0.7
		Sc. PD:	0.13
		Sc. rank:	302.3

PB2			
Pos.	397	obs:	exp:
gct	A	0	230.10
gcc	A	0	168.60
gca	A	656	419.90
gcg	A	242	79.43
---	--	------	------
mPD		0.39	0.67
		nPD:	0.59
		N. weight:	0.79
		Sc. PD:	0.3
		Sc. rank:	447.9

PB2			
Pos.	398	obs:	exp:
att	I	14	327.30
atc	I	835	217.00
ata	I	48	352.70
gtt	V	0	0.21
gtc	V	1	0.20
gta	V	0	0.21
gtg	V	0	0.39
---	--	------	------
mPD		0.13	0.66
		nPD:	0.2
		N. weight:	1.4
		Sc. PD:	0.015
		Sc. rank:	244.2

PB2			
Pos.	399	obs:	exp:
att	I	64	327.70
atc	I	10	217.20
ata	I	824	353.10
---	--	------	------
mPD		0.15	0.65
		nPD:	0.23
		N. weight:	0.79
		Sc. PD:	0.033
		Sc. rank:	208.8

PB2			
Pos.	400	obs:	exp:
gtt	V	0	185.40
gtc	V	0	179.90
gta	V	28	185.90
gtg	V	870	346.80
---	--	------	------
mPD		0.060	0.73
		nPD:	0.08
		N. weight:	1.
		Sc. PD:	-0.11
		Sc. rank:	-235.4

PB2			
Pos.	401	obs:	exp:
gct	A	13	230.10
gcc	A	41	168.60
gca	A	837	419.90
gcg	A	7	79.43
---	--	------	------
mPD		0.13	0.67
		nPD:	0.19
		N. weight:	0.66
		Sc. PD:	0.00068
		Sc. rank:	97.7

PB2			
Pos.	402	obs:	exp:
att	I	0	0.36
atc	I	0	0.24
ata	I	1	0.39
atg	M	896	896.00
act	T	0	0.26
acc	T	0	0.20
aca	T	0	0.46
acg	T	1	0.08
---	--	------	------
mPD		0.0045	0.0065
		nPD:	0.69
		N. weight:	0.0049
		Sc. PD:	0.0023
		Sc. rank:	2.9

PB2			
Pos.	403	obs:	exp:
gtt	V	17	185.40
gtc	V	1	179.90
gta	V	281	185.90
gtg	V	599	346.80
---	--	------	------
mPD		0.46	0.73
		nPD:	0.63
		N. weight:	0.57
		Sc. PD:	0.24
		Sc. rank:	327.3

PB2			
Pos.	404	obs:	exp:
ttt	F	22	354.40
ttc	F	876	543.60
---	--	------	------
mPD		0.048	0.48
		nPD:	0.1
		N. weight:	0.5
		Sc. PD:	-0.044
		Sc. rank:	-72.6

PB2			
Pos.	405	obs:	exp:
tct	S	0	143.70
tcc	S	4	116.20
tca	S	881	226.60
tcg	S	13	52.26
agt	S	0	184.00
agc	S	0	175.10
---	--	------	------
mPD		0.037	1.7
		nPD:	0.02
		N. weight:	1.6
		Sc. PD:	-0.27
		Sc. rank:	-748.5

PB2			
Pos.	406	obs:	exp:
caa	Q	875	461.30
cag	Q	23	436.70
---	--	------	------
mPD		0.050	0.50
		nPD:	0.1
		N. weight:	0.69
		Sc. PD:	-0.061
		Sc. rank:	-101.4

PB2			
Pos.	407	obs:	exp:
gaa	E	160	540.20
gag	E	738	357.80
---	--	------	------
mPD		0.29	0.48
		nPD:	0.61
		N. weight:	0.48
		Sc. PD:	0.19
		Sc. rank:	274.1

PB2			
Pos.	408	obs:	exp:
gat	D	805	484.80
gac	D	93	413.20
---	--	------	------
mPD		0.19	0.50
		nPD:	0.37
		N. weight:	0.38
		Sc. PD:	0.067
		Sc. rank:	163.8

PB2			
Pos.	409	obs:	exp:
tgt	C	727	363.70
tgc	C	159	522.30
cgt	R	12	0.51
cgc	R	0	0.61
cga	R	0	1.20
cgg	R	0	1.00
aga	R	0	5.48
agg	R	0	3.20
---	--	------	------
mPD		0.32	0.52
		nPD:	0.61
		N. weight:	0.5
		Sc. PD:	0.2
		Sc. rank:	283.8

PB2			
Pos.	410	obs:	exp:
atg	M	897	897.00
gtt	V	0	0.21
gtc	V	0	0.20
gta	V	0	0.21
gtg	V	1	0.39
---	--	------	------
mPD		0.0022	0.0036
		nPD:	0.62
		N. weight:	0.0013
		Sc. PD:	0.00055
		Sc. rank:	0.8

PB2			
Pos.	411	obs:	exp:
att	I	23	322.20
atc	I	16	213.60
ata	I	844	347.20
act	T	0	0.26
acc	T	0	0.20
aca	T	1	0.46
acg	T	0	0.08
aaa	K	1	0.59
aag	K	0	0.41
gtt	V	0	2.68
gtc	V	0	2.60
gta	V	13	2.69
gtg	V	0	5.02
---	--	------	------
mPD		0.12	0.69
		nPD:	0.17
		N. weight:	0.94
		Sc. PD:	-0.019
		Sc. rank:	77.2

PB2			
Pos.	412	obs:	exp:
aaa	K	49	526.00
aag	K	849	372.00
---	--	------	------
mPD		0.10	0.49
		nPD:	0.21
		N. weight:	0.82
		Sc. PD:	0.018
		Sc. rank:	165.7

PB2			
Pos.	413	obs:	exp:
gct	A	3	230.10
gcc	A	5	168.60
gca	A	886	419.90
gcg	A	4	79.43
---	--	------	------
mPD		0.027	0.67
		nPD:	0.04
		N. weight:	0.87
		Sc. PD:	-0.13
		Sc. rank:	-324.6

PB2			
Pos.	414	obs:	exp:
gtt	V	42	185.40
gtc	V	24	179.90
gta	V	30	185.90
gtg	V	802	346.80
---	--	------	------
mPD		0.20	0.73
		nPD:	0.27
		N. weight:	0.72
		Sc. PD:	0.058
		Sc. rank:	241.8

PB2			
Pos.	415	obs:	exp:
cgt	R	0	38.36
cgc	R	0	45.62
cga	R	89	89.54
cgg	R	0	74.79
aga	R	806	410.00
agg	R	3	239.70
---	--	------	------
mPD		0.19	0.96
		nPD:	0.19
		N. weight:	0.75
		Sc. PD:	0.0012
		Sc. rank:	112.3

PB2			
Pos. 416		obs:	exp:
tct	S	0	0.16
tcc	S	0	0.13
tca	S	0	0.25
tcg	S	0	0.06
agt	S	1	0.20
agc	S	0	0.20
ggt	G	724	120.30
ggc	G	172	114.00
gga	G	0	414.70
ggg	G	1	248.00
---	--	------	------
mPD		0.31	0.68
		nPD:	0.46
		N. weight:	1.9
		Sc. PD:	0.5
		Sc. rank:	963.6

PB2			
Pos. 417		obs:	exp:
gat	D	883	484.20
gac	D	14	412.80
ggt	G	1	0.13
ggc	G	0	0.13
gga	G	0	0.46
ggg	G	0	0.28
---	--	------	------
mPD		0.033	0.50
		nPD:	0.07
		N. weight:	0.68
		Sc. PD:	-0.082
		Sc. rank:	-188.5

PB2			
Pos. 418		obs:	exp:
tta	L	1	78.92
ttg	L	836	167.60
ctt	L	0	152.20
ctc	L	0	150.40
cta	L	2	149.70
ctg	L	59	199.20
---	--	------	------
mPD		0.13	1.1
		nPD:	0.12
		N. weight:	1.8
		Sc. PD:	-0.12
		Sc. rank:	-99.4

PB2			
Pos. 419		obs:	exp:
aat	N	83	456.90
aac	N	815	441.10
---	--	------	------
mPD		0.17	0.50
		nPD:	0.34
		N. weight:	0.51
		Sc. PD:	0.071
		Sc. rank:	203.4

PB2			
Pos. 420		obs:	exp:
ttt	F	872	354.40
ttc	F	26	543.60
---	--	------	------
mPD		0.056	0.48
		nPD:	0.12
		N. weight:	0.99
		Sc. PD:	-0.07
		Sc. rank:	-67.2

PB2			
Pos. 421		obs:	exp:
gtt	V	17	185.40
gtc	V	855	179.90
gta	V	23	185.90
gtg	V	3	346.80
---	--	------	------
mPD		0.093	0.73
		nPD:	0.13
		N. weight:	1.7
		Sc. PD:	-0.11
		Sc. rank:	-71.6

PB2			
Pos. 422		obs:	exp:
aat	N	65	456.90
aac	N	833	441.10
---	--	------	------
mPD		0.13	0.50
		nPD:	0.27
		N. weight:	0.57
		Sc. PD:	0.043
		Sc. rank:	186.7

PB2			
Pos. 423		obs:	exp:
cgt	R	0	38.36
cgc	R	0	45.62
cga	R	0	89.54
cgg	R	0	74.79
aga	R	874	410.00
agg	R	24	239.70
---	--	------	------
mPD		0.052	0.96
		nPD:	0.05
		N. weight:	0.85
		Sc. PD:	-0.11
		Sc. rank:	-274.3

PB2			
Pos. 424		obs:	exp:
tct	S	0	0.16
tcc	S	0	0.13
tca	S	1	0.25
tcg	S	0	0.06
agt	S	0	0.20
agc	S	0	0.20
gct	A	5	229.80
gcc	A	1	168.40
gca	A	304	419.40
gcg	A	587	79.34
---	--	------	------
mPD		0.46	0.68
		nPD:	0.68
		N. weight:	1.5
		Sc. PD:	0.7
		Sc. rank:	883.0

PB2			
Pos. 425		obs:	exp:
aat	N	147	456.90
aac	N	751	441.10
---	--	------	------
mPD		0.27	0.50
		nPD:	0.55
		N. weight:	0.33
		Sc. PD:	0.11
		Sc. rank:	181.3

PB2			
Pos. 426		obs:	exp:
caa	Q	29	461.30
cag	Q	869	436.70
---	--	------	------
mPD		0.063	0.50
		nPD:	0.13
		N. weight:	0.73
		Sc. PD:	-0.046
		Sc. rank:	-32.2

PB2			
Pos. 427		obs:	exp:
cgt	R	0	38.36
cgc	R	0	45.62
cga	R	219	89.54
cgg	R	520	74.79
aga	R	25	410.00
agg	R	134	239.70
---	--	------	------
mPD		0.69	0.96
		nPD:	0.71
		N. weight:	1.5
		Sc. PD:	0.75
		Sc. rank:	898.5

PB2			
Pos. 428		obs:	exp:
tta	L	19	78.92
ttg	L	7	167.60
ctt	L	1	152.20
ctc	L	2	150.40
cta	L	813	149.70
ctg	L	56	199.20
---	--	------	------
mPD		0.19	1.1
		nPD:	0.17
		N. weight:	1.7
		Sc. PD:	-0.028
		Sc. rank:	170.7

PB2			
Pos. 429		obs:	exp:
aat	N	822	456.90
aac	N	76	441.10
---	--	------	------
mPD		0.16	0.50
		nPD:	0.31
		N. weight:	0.49
		Sc. PD:	0.057
		Sc. rank:	184.2

PB2			
Pos. 430		obs:	exp:
cct	P	24	227.80
ccc	P	830	159.80
cca	P	42	366.10
ccg	P	0	142.40
act	T	0	0.52
acc	T	2	0.41
aca	T	0	0.92
acg	T	0	0.16
---	--	------	------
mPD		0.14	0.72
		nPD:	0.2
		N. weight:	1.7
		Sc. PD:	0.016
		Sc. rank:	299.2

PB2			
Pos. 431		obs:	exp:
atg	M	898	898.00
---	--	------	------
mPD		0	0
		nPD:	1.
		N. weight:	0.
		Sc. PD:	0
		Sc. rank:	0

PB2			
Pos. 432		obs:	exp:
cat	H	862	515.90
cac	H	36	382.10
---	--	------	------
mPD		0.077	0.49
		nPD:	0.16
		N. weight:	0.5
		Sc. PD:	-0.016
		Sc. rank:	14.1

PB2			
Pos. 433		obs:	exp:
caa	Q	864	461.30
cag	Q	34	436.70
---	--	------	------
mPD		0.073	0.50
		nPD:	0.15
		N. weight:	0.64
		Sc. PD:	-0.028
		Sc. rank:	-5.6

PB2			
Pos. 434		obs:	exp:
tta	L	0	78.92
ttg	L	1	167.60
ctt	L	33	152.20
ctc	L	851	150.40
cta	L	3	149.70
ctg	L	10	199.20
---	--	------	------
mPD		0.10	1.1
		nPD:	0.09
		N. weight:	1.9
		Sc. PD:	-0.18
		Sc. rank:	-356.4

PB2			
Pos. 435		obs:	exp:
tta	L	4	78.75
ttg	L	54	167.20
ctt	L	0	151.80
ctc	L	0	150.10
cta	L	4	149.40
ctg	L	834	198.70
caa	Q	1	0.51
cag	Q	0	0.49
aaa	K	1	0.59
aag	K	0	0.41
---	--	------	------
mPD		0.15	1.1
		nPD:	0.13
		N. weight:	1.6
		Sc. PD:	-0.085
		Sc. rank:	-38.0

PB2			
Pos. 436		obs:	exp:
cgt	R	0	38.36
cgc	R	0	45.62
cga	R	0	89.54
cgg	R	1	74.79
aga	R	207	410.00
agg	R	690	239.70
---	--	------	------
mPD		0.36	0.96
		nPD:	0.37
		N. weight:	0.82
		Sc. PD:	0.14
		Sc. rank:	351.8

PB2			
Pos. 437		obs:	exp:
cat	H	844	515.90
cac	H	54	382.10
---	--	------	------
mPD		0.11	0.49
		nPD:	0.23
		N. weight:	0.44
		Sc. PD:	0.017
		Sc. rank:	110.2

PB2			
Pos. 438		obs:	exp:
ttt	F	61	354.40
ttc	F	837	543.60
---	--	------	------
mPD		0.13	0.48
		nPD:	0.27
		N. weight:	0.36
		Sc. PD:	0.026
		Sc. rank:	114.5

PB2			
Pos. 439		obs:	exp:
caa	Q	880	460.70
cag	Q	17	436.30
cgt	R	0	0.04
cgc	R	0	0.05
cga	R	1	0.10
cgg	R	0	0.08
aga	R	0	0.46
agg	R	0	0.27
---	--	------	------
mPD		0.039	0.50
		nPD:	0.08
		N. weight:	0.73
		Sc. PD:	-0.079
		Sc. rank:	-168.5

PB2			
Pos. 440		obs:	exp:
cgt	R	0	0.04
cgc	R	0	0.05
cga	R	0	0.10
cgg	R	0	0.08
aaa	K	39	525.50
aag	K	858	371.50
aga	R	0	0.46
agg	R	1	0.27
---	--	------	------
mPD		0.085	0.49
		nPD:	0.17
		N. weight:	0.87
		Sc. PD:	-0.013
		Sc. rank:	86.8

PB2			
Pos. 441		obs:	exp:
gat	D	728	483.20
gac	D	167	411.80
gaa	E	2	1.20
gag	E	0	0.80
ggt	G	1	0.13
ggc	G	0	0.13
gga	G	0	0.46
ggg	G	0	0.28
---	--	------	------
mPD		0.31	0.50
		nPD:	0.62
		N. weight:	0.22
		Sc. PD:	0.088
		Sc. rank:	123.2

,

PB2			
Pos. 442		obs:	exp:
tct	S	0	0.16
tcc	S	0	0.13
tca	S	1	0.25
tcg	S	0	0.06
act	T	0	1.04
acc	T	0	0.81
aca	T	4	1.84
acg	T	0	0.31
agt	S	0	0.20
agc	S	0	0.20
gct	A	0	228.80
gcc	A	25	167.70
gca	A	864	417.50
gcg	A	4	78.98
---	--	------	------
mPD		0.074	0.68
		nPD:	0.11
		N. weight:	0.81
		Sc. PD:	-0.065
		Sc. rank:	-90.7

,

PB2			
Pos. 443		obs:	exp:
cgt	R	0	1.71
cgc	R	0	2.03
cga	R	0	3.99
cgg	R	0	3.33
aaa	K	85	502.60
aag	K	773	355.40
aga	R	0	18.26
agg	R	40	10.68
---	--	------	------
mPD		0.26	0.60
		nPD:	0.43
		N. weight:	0.71
		Sc. PD:	0.16
		Sc. rank:	335.9

,

PB2			
Pos. 444		obs:	exp:
att	I	0	2.92
atc	I	8	1.93
ata	I	0	3.15
gtt	V	213	183.70
gtc	V	586	178.30
gta	V	5	184.30
gtg	V	86	343.70
---	--	------	------
mPD		0.52	0.74
		nPD:	0.69
		N. weight:	0.85
		Sc. PD:	0.41
		Sc. rank:	511.5

,

PB2			
Pos. 445		obs:	exp:
tta	L	0	78.92
ttg	L	9	167.60
ctt	L	42	152.20
ctc	L	0	150.40
cta	L	3	149.70
ctg	L	844	199.20
---	--	------	------
mPD		0.12	1.1
		nPD:	0.1
		N. weight:	1.6
		Sc. PD:	-0.13
		Sc. rank:	-210.0

,

PB2			
Pos. 446		obs:	exp:
ttt	F	855	354.00
ttc	F	42	543.00
tta	L	0	0.09
ttg	L	1	0.19
ctt	L	0	0.17
ctc	L	0	0.17
cta	L	0	0.17
ctg	L	0	0.22
---	--	------	------
mPD		0.091	0.48
		nPD:	0.19
		N. weight:	0.91
		Sc. PD:	-0.00039
		Sc. rank:	132.0

,

PB2			
Pos. 447		obs:	exp:
cat	H	0	4.60
cac	H	8	3.40
caa	Q	853	455.60
cag	Q	34	431.40
cgt	R	0	0.13
cgc	R	0	0.15
cga	R	3	0.30
cgg	R	0	0.25
aga	R	0	1.37
agg	R	0	0.80
---	--	------	------
mPD		0.097	0.52
		nPD:	0.19
		N. weight:	0.65
		Sc. PD:	-0.0032
		Sc. rank:	82.2

,

PB2			
Pos. 448		obs:	exp:
tct	S	0	0.64
tcc	S	0	0.52
tca	S	0	1.01
tcg	S	0	0.23
act	T	0	0.78
acc	T	3	0.61
aca	T	0	1.38
acg	T	0	0.23
aat	N	45	453.30
aac	N	846	437.70
agt	S	1	0.82
agc	S	3	0.78
---	--	------	------
mPD		0.11	0.52
		nPD:	0.22
		N. weight:	0.65
		Sc. PD:	0.015
		Sc. rank:	135.9

,

PB2			
Pos. 449		obs:	exp:
tgg	W	898	898.00
---	--	------	------
mPD		0	0
		nPD:	1.
		N. weight:	0.
		Sc. PD:	0
		Sc. rank:	0

,

PB2			
Pos. 450		obs:	exp:
ggt	G	2	120.40
ggc	G	0	114.10
gga	G	887	415.20
ggg	G	9	248.30
---	--	------	------
mPD		0.024	0.68
		nPD:	0.04
		N. weight:	0.89
		Sc. PD:	-0.13
		Sc. rank:	-347.6

,

PB2			
Pos. 451		obs:	exp:
tta	L	0	0.09
ttg	L	1	0.19
ctt	L	0	0.17
ctc	L	0	0.17
cta	L	0	0.17
ctg	L	0	0.22
att	I	837	312.30
atc	I	19	207.00
ata	I	0	336.60
act	T	19	4.92
acc	T	0	3.87
aca	T	0	8.73
acg	T	0	1.48
gtt	V	22	4.54
gtc	V	0	4.41
gta	V	0	4.56
gtg	V	0	8.50
---	--	------	------
mPD		0.14	0.75
		nPD:	0.18
		N. weight:	1.2
		Sc. PD:	-0.013
		Sc. rank:	126.9

,

PB2			
Pos. 452		obs:	exp:
gaa	E	490	539.60
gag	E	407	357.40
ggt	G	0	0.13
ggc	G	0	0.13
gga	G	0	0.46
ggg	G	1	0.28
---	--	------	------
mPD		0.50	0.48
		nPD:	1.03
		N. weight:	0.015
		Sc. PD:	0.012
		Sc. rank:	9.1

,

PB2			
Pos. 453		obs:	exp:
cct	P	15	226.80
ccc	P	864	159.00
cca	P	9	364.40
ccg	P	4	141.70
cat	H	0	2.87
cac	H	5	2.13
caa	Q	0	0.51
cag	Q	1	0.49
---	--	------	------
mPD		0.076	0.73
		nPD:	0.11
		N. weight:	1.9
		Sc. PD:	-0.16
		Sc. rank:	-250.5

,

PB2			
Pos. 454		obs:	exp:
att	I	891	327.70
atc	I	6	217.20
ata	I	1	353.10
---	--	------	------
mPD		0.016	0.65
		nPD:	0.02
		N. weight:	1.2
		Sc. PD:	-0.2
		Sc. rank:	-539.7

,

PB2			
Pos. 455		obs:	exp:
gat	D	17	484.80
gac	D	881	413.20
---	--	------	------
mPD		0.037	0.50
		nPD:	0.07
		N. weight:	0.86
		Sc. PD:	-0.096
		Sc. rank:	-212.8

,

PB2			
Pos. 456		obs:	exp:
tct	S	0	0.16
tcc	S	0	0.13
tca	S	0	0.25
tcg	S	0	0.06
aat	N	895	456.30
aac	N	2	440.70
agt	S	1	0.20
agc	S	0	0.20
---	--	------	------
mPD		0.0067	0.50
		nPD:	0.01
		N. weight:	0.83
		Sc. PD:	-0.14
		Sc. rank:	-412.6

,

PB2			
Pos. 457		obs:	exp:
att	I	0	0.73
atc	I	0	0.48
ata	I	2	0.79
gtt	V	4	185.00
gtc	V	42	179.50
gta	V	805	185.50
gtg	V	45	346.00
---	--	------	------
mPD		0.19	0.73
		nPD:	0.26
		N. weight:	1.4
		Sc. PD:	0.1
		Sc. rank:	449.1

,

PB2			
Pos. 458		obs:	exp:
atg	M	898	898.00
---	--	------	------
mPD		0	0
		nPD:	1.
		N. weight:	0.
		Sc. PD:	0
		Sc. rank:	0

,

PB2			
Pos. 459		obs:	exp:
ggt	G	0	120.40
ggc	G	0	114.10
gga	G	207	415.20
ggg	G	691	248.30
---	--	------	------
mPD		0.36	0.68
		nPD:	0.53
		N. weight:	0.79
		Sc. PD:	0.26
		Sc. rank:	423.7

,

PB2			
Pos. 460		obs:	exp:
atg	M	898	898.00
---	--	------	------
mPD		0	0
		nPD:	1.
		N. weight:	0.
		Sc. PD:	0
		Sc. rank:	0

,

PB2

Pos.	461	obs:	exp:
att	I	21	324.70
atc	I	865	215.30
ata	I	4	350.00
gtt	V	4	1.65
gtc	V	4	1.60
gta	V	0	1.66
gtg	V	0	3.09
mPD		0.080	0.67
		nPD:	0.12
		N. weight:	1.6
		Sc. PD:	-0.11
		Sc. rank:	-94.2

,

PB2

Pos.	462	obs:	exp:
ggt	G	2	120.40
ggc	G	0	114.10
gga	G	690	415.20
ggg	G	206	248.30
mPD		0.36	0.68
		nPD:	0.53
		N. weight:	0.43
		Sc. PD:	0.14
		Sc. rank:	231.0

,

PB2

Pos.	463	obs:	exp:
att	I	0	325.80
atc	I	3	216.00
ata	I	890	351.20
gtt	V	0	1.03
gtc	V	0	1.00
gta	V	5	1.04
gtg	V	0	1.93
mPD		0.018	0.67
		nPD:	0.03
		N. weight:	1.2
		Sc. PD:	-0.18
		Sc. rank:	-504.9

,

PB2

Pos.	464	obs:	exp:
tta	L	124	78.92
ttg	L	752	167.60
ctt	L	0	152.20
ctc	L	0	150.40
cta	L	6	149.70
ctg	L	16	199.20
mPD		0.30	1.1
		nPD:	0.27
		N. weight:	1.6
		Sc. PD:	0.12
		Sc. rank:	514.9

,

PB2

Pos.	465	obs:	exp:
cct	P	887	228.30
ccc	P	10	160.10
cca	P	1	366.90
ccg	P	0	142.70
mPD		0.024	0.71
		nPD:	0.03
		N. weight:	1.6
		Sc. PD:	-0.25
		Sc. rank:	-645.5

,

PB2

Pos.	466	obs:	exp:
gat	D	39	484.80
gac	D	859	413.20
mPD		0.083	0.50
		nPD:	0.17
		N. weight:	0.75
		Sc. PD:	-0.017
		Sc. rank:	50.0

,

PB2

Pos.	467	obs:	exp:
tta	L	0	0.18
ttg	L	2	0.37
ctt	L	0	0.34
ctc	L	0	0.34
cta	L	0	0.33
ctg	L	0	0.44
atg	M	896	896.00
mPD		0.0044	0.0071
		nPD:	0.63
		N. weight:	0.0047
		Sc. PD:	0.002
		Sc. rank:	2.7

,

PB2

Pos.	468	obs:	exp:
act	T	26	231.80
acc	T	869	182.30
aca	T	0	411.20
acg	T	0	69.72
gct	A	0	0.77
gcc	A	3	0.56
gca	A	0	1.40
gcg	A	0	0.27
mPD		0.063	0.68
		nPD:	0.09
		N. weight:	1.8
		Sc. PD:	-0.17
		Sc. rank:	-342.4

,

PB2

Pos.	469	obs:	exp:
tta	L	0	0.09
ttg	L	0	0.19
ctt	L	0	0.17
ctc	L	1	0.17
cta	L	0	0.17
ctg	L	0	0.22
cct	P	7	228.10
ccc	P	826	159.90
cca	P	64	366.50
ccg	P	0	142.50
mPD		0.15	0.71
		nPD:	0.21
		N. weight:	1.7
		Sc. PD:	0.03
		Sc. rank:	330.3

,

PB2

Pos.	470	obs:	exp:
tct	S	0	143.40
tcc	S	0	116.00
tca	S	0	226.10
tcg	S	0	52.14
act	T	0	0.26
acc	T	1	0.20
aca	T	0	0.46
acg	T	0	0.08
aat	N	1	0.51
aac	N	0	0.49
agt	S	45	183.60
agc	S	851	174.70
mPD		0.10	1.7
		nPD:	0.06
		N. weight:	1.8
		Sc. PD:	-0.22
		Sc. rank:	-533.8

,

PB2

Pos.	471	obs:	exp:
cct	P	0	0.76
ccc	P	0	0.53
cca	P	3	1.23
ccg	P	0	0.48
act	T	52	231.30
acc	T	1	181.80
aca	T	837	410.30
acg	T	3	69.56
gct	A	0	0.51
gcc	A	0	0.38
gca	A	2	0.94
gcg	A	0	0.18
mPD		0.13	0.69
		nPD:	0.19
		N. weight:	0.72
		Sc. PD:	-0.0021
		Sc. rank:	100.4

,

PB2

Pos.	472	obs:	exp:
gat	D	4	2.16
gac	D	0	1.84
gaa	E	7	537.80
gag	E	887	356.20
mPD		0.024	0.48
		nPD:	0.05
		N. weight:	1.1
		Sc. PD:	-0.15
		Sc. rank:	-362.7

,

PB2

Pos.	473	obs:	exp:
tta	L	0	0.18
ttg	L	2	0.37
ctt	L	0	0.34
ctc	L	0	0.34
cta	L	0	0.33
ctg	L	0	0.44
att	I	0	0.36
atc	I	0	0.24
ata	I	1	0.39
atg	M	846	846.00
gtt	V	0	10.12
gtc	V	0	9.82
gta	V	0	10.15
gtg	V	49	18.92
mPD		0.11	0.18
		nPD:	0.62
		N. weight:	0.071
		Sc. PD:	0.029
		Sc. rank:	40.8

,

PB2

Pos.	474	obs:	exp:
tct	S	45	143.70
tcc	S	308	116.20
tca	S	512	226.60
tcg	S	33	52.26
agt	S	0	184.00
agc	S	0	175.10
mPD		0.55	1.7
		nPD:	0.33
		N. weight:	0.92
		Sc. PD:	0.13
		Sc. rank:	369.7

,

PB2

Pos.	475	obs:	exp:
tta	L	8	78.75
ttg	L	772	167.20
ctt	L	3	151.80
ctc	L	0	150.10
cta	L	8	149.40
ctg	L	105	198.70
atg	M	2	2.00
mPD		0.27	1.1
		nPD:	0.24
		N. weight:	1.5
		Sc. PD:	0.076
		Sc. rank:	417.9

,

PB2

Pos.	476	obs:	exp:
cgt	R	0	38.32
cgc	R	0	45.57
cga	R	0	89.44
cgg	R	0	74.71
aaa	K	1	0.59
aag	K	0	0.41
aga	R	858	409.60
agg	R	39	239.40
mPD		0.085	0.97
		nPD:	0.09
		N. weight:	0.79
		Sc. PD:	-0.078
		Sc. rank:	-162.3

,

PB2

Pos.	477	obs:	exp:
ggt	G	0	120.40
ggc	G	0	114.10
gga	G	863	415.20
ggg	G	35	248.30
mPD		0.075	0.68
		nPD:	0.11
		N. weight:	0.79
		Sc. PD:	-0.061
		Sc. rank:	-70.8

,

PB2

Pos.	478	obs:	exp:
att	I	0	15.69
atc	I	0	10.40
ata	I	43	16.91
atg	M	1	1.00
gtt	V	0	176.30
gtc	V	0	171.10
gta	V	16	176.80
gtg	V	838	329.80
mPD		0.22	0.82
		nPD:	0.26
		N. weight:	1.1
		Sc. PD:	0.076
		Sc. rank:	342.4

,

PB2

Pos.	479	obs:	exp:
cgt	R	0	38.32
cgc	R	0	45.57
cga	R	0	89.44
cgg	R	0	74.71
aaa	K	1	0.59
aag	K	0	0.41
aga	R	882	409.60
agg	R	15	239.40
mPD		0.035	0.97
		nPD:	0.04
		N. weight:	0.89
		Sc. PD:	-0.13
		Sc. rank:	-345.9

,

PB2

Pos.	480	obs:	exp:
gtt	V	816	185.20
gtc	V	79	179.70
gta	V	1	185.70
gtg	V	1	346.40
gct	A	0	0.26
gcc	A	1	0.19
gca	A	0	0.47
gcg	A	0	0.09
mPD		0.17	0.73
		nPD:	0.23
		N. weight:	1.6
		Sc. PD:	0.063
		Sc. rank:	410.4

,

PB2			
Pos.	481	obs:	exp:
tct	S	0	143.70
tcc	S	0	116.20
tca	S	0	226.60
tcg	S	0	52.26
agt	S	848	184.00
agc	S	50	175.10
---	--	------	------
mPD		0.11	1.7
		nPD:	0.06
		N. weight:	1.7
		Sc. PD:	-0.21
		Sc. rank:	-492.7

PB2			
Pos.	482	obs:	exp:
cgt	R	0	0.04
cgc	R	0	0.05
cga	R	0	0.10
cgg	R	0	0.08
aaa	K	842	525.50
aag	K	55	371.50
aga	R	1	0.46
agg	R	0	0.27
---	--	------	------
mPD		0.12	0.49
		nPD:	0.24
		N. weight:	0.41
		Sc. PD:	0.02
		Sc. rank:	113.7

PB2			
Pos.	483	obs:	exp:
cgt	R	0	0.04
cgc	R	0	0.05
cga	R	0	0.10
cgg	R	0	0.08
atg	M	893	893.00
act	T	0	0.78
acc	T	0	0.61
aca	T	0	1.38
acg	T	3	0.23
aga	R	0	0.46
agg	R	1	0.27
gtt	V	0	0.21
gtc	V	0	0.20
gta	V	0	0.21
gtg	V	1	0.39
---	--	------	------
mPD		0.011	0.021
		nPD:	0.54
		N. weight:	0.014
		Sc. PD:	0.0047
		Sc. rank:	7.5

PB2			
Pos.	484	obs:	exp:
ggt	G	4	120.40
ggc	G	0	114.10
gga	G	891	415.20
ggg	G	3	248.30
---	--	------	------
mPD		0.016	0.68
		nPD:	0.02
		N. weight:	0.92
		Sc. PD:	-0.15
		Sc. rank:	-416.8

PB2			
Pos.	485	obs:	exp:
gtt	V	8	185.40
gtc	V	2	179.90
gta	V	847	185.90
gtg	V	41	346.80
---	--	------	------
mPD		0.11	0.73
		nPD:	0.15
		N. weight:	1.6
		Sc. PD:	-0.065
		Sc. rank:	2.9

PB2			
Pos.	486	obs:	exp:
gat	D	883	484.80
gac	D	15	413.20
---	--	------	------
mPD		0.033	0.50
		nPD:	0.07
		N. weight:	0.67
		Sc. PD:	-0.081
		Sc. rank:	-183.8

PB2			
Pos.	487	obs:	exp:
gaa	E	871	539.00
gag	E	25	357.00
ggt	G	0	0.27
ggc	G	0	0.25
gga	G	2	0.92
ggg	G	0	0.55
---	--	------	------
mPD		0.059	0.48
		nPD:	0.12
		N. weight:	0.5
		Sc. PD:	-0.033
		Sc. rank:	-26.5

PB2			
Pos.	488	obs:	exp:
tat	Y	641	476.10
tac	Y	257	421.90
---	--	------	------
mPD		0.41	0.50
		nPD:	0.82
		N. weight:	0.093
		Sc. PD:	0.057
		Sc. rank:	57.1

PB2			
Pos.	489	obs:	exp:
tct	S	141	143.60
tcc	S	753	116.10
tca	S	3	226.40
tcg	S	0	52.20
tat	Y	0	0.53
tac	Y	1	0.47
agt	S	0	183.80
agc	S	0	174.90
---	--	------	------
mPD		0.27	1.7
		nPD:	0.16
		N. weight:	2.
		Sc. PD:	-0.049
		Sc. rank:	100.1

PB2			
Pos.	490	obs:	exp:
tct	S	0	143.60
tcc	S	0	116.20
tca	S	0	226.40
tcg	S	0	52.20
aat	N	0	0.51
aac	N	1	0.49
agt	S	768	183.80
agc	S	129	174.90
---	--	------	------
mPD		0.25	1.7
		nPD:	0.15
		N. weight:	1.5
		Sc. PD:	-0.057
		Sc. rank:	6.3

PB2			
Pos.	491	obs:	exp:
act	T	720	231.10
acc	T	19	181.60
aca	T	151	409.80
acg	T	2	69.48
gct	A	3	1.54
gcc	A	0	1.13
gca	A	3	2.81
gcg	A	0	0.53
---	--	------	------
mPD		0.34	0.69
		nPD:	0.49
		N. weight:	0.88
		Sc. PD:	0.25
		Sc. rank:	456.2

PB2			
Pos.	492	obs:	exp:
gaa	E	65	540.20
gag	E	833	357.80
---	--	------	------
mPD		0.13	0.48
		nPD:	0.28
		N. weight:	0.8
		Sc. PD:	0.069
		Sc. rank:	273.6

PB2			
Pos.	493	obs:	exp:
cgt	R	0	38.36
cgc	R	0	45.62
cga	R	3	89.54
cgg	R	22	74.79
aga	R	863	410.00
agg	R	10	239.70
---	--	------	------
mPD		0.12	0.96
		nPD:	0.13
		N. weight:	0.81
		Sc. PD:	-0.049
		Sc. rank:	-32.5

PB2			
Pos.	494	obs:	exp:
att	I	0	1.09
atc	I	0	0.73
ata	I	3	1.18
gtt	V	0	184.80
gtc	V	0	179.30
gta	V	52	185.30
gtg	V	843	345.60
---	--	------	------
mPD		0.12	0.73
		nPD:	0.17
		N. weight:	0.97
		Sc. PD:	-0.023
		Sc. rank:	60.2

PB2			
Pos.	495	obs:	exp:
att	I	0	0.36
atc	I	1	0.24
ata	I	0	0.39
gtt	V	43	184.80
gtc	V	780	179.30
gta	V	66	185.30
gtg	V	6	345.60
gct	A	0	0.51
gcc	A	2	0.38
gca	A	0	0.94
gcg	A	0	0.18
---	--	------	------
mPD		0.24	0.73
		nPD:	0.33
		N. weight:	1.4
		Sc. PD:	0.18
		Sc. rank:	551.7

PB2			
Pos.	496	obs:	exp:
gtt	V	15	185.40
gtc	V	0	179.90
gta	V	39	185.90
gtg	V	844	346.80
---	--	------	------
mPD		0.11	0.73
		nPD:	0.16
		N. weight:	0.92
		Sc. PD:	-0.029
		Sc. rank:	27.2

PB2			
Pos.	497	obs:	exp:
tct	S	0	143.60
tcc	S	0	116.10
tca	S	0	226.40
tcg	S	0	52.20
aat	N	0	0.51
aac	N	1	0.49
agt	S	679	183.80
agc	S	218	174.90
---	--	------	------
mPD		0.37	1.7
		nPD:	0.22
		N. weight:	1.3
		Sc. PD:	0.043
		Sc. rank:	318.6

PB2			
Pos.	498	obs:	exp:
att	I	871	327.70
atc	I	27	217.20
ata	I	0	353.10
---	--	------	------
mPD		0.058	0.65
		nPD:	0.09
		N. weight:	1.1
		Sc. PD:	-0.11
		Sc. rank:	-226.3

PB2			
Pos.	499	obs:	exp:
gat	D	831	484.80
gac	D	67	413.20
---	--	------	------
mPD		0.14	0.50
		nPD:	0.28
		N. weight:	0.46
		Sc. PD:	0.039
		Sc. rank:	157.3

PB2			
Pos.	500	obs:	exp:
tct	S	0	0.32
tcc	S	0	0.26
tca	S	0	0.50
tcg	S	0	0.12
cgt	R	848	38.28
cgc	R	5	45.52
cga	R	40	89.34
cgg	R	3	74.63
agt	S	2	0.41
agc	S	0	0.39
aga	R	0	409.10
agg	R	0	239.10
---	--	------	------
mPD		0.11	0.97
		nPD:	0.11
		N. weight:	3.6
		Sc. PD:	-0.28
		Sc. rank:	-328.2

PB2			
Pos.	501	obs:	exp:
ttt	F	77	354.40
ttc	F	821	543.60
---	--	------	------
mPD		0.16	0.48
		nPD:	0.33
		N. weight:	0.31
		Sc. PD:	0.042
		Sc. rank:	123.4

PB2			
Pos.	502	obs:	exp:
tta	L	146	78.92
ttg	L	732	167.60
ctt	L	0	152.20
ctc	L	0	150.40
cta	L	3	149.70
ctg	L	17	199.20
---	--	------	------
mPD		0.32	1.1
		nPD:	0.29
		N. weight:	1.6
		Sc. PD:	0.15
		Sc. rank:	574.5

PB2			
Pos.	503	obs:	exp:
cgt	R	0	38.36
cgc	R	0	45.62
cga	R	0	89.54
cgg	R	1	74.79
aga	R	68	410.00
agg	R	829	239.70
---	--	------	------
mPD		0.14	0.96
		nPD:	0.15
		N. weight:	1.3
		Sc. PD:	-0.053
		Sc. rank:	-3.0

PB2			
Pos.	504	obs:	exp:
gtt	V	172	185.40
gtc	V	726	179.90
gta	V	0	185.90
gtg	V	0	346.80
---	--	------	------
mPD		0.31	0.73
		nPD:	0.43
		N. weight:	1.4
		Sc. PD:	0.32
		Sc. rank:	662.3

PB2			
Pos.	505	obs:	exp:
cgt	R	0	38.36
cgc	R	0	45.62
cga	R	661	89.54
cgg	R	231	74.79
aga	R	3	410.00
agg	R	3	239.70
---	--	------	------
mPD		0.40	0.96
		nPD:	0.41
		N. weight:	2.2
		Sc. PD:	0.47
		Sc. rank:	1008.5

PB2			
Pos.	506	obs:	exp:
gat	D	102	484.80
gac	D	796	413.20
---	--	------	------
mPD		0.20	0.50
		nPD:	0.41
		N. weight:	0.51
		Sc. PD:	0.11
		Sc. rank:	232.7

PB2			
Pos.	507	obs:	exp:
caa	Q	115	461.30
cag	Q	783	436.70
---	--	------	------
mPD		0.22	0.50
		nPD:	0.45
		N. weight:	0.42
		Sc. PD:	0.1
		Sc. rank:	206.2

PB2			
Pos.	508	obs:	exp:
caa	Q	0	9.25
cag	Q	18	8.75
cgt	R	1	37.12
cgc	R	0	44.15
cga	R	15	86.65
cgg	R	28	72.38
aaa	K	0	6.44
aag	K	11	4.56
aga	R	358	396.80
agg	R	467	231.90
---	--	------	------
mPD		0.68	1.0
		nPD:	0.66
		N. weight:	0.37
		Sc. PD:	0.17
		Sc. rank:	217.4

PB2			
Pos.	509	obs:	exp:
ggt	G	2	120.40
ggc	G	0	114.10
gga	G	797	415.20
ggg	G	99	248.30
---	--	------	------
mPD		0.20	0.68
		nPD:	0.3
		N. weight:	0.59
		Sc. PD:	0.061
		Sc. rank:	218.9

PB2			
Pos.	510	obs:	exp:
act	T	0	0.52
acc	T	2	0.41
aca	T	0	0.92
acg	T	0	0.16
aat	N	52	455.80
aac	N	844	440.20
---	--	------	------
mPD		0.11	0.51
		nPD:	0.22
		N. weight:	0.62
		Sc. PD:	0.02
		Sc. rank:	150.7

PB2			
Pos.	511	obs:	exp:
att	I	0	134.30
atc	I	5	89.01
ata	I	363	144.70
gtt	V	0	109.40
gtc	V	2	106.20
gta	V	325	109.70
gtg	V	203	204.70
---	--	------	------
mPD		0.85	1.2
		nPD:	0.69
		N. weight:	0.94
		Sc. PD:	0.45
		Sc. rank:	558.6

PB2			
Pos.	512	obs:	exp:
tta	L	4	78.83
ttg	L	0	167.40
ctt	L	7	152.00
ctc	L	848	150.30
cta	L	38	149.50
ctg	L	0	198.90
cct	P	0	0.25
ccc	P	1	0.18
cca	P	0	0.41
ccg	P	0	0.16
---	--	------	------
mPD		0.12	1.1
		nPD:	0.1
		N. weight:	1.9
		Sc. PD:	-0.16
		Sc. rank:	-262.6

PB2			
Pos.	513	obs:	exp:
tta	L	20	78.92
ttg	L	52	167.60
ctt	L	1	152.20
ctc	L	0	150.40
cta	L	6	149.70
ctg	L	819	199.20
---	--	------	------
mPD		0.21	1.1
		nPD:	0.19
		N. weight:	1.5
		Sc. PD:	-0.0072
		Sc. rank:	189.8

PB2			
Pos.	514	obs:	exp:
tct	S	839	143.70
tcc	S	18	116.20
tca	S	33	226.60
tcg	S	8	52.26
agt	S	0	184.00
agc	S	0	175.10
---	--	------	------
mPD		0.13	1.7
		nPD:	0.08
		N. weight:	1.9
		Sc. PD:	-0.21
		Sc. rank:	-467.2

PB2			
Pos.	515	obs:	exp:
cct	P	839	228.10
ccc	P	56	159.90
cca	P	2	366.50
ccg	P	0	142.50
cat	H	1	0.57
cac	H	0	0.43
---	--	------	------
mPD		0.12	0.71
		nPD:	0.17
		N. weight:	1.4
		Sc. PD:	-0.025
		Sc. rank:	130.1

PB2			
Pos.	516	obs:	exp:
gaa	E	698	540.20
gag	E	200	357.80
---	--	------	------
mPD		0.35	0.48
		nPD:	0.72
		N. weight:	0.092
		Sc. PD:	0.047
		Sc. rank:	55.8

PB2			
Pos.	517	obs:	exp:
gaa	E	65	540.20
gag	E	833	357.80
---	--	------	------
mPD		0.13	0.48
		nPD:	0.28
		N. weight:	0.8
		Sc. PD:	0.069
		Sc. rank:	273.6

PB2			
Pos.	518	obs:	exp:
gtt	V	844	185.40
gtc	V	53	179.90
gta	V	1	185.90
gtg	V	0	346.80
---	--	------	------
mPD		0.11	0.73
		nPD:	0.16
		N. weight:	1.7
		Sc. PD:	-0.057
		Sc. rank:	40.0

PB2			
Pos.	519	obs:	exp:
tct	S	0	143.70
tcc	S	0	116.20
tca	S	0	226.60
tcg	S	0	52.26
agt	S	846	184.00
agc	S	52	175.10
---	--	------	------
mPD		0.11	1.7
		nPD:	0.07
		N. weight:	1.7
		Sc. PD:	-0.21
		Sc. rank:	-471.1

PB2			
Pos.	520	obs:	exp:
gaa	E	868	540.20
gag	E	30	357.80
---	--	------	------
mPD		0.065	0.48
		nPD:	0.13
		N. weight:	0.48
		Sc. PD:	-0.026
		Sc. rank:	-11.2

PB2			
Pos.	521	obs:	exp:
act	T	0	232.60
acc	T	1	182.90
aca	T	897	412.60
acg	T	0	69.95
---	--	------	------
mPD		0.0022	0.67
		nPD:	0.
		N. weight:	0.97
		Sc. PD:	-0.18
		Sc. rank:	-539.9

PB2			
Pos.	522	obs:	exp:
caa	Q	53	461.30
cag	Q	845	436.70
---	--	------	------
mPD		0.11	0.50
		nPD:	0.22
		N. weight:	0.62
		Sc. PD:	0.019
		Sc. rank:	146.5

PB2			
Pos.	523	obs:	exp:
ggt	G	0	120.40
ggc	G	0	114.10
gga	G	875	415.20
ggg	G	23	248.30
---	--	------	------
mPD		0.050	0.68
		nPD:	0.07
		N. weight:	0.84
		Sc. PD:	-0.095
		Sc. rank:	-209.7

PB2			
Pos.	524	obs:	exp:
att	I	0	1.46
atc	I	0	0.97
ata	I	4	1.57
act	T	2	231.30
acc	T	149	181.80
aca	T	720	410.30
acg	T	22	69.56
aat	N	0	0.51
aac	N	1	0.49
---	--	------	------
mPD		0.33	0.69
		nPD:	0.49
		N. weight:	0.49
		Sc. PD:	0.14
		Sc. rank:	254.8

PB2			
Pos.	525	obs:	exp:
gaa	E	816	540.20
gag	E	82	357.80
---	--	------	------
mPD		0.17	0.48
		nPD:	0.35
		N. weight:	0.31
		Sc. PD:	0.046
		Sc. rank:	124.8

PB2			
Pos.	526	obs:	exp:
cgt	R	0	4.91
cgc	R	0	5.84
cga	R	0	11.47
cgg	R	0	9.58
aaa	K	59	458.70
aag	K	724	324.30
aga	R	7	52.51
agg	R	108	30.69
---	--	------	------
mPD		0.36	0.79
		nPD:	0.46
		N. weight:	0.82
		Sc. PD:	0.21
		Sc. rank:	406.1

PB2			
Pos.	527	obs:	exp:
tta	L	5	78.92
ttg	L	20	167.60
ctt	L	0	152.20
ctc	L	0	150.40
cta	L	6	149.70
ctg	L	867	199.20
---	--	------	------
mPD		0.078	1.1
		nPD:	0.07
		N. weight:	1.7
		Sc. PD:	-0.2
		Sc. rank:	-441.9

PB2			
Pos.	528	obs:	exp:
act	T	804	232.60
acc	T	1	182.90
aca	T	66	412.60
acg	T	27	69.95
---	--	------	------
mPD		0.19	0.67
		nPD:	0.29
		N. weight:	1.2
		Sc. PD:	0.11
		Sc. rank:	426.8

PB2			
Pos.	529	obs:	exp:
att	I	1	326.20
atc	I	24	216.20
ata	I	869	351.60
gtt	V	0	0.83
gtc	V	2	0.80
gta	V	2	0.83
gtg	V	0	1.54
---	--	------	------
mPD		0.067	0.66
		nPD:	0.1
		N. weight:	1.
		Sc. PD:	-0.089
		Sc. rank:	-146.9

PB2			
Pos.	530	obs:	exp:
act	T	42	232.60
acc	T	1	182.90
aca	T	846	412.60
acg	T	9	69.95
---	--	------	------
mPD		0.11	0.67
		nPD:	0.16
		N. weight:	0.72
		Sc. PD:	-0.019
		Sc. rank:	32.6

PB2			
Pos.	531	obs:	exp:
tat	Y	683	476.10
tac	Y	215	421.90
---	--	------	------
mPD		0.36	0.50
		nPD:	0.73
		N. weight:	0.15
		Sc. PD:	0.077
		Sc. rank:	88.8

PB2			
Pos.	532	obs:	exp:
tct	S	0	143.70
tcc	S	2	116.20
tca	S	797	226.60
tcg	S	99	52.26
agt	S	0	184.00
agc	S	0	175.10
---	--	------	------
mPD		0.20	1.7
		nPD:	0.12
		N. weight:	1.5
		Sc. PD:	-0.1
		Sc. rank:	-75.4

PB2			
Pos.	533	obs:	exp:
tct	S	0	143.70
tcc	S	10	116.20
tca	S	57	226.60
tcg	S	831	52.26
agt	S	0	184.00
agc	S	0	175.10
---	--	------	------
mPD		0.14	1.7
		nPD:	0.08
		N. weight:	3.1
		Sc. PD:	-0.32
		Sc. rank:	-675.5

PB2			
Pos.	534	obs:	exp:
ttt	F	0	0.79
ttc	F	2	1.21
tct	S	16	143.40
tcc	S	821	116.00
tca	S	56	226.10
tcg	S	3	52.14
agt	S	0	183.60
agc	S	0	174.70
---	--	------	------
mPD		0.16	1.7
		nPD:	0.1
		N. weight:	2.1
		Sc. PD:	-0.19
		Sc. rank:	-337.3

PB2			
Pos.	535	obs:	exp:
tta	L	0	31.02
ttg	L	346	65.87
ctt	L	0	59.82
ctc	L	0	59.14
cta	L	0	58.85
ctg	L	7	78.29
atg	M	545	545.00
---	--	------	------
mPD		0.48	0.93
		nPD:	0.52
		N. weight:	0.78
		Sc. PD:	0.25
		Sc. rank:	415.0

PB2			
Pos.	536	obs:	exp:
atg	M	898	898.00
---	--	------	------
mPD		0	0
		nPD:	1.
		N. weight:	0.
		Sc. PD:	0
		Sc. rank:	0

PB2			
Pos.	537	obs:	exp:
tgg	W	898	898.00
---	--	------	------
mPD		0	0
		nPD:	1.
		N. weight:	0.
		Sc. PD:	0
		Sc. rank:	0

PB2			
Pos.	538	obs:	exp:
gaa	E	399	540.20
gag	E	499	357.80
---	--	------	------
mPD		0.49	0.48
		nPD:	1.03
		N. weight:	0.068
		Sc. PD:	0.055
		Sc. rank:	42.1

PB2			
Pos.	539	obs:	exp:
att	I	4	326.60
atc	I	889	216.50
ata	I	2	352.00
act	T	0	0.26
acc	T	1	0.20
aca	T	0	0.46
acg	T	0	0.08
gtt	V	1	0.41
gtc	V	1	0.40
gta	V	0	0.41
gtg	V	0	0.77
---	--	------	------
mPD		0.022	0.66
		nPD:	0.03
		N. weight:	1.7
		Sc. PD:	-0.26
		Sc. rank:	-689.5

PB2			
Pos.	540	obs:	exp:
aat	N	836	456.90
aac	N	62	441.10
---	--	------	------
mPD		0.13	0.50
		nPD:	0.26
		N. weight:	0.54
		Sc. PD:	0.035
		Sc. rank:	160.7

PB2			
Pos.	541	obs:	exp:
ggt	G	802	120.40
ggc	G	91	114.10
gga	G	4	415.20
ggg	G	1	248.30
---	--	------	------
mPD		0.19	0.68
		nPD:	0.28
		N. weight:	2.1
		Sc. PD:	0.19
		Sc. rank:	738.4

PB2			
Pos.	542	obs:	exp:
cct	P	67	228.30
ccc	P	1	160.10
cca	P	21	366.90
ccg	P	809	142.70
---	--	------	------
mPD		0.18	0.71
		nPD:	0.26
		N. weight:	1.8
		Sc. PD:	0.11
		Sc. rank:	523.5

PB2			
Pos.	543	obs:	exp:
gaa	E	851	540.20
gag	E	47	357.80
---	--	------	------
mPD		0.099	0.48
		nPD:	0.21
		N. weight:	0.41
		Sc. PD:	0.0065
		Sc. rank:	75.2

PB2			
Pos.	544	obs:	exp:
tct	S	3	143.70
tcc	S	0	116.20
tca	S	876	226.60
tcg	S	19	52.26
agt	S	0	184.00
agc	S	0	175.10
---	--	------	------
mPD		0.048	1.7
		nPD:	0.03
		N. weight:	1.6
		Sc. PD:	-0.25
		Sc. rank:	-686.8

PB2			
Pos.	545	obs:	exp:
gtt	V	14	185.40
gtc	V	0	179.90
gta	V	27	185.90
gtg	V	857	346.80
---	--	------	------
mPD		0.088	0.73
		nPD:	0.12
		N. weight:	0.97
		Sc. PD:	-0.065
		Sc. rank:	-47.6

PB2			
Pos.	546	obs:	exp:
tta	L	113	78.92
ttg	L	42	167.60
ctt	L	20	152.20
ctc	L	4	150.40
cta	L	564	149.70
ctg	L	155	199.20
---	--	------	------
mPD		0.67	1.1
		nPD:	0.6
		N. weight:	0.91
		Sc. PD:	0.36
		Sc. rank:	517.7

PB2			
Pos.	547	obs:	exp:
ttt	F	1	0.39
ttc	F	0	0.61
att	I	2	0.73
atc	I	0	0.48
ata	I	0	0.79
gtt	V	709	184.80
gtc	V	184	179.30
gta	V	0	185.30
gtg	V	2	345.60
---	--	------	------
mPD		0.34	0.73
		nPD:	0.46
		N. weight:	1.3
		Sc. PD:	0.35
		Sc. rank:	667.6

PB2			
Pos.	548	obs:	exp:
aat	N	54	456.90
aac	N	844	441.10
---	--	------	------
mPD		0.11	0.50
		nPD:	0.23
		N. weight:	0.61
		Sc. PD:	0.021
		Sc. rank:	150.6

PB2			
Pos.	549	obs:	exp:
act	T	48	232.60
acc	T	23	182.90
aca	T	825	412.60
acg	T	2	69.95
---	--	------	------
mPD		0.15	0.67
		nPD:	0.23
		N. weight:	0.63
		Sc. PD:	0.022
		Sc. rank:	154.3

PB2			
Pos.	550	obs:	exp:
tat	Y	860	476.10
tac	Y	38	421.90
---	--	------	------
mPD		0.081	0.50
		nPD:	0.16
		N. weight:	0.59
		Sc. PD:	-0.016
		Sc. rank:	25.3

PB2			
Pos.	551	obs:	exp:
cat	H	1	0.57
cac	H	0	0.43
caa	Q	785	460.70
cag	Q	112	436.30
---	--	------	------
mPD		0.22	0.50
		nPD:	0.44
		N. weight:	0.38
		Sc. PD:	0.091
		Sc. rank:	181.7

PB2			
Pos.	552	obs:	exp:
tgg	W	898	898.00
---	--	------	------
mPD		0	0
		nPD:	1.
		N. weight:	0.
		Sc. PD:	0
		Sc. rank:	0

PB2			
Pos.	553	obs:	exp:
att	I	1	327.70
atc	I	862	217.20
ata	I	35	353.10
---	--	------	------
mPD		0.077	0.65
		nPD:	0.12
		N. weight:	1.5
		Sc. PD:	-0.11
		Sc. rank:	-100.9

PB2			
Pos.	554	obs:	exp:
att	I	815	326.60
atc	I	80	216.50
ata	I	0	352.00
gtt	V	3	0.62
gtc	V	0	0.60
gta	V	0	0.62
gtg	V	0	1.16
---	--	------	------
mPD		0.17	0.66
		nPD:	0.26
		N. weight:	0.94
		Sc. PD:	0.059
		Sc. rank:	276.5

PB2			
Pos.	555	obs:	exp:
cgt	R	0	38.36
cgc	R	0	45.62
cga	R	0	89.54
cgg	R	0	74.79
aga	R	871	410.00
agg	R	27	239.70
---	--	------	------
mPD		0.058	0.96
		nPD:	0.06
		N. weight:	0.84
		Sc. PD:	-0.11
		Sc. rank:	-259.0

PB2			
Pos.	556	obs:	exp:
aat	N	744	456.90
aac	N	154	441.10
---	--	------	------
mPD		0.28	0.50
		nPD:	0.57
		N. weight:	0.29
		Sc. PD:	0.1
		Sc. rank:	160.7

PB2			
Pos.	557	obs:	exp:
tgg	W	898	898.00
---	--	------	------
mPD		0	0
		nPD:	1.
		N. weight:	0.
		Sc. PD:	0
		Sc. rank:	0

PB2			
Pos.	558	obs:	exp:
gaa	E	855	540.20
gag	E	43	357.80
---	--	------	------
mPD		0.091	0.48
		nPD:	0.19
		N. weight:	0.43
		Sc. PD:	-0.00014
		Sc. rank:	62.0

PB2			
Pos.	559	obs:	exp:
tct	S	1	0.16
tcc	S	0	0.13
tca	S	0	0.25
tcg	S	0	0.06
att	I	1	0.36
atc	I	0	0.24
ata	I	0	0.39
act	T	789	211.90
acc	T	21	166.60
aca	T	7	375.80
acg	T	1	63.72
aat	N	69	35.10
aac	N	0	33.90
agt	S	0	0.20
agc	S	0	0.20
gct	A	9	2.31
gcc	A	0	1.69
gca	A	0	4.21
gcg	A	0	0.80
---	--	------	------
mPD		0.23	0.85
		nPD:	0.27
		N. weight:	1.4
		Sc. PD:	0.11
		Sc. rank:	472.0

PB2			
Pos.	560	obs:	exp:
tta	L	0	0.09
ttg	L	1	0.19
ctt	L	0	0.17
ctc	L	0	0.17
cta	L	0	0.17
ctg	L	0	0.22
att	I	0	2.19
atc	I	0	1.45
ata	I	6	2.36
atg	M	1	1.00
gtt	V	150	183.70
gtc	V	1	178.30
gta	V	676	184.30
gtg	V	63	343.70
---	--	------	------
mPD		0.41	0.74
		nPD:	0.55
		N. weight:	1.
		Sc. PD:	0.36
		Sc. rank:	578.0

PB2			
Pos.	561	obs:	exp:
aaa	K	208	526.00
aag	K	690	372.00
---	--	------	------
mPD		0.36	0.49
		nPD:	0.73
		N. weight:	0.33
		Sc. PD:	0.17
		Sc. rank:	200.5

PB2			
Pos.	562	obs:	exp:
tta	L	0	0.09
ttg	L	0	0.19
ctt	L	1	0.17
ctc	L	0	0.17
cta	L	0	0.17
ctg	L	0	0.22
att	I	891	327.30
atc	I	6	217.00
ata	I	0	352.70
---	--	------	------
mPD		0.016	0.66
		nPD:	0.02
		N. weight:	1.2
		Sc. PD:	-0.2
		Sc. rank:	-549.2

PB2			
Pos.	563	obs:	exp:
caa	Q	870	460.70
cag	Q	27	436.30
cgt	R	0	0.04
cgc	R	0	0.05
cga	R	1	0.10
cgg	R	0	0.08
aga	R	0	0.46
agg	R	0	0.27
---	--	------	------
mPD		0.061	0.50
		nPD:	0.12
		N. weight:	0.68
		Sc. PD:	-0.046
		Sc. rank:	-37.2

PB2			
Pos.	564	obs:	exp:
tgg	W	898	898.00
---	--	------	------
mPD		0	0
		nPD:	1.
		N. weight:	0.
		Sc. PD:	0
		Sc. rank:	0

PB2			
Pos.	565	obs:	exp:
tct	S	121	143.70
tcc	S	734	116.20
tca	S	42	226.60
tcg	S	1	52.26
agt	S	0	184.00
agc	S	0	175.10
---	--	------	------
mPD		0.31	1.7
		nPD:	0.19
		N. weight:	1.8
		Sc. PD:	-0.0038
		Sc. rank:	251.9

PB2			
Pos.	566	obs:	exp:
cat	H	12	6.89
cac	H	0	5.11
caa	Q	539	455.10
cag	Q	347	430.90
---	--	------	------
mPD		0.49	0.51
		nPD:	0.96
		N. weight:	0.037
		Sc. PD:	0.027
		Sc. rank:	22.7

PB2			
Pos.	567	obs:	exp:
aat	N	2	3.56
aac	N	5	3.44
gat	D	267	481.00
gac	D	624	410.00
---	--	------	------
mPD		0.44	0.51
		nPD:	0.85
		N. weight:	0.15
		Sc. PD:	0.098
		Sc. rank:	94.9

PB2			
Pos.	568	obs:	exp:
cct	P	839	228.30
ccc	P	58	160.10
cca	P	0	366.90
ccg	P	1	142.70
---	--	------	------
mPD		0.12	0.71
		nPD:	0.17
		N. weight:	1.4
		Sc. PD:	-0.025
		Sc. rank:	127.2

PB2			
Pos.	569	obs:	exp:
act	T	1	227.70
acc	T	0	179.00
aca	T	860	403.80
acg	T	18	68.47
gct	A	0	4.87
gcc	A	0	3.57
gca	A	18	8.88
gcg	A	1	1.68
---	--	------	------
mPD		0.085	0.72
		nPD:	0.12
		N. weight:	0.89
		Sc. PD:	-0.062
		Sc. rank:	-55.8

PB2			
Pos.	570	obs:	exp:
att	I	349	182.40
atc	I	1	120.90
ata	I	150	196.60
atg	M	398	398.00
---	--	------	------
mPD		0.63	0.70
		nPD:	0.9
		N. weight:	0.26
		Sc. PD:	0.18
		Sc. rank:	160.0

PB2			
Pos.	571	obs:	exp:
tta	L	19	78.92
ttg	L	42	167.60
ctt	L	1	152.20
ctc	L	1	150.40
cta	L	487	149.70
ctg	L	348	199.20
---	--	------	------
mPD		0.62	1.1
		nPD:	0.56
		N. weight:	0.96
		Sc. PD:	0.34
		Sc. rank:	537.3

PB2			
Pos.	572	obs:	exp:
tat	Y	42	476.10
tac	Y	856	421.90
---	--	------	------
mPD		0.089	0.50
		nPD:	0.18
		N. weight:	0.71
		Sc. PD:	-0.0079
		Sc. rank:	74.8

PB2			
Pos.	573	obs:	exp:
aat	N	875	456.90
aac	N	23	441.10
---	--	------	------
mPD		0.050	0.50
		nPD:	0.1
		N. weight:	0.7
		Sc. PD:	-0.062
		Sc. rank:	-104.7

PB2			
Pos.	574	obs:	exp:
cgt	R	0	0.09
cgc	R	0	0.10
cga	R	0	0.20
cgg	R	0	0.17
aaa	K	17	524.90
aag	K	879	371.10
aga	R	0	0.91
agg	R	2	0.53
---	--	------	------
mPD		0.042	0.49
		nPD:	0.08
		N. weight:	0.99
		Sc. PD:	-0.1
		Sc. rank:	-214.7

PB2			
Pos.	575	obs:	exp:
att	I	0	1.09
atc	I	0	0.73
ata	I	3	1.18
atg	M	895	895.00
---	--	------	------
mPD		0.0067	0.0067
		nPD:	1.
		N. weight:	0.0039
		Sc. PD:	0.0031
		Sc. rank:	2.4

PB2			
Pos.	576	obs:	exp:
gaa	E	653	540.20
gag	E	245	357.80
---	--	------	------
mPD		0.40	0.48
		nPD:	0.83
		N. weight:	0.048
		Sc. PD:	0.03
		Sc. rank:	29.7

PB2			
Pos.	577	obs:	exp:
ttt	F	857	354.40
ttc	F	41	543.60
---	--	------	------
mPD		0.087	0.48
		nPD:	0.18
		N. weight:	0.91
		Sc. PD:	-0.0072
		Sc. rank:	103.7

PB2			
Pos.	578	obs:	exp:
gaa	E	827	540.20
gag	E	71	357.80
---	--	------	------
mPD		0.15	0.48
		nPD:	0.3
		N. weight:	0.34
		Sc. PD:	0.037
		Sc. rank:	125.0

PB2			
Pos.	579	obs:	exp:
cct	P	7	228.30
ccc	P	824	160.10
cca	P	52	366.90
ccg	P	15	142.70
---	--	------	------
mPD		0.15	0.71
		nPD:	0.22
		N. weight:	1.7
		Sc. PD:	0.043
		Sc. rank:	366.2

PB2			
Pos.	580	obs:	exp:
ttt	F	854	354.40
ttc	F	44	543.60
---	--	------	------
mPD		0.093	0.48
		nPD:	0.2
		N. weight:	0.9
		Sc. PD:	0.0039
		Sc. rank:	143.0

PB2			
Pos.	581	obs:	exp:
caa	Q	839	461.30
cag	Q	59	436.70
---	--	------	------
mPD		0.12	0.50
		nPD:	0.25
		N. weight:	0.54
		Sc. PD:	0.029
		Sc. rank:	153.7

PB2			
Pos.	582	obs:	exp:
tct	S	46	143.60
tcc	S	850	116.10
tca	S	0	226.40
tcg	S	1	52.20
agt	S	0	183.80
agc	S	0	174.90
gct	A	0	0.26
gcc	A	1	0.19
gca	A	0	0.47
gcg	A	0	0.09
---	--	------	------
mPD		0.10	1.7
		nPD:	0.06
		N. weight:	2.3
		Sc. PD:	-0.29
		Sc. rank:	-686.4

PB2			
Pos.	583	obs:	exp:
tta	L	21	78.92
ttg	L	36	167.60
ctt	L	1	152.20
ctc	L	0	150.40
cta	L	787	149.70
ctg	L	53	199.20
---	--	------	------
mPD		0.30	1.1
		nPD:	0.27
		N. weight:	1.6
		Sc. PD:	0.12
		Sc. rank:	536.4

PB2			
Pos.	584	obs:	exp:
att	I	0	1.46
atc	I	0	0.97
ata	I	4	1.57
gtt	V	3	184.60
gtc	V	42	179.10
gta	V	25	185.10
gtg	V	824	345.30
---	--	------	------
mPD		0.16	0.73
		nPD:	0.22
		N. weight:	0.84
		Sc. PD:	0.026
		Sc. rank:	199.6

PB2			
Pos.	585	obs:	exp:
tct	S	2	0.32
tcc	S	0	0.26
tca	S	0	0.50
tcg	S	0	0.12
cct	P	738	227.80
ccc	P	158	159.80
cca	P	0	366.10
ccg	P	0	142.40
agt	S	0	0.41
agc	S	0	0.39
---	--	------	------
mPD		0.29	0.72
		nPD:	0.41
		N. weight:	1.2
		Sc. PD:	0.26
		Sc. rank:	557.2

PB2			
Pos.	586	obs:	exp:
cgt	R	0	0.04
cgc	R	0	0.05
cga	R	0	0.10
cgg	R	0	0.08
aaa	K	827	525.50
aag	K	70	371.50
aga	R	1	0.46
agg	R	0	0.27
---	--	------	------
mPD		0.15	0.49
		nPD:	0.3
		N. weight:	0.37
		Sc. PD:	0.038
		Sc. rank:	135.2

PB2			
Pos.	587	obs:	exp:
gct	A	845	230.10
gcc	A	11	168.60
gca	A	41	419.90
gcg	A	1	79.43
---	--	------	------
mPD		0.11	0.67
		nPD:	0.17
		N. weight:	1.4
		Sc. PD:	-0.031
		Sc. rank:	88.0

PB2			
Pos.	588	obs:	exp:
att	I	1	1.46
atc	I	3	0.97
ata	I	0	1.57
act	T	0	2.33
acc	T	3	1.83
aca	T	6	4.13
acg	T	0	0.70
gtt	V	0	36.33
gtc	V	176	35.26
gta	V	0	36.44
gtg	V	0	67.97
gct	A	10	181.70
gcc	A	699	133.10
gca	A	0	331.50
gcg	A	0	62.71
---	--	------	------
mPD		0.39	1.1
		nPD:	0.37
		N. weight:	2.
		Sc. PD:	0.34
		Sc. rank:	849.1

PB2			
Pos.	589	obs:	exp:
cgt	R	0	38.36
cgc	R	0	45.62
cga	R	2	89.54
cgg	R	0	74.79
aga	R	890	410.00
agg	R	6	239.70
---	--	------	------
mPD		0.018	0.96
		nPD:	0.02
		N. weight:	0.93
		Sc. PD:	-0.15
		Sc. rank:	-440.1

PB2			
Pos.	590	obs:	exp:
tct	S	0	2.40
tcc	S	0	1.94
tca	S	0	3.79
tcg	S	0	0.87
agt	S	13	3.07
agc	S	2	2.93
ggt	G	194	118.40
ggc	G	689	112.20
gga	G	0	408.30
ggg	G	0	244.10
---	--	------	------
mPD		0.39	0.73
		nPD:	0.53
		N. weight:	1.9
		Sc. PD:	0.63
		Sc. rank:	1030.9

PB2			
Pos.	591	obs:	exp:
cct	P	0	0.51
ccc	P	0	0.36
cca	P	2	0.82
ccg	P	0	0.32
caa	Q	855	460.20
cag	Q	41	435.80
---	--	------	------
mPD		0.092	0.51
		nPD:	0.18
		N. weight:	0.61
		Sc. PD:	-0.0054
		Sc. rank:	68.4

PB2			
Pos.	592	obs:	exp:
tat	Y	771	476.10
tac	Y	127	421.90
---	--	------	------
mPD		0.24	0.50
		nPD:	0.49
		N. weight:	0.31
		Sc. PD:	0.089
		Sc. rank:	161.1

PB2			
Pos.	593	obs:	exp:
tct	S	0	143.70
tcc	S	0	116.20
tca	S	0	226.60
tcg	S	0	52.26
agt	S	889	184.00
agc	S	9	175.10
---	--	------	------
mPD		0.020	1.7
		nPD:	0.01
		N. weight:	1.9
		Sc. PD:	-0.33
		Sc. rank:	-979.7

PB2			
Pos.	594	obs:	exp:
ggt	G	1	120.40
ggc	G	1	114.10
gga	G	624	415.20
ggg	G	272	248.30
---	--	------	------
mPD		0.43	0.68
		nPD:	0.63
		N. weight:	0.38
		Sc. PD:	0.16
		Sc. rank:	222.2

PB2			
Pos.	595	obs:	exp:
ttt	F	72	354.40
ttc	F	826	543.60
---	--	------	------
mPD		0.15	0.48
		nPD:	0.31
		N. weight:	0.33
		Sc. PD:	0.037
		Sc. rank:	122.0

PB2			
Pos.	596	obs:	exp:
gtt	V	0	185.40
gtc	V	1	179.90
gta	V	726	185.90
gtg	V	171	346.80
---	--	------	------
mPD		0.31	0.73
		nPD:	0.43
		N. weight:	1.2
		Sc. PD:	0.28
		Sc. rank:	574.8

PB2			
Pos.	597	obs:	exp:
cgt	R	0	38.36
cgc	R	0	45.62
cga	R	0	89.54
cgg	R	0	74.79
aga	R	40	410.00
agg	R	858	239.70
---	--	------	------
mPD		0.085	0.96
		nPD:	0.09
		N. weight:	1.4
		Sc. PD:	-0.14
		Sc. rank:	-289.1

PB2			
Pos.	598	obs:	exp:
att	I	8	2.92
atc	I	0	1.93
ata	I	0	3.15
act	T	1	23.83
acc	T	0	18.73
aca	T	67	42.27
acg	T	24	7.17
gtt	V	447	164.70
gtc	V	350	159.90
gta	V	0	165.20
gtg	V	1	308.20
---	--	------	------
mPD		0.97	1.1
		nPD:	0.87
		N. weight:	1.1
		Sc. PD:	0.72
		Sc. rank:	679.5

PB2			
Pos.	599	obs:	exp:
tta	L	14	78.92
ttg	L	11	167.60
ctt	L	0	152.20
ctc	L	0	150.40
cta	L	857	149.70
ctg	L	16	199.20
---	--	------	------
mPD		0.11	1.1
		nPD:	0.1
		N. weight:	2.
		Sc. PD:	-0.17
		Sc. rank:	-274.7

PB2			
Pos.	600	obs:	exp:
ttt	F	20	354.40
ttc	F	878	543.60
---	--	------	------
mPD		0.044	0.48
		nPD:	0.09
		N. weight:	0.51
		Sc. PD:	-0.049
		Sc. rank:	-101.5

PB2			
Pos.	601	obs:	exp:
caa	Q	274	461.30
cag	Q	624	436.70
---	--	------	------
mPD		0.42	0.50
		nPD:	0.85
		N. weight:	0.12
		Sc. PD:	0.074
		Sc. rank:	71.7

PB2			
Pos.	602	obs:	exp:
caa	Q	46	461.30
cag	Q	852	436.70
---	--	------	------
mPD		0.097	0.50
		nPD:	0.19
		N. weight:	0.65
		Sc. PD:	0.0025
		Sc. rank:	102.7

PB2			
Pos.	603	obs:	exp:
atg	M	898	898.00
---	--	------	------
mPD		0	0
		nPD:	1.
		N. weight:	0.
		Sc. PD:	0
		Sc. rank:	0

PB2			
Pos.	604	obs:	exp:
cgt	R	851	38.36
cgc	R	3	45.62
cga	R	30	89.54
cgg	R	12	74.79
aga	R	2	410.00
agg	R	0	239.70
---	--	------	------
mPD		0.11	0.96
		nPD:	0.11
		N. weight:	3.6
		Sc. PD:	-0.28
		Sc. rank:	-363.6

PB2			
Pos.	605	obs:	exp:
gat	D	318	484.80
gac	D	580	413.20
---	--	------	------
mPD		0.46	0.50
		nPD:	0.92
		N. weight:	0.092
		Sc. PD:	0.065
		Sc. rank:	57.1

PB2			
Pos.	606	obs:	exp:
gtt	V	0	185.40
gtc	V	10	179.90
gta	V	755	185.90
gtg	V	133	346.80
mPD		0.27	0.73
		nPD:	0.37
		N. weight:	1.3
		Sc. PD:	0.22
		Sc. rank:	547.4

PB2			
Pos.	607	obs:	exp:
tta	L	0	78.92
ttg	L	12	167.60
ctt	L	42	152.20
ctc	L	1	150.40
cta	L	5	149.70
ctg	L	838	199.20
mPD		0.13	1.1
		nPD:	0.12
		N. weight:	1.5
		Sc. PD:	-0.11
		Sc. rank:	-120.5

PB2			
Pos.	608	obs:	exp:
cgt	R	0	0.04
cgc	R	0	0.05
cga	R	0	0.10
cgg	R	1	0.08
aga	R	0	0.46
agg	R	0	0.27
ggt	G	0	120.30
ggc	G	0	114.00
gga	G	141	414.70
ggg	G	756	248.00
mPD		0.27	0.68
		nPD:	0.39
		N. weight:	0.97
		Sc. PD:	0.19
		Sc. rank:	436.4

PB2			
Pos.	609	obs:	exp:
act	T	12	232.60
acc	T	3	182.90
aca	T	679	412.60
acg	T	204	69.95
mPD		0.38	0.67
		nPD:	0.56
		N. weight:	0.72
		Sc. PD:	0.26
		Sc. rank:	403.6

PB2			
Pos.	610	obs:	exp:
ttt	F	575	353.60
ttc	F	321	542.40
tta	L	0	0.18
ttg	L	1	0.37
ctt	L	0	0.34
ctc	L	1	0.34
cta	L	0	0.33
ctg	L	0	0.44
mPD		0.46	0.48
		nPD:	0.96
		N. weight:	0.16
		Sc. PD:	0.12
		Sc. rank:	101.6

PB2			
Pos.	611	obs:	exp:
act	T	0	0.26
acc	T	1	0.20
aca	T	0	0.46
acg	T	0	0.08
gat	D	23	484.20
gac	D	874	412.80
mPD		0.054	0.50
		nPD:	0.11
		N. weight:	0.82
		Sc. PD:	-0.066
		Sc. rank:	-89.3

PB2			
Pos.	612	obs:	exp:
tct	S	0	0.16
tcc	S	0	0.13
tca	S	0	0.25
tcg	S	0	0.06
act	T	734	232.40
acc	T	161	182.70
aca	T	0	412.10
acg	T	2	69.87
agt	S	0	0.20
agc	S	1	0.20
mPD		0.30	0.68
		nPD:	0.45
		N. weight:	1.2
		Sc. PD:	0.28
		Sc. rank:	558.1

PB2			
Pos.	613	obs:	exp:
ttt	F	0	0.39
ttc	F	1	0.61
act	T	0	0.26
acc	T	1	0.20
aca	T	0	0.46
acg	T	0	0.08
gtt	V	210	184.80
gtc	V	682	179.30
gta	V	3	185.30
gtg	V	0	345.60
gct	A	0	0.26
gcc	A	1	0.19
gca	A	0	0.47
gcg	A	0	0.09
mPD		0.37	0.73
		nPD:	0.51
		N. weight:	1.3
		Sc. PD:	0.4
		Sc. rank:	689.2

PB2			
Pos.	614	obs:	exp:
caa	Q	838	461.30
cag	Q	60	436.70
mPD		0.12	0.50
		nPD:	0.25
		N. weight:	0.54
		Sc. PD:	0.031
		Sc. rank:	154.3

PB2			
Pos.	615	obs:	exp:
att	I	0	320.70
atc	I	1	212.60
ata	I	878	345.70
gtt	V	0	3.92
gtc	V	0	3.81
gta	V	19	3.93
gtg	V	0	7.34
mPD		0.044	0.70
		nPD:	0.06
		N. weight:	1.2
		Sc. PD:	-0.15
		Sc. rank:	-344.2

PB2			
Pos.	616	obs:	exp:
att	I	0	320.40
atc	I	2	212.40
ata	I	876	345.30
gtt	V	0	4.13
gtc	V	0	4.01
gta	V	20	4.14
gtg	V	0	7.72
mPD		0.048	0.70
		nPD:	0.07
		N. weight:	1.2
		Sc. PD:	-0.14
		Sc. rank:	-312.8

PB2			
Pos.	617	obs:	exp:
cgt	R	0	0.04
cgc	R	0	0.05
cga	R	0	0.10
cgg	R	0	0.08
aaa	K	48	525.50
aag	K	849	371.50
aga	R	0	0.46
agg	R	1	0.27
mPD		0.10	0.49
		nPD:	0.21
		N. weight:	0.83
		Sc. PD:	0.017
		Sc. rank:	164.9

PB2			
Pos.	618	obs:	exp:
tta	L	25	78.83
ttg	L	1	167.40
ctt	L	19	152.00
ctc	L	40	150.30
cta	L	783	149.50
ctg	L	29	198.90
cgt	R	0	0.04
cgc	R	0	0.05
cga	R	0	0.10
cgg	R	0	0.08
aga	R	1	0.46
agg	R	0	0.27
mPD		0.25	1.1
		nPD:	0.22
		N. weight:	1.6
		Sc. PD:	0.046
		Sc. rank:	355.6

PB2			
Pos.	619	obs:	exp:
ttt	F	0	0.39
ttc	F	1	0.61
tta	L	661	78.75
ttg	L	14	167.20
ctt	L	22	151.80
ctc	L	29	150.10
cta	L	170	149.40
ctg	L	0	198.70
gtt	V	0	0.21
gtc	V	1	0.20
gta	V	0	0.21
gtg	V	0	0.39
mPD		0.51	1.1
		nPD:	0.46
		N. weight:	1.8
		Sc. PD:	0.48
		Sc. rank:	936.6

PB2			
Pos.	620	obs:	exp:
cct	P	2	228.10
ccc	P	41	159.90
cca	P	843	366.50
ccg	P	11	142.50
act	T	0	0.26
acc	T	0	0.20
aca	T	1	0.46
acg	T	0	0.08
mPD		0.12	0.71
		nPD:	0.16
		N. weight:	0.86
		Sc. PD:	-0.023
		Sc. rank:	40.0

PB2			
Pos.	621	obs:	exp:
ttt	F	897	354.00
ttc	F	0	543.00
gtt	V	0	0.21
gtc	V	0	0.20
gta	V	1	0.21
gtg	V	0	0.39
mPD		0.0045	0.48
		nPD:	0.01
		N. weight:	1.2
		Sc. PD:	-0.2
		Sc. rank:	-602.2

PB2			
Pos.	622	obs:	exp:
gct	A	42	230.10
gcc	A	0	168.60
gca	A	836	419.90
gcg	A	20	79.43
mPD		0.13	0.67
		nPD:	0.19
		N. weight:	0.67
		Sc. PD:	0.0024
		Sc. rank:	103.6

PB2			
Pos.	623	obs:	exp:
gct	A	41	230.10
gcc	A	3	168.60
gca	A	854	419.90
gcg	A	0	79.43
mPD		0.094	0.67
		nPD:	0.14
		N. weight:	0.74
		Sc. PD:	-0.037
		Sc. rank:	-13.0

PB2			
Pos.	624	obs:	exp:
tct	S	0	0.64
tcc	S	4	0.52
tca	S	0	1.01
tcg	S	0	0.23
agt	S	0	0.82
agc	S	0	0.78
gct	A	21	228.80
gcc	A	869	167.70
gca	A	2	417.50
gcg	A	1	78.98
gat	D	0	0.54
gac	D	1	0.46
mPD		0.063	0.69
		nPD:	0.09
		N. weight:	1.9
		Sc. PD:	-0.18
		Sc. rank:	-371.7

PB2			
Pos.	625	obs:	exp:
cct	P	5	228.30
ccc	P	1	160.10
cca	P	111	366.90
ccg	P	781	142.70
mPD		0.23	0.71
		nPD:	0.32
		N. weight:	1.6
		Sc. PD:	0.21
		Sc. rank:	641.2

PB2			
Pos.	626	obs:	exp:
cct	P	0	228.30
ccc	P	4	160.10
cca	P	25	366.90
ccg	P	869	142.70
---	--	------	------
mPD		0.063	0.71
		nPD:	0.09
		N. weight:	2.1
		Sc. PD:	-0.21
		Sc. rank:	-434.6

PB2			
Pos.	627	obs:	exp:
aaa	K	2	4.10
aag	K	5	2.90
gtt	V	0	0.62
gtc	V	0	0.60
gta	V	1	0.62
gtg	V	2	1.16
gaa	E	252	534.20
gag	E	636	353.80
---	--	------	------
mPD		0.43	0.50
		nPD:	0.85
		N. weight:	0.27
		Sc. PD:	0.17
		Sc. rank:	165.1

PB2			
Pos.	628	obs:	exp:
caa	Q	57	461.30
cag	Q	841	436.70
---	--	------	------
mPD		0.12	0.50
		nPD:	0.24
		N. weight:	0.61
		Sc. PD:	0.028
		Sc. rank:	163.3

PB2			
Pos.	629	obs:	exp:
tct	S	0	143.30
tcc	S	0	115.80
tca	S	0	225.90
tcg	S	0	52.08
act	T	2	0.52
acc	T	0	0.41
aca	T	0	0.92
acg	T	0	0.16
aat	N	1	0.51
aac	N	0	0.49
agt	S	867	183.40
agc	S	28	174.50
---	--	------	------
mPD		0.067	1.7
		nPD:	0.04
		N. weight:	1.8
		Sc. PD:	-0.26
		Sc. rank:	-662.8

PB2			
Pos.	630	obs:	exp:
cgt	R	0	38.36
cgc	R	0	45.62
cga	R	0	89.54
cgg	R	0	74.79
aga	R	35	410.00
agg	R	863	239.70
---	--	------	------
mPD		0.075	0.96
		nPD:	0.08
		N. weight:	1.4
		Sc. PD:	-0.16
		Sc. rank:	-340.0

PB2			
Pos.	631	obs:	exp:
atg	M	898	898.00
---	--	------	------
mPD		0	0
		nPD:	1.
		N. weight:	0.
		Sc. PD:	0
		Sc. rank:	0

PB2			
Pos.	632	obs:	exp:
caa	Q	30	461.30
cag	Q	868	436.70
---	--	------	------
mPD		0.065	0.50
		nPD:	0.13
		N. weight:	0.72
		Sc. PD:	-0.043
		Sc. rank:	-28.1

PB2			
Pos.	633	obs:	exp:
ttt	F	66	354.40
ttc	F	832	543.60
---	--	------	------
mPD		0.14	0.48
		nPD:	0.29
		N. weight:	0.34
		Sc. PD:	0.031
		Sc. rank:	124.7

PB2			
Pos.	634	obs:	exp:
ttt	F	1	0.39
ttc	F	0	0.61
tct	S	842	143.60
tcc	S	48	116.10
tca	S	7	226.40
tcg	S	0	52.20
agt	S	0	183.80
agc	S	0	174.90
---	--	------	------
mPD		0.12	1.7
		nPD:	0.07
		N. weight:	2.
		Sc. PD:	-0.23
		Sc. rank:	-509.2

PB2			
Pos.	635	obs:	exp:
tct	S	870	143.70
tcc	S	27	116.20
tca	S	1	226.60
tcg	S	0	52.26
agt	S	0	184.00
agc	S	0	175.10
---	--	------	------
mPD		0.061	1.7
		nPD:	0.04
		N. weight:	2.1
		Sc. PD:	-0.32
		Sc. rank:	-819.7

PB2			
Pos.	636	obs:	exp:
tta	L	6	78.92
ttg	L	15	167.60
ctt	L	0	152.20
ctc	L	0	150.40
cta	L	824	149.70
ctg	L	53	199.20
---	--	------	------
mPD		0.19	1.1
		nPD:	0.17
		N. weight:	1.8
		Sc. PD:	-0.041
		Sc. rank:	126.4

PB2			
Pos.	637	obs:	exp:
att	I	11	6.57
atc	I	7	4.35
ata	I	0	7.08
act	T	870	227.70
acc	T	5	179.00
aca	T	2	403.80
acg	T	2	68.47
gct	A	1	0.26
gcc	A	0	0.19
gca	A	0	0.47
gcg	A	0	0.09
---	--	------	------
mPD		0.077	0.72
		nPD:	0.11
		N. weight:	1.6
		Sc. PD:	-0.13
		Sc. rank:	-184.2

PB2			
Pos.	638	obs:	exp:
gtt	V	2	185.40
gtc	V	0	179.90
gta	V	11	185.90
gtg	V	885	346.80
---	--	------	------
mPD		0.029	0.73
		nPD:	0.04
		N. weight:	1.1
		Sc. PD:	-0.16
		Sc. rank:	-410.2

PB2			
Pos.	639	obs:	exp:
aat	N	318	456.90
aac	N	580	441.10
---	--	------	------
mPD		0.46	0.50
		nPD:	0.92
		N. weight:	0.066
		Sc. PD:	0.046
		Sc. rank:	40.7

PB2			
Pos.	640	obs:	exp:
att	I	0	39.41
atc	I	0	26.12
ata	I	108	42.47
gtt	V	18	163.10
gtc	V	0	158.30
gta	V	133	163.60
gtg	V	639	305.10
---	--	------	------
mPD		0.63	0.95
		nPD:	0.67
		N. weight:	0.72
		Sc. PD:	0.33
		Sc. rank:	423.0

PB2			
Pos.	641	obs:	exp:
cgt	R	0	38.36
cgc	R	0	45.62
cga	R	0	89.54
cgg	R	0	74.79
aga	R	445	410.00
agg	R	453	239.70
---	--	------	------
mPD		0.50	0.96
		nPD:	0.52
		N. weight:	0.46
		Sc. PD:	0.15
		Sc. rank:	243.3

PB2			
Pos.	642	obs:	exp:
ggt	G	835	120.40
ggc	G	19	114.10
gga	G	43	415.20
ggg	G	1	248.30
---	--	------	------
mPD		0.13	0.68
		nPD:	0.2
		N. weight:	2.1
		Sc. PD:	0.012
		Sc. rank:	343.4

PB2			
Pos.	643	obs:	exp:
tct	S	0	143.70
tcc	S	71	116.20
tca	S	824	226.60
tcg	S	3	52.26
agt	S	0	184.00
agc	S	0	175.10
---	--	------	------
mPD		0.15	1.7
		nPD:	0.09
		N. weight:	1.4
		Sc. PD:	-0.14
		Sc. rank:	-280.8

PB2			
Pos.	644	obs:	exp:
ggt	G	0	120.40
ggc	G	52	114.10
gga	G	841	415.20
ggg	G	5	248.30
---	--	------	------
mPD		0.12	0.68
		nPD:	0.18
		N. weight:	0.75
		Sc. PD:	-0.0099
		Sc. rank:	78.5

PB2			
Pos.	645	obs:	exp:
atg	M	897	897.00
act	T	0	0.26
acc	T	0	0.20
aca	T	0	0.46
acg	T	1	0.08
---	--	------	------
mPD		0.0022	0.0043
		nPD:	0.52
		N. weight:	0.0036
		Sc. PD:	0.0011
		Sc. rank:	1.9

PB2			
Pos.	646	obs:	exp:
cgt	R	0	38.36
cgc	R	0	45.62
cga	R	0	89.54
cgg	R	0	74.79
aga	R	889	410.00
agg	R	9	239.70
---	--	------	------
mPD		0.020	0.96
		nPD:	0.02
		N. weight:	0.92
		Sc. PD:	-0.15
		Sc. rank:	-431.2

PB2			
Pos.	647	obs:	exp:
att	I	0	201.80
atc	I	3	133.80
ata	I	550	217.50
atg	M	2	2.00
gtt	V	1	70.81
gtc	V	0	68.71
gta	V	155	71.02
gtg	V	187	132.50
---	--	------	------
mPD		0.81	1.2
		nPD:	0.67
		N. weight:	0.96
		Sc. PD:	0.45
		Sc. rank:	568.4

PB2			
Pos.	648	obs:	exp:
tta	L	0	8.09
ttg	L	0	17.17
ctt	L	28	15.59
ctc	L	23	15.41
cta	L	12	15.34
ctg	L	29	20.40
att	I	2	0.73
atc	I	0	0.48
ata	I	0	0.79
gtt	V	802	165.60
gtc	V	0	160.70
gta	V	0	166.10
gtg	V	0	309.70
gct	A	1	0.26
gcc	A	0	0.19
gca	A	0	0.47
gcg	A	0	0.09
ggt	G	1	0.13
ggc	G	0	0.13
gga	G	0	0.46
ggg	G	0	0.28
---	--	------	------
mPD		0.33	0.92
		nPD:	0.36
		N. weight:	1.8
		Sc. PD:	0.29
		Sc. rank:	765.1

PB2			
Pos.	649	obs:	exp:
att	I	0	0.36
atc	I	0	0.24
ata	I	1	0.39
gtt	V	0	185.20
gtc	V	0	179.70
gta	V	52	185.70
gtg	V	845	346.40
---	--	------	------
mPD		0.11	0.73
		nPD:	0.16
		N. weight:	0.97
		Sc. PD:	-0.033
		Sc. rank:	20.1

PB2			
Pos.	650	obs:	exp:
cgt	R	0	38.36
cgc	R	0	45.62
cga	R	0	89.54
cgg	R	0	74.79
aga	R	865	410.00
agg	R	33	239.70
---	--	------	------
mPD		0.071	0.96
		nPD:	0.07
		N. weight:	0.82
		Sc. PD:	-0.092
		Sc. rank:	-206.7

PB2			
Pos.	651	obs:	exp:
ggt	G	165	120.40
ggc	G	732	114.10
gga	G	1	415.20
ggg	G	0	248.30
---	--	------	------
mPD		0.30	0.68
		nPD:	0.45
		N. weight:	2.
		Sc. PD:	0.49
		Sc. rank:	966.6

PB2			
Pos.	652	obs:	exp:
aat	N	705	456.90
aac	N	193	441.10
---	--	------	------
mPD		0.34	0.50
		nPD:	0.68
		N. weight:	0.21
		Sc. PD:	0.098
		Sc. rank:	124.2

PB2			
Pos.	653	obs:	exp:
tct	S	808	143.70
tcc	S	90	116.20
tca	S	0	226.60
tcg	S	0	52.26
agt	S	0	184.00
agc	S	0	175.10
---	--	------	------
mPD		0.18	1.7
		nPD:	0.11
		N. weight:	1.9
		Sc. PD:	-0.15
		Sc. rank:	-188.4

PB2			
Pos.	654	obs:	exp:
tta	L	0	0.09
ttg	L	0	0.19
tct	S	1	0.16
tcc	S	0	0.13
tca	S	0	0.25
tcg	S	0	0.06
ctt	L	1	0.17
ctc	L	0	0.17
cta	L	0	0.17
ctg	L	0	0.22
cct	P	737	227.80
ccc	P	5	159.80
cca	P	153	366.10
ccg	P	1	142.40
agt	S	0	0.20
agc	S	0	0.20
---	--	------	------
mPD		0.30	0.72
		nPD:	0.42
		N. weight:	1.
		Sc. PD:	0.22
		Sc. rank:	466.4

PB2			
Pos.	655	obs:	exp:
tta	L	0	0.09
ttg	L	1	0.19
ctt	L	0	0.17
ctc	L	0	0.17
cta	L	0	0.17
ctg	L	0	0.22
gtt	V	3	185.20
gtc	V	0	179.70
gta	V	75	185.70
gtg	V	819	346.40
---	--	------	------
mPD		0.16	0.73
		nPD:	0.22
		N. weight:	0.88
		Sc. PD:	0.027
		Sc. rank:	206.2

PB2			
Pos.	656	obs:	exp:
ttt	F	58	354.40
ttc	F	840	543.60
---	--	------	------
mPD		0.12	0.48
		nPD:	0.25
		N. weight:	0.37
		Sc. PD:	0.022
		Sc. rank:	107.6

PB2			
Pos.	657	obs:	exp:
tct	S	0	0.16
tcc	S	0	0.13
tca	S	0	0.25
tcg	S	0	0.06
aat	N	44	456.30
aac	N	853	440.70
agt	S	0	0.20
agc	S	1	0.20
---	--	------	------
mPD		0.096	0.50
		nPD:	0.19
		N. weight:	0.65
		Sc. PD:	-0.00071
		Sc. rank:	93.3

PB2			
Pos.	658	obs:	exp:
tat	Y	27	476.10
tac	Y	871	421.90
---	--	------	------
mPD		0.058	0.50
		nPD:	0.12
		N. weight:	0.78
		Sc. PD:	-0.055
		Sc. rank:	-57.3

PB2			
Pos.	659	obs:	exp:
tct	S	0	0.16
tcc	S	0	0.13
tca	S	0	0.25
tcg	S	0	0.06
act	T	1	0.26
acc	T	0	0.20
aca	T	0	0.46
acg	T	0	0.08
aat	N	114	455.80
aac	N	782	440.20
agt	S	0	0.20
agc	S	1	0.20
---	--	------	------
mPD		0.23	0.51
		nPD:	0.45
		N. weight:	0.42
		Sc. PD:	0.1
		Sc. rank:	204.5

PB2			
Pos.	660	obs:	exp:
cgt	R	0	0.09
cgc	R	0	0.10
cga	R	0	0.20
cgg	R	0	0.17
aaa	K	11	524.90
aag	K	885	371.10
aga	R	0	0.91
agg	R	2	0.53
---	--	------	------
mPD		0.029	0.49
		nPD:	0.06
		N. weight:	1.
		Sc. PD:	-0.13
		Sc. rank:	-322.3

PB2			
Pos.	661	obs:	exp:
act	T	1	3.11
acc	T	2	2.44
aca	T	8	5.51
acg	T	1	0.93
gtt	V	0	2.06
gtc	V	0	2.00
gta	V	10	2.07
gtg	V	0	3.86
gct	A	2	224.50
gcc	A	40	164.50
gca	A	832	409.60
gcg	A	2	77.48
---	--	------	------
mPD		0.15	0.72
		nPD:	0.21
		N. weight:	0.76
		Sc. PD:	0.013
		Sc. rank:	143.8

PB2			
Pos.	662	obs:	exp:
act	T	17	232.10
acc	T	751	182.50
aca	T	128	411.60
acg	T	0	69.80
aat	N	0	1.02
aac	N	2	0.98
---	--	------	------
mPD		0.28	0.68
		nPD:	0.41
		N. weight:	1.2
		Sc. PD:	0.26
		Sc. rank:	561.6

PB2			
Pos.	663	obs:	exp:
cgt	R	0	0.09
cgc	R	0	0.10
cga	R	0	0.20
cgg	R	0	0.17
aat	N	1	0.51
aac	N	0	0.49
aaa	K	387	524.30
aag	K	508	370.70
aga	R	1	0.91
agg	R	1	0.53
---	--	------	------
mPD		0.50	0.49
		nPD:	1.01
		N. weight:	0.067
		Sc. PD:	0.053
		Sc. rank:	41.7

PB2			
Pos.	664	obs:	exp:
cgt	R	1	38.36
cgc	R	0	45.62
cga	R	0	89.54
cgg	R	0	74.79
aga	R	22	410.00
agg	R	875	239.70
---	--	------	------
mPD		0.052	0.96
		nPD:	0.05
		N. weight:	1.5
		Sc. PD:	-0.2
		Sc. rank:	-477.8

PB2			
Pos.	665	obs:	exp:
ttt	F	1	0.39
ttc	F	0	0.61
tta	L	0	78.83
ttg	L	0	167.40
ctt	L	846	152.00
ctc	L	49	150.30
cta	L	2	149.50
ctg	L	0	198.90
---	--	------	------
mPD		0.11	1.1
		nPD:	0.1
		N. weight:	1.9
		Sc. PD:	-0.17
		Sc. rank:	-299.7

PB2			
Pos.	666	obs:	exp:
tct	S	0	1.60
tcc	S	0	1.29
tca	S	10	2.52
tcg	S	0	0.58
atg	M	2	2.00
act	T	2	229.50
acc	T	11	180.40
aca	T	632	407.00
acg	T	241	69.02
agt	S	0	2.05
agc	S	0	1.95
---	--	------	------
mPD		0.44	0.70
		nPD:	0.63
		N. weight:	0.78
		Sc. PD:	0.33
		Sc. rank:	451.8

PB2			
Pos.	667	obs:	exp:
att	I	3	1.09
atc	I	0	0.73
ata	I	0	1.18
atg	M	1	1.00
gtt	V	62	184.60
gtc	V	26	179.10
gta	V	2	185.10
gtg	V	804	345.30
---	--	------	------
mPD		0.20	0.73
		nPD:	0.27
		N. weight:	0.79
		Sc. PD:	0.061
		Sc. rank:	262.5

PB2			
Pos.	668	obs:	exp:
tta	L	0	78.92
ttg	L	0	167.60
ctt	L	836	152.20
ctc	L	61	150.40
cta	L	1	149.70
ctg	L	0	199.20
---	--	------	------
mPD		0.13	1.1
		nPD:	0.12
		N. weight:	1.9
		Sc. PD:	-0.14
		Sc. rank:	-143.3

PB2			
Pos.	669	obs:	exp:
ggt	G	37	120.40
ggc	G	0	114.10
gga	G	344	415.20
ggg	G	517	248.30
---	--	------	------
mPD		0.52	0.68
		nPD:	0.77
		N. weight:	0.39
		Sc. PD:	0.22
		Sc. rank:	237.1

PB2			
Pos.	670	obs:	exp:
aaa	K	47	526.00
aag	K	851	372.00
---	--	------	------
mPD		0.099	0.49
		nPD:	0.2
		N. weight:	0.83
		Sc. PD:	0.011
		Sc. rank:	147.3

PB2			
Pos.	671	obs:	exp:
gat	D	849	484.20
gac	D	48	412.80
gaa	E	0	0.60
gag	E	1	0.40
---	--	------	------
mPD		0.10	0.50
		nPD:	0.21
		N. weight:	0.53
		Sc. PD:	0.0086
		Sc. rank:	99.4

PB2			
Pos.	672	obs:	exp:
gct	A	12	230.10
gcc	A	1	168.60
gca	A	884	419.90
gcg	A	1	79.43
---	--	------	------
mPD		0.031	0.67
		nPD:	0.05
		N. weight:	0.86
		Sc. PD:	-0.12
		Sc. rank:	-303.8

PB2			
Pos.	673	obs:	exp:
tgt	C	2	0.82
tgc	C	0	1.18
ggt	G	888	120.20
ggc	G	4	113.80
gga	G	4	414.30
ggg	G	0	247.70
---	--	------	------
mPD		0.022	0.68
		nPD:	0.03
		N. weight:	2.4
		Sc. PD:	-0.37
		Sc. rank:	-988.0

PB2			
Pos.	674	obs:	exp:
act	T	1	1.81
acc	T	0	1.43
aca	T	0	3.22
acg	T	6	0.55
gtt	V	0	0.41
gtc	V	0	0.40
gta	V	1	0.41
gtg	V	1	0.77
gct	A	2	227.30
gcc	A	156	166.50
gca	A	543	414.70
gcg	A	186	78.45
gaa	E	0	1.20
gag	E	2	0.80
---	--	------	------
mPD		0.58	0.70
		nPD:	0.83
		N. weight:	0.44
		Sc. PD:	0.27
		Sc. rank:	269.4

PB2			
Pos.	675	obs:	exp:
tta	L	265	78.92
ttg	L	627	167.60
ctt	L	0	152.20
ctc	L	2	150.40
cta	L	2	149.70
ctg	L	2	199.20
---	--	------	------
mPD		0.43	1.1
		nPD:	0.39
		N. weight:	1.6
		Sc. PD:	0.31
		Sc. rank:	700.7

PB2			
Pos.	676	obs:	exp:
tta	L	2	0.18
ttg	L	0	0.37
ctt	L	0	0.34
ctc	L	0	0.34
cta	L	0	0.33
ctg	L	0	0.44
att	I	2	9.85
atc	I	0	6.53
ata	I	25	10.62
atg	M	504	504.00
act	T	1	22.02
acc	T	40	17.31
aca	T	38	39.05
acg	T	6	6.62
aaa	K	0	2.93
aag	K	5	2.07
gtt	V	1	56.77
gtc	V	0	55.09
gta	V	1	56.94
gtg	V	273	106.20
---	--	------	------
mPD		0.83	1.1
		nPD:	0.76
		N. weight:	0.44
		Sc. PD:	0.24
		Sc. rank:	267.8

PB2			
Pos.	677	obs:	exp:
aaa	K	31	18.16
aag	K	0	12.84
gat	D	0	1.08
gac	D	2	0.92
gaa	E	808	498.70
gag	E	21	330.30
ggt	G	0	4.83
ggc	G	0	4.57
gga	G	0	16.64
ggg	G	36	9.95
---	--	------	------
mPD		0.27	0.64
		nPD:	0.42
		N. weight:	0.56
		Sc. PD:	0.12
		Sc. rank:	260.5

PB2			
Pos.	678	obs:	exp:
aat	N	0	0.51
aac	N	1	0.49
gat	D	86	484.20
gac	D	811	412.80
---	--	------	------
mPD		0.18	0.50
		nPD:	0.35
		N. weight:	0.56
		Sc. PD:	0.088
		Sc. rank:	231.1

PB2			
Pos.	679	obs:	exp:
tta	L	0	0.09
ttg	L	0	0.19
ctt	L	0	0.17
ctc	L	1	0.17
cta	L	0	0.17
ctg	L	0	0.22
cct	P	3	228.10
ccc	P	74	159.90
cca	P	817	366.50
ccg	P	3	142.50
---	--	------	------
mPD		0.17	0.71
		nPD:	0.23
		N. weight:	0.81
		Sc. PD:	0.034
		Sc. rank:	214.7

PB2			
Pos.	680	obs:	exp:
aat	N	1	0.51
aac	N	0	0.49
gat	D	551	483.70
gac	D	345	412.30
gaa	E	1	0.60
gag	E	0	0.40
---	--	------	------
mPD		0.48	0.50
		nPD:	0.95
		N. weight:	0.021
		Sc. PD:	0.016
		Sc. rank:	13.0

PB2			
Pos.	681	obs:	exp:
gaa	E	53	539.60
gag	E	844	357.40
ggt	G	0	0.13
ggc	G	0	0.13
gga	G	1	0.46
ggg	G	0	0.28
---	--	------	------
mPD		0.12	0.48
		nPD:	0.24
		N. weight:	0.85
		Sc. PD:	0.04
		Sc. rank:	230.8

PB2			
Pos.	682	obs:	exp:
tct	S	0	0.16
tcc	S	0	0.13
tca	S	0	0.25
tcg	S	0	0.06
agt	S	0	0.20
agc	S	1	0.20
ggt	G	3	120.30
ggc	G	42	114.00
gga	G	688	414.70
ggg	G	164	248.00
---	--	------	------
mPD		0.38	0.68
		nPD:	0.56
		N. weight:	0.33
		Sc. PD:	0.12
		Sc. rank:	185.3

PB2			
Pos.	683	obs:	exp:
att	I	0	1.82
atc	I	0	1.21
ata	I	5	1.97
act	T	1	231.10
acc	T	0	181.60
aca	T	887	409.80
acg	T	4	69.48
aaa	K	1	0.59
aag	K	0	0.41
---	--	------	------
mPD		0.024	0.69
		nPD:	0.04
		N. weight:	0.95
		Sc. PD:	-0.14
		Sc. rank:	-369.3

PB2			
Pos.	684	obs:	exp:
tct	S	0	0.32
tcc	S	1	0.26
tca	S	1	0.50
tcg	S	0	0.12
act	T	0	0.52
acc	T	0	0.41
aca	T	2	0.92
acg	T	0	0.16
agt	S	0	0.41
agc	S	0	0.39
gtt	V	0	8.88
gtc	V	0	8.61
gta	V	40	8.90
gtg	V	3	16.61
gct	A	47	218.10
gcc	A	17	159.80
gca	A	781	397.90
gcg	A	6	75.27
---	--	------	------
mPD		0.26	0.78
		nPD:	0.33
		N. weight:	0.66
		Sc. PD:	0.085
		Sc. rank:	256.2

PB2			
Pos.	685	obs:	exp:
ggt	G	0	120.40
ggc	G	1	114.10
gga	G	864	415.20
ggg	G	33	248.30
---	--	------	------
mPD		0.073	0.68
		nPD:	0.11
		N. weight:	0.79
		Sc. PD:	-0.063
		Sc. rank:	-87.8

PB2			
Pos.	686	obs:	exp:
gtt	V	11	185.40
gtc	V	0	179.90
gta	V	138	185.90
gtg	V	749	346.80
---	--	------	------
mPD		0.28	0.73
		nPD:	0.39
		N. weight:	0.71
		Sc. PD:	0.14
		Sc. rank:	312.9

PB2			
Pos.	687	obs:	exp:
aaa	K	1	0.59
aag	K	0	0.41
gat	D	1	0.54
gac	D	0	0.46
gaa	E	833	539.00
gag	E	63	357.00
---	--	------	------
mPD		0.13	0.48
		nPD:	0.28
		N. weight:	0.36
		Sc. PD:	0.031
		Sc. rank:	123.3

PB2			
Pos.	688	obs:	exp:
ttt	F	2	0.79
ttc	F	0	1.21
tct	S	869	143.40
tcc	S	23	116.00
tca	S	4	226.10
tcg	S	0	52.14
agt	S	0	183.60
agc	S	0	174.70
---	--	------	------
mPD		0.063	1.7
		nPD:	0.04
		N. weight:	2.1
		Sc. PD:	-0.31
		Sc. rank:	-801.3

PB2			
Pos.	689	obs:	exp:
gct	A	42	230.10
gcc	A	3	168.60
gca	A	224	419.90
gcg	A	629	79.43
---	--	------	------
mPD		0.45	0.67
		nPD:	0.66
		N. weight:	1.5
		Sc. PD:	0.69
		Sc. rank:	893.8

PB2			
Pos.	690	obs:	exp:
gtt	V	37	185.40
gtc	V	5	179.90
gta	V	844	185.90
gtg	V	12	346.80
---	--	------	------
mPD		0.11	0.73
		nPD:	0.16
		N. weight:	1.6
		Sc. PD:	-0.051
		Sc. rank:	52.5

PB2			
Pos.	691	obs:	exp:
tta	L	65	78.92
ttg	L	798	167.60
ctt	L	2	152.20
ctc	L	0	150.40
cta	L	15	149.70
ctg	L	18	199.20
---	--	------	------
mPD		0.24	1.1
		nPD:	0.22
		N. weight:	1.6
		Sc. PD:	0.042
		Sc. rank:	357.7

PB2			
Pos.	692	obs:	exp:
cgt	R	0	38.32
cgc	R	0	45.57
cga	R	1	89.44
cgg	R	0	74.71
aaa	K	1	0.59
aag	K	0	0.41
aga	R	626	409.60
agg	R	270	239.40
---	--	------	------
mPD		0.43	0.97
		nPD:	0.44
		N. weight:	0.42
		Sc. PD:	0.1
		Sc. rank:	201.4

PB2			
Pos.	693	obs:	exp:
ggt	G	1	120.40
ggc	G	0	114.10
gga	G	819	415.20
ggg	G	78	248.30
---	--	------	------
mPD		0.16	0.68
		nPD:	0.24
		N. weight:	0.65
		Sc. PD:	0.03
		Sc. rank:	174.2

PB2			
Pos.	694	obs:	exp:
ttt	F	838	354.40
ttc	F	60	543.60
---	--	------	------
mPD		0.12	0.48
		nPD:	0.26
		N. weight:	0.83
		Sc. PD:	0.056
		Sc. rank:	253.4

PB2			
Pos.	695	obs:	exp:
tta	L	1	78.92
ttg	L	4	167.60
ctt	L	4	152.20
ctc	L	40	150.40
cta	L	51	149.70
ctg	L	798	199.20
---	--	------	------
mPD		0.21	1.1
		nPD:	0.19
		N. weight:	1.4
		Sc. PD:	-0.004
		Sc. rank:	188.9

PB2			
Pos.	696	obs:	exp:
att	I	874	327.30
atc	I	23	217.00
ata	I	0	352.70
gtt	V	1	0.21
gtc	V	0	0.20
gta	V	0	0.21
gtg	V	0	0.39
---	--	------	------
mPD		0.052	0.66
		nPD:	0.08
		N. weight:	1.1
		Sc. PD:	-0.12
		Sc. rank:	-259.9

PB2			
Pos.	697	obs:	exp:
tta	L	6	78.83
ttg	L	44	167.40
ctt	L	1	152.00
ctc	L	0	150.30
cta	L	358	149.50
ctg	L	488	198.90
att	I	0	0.36
atc	I	0	0.24
ata	I	1	0.39
---	--	------	------
mPD		0.59	1.1
		nPD:	0.53
		N. weight:	0.95
		Sc. PD:	0.31
		Sc. rank:	515.4

PB2			
Pos.	698	obs:	exp:
ggt	G	3	120.40
ggc	G	876	114.10
gga	G	19	415.20
ggg	G	0	248.30
---	--	------	------
mPD		0.048	0.68
		nPD:	0.07
		N. weight:	2.4
		Sc. PD:	-0.28
		Sc. rank:	-620.9

PB2			
Pos.	699	obs:	exp:
aat	N	0	0.51
aac	N	1	0.49
aaa	K	769	524.90
aag	K	127	371.10
gaa	E	1	0.60
gag	E	0	0.40
---	--	------	------
mPD		0.25	0.49
		nPD:	0.51
		N. weight:	0.23
		Sc. PD:	0.069
		Sc. rank:	119.0

PB2			
Pos.	700	obs:	exp:
gaa	E	867	540.20
gag	E	31	357.80
---	--	------	------
mPD		0.067	0.48
		nPD:	0.14
		N. weight:	0.47
		Sc. PD:	-0.023
		Sc. rank:	-7.9

PB2			
Pos.	701	obs:	exp:
aat	N	0	1.53
aac	N	3	1.47
gat	D	23	483.20
gac	D	872	411.80
---	--	------	------
mPD		0.057	0.50
		nPD:	0.11
		N. weight:	0.82
		Sc. PD:	-0.062
		Sc. rank:	-70.7

PB2			
Pos.	702	obs:	exp:
cgt	R	0	0.21
cgc	R	0	0.25
cga	R	0	0.50
cgg	R	0	0.42
aaa	K	685	523.10
aag	K	208	369.90
aga	R	2	2.28
agg	R	3	1.33
---	--	------	------
mPD		0.37	0.50
		nPD:	0.74
		N. weight:	0.1
		Sc. PD:	0.053
		Sc. rank:	60.8

PB2			
Pos.	703	obs:	exp:
cgt	R	0	38.36
cgc	R	0	45.62
cga	R	0	89.54
cgg	R	0	74.79
aga	R	798	410.00
agg	R	100	239.70
---	--	------	------
mPD		0.20	0.96
		nPD:	0.21
		N. weight:	0.63
		Sc. PD:	0.009
		Sc. rank:	112.4

PB2			
Pos.	704	obs:	exp:
tat	Y	848	476.10
tac	Y	50	421.90
---	--	------	------
mPD		0.11	0.50
		nPD:	0.21
		N. weight:	0.54
		Sc. PD:	0.011
		Sc. rank:	105.8

PB2			
Pos.	705	obs:	exp:
ggt	G	18	120.40
ggc	G	40	114.10
gga	G	60	415.20
ggg	G	780	248.30
---	--	------	------
mPD		0.24	0.68
		nPD:	0.35
		N. weight:	0.99
		Sc. PD:	0.16
		Sc. rank:	408.0

PB2			
Pos.	706	obs:	exp:
cct	P	0	228.30
ccc	P	2	160.10
cca	P	894	366.90
ccg	P	2	142.70
---	--	------	------
mPD		0.0089	0.71
		nPD:	0.01
		N. weight:	1.1
		Sc. PD:	-0.19
		Sc. rank:	-554.7

PB2			
Pos.	707	obs:	exp:
tct	S	0	0.32
tcc	S	0	0.26
tca	S	2	0.50
tcg	S	0	0.12
cct	P	0	0.25
ccc	P	0	0.18
cca	P	1	0.41
ccg	P	0	0.16
agt	S	0	0.41
agc	S	0	0.39
gtt	V	0	0.21
gtc	V	0	0.20
gta	V	1	0.21
gtg	V	0	0.39
gct	A	0	229.10
gcc	A	1	167.90
gca	A	888	418.00
gcg	A	5	79.07
---	--	------	------
mPD		0.022	0.68
		nPD:	0.03
		N. weight:	0.92
		Sc. PD:	-0.14
		Sc. rank:	-375.4

PB2			
Pos.	708	obs:	exp:
ttt	F	2	0.79
ttc	F	0	1.21
tta	L	2	78.75
ttg	L	880	167.20
ctt	L	0	151.80
ctc	L	0	150.10
cta	L	5	149.40
ctg	L	9	198.70
---	--	------	------
mPD		0.051	1.1
		nPD:	0.05
		N. weight:	2.
		Sc. PD:	-0.28
		Sc. rank:	-699.6

PB2			
Pos.	709	obs:	exp:
tct	S	0	143.70
tcc	S	0	116.20
tca	S	0	226.60
tcg	S	0	52.26
agt	S	11	184.00
agc	S	887	175.10
---	--	------	------
mPD		0.024	1.7
		nPD:	0.01
		N. weight:	2.
		Sc. PD:	-0.34
		Sc. rank:	-969.5

PB2			
Pos.	710	obs:	exp:
att	I	5	327.70
atc	I	893	217.20
ata	I	0	353.10
---	--	------	------
mPD		0.011	0.65
		nPD:	0.02
		N. weight:	1.7
		Sc. PD:	-0.29
		Sc. rank:	-837.1

PB2

Pos.	711	obs:	exp:
tct	S	0	5.76
tcc	S	0	4.66
tca	S	0	9.09
tcg	S	0	2.09
aat	N	198	438.50
aac	N	664	423.50
agt	S	0	7.38
agc	S	36	7.02
mPD		0.42	0.64
		nPD:	0.66
		N. weight:	0.28
		Sc. PD:	0.13
		Sc. rank:	166.9

PB2

Pos.	712	obs:	exp:
gaa	E	512	540.20
gag	E	386	357.80
mPD		0.49	0.48
		nPD:	1.02
		N. weight:	0.0076
		Sc. PD:	0.0061
		Sc. rank:	4.7

PB2

Pos.	713	obs:	exp:
tta	L	16	78.92
ttg	L	827	167.60
ctt	L	0	152.20
ctc	L	0	150.40
cta	L	1	149.70
ctg	L	54	199.20
mPD		0.15	1.1
		nPD:	0.14
		N. weight:	1.7
		Sc. PD:	-0.089
		Sc. rank:	-34.3

PB2

Pos.	714	obs:	exp:
tct	S	0	142.30
tcc	S	0	115.10
tca	S	0	224.40
tcg	S	0	51.73
tgt	C	0	2.05
tgc	C	5	2.95
aat	N	0	0.51
aac	N	1	0.49
agt	S	4	182.20
agc	S	885	173.40
ggt	G	0	0.40
ggc	G	3	0.38
gga	G	0	1.39
ggg	G	0	0.83
mPD		0.029	1.7
		nPD:	0.02
		N. weight:	2.
		Sc. PD:	-0.34
		Sc. rank:	-962.0

PB2

Pos.	715	obs:	exp:
tct	S	0	0.16
tcc	S	0	0.13
tca	S	0	0.25
tcg	S	0	0.06
act	T	0	0.26
acc	T	1	0.20
aca	T	0	0.46
acg	T	0	0.08
aat	N	544	455.80
aac	N	352	440.20
agt	S	1	0.20
agc	S	0	0.20
mPD		0.48	0.51
		nPD:	0.95
		N. weight:	0.034
		Sc. PD:	0.025
		Sc. rank:	21.0

PB2

Pos.	716	obs:	exp:
tta	L	0	78.83
ttg	L	0	167.40
ctt	L	895	152.00
ctc	L	2	150.30
cta	L	0	149.50
ctg	L	0	198.90
cct	P	1	0.25
ccc	P	0	0.18
cca	P	0	0.41
ccg	P	0	0.16
mPD		0.0067	1.1
		nPD:	0.01
		N. weight:	2.2
		Sc. PD:	-0.39
		Sc. rank:	-1199.5

PB2

Pos.	717	obs:	exp:
gct	A	2	230.10
gcc	A	1	168.60
gca	A	70	419.90
gcg	A	825	79.43
mPD		0.15	0.67
		nPD:	0.22
		N. weight:	2.5
		Sc. PD:	0.078
		Sc. rank:	603.8

PB2

Pos.	718	obs:	exp:
aaa	K	861	526.00
aag	K	37	372.00
mPD		0.079	0.49
		nPD:	0.16
		N. weight:	0.48
		Sc. PD:	-0.013
		Sc. rank:	21.0

PB2

Pos.	719	obs:	exp:
ggt	G	1	120.40
ggc	G	0	114.10
gga	G	272	415.20
ggg	G	625	248.30
mPD		0.42	0.68
		nPD:	0.63
		N. weight:	0.65
		Sc. PD:	0.27
		Sc. rank:	371.8

PB2

Pos.	720	obs:	exp:
gaa	E	374	540.20
gag	E	524	357.80
mPD		0.49	0.48
		nPD:	1.02
		N. weight:	0.092
		Sc. PD:	0.073
		Sc. rank:	57.1

PB2

Pos.	721	obs:	exp:
aaa	K	20	526.00
aag	K	878	372.00
mPD		0.044	0.49
		nPD:	0.09
		N. weight:	0.97
		Sc. PD:	-0.094
		Sc. rank:	-194.5

PB2

Pos.	722	obs:	exp:
gct	A	882	230.10
gcc	A	11	168.60
gca	A	4	419.90
gcg	A	1	79.43
mPD		0.035	0.67
		nPD:	0.05
		N. weight:	1.6
		Sc. PD:	-0.21
		Sc. rank:	-518.5

PB2

Pos.	723	obs:	exp:
aat	N	896	456.90
aac	N	2	441.10
mPD		0.0044	0.50
		nPD:	0.01
		N. weight:	0.83
		Sc. PD:	-0.15
		Sc. rank:	-431.0

PB2

Pos.	724	obs:	exp:
gtt	V	23	185.40
gtc	V	0	179.90
gta	V	8	185.90
gtg	V	867	346.80
mPD		0.067	0.73
		nPD:	0.09
		N. weight:	1.
		Sc. PD:	-0.096
		Sc. rank:	-185.1

PB2

Pos.	725	obs:	exp:
tta	L	4	78.92
ttg	L	831	167.60
ctt	L	0	152.20
ctc	L	0	150.40
cta	L	36	149.70
ctg	L	27	199.20
mPD		0.22	1.1
		nPD:	0.19
		N. weight:	1.7
		Sc. PD:	0.0061
		Sc. rank:	267.0

PB2

Pos.	726	obs:	exp:
att	I	40	327.30
atc	I	2	217.00
ata	I	855	352.70
atg	M	1	1.00
mPD		0.092	0.65
		nPD:	0.14
		N. weight:	0.93
		Sc. PD:	-0.046
		Sc. rank:	-14.3

PB2

Pos.	727	obs:	exp:
ggt	G	0	120.40
ggc	G	1	114.10
gga	G	8	415.20
ggg	G	889	248.30
mPD		0.020	0.68
		nPD:	0.03
		N. weight:	1.5
		Sc. PD:	-0.24
		Sc. rank:	-649.2

PB2

Pos.	728	obs:	exp:
caa	Q	897	461.30
cag	Q	1	436.70
mPD		0.0022	0.50
		nPD:	0.
		N. weight:	0.83
		Sc. PD:	-0.15
		Sc. rank:	-456.8

PB2

Pos.	729	obs:	exp:
ggt	G	1	120.40
ggc	G	1	114.10
gga	G	889	415.20
ggg	G	7	248.30
mPD		0.020	0.68
		nPD:	0.03
		N. weight:	0.9
		Sc. PD:	-0.14
		Sc. rank:	-379.9

PB2

Pos.	730	obs:	exp:
aat	N	0	0.51
aac	N	1	0.49
gat	D	0	484.20
gac	D	897	412.80
mPD		0.0022	0.50
		nPD:	0.
		N. weight:	0.97
		Sc. PD:	-0.18
		Sc. rank:	-536.7

PB2

Pos.	731	obs:	exp:
gtt	V	92	185.40
gtc	V	0	179.90
gta	V	10	185.90
gtg	V	796	346.80
mPD		0.20	0.73
		nPD:	0.28
		N. weight:	0.8
		Sc. PD:	0.07
		Sc. rank:	277.5

PB2

Pos.	732	obs:	exp:
tta	L	0	0.09
ttg	L	1	0.19
ctt	L	0	0.17
ctc	L	0	0.17
cta	L	0	0.17
ctg	L	0	0.22
atg	M	1	1.00
gtt	V	0	185.00
gtc	V	0	179.50
gta	V	0	185.50
gtg	V	896	346.00
mPD		0.0045	0.73
		nPD:	0.01
		N. weight:	1.2
		Sc. PD:	-0.21
		Sc. rank:	-645.8

PB2

Pos.	733	obs:	exp:
tta	L	0	78.92
ttg	L	898	167.60
ctt	L	0	152.20
ctc	L	0	150.40
cta	L	0	149.70
ctg	L	0	199.20
mPD		0	1.1
		nPD:	0.
		N. weight:	2.1
		Sc. PD:	-0.39
		Sc. rank:	-1300.6

PB2

Pos.	734	obs:	exp:
gtt	V	1	185.40
gtc	V	0	179.90
gta	V	781	185.90
gtg	V	116	346.80
mPD		0.23	0.73
		nPD:	0.31
		N. weight:	1.4
		Sc. PD:	0.16
		Sc. rank:	527.0

PB2

Pos.	735	obs:	exp:
atg	M	898	898.00
mPD		0	0
		nPD:	1.
		N. weight:	0.
		Sc. PD:	0
		Sc. rank:	0

PB2			
Pos.	736	obs:	exp:
aaa	K	898	526.00
aag	K	0	372.00
---	--	------	------
mPD		0	0.49
		nPD:	0.
		N. weight:	0.67
		Sc. PD:	-0.12
		Sc. rank:	-414.3

,

PB2			
Pos.	737	obs:	exp:
cgt	R	0	38.36
cgc	R	0	45.62
cga	R	14	89.54
cgg	R	884	74.79
aga	R	0	410.00
agg	R	0	239.70
---	--	------	------
mPD		0.031	0.96
		nPD:	0.03
		N. weight:	3.
		Sc. PD:	-0.46
		Sc. rank:	-1236.3

,

PB2			
Pos.	738	obs:	exp:
aaa	K	898	526.00
aag	K	0	372.00
---	--	------	------
mPD		0	0.49
		nPD:	0.
		N. weight:	0.67
		Sc. PD:	-0.12
		Sc. rank:	-414.3

,

PB2			
Pos.	739	obs:	exp:
tta	L	0	0.09
ttg	L	0	0.19
ctt	L	0	0.17
ctc	L	0	0.17
cta	L	0	0.17
ctg	L	1	0.22
cgt	R	4	38.32
cgc	R	0	45.57
cga	R	24	89.44
cgg	R	869	74.71
aga	R	0	409.60
agg	R	0	239.40
---	--	------	------
mPD		0.063	0.97
		nPD:	0.07
		N. weight:	2.9
		Sc. PD:	-0.36
		Sc. rank:	-814.5

,

PB2			
Pos.	740	obs:	exp:
aat	N	0	1.02
aac	N	2	0.98
gat	D	0	483.70
gac	D	896	412.30
---	--	------	------
mPD		0.0044	0.50
		nPD:	0.01
		N. weight:	0.97
		Sc. PD:	-0.17
		Sc. rank:	-506.9

,

PB2			
Pos.	741	obs:	exp:
tct	S	892	143.70
tcc	S	4	116.20
tca	S	2	226.60
tcg	S	0	52.26
agt	S	0	184.00
agc	S	0	175.10
---	--	------	------
mPD		0.013	1.7
		nPD:	0.01
		N. weight:	2.2
		Sc. PD:	-0.4
		Sc. rank:	-1173.5

,

PB2			
Pos.	742	obs:	exp:
tct	S	0	143.70
tcc	S	0	116.20
tca	S	0	226.60
tcg	S	0	52.26
agt	S	2	184.00
agc	S	896	175.10
---	--	------	------
mPD		0.0044	1.7
		nPD:	0.
		N. weight:	2.
		Sc. PD:	-0.37
		Sc. rank:	-1143.2

,

PB2			
Pos.	743	obs:	exp:
att	I	0	327.70
atc	I	0	217.20
ata	I	898	353.10
---	--	------	------
mPD		0	0.65
		nPD:	0.
		N. weight:	1.2
		Sc. PD:	-0.22
		Sc. rank:	-723.1

,

PB2			
Pos.	744	obs:	exp:
tta	L	0	78.92
ttg	L	0	167.60
ctt	L	898	152.20
ctc	L	0	150.40
cta	L	0	149.70
ctg	L	0	199.20
---	--	------	------
mPD		0	1.1
		nPD:	0.
		N. weight:	2.2
		Sc. PD:	-0.41
		Sc. rank:	-1375.3

,

PB2			
Pos.	745	obs:	exp:
act	T	897	232.40
acc	T	0	182.70
aca	T	0	412.10
acg	T	0	69.87
gct	A	1	0.26
gcc	A	0	0.19
gca	A	0	0.47
gcg	A	0	0.09
---	--	------	------
mPD		0.0022	0.68
		nPD:	0.
		N. weight:	1.7
		Sc. PD:	-0.31
		Sc. rank:	-948.4

,

PB2			
Pos.	746	obs:	exp:
gat	D	2	484.80
gac	D	896	413.20
---	--	------	------
mPD		0.0044	0.50
		nPD:	0.01
		N. weight:	0.96
		Sc. PD:	-0.17
		Sc. rank:	-494.6

,

PB2			
Pos.	747	obs:	exp:
tct	S	0	143.70
tcc	S	0	116.20
tca	S	0	226.60
tcg	S	0	52.26
agt	S	804	184.00
agc	S	94	175.10
---	--	------	------
mPD		0.19	1.7
		nPD:	0.11
		N. weight:	1.6
		Sc. PD:	-0.12
		Sc. rank:	-131.6

,

PB2			
Pos.	748	obs:	exp:
caa	Q	0	461.30
cag	Q	898	436.70
---	--	------	------
mPD		0	0.50
		nPD:	0.
		N. weight:	0.91
		Sc. PD:	-0.17
		Sc. rank:	-558.5

,

PB2			
Pos.	749	obs:	exp:
act	T	0	232.60
acc	T	0	182.90
aca	T	898	412.60
acg	T	0	69.95
---	--	------	------
mPD		0	0.67
		nPD:	0.
		N. weight:	0.98
		Sc. PD:	-0.18
		Sc. rank:	-602.6

,

PB2			
Pos.	750	obs:	exp:
gct	A	1	230.10
gcc	A	0	168.60
gca	A	1	419.90
gcg	A	896	79.43
---	--	------	------
mPD		0.0045	0.67
		nPD:	0.01
		N. weight:	3.
		Sc. PD:	-0.54
		Sc. rank:	-1619.4

,

PB2			
Pos.	751	obs:	exp:
act	T	0	232.60
acc	T	898	182.90
aca	T	0	412.60
acg	T	0	69.95
---	--	------	------
mPD		0	0.67
		nPD:	0.
		N. weight:	2.
		Sc. PD:	-0.37
		Sc. rank:	-1233.0

,

PB2			
Pos.	752	obs:	exp:
aaa	K	898	526.00
aag	K	0	372.00
---	--	------	------
mPD		0	0.49
		nPD:	0.
		N. weight:	0.67
		Sc. PD:	-0.12
		Sc. rank:	-414.3

,

PB2			
Pos.	753	obs:	exp:
cgt	R	0	38.36
cgc	R	0	45.62
cga	R	0	89.54
cgg	R	0	74.79
aga	R	382	410.00
agg	R	516	239.70
---	--	------	------
mPD		0.49	0.96
		nPD:	0.51
		N. weight:	0.52
		Sc. PD:	0.16
		Sc. rank:	275.0

,

PB2			
Pos.	754	obs:	exp:
att	I	897	327.70
atc	I	1	217.20
ata	I	0	353.10
---	--	------	------
mPD		0.0022	0.65
		nPD:	0.
		N. weight:	1.3
		Sc. PD:	-0.23
		Sc. rank:	-695.9

,

PB2			
Pos.	755	obs:	exp:
cgt	R	0	38.36
cgc	R	0	45.62
cga	R	1	89.54
cgg	R	897	74.79
aga	R	0	410.00
agg	R	0	239.70
---	--	------	------
mPD		0.0022	0.96
		nPD:	0.
		N. weight:	3.1
		Sc. PD:	-0.57
		Sc. rank:	-1758.5

,

PB2			
Pos.	756	obs:	exp:
atg	M	898	898.00
---	--	------	------
mPD		0	0
		nPD:	1.
		N. weight:	0.
		Sc. PD:	0
		Sc. rank:	0

,

PB2			
Pos.	757	obs:	exp:
gct	A	0	230.10
gcc	A	898	168.60
gca	A	0	419.90
gcg	A	0	79.43
---	--	------	------
mPD		0	0.67
		nPD:	0.
		N. weight:	2.1
		Sc. PD:	-0.39
		Sc. rank:	-1295.9

,

PB2			
Pos.	758	obs:	exp:
att	I	0	327.70
atc	I	898	217.20
ata	I	0	353.10
---	--	------	------
mPD		0	0.65
		nPD:	0.
		N. weight:	1.8
		Sc. PD:	-0.33
		Sc. rank:	-1099.7

,

PB2			
Pos.	759	obs:	exp:
tct	S	0	0.16
tcc	S	0	0.13
tca	S	0	0.25
tcg	S	0	0.06
tat	Y	1	0.53
tac	Y	0	0.47
aat	N	895	455.30
aac	N	0	439.70
agt	S	1	0.20
agc	S	0	0.20
gat	D	1	0.54
gac	D	0	0.46
---	--	------	------
mPD		0.0067	0.51
		nPD:	0.01
		N. weight:	0.85
		Sc. PD:	-0.15
		Sc. rank:	-430.1

,

PB2			
Pos.	760	obs:	exp:
taa	.	804	386.20
tag	.	94	227.10
tga	.	0	284.70
---	--	------	------
mPD		0.19	0.81
		nPD:	0.23
		N. weight:	0.71
		Sc. PD:	0.028
		Sc. rank:	178.1

}

Amino acid level conservation per codon

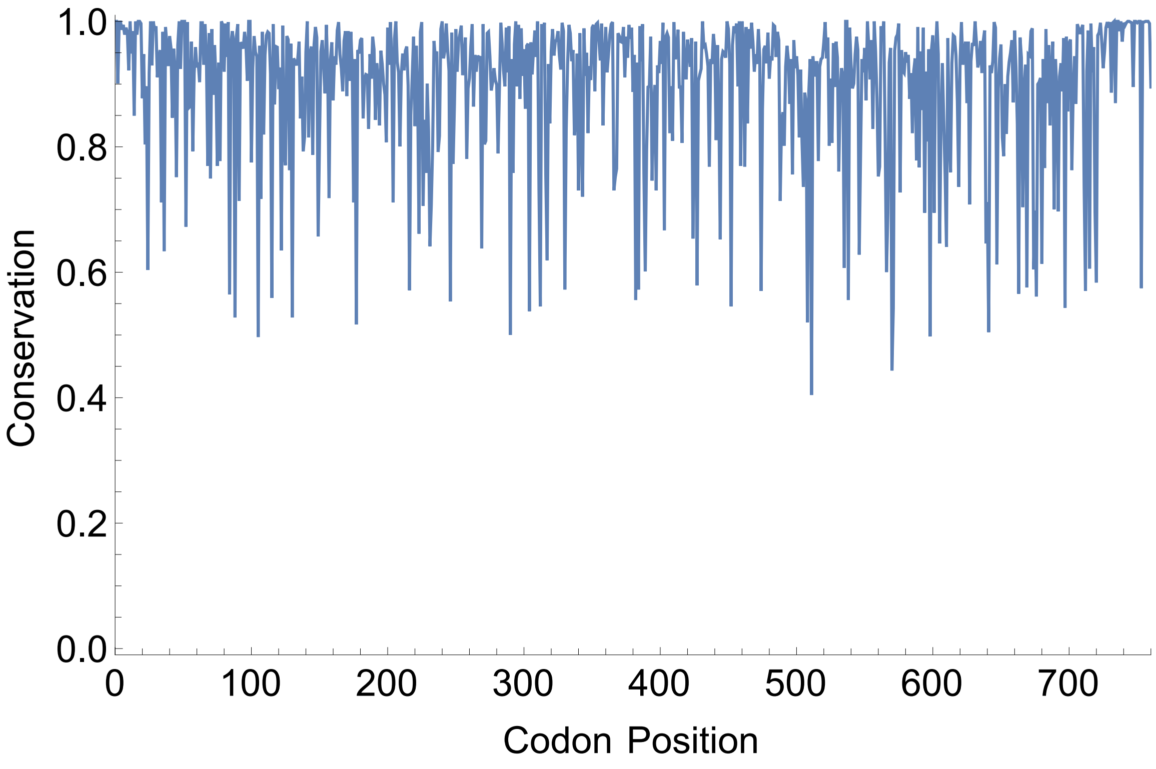

Conservation analysis plots

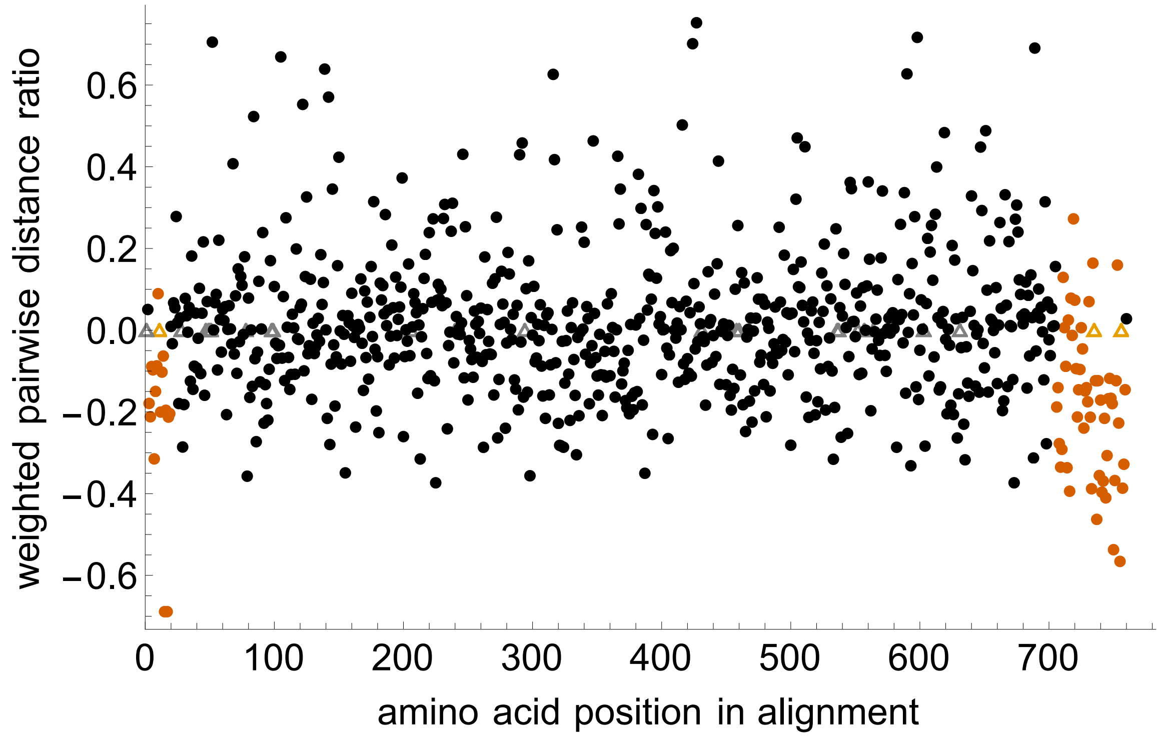

PB2

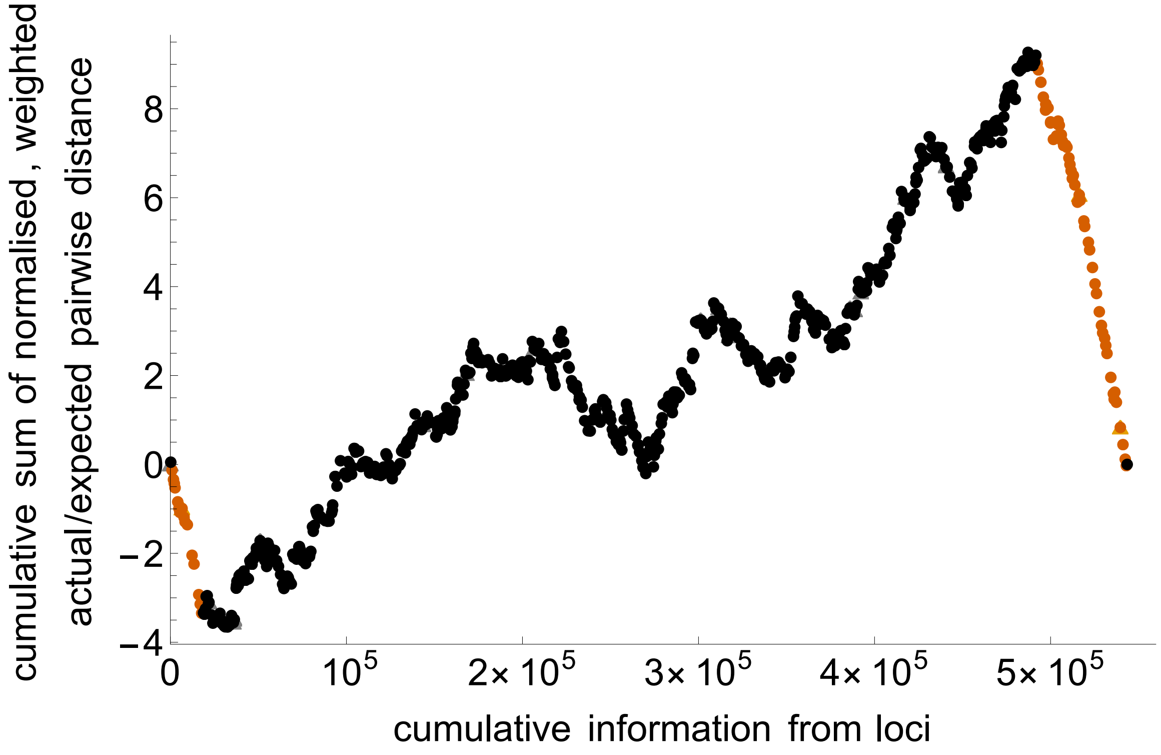

PB2

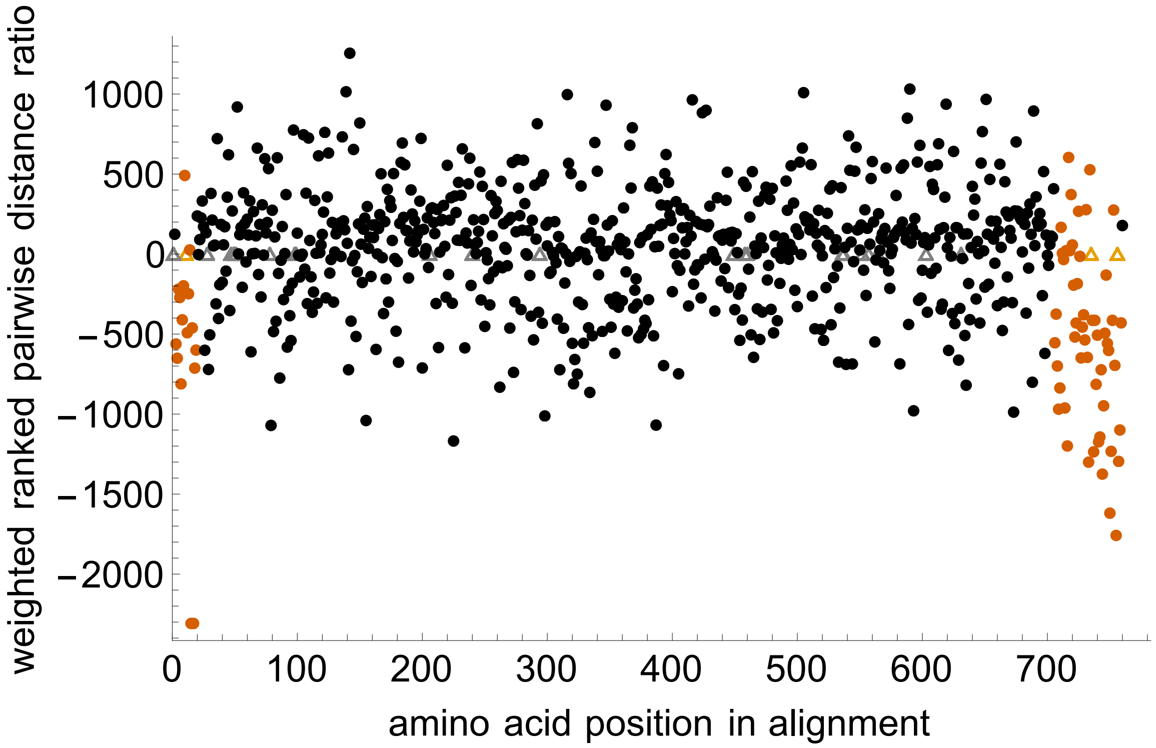
weighted ranked pairwise distance ratio
1000
500
0
−500
−1000
−1500
−2000
0
100
200
300
400
500
600
700
amino acid position in alignment

PB2

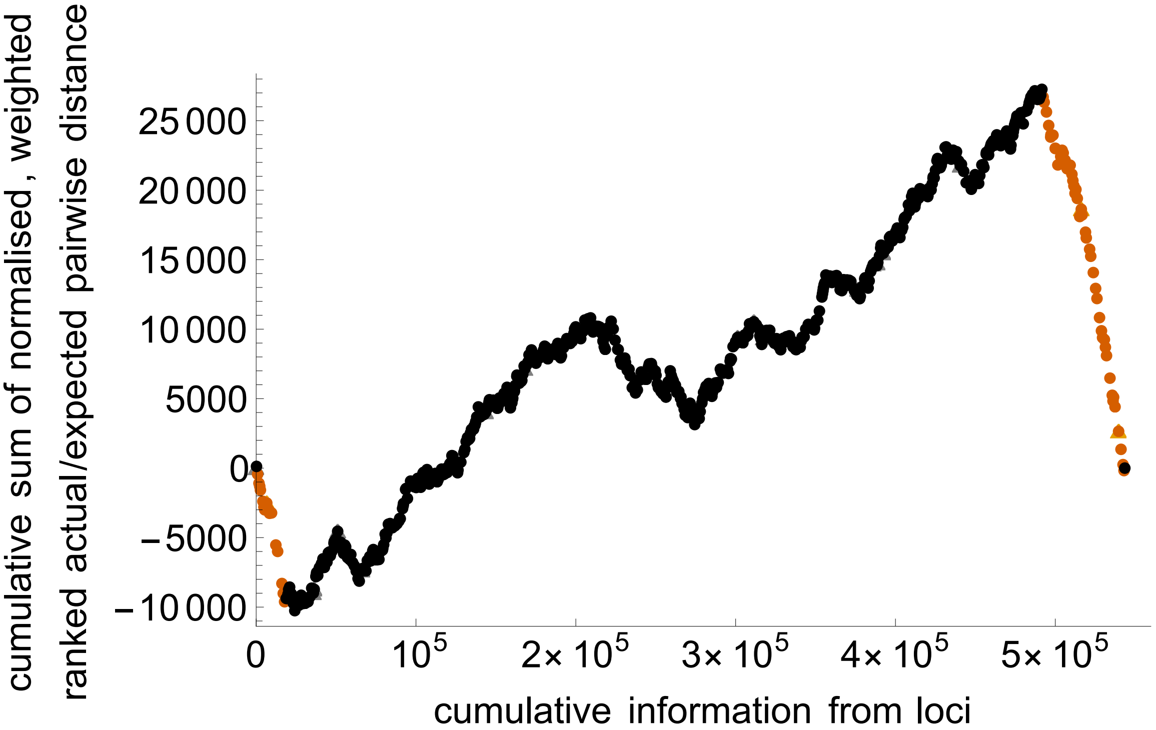
cumulative sum of normalised, weighted
ranked actual/expected pairwise distance
25 000
20 000
15 000
10 000
5000
0
−5000
−10 000
0
10^5
2×10^5
3×10^5
4×10^5
5×10^5
cumulative information from loci

PB2

PB1

Gene length histogram

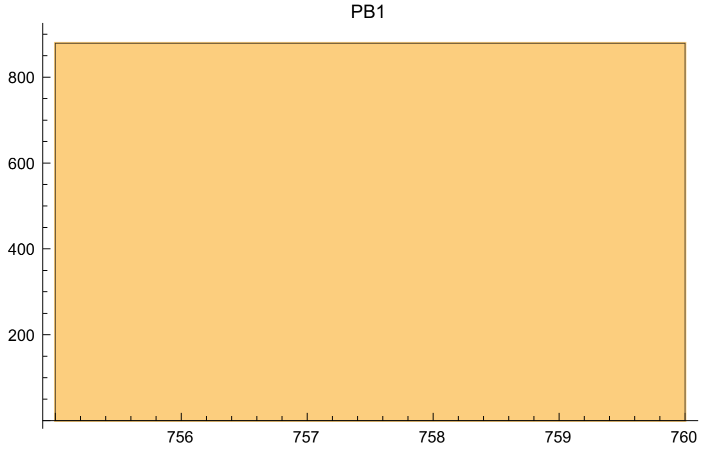

Information vs. nPD

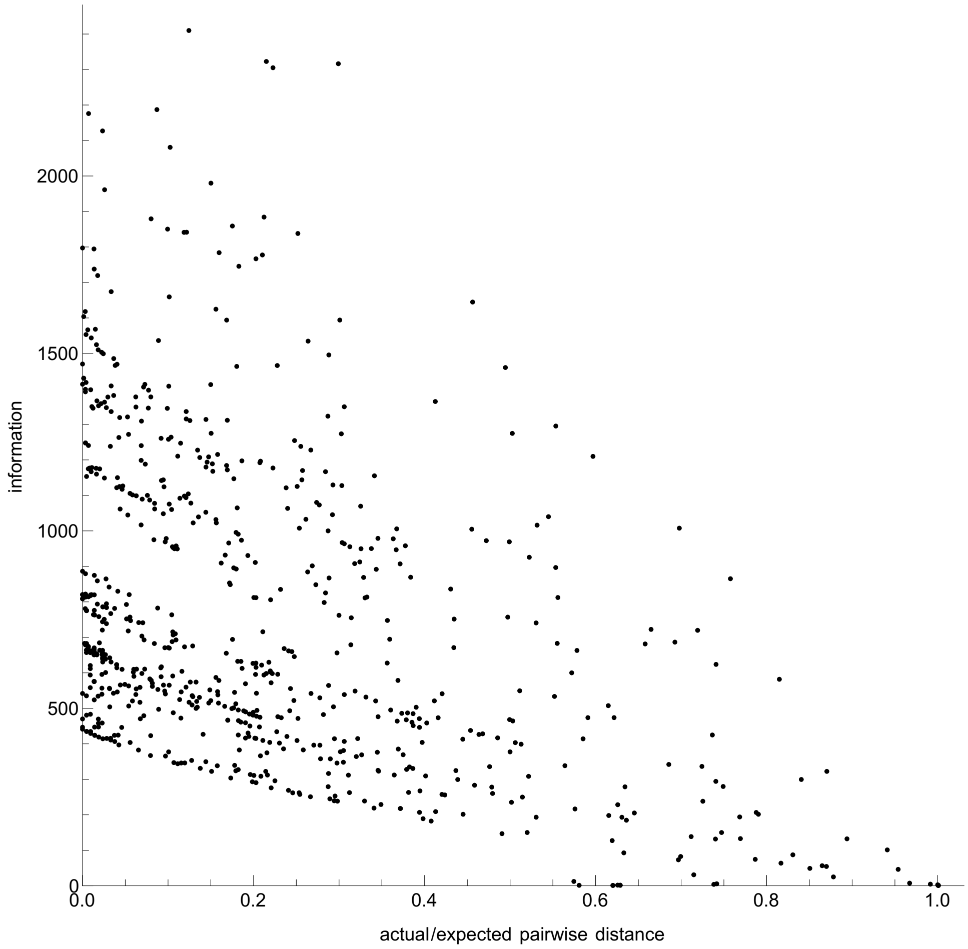

PB1

Example sequences highlighted by regions found in analyses to be conserved

Interesting points (by weighted raw PD) highlighted for gene PB1:

ATGGATGTCAATCCGACTTTACTTTTCTTAAAAGTGCCAGCGCAAAATGCTATAAGCACTACATTCCCTTACACTG⋱
GAGATCCTCCATACAGCCATGGAACAGGAACAGGGTACACCATGGATACAGTCAACAGAACACATCAATACTCAG⋱
AAAAGGGAAAGTGGACAACAAACACAGAGACCGGAGCACCCCAACTCAACCCAATTGATGGACCATTACCTGAGG⋱
ACAACGAGCCAAGTGGATATGCACAAACGGATTGCGTATTGGAAGCAATGGCTTTCCTTGAAGAATCCCACCCAG⋱
GGATCTTTGAAAACTCTTGTCTTGAAACGATGGAAGTTGTTCAGCAAACAAGAGTGGACAAACTAACCCAAGGTC⋱
GCCAGACTTATGACTGGACACTGAATAGAAACCAACCAGCTGCAACTGCTTTGGCCAACACTATAGAGGTCTTCA⋱
GATCGAACGGTCTGACAGCCAATGAATCAGGGAGACTAATAGATTTCCTCAAGGATGTGATGGAATCAATGGATA⋱
AAGAAGAAATGGAAATAACAACACATTTCCAGAGAAAGAGAAGAGTAAGGGACAACATGACCAAGAAGATGGTCA⋱
CACAAAGAACAATAGGGAAAAAGAAACAGAGGCTGAACAAGAGGAGCTACTTAATAAGAGCACTTACACTGAACA⋱
CAATGACAAAAGATGCAGAAAGAGGCAAATTGAAAAGGCGGGCAATTGCAACACCCGGGATGCAGATTAGAGGAT⋱
TCGTGTACTTTGTTGAAACACTGGCGAGGAGCATCTGTGAGAAACTTGAGCAATCCGGACTTCCTGTTGGAGGGA⋱
ATGAGAAGAAGGCTAAATTGGCAAATGTTGTGAGAAAAATGATGACTAACTCACAAGATACAGAACTCTCCTTTA⋱
CAATTACTGGAGACAACACCAAATGGAATGAGAATCAAAACCCTCGGATGTTCCTGGCAATGATAACATACATCA⋱
CAAGAAACCAACCTGAATGGTTTAGAAATGTTTTGAGCATTGCCCCTATAATGTTCTCAAACAAAATGGCGAGAT⋱
TAGGAAAAGGATACATGTTTGAAAGTAAGAGTATGAAGCTACGGACACAAATACCGGCAGAACTGCTTGCAAACA⋱
TTGACTTGAAATATTTCAACGAATCAACAAGAAAGAAAATCGAGAAAATAAGACCTCTGCTAATTGATGGCACAG⋱
CCTCATTGAGTCCTGGAATGATGATGGGCATGTTCAATATGCTGAGCACAGTATTAGGAGTCTCAATCCTGAATC⋱
TTGGGCAAAAGAAGTACACCAAAACCACATACTGGTGGGATGGACTCCAATCCTCTGATGATTTCGCTCTCATAG⋱
TAAATGCACCGAATCATGAGGGGATACAAGCAGGAGTGGATAGGTTCTATAGGACCTGCAAACTAGTTGGGATCA⋱
ACATGAGCAAAAAGAAGTCTTACATAAACCGAACAGGAACTTTTGAGTTCACAAGCTTTTTCTACCGCTATGGGT⋱
TTGTAGCCAATTTCAGTATGGAGTTACCCAGTTTTGGAGTGTCTGGAATCAATGAATCGGCTGACATGAGCATTG⋱
GAGTCACAGTGATAAAGAACAATATGATAAACAATGACCTTGGACCAGCAACAGCTCAGATGGCTCTTCAGCTAT⋱
TCATCAAAGACTATAGATACACATACCGGTGCCACAGGGGTGATACACAAATTCAAACGAGGAGATCATTTGAGC⋱
TGAAAAAGCTGTGGGAGCAGACCCGTTCAAAAGCAGGGCTGTTGGTATCAGATGGAGGACCAAATCTATACAACA⋱
TTCGGAATCTCCACATCCCAGAGGTATGCTTGAAATGGGAACTGATGGATGAAGATTACCAGGGCAGGCTGTGTA⋱
ATCCTCTGAACCCGTTTGTCAGTCATAAGGAAATTGAGTCCGTAAACAATGCTGTGGTAATGCCAGCCCATGGCC⋱
CAGCCAAGAGCATGGAATATGATGCTGTTGCGACTACACACTCATGGATTCCTAAGAGGAACCGTTCCATTCTCA⋱
ATACCAGCCAAAGGGGAATTCTTGAAGATGAGCAAATGTACCAGAAGTGCTGCAGTCTATTCGAGAAATTCTTCC⋱
CCAGTAGTTCATACAGG
AGGCCAGTTGGAATTTCCAGCATGGTGGAGGCCATGGTGTCTAGGGCCCGAATTG
ATGCACGCATTGATTTCGAATCTGGAAGGATTAAGAAGGAAGAGTTTGCTGA
GATCATGAAGATCTGTTCCACCATTGAAGAGCTCAGACGG---CAAAAATAG

Interesting points (by weighted ranked PD) highlighted for gene PB1:

ATG
GATGTCAATCCGACTTTACTTTTCTTAAAAGTGCCAGCGCAAAATGCTATAAGCA
CTACATTCCCTTACACTGGAGATCCTCCATACAGCCATGGAACAGGAACAGG
GTACACCATGGATACAGTCAACAGAACACATCAATACTCAGAAAAGGGAAAGTGGA⋱
CAACAAACACAGAGACCGGAGCACCCCAACTCAACCCAATTGATGGACCATTACCTGAGGACAACGAGCC⋱
AAGTGGATATGCACAAACGGATTGCGTATTGGAAGCAATGGCTTTCCTTGAAGAATCCCACCCAGGGATC⋱
TTTGAAAACTCTTGTCTTGAAACGATGGAAGTTGTTCAGCAAACAAGAGTGGACAAACTAACCCAAGGTC⋱
GCCAGACTTATGACTGGACACTGAATAGAAACCAACCAGCTGCAACTGCTTTGGCCAACACTATAGAGGT⋱
CTTCAGATCGAACGGTCTGACAGCCAATGAATCAGGGAGACTAATAGATTTCCTCAAGGATGTGATGGAA⋱

TCAATGGATAAAGAAGAAATGGAAATAACAACACATTTCCAGAGAAAGAGAAGAGTAAGGGACAACATGA⋱.
CCAAGAAGATGGTCACACAAAGAACAATAGGGAAAAAGAAACAGAGGCTGAACAAGAGGAGCTACTTAAT⋱.
AAGAGCACTTACACTGAACACAATGACAAAAGATGCAGAAAGAGGCAAATTGAAAAGGCGGGCAATTGCA⋱.
ACACCCGGGATGCAGATTAGAGGATTCGTGTACTTTGTTGAAACACTGGCGAGGAGCATCTGTGAGAAAC⋱.
TTGAGCAATCCGGACTTCCTGTTGGAGGGAATGAGAAGAAGGCTAAATTGGCAAATGTTGTGAGAAAAAT⋱.
GATGACTAACTCACAAGATACAGAACTCTCCTTTACAATTACTGGAGACAACACCAAATGGAATGAGAAT⋱.
CAAAACCCTCGGATGTTCCTGGCAATGATAACATACATCACAAGAAACCAACCTGAATGGTTTAGAAATG⋱.
TTTTGAGCATTGCCCCTATAATGTTCTCAAACAAAATGGCGAGATTAGGAAAAGGATACATGTTTGAAAG⋱.
TAAGAGTATGAAGCTACGGACACAAATACCGGCAGAACTGCTTGCAAACATTGACTTGAAATATTTCAAC⋱.
GAATCAACAAGAAAGAAAATCGAGAAAATAAGACCTCTGCTAATTGATGGCACAGCCTCATTGAGTCCTG⋱.
GAATGATGATGGGCATGTTCAATATGCTGAGCACAGTATTAGGAGTCTCAATCCTGAATCTTGGGCAAAA⋱.
GAAGTACACCAAAACCACATACTGGTGGGATGGACTCCAATCCTCTGATGATTTCGCTCTCATAGTAAAT⋱.
GCACCGAATCATGAGGGGATACAAGCAGGAGTGGATAGGTTCTATAGGACCTGCAAACTAGTTGGGATCA⋱.
ACATGAGCAAAAAGAAGTCTTACATAAACCGAACAGGAACTTTTGAGTTCACAAGCTTTTTCTACCGCTA⋱.
TGGGTTTGTAGCCAATTTCAGTATGGAGTTACCCAGTTTTGGAGTGTCTGGAATCAATGAATCGGCTGAC⋱.
ATGAGCATTGGAGTCACAGTGATAAAGAACAATATGATAAACAATGACCTTGGACCAGCAACAGCTCAGA⋱.
TGGCTCTTCAGCTATTCATCAAAGACTATAGATACACATACCGGTGCCACAGGGGTGATACACAAATTCA⋱.
AACGAGGAGATCATTTGAGCTGAAAAAGCTGTGGGAGCAGACCCGTTCAAAAGCAGGGCTGTTGGTATCA⋱.
GATGGAGGACCAAATCTATACAACATTCGGAATCTCCACATCCCAGAGGTATGCTTGAAATGGGAACTGA⋱.
TGGATGAAGATTACCAGGGCAGGCTGTGTAATCCTCTGAACCCGTTTGTCAGTCATAAGGAAATTGAGTC⋱.
CGTAAACAATGCTGTGGTAATGCCAGCCCATGGCCCAGCCAAGAGCATGGAATATGATGCTGTTGCGACT⋱.
ACACACTCATGGATTCCTAAGAGGAACCGTTCCATTCTCAATACCAGCCAAAGGGGAATTCTTGAAGATG⋱.
AGCAAATGTACCAGAAGTGCTGCAGTCTATTCGAGAAATTCTTCCCCAGTAGTTCATACAGG
AGGCCAGTTGGAATTTCCAGCATGGTGGAGGCCATGGTGTCTAGGGCCCGAATTG
ATGCACGCATTGATTTCGAATCTGGAAGGATTAAGAAGGAAGAGTTTGCTGA
GATCATGAAGATCTGTTCCACCATTGAAGAGCTCAGACGG---CAAAAATAG

Per codon conservation report

PB1			
Pos.	1	obs:	exp:
atg	M	879	879.00
---	--	------	------
mPD		0	0
		nPD:	1.
		N. weight:	0.
		Sc. PD:	0
		Sc. rank:	0

PB1			
Pos.	2	obs:	exp:
gat	D	879	474.50
gac	D	0	404.50
---	--	------	------
mPD		0	0.50
		nPD:	0.
		N. weight:	0.76
		Sc. PD:	-0.12
		Sc. rank:	-468.3

PB1			
Pos.	3	obs:	exp:
gtt	V	0	181.50
gtc	V	879	176.10
gta	V	0	182.00
gtg	V	0	339.50
---	--	------	------
mPD		0	0.73
		nPD:	0.
		N. weight:	2.
		Sc. PD:	-0.31
		Sc. rank:	-1221.3

PB1			
Pos.	4	obs:	exp:
act	T	0	0.26
acc	T	0	0.20
aca	T	1	0.46
acg	T	0	0.08
aat	N	819	446.70
aac	N	59	431.30
---	--	------	------
mPD		0.13	0.50
		nPD:	0.26
		N. weight:	0.54
		Sc. PD:	0.049
		Sc. rank:	182.3

PB1			
Pos.	5	obs:	exp:
tct	S	0	0.16
tcc	S	0	0.13
tca	S	0	0.25
tcg	S	1	0.06
cct	P	0	223.30
ccc	P	0	156.50
cca	P	0	358.70
ccg	P	878	139.50
agt	S	0	0.20
agc	S	0	0.20
---	--	------	------
mPD		0.0023	0.71
		nPD:	0.
		N. weight:	2.3
		Sc. PD:	-0.35
		Sc. rank:	-1312.3

PB1			
Pos.	6	obs:	exp:
act	T	877	227.70
acc	T	2	179.00
aca	T	0	403.80
acg	T	0	68.47
---	--	------	------
mPD		0.0045	0.67
		nPD:	0.01
		N. weight:	1.6
		Sc. PD:	-0.25
		Sc. rank:	-858.5

PB1			
Pos.	7	obs:	exp:
tta	L	819	77.25
ttg	L	37	164.00
ctt	L	0	149.00
ctc	L	0	147.30
cta	L	23	146.50
ctg	L	0	194.90
---	--	------	------
mPD		0.13	1.1
		nPD:	0.12
		N. weight:	2.6
		Sc. PD:	-0.11
		Sc. rank:	19.0

PB1			
Pos.	8	obs:	exp:
tta	L	0	77.25
ttg	L	0	164.00
ctt	L	645	149.00
ctc	L	212	147.30
cta	L	1	146.50
ctg	L	21	194.90
---	--	------	------
mPD		0.40	1.1
		nPD:	0.36
		N. weight:	1.4
		Sc. PD:	0.26
		Sc. rank:	652.6

PB1			
Pos.	9	obs:	exp:
ttt	F	4	344.90
ttc	F	870	529.10
tta	L	1	0.44
ttg	L	0	0.93
ctt	L	0	0.85
ctc	L	4	0.84
cta	L	0	0.83
ctg	L	0	1.11
---	--	------	------
mPD		0.020	0.49
		nPD:	0.04
		N. weight:	0.6
		Sc. PD:	-0.07
		Sc. rank:	-148.5

PB1			
Pos.	10	obs:	exp:
ttt	F	1	0.39
ttc	F	0	0.61
tta	L	34	77.16
ttg	L	842	163.80
ctt	L	1	148.80
ctc	L	0	147.10
cta	L	1	146.40
ctg	L	0	194.70
---	--	------	------
mPD		0.085	1.1
		nPD:	0.08
		N. weight:	1.9
		Sc. PD:	-0.16
		Sc. rank:	-263.3

PB1			
Pos.	11	obs:	exp:
aaa	K	875	513.70
aag	K	2	363.30
gaa	E	2	1.20
gag	E	0	0.80
---	--	------	------
mPD		0.0091	0.49
		nPD:	0.02
		N. weight:	0.64
		Sc. PD:	-0.089
		Sc. rank:	-249.6

PB1			
Pos.	12	obs:	exp:
gtt	V	46	181.50
gtc	V	2	176.10
gta	V	15	182.00
gtg	V	816	339.50
---	--	------	------
mPD		0.14	0.73
		nPD:	0.19
		N. weight:	0.86
		Sc. PD:	0.019
		Sc. rank:	171.0

PB1			
Pos.	13	obs:	exp:
cct	P	0	223.50
ccc	P	0	156.70
cca	P	871	359.10
ccg	P	8	139.70
---	--	------	------
mPD		0.018	0.71
		nPD:	0.03
		N. weight:	1.1
		Sc. PD:	-0.14
		Sc. rank:	-354.4

PB1			
Pos.	14	obs:	exp:
gtt	V	0	149.30
gtc	V	0	144.80
gta	V	4	149.70
gtg	V	719	279.20
gct	A	0	39.97
gcc	A	0	29.29
gca	A	0	72.94
gcg	A	156	13.80
---	--	------	------
mPD		0.30	1.0
		nPD:	0.29
		N. weight:	1.5
		Sc. PD:	0.18
		Sc. rank:	571.0

PB1			
Pos.	15	obs:	exp:
caa	Q	874	451.50
cag	Q	5	427.50
---	--	------	------
mPD		0.011	0.50
		nPD:	0.02
		N. weight:	0.78
		Sc. PD:	-0.11
		Sc. rank:	-286.6

PB1			
Pos.	16	obs:	exp:
aat	N	877	446.20
aac	N	0	430.80
gat	D	2	1.08
gac	D	0	0.92
---	--	------	------
mPD		0.0045	0.50
		nPD:	0.01
		N. weight:	0.83
		Sc. PD:	-0.12
		Sc. rank:	-416.7

PB1			
Pos.	17	obs:	exp:
gct	A	772	225.20
gcc	A	107	165.00
gca	A	0	411.00
gcg	A	0	77.75
---	--	------	------
mPD		0.21	0.67
		nPD:	0.32
		N. weight:	1.3
		Sc. PD:	0.19
		Sc. rank:	554.1

PB1			
Pos.	18	obs:	exp:
att	I	0	320.70
atc	I	0	212.60
ata	I	879	345.70
---	--	------	------
mPD		0	0.65
		nPD:	0.
		N. weight:	1.2
		Sc. PD:	-0.18
		Sc. rank:	-708.9

PB1			
Pos.	19	obs:	exp:
tct	S	0	140.70
tcc	S	0	113.80
tca	S	0	221.80
tcg	S	0	51.15
agt	S	826	180.10
agc	S	53	171.40
---	--	------	------
mPD		0.11	1.7
		nPD:	0.07
		N. weight:	1.7
		Sc. PD:	-0.15
		Sc. rank:	-287.9

PB1			
Pos.	20	obs:	exp:
act	T	49	227.70
acc	T	773	179.00
aca	T	57	403.80
acg	T	0	68.47
---	--	------	------
mPD		0.22	0.67
		nPD:	0.33
		N. weight:	1.3
		Sc. PD:	0.21
		Sc. rank:	592.6

PB1			
Pos.	21	obs:	exp:
tct	S	0	0.16
tcc	S	0	0.13
tca	S	0	0.25
tcg	S	0	0.06
act	T	475	227.40
acc	T	1	178.80
aca	T	280	403.40
acg	T	122	68.39
agt	S	1	0.20
agc	S	0	0.20
---	--	------	------
mPD		0.59	0.68
		nPD:	0.87
		N. weight:	0.45
		Sc. PD:	0.31
		Sc. rank:	278.4

PB1			
Pos.	22	obs:	exp:
ttt	F	17	346.90
ttc	F	862	532.10
---	--	------	------
mPD		0.038	0.48
		nPD:	0.08
		N. weight:	0.51
		Sc. PD:	-0.042
		Sc. rank:	-67.7

PB1			
Pos.	23	obs:	exp:
cct	P	837	223.50
ccc	P	2	156.70
cca	P	9	359.10
ccg	P	31	139.70
---	--	------	------
mPD		0.092	0.71
		nPD:	0.13
		N. weight:	1.4
		Sc. PD:	-0.047
		Sc. rank:	72.3

PB1			
Pos.	24	obs:	exp:
tat	Y	829	466.10
tac	Y	50	412.90
---	--	------	------
mPD		0.11	0.50
		nPD:	0.22
		N. weight:	0.53
		Sc. PD:	0.026
		Sc. rank:	140.6

PB1			
Pos.	25	obs:	exp:
act	T	847	227.70
acc	T	32	179.00
aca	T	0	403.80
acg	T	0	68.47
---	--	------	------
mPD		0.070	0.67
		nPD:	0.1
		N. weight:	1.5
		Sc. PD:	-0.085
		Sc. rank:	-52.5

PB1			
Pos.	26	obs:	exp:
ggt	G	0	117.90
ggc	G	0	111.70
gga	G	877	406.40
ggg	G	2	243.00
---	--	------	------
mPD		0.0045	0.68
		nPD:	0.01
		N. weight:	0.93
		Sc. PD:	-0.14
		Sc. rank:	-488.5

PB1			
Pos.	27	obs:	exp:
aat	N	0	0.51
aac	N	1	0.49
gat	D	88	473.40
gac	D	789	403.60
gaa	E	1	0.60
gag	E	0	0.40
---	--	------	------
mPD		0.18	0.50
		nPD:	0.37
		N. weight:	0.54
		Sc. PD:	0.11
		Sc. rank:	261.7

PB1			
Pos.	28	obs:	exp:
cct	P	874	223.50
ccc	P	5	156.70
cca	P	0	359.10
ccg	P	0	139.70
---	--	------	------
mPD		0.011	0.71
		nPD:	0.02
		N. weight:	1.7
		Sc. PD:	-0.23
		Sc. rank:	-687.8

PB1			
Pos.	29	obs:	exp:
cct	P	0	223.50
ccc	P	0	156.70
cca	P	873	359.10
ccg	P	6	139.70
---	--	------	------
mPD		0.014	0.71
		nPD:	0.02
		N. weight:	1.1
		Sc. PD:	-0.15
		Sc. rank:	-406.2

PB1			
Pos.	30	obs:	exp:
tat	Y	0	465.50
tac	Y	878	412.50
cat	H	0	0.57
cac	H	1	0.43
---	--	------	------
mPD		0.0023	0.50
		nPD:	0.
		N. weight:	0.93
		Sc. PD:	-0.14
		Sc. rank:	-506.0

PB1			
Pos.	31	obs:	exp:
tct	S	0	140.70
tcc	S	0	113.80
tca	S	0	221.80
tcg	S	0	51.15
agt	S	1	180.10
agc	S	878	171.40
---	--	------	------
mPD		0.0023	1.7
		nPD:	0.
		N. weight:	2.
		Sc. PD:	-0.31
		Sc. rank:	-1184.0

PB1			
Pos.	32	obs:	exp:
cat	H	877	504.40
cac	H	1	373.60
cgt	R	1	0.04
cgc	R	0	0.05
cga	R	0	0.10
cgg	R	0	0.08
aga	R	0	0.46
agg	R	0	0.27
---	--	------	------
mPD		0.0046	0.49
		nPD:	0.01
		N. weight:	0.68
		Sc. PD:	-0.1
		Sc. rank:	-334.6

PB1			
Pos.	33	obs:	exp:
ggt	G	0	117.90
ggc	G	0	111.70
gga	G	876	406.40
ggg	G	3	243.00
---	--	------	------
mPD		0.0068	0.68
		nPD:	0.01
		N. weight:	0.93
		Sc. PD:	-0.14
		Sc. rank:	-448.0

PB1			
Pos.	34	obs:	exp:
act	T	0	227.70
acc	T	10	179.00
aca	T	861	403.80
acg	T	8	68.47
---	--	------	------
mPD		0.040	0.67
		nPD:	0.06
		N. weight:	0.86
		Sc. PD:	-0.085
		Sc. rank:	-167.9

PB1			
Pos.	35	obs:	exp:
ggt	G	0	117.90
ggc	G	0	111.70
gga	G	876	406.40
ggg	G	3	243.00
---	--	------	------
mPD		0.0068	0.68
		nPD:	0.01
		N. weight:	0.93
		Sc. PD:	-0.14
		Sc. rank:	-448.0

PB1			
Pos.	36	obs:	exp:
act	T	45	227.70
acc	T	0	179.00
aca	T	822	403.80
acg	T	12	68.47
---	--	------	------
mPD		0.12	0.67
		nPD:	0.18
		N. weight:	0.69
		Sc. PD:	0.013
		Sc. rank:	128.0

PB1			
Pos.	37	obs:	exp:
ggt	G	0	117.90
ggc	G	0	111.70
gga	G	861	406.40
ggg	G	18	243.00
---	--	------	------
mPD		0.040	0.68
		nPD:	0.06
		N. weight:	0.84
		Sc. PD:	-0.084
		Sc. rank:	-166.9

PB1			
Pos.	38	obs:	exp:
tat	Y	29	466.10
tac	Y	850	412.90
---	--	------	------
mPD		0.064	0.50
		nPD:	0.13
		N. weight:	0.75
		Sc. PD:	-0.025
		Sc. rank:	34.1

PB1			
Pos.	39	obs:	exp:
tct	S	0	0.48
tcc	S	0	0.39
tca	S	0	0.76
tcg	S	0	0.17
act	T	18	226.90
acc	T	858	178.40
aca	T	0	402.50
acg	T	0	68.24
agt	S	0	0.61
agc	S	3	0.59
---	--	------	------
mPD		0.047	0.68
		nPD:	0.07
		N. weight:	1.8
		Sc. PD:	-0.17
		Sc. rank:	-303.7

PB1			
Pos.	40	obs:	exp:
tta	L	0	0.09
ttg	L	0	0.19
ctt	L	0	0.17
ctc	L	0	0.17
cta	L	0	0.17
ctg	L	1	0.22
atg	M	878	878.00
---	--	------	------
mPD		0.0023	0.0036
		nPD:	0.63
		N. weight:	0.0021
		Sc. PD:	0.00094
		Sc. rank:	1.2

PB1			
Pos.	41	obs:	exp:
gat	D	7	474.50
gac	D	872	404.50
---	--	------	------
mPD		0.016	0.50
		nPD:	0.03
		N. weight:	0.9
		Sc. PD:	-0.11
		Sc. rank:	-270.5

PB1			
Pos.	42	obs:	exp:
act	T	13	227.70
acc	T	1	179.00
aca	T	830	403.80
acg	T	35	68.47
---	--	------	------
mPD		0.11	0.67
		nPD:	0.16
		N. weight:	0.75
		Sc. PD:	-0.0037
		Sc. rank:	88.0

PB1			
Pos.	43	obs:	exp:
gtt	V	34	181.50
gtc	V	841	176.10
gta	V	4	182.00
gtg	V	0	339.50
---	--	------	------
mPD		0.083	0.73
		nPD:	0.11
		N. weight:	1.7
		Sc. PD:	-0.082
		Sc. rank:	-2.4

PB1			
Pos.	44	obs:	exp:
aat	N	12	447.20
aac	N	867	431.80
---	--	------	------
mPD		0.027	0.50
		nPD:	0.05
		N. weight:	0.79
		Sc. PD:	-0.083
		Sc. rank:	-168.0

PB1			
Pos.	45	obs:	exp:
cgt	R	0	37.55
cgc	R	0	44.66
cga	R	0	87.65
cgg	R	0	73.21
aga	R	869	401.30
agg	R	10	234.60
---	--	------	------
mPD		0.023	0.96
		nPD:	0.02
		N. weight:	0.9
		Sc. PD:	-0.12
		Sc. rank:	-322.7

PB1			
Pos.	46	obs:	exp:
act	T	0	227.70
acc	T	0	179.00
aca	T	875	403.80
acg	T	4	68.47
---	--	------	------
mPD		0.0091	0.67
		nPD:	0.01
		N. weight:	0.94
		Sc. PD:	-0.13
		Sc. rank:	-423.6

PB1			
Pos.	47	obs:	exp:
cat	H	839	505.00
cac	H	40	374.00
---	--	------	------
mPD		0.087	0.49
		nPD:	0.18
		N. weight:	0.48
		Sc. PD:	0.0066
		Sc. rank:	78.9

PB1			
Pos.	48	obs:	exp:
caa	Q	94	48.28
cag	Q	0	45.72
aaa	K	785	459.80
aag	K	0	325.20
---	--	------	------
mPD		0.19	0.68
		nPD:	0.28
		N. weight:	0.68
		Sc. PD:	0.077
		Sc. rank:	247.6

PB1			
Pos.	49	obs:	exp:
tat	Y	43	465.50
tac	Y	835	412.50
cat	H	0	0.57
cac	H	1	0.43
---	--	------	------
mPD		0.095	0.50
		nPD:	0.19
		N. weight:	0.69
		Sc. PD:	0.018
		Sc. rank:	140.6

PB1			
Pos.	50	obs:	exp:
tct	S	0	140.70
tcc	S	0	113.80
tca	S	871	221.80
tcg	S	8	51.15
agt	S	0	180.10
agc	S	0	171.40
---	--	------	------
mPD		0.018	1.7
		nPD:	0.01
		N. weight:	1.7
		Sc. PD:	-0.24
		Sc. rank:	-773.1

PB1			
Pos.	51	obs:	exp:
gaa	E	846	528.70
gag	E	33	350.30
---	--	------	------
mPD		0.072	0.48
		nPD:	0.15
		N. weight:	0.45
		Sc. PD:	-0.0054
		Sc. rank:	43.2

,

PB1			
Pos.	52	obs:	exp:
cgt	R	0	0.04
cgc	R	0	0.05
cga	R	0	0.10
cgg	R	0	0.08
aat	N	56	28.49
aac	N	0	27.51
aaa	K	749	481.50
aag	K	73	340.50
aga	R	1	0.46
agg	R	0	0.27
---	--	------	------
mPD		0.26	0.55
		nPD:	0.48
		N. weight:	0.36
		Sc. PD:	0.11
		Sc. rank:	196.9

,

PB1			
Pos.	53	obs:	exp:
ggt	G	0	117.90
ggc	G	0	111.70
gga	G	819	406.40
ggg	G	60	243.00
---	--	------	------
mPD		0.13	0.68
		nPD:	0.19
		N. weight:	0.69
		Sc. PD:	0.017
		Sc. rank:	138.4

,

PB1			
Pos.	54	obs:	exp:
cgt	R	0	0.47
cgc	R	0	0.56
cga	R	0	1.10
cgg	R	0	0.92
atg	M	2	2.00
aaa	K	86	507.30
aag	K	780	358.70
aga	R	0	5.02
agg	R	11	2.94
---	--	------	------
mPD		0.21	0.52
		nPD:	0.39
		N. weight:	0.66
		Sc. PD:	0.15
		Sc. rank:	332.9

,

PB1			
Pos.	55	obs:	exp:
tgg	W	879	879.00
---	--	------	------
mPD		0	0
		nPD:	1.
		N. weight:	0.
		Sc. PD:	0
		Sc. rank:	0

,

PB1			
Pos.	56	obs:	exp:
att	I	0	0.36
atc	I	0	0.24
ata	I	1	0.39
act	T	0	219.70
acc	T	0	172.70
aca	T	845	389.60
acg	T	3	66.06
aaa	K	2	1.17
aag	K	0	0.83
gct	A	0	7.17
gcc	A	0	5.26
gca	A	28	13.09
gcg	A	0	2.48
---	--	------	------
mPD		0.075	0.74
		nPD:	0.1
		N. weight:	0.94
		Sc. PD:	-0.056
		Sc. rank:	-42.6

,

PB1			
Pos.	57	obs:	exp:
att	I	0	7.66
atc	I	0	5.08
ata	I	21	8.26
atg	M	23	23.00
act	T	0	214.20
acc	T	0	168.40
aca	T	105	379.90
acg	T	722	64.42
aaa	K	3	1.76
aag	K	0	1.24
gtt	V	0	0.21
gtc	V	0	0.20
gta	V	0	0.21
gtg	V	1	0.39
gct	A	0	1.02
gcc	A	0	0.75
gca	A	0	1.87
gcg	A	4	0.35
---	--	------	------
mPD		0.37	0.80
		nPD:	0.46
		N. weight:	2.3
		Sc. PD:	0.65
		Sc. rank:	1229.4

,

PB1			
Pos.	58	obs:	exp:
aat	N	11	447.20
aac	N	868	431.80
---	--	------	------
mPD		0.025	0.50
		nPD:	0.05
		N. weight:	0.8
		Sc. PD:	-0.087
		Sc. rank:	-181.0

,

PB1			
Pos.	59	obs:	exp:
tct	S	0	5.60
tcc	S	0	4.53
tca	S	35	8.83
tcg	S	0	2.04
act	T	1	218.60
acc	T	3	171.90
aca	T	838	387.70
acg	T	2	65.75
agt	S	0	7.17
agc	S	0	6.83
---	--	------	------
mPD		0.090	0.76
		nPD:	0.12
		N. weight:	0.94
		Sc. PD:	-0.04
		Sc. rank:	10.7

,

PB1			
Pos.	60	obs:	exp:
gaa	E	124	528.70
gag	E	755	350.30
---	--	------	------
mPD		0.24	0.48
		nPD:	0.51
		N. weight:	0.57
		Sc. PD:	0.19
		Sc. rank:	314.3

,

PB1			
Pos.	61	obs:	exp:
act	T	860	227.70
acc	T	18	179.00
aca	T	1	403.80
acg	T	0	68.47
---	--	------	------
mPD		0.042	0.67
		nPD:	0.06
		N. weight:	1.5
		Sc. PD:	-0.15
		Sc. rank:	-283.2

,

PB1			
Pos.	62	obs:	exp:
ggt	G	1	117.90
ggc	G	0	111.70
gga	G	850	406.40
ggg	G	28	243.00
---	--	------	------
mPD		0.064	0.68
		nPD:	0.09
		N. weight:	0.79
		Sc. PD:	-0.052
		Sc. rank:	-65.1

,

PB1			
Pos.	63	obs:	exp:
gct	A	0	225.20
gcc	A	0	165.00
gca	A	875	411.00
gcg	A	4	77.75
---	--	------	------
mPD		0.0091	0.67
		nPD:	0.01
		N. weight:	0.91
		Sc. PD:	-0.13
		Sc. rank:	-409.4

,

PB1			
Pos.	64	obs:	exp:
tta	L	0	1.05
ttg	L	0	2.24
ctt	L	0	2.03
ctc	L	12	2.01
cta	L	0	2.00
ctg	L	0	2.66
cct	P	41	220.20
ccc	P	821	154.40
cca	P	4	353.80
ccg	P	0	137.60
cat	H	0	0.57
cac	H	1	0.43
---	--	------	------
mPD		0.13	0.75
		nPD:	0.17
		N. weight:	1.8
		Sc. PD:	0.01
		Sc. rank:	261.3

,

PB1			
Pos.	65	obs:	exp:
caa	Q	871	451.50
cag	Q	8	427.50
---	--	------	------
mPD		0.018	0.50
		nPD:	0.04
		N. weight:	0.76
		Sc. PD:	-0.093
		Sc. rank:	-212.9

,

PB1			
Pos.	66	obs:	exp:
tta	L	0	76.99
ttg	L	0	163.50
ctt	L	18	148.40
ctc	L	800	146.80
cta	L	58	146.00
ctg	L	0	194.30
cct	P	0	0.25
ccc	P	1	0.18
cca	P	0	0.41
ccg	P	0	0.16
att	I	0	0.73
atc	I	2	0.48
ata	I	0	0.79
---	--	------	------
mPD		0.17	1.1
		nPD:	0.15
		N. weight:	1.8
		Sc. PD:	-0.022
		Sc. rank:	169.4

,

PB1			
Pos.	67	obs:	exp:
aat	N	828	447.20
aac	N	51	431.80
---	--	------	------
mPD		0.11	0.50
		nPD:	0.22
		N. weight:	0.57
		Sc. PD:	0.03
		Sc. rank:	153.4

,

PB1			
Pos.	68	obs:	exp:
tct	S	0	0.16
tcc	S	0	0.13
tca	S	0	0.25
tcg	S	1	0.06
cct	P	0	223.30
ccc	P	0	156.50
cca	P	596	358.70
ccg	P	282	139.50
agt	S	0	0.20
agc	S	0	0.20
---	--	------	------
mPD		0.44	0.71
		nPD:	0.61
		N. weight:	0.71
		Sc. PD:	0.31
		Sc. rank:	418.6

,

PB1			
Pos.	69	obs:	exp:
att	I	872	319.60
atc	I	4	211.90
ata	I	0	344.50
act	T	1	0.52
acc	T	1	0.41
aca	T	0	0.92
acg	T	0	0.16
gtt	V	1	0.21
gtc	V	0	0.20
gta	V	0	0.21
gtg	V	0	0.39
---	--	------	------
mPD		0.018	0.66
		nPD:	0.03
		N. weight:	1.2
		Sc. PD:	-0.16
		Sc. rank:	-386.4

,

PB1			
Pos.	70	obs:	exp:
gat	D	864	474.50
gac	D	15	404.50
---	--	------	------
mPD		0.034	0.50
		nPD:	0.07
		N. weight:	0.66
		Sc. PD:	-0.061
		Sc. rank:	-116.4

,

PB1

Pos.	71	obs:	exp:
ggt	G	0	117.90
ggc	G	0	111.70
gga	G	829	406.40
ggg	G	50	243.00
---	--	------	------
mPD		0.11	0.68
		nPD:	0.16
		N. weight:	0.72
		Sc. PD:	-0.0031
		Sc. rank:	85.0

PB1

Pos.	72	obs:	exp:
cct	P	1	223.50
ccc	P	0	156.70
cca	P	723	359.10
ccg	P	155	139.70
---	--	------	------
mPD		0.29	0.71
		nPD:	0.41
		N. weight:	0.73
		Sc. PD:	0.17
		Sc. rank:	373.3

PB1

Pos.	73	obs:	exp:
tta	L	472	77.25
ttg	L	352	164.00
ctt	L	0	149.00
ctc	L	0	147.30
cta	L	39	146.50
ctg	L	16	194.90
---	--	------	------
mPD		0.60	1.1
		nPD:	0.54
		N. weight:	1.5
		Sc. PD:	0.53
		Sc. rank:	829.1

PB1

Pos.	74	obs:	exp:
cct	P	823	223.50
ccc	P	35	156.70
cca	P	8	359.10
ccg	P	13	139.70
---	--	------	------
mPD		0.12	0.71
		nPD:	0.17
		N. weight:	1.4
		Sc. PD:	0.0098
		Sc. rank:	198.3

PB1

Pos.	75	obs:	exp:
aaa	K	0	0.59
aag	K	1	0.41
gaa	E	10	528.10
gag	E	868	349.90
---	--	------	------
mPD		0.025	0.48
		nPD:	0.05
		N. weight:	1.1
		Sc. PD:	-0.11
		Sc. rank:	-236.2

PB1

Pos.	76	obs:	exp:
aat	N	0	2.03
aac	N	4	1.97
gat	D	19	471.80
gac	D	855	402.20
ggt	G	0	0.13
ggc	G	1	0.13
gga	G	0	0.46
ggg	G	0	0.28
---	--	------	------
mPD		0.054	0.51
		nPD:	0.11
		N. weight:	0.83
		Sc. PD:	-0.046
		Sc. rank:	-23.6

PB1

Pos.	77	obs:	exp:
aat	N	258	447.20
aac	N	621	431.80
---	--	------	------
mPD		0.42	0.50
		nPD:	0.83
		N. weight:	0.12
		Sc. PD:	0.078
		Sc. rank:	75.1

PB1

Pos.	78	obs:	exp:
gaa	E	45	528.70
gag	E	834	350.30
---	--	------	------
mPD		0.097	0.48
		nPD:	0.2
		N. weight:	0.86
		Sc. PD:	0.033
		Sc. rank:	200.3

PB1

Pos.	79	obs:	exp:
cct	P	1	223.50
ccc	P	0	156.70
cca	P	177	359.10
ccg	P	701	139.70
---	--	------	------
mPD		0.32	0.71
		nPD:	0.46
		N. weight:	1.4
		Sc. PD:	0.4
		Sc. rank:	745.7

PB1

Pos.	80	obs:	exp:
tct	S	0	140.40
tcc	S	0	113.50
tca	S	0	221.30
tcg	S	0	51.04
aat	N	2	1.02
aac	N	0	0.98
agt	S	758	179.70
agc	S	119	171.00
---	--	------	------
mPD		0.24	1.7
		nPD:	0.14
		N. weight:	1.5
		Sc. PD:	-0.027
		Sc. rank:	101.1

PB1

Pos.	81	obs:	exp:
ggt	G	3	117.90
ggc	G	0	111.70
gga	G	97	406.40
ggg	G	779	243.00
---	--	------	------
mPD		0.20	0.68
		nPD:	0.3
		N. weight:	1.1
		Sc. PD:	0.14
		Sc. rank:	432.9

PB1

Pos.	82	obs:	exp:
tat	Y	875	466.10
tac	Y	4	412.90
---	--	------	------
mPD		0.0091	0.50
		nPD:	0.02
		N. weight:	0.75
		Sc. PD:	-0.1
		Sc. rank:	-295.4

PB1

Pos.	83	obs:	exp:
gct	A	2	225.20
gcc	A	8	165.00
gca	A	861	411.00
gcg	A	8	77.75
---	--	------	------
mPD		0.040	0.67
		nPD:	0.06
		N. weight:	0.83
		Sc. PD:	-0.082
		Sc. rank:	-161.4

PB1

Pos.	84	obs:	exp:
caa	Q	853	451.50
cag	Q	26	427.50
---	--	------	------
mPD		0.057	0.50
		nPD:	0.12
		N. weight:	0.66
		Sc. PD:	-0.031
		Sc. rank:	-0.2

PB1

Pos.	85	obs:	exp:
cgt	R	0	0.04
cgc	R	0	0.05
cga	R	0	0.10
cgg	R	0	0.08
act	T	1	227.40
acc	T	0	178.80
aca	T	45	403.40
acg	T	832	68.39
aga	R	0	0.46
agg	R	1	0.27
---	--	------	------
mPD		0.10	0.68
		nPD:	0.15
		N. weight:	2.8
		Sc. PD:	-0.035
		Sc. rank:	255.0

PB1

Pos.	86	obs:	exp:
gat	D	837	474.50
gac	D	42	404.50
---	--	------	------
mPD		0.091	0.50
		nPD:	0.18
		N. weight:	0.54
		Sc. PD:	0.01
		Sc. rank:	102.1

PB1

Pos.	87	obs:	exp:
tgt	C	789	360.40
tgc	C	89	517.60
cgt	R	1	0.04
cgc	R	0	0.05
cga	R	0	0.10
cgg	R	0	0.08
aga	R	0	0.46
agg	R	0	0.27
---	--	------	------
mPD		0.18	0.49
		nPD:	0.38
		N. weight:	0.66
		Sc. PD:	0.14
		Sc. rank:	323.2

PB1

Pos.	88	obs:	exp:
gtt	V	11	181.20
gtc	V	40	175.90
gta	V	765	181.80
gtg	V	62	339.10
gct	A	0	0.26
gcc	A	0	0.19
gca	A	1	0.47
gcg	A	0	0.09
---	--	------	------
mPD		0.24	0.73
		nPD:	0.32
		N. weight:	1.3
		Sc. PD:	0.2
		Sc. rank:	563.5

PB1

Pos.	89	obs:	exp:
tta	L	44	77.16
ttg	L	784	163.80
tct	S	0	0.16
tcc	S	0	0.13
tca	S	0	0.25
tcg	S	1	0.06
ctt	L	36	148.80
ctc	L	0	147.10
cta	L	0	146.40
ctg	L	14	194.70
agt	S	0	0.20
agc	S	0	0.20
---	--	------	------
mPD		0.28	1.1
		nPD:	0.25
		N. weight:	1.6
		Sc. PD:	0.13
		Sc. rank:	507.9

PB1

Pos.	90	obs:	exp:
gaa	E	693	528.70
gag	E	186	350.30
---	--	------	------
mPD		0.33	0.48
		nPD:	0.7
		N. weight:	0.1
		Sc. PD:	0.053
		Sc. rank:	61.4

PB1

Pos.	91	obs:	exp:
gct	A	12	225.20
gcc	A	0	165.00
gca	A	867	411.00
gcg	A	0	77.75
---	--	------	------
mPD		0.027	0.67
		nPD:	0.04
		N. weight:	0.86
		Sc. PD:	-0.1
		Sc. rank:	-223.5

PB1

Pos.	92	obs:	exp:
atg	M	879	879.00
---	--	------	------
mPD		0	0
		nPD:	1.
		N. weight:	0.
		Sc. PD:	0
		Sc. rank:	0

PB1

Pos.	93	obs:	exp:
gct	A	762	225.20
gcc	A	117	165.00
gca	A	0	411.00
gcg	A	0	77.75
---	--	------	------
mPD		0.23	0.67
		nPD:	0.34
		N. weight:	1.3
		Sc. PD:	0.22
		Sc. rank:	577.4

PB1

Pos.	94	obs:	exp:
ttt	F	13	346.50
ttc	F	865	531.50
tta	L	0	0.09
ttg	L	1	0.19
ctt	L	0	0.17
ctc	L	0	0.17
cta	L	0	0.17
ctg	L	0	0.22
---	--	------	------
mPD		0.031	0.48
		nPD:	0.07
		N. weight:	0.54
		Sc. PD:	-0.051
		Sc. rank:	-97.4

PB1

Pos.	95	obs:	exp:
tta	L	0	77.16
ttg	L	0	163.80
ctt	L	877	148.80
ctc	L	1	147.10
cta	L	0	146.40
ctg	L	0	194.70
cct	P	1	0.25
ccc	P	0	0.18
cca	P	0	0.41
ccg	P	0	0.16
---	--	------	------
mPD		0.0046	1.1
		nPD:	0.
		N. weight:	2.2
		Sc. PD:	-0.33
		Sc. rank:	-1193.7

PB1			
Pos.	96	obs:	exp:
gct	A	0	4.61
gcc	A	0	3.38
gca	A	18	8.42
gcg	A	0	1.59
gaa	E	809	517.90
gag	E	52	343.10
---	--	------	------
mPD		0.15	0.53
		nPD:	0.29
		N. weight:	0.39
		Sc. PD:	0.047
		Sc. rank:	148.1

,

PB1			
Pos.	97	obs:	exp:
gaa	E	825	528.70
gag	E	54	350.30
---	--	------	------
mPD		0.12	0.48
		nPD:	0.24
		N. weight:	0.38
		Sc. PD:	0.028
		Sc. rank:	115.7

,

PB1			
Pos.	98	obs:	exp:
tct	S	780	140.70
tcc	S	97	113.80
tca	S	2	221.80
tcg	S	0	51.15
agt	S	0	180.10
agc	S	0	171.40
---	--	------	------
mPD		0.20	1.7
		nPD:	0.12
		N. weight:	1.8
		Sc. PD:	-0.075
		Sc. rank:	40.6

,

PB1			
Pos.	99	obs:	exp:
cat	H	220	505.00
cac	H	659	374.00
---	--	------	------
mPD		0.38	0.49
		nPD:	0.77
		N. weight:	0.27
		Sc. PD:	0.16
		Sc. rank:	166.2

,

PB1			
Pos.	100	obs:	exp:
cct	P	1	223.50
ccc	P	1	156.70
cca	P	806	359.10
ccg	P	71	139.70
---	--	------	------
mPD		0.15	0.71
		nPD:	0.21
		N. weight:	0.84
		Sc. PD:	0.042
		Sc. rank:	219.3

,

PB1			
Pos.	101	obs:	exp:
ggt	G	1	117.90
ggc	G	0	111.70
gga	G	50	406.40
ggg	G	828	243.00
---	--	------	------
mPD		0.11	0.68
		nPD:	0.16
		N. weight:	1.3
		Sc. PD:	-0.0015
		Sc. rank:	159.7

,

PB1			
Pos.	102	obs:	exp:
att	I	209	320.70
atc	I	670	212.60
ata	I	0	345.70
---	--	------	------
mPD		0.36	0.65
		nPD:	0.56
		N. weight:	0.96
		Sc. PD:	0.36
		Sc. rank:	551.9

,

PB1			
Pos.	103	obs:	exp:
ttt	F	878	346.50
ttc	F	0	531.50
cct	P	1	0.25
ccc	P	0	0.18
cca	P	0	0.41
ccg	P	0	0.16
---	--	------	------
mPD		0.0046	0.48
		nPD:	0.01
		N. weight:	1.1
		Sc. PD:	-0.17
		Sc. rank:	-561.8

,

PB1			
Pos.	104	obs:	exp:
gct	A	0	0.26
gcc	A	0	0.19
gca	A	1	0.47
gcg	A	0	0.09
gaa	E	872	528.10
gag	E	6	349.90
---	--	------	------
mPD		0.016	0.48
		nPD:	0.03
		N. weight:	0.58
		Sc. PD:	-0.073
		Sc. rank:	-172.0

,

PB1			
Pos.	105	obs:	exp:
tct	S	0	0.32
tcc	S	0	0.26
tca	S	0	0.50
tcg	S	0	0.12
cat	H	0	1.15
cac	H	2	0.85
aat	N	76	445.20
aac	N	799	429.80
agt	S	0	0.41
agc	S	2	0.39
---	--	------	------
mPD		0.17	0.51
		nPD:	0.33
		N. weight:	0.52
		Sc. PD:	0.081
		Sc. rank:	230.7

,

PB1			
Pos.	106	obs:	exp:
tct	S	29	140.70
tcc	S	0	113.80
tca	S	51	221.80
tcg	S	799	51.15
agt	S	0	180.10
agc	S	0	171.40
---	--	------	------
mPD		0.17	1.7
		nPD:	0.1
		N. weight:	2.9
		Sc. PD:	-0.17
		Sc. rank:	-118.6

,

PB1			
Pos.	107	obs:	exp:
tgt	C	876	360.90
tgc	C	3	518.10
---	--	------	------
mPD		0.0068	0.48
		nPD:	0.01
		N. weight:	1.1
		Sc. PD:	-0.15
		Sc. rank:	-456.4

,

PB1			
Pos.	108	obs:	exp:
ttt	F	1	0.39
ttc	F	0	0.61
tta	L	1	76.46
ttg	L	0	162.40
ctt	L	105	147.40
ctc	L	679	145.80
cta	L	85	145.00
ctg	L	0	192.90
att	I	0	2.92
atc	I	8	1.93
ata	I	0	3.15
---	--	------	------
mPD		0.39	1.1
		nPD:	0.35
		N. weight:	1.4
		Sc. PD:	0.24
		Sc. rank:	638.1

,

PB1			
Pos.	109	obs:	exp:
gaa	E	834	528.70
gag	E	45	350.30
---	--	------	------
mPD		0.097	0.48
		nPD:	0.2
		N. weight:	0.41
		Sc. PD:	0.015
		Sc. rank:	94.6

,

PB1			
Pos.	110	obs:	exp:
act	T	68	227.70
acc	T	0	179.00
aca	T	13	403.80
acg	T	798	68.47
---	--	------	------
mPD		0.17	0.67
		nPD:	0.25
		N. weight:	2.6
		Sc. PD:	0.22
		Sc. rank:	840.5

,

PB1			
Pos.	111	obs:	exp:
att	I	0	0.36
atc	I	0	0.24
ata	I	1	0.39
atg	M	878	878.00
---	--	------	------
mPD		0.0023	0.0023
		nPD:	1.
		N. weight:	0.0013
		Sc. PD:	0.0011
		Sc. rank:	0.8

,

PB1			
Pos.	112	obs:	exp:
gaa	E	871	528.70
gag	E	8	350.30
---	--	------	------
mPD		0.018	0.48
		nPD:	0.04
		N. weight:	0.57
		Sc. PD:	-0.069
		Sc. rank:	-153.7

,

PB1			
Pos.	113	obs:	exp:
ttt	F	3	1.18
ttc	F	0	1.82
att	I	791	289.00
atc	I	1	191.60
ata	I	0	311.50
gtt	V	64	17.34
gtc	V	20	16.83
gta	V	0	17.39
gtg	V	0	32.44
---	--	------	------
mPD		0.23	0.86
		nPD:	0.26
		N. weight:	1.2
		Sc. PD:	0.12
		Sc. rank:	426.6

,

PB1			
Pos.	114	obs:	exp:
att	I	4	1.46
atc	I	0	0.97
ata	I	0	1.57
gtt	V	829	180.60
gtc	V	46	175.30
gta	V	0	181.20
gtg	V	0	337.90
---	--	------	------
mPD		0.11	0.73
		nPD:	0.15
		N. weight:	1.7
		Sc. PD:	-0.026
		Sc. rank:	136.4

,

PB1			
Pos.	115	obs:	exp:
caa	Q	44	451.50
cag	Q	835	427.50
---	--	------	------
mPD		0.095	0.50
		nPD:	0.19
		N. weight:	0.64
		Sc. PD:	0.017
		Sc. rank:	130.4

,

PB1			
Pos.	116	obs:	exp:
caa	Q	813	451.50
cag	Q	66	427.50
---	--	------	------
mPD		0.14	0.50
		nPD:	0.28
		N. weight:	0.5
		Sc. PD:	0.055
		Sc. rank:	183.1

,

PB1			
Pos.	117	obs:	exp:
act	T	28	227.70
acc	T	0	179.00
aca	T	848	403.80
acg	T	3	68.47
---	--	------	------
mPD		0.068	0.67
		nPD:	0.1
		N. weight:	0.79
		Sc. PD:	-0.047
		Sc. rank:	-42.1

,

PB1			
Pos.	118	obs:	exp:
cgt	R	0	37.55
cgc	R	0	44.66
cga	R	1	87.65
cgg	R	0	73.21
aga	R	874	401.30
agg	R	4	234.60
---	--	------	------
mPD		0.011	0.96
		nPD:	0.01
		N. weight:	0.93
		Sc. PD:	-0.14
		Sc. rank:	-430.3

,

PB1			
Pos.	119	obs:	exp:
atg	M	11	11.00
gtt	V	3	179.20
gtc	V	0	173.90
gta	V	3	179.70
gtg	V	862	335.20
---	--	------	------
mPD		0.038	0.75
		nPD:	0.05
		N. weight:	1.1
		Sc. PD:	-0.12
		Sc. rank:	-250.8

,

PB1			
Pos.	120	obs:	exp:
gat	D	645	474.00
gac	D	233	404.00
gaa	E	1	0.60
gag	E	0	0.40
---	--	------	------
mPD		0.39	0.50
		nPD:	0.79
		N. weight:	0.1
		Sc. PD:	0.063
		Sc. rank:	63.9

,

PB1

Pos.	121	obs:	exp:
aaa	K	857	514.90
aag	K	22	364.10
---	--	------	------
mPD		0.049	0.49
		nPD:	0.1
		N. weight:	0.53
		Sc. PD:	-0.032
		Sc. rank:	-30.5

PB1

Pos.	122	obs:	exp:
tta	L	0	77.25
ttg	L	36	164.00
ctt	L	0	149.00
ctc	L	0	147.30
cta	L	58	146.50
ctg	L	785	194.90
---	--	------	------
mPD		0.20	1.1
		nPD:	0.18
		N. weight:	1.4
		Sc. PD:	0.025
		Sc. rank:	252.1

PB1

Pos.	123	obs:	exp:
act	T	25	227.70
acc	T	851	179.00
aca	T	3	403.80
acg	T	0	68.47
---	--	------	------
mPD		0.062	0.67
		nPD:	0.09
		N. weight:	1.8
		Sc. PD:	-0.12
		Sc. rank:	-165.7

PB1

Pos.	124	obs:	exp:
caa	Q	872	450.50
cag	Q	5	426.50
gaa	E	2	1.20
gag	E	0	0.80
---	--	------	------
mPD		0.016	0.50
		nPD:	0.03
		N. weight:	0.78
		Sc. PD:	-0.099
		Sc. rank:	-236.4

PB1

Pos.	125	obs:	exp:
ggt	G	49	117.90
ggc	G	830	111.70
gga	G	0	406.40
ggg	G	0	243.00
---	--	------	------
mPD		0.11	0.68
		nPD:	0.16
		N. weight:	2.3
		Sc. PD:	-0.016
		Sc. rank:	246.7

PB1

Pos.	126	obs:	exp:
cgt	R	103	37.55
cgc	R	776	44.66
cga	R	0	87.65
cgg	R	0	73.21
aga	R	0	401.30
agg	R	0	234.60
---	--	------	------
mPD		0.21	0.96
		nPD:	0.21
		N. weight:	3.3
		Sc. PD:	0.16
		Sc. rank:	868.9

PB1

Pos.	127	obs:	exp:
caa	Q	2	451.50
cag	Q	877	427.50
---	--	------	------
mPD		0.0045	0.50
		nPD:	0.01
		N. weight:	0.87
		Sc. PD:	-0.13
		Sc. rank:	-430.6

PB1

Pos.	128	obs:	exp:
act	T	52	227.70
acc	T	826	179.00
aca	T	1	403.80
acg	T	0	68.47
---	--	------	------
mPD		0.11	0.67
		nPD:	0.17
		N. weight:	1.7
		Sc. PD:	0.0081
		Sc. rank:	220.4

PB1

Pos.	129	obs:	exp:
tat	Y	875	465.50
tac	Y	3	412.50
cat	H	0	0.57
cac	H	1	0.43
---	--	------	------
mPD		0.011	0.50
		nPD:	0.02
		N. weight:	0.76
		Sc. PD:	-0.1
		Sc. rank:	-276.9

PB1

Pos.	130	obs:	exp:
gat	D	54	474.50
gac	D	825	404.50
---	--	------	------
mPD		0.12	0.50
		nPD:	0.23
		N. weight:	0.66
		Sc. PD:	0.044
		Sc. rank:	197.8

PB1

Pos.	131	obs:	exp:
tgg	W	879	879.00
---	--	------	------
mPD		0	0
		nPD:	1.
		N. weight:	0.
		Sc. PD:	0
		Sc. rank:	0

PB1

Pos.	132	obs:	exp:
act	T	12	227.20
acc	T	0	179.00
aca	T	399	403.80
acg	T	468	68.47
---	--	------	------
mPD		0.51	0.67
		nPD:	0.76
		N. weight:	1.2
		Sc. PD:	0.69
		Sc. rank:	740.9

PB1

Pos.	133	obs:	exp:
tta	L	39	77.08
ttg	L	722	163.70
ctt	L	0	148.60
ctc	L	0	146.90
cta	L	10	146.20
ctg	L	106	194.50
cct	P	0	0.25
ccc	P	0	0.18
cca	P	0	0.41
ccg	P	1	0.16
atg	M	1	1.00
---	--	------	------
mPD		0.34	1.1
		nPD:	0.31
		N. weight:	1.4
		Sc. PD:	0.19
		Sc. rank:	569.2

PB1

Pos.	134	obs:	exp:
att	I	0	0.36
atc	I	1	0.24
ata	I	0	0.39
aat	N	835	446.20
aac	N	42	430.80
gat	D	1	0.54
gac	D	0	0.46
---	--	------	------
mPD		0.098	0.50
		nPD:	0.19
		N. weight:	0.6
		Sc. PD:	0.017
		Sc. rank:	125.3

PB1

Pos.	135	obs:	exp:
cgt	R	0	37.55
cgc	R	0	44.66
cga	R	0	87.65
cgg	R	0	73.21
aga	R	840	401.30
agg	R	39	234.60
---	--	------	------
mPD		0.085	0.96
		nPD:	0.09
		N. weight:	0.78
		Sc. PD:	-0.056
		Sc. rank:	-79.6

PB1

Pos.	136	obs:	exp:
aat	N	795	447.20
aac	N	84	431.80
---	--	------	------
mPD		0.17	0.50
		nPD:	0.35
		N. weight:	0.45
		Sc. PD:	0.08
		Sc. rank:	211.7

PB1

Pos.	137	obs:	exp:
caa	Q	73	451.50
cag	Q	806	427.50
---	--	------	------
mPD		0.15	0.50
		nPD:	0.31
		N. weight:	0.53
		Sc. PD:	0.073
		Sc. rank:	223.9

PB1

Pos.	138	obs:	exp:
cct	P	7	223.00
ccc	P	0	156.40
cca	P	79	358.30
ccg	P	791	139.30
caa	Q	0	0.51
cag	Q	1	0.49
act	T	0	0.26
acc	T	0	0.20
aca	T	0	0.46
acg	T	1	0.08
---	--	------	------
mPD		0.18	0.72
		nPD:	0.26
		N. weight:	1.7
		Sc. PD:	0.15
		Sc. rank:	579.0

PB1

Pos.	139	obs:	exp:
gct	A	837	225.20
gcc	A	41	165.00
gca	A	1	411.00
gcg	A	0	77.75
---	--	------	------
mPD		0.091	0.67
		nPD:	0.14
		N. weight:	1.5
		Sc. PD:	-0.039
		Sc. rank:	88.0

PB1

Pos.	140	obs:	exp:
gct	A	732	225.20
gcc	A	56	165.00
gca	A	91	411.00
gcg	A	0	77.75
---	--	------	------
mPD		0.29	0.67
		nPD:	0.43
		N. weight:	0.94
		Sc. PD:	0.24
		Sc. rank:	490.7

PB1

Pos.	141	obs:	exp:
act	T	362	227.70
acc	T	517	179.00
aca	T	0	403.80
acg	T	0	68.47
---	--	------	------
mPD		0.49	0.67
		nPD:	0.72
		N. weight:	1.
		Sc. PD:	0.54
		Sc. rank:	608.7

PB1

Pos.	142	obs:	exp:
gct	A	41	225.20
gcc	A	13	165.00
gca	A	818	411.00
gcg	A	7	77.75
---	--	------	------
mPD		0.13	0.67
		nPD:	0.2
		N. weight:	0.63
		Sc. PD:	0.02
		Sc. rank:	133.1

PB1

Pos.	143	obs:	exp:
tta	L	52	77.25
ttg	L	793	164.00
ctt	L	0	149.00
ctc	L	0	147.30
cta	L	1	146.50
ctg	L	33	194.90
---	--	------	------
mPD		0.19	1.1
		nPD:	0.17
		N. weight:	1.6
		Sc. PD:	0.0093
		Sc. rank:	228.5

PB1

Pos.	144	obs:	exp:
gct	A	60	225.20
gcc	A	813	165.00
gca	A	6	411.00
gcg	A	0	77.75
---	--	------	------
mPD		0.14	0.67
		nPD:	0.21
		N. weight:	1.7
		Sc. PD:	0.072
		Sc. rank:	411.0

PB1

Pos.	145	obs:	exp:
aat	N	93	447.20
aac	N	786	431.80
---	--	------	------
mPD		0.19	0.50
		nPD:	0.38
		N. weight:	0.46
		Sc. PD:	0.095
		Sc. rank:	226.9

PB1			
Pos.	146	obs:	exp:
act	T	866	227.70
acc	T	13	179.00
aca	T	0	403.80
acg	T	0	68.47
---	--	------	------
mPD		0.029	0.67
		nPD:	0.04
		N. weight:	1.6
		Sc. PD:	-0.18
		Sc. rank:	-383.4

PB1			
Pos.	147	obs:	exp:
att	I	0	320.40
atc	I	0	212.40
ata	I	878	345.30
act	T	0	0.26
acc	T	0	0.20
aca	T	1	0.46
acg	T	0	0.08
---	--	------	------
mPD		0.0023	0.66
		nPD:	0.
		N. weight:	1.2
		Sc. PD:	-0.18
		Sc. rank:	-646.8

PB1			
Pos.	148	obs:	exp:
gaa	E	118	528.70
gag	E	761	350.30
---	--	------	------
mPD		0.23	0.48
		nPD:	0.49
		N. weight:	0.58
		Sc. PD:	0.18
		Sc. rank:	316.0

PB1			
Pos.	149	obs:	exp:
att	I	0	3.65
atc	I	0	2.42
ata	I	10	3.93
gtt	V	4	179.40
gtc	V	81	174.10
gta	V	743	179.90
gtg	V	41	335.60
---	--	------	------
mPD		0.28	0.75
		nPD:	0.37
		N. weight:	1.3
		Sc. PD:	0.25
		Sc. rank:	618.9

PB1			
Pos.	150	obs:	exp:
ttt	F	96	346.90
ttc	F	783	532.10
---	--	------	------
mPD		0.19	0.48
		nPD:	0.41
		N. weight:	0.26
		Sc. PD:	0.06
		Sc. rank:	130.3

PB1			
Pos.	151	obs:	exp:
cgt	R	0	37.55
cgc	R	0	44.66
cga	R	0	87.65
cgg	R	0	73.21
aga	R	872	401.30
agg	R	7	234.60
---	--	------	------
mPD		0.016	0.96
		nPD:	0.02
		N. weight:	0.92
		Sc. PD:	-0.13
		Sc. rank:	-375.4

PB1			
Pos.	152	obs:	exp:
tta	L	0	0.70
ttg	L	8	1.49
tct	S	0	139.40
tcc	S	0	112.70
tca	S	60	219.80
tcg	S	811	50.69
ctt	L	0	1.36
ctc	L	0	1.34
cta	L	0	1.33
ctg	L	0	1.77
agt	S	0	178.50
agc	S	0	169.90
---	--	------	------
mPD		0.15	1.7
		nPD:	0.09
		N. weight:	3.1
		Sc. PD:	-0.23
		Sc. rank:	-324.1

PB1			
Pos.	153	obs:	exp:
aat	N	790	447.20
aac	N	89	431.80
---	--	------	------
mPD		0.18	0.50
		nPD:	0.36
		N. weight:	0.44
		Sc. PD:	0.085
		Sc. rank:	208.7

PB1			
Pos.	154	obs:	exp:
gat	D	0	1.62
gac	D	3	1.38
ggt	G	90	117.50
ggc	G	783	111.30
gga	G	1	405.00
ggg	G	2	242.20
---	--	------	------
mPD		0.20	0.68
		nPD:	0.29
		N. weight:	2.1
		Sc. PD:	0.25
		Sc. rank:	804.8

PB1			
Pos.	155	obs:	exp:
tta	L	0	77.16
ttg	L	6	163.80
ctt	L	3	148.80
ctc	L	22	147.10
cta	L	165	146.40
ctg	L	682	194.70
cct	P	0	0.25
ccc	P	0	0.18
cca	P	1	0.41
ccg	P	0	0.16
---	--	------	------
mPD		0.37	1.1
		nPD:	0.33
		N. weight:	1.1
		Sc. PD:	0.18
		Sc. rank:	512.1

PB1			
Pos.	156	obs:	exp:
atg	M	5	5.00
act	T	0	225.90
acc	T	62	177.60
aca	T	806	400.60
acg	T	4	67.93
gct	A	0	0.51
gcc	A	1	0.38
gca	A	1	0.94
gcg	A	0	0.18
---	--	------	------
mPD		0.17	0.69
		nPD:	0.24
		N. weight:	0.69
		Sc. PD:	0.052
		Sc. rank:	214.1

PB1			
Pos.	157	obs:	exp:
act	T	0	0.26
acc	T	1	0.20
aca	T	0	0.46
acg	T	0	0.08
gct	A	240	225.00
gcc	A	594	164.90
gca	A	44	410.50
gcg	A	0	77.66
---	--	------	------
mPD		0.47	0.67
		nPD:	0.69
		N. weight:	0.96
		Sc. PD:	0.49
		Sc. rank:	576.0

PB1			
Pos.	158	obs:	exp:
tct	S	0	2.40
tcc	S	0	1.94
tca	S	0	3.79
tcg	S	0	0.87
aat	N	859	439.60
aac	N	5	424.40
agt	S	15	3.07
agc	S	0	2.93
---	--	------	------
mPD		0.045	0.56
		nPD:	0.08
		N. weight:	0.81
		Sc. PD:	-0.065
		Sc. rank:	-99.0

PB1			
Pos.	159	obs:	exp:
gat	D	3	3.78
gac	D	4	3.22
gaa	E	844	523.90
gag	E	27	347.10
ggt	G	0	0.13
ggc	G	0	0.13
gga	G	1	0.46
ggg	G	0	0.28
---	--	------	------
mPD		0.077	0.49
		nPD:	0.16
		N. weight:	0.47
		Sc. PD:	-0.0026
		Sc. rank:	52.8

PB1			
Pos.	160	obs:	exp:
tct	S	0	140.70
tcc	S	0	113.80
tca	S	844	221.80
tcg	S	35	51.15
agt	S	0	180.10
agc	S	0	171.40
---	--	------	------
mPD		0.077	1.7
		nPD:	0.05
		N. weight:	1.6
		Sc. PD:	-0.18
		Sc. rank:	-362.5

PB1			
Pos.	161	obs:	exp:
ggt	G	0	117.90
ggc	G	0	111.70
gga	G	823	406.40
ggg	G	56	243.00
---	--	------	------
mPD		0.12	0.68
		nPD:	0.18
		N. weight:	0.7
		Sc. PD:	0.009
		Sc. rank:	112.2

PB1			
Pos.	162	obs:	exp:
cgt	R	0	37.55
cgc	R	0	44.66
cga	R	0	87.65
cgg	R	2	73.21
aga	R	78	401.30
agg	R	799	234.60
---	--	------	------
mPD		0.17	0.96
		nPD:	0.17
		N. weight:	1.2
		Sc. PD:	0.011
		Sc. rank:	178.8

PB1			
Pos.	163	obs:	exp:
tta	L	3	77.25
ttg	L	782	164.00
ctt	L	0	149.00
ctc	L	0	147.30
cta	L	89	146.50
ctg	L	5	194.90
---	--	------	------
mPD		0.38	1.1
		nPD:	0.34
		N. weight:	1.6
		Sc. PD:	0.28
		Sc. rank:	742.8

PB1			
Pos.	164	obs:	exp:
att	I	269	320.40
atc	I	517	212.40
ata	I	92	345.30
gtt	V	1	0.21
gtc	V	0	0.20
gta	V	0	0.21
gtg	V	0	0.39
---	--	------	------
mPD		0.55	0.66
		nPD:	0.84
		N. weight:	0.42
		Sc. PD:	0.27
		Sc. rank:	258.3

PB1			
Pos.	165	obs:	exp:
gat	D	876	474.50
gac	D	3	404.50
---	--	------	------
mPD		0.0068	0.50
		nPD:	0.01
		N. weight:	0.73
		Sc. PD:	-0.11
		Sc. rank:	-318.5

PB1			
Pos.	166	obs:	exp:
ttt	F	49	346.90
ttc	F	830	532.10
---	--	------	------
mPD		0.11	0.48
		nPD:	0.22
		N. weight:	0.39
		Sc. PD:	0.021
		Sc. rank:	107.3

PB1			
Pos.	167	obs:	exp:
tta	L	0	77.25
ttg	L	0	164.00
ctt	L	7	149.00
ctc	L	871	147.30
cta	L	1	146.50
ctg	L	0	194.90
---	--	------	------
mPD		0.018	1.1
		nPD:	0.02
		N. weight:	2.1
		Sc. PD:	-0.3
		Sc. rank:	-884.0

PB1			
Pos.	168	obs:	exp:
aaa	K	5	514.90
aag	K	874	364.10
---	--	------	------
mPD		0.011	0.49
		nPD:	0.02
		N. weight:	1.
		Sc. PD:	-0.14
		Sc. rank:	-375.5

PB1			
Pos.	169	obs:	exp:
gat	D	495	474.50
gac	D	384	404.50
---	--	------	------
mPD		0.49	0.50
		nPD:	0.99
		N. weight:	0.0064
		Sc. PD:	0.0051
		Sc. rank:	4.0

PB1			
Pos.	170	obs:	exp:
gtt	V	9	181.50
gtc	V	0	176.10
gta	V	27	182.00
gtg	V	843	339.50
---	--	------	------
mPD		0.079	0.73
		nPD:	0.11
		N. weight:	0.97
		Sc. PD:	-0.051
		Sc. rank:	-15.3

PB1			
Pos.	171	obs:	exp:
tta	L	0	0.09
ttg	L	1	0.19
ctt	L	0	0.17
ctc	L	0	0.17
cta	L	0	0.17
ctg	L	0	0.22
att	I	2	12.41
atc	I	0	8.22
ata	I	32	13.37
atg	M	657	657.00
gtt	V	0	38.60
gtc	V	0	37.46
gta	V	0	38.72
gtg	V	187	72.22
---	--	------	------
mPD		0.41	0.64
		nPD:	0.65
		N. weight:	0.29
		Sc. PD:	0.13
		Sc. rank:	170.5

,

PB1			
Pos.	172	obs:	exp:
aat	N	2	1.02
aac	N	0	0.98
gat	D	779	425.40
gac	D	9	362.60
gaa	E	76	49.93
gag	E	7	33.07
ggt	G	6	0.80
ggc	G	0	0.76
gga	G	0	2.77
ggg	G	0	1.66
---	--	------	------
mPD		0.21	0.60
		nPD:	0.35
		N. weight:	0.67
		Sc. PD:	0.12
		Sc. rank:	311.8

,

PB1			
Pos.	173	obs:	exp:
tct	S	0	140.70
tcc	S	0	113.80
tca	S	825	221.80
tcg	S	54	51.15
agt	S	0	180.10
agc	S	0	171.40
---	--	------	------
mPD		0.12	1.7
		nPD:	0.07
		N. weight:	1.5
		Sc. PD:	-0.14
		Sc. rank:	-244.4

,

PB1			
Pos.	174	obs:	exp:
atg	M	879	879.00
---	--	------	------
mPD		0	0
		nPD:	1.
		N. weight:	0.
		Sc. PD:	0
		Sc. rank:	0

,

PB1			
Pos.	175	obs:	exp:
aat	N	7	3.56
aac	N	0	3.44
gat	D	773	469.70
gac	D	97	400.30
gaa	E	0	1.20
gag	E	2	0.80
---	--	------	------
mPD		0.22	0.51
		nPD:	0.42
		N. weight:	0.36
		Sc. PD:	0.089
		Sc. rank:	186.3

,

PB1			
Pos.	176	obs:	exp:
cgt	R	0	0.13
cgc	R	0	0.15
cga	R	0	0.30
cgg	R	0	0.25
aaa	K	89	512.60
aag	K	786	362.40
aga	R	0	1.37
agg	R	3	0.80
gaa	E	0	0.60
gag	E	1	0.40
---	--	------	------
mPD		0.19	0.50
		nPD:	0.39
		N. weight:	0.65
		Sc. PD:	0.14
		Sc. rank:	322.3

,

PB1			
Pos.	177	obs:	exp:
aaa	K	2	1.76
aag	K	1	1.24
gat	D	3	1.62
gac	D	0	1.38
gaa	E	829	524.50
gag	E	43	347.50
ggt	G	0	0.13
ggc	G	0	0.13
gga	G	1	0.46
ggg	G	0	0.28
---	--	------	------
mPD		0.11	0.49
		nPD:	0.22
		N. weight:	0.41
		Sc. PD:	0.025
		Sc. rank:	119.2

,

PB1			
Pos.	178	obs:	exp:
gat	D	2	1.08
gac	D	0	0.92
gaa	E	829	521.50
gag	E	38	345.50
ggt	G	0	1.34
ggc	G	0	1.27
gga	G	10	4.62
ggg	G	0	2.76
---	--	------	------
mPD		0.11	0.51
		nPD:	0.22
		N. weight:	0.44
		Sc. PD:	0.022
		Sc. rank:	117.4

,

PB1			
Pos.	179	obs:	exp:
att	I	9	6.20
atc	I	0	4.11
ata	I	8	6.69
atg	M	861	861.00
gtt	V	0	0.21
gtc	V	0	0.20
gta	V	0	0.21
gtg	V	1	0.39
---	--	------	------
mPD		0.040	0.042
		nPD:	0.97
		N. weight:	0.01
		Sc. PD:	0.008
		Sc. rank:	6.4

,

PB1			
Pos.	180	obs:	exp:
gat	D	1	0.54
gac	D	0	0.46
gaa	E	95	525.70
gag	E	779	348.30
ggt	G	0	0.54
ggc	G	0	0.51
gga	G	0	1.85
ggg	G	4	1.11
---	--	------	------
mPD		0.20	0.49
		nPD:	0.42
		N. weight:	0.66
		Sc. PD:	0.16
		Sc. rank:	342.3

,

PB1			
Pos.	181	obs:	exp:
att	I	776	319.60
atc	I	9	211.90
ata	I	91	344.50
gtt	V	3	0.62
gtc	V	0	0.60
gta	V	0	0.62
gtg	V	0	1.16
---	--	------	------
mPD		0.21	0.66
		nPD:	0.32
		N. weight:	0.77
		Sc. PD:	0.11
		Sc. rank:	335.8

,

PB1			
Pos.	182	obs:	exp:
att	I	0	2.19
atc	I	0	1.45
ata	I	6	2.36
act	T	0	226.10
acc	T	0	177.80
aca	T	873	401.10
acg	T	0	68.00
---	--	------	------
mPD		0.014	0.69
		nPD:	0.02
		N. weight:	0.96
		Sc. PD:	-0.13
		Sc. rank:	-365.2

,

PB1			
Pos.	183	obs:	exp:
att	I	0	0.73
atc	I	0	0.48
ata	I	2	0.79
act	T	0	227.20
acc	T	0	178.60
aca	T	830	402.90
acg	T	47	68.32
---	--	------	------
mPD		0.11	0.68
		nPD:	0.16
		N. weight:	0.82
		Sc. PD:	-0.0059
		Sc. rank:	84.1

,

PB1			
Pos.	184	obs:	exp:
tat	Y	2	1.06
tac	Y	0	0.94
cat	H	869	503.80
cac	H	8	373.20
---	--	------	------
mPD		0.023	0.49
		nPD:	0.05
		N. weight:	0.63
		Sc. PD:	-0.071
		Sc. rank:	-147.2

,

PB1			
Pos.	185	obs:	exp:
ttt	F	3	346.90
ttc	F	876	532.10
---	--	------	------
mPD		0.0068	0.48
		nPD:	0.01
		N. weight:	0.59
		Sc. PD:	-0.085
		Sc. rank:	-252.2

,

PB1			
Pos.	186	obs:	exp:
caa	Q	48	451.50
cag	Q	831	427.50
---	--	------	------
mPD		0.10	0.50
		nPD:	0.21
		N. weight:	0.63
		Sc. PD:	0.026
		Sc. rank:	149.1

,

PB1			
Pos.	187	obs:	exp:
cgt	R	0	37.51
cgc	R	0	44.61
cga	R	1	87.55
cgg	R	0	73.13
aga	R	93	400.90
agg	R	784	234.30
ggt	G	0	0.13
ggc	G	0	0.13
gga	G	0	0.46
ggg	G	1	0.28
---	--	------	------
mPD		0.20	0.97
		nPD:	0.2
		N. weight:	1.1
		Sc. PD:	0.043
		Sc. rank:	263.0

,

PB1			
Pos.	188	obs:	exp:
cgt	R	0	0.04
cgc	R	0	0.05
cga	R	0	0.10
cgg	R	0	0.08
aaa	K	49	514.30
aag	K	829	363.70
aga	R	0	0.46
agg	R	1	0.27
---	--	------	------
mPD		0.11	0.49
		nPD:	0.22
		N. weight:	0.8
		Sc. PD:	0.044
		Sc. rank:	221.9

,

PB1			
Pos.	189	obs:	exp:
cgt	R	0	37.55
cgc	R	0	44.66
cga	R	0	87.65
cgg	R	0	73.21
aga	R	95	401.30
agg	R	784	234.60
---	--	------	------
mPD		0.19	0.96
		nPD:	0.2
		N. weight:	1.1
		Sc. PD:	0.04
		Sc. rank:	247.9

,

PB1			
Pos.	190	obs:	exp:
cgt	R	0	37.51
cgc	R	0	44.61
cga	R	0	87.55
cgg	R	0	73.13
aaa	K	1	0.59
aag	K	0	0.41
aga	R	877	400.90
agg	R	1	234.30
---	--	------	------
mPD		0.0046	0.97
		nPD:	0.
		N. weight:	0.96
		Sc. PD:	-0.15
		Sc. rank:	-509.4

,

PB1			
Pos.	191	obs:	exp:
att	I	0	0.36
atc	I	0	0.24
ata	I	1	0.39
atg	M	44	44.00
gtt	V	0	172.20
gtc	V	0	167.10
gta	V	47	172.70
gtg	V	787	322.10
mPD		0.20	0.81
		nPD:	0.25
		N. weight:	0.91
		Sc. PD:	0.073
		Sc. rank:	285.7

PB1			
Pos.	192	obs:	exp:
cgt	R	0	37.55
cgc	R	0	44.66
cga	R	0	87.65
cgg	R	0	73.21
aga	R	43	401.30
agg	R	836	234.60
mPD		0.093	0.96
		nPD:	0.1
		N. weight:	1.4
		Sc. PD:	-0.087
		Sc. rank:	-101.3

PB1			
Pos.	193	obs:	exp:
gat	D	5	474.50
gac	D	874	404.50
mPD		0.011	0.50
		nPD:	0.02
		N. weight:	0.92
		Sc. PD:	-0.12
		Sc. rank:	-333.3

PB1			
Pos.	194	obs:	exp:
aat	N	64	446.20
aac	N	813	430.80
aaa	K	2	1.17
aag	K	0	0.83
mPD		0.14	0.50
		nPD:	0.28
		N. weight:	0.56
		Sc. PD:	0.061
		Sc. rank:	202.2

PB1			
Pos.	195	obs:	exp:
tta	L	0	0.09
ttg	L	1	0.19
ctt	L	0	0.17
ctc	L	0	0.17
cta	L	0	0.17
ctg	L	0	0.22
atg	M	872	872.00
act	T	0	0.78
acc	T	0	0.61
aca	T	0	1.38
acg	T	3	0.23
gtt	V	0	0.62
gtc	V	0	0.60
gta	V	0	0.62
gtg	V	3	1.16
mPD		0.016	0.028
		nPD:	0.57
		N. weight:	0.017
		Sc. PD:	0.0068
		Sc. rank:	9.9

PB1			
Pos.	196	obs:	exp:
act	T	1	227.70
acc	T	878	179.00
aca	T	0	403.80
acg	T	0	68.47
mPD		0.0023	0.67
		nPD:	0.
		N. weight:	2.
		Sc. PD:	-0.3
		Sc. rank:	-1114.5

PB1			
Pos.	197	obs:	exp:
cgt	R	0	3.20
cgc	R	0	3.81
cga	R	0	7.48
cgg	R	0	6.25
aaa	K	28	471.00
aag	K	776	333.00
aga	R	0	34.24
agg	R	75	20.02
mPD		0.22	0.69
		nPD:	0.31
		N. weight:	0.95
		Sc. PD:	0.14
		Sc. rank:	410.8

PB1			
Pos.	198	obs:	exp:
aaa	K	834	514.90
aag	K	45	364.10
mPD		0.097	0.49
		nPD:	0.2
		N. weight:	0.44
		Sc. PD:	0.016
		Sc. rank:	95.2

PB1			
Pos.	199	obs:	exp:
att	I	0	1.09
atc	I	0	0.73
ata	I	3	1.18
atg	M	876	876.00
mPD		0.0068	0.0068
		nPD:	1.
		N. weight:	0.0039
		Sc. PD:	0.0032
		Sc. rank:	2.4

PB1			
Pos.	200	obs:	exp:
att	I	0	5.11
atc	I	11	3.39
ata	I	3	5.51
gtt	V	2	178.60
gtc	V	861	173.30
gta	V	2	179.10
gtg	V	0	334.10
mPD		0.047	0.76
		nPD:	0.06
		N. weight:	1.9
		Sc. PD:	-0.19
		Sc. rank:	-370.6

PB1			
Pos.	201	obs:	exp:
act	T	0	227.70
acc	T	0	179.00
aca	T	878	403.80
acg	T	1	68.47
mPD		0.0023	0.67
		nPD:	0.
		N. weight:	0.95
		Sc. PD:	-0.15
		Sc. rank:	-543.4

PB1			
Pos.	202	obs:	exp:
cat	H	1	0.57
cac	H	0	0.43
caa	Q	93	451.00
cag	Q	785	427.00
mPD		0.19	0.50
		nPD:	0.38
		N. weight:	0.47
		Sc. PD:	0.099
		Sc. rank:	232.8

PB1			
Pos.	203	obs:	exp:
tct	S	0	0.16
tcc	S	0	0.13
tca	S	0	0.25
tcg	S	0	0.06
cgt	R	0	37.51
cgc	R	0	44.61
cga	R	1	87.55
cgg	R	0	73.13
agt	S	1	0.20
agc	S	0	0.20
aga	R	843	400.90
agg	R	34	234.30
mPD		0.079	0.97
		nPD:	0.08
		N. weight:	0.79
		Sc. PD:	-0.062
		Sc. rank:	-94.0

PB1			
Pos.	204	obs:	exp:
act	T	0	227.70
acc	T	0	179.00
aca	T	877	403.80
acg	T	2	68.47
mPD		0.0045	0.67
		nPD:	0.01
		N. weight:	0.95
		Sc. PD:	-0.14
		Sc. rank:	-492.8

PB1			
Pos.	205	obs:	exp:
att	I	0	319.30
atc	I	0	211.60
ata	I	875	344.10
atg	M	2	2.00
gtt	V	0	0.41
gtc	V	0	0.40
gta	V	2	0.41
gtg	V	0	0.77
mPD		0.0091	0.66
		nPD:	0.01
		N. weight:	1.1
		Sc. PD:	-0.17
		Sc. rank:	-496.3

PB1			
Pos.	206	obs:	exp:
ggt	G	5	117.90
ggc	G	0	111.70
gga	G	433	406.40
ggg	G	441	243.00
mPD		0.51	0.68
		nPD:	0.75
		N. weight:	0.39
		Sc. PD:	0.22
		Sc. rank:	238.8

PB1			
Pos.	207	obs:	exp:
aaa	K	6	514.90
aag	K	873	364.10
mPD		0.014	0.49
		nPD:	0.03
		N. weight:	1.
		Sc. PD:	-0.13
		Sc. rank:	-325.1

PB1			
Pos.	208	obs:	exp:
aaa	K	749	514.90
aag	K	130	364.10
mPD		0.25	0.49
		nPD:	0.52
		N. weight:	0.21
		Sc. PD:	0.072
		Sc. rank:	117.6

PB1			
Pos.	209	obs:	exp:
aat	N	0	0.51
aac	N	1	0.49
aaa	K	832	514.30
aag	K	46	363.70
mPD		0.10	0.49
		nPD:	0.21
		N. weight:	0.43
		Sc. PD:	0.019
		Sc. rank:	106.3

PB1			
Pos.	210	obs:	exp:
caa	Q	808	451.50
cag	Q	71	427.50
mPD		0.15	0.50
		nPD:	0.3
		N. weight:	0.49
		Sc. PD:	0.063
		Sc. rank:	192.0

PB1			
Pos.	211	obs:	exp:
cgt	R	0	37.08
cgc	R	0	44.10
cga	R	1	86.55
cgg	R	0	72.29
aaa	K	6	5.86
aag	K	4	4.14
aga	R	731	396.30
agg	R	136	231.70
ggt	G	0	0.13
ggc	G	0	0.13
gga	G	0	0.46
ggg	G	1	0.28
mPD		0.30	0.98
		nPD:	0.3
		N. weight:	0.53
		Sc. PD:	0.07
		Sc. rank:	216.9

PB1			
Pos.	212	obs:	exp:
tta	L	3	77.16
ttg	L	7	163.80
ctt	L	2	148.80
ctc	L	0	147.10
cta	L	46	146.40
ctg	L	820	194.70
gtt	V	0	0.21
gtc	V	0	0.20
gta	V	0	0.21
gtg	V	1	0.39
mPD		0.13	1.1
		nPD:	0.12
		N. weight:	1.5
		Sc. PD:	-0.063
		Sc. rank:	24.5

PB1			
Pos.	213	obs:	exp:
act	T	0	15.28
acc	T	59	12.01
aca	T	0	27.11
acg	T	0	4.60
aat	N	48	415.10
aac	N	768	400.90
gct	A	0	0.26
gcc	A	1	0.19
gca	A	0	0.47
gcg	A	0	0.09
gat	D	1	1.62
gac	D	2	1.38
mPD		0.24	0.67
		nPD:	0.36
		N. weight:	0.69
		Sc. PD:	0.13
		Sc. rank:	329.0

PB1			
Pos.	214	obs:	exp:
cgt	R	0	0.21
cgc	R	0	0.25
cga	R	0	0.50
cgg	R	0	0.42
aaa	K	808	512.00
aag	K	66	362.00
aga	R	2	2.28
agg	R	3	1.33
mPD		0.16	0.50
		nPD:	0.31
		N. weight:	0.37
		Sc. PD:	0.053
		Sc. rank:	157.5

PB1			
Pos.	215	obs:	exp:
tgg	W	1	1.00
cgt	R	0	33.92
cgc	R	0	40.34
cga	R	0	79.17
cgg	R	1	66.13
aaa	K	4	49.21
aag	K	80	34.79
aga	R	6	362.50
agg	R	787	211.90
mPD		0.20	1.1
		nPD:	0.18
		N. weight:	1.5
		Sc. PD:	0.025
		Sc. rank:	265.4

PB1

Pos.	216	obs:	exp:
tct	S	0	136.20
tcc	S	0	110.20
tca	S	0	214.80
tcg	S	0	49.52
cat	H	0	1.15
cac	H	2	0.85
cgt	R	0	0.81
cgc	R	19	0.97
cga	R	0	1.89
cgg	R	0	1.58
aat	N	0	2.03
aac	N	4	1.97
agt	S	85	174.40
agc	S	766	166.00
aga	R	0	8.68
agg	R	0	5.07
ggt	G	1	0.40
ggc	G	2	0.38
gga	G	0	1.39
ggg	G	0	0.83
---	--	------	------
mPD		0.24	1.7
		nPD:	0.14
		N. weight:	1.7
		Sc. PD:	-0.03
		Sc. rank:	119.1

PB1

Pos.	217	obs:	exp:
tat	Y	48	466.10
tac	Y	831	412.90
---	--	------	------
mPD		0.10	0.50
		nPD:	0.21
		N. weight:	0.67
		Sc. PD:	0.028
		Sc. rank:	160.7

PB1

Pos.	218	obs:	exp:
tta	L	48	77.16
ttg	L	1	163.80
ctt	L	0	148.80
ctc	L	1	147.10
cta	L	828	146.40
ctg	L	0	194.70
att	I	0	0.36
atc	I	0	0.24
ata	I	1	0.39
---	--	------	------
mPD		0.11	1.1
		nPD:	0.1
		N. weight:	2.
		Sc. PD:	-0.12
		Sc. rank:	-107.5

PB1

Pos.	219	obs:	exp:
tta	L	0	0.09
ttg	L	0	0.19
ctt	L	0	0.17
ctc	L	0	0.17
cta	L	1	0.17
ctg	L	0	0.22
att	I	0	320.40
atc	I	0	212.40
ata	I	878	345.30
---	--	------	------
mPD		0.0023	0.66
		nPD:	0.
		N. weight:	1.2
		Sc. PD:	-0.18
		Sc. rank:	-652.7

PB1

Pos.	220	obs:	exp:
cgt	R	0	37.55
cgc	R	0	44.66
cga	R	1	87.65
cgg	R	0	73.21
aga	R	872	401.30
agg	R	6	234.60
---	--	------	------
mPD		0.016	0.96
		nPD:	0.02
		N. weight:	0.92
		Sc. PD:	-0.13
		Sc. rank:	-373.5

PB1

Pos.	221	obs:	exp:
act	T	0	0.52
acc	T	0	0.41
aca	T	2	0.92
acg	T	0	0.16
gct	A	3	224.70
gcc	A	0	164.70
gca	A	823	410.00
gcg	A	51	77.57
---	--	------	------
mPD		0.12	0.68
		nPD:	0.18
		N. weight:	0.76
		Sc. PD:	0.011
		Sc. rank:	126.1

PB1

Pos.	222	obs:	exp:
tta	L	12	77.25
ttg	L	280	164.00
ctt	L	3	149.00
ctc	L	1	147.30
cta	L	1	146.50
ctg	L	582	194.90
---	--	------	------
mPD		0.48	1.1
		nPD:	0.43
		N. weight:	1.1
		Sc. PD:	0.27
		Sc. rank:	551.3

PB1

Pos.	223	obs:	exp:
act	T	1	227.70
acc	T	0	179.00
aca	T	800	403.80
acg	T	78	68.47
---	--	------	------
mPD		0.16	0.67
		nPD:	0.24
		N. weight:	0.78
		Sc. PD:	0.06
		Sc. rank:	242.2

PB1

Pos.	224	obs:	exp:
tta	L	5	77.25
ttg	L	809	164.00
ctt	L	0	149.00
ctc	L	0	147.30
cta	L	9	146.50
ctg	L	56	194.90
---	--	------	------
mPD		0.17	1.1
		nPD:	0.15
		N. weight:	1.7
		Sc. PD:	-0.019
		Sc. rank:	163.7

PB1

Pos.	225	obs:	exp:
aat	N	5	447.20
aac	N	874	431.80
---	--	------	------
mPD		0.011	0.50
		nPD:	0.02
		N. weight:	0.84
		Sc. PD:	-0.11
		Sc. rank:	-307.6

PB1

Pos.	226	obs:	exp:
act	T	0	227.70
acc	T	1	179.00
aca	T	877	403.80
acg	T	1	68.47
---	--	------	------
mPD		0.0045	0.67
		nPD:	0.01
		N. weight:	0.94
		Sc. PD:	-0.14
		Sc. rank:	-485.0

PB1

Pos.	227	obs:	exp:
atg	M	879	879.00
---	--	------	------
mPD		0	0
		nPD:	1.
		N. weight:	0.
		Sc. PD:	0
		Sc. rank:	0

PB1

Pos.	228	obs:	exp:
act	T	2	227.70
acc	T	19	179.00
aca	T	858	403.80
acg	T	0	68.47
---	--	------	------
mPD		0.047	0.67
		nPD:	0.07
		N. weight:	0.84
		Sc. PD:	-0.076
		Sc. rank:	-136.3

PB1

Pos.	229	obs:	exp:
aaa	K	108	514.90
aag	K	771	364.10
---	--	------	------
mPD		0.22	0.49
		nPD:	0.44
		N. weight:	0.58
		Sc. PD:	0.16
		Sc. rank:	304.4

PB1

Pos.	230	obs:	exp:
gat	D	811	474.50
gac	D	68	404.50
---	--	------	------
mPD		0.14	0.50
		nPD:	0.29
		N. weight:	0.44
		Sc. PD:	0.053
		Sc. rank:	168.2

PB1

Pos.	231	obs:	exp:
gct	A	788	225.20
gcc	A	37	165.00
gca	A	53	411.00
gcg	A	1	77.75
---	--	------	------
mPD		0.19	0.67
		nPD:	0.28
		N. weight:	1.2
		Sc. PD:	0.13
		Sc. rank:	430.9

PB1

Pos.	232	obs:	exp:
gaa	E	872	528.70
gag	E	7	350.30
---	--	------	------
mPD		0.016	0.48
		nPD:	0.03
		N. weight:	0.58
		Sc. PD:	-0.072
		Sc. rank:	-169.3

PB1

Pos.	233	obs:	exp:
cgt	R	0	37.55
cgc	R	0	44.66
cga	R	2	87.65
cgg	R	0	73.21
aga	R	819	401.30
agg	R	58	234.60
---	--	------	------
mPD		0.13	0.96
		nPD:	0.13
		N. weight:	0.7
		Sc. PD:	-0.021
		Sc. rank:	37.1

PB1

Pos.	234	obs:	exp:
tct	S	0	0.16
tcc	S	0	0.13
tca	S	0	0.25
tcg	S	0	0.06
agt	S	0	0.20
agc	S	1	0.20
ggt	G	3	117.70
ggc	G	875	111.60
gga	G	0	405.90
ggg	G	0	242.80
---	--	------	------
mPD		0.0091	0.68
		nPD:	0.01
		N. weight:	2.5
		Sc. PD:	-0.36
		Sc. rank:	-1143.7

PB1

Pos.	235	obs:	exp:
aaa	K	124	514.90
aag	K	755	364.10
---	--	------	------
mPD		0.24	0.49
		nPD:	0.5
		N. weight:	0.53
		Sc. PD:	0.17
		Sc. rank:	291.2

PB1

Pos.	236	obs:	exp:
tta	L	231	77.25
ttg	L	246	164.00
ctt	L	0	149.00
ctc	L	0	147.30
cta	L	19	146.50
ctg	L	383	194.90
---	--	------	------
mPD		0.90	1.1
		nPD:	0.81
		N. weight:	0.82
		Sc. PD:	0.51
		Sc. rank:	500.8

PB1

Pos.	237	obs:	exp:
aaa	K	802	514.90
aag	K	77	364.10
---	--	------	------
mPD		0.16	0.49
		nPD:	0.33
		N. weight:	0.33
		Sc. PD:	0.054
		Sc. rank:	150.1

PB1

Pos.	238	obs:	exp:
cgt	R	0	37.55
cgc	R	0	44.66
cga	R	0	87.65
cgg	R	0	73.21
aga	R	47	401.30
agg	R	832	234.60
---	--	------	------
mPD		0.10	0.96
		nPD:	0.11
		N. weight:	1.3
		Sc. PD:	-0.075
		Sc. rank:	-40.0

PB1

Pos.	239	obs:	exp:
cgt	R	0	37.55
cgc	R	0	44.66
cga	R	13	87.65
cgg	R	39	73.21
aga	R	144	401.30
agg	R	683	234.60
---	--	------	------
mPD		0.41	0.96
		nPD:	0.42
		N. weight:	0.76
		Sc. PD:	0.19
		Sc. rank:	392.6

PB1

Pos.	240	obs:	exp:
gct	A	1	225.20
gcc	A	0	165.00
gca	A	872	411.00
gcg	A	6	77.75
---	--	------	------
mPD		0.016	0.67
		nPD:	0.02
		N. weight:	0.89
		Sc. PD:	-0.12
		Sc. rank:	-311.1

PB1

Pos.	241	obs :	exp :
att	I	104	320.70
atc	I	772	212.60
ata	I	3	345.70
mPD		0.21	0.65
		nPD :	0.33
		N. weight :	1.2
		Sc. PD :	0.19
		Sc. rank :	545.6

PB1

Pos.	242	obs :	exp :
gct	A	1	225.20
gcc	A	0	165.00
gca	A	851	411.00
gcg	A	27	77.75
mPD		0.062	0.67
		nPD :	0.09
		N. weight :	0.83
		Sc. PD :	-0.057
		Sc. rank :	-76.3

PB1

Pos.	243	obs :	exp :
act	T	3	227.70
acc	T	9	179.00
aca	T	842	403.80
acg	T	25	68.47
mPD		0.082	0.67
		nPD :	0.12
		N. weight :	0.78
		Sc. PD :	-0.032
		Sc. rank :	10.6

PB1

Pos.	244	obs :	exp :
cct	P	3	223.50
ccc	P	838	156.70
cca	P	38	359.10
ccg	P	0	139.70
mPD		0.089	0.71
		nPD :	0.13
		N. weight :	1.8
		Sc. PD :	-0.067
		Sc. rank :	70.6

PB1

Pos.	245	obs :	exp :
ggt	G	0	117.90
ggc	G	0	111.70
gga	G	56	406.40
ggg	G	823	243.00
mPD		0.12	0.68
		nPD :	0.18
		N. weight :	1.3
		Sc. PD :	0.016
		Sc. rank :	200.4

PB1

Pos.	246	obs :	exp :
att	I	1	0.73
atc	I	0	0.48
ata	I	1	0.79
atg	M	874	874.00
aaa	K	0	1.17
aag	K	2	0.83
gtt	V	0	0.21
gtc	V	0	0.20
gta	V	0	0.21
gtg	V	1	0.39
mPD		0.011	0.015
		nPD :	0.74
		N. weight :	0.0056
		Sc. PD :	0.0031
		Sc. rank :	3.4

PB1

Pos.	247	obs :	exp :
cat	H	1	0.57
cac	H	0	0.43
caa	Q	837	451.00
cag	Q	41	427.00
mPD		0.091	0.50
		nPD :	0.18
		N. weight :	0.6
		Sc. PD :	0.011
		Sc. rank :	109.1

PB1

Pos.	248	obs :	exp :
att	I	36	320.40
atc	I	841	212.40
ata	I	1	345.30
act	T	0	0.26
acc	T	1	0.20
aca	T	0	0.46
acg	T	0	0.08
mPD		0.083	0.66
		nPD :	0.13
		N. weight :	1.5
		Sc. PD :	-0.053
		Sc. rank :	63.8

PB1

Pos.	249	obs :	exp :
cgt	R	0	37.51
cgc	R	0	44.61
cga	R	0	87.55
cgg	R	0	73.13
aaa	K	1	0.59
aag	K	0	0.41
aga	R	859	400.90
agg	R	19	234.30
mPD		0.045	0.97
		nPD :	0.05
		N. weight :	0.86
		Sc. PD :	-0.096
		Sc. rank :	-200.0

PB1

Pos.	250	obs :	exp :
ggt	G	4	117.90
ggc	G	0	111.70
gga	G	833	406.40
ggg	G	42	243.00
mPD		0.10	0.68
		nPD :	0.15
		N. weight :	0.72
		Sc. PD :	-0.011
		Sc. rank :	59.0

PB1

Pos.	251	obs :	exp :
ttt	F	57	346.90
ttc	F	822	532.10
mPD		0.12	0.48
		nPD :	0.25
		N. weight :	0.36
		Sc. PD :	0.031
		Sc. rank :	119.4

PB1

Pos.	252	obs :	exp :
gtt	V	0	181.50
gtc	V	2	176.10
gta	V	21	182.00
gtg	V	856	339.50
mPD		0.051	0.73
		nPD :	0.07
		N. weight :	1.
		Sc. PD :	-0.093
		Sc. rank :	-164.5

PB1

Pos.	253	obs :	exp :
tat	Y	773	464.50
tac	Y	103	411.50
cat	H	3	1.72
cac	H	0	1.28
mPD		0.21	0.50
		nPD :	0.42
		N. weight :	0.36
		Sc. PD :	0.09
		Sc. rank :	185.9

PB1

Pos.	254	obs :	exp :
ttt	F	834	346.50
ttc	F	44	531.50
tta	L	0	0.09
ttg	L	1	0.19
ctt	L	0	0.17
ctc	L	0	0.17
cta	L	0	0.17
ctg	L	0	0.22
mPD		0.097	0.48
		nPD :	0.2
		N. weight :	0.88
		Sc. PD :	0.033
		Sc. rank :	198.1

PB1

Pos.	255	obs :	exp :
gtt	V	58	181.20
gtc	V	37	175.90
gta	V	779	181.80
gtg	V	4	339.10
gct	A	0	0.26
gcc	A	0	0.19
gca	A	1	0.47
gcg	A	0	0.09
mPD		0.21	0.73
		nPD :	0.29
		N. weight :	1.4
		Sc. PD :	0.17
		Sc. rank :	529.6

PB1

Pos.	256	obs :	exp :
gaa	E	873	528.70
gag	E	6	350.30
mPD		0.014	0.48
		nPD :	0.03
		N. weight :	0.58
		Sc. PD :	-0.076
		Sc. rank :	-180.1

PB1

Pos.	257	obs :	exp :
act	T	1	26.42
acc	T	0	20.77
aca	T	101	46.86
acg	T	0	7.95
gtt	V	0	0.21
gtc	V	0	0.20
gta	V	1	0.21
gtg	V	0	0.39
gct	A	0	198.80
gcc	A	0	145.70
gca	A	775	362.80
gcg	A	1	68.64
mPD		0.21	0.88
		nPD :	0.24
		N. weight :	0.93
		Sc. PD :	0.069
		Sc. rank :	286.2

PB1

Pos.	258	obs :	exp :
tta	L	45	77.25
ttg	L	8	164.00
ctt	L	0	149.00
ctc	L	1	147.30
cta	L	813	146.50
ctg	L	12	194.90
mPD		0.16	1.1
		nPD :	0.14
		N. weight :	1.8
		Sc. PD :	-0.034
		Sc. rank :	138.2

PB1

Pos.	259	obs :	exp :
gct	A	3	225.20
gcc	A	0	165.00
gca	A	64	411.00
gcg	A	812	77.75
mPD		0.14	0.67
		nPD :	0.21
		N. weight :	2.5
		Sc. PD :	0.11
		Sc. rank :	626.0

PB1

Pos.	260	obs :	exp :
cgt	R	0	37.55
cgc	R	0	44.66
cga	R	0	87.65
cgg	R	0	73.21
aga	R	19	401.30
agg	R	860	234.60
mPD		0.042	0.96
		nPD :	0.04
		N. weight :	1.5
		Sc. PD :	-0.17
		Sc. rank :	-353.7

PB1

Pos.	261	obs :	exp :
tct	S	0	139.90
tcc	S	0	113.10
tca	S	0	220.60
tcg	S	0	50.86
tgt	C	0	0.41
tgc	C	1	0.59
cgt	R	0	0.04
cgc	R	0	0.05
cga	R	0	0.10
cgg	R	0	0.08
att	I	0	0.36
atc	I	1	0.24
ata	I	0	0.39
aat	N	0	1.02
aac	N	2	0.98
agt	S	14	179.10
agc	S	860	170.40
aga	R	1	0.46
agg	R	0	0.27
mPD		0.043	1.7
		nPD :	0.03
		N. weight :	1.9
		Sc. PD :	-0.25
		Sc. rank :	-632.2

PB1

Pos.	262	obs :	exp :
att	I	43	320.00
atc	I	789	212.10
ata	I	45	344.90
gtt	V	0	0.41
gtc	V	2	0.40
gta	V	0	0.41
gtg	V	0	0.77
mPD		0.19	0.66
		nPD :	0.29
		N. weight :	1.2
		Sc. PD :	0.15
		Sc. rank :	469.1

PB1

Pos.	263	obs :	exp :
tgt	C	878	360.90
tgc	C	1	518.10
mPD		0.0023	0.48
		nPD :	0.
		N. weight :	1.1
		Sc. PD :	-0.17
		Sc. rank :	-582.8

PB1

Pos.	264	obs :	exp :
gaa	E	89	528.70
gag	E	790	350.30
mPD		0.18	0.48
		nPD :	0.38
		N. weight :	0.68
		Sc. PD :	0.14
		Sc. rank :	337.8

PB1

Pos.	265	obs :	exp :
aaa	K	839	514.90
aag	K	40	364.10
mPD		0.087	0.49
		nPD :	0.18
		N. weight :	0.45
		Sc. PD :	0.0069
		Sc. rank :	76.2

PB1			
Pos.	266	obs:	exp:
tta	L	0	77.25
ttg	L	0	164.00
ctt	L	867	149.00
ctc	L	12	147.30
cta	L	0	146.50
ctg	L	0	194.90
---	--	------	------
mPD		0.027	1.1
		nPD:	0.02
		N. weight:	2.1
		Sc. PD:	-0.28
		Sc. rank:	-711.2

,

PB1			
Pos.	267	obs:	exp:
gaa	E	49	528.70
gag	E	830	350.30
---	--	------	------
mPD		0.11	0.48
		nPD:	0.22
		N. weight:	0.84
		Sc. PD:	0.046
		Sc. rank:	231.0

,

PB1			
Pos.	268	obs:	exp:
caa	Q	602	451.50
cag	Q	277	427.50
---	--	------	------
mPD		0.43	0.50
		nPD:	0.86
		N. weight:	0.079
		Sc. PD:	0.053
		Sc. rank:	48.8

,

PB1			
Pos.	269	obs:	exp:
tct	S	870	140.50
tcc	S	8	113.60
tca	S	0	221.60
tcg	S	0	51.09
att	I	0	0.36
atc	I	1	0.24
ata	I	0	0.39
agt	S	0	179.90
agc	S	0	171.20
---	--	------	------
mPD		0.025	1.7
		nPD:	0.02
		N. weight:	2.2
		Sc. PD:	-0.31
		Sc. rank:	-922.8

,

PB1			
Pos.	270	obs:	exp:
tgt	C	0	0.41
tgc	C	1	0.59
ggt	G	2	117.70
ggc	G	777	111.60
gga	G	94	405.90
ggg	G	5	242.80
---	--	------	------
mPD		0.21	0.68
		nPD:	0.31
		N. weight:	1.9
		Sc. PD:	0.26
		Sc. rank:	804.7

,

PB1			
Pos.	271	obs:	exp:
ttt	F	0	0.39
ttc	F	1	0.61
tta	L	0	77.16
ttg	L	0	163.80
ctt	L	9	148.80
ctc	L	840	147.10
cta	L	29	146.40
ctg	L	0	194.70
---	--	------	------
mPD		0.086	1.1
		nPD:	0.08
		N. weight:	2.
		Sc. PD:	-0.16
		Sc. rank:	-266.9

,

PB1			
Pos.	272	obs:	exp:
cct	P	812	223.50
ccc	P	21	156.70
cca	P	44	359.10
ccg	P	2	139.70
---	--	------	------
mPD		0.14	0.71
		nPD:	0.2
		N. weight:	1.3
		Sc. PD:	0.047
		Sc. rank:	282.9

,

PB1			
Pos.	273	obs:	exp:
gtt	V	106	181.50
gtc	V	741	176.10
gta	V	32	182.00
gtg	V	0	339.50
---	--	------	------
mPD		0.27	0.73
		nPD:	0.38
		N. weight:	1.3
		Sc. PD:	0.28
		Sc. rank:	660.5

,

PB1			
Pos.	274	obs:	exp:
ggt	G	0	117.90
ggc	G	0	111.70
gga	G	832	406.40
ggg	G	47	243.00
---	--	------	------
mPD		0.10	0.68
		nPD:	0.15
		N. weight:	0.73
		Sc. PD:	-0.0094
		Sc. rank:	63.9

,

PB1			
Pos.	275	obs:	exp:
cgt	R	0	0.04
cgc	R	0	0.05
cga	R	0	0.10
cgg	R	1	0.08
aga	R	0	0.46
agg	R	0	0.27
ggt	G	1	117.70
ggc	G	37	111.60
gga	G	13	405.90
ggg	G	827	242.80
---	--	------	------
mPD		0.11	0.68
		nPD:	0.17
		N. weight:	1.3
		Sc. PD:	0.0043
		Sc. rank:	168.0

,

PB1			
Pos.	276	obs:	exp:
aat	N	876	447.20
aac	N	3	431.80
---	--	------	------
mPD		0.0068	0.50
		nPD:	0.01
		N. weight:	0.81
		Sc. PD:	-0.12
		Sc. rank:	-359.7

,

PB1			
Pos.	277	obs:	exp:
caa	Q	0	0.51
cag	Q	1	0.49
gaa	E	40	528.10
gag	E	838	349.90
---	--	------	------
mPD		0.089	0.48
		nPD:	0.19
		N. weight:	0.89
		Sc. PD:	0.019
		Sc. rank:	170.9

,

PB1			
Pos.	278	obs:	exp:
aaa	K	94	514.90
aag	K	785	364.10
---	--	------	------
mPD		0.19	0.49
		nPD:	0.39
		N. weight:	0.63
		Sc. PD:	0.14
		Sc. rank:	316.0

,

PB1			
Pos.	279	obs:	exp:
aaa	K	609	514.90
aag	K	270	364.10
---	--	------	------
mPD		0.43	0.49
		nPD:	0.88
		N. weight:	0.035
		Sc. PD:	0.024
		Sc. rank:	21.6

,

PB1			
Pos.	280	obs:	exp:
gct	A	856	225.20
gcc	A	23	165.00
gca	A	0	411.00
gcg	A	0	77.75
---	--	------	------
mPD		0.051	0.67
		nPD:	0.08
		N. weight:	1.5
		Sc. PD:	-0.13
		Sc. rank:	-219.9

,

PB1			
Pos.	281	obs:	exp:
aaa	K	879	514.90
aag	K	0	364.10
---	--	------	------
mPD		0	0.49
		nPD:	0.
		N. weight:	0.66
		Sc. PD:	-0.1
		Sc. rank:	-406.2

,

PB1			
Pos.	282	obs:	exp:
tta	L	1	77.25
ttg	L	745	164.00
ctt	L	2	149.00
ctc	L	0	147.30
cta	L	1	146.50
ctg	L	130	194.90
---	--	------	------
mPD		0.27	1.1
		nPD:	0.24
		N. weight:	1.5
		Sc. PD:	0.11
		Sc. rank:	456.7

,

PB1			
Pos.	283	obs:	exp:
gct	A	0	225.00
gcc	A	0	164.90
gca	A	876	410.50
gcg	A	2	77.66
ggt	G	0	0.13
ggc	G	0	0.13
gga	G	1	0.46
ggg	G	0	0.28
---	--	------	------
mPD		0.0068	0.67
		nPD:	0.01
		N. weight:	0.92
		Sc. PD:	-0.14
		Sc. rank:	-442.3

,

PB1			
Pos.	284	obs:	exp:
aat	N	872	447.20
aac	N	7	431.80
---	--	------	------
mPD		0.016	0.50
		nPD:	0.03
		N. weight:	0.78
		Sc. PD:	-0.099
		Sc. rank:	-235.0

,

PB1			
Pos.	285	obs:	exp:
gtt	V	833	181.50
gtc	V	45	176.10
gta	V	1	182.00
gtg	V	0	339.50
---	--	------	------
mPD		0.099	0.73
		nPD:	0.14
		N. weight:	1.7
		Sc. PD:	-0.043
		Sc. rank:	108.9

,

PB1			
Pos.	286	obs:	exp:
gtt	V	2	181.50
gtc	V	0	176.10
gta	V	16	182.00
gtg	V	861	339.50
---	--	------	------
mPD		0.040	0.73
		nPD:	0.06
		N. weight:	1.1
		Sc. PD:	-0.11
		Sc. rank:	-220.8

,

PB1			
Pos.	287	obs:	exp:
cgt	R	0	37.55
cgc	R	0	44.66
cga	R	0	87.65
cgg	R	1	73.21
aga	R	201	401.30
agg	R	677	234.60
---	--	------	------
mPD		0.36	0.96
		nPD:	0.37
		N. weight:	0.81
		Sc. PD:	0.16
		Sc. rank:	392.4

,

PB1			
Pos.	288	obs:	exp:
aaa	K	87	514.90
aag	K	792	364.10
---	--	------	------
mPD		0.18	0.49
		nPD:	0.37
		N. weight:	0.65
		Sc. PD:	0.13
		Sc. rank:	313.7

,

PB1			
Pos.	289	obs:	exp:
atg	M	879	879.00
---	--	------	------
mPD		0	0
		nPD:	1.
		N. weight:	0.
		Sc. PD:	0
		Sc. rank:	0

,

PB1			
Pos.	290	obs:	exp:
atg	M	879	879.00
---	--	------	------
mPD		0	0
		nPD:	1.
		N. weight:	0.
		Sc. PD:	0
		Sc. rank:	0

,

PB1			
Pos.	291	obs:	exp:
att	I	3	1.09
atc	I	0	0.73
ata	I	0	1.18
act	T	855	226.40
acc	T	14	178.00
aca	T	5	401.50
acg	T	0	68.08
aat	N	2	1.02
aac	N	0	0.98
---	--	------	------
mPD		0.054	0.69
		nPD:	0.08
		N. weight:	1.5
		Sc. PD:	-0.12
		Sc. rank:	-204.0

PB1			
Pos.	292	obs:	exp:
att	I	0	0.36
atc	I	1	0.24
ata	I	0	0.39
aat	N	826	446.70
aac	N	52	431.30
---	--	------	------
mPD		0.12	0.50
		nPD:	0.23
		N. weight:	0.56
		Sc. PD:	0.036
		Sc. rank:	166.3

PB1			
Pos.	293	obs:	exp:
tct	S	1	140.70
tcc	S	0	113.80
tca	S	849	221.80
tcg	S	29	51.15
agt	S	0	180.10
agc	S	0	171.40
---	--	------	------
mPD		0.066	1.7
		nPD:	0.04
		N. weight:	1.6
		Sc. PD:	-0.19
		Sc. rank:	-410.4

PB1			
Pos.	294	obs:	exp:
cat	H	1	0.57
cac	H	0	0.43
caa	Q	859	451.00
cag	Q	19	427.00
---	--	------	------
mPD		0.045	0.50
		nPD:	0.09
		N. weight:	0.7
		Sc. PD:	-0.05
		Sc. rank:	-68.3

PB1			
Pos.	295	obs:	exp:
gat	D	613	474.50
gac	D	266	404.50
---	--	------	------
mPD		0.42	0.50
		nPD:	0.85
		N. weight:	0.069
		Sc. PD:	0.045
		Sc. rank:	42.3

PB1			
Pos.	296	obs:	exp:
act	T	2	227.70
acc	T	0	179.00
aca	T	875	403.80
acg	T	2	68.47
---	--	------	------
mPD		0.0091	0.67
		nPD:	0.01
		N. weight:	0.93
		Sc. PD:	-0.13
		Sc. rank:	-418.1

PB1			
Pos.	297	obs:	exp:
gaa	E	40	528.70
gag	E	839	350.30
---	--	------	------
mPD		0.087	0.48
		nPD:	0.18
		N. weight:	0.89
		Sc. PD:	0.015
		Sc. rank:	158.9

PB1			
Pos.	298	obs:	exp:
tta	L	0	76.99
ttg	L	0	163.50
ctt	L	53	148.40
ctc	L	817	146.80
cta	L	6	146.00
ctg	L	0	194.30
att	I	0	1.09
atc	I	2	0.73
ata	I	1	1.18
---	--	------	------
mPD		0.14	1.1
		nPD:	0.12
		N. weight:	1.9
		Sc. PD:	-0.076
		Sc. rank:	35.6

PB1			
Pos.	299	obs:	exp:
tct	S	126	140.50
tcc	S	752	113.60
tca	S	0	221.60
tcg	S	0	51.09
cct	P	0	0.25
ccc	P	1	0.18
cca	P	0	0.41
ccg	P	0	0.16
agt	S	0	179.90
agc	S	0	171.20
---	--	------	------
mPD		0.25	1.7
		nPD:	0.15
		N. weight:	2.
		Sc. PD:	-0.026
		Sc. rank:	170.6

PB1			
Pos.	300	obs:	exp:
ttt	F	60	346.90
ttc	F	819	532.10
---	--	------	------
mPD		0.13	0.48
		nPD:	0.27
		N. weight:	0.35
		Sc. PD:	0.035
		Sc. rank:	122.4

PB1			
Pos.	301	obs:	exp:
act	T	11	227.70
acc	T	0	179.00
aca	T	830	403.80
acg	T	38	68.47
---	--	------	------
mPD		0.11	0.67
		nPD:	0.16
		N. weight:	0.77
		Sc. PD:	-0.004
		Sc. rank:	86.4

PB1			
Pos.	302	obs:	exp:
att	I	802	320.70
atc	I	77	212.60
ata	I	0	345.70
---	--	------	------
mPD		0.16	0.65
		nPD:	0.24
		N. weight:	0.93
		Sc. PD:	0.072
		Sc. rank:	289.0

PB1			
Pos.	303	obs:	exp:
act	T	876	227.70
acc	T	1	179.00
aca	T	2	403.80
acg	T	0	68.47
---	--	------	------
mPD		0.0068	0.67
		nPD:	0.01
		N. weight:	1.6
		Sc. PD:	-0.24
		Sc. rank:	-779.0

PB1			
Pos.	304	obs:	exp:
ggt	G	0	117.90
ggc	G	0	111.70
gga	G	448	406.40
ggg	G	431	243.00
---	--	------	------
mPD		0.50	0.68
		nPD:	0.74
		N. weight:	0.41
		Sc. PD:	0.23
		Sc. rank:	250.1

PB1			
Pos.	305	obs:	exp:
gat	D	14	474.50
gac	D	865	404.50
---	--	------	------
mPD		0.031	0.50
		nPD:	0.06
		N. weight:	0.86
		Sc. PD:	-0.082
		Sc. rank:	-156.1

PB1			
Pos.	306	obs:	exp:
aat	N	14	447.20
aac	N	865	431.80
---	--	------	------
mPD		0.031	0.50
		nPD:	0.06
		N. weight:	0.78
		Sc. PD:	-0.075
		Sc. rank:	-144.9

PB1			
Pos.	307	obs:	exp:
act	T	6	227.70
acc	T	869	179.00
aca	T	4	403.80
acg	T	0	68.47
---	--	------	------
mPD		0.023	0.67
		nPD:	0.03
		N. weight:	1.9
		Sc. PD:	-0.23
		Sc. rank:	-533.9

PB1			
Pos.	308	obs:	exp:
aaa	K	875	514.90
aag	K	4	364.10
---	--	------	------
mPD		0.0091	0.49
		nPD:	0.02
		N. weight:	0.63
		Sc. PD:	-0.087
		Sc. rank:	-243.3

PB1			
Pos.	309	obs:	exp:
tgg	W	879	879.00
---	--	------	------
mPD		0	0
		nPD:	1.
		N. weight:	0.
		Sc. PD:	0
		Sc. rank:	0

PB1			
Pos.	310	obs:	exp:
aat	N	876	447.20
aac	N	3	431.80
---	--	------	------
mPD		0.0068	0.50
		nPD:	0.01
		N. weight:	0.81
		Sc. PD:	-0.12
		Sc. rank:	-359.7

PB1			
Pos.	311	obs:	exp:
gaa	E	109	528.70
gag	E	770	350.30
---	--	------	------
mPD		0.22	0.48
		nPD:	0.45
		N. weight:	0.61
		Sc. PD:	0.17
		Sc. rank:	323.4

PB1			
Pos.	312	obs:	exp:
aat	N	830	447.20
aac	N	49	431.80
---	--	------	------
mPD		0.11	0.50
		nPD:	0.21
		N. weight:	0.57
		Sc. PD:	0.026
		Sc. rank:	145.7

PB1			
Pos.	313	obs:	exp:
caa	Q	824	451.50
cag	Q	55	427.50
---	--	------	------
mPD		0.12	0.50
		nPD:	0.24
		N. weight:	0.54
		Sc. PD:	0.037
		Sc. rank:	161.7

PB1			
Pos.	314	obs:	exp:
tct	S	0	0.48
tcc	S	0	0.39
tca	S	0	0.76
tcg	S	0	0.17
aat	N	51	445.70
aac	N	825	430.30
agt	S	0	0.61
agc	S	3	0.59
---	--	------	------
mPD		0.12	0.51
		nPD:	0.23
		N. weight:	0.61
		Sc. PD:	0.037
		Sc. rank:	175.5

PB1			
Pos.	315	obs:	exp:
cct	P	99	223.50
ccc	P	779	156.70
cca	P	1	359.10
ccg	P	0	139.70
---	--	------	------
mPD		0.20	0.71
		nPD:	0.28
		N. weight:	1.6
		Sc. PD:	0.19
		Sc. rank:	606.1

PB1			
Pos.	316	obs:	exp:
cgt	R	10	37.55
cgc	R	0	44.66
cga	R	37	87.65
cgg	R	792	73.21
aga	R	3	401.30
agg	R	37	234.60
---	--	------	------
mPD		0.20	0.96
		nPD:	0.2
		N. weight:	2.5
		Sc. PD:	0.094
		Sc. rank:	567.8

PB1			
Pos.	317	obs:	exp:
att	I	2	1.09
atc	I	0	0.73
ata	I	1	1.18
atg	M	872	872.00
gtt	V	0	0.83
gtc	V	0	0.80
gta	V	0	0.83
gtg	V	4	1.54
---	--	------	------
mPD		0.016	0.021
		nPD:	0.74
		N. weight:	0.0079
		Sc. PD:	0.0044
		Sc. rank:	4.8

PB1			
Pos.	318	obs:	exp:
ttt	F	842	346.50
ttc	F	36	531.50
tct	S	1	0.16
tcc	S	0	0.13
tca	S	0	0.25
tcg	S	0	0.06
agt	S	0	0.20
agc	S	0	0.20
---	--	------	------
mPD		0.081	0.48
		nPD:	0.17
		N. weight:	0.92
		Sc. PD:	0.0042
		Sc. rank:	119.5

PB1			
Pos.	319	obs:	exp:
tta	L	1	77.25
ttg	L	19	164.00
ctt	L	1	149.00
ctc	L	2	147.30
cta	L	778	146.50
ctg	L	78	194.90
---	--	------	------
mPD		0.25	1.1
		nPD:	0.22
		N. weight:	1.7
		Sc. PD:	0.094
		Sc. rank:	464.3

PB1			
Pos.	320	obs:	exp:
gct	A	3	225.20
gcc	A	0	165.00
gca	A	799	411.00
gcg	A	77	77.75
---	--	------	------
mPD		0.17	0.67
		nPD:	0.25
		N. weight:	0.73
		Sc. PD:	0.059
		Sc. rank:	229.9

PB1			
Pos.	321	obs:	exp:
atg	M	879	879.00
---	--	------	------
mPD		0	0
		nPD:	1.
		N. weight:	0.
		Sc. PD:	0
		Sc. rank:	0

,

PB1			
Pos.	322	obs:	exp:
att	I	1	320.40
atc	I	0	212.40
ata	I	877	345.30
gtt	V	0	0.21
gtc	V	0	0.20
gta	V	1	0.21
gtg	V	0	0.39
---	--	------	------
mPD		0.0046	0.66
		nPD:	0.01
		N. weight:	1.1
		Sc. PD:	-0.17
		Sc. rank:	-584.4

,

PB1			
Pos.	323	obs:	exp:
act	T	0	227.70
acc	T	0	179.00
aca	T	877	403.80
acg	T	2	68.47
---	--	------	------
mPD		0.0045	0.67
		nPD:	0.01
		N. weight:	0.95
		Sc. PD:	-0.14
		Sc. rank:	-492.8

,

PB1			
Pos.	324	obs:	exp:
tat	Y	47	466.10
tac	Y	832	412.90
---	--	------	------
mPD		0.10	0.50
		nPD:	0.2
		N. weight:	0.67
		Sc. PD:	0.026
		Sc. rank:	157.8

,

PB1			
Pos.	325	obs:	exp:
att	I	41	320.70
atc	I	774	212.60
ata	I	64	345.70
---	--	------	------
mPD		0.22	0.65
		nPD:	0.33
		N. weight:	1.1
		Sc. PD:	0.19
		Sc. rank:	517.1

,

PB1			
Pos.	326	obs:	exp:
act	T	0	227.40
acc	T	0	178.80
aca	T	873	403.40
acg	T	5	68.39
aat	N	0	0.51
aac	N	1	0.49
---	--	------	------
mPD		0.016	0.68
		nPD:	0.02
		N. weight:	0.93
		Sc. PD:	-0.13
		Sc. rank:	-327.6

,

PB1			
Pos.	327	obs:	exp:
cgt	R	0	37.21
cgc	R	0	44.25
cga	R	3	86.85
cgg	R	0	72.54
aaa	K	8	4.69
aag	K	0	3.31
aga	R	793	397.70
agg	R	75	232.50
---	--	------	------
mPD		0.18	0.98
		nPD:	0.19
		N. weight:	0.65
		Sc. PD:	0.013
		Sc. rank:	123.7

,

PB1			
Pos.	328	obs:	exp:
tct	S	0	1.92
tcc	S	0	1.55
tca	S	0	3.03
tcg	S	0	0.70
aat	N	77	440.60
aac	N	789	425.40
aaa	K	1	0.59
aag	K	0	0.41
agt	S	0	2.46
agc	S	12	2.34
---	--	------	------
mPD		0.19	0.55
		nPD:	0.34
		N. weight:	0.53
		Sc. PD:	0.092
		Sc. rank:	244.1

,

PB1			
Pos.	329	obs:	exp:
caa	Q	110	451.50
cag	Q	769	427.50
---	--	------	------
mPD		0.22	0.50
		nPD:	0.44
		N. weight:	0.42
		Sc. PD:	0.11
		Sc. rank:	220.2

,

PB1			
Pos.	330	obs:	exp:
cct	P	160	223.50
ccc	P	2	156.70
cca	P	528	359.10
ccg	P	189	139.70
---	--	------	------
mPD		0.56	0.71
		nPD:	0.79
		N. weight:	0.29
		Sc. PD:	0.17
		Sc. rank:	177.3

,

PB1			
Pos.	331	obs:	exp:
gaa	E	813	528.10
gag	E	65	349.90
ggt	G	0	0.13
ggc	G	0	0.13
gga	G	1	0.46
ggg	G	0	0.28
---	--	------	------
mPD		0.14	0.48
		nPD:	0.29
		N. weight:	0.34
		Sc. PD:	0.042
		Sc. rank:	132.7

,

PB1			
Pos.	332	obs:	exp:
tgg	W	879	879.00
---	--	------	------
mPD		0	0
		nPD:	1.
		N. weight:	0.
		Sc. PD:	0
		Sc. rank:	0

,

PB1			
Pos.	333	obs:	exp:
ttt	F	835	346.90
ttc	F	44	532.10
---	--	------	------
mPD		0.095	0.48
		nPD:	0.2
		N. weight:	0.88
		Sc. PD:	0.03
		Sc. rank:	187.7

,

PB1			
Pos.	334	obs:	exp:
cgt	R	0	37.55
cgc	R	0	44.66
cga	R	0	87.65
cgg	R	0	73.21
aga	R	818	401.30
agg	R	61	234.60
---	--	------	------
mPD		0.13	0.96
		nPD:	0.13
		N. weight:	0.71
		Sc. PD:	-0.02
		Sc. rank:	39.0

,

PB1			
Pos.	335	obs:	exp:
aat	N	876	447.20
aac	N	3	431.80
---	--	------	------
mPD		0.0068	0.50
		nPD:	0.01
		N. weight:	0.81
		Sc. PD:	-0.12
		Sc. rank:	-359.7

,

PB1			
Pos.	336	obs:	exp:
att	I	0	0.36
atc	I	1	0.24
ata	I	0	0.39
gtt	V	20	181.20
gtc	V	846	175.90
gta	V	12	181.80
gtg	V	0	339.10
---	--	------	------
mPD		0.073	0.73
		nPD:	0.1
		N. weight:	1.8
		Sc. PD:	-0.11
		Sc. rank:	-103.2

,

PB1			
Pos.	337	obs:	exp:
tta	L	806	77.25
ttg	L	55	164.00
ctt	L	0	149.00
ctc	L	0	147.30
cta	L	8	146.50
ctg	L	10	194.90
---	--	------	------
mPD		0.18	1.1
		nPD:	0.16
		N. weight:	2.5
		Sc. PD:	-0.0088
		Sc. rank:	308.2

,

PB1			
Pos.	338	obs:	exp:
tct	S	0	140.70
tcc	S	0	113.80
tca	S	0	221.80
tcg	S	0	51.15
agt	S	7	180.10
agc	S	872	171.40
---	--	------	------
mPD		0.016	1.7
		nPD:	0.01
		N. weight:	2.
		Sc. PD:	-0.29
		Sc. rank:	-950.2

,

PB1			
Pos.	339	obs:	exp:
att	I	863	320.00
atc	I	13	212.10
ata	I	1	344.90
gtt	V	2	0.41
gtc	V	0	0.40
gta	V	0	0.41
gtg	V	0	0.77
---	--	------	------
mPD		0.036	0.66
		nPD:	0.05
		N. weight:	1.2
		Sc. PD:	-0.12
		Sc. rank:	-241.3

,

PB1			
Pos.	340	obs:	exp:
gct	A	792	225.20
gcc	A	48	165.00
gca	A	38	411.00
gcg	A	1	77.75
---	--	------	------
mPD		0.18	0.67
		nPD:	0.27
		N. weight:	1.2
		Sc. PD:	0.13
		Sc. rank:	424.7

,

PB1			
Pos.	341	obs:	exp:
cct	P	852	223.50
ccc	P	21	156.70
cca	P	6	359.10
ccg	P	0	139.70
---	--	------	------
mPD		0.060	0.71
		nPD:	0.08
		N. weight:	1.5
		Sc. PD:	-0.11
		Sc. rank:	-170.9

,

PB1			
Pos.	342	obs:	exp:
att	I	0	320.70
atc	I	5	212.60
ata	I	874	345.70
---	--	------	------
mPD		0.011	0.65
		nPD:	0.02
		N. weight:	1.1
		Sc. PD:	-0.16
		Sc. rank:	-446.7

,

PB1			
Pos.	343	obs:	exp:
tta	L	0	0.09
ttg	L	0	0.19
ctt	L	0	0.17
ctc	L	0	0.17
cta	L	0	0.17
ctg	L	1	0.22
atg	M	878	878.00
---	--	------	------
mPD		0.0023	0.0036
		nPD:	0.63
		N. weight:	0.0021
		Sc. PD:	0.00094
		Sc. rank:	1.2

,

PB1			
Pos.	344	obs:	exp:
ttt	F	1	346.90
ttc	F	878	532.10
---	--	------	------
mPD		0.0023	0.48
		nPD:	0.
		N. weight:	0.61
		Sc. PD:	-0.093
		Sc. rank:	-322.6

,

PB1			
Pos.	345	obs:	exp:
tct	S	0	140.70
tcc	S	0	113.80
tca	S	860	221.80
tcg	S	19	51.15
agt	S	0	180.10
agc	S	0	171.40
---	--	------	------
mPD		0.042	1.7
		nPD:	0.03
		N. weight:	1.6
		Sc. PD:	-0.21
		Sc. rank:	-538.0

,

PB1			
Pos.	346	obs:	exp:
aat	N	39	447.20
aac	N	840	431.80
---	--	------	------
mPD		0.085	0.50
		nPD:	0.17
		N. weight:	0.65
		Sc. PD:	0.0041
		Sc. rank:	93.6

PB1			
Pos.	347	obs:	exp:
aaa	K	279	514.90
aag	K	600	364.10
---	--	------	------
mPD		0.43	0.49
		nPD:	0.89
		N. weight:	0.19
		Sc. PD:	0.13
		Sc. rank:	114.3

PB1			
Pos.	348	obs:	exp:
att	I	0	0.36
atc	I	0	0.24
ata	I	1	0.39
atg	M	878	878.00
---	--	------	------
mPD		0.0023	0.0023
		nPD:	1.
		N. weight:	0.0013
		Sc. PD:	0.0011
		Sc. rank:	0.8

PB1			
Pos.	349	obs:	exp:
gct	A	0	225.20
gcc	A	0	165.00
gca	A	244	411.00
gcg	A	635	77.75
---	--	------	------
mPD		0.40	0.67
		nPD:	0.6
		N. weight:	1.7
		Sc. PD:	0.71
		Sc. rank:	996.1

PB1			
Pos.	350	obs:	exp:
cgt	R	0	37.38
cgc	R	0	44.46
cga	R	0	87.25
cgg	R	0	72.88
aaa	K	4	2.34
aag	K	0	1.66
aga	R	843	399.50
agg	R	32	233.50
---	--	------	------
mPD		0.079	0.97
		nPD:	0.08
		N. weight:	0.8
		Sc. PD:	-0.063
		Sc. rank:	-96.5

PB1			
Pos.	351	obs:	exp:
tta	L	817	77.25
ttg	L	16	164.00
ctt	L	0	149.00
ctc	L	0	147.30
cta	L	46	146.50
ctg	L	0	194.90
---	--	------	------
mPD		0.14	1.1
		nPD:	0.12
		N. weight:	2.6
		Sc. PD:	-0.1
		Sc. rank:	67.7

PB1			
Pos.	352	obs:	exp:
ggt	G	1	117.90
ggc	G	0	111.70
gga	G	679	406.40
ggg	G	199	243.00
---	--	------	------
mPD		0.35	0.68
		nPD:	0.52
		N. weight:	0.43
		Sc. PD:	0.15
		Sc. rank:	242.0

PB1			
Pos.	353	obs:	exp:
cgt	R	0	0.13
cgc	R	0	0.15
cga	R	0	0.30
cgg	R	0	0.25
aaa	K	874	513.20
aag	K	2	362.80
aga	R	3	1.37
agg	R	0	0.80
---	--	------	------
mPD		0.011	0.49
		nPD:	0.02
		N. weight:	0.64
		Sc. PD:	-0.087
		Sc. rank:	-233.7

PB1			
Pos.	354	obs:	exp:
ggt	G	3	117.90
ggc	G	0	111.70
gga	G	363	406.40
ggg	G	513	243.00
---	--	------	------
mPD		0.49	0.68
		nPD:	0.72
		N. weight:	0.47
		Sc. PD:	0.25
		Sc. rank:	284.8

PB1			
Pos.	355	obs:	exp:
tat	Y	1	465.50
tac	Y	877	412.50
tgt	C	0	0.41
tgc	C	1	0.59
---	--	------	------
mPD		0.0046	0.50
		nPD:	0.01
		N. weight:	0.92
		Sc. PD:	-0.14
		Sc. rank:	-458.4

PB1			
Pos.	356	obs:	exp:
atg	M	879	879.00
---	--	------	------
mPD		0	0
		nPD:	1.
		N. weight:	0.
		Sc. PD:	0
		Sc. rank:	0

PB1			
Pos.	357	obs:	exp:
ttt	F	66	346.50
ttc	F	812	531.50
tta	L	0	0.09
ttg	L	0	0.19
ctt	L	0	0.17
ctc	L	1	0.17
cta	L	0	0.17
ctg	L	0	0.22
---	--	------	------
mPD		0.14	0.48
		nPD:	0.29
		N. weight:	0.34
		Sc. PD:	0.042
		Sc. rank:	132.2

PB1			
Pos.	358	obs:	exp:
tat	Y	1	0.53
tac	Y	0	0.47
gaa	E	859	528.10
gag	E	19	349.90
---	--	------	------
mPD		0.047	0.48
		nPD:	0.1
		N. weight:	0.51
		Sc. PD:	-0.033
		Sc. rank:	-37.1

PB1			
Pos.	359	obs:	exp:
tct	S	0	140.70
tcc	S	0	113.80
tca	S	0	221.80
tcg	S	0	51.15
agt	S	870	180.10
agc	S	9	171.40
---	--	------	------
mPD		0.020	1.7
		nPD:	0.01
		N. weight:	1.9
		Sc. PD:	-0.27
		Sc. rank:	-868.7

PB1			
Pos.	360	obs:	exp:
cgt	R	0	0.04
cgc	R	0	0.05
cga	R	0	0.10
cgg	R	0	0.08
aaa	K	63	514.30
aag	K	815	363.70
aga	R	0	0.46
agg	R	1	0.27
---	--	------	------
mPD		0.14	0.49
		nPD:	0.28
		N. weight:	0.74
		Sc. PD:	0.081
		Sc. rank:	269.7

PB1			
Pos.	361	obs:	exp:
tct	S	0	140.10
tcc	S	0	113.30
tca	S	0	220.80
tcg	S	0	50.92
cgt	R	0	0.04
cgc	R	0	0.05
cga	R	0	0.10
cgg	R	0	0.08
aat	N	2	1.02
aac	N	0	0.98
agt	S	721	179.30
agc	S	154	170.60
aga	R	0	0.46
agg	R	1	0.27
ggt	G	1	0.13
ggc	G	0	0.13
gga	G	0	0.46
ggg	G	0	0.28
---	--	------	------
mPD		0.30	1.7
		nPD:	0.18
		N. weight:	1.4
		Sc. PD:	0.022
		Sc. rank:	237.2

PB1			
Pos.	362	obs:	exp:
atg	M	879	879.00
---	--	------	------
mPD		0	0
		nPD:	1.
		N. weight:	0.
		Sc. PD:	0
		Sc. rank:	0

PB1			
Pos.	363	obs:	exp:
cgt	R	0	0.51
cgc	R	0	0.61
cga	R	0	1.20
cgg	R	0	1.00
aaa	K	81	507.90
aag	K	786	359.10
aga	R	0	5.48
agg	R	12	3.20
---	--	------	------
mPD		0.19	0.52
		nPD:	0.37
		N. weight:	0.68
		Sc. PD:	0.14
		Sc. rank:	332.9

PB1			
Pos.	364	obs:	exp:
tta	L	705	77.25
ttg	L	59	164.00
ctt	L	39	149.00
ctc	L	1	147.30
cta	L	62	146.50
ctg	L	13	194.90
---	--	------	------
mPD		0.46	1.1
		nPD:	0.41
		N. weight:	1.9
		Sc. PD:	0.46
		Sc. rank:	983.6

PB1			
Pos.	365	obs:	exp:
tta	L	0	0.09
ttg	L	0	0.19
ctt	L	1	0.17
ctc	L	0	0.17
cta	L	0	0.17
ctg	L	0	0.22
cgt	R	0	37.51
cgc	R	0	44.61
cga	R	59	87.55
cgg	R	803	73.13
aga	R	2	400.90
agg	R	14	234.30
---	--	------	------
mPD		0.17	0.97
		nPD:	0.18
		N. weight:	2.6
		Sc. PD:	0.03
		Sc. rank:	407.6

PB1			
Pos.	366	obs:	exp:
act	T	0	226.90
acc	T	0	178.40
aca	T	843	402.50
acg	T	33	68.24
gct	A	0	0.77
gcc	A	0	0.56
gca	A	3	1.40
gcg	A	0	0.27
---	--	------	------
mPD		0.079	0.68
		nPD:	0.12
		N. weight:	0.85
		Sc. PD:	-0.038
		Sc. rank:	2.7

PB1			
Pos.	367	obs:	exp:
tta	L	0	0.09
ttg	L	0	0.19
ctt	L	1	0.17
ctc	L	0	0.17
cta	L	0	0.17
ctg	L	0	0.22
caa	Q	856	451.00
cag	Q	22	427.00
---	--	------	------
mPD		0.053	0.50
		nPD:	0.11
		N. weight:	0.68
		Sc. PD:	-0.038
		Sc. rank:	-15.0

PB1			
Pos.	368	obs:	exp:
att	I	0	38.31
atc	I	0	25.40
ata	I	105	41.29
gtt	V	0	159.80
gtc	V	0	155.10
gta	V	763	160.30
gtg	V	11	298.90
---	--	------	------
mPD		0.24	0.95
		nPD:	0.25
		N. weight:	1.8
		Sc. PD:	0.14
		Sc. rank:	560.1

PB1			
Pos.	369	obs:	exp:
tct	S	0	2.24
tcc	S	0	1.81
tca	S	14	3.53
tcg	S	0	0.81
cct	P	7	219.70
ccc	P	3	154.10
cca	P	802	353.00
ccg	P	52	137.30
caa	Q	0	0.51
cag	Q	1	0.49
agt	S	0	2.87
agc	S	0	2.73
---	--	------	------
mPD		0.17	0.76
		nPD:	0.22
		N. weight:	0.84
		Sc. PD:	0.047
		Sc. rank:	233.6

PB1			
Pos.	370	obs:	exp:
tct	S	0	0.16
tcc	S	0	0.13
tca	S	1	0.25
tcg	S	0	0.06
act	T	0	0.26
acc	T	0	0.20
aca	T	0	0.46
acg	T	1	0.08
aaa	K	1	0.59
aag	K	0	0.41
agt	S	0	0.20
agc	S	0	0.20
gct	A	23	224.50
gcc	A	0	164.50
gca	A	276	409.60
gcg	A	577	77.48
---	--	------	------
mPD		0.48	0.68
		nPD:	0.7
		N. weight:	1.4
		Sc. PD:	0.73
		Sc. rank:	848.9

PB1			
Pos.	371	obs:	exp:
gaa	E	877	528.70
gag	E	2	350.30
---	--	------	------
mPD		0.0045	0.48
		nPD:	0.01
		N. weight:	0.61
		Sc. PD:	-0.09
		Sc. rank:	-298.1

PB1			
Pos.	372	obs:	exp:
tta	L	0	5.98
ttg	L	4	12.69
ctt	L	0	11.52
ctc	L	0	11.39
cta	L	3	11.34
ctg	L	61	15.08
att	I	0	20.07
atc	I	0	13.30
ata	I	55	21.63
atg	M	754	754.00
aaa	K	0	0.59
aag	K	1	0.41
gtt	V	0	0.21
gtc	V	0	0.20
gta	V	0	0.21
gtg	V	1	0.39
---	--	------	------
mPD		0.27	0.35
		nPD:	0.77
		N. weight:	0.19
		Sc. PD:	0.11
		Sc. rank:	113.9

PB1			
Pos.	373	obs:	exp:
tta	L	1	77.25
ttg	L	0	164.00
ctt	L	51	149.00
ctc	L	781	147.30
cta	L	46	146.50
ctg	L	0	194.90
---	--	------	------
mPD		0.21	1.1
		nPD:	0.19
		N. weight:	1.7
		Sc. PD:	0.037
		Sc. rank:	331.9

PB1			
Pos.	374	obs:	exp:
tct	S	0	0.16
tcc	S	0	0.13
tca	S	1	0.25
tcg	S	0	0.06
act	T	0	2.59
acc	T	0	2.04
aca	T	9	4.59
acg	T	1	0.78
agt	S	0	0.20
agc	S	0	0.20
gtt	V	0	0.21
gtc	V	0	0.20
gta	V	1	0.21
gtg	V	0	0.39
gct	A	1	222.20
gcc	A	0	162.80
gca	A	662	405.40
gcg	A	204	76.68
---	--	------	------
mPD		0.39	0.70
		nPD:	0.55
		N. weight:	0.75
		Sc. PD:	0.28
		Sc. rank:	426.2

PB1			
Pos.	375	obs:	exp:
tct	S	0	6.08
tcc	S	0	4.92
tca	S	0	9.59
tcg	S	0	2.21
aat	N	782	426.80
aac	N	57	412.20
aaa	K	1	0.59
aag	K	0	0.41
agt	S	13	7.79
agc	S	25	7.41
gat	D	0	0.54
gac	D	1	0.46
---	--	------	------
mPD		0.26	0.65
		nPD:	0.4
		N. weight:	0.57
		Sc. PD:	0.13
		Sc. rank:	287.3

PB1			
Pos.	376	obs:	exp:
ttt	F	1	0.39
ttc	F	0	0.61
att	I	875	320.00
atc	I	2	212.10
ata	I	0	344.90
gtt	V	1	0.21
gtc	V	0	0.20
gta	V	0	0.21
gtg	V	0	0.39
---	--	------	------
mPD		0.0091	0.66
		nPD:	0.01
		N. weight:	1.2
		Sc. PD:	-0.18
		Sc. rank:	-524.9

PB1			
Pos.	377	obs:	exp:
gat	D	53	474.50
gac	D	826	404.50
---	--	------	------
mPD		0.11	0.50
		nPD:	0.23
		N. weight:	0.67
		Sc. PD:	0.042
		Sc. rank:	196.4

PB1			
Pos.	378	obs:	exp:
tta	L	5	77.08
ttg	L	50	163.70
ctt	L	0	148.60
ctc	L	0	146.90
cta	L	2	146.20
ctg	L	820	194.50
atg	M	2	2.00
---	--	------	------
mPD		0.14	1.1
		nPD:	0.12
		N. weight:	1.5
		Sc. PD:	-0.059
		Sc. rank:	44.5

PB1			
Pos.	379	obs:	exp:
cgt	R	0	0.04
cgc	R	0	0.05
cga	R	0	0.10
cgg	R	0	0.08
aaa	K	847	514.30
aag	K	31	363.70
aga	R	1	0.46
agg	R	0	0.27
---	--	------	------
mPD		0.070	0.49
		nPD:	0.14
		N. weight:	0.49
		Sc. PD:	-0.0091
		Sc. rank:	34.0

PB1			
Pos.	380	obs:	exp:
tat	Y	217	466.10
tac	Y	662	412.90
---	--	------	------
mPD		0.37	0.50
		nPD:	0.75
		N. weight:	0.21
		Sc. PD:	0.12
		Sc. rank:	127.9

PB1			
Pos.	381	obs:	exp:
ttt	F	3	346.90
ttc	F	876	532.10
---	--	------	------
mPD		0.0068	0.48
		nPD:	0.01
		N. weight:	0.59
		Sc. PD:	-0.085
		Sc. rank:	-252.2

PB1			
Pos.	382	obs:	exp:
aat	N	59	447.20
aac	N	820	431.80
---	--	------	------
mPD		0.13	0.50
		nPD:	0.25
		N. weight:	0.57
		Sc. PD:	0.048
		Sc. rank:	183.3

PB1			
Pos.	383	obs:	exp:
caa	Q	1	0.51
cag	Q	0	0.49
cgt	R	0	0.04
cgc	R	0	0.05
cga	R	1	0.10
cgg	R	0	0.08
aaa	K	764	460.40
aag	K	22	325.60
aga	R	0	0.46
agg	R	0	0.27
gaa	E	52	54.74
gag	E	39	36.26
---	--	------	------
mPD		0.32	0.68
		nPD:	0.48
		N. weight:	0.47
		Sc. PD:	0.14
		Sc. rank:	253.5

PB1			
Pos.	384	obs:	exp:
tct	S	1	140.70
tcc	S	1	113.80
tca	S	867	221.80
tcg	S	10	51.15
agt	S	0	180.10
agc	S	0	171.40
---	--	------	------
mPD		0.027	1.7
		nPD:	0.02
		N. weight:	1.6
		Sc. PD:	-0.23
		Sc. rank:	-668.2

PB1			
Pos.	385	obs:	exp:
tct	S	0	0.16
tcc	S	0	0.13
tca	S	1	0.25
tcg	S	0	0.06
act	T	0	227.40
acc	T	0	178.80
aca	T	851	403.40
acg	T	27	68.39
agt	S	0	0.20
agc	S	0	0.20
---	--	------	------
mPD		0.062	0.68
		nPD:	0.09
		N. weight:	0.86
		Sc. PD:	-0.06
		Sc. rank:	-83.2

PB1

Pos. 386		obs:	exp:
tct	S	0	0.16
tcc	S	0	0.13
tca	S	0	0.25
tcg	S	0	0.06
cgt	R	0	36.44
cgc	R	0	43.34
cga	R	0	85.05
cgg	R	0	71.05
aaa	K	23	14.64
aag	K	2	10.36
agt	S	1	0.20
agc	S	0	0.20
aga	R	839	389.50
agg	R	14	227.70
---	--	------	------
mPD		0.093	1.0
		nPD:	0.09
		N. weight:	0.87
		Sc. PD:	-0.059
		Sc. rank:	-78.7

PB1

Pos. 387		obs:	exp:
aaa	K	0	53.31
aag	K	91	37.69
gaa	E	1	474.00
gag	E	787	314.00
---	--	------	------
mPD		0.19	0.67
		nPD:	0.28
		N. weight:	1.1
		Sc. PD:	0.13
		Sc. rank:	411.7

PB1

Pos. 388		obs:	exp:
cgt	R	0	0.04
cgc	R	0	0.05
cga	R	0	0.10
cgg	R	0	0.08
aaa	K	689	514.30
aag	K	189	363.70
aga	R	0	0.46
agg	R	1	0.27
---	--	------	------
mPD		0.34	0.49
		nPD:	0.7
		N. weight:	0.12
		Sc. PD:	0.06
		Sc. rank:	69.5

PB1

Pos. 389		obs:	exp:
att	I	10	320.70
atc	I	832	212.60
ata	I	37	345.70
---	--	------	------
mPD		0.10	0.65
		nPD:	0.16
		N. weight:	1.4
		Sc. PD:	-0.0095
		Sc. rank:	158.6

PB1

Pos. 390		obs:	exp:
caa	Q	0	0.51
cag	Q	1	0.49
gct	A	0	0.51
gcc	A	0	0.38
gca	A	0	0.94
gcg	A	2	0.18
gaa	E	44	526.90
gag	E	832	349.10
---	--	------	------
mPD		0.10	0.49
		nPD:	0.21
		N. weight:	0.87
		Sc. PD:	0.039
		Sc. rank:	215.4

PB1

Pos. 391		obs:	exp:
cgt	R	0	0.09
cgc	R	0	0.10
cga	R	0	0.20
cgg	R	0	0.17
aaa	K	838	513.70
aag	K	39	363.30
aga	R	2	0.91
agg	R	0	0.53
---	--	------	------
mPD		0.089	0.49
		nPD:	0.18
		N. weight:	0.46
		Sc. PD:	0.0082
		Sc. rank:	83.6

PB1

Pos. 392		obs:	exp:
att	I	0	320.00
atc	I	0	212.10
ata	I	877	344.90
gtt	V	0	0.41
gtc	V	0	0.40
gta	V	1	0.41
gtg	V	1	0.77
---	--	------	------
mPD		0.0068	0.66
		nPD:	0.01
		N. weight:	1.2
		Sc. PD:	-0.17
		Sc. rank:	-541.5

PB1

Pos. 393		obs:	exp:
cgt	R	0	37.46
cgc	R	0	44.56
cga	R	0	87.45
cgg	R	1	73.04
aaa	K	1	1.17
aag	K	1	0.83
aga	R	653	400.40
agg	R	223	234.10
---	--	------	------
mPD		0.39	0.97
		nPD:	0.4
		N. weight:	0.43
		Sc. PD:	0.099
		Sc. rank:	220.6

PB1

Pos. 394		obs:	exp:
cct	P	866	222.70
ccc	P	9	156.20
cca	P	0	357.90
ccg	P	1	139.20
cat	H	3	1.72
cac	H	0	1.28
---	--	------	------
mPD		0.029	0.72
		nPD:	0.04
		N. weight:	1.6
		Sc. PD:	-0.19
		Sc. rank:	-406.8

PB1

Pos. 395		obs:	exp:
tta	L	2	77.16
ttg	L	0	163.80
ctt	L	38	148.80
ctc	L	13	147.10
cta	L	797	146.40
ctg	L	28	194.70
att	I	0	0.36
atc	I	0	0.24
ata	I	1	0.39
---	--	------	------
mPD		0.18	1.1
		nPD:	0.16
		N. weight:	1.7
		Sc. PD:	-0.0089
		Sc. rank:	196.7

PB1

Pos. 396		obs:	exp:
tta	L	59	76.99
ttg	L	1	163.50
ctt	L	0	148.40
ctc	L	0	146.80
cta	L	204	146.00
ctg	L	612	194.30
atg	M	3	3.00
---	--	------	------
mPD		0.55	1.1
		nPD:	0.5
		N. weight:	1.1
		Sc. PD:	0.34
		Sc. rank:	579.8

PB1

Pos. 397		obs:	exp:
tta	L	0	0.26
ttg	L	0	0.56
ctt	L	0	0.51
ctc	L	0	0.50
cta	L	3	0.50
ctg	L	0	0.67
att	I	21	251.40
atc	I	2	166.70
ata	I	666	271.00
atg	M	185	185.00
act	T	0	0.26
acc	T	0	0.20
aca	T	1	0.46
acg	T	0	0.08
gtt	V	0	0.21
gtc	V	0	0.20
gta	V	1	0.21
gtg	V	0	0.39
---	--	------	------
mPD		0.38	0.75
		nPD:	0.51
		N. weight:	0.77
		Sc. PD:	0.26
		Sc. rank:	429.2

PB1

Pos. 398		obs:	exp:
tat	Y	1	0.53
tac	Y	0	0.47
gct	A	3	0.77
gcc	A	0	0.56
gca	A	0	1.40
gcg	A	0	0.27
gat	D	847	459.90
gac	D	5	392.10
gaa	E	1	13.84
gag	E	22	9.16
---	--	------	------
mPD		0.071	0.53
		nPD:	0.13
		N. weight:	0.73
		Sc. PD:	-0.021
		Sc. rank:	39.4

PB1

Pos. 399		obs:	exp:
gat	D	0	0.54
gac	D	1	0.46
ggt	G	47	117.70
ggc	G	826	111.60
gga	G	5	405.90
ggg	G	0	242.80
---	--	------	------
mPD		0.11	0.68
		nPD:	0.17
		N. weight:	2.2
		Sc. PD:	0.011
		Sc. rank:	304.8

PB1

Pos. 400		obs:	exp:
act	T	1	227.70
acc	T	3	179.00
aca	T	872	403.80
acg	T	3	68.47
---	--	------	------
mPD		0.016	0.67
		nPD:	0.02
		N. weight:	0.91
		Sc. PD:	-0.12
		Sc. rank:	-317.3

PB1

Pos. 401		obs:	exp:
gct	A	12	225.20
gcc	A	803	165.00
gca	A	64	411.00
gcg	A	0	77.75
---	--	------	------
mPD		0.16	0.67
		nPD:	0.24
		N. weight:	1.6
		Sc. PD:	0.11
		Sc. rank:	476.3

PB1

Pos. 402		obs:	exp:
tct	S	2	140.70
tcc	S	2	113.80
tca	S	873	221.80
tcg	S	2	51.15
agt	S	0	180.10
agc	S	0	171.40
---	--	------	------
mPD		0.014	1.7
		nPD:	0.01
		N. weight:	1.7
		Sc. PD:	-0.25
		Sc. rank:	-828.0

PB1

Pos. 403		obs:	exp:
tta	L	27	77.25
ttg	L	828	164.00
ctt	L	0	149.00
ctc	L	0	147.30
cta	L	1	146.50
ctg	L	23	194.90
---	--	------	------
mPD		0.11	1.1
		nPD:	0.1
		N. weight:	1.8
		Sc. PD:	-0.1
		Sc. rank:	-66.9

PB1

Pos. 404		obs:	exp:
tct	S	0	140.50
tcc	S	0	113.60
tca	S	0	221.60
tcg	S	0	51.09
act	T	1	0.26
acc	T	0	0.20
aca	T	0	0.46
acg	T	0	0.08
agt	S	782	179.90
agc	S	96	171.20
---	--	------	------
mPD		0.20	1.7
		nPD:	0.12
		N. weight:	1.5
		Sc. PD:	-0.066
		Sc. rank:	15.2

PB1

Pos. 405		obs:	exp:
cct	P	852	223.50
ccc	P	3	156.70
cca	P	20	359.10
ccg	P	4	139.70
---	--	------	------
mPD		0.060	0.71
		nPD:	0.08
		N. weight:	1.5
		Sc. PD:	-0.11
		Sc. rank:	-164.8

PB1			
Pos.	406	obs:	exp:
gtt	V	0	0.21
gtc	V	0	0.20
gta	V	1	0.21
gtg	V	0	0.39
ggt	G	0	117.70
ggc	G	0	111.60
gga	G	606	405.90
ggg	G	272	242.80
---	--	------	------
mPD		0.43	0.68
		nPD:	0.63
		N. weight:	0.39
		Sc. PD:	0.18
		Sc. rank:	231.4

PB1			
Pos.	407	obs:	exp:
atg	M	878	878.00
gtt	V	0	0.21
gtc	V	0	0.20
gta	V	0	0.21
gtg	V	1	0.39
---	--	------	------
mPD		0.0023	0.0037
		nPD:	0.62
		N. weight:	0.0013
		Sc. PD:	0.00059
		Sc. rank:	0.8

PB1			
Pos.	408	obs:	exp:
atg	M	879	879.00
---	--	------	------
mPD		0	0
		nPD:	1.
		N. weight:	0.
		Sc. PD:	0
		Sc. rank:	0

PB1			
Pos.	409	obs:	exp:
atg	M	879	879.00
---	--	------	------
mPD		0	0
		nPD:	1.
		N. weight:	0.
		Sc. PD:	0
		Sc. rank:	0

PB1			
Pos.	410	obs:	exp:
ggt	G	214	117.90
ggc	G	662	111.70
gga	G	2	406.40
ggg	G	1	243.00
---	--	------	------
mPD		0.37	0.68
		nPD:	0.55
		N. weight:	1.8
		Sc. PD:	0.68
		Sc. rank:	1043.9

PB1			
Pos.	411	obs:	exp:
atg	M	879	879.00
---	--	------	------
mPD		0	0
		nPD:	1.
		N. weight:	0.
		Sc. PD:	0
		Sc. rank:	0

PB1			
Pos.	412	obs:	exp:
ttt	F	1	346.90
ttc	F	878	532.10
---	--	------	------
mPD		0.0023	0.48
		nPD:	0.
		N. weight:	0.61
		Sc. PD:	-0.093
		Sc. rank:	-322.6

PB1			
Pos.	413	obs:	exp:
aat	N	215	447.20
aac	N	664	431.80
---	--	------	------
mPD		0.37	0.50
		nPD:	0.74
		N. weight:	0.18
		Sc. PD:	0.1
		Sc. rank:	111.9

PB1			
Pos.	414	obs:	exp:
att	I	0	0.36
atc	I	0	0.24
ata	I	1	0.39
atg	M	878	878.00
---	--	------	------
mPD		0.0023	0.0023
		nPD:	1.
		N. weight:	0.0013
		Sc. PD:	0.0011
		Sc. rank:	0.8

PB1			
Pos.	415	obs:	exp:
tta	L	12	77.16
ttg	L	721	163.80
ctt	L	0	148.80
ctc	L	0	147.10
cta	L	15	146.40
ctg	L	130	194.70
atg	M	1	1.00
---	--	------	------
mPD		0.34	1.1
		nPD:	0.3
		N. weight:	1.4
		Sc. PD:	0.18
		Sc. rank:	565.8

PB1			
Pos.	416	obs:	exp:
tct	S	0	140.50
tcc	S	0	113.60
tca	S	0	221.60
tcg	S	0	51.09
cgt	R	0	0.04
cgc	R	0	0.05
cga	R	0	0.10
cgg	R	0	0.08
agt	S	847	179.90
agc	S	31	171.20
aga	R	0	0.46
agg	R	1	0.27
---	--	------	------
mPD		0.070	1.7
		nPD:	0.04
		N. weight:	1.8
		Sc. PD:	-0.21
		Sc. rank:	-435.1

PB1			
Pos.	417	obs:	exp:
act	T	2	227.70
acc	T	1	179.00
aca	T	857	403.80
acg	T	19	68.47
---	--	------	------
mPD		0.049	0.67
		nPD:	0.07
		N. weight:	0.86
		Sc. PD:	-0.075
		Sc. rank:	-129.3

PB1			
Pos.	418	obs:	exp:
gtt	V	68	181.50
gtc	V	596	176.10
gta	V	211	182.00
gtg	V	4	339.50
---	--	------	------
mPD		0.48	0.73
		nPD:	0.66
		N. weight:	0.96
		Sc. PD:	0.45
		Sc. rank:	567.9

PB1			
Pos.	419	obs:	exp:
tta	L	779	77.25
ttg	L	98	164.00
ctt	L	0	149.00
ctc	L	0	147.30
cta	L	2	146.50
ctg	L	0	194.90
---	--	------	------
mPD		0.20	1.1
		nPD:	0.18
		N. weight:	2.4
		Sc. PD:	0.046
		Sc. rank:	463.5

PB1			
Pos.	420	obs:	exp:
ggt	G	0	117.90
ggc	G	0	111.70
gga	G	855	406.40
ggg	G	24	243.00
---	--	------	------
mPD		0.053	0.68
		nPD:	0.08
		N. weight:	0.82
		Sc. PD:	-0.067
		Sc. rank:	-108.3

PB1			
Pos.	421	obs:	exp:
gtt	V	819	181.50
gtc	V	53	176.10
gta	V	1	182.00
gtg	V	6	339.50
---	--	------	------
mPD		0.13	0.73
		nPD:	0.18
		N. weight:	1.6
		Sc. PD:	0.021
		Sc. rank:	263.3

PB1			
Pos.	422	obs:	exp:
tct	S	1	140.10
tcc	S	0	113.30
tca	S	837	220.80
tcg	S	37	50.92
cct	P	0	0.51
ccc	P	0	0.36
cca	P	2	0.82
ccg	P	0	0.32
act	T	0	0.52
acc	T	0	0.41
aca	T	2	0.92
acg	T	0	0.16
agt	S	0	179.30
agc	S	0	170.60
---	--	------	------
mPD		0.092	1.7
		nPD:	0.06
		N. weight:	1.6
		Sc. PD:	-0.16
		Sc. rank:	-318.5

PB1			
Pos.	423	obs:	exp:
att	I	848	320.70
atc	I	30	212.60
ata	I	1	345.70
---	--	------	------
mPD		0.068	0.65
		nPD:	0.1
		N. weight:	1.1
		Sc. PD:	-0.061
		Sc. rank:	-36.0

PB1			
Pos.	424	obs:	exp:
tta	L	0	77.25
ttg	L	5	164.00
ctt	L	0	149.00
ctc	L	0	147.30
cta	L	42	146.50
ctg	L	832	194.90
---	--	------	------
mPD		0.10	1.1
		nPD:	0.09
		N. weight:	1.6
		Sc. PD:	-0.11
		Sc. rank:	-141.5

PB1			
Pos.	425	obs:	exp:
aat	N	870	447.20
aac	N	9	431.80
---	--	------	------
mPD		0.020	0.50
		nPD:	0.04
		N. weight:	0.77
		Sc. PD:	-0.09
		Sc. rank:	-195.2

PB1			
Pos.	426	obs:	exp:
tta	L	0	77.25
ttg	L	1	164.00
ctt	L	183	149.00
ctc	L	652	147.30
cta	L	40	146.50
ctg	L	3	194.90
---	--	------	------
mPD		0.41	1.1
		nPD:	0.37
		N. weight:	1.3
		Sc. PD:	0.26
		Sc. rank:	635.8

PB1			
Pos.	427	obs:	exp:
ggt	G	77	117.90
ggc	G	0	111.70
gga	G	41	406.40
ggg	G	761	243.00
---	--	------	------
mPD		0.24	0.68
		nPD:	0.36
		N. weight:	1.
		Sc. PD:	0.19
		Sc. rank:	493.9

PB1			
Pos.	428	obs:	exp:
caa	Q	95	451.50
cag	Q	784	427.50
---	--	------	------
mPD		0.19	0.50
		nPD:	0.39
		N. weight:	0.46
		Sc. PD:	0.099
		Sc. rank:	231.6

PB1			
Pos.	429	obs:	exp:
cgt	R	0	0.04
cgc	R	0	0.05
cga	R	0	0.10
cgg	R	0	0.08
aaa	K	21	514.30
aag	K	857	363.70
aga	R	0	0.46
agg	R	1	0.27
---	--	------	------
mPD		0.049	0.49
		nPD:	0.1
		N. weight:	0.94
		Sc. PD:	-0.057
		Sc. rank:	-57.8

PB1			
Pos.	430	obs:	exp:
cgt	R	0	3.55
cgc	R	0	4.22
cga	R	0	8.28
cgg	R	0	6.91
aaa	K	124	466.30
aag	K	672	329.70
aga	R	3	37.90
agg	R	80	22.15
---	--	------	------
mPD		0.42	0.72
		nPD:	0.59
		N. weight:	0.58
		Sc. PD:	0.24
		Sc. rank:	338.7

PB1			
Pos.	431	obs:	exp:
tat	Y	2	465.50
tac	Y	876	412.50
cat	H	0	0.57
cac	H	1	0.43
---	--	------	------
mPD		0.0068	0.50
		nPD:	0.01
		N. weight:	0.91
		Sc. PD:	-0.13
		Sc. rank:	-401.9

,

PB1			
Pos.	432	obs:	exp:
att	I	0	0.73
atc	I	2	0.48
ata	I	0	0.79
act	T	70	227.20
acc	T	733	178.60
aca	T	74	402.90
acg	T	0	68.32
---	--	------	------
mPD		0.29	0.68
		nPD:	0.43
		N. weight:	1.2
		Sc. PD:	0.3
		Sc. rank:	609.6

,

PB1			
Pos.	433	obs:	exp:
cgt	R	0	0.34
cgc	R	0	0.41
cga	R	0	0.80
cgg	R	0	0.67
aaa	K	861	510.20
aag	K	10	360.80
aga	R	8	3.65
agg	R	0	2.14
---	--	------	------
mPD		0.041	0.51
		nPD:	0.08
		N. weight:	0.59
		Sc. PD:	-0.048
		Sc. rank:	-77.5

,

PB1			
Pos.	434	obs:	exp:
act	T	19	219.40
acc	T	787	172.50
aca	T	41	389.10
acg	T	0	65.98
aat	N	4	16.28
aac	N	28	15.72
---	--	------	------
mPD		0.21	0.75
		nPD:	0.28
		N. weight:	1.5
		Sc. PD:	0.16
		Sc. rank:	544.7

,

PB1			
Pos.	435	obs:	exp:
att	I	0	0.36
atc	I	0	0.24
ata	I	1	0.39
act	T	0	227.40
acc	T	0	178.80
aca	T	873	403.40
acg	T	5	68.39
---	--	------	------
mPD		0.014	0.68
		nPD:	0.02
		N. weight:	0.93
		Sc. PD:	-0.13
		Sc. rank:	-350.1

,

PB1			
Pos.	436	obs:	exp:
tat	Y	782	466.10
tac	Y	97	412.90
---	--	------	------
mPD		0.20	0.50
		nPD:	0.39
		N. weight:	0.38
		Sc. PD:	0.083
		Sc. rank:	189.8

,

PB1			
Pos.	437	obs:	exp:
tgg	W	879	879.00
---	--	------	------
mPD		0	0
		nPD:	1.
		N. weight:	0.
		Sc. PD:	0
		Sc. rank:	0

,

PB1			
Pos.	438	obs:	exp:
tgg	W	878	878.00
ggt	G	0	0.13
ggc	G	0	0.13
gga	G	0	0.46
ggg	G	1	0.28
---	--	------	------
mPD		0.0023	0.0039
		nPD:	0.58
		N. weight:	0.0018
		Sc. PD:	0.00072
		Sc. rank:	1.0

,

PB1			
Pos.	439	obs:	exp:
gat	D	169	474.50
gac	D	710	404.50
---	--	------	------
mPD		0.31	0.50
		nPD:	0.63
		N. weight:	0.32
		Sc. PD:	0.14
		Sc. rank:	189.2

,

PB1			
Pos.	440	obs:	exp:
ggt	G	0	117.90
ggc	G	0	111.70
gga	G	871	406.40
ggg	G	8	243.00
---	--	------	------
mPD		0.018	0.68
		nPD:	0.03
		N. weight:	0.9
		Sc. PD:	-0.12
		Sc. rank:	-291.0

,

PB1			
Pos.	441	obs:	exp:
tta	L	0	77.25
ttg	L	0	164.00
ctt	L	35	149.00
ctc	L	842	147.30
cta	L	2	146.50
ctg	L	0	194.90
---	--	------	------
mPD		0.081	1.1
		nPD:	0.07
		N. weight:	2.
		Sc. PD:	-0.17
		Sc. rank:	-292.8

,

PB1			
Pos.	442	obs:	exp:
caa	Q	866	451.50
cag	Q	13	427.50
---	--	------	------
mPD		0.029	0.50
		nPD:	0.06
		N. weight:	0.73
		Sc. PD:	-0.074
		Sc. rank:	-147.3

,

PB1			
Pos.	443	obs:	exp:
tct	S	8	140.70
tcc	S	866	113.80
tca	S	5	221.80
tcg	S	0	51.15
agt	S	0	180.10
agc	S	0	171.40
---	--	------	------
mPD		0.029	1.7
		nPD:	0.02
		N. weight:	2.4
		Sc. PD:	-0.34
		Sc. rank:	-953.8

,

PB1			
Pos.	444	obs:	exp:
tct	S	84	140.70
tcc	S	5	113.80
tca	S	790	221.80
tcg	S	0	51.15
agt	S	0	180.10
agc	S	0	171.40
---	--	------	------
mPD		0.18	1.7
		nPD:	0.11
		N. weight:	1.3
		Sc. PD:	-0.067
		Sc. rank:	-15.2

,

PB1			
Pos.	445	obs:	exp:
aat	N	1	0.51
aac	N	0	0.49
gat	D	863	472.90
gac	D	13	403.10
gaa	E	0	1.20
gag	E	2	0.80
---	--	------	------
mPD		0.036	0.50
		nPD:	0.07
		N. weight:	0.67
		Sc. PD:	-0.059
		Sc. rank:	-103.5

,

PB1			
Pos.	446	obs:	exp:
gat	D	47	474.50
gac	D	832	404.50
---	--	------	------
mPD		0.10	0.50
		nPD:	0.2
		N. weight:	0.69
		Sc. PD:	0.027
		Sc. rank:	164.0

,

PB1			
Pos.	447	obs:	exp:
ttt	F	55	346.90
ttc	F	824	532.10
---	--	------	------
mPD		0.12	0.48
		nPD:	0.25
		N. weight:	0.37
		Sc. PD:	0.029
		Sc. rank:	114.9

,

PB1			
Pos.	448	obs:	exp:
act	T	0	0.52
acc	T	2	0.41
aca	T	0	0.92
acg	T	0	0.16
gct	A	94	224.70
gcc	A	756	164.70
gca	A	27	410.00
gcg	A	0	77.57
---	--	------	------
mPD		0.25	0.68
		nPD:	0.37
		N. weight:	1.4
		Sc. PD:	0.28
		Sc. rank:	678.6

,

PB1			
Pos.	449	obs:	exp:
ttt	F	0	0.39
ttc	F	1	0.61
tta	L	0	77.16
ttg	L	0	163.80
ctt	L	3	148.80
ctc	L	874	147.10
cta	L	1	146.40
ctg	L	0	194.70
---	--	------	------
mPD		0.011	1.1
		nPD:	0.01
		N. weight:	2.2
		Sc. PD:	-0.32
		Sc. rank:	-1023.1

,

PB1			
Pos.	450	obs:	exp:
att	I	1	320.70
atc	I	0	212.60
ata	I	878	345.70
---	--	------	------
mPD		0.0023	0.65
		nPD:	0.
		N. weight:	1.1
		Sc. PD:	-0.18
		Sc. rank:	-633.7

,

PB1			
Pos.	451	obs:	exp:
atg	M	1	1.00
gtt	V	1	181.20
gtc	V	0	175.90
gta	V	7	181.80
gtg	V	870	339.10
---	--	------	------
mPD		0.020	0.73
		nPD:	0.03
		N. weight:	1.1
		Sc. PD:	-0.15
		Sc. rank:	-347.7

,

PB1			
Pos.	452	obs:	exp:
aat	N	876	447.20
aac	N	3	431.80
---	--	------	------
mPD		0.0068	0.50
		nPD:	0.01
		N. weight:	0.81
		Sc. PD:	-0.12
		Sc. rank:	-359.7

,

PB1			
Pos.	453	obs:	exp:
gct	A	18	225.20
gcc	A	19	165.00
gca	A	841	411.00
gcg	A	1	77.75
---	--	------	------
mPD		0.084	0.67
		nPD:	0.12
		N. weight:	0.73
		Sc. PD:	-0.027
		Sc. rank:	25.7

,

PB1			
Pos.	454	obs:	exp:
tta	L	0	0.09
ttg	L	0	0.19
tct	S	0	0.64
tcc	S	0	0.52
tca	S	1	1.01
tcg	S	3	0.23
ctt	L	0	0.17
ctc	L	0	0.17
cta	L	1	0.17
ctg	L	0	0.22
cct	P	0	222.20
ccc	P	0	155.80
cca	P	232	357.10
ccg	P	642	138.90
agt	S	0	0.82
agc	S	0	0.78
---	--	------	------
mPD		0.40	0.73
		nPD:	0.55
		N. weight:	1.3
		Sc. PD:	0.47
		Sc. rank:	719.0

,

PB1			
Pos.	455	obs:	exp:
tct	S	0	0.16
tcc	S	0	0.13
tca	S	0	0.25
tcg	S	0	0.06
aat	N	869	444.60
aac	N	5	429.40
agt	S	1	0.20
agc	S	0	0.20
gat	D	4	2.16
gac	D	0	1.84
---	--	------	------
mPD		0.023	0.51
		nPD:	0.04
		N. weight:	0.79
		Sc. PD:	-0.091
		Sc. rank:	-187.5

,

PB1			
Pos.	456	obs:	exp:
cat	H	878	505.00
cac	H	1	374.00
---	--	------	------
mPD		0.0023	0.49
		nPD:	0.
		N. weight:	0.67
		Sc. PD:	-0.1
		Sc. rank:	-363.7

PB1			
Pos.	457	obs:	exp:
gaa	E	7	528.70
gag	E	872	350.30
---	--	------	------
mPD		0.016	0.48
		nPD:	0.03
		N. weight:	1.1
		Sc. PD:	-0.13
		Sc. rank:	-316.2

PB1			
Pos.	458	obs:	exp:
gaa	E	4	2.41
gag	E	0	1.59
ggt	G	1	117.30
ggc	G	0	111.20
gga	G	815	404.60
ggg	G	59	241.90
---	--	------	------
mPD		0.14	0.68
		nPD:	0.2
		N. weight:	0.69
		Sc. PD:	0.024
		Sc. rank:	147.9

PB1			
Pos.	459	obs:	exp:
att	I	0	320.70
atc	I	0	212.60
ata	I	879	345.70
---	--	------	------
mPD		0	0.65
		nPD:	0.
		N. weight:	1.2
		Sc. PD:	-0.18
		Sc. rank:	-708.9

PB1			
Pos.	460	obs:	exp:
caa	Q	172	451.50
cag	Q	707	427.50
---	--	------	------
mPD		0.32	0.50
		nPD:	0.63
		N. weight:	0.27
		Sc. PD:	0.12
		Sc. rank:	160.0

PB1			
Pos.	461	obs:	exp:
gct	A	3	225.20
gcc	A	3	165.00
gca	A	872	411.00
gcg	A	1	77.75
---	--	------	------
mPD		0.016	0.67
		nPD:	0.02
		N. weight:	0.88
		Sc. PD:	-0.12
		Sc. rank:	-306.2

PB1			
Pos.	462	obs:	exp:
ggt	G	1	117.90
ggc	G	1	111.70
gga	G	839	406.40
ggg	G	38	243.00
---	--	------	------
mPD		0.087	0.68
		nPD:	0.13
		N. weight:	0.75
		Sc. PD:	-0.025
		Sc. rank:	36.0

PB1			
Pos.	463	obs:	exp:
gtt	V	36	181.50
gtc	V	0	176.10
gta	V	465	182.00
gtg	V	378	339.50
---	--	------	------
mPD		0.53	0.73
		nPD:	0.74
		N. weight:	0.6
		Sc. PD:	0.33
		Sc. rank:	360.6

PB1			
Pos.	464	obs:	exp:
aat	N	2	1.53
aac	N	1	1.47
gat	D	630	472.90
gac	D	246	403.10
---	--	------	------
mPD		0.41	0.50
		nPD:	0.82
		N. weight:	0.089
		Sc. PD:	0.056
		Sc. rank:	54.8

PB1			
Pos.	465	obs:	exp:
cgt	R	0	37.42
cgc	R	0	44.51
cga	R	0	87.35
cgg	R	0	72.96
aaa	K	0	1.76
aag	K	3	1.24
aga	R	46	400.00
agg	R	830	233.80
---	--	------	------
mPD		0.11	0.97
		nPD:	0.11
		N. weight:	1.3
		Sc. PD:	-0.07
		Sc. rank:	-18.3

PB1			
Pos.	466	obs:	exp:
ttt	F	24	346.90
ttc	F	855	532.10
---	--	------	------
mPD		0.053	0.48
		nPD:	0.11
		N. weight:	0.48
		Sc. PD:	-0.024
		Sc. rank:	-5.1

PB1			
Pos.	467	obs:	exp:
tat	Y	847	466.10
tac	Y	32	412.90
---	--	------	------
mPD		0.070	0.50
		nPD:	0.14
		N. weight:	0.6
		Sc. PD:	-0.013
		Sc. rank:	39.6

PB1			
Pos.	468	obs:	exp:
cgt	R	0	37.55
cgc	R	0	44.66
cga	R	0	87.65
cgg	R	0	73.21
aga	R	821	401.30
agg	R	58	234.60
---	--	------	------
mPD		0.12	0.96
		nPD:	0.13
		N. weight:	0.71
		Sc. PD:	-0.024
		Sc. rank:	31.5

PB1			
Pos.	469	obs:	exp:
act	T	7	227.70
acc	T	866	179.00
aca	T	6	403.80
acg	T	0	68.47
---	--	------	------
mPD		0.029	0.67
		nPD:	0.04
		N. weight:	1.9
		Sc. PD:	-0.21
		Sc. rank:	-444.1

PB1			
Pos.	470	obs:	exp:
tgt	C	3	360.90
tgc	C	876	518.10
---	--	------	------
mPD		0.0068	0.48
		nPD:	0.01
		N. weight:	0.63
		Sc. PD:	-0.09
		Sc. rank:	-267.4

PB1			
Pos.	471	obs:	exp:
aaa	K	835	514.90
aag	K	44	364.10
---	--	------	------
mPD		0.095	0.49
		nPD:	0.2
		N. weight:	0.44
		Sc. PD:	0.014
		Sc. rank:	93.0

PB1			
Pos.	472	obs:	exp:
tta	L	668	77.25
ttg	L	38	164.00
ctt	L	1	149.00
ctc	L	0	147.30
cta	L	88	146.50
ctg	L	84	194.90
---	--	------	------
mPD		0.56	1.1
		nPD:	0.5
		N. weight:	1.8
		Sc. PD:	0.58
		Sc. rank:	989.9

PB1			
Pos.	473	obs:	exp:
gtt	V	830	181.50
gtc	V	47	176.10
gta	V	0	182.00
gtg	V	2	339.50
---	--	------	------
mPD		0.11	0.73
		nPD:	0.15
		N. weight:	1.7
		Sc. PD:	-0.029
		Sc. rank:	130.1

PB1			
Pos.	474	obs:	exp:
ggt	G	0	117.90
ggc	G	0	111.70
gga	G	57	406.40
ggg	G	822	243.00
---	--	------	------
mPD		0.12	0.68
		nPD:	0.18
		N. weight:	1.3
		Sc. PD:	0.02
		Sc. rank:	215.3

PB1			
Pos.	475	obs:	exp:
att	I	2	320.70
atc	I	90	212.60
ata	I	787	345.70
---	--	------	------
mPD		0.19	0.65
		nPD:	0.29
		N. weight:	0.79
		Sc. PD:	0.095
		Sc. rank:	301.0

PB1			
Pos.	476	obs:	exp:
aat	N	77	447.20
aac	N	802	431.80
---	--	------	------
mPD		0.16	0.50
		nPD:	0.32
		N. weight:	0.51
		Sc. PD:	0.077
		Sc. rank:	223.3

PB1			
Pos.	477	obs:	exp:
atg	M	879	879.00
---	--	------	------
mPD		0	0
		nPD:	1.
		N. weight:	0.
		Sc. PD:	0
		Sc. rank:	0

PB1			
Pos.	478	obs:	exp:
tct	S	0	140.70
tcc	S	0	113.80
tca	S	0	221.80
tcg	S	0	51.15
agt	S	1	180.10
agc	S	878	171.40
---	--	------	------
mPD		0.0023	1.7
		nPD:	0.
		N. weight:	2.
		Sc. PD:	-0.31
		Sc. rank:	-1184.0

PB1			
Pos.	479	obs:	exp:
aaa	K	92	514.90
aag	K	787	364.10
---	--	------	------
mPD		0.19	0.49
		nPD:	0.39
		N. weight:	0.63
		Sc. PD:	0.14
		Sc. rank:	317.6

PB1			
Pos.	480	obs:	exp:
cgt	R	0	0.04
cgc	R	0	0.05
cga	R	0	0.10
cgg	R	0	0.08
aaa	K	0	514.30
aag	K	878	363.70
aga	R	0	0.46
agg	R	1	0.27
---	--	------	------
mPD		0.0023	0.49
		nPD:	0.
		N. weight:	1.1
		Sc. PD:	-0.17
		Sc. rank:	-584.5

PB1			
Pos.	481	obs:	exp:
aaa	K	791	514.90
aag	K	88	364.10
---	--	------	------
mPD		0.18	0.49
		nPD:	0.37
		N. weight:	0.31
		Sc. PD:	0.061
		Sc. rank:	148.7

PB1			
Pos.	482	obs:	exp:
tct	S	69	140.70
tcc	S	809	113.80
tca	S	1	221.80
tcg	S	0	51.15
agt	S	0	180.10
agc	S	0	171.40
---	--	------	------
mPD		0.15	1.7
		nPD:	0.09
		N. weight:	2.2
		Sc. PD:	-0.15
		Sc. rank:	-213.6

PB1			
Pos.	483	obs:	exp:
tat	Y	45	466.10
tac	Y	834	412.90
---	--	------	------
mPD		0.097	0.50
		nPD:	0.2
		N. weight:	0.68
		Sc. PD:	0.021
		Sc. rank:	142.3

PB1			
Pos.	484	obs:	exp:
tta	L	1	0.09
ttg	L	0	0.19
ctt	L	0	0.17
ctc	L	0	0.17
cta	L	0	0.17
ctg	L	0	0.22
att	I	0	320.40
atc	I	0	212.40
ata	I	878	345.30
---	--	------	------
mPD		0.0023	0.66
		nPD:	0.
		N. weight:	1.2
		Sc. PD:	-0.18
		Sc. rank:	-653.2

PB1			
Pos.	485	obs:	exp:
aat	N	806	447.20
aac	N	73	431.80
---	--	------	------
mPD		0.15	0.50
		nPD:	0.31
		N. weight:	0.49
		Sc. PD:	0.066
		Sc. rank:	204.1

PB1			
Pos.	486	obs:	exp:
cgt	R	0	37.51
cgc	R	0	44.61
cga	R	23	87.55
cgg	R	743	73.13
aaa	K	1	0.59
aag	K	0	0.41
aga	R	5	400.90
agg	R	107	234.30
---	--	------	------
mPD		0.29	0.97
		nPD:	0.3
		N. weight:	2.2
		Sc. PD:	0.3
		Sc. rank:	913.7

,

PB1			
Pos.	487	obs:	exp:
act	T	1	227.70
acc	T	0	179.00
aca	T	875	403.80
acg	T	3	68.47
---	--	------	------
mPD		0.0091	0.67
		nPD:	0.01
		N. weight:	0.93
		Sc. PD:	-0.13
		Sc. rank:	-420.6

,

PB1			
Pos.	488	obs:	exp:
ggt	G	0	117.90
ggc	G	0	111.70
gga	G	871	406.40
ggg	G	8	243.00
---	--	------	------
mPD		0.018	0.68
		nPD:	0.03
		N. weight:	0.9
		Sc. PD:	-0.12
		Sc. rank:	-291.0

,

PB1			
Pos.	489	obs:	exp:
act	T	5	227.70
acc	T	0	179.00
aca	T	838	403.80
acg	T	36	68.47
---	--	------	------
mPD		0.090	0.67
		nPD:	0.13
		N. weight:	0.81
		Sc. PD:	-0.024
		Sc. rank:	41.7

,

PB1			
Pos.	490	obs:	exp:
ttt	F	119	346.90
ttc	F	760	532.10
---	--	------	------
mPD		0.23	0.48
		nPD:	0.49
		N. weight:	0.21
		Sc. PD:	0.065
		Sc. rank:	111.4

,

PB1			
Pos.	491	obs:	exp:
gaa	E	835	528.70
gag	E	44	350.30
---	--	------	------
mPD		0.095	0.48
		nPD:	0.2
		N. weight:	0.41
		Sc. PD:	0.014
		Sc. rank:	87.2

,

PB1			
Pos.	492	obs:	exp:
ttt	F	2	346.90
ttc	F	877	532.10
---	--	------	------
mPD		0.0045	0.48
		nPD:	0.01
		N. weight:	0.6
		Sc. PD:	-0.089
		Sc. rank:	-293.7

,

PB1			
Pos.	493	obs:	exp:
act	T	0	227.70
acc	T	2	179.00
aca	T	877	403.80
acg	T	0	68.47
---	--	------	------
mPD		0.0045	0.67
		nPD:	0.01
		N. weight:	0.94
		Sc. PD:	-0.14
		Sc. rank:	-491.4

,

PB1			
Pos.	494	obs:	exp:
tct	S	0	140.70
tcc	S	0	113.80
tca	S	0	221.80
tcg	S	0	51.15
agt	S	41	180.10
agc	S	838	171.40
---	--	------	------
mPD		0.089	1.7
		nPD:	0.05
		N. weight:	1.8
		Sc. PD:	-0.19
		Sc. rank:	-381.7

,

PB1			
Pos.	495	obs:	exp:
ttt	F	865	346.90
ttc	F	14	532.10
---	--	------	------
mPD		0.031	0.48
		nPD:	0.07
		N. weight:	1.
		Sc. PD:	-0.098
		Sc. rank:	-187.0

,

PB1			
Pos.	496	obs:	exp:
ttt	F	9	346.90
ttc	F	870	532.10
---	--	------	------
mPD		0.020	0.48
		nPD:	0.04
		N. weight:	0.56
		Sc. PD:	-0.065
		Sc. rank:	-138.3

,

PB1			
Pos.	497	obs:	exp:
tat	Y	38	466.10
tac	Y	841	412.90
---	--	------	------
mPD		0.083	0.50
		nPD:	0.17
		N. weight:	0.71
		Sc. PD:	0.0019
		Sc. rank:	89.6

,

PB1			
Pos.	498	obs:	exp:
cgt	R	105	37.55
cgc	R	772	44.66
cga	R	2	87.65
cgg	R	0	73.21
aga	R	0	401.30
agg	R	0	234.60
---	--	------	------
mPD		0.21	0.96
		nPD:	0.22
		N. weight:	3.2
		Sc. PD:	0.18
		Sc. rank:	926.2

,

PB1			
Pos.	499	obs:	exp:
tat	Y	868	465.50
tac	Y	10	412.50
cat	H	0	0.57
cac	H	1	0.43
---	--	------	------
mPD		0.027	0.50
		nPD:	0.05
		N. weight:	0.71
		Sc. PD:	-0.075
		Sc. rank:	-150.3

,

PB1			
Pos.	500	obs:	exp:
ggt	G	3	117.90
ggc	G	0	111.70
gga	G	749	406.40
ggg	G	127	243.00
---	--	------	------
mPD		0.25	0.68
		nPD:	0.37
		N. weight:	0.52
		Sc. PD:	0.11
		Sc. rank:	253.4

,

PB1			
Pos.	501	obs:	exp:
ttt	F	163	346.90
ttc	F	716	532.10
---	--	------	------
mPD		0.30	0.48
		nPD:	0.63
		N. weight:	0.13
		Sc. PD:	0.059
		Sc. rank:	76.9

,

PB1			
Pos.	502	obs:	exp:
gtt	V	0	181.50
gtc	V	0	176.10
gta	V	842	182.00
gtg	V	37	339.50
---	--	------	------
mPD		0.081	0.73
		nPD:	0.11
		N. weight:	1.7
		Sc. PD:	-0.085
		Sc. rank:	-13.4

,

PB1			
Pos.	503	obs:	exp:
tct	S	0	0.16
tcc	S	0	0.13
tca	S	0	0.25
tcg	S	0	0.06
agt	S	1	0.20
agc	S	0	0.20
gct	A	587	224.70
gcc	A	288	164.70
gca	A	2	410.00
gcg	A	0	77.57
ggt	G	1	0.13
ggc	G	0	0.13
gga	G	0	0.46
ggg	G	0	0.28
---	--	------	------
mPD		0.45	0.68
		nPD:	0.66
		N. weight:	1.
		Sc. PD:	0.49
		Sc. rank:	603.7

,

PB1			
Pos.	504	obs:	exp:
aat	N	133	447.20
aac	N	746	431.80
---	--	------	------
mPD		0.26	0.50
		nPD:	0.51
		N. weight:	0.35
		Sc. PD:	0.12
		Sc. rank:	195.7

,

PB1			
Pos.	505	obs:	exp:
ttt	F	23	346.90
ttc	F	856	532.10
---	--	------	------
mPD		0.051	0.48
		nPD:	0.11
		N. weight:	0.49
		Sc. PD:	-0.026
		Sc. rank:	-10.3

,

PB1			
Pos.	506	obs:	exp:
tct	S	0	140.70
tcc	S	0	113.80
tca	S	0	221.80
tcg	S	0	51.15
agt	S	822	180.10
agc	S	57	171.40
---	--	------	------
mPD		0.12	1.7
		nPD:	0.07
		N. weight:	1.7
		Sc. PD:	-0.14
		Sc. rank:	-241.7

,

PB1			
Pos.	507	obs:	exp:
atg	M	879	879.00
---	--	------	------
mPD		0	0
		nPD:	1.
		N. weight:	0.
		Sc. PD:	0
		Sc. rank:	0

,

PB1			
Pos.	508	obs:	exp:
gaa	E	28	528.70
gag	E	851	350.30
---	--	------	------
mPD		0.062	0.48
		nPD:	0.13
		N. weight:	0.95
		Sc. PD:	-0.032
		Sc. rank:	44.4

,

PB1			
Pos.	509	obs:	exp:
tta	L	52	77.25
ttg	L	711	164.00
ctt	L	1	149.00
ctc	L	0	147.30
cta	L	2	146.50
ctg	L	113	194.90
---	--	------	------
mPD		0.35	1.1
		nPD:	0.31
		N. weight:	1.3
		Sc. PD:	0.19
		Sc. rank:	575.9

,

PB1			
Pos.	510	obs:	exp:
tct	S	0	0.16
tcc	S	1	0.13
tca	S	0	0.25
tcg	S	0	0.06
cct	P	5	223.30
ccc	P	847	156.50
cca	P	24	358.70
ccg	P	2	139.50
agt	S	0	0.20
agc	S	0	0.20
---	--	------	------
mPD		0.071	0.71
		nPD:	0.1
		N. weight:	1.9
		Sc. PD:	-0.12
		Sc. rank:	-126.7

,

PB1			
Pos.	511	obs:	exp:
tct	S	0	140.70
tcc	S	0	113.80
tca	S	0	221.80
tcg	S	0	51.15
agt	S	804	180.10
agc	S	75	171.40
---	--	------	------
mPD		0.16	1.7
		nPD:	0.09
		N. weight:	1.6
		Sc. PD:	-0.11
		Sc. rank:	-137.5

PB1			
Pos.	512	obs:	exp:
ttt	F	874	346.90
ttc	F	5	532.10
---	--	------	------
mPD		0.011	0.48
		nPD:	0.02
		N. weight:	1.1
		Sc. PD:	-0.15
		Sc. rank:	-380.0

PB1			
Pos.	513	obs:	exp:
ggt	G	0	117.90
ggc	G	0	111.70
gga	G	867	406.40
ggg	G	12	243.00
---	--	------	------
mPD		0.027	0.68
		nPD:	0.04
		N. weight:	0.87
		Sc. PD:	-0.1
		Sc. rank:	-231.4

PB1			
Pos.	514	obs:	exp:
atg	M	1	1.00
gtt	V	0	181.20
gtc	V	0	175.90
gta	V	34	181.80
gtg	V	844	339.10
---	--	------	------
mPD		0.077	0.73
		nPD:	0.11
		N. weight:	1.
		Sc. PD:	-0.056
		Sc. rank:	-26.9

PB1			
Pos.	515	obs:	exp:
tct	S	314	138.00
tcc	S	542	111.60
tca	S	6	217.50
tcg	S	0	50.16
cct	P	0	4.07
ccc	P	16	2.85
cca	P	0	6.54
ccg	P	0	2.54
agt	S	0	176.60
agc	S	0	168.10
gct	A	1	0.26
gcc	A	0	0.19
gca	A	0	0.47
gcg	A	0	0.09
---	--	------	------
mPD		0.51	1.7
		nPD:	0.3
		N. weight:	1.6
		Sc. PD:	0.21
		Sc. rank:	656.4

PB1			
Pos.	516	obs:	exp:
ggt	G	1	117.90
ggc	G	0	111.70
gga	G	74	406.40
ggg	G	804	243.00
---	--	------	------
mPD		0.16	0.68
		nPD:	0.23
		N. weight:	1.2
		Sc. PD:	0.077
		Sc. rank:	347.6

PB1			
Pos.	517	obs:	exp:
att	I	724	319.30
atc	I	94	211.60
ata	I	57	344.10
gtt	V	4	0.83
gtc	V	0	0.80
gta	V	0	0.83
gtg	V	0	1.54
---	--	------	------
mPD		0.31	0.66
		nPD:	0.46
		N. weight:	0.6
		Sc. PD:	0.17
		Sc. rank:	319.7

PB1			
Pos.	518	obs:	exp:
aat	N	834	447.20
aac	N	45	431.80
---	--	------	------
mPD		0.097	0.50
		nPD:	0.19
		N. weight:	0.59
		Sc. PD:	0.018
		Sc. rank:	123.1

PB1			
Pos.	519	obs:	exp:
gaa	E	126	528.70
gag	E	753	350.30
---	--	------	------
mPD		0.25	0.48
		nPD:	0.51
		N. weight:	0.56
		Sc. PD:	0.19
		Sc. rank:	312.1

PB1			
Pos.	520	obs:	exp:
tct	S	37	140.70
tcc	S	0	113.80
tca	S	803	221.80
tcg	S	39	51.15
agt	S	0	180.10
agc	S	0	171.40
---	--	------	------
mPD		0.16	1.7
		nPD:	0.1
		N. weight:	1.4
		Sc. PD:	-0.086
		Sc. rank:	-96.3

PB1			
Pos.	521	obs:	exp:
gct	A	818	225.20
gcc	A	5	165.00
gca	A	56	411.00
gcg	A	0	77.75
---	--	------	------
mPD		0.13	0.67
		nPD:	0.19
		N. weight:	1.3
		Sc. PD:	0.038
		Sc. rank:	269.8

PB1			
Pos.	522	obs:	exp:
gat	D	2	474.00
gac	D	876	404.00
ggt	G	0	0.13
ggc	G	1	0.13
gga	G	0	0.46
ggg	G	0	0.28
---	--	------	------
mPD		0.0068	0.50
		nPD:	0.01
		N. weight:	0.94
		Sc. PD:	-0.14
		Sc. rank:	-408.7

PB1			
Pos.	523	obs:	exp:
atg	M	879	879.00
---	--	------	------
mPD		0	0
		nPD:	1.
		N. weight:	0.
		Sc. PD:	0
		Sc. rank:	0

PB1			
Pos.	524	obs:	exp:
tct	S	0	140.70
tcc	S	0	113.80
tca	S	0	221.80
tcg	S	0	51.15
agt	S	3	180.10
agc	S	876	171.40
---	--	------	------
mPD		0.0068	1.7
		nPD:	0.
		N. weight:	2.
		Sc. PD:	-0.3
		Sc. rank:	-1083.5

PB1			
Pos.	525	obs:	exp:
att	I	420	173.70
atc	I	56	115.10
ata	I	0	187.20
gtt	V	403	83.19
gtc	V	0	80.73
gta	V	0	83.44
gtg	V	0	155.60
---	--	------	------
mPD		0.62	1.2
		nPD:	0.5
		N. weight:	1.4
		Sc. PD:	0.44
		Sc. rank:	745.4

PB1			
Pos.	526	obs:	exp:
ggt	G	785	117.90
ggc	G	4	111.70
gga	G	82	406.40
ggg	G	8	243.00
---	--	------	------
mPD		0.19	0.68
		nPD:	0.29
		N. weight:	1.9
		Sc. PD:	0.22
		Sc. rank:	696.5

PB1			
Pos.	527	obs:	exp:
att	I	1	0.36
atc	I	0	0.24
ata	I	0	0.39
gtt	V	828	181.20
gtc	V	9	175.90
gta	V	39	181.80
gtg	V	2	339.10
---	--	------	------
mPD		0.11	0.73
		nPD:	0.15
		N. weight:	1.6
		Sc. PD:	-0.017
		Sc. rank:	165.2

PB1			
Pos.	528	obs:	exp:
act	T	0	227.70
acc	T	0	179.00
aca	T	872	403.80
acg	T	7	68.47
---	--	------	------
mPD		0.016	0.67
		nPD:	0.02
		N. weight:	0.92
		Sc. PD:	-0.12
		Sc. rank:	-323.1

PB1			
Pos.	529	obs:	exp:
gtt	V	0	181.50
gtc	V	0	176.10
gta	V	786	182.00
gtg	V	93	339.50
---	--	------	------
mPD		0.19	0.73
		nPD:	0.26
		N. weight:	1.4
		Sc. PD:	0.14
		Sc. rank:	495.2

PB1			
Pos.	530	obs:	exp:
att	I	0	320.70
atc	I	1	212.60
ata	I	878	345.70
---	--	------	------
mPD		0.0023	0.65
		nPD:	0.
		N. weight:	1.1
		Sc. PD:	-0.18
		Sc. rank:	-634.1

PB1			
Pos.	531	obs:	exp:
aaa	K	143	514.30
aag	K	735	363.70
gaa	E	0	0.60
gag	E	1	0.40
---	--	------	------
mPD		0.28	0.49
		nPD:	0.56
		N. weight:	0.47
		Sc. PD:	0.18
		Sc. rank:	274.7

PB1			
Pos.	532	obs:	exp:
aat	N	4	446.20
aac	N	873	430.80
gat	D	0	1.08
gac	D	2	0.92
---	--	------	------
mPD		0.014	0.50
		nPD:	0.03
		N. weight:	0.84
		Sc. PD:	-0.11
		Sc. rank:	-270.1

PB1			
Pos.	533	obs:	exp:
aat	N	750	447.20
aac	N	129	431.80
---	--	------	------
mPD		0.25	0.50
		nPD:	0.5
		N. weight:	0.33
		Sc. PD:	0.11
		Sc. rank:	181.7

PB1			
Pos.	534	obs:	exp:
atg	M	879	879.00
---	--	------	------
mPD		0	0
		nPD:	1.
		N. weight:	0.
		Sc. PD:	0
		Sc. rank:	0

PB1			
Pos.	535	obs:	exp:
att	I	6	320.70
atc	I	25	212.60
ata	I	848	345.70
---	--	------	------
mPD		0.069	0.65
		nPD:	0.1
		N. weight:	0.96
		Sc. PD:	-0.054
		Sc. rank:	-30.9

PB1			
Pos.	536	obs:	exp:
aat	N	2	447.20
aac	N	877	431.80
---	--	------	------
mPD		0.0045	0.50
		nPD:	0.01
		N. weight:	0.86
		Sc. PD:	-0.13
		Sc. rank:	-425.8

PB1			
Pos.	537	obs:	exp:
aat	N	153	447.20
aac	N	726	431.80
---	--	------	------
mPD		0.29	0.50
		nPD:	0.58
		N. weight:	0.3
		Sc. PD:	0.12
		Sc. rank:	176.3

PB1			
Pos.	538	obs:	exp:
gat	D	831	474.50
gac	D	48	404.50
---	--	------	------
mPD		0.10	0.50
		nPD:	0.21
		N. weight:	0.51
		Sc. PD:	0.022
		Sc. rank:	124.4

PB1			
Pos.	539	obs:	exp:
tta	L	0	77.25
ttg	L	0	164.00
ctt	L	870	149.00
ctc	L	1	147.30
cta	L	8	146.50
ctg	L	0	194.90
---	--	------	------
mPD		0.020	1.1
		nPD:	0.02
		N. weight:	2.1
		Sc. PD:	-0.3
		Sc. rank:	-827.8

PB1			
Pos.	540	obs:	exp:
ggt	G	0	117.90
ggc	G	1	111.70
gga	G	876	406.40
ggg	G	2	243.00
---	--	------	------
mPD		0.0068	0.68
		nPD:	0.01
		N. weight:	0.93
		Sc. PD:	-0.14
		Sc. rank:	-445.0

PB1			
Pos.	541	obs:	exp:
cct	P	0	223.50
ccc	P	1	156.70
cca	P	878	359.10
ccg	P	0	139.70
---	--	------	------
mPD		0.0023	0.71
		nPD:	0.
		N. weight:	1.1
		Sc. PD:	-0.17
		Sc. rank:	-631.4

PB1			
Pos.	542	obs:	exp:
gct	A	0	225.20
gcc	A	0	165.00
gca	A	875	411.00
gcg	A	4	77.75
---	--	------	------
mPD		0.0091	0.67
		nPD:	0.01
		N. weight:	0.91
		Sc. PD:	-0.13
		Sc. rank:	-409.4

PB1			
Pos.	543	obs:	exp:
act	T	0	227.70
acc	T	1	179.00
aca	T	871	403.80
acg	T	7	68.47
---	--	------	------
mPD		0.018	0.67
		nPD:	0.03
		N. weight:	0.91
		Sc. PD:	-0.12
		Sc. rank:	-294.0

PB1			
Pos.	544	obs:	exp:
gct	A	168	225.00
gcc	A	707	164.90
gca	A	3	410.50
gcg	A	0	77.66
ggt	G	1	0.13
ggc	G	0	0.13
gga	G	0	0.46
ggg	G	0	0.28
---	--	------	------
mPD		0.32	0.67
		nPD:	0.47
		N. weight:	1.4
		Sc. PD:	0.4
		Sc. rank:	733.4

PB1			
Pos.	545	obs:	exp:
caa	Q	599	451.50
cag	Q	280	427.50
---	--	------	------
mPD		0.43	0.50
		nPD:	0.87
		N. weight:	0.076
		Sc. PD:	0.052
		Sc. rank:	46.9

PB1			
Pos.	546	obs:	exp:
atg	M	879	879.00
---	--	------	------
mPD		0	0
		nPD:	1.
		N. weight:	0.
		Sc. PD:	0
		Sc. rank:	0

PB1			
Pos.	547	obs:	exp:
gct	A	84	225.20
gcc	A	795	165.00
gca	A	0	411.00
gcg	A	0	77.75
---	--	------	------
mPD		0.17	0.67
		nPD:	0.26
		N. weight:	1.6
		Sc. PD:	0.15
		Sc. rank:	555.8

PB1			
Pos.	548	obs:	exp:
tta	L	0	77.25
ttg	L	0	164.00
ctt	L	860	149.00
ctc	L	17	147.30
cta	L	2	146.50
ctg	L	0	194.90
---	--	------	------
mPD		0.042	1.1
		nPD:	0.04
		N. weight:	2.1
		Sc. PD:	-0.25
		Sc. rank:	-547.5

PB1			
Pos.	549	obs:	exp:
caa	Q	15	451.50
cag	Q	864	427.50
---	--	------	------
mPD		0.034	0.50
		nPD:	0.07
		N. weight:	0.78
		Sc. PD:	-0.072
		Sc. rank:	-139.1

PB1			
Pos.	550	obs:	exp:
tta	L	2	77.25
ttg	L	2	164.00
ctt	L	2	149.00
ctc	L	15	147.30
cta	L	841	146.50
ctg	L	17	194.90
---	--	------	------
mPD		0.089	1.1
		nPD:	0.08
		N. weight:	1.9
		Sc. PD:	-0.16
		Sc. rank:	-246.4

PB1			
Pos.	551	obs:	exp:
ttt	F	800	346.90
ttc	F	79	532.10
---	--	------	------
mPD		0.16	0.48
		nPD:	0.34
		N. weight:	0.73
		Sc. PD:	0.13
		Sc. rank:	335.8

PB1			
Pos.	552	obs:	exp:
att	I	1	320.40
atc	I	877	212.40
ata	I	0	345.30
gtt	V	0	0.21
gtc	V	1	0.20
gta	V	0	0.21
gtg	V	0	0.39
---	--	------	------
mPD		0.0046	0.66
		nPD:	0.01
		N. weight:	1.7
		Sc. PD:	-0.26
		Sc. rank:	-890.3

PB1			
Pos.	553	obs:	exp:
aaa	K	797	514.90
aag	K	82	364.10
---	--	------	------
mPD		0.17	0.49
		nPD:	0.35
		N. weight:	0.32
		Sc. PD:	0.057
		Sc. rank:	150.3

PB1			
Pos.	554	obs:	exp:
gat	D	41	474.50
gac	D	838	404.50
---	--	------	------
mPD		0.089	0.50
		nPD:	0.18
		N. weight:	0.72
		Sc. PD:	0.011
		Sc. rank:	120.0

PB1			
Pos.	555	obs:	exp:
tat	Y	26	466.10
tac	Y	853	412.90
---	--	------	------
mPD		0.057	0.50
		nPD:	0.12
		N. weight:	0.77
		Sc. PD:	-0.035
		Sc. rank:	0.7

PB1			
Pos.	556	obs:	exp:
cgt	R	0	37.55
cgc	R	0	44.66
cga	R	1	87.65
cgg	R	0	73.21
aga	R	836	401.30
agg	R	42	234.60
---	--	------	------
mPD		0.093	0.96
		nPD:	0.1
		N. weight:	0.76
		Sc. PD:	-0.048
		Sc. rank:	-55.5

PB1			
Pos.	557	obs:	exp:
tat	Y	1	466.10
tac	Y	878	412.90
---	--	------	------
mPD		0.0023	0.50
		nPD:	0.
		N. weight:	0.92
		Sc. PD:	-0.14
		Sc. rank:	-499.3

PB1			
Pos.	558	obs:	exp:
act	T	0	227.70
acc	T	1	179.00
aca	T	829	403.80
acg	T	49	68.47
---	--	------	------
mPD		0.11	0.67
		nPD:	0.16
		N. weight:	0.81
		Sc. PD:	-0.003
		Sc. rank:	96.6

PB1			
Pos.	559	obs:	exp:
tat	Y	109	466.10
tac	Y	770	412.90
---	--	------	------
mPD		0.22	0.50
		nPD:	0.44
		N. weight:	0.46
		Sc. PD:	0.12
		Sc. rank:	238.5

PB1			
Pos.	560	obs:	exp:
cgt	R	0	37.55
cgc	R	0	44.66
cga	R	836	87.65
cgg	R	40	73.21
aga	R	2	401.30
agg	R	1	234.60
---	--	------	------
mPD		0.096	0.96
		nPD:	0.1
		N. weight:	2.6
		Sc. PD:	-0.16
		Sc. rank:	-163.2

PB1			
Pos.	561	obs:	exp:
tgt	C	818	360.90
tgc	C	61	518.10
---	--	------	------
mPD		0.13	0.48
		nPD:	0.27
		N. weight:	0.76
		Sc. PD:	0.076
		Sc. rank:	267.6

PB1			
Pos.	562	obs:	exp:
cat	H	43	505.00
cac	H	836	374.00
---	--	------	------
mPD		0.093	0.49
		nPD:	0.19
		N. weight:	0.8
		Sc. PD:	0.021
		Sc. rank:	160.8

PB1			
Pos.	563	obs:	exp:
cgt	R	0	37.55
cgc	R	0	44.66
cga	R	1	87.65
cgg	R	2	73.21
aga	R	43	401.30
agg	R	833	234.60
---	--	------	------
mPD		0.10	0.96
		nPD:	0.11
		N. weight:	1.3
		Sc. PD:	-0.074
		Sc. rank:	-33.0

PB1			
Pos.	564	obs:	exp:
ggt	G	823	117.90
ggc	G	14	111.70
gga	G	31	406.40
ggg	G	11	243.00
---	--	------	------
mPD		0.12	0.68
		nPD:	0.18
		N. weight:	2.1
		Sc. PD:	0.033
		Sc. rank:	359.8

PB1			
Pos.	565	obs:	exp:
aat	N	0	0.51
aac	N	1	0.49
gat	D	743	473.40
gac	D	134	403.60
ggt	G	0	0.13
ggc	G	1	0.13
gga	G	0	0.46
ggg	G	0	0.28
---	--	------	------
mPD		0.27	0.50
		nPD:	0.53
		N. weight:	0.27
		Sc. PD:	0.096
		Sc. rank:	152.7

PB1			
Pos.	566	obs:	exp:
atg	M	3	3.00
act	T	2	226.70
acc	T	0	178.20
aca	T	180	402.00
acg	T	693	68.16
gct	A	0	0.26
gcc	A	0	0.19
gca	A	0	0.47
gcg	A	1	0.09
---	--	------	------
mPD		0.34	0.69
		nPD:	0.49
		N. weight:	2.
		Sc. PD:	0.65
		Sc. rank:	1114.9

PB1			
Pos.	567	obs:	exp:
cat	H	0	2.87
cac	H	5	2.13
caa	Q	865	448.90
cag	Q	9	425.10
---	--	------	------
mPD		0.031	0.51
		nPD:	0.06
		N. weight:	0.76
		Sc. PD:	-0.073
		Sc. rank:	-144.1

PB1			
Pos.	568	obs:	exp:
tta	L	0	0.09
ttg	L	0	0.19
ctt	L	1	0.17
ctc	L	0	0.17
cta	L	0	0.17
ctg	L	0	0.22
att	I	853	320.40
atc	I	24	212.40
ata	I	1	345.30
---	--	------	------
mPD		0.058	0.66
		nPD:	0.09
		N. weight:	1.1
		Sc. PD:	-0.08
		Sc. rank:	-114.0

PB1			
Pos.	569	obs:	exp:
caa	Q	835	451.50
cag	Q	44	427.50
---	--	------	------
mPD		0.095	0.50
		nPD:	0.19
		N. weight:	0.58
		Sc. PD:	0.015
		Sc. rank:	118.2

PB1			
Pos.	570	obs:	exp:
act	T	2	227.40
acc	T	0	178.80
aca	T	65	403.40
acg	T	811	68.39
gct	A	0	0.26
gcc	A	0	0.19
gca	A	0	0.47
gcg	A	1	0.09
---	--	------	------
mPD		0.14	0.68
		nPD:	0.21
		N. weight:	2.6
		Sc. PD:	0.12
		Sc. rank:	685.3

PB1			
Pos.	571	obs:	exp:
cgt	R	0	37.55
cgc	R	0	44.66
cga	R	0	87.65
cgg	R	0	73.21
aga	R	80	401.30
agg	R	799	234.60
---	--	------	------
mPD		0.17	0.96
		nPD:	0.17
		N. weight:	1.2
		Sc. PD:	0.0098
		Sc. rank:	177.5

PB1			
Pos.	572	obs:	exp:
cgt	R	0	37.55
cgc	R	0	44.66
cga	R	0	87.65
cgg	R	0	73.21
aga	R	828	401.30
agg	R	51	234.60
---	--	------	------
mPD		0.11	0.96
		nPD:	0.11
		N. weight:	0.74
		Sc. PD:	-0.035
		Sc. rank:	-4.9

PB1			
Pos.	573	obs:	exp:
tct	S	0	15.05
tcc	S	0	12.17
tca	S	89	23.72
tcg	S	5	5.47
agt	S	0	19.26
agc	S	0	18.33
gct	A	0	201.10
gcc	A	0	147.40
gca	A	785	367.00
gcg	A	0	69.43
---	--	------	------
mPD		0.20	0.96
		nPD:	0.21
		N. weight:	1.
		Sc. PD:	0.046
		Sc. rank:	253.7

PB1			
Pos.	574	obs:	exp:
ttt	F	93	346.50
ttc	F	785	531.50
tct	S	0	0.16
tcc	S	1	0.13
tca	S	0	0.25
tcg	S	0	0.06
agt	S	0	0.20
agc	S	0	0.20
---	--	------	------
mPD		0.19	0.48
		nPD:	0.4
		N. weight:	0.27
		Sc. PD:	0.06
		Sc. rank:	134.5

PB1			
Pos.	575	obs:	exp:
gaa	E	113	528.70
gag	E	766	350.30
---	--	------	------
mPD		0.22	0.48
		nPD:	0.47
		N. weight:	0.6
		Sc. PD:	0.18
		Sc. rank:	321.7

PB1			
Pos.	576	obs:	exp:
tta	L	0	77.25
ttg	L	35	164.00
ctt	L	0	149.00
ctc	L	0	147.30
cta	L	13	146.50
ctg	L	831	194.90
---	--	------	------
mPD		0.11	1.1
		nPD:	0.1
		N. weight:	1.6
		Sc. PD:	-0.1
		Sc. rank:	-125.3

PB1			
Pos.	577	obs:	exp:
cgt	R	0	0.30
cgc	R	0	0.36
cga	R	0	0.70
cgg	R	0	0.58
aaa	K	12	510.20
aag	K	859	360.80
aga	R	4	3.20
agg	R	3	1.87
gaa	E	0	0.60
gag	E	1	0.40
---	--	------	------
mPD		0.054	0.51
		nPD:	0.11
		N. weight:	0.99
		Sc. PD:	-0.055
		Sc. rank:	-22.9

PB1			
Pos.	578	obs:	exp:
cgt	R	0	0.21
cgc	R	0	0.25
cga	R	0	0.50
cgg	R	0	0.42
aaa	K	227	511.40
aag	K	646	361.60
aga	R	0	2.28
agg	R	5	1.33
gaa	E	0	0.60
gag	E	1	0.40
---	--	------	------
mPD		0.40	0.50
		nPD:	0.79
		N. weight:	0.28
		Sc. PD:	0.17
		Sc. rank:	173.1

PB1			
Pos.	579	obs:	exp:
tta	L	1	77.25
ttg	L	45	164.00
ctt	L	4	149.00
ctc	L	0	147.30
cta	L	7	146.50
ctg	L	822	194.90
---	--	------	------
mPD		0.13	1.1
		nPD:	0.11
		N. weight:	1.5
		Sc. PD:	-0.073
		Sc. rank:	-6.4

PB1			
Pos.	580	obs:	exp:
tgg	W	879	879.00
---	--	------	------
mPD		0	0
		nPD:	1.
		N. weight:	0.
		Sc. PD:	0
		Sc. rank:	0

PB1			
Pos.	581	obs:	exp:
gat	D	1	0.54
gac	D	0	0.46
gaa	E	48	528.10
gag	E	830	349.90
---	--	------	------
mPD		0.11	0.48
		nPD:	0.22
		N. weight:	0.85
		Sc. PD:	0.046
		Sc. rank:	231.0

PB1			
Pos.	582	obs:	exp:
caa	Q	42	451.50
cag	Q	837	427.50
---	--	------	------
mPD		0.091	0.50
		nPD:	0.18
		N. weight:	0.65
		Sc. PD:	0.012
		Sc. rank:	121.0

PB1			
Pos.	583	obs:	exp:
act	T	4	227.70
acc	T	874	179.00
aca	T	1	403.80
acg	T	0	68.47
---	--	------	------
mPD		0.011	0.67
		nPD:	0.02
		N. weight:	1.9
		Sc. PD:	-0.27
		Sc. rank:	-772.7

PB1			
Pos.	584	obs:	exp:
cat	H	7	4.02
cac	H	0	2.98
caa	Q	1	0.51
cag	Q	0	0.49
cgt	R	726	37.21
cgc	R	141	44.25
cga	R	3	86.85
cgg	R	1	72.54
aga	R	0	397.70
agg	R	0	232.50
---	--	------	------
mPD		0.30	0.99
		nPD:	0.3
		N. weight:	3.2
		Sc. PD:	0.42
		Sc. rank:	1310.2

PB1			
Pos.	585	obs:	exp:
tct	S	4	140.50
tcc	S	0	113.60
tca	S	271	221.60
tcg	S	603	51.09
act	T	0	0.26
acc	T	0	0.20
aca	T	1	0.46
acg	T	0	0.08
agt	S	0	179.90
agc	S	0	171.20
---	--	------	------
mPD		0.44	1.7
		nPD:	0.26
		N. weight:	2.2
		Sc. PD:	0.21
		Sc. rank:	747.9

PB1			
Pos.	586	obs:	exp:
cgt	R	0	0.09
cgc	R	0	0.10
cga	R	0	0.20
cgg	R	0	0.17
aaa	K	311	513.70
aag	K	566	363.30
aga	R	0	0.91
agg	R	2	0.53
---	--	------	------
mPD		0.46	0.49
		nPD:	0.94
		N. weight:	0.14
		Sc. PD:	0.11
		Sc. rank:	87.4

,

PB1			
Pos.	587	obs:	exp:
tct	S	0	0.16
tcc	S	0	0.13
tca	S	1	0.25
tcg	S	0	0.06
act	T	0	0.52
acc	T	0	0.41
aca	T	0	0.92
acg	T	2	0.16
agt	S	0	0.20
agc	S	0	0.20
gtt	V	1	0.62
gtc	V	0	0.60
gta	V	0	0.62
gtg	V	2	1.16
gct	A	76	223.70
gcc	A	0	163.90
gca	A	614	408.20
gcg	A	183	77.22
---	--	------	------
mPD		0.47	0.69
		nPD:	0.69
		N. weight:	0.48
		Sc. PD:	0.24
		Sc. rank:	286.1

,

PB1			
Pos.	588	obs:	exp:
gct	A	0	0.51
gcc	A	0	0.38
gca	A	2	0.94
gcg	A	0	0.18
gat	D	0	2.70
gac	D	5	2.30
ggt	G	0	116.90
ggc	G	0	110.80
gga	G	837	403.20
ggg	G	35	241.10
---	--	------	------
mPD		0.10	0.69
		nPD:	0.15
		N. weight:	0.77
		Sc. PD:	-0.011
		Sc. rank:	64.2

,

PB1			
Pos.	589	obs:	exp:
tta	L	0	77.25
ttg	L	8	164.00
ctt	L	7	149.00
ctc	L	0	147.30
cta	L	1	146.50
ctg	L	863	194.90
---	--	------	------
mPD		0.036	1.1
		nPD:	0.03
		N. weight:	1.7
		Sc. PD:	-0.22
		Sc. rank:	-514.4

,

PB1			
Pos.	590	obs:	exp:
tta	L	17	77.25
ttg	L	631	164.00
ctt	L	0	149.00
ctc	L	0	147.30
cta	L	0	146.50
ctg	L	231	194.90
---	--	------	------
mPD		0.43	1.1
		nPD:	0.38
		N. weight:	1.2
		Sc. PD:	0.26
		Sc. rank:	607.1

,

PB1			
Pos.	591	obs:	exp:
att	I	38	13.87
atc	I	0	9.19
ata	I	0	14.94
gtt	V	776	173.40
gtc	V	34	168.30
gta	V	22	173.90
gtg	V	8	324.40
gct	A	1	0.26
gcc	A	0	0.19
gca	A	0	0.47
gcg	A	0	0.09
---	--	------	------
mPD		0.22	0.82
		nPD:	0.27
		N. weight:	1.5
		Sc. PD:	0.16
		Sc. rank:	544.5

,

PB1			
Pos.	592	obs:	exp:
tct	S	3	139.10
tcc	S	0	112.50
tca	S	835	219.30
tcg	S	31	50.57
agt	S	0	178.10
agc	S	0	169.50
gct	A	0	2.56
gcc	A	0	1.88
gca	A	10	4.68
gcg	A	0	0.88
---	--	------	------
mPD		0.097	1.7
		nPD:	0.06
		N. weight:	1.5
		Sc. PD:	-0.16
		Sc. rank:	-307.6

,

PB1			
Pos.	593	obs:	exp:
gat	D	872	474.50
gac	D	7	404.50
---	--	------	------
mPD		0.016	0.50
		nPD:	0.03
		N. weight:	0.71
		Sc. PD:	-0.089
		Sc. rank:	-212.3

,

PB1			
Pos.	594	obs:	exp:
ggt	G	0	117.90
ggc	G	0	111.70
gga	G	867	406.40
ggg	G	12	243.00
---	--	------	------
mPD		0.027	0.68
		nPD:	0.04
		N. weight:	0.87
		Sc. PD:	-0.1
		Sc. rank:	-231.4

,

PB1			
Pos.	595	obs:	exp:
ggt	G	0	117.90
ggc	G	28	111.70
gga	G	92	406.40
ggg	G	759	243.00
---	--	------	------
mPD		0.24	0.68
		nPD:	0.36
		N. weight:	0.97
		Sc. PD:	0.18
		Sc. rank:	460.4

,

PB1			
Pos.	596	obs:	exp:
cct	P	2	223.50
ccc	P	0	156.70
cca	P	875	359.10
ccg	P	2	139.70
---	--	------	------
mPD		0.0091	0.71
		nPD:	0.01
		N. weight:	1.1
		Sc. PD:	-0.16
		Sc. rank:	-491.3

,

PB1			
Pos.	597	obs:	exp:
aat	N	39	447.20
aac	N	840	431.80
---	--	------	------
mPD		0.085	0.50
		nPD:	0.17
		N. weight:	0.65
		Sc. PD:	0.0041
		Sc. rank:	93.6

,

PB1			
Pos.	598	obs:	exp:
tta	L	19	77.25
ttg	L	29	164.00
ctt	L	0	149.00
ctc	L	0	147.30
cta	L	77	146.50
ctg	L	754	194.90
---	--	------	------
mPD		0.30	1.1
		nPD:	0.27
		N. weight:	1.3
		Sc. PD:	0.13
		Sc. rank:	447.7

,

PB1			
Pos.	599	obs:	exp:
tat	Y	46	466.10
tac	Y	833	412.90
---	--	------	------
mPD		0.099	0.50
		nPD:	0.2
		N. weight:	0.67
		Sc. PD:	0.023
		Sc. rank:	144.8

,

PB1			
Pos.	600	obs:	exp:
aat	N	832	447.20
aac	N	47	431.80
---	--	------	------
mPD		0.10	0.50
		nPD:	0.2
		N. weight:	0.58
		Sc. PD:	0.022
		Sc. rank:	130.2

,

PB1			
Pos.	601	obs:	exp:
att	I	222	320.70
atc	I	657	212.60
ata	I	0	345.70
---	--	------	------
mPD		0.38	0.65
		nPD:	0.58
		N. weight:	0.93
		Sc. PD:	0.37
		Sc. rank:	541.4

,

PB1			
Pos.	602	obs:	exp:
cgt	R	0	37.55
cgc	R	0	44.66
cga	R	9	87.65
cgg	R	869	73.21
aga	R	0	401.30
agg	R	1	234.60
---	--	------	------
mPD		0.023	0.96
		nPD:	0.02
		N. weight:	3.
		Sc. PD:	-0.4
		Sc. rank:	-1054.8

,

PB1			
Pos.	603	obs:	exp:
aat	N	115	446.70
aac	N	763	431.30
aaa	K	0	0.59
aag	K	1	0.41
---	--	------	------
mPD		0.23	0.50
		nPD:	0.46
		N. weight:	0.4
		Sc. PD:	0.11
		Sc. rank:	212.0

,

PB1			
Pos.	604	obs:	exp:
tta	L	0	77.25
ttg	L	0	164.00
ctt	L	28	149.00
ctc	L	843	147.30
cta	L	8	146.50
ctg	L	0	194.90
---	--	------	------
mPD		0.079	1.1
		nPD:	0.07
		N. weight:	2.
		Sc. PD:	-0.17
		Sc. rank:	-305.6

,

PB1			
Pos.	605	obs:	exp:
cat	H	5	505.00
cac	H	874	374.00
---	--	------	------
mPD		0.011	0.49
		nPD:	0.02
		N. weight:	1.
		Sc. PD:	-0.14
		Sc. rank:	-365.4

,

PB1			
Pos.	606	obs:	exp:
att	I	650	320.70
atc	I	228	212.60
ata	I	1	345.70
---	--	------	------
mPD		0.39	0.65
		nPD:	0.59
		N. weight:	0.66
		Sc. PD:	0.27
		Sc. rank:	388.2

,

PB1			
Pos.	607	obs:	exp:
cct	P	1	223.50
ccc	P	0	156.70
cca	P	862	359.10
ccg	P	16	139.70
---	--	------	------
mPD		0.038	0.71
		nPD:	0.05
		N. weight:	1.
		Sc. PD:	-0.11
		Sc. rank:	-218.4

,

PB1			
Pos.	608	obs:	exp:
gaa	E	51	528.70
gag	E	828	350.30
---	--	------	------
mPD		0.11	0.48
		nPD:	0.23
		N. weight:	0.84
		Sc. PD:	0.052
		Sc. rank:	244.8

,

PB1			
Pos.	609	obs:	exp:
gtt	V	8	181.50
gtc	V	862	176.10
gta	V	9	182.00
gtg	V	0	339.50
---	--	------	------
mPD		0.038	0.73
		nPD:	0.05
		N. weight:	1.9
		Sc. PD:	-0.2
		Sc. rank:	-409.3

,

PB1			
Pos.	610	obs:	exp:
tgt	C	68	360.90
tgc	C	811	518.10
---	--	------	------
mPD		0.14	0.48
		nPD:	0.3
		N. weight:	0.35
		Sc. PD:	0.045
		Sc. rank:	140.1

,

PB1			
Pos.	611	obs:	exp:
tta	L	5	77.25
ttg	L	855	164.00
ctt	L	0	149.00
ctc	L	0	147.30
cta	L	7	146.50
ctg	L	12	194.90
---	--	------	------
mPD		0.069	1.1
		nPD:	0.06
		N. weight:	1.9
		Sc. PD:	-0.18
		Sc. rank:	-354.8

PB1			
Pos.	612	obs:	exp:
aaa	K	785	514.90
aag	K	94	364.10
---	--	------	------
mPD		0.19	0.49
		nPD:	0.39
		N. weight:	0.29
		Sc. PD:	0.064
		Sc. rank:	146.5

PB1			
Pos.	613	obs:	exp:
tgt	C	1	0.41
tgc	C	0	0.59
tgg	W	878	878.00
---	--	------	------
mPD		0.0023	0.0023
		nPD:	1.
		N. weight:	0.0012
		Sc. PD:	0.001
		Sc. rank:	0.8

PB1			
Pos.	614	obs:	exp:
gaa	E	822	528.70
gag	E	57	350.30
---	--	------	------
mPD		0.12	0.48
		nPD:	0.25
		N. weight:	0.37
		Sc. PD:	0.032
		Sc. rank:	120.5

PB1			
Pos.	615	obs:	exp:
tta	L	2	77.25
ttg	L	787	164.00
ctt	L	0	149.00
ctc	L	0	147.30
cta	L	65	146.50
ctg	L	25	194.90
---	--	------	------
mPD		0.33	1.1
		nPD:	0.29
		N. weight:	1.6
		Sc. PD:	0.2
		Sc. rank:	620.9

PB1			
Pos.	616	obs:	exp:
atg	M	879	879.00
---	--	------	------
mPD		0	0
		nPD:	1.
		N. weight:	0.
		Sc. PD:	0
		Sc. rank:	0

PB1			
Pos.	617	obs:	exp:
gat	D	786	474.50
gac	D	93	404.50
---	--	------	------
mPD		0.19	0.50
		nPD:	0.38
		N. weight:	0.37
		Sc. PD:	0.077
		Sc. rank:	182.7

PB1			
Pos.	618	obs:	exp:
aaa	K	1	0.59
aag	K	0	0.41
gtt	V	0	0.21
gtc	V	0	0.20
gta	V	1	0.21
gtg	V	0	0.39
gaa	E	867	526.90
gag	E	9	349.10
ggt	G	0	0.13
ggc	G	0	0.13
gga	G	1	0.46
ggg	G	0	0.28
---	--	------	------
mPD		0.027	0.49
		nPD:	0.06
		N. weight:	0.57
		Sc. PD:	-0.059
		Sc. rank:	-115.8

PB1			
Pos.	619	obs:	exp:
aat	N	3	1.53
aac	N	0	1.47
gat	D	30	466.40
gac	D	834	397.60
gaa	E	5	7.22
gag	E	7	4.78
---	--	------	------
mPD		0.11	0.52
		nPD:	0.2
		N. weight:	0.76
		Sc. PD:	0.029
		Sc. rank:	178.3

PB1			
Pos.	620	obs:	exp:
tat	Y	73	466.10
tac	Y	806	412.90
---	--	------	------
mPD		0.15	0.50
		nPD:	0.31
		N. weight:	0.57
		Sc. PD:	0.078
		Sc. rank:	240.7

PB1			
Pos.	621	obs:	exp:
caa	Q	666	444.80
cag	Q	200	421.20
cgt	R	0	0.26
cgc	R	0	0.30
cga	R	4	0.60
cgg	R	2	0.50
aaa	K	7	4.10
aag	K	0	2.90
aga	R	0	2.74
agg	R	0	1.60
---	--	------	------
mPD		0.38	0.54
		nPD:	0.71
		N. weight:	0.19
		Sc. PD:	0.1
		Sc. rank:	116.8

PB1			
Pos.	622	obs:	exp:
ggt	G	31	117.90
ggc	G	807	111.70
gga	G	41	406.40
ggg	G	0	243.00
---	--	------	------
mPD		0.15	0.68
		nPD:	0.23
		N. weight:	2.1
		Sc. PD:	0.13
		Sc. rank:	599.3

PB1			
Pos.	623	obs:	exp:
cgt	R	0	37.55
cgc	R	0	44.66
cga	R	0	87.65
cgg	R	1	73.21
aga	R	47	401.30
agg	R	831	234.60
---	--	------	------
mPD		0.10	0.96
		nPD:	0.11
		N. weight:	1.3
		Sc. PD:	-0.072
		Sc. rank:	-25.3

PB1			
Pos.	624	obs:	exp:
tta	L	10	77.16
ttg	L	475	163.80
ctt	L	45	148.80
ctc	L	1	147.10
cta	L	9	146.40
ctg	L	338	194.70
gtt	V	0	0.21
gtc	V	0	0.20
gta	V	0	0.21
gtg	V	1	0.39
---	--	------	------
mPD		0.64	1.1
		nPD:	0.57
		N. weight:	0.84
		Sc. PD:	0.33
		Sc. rank:	488.2

PB1			
Pos.	625	obs:	exp:
tgt	C	864	360.90
tgc	C	15	518.10
---	--	------	------
mPD		0.034	0.48
		nPD:	0.07
		N. weight:	0.99
		Sc. PD:	-0.089
		Sc. rank:	-161.6

PB1			
Pos.	626	obs:	exp:
aat	N	810	447.20
aac	N	69	431.80
---	--	------	------
mPD		0.14	0.50
		nPD:	0.29
		N. weight:	0.5
		Sc. PD:	0.061
		Sc. rank:	194.1

PB1			
Pos.	627	obs:	exp:
cct	P	817	223.30
ccc	P	51	156.50
cca	P	9	358.70
ccg	P	1	139.50
cgt	R	1	0.04
cgc	R	0	0.05
cga	R	0	0.10
cgg	R	0	0.08
aga	R	0	0.46
agg	R	0	0.27
---	--	------	------
mPD		0.13	0.72
		nPD:	0.19
		N. weight:	1.4
		Sc. PD:	0.029
		Sc. rank:	266.2

PB1			
Pos.	628	obs:	exp:
tta	L	0	8.09
ttg	L	2	17.17
ctt	L	0	15.59
ctc	L	0	15.41
cta	L	2	15.34
ctg	L	88	20.40
atg	M	784	784.00
act	T	0	0.26
acc	T	0	0.20
aca	T	0	0.46
acg	T	1	0.08
gtt	V	0	0.41
gtc	V	0	0.40
gta	V	0	0.41
gtg	V	2	0.77
---	--	------	------
mPD		0.20	0.32
		nPD:	0.62
		N. weight:	0.18
		Sc. PD:	0.078
		Sc. rank:	105.1

PB1			
Pos.	629	obs:	exp:
aat	N	174	447.20
aac	N	705	431.80
---	--	------	------
mPD		0.32	0.50
		nPD:	0.64
		N. weight:	0.26
		Sc. PD:	0.12
		Sc. rank:	153.4

PB1			
Pos.	630	obs:	exp:
cct	P	1	223.50
ccc	P	1	156.70
cca	P	91	359.10
ccg	P	786	139.70
---	--	------	------
mPD		0.19	0.71
		nPD:	0.27
		N. weight:	1.7
		Sc. PD:	0.17
		Sc. rank:	604.1

PB1			
Pos.	631	obs:	exp:
ttt	F	874	346.90
ttc	F	5	532.10
---	--	------	------
mPD		0.011	0.48
		nPD:	0.02
		N. weight:	1.1
		Sc. PD:	-0.15
		Sc. rank:	-380.0

PB1			
Pos.	632	obs:	exp:
gtt	V	42	181.50
gtc	V	834	176.10
gta	V	3	182.00
gtg	V	0	339.50
---	--	------	------
mPD		0.098	0.73
		nPD:	0.13
		N. weight:	1.7
		Sc. PD:	-0.048
		Sc. rank:	99.9

PB1			
Pos.	633	obs:	exp:
tct	S	0	140.40
tcc	S	0	113.50
tca	S	0	221.30
tcg	S	0	51.04
att	I	1	0.36
atc	I	0	0.24
ata	I	0	0.39
aat	N	1	0.51
aac	N	0	0.49
agt	S	746	179.70
agc	S	131	171.00
---	--	------	------
mPD		0.26	1.7
		nPD:	0.16
		N. weight:	1.4
		Sc. PD:	-0.01
		Sc. rank:	151.3

PB1			
Pos.	634	obs:	exp:
cat	H	831	504.40
cac	H	47	373.60
ggt	G	1	0.13
ggc	G	0	0.13
gga	G	0	0.46
ggg	G	0	0.28
---	--	------	------
mPD		0.11	0.49
		nPD:	0.21
		N. weight:	0.45
		Sc. PD:	0.022
		Sc. rank:	117.5

PB1			
Pos.	635	obs:	exp:
cgt	R	0	0.26
cgc	R	0	0.30
cga	R	0	0.60
cgg	R	0	0.50
aaa	K	43	511.40
aag	K	830	361.60
aga	R	0	2.74
agg	R	6	1.60
---	--	------	------
mPD		0.11	0.50
		nPD:	0.21
		N. weight:	0.83
		Sc. PD:	0.039
		Sc. rank:	212.4

PB1			
Pos.	636	obs:	exp:
gaa	E	849	528.70
gag	E	30	350.30
---	--	------	------
mPD		0.066	0.48
		nPD:	0.14
		N. weight:	0.46
		Sc. PD:	-0.011
		Sc. rank:	30.2

,

PB1			
Pos.	637	obs:	exp:
ttt	F	4	1.58
ttc	F	0	2.42
att	I	825	317.40
atc	I	8	210.40
ata	I	37	342.10
atg	M	1	1.00
gtt	V	4	0.83
gtc	V	0	0.80
gta	V	0	0.83
gtg	V	0	1.54
---	--	------	------
mPD		0.12	0.67
		nPD:	0.18
		N. weight:	0.97
		Sc. PD:	0.011
		Sc. rank:	153.7

,

PB1			
Pos.	638	obs:	exp:
tat	Y	3	1.59
tac	Y	0	1.41
gat	D	761	416.80
gac	D	11	355.20
gaa	E	6	58.95
gag	E	92	39.05
ggt	G	6	0.80
ggc	G	0	0.76
gga	G	0	2.77
ggg	G	0	1.66
---	--	------	------
mPD		0.24	0.62
		nPD:	0.39
		N. weight:	0.71
		Sc. PD:	0.15
		Sc. rank:	354.8

,

PB1			
Pos.	639	obs:	exp:
tct	S	54	140.70
tcc	S	228	113.80
tca	S	581	221.80
tcg	S	16	51.15
agt	S	0	180.10
agc	S	0	171.40
---	--	------	------
mPD		0.49	1.7
		nPD:	0.3
		N. weight:	0.92
		Sc. PD:	0.12
		Sc. rank:	365.8

,

PB1			
Pos.	640	obs:	exp:
att	I	0	0.36
atc	I	1	0.24
ata	I	0	0.39
gtt	V	46	181.20
gtc	V	675	175.90
gta	V	129	181.80
gtg	V	28	339.10
---	--	------	------
mPD		0.39	0.73
		nPD:	0.53
		N. weight:	1.
		Sc. PD:	0.37
		Sc. rank:	585.1

,

PB1			
Pos.	641	obs:	exp:
aat	N	64	447.20
aac	N	815	431.80
---	--	------	------
mPD		0.14	0.50
		nPD:	0.27
		N. weight:	0.56
		Sc. PD:	0.057
		Sc. rank:	197.3

,

PB1			
Pos.	642	obs:	exp:
tct	S	0	9.28
tcc	S	0	7.51
tca	S	0	14.64
tcg	S	0	3.38
aat	N	812	417.70
aac	N	9	403.30
agt	S	57	11.88
agc	S	1	11.31
---	--	------	------
mPD		0.15	0.72
		nPD:	0.2
		N. weight:	0.84
		Sc. PD:	0.031
		Sc. rank:	186.0

,

PB1			
Pos.	643	obs:	exp:
act	T	2	0.52
acc	T	0	0.41
aca	T	0	0.92
acg	T	0	0.16
gct	A	680	224.20
gcc	A	1	164.30
gca	A	2	409.10
gcg	A	192	77.39
ggt	G	2	0.27
ggc	G	0	0.25
gga	G	0	0.92
ggg	G	0	0.55
---	--	------	------
mPD		0.36	0.68
		nPD:	0.52
		N. weight:	1.3
		Sc. PD:	0.45
		Sc. rank:	729.0

,

PB1			
Pos.	644	obs:	exp:
gtt	V	1	181.20
gtc	V	0	175.90
gta	V	34	181.80
gtg	V	843	339.10
gaa	E	0	0.60
gag	E	1	0.40
---	--	------	------
mPD		0.079	0.73
		nPD:	0.11
		N. weight:	1.
		Sc. PD:	-0.053
		Sc. rank:	-17.3

,

PB1			
Pos.	645	obs:	exp:
atg	M	2	2.00
gtt	V	0	181.00
gtc	V	0	175.70
gta	V	74	181.60
gtg	V	803	338.70
---	--	------	------
mPD		0.16	0.73
		nPD:	0.22
		N. weight:	0.88
		Sc. PD:	0.046
		Sc. rank:	238.3

,

PB1			
Pos.	646	obs:	exp:
atg	M	877	877.00
aaa	K	0	0.59
aag	K	1	0.41
gtt	V	0	0.21
gtc	V	0	0.20
gta	V	0	0.21
gtg	V	1	0.39
---	--	------	------
mPD		0.0046	0.0073
		nPD:	0.63
		N. weight:	0.0026
		Sc. PD:	0.0011
		Sc. rank:	1.5

,

PB1			
Pos.	647	obs:	exp:
tct	S	0	0.16
tcc	S	0	0.13
tca	S	1	0.25
tcg	S	0	0.06
cct	P	4	223.30
ccc	P	1	156.50
cca	P	852	358.70
ccg	P	21	139.50
agt	S	0	0.20
agc	S	0	0.20
---	--	------	------
mPD		0.060	0.71
		nPD:	0.08
		N. weight:	0.96
		Sc. PD:	-0.073
		Sc. rank:	-110.6

,

PB1			
Pos.	648	obs:	exp:
tct	S	25	4.00
tcc	S	0	3.24
tca	S	0	6.31
tcg	S	0	1.45
act	T	4	1.04
acc	T	0	0.81
aca	T	0	1.84
acg	T	0	0.31
agt	S	0	5.12
agc	S	0	4.88
gct	A	789	217.50
gcc	A	59	159.40
gca	A	1	396.90
gcg	A	0	75.09
gat	D	1	0.54
gac	D	0	0.46
---	--	------	------
mPD		0.19	0.76
		nPD:	0.25
		N. weight:	1.4
		Sc. PD:	0.12
		Sc. rank:	466.4

,

PB1			
Pos.	649	obs:	exp:
cat	H	875	504.40
cac	H	3	373.60
caa	Q	0	0.51
cag	Q	1	0.49
---	--	------	------
mPD		0.0091	0.49
		nPD:	0.02
		N. weight:	0.66
		Sc. PD:	-0.092
		Sc. rank:	-256.8

,

PB1			
Pos.	650	obs:	exp:
ggt	G	25	117.90
ggc	G	848	111.70
gga	G	6	406.40
ggg	G	0	243.00
---	--	------	------
mPD		0.069	0.68
		nPD:	0.1
		N. weight:	2.3
		Sc. PD:	-0.14
		Sc. rank:	-109.2

,

PB1			
Pos.	651	obs:	exp:
cct	P	1	223.50
ccc	P	75	156.70
cca	P	778	359.10
ccg	P	25	139.70
---	--	------	------
mPD		0.21	0.71
		nPD:	0.29
		N. weight:	0.71
		Sc. PD:	0.088
		Sc. rank:	278.1

,

PB1			
Pos.	652	obs:	exp:
tct	S	29	4.64
tcc	S	0	3.75
tca	S	0	7.32
tcg	S	0	1.69
act	T	0	1.04
acc	T	4	0.81
aca	T	0	1.84
acg	T	0	0.31
agt	S	0	5.94
agc	S	0	5.66
gct	A	161	216.80
gcc	A	685	158.80
gca	A	0	395.50
gcg	A	0	74.83
---	--	------	------
mPD		0.41	0.77
		nPD:	0.53
		N. weight:	1.4
		Sc. PD:	0.5
		Sc. rank:	806.6

,

PB1			
Pos.	653	obs:	exp:
aaa	K	791	514.90
aag	K	88	364.10
---	--	------	------
mPD		0.18	0.49
		nPD:	0.37
		N. weight:	0.31
		Sc. PD:	0.061
		Sc. rank:	148.7

,

PB1			
Pos.	654	obs:	exp:
tct	S	0	138.60
tcc	S	0	112.10
tca	S	0	218.60
tcg	S	0	50.40
att	I	0	0.36
atc	I	1	0.24
ata	I	0	0.39
aat	N	0	6.10
aac	N	12	5.90
agt	S	9	177.50
agc	S	857	168.90
---	--	------	------
mPD		0.049	1.7
		nPD:	0.03
		N. weight:	1.9
		Sc. PD:	-0.25
		Sc. rank:	-591.2

,

PB1			
Pos.	655	obs:	exp:
atg	M	878	878.00
gtt	V	0	0.21
gtc	V	0	0.20
gta	V	0	0.21
gtg	V	1	0.39
---	--	------	------
mPD		0.0023	0.0037
		nPD:	0.62
		N. weight:	0.0013
		Sc. PD:	0.00059
		Sc. rank:	0.8

,

PB1

Pos.	656	obs:	exp:
gaa	E	90	528.10
gag	E	788	349.90
ggt	G	0	0.13
ggc	G	0	0.13
gga	G	0	0.46
ggg	G	1	0.28
mPD		0.19	0.48
		nPD:	0.39
		N. weight:	0.68
		Sc. PD:	0.15
		Sc. rank:	340.6

PB1

Pos.	657	obs:	exp:
ttt	F	1	0.39
ttc	F	0	0.61
tat	Y	877	465.00
tac	Y	0	412.00
tgt	C	1	0.41
tgc	C	0	0.59
mPD		0.0045	0.50
		nPD:	0.01
		N. weight:	0.78
		Sc. PD:	-0.12
		Sc. rank:	-390.7

PB1

Pos.	658	obs:	exp:
gat	D	878	474.50
gac	D	1	404.50
mPD		0.0023	0.50
		nPD:	0.
		N. weight:	0.75
		Sc. PD:	-0.11
		Sc. rank:	-405.8

PB1

Pos.	659	obs:	exp:
gct	A	96	225.20
gcc	A	771	165.00
gca	A	12	411.00
gcg	A	0	77.75
mPD		0.22	0.67
		nPD:	0.33
		N. weight:	1.5
		Sc. PD:	0.23
		Sc. rank:	664.1

PB1

Pos.	660	obs:	exp:
att	I	1	0.36
atc	I	0	0.24
ata	I	0	0.39
gtt	V	873	181.20
gtc	V	2	175.90
gta	V	1	181.80
gtg	V	2	339.10
mPD		0.014	0.73
		nPD:	0.02
		N. weight:	1.9
		Sc. PD:	-0.26
		Sc. rank:	-729.6

PB1

Pos.	661	obs:	exp:
gtt	V	0	0.21
gtc	V	0	0.20
gta	V	1	0.21
gtg	V	0	0.39
gct	A	23	225.00
gcc	A	26	164.90
gca	A	804	410.50
gcg	A	25	77.66
mPD		0.16	0.68
		nPD:	0.24
		N. weight:	0.59
		Sc. PD:	0.043
		Sc. rank:	179.3

PB1

Pos.	662	obs:	exp:
act	T	320	227.70
acc	T	533	179.00
aca	T	25	403.80
acg	T	1	68.47
mPD		0.50	0.67
		nPD:	0.74
		N. weight:	0.87
		Sc. PD:	0.49
		Sc. rank:	531.6

PB1

Pos.	663	obs:	exp:
act	T	27	227.70
acc	T	1	179.00
aca	T	837	403.80
acg	T	14	68.47
mPD		0.092	0.67
		nPD:	0.14
		N. weight:	0.74
		Sc. PD:	-0.019
		Sc. rank:	45.9

PB1

Pos.	664	obs:	exp:
cat	H	779	505.00
cac	H	100	374.00
mPD		0.20	0.49
		nPD:	0.41
		N. weight:	0.29
		Sc. PD:	0.07
		Sc. rank:	150.7

PB1

Pos.	665	obs:	exp:
tct	S	17	140.70
tcc	S	39	113.80
tca	S	815	221.80
tcg	S	8	51.15
agt	S	0	180.10
agc	S	0	171.40
mPD		0.14	1.7
		nPD:	0.08
		N. weight:	1.4
		Sc. PD:	-0.11
		Sc. rank:	-160.1

PB1

Pos.	666	obs:	exp:
tgg	W	879	879.00
mPD		0	0
		nPD:	1.
		N. weight:	0.
		Sc. PD:	0
		Sc. rank:	0

PB1

Pos.	667	obs:	exp:
att	I	630	318.90
atc	I	244	211.40
ata	I	0	343.70
act	T	2	0.78
acc	T	1	0.61
aca	T	0	1.38
acg	T	0	0.23
gtt	V	2	0.41
gtc	V	0	0.40
gta	V	0	0.41
gtg	V	0	0.77
mPD		0.41	0.67
		nPD:	0.62
		N. weight:	0.66
		Sc. PD:	0.29
		Sc. rank:	392.0

PB1

Pos.	668	obs:	exp:
tct	S	2	0.32
tcc	S	0	0.26
tca	S	0	0.50
tcg	S	0	0.12
cct	P	846	222.70
ccc	P	27	156.20
cca	P	3	357.90
ccg	P	0	139.20
cat	H	1	0.57
cac	H	0	0.43
agt	S	0	0.41
agc	S	0	0.39
mPD		0.073	0.72
		nPD:	0.1
		N. weight:	1.5
		Sc. PD:	-0.09
		Sc. rank:	-76.5

PB1

Pos.	669	obs:	exp:
aaa	K	21	514.90
aag	K	858	364.10
mPD		0.047	0.49
		nPD:	0.1
		N. weight:	0.94
		Sc. PD:	-0.061
		Sc. rank:	-72.2

PB1

Pos.	670	obs:	exp:
cgt	R	0	37.55
cgc	R	0	44.66
cga	R	0	87.65
cgg	R	0	73.21
aga	R	30	401.30
agg	R	849	234.60
mPD		0.066	0.96
		nPD:	0.07
		N. weight:	1.4
		Sc. PD:	-0.13
		Sc. rank:	-248.1

PB1

Pos.	671	obs:	exp:
aat	N	795	446.70
aac	N	83	431.30
gat	D	1	0.54
gac	D	0	0.46
mPD		0.17	0.50
		nPD:	0.35
		N. weight:	0.46
		Sc. PD:	0.08
		Sc. rank:	212.3

PB1

Pos.	672	obs:	exp:
cgt	R	53	37.55
cgc	R	823	44.66
cga	R	1	87.65
cgg	R	2	73.21
aga	R	0	401.30
agg	R	0	234.60
mPD		0.12	0.96
		nPD:	0.12
		N. weight:	3.4
		Sc. PD:	-0.13
		Sc. rank:	115.7

PB1

Pos.	673	obs:	exp:
tct	S	8	140.70
tcc	S	869	113.80
tca	S	2	221.80
tcg	S	0	51.15
agt	S	0	180.10
agc	S	0	171.40
mPD		0.023	1.7
		nPD:	0.01
		N. weight:	2.4
		Sc. PD:	-0.35
		Sc. rank:	-1063.1

PB1

Pos.	674	obs:	exp:
att	I	805	320.40
atc	I	73	212.40
ata	I	0	345.30
gtt	V	1	0.21
gtc	V	0	0.20
gta	V	0	0.21
gtg	V	0	0.39
mPD		0.15	0.66
		nPD:	0.24
		N. weight:	0.94
		Sc. PD:	0.065
		Sc. rank:	281.5

PB1

Pos.	675	obs:	exp:
ttt	F	0	0.39
ttc	F	1	0.61
tta	L	0	77.16
ttg	L	0	163.80
ctt	L	13	148.80
ctc	L	859	147.10
cta	L	6	146.40
ctg	L	0	194.70
mPD		0.045	1.1
		nPD:	0.04
		N. weight:	2.1
		Sc. PD:	-0.24
		Sc. rank:	-527.8

PB1

Pos.	676	obs:	exp:
aat	N	165	446.20
aac	N	712	430.80
aaa	K	2	1.17
aag	K	0	0.83
mPD		0.31	0.50
		nPD:	0.62
		N. weight:	0.28
		Sc. PD:	0.12
		Sc. rank:	163.4

PB1

Pos.	677	obs:	exp:
tct	S	0	0.16
tcc	S	1	0.13
tca	S	0	0.25
tcg	S	0	0.06
cct	P	0	0.51
ccc	P	2	0.36
cca	P	0	0.82
ccg	P	0	0.32
act	T	62	226.90
acc	T	768	178.40
aca	T	45	402.50
acg	T	1	68.24
agt	S	0	0.20
agc	S	0	0.20
mPD		0.23	0.68
		nPD:	0.34
		N. weight:	1.3
		Sc. PD:	0.22
		Sc. rank:	606.8

PB1

Pos.	678	obs:	exp:
tct	S	0	137.80
tcc	S	0	111.40
tca	S	0	217.30
tcg	S	0	50.10
act	T	0	2.85
acc	T	11	2.24
aca	T	0	5.05
acg	T	0	0.86
aat	N	0	3.56
aac	N	7	3.44
agt	S	9	176.40
agc	S	852	167.90
mPD		0.061	1.7
		nPD:	0.04
		N. weight:	1.9
		Sc. PD:	-0.24
		Sc. rank:	-536.1

PB1

Pos.	679	obs:	exp:
cat	H	0	0.57
cac	H	1	0.43
caa	Q	864	451.00
cag	Q	14	427.00
mPD		0.034	0.50
		nPD:	0.07
		N. weight:	0.73
		Sc. PD:	-0.067
		Sc. rank:	-129.5

PB1

Pos.	680	obs:	exp:
cgt	R	0	37.46
cgc	R	0	44.56
cga	R	0	87.45
cgg	R	68	73.04
aga	R	190	400.40
agg	R	619	234.10
ggt	G	0	0.27
ggc	G	0	0.25
gga	G	0	0.92
ggg	G	2	0.55
mPD		0.49	0.97
		nPD:	0.5
		N. weight:	0.65
		Sc. PD:	0.21
		Sc. rank:	361.4

PB1			
Pos.	681	obs:	exp:
ggt	G	0	117.90
ggc	G	0	111.70
gga	G	106	406.40
ggg	G	773	243.00
---	--	------	------
mPD		0.21	0.68
		nPD:	0.31
		N. weight:	1.1
		Sc. PD:	0.15
		Sc. rank:	458.7

PB1			
Pos.	682	obs:	exp:
att	I	756	312.70
atc	I	100	207.30
ata	I	1	337.00
gtt	V	22	4.54
gtc	V	0	4.41
gta	V	0	4.56
gtg	V	0	8.50
---	--	------	------
mPD		0.25	0.71
		nPD:	0.36
		N. weight:	0.88
		Sc. PD:	0.16
		Sc. rank:	413.1

PB1			
Pos.	683	obs:	exp:
ttt	F	1	0.39
ttc	F	0	0.61
tta	L	0	77.16
ttg	L	0	163.80
ctt	L	868	148.80
ctc	L	8	147.10
cta	L	2	146.40
ctg	L	0	194.70
---	--	------	------
mPD		0.025	1.1
		nPD:	0.02
		N. weight:	2.1
		Sc. PD:	-0.29
		Sc. rank:	-779.3

PB1			
Pos.	684	obs:	exp:
gaa	E	70	528.70
gag	E	809	350.30
---	--	------	------
mPD		0.15	0.48
		nPD:	0.31
		N. weight:	0.76
		Sc. PD:	0.1
		Sc. rank:	322.1

PB1			
Pos.	685	obs:	exp:
gat	D	338	474.50
gac	D	541	404.50
---	--	------	------
mPD		0.47	0.50
		nPD:	0.95
		N. weight:	0.065
		Sc. PD:	0.049
		Sc. rank:	40.0

PB1			
Pos.	686	obs:	exp:
caa	Q	1	0.51
cag	Q	0	0.49
gaa	E	853	528.10
gag	E	25	349.90
---	--	------	------
mPD		0.058	0.48
		nPD:	0.12
		N. weight:	0.49
		Sc. PD:	-0.02
		Sc. rank:	5.9

PB1			
Pos.	687	obs:	exp:
caa	Q	19	450.00
cag	Q	857	426.00
cgt	R	0	0.13
cgc	R	0	0.15
cga	R	0	0.30
cgg	R	3	0.25
aga	R	0	1.37
agg	R	0	0.80
---	--	------	------
mPD		0.049	0.51
		nPD:	0.1
		N. weight:	0.77
		Sc. PD:	-0.05
		Sc. rank:	-60.2

PB1			
Pos.	688	obs:	exp:
atg	M	879	879.00
---	--	------	------
mPD		0	0
		nPD:	1.
		N. weight:	0.
		Sc. PD:	0
		Sc. rank:	0

PB1			
Pos.	689	obs:	exp:
tat	Y	70	466.10
tac	Y	809	412.90
---	--	------	------
mPD		0.15	0.50
		nPD:	0.29
		N. weight:	0.58
		Sc. PD:	0.073
		Sc. rank:	229.3

PB1			
Pos.	690	obs:	exp:
cat	H	1	0.57
cac	H	0	0.43
caa	Q	121	451.00
cag	Q	757	427.00
---	--	------	------
mPD		0.24	0.50
		nPD:	0.48
		N. weight:	0.39
		Sc. PD:	0.12
		Sc. rank:	210.2

PB1			
Pos.	691	obs:	exp:
cgt	R	0	0.04
cgc	R	0	0.05
cga	R	0	0.10
cgg	R	0	0.08
aaa	K	200	514.30
aag	K	678	363.70
aga	R	0	0.46
agg	R	1	0.27
---	--	------	------
mPD		0.35	0.49
		nPD:	0.73
		N. weight:	0.33
		Sc. PD:	0.18
		Sc. rank:	201.8

PB1			
Pos.	692	obs:	exp:
tgt	C	28	360.90
tgc	C	851	518.10
---	--	------	------
mPD		0.062	0.48
		nPD:	0.13
		N. weight:	0.5
		Sc. PD:	-0.017
		Sc. rank:	21.3

PB1			
Pos.	693	obs:	exp:
tgt	C	8	360.90
tgc	C	871	518.10
---	--	------	------
mPD		0.018	0.48
		nPD:	0.04
		N. weight:	0.59
		Sc. PD:	-0.072
		Sc. rank:	-161.9

PB1			
Pos.	694	obs:	exp:
tct	S	0	26.09
tcc	S	0	21.10
tca	S	0	41.14
tcg	S	0	9.49
cat	H	0	0.57
cac	H	1	0.43
cgt	R	0	0.30
cgc	R	7	0.36
cga	R	0	0.70
cgg	R	0	0.58
aat	N	80	360.20
aac	N	628	347.80
agt	S	28	33.40
agc	S	135	31.79
aga	R	0	3.20
agg	R	0	1.87
---	--	------	------
mPD		0.55	1.1
		nPD:	0.5
		N. weight:	0.66
		Sc. PD:	0.21
		Sc. rank:	360.8

PB1			
Pos.	695	obs:	exp:
tta	L	4	77.16
ttg	L	1	163.80
ctt	L	0	148.80
ctc	L	0	147.10
cta	L	862	146.40
ctg	L	11	194.70
att	I	0	0.36
atc	I	0	0.24
ata	I	1	0.39
---	--	------	------
mPD		0.041	1.1
		nPD:	0.04
		N. weight:	2.1
		Sc. PD:	-0.25
		Sc. rank:	-569.4

PB1			
Pos.	696	obs:	exp:
ttt	F	38	346.90
ttc	F	841	532.10
---	--	------	------
mPD		0.083	0.48
		nPD:	0.17
		N. weight:	0.43
		Sc. PD:	0.0041
		Sc. rank:	64.3

PB1			
Pos.	697	obs:	exp:
caa	Q	1	0.51
cag	Q	0	0.49
gaa	E	800	528.10
gag	E	78	349.90
---	--	------	------
mPD		0.16	0.48
		nPD:	0.34
		N. weight:	0.31
		Sc. PD:	0.052
		Sc. rank:	139.9

PB1			
Pos.	698	obs:	exp:
cgt	R	0	0.38
cgc	R	0	0.46
cga	R	0	0.90
cgg	R	0	0.75
aaa	K	91	509.60
aag	K	779	360.40
aga	R	0	4.11
agg	R	9	2.40
---	--	------	------
mPD		0.21	0.51
		nPD:	0.4
		N. weight:	0.64
		Sc. PD:	0.15
		Sc. rank:	327.7

PB1			
Pos.	699	obs:	exp:
ttt	F	4	346.90
ttc	F	875	532.10
---	--	------	------
mPD		0.0091	0.48
		nPD:	0.02
		N. weight:	0.59
		Sc. PD:	-0.082
		Sc. rank:	-225.5

PB1			
Pos.	700	obs:	exp:
ttt	F	5	346.90
ttc	F	874	532.10
---	--	------	------
mPD		0.011	0.48
		nPD:	0.02
		N. weight:	0.58
		Sc. PD:	-0.078
		Sc. rank:	-200.3

PB1			
Pos.	701	obs:	exp:
cct	P	63	223.50
ccc	P	792	156.70
cca	P	24	359.10
ccg	P	0	139.70
---	--	------	------
mPD		0.18	0.71
		nPD:	0.26
		N. weight:	1.6
		Sc. PD:	0.14
		Sc. rank:	539.1

PB1			
Pos.	702	obs:	exp:
tct	S	0	140.50
tcc	S	0	113.60
tca	S	0	221.60
tcg	S	0	51.09
agt	S	52	179.90
agc	S	826	171.20
ggt	G	0	0.13
ggc	G	1	0.13
gga	G	0	0.46
ggg	G	0	0.28
---	--	------	------
mPD		0.11	1.7
		nPD:	0.07
		N. weight:	1.7
		Sc. PD:	-0.16
		Sc. rank:	-292.9

PB1			
Pos.	703	obs:	exp:
tct	S	0	140.70
tcc	S	0	113.80
tca	S	0	221.80
tcg	S	0	51.15
agt	S	871	180.10
agc	S	8	171.40
---	--	------	------
mPD		0.018	1.7
		nPD:	0.01
		N. weight:	1.9
		Sc. PD:	-0.28
		Sc. rank:	-885.8

PB1			
Pos.	704	obs:	exp:
tct	S	0	140.70
tcc	S	1	113.80
tca	S	419	221.80
tcg	S	459	51.15
agt	S	0	180.10
agc	S	0	171.40
---	--	------	------
mPD		0.50	1.7
		nPD:	0.3
		N. weight:	1.8
		Sc. PD:	0.24
		Sc. rank:	736.7

PB1			
Pos.	705	obs:	exp:
tat	Y	40	466.10
tac	Y	839	412.90
---	--	------	------
mPD		0.087	0.50
		nPD:	0.17
		N. weight:	0.7
		Sc. PD:	0.0075
		Sc. rank:	106.5

PB1			
Pos.	706	obs:	exp:
cgt	R	0	37.51
cgc	R	0	44.61
cga	R	6	87.55
cgg	R	312	73.13
act	T	0	0.26
acc	T	0	0.20
aca	T	0	0.46
acg	T	1	0.08
aga	R	27	400.90
agg	R	533	234.30
---	--	------	------
mPD		0.54	0.97
		nPD:	0.56
		N. weight:	1.1
		Sc. PD:	0.43
		Sc. rank:	658.4

,

PB1			
Pos.	707	obs:	exp:
cgt	R	0	37.55
cgc	R	0	44.66
cga	R	0	87.65
cgg	R	0	73.21
aga	R	23	401.30
agg	R	856	234.60
---	--	------	------
mPD		0.051	0.96
		nPD:	0.05
		N. weight:	1.5
		Sc. PD:	-0.16
		Sc. rank:	-320.2

,

PB1			
Pos.	708	obs:	exp:
tta	L	0	0.09
ttg	L	0	0.19
ctt	L	0	0.17
ctc	L	0	0.17
cta	L	1	0.17
ctg	L	0	0.22
cct	P	2	223.30
ccc	P	4	156.50
cca	P	856	358.70
ccg	P	16	139.50
---	--	------	------
mPD		0.051	0.71
		nPD:	0.07
		N. weight:	0.97
		Sc. PD:	-0.085
		Sc. rank:	-148.1

,

PB1			
Pos.	709	obs:	exp:
att	I	3	1.09
atc	I	0	0.73
ata	I	0	1.18
gtt	V	870	180.80
gtc	V	4	175.50
gta	V	0	181.40
gtg	V	2	338.30
---	--	------	------
mPD		0.020	0.73
		nPD:	0.03
		N. weight:	1.9
		Sc. PD:	-0.25
		Sc. rank:	-596.1

,

PB1			
Pos.	710	obs:	exp:
ggt	G	0	117.90
ggc	G	0	111.70
gga	G	869	406.40
ggg	G	10	243.00
---	--	------	------
mPD		0.023	0.68
		nPD:	0.03
		N. weight:	0.88
		Sc. PD:	-0.11
		Sc. rank:	-257.5

,

PB1			
Pos.	711	obs:	exp:
att	I	867	320.70
atc	I	12	212.60
ata	I	0	345.70
---	--	------	------
mPD		0.027	0.65
		nPD:	0.04
		N. weight:	1.2
		Sc. PD:	-0.14
		Sc. rank:	-291.4

,

PB1			
Pos.	712	obs:	exp:
tct	S	23	140.70
tcc	S	854	113.80
tca	S	2	221.80
tcg	S	0	51.15
agt	S	0	180.10
agc	S	0	171.40
---	--	------	------
mPD		0.055	1.7
		nPD:	0.03
		N. weight:	2.3
		Sc. PD:	-0.29
		Sc. rank:	-659.9

,

PB1			
Pos.	713	obs:	exp:
tct	S	0	140.70
tcc	S	0	113.80
tca	S	0	221.80
tcg	S	0	51.15
agt	S	16	180.10
agc	S	863	171.40
---	--	------	------
mPD		0.036	1.7
		nPD:	0.02
		N. weight:	1.9
		Sc. PD:	-0.26
		Sc. rank:	-710.8

,

PB1			
Pos.	714	obs:	exp:
atg	M	879	879.00
---	--	------	------
mPD		0	0
		nPD:	1.
		N. weight:	0.
		Sc. PD:	0
		Sc. rank:	0

,

PB1			
Pos.	715	obs:	exp:
gtt	V	0	181.50
gtc	V	0	176.10
gta	V	3	182.00
gtg	V	876	339.50
---	--	------	------
mPD		0.0068	0.73
		nPD:	0.01
		N. weight:	1.1
		Sc. PD:	-0.17
		Sc. rank:	-565.3

,

PB1			
Pos.	716	obs:	exp:
gaa	E	0	528.70
gag	E	879	350.30
---	--	------	------
mPD		0	0.48
		nPD:	0.
		N. weight:	1.1
		Sc. PD:	-0.18
		Sc. rank:	-698.9

,

PB1			
Pos.	717	obs:	exp:
gct	A	0	225.20
gcc	A	869	165.00
gca	A	10	411.00
gcg	A	0	77.75
---	--	------	------
mPD		0.023	0.67
		nPD:	0.03
		N. weight:	2.
		Sc. PD:	-0.25
		Sc. rank:	-568.8

,

PB1			
Pos.	718	obs:	exp:
atg	M	879	879.00
---	--	------	------
mPD		0	0
		nPD:	1.
		N. weight:	0.
		Sc. PD:	0
		Sc. rank:	0

,

PB1			
Pos.	719	obs:	exp:
att	I	1	0.36
atc	I	0	0.24
ata	I	0	0.39
atg	M	5	5.00
gtt	V	102	180.20
gtc	V	0	174.90
gta	V	12	180.80
gtg	V	759	337.10
---	--	------	------
mPD		0.24	0.74
		nPD:	0.33
		N. weight:	0.75
		Sc. PD:	0.12
		Sc. rank:	336.5

,

PB1			
Pos.	720	obs:	exp:
tct	S	686	140.70
tcc	S	193	113.80
tca	S	0	221.80
tcg	S	0	51.15
agt	S	0	180.10
agc	S	0	171.40
---	--	------	------
mPD		0.34	1.7
		nPD:	0.21
		N. weight:	1.7
		Sc. PD:	0.071
		Sc. rank:	401.8

PB1			
Pos.	721	obs:	exp:
cgt	R	0	37.51
cgc	R	0	44.61
cga	R	0	87.55
cgg	R	0	73.13
aaa	K	0	0.59
aag	K	1	0.41
aga	R	1	400.90
agg	R	877	234.30
---	--	------	------
mPD		0.0046	0.97
		nPD:	0.
		N. weight:	1.6
		Sc. PD:	-0.25
		Sc. rank:	-860.6

,

PB1			
Pos.	722	obs:	exp:
gct	A	0	225.20
gcc	A	879	165.00
gca	A	0	411.00
gcg	A	0	77.75
---	--	------	------
mPD		0	0.67
		nPD:	0.
		N. weight:	2.1
		Sc. PD:	-0.32
		Sc. rank:	-1270.4

,

PB1			
Pos.	723	obs:	exp:
cgt	R	0	37.55
cgc	R	0	44.66
cga	R	868	87.65
cgg	R	8	73.21
aga	R	3	401.30
agg	R	0	234.60
---	--	------	------
mPD		0.025	0.96
		nPD:	0.03
		N. weight:	2.8
		Sc. PD:	-0.36
		Sc. rank:	-897.6

,

PB1			
Pos.	724	obs:	exp:
att	I	870	320.70
atc	I	9	212.60
ata	I	0	345.70
---	--	------	------
mPD		0.020	0.65
		nPD:	0.03
		N. weight:	1.2
		Sc. PD:	-0.15
		Sc. rank:	-359.1

,

PB1			
Pos.	725	obs:	exp:
gat	D	804	472.90
gac	D	72	403.10
ggt	G	3	0.40
ggc	G	0	0.38
gga	G	0	1.39
ggg	G	0	0.83
---	--	------	------
mPD		0.16	0.51
		nPD:	0.31
		N. weight:	0.44
		Sc. PD:	0.062
		Sc. rank:	186.7

,

PB1			
Pos.	726	obs:	exp:
tct	S	0	0.16
tcc	S	0	0.13
tca	S	1	0.25
tcg	S	0	0.06
agt	S	0	0.20
agc	S	0	0.20
gct	A	0	225.00
gcc	A	1	164.90
gca	A	840	410.50
gcg	A	37	77.66
---	--	------	------
mPD		0.085	0.68
		nPD:	0.13
		N. weight:	0.81
		Sc. PD:	-0.029
		Sc. rank:	32.0

,

PB1			
Pos.	727	obs:	exp:
cgt	R	2	37.55
cgc	R	27	44.66
cga	R	844	87.65
cgg	R	5	73.21
aga	R	1	401.30
agg	R	0	234.60
---	--	------	------
mPD		0.077	0.96
		nPD:	0.08
		N. weight:	2.6
		Sc. PD:	-0.21
		Sc. rank:	-324.8

,

PB1			
Pos.	728	obs:	exp:
att	I	878	320.70
atc	I	0	212.60
ata	I	1	345.70
---	--	------	------
mPD		0.0023	0.65
		nPD:	0.
		N. weight:	1.2
		Sc. PD:	-0.19
		Sc. rank:	-684.9

,

PB1			
Pos.	729	obs:	exp:
gat	D	76	474.00
gac	D	802	404.00
gaa	E	0	0.60
gag	E	1	0.40
---	--	------	------
mPD		0.16	0.50
		nPD:	0.32
		N. weight:	0.58
		Sc. PD:	0.089
		Sc. rank:	254.9

,

PB1			
Pos.	730	obs:	exp:
ttt	F	0	346.90
ttc	F	879	532.10
---	--	------	------
mPD		0	0.48
		nPD:	0.
		N. weight:	0.62
		Sc. PD:	-0.097
		Sc. rank:	-381.3

,

PB1			
Pos.	731	obs:	exp:
gaa	E	854	528.70
gag	E	25	350.30
---	--	------	------
mPD		0.055	0.48
		nPD:	0.12
		N. weight:	0.49
		Sc. PD:	-0.022
		Sc. rank:	0.9

PB1			
Pos.	732	obs:	exp:
tct	S	878	140.70
tcc	S	1	113.80
tca	S	0	221.80
tcg	S	0	51.15
agt	S	0	180.10
agc	S	0	171.40
---	--	------	------
mPD		0.0023	1.7
		nPD:	0.
		N. weight:	2.2
		Sc. PD:	-0.35
		Sc. rank:	-1327.8

PB1			
Pos.	733	obs:	exp:
ggt	G	0	117.90
ggc	G	1	111.70
gga	G	874	406.40
ggg	G	4	243.00
---	--	------	------
mPD		0.011	0.68
		nPD:	0.02
		N. weight:	0.91
		Sc. PD:	-0.13
		Sc. rank:	-370.8

PB1			
Pos.	734	obs:	exp:
cgt	R	0	37.42
cgc	R	0	44.51
cga	R	0	87.35
cgg	R	39	72.96
atg	M	1	1.00
aga	R	0	400.00
agg	R	837	233.80
ggt	G	0	0.27
ggc	G	0	0.25
gga	G	0	0.92
ggg	G	2	0.55
---	--	------	------
mPD		0.092	0.97
		nPD:	0.09
		N. weight:	1.5
		Sc. PD:	-0.097
		Sc. rank:	-122.6

PB1			
Pos.	735	obs:	exp:
att	I	874	320.70
atc	I	5	212.60
ata	I	0	345.70
---	--	------	------
mPD		0.011	0.65
		nPD:	0.02
		N. weight:	1.2
		Sc. PD:	-0.17
		Sc. rank:	-483.6

PB1			
Pos.	736	obs:	exp:
cgt	R	0	2.99
cgc	R	0	3.56
cga	R	0	6.98
cgg	R	0	5.83
aaa	K	0	473.30
aag	K	808	334.70
aga	R	0	31.96
agg	R	70	18.68
ggt	G	0	0.13
ggc	G	0	0.13
gga	G	0	0.46
ggg	G	1	0.28
---	--	------	------
mPD		0.15	0.69
		nPD:	0.22
		N. weight:	1.1
		Sc. PD:	0.061
		Sc. rank:	311.1

PB1			
Pos.	737	obs:	exp:
cgt	R	0	0.51
cgc	R	0	0.61
cga	R	0	1.20
cgg	R	0	1.00
aat	N	2	1.02
aac	N	0	0.98
aaa	K	765	506.70
aag	K	100	358.30
aga	R	12	5.48
agg	R	0	3.20
---	--	------	------
mPD		0.23	0.52
		nPD:	0.45
		N. weight:	0.28
		Sc. PD:	0.076
		Sc. rank:	148.6

PB1			
Pos.	738	obs:	exp:
gaa	E	811	528.70
gag	E	68	350.30
---	--	------	------
mPD		0.14	0.48
		nPD:	0.3
		N. weight:	0.33
		Sc. PD:	0.043
		Sc. rank:	133.1

PB1			
Pos.	739	obs:	exp:
gat	D	0	0.54
gac	D	1	0.46
gaa	E	11	528.10
gag	E	867	349.90
---	--	------	------
mPD		0.027	0.48
		nPD:	0.06
		N. weight:	1.
		Sc. PD:	-0.11
		Sc. rank:	-212.5

PB1			
Pos.	740	obs:	exp:
ttt	F	871	346.10
ttc	F	6	530.90
tta	L	0	0.18
ttg	L	1	0.37
ctt	L	1	0.34
ctc	L	0	0.34
cta	L	0	0.33
ctg	L	0	0.44
---	--	------	------
mPD		0.018	0.48
		nPD:	0.04
		N. weight:	1.1
		Sc. PD:	-0.13
		Sc. rank:	-296.5

PB1			
Pos.	741	obs:	exp:
tct	S	1	0.16
tcc	S	0	0.13
tca	S	0	0.25
tcg	S	0	0.06
act	T	1	0.26
acc	T	0	0.20
aca	T	0	0.46
acg	T	0	0.08
agt	S	0	0.20
agc	S	0	0.20
gct	A	873	224.70
gcc	A	4	164.70
gca	A	0	410.00
gcg	A	0	77.57
---	--	------	------
mPD		0.014	0.68
		nPD:	0.02
		N. weight:	1.6
		Sc. PD:	-0.23
		Sc. rank:	-622.2

PB1			
Pos.	742	obs:	exp:
gaa	E	0	528.70
gag	E	879	350.30
---	--	------	------
mPD		0	0.48
		nPD:	0.
		N. weight:	1.1
		Sc. PD:	-0.18
		Sc. rank:	-698.9

PB1			
Pos.	743	obs:	exp:
att	I	0	320.40
atc	I	878	212.40
ata	I	0	345.30
act	T	0	0.26
acc	T	1	0.20
aca	T	0	0.46
acg	T	0	0.08
---	--	------	------
mPD		0.0023	0.66
		nPD:	0.
		N. weight:	1.8
		Sc. PD:	-0.27
		Sc. rank:	-984.0

PB1			
Pos.	744	obs:	exp:
tta	L	0	0.09
ttg	L	1	0.19
ctt	L	0	0.17
ctc	L	0	0.17
cta	L	0	0.17
ctg	L	0	0.22
att	I	0	4.01
atc	I	0	2.66
ata	I	11	4.33
atg	M	847	847.00
gtt	V	0	4.13
gtc	V	0	4.01
gta	V	0	4.14
gtg	V	20	7.72
---	--	------	------
mPD		0.071	0.10
		nPD:	0.71
		N. weight:	0.043
		Sc. PD:	0.023
		Sc. rank:	26.1

PB1			
Pos.	745	obs:	exp:
cgt	R	0	0.13
cgc	R	0	0.15
cga	R	0	0.30
cgg	R	0	0.25
aaa	K	0	513.20
aag	K	876	362.80
aga	R	0	1.37
agg	R	3	0.80
---	--	------	------
mPD		0.0068	0.49
		nPD:	0.01
		N. weight:	1.1
		Sc. PD:	-0.16
		Sc. rank:	-467.9

PB1			
Pos.	746	obs:	exp:
att	I	0	320.40
atc	I	878	212.40
ata	I	0	345.30
act	T	0	0.26
acc	T	1	0.20
aca	T	0	0.46
acg	T	0	0.08
---	--	------	------
mPD		0.0023	0.66
		nPD:	0.
		N. weight:	1.8
		Sc. PD:	-0.27
		Sc. rank:	-984.0

PB1			
Pos.	747	obs:	exp:
tgt	C	878	360.90
tgc	C	1	518.10
---	--	------	------
mPD		0.0023	0.48
		nPD:	0.
		N. weight:	1.1
		Sc. PD:	-0.17
		Sc. rank:	-582.8

PB1			
Pos.	748	obs:	exp:
tct	S	0	140.70
tcc	S	879	113.80
tca	S	0	221.80
tcg	S	0	51.15
agt	S	0	180.10
agc	S	0	171.40
---	--	------	------
mPD		0	1.7
		nPD:	0.
		N. weight:	2.5
		Sc. PD:	-0.4
		Sc. rank:	-1553.0

PB1			
Pos.	749	obs:	exp:
tct	S	0	0.16
tcc	S	0	0.13
tca	S	0	0.25
tcg	S	0	0.06
act	T	0	227.40
acc	T	878	178.80
aca	T	0	403.40
acg	T	0	68.39
agt	S	0	0.20
agc	S	1	0.20
---	--	------	------
mPD		0.0023	0.68
		nPD:	0.
		N. weight:	2.
		Sc. PD:	-0.3
		Sc. rank:	-1124.8

PB1			
Pos.	750	obs:	exp:
att	I	879	320.70
atc	I	0	212.60
ata	I	0	345.70
---	--	------	------
mPD		0	0.65
		nPD:	0.
		N. weight:	1.2
		Sc. PD:	-0.2
		Sc. rank:	-765.8

PB1			
Pos.	751	obs:	exp:
gaa	E	879	528.70
gag	E	0	350.30
---	--	------	------
mPD		0	0.48
		nPD:	0.
		N. weight:	0.63
		Sc. PD:	-0.098
		Sc. rank:	-386.1

PB1			
Pos.	752	obs:	exp:
gaa	E	4	527.50
gag	E	873	349.50
ggt	G	0	0.27
ggc	G	0	0.25
gga	G	0	0.92
ggg	G	2	0.55
---	--	------	------
mPD		0.014	0.48
		nPD:	0.03
		N. weight:	1.1
		Sc. PD:	-0.14
		Sc. rank:	-341.1

PB1			
Pos.	753	obs:	exp:
ttt	F	0	1.18
ttc	F	3	1.82
tta	L	0	76.99
ttg	L	0	163.50
ctt	L	0	148.40
ctc	L	876	146.80
cta	L	0	146.00
ctg	L	0	194.30
---	--	------	------
mPD		0.0068	1.1
		nPD:	0.01
		N. weight:	2.2
		Sc. PD:	-0.33
		Sc. rank:	-1152.0

PB1			
Pos.	754	obs:	exp:
cgt	R	0	37.55
cgc	R	0	44.66
cga	R	0	87.65
cgg	R	0	73.21
aga	R	878	401.30
agg	R	1	234.60
---	--	------	------
mPD		0.0023	0.96
		nPD:	0.
		N. weight:	0.96
		Sc. PD:	-0.15
		Sc. rank:	-557.4

PB1			
Pos.	755	obs:	exp:
caa	Q	1	0.51
cag	Q	0	0.49
cgt	R	0	37.51
cgc	R	0	44.61
cga	R	1	87.55
cgg	R	877	73.13
aga	R	0	400.90
agg	R	0	234.30
---	--	------	------
mPD		0.0068	0.97
		nPD:	0.01
		N. weight:	3.1
		Sc. PD:	-0.46
		Sc. rank:	-1539.2

PB1			
Pos.	756	obs:	exp:
caa	Q	3	1.54
cag	Q	0	1.46
---	-	876	876.00
---	--	------	------
mPD		0	5.8×10^{-6}
		nPD:	0.
		N. weight:	0.
		Sc. PD:	0
		Sc. rank:	0

PB1			
Pos.	757	obs:	exp:
caa	Q	832	450.00
cag	Q	44	426.00
aaa	K	2	1.17
aag	K	0	0.83
gaa	E	1	0.60
gag	E	0	0.40
---	--	------	------
mPD		0.10	0.51
		nPD:	0.2
		N. weight:	0.58
		Sc. PD:	0.021
		Sc. rank:	127.9

PB1			
Pos.	758	obs:	exp:
caa	Q	4	3.60
cag	Q	3	3.40
aat	N	55	28.49
aac	N	1	27.51
aaa	K	816	478.00
aag	K	0	338.00
---	--	------	------
mPD		0.14	0.56
		nPD:	0.25
		N. weight:	0.66
		Sc. PD:	0.056
		Sc. rank:	216.3

PB1			
Pos.	759	obs:	exp:
taa	.	11	378.00
tag	.	865	222.30
tga	.	3	278.70
---	--	------	------
mPD		0.038	0.81
		nPD:	0.05
		N. weight:	1.6
		Sc. PD:	-0.18
		Sc. rank:	-361.4

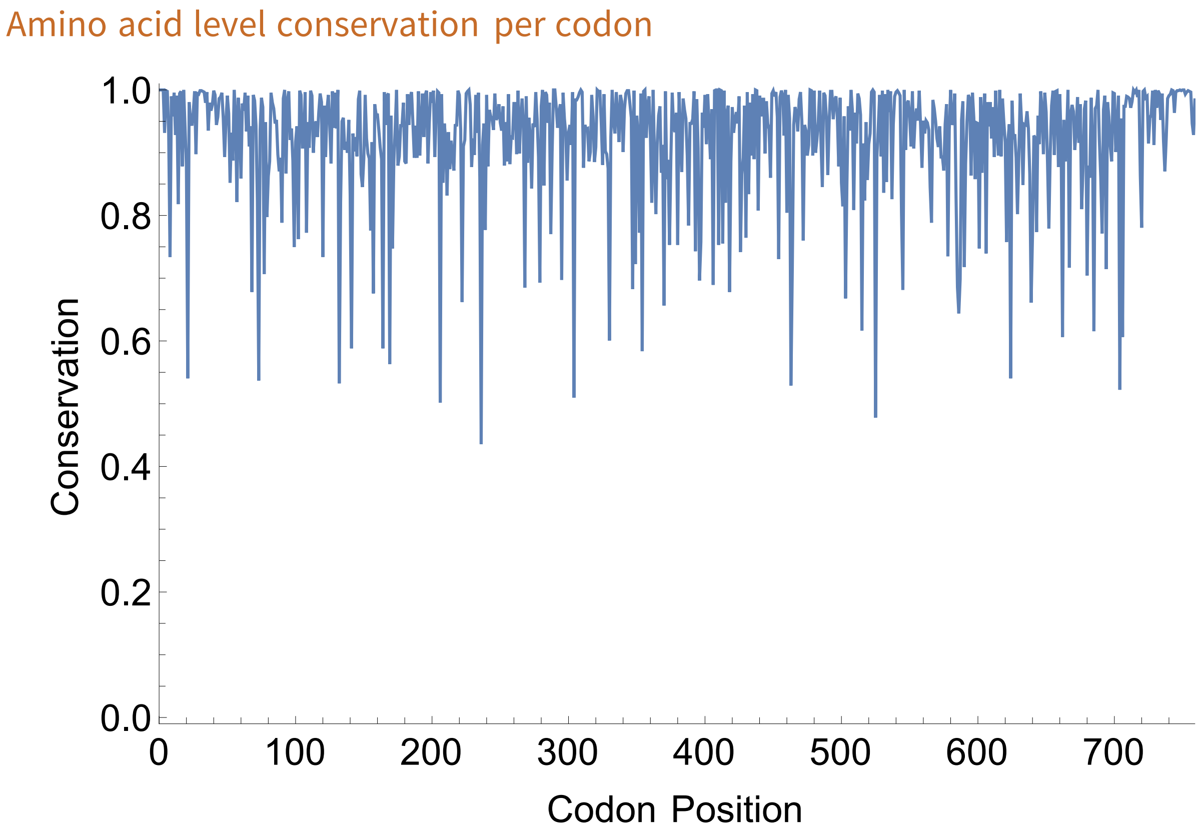
Amino acid level conservation per codon
Conservation
Codon Position
1.0
0.8
0.6
0.4
0.2
0.0
0
100
200
300
400
500
600
700

Conservation analysis plots

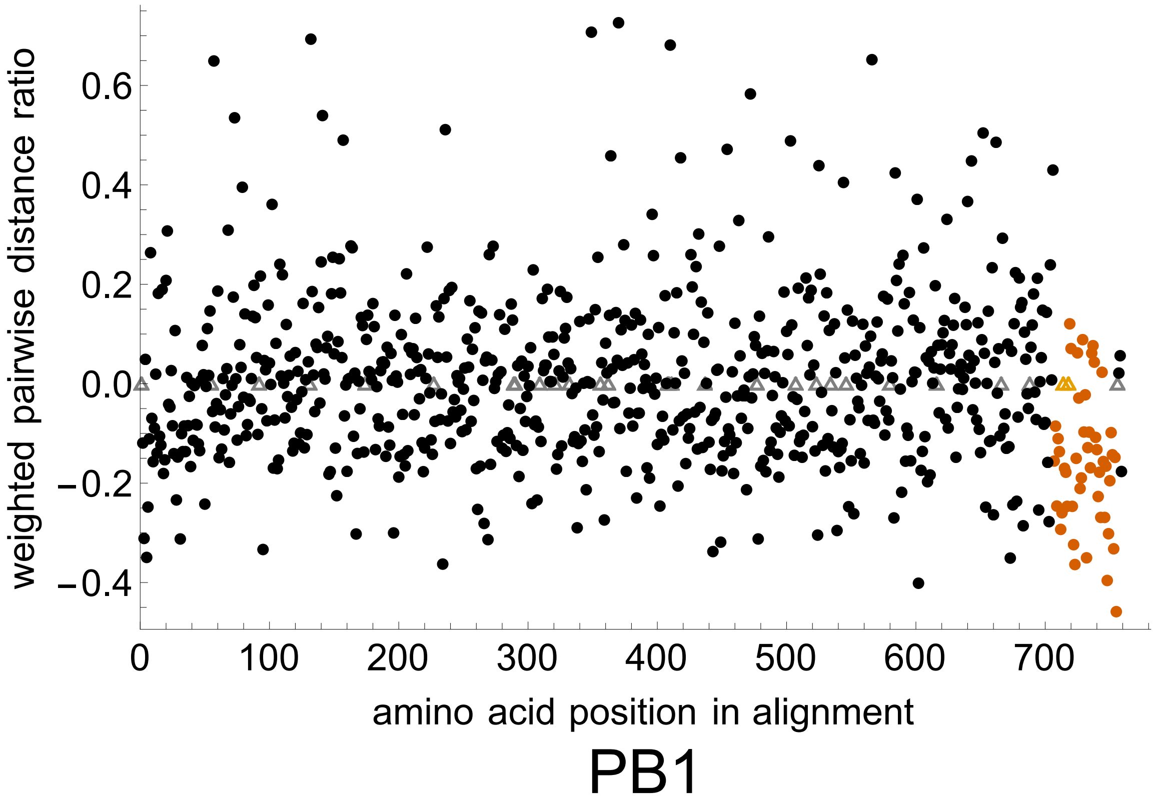

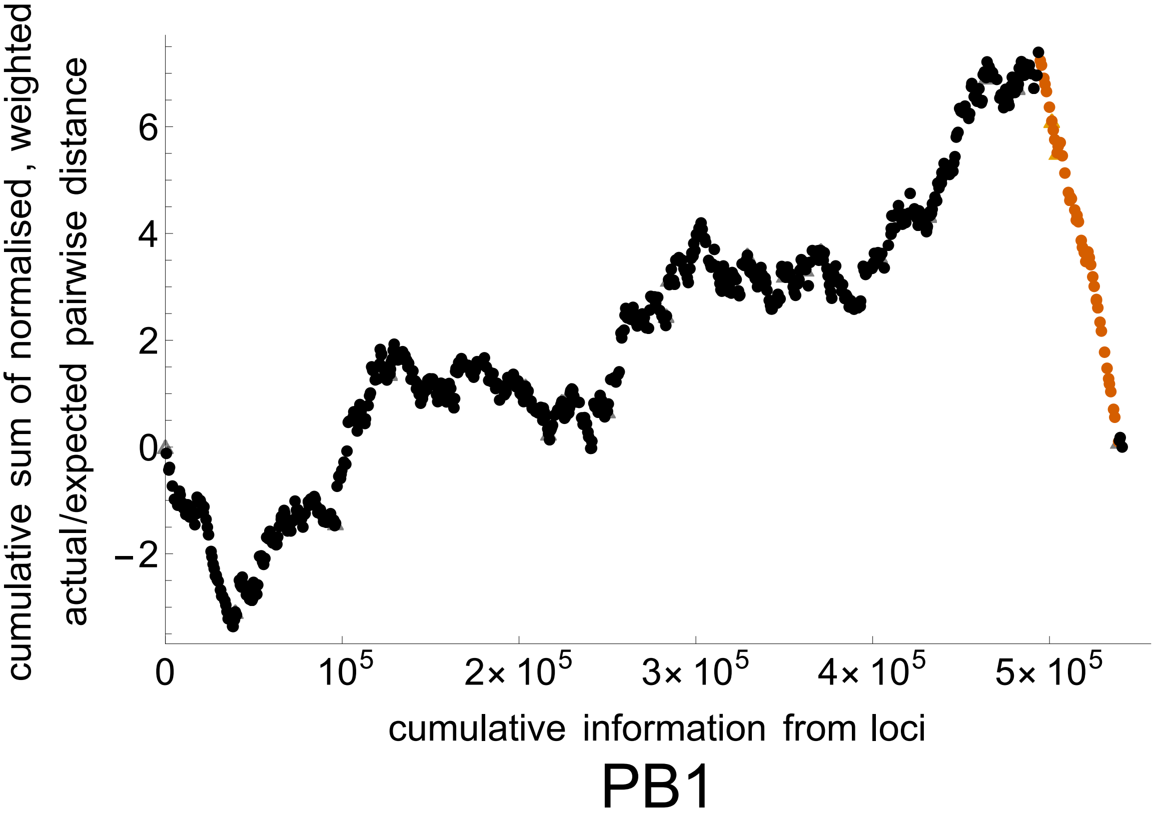

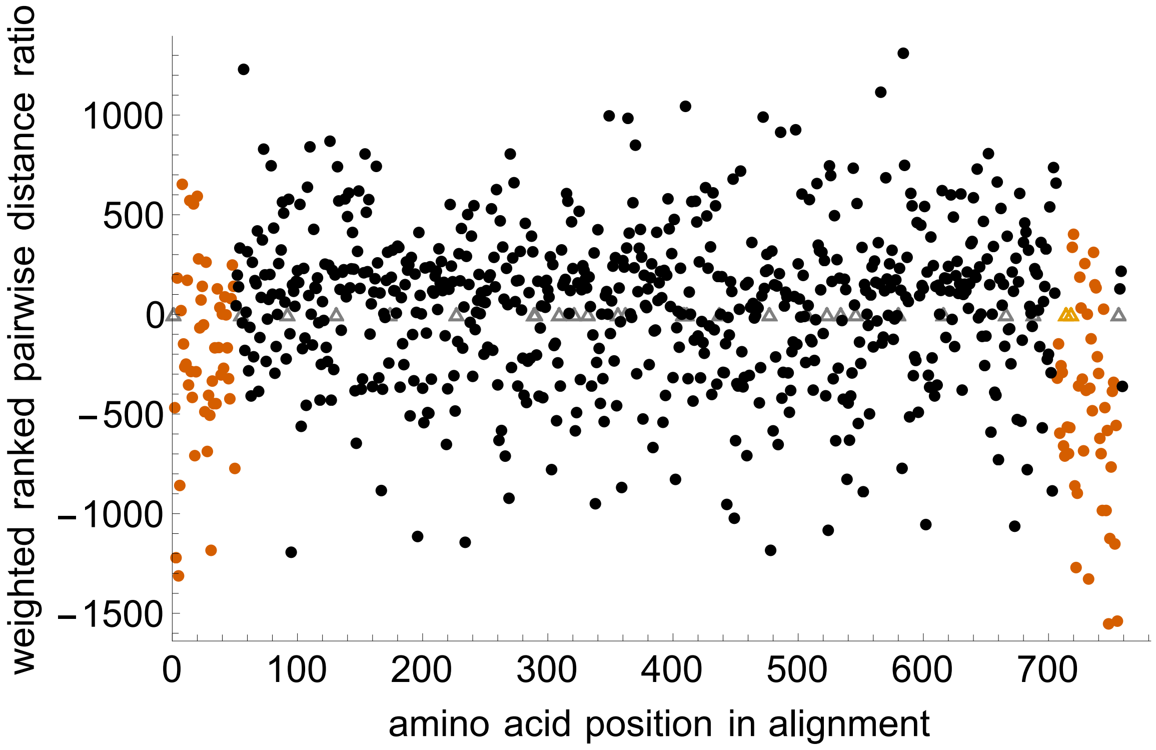
weighted ranked pairwise distance ratio
1000
500
0
−500
−1000
−1500
0
100
200
300
400
500
600
700
amino acid position in alignment

PB1

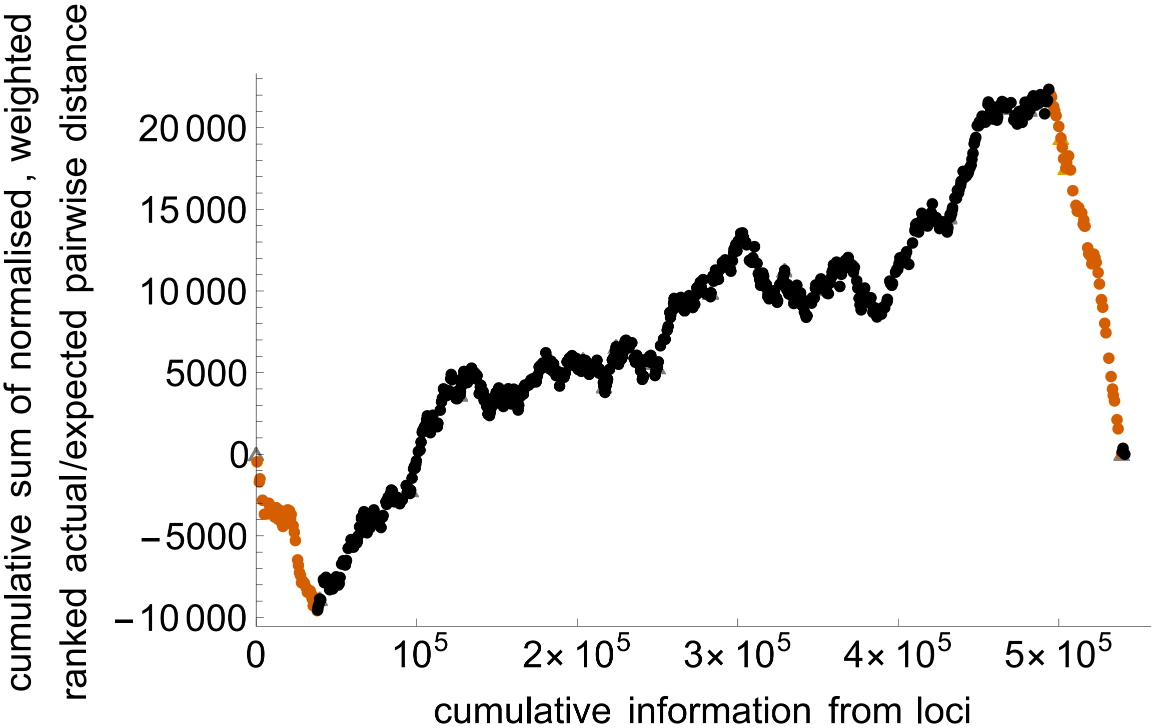
cumulative sum of normalised, weighted ranked actual/expected pairwise distance
20 000
15 000
10 000
5000
0
−5000
−10 000
0
10^5
2×10^5
3×10^5
4×10^5
5×10^5
cumulative information from loci

PB1

PB1-F2

Gene length histogram

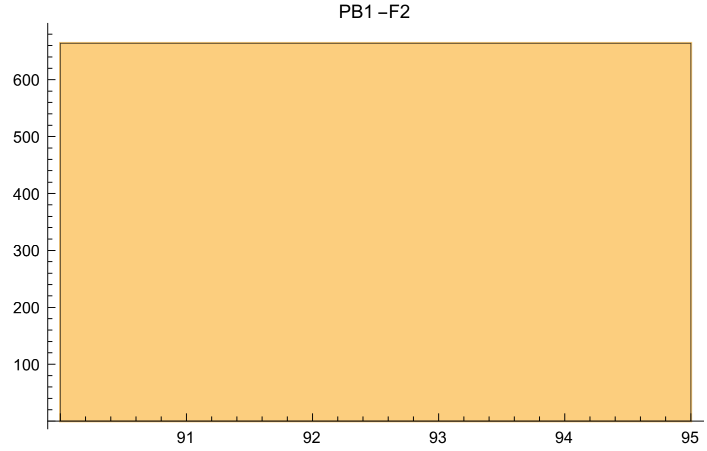

Information vs. nPD

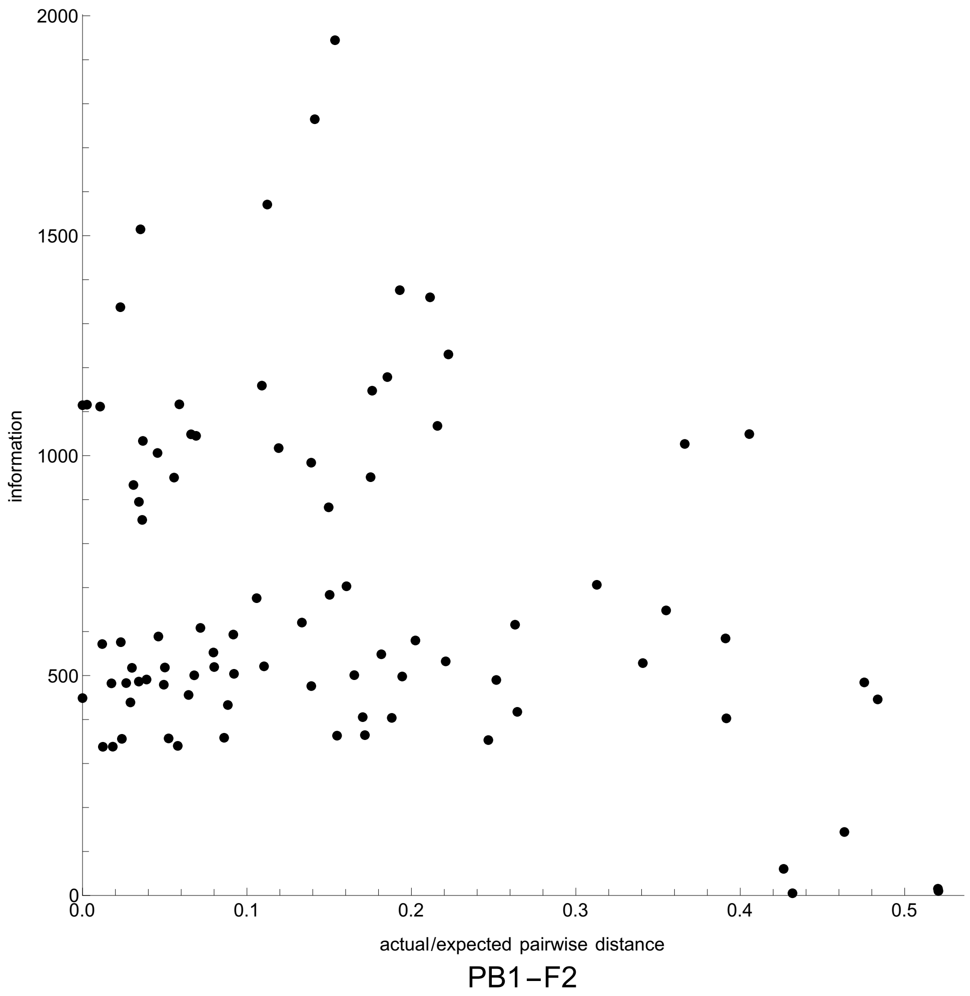

Example sequences highlighted by regions found in analyses to be conserved

```
Interesting points (by weighted raw PD) highlighted for gene PB1-F2:
ATGGAACAGGAACAGGGTACACCATGGATACAGTCAACAGAACACATCAATACTCAGAAAAGGGAAAGTGGACAACAA∵.
    ACACAGAGACCGGAGCACCCCAACTCAACCCAATTGATGGACCATTACCTGAGGACAACGAGCCAAGTGGATATGCA∵.
    CAAACGGATTGCGTATTGGAAGCAATGGCTTTCCTTGAAGAATCCCACCCAGGGATCTTTGAAAACTCTTGTCTTGA∵.
    AACGATGGAAGTTGTTCAGCAAACAAGAGTGGACAAACTAA
```

```
Interesting points (by weighted ranked PD) highlighted for gene PB1-F2:
ATGGAACAGGAACAGGGTACACCATGGATACAGTCAACAGAACACATCAATACTCAGAAAAGGGAAAGTGGACAACAA∵.
    ACACAGAGACCGGAGCACCCCAACTCAACCCAATTGATGGACCATTACCTGAGGACAACGAGCCAAGTGGATATGCA∵.
    CAAACGGATTGCGTATTGGAAGCAATGGCTT
```
TCCTTGAAGAATCCCACCCAGGGATCTTTGAAAACTCTTGTCTTGAAACGATGGAAGTTGTTCAGCAAACAA∵.
```
    GAGTGGACAAACTAA
```

Per codon conservation report

PB1-F2

Pos.	1	obs:	exp:
atg	M	664	664.00
---	--	------	------
mPD		0	0
		nPD:	1.
		N. weight:	0.
		Sc. PD:	0
		Sc. rank:	0

PB1-F2

Pos.	2	obs:	exp:
gaa	E	661	397.60
gag	E	0	263.40
ggt	G	0	0.40
ggc	G	0	0.38
gga	G	3	1.39
ggg	G	0	0.83
---	--	------	------
mPD		0.0090	0.49
		nPD:	0.02
		N. weight:	0.51
		Sc. PD:	-0.055
		Sc. rank:	-243.8

PB1-F2

Pos.	3	obs:	exp:
cct	P	0	2.54
ccc	P	0	1.78
cca	P	0	4.09
ccg	P	10	1.59
caa	Q	0	331.80
cag	Q	646	314.20
cgt	R	0	0.34
cgc	R	0	0.41
cga	R	0	0.80
cgg	R	8	0.67
aga	R	0	3.65
agg	R	0	2.14
---	--	------	------
mPD		0.053	0.58
		nPD:	0.09
		N. weight:	0.75
		Sc. PD:	-0.03
		Sc. rank:	-49.9

PB1-F2

Pos.	4	obs:	exp:
gaa	E	662	398.20
gag	E	0	263.80
ggt	G	0	0.27
ggc	G	0	0.25
gga	G	2	0.92
ggg	G	0	0.55
---	--	------	------
mPD		0.0060	0.49
		nPD:	0.01
		N. weight:	0.51
		Sc. PD:	-0.058
		Sc. rank:	-251.4

PB1-F2

Pos.	5	obs:	exp:
tta	L	0	0.09
ttg	L	0	0.19
ctt	L	0	0.17
ctc	L	0	0.17
cta	L	0	0.17
ctg	L	1	0.22
caa	Q	0	334.40
cag	Q	651	316.60
cgt	R	0	0.51
cgc	R	0	0.61
cga	R	0	1.20
cgg	R	12	1.00
aga	R	0	5.48
agg	R	0	3.20
---	--	------	------
mPD		0.039	0.57
		nPD:	0.07
		N. weight:	0.75
		Sc. PD:	-0.047
		Sc. rank:	-115.1

PB1-F2

Pos.	6	obs:	exp:
gat	D	647	349.30
gac	D	0	297.70
ggt	G	17	2.28
ggc	G	0	2.16
gga	G	0	7.86
ggg	G	0	4.70
---	--	------	------
mPD		0.050	0.57
		nPD:	0.09
		N. weight:	0.65
		Sc. PD:	-0.028
		Sc. rank:	-56.4

PB1-F2

Pos.	7	obs:	exp:
att	I	0	10.58
atc	I	0	7.01
ata	I	29	11.40
act	T	0	164.50
acc	T	0	129.30
aca	T	635	291.70
acg	T	0	49.46
---	--	------	------
mPD		0.084	0.76
		nPD:	0.11
		N. weight:	0.78
		Sc. PD:	-0.017
		Sc. rank:	-16.7

PB1-F2

Pos.	8	obs:	exp:
tta	L	0	1.41
ttg	L	0	2.99
ctt	L	0	2.71
ctc	L	0	2.68
cta	L	16	2.67
ctg	L	0	3.55
cct	P	0	164.00
ccc	P	0	115.00
cca	P	645	263.50
ccg	P	0	102.50
gct	A	0	0.77
gcc	A	0	0.56
gca	A	3	1.40
gcg	A	0	0.27
---	--	------	------
mPD		0.056	0.78
		nPD:	0.07
		N. weight:	0.91
		Sc. PD:	-0.054
		Sc. rank:	-111.4

PB1-F2

Pos.	9	obs:	exp:
tgg	W	664	664.00
---	--	------	------
mPD		0	0
		nPD:	1.
		N. weight:	0.
		Sc. PD:	0
		Sc. rank:	0

PB1-F2

Pos.	10	obs:	exp:
att	I	0	2.55
atc	I	0	1.69
ata	I	7	2.75
act	T	0	170.20
acc	T	0	133.80
aca	T	657	301.80
acg	T	0	51.18
---	--	------	------
mPD		0.021	0.70
		nPD:	0.03
		N. weight:	0.77
		Sc. PD:	-0.076
		Sc. rank:	-318.4

PB1-F2

Pos.	11	obs:	exp:
tta	L	0	0.97
ttg	L	0	2.05
ctt	L	0	1.86
ctc	L	0	1.84
cta	L	0	1.83
ctg	L	11	2.44
cct	P	0	0.25
ccc	P	0	0.18
cca	P	0	0.41
ccg	P	1	0.16
caa	Q	0	317.40
cag	Q	618	300.60
cgt	R	0	1.45
cgc	R	0	1.73
cga	R	0	3.39
cgg	R	34	2.83
aga	R	0	15.52
agg	R	0	9.07
---	--	------	------
mPD		0.13	0.72
		nPD:	0.18
		N. weight:	0.82
		Sc. PD:	0.037
		Sc. rank:	214.8

PB1-F2

Pos.	12	obs:	exp:
tta	L	34	2.99
ttg	L	0	6.34
tct	S	0	100.80
tcc	S	0	81.55
tca	S	630	159.00
tcg	S	0	36.66
ctt	L	0	5.76
ctc	L	0	5.70
cta	L	0	5.67
ctg	L	0	7.54
agt	S	0	129.10
agc	S	0	122.90
---	--	------	------
mPD		0.097	1.7
		nPD:	0.06
		N. weight:	1.4
		Sc. PD:	-0.11
		Sc. rank:	-311.9

PB1-F2

Pos.	13	obs:	exp:
att	I	0	4.38
atc	I	0	2.90
ata	I	12	4.72
act	T	0	168.90
acc	T	0	132.80
aca	T	652	299.50
acg	T	0	50.79
---	--	------	------
mPD		0.036	0.71
		nPD:	0.05
		N. weight:	0.77
		Sc. PD:	-0.062
		Sc. rank:	-189.4

PB1-F2

Pos.	14	obs:	exp:
gaa	E	654	393.40
gag	E	0	260.60
ggt	G	0	1.34
ggc	G	0	1.27
gga	G	10	4.62
ggg	G	0	2.76
---	--	------	------
mPD		0.030	0.51
		nPD:	0.06
		N. weight:	0.51
		Sc. PD:	-0.037
		Sc. rank:	-108.4

PB1-F2

Pos.	15	obs:	exp:
cat	H	0	379.20
cac	H	660	280.80
cgt	R	0	0.17
cgc	R	4	0.20
cga	R	0	0.40
cgg	R	0	0.33
aga	R	0	1.83
agg	R	0	1.07
---	--	------	------
mPD		0.012	0.52
		nPD:	0.02
		N. weight:	0.86
		Sc. PD:	-0.09
		Sc. rank:	-383.7

PB1-F2

Pos.	16	obs:	exp:
att	I	0	239.70
atc	I	90	158.90
ata	I	567	258.40
act	T	0	1.81
acc	T	4	1.43
aca	T	3	3.22
acg	T	0	0.55
---	--	------	------
mPD		0.26	0.68
		nPD:	0.39
		N. weight:	0.6
		Sc. PD:	0.15
		Sc. rank:	317.8

PB1-F2

Pos.	17	obs:	exp:
aat	N	664	337.80
aac	N	0	326.20
---	--	------	------
mPD		0	0.50
		nPD:	0.
		N. weight:	0.67
		Sc. PD:	-0.085
		Sc. rank:	-383.7

PB1-F2

Pos.	18	obs:	exp:
att	I	42	15.32
atc	I	0	10.16
ata	I	0	16.52
act	T	622	161.10
acc	T	0	126.70
aca	T	0	285.80
acg	T	0	48.45
---	--	------	------
mPD		0.12	0.79
		nPD:	0.15
		N. weight:	1.3
		Sc. PD:	0.02
		Sc. rank:	154.9

PB1-F2

Pos.	19	obs:	exp:
caa	Q	0	337.50
cag	Q	657	319.50
cgt	R	0	0.30
cgc	R	0	0.36
cga	R	0	0.70
cgg	R	7	0.58
aga	R	0	3.20
agg	R	0	1.87
---	--	------	------
mPD		0.021	0.54
		nPD:	0.04
		N. weight:	0.73
		Sc. PD:	-0.066
		Sc. rank:	-215.5

PB1-F2

Pos.	20	obs:	exp:
cgt	R	0	1.41
cgc	R	0	1.68
cga	R	0	3.29
cgg	R	0	2.75
aaa	K	631	369.60
aag	K	0	261.40
aga	R	33	15.07
agg	R	0	8.81
---	--	------	------
mPD		0.095	0.61
		nPD:	0.15
		N. weight:	0.54
		Sc. PD:	0.011
		Sc. rank:	94.6

PB1-F2			
Pos.	21	obs:	exp:
cgt	R	0	3.03
cgc	R	0	3.61
cga	R	0	7.08
cgg	R	0	5.91
atg	M	1	1.00
aaa	K	0	346.20
aag	K	591	244.80
aga	R	0	32.42
agg	R	71	18.95
gaa	E	0	0.60
gag	E	1	0.40
---	--	------	------
mPD		0.20	0.75
		nPD:	0.26
		N. weight:	0.92
		Sc. PD:	0.11
		Sc. rank:	410.4

,

PB1-F2			
Pos.	22	obs:	exp:
gaa	E	604	363.30
gag	E	0	240.70
ggt	G	0	8.05
ggc	G	0	7.62
gga	G	60	27.74
ggg	G	0	16.59
---	--	------	------
mPD		0.16	0.67
		nPD:	0.25
		N. weight:	0.53
		Sc. PD:	0.057
		Sc. rank:	224.4

,

PB1-F2			
Pos.	23	obs:	exp:
tct	S	0	91.56
tcc	S	0	74.04
tca	S	0	144.40
tcg	S	0	33.29
tgt	C	2	0.82
tgc	C	0	1.18
aat	N	79	40.19
aac	N	0	38.81
agt	S	572	117.20
agc	S	0	111.50
ggt	G	11	1.48
ggc	G	0	1.40
gga	G	0	5.09
ggg	G	0	3.04
---	--	------	------
mPD		0.25	1.8
		nPD:	0.14
		N. weight:	1.5
		Sc. PD:	0.0074
		Sc. rank:	85.5

,

PB1-F2			
Pos.	24	obs:	exp:
ggt	G	0	89.04
ggc	G	0	84.36
gga	G	658	307.00
ggg	G	6	183.60
---	--	------	------
mPD		0.018	0.68
		nPD:	0.03
		N. weight:	0.72
		Sc. PD:	-0.073
		Sc. rank:	-310.2

,

PB1-F2			
Pos.	25	obs:	exp:
caa	Q	659	339.00
cag	Q	1	321.00
cgt	R	0	0.09
cgc	R	0	0.10
cga	R	2	0.20
cgg	R	0	0.17
aaa	K	2	1.17
aag	K	0	0.83
aga	R	0	0.91
agg	R	0	0.53
---	--	------	------
mPD		0.015	0.52
		nPD:	0.03
		N. weight:	0.66
		Sc. PD:	-0.065
		Sc. rank:	-276.5

,

PB1-F2			
Pos.	26	obs:	exp:
caa	Q	101	51.88
cag	Q	0	49.12
cgt	R	0	23.92
cgc	R	0	28.45
cga	R	560	55.84
cgg	R	0	46.64
aaa	K	3	1.76
aag	K	0	1.24
aga	R	0	255.70
agg	R	0	149.50
---	--	------	------
mPD		0.27	1.3
		nPD:	0.21
		N. weight:	2.
		Sc. PD:	0.15
		Sc. rank:	741.0

,

PB1-F2			
Pos.	27	obs:	exp:
att	I	2	2.19
atc	I	0	1.45
ata	I	4	2.36
act	T	33	170.50
acc	T	0	134.00
aca	T	625	302.30
acg	T	0	51.26
---	--	------	------
mPD		0.12	0.69
		nPD:	0.17
		N. weight:	0.61
		Sc. PD:	0.021
		Sc. rank:	124.1

,

PB1-F2			
Pos.	28	obs:	exp:
tta	L	0	0.09
ttg	L	0	0.19
ctt	L	0	0.17
ctc	L	0	0.17
cta	L	0	0.17
ctg	L	1	0.22
cct	P	0	0.76
ccc	P	0	0.53
cca	P	0	1.23
ccg	P	3	0.48
caa	Q	0	338.00
cag	Q	658	320.00
cgt	R	0	0.09
cgc	R	0	0.10
cga	R	0	0.20
cgg	R	2	0.17
aga	R	0	0.91
agg	R	0	0.53
---	--	------	------
mPD		0.018	0.53
		nPD:	0.03
		N. weight:	0.73
		Sc. PD:	-0.069
		Sc. rank:	-279.6

,

PB1-F2			
Pos.	29	obs:	exp:
cgt	R	0	23.11
cgc	R	0	27.49
cga	R	0	53.94
cgg	R	0	45.06
aaa	K	123	72.05
aag	K	0	50.95
aga	R	541	247.00
agg	R	0	144.40
---	--	------	------
mPD		0.30	1.2
		nPD:	0.25
		N. weight:	0.73
		Sc. PD:	0.082
		Sc. rank:	318.0

,

PB1-F2			
Pos.	30	obs:	exp:
tta	L	0	56.78
ttg	L	0	120.60
ctt	L	0	109.50
ctc	L	0	108.20
cta	L	0	107.70
ctg	L	646	143.30
cct	P	0	4.58
ccc	P	0	3.21
cca	P	0	7.35
ccg	P	18	2.86
---	--	------	------
mPD		0.053	1.2
		nPD:	0.05
		N. weight:	1.5
		Sc. PD:	-0.13
		Sc. rank:	-412.9

,

PB1-F2			
Pos.	31	obs:	exp:
gaa	E	0	383.80
gag	E	638	254.20
ggt	G	0	3.49
ggc	G	0	3.30
gga	G	0	12.02
ggg	G	26	7.19
---	--	------	------
mPD		0.075	0.56
		nPD:	0.13
		N. weight:	0.93
		Sc. PD:	-0.00032
		Sc. rank:	36.6

,

PB1-F2			
Pos.	32	obs:	exp:
cat	H	0	380.30
cac	H	662	281.70
cgt	R	0	0.09
cgc	R	2	0.10
cga	R	0	0.20
cgg	R	0	0.17
aga	R	0	0.91
agg	R	0	0.53
---	--	------	------
mPD		0.0060	0.50
		nPD:	0.01
		N. weight:	0.85
		Sc. PD:	-0.099
		Sc. rank:	-430.7

,

PB1-F2			
Pos.	33	obs:	exp:
tta	L	0	3.52
ttg	L	0	7.46
tct	S	0	1.92
tcc	S	12	1.55
tca	S	0	3.03
tcg	S	0	0.70
ctt	L	0	6.78
ctc	L	40	6.70
cta	L	0	6.67
ctg	L	0	8.87
cct	P	0	154.30
ccc	P	607	108.20
cca	P	0	248.00
ccg	P	0	96.44
cat	H	0	2.30
cac	H	4	1.70
act	T	0	0.26
acc	T	1	0.20
aca	T	0	0.46
acg	T	0	0.08
agt	S	0	2.46
agc	S	0	2.34
---	--	------	------
mPD		0.16	0.93
		nPD:	0.18
		N. weight:	1.7
		Sc. PD:	0.069
		Sc. rank:	431.7

,

PB1-F2			
Pos.	34	obs:	exp:
tct	S	0	1.28
tcc	S	0	1.04
tca	S	0	2.02
tcg	S	0	0.47
aat	N	0	333.20
aac	N	655	321.80
aaa	K	1	0.59
aag	K	0	0.41
agt	S	0	1.64
agc	S	8	1.56
---	--	------	------
mPD		0.027	0.54
		nPD:	0.05
		N. weight:	0.72
		Sc. PD:	-0.057
		Sc. rank:	-182.2

,

PB1-F2

Pos.	35	obs:	exp:
tta	L	18	1.58
ttg	L	0	3.36
tct	S	0	103.40
tcc	S	0	83.62
tca	S	646	163.00
tcg	S	0	37.59
ctt	L	0	3.05
ctc	L	0	3.02
cta	L	0	3.00
ctg	L	0	3.99
agt	S	0	132.40
agc	S	0	126.00
---	--	------	------
mPD		0.053	1.7
		nPD:	0.03
		N. weight:	1.4
		Sc. PD:	-0.14
		Sc. rank:	-549.4

PB1-F2

Pos.	36	obs:	exp:
att	I	0	224.00
atc	I	614	148.50
ata	I	0	241.50
act	T	0	12.95
acc	T	50	10.18
aca	T	0	22.97
acg	T	0	3.89
---	--	------	------
mPD		0.14	0.80
		nPD:	0.18
		N. weight:	1.4
		Sc. PD:	0.056
		Sc. rank:	326.7

PB1-F2

Pos.	37	obs:	exp:
caa	Q	503	258.40
cag	Q	0	244.60
cgt	R	0	6.88
cgc	R	0	8.18
cga	R	161	16.05
cgg	R	0	13.41
aga	R	0	73.51
agg	R	0	42.97
---	--	------	------
mPD		0.37	1.2
		nPD:	0.31
		N. weight:	1.1
		Sc. PD:	0.18
		Sc. rank:	493.4

PB1-F2

Pos.	38	obs:	exp:
tta	L	0	58.09
ttg	L	660	123.40
tct	S	0	0.48
tcc	S	0	0.39
tca	S	0	0.76
tcg	S	3	0.17
ctt	L	0	112.00
ctc	L	0	110.70
cta	L	0	110.20
ctg	L	1	146.60
agt	S	0	0.61
agc	S	0	0.59
---	--	------	------
mPD		0.012	1.1
		nPD:	0.01
		N. weight:	1.7
		Sc. PD:	-0.19
		Sc. rank:	-855.5

PB1-F2

Pos.	39	obs:	exp:
atg	M	658	658.00
act	T	0	1.55
acc	T	0	1.22
aca	T	0	2.76
acg	T	6	0.47
---	--	------	------
mPD		0.018	0.034
		nPD:	0.52
		N. weight:	0.023
		Sc. PD:	0.0084
		Sc. rank:	13.0

PB1-F2

Pos.	40	obs:	exp:
gat	D	0	331.50
gac	D	614	282.50
ggt	G	0	6.71
ggc	G	50	6.35
gga	G	0	23.12
ggg	G	0	13.82
---	--	------	------
mPD		0.14	0.69
		nPD:	0.2
		N. weight:	0.87
		Sc. PD:	0.056
		Sc. rank:	293.5

PB1-F2

Pos.	41	obs:	exp:
tta	L	0	0.09
ttg	L	0	0.19
ctt	L	1	0.17
ctc	L	0	0.17
cta	L	0	0.17
ctg	L	0	0.22
cat	H	522	318.80
cac	H	33	236.20
cgt	R	106	4.61
cgc	R	2	5.49
cga	R	0	10.77
cgg	R	0	9.00
aga	R	0	49.31
agg	R	0	28.82
---	--	------	------
mPD		0.38	1.1
		nPD:	0.34
		N. weight:	0.79
		Sc. PD:	0.16
		Sc. rank:	377.0

PB1-F2

Pos.	42	obs:	exp:
tat	Y	0	221.60
tac	Y	418	196.40
tgt	C	0	101.00
tgc	C	246	145.00
---	--	------	------
mPD		0.47	0.97
		nPD:	0.48
		N. weight:	0.67
		Sc. PD:	0.22
		Sc. rank:	379.5

PB1-F2

Pos.	43	obs:	exp:
tta	L	0	53.70
ttg	L	0	114.00
ctt	L	0	103.50
ctc	L	0	102.40
cta	L	0	101.90
ctg	L	611	135.50
cct	P	0	8.14
ccc	P	0	5.71
cca	P	0	13.07
ccg	P	32	5.08
caa	Q	0	4.11
cag	Q	8	3.89
cgt	R	0	0.56
cgc	R	0	0.66
cga	R	0	1.30
cgg	R	13	1.08
aga	R	0	5.94
agg	R	0	3.47
---	--	------	------
mPD		0.15	1.3
		nPD:	0.12
		N. weight:	1.5
		Sc. PD:	-0.021
		Sc. rank:	42.0

PB1-F2

Pos.	44	obs:	exp:
cgt	R	0	27.98
cgc	R	0	33.28
cga	R	0	65.31
cgg	R	0	54.55
aaa	K	0	5.27
aag	K	9	3.73
aga	R	3	299.10
agg	R	652	174.80
---	--	------	------
mPD		0.036	0.99
		nPD:	0.04
		N. weight:	1.3
		Sc. PD:	-0.12
		Sc. rank:	-411.8

PB1-F2

Pos.	45	obs:	exp:
att	I	0	6.93
atc	I	0	4.60
ata	I	19	7.47
act	T	0	166.80
acc	T	0	131.10
aca	T	644	295.90
acg	T	0	50.17
gct	A	0	0.26
gcc	A	0	0.19
gca	A	1	0.47
gcg	A	0	0.09
---	--	------	------
mPD		0.059	0.73
		nPD:	0.08
		N. weight:	0.78
		Sc. PD:	-0.04
		Sc. rank:	-79.3

PB1-F2

Pos.	46	obs:	exp:
atg	M	253	253.00
act	T	0	106.50
acc	T	0	83.69
aca	T	0	188.80
acg	T	411	32.02
---	--	------	------
mPD		0.47	1.2
		nPD:	0.41
		N. weight:	1.6
		Sc. PD:	0.4
		Sc. rank:	859.1

PB1-F2

Pos.	47	obs:	exp:
tct	S	0	99.89
tcc	S	0	80.77
tca	S	0	157.50
tcg	S	0	36.31
aat	N	0	20.35
aac	N	40	19.65
agt	S	0	127.90
agc	S	624	121.70
---	--	------	------
mPD		0.11	1.7
		nPD:	0.07
		N. weight:	1.6
		Sc. PD:	-0.1
		Sc. rank:	-256.1

PB1-F2

Pos.	48	obs:	exp:
tta	L	0	0.09
ttg	L	0	0.19
ctt	L	0	0.17
ctc	L	0	0.17
cta	L	1	0.17
ctg	L	0	0.22
caa	Q	94	48.28
cag	Q	0	45.72
cgt	R	0	24.31
cgc	R	0	28.91
cga	R	569	56.74
cgg	R	0	47.39
aga	R	0	259.80
agg	R	0	151.90
---	--	------	------
mPD		0.25	1.3
		nPD:	0.19
		N. weight:	2.1
		Sc. PD:	0.12
		Sc. rank:	654.7

PB1-F2

Pos.	49	obs:	exp:
atg	M	1	1.00
gtt	V	0	126.80
gtc	V	0	123.00
gta	V	0	127.10
gtg	V	614	237.10
gct	A	0	12.56
gcc	A	0	9.20
gca	A	0	22.91
gcg	A	49	4.33
---	--	------	------
mPD		0.14	0.87
		nPD:	0.16
		N. weight:	1.1
		Sc. PD:	0.027
		Sc. rank:	197.2

PB1-F2

Pos.	50	obs:	exp:
gtt	V	2	0.41
gtc	V	0	0.40
gta	V	0	0.41
gtg	V	0	0.77
gat	D	74	39.95
gac	D	0	34.05
ggt	G	588	78.85
ggc	G	0	74.71
gga	G	0	271.90
ggg	G	0	162.60
---	--	------	------
mPD		0.20	0.92
		nPD:	0.22
		N. weight:	1.8
		Sc. PD:	0.15
		Sc. rank:	769.1

PB1-F2

Pos.	51	obs:	exp:
atg	M	660	660.00
act	T	0	1.04
acc	T	0	0.81
aca	T	0	1.84
acg	T	4	0.31
---	--	------	------
mPD		0.012	0.023
		nPD:	0.52
		N. weight:	0.015
		Sc. PD:	0.0056
		Sc. rank:	8.7

PB1-F2

Pos.	52	obs:	exp:
tta	L	0	0.18
ttg	L	0	0.37
ctt	L	0	0.34
ctc	L	2	0.34
cta	L	0	0.33
ctg	L	0	0.44
cct	P	0	2.03
ccc	P	8	1.43
cca	P	0	3.27
ccg	P	0	1.27
cat	H	0	371.10
cac	H	646	274.90
cgt	R	0	0.34
cgc	R	8	0.41
cga	R	0	0.80
cgg	R	0	0.67
aga	R	0	3.65
agg	R	0	2.14
---	--	------	------
mPD		0.053	0.58
		nPD:	0.09
		N. weight:	0.89
		Sc. PD:	-0.035
		Sc. rank:	-67.2

PB1-F2

Pos.	53	obs:	exp:
cgt	R	0	0.17
cgc	R	0	0.20
cga	R	0	0.40
cgg	R	0	0.33
aaa	K	660	386.60
aag	K	0	273.40
aga	R	4	1.83
agg	R	0	1.07
---	--	------	------
mPD		0.012	0.50
		nPD:	0.02
		N. weight:	0.53
		Sc. PD:	-0.055
		Sc. rank:	-233.6

PB1-F2

Pos.	54	obs:	exp:
caa	Q	0	23.11
cag	Q	45	21.89
cgt	R	0	26.44
cgc	R	0	31.45
cga	R	0	61.72
cgg	R	619	51.56
aga	R	0	282.60
agg	R	0	165.20
---	--	------	------
mPD		0.13	1.1
		nPD:	0.11
		N. weight:	2.3
		Sc. PD:	-0.048
		Sc. rank:	19.6

PB1-F2

Pos.	55	obs:	exp:
att	I	623	227.30
atc	I	0	150.70
ata	I	0	245.00
act	T	41	10.62
acc	T	0	8.35
aca	T	0	18.84
acg	T	0	3.19
mPD		0.12	0.77
		nPD:	0.15
		N. weight:	1.
		Sc. PD:	0.016
		Sc. rank:	133.3

PB1-F2

Pos.	56	obs:	exp:
gtt	V	0	130.90
gtc	V	0	127.00
gta	V	0	131.30
gtg	V	634	244.80
gct	A	0	7.69
gcc	A	0	5.63
gca	A	0	14.03
gcg	A	30	2.65
mPD		0.086	0.82
		nPD:	0.11
		N. weight:	1.
		Sc. PD:	-0.027
		Sc. rank:	-54.0

PB1-F2

Pos.	57	obs:	exp:
ttt	F	10	4.34
ttc	F	1	6.66
tct	S	1	6.40
tcc	S	39	5.18
tca	S	0	10.09
tcg	S	0	2.33
tat	Y	556	294.80
tac	Y	0	261.20
tgt	C	56	23.40
tgc	C	1	33.60
agt	S	0	8.20
agc	S	0	7.80
mPD		0.40	0.85
		nPD:	0.48
		N. weight:	0.72
		Sc. PD:	0.23
		Sc. rank:	406.2

PB1-F2

Pos.	58	obs:	exp:
tta	L	0	3.16
ttg	L	36	6.72
tgg	W	628	628.00
ctt	L	0	6.10
ctc	L	0	6.03
cta	L	0	6.00
ctg	L	0	7.98
mPD		0.10	0.24
		nPD:	0.43
		N. weight:	0.09
		Sc. PD:	0.025
		Sc. rank:	49.6

PB1-F2

Pos.	59	obs:	exp:
cgt	R	0	6.11
cgc	R	0	7.27
cga	R	0	14.26
cgg	R	0	11.91
aaa	K	0	305.20
aag	K	521	215.80
aga	R	0	65.29
agg	R	143	38.17
mPD		0.34	0.95
		nPD:	0.35
		N. weight:	0.97
		Sc. PD:	0.2
		Sc. rank:	474.4

PB1-F2

Pos.	60	obs:	exp:
tta	L	0	1.05
ttg	L	0	2.24
ctt	L	0	2.03
ctc	L	0	2.01
cta	L	12	2.00
ctg	L	0	2.66
caa	Q	652	334.90
cag	Q	0	317.10
mPD		0.036	0.55
		nPD:	0.06
		N. weight:	0.68
		Sc. PD:	-0.045
		Sc. rank:	-124.9

PB1-F2

Pos.	61	obs:	exp:
tgg	W	664	664.00
mPD		0	0
		nPD:	1.
		N. weight:	0.
		Sc. PD:	0
		Sc. rank:	0

PB1-F2

Pos.	62	obs:	exp:
tta	L	0	54.14
ttg	L	0	115.00
ctt	L	616	104.40
ctc	L	0	103.20
cta	L	0	102.70
ctg	L	0	136.60
cct	P	48	12.21
ccc	P	0	8.56
cca	P	0	19.61
ccg	P	0	7.63
mPD		0.13	1.2
		nPD:	0.11
		N. weight:	1.7
		Sc. PD:	-0.041
		Sc. rank:	-54.4

PB1-F2

Pos.	63	obs:	exp:
ttt	F	0	5.13
ttc	F	13	7.87
tct	S	0	104.20
tcc	S	651	84.27
tca	S	0	164.30
tcg	S	0	37.88
agt	S	0	133.40
agc	S	0	127.00
mPD		0.038	1.7
		nPD:	0.02
		N. weight:	2.
		Sc. PD:	-0.21
		Sc. rank:	-913.0

PB1-F2

Pos.	64	obs:	exp:
tta	L	0	58.27
ttg	L	663	123.70
tct	S	0	0.16
tcc	S	0	0.13
tca	S	0	0.25
tcg	S	1	0.06
ctt	L	0	112.40
ctc	L	0	111.10
cta	L	0	110.50
ctg	L	0	147.00
agt	S	0	0.20
agc	S	0	0.20
mPD		0.0030	1.1
		nPD:	0.
		N. weight:	1.7
		Sc. PD:	-0.21
		Sc. rank:	-894.0

PB1-F2

Pos.	65	obs:	exp:
cgt	R	0	0.34
cgc	R	0	0.41
cga	R	0	0.80
cgg	R	0	0.67
aaa	K	0	384.30
aag	K	656	271.70
aga	R	0	3.65
agg	R	8	2.14
mPD		0.024	0.52
		nPD:	0.05
		N. weight:	0.88
		Sc. PD:	-0.073
		Sc. rank:	-231.8

PB1-F2

Pos.	66	obs:	exp:
tct	S	0	8.64
tcc	S	0	6.99
tca	S	0	13.63
tcg	S	0	3.14
aat	N	610	310.30
aac	N	0	299.70
agt	S	54	11.07
agc	S	0	10.53
mPD		0.15	0.77
		nPD:	0.19
		N. weight:	0.74
		Sc. PD:	0.043
		Sc. rank:	243.9

PB1-F2

Pos.	67	obs:	exp:
tta	L	0	50.01
ttg	L	0	106.20
ctt	L	0	96.42
ctc	L	569	95.33
cta	L	0	94.86
ctg	L	0	126.20
cct	P	0	23.65
ccc	P	93	16.58
cca	P	0	37.99
ccg	P	0	14.78
cat	H	0	1.15
cac	H	2	0.85
mPD		0.25	1.3
		nPD:	0.19
		N. weight:	1.8
		Sc. PD:	0.086
		Sc. rank:	501.1

PB1-F2

Pos.	68	obs:	exp:
att	I	0	63.85
atc	I	175	42.33
ata	I	0	68.82
act	T	0	126.70
acc	T	489	99.58
aca	T	0	224.70
acg	T	0	38.09
mPD		0.39	1.1
		nPD:	0.37
		N. weight:	1.5
		Sc. PD:	0.34
		Sc. rank:	781.3

PB1-F2

Pos.	69	obs:	exp:
tta	L	0	0.09
ttg	L	0	0.19
ctt	L	0	0.17
ctc	L	0	0.17
cta	L	0	0.17
ctg	L	1	0.22
cct	P	0	0.25
ccc	P	0	0.18
cca	P	0	0.41
ccg	P	1	0.16
caa	Q	0	339.50
cag	Q	661	321.50
cgt	R	0	0.04
cgc	R	0	0.05
cga	R	0	0.10
cgg	R	1	0.08
aga	R	0	0.46
agg	R	0	0.27
mPD		0.0090	0.51
		nPD:	0.02
		N. weight:	0.72
		Sc. PD:	-0.079
		Sc. rank:	-352.1

PB1-F2

Pos.	70	obs:	exp:
gtt	V	0	0.21
gtc	V	0	0.20
gta	V	1	0.21
gtg	V	0	0.39
gaa	E	46	27.67
gag	E	0	18.33
ggt	G	0	82.74
ggc	G	0	78.39
gga	G	617	285.30
ggg	G	0	170.60
mPD		0.13	0.80
		nPD:	0.17
		N. weight:	0.75
		Sc. PD:	0.022
		Sc. rank:	147.6

PB1-F2

Pos.	71	obs:	exp:
ttt	F	165	65.12
ttc	F	0	99.88
tct	S	499	79.88
tcc	S	0	64.59
tca	S	0	125.90
tcg	S	0	29.04
agt	S	0	102.30
agc	S	0	97.31
mPD		0.37	1.7
		nPD:	0.22
		N. weight:	1.6
		Sc. PD:	0.12
		Sc. rank:	614.1

PB1-F2

Pos.	72	obs:	exp:
tta	L	0	58.36
ttg	L	664	123.90
ctt	L	0	112.50
ctc	L	0	111.20
cta	L	0	110.70
ctg	L	0	147.30
mPD		0	1.1
		nPD:	0.
		N. weight:	1.7
		Sc. PD:	-0.21
		Sc. rank:	-953.2

PB1-F2

Pos.	73	obs:	exp:
caa	Q	1	0.51
cag	Q	0	0.49
cgt	R	0	0.26
cgc	R	0	0.30
cga	R	0	0.60
cgg	R	0	0.50
aat	N	0	1.02
aac	N	2	0.98
aaa	K	655	383.70
aag	K	0	271.30
aga	R	6	2.74
agg	R	0	1.60
mPD		0.027	0.52
		nPD:	0.05
		N. weight:	0.53
		Sc. PD:	-0.041
		Sc. rank:	-126.9

PB1-F2

Pos.	74	obs:	exp:
att	I	6	2.19
atc	I	0	1.45
ata	I	0	2.36
act	T	656	169.90
acc	T	0	133.60
aca	T	0	301.40
acg	T	0	51.10
gct	A	2	0.51
gcc	A	0	0.38
gca	A	0	0.94
gcg	A	0	0.18
mPD		0.024	0.70
		nPD:	0.03
		N. weight:	1.3
		Sc. PD:	-0.13
		Sc. rank:	-491.8

PB1-F2

Pos.	75	obs:	exp:
tta	L	0	2.55
ttg	L	0	5.41
ctt	L	29	4.91
ctc	L	0	4.86
cta	L	0	4.83
ctg	L	0	6.43
cat	H	42	24.13
cac	H	0	17.87
cgt	R	593	25.33
cgc	R	0	30.13
cga	R	0	59.13
cgg	R	0	49.39
aga	R	0	270.80
agg	R	0	158.30
---	--	------	------
mPD		0.20	1.3
		nPD:	0.15
		N. weight:	2.9
		Sc. PD:	0.055
		Sc. rank:	486.5

,

PB1-F2

Pos.	76	obs:	exp:
gtt	V	1	136.50
gtc	V	655	132.40
gta	V	5	136.90
gtg	V	0	255.30
gct	A	0	0.77
gcc	A	3	0.56
gca	A	0	1.40
gcg	A	0	0.27
---	--	------	------
mPD		0.027	0.73
		nPD:	0.04
		N. weight:	1.5
		Sc. PD:	-0.14
		Sc. rank:	-468.1

,

PB1-F2

Pos.	77	obs:	exp:
tta	L	0	9.32
ttg	L	106	19.78
tct	S	0	89.32
tcc	S	0	72.23
tca	S	0	140.80
tcg	S	558	32.47
ctt	L	0	17.96
ctc	L	0	17.76
cta	L	0	17.67
ctg	L	0	23.51
agt	S	0	114.30
agc	S	0	108.80
---	--	------	------
mPD		0.27	1.9
		nPD:	0.14
		N. weight:	2.6
		Sc. PD:	0.019
		Sc. rank:	265.6

,

PB1-F2

Pos.	78	obs:	exp:
cgt	R	0	1.62
cgc	R	0	1.93
cga	R	0	3.79
cgg	R	0	3.16
aaa	K	626	366.70
aag	K	0	259.30
aga	R	38	17.35
agg	R	0	10.14
---	--	------	------
mPD		0.11	0.63
		nPD:	0.17
		N. weight:	0.54
		Sc. PD:	0.02
		Sc. rank:	115.4

,

PB1-F2

Pos.	79	obs:	exp:
tta	L	0	0.26
ttg	L	0	0.56
ctt	L	0	0.51
ctc	L	0	0.50
cta	L	3	0.50
ctg	L	0	0.67
caa	Q	9	4.62
cag	Q	0	4.38
cgt	R	0	27.85
cgc	R	0	33.13
cga	R	652	65.01
cgg	R	0	54.30
aga	R	0	297.70
agg	R	0	174.00
---	--	------	------
mPD		0.036	1.0
		nPD:	0.04
		N. weight:	2.3
		Sc. PD:	-0.21
		Sc. rank:	-767.2

,

PB1-F2

Pos.	80	obs:	exp:
tgg	W	663	663.00
---	-	1	1.00
---	--	------	------
mPD		0	0
		nPD:	1.
		N. weight:	0.
		Sc. PD:	0
		Sc. rank:	0

,

PB1-F2

Pos.	81	obs:	exp:
cgt	R	0	0.21
cgc	R	0	0.25
cga	R	0	0.50
cgg	R	0	0.42
aat	N	1	0.51
aac	N	0	0.49
aaa	K	578	384.90
aag	K	79	272.10
aga	R	2	2.28
agg	R	3	1.33
---	-	1	1.00
---	--	------	------
mPD		0.23	0.51
		nPD:	0.46
		N. weight:	0.22
		Sc. PD:	0.067
		Sc. rank:	119.0

,

PB1-F2

Pos.	82	obs:	exp:
tta	L	4	56.51
ttg	L	639	120.00
tct	S	0	3.20
tcc	S	0	2.59
tca	S	0	5.05
tcg	S	20	1.16
ctt	L	0	109.00
ctc	L	0	107.70
cta	L	0	107.20
ctg	L	0	142.60
agt	S	0	4.10
agc	S	0	3.90
---	-	1	1.00
---	--	------	------
mPD		0.070	1.2
		nPD:	0.06
		N. weight:	1.7
		Sc. PD:	-0.12
		Sc. rank:	-320.4

,

PB1-F2

Pos.	83	obs:	exp:
ttt	F	0	243.50
ttc	F	617	373.50
tct	S	0	7.36
tcc	S	46	5.95
tca	S	0	11.61
tcg	S	0	2.68
agt	S	0	9.43
agc	S	0	8.97
---	-	1	1.00
---	--	------	------
mPD		0.13	0.69
		nPD:	0.19
		N. weight:	0.6
		Sc. PD:	0.031
		Sc. rank:	176.3

,

PB1-F2

Pos.	84	obs:	exp:
tct	S	0	99.41
tcc	S	0	80.38
tca	S	0	156.70
tcg	S	0	36.14
aat	N	0	21.37
aac	N	42	20.63
agt	S	0	127.30
agc	S	621	121.10
---	-	1	1.00
---	--	------	------
mPD		0.12	1.7
		nPD:	0.07
		N. weight:	1.6
		Sc. PD:	-0.096
		Sc. rank:	-209.4

,

PB1-F2

Pos.	85	obs:	exp:
cgt	R	0	0.68
cgc	R	0	0.81
cga	R	0	1.60
cgg	R	0	1.33
aaa	K	647	379.00
aag	K	0	268.00
aga	R	16	7.31
agg	R	0	4.27
---	-	1	1.00
---	--	------	------
mPD		0.047	0.55
		nPD:	0.09
		N. weight:	0.54
		Sc. PD:	-0.024
		Sc. rank:	-51.1

,

PB1-F2

Pos.	86	obs:	exp:
tta	L	0	2.29
ttg	L	0	4.85
ctt	L	0	4.41
ctc	L	0	4.36
cta	L	26	4.33
ctg	L	0	5.77
cat	H	0	0.57
cac	H	1	0.43
caa	Q	633	325.10
cag	Q	0	307.90
cgt	R	0	0.13
cgc	R	0	0.15
cga	R	3	0.30
cgg	R	0	0.25
aga	R	0	1.37
agg	R	0	0.80
---	-	1	1.00
---	--	------	------
mPD		0.087	0.62
		nPD:	0.14
		N. weight:	0.71
		Sc. PD:	0.0036
		Sc. rank:	47.8

,

PB1-F2

Pos.	87	obs:	exp:
gaa	E	11	397.60
gag	E	650	263.40
ggt	G	0	0.27
ggc	G	0	0.25
gga	G	0	0.92
ggg	G	2	0.55
---	-	1	1.00
---	--	------	------
mPD		0.039	0.48
		nPD:	0.08
		N. weight:	0.83
		Sc. PD:	-0.042
		Sc. rank:	-92.5

,

PB1-F2

Pos.	88	obs:	exp:
tta	L	0	0.26
ttg	L	3	0.56
tgg	W	657	657.00
ctt	L	0	0.51
ctc	L	0	0.50
cta	L	0	0.50
ctg	L	0	0.67
---	-	4	4.00
---	--	------	------
mPD		0.0090	0.021
		nPD:	0.43
		N. weight:	0.0075
		Sc. PD:	0.0021
		Sc. rank:	4.1

,

PB1-F2

Pos.	89	obs:	exp:
att	I	0	166.70
atc	I	0	110.50
ata	I	457	179.70
act	T	0	52.59
acc	T	0	41.34
aca	T	203	93.26
acg	T	0	15.81
---	-	4	4.00
---	--	------	------
mPD		0.42	1.1
		nPD:	0.39
		N. weight:	0.87
		Sc. PD:	0.21
		Sc. rank:	454.5

,

PB1-F2

Pos.	90	obs:	exp:
tct	S	0	3.52
tcc	S	0	2.85
tca	S	0	5.55
tcg	S	0	1.28
aat	N	14	324.60
aac	N	624	313.40
agt	S	20	4.51
agc	S	2	4.29
---	-	4	4.00
---	--	------	------
mPD		0.16	0.61
		nPD:	0.26
		N. weight:	0.62
		Sc. PD:	0.077
		Sc. rank:	283.2

,

PB1-F2

Pos.	91	obs:	exp:
taa	.	57	285.60
tag	.	4	167.90
tga	.	603	210.50
---	--	------	------
mPD		0.18	0.81
		nPD:	0.22
		N. weight:	0.8
		Sc. PD:	0.066
		Sc. rank:	314.2

}

Amino acid level conservation per codon

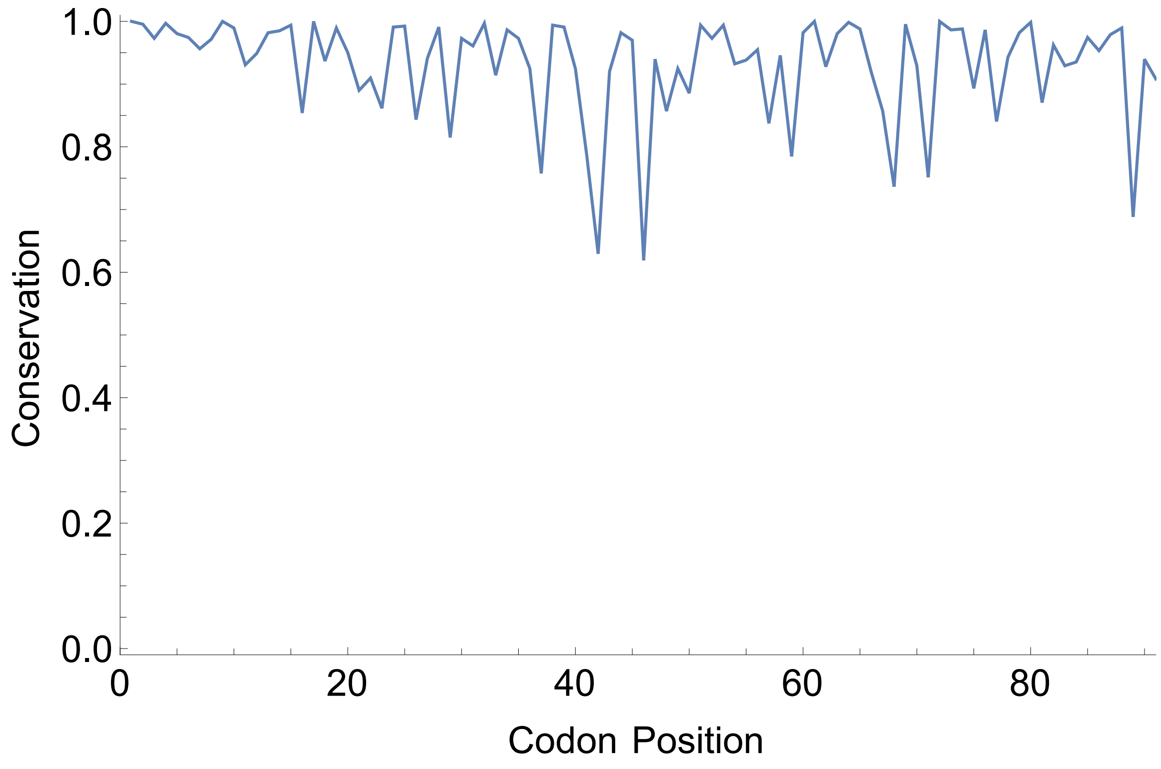

Conservation analysis plots

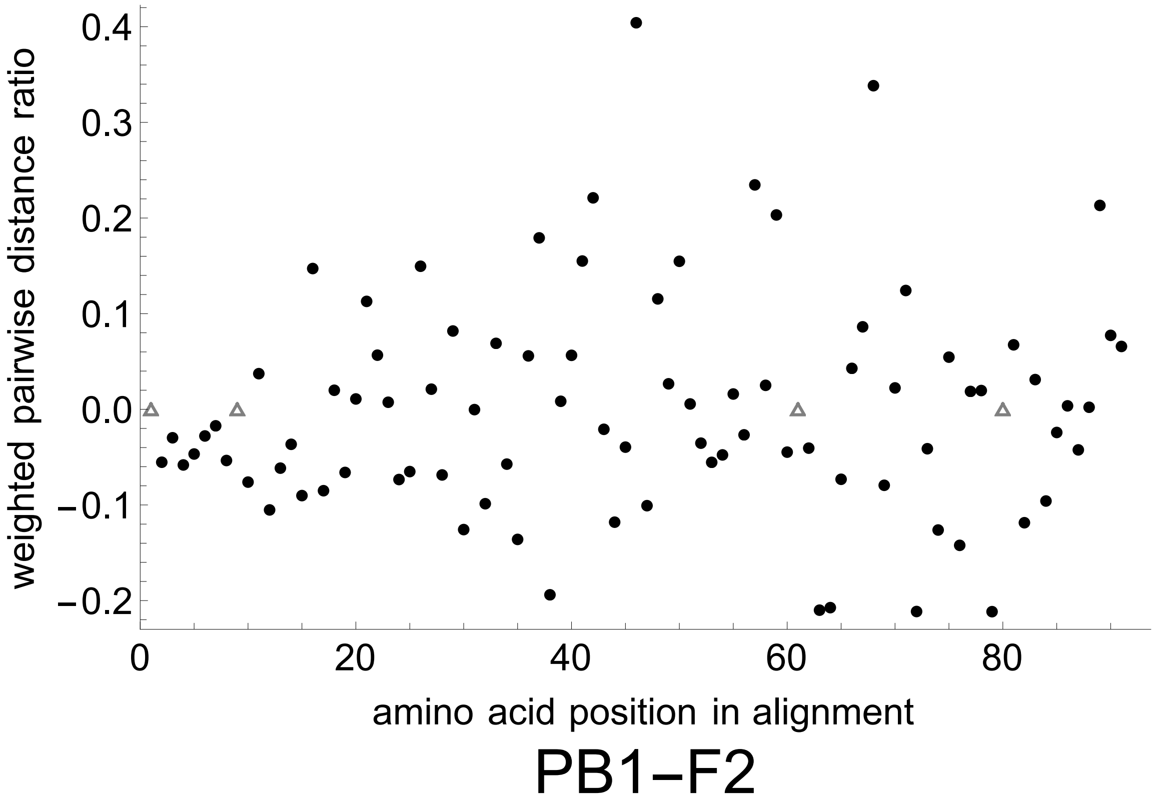

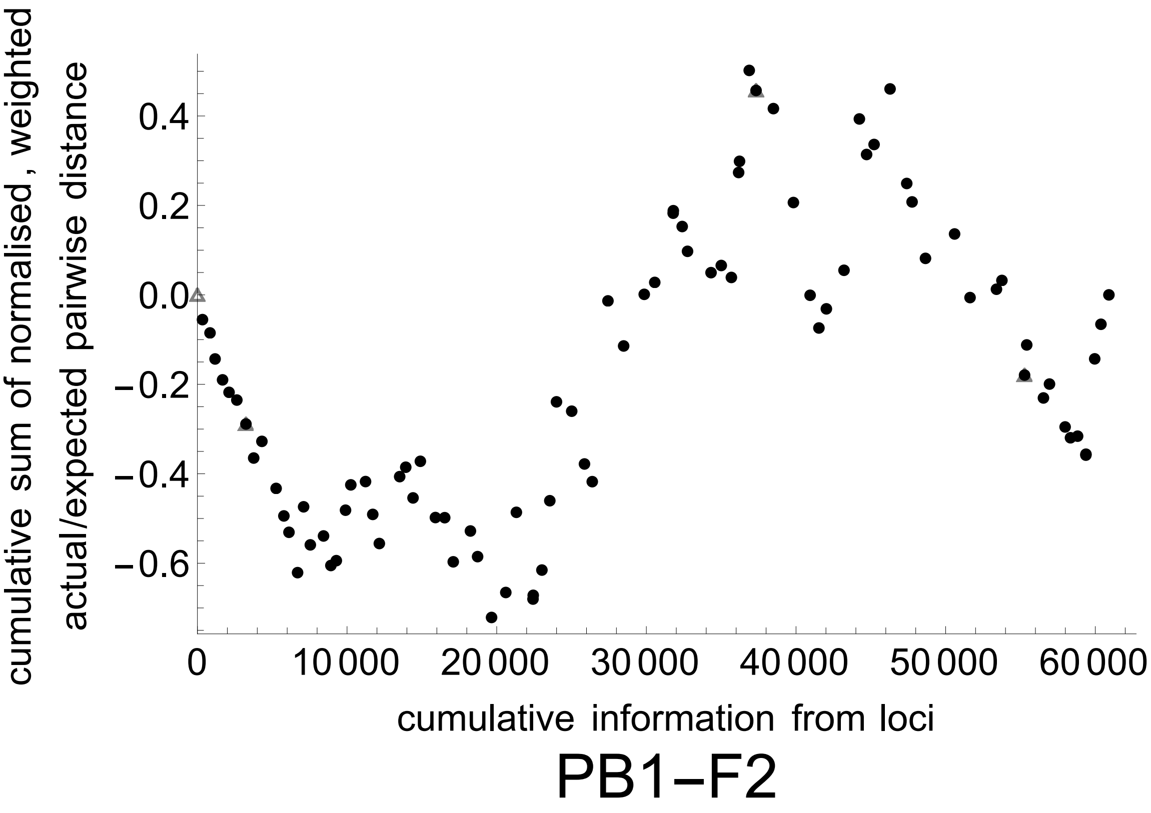

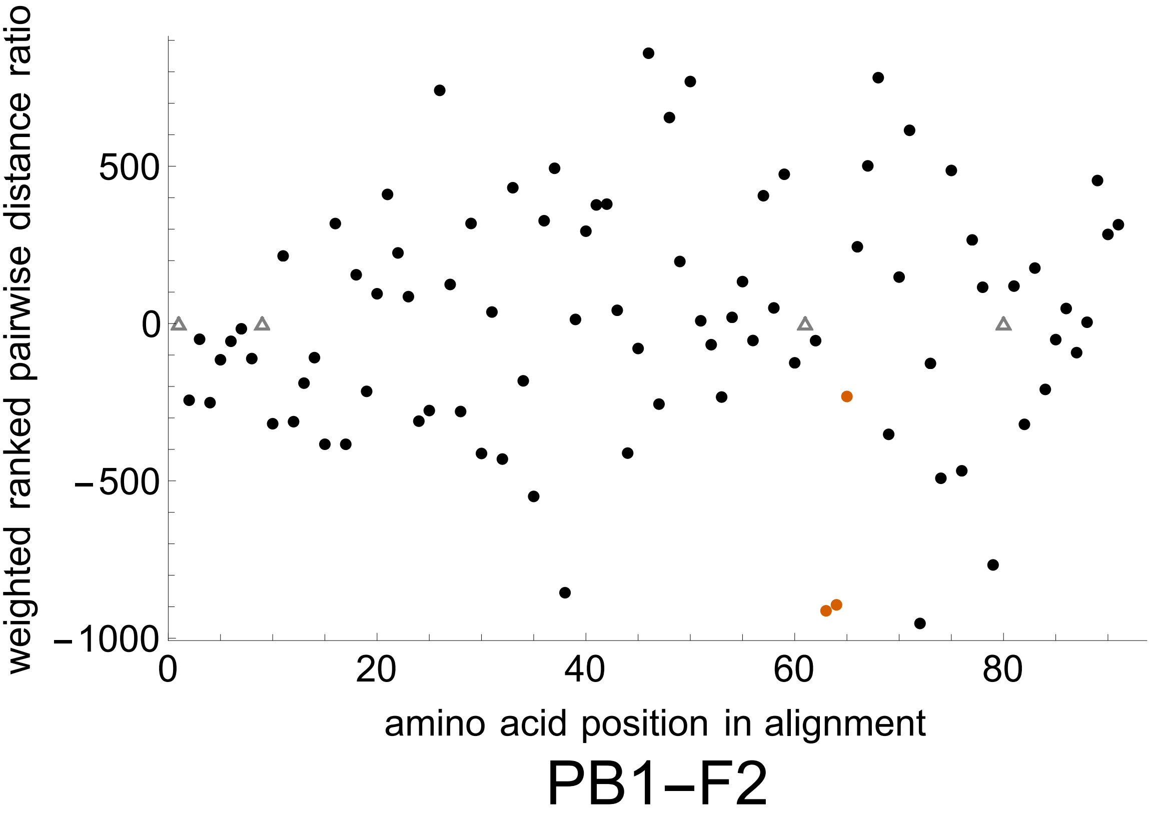
weighted ranked pairwise distance ratio
500
0
−500
−1000
0
20
40
60
80
amino acid position in alignment
PB1−F2

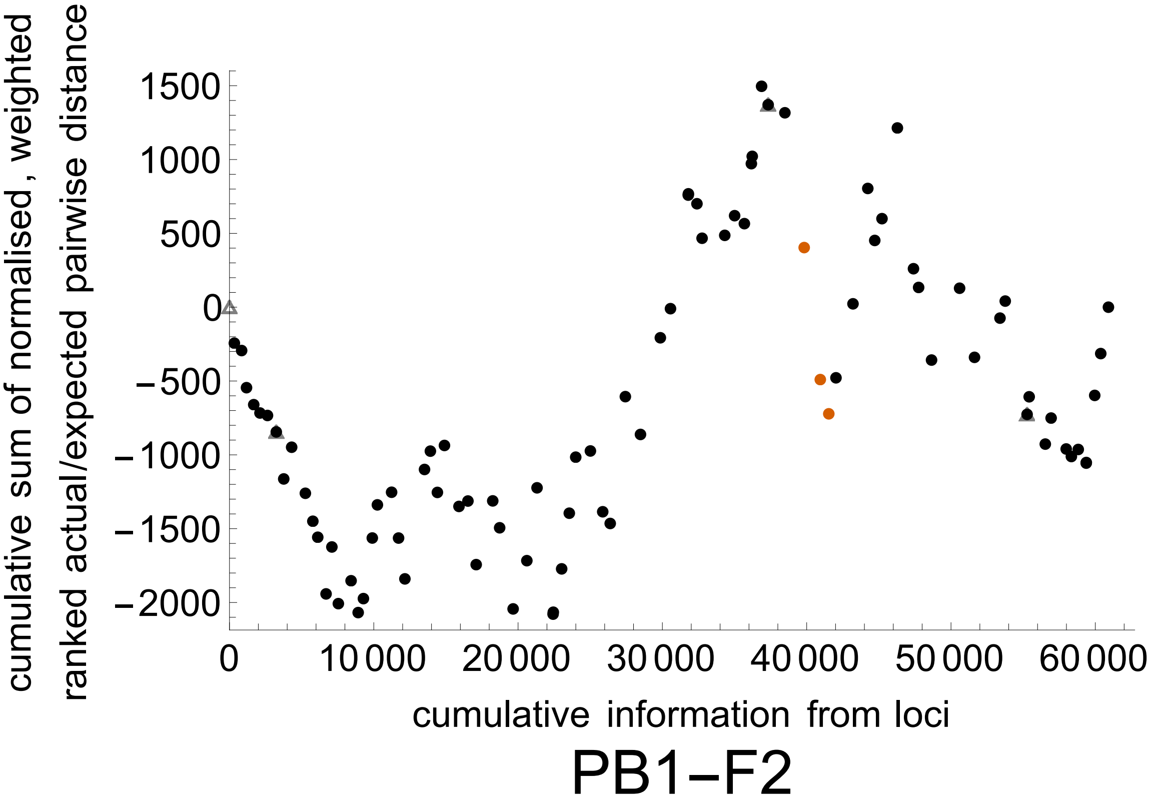
cumulative sum of normalised, weighted ranked actual/expected pairwise distance
1500
1000
500
0
−500
−1000
−1500
−2000
0
10 000
20 000
30 000
40 000
50 000
60 000
cumulative information from loci
PB1−F2

PA

Gene length histogram

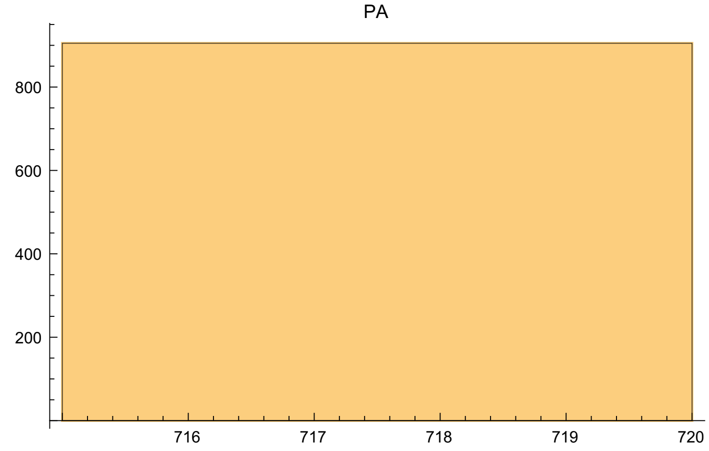

Information vs. nPD

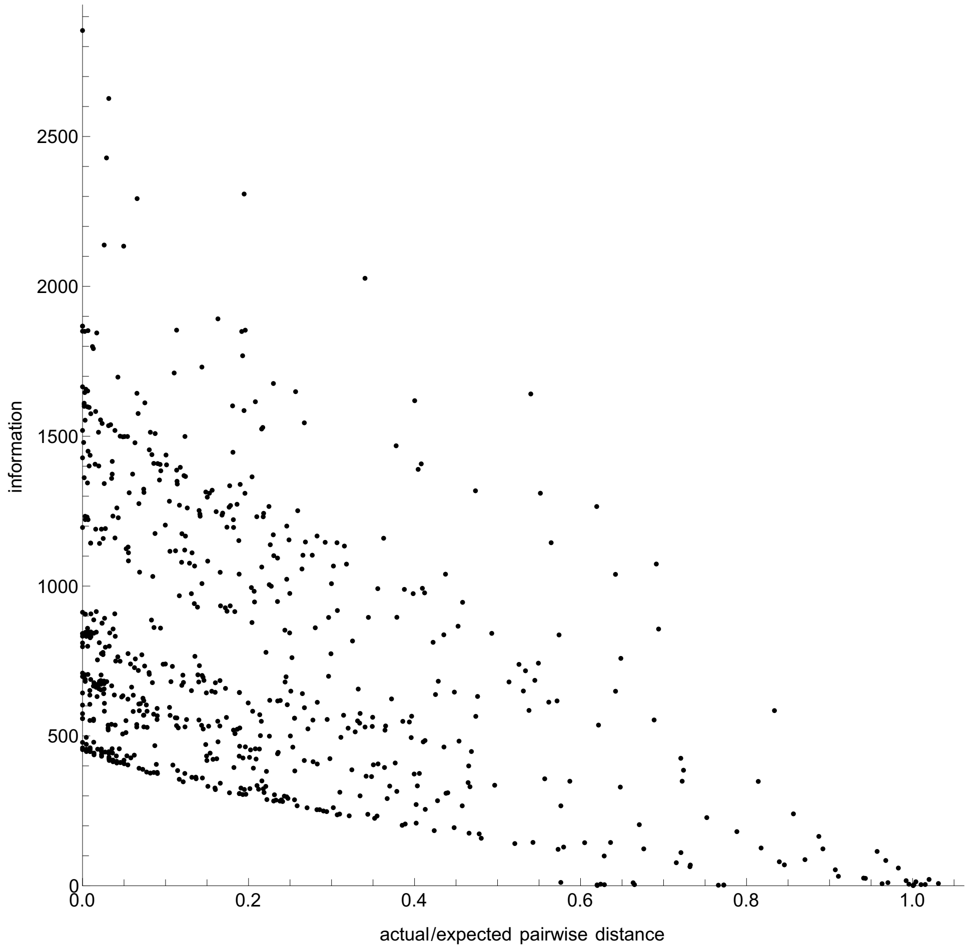

PA

Example sequences highlighted by regions found in analyses to be conserved

Interesting points (by weighted raw PD) highlighted for gene PA:

ATG
GAAGATTTTGTGCGACAATGCTTCAATCCAATGATTGTCGAGCTTGCGGAAAAGG
CAATGAAAGAATATGGGGAAGATCCGAAAATCGAAACGAACAAATTTGCCGC
AATATGCACACACTTAGAGGTCTGTTTCATGTATTCGGATTTCCACTTTATTGATGAACGGGGCGA⋱
ATCAATAATTGTAGAATCTGGCGATCCAAATGCATTATTGAAACACCGATTTGAGATAATTGAAGGGAGA⋱
GACCGAACGATGGCCTGGACAGTGGTGAATAGTATCTGCAACACCACAGGAGTCGAGAAACCTAAATTTC⋱
TCCCAGATTTGTATGACTACAAAGAGAATCGATTCATTGAAATTGGAGTAACACGGAGGGAAGTTCATAT⋱
ATACTATCTAGAAAAGGCCAACAAGATAAAATCAGAGAAGACACACATTCACATATTCTCATTCACTGGA⋱
GAGGAAATGGCCACCAAAGCGGACTACACTCTTGACGAAGAGAGTAGGGCAAGAATCAAAACCAGGCTGT⋱
TCACTATAAGGCAGGAAATGGCCAGTAGGGGTCTATGG
GATTCCTTTCGTCAGTCCGAGAGAGGCGAAGAGACAGTTGAAGAAAGATTTGAAA
TCACAGGAACCATGCGCAGGCTTGCCGACCAAAGTCTCCCACCGAACTTCTC
CAGCCTTGAAAACTTTAGAGCCTATGTGGATGGATTCGAACCGAACGGCTGC
ATTGAGGGCAAGCTTTCTCAAATGTCAAAAGAAGTGAACGCCCGAATTGAGCCATTTCT⋱
GAAGACAACACCACGCCCTCTCAAACTACCTGACGGGCCTCCCTGCTCTCAACGGTCGAAGTTCCTGCTG⋱
ATGGATGCCCTTAAATTAAGCATCGAAGACCCGAGTCATGAGGGGGAGGGTATACCGCTATATGATGCAA⋱
TCAAATGCATGAAGACATTTTTCGGCTGGAAAGAGCCCAACATTGTAAAACCACATGAAAAGGGCATAAA⋱
CCCCAATTACCTCCTGGCTTGGAAGCAAGTGCTGGCAGAACTCCAAGATATTGAAAATGAGGAGAAAATC⋱
CCAAAAACAAAGAACATGAAGAAAACGAGCCAGTTGAAGTGGGCACTTGGTGAGAATATGGCACCGGAGA⋱
AGGTAGACTTTGAGGATTGCAAGGATGTTAGCGATCTGAGACAGTATGACAGTGATGAACCAGAGTCTAG⋱
ATCGCTAGCAAGCTGGATCCAGAGTGAATTCAACAAGGCATGTGAATTGACAGATTCAAGTTGGATTGAG⋱
CTTGATGAAATAGGGGAAGACATTGCTCCAATTGAGCACATTGCGAGTATGAGAAGAAACTACTTCACAG⋱
CGGAAGTATCCCATTGCAGGGCTACTGAATACATAATGAAAGGAGTGTACATAAACACAGCCTTGTTGAA⋱
TGCATCCTGTGCAGCCATGGATGACTTCCAACTGATTCCAATGATAAGCAAATGCAGGACCAAAGAAGGG⋱
AGGCGGAAGACTAATCTGTATGGATTCATTATAAAAGGAAGATCCCATTTGAGAAATGACACCGATGTAG⋱
TAAACTTTGTGAGCATGGAATTCTCTCTTACTGACCCGAGGCTGGAG
CCACACAAGTGGGAAAAGTACTGTGTTCTCGAGATAGGAGACATGCTCCTACGGA
CTGCAATAGGCCAAGTGTCAAGGCCCATGTTCCTGTATGTGAGAACCAATGG
GACTTCCAAGATCAAGATGAAGTGGGGCATGGAAATGAGGCGATGCCTTCTT
CAATCCCTTCAACAAATTGAGAGCATGATTGAAGCCGAGTCTTCTGTCAAAG
AGAAGGACATGACCAAAGAATTCTTTGAAAACAAATCAGAAACATGGCCAAT
TGGAGAGTCACCCAAAGGGGTGGAGGAAGGCTCCATTGGGAAGGTGTGCAGA
ACCTTACTGGCAAAATCTGTATTCAACAGCCTATATGCATCTCCACAACTCG
AGGGATTTTCAGCTGAATCAAGAAAGTTGCTTCTCATTGTCCAGGCA
CTTAGGGACAACCTGGAACCTGGGACCTTCGATCTTGGGGGGCTATATGAAGCAA
TTGAGGAGTGCCTGATTAATGATCCCTGGGTTTTGCTTAATGCGTCTTGGTT
CAACTCCTTCCTCACACATGCACTGAAATAG

Interesting points (by weighted ranked PD) highlighted for gene PA:

ATG
GAAGATTTTGTGCGACAATGCTTCAATCCAATGATTGTCGAGCTTGCGGAAAAGG
CAATGAAAGAATATGGGGAAGATCCGAAAATCGAAACGAACAAATTTGCCGCAATATGCACA⋱
CACTTAGAGGTCTGTTTCATGTATTCGGATTTCCACTTTATTGATGAACGGGGCGAATCAATAATTGTAG⋱

AATCTGGCGATCCAAATGCATTATTGAAACACCGATTTGAGATAATTGAAGGGAGAGACCGAACGATGGC⋱
CTGGACAGTGGTGAATAGTATCTGCAACACCACAGGAGTCGAGAAACCTAAATTTCTCCCAGATTTGTAT⋱
GACTACAAAGAGAATCGATTCATTGAAATTGGAGTAACACGGAGGGAAGTTCATATATACTATCTAGAAA⋱
AGGCCAACAAGATAAAATCAGAGAAGACACACATTCACATATTCTCATTCACTGGAGAGGAAATGGCCAC⋱
CAAAGCGGACTACACTCTTGACGAAGAGAGTAGGGCAAGAATCAAAACCAGGCTGTTCACTATAAGGCAG⋱
GAAATGGCCAGTAGGGGTCTATGG
GATTCCTTTCGTCAGTCCGAGAGAGGCGAAGAGACAGTTGAAGAAAGATTTGAAA
TCACAGGAACCATGCGCAGGCTTGCCGACCAAAGTCTCCCACCGAACTTCTC
CAGCCTTGAAAACTTTAGAGCCTATGTGGATGGATTCGAACCGAACGGCTGC
ATTGAGGGCAAGCTTTCTCAAATGTCAAAAGAAGTGAACGCCCGAATTGAGCCATTTCT⋱
GAAGACAACACCACGCCCTCTCAAACTACCTGACGGGCCTCCCTGCTCTCAACGGTCGAAGTTCCTGCTG⋱
ATGGATGCCCTTAAATTAAGCATCGAAGACCCGAGTCATGAGGGGGAGGGTATACCGCTATATGATGCAA⋱
TCAAATGCATGAAGACATTTTTCGGCTGGAAAGAGCCCAACATTGTAAAACCACATGAAAAGGGCATAAA⋱
CCCCAATTACCTCCTGGCTTGGAAGCAAGTGCTGGCAGAACTCCAAGATATTGAAAATGAGGAGAAAATC⋱
CCAAAAACAAAGAACATGAAGAAAACGAGCCAGTTGAAGTGGGCACTTGGTGAGAATATGGCACCGGAGA⋱
AGGTAGACTTTGAGGATTGCAAGGATGTTAGCGATCTGAGACAGTATGACAGTGATGAACCAGAGTCTAG⋱
ATCGCTAGCAAGCTGGATCCAGAGTGAATTCAACAAGGCATGTGAATTGACAGATTCAAGTTGGATTGAG⋱
CTTGATGAAATAGGGGAAGACATTGCTCCAATTGAGCACATTGCGAGTATGAGAAGAAACTACTTCACAG⋱
CGGAAGTATCCCATTGCAGGGCTACTGAATACATAATGAAAGGAGTGTACATAAACACAGCCTTGTTGAA⋱
TGCATCCTGTGCAGCCATGGATGACTTCCAACTGATTCCAATGATAAGCAAATGCAGGACCAAAGAAGGG⋱
AGGCGGAAGACTAATCTGTATGGATTCATTATAAAAGGAAGATCCCATTTGAGAAATGACACCGATGTAG⋱
TAAACTTTGTGAGCATGGAATTCTCTCTTACTGACCCGAGGCTGGAGCCACACAAGTGGGAAAAGTACTG⋱
TGTTCTCGAGATAGGAGACATGCTCCTACGGACTGCAATAGGCCAAGTGTCAAGGCCCATGTTCCTGTAT⋱
GTGAGAACCAATGGGACTTCCAAGATCAAGATGAAGTGGGGCATGGAAATGAGGCGATGCCTTCTTCAAT⋱
CCCTTCAACAAATTGAGAGCATGATTGAAGCCGAGTCTTCTGTCAAAGAGAAGGACATGACCAAAGAATT⋱
CTTTGAAAACAAATCAGAAACATGGCCAATTGGAGAGTCACCCAAAGGGGTGGAGGAAGGCTCCATTGGG⋱
AAGGTGTGCAGAACCTTACTGGCAAAATCTGTATTCAACAGCCTATATGCATCTCCACAACTCGAGGGAT⋱
TTTCAGCTGAATCAAGAAAGTTGCTT
CTCATTGTCCAGGCACTTAGGGACAACCTGGAACCTGGGACCTTCGATCTTGGGG
GGCTATATGAAGCAATTGAGGAGTGCCTGATTAATGATCCCTGGGTTTTGCT
TAATGCGTCTTGGTTCAACTCCTTCCTCACACATGCACTGAAATAG

Per codon conservation report

{

PA			
Pos.	1	obs:	exp:
atg	M	905	905.00
---	--	------	------
mPD		0	0
		nPD:	1.
		N. weight:	0.
		Sc. PD:	0
		Sc. rank:	0

,

PA			
Pos.	2	obs:	exp:
gaa	E	905	544.40
gag	E	0	360.60
---	--	------	------
mPD		0	0.48
		nPD:	0.
		N. weight:	0.62
		Sc. PD:	-0.092
		Sc. rank:	-396.6

,

PA			
Pos.	3	obs:	exp:
gat	D	43	487.50
gac	D	860	415.50
gaa	E	1	0.60
gag	E	0	0.40
ggt	G	0	0.13
ggc	G	1	0.13
gga	G	0	0.46
ggg	G	0	0.28
---	--	------	------
mPD		0.095	0.50
		nPD:	0.19
		N. weight:	0.71
		Sc. PD:	0.025
		Sc. rank:	165.7

,

PA			
Pos.	4	obs:	exp:
ttt	F	902	356.00
ttc	F	0	546.00
tta	L	0	0.26
ttg	L	1	0.56
ctt	L	2	0.51
ctc	L	0	0.50
cta	L	0	0.50
ctg	L	0	0.67
---	--	------	------
mPD		0.0066	0.48
		nPD:	0.01
		N. weight:	1.1
		Sc. PD:	-0.15
		Sc. rank:	-475.0

,

PA			
Pos.	5	obs:	exp:
atg	M	2	2.00
gtt	V	0	186.40
gtc	V	0	180.90
gta	V	0	187.00
gtg	V	903	348.70
---	--	------	------
mPD		0.0044	0.73
		nPD:	0.01
		N. weight:	1.2
		Sc. PD:	-0.17
		Sc. rank:	-583.3

,

PA			
Pos.	6	obs:	exp:
cgt	R	0	38.62
cgc	R	0	45.93
cga	R	803	90.14
cgg	R	1	75.29
aga	R	100	412.70
agg	R	0	241.30
ggt	G	0	0.13
ggc	G	0	0.13
gga	G	1	0.46
ggg	G	0	0.28
---	--	------	------
mPD		0.20	0.97
		nPD:	0.21
		N. weight:	2.2
		Sc. PD:	0.12
		Sc. rank:	636.0

,

PA			
Pos.	7	obs:	exp:
caa	Q	564	464.80
cag	Q	341	440.20
---	--	------	------
mPD		0.47	0.50
		nPD:	0.94
		N. weight:	0.035
		Sc. PD:	0.026
		Sc. rank:	22.1

,

PA			
Pos.	8	obs:	exp:
tgt	C	4	371.50
tgc	C	901	533.50
---	--	------	------
mPD		0.0088	0.48
		nPD:	0.02
		N. weight:	0.62
		Sc. PD:	-0.081
		Sc. rank:	-238.2

,

PA			
Pos.	9	obs:	exp:
ttt	F	4	356.80
ttc	F	900	547.20
tat	Y	0	0.53
tac	Y	1	0.47
---	--	------	------
mPD		0.011	0.48
		nPD:	0.02
		N. weight:	0.59
		Sc. PD:	-0.074
		Sc. rank:	-203.9

,

PA			
Pos.	10	obs:	exp:
cat	H	1	0.57
cac	H	0	0.43
aat	N	899	459.90
aac	N	5	444.10
---	--	------	------
mPD		0.013	0.50
		nPD:	0.03
		N. weight:	0.79
		Sc. PD:	-0.097
		Sc. rank:	-250.5

,

PA			
Pos.	11	obs:	exp:
tta	L	0	0.09
ttg	L	0	0.19
ctt	L	0	0.17
ctc	L	0	0.17
cta	L	1	0.17
ctg	L	0	0.22
cct	P	0	229.90
ccc	P	0	161.20
cca	P	897	369.30
ccg	P	7	143.60
---	--	------	------
mPD		0.018	0.71
		nPD:	0.02
		N. weight:	1.1
		Sc. PD:	-0.13
		Sc. rank:	-356.9

,

PA			
Pos.	12	obs:	exp:
att	I	0	0.36
atc	I	0	0.24
ata	I	1	0.39
atg	M	904	904.00
---	--	------	------
mPD		0.0022	0.0022
		nPD:	1.
		N. weight:	0.0013
		Sc. PD:	0.001
		Sc. rank:	0.8

,

PA			
Pos.	13	obs:	exp:
att	I	194	328.00
atc	I	705	217.40
ata	I	0	353.50
gtt	V	1	1.24
gtc	V	5	1.20
gta	V	0	1.24
gtg	V	0	2.32
---	--	------	------
mPD		0.35	0.67
		nPD:	0.53
		N. weight:	1.
		Sc. PD:	0.36
		Sc. rank:	584.3

,

PA			
Pos.	14	obs:	exp:
gtt	V	0	186.80
gtc	V	902	181.30
gta	V	3	187.40
gtg	V	0	349.50
---	--	------	------
mPD		0.0066	0.73
		nPD:	0.01
		N. weight:	1.9
		Sc. PD:	-0.27
		Sc. rank:	-894.9

,

PA			
Pos.	15	obs:	exp:
gaa	E	14	544.40
gag	E	891	360.60
---	--	------	------
mPD		0.030	0.48
		nPD:	0.06
		N. weight:	1.
		Sc. PD:	-0.089
		Sc. rank:	-151.4

,

PA			
Pos.	16	obs:	exp:
tta	L	0	79.54
ttg	L	0	168.90
ctt	L	900	153.40
ctc	L	3	151.60
cta	L	2	150.90
ctg	L	0	200.70
---	--	------	------
mPD		0.011	1.1
		nPD:	0.01
		N. weight:	2.1
		Sc. PD:	-0.3
		Sc. rank:	-940.0

,

PA			
Pos.	17	obs:	exp:
gct	A	0	231.90
gcc	A	0	169.90
gca	A	8	423.10
gcg	A	897	80.05
---	--	------	------
mPD		0.018	0.67
		nPD:	0.03
		N. weight:	2.9
		Sc. PD:	-0.36
		Sc. rank:	-932.9

,

PA			
Pos.	18	obs:	exp:
gat	D	1	1.62
gac	D	2	1.38
gaa	E	894	542.60
gag	E	8	359.40
---	--	------	------
mPD		0.024	0.48
		nPD:	0.05
		N. weight:	0.57
		Sc. PD:	-0.057
		Sc. rank:	-111.6

,

PA			
Pos.	19	obs:	exp:
cgt	R	0	0.04
cgc	R	0	0.05
cga	R	0	0.10
cgg	R	0	0.08
atg	M	2	2.00
aaa	K	10	528.40
aag	K	892	373.60
aga	R	0	0.46
agg	R	1	0.27
---	--	------	------
mPD		0.028	0.49
		nPD:	0.06
		N. weight:	1.
		Sc. PD:	-0.093
		Sc. rank:	-165.1

,

PA			
Pos.	20	obs:	exp:
act	T	0	27.20
acc	T	0	21.38
aca	T	105	48.24
acg	T	0	8.18
gtt	V	0	0.21
gtc	V	0	0.20
gta	V	1	0.21
gtg	V	0	0.39
gct	A	11	204.50
gcc	A	0	149.80
gca	A	787	373.10
gcg	A	0	70.58
gaa	E	1	0.60
gag	E	0	0.40
---	--	------	------
mPD		0.23	0.88
		nPD:	0.26
		N. weight:	0.87
		Sc. PD:	0.093
		Sc. rank:	351.1

,

PA			
Pos.	21	obs:	exp:
atg	M	905	905.00
---	--	------	------
mPD		0	0
		nPD:	1.
		N. weight:	0.
		Sc. PD:	0
		Sc. rank:	0

,

PA			
Pos.	22	obs:	exp:
cgt	R	0	0.73
cgc	R	0	0.86
cga	R	0	1.70
cgg	R	0	1.42
aaa	K	851	520.20
aag	K	37	367.80
aga	R	17	7.76
agg	R	0	4.54
---	--	------	------
mPD		0.12	0.53
		nPD:	0.22
		N. weight:	0.47
		Sc. PD:	0.029
		Sc. rank:	146.2

,

PA			
Pos.	23	obs:	exp:
gaa	E	869	544.40
gag	E	36	360.60
---	--	------	------
mPD		0.076	0.48
		nPD:	0.16
		N. weight:	0.44
		Sc. PD:	0.0025
		Sc. rank:	62.5

,

PA			
Pos.	24	obs:	exp:
tat	Y	902	479.80
tac	Y	3	425.20
---	--	------	------
mPD		0.0066	0.50
		nPD:	0.01
		N. weight:	0.75
		Sc. PD:	-0.1
		Sc. rank:	-317.3

,

PA			
Pos.	25	obs:	exp:
ggt	G	0	121.40
ggc	G	0	115.00
gga	G	3	418.40
ggg	G	902	250.20
---	--	------	------
mPD		0.0066	0.68
		nPD:	0.01
		N. weight:	1.5
		Sc. PD:	-0.22
		Sc. rank:	-695.1

,

PA			
Pos.	26	obs:	exp:
gaa	E	895	544.40
gag	E	10	360.60
---	--	------	------
mPD		0.022	0.48
		nPD:	0.05
		N. weight:	0.56
		Sc. PD:	-0.058
		Sc. rank:	-117.9

,

PA			
Pos.	27	obs:	exp:
aat	N	3	2.03
aac	N	1	1.97
gat	D	898	486.40
gac	D	3	414.60
---	--	------	------
mPD		0.018	0.51
		nPD:	0.03
		N. weight:	0.73
		Sc. PD:	-0.084
		Sc. rank:	-197.3

,

PA			
Pos.	28	obs:	exp:
tta	L	0	0.44
ttg	L	0	0.93
ctt	L	0	0.85
ctc	L	0	0.84
cta	L	0	0.83
ctg	L	5	1.11
cct	P	0	228.80
ccc	P	0	160.50
cca	P	67	367.70
ccg	P	833	143.00
---	--	------	------
mPD		0.15	0.73
		nPD:	0.2
		N. weight:	1.8
		Sc. PD:	0.09
		Sc. rank:	515.1

,

PA			
Pos.	29	obs:	exp:
cgt	R	0	0.17
cgc	R	0	0.20
cga	R	0	0.40
cgg	R	0	0.33
aaa	K	855	527.80
aag	K	46	373.20
aga	R	4	1.83
agg	R	0	1.07
---	--	------	------
mPD		0.11	0.50
		nPD:	0.21
		N. weight:	0.44
		Sc. PD:	0.025
		Sc. rank:	129.3

,

PA			
Pos.	30	obs:	exp:
att	I	37	329.10
atc	I	864	218.20
ata	I	1	354.70
gtt	V	0	0.62
gtc	V	3	0.60
gta	V	0	0.62
gtg	V	0	1.16
---	--	------	------
mPD		0.087	0.66
		nPD:	0.13
		N. weight:	1.5
		Sc. PD:	-0.031
		Sc. rank:	111.1

,

PA			
Pos.	31	obs:	exp:
aaa	K	1	0.59
aag	K	0	0.41
gaa	E	838	543.80
gag	E	66	360.20
---	--	------	------
mPD		0.14	0.48
		nPD:	0.29
		N. weight:	0.34
		Sc. PD:	0.044
		Sc. rank:	148.4

,

PA			
Pos.	32	obs:	exp:
act	T	6	234.40
acc	T	8	184.30
aca	T	853	415.80
acg	T	38	70.50
---	--	------	------
mPD		0.11	0.67
		nPD:	0.16
		N. weight:	0.74
		Sc. PD:	0.0067
		Sc. rank:	112.2

,

PA			
Pos.	33	obs:	exp:
aat	N	6	460.40
aac	N	899	444.60
---	--	------	------
mPD		0.013	0.50
		nPD:	0.03
		N. weight:	0.82
		Sc. PD:	-0.1
		Sc. rank:	-260.6

,

PA			
Pos.	34	obs:	exp:
aaa	K	900	530.10
aag	K	5	374.90
---	--	------	------
mPD		0.011	0.49
		nPD:	0.02
		N. weight:	0.62
		Sc. PD:	-0.078
		Sc. rank:	-219.6

,

PA			
Pos.	35	obs:	exp:
ttt	F	131	356.80
ttc	F	773	547.20
tta	L	0	0.09
ttg	L	0	0.19
ctt	L	0	0.17
ctc	L	1	0.17
cta	L	0	0.17
ctg	L	0	0.22
---	--	------	------
mPD		0.25	0.48
		nPD:	0.52
		N. weight:	0.19
		Sc. PD:	0.068
		Sc. rank:	111.1

,

PA			
Pos.	36	obs:	exp:
tct	S	0	0.16
tcc	S	0	0.13
tca	S	1	0.25
tcg	S	0	0.06
agt	S	0	0.20
agc	S	0	0.20
gtt	V	0	0.21
gtc	V	0	0.20
gta	V	1	0.21
gtg	V	0	0.39
gct	A	55	231.40
gcc	A	18	169.60
gca	A	828	422.20
gcg	A	2	79.87
---	--	------	------
mPD		0.16	0.68
		nPD:	0.23
		N. weight:	0.6
		Sc. PD:	0.047
		Sc. rank:	211.7

,

PA			
Pos.	37	obs:	exp:
tct	S	0	129.50
tcc	S	0	104.70
tca	S	809	204.20
tcg	S	0	47.08
agt	S	0	165.80
agc	S	0	157.80
gct	A	0	24.60
gcc	A	0	18.03
gca	A	88	44.88
gcg	A	8	8.49
---	--	------	------
mPD		0.21	1.7
		nPD:	0.12
		N. weight:	1.6
		Sc. PD:	-0.052
		Sc. rank:	59.2

,

PA			
Pos.	38	obs:	exp:
att	I	0	319.60
atc	I	0	211.90
ata	I	876	344.50
atg	M	1	1.00
gtt	V	0	5.78
gtc	V	0	5.61
gta	V	28	5.80
gtg	V	0	10.81
---	--	------	------
mPD		0.062	0.72
		nPD:	0.09
		N. weight:	1.2
		Sc. PD:	-0.076
		Sc. rank:	-73.7

,

PA			
Pos.	39	obs:	exp:
tgt	C	6	371.50
tgc	C	899	533.50
---	--	------	------
mPD		0.013	0.48
		nPD:	0.03
		N. weight:	0.6
		Sc. PD:	-0.074
		Sc. rank:	-189.1

,

PA			
Pos.	40	obs:	exp:
act	T	10	234.40
acc	T	7	184.30
aca	T	884	415.80
acg	T	4	70.50
---	--	------	------
mPD		0.046	0.67
		nPD:	0.07
		N. weight:	0.82
		Sc. PD:	-0.068
		Sc. rank:	-105.6

,

PA			
Pos.	41	obs:	exp:
cat	H	19	519.90
cac	H	886	385.10
---	--	------	------
mPD		0.041	0.49
		nPD:	0.08
		N. weight:	0.92
		Sc. PD:	-0.062
		Sc. rank:	-67.4

,

PA			
Pos.	42	obs:	exp:
tta	L	807	79.54
ttg	L	82	168.90
ctt	L	0	153.40
ctc	L	0	151.60
cta	L	8	150.90
ctg	L	8	200.70
---	--	------	------
mPD		0.21	1.1
		nPD:	0.19
		N. weight:	2.4
		Sc. PD:	0.091
		Sc. rank:	587.0

,

PA			
Pos.	43	obs:	exp:
gaa	E	885	544.40
gag	E	20	360.60
---	--	------	------
mPD		0.043	0.48
		nPD:	0.09
		N. weight:	0.51
		Sc. PD:	-0.031
		Sc. rank:	-24.7

,

PA			
Pos.	44	obs:	exp:
att	I	0	1.82
atc	I	5	1.21
ata	I	0	1.97
gtt	V	3	185.80
gtc	V	856	180.30
gta	V	6	186.30
gtg	V	35	347.60
---	--	------	------
mPD		0.10	0.74
		nPD:	0.14
		N. weight:	1.7
		Sc. PD:	-0.019
		Sc. rank:	166.9

,

PA			
Pos.	45	obs:	exp:
tgt	C	117	371.50
tgc	C	788	533.50
---	--	------	------
mPD		0.23	0.48
		nPD:	0.47
		N. weight:	0.24
		Sc. PD:	0.072
		Sc. rank:	135.1

,

PA			
Pos.	46	obs:	exp:
ttt	F	0	356.80
ttc	F	904	547.20
tta	L	0	0.09
ttg	L	0	0.19
ctt	L	0	0.17
ctc	L	1	0.17
cta	L	0	0.17
ctg	L	0	0.22
---	--	------	------
mPD		0.0022	0.48
		nPD:	0.
		N. weight:	0.62
		Sc. PD:	-0.089
		Sc. rank:	-319.3

PA			
Pos.	47	obs:	exp:
atg	M	905	905.00
---	--	------	------
mPD		0	0
		nPD:	1.
		N. weight:	0.
		Sc. PD:	0
		Sc. rank:	0

PA			
Pos.	48	obs:	exp:
tat	Y	287	479.80
tac	Y	618	425.20
---	--	------	------
mPD		0.43	0.50
		nPD:	0.87
		N. weight:	0.12
		Sc. PD:	0.082
		Sc. rank:	75.3

PA			
Pos.	49	obs:	exp:
ttt	F	1	0.39
ttc	F	0	0.61
tct	S	789	144.70
tcc	S	2	117.00
tca	S	65	228.10
tcg	S	48	52.61
agt	S	0	185.20
agc	S	0	176.30
---	--	------	------
mPD		0.23	1.7
		nPD:	0.14
		N. weight:	1.7
		Sc. PD:	-0.022
		Sc. rank:	156.4

PA			
Pos.	50	obs:	exp:
gat	D	870	488.60
gac	D	35	416.40
---	--	------	------
mPD		0.074	0.50
		nPD:	0.15
		N. weight:	0.57
		Sc. PD:	-0.0021
		Sc. rank:	69.6

PA			
Pos.	51	obs:	exp:
ttt	F	18	357.20
ttc	F	887	547.80
---	--	------	------
mPD		0.039	0.48
		nPD:	0.08
		N. weight:	0.51
		Sc. PD:	-0.035
		Sc. rank:	-43.0

PA			
Pos.	52	obs:	exp:
cat	H	45	518.20
cac	H	857	383.80
caa	Q	1	1.54
cag	Q	2	1.46
---	--	------	------
mPD		0.10	0.49
		nPD:	0.2
		N. weight:	0.79
		Sc. PD:	0.039
		Sc. rank:	220.9

PA			
Pos.	53	obs:	exp:
ttt	F	40	357.20
ttc	F	865	547.80
---	--	------	------
mPD		0.085	0.48
		nPD:	0.18
		N. weight:	0.42
		Sc. PD:	0.0095
		Sc. rank:	77.9

PA			
Pos.	54	obs:	exp:
att	I	202	330.20
atc	I	702	218.90
ata	I	1	355.90
---	--	------	------
mPD		0.35	0.65
		nPD:	0.53
		N. weight:	0.97
		Sc. PD:	0.36
		Sc. rank:	570.7

PA			
Pos.	55	obs:	exp:
aat	N	3	3.05
aac	N	3	2.95
gat	D	113	485.30
gac	D	786	413.70
---	--	------	------
mPD		0.24	0.51
		nPD:	0.46
		N. weight:	0.47
		Sc. PD:	0.14
		Sc. rank:	264.5

PA			
Pos.	56	obs:	exp:
gaa	E	898	544.40
gag	E	7	360.60
---	--	------	------
mPD		0.015	0.48
		nPD:	0.03
		N. weight:	0.57
		Sc. PD:	-0.067
		Sc. rank:	-162.1

PA			
Pos.	57	obs:	exp:
tgg	W	1	1.00
caa	Q	20	10.79
cag	Q	1	10.21
cgt	R	38	37.72
cgc	R	2	44.86
cga	R	548	88.05
cgg	R	184	73.54
aga	R	110	403.20
agg	R	1	235.70
---	--	------	------
mPD		0.66	1.0
		nPD:	0.64
		N. weight:	1.4
		Sc. PD:	0.66
		Sc. rank:	870.2

PA			
Pos.	58	obs:	exp:
ggt	G	84	121.40
ggc	G	815	115.00
gga	G	6	418.40
ggg	G	0	250.20
---	--	------	------
mPD		0.18	0.68
		nPD:	0.27
		N. weight:	2.1
		Sc. PD:	0.23
		Sc. rank:	861.5

PA			
Pos.	59	obs:	exp:
aaa	K	6	3.51
aag	K	0	2.49
gaa	E	875	540.80
gag	E	24	358.20
---	--	------	------
mPD		0.065	0.49
		nPD:	0.13
		N. weight:	0.49
		Sc. PD:	-0.01
		Sc. rank:	34.9

PA			
Pos.	60	obs:	exp:
tta	L	3	0.26
ttg	L	0	0.56
tct	S	0	144.20
tcc	S	0	116.60
tca	S	884	227.40
tcg	S	17	52.43
ctt	L	0	0.51
ctc	L	0	0.50
cta	L	0	0.50
ctg	L	0	0.67
cct	P	0	0.25
ccc	P	0	0.18
cca	P	1	0.41
ccg	P	0	0.16
agt	S	0	184.60
agc	S	0	175.70
---	--	------	------
mPD		0.046	1.7
		nPD:	0.03
		N. weight:	1.6
		Sc. PD:	-0.2
		Sc. rank:	-497.2

PA			
Pos.	61	obs:	exp:
att	I	0	33.57
atc	I	0	22.25
ata	I	92	36.18
atg	M	2	2.00
act	T	676	209.10
acc	T	131	164.30
aca	T	0	370.80
acg	T	0	62.86
gtt	V	0	0.41
gtc	V	0	0.40
gta	V	1	0.41
gtg	V	1	0.77
gct	A	1	0.26
gcc	A	0	0.19
gca	A	0	0.47
gcg	A	0	0.09
gat	D	1	0.54
gac	D	0	0.46
---	--	------	------
mPD		0.61	0.88
		nPD:	0.69
		N. weight:	1.2
		Sc. PD:	0.61
		Sc. rank:	730.2

PA			
Pos.	62	obs:	exp:
att	I	528	329.10
atc	I	34	218.20
ata	I	340	354.70
atg	M	1	1.00
gtt	V	1	0.41
gtc	V	0	0.40
gta	V	1	0.41
gtg	V	0	0.77
---	--	------	------
mPD		0.52	0.66
		nPD:	0.79
		N. weight:	0.24
		Sc. PD:	0.15
		Sc. rank:	155.1

PA			
Pos.	63	obs:	exp:
att	I	0	294.10
atc	I	0	195.00
ata	I	806	317.00
gtt	V	0	20.23
gtc	V	0	19.63
gta	V	56	20.29
gtg	V	42	37.85
gct	A	0	0.26
gcc	A	0	0.19
gca	A	1	0.47
gcg	A	0	0.09
---	--	------	------
mPD		0.29	0.88
		nPD:	0.33
		N. weight:	1.1
		Sc. PD:	0.18
		Sc. rank:	518.5

PA			
Pos.	64	obs:	exp:
gaa	E	896	544.40
gag	E	9	360.60
---	--	------	------
mPD		0.020	0.48
		nPD:	0.04
		N. weight:	0.56
		Sc. PD:	-0.061
		Sc. rank:	-130.6

PA			
Pos.	65	obs:	exp:
ttt	F	1	0.39
ttc	F	0	0.61
tta	L	0	0.18
ttg	L	0	0.37
tct	S	865	144.40
tcc	S	32	116.80
tca	S	2	227.60
tcg	S	3	52.49
ctt	L	2	0.34
ctc	L	0	0.34
cta	L	0	0.33
ctg	L	0	0.44
agt	S	0	184.80
agc	S	0	175.90
---	--	------	------
mPD		0.090	1.7
		nPD:	0.05
		N. weight:	2.
		Sc. PD:	-0.19
		Sc. rank:	-369.7

PA			
Pos.	66	obs:	exp:
tct	S	0	2.88
tcc	S	0	2.33
tca	S	0	4.54
tcg	S	0	1.05
agt	S	0	3.69
agc	S	18	3.51
gat	D	3	1.62
gac	D	0	1.38
ggt	G	24	118.50
ggc	G	858	112.30
gga	G	2	408.70
ggg	G	0	244.40
---	--	------	------
mPD		0.11	0.75
		nPD:	0.14
		N. weight:	2.3
		Sc. PD:	-0.022
		Sc. rank:	247.1

,

PA			
Pos.	67	obs:	exp:
aat	N	3	1.53
aac	N	0	1.47
gat	D	893	486.90
gac	D	9	415.10
---	--	------	------
mPD		0.026	0.50
		nPD:	0.05
		N. weight:	0.69
		Sc. PD:	-0.068
		Sc. rank:	-132.1

,

PA			
Pos.	68	obs:	exp:
tta	L	0	0.26
ttg	L	0	0.56
ctt	L	0	0.51
ctc	L	0	0.50
cta	L	3	0.50
ctg	L	0	0.67
cct	P	5	229.40
ccc	P	0	160.80
cca	P	864	368.50
ccg	P	33	143.30
---	--	------	------
mPD		0.088	0.72
		nPD:	0.12
		N. weight:	0.92
		Sc. PD:	-0.028
		Sc. rank:	38.0

,

PA			
Pos.	69	obs:	exp:
aat	N	890	460.40
aac	N	15	444.60
---	--	------	------
mPD		0.033	0.50
		nPD:	0.07
		N. weight:	0.73
		Sc. PD:	-0.062
		Sc. rank:	-106.7

,

PA			
Pos.	70	obs:	exp:
gtt	V	0	32.62
gtc	V	0	31.65
gta	V	1	32.71
gtg	V	157	61.02
gct	A	0	191.40
gcc	A	1	140.30
gca	A	245	349.30
gcg	A	501	66.07
---	--	------	------
mPD		0.69	0.99
		nPD:	0.69
		N. weight:	1.5
		Sc. PD:	0.76
		Sc. rank:	912.0

,

PA			
Pos.	71	obs:	exp:
tta	L	50	79.54
ttg	L	341	168.90
ctt	L	0	153.40
ctc	L	34	151.60
cta	L	14	150.90
ctg	L	466	200.70
---	--	------	------
mPD		0.69	1.1
		nPD:	0.62
		N. weight:	0.73
		Sc. PD:	0.33
		Sc. rank:	446.7

,

PA			
Pos.	72	obs:	exp:
tta	L	9	79.54
ttg	L	458	168.90
ctt	L	7	153.40
ctc	L	0	151.60
cta	L	49	150.90
ctg	L	382	200.70
---	--	------	------
mPD		0.63	1.1
		nPD:	0.57
		N. weight:	0.84
		Sc. PD:	0.34
		Sc. rank:	506.5

,

PA			
Pos.	73	obs:	exp:
cgt	R	0	0.04
cgc	R	0	0.05
cga	R	0	0.10
cgg	R	0	0.08
aat	N	0	0.51
aac	N	1	0.49
aaa	K	879	529.00
aag	K	24	374.00
aga	R	1	0.46
agg	R	0	0.27
---	--	------	------
mPD		0.056	0.49
		nPD:	0.11
		N. weight:	0.52
		Sc. PD:	-0.02
		Sc. rank:	10.0

,

PA			
Pos.	74	obs:	exp:
cat	H	290	519.30
cac	H	614	384.70
att	I	0	0.36
atc	I	1	0.24
ata	I	0	0.39
---	--	------	------
mPD		0.44	0.49
		nPD:	0.89
		N. weight:	0.17
		Sc. PD:	0.12
		Sc. rank:	106.5

,

PA			
Pos.	75	obs:	exp:
cgt	R	1	38.66
cgc	R	0	45.98
cga	R	732	90.24
cgg	R	100	75.38
aga	R	43	413.20
agg	R	29	241.50
---	--	------	------
mPD		0.39	0.96
		nPD:	0.41
		N. weight:	1.9
		Sc. PD:	0.47
		Sc. rank:	1024.2

,

PA			
Pos.	76	obs:	exp:
ttt	F	898	357.20
ttc	F	7	547.80
---	--	------	------
mPD		0.015	0.48
		nPD:	0.03
		N. weight:	1.1
		Sc. PD:	-0.13
		Sc. rank:	-302.0

,

PA			
Pos.	77	obs:	exp:
gaa	E	837	544.40
gag	E	68	360.60
---	--	------	------
mPD		0.14	0.48
		nPD:	0.29
		N. weight:	0.34
		Sc. PD:	0.045
		Sc. rank:	146.1

,

PA			
Pos.	78	obs:	exp:
tta	L	28	2.46
ttg	L	0	5.23
ctt	L	0	4.74
ctc	L	0	4.69
cta	L	0	4.67
ctg	L	0	6.21
att	I	0	320.00
atc	I	0	212.10
ata	I	877	344.90
---	--	------	------
mPD		0.060	0.72
		nPD:	0.08
		N. weight:	1.2
		Sc. PD:	-0.082
		Sc. rank:	-94.4

,

PA			
Pos.	79	obs:	exp:
att	I	100	330.20
atc	I	803	218.90
ata	I	2	355.90
---	--	------	------
mPD		0.20	0.65
		nPD:	0.31
		N. weight:	1.2
		Sc. PD:	0.18
		Sc. rank:	565.6

,

PA			
Pos.	80	obs:	exp:
gaa	E	898	544.40
gag	E	7	360.60
---	--	------	------
mPD		0.015	0.48
		nPD:	0.03
		N. weight:	0.57
		Sc. PD:	-0.067
		Sc. rank:	-162.1

,

PA			
Pos.	81	obs:	exp:
ggt	G	0	121.40
ggc	G	0	115.00
gga	G	84	418.40
ggg	G	821	250.20
---	--	------	------
mPD		0.17	0.68
		nPD:	0.25
		N. weight:	1.1
		Sc. PD:	0.11
		Sc. rank:	434.5

,

PA			
Pos.	82	obs:	exp:
cgt	R	0	38.66
cgc	R	0	45.98
cga	R	1	90.24
cgg	R	0	75.38
aga	R	899	413.20
agg	R	5	241.50
---	--	------	------
mPD		0.013	0.96
		nPD:	0.01
		N. weight:	0.92
		Sc. PD:	-0.12
		Sc. rank:	-380.4

,

PA			
Pos.	83	obs:	exp:
gat	D	13	488.00
gac	D	891	416.00
ggt	G	0	0.13
ggc	G	1	0.13
gga	G	0	0.46
ggg	G	0	0.28
---	--	------	------
mPD		0.031	0.50
		nPD:	0.06
		N. weight:	0.86
		Sc. PD:	-0.077
		Sc. rank:	-134.2

,

PA			
Pos.	84	obs:	exp:
cgt	R	5	38.66
cgc	R	28	45.98
cga	R	854	90.24
cgg	R	11	75.38
aga	R	7	413.20
agg	R	0	241.50
---	--	------	------
mPD		0.11	0.96
		nPD:	0.11
		N. weight:	2.5
		Sc. PD:	-0.098
		Sc. rank:	17.9

,

PA			
Pos.	85	obs:	exp:
act	T	31	232.90
acc	T	3	183.10
aca	T	860	413.00
acg	T	5	70.03
gct	A	0	1.54
gcc	A	0	1.13
gca	A	6	2.81
gcg	A	0	0.53
---	--	------	------
mPD		0.096	0.69
		nPD:	0.14
		N. weight:	0.75
		Sc. PD:	-0.0097
		Sc. rank:	66.8

,

PA			
Pos.	86	obs:	exp:
atg	M	902	902.00
gtt	V	0	0.62
gtc	V	0	0.60
gta	V	0	0.62
gtg	V	3	1.16
---	--	------	------
mPD		0.0066	0.011
		nPD:	0.62
		N. weight:	0.0039
		Sc. PD:	0.0017
		Sc. rank:	2.4

,

PA			
Pos.	87	obs:	exp:
gct	A	4	231.90
gcc	A	876	169.90
gca	A	25	423.10
gcg	A	0	80.05
---	--	------	------
mPD		0.062	0.67
		nPD:	0.09
		N. weight:	1.8
		Sc. PD:	-0.11
		Sc. rank:	-77.0

,

PA			
Pos.	88	obs:	exp:
tgg	W	905	905.00
---	--	------	------
mPD		0	0
		nPD:	1.
		N. weight:	0.
		Sc. PD:	0
		Sc. rank:	0

,

PA			
Pos.	89	obs:	exp:
act	T	11	234.40
acc	T	3	184.30
aca	T	877	415.80
acg	T	14	70.50
---	--	------	------
mPD		0.061	0.67
		nPD:	0.09
		N. weight:	0.8
		Sc. PD:	-0.049
		Sc. rank:	-41.5

,

PA			
Pos.	90	obs:	exp:
tta	L	0	0.18
ttg	L	1	0.37
ctt	L	0	0.34
ctc	L	0	0.34
cta	L	0	0.33
ctg	L	1	0.44
att	I	1	0.36
atc	I	0	0.24
ata	I	0	0.39
atg	M	1	1.00
gtt	V	0	186.00
gtc	V	0	180.50
gta	V	100	186.50
gtg	V	801	348.00
---	--	------	------
mPD		0.21	0.73
		nPD:	0.28
		N. weight:	0.83
		Sc. PD:	0.1
		Sc. rank:	357.2

,

PA			
Pos.	91	obs:	exp:
gtt	V	0	186.80
gtc	V	0	181.30
gta	V	50	187.40
gtg	V	855	349.50
---	--	------	------
mPD		0.10	0.73
		nPD:	0.14
		N. weight:	0.95
		Sc. PD:	-0.0087
		Sc. rank:	104.1

,

PA			
Pos.	92	obs:	exp:
aat	N	857	460.40
aac	N	48	444.60
---	--	------	------
mPD		0.10	0.50
		nPD:	0.2
		N. weight:	0.58
		Sc. PD:	0.027
		Sc. rank:	156.1

,

PA			
Pos.	93	obs:	exp:
tct	S	0	144.70
tcc	S	0	117.00
tca	S	0	228.10
tcg	S	0	52.61
agt	S	758	185.20
agc	S	146	176.30
ggt	G	0	0.13
ggc	G	1	0.13
gga	G	0	0.46
ggg	G	0	0.28
---	--	------	------
mPD		0.27	1.7
		nPD:	0.17
		N. weight:	1.4
		Sc. PD:	0.017
		Sc. rank:	229.6

,

PA			
Pos.	94	obs:	exp:
att	I	89	303.60
atc	I	742	201.20
ata	I	1	327.20
gtt	V	0	15.07
gtc	V	73	14.62
gta	V	0	15.11
gtg	V	0	28.19
---	--	------	------
mPD		0.33	0.82
		nPD:	0.4
		N. weight:	1.3
		Sc. PD:	0.31
		Sc. rank:	690.1

,

PA			
Pos.	95	obs:	exp:
tgt	C	1	371.50
tgc	C	904	533.50
---	--	------	------
mPD		0.0022	0.48
		nPD:	0.
		N. weight:	0.64
		Sc. PD:	-0.092
		Sc. rank:	-334.0

,

PA			
Pos.	96	obs:	exp:
cat	H	0	58.02
cac	H	101	42.98
aat	N	21	409.00
aac	N	783	395.00
---	--	------	------
mPD		0.24	0.70
		nPD:	0.35
		N. weight:	0.76
		Sc. PD:	0.14
		Sc. rank:	372.8

,

PA			
Pos.	97	obs:	exp:
act	T	126	233.90
acc	T	767	183.90
aca	T	10	414.90
acg	T	0	70.34
gct	A	0	0.51
gcc	A	2	0.38
gca	A	0	0.94
gcg	A	0	0.18
---	--	------	------
mPD		0.26	0.68
		nPD:	0.39
		N. weight:	1.3
		Sc. PD:	0.3
		Sc. rank:	691.1

,

PA			
Pos.	98	obs:	exp:
act	T	66	234.40
acc	T	2	184.30
aca	T	466	415.80
acg	T	371	70.50
---	--	------	------
mPD		0.56	0.67
		nPD:	0.83
		N. weight:	0.79
		Sc. PD:	0.52
		Sc. rank:	503.8

,

PA			
Pos.	99	obs:	exp:
tct	S	0	0.16
tcc	S	0	0.13
tca	S	0	0.25
tcg	S	0	0.06
agt	S	1	0.20
agc	S	0	0.20
ggt	G	32	121.20
ggc	G	0	114.90
gga	G	796	418.00
ggg	G	76	249.90
---	--	------	------
mPD		0.22	0.68
		nPD:	0.32
		N. weight:	0.52
		Sc. PD:	0.086
		Sc. rank:	244.6

,

PA			
Pos.	100	obs:	exp:
ttt	F	0	0.39
ttc	F	1	0.61
att	I	0	5.47
atc	I	15	3.63
ata	I	0	5.90
act	T	0	0.52
acc	T	2	0.41
aca	T	0	0.92
acg	T	0	0.16
gtt	V	34	112.70
gtc	V	511	109.40
gta	V	1	113.00
gtg	V	0	210.90
gct	A	3	87.38
gcc	A	337	64.03
gca	A	1	159.40
gcg	A	0	30.16
---	--	------	------
mPD		0.59	1.3
		nPD:	0.47
		N. weight:	1.8
		Sc. PD:	0.55
		Sc. rank:	1024.2

,

PA			
Pos.	101	obs:	exp:
gat	D	27	14.58
gac	D	0	12.42
gaa	E	738	526.30
gag	E	137	348.70
ggt	G	0	0.40
ggc	G	0	0.38
gga	G	3	1.39
ggg	G	0	0.83
---	--	------	------
mPD		0.31	0.52
		nPD:	0.6
		N. weight:	0.19
		Sc. PD:	0.085
		Sc. rank:	118.9

,

PA			
Pos.	102	obs:	exp:
aaa	K	845	530.10
aag	K	60	374.90
---	--	------	------
mPD		0.12	0.49
		nPD:	0.26
		N. weight:	0.39
		Sc. PD:	0.038
		Sc. rank:	151.3

,

PA			
Pos.	103	obs:	exp:
tct	S	0	0.16
tcc	S	1	0.13
tca	S	0	0.25
tcg	S	0	0.06
cct	P	167	229.90
ccc	P	732	161.20
cca	P	3	369.30
ccg	P	2	143.60
agt	S	0	0.20
agc	S	0	0.20
---	--	------	------
mPD		0.31	0.71
		nPD:	0.44
		N. weight:	1.4
		Sc. PD:	0.39
		Sc. rank:	784.0

,

PA			
Pos.	104	obs:	exp:
cgt	R	0	0.26
cgc	R	0	0.30
cga	R	0	0.60
cgg	R	0	0.50
aat	N	1	0.51
aac	N	0	0.49
aaa	K	857	526.00
aag	K	41	372.00
aga	R	4	2.74
agg	R	2	1.60
---	--	------	------
mPD		0.11	0.50
		nPD:	0.21
		N. weight:	0.45
		Sc. PD:	0.025
		Sc. rank:	133.8

,

PA			
Pos.	105	obs:	exp:
ttt	F	826	356.40
ttc	F	77	546.60
tta	L	0	0.09
ttg	L	0	0.19
tct	S	1	0.16
tcc	S	0	0.13
tca	S	0	0.25
tcg	S	0	0.06
ctt	L	1	0.17
ctc	L	0	0.17
cta	L	0	0.17
ctg	L	0	0.22
agt	S	0	0.20
agc	S	0	0.20
---	--	------	------
mPD		0.16	0.48
		nPD:	0.33
		N. weight:	0.75
		Sc. PD:	0.13
		Sc. rank:	351.1

,

PA			
Pos.	106	obs:	exp:
tta	L	0	79.54
ttg	L	0	168.90
ctt	L	125	153.40
ctc	L	778	151.60
cta	L	2	150.90
ctg	L	0	200.70
---	--	------	------
mPD		0.24	1.1
		nPD:	0.22
		N. weight:	1.7
		Sc. PD:	0.1
		Sc. rank:	536.7

,

PA			
Pos.	107	obs:	exp:
cct	P	19	230.10
ccc	P	0	161.40
cca	P	72	369.70
ccg	P	814	143.80
---	--	------	------
mPD		0.18	0.71
		nPD:	0.26
		N. weight:	1.7
		Sc. PD:	0.17
		Sc. rank:	678.4

,

PA			
Pos.	108	obs:	exp:
att	I	0	0.36
atc	I	1	0.24
ata	I	0	0.39
gat	D	870	488.00
gac	D	34	416.00
---	--	------	------
mPD		0.079	0.50
		nPD:	0.16
		N. weight:	0.57
		Sc. PD:	0.002
		Sc. rank:	80.5

,

PA			
Pos.	109	obs:	exp:
tta	L	33	79.45
ttg	L	47	168.70
ctt	L	1	153.20
ctc	L	0	151.50
cta	L	2	150.70
ctg	L	821	200.50
gtt	V	0	0.21
gtc	V	0	0.20
gta	V	0	0.21
gtg	V	1	0.39
---	--	------	------
mPD		0.24	1.1
		nPD:	0.22
		N. weight:	1.4
		Sc. PD:	0.087
		Sc. rank:	443.0

,

PA			
Pos.	110	obs:	exp:
tat	Y	867	479.80
tac	Y	38	425.20
---	--	------	------
mPD		0.081	0.50
		nPD:	0.16
		N. weight:	0.57
		Sc. PD:	0.0045
		Sc. rank:	85.9

,

PA			
Pos.	111	obs:	exp:
gat	D	51	488.60
gac	D	854	416.40
---	--	------	------
mPD		0.11	0.50
		nPD:	0.21
		N. weight:	0.68
		Sc. PD:	0.04
		Sc. rank:	203.6

,

PA			
Pos.	112	obs:	exp:
tat	Y	5	479.80
tac	Y	900	425.20
---	--	------	------
mPD		0.011	0.50
		nPD:	0.02
		N. weight:	0.89
		Sc. PD:	-0.11
		Sc. rank:	-319.4

,

PA			
Pos.	113	obs:	exp:
caa	Q	0	0.51
cag	Q	1	0.49
cgt	R	0	0.09
cgc	R	0	0.10
cga	R	0	0.20
cgg	R	0	0.17
atg	M	1	1.00
aaa	K	76	527.80
aag	K	825	373.20
aga	R	0	0.91
agg	R	2	0.53
---	--	------	------
mPD		0.16	0.50
		nPD:	0.33
		N. weight:	0.7
		Sc. PD:	0.12
		Sc. rank:	326.9

,

PA			
Pos.	114	obs:	exp:
gct	A	0	0.26
gcc	A	0	0.19
gca	A	1	0.47
gcg	A	0	0.09
gat	D	0	0.54
gac	D	1	0.46
gaa	E	820	542.00
gag	E	81	359.00
ggt	G	0	0.27
ggc	G	0	0.25
gga	G	2	0.92
ggg	G	0	0.55
---	--	------	------
mPD		0.17	0.49
		nPD:	0.35
		N. weight:	0.3
		Sc. PD:	0.058
		Sc. rank:	149.7

,

PA			
Pos.	115	obs:	exp:
tct	S	0	16.49
tcc	S	0	13.33
tca	S	0	25.99
tcg	S	0	5.99
aat	N	42	408.00
aac	N	760	394.00
agt	S	0	21.11
agc	S	103	20.09
---	--	------	------
mPD		0.29	0.87
		nPD:	0.33
		N. weight:	0.78
		Sc. PD:	0.14
		Sc. rank:	370.9

,

PA			
Pos.	116	obs:	exp:
cgt	R	341	38.66
cgc	R	26	45.98
cga	R	528	90.24
cgg	R	8	75.38
aga	R	1	413.20
agg	R	1	241.50
---	--	------	------
mPD		0.52	0.96
		nPD:	0.54
		N. weight:	2.2
		Sc. PD:	0.83
		Sc. rank:	1317.5

,

PA			
Pos.	117	obs:	exp:
ttt	F	27	357.20
ttc	F	878	547.80
---	--	------	------
mPD		0.058	0.48
		nPD:	0.12
		N. weight:	0.47
		Sc. PD:	-0.015
		Sc. rank:	18.7

,

PA			
Pos.	118	obs:	exp:
tta	L	0	0.09
ttg	L	0	0.19
ctt	L	1	0.17
ctc	L	0	0.17
cta	L	0	0.17
ctg	L	0	0.22
att	I	897	328.80
atc	I	4	217.90
ata	I	0	354.30
gtt	V	3	0.62
gtc	V	0	0.60
gta	V	0	0.62
gtg	V	0	1.16
---	--	------	------
mPD		0.018	0.66
		nPD:	0.03
		N. weight:	1.2
		Sc. PD:	-0.15
		Sc. rank:	-379.5

,

PA			
Pos.	119	obs:	exp:
gaa	E	903	544.40
gag	E	2	360.60
---	--	------	------
mPD		0.0044	0.48
		nPD:	0.01
		N. weight:	0.61
		Sc. PD:	-0.085
		Sc. rank:	-275.9

,

PA			
Pos.	120	obs:	exp:
att	I	904	330.20
atc	I	1	218.90
ata	I	0	355.90
---	--	------	------
mPD		0.0022	0.65
		nPD:	0.
		N. weight:	1.2
		Sc. PD:	-0.18
		Sc. rank:	-651.8

,

PA			
Pos.	121	obs:	exp:
ggt	G	1	121.40
ggc	G	0	115.00
gga	G	888	418.40
ggg	G	16	250.20
---	--	------	------
mPD		0.037	0.68
		nPD:	0.05
		N. weight:	0.84
		Sc. PD:	-0.081
		Sc. rank:	-151.5

,

PA			
Pos.	122	obs:	exp:
gtt	V	11	186.80
gtc	V	0	181.30
gta	V	173	187.40
gtg	V	721	349.50
---	--	------	------
mPD		0.33	0.73
		nPD:	0.45
		N. weight:	0.65
		Sc. PD:	0.19
		Sc. rank:	368.4

,

PA			
Pos.	123	obs:	exp:
act	T	4	234.40
acc	T	33	184.30
aca	T	102	415.80
acg	T	766	70.50
---	--	------	------
mPD		0.27	0.67
		nPD:	0.4
		N. weight:	2.2
		Sc. PD:	0.52
		Sc. rank:	1156.4

,

PA			
Pos.	124	obs:	exp:
cgt	R	0	38.62
cgc	R	0	45.93
cga	R	39	90.14
cgg	R	20	75.29
aaa	K	0	0.59
aag	K	1	0.41
aga	R	10	412.70
agg	R	835	241.30
---	--	------	------
mPD		0.23	0.97
		nPD:	0.23
		N. weight:	1.3
		Sc. PD:	0.1
		Sc. rank:	451.1

,

PA			
Pos.	125	obs:	exp:
cgt	R	0	38.66
cgc	R	0	45.98
cga	R	0	90.24
cgg	R	0	75.38
aga	R	9	413.20
agg	R	896	241.50
---	--	------	------
mPD		0.020	0.96
		nPD:	0.02
		N. weight:	1.5
		Sc. PD:	-0.2
		Sc. rank:	-568.7

,

PA			
Pos.	126	obs:	exp:
gaa	E	889	544.40
gag	E	16	360.60
---	--	------	------
mPD		0.035	0.48
		nPD:	0.07
		N. weight:	0.53
		Sc. PD:	-0.041
		Sc. rank:	-61.2

,

PA			
Pos.	127	obs:	exp:
att	I	0	2.19
atc	I	6	1.45
ata	I	0	2.36
gtt	V	47	185.60
gtc	V	846	180.10
gta	V	6	186.10
gtg	V	0	347.20
---	--	------	------
mPD		0.12	0.74
		nPD:	0.17
		N. weight:	1.7
		Sc. PD:	0.023
		Sc. rank:	281.7

,

PA			
Pos.	128	obs:	exp:
cat	H	49	519.30
cac	H	855	384.70
caa	Q	1	0.51
cag	Q	0	0.49
---	--	------	------
mPD		0.10	0.49
		nPD:	0.21
		N. weight:	0.77
		Sc. PD:	0.045
		Sc. rank:	231.0

,

PA			
Pos.	129	obs:	exp:
att	I	1	326.20
atc	I	0	216.20
ata	I	893	351.60
atg	M	6	6.00
act	T	0	1.04
acc	T	0	0.81
aca	T	4	1.84
acg	T	0	0.31
gtt	V	0	0.21
gtc	V	0	0.20
gta	V	1	0.21
gtg	V	0	0.39
---	--	------	------
mPD		0.026	0.67
		nPD:	0.04
		N. weight:	1.1
		Sc. PD:	-0.12
		Sc. rank:	-266.1

,

PA			
Pos.	130	obs:	exp:
tat	Y	591	479.80
tac	Y	314	425.20
---	--	------	------
mPD		0.45	0.50
		nPD:	0.91
		N. weight:	0.043
		Sc. PD:	0.031
		Sc. rank:	27.3

,

PA			
Pos.	131	obs:	exp:
tat	Y	73	479.80
tac	Y	832	425.20
---	--	------	------
mPD		0.15	0.50
		nPD:	0.3
		N. weight:	0.57
		Sc. PD:	0.08
		Sc. rank:	253.7

,

PA			
Pos.	132	obs:	exp:
tta	L	49	79.54
ttg	L	1	168.90
ctt	L	1	153.40
ctc	L	0	151.60
cta	L	844	150.90
ctg	L	10	200.70
---	--	------	------
mPD		0.13	1.1
		nPD:	0.12
		N. weight:	1.9
		Sc. PD:	-0.065
		Sc. rank:	57.1

,

PA			
Pos.	133	obs:	exp:
gat	D	3	1.62
gac	D	0	1.38
gaa	E	98	542.60
gag	E	804	359.40
---	--	------	------
mPD		0.20	0.48
		nPD:	0.41
		N. weight:	0.66
		Sc. PD:	0.16
		Sc. rank:	357.2

,

PA			
Pos.	134	obs:	exp:
aaa	K	833	530.10
aag	K	72	374.90
---	--	------	------
mPD		0.15	0.49
		nPD:	0.3
		N. weight:	0.35
		Sc. PD:	0.051
		Sc. rank:	157.7

,

PA			
Pos.	135	obs:	exp:
gct	A	35	231.90
gcc	A	869	169.90
gca	A	1	423.10
gcg	A	0	80.05
---	--	------	------
mPD		0.077	0.67
		nPD:	0.11
		N. weight:	1.8
		Sc. PD:	-0.07
		Sc. rank:	27.6

,

PA			
Pos.	136	obs:	exp:
act	T	1	0.26
acc	T	0	0.20
aca	T	0	0.46
acg	T	0	0.08
aat	N	809	459.90
aac	N	95	444.10
---	--	------	------
mPD		0.19	0.50
		nPD:	0.38
		N. weight:	0.43
		Sc. PD:	0.093
		Sc. rank:	217.4

,

PA			
Pos.	137	obs:	exp:
aaa	K	784	530.10
aag	K	121	374.90
---	--	------	------
mPD		0.23	0.49
		nPD:	0.48
		N. weight:	0.23
		Sc. PD:	0.073
		Sc. rank:	135.4

,

PA			
Pos.	138	obs:	exp:
tta	L	2	2.72
ttg	L	0	5.79
ctt	L	0	5.25
ctc	L	0	5.19
cta	L	29	5.17
ctg	L	0	6.88
att	I	1	318.90
atc	I	0	211.40
ata	I	873	343.70
---	--	------	------
mPD		0.069	0.73
		nPD:	0.09
		N. weight:	1.2
		Sc. PD:	-0.067
		Sc. rank:	-42.5

,

PA			
Pos.	139	obs:	exp:
cgt	R	0	0.09
cgc	R	0	0.10
cga	R	0	0.20
cgg	R	0	0.17
aaa	K	858	529.00
aag	K	45	374.00
aga	R	2	0.91
agg	R	0	0.53
---	--	------	------
mPD		0.099	0.49
		nPD:	0.2
		N. weight:	0.44
		Sc. PD:	0.02
		Sc. rank:	118.4

,

PA			
Pos.	140	obs:	exp:
tct	S	47	144.90
tcc	S	696	117.10
tca	S	162	228.40
tcg	S	0	52.67
agt	S	0	185.40
agc	S	0	176.50
---	--	------	------
mPD		0.37	1.7
		nPD:	0.23
		N. weight:	1.5
		Sc. PD:	0.11
		Sc. rank:	516.1

,

PA			
Pos.	141	obs:	exp:
gat	D	1	0.54
gac	D	0	0.46
gaa	E	29	542.00
gag	E	872	359.00
ggt	G	0	0.40
ggc	G	0	0.38
gga	G	0	1.39
ggg	G	3	0.83
---	--	------	------
mPD		0.071	0.49
		nPD:	0.15
		N. weight:	0.94
		Sc. PD:	-0.0076
		Sc. rank:	105.7

,

PA			
Pos.	142	obs:	exp:
cgt	R	0	0.13
cgc	R	0	0.15
cga	R	0	0.30
cgg	R	0	0.25
aat	N	0	0.51
aac	N	1	0.49
aaa	K	12	527.20
aag	K	888	372.80
aga	R	0	1.37
agg	R	3	0.80
gaa	E	0	0.60
gag	E	1	0.40
---	--	------	------
mPD		0.037	0.50
		nPD:	0.07
		N. weight:	0.99
		Sc. PD:	-0.076
		Sc. rank:	-100.5

,

PA			
Pos.	143	obs:	exp:
act	T	0	233.40
acc	T	4	183.50
aca	T	897	413.90
acg	T	0	70.19
gct	A	0	1.02
gcc	A	0	0.75
gca	A	4	1.87
gcg	A	0	0.35
---	--	------	------
mPD		0.018	0.68
		nPD:	0.03
		N. weight:	0.93
		Sc. PD:	-0.11
		Sc. rank:	-305.8

,

PA			
Pos.	144	obs:	exp:
cat	H	3	519.30
cac	H	901	384.70
aat	N	0	0.51
aac	N	1	0.49
---	--	------	------
mPD		0.0088	0.49
		nPD:	0.02
		N. weight:	1.
		Sc. PD:	-0.13
		Sc. rank:	-395.0

,

PA			
Pos.	145	obs:	exp:
att	I	84	330.20
atc	I	622	218.90
ata	I	199	355.90
---	--	------	------
mPD		0.47	0.65
		nPD:	0.72
		N. weight:	0.58
		Sc. PD:	0.32
		Sc. rank:	363.3

,

PA			
Pos.	146	obs:	exp:
cat	H	362	519.90
cac	H	543	385.10
---	--	------	------
mPD		0.48	0.49
		nPD:	0.98
		N. weight:	0.08
		Sc. PD:	0.064
		Sc. rank:	51.2

,

PA			
Pos.	147	obs:	exp:
att	I	820	330.20
atc	I	47	218.90
ata	I	38	355.90
---	--	------	------
mPD		0.17	0.65
		nPD:	0.27
		N. weight:	0.8
		Sc. PD:	0.088
		Sc. rank:	332.5

,

PA			
Pos.	148	obs:	exp:
ttt	F	367	357.20
ttc	F	538	547.80
---	--	------	------
mPD		0.48	0.48
		nPD:	1.01
		N. weight:	0.0052
		Sc. PD:	0.0043
		Sc. rank:	3.3

,

PA			
Pos.	149	obs:	exp:
tct	S	1	144.90
tcc	S	0	117.10
tca	S	900	228.40
tcg	S	4	52.67
agt	S	0	185.40
agc	S	0	176.50
---	--	------	------
mPD		0.011	1.7
		nPD:	0.01
		N. weight:	1.7
		Sc. PD:	-0.23
		Sc. rank:	-795.3

,

PA			
Pos.	150	obs:	exp:
ttt	F	17	357.20
ttc	F	888	547.80
---	--	------	------
mPD		0.037	0.48
		nPD:	0.08
		N. weight:	0.51
		Sc. PD:	-0.038
		Sc. rank:	-48.8

,

PA			
Pos.	151	obs:	exp:
act	T	904	234.20
acc	T	0	184.10
aca	T	0	415.30
acg	T	0	70.42
aat	N	1	0.51
aac	N	0	0.49
---	--	------	------
mPD		0.0022	0.68
		nPD:	0.
		N. weight:	1.7
		Sc. PD:	-0.24
		Sc. rank:	-894.2

,

PA			
Pos.	152	obs:	exp:
ggt	G	1	121.40
ggc	G	0	115.00
gga	G	856	418.40
ggg	G	48	250.20
---	--	------	------
mPD		0.10	0.68
		nPD:	0.15
		N. weight:	0.72
		Sc. PD:	-0.0012
		Sc. rank:	94.3

,

PA			
Pos.	153	obs:	exp:
gaa	E	851	543.20
gag	E	52	359.80
ggt	G	0	0.27
ggc	G	0	0.25
gga	G	2	0.92
ggg	G	0	0.55
---	--	------	------
mPD		0.11	0.48
		nPD:	0.23
		N. weight:	0.39
		Sc. PD:	0.03
		Sc. rank:	135.1

,

PA			
Pos.	154	obs:	exp:
gaa	E	100	544.40
gag	E	805	360.60
---	--	------	------
mPD		0.20	0.48
		nPD:	0.41
		N. weight:	0.65
		Sc. PD:	0.16
		Sc. rank:	351.6

,

PA			
Pos.	155	obs:	exp:
atg	M	905	905.00
---	--	------	------
mPD		0	0
		nPD:	1.
		N. weight:	0.
		Sc. PD:	0
		Sc. rank:	0

,

PA			
Pos.	156	obs:	exp:
act	T	0	0.26
acc	T	1	0.20
aca	T	0	0.46
acg	T	0	0.08
gct	A	43	231.60
gcc	A	814	169.70
gca	A	44	422.70
gcg	A	3	79.96
---	--	------	------
mPD		0.19	0.67
		nPD:	0.28
		N. weight:	1.5
		Sc. PD:	0.18
		Sc. rank:	628.2

,

PA			
Pos.	157	obs:	exp:
tct	S	0	0.16
tcc	S	0	0.13
tca	S	0	0.25
tcg	S	0	0.06
act	T	32	234.20
acc	T	868	184.10
aca	T	4	415.30
acg	T	0	70.42
agt	S	0	0.20
agc	S	1	0.20
---	--	------	------
mPD		0.079	0.68
		nPD:	0.12
		N. weight:	1.7
		Sc. PD:	-0.061
		Sc. rank:	46.1

,

PA			
Pos.	158	obs:	exp:
caa	Q	1	0.51
cag	Q	0	0.49
cgt	R	0	0.04
cgc	R	0	0.05
cga	R	0	0.10
cgg	R	0	0.08
aaa	K	847	529.00
aag	K	56	374.00
aga	R	1	0.46
agg	R	0	0.27
---	--	------	------
mPD		0.12	0.49
		nPD:	0.25
		N. weight:	0.4
		Sc. PD:	0.036
		Sc. rank:	148.8

,

PA			
Pos.	159	obs:	exp:
tct	S	0	0.16
tcc	S	0	0.13
tca	S	1	0.25
tcg	S	0	0.06
agt	S	0	0.20
agc	S	0	0.20
gct	A	0	231.60
gcc	A	3	169.70
gca	A	834	422.70
gcg	A	67	79.96
---	--	------	------
mPD		0.15	0.68
		nPD:	0.22
		N. weight:	0.74
		Sc. PD:	0.044
		Sc. rank:	224.0

,

PA			
Pos.	160	obs:	exp:
gat	D	800	488.00
gac	D	104	416.00
gaa	E	1	0.60
gag	E	0	0.40
---	--	------	------
mPD		0.21	0.50
		nPD:	0.41
		N. weight:	0.35
		Sc. PD:	0.086
		Sc. rank:	188.1

,

PA			
Pos.	161	obs:	exp:
tat	Y	15	479.30
tac	Y	889	424.70
tgt	C	0	0.41
tgc	C	1	0.59
mPD		0.035	0.50
		nPD:	0.07
		N. weight:	0.82
		Sc. PD:	-0.067
		Sc. rank:	-98.0

PA			
Pos.	162	obs:	exp:
act	T	735	234.40
acc	T	55	184.30
aca	T	115	415.80
acg	T	0	70.50
mPD		0.32	0.67
		nPD:	0.48
		N. weight:	0.86
		Sc. PD:	0.27
		Sc. rank:	493.4

PA			
Pos.	163	obs:	exp:
tta	L	0	79.36
ttg	L	0	168.50
ctt	L	894	153.00
ctc	L	8	151.30
cta	L	1	150.50
ctg	L	0	200.30
att	I	2	0.73
atc	I	0	0.48
ata	I	0	0.79
mPD		0.024	1.1
		nPD:	0.02
		N. weight:	2.1
		Sc. PD:	-0.27
		Sc. rank:	-766.1

PA			
Pos.	164	obs:	exp:
gat	D	256	488.60
gac	D	649	416.40
mPD		0.41	0.50
		nPD:	0.82
		N. weight:	0.17
		Sc. PD:	0.11
		Sc. rank:	108.4

PA			
Pos.	165	obs:	exp:
gaa	E	879	544.40
gag	E	26	360.60
mPD		0.056	0.48
		nPD:	0.12
		N. weight:	0.48
		Sc. PD:	-0.017
		Sc. rank:	10.3

PA			
Pos.	166	obs:	exp:
gaa	E	700	544.40
gag	E	205	360.60
mPD		0.35	0.48
		nPD:	0.73
		N. weight:	0.086
		Sc. PD:	0.048
		Sc. rank:	54.6

PA			
Pos.	167	obs:	exp:
tct	S	0	144.90
tcc	S	0	117.10
tca	S	0	228.40
tcg	S	0	52.67
agt	S	82	185.40
agc	S	823	176.50
mPD		0.16	1.7
		nPD:	0.1
		N. weight:	1.6
		Sc. PD:	-0.085
		Sc. rank:	-46.4

PA			
Pos.	168	obs:	exp:
cgt	R	0	38.66
cgc	R	0	45.98
cga	R	18	90.24
cgg	R	0	75.38
aga	R	60	413.20
agg	R	827	241.50
mPD		0.20	0.96
		nPD:	0.2
		N. weight:	1.2
		Sc. PD:	0.058
		Sc. rank:	327.7

PA			
Pos.	169	obs:	exp:
gct	A	1	231.90
gcc	A	1	169.90
gca	A	872	423.10
gcg	A	31	80.05
mPD		0.071	0.67
		nPD:	0.1
		N. weight:	0.81
		Sc. PD:	-0.038
		Sc. rank:	-12.3

PA			
Pos.	170	obs:	exp:
cgt	R	0	38.66
cgc	R	0	45.98
cga	R	0	90.24
cgg	R	0	75.38
aga	R	866	413.20
agg	R	39	241.50
mPD		0.083	0.96
		nPD:	0.09
		N. weight:	0.78
		Sc. PD:	-0.051
		Sc. rank:	-53.9

PA			
Pos.	171	obs:	exp:
att	I	10	330.20
atc	I	873	218.90
ata	I	22	355.90
mPD		0.069	0.65
		nPD:	0.11
		N. weight:	1.5
		Sc. PD:	-0.071
		Sc. rank:	-19.0

PA			
Pos.	172	obs:	exp:
aaa	K	897	530.10
aag	K	8	374.90
mPD		0.018	0.49
		nPD:	0.04
		N. weight:	0.6
		Sc. PD:	-0.068
		Sc. rank:	-153.9

PA			
Pos.	173	obs:	exp:
act	T	8	234.40
acc	T	894	184.30
aca	T	3	415.80
acg	T	0	70.50
mPD		0.024	0.67
		nPD:	0.04
		N. weight:	1.9
		Sc. PD:	-0.21
		Sc. rank:	-485.7

PA			
Pos.	174	obs:	exp:
cgt	R	0	38.66
cgc	R	0	45.98
cga	R	0	90.24
cgg	R	0	75.38
aga	R	65	413.20
agg	R	840	241.50
mPD		0.13	0.96
		nPD:	0.14
		N. weight:	1.3
		Sc. PD:	-0.018
		Sc. rank:	106.4

PA			
Pos.	175	obs:	exp:
tta	L	0	79.54
ttg	L	70	168.90
ctt	L	0	153.40
ctc	L	0	151.60
cta	L	41	150.90
ctg	L	794	200.70
mPD		0.23	1.1
		nPD:	0.21
		N. weight:	1.3
		Sc. PD:	0.068
		Sc. rank:	377.2

PA			
Pos.	176	obs:	exp:
ttt	F	6	356.80
ttc	F	898	547.20
tat	Y	0	0.53
tac	Y	1	0.47
mPD		0.015	0.48
		nPD:	0.03
		N. weight:	0.57
		Sc. PD:	-0.067
		Sc. rank:	-160.6

PA			
Pos.	177	obs:	exp:
act	T	147	234.40
acc	T	756	184.30
aca	T	2	415.80
acg	T	0	70.50
mPD		0.28	0.67
		nPD:	0.41
		N. weight:	1.3
		Sc. PD:	0.33
		Sc. rank:	725.3

PA			
Pos.	178	obs:	exp:
att	I	1	330.20
atc	I	104	218.90
ata	I	800	355.90
mPD		0.21	0.65
		nPD:	0.31
		N. weight:	0.77
		Sc. PD:	0.12
		Sc. rank:	353.4

PA			
Pos.	179	obs:	exp:
cgt	R	0	38.66
cgc	R	0	45.98
cga	R	0	90.24
cgg	R	0	75.38
aga	R	156	413.20
agg	R	749	241.50
mPD		0.29	0.96
		nPD:	0.3
		N. weight:	0.95
		Sc. PD:	0.13
		Sc. rank:	415.1

PA			
Pos.	180	obs:	exp:
cat	H	2	1.15
cac	H	0	0.85
caa	Q	101	463.80
cag	Q	802	439.20
mPD		0.20	0.50
		nPD:	0.4
		N. weight:	0.45
		Sc. PD:	0.11
		Sc. rank:	239.0

PA			
Pos.	181	obs:	exp:
gaa	E	836	544.40
gag	E	69	360.60
mPD		0.14	0.48
		nPD:	0.29
		N. weight:	0.34
		Sc. PD:	0.046
		Sc. rank:	146.0

PA			
Pos.	182	obs:	exp:
atg	M	905	905.00
mPD		0	0
		nPD:	1.
		N. weight:	0.
		Sc. PD:	0
		Sc. rank:	0

PA			
Pos.	183	obs:	exp:
gct	A	32	231.90
gcc	A	869	169.90
gca	A	4	423.10
gcg	A	0	80.05
mPD		0.077	0.67
		nPD:	0.11
		N. weight:	1.8
		Sc. PD:	-0.069
		Sc. rank:	33.3

PA			
Pos.	184	obs:	exp:
tct	S	0	144.10
tcc	S	0	116.50
tca	S	0	227.10
tcg	S	0	52.37
aat	N	1	1.02
aac	N	1	0.98
agt	S	98	184.40
agc	S	802	175.50
ggt	G	0	0.40
ggc	G	3	0.38
gga	G	0	1.39
ggg	G	0	0.83
mPD		0.21	1.7
		nPD:	0.12
		N. weight:	1.6
		Sc. PD:	-0.045
		Sc. rank:	88.6

PA			
Pos.	185	obs:	exp:
cgt	R	0	38.58
cgc	R	0	45.88
cga	R	0	90.04
cgg	R	0	75.21
aaa	K	0	1.17
aag	K	2	0.83
aga	R	61	412.30
agg	R	842	241.00
mPD		0.13	0.97
		nPD:	0.13
		N. weight:	1.3
		Sc. PD:	-0.023
		Sc. rank:	97.0

PA

Pos.	186	obs:	exp:
tct	S	0	0.16
tcc	S	0	0.13
tca	S	0	0.25
tcg	S	0	0.06
agt	S	1	0.20
agc	S	0	0.20
ggt	G	841	121.20
ggc	G	61	114.90
gga	G	2	418.00
ggg	G	0	249.90
mPD		0.13	0.68
		nPD:	0.19
		N. weight:	2.1
		Sc. PD:	0.085
		Sc. rank:	538.3

PA

Pos.	187	obs:	exp:
tta	L	3	79.54
ttg	L	8	168.90
ctt	L	0	153.40
ctc	L	41	151.60
cta	L	848	150.90
ctg	L	5	200.70
mPD		0.14	1.1
		nPD:	0.12
		N. weight:	1.8
		Sc. PD:	-0.053
		Sc. rank:	109.8

PA

Pos.	188	obs:	exp:
tgg	W	905	905.00
mPD		0	0
		nPD:	1.
		N. weight:	0.
		Sc. PD:	0
		Sc. rank:	0

PA

Pos.	189	obs:	exp:
gat	D	903	488.00
gac	D	1	416.00
gaa	E	0	0.60
gag	E	1	0.40
mPD		0.0044	0.50
		nPD:	0.01
		N. weight:	0.75
		Sc. PD:	-0.1
		Sc. rank:	-346.3

PA

Pos.	190	obs:	exp:
tct	S	0	144.50
tcc	S	903	116.90
tca	S	0	227.90
tcg	S	0	52.55
cct	P	0	0.51
ccc	P	2	0.36
cca	P	0	0.82
ccg	P	0	0.32
agt	S	0	185.00
agc	S	0	176.10
mPD		0.0044	1.7
		nPD:	0.
		N. weight:	2.5
		Sc. PD:	-0.37
		Sc. rank:	-1388.0

PA

Pos.	191	obs:	exp:
ttt	F	903	356.80
ttc	F	1	547.20
tat	Y	1	0.53
tac	Y	0	0.47
mPD		0.0044	0.48
		nPD:	0.01
		N. weight:	1.1
		Sc. PD:	-0.16
		Sc. rank:	-514.8

PA

Pos.	192	obs:	exp:
cgt	R	905	38.66
cgc	R	0	45.98
cga	R	0	90.24
cgg	R	0	75.38
aga	R	0	413.20
agg	R	0	241.50
mPD		0	0.96
		nPD:	0.
		N. weight:	3.9
		Sc. PD:	-0.57
		Sc. rank:	-2460.5

PA

Pos.	193	obs:	exp:
caa	Q	14	464.80
cag	Q	891	440.20
mPD		0.030	0.50
		nPD:	0.06
		N. weight:	0.79
		Sc. PD:	-0.07
		Sc. rank:	-124.0

PA

Pos.	194	obs:	exp:
tct	S	164	144.90
tcc	S	726	117.10
tca	S	15	228.40
tcg	S	0	52.67
agt	S	0	185.40
agc	S	0	176.50
mPD		0.32	1.7
		nPD:	0.2
		N. weight:	1.8
		Sc. PD:	0.072
		Sc. rank:	461.4

PA

Pos.	195	obs:	exp:
gaa	E	829	544.40
gag	E	76	360.60
mPD		0.15	0.48
		nPD:	0.32
		N. weight:	0.32
		Sc. PD:	0.051
		Sc. rank:	147.2

PA

Pos.	196	obs:	exp:
cgt	R	0	38.66
cgc	R	0	45.98
cga	R	0	90.24
cgg	R	0	75.38
aga	R	905	413.20
agg	R	0	241.50
mPD		0	0.96
		nPD:	0.
		N. weight:	0.96
		Sc. PD:	-0.14
		Sc. rank:	-611.8

PA

Pos.	197	obs:	exp:
ggt	G	0	121.40
ggc	G	905	115.00
gga	G	0	418.40
ggg	G	0	250.20
mPD		0	0.68
		nPD:	0.
		N. weight:	2.5
		Sc. PD:	-0.38
		Sc. rank:	-1610.0

PA

Pos.	198	obs:	exp:
gaa	E	845	544.40
gag	E	60	360.60
mPD		0.12	0.48
		nPD:	0.26
		N. weight:	0.36
		Sc. PD:	0.037
		Sc. rank:	143.6

PA

Pos.	199	obs:	exp:
gaa	E	812	544.40
gag	E	93	360.60
mPD		0.18	0.48
		nPD:	0.39
		N. weight:	0.27
		Sc. PD:	0.061
		Sc. rank:	140.0

PA

Pos.	200	obs:	exp:
act	T	0	234.20
acc	T	0	184.10
aca	T	904	415.30
acg	T	0	70.42
gct	A	0	0.26
gcc	A	0	0.19
gca	A	1	0.47
gcg	A	0	0.09
mPD		0.0022	0.68
		nPD:	0.
		N. weight:	0.95
		Sc. PD:	-0.14
		Sc. rank:	-513.6

PA

Pos.	201	obs:	exp:
att	I	900	328.40
atc	I	0	217.70
ata	I	0	353.90
act	T	2	0.52
acc	T	0	0.41
aca	T	0	0.92
acg	T	0	0.16
gtt	V	3	0.62
gtc	V	0	0.60
gta	V	0	0.62
gtg	V	0	1.16
mPD		0.011	0.67
		nPD:	0.02
		N. weight:	1.2
		Sc. PD:	-0.16
		Sc. rank:	-491.8

PA

Pos.	202	obs:	exp:
gaa	E	903	544.40
gag	E	2	360.60
mPD		0.0044	0.48
		nPD:	0.01
		N. weight:	0.61
		Sc. PD:	-0.085
		Sc. rank:	-275.9

PA

Pos.	203	obs:	exp:
gaa	E	871	544.40
gag	E	34	360.60
mPD		0.072	0.48
		nPD:	0.15
		N. weight:	0.45
		Sc. PD:	-0.0011
		Sc. rank:	58.2

PA

Pos.	204	obs:	exp:
cgt	R	0	38.49
cgc	R	0	45.78
cga	R	0	89.84
cgg	R	0	75.04
aaa	K	4	2.34
aag	K	0	1.66
aga	R	896	411.40
agg	R	5	240.50
mPD		0.020	0.97
		nPD:	0.02
		N. weight:	0.92
		Sc. PD:	-0.12
		Sc. rank:	-342.1

PA

Pos.	205	obs:	exp:
ttt	F	905	357.20
ttc	F	0	547.80
mPD		0	0.48
		nPD:	0.
		N. weight:	1.1
		Sc. PD:	-0.17
		Sc. rank:	-725.5

PA

Pos.	206	obs:	exp:
aaa	K	2	1.17
aag	K	0	0.83
gat	D	0	3.24
gac	D	6	2.76
gaa	E	885	539.60
gag	E	12	357.40
mPD		0.044	0.49
		nPD:	0.09
		N. weight:	0.55
		Sc. PD:	-0.034
		Sc. rank:	-30.0

PA

Pos.	207	obs:	exp:
att	I	47	330.20
atc	I	858	218.90
ata	I	0	355.90
mPD		0.099	0.65
		nPD:	0.15
		N. weight:	1.5
		Sc. PD:	-0.0039
		Sc. rank:	188.8

PA

Pos.	208	obs:	exp:
act	T	0	232.60
acc	T	0	182.90
aca	T	898	412.60
acg	T	0	69.95
aaa	K	2	1.17
aag	K	0	0.83
gct	A	0	1.28
gcc	A	0	0.94
gca	A	5	2.34
gcg	A	0	0.44
mPD		0.015	0.69
		nPD:	0.02
		N. weight:	0.95
		Sc. PD:	-0.12
		Sc. rank:	-340.4

PA

Pos.	209	obs:	exp:
ggt	G	0	121.40
ggc	G	3	115.00
gga	G	897	418.40
ggg	G	5	250.20
mPD		0.018	0.68
		nPD:	0.03
		N. weight:	0.89
		Sc. PD:	-0.11
		Sc. rank:	-291.2

PA

Pos.	210	obs:	exp:
act	T	4	234.40
acc	T	899	184.30
aca	T	2	415.80
acg	T	0	70.50
mPD		0.013	0.67
		nPD:	0.02
		N. weight:	1.9
		Sc. PD:	-0.25
		Sc. rank:	-712.5

PA			
Pos.	211	obs:	exp:
tta	L	0	0.53
ttg	L	0	1.12
ctt	L	0	1.02
ctc	L	0	1.01
cta	L	0	1.00
ctg	L	6	1.33
att	I	0	0.36
atc	I	0	0.24
ata	I	1	0.39
atg	M	898	898.00
---	--	------	------
mPD		0.015	0.023
		nPD:	0.66
		N. weight:	0.014
		Sc. PD:	0.0066
		Sc. rank:	8.4

PA			
Pos.	212	obs:	exp:
tta	L	0	0.18
ttg	L	0	0.37
ctt	L	0	0.34
ctc	L	2	0.34
cta	L	0	0.33
ctg	L	0	0.44
cgt	R	9	38.58
cgc	R	891	45.88
cga	R	3	90.04
cgg	R	0	75.21
aga	R	0	412.30
agg	R	0	241.00
---	--	------	------
mPD		0.031	0.97
		nPD:	0.03
		N. weight:	3.6
		Sc. PD:	-0.42
		Sc. rank:	-1019.9

PA			
Pos.	213	obs:	exp:
cgt	R	0	38.66
cgc	R	0	45.98
cga	R	0	90.24
cgg	R	0	75.38
aga	R	0	413.20
agg	R	905	241.50
---	--	------	------
mPD		0	0.96
		nPD:	0.
		N. weight:	1.6
		Sc. PD:	-0.24
		Sc. rank:	-1030.8

PA			
Pos.	214	obs:	exp:
ttt	F	0	0.39
ttc	F	1	0.61
tta	L	0	79.45
ttg	L	0	168.70
ctt	L	862	153.20
ctc	L	42	151.50
cta	L	0	150.70
ctg	L	0	200.50
---	--	------	------
mPD		0.093	1.1
		nPD:	0.08
		N. weight:	1.9
		Sc. PD:	-0.13
		Sc. rank:	-146.3

PA			
Pos.	215	obs:	exp:
gct	A	43	231.90
gcc	A	857	169.90
gca	A	5	423.10
gcg	A	0	80.05
---	--	------	------
mPD		0.10	0.67
		nPD:	0.15
		N. weight:	1.8
		Sc. PD:	-0.0056
		Sc. rank:	221.4

PA			
Pos.	216	obs:	exp:
aat	N	0	1.02
aac	N	2	0.98
gat	D	6	487.50
gac	D	897	415.50
---	--	------	------
mPD		0.018	0.50
		nPD:	0.04
		N. weight:	0.9
		Sc. PD:	-0.1
		Sc. rank:	-239.9

PA			
Pos.	217	obs:	exp:
caa	Q	905	464.80
cag	Q	0	440.20
---	--	------	------
mPD		0	0.50
		nPD:	0.
		N. weight:	0.82
		Sc. PD:	-0.12
		Sc. rank:	-519.9

PA			
Pos.	218	obs:	exp:
tct	S	0	144.90
tcc	S	0	117.10
tca	S	0	228.40
tcg	S	0	52.67
agt	S	899	185.40
agc	S	6	176.50
---	--	------	------
mPD		0.013	1.7
		nPD:	0.01
		N. weight:	1.9
		Sc. PD:	-0.27
		Sc. rank:	-890.5

PA			
Pos.	219	obs:	exp:
tta	L	0	79.54
ttg	L	0	168.90
ctt	L	1	153.40
ctc	L	904	151.60
cta	L	0	150.90
ctg	L	0	200.70
---	--	------	------
mPD		0.0022	1.1
		nPD:	0.
		N. weight:	2.2
		Sc. PD:	-0.32
		Sc. rank:	-1230.1

PA			
Pos.	220	obs:	exp:
cct	P	0	230.10
ccc	P	0	161.40
cca	P	905	369.70
ccg	P	0	143.80
---	--	------	------
mPD		0	0.71
		nPD:	0.
		N. weight:	1.1
		Sc. PD:	-0.16
		Sc. rank:	-698.5

PA			
Pos.	221	obs:	exp:
cct	P	2	230.10
ccc	P	0	161.40
cca	P	0	369.70
ccg	P	903	143.80
---	--	------	------
mPD		0.0044	0.71
		nPD:	0.01
		N. weight:	2.2
		Sc. PD:	-0.32
		Sc. rank:	-1109.8

PA			
Pos.	222	obs:	exp:
aat	N	16	458.90
aac	N	886	443.10
ggt	G	3	0.40
ggc	G	0	0.38
gga	G	0	1.39
ggg	G	0	0.83
---	--	------	------
mPD		0.054	0.52
		nPD:	0.11
		N. weight:	0.77
		Sc. PD:	-0.036
		Sc. rank:	-8.6

PA			
Pos.	223	obs:	exp:
ttt	F	0	356.80
ttc	F	904	547.20
tct	S	1	0.16
tcc	S	0	0.13
tca	S	0	0.25
tcg	S	0	0.06
agt	S	0	0.20
agc	S	0	0.20
---	--	------	------
mPD		0.0044	0.48
		nPD:	0.01
		N. weight:	0.62
		Sc. PD:	-0.086
		Sc. rank:	-282.5

PA			
Pos.	224	obs:	exp:
tct	S	0	142.80
tcc	S	892	115.50
tca	S	0	225.10
tcg	S	0	51.91
cct	P	0	0.25
ccc	P	1	0.18
cca	P	0	0.41
ccg	P	0	0.16
act	T	0	3.11
acc	T	12	2.44
aca	T	0	5.51
acg	T	0	0.93
agt	S	0	182.80
agc	S	0	173.90
---	--	------	------
mPD		0.028	1.7
		nPD:	0.02
		N. weight:	2.5
		Sc. PD:	-0.33
		Sc. rank:	-975.1

PA			
Pos.	225	obs:	exp:
tct	S	0	144.70
tcc	S	0	117.00
tca	S	0	228.10
tcg	S	0	52.61
cgt	R	0	0.04
cgc	R	0	0.05
cga	R	0	0.10
cgg	R	0	0.08
agt	S	0	185.20
agc	S	904	176.30
aga	R	0	0.46
agg	R	1	0.27
---	--	------	------
mPD		0.0022	1.7
		nPD:	0.
		N. weight:	2.
		Sc. PD:	-0.29
		Sc. rank:	-1152.2

PA			
Pos.	226	obs:	exp:
ttt	F	35	13.81
ttc	F	0	21.19
tta	L	0	76.37
ttg	L	0	162.20
ctt	L	869	147.30
ctc	L	0	145.60
cta	L	0	144.90
ctg	L	0	192.70
cat	H	1	0.57
cac	H	0	0.43
---	--	------	------
mPD		0.077	1.1
		nPD:	0.07
		N. weight:	2.1
		Sc. PD:	-0.18
		Sc. rank:	-281.2

PA			
Pos.	227	obs:	exp:
gaa	E	902	544.40
gag	E	3	360.60
---	--	------	------
mPD		0.0066	0.48
		nPD:	0.01
		N. weight:	0.6
		Sc. PD:	-0.081
		Sc. rank:	-247.8

PA			
Pos.	228	obs:	exp:
tct	S	0	0.80
tcc	S	0	0.65
tca	S	0	1.26
tcg	S	0	0.29
aat	N	1	457.90
aac	N	899	442.10
agt	S	0	1.02
agc	S	5	0.98
---	--	------	------
mPD		0.013	0.52
		nPD:	0.03
		N. weight:	0.87
		Sc. PD:	-0.11
		Sc. rank:	-288.5

PA			
Pos.	229	obs:	exp:
ttt	F	905	357.20
ttc	F	0	547.80
---	--	------	------
mPD		0	0.48
		nPD:	0.
		N. weight:	1.1
		Sc. PD:	-0.17
		Sc. rank:	-725.5

PA			
Pos.	230	obs:	exp:
cgt	R	0	38.66
cgc	R	0	45.98
cga	R	0	90.24
cgg	R	0	75.38
aga	R	904	413.20
agg	R	1	241.50
---	--	------	------
mPD		0.0022	0.96
		nPD:	0.
		N. weight:	0.95
		Sc. PD:	-0.14
		Sc. rank:	-532.0

PA			
Pos.	231	obs:	exp:
act	T	0	1.55
acc	T	6	1.22
aca	T	0	2.76
acg	T	0	0.47
gct	A	0	230.40
gcc	A	899	168.80
gca	A	0	420.30
gcg	A	0	79.51
---	--	------	------
mPD		0.013	0.69
		nPD:	0.02
		N. weight:	2.
		Sc. PD:	-0.27
		Sc. rank:	-783.3

PA			
Pos.	232	obs:	exp:
tat	Y	905	479.80
tac	Y	0	425.20
---	--	------	------
mPD		0	0.50
		nPD:	0.
		N. weight:	0.78
		Sc. PD:	-0.12
		Sc. rank:	-495.1

PA			
Pos.	233	obs:	exp:
gtt	V	0	186.80
gtc	V	0	181.30
gta	V	2	187.40
gtg	V	903	349.50
---	--	------	------
mPD		0.0044	0.73
		nPD:	0.01
		N. weight:	1.2
		Sc. PD:	-0.16
		Sc. rank:	-574.3

PA			
Pos.	234	obs:	exp:
gat	D	905	488.60
gac	D	0	416.40
---	--	------	------
mPD		0	0.50
		nPD:	0.
		N. weight:	0.76
		Sc. PD:	-0.11
		Sc. rank:	-481.1

PA			
Pos.	235	obs:	exp:
ggt	G	0	121.40
ggc	G	0	115.00
gga	G	905	418.40
ggg	G	0	250.20
---	--	------	------
mPD		0	0.68
		nPD:	0.
		N. weight:	0.95
		Sc. PD:	-0.14
		Sc. rank:	-602.0

PA

Pos.	236	obs:	exp:
ttt	F	7	357.20
ttc	F	898	547.80
---	--	------	------
mPD		0.015	0.48
		nPD:	0.03
		N. weight:	0.57
		Sc. PD:	-0.066
		Sc. rank:	-158.0

PA

Pos.	237	obs:	exp:
aaa	K	5	2.93
aag	K	0	2.07
gat	D	0	0.54
gac	D	1	0.46
gaa	E	893	540.20
gag	E	5	357.80
ggt	G	0	0.13
ggc	G	0	0.13
gga	G	1	0.46
ggg	G	0	0.28
---	--	------	------
mPD		0.026	0.49
		nPD:	0.05
		N. weight:	0.59
		Sc. PD:	-0.057
		Sc. rank:	-109.0

PA

Pos.	238	obs:	exp:
cct	P	0	230.10
ccc	P	0	161.40
cca	P	0	369.70
ccg	P	905	143.80
---	--	------	------
mPD		0	0.71
		nPD:	0.
		N. weight:	2.3
		Sc. PD:	-0.33
		Sc. rank:	-1435.5

PA

Pos.	239	obs:	exp:
aat	N	1	459.90
aac	N	903	444.10
aaa	K	1	0.59
aag	K	0	0.41
---	--	------	------
mPD		0.0044	0.50
		nPD:	0.01
		N. weight:	0.86
		Sc. PD:	-0.12
		Sc. rank:	-400.6

PA

Pos.	240	obs:	exp:
ggt	G	0	121.40
ggc	G	905	115.00
gga	G	0	418.40
ggg	G	0	250.20
---	--	------	------
mPD		0	0.68
		nPD:	0.
		N. weight:	2.5
		Sc. PD:	-0.38
		Sc. rank:	-1610.0

PA

Pos.	241	obs:	exp:
tat	Y	0	1.59
tac	Y	3	1.41
tgt	C	0	370.30
tgc	C	902	531.70
---	--	------	------
mPD		0.0066	0.49
		nPD:	0.01
		N. weight:	0.65
		Sc. PD:	-0.088
		Sc. rank:	-272.7

PA

Pos.	242	obs:	exp:
att	I	904	330.20
atc	I	1	218.90
ata	I	0	355.90
---	--	------	------
mPD		0.0022	0.65
		nPD:	0.
		N. weight:	1.2
		Sc. PD:	-0.18
		Sc. rank:	-651.8

PA

Pos.	243	obs:	exp:
gaa	E	0	544.40
gag	E	905	360.60
---	--	------	------
mPD		0	0.48
		nPD:	0.
		N. weight:	1.1
		Sc. PD:	-0.17
		Sc. rank:	-718.0

PA

Pos.	244	obs:	exp:
ggt	G	1	121.40
ggc	G	903	115.00
gga	G	1	418.40
ggg	G	0	250.20
---	--	------	------
mPD		0.0044	0.68
		nPD:	0.01
		N. weight:	2.5
		Sc. PD:	-0.36
		Sc. rank:	-1222.1

PA

Pos.	245	obs:	exp:
aaa	K	0	530.10
aag	K	905	374.90
---	--	------	------
mPD		0	0.49
		nPD:	0.
		N. weight:	1.1
		Sc. PD:	-0.16
		Sc. rank:	-687.8

PA

Pos.	246	obs:	exp:
tta	L	0	79.45
ttg	L	0	168.70
ctt	L	904	153.20
ctc	L	0	151.50
cta	L	0	150.70
ctg	L	0	200.50
att	I	1	0.36
atc	I	0	0.24
ata	I	0	0.39
---	--	------	------
mPD		0.0022	1.1
		nPD:	0.
		N. weight:	2.2
		Sc. PD:	-0.32
		Sc. rank:	-1242.4

PA

Pos.	247	obs:	exp:
tct	S	902	144.40
tcc	S	0	116.80
tca	S	0	227.60
tcg	S	0	52.49
agt	S	0	184.80
agc	S	0	175.90
gct	A	3	0.77
gcc	A	0	0.56
gca	A	0	1.40
gcg	A	0	0.27
---	--	------	------
mPD		0.0066	1.7
		nPD:	0.
		N. weight:	2.2
		Sc. PD:	-0.32
		Sc. rank:	-1177.9

PA

Pos.	248	obs:	exp:
caa	Q	562	464.80
cag	Q	343	440.20
---	--	------	------
mPD		0.47	0.50
		nPD:	0.94
		N. weight:	0.033
		Sc. PD:	0.026
		Sc. rank:	21.4

PA

Pos.	249	obs:	exp:
atg	M	905	905.00
---	--	------	------
mPD		0	0
		nPD:	1.
		N. weight:	0.
		Sc. PD:	0
		Sc. rank:	0

PA

Pos.	250	obs:	exp:
tta	L	0	0.09
ttg	L	0	0.19
tct	S	0	144.20
tcc	S	34	116.60
tca	S	864	227.40
tcg	S	3	52.43
ctt	L	0	0.17
ctc	L	1	0.17
cta	L	0	0.17
ctg	L	0	0.22
agt	S	0	184.60
agc	S	0	175.70
gct	A	0	0.77
gcc	A	0	0.56
gca	A	3	1.40
gcg	A	0	0.27
---	--	------	------
mPD		0.092	1.7
		nPD:	0.06
		N. weight:	1.5
		Sc. PD:	-0.14
		Sc. rank:	-255.4

PA

Pos.	251	obs:	exp:
caa	Q	1	0.51
cag	Q	0	0.49
cgt	R	0	0.09
cgc	R	0	0.10
cga	R	0	0.20
cgg	R	0	0.17
aaa	K	895	527.20
aag	K	5	372.80
aga	R	2	0.91
agg	R	0	0.53
gaa	E	2	1.20
gag	E	0	0.80
---	--	------	------
mPD		0.022	0.50
		nPD:	0.04
		N. weight:	0.62
		Sc. PD:	-0.065
		Sc. rank:	-135.0

PA

Pos.	252	obs:	exp:
aaa	K	1	0.59
aag	K	0	0.41
gaa	E	903	543.20
gag	E	0	359.80
ggt	G	0	0.13
ggc	G	0	0.13
gga	G	1	0.46
ggg	G	0	0.28
---	--	------	------
mPD		0.0044	0.48
		nPD:	0.01
		N. weight:	0.62
		Sc. PD:	-0.087
		Sc. rank:	-286.1

PA

Pos.	253	obs:	exp:
gtt	V	0	186.80
gtc	V	0	181.30
gta	V	34	187.40
gtg	V	871	349.50
---	--	------	------
mPD		0.072	0.73
		nPD:	0.1
		N. weight:	1.
		Sc. PD:	-0.052
		Sc. rank:	-26.7

PA

Pos.	254	obs:	exp:
tct	S	0	0.32
tcc	S	0	0.26
tca	S	0	0.50
tcg	S	0	0.12
aat	N	67	459.40
aac	N	836	443.60
agt	S	1	0.41
agc	S	1	0.39
---	--	------	------
mPD		0.14	0.51
		nPD:	0.28
		N. weight:	0.55
		Sc. PD:	0.069
		Sc. rank:	236.3

PA

Pos.	255	obs:	exp:
gct	A	12	231.90
gcc	A	851	169.90
gca	A	42	423.10
gcg	A	0	80.05
---	--	------	------
mPD		0.11	0.67
		nPD:	0.17
		N. weight:	1.7
		Sc. PD:	0.025
		Sc. rank:	288.3

PA

Pos.	256	obs:	exp:
cgt	R	0	38.58
cgc	R	0	45.88
cga	R	3	90.04
cgg	R	0	75.21
act	T	0	0.26
acc	T	0	0.20
aca	T	1	0.46
acg	T	0	0.08
aaa	K	1	0.59
aag	K	0	0.41
aga	R	870	412.30
agg	R	30	241.00
---	--	------	------
mPD		0.075	0.97
		nPD:	0.08
		N. weight:	0.79
		Sc. PD:	-0.058
		Sc. rank:	-73.7

PA

Pos.	257	obs:	exp:
att	I	456	330.20
atc	I	418	218.90
ata	I	31	355.90
---	--	------	------
mPD		0.53	0.65
		nPD:	0.81
		N. weight:	0.47
		Sc. PD:	0.3
		Sc. rank:	299.3

PA

Pos.	258	obs:	exp:
aaa	K	0	0.59
aag	K	1	0.41
gat	D	1	0.54
gac	D	0	0.46
gaa	E	85	542.60
gag	E	817	359.40
ggt	G	0	0.13
ggc	G	0	0.13
gga	G	0	0.46
ggg	G	1	0.28
---	--	------	------
mPD		0.18	0.49
		nPD:	0.36
		N. weight:	0.7
		Sc. PD:	0.14
		Sc. rank:	351.6

PA

Pos.	259	obs:	exp:
cct	P	1	229.90
ccc	P	6	161.20
cca	P	892	369.30
ccg	P	5	143.60
caa	Q	1	0.51
cag	Q	0	0.49
---	--	------	------
mPD		0.029	0.71
		nPD:	0.04
		N. weight:	1.
		Sc. PD:	-0.11
		Sc. rank:	-237.9

PA

Pos.	260	obs:	exp:
ttt	F	893	357.20
ttc	F	12	547.80
---	--	------	------
mPD		0.026	0.48
		nPD:	0.05
		N. weight:	1.
		Sc. PD:	-0.1
		Sc. rank:	-186.5

PA

Pos.	261	obs:	exp:
tta	L	7	79.27
ttg	L	11	168.30
ctt	L	0	152.90
ctc	L	1	151.10
cta	L	803	150.40
ctg	L	80	200.00
att	I	0	0.36
atc	I	0	0.24
ata	I	1	0.39
atg	M	1	1.00
act	T	0	0.26
acc	T	0	0.20
aca	T	0	0.46
acg	T	1	0.08
---	--	------	------
mPD		0.23	1.1
		nPD:	0.21
		N. weight:	1.7
		Sc. PD:	0.091
		Sc. rank:	491.6

,

PA

Pos.	262	obs:	exp:
cgt	R	0	26.61
cgc	R	0	31.65
cga	R	0	62.12
cgg	R	0	51.89
atg	M	1	1.00
aaa	K	31	164.60
aag	K	250	116.40
aga	R	370	284.40
agg	R	253	166.30
---	--	------	------
mPD		0.92	1.3
		nPD:	0.72
		N. weight:	0.47
		Sc. PD:	0.26
		Sc. rank:	298.5

,

PA

Pos.	263	obs:	exp:
act	T	102	226.40
acc	T	14	178.00
aca	T	756	401.50
acg	T	2	68.08
aat	N	0	0.51
aac	N	1	0.49
aaa	K	29	17.57
aag	K	1	12.43
---	--	------	------
mPD		0.30	0.74
		nPD:	0.41
		N. weight:	0.51
		Sc. PD:	0.12
		Sc. rank:	271.0

,

PA

Pos.	264	obs:	exp:
act	T	1	234.40
acc	T	0	184.30
aca	T	902	415.80
acg	T	2	70.50
---	--	------	------
mPD		0.0066	0.67
		nPD:	0.01
		N. weight:	0.93
		Sc. PD:	-0.13
		Sc. rank:	-414.3

,

PA

Pos.	265	obs:	exp:
cct	P	1	230.10
ccc	P	0	161.40
cca	P	897	369.70
ccg	P	7	143.80
---	--	------	------
mPD		0.018	0.71
		nPD:	0.02
		N. weight:	1.
		Sc. PD:	-0.13
		Sc. rank:	-351.6

,

PA

Pos.	266	obs:	exp:
cct	P	0	0.25
ccc	P	1	0.18
cca	P	0	0.41
ccg	P	0	0.16
cgt	R	10	38.62
cgc	R	733	45.93
cga	R	121	90.14
cgg	R	38	75.29
aga	R	1	412.70
agg	R	1	241.30
---	--	------	------
mPD		0.33	0.97
		nPD:	0.34
		N. weight:	2.7
		Sc. PD:	0.5
		Sc. rank:	1320.8

,

PA

Pos.	267	obs:	exp:
tta	L	0	0.09
ttg	L	0	0.19
tct	S	8	1.28
tcc	S	0	1.04
tca	S	0	2.02
tcg	S	0	0.47
ctt	L	0	0.17
ctc	L	0	0.17
cta	L	0	0.17
ctg	L	1	0.22
cct	P	682	227.80
ccc	P	15	159.80
cca	P	32	366.10
ccg	P	167	142.40
agt	S	0	1.64
agc	S	0	1.56
---	--	------	------
mPD		0.40	0.74
		nPD:	0.54
		N. weight:	0.93
		Sc. PD:	0.35
		Sc. rank:	552.2

,

PA

Pos.	268	obs:	exp:
ttt	F	0	3.95
ttc	F	10	6.05
tta	L	0	78.57
ttg	L	0	166.80
ctt	L	9	151.50
ctc	L	852	149.80
cta	L	1	149.00
ctg	L	32	198.30
att	I	0	0.36
atc	I	1	0.24
ata	I	0	0.39
---	--	------	------
mPD		0.11	1.1
		nPD:	0.1
		N. weight:	1.9
		Sc. PD:	-0.096
		Sc. rank:	-37.7

,

PA

Pos.	269	obs:	exp:
cgt	R	0	37.93
cgc	R	0	45.12
cga	R	0	88.54
cgg	R	0	73.96
att	I	0	0.73
atc	I	0	0.48
ata	I	2	0.79
aaa	K	13	8.79
aag	K	2	6.21
aga	R	880	405.40
agg	R	8	237.00
---	--	------	------
mPD		0.059	1.0
		nPD:	0.06
		N. weight:	0.9
		Sc. PD:	-0.082
		Sc. rank:	-145.5

,

PA

Pos.	270	obs:	exp:
ttt	F	6	2.37
ttc	F	0	3.63
tta	L	55	77.08
ttg	L	814	163.70
ctt	L	1	148.60
ctc	L	0	146.90
cta	L	5	146.20
ctg	L	2	194.50
atg	M	22	22.00
---	--	------	------
mPD		0.20	1.1
		nPD:	0.18
		N. weight:	1.7
		Sc. PD:	0.041
		Sc. rank:	333.0

,

PA

Pos.	271	obs:	exp:
tta	L	0	0.09
ttg	L	0	0.19
ctt	L	1	0.17
ctc	L	0	0.17
cta	L	0	0.17
ctg	L	0	0.22
cct	P	865	229.90
ccc	P	0	161.20
cca	P	6	369.30
ccg	P	33	143.60
---	--	------	------
mPD		0.085	0.71
		nPD:	0.12
		N. weight:	1.5
		Sc. PD:	-0.048
		Sc. rank:	47.9

,

PA

Pos.	272	obs:	exp:
tct	S	0	0.80
tcc	S	0	0.65
tca	S	0	1.26
tcg	S	0	0.29
act	T	1	0.26
acc	T	0	0.20
aca	T	0	0.46
acg	T	0	0.08
aat	N	734	373.90
aac	N	1	361.10
agt	S	5	1.02
agc	S	0	0.98
gat	D	121	70.72
gac	D	10	60.28
gaa	E	33	19.85
gag	E	0	13.15
---	--	------	------
mPD		0.40	0.85
		nPD:	0.47
		N. weight:	0.77
		Sc. PD:	0.24
		Sc. rank:	440.4

,

PA

Pos.	273	obs:	exp:
ggt	G	0	121.40
ggc	G	13	115.00
gga	G	43	418.40
ggg	G	849	250.20
---	--	------	------
mPD		0.12	0.68
		nPD:	0.17
		N. weight:	1.2
		Sc. PD:	0.025
		Sc. rank:	225.2

,

PA

Pos.	274	obs:	exp:
tta	L	0	0.09
ttg	L	0	0.19
ctt	L	1	0.17
ctc	L	0	0.17
cta	L	0	0.17
ctg	L	0	0.22
cct	P	745	229.60
ccc	P	50	161.00
cca	P	104	368.90
ccg	P	4	143.50
cat	H	1	0.57
cac	H	0	0.43
---	--	------	------
mPD		0.31	0.72
		nPD:	0.43
		N. weight:	0.92
		Sc. PD:	0.25
		Sc. rank:	509.4

,

PA

Pos.	275	obs:	exp:
tta	L	0	0.35
ttg	L	0	0.75
tct	S	0	0.48
tcc	S	3	0.39
tca	S	0	0.76
tcg	S	0	0.17
ctt	L	0	0.68
ctc	L	4	0.67
cta	L	0	0.67
ctg	L	0	0.89
cct	P	54	228.30
ccc	P	843	160.10
cca	P	0	366.90
ccg	P	1	142.70
agt	S	0	0.61
agc	S	0	0.59
---	--	------	------
mPD		0.13	0.73
		nPD:	0.18
		N. weight:	1.8
		Sc. PD:	0.041
		Sc. rank:	340.7

,

PA

Pos.	276	obs:	exp:
tgt	C	334	371.50
tgc	C	571	533.50
---	--	------	------
mPD		0.47	0.48
		nPD:	0.96
		N. weight:	0.0093
		Sc. PD:	0.0073
		Sc. rank:	5.9

,

PA

Pos.	277	obs:	exp:
ttt	F	5	1.97
ttc	F	0	3.03
tct	S	841	143.70
tcc	S	57	116.20
tca	S	0	226.60
tcg	S	0	52.26
tat	Y	1	0.53
tac	Y	0	0.47
cct	P	1	0.25
ccc	P	0	0.18
cca	P	0	0.41
ccg	P	0	0.16
agt	S	0	184.00
agc	S	0	175.10
---	--	------	------
mPD		0.13	1.7
		nPD:	0.08
		N. weight:	2.
		Sc. PD:	-0.14
		Sc. rank:	-171.4

,

PA

Pos.	278	obs:	exp:
caa	Q	87	464.80
cag	Q	818	440.20
---	--	------	------
mPD		0.17	0.50
		nPD:	0.35
		N. weight:	0.49
		Sc. PD:	0.093
		Sc. rank:	240.2

,

PA

Pos.	279	obs:	exp:
cgt	R	0	38.66
cgc	R	2	45.98
cga	R	34	90.24
cgg	R	832	75.38
aga	R	1	413.20
agg	R	36	241.50
---	--	------	------
mPD		0.16	0.96
		nPD:	0.16
		N. weight:	2.6
		Sc. PD:	0.024
		Sc. rank:	398.3

,

PA

Pos.	280	obs:	exp:
tct	S	6	144.90
tcc	S	2	117.10
tca	S	14	228.40
tcg	S	883	52.67
agt	S	0	185.40
agc	S	0	176.50
---	--	------	------
mPD		0.048	1.7
		nPD:	0.03
		N. weight:	3.3
		Sc. PD:	-0.4
		Sc. rank:	-994.0

,

PA			
Pos.	281	obs:	exp:
aaa	K	752	530.10
aag	K	153	374.90
---	--	------	------
mPD		0.28	0.49
		nPD:	0.58
		N. weight:	0.17
		Sc. PD:	0.072
		Sc. rank:	106.6

,

PA			
Pos.	282	obs:	exp:
ttt	F	36	357.20
ttc	F	869	547.80
---	--	------	------
mPD		0.076	0.48
		nPD:	0.16
		N. weight:	0.43
		Sc. PD:	0.0027
		Sc. rank:	62.8

,

PA			
Pos.	283	obs:	exp:
tta	L	52	79.54
ttg	L	814	168.90
ctt	L	0	153.40
ctc	L	1	151.60
cta	L	0	150.90
ctg	L	38	200.70
---	--	------	------
mPD		0.19	1.1
		nPD:	0.17
		N. weight:	1.6
		Sc. PD:	0.032
		Sc. rank:	290.5

,

PA			
Pos.	284	obs:	exp:
tta	L	0	79.27
ttg	L	7	168.30
ctt	L	4	152.90
ctc	L	0	151.10
cta	L	7	150.40
ctg	L	884	200.00
atg	M	3	3.00
---	--	------	------
mPD		0.046	1.1
		nPD:	0.04
		N. weight:	1.7
		Sc. PD:	-0.19
		Sc. rank:	-389.6

,

PA			
Pos.	285	obs:	exp:
atg	M	905	905.00
---	--	------	------
mPD		0	0
		nPD:	1.
		N. weight:	0.
		Sc. PD:	0
		Sc. rank:	0

,

PA			
Pos.	286	obs:	exp:
gat	D	898	488.60
gac	D	7	416.40
---	--	------	------
mPD		0.015	0.50
		nPD:	0.03
		N. weight:	0.7
		Sc. PD:	-0.083
		Sc. rank:	-208.9

,

PA			
Pos.	287	obs:	exp:
gct	A	864	231.90
gcc	A	40	169.90
gca	A	1	423.10
gcg	A	0	80.05
---	--	------	------
mPD		0.087	0.67
		nPD:	0.13
		N. weight:	1.5
		Sc. PD:	-0.035
		Sc. rank:	96.7

,

PA			
Pos.	288	obs:	exp:
tta	L	0	79.45
ttg	L	94	168.70
ctt	L	71	153.20
ctc	L	1	151.50
cta	L	74	150.70
ctg	L	664	200.50
atg	M	1	1.00
---	--	------	------
mPD		0.47	1.1
		nPD:	0.43
		N. weight:	0.86
		Sc. PD:	0.23
		Sc. rank:	474.3

,

PA			
Pos.	289	obs:	exp:
aat	N	0	0.51
aac	N	1	0.49
aaa	K	851	529.60
aag	K	53	374.40
---	--	------	------
mPD		0.11	0.49
		nPD:	0.23
		N. weight:	0.41
		Sc. PD:	0.031
		Sc. rank:	143.2

,

PA			
Pos.	290	obs:	exp:
tta	L	717	79.54
ttg	L	21	168.90
ctt	L	34	153.40
ctc	L	0	151.60
cta	L	132	150.90
ctg	L	1	200.70
---	--	------	------
mPD		0.42	1.1
		nPD:	0.38
		N. weight:	2.
		Sc. PD:	0.43
		Sc. rank:	1011.8

,

PA			
Pos.	291	obs:	exp:
tct	S	0	144.90
tcc	S	0	117.10
tca	S	0	228.40
tcg	S	0	52.67
agt	S	27	185.40
agc	S	878	176.50
---	--	------	------
mPD		0.058	1.7
		nPD:	0.04
		N. weight:	1.8
		Sc. PD:	-0.21
		Sc. rank:	-491.8

,

PA			
Pos.	292	obs:	exp:
att	I	863	329.80
atc	I	41	218.70
ata	I	0	355.50
act	T	1	0.26
acc	T	0	0.20
aca	T	0	0.46
acg	T	0	0.08
---	--	------	------
mPD		0.089	0.66
		nPD:	0.14
		N. weight:	1.
		Sc. PD:	-0.018
		Sc. rank:	83.5

,

PA			
Pos.	293	obs:	exp:
gaa	E	322	544.40
gag	E	583	360.60
---	--	------	------
mPD		0.46	0.48
		nPD:	0.96
		N. weight:	0.16
		Sc. PD:	0.12
		Sc. rank:	99.1

,

PA			
Pos.	294	obs:	exp:
aat	N	0	0.51
aac	N	1	0.49
gat	D	3	487.50
gac	D	900	415.50
ggt	G	0	0.13
ggc	G	1	0.13
gga	G	0	0.46
ggg	G	0	0.28
---	--	------	------
mPD		0.011	0.50
		nPD:	0.02
		N. weight:	0.93
		Sc. PD:	-0.12
		Sc. rank:	-335.9

,

PA			
Pos.	295	obs:	exp:
tta	L	0	0.18
ttg	L	0	0.37
ctt	L	0	0.34
ctc	L	0	0.34
cta	L	0	0.33
ctg	L	2	0.44
cct	P	9	229.60
ccc	P	0	161.00
cca	P	18	368.90
ccg	P	876	143.50
---	--	------	------
mPD		0.063	0.72
		nPD:	0.09
		N. weight:	2.
		Sc. PD:	-0.13
		Sc. rank:	-113.6

,

PA			
Pos.	296	obs:	exp:
tct	S	0	143.70
tcc	S	0	116.20
tca	S	0	226.60
tcg	S	0	52.26
aat	N	0	3.56
aac	N	7	3.44
agt	S	165	184.00
agc	S	733	175.10
---	--	------	------
mPD		0.31	1.7
		nPD:	0.19
		N. weight:	1.4
		Sc. PD:	0.048
		Sc. rank:	314.8

,

PA			
Pos.	297	obs:	exp:
tat	Y	0	3.18
tac	Y	6	2.82
cat	H	114	516.50
cac	H	785	382.50
---	--	------	------
mPD		0.23	0.50
		nPD:	0.47
		N. weight:	0.54
		Sc. PD:	0.16
		Sc. rank:	307.8

,

PA			
Pos.	298	obs:	exp:
gaa	E	849	543.20
gag	E	54	359.80
ggt	G	0	0.27
ggc	G	0	0.25
gga	G	2	0.92
ggg	G	0	0.55
---	--	------	------
mPD		0.12	0.48
		nPD:	0.24
		N. weight:	0.38
		Sc. PD:	0.032
		Sc. rank:	137.5

,

PA			
Pos.	299	obs:	exp:
cgt	R	0	0.09
cgc	R	0	0.10
cga	R	0	0.20
cgg	R	0	0.17
aga	R	0	0.91
agg	R	2	0.53
ggt	G	0	121.10
ggc	G	33	114.70
gga	G	45	417.50
ggg	G	825	249.70
---	--	------	------
mPD		0.17	0.68
		nPD:	0.24
		N. weight:	1.2
		Sc. PD:	0.1
		Sc. rank:	420.2

,

PA			
Pos.	300	obs:	exp:
gct	A	0	0.26
gcc	A	0	0.19
gca	A	0	0.47
gcg	A	1	0.09
gaa	E	32	543.80
gag	E	872	360.20
---	--	------	------
mPD		0.071	0.48
		nPD:	0.15
		N. weight:	0.93
		Sc. PD:	-0.0066
		Sc. rank:	106.8

,

PA			
Pos.	301	obs:	exp:
ggt	G	37	121.40
ggc	G	8	115.00
gga	G	12	418.40
ggg	G	848	250.20
---	--	------	------
mPD		0.12	0.68
		nPD:	0.18
		N. weight:	1.3
		Sc. PD:	0.03
		Sc. rank:	241.3

,

PA			
Pos.	302	obs:	exp:
att	I	1	329.80
atc	I	0	218.70
ata	I	903	355.50
gtt	V	0	0.21
gtc	V	0	0.20
gta	V	1	0.21
gtg	V	0	0.39
---	--	------	------
mPD		0.0044	0.66
		nPD:	0.01
		N. weight:	1.1
		Sc. PD:	-0.16
		Sc. rank:	-541.3

,

PA			
Pos.	303	obs:	exp:
cct	P	2	230.10
ccc	P	1	161.40
cca	P	24	369.70
ccg	P	878	143.80
---	--	------	------
mPD		0.058	0.71
		nPD:	0.08
		N. weight:	2.
		Sc. PD:	-0.14
		Sc. rank:	-165.4

,

PA			
Pos.	304	obs:	exp:
tta	L	4	79.54
ttg	L	0	168.90
ctt	L	1	153.40
ctc	L	0	151.60
cta	L	824	150.90
ctg	L	76	200.70
---	--	------	------
mPD		0.16	1.1
		nPD:	0.15
		N. weight:	1.8
		Sc. PD:	-0.0087
		Sc. rank:	212.3

,

PA			
Pos.	305	obs:	exp:
tat	Y	896	478.80
tac	Y	7	424.20
tgt	C	1	0.41
tgc	C	0	0.59
cat	H	1	0.57
cac	H	0	0.43
---	--	------	------
mPD		0.020	0.50
		nPD:	0.04
		N. weight:	0.73
		Sc. PD:	-0.08
		Sc. rank:	-172.9

,

PA

Pos.	306	obs:	exp:
gat	D	905	488.60
gac	D	0	416.40
---	--	------	------
mPD		0	0.50
		nPD:	0.
		N. weight:	0.76
		Sc. PD:	-0.11
		Sc. rank:	-481.1

,

PA

Pos.	307	obs:	exp:
gct	A	2	231.90
gcc	A	0	169.90
gca	A	62	423.10
gcg	A	841	80.05
---	--	------	------
mPD		0.13	0.67
		nPD:	0.2
		N. weight:	2.5
		Sc. PD:	0.1
		Sc. rank:	664.5

,

PA

Pos.	308	obs:	exp:
att	I	5	328.80
atc	I	892	217.90
ata	I	4	354.30
gtt	V	0	0.83
gtc	V	4	0.80
gta	V	0	0.83
gtg	V	0	1.54
---	--	------	------
mPD		0.029	0.66
		nPD:	0.04
		N. weight:	1.7
		Sc. PD:	-0.18
		Sc. rank:	-364.6

,

PA

Pos.	309	obs:	exp:
cgt	R	0	0.13
cgc	R	0	0.15
cga	R	0	0.30
cgg	R	0	0.25
aaa	K	897	528.40
aag	K	5	373.60
aga	R	3	1.37
agg	R	0	0.80
---	--	------	------
mPD		0.018	0.49
		nPD:	0.04
		N. weight:	0.62
		Sc. PD:	-0.071
		Sc. rank:	-163.8

,

PA

Pos.	310	obs:	exp:
tgt	C	9	371.50
tgc	C	896	533.50
---	--	------	------
mPD		0.020	0.48
		nPD:	0.04
		N. weight:	0.59
		Sc. PD:	-0.064
		Sc. rank:	-136.8

,

PA

Pos.	311	obs:	exp:
att	I	0	0.36
atc	I	0	0.24
ata	I	1	0.39
atg	M	904	904.00
---	--	------	------
mPD		0.0022	0.0022
		nPD:	1.
		N. weight:	0.0013
		Sc. PD:	0.001
		Sc. rank:	0.8

,

PA

Pos.	312	obs:	exp:
aaa	K	819	530.10
aag	K	86	374.90
---	--	------	------
mPD		0.17	0.49
		nPD:	0.35
		N. weight:	0.32
		Sc. PD:	0.061
		Sc. rank:	155.0

,

PA

Pos.	313	obs:	exp:
act	T	1	234.40
acc	T	34	184.30
aca	T	214	415.80
acg	T	656	70.50
---	--	------	------
mPD		0.42	0.67
		nPD:	0.62
		N. weight:	1.7
		Sc. PD:	0.77
		Sc. rank:	1052.1

,

PA

Pos.	314	obs:	exp:
ttt	F	72	357.20
ttc	F	833	547.80
---	--	------	------
mPD		0.15	0.48
		nPD:	0.31
		N. weight:	0.32
		Sc. PD:	0.047
		Sc. rank:	145.1

,

PA

Pos.	315	obs:	exp:
ttt	F	43	356.80
ttc	F	861	547.20
tta	L	1	0.09
ttg	L	0	0.19
ctt	L	0	0.17
ctc	L	0	0.17
cta	L	0	0.17
ctg	L	0	0.22
---	--	------	------
mPD		0.093	0.48
		nPD:	0.19
		N. weight:	0.41
		Sc. PD:	0.016
		Sc. rank:	101.4

,

PA

Pos.	316	obs:	exp:
gaa	E	0	0.60
gag	E	1	0.40
ggt	G	3	121.20
ggc	G	101	114.90
gga	G	91	418.00
ggg	G	709	249.90
---	--	------	------
mPD		0.36	0.68
		nPD:	0.54
		N. weight:	0.79
		Sc. PD:	0.29
		Sc. rank:	466.6

,

PA

Pos.	317	obs:	exp:
tgg	W	905	905.00
---	--	------	------
mPD		0	0
		nPD:	1.
		N. weight:	0.
		Sc. PD:	0
		Sc. rank:	0

,

PA

Pos.	318	obs:	exp:
aaa	K	764	530.10
aag	K	141	374.90
---	--	------	------
mPD		0.26	0.49
		nPD:	0.54
		N. weight:	0.2
		Sc. PD:	0.074
		Sc. rank:	116.2

,

PA

Pos.	319	obs:	exp:
aaa	K	0	2.93
aag	K	5	2.07
gaa	E	11	541.40
gag	E	889	358.60
---	--	------	------
mPD		0.035	0.49
		nPD:	0.07
		N. weight:	1.
		Sc. PD:	-0.083
		Sc. rank:	-122.4

,

PA

Pos.	320	obs:	exp:
cct	P	37	230.10
ccc	P	864	161.40
cca	P	2	369.70
ccg	P	2	143.80
---	--	------	------
mPD		0.087	0.71
		nPD:	0.12
		N. weight:	1.9
		Sc. PD:	-0.056
		Sc. rank:	82.9

,

PA

Pos.	321	obs:	exp:
tct	S	0	1.44
tcc	S	0	1.16
tca	S	0	2.27
tcg	S	0	0.52
tat	Y	1	0.53
tac	Y	0	0.47
att	I	9	4.01
atc	I	2	2.66
ata	I	0	4.33
aat	N	101	448.20
aac	N	780	432.80
aaa	K	2	1.17
aag	K	0	0.83
agt	S	8	1.84
agc	S	1	1.76
gtt	V	1	0.21
gtc	V	0	0.20
gta	V	0	0.21
gtg	V	0	0.39
---	--	------	------
mPD		0.28	0.57
		nPD:	0.5
		N. weight:	0.45
		Sc. PD:	0.15
		Sc. rank:	263.6

,

PA

Pos.	322	obs:	exp:
att	I	824	328.80
atc	I	65	217.90
ata	I	12	354.30
gtt	V	4	0.83
gtc	V	0	0.80
gta	V	0	0.83
gtg	V	0	1.54
---	--	------	------
mPD		0.17	0.66
		nPD:	0.25
		N. weight:	0.88
		Sc. PD:	0.083
		Sc. rank:	338.9

,

PA

Pos.	323	obs:	exp:
att	I	3	303.60
atc	I	814	201.20
ata	I	15	327.20
gtt	V	0	15.07
gtc	V	38	14.62
gta	V	30	15.11
gtg	V	5	28.19
---	--	------	------
mPD		0.26	0.82
		nPD:	0.32
		N. weight:	1.5
		Sc. PD:	0.24
		Sc. rank:	708.2

,

PA

Pos.	324	obs:	exp:
act	T	0	0.26
acc	T	0	0.20
aca	T	1	0.46
acg	T	0	0.08
aaa	K	854	529.60
aag	K	50	374.40
---	--	------	------
mPD		0.11	0.49
		nPD:	0.22
		N. weight:	0.42
		Sc. PD:	0.026
		Sc. rank:	134.7

,

PA

Pos.	325	obs:	exp:
tta	L	0	0.09
ttg	L	0	0.19
tct	S	0	0.48
tcc	S	0	0.39
tca	S	3	0.76
tcg	S	0	0.17
ctt	L	0	0.17
ctc	L	0	0.17
cta	L	1	0.17
ctg	L	0	0.22
cct	P	0	229.10
ccc	P	3	160.60
cca	P	891	368.10
ccg	P	7	143.10
agt	S	0	0.61
agc	S	0	0.59
---	--	------	------
mPD		0.031	0.72
		nPD:	0.04
		N. weight:	1.
		Sc. PD:	-0.11
		Sc. rank:	-234.1

PA

Pos.	326	obs:	exp:
cat	H	891	519.90
cac	H	14	385.10
---	--	------	------
mPD		0.030	0.49
		nPD:	0.06
		N. weight:	0.59
		Sc. PD:	-0.052
		Sc. rank:	-91.4

,

PA

Pos.	327	obs:	exp:
gat	D	1	0.54
gac	D	0	0.46
gaa	E	80	539.00
gag	E	816	357.00
ggt	G	0	1.07
ggc	G	0	1.02
gga	G	1	3.70
ggg	G	7	2.21
---	--	------	------
mPD		0.18	0.50
		nPD:	0.37
		N. weight:	0.72
		Sc. PD:	0.15
		Sc. rank:	362.1

,

PA

Pos.	328	obs:	exp:
aaa	K	703	530.10
aag	K	202	374.90
---	--	------	------
mPD		0.35	0.49
		nPD:	0.72
		N. weight:	0.1
		Sc. PD:	0.057
		Sc. rank:	65.6

,

PA

Pos.	329	obs:	exp:
ggt	G	29	121.40
ggc	G	835	115.00
gga	G	41	418.40
ggg	G	0	250.20
---	--	------	------
mPD		0.15	0.68
		nPD:	0.22
		N. weight:	2.1
		Sc. PD:	0.12
		Sc. rank:	629.6

,

PA

Pos.	330	obs:	exp:
att	I	0	328.40
atc	I	0	217.70
ata	I	900	353.90
act	T	0	0.52
acc	T	0	0.41
aca	T	2	0.92
acg	T	0	0.16
gtt	V	0	0.62
gtc	V	0	0.60
gta	V	3	0.62
gtg	V	0	1.16
---	--	------	------
mPD		0.011	0.67
		nPD:	0.02
		N. weight:	1.1
		Sc. PD:	-0.15
		Sc. rank:	-454.9

,

PA

Pos.	331	obs:	exp:
tct	S	0	0.32
tcc	S	0	0.26
tca	S	0	0.50
tcg	S	0	0.12
aat	N	19	459.40
aac	N	884	443.60
agt	S	0	0.41
agc	S	2	0.39
---	--	------	------
mPD		0.046	0.51
		nPD:	0.09
		N. weight:	0.75
		Sc. PD:	-0.046
		Sc. rank:	-40.0

PA

Pos.	332	obs:	exp:
tct	S	0	0.48
tcc	S	2	0.39
tca	S	1	0.76
tcg	S	0	0.17
cct	P	24	228.30
ccc	P	874	160.10
cca	P	0	366.90
ccg	P	0	142.70
cat	H	2	2.30
cac	H	2	1.70
agt	S	0	0.61
agc	S	0	0.59
---	--	------	------
mPD		0.073	0.73
		nPD:	0.1
		N. weight:	1.9
		Sc. PD:	-0.1
		Sc. rank:	-45.2

PA

Pos.	333	obs:	exp:
aat	N	631	460.40
aac	N	274	444.60
---	--	------	------
mPD		0.42	0.50
		nPD:	0.85
		N. weight:	0.095
		Sc. PD:	0.063
		Sc. rank:	60.2

PA

Pos.	334	obs:	exp:
tat	Y	813	479.80
tac	Y	92	425.20
---	--	------	------
mPD		0.18	0.50
		nPD:	0.37
		N. weight:	0.39
		Sc. PD:	0.081
		Sc. rank:	197.9

PA

Pos.	335	obs:	exp:
ttt	F	0	0.39
ttc	F	1	0.61
tta	L	0	79.45
ttg	L	0	168.70
ctt	L	21	153.20
ctc	L	880	151.50
cta	L	2	150.70
ctg	L	1	200.50
---	--	------	------
mPD		0.054	1.1
		nPD:	0.05
		N. weight:	2.
		Sc. PD:	-0.21
		Sc. rank:	-411.5

PA

Pos.	336	obs:	exp:
tta	L	0	69.78
ttg	L	33	148.20
ctt	L	3	134.60
ctc	L	0	133.00
cta	L	5	132.40
ctg	L	753	176.10
atg	M	111	111.00
---	--	------	------
mPD		0.29	1.2
		nPD:	0.25
		N. weight:	1.4
		Sc. PD:	0.12
		Sc. rank:	512.0

PA

Pos.	337	obs:	exp:
tct	S	0	0.32
tcc	S	0	0.26
tca	S	1	0.50
tcg	S	0	0.12
act	T	790	209.10
acc	T	17	164.30
aca	T	0	370.80
acg	T	0	62.86
agt	S	1	0.41
agc	S	0	0.39
gct	A	96	24.60
gcc	A	0	18.03
gca	A	0	44.88
gcg	A	0	8.49
---	--	------	------
mPD		0.23	0.87
		nPD:	0.27
		N. weight:	1.6
		Sc. PD:	0.17
		Sc. rank:	646.2

PA

Pos.	338	obs:	exp:
tgg	W	905	905.00
---	--	------	------
mPD		0	0
		nPD:	1.
		N. weight:	0.
		Sc. PD:	0
		Sc. rank:	0

PA

Pos.	339	obs:	exp:
aaa	K	24	530.10
aag	K	881	374.90
---	--	------	------
mPD		0.052	0.49
		nPD:	0.11
		N. weight:	0.92
		Sc. PD:	-0.042
		Sc. rank:	-8.8

PA

Pos.	340	obs:	exp:
caa	Q	65	464.80
cag	Q	840	440.20
---	--	------	------
mPD		0.13	0.50
		nPD:	0.27
		N. weight:	0.57
		Sc. PD:	0.062
		Sc. rank:	231.0

PA

Pos.	341	obs:	exp:
atg	M	2	2.00
gtt	V	1	186.40
gtc	V	0	180.90
gta	V	34	187.00
gtg	V	868	348.70
---	--	------	------
mPD		0.079	0.73
		nPD:	0.11
		N. weight:	0.99
		Sc. PD:	-0.043
		Sc. rank:	-7.7

PA

Pos.	342	obs:	exp:
tta	L	32	79.45
ttg	L	2	168.70
ctt	L	0	153.20
ctc	L	0	151.50
cta	L	769	150.70
ctg	L	101	200.50
att	I	0	0.36
atc	I	0	0.24
ata	I	1	0.39
---	--	------	------
mPD		0.28	1.1
		nPD:	0.25
		N. weight:	1.6
		Sc. PD:	0.14
		Sc. rank:	591.1

PA

Pos.	343	obs:	exp:
tct	S	0	32.02
tcc	S	0	25.89
tca	S	200	50.47
tcg	S	0	11.64
act	T	0	0.26
acc	T	0	0.20
aca	T	1	0.46
acg	T	0	0.08
agt	S	0	40.98
agc	S	0	39.00
gtt	V	0	0.21
gtc	V	0	0.20
gta	V	1	0.21
gtg	V	0	0.39
gct	A	1	180.10
gcc	A	6	132.00
gca	A	695	328.70
gcg	A	1	62.18
---	--	------	------
mPD		0.37	1.2
		nPD:	0.3
		N. weight:	1.
		Sc. PD:	0.15
		Sc. rank:	465.0

PA

Pos.	344	obs:	exp:
gaa	E	894	544.40
gag	E	11	360.60
---	--	------	------
mPD		0.024	0.48
		nPD:	0.05
		N. weight:	0.55
		Sc. PD:	-0.055
		Sc. rank:	-107.9

PA

Pos.	345	obs:	exp:
tta	L	0	79.45
ttg	L	1	168.70
ctt	L	807	153.20
ctc	L	59	151.50
cta	L	32	150.70
ctg	L	5	200.50
att	I	1	0.36
atc	I	0	0.24
ata	I	0	0.39
---	--	------	------
mPD		0.20	1.1
		nPD:	0.18
		N. weight:	1.7
		Sc. PD:	0.045
		Sc. rank:	337.7

PA

Pos.	346	obs:	exp:
cat	H	1	0.57
cac	H	0	0.43
caa	Q	55	463.80
cag	Q	848	439.20
aaa	K	0	0.59
aag	K	1	0.41
---	--	------	------
mPD		0.12	0.50
		nPD:	0.24
		N. weight:	0.6
		Sc. PD:	0.048
		Sc. rank:	215.5

PA

Pos.	347	obs:	exp:
gat	D	87	488.00
gac	D	817	416.00
ggt	G	1	0.13
ggc	G	0	0.13
gga	G	0	0.46
ggg	G	0	0.28
---	--	------	------
mPD		0.18	0.50
		nPD:	0.36
		N. weight:	0.55
		Sc. PD:	0.11
		Sc. rank:	272.6

PA

Pos.	348	obs:	exp:
tta	L	0	0.70
ttg	L	0	1.49
ctt	L	8	1.36
ctc	L	0	1.34
cta	L	0	1.33
ctg	L	0	1.77
att	I	893	326.60
atc	I	2	216.50
ata	I	0	352.00
act	T	1	0.26
acc	T	0	0.20
aca	T	0	0.46
acg	T	0	0.08
gtt	V	1	0.21
gtc	V	0	0.20
gta	V	0	0.21
gtg	V	0	0.39
---	--	------	------
mPD		0.026	0.68
		nPD:	0.04
		N. weight:	1.2
		Sc. PD:	-0.14
		Sc. rank:	-302.1

PA

Pos.	349	obs:	exp:
gaa	E	885	543.80
gag	E	19	360.20
ggt	G	0	0.13
ggc	G	0	0.13
gga	G	1	0.46
ggg	G	0	0.28
---	--	------	------
mPD		0.043	0.48
		nPD:	0.09
		N. weight:	0.51
		Sc. PD:	-0.031
		Sc. rank:	-26.1

PA

Pos.	350	obs:	exp:
tct	S	0	0.16
tcc	S	0	0.13
tca	S	0	0.25
tcg	S	0	0.06
att	I	1	0.36
atc	I	0	0.24
ata	I	0	0.39
act	T	0	2.59
acc	T	10	2.04
aca	T	0	4.59
acg	T	0	0.78
aat	N	874	454.30
aac	N	19	438.70
agt	S	1	0.20
agc	S	0	0.20
---	--	------	------
mPD		0.088	0.53
		nPD:	0.17
		N. weight:	0.72
		Sc. PD:	0.0083
		Sc. rank:	114.0

PA			
Pos.	351	obs:	exp:
aaa	K	3	1.76
aag	K	0	1.24
gaa	E	810	542.00
gag	E	91	359.00
ggt	G	0	0.13
ggc	G	0	0.13
gga	G	1	0.46
ggg	G	0	0.28
mPD		0.19	0.49
		nPD:	0.39
		N. weight:	0.28
		Sc. PD:	0.063
		Sc. rank:	144.2

,

PA			
Pos.	352	obs:	exp:
gaa	E	6	544.40
gag	E	899	360.60
mPD		0.013	0.48
		nPD:	0.03
		N. weight:	1.1
		Sc. PD:	-0.13
		Sc. rank:	-335.2

,

PA			
Pos.	353	obs:	exp:
cgt	R	0	0.13
cgc	R	0	0.15
cga	R	0	0.30
cgg	R	0	0.25
aat	N	2	1.02
aac	N	0	0.98
aaa	K	72	526.60
aag	K	827	372.40
aga	R	0	1.37
agg	R	3	0.80
gaa	E	0	0.60
gag	E	1	0.40
mPD		0.16	0.50
		nPD:	0.32
		N. weight:	0.71
		Sc. PD:	0.11
		Sc. rank:	331.3

,

PA			
Pos.	354	obs:	exp:
att	I	774	328.40
atc	I	108	217.70
ata	I	18	353.90
act	T	0	0.26
acc	T	1	0.20
aca	T	0	0.46
acg	T	0	0.08
gtt	V	1	0.83
gtc	V	3	0.80
gta	V	0	0.83
gtg	V	0	1.54
mPD		0.26	0.67
		nPD:	0.39
		N. weight:	0.74
		Sc. PD:	0.17
		Sc. rank:	383.3

,

PA			
Pos.	355	obs:	exp:
tta	L	0	0.35
ttg	L	0	0.75
ctt	L	0	0.68
ctc	L	0	0.67
cta	L	0	0.67
ctg	L	4	0.89
cct	P	2	229.10
ccc	P	18	160.60
cca	P	810	368.10
ccg	P	71	143.10
mPD		0.20	0.72
		nPD:	0.28
		N. weight:	0.75
		Sc. PD:	0.09
		Sc. rank:	315.9

,

PA			
Pos.	356	obs:	exp:
cgt	R	0	29.90
cgc	R	0	35.57
cga	R	0	69.80
cgg	R	0	58.30
aaa	K	70	120.10
aag	K	135	84.91
aga	R	24	319.60
agg	R	676	186.80
mPD		0.54	1.2
		nPD:	0.44
		N. weight:	1.1
		Sc. PD:	0.31
		Sc. rank:	627.4

,

PA			
Pos.	357	obs:	exp:
tct	S	0	14.25
tcc	S	1	11.52
tca	S	86	22.46
tcg	S	2	5.18
act	T	1	210.90
acc	T	2	165.80
aca	T	747	374.00
acg	T	64	63.41
agt	S	0	18.24
agc	S	0	17.36
gct	A	0	0.51
gcc	A	0	0.38
gca	A	2	0.94
gcg	A	0	0.18
mPD		0.33	0.87
		nPD:	0.37
		N. weight:	0.84
		Sc. PD:	0.18
		Sc. rank:	425.9

,

PA			
Pos.	358	obs:	exp:
aaa	K	340	530.10
aag	K	565	374.90
mPD		0.47	0.49
		nPD:	0.97
		N. weight:	0.11
		Sc. PD:	0.09
		Sc. rank:	73.0

,

PA			
Pos.	359	obs:	exp:
cat	H	0	0.57
cac	H	1	0.43
aat	N	15	459.40
aac	N	888	443.60
gat	D	0	0.54
gac	D	1	0.46
mPD		0.037	0.50
		nPD:	0.07
		N. weight:	0.77
		Sc. PD:	-0.06
		Sc. rank:	-87.4

,

PA			
Pos.	360	obs:	exp:
tta	L	0	0.18
ttg	L	2	0.37
ctt	L	0	0.34
ctc	L	0	0.34
cta	L	0	0.33
ctg	L	0	0.44
atg	M	901	901.00
gtt	V	0	0.41
gtc	V	0	0.40
gta	V	0	0.41
gtg	V	2	0.77
mPD		0.0088	0.014
		nPD:	0.62
		N. weight:	0.0071
		Sc. PD:	0.0032
		Sc. rank:	4.4

,

PA			
Pos.	361	obs:	exp:
act	T	0	0.26
acc	T	0	0.20
aca	T	0	0.46
acg	T	1	0.08
aaa	K	17	529.60
aag	K	887	374.40
mPD		0.039	0.49
		nPD:	0.08
		N. weight:	0.96
		Sc. PD:	-0.068
		Sc. rank:	-87.2

,

PA			
Pos.	362	obs:	exp:
cgt	R	0	0.04
cgc	R	0	0.05
cga	R	0	0.10
cgg	R	0	0.08
aaa	K	872	529.60
aag	K	32	374.40
aga	R	1	0.46
agg	R	0	0.27
mPD		0.071	0.49
		nPD:	0.14
		N. weight:	0.49
		Sc. PD:	-0.0043
		Sc. rank:	53.8

,

PA			
Pos.	363	obs:	exp:
act	T	0	234.40
acc	T	0	184.30
aca	T	897	415.80
acg	T	8	70.50
mPD		0.018	0.67
		nPD:	0.03
		N. weight:	0.91
		Sc. PD:	-0.11
		Sc. rank:	-300.4

,

PA			
Pos.	364	obs:	exp:
tct	S	0	128.40
tcc	S	0	103.80
tca	S	0	202.40
tcg	S	0	46.67
att	I	0	0.73
atc	I	2	0.48
ata	I	0	0.79
agt	S	10	164.30
agc	S	792	156.40
gtt	V	0	10.73
gtc	V	52	10.42
gta	V	0	10.77
gtg	V	0	20.08
ggt	G	0	6.57
ggc	G	49	6.23
gga	G	0	22.66
ggg	G	0	13.55
mPD		0.33	1.8
		nPD:	0.18
		N. weight:	2.
		Sc. PD:	0.052
		Sc. rank:	394.1

,

PA			
Pos.	365	obs:	exp:
cat	H	2	1.72
cac	H	1	1.28
caa	Q	887	463.30
cag	Q	15	438.70
mPD		0.039	0.50
		nPD:	0.08
		N. weight:	0.72
		Sc. PD:	-0.052
		Sc. rank:	-66.0

,

PA			
Pos.	366	obs:	exp:
tta	L	780	79.54
ttg	L	91	168.90
ctt	L	0	153.40
ctc	L	0	151.60
cta	L	33	150.90
ctg	L	1	200.70
mPD		0.26	1.1
		nPD:	0.23
		N. weight:	2.3
		Sc. PD:	0.17
		Sc. rank:	781.1

,

PA			
Pos.	367	obs:	exp:
aaa	K	34	530.10
aag	K	871	374.90
mPD		0.072	0.49
		nPD:	0.15
		N. weight:	0.87
		Sc. PD:	-0.0037
		Sc. rank:	105.4

,

PA			
Pos.	368	obs:	exp:
tgg	W	905	905.00
mPD		0	0
		nPD:	1.
		N. weight:	0.
		Sc. PD:	0
		Sc. rank:	0

,

PA			
Pos.	369	obs:	exp:
act	T	0	0.26
acc	T	0	0.20
aca	T	1	0.46
acg	T	0	0.08
gtt	V	0	0.41
gtc	V	0	0.40
gta	V	2	0.41
gtg	V	0	0.77
gct	A	0	231.10
gcc	A	1	169.40
gca	A	899	421.70
gcg	A	2	79.78
mPD		0.013	0.68
		nPD:	0.02
		N. weight:	0.91
		Sc. PD:	-0.12
		Sc. rank:	-346.1

,

PA			
Pos.	370	obs:	exp:
tta	L	1	79.54
ttg	L	0	168.90
ctt	L	174	153.40
ctc	L	708	151.60
cta	L	22	150.90
ctg	L	0	200.70
mPD		0.35	1.1
		nPD:	0.32
		N. weight:	1.5
		Sc. PD:	0.23
		Sc. rank:	674.3

,

PA			
Pos.	371	obs:	exp:
ggt	G	901	121.40
ggc	G	3	115.00
gga	G	0	418.40
ggg	G	1	250.20
---	--	------	------
mPD		0.0088	0.68
		nPD:	0.01
		N. weight:	2.4
		Sc. PD:	-0.33
		Sc. rank:	-1033.5

PA			
Pos.	372	obs:	exp:
gaa	E	277	544.40
gag	E	628	360.60
---	--	------	------
mPD		0.43	0.48
		nPD:	0.89
		N. weight:	0.22
		Sc. PD:	0.16
		Sc. rank:	142.4

PA			
Pos.	373	obs:	exp:
tct	S	0	0.16
tcc	S	0	0.13
tca	S	0	0.25
tcg	S	0	0.06
aat	N	46	459.90
aac	N	858	444.10
agt	S	0	0.20
agc	S	1	0.20
---	--	------	------
mPD		0.099	0.50
		nPD:	0.2
		N. weight:	0.63
		Sc. PD:	0.026
		Sc. rank:	166.9

PA			
Pos.	374	obs:	exp:
tta	L	0	0.09
ttg	L	0	0.19
ctt	L	0	0.17
ctc	L	0	0.17
cta	L	0	0.17
ctg	L	1	0.22
cgt	R	0	0.04
cgc	R	0	0.05
cga	R	0	0.10
cgg	R	0	0.08
att	I	0	0.36
atc	I	0	0.24
ata	I	1	0.39
atg	M	902	902.00
aga	R	0	0.46
agg	R	1	0.27
---	--	------	------
mPD		0.0066	0.010
		nPD:	0.66
		N. weight:	0.0051
		Sc. PD:	0.0025
		Sc. rank:	3.2

PA			
Pos.	375	obs:	exp:
gct	A	14	231.90
gcc	A	1	169.90
gca	A	870	423.10
gcg	A	20	80.05
---	--	------	------
mPD		0.075	0.67
		nPD:	0.11
		N. weight:	0.76
		Sc. PD:	-0.031
		Sc. rank:	-1.0

PA			
Pos.	376	obs:	exp:
cct	P	34	230.10
ccc	P	0	161.40
cca	P	207	369.70
ccg	P	664	143.80
---	--	------	------
mPD		0.41	0.71
		nPD:	0.57
		N. weight:	1.1
		Sc. PD:	0.46
		Sc. rank:	690.0

PA			
Pos.	377	obs:	exp:
gaa	E	81	544.40
gag	E	824	360.60
---	--	------	------
mPD		0.16	0.48
		nPD:	0.34
		N. weight:	0.72
		Sc. PD:	0.13
		Sc. rank:	346.1

PA			
Pos.	378	obs:	exp:
aaa	K	217	530.10
aag	K	688	374.90
---	--	------	------
mPD		0.36	0.49
		nPD:	0.75
		N. weight:	0.31
		Sc. PD:	0.18
		Sc. rank:	195.1

PA			
Pos.	379	obs:	exp:
tta	L	0	0.09
ttg	L	0	0.19
ctt	L	0	0.17
ctc	L	0	0.17
cta	L	0	0.17
ctg	L	1	0.22
att	I	0	0.36
atc	I	0	0.24
ata	I	1	0.39
atg	M	1	1.00
gtt	V	1	186.20
gtc	V	0	180.70
gta	V	147	186.80
gtg	V	754	348.30
---	--	------	------
mPD		0.28	0.73
		nPD:	0.39
		N. weight:	0.74
		Sc. PD:	0.17
		Sc. rank:	381.6

PA			
Pos.	380	obs:	exp:
gat	D	44	488.60
gac	D	861	416.40
---	--	------	------
mPD		0.093	0.50
		nPD:	0.19
		N. weight:	0.71
		Sc. PD:	0.022
		Sc. rank:	154.4

PA			
Pos.	381	obs:	exp:
ttt	F	903	357.20
ttc	F	2	547.80
---	--	------	------
mPD		0.0044	0.48
		nPD:	0.01
		N. weight:	1.1
		Sc. PD:	-0.16
		Sc. rank:	-506.3

PA			
Pos.	382	obs:	exp:
gct	A	0	0.26
gcc	A	0	0.19
gca	A	0	0.47
gcg	A	1	0.09
gat	D	4	2.16
gac	D	0	1.84
gaa	E	73	523.90
gag	E	798	347.10
ggt	G	0	3.89
ggc	G	0	3.68
gga	G	0	13.41
ggg	G	29	8.02
---	--	------	------
mPD		0.22	0.56
		nPD:	0.4
		N. weight:	0.77
		Sc. PD:	0.18
		Sc. rank:	398.7

PA			
Pos.	383	obs:	exp:
aat	N	3	1.53
aac	N	0	1.47
gat	D	726	486.90
gac	D	176	415.10
---	--	------	------
mPD		0.32	0.50
		nPD:	0.64
		N. weight:	0.2
		Sc. PD:	0.091
		Sc. rank:	120.4

PA			
Pos.	384	obs:	exp:
tgt	C	208	371.50
tgc	C	697	533.50
---	--	------	------
mPD		0.35	0.48
		nPD:	0.73
		N. weight:	0.094
		Sc. PD:	0.052
		Sc. rank:	59.3

PA			
Pos.	385	obs:	exp:
cgt	R	0	1.45
cgc	R	0	1.73
cga	R	0	3.39
cgg	R	0	2.83
aaa	K	812	510.20
aag	K	59	360.80
aga	R	34	15.52
agg	R	0	9.07
---	--	------	------
mPD		0.19	0.58
		nPD:	0.33
		N. weight:	0.41
		Sc. PD:	0.071
		Sc. rank:	193.0

PA			
Pos.	386	obs:	exp:
gct	A	1	0.26
gcc	A	0	0.19
gca	A	0	0.47
gcg	A	0	0.09
gat	D	855	471.30
gac	D	18	401.70
ggt	G	31	4.16
ggc	G	0	3.94
gga	G	0	14.33
ggg	G	0	8.57
---	--	------	------
mPD		0.11	0.59
		nPD:	0.18
		N. weight:	0.7
		Sc. PD:	0.019
		Sc. rank:	144.5

PA			
Pos.	387	obs:	exp:
att	I	6	10.22
atc	I	20	6.77
ata	I	2	11.01
gtt	V	81	181.00
gtc	V	794	175.70
gta	V	1	181.60
gtg	V	1	338.70
---	--	------	------
mPD		0.24	0.79
		nPD:	0.31
		N. weight:	1.6
		Sc. PD:	0.23
		Sc. rank:	700.6

PA			
Pos.	388	obs:	exp:
tct	S	0	14.09
tcc	S	0	11.39
tca	S	0	22.21
tcg	S	0	5.12
aat	N	108	409.50
aac	N	697	395.50
aaa	K	2	1.17
aag	K	0	0.83
agt	S	48	18.03
agc	S	40	17.16
gat	D	0	1.08
gac	D	2	0.92
ggt	G	0	1.07
ggc	G	5	1.02
gga	G	3	3.70
ggg	G	0	2.21
---	--	------	------
mPD		0.51	0.86
		nPD:	0.59
		N. weight:	0.47
		Sc. PD:	0.2
		Sc. rank:	288.4

PA			
Pos.	389	obs:	exp:
gat	D	101	488.60
gac	D	804	416.40
---	--	------	------
mPD		0.20	0.50
		nPD:	0.4
		N. weight:	0.51
		Sc. PD:	0.12
		Sc. rank:	264.5

PA			
Pos.	390	obs:	exp:
tta	L	2	79.45
ttg	L	368	168.70
ctt	L	4	153.20
ctc	L	1	151.50
cta	L	42	150.70
ctg	L	487	200.50
att	I	0	0.36
atc	I	0	0.24
ata	I	1	0.39
---	--	------	------
mPD		0.59	1.1
		nPD:	0.53
		N. weight:	0.88
		Sc. PD:	0.32
		Sc. rank:	515.5

PA			
Pos.	391	obs:	exp:
cgt	R	0	2.90
cgc	R	0	3.45
cga	R	2	6.78
cgg	R	0	5.66
aat	N	0	3.05
aac	N	6	2.95
aaa	K	814	486.80
aag	K	17	344.20
aga	R	40	31.05
agg	R	26	18.15
---	--	------	------
mPD		0.25	0.68
		nPD:	0.36
		N. weight:	0.53
		Sc. PD:	0.11
		Sc. rank:	266.6

,

PA			
Pos.	392	obs:	exp:
caa	Q	17	464.80
cag	Q	888	440.20
---	--	------	------
mPD		0.037	0.50
		nPD:	0.07
		N. weight:	0.77
		Sc. PD:	-0.059
		Sc. rank:	-81.8

,

PA			
Pos.	393	obs:	exp:
tct	S	0	0.80
tcc	S	5	0.65
tca	S	0	1.26
tcg	S	0	0.29
tat	Y	192	477.20
tac	Y	708	422.80
agt	S	0	1.02
agc	S	0	0.98
---	--	------	------
mPD		0.35	0.52
		nPD:	0.67
		N. weight:	0.28
		Sc. PD:	0.14
		Sc. rank:	172.0

,

PA			
Pos.	394	obs:	exp:
cat	H	0	0.57
cac	H	1	0.43
act	T	0	0.78
acc	T	3	0.61
aca	T	0	1.38
acg	T	0	0.23
aat	N	0	172.00
aac	N	338	166.00
gat	D	116	301.80
gac	D	443	257.20
gaa	E	1	0.60
gag	E	0	0.40
ggt	G	3	0.40
ggc	G	0	0.38
gga	G	0	1.39
ggg	G	0	0.83
---	--	------	------
mPD		0.72	0.99
		nPD:	0.72
		N. weight:	0.52
		Sc. PD:	0.29
		Sc. rank:	330.3

,

PA			
Pos.	395	obs:	exp:
tct	S	0	144.90
tcc	S	0	117.10
tca	S	0	228.40
tcg	S	0	52.67
agt	S	885	185.40
agc	S	20	176.50
---	--	------	------
mPD		0.043	1.7
		nPD:	0.03
		N. weight:	1.8
		Sc. PD:	-0.22
		Sc. rank:	-579.7

,

PA			
Pos.	396	obs:	exp:
gct	A	37	9.48
gcc	A	0	6.95
gca	A	0	17.30
gcg	A	0	3.27
gat	D	775	468.00
gac	D	92	399.00
gaa	E	0	0.60
gag	E	1	0.40
---	--	------	------
mPD		0.26	0.60
		nPD:	0.44
		N. weight:	0.42
		Sc. PD:	0.12
		Sc. rank:	234.6

,

PA			
Pos.	397	obs:	exp:
gaa	E	51	543.80
gag	E	853	360.20
ggt	G	0	0.13
ggc	G	0	0.13
gga	G	0	0.46
ggg	G	1	0.28
---	--	------	------
mPD		0.11	0.48
		nPD:	0.23
		N. weight:	0.84
		Sc. PD:	0.058
		Sc. rank:	278.3

,

PA			
Pos.	398	obs:	exp:
cct	P	49	229.90
ccc	P	74	161.20
cca	P	769	369.30
ccg	P	12	143.60
act	T	0	0.26
acc	T	0	0.20
aca	T	1	0.46
acg	T	0	0.08
---	--	------	------
mPD		0.27	0.71
		nPD:	0.38
		N. weight:	0.55
		Sc. PD:	0.12
		Sc. rank:	280.4

,

PA			
Pos.	399	obs:	exp:
cgt	R	0	0.04
cgc	R	0	0.05
cga	R	0	0.10
cgg	R	0	0.08
aga	R	0	0.46
agg	R	1	0.27
gct	A	0	0.77
gcc	A	0	0.56
gca	A	0	1.40
gcg	A	3	0.27
gaa	E	21	537.80
gag	E	873	356.20
ggt	G	0	0.94
ggc	G	0	0.89
gga	G	0	3.24
ggg	G	7	1.94
---	--	------	------
mPD		0.072	0.51
		nPD:	0.14
		N. weight:	0.99
		Sc. PD:	-0.012
		Sc. rank:	93.5

,

PA			
Pos.	400	obs:	exp:
tta	L	1	0.35
ttg	L	0	0.75
tct	S	36	9.92
tcc	S	26	8.03
tca	S	0	15.65
tcg	S	0	3.61
ctt	L	1	0.68
ctc	L	2	0.67
cta	L	0	0.67
ctg	L	0	0.89
cct	P	14	204.40
ccc	P	785	143.40
cca	P	5	328.50
ccg	P	0	127.70
cat	H	0	0.57
cac	H	1	0.43
caa	Q	33	16.95
cag	Q	0	16.05
agt	S	0	12.70
agc	S	0	12.09
gct	A	0	0.26
gcc	A	1	0.19
gca	A	0	0.47
gcg	A	0	0.09
---	--	------	------
mPD		0.40	0.98
		nPD:	0.4
		N. weight:	1.9
		Sc. PD:	0.46
		Sc. rank:	1002.7

,

PA			
Pos.	401	obs:	exp:
cgt	R	0	38.66
cgc	R	0	45.98
cga	R	15	90.24
cgg	R	0	75.38
aga	R	853	413.20
agg	R	37	241.50
---	--	------	------
mPD		0.11	0.96
		nPD:	0.12
		N. weight:	0.71
		Sc. PD:	-0.026
		Sc. rank:	14.7

,

PA			
Pos.	402	obs:	exp:
tct	S	2	144.90
tcc	S	0	117.10
tca	S	862	228.40
tcg	S	41	52.67
agt	S	0	185.40
agc	S	0	176.50
---	--	------	------
mPD		0.091	1.7
		nPD:	0.05
		N. weight:	1.5
		Sc. PD:	-0.15
		Sc. rank:	-267.9

,

PA			
Pos.	403	obs:	exp:
tta	L	9	79.45
ttg	L	0	168.70
ctt	L	2	153.20
ctc	L	6	151.50
cta	L	882	150.70
ctg	L	5	200.50
att	I	0	0.36
atc	I	0	0.24
ata	I	1	0.39
---	--	------	------
mPD		0.050	1.1
		nPD:	0.05
		N. weight:	2.
		Sc. PD:	-0.21
		Sc. rank:	-432.1

,

PA			
Pos.	404	obs:	exp:
tct	S	0	0.32
tcc	S	0	0.26
tca	S	2	0.50
tcg	S	0	0.12
agt	S	0	0.41
agc	S	0	0.39
gct	A	0	231.40
gcc	A	0	169.60
gca	A	899	422.20
gcg	A	4	79.87
---	--	------	------
mPD		0.013	0.68
		nPD:	0.02
		N. weight:	0.91
		Sc. PD:	-0.12
		Sc. rank:	-346.3

,

PA			
Pos.	405	obs:	exp:
tct	S	0	15.05
tcc	S	0	12.17
tca	S	0	23.72
tcg	S	0	5.47
tgt	C	761	332.90
tgc	C	50	478.10
agt	S	33	19.26
agc	S	61	18.33
---	--	------	------
mPD		0.40	0.72
		nPD:	0.56
		N. weight:	0.83
		Sc. PD:	0.33
		Sc. rank:	499.6

,

PA			
Pos.	406	obs:	exp:
tgg	W	905	905.00
---	--	------	------
mPD		0	0
		nPD:	1.
		N. weight:	0.
		Sc. PD:	0
		Sc. rank:	0

,

PA			
Pos.	407	obs:	exp:
att	I	147	330.20
atc	I	756	218.90
ata	I	2	355.90
---	--	------	------
mPD		0.28	0.65
		nPD:	0.42
		N. weight:	1.1
		Sc. PD:	0.29
		Sc. rank:	601.6

,

PA			
Pos.	408	obs:	exp:
caa	Q	51	464.80
cag	Q	854	440.20
---	--	------	------
mPD		0.11	0.50
		nPD:	0.21
		N. weight:	0.62
		Sc. PD:	0.036
		Sc. rank:	183.8

,

PA			
Pos.	409	obs:	exp:
tct	S	0	25.77
tcc	S	0	20.84
tca	S	0	40.63
tcg	S	0	9.37
aat	N	733	378.50
aac	N	11	365.50
agt	S	146	32.99
agc	S	15	31.40
---	--	------	------
mPD		0.35	1.0
		nPD:	0.33
		N. weight:	0.89
		Sc. PD:	0.15
		Sc. rank:	420.3

,

PA			
Pos.	410	obs:	exp:
gaa	E	862	544.40
gag	E	43	360.60
---	--	------	------
mPD		0.091	0.48
		nPD:	0.19
		N. weight:	0.42
		Sc. PD:	0.014
		Sc. rank:	94.0

,

PA			
Pos.	411	obs:	exp:
ttt	F	9	357.20
ttc	F	896	547.80
---	--	------	------
mPD		0.020	0.48
		nPD:	0.04
		N. weight:	0.55
		Sc. PD:	-0.06
		Sc. rank:	-128.3

PA			
Pos.	412	obs:	exp:
aat	N	45	460.40
aac	N	860	444.60
---	--	------	------
mPD		0.095	0.50
		nPD:	0.19
		N. weight:	0.63
		Sc. PD:	0.022
		Sc. rank:	145.7

PA			
Pos.	413	obs:	exp:
cgt	R	0	0.17
cgc	R	0	0.20
cga	R	0	0.40
cgg	R	0	0.33
aaa	K	144	527.80
aag	K	757	373.20
aga	R	0	1.83
agg	R	4	1.07
---	--	------	------
mPD		0.28	0.50
		nPD:	0.56
		N. weight:	0.48
		Sc. PD:	0.19
		Sc. rank:	290.4

PA			
Pos.	414	obs:	exp:
gct	A	40	231.90
gcc	A	110	169.90
gca	A	534	423.10
gcg	A	221	80.05
---	--	------	------
mPD		0.58	0.67
		nPD:	0.86
		N. weight:	0.33
		Sc. PD:	0.22
		Sc. rank:	207.2

PA			
Pos.	415	obs:	exp:
tgt	C	859	371.50
tgc	C	46	533.50
---	--	------	------
mPD		0.097	0.48
		nPD:	0.2
		N. weight:	0.83
		Sc. PD:	0.037
		Sc. rank:	221.4

PA			
Pos.	416	obs:	exp:
gaa	E	897	544.40
gag	E	8	360.60
---	--	------	------
mPD		0.018	0.48
		nPD:	0.04
		N. weight:	0.57
		Sc. PD:	-0.064
		Sc. rank:	-143.5

PA			
Pos.	417	obs:	exp:
tta	L	125	79.54
ttg	L	440	168.90
ctt	L	0	153.40
ctc	L	0	151.60
cta	L	3	150.90
ctg	L	337	200.70
---	--	------	------
mPD		0.71	1.1
		nPD:	0.64
		N. weight:	0.88
		Sc. PD:	0.41
		Sc. rank:	544.9

PA			
Pos.	418	obs:	exp:
att	I	1	0.36
atc	I	0	0.24
ata	I	0	0.39
act	T	715	234.20
acc	T	149	184.10
aca	T	40	415.30
acg	T	0	70.42
---	--	------	------
mPD		0.35	0.68
		nPD:	0.51
		N. weight:	0.92
		Sc. PD:	0.32
		Sc. rank:	536.0

PA			
Pos.	419	obs:	exp:
gat	D	539	488.00
gac	D	365	416.00
gaa	E	0	0.60
gag	E	1	0.40
---	--	------	------
mPD		0.48	0.50
		nPD:	0.97
		N. weight:	0.014
		Sc. PD:	0.011
		Sc. rank:	9.0

PA			
Pos.	420	obs:	exp:
tct	S	0	144.90
tcc	S	5	117.10
tca	S	864	228.40
tcg	S	36	52.67
agt	S	0	185.40
agc	S	0	176.50
---	--	------	------
mPD		0.087	1.7
		nPD:	0.05
		N. weight:	1.5
		Sc. PD:	-0.15
		Sc. rank:	-284.5

PA			
Pos.	421	obs:	exp:
tct	S	0	142.50
tcc	S	0	115.20
tca	S	0	224.60
tcg	S	0	51.79
att	I	0	0.36
atc	I	1	0.24
ata	I	0	0.39
agt	S	200	182.40
agc	S	690	173.60
ggt	G	0	1.88
ggc	G	14	1.78
gga	G	0	6.47
ggg	G	0	3.87
---	--	------	------
mPD		0.38	1.7
		nPD:	0.22
		N. weight:	1.4
		Sc. PD:	0.094
		Sc. rank:	448.2

PA			
Pos.	422	obs:	exp:
tgg	W	905	905.00
---	--	------	------
mPD		0	0
		nPD:	1.
		N. weight:	0.
		Sc. PD:	0
		Sc. rank:	0

PA			
Pos.	423	obs:	exp:
att	I	38	37.95
atc	I	1	25.15
ata	I	65	40.90
gtt	V	0	165.40
gtc	V	0	160.50
gta	V	774	165.80
gtg	V	27	309.30
---	--	------	------
mPD		0.34	0.94
		nPD:	0.36
		N. weight:	1.6
		Sc. PD:	0.32
		Sc. rank:	781.8

PA			
Pos.	424	obs:	exp:
gaa	E	842	544.40
gag	E	63	360.60
---	--	------	------
mPD		0.13	0.48
		nPD:	0.27
		N. weight:	0.35
		Sc. PD:	0.04
		Sc. rank:	146.9

PA			
Pos.	425	obs:	exp:
ttt	F	1	0.39
ttc	F	0	0.61
tta	L	0	79.45
ttg	L	2	168.70
ctt	L	858	153.20
ctc	L	40	151.50
cta	L	2	150.70
ctg	L	2	200.50
---	--	------	------
mPD		0.10	1.1
		nPD:	0.09
		N. weight:	1.9
		Sc. PD:	-0.11
		Sc. rank:	-73.4

PA			
Pos.	426	obs:	exp:
gat	D	894	488.00
gac	D	10	416.00
gaa	E	1	0.60
gag	E	0	0.40
---	--	------	------
mPD		0.024	0.50
		nPD:	0.05
		N. weight:	0.69
		Sc. PD:	-0.07
		Sc. rank:	-142.7

PA			
Pos.	427	obs:	exp:
gaa	E	886	544.40
gag	E	19	360.60
---	--	------	------
mPD		0.041	0.48
		nPD:	0.09
		N. weight:	0.51
		Sc. PD:	-0.033
		Sc. rank:	-33.4

PA			
Pos.	428	obs:	exp:
att	I	1	330.20
atc	I	5	218.90
ata	I	899	355.90
---	--	------	------
mPD		0.013	0.65
		nPD:	0.02
		N. weight:	1.1
		Sc. PD:	-0.14
		Sc. rank:	-408.7

PA			
Pos.	429	obs:	exp:
ggt	G	0	121.40
ggc	G	0	115.00
gga	G	42	418.40
ggg	G	863	250.20
---	--	------	------
mPD		0.089	0.68
		nPD:	0.13
		N. weight:	1.3
		Sc. PD:	-0.029
		Sc. rank:	90.7

PA			
Pos.	430	obs:	exp:
gat	D	10	5.40
gac	D	0	4.60
gaa	E	848	538.40
gag	E	47	356.60
---	--	------	------
mPD		0.12	0.49
		nPD:	0.24
		N. weight:	0.41
		Sc. PD:	0.035
		Sc. rank:	146.5

PA			
Pos.	431	obs:	exp:
gat	D	829	488.60
gac	D	76	416.40
---	--	------	------
mPD		0.15	0.50
		nPD:	0.31
		N. weight:	0.42
		Sc. PD:	0.064
		Sc. rank:	192.8

PA			
Pos.	432	obs:	exp:
att	I	31	11.31
atc	I	0	7.50
ata	I	0	12.19
gtt	V	870	180.40
gtc	V	2	175.10
gta	V	2	181.00
gtg	V	0	337.50
---	--	------	------
mPD		0.075	0.80
		nPD:	0.09
		N. weight:	1.9
		Sc. PD:	-0.11
		Sc. rank:	-62.2

PA			
Pos.	433	obs:	exp:
gct	A	92	231.90
gcc	A	806	169.90
gca	A	7	423.10
gcg	A	0	80.05
---	--	------	------
mPD		0.20	0.67
		nPD:	0.29
		N. weight:	1.6
		Sc. PD:	0.21
		Sc. rank:	674.9

PA			
Pos.	434	obs:	exp:
cct	P	6	230.10
ccc	P	0	161.40
cca	P	865	369.70
ccg	P	34	143.80
---	--	------	------
mPD		0.085	0.71
		nPD:	0.12
		N. weight:	0.91
		Sc. PD:	-0.03
		Sc. rank:	31.2

PA			
Pos.	435	obs:	exp:
att	I	72	330.20
atc	I	832	218.90
ata	I	1	355.90
---	--	------	------
mPD		0.15	0.65
		nPD:	0.23
		N. weight:	1.4
		Sc. PD:	0.096
		Sc. rank:	456.5

PA

Pos.	436	obs:	exp:
gaa	E	862	544.40
gag	E	43	360.60
---	--	------	------
mPD		0.091	0.48
		nPD:	0.19
		N. weight:	0.42
		Sc. PD:	0.014
		Sc. rank:	94.0

,

PA

Pos.	437	obs:	exp:
tat	Y	0	5.83
tac	Y	11	5.17
cat	H	27	513.60
cac	H	867	380.40
---	--	------	------
mPD		0.082	0.51
		nPD:	0.16
		N. weight:	0.87
		Sc. PD:	0.0053
		Sc. rank:	125.2

,

PA

Pos.	438	obs:	exp:
att	I	898	330.20
atc	I	7	218.90
ata	I	0	355.90
---	--	------	------
mPD		0.015	0.65
		nPD:	0.02
		N. weight:	1.2
		Sc. PD:	-0.15
		Sc. rank:	-410.8

,

PA

Pos.	439	obs:	exp:
gct	A	1	231.90
gcc	A	0	169.90
gca	A	882	423.10
gcg	A	22	80.05
---	--	------	------
mPD		0.050	0.67
		nPD:	0.07
		N. weight:	0.84
		Sc. PD:	-0.064
		Sc. rank:	-91.0

,

PA

Pos.	440	obs:	exp:
tct	S	0	144.90
tcc	S	0	117.10
tca	S	0	228.40
tcg	S	0	52.67
agt	S	54	185.40
agc	S	851	176.50
---	--	------	------
mPD		0.11	1.7
		nPD:	0.07
		N. weight:	1.7
		Sc. PD:	-0.14
		Sc. rank:	-216.7

,

PA

Pos.	441	obs:	exp:
atg	M	905	905.00
---	--	------	------
mPD		0	0
		nPD:	1.
		N. weight:	0.
		Sc. PD:	0
		Sc. rank:	0

,

PA

Pos.	442	obs:	exp:
cgt	R	0	38.66
cgc	R	0	45.98
cga	R	0	90.24
cgg	R	3	75.38
aga	R	851	413.20
agg	R	51	241.50
---	--	------	------
mPD		0.12	0.96
		nPD:	0.12
		N. weight:	0.72
		Sc. PD:	-0.021
		Sc. rank:	38.2

,

PA

Pos.	443	obs:	exp:
cgt	R	0	38.66
cgc	R	0	45.98
cga	R	17	90.24
cgg	R	596	75.38
aga	R	34	413.20
agg	R	258	241.50
---	--	------	------
mPD		0.54	0.96
		nPD:	0.56
		N. weight:	1.6
		Sc. PD:	0.62
		Sc. rank:	937.7

,

PA

Pos.	444	obs:	exp:
aat	N	48	459.40
aac	N	855	443.60
gat	D	0	1.08
gac	D	2	0.92
---	--	------	------
mPD		0.10	0.50
		nPD:	0.21
		N. weight:	0.62
		Sc. PD:	0.033
		Sc. rank:	177.5

,

PA

Pos.	445	obs:	exp:
tat	Y	856	479.80
tac	Y	49	425.20
---	--	------	------
mPD		0.10	0.50
		nPD:	0.21
		N. weight:	0.53
		Sc. PD:	0.027
		Sc. rank:	149.4

,

PA

Pos.	446	obs:	exp:
ttt	F	732	357.20
ttc	F	173	547.80
---	--	------	------
mPD		0.31	0.48
		nPD:	0.65
		N. weight:	0.45
		Sc. PD:	0.21
		Sc. rank:	276.8

,

PA

Pos.	447	obs:	exp:
act	T	20	234.40
acc	T	23	184.30
aca	T	859	415.80
acg	T	3	70.50
---	--	------	------
mPD		0.098	0.67
		nPD:	0.15
		N. weight:	0.71
		Sc. PD:	-0.0056
		Sc. rank:	80.3

,

PA

Pos.	448	obs:	exp:
act	T	0	0.26
acc	T	0	0.20
aca	T	1	0.46
acg	T	0	0.08
gct	A	32	231.40
gcc	A	1	169.60
gca	A	822	422.20
gcg	A	48	79.87
gaa	E	1	0.60
gag	E	0	0.40
---	--	------	------
mPD		0.17	0.68
		nPD:	0.25
		N. weight:	0.63
		Sc. PD:	0.06
		Sc. rank:	243.3

,

PA

Pos.	449	obs:	exp:
gaa	E	107	544.40
gag	E	798	360.60
---	--	------	------
mPD		0.21	0.48
		nPD:	0.44
		N. weight:	0.63
		Sc. PD:	0.17
		Sc. rank:	347.6

,

PA

Pos.	450	obs:	exp:
att	I	0	0.36
atc	I	0	0.24
ata	I	1	0.39
gtt	V	5	186.60
gtc	V	10	181.10
gta	V	91	187.20
gtg	V	798	349.10
---	--	------	------
mPD		0.21	0.73
		nPD:	0.29
		N. weight:	0.75
		Sc. PD:	0.1
		Sc. rank:	328.2

,

PA

Pos.	451	obs:	exp:
tct	S	52	144.90
tcc	S	853	117.10
tca	S	0	228.40
tcg	S	0	52.67
agt	S	0	185.40
agc	S	0	176.50
---	--	------	------
mPD		0.11	1.7
		nPD:	0.07
		N. weight:	2.2
		Sc. PD:	-0.19
		Sc. rank:	-314.0

,

PA

Pos.	452	obs:	exp:
cat	H	75	519.90
cac	H	830	385.10
---	--	------	------
mPD		0.15	0.49
		nPD:	0.31
		N. weight:	0.67
		Sc. PD:	0.1
		Sc. rank:	306.7

,

PA

Pos.	453	obs:	exp:
tgt	C	7	371.50
tgc	C	898	533.50
---	--	------	------
mPD		0.015	0.48
		nPD:	0.03
		N. weight:	0.6
		Sc. PD:	-0.07
		Sc. rank:	-170.2

,

PA

Pos.	454	obs:	exp:
cgt	R	0	38.66
cgc	R	0	45.98
cga	R	0	90.24
cgg	R	0	75.38
aga	R	128	413.20
agg	R	777	241.50
---	--	------	------
mPD		0.24	0.96
		nPD:	0.25
		N. weight:	1.
		Sc. PD:	0.098
		Sc. rank:	399.1

,

PA

Pos.	455	obs:	exp:
tct	S	1	0.16
tcc	S	0	0.13
tca	S	0	0.25
tcg	S	0	0.06
agt	S	0	0.20
agc	S	0	0.20
gtt	V	1	0.21
gtc	V	0	0.20
gta	V	0	0.21
gtg	V	0	0.39
gct	A	838	231.40
gcc	A	31	169.60
gca	A	33	422.20
gcg	A	1	79.87
---	--	------	------
mPD		0.14	0.68
		nPD:	0.21
		N. weight:	1.3
		Sc. PD:	0.066
		Sc. rank:	366.5

,

PA

Pos.	456	obs:	exp:
act	T	859	234.40
acc	T	4	184.30
aca	T	42	415.80
acg	T	0	70.50
---	--	------	------
mPD		0.097	0.67
		nPD:	0.14
		N. weight:	1.4
		Sc. PD:	-0.013
		Sc. rank:	147.3

,

PA

Pos.	457	obs:	exp:
gaa	E	738	544.40
gag	E	167	360.60
---	--	------	------
mPD		0.30	0.48
		nPD:	0.63
		N. weight:	0.13
		Sc. PD:	0.062
		Sc. rank:	82.8

,

PA

Pos.	458	obs:	exp:
tat	Y	402	479.80
tac	Y	503	425.20
---	--	------	------
mPD		0.49	0.50
		nPD:	0.99
		N. weight:	0.023
		Sc. PD:	0.019
		Sc. rank:	14.8

,

PA

Pos.	459	obs:	exp:
tta	L	0	0.09
ttg	L	0	0.19
ctt	L	0	0.17
ctc	L	0	0.17
cta	L	1	0.17
ctg	L	0	0.22
att	I	0	329.10
atc	I	0	218.20
ata	I	902	354.70
gtt	V	0	0.41
gtc	V	0	0.40
gta	V	2	0.41
gtg	V	0	0.77
---	--	------	------
mPD		0.0066	0.66
		nPD:	0.01
		N. weight:	1.1
		Sc. PD:	-0.16
		Sc. rank:	-503.0

,

PA

Pos.	460	obs:	exp:
atg	M	905	905.00
---	--	------	------
mPD		0	0
		nPD:	1.
		N. weight:	0.
		Sc. PD:	0
		Sc. rank:	0

,

PA			
Pos.	461	obs:	exp:
cgt	R	0	0.04
cgc	R	0	0.05
cga	R	0	0.10
cgg	R	0	0.08
aaa	K	13	529.60
aag	K	891	374.40
aga	R	0	0.46
agg	R	1	0.27
---	--	------	------
mPD		0.031	0.49
		nPD:	0.06
		N. weight:	0.99
		Sc. PD:	-0.087
		Sc. rank:	-150.9

PA			
Pos.	462	obs:	exp:
ggt	G	0	121.40
ggc	G	0	115.00
gga	G	900	418.40
ggg	G	5	250.20
---	--	------	------
mPD		0.011	0.68
		nPD:	0.02
		N. weight:	0.91
		Sc. PD:	-0.12
		Sc. rank:	-364.3

PA			
Pos.	463	obs:	exp:
atg	M	1	1.00
gtt	V	2	186.60
gtc	V	5	181.10
gta	V	81	187.20
gtg	V	816	349.10
---	--	------	------
mPD		0.18	0.73
		nPD:	0.25
		N. weight:	0.82
		Sc. PD:	0.074
		Sc. rank:	306.5

PA			
Pos.	464	obs:	exp:
tat	Y	11	476.70
tac	Y	888	422.30
cat	H	0	3.45
cac	H	6	2.55
---	--	------	------
mPD		0.037	0.51
		nPD:	0.07
		N. weight:	0.85
		Sc. PD:	-0.066
		Sc. rank:	-97.3

PA			
Pos.	465	obs:	exp:
att	I	1	329.80
atc	I	1	218.70
ata	I	902	355.50
act	T	0	0.26
acc	T	0	0.20
aca	T	1	0.46
acg	T	0	0.08
---	--	------	------
mPD		0.0066	0.66
		nPD:	0.01
		N. weight:	1.1
		Sc. PD:	-0.16
		Sc. rank:	-489.2

PA			
Pos.	466	obs:	exp:
aat	N	786	460.40
aac	N	119	444.60
---	--	------	------
mPD		0.23	0.50
		nPD:	0.46
		N. weight:	0.36
		Sc. PD:	0.11
		Sc. rank:	203.9

PA			
Pos.	467	obs:	exp:
act	T	21	234.40
acc	T	1	184.30
aca	T	881	415.80
acg	T	2	70.50
---	--	------	------
mPD		0.052	0.67
		nPD:	0.08
		N. weight:	0.82
		Sc. PD:	-0.061
		Sc. rank:	-78.3

PA			
Pos.	468	obs:	exp:
gct	A	862	231.90
gcc	A	41	169.90
gca	A	2	423.10
gcg	A	0	80.05
---	--	------	------
mPD		0.091	0.67
		nPD:	0.14
		N. weight:	1.4
		Sc. PD:	-0.026
		Sc. rank:	113.7

PA			
Pos.	469	obs:	exp:
tta	L	5	79.54
ttg	L	843	168.90
ctt	L	0	153.40
ctc	L	0	151.60
cta	L	5	150.90
ctg	L	52	200.70
---	--	------	------
mPD		0.14	1.1
		nPD:	0.13
		N. weight:	1.7
		Sc. PD:	-0.045
		Sc. rank:	108.8

PA			
Pos.	470	obs:	exp:
tta	L	0	79.54
ttg	L	36	168.90
ctt	L	76	153.40
ctc	L	786	151.60
cta	L	5	150.90
ctg	L	2	200.70
---	--	------	------
mPD		0.31	1.1
		nPD:	0.28
		N. weight:	1.6
		Sc. PD:	0.2
		Sc. rank:	677.1

PA			
Pos.	471	obs:	exp:
aat	N	795	460.40
aac	N	110	444.60
---	--	------	------
mPD		0.21	0.50
		nPD:	0.43
		N. weight:	0.38
		Sc. PD:	0.1
		Sc. rank:	211.2

PA			
Pos.	472	obs:	exp:
tct	S	0	0.16
tcc	S	0	0.13
tca	S	1	0.25
tcg	S	0	0.06
agt	S	0	0.20
agc	S	0	0.20
gct	A	33	231.60
gcc	A	0	169.70
gca	A	869	422.70
gcg	A	2	79.96
---	--	------	------
mPD		0.077	0.68
		nPD:	0.11
		N. weight:	0.76
		Sc. PD:	-0.029
		Sc. rank:	9.0

PA			
Pos.	473	obs:	exp:
tct	S	836	144.90
tcc	S	63	117.10
tca	S	6	228.40
tcg	S	0	52.67
agt	S	0	185.40
agc	S	0	176.50
---	--	------	------
mPD		0.14	1.7
		nPD:	0.09
		N. weight:	1.9
		Sc. PD:	-0.12
		Sc. rank:	-126.3

PA			
Pos.	474	obs:	exp:
tgt	C	895	371.50
tgc	C	10	533.50
---	--	------	------
mPD		0.022	0.48
		nPD:	0.05
		N. weight:	1.
		Sc. PD:	-0.11
		Sc. rank:	-219.5

PA			
Pos.	475	obs:	exp:
gct	A	23	231.90
gcc	A	0	169.90
gca	A	734	423.10
gcg	A	148	80.05
---	--	------	------
mPD		0.32	0.67
		nPD:	0.47
		N. weight:	0.61
		Sc. PD:	0.18
		Sc. rank:	346.2

PA			
Pos.	476	obs:	exp:
gct	A	109	231.90
gcc	A	761	169.90
gca	A	35	423.10
gcg	A	0	80.05
---	--	------	------
mPD		0.28	0.67
		nPD:	0.41
		N. weight:	1.3
		Sc. PD:	0.33
		Sc. rank:	718.9

PA			
Pos.	477	obs:	exp:
atg	M	904	904.00
gtt	V	0	0.21
gtc	V	0	0.20
gta	V	0	0.21
gtg	V	1	0.39
---	--	------	------
mPD		0.0022	0.0036
		nPD:	0.62
		N. weight:	0.0013
		Sc. PD:	0.00058
		Sc. rank:	0.8

PA			
Pos.	478	obs:	exp:
gat	D	885	488.60
gac	D	20	416.40
---	--	------	------
mPD		0.043	0.50
		nPD:	0.09
		N. weight:	0.63
		Sc. PD:	-0.041
		Sc. rank:	-39.3

PA			
Pos.	479	obs:	exp:
aat	N	0	0.51
aac	N	1	0.49
gat	D	155	487.50
gac	D	748	415.50
gaa	E	1	0.60
gag	E	0	0.40
---	--	------	------
mPD		0.29	0.50
		nPD:	0.58
		N. weight:	0.36
		Sc. PD:	0.15
		Sc. rank:	220.1

PA			
Pos.	480	obs:	exp:
ttt	F	822	357.20
ttc	F	83	547.80
---	--	------	------
mPD		0.17	0.48
		nPD:	0.35
		N. weight:	0.72
		Sc. PD:	0.14
		Sc. rank:	351.5

PA			
Pos.	481	obs:	exp:
caa	Q	843	464.80
cag	Q	62	440.20
---	--	------	------
mPD		0.13	0.50
		nPD:	0.26
		N. weight:	0.52
		Sc. PD:	0.051
		Sc. rank:	203.8

PA			
Pos.	482	obs:	exp:
tta	L	3	79.54
ttg	L	14	168.90
ctt	L	0	153.40
ctc	L	0	151.60
cta	L	25	150.90
ctg	L	863	200.70
---	--	------	------
mPD		0.097	1.1
		nPD:	0.09
		N. weight:	1.6
		Sc. PD:	-0.1
		Sc. rank:	-94.3

PA			
Pos.	483	obs:	exp:
att	I	826	330.20
atc	I	79	218.90
ata	I	0	355.90
---	--	------	------
mPD		0.16	0.65
		nPD:	0.24
		N. weight:	0.92
		Sc. PD:	0.08
		Sc. rank:	336.5

PA			
Pos.	484	obs:	exp:
cct	P	2	230.10
ccc	P	1	161.40
cca	P	852	369.70
ccg	P	50	143.80
---	--	------	------
mPD		0.11	0.71
		nPD:	0.16
		N. weight:	0.88
		Sc. PD:	0.0018
		Sc. rank:	120.1

PA			
Pos.	485	obs:	exp:
atg	M	905	905.00
---	--	------	------
mPD		0	0
		nPD:	1.
		N. weight:	0.
		Sc. PD:	0
		Sc. rank:	0

PA	PA	PA	PA
Pos.	486	obs:	exp:
att	I	0	329.80
atc	I	0	218.70
ata	I	904	355.50
gtt	V	0	0.21
gtc	V	0	0.20
gta	V	1	0.21
gtg	V	0	0.39
mPD		0.0022	0.66
		nPD:	0.
		N. weight:	1.1
		Sc. PD:	-0.17
		Sc. rank:	-610.6

PA	PA	PA	PA
Pos.	487	obs:	exp:
tct	S	0	144.90
tcc	S	0	117.10
tca	S	0	228.40
tcg	S	0	52.67
agt	S	575	185.40
agc	S	330	176.50
mPD		0.46	1.7
		nPD:	0.28
		N. weight:	1.2
		Sc. PD:	0.14
		Sc. rank:	495.4

PA	PA	PA	PA
Pos.	488	obs:	exp:
aaa	K	822	530.10
aag	K	83	374.90
mPD		0.17	0.49
		nPD:	0.34
		N. weight:	0.32
		Sc. PD:	0.059
		Sc. rank:	156.3

PA	PA	PA	PA
Pos.	489	obs:	exp:
tct	S	0	0.16
tcc	S	0	0.13
tca	S	0	0.25
tcg	S	0	0.06
tgt	C	366	371.10
tgc	C	538	532.90
agt	S	0	0.20
agc	S	1	0.20
mPD		0.48	0.49
		nPD:	1.
		N. weight:	0.0072
		Sc. PD:	0.0058
		Sc. rank:	4.6

PA	PA	PA	PA
Pos.	490	obs:	exp:
cgt	R	0	38.58
cgc	R	0	45.88
cga	R	0	90.04
cgg	R	0	75.21
aga	R	884	412.30
agg	R	19	241.00
ggt	G	0	0.27
ggc	G	0	0.25
gga	G	2	0.92
ggg	G	0	0.55
mPD		0.046	0.97
		nPD:	0.05
		N. weight:	0.85
		Sc. PD:	-0.088
		Sc. rank:	-178.5

PA	PA	PA	PA
Pos.	491	obs:	exp:
act	T	38	234.20
acc	T	805	184.10
aca	T	61	415.30
acg	T	0	70.42
aat	N	0	0.51
aac	N	1	0.49
mPD		0.20	0.68
		nPD:	0.3
		N. weight:	1.4
		Sc. PD:	0.19
		Sc. rank:	608.8

PA	PA	PA	PA
Pos.	492	obs:	exp:
cgt	R	0	0.13
cgc	R	0	0.15
cga	R	1	0.30
cgg	R	0	0.25
att	I	0	0.73
atc	I	0	0.48
ata	I	2	0.79
aaa	K	881	527.20
aag	K	19	372.80
aga	R	2	1.37
agg	R	0	0.80
mPD		0.054	0.50
		nPD:	0.11
		N. weight:	0.55
		Sc. PD:	-0.024
		Sc. rank:	-3.7

PA	PA	PA	PA
Pos.	493	obs:	exp:
gaa	E	890	544.40
gag	E	15	360.60
mPD		0.033	0.48
		nPD:	0.07
		N. weight:	0.53
		Sc. PD:	-0.044
		Sc. rank:	-66.1

PA	PA	PA	PA
Pos.	494	obs:	exp:
ggt	G	0	121.40
ggc	G	0	115.00
gga	G	781	418.40
ggg	G	124	250.20
mPD		0.24	0.68
		nPD:	0.35
		N. weight:	0.55
		Sc. PD:	0.1
		Sc. rank:	268.2

PA	PA	PA	PA
Pos.	495	obs:	exp:
cgt	R	0	38.49
cgc	R	0	45.78
cga	R	4	89.84
cgg	R	34	75.04
att	I	0	1.09
atc	I	0	0.73
ata	I	3	1.18
act	T	0	0.26
acc	T	0	0.20
aca	T	1	0.46
acg	T	0	0.08
aga	R	834	411.40
agg	R	29	240.50
mPD		0.22	0.97
		nPD:	0.23
		N. weight:	0.68
		Sc. PD:	0.047
		Sc. rank:	223.5

PA	PA	PA	PA
Pos.	496	obs:	exp:
cgt	R	0	38.66
cgc	R	0	45.98
cga	R	19	90.24
cgg	R	883	75.38
aga	R	0	413.20
agg	R	3	241.50
mPD		0.048	0.96
		nPD:	0.05
		N. weight:	2.9
		Sc. PD:	-0.29
		Sc. rank:	-571.3

PA	PA	PA	PA
Pos.	497	obs:	exp:
cgt	R	0	0.09
cgc	R	0	0.10
cga	R	0	0.20
cgg	R	0	0.17
aaa	K	794	529.00
aag	K	109	374.00
aga	R	0	0.91
agg	R	2	0.53
mPD		0.22	0.49
		nPD:	0.45
		N. weight:	0.26
		Sc. PD:	0.075
		Sc. rank:	146.8

PA	PA	PA	PA
Pos.	498	obs:	exp:
act	T	7	234.40
acc	T	2	184.30
aca	T	890	415.80
acg	T	6	70.50
mPD		0.033	0.67
		nPD:	0.05
		N. weight:	0.86
		Sc. PD:	-0.087
		Sc. rank:	-177.3

PA	PA	PA	PA
Pos.	499	obs:	exp:
tct	S	0	0.16
tcc	S	0	0.13
tca	S	0	0.25
tcg	S	0	0.06
att	I	0	0.36
atc	I	1	0.24
ata	I	0	0.39
aat	N	84	459.40
aac	N	819	443.60
agt	S	0	0.20
agc	S	1	0.20
mPD		0.17	0.51
		nPD:	0.34
		N. weight:	0.5
		Sc. PD:	0.09
		Sc. rank:	239.3

PA	PA	PA	PA
Pos.	500	obs:	exp:
tta	L	1	79.54
ttg	L	1	168.90
ctt	L	1	153.40
ctc	L	0	151.60
cta	L	491	150.90
ctg	L	411	200.70
mPD		0.50	1.1
		nPD:	0.45
		N. weight:	1.2
		Sc. PD:	0.34
		Sc. rank:	659.7

PA	PA	PA	PA
Pos.	501	obs:	exp:
tat	Y	852	479.80
tac	Y	53	425.20
mPD		0.11	0.50
		nPD:	0.22
		N. weight:	0.52
		Sc. PD:	0.034
		Sc. rank:	167.1

PA	PA	PA	PA
Pos.	502	obs:	exp:
ggt	G	1	121.40
ggc	G	0	115.00
gga	G	845	418.40
ggg	G	59	250.20
mPD		0.12	0.68
		nPD:	0.18
		N. weight:	0.69
		Sc. PD:	0.02
		Sc. rank:	146.3

PA	PA	PA	PA
Pos.	503	obs:	exp:
ttt	F	5	356.40
ttc	F	898	546.60
att	I	0	0.73
atc	I	2	0.48
ata	I	0	0.79
mPD		0.015	0.48
		nPD:	0.03
		N. weight:	0.58
		Sc. PD:	-0.068
		Sc. rank:	-164.7

PA	PA	PA	PA
Pos.	504	obs:	exp:
tta	L	18	1.76
ttg	L	0	3.73
ctt	L	2	3.39
ctc	L	0	3.35
cta	L	0	3.33
ctg	L	0	4.44
att	I	850	322.90
atc	I	24	214.10
ata	I	11	348.00
mPD		0.16	0.70
		nPD:	0.22
		N. weight:	1.1
		Sc. PD:	0.069
		Sc. rank:	340.0

PA	PA	PA	PA
Pos.	505	obs:	exp:
att	I	0	329.50
atc	I	0	218.40
ata	I	903	355.10
gtt	V	0	0.41
gtc	V	0	0.40
gta	V	2	0.41
gtg	V	0	0.77
mPD		0.0044	0.66
		nPD:	0.01
		N. weight:	1.1
		Sc. PD:	-0.16
		Sc. rank:	-548.4

PA	PA	PA	PA
Pos.	506	obs:	exp:
aaa	K	861	530.10
aag	K	44	374.90
mPD		0.093	0.49
		nPD:	0.19
		N. weight:	0.44
		Sc. PD:	0.016
		Sc. rank:	104.4

PA	PA	PA	PA
Pos.	507	obs:	exp:
ggt	G	1	121.40
ggc	G	0	115.00
gga	G	865	418.40
ggg	G	39	250.20
mPD		0.085	0.68
		nPD:	0.13
		N. weight:	0.75
		Sc. PD:	-0.021
		Sc. rank:	45.6

PA	PA	PA	PA
Pos.	508	obs:	exp:
cgt	R	0	38.66
cgc	R	0	45.98
cga	R	4	90.24
cgg	R	0	75.38
aga	R	828	413.20
agg	R	73	241.50
mPD		0.16	0.96
		nPD:	0.16
		N. weight:	0.65
		Sc. PD:	0.0061
		Sc. rank:	101.9

PA	PA	PA	PA
Pos.	509	obs:	exp:
tct	S	855	144.90
tcc	S	50	117.10
tca	S	0	228.40
tcg	S	0	52.67
agt	S	0	185.40
agc	S	0	176.50
mPD		0.10	1.7
		nPD:	0.06
		N. weight:	2.
		Sc. PD:	-0.17
		Sc. rank:	-299.3

PA	PA	PA	PA
Pos.	510	obs:	exp:
cat	H	854	519.90
cac	H	51	385.10
mPD		0.11	0.49
		nPD:	0.22
		N. weight:	0.44
		Sc. PD:	0.027
		Sc. rank:	138.4

PA			
Pos.	511	obs:	exp:
tta	L	6	79.54
ttg	L	874	168.90
ctt	L	0	153.40
ctc	L	0	151.60
cta	L	0	150.90
ctg	L	25	200.70
———	——	———	———
mPD		0.067	1.1
		nPD:	0.06
		N. weight:	1.9
		Sc. PD:	-0.17
		Sc. rank:	-295.4

PA			
Pos.	512	obs:	exp:
cgt	R	0	38.66
cgc	R	0	45.98
cga	R	0	90.24
cgg	R	0	75.38
aga	R	31	413.20
agg	R	874	241.50
———	——	———	———
mPD		0.066	0.96
		nPD:	0.07
		N. weight:	1.4
		Sc. PD:	-0.12
		Sc. rank:	-170.9

PA			
Pos.	513	obs:	exp:
aat	N	888	460.40
aac	N	17	444.60
———	——	———	———
mPD		0.037	0.50
		nPD:	0.07
		N. weight:	0.72
		Sc. PD:	-0.055
		Sc. rank:	-77.2

PA			
Pos.	514	obs:	exp:
gat	D	862	488.60
gac	D	43	416.40
———	——	———	———
mPD		0.091	0.50
		nPD:	0.18
		N. weight:	0.53
		Sc. PD:	0.015
		Sc. rank:	111.8

PA			
Pos.	515	obs:	exp:
act	T	95	234.40
acc	T	802	184.30
aca	T	7	415.80
acg	T	1	70.50
———	——	———	———
mPD		0.20	0.67
		nPD:	0.3
		N. weight:	1.4
		Sc. PD:	0.21
		Sc. rank:	649.0

PA			
Pos.	516	obs:	exp:
gat	D	487	488.60
gac	D	418	416.40
———	——	———	———
mPD		0.50	0.50
		nPD:	1.
		N. weight:	0.0049
		Sc. PD:	0.004
		Sc. rank:	3.1

PA			
Pos.	517	obs:	exp:
gtt	V	2	186.80
gtc	V	8	181.30
gta	V	38	187.40
gtg	V	857	349.50
———	——	———	———
mPD		0.10	0.73
		nPD:	0.14
		N. weight:	0.92
		Sc. PD:	-0.012
		Sc. rank:	80.9

PA			
Pos.	518	obs:	exp:
gtt	V	0	186.80
gtc	V	0	181.30
gta	V	657	187.40
gtg	V	248	349.50
———	——	———	———
mPD		0.40	0.73
		nPD:	0.55
		N. weight:	1.
		Sc. PD:	0.38
		Sc. rank:	600.1

PA			
Pos.	519	obs:	exp:
tct	S	0	4.64
tcc	S	0	3.75
tca	S	0	7.32
tcg	S	0	1.69
aat	N	43	445.70
aac	N	833	430.30
agt	S	0	5.94
agc	S	29	5.66
———	——	———	———
mPD		0.15	0.61
		nPD:	0.25
		N. weight:	0.68
		Sc. PD:	0.063
		Sc. rank:	259.8

PA			
Pos.	520	obs:	exp:
ttt	F	851	357.20
ttc	F	54	547.80
———	——	———	———
mPD		0.11	0.48
		nPD:	0.24
		N. weight:	0.84
		Sc. PD:	0.066
		Sc. rank:	297.7

PA			
Pos.	521	obs:	exp:
att	I	0	0.73
atc	I	0	0.48
ata	I	2	0.79
gtt	V	0	186.40
gtc	V	2	180.90
gta	V	808	187.00
gtg	V	93	348.70
———	——	———	———
mPD		0.19	0.73
		nPD:	0.26
		N. weight:	1.4
		Sc. PD:	0.15
		Sc. rank:	576.7

PA			
Pos.	522	obs:	exp:
tct	S	0	144.90
tcc	S	0	117.10
tca	S	0	228.40
tcg	S	0	52.67
agt	S	801	185.40
agc	S	104	176.50
———	——	———	———
mPD		0.20	1.7
		nPD:	0.12
		N. weight:	1.5
		Sc. PD:	-0.045
		Sc. rank:	71.9

PA			
Pos.	523	obs:	exp:
atg	M	905	905.00
———	——	———	———
mPD		0	0
		nPD:	1.
		N. weight:	0.
		Sc. PD:	0
		Sc. rank:	0

PA			
Pos.	524	obs:	exp:
gaa	E	854	544.40
gag	E	51	360.60
———	——	———	———
mPD		0.11	0.48
		nPD:	0.22
		N. weight:	0.39
		Sc. PD:	0.026
		Sc. rank:	126.2

PA			
Pos.	525	obs:	exp:
ttt	F	850	356.80
ttc	F	54	547.20
tta	L	1	0.09
ttg	L	0	0.19
ctt	L	0	0.17
ctc	L	0	0.17
cta	L	0	0.17
ctg	L	0	0.22
———	——	———	———
mPD		0.11	0.48
		nPD:	0.24
		N. weight:	0.84
		Sc. PD:	0.068
		Sc. rank:	301.1

PA			
Pos.	526	obs:	exp:
tct	S	102	144.90
tcc	S	801	117.10
tca	S	2	228.40
tcg	S	0	52.67
agt	S	0	185.40
agc	S	0	176.50
———	——	———	———
mPD		0.20	1.7
		nPD:	0.12
		N. weight:	2.
		Sc. PD:	-0.059
		Sc. rank:	105.5

PA			
Pos.	527	obs:	exp:
tta	L	0	79.54
ttg	L	0	168.90
ctt	L	844	153.40
ctc	L	61	151.60
cta	L	0	150.90
ctg	L	0	200.70
———	——	———	———
mPD		0.13	1.1
		nPD:	0.11
		N. weight:	1.9
		Sc. PD:	-0.073
		Sc. rank:	19.7

PA			
Pos.	528	obs:	exp:
act	T	148	234.40
acc	T	724	184.30
aca	T	31	415.80
acg	T	2	70.50
———	——	———	———
mPD		0.33	0.67
		nPD:	0.49
		N. weight:	1.1
		Sc. PD:	0.37
		Sc. rank:	661.1

PA			
Pos.	529	obs:	exp:
gat	D	121	488.60
gac	D	784	416.40
———	——	———	———
mPD		0.23	0.50
		nPD:	0.47
		N. weight:	0.45
		Sc. PD:	0.14
		Sc. rank:	254.9

PA			
Pos.	530	obs:	exp:
cct	P	34	230.10
ccc	P	0	161.40
cca	P	810	369.70
ccg	P	61	143.80
———	——	———	———
mPD		0.19	0.71
		nPD:	0.27
		N. weight:	0.71
		Sc. PD:	0.081
		Sc. rank:	296.3

PA			
Pos.	531	obs:	exp:
cgt	R	0	38.62
cgc	R	0	45.93
cga	R	0	90.14
cgg	R	0	75.29
aaa	K	0	0.59
aag	K	1	0.41
aga	R	53	412.70
agg	R	851	241.30
———	——	———	———
mPD		0.11	0.97
		nPD:	0.12
		N. weight:	1.3
		Sc. PD:	-0.047
		Sc. rank:	31.1

PA			
Pos.	532	obs:	exp:
tta	L	1	79.54
ttg	L	819	168.90
ctt	L	0	153.40
ctc	L	1	151.60
cta	L	38	150.90
ctg	L	46	200.70
———	——	———	———
mPD		0.26	1.1
		nPD:	0.23
		N. weight:	1.6
		Sc. PD:	0.12
		Sc. rank:	539.4

PA			
Pos.	533	obs:	exp:
gat	D	1	1.08
gac	D	1	0.92
gaa	E	850	543.20
gag	E	53	359.80
———	——	———	———
mPD		0.11	0.48
		nPD:	0.24
		N. weight:	0.38
		Sc. PD:	0.031
		Sc. rank:	137.1

PA			
Pos.	534	obs:	exp:
cct	P	0	230.10
ccc	P	0	161.40
cca	P	903	369.70
ccg	P	2	143.80
———	——	———	———
mPD		0.0044	0.71
		nPD:	0.01
		N. weight:	1.1
		Sc. PD:	-0.15
		Sc. rank:	-537.1

PA			
Pos.	535	obs:	exp:
cat	H	855	518.80
cac	H	48	384.20
aat	N	0	1.02
aac	N	2	0.98
———	——	———	———
mPD		0.11	0.49
		nPD:	0.22
		N. weight:	0.45
		Sc. PD:	0.029
		Sc. rank:	143.9

PA			
Pos.	536	obs:	exp:
aaa	K	847	530.10
aag	K	58	374.90
———	——	———	———
mPD		0.12	0.49
		nPD:	0.25
		N. weight:	0.39
		Sc. PD:	0.036
		Sc. rank:	148.2

PA			
Pos.	537	obs:	exp:
tgg	W	905	905.00
———	——	———	———
mPD		0	0
		nPD:	1.
		N. weight:	0.
		Sc. PD:	0
		Sc. rank:	0

PA			
Pos.	538	obs:	exp:
gaa	E	860	543.80
gag	E	44	360.20
ggt	G	0	0.13
ggc	G	0	0.13
gga	G	1	0.46
ggg	G	0	0.28
———	——	———	———
mPD		0.095	0.48
		nPD:	0.2
		N. weight:	0.41
		Sc. PD:	0.017
		Sc. rank:	110.2

PA			
Pos.	539	obs:	exp:
aaa	K	18	530.10
aag	K	887	374.90
———	——	———	———
mPD		0.039	0.49
		nPD:	0.08
		N. weight:	0.96
		Sc. PD:	-0.068
		Sc. rank:	-81.5

PA			
Pos.	540	obs:	exp:
tat	Y	803	479.80
tac	Y	102	425.20
———	——	———	———
mPD		0.20	0.50
		nPD:	0.4
		N. weight:	0.37
		Sc. PD:	0.088
		Sc. rank:	193.9

PA

Pos.	541	obs:	exp:
tgt	C	900	371.50
tgc	C	5	533.50
mPD		0.011	0.48
		nPD:	0.02
		N. weight:	1.
		Sc. PD:	-0.13
		Sc. rank:	-370.7

PA

Pos.	542	obs:	exp:
att	I	1	0.36
atc	I	0	0.24
ata	I	0	0.39
gtt	V	856	186.60
gtc	V	47	181.10
gta	V	1	187.20
gtg	V	0	349.10
mPD		0.10	0.73
		nPD:	0.14
		N. weight:	1.7
		Sc. PD:	-0.02
		Sc. rank:	162.9

PA

Pos.	543	obs:	exp:
tta	L	0	79.54
ttg	L	0	168.90
ctt	L	758	153.40
ctc	L	145	151.60
cta	L	0	150.90
ctg	L	2	200.70
mPD		0.27	1.1
		nPD:	0.25
		N. weight:	1.6
		Sc. PD:	0.15
		Sc. rank:	606.7

PA

Pos.	544	obs:	exp:
gaa	E	862	544.40
gag	E	43	360.60
mPD		0.091	0.48
		nPD:	0.19
		N. weight:	0.42
		Sc. PD:	0.014
		Sc. rank:	94.0

PA

Pos.	545	obs:	exp:
att	I	0	327.70
atc	I	0	217.20
ata	I	898	353.10
gtt	V	0	1.45
gtc	V	0	1.40
gta	V	4	1.45
gtg	V	3	2.70
mPD		0.022	0.67
		nPD:	0.03
		N. weight:	1.1
		Sc. PD:	-0.13
		Sc. rank:	-314.7

PA

Pos.	546	obs:	exp:
ggt	G	0	121.40
ggc	G	0	115.00
gga	G	56	418.40
ggg	G	849	250.20
mPD		0.12	0.68
		nPD:	0.17
		N. weight:	1.3
		Sc. PD:	0.022
		Sc. rank:	217.9

PA

Pos.	547	obs:	exp:
gat	D	425	487.50
gac	D	478	415.50
gaa	E	2	1.20
gag	E	0	0.80
mPD		0.50	0.50
		nPD:	1.
		N. weight:	0.018
		Sc. PD:	0.015
		Sc. rank:	11.5

PA

Pos.	548	obs:	exp:
att	I	0	0.36
atc	I	0	0.24
ata	I	1	0.39
atg	M	903	903.00
gtt	V	0	0.21
gtc	V	0	0.20
gta	V	0	0.21
gtg	V	1	0.39
mPD		0.0044	0.0058
		nPD:	0.77
		N. weight:	0.0026
		Sc. PD:	0.0015
		Sc. rank:	1.6

PA

Pos.	549	obs:	exp:
tta	L	1	79.45
ttg	L	0	168.70
ctt	L	21	153.20
ctc	L	880	151.50
cta	L	1	150.70
ctg	L	1	200.50
att	I	0	0.36
atc	I	1	0.24
ata	I	0	0.39
mPD		0.056	1.1
		nPD:	0.05
		N. weight:	2.
		Sc. PD:	-0.2
		Sc. rank:	-389.9

PA

Pos.	550	obs:	exp:
tta	L	11	79.54
ttg	L	28	168.90
ctt	L	1	153.40
ctc	L	1	151.60
cta	L	57	150.90
ctg	L	807	200.70
mPD		0.23	1.1
		nPD:	0.2
		N. weight:	1.3
		Sc. PD:	0.065
		Sc. rank:	368.3

PA

Pos.	551	obs:	exp:
cgt	R	3	38.66
cgc	R	0	45.98
cga	R	797	90.24
cgg	R	76	75.38
aga	R	15	413.20
agg	R	14	241.50
mPD		0.25	0.96
		nPD:	0.26
		N. weight:	2.2
		Sc. PD:	0.22
		Sc. rank:	885.5

PA

Pos.	552	obs:	exp:
tct	S	0	0.16
tcc	S	0	0.13
tca	S	0	0.25
tcg	S	0	0.06
act	T	898	234.20
acc	T	6	184.10
aca	T	0	415.30
acg	T	0	70.42
agt	S	1	0.20
agc	S	0	0.20
mPD		0.015	0.68
		nPD:	0.02
		N. weight:	1.6
		Sc. PD:	-0.2
		Sc. rank:	-564.7

PA

Pos.	553	obs:	exp:
gct	A	1	231.90
gcc	A	1	169.90
gca	A	900	423.10
gcg	A	3	80.05
mPD		0.011	0.67
		nPD:	0.02
		N. weight:	0.9
		Sc. PD:	-0.12
		Sc. rank:	-357.9

PA

Pos.	554	obs:	exp:
tta	L	2	0.18
ttg	L	0	0.37
ctt	L	0	0.34
ctc	L	0	0.34
cta	L	0	0.33
ctg	L	0	0.44
att	I	2	35.76
atc	I	0	23.70
ata	I	96	38.54
atg	M	1	1.00
gtt	V	2	166.00
gtc	V	0	161.10
gta	V	688	166.50
gtg	V	114	310.50
mPD		0.43	0.94
		nPD:	0.46
		N. weight:	1.3
		Sc. PD:	0.38
		Sc. rank:	725.7

PA

Pos.	555	obs:	exp:
ggt	G	33	121.40
ggc	G	870	115.00
gga	G	2	418.40
ggg	G	0	250.20
mPD		0.075	0.68
		nPD:	0.11
		N. weight:	2.3
		Sc. PD:	-0.097
		Sc. rank:	-6.2

PA

Pos.	556	obs:	exp:
cat	H	0	1.15
cac	H	2	0.85
caa	Q	893	463.80
cag	Q	10	439.20
mPD		0.026	0.50
		nPD:	0.05
		N. weight:	0.75
		Sc. PD:	-0.073
		Sc. rank:	-141.4

PA

Pos.	557	obs:	exp:
att	I	1	0.36
atc	I	0	0.24
ata	I	0	0.39
gtt	V	2	186.60
gtc	V	0	181.10
gta	V	44	187.20
gtg	V	858	349.10
mPD		0.10	0.73
		nPD:	0.14
		N. weight:	0.95
		Sc. PD:	-0.013
		Sc. rank:	82.2

PA

Pos.	558	obs:	exp:
tct	S	32	144.90
tcc	S	0	117.10
tca	S	838	228.40
tcg	S	35	52.67
agt	S	0	185.40
agc	S	0	176.50
mPD		0.14	1.7
		nPD:	0.08
		N. weight:	1.4
		Sc. PD:	-0.093
		Sc. rank:	-99.2

PA

Pos.	559	obs:	exp:
cgt	R	0	38.66
cgc	R	0	45.98
cga	R	0	90.24
cgg	R	1	75.38
aga	R	697	413.20
agg	R	207	241.50
mPD		0.36	0.96
		nPD:	0.37
		N. weight:	0.45
		Sc. PD:	0.094
		Sc. rank:	226.5

PA

Pos.	560	obs:	exp:
cct	P	26	230.10
ccc	P	875	161.40
cca	P	4	369.70
ccg	P	0	143.80
mPD		0.064	0.71
		nPD:	0.09
		N. weight:	1.9
		Sc. PD:	-0.12
		Sc. rank:	-86.3

PA

Pos.	561	obs:	exp:
tta	L	0	0.26
ttg	L	1	0.56
ctt	L	0	0.51
ctc	L	0	0.50
cta	L	0	0.50
ctg	L	2	0.67
atg	M	902	902.00
mPD		0.0066	0.011
		nPD:	0.63
		N. weight:	0.0049
		Sc. PD:	0.0022
		Sc. rank:	3.0

PA

Pos.	562	obs:	exp:
ttt	F	826	357.20
ttc	F	79	547.80
mPD		0.16	0.48
		nPD:	0.33
		N. weight:	0.73
		Sc. PD:	0.13
		Sc. rank:	348.5

PA

Pos.	563	obs:	exp:
tta	L	0	79.54
ttg	L	31	168.90
ctt	L	3	153.40
ctc	L	0	151.60
cta	L	24	150.90
ctg	L	847	200.70
mPD		0.12	1.1
		nPD:	0.11
		N. weight:	1.5
		Sc. PD:	-0.061
		Sc. rank:	2.1

PA

Pos.	564	obs:	exp:
tat	Y	870	479.80
tac	Y	35	425.20
mPD		0.074	0.50
		nPD:	0.15
		N. weight:	0.59
		Sc. PD:	-0.0024
		Sc. rank:	71.5

PA

Pos.	565	obs:	exp:
att	I	0	1.46
atc	I	0	0.97
ata	I	4	1.57
atg	M	3	3.00
gtt	V	115	185.40
gtc	V	0	179.90
gta	V	26	185.90
gtg	V	757	346.80
mPD		0.29	0.74
		nPD:	0.4
		N. weight:	0.67
		Sc. PD:	0.16
		Sc. rank:	347.5

PA

Pos.	566	obs:	exp:
cgt	R	0	38.66
cgc	R	0	45.98
cga	R	3	90.24
cgg	R	0	75.38
aga	R	853	413.20
agg	R	49	241.50
mPD		0.11	0.96
		nPD:	0.11
		N. weight:	0.72
		Sc. PD:	-0.028
		Sc. rank:	1.9

PA

Pos.	567	obs:	exp:
act	T	30	234.40
acc	T	872	184.30
aca	T	3	415.80
acg	T	0	70.50
mPD		0.071	0.67
		nPD:	0.1
		N. weight:	1.7
		Sc. PD:	-0.082
		Sc. rank:	-29.0

PA

Pos.	568	obs:	exp:
aat	N	855	460.40
aac	N	50	444.60
mPD		0.10	0.50
		nPD:	0.21
		N. weight:	0.57
		Sc. PD:	0.031
		Sc. rank:	167.1

PA

Pos.	569	obs:	exp:
ggt	G	0	121.40
ggc	G	0	115.00
gga	G	60	418.40
ggg	G	845	250.20
mPD		0.12	0.68
		nPD:	0.18
		N. weight:	1.2
		Sc. PD:	0.036
		Sc. rank:	262.0

PA

Pos.	570	obs:	exp:
act	T	54	234.40
acc	T	846	184.30
aca	T	2	415.80
acg	T	3	70.50
mPD		0.12	0.67
		nPD:	0.18
		N. weight:	1.6
		Sc. PD:	0.044
		Sc. rank:	337.3

PA

Pos.	571	obs:	exp:
tct	S	31	144.90
tcc	S	872	117.10
tca	S	2	228.40
tcg	S	0	52.67
agt	S	0	185.40
agc	S	0	176.50
mPD		0.070	1.7
		nPD:	0.04
		N. weight:	2.3
		Sc. PD:	-0.25
		Sc. rank:	-510.7

PA

Pos.	572	obs:	exp:
aaa	K	60	530.10
aag	K	845	374.90
mPD		0.12	0.49
		nPD:	0.26
		N. weight:	0.76
		Sc. PD:	0.074
		Sc. rank:	294.9

PA

Pos.	573	obs:	exp:
att	I	4	329.10
atc	I	894	218.20
ata	I	4	354.70
gtt	V	0	0.62
gtc	V	3	0.60
gta	V	0	0.62
gtg	V	0	1.16
mPD		0.024	0.66
		nPD:	0.04
		N. weight:	1.7
		Sc. PD:	-0.19
		Sc. rank:	-423.7

PA

Pos.	574	obs:	exp:
aaa	K	60	530.10
aag	K	845	374.90
mPD		0.12	0.49
		nPD:	0.26
		N. weight:	0.76
		Sc. PD:	0.074
		Sc. rank:	294.9

PA

Pos.	575	obs:	exp:
atg	M	905	905.00
mPD		0	0
		nPD:	1.
		N. weight:	0.
		Sc. PD:	0
		Sc. rank:	0

PA

Pos.	576	obs:	exp:
aaa	K	898	530.10
aag	K	7	374.90
mPD		0.015	0.49
		nPD:	0.03
		N. weight:	0.61
		Sc. PD:	-0.071
		Sc. rank:	-173.0

PA

Pos.	577	obs:	exp:
tgg	W	905	905.00
mPD		0	0
		nPD:	1.
		N. weight:	0.
		Sc. PD:	0
		Sc. rank:	0

PA

Pos.	578	obs:	exp:
tct	S	0	0.64
tcc	S	0	0.52
tca	S	0	1.01
tcg	S	0	0.23
agt	S	4	0.82
agc	S	0	0.78
ggt	G	833	120.80
ggc	G	53	114.50
gga	G	3	416.60
ggg	G	12	249.10
mPD		0.15	0.69
		nPD:	0.22
		N. weight:	2.1
		Sc. PD:	0.13
		Sc. rank:	646.4

PA

Pos.	579	obs:	exp:
att	I	0	0.36
atc	I	0	0.24
ata	I	1	0.39
atg	M	900	900.00
act	T	0	1.04
acc	T	0	0.81
aca	T	0	1.84
acg	T	4	0.31
mPD		0.011	0.019
		nPD:	0.58
		N. weight:	0.015
		Sc. PD:	0.0062
		Sc. rank:	9.2

PA

Pos.	580	obs:	exp:
gaa	E	839	544.40
gag	E	66	360.60
mPD		0.14	0.48
		nPD:	0.28
		N. weight:	0.34
		Sc. PD:	0.043
		Sc. rank:	146.3

PA

Pos.	581	obs:	exp:
tta	L	0	0.09
ttg	L	0	0.19
ctt	L	0	0.17
ctc	L	0	0.17
cta	L	0	0.17
ctg	L	1	0.22
att	I	0	0.36
atc	I	0	0.24
ata	I	1	0.39
atg	M	903	903.00
mPD		0.0044	0.0057
		nPD:	0.77
		N. weight:	0.0033
		Sc. PD:	0.002
		Sc. rank:	2.1

PA

Pos.	582	obs:	exp:
cgt	R	0	38.66
cgc	R	0	45.98
cga	R	0	90.24
cgg	R	0	75.38
aga	R	806	413.20
agg	R	99	241.50
mPD		0.20	0.96
		nPD:	0.2
		N. weight:	0.61
		Sc. PD:	0.029
		Sc. rank:	166.3

PA

Pos.	583	obs:	exp:
cat	H	0	1.15
cac	H	2	0.85
cgt	R	44	38.58
cgc	R	813	45.88
cga	R	42	90.04
cgg	R	4	75.21
aga	R	0	412.30
agg	R	0	241.00
mPD		0.19	0.97
		nPD:	0.19
		N. weight:	3.1
		Sc. PD:	0.12
		Sc. rank:	801.0

PA

Pos.	584	obs:	exp:
tat	Y	0	0.53
tac	Y	1	0.47
tgt	C	44	371.10
tgc	C	860	532.90
mPD		0.095	0.49
		nPD:	0.2
		N. weight:	0.44
		Sc. PD:	0.017
		Sc. rank:	111.9

PA

Pos.	585	obs:	exp:
tta	L	0	79.54
ttg	L	0	168.90
ctt	L	893	153.40
ctc	L	11	151.60
cta	L	1	150.90
ctg	L	0	200.70
mPD		0.026	1.1
		nPD:	0.02
		N. weight:	2.1
		Sc. PD:	-0.26
		Sc. rank:	-711.0

PA

Pos.	586	obs:	exp:
tta	L	0	79.54
ttg	L	0	168.90
ctt	L	680	153.40
ctc	L	177	151.60
cta	L	48	150.90
ctg	L	0	200.70
mPD		0.39	1.1
		nPD:	0.36
		N. weight:	1.3
		Sc. PD:	0.26
		Sc. rank:	663.4

PA

Pos.	587	obs:	exp:
caa	Q	859	464.80
cag	Q	46	440.20
mPD		0.097	0.50
		nPD:	0.19
		N. weight:	0.58
		Sc. PD:	0.022
		Sc. rank:	142.5

PA

Pos.	588	obs:	exp:
tct	S	745	144.90
tcc	S	158	117.10
tca	S	1	228.40
tcg	S	1	52.67
agt	S	0	185.40
agc	S	0	176.50
mPD		0.29	1.7
		nPD:	0.18
		N. weight:	1.7
		Sc. PD:	0.038
		Sc. rank:	315.7

PA

Pos.	589	obs:	exp:
tta	L	0	79.54
ttg	L	0	168.90
ctt	L	100	153.40
ctc	L	800	151.60
cta	L	3	150.90
ctg	L	2	200.70
mPD		0.21	1.1
		nPD:	0.19
		N. weight:	1.7
		Sc. PD:	0.054
		Sc. rank:	374.0

PA

Pos.	590	obs:	exp:
cct	P	0	0.25
ccc	P	0	0.18
cca	P	1	0.41
ccg	P	0	0.16
caa	Q	860	464.30
cag	Q	44	439.70
mPD		0.095	0.50
		nPD:	0.19
		N. weight:	0.59
		Sc. PD:	0.02
		Sc. rank:	131.5

PA			
Pos.	591	obs:	exp:
caa	Q	113	464.80
cag	Q	792	440.20
---	--	------	------
mPD		0.22	0.50
		nPD:	0.44
		N. weight:	0.42
		Sc. PD:	0.11
		Sc. rank:	233.1

PA			
Pos.	592	obs:	exp:
att	I	898	330.20
atc	I	7	218.90
ata	I	0	355.90
---	--	------	------
mPD		0.015	0.65
		nPD:	0.02
		N. weight:	1.2
		Sc. PD:	-0.15
		Sc. rank:	-410.8

PA			
Pos.	593	obs:	exp:
gaa	E	39	544.40
gag	E	866	360.60
---	--	------	------
mPD		0.083	0.48
		nPD:	0.17
		N. weight:	0.89
		Sc. PD:	0.016
		Sc. rank:	156.1

PA			
Pos.	594	obs:	exp:
tct	S	0	144.90
tcc	S	0	117.10
tca	S	0	228.40
tcg	S	0	52.67
agt	S	5	185.40
agc	S	900	176.50
---	--	------	------
mPD		0.011	1.7
		nPD:	0.01
		N. weight:	2.
		Sc. PD:	-0.28
		Sc. rank:	-949.8

PA			
Pos.	595	obs:	exp:
tta	L	0	0.09
ttg	L	0	0.19
ctt	L	0	0.17
ctc	L	0	0.17
cta	L	0	0.17
ctg	L	1	0.22
att	I	0	0.36
atc	I	0	0.24
ata	I	1	0.39
atg	M	903	903.00
---	--	------	------
mPD		0.0044	0.0057
		nPD:	0.77
		N. weight:	0.0033
		Sc. PD:	0.002
		Sc. rank:	2.1

PA			
Pos.	596	obs:	exp:
att	I	901	330.20
atc	I	2	218.90
ata	I	2	355.90
---	--	------	------
mPD		0.0088	0.65
		nPD:	0.01
		N. weight:	1.2
		Sc. PD:	-0.16
		Sc. rank:	-502.5

PA			
Pos.	597	obs:	exp:
gaa	E	832	544.40
gag	E	73	360.60
---	--	------	------
mPD		0.15	0.48
		nPD:	0.31
		N. weight:	0.32
		Sc. PD:	0.049
		Sc. rank:	147.6

PA			
Pos.	598	obs:	exp:
tct	S	3	0.48
tcc	S	0	0.39
tca	S	0	0.76
tcg	S	0	0.17
agt	S	0	0.61
agc	S	0	0.59
gct	A	821	231.10
gcc	A	78	169.40
gca	A	3	421.70
gcg	A	0	79.78
---	--	------	------
mPD		0.17	0.68
		nPD:	0.25
		N. weight:	1.3
		Sc. PD:	0.12
		Sc. rank:	505.4

PA			
Pos.	599	obs:	exp:
gaa	E	849	542.00
gag	E	52	359.00
ggt	G	0	0.54
ggc	G	0	0.51
gga	G	4	1.85
ggg	G	0	1.11
---	--	------	------
mPD		0.12	0.49
		nPD:	0.24
		N. weight:	0.39
		Sc. PD:	0.032
		Sc. rank:	139.9

PA			
Pos.	600	obs:	exp:
tct	S	58	144.90
tcc	S	845	117.10
tca	S	2	228.40
tcg	S	0	52.67
agt	S	0	185.40
agc	S	0	176.50
---	--	------	------
mPD		0.12	1.7
		nPD:	0.08
		N. weight:	2.2
		Sc. PD:	-0.17
		Sc. rank:	-212.3

PA			
Pos.	601	obs:	exp:
tct	S	166	144.90
tcc	S	731	117.10
tca	S	6	228.40
tcg	S	2	52.67
agt	S	0	185.40
agc	S	0	176.50
---	--	------	------
mPD		0.31	1.7
		nPD:	0.19
		N. weight:	1.8
		Sc. PD:	0.064
		Sc. rank:	427.2

PA			
Pos.	602	obs:	exp:
att	I	1	1.82
atc	I	4	1.21
ata	I	0	1.97
gtt	V	5	185.80
gtc	V	894	180.30
gta	V	1	186.30
gtg	V	0	347.60
---	--	------	------
mPD		0.026	0.74
		nPD:	0.04
		N. weight:	1.9
		Sc. PD:	-0.22
		Sc. rank:	-494.2

PA			
Pos.	603	obs:	exp:
cgt	R	0	1.32
cgc	R	0	1.58
cga	R	0	3.09
cgg	R	0	2.58
aaa	K	864	512.00
aag	K	10	362.00
aga	R	31	14.15
agg	R	0	8.27
---	--	------	------
mPD		0.088	0.57
		nPD:	0.15
		N. weight:	0.6
		Sc. PD:	0.000079
		Sc. rank:	80.9

PA			
Pos.	604	obs:	exp:
gaa	E	27	544.40
gag	E	878	360.60
---	--	------	------
mPD		0.058	0.48
		nPD:	0.12
		N. weight:	0.95
		Sc. PD:	-0.03
		Sc. rank:	37.1

PA			
Pos.	605	obs:	exp:
aaa	K	806	530.10
aag	K	99	374.90
---	--	------	------
mPD		0.20	0.49
		nPD:	0.4
		N. weight:	0.28
		Sc. PD:	0.068
		Sc. rank:	149.7

PA			
Pos.	606	obs:	exp:
gat	D	5	488.60
gac	D	900	416.40
---	--	------	------
mPD		0.011	0.50
		nPD:	0.02
		N. weight:	0.91
		Sc. PD:	-0.12
		Sc. rank:	-327.0

PA			
Pos.	607	obs:	exp:
tta	L	0	70.92
ttg	L	0	150.60
ctt	L	0	136.80
ctc	L	0	135.20
cta	L	14	134.50
ctg	L	793	179.00
caa	Q	0	0.51
cag	Q	1	0.49
atg	M	97	97.00
---	--	------	------
mPD		0.22	1.2
		nPD:	0.19
		N. weight:	1.6
		Sc. PD:	0.052
		Sc. rank:	344.7

PA			
Pos.	608	obs:	exp:
act	T	15	234.40
acc	T	882	184.30
aca	T	8	415.80
acg	T	0	70.50
---	--	------	------
mPD		0.050	0.67
		nPD:	0.07
		N. weight:	1.8
		Sc. PD:	-0.14
		Sc. rank:	-183.0

PA			
Pos.	609	obs:	exp:
cgt	R	0	0.04
cgc	R	0	0.05
cga	R	0	0.10
cgg	R	0	0.08
aaa	K	877	529.60
aag	K	27	374.40
aga	R	1	0.46
agg	R	0	0.27
---	--	------	------
mPD		0.060	0.49
		nPD:	0.12
		N. weight:	0.51
		Sc. PD:	-0.015
		Sc. rank:	24.5

PA			
Pos.	610	obs:	exp:
gaa	E	895	544.40
gag	E	10	360.60
---	--	------	------
mPD		0.022	0.48
		nPD:	0.05
		N. weight:	0.56
		Sc. PD:	-0.058
		Sc. rank:	-117.9

PA			
Pos.	611	obs:	exp:
ttt	F	8	357.20
ttc	F	897	547.80
---	--	------	------
mPD		0.018	0.48
		nPD:	0.04
		N. weight:	0.56
		Sc. PD:	-0.063
		Sc. rank:	-141.0

PA			
Pos.	612	obs:	exp:
ttt	F	869	357.20
ttc	F	36	547.80
---	--	------	------
mPD		0.076	0.48
		nPD:	0.16
		N. weight:	0.92
		Sc. PD:	0.0057
		Sc. rank:	132.4

PA			
Pos.	613	obs:	exp:
gaa	E	852	544.40
gag	E	53	360.60
---	--	------	------
mPD		0.11	0.48
		nPD:	0.23
		N. weight:	0.38
		Sc. PD:	0.028
		Sc. rank:	132.1

PA			
Pos.	614	obs:	exp:
act	T	0	0.26
acc	T	1	0.20
aca	T	0	0.46
acg	T	0	0.08
aat	N	2	459.40
aac	N	901	443.60
gat	D	0	0.54
gac	D	1	0.46
---	--	------	------
mPD		0.0088	0.50
		nPD:	0.02
		N. weight:	0.85
		Sc. PD:	-0.11
		Sc. rank:	-332.4

PA			
Pos.	615	obs:	exp:
cgt	R	0	0.04
cgc	R	0	0.05
cga	R	0	0.10
cgg	R	0	0.08
aaa	K	892	529.60
aag	K	12	374.40
aga	R	1	0.46
agg	R	0	0.27
---	--	------	------
mPD		0.028	0.49
		nPD:	0.06
		N. weight:	0.58
		Sc. PD:	-0.053
		Sc. rank:	-94.6

PA			
Pos.	616	obs:	exp:
tct	S	32	144.90
tcc	S	0	117.10
tca	S	862	228.40
tcg	S	11	52.67
agt	S	0	185.40
agc	S	0	176.50
---	--	------	------
mPD		0.091	1.7
		nPD:	0.06
		N. weight:	1.5
		Sc. PD:	-0.14
		Sc. rank:	-253.1

PA			
Pos.	617	obs:	exp:
gaa	E	785	544.40
gag	E	120	360.60
---	--	------	------
mPD		0.23	0.48
		nPD:	0.48
		N. weight:	0.21
		Sc. PD:	0.068
		Sc. rank:	124.0

PA			
Pos.	618	obs:	exp:
act	T	0	233.90
acc	T	0	183.90
aca	T	897	414.90
acg	T	6	70.34
gct	A	0	0.51
gcc	A	0	0.38
gca	A	1	0.94
gcg	A	1	0.18
---	--	------	------
mPD		0.020	0.68
		nPD:	0.03
		N. weight:	0.92
		Sc. PD:	-0.11
		Sc. rank:	-277.3

PA			
Pos.	619	obs:	exp:
tgg	W	905	905.00
---	--	------	------
mPD		0	0
		nPD:	1.
		N. weight:	0.
		Sc. PD:	0
		Sc. rank:	0

PA			
Pos.	620	obs:	exp:
cct	P	2	230.10
ccc	P	36	161.40
cca	P	861	369.70
ccg	P	6	143.80
---	--	------	------
mPD		0.093	0.71
		nPD:	0.13
		N. weight:	0.88
		Sc. PD:	-0.019
		Sc. rank:	62.0

PA			
Pos.	621	obs:	exp:
att	I	894	330.20
atc	I	10	218.90
ata	I	1	355.90
---	--	------	------
mPD		0.024	0.65
		nPD:	0.04
		N. weight:	1.2
		Sc. PD:	-0.13
		Sc. rank:	-289.6

PA			
Pos.	622	obs:	exp:
ggt	G	3	121.40
ggc	G	0	115.00
gga	G	859	418.40
ggg	G	43	250.20
---	--	------	------
mPD		0.097	0.68
		nPD:	0.14
		N. weight:	0.73
		Sc. PD:	-0.0071
		Sc. rank:	73.4

PA			
Pos.	623	obs:	exp:
gaa	E	503	544.40
gag	E	402	360.60
---	--	------	------
mPD		0.49	0.48
		nPD:	1.03
		N. weight:	0.01
		Sc. PD:	0.0086
		Sc. rank:	6.5

PA			
Pos.	624	obs:	exp:
tct	S	1	144.90
tcc	S	11	117.10
tca	S	893	228.40
tcg	S	0	52.67
agt	S	0	185.40
agc	S	0	176.50
---	--	------	------
mPD		0.026	1.7
		nPD:	0.02
		N. weight:	1.6
		Sc. PD:	-0.21
		Sc. rank:	-648.2

PA			
Pos.	625	obs:	exp:
cct	P	760	230.10
ccc	P	145	161.40
cca	P	0	369.70
ccg	P	0	143.80
---	--	------	------
mPD		0.27	0.71
		nPD:	0.38
		N. weight:	1.2
		Sc. PD:	0.26
		Sc. rank:	621.0

PA			
Pos.	626	obs:	exp:
caa	Q	0	0.51
cag	Q	1	0.49
cgt	R	0	0.26
cgc	R	0	0.30
cga	R	0	0.60
cgg	R	0	0.50
atg	M	1	1.00
aat	N	4	2.03
aac	N	0	1.97
aaa	K	459	523.10
aag	K	434	369.90
aga	R	6	2.74
agg	R	0	1.60
---	--	------	------
mPD		0.52	0.51
		nPD:	1.02
		N. weight:	0.029
		Sc. PD:	0.024
		Sc. rank:	18.3

PA			
Pos.	627	obs:	exp:
ggt	G	1	121.40
ggc	G	0	115.00
gga	G	845	418.40
ggg	G	59	250.20
---	--	------	------
mPD		0.12	0.68
		nPD:	0.18
		N. weight:	0.69
		Sc. PD:	0.02
		Sc. rank:	146.3

PA			
Pos.	628	obs:	exp:
gtt	V	0	186.80
gtc	V	0	181.30
gta	V	58	187.40
gtg	V	847	349.50
---	--	------	------
mPD		0.12	0.73
		nPD:	0.17
		N. weight:	0.93
		Sc. PD:	0.011
		Sc. rank:	148.0

PA			
Pos.	629	obs:	exp:
gaa	E	43	544.40
gag	E	862	360.60
---	--	------	------
mPD		0.091	0.48
		nPD:	0.19
		N. weight:	0.87
		Sc. PD:	0.03
		Sc. rank:	197.0

PA			
Pos.	630	obs:	exp:
gat	D	0	0.54
gac	D	1	0.46
gaa	E	900	543.80
gag	E	4	360.20
---	--	------	------
mPD		0.011	0.48
		nPD:	0.02
		N. weight:	0.59
		Sc. PD:	-0.075
		Sc. rank:	-207.2

PA			
Pos.	631	obs:	exp:
gtt	V	0	0.21
gtc	V	1	0.20
gta	V	0	0.21
gtg	V	0	0.39
ggt	G	847	121.20
ggc	G	55	114.90
gga	G	2	418.00
ggg	G	0	249.90
---	--	------	------
mPD		0.12	0.68
		nPD:	0.18
		N. weight:	2.2
		Sc. PD:	0.057
		Sc. rank:	428.7

PA			
Pos.	632	obs:	exp:
tct	S	5	144.70
tcc	S	896	117.00
tca	S	3	228.10
tcg	S	0	52.61
cct	P	0	0.25
ccc	P	1	0.18
cca	P	0	0.41
ccg	P	0	0.16
agt	S	0	185.20
agc	S	0	176.30
---	--	------	------
mPD		0.020	1.7
		nPD:	0.01
		N. weight:	2.4
		Sc. PD:	-0.33
		Sc. rank:	-1047.9

PA			
Pos.	633	obs:	exp:
att	I	293	330.20
atc	I	604	218.90
ata	I	8	355.90
---	--	------	------
mPD		0.45	0.65
		nPD:	0.69
		N. weight:	0.75
		Sc. PD:	0.39
		Sc. rank:	468.0

PA			
Pos.	634	obs:	exp:
ggt	G	0	121.40
ggc	G	0	115.00
gga	G	168	418.40
ggg	G	737	250.20
---	--	------	------
mPD		0.30	0.68
		nPD:	0.45
		N. weight:	0.88
		Sc. PD:	0.25
		Sc. rank:	490.4

PA			
Pos.	635	obs:	exp:
aaa	K	15	530.10
aag	K	890	374.90
---	--	------	------
mPD		0.033	0.49
		nPD:	0.07
		N. weight:	0.97
		Sc. PD:	-0.081
		Sc. rank:	-126.5

PA			
Pos.	636	obs:	exp:
gtt	V	0	186.80
gtc	V	1	181.30
gta	V	32	187.40
gtg	V	872	349.50
---	--	------	------
mPD		0.070	0.73
		nPD:	0.1
		N. weight:	1.
		Sc. PD:	-0.055
		Sc. rank:	-31.4

PA			
Pos.	637	obs:	exp:
tgt	C	31	371.50
tgc	C	874	533.50
---	--	------	------
mPD		0.066	0.48
		nPD:	0.14
		N. weight:	0.48
		Sc. PD:	-0.0078
		Sc. rank:	39.8

PA			
Pos.	638	obs:	exp:
cgt	R	0	38.62
cgc	R	0	45.93
cga	R	1	90.14
cgg	R	0	75.29
act	T	0	0.26
acc	T	0	0.20
aca	T	1	0.46
acg	T	0	0.08
aga	R	864	412.70
agg	R	39	241.30
---	--	------	------
mPD		0.087	0.97
		nPD:	0.09
		N. weight:	0.77
		Sc. PD:	-0.047
		Sc. rank:	-38.1

PA			
Pos.	639	obs:	exp:
act	T	40	234.20
acc	T	830	184.10
aca	T	34	415.30
acg	T	0	70.42
gct	A	0	0.26
gcc	A	1	0.19
gca	A	0	0.47
gcg	A	0	0.09
---	--	------	------
mPD		0.16	0.68
		nPD:	0.23
		N. weight:	1.5
		Sc. PD:	0.11
		Sc. rank:	518.3

PA

Pos.	640	obs:	exp:
ttt	F	1	0.39
ttc	F	0	0.61
tta	L	838	79.45
ttg	L	14	168.70
ctt	L	0	153.20
ctc	L	0	151.50
cta	L	17	150.70
ctg	L	35	200.50
mPD		0.21	1.1
		nPD:	0.19
		N. weight:	2.5
		Sc. PD:	0.092
		Sc. rank:	603.1

PA

Pos.	641	obs:	exp:
tta	L	3	79.45
ttg	L	4	168.70
ctt	L	1	153.20
ctc	L	0	151.50
cta	L	826	150.70
ctg	L	70	200.50
att	I	0	0.36
atc	I	0	0.24
ata	I	1	0.39
mPD		0.17	1.1
		nPD:	0.15
		N. weight:	1.8
		Sc. PD:	-0.0012
		Sc. rank:	237.6

PA

Pos.	642	obs:	exp:
gct	A	3	231.90
gcc	A	0	169.90
gca	A	899	423.10
gcg	A	3	80.05
mPD		0.013	0.67
		nPD:	0.02
		N. weight:	0.89
		Sc. PD:	-0.12
		Sc. rank:	-333.1

PA

Pos.	643	obs:	exp:
aaa	K	874	530.10
aag	K	31	374.90
mPD		0.066	0.49
		nPD:	0.14
		N. weight:	0.49
		Sc. PD:	-0.0081
		Sc. rank:	39.9

PA

Pos.	644	obs:	exp:
tct	S	772	144.90
tcc	S	25	117.10
tca	S	3	228.40
tcg	S	105	52.67
agt	S	0	185.40
agc	S	0	176.50
mPD		0.26	1.7
		nPD:	0.16
		N. weight:	1.8
		Sc. PD:	0.0045
		Sc. rank:	249.8

PA

Pos.	645	obs:	exp:
gtt	V	0	186.80
gtc	V	0	181.30
gta	V	820	187.40
gtg	V	85	349.50
mPD		0.17	0.73
		nPD:	0.23
		N. weight:	1.5
		Sc. PD:	0.12
		Sc. rank:	524.0

PA

Pos.	646	obs:	exp:
ttt	F	3	357.20
ttc	F	902	547.80
mPD		0.0066	0.48
		nPD:	0.01
		N. weight:	0.59
		Sc. PD:	-0.08
		Sc. rank:	-244.0

PA

Pos.	647	obs:	exp:
act	T	1	0.26
acc	T	0	0.20
aca	T	0	0.46
acg	T	0	0.08
aat	N	313	459.90
aac	N	591	444.10
mPD		0.46	0.50
		nPD:	0.91
		N. weight:	0.072
		Sc. PD:	0.053
		Sc. rank:	46.2

PA

Pos.	648	obs:	exp:
tct	S	0	144.40
tcc	S	0	116.80
tca	S	0	227.60
tcg	S	0	52.49
att	I	0	0.36
atc	I	1	0.24
ata	I	0	0.39
aat	N	0	1.02
aac	N	2	0.98
agt	S	41	184.80
agc	S	861	175.90
mPD		0.093	1.7
		nPD:	0.06
		N. weight:	1.8
		Sc. PD:	-0.17
		Sc. rank:	-295.8

PA

Pos.	649	obs:	exp:
tta	L	34	79.45
ttg	L	34	168.70
ctt	L	0	153.20
ctc	L	0	151.50
cta	L	818	150.70
ctg	L	18	200.50
att	I	0	0.36
atc	I	0	0.24
ata	I	1	0.39
mPD		0.25	1.1
		nPD:	0.22
		N. weight:	1.7
		Sc. PD:	0.12
		Sc. rank:	560.5

PA

Pos.	650	obs:	exp:
tat	Y	693	479.80
tac	Y	212	425.20
mPD		0.36	0.50
		nPD:	0.72
		N. weight:	0.15
		Sc. PD:	0.082
		Sc. rank:	94.6

PA

Pos.	651	obs:	exp:
gct	A	1	231.90
gcc	A	1	169.90
gca	A	901	423.10
gcg	A	2	80.05
mPD		0.0088	0.67
		nPD:	0.01
		N. weight:	0.9
		Sc. PD:	-0.12
		Sc. rank:	-382.8

PA

Pos.	652	obs:	exp:
tct	S	879	144.90
tcc	S	23	117.10
tca	S	3	228.40
tcg	S	0	52.67
agt	S	0	185.40
agc	S	0	176.50
mPD		0.056	1.7
		nPD:	0.03
		N. weight:	2.1
		Sc. PD:	-0.24
		Sc. rank:	-566.1

PA

Pos.	653	obs:	exp:
cct	P	4	230.10
ccc	P	0	161.40
cca	P	317	369.70
ccg	P	584	143.80
mPD		0.46	0.71
		nPD:	0.65
		N. weight:	1.
		Sc. PD:	0.49
		Sc. rank:	639.7

PA

Pos.	654	obs:	exp:
caa	Q	890	464.80
cag	Q	15	440.20
mPD		0.033	0.50
		nPD:	0.07
		N. weight:	0.72
		Sc. PD:	-0.061
		Sc. rank:	-104.1

PA

Pos.	655	obs:	exp:
tta	L	0	79.45
ttg	L	3	168.70
ctt	L	73	153.20
ctc	L	757	151.50
cta	L	70	150.70
ctg	L	1	200.50
att	I	0	0.36
atc	I	1	0.24
ata	I	0	0.39
mPD		0.30	1.1
		nPD:	0.27
		N. weight:	1.5
		Sc. PD:	0.16
		Sc. rank:	608.0

PA

Pos.	656	obs:	exp:
gaa	E	43	544.40
gag	E	862	360.60
mPD		0.091	0.48
		nPD:	0.19
		N. weight:	0.87
		Sc. PD:	0.03
		Sc. rank:	197.0

PA

Pos.	657	obs:	exp:
ggt	G	0	121.40
ggc	G	0	115.00
gga	G	54	418.40
ggg	G	851	250.20
mPD		0.11	0.68
		nPD:	0.17
		N. weight:	1.3
		Sc. PD:	0.015
		Sc. rank:	209.0

PA

Pos.	658	obs:	exp:
ttt	F	102	356.40
ttc	F	801	546.60
tat	Y	0	0.53
tac	Y	1	0.47
gtt	V	0	0.21
gtc	V	1	0.20
gta	V	0	0.21
gtg	V	0	0.39
mPD		0.20	0.48
		nPD:	0.42
		N. weight:	0.25
		Sc. PD:	0.065
		Sc. rank:	136.4

PA

Pos.	659	obs:	exp:
tct	S	0	144.90
tcc	S	0	117.10
tca	S	901	228.40
tcg	S	4	52.67
agt	S	0	185.40
agc	S	0	176.50
mPD		0.0088	1.7
		nPD:	0.01
		N. weight:	1.7
		Sc. PD:	-0.24
		Sc. rank:	-853.9

PA

Pos.	660	obs:	exp:
gct	A	893	231.90
gcc	A	4	169.90
gca	A	7	423.10
gcg	A	1	80.05
mPD		0.026	0.67
		nPD:	0.04
		N. weight:	1.6
		Sc. PD:	-0.17
		Sc. rank:	-376.2

PA

Pos.	661	obs:	exp:
gaa	E	756	543.80
gag	E	148	360.20
ggt	G	0	0.13
ggc	G	0	0.13
gga	G	1	0.46
ggg	G	0	0.28
mPD		0.28	0.48
		nPD:	0.57
		N. weight:	0.16
		Sc. PD:	0.067
		Sc. rank:	99.9

PA

Pos.	662	obs:	exp:
tct	S	3	144.90
tcc	S	0	117.10
tca	S	49	228.40
tcg	S	853	52.67
agt	S	0	185.40
agc	S	0	176.50
mPD		0.11	1.7
		nPD:	0.07
		N. weight:	3.1
		Sc. PD:	-0.26
		Sc. rank:	-421.0

PA

Pos.	663	obs:	exp:
cgt	R	0	38.66
cgc	R	0	45.98
cga	R	0	90.24
cgg	R	0	75.38
aga	R	888	413.20
agg	R	17	241.50
mPD		0.037	0.96
		nPD:	0.04
		N. weight:	0.86
		Sc. PD:	-0.096
		Sc. rank:	-213.8

PA

Pos.	664	obs:	exp:
aaa	K	867	530.10
aag	K	38	374.90
mPD		0.081	0.49
		nPD:	0.17
		N. weight:	0.46
		Sc. PD:	0.0055
		Sc. rank:	75.5

PA			
Pos.	665	obs:	exp:
tta	L	2	79.54
ttg	L	160	168.90
ctt	L	0	153.40
ctc	L	1	151.60
cta	L	13	150.90
ctg	L	729	200.70
---	--	------	------
mPD		0.33	1.1
		nPD:	0.3
		N. weight:	1.2
		Sc. PD:	0.17
		Sc. rank:	534.3

PA			
Pos.	666	obs:	exp:
tta	L	5	79.54
ttg	L	0	168.90
ctt	L	48	153.40
ctc	L	19	151.60
cta	L	819	150.90
ctg	L	14	200.70
---	--	------	------
mPD		0.18	1.1
		nPD:	0.16
		N. weight:	1.7
		Sc. PD:	0.012
		Sc. rank:	251.2

PA			
Pos.	667	obs:	exp:
tta	L	0	79.54
ttg	L	0	168.90
ctt	L	3	153.40
ctc	L	902	151.60
cta	L	0	150.90
ctg	L	0	200.70
---	--	------	------
mPD		0.0066	1.1
		nPD:	0.01
		N. weight:	2.2
		Sc. PD:	-0.31
		Sc. rank:	-1096.6

PA			
Pos.	668	obs:	exp:
att	I	902	329.50
atc	I	1	218.40
ata	I	0	355.10
gtt	V	2	0.41
gtc	V	0	0.40
gta	V	0	0.41
gtg	V	0	0.77
---	--	------	------
mPD		0.0066	0.66
		nPD:	0.01
		N. weight:	1.2
		Sc. PD:	-0.17
		Sc. rank:	-536.2

PA			
Pos.	669	obs:	exp:
att	I	2	0.73
atc	I	0	0.48
ata	I	0	0.79
gtt	V	900	186.40
gtc	V	2	180.90
gta	V	0	187.00
gtg	V	1	348.70
---	--	------	------
mPD		0.011	0.73
		nPD:	0.02
		N. weight:	1.9
		Sc. PD:	-0.25
		Sc. rank:	-779.0

PA			
Pos.	670	obs:	exp:
cat	H	1	1.15
cac	H	1	0.85
caa	Q	0	463.80
cag	Q	903	439.20
---	--	------	------
mPD		0.0044	0.50
		nPD:	0.01
		N. weight:	0.88
		Sc. PD:	-0.12
		Sc. rank:	-411.6

PA			
Pos.	671	obs:	exp:
gct	A	4	231.90
gcc	A	1	169.90
gca	A	215	423.10
gcg	A	685	80.05
---	--	------	------
mPD		0.37	0.67
		nPD:	0.55
		N. weight:	1.8
		Sc. PD:	0.68
		Sc. rank:	1063.9

PA			
Pos.	672	obs:	exp:
tta	L	0	79.36
ttg	L	0	168.50
ctt	L	885	153.00
ctc	L	18	151.30
cta	L	0	150.50
ctg	L	0	200.30
att	I	2	0.73
atc	I	0	0.48
ata	I	0	0.79
---	--	------	------
mPD		0.043	1.1
		nPD:	0.04
		N. weight:	2.1
		Sc. PD:	-0.23
		Sc. rank:	-498.3

PA			
Pos.	673	obs:	exp:
cgt	R	0	38.58
cgc	R	0	45.88
cga	R	0	90.04
cgg	R	9	75.21
act	T	0	0.26
acc	T	0	0.20
aca	T	0	0.46
acg	T	1	0.08
aga	R	0	412.30
agg	R	894	241.00
ggt	G	0	0.13
ggc	G	0	0.13
gga	G	0	0.46
ggg	G	1	0.28
---	--	------	------
mPD		0.024	0.97
		nPD:	0.02
		N. weight:	1.6
		Sc. PD:	-0.2
		Sc. rank:	-523.8

PA			
Pos.	674	obs:	exp:
gat	D	712	488.60
gac	D	193	416.40
---	--	------	------
mPD		0.34	0.50
		nPD:	0.68
		N. weight:	0.17
		Sc. PD:	0.084
		Sc. rank:	104.0

PA			
Pos.	675	obs:	exp:
tct	S	0	0.32
tcc	S	0	0.26
tca	S	0	0.50
tcg	S	0	0.12
act	T	0	0.26
acc	T	1	0.20
aca	T	0	0.46
acg	T	0	0.08
aat	N	12	458.40
aac	N	889	442.60
aaa	K	0	0.59
aag	K	1	0.41
agt	S	0	0.41
agc	S	2	0.39
---	--	------	------
mPD		0.035	0.51
		nPD:	0.07
		N. weight:	0.79
		Sc. PD:	-0.065
		Sc. rank:	-97.4

PA			
Pos.	676	obs:	exp:
tta	L	0	79.45
ttg	L	0	168.70
ctt	L	0	153.20
ctc	L	0	151.50
cta	L	0	150.70
ctg	L	904	200.50
atg	M	1	1.00
---	--	------	------
mPD		0.0022	1.1
		nPD:	0.
		N. weight:	1.8
		Sc. PD:	-0.27
		Sc. rank:	-1047.4

PA			
Pos.	677	obs:	exp:
gaa	E	893	544.40
gag	E	12	360.60
---	--	------	------
mPD		0.026	0.48
		nPD:	0.05
		N. weight:	0.55
		Sc. PD:	-0.052
		Sc. rank:	-98.9

PA			
Pos.	678	obs:	exp:
cct	P	904	230.10
ccc	P	1	161.40
cca	P	0	369.70
ccg	P	0	143.80
---	--	------	------
mPD		0.0022	0.71
		nPD:	0.
		N. weight:	1.7
		Sc. PD:	-0.24
		Sc. rank:	-912.9

PA			
Pos.	679	obs:	exp:
ggt	G	32	121.40
ggc	G	1	115.00
gga	G	813	418.40
ggg	G	59	250.20
---	--	------	------
mPD		0.19	0.68
		nPD:	0.28
		N. weight:	0.56
		Sc. PD:	0.067
		Sc. rank:	237.1

PA			
Pos.	680	obs:	exp:
act	T	21	234.40
acc	T	882	184.30
aca	T	2	415.80
acg	T	0	70.50
---	--	------	------
mPD		0.050	0.67
		nPD:	0.07
		N. weight:	1.8
		Sc. PD:	-0.14
		Sc. rank:	-197.6

PA			
Pos.	681	obs:	exp:
ttt	F	373	357.20
ttc	F	532	547.80
---	--	------	------
mPD		0.49	0.48
		nPD:	1.02
		N. weight:	0.0057
		Sc. PD:	0.0047
		Sc. rank:	3.6

PA			
Pos.	682	obs:	exp:
aat	N	2	1.02
aac	N	0	0.98
gat	D	902	487.50
gac	D	1	415.50
---	--	------	------
mPD		0.0066	0.50
		nPD:	0.01
		N. weight:	0.75
		Sc. PD:	-0.1
		Sc. rank:	-315.7

PA			
Pos.	683	obs:	exp:
tta	L	0	79.36
ttg	L	0	168.50
ctt	L	889	153.00
ctc	L	14	151.30
cta	L	0	150.50
ctg	L	0	200.30
att	I	2	0.73
atc	I	0	0.48
ata	I	0	0.79
---	--	------	------
mPD		0.035	1.1
		nPD:	0.03
		N. weight:	2.1
		Sc. PD:	-0.25
		Sc. rank:	-609.2

PA			
Pos.	684	obs:	exp:
gaa	E	603	481.20
gag	E	197	318.80
ggt	G	0	14.08
ggc	G	0	13.34
gga	G	58	48.55
ggg	G	47	29.03
---	--	------	------
mPD		0.60	0.71
		nPD:	0.84
		N. weight:	0.11
		Sc. PD:	0.072
		Sc. rank:	69.1

PA			
Pos.	685	obs:	exp:
ggt	G	0	121.40
ggc	G	0	115.00
gga	G	3	418.40
ggg	G	902	250.20
---	--	------	------
mPD		0.0066	0.68
		nPD:	0.01
		N. weight:	1.5
		Sc. PD:	-0.22
		Sc. rank:	-695.1

PA			
Pos.	686	obs:	exp:
tta	L	2	79.54
ttg	L	0	168.90
ctt	L	0	153.40
ctc	L	0	151.60
cta	L	901	150.90
ctg	L	2	200.70
---	--	------	------
mPD		0.0088	1.1
		nPD:	0.01
		N. weight:	2.2
		Sc. PD:	-0.3
		Sc. rank:	-1022.1

PA			
Pos.	687	obs:	exp:
tat	Y	898	479.80
tac	Y	7	425.20
---	--	------	------
mPD		0.015	0.50
		nPD:	0.03
		N. weight:	0.73
		Sc. PD:	-0.086
		Sc. rank:	-216.3

PA			
Pos.	688	obs:	exp:
gaa	E	900	542.60
gag	E	2	359.40
ggt	G	0	0.40
ggc	G	0	0.38
gga	G	3	1.39
ggg	G	0	0.83
---	--	------	------
mPD		0.011	0.49
		nPD:	0.02
		N. weight:	0.61
		Sc. PD:	-0.077
		Sc. rank:	-216.5

PA			
Pos.	689	obs:	exp:
tct	S	0	0.16
tcc	S	0	0.13
tca	S	1	0.25
tcg	S	0	0.06
agt	S	0	0.20
agc	S	0	0.20
gct	A	0	231.60
gcc	A	0	169.70
gca	A	904	422.70
gcg	A	0	79.96
---	--	------	------
mPD		0.0022	0.68
		nPD:	0.
		N. weight:	0.93
		Sc. PD:	-0.14
		Sc. rank:	-500.9

PA			
Pos.	690	obs:	exp:
ttt	F	1	0.39
ttc	F	0	0.61
att	I	115	329.80
atc	I	789	218.70
ata	I	0	355.50
---	--	------	------
mPD		0.23	0.66
		nPD:	0.34
		N. weight:	1.2
		Sc. PD:	0.22
		Sc. rank:	589.5

,

PA			
Pos.	691	obs:	exp:
gat	D	0	0.54
gac	D	1	0.46
gaa	E	0	543.80
gag	E	904	360.20
---	--	------	------
mPD		0.0022	0.48
		nPD:	0.
		N. weight:	1.1
		Sc. PD:	-0.16
		Sc. rank:	-587.1

,

PA			
Pos.	692	obs:	exp:
gat	D	1	0.54
gac	D	0	0.46
gaa	E	0	543.80
gag	E	904	360.20
---	--	------	------
mPD		0.0022	0.48
		nPD:	0.
		N. weight:	1.1
		Sc. PD:	-0.16
		Sc. rank:	-587.0

,

PA			
Pos.	693	obs:	exp:
tgt	C	0	371.50
tgc	C	905	533.50
---	--	------	------
mPD		0	0.48
		nPD:	0.
		N. weight:	0.65
		Sc. PD:	-0.096
		Sc. rank:	-412.4

,

PA			
Pos.	694	obs:	exp:
tta	L	0	79.54
ttg	L	0	168.90
ctt	L	1	153.40
ctc	L	0	151.60
cta	L	2	150.90
ctg	L	902	200.70
---	--	------	------
mPD		0.0066	1.1
		nPD:	0.01
		N. weight:	1.8
		Sc. PD:	-0.26
		Sc. rank:	-916.4

,

PA			
Pos.	695	obs:	exp:
att	I	905	330.20
atc	I	0	218.90
ata	I	0	355.90
---	--	------	------
mPD		0	0.65
		nPD:	0.
		N. weight:	1.2
		Sc. PD:	-0.18
		Sc. rank:	-786.7

,

PA			
Pos.	696	obs:	exp:
act	T	1	0.26
acc	T	0	0.20
aca	T	0	0.46
acg	T	0	0.08
aat	N	870	459.90
aac	N	34	444.10
---	--	------	------
mPD		0.075	0.50
		nPD:	0.15
		N. weight:	0.64
		Sc. PD:	-0.0032
		Sc. rank:	74.1

,

PA			
Pos.	697	obs:	exp:
gat	D	898	488.00
gac	D	6	416.00
ggt	G	1	0.13
ggc	G	0	0.13
gga	G	0	0.46
ggg	G	0	0.28
---	--	------	------
mPD		0.015	0.50
		nPD:	0.03
		N. weight:	0.71
		Sc. PD:	-0.084
		Sc. rank:	-213.2

,

PA			
Pos.	698	obs:	exp:
cct	P	1	230.10
ccc	P	904	161.40
cca	P	0	369.70
ccg	P	0	143.80
---	--	------	------
mPD		0.0022	0.71
		nPD:	0.
		N. weight:	2.1
		Sc. PD:	-0.31
		Sc. rank:	-1150.2

,

PA			
Pos.	699	obs:	exp:
tgg	W	905	905.00
---	--	------	------
mPD		0	0
		nPD:	1.
		N. weight:	0.
		Sc. PD:	0
		Sc. rank:	0

,

PA			
Pos.	700	obs:	exp:
gtt	V	905	186.80
gtc	V	0	181.30
gta	V	0	187.40
gtg	V	0	349.50
---	--	------	------
mPD		0	0.73
		nPD:	0.
		N. weight:	1.9
		Sc. PD:	-0.29
		Sc. rank:	-1231.2

,

PA			
Pos.	701	obs:	exp:
tta	L	0	79.54
ttg	L	905	168.90
ctt	L	0	153.40
ctc	L	0	151.60
cta	L	0	150.90
ctg	L	0	200.70
---	--	------	------
mPD		0	1.1
		nPD:	0.
		N. weight:	2.1
		Sc. PD:	-0.31
		Sc. rank:	-1310.0

,

PA			
Pos.	702	obs:	exp:
tta	L	0	79.54
ttg	L	0	168.90
ctt	L	904	153.40
ctc	L	1	151.60
cta	L	0	150.90
ctg	L	0	200.70
---	--	------	------
mPD		0.0022	1.1
		nPD:	0.
		N. weight:	2.2
		Sc. PD:	-0.32
		Sc. rank:	-1222.2

,

PA			
Pos.	703	obs:	exp:
act	T	1	0.26
acc	T	0	0.20
aca	T	0	0.46
acg	T	0	0.08
aat	N	903	459.90
aac	N	1	444.10
---	--	------	------
mPD		0.0044	0.50
		nPD:	0.01
		N. weight:	0.82
		Sc. PD:	-0.11
		Sc. rank:	-383.8

,

PA			
Pos.	704	obs:	exp:
gct	A	0	231.90
gcc	A	3	169.90
gca	A	849	423.10
gcg	A	53	80.05
---	--	------	------
mPD		0.12	0.67
		nPD:	0.17
		N. weight:	0.76
		Sc. PD:	0.015
		Sc. rank:	134.2

,

PA			
Pos.	705	obs:	exp:
tct	S	903	144.90
tcc	S	2	117.10
tca	S	0	228.40
tcg	S	0	52.67
agt	S	0	185.40
agc	S	0	176.50
---	--	------	------
mPD		0.0044	1.7
		nPD:	0.
		N. weight:	2.2
		Sc. PD:	-0.32
		Sc. rank:	-1226.0

,

PA			
Pos.	706	obs:	exp:
tgg	W	905	905.00
---	--	------	------
mPD		0	0
		nPD:	1.
		N. weight:	0.
		Sc. PD:	0
		Sc. rank:	0

,

PA			
Pos.	707	obs:	exp:
ttt	F	1	357.20
ttc	F	904	547.80
---	--	------	------
mPD		0.0022	0.48
		nPD:	0.
		N. weight:	0.61
		Sc. PD:	-0.087
		Sc. rank:	-313.3

,

PA			
Pos.	708	obs:	exp:
aat	N	0	460.40
aac	N	905	444.60
---	--	------	------
mPD		0	0.50
		nPD:	0.
		N. weight:	0.87
		Sc. PD:	-0.13
		Sc. rank:	-554.7

,

PA			
Pos.	709	obs:	exp:
tct	S	0	144.90
tcc	S	905	117.10
tca	S	0	228.40
tcg	S	0	52.67
agt	S	0	185.40
agc	S	0	176.50
---	--	------	------
mPD		0	1.7
		nPD:	0.
		N. weight:	2.5
		Sc. PD:	-0.37
		Sc. rank:	-1595.4

,

PA			
Pos.	710	obs:	exp:
ttt	F	0	357.20
ttc	F	905	547.80
---	--	------	------
mPD		0	0.48
		nPD:	0.
		N. weight:	0.62
		Sc. PD:	-0.091
		Sc. rank:	-391.7

,

PA			
Pos.	711	obs:	exp:
tta	L	0	79.36
ttg	L	0	168.50
ctt	L	6	153.00
ctc	L	897	151.30
cta	L	0	150.50
ctg	L	0	200.30
cct	P	0	0.51
ccc	P	2	0.36
cca	P	0	0.82
ccg	P	0	0.32
---	--	------	------
mPD		0.018	1.1
		nPD:	0.02
		N. weight:	2.1
		Sc. PD:	-0.29
		Sc. rank:	-868.3

,

PA			
Pos.	712	obs:	exp:
tct	S	0	0.32
tcc	S	0	0.26
tca	S	2	0.50
tcg	S	0	0.12
act	T	0	233.90
acc	T	0	183.90
aca	T	903	414.90
acg	T	0	70.34
agt	S	0	0.41
agc	S	0	0.39
---	--	------	------
mPD		0.0044	0.68
		nPD:	0.01
		N. weight:	0.96
		Sc. PD:	-0.14
		Sc. rank:	-469.5

,

PA			
Pos.	713	obs:	exp:
cat	H	904	519.90
cac	H	1	385.10
---	--	------	------
mPD		0.0022	0.49
		nPD:	0.
		N. weight:	0.67
		Sc. PD:	-0.097
		Sc. rank:	-351.2

PA			
Pos.	714	obs:	exp:
gct	A	1	231.90
gcc	A	0	169.90
gca	A	904	423.10
gcg	A	0	80.05
---	--	------	------
mPD		0.0022	0.67
		nPD:	0.
		N. weight:	0.92
		Sc. PD:	-0.13
		Sc. rank:	-494.4

PA			
Pos.	715	obs:	exp:
tta	L	1	79.27
ttg	L	0	168.30
tct	S	0	0.16
tcc	S	0	0.13
tca	S	0	0.25
tcg	S	1	0.06
ctt	L	0	152.90
ctc	L	0	151.10
cta	L	781	150.40
ctg	L	120	200.00
agt	S	0	0.20
agc	S	0	0.20
gtt	V	0	0.41
gtc	V	0	0.40
gta	V	2	0.41
gtg	V	0	0.77
---	--	------	------
mPD		0.24	1.1
		nPD:	0.22
		N. weight:	1.7
		Sc. PD:	0.1
		Sc. rank:	525.4

PA			
Pos.	716	obs:	exp:
tct	S	0	4.32
tcc	S	0	3.49
tca	S	0	6.81
tcg	S	0	1.57
cgt	R	0	32.64
cgc	R	0	38.82
cga	R	0	76.18
cgg	R	0	63.63
att	I	0	0.36
atc	I	0	0.24
ata	I	1	0.39
aaa	K	84	50.38
aag	K	2	35.62
agt	S	0	5.53
agc	S	27	5.27
aga	R	763	348.80
agg	R	1	203.90
ggt	G	0	3.62
ggc	G	0	3.43
gga	G	27	12.48
ggg	G	0	7.47
---	--	------	------
mPD		0.30	1.2
		nPD:	0.25
		N. weight:	0.94
		Sc. PD:	0.084
		Sc. rank:	346.7

PA			
Pos.	717	obs:	exp:
taa	.	2	389.20
tag	.	903	228.80
tga	.	0	286.90
---	--	------	------
mPD		0.0044	0.81
		nPD:	0.01
		N. weight:	1.7
		Sc. PD:	-0.24
		Sc. rank:	-850.7

Amino acid level conservation per codon

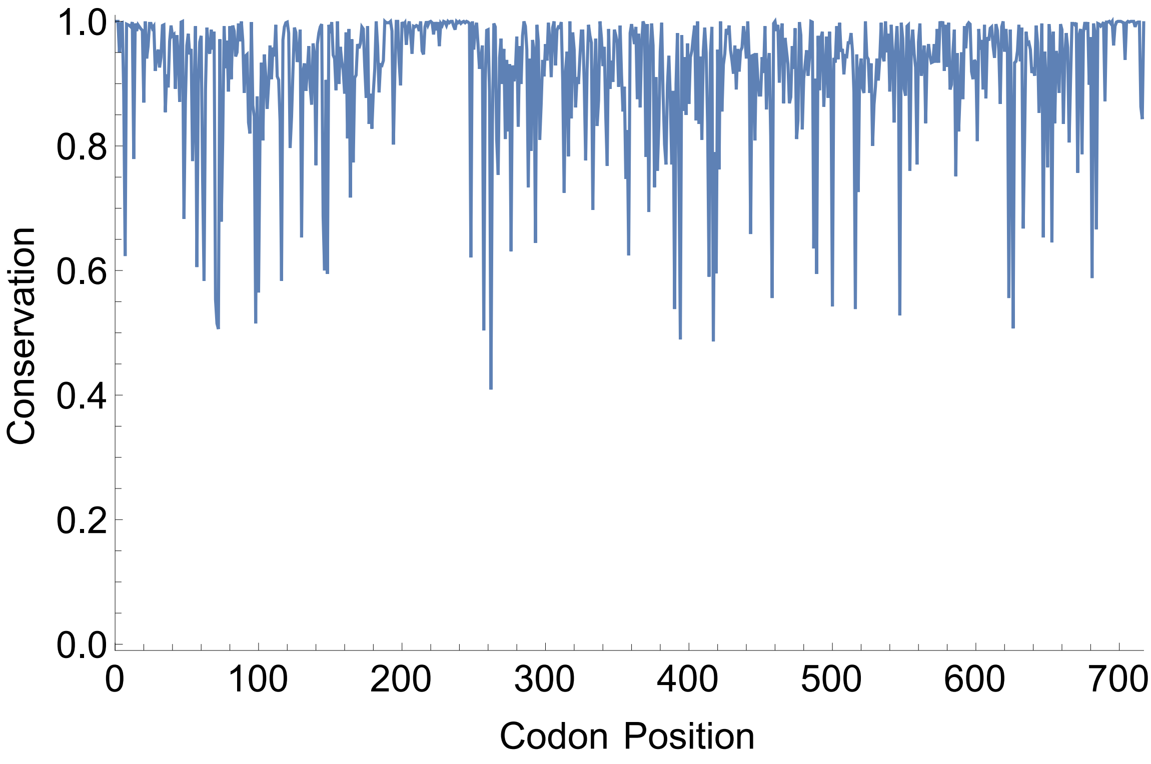

Conservation analysis plots

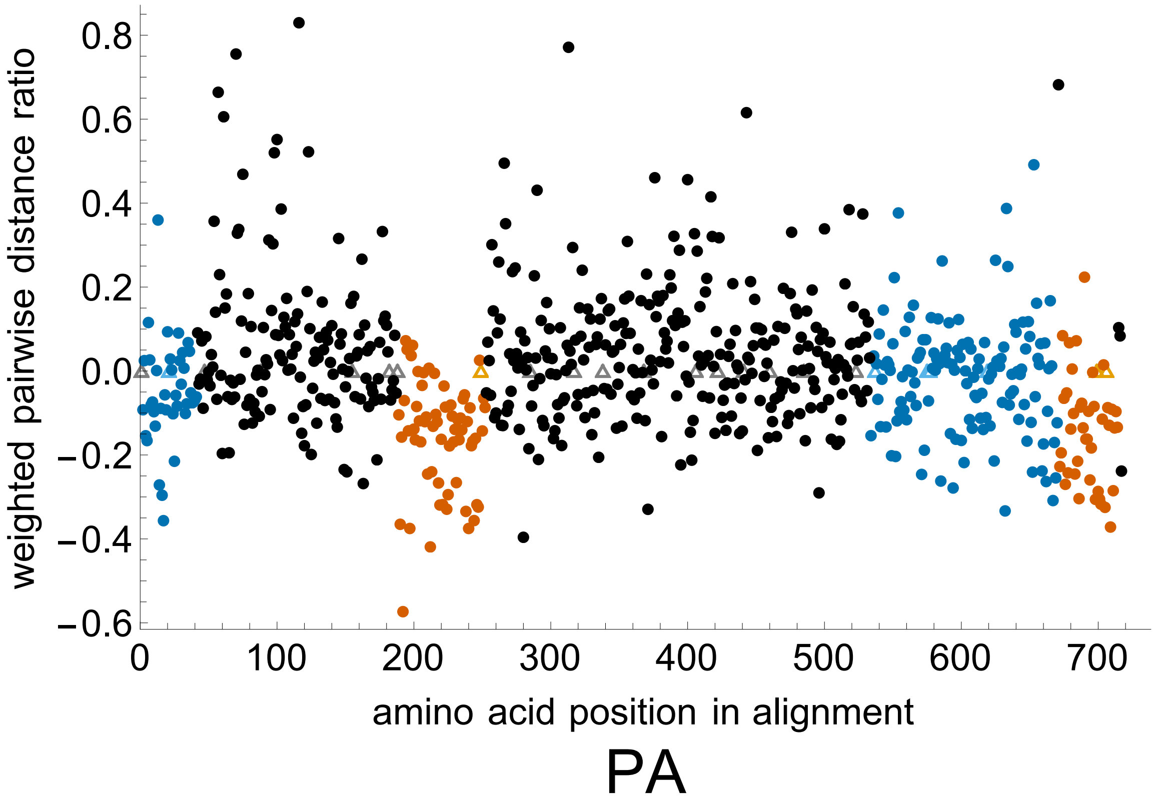

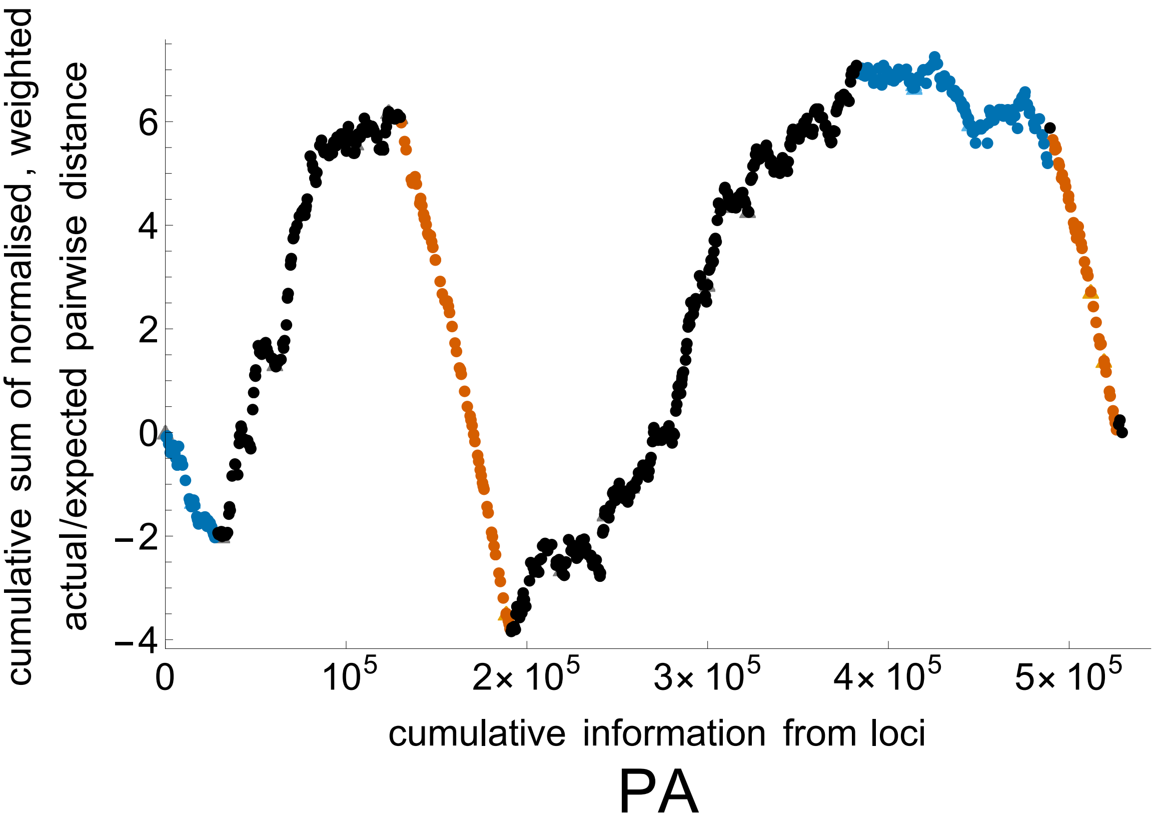

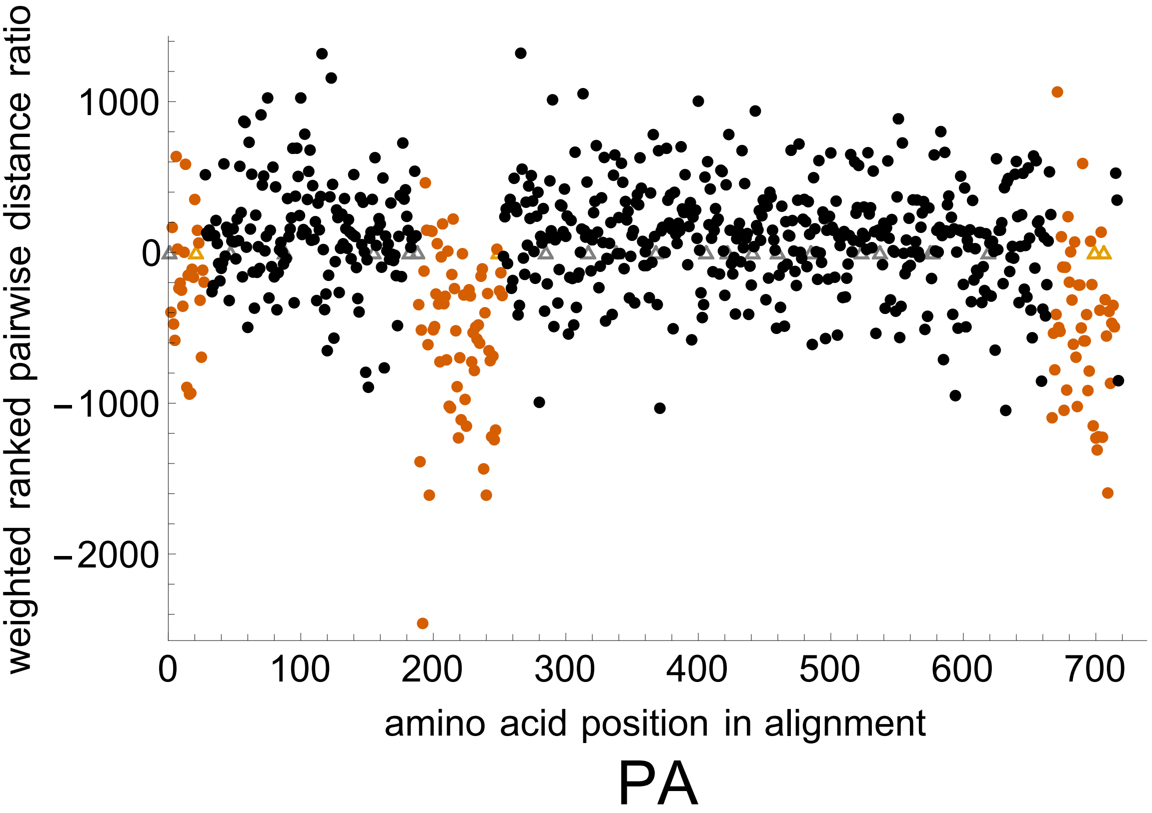
weighted ranked pairwise distance ratio
1000
0
−1000
−2000
0
100
200
300
400
500
600
700
amino acid position in alignment
PA

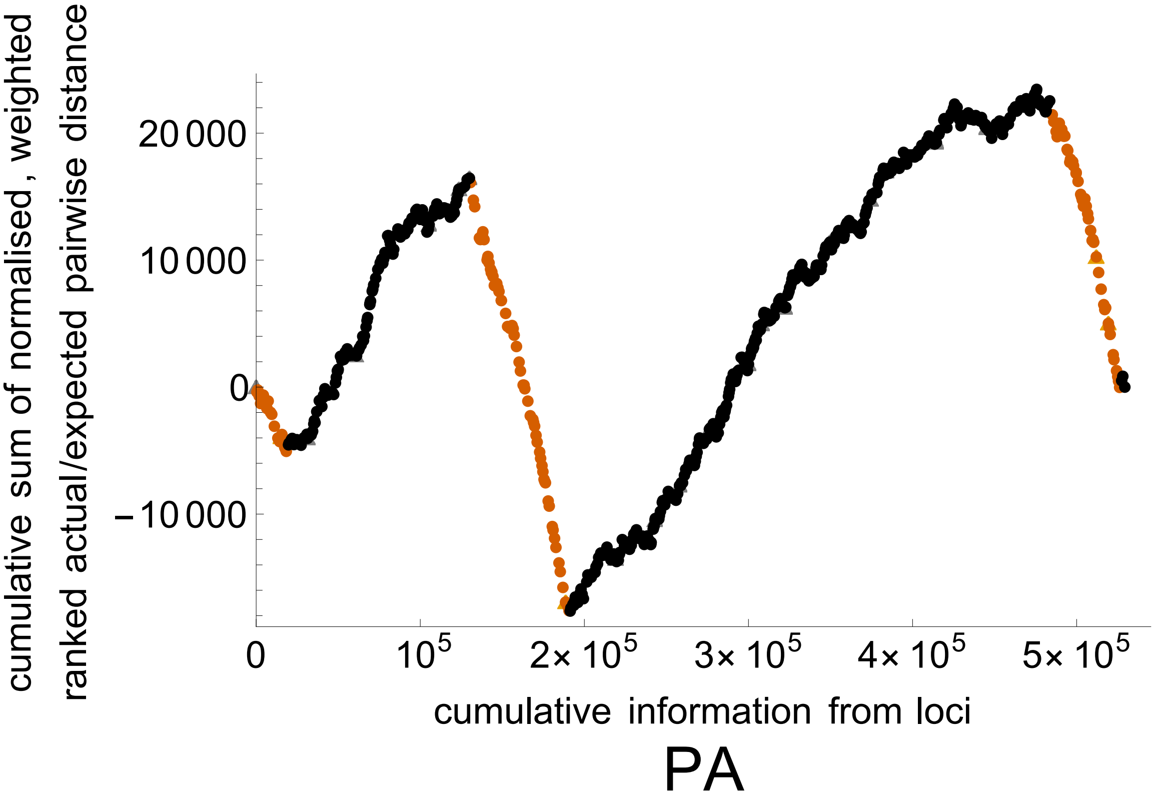
cumulative sum of normalised, weighted ranked actual/expected pairwise distance
20 000
10 000
0
−10 000
0
10^5
2×10^5
3×10^5
4×10^5
5×10^5
cumulative information from loci
PA

PA-X

Gene length histogram

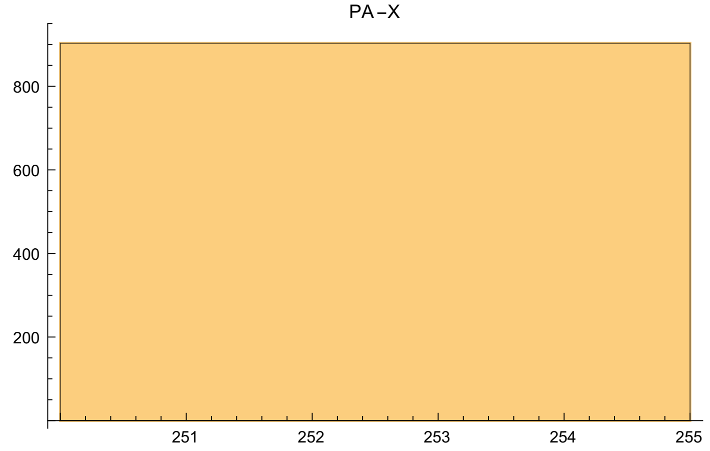

Information vs. nPD

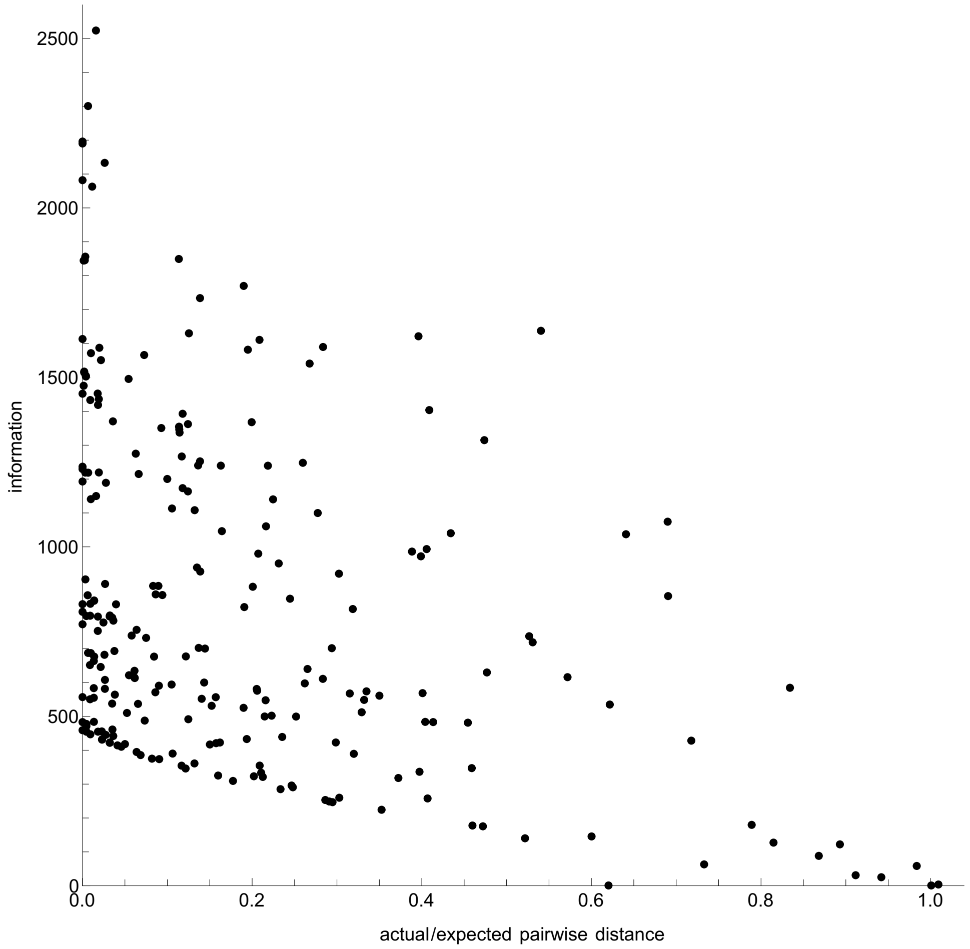

PA–X

Example sequences highlighted by regions found in analyses to be conserved

Interesting points (by weighted raw PD) highlighted for gene PA-X:

ATG

GAAGATTTTGTGCGACAATGCTTCAATCCAATGATTGTCGAGCTTGCGGAAAAGG
CAATGAAAGAATATGGGGAAGATCCGAAAATCGAAACGAACAAATTTGCCGC
AATATGCACACACTTAGAGGTCTGTTTCATGTATTCGGATTTCCACTTTATTGATGAACGGGGCGA⋱
ATCAATAATTGTAGAATCTGGCGATCCAAATGCATTATTGAAACACCGATTTGAGATAATTGAAGGGAGA⋱
GACCGAACGATGGCCTGGACAGTGGTGAATAGTATCTGCAACACCACAGGAGTCGAGAAACCTAAATTTC⋱
TCCCAGATTTGTATGACTACAAAGAGAATCGATTCATTGAAATTGGAGTAACACGGAGGGAAGTTCATAT⋱
ATACTATCTAGAAAAGGCCAACAAGATAAAATCAGAGAAGACACACATTCACATATTCTCATTCACTGGA⋱
GAGGAAATGGCCACCAAAGCGGACTACACTCTTGACGAAGAGAGTAGGGCAAGAATCAAAACCAGGCTGT⋱
TCACTATAAGGCAGGAAATGGCCAGTAGGGGTCTATGG
GATTCCTTTGTCAGTCCGAGAGAGGCGAAGAGACAGTTGAAGAAAGATTTGAAAT
CACAGGAACCATGCGCAGGCTTGCCGACCAAAGTCTCCCACCGAACTTCTCC
AGCCTTGAAAACTTTAGAGCCTATGTGGATGGATTCGAACCGAACGGCTGCA
TTGAGGGCAAGCTTTCTCAAATGTCAAAAGAAGTGA

Interesting points (by weighted ranked PD) highlighted for gene PA-X:

ATG

GAAGATTTTGTGCGACAATGCTTCAATCCAATGATTGTCGAGCTTGCGGAAAAGG
CAATGAAAGAATATGGGGAAGATCCGAAAATCGAAACGAACAAATTTGCCGCAATATGCACA⋱
CACTTAGAGGTCTGTTTCATGTATTCGGATTTCCACTTTATTGATGAACGGGGCGAATCAATAATTGTAG⋱
AATCTGGCGATCCAAATGCATTATTGAAACACCGATTTGAGATAATTGAAGGGAGAGACCGAACGATGGC⋱
CTGGACAGTGGTGAATAGTATCTGCAACACCACAGGAGTCGAGAAACCTAAATTTCTCCCAGATTTGTAT⋱
GACTACAAAGAGAATCGATTCATTGAAATTGGAGTAACACGGAGGGAAGTTCATATATACTATCTAGAAA⋱
AGGCCAACAAGATAAAATCAGAGAAGACACACATTCACATATTC
TCATTCACTGGAGAGGAAATGGCCACCAAAGCGGACTACACTCTTGACGAAGAGAGTAGGGCAAGAATCAAA⋱
ACCAGGCTGTTCACTATAAGGCAGGAAATGGCCAGTAGGGGTCTATGG
GATTCCTTTGTCAGTCCGAGAGAGGCGAAGAGACAGTTGAAGAAAGATTTGAAAT
CACAGGAACCATGCGCAGGCTTGCCGACCAAAGTCTCCCACCGAACTTCTCC
AGCCTTGAAAACTTTAGAGCCTATGTGGATGGATTCGAACCGAACGGCTGCA
TTGAGGGCAAGCTTTCTCAAATGTCAAAAGAAGTGA

Per codon conservation report

PA-X

Pos.	1	obs:	exp:
atg	M	903	903.00
---	--	------	------
mPD		0	0
		nPD:	1.
		N. weight:	0.
		Sc. PD:	0
		Sc. rank:	0

PA-X

Pos.	2	obs:	exp:
gaa	E	903	543.20
gag	E	0	359.80
---	--	------	------
mPD		0	0.48
		nPD:	0.
		N. weight:	0.58
		Sc. PD:	-0.075
		Sc. rank:	-392.0

PA-X

Pos.	3	obs:	exp:
gat	D	43	486.40
gac	D	858	414.60
gaa	E	1	0.60
gag	E	0	0.40
ggt	G	0	0.13
ggc	G	1	0.13
gga	G	0	0.46
ggg	G	0	0.28
---	--	------	------
mPD		0.095	0.50
		nPD:	0.19
		N. weight:	0.66
		Sc. PD:	0.035
		Sc. rank:	196.6

PA-X

Pos.	4	obs:	exp:
ttt	F	900	355.20
ttc	F	0	544.80
tta	L	0	0.26
ttg	L	1	0.56
ctt	L	2	0.51
ctc	L	0	0.50
cta	L	0	0.50
ctg	L	0	0.67
---	--	------	------
mPD		0.0066	0.48
		nPD:	0.01
		N. weight:	1.1
		Sc. PD:	-0.12
		Sc. rank:	-336.1

PA-X

Pos.	5	obs:	exp:
atg	M	2	2.00
gtt	V	0	186.00
gtc	V	0	180.50
gta	V	0	186.50
gtg	V	901	348.00
---	--	------	------
mPD		0.0044	0.73
		nPD:	0.01
		N. weight:	1.1
		Sc. PD:	-0.13
		Sc. rank:	-478.9

PA-X

Pos.	6	obs:	exp:
cgt	R	0	38.53
cgc	R	0	45.83
cga	R	801	89.94
cgg	R	1	75.13
aga	R	100	411.80
agg	R	0	240.70
ggt	G	0	0.13
ggc	G	0	0.13
gga	G	1	0.46
ggg	G	0	0.28
---	--	------	------
mPD		0.20	0.97
		nPD:	0.21
		N. weight:	2.
		Sc. PD:	0.14
		Sc. rank:	755.4

PA-X

Pos.	7	obs:	exp:
caa	Q	562	463.80
cag	Q	341	439.20
---	--	------	------
mPD		0.47	0.50
		nPD:	0.94
		N. weight:	0.032
		Sc. PD:	0.025
		Sc. rank:	21.9

PA-X

Pos.	8	obs:	exp:
tgt	C	4	370.70
tgc	C	899	532.30
---	--	------	------
mPD		0.0088	0.48
		nPD:	0.02
		N. weight:	0.58
		Sc. PD:	-0.064
		Sc. rank:	-151.0

PA-X

Pos.	9	obs:	exp:
ttt	F	4	356.00
ttc	F	898	546.00
tat	Y	0	0.53
tac	Y	1	0.47
---	--	------	------
mPD		0.011	0.48
		nPD:	0.02
		N. weight:	0.55
		Sc. PD:	-0.058
		Sc. rank:	-109.0

PA-X

Pos.	10	obs:	exp:
cat	H	1	0.57
cac	H	0	0.43
aat	N	897	458.90
aac	N	5	443.10
---	--	------	------
mPD		0.013	0.50
		nPD:	0.03
		N. weight:	0.74
		Sc. PD:	-0.076
		Sc. rank:	-125.8

PA-X

Pos.	11	obs:	exp:
tta	L	0	0.09
ttg	L	0	0.19
ctt	L	0	0.17
ctc	L	0	0.17
cta	L	1	0.17
ctg	L	0	0.22
cct	P	0	229.40
ccc	P	0	160.80
cca	P	895	368.50
ccg	P	7	143.30
---	--	------	------
mPD		0.018	0.71
		nPD:	0.02
		N. weight:	0.98
		Sc. PD:	-0.1
		Sc. rank:	-191.0

PA-X

Pos.	12	obs:	exp:
att	I	0	0.36
atc	I	0	0.24
ata	I	1	0.39
atg	M	902	902.00
---	--	------	------
mPD		0.0022	0.0022
		nPD:	1.
		N. weight:	0.0012
		Sc. PD:	0.00098
		Sc. rank:	0.8

PA-X

Pos.	13	obs:	exp:
att	I	194	327.30
atc	I	703	217.00
ata	I	0	352.70
gtt	V	1	1.24
gtc	V	5	1.20
gta	V	0	1.24
gtg	V	0	2.32
---	--	------	------
mPD		0.35	0.67
		nPD:	0.53
		N. weight:	0.93
		Sc. PD:	0.35
		Sc. rank:	585.3

PA-X

Pos.	14	obs:	exp:
gtt	V	0	186.40
gtc	V	900	180.90
gta	V	3	187.00
gtg	V	0	348.70
---	--	------	------
mPD		0.0066	0.73
		nPD:	0.01
		N. weight:	1.8
		Sc. PD:	-0.22
		Sc. rank:	-705.8

PA-X

Pos.	15	obs:	exp:
gaa	E	14	543.20
gag	E	889	359.80
---	--	------	------
mPD		0.031	0.48
		nPD:	0.06
		N. weight:	0.96
		Sc. PD:	-0.065
		Sc. rank:	-30.4

PA-X

Pos.	16	obs:	exp:
tta	L	0	79.36
ttg	L	0	168.50
ctt	L	898	153.00
ctc	L	3	151.30
cta	L	2	150.50
ctg	L	0	200.30
---	--	------	------
mPD		0.011	1.1
		nPD:	0.01
		N. weight:	2.
		Sc. PD:	-0.24
		Sc. rank:	-710.2

PA-X

Pos.	17	obs:	exp:
gct	A	0	231.40
gcc	A	0	169.60
gca	A	8	422.20
gcg	A	895	79.87
---	--	------	------
mPD		0.018	0.67
		nPD:	0.03
		N. weight:	2.7
		Sc. PD:	-0.28
		Sc. rank:	-472.6

PA-X

Pos.	18	obs:	exp:
gat	D	1	1.62
gac	D	2	1.38
gaa	E	892	541.40
gag	E	8	358.60
---	--	------	------
mPD		0.024	0.48
		nPD:	0.05
		N. weight:	0.53
		Sc. PD:	-0.043
		Sc. rank:	-42.4

PA-X

Pos.	19	obs:	exp:
cgt	R	0	0.04
cgc	R	0	0.05
cga	R	0	0.10
cgg	R	0	0.08
atg	M	2	2.00
aaa	K	10	527.20
aag	K	890	372.80
aga	R	0	0.46
agg	R	1	0.27
---	--	------	------
mPD		0.029	0.49
		nPD:	0.06
		N. weight:	0.93
		Sc. PD:	-0.069
		Sc. rank:	-53.4

PA-X

Pos.	20	obs:	exp:
act	T	0	27.20
acc	T	0	21.38
aca	T	105	48.24
acg	T	0	8.18
gtt	V	0	0.21
gtc	V	0	0.20
gta	V	1	0.21
gtg	V	0	0.39
gct	A	11	204.00
gcc	A	0	149.50
gca	A	785	372.20
gcg	A	0	70.40
gaa	E	1	0.60
gag	E	0	0.40
---	--	------	------
mPD		0.23	0.88
		nPD:	0.27
		N. weight:	0.81
		Sc. PD:	0.1
		Sc. rank:	367.0

PA-X			
Pos.	21	obs:	exp:
atg	M	903	903.00
---	--	------	------
mPD		0	0
		nPD:	1.
		N. weight:	0.
		Sc. PD:	0
		Sc. rank:	0

,

PA-X			
Pos.	22	obs:	exp:
cgt	R	0	0.73
cgc	R	0	0.86
cga	R	0	1.70
cgg	R	0	1.42
aaa	K	851	519.00
aag	K	35	367.00
aga	R	17	7.76
agg	R	0	4.54
---	--	------	------
mPD		0.11	0.53
		nPD:	0.21
		N. weight:	0.45
		Sc. PD:	0.032
		Sc. rank:	167.4

,

PA-X			
Pos.	23	obs:	exp:
gaa	E	867	543.20
gag	E	36	359.80
---	--	------	------
mPD		0.077	0.48
		nPD:	0.16
		N. weight:	0.41
		Sc. PD:	0.01
		Sc. rank:	111.8

,

PA-X			
Pos.	24	obs:	exp:
tat	Y	900	478.80
tac	Y	3	424.20
---	--	------	------
mPD		0.0066	0.50
		nPD:	0.01
		N. weight:	0.7
		Sc. PD:	-0.082
		Sc. rank:	-235.0

,

PA-X			
Pos.	25	obs:	exp:
ggt	G	0	121.10
ggc	G	0	114.70
gga	G	3	417.50
ggg	G	900	249.70
---	--	------	------
mPD		0.0066	0.68
		nPD:	0.01
		N. weight:	1.4
		Sc. PD:	-0.17
		Sc. rank:	-531.1

,

PA-X			
Pos.	26	obs:	exp:
gaa	E	893	543.20
gag	E	10	359.80
---	--	------	------
mPD		0.022	0.48
		nPD:	0.05
		N. weight:	0.52
		Sc. PD:	-0.044
		Sc. rank:	-43.1

,

PA-X			
Pos.	27	obs:	exp:
aat	N	3	2.03
aac	N	1	1.97
gat	D	896	485.30
gac	D	3	413.70
---	--	------	------
mPD		0.018	0.51
		nPD:	0.03
		N. weight:	0.68
		Sc. PD:	-0.065
		Sc. rank:	-88.0

,

PA-X			
Pos.	28	obs:	exp:
tta	L	0	0.44
ttg	L	0	0.93
ctt	L	0	0.85
ctc	L	0	0.84
cta	L	0	0.83
ctg	L	5	1.11
cct	P	0	228.30
ccc	P	0	160.10
cca	P	65	366.90
ccg	P	833	142.70
---	--	------	------
mPD		0.14	0.73
		nPD:	0.2
		N. weight:	1.7
		Sc. PD:	0.11
		Sc. rank:	582.9

,

PA-X			
Pos.	29	obs:	exp:
cgt	R	0	0.17
cgc	R	0	0.20
cga	R	0	0.40
cgg	R	0	0.33
aaa	K	853	526.60
aag	K	46	372.40
aga	R	4	1.83
agg	R	0	1.07
---	--	------	------
mPD		0.11	0.50
		nPD:	0.21
		N. weight:	0.41
		Sc. PD:	0.03
		Sc. rank:	153.4

,

PA-X			
Pos.	30	obs:	exp:
att	I	37	328.40
atc	I	862	217.70
ata	I	1	353.90
gtt	V	0	0.62
gtc	V	3	0.60
gta	V	0	0.62
gtg	V	0	1.16
---	--	------	------
mPD		0.087	0.66
		nPD:	0.13
		N. weight:	1.4
		Sc. PD:	-0.0032
		Sc. rank:	276.3

,

PA-X			
Pos.	31	obs:	exp:
aaa	K	1	0.59
aag	K	0	0.41
gaa	E	836	542.60
gag	E	66	359.40
---	--	------	------
mPD		0.14	0.48
		nPD:	0.29
		N. weight:	0.32
		Sc. PD:	0.047
		Sc. rank:	156.3

,

PA-X			
Pos.	32	obs:	exp:
act	T	4	233.90
acc	T	8	183.90
aca	T	853	414.90
acg	T	38	70.34
---	--	------	------
mPD		0.11	0.67
		nPD:	0.16
		N. weight:	0.7
		Sc. PD:	0.015
		Sc. rank:	187.6

,

PA-X			
Pos.	33	obs:	exp:
aat	N	6	459.40
aac	N	897	443.60
---	--	------	------
mPD		0.013	0.50
		nPD:	0.03
		N. weight:	0.77
		Sc. PD:	-0.08
		Sc. rank:	-128.3

,

PA-X			
Pos.	34	obs:	exp:
aaa	K	898	529.00
aag	K	5	374.00
---	--	------	------
mPD		0.011	0.49
		nPD:	0.02
		N. weight:	0.58
		Sc. PD:	-0.062
		Sc. rank:	-118.5

,

PA-X			
Pos.	35	obs:	exp:
ttt	F	131	356.00
ttc	F	771	546.00
tta	L	0	0.09
ttg	L	0	0.19
ctt	L	0	0.17
ctc	L	1	0.17
cta	L	0	0.17
ctg	L	0	0.22
---	--	------	------
mPD		0.25	0.48
		nPD:	0.52
		N. weight:	0.18
		Sc. PD:	0.066
		Sc. rank:	110.5

,

PA-X			
Pos.	36	obs:	exp:
tct	S	0	0.16
tcc	S	0	0.13
tca	S	1	0.25
tcg	S	0	0.06
agt	S	0	0.20
agc	S	0	0.20
gtt	V	0	0.21
gtc	V	0	0.20
gta	V	1	0.21
gtg	V	0	0.39
gct	A	55	230.90
gcc	A	18	169.20
gca	A	826	421.30
gcg	A	2	79.69
---	--	------	------
mPD		0.16	0.68
		nPD:	0.24
		N. weight:	0.56
		Sc. PD:	0.054
		Sc. rank:	235.1

,

PA-X			
Pos.	37	obs:	exp:
tct	S	0	129.50
tcc	S	0	104.70
tca	S	809	204.20
tcg	S	0	47.08
agt	S	0	165.80
agc	S	0	157.80
gct	A	0	24.09
gcc	A	0	17.65
gca	A	86	43.95
gcg	A	8	8.31
---	--	------	------
mPD		0.20	1.7
		nPD:	0.12
		N. weight:	1.5
		Sc. PD:	-0.024
		Sc. rank:	205.8

,

PA-X			
Pos.	38	obs:	exp:
att	I	0	318.90
atc	I	0	211.40
ata	I	874	343.70
atg	M	1	1.00
gtt	V	0	5.78
gtc	V	0	5.61
gta	V	28	5.80
gtg	V	0	10.81
---	--	------	------
mPD		0.062	0.72
		nPD:	0.09
		N. weight:	1.1
		Sc. PD:	-0.05
		Sc. rank:	31.4

,

PA-X			
Pos.	39	obs:	exp:
tgt	C	6	370.70
tgc	C	897	532.30
---	--	------	------
mPD		0.013	0.48
		nPD:	0.03
		N. weight:	0.56
		Sc. PD:	-0.058
		Sc. rank:	-88.9

,

PA-X			
Pos.	40	obs:	exp:
act	T	8	233.90
acc	T	7	183.90
aca	T	884	414.90
acg	T	4	70.34
---	--	------	------
mPD		0.042	0.67
		nPD:	0.06
		N. weight:	0.78
		Sc. PD:	-0.054
		Sc. rank:	-34.5

,

PA-X			
Pos.	41	obs:	exp:
cat	H	19	518.80
cac	H	884	384.20
---	--	------	------
mPD		0.041	0.49
		nPD:	0.08
		N. weight:	0.86
		Sc. PD:	-0.041
		Sc. rank:	16.3

,

PA-X			
Pos.	42	obs:	exp:
tta	L	807	79.36
ttg	L	80	168.50
ctt	L	0	153.00
ctc	L	0	151.30
cta	L	8	150.50
ctg	L	8	200.30
---	--	------	------
mPD		0.21	1.1
		nPD:	0.19
		N. weight:	2.2
		Sc. PD:	0.12
		Sc. rank:	689.7

,

PA-X			
Pos.	43	obs:	exp:
gaa	E	883	543.20
gag	E	20	359.80
---	--	------	------
mPD		0.043	0.48
		nPD:	0.09
		N. weight:	0.47
		Sc. PD:	-0.02
		Sc. rank:	19.6

,

PA-X			
Pos.	44	obs:	exp:
att	I	0	1.82
atc	I	5	1.21
ata	I	0	1.97
gtt	V	3	185.40
gtc	V	856	179.90
gta	V	4	185.90
gtg	V	35	346.80
---	--	------	------
mPD		0.10	0.74
		nPD:	0.14
		N. weight:	1.6
		Sc. PD:	0.0025
		Sc. rank:	332.6

,

PA-X			
Pos.	45	obs:	exp:
tgt	C	115	370.70
tgc	C	788	532.30
---	--	------	------
mPD		0.22	0.48
		nPD:	0.46
		N. weight:	0.22
		Sc. PD:	0.07
		Sc. rank:	136.7

,

PA-X			
Pos.	46	obs:	exp:
ttt	F	0	356.00
ttc	F	902	546.00
tta	L	0	0.09
ttg	L	0	0.19
ctt	L	0	0.17
ctc	L	1	0.17
cta	L	0	0.17
ctg	L	0	0.22
---	--	------	------
mPD		0.0022	0.48
		nPD:	0.
		N. weight:	0.58
		Sc. PD:	-0.072
		Sc. rank:	-257.3

,

PA-X			
Pos.	47	obs:	exp:
atg	M	903	903.00
---	--	------	------
mPD		0	0
		nPD:	1.
		N. weight:	0.
		Sc. PD:	0
		Sc. rank:	0

,

PA-X			
Pos.	48	obs:	exp:
tat	Y	285	478.80
tac	Y	618	424.20
---	--	------	------
mPD		0.43	0.50
		nPD:	0.87
		N. weight:	0.11
		Sc. PD:	0.078
		Sc. rank:	76.3

,

PA-X			
Pos.	49	obs:	exp:
ttt	F	1	0.39
ttc	F	0	0.61
tct	S	789	144.40
tcc	S	2	116.80
tca	S	65	227.60
tcg	S	46	52.49
agt	S	0	184.80
agc	S	0	175.90
---	--	------	------
mPD		0.23	1.7
		nPD:	0.14
		N. weight:	1.6
		Sc. PD:	0.0059
		Sc. rank:	357.0

,

PA-X			
Pos.	50	obs:	exp:
gat	D	868	487.50
gac	D	35	415.50
---	--	------	------
mPD		0.075	0.50
		nPD:	0.15
		N. weight:	0.53
		Sc. PD:	0.0078
		Sc. rank:	136.7

,

PA-X			
Pos.	51	obs:	exp:
ttt	F	18	356.40
ttc	F	885	546.60
---	--	------	------
mPD		0.039	0.48
		nPD:	0.08
		N. weight:	0.47
		Sc. PD:	-0.024
		Sc. rank:	4.0

,

PA-X			
Pos.	52	obs:	exp:
cat	H	45	517.00
cac	H	855	383.00
caa	Q	1	1.54
cag	Q	2	1.46
---	--	------	------
mPD		0.10	0.49
		nPD:	0.21
		N. weight:	0.73
		Sc. PD:	0.05
		Sc. rank:	256.4

,

PA-X			
Pos.	53	obs:	exp:
ttt	F	40	356.40
ttc	F	863	546.60
---	--	------	------
mPD		0.085	0.48
		nPD:	0.18
		N. weight:	0.39
		Sc. PD:	0.016
		Sc. rank:	114.4

,

PA-X			
Pos.	54	obs:	exp:
att	I	200	329.50
atc	I	702	218.40
ata	I	1	355.10
---	--	------	------
mPD		0.35	0.65
		nPD:	0.53
		N. weight:	0.91
		Sc. PD:	0.34
		Sc. rank:	575.6

,

PA-X			
Pos.	55	obs:	exp:
aat	N	3	3.05
aac	N	3	2.95
gat	D	111	484.20
gac	D	786	412.80
---	--	------	------
mPD		0.23	0.51
		nPD:	0.46
		N. weight:	0.44
		Sc. PD:	0.14
		Sc. rank:	266.4

,

PA-X			
Pos.	56	obs:	exp:
gaa	E	898	543.20
gag	E	5	359.80
---	--	------	------
mPD		0.011	0.48
		nPD:	0.02
		N. weight:	0.55
		Sc. PD:	-0.058
		Sc. rank:	-110.7

,

PA-X			
Pos.	57	obs:	exp:
tgg	W	1	1.00
caa	Q	20	10.79
cag	Q	1	10.21
cgt	R	38	37.64
cgc	R	2	44.76
cga	R	548	87.85
cgg	R	182	73.38
aga	R	110	402.20
agg	R	1	235.10
---	--	------	------
mPD		0.66	1.0
		nPD:	0.64
		N. weight:	1.3
		Sc. PD:	0.64
		Sc. rank:	866.7

,

PA-X			
Pos.	58	obs:	exp:
ggt	G	84	121.10
ggc	G	813	114.70
gga	G	6	417.50
ggg	G	0	249.70
---	--	------	------
mPD		0.18	0.68
		nPD:	0.27
		N. weight:	1.9
		Sc. PD:	0.25
		Sc. rank:	904.5

,

PA-X			
Pos.	59	obs:	exp:
aaa	K	6	3.51
aag	K	0	2.49
gaa	E	873	539.60
gag	E	24	357.40
---	--	------	------
mPD		0.065	0.49
		nPD:	0.13
		N. weight:	0.46
		Sc. PD:	-0.0012
		Sc. rank:	86.5

,

PA-X			
Pos.	60	obs:	exp:
tta	L	3	0.26
ttg	L	0	0.56
tct	S	0	143.90
tcc	S	0	116.40
tca	S	882	226.90
tcg	S	17	52.32
ctt	L	0	0.51
ctc	L	0	0.50
cta	L	0	0.50
ctg	L	0	0.67
cct	P	0	0.25
ccc	P	0	0.18
cca	P	1	0.41
ccg	P	0	0.16
agt	S	0	184.20
agc	S	0	175.30
---	--	------	------
mPD		0.046	1.7
		nPD:	0.03
		N. weight:	1.5
		Sc. PD:	-0.15
		Sc. rank:	-225.2

,

PA-X			
Pos.	61	obs:	exp:
att	I	0	32.84
atc	I	0	21.77
ata	I	90	35.39
atg	M	2	2.00
act	T	676	209.10
acc	T	131	164.30
aca	T	0	370.80
acg	T	0	62.86
gtt	V	0	0.41
gtc	V	0	0.40
gta	V	1	0.41
gtg	V	1	0.77
gct	A	1	0.26
gcc	A	0	0.19
gca	A	0	0.47
gcg	A	0	0.09
gat	D	1	0.54
gac	D	0	0.46
---	--	------	------
mPD		0.60	0.87
		nPD:	0.69
		N. weight:	1.1
		Sc. PD:	0.58
		Sc. rank:	728.5

,

PA-X			
Pos.	62	obs:	exp:
att	I	526	328.40
atc	I	34	217.70
ata	I	340	353.90
atg	M	1	1.00
gtt	V	1	0.41
gtc	V	0	0.40
gta	V	1	0.41
gtg	V	0	0.77
---	--	------	------
mPD		0.52	0.66
		nPD:	0.79
		N. weight:	0.23
		Sc. PD:	0.14
		Sc. rank:	154.2

,

PA-X			
Pos.	63	obs:	exp:
att	I	0	294.10
atc	I	0	195.00
ata	I	806	317.00
gtt	V	0	19.82
gtc	V	0	19.23
gta	V	56	19.88
gtg	V	40	37.07
gct	A	0	0.26
gcc	A	0	0.19
gca	A	1	0.47
gcg	A	0	0.09
---	--	------	------
mPD		0.28	0.88
		nPD:	0.32
		N. weight:	1.
		Sc. PD:	0.18
		Sc. rank:	534.1

,

PA-X			
Pos.	64	obs:	exp:
gaa	E	894	543.20
gag	E	9	359.80
---	--	------	------
mPD		0.020	0.48
		nPD:	0.04
		N. weight:	0.52
		Sc. PD:	-0.047
		Sc. rank:	-45.0

,

PA-X			
Pos.	65	obs:	exp:
ttt	F	1	0.39
ttc	F	0	0.61
tta	L	0	0.18
ttg	L	0	0.37
tct	S	863	144.10
tcc	S	32	116.50
tca	S	2	227.10
tcg	S	3	52.37
ctt	L	2	0.34
ctc	L	0	0.34
cta	L	0	0.33
ctg	L	0	0.44
agt	S	0	184.40
agc	S	0	175.50
---	--	------	------
mPD		0.090	1.7
		nPD:	0.05
		N. weight:	1.9
		Sc. PD:	-0.15
		Sc. rank:	-125.8

,

PA-X			
Pos. 66		obs:	exp:
tct	S	0	2.88
tcc	S	0	2.33
tca	S	0	4.54
tcg	S	0	1.05
agt	S	0	3.69
agc	S	18	3.51
gat	D	3	1.62
gac	D	0	1.38
ggt	G	22	118.30
ggc	G	858	112.10
gga	G	2	407.80
ggg	G	0	243.90
---	--	------	------
mPD		0.10	0.75
		nPD:	0.14
		N. weight:	2.2
		Sc. PD:	0.0082
		Sc. rank:	520.4

PA-X			
Pos. 67		obs:	exp:
aat	N	3	1.53
aac	N	0	1.47
gat	D	891	485.90
gac	D	9	414.10
---	--	------	------
mPD		0.026	0.50
		nPD:	0.05
		N. weight:	0.65
		Sc. PD:	-0.051
		Sc. rank:	-49.5

PA-X			
Pos. 68		obs:	exp:
tta	L	0	0.26
ttg	L	0	0.56
ctt	L	0	0.51
ctc	L	0	0.50
cta	L	3	0.50
ctg	L	0	0.67
cct	P	5	228.80
ccc	P	0	160.50
cca	P	862	367.70
ccg	P	33	143.00
---	--	------	------
mPD		0.088	0.72
		nPD:	0.12
		N. weight:	0.86
		Sc. PD:	-0.01
		Sc. rank:	132.9

PA-X			
Pos. 69		obs:	exp:
aat	N	888	459.40
aac	N	15	443.60
---	--	------	------
mPD		0.033	0.50
		nPD:	0.07
		N. weight:	0.68
		Sc. PD:	-0.045
		Sc. rank:	-16.4

PA-X			
Pos. 70		obs:	exp:
gtt	V	0	32.62
gtc	V	0	31.65
gta	V	1	32.71
gtg	V	157	61.02
gct	A	0	190.90
gcc	A	1	139.90
gca	A	243	348.30
gcg	A	501	65.89
---	--	------	------
mPD		0.69	0.99
		nPD:	0.69
		N. weight:	1.4
		Sc. PD:	0.72
		Sc. rank:	907.7

PA-X			
Pos. 71		obs:	exp:
tta	L	50	79.36
ttg	L	339	168.50
ctt	L	0	153.00
ctc	L	34	151.30
cta	L	14	150.50
ctg	L	466	200.30
---	--	------	------
mPD		0.69	1.1
		nPD:	0.62
		N. weight:	0.68
		Sc. PD:	0.32
		Sc. rank:	442.0

PA-X			
Pos. 72		obs:	exp:
tta	L	9	79.36
ttg	L	458	168.50
ctt	L	7	153.00
ctc	L	0	151.30
cta	L	49	150.50
ctg	L	380	200.30
---	--	------	------
mPD		0.63	1.1
		nPD:	0.57
		N. weight:	0.78
		Sc. PD:	0.33
		Sc. rank:	505.2

PA-X			
Pos. 73		obs:	exp:
cgt	R	0	0.04
cgc	R	0	0.05
cga	R	0	0.10
cgg	R	0	0.08
aat	N	0	0.51
aac	N	1	0.49
aaa	K	879	527.80
aag	K	22	373.20
aga	R	1	0.46
agg	R	0	0.27
---	--	------	------
mPD		0.052	0.49
		nPD:	0.11
		N. weight:	0.49
		Sc. PD:	-0.013
		Sc. rank:	39.1

PA-X			
Pos. 74		obs:	exp:
cat	H	290	518.20
cac	H	612	383.80
att	I	0	0.36
atc	I	1	0.24
ata	I	0	0.39
---	--	------	------
mPD		0.44	0.49
		nPD:	0.89
		N. weight:	0.15
		Sc. PD:	0.11
		Sc. rank:	105.8

PA-X			
Pos. 75		obs:	exp:
cgt	R	1	38.58
cgc	R	0	45.88
cga	R	730	90.04
cgg	R	100	75.21
aga	R	43	412.30
agg	R	29	241.00
---	--	------	------
mPD		0.39	0.96
		nPD:	0.41
		N. weight:	1.8
		Sc. PD:	0.47
		Sc. rank:	1048.6

PA-X			
Pos. 76		obs:	exp:
ttt	F	896	356.40
ttc	F	7	546.60
---	--	------	------
mPD		0.015	0.48
		nPD:	0.03
		N. weight:	1.
		Sc. PD:	-0.099
		Sc. rank:	-134.2

PA-X			
Pos. 77		obs:	exp:
gaa	E	835	543.20
gag	E	68	359.80
---	--	------	------
mPD		0.14	0.48
		nPD:	0.29
		N. weight:	0.31
		Sc. PD:	0.047
		Sc. rank:	154.2

PA-X			
Pos. 78		obs:	exp:
tta	L	28	2.46
ttg	L	0	5.23
ctt	L	0	4.74
ctc	L	0	4.69
cta	L	0	4.67
ctg	L	0	6.21
att	I	0	319.30
atc	I	0	211.60
ata	I	875	344.10
---	--	------	------
mPD		0.060	0.72
		nPD:	0.08
		N. weight:	1.1
		Sc. PD:	-0.055
		Sc. rank:	16.2

PA-X			
Pos. 79		obs:	exp:
att	I	98	329.50
atc	I	803	218.40
ata	I	2	355.10
---	--	------	------
mPD		0.20	0.65
		nPD:	0.3
		N. weight:	1.2
		Sc. PD:	0.19
		Sc. rank:	589.1

PA-X			
Pos. 80		obs:	exp:
gaa	E	896	543.20
gag	E	7	359.80
---	--	------	------
mPD		0.015	0.48
		nPD:	0.03
		N. weight:	0.53
		Sc. PD:	-0.053
		Sc. rank:	-75.6

PA-X			
Pos. 81		obs:	exp:
ggt	G	0	121.10
ggc	G	0	114.70
gga	G	82	417.50
ggg	G	821	249.70
---	--	------	------
mPD		0.17	0.68
		nPD:	0.24
		N. weight:	1.1
		Sc. PD:	0.11
		Sc. rank:	459.7

PA-X			
Pos. 82		obs:	exp:
cgt	R	0	38.58
cgc	R	0	45.88
cga	R	1	90.04
cgg	R	0	75.21
aga	R	897	412.30
agg	R	5	241.00
---	--	------	------
mPD		0.013	0.96
		nPD:	0.01
		N. weight:	0.86
		Sc. PD:	-0.099
		Sc. rank:	-266.3

PA-X			
Pos. 83		obs:	exp:
gat	D	13	486.90
gac	D	889	415.10
ggt	G	0	0.13
ggc	G	1	0.13
gga	G	0	0.46
ggg	G	0	0.28
---	--	------	------
mPD		0.031	0.50
		nPD:	0.06
		N. weight:	0.8
		Sc. PD:	-0.056
		Sc. rank:	-39.0

PA-X			
Pos. 84		obs:	exp:
cgt	R	5	38.58
cgc	R	28	45.88
cga	R	852	90.04
cgg	R	11	75.21
aga	R	7	412.30
agg	R	0	241.00
---	--	------	------
mPD		0.11	0.96
		nPD:	0.11
		N. weight:	2.3
		Sc. PD:	-0.047
		Sc. rank:	215.0

PA-X			
Pos. 85		obs:	exp:
act	T	31	232.40
acc	T	3	182.70
aca	T	858	412.10
acg	T	5	69.87
gct	A	0	1.54
gcc	A	0	1.13
gca	A	6	2.81
gcg	A	0	0.53
---	--	------	------
mPD		0.097	0.69
		nPD:	0.14
		N. weight:	0.7
		Sc. PD:	0.0039
		Sc. rank:	172.6

PA-X			
Pos. 86		obs:	exp:
atg	M	902	902.00
gtt	V	0	0.21
gtc	V	0	0.20
gta	V	0	0.21
gtg	V	1	0.39
---	--	------	------
mPD		0.0022	0.0036
		nPD:	0.62
		N. weight:	0.0012
		Sc. PD:	0.00056
		Sc. rank:	0.8

PA-X			
Pos. 87		obs:	exp:
gct	A	4	231.40
gcc	A	874	169.60
gca	A	25	422.20
gcg	A	0	79.87
---	--	------	------
mPD		0.062	0.67
		nPD:	0.09
		N. weight:	1.7
		Sc. PD:	-0.068
		Sc. rank:	86.8

PA-X			
Pos. 88		obs:	exp:
tgg	W	903	903.00
---	--	------	------
mPD		0	0
		nPD:	1.
		N. weight:	0.
		Sc. PD:	0
		Sc. rank:	0

PA-X			
Pos. 89		obs:	exp:
act	T	11	233.90
acc	T	3	183.90
aca	T	875	414.90
acg	T	14	70.34
---	--	------	------
mPD		0.061	0.67
		nPD:	0.09
		N. weight:	0.75
		Sc. PD:	-0.032
		Sc. rank:	29.1

PA-X			
Pos. 90		obs:	exp:
tta	L	0	0.18
ttg	L	1	0.37
ctt	L	0	0.34
ctc	L	0	0.34
cta	L	0	0.33
ctg	L	1	0.44
att	I	1	0.36
atc	I	0	0.24
ata	I	0	0.39
atg	M	1	1.00
gtt	V	0	185.60
gtc	V	0	180.10
gta	V	100	186.10
gtg	V	799	347.20
---	--	------	------
mPD		0.21	0.73
		nPD:	0.28
		N. weight:	0.77
		Sc. PD:	0.11
		Sc. rank:	367.4

PA-X			
Pos.	91	obs:	exp:
gtt	V	0	186.40
gtc	V	0	180.90
gta	V	50	187.00
gtg	V	853	348.70
---	--	------	------
mPD		0.10	0.73
		nPD:	0.14
		N. weight:	0.89
		Sc. PD:	0.0083
		Sc. rank:	226.9

,

PA-X			
Pos.	92	obs:	exp:
aat	N	857	459.40
aac	N	46	443.60
---	--	------	------
mPD		0.097	0.50
		nPD:	0.19
		N. weight:	0.55
		Sc. PD:	0.031
		Sc. rank:	173.4

,

PA-X			
Pos.	93	obs:	exp:
tct	S	0	144.40
tcc	S	0	116.80
tca	S	0	227.60
tcg	S	0	52.49
agt	S	758	184.80
agc	S	144	175.90
ggt	G	0	0.13
ggc	G	1	0.13
gga	G	0	0.46
ggg	G	0	0.28
---	--	------	------
mPD		0.27	1.7
		nPD:	0.16
		N. weight:	1.3
		Sc. PD:	0.038
		Sc. rank:	384.1

,

PA-X			
Pos.	94	obs:	exp:
att	I	89	302.80
atc	I	740	200.80
ata	I	1	326.40
gtt	V	0	15.07
gtc	V	73	14.62
gta	V	0	15.11
gtg	V	0	28.19
---	--	------	------
mPD		0.33	0.82
		nPD:	0.4
		N. weight:	1.2
		Sc. PD:	0.31
		Sc. rank:	695.1

,

PA-X			
Pos.	95	obs:	exp:
tgt	C	1	370.70
tgc	C	902	532.30
---	--	------	------
mPD		0.0022	0.48
		nPD:	0.
		N. weight:	0.6
		Sc. PD:	-0.074
		Sc. rank:	-268.4

,

PA-X			
Pos.	96	obs:	exp:
cat	H	0	58.02
cac	H	101	42.98
aat	N	21	408.00
aac	N	781	394.00
---	--	------	------
mPD		0.24	0.70
		nPD:	0.35
		N. weight:	0.71
		Sc. PD:	0.15
		Sc. rank:	379.4

,

PA-X			
Pos.	97	obs:	exp:
act	T	126	233.40
acc	T	765	183.50
aca	T	10	413.90
acg	T	0	70.19
gct	A	0	0.51
gcc	A	2	0.38
gca	A	0	0.94
gcg	A	0	0.18
---	--	------	------
mPD		0.26	0.68
		nPD:	0.39
		N. weight:	1.2
		Sc. PD:	0.3
		Sc. rank:	680.1

,

PA-X			
Pos.	98	obs:	exp:
act	T	66	233.90
acc	T	2	183.90
aca	T	464	414.90
acg	T	371	70.34
---	--	------	------
mPD		0.56	0.67
		nPD:	0.83
		N. weight:	0.74
		Sc. PD:	0.5
		Sc. rank:	504.9

,

PA-X			
Pos.	99	obs:	exp:
tct	S	0	0.16
tcc	S	0	0.13
tca	S	0	0.25
tcg	S	0	0.06
agt	S	1	0.20
agc	S	0	0.20
ggt	G	32	121.00
ggc	G	0	114.60
gga	G	796	417.00
ggg	G	74	249.40
---	--	------	------
mPD		0.22	0.68
		nPD:	0.32
		N. weight:	0.49
		Sc. PD:	0.087
		Sc. rank:	256.0

,

PA-X			
Pos.	100	obs:	exp:
ttt	F	0	0.39
ttc	F	1	0.61
att	I	0	5.47
atc	I	15	3.63
ata	I	0	5.90
act	T	0	0.52
acc	T	2	0.41
aca	T	0	0.92
acg	T	0	0.16
gtt	V	34	112.30
gtc	V	509	109.00
gta	V	1	112.60
gtg	V	0	210.10
gct	A	3	87.38
gcc	A	337	64.03
gca	A	1	159.40
gcg	A	0	30.16
---	--	------	------
mPD		0.59	1.3
		nPD:	0.47
		N. weight:	1.7
		Sc. PD:	0.54
		Sc. rank:	1028.1

,

PA-X			
Pos.	101	obs:	exp:
gat	D	27	14.58
gac	D	0	12.42
gaa	E	738	525.10
gag	E	135	347.90
ggt	G	0	0.40
ggc	G	0	0.38
gga	G	3	1.39
ggg	G	0	0.83
---	--	------	------
mPD		0.31	0.52
		nPD:	0.6
		N. weight:	0.18
		Sc. PD:	0.082
		Sc. rank:	119.7

,

PA-X			
Pos.	102	obs:	exp:
aaa	K	845	529.00
aag	K	58	374.00
---	--	------	------
mPD		0.12	0.49
		nPD:	0.25
		N. weight:	0.37
		Sc. PD:	0.04
		Sc. rank:	159.2

,

PA-X			
Pos.	103	obs:	exp:
tct	S	0	0.16
tcc	S	1	0.13
tca	S	0	0.25
tcg	S	0	0.06
cct	P	165	229.40
ccc	P	732	160.80
cca	P	3	368.50
ccg	P	2	143.30
agt	S	0	0.20
agc	S	0	0.20
---	--	------	------
mPD		0.31	0.71
		nPD:	0.43
		N. weight:	1.3
		Sc. PD:	0.38
		Sc. rank:	790.9

,

PA-X			
Pos.	104	obs:	exp:
cgt	R	0	0.26
cgc	R	0	0.30
cga	R	0	0.60
cgg	R	0	0.50
aat	N	1	0.51
aac	N	0	0.49
aaa	K	855	524.90
aag	K	41	371.10
aga	R	4	2.74
agg	R	2	1.60
---	--	------	------
mPD		0.11	0.50
		nPD:	0.21
		N. weight:	0.42
		Sc. PD:	0.031
		Sc. rank:	158.3

,

PA-X			
Pos.	105	obs:	exp:
ttt	F	824	355.60
ttc	F	77	545.40
tta	L	0	0.09
ttg	L	0	0.19
tct	S	1	0.16
tcc	S	0	0.13
tca	S	0	0.25
tcg	S	0	0.06
ctt	L	1	0.17
ctc	L	0	0.17
cta	L	0	0.17
ctg	L	0	0.22
agt	S	0	0.20
agc	S	0	0.20
---	--	------	------
mPD		0.16	0.48
		nPD:	0.33
		N. weight:	0.69
		Sc. PD:	0.13
		Sc. rank:	365.6

,

PA-X			
Pos.	106	obs:	exp:
tta	L	0	79.36
ttg	L	0	168.50
ctt	L	125	153.00
ctc	L	776	151.30
cta	L	2	150.50
ctg	L	0	200.30
---	--	------	------
mPD		0.24	1.1
		nPD:	0.22
		N. weight:	1.6
		Sc. PD:	0.13
		Sc. rank:	628.0

,

PA-X			
Pos.	107	obs:	exp:
cct	P	19	229.60
ccc	P	0	161.00
cca	P	72	368.90
ccg	P	812	143.50
---	--	------	------
mPD		0.18	0.71
		nPD:	0.26
		N. weight:	1.6
		Sc. PD:	0.19
		Sc. rank:	702.6

,

PA-X			
Pos.	108	obs:	exp:
att	I	0	0.36
atc	I	1	0.24
ata	I	0	0.39
gat	D	868	486.90
gac	D	34	415.10
---	--	------	------
mPD		0.079	0.50
		nPD:	0.16
		N. weight:	0.53
		Sc. PD:	0.012
		Sc. rank:	143.4

,

PA-X			
Pos.	109	obs:	exp:
tta	L	33	79.27
ttg	L	47	168.30
ctt	L	1	152.90
ctc	L	0	151.10
cta	L	2	150.40
ctg	L	819	200.00
gtt	V	0	0.21
gtc	V	0	0.20
gta	V	0	0.21
gtg	V	1	0.39
---	--	------	------
mPD		0.24	1.1
		nPD:	0.22
		N. weight:	1.3
		Sc. PD:	0.11
		Sc. rank:	526.1

,

PA-X			
Pos.	110	obs:	exp:
tat	Y	865	478.80
tac	Y	38	424.20
---	--	------	------
mPD		0.081	0.50
		nPD:	0.16
		N. weight:	0.53
		Sc. PD:	0.014
		Sc. rank:	146.8

,

PA-X

Pos.	111	obs:	exp:
gat	D	51	487.50
gac	D	852	415.50
---	--	------	------
mPD		0.11	0.50
		nPD:	0.21
		N. weight:	0.63
		Sc. PD:	0.049
		Sc. rank:	240.8

,

PA-X

Pos.	112	obs:	exp:
tat	Y	3	478.80
tac	Y	900	424.20
---	--	------	------
mPD		0.0066	0.50
		nPD:	0.01
		N. weight:	0.84
		Sc. PD:	-0.098
		Sc. rank:	-281.0

,

PA-X

Pos.	113	obs:	exp:
caa	Q	0	0.51
cag	Q	1	0.49
cgt	R	0	0.09
cgc	R	0	0.10
cga	R	0	0.20
cgg	R	0	0.17
atg	M	1	1.00
aaa	K	76	526.60
aag	K	823	372.40
aga	R	0	0.91
agg	R	2	0.53
---	--	------	------
mPD		0.16	0.50
		nPD:	0.33
		N. weight:	0.65
		Sc. PD:	0.12
		Sc. rank:	338.9

,

PA-X

Pos.	114	obs:	exp:
gct	A	0	0.26
gcc	A	0	0.19
gca	A	1	0.47
gcg	A	0	0.09
gat	D	0	0.54
gac	D	1	0.46
gaa	E	818	540.80
gag	E	81	358.20
ggt	G	0	0.27
ggc	G	0	0.25
gga	G	2	0.92
ggg	G	0	0.55
---	--	------	------
mPD		0.17	0.49
		nPD:	0.35
		N. weight:	0.28
		Sc. PD:	0.059
		Sc. rank:	152.1

,

PA-X

Pos.	115	obs:	exp:
tct	S	0	16.49
tcc	S	0	13.33
tca	S	0	25.99
tcg	S	0	5.99
aat	N	42	407.00
aac	N	758	393.00
agt	S	0	21.11
agc	S	103	20.09
---	--	------	------
mPD		0.29	0.87
		nPD:	0.33
		N. weight:	0.73
		Sc. PD:	0.14
		Sc. rank:	385.3

,

PA-X

Pos.	116	obs:	exp:
cgt	R	341	38.58
cgc	R	26	45.88
cga	R	526	90.04
cgg	R	8	75.21
aga	R	1	412.30
agg	R	1	241.00
---	--	------	------
mPD		0.52	0.96
		nPD:	0.54
		N. weight:	2.1
		Sc. PD:	0.81
		Sc. rank:	1335.2

,

PA-X

Pos.	117	obs:	exp:
ttt	F	27	356.40
ttc	F	876	546.60
---	--	------	------
mPD		0.058	0.48
		nPD:	0.12
		N. weight:	0.44
		Sc. PD:	-0.0055
		Sc. rank:	65.9

,

PA-X

Pos.	118	obs:	exp:
tta	L	0	0.09
ttg	L	0	0.19
ctt	L	1	0.17
ctc	L	0	0.17
cta	L	0	0.17
ctg	L	0	0.22
att	I	895	328.00
atc	I	4	217.40
ata	I	0	353.50
gtt	V	3	0.62
gtc	V	0	0.60
gta	V	0	0.62
gtg	V	0	1.16
---	--	------	------
mPD		0.018	0.66
		nPD:	0.03
		N. weight:	1.1
		Sc. PD:	-0.12
		Sc. rank:	-181.3

,

PA-X

Pos.	119	obs:	exp:
gaa	E	901	543.20
gag	E	2	359.80
---	--	------	------
mPD		0.0044	0.48
		nPD:	0.01
		N. weight:	0.57
		Sc. PD:	-0.068
		Sc. rank:	-215.1

,

PA-X

Pos.	120	obs:	exp:
att	I	902	329.50
atc	I	1	218.40
ata	I	0	355.10
---	--	------	------
mPD		0.0022	0.65
		nPD:	0.
		N. weight:	1.1
		Sc. PD:	-0.14
		Sc. rank:	-552.5

,

PA-X

Pos.	121	obs:	exp:
ggt	G	1	121.10
ggc	G	0	114.70
gga	G	886	417.50
ggg	G	16	249.70
---	--	------	------
mPD		0.037	0.68
		nPD:	0.05
		N. weight:	0.79
		Sc. PD:	-0.06
		Sc. rank:	-48.9

,

PA-X

Pos.	122	obs:	exp:
gtt	V	11	186.40
gtc	V	0	180.90
gta	V	173	187.00
gtg	V	719	348.70
---	--	------	------
mPD		0.33	0.73
		nPD:	0.45
		N. weight:	0.61
		Sc. PD:	0.19
		Sc. rank:	367.9

,

PA-X

Pos.	123	obs:	exp:
act	T	4	233.90
acc	T	33	183.90
aca	T	100	414.90
acg	T	766	70.34
---	--	------	------
mPD		0.27	0.67
		nPD:	0.4
		N. weight:	2.1
		Sc. PD:	0.51
		Sc. rank:	1140.9

,

PA-X

Pos.	124	obs:	exp:
cgt	R	0	38.53
cgc	R	0	45.83
cga	R	39	89.94
cgg	R	18	75.13
aaa	K	0	0.59
aag	K	1	0.41
aga	R	10	411.80
agg	R	835	240.70
---	--	------	------
mPD		0.22	0.97
		nPD:	0.23
		N. weight:	1.2
		Sc. PD:	0.11
		Sc. rank:	503.3

,

PA-X

Pos.	125	obs:	exp:
cgt	R	0	38.58
cgc	R	0	45.88
cga	R	0	90.04
cgg	R	0	75.21
aga	R	7	412.30
agg	R	896	241.00
---	--	------	------
mPD		0.015	0.96
		nPD:	0.02
		N. weight:	1.5
		Sc. PD:	-0.17
		Sc. rank:	-416.3

,

PA-X

Pos.	126	obs:	exp:
gaa	E	889	543.20
gag	E	14	359.80
---	--	------	------
mPD		0.031	0.48
		nPD:	0.06
		N. weight:	0.5
		Sc. PD:	-0.034
		Sc. rank:	-15.9

,

PA-X

Pos.	127	obs:	exp:
att	I	0	2.19
atc	I	6	1.45
ata	I	0	2.36
gtt	V	45	185.20
gtc	V	846	179.70
gta	V	6	185.70
gtg	V	0	346.40
---	--	------	------
mPD		0.12	0.74
		nPD:	0.16
		N. weight:	1.6
		Sc. PD:	0.043
		Sc. rank:	443.9

,

PA-X

Pos.	128	obs:	exp:
cat	H	47	518.20
cac	H	855	383.80
caa	Q	1	0.51
cag	Q	0	0.49
---	--	------	------
mPD		0.10	0.49
		nPD:	0.21
		N. weight:	0.73
		Sc. PD:	0.05
		Sc. rank:	257.0

,

PA-X

Pos.	129	obs:	exp:
att	I	1	325.50
atc	I	0	215.80
ata	I	891	350.80
atg	M	6	6.00
act	T	0	1.04
acc	T	0	0.81
aca	T	4	1.84
acg	T	0	0.31
gtt	V	0	0.21
gtc	V	0	0.20
gta	V	1	0.21
gtg	V	0	0.39
---	--	------	------
mPD		0.026	0.67
		nPD:	0.04
		N. weight:	1.1
		Sc. PD:	-0.096
		Sc. rank:	-93.2

,

PA-X

Pos.	130	obs:	exp:
tat	Y	589	478.80
tac	Y	314	424.20
---	--	------	------
mPD		0.45	0.50
		nPD:	0.91
		N. weight:	0.039
		Sc. PD:	0.029
		Sc. rank:	27.0

,

PA-X

Pos.	131	obs:	exp:
tat	Y	73	478.80
tac	Y	830	424.20
---	--	------	------
mPD		0.15	0.50
		nPD:	0.3
		N. weight:	0.53
		Sc. PD:	0.084
		Sc. rank:	267.2

,

PA-X

Pos.	132	obs:	exp:
tta	L	49	79.36
ttg	L	1	168.50
ctt	L	1	153.00
ctc	L	0	151.30
cta	L	842	150.50
ctg	L	10	200.30
---	--	------	------
mPD		0.13	1.1
		nPD:	0.12
		N. weight:	1.8
		Sc. PD:	-0.028
		Sc. rank:	261.1

,

PA-X

Pos.	133	obs:	exp:
gat	D	3	1.62
gac	D	0	1.38
gaa	E	98	541.40
gag	E	802	358.60
---	--	------	------
mPD		0.20	0.48
		nPD:	0.41
		N. weight:	0.61
		Sc. PD:	0.16
		Sc. rank:	362.9

,

PA-X

Pos.	134	obs:	exp:
aaa	K	831	529.00
aag	K	72	374.00
---	--	------	------
mPD		0.15	0.49
		nPD:	0.3
		N. weight:	0.33
		Sc. PD:	0.053
		Sc. rank:	166.8

,

PA-X

Pos.	135	obs:	exp:
gct	A	35	231.40
gcc	A	867	169.60
gca	A	1	422.20
gcg	A	0	79.87
---	--	------	------
mPD		0.077	0.67
		nPD:	0.11
		N. weight:	1.7
		Sc. PD:	-0.034
		Sc. rank:	188.0

,

PA-X			
Pos.	136	obs:	exp:
act	T	1	0.26
acc	T	0	0.20
aca	T	0	0.46
acg	T	0	0.08
aat	N	809	458.90
aac	N	93	443.10
---	--	------	------
mPD		0.19	0.50
		nPD:	0.37
		N. weight:	0.4
		Sc. PD:	0.092
		Sc. rank:	216.6

PA-X			
Pos.	137	obs:	exp:
aaa	K	784	529.00
aag	K	119	374.00
---	--	------	------
mPD		0.23	0.49
		nPD:	0.47
		N. weight:	0.22
		Sc. PD:	0.072
		Sc. rank:	135.2

PA-X			
Pos.	138	obs:	exp:
tta	L	2	2.72
ttg	L	0	5.79
ctt	L	0	5.25
ctc	L	0	5.19
cta	L	29	5.17
ctg	L	0	6.88
att	I	1	318.20
atc	I	0	210.90
ata	I	871	342.90
---	--	------	------
mPD		0.069	0.73
		nPD:	0.09
		N. weight:	1.1
		Sc. PD:	-0.042
		Sc. rank:	61.5

PA-X			
Pos.	139	obs:	exp:
cgt	R	0	0.09
cgc	R	0	0.10
cga	R	0	0.20
cgg	R	0	0.17
aaa	K	856	527.80
aag	K	45	373.20
aga	R	2	0.91
agg	R	0	0.53
---	--	------	------
mPD		0.099	0.49
		nPD:	0.2
		N. weight:	0.41
		Sc. PD:	0.026
		Sc. rank:	141.0

PA-X			
Pos.	140	obs:	exp:
tct	S	45	144.50
tcc	S	696	116.90
tca	S	162	227.90
tcg	S	0	52.55
agt	S	0	185.00
agc	S	0	176.10
---	--	------	------
mPD		0.37	1.7
		nPD:	0.22
		N. weight:	1.4
		Sc. PD:	0.12
		Sc. rank:	594.0

PA-X			
Pos.	141	obs:	exp:
gat	D	1	0.54
gac	D	0	0.46
gaa	E	27	540.80
gag	E	872	358.20
ggt	G	0	0.40
ggc	G	0	0.38
gga	G	0	1.39
ggg	G	3	0.83
---	--	------	------
mPD		0.067	0.49
		nPD:	0.14
		N. weight:	0.89
		Sc. PD:	0.002
		Sc. rank:	192.5

PA-X			
Pos.	142	obs:	exp:
cgt	R	0	0.13
cgc	R	0	0.15
cga	R	0	0.30
cgg	R	0	0.25
aat	N	0	0.51
aac	N	1	0.49
aaa	K	12	526.00
aag	K	886	372.00
aga	R	0	1.37
agg	R	3	0.80
gaa	E	0	0.60
gag	E	1	0.40
---	--	------	------
mPD		0.037	0.50
		nPD:	0.07
		N. weight:	0.93
		Sc. PD:	-0.053
		Sc. rank:	5.4

PA-X			
Pos.	143	obs:	exp:
act	T	0	232.90
acc	T	4	183.10
aca	T	895	413.00
acg	T	0	70.03
gct	A	0	1.02
gcc	A	0	0.75
gca	A	4	1.87
gcg	A	0	0.35
---	--	------	------
mPD		0.018	0.68
		nPD:	0.03
		N. weight:	0.86
		Sc. PD:	-0.09
		Sc. rank:	-163.6

PA-X			
Pos.	144	obs:	exp:
cat	H	3	518.20
cac	H	899	383.80
aat	N	0	0.51
aac	N	1	0.49
---	--	------	------
mPD		0.0088	0.49
		nPD:	0.02
		N. weight:	0.95
		Sc. PD:	-0.11
		Sc. rank:	-257.9

PA-X			
Pos.	145	obs:	exp:
att	I	82	329.50
atc	I	622	218.40
ata	I	199	355.10
---	--	------	------
mPD		0.47	0.65
		nPD:	0.72
		N. weight:	0.54
		Sc. PD:	0.3
		Sc. rank:	366.5

PA-X			
Pos.	146	obs:	exp:
cat	H	362	518.80
cac	H	541	384.20
---	--	------	------
mPD		0.48	0.49
		nPD:	0.98
		N. weight:	0.074
		Sc. PD:	0.06
		Sc. rank:	50.7

PA-X			
Pos.	147	obs:	exp:
att	I	820	329.50
atc	I	45	218.40
ata	I	38	355.10
---	--	------	------
mPD		0.17	0.65
		nPD:	0.26
		N. weight:	0.76
		Sc. PD:	0.092
		Sc. rank:	339.3

PA-X			
Pos.	148	obs:	exp:
ttt	F	365	356.40
ttc	F	538	546.60
---	--	------	------
mPD		0.48	0.48
		nPD:	1.01
		N. weight:	0.0048
		Sc. PD:	0.004
		Sc. rank:	3.3

PA-X			
Pos.	149	obs:	exp:
tct	S	1	144.50
tcc	S	0	116.90
tca	S	898	227.90
tcg	S	4	52.55
agt	S	0	185.00
agc	S	0	176.10
---	--	------	------
mPD		0.011	1.7
		nPD:	0.01
		N. weight:	1.5
		Sc. PD:	-0.19
		Sc. rank:	-636.4

PA-X			
Pos.	150	obs:	exp:
ttt	F	15	356.40
ttc	F	888	546.60
---	--	------	------
mPD		0.033	0.48
		nPD:	0.07
		N. weight:	0.49
		Sc. PD:	-0.031
		Sc. rank:	-6.4

PA-X			
Pos.	151	obs:	exp:
act	T	902	233.70
acc	T	0	183.70
aca	T	0	414.40
acg	T	0	70.26
aat	N	1	0.51
aac	N	0	0.49
---	--	------	------
mPD		0.0022	0.68
		nPD:	0.
		N. weight:	1.5
		Sc. PD:	-0.19
		Sc. rank:	-774.1

PA-X			
Pos.	152	obs:	exp:
ggt	G	1	121.10
ggc	G	0	114.70
gga	G	854	417.50
ggg	G	48	249.70
---	--	------	------
mPD		0.10	0.68
		nPD:	0.15
		N. weight:	0.67
		Sc. PD:	0.011
		Sc. rank:	176.5

PA-X			
Pos.	153	obs:	exp:
gaa	E	849	542.00
gag	E	52	359.00
ggt	G	0	0.27
ggc	G	0	0.25
gga	G	2	0.92
ggg	G	0	0.55
---	--	------	------
mPD		0.11	0.48
		nPD:	0.23
		N. weight:	0.36
		Sc. PD:	0.034
		Sc. rank:	151.5

PA-X			
Pos.	154	obs:	exp:
gaa	E	98	543.20
gag	E	805	359.80
---	--	------	------
mPD		0.19	0.48
		nPD:	0.4
		N. weight:	0.61
		Sc. PD:	0.16
		Sc. rank:	350.2

PA-X			
Pos.	155	obs:	exp:
atg	M	903	903.00
---	--	------	------
mPD		0	0
		nPD:	1.
		N. weight:	0.
		Sc. PD:	0
		Sc. rank:	0

PA-X			
Pos.	156	obs:	exp:
act	T	0	0.26
acc	T	1	0.20
aca	T	0	0.46
acg	T	0	0.08
gct	A	43	231.10
gcc	A	812	169.40
gca	A	44	421.70
gcg	A	3	79.78
---	--	------	------
mPD		0.19	0.67
		nPD:	0.28
		N. weight:	1.4
		Sc. PD:	0.19
		Sc. rank:	656.1

PA-X			
Pos.	157	obs:	exp:
tct	S	0	0.16
tcc	S	0	0.13
tca	S	0	0.25
tcg	S	0	0.06
act	T	32	233.70
acc	T	866	183.70
aca	T	4	414.40
acg	T	0	70.26
agt	S	0	0.20
agc	S	1	0.20
---	--	------	------
mPD		0.079	0.68
		nPD:	0.12
		N. weight:	1.6
		Sc. PD:	-0.027
		Sc. rank:	209.3

PA-X			
Pos.	158	obs:	exp:
caa	Q	1	0.51
cag	Q	0	0.49
cgt	R	0	0.04
cgc	R	0	0.05
cga	R	0	0.10
cgg	R	0	0.08
aaa	K	845	527.80
aag	K	56	373.20
aga	R	1	0.46
agg	R	0	0.27
---	--	------	------
mPD		0.12	0.49
		nPD:	0.25
		N. weight:	0.37
		Sc. PD:	0.04
		Sc. rank:	161.3

PA-X			
Pos.	159	obs:	exp:
tct	S	0	0.16
tcc	S	0	0.13
tca	S	1	0.25
tcg	S	0	0.06
agt	S	0	0.20
agc	S	0	0.20
gct	A	0	231.10
gcc	A	3	169.40
gca	A	832	421.70
gcg	A	67	79.78
---	--	------	------
mPD		0.15	0.68
		nPD:	0.22
		N. weight:	0.69
		Sc. PD:	0.054
		Sc. rank:	266.5

PA-X			
Pos.	160	obs:	exp:
gat	D	800	486.90
gac	D	102	415.10
gaa	E	1	0.60
gag	E	0	0.40
---	--	------	------
mPD		0.20	0.50
		nPD:	0.41
		N. weight:	0.33
		Sc. PD:	0.085
		Sc. rank:	189.4

PA-X			
Pos.	161	obs:	exp:
tat	Y	13	478.30
tac	Y	889	423.70
tgt	C	0	0.41
tgc	C	1	0.59
mPD		0.031	0.50
		nPD:	0.06
		N. weight:	0.78
		Sc. PD:	-0.055
		Sc. rank:	-41.2

PA-X			
Pos.	162	obs:	exp:
act	T	733	233.90
acc	T	55	183.90
aca	T	115	414.90
acg	T	0	70.34
mPD		0.32	0.67
		nPD:	0.48
		N. weight:	0.8
		Sc. PD:	0.26
		Sc. rank:	495.6

PA-X			
Pos.	163	obs:	exp:
tta	L	0	79.19
ttg	L	0	168.10
ctt	L	892	152.70
ctc	L	8	151.00
cta	L	1	150.20
ctg	L	0	199.80
att	I	2	0.73
atc	I	0	0.48
ata	I	0	0.79
mPD		0.024	1.1
		nPD:	0.02
		N. weight:	2.
		Sc. PD:	-0.21
		Sc. rank:	-409.6

PA-X			
Pos.	164	obs:	exp:
gat	D	254	487.50
gac	D	649	415.50
mPD		0.40	0.50
		nPD:	0.81
		N. weight:	0.16
		Sc. PD:	0.1
		Sc. rank:	109.4

PA-X			
Pos.	165	obs:	exp:
gaa	E	877	543.20
gag	E	26	359.80
mPD		0.056	0.48
		nPD:	0.12
		N. weight:	0.45
		Sc. PD:	-0.0077
		Sc. rank:	54.7

PA-X			
Pos.	166	obs:	exp:
gaa	E	698	543.20
gag	E	205	359.80
mPD		0.35	0.48
		nPD:	0.73
		N. weight:	0.08
		Sc. PD:	0.046
		Sc. rank:	54.1

PA-X			
Pos.	167	obs:	exp:
tct	S	0	144.50
tcc	S	0	116.90
tca	S	0	227.90
tcg	S	0	52.55
agt	S	82	185.00
agc	S	821	176.10
mPD		0.17	1.7
		nPD:	0.1
		N. weight:	1.5
		Sc. PD:	-0.05
		Sc. rank:	98.5

PA-X			
Pos.	168	obs:	exp:
cgt	R	0	38.58
cgc	R	0	45.88
cga	R	18	90.04
cgg	R	0	75.21
aga	R	58	412.30
agg	R	827	241.00
mPD		0.19	0.96
		nPD:	0.2
		N. weight:	1.1
		Sc. PD:	0.071
		Sc. rank:	382.7

PA-X			
Pos.	169	obs:	exp:
gct	A	1	231.40
gcc	A	1	169.60
gca	A	870	422.20
gcg	A	31	79.87
mPD		0.071	0.67
		nPD:	0.11
		N. weight:	0.75
		Sc. PD:	-0.021
		Sc. rank:	51.8

PA-X			
Pos.	170	obs:	exp:
cgt	R	0	38.58
cgc	R	0	45.88
cga	R	0	90.04
cgg	R	0	75.21
aga	R	864	412.30
agg	R	39	241.00
mPD		0.083	0.96
		nPD:	0.09
		N. weight:	0.72
		Sc. PD:	-0.034
		Sc. rank:	16.6

PA-X			
Pos.	171	obs:	exp:
att	I	10	329.50
atc	I	871	218.40
ata	I	22	355.10
mPD		0.069	0.65
		nPD:	0.11
		N. weight:	1.4
		Sc. PD:	-0.039
		Sc. rank:	107.8

PA-X			
Pos.	172	obs:	exp:
aaa	K	895	529.00
aag	K	8	374.00
mPD		0.018	0.49
		nPD:	0.04
		N. weight:	0.56
		Sc. PD:	-0.053
		Sc. rank:	-60.6

PA-X			
Pos.	173	obs:	exp:
act	T	8	233.90
acc	T	892	183.90
aca	T	3	414.90
acg	T	0	70.34
mPD		0.024	0.67
		nPD:	0.04
		N. weight:	1.7
		Sc. PD:	-0.16
		Sc. rank:	-193.1

PA-X			
Pos.	174	obs:	exp:
cgt	R	0	38.58
cgc	R	0	45.88
cga	R	0	90.04
cgg	R	0	75.21
aga	R	65	412.30
agg	R	838	241.00
mPD		0.13	0.96
		nPD:	0.14
		N. weight:	1.2
		Sc. PD:	0.0047
		Sc. rank:	285.7

PA-X			
Pos.	175	obs:	exp:
tta	L	0	79.36
ttg	L	70	168.50
ctt	L	0	153.00
ctc	L	0	151.30
cta	L	41	150.50
ctg	L	792	200.30
mPD		0.23	1.1
		nPD:	0.21
		N. weight:	1.2
		Sc. PD:	0.086
		Sc. rank:	445.9

PA-X			
Pos.	176	obs:	exp:
ttt	F	6	356.00
ttc	F	896	546.00
tat	Y	0	0.53
tac	Y	1	0.47
mPD		0.015	0.48
		nPD:	0.03
		N. weight:	0.53
		Sc. PD:	-0.052
		Sc. rank:	-73.9

PA-X			
Pos.	177	obs:	exp:
act	T	145	233.90
acc	T	756	183.90
aca	T	2	414.90
acg	T	0	70.34
mPD		0.27	0.67
		nPD:	0.41
		N. weight:	1.3
		Sc. PD:	0.33
		Sc. rank:	727.9

PA-X			
Pos.	178	obs:	exp:
att	I	1	329.50
atc	I	104	218.40
ata	I	798	355.10
mPD		0.21	0.65
		nPD:	0.32
		N. weight:	0.72
		Sc. PD:	0.12
		Sc. rank:	367.1

PA-X			
Pos.	179	obs:	exp:
cgt	R	0	38.58
cgc	R	0	45.88
cga	R	0	90.04
cgg	R	0	75.21
aga	R	154	412.30
agg	R	749	241.00
mPD		0.28	0.96
		nPD:	0.29
		N. weight:	0.89
		Sc. PD:	0.14
		Sc. rank:	438.9

PA-X			
Pos.	180	obs:	exp:
cat	H	2	1.15
cac	H	0	0.85
caa	Q	99	462.80
cag	Q	802	438.20
mPD		0.20	0.50
		nPD:	0.4
		N. weight:	0.43
		Sc. PD:	0.11
		Sc. rank:	237.7

PA-X			
Pos.	181	obs:	exp:
gaa	E	834	543.20
gag	E	69	359.80
mPD		0.14	0.48
		nPD:	0.29
		N. weight:	0.31
		Sc. PD:	0.048
		Sc. rank:	155.0

PA-X			
Pos.	182	obs:	exp:
atg	M	903	903.00
mPD		0	0
		nPD:	1.
		N. weight:	0.
		Sc. PD:	0
		Sc. rank:	0

PA-X			
Pos.	183	obs:	exp:
gct	A	32	231.40
gcc	A	867	169.60
gca	A	4	422.20
gcg	A	0	79.87
mPD		0.077	0.67
		nPD:	0.11
		N. weight:	1.7
		Sc. PD:	-0.033
		Sc. rank:	202.2

PA-X			
Pos.	184	obs:	exp:
tct	S	0	143.70
tcc	S	0	116.20
tca	S	0	226.60
tcg	S	0	52.26
aat	N	1	1.02
aac	N	1	0.98
agt	S	98	184.00
agc	S	800	175.10
ggt	G	0	0.40
ggc	G	3	0.38
gga	G	0	1.39
ggg	G	0	0.83
mPD		0.21	1.7
		nPD:	0.12
		N. weight:	1.5
		Sc. PD:	-0.015
		Sc. rank:	240.2

PA-X			
Pos.	185	obs:	exp:
cgt	R	0	38.49
cgc	R	0	45.78
cga	R	0	89.84
cgg	R	0	75.04
aaa	K	0	1.17
aag	K	2	0.83
aga	R	61	411.40
agg	R	840	240.50
mPD		0.13	0.97
		nPD:	0.13
		N. weight:	1.2
		Sc. PD:	0.00037
		Sc. rank:	241.7

PA-X

Pos.	186	obs:	exp:
tct	S	0	0.16
tcc	S	0	0.13
tca	S	0	0.25
tcg	S	0	0.06
agt	S	1	0.20
agc	S	0	0.20
ggt	G	839	121.00
ggc	G	61	114.60
gga	G	2	417.00
ggg	G	0	249.40
mPD		0.13	0.68
		nPD:	0.19
		N. weight:	2.
		Sc. PD:	0.12
		Sc. rank:	655.2

PA-X

Pos.	187	obs:	exp:
tta	L	3	79.36
ttg	L	8	168.50
ctt	L	0	153.00
ctc	L	41	151.30
cta	L	846	150.50
ctg	L	5	200.30
mPD		0.14	1.1
		nPD:	0.12
		N. weight:	1.7
		Sc. PD:	-0.017
		Sc. rank:	297.3

PA-X

Pos.	188	obs:	exp:
tgg	W	903	903.00
mPD		0	0
		nPD:	1.
		N. weight:	0.
		Sc. PD:	0
		Sc. rank:	0

PA-X

Pos.	189	obs:	exp:
gat	D	901	486.90
gac	D	1	415.10
gaa	E	0	0.60
gag	E	1	0.40
mPD		0.0044	0.50
		nPD:	0.01
		N. weight:	0.7
		Sc. PD:	-0.084
		Sc. rank:	-281.6

PA-X

Pos.	190	obs:	exp:
tct	S	0	144.20
tcc	S	901	116.60
tca	S	0	227.40
tcg	S	0	52.43
cct	P	0	0.51
ccc	P	2	0.36
cca	P	0	0.82
ccg	P	0	0.32
agt	S	0	184.60
agc	S	0	175.70
mPD		0.0044	1.7
		nPD:	0.
		N. weight:	2.3
		Sc. PD:	-0.3
		Sc. rank:	-1191.5

PA-X

Pos.	191	obs:	exp:
ttt	F	901	356.00
ttc	F	1	546.00
tat	Y	1	0.53
tac	Y	0	0.47
mPD		0.0044	0.48
		nPD:	0.01
		N. weight:	1.1
		Sc. PD:	-0.13
		Sc. rank:	-403.8

PA-X

Pos.	192	obs:	exp:
gtt	V	0	186.40
gtc	V	903	180.90
gta	V	0	187.00
gtg	V	0	348.70
mPD		0	0.73
		nPD:	0.
		N. weight:	1.8
		Sc. PD:	-0.24
		Sc. rank:	-1240.0

PA-X

Pos.	193	obs:	exp:
tct	S	0	142.30
tcc	S	0	115.10
tca	S	0	224.40
tcg	S	0	51.73
aat	N	14	7.12
aac	N	0	6.88
agt	S	889	182.20
agc	S	0	173.40
mPD		0.031	1.7
		nPD:	0.02
		N. weight:	1.8
		Sc. PD:	-0.2
		Sc. rank:	-453.8

PA-X

Pos.	194	obs:	exp:
tta	L	0	14.41
ttg	L	0	30.60
ctt	L	0	27.79
ctc	L	0	27.48
cta	L	0	27.34
ctg	L	164	36.37
cct	P	0	184.10
ccc	P	0	129.10
cca	P	0	295.80
ccg	P	724	115.00
caa	Q	0	7.70
cag	Q	15	7.30
mPD		0.32	1.1
		nPD:	0.28
		N. weight:	2.
		Sc. PD:	0.29
		Sc. rank:	978.7

PA-X

Pos.	195	obs:	exp:
cgt	R	0	3.25
cgc	R	0	3.86
cga	R	0	7.58
cgg	R	0	6.33
aaa	K	827	484.50
aag	K	0	342.50
aga	R	76	34.70
agg	R	0	20.28
mPD		0.15	0.69
		nPD:	0.22
		N. weight:	0.63
		Sc. PD:	0.054
		Sc. rank:	256.5

PA-X

Pos.	196	obs:	exp:
gaa	E	0	543.20
gag	E	903	359.80
mPD		0	0.48
		nPD:	0.
		N. weight:	1.1
		Sc. PD:	-0.14
		Sc. rank:	-709.6

PA-X

Pos.	197	obs:	exp:
gct	A	0	231.40
gcc	A	0	169.60
gca	A	0	422.20
gcg	A	903	79.87
mPD		0	0.67
		nPD:	0.
		N. weight:	2.8
		Sc. PD:	-0.36
		Sc. rank:	-1870.6

PA-X

Pos.	198	obs:	exp:
cgt	R	0	2.56
cgc	R	0	3.05
cga	R	0	5.98
cgg	R	0	5.00
aaa	K	0	493.80
aag	K	843	349.20
aga	R	0	27.39
agg	R	60	16.01
mPD		0.12	0.65
		nPD:	0.19
		N. weight:	1.
		Sc. PD:	0.056
		Sc. rank:	326.3

PA-X

Pos.	199	obs:	exp:
cgt	R	0	3.89
cgc	R	0	4.62
cga	R	0	9.07
cgg	R	0	7.58
aaa	K	811	475.70
aag	K	1	336.30
aga	R	91	41.55
agg	R	0	24.29
mPD		0.18	0.73
		nPD:	0.25
		N. weight:	0.63
		Sc. PD:	0.071
		Sc. rank:	275.7

PA-X

Pos.	200	obs:	exp:
caa	Q	900	463.80
cag	Q	3	439.20
mPD		0.0066	0.50
		nPD:	0.01
		N. weight:	0.74
		Sc. PD:	-0.086
		Sc. rank:	-250.2

PA-X

Pos.	201	obs:	exp:
tta	L	0	79.36
ttg	L	901	168.50
ctt	L	0	153.00
ctc	L	0	151.30
cta	L	0	150.50
ctg	L	2	200.30
mPD		0.0044	1.1
		nPD:	0.
		N. weight:	1.9
		Sc. PD:	-0.24
		Sc. rank:	-898.7

PA-X

Pos.	202	obs:	exp:
cgt	R	0	0.09
cgc	R	0	0.10
cga	R	0	0.20
cgg	R	0	0.17
aaa	K	0	527.80
aag	K	901	373.20
aga	R	0	0.91
agg	R	2	0.53
mPD		0.0044	0.49
		nPD:	0.01
		N. weight:	1.
		Sc. PD:	-0.12
		Sc. rank:	-402.3

PA-X

Pos.	203	obs:	exp:
cgt	R	0	1.45
cgc	R	0	1.73
cga	R	0	3.39
cgg	R	0	2.83
aaa	K	869	509.10
aag	K	0	359.90
aga	R	34	15.52
agg	R	0	9.07
mPD		0.073	0.58
		nPD:	0.12
		N. weight:	0.62
		Sc. PD:	-0.0059
		Sc. rank:	109.3

PA-X

Pos.	204	obs:	exp:
aat	N	4	2.03
aac	N	0	1.97
gat	D	894	482.60
gac	D	0	411.40
ggt	G	5	0.67
ggc	G	0	0.64
gga	G	0	2.31
ggg	G	0	1.38
mPD		0.020	0.52
		nPD:	0.04
		N. weight:	0.71
		Sc. PD:	-0.066
		Sc. rank:	-67.4

PA-X

Pos.	205	obs:	exp:
tta	L	2	79.36
ttg	L	901	168.50
ctt	L	0	153.00
ctc	L	0	151.30
cta	L	0	150.50
ctg	L	0	200.30
mPD		0.0044	1.1
		nPD:	0.
		N. weight:	1.9
		Sc. PD:	-0.24
		Sc. rank:	-899.8

PA-X

Pos.	206	obs:	exp:
cgt	R	0	0.51
cgc	R	0	0.61
cga	R	0	1.20
cgg	R	0	1.00
act	T	0	1.55
acc	T	0	1.22
aca	T	6	2.76
acg	T	0	0.47
aaa	K	885	518.40
aag	K	0	366.60
aga	R	12	5.48
agg	R	0	3.20
mPD		0.039	0.54
		nPD:	0.07
		N. weight:	0.62
		Sc. PD:	-0.036
		Sc. rank:	0.5

PA-X

Pos.	207	obs:	exp:
tta	L	42	4.13
ttg	L	5	8.77
tct	S	0	137.00
tcc	S	0	110.80
tca	S	856	216.00
tcg	S	0	49.81
ctt	L	0	7.96
ctc	L	0	7.87
cta	L	0	7.84
ctg	L	0	10.42
agt	S	0	175.40
agc	S	0	166.90
mPD		0.11	1.7
		nPD:	0.06
		N. weight:	1.6
		Sc. PD:	-0.11
		Sc. rank:	-57.6

PA-X

Pos.	208	obs:	exp:
caa	Q	0	462.80
cag	Q	901	438.20
aaa	K	0	1.17
aag	K	2	0.83
mPD		0.0044	0.50
		nPD:	0.01
		N. weight:	0.82
		Sc. PD:	-0.099
		Sc. rank:	-336.4

PA-X

Pos.	209	obs:	exp:
gct	A	0	0.77
gcc	A	0	0.56
gca	A	3	1.40
gcg	A	0	0.27
gaa	E	895	538.40
gag	E	0	356.60
ggt	G	0	0.67
ggc	G	0	0.64
gga	G	5	2.31
ggg	G	0	1.38
mPD		0.018	0.50
		nPD:	0.04
		N. weight:	0.58
		Sc. PD:	-0.056
		Sc. rank:	-70.5

PA-X

Pos.	210	obs:	exp:
tta	L	0	0.35
ttg	L	0	0.75
ctt	L	0	0.68
ctc	L	0	0.67
cta	L	4	0.67
ctg	L	0	0.89
cct	P	0	228.10
ccc	P	6	159.90
cca	P	891	366.50
ccg	P	0	142.50
caa	Q	2	1.03
cag	Q	0	0.97
mPD		0.026	0.73
		nPD:	0.04
		N. weight:	0.99
		Sc. PD:	-0.093
		Sc. rank:	-102.0

PA-X

Pos. 211		obs:	exp:
tat	Y	0	0.53
tac	Y	1	0.47
tgt	C	0	370.30
tgc	C	902	531.70
mPD		0.0022	0.49
		nPD:	0.
		N. weight:	0.6
		Sc. PD:	-0.075
		Sc. rank:	-274.1

PA-X

Pos. 212		obs:	exp:
tct	S	0	0.32
tcc	S	0	0.26
tca	S	2	0.50
tcg	S	0	0.12
agt	S	0	0.41
agc	S	0	0.39
gtt	V	0	1.45
gtc	V	0	1.40
gta	V	7	1.45
gtg	V	0	2.70
gct	A	0	228.30
gcc	A	0	167.30
gca	A	891	416.60
gcg	A	0	78.81
gaa	E	3	1.80
gag	E	0	1.20
mPD		0.026	0.70
		nPD:	0.04
		N. weight:	0.88
		Sc. PD:	-0.081
		Sc. rank:	-86.1

PA-X

Pos. 213		obs:	exp:
ggt	G	1	121.10
ggc	G	902	114.70
gga	G	0	417.50
ggg	G	0	249.70
mPD		0.0022	0.68
		nPD:	0.
		N. weight:	2.3
		Sc. PD:	-0.3
		Sc. rank:	-1148.9

PA-X

Pos. 214		obs:	exp:
tta	L	0	75.58
ttg	L	860	160.50
tct	S	0	6.88
tcc	S	0	5.57
tca	S	0	10.85
tcg	S	43	2.50
ctt	L	0	145.70
ctc	L	0	144.10
cta	L	0	143.40
ctg	L	0	190.70
agt	S	0	8.81
agc	S	0	8.39
mPD		0.091	1.2
		nPD:	0.07
		N. weight:	2.
		Sc. PD:	-0.12
		Sc. rank:	-5.0

PA-X

Pos. 215		obs:	exp:
tta	L	0	3.78
ttg	L	0	8.02
ctt	L	0	7.29
ctc	L	0	7.20
cta	L	1	7.17
ctg	L	42	9.54
cct	P	0	217.40
ccc	P	0	152.40
cca	P	1	349.30
ccg	P	854	135.80
caa	Q	0	2.57
cag	Q	5	2.43
mPD		0.11	0.84
		nPD:	0.13
		N. weight:	2.1
		Sc. PD:	-0.018
		Sc. rank:	385.7

PA-X

Pos. 216		obs:	exp:
att	I	0	2.19
atc	I	6	1.45
ata	I	0	2.36
act	T	0	232.40
acc	T	897	182.70
aca	T	0	412.10
acg	T	0	69.87
mPD		0.013	0.69
		nPD:	0.02
		N. weight:	1.8
		Sc. PD:	-0.2
		Sc. rank:	-441.5

PA-X

Pos. 217		obs:	exp:
aaa	K	903	529.00
aag	K	0	374.00
mPD		0	0.49
		nPD:	0.
		N. weight:	0.61
		Sc. PD:	-0.079
		Sc. rank:	-412.5

PA-X

Pos. 218		obs:	exp:
gtt	V	0	185.20
gtc	V	897	179.70
gta	V	0	185.70
gtg	V	0	346.40
gct	A	0	1.54
gcc	A	6	1.13
gca	A	0	2.81
gcg	A	0	0.53
mPD		0.013	0.74
		nPD:	0.02
		N. weight:	1.8
		Sc. PD:	-0.21
		Sc. rank:	-507.5

PA-X

Pos. 219		obs:	exp:
ttt	F	0	0.39
ttc	F	1	0.61
tct	S	0	144.40
tcc	S	902	116.80
tca	S	0	227.60
tcg	S	0	52.49
agt	S	0	184.80
agc	S	0	175.90
mPD		0.0022	1.7
		nPD:	0.
		N. weight:	2.3
		Sc. PD:	-0.3
		Sc. rank:	-1268.8

PA-X

Pos. 220		obs:	exp:
cat	H	0	518.80
cac	H	903	384.20
mPD		0	0.49
		nPD:	0.
		N. weight:	0.98
		Sc. PD:	-0.13
		Sc. rank:	-659.0

PA-X

Pos. 221		obs:	exp:
tta	L	0	0.18
ttg	L	0	0.37
ctt	L	0	0.34
ctc	L	0	0.34
cta	L	2	0.33
ctg	L	0	0.44
cgt	R	0	38.49
cgc	R	0	45.78
cga	R	898	89.84
cgg	R	3	75.04
aga	R	0	411.40
agg	R	0	240.50
mPD		0.011	0.97
		nPD:	0.01
		N. weight:	2.6
		Sc. PD:	-0.31
		Sc. rank:	-895.4

PA-X

Pos. 222		obs:	exp:
att	I	16	5.84
atc	I	0	3.87
ata	I	0	6.29
act	T	884	229.00
acc	T	0	180.00
aca	T	0	406.10
acg	T	0	68.86
gtt	V	3	0.62
gtc	V	0	0.60
gta	V	0	0.62
gtg	V	0	1.16
mPD		0.048	0.72
		nPD:	0.07
		N. weight:	1.5
		Sc. PD:	-0.1
		Sc. rank:	-24.4

PA-X

Pos. 223		obs:	exp:
tta	L	0	0.09
ttg	L	0	0.19
tct	S	890	144.40
tcc	S	0	116.80
tca	S	12	227.60
tcg	S	0	52.49
ctt	L	0	0.17
ctc	L	1	0.17
cta	L	0	0.17
ctg	L	0	0.22
agt	S	0	184.80
agc	S	0	175.90
mPD		0.033	1.7
		nPD:	0.02
		N. weight:	2.
		Sc. PD:	-0.22
		Sc. rank:	-449.4

PA-X

Pos. 224		obs:	exp:
cct	P	0	229.60
ccc	P	0	161.00
cca	P	903	368.90
ccg	P	0	143.50
mPD		0	0.71
		nPD:	0.
		N. weight:	1.
		Sc. PD:	-0.13
		Sc. rank:	-690.4

PA-X

Pos. 225		obs:	exp:
gct	A	35	231.10
gcc	A	867	169.40
gca	A	0	421.70
gcg	A	0	79.78
ggt	G	0	0.13
ggc	G	1	0.13
gga	G	0	0.46
ggg	G	0	0.28
mPD		0.077	0.67
		nPD:	0.11
		N. weight:	1.7
		Sc. PD:	-0.034
		Sc. rank:	173.4

PA-X

Pos. 226		obs:	exp:
tta	L	0	79.27
ttg	L	902	168.30
ctt	L	0	152.90
ctc	L	0	151.10
cta	L	0	150.40
ctg	L	0	200.00
atg	M	1	1.00
mPD		0.0022	1.1
		nPD:	0.
		N. weight:	1.9
		Sc. PD:	-0.24
		Sc. rank:	-1001.8

PA-X

Pos. 227		obs:	exp:
cgt	R	0	0.13
cgc	R	0	0.15
cga	R	0	0.30
cgg	R	0	0.25
aaa	K	900	527.20
aag	K	0	372.80
aga	R	3	1.37
agg	R	0	0.80
mPD		0.0066	0.49
		nPD:	0.01
		N. weight:	0.61
		Sc. PD:	-0.071
		Sc. rank:	-200.4

PA-X

Pos. 228		obs:	exp:
att	I	1	0.36
atc	I	0	0.24
ata	I	0	0.39
act	T	897	232.40
acc	T	0	182.70
aca	T	0	412.10
acg	T	0	69.87
gct	A	5	1.28
gcc	A	0	0.94
gca	A	0	2.34
gcg	A	0	0.44
mPD		0.013	0.69
		nPD:	0.02
		N. weight:	1.5
		Sc. PD:	-0.17
		Sc. rank:	-362.0

PA-X

Pos. 229		obs:	exp:
tta	L	903	79.36
ttg	L	0	168.50
ctt	L	0	153.00
ctc	L	0	151.30
cta	L	0	150.50
ctg	L	0	200.30
mPD		0	1.1
		nPD:	0.
		N. weight:	2.8
		Sc. PD:	-0.36
		Sc. rank:	-1875.5

PA-X

Pos. 230		obs:	exp:
gaa	E	6	542.60
gag	E	896	359.40
ggt	G	0	0.13
ggc	G	0	0.13
gga	G	0	0.46
ggg	G	1	0.28
mPD		0.015	0.48
		nPD:	0.03
		N. weight:	1.
		Sc. PD:	-0.099
		Sc. rank:	-145.0

PA-X

Pos. 231		obs:	exp:
cct	P	903	229.60
ccc	P	0	161.00
cca	P	0	368.90
ccg	P	0	143.50
mPD		0	0.71
		nPD:	0.
		N. weight:	1.6
		Sc. PD:	-0.2
		Sc. rank:	-1056.1

PA-X

Pos. 232		obs:	exp:
atg	M	903	903.00
mPD		0	0
		nPD:	1.
		N. weight:	0.
		Sc. PD:	0
		Sc. rank:	0

PA-X

Pos. 233		obs:	exp:
tgg	W	903	903.00
mPD		0	0
		nPD:	1.
		N. weight:	0.
		Sc. PD:	0
		Sc. rank:	0

PA-X

Pos. 234		obs:	exp:
atg	M	903	903.00
mPD		0	0
		nPD:	1.
		N. weight:	0.
		Sc. PD:	0
		Sc. rank:	0

PA-X

Pos. 235		obs:	exp:
gat	D	903	487.50
gac	D	0	415.50
mPD		0	0.50
		nPD:	0.
		N. weight:	0.7
		Sc. PD:	-0.091
		Sc. rank:	-475.5

PA-X

Pos. 236		obs:	exp:
tta	L	0	0.62
ttg	L	7	1.31
tct	S	0	143.40
tcc	S	0	116.00
tca	S	5	226.10
tcg	S	891	52.14
ctt	L	0	1.19
ctc	L	0	1.17
cta	L	0	1.17
ctg	L	0	1.55
agt	S	0	183.60
agc	S	0	174.70
---	--	------	------
mPD		0.026	1.7
		nPD:	0.02
		N. weight:	3.2
		Sc. PD:	-0.36
		Sc. rank:	-938.7

,

PA-X

Pos. 237		obs:	exp:
tct	S	0	0.48
tcc	S	0	0.39
tca	S	0	0.76
tcg	S	0	0.17
act	T	0	0.26
acc	T	1	0.20
aca	T	0	0.46
acg	T	0	0.08
aat	N	0	456.90
aac	N	898	441.10
agt	S	0	0.61
agc	S	3	0.59
gat	D	0	0.54
gac	D	1	0.46
---	--	------	------
mPD		0.011	0.52
		nPD:	0.02
		N. weight:	0.82
		Sc. PD:	-0.089
		Sc. rank:	-179.2

,

PA-X

Pos. 238		obs:	exp:
cgt	R	0	38.58
cgc	R	0	45.88
cga	R	903	90.04
cgg	R	0	75.21
aga	R	0	412.30
agg	R	0	241.00
---	--	------	------
mPD		0	0.96
		nPD:	0.
		N. weight:	2.6
		Sc. PD:	-0.34
		Sc. rank:	-1778.1

,

PA-X

Pos. 239		obs:	exp:
atg	M	1	1.00
act	T	0	233.40
acc	T	0	183.50
aca	T	0	413.90
acg	T	901	70.19
aaa	K	0	0.59
aag	K	1	0.41
---	--	------	------
mPD		0.0044	0.68
		nPD:	0.01
		N. weight:	2.9
		Sc. PD:	-0.36
		Sc. rank:	-1239.3

,

PA-X

Pos. 240		obs:	exp:
gct	A	903	231.40
gcc	A	0	169.60
gca	A	0	422.20
gcg	A	0	79.87
---	--	------	------
mPD		0	0.67
		nPD:	0.
		N. weight:	1.6
		Sc. PD:	-0.2
		Sc. rank:	-1050.2

,

PA-X

Pos. 241		obs:	exp:
act	T	0	0.78
acc	T	0	0.61
aca	T	3	1.38
acg	T	0	0.23
gct	A	0	230.60
gcc	A	0	169.00
gca	A	900	420.80
gcg	A	0	79.60
---	--	------	------
mPD		0.0066	0.68
		nPD:	0.01
		N. weight:	0.87
		Sc. PD:	-0.1
		Sc. rank:	-326.4

,

PA-X

Pos. 242		obs:	exp:
tta	L	0	79.27
ttg	L	902	168.30
tct	S	0	0.16
tcc	S	0	0.13
tca	S	0	0.25
tcg	S	1	0.06
ctt	L	0	152.90
ctc	L	0	151.10
cta	L	0	150.40
ctg	L	0	200.00
agt	S	0	0.20
agc	S	0	0.20
---	--	------	------
mPD		0.0022	1.1
		nPD:	0.
		N. weight:	1.9
		Sc. PD:	-0.24
		Sc. rank:	-1023.5

,

PA-X

Pos. 243		obs:	exp:
cgt	R	0	38.58
cgc	R	0	45.88
cga	R	0	90.04
cgg	R	0	75.21
aga	R	0	412.30
agg	R	903	241.00
---	--	------	------
mPD		0	0.96
		nPD:	0.
		N. weight:	1.5
		Sc. PD:	-0.19
		Sc. rank:	-1018.8

,

PA-X

Pos. 244		obs:	exp:
gtt	V	0	0.21
gtc	V	0	0.20
gta	V	1	0.21
gtg	V	0	0.39
gct	A	0	230.90
gcc	A	0	169.20
gca	A	901	421.30
gcg	A	0	79.69
gaa	E	1	0.60
gag	E	0	0.40
---	--	------	------
mPD		0.0044	0.68
		nPD:	0.01
		N. weight:	0.87
		Sc. PD:	-0.11
		Sc. rank:	-366.0

,

PA-X

Pos. 245		obs:	exp:
tct	S	0	144.40
tcc	S	0	116.80
tca	S	0	227.60
tcg	S	0	52.49
cgt	R	0	0.04
cgc	R	0	0.05
cga	R	0	0.10
cgg	R	0	0.08
agt	S	0	184.80
agc	S	902	175.90
aga	R	1	0.46
agg	R	0	0.27
---	--	------	------
mPD		0.0022	1.7
		nPD:	0.
		N. weight:	1.9
		Sc. PD:	-0.24
		Sc. rank:	-1038.3

,

PA-X

Pos. 246		obs:	exp:
ttt	F	900	355.20
ttc	F	0	544.80
tta	L	0	0.26
ttg	L	3	0.56
ctt	L	0	0.51
ctc	L	0	0.50
cta	L	0	0.50
ctg	L	0	0.67
---	--	------	------
mPD		0.0066	0.48
		nPD:	0.01
		N. weight:	1.1
		Sc. PD:	-0.12
		Sc. rank:	-342.6

,

PA-X

Pos. 247		obs:	exp:
tta	L	0	79.36
ttg	L	0	168.50
ctt	L	0	153.00
ctc	L	903	151.30
cta	L	0	150.50
ctg	L	0	200.30
---	--	------	------
mPD		0	1.1
		nPD:	0.
		N. weight:	2.
		Sc. PD:	-0.26
		Sc. rank:	-1377.9

,

PA-X

Pos. 248		obs:	exp:
cgt	R	0	14.65
cgc	R	0	17.43
cga	R	0	34.20
cgg	R	0	28.57
aaa	K	560	328.00
aag	K	0	232.00
aga	R	343	156.60
agg	R	0	91.54
---	--	------	------
mPD		0.47	1.2
		nPD:	0.4
		N. weight:	0.72
		Sc. PD:	0.18
		Sc. rank:	409.2

,

PA-X

Pos. 249		obs:	exp:
tgt	C	899	369.50
tgc	C	1	530.50
tgg	W	3	3.00
---	--	------	------
mPD		0.0088	0.49
		nPD:	0.02
		N. weight:	1.
		Sc. PD:	-0.11
		Sc. rank:	-266.9

,

PA-X

Pos. 250		obs:	exp:
tct	S	0	0.16
tcc	S	1	0.13
tca	S	0	0.25
tcg	S	0	0.06
cct	P	0	8.65
ccc	P	0	6.06
cca	P	34	13.89
ccg	P	0	5.40
caa	Q	865	445.30
cag	Q	2	421.70
cgt	R	0	0.04
cgc	R	0	0.05
cga	R	1	0.10
cgg	R	0	0.08
agt	S	0	0.20
agc	S	0	0.20
aga	R	0	0.46
agg	R	0	0.27
---	--	------	------
mPD		0.086	0.60
		nPD:	0.14
		N. weight:	0.76
		Sc. PD:	0.0064
		Sc. rank:	190.8

,

PA-X

Pos. 251		obs:	exp:
cgt	R	0	0.21
cgc	R	0	0.25
cga	R	0	0.50
cgg	R	0	0.42
aaa	K	1	524.90
aag	K	895	371.10
aga	R	0	2.28
agg	R	5	1.33
gaa	E	0	1.20
gag	E	2	0.80
---	--	------	------
mPD		0.018	0.50
		nPD:	0.04
		N. weight:	1.
		Sc. PD:	-0.095
		Sc. rank:	-124.0

,

PA-X

Pos. 252		obs:	exp:
aaa	K	0	528.40
aag	K	902	373.60
gaa	E	0	0.60
gag	E	1	0.40
---	--	------	------
mPD		0.0022	0.49
		nPD:	0.
		N. weight:	1.
		Sc. PD:	-0.13
		Sc. rank:	-460.2

,

PA-X

Pos. 253		obs:	exp:
taa	.	34	388.40
tag	.	0	228.30
tga	.	869	286.30
---	--	------	------
mPD		0.073	0.81
		nPD:	0.09
		N. weight:	1.1
		Sc. PD:	-0.048
		Sc. rank:	39.1

}

Amino acid level conservation per codon

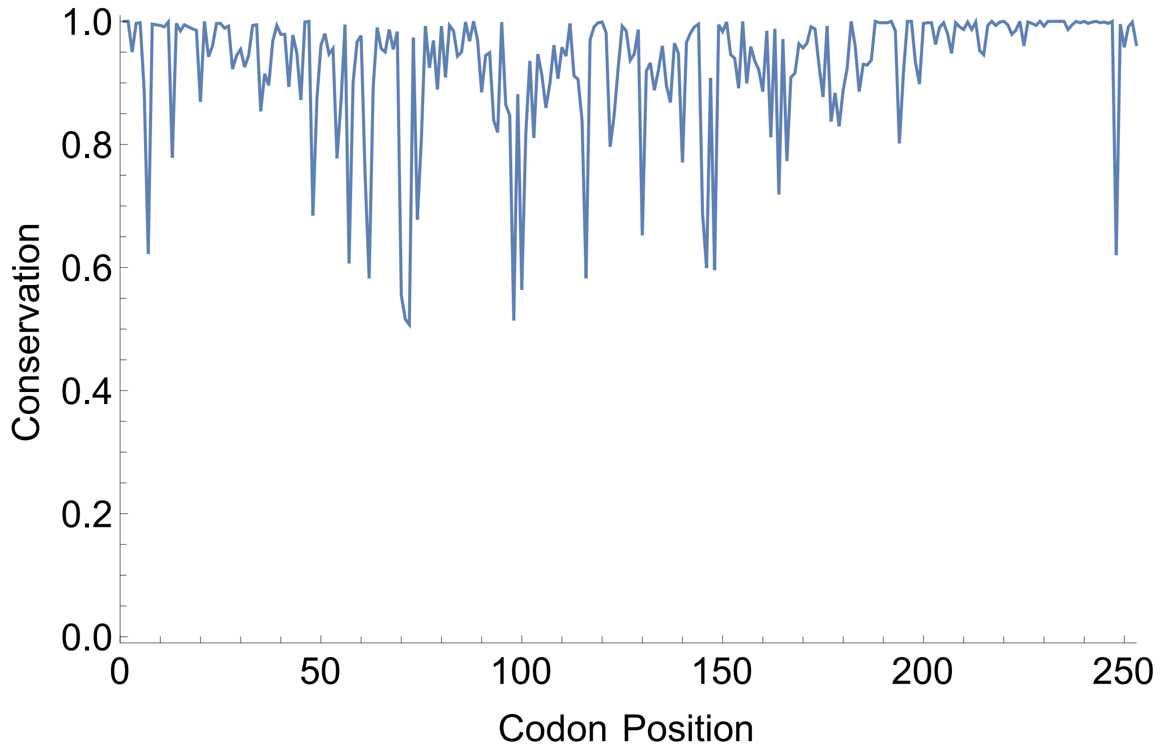

Conservation analysis plots

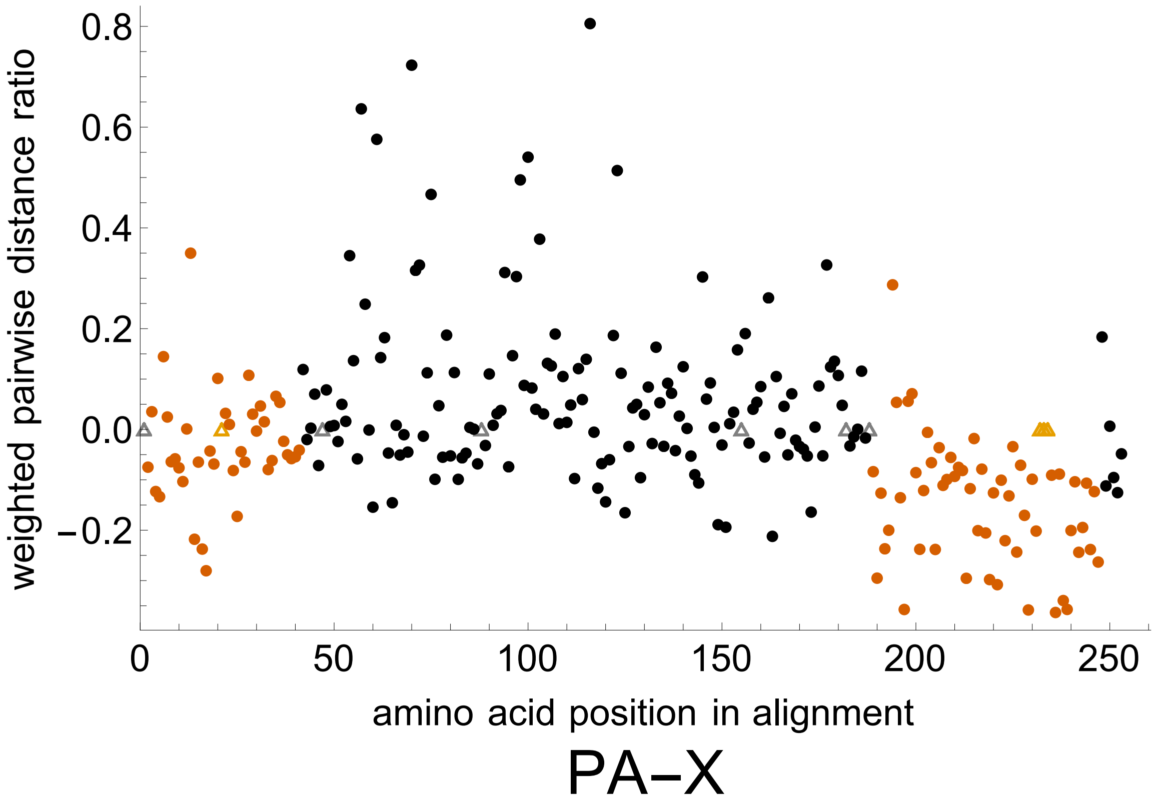

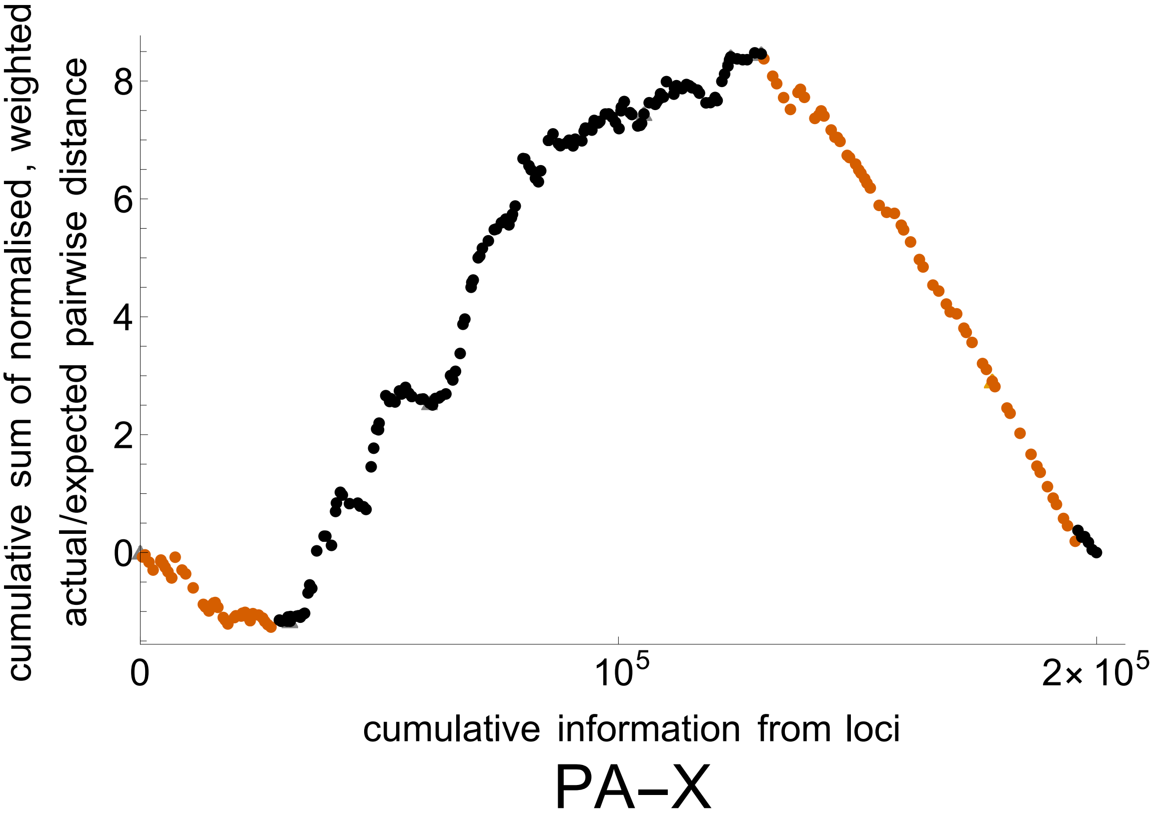

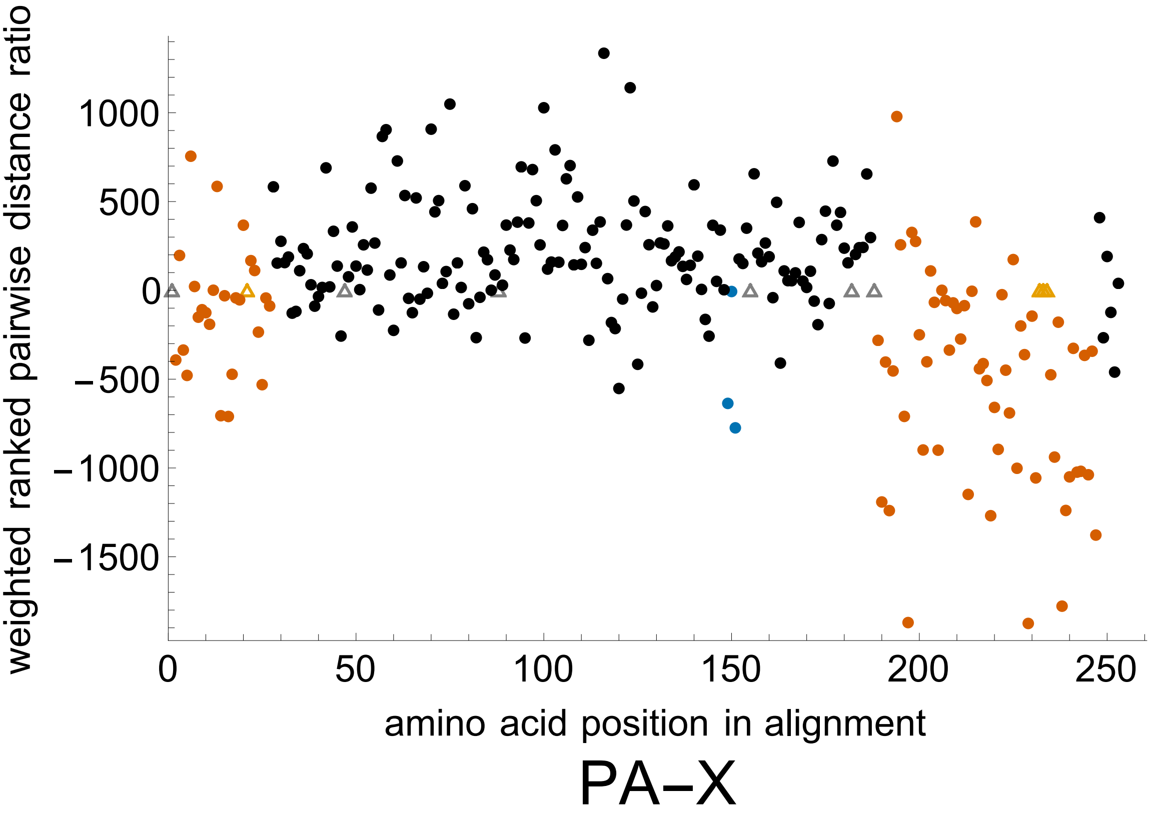
weighted ranked pairwise distance ratio
1000
500
0
−500
−1000
−1500
0
50
100
150
200
250
amino acid position in alignment
PA−X

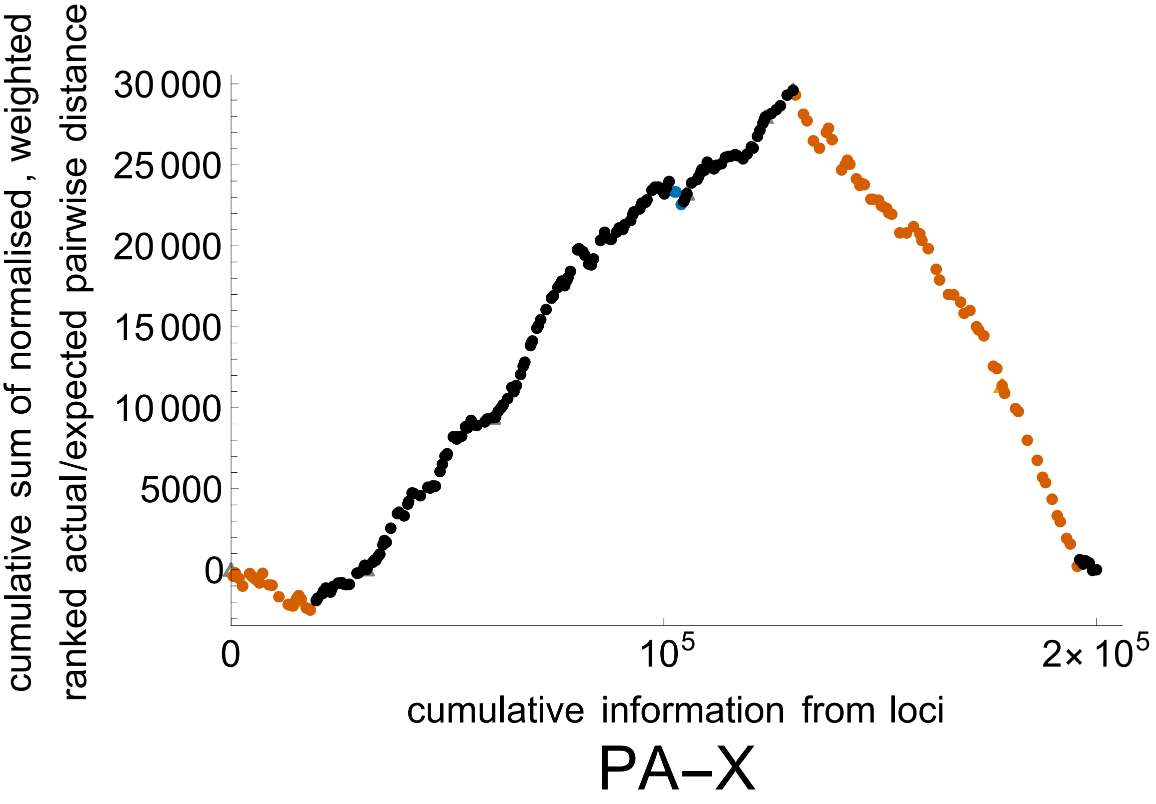
cumulative sum of normalised, weighted
ranked actual/expected pairwise distance
30 000
25 000
20 000
15 000
10 000
5000
0
0
10^5
2×10^5
cumulative information from loci
PA−X

HA

Gene length histogram

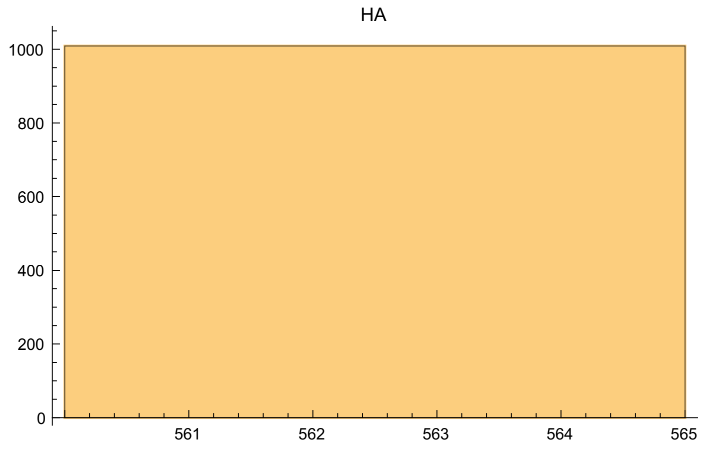

Information vs. nPD

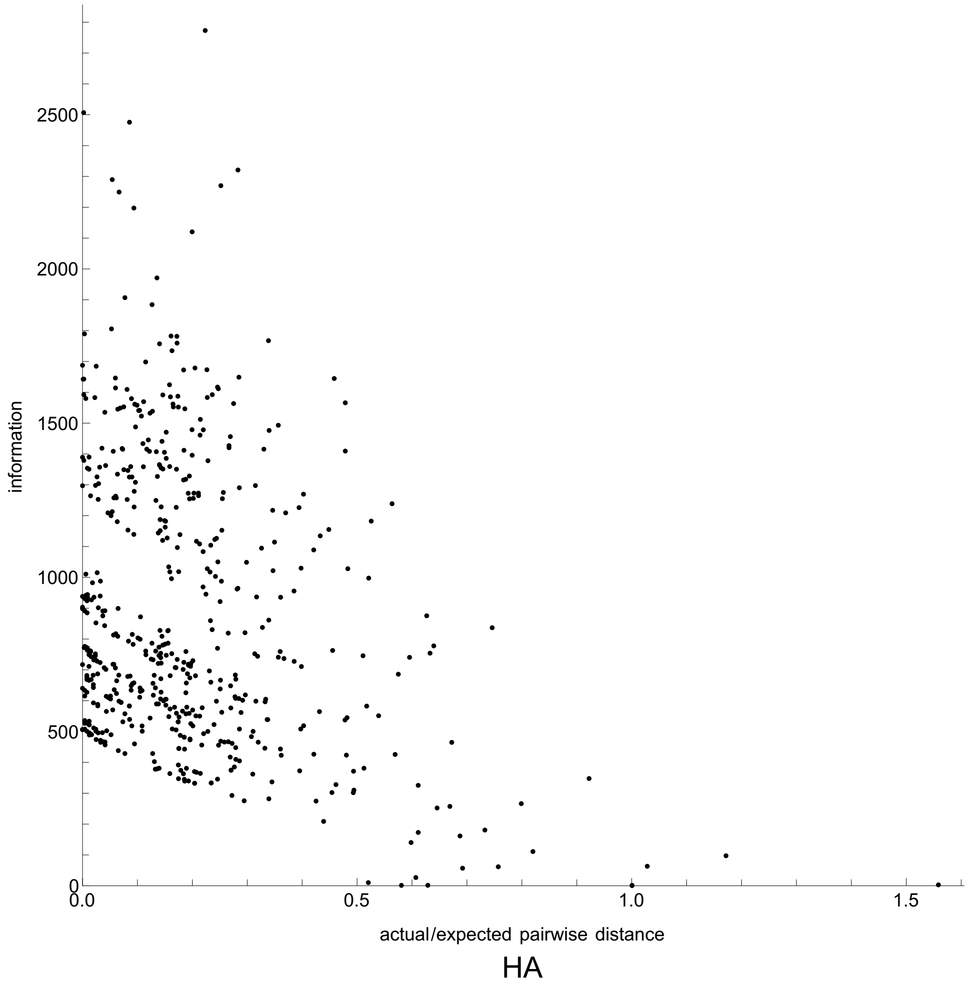

Example sequences highlighted by regions found in analyses to be conserved

Interesting points (by weighted raw PD) highlighted for gene HA:

ATGAATATTCAAATCTTGGTATTCGCTCTGGTGGCGATCATTCCAACAAATGCAGACAAGATCTGCCTTGGGCACC
ATGCCGTGTCAAACGGAACTAAAGTAAACACATTAACTGAAAGGGGAGTGGAAGTCGTTAATGCAACTGAAACGG
TGGAACGAACAAATGTCCCCAGGATCTGCTCAAAAGGGAAAAGAACAGTTGACCTCGGTCAATGTGGACTTCTGG
GAACGATCACTGGACCACCCCAATGTGACCAATTCCTAGAATTTTCAGCCGATTTAATTATTGAAAGGCGAGGAG
GAAGTGATGTTTGTTATCCTGGGAAATTCGTGAATGAAGAAGCTCTGAGGCAAATTCTCAGGGAGTCGGGCGGAA
TTGACAAGGAGACGATGGGATTCACATACAGCGGGATAAGAACTAATGGAGCAACCAGTGCATGTAGGAGATCAG
GA---TCTTCATTCTATGCAGAGATGAAATGGCTCCTTTCAAACACAGACAATGCTGCTTTCCCACAGATGACTAA
GTCATACAAAAACACAAGGAAAGATCCAGCTCTGATAATATGGGGAATCCACCATTCCGGATCAACCACAGAACA
GACCAAGCTATACGGGAGTGGAAGCAAATTGATAACAGTTGGGAGTTCTAATTACCAACAGTCCTTTGTACCGAG
TCCAGGAGCGAGGCCACAAGTGAATGGCCAATCTGGAAGGATTGACTTTCATTGGCTGATGCTAAACCCCAATGA
CACAGTTACTTTCAGTTTCAATGGGGCCTTCATAGCTCCAGACCGTGCAAGCTTTTTGAAAGGGAAGTCTATGGG
AATTCAGAGTGGAGTACAG ------GTTGATGCAAATTGTGAAGGAGATTGCTATCATAGTGGAGGGACAATAATA
AGTAATTTGCCCTTTCAGAACATAAATAGCAGGGCAGTAGGAAAATGCCCGAGATATGTCAAGCAGGAAAGTCTG
ATGCTGGCAACAGGAATGAAAAATGTTCCCGAACTCCCAAAGGGA --------------------------AGAGGC
CTATTTGGTGCTATAGCGGGTTTCATTGAAAATGGATGGGAAGGTCTGATTGACGGGTGGTATGGCTTCAGGCAC
CAAAATGCACAAGGGGAGGGAACTGCTGCAGATTACAAAAGCACCCAATCAGCAATTGATCAAATAACAGGAAAA
TTAAACCGGCTTATAGAAAAAACCAACCAACAATTTGAGTTGATAGACAATGAATTCACTGAGGTTGAAAAGCAA
ATTGGCAATGTGATAAATTGGACCAGAGATTCCATGACAGAAGTGTGGTCCTATAATGCTGAACTCTTGGTAGCA
ATGGAGAATCAACACACAATTGATCTGGCCGACTCAGAAATGAACAAACTATACGAACGAGTGAAGAGGCAACTG
AGAGAGAATGCTGAAGAAGATGGCACTGGCTGTTTTGAAATATTCCACAAGTGTGATGACGACTGTATGGCCAGT
ATTAGAAACAACACTTATGATCACAGCAAATACAGGGAAGAGGCAATGCAAAATAGAATACAGATTAACCCGGTC
AAACTAAGCAGCGGTTATAAAGATGTGATACTCTGG
TTTAGCTTCGGGGCATCATGTTTCATACTTCTTGCCATTGCAATGGGCCTTGTCT
TCATATGCGTGAAGAATGGAAACATGCGGTGCACTATTTGTATATAA

Interesting points (by weighted ranked PD) highlighted for gene HA:

ATGAATATTCAAATCTTGGTATTCGCTCTGGTGGCGATCATTCCAACAAATGCAGACAAGATCTGCCTTGGGCACCAT
GCCGTGTCAAACGGAACTAAAGTAAACACATTAACTGAAAGGGGAGTGGAAGTCGTTAATGCAACTGAAACGGTGGA
ACGAACAAATGTCCCCAGGATCTGCTCAAAAGGGAAAAGAACAGTTGACCTCGGTCAATGTGGACTTCTGGGAACGA
TCACTGGACCACCCCAATGTGACCAATTCCTAGAATTTTCAGCCGATTTAATTATTGAAAGGCGAGGAGGAAGTGAT
GTTTGTTATCCTGGGAAATTCGTGAATGAAGAAGCTCTGAGGCAAATTCTCAGGGAGTCGGGCGGAATTGACAAGGA
GACGATGGGATTCACATACAGCGGGATAAGAACTAATGGAGCAACCAGTGCATGTAGGAGATCAGGA ---TCTTCAT
CTATGCAGAGATGAAATGGCTCCTTTCAAACACAGACAATGCTGCTTTCCCACAGATGACTAAGTCATACAAAAA
CACAAGGAAAGATCCAGCTCTGATAATATGGGGAATCCACCATTCCGGATCAACCACAGAACAGACCAAGCTATA
CGGGAGTGGAAGCAAATTGATAACAGTTGGGAGTTCTAATTACCAACAGTCCTTTGTACCGAGTCCAGGAGCGAG
GCCACAAGTGAATGGCCAATCTGGAAGGATTGACTTTCATTGGCTGATGCTAAACCCCAATGACACAGTTACTTT
CAGTTTCAATGGGGCCTTCATAGCTCCAGACCGTGCAAGCTTTTTGAAAGGGAAGTCTATGGGAATTCAGAGTGG
AGTACAG------GTTGATGCAAATTGTGAAGGAGATTGCTATCATAGTGGAGGGACAATAATAAGTAATTTGCCC
TTTCAGAACATAAATAGCAGGGCAGTAGGAAAATGCCCGAGATATGTCAAGCAGGAAAGTCTGATGCTGGCAACA
GGAATGAAAAATGTTCCCGAACTCCCAAAGGGA --------------------------AGAGGCCTATTTGGTGCT
ATAGCGGGTTTCATTGAAAATGGATGGGAAGGTCTGATTGACGGGTGGTATGGCTTCAGGCACCAAAATGCACAA
GGGGAGGGAACTGCTGCAGATTACAAAAGCACCCAATCAGCAATTGATCAAATAACAGGAAAATTAAACCGGCTT
ATAGAAAAAACCAACCAACAATTTGAGTTGATAGACAATGAATTCACTGAGGTTGAAAAGCAAATTGGCAATGTG

ATAAATTGGACCAGAGATTCCATGACAGAAGTGTGGTCCTATAATGCTGAACTCTTGGTAGCAATGGAGAATCAA⋱.

CACACAATTGATCTGGCCGACTCAGAAATGAACAAACTATACGAACGAGTGAAGAGGCAACTGAGAGAGAATGCT⋱.

GAAGAAGATGGCACTGGCTGTTTTGAAATATTCCACAAGTGTGATGACGACTGTATGGCCAGTATTAGAAACAAC⋱.

ACTTATGATCACAGCAAATACAGGGAAGAGGCAATGCAAAATAGAATACAGATTAACCCGGTCAAACTAAGCAGC⋱.

GGTTATAAAGATGTGATACTCTGG

TTTAGCTTCGGGGCATCATGTTTCATACTTCTTGCCATT

GCAATGGGCCTTGTCTTCATATGCGTG

AAGAATGGAAACATGCGGTGCACTATTTGTATATAA

Per codon conservation report

HA

Pos.	1	obs:	exp:
atg	M	1009	1009.00
mPD		0	0
		nPD:	1.
		N. weight:	0.
		Sc. PD:	0
		Sc. rank:	0

HA

Pos.	2	obs:	exp:
tct	S	0	0.16
tcc	S	0	0.13
tca	S	0	0.25
tcg	S	0	0.06
aat	N	1	512.30
aac	N	1006	494.70
agt	S	0	0.20
agc	S	1	0.20
gat	D	0	0.54
gac	D	1	0.46
mPD		0.0059	0.51
		nPD:	0.01
		N. weight:	0.84
		Sc. PD:	-0.12
		Sc. rank:	-494.8

HA

Pos.	3	obs:	exp:
att	I	7	2.92
atc	I	1	1.93
ata	I	0	3.15
act	T	999	258.80
acc	T	0	203.40
aca	T	0	459.00
acg	T	0	77.82
aat	N	2	1.02
aac	N	0	0.98
mPD		0.022	0.69
		nPD:	0.03
		N. weight:	1.6
		Sc. PD:	-0.2
		Sc. rank:	-761.6

HA

Pos.	4	obs:	exp:
tct	S	0	0.16
tcc	S	0	0.13
tca	S	1	0.25
tcg	S	0	0.06
caa	Q	1007	517.80
cag	Q	1	490.20
agt	S	0	0.20
agc	S	0	0.20
mPD		0.0059	0.50
		nPD:	0.01
		N. weight:	0.79
		Sc. PD:	-0.11
		Sc. rank:	-461.8

HA

Pos.	5	obs:	exp:
att	I	47	363.00
atc	I	948	240.70
ata	I	0	391.30
act	T	0	2.85
acc	T	11	2.24
aca	T	0	5.05
acg	T	0	0.86
aat	N	0	1.02
aac	N	2	0.98
gtt	V	0	0.21
gtc	V	1	0.20
gta	V	0	0.21
gtg	V	0	0.39
mPD		0.12	0.68
		nPD:	0.17
		N. weight:	1.5
		Sc. PD:	0.0096
		Sc. rank:	194.6

HA

Pos.	6	obs:	exp:
tta	L	0	88.68
ttg	L	44	188.30
ctt	L	0	171.00
ctc	L	0	169.00
cta	L	0	168.20
ctg	L	965	223.80
mPD		0.083	1.1
		nPD:	0.08
		N. weight:	1.6
		Sc. PD:	-0.14
		Sc. rank:	-468.1

HA

Pos.	7	obs:	exp:
att	I	0	20.07
atc	I	0	13.30
ata	I	55	21.63
act	T	0	0.26
acc	T	0	0.20
aca	T	1	0.46
acg	T	0	0.08
gtt	V	0	186.40
gtc	V	0	180.90
gta	V	903	187.00
gtg	V	0	348.70
gct	A	0	12.81
gcc	A	0	9.39
gca	A	50	23.38
gcg	A	0	4.42
mPD		0.20	0.94
		nPD:	0.21
		N. weight:	1.8
		Sc. PD:	0.087
		Sc. rank:	588.0

HA

Pos.	8	obs:	exp:
ttt	F	3	376.90
ttc	F	952	578.10
tta	L	0	4.57
ttg	L	0	9.70
ctt	L	0	8.81
ctc	L	52	8.71
cta	L	0	8.67
ctg	L	0	11.53
att	I	0	0.36
atc	I	0	0.24
ata	I	1	0.39
gct	A	0	0.26
gcc	A	1	0.19
gca	A	0	0.47
gcg	A	0	0.09
mPD		0.11	0.59
		nPD:	0.19
		N. weight:	0.66
		Sc. PD:	0.016
		Sc. rank:	148.9

HA

Pos.	9	obs:	exp:
att	I	43	16.05
atc	I	1	10.64
ata	I	0	17.30
act	T	2	0.52
acc	T	0	0.41
aca	T	0	0.92
acg	T	0	0.16
gtt	V	4	0.83
gtc	V	0	0.80
gta	V	0	0.83
gtg	V	0	1.54
gct	A	940	245.70
gcc	A	13	180.10
gca	A	6	448.40
gcg	A	0	84.82
mPD		0.22	0.85
		nPD:	0.25
		N. weight:	1.5
		Sc. PD:	0.13
		Sc. rank:	676.1

HA

Pos.	10	obs:	exp:
tta	L	0	84.55
ttg	L	0	179.50
ctt	L	0	163.00
ctc	L	0	161.20
cta	L	5	160.40
ctg	L	957	213.40
gct	A	45	12.04
gcc	A	2	8.82
gca	A	0	21.97
gcg	A	0	4.16
mPD		0.28	1.3
		nPD:	0.22
		N. weight:	1.8
		Sc. PD:	0.094
		Sc. rank:	600.9

HA

Pos.	11	obs:	exp:
tgt	C	28	19.29
tgc	C	19	27.71
att	I	916	334.20
atc	I	0	221.60
ata	I	0	360.20
atg	M	1	1.00
act	T	2	2.33
acc	T	7	1.83
aca	T	0	4.13
acg	T	0	0.70
gtt	V	12	7.43
gtc	V	0	7.21
gta	V	2	7.45
gtg	V	22	13.90
mPD		0.36	0.93
		nPD:	0.39
		N. weight:	1.1
		Sc. PD:	0.24
		Sc. rank:	707.9

HA

Pos.	12	obs:	exp:
tct	S	0	0.48
tcc	S	0	0.39
tca	S	0	0.76
tcg	S	3	0.17
atg	M	46	46.00
act	T	0	12.43
acc	T	0	9.77
aca	T	0	22.05
acg	T	48	3.74
agt	S	0	0.61
agc	S	0	0.59
gtt	V	0	0.83
gtc	V	0	0.80
gta	V	0	0.83
gtg	V	4	1.54
gct	A	0	232.70
gcc	A	0	170.50
gca	A	2	424.50
gcg	A	906	80.31
mPD		0.27	0.96
		nPD:	0.28
		N. weight:	2.7
		Sc. PD:	0.31
		Sc. rank:	1417.1

HA

Pos.	13	obs:	exp:
tta	L	0	4.13
ttg	L	0	8.77
ctt	L	0	7.96
ctc	L	1	7.87
cta	L	0	7.84
ctg	L	46	10.42
att	I	0	332.00
atc	I	906	220.10
ata	I	4	357.90
act	T	0	12.43
acc	T	48	9.77
aca	T	0	22.05
acg	T	0	3.74
gtt	V	0	0.62
gtc	V	3	0.60
gta	V	0	0.62
gtg	V	0	1.16
gct	A	0	0.26
gcc	A	1	0.19
gca	A	0	0.47
gcg	A	0	0.09
mPD		0.28	0.86
		nPD:	0.33
		N. weight:	1.7
		Sc. PD:	0.27
		Sc. rank:	955.0

HA

Pos.	14	obs:	exp:
ttt	F	2	0.79
ttc	F	0	1.21
att	I	943	346.30
atc	I	6	229.50
ata	I	0	373.20
act	T	1	0.26
acc	T	0	0.20
aca	T	0	0.46
acg	T	0	0.08
gtt	V	57	11.77
gtc	V	0	11.42
gta	V	0	11.80
gtg	V	0	22.01
mPD		0.12	0.78
		nPD:	0.16
		N. weight:	1.2
		Sc. PD:	-0.0055
		Sc. rank:	103.8

HA

Pos.	15	obs:	exp:
tta	L	0	0.18
ttg	L	0	0.37
tct	S	0	0.96
tcc	S	1	0.78
tca	S	5	1.51
tcg	S	0	0.35
ctt	L	0	0.34
ctc	L	2	0.34
cta	L	0	0.33
ctg	L	0	0.44
cct	P	0	242.60
ccc	P	0	170.10
cca	P	934	389.80
ccg	P	20	151.60
agt	S	0	1.23
agc	S	0	1.17
ggt	G	0	6.30
ggc	G	0	5.97
gga	G	45	21.73
ggg	G	2	12.99
mPD		0.24	0.91
		nPD:	0.27
		N. weight:	0.97
		Sc. PD:	0.094
		Sc. rank:	448.8

HA			
Pos.	16	obs:	exp:
att	I	1	2.92
atc	I	0	1.93
ata	I	7	3.15
act	T	4	247.40
acc	T	0	194.50
aca	T	951	438.70
acg	T	0	74.39
gtt	V	2	0.41
gtc	V	0	0.40
gta	V	0	0.41
gtg	V	0	0.77
gct	A	38	11.27
gcc	A	0	8.26
gca	A	6	20.57
gcg	A	0	3.89
---	--	------	------
mPD		0.19	0.78
		nPD:	0.25
		N. weight:	0.91
		Sc. PD:	0.072
		Sc. rank:	376.2

HA			
Pos.	17	obs:	exp:
tct	S	0	0.96
tcc	S	0	0.78
tca	S	0	1.51
tcg	S	0	0.35
cgt	R	0	0.09
cgc	R	0	0.10
cga	R	0	0.20
cgg	R	0	0.17
aat	N	952	484.80
aac	N	1	468.20
aaa	K	45	26.36
aag	K	0	18.64
agt	S	6	1.23
agc	S	0	1.17
aga	R	2	0.91
agg	R	0	0.53
gat	D	3	1.62
gac	D	0	1.38
---	--	------	------
mPD		0.11	0.58
		nPD:	0.2
		N. weight:	0.8
		Sc. PD:	0.024
		Sc. rank:	202.2

HA			
Pos.	18	obs:	exp:
gct	A	0	247.00
gcc	A	1	181.00
gca	A	952	450.70
gcg	A	11	85.26
ggt	G	0	6.03
ggc	G	0	5.72
gga	G	45	20.81
ggg	G	0	12.44
---	--	------	------
mPD		0.11	0.76
		nPD:	0.14
		N. weight:	0.85
		Sc. PD:	-0.017
		Sc. rank:	-1.8

HA			
Pos.	19	obs:	exp:
gat	D	201	544.20
gac	D	807	463.80
ggt	G	1	0.13
ggc	G	0	0.13
gga	G	0	0.46
ggg	G	0	0.28
---	--	------	------
mPD		0.32	0.50
		nPD:	0.65
		N. weight:	0.3
		Sc. PD:	0.14
		Sc. rank:	215.3

HA			
Pos.	20	obs:	exp:
cgt	R	0	0.26
cgc	R	0	0.30
cga	R	0	0.60
cgg	R	0	0.50
aaa	K	976	587.60
aag	K	27	415.40
aga	R	6	2.74
agg	R	0	1.60
---	--	------	------
mPD		0.064	0.50
		nPD:	0.13
		N. weight:	0.51
		Sc. PD:	-0.018
		Sc. rank:	-46.0

HA			
Pos.	21	obs:	exp:
tta	L	0	0.09
ttg	L	0	0.19
ctt	L	0	0.17
ctc	L	1	0.17
cta	L	0	0.17
ctg	L	0	0.22
att	I	28	367.80
atc	I	932	243.80
ata	I	48	396.40
---	--	------	------
mPD		0.14	0.66
		nPD:	0.22
		N. weight:	1.3
		Sc. PD:	0.069
		Sc. rank:	434.7

HA			
Pos.	22	obs:	exp:
tgt	C	47	414.20
tgc	C	962	594.80
---	--	------	------
mPD		0.089	0.48
		nPD:	0.18
		N. weight:	0.43
		Sc. PD:	0.0082
		Sc. rank:	81.4

HA			
Pos.	23	obs:	exp:
tta	L	0	88.68
ttg	L	0	188.30
ctt	L	72	171.00
ctc	L	935	169.00
cta	L	2	168.20
ctg	L	0	223.80
---	--	------	------
mPD		0.14	1.1
		nPD:	0.12
		N. weight:	1.8
		Sc. PD:	-0.071
		Sc. rank:	-196.7

HA			
Pos.	24	obs:	exp:
ggt	G	0	135.30
ggc	G	0	128.20
gga	G	933	466.50
ggg	G	76	279.00
---	--	------	------
mPD		0.14	0.68
		nPD:	0.21
		N. weight:	0.65
		Sc. PD:	0.027
		Sc. rank:	197.1

HA			
Pos.	25	obs:	exp:
cat	H	917	579.70
cac	H	92	429.30
---	--	------	------
mPD		0.17	0.49
		nPD:	0.34
		N. weight:	0.33
		Sc. PD:	0.056
		Sc. rank:	195.9

HA			
Pos.	26	obs:	exp:
cat	H	956	579.70
cac	H	53	429.30
---	--	------	------
mPD		0.10	0.49
		nPD:	0.2
		N. weight:	0.44
		Sc. PD:	0.017
		Sc. rank:	127.8

HA			
Pos.	27	obs:	exp:
tct	S	0	8.80
tcc	S	55	7.12
tca	S	0	13.88
tcg	S	0	3.20
agt	S	0	11.27
agc	S	0	10.73
gct	A	64	244.50
gcc	A	890	179.10
gca	A	0	446.00
gcg	A	0	84.38
---	--	------	------
mPD		0.22	0.83
		nPD:	0.27
		N. weight:	1.7
		Sc. PD:	0.17
		Sc. rank:	824.9

HA			
Pos.	28	obs:	exp:
gtt	V	1	208.30
gtc	V	0	202.10
gta	V	5	208.90
gtg	V	1003	389.70
---	--	------	------
mPD		0.012	0.73
		nPD:	0.02
		N. weight:	1.1
		Sc. PD:	-0.16
		Sc. rank:	-605.1

HA			
Pos.	29	obs:	exp:
tct	S	3	153.00
tcc	S	0	123.70
tca	S	952	241.30
tcg	S	1	55.63
cct	P	0	1.53
ccc	P	0	1.07
cca	P	6	2.45
ccg	P	0	0.95
agt	S	0	195.90
agc	S	0	186.40
gct	A	0	12.04
gcc	A	0	8.82
gca	A	47	21.97
gcg	A	0	4.16
---	--	------	------
mPD		0.11	1.7
		nPD:	0.06
		N. weight:	1.6
		Sc. PD:	-0.15
		Sc. rank:	-540.5

HA			
Pos.	30	obs:	exp:
aat	N	69	512.80
aac	N	939	495.20
aaa	K	1	0.59
aag	K	0	0.41
---	--	------	------
mPD		0.13	0.50
		nPD:	0.26
		N. weight:	0.55
		Sc. PD:	0.05
		Sc. rank:	253.9

HA			
Pos.	31	obs:	exp:
ggt	G	0	135.30
ggc	G	0	128.20
gga	G	866	466.50
ggg	G	143	279.00
---	--	------	------
mPD		0.24	0.68
		nPD:	0.36
		N. weight:	0.52
		Sc. PD:	0.099
		Sc. rank:	321.7

HA			
Pos.	32	obs:	exp:
att	I	0	1.46
atc	I	4	0.97
ata	I	0	1.57
act	T	19	259.60
acc	T	935	204.00
aca	T	45	460.30
acg	T	3	78.05
aaa	K	3	1.76
aag	K	0	1.24
---	--	------	------
mPD		0.15	0.69
		nPD:	0.21
		N. weight:	1.5
		Sc. PD:	0.068
		Sc. rank:	468.2

HA			
Pos.	33	obs:	exp:
cgt	R	0	0.13
cgc	R	0	0.15
cga	R	0	0.30
cgg	R	0	0.25
aaa	K	996	589.30
aag	K	10	416.70
aga	R	3	1.37
agg	R	0	0.80
---	--	------	------
mPD		0.026	0.49
		nPD:	0.05
		N. weight:	0.58
		Sc. PD:	-0.062
		Sc. rank:	-240.0

HA			
Pos.	34	obs:	exp:
att	I	0	2.19
atc	I	0	1.45
ata	I	6	2.36
gtt	V	0	207.10
gtc	V	1	200.90
gta	V	952	207.70
gtg	V	50	387.40
---	--	------	------
mPD		0.11	0.74
		nPD:	0.15
		N. weight:	1.6
		Sc. PD:	-0.027
		Sc. rank:	36.9

HA			
Pos.	35	obs:	exp:
aat	N	0	505.70
aac	N	994	488.30
gat	D	0	8.10
gac	D	15	6.90
---	--	------	------
mPD		0.029	0.53
		nPD:	0.06
		N. weight:	0.85
		Sc. PD:	-0.088
		Sc. rank:	-331.8

HA			
Pos.	36	obs:	exp:
act	T	1	261.40
acc	T	1	205.50
aca	T	1006	463.60
acg	T	1	78.60
---	--	------	------
mPD		0.0059	0.67
		nPD:	0.01
		N. weight:	0.91
		Sc. PD:	-0.13
		Sc. rank:	-552.4

HA			
Pos.	37	obs:	exp:
tta	L	979	88.68
ttg	L	3	188.30
ctt	L	0	171.00
ctc	L	0	169.00
cta	L	26	168.20
ctg	L	1	223.80
---	--	------	------
mPD		0.060	1.1
		nPD:	0.05
		N. weight:	2.7
		Sc. PD:	-0.29
		Sc. rank:	-1068.6

HA			
Pos.	38	obs:	exp:
act	T	957	261.40
acc	T	4	205.50
aca	T	47	463.60
acg	T	1	78.60
---	--	------	------
mPD		0.098	0.67
		nPD:	0.15
		N. weight:	1.3
		Sc. PD:	-0.023
		Sc. rank:	15.7

HA			
Pos.	39	obs:	exp:
gaa	E	957	606.90
gag	E	52	402.10
---	--	------	------
mPD		0.098	0.48
		nPD:	0.2
		N. weight:	0.39
		Sc. PD:	0.015
		Sc. rank:	115.1

HA			
Pos.	40	obs:	exp:
cgt	R	0	37.76
cgc	R	0	44.91
cga	R	0	88.14
cgg	R	0	73.63
aaa	K	122	72.64
aag	K	2	51.36
aga	R	861	403.60
agg	R	23	235.90
ggt	G	0	0.13
ggc	G	0	0.13
gga	G	1	0.46
ggg	G	0	0.28
---	--	------	------
mPD		0.27	1.1
		nPD:	0.23
		N. weight:	0.78
		Sc. PD:	0.052
		Sc. rank:	303.1

HA			
Pos.	41	obs:	exp:
cgt	R	0	0.04
cgc	R	0	0.05
cga	R	0	0.10
cgg	R	0	0.08
aga	R	1	0.46
agg	R	0	0.27
gaa	E	7	4.21
gag	E	0	2.79
ggt	G	1	134.20
ggc	G	0	127.20
gga	G	989	462.80
ggg	G	11	276.80
---	--	------	------
mPD		0.039	0.69
		nPD:	0.06
		N. weight:	0.85
		Sc. PD:	-0.087
		Sc. rank:	-324.2

HA			
Pos.	42	obs:	exp:
att	I	12	16.78
atc	I	34	11.13
ata	I	0	18.09
gtt	V	1	198.60
gtc	V	0	192.70
gta	V	35	199.20
gtg	V	926	371.50
ggt	G	0	0.13
ggc	G	0	0.13
gga	G	0	0.46
ggg	G	1	0.28
---	--	------	------
mPD		0.24	0.82
		nPD:	0.3
		N. weight:	0.97
		Sc. PD:	0.12
		Sc. rank:	520.7

HA			
Pos.	43	obs:	exp:
gaa	E	1001	606.90
gag	E	8	402.10
---	--	------	------
mPD		0.016	0.48
		nPD:	0.03
		N. weight:	0.56
		Sc. PD:	-0.07
		Sc. rank:	-259.3

HA			
Pos.	44	obs:	exp:
gtt	V	1	208.30
gtc	V	950	202.10
gta	V	56	208.90
gtg	V	2	389.70
---	--	------	------
mPD		0.11	0.73
		nPD:	0.15
		N. weight:	1.6
		Sc. PD:	-0.018
		Sc. rank:	92.3

HA			
Pos.	45	obs:	exp:
gtt	V	35	208.30
gtc	V	927	202.10
gta	V	41	208.90
gtg	V	6	389.70
---	--	------	------
mPD		0.15	0.73
		nPD:	0.21
		N. weight:	1.5
		Sc. PD:	0.068
		Sc. rank:	470.9

HA			
Pos.	46	obs:	exp:
tct	S	0	0.16
tcc	S	0	0.13
tca	S	0	0.25
tcg	S	0	0.06
aat	N	1007	512.80
aac	N	1	495.20
agt	S	1	0.20
agc	S	0	0.20
---	--	------	------
mPD		0.0040	0.50
		nPD:	0.01
		N. weight:	0.8
		Sc. PD:	-0.12
		Sc. rank:	-502.2

HA			
Pos.	47	obs:	exp:
tct	S	0	0.16
tcc	S	0	0.13
tca	S	1	0.25
tcg	S	0	0.06
agt	S	0	0.20
agc	S	0	0.20
gct	A	4	258.30
gcc	A	43	189.30
gca	A	955	471.30
gcg	A	6	89.16
---	--	------	------
mPD		0.10	0.68
		nPD:	0.15
		N. weight:	0.69
		Sc. PD:	-0.008
		Sc. rank:	31.7

HA			
Pos.	48	obs:	exp:
act	T	906	261.40
acc	T	56	205.50
aca	T	24	463.60
acg	T	23	78.60
---	--	------	------
mPD		0.19	0.67
		nPD:	0.28
		N. weight:	1.1
		Sc. PD:	0.13
		Sc. rank:	576.1

HA			
Pos.	49	obs:	exp:
gaa	E	1000	606.90
gag	E	9	402.10
---	--	------	------
mPD		0.018	0.48
		nPD:	0.04
		N. weight:	0.55
		Sc. PD:	-0.067
		Sc. rank:	-251.8

HA			
Pos.	50	obs:	exp:
att	I	0	0.73
atc	I	0	0.48
ata	I	2	0.79
act	T	0	260.90
acc	T	1	205.10
aca	T	915	462.60
acg	T	91	78.44
---	--	------	------
mPD		0.17	0.68
		nPD:	0.25
		N. weight:	0.76
		Sc. PD:	0.063
		Sc. rank:	327.0

HA			
Pos.	51	obs:	exp:
gtt	V	12	208.30
gtc	V	0	202.10
gta	V	22	208.90
gtg	V	975	389.70
---	--	------	------
mPD		0.066	0.73
		nPD:	0.09
		N. weight:	0.96
		Sc. PD:	-0.068
		Sc. rank:	-216.3

HA			
Pos.	52	obs:	exp:
gaa	E	969	606.90
gag	E	40	402.10
---	--	------	------
mPD		0.076	0.48
		nPD:	0.16
		N. weight:	0.43
		Sc. PD:	-0.002
		Sc. rank:	37.6

HA			
Pos.	53	obs:	exp:
tct	S	0	0.16
tcc	S	0	0.13
tca	S	0	0.25
tcg	S	0	0.06
cgt	R	0	41.10
cgc	R	0	48.88
cga	R	962	95.92
cgg	R	0	80.12
act	T	41	11.66
acc	T	4	9.16
aca	T	0	20.67
acg	T	0	3.51
aat	N	1	0.51
aac	N	0	0.49
agt	S	1	0.20
agc	S	0	0.20
aga	R	0	439.20
agg	R	0	256.80
---	--	------	------
mPD		0.27	1.1
		nPD:	0.25
		N. weight:	2.7
		Sc. PD:	0.23
		Sc. rank:	1190.6

HA			
Pos.	54	obs:	exp:
att	I	0	0.36
atc	I	0	0.24
ata	I	1	0.39
act	T	0	248.90
acc	T	0	195.70
aca	T	960	441.50
acg	T	1	74.86
aaa	K	1	0.59
aag	K	0	0.41
gtt	V	0	0.21
gtc	V	0	0.20
gta	V	1	0.21
gtg	V	0	0.39
gct	A	0	11.53
gcc	A	0	8.45
gca	A	39	21.04
gcg	A	6	3.98
---	--	------	------
mPD		0.11	0.77
		nPD:	0.14
		N. weight:	0.92
		Sc. PD:	-0.022
		Sc. rank:	-39.3

HA			
Pos.	55	obs:	exp:
tct	S	0	2.24
tcc	S	0	1.81
tca	S	0	3.53
tcg	S	0	0.81
act	T	0	4.14
acc	T	16	3.26
aca	T	0	7.35
acg	T	0	1.25
aat	N	67	498.10
aac	N	912	480.90
agt	S	0	2.87
agc	S	14	2.73
---	--	------	------
mPD		0.18	0.59
		nPD:	0.31
		N. weight:	0.59
		Sc. PD:	0.083
		Sc. rank:	323.1

HA			
Pos.	56	obs:	exp:
att	I	37	305.40
atc	I	799	202.40
ata	I	1	329.20
act	T	1	36.53
acc	T	140	28.71
aca	T	0	64.78
acg	T	0	10.98
gtt	V	8	6.40
gtc	V	23	6.21
gta	V	0	6.42
gtg	V	0	11.97
---	--	------	------
mPD		0.39	0.97
		nPD:	0.4
		N. weight:	1.5
		Sc. PD:	0.34
		Sc. rank:	967.1

,

HA			
Pos.	57	obs:	exp:
cct	P	144	244.60
ccc	P	817	171.50
cca	P	1	393.00
ccg	P	0	152.80
aaa	K	0	26.95
aag	K	46	19.05
gaa	E	0	0.60
gag	E	1	0.40
---	--	------	------
mPD		0.50	0.89
		nPD:	0.56
		N. weight:	1.5
		Sc. PD:	0.56
		Sc. rank:	1034.8

,

HA			
Pos.	58	obs:	exp:
cgt	R	0	41.10
cgc	R	0	48.88
cga	R	1	95.92
cgg	R	26	80.12
aaa	K	46	27.53
aag	K	1	19.47
aga	R	13	439.20
agg	R	922	256.80
---	--	------	------
mPD		0.25	1.0
		nPD:	0.24
		N. weight:	1.3
		Sc. PD:	0.1
		Sc. rank:	547.3

,

HA			
Pos.	59	obs:	exp:
tta	L	1	0.09
ttg	L	0	0.19
ctt	L	0	0.17
ctc	L	0	0.17
cta	L	0	0.17
ctg	L	0	0.22
att	I	39	367.80
atc	I	923	243.80
ata	I	46	396.40
---	--	------	------
mPD		0.16	0.66
		nPD:	0.25
		N. weight:	1.2
		Sc. PD:	0.099
		Sc. rank:	524.4

,

HA			
Pos.	60	obs:	exp:
tgt	C	1	414.20
tgc	C	1008	594.80
---	--	------	------
mPD		0.0020	0.48
		nPD:	0.
		N. weight:	0.62
		Sc. PD:	-0.095
		Sc. rank:	-407.9

,

HA			
Pos.	61	obs:	exp:
tct	S	0	154.00
tcc	S	0	124.50
tca	S	958	242.80
tcg	S	4	55.98
act	T	44	12.18
acc	T	2	9.57
aca	T	1	21.59
acg	T	0	3.66
agt	S	0	197.10
agc	S	0	187.60
---	--	------	------
mPD		0.18	1.7
		nPD:	0.11
		N. weight:	1.6
		Sc. PD:	-0.082
		Sc. rank:	-225.8

,

HA			
Pos.	62	obs:	exp:
caa	Q	12	22.60
cag	Q	32	21.40
cgt	R	0	0.17
cgc	R	0	0.20
cga	R	0	0.40
cgg	R	2	0.33
aaa	K	958	562.90
aag	K	3	398.10
aga	R	2	1.83
agg	R	0	1.07
---	--	------	------
mPD		0.17	0.58
		nPD:	0.29
		N. weight:	0.6
		Sc. PD:	0.07
		Sc. rank:	317.7

,

HA			
Pos.	63	obs:	exp:
ggt	G	0	135.30
ggc	G	0	128.20
gga	G	18	466.50
ggg	G	991	279.00
---	--	------	------
mPD		0.035	0.68
		nPD:	0.05
		N. weight:	1.4
		Sc. PD:	-0.15
		Sc. rank:	-582.8

,

HA			
Pos.	64	obs:	exp:
cgt	R	0	0.77
cgc	R	0	0.91
cga	R	0	1.79
cgg	R	0	1.50
aaa	K	989	580.50
aag	K	2	410.50
aga	R	18	8.22
agg	R	0	4.80
---	--	------	------
mPD		0.039	0.53
		nPD:	0.07
		N. weight:	0.63
		Sc. PD:	-0.055
		Sc. rank:	-190.0

,

HA			
Pos.	65	obs:	exp:
tta	L	0	1.05
ttg	L	0	2.24
ctt	L	0	2.03
ctc	L	0	2.01
cta	L	0	2.00
ctg	L	12	2.66
cgt	R	0	26.57
cgc	R	0	31.60
cga	R	1	62.02
cgg	R	130	51.81
atg	M	7	7.00
aat	N	1	0.51
aac	N	0	0.49
aaa	K	0	215.00
aag	K	367	152.00
aga	R	62	284.00
agg	R	429	166.00
---	--	------	------
mPD		0.85	1.3
		nPD:	0.64
		N. weight:	0.92
		Sc. PD:	0.42
		Sc. rank:	663.4

,

HA			
Pos.	66	obs:	exp:
tct	S	0	0.64
tcc	S	0	0.52
tca	S	4	1.01
tcg	S	0	0.23
cct	P	0	10.93
ccc	P	0	7.67
cca	P	43	17.57
ccg	P	0	6.83
att	I	0	0.36
atc	I	0	0.24
ata	I	1	0.39
act	T	4	246.40
acc	T	0	193.70
aca	T	933	436.90
acg	T	14	74.08
agt	S	0	0.82
agc	S	0	0.78
gct	A	0	2.56
gcc	A	0	1.88
gca	A	10	4.68
gcg	A	0	0.88
---	--	------	------
mPD		0.15	0.79
		nPD:	0.18
		N. weight:	0.86
		Sc. PD:	0.017
		Sc. rank:	172.0

,

HA			
Pos.	67	obs:	exp:
att	I	43	16.42
atc	I	1	10.88
ata	I	1	17.70
act	T	0	11.92
acc	T	0	9.37
aca	T	46	21.13
acg	T	0	3.58
gtt	V	913	189.50
gtc	V	5	183.90
gta	V	0	190.10
gtg	V	0	354.50
---	--	------	------
mPD		0.35	0.99
		nPD:	0.36
		N. weight:	1.8
		Sc. PD:	0.33
		Sc. rank:	1072.9

,

HA			
Pos.	68	obs:	exp:
gat	D	84	544.70
gac	D	925	464.30
---	--	------	------
mPD		0.15	0.50
		nPD:	0.31
		N. weight:	0.57
		Sc. PD:	0.079
		Sc. rank:	310.7

,

HA			
Pos.	69	obs:	exp:
tta	L	0	88.68
ttg	L	1	188.30
ctt	L	69	171.00
ctc	L	891	169.00
cta	L	8	168.20
ctg	L	40	223.80
---	--	------	------
mPD		0.22	1.1
		nPD:	0.19
		N. weight:	1.6
		Sc. PD:	0.047
		Sc. rank:	389.3

,

HA			
Pos.	70	obs:	exp:
tgt	C	1	0.41
tgc	C	0	0.59
gat	D	3	1.62
gac	D	0	1.38
ggt	G	950	134.80
ggc	G	5	127.70
gga	G	50	464.70
ggg	G	0	277.90
---	--	------	------
mPD		0.11	0.69
		nPD:	0.16
		N. weight:	2.1
		Sc. PD:	-0.0017
		Sc. rank:	229.5

,

HA			
Pos.	71	obs:	exp:
cat	H	0	1.15
cac	H	2	0.85
caa	Q	992	511.60
cag	Q	4	484.40
cgt	R	0	0.43
cgc	R	0	0.51
cga	R	10	1.00
cgg	R	0	0.83
aaa	K	1	0.59
aag	K	0	0.41
aga	R	0	4.57
agg	R	0	2.67
---	--	------	------
mPD		0.033	0.54
		nPD:	0.06
		N. weight:	0.79
		Sc. PD:	-0.077
		Sc. rank:	-275.2

,

HA			
Pos.	72	obs:	exp:
tgt	C	963	413.80
tgc	C	45	594.20
ggt	G	0	0.13
ggc	G	0	0.13
gga	G	0	0.46
ggg	G	1	0.28
---	--	------	------
mPD		0.089	0.49
		nPD:	0.18
		N. weight:	0.83
		Sc. PD:	0.016
		Sc. rank:	156.3

,

HA			
Pos.	73	obs:	exp:
ggt	G	0	135.30
ggc	G	0	128.20
gga	G	972	466.50
ggg	G	37	279.00
---	--	------	------
mPD		0.071	0.68
		nPD:	0.1
		N. weight:	0.76
		Sc. PD:	-0.043
		Sc. rank:	-125.6

,

HA			
Pos.	74	obs:	exp:
tta	L	0	88.50
ttg	L	0	187.90
ctt	L	69	170.60
ctc	L	769	168.70
cta	L	169	167.90
ctg	L	0	223.30
cct	P	0	0.25
ccc	P	1	0.18
cca	P	0	0.41
ccg	P	0	0.16
att	I	1	0.36
atc	I	0	0.24
ata	I	0	0.39
---	--	------	------
mPD		0.39	1.1
		nPD:	0.35
		N. weight:	1.3
		Sc. PD:	0.24
		Sc. rank:	794.7

,

HA			
Pos.	75	obs:	exp:
tta	L	0	88.59
ttg	L	1	188.10
ctt	L	1	170.80
ctc	L	0	168.90
cta	L	50	168.10
ctg	L	956	223.60
cgt	R	0	0.04
cgc	R	0	0.05
cga	R	0	0.10
cgg	R	1	0.08
aga	R	0	0.46
agg	R	0	0.27
---	--	------	------
mPD		0.10	1.1
		nPD:	0.09
		N. weight:	1.6
		Sc. PD:	-0.11
		Sc. rank:	-355.7

,

HA

Pos.	76	obs:	exp:
ggt	G	11	135.30
ggc	G	0	128.20
gga	G	52	466.50
ggg	G	946	279.00
---	--	------	------
mPD		0.12	0.68
		nPD:	0.18
		N. weight:	1.2
		Sc. PD:	0.013
		Sc. rank:	209.8

,

HA

Pos.	77	obs:	exp:
att	I	0	0.36
atc	I	0	0.24
ata	I	1	0.39
act	T	6	260.60
acc	T	47	204.90
aca	T	939	462.20
acg	T	14	78.36
gct	A	0	0.51
gcc	A	0	0.38
gca	A	2	0.94
gcg	A	0	0.18
---	--	------	------
mPD		0.13	0.68
		nPD:	0.19
		N. weight:	0.66
		Sc. PD:	0.019
		Sc. rank:	161.2

,

HA

Pos.	78	obs:	exp:
tta	L	0	4.13
ttg	L	1	8.77
ctt	L	0	7.96
ctc	L	0	7.87
cta	L	46	7.84
ctg	L	0	10.42
att	I	2	345.90
atc	I	945	229.30
ata	I	1	372.80
gtt	V	0	2.89
gtc	V	14	2.80
gta	V	0	2.90
gtg	V	0	5.41
---	--	------	------
mPD		0.21	0.79
		nPD:	0.27
		N. weight:	1.7
		Sc. PD:	0.17
		Sc. rank:	798.7

,

HA

Pos.	79	obs:	exp:
att	I	2	17.51
atc	I	0	11.61
ata	I	46	18.88
act	T	951	248.90
acc	T	5	195.70
aca	T	5	441.50
acg	T	0	74.86
---	--	------	------
mPD		0.20	0.76
		nPD:	0.26
		N. weight:	1.5
		Sc. PD:	0.13
		Sc. rank:	692.8

,

HA

Pos.	80	obs:	exp:
ggt	G	1	135.30
ggc	G	4	128.20
gga	G	978	466.50
ggg	G	26	279.00
---	--	------	------
mPD		0.060	0.68
		nPD:	0.09
		N. weight:	0.77
		Sc. PD:	-0.055
		Sc. rank:	-182.0

,

HA

Pos.	81	obs:	exp:
cct	P	46	256.60
ccc	P	1	179.90
cca	P	962	412.20
ccg	P	0	160.30
---	--	------	------
mPD		0.089	0.71
		nPD:	0.13
		N. weight:	0.87
		Sc. PD:	-0.032
		Sc. rank:	-91.0

,

HA

Pos.	82	obs:	exp:
cct	P	917	256.60
ccc	P	91	179.90
cca	P	1	412.20
ccg	P	0	160.30
---	--	------	------
mPD		0.17	0.71
		nPD:	0.23
		N. weight:	1.3
		Sc. PD:	0.087
		Sc. rank:	504.3

,

HA

Pos.	83	obs:	exp:
caa	Q	996	518.30
cag	Q	13	490.70
---	--	------	------
mPD		0.025	0.50
		nPD:	0.05
		N. weight:	0.72
		Sc. PD:	-0.078
		Sc. rank:	-297.8

,

HA

Pos.	84	obs:	exp:
tgt	C	966	414.20
tgc	C	43	594.80
---	--	------	------
mPD		0.082	0.48
		nPD:	0.17
		N. weight:	0.84
		Sc. PD:	0.004
		Sc. rank:	106.1

,

HA

Pos.	85	obs:	exp:
gat	D	50	544.70
gac	D	959	464.30
---	--	------	------
mPD		0.094	0.50
		nPD:	0.19
		N. weight:	0.69
		Sc. PD:	0.017
		Sc. rank:	158.0

,

HA

Pos.	86	obs:	exp:
caa	Q	1004	518.30
cag	Q	5	490.70
---	--	------	------
mPD		0.0099	0.50
		nPD:	0.02
		N. weight:	0.76
		Sc. PD:	-0.11
		Sc. rank:	-411.6

,

HA

Pos.	87	obs:	exp:
ttt	F	33	397.80
ttc	F	975	610.20
tta	L	0	0.09
ttg	L	0	0.19
ctt	L	0	0.17
ctc	L	1	0.17
cta	L	0	0.17
ctg	L	0	0.22
---	--	------	------
mPD		0.065	0.48
		nPD:	0.14
		N. weight:	0.45
		Sc. PD:	-0.012
		Sc. rank:	-26.4

,

HA

Pos.	88	obs:	exp:
tta	L	1	88.68
ttg	L	0	188.30
ctt	L	5	171.00
ctc	L	0	169.00
cta	L	953	168.20
ctg	L	50	223.80
---	--	------	------
mPD		0.11	1.1
		nPD:	0.1
		N. weight:	1.8
		Sc. PD:	-0.12
		Sc. rank:	-358.8

,

HA

Pos.	89	obs:	exp:
aaa	K	55	32.22
aag	K	0	22.78
gaa	E	902	573.90
gag	E	52	380.10
---	--	------	------
mPD		0.20	0.58
		nPD:	0.34
		N. weight:	0.4
		Sc. PD:	0.069
		Sc. rank:	236.1

,

HA

Pos.	90	obs:	exp:
ttt	F	1008	398.20
ttc	F	1	610.80
---	--	------	------
mPD		0.0020	0.48
		nPD:	0.
		N. weight:	1.1
		Sc. PD:	-0.17
		Sc. rank:	-713.6

,

HA

Pos.	91	obs:	exp:
tct	S	1	154.00
tcc	S	0	124.50
tca	S	617	242.80
tcg	S	344	55.98
aat	N	0	1.02
aac	N	2	0.98
agt	S	0	197.10
agc	S	0	187.60
gat	D	3	23.75
gac	D	41	20.25
gaa	E	0	0.60
gag	E	1	0.40
---	--	------	------
mPD		0.69	1.7
		nPD:	0.39
		N. weight:	1.5
		Sc. PD:	0.32
		Sc. rank:	916.9

,

HA

Pos.	92	obs:	exp:
tct	S	3	0.64
tcc	S	1	0.52
tca	S	0	1.01
tcg	S	0	0.23
agt	S	0	0.82
agc	S	0	0.78
gct	A	45	257.50
gcc	A	957	188.70
gca	A	3	469.90
gcg	A	0	88.89
---	--	------	------
mPD		0.10	0.68
		nPD:	0.15
		N. weight:	1.7
		Sc. PD:	-0.019
		Sc. rank:	90.6

,

HA

Pos.	93	obs:	exp:
gat	D	963	544.70
gac	D	46	464.30
---	--	------	------
mPD		0.087	0.50
		nPD:	0.18
		N. weight:	0.53
		Sc. PD:	0.0059
		Sc. rank:	92.4

,

HA

Pos.	94	obs:	exp:
tta	L	972	88.59
ttg	L	16	188.10
ctt	L	0	170.80
ctc	L	0	168.90
cta	L	19	168.10
ctg	L	1	223.60
gtt	V	0	0.21
gtc	V	0	0.20
gta	V	1	0.21
gtg	V	0	0.39
---	--	------	------
mPD		0.074	1.1
		nPD:	0.07
		N. weight:	2.7
		Sc. PD:	-0.25
		Sc. rank:	-852.9

,

HA

Pos.	95	obs:	exp:
att	I	939	368.20
atc	I	23	244.10
ata	I	47	396.80
---	--	------	------
mPD		0.13	0.65
		nPD:	0.2
		N. weight:	0.86
		Sc. PD:	0.031
		Sc. rank:	244.3

,

HA

Pos.	96	obs:	exp:
att	I	943	345.20
atc	I	3	228.80
ata	I	0	372.00
atg	M	1	1.00
gtt	V	62	12.80
gtc	V	0	12.42
gta	V	0	12.84
gtg	V	0	23.94
---	--	------	------
mPD		0.12	0.79
		nPD:	0.16
		N. weight:	1.2
		Sc. PD:	-0.008
		Sc. rank:	88.6

,

HA

Pos.	97	obs:	exp:
gaa	E	70	606.90
gag	E	939	402.10
---	--	------	------
mPD		0.13	0.48
		nPD:	0.27
		N. weight:	0.77
		Sc. PD:	0.078
		Sc. rank:	368.7

,

HA

Pos.	98	obs:	exp:
cgt	R	0	43.10
cgc	R	0	51.26
cga	R	25	100.60
cgg	R	22	84.04
aga	R	6	460.70
agg	R	956	269.30
---	--	------	------
mPD		0.15	0.96
		nPD:	0.15
		N. weight:	1.3
		Sc. PD:	-0.012
		Sc. rank:	82.6

,

HA

Pos.	99	obs:	exp:
caa	Q	0	0.51
cag	Q	1	0.49
cgt	R	0	43.06
cgc	R	0	51.21
cga	R	941	100.50
cgg	R	14	83.96
aga	R	53	460.20
agg	R	0	269.00
---	--	------	------
mPD		0.13	0.97
		nPD:	0.14
		N. weight:	2.3
		Sc. PD:	-0.063
		Sc. rank:	-140.1

,

HA

Pos.	100	obs:	exp:
gat	D	2	1.08
gac	D	0	0.92
gaa	E	1003	604.50
gag	E	2	400.50
ggt	G	0	0.27
ggc	G	0	0.25
gga	G	2	0.92
ggg	G	0	0.55
---	--	------	------
mPD		0.012	0.49
		nPD:	0.02
		N. weight:	0.59
		Sc. PD:	-0.079
		Sc. rank:	-303.6

,

HA

Pos.	101	obs:	exp:
ggt	G	0	135.30
ggc	G	0	128.20
gga	G	1008	466.50
ggg	G	1	279.00
---	--	------	------
mPD		0.0020	0.68
		nPD:	0.
		N. weight:	0.91
		Sc. PD:	-0.14
		Sc. rank:	-615.8

,

HA

Pos.	102	obs:	exp:
tct	S	0	153.80
tcc	S	0	124.40
tca	S	0	242.50
tcg	S	0	55.92
cgt	R	1	0.04
cgc	R	0	0.05
cga	R	0	0.10
cgg	R	0	0.08
act	T	1	11.92
acc	T	23	9.37
aca	T	0	21.13
acg	T	22	3.58
aaa	K	1	0.59
aag	K	0	0.41
agt	S	958	196.90
agc	S	3	187.40
aga	R	0	0.46
agg	R	0	0.27
---	--	------	------
mPD		0.18	1.7
		nPD:	0.11
		N. weight:	1.9
		Sc. PD:	-0.094
		Sc. rank:	-252.1

,

HA

Pos.	103	obs:	exp:
gat	D	1008	544.70
gac	D	1	464.30
---	--	------	------
mPD		0.0020	0.50
		nPD:	0.
		N. weight:	0.73
		Sc. PD:	-0.11
		Sc. rank:	-481.9

,

HA

Pos.	104	obs:	exp:
ttt	F	0	0.79
ttc	F	2	1.21
att	I	0	2.19
atc	I	6	1.45
ata	I	0	2.36
ggt	V	17	206.20
gtc	V	935	200.10
gta	V	0	206.80
gtg	V	47	385.80
gct	A	0	0.51
gcc	A	2	0.38
gca	A	0	0.94
gcg	A	0	0.18
---	--	------	------
mPD		0.14	0.75
		nPD:	0.19
		N. weight:	1.6
		Sc. PD:	0.036
		Sc. rank:	342.9

,

HA

Pos.	105	obs:	exp:
tgt	C	994	414.20
tgc	C	15	594.80
---	--	------	------
mPD		0.029	0.48
		nPD:	0.06
		N. weight:	0.97
		Sc. PD:	-0.096
		Sc. rank:	-345.8

,

HA

Pos.	106	obs:	exp:
tat	Y	995	535.00
tac	Y	14	474.00
---	--	------	------
mPD		0.027	0.50
		nPD:	0.05
		N. weight:	0.68
		Sc. PD:	-0.07
		Sc. rank:	-265.0

,

HA

Pos.	107	obs:	exp:
cct	P	979	256.60
ccc	P	28	179.90
cca	P	1	412.20
ccg	P	1	160.30
---	--	------	------
mPD		0.058	0.71
		nPD:	0.08
		N. weight:	1.5
		Sc. PD:	-0.12
		Sc. rank:	-408.8

,

HA

Pos.	108	obs:	exp:
ggt	G	0	135.30
ggc	G	1	128.20
gga	G	349	466.50
ggg	G	659	279.00
---	--	------	------
mPD		0.45	0.68
		nPD:	0.67
		N. weight:	0.55
		Sc. PD:	0.27
		Sc. rank:	398.2

,

HA

Pos.	109	obs:	exp:
aaa	K	948	591.10
aag	K	61	417.90
---	--	------	------
mPD		0.11	0.49
		nPD:	0.23
		N. weight:	0.39
		Sc. PD:	0.027
		Sc. rank:	153.5

,

HA

Pos.	110	obs:	exp:
ttt	F	15	397.80
ttc	F	993	610.20
gtt	V	0	0.21
gtc	V	1	0.20
gta	V	0	0.21
gtg	V	0	0.39
---	--	------	------
mPD		0.031	0.48
		nPD:	0.07
		N. weight:	0.52
		Sc. PD:	-0.049
		Sc. rank:	-171.7

,

HA

Pos.	111	obs:	exp:
tct	S	0	0.16
tcc	S	0	0.13
tca	S	0	0.25
tcg	S	1	0.06
att	I	0	0.36
atc	I	0	0.24
ata	I	1	0.39
atg	M	1	1.00
act	T	0	11.92
acc	T	0	9.37
aca	T	43	21.13
acg	T	3	3.58
agt	S	0	0.20
agc	S	0	0.20
gtt	V	0	197.60
gtc	V	0	191.70
gta	V	23	198.10
gtg	V	934	369.60
gct	A	0	0.77
gcc	A	0	0.56
gca	A	0	1.40
gcg	A	3	0.27
---	--	------	------
mPD		0.31	0.92
		nPD:	0.34
		N. weight:	1.
		Sc. PD:	0.17
		Sc. rank:	597.6

,

HA

Pos.	112	obs:	exp:
aat	N	993	510.80
aac	N	11	493.20
aaa	K	1	0.59
aag	K	0	0.41
gat	D	4	2.16
gac	D	0	1.84
---	--	------	------
mPD		0.031	0.51
		nPD:	0.06
		N. weight:	0.74
		Sc. PD:	-0.072
		Sc. rank:	-259.6

,

HA

Pos.	113	obs:	exp:
gaa	E	974	606.30
gag	E	34	401.70
ggt	G	0	0.13
ggc	G	0	0.13
gga	G	1	0.46
ggg	G	0	0.28
---	--	------	------
mPD		0.067	0.48
		nPD:	0.14
		N. weight:	0.45
		Sc. PD:	-0.011
		Sc. rank:	-18.8

,

HA

Pos.	114	obs:	exp:
aaa	K	1	0.59
aag	K	0	0.41
gaa	E	1003	603.90
gag	E	1	400.10
ggt	G	0	0.54
ggc	G	0	0.51
gga	G	4	1.85
ggg	G	0	1.11
---	--	------	------
mPD		0.012	0.49
		nPD:	0.02
		N. weight:	0.6
		Sc. PD:	-0.08
		Sc. rank:	-309.7

,

HA

Pos.	115	obs:	exp:
tct	S	0	7.20
tcc	S	0	5.82
tca	S	45	11.36
tcg	S	0	2.62
cct	P	0	0.51
ccc	P	0	0.36
cca	P	2	0.82
ccg	P	0	0.32
act	T	41	10.62
acc	T	0	8.35
aca	T	0	18.84
acg	T	0	3.19
agt	S	0	9.22
agc	S	0	8.78
gct	A	908	236.00
gcc	A	1	172.90
gca	A	12	430.60
gcg	A	0	81.46
---	--	------	------
mPD		0.28	0.87
		nPD:	0.31
		N. weight:	1.5
		Sc. PD:	0.22
		Sc. rank:	847.1

,

HA

Pos.	116	obs:	exp:
tta	L	1	88.68
ttg	L	13	188.30
ctt	L	1	171.00
ctc	L	0	169.00
cta	L	4	168.20
ctg	L	990	223.80
---	--	------	------
mPD		0.039	1.1
		nPD:	0.04
		N. weight:	1.7
		Sc. PD:	-0.21
		Sc. rank:	-770.8

,

HA

Pos.	117	obs:	exp:
cgt	R	0	43.10
cgc	R	0	51.26
cga	R	0	100.60
cgg	R	1	84.04
aga	R	13	460.70
agg	R	995	269.30
---	--	------	------
mPD		0.027	0.96
		nPD:	0.03
		N. weight:	1.5
		Sc. PD:	-0.19
		Sc. rank:	-721.3

,

HA

Pos.	118	obs:	exp:
caa	Q	960	518.30
cag	Q	49	490.70
---	--	------	------
mPD		0.093	0.50
		nPD:	0.19
		N. weight:	0.57
		Sc. PD:	0.012
		Sc. rank:	117.6

,

HA

Pos.	119	obs:	exp:
att	I	955	367.80
atc	I	51	243.80
ata	I	2	396.40
gtt	V	1	0.21
gtc	V	0	0.20
gta	V	0	0.21
gtg	V	0	0.39
---	--	------	------
mPD		0.10	0.66
		nPD:	0.16
		N. weight:	0.98
		Sc. PD:	-0.008
		Sc. rank:	63.0

,

HA

Pos.	120	obs:	exp:
tta	L	0	88.50
ttg	L	0	187.90
ctt	L	26	170.60
ctc	L	953	168.70
cta	L	28	167.90
ctg	L	0	223.30
att	I	2	0.73
atc	I	0	0.48
ata	I	0	0.79
---	--	------	------
mPD		0.11	1.1
		nPD:	0.1
		N. weight:	1.8
		Sc. PD:	-0.11
		Sc. rank:	-333.7

,

HA

Pos.	121	obs:	exp:
cgt	R	0	43.10
cgc	R	0	51.26
cga	R	47	100.60
cgg	R	14	84.04
aga	R	922	460.70
agg	R	26	269.30
---	--	------	------
mPD		0.19	0.96
		nPD:	0.2
		N. weight:	0.62
		Sc. PD:	0.02
		Sc. rank:	161.8

HA

Pos.	122	obs:	exp:
aat	N	0	0.51
aac	N	1	0.49
aaa	K	104	60.92
aag	K	0	43.08
gaa	E	788	514.30
gag	E	67	340.70
ggt	G	0	6.57
ggc	G	0	6.23
gga	G	8	22.66
ggg	G	41	13.55
---	--	------	------
mPD		0.47	0.77
		nPD:	0.61
		N. weight:	0.39
		Sc. PD:	0.17
		Sc. rank:	274.7

HA

Pos.	123	obs:	exp:
tct	S	14	161.50
tcc	S	0	130.60
tca	S	989	254.60
tcg	S	6	58.72
agt	S	0	206.80
agc	S	0	196.80
---	--	------	------
mPD		0.039	1.7
		nPD:	0.02
		N. weight:	1.5
		Sc. PD:	-0.21
		Sc. rank:	-794.7

HA

Pos.	124	obs:	exp:
ggt	G	8	135.30
ggc	G	947	128.20
gga	G	54	466.50
ggg	G	0	279.00
---	--	------	------
mPD		0.12	0.68
		nPD:	0.17
		N. weight:	2.1
		Sc. PD:	0.017
		Sc. rank:	313.4

HA

Pos.	125	obs:	exp:
ggt	G	1	135.30
ggc	G	0	128.20
gga	G	998	466.50
ggg	G	10	279.00
---	--	------	------
mPD		0.022	0.68
		nPD:	0.03
		N. weight:	0.86
		Sc. PD:	-0.11
		Sc. rank:	-404.3

HA

Pos.	126	obs:	exp:
att	I	973	368.20
atc	I	34	244.10
ata	I	2	396.80
---	--	------	------
mPD		0.069	0.65
		nPD:	0.11
		N. weight:	1.
		Sc. PD:	-0.058
		Sc. rank:	-163.6

HA

Pos.	127	obs:	exp:
aat	N	0	0.51
aac	N	1	0.49
gtt	V	0	0.21
gtc	V	1	0.20
gta	V	0	0.21
gtg	V	0	0.39
gat	D	51	531.20
gac	D	933	452.80
gaa	E	20	12.03
gag	E	0	7.97
ggt	G	0	0.40
ggc	G	3	0.38
gga	G	0	1.39
ggg	G	0	0.83
---	--	------	------
mPD		0.14	0.53
		nPD:	0.27
		N. weight:	0.68
		Sc. PD:	0.07
		Sc. rank:	329.1

HA

Pos.	128	obs:	exp:
aaa	K	6	591.10
aag	K	1003	417.90
---	--	------	------
mPD		0.012	0.49
		nPD:	0.02
		N. weight:	1.
		Sc. PD:	-0.13
		Sc. rank:	-517.7

HA

Pos.	129	obs:	exp:
aaa	K	2	1.17
aag	K	0	0.83
gaa	E	938	605.70
gag	E	69	401.30
---	--	------	------
mPD		0.13	0.48
		nPD:	0.27
		N. weight:	0.35
		Sc. PD:	0.036
		Sc. rank:	168.2

HA

Pos.	130	obs:	exp:
tct	S	0	12.33
tcc	S	27	9.97
tca	S	50	19.43
tcg	S	0	4.48
cct	P	0	29.50
ccc	P	116	20.68
cca	P	0	47.39
ccg	P	0	18.43
act	T	0	46.89
acc	T	2	36.86
aca	T	177	83.15
acg	T	2	14.10
agt	S	0	15.78
agc	S	0	15.02
gtt	V	0	0.21
gtc	V	0	0.20
gta	V	1	0.21
gtg	V	0	0.39
gct	A	2	162.20
gcc	A	0	118.90
gca	A	631	296.00
gcg	A	0	55.99
gaa	E	1	0.60
gag	E	0	0.40
---	--	------	------
mPD		0.81	1.3
		nPD:	0.63
		N. weight:	1.
		Sc. PD:	0.46
		Sc. rank:	741.9

HA

Pos.	131	obs:	exp:
att	I	0	0.36
atc	I	0	0.24
ata	I	1	0.39
atg	M	1008	1008.00
---	--	------	------
mPD		0.0020	0.0020
		nPD:	1.
		N. weight:	0.0011
		Sc. PD:	0.00089
		Sc. rank:	0.8

HA

Pos.	132	obs:	exp:
cgt	R	0	0.17
cgc	R	0	0.20
cga	R	0	0.40
cgg	R	0	0.33
aga	R	4	1.83
agg	R	0	1.07
ggt	G	44	134.80
ggc	G	4	127.70
gga	G	808	464.70
ggg	G	149	277.90
---	--	------	------
mPD		0.34	0.68
		nPD:	0.49
		N. weight:	0.36
		Sc. PD:	0.11
		Sc. rank:	245.3

HA

Pos.	133	obs:	exp:
ttt	F	3	395.80
ttc	F	1000	607.20
tta	L	0	0.53
ttg	L	0	1.12
ctt	L	0	1.02
ctc	L	6	1.01
cta	L	0	1.00
ctg	L	0	1.33
---	--	------	------
mPD		0.018	0.49
		nPD:	0.04
		N. weight:	0.59
		Sc. PD:	-0.072
		Sc. rank:	-268.9

HA

Pos.	134	obs:	exp:
act	T	2	247.70
acc	T	46	194.70
aca	T	890	439.20
acg	T	18	74.47
aaa	K	53	31.05
aag	K	0	21.95
---	--	------	------
mPD		0.22	0.78
		nPD:	0.29
		N. weight:	0.67
		Sc. PD:	0.08
		Sc. rank:	353.1

HA

Pos.	135	obs:	exp:
tat	Y	48	535.00
tac	Y	961	474.00
---	--	------	------
mPD		0.091	0.50
		nPD:	0.18
		N. weight:	0.67
		Sc. PD:	0.012
		Sc. rank:	124.8

HA

Pos.	136	obs:	exp:
tct	S	0	98.45
tcc	S	0	79.61
tca	S	0	155.20
tcg	S	0	35.79
tat	Y	1	0.53
tac	Y	0	0.47
aat	N	381	193.80
aac	N	0	187.20
agt	S	574	126.00
agc	S	41	119.90
gat	D	12	6.48
gac	D	0	5.52
---	--	------	------
mPD		0.58	1.8
		nPD:	0.33
		N. weight:	1.3
		Sc. PD:	0.2
		Sc. rank:	729.5

HA

Pos.	137	obs:	exp:
gaa	E	5	3.01
gag	E	0	1.99
ggt	G	0	134.60
ggc	G	0	127.60
gga	G	980	464.20
ggg	G	24	277.60
---	--	------	------
mPD		0.056	0.68
		nPD:	0.08
		N. weight:	0.8
		Sc. PD:	-0.063
		Sc. rank:	-219.8

HA

Pos.	138	obs:	exp:
att	I	0	367.40
atc	I	0	243.60
ata	I	1007	396.00
atg	M	1	1.00
gtt	V	0	0.21
gtc	V	0	0.20
gta	V	1	0.21
gtg	V	0	0.39
---	--	------	------
mPD		0.0040	0.66
		nPD:	0.01
		N. weight:	1.1
		Sc. PD:	-0.17
		Sc. rank:	-713.2

HA

Pos.	139	obs:	exp:
cgt	R	0	43.06
cgc	R	0	51.21
cga	R	3	100.50
cgg	R	0	83.96
aaa	K	1	0.59
aag	K	0	0.41
aga	R	988	460.20
agg	R	17	269.00
---	--	------	------
mPD		0.041	0.97
		nPD:	0.04
		N. weight:	0.83
		Sc. PD:	-0.097
		Sc. rank:	-356.9

HA

Pos.	140	obs:	exp:
att	I	1	0.36
atc	I	0	0.24
ata	I	0	0.39
act	T	801	231.10
acc	T	90	181.60
aca	T	1	409.80
acg	T	0	69.48
aat	N	1	0.51
aac	N	0	0.49
gct	A	115	29.47
gcc	A	0	21.59
gca	A	0	53.77
gcg	A	0	10.17
---	--	------	------
mPD		0.37	0.88
		nPD:	0.42
		N. weight:	1.3
		Sc. PD:	0.32
		Sc. rank:	835.7

HA			
Pos.	141	obs:	exp:
aat	N	1005	512.30
aac	N	2	494.70
gat	D	2	1.08
gac	D	0	0.92
---	--	------	------
mPD		0.0079	0.50
		nPD:	0.02
		N. weight:	0.79
		Sc. PD:	-0.11
		Sc. rank:	-440.5

,

HA			
Pos.	142	obs:	exp:
ggt	G	0	135.30
ggc	G	0	128.20
gga	G	796	466.50
ggg	G	213	279.00
---	--	------	------
mPD		0.33	0.68
		nPD:	0.49
		N. weight:	0.44
		Sc. PD:	0.14
		Sc. rank:	302.2

,

HA			
Pos.	143	obs:	exp:
act	T	0	15.28
acc	T	0	12.01
aca	T	55	27.11
acg	T	4	4.60
gtt	V	0	73.90
gtc	V	0	71.72
gta	V	268	74.12
gtg	V	90	138.30
gct	A	0	151.40
gcc	A	2	111.00
gca	A	571	276.30
gcg	A	18	52.27
gaa	E	0	0.60
gag	E	1	0.40
---	--	------	------
mPD		0.77	1.3
		nPD:	0.6
		N. weight:	0.88
		Sc. PD:	0.36
		Sc. rank:	623.6

,

HA			
Pos.	144	obs:	exp:
act	T	1	261.40
acc	T	956	205.50
aca	T	10	463.60
acg	T	42	78.60
---	--	------	------
mPD		0.10	0.67
		nPD:	0.15
		N. weight:	1.7
		Sc. PD:	-0.023
		Sc. rank:	48.5

,

HA			
Pos.	145	obs:	exp:
tct	S	0	158.80
tcc	S	0	128.40
tca	S	0	250.30
tcg	S	0	57.73
cgt	R	0	0.09
cgc	R	0	0.10
cga	R	0	0.20
cgg	R	0	0.17
aat	N	7	3.56
aac	N	0	3.44
agt	S	990	203.30
agc	S	2	193.40
aga	R	0	0.91
agg	R	2	0.53
ggt	G	8	1.07
ggc	G	0	1.02
gga	G	0	3.70
ggg	G	0	2.21
---	--	------	------
mPD		0.037	1.7
		nPD:	0.02
		N. weight:	1.9
		Sc. PD:	-0.25
		Sc. rank:	-989.1

,

HA			
Pos.	146	obs:	exp:
act	T	2	0.52
acc	T	0	0.41
aca	T	0	0.92
acg	T	0	0.16
gct	A	40	258.00
gcc	A	5	189.10
gca	A	950	470.80
gcg	A	12	89.07
---	--	------	------
mPD		0.12	0.68
		nPD:	0.17
		N. weight:	0.66
		Sc. PD:	0.0046
		Sc. rank:	89.8

,

HA			
Pos.	147	obs:	exp:
tgt	C	934	414.20
tgc	C	75	594.80
---	--	------	------
mPD		0.14	0.48
		nPD:	0.28
		N. weight:	0.72
		Sc. PD:	0.083
		Sc. rank:	372.0

,

HA			
Pos.	148	obs:	exp:
cgt	R	0	34.60
cgc	R	0	41.15
cga	R	0	80.77
cgg	R	0	67.46
att	I	0	0.36
atc	I	0	0.24
ata	I	1	0.39
atg	M	10	10.00
aat	N	2	1.02
aac	N	0	0.98
aaa	K	0	108.40
aag	K	185	76.63
aga	R	51	369.80
agg	R	759	216.20
ggt	G	0	0.13
ggc	G	0	0.13
gga	G	0	0.46
ggg	G	1	0.28
---	--	------	------
mPD		0.42	1.2
		nPD:	0.35
		N. weight:	1.2
		Sc. PD:	0.21
		Sc. rank:	724.6

,

HA			
Pos.	149	obs:	exp:
cgt	R	0	43.10
cgc	R	0	51.26
cga	R	0	100.60
cgg	R	0	84.04
aga	R	1003	460.70
agg	R	6	269.30
---	--	------	------
mPD		0.012	0.96
		nPD:	0.01
		N. weight:	0.9
		Sc. PD:	-0.13
		Sc. rank:	-515.7

,

HA			
Pos.	150	obs:	exp:
tta	L	7	0.62
ttg	L	0	1.31
tct	S	0	160.10
tcc	S	0	129.40
tca	S	999	252.40
tcg	S	1	58.19
ctt	L	0	1.19
ctc	L	0	1.17
cta	L	0	1.17
ctg	L	0	1.55
act	T	0	0.52
acc	T	0	0.41
aca	T	2	0.92
acg	T	0	0.16
agt	S	0	204.90
agc	S	0	195.00
---	--	------	------
mPD		0.020	1.7
		nPD:	0.01
		N. weight:	1.6
		Sc. PD:	-0.24
		Sc. rank:	-953.0

,

HA			
Pos.	151	obs:	exp:
cgt	R	0	0.43
cgc	R	0	0.51
cga	R	0	1.00
cgg	R	0	0.83
aga	R	10	4.57
agg	R	0	2.67
gaa	E	9	5.41
gag	E	0	3.59
ggt	G	47	132.80
ggc	G	0	125.80
gga	G	939	457.70
ggg	G	4	273.70
---	--	------	------
mPD		0.13	0.71
		nPD:	0.19
		N. weight:	0.74
		Sc. PD:	0.017
		Sc. rank:	164.2

,

HA			
Pos.	152	obs:	exp:
tct	S	2	0.32
tcc	S	0	0.26
tca	S	0	0.50
tcg	S	0	0.12
agt	S	0	0.41
agc	S	0	0.39
---	-	1007	1007.00
---	--	------	------
mPD		0	6.5×10^{-6}
		nPD:	0.
		N. weight:	0.
		Sc. PD:	0
		Sc. rank:	0

,

HA			
Pos.	153	obs:	exp:
tct	S	974	156.20
tcc	S	1	126.30
tca	S	0	246.30
tcg	S	1	56.80
cct	P	22	8.39
ccc	P	11	5.88
cca	P	0	13.48
ccg	P	0	5.24
agt	S	0	200.00
agc	S	0	190.30
---	--	------	------
mPD		0.089	1.7
		nPD:	0.05
		N. weight:	2.1
		Sc. PD:	-0.23
		Sc. rank:	-865.3

,

HA			
Pos.	154	obs:	exp:
tct	S	6	161.50
tcc	S	40	130.60
tca	S	962	254.60
tcg	S	1	58.72
agt	S	0	206.80
agc	S	0	196.80
---	--	------	------
mPD		0.089	1.7
		nPD:	0.05
		N. weight:	1.4
		Sc. PD:	-0.15
		Sc. rank:	-576.0

,

HA			
Pos.	155	obs:	exp:
ttt	F	8	397.80
ttc	F	1000	610.20
tct	S	0	0.16
tcc	S	0	0.13
tca	S	1	0.25
tcg	S	0	0.06
agt	S	0	0.20
agc	S	0	0.20
---	--	------	------
mPD		0.020	0.48
		nPD:	0.04
		N. weight:	0.55
		Sc. PD:	-0.065
		Sc. rank:	-241.3

,

HA			
Pos.	156	obs:	exp:
tat	Y	1008	535.00
tac	Y	1	474.00
---	--	------	------
mPD		0.0020	0.50
		nPD:	0.
		N. weight:	0.75
		Sc. PD:	-0.11
		Sc. rank:	-497.6

,

HA			
Pos.	157	obs:	exp:
gct	A	4	258.50
gcc	A	0	189.50
gca	A	962	471.80
gcg	A	43	89.24
---	--	------	------
mPD		0.089	0.67
		nPD:	0.13
		N. weight:	0.76
		Sc. PD:	-0.023
		Sc. rank:	-59.4

,

HA			
Pos.	158	obs:	exp:
gaa	E	932	606.90
gag	E	77	402.10
---	--	------	------
mPD		0.14	0.48
		nPD:	0.29
		N. weight:	0.33
		Sc. PD:	0.041
		Sc. rank:	174.1

,

HA			
Pos.	159	obs:	exp:
tta	L	0	0.09
ttg	L	0	0.19
ctt	L	0	0.17
ctc	L	0	0.17
cta	L	0	0.17
ctg	L	1	0.22
atg	M	1008	1008.00
---	--	------	------
mPD		0.0020	0.0032
		nPD:	0.63
		N. weight:	0.0018
		Sc. PD:	0.00079
		Sc. rank:	1.3

,

HA			
Pos.	160	obs:	exp:
cgt	R	0	0.04
cgc	R	0	0.05
cga	R	0	0.10
cgg	R	0	0.08
aaa	K	963	590.50
aag	K	45	417.50
aga	R	1	0.46
agg	R	0	0.27
---	--	------	------
mPD		0.087	0.49
		nPD:	0.18
		N. weight:	0.44
		Sc. PD:	0.0064
		Sc. rank:	81.7

,

HA			
Pos.	161	obs:	exp:
tgg	W	1009	1009.00
---	--	------	------
mPD		0	0
		nPD:	1.
		N. weight:	0.
		Sc. PD:	0
		Sc. rank:	0

,

HA			
Pos.	162	obs:	exp:
tta	L	25	88.59
ttg	L	21	188.10
ctt	L	3	170.80
ctc	L	954	168.90
cta	L	4	168.10
ctg	L	1	223.60
cct	P	1	0.25
ccc	P	0	0.18
cca	P	0	0.41
ccg	P	0	0.16
---	--	------	------
mPD		0.19	1.1
		nPD:	0.17
		N. weight:	1.8
		Sc. PD:	0.019
		Sc. rank:	309.9

,

HA			
Pos.	163	obs:	exp:
tta	L	0	88.59
ttg	L	27	188.10
ctt	L	20	170.80
ctc	L	0	168.90
cta	L	6	168.10
ctg	L	955	223.60
caa	Q	0	0.51
cag	Q	1	0.49
---	--	------	------
mPD		0.10	1.1
		nPD:	0.09
		N. weight:	1.5
		Sc. PD:	-0.1
		Sc. rank:	-303.1

,

HA			
Pos.	164	obs:	exp:
tct	S	0	161.50
tcc	S	2	130.60
tca	S	959	254.60
tcg	S	48	58.72
agt	S	0	206.80
agc	S	0	196.80
---	--	------	------
mPD		0.094	1.7
		nPD:	0.06
		N. weight:	1.5
		Sc. PD:	-0.15
		Sc. rank:	-562.3

,

HA			
Pos.	165	obs:	exp:
tct	S	0	0.16
tcc	S	0	0.13
tca	S	0	0.25
tcg	S	0	0.06
act	T	0	0.26
acc	T	1	0.20
aca	T	0	0.46
acg	T	0	0.08
aat	N	51	511.80
aac	N	955	494.20
agt	S	0	0.20
agc	S	1	0.20
gat	D	0	0.54
gac	D	1	0.46
---	--	------	------
mPD		0.10	0.51
		nPD:	0.2
		N. weight:	0.61
		Sc. PD:	0.022
		Sc. rank:	172.2

,

HA			
Pos.	166	obs:	exp:
tct	S	0	7.36
tcc	S	0	5.95
tca	S	46	11.61
tcg	S	0	2.68
act	T	0	249.50
acc	T	3	196.10
aca	T	958	442.40
acg	T	2	75.01
agt	S	0	9.43
agc	S	0	8.97
---	--	------	------
mPD		0.097	0.77
		nPD:	0.13
		N. weight:	0.93
		Sc. PD:	-0.033
		Sc. rank:	-95.1

,

HA			
Pos.	167	obs:	exp:
aat	N	0	12.72
aac	N	25	12.28
gat	D	937	531.20
gac	D	47	452.80
---	--	------	------
mPD		0.18	0.55
		nPD:	0.33
		N. weight:	0.53
		Sc. PD:	0.085
		Sc. rank:	301.5

,

HA			
Pos.	168	obs:	exp:
tct	S	0	0.16
tcc	S	0	0.13
tca	S	0	0.25
tcg	S	0	0.06
aat	N	1004	510.80
aac	N	0	493.20
agt	S	1	0.20
agc	S	0	0.20
gat	D	4	2.16
gac	D	0	1.84
---	--	------	------
mPD		0.0099	0.51
		nPD:	0.02
		N. weight:	0.81
		Sc. PD:	-0.11
		Sc. rank:	-440.3

,

HA			
Pos.	169	obs:	exp:
gct	A	959	258.30
gcc	A	3	189.30
gca	A	8	471.30
gcg	A	38	89.16
gat	D	1	0.54
gac	D	0	0.46
---	--	------	------
mPD		0.095	0.67
		nPD:	0.14
		N. weight:	1.4
		Sc. PD:	-0.03
		Sc. rank:	-31.0

,

HA			
Pos.	170	obs:	exp:
act	T	0	15.02
acc	T	0	11.81
aca	T	58	26.65
acg	T	0	4.52
gtt	V	0	0.62
gtc	V	0	0.60
gta	V	3	0.62
gtg	V	0	1.16
gct	A	82	242.90
gcc	A	0	178.00
gca	A	865	443.20
gcg	A	1	83.85
---	--	------	------
mPD		0.27	0.79
		nPD:	0.34
		N. weight:	0.64
		Sc. PD:	0.11
		Sc. rank:	368.7

,

HA			
Pos.	171	obs:	exp:
ttt	F	47	398.20
ttc	F	962	610.80
---	--	------	------
mPD		0.089	0.48
		nPD:	0.19
		N. weight:	0.4
		Sc. PD:	0.0086
		Sc. rank:	84.8

,

HA			
Pos.	172	obs:	exp:
cct	P	8	256.60
ccc	P	44	179.90
cca	P	10	412.20
ccg	P	947	160.30
---	--	------	------
mPD		0.12	0.71
		nPD:	0.16
		N. weight:	1.8
		Sc. PD:	0.0017
		Sc. rank:	218.2

,

HA			
Pos.	173	obs:	exp:
cat	H	1	0.57
cac	H	0	0.43
caa	Q	289	517.80
cag	Q	719	490.20
---	--	------	------
mPD		0.41	0.50
		nPD:	0.82
		N. weight:	0.13
		Sc. PD:	0.083
		Sc. rank:	95.7

,

HA			
Pos.	174	obs:	exp:
att	I	0	0.73
atc	I	0	0.48
ata	I	2	0.79
atg	M	995	995.00
act	T	0	2.85
acc	T	0	2.24
aca	T	1	5.05
acg	T	10	0.86
gtt	V	0	0.21
gtc	V	0	0.20
gta	V	0	0.21
gtg	V	1	0.39
---	--	------	------
mPD		0.030	0.049
		nPD:	0.61
		N. weight:	0.032
		Sc. PD:	0.013
		Sc. rank:	22.5

,

HA			
Pos.	175	obs:	exp:
act	T	930	259.80
acc	T	71	204.20
aca	T	2	460.80
acg	T	0	78.13
aat	N	6	3.05
aac	N	0	2.95
---	--	------	------
mPD		0.15	0.69
		nPD:	0.21
		N. weight:	1.3
		Sc. PD:	0.062
		Sc. rank:	417.0

,

HA			
Pos.	176	obs:	exp:
cgt	R	0	0.04
cgc	R	0	0.05
cga	R	0	0.10
cgg	R	0	0.08
atg	M	1	1.00
aaa	K	72	589.90
aag	K	935	417.10
aga	R	0	0.46
agg	R	1	0.27
---	--	------	------
mPD		0.14	0.49
		nPD:	0.28
		N. weight:	0.72
		Sc. PD:	0.079
		Sc. rank:	358.5

,

HA			
Pos.	177	obs:	exp:
tct	S	0	161.20
tcc	S	1	130.30
tca	S	955	254.10
tcg	S	51	58.60
act	T	0	0.26
acc	T	0	0.20
aca	T	1	0.46
acg	T	0	0.08
agt	S	0	206.30
agc	S	0	196.40
gct	A	0	0.26
gcc	A	0	0.19
gca	A	1	0.47
gcg	A	0	0.09
---	--	------	------
mPD		0.10	1.7
		nPD:	0.06
		N. weight:	1.5
		Sc. PD:	-0.15
		Sc. rank:	-526.5

,

HA			
Pos.	178	obs:	exp:
tat	Y	924	535.00
tac	Y	85	474.00
---	--	------	------
mPD		0.15	0.50
		nPD:	0.31
		N. weight:	0.43
		Sc. PD:	0.06
		Sc. rank:	233.0

,

HA			
Pos.	179	obs:	exp:
cgt	R	0	2.26
cgc	R	0	2.69
cga	R	0	5.28
cgg	R	0	4.41
aaa	K	953	560.00
aag	K	3	396.00
aga	R	52	24.20
agg	R	1	14.15
---	--	------	------
mPD		0.11	0.62
		nPD:	0.17
		N. weight:	0.63
		Sc. PD:	0.0062
		Sc. rank:	102.2

,

HA			
Pos.	180	obs:	exp:
aat	N	977	513.30
aac	N	32	495.70
---	--	------	------
mPD		0.061	0.50
		nPD:	0.12
		N. weight:	0.64
		Sc. PD:	-0.025
		Sc. rank:	-68.7

,

HA			
Pos.	181	obs:	exp:
tct	S	0	0.32
tcc	S	0	0.26
tca	S	2	0.50
tcg	S	0	0.12
cct	P	0	11.95
ccc	P	47	8.38
cca	P	0	19.20
ccg	P	0	7.47
act	T	0	248.70
acc	T	0	195.50
aca	T	960	441.00
acg	T	0	74.78
agt	S	0	0.41
agc	S	0	0.39
---	--	------	------
mPD		0.18	0.77
		nPD:	0.24
		N. weight:	0.98
		Sc. PD:	0.068
		Sc. rank:	385.7

,

HA			
Pos.	182	obs:	exp:
cgt	R	0	42.93
cgc	R	0	51.06
cga	R	0	100.20
cgg	R	0	83.71
aaa	K	0	2.34
aag	K	4	1.66
aga	R	919	458.90
agg	R	86	268.20
---	--	------	------
mPD		0.17	0.97
		nPD:	0.18
		N. weight:	0.65
		Sc. PD:	0.0075
		Sc. rank:	115.5

,

HA			
Pos.	183	obs:	exp:
tct	S	0	3.52
tcc	S	0	2.85
tca	S	0	5.55
tcg	S	0	1.28
cgt	R	0	0.68
cgc	R	0	0.81
cga	R	0	1.60
cgg	R	0	1.33
att	I	0	0.36
atc	I	1	0.24
ata	I	0	0.39
aat	N	2	10.17
aac	N	18	9.83
aaa	K	799	468.60
aag	K	1	331.40
agt	S	10	4.51
agc	S	12	4.29
aga	R	16	7.31
agg	R	0	4.27
gat	D	0	3.24
gac	D	6	2.76
gaa	E	141	84.82
gag	E	0	56.18
ggt	G	0	0.40
ggc	G	0	0.38
gga	G	3	1.39
ggg	G	0	0.83
---	--	------	------
mPD		0.43	0.89
		nPD:	0.48
		N. weight:	0.65
		Sc. PD:	0.2
		Sc. rank:	440.3

,

HA			
Pos.	184	obs:	exp:
tct	S	0	147.10
tcc	S	0	119.00
tca	S	0	231.90
tcg	S	0	53.48
cgt	R	0	0.04
cgc	R	1	0.05
cga	R	0	0.10
cgg	R	0	0.08
aat	N	1	0.51
aac	N	0	0.49
aaa	K	42	27.53
aag	K	5	19.47
agt	S	0	188.30
agc	S	919	179.20
aga	R	0	0.46
agg	R	0	0.27
gat	D	1	22.13
gac	D	40	18.87
---	--	------	------
mPD		0.33	1.8
		nPD:	0.19
		N. weight:	1.8
		Sc. PD:	0.04
		Sc. rank:	395.1

,

HA			
Pos.	185	obs:	exp:
cct	P	2	256.60
ccc	P	1	179.90
cca	P	1006	412.20
ccg	P	0	160.30
---	--	------	------
mPD		0.0059	0.71
		nPD:	0.01
		N. weight:	1.
		Sc. PD:	-0.16
		Sc. rank:	-644.5

,

HA			
Pos.	186	obs:	exp:
gct	A	1000	258.50
gcc	A	8	189.50
gca	A	0	471.80
gcg	A	1	89.24
---	--	------	------
mPD		0.018	0.67
		nPD:	0.03
		N. weight:	1.6
		Sc. PD:	-0.21
		Sc. rank:	-786.3

,

HA			
Pos.	187	obs:	exp:
tta	L	13	44.56
ttg	L	7	94.61
ctt	L	0	85.92
ctc	L	0	84.94
cta	L	451	84.53
ctg	L	36	112.40
att	I	0	183.20
atc	I	0	121.40
ata	I	502	197.40
---	--	------	------
mPD		0.60	1.3
		nPD:	0.45
		N. weight:	1.4
		Sc. PD:	0.37
		Sc. rank:	902.1

,

HA			
Pos.	188	obs:	exp:
att	I	0	325.10
atc	I	0	215.50
ata	I	891	350.40
gtt	V	0	24.36
gtc	V	0	23.64
gta	V	118	24.43
gtg	V	0	45.57
---	--	------	------
mPD		0.21	0.89
		nPD:	0.23
		N. weight:	1.2
		Sc. PD:	0.079
		Sc. rank:	457.6

,

HA			
Pos.	189	obs:	exp:
att	I	40	24.81
atc	I	0	16.45
ata	I	28	26.74
gtt	V	8	194.00
gtc	V	1	188.30
gta	V	926	194.60
gtg	V	5	363.00
gct	A	0	0.26
gcc	A	0	0.19
gca	A	1	0.47
gcg	A	0	0.09
---	--	------	------
mPD		0.23	0.86
		nPD:	0.27
		N. weight:	1.7
		Sc. PD:	0.17
		Sc. rank:	787.7

,

HA			
Pos.	190	obs:	exp:
tgg	W	1009	1009.00
---	--	------	------
mPD		0	0
		nPD:	1.
		N. weight:	0.
		Sc. PD:	0
		Sc. rank:	0

,

HA			
Pos.	191	obs:	exp:
ggt	G	3	135.30
ggc	G	0	128.20
gga	G	78	466.50
ggg	G	928	279.00
---	--	------	------
mPD		0.15	0.68
		nPD:	0.22
		N. weight:	1.1
		Sc. PD:	0.061
		Sc. rank:	385.0

,

HA			
Pos.	192	obs:	exp:
att	I	20	350.30
atc	I	940	232.20
ata	I	0	377.50
gtt	V	3	10.12
gtc	V	3	9.82
gta	V	0	10.15
gtg	V	43	18.92
---	--	------	------
mPD		0.22	0.76
		nPD:	0.29
		N. weight:	1.5
		Sc. PD:	0.18
		Sc. rank:	804.4

,

HA			
Pos.	193	obs:	exp:
cat	H	964	579.70
cac	H	45	429.30
---	--	------	------
mPD		0.085	0.49
		nPD:	0.17
		N. weight:	0.46
		Sc. PD:	0.0048
		Sc. rank:	78.8

,

HA			
Pos.	194	obs:	exp:
cat	H	960	579.70
cac	H	49	429.30
---	--	------	------
mPD		0.093	0.49
		nPD:	0.19
		N. weight:	0.45
		Sc. PD:	0.011
		Sc. rank:	103.1

,

HA			
Pos.	195	obs:	exp:
ttt	F	0	0.39
ttc	F	1	0.61
tct	S	99	160.90
tcc	S	903	130.10
tca	S	3	253.60
tcg	S	0	58.48
cct	P	0	0.51
ccc	P	2	0.36
cca	P	0	0.82
ccg	P	0	0.32
act	T	0	0.26
acc	T	1	0.20
aca	T	0	0.46
acg	T	0	0.08
agt	S	0	205.90
agc	S	0	196.00
---	--	------	------
mPD		0.19	1.7
		nPD:	0.11
		N. weight:	2.
		Sc. PD:	-0.094
		Sc. rank:	-262.4

,

HA			
Pos. 196		obs:	exp:
att	I	2	0.73
atc	I	0	0.48
ata	I	0	0.79
gtt	V	405	186.60
gtc	V	0	181.10
gta	V	493	187.20
gtg	V	6	349.10
gct	A	0	4.36
gcc	A	0	3.19
gca	A	17	7.95
gcg	A	0	1.50
ggt	G	0	11.53
ggc	G	0	10.93
gga	G	80	39.76
ggg	G	6	23.78
mPD		0.69	0.92
		nPD:	0.75
		N. weight:	0.99
		Sc. PD:	0.55
		Sc. rank:	720.3

HA			
Pos. 197		obs:	exp:
tct	S	0	161.40
tcc	S	0	130.50
tca	S	956	254.40
tcg	S	52	58.66
cct	P	0	0.25
ccc	P	0	0.18
cca	P	1	0.41
ccg	P	0	0.16
agt	S	0	206.60
agc	S	0	196.60
mPD		0.10	1.7
		nPD:	0.06
		N. weight:	1.5
		Sc. PD:	-0.15
		Sc. rank:	-538.2

HA			
Pos. 198		obs:	exp:
tct	S	0	0.16
tcc	S	0	0.13
tca	S	0	0.25
tcg	S	0	0.06
att	I	5	1.82
atc	I	0	1.21
ata	I	0	1.97
act	T	930	246.10
acc	T	19	193.50
aca	T	1	436.40
acg	T	0	74.00
aat	N	5	2.54
aac	N	0	2.46
agt	S	0	0.20
agc	S	1	0.20
gct	A	45	12.30
gcc	A	3	9.01
gca	A	0	22.44
gcg	A	0	4.25
mPD		0.16	0.79
		nPD:	0.2
		N. weight:	1.5
		Sc. PD:	0.054
		Sc. rank:	426.1

HA			
Pos. 199		obs:	exp:
act	T	22	16.06
acc	T	1	12.63
aca	T	39	28.48
acg	T	0	4.83
aat	N	3	2.03
aac	N	1	1.97
gct	A	19	241.10
gcc	A	1	176.70
gca	A	904	440.00
gcg	A	17	83.23
gaa	E	2	1.20
gag	E	0	0.80
mPD		0.25	0.81
		nPD:	0.32
		N. weight:	0.71
		Sc. PD:	0.1
		Sc. rank:	392.0

HA			
Pos. 200		obs:	exp:
aaa	K	0	0.59
aag	K	1	0.41
gaa	E	64	606.30
gag	E	944	401.70
mPD		0.12	0.48
		nPD:	0.25
		N. weight:	0.79
		Sc. PD:	0.066
		Sc. rank:	343.1

HA			
Pos. 201		obs:	exp:
caa	Q	931	518.30
cag	Q	78	490.70
mPD		0.14	0.50
		nPD:	0.29
		N. weight:	0.48
		Sc. PD:	0.056
		Sc. rank:	253.7

HA			
Pos. 202		obs:	exp:
att	I	0	0.36
atc	I	1	0.24
ata	I	0	0.39
act	T	13	261.10
acc	T	995	205.30
aca	T	0	463.10
acg	T	0	78.52
mPD		0.027	0.68
		nPD:	0.04
		N. weight:	1.8
		Sc. PD:	-0.21
		Sc. rank:	-802.1

HA			
Pos. 203		obs:	exp:
caa	Q	0	0.51
cag	Q	1	0.49
cgt	R	0	0.17
cgc	R	0	0.20
cga	R	0	0.40
cgg	R	0	0.33
aaa	K	49	588.10
aag	K	955	415.90
aga	R	0	1.83
agg	R	4	1.07
mPD		0.10	0.50
		nPD:	0.21
		N. weight:	0.81
		Sc. PD:	0.032
		Sc. rank:	242.4

HA			
Pos. 204		obs:	exp:
tta	L	1	88.68
ttg	L	0	188.30
ctt	L	2	171.00
ctc	L	45	169.00
cta	L	949	168.20
ctg	L	12	223.80
mPD		0.11	1.1
		nPD:	0.1
		N. weight:	1.8
		Sc. PD:	-0.11
		Sc. rank:	-317.2

HA			
Pos. 205		obs:	exp:
tat	Y	987	535.00
tac	Y	22	474.00
mPD		0.043	0.50
		nPD:	0.09
		N. weight:	0.64
		Sc. PD:	-0.048
		Sc. rank:	-157.7

HA			
Pos. 206		obs:	exp:
ggt	G	0	135.30
ggc	G	0	128.20
gga	G	16	466.50
ggg	G	993	279.00
mPD		0.031	0.68
		nPD:	0.05
		N. weight:	1.4
		Sc. PD:	-0.16
		Sc. rank:	-602.4

HA			
Pos. 207		obs:	exp:
tct	S	0	161.40
tcc	S	0	130.50
tca	S	0	254.40
tcg	S	0	58.66
act	T	1	0.26
acc	T	0	0.20
aca	T	0	0.46
acg	T	0	0.08
agt	S	1007	206.60
agc	S	1	196.60
mPD		0.0040	1.7
		nPD:	0.
		N. weight:	1.9
		Sc. PD:	-0.29
		Sc. rank:	-1274.6

HA			
Pos. 208		obs:	exp:
gaa	E	2	1.20
gag	E	0	0.80
ggt	G	0	135.00
ggc	G	0	127.90
gga	G	976	465.60
ggg	G	31	278.40
mPD		0.064	0.68
		nPD:	0.09
		N. weight:	0.78
		Sc. PD:	-0.052
		Sc. rank:	-161.8

HA			
Pos. 209		obs:	exp:
tct	S	0	4.80
tcc	S	0	3.88
tca	S	0	7.57
tcg	S	0	1.75
aat	N	2	494.50
aac	N	970	477.50
agt	S	0	6.15
agc	S	30	5.85
gct	A	1	0.26
gcc	A	0	0.19
gca	A	0	0.47
gcg	A	0	0.09
gat	D	0	3.24
gac	D	6	2.76
mPD		0.079	0.62
		nPD:	0.13
		N. weight:	0.87
		Sc. PD:	-0.029
		Sc. rank:	-74.9

HA			
Pos. 210		obs:	exp:
aaa	K	948	591.10
aag	K	61	417.90
mPD		0.11	0.49
		nPD:	0.23
		N. weight:	0.39
		Sc. PD:	0.027
		Sc. rank:	153.5

HA			
Pos. 211		obs:	exp:
tta	L	2	88.06
ttg	L	67	187.00
ctt	L	0	169.80
ctc	L	0	167.90
cta	L	8	167.10
ctg	L	925	222.20
atg	M	3	3.00
gtt	V	0	0.83
gtc	V	0	0.80
gta	V	0	0.83
gtg	V	4	1.54
mPD		0.16	1.1
		nPD:	0.14
		N. weight:	1.5
		Sc. PD:	-0.029
		Sc. rank:	2.4

HA			
Pos. 212		obs:	exp:
att	I	0	31.74
atc	I	0	21.04
ata	I	87	34.21
gtt	V	1	190.30
gtc	V	0	184.70
gta	V	3	190.90
gtg	V	918	356.10
mPD		0.32	0.89
		nPD:	0.36
		N. weight:	1.1
		Sc. PD:	0.21
		Sc. rank:	681.8

HA			
Pos. 213		obs:	exp:
act	T	0	261.40
acc	T	0	205.50
aca	T	1006	463.60
acg	T	3	78.60
mPD		0.0059	0.67
		nPD:	0.01
		N. weight:	0.91
		Sc. PD:	-0.14
		Sc. rank:	-557.4

HA			
Pos. 214		obs:	exp:
att	I	2	0.73
atc	I	0	0.48
ata	I	0	0.79
gtt	V	955	207.90
gtc	V	5	201.70
gta	V	44	208.50
gtg	V	3	388.90
mPD		0.10	0.73
		nPD:	0.14
		N. weight:	1.6
		Sc. PD:	-0.036
		Sc. rank:	-45.5

HA			
Pos. 215		obs:	exp:
gaa	E	1	15.04
gag	E	24	9.96
ggt	G	2	132.00
ggc	G	0	125.00
gga	G	65	455.00
ggg	G	917	272.10
mPD		0.17	0.72
		nPD:	0.24
		N. weight:	1.2
		Sc. PD:	0.089
		Sc. rank:	481.1

HA

Pos.	216	obs:	exp:
tct	S	0	161.50
tcc	S	0	130.60
tca	S	0	254.60
tcg	S	0	58.72
agt	S	921	206.80
agc	S	88	196.80
---	--	------	------
mPD		0.16	1.7
		nPD:	0.1
		N. weight:	1.5
		Sc. PD:	-0.1
		Sc. rank:	-294.5

,

HA

Pos.	217	obs:	exp:
tct	S	957	161.50
tcc	S	5	130.60
tca	S	9	254.60
tcg	S	38	58.72
agt	S	0	206.80
agc	S	0	196.80
---	--	------	------
mPD		0.099	1.7
		nPD:	0.06
		N. weight:	1.9
		Sc. PD:	-0.19
		Sc. rank:	-718.3

,

HA

Pos.	218	obs:	exp:
aat	N	935	488.40
aac	N	25	471.60
aaa	K	48	28.12
aag	K	0	19.88
gat	D	1	0.54
gac	D	0	0.46
---	--	------	------
mPD		0.14	0.55
		nPD:	0.25
		N. weight:	0.67
		Sc. PD:	0.057
		Sc. rank:	298.2

,

HA

Pos.	219	obs:	exp:
tat	Y	934	535.00
tac	Y	75	474.00
---	--	------	------
mPD		0.14	0.50
		nPD:	0.28
		N. weight:	0.46
		Sc. PD:	0.049
		Sc. rank:	224.6

,

HA

Pos.	220	obs:	exp:
caa	Q	933	518.30
cag	Q	76	490.70
---	--	------	------
mPD		0.14	0.50
		nPD:	0.28
		N. weight:	0.49
		Sc. PD:	0.054
		Sc. rank:	243.0

,

HA

Pos.	221	obs:	exp:
caa	Q	936	518.30
cag	Q	73	490.70
---	--	------	------
mPD		0.13	0.50
		nPD:	0.27
		N. weight:	0.49
		Sc. PD:	0.05
		Sc. rank:	234.1

,

HA

Pos.	222	obs:	exp:
tct	S	943	161.50
tcc	S	20	130.60
tca	S	46	254.60
tcg	S	0	58.72
agt	S	0	206.80
agc	S	0	196.80
---	--	------	------
mPD		0.12	1.7
		nPD:	0.08
		N. weight:	1.8
		Sc. PD:	-0.16
		Sc. rank:	-546.3

,

HA

Pos.	223	obs:	exp:
ttt	F	562	397.40
ttc	F	445	609.60
tta	L	2	0.18
ttg	L	0	0.37
ctt	L	0	0.34
ctc	L	0	0.34
cta	L	0	0.33
ctg	L	0	0.44
---	--	------	------
mPD		0.50	0.48
		nPD:	1.03
		N. weight:	0.075
		Sc. PD:	0.062
		Sc. rank:	54.6

,

HA

Pos.	224	obs:	exp:
att	I	0	1.82
atc	I	0	1.21
ata	I	5	1.97
act	T	3	12.18
acc	T	44	9.57
aca	T	0	21.59
acg	T	0	3.66
gtt	V	0	197.10
gtc	V	0	191.30
gta	V	937	197.70
gtg	V	18	368.80
gct	A	0	0.51
gcc	A	0	0.38
gca	A	2	0.94
gcg	A	0	0.18
---	--	------	------
mPD		0.31	0.92
		nPD:	0.34
		N. weight:	1.7
		Sc. PD:	0.3
		Sc. rank:	1033.5

,

HA

Pos.	225	obs:	exp:
cct	P	4	256.60
ccc	P	0	179.90
cca	P	59	412.20
ccg	P	946	160.30
---	--	------	------
mPD		0.12	0.71
		nPD:	0.17
		N. weight:	1.8
		Sc. PD:	0.0029
		Sc. rank:	225.5

,

HA

Pos.	226	obs:	exp:
tct	S	0	159.10
tcc	S	0	128.70
tca	S	0	250.90
tcg	S	0	57.84
att	I	2	0.73
atc	I	0	0.48
ata	I	0	0.79
aat	N	12	6.10
aac	N	0	5.90
agt	S	931	203.70
agc	S	63	193.80
ggt	G	1	0.13
ggc	G	0	0.13
gga	G	0	0.46
ggg	G	0	0.28
---	--	------	------
mPD		0.15	1.7
		nPD:	0.09
		N. weight:	1.6
		Sc. PD:	-0.12
		Sc. rank:	-387.6

,

HA

Pos.	227	obs:	exp:
cct	P	2	256.60
ccc	P	0	179.90
cca	P	966	412.20
ccg	P	41	160.30
---	--	------	------
mPD		0.082	0.71
		nPD:	0.12
		N. weight:	0.9
		Sc. PD:	-0.042
		Sc. rank:	-115.5

,

HA

Pos.	228	obs:	exp:
gaa	E	1	0.60
gag	E	0	0.40
ggt	G	1	135.20
ggc	G	0	128.10
gga	G	956	466.10
ggg	G	51	278.70
---	--	------	------
mPD		0.10	0.68
		nPD:	0.15
		N. weight:	0.71
		Sc. PD:	-0.011
		Sc. rank:	17.7

,

HA

Pos.	229	obs:	exp:
act	T	0	0.26
acc	T	0	0.20
aca	T	1	0.46
acg	T	0	0.08
gct	A	0	258.30
gcc	A	0	189.30
gca	A	190	471.30
gcg	A	818	89.16
---	--	------	------
mPD		0.31	0.67
		nPD:	0.46
		N. weight:	1.9
		Sc. PD:	0.55
		Sc. rank:	1300.3

,

HA

Pos.	230	obs:	exp:
cgt	R	0	43.06
cgc	R	0	51.21
cga	R	41	100.50
cgg	R	4	83.96
aaa	K	1	0.59
aag	K	0	0.41
aga	R	945	460.20
agg	R	18	269.00
---	--	------	------
mPD		0.13	0.97
		nPD:	0.13
		N. weight:	0.7
		Sc. PD:	-0.019
		Sc. rank:	-46.1

,

HA

Pos.	231	obs:	exp:
cct	P	0	256.60
ccc	P	0	179.90
cca	P	1009	412.20
ccg	P	0	160.30
---	--	------	------
mPD		0	0.71
		nPD:	0.
		N. weight:	1.1
		Sc. PD:	-0.17
		Sc. rank:	-780.6

,

HA

Pos.	232	obs:	exp:
tta	L	0	0.09
ttg	L	0	0.19
ctt	L	0	0.17
ctc	L	0	0.17
cta	L	1	0.17
ctg	L	0	0.22
cct	P	0	3.05
ccc	P	0	2.14
cca	P	12	4.90
ccg	P	0	1.91
caa	Q	944	507.50
cag	Q	44	480.50
aaa	K	8	4.69
aag	K	0	3.31
---	--	------	------
mPD		0.12	0.55
		nPD:	0.23
		N. weight:	0.59
		Sc. PD:	0.037
		Sc. rank:	219.5

,

HA

Pos.	233	obs:	exp:
att	I	3	1.09
atc	I	0	0.73
ata	I	0	1.18
gtt	V	933	207.70
gtc	V	2	201.50
gta	V	2	208.30
gtg	V	69	388.50
---	--	------	------
mPD		0.14	0.73
		nPD:	0.19
		N. weight:	1.5
		Sc. PD:	0.042
		Sc. rank:	356.3

,

HA

Pos.	234	obs:	exp:
aat	N	996	513.30
aac	N	13	495.70
---	--	------	------
mPD		0.025	0.50
		nPD:	0.05
		N. weight:	0.73
		Sc. PD:	-0.079
		Sc. rank:	-303.8

,

HA

Pos.	235	obs:	exp:
cgt	R	1	0.04
cgc	R	0	0.05
cga	R	0	0.10
cgg	R	0	0.08
aga	R	0	0.46
agg	R	0	0.27
ggt	G	919	135.20
ggc	G	39	128.10
gga	G	9	466.10
ggg	G	41	278.70
---	--	------	------
mPD		0.17	0.68
		nPD:	0.25
		N. weight:	1.9
		Sc. PD:	0.15
		Sc. rank:	814.1

,

HA

Pos.	236	obs:	exp:
tta	L	216	65.56
ttg	L	0	139.20
ctt	L	1	126.40
ctc	L	0	125.00
cta	L	528	124.40
ctg	L	1	165.40
caa	Q	257	133.00
cag	Q	2	126.00
att	I	0	1.46
atc	I	0	0.97
ata	I	4	1.57
---	--	------	------
mPD		0.73	1.4
		nPD:	0.53
		N. weight:	1.4
		Sc. PD:	0.49
		Sc. rank:	980.1

,

HA

Pos.	237	obs:	exp:
ttt	F	0	0.39
ttc	F	1	0.61
tct	S	819	161.40
tcc	S	137	130.50
tca	S	48	254.40
tcg	S	4	58.66
agt	S	0	206.60
agc	S	0	196.60
---	--	------	------
mPD		0.32	1.7
		nPD:	0.19
		N. weight:	1.5
		Sc. PD:	0.044
		Sc. rank:	373.3

,

HA

Pos.	238	obs:	exp:
ggt	G	0	135.30
ggc	G	0	128.20
gga	G	958	466.50
ggg	G	51	279.00
---	--	------	------
mPD		0.096	0.68
		nPD:	0.14
		N. weight:	0.72
		Sc. PD:	-0.015
		Sc. rank:	-12.0

,

HA

Pos.	239	obs:	exp:
cgt	R	0	43.10
cgc	R	0	51.26
cga	R	1	100.60
cgg	R	0	84.04
aga	R	926	460.70
agg	R	82	269.30
---	--	------	------
mPD		0.15	0.96
		nPD:	0.16
		N. weight:	0.65
		Sc. PD:	-0.0041
		Sc. rank:	48.1

,

HA

Pos.	240	obs:	exp:
att	I	1003	368.20
atc	I	5	244.10
ata	I	1	396.80
---	--	------	------
mPD		0.012	0.65
		nPD:	0.02
		N. weight:	1.2
		Sc. PD:	-0.16
		Sc. rank:	-636.2

,

HA

Pos.	241	obs:	exp:
gat	D	53	544.70
gac	D	956	464.30
---	--	------	------
mPD		0.10	0.50
		nPD:	0.2
		N. weight:	0.67
		Sc. PD:	0.024
		Sc. rank:	188.2

,

HA

Pos.	242	obs:	exp:
ttt	F	999	397.40
ttc	F	8	609.60
tta	L	0	0.09
ttg	L	0	0.19
tct	S	1	0.16
tcc	S	0	0.13
tca	S	0	0.25
tcg	S	0	0.06
ctt	L	1	0.17
ctc	L	0	0.17
cta	L	0	0.17
ctg	L	0	0.22
agt	S	0	0.20
agc	S	0	0.20
---	--	------	------
mPD		0.020	0.48
		nPD:	0.04
		N. weight:	1.1
		Sc. PD:	-0.12
		Sc. rank:	-463.1

,

HA

Pos.	243	obs:	exp:
tta	L	0	0.09
ttg	L	0	0.19
ctt	L	1	0.17
ctc	L	0	0.17
cta	L	0	0.17
ctg	L	0	0.22
cat	H	985	579.10
cac	H	23	428.90
---	--	------	------
mPD		0.047	0.49
		nPD:	0.09
		N. weight:	0.54
		Sc. PD:	-0.036
		Sc. rank:	-108.3

,

HA

Pos.	244	obs:	exp:
tgg	W	1008	1008.00
ggt	G	0	0.13
ggc	G	0	0.13
gga	G	0	0.46
ggg	G	1	0.28
---	--	------	------
mPD		0.0020	0.0034
		nPD:	0.58
		N. weight:	0.0015
		Sc. PD:	0.00061
		Sc. rank:	1.1

,

HA

Pos.	245	obs:	exp:
tta	L	6	88.68
ttg	L	0	188.30
ctt	L	0	171.00
ctc	L	0	169.00
cta	L	957	168.20
ctg	L	46	223.80
---	--	------	------
mPD		0.099	1.1
		nPD:	0.09
		N. weight:	1.9
		Sc. PD:	-0.13
		Sc. rank:	-434.2

,

HA

Pos.	246	obs:	exp:
tta	L	0	6.50
ttg	L	2	13.81
ctt	L	1	12.54
ctc	L	44	12.40
cta	L	1	12.34
ctg	L	26	16.41
att	I	0	111.70
atc	I	1	74.01
ata	I	305	120.30
atg	M	625	625.00
gtt	V	0	0.83
gtc	V	0	0.80
gta	V	1	0.83
gtg	V	3	1.54
---	--	------	------
mPD		0.63	0.68
		nPD:	0.92
		N. weight:	0.41
		Sc. PD:	0.3
		Sc. rank:	300.4

,

HA

Pos.	247	obs:	exp:
tta	L	57	88.68
ttg	L	0	188.30
ctt	L	47	171.00
ctc	L	0	169.00
cta	L	900	168.20
ctg	L	5	223.80
---	--	------	------
mPD		0.21	1.1
		nPD:	0.18
		N. weight:	1.7
		Sc. PD:	0.034
		Sc. rank:	342.3

,

HA

Pos.	248	obs:	exp:
tct	S	0	1.12
tcc	S	0	0.91
tca	S	0	1.77
tcg	S	0	0.41
aat	N	923	482.80
aac	N	26	466.20
agt	S	7	1.43
agc	S	0	1.37
gat	D	28	28.61
gac	D	25	24.39
---	--	------	------
mPD		0.21	0.62
		nPD:	0.34
		N. weight:	0.64
		Sc. PD:	0.11
		Sc. rank:	367.8

,

HA

Pos.	249	obs:	exp:
cct	P	8	256.60
ccc	P	1000	179.90
cca	P	1	412.20
ccg	P	0	160.30
---	--	------	------
mPD		0.018	0.71
		nPD:	0.02
		N. weight:	2.
		Sc. PD:	-0.27
		Sc. rank:	-1007.1

,

HA

Pos.	250	obs:	exp:
aat	N	987	512.80
aac	N	21	495.20
aaa	K	1	0.59
aag	K	0	0.41
---	--	------	------
mPD		0.043	0.50
		nPD:	0.09
		N. weight:	0.69
		Sc. PD:	-0.052
		Sc. rank:	-176.7

,

HA

Pos.	251	obs:	exp:
gat	D	936	544.70
gac	D	73	464.30
---	--	------	------
mPD		0.13	0.50
		nPD:	0.27
		N. weight:	0.44
		Sc. PD:	0.045
		Sc. rank:	214.4

,

HA

Pos.	252	obs:	exp:
act	T	0	261.40
acc	T	1	205.50
aca	T	1004	463.60
acg	T	4	78.60
---	--	------	------
mPD		0.0099	0.67
		nPD:	0.01
		N. weight:	0.9
		Sc. PD:	-0.13
		Sc. rank:	-507.5

,

HA

Pos.	253	obs:	exp:
att	I	0	2.55
atc	I	7	1.69
ata	I	0	2.75
gtt	V	18	206.80
gtc	V	937	200.70
gta	V	0	207.50
gtg	V	47	387.00
---	--	------	------
mPD		0.14	0.74
		nPD:	0.18
		N. weight:	1.6
		Sc. PD:	0.03
		Sc. rank:	301.1

,

HA

Pos.	254	obs:	exp:
att	I	1	0.36
atc	I	0	0.24
ata	I	0	0.39
act	T	955	261.10
acc	T	51	205.30
aca	T	1	463.10
acg	T	1	78.52
---	--	------	------
mPD		0.10	0.68
		nPD:	0.15
		N. weight:	1.4
		Sc. PD:	-0.017
		Sc. rank:	53.2

,

HA

Pos.	255	obs:	exp:
ttt	F	8	398.20
ttc	F	1001	610.80
---	--	------	------
mPD		0.016	0.48
		nPD:	0.03
		N. weight:	0.55
		Sc. PD:	-0.069
		Sc. rank:	-255.1

,

HA			
Pos.	256	obs:	exp:
tct	S	0	153.20
tcc	S	0	123.90
tca	S	0	241.50
tcg	S	0	55.69
act	T	43	11.92
acc	T	2	9.37
aca	T	0	21.13
acg	T	1	3.58
aat	N	3	1.53
aac	N	0	1.47
agt	S	952	196.10
agc	S	5	186.60
ggt	G	3	0.40
ggc	G	0	0.38
gga	G	0	1.39
ggg	G	0	0.83
---	--	------	------
mPD		0.11	1.7
		nPD:	0.07
		N. weight:	1.8
		Sc. PD:	-0.17
		Sc. rank:	-578.7

,

HA			
Pos.	257	obs:	exp:
ttt	F	6	398.20
ttc	F	1003	610.80
---	--	------	------
mPD		0.012	0.48
		nPD:	0.02
		N. weight:	0.56
		Sc. PD:	-0.075
		Sc. rank:	-286.3

,

HA			
Pos.	258	obs:	exp:
aat	N	1007	512.30
aac	N	0	494.70
gat	D	2	1.08
gac	D	0	0.92
---	--	------	------
mPD		0.0040	0.50
		nPD:	0.01
		N. weight:	0.81
		Sc. PD:	-0.12
		Sc. rank:	-508.2

,

HA			
Pos.	259	obs:	exp:
ggt	G	1	135.30
ggc	G	0	128.20
gga	G	4	466.50
ggg	G	1004	279.00
---	--	------	------
mPD		0.0099	0.68
		nPD:	0.01
		N. weight:	1.5
		Sc. PD:	-0.21
		Sc. rank:	-846.3

,

HA			
Pos.	260	obs:	exp:
gct	A	917	258.30
gcc	A	39	189.30
gca	A	48	471.30
gcg	A	4	89.16
ggt	G	1	0.13
ggc	G	0	0.13
gga	G	0	0.46
ggg	G	0	0.28
---	--	------	------
mPD		0.17	0.67
		nPD:	0.25
		N. weight:	1.2
		Sc. PD:	0.1
		Sc. rank:	521.4

,

HA			
Pos.	261	obs:	exp:
ttt	F	10	398.20
ttc	F	999	610.80
---	--	------	------
mPD		0.020	0.48
		nPD:	0.04
		N. weight:	0.54
		Sc. PD:	-0.064
		Sc. rank:	-235.7

,

HA			
Pos.	262	obs:	exp:
att	I	1	368.20
atc	I	0	244.10
ata	I	1008	396.80
---	--	------	------
mPD		0.0020	0.65
		nPD:	0.
		N. weight:	1.1
		Sc. PD:	-0.17
		Sc. rank:	-742.6

,

HA			
Pos.	263	obs:	exp:
gct	A	1005	258.50
gcc	A	4	189.50
gca	A	0	471.80
gcg	A	0	89.24
---	--	------	------
mPD		0.0079	0.67
		nPD:	0.01
		N. weight:	1.6
		Sc. PD:	-0.23
		Sc. rank:	-948.2

,

HA			
Pos.	264	obs:	exp:
cct	P	46	256.60
ccc	P	0	179.90
cca	P	915	412.20
ccg	P	48	160.30
---	--	------	------
mPD		0.17	0.71
		nPD:	0.24
		N. weight:	0.71
		Sc. PD:	0.054
		Sc. rank:	288.2

,

HA			
Pos.	265	obs:	exp:
aat	N	0	0.51
aac	N	1	0.49
gtt	V	0	0.21
gtc	V	1	0.20
gta	V	0	0.21
gtg	V	0	0.39
gat	D	189	528.00
gac	D	789	450.00
gaa	E	21	17.44
gag	E	8	11.56
---	--	------	------
mPD		0.35	0.53
		nPD:	0.67
		N. weight:	0.3
		Sc. PD:	0.15
		Sc. rank:	220.0

,

HA			
Pos.	266	obs:	exp:
cgt	R	942	43.10
cgc	R	20	51.26
cga	R	0	100.60
cgg	R	0	84.04
aga	R	42	460.70
agg	R	5	269.30
---	--	------	------
mPD		0.22	0.96
		nPD:	0.22
		N. weight:	3.3
		Sc. PD:	0.19
		Sc. rank:	1159.6

,

HA			
Pos.	267	obs:	exp:
gtt	V	0	0.41
gtc	V	0	0.40
gta	V	2	0.41
gtg	V	0	0.77
gct	A	0	258.00
gcc	A	0	189.10
gca	A	1006	470.80
gcg	A	1	89.07
---	--	------	------
mPD		0.0059	0.68
		nPD:	0.01
		N. weight:	0.9
		Sc. PD:	-0.13
		Sc. rank:	-553.9

,

HA			
Pos.	268	obs:	exp:
tct	S	0	161.20
tcc	S	0	130.30
tca	S	0	254.10
tcg	S	0	58.60
tgt	C	0	0.41
tgc	C	1	0.59
act	T	1	0.26
acc	T	0	0.20
aca	T	0	0.46
acg	T	0	0.08
agt	S	61	206.30
agc	S	946	196.40
---	--	------	------
mPD		0.12	1.7
		nPD:	0.07
		N. weight:	1.7
		Sc. PD:	-0.15
		Sc. rank:	-516.5

,

HA			
Pos.	269	obs:	exp:
ttt	F	33	398.20
ttc	F	976	610.80
---	--	------	------
mPD		0.063	0.48
		nPD:	0.13
		N. weight:	0.45
		Sc. PD:	-0.013
		Sc. rank:	-35.8

,

HA			
Pos.	270	obs:	exp:
ttt	F	26	10.26
ttc	F	0	15.74
tta	L	0	85.95
ttg	L	17	182.50
ctt	L	20	165.70
ctc	L	43	163.90
cta	L	9	163.10
ctg	L	889	216.90
cct	P	1	0.25
ccc	P	0	0.18
cca	P	0	0.41
ccg	P	0	0.16
atg	M	4	4.00
---	--	------	------
mPD		0.27	1.1
		nPD:	0.24
		N. weight:	1.3
		Sc. PD:	0.098
		Sc. rank:	534.6

,

HA			
Pos.	271	obs:	exp:
cgt	R	0	42.76
cgc	R	0	50.86
cga	R	0	99.81
cgg	R	0	83.37
aaa	K	6	4.10
aag	K	1	2.90
aga	R	982	457.00
agg	R	19	267.20
gaa	E	0	0.60
gag	E	1	0.40
---	--	------	------
mPD		0.059	0.98
		nPD:	0.06
		N. weight:	0.84
		Sc. PD:	-0.083
		Sc. rank:	-312.3

,

HA			
Pos.	272	obs:	exp:
cgt	R	0	0.04
cgc	R	0	0.05
cga	R	0	0.10
cgg	R	0	0.08
aga	R	0	0.46
agg	R	1	0.27
ggt	G	2	135.20
ggc	G	0	128.10
gga	G	886	466.10
ggg	G	120	278.70
---	--	------	------
mPD		0.22	0.68
		nPD:	0.32
		N. weight:	0.55
		Sc. PD:	0.083
		Sc. rank:	308.4

,

HA			
Pos.	273	obs:	exp:
caa	Q	1	0.51
cag	Q	0	0.49
aaa	K	891	563.50
aag	K	71	398.50
gaa	E	8	26.47
gag	E	36	17.53
ggt	G	0	0.27
ggc	G	0	0.25
gga	G	0	0.92
ggg	G	2	0.55
---	--	------	------
mPD		0.29	0.58
		nPD:	0.49
		N. weight:	0.37
		Sc. PD:	0.12
		Sc. rank:	252.8

,

HA			
Pos.	274	obs:	exp:
tct	S	958	161.40
tcc	S	3	130.50
tca	S	34	254.40
tcg	S	13	58.66
aat	N	1	0.51
aac	N	0	0.49
agt	S	0	206.60
agc	S	0	196.60
---	--	------	------
mPD		0.099	1.7
		nPD:	0.06
		N. weight:	1.9
		Sc. PD:	-0.19
		Sc. rank:	-695.0

,

HA			
Pos.	275	obs:	exp:
tta	L	0	4.22
ttg	L	0	8.96
ctt	L	0	8.13
ctc	L	1	8.04
cta	L	47	8.00
ctg	L	0	10.65
att	I	2	8.03
atc	I	0	5.32
ata	I	20	8.65
atg	M	939	939.00
---	--	------	------
mPD		0.22	0.19
		nPD:	1.17
		N. weight:	0.12
		Sc. PD:	0.11
		Sc. rank:	84.1

,

HA

Pos.	276	obs:	exp:
tgg	W	1	1.00
ggt	G	0	135.20
ggc	G	0	128.10
gga	G	1002	466.10
ggg	G	6	278.70
---	--	------	------
mPD		0.016	0.68
		nPD:	0.02
		N. weight:	0.88
		Sc. PD:	-0.12
		Sc. rank:	-464.0

,

HA

Pos.	277	obs:	exp:
att	I	41	343.30
atc	I	900	227.60
ata	I	0	370.10
gtt	V	42	13.83
gtc	V	25	13.42
gta	V	0	13.87
gtg	V	0	25.87
---	-	1	1.00
---	--	------	------
mPD		0.27	0.79
		nPD:	0.35
		N. weight:	1.4
		Sc. PD:	0.25
		Sc. rank:	859.0

,

HA

Pos.	278	obs:	exp:
caa	Q	25	517.80
cag	Q	983	490.20
gaa	E	1	0.60
gag	E	0	0.40
---	--	------	------
mPD		0.052	0.50
		nPD:	0.1
		N. weight:	0.72
		Sc. PD:	-0.041
		Sc. rank:	-121.1

,

HA

Pos.	279	obs:	exp:
tct	S	0	161.50
tcc	S	1	130.60
tca	S	0	254.60
tcg	S	0	58.72
agt	S	1006	206.80
agc	S	2	196.80
---	--	------	------
mPD		0.0099	1.7
		nPD:	0.01
		N. weight:	1.9
		Sc. PD:	-0.28
		Sc. rank:	-1202.3

,

HA

Pos.	280	obs:	exp:
cgt	R	0	0.04
cgc	R	0	0.05
cga	R	0	0.10
cgg	R	0	0.08
aat	N	1	0.51
aac	N	0	0.49
aga	R	1	0.46
agg	R	0	0.27
gat	D	19	24.83
gac	D	27	21.17
gaa	E	5	3.01
gag	E	0	1.99
ggt	G	4	128.20
ggc	G	0	121.50
gga	G	940	442.00
ggg	G	12	264.30
---	--	------	------
mPD		0.22	0.79
		nPD:	0.28
		N. weight:	0.79
		Sc. PD:	0.088
		Sc. rank:	398.7

,

HA

Pos.	281	obs:	exp:
att	I	0	0.73
atc	I	0	0.48
ata	I	2	0.79
atg	M	1	1.00
gtt	V	46	206.40
gtc	V	0	200.30
gta	V	948	207.00
gtg	V	6	386.20
gct	A	0	1.54
gcc	A	0	1.13
gca	A	6	2.81
gcg	A	0	0.53
---	--	------	------
mPD		0.12	0.74
		nPD:	0.16
		N. weight:	1.6
		Sc. PD:	-0.0078
		Sc. rank:	133.7

,

HA

Pos.	282	obs:	exp:
tct	S	1	0.16
tcc	S	0	0.13
tca	S	0	0.25
tcg	S	0	0.06
cct	P	46	11.70
ccc	P	0	8.20
cca	P	0	18.79
ccg	P	0	7.31
cat	H	1	0.57
cac	H	0	0.43
caa	Q	1	490.50
cag	Q	954	464.50
aaa	K	0	2.34
aag	K	4	1.66
agt	S	0	0.20
agc	S	0	0.20
gaa	E	0	1.20
gag	E	2	0.80
---	--	------	------
mPD		0.20	0.62
		nPD:	0.31
		N. weight:	0.89
		Sc. PD:	0.13
		Sc. rank:	487.5

,

HA

Pos.	283	obs:	exp:
tat	Y	0	0.53
tac	Y	1	0.47
---	-	1008	1008.00
---	--	------	------
mPD		0	4.9×10^{-7}
		nPD:	0.
		N. weight:	0.
		Sc. PD:	0
		Sc. rank:	0

,

HA

Pos.	284	obs:	exp:
cgt	R	0	0.04
cgc	R	0	0.05
cga	R	0	0.10
cgg	R	0	0.08
aga	R	0	0.46
agg	R	1	0.27
---	-	1008	1008.00
---	--	------	------
mPD		0	9.5×10^{-7}
		nPD:	0.
		N. weight:	0.
		Sc. PD:	0
		Sc. rank:	0

,

HA

Pos.	285	obs:	exp:
ttt	F	1	0.39
ttc	F	0	0.61
tta	L	1	4.04
ttg	L	45	8.58
ctt	L	0	7.80
ctc	L	0	7.71
cta	L	0	7.67
ctg	L	0	10.20
att	I	5	1.82
atc	I	0	1.21
ata	I	0	1.97
gtt	V	956	197.60
gtc	V	1	191.70
gta	V	0	198.10
gtg	V	0	369.60
---	--	------	------
mPD		0.19	0.82
		nPD:	0.23
		N. weight:	1.9
		Sc. PD:	0.11
		Sc. rank:	691.7

,

HA

Pos.	286	obs:	exp:
gat	D	1004	544.70
gac	D	5	464.30
---	--	------	------
mPD		0.0099	0.50
		nPD:	0.02
		N. weight:	0.7
		Sc. PD:	-0.097
		Sc. rank:	-378.5

,

HA

Pos.	287	obs:	exp:
tct	S	46	7.52
tcc	S	1	6.08
tca	S	0	11.86
tcg	S	0	2.74
act	T	0	2.07
acc	T	8	1.63
aca	T	0	3.68
acg	T	0	0.62
agt	S	0	9.63
agc	S	0	9.17
gtt	V	0	3.10
gtc	V	15	3.00
gta	V	0	3.11
gtg	V	0	5.79
gct	A	21	229.10
gcc	A	851	167.90
gca	A	19	418.00
gcg	A	3	79.07
gat	D	43	24.29
gac	D	2	20.71
---	--	------	------
mPD		0.45	0.94
		nPD:	0.48
		N. weight:	1.7
		Sc. PD:	0.5
		Sc. rank:	1133.7

,

HA

Pos.	288	obs:	exp:
tct	S	0	6.40
tcc	S	0	5.18
tca	S	0	10.09
tcg	S	0	2.33
cgt	R	0	0.04
cgc	R	0	0.05
cga	R	0	0.10
cgg	R	0	0.08
aat	N	856	435.50
aac	N	0	420.50
agt	S	40	8.20
agc	S	0	7.80
aga	R	1	0.46
agg	R	0	0.27
gat	D	91	49.13
gac	D	0	41.87
ggt	G	21	2.82
ggc	G	0	2.67
gga	G	0	9.71
ggg	G	0	5.81
---	--	------	------
mPD		0.31	0.88
		nPD:	0.36
		N. weight:	0.88
		Sc. PD:	0.16
		Sc. rank:	534.4

,

HA

Pos.	289	obs:	exp:
tgt	C	986	414.20
tgc	C	23	594.80
---	--	------	------
mPD		0.045	0.48
		nPD:	0.09
		N. weight:	0.93
		Sc. PD:	-0.064
		Sc. rank:	-203.7

,

HA			
Pos.	290	obs:	exp:
aaa	K	1	1.76
aag	K	2	1.24
gtt	V	0	0.21
gtc	V	0	0.20
gta	V	0	0.21
gtg	V	1	0.39
gat	D	0	1.08
gac	D	2	0.92
gaa	E	959	601.50
gag	E	41	398.50
ggt	G	0	0.40
ggc	G	0	0.38
gga	G	0	1.39
ggg	G	3	0.83
---	--	------	------
mPD		0.11	0.50
		nPD:	0.21
		N. weight:	0.43
		Sc. PD:	0.021
		Sc. rank:	142.2

,

HA			
Pos.	291	obs:	exp:
ggt	G	0	135.30
ggc	G	0	128.20
gga	G	29	466.50
ggg	G	980	279.00
---	--	------	------
mPD		0.056	0.68
		nPD:	0.08
		N. weight:	1.4
		Sc. PD:	-0.11
		Sc. rank:	-363.3

,

HA			
Pos.	292	obs:	exp:
aat	N	0	4.58
aac	N	9	4.42
gat	D	109	538.20
gac	D	888	458.80
gaa	E	1	0.60
gag	E	0	0.40
ggt	G	2	0.27
ggc	G	0	0.25
gga	G	0	0.92
ggg	G	0	0.55
---	--	------	------
mPD		0.22	0.52
		nPD:	0.42
		N. weight:	0.5
		Sc. PD:	0.12
		Sc. rank:	328.0

,

HA			
Pos.	293	obs:	exp:
tgt	C	7	414.20
tgc	C	1002	594.80
---	--	------	------
mPD		0.014	0.48
		nPD:	0.03
		N. weight:	0.59
		Sc. PD:	-0.076
		Sc. rank:	-284.6

,

HA			
Pos.	294	obs:	exp:
ttt	F	3	18.55
ttc	F	44	28.45
tat	Y	961	510.10
tac	Y	1	451.90
---	--	------	------
mPD		0.17	0.59
		nPD:	0.3
		N. weight:	0.73
		Sc. PD:	0.093
		Sc. rank:	394.1

,

HA			
Pos.	295	obs:	exp:
tat	Y	2	1.06
tac	Y	0	0.94
cat	H	944	578.50
cac	H	63	428.50
---	--	------	------
mPD		0.12	0.49
		nPD:	0.25
		N. weight:	0.41
		Sc. PD:	0.032
		Sc. rank:	169.3

,

HA			
Pos.	296	obs:	exp:
tct	S	0	153.40
tcc	S	0	124.00
tca	S	0	241.80
tcg	S	0	55.75
aat	N	45	22.89
aac	N	0	22.11
aaa	K	6	3.51
aag	K	0	2.49
agt	S	957	196.30
agc	S	1	186.80
---	--	------	------
mPD		0.11	1.7
		nPD:	0.06
		N. weight:	1.8
		Sc. PD:	-0.17
		Sc. rank:	-612.9

,

HA			
Pos.	297	obs:	exp:
cgt	R	0	0.98
cgc	R	0	1.17
cga	R	0	2.29
cgg	R	0	1.92
aaa	K	1	0.59
aag	K	0	0.41
aga	R	23	10.50
agg	R	0	6.14
ggt	G	200	132.10
ggc	G	0	125.10
gga	G	756	455.40
ggg	G	29	272.30
---	--	------	------
mPD		0.41	0.72
		nPD:	0.57
		N. weight:	0.5
		Sc. PD:	0.2
		Sc. rank:	356.3

,

HA			
Pos.	298	obs:	exp:
ggt	G	45	135.30
ggc	G	1	128.20
gga	G	77	466.50
ggg	G	886	279.00
---	--	------	------
mPD		0.22	0.68
		nPD:	0.33
		N. weight:	0.99
		Sc. PD:	0.16
		Sc. rank:	560.8

,

HA			
Pos.	299	obs:	exp:
act	T	0	261.40
acc	T	1	205.50
aca	T	958	463.60
acg	T	50	78.60
---	--	------	------
mPD		0.096	0.67
		nPD:	0.14
		N. weight:	0.79
		Sc. PD:	-0.016
		Sc. rank:	-8.1

,

HA			
Pos.	300	obs:	exp:
tta	L	1	0.09
ttg	L	0	0.19
ctt	L	0	0.17
ctc	L	0	0.17
cta	L	0	0.17
ctg	L	0	0.22
att	I	1	367.40
atc	I	0	243.60
ata	I	1006	396.00
atg	M	1	1.00
---	--	------	------
mPD		0.0059	0.66
		nPD:	0.01
		N. weight:	1.1
		Sc. PD:	-0.16
		Sc. rank:	-662.7

,

HA			
Pos.	301	obs:	exp:
tta	L	0	0.09
ttg	L	0	0.19
ctt	L	0	0.17
ctc	L	0	0.17
cta	L	1	0.17
ctg	L	0	0.22
att	I	0	346.60
atc	I	0	229.80
ata	I	950	373.60
atg	M	2	2.00
act	T	0	0.78
acc	T	0	0.61
aca	T	3	1.38
acg	T	0	0.23
gtt	V	0	10.94
gtc	V	1	10.62
gta	V	9	10.97
gtg	V	43	20.47
---	--	------	------
mPD		0.19	0.78
		nPD:	0.25
		N. weight:	1.1
		Sc. PD:	0.091
		Sc. rank:	470.0

,

HA			
Pos.	302	obs:	exp:
tct	S	0	161.00
tcc	S	0	130.20
tca	S	0	253.90
tcg	S	0	58.54
aat	N	3	1.53
aac	N	0	1.47
agt	S	935	206.10
agc	S	71	196.20
---	--	------	------
mPD		0.14	1.7
		nPD:	0.08
		N. weight:	1.6
		Sc. PD:	-0.12
		Sc. rank:	-429.7

,

HA			
Pos.	303	obs:	exp:
tct	S	2	8.48
tcc	S	44	6.86
tca	S	0	13.38
tcg	S	0	3.08
cct	P	0	0.25
ccc	P	0	0.18
cca	P	1	0.41
ccg	P	0	0.16
att	I	0	0.36
atc	I	1	0.24
ata	I	0	0.39
aat	N	50	484.30
aac	N	902	467.70
aaa	K	1	0.59
aag	K	0	0.41
agt	S	0	10.86
agc	S	7	10.34
gat	D	0	0.54
gac	D	1	0.46
---	--	------	------
mPD		0.30	0.69
		nPD:	0.43
		N. weight:	0.67
		Sc. PD:	0.17
		Sc. rank:	435.9

,

HA			
Pos.	304	obs:	exp:
tta	L	4	88.68
ttg	L	946	188.30
ctt	L	0	171.00
ctc	L	0	169.00
cta	L	2	168.20
ctg	L	57	223.80
---	--	------	------
mPD		0.12	1.1
		nPD:	0.11
		N. weight:	1.7
		Sc. PD:	-0.088
		Sc. rank:	-245.3

,

HA			
Pos.	305	obs:	exp:
cct	P	5	256.10
ccc	P	33	179.50
cca	P	967	411.40
ccg	P	2	160.00
cat	H	1	0.57
cac	H	0	0.43
gct	A	0	0.26
gcc	A	0	0.19
gca	A	1	0.47
gcg	A	0	0.09
---	--	------	------
mPD		0.083	0.72
		nPD:	0.12
		N. weight:	0.89
		Sc. PD:	-0.041
		Sc. rank:	-111.7

,

HA			
Pos.	306	obs:	exp:
ttt	F	959	398.20
ttc	F	50	610.80
---	--	------	------
mPD		0.094	0.48
		nPD:	0.2
		N. weight:	0.85
		Sc. PD:	0.027
		Sc. rank:	223.6

,

HA			
Pos.	307	obs:	exp:
caa	Q	7	518.30
cag	Q	1002	490.70
---	--	------	------
mPD		0.014	0.50
		nPD:	0.03
		N. weight:	0.81
		Sc. PD:	-0.11
		Sc. rank:	-401.5

,

HA			
Pos.	308	obs:	exp:
aat	N	46	513.30
aac	N	963	495.70
---	--	------	------
mPD		0.087	0.50
		nPD:	0.17
		N. weight:	0.63
		Sc. PD:	0.0063
		Sc. rank:	103.2

,

HA			
Pos.	309	obs:	exp:
att	I	0	365.20
atc	I	44	242.10
ata	I	957	393.60
gtt	V	0	1.65
gtc	V	3	1.60
gta	V	5	1.66
gtg	V	0	3.09
---	--	------	------
mPD		0.10	0.67
		nPD:	0.16
		N. weight:	0.93
		Sc. PD:	-0.0071
		Sc. rank:	62.2

,

HA				
Pos.	310	obs:	exp:	
aat	N	42	44.77	
aac	N	46	43.23	
gat	D	911	497.20	
gac	D	10	423.80	
---	--	------	------	
mPD		0.26	0.66	
		nPD:	0.4	
		N. weight:	0.61	
		Sc. PD:	0.14	
		Sc. rank:	396.2	,

HA				
Pos.	311	obs:	exp:	
tct	S	0	153.70	
tcc	S	1	124.30	
tca	S	0	242.30	
tcg	S	0	55.87	
cct	P	45	11.70	
ccc	P	1	8.20	
cca	P	0	18.79	
ccg	P	0	7.31	
aat	N	0	1.53	
aac	N	3	1.47	
agt	S	0	196.70	
agc	S	959	187.20	
---	--	------	------	
mPD		0.27	1.7	
		nPD:	0.16	
		N. weight:	1.9	
		Sc. PD:	-0.01	
		Sc. rank:	151.9	,

HA				
Pos.	312	obs:	exp:	
cgt	R	0	43.10	
cgc	R	0	51.26	
cga	R	0	100.60	
cgg	R	0	84.04	
aga	R	86	460.70	
agg	R	923	269.30	
---	--	------	------	
mPD		0.16	0.96	
		nPD:	0.16	
		N. weight:	1.2	
		Sc. PD:	-0.002	
		Sc. rank:	122.9	,

HA				
Pos.	313	obs:	exp:	
act	T	1	21.50	
acc	T	0	16.90	
aca	T	82	38.13	
acg	T	0	6.47	
gct	A	0	237.30	
gcc	A	0	173.90	
gca	A	925	432.90	
gcg	A	1	81.90	
---	--	------	------	
mPD		0.16	0.82	
		nPD:	0.19	
		N. weight:	0.9	
		Sc. PD:	0.021	
		Sc. rank:	201.4	,

HA				
Pos.	314	obs:	exp:	
att	I	2	0.73	
atc	I	0	0.48	
ata	I	0	0.79	
gtt	V	909	207.90	
gtc	V	11	201.70	
gta	V	38	208.50	
gtg	V	49	388.90	
---	--	------	------	
mPD		0.19	0.73	
		nPD:	0.25	
		N. weight:	1.4	
		Sc. PD:	0.12	
		Sc. rank:	615.7	,

HA				
Pos.	315	obs:	exp:	
ggt	G	0	135.30	
ggc	G	0	128.20	
gga	G	963	466.50	
ggg	G	46	279.00	
---	--	------	------	
mPD		0.087	0.68	
		nPD:	0.13	
		N. weight:	0.73	
		Sc. PD:	-0.024	
		Sc. rank:	-61.8	,

HA				
Pos.	316	obs:	exp:	
cgt	R	0	0.09	
cgc	R	0	0.10	
cga	R	0	0.20	
cgg	R	0	0.17	
aaa	K	999	589.30	
aag	K	7	416.70	
aga	R	1	0.91	
agg	R	1	0.53	
gaa	E	1	0.60	
gag	E	0	0.40	
---	--	------	------	
mPD		0.022	0.49	
		nPD:	0.04	
		N. weight:	0.59	
		Sc. PD:	-0.068	
		Sc. rank:	-252.5	,

HA				
Pos.	317	obs:	exp:	
tgt	C	932	414.20	
tgc	C	77	594.80	
---	--	------	------	
mPD		0.14	0.48	
		nPD:	0.29	
		N. weight:	0.71	
		Sc. PD:	0.087	
		Sc. rank:	379.3	,

HA				
Pos.	318	obs:	exp:	
cct	P	46	256.60	
ccc	P	1	179.90	
cca	P	8	412.20	
ccg	P	954	160.30	
---	--	------	------	
mPD		0.10	0.71	
		nPD:	0.15	
		N. weight:	1.9	
		Sc. PD:	-0.032	
		Sc. rank:	35.8	,

HA				
Pos.	319	obs:	exp:	
cgt	R	0	43.06	
cgc	R	0	51.21	
cga	R	24	100.50	
cgg	R	21	83.96	
aaa	K	1	0.59	
aag	K	0	0.41	
aga	R	953	460.20	
agg	R	10	269.00	
---	--	------	------	
mPD		0.15	0.97	
		nPD:	0.15	
		N. weight:	0.72	
		Sc. PD:	-0.0079	
		Sc. rank:	34.1	,

HA				
Pos.	320	obs:	exp:	
tat	Y	1007	535.00	
tac	Y	2	474.00	
---	--	------	------	
mPD		0.0040	0.50	
		nPD:	0.01	
		N. weight:	0.74	
		Sc. PD:	-0.11	
		Sc. rank:	-463.2	,

HA				
Pos.	321	obs:	exp:	
gtt	V	942	208.30	
gtc	V	67	202.10	
gta	V	0	208.90	
gtg	V	0	389.70	
---	--	------	------	
mPD		0.12	0.73	
		nPD:	0.17	
		N. weight:	1.6	
		Sc. PD:	0.011	
		Sc. rank:	223.4	,

HA				
Pos.	322	obs:	exp:	
cgt	R	0	2.35	
cgc	R	0	2.79	
cga	R	0	5.48	
cgg	R	0	4.58	
aaa	K	110	558.80	
aag	K	844	395.20	
aga	R	0	25.11	
agg	R	55	14.68	
---	--	------	------	
mPD		0.30	0.62	
		nPD:	0.48	
		N. weight:	0.64	
		Sc. PD:	0.19	
		Sc. rank:	426.7	,

HA				
Pos.	323	obs:	exp:	
caa	Q	957	518.30	
cag	Q	52	490.70	
---	--	------	------	
mPD		0.098	0.50	
		nPD:	0.2	
		N. weight:	0.56	
		Sc. PD:	0.017	
		Sc. rank:	143.5	,

HA				
Pos.	324	obs:	exp:	
cct	P	0	0.25	
ccc	P	0	0.18	
cca	P	0	0.41	
ccg	P	1	0.16	
cgt	R	0	32.85	
cgc	R	0	39.07	
cga	R	0	76.68	
cgg	R	0	64.05	
act	T	0	11.92	
acc	T	0	9.37	
aca	T	46	21.13	
acg	T	0	3.58	
aaa	K	0	88.46	
aag	K	151	62.54	
aga	R	15	351.10	
agg	R	754	205.20	
gaa	E	4	22.26	
gag	E	33	14.74	
ggt	G	0	0.67	
ggc	G	0	0.64	
gga	G	0	2.31	
ggg	G	5	1.38	
---	--	------	------	
mPD		0.58	1.3	
		nPD:	0.43	
		N. weight:	1.3	
		Sc. PD:	0.35	
		Sc. rank:	880.4	,

HA				
Pos.	325	obs:	exp:	
tct	S	0	161.50	
tcc	S	0	130.60	
tca	S	0	254.60	
tcg	S	0	58.72	
agt	S	960	206.80	
agc	S	49	196.80	
---	--	------	------	
mPD		0.093	1.7	
		nPD:	0.06	
		N. weight:	1.7	
		Sc. PD:	-0.17	
		Sc. rank:	-643.8	,

HA				
Pos.	326	obs:	exp:	
tta	L	3	88.68	
ttg	L	2	188.30	
ctt	L	158	171.00	
ctc	L	46	169.00	
cta	L	0	168.20	
ctg	L	800	223.80	
---	--	------	------	
mPD		0.35	1.1	
		nPD:	0.32	
		N. weight:	1.1	
		Sc. PD:	0.16	
		Sc. rank:	616.5	,

HA				
Pos.	327	obs:	exp:	
tta	L	0	86.22	
ttg	L	3	183.10	
ctt	L	25	166.20	
ctc	L	22	164.40	
cta	L	1	163.60	
ctg	L	930	217.60	
atg	M	28	28.00	
---	--	------	------	
mPD		0.15	1.1	
		nPD:	0.13	
		N. weight:	1.5	
		Sc. PD:	-0.043	
		Sc. rank:	-106.6	,

HA				
Pos.	328	obs:	exp:	
tta	L	4	88.68	
ttg	L	52	188.30	
ctt	L	39	171.00	
ctc	L	0	169.00	
cta	L	524	168.20	
ctg	L	390	223.80	
---	--	------	------	
mPD		0.64	1.1	
		nPD:	0.58	
		N. weight:	0.81	
		Sc. PD:	0.32	
		Sc. rank:	575.7	,

HA				
Pos.	329	obs:	exp:	
gct	A	44	258.50	
gcc	A	3	189.50	
gca	A	960	471.80	
gcg	A	2	89.24	
---	--	------	------	
mPD		0.093	0.67	
		nPD:	0.14	
		N. weight:	0.7	
		Sc. PD:	-0.017	
		Sc. rank:	-33.1	,

HA			
Pos.	330	obs:	exp:
act	T	0	261.40
acc	T	2	205.50
aca	T	1004	463.60
acg	T	3	78.60
---	--	------	------
mPD		0.0099	0.67
		nPD:	0.01
		N. weight:	0.9
		Sc. PD:	-0.13
		Sc. rank:	-504.3

,

HA			
Pos.	331	obs:	exp:
ggt	G	1	135.30
ggc	G	0	128.20
gga	G	21	466.50
ggg	G	987	279.00
---	--	------	------
mPD		0.043	0.68
		nPD:	0.06
		N. weight:	1.4
		Sc. PD:	-0.13
		Sc. rank:	-483.8

,

HA			
Pos.	332	obs:	exp:
atg	M	1009	1009.00
---	--	------	------
mPD		0	0
		nPD:	1.
		N. weight:	0.
		Sc. PD:	0
		Sc. rank:	0

,

HA			
Pos.	333	obs:	exp:
cgt	R	0	1.97
cgc	R	0	2.34
cga	R	0	4.59
cgg	R	0	3.83
aaa	K	25	564.10
aag	K	938	398.90
aga	R	45	21.00
agg	R	1	12.28
---	--	------	------
mPD		0.22	0.60
		nPD:	0.36
		N. weight:	0.9
		Sc. PD:	0.17
		Sc. rank:	549.8

,

HA			
Pos.	334	obs:	exp:
aat	N	966	513.30
aac	N	43	495.70
---	--	------	------
mPD		0.082	0.50
		nPD:	0.16
		N. weight:	0.6
		Sc. PD:	-0.00019
		Sc. rank:	68.2

,

HA			
Pos.	335	obs:	exp:
gtt	V	930	208.10
gtc	V	27	201.90
gta	V	51	208.70
gtg	V	0	389.30
gct	A	1	0.26
gcc	A	0	0.19
gca	A	0	0.47
gcg	A	0	0.09
---	--	------	------
mPD		0.15	0.73
		nPD:	0.2
		N. weight:	1.5
		Sc. PD:	0.056
		Sc. rank:	437.6

,

HA			
Pos.	336	obs:	exp:
cct	P	921	255.80
ccc	P	30	179.40
cca	P	55	411.00
ccg	P	0	159.80
cat	H	1	0.57
cac	H	0	0.43
cgt	R	2	0.09
cgc	R	0	0.10
cga	R	0	0.20
cgg	R	0	0.17
aga	R	0	0.91
agg	R	0	0.53
---	--	------	------
mPD		0.16	0.72
		nPD:	0.23
		N. weight:	1.2
		Sc. PD:	0.074
		Sc. rank:	443.3

,

HA			
Pos.	337	obs:	exp:
gaa	E	26	606.90
gag	E	983	402.10
---	--	------	------
mPD		0.050	0.48
		nPD:	0.1
		N. weight:	0.95
		Sc. PD:	-0.053
		Sc. rank:	-154.2

,

HA			
Pos.	338	obs:	exp:
tta	L	0	1.49
ttg	L	0	3.17
ctt	L	1	2.88
ctc	L	16	2.85
cta	L	0	2.83
ctg	L	0	3.77
cct	P	0	0.76
ccc	P	3	0.53
cca	P	0	1.23
ccg	P	0	0.48
att	I	783	287.50
atc	I	5	190.60
ata	I	0	309.90
act	T	3	0.78
acc	T	0	0.61
aca	T	0	1.38
acg	T	0	0.23
aat	N	6	20.35
aac	N	34	19.65
aaa	K	7	4.10
aag	K	0	2.90
gtt	V	142	31.17
gtc	V	9	30.25
gta	V	0	31.26
gtg	V	0	58.32
---	--	------	------
mPD		0.52	1.1
		nPD:	0.48
		N. weight:	1.2
		Sc. PD:	0.37
		Sc. rank:	834.1

,

HA			
Pos.	339	obs:	exp:
cct	P	1	256.60
ccc	P	62	179.90
cca	P	944	412.20
ccg	P	2	160.30
---	--	------	------
mPD		0.12	0.71
		nPD:	0.17
		N. weight:	0.84
		Sc. PD:	0.0051
		Sc. rank:	107.8

,

HA			
Pos.	340	obs:	exp:
cgt	R	0	0.04
cgc	R	0	0.05
cga	R	0	0.10
cgg	R	0	0.08
aaa	K	15	590.50
aag	K	993	417.50
aga	R	0	0.46
agg	R	1	0.27
---	--	------	------
mPD		0.031	0.49
		nPD:	0.06
		N. weight:	0.96
		Sc. PD:	-0.091
		Sc. rank:	-325.3

,

HA			
Pos.	341	obs:	exp:
cgt	R	0	5.21
cgc	R	0	6.20
cga	R	0	12.16
cgg	R	0	10.16
act	T	0	12.18
acc	T	46	9.57
aca	T	1	21.59
acg	T	0	3.66
aga	R	122	55.70
agg	R	0	32.56
ggt	G	0	112.60
ggc	G	0	106.70
gga	G	829	388.40
ggg	G	11	232.30
---	--	------	------
mPD		0.48	1.0
		nPD:	0.46
		N. weight:	0.9
		Sc. PD:	0.25
		Sc. rank:	598.7

,

HA			
Pos.	342	obs:	exp:
cgt	R	0	0.04
cgc	R	0	0.05
cga	R	0	0.10
cgg	R	0	0.08
aaa	K	141	82.60
aag	K	0	58.40
aga	R	1	0.46
agg	R	0	0.27
gat	D	10	5.40
gac	D	0	4.60
---	-	857	857.00
---	--	------	------
mPD		0.0059	0.016
		nPD:	0.38
		N. weight:	0.
		Sc. PD:	0
		Sc. rank:	0

,

HA			
Pos.	343	obs:	exp:
cgt	R	0	6.49
cgc	R	0	7.72
cga	R	0	15.16
cgg	R	142	12.66
aga	R	0	69.40
agg	R	10	40.57
---	-	857	857.00
---	--	------	------
mPD		0.0028	0.022
		nPD:	0.13
		N. weight:	0.
		Sc. PD:	0
		Sc. rank:	0

,

HA			
Pos.	344	obs:	exp:
att	I	10	3.65
atc	I	0	2.42
ata	I	0	3.93
act	T	132	34.19
acc	T	0	26.88
aca	T	0	60.64
acg	T	0	10.28
aaa	K	0	5.86
aag	K	10	4.14
---	-	857	857.00
---	--	------	------
mPD		0.0082	0.021
		nPD:	0.39
		N. weight:	0.
		Sc. PD:	0
		Sc. rank:	0

,

HA			
Pos.	345	obs:	exp:
tct	S	0	1.60
tcc	S	0	1.29
tca	S	0	2.52
tcg	S	0	0.58
agt	S	0	2.05
agc	S	10	1.95
gct	A	1	36.39
gcc	A	0	26.66
gca	A	1	66.39
gcg	A	140	12.56
---	-	857	857.00
---	--	------	------
mPD		0.0089	0.019
		nPD:	0.46
		N. weight:	0.
		Sc. PD:	0
		Sc. rank:	0

,

HA			
Pos.	346	obs:	exp:
cgt	R	0	0.43
cgc	R	0	0.51
cga	R	10	1.00
cgg	R	0	0.83
aga	R	0	4.57
agg	R	0	2.67
---	-	999	999.00
---	--	------	------
mPD		0	0.000095
		nPD:	0.
		N. weight:	0.
		Sc. PD:	0
		Sc. rank:	0

,

HA			
Pos.	347	obs:	exp:
cat	H	10	5.74
cac	H	0	4.26
---	-	999	999.00
---	--	------	------
mPD		0	0.000048
		nPD:	0.
		N. weight:	0.
		Sc. PD:	0
		Sc. rank:	0

,

HA			
Pos.	348	obs:	exp:
cgt	R	0	0.43
cgc	R	0	0.51
cga	R	10	1.00
cgg	R	0	0.83
aga	R	0	4.57
agg	R	0	2.67
---	-	999	999.00
---	--	------	------
mPD		0	0.000095
		nPD:	0.
		N. weight:	0.
		Sc. PD:	0
		Sc. rank:	0

,

HA			
Pos.	349	obs:	exp:
cgt	R	0	0.43
cgc	R	0	0.51
cga	R	0	1.00
cgg	R	0	0.83
aga	R	0	4.57
agg	R	10	2.67
---	-	999	999.00
---	--	------	------
mPD		0	0.000095
		nPD:	0.
		N. weight:	0.
		Sc. PD:	0
		Sc. rank:	0

HA			
Pos.	350	obs:	exp:
att	I	0	3.65
atc	I	10	2.42
ata	I	0	3.93
---	-	999	999.00
---	--	------	------
mPD		0	0.000064
		nPD:	0.
		N. weight:	0.
		Sc. PD:	0
		Sc. rank:	0

HA			
Pos.	351	obs:	exp:
cgt	R	0	43.10
cgc	R	0	51.26
cga	R	0	100.60
cgg	R	0	84.04
aga	R	1006	460.70
agg	R	3	269.30
---	--	------	------
mPD		0.0059	0.96
		nPD:	0.01
		N. weight:	0.92
		Sc. PD:	-0.14
		Sc. rank:	-578.7

HA			
Pos.	352	obs:	exp:
ggt	G	42	135.30
ggc	G	964	128.20
gga	G	2	466.50
ggg	G	1	279.00
---	--	------	------
mPD		0.086	0.68
		nPD:	0.13
		N. weight:	2.2
		Sc. PD:	-0.079
		Sc. rank:	-215.2

HA			
Pos.	353	obs:	exp:
tta	L	0	88.68
ttg	L	0	188.30
ctt	L	43	171.00
ctc	L	5	169.00
cta	L	946	168.20
ctg	L	15	223.80
---	--	------	------
mPD		0.12	1.1
		nPD:	0.11
		N. weight:	1.8
		Sc. PD:	-0.098
		Sc. rank:	-277.4

HA			
Pos.	354	obs:	exp:
ttt	F	1000	397.80
ttc	F	8	610.20
tta	L	0	0.09
ttg	L	0	0.19
ctt	L	1	0.17
ctc	L	0	0.17
cta	L	0	0.17
ctg	L	0	0.22
---	--	------	------
mPD		0.018	0.48
		nPD:	0.04
		N. weight:	1.1
		Sc. PD:	-0.13
		Sc. rank:	-475.3

HA			
Pos.	355	obs:	exp:
ggt	G	959	135.30
ggc	G	3	128.20
gga	G	45	466.50
ggg	G	2	279.00
---	--	------	------
mPD		0.095	0.68
		nPD:	0.14
		N. weight:	2.1
		Sc. PD:	-0.047
		Sc. rank:	-67.2

HA			
Pos.	356	obs:	exp:
gct	A	956	258.50
gcc	A	5	189.50
gca	A	5	471.80
gcg	A	43	89.24
---	--	------	------
mPD		0.10	0.67
		nPD:	0.15
		N. weight:	1.4
		Sc. PD:	-0.019
		Sc. rank:	45.9

HA			
Pos.	357	obs:	exp:
att	I	45	368.20
atc	I	3	244.10
ata	I	961	396.80
---	--	------	------
mPD		0.091	0.65
		nPD:	0.14
		N. weight:	0.88
		Sc. PD:	-0.021
		Sc. rank:	-40.0

HA			
Pos.	358	obs:	exp:
gct	A	64	258.50
gcc	A	0	189.50
gca	A	126	471.80
gcg	A	819	89.24
---	--	------	------
mPD		0.32	0.67
		nPD:	0.48
		N. weight:	1.9
		Sc. PD:	0.56
		Sc. rank:	1251.7

HA			
Pos.	359	obs:	exp:
ggt	G	943	135.30
ggc	G	2	128.20
gga	G	44	466.50
ggg	G	20	279.00
---	--	------	------
mPD		0.12	0.68
		nPD:	0.18
		N. weight:	2.
		Sc. PD:	0.039
		Sc. rank:	392.7

HA			
Pos.	360	obs:	exp:
ttt	F	3	398.20
ttc	F	1006	610.80
---	--	------	------
mPD		0.0059	0.48
		nPD:	0.01
		N. weight:	0.58
		Sc. PD:	-0.084
		Sc. rank:	-329.4

HA			
Pos.	361	obs:	exp:
att	I	959	368.20
atc	I	3	244.10
ata	I	47	396.80
---	--	------	------
mPD		0.095	0.65
		nPD:	0.14
		N. weight:	0.96
		Sc. PD:	-0.018
		Sc. rank:	3.9

HA			
Pos.	362	obs:	exp:
gaa	E	960	606.90
gag	E	49	402.10
---	--	------	------
mPD		0.093	0.48
		nPD:	0.19
		N. weight:	0.4
		Sc. PD:	0.011
		Sc. rank:	96.7

HA			
Pos.	363	obs:	exp:
aat	N	964	512.80
aac	N	44	495.20
gat	D	1	0.54
gac	D	0	0.46
---	--	------	------
mPD		0.085	0.50
		nPD:	0.17
		N. weight:	0.6
		Sc. PD:	0.0038
		Sc. rank:	77.9

HA			
Pos.	364	obs:	exp:
ggt	G	0	135.30
ggc	G	0	128.20
gga	G	977	466.50
ggg	G	32	279.00
---	--	------	------
mPD		0.061	0.68
		nPD:	0.09
		N. weight:	0.78
		Sc. PD:	-0.054
		Sc. rank:	-172.5

HA			
Pos.	365	obs:	exp:
tgg	W	1009	1009.00
---	--	------	------
mPD		0	0
		nPD:	1.
		N. weight:	0.
		Sc. PD:	0
		Sc. rank:	0

HA			
Pos.	366	obs:	exp:
gaa	E	1005	606.90
gag	E	4	402.10
---	--	------	------
mPD		0.0079	0.48
		nPD:	0.02
		N. weight:	0.58
		Sc. PD:	-0.082
		Sc. rank:	-319.0

HA			
Pos.	367	obs:	exp:
ggt	G	79	135.30
ggc	G	903	128.20
gga	G	27	466.50
ggg	G	0	279.00
---	--	------	------
mPD		0.19	0.68
		nPD:	0.28
		N. weight:	2.
		Sc. PD:	0.23
		Sc. rank:	1020.2

HA			
Pos.	368	obs:	exp:
tta	L	4	88.68
ttg	L	0	188.30
ctt	L	0	171.00
ctc	L	47	169.00
cta	L	923	168.20
ctg	L	35	223.80
---	--	------	------
mPD		0.16	1.1
		nPD:	0.14
		N. weight:	1.7
		Sc. PD:	-0.031
		Sc. rank:	14.4

HA			
Pos.	369	obs:	exp:
att	I	998	366.30
atc	I	6	242.80
ata	I	0	394.80
gtt	V	5	1.03
gtc	V	0	1.00
gta	V	0	1.04
gtg	V	0	1.93
---	--	------	------
mPD		0.022	0.66
		nPD:	0.03
		N. weight:	1.2
		Sc. PD:	-0.15
		Sc. rank:	-544.9

HA			
Pos.	370	obs:	exp:
gat	D	986	544.70
gac	D	23	464.30
---	--	------	------
mPD		0.045	0.50
		nPD:	0.09
		N. weight:	0.61
		Sc. PD:	-0.044
		Sc. rank:	-141.6

HA			
Pos.	371	obs:	exp:
ggt	G	923	135.30
ggc	G	0	128.20
gga	G	50	466.50
ggg	G	36	279.00
---	--	------	------
mPD		0.16	0.68
		nPD:	0.24
		N. weight:	1.9
		Sc. PD:	0.13
		Sc. rank:	748.7

HA			
Pos.	372	obs:	exp:
tgg	W	1009	1009.00
---	--	------	------
mPD		0	0
		nPD:	1.
		N. weight:	0.
		Sc. PD:	0
		Sc. rank:	0

HA			
Pos.	373	obs:	exp:
tat	Y	981	535.00
tac	Y	28	474.00
---	--	------	------
mPD		0.054	0.50
		nPD:	0.11
		N. weight:	0.61
		Sc. PD:	-0.033
		Sc. rank:	-92.2

HA			
Pos.	374	obs:	exp:
ggt	G	982	135.30
ggc	G	27	128.20
gga	G	0	466.50
ggg	G	0	279.00
---	--	------	------
mPD		0.052	0.68
		nPD:	0.08
		N. weight:	2.3
		Sc. PD:	-0.19
		Sc. rank:	-641.9

HA			
Pos.	375	obs:	exp:
ttt	F	0	398.20
ttc	F	1009	610.80
---	--	------	------
mPD		0	0.48
		nPD:	0.
		N. weight:	0.6
		Sc. PD:	-0.094
		Sc. rank:	-437.7

HA			
Pos.	376	obs:	exp:
cgt	R	0	41.78
cgc	R	0	49.69
cga	R	0	97.52
cgg	R	0	81.46
aaa	K	31	18.16
aag	K	0	12.84
aga	R	933	446.50
agg	R	45	261.00
---	--	------	------
mPD		0.14	1.0
		nPD:	0.14
		N. weight:	0.74
		Sc. PD:	-0.015
		Sc. rank:	-6.2

HA			
Pos.	377	obs:	exp:
cat	H	137	579.70
cac	H	872	429.30
---	--	------	------
mPD		0.23	0.49
		nPD:	0.48
		N. weight:	0.5
		Sc. PD:	0.15
		Sc. rank:	341.3

HA			
Pos.	378	obs:	exp:
caa	Q	134	518.30
cag	Q	875	490.70
---	--	------	------
mPD		0.23	0.50
		nPD:	0.46
		N. weight:	0.39
		Sc. PD:	0.11
		Sc. rank:	259.8

HA			
Pos.	379	obs:	exp:
aat	N	992	513.30
aac	N	17	495.70
---	--	------	------
mPD		0.033	0.50
		nPD:	0.07
		N. weight:	0.71
		Sc. PD:	-0.066
		Sc. rank:	-231.8

,

HA			
Pos.	380	obs:	exp:
gct	A	0	258.50
gcc	A	1	189.50
gca	A	1001	471.80
gcg	A	7	89.24
---	--	------	------
mPD		0.016	0.67
		nPD:	0.02
		N. weight:	0.87
		Sc. PD:	-0.12
		Sc. rank:	-452.1

,

HA			
Pos.	381	obs:	exp:
caa	Q	72	518.30
cag	Q	937	490.70
---	--	------	------
mPD		0.13	0.50
		nPD:	0.27
		N. weight:	0.55
		Sc. PD:	0.054
		Sc. rank:	256.6

,

HA			
Pos.	382	obs:	exp:
ggt	G	0	135.30
ggc	G	0	128.20
gga	G	986	466.50
ggg	G	23	279.00
---	--	------	------
mPD		0.045	0.68
		nPD:	0.07
		N. weight:	0.81
		Sc. PD:	-0.076
		Sc. rank:	-265.9

,

HA			
Pos.	383	obs:	exp:
gat	D	1	0.54
gac	D	0	0.46
gaa	E	48	606.30
gag	E	960	401.70
---	--	------	------
mPD		0.093	0.48
		nPD:	0.19
		N. weight:	0.85
		Sc. PD:	0.024
		Sc. rank:	202.9

,

HA			
Pos.	384	obs:	exp:
ggt	G	24	135.30
ggc	G	0	128.20
gga	G	971	466.50
ggg	G	14	279.00
---	--	------	------
mPD		0.073	0.68
		nPD:	0.11
		N. weight:	0.75
		Sc. PD:	-0.04
		Sc. rank:	-113.9

,

HA			
Pos.	385	obs:	exp:
act	T	999	261.40
acc	T	1	205.50
aca	T	5	463.60
acg	T	4	78.60
---	--	------	------
mPD		0.020	0.67
		nPD:	0.03
		N. weight:	1.5
		Sc. PD:	-0.2
		Sc. rank:	-738.1

,

HA			
Pos.	386	obs:	exp:
gct	A	960	258.50
gcc	A	2	189.50
gca	A	46	471.80
gcg	A	1	89.24
---	--	------	------
mPD		0.093	0.67
		nPD:	0.14
		N. weight:	1.4
		Sc. PD:	-0.034
		Sc. rank:	-66.7

,

HA			
Pos.	387	obs:	exp:
gct	A	53	258.50
gcc	A	2	189.50
gca	A	952	471.80
gcg	A	2	89.24
---	--	------	------
mPD		0.11	0.67
		nPD:	0.16
		N. weight:	0.68
		Sc. PD:	-0.0029
		Sc. rank:	60.4

,

HA			
Pos.	388	obs:	exp:
gat	D	998	544.70
gac	D	11	464.30
---	--	------	------
mPD		0.022	0.50
		nPD:	0.04
		N. weight:	0.67
		Sc. PD:	-0.077
		Sc. rank:	-285.1

,

HA			
Pos.	389	obs:	exp:
ttt	F	0	0.39
ttc	F	1	0.61
tat	Y	3	534.50
tac	Y	1005	473.50
---	--	------	------
mPD		0.0079	0.50
		nPD:	0.02
		N. weight:	0.88
		Sc. PD:	-0.12
		Sc. rank:	-487.3

,

HA			
Pos.	390	obs:	exp:
aaa	K	1006	591.10
aag	K	3	417.90
---	--	------	------
mPD		0.0059	0.49
		nPD:	0.01
		N. weight:	0.62
		Sc. PD:	-0.09
		Sc. rank:	-355.7

,

HA			
Pos.	391	obs:	exp:
tct	S	0	161.50
tcc	S	0	130.60
tca	S	0	254.60
tcg	S	0	58.72
agt	S	1	206.80
agc	S	1008	196.80
---	--	------	------
mPD		0.0020	1.7
		nPD:	0.
		N. weight:	1.9
		Sc. PD:	-0.3
		Sc. rank:	-1356.5

,

HA			
Pos.	392	obs:	exp:
act	T	976	261.40
acc	T	33	205.50
aca	T	0	463.60
acg	T	0	78.60
---	--	------	------
mPD		0.063	0.67
		nPD:	0.09
		N. weight:	1.5
		Sc. PD:	-0.097
		Sc. rank:	-296.7

,

HA			
Pos.	393	obs:	exp:
caa	Q	998	518.30
cag	Q	11	490.70
---	--	------	------
mPD		0.022	0.50
		nPD:	0.04
		N. weight:	0.73
		Sc. PD:	-0.084
		Sc. rank:	-311.1

,

HA			
Pos.	394	obs:	exp:
tct	S	59	161.50
tcc	S	3	130.60
tca	S	14	254.60
tcg	S	933	58.72
agt	S	0	206.80
agc	S	0	196.80
---	--	------	------
mPD		0.14	1.7
		nPD:	0.09
		N. weight:	2.9
		Sc. PD:	-0.22
		Sc. rank:	-728.8

,

HA			
Pos.	395	obs:	exp:
gct	A	3	258.50
gcc	A	0	189.50
gca	A	1002	471.80
gcg	A	4	89.24
---	--	------	------
mPD		0.014	0.67
		nPD:	0.02
		N. weight:	0.87
		Sc. PD:	-0.12
		Sc. rank:	-464.1

,

HA			
Pos.	396	obs:	exp:
att	I	908	367.80
atc	I	1	243.80
ata	I	99	396.40
gtt	V	0	0.21
gtc	V	0	0.20
gta	V	1	0.21
gtg	V	0	0.39
---	--	------	------
mPD		0.18	0.66
		nPD:	0.28
		N. weight:	0.81
		Sc. PD:	0.089
		Sc. rank:	401.7

,

HA			
Pos.	397	obs:	exp:
tat	Y	1	0.53
tac	Y	0	0.47
aat	N	4	2.03
aac	N	0	1.97
gat	D	989	540.90
gac	D	13	461.10
gaa	E	0	1.20
gag	E	2	0.80
---	--	------	------
mPD		0.039	0.51
		nPD:	0.08
		N. weight:	0.66
		Sc. PD:	-0.055
		Sc. rank:	-192.4

,

HA			
Pos.	398	obs:	exp:
caa	Q	962	518.30
cag	Q	47	490.70
---	--	------	------
mPD		0.089	0.50
		nPD:	0.18
		N. weight:	0.58
		Sc. PD:	0.0079
		Sc. rank:	105.8

,

HA			
Pos.	399	obs:	exp:
att	I	2	364.90
atc	I	42	241.90
ata	I	956	393.30
gtt	V	0	1.86
gtc	V	0	1.80
gta	V	9	1.86
gtg	V	0	3.48
---	--	------	------
mPD		0.10	0.67
		nPD:	0.15
		N. weight:	0.93
		Sc. PD:	-0.012
		Sc. rank:	32.6

,

HA			
Pos.	400	obs:	exp:
act	T	2	261.10
acc	T	0	205.30
aca	T	1001	463.10
acg	T	5	78.52
gct	A	0	0.26
gcc	A	0	0.19
gca	A	1	0.47
gcg	A	0	0.09
---	--	------	------
mPD		0.016	0.68
		nPD:	0.02
		N. weight:	0.89
		Sc. PD:	-0.12
		Sc. rank:	-465.8

,

HA			
Pos.	401	obs:	exp:
tct	S	0	0.48
tcc	S	0	0.39
tca	S	0	0.76
tcg	S	0	0.17
aat	N	0	0.51
aac	N	1	0.49
agt	S	0	0.61
agc	S	3	0.59
ggt	G	0	134.80
ggc	G	43	127.70
gga	G	446	464.70
ggg	G	516	277.90
---	--	------	------
mPD		0.55	0.69
		nPD:	0.8
		N. weight:	0.32
		Sc. PD:	0.19
		Sc. rank:	229.6

,

HA			
Pos.	402	obs:	exp:
aaa	K	1007	591.10
aag	K	2	417.90
---	--	------	------
mPD		0.0040	0.49
		nPD:	0.01
		N. weight:	0.62
		Sc. PD:	-0.093
		Sc. rank:	-388.3

,

HA			
Pos.	403	obs:	exp:
tta	L	956	88.68
ttg	L	50	188.30
ctt	L	0	171.00
ctc	L	0	169.00
cta	L	1	168.20
ctg	L	2	223.80
---	--	------	------
mPD		0.10	1.1
		nPD:	0.09
		N. weight:	2.6
		Sc. PD:	-0.18
		Sc. rank:	-545.7

,

HA			
Pos.	404	obs:	exp:
aat	N	59	513.30
aac	N	950	495.70
---	--	------	------
mPD		0.11	0.50
		nPD:	0.22
		N. weight:	0.58
		Sc. PD:	0.032
		Sc. rank:	201.4

HA			
Pos.	405	obs:	exp:
cgt	R	47	43.10
cgc	R	0	51.26
cga	R	57	100.60
cgg	R	904	84.04
aga	R	0	460.70
agg	R	1	269.30
---	--	------	------
mPD		0.19	0.96
		nPD:	0.2
		N. weight:	2.5
		Sc. PD:	0.086
		Sc. rank:	685.3

HA			
Pos.	406	obs:	exp:
tta	L	1	88.24
ttg	L	0	187.40
ctt	L	949	170.10
ctc	L	5	168.20
cta	L	43	167.40
ctg	L	6	222.70
att	I	5	1.82
atc	I	0	1.21
ata	I	0	1.97
---	--	------	------
mPD		0.12	1.1
		nPD:	0.1
		N. weight:	1.8
		Sc. PD:	-0.1
		Sc. rank:	-308.7

HA			
Pos.	407	obs:	exp:
att	I	37	367.40
atc	I	10	243.60
ata	I	960	396.00
atg	M	1	1.00
gtt	V	0	0.21
gtc	V	0	0.20
gta	V	1	0.21
gtg	V	0	0.39
---	--	------	------
mPD		0.094	0.66
		nPD:	0.14
		N. weight:	0.88
		Sc. PD:	-0.018
		Sc. rank:	-10.7

HA			
Pos.	408	obs:	exp:
gtt	V	0	0.21
gtc	V	0	0.20
gta	V	1	0.21
gtg	V	0	0.39
gct	A	0	103.30
gcc	A	8	75.67
gca	A	391	188.40
gcg	A	4	35.64
gat	D	1	25.37
gac	D	46	21.63
gaa	E	551	335.00
gag	E	6	222.00
ggt	G	0	0.13
ggc	G	0	0.13
gga	G	1	0.46
ggg	G	0	0.28
---	--	------	------
mPD		0.60	1.1
		nPD:	0.54
		N. weight:	0.65
		Sc. PD:	0.23
		Sc. rank:	458.1

HA			
Pos.	409	obs:	exp:
cgt	R	0	0.09
cgc	R	0	0.10
cga	R	0	0.20
cgg	R	0	0.17
aaa	K	1006	589.90
aag	K	1	417.10
aga	R	2	0.91
agg	R	0	0.53
---	--	------	------
mPD		0.0059	0.49
		nPD:	0.01
		N. weight:	0.63
		Sc. PD:	-0.092
		Sc. rank:	-364.8

HA			
Pos.	410	obs:	exp:
act	T	4	261.40
acc	T	958	205.50
aca	T	47	463.60
acg	T	0	78.60
---	--	------	------
mPD		0.096	0.67
		nPD:	0.14
		N. weight:	1.6
		Sc. PD:	-0.032
		Sc. rank:	-6.8

HA			
Pos.	411	obs:	exp:
aat	N	68	513.30
aac	N	941	495.70
---	--	------	------
mPD		0.13	0.50
		nPD:	0.25
		N. weight:	0.55
		Sc. PD:	0.047
		Sc. rank:	242.1

HA			
Pos.	412	obs:	exp:
caa	Q	960	518.30
cag	Q	49	490.70
---	--	------	------
mPD		0.093	0.50
		nPD:	0.19
		N. weight:	0.57
		Sc. PD:	0.012
		Sc. rank:	117.6

HA			
Pos.	413	obs:	exp:
cat	H	1	0.57
cac	H	0	0.43
caa	Q	957	517.80
cag	Q	51	490.20
---	--	------	------
mPD		0.098	0.50
		nPD:	0.2
		N. weight:	0.56
		Sc. PD:	0.017
		Sc. rank:	143.5

HA			
Pos.	414	obs:	exp:
ttt	F	1002	397.80
ttc	F	6	610.20
tta	L	0	0.09
ttg	L	0	0.19
ctt	L	1	0.17
ctc	L	0	0.17
cta	L	0	0.17
ctg	L	0	0.22
---	--	------	------
mPD		0.014	0.48
		nPD:	0.03
		N. weight:	1.1
		Sc. PD:	-0.14
		Sc. rank:	-514.5

HA			
Pos.	415	obs:	exp:
aaa	K	0	49.21
aag	K	84	34.79
gaa	E	51	554.60
gag	E	871	367.40
ggt	G	0	0.40
ggc	G	0	0.38
gga	G	0	1.39
ggg	G	3	0.83
---	--	------	------
mPD		0.25	0.64
		nPD:	0.4
		N. weight:	0.84
		Sc. PD:	0.19
		Sc. rank:	538.2

HA			
Pos.	416	obs:	exp:
tta	L	21	88.50
ttg	L	940	187.90
ctt	L	1	170.60
ctc	L	0	168.70
cta	L	1	167.90
ctg	L	44	223.30
atg	M	2	2.00
---	--	------	------
mPD		0.14	1.1
		nPD:	0.12
		N. weight:	1.7
		Sc. PD:	-0.067
		Sc. rank:	-188.5

HA			
Pos.	417	obs:	exp:
att	I	1	366.00
atc	I	0	242.60
ata	I	1002	394.40
atg	M	2	2.00
gtt	V	0	0.83
gtc	V	0	0.80
gta	V	4	0.83
gtg	V	0	1.54
---	--	------	------
mPD		0.014	0.66
		nPD:	0.02
		N. weight:	1.1
		Sc. PD:	-0.15
		Sc. rank:	-589.9

HA			
Pos.	418	obs:	exp:
gat	D	1	544.70
gac	D	1008	464.30
---	--	------	------
mPD		0.0020	0.50
		nPD:	0.
		N. weight:	0.92
		Sc. PD:	-0.14
		Sc. rank:	-607.8

HA			
Pos.	419	obs:	exp:
cat	H	1	0.57
cac	H	0	0.43
aat	N	987	512.80
aac	N	21	495.20
---	--	------	------
mPD		0.043	0.50
		nPD:	0.09
		N. weight:	0.69
		Sc. PD:	-0.052
		Sc. rank:	-180.7

HA			
Pos.	420	obs:	exp:
gaa	E	990	606.90
gag	E	19	402.10
---	--	------	------
mPD		0.037	0.48
		nPD:	0.08
		N. weight:	0.51
		Sc. PD:	-0.042
		Sc. rank:	-147.3

HA			
Pos.	421	obs:	exp:
ttt	F	0	398.20
ttc	F	1009	610.80
---	--	------	------
mPD		0	0.48
		nPD:	0.
		N. weight:	0.6
		Sc. PD:	-0.094
		Sc. rank:	-437.7

HA			
Pos.	422	obs:	exp:
tct	S	0	6.88
tcc	S	0	5.57
tca	S	0	10.85
tcg	S	0	2.50
act	T	39	10.10
acc	T	0	7.94
aca	T	0	17.92
acg	T	0	3.04
aat	N	798	471.60
aac	N	129	455.40
agt	S	43	8.81
agc	S	0	8.39
---	--	------	------
mPD		0.38	0.73
		nPD:	0.51
		N. weight:	0.45
		Sc. PD:	0.15
		Sc. rank:	312.1

HA			
Pos.	423	obs:	exp:
gaa	E	49	606.90
gag	E	960	402.10
---	--	------	------
mPD		0.093	0.48
		nPD:	0.19
		N. weight:	0.85
		Sc. PD:	0.024
		Sc. rank:	203.3

HA			
Pos.	424	obs:	exp:
att	I	0	16.78
atc	I	0	11.13
ata	I	46	18.09
gtt	V	40	198.80
gtc	V	0	192.90
gta	V	923	199.40
gtg	V	0	371.90
---	--	------	------
mPD		0.16	0.82
		nPD:	0.2
		N. weight:	1.7
		Sc. PD:	0.057
		Sc. rank:	458.1

,

HA			
Pos.	425	obs:	exp:
gaa	E	88	606.90
gag	E	921	402.10
---	--	------	------
mPD		0.16	0.48
		nPD:	0.33
		N. weight:	0.71
		Sc. PD:	0.11
		Sc. rank:	404.6

,

HA			
Pos.	426	obs:	exp:
caa	Q	47	24.14
cag	Q	0	22.86
cgt	R	0	0.47
cgc	R	0	0.56
cga	R	0	1.10
cgg	R	0	0.92
act	T	1	9.58
acc	T	0	7.53
aca	T	0	17.00
acg	T	36	2.88
aaa	K	46	534.20
aag	K	866	377.80
aga	R	0	5.02
agg	R	11	2.94
gaa	E	0	1.20
gag	E	2	0.80
---	--	------	------
mPD		0.35	0.69
		nPD:	0.51
		N. weight:	0.88
		Sc. PD:	0.29
		Sc. rank:	609.8

,

HA			
Pos.	427	obs:	exp:
caa	Q	997	518.30
cag	Q	12	490.70
---	--	------	------
mPD		0.024	0.50
		nPD:	0.05
		N. weight:	0.72
		Sc. PD:	-0.081
		Sc. rank:	-302.6

,

HA			
Pos.	428	obs:	exp:
tta	L	0	0.18
ttg	L	0	0.37
ctt	L	1	0.34
ctc	L	1	0.34
cta	L	0	0.33
ctg	L	0	0.44
att	I	105	366.70
atc	I	899	243.10
ata	I	1	395.20
gtt	V	0	0.41
gtc	V	2	0.40
gta	V	0	0.41
gtg	V	0	0.77
---	--	------	------
mPD		0.20	0.66
		nPD:	0.3
		N. weight:	1.2
		Sc. PD:	0.16
		Sc. rank:	671.9

,

HA			
Pos.	429	obs:	exp:
tct	S	0	0.16
tcc	S	0	0.13
tca	S	0	0.25
tcg	S	0	0.06
agt	S	1	0.20
agc	S	0	0.20
ggt	G	935	135.20
ggc	G	26	128.10
gga	G	3	466.10
ggg	G	44	278.70
---	--	------	------
mPD		0.14	0.68
		nPD:	0.2
		N. weight:	2.
		Sc. PD:	0.078
		Sc. rank:	593.4

,

HA			
Pos.	430	obs:	exp:
aat	N	991	513.30
aac	N	18	495.70
---	--	------	------
mPD		0.035	0.50
		nPD:	0.07
		N. weight:	0.7
		Sc. PD:	-0.063
		Sc. rank:	-220.9

,

HA			
Pos.	431	obs:	exp:
tta	L	0	0.18
ttg	L	2	0.37
ctt	L	0	0.34
ctc	L	0	0.34
cta	L	0	0.33
ctg	L	0	0.44
att	I	0	0.36
atc	I	1	0.24
ata	I	0	0.39
atg	M	2	2.00
gtt	V	1	207.30
gtc	V	46	201.10
gta	V	10	207.90
gtg	V	947	387.70
---	--	------	------
mPD		0.12	0.73
		nPD:	0.16
		N. weight:	0.89
		Sc. PD:	-0.0011
		Sc. rank:	94.9

,

HA			
Pos.	432	obs:	exp:
att	I	46	368.20
atc	I	0	244.10
ata	I	963	396.80
---	--	------	------
mPD		0.087	0.65
		nPD:	0.13
		N. weight:	0.9
		Sc. PD:	-0.026
		Sc. rank:	-68.3

,

HA			
Pos.	433	obs:	exp:
tct	S	0	0.32
tcc	S	0	0.26
tca	S	0	0.50
tcg	S	0	0.12
aat	N	876	512.30
aac	N	131	494.70
agt	S	2	0.41
agc	S	0	0.39
---	--	------	------
mPD		0.23	0.51
		nPD:	0.45
		N. weight:	0.36
		Sc. PD:	0.1
		Sc. rank:	236.4

,

HA			
Pos.	434	obs:	exp:
tgg	W	1009	1009.00
---	--	------	------
mPD		0	0
		nPD:	1.
		N. weight:	0.
		Sc. PD:	0
		Sc. rank:	0

,

HA			
Pos.	435	obs:	exp:
act	T	1	261.40
acc	T	959	205.50
aca	T	49	463.60
acg	T	0	78.60
---	--	------	------
mPD		0.094	0.67
		nPD:	0.14
		N. weight:	1.6
		Sc. PD:	-0.037
		Sc. rank:	-60.8

,

HA			
Pos.	436	obs:	exp:
cgt	R	0	43.10
cgc	R	0	51.26
cga	R	44	100.60
cgg	R	0	84.04
aga	R	965	460.70
agg	R	0	269.30
---	--	------	------
mPD		0.083	0.96
		nPD:	0.09
		N. weight:	0.8
		Sc. PD:	-0.06
		Sc. rank:	-197.2

,

HA			
Pos.	437	obs:	exp:
gat	D	981	544.70
gac	D	28	464.30
---	--	------	------
mPD		0.054	0.50
		nPD:	0.11
		N. weight:	0.59
		Sc. PD:	-0.031
		Sc. rank:	-88.3

,

HA			
Pos.	438	obs:	exp:
tct	S	923	161.50
tcc	S	39	130.60
tca	S	47	254.60
tcg	S	0	58.72
agt	S	0	206.80
agc	S	0	196.80
---	--	------	------
mPD		0.16	1.7
		nPD:	0.1
		N. weight:	1.8
		Sc. PD:	-0.11
		Sc. rank:	-327.0

,

HA			
Pos.	439	obs:	exp:
att	I	0	335.30
atc	I	0	222.30
ata	I	919	361.40
atg	M	88	88.00
gtt	V	0	0.41
gtc	V	0	0.40
gta	V	1	0.41
gtg	V	1	0.77
---	--	------	------
mPD		0.16	0.71
		nPD:	0.23
		N. weight:	1.
		Sc. PD:	0.068
		Sc. rank:	389.2

,

HA			
Pos.	440	obs:	exp:
att	I	0	0.73
atc	I	0	0.48
ata	I	2	0.79
act	T	47	260.90
acc	T	0	205.10
aca	T	955	462.60
acg	T	5	78.44
---	--	------	------
mPD		0.10	0.68
		nPD:	0.15
		N. weight:	0.72
		Sc. PD:	-0.0089
		Sc. rank:	28.9

,

HA			
Pos.	441	obs:	exp:
gaa	E	962	606.90
gag	E	47	402.10
---	--	------	------
mPD		0.089	0.48
		nPD:	0.19
		N. weight:	0.41
		Sc. PD:	0.0085
		Sc. rank:	85.3

,

HA			
Pos.	442	obs:	exp:
tta	L	0	3.34
ttg	L	21	7.09
ctt	L	0	6.44
ctc	L	0	6.37
cta	L	1	6.34
ctg	L	16	8.43
att	I	0	2.55
atc	I	0	1.69
ata	I	7	2.75
atg	M	26	26.00
gtt	V	1	193.60
gtc	V	0	187.90
gta	V	182	194.20
gtg	V	755	362.20
---	--	------	------
mPD		0.44	0.85
		nPD:	0.52
		N. weight:	0.69
		Sc. PD:	0.23
		Sc. rank:	478.3

,

HA			
Pos.	443	obs:	exp:
tgg	W	1009	1009.00
---	--	------	------
mPD		0	0
		nPD:	1.
		N. weight:	0.
		Sc. PD:	0
		Sc. rank:	0

,

HA			
Pos.	444	obs:	exp:
tct	S	1	161.50
tcc	S	36	130.60
tca	S	926	254.60
tcg	S	46	58.72
agt	S	0	206.80
agc	S	0	196.80
---	--	------	------
mPD		0.15	1.7
		nPD:	0.09
		N. weight:	1.3
		Sc. PD:	-0.091
		Sc. rank:	-291.9

,

HA			
Pos.	445	obs:	exp:
tat	Y	44	535.00
tac	Y	965	474.00
---	--	------	------
mPD		0.083	0.50
		nPD:	0.17
		N. weight:	0.69
		Sc. PD:	0.0025
		Sc. rank:	85.3

,

HA			
Pos.	446	obs:	exp:
aat	N	1004	513.30
aac	N	5	495.70
---	--	------	------
mPD		0.0099	0.50
		nPD:	0.02
		N. weight:	0.77
		Sc. PD:	-0.11
		Sc. rank:	-419.2

,

HA			
Pos.	447	obs:	exp:
gtt	V	2	0.41
gtc	V	0	0.40
gta	V	0	0.41
gtg	V	0	0.77
gct	A	959	258.00
gcc	A	30	189.10
gca	A	16	470.80
gcg	A	2	89.07
---	--	------	------
mPD		0.096	0.68
		nPD:	0.14
		N. weight:	1.4
		Sc. PD:	-0.029
		Sc. rank:	-25.4

,

HA			
Pos.	448	obs:	exp:
gaa	E	1006	606.90
gag	E	3	402.10
---	--	------	------
mPD		0.0059	0.48
		nPD:	0.01
		N. weight:	0.59
		Sc. PD:	-0.085
		Sc. rank:	-335.4

,

HA			
Pos.	449	obs:	exp:
ttt	F	0	21.31
ttc	F	54	32.69
tta	L	0	83.93
ttg	L	24	178.20
ctt	L	7	161.80
ctc	L	900	160.00
cta	L	2	159.20
ctg	L	22	211.80
---	--	------	------
mPD		0.25	1.2
		nPD:	0.21
		N. weight:	1.7
		Sc. PD:	0.083
		Sc. rank:	560.2

,

HA			
Pos.	450	obs:	exp:
tta	L	20	88.68
ttg	L	943	188.30
ctt	L	0	171.00
ctc	L	0	169.00
cta	L	2	168.20
ctg	L	44	223.80
---	--	------	------
mPD		0.13	1.1
		nPD:	0.12
		N. weight:	1.7
		Sc. PD:	-0.075
		Sc. rank:	-203.9

,

HA			
Pos.	451	obs:	exp:
att	I	0	62.39
atc	I	0	41.36
ata	I	171	67.25
gtt	V	0	173.00
gtc	V	0	167.90
gta	V	689	173.50
gtg	V	149	323.60
---	--	------	------
mPD		0.53	1.0
		nPD:	0.52
		N. weight:	1.2
		Sc. PD:	0.4
		Sc. rank:	822.6

,

HA			
Pos.	452	obs:	exp:
gct	A	36	258.50
gcc	A	1	189.50
gca	A	972	471.80
gcg	A	0	89.24
---	--	------	------
mPD		0.071	0.67
		nPD:	0.11
		N. weight:	0.75
		Sc. PD:	-0.042
		Sc. rank:	-120.2

,

HA			
Pos.	453	obs:	exp:
atg	M	1005	1005.00
act	T	0	1.04
acc	T	0	0.81
aca	T	0	1.84
acg	T	4	0.31
---	--	------	------
mPD		0.0079	0.015
		nPD:	0.52
		N. weight:	0.012
		Sc. PD:	0.0041
		Sc. rank:	8.4

,

HA			
Pos.	454	obs:	exp:
gaa	E	50	606.90
gag	E	959	402.10
---	--	------	------
mPD		0.094	0.48
		nPD:	0.2
		N. weight:	0.84
		Sc. PD:	0.027
		Sc. rank:	217.7

,

HA			
Pos.	455	obs:	exp:
aat	N	76	513.30
aac	N	933	495.70
---	--	------	------
mPD		0.14	0.50
		nPD:	0.28
		N. weight:	0.53
		Sc. PD:	0.059
		Sc. rank:	265.0

,

HA			
Pos.	456	obs:	exp:
caa	Q	74	518.30
cag	Q	935	490.70
---	--	------	------
mPD		0.14	0.50
		nPD:	0.27
		N. weight:	0.55
		Sc. PD:	0.057
		Sc. rank:	266.0

,

HA			
Pos.	457	obs:	exp:
cat	H	955	579.70
cac	H	54	429.30
---	--	------	------
mPD		0.10	0.49
		nPD:	0.21
		N. weight:	0.44
		Sc. PD:	0.018
		Sc. rank:	132.1

,

HA			
Pos.	458	obs:	exp:
act	T	0	261.40
acc	T	3	205.50
aca	T	1004	463.60
acg	T	2	78.60
---	--	------	------
mPD		0.0099	0.67
		nPD:	0.01
		N. weight:	0.9
		Sc. PD:	-0.13
		Sc. rank:	-503.7

,

HA			
Pos.	459	obs:	exp:
tta	L	0	0.09
ttg	L	0	0.19
ctt	L	1	0.17
ctc	L	0	0.17
cta	L	0	0.17
ctg	L	0	0.22
att	I	960	367.40
atc	I	0	243.60
ata	I	47	396.00
gtt	V	1	0.21
gtc	V	0	0.20
gta	V	0	0.21
gtg	V	0	0.39
---	--	------	------
mPD		0.093	0.66
		nPD:	0.14
		N. weight:	0.98
		Sc. PD:	-0.021
		Sc. rank:	-25.2

,

HA			
Pos.	460	obs:	exp:
gat	D	1002	544.70
gac	D	7	464.30
---	--	------	------
mPD		0.014	0.50
		nPD:	0.03
		N. weight:	0.69
		Sc. PD:	-0.09
		Sc. rank:	-338.4

,

HA			
Pos.	461	obs:	exp:
tta	L	0	88.68
ttg	L	1	188.30
ctt	L	47	171.00
ctc	L	1	169.00
cta	L	1	168.20
ctg	L	959	223.80
---	--	------	------
mPD		0.095	1.1
		nPD:	0.09
		N. weight:	1.6
		Sc. PD:	-0.12
		Sc. rank:	-404.6

,

HA			
Pos.	462	obs:	exp:
act	T	2	0.52
acc	T	0	0.41
aca	T	0	0.92
acg	T	0	0.16
gct	A	947	258.00
gcc	A	8	189.10
gca	A	51	470.80
gcg	A	1	89.07
---	--	------	------
mPD		0.12	0.68
		nPD:	0.17
		N. weight:	1.3
		Sc. PD:	0.011
		Sc. rank:	202.5

,

HA			
Pos.	463	obs:	exp:
gat	D	936	544.70
gac	D	73	464.30
---	--	------	------
mPD		0.13	0.50
		nPD:	0.27
		N. weight:	0.44
		Sc. PD:	0.045
		Sc. rank:	214.4

,

HA			
Pos.	464	obs:	exp:
tct	S	0	161.50
tcc	S	0	130.60
tca	S	1007	254.60
tcg	S	2	58.72
agt	S	0	206.80
agc	S	0	196.80
---	--	------	------
mPD		0.0040	1.7
		nPD:	0.
		N. weight:	1.6
		Sc. PD:	-0.25
		Sc. rank:	-1119.8

,

HA			
Pos.	465	obs:	exp:
gaa	E	1006	606.90
gag	E	3	402.10
---	--	------	------
mPD		0.0059	0.48
		nPD:	0.01
		N. weight:	0.59
		Sc. PD:	-0.085
		Sc. rank:	-335.4

,

HA			
Pos.	466	obs:	exp:
atg	M	1009	1009.00
---	--	------	------
mPD		0	0
		nPD:	1.
		N. weight:	0.
		Sc. PD:	0
		Sc. rank:	0

,

HA			
Pos.	467	obs:	exp:
aat	N	16	75.29
aac	N	132	72.71
aaa	K	1	0.59
aag	K	0	0.41
gtt	V	0	0.83
gtc	V	4	0.80
gta	V	0	0.83
gtg	V	0	1.54
gat	D	0	462.10
gac	D	856	393.90
---	--	------	------
mPD		0.29	0.76
		nPD:	0.39
		N. weight:	0.86
		Sc. PD:	0.18
		Sc. rank:	540.9

,

HA			
Pos.	468	obs:	exp:
aaa	K	1004	591.10
aag	K	5	417.90
---	--	------	------
mPD		0.0099	0.49
		nPD:	0.02
		N. weight:	0.61
		Sc. PD:	-0.083
		Sc. rank:	-325.6

,

HA			
Pos.	469	obs:	exp:
tta	L	3	88.50
ttg	L	2	187.90
ctt	L	46	170.60
ctc	L	1	168.70
cta	L	33	167.90
ctg	L	922	223.30
cct	P	0	0.51
ccc	P	0	0.36
cca	P	0	0.82
ccg	P	2	0.32
mPD		0.17	1.1
		nPD:	0.15
		N. weight:	1.4
		Sc. PD:	-0.017
		Sc. rank:	61.6

HA			
Pos.	470	obs:	exp:
tat	Y	242	535.00
tac	Y	767	474.00
mPD		0.36	0.50
		nPD:	0.73
		N. weight:	0.21
		Sc. PD:	0.12
		Sc. rank:	155.0

HA			
Pos.	471	obs:	exp:
aaa	K	8	4.69
aag	K	0	3.31
gaa	E	886	601.50
gag	E	114	398.50
ggt	G	0	0.13
ggc	G	0	0.13
gga	G	1	0.46
ggg	G	0	0.28
mPD		0.22	0.50
		nPD:	0.44
		N. weight:	0.25
		Sc. PD:	0.065
		Sc. rank:	162.1

HA			
Pos.	472	obs:	exp:
cgt	R	41	43.10
cgc	R	6	51.26
cga	R	815	100.60
cgg	R	145	84.04
aga	R	2	460.70
agg	R	0	269.30
mPD		0.33	0.96
		nPD:	0.34
		N. weight:	2.1
		Sc. PD:	0.35
		Sc. rank:	1220.7

HA			
Pos.	473	obs:	exp:
gtt	V	1	208.30
gtc	V	7	202.10
gta	V	59	208.90
gtg	V	942	389.70
mPD		0.13	0.73
		nPD:	0.17
		N. weight:	0.87
		Sc. PD:	0.0072
		Sc. rank:	132.6

HA			
Pos.	474	obs:	exp:
cgt	R	0	3.25
cgc	R	0	3.86
cga	R	0	7.58
cgg	R	0	6.33
aaa	K	924	546.50
aag	K	9	386.50
aga	R	16	34.70
agg	R	60	20.28
mPD		0.27	0.67
		nPD:	0.4
		N. weight:	0.6
		Sc. PD:	0.13
		Sc. rank:	381.6

HA			
Pos.	475	obs:	exp:
cgt	R	0	41.10
cgc	R	0	48.88
cga	R	0	95.92
cgg	R	0	80.12
aaa	K	47	27.53
aag	K	0	19.47
aga	R	938	439.20
agg	R	24	256.80
mPD		0.14	1.0
		nPD:	0.13
		N. weight:	0.81
		Sc. PD:	-0.026
		Sc. rank:	-66.6

HA			
Pos.	476	obs:	exp:
caa	Q	112	518.30
cag	Q	897	490.70
mPD		0.20	0.50
		nPD:	0.4
		N. weight:	0.44
		Sc. PD:	0.098
		Sc. rank:	279.2

HA			
Pos.	477	obs:	exp:
tta	L	0	88.68
ttg	L	118	188.30
ctt	L	2	171.00
ctc	L	0	169.00
cta	L	10	168.20
ctg	L	879	223.80
mPD		0.23	1.1
		nPD:	0.21
		N. weight:	1.3
		Sc. PD:	0.055
		Sc. rank:	405.7

HA			
Pos.	478	obs:	exp:
cgt	R	0	43.10
cgc	R	0	51.26
cga	R	0	100.60
cgg	R	0	84.04
aga	R	964	460.70
agg	R	45	269.30
mPD		0.085	0.96
		nPD:	0.09
		N. weight:	0.75
		Sc. PD:	-0.054
		Sc. rank:	-179.4

HA			
Pos.	479	obs:	exp:
gaa	E	9	606.90
gag	E	1000	402.10
mPD		0.018	0.48
		nPD:	0.04
		N. weight:	1.
		Sc. PD:	-0.13
		Sc. rank:	-471.9

HA			
Pos.	480	obs:	exp:
aat	N	991	513.30
aac	N	18	495.70
mPD		0.035	0.50
		nPD:	0.07
		N. weight:	0.7
		Sc. PD:	-0.063
		Sc. rank:	-220.9

HA			
Pos.	481	obs:	exp:
gct	A	1006	258.50
gcc	A	2	189.50
gca	A	1	471.80
gcg	A	0	89.24
mPD		0.0059	0.67
		nPD:	0.01
		N. weight:	1.6
		Sc. PD:	-0.24
		Sc. rank:	-968.3

HA			
Pos.	482	obs:	exp:
gaa	E	1007	606.90
gag	E	2	402.10
mPD		0.0040	0.48
		nPD:	0.01
		N. weight:	0.59
		Sc. PD:	-0.088
		Sc. rank:	-367.8

HA			
Pos.	483	obs:	exp:
gaa	E	769	606.90
gag	E	240	402.10
mPD		0.36	0.48
		nPD:	0.76
		N. weight:	0.073
		Sc. PD:	0.042
		Sc. rank:	53.1

HA			
Pos.	484	obs:	exp:
gat	D	888	544.70
gac	D	121	464.30
mPD		0.21	0.50
		nPD:	0.43
		N. weight:	0.32
		Sc. PD:	0.082
		Sc. rank:	211.4

HA			
Pos.	485	obs:	exp:
gat	D	0	0.54
gac	D	1	0.46
ggt	G	1	135.20
ggc	G	947	128.10
gga	G	6	466.10
ggg	G	54	278.70
mPD		0.12	0.68
		nPD:	0.17
		N. weight:	2.1
		Sc. PD:	0.016
		Sc. rank:	306.1

HA			
Pos.	486	obs:	exp:
act	T	852	261.40
acc	T	13	205.50
aca	T	6	463.60
acg	T	138	78.60
mPD		0.27	0.67
		nPD:	0.4
		N. weight:	1.2
		Sc. PD:	0.27
		Sc. rank:	777.1

HA			
Pos.	487	obs:	exp:
ggt	G	908	135.30
ggc	G	52	128.20
gga	G	43	466.50
ggg	G	6	279.00
mPD		0.19	0.68
		nPD:	0.28
		N. weight:	1.9
		Sc. PD:	0.2
		Sc. rank:	909.6

HA			
Pos.	488	obs:	exp:
tgt	C	33	414.20
tgc	C	976	594.80
mPD		0.063	0.48
		nPD:	0.13
		N. weight:	0.48
		Sc. PD:	-0.015
		Sc. rank:	-38.7

HA			
Pos.	489	obs:	exp:
ttt	F	1007	398.20
ttc	F	2	610.80
mPD		0.0040	0.48
		nPD:	0.01
		N. weight:	1.1
		Sc. PD:	-0.16
		Sc. rank:	-675.9

HA			
Pos.	490	obs:	exp:
gaa	E	957	606.90
gag	E	52	402.10
mPD		0.098	0.48
		nPD:	0.2
		N. weight:	0.39
		Sc. PD:	0.015
		Sc. rank:	115.1

HA			
Pos.	491	obs:	exp:
tta	L	0	0.26
ttg	L	0	0.56
ctt	L	0	0.51
ctc	L	0	0.50
cta	L	3	0.50
ctg	L	0	0.67
att	I	0	367.10
atc	I	0	243.30
ata	I	1006	395.60
mPD		0.0059	0.66
		nPD:	0.01
		N. weight:	1.1
		Sc. PD:	-0.17
		Sc. rank:	-672.2

HA			
Pos.	492	obs:	exp:
ttt	F	921	398.20
ttc	F	88	610.80
mPD		0.16	0.48
		nPD:	0.33
		N. weight:	0.72
		Sc. PD:	0.12
		Sc. rank:	411.6

HA			
Pos.	493	obs:	exp:
cat	H	48	579.10
cac	H	960	428.90
caa	Q	1	0.51
cag	Q	0	0.49
mPD		0.093	0.49
		nPD:	0.19
		N. weight:	0.78
		Sc. PD:	0.019
		Sc. rank:	177.2

HA			
Pos.	494	obs:	exp:
act	T	0	0.26
acc	T	0	0.20
aca	T	0	0.46
acg	T	1	0.08
aat	N	1	0.51
aac	N	0	0.49
aaa	K	8	589.90
aag	K	999	417.10
---	--	------	------
mPD		0.020	0.49
		nPD:	0.04
		N. weight:	1.
		Sc. PD:	-0.12
		Sc. rank:	-445.3

HA			
Pos.	495	obs:	exp:
tgt	C	1009	414.20
tgc	C	0	594.80
---	--	------	------
mPD		0	0.48
		nPD:	0.
		N. weight:	1.1
		Sc. PD:	-0.17
		Sc. rank:	-776.4

HA			
Pos.	496	obs:	exp:
aat	N	1	0.51
aac	N	0	0.49
gat	D	1002	544.20
gac	D	6	463.80
---	--	------	------
mPD		0.014	0.50
		nPD:	0.03
		N. weight:	0.7
		Sc. PD:	-0.091
		Sc. rank:	-342.5

HA			
Pos.	497	obs:	exp:
aat	N	52	26.45
aac	N	0	25.55
gat	D	934	516.60
gac	D	23	440.40
---	--	------	------
mPD		0.14	0.59
		nPD:	0.24
		N. weight:	0.62
		Sc. PD:	0.045
		Sc. rank:	246.8

HA			
Pos.	498	obs:	exp:
caa	Q	3	24.14
cag	Q	44	22.86
aat	N	5	16.79
aac	N	28	16.21
gat	D	3	501.50
gac	D	926	427.50
---	--	------	------
mPD		0.25	0.69
		nPD:	0.37
		N. weight:	0.87
		Sc. PD:	0.17
		Sc. rank:	540.1

HA			
Pos.	499	obs:	exp:
tgt	C	936	414.20
tgc	C	73	594.80
---	--	------	------
mPD		0.13	0.48
		nPD:	0.28
		N. weight:	0.73
		Sc. PD:	0.079
		Sc. rank:	358.8

HA			
Pos.	500	obs:	exp:
atg	M	1009	1009.00
---	--	------	------
mPD		0	0
		nPD:	1.
		N. weight:	0.
		Sc. PD:	0
		Sc. rank:	0

HA			
Pos.	501	obs:	exp:
gct	A	5	246.50
gcc	A	957	180.60
gca	A	0	449.80
gcg	A	0	85.09
gaa	E	1	28.27
gag	E	46	18.73
---	--	------	------
mPD		0.19	0.76
		nPD:	0.25
		N. weight:	1.9
		Sc. PD:	0.15
		Sc. rank:	801.3

HA			
Pos.	502	obs:	exp:
tct	S	0	161.50
tcc	S	0	130.60
tca	S	0	254.60
tcg	S	0	58.72
agt	S	829	206.80
agc	S	180	196.80
---	--	------	------
mPD		0.29	1.7
		nPD:	0.18
		N. weight:	1.3
		Sc. PD:	0.018
		Sc. rank:	245.0

HA			
Pos.	503	obs:	exp:
att	I	928	368.20
atc	I	36	244.10
ata	I	45	396.80
---	--	------	------
mPD		0.15	0.65
		nPD:	0.23
		N. weight:	0.82
		Sc. PD:	0.053
		Sc. rank:	310.7

HA			
Pos.	504	obs:	exp:
cgt	R	0	43.02
cgc	R	0	51.16
cga	R	4	100.40
cgg	R	0	83.87
aaa	K	2	1.17
aag	K	0	0.83
aga	R	871	459.80
agg	R	132	268.80
---	--	------	------
mPD		0.24	0.97
		nPD:	0.25
		N. weight:	0.54
		Sc. PD:	0.043
		Sc. rank:	230.9

HA			
Pos.	505	obs:	exp:
aat	N	787	513.30
aac	N	222	495.70
---	--	------	------
mPD		0.34	0.50
		nPD:	0.69
		N. weight:	0.19
		Sc. PD:	0.096
		Sc. rank:	138.4

HA			
Pos.	506	obs:	exp:
aat	N	0	513.30
aac	N	1009	495.70
---	--	------	------
mPD		0	0.50
		nPD:	0.
		N. weight:	0.85
		Sc. PD:	-0.13
		Sc. rank:	-619.9

HA			
Pos.	507	obs:	exp:
cct	P	0	0.25
ccc	P	1	0.18
cca	P	0	0.41
ccg	P	0	0.16
act	T	47	261.10
acc	T	961	205.30
aca	T	0	463.10
acg	T	0	78.52
---	--	------	------
mPD		0.091	0.68
		nPD:	0.13
		N. weight:	1.7
		Sc. PD:	-0.047
		Sc. rank:	-113.0

HA			
Pos.	508	obs:	exp:
tat	Y	1009	535.00
tac	Y	0	474.00
---	--	------	------
mPD		0	0.50
		nPD:	0.
		N. weight:	0.76
		Sc. PD:	-0.12
		Sc. rank:	-553.3

HA			
Pos.	509	obs:	exp:
gat	D	963	544.70
gac	D	46	464.30
---	--	------	------
mPD		0.087	0.50
		nPD:	0.18
		N. weight:	0.53
		Sc. PD:	0.0059
		Sc. rank:	92.4

HA			
Pos.	510	obs:	exp:
cat	H	41	579.70
cac	H	968	429.30
---	--	------	------
mPD		0.078	0.49
		nPD:	0.16
		N. weight:	0.81
		Sc. PD:	-0.0032
		Sc. rank:	73.4

HA			
Pos.	511	obs:	exp:
tct	S	0	83.40
tcc	S	0	67.44
tca	S	0	131.50
tcg	S	0	30.32
cgt	R	0	17.39
cgc	R	0	20.68
cga	R	0	40.58
cgg	R	0	33.90
act	T	19	12.18
acc	T	28	9.57
aca	T	0	21.59
acg	T	0	3.66
aat	N	0	16.79
aac	N	33	16.21
agt	S	2	106.80
agc	S	519	101.60
aga	R	404	185.80
agg	R	3	108.60
ggt	G	0	0.13
ggc	G	0	0.13
gga	G	1	0.46
ggg	G	0	0.28
---	--	------	------
mPD		0.66	1.8
		nPD:	0.37
		N. weight:	1.4
		Sc. PD:	0.28
		Sc. rank:	891.5

HA			
Pos.	512	obs:	exp:
caa	Q	45	24.14
cag	Q	2	22.86
aat	N	0	0.51
aac	N	1	0.49
aaa	K	807	562.90
aag	K	154	398.10
---	--	------	------
mPD		0.35	0.58
		nPD:	0.61
		N. weight:	0.2
		Sc. PD:	0.088
		Sc. rank:	146.0

HA			
Pos.	513	obs:	exp:
ttt	F	0	0.39
ttc	F	1	0.61
tct	S	0	0.16
tcc	S	0	0.13
tca	S	0	0.25
tcg	S	0	0.06
tat	Y	0	533.90
tac	Y	1007	473.10
agt	S	0	0.20
agc	S	1	0.20
---	--	------	------
mPD		0.0059	0.50
		nPD:	0.01
		N. weight:	0.9
		Sc. PD:	-0.13
		Sc. rank:	-526.8

HA			
Pos.	514	obs:	exp:
cgt	R	0	43.10
cgc	R	0	51.26
cga	R	0	100.60
cgg	R	0	84.04
aga	R	191	460.70
agg	R	818	269.30
---	--	------	------
mPD		0.31	0.96
		nPD:	0.32
		N. weight:	0.88
		Sc. PD:	0.13
		Sc. rank:	491.8

,

HA			
Pos.	515	obs:	exp:
act	T	0	1.81
acc	T	0	1.43
aca	T	7	3.22
acg	T	0	0.55
aaa	K	7	4.10
aag	K	0	2.90
gct	A	0	8.71
gcc	A	19	6.38
gca	A	8	15.90
gcg	A	7	3.01
gat	D	0	0.54
gac	D	1	0.46
gaa	E	923	570.20
gag	E	25	377.80
ggt	G	0	1.61
ggc	G	0	1.52
gga	G	12	5.55
ggg	G	0	3.32
---	--	------	------
mPD		0.23	0.63
		nPD:	0.36
		N. weight:	0.5
		Sc. PD:	0.095
		Sc. rank:	309.0

,

HA			
Pos.	516	obs:	exp:
gaa	E	25	606.90
gag	E	984	402.10
---	--	------	------
mPD		0.048	0.48
		nPD:	0.1
		N. weight:	0.95
		Sc. PD:	-0.057
		Sc. rank:	-169.8

,

HA			
Pos.	517	obs:	exp:
tct	S	0	7.52
tcc	S	0	6.08
tca	S	44	11.86
tcg	S	3	2.74
agt	S	0	9.63
agc	S	0	9.17
gct	A	0	246.50
gcc	A	0	180.60
gca	A	952	449.80
gcg	A	10	85.09
---	--	------	------
mPD		0.11	0.80
		nPD:	0.14
		N. weight:	0.89
		Sc. PD:	-0.018
		Sc. rank:	-12.9

,

HA			
Pos.	518	obs:	exp:
tta	L	2	4.13
ttg	L	35	8.77
ctt	L	1	7.96
ctc	L	0	7.87
cta	L	0	7.84
ctg	L	9	10.42
att	I	0	2.55
atc	I	0	1.69
ata	I	7	2.75
atg	M	954	954.00
act	T	0	0.26
acc	T	0	0.20
aca	T	0	0.46
acg	T	1	0.08
---	--	------	------
mPD		0.11	0.16
		nPD:	0.69
		N. weight:	0.067
		Sc. PD:	0.034
		Sc. rank:	48.7

,

HA			
Pos.	519	obs:	exp:
caa	Q	951	518.30
cag	Q	58	490.70
---	--	------	------
mPD		0.11	0.50
		nPD:	0.22
		N. weight:	0.54
		Sc. PD:	0.028
		Sc. rank:	178.8

,

HA			
Pos.	520	obs:	exp:
tct	S	0	0.48
tcc	S	0	0.39
tca	S	0	0.76
tcg	S	0	0.17
aat	N	997	511.80
aac	N	9	494.20
agt	S	0	0.61
agc	S	3	0.59
---	--	------	------
mPD		0.029	0.51
		nPD:	0.06
		N. weight:	0.75
		Sc. PD:	-0.076
		Sc. rank:	-282.7

,

HA			
Pos.	521	obs:	exp:
cgt	R	0	43.10
cgc	R	0	51.26
cga	R	4	100.60
cgg	R	0	84.04
aga	R	996	460.70
agg	R	9	269.30
---	--	------	------
mPD		0.026	0.96
		nPD:	0.03
		N. weight:	0.86
		Sc. PD:	-0.11
		Sc. rank:	-429.9

,

HA			
Pos.	522	obs:	exp:
att	I	0	368.20
atc	I	2	244.10
ata	I	1007	396.80
---	--	------	------
mPD		0.0040	0.65
		nPD:	0.01
		N. weight:	1.1
		Sc. PD:	-0.17
		Sc. rank:	-697.8

,

HA			
Pos.	523	obs:	exp:
caa	Q	44	510.60
cag	Q	950	483.40
aaa	K	0	8.79
aag	K	15	6.21
---	--	------	------
mPD		0.11	0.53
		nPD:	0.21
		N. weight:	0.65
		Sc. PD:	0.031
		Sc. rank:	208.0

,

HA			
Pos.	524	obs:	exp:
att	I	954	365.60
atc	I	0	242.40
ata	I	48	394.00
gtt	V	7	1.45
gtc	V	0	1.40
gta	V	0	1.45
gtg	V	0	2.70
---	--	------	------
mPD		0.10	0.67
		nPD:	0.16
		N. weight:	0.98
		Sc. PD:	-0.0072
		Sc. rank:	67.9

,

HA			
Pos.	525	obs:	exp:
aat	N	0	3.56
aac	N	7	3.44
gat	D	51	540.40
gac	D	950	460.60
ggt	G	0	0.13
ggc	G	1	0.13
gga	G	0	0.46
ggg	G	0	0.28
---	--	------	------
mPD		0.11	0.51
		nPD:	0.22
		N. weight:	0.68
		Sc. PD:	0.035
		Sc. rank:	227.3

,

HA			
Pos.	526	obs:	exp:
tct	S	0	0.32
tcc	S	0	0.26
tca	S	2	0.50
tcg	S	0	0.12
cct	P	3	256.10
ccc	P	0	179.50
cca	P	978	411.40
ccg	P	26	160.00
agt	S	0	0.41
agc	S	0	0.39
---	--	------	------
mPD		0.060	0.72
		nPD:	0.08
		N. weight:	0.94
		Sc. PD:	-0.072
		Sc. rank:	-247.6

,

HA			
Pos.	527	obs:	exp:
att	I	1	18.61
atc	I	50	12.34
ata	I	0	20.06
gtt	V	3	197.80
gtc	V	910	191.90
gta	V	1	198.40
gtg	V	44	370.00
---	--	------	------
mPD		0.19	0.83
		nPD:	0.23
		N. weight:	1.6
		Sc. PD:	0.1
		Sc. rank:	611.3

,

HA			
Pos.	528	obs:	exp:
cgt	R	0	0.13
cgc	R	0	0.15
cga	R	0	0.30
cgg	R	0	0.25
aat	N	0	1.02
aac	N	2	0.98
aaa	K	829	588.10
aag	K	175	415.90
aga	R	3	1.37
agg	R	0	0.80
---	--	------	------
mPD		0.30	0.50
		nPD:	0.6
		N. weight:	0.17
		Sc. PD:	0.069
		Sc. rank:	118.2

,

HA			
Pos.	529	obs:	exp:
tta	L	16	88.59
ttg	L	17	188.10
ctt	L	0	170.80
ctc	L	0	168.90
cta	L	952	168.10
ctg	L	23	223.60
att	I	0	0.36
atc	I	0	0.24
ata	I	1	0.39
---	--	------	------
mPD		0.14	1.1
		nPD:	0.13
		N. weight:	1.8
		Sc. PD:	-0.064
		Sc. rank:	-167.3

,

HA			
Pos.	530	obs:	exp:
tct	S	0	161.20
tcc	S	0	130.30
tca	S	0	254.10
tcg	S	0	58.60
att	I	0	0.36
atc	I	1	0.24
ata	I	0	0.39
aat	N	1	0.51
aac	N	0	0.49
agt	S	62	206.30
agc	S	945	196.40
---	--	------	------
mPD		0.12	1.7
		nPD:	0.07
		N. weight:	1.7
		Sc. PD:	-0.15
		Sc. rank:	-508.4

,

HA			
Pos.	531	obs:	exp:
tct	S	0	157.00
tcc	S	0	127.00
tca	S	0	247.60
tcg	S	0	57.09
cgt	R	0	0.43
cgc	R	0	0.51
cga	R	0	1.00
cgg	R	0	0.83
aat	N	2	1.02
aac	N	0	0.98
agt	S	96	201.00
agc	S	885	191.30
aga	R	10	4.57
agg	R	0	2.67
ggt	G	1	2.15
ggc	G	15	2.03
gga	G	0	7.40
ggg	G	0	4.42
---	--	------	------
mPD		0.23	1.7
		nPD:	0.14
		N. weight:	1.6
		Sc. PD:	-0.041
		Sc. rank:	-86.2

,

HA			
Pos.	532	obs:	exp:
ggt	G	34	135.30
ggc	G	927	128.20
gga	G	44	466.50
ggg	G	4	279.00
---	--	------	------
mPD		0.15	0.68
		nPD:	0.23
		N. weight:	2.
		Sc. PD:	0.12
		Sc. rank:	715.4

,

HA			
Pos.	533	obs:	exp:
tat	Y	10	535.00
tac	Y	999	474.00
---	--	------	------
mPD		0.020	0.50
		nPD:	0.04
		N. weight:	0.84
		Sc. PD:	-0.1
		Sc. rank:	-375.4

,

HA			
Pos.	534	obs:	exp:
aaa	K	1008	591.10
aag	K	1	417.90
---	--	------	------
mPD		0.0020	0.49
		nPD:	0.
		N. weight:	0.63
		Sc. PD:	-0.097
		Sc. rank:	-414.1

,

HA			
Pos.	535	obs:	exp:
gat	D	960	543.60
gac	D	47	463.40
gaa	E	0	1.20
gag	E	2	0.80
---	--	------	------
mPD		0.093	0.50
		nPD:	0.19
		N. weight:	0.52
		Sc. PD:	0.011
		Sc. rank:	110.2

,

HA			
Pos.	536	obs:	exp:
att	I	0	17.15
atc	I	0	11.37
ata	I	47	18.48
atg	M	2	2.00
gtt	V	0	198.20
gtc	V	0	192.30
gta	V	2	198.80
gtg	V	958	370.70
---	--	------	------
mPD		0.19	0.82
		nPD:	0.22
		N. weight:	1.1
		Sc. PD:	0.065
		Sc. rank:	398.5

,

HA			
Pos.	537	obs:	exp:
att	I	0	366.70
atc	I	47	243.10
ata	I	958	395.20
gtt	V	0	0.83
gtc	V	0	0.80
gta	V	4	0.83
gtg	V	0	1.54
---	--	------	------
mPD		0.097	0.66
		nPD:	0.15
		N. weight:	0.92
		Sc. PD:	-0.016
		Sc. rank:	13.1

,

HA			
Pos.	538	obs:	exp:
tta	L	47	88.68
ttg	L	0	188.30
ctt	L	954	171.00
ctc	L	6	169.00
cta	L	2	168.20
ctg	L	0	223.80
---	--	------	------
mPD		0.19	1.1
		nPD:	0.17
		N. weight:	1.9
		Sc. PD:	0.018
		Sc. rank:	302.1

,

HA			
Pos.	539	obs:	exp:
tgg	W	1009	1009.00
---	--	------	------
mPD		0	0
		nPD:	1.
		N. weight:	0.
		Sc. PD:	0
		Sc. rank:	0

,

HA			
Pos.	540	obs:	exp:
ttt	F	1009	398.20
ttc	F	0	610.80
---	--	------	------
mPD		0	0.48
		nPD:	0.
		N. weight:	1.1
		Sc. PD:	-0.17
		Sc. rank:	-810.8

,

HA			
Pos.	541	obs:	exp:
tct	S	0	161.40
tcc	S	0	130.50
tca	S	0	254.40
tcg	S	0	58.66
agt	S	1	206.60
agc	S	1007	196.60
ggt	G	0	0.13
ggc	G	1	0.13
gga	G	0	0.46
ggg	G	0	0.28
---	--	------	------
mPD		0.0040	1.7
		nPD:	0.
		N. weight:	1.9
		Sc. PD:	-0.3
		Sc. rank:	-1324.4

,

HA			
Pos.	542	obs:	exp:
ttt	F	0	398.20
ttc	F	1009	610.80
---	--	------	------
mPD		0	0.48
		nPD:	0.
		N. weight:	0.6
		Sc. PD:	-0.094
		Sc. rank:	-437.7

,

HA			
Pos.	543	obs:	exp:
ggt	G	0	135.30
ggc	G	0	128.20
gga	G	0	466.50
ggg	G	1009	279.00
---	--	------	------
mPD		0	0.68
		nPD:	0.
		N. weight:	1.5
		Sc. PD:	-0.24
		Sc. rank:	-1121.1

,

HA			
Pos.	544	obs:	exp:
gct	A	0	258.50
gcc	A	0	189.50
gca	A	1005	471.80
gcg	A	4	89.24
---	--	------	------
mPD		0.0079	0.67
		nPD:	0.01
		N. weight:	0.89
		Sc. PD:	-0.13
		Sc. rank:	-526.0

,

HA			
Pos.	545	obs:	exp:
tct	S	0	161.50
tcc	S	0	130.60
tca	S	1009	254.60
tcg	S	0	58.72
agt	S	0	206.80
agc	S	0	196.80
---	--	------	------
mPD		0	1.7
		nPD:	0.
		N. weight:	1.6
		Sc. PD:	-0.26
		Sc. rank:	-1200.7

,

HA			
Pos.	546	obs:	exp:
tgt	C	1008	414.20
tgc	C	1	594.80
---	--	------	------
mPD		0.0020	0.48
		nPD:	0.
		N. weight:	1.1
		Sc. PD:	-0.16
		Sc. rank:	-690.0

,

HA			
Pos.	547	obs:	exp:
ttt	F	44	398.20
ttc	F	965	610.80
---	--	------	------
mPD		0.083	0.48
		nPD:	0.17
		N. weight:	0.41
		Sc. PD:	0.0043
		Sc. rank:	70.3

,

HA			
Pos.	548	obs:	exp:
tta	L	0	4.13
ttg	L	0	8.77
ctt	L	46	7.96
ctc	L	1	7.87
cta	L	0	7.84
ctg	L	0	10.42
att	I	0	342.60
atc	I	0	227.10
ata	I	939	369.30
atg	M	14	14.00
act	T	0	1.81
acc	T	0	1.43
aca	T	7	3.22
acg	T	0	0.55
gtt	V	0	0.41
gtc	V	0	0.40
gta	V	2	0.41
gtg	V	0	0.77
---	--	------	------
mPD		0.22	0.78
		nPD:	0.28
		N. weight:	1.1
		Sc. PD:	0.13
		Sc. rank:	581.0

,

HA			
Pos.	549	obs:	exp:
tta	L	0	88.50
ttg	L	0	187.90
ctt	L	1007	170.60
ctc	L	0	168.70
cta	L	0	167.90
ctg	L	0	223.30
att	I	2	0.73
atc	I	0	0.48
ata	I	0	0.79
---	--	------	------
mPD		0.0040	1.1
		nPD:	0.
		N. weight:	2.1
		Sc. PD:	-0.33
		Sc. rank:	-1410.1

,

HA			
Pos.	550	obs:	exp:
tta	L	0	87.98
ttg	L	0	186.80
ctt	L	23	169.60
ctc	L	0	167.70
cta	L	962	166.90
ctg	L	16	222.00
att	I	0	2.92
atc	I	0	1.93
ata	I	8	3.15
---	--	------	------
mPD		0.091	1.1
		nPD:	0.08
		N. weight:	1.9
		Sc. PD:	-0.15
		Sc. rank:	-532.5

,

HA			
Pos.	551	obs:	exp:
gct	A	0	258.50
gcc	A	1009	189.50
gca	A	0	471.80
gcg	A	0	89.24
---	--	------	------
mPD		0	0.67
		nPD:	0.
		N. weight:	2.
		Sc. PD:	-0.31
		Sc. rank:	-1458.6

,

HA			
Pos.	552	obs:	exp:
att	I	1007	367.80
atc	I	1	243.80
ata	I	0	396.40
aat	N	1	0.51
aac	N	0	0.49
---	--	------	------
mPD		0.0040	0.66
		nPD:	0.01
		N. weight:	1.2
		Sc. PD:	-0.18
		Sc. rank:	-761.9

,

HA			
Pos.	553	obs:	exp:
tta	L	1	0.09
ttg	L	0	0.19
ctt	L	0	0.17
ctc	L	0	0.17
cta	L	0	0.17
ctg	L	0	0.22
gtt	V	0	189.10
gtc	V	1	183.50
gta	V	911	189.70
gtg	V	4	353.80
gct	A	0	23.57
gcc	A	0	17.27
gca	A	91	43.01
gcg	A	1	8.14
---	--	------	------
mPD		0.18	0.90
		nPD:	0.2
		N. weight:	1.8
		Sc. PD:	0.06
		Sc. rank:	466.7

,

HA	Pos. 554	obs:	exp:
att	I	0	0.36
atc	I	0	0.24
ata	I	1	0.39
atg	M	1008	1008.00
---	--	------	------
mPD		0.0020	0.0020
		nPD:	1.
		N. weight:	0.0011
		Sc. PD:	0.00089
		Sc. rank:	0.8

HA	Pos. 555	obs:	exp:
gct	A	0	0.26
gcc	A	1	0.19
gca	A	0	0.47
gcg	A	0	0.09
ggt	G	5	135.20
ggc	G	951	128.10
gga	G	52	466.10
ggg	G	0	278.70
---	--	------	------
mPD		0.11	0.68
		nPD:	0.16
		N. weight:	2.1
		Sc. PD:	-0.0053
		Sc. rank:	213.6

HA	Pos. 556	obs:	exp:
tta	L	0	88.59
ttg	L	45	188.10
ctt	L	960	170.80
ctc	L	0	168.90
cta	L	0	168.10
ctg	L	3	223.60
att	I	1	0.36
atc	I	0	0.24
ata	I	0	0.39
---	--	------	------
mPD		0.18	1.1
		nPD:	0.16
		N. weight:	1.9
		Sc. PD:	-0.0066
		Sc. rank:	179.9

HA	Pos. 557	obs:	exp:
att	I	0	1.09
atc	I	3	0.73
ata	I	0	1.18
gtt	V	43	207.70
gtc	V	963	201.50
gta	V	0	208.30
gtg	V	0	388.50
---	--	------	------
mPD		0.088	0.73
		nPD:	0.12
		N. weight:	1.7
		Sc. PD:	-0.072
		Sc. rank:	-200.8

HA	Pos. 558	obs:	exp:
ttt	F	0	397.80
ttc	F	1008	610.20
tta	L	0	0.09
ttg	L	0	0.19
ctt	L	0	0.17
ctc	L	1	0.17
cta	L	0	0.17
ctg	L	0	0.22
---	--	------	------
mPD		0.0020	0.48
		nPD:	0.
		N. weight:	0.6
		Sc. PD:	-0.092
		Sc. rank:	-391.0

HA	Pos. 559	obs:	exp:
att	I	47	366.30
atc	I	1	242.80
ata	I	956	394.80
atg	M	5	5.00
---	--	------	------
mPD		0.10	0.66
		nPD:	0.15
		N. weight:	0.88
		Sc. PD:	-0.0096
		Sc. rank:	51.7

HA	Pos. 560	obs:	exp:
tgt	C	960	414.20
tgc	C	49	594.80
---	--	------	------
mPD		0.093	0.48
		nPD:	0.19
		N. weight:	0.81
		Sc. PD:	0.021
		Sc. rank:	189.3

HA	Pos. 561	obs:	exp:
att	I	0	17.88
atc	I	1	11.85
ata	I	48	19.27
gtt	V	1	198.20
gtc	V	0	192.30
gta	V	649	198.80
gtg	V	310	370.70
---	--	------	------
mPD		0.52	0.82
		nPD:	0.63
		N. weight:	0.89
		Sc. PD:	0.4
		Sc. rank:	641.2

HA	Pos. 562	obs:	exp:
aaa	K	14	591.10
aag	K	995	417.90
---	--	------	------
mPD		0.027	0.49
		nPD:	0.06
		N. weight:	0.96
		Sc. PD:	-0.099
		Sc. rank:	-369.2

HA	Pos. 563	obs:	exp:
aat	N	1007	513.30
aac	N	2	495.70
---	--	------	------
mPD		0.0040	0.50
		nPD:	0.01
		N. weight:	0.79
		Sc. PD:	-0.12
		Sc. rank:	-495.4

HA	Pos. 564	obs:	exp:
gaa	E	1	0.60
gag	E	0	0.40
ggt	G	0	135.20
ggc	G	0	128.10
gga	G	1005	466.10
ggg	G	3	278.70
---	--	------	------
mPD		0.0079	0.68
		nPD:	0.01
		N. weight:	0.9
		Sc. PD:	-0.13
		Sc. rank:	-536.9

HA	Pos. 565	obs:	exp:
act	T	0	0.26
acc	T	1	0.20
aca	T	0	0.46
acg	T	0	0.08
aat	N	0	492.00
aac	N	967	475.00
gat	D	0	22.13
gac	D	41	18.87
---	--	------	------
mPD		0.080	0.58
		nPD:	0.14
		N. weight:	0.85
		Sc. PD:	-0.021
		Sc. rank:	-45.0

HA	Pos. 566	obs:	exp:
cgt	R	0	0.04
cgc	R	1	0.05
cga	R	0	0.10
cgg	R	0	0.08
atg	M	1008	1008.00
aga	R	0	0.46
agg	R	0	0.27
---	--	------	------
mPD		0.0059	0.0038
		nPD:	1.56
		N. weight:	0.0035
		Sc. PD:	0.0047
		Sc. rank:	2.6

HA	Pos. 567	obs:	exp:
tta	L	0	0.09
ttg	L	0	0.19
ctt	L	0	0.17
ctc	L	0	0.17
cta	L	0	0.17
ctg	L	1	0.22
cgt	R	0	43.06
cgc	R	0	51.21
cga	R	0	100.50
cgg	R	1008	83.96
aga	R	0	460.20
agg	R	0	269.00
---	--	------	------
mPD		0.0020	0.97
		nPD:	0.
		N. weight:	3.
		Sc. PD:	-0.46
		Sc. rank:	-2048.0

HA	Pos. 568	obs:	exp:
tgt	C	0	413.80
tgc	C	1008	594.20
cgt	R	0	0.04
cgc	R	1	0.05
cga	R	0	0.10
cgg	R	0	0.08
aga	R	0	0.46
agg	R	0	0.27
---	--	------	------
mPD		0.0020	0.49
		nPD:	0.
		N. weight:	0.63
		Sc. PD:	-0.097
		Sc. rank:	-417.2

HA	Pos. 569	obs:	exp:
cct	P	10	2.54
ccc	P	0	1.78
cca	P	0	4.09
ccg	P	0	1.59
att	I	2	0.73
atc	I	0	0.48
ata	I	0	0.79
act	T	996	258.00
acc	T	0	202.80
aca	T	0	457.60
acg	T	0	77.59
gtt	V	0	0.21
gtc	V	0	0.20
gta	V	1	0.21
gtg	V	0	0.39
---	--	------	------
mPD		0.030	0.70
		nPD:	0.04
		N. weight:	1.6
		Sc. PD:	-0.19
		Sc. rank:	-696.5

HA	Pos. 570	obs:	exp:
ttt	F	4	1.58
ttc	F	0	2.42
tct	S	1	0.16
tcc	S	0	0.13
tca	S	0	0.25
tcg	S	0	0.06
att	I	1000	364.90
atc	I	0	241.90
ata	I	0	393.30
atg	M	2	2.00
act	T	1	0.26
acc	T	0	0.20
aca	T	0	0.46
acg	T	0	0.08
agt	S	0	0.20
agc	S	0	0.20
---	-	1	1.00
---	--	------	------
mPD		0.018	0.67
		nPD:	0.03
		N. weight:	1.2
		Sc. PD:	-0.16
		Sc. rank:	-595.7

HA	Pos. 571	obs:	exp:
ttt	F	2	0.79
ttc	F	0	1.21
tgt	C	998	409.70
tgc	C	0	588.30
gtt	V	4	1.03
gtc	V	0	1.00
gta	V	1	1.04
gtg	V	0	1.93
gaa	E	1	0.60
gag	E	0	0.40
ggt	G	1	0.13
ggc	G	0	0.13
gga	G	0	0.46
ggg	G	0	0.28
---	-	2	2.00
---	--	------	------
mPD		0.033	0.52
		nPD:	0.06
		N. weight:	1.1
		Sc. PD:	-0.1
		Sc. rank:	-353.7

HA	Pos. 572	obs:	exp:
tta	L	5	0.44
ttg	L	0	0.93
ctt	L	0	0.85
ctc	L	0	0.84
cta	L	0	0.83
ctg	L	0	1.11
att	I	1	364.10
atc	I	0	241.40
ata	I	997	392.50
aaa	K	5	2.93
aag	K	0	2.07
---	-	1	1.00
---	--	------	------
mPD		0.022	0.67
		nPD:	0.03
		N. weight:	1.1
		Sc. PD:	-0.14
		Sc. rank:	-521.6

HA	Pos. 573	obs:	exp:
taa	.	954	433.90
tag	.	51	255.10
tga	.	4	319.90
---	--	------	------
mPD		0.10	0.81
		nPD:	0.13
		N. weight:	0.78
		Sc. PD:	-0.026
		Sc. rank:	-68.8

Amino acid level conservation per codon

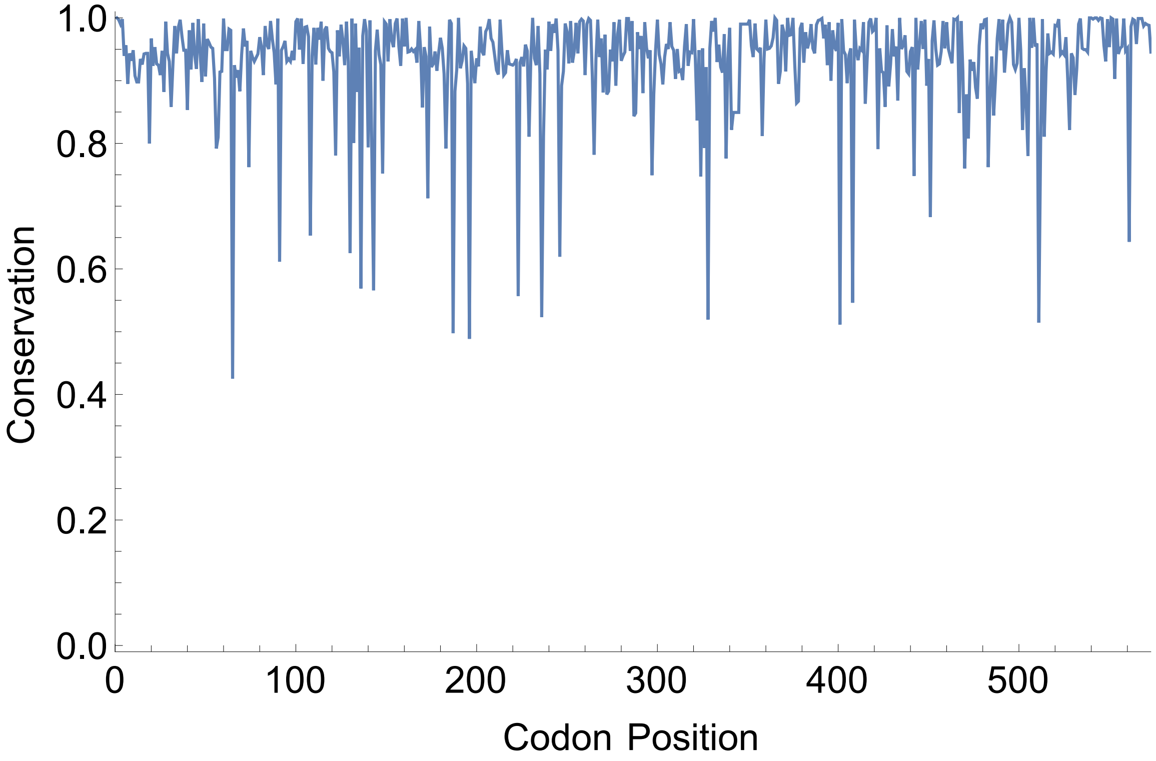

Conservation analysis plots

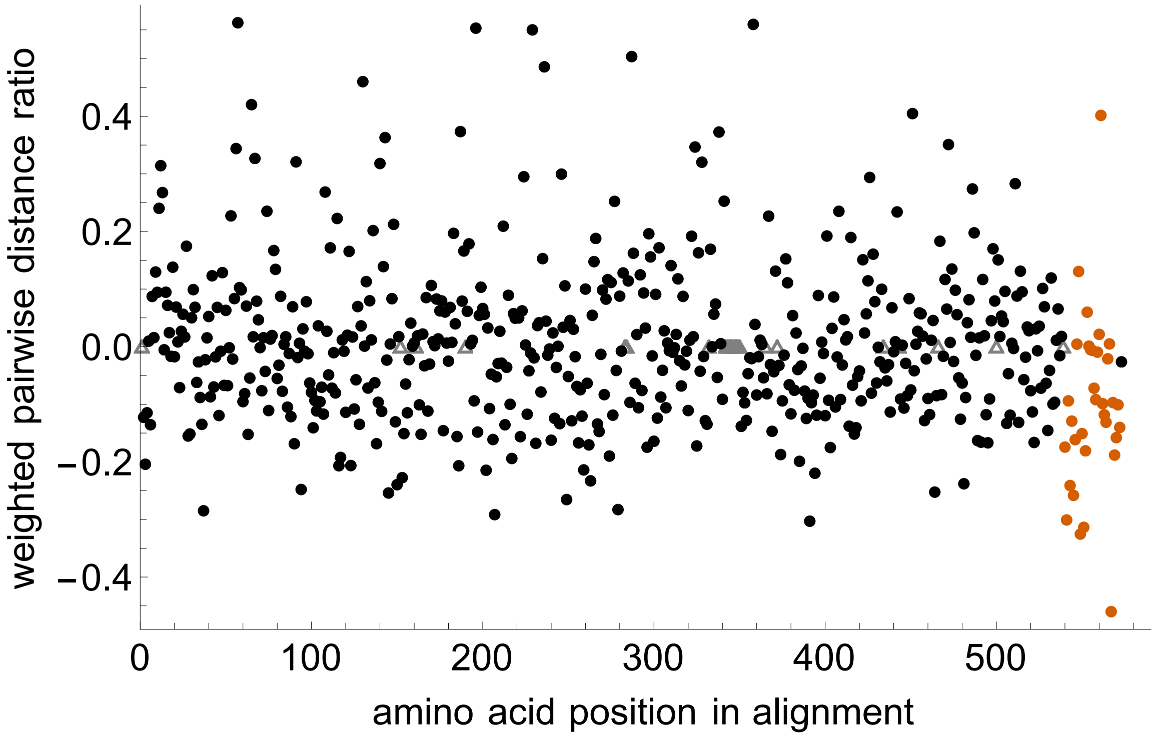

HA

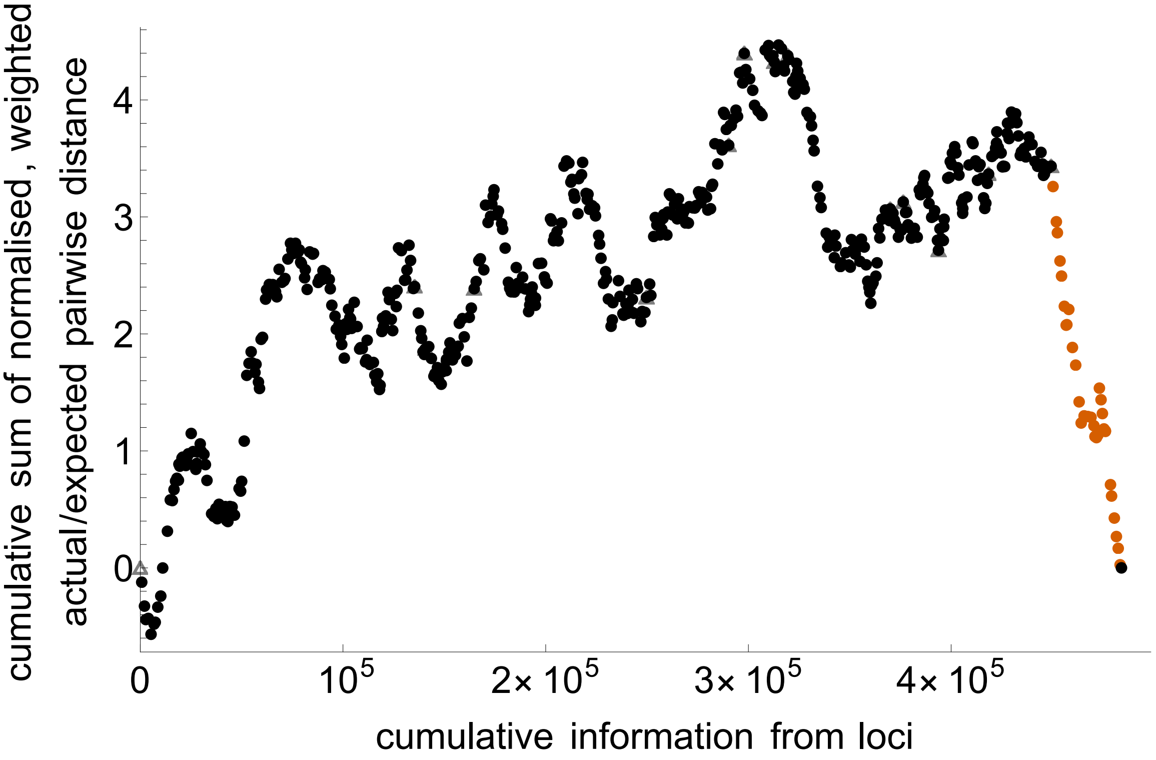

HA

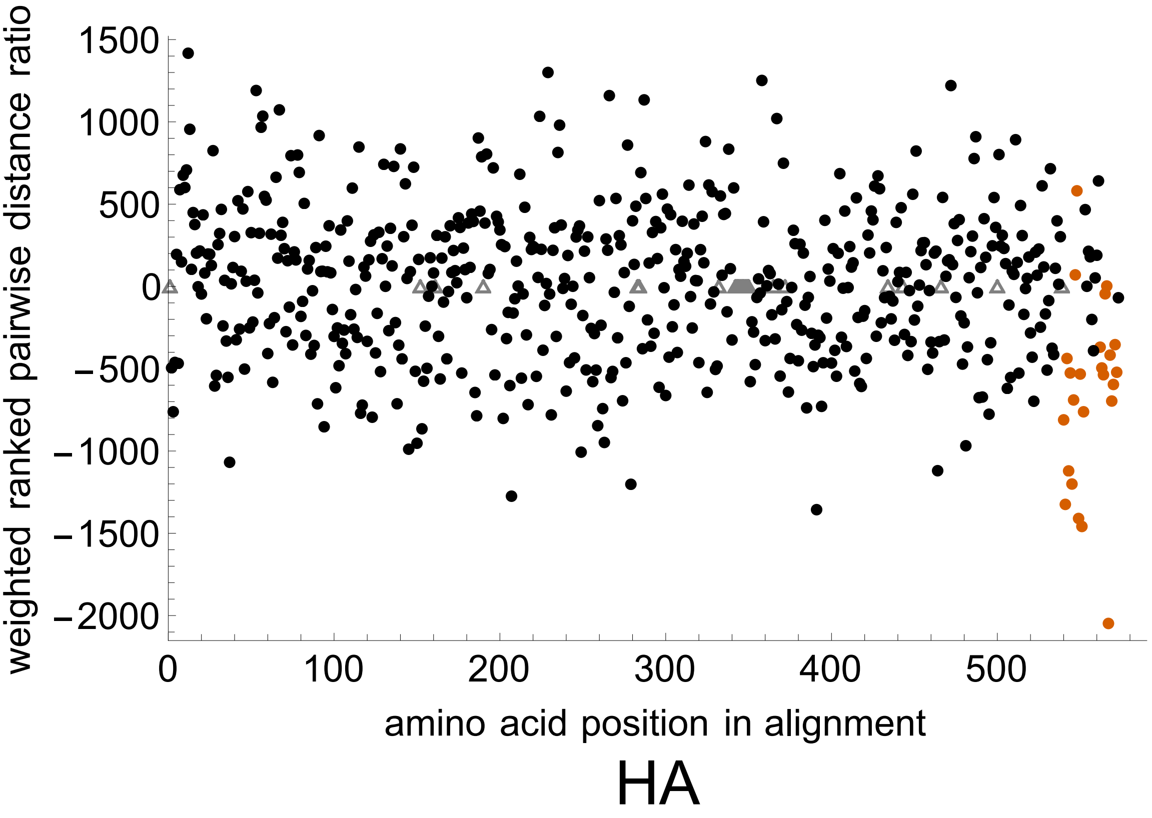
weighted ranked pairwise distance ratio
1500
1000
500
0
−500
−1000
−1500
−2000
0
100
200
300
400
500
amino acid position in alignment
HA

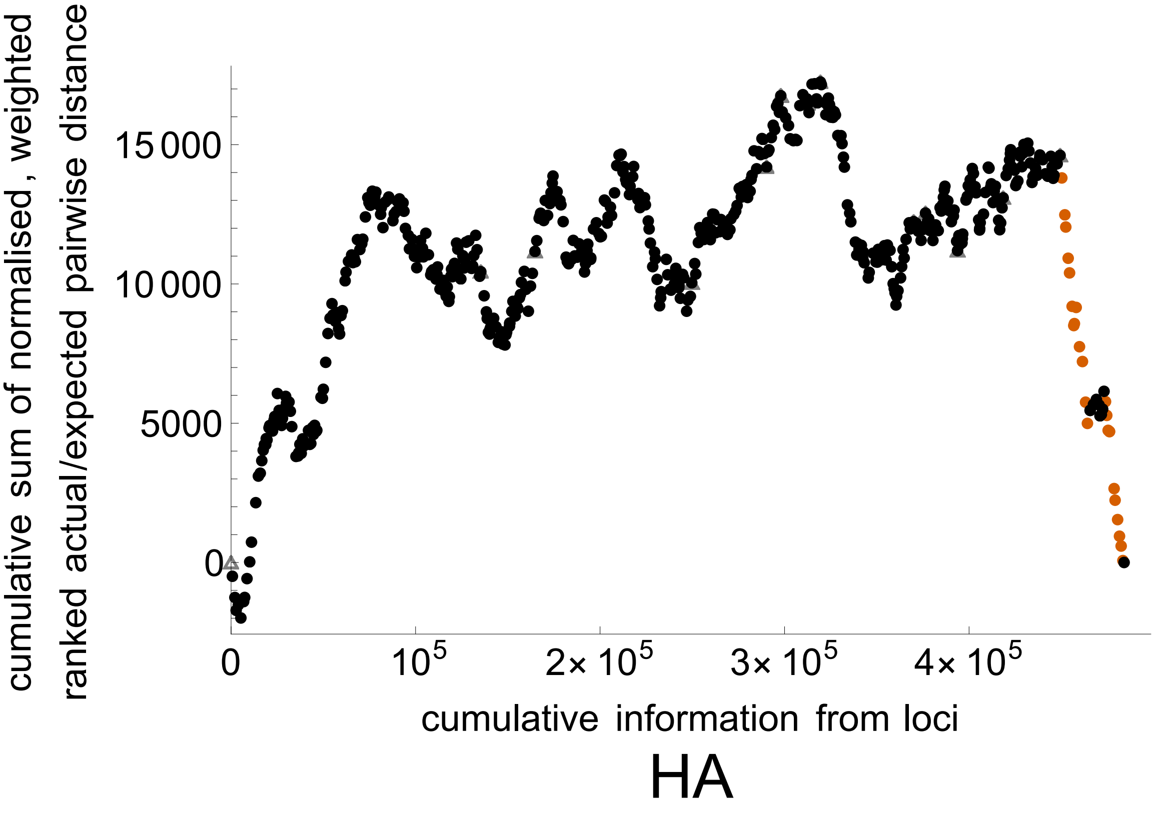
cumulative sum of normalised, weighted ranked actual/expected pairwise distance
15 000
10 000
5000
0
0
10^5
2×10^5
3×10^5
4×10^5
cumulative information from loci
HA

NP

Gene length histogram

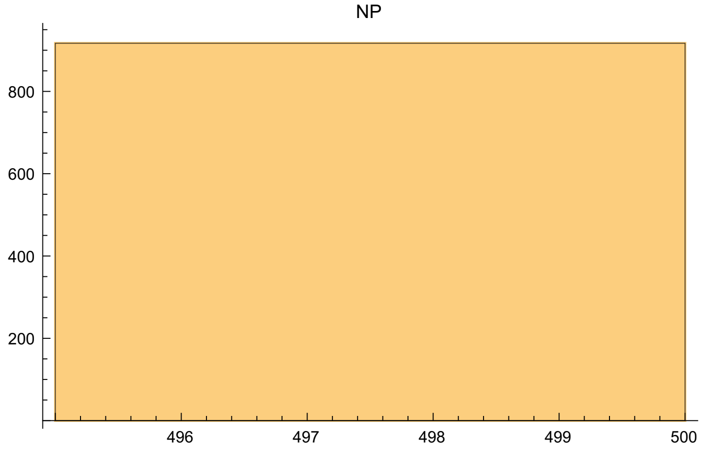

Information vs. nPD

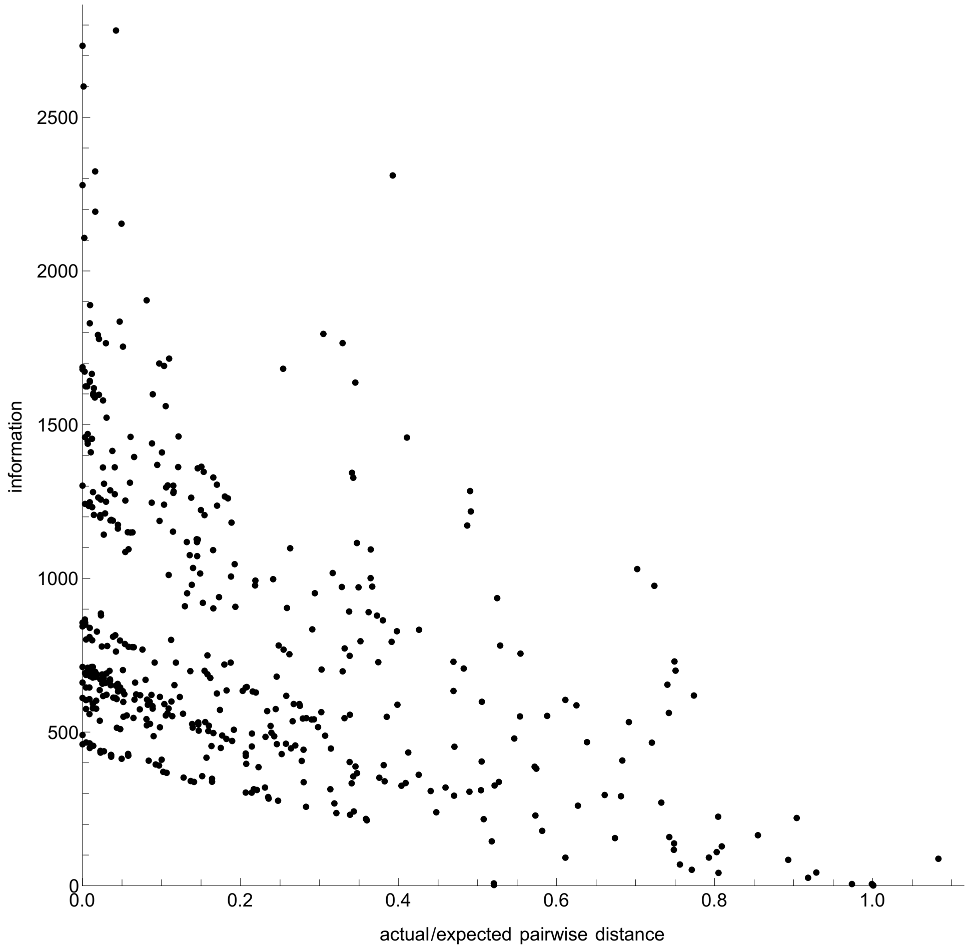

NP

Example sequences highlighted by regions found in analyses to be conserved

Interesting points (by weighted raw PD) highlighted for gene NP:

ATG
GCGTCTCAAGGCACCAAACGATCTTATGAGCAGATGGAGACTGGTGGAGAACGCC
AGAATGCCACTGAGATCAGAGCATCTGTTGGAAGAATGGTTGGTGGAATTGGGAGGTTTTACATACAGA⋱
TGTGCACTGAACTCAAACTCAGCGACTATGAAGGAAGGCTGGTCCAGAACAGCATAACAATAGAGAGAAT⋱
GGTTCTCTCTGCATTTGATGAAAGGAGGAATAAATACCTGGAAGAACATCCCAGTGCGGGGAAGGACCCG⋱
AAGAAGACTGGAGGTCCAATTTATCGAAGGAGAGATGGGAAGTGGGTGAGAGAGTTGATCCTGTATGACA⋱
AAGAGGAGATCAGGAGGATCTGGCGTCAAGCAAATAATGGAGAAGACGCAACTGCTGGTCTCACTCACCT⋱
GATGATCTGGCATTCCAACCTAAATGATGCCACATACCAGAGAACAAGAGCTCTCGTGCGTACCGGGATG⋱
GACCCCAGAATGTGCTCTCTTATGCAAGGATCAACTCTCCCGAGGAGATCTGGAGCAGCTGGTGCAGCAG⋱
TAAAGGGAGTCGGGACGATGGTGATGGAACTGATTCGGATGATAAAGAGAGGGATTAATGATCGGAATTT⋱
CTGGAGAGGCGAAAATGGACGAAGAACAAGGATTGCATATGAGAGAATGTGCAACATCCTCAAAGGGAAA⋱
TTCCAAACAGCAGCACAAAGAGCAATGATGGACCAAGTGCGAGAAAGCAGGAATCCTGGGAATGCTGAAA⋱
TTGAAGATCTCATCTTCCTGGCACGATCTGCACTCATCCTGAGAGGATCAGTGGCCCATAAGTCCTGCTT⋱
GCCTGCTTGTGTGTATGGGCTTGCTGTGGCCAGTGGATATGACTTTGAAAGAGAAGGGTATTCTCTAGTT⋱
GGAATAGATCCTTTCCGTCTGCTTCAAAACAGCCAGGTCTTCAGTCTCATTAGACCAAATGAGAATCCAG⋱
CACACAAGAGTCAATTGGTGTGGATGGCATGCCATTCTGCCGCATTCGAGGACCTGAGAGTCTCAAGTTT⋱
CATCAGAGGGACAAGAGTAGTTCCAAGAGGACAACTATCCACCAGAGGAGTTCAAATTGCTTCAAACGAG⋱
AATATGGAAACCATGGACTCCAGCACTCTTGAACTGAGAAGCAGATATTGGGCTATAAGAACCAGGAGTG⋱
GAGGAAACACCAATCAACAGAGAGCATCTGCAGGACAAATCAGTGTACAGCCCACTTTCTCGGTACAGAG⋱
AAATCTTCCCTTCGAAAGAGCGACCATTATGGCGGCGTTCACAGGGAATACTGAGGGCAGAACATCTGAC⋱
ATGAGGACTGAAATCATAAGAATGATGGAAAGTGCCAGACCAGAGGATGTGTCT
TTCCAGGGGCGGGGAGTCTTCGAGCTCTCGGACGAAAAGGCAACGAACCCGATCG
TGCCTTCCTTTGAC------ATGAGTAATGAAGGATCTTATTTCTTCGGAG
ACAATGCAGAGGAGTATGACAATTAA

Interesting points (by weighted ranked PD) highlighted for gene NP:

ATG
GCGTCTCAAGGCACCAAACGATCTTATGAGCAGATGGAGACTGGTGGAGAACGCC
AGAATGCCACTGAGATCAGAGCATCTGTTGGAAGAATGGTTGGTGGAATTGGGAGGTTTTACATACAGA⋱
TGTGCACTGAACTCAAACTCAGCGACTATGAAGGAAGGCTGGTCCAGAACAGCATAACAATAGAGAGAAT⋱
GGTTCTCTCTGCATTTGATGAAAGGAGGAATAAATACCTGGAAGAACATCCCAGTGCGGGGAAGGACCCG⋱
AAGAAGACTGGAGGTCCAATTTATCGAAGGAGAGATGGGAAGTGGGTGAGAGAGTTGATCCTGTATGACA⋱
AAGAGGAGATCAGGAGGATCTGGCGTCAAGCAAATAATGGAGAAGACGCAACTGCTGGTCTCACTCACCT⋱
GATGATCTGGCATTCCAACCTAAATGATGCCACATACCAGAGAACAAGAGCTCTCGTGCGTACCGGGATG⋱
GACCCCAGAATGTGCTCTCTTATGCAAGGATCAACTCTCCCGAGGAGATCTGGAGCAGCTGGTGCAGCAG⋱
TAAAGGGAGTCGGGACGATGGTGATGGAACTGATTCGGATGATAAAGAGAGGGATTAATGATCGGAATTT⋱
CTGGAGAGGCGAAAATGGACGAAGAACAAGGATTGCATATGAGAGAATGTGCAACATCCTCAAAGGGAAA⋱
TTCCAAACAGCAGCACAAAGAGCAATGATGGACCAAGTGCGAGAAAGCAGGAATCCTGGGAATGCTGAAA⋱
TTGAAGATCTCATCTTCCTGGCACGATCTGCACTCATCCTGAGAGGATCAGTGGCCCATAAGTCCTGCTT⋱
GCCTGCTTGTGTGTATGGGCTTGCTGTGGCCAGTGGATATGACTTTGAAAGAGAAGGGTATTCTCTAGTT⋱
GGAATAGATCCTTTCCGTCTGCTTCAAAACAGCCAGGTCTTCAGTCTCATTAGACCAAATGAGAATCCAG⋱
CACACAAGAGTCAATTGGTGTGGATGGCATGCCATTCTGCCGCATTCGAGGACCTGAGAGTCTCAAGTTT⋱
CATCAGAGGGACAAGAGTAGTTCCAAGAGGACAACTATCCACCAGAGGAGTTCAAATTGCTTCAAACGAG⋱

AATATGGAAACCATGGACTCCAGCACTCTTGAACTGAGAAGCAGATATTGGGCTATAAGAACCAGGAGTG∵.
GAGGAAACACCAATCAACAGAGAGCATCTGCAGGACAAATCAGTGTACAGCCCACTTTCTCGGTACAGAG∵.
AAATCTTCCCTTCGAAAGAGCGACCATTATGGCGGCGTTCACAGGGAATACTGAGGGCAGAACATCTGAC∵.
ATGAGGACTGAAATCATAAGAATGATGGAAAGTGCCAGACCAGAGGATGTGTCT
TTCCAGGGGCGGGGAGTCTTCGAGCTCTCGGACGAAAAGGCAACGAACCCGATCG
TGCCTTCCTTTGAC––––––ATGAGTAATGAAGGATCTTATTTCTTCGGAG
ACAATGCAGAGGAGTATGACAATTAA

Per codon conservation report

NP			
Pos.	1	obs:	exp:
atg	M	917	917.00
---	--	------	------
mPD		0	0
		nPD:	1.
		N. weight:	0.
		Sc. PD:	0
		Sc. rank:	0

NP			
Pos.	2	obs:	exp:
act	T	0	0.26
acc	T	0	0.20
aca	T	0	0.46
acg	T	1	0.08
gct	A	2	234.70
gcc	A	0	172.00
gca	A	2	428.30
gcg	A	912	81.02
---	--	------	------
mPD		0.011	0.67
		nPD:	0.02
		N. weight:	2.9
		Sc. PD:	-0.36
		Sc. rank:	-1042.0

NP			
Pos.	3	obs:	exp:
ttt	F	3	1.58
ttc	F	1	2.42
tct	S	909	145.70
tcc	S	1	117.80
tca	S	0	229.70
tcg	S	0	52.96
tat	Y	2	1.06
tac	Y	0	0.94
act	T	1	0.26
acc	T	0	0.20
aca	T	0	0.46
acg	T	0	0.08
agt	S	0	186.50
agc	S	0	177.50
---	--	------	------
mPD		0.020	1.7
		nPD:	0.01
		N. weight:	2.2
		Sc. PD:	-0.28
		Sc. rank:	-981.0

NP			
Pos.	4	obs:	exp:
caa	Q	914	471.00
cag	Q	3	446.00
---	--	------	------
mPD		0.0065	0.50
		nPD:	0.01
		N. weight:	0.79
		Sc. PD:	-0.099
		Sc. rank:	-332.6

NP			
Pos.	5	obs:	exp:
gct	A	0	0.26
gcc	A	1	0.19
gca	A	0	0.47
gcg	A	0	0.09
gat	D	0	1.08
gac	D	2	0.92
ggt	G	0	122.60
ggc	G	914	116.10
gga	G	0	422.60
ggg	G	0	252.70
---	--	------	------
mPD		0.0065	0.68
		nPD:	0.01
		N. weight:	2.5
		Sc. PD:	-0.33
		Sc. rank:	-1157.5

NP			
Pos.	6	obs:	exp:
att	I	0	0.36
atc	I	1	0.24
ata	I	0	0.39
act	T	0	237.30
acc	T	916	186.50
aca	T	0	420.80
acg	T	0	71.35
---	--	------	------
mPD		0.0022	0.68
		nPD:	0.
		N. weight:	1.9
		Sc. PD:	-0.26
		Sc. rank:	-1111.2

NP			
Pos.	7	obs:	exp:
aaa	K	917	537.20
aag	K	0	379.80
---	--	------	------
mPD		0	0.49
		nPD:	0.
		N. weight:	0.65
		Sc. PD:	-0.09
		Sc. rank:	-422.6

NP			
Pos.	8	obs:	exp:
cgt	R	0	39.17
cgc	R	0	46.59
cga	R	916	91.44
cgg	R	1	76.38
aga	R	0	418.70
agg	R	0	244.70
---	--	------	------
mPD		0.0022	0.96
		nPD:	0.
		N. weight:	2.8
		Sc. PD:	-0.38
		Sc. rank:	-1643.6

NP			
Pos.	9	obs:	exp:
tct	S	86	146.80
tcc	S	829	118.70
tca	S	2	231.40
tcg	S	0	53.36
agt	S	0	187.90
agc	S	0	178.80
---	--	------	------
mPD		0.17	1.7
		nPD:	0.11
		N. weight:	2.1
		Sc. PD:	-0.08
		Sc. rank:	146.9

NP			
Pos.	10	obs:	exp:
tat	Y	896	486.20
tac	Y	21	430.80
---	--	------	------
mPD		0.045	0.50
		nPD:	0.09
		N. weight:	0.65
		Sc. PD:	-0.034
		Sc. rank:	14.2

NP			
Pos.	11	obs:	exp:
gaa	E	894	551.60
gag	E	23	365.40
---	--	------	------
mPD		0.049	0.48
		nPD:	0.1
		N. weight:	0.49
		Sc. PD:	-0.021
		Sc. rank:	26.3

NP			
Pos.	12	obs:	exp:
caa	Q	38	471.00
cag	Q	879	446.00
---	--	------	------
mPD		0.080	0.50
		nPD:	0.16
		N. weight:	0.67
		Sc. PD:	0.0084
		Sc. rank:	158.7

NP			
Pos.	13	obs:	exp:
atg	M	917	917.00
---	--	------	------
mPD		0	0
		nPD:	1.
		N. weight:	0.
		Sc. PD:	0
		Sc. rank:	0

NP			
Pos.	14	obs:	exp:
caa	Q	1	0.51
cag	Q	0	0.49
aaa	K	1	0.59
aag	K	0	0.41
gaa	E	835	550.40
gag	E	80	364.60
---	--	------	------
mPD		0.16	0.48
		nPD:	0.34
		N. weight:	0.31
		Sc. PD:	0.056
		Sc. rank:	144.2

NP			
Pos.	15	obs:	exp:
act	T	910	237.30
acc	T	6	186.50
aca	T	0	420.80
acg	T	0	71.35
gct	A	1	0.26
gcc	A	0	0.19
gca	A	0	0.47
gcg	A	0	0.09
---	--	------	------
mPD		0.015	0.68
		nPD:	0.02
		N. weight:	1.6
		Sc. PD:	-0.19
		Sc. rank:	-505.6

NP			
Pos.	16	obs:	exp:
tct	S	0	2.24
tcc	S	0	1.81
tca	S	0	3.53
tcg	S	0	0.81
aat	N	1	0.51
aac	N	0	0.49
agt	S	14	2.87
agc	S	0	2.73
ggt	G	902	121.00
ggc	G	0	114.60
gga	G	0	417.00
ggg	G	0	249.40
---	--	------	------
mPD		0.034	0.73
		nPD:	0.05
		N. weight:	2.4
		Sc. PD:	-0.23
		Sc. rank:	-316.2

NP			
Pos.	17	obs:	exp:
gtt	V	0	0.83
gtc	V	0	0.80
gta	V	0	0.83
gtg	V	4	1.54
ggt	G	2	122.40
ggc	G	0	116.00
gga	G	112	422.10
ggg	G	799	252.40
---	--	------	------
mPD		0.23	0.68
		nPD:	0.33
		N. weight:	1.
		Sc. PD:	0.18
		Sc. rank:	472.2

NP			
Pos.	18	obs:	exp:
aaa	K	1	0.59
aag	K	0	0.41
gct	A	0	0.26
gcc	A	0	0.19
gca	A	1	0.47
gcg	A	0	0.09
gaa	E	879	549.20
gag	E	34	363.80
ggt	G	0	0.27
ggc	G	0	0.25
gga	G	2	0.92
ggg	G	0	0.55
---	--	------	------
mPD		0.080	0.49
		nPD:	0.16
		N. weight:	0.45
		Sc. PD:	0.0077
		Sc. rank:	110.3

NP			
Pos.	19	obs:	exp:
cgt	R	0	39.17
cgc	R	917	46.59
cga	R	0	91.44
cgg	R	0	76.38
aga	R	0	418.70
agg	R	0	244.70
---	--	------	------
mPD		0	0.96
		nPD:	0.
		N. weight:	3.6
		Sc. PD:	-0.5
		Sc. rank:	-2354.4

NP			
Pos.	20	obs:	exp:
caa	Q	0	471.00
cag	Q	917	446.00
---	--	------	------
mPD		0	0.50
		nPD:	0.
		N. weight:	0.88
		Sc. PD:	-0.12
		Sc. rank:	-569.6

NP			
Pos.	21	obs:	exp:
aat	N	915	466.50
aac	N	2	450.50
---	--	------	------
mPD		0.0044	0.50
		nPD:	0.01
		N. weight:	0.81
		Sc. PD:	-0.11
		Sc. rank:	-405.2

NP			
Pos.	22	obs:	exp:
act	T	62	16.06
acc	T	0	12.63
aca	T	0	28.48
acg	T	0	4.83
gct	A	772	219.10
gcc	A	82	160.50
gca	A	0	399.80
gcg	A	1	75.62
---	--	------	------
mPD		0.29	0.80
		nPD:	0.36
		N. weight:	1.3
		Sc. PD:	0.28
		Sc. rank:	676.4

NP			
Pos.	23	obs:	exp:
act	T	910	237.50
acc	T	5	186.70
aca	T	1	421.30
acg	T	1	71.43
---	--	------	------
mPD		0.015	0.67
		nPD:	0.02
		N. weight:	1.6
		Sc. PD:	-0.19
		Sc. rank:	-489.0

NP			
Pos.	24	obs:	exp:
gtt	V	0	0.21
gtc	V	0	0.20
gta	V	0	0.21
gtg	V	1	0.39
gaa	E	79	551.00
gag	E	837	365.00
---	--	------	------
mPD		0.16	0.48
		nPD:	0.33
		N. weight:	0.72
		Sc. PD:	0.13
		Sc. rank:	331.5

NP			
Pos.	25	obs:	exp:
att	I	2	334.20
atc	I	913	221.60
ata	I	1	360.20
gtt	V	0	0.21
gtc	V	1	0.20
gta	V	0	0.21
gtg	V	0	0.39
---	--	------	------
mPD		0.0087	0.66
		nPD:	0.01
		N. weight:	1.7
		Sc. PD:	-0.21
		Sc. rank:	-707.9

NP			
Pos.	26	obs:	exp:
cgt	R	0	39.17
cgc	R	0	46.59
cga	R	0	91.44
cgg	R	0	76.38
aga	R	318	418.70
agg	R	599	244.70
---	--	------	------
mPD		0.45	0.96
		nPD:	0.47
		N. weight:	0.6
		Sc. PD:	0.19
		Sc. rank:	341.8

,

NP			
Pos.	27	obs:	exp:
tct	S	0	0.16
tcc	S	0	0.13
tca	S	1	0.25
tcg	S	0	0.06
act	T	0	0.26
acc	T	0	0.20
aca	T	1	0.46
acg	T	0	0.08
agt	S	0	0.20
agc	S	0	0.20
gct	A	2	234.50
gcc	A	0	171.80
gca	A	821	427.80
gcg	A	92	80.93
---	--	------	------
mPD		0.19	0.68
		nPD:	0.28
		N. weight:	0.72
		Sc. PD:	0.091
		Sc. rank:	293.9

,

NP			
Pos.	28	obs:	exp:
ttt	F	1	0.39
ttc	F	0	0.61
tct	S	906	146.60
tcc	S	4	118.60
tca	S	5	231.20
tcg	S	1	53.31
agt	S	0	187.70
agc	S	0	178.60
---	--	------	------
mPD		0.024	1.7
		nPD:	0.01
		N. weight:	2.1
		Sc. PD:	-0.27
		Sc. rank:	-834.8

,

NP			
Pos.	29	obs:	exp:
gtt	V	877	189.30
gtc	V	39	183.70
gta	V	1	189.90
gtg	V	0	354.10
---	--	------	------
mPD		0.084	0.73
		nPD:	0.12
		N. weight:	1.7
		Sc. PD:	-0.05
		Sc. rank:	205.2

,

NP			
Pos.	30	obs:	exp:
ggt	G	0	123.00
ggc	G	0	116.50
gga	G	708	424.00
ggg	G	209	253.50
---	--	------	------
mPD		0.35	0.68
		nPD:	0.52
		N. weight:	0.43
		Sc. PD:	0.15
		Sc. rank:	256.5

,

NP			
Pos.	31	obs:	exp:
cgt	R	0	39.05
cgc	R	0	46.44
cga	R	0	91.14
cgg	R	0	76.13
aaa	K	2	1.76
aag	K	1	1.24
aga	R	899	417.30
agg	R	15	243.90
---	--	------	------
mPD		0.041	0.97
		nPD:	0.04
		N. weight:	0.86
		Sc. PD:	-0.085
		Sc. rank:	-144.4

,

NP			
Pos.	32	obs:	exp:
atg	M	917	917.00
---	--	------	------
mPD		0	0
		nPD:	1.
		N. weight:	0.
		Sc. PD:	0
		Sc. rank:	0

,

NP			
Pos.	33	obs:	exp:
att	I	10	3.65
atc	I	0	2.42
ata	I	0	3.93
gtt	V	880	187.20
gtc	V	8	181.70
gta	V	0	187.80
gtg	V	19	350.30
---	--	------	------
mPD		0.079	0.75
		nPD:	0.11
		N. weight:	1.7
		Sc. PD:	-0.066
		Sc. rank:	133.0

,

NP			
Pos.	34	obs:	exp:
tct	S	0	128.90
tcc	S	0	104.20
tca	S	0	203.20
tcg	S	0	46.85
aat	N	0	1.02
aac	N	2	0.98
agt	S	231	165.00
agc	S	574	157.00
gat	D	1	0.54
gac	D	0	0.46
ggt	G	66	14.62
ggc	G	42	13.85
gga	G	1	50.40
ggg	G	0	30.14
---	--	------	------
mPD		0.66	1.8
		nPD:	0.37
		N. weight:	1.3
		Sc. PD:	0.27
		Sc. rank:	667.0

,

NP			
Pos.	35	obs:	exp:
ggt	G	1	123.00
ggc	G	728	116.50
gga	G	186	424.00
ggg	G	2	253.50
---	--	------	------
mPD		0.33	0.68
		nPD:	0.49
		N. weight:	1.6
		Sc. PD:	0.5
		Sc. rank:	895.8

,

NP			
Pos.	36	obs:	exp:
att	I	867	334.60
atc	I	49	221.80
ata	I	1	360.60
---	--	------	------
mPD		0.10	0.65
		nPD:	0.16
		N. weight:	0.99
		Sc. PD:	0.011
		Sc. rank:	234.6

,

NP			
Pos.	37	obs:	exp:
tta	L	0	0.09
ttg	L	1	0.19
ctt	L	0	0.17
ctc	L	0	0.17
cta	L	0	0.17
ctg	L	0	0.22
ggt	G	201	122.80
ggc	G	0	116.40
gga	G	18	423.50
ggg	G	697	253.30
---	--	------	------
mPD		0.38	0.68
		nPD:	0.55
		N. weight:	1.
		Sc. PD:	0.39
		Sc. rank:	607.3

,

NP			
Pos.	38	obs:	exp:
cgt	R	0	39.09
cgc	R	1	46.49
cga	R	0	91.24
cgg	R	0	76.21
aaa	K	1	1.17
aag	K	1	0.83
aga	R	869	417.80
agg	R	45	244.20
---	--	------	------
mPD		0.10	0.97
		nPD:	0.11
		N. weight:	0.74
		Sc. PD:	-0.027
		Sc. rank:	60.0

,

NP			
Pos.	39	obs:	exp:
ttt	F	32	361.90
ttc	F	885	555.10
---	--	------	------
mPD		0.067	0.48
		nPD:	0.14
		N. weight:	0.45
		Sc. PD:	-0.0021
		Sc. rank:	78.0

,

NP			
Pos.	40	obs:	exp:
tat	Y	1	486.20
tac	Y	916	430.80
---	--	------	------
mPD		0.0022	0.50
		nPD:	0.
		N. weight:	0.91
		Sc. PD:	-0.12
		Sc. rank:	-497.1

,

NP			
Pos.	41	obs:	exp:
att	I	1	334.60
atc	I	0	221.80
ata	I	916	360.60
---	--	------	------
mPD		0.0022	0.65
		nPD:	0.
		N. weight:	1.1
		Sc. PD:	-0.15
		Sc. rank:	-632.4

,

NP			
Pos.	42	obs:	exp:
caa	Q	7	471.00
cag	Q	910	446.00
---	--	------	------
mPD		0.015	0.50
		nPD:	0.03
		N. weight:	0.82
		Sc. PD:	-0.091
		Sc. rank:	-191.0

,

NP			
Pos.	43	obs:	exp:
atg	M	916	916.00
act	T	0	0.26
acc	T	0	0.20
aca	T	0	0.46
acg	T	1	0.08
---	--	------	------
mPD		0.0022	0.0042
		nPD:	0.52
		N. weight:	0.0034
		Sc. PD:	0.0012
		Sc. rank:	2.0

,

NP			
Pos.	44	obs:	exp:
tgt	C	734	376.50
tgc	C	183	540.50
---	--	------	------
mPD		0.32	0.48
		nPD:	0.66
		N. weight:	0.39
		Sc. PD:	0.19
		Sc. rank:	242.9

,

NP			
Pos.	45	obs:	exp:
act	T	88	237.50
acc	T	4	186.70
aca	T	825	421.30
acg	T	0	71.43
---	--	------	------
mPD		0.18	0.67
		nPD:	0.27
		N. weight:	0.61
		Sc. PD:	0.071
		Sc. rank:	242.1

,

NP			
Pos.	46	obs:	exp:
gaa	E	850	551.60
gag	E	67	365.40
---	--	------	------
mPD		0.14	0.48
		nPD:	0.28
		N. weight:	0.34
		Sc. PD:	0.044
		Sc. rank:	140.0

,

NP			
Pos.	47	obs:	exp:
tta	L	0	80.59
ttg	L	0	171.10
ctt	L	4	155.40
ctc	L	910	153.60
cta	L	3	152.90
ctg	L	0	203.40
---	--	------	------
mPD		0.015	1.1
		nPD:	0.01
		N. weight:	2.1
		Sc. PD:	-0.27
		Sc. rank:	-843.6

,

NP			
Pos.	48	obs:	exp:
aaa	K	898	537.20
aag	K	19	379.80
---	--	------	------
mPD		0.041	0.49
		nPD:	0.08
		N. weight:	0.54
		Sc. PD:	-0.032
		Sc. rank:	-3.6

,

NP			
Pos.	49	obs:	exp:
tta	L	0	80.50
ttg	L	0	170.90
ctt	L	5	155.20
ctc	L	910	153.50
cta	L	1	152.70
ctg	L	0	203.10
att	I	0	0.36
atc	I	1	0.24
ata	I	0	0.39
---	--	------	------
mPD		0.015	1.1
		nPD:	0.01
		N. weight:	2.1
		Sc. PD:	-0.27
		Sc. rank:	-858.6

,

NP			
Pos.	50	obs:	exp:
tct	S	0	141.20
tcc	S	0	114.20
tca	S	0	222.60
tcg	S	0	51.33
act	T	1	0.26
acc	T	0	0.20
aca	T	0	0.46
acg	T	0	0.08
aat	N	33	16.79
aac	N	0	16.21
agt	S	833	180.70
agc	S	49	172.00
ggt	G	1	0.13
ggc	G	0	0.13
gga	G	0	0.46
ggg	G	0	0.28
---	--	------	------
mPD		0.18	1.7
		nPD:	0.1
		N. weight:	1.6
		Sc. PD:	-0.067
		Sc. rank:	94.9

,

NP			
Pos.	51	obs:	exp:
gat	D	8	495.00
gac	D	909	422.00
---	--	------	------
mPD		0.017	0.50
		nPD:	0.03
		N. weight:	0.88
		Sc. PD:	-0.094
		Sc. rank:	-191.6

,

NP			
Pos.	52	obs:	exp:
tat	Y	67	47.72
tac	Y	23	42.28
cat	H	10	8.04
cac	H	4	5.96
act	T	1	0.26
acc	T	0	0.20
aca	T	0	0.46
acg	T	0	0.08
aat	N	619	413.10
aac	N	193	398.90
---	--	------	------
mPD		0.57	0.71
		nPD:	0.81
		N. weight:	0.17
		Sc. PD:	0.11
		Sc. rank:	110.4

,

NP			
Pos.	53	obs:	exp:
gaa	E	909	551.60
gag	E	8	365.40
---	--	------	------
mPD		0.017	0.48
		nPD:	0.04
		N. weight:	0.56
		Sc. PD:	-0.059
		Sc. rank:	-115.4

,

NP			
Pos.	54	obs:	exp:
ggt	G	0	123.00
ggc	G	0	116.50
gga	G	45	424.00
ggg	G	872	253.50
---	--	------	------
mPD		0.093	0.68
		nPD:	0.14
		N. weight:	1.3
		Sc. PD:	-0.0096
		Sc. rank:	215.3

,

NP			
Pos.	55	obs:	exp:
cgt	R	0	39.13
cgc	R	0	46.54
cga	R	0	91.34
cgg	R	0	76.29
aaa	K	0	0.59
aag	K	1	0.41
aga	R	79	418.20
agg	R	837	244.50
---	--	------	------
mPD		0.16	0.97
		nPD:	0.17
		N. weight:	1.2
		Sc. PD:	0.022
		Sc. rank:	308.0

,

NP			
Pos.	56	obs:	exp:
tta	L	0	80.59
ttg	L	7	171.10
ctt	L	0	155.40
ctc	L	1	153.60
cta	L	10	152.90
ctg	L	899	203.40
---	--	------	------
mPD		0.039	1.1
		nPD:	0.04
		N. weight:	1.7
		Sc. PD:	-0.18
		Sc. rank:	-362.2

,

NP			
Pos.	57	obs:	exp:
att	I	778	333.90
atc	I	135	221.30
ata	I	2	359.80
gtt	V	0	0.41
gtc	V	2	0.40
gta	V	0	0.41
gtg	V	0	0.77
---	--	------	------
mPD		0.26	0.66
		nPD:	0.4
		N. weight:	0.78
		Sc. PD:	0.19
		Sc. rank:	426.9

,

NP			
Pos.	58	obs:	exp:
caa	Q	228	471.00
cag	Q	689	446.00
---	--	------	------
mPD		0.37	0.50
		nPD:	0.75
		N. weight:	0.18
		Sc. PD:	0.1
		Sc. rank:	117.0

,

NP			
Pos.	59	obs:	exp:
aat	N	1	466.50
aac	N	916	450.50
---	--	------	------
mPD		0.0022	0.50
		nPD:	0.
		N. weight:	0.86
		Sc. PD:	-0.12
		Sc. rank:	-471.2

,

NP			
Pos.	60	obs:	exp:
tct	S	0	146.80
tcc	S	0	118.70
tca	S	0	231.40
tcg	S	0	53.36
agt	S	820	187.90
agc	S	97	178.80
---	--	------	------
mPD		0.19	1.7
		nPD:	0.11
		N. weight:	1.5
		Sc. PD:	-0.046
		Sc. rank:	163.9

,

NP			
Pos.	61	obs:	exp:
att	I	18	334.20
atc	I	9	221.60
ata	I	889	360.20
atg	M	1	1.00
---	--	------	------
mPD		0.060	0.65
		nPD:	0.09
		N. weight:	0.96
		Sc. PD:	-0.05
		Sc. rank:	23.6

,

NP			
Pos.	62	obs:	exp:
act	T	6	237.50
acc	T	33	186.70
aca	T	867	421.30
acg	T	11	71.43
---	--	------	------
mPD		0.10	0.67
		nPD:	0.16
		N. weight:	0.71
		Sc. PD:	0.0062
		Sc. rank:	162.1

,

NP			
Pos.	63	obs:	exp:
att	I	0	334.20
atc	I	0	221.60
ata	I	916	360.20
gtt	V	0	0.21
gtc	V	0	0.20
gta	V	1	0.21
gtg	V	0	0.39
---	--	------	------
mPD		0.0022	0.66
		nPD:	0.
		N. weight:	1.1
		Sc. PD:	-0.15
		Sc. rank:	-641.3

,

NP			
Pos.	64	obs:	exp:
aaa	K	0	1.17
aag	K	2	0.83
gaa	E	10	550.40
gag	E	905	364.60
---	--	------	------
mPD		0.026	0.48
		nPD:	0.05
		N. weight:	1.
		Sc. PD:	-0.092
		Sc. rank:	-96.5

,

NP			
Pos.	65	obs:	exp:
cgt	R	0	39.17
cgc	R	0	46.59
cga	R	0	91.44
cgg	R	0	76.38
aga	R	887	418.70
agg	R	30	244.70
---	--	------	------
mPD		0.063	0.96
		nPD:	0.07
		N. weight:	0.8
		Sc. PD:	-0.061
		Sc. rank:	-28.9

,

NP			
Pos.	66	obs:	exp:
atg	M	917	917.00
---	--	------	------
mPD		0	0
		nPD:	1.
		N. weight:	0.
		Sc. PD:	0
		Sc. rank:	0

,

NP			
Pos.	67	obs:	exp:
tta	L	0	0.18
ttg	L	0	0.37
ctt	L	2	0.34
ctc	L	0	0.34
cta	L	0	0.33
ctg	L	0	0.44
att	I	0	0.36
atc	I	0	0.24
ata	I	1	0.39
gtt	V	73	188.70
gtc	V	14	183.10
gta	V	823	189.20
gtg	V	4	353.00
---	--	------	------
mPD		0.19	0.73
		nPD:	0.26
		N. weight:	1.5
		Sc. PD:	0.16
		Sc. rank:	572.2

,

NP			
Pos.	68	obs:	exp:
tta	L	0	80.59
ttg	L	0	171.10
ctt	L	6	155.40
ctc	L	910	153.60
cta	L	1	152.90
ctg	L	0	203.40
---	--	------	------
mPD		0.015	1.1
		nPD:	0.01
		N. weight:	2.1
		Sc. PD:	-0.27
		Sc. rank:	-867.8

,

NP			
Pos.	69	obs:	exp:
tct	S	869	146.80
tcc	S	2	118.70
tca	S	34	231.40
tcg	S	12	53.36
agt	S	0	187.90
agc	S	0	178.80
---	--	------	------
mPD		0.10	1.7
		nPD:	0.06
		N. weight:	1.9
		Sc. PD:	-0.16
		Sc. rank:	-112.5

,

NP			
Pos.	70	obs:	exp:
gct	A	4	235.00
gcc	A	3	172.20
gca	A	903	428.70
gcg	A	7	81.11
---	--	------	------
mPD		0.030	0.67
		nPD:	0.04
		N. weight:	0.84
		Sc. PD:	-0.081
		Sc. rank:	-114.2

NP			
Pos.	71	obs:	exp:
ttt	F	913	361.90
ttc	F	4	555.10
---	--	------	------
mPD		0.0087	0.48
		nPD:	0.02
		N. weight:	1.1
		Sc. PD:	-0.13
		Sc. rank:	-382.7

,

NP			
Pos.	72	obs:	exp:
gat	D	907	495.00
gac	D	10	422.00
---	--	------	------
mPD		0.022	0.50
		nPD:	0.04
		N. weight:	0.68
		Sc. PD:	-0.066
		Sc. rank:	-102.9

,

NP			
Pos.	73	obs:	exp:
gaa	E	678	551.60
gag	E	239	365.40
---	--	------	------
mPD		0.39	0.48
		nPD:	0.8
		N. weight:	0.056
		Sc. PD:	0.035
		Sc. rank:	36.1

,

NP			
Pos.	74	obs:	exp:
cgt	R	0	39.17
cgc	R	0	46.59
cga	R	0	91.44
cgg	R	0	76.38
aga	R	824	418.70
agg	R	93	244.70
---	--	------	------
mPD		0.18	0.96
		nPD:	0.19
		N. weight:	0.63
		Sc. PD:	0.026
		Sc. rank:	190.7

,

NP			
Pos.	75	obs:	exp:
cgt	R	0	39.17
cgc	R	0	46.59
cga	R	189	91.44
cgg	R	12	76.38
aga	R	45	418.70
agg	R	671	244.70
---	--	------	------
mPD		0.72	0.96
		nPD:	0.75
		N. weight:	0.93
		Sc. PD:	0.53
		Sc. rank:	598.7

,

NP			
Pos.	76	obs:	exp:
aat	N	10	465.50
aac	N	905	449.50
aaa	K	2	1.17
aag	K	0	0.83
---	--	------	------
mPD		0.026	0.50
		nPD:	0.05
		N. weight:	0.79
		Sc. PD:	-0.071
		Sc. rank:	-78.8

,

NP			
Pos.	77	obs:	exp:
cgt	R	0	35.67
cgc	R	0	42.42
cga	R	0	83.26
cgg	R	0	69.55
aaa	K	59	48.04
aag	K	23	33.96
aga	R	830	381.20
agg	R	5	222.90
---	--	------	------
mPD		0.22	1.1
		nPD:	0.2
		N. weight:	0.84
		Sc. PD:	0.045
		Sc. rank:	265.7

,

NP			
Pos.	78	obs:	exp:
tat	Y	85	486.20
tac	Y	832	430.80
---	--	------	------
mPD		0.17	0.50
		nPD:	0.34
		N. weight:	0.53
		Sc. PD:	0.098
		Sc. rank:	248.5

,

NP			
Pos.	79	obs:	exp:
tta	L	0	80.59
ttg	L	277	171.10
ctt	L	0	155.40
ctc	L	0	153.60
cta	L	9	152.90
ctg	L	631	203.40
---	--	------	------
mPD		0.44	1.1
		nPD:	0.4
		N. weight:	1.1
		Sc. PD:	0.26
		Sc. rank:	598.0

,

NP			
Pos.	80	obs:	exp:
gaa	E	859	551.60
gag	E	58	365.40
---	--	------	------
mPD		0.12	0.48
		nPD:	0.25
		N. weight:	0.37
		Sc. PD:	0.035
		Sc. rank:	134.7

,

NP			
Pos.	81	obs:	exp:
aaa	K	0	1.17
aag	K	2	0.83
gaa	E	238	550.40
gag	E	677	364.60
---	--	------	------
mPD		0.39	0.48
		nPD:	0.8
		N. weight:	0.3
		Sc. PD:	0.19
		Sc. rank:	193.5

,

NP			
Pos.	82	obs:	exp:
cat	H	104	526.20
cac	H	812	389.80
cgt	R	0	0.04
cgc	R	1	0.05
cga	R	0	0.10
cgg	R	0	0.08
aga	R	0	0.46
agg	R	0	0.27
---	--	------	------
mPD		0.20	0.49
		nPD:	0.41
		N. weight:	0.58
		Sc. PD:	0.15
		Sc. rank:	319.4

,

NP			
Pos.	83	obs:	exp:
cct	P	203	233.20
ccc	P	697	163.50
cca	P	17	374.60
ccg	P	0	145.70
---	--	------	------
mPD		0.37	0.71
		nPD:	0.52
		N. weight:	1.2
		Sc. PD:	0.45
		Sc. rank:	740.0

,

NP			
Pos.	84	obs:	exp:
tct	S	0	146.30
tcc	S	0	118.30
tca	S	0	230.70
tcg	S	0	53.19
aat	N	3	1.53
aac	N	0	1.47
agt	S	912	187.30
agc	S	2	178.20
---	--	------	------
mPD		0.011	1.7
		nPD:	0.01
		N. weight:	1.9
		Sc. PD:	-0.25
		Sc. rank:	-976.6

,

NP			
Pos.	85	obs:	exp:
tct	S	0	0.48
tcc	S	0	0.39
tca	S	3	0.76
tcg	S	0	0.17
act	T	0	0.26
acc	T	0	0.20
aca	T	0	0.46
acg	T	1	0.08
agt	S	0	0.61
agc	S	0	0.59
gct	A	119	233.90
gcc	A	0	171.40
gca	A	732	426.90
gcg	A	62	80.75
---	--	------	------
mPD		0.35	0.68
		nPD:	0.5
		N. weight:	0.41
		Sc. PD:	0.14
		Sc. rank:	242.0

,

NP			
Pos.	86	obs:	exp:
aaa	K	0	1.17
aag	K	2	0.83
ggt	G	0	122.70
ggc	G	2	116.30
gga	G	782	423.10
ggg	G	131	253.00
---	--	------	------
mPD		0.26	0.68
		nPD:	0.38
		N. weight:	0.52
		Sc. PD:	0.12
		Sc. rank:	274.8

,

NP			
Pos.	87	obs:	exp:
cgt	R	0	0.04
cgc	R	0	0.05
cga	R	0	0.10
cgg	R	0	0.08
aaa	K	269	536.60
aag	K	647	379.40
aga	R	0	0.46
agg	R	1	0.27
---	--	------	------
mPD		0.42	0.49
		nPD:	0.85
		N. weight:	0.22
		Sc. PD:	0.15
		Sc. rank:	141.8

,

NP			
Pos.	88	obs:	exp:
gat	D	304	495.00
gac	D	613	422.00
---	--	------	------
mPD		0.44	0.50
		nPD:	0.89
		N. weight:	0.11
		Sc. PD:	0.079
		Sc. rank:	72.7

,

NP			
Pos.	89	obs:	exp:
cct	P	733	233.20
ccc	P	100	163.50
cca	P	12	374.60
ccg	P	72	145.70
---	--	------	------
mPD		0.34	0.71
		nPD:	0.48
		N. weight:	0.94
		Sc. PD:	0.3
		Sc. rank:	536.4

,

NP			
Pos.	90	obs:	exp:
aaa	K	1	537.20
aag	K	916	379.80
---	--	------	------
mPD		0.0022	0.49
		nPD:	0.
		N. weight:	1.1
		Sc. PD:	-0.14
		Sc. rank:	-575.4

,

NP			
Pos.	91	obs:	exp:
aaa	K	865	537.20
aag	K	52	379.80
---	--	------	------
mPD		0.11	0.49
		nPD:	0.22
		N. weight:	0.41
		Sc. PD:	0.029
		Sc. rank:	141.9

,

NP			
Pos.	92	obs:	exp:
act	T	910	237.50
acc	T	7	186.70
aca	T	0	421.30
acg	T	0	71.43
---	--	------	------
mPD		0.015	0.67
		nPD:	0.02
		N. weight:	1.6
		Sc. PD:	-0.19
		Sc. rank:	-497.1

,

NP			
Pos.	93	obs:	exp:
ggt	G	0	123.00
ggc	G	0	116.50
gga	G	910	424.00
ggg	G	7	253.50
---	--	------	------
mPD		0.015	0.68
		nPD:	0.02
		N. weight:	0.89
		Sc. PD:	-0.1
		Sc. rank:	-285.4

,

NP			
Pos.	94	obs:	exp:
ggt	G	884	123.00
ggc	G	29	116.50
gga	G	0	424.00
ggg	G	4	253.50
---	--	------	------
mPD		0.070	0.68
		nPD:	0.1
		N. weight:	2.2
		Sc. PD:	-0.091
		Sc. rank:	142.6

,

NP			
Pos.	95	obs:	exp:
tct	S	1	0.32
tcc	S	0	0.26
tca	S	1	0.50
tcg	S	0	0.12
cct	P	203	232.70
ccc	P	1	163.10
cca	P	705	373.80
ccg	P	6	145.40
agt	S	0	0.41
agc	S	0	0.39
---	--	------	------
mPD		0.36	0.72
		nPD:	0.51
		N. weight:	0.54
		Sc. PD:	0.18
		Sc. rank:	315.5

NP			
Pos.	96	obs:	exp:
att	I	860	334.60
atc	I	52	221.80
ata	I	5	360.60
---	--	------	------
mPD		0.12	0.65
		nPD:	0.18
		N. weight:	0.96
		Sc. PD:	0.03
		Sc. rank:	268.2

NP			
Pos.	97	obs:	exp:
tat	Y	46	486.20
tac	Y	871	430.80
---	--	------	------
mPD		0.095	0.50
		nPD:	0.19
		N. weight:	0.67
		Sc. PD:	0.029
		Sc. rank:	206.6

NP			
Pos.	98	obs:	exp:
cgt	R	0	39.13
cgc	R	0	46.54
cga	R	75	91.34
cgg	R	13	76.29
aaa	K	0	0.59
aag	K	1	0.41
aga	R	23	418.20
agg	R	805	244.50
---	--	------	------
mPD		0.37	0.97
		nPD:	0.38
		N. weight:	1.1
		Sc. PD:	0.26
		Sc. rank:	603.0

NP			
Pos.	99	obs:	exp:
cgt	R	0	39.05
cgc	R	0	46.44
cga	R	0	91.14
cgg	R	0	76.13
atg	M	1	1.00
aaa	K	2	1.17
aag	K	0	0.83
aga	R	832	417.30
agg	R	82	243.90
---	--	------	------
mPD		0.17	0.97
		nPD:	0.18
		N. weight:	0.65
		Sc. PD:	0.019
		Sc. rank:	180.5

NP			
Pos.	100	obs:	exp:
cgt	R	0	39.13
cgc	R	0	46.54
cga	R	1	91.34
cgg	R	0	76.29
aga	R	910	418.20
agg	R	5	244.50
gtt	V	0	0.21
gtc	V	0	0.20
gta	V	1	0.21
gtg	V	0	0.39
---	--	------	------
mPD		0.017	0.97
		nPD:	0.02
		N. weight:	0.91
		Sc. PD:	-0.11
		Sc. rank:	-321.0

NP			
Pos.	101	obs:	exp:
gat	D	75	492.90
gac	D	838	420.10
gaa	E	0	1.20
gag	E	2	0.80
ggt	G	0	0.27
ggc	G	2	0.25
gga	G	0	0.92
ggg	G	0	0.55
---	--	------	------
mPD		0.16	0.50
		nPD:	0.31
		N. weight:	0.59
		Sc. PD:	0.095
		Sc. rank:	260.2

NP			
Pos.	102	obs:	exp:
cgt	R	0	0.04
cgc	R	0	0.05
cga	R	0	0.10
cgg	R	0	0.08
aga	R	1	0.46
agg	R	0	0.27
ggt	G	4	122.80
ggc	G	26	116.40
gga	G	757	423.50
ggg	G	129	253.30
---	--	------	------
mPD		0.30	0.68
		nPD:	0.44
		N. weight:	0.41
		Sc. PD:	0.11
		Sc. rank:	229.1

NP			
Pos.	103	obs:	exp:
aaa	K	904	537.20
aag	K	13	379.80
---	--	------	------
mPD		0.028	0.49
		nPD:	0.06
		N. weight:	0.57
		Sc. PD:	-0.048
		Sc. rank:	-43.8

NP			
Pos.	104	obs:	exp:
tgg	W	917	917.00
---	--	------	------
mPD		0	0
		nPD:	1.
		N. weight:	0.
		Sc. PD:	0
		Sc. rank:	0

NP			
Pos.	105	obs:	exp:
att	I	10	9.49
atc	I	0	6.29
ata	I	16	10.22
atg	M	45	45.00
gtt	V	2	174.60
gtc	V	0	169.50
gta	V	11	175.20
gtg	V	833	326.70
---	--	------	------
mPD		0.23	0.86
		nPD:	0.26
		N. weight:	1.
		Sc. PD:	0.11
		Sc. rank:	388.8

NP			
Pos.	106	obs:	exp:
cgt	R	0	39.17
cgc	R	0	46.59
cga	R	0	91.44
cgg	R	0	76.38
aga	R	893	418.70
agg	R	24	244.70
---	--	------	------
mPD		0.051	0.96
		nPD:	0.05
		N. weight:	0.83
		Sc. PD:	-0.073
		Sc. rank:	-78.7

NP			
Pos.	107	obs:	exp:
gaa	E	31	551.60
gag	E	886	365.40
---	--	------	------
mPD		0.065	0.48
		nPD:	0.14
		N. weight:	0.93
		Sc. PD:	-0.0085
		Sc. rank:	145.3

NP			
Pos.	108	obs:	exp:
tta	L	3	80.59
ttg	L	56	171.10
ctt	L	0	155.40
ctc	L	2	153.60
cta	L	7	152.90
ctg	L	849	203.40
---	--	------	------
mPD		0.15	1.1
		nPD:	0.13
		N. weight:	1.5
		Sc. PD:	-0.02
		Sc. rank:	218.7

NP			
Pos.	109	obs:	exp:
att	I	38	332.40
atc	I	871	220.30
ata	I	2	358.30
act	T	0	0.78
acc	T	3	0.61
aca	T	0	1.38
acg	T	0	0.23
gtt	V	0	0.62
gtc	V	3	0.60
gta	V	0	0.62
gtg	V	0	1.16
---	--	------	------
mPD		0.097	0.67
		nPD:	0.14
		N. weight:	1.5
		Sc. PD:	-0.0016
		Sc. rank:	269.7

NP			
Pos.	110	obs:	exp:
tta	L	0	80.59
ttg	L	39	171.10
ctt	L	5	155.40
ctc	L	1	153.60
cta	L	56	152.90
ctg	L	816	203.40
---	--	------	------
mPD		0.21	1.1
		nPD:	0.19
		N. weight:	1.3
		Sc. PD:	0.053
		Sc. rank:	399.6

NP			
Pos.	111	obs:	exp:
tct	S	2	0.32
tcc	S	0	0.26
tca	S	0	0.50
tcg	S	0	0.12
tat	Y	914	485.10
tac	Y	1	429.90
agt	S	0	0.41
agc	S	0	0.39
---	--	------	------
mPD		0.0065	0.50
		nPD:	0.01
		N. weight:	0.77
		Sc. PD:	-0.097
		Sc. rank:	-327.7

NP			
Pos.	112	obs:	exp:
aat	N	0	1.02
aac	N	2	0.98
gat	D	4	494.00
gac	D	911	421.00
---	--	------	------
mPD		0.013	0.50
		nPD:	0.03
		N. weight:	0.91
		Sc. PD:	-0.1
		Sc. rank:	-244.0

NP			
Pos.	113	obs:	exp:
aaa	K	866	537.20
aag	K	51	379.80
---	--	------	------
mPD		0.11	0.49
		nPD:	0.22
		N. weight:	0.42
		Sc. PD:	0.028
		Sc. rank:	138.8

NP			
Pos.	114	obs:	exp:
aaa	K	0	0.59
aag	K	1	0.41
gat	D	1	0.54
gac	D	0	0.46
gaa	E	46	550.40
gag	E	869	364.60
---	--	------	------
mPD		0.10	0.48
		nPD:	0.21
		N. weight:	0.86
		Sc. PD:	0.049
		Sc. rank:	272.1

NP			
Pos.	115	obs:	exp:
gaa	E	830	551.60
gag	E	87	365.40
---	--	------	------
mPD		0.17	0.48
		nPD:	0.36
		N. weight:	0.29
		Sc. PD:	0.058
		Sc. rank:	144.8

NP			
Pos.	116	obs:	exp:
att	I	5	334.60
atc	I	910	221.80
ata	I	2	360.60
---	--	------	------
mPD		0.015	0.65
		nPD:	0.02
		N. weight:	1.7
		Sc. PD:	-0.19
		Sc. rank:	-495.6

NP			
Pos.	117	obs:	exp:
cgt	R	0	39.13
cgc	R	0	46.54
cga	R	0	91.34
cgg	R	5	76.29
act	T	0	0.26
acc	T	0	0.20
aca	T	0	0.46
acg	T	1	0.08
aga	R	56	418.20
agg	R	855	244.50
---	--	------	------
mPD		0.13	0.97
		nPD:	0.13
		N. weight:	1.3
		Sc. PD:	-0.016
		Sc. rank:	190.2

NP			
Pos.	118	obs:	exp:
cgt	R	0	39.09
cgc	R	0	46.49
cga	R	0	91.24
cgg	R	0	76.21
aaa	K	2	1.17
aag	K	0	0.83
aga	R	837	417.80
agg	R	78	244.20
---	--	------	------
mPD		0.16	0.97
		nPD:	0.17
		N. weight:	0.66
		Sc. PD:	0.012
		Sc. rank:	170.7

NP			
Pos.	119	obs:	exp:
tta	L	0	0.09
ttg	L	0	0.19
ctt	L	1	0.17
ctc	L	0	0.17
cta	L	0	0.17
ctg	L	0	0.22
att	I	882	333.90
atc	I	33	221.30
ata	I	0	359.80
act	T	1	0.26
acc	T	0	0.20
aca	T	0	0.46
acg	T	0	0.08
---	--	------	------
mPD		0.074	0.66
		nPD:	0.11
		N. weight:	1.1
		Sc. PD:	-0.034
		Sc. rank:	105.3

NP			
Pos.	120	obs:	exp:
tgg	W	917	917.00
---	--	------	------
mPD		0	0
		nPD:	1.
		N. weight:	0.
		Sc. PD:	0
		Sc. rank:	0

NP			
Pos.	121	obs:	exp:
cgt	R	112	39.17
cgc	R	23	46.59
cga	R	671	91.44
cgg	R	17	76.38
aga	R	94	418.70
agg	R	0	244.70
---	--	------	------
mPD		0.47	0.96
		nPD:	0.49
		N. weight:	1.7
		Sc. PD:	0.56
		Sc. rank:	990.9

,

NP			
Pos.	122	obs:	exp:
caa	Q	905	471.00
cag	Q	12	446.00
---	--	------	------
mPD		0.026	0.50
		nPD:	0.05
		N. weight:	0.73
		Sc. PD:	-0.065
		Sc. rank:	-71.0

,

NP			
Pos.	123	obs:	exp:
gct	A	27	235.00
gcc	A	1	172.20
gca	A	289	428.70
gcg	A	600	81.11
---	--	------	------
mPD		0.47	0.67
		nPD:	0.7
		N. weight:	1.4
		Sc. PD:	0.72
		Sc. rank:	858.9

,

NP			
Pos.	124	obs:	exp:
aat	N	52	466.50
aac	N	865	450.50
---	--	------	------
mPD		0.11	0.50
		nPD:	0.21
		N. weight:	0.6
		Sc. PD:	0.039
		Sc. rank:	197.4

,

NP			
Pos.	125	obs:	exp:
aat	N	902	466.50
aac	N	15	450.50
---	--	------	------
mPD		0.032	0.50
		nPD:	0.06
		N. weight:	0.72
		Sc. PD:	-0.056
		Sc. rank:	-33.0

,

NP			
Pos.	126	obs:	exp:
gct	A	0	0.26
gcc	A	0	0.19
gca	A	1	0.47
gcg	A	0	0.09
ggt	G	0	122.80
ggc	G	1	116.40
gga	G	891	423.50
ggg	G	24	253.30
---	--	------	------
mPD		0.055	0.68
		nPD:	0.08
		N. weight:	0.8
		Sc. PD:	-0.049
		Sc. rank:	-8.1

,

NP			
Pos.	127	obs:	exp:
gat	D	1	2.16
gac	D	3	1.84
gaa	E	75	546.80
gag	E	834	362.20
ggt	G	0	0.54
ggc	G	0	0.51
gga	G	0	1.85
ggg	G	4	1.11
---	--	------	------
mPD		0.17	0.49
		nPD:	0.34
		N. weight:	0.74
		Sc. PD:	0.14
		Sc. rank:	346.9

,

NP			
Pos.	128	obs:	exp:
aat	N	1	0.51
aac	N	0	0.49
gat	D	800	494.50
gac	D	116	421.50
---	--	------	------
mPD		0.22	0.50
		nPD:	0.45
		N. weight:	0.32
		Sc. PD:	0.091
		Sc. rank:	178.0

,

NP			
Pos.	129	obs:	exp:
gct	A	32	235.00
gcc	A	0	172.20
gca	A	870	428.70
gcg	A	15	81.11
---	--	------	------
mPD		0.099	0.67
		nPD:	0.15
		N. weight:	0.71
		Sc. PD:	0.00028
		Sc. rank:	138.2

,

NP			
Pos.	130	obs:	exp:
act	T	909	236.50
acc	T	3	185.90
aca	T	1	419.40
acg	T	0	71.12
aat	N	2	1.02
aac	N	0	0.98
gct	A	1	0.26
gcc	A	0	0.19
gca	A	0	0.47
gcg	A	0	0.09
gat	D	1	0.54
gac	D	0	0.46
---	--	------	------
mPD		0.020	0.69
		nPD:	0.03
		N. weight:	1.6
		Sc. PD:	-0.18
		Sc. rank:	-400.0

,

NP			
Pos.	131	obs:	exp:
act	T	1	0.26
acc	T	0	0.20
aca	T	0	0.46
acg	T	0	0.08
gct	A	906	234.70
gcc	A	5	172.00
gca	A	5	428.30
gcg	A	0	81.02
---	--	------	------
mPD		0.024	0.67
		nPD:	0.04
		N. weight:	1.6
		Sc. PD:	-0.17
		Sc. rank:	-328.1

,

NP			
Pos.	132	obs:	exp:
ggt	G	886	123.00
ggc	G	28	116.50
gga	G	3	424.00
ggg	G	0	253.50
---	--	------	------
mPD		0.066	0.68
		nPD:	0.1
		N. weight:	2.3
		Sc. PD:	-0.1
		Sc. rank:	85.3

,

NP			
Pos.	133	obs:	exp:
tta	L	0	80.59
ttg	L	0	171.10
ctt	L	821	155.40
ctc	L	91	153.60
cta	L	4	152.90
ctg	L	1	203.40
---	--	------	------
mPD		0.19	1.1
		nPD:	0.17
		N. weight:	1.7
		Sc. PD:	0.04
		Sc. rank:	459.8

,

NP			
Pos.	134	obs:	exp:
act	T	54	237.30
acc	T	861	186.50
aca	T	1	420.80
acg	T	0	71.35
gct	A	0	0.26
gcc	A	1	0.19
gca	A	0	0.47
gcg	A	0	0.09
---	--	------	------
mPD		0.12	0.68
		nPD:	0.17
		N. weight:	1.6
		Sc. PD:	0.038
		Sc. rank:	442.8

,

NP			
Pos.	135	obs:	exp:
cat	H	839	526.80
cac	H	78	390.20
---	--	------	------
mPD		0.16	0.49
		nPD:	0.32
		N. weight:	0.36
		Sc. PD:	0.058
		Sc. rank:	157.7

,

NP			
Pos.	136	obs:	exp:
tta	L	1	78.31
ttg	L	27	166.30
ctt	L	0	151.00
ctc	L	0	149.30
cta	L	23	148.50
ctg	L	840	197.60
att	I	0	0.36
atc	I	0	0.24
ata	I	1	0.39
atg	M	25	25.00
---	--	------	------
mPD		0.17	1.1
		nPD:	0.15
		N. weight:	1.5
		Sc. PD:	-0.00012
		Sc. rank:	290.1

,

NP			
Pos.	137	obs:	exp:
atg	M	917	917.00
---	--	------	------
mPD		0	0
		nPD:	1.
		N. weight:	0.
		Sc. PD:	0
		Sc. rank:	0

,

NP			
Pos.	138	obs:	exp:
att	I	41	334.60
atc	I	56	221.80
ata	I	820	360.60
---	--	------	------
mPD		0.19	0.65
		nPD:	0.3
		N. weight:	0.68
		Sc. PD:	0.099
		Sc. rank:	290.6

,

NP			
Pos.	139	obs:	exp:
tgg	W	917	917.00
---	--	------	------
mPD		0	0
		nPD:	1.
		N. weight:	0.
		Sc. PD:	0
		Sc. rank:	0

,

NP			
Pos.	140	obs:	exp:
cat	H	894	526.80
cac	H	23	390.20
---	--	------	------
mPD		0.049	0.49
		nPD:	0.1
		N. weight:	0.54
		Sc. PD:	-0.024
		Sc. rank:	25.7

,

NP			
Pos.	141	obs:	exp:
tct	S	23	146.80
tcc	S	894	118.70
tca	S	0	231.40
tcg	S	0	53.36
agt	S	0	187.90
agc	S	0	178.80
---	--	------	------
mPD		0.049	1.7
		nPD:	0.03
		N. weight:	2.3
		Sc. PD:	-0.26
		Sc. rank:	-563.2

,

NP			
Pos.	142	obs:	exp:
aat	N	98	466.50
aac	N	819	450.50
---	--	------	------
mPD		0.19	0.50
		nPD:	0.38
		N. weight:	0.45
		Sc. PD:	0.1
		Sc. rank:	238.5

,

NP			
Pos.	143	obs:	exp:
tta	L	5	80.59
ttg	L	3	171.10
ctt	L	1	155.40
ctc	L	1	153.60
cta	L	127	152.90
ctg	L	780	203.40
---	--	------	------
mPD		0.27	1.1
		nPD:	0.24
		N. weight:	1.3
		Sc. PD:	0.12
		Sc. rank:	474.2

,

NP			
Pos.	144	obs:	exp:
aat	N	915	466.50
aac	N	2	450.50
---	--	------	------
mPD		0.0044	0.50
		nPD:	0.01
		N. weight:	0.81
		Sc. PD:	-0.11
		Sc. rank:	-405.2

,

NP			
Pos.	145	obs:	exp:
gat	D	912	495.00
gac	D	5	422.00
---	--	------	------
mPD		0.011	0.50
		nPD:	0.02
		N. weight:	0.71
		Sc. PD:	-0.084
		Sc. rank:	-229.7

,

NP			
Pos.	146	obs:	exp:
act	T	1	1.04
acc	T	3	0.81
aca	T	0	1.84
acg	T	0	0.31
gct	A	517	233.90
gcc	A	366	171.40
gca	A	30	426.90
gcg	A	0	80.75
---	--	------	------
mPD		0.53	0.68
		nPD:	0.77
		N. weight:	0.82
		Sc. PD:	0.49
		Sc. rank:	531.6

NP			
Pos.	147	obs:	exp:
act	T	8	237.50
acc	T	794	186.70
aca	T	112	421.30
acg	T	3	71.43
---	--	------	------
mPD		0.24	0.67
		nPD:	0.35
		N. weight:	1.3
		Sc. PD:	0.25
		Sc. rank:	642.4

NP			
Pos.	148	obs:	exp:
tat	Y	756	486.20
tac	Y	161	430.80
---	--	------	------
mPD		0.29	0.50
		nPD:	0.58
		N. weight:	0.24
		Sc. PD:	0.098
		Sc. rank:	144.6

NP			
Pos.	149	obs:	exp:
caa	Q	10	471.00
cag	Q	907	446.00
---	--	------	------
mPD		0.022	0.50
		nPD:	0.04
		N. weight:	0.81
		Sc. PD:	-0.079
		Sc. rank:	-123.1

NP			
Pos.	150	obs:	exp:
cgt	R	0	39.13
cgc	R	0	46.54
cga	R	0	91.34
cgg	R	0	76.29
aaa	K	1	0.59
aag	K	0	0.41
aga	R	901	418.20
agg	R	15	244.50
---	--	------	------
mPD		0.034	0.97
		nPD:	0.04
		N. weight:	0.87
		Sc. PD:	-0.091
		Sc. rank:	-178.2

NP			
Pos.	151	obs:	exp:
act	T	6	237.50
acc	T	3	186.70
aca	T	106	421.30
acg	T	802	71.43
---	--	------	------
mPD		0.22	0.67
		nPD:	0.33
		N. weight:	2.3
		Sc. PD:	0.41
		Sc. rank:	1063.3

NP			
Pos.	152	obs:	exp:
cgt	R	0	39.17
cgc	R	0	46.59
cga	R	0	91.44
cgg	R	0	76.38
aga	R	876	418.70
agg	R	41	244.70
---	--	------	------
mPD		0.086	0.96
		nPD:	0.09
		N. weight:	0.76
		Sc. PD:	-0.042
		Sc. rank:	11.3

NP			
Pos.	153	obs:	exp:
gct	A	747	235.00
gcc	A	125	172.20
gca	A	45	428.70
gcg	A	0	81.11
---	--	------	------
mPD		0.32	0.67
		nPD:	0.47
		N. weight:	0.97
		Sc. PD:	0.3
		Sc. rank:	546.0

NP			
Pos.	154	obs:	exp:
tta	L	0	80.50
ttg	L	0	170.90
ctt	L	66	155.20
ctc	L	817	153.50
cta	L	33	152.70
ctg	L	0	203.10
att	I	0	0.36
atc	I	1	0.24
ata	I	0	0.39
---	--	------	------
mPD		0.20	1.1
		nPD:	0.18
		N. weight:	1.7
		Sc. PD:	0.054
		Sc. rank:	479.2

NP			
Pos.	155	obs:	exp:
gtt	V	2	189.30
gtc	V	0	183.70
gta	V	11	189.90
gtg	V	904	354.10
---	--	------	------
mPD		0.028	0.73
		nPD:	0.04
		N. weight:	1.1
		Sc. PD:	-0.11
		Sc. rank:	-199.4

NP			
Pos.	156	obs:	exp:
cgt	R	898	39.17
cgc	R	13	46.59
cga	R	5	91.44
cgg	R	1	76.38
aga	R	0	418.70
agg	R	0	244.70
---	--	------	------
mPD		0.041	0.96
		nPD:	0.04
		N. weight:	3.7
		Sc. PD:	-0.36
		Sc. rank:	-571.6

NP			
Pos.	157	obs:	exp:
act	T	899	237.50
acc	T	16	186.70
aca	T	2	421.30
acg	T	0	71.43
---	--	------	------
mPD		0.039	0.67
		nPD:	0.06
		N. weight:	1.5
		Sc. PD:	-0.13
		Sc. rank:	-119.8

NP			
Pos.	158	obs:	exp:
ggt	G	0	123.00
ggc	G	0	116.50
gga	G	845	424.00
ggg	G	72	253.50
---	--	------	------
mPD		0.14	0.68
		nPD:	0.21
		N. weight:	0.66
		Sc. PD:	0.043
		Sc. rank:	216.8

NP			
Pos.	159	obs:	exp:
atg	M	917	917.00
---	--	------	------
mPD		0	0
		nPD:	1.
		N. weight:	0.
		Sc. PD:	0
		Sc. rank:	0

NP			
Pos.	160	obs:	exp:
gat	D	595	495.00
gac	D	322	422.00
---	--	------	------
mPD		0.46	0.50
		nPD:	0.92
		N. weight:	0.034
		Sc. PD:	0.025
		Sc. rank:	22.4

NP			
Pos.	161	obs:	exp:
cct	P	9	233.20
ccc	P	907	163.50
cca	P	0	374.60
ccg	P	1	145.70
---	--	------	------
mPD		0.022	0.71
		nPD:	0.03
		N. weight:	2.
		Sc. PD:	-0.22
		Sc. rank:	-457.4

NP			
Pos.	162	obs:	exp:
cgt	R	0	39.17
cgc	R	0	46.59
cga	R	3	91.44
cgg	R	783	76.38
aga	R	39	418.70
agg	R	92	244.70
---	--	------	------
mPD		0.33	0.96
		nPD:	0.35
		N. weight:	2.2
		Sc. PD:	0.41
		Sc. rank:	1061.7

NP			
Pos.	163	obs:	exp:
atg	M	917	917.00
---	--	------	------
mPD		0	0
		nPD:	1.
		N. weight:	0.
		Sc. PD:	0
		Sc. rank:	0

NP			
Pos.	164	obs:	exp:
tgt	C	22	376.50
tgc	C	895	540.50
---	--	------	------
mPD		0.047	0.48
		nPD:	0.1
		N. weight:	0.52
		Sc. PD:	-0.024
		Sc. rank:	16.6

NP			
Pos.	165	obs:	exp:
tct	S	910	146.80
tcc	S	7	118.70
tca	S	0	231.40
tcg	S	0	53.36
agt	S	0	187.90
agc	S	0	178.80
---	--	------	------
mPD		0.015	1.7
		nPD:	0.01
		N. weight:	2.2
		Sc. PD:	-0.28
		Sc. rank:	-1046.8

NP			
Pos.	166	obs:	exp:
tta	L	0	80.59
ttg	L	0	171.10
ctt	L	10	155.40
ctc	L	0	153.60
cta	L	4	152.90
ctg	L	903	203.40
---	--	------	------
mPD		0.030	1.1
		nPD:	0.03
		N. weight:	1.7
		Sc. PD:	-0.2
		Sc. rank:	-439.1

NP			
Pos.	167	obs:	exp:
atg	M	917	917.00
---	--	------	------
mPD		0	0
		nPD:	1.
		N. weight:	0.
		Sc. PD:	0
		Sc. rank:	0

NP			
Pos.	168	obs:	exp:
caa	Q	914	471.00
cag	Q	3	446.00
---	--	------	------
mPD		0.0065	0.50
		nPD:	0.01
		N. weight:	0.79
		Sc. PD:	-0.099
		Sc. rank:	-332.6

NP			
Pos.	169	obs:	exp:
ggt	G	4	123.00
ggc	G	40	116.50
gga	G	775	424.00
ggg	G	98	253.50
---	--	------	------
mPD		0.27	0.68
		nPD:	0.4
		N. weight:	0.43
		Sc. PD:	0.11
		Sc. rank:	236.4

NP			
Pos.	170	obs:	exp:
tct	S	2	146.60
tcc	S	0	118.60
tca	S	911	231.20
tcg	S	3	53.31
act	T	0	0.26
acc	T	0	0.20
aca	T	1	0.46
acg	T	0	0.08
agt	S	0	187.70
agc	S	0	178.60
---	--	------	------
mPD		0.013	1.7
		nPD:	0.01
		N. weight:	1.6
		Sc. PD:	-0.22
		Sc. rank:	-832.2

NP			
Pos.	171	obs:	exp:
act	T	873	237.50
acc	T	44	186.70
aca	T	0	421.30
acg	T	0	71.43
---	--	------	------
mPD		0.091	0.67
		nPD:	0.14
		N. weight:	1.4
		Sc. PD:	-0.014
		Sc. rank:	220.3

NP			
Pos.	172	obs:	exp:
tta	L	0	80.59
ttg	L	0	171.10
ctt	L	258	155.40
ctc	L	659	153.60
cta	L	0	152.90
ctg	L	0	203.40
---	--	------	------
mPD		0.40	1.1
		nPD:	0.36
		N. weight:	1.5
		Sc. PD:	0.3
		Sc. rank:	744.9

NP			
Pos.	173	obs:	exp:
cct	P	8	233.20
ccc	P	4	163.50
cca	P	29	374.60
ccg	P	876	145.70
---	--	------	------
mPD		0.086	0.71
		nPD:	0.12
		N. weight:	1.9
		Sc. PD:	-0.045
		Sc. rank:	262.0

NP			
Pos.	174	obs:	exp:
cgt	R	0	39.17
cgc	R	0	46.59
cga	R	0	91.44
cgg	R	1	76.38
aga	R	11	418.70
agg	R	905	244.70
---	--	------	------
mPD		0.026	0.96
		nPD:	0.03
		N. weight:	1.5
		Sc. PD:	-0.17
		Sc. rank:	-397.4

NP			
Pos.	175	obs:	exp:
cgt	R	0	39.17
cgc	R	0	46.59
cga	R	1	91.44
cgg	R	0	76.38
aga	R	868	418.70
agg	R	48	244.70
---	--	------	------
mPD		0.10	0.96
		nPD:	0.11
		N. weight:	0.74
		Sc. PD:	-0.028
		Sc. rank:	53.6

NP			
Pos.	176	obs:	exp:
tct	S	901	146.80
tcc	S	13	118.70
tca	S	2	231.40
tcg	S	1	53.36
agt	S	0	187.90
agc	S	0	178.80
---	--	------	------
mPD		0.034	1.7
		nPD:	0.02
		N. weight:	2.1
		Sc. PD:	-0.25
		Sc. rank:	-705.1

NP			
Pos.	177	obs:	exp:
ggt	G	1	123.00
ggc	G	0	116.50
gga	G	882	424.00
ggg	G	34	253.50
---	--	------	------
mPD		0.074	0.68
		nPD:	0.11
		N. weight:	0.77
		Sc. PD:	-0.027
		Sc. rank:	66.6

NP			
Pos.	178	obs:	exp:
gct	A	898	235.00
gcc	A	8	172.20
gca	A	4	428.70
gcg	A	7	81.11
---	--	------	------
mPD		0.041	0.67
		nPD:	0.06
		N. weight:	1.5
		Sc. PD:	-0.12
		Sc. rank:	-82.5

NP			
Pos.	179	obs:	exp:
gct	A	80	235.00
gcc	A	2	172.20
gca	A	834	428.70
gcg	A	1	81.11
---	--	------	------
mPD		0.17	0.67
		nPD:	0.25
		N. weight:	0.61
		Sc. PD:	0.058
		Sc. rank:	223.7

NP			
Pos.	180	obs:	exp:
ggt	G	795	123.00
ggc	G	1	116.50
gga	G	120	424.00
ggg	G	1	253.50
---	--	------	------
mPD		0.23	0.68
		nPD:	0.34
		N. weight:	1.8
		Sc. PD:	0.33
		Sc. rank:	849.4

NP			
Pos.	181	obs:	exp:
gct	A	3	235.00
gcc	A	0	172.20
gca	A	894	428.70
gcg	A	20	81.11
---	--	------	------
mPD		0.049	0.67
		nPD:	0.07
		N. weight:	0.82
		Sc. PD:	-0.057
		Sc. rank:	-22.5

NP			
Pos.	182	obs:	exp:
tct	S	0	2.08
tcc	S	0	1.68
tca	S	13	3.28
tcg	S	0	0.76
agt	S	0	2.66
agc	S	0	2.54
gct	A	6	231.60
gcc	A	0	169.70
gca	A	895	422.70
gcg	A	3	79.96
---	--	------	------
mPD		0.047	0.71
		nPD:	0.07
		N. weight:	0.88
		Sc. PD:	-0.066
		Sc. rank:	-29.5

NP			
Pos.	183	obs:	exp:
att	I	0	0.36
atc	I	0	0.24
ata	I	1	0.39
gtt	V	0	189.10
gtc	V	1	183.50
gta	V	475	189.70
gtg	V	440	353.80
---	--	------	------
mPD		0.50	0.73
		nPD:	0.69
		N. weight:	0.71
		Sc. PD:	0.37
		Sc. rank:	441.4

NP			
Pos.	184	obs:	exp:
aaa	K	68	537.20
aag	K	849	379.80
---	--	------	------
mPD		0.14	0.49
		nPD:	0.28
		N. weight:	0.72
		Sc. PD:	0.094
		Sc. rank:	298.6

NP			
Pos.	185	obs:	exp:
ggt	G	0	123.00
ggc	G	0	116.50
gga	G	228	424.00
ggg	G	689	253.50
---	--	------	------
mPD		0.37	0.68
		nPD:	0.55
		N. weight:	0.73
		Sc. PD:	0.28
		Sc. rank:	441.1

NP			
Pos.	186	obs:	exp:
att	I	0	300.30
atc	I	1	199.10
ata	I	822	323.60
gtt	V	50	19.40
gtc	V	39	18.83
gta	V	4	19.46
gtg	V	1	36.30
---	--	------	------
mPD		0.37	0.86
		nPD:	0.43
		N. weight:	1.1
		Sc. PD:	0.29
		Sc. rank:	618.0

NP			
Pos.	187	obs:	exp:
ggt	G	0	123.00
ggc	G	0	116.50
gga	G	136	424.00
ggg	G	781	253.50
---	--	------	------
mPD		0.25	0.68
		nPD:	0.37
		N. weight:	0.97
		Sc. PD:	0.21
		Sc. rank:	503.9

NP			
Pos.	188	obs:	exp:
act	T	190	237.50
acc	T	0	186.70
aca	T	668	421.30
acg	T	59	71.43
---	--	------	------
mPD		0.42	0.67
		nPD:	0.63
		N. weight:	0.35
		Sc. PD:	0.16
		Sc. rank:	213.4

NP			
Pos.	189	obs:	exp:
atg	M	917	917.00
---	--	------	------
mPD		0	0
		nPD:	1.
		N. weight:	0.
		Sc. PD:	0
		Sc. rank:	0

NP			
Pos.	190	obs:	exp:
gtt	V	0	189.10
gtc	V	0	183.50
gta	V	13	189.70
gtg	V	903	353.80
ggt	G	0	0.13
ggc	G	0	0.13
gga	G	0	0.46
ggg	G	1	0.28
---	--	------	------
mPD		0.030	0.73
		nPD:	0.04
		N. weight:	1.1
		Sc. PD:	-0.11
		Sc. rank:	-185.4

NP			
Pos.	191	obs:	exp:
atg	M	917	917.00
---	--	------	------
mPD		0	0
		nPD:	1.
		N. weight:	0.
		Sc. PD:	0
		Sc. rank:	0

NP			
Pos.	192	obs:	exp:
gaa	E	840	551.60
gag	E	77	365.40
---	--	------	------
mPD		0.15	0.48
		nPD:	0.32
		N. weight:	0.31
		Sc. PD:	0.052
		Sc. rank:	139.4

NP			
Pos.	193	obs:	exp:
tta	L	18	80.59
ttg	L	22	171.10
ctt	L	0	155.40
ctc	L	1	153.60
cta	L	45	152.90
ctg	L	831	203.40
---	--	------	------
mPD		0.21	1.1
		nPD:	0.19
		N. weight:	1.4
		Sc. PD:	0.061
		Sc. rank:	431.0

NP			
Pos.	194	obs:	exp:
att	I	782	310.10
atc	I	66	205.60
ata	I	2	334.30
gtt	V	67	13.83
gtc	V	0	13.42
gta	V	0	13.87
gtg	V	0	25.87
---	--	------	------
mPD		0.27	0.81
		nPD:	0.34
		N. weight:	0.99
		Sc. PD:	0.18
		Sc. rank:	464.5

NP			
Pos.	195	obs:	exp:
caa	Q	0	0.51
cag	Q	1	0.49
cgt	R	0	39.09
cgc	R	0	46.49
cga	R	23	91.24
cgg	R	694	76.21
act	T	0	0.26
acc	T	0	0.20
aca	T	0	0.46
acg	T	1	0.08
aga	R	2	417.80
agg	R	196	244.20
---	--	------	------
mPD		0.40	0.97
		nPD:	0.41
		N. weight:	1.9
		Sc. PD:	0.49
		Sc. rank:	1071.2

NP			
Pos.	196	obs:	exp:
atg	M	917	917.00
mPD		0	0
		nPD:	1.
		N. weight:	0.
		Sc. PD:	0
		Sc. rank:	0

NP			
Pos.	197	obs:	exp:
att	I	0	333.90
atc	I	46	221.30
ata	I	869	359.80
gtt	V	0	0.41
gtc	V	1	0.40
gta	V	1	0.41
gtg	V	0	0.77
mPD		0.10	0.66
		nPD:	0.15
		N. weight:	0.93
		Sc. PD:	0.0074
		Sc. rank:	211.2

NP			
Pos.	198	obs:	exp:
aaa	K	833	537.20
aag	K	84	379.80
mPD		0.17	0.49
		nPD:	0.34
		N. weight:	0.32
		Sc. PD:	0.06
		Sc. rank:	155.0

NP			
Pos.	199	obs:	exp:
tta	L	0	0.09
ttg	L	0	0.19
ctt	L	0	0.17
ctc	L	0	0.17
cta	L	1	0.17
ctg	L	0	0.22
cgt	R	1	39.13
cgc	R	0	46.54
cga	R	880	91.34
cgg	R	4	76.29
aga	R	31	418.20
agg	R	0	244.50
mPD		0.078	0.97
		nPD:	0.08
		N. weight:	2.5
		Sc. PD:	-0.16
		Sc. rank:	-35.3

NP			
Pos.	200	obs:	exp:
ggt	G	0	123.00
ggc	G	0	116.50
gga	G	57	424.00
ggg	G	860	253.50
mPD		0.12	0.68
		nPD:	0.17
		N. weight:	1.2
		Sc. PD:	0.032
		Sc. rank:	340.3

NP			
Pos.	201	obs:	exp:
att	I	25	333.50
atc	I	847	221.10
ata	I	42	359.40
gtt	V	0	0.62
gtc	V	3	0.60
gta	V	0	0.62
gtg	V	0	1.16
mPD		0.14	0.66
		nPD:	0.22
		N. weight:	1.3
		Sc. PD:	0.091
		Sc. rank:	448.0

NP			
Pos.	202	obs:	exp:
aat	N	158	466.00
aac	N	758	450.00
gat	D	0	0.54
gac	D	1	0.46
mPD		0.29	0.50
		nPD:	0.57
		N. weight:	0.3
		Sc. PD:	0.12
		Sc. rank:	184.4

NP			
Pos.	203	obs:	exp:
gat	D	110	495.00
gac	D	807	422.00
mPD		0.21	0.50
		nPD:	0.43
		N. weight:	0.48
		Sc. PD:	0.13
		Sc. rank:	266.6

NP			
Pos.	204	obs:	exp:
cgt	R	4	39.17
cgc	R	0	46.59
cga	R	7	91.44
cgg	R	897	76.38
aga	R	2	418.70
agg	R	7	244.70
mPD		0.048	0.96
		nPD:	0.05
		N. weight:	2.9
		Sc. PD:	-0.26
		Sc. rank:	-324.2

NP			
Pos.	205	obs:	exp:
aat	N	894	466.50
aac	N	23	450.50
mPD		0.049	0.50
		nPD:	0.1
		N. weight:	0.68
		Sc. PD:	-0.031
		Sc. rank:	29.9

NP			
Pos.	206	obs:	exp:
ttt	F	5	361.90
ttc	F	912	555.10
mPD		0.011	0.48
		nPD:	0.02
		N. weight:	0.57
		Sc. PD:	-0.067
		Sc. rank:	-175.1

NP			
Pos.	207	obs:	exp:
tgg	W	917	917.00
mPD		0	0
		nPD:	1.
		N. weight:	0.
		Sc. PD:	0
		Sc. rank:	0

NP			
Pos.	208	obs:	exp:
cgt	R	0	39.17
cgc	R	0	46.59
cga	R	0	91.44
cgg	R	0	76.38
aga	R	906	418.70
agg	R	11	244.70
mPD		0.024	0.96
		nPD:	0.02
		N. weight:	0.89
		Sc. PD:	-0.1
		Sc. rank:	-256.2

NP			
Pos.	209	obs:	exp:
ggt	G	26	123.00
ggc	G	882	116.50
gga	G	9	424.00
ggg	G	0	253.50
mPD		0.074	0.68
		nPD:	0.11
		N. weight:	2.3
		Sc. PD:	-0.079
		Sc. rank:	219.6

NP			
Pos.	210	obs:	exp:
gaa	E	893	551.60
gag	E	24	365.40
mPD		0.051	0.48
		nPD:	0.11
		N. weight:	0.49
		Sc. PD:	-0.018
		Sc. rank:	38.3

NP			
Pos.	211	obs:	exp:
aat	N	904	466.50
aac	N	13	450.50
mPD		0.028	0.50
		nPD:	0.06
		N. weight:	0.74
		Sc. PD:	-0.063
		Sc. rank:	-60.7

NP			
Pos.	212	obs:	exp:
ggt	G	9	123.00
ggc	G	5	116.50
gga	G	889	424.00
ggg	G	14	253.50
mPD		0.060	0.68
		nPD:	0.09
		N. weight:	0.78
		Sc. PD:	-0.042
		Sc. rank:	9.9

NP			
Pos.	213	obs:	exp:
cgt	R	0	39.13
cgc	R	0	46.54
cga	R	35	91.34
cgg	R	53	76.29
aaa	K	1	0.59
aag	K	0	0.41
aga	R	822	418.20
agg	R	6	244.50
mPD		0.30	0.97
		nPD:	0.31
		N. weight:	0.65
		Sc. PD:	0.099
		Sc. rank:	282.8

NP			
Pos.	214	obs:	exp:
cgt	R	0	39.09
cgc	R	0	46.49
cga	R	3	91.24
cgg	R	0	76.21
aaa	K	1	1.17
aag	K	1	0.83
aga	R	427	417.80
agg	R	485	244.20
mPD		0.51	0.97
		nPD:	0.53
		N. weight:	0.45
		Sc. PD:	0.16
		Sc. rank:	267.6

NP			
Pos.	215	obs:	exp:
act	T	23	237.50
acc	T	4	186.70
aca	T	838	421.30
acg	T	52	71.43
mPD		0.16	0.67
		nPD:	0.24
		N. weight:	0.66
		Sc. PD:	0.058
		Sc. rank:	234.3

NP			
Pos.	216	obs:	exp:
cgt	R	0	38.32
cgc	R	0	45.57
cga	R	0	89.44
cgg	R	0	74.71
aaa	K	20	11.72
aag	K	0	8.28
aga	R	589	409.60
agg	R	308	239.40
mPD		0.49	1.0
		nPD:	0.49
		N. weight:	0.41
		Sc. PD:	0.13
		Sc. rank:	234.3

NP			
Pos.	217	obs:	exp:
tct	S	0	0.16
tcc	S	0	0.13
tca	S	0	0.25
tcg	S	0	0.06
att	I	872	320.40
atc	I	5	212.40
ata	I	1	345.30
agt	S	1	0.20
agc	S	0	0.20
gtt	V	37	7.84
gtc	V	1	7.61
gta	V	0	7.87
gtg	V	0	14.68
mPD		0.097	0.75
		nPD:	0.13
		N. weight:	1.2
		Sc. PD:	-0.019
		Sc. rank:	173.2

NP			
Pos.	218	obs:	exp:
act	T	0	0.26
acc	T	1	0.20
aca	T	0	0.46
acg	T	0	0.08
gct	A	20	234.70
gcc	A	24	172.00
gca	A	871	428.30
gcg	A	1	81.02
mPD		0.099	0.67
		nPD:	0.15
		N. weight:	0.7
		Sc. PD:	0.00033
		Sc. rank:	137.8

NP			
Pos.	219	obs:	exp:
tat	Y	781	486.20
tac	Y	136	430.80
mPD		0.25	0.50
		nPD:	0.51
		N. weight:	0.29
		Sc. PD:	0.099
		Sc. rank:	169.9

NP			
Pos.	220	obs:	exp:
gaa	E	51	551.60
gag	E	866	365.40
mPD		0.11	0.48
		nPD:	0.22
		N. weight:	0.83
		Sc. PD:	0.058
		Sc. rank:	285.5

NP			
Pos.	221	obs:	exp:
cgt	R	0	39.17
cgc	R	0	46.59
cga	R	2	91.44
cgg	R	0	76.38
aga	R	604	418.70
agg	R	311	244.70
---	--	------	------
mPD		0.45	0.96
		nPD:	0.47
		N. weight:	0.39
		Sc. PD:	0.12
		Sc. rank:	220.9

NP			
Pos.	222	obs:	exp:
atg	M	917	917.00
---	--	------	------
mPD		0	0
		nPD:	1.
		N. weight:	0.
		Sc. PD:	0
		Sc. rank:	0

NP			
Pos.	223	obs:	exp:
tgt	C	21	376.50
tgc	C	896	540.50
---	--	------	------
mPD		0.045	0.48
		nPD:	0.09
		N. weight:	0.52
		Sc. PD:	-0.027
		Sc. rank:	13.5

NP			
Pos.	224	obs:	exp:
aat	N	61	466.00
aac	N	855	450.00
gat	D	0	0.54
gac	D	1	0.46
---	--	------	------
mPD		0.13	0.50
		nPD:	0.25
		N. weight:	0.57
		Sc. PD:	0.057
		Sc. rank:	211.0

NP			
Pos.	225	obs:	exp:
att	I	9	334.60
atc	I	908	221.80
ata	I	0	360.60
---	--	------	------
mPD		0.019	0.65
		nPD:	0.03
		N. weight:	1.7
		Sc. PD:	-0.18
		Sc. rank:	-391.5

NP			
Pos.	226	obs:	exp:
tta	L	0	80.59
ttg	L	0	171.10
ctt	L	1	155.40
ctc	L	915	153.60
cta	L	1	152.90
ctg	L	0	203.40
---	--	------	------
mPD		0.0044	1.1
		nPD:	0.
		N. weight:	2.2
		Sc. PD:	-0.29
		Sc. rank:	-1191.6

NP			
Pos.	227	obs:	exp:
aaa	K	879	537.20
aag	K	38	379.80
---	--	------	------
mPD		0.080	0.49
		nPD:	0.16
		N. weight:	0.46
		Sc. PD:	0.0078
		Sc. rank:	113.0

NP			
Pos.	228	obs:	exp:
ggt	G	2	123.00
ggc	G	0	116.50
gga	G	33	424.00
ggg	G	882	253.50
---	--	------	------
mPD		0.074	0.68
		nPD:	0.11
		N. weight:	1.3
		Sc. PD:	-0.047
		Sc. rank:	121.5

NP			
Pos.	229	obs:	exp:
aaa	K	882	537.20
aag	K	35	379.80
---	--	------	------
mPD		0.074	0.49
		nPD:	0.15
		N. weight:	0.47
		Sc. PD:	0.0025
		Sc. rank:	100.9

NP			
Pos.	230	obs:	exp:
ttt	F	17	353.20
ttc	F	878	541.80
tta	L	18	1.93
ttg	L	0	4.11
ctt	L	0	3.73
ctc	L	4	3.69
cta	L	0	3.67
ctg	L	0	4.88
---	--	------	------
mPD		0.083	0.53
		nPD:	0.16
		N. weight:	0.55
		Sc. PD:	0.0057
		Sc. rank:	127.7

NP			
Pos.	231	obs:	exp:
caa	Q	916	471.00
cag	Q	1	446.00
---	--	------	------
mPD		0.0022	0.50
		nPD:	0.
		N. weight:	0.8
		Sc. PD:	-0.11
		Sc. rank:	-439.7

NP			
Pos.	232	obs:	exp:
act	T	0	237.30
acc	T	0	186.50
aca	T	912	420.80
acg	T	4	71.35
aaa	K	1	0.59
aag	K	0	0.41
---	--	------	------
mPD		0.011	0.68
		nPD:	0.02
		N. weight:	0.92
		Sc. PD:	-0.11
		Sc. rank:	-342.1

NP			
Pos.	233	obs:	exp:
tct	S	0	0.48
tcc	S	0	0.39
tca	S	3	0.76
tcg	S	0	0.17
agt	S	0	0.61
agc	S	0	0.59
gct	A	5	234.20
gcc	A	0	171.60
gca	A	902	427.30
gcg	A	7	80.84
---	--	------	------
mPD		0.032	0.68
		nPD:	0.05
		N. weight:	0.86
		Sc. PD:	-0.08
		Sc. rank:	-106.9

NP			
Pos.	234	obs:	exp:
tct	S	0	0.32
tcc	S	0	0.26
tca	S	2	0.50
tcg	S	0	0.12
agt	S	0	0.41
agc	S	0	0.39
gct	A	0	234.50
gcc	A	0	171.80
gca	A	903	427.80
gcg	A	12	80.93
---	--	------	------
mPD		0.030	0.68
		nPD:	0.04
		N. weight:	0.87
		Sc. PD:	-0.084
		Sc. rank:	-129.5

NP			
Pos.	235	obs:	exp:
caa	Q	917	471.00
cag	Q	0	446.00
---	--	------	------
mPD		0	0.50
		nPD:	0.
		N. weight:	0.81
		Sc. PD:	-0.11
		Sc. rank:	-526.4

NP			
Pos.	236	obs:	exp:
cgt	R	0	39.17
cgc	R	0	46.59
cga	R	45	91.44
cgg	R	0	76.38
aga	R	283	418.70
agg	R	589	244.70
---	--	------	------
mPD		0.55	0.96
		nPD:	0.57
		N. weight:	0.51
		Sc. PD:	0.21
		Sc. rank:	308.0

NP			
Pos.	237	obs:	exp:
gct	A	0	235.00
gcc	A	2	172.20
gca	A	817	428.70
gcg	A	98	81.11
---	--	------	------
mPD		0.19	0.67
		nPD:	0.29
		N. weight:	0.72
		Sc. PD:	0.098
		Sc. rank:	297.5

NP			
Pos.	238	obs:	exp:
atg	M	917	917.00
---	--	------	------
mPD		0	0
		nPD:	1.
		N. weight:	0.
		Sc. PD:	0
		Sc. rank:	0

NP			
Pos.	239	obs:	exp:
att	I	2	0.73
atc	I	0	0.48
ata	I	0	0.79
atg	M	821	821.00
gtt	V	0	19.40
gtc	V	0	18.83
gta	V	0	19.46
gtg	V	94	36.30
---	--	------	------
mPD		0.19	0.31
		nPD:	0.61
		N. weight:	0.12
		Sc. PD:	0.054
		Sc. rank:	74.6

NP			
Pos.	240	obs:	exp:
gat	D	839	495.00
gac	D	78	422.00
---	--	------	------
mPD		0.16	0.50
		nPD:	0.31
		N. weight:	0.42
		Sc. PD:	0.066
		Sc. rank:	182.4

NP			
Pos.	241	obs:	exp:
caa	Q	878	471.00
cag	Q	39	446.00
---	--	------	------
mPD		0.082	0.50
		nPD:	0.16
		N. weight:	0.6
		Sc. PD:	0.0098
		Sc. rank:	146.7

NP			
Pos.	242	obs:	exp:
gtt	V	4	189.30
gtc	V	0	183.70
gta	V	18	189.90
gtg	V	895	354.10
---	--	------	------
mPD		0.047	0.73
		nPD:	0.06
		N. weight:	1.
		Sc. PD:	-0.079
		Sc. rank:	-44.1

NP			
Pos.	243	obs:	exp:
cgt	R	18	39.17
cgc	R	0	46.59
cga	R	833	91.44
cgg	R	5	76.38
aga	R	26	418.70
agg	R	35	244.70
---	--	------	------
mPD		0.24	0.96
		nPD:	0.25
		N. weight:	2.2
		Sc. PD:	0.23
		Sc. rank:	841.0

NP			
Pos.	244	obs:	exp:
gaa	E	208	551.60
gag	E	709	365.40
---	--	------	------
mPD		0.35	0.48
		nPD:	0.73
		N. weight:	0.36
		Sc. PD:	0.2
		Sc. rank:	227.7

NP			
Pos.	245	obs:	exp:
tct	S	0	146.80
tcc	S	0	118.70
tca	S	0	231.40
tcg	S	0	53.36
agt	S	5	187.90
agc	S	912	178.80
---	--	------	------
mPD		0.011	1.7
		nPD:	0.01
		N. weight:	2.
		Sc. PD:	-0.26
		Sc. rank:	-1007.6

NP			
Pos.	246	obs:	exp:
cgt	R	0	39.13
cgc	R	1	46.54
cga	R	4	91.34
cgg	R	43	76.29
aaa	K	1	0.59
aag	K	0	0.41
aga	R	849	418.20
agg	R	19	244.50
---	--	------	------
mPD		0.23	0.97
		nPD:	0.24
		N. weight:	0.69
		Sc. PD:	0.06
		Sc. rank:	243.6

NP			
Pos.	247	obs:	exp:
act	T	1	0.26
acc	T	0	0.20
aca	T	0	0.46
acg	T	0	0.08
aat	N	720	466.00
aac	N	196	450.00
---	--	------	------
mPD		0.34	0.50
		nPD:	0.67
		N. weight:	0.21
		Sc. PD:	0.1
		Sc. rank:	127.7

NP			
Pos.	248	obs:	exp:
cct	P	896	233.20
ccc	P	15	163.50
cca	P	6	374.60
ccg	P	0	145.70
---	--	------	------
mPD		0.045	0.71
		nPD:	0.06
		N. weight:	1.5
		Sc. PD:	-0.12
		Sc. rank:	-72.4

NP			
Pos.	249	obs:	exp:
ggt	G	0	123.00
ggc	G	5	116.50
gga	G	59	424.00
ggg	G	853	253.50
---	--	------	------
mPD		0.13	0.68
		nPD:	0.19
		N. weight:	1.2
		Sc. PD:	0.054
		Sc. rank:	377.6

NP			
Pos.	250	obs:	exp:
aat	N	897	466.50
aac	N	20	450.50
---	--	------	------
mPD		0.043	0.50
		nPD:	0.09
		N. weight:	0.7
		Sc. PD:	-0.04
		Sc. rank:	-0.5

NP			
Pos.	251	obs:	exp:
gct	A	916	235.00
gcc	A	1	172.20
gca	A	0	428.70
gcg	A	0	81.11
---	--	------	------
mPD		0.0022	0.67
		nPD:	0.
		N. weight:	1.6
		Sc. PD:	-0.22
		Sc. rank:	-940.4

NP			
Pos.	252	obs:	exp:
gaa	E	906	551.60
gag	E	11	365.40
---	--	------	------
mPD		0.024	0.48
		nPD:	0.05
		N. weight:	0.55
		Sc. PD:	-0.05
		Sc. rank:	-61.4

NP			
Pos.	253	obs:	exp:
att	I	71	333.10
atc	I	18	220.80
ata	I	824	359.00
gtt	V	4	0.83
gtc	V	0	0.80
gta	V	0	0.83
gtg	V	0	1.54
---	--	------	------
mPD		0.19	0.66
		nPD:	0.29
		N. weight:	0.72
		Sc. PD:	0.1
		Sc. rank:	301.1

NP			
Pos.	254	obs:	exp:
gaa	E	912	551.60
gag	E	5	365.40
---	--	------	------
mPD		0.011	0.48
		nPD:	0.02
		N. weight:	0.58
		Sc. PD:	-0.068
		Sc. rank:	-178.3

NP			
Pos.	255	obs:	exp:
gat	D	906	495.00
gac	D	11	422.00
---	--	------	------
mPD		0.024	0.50
		nPD:	0.05
		N. weight:	0.68
		Sc. PD:	-0.063
		Sc. rank:	-83.2

NP			
Pos.	256	obs:	exp:
tta	L	0	80.50
ttg	L	0	170.90
ctt	L	237	155.20
ctc	L	679	153.50
cta	L	0	152.70
ctg	L	0	203.10
att	I	0	0.36
atc	I	1	0.24
ata	I	0	0.39
---	--	------	------
mPD		0.39	1.1
		nPD:	0.35
		N. weight:	1.5
		Sc. PD:	0.28
		Sc. rank:	730.9

NP			
Pos.	257	obs:	exp:
tta	L	0	0.35
ttg	L	0	0.75
ctt	L	1	0.68
ctc	L	3	0.67
cta	L	0	0.67
ctg	L	0	0.89
att	I	827	329.10
atc	I	74	218.20
ata	I	1	354.70
act	T	0	2.85
acc	T	11	2.24
aca	T	0	5.05
acg	T	0	0.86
---	--	------	------
mPD		0.21	0.69
		nPD:	0.3
		N. weight:	0.93
		Sc. PD:	0.14
		Sc. rank:	400.1

NP			
Pos.	258	obs:	exp:
ttt	F	881	361.90
ttc	F	36	555.10
---	--	------	------
mPD		0.076	0.48
		nPD:	0.16
		N. weight:	0.91
		Sc. PD:	0.01
		Sc. rank:	213.0

NP			
Pos.	259	obs:	exp:
tta	L	0	80.24
ttg	L	16	170.40
ctt	L	11	154.70
ctc	L	31	153.00
cta	L	11	152.20
ctg	L	844	202.50
att	I	0	1.46
atc	I	0	0.97
ata	I	4	1.57
---	--	------	------
mPD		0.16	1.1
		nPD:	0.15
		N. weight:	1.4
		Sc. PD:	-0.0013
		Sc. rank:	264.0

NP			
Pos.	260	obs:	exp:
gtt	V	0	0.21
gtc	V	0	0.20
gta	V	1	0.21
gtg	V	0	0.39
gct	A	0	234.70
gcc	A	0	172.00
gca	A	915	428.30
gcg	A	1	81.02
---	--	------	------
mPD		0.0044	0.68
		nPD:	0.01
		N. weight:	0.92
		Sc. PD:	-0.12
		Sc. rank:	-484.3

NP			
Pos.	261	obs:	exp:
cgt	R	0	39.17
cgc	R	0	46.59
cga	R	17	91.44
cgg	R	70	76.38
aga	R	1	418.70
agg	R	829	244.70
---	--	------	------
mPD		0.21	0.96
		nPD:	0.22
		N. weight:	1.3
		Sc. PD:	0.089
		Sc. rank:	436.4

NP			
Pos.	262	obs:	exp:
tct	S	910	146.80
tcc	S	1	118.70
tca	S	2	231.40
tcg	S	4	53.36
agt	S	0	187.90
agc	S	0	178.80
---	--	------	------
mPD		0.015	1.7
		nPD:	0.01
		N. weight:	2.2
		Sc. PD:	-0.28
		Sc. rank:	-1018.9

NP			
Pos.	263	obs:	exp:
tct	S	0	0.16
tcc	S	0	0.13
tca	S	1	0.25
tcg	S	0	0.06
agt	S	0	0.20
agc	S	0	0.20
gct	A	44	234.70
gcc	A	1	172.00
gca	A	865	428.30
gcg	A	6	81.02
---	--	------	------
mPD		0.11	0.68
		nPD:	0.16
		N. weight:	0.69
		Sc. PD:	0.0091
		Sc. rank:	165.4

NP			
Pos.	264	obs:	exp:
tta	L	0	80.59
ttg	L	1	171.10
ctt	L	1	155.40
ctc	L	915	153.60
cta	L	0	152.90
ctg	L	0	203.40
---	--	------	------
mPD		0.0065	1.1
		nPD:	0.01
		N. weight:	2.2
		Sc. PD:	-0.29
		Sc. rank:	-1151.3

NP			
Pos.	265	obs:	exp:
att	I	39	331.70
atc	I	864	219.90
ata	I	6	357.50
gtt	V	0	1.65
gtc	V	8	1.60
gta	V	0	1.66
gtg	V	0	3.09
---	--	------	------
mPD		0.11	0.67
		nPD:	0.17
		N. weight:	1.4
		Sc. PD:	0.027
		Sc. rank:	361.4

NP			
Pos.	266	obs:	exp:
tta	L	0	80.50
ttg	L	5	170.90
ctt	L	10	155.20
ctc	L	0	153.50
cta	L	5	152.70
ctg	L	896	203.10
caa	Q	0	0.51
cag	Q	1	0.49
---	--	------	------
mPD		0.045	1.1
		nPD:	0.04
		N. weight:	1.7
		Sc. PD:	-0.17
		Sc. rank:	-294.6

NP			
Pos.	267	obs:	exp:
cgt	R	0	39.17
cgc	R	0	46.59
cga	R	0	91.44
cgg	R	0	76.38
aga	R	726	418.70
agg	R	191	244.70
---	--	------	------
mPD		0.33	0.96
		nPD:	0.34
		N. weight:	0.47
		Sc. PD:	0.088
		Sc. rank:	225.5

NP			
Pos.	268	obs:	exp:
gaa	E	1	0.60
gag	E	0	0.40
ggt	G	2	122.80
ggc	G	0	116.40
gga	G	903	423.50
ggg	G	11	253.30
---	--	------	------
mPD		0.030	0.68
		nPD:	0.04
		N. weight:	0.86
		Sc. PD:	-0.083
		Sc. rank:	-125.3

NP			
Pos.	269	obs:	exp:
tct	S	37	146.60
tcc	S	0	118.60
tca	S	871	231.20
tcg	S	8	53.31
act	T	0	0.26
acc	T	0	0.20
aca	T	1	0.46
acg	T	0	0.08
agt	S	0	187.70
agc	S	0	178.60
---	--	------	------
mPD		0.096	1.7
		nPD:	0.06
		N. weight:	1.5
		Sc. PD:	-0.12
		Sc. rank:	-98.3

NP			
Pos.	270	obs:	exp:
gtt	V	3	189.30
gtc	V	0	183.70
gta	V	13	189.90
gtg	V	901	354.10
---	--	------	------
mPD		0.034	0.73
		nPD:	0.05
		N. weight:	1.1
		Sc. PD:	-0.099
		Sc. rank:	-134.5

NP

Pos.	271	obs:	exp:
tct	S	1	0.16
tcc	S	0	0.13
tca	S	0	0.25
tcg	S	0	0.06
agt	S	0	0.20
agc	S	0	0.20
gct	A	788	234.70
gcc	A	121	172.00
gca	A	7	428.30
gcg	A	0	81.02
mPD		0.24	0.68
		nPD:	0.36
		N. weight:	1.2
		Sc. PD:	0.24
		Sc. rank:	597.5

NP

Pos.	272	obs:	exp:
cat	H	862	526.80
cac	H	55	390.20
mPD		0.11	0.49
		nPD:	0.23
		N. weight:	0.42
		Sc. PD:	0.034
		Sc. rank:	146.6

NP

Pos.	273	obs:	exp:
aaa	K	567	537.20
aag	K	350	379.80
mPD		0.47	0.49
		nPD:	0.97
		N. weight:	0.0075
		Sc. PD:	0.0059
		Sc. rank:	4.9

NP

Pos.	274	obs:	exp:
tct	S	13	146.80
tcc	S	902	118.70
tca	S	2	231.40
tcg	S	0	53.36
agt	S	0	187.90
agc	S	0	178.80
mPD		0.032	1.7
		nPD:	0.02
		N. weight:	2.4
		Sc. PD:	-0.29
		Sc. rank:	-814.6

NP

Pos.	275	obs:	exp:
tgt	C	13	376.50
tgc	C	904	540.50
mPD		0.028	0.48
		nPD:	0.06
		N. weight:	0.56
		Sc. PD:	-0.047
		Sc. rank:	-42.5

NP

Pos.	276	obs:	exp:
tta	L	1	80.59
ttg	L	848	171.10
ctt	L	3	155.40
ctc	L	0	153.60
cta	L	3	152.90
ctg	L	62	203.40
mPD		0.15	1.1
		nPD:	0.14
		N. weight:	1.7
		Sc. PD:	-0.014
		Sc. rank:	272.0

NP

Pos.	277	obs:	exp:
cct	P	789	233.20
ccc	P	65	163.50
cca	P	63	374.60
ccg	P	0	145.70
mPD		0.25	0.71
		nPD:	0.35
		N. weight:	1.1
		Sc. PD:	0.21
		Sc. rank:	529.2

NP

Pos.	278	obs:	exp:
gct	A	785	235.00
gcc	A	118	172.20
gca	A	1	428.70
gcg	A	13	81.11
mPD		0.25	0.67
		nPD:	0.37
		N. weight:	1.2
		Sc. PD:	0.25
		Sc. rank:	605.9

NP

Pos.	279	obs:	exp:
ttt	F	1	0.39
ttc	F	0	0.61
tgt	C	910	376.00
tgc	C	6	540.00
mPD		0.015	0.49
		nPD:	0.03
		N. weight:	1.
		Sc. PD:	-0.11
		Sc. rank:	-231.5

NP

Pos.	280	obs:	exp:
gtt	V	4	189.10
gtc	V	0	183.50
gta	V	242	189.70
gtg	V	670	353.80
gct	A	0	0.26
gcc	A	0	0.19
gca	A	0	0.47
gcg	A	1	0.09
mPD		0.40	0.73
		nPD:	0.55
		N. weight:	0.64
		Sc. PD:	0.24
		Sc. rank:	382.3

NP

Pos.	281	obs:	exp:
tat	Y	69	486.20
tac	Y	848	430.80
mPD		0.14	0.50
		nPD:	0.28
		N. weight:	0.59
		Sc. PD:	0.075
		Sc. rank:	240.0

NP

Pos.	282	obs:	exp:
ggt	G	8	123.00
ggc	G	1	116.50
gga	G	795	424.00
ggg	G	113	253.50
mPD		0.23	0.68
		nPD:	0.35
		N. weight:	0.51
		Sc. PD:	0.098
		Sc. rank:	252.4

NP

Pos.	283	obs:	exp:
tta	L	0	80.50
ttg	L	0	170.90
ctt	L	909	155.20
ctc	L	7	153.50
cta	L	0	152.70
ctg	L	0	203.10
cct	P	1	0.25
ccc	P	0	0.18
cca	P	0	0.41
ccg	P	0	0.16
mPD		0.017	1.1
		nPD:	0.02
		N. weight:	2.1
		Sc. PD:	-0.26
		Sc. rank:	-807.6

NP

Pos.	284	obs:	exp:
act	T	0	0.26
acc	T	0	0.20
aca	T	1	0.46
acg	T	0	0.08
gct	A	66	234.70
gcc	A	22	172.00
gca	A	824	428.30
gcg	A	4	81.02
mPD		0.19	0.67
		nPD:	0.28
		N. weight:	0.54
		Sc. PD:	0.067
		Sc. rank:	218.4

NP

Pos.	285	obs:	exp:
gtt	V	1	189.30
gtc	V	1	183.70
gta	V	39	189.90
gtg	V	876	354.10
mPD		0.086	0.73
		nPD:	0.12
		N. weight:	0.96
		Sc. PD:	-0.026
		Sc. rank:	120.7

NP

Pos.	286	obs:	exp:
tct	S	0	0.16
tcc	S	1	0.13
tca	S	0	0.25
tcg	S	0	0.06
agt	S	0	0.20
agc	S	0	0.20
gtt	V	3	1.03
gtc	V	2	1.00
gta	V	0	1.04
gtg	V	0	1.93
gct	A	487	233.40
gcc	A	420	171.10
gca	A	4	425.90
gcg	A	0	80.58
mPD		0.52	0.69
		nPD:	0.75
		N. weight:	0.97
		Sc. PD:	0.56
		Sc. rank:	622.0

NP

Pos.	287	obs:	exp:
tct	S	0	146.80
tcc	S	0	118.70
tca	S	0	231.40
tcg	S	0	53.36
agt	S	909	187.90
agc	S	8	178.80
mPD		0.017	1.7
		nPD:	0.01
		N. weight:	1.9
		Sc. PD:	-0.24
		Sc. rank:	-850.5

NP

Pos.	288	obs:	exp:
ggt	G	3	123.00
ggc	G	0	116.50
gga	G	901	424.00
ggg	G	13	253.50
mPD		0.034	0.68
		nPD:	0.05
		N. weight:	0.84
		Sc. PD:	-0.076
		Sc. rank:	-92.3

NP

Pos.	289	obs:	exp:
tat	Y	863	486.20
tac	Y	54	430.80
mPD		0.11	0.50
		nPD:	0.22
		N. weight:	0.51
		Sc. PD:	0.037
		Sc. rank:	176.5

NP

Pos.	290	obs:	exp:
aat	N	0	1.02
aac	N	2	0.98
gat	D	20	493.40
gac	D	894	420.60
gaa	E	1	0.60
gag	E	0	0.40
mPD		0.049	0.50
		nPD:	0.1
		N. weight:	0.82
		Sc. PD:	-0.037
		Sc. rank:	37.4

NP

Pos.	291	obs:	exp:
ttt	F	903	361.90
ttc	F	14	555.10
mPD		0.030	0.48
		nPD:	0.06
		N. weight:	1.
		Sc. PD:	-0.081
		Sc. rank:	-53.0

NP

Pos.	292	obs:	exp:
gaa	E	63	551.60
gag	E	854	365.40
mPD		0.13	0.48
		nPD:	0.27
		N. weight:	0.78
		Sc. PD:	0.09
		Sc. rank:	312.4

NP

Pos.	293	obs:	exp:
cgt	R	0	39.09
cgc	R	0	46.49
cga	R	0	91.24
cgg	R	0	76.21
aaa	K	1	1.17
aag	K	1	0.83
aga	R	906	417.80
agg	R	9	244.20
mPD		0.026	0.97
		nPD:	0.03
		N. weight:	0.89
		Sc. PD:	-0.1
		Sc. rank:	-237.4

NP

Pos.	294	obs:	exp:
gaa	E	862	551.60
gag	E	55	365.40
mPD		0.11	0.48
		nPD:	0.24
		N. weight:	0.38
		Sc. PD:	0.032
		Sc. rank:	132.2

NP

Pos.	295	obs:	exp:
ggt	G	2	123.00
ggc	G	0	116.50
gga	G	326	424.00
ggg	G	589	253.50
mPD		0.46	0.68
		nPD:	0.68
		N. weight:	0.54
		Sc. PD:	0.28
		Sc. rank:	336.8

NP			
Pos.	296	obs:	exp:
tat	Y	30	486.20
tac	Y	887	430.80
---	--	------	------
mPD		0.063	0.50
		nPD:	0.13
		N. weight:	0.74
		Sc. PD:	-0.013
		Sc. rank:	103.3

,

NP			
Pos.	297	obs:	exp:
tct	S	71	146.80
tcc	S	844	118.70
tca	S	2	231.40
tcg	S	0	53.36
agt	S	0	187.90
agc	S	0	178.80
---	--	------	------
mPD		0.15	1.7
		nPD:	0.09
		N. weight:	2.1
		Sc. PD:	-0.12
		Sc. rank:	43.1

,

NP			
Pos.	298	obs:	exp:
tta	L	20	80.59
ttg	L	526	171.10
ctt	L	6	155.40
ctc	L	0	153.60
cta	L	74	152.90
ctg	L	291	203.40
---	--	------	------
mPD		0.68	1.1
		nPD:	0.61
		N. weight:	0.8
		Sc. PD:	0.35
		Sc. rank:	492.7

,

NP			
Pos.	299	obs:	exp:
gtt	V	796	189.30
gtc	V	55	183.70
gta	V	0	189.90
gtg	V	66	354.10
---	--	------	------
mPD		0.24	0.73
		nPD:	0.33
		N. weight:	1.3
		Sc. PD:	0.22
		Sc. rank:	577.6

,

NP			
Pos.	300	obs:	exp:
ggt	G	2	123.00
ggc	G	0	116.50
gga	G	902	424.00
ggg	G	13	253.50
---	--	------	------
mPD		0.032	0.68
		nPD:	0.05
		N. weight:	0.85
		Sc. PD:	-0.079
		Sc. rank:	-102.3

,

NP			
Pos.	301	obs:	exp:
tta	L	0	0.09
ttg	L	0	0.19
ctt	L	0	0.17
ctc	L	0	0.17
cta	L	1	0.17
ctg	L	0	0.22
att	I	51	334.20
atc	I	6	221.60
ata	I	859	360.20
---	--	------	------
mPD		0.12	0.66
		nPD:	0.18
		N. weight:	0.84
		Sc. PD:	0.029
		Sc. rank:	243.7

,

NP			
Pos.	302	obs:	exp:
aat	N	1	0.51
aac	N	0	0.49
gat	D	848	494.50
gac	D	68	421.50
---	--	------	------
mPD		0.14	0.50
		nPD:	0.28
		N. weight:	0.45
		Sc. PD:	0.057
		Sc. rank:	183.3

,

NP			
Pos.	303	obs:	exp:
cct	P	905	233.20
ccc	P	9	163.50
cca	P	3	374.60
ccg	P	0	145.70
---	--	------	------
mPD		0.026	0.71
		nPD:	0.04
		N. weight:	1.6
		Sc. PD:	-0.16
		Sc. rank:	-311.6

,

NP			
Pos.	304	obs:	exp:
ttt	F	87	361.90
ttc	F	830	555.10
---	--	------	------
mPD		0.17	0.48
		nPD:	0.36
		N. weight:	0.28
		Sc. PD:	0.057
		Sc. rank:	142.1

,

NP			
Pos.	305	obs:	exp:
tgt	C	1	0.41
tgc	C	0	0.59
cgt	R	696	39.05
cgc	R	211	46.44
cga	R	5	91.14
cgg	R	2	76.13
aaa	K	1	1.17
aag	K	1	0.83
aga	R	0	417.30
agg	R	0	243.90
---	--	------	------
mPD		0.38	0.97
		nPD:	0.39
		N. weight:	3.1
		Sc. PD:	0.72
		Sc. rank:	1660.1

,

NP			
Pos.	306	obs:	exp:
tta	L	2	80.59
ttg	L	9	171.10
ctt	L	0	155.40
ctc	L	0	153.60
cta	L	15	152.90
ctg	L	891	203.40
---	--	------	------
mPD		0.060	1.1
		nPD:	0.05
		N. weight:	1.7
		Sc. PD:	-0.15
		Sc. rank:	-140.4

,

NP			
Pos.	307	obs:	exp:
tta	L	0	80.59
ttg	L	0	171.10
ctt	L	863	155.40
ctc	L	49	153.60
cta	L	0	152.90
ctg	L	5	203.40
---	--	------	------
mPD		0.11	1.1
		nPD:	0.1
		N. weight:	1.9
		Sc. PD:	-0.081
		Sc. rank:	97.5

,

NP			
Pos.	308	obs:	exp:
caa	Q	898	471.00
cag	Q	19	446.00
---	--	------	------
mPD		0.041	0.50
		nPD:	0.08
		N. weight:	0.69
		Sc. PD:	-0.043
		Sc. rank:	-8.4

,

NP			
Pos.	309	obs:	exp:
tat	Y	0	0.53
tac	Y	1	0.47
aat	N	1	466.00
aac	N	915	450.00
---	--	------	------
mPD		0.0044	0.50
		nPD:	0.01
		N. weight:	0.86
		Sc. PD:	-0.11
		Sc. rank:	-432.5

,

NP			
Pos.	310	obs:	exp:
tct	S	0	146.80
tcc	S	0	118.70
tca	S	0	231.40
tcg	S	0	53.36
agt	S	32	187.90
agc	S	885	178.80
---	--	------	------
mPD		0.067	1.7
		nPD:	0.04
		N. weight:	1.8
		Sc. PD:	-0.18
		Sc. rank:	-322.7

,

NP			
Pos.	311	obs:	exp:
caa	Q	254	471.00
cag	Q	663	446.00
---	--	------	------
mPD		0.40	0.50
		nPD:	0.8
		N. weight:	0.15
		Sc. PD:	0.091
		Sc. rank:	94.2

,

NP			
Pos.	312	obs:	exp:
att	I	0	0.36
atc	I	0	0.24
ata	I	1	0.39
gtt	V	22	189.10
gtc	V	881	183.50
gta	V	12	189.70
gtg	V	1	353.80
---	--	------	------
mPD		0.078	0.73
		nPD:	0.11
		N. weight:	1.7
		Sc. PD:	-0.063
		Sc. rank:	147.0

,

NP			
Pos.	313	obs:	exp:
ttt	F	768	361.50
ttc	F	148	554.50
tat	Y	0	0.53
tac	Y	1	0.47
---	--	------	------
mPD		0.27	0.48
		nPD:	0.57
		N. weight:	0.51
		Sc. PD:	0.21
		Sc. rank:	312.1

,

NP			
Pos.	314	obs:	exp:
tct	S	0	146.80
tcc	S	0	118.70
tca	S	0	231.40
tcg	S	0	53.36
agt	S	897	187.90
agc	S	20	178.80
---	--	------	------
mPD		0.043	1.7
		nPD:	0.03
		N. weight:	1.8
		Sc. PD:	-0.21
		Sc. rank:	-501.2

,

NP			
Pos.	315	obs:	exp:
ttt	F	0	0.39
ttc	F	1	0.61
tta	L	0	80.42
ttg	L	0	170.80
ctt	L	13	155.10
ctc	L	852	153.30
cta	L	44	152.50
ctg	L	6	202.90
att	I	0	0.36
atc	I	1	0.24
ata	I	0	0.39
---	--	------	------
mPD		0.13	1.1
		nPD:	0.12
		N. weight:	1.8
		Sc. PD:	-0.043
		Sc. rank:	235.0

,

NP			
Pos.	316	obs:	exp:
att	I	834	334.60
atc	I	54	221.80
ata	I	29	360.60
---	--	------	------
mPD		0.17	0.65
		nPD:	0.26
		N. weight:	0.82
		Sc. PD:	0.087
		Sc. rank:	314.2

,

NP			
Pos.	317	obs:	exp:
cgt	R	0	39.17
cgc	R	0	46.59
cga	R	0	91.44
cgg	R	0	76.38
aga	R	904	418.70
agg	R	13	244.70
---	--	------	------
mPD		0.028	0.96
		nPD:	0.03
		N. weight:	0.88
		Sc. PD:	-0.097
		Sc. rank:	-215.9

,

NP			
Pos.	318	obs:	exp:
tta	L	0	0.09
ttg	L	0	0.19
ctt	L	0	0.17
ctc	L	0	0.17
cta	L	1	0.17
ctg	L	0	0.22
cct	P	2	232.70
ccc	P	2	163.10
cca	P	903	373.80
ccg	P	8	145.40
caa	Q	1	0.51
cag	Q	0	0.49
---	--	------	------
mPD		0.030	0.72
		nPD:	0.04
		N. weight:	1.
		Sc. PD:	-0.1
		Sc. rank:	-166.3

,

NP			
Pos.	319	obs:	exp:
aat	N	689	466.50
aac	N	228	450.50
---	--	------	------
mPD		0.37	0.50
		nPD:	0.75
		N. weight:	0.16
		Sc. PD:	0.089
		Sc. rank:	99.2

,

NP			
Pos.	320	obs:	exp:
gaa	E	50	551.60
gag	E	867	365.40
---	--	------	------
mPD		0.10	0.48
		nPD:	0.22
		N. weight:	0.84
		Sc. PD:	0.055
		Sc. rank:	278.5

,

NP			
Pos.	321	obs:	exp:
tct	S	0	0.32
tcc	S	0	0.26
tca	S	0	0.50
tcg	S	0	0.12
aat	N	87	465.50
aac	N	828	449.50
agt	S	0	0.41
agc	S	2	0.39
---	--	------	------
mPD		0.18	0.51
		nPD:	0.35
		N. weight:	0.49
		Sc. PD:	0.093
		Sc. rank:	240.7

NP			
Pos.	322	obs:	exp:
cct	P	1	233.20
ccc	P	0	163.50
cca	P	913	374.60
ccg	P	3	145.70
---	--	------	------
mPD		0.0087	0.71
		nPD:	0.01
		N. weight:	1.1
		Sc. PD:	-0.13
		Sc. rank:	-462.3

NP			
Pos.	323	obs:	exp:
tct	S	0	0.16
tcc	S	0	0.13
tca	S	1	0.25
tcg	S	0	0.06
agt	S	0	0.20
agc	S	0	0.20
gtt	V	0	0.21
gtc	V	0	0.20
gta	V	1	0.21
gtg	V	0	0.39
gct	A	9	234.50
gcc	A	0	171.80
gca	A	884	427.80
gcg	A	22	80.93
---	--	------	------
mPD		0.070	0.68
		nPD:	0.1
		N. weight:	0.78
		Sc. PD:	-0.032
		Sc. rank:	51.4

NP			
Pos.	324	obs:	exp:
cat	H	693	526.80
cac	H	224	390.20
---	--	------	------
mPD		0.37	0.49
		nPD:	0.76
		N. weight:	0.092
		Sc. PD:	0.053
		Sc. rank:	59.2

NP			
Pos.	325	obs:	exp:
aaa	K	58	537.20
aag	K	859	379.80
---	--	------	------
mPD		0.12	0.49
		nPD:	0.24
		N. weight:	0.76
		Sc. PD:	0.071
		Sc. rank:	276.1

NP			
Pos.	326	obs:	exp:
tct	S	0	146.80
tcc	S	0	118.70
tca	S	0	231.40
tcg	S	0	53.36
agt	S	127	187.90
agc	S	790	178.80
---	--	------	------
mPD		0.24	1.7
		nPD:	0.14
		N. weight:	1.5
		Sc. PD:	-0.0023
		Sc. rank:	266.2

NP			
Pos.	327	obs:	exp:
caa	Q	668	471.00
cag	Q	249	446.00
---	--	------	------
mPD		0.40	0.50
		nPD:	0.79
		N. weight:	0.12
		Sc. PD:	0.075
		Sc. rank:	78.9

NP			
Pos.	328	obs:	exp:
tta	L	198	80.59
ttg	L	116	171.10
ctt	L	0	155.40
ctc	L	0	153.60
cta	L	494	152.90
ctg	L	109	203.40
---	--	------	------
mPD		0.82	1.1
		nPD:	0.74
		N. weight:	0.87
		Sc. PD:	0.49
		Sc. rank:	552.4

NP			
Pos.	329	obs:	exp:
att	I	0	0.73
atc	I	0	0.48
ata	I	2	0.79
gtt	V	202	188.90
gtc	V	1	183.30
gta	V	99	189.40
gtg	V	613	353.40
---	--	------	------
mPD		0.50	0.73
		nPD:	0.68
		N. weight:	0.39
		Sc. PD:	0.2
		Sc. rank:	240.2

NP			
Pos.	330	obs:	exp:
tgg	W	917	917.00
---	--	------	------
mPD		0	0
		nPD:	1.
		N. weight:	0.
		Sc. PD:	0
		Sc. rank:	0

NP			
Pos.	331	obs:	exp:
atg	M	917	917.00
---	--	------	------
mPD		0	0
		nPD:	1.
		N. weight:	0.
		Sc. PD:	0
		Sc. rank:	0

NP			
Pos.	332	obs:	exp:
gct	A	1	235.00
gcc	A	0	172.20
gca	A	913	428.70
gcg	A	3	81.11
---	--	------	------
mPD		0.0087	0.67
		nPD:	0.01
		N. weight:	0.9
		Sc. PD:	-0.11
		Sc. rank:	-386.1

NP			
Pos.	333	obs:	exp:
tgt	C	13	376.50
tgc	C	904	540.50
---	--	------	------
mPD		0.028	0.48
		nPD:	0.06
		N. weight:	0.56
		Sc. PD:	-0.047
		Sc. rank:	-42.5

NP			
Pos.	334	obs:	exp:
cat	H	64	526.80
cac	H	853	390.20
---	--	------	------
mPD		0.13	0.49
		nPD:	0.27
		N. weight:	0.71
		Sc. PD:	0.081
		Sc. rank:	281.4

NP			
Pos.	335	obs:	exp:
ttt	F	1	0.39
ttc	F	0	0.61
tct	S	915	146.60
tcc	S	1	118.60
tca	S	0	231.20
tcg	S	0	53.31
agt	S	0	187.70
agc	S	0	178.60
---	--	------	------
mPD		0.0044	1.7
		nPD:	0.
		N. weight:	2.2
		Sc. PD:	-0.3
		Sc. rank:	-1291.5

NP			
Pos.	336	obs:	exp:
act	T	0	0.26
acc	T	0	0.20
aca	T	1	0.46
acg	T	0	0.08
gct	A	13	234.70
gcc	A	13	172.00
gca	A	881	428.30
gcg	A	9	81.02
---	--	------	------
mPD		0.077	0.67
		nPD:	0.11
		N. weight:	0.73
		Sc. PD:	-0.023
		Sc. rank:	75.6

NP			
Pos.	337	obs:	exp:
tct	S	0	0.32
tcc	S	0	0.26
tca	S	0	0.50
tcg	S	2	0.12
act	T	0	0.26
acc	T	0	0.20
aca	T	1	0.46
acg	T	0	0.08
agt	S	0	0.41
agc	S	0	0.39
gct	A	9	234.20
gcc	A	0	171.60
gca	A	353	427.30
gcg	A	552	80.84
---	--	------	------
mPD		0.49	0.68
		nPD:	0.72
		N. weight:	1.3
		Sc. PD:	0.71
		Sc. rank:	819.9

NP			
Pos.	338	obs:	exp:
ttt	F	900	361.50
ttc	F	16	554.50
tct	S	1	0.16
tcc	S	0	0.13
tca	S	0	0.25
tcg	S	0	0.06
agt	S	0	0.20
agc	S	0	0.20
---	--	------	------
mPD		0.037	0.48
		nPD:	0.08
		N. weight:	1.
		Sc. PD:	-0.068
		Sc. rank:	-25.2

NP			
Pos.	339	obs:	exp:
gaa	E	65	551.60
gag	E	852	365.40
---	--	------	------
mPD		0.13	0.48
		nPD:	0.28
		N. weight:	0.78
		Sc. PD:	0.095
		Sc. rank:	313.6

NP			
Pos.	340	obs:	exp:
gat	D	59	495.00
gac	D	858	422.00
---	--	------	------
mPD		0.12	0.50
		nPD:	0.24
		N. weight:	0.65
		Sc. PD:	0.059
		Sc. rank:	232.3

NP			
Pos.	341	obs:	exp:
tta	L	1	80.50
ttg	L	6	170.90
ctt	L	821	155.20
ctc	L	5	153.50
cta	L	0	152.70
ctg	L	83	203.10
att	I	1	0.36
atc	I	0	0.24
ata	I	0	0.39
---	--	------	------
mPD		0.20	1.1
		nPD:	0.18
		N. weight:	1.7
		Sc. PD:	0.06
		Sc. rank:	490.6

,

NP			
Pos.	342	obs:	exp:
cgt	R	0	39.17
cgc	R	0	46.59
cga	R	0	91.44
cgg	R	0	76.38
aga	R	131	418.70
agg	R	786	244.70
---	--	------	------
mPD		0.25	0.96
		nPD:	0.25
		N. weight:	1.
		Sc. PD:	0.11
		Sc. rank:	386.8

,

NP			
Pos.	343	obs:	exp:
tta	L	0	0.09
ttg	L	1	0.19
ctt	L	0	0.17
ctc	L	0	0.17
cta	L	0	0.17
ctg	L	0	0.22
att	I	0	1.46
atc	I	4	0.97
ata	I	0	1.57
gtt	V	8	188.30
gtc	V	851	182.70
gta	V	7	188.80
gtg	V	46	352.20
---	--	------	------
mPD		0.14	0.74
		nPD:	0.19
		N. weight:	1.6
		Sc. PD:	0.063
		Sc. rank:	475.7

,

NP			
Pos.	344	obs:	exp:
tta	L	3	0.26
ttg	L	0	0.56
tct	S	0	146.30
tcc	S	0	118.30
tca	S	910	230.70
tcg	S	4	53.19
ctt	L	0	0.51
ctc	L	0	0.50
cta	L	0	0.50
ctg	L	0	0.67
agt	S	0	187.30
agc	S	0	178.20
---	--	------	------
mPD		0.015	1.7
		nPD:	0.01
		N. weight:	1.7
		Sc. PD:	-0.22
		Sc. rank:	-804.5

,

NP			
Pos.	345	obs:	exp:
tct	S	0	146.80
tcc	S	0	118.70
tca	S	0	231.40
tcg	S	0	53.36
agt	S	836	187.90
agc	S	81	178.80
---	--	------	------
mPD		0.16	1.7
		nPD:	0.1
		N. weight:	1.6
		Sc. PD:	-0.073
		Sc. rank:	66.0

,

NP			
Pos.	346	obs:	exp:
ttt	F	8	361.90
ttc	F	909	555.10
---	--	------	------
mPD		0.017	0.48
		nPD:	0.04
		N. weight:	0.56
		Sc. PD:	-0.058
		Sc. rank:	-112.2

,

NP			
Pos.	347	obs:	exp:
tta	L	0	0.26
ttg	L	0	0.56
ctt	L	1	0.51
ctc	L	2	0.50
cta	L	0	0.50
ctg	L	0	0.67
att	I	571	333.50
atc	I	343	221.10
ata	I	0	359.40
---	--	------	------
mPD		0.48	0.66
		nPD:	0.72
		N. weight:	0.62
		Sc. PD:	0.34
		Sc. rank:	389.2

,

NP			
Pos.	348	obs:	exp:
cgt	R	0	39.00
cgc	R	0	46.39
cga	R	0	91.04
cgg	R	0	76.04
aaa	K	4	2.34
aag	K	0	1.66
aga	R	912	416.90
agg	R	1	243.70
---	--	------	------
mPD		0.011	0.97
		nPD:	0.01
		N. weight:	0.94
		Sc. PD:	-0.12
		Sc. rank:	-426.9

,

NP			
Pos.	349	obs:	exp:
ggt	G	0	123.00
ggc	G	0	116.50
gga	G	181	424.00
ggg	G	736	253.50
---	--	------	------
mPD		0.32	0.68
		nPD:	0.47
		N. weight:	0.84
		Sc. PD:	0.26
		Sc. rank:	476.8

,

NP			
Pos.	350	obs:	exp:
att	I	0	1.46
atc	I	0	0.97
ata	I	4	1.57
act	T	0	233.90
acc	T	1	183.90
aca	T	891	414.90
acg	T	11	70.34
gct	A	0	2.56
gcc	A	0	1.88
gca	A	10	4.68
gcg	A	0	0.88
---	--	------	------
mPD		0.056	0.70
		nPD:	0.08
		N. weight:	0.89
		Sc. PD:	-0.056
		Sc. rank:	-19.9

,

NP			
Pos.	351	obs:	exp:
cgt	R	0	37.21
cgc	R	0	44.25
cga	R	0	86.85
cgg	R	0	72.54
aaa	K	46	26.95
aag	K	0	19.05
aga	R	859	397.70
agg	R	12	232.50
---	--	------	------
mPD		0.12	1.0
		nPD:	0.12
		N. weight:	0.87
		Sc. PD:	-0.024
		Sc. rank:	106.4

,

NP			
Pos.	352	obs:	exp:
att	I	0	0.73
atc	I	0	0.48
ata	I	2	0.79
atg	M	822	822.00
gtt	V	0	19.20
gtc	V	0	18.63
gta	V	72	19.26
gtg	V	21	35.92
---	--	------	------
mPD		0.33	0.31
		nPD:	1.08
		N. weight:	0.12
		Sc. PD:	0.1
		Sc. rank:	76.0

,

NP			
Pos.	353	obs:	exp:
tta	L	0	0.35
ttg	L	0	0.75
tct	S	1	0.16
tcc	S	0	0.13
tca	S	0	0.25
tcg	S	0	0.06
ctt	L	3	0.68
ctc	L	1	0.67
cta	L	0	0.67
ctg	L	0	0.89
att	I	2	6.57
atc	I	16	4.35
ata	I	0	7.08
agt	S	0	0.20
agc	S	0	0.20
gtt	V	30	184.60
gtc	V	863	179.10
gta	V	1	185.10
gtg	V	0	345.30
---	--	------	------
mPD		0.13	0.78
		nPD:	0.17
		N. weight:	1.8
		Sc. PD:	0.033
		Sc. rank:	447.8

,

NP			
Pos.	354	obs:	exp:
cct	P	2	233.20
ccc	P	1	163.50
cca	P	909	374.60
ccg	P	5	145.70
---	--	------	------
mPD		0.017	0.71
		nPD:	0.02
		N. weight:	1.
		Sc. PD:	-0.12
		Sc. rank:	-301.1

,

NP			
Pos.	355	obs:	exp:
cgt	R	0	39.17
cgc	R	0	46.59
cga	R	0	91.44
cgg	R	1	76.38
aga	R	907	418.70
agg	R	9	244.70
---	--	------	------
mPD		0.024	0.96
		nPD:	0.02
		N. weight:	0.89
		Sc. PD:	-0.1
		Sc. rank:	-253.0

,

NP			
Pos.	356	obs:	exp:
ggt	G	1	123.00
ggc	G	0	116.50
gga	G	829	424.00
ggg	G	87	253.50
---	--	------	------
mPD		0.17	0.68
		nPD:	0.26
		N. weight:	0.61
		Sc. PD:	0.065
		Sc. rank:	233.7

,

NP			
Pos.	357	obs:	exp:
caa	Q	104	470.50
cag	Q	812	445.50
aaa	K	1	0.59
aag	K	0	0.41
---	--	------	------
mPD		0.21	0.50
		nPD:	0.41
		N. weight:	0.44
		Sc. PD:	0.11
		Sc. rank:	243.3

,

NP			
Pos.	358	obs:	exp:
tta	L	49	80.59
ttg	L	2	171.10
ctt	L	0	155.40
ctc	L	0	153.60
cta	L	499	152.90
ctg	L	367	203.40
---	--	------	------
mPD		0.59	1.1
		nPD:	0.53
		N. weight:	1.
		Sc. PD:	0.38
		Sc. rank:	622.0

,

NP			
Pos.	359	obs:	exp:
tct	S	19	144.10
tcc	S	879	116.50
tca	S	2	227.10
tcg	S	0	52.37
agt	S	0	184.40
agc	S	0	175.50
gct	A	1	4.36
gcc	A	15	3.19
gca	A	1	7.95
gcg	A	0	1.50
---	--	------	------
mPD		0.086	1.7
		nPD:	0.05
		N. weight:	2.3
		Sc. PD:	-0.21
		Sc. rank:	-235.9

,

NP			
Pos.	360	obs:	exp:
tct	S	1	0.16
tcc	S	0	0.13
tca	S	0	0.25
tcg	S	0	0.06
act	T	798	237.30
acc	T	112	186.50
aca	T	6	420.80
acg	T	0	71.35
agt	S	0	0.20
agc	S	0	0.20
---	--	------	------
mPD		0.23	0.68
		nPD:	0.34
		N. weight:	1.2
		Sc. PD:	0.22
		Sc. rank:	549.2

,

NP			
Pos.	361	obs:	exp:
cgt	R	0	39.17
cgc	R	0	46.59
cga	R	1	91.44
cgg	R	0	76.38
aga	R	905	418.70
agg	R	11	244.70
---	--	------	------
mPD		0.026	0.96
		nPD:	0.03
		N. weight:	0.88
		Sc. PD:	-0.1
		Sc. rank:	-230.3

,

NP			
Pos.	362	obs:	exp:
ggt	G	0	123.00
ggc	G	0	116.50
gga	G	200	424.00
ggg	G	717	253.50
---	--	------	------
mPD		0.34	0.68
		nPD:	0.51
		N. weight:	0.79
		Sc. PD:	0.27
		Sc. rank:	468.8

,

NP			
Pos.	363	obs:	exp:
att	I	9	3.28
atc	I	0	2.18
ata	I	0	3.54
gtt	V	904	187.40
gtc	V	1	181.90
gta	V	2	188.00
gtg	V	1	350.70
---	--	------	------
mPD		0.028	0.75
		nPD:	0.04
		N. weight:	1.9
		Sc. PD:	-0.19
		Sc. rank:	-361.1

,

NP			
Pos.	364	obs:	exp:
caa	Q	867	471.00
cag	Q	50	446.00
---	--	------	------
mPD		0.10	0.50
		nPD:	0.21
		N. weight:	0.56
		Sc. PD:	0.032
		Sc. rank:	180.5

,

NP			
Pos.	365	obs:	exp:
att	I	910	334.60
atc	I	6	221.80
ata	I	1	360.60
---	--	------	------
mPD		0.015	0.65
		nPD:	0.02
		N. weight:	1.2
		Sc. PD:	-0.14
		Sc. rank:	-354.6

,

NP			
Pos.	366	obs:	exp:
tct	S	1	0.16
tcc	S	0	0.13
tca	S	0	0.25
tcg	S	0	0.06
agt	S	0	0.20
agc	S	0	0.20
gct	A	905	234.70
gcc	A	8	172.00
gca	A	2	428.30
gcg	A	1	81.02
---	--	------	------
mPD		0.026	0.68
		nPD:	0.04
		N. weight:	1.6
		Sc. PD:	-0.16
		Sc. rank:	-296.8

,

NP			
Pos.	367	obs:	exp:
tct	S	2	146.80
tcc	S	5	118.70
tca	S	906	231.40
tcg	S	4	53.36
agt	S	0	187.90
agc	S	0	178.80
---	--	------	------
mPD		0.024	1.7
		nPD:	0.01
		N. weight:	1.6
		Sc. PD:	-0.2
		Sc. rank:	-631.2

,

NP			
Pos.	368	obs:	exp:
tct	S	0	0.16
tcc	S	0	0.13
tca	S	0	0.25
tcg	S	0	0.06
aat	N	913	466.00
aac	N	3	450.00
agt	S	1	0.20
agc	S	0	0.20
---	--	------	------
mPD		0.0087	0.50
		nPD:	0.02
		N. weight:	0.8
		Sc. PD:	-0.098
		Sc. rank:	-282.8

,

NP			
Pos.	369	obs:	exp:
gaa	E	37	551.60
gag	E	880	365.40
---	--	------	------
mPD		0.078	0.48
		nPD:	0.16
		N. weight:	0.9
		Sc. PD:	0.013
		Sc. rank:	216.9

,

NP			
Pos.	370	obs:	exp:
tct	S	0	0.16
tcc	S	0	0.13
tca	S	0	0.25
tcg	S	0	0.06
aat	N	16	466.00
aac	N	900	450.00
agt	S	0	0.20
agc	S	1	0.20
---	--	------	------
mPD		0.037	0.50
		nPD:	0.07
		N. weight:	0.76
		Sc. PD:	-0.053
		Sc. rank:	-22.5

,

NP			
Pos.	371	obs:	exp:
att	I	0	84.65
atc	I	0	56.11
ata	I	232	91.23
atg	M	682	682.00
act	T	0	0.26
acc	T	0	0.20
aca	T	1	0.46
acg	T	0	0.08
gtt	V	0	0.41
gtc	V	0	0.40
gta	V	2	0.41
gtg	V	0	0.77
---	--	------	------
mPD		0.39	0.43
		nPD:	0.9
		N. weight:	0.29
		Sc. PD:	0.21
		Sc. rank:	190.4

,

NP			
Pos.	372	obs:	exp:
gct	A	0	0.26
gcc	A	0	0.19
gca	A	1	0.47
gcg	A	0	0.09
gat	D	1	1.08
gac	D	1	0.92
gaa	E	867	549.80
gag	E	47	364.20
---	--	------	------
mPD		0.10	0.48
		nPD:	0.21
		N. weight:	0.4
		Sc. PD:	0.026
		Sc. rank:	131.3

,

NP			
Pos.	373	obs:	exp:
act	T	0	24.87
acc	T	2	19.55
aca	T	89	44.10
acg	T	5	7.48
gtt	V	0	0.83
gtc	V	0	0.80
gta	V	4	0.83
gtg	V	0	1.54
gct	A	1	209.30
gcc	A	0	153.40
gca	A	816	382.00
gcg	A	0	72.26
---	--	------	------
mPD		0.21	0.87
		nPD:	0.25
		N. weight:	0.9
		Sc. PD:	0.086
		Sc. rank:	328.8

,

NP			
Pos.	374	obs:	exp:
att	I	0	2.19
atc	I	0	1.45
ata	I	6	2.36
atg	M	911	911.00
---	--	------	------
mPD		0.013	0.013
		nPD:	1.
		N. weight:	0.0074
		Sc. PD:	0.006
		Sc. rank:	4.8

,

NP			
Pos.	375	obs:	exp:
cat	H	0	0.57
cac	H	1	0.43
aat	N	0	0.51
aac	N	1	0.49
gat	D	23	379.00
gac	D	679	323.00
gaa	E	212	127.50
gag	E	0	84.48
ggt	G	0	0.13
ggc	G	0	0.13
gga	G	1	0.46
ggg	G	0	0.28
---	--	------	------
mPD		0.40	0.68
		nPD:	0.59
		N. weight:	0.73
		Sc. PD:	0.31
		Sc. rank:	448.7

,

NP			
Pos.	376	obs:	exp:
tct	S	5	146.80
tcc	S	901	118.70
tca	S	10	231.40
tcg	S	1	53.36
agt	S	0	187.90
agc	S	0	178.80
---	--	------	------
mPD		0.034	1.7
		nPD:	0.02
		N. weight:	2.4
		Sc. PD:	-0.28
		Sc. rank:	-770.8

,

NP			
Pos.	377	obs:	exp:
tct	S	0	14.41
tcc	S	0	11.65
tca	S	0	22.71
tcg	S	0	5.24
aat	N	587	420.70
aac	N	240	406.30
agt	S	40	18.44
agc	S	50	17.55
---	--	------	------
mPD		0.61	0.82
		nPD:	0.74
		N. weight:	0.21
		Sc. PD:	0.12
		Sc. rank:	134.3

,

NP			
Pos.	378	obs:	exp:
act	T	903	237.30
acc	T	6	186.50
aca	T	7	420.80
acg	T	0	71.35
aat	N	1	0.51
aac	N	0	0.49
---	--	------	------
mPD		0.030	0.68
		nPD:	0.04
		N. weight:	1.5
		Sc. PD:	-0.15
		Sc. rank:	-219.6

,

NP			
Pos.	379	obs:	exp:
tta	L	0	80.59
ttg	L	0	171.10
ctt	L	870	155.40
ctc	L	2	153.60
cta	L	45	152.90
ctg	L	0	203.40
---	--	------	------
mPD		0.098	1.1
		nPD:	0.09
		N. weight:	1.9
		Sc. PD:	-0.11
		Sc. rank:	20.6

,

NP			
Pos.	380	obs:	exp:
gaa	E	693	551.60
gag	E	224	365.40
---	--	------	------
mPD		0.37	0.48
		nPD:	0.77
		N. weight:	0.069
		Sc. PD:	0.041
		Sc. rank:	44.5

,

NP			
Pos.	381	obs:	exp:
tta	L	20	80.59
ttg	L	181	171.10
ctt	L	0	155.40
ctc	L	0	153.60
cta	L	24	152.90
ctg	L	692	203.40
---	--	------	------
mPD		0.43	1.1
		nPD:	0.39
		N. weight:	1.1
		Sc. PD:	0.25
		Sc. rank:	561.7

,

NP			
Pos.	382	obs:	exp:
cgt	R	0	39.17
cgc	R	0	46.59
cga	R	2	91.44
cgg	R	0	76.38
aga	R	851	418.70
agg	R	64	244.70
---	--	------	------
mPD		0.13	0.96
		nPD:	0.14
		N. weight:	0.68
		Sc. PD:	-0.0043
		Sc. rank:	115.6

,

NP			
Pos.	383	obs:	exp:
tct	S	0	146.80
tcc	S	0	118.70
tca	S	0	231.40
tcg	S	0	53.36
agt	S	633	187.90
agc	S	284	178.80
---	--	------	------
mPD		0.43	1.7
		nPD:	0.26
		N. weight:	1.2
		Sc. PD:	0.13
		Sc. rank:	463.3

,

NP			
Pos.	384	obs:	exp:
cgt	R	0	38.92
cgc	R	0	46.29
cga	R	0	90.84
cgg	R	0	75.88
aaa	K	5	2.93
aag	K	0	2.07
aga	R	887	415.90
agg	R	24	243.10
ggt	G	0	0.13
ggc	G	0	0.13
gga	G	0	0.46
ggg	G	1	0.28
---	--	------	------
mPD		0.066	0.97
		nPD:	0.07
		N. weight:	0.83
		Sc. PD:	-0.061
		Sc. rank:	-26.0

,

NP			
Pos.	385	obs:	exp:
tat	Y	867	486.20
tac	Y	50	430.80
---	--	------	------
mPD		0.10	0.50
		nPD:	0.21
		N. weight:	0.53
		Sc. PD:	0.031
		Sc. rank:	170.2

,

NP			
Pos.	386	obs:	exp:
tgg	W	917	917.00
---	--	------	------
mPD		0	0
		nPD:	1.
		N. weight:	0.
		Sc. PD:	0
		Sc. rank:	0

,

NP			
Pos.	387	obs:	exp:
gct	A	903	235.00
gcc	A	11	172.20
gca	A	3	428.70
gcg	A	0	81.11
---	--	------	------
mPD		0.030	0.67
		nPD:	0.04
		N. weight:	1.6
		Sc. PD:	-0.15
		Sc. rank:	-215.6

,

NP			
Pos.	388	obs:	exp:
att	I	0	334.60
atc	I	0	221.80
ata	I	917	360.60
---	--	------	------
mPD		0	0.65
		nPD:	0.
		N. weight:	1.1
		Sc. PD:	-0.16
		Sc. rank:	-737.4

,

NP			
Pos.	389	obs:	exp:
cgt	R	0	39.09
cgc	R	0	46.49
cga	R	0	91.24
cgg	R	0	76.21
aaa	K	2	1.17
aag	K	0	0.83
aga	R	879	417.80
agg	R	36	244.20
---	--	------	------
mPD		0.080	0.97
		nPD:	0.08
		N. weight:	0.78
		Sc. PD:	-0.047
		Sc. rank:	-6.3

,

NP			
Pos.	390	obs:	exp:
act	T	1	237.30
acc	T	880	186.50
aca	T	35	420.80
acg	T	0	71.35
gct	A	0	0.26
gcc	A	1	0.19
gca	A	0	0.47
gcg	A	0	0.09
---	--	------	------
mPD		0.078	0.68
		nPD:	0.11
		N. weight:	1.7
		Sc. PD:	-0.05
		Sc. rank:	197.0

,

NP			
Pos.	391	obs:	exp:
cgt	R	0	39.17
cgc	R	0	46.59
cga	R	0	91.44
cgg	R	0	76.38
aga	R	828	418.70
agg	R	89	244.70
---	--	------	------
mPD		0.18	0.96
		nPD:	0.18
		N. weight:	0.63
		Sc. PD:	0.022
		Sc. rank:	181.8

,

NP			
Pos.	392	obs:	exp:
tct	S	0	146.80
tcc	S	0	118.70
tca	S	0	231.40
tcg	S	0	53.36
agt	S	72	187.90
agc	S	845	178.80
---	--	------	------
mPD		0.14	1.7
		nPD:	0.09
		N. weight:	1.7
		Sc. PD:	-0.092
		Sc. rank:	9.6

,

NP			
Pos.	393	obs:	exp:
ggt	G	1	123.00
ggc	G	0	116.50
gga	G	912	424.00
ggg	G	4	253.50
---	--	------	------
mPD		0.011	0.68
		nPD:	0.02
		N. weight:	0.9
		Sc. PD:	-0.11
		Sc. rank:	-332.0

,

NP			
Pos.	394	obs:	exp:
aaa	K	1	0.59
aag	K	0	0.41
ggt	G	0	122.80
ggc	G	0	116.40
gga	G	285	423.50
ggg	G	631	253.30
---	--	------	------
mPD		0.43	0.68
		nPD:	0.64
		N. weight:	0.62
		Sc. PD:	0.29
		Sc. rank:	383.7

,

NP			
Pos.	395	obs:	exp:
aat	N	6	466.50
aac	N	911	450.50
---	--	------	------
mPD		0.013	0.50
		nPD:	0.03
		N. weight:	0.82
		Sc. PD:	-0.093
		Sc. rank:	-221.2

,

NP			
Pos.	396	obs:	exp:
act	T	36	237.30
acc	T	880	186.50
aca	T	0	420.80
acg	T	0	71.35
gct	A	0	0.26
gcc	A	1	0.19
gca	A	0	0.47
gcg	A	0	0.09
---	--	------	------
mPD		0.078	0.68
		nPD:	0.11
		N. weight:	1.7
		Sc. PD:	-0.051
		Sc. rank:	193.0

,

NP			
Pos.	397	obs:	exp:
aat	N	35	466.50
aac	N	882	450.50
---	--	------	------
mPD		0.074	0.50
		nPD:	0.15
		N. weight:	0.67
		Sc. PD:	0.00066
		Sc. rank:	133.6

,

NP			
Pos.	398	obs:	exp:
cat	H	1	0.57
cac	H	0	0.43
caa	Q	875	470.50
cag	Q	41	445.50
---	--	------	------
mPD		0.088	0.50
		nPD:	0.17
		N. weight:	0.6
		Sc. PD:	0.016
		Sc. rank:	164.6

,

NP			
Pos.	399	obs:	exp:
cat	H	2	1.15
cac	H	0	0.85
caa	Q	31	470.00
cag	Q	884	445.00
---	--	------	------
mPD		0.070	0.50
		nPD:	0.14
		N. weight:	0.7
		Sc. PD:	-0.0049
		Sc. rank:	117.0

,

NP			
Pos.	400	obs:	exp:
cgt	R	0	39.09
cgc	R	0	46.49
cga	R	0	91.24
cgg	R	0	76.21
aaa	K	0	1.17
aag	K	2	0.83
aga	R	125	417.80
agg	R	790	244.20
---	--	------	------
mPD		0.24	0.97
		nPD:	0.25
		N. weight:	1.
		Sc. PD:	0.1
		Sc. rank:	383.3

,

NP			
Pos.	401	obs:	exp:
gct	A	0	235.00
gcc	A	1	172.20
gca	A	914	428.70
gcg	A	2	81.11
---	--	------	------
mPD		0.0065	0.67
		nPD:	0.01
		N. weight:	0.9
		Sc. PD:	-0.12
		Sc. rank:	-415.6

,

NP			
Pos.	402	obs:	exp:
tct	S	897	146.80
tcc	S	12	118.70
tca	S	2	231.40
tcg	S	6	53.36
agt	S	0	187.90
agc	S	0	178.80
---	--	------	------
mPD		0.043	1.7
		nPD:	0.03
		N. weight:	2.1
		Sc. PD:	-0.24
		Sc. rank:	-570.1

,

NP			
Pos.	403	obs:	exp:
gct	A	2	235.00
gcc	A	0	172.20
gca	A	881	428.70
gcg	A	34	81.11
---	--	------	------
mPD		0.076	0.67
		nPD:	0.11
		N. weight:	0.8
		Sc. PD:	-0.025
		Sc. rank:	80.6

,

NP			
Pos.	404	obs:	exp:
ggt	G	0	123.00
ggc	G	1	116.50
gga	G	890	424.00
ggg	G	26	253.50
---	--	------	------
mPD		0.057	0.68
		nPD:	0.08
		N. weight:	0.8
		Sc. PD:	-0.046
		Sc. rank:	-3.6

,

NP			
Pos.	405	obs:	exp:
caa	Q	96	471.00
cag	Q	821	446.00
---	--	------	------
mPD		0.19	0.50
		nPD:	0.38
		N. weight:	0.47
		Sc. PD:	0.1
		Sc. rank:	244.0

,

NP			
Pos.	406	obs:	exp:
att	I	38	49.62
atc	I	97	32.89
ata	I	1	53.48
act	T	0	0.26
acc	T	1	0.20
aca	T	0	0.46
acg	T	0	0.08
gtt	V	1	161.00
gtc	V	779	156.30
gta	V	0	161.50
gtg	V	0	301.20
---	--	------	------
mPD		0.34	1.0
		nPD:	0.34
		N. weight:	1.8
		Sc. PD:	0.33
		Sc. rank:	849.4

,

NP			
Pos.	407	obs:	exp:
tct	S	0	146.80
tcc	S	0	118.70
tca	S	0	231.40
tcg	S	0	53.36
agt	S	48	187.90
agc	S	869	178.80
---	--	------	------
mPD		0.099	1.7
		nPD:	0.06
		N. weight:	1.7
		Sc. PD:	-0.14
		Sc. rank:	-109.8

,

NP			
Pos.	408	obs:	exp:
att	I	8	2.92
atc	I	0	1.93
ata	I	0	3.15
gtt	V	799	187.60
gtc	V	22	182.10
gta	V	76	188.20
gtg	V	12	351.00
---	--	------	------
mPD		0.24	0.74
		nPD:	0.32
		N. weight:	1.4
		Sc. PD:	0.22
		Sc. rank:	597.7

,

NP			
Pos.	409	obs:	exp:
caa	Q	831	471.00
cag	Q	86	446.00
---	--	------	------
mPD		0.17	0.50
		nPD:	0.34
		N. weight:	0.44
		Sc. PD:	0.082
		Sc. rank:	208.7

,

NP			
Pos.	410	obs:	exp:
cct	P	46	233.20
ccc	P	867	163.50
cca	P	4	374.60
ccg	P	0	145.70
---	--	------	------
mPD		0.10	0.71
		nPD:	0.15
		N. weight:	1.8
		Sc. PD:	-0.00054
		Sc. rank:	342.8

,

NP			
Pos.	411	obs:	exp:
act	T	869	237.50
acc	T	2	186.70
aca	T	36	421.30
acg	T	10	71.43
---	--	------	------
mPD		0.10	0.67
		nPD:	0.15
		N. weight:	1.3
		Sc. PD:	0.0037
		Sc. rank:	273.7

,

NP			
Pos.	412	obs:	exp:
ttt	F	4	361.50
ttc	F	912	554.50
atg	M	1	1.00
---	--	------	------
mPD		0.013	0.48
		nPD:	0.03
		N. weight:	0.58
		Sc. PD:	-0.066
		Sc. rank:	-149.5

,

NP			
Pos.	413	obs:	exp:
tct	S	49	146.80
tcc	S	1	118.70
tca	S	598	231.40
tcg	S	269	53.36
agt	S	0	187.90
agc	S	0	178.80
---	--	------	------
mPD		0.49	1.7
		nPD:	0.29
		N. weight:	1.3
		Sc. PD:	0.18
		Sc. rank:	533.6

,

NP			
Pos.	414	obs:	exp:
tta	L	1	0.09
ttg	L	0	0.19
ctt	L	0	0.17
ctc	L	0	0.17
cta	L	0	0.17
ctg	L	0	0.22
att	I	0	2.19
atc	I	0	1.45
ata	I	6	2.36
gtt	V	5	187.90
gtc	V	0	182.30
gta	V	864	188.40
gtg	V	41	351.40
---	--	------	------
mPD		0.11	0.74
		nPD:	0.15
		N. weight:	1.6
		Sc. PD:	0.0059
		Sc. rank:	336.1

,

NP			
Pos.	415	obs:	exp:
caa	Q	9	471.00
cag	Q	908	446.00
---	--	------	------
mPD		0.019	0.50
		nPD:	0.04
		N. weight:	0.81
		Sc. PD:	-0.083
		Sc. rank:	-148.9

,

NP			
Pos.	416	obs:	exp:
cgt	R	0	39.17
cgc	R	0	46.59
cga	R	0	91.44
cgg	R	0	76.38
aga	R	910	418.70
agg	R	7	244.70
---	--	------	------
mPD		0.015	0.96
		nPD:	0.02
		N. weight:	0.91
		Sc. PD:	-0.11
		Sc. rank:	-345.4

,

NP			
Pos.	417	obs:	exp:
tct	S	0	0.64
tcc	S	0	0.52
tca	S	0	1.01
tcg	S	0	0.23
aat	N	340	463.50
aac	N	571	447.50
agt	S	3	0.82
agc	S	1	0.78
gat	D	1	1.08
gac	D	1	0.92
---	--	------	------
mPD		0.48	0.52
		nPD:	0.93
		N. weight:	0.057
		Sc. PD:	0.043
		Sc. rank:	37.2

,

NP			
Pos.	418	obs:	exp:
tta	L	0	80.59
ttg	L	0	171.10
ctt	L	831	155.40
ctc	L	86	153.60
cta	L	0	152.90
ctg	L	0	203.40
---	--	------	------
mPD		0.17	1.1
		nPD:	0.15
		N. weight:	1.8
		Sc. PD:	0.012
		Sc. rank:	394.9

,

NP			
Pos.	419	obs:	exp:
cct	P	542	233.20
ccc	P	317	163.50
cca	P	57	374.60
ccg	P	1	145.70
---	--	------	------
mPD		0.53	0.71
		nPD:	0.74
		N. weight:	0.75
		Sc. PD:	0.42
		Sc. rank:	476.3

,

NP			
Pos.	420	obs:	exp:
ttt	F	31	361.90
ttc	F	886	555.10
---	--	------	------
mPD		0.065	0.48
		nPD:	0.14
		N. weight:	0.45
		Sc. PD:	-0.0039
		Sc. rank:	71.4

,

NP			
Pos.	421	obs:	exp:
gct	A	0	0.26
gcc	A	0	0.19
gca	A	1	0.47
gcg	A	0	0.09
gaa	E	869	551.00
gag	E	47	365.00
---	--	------	------
mPD		0.10	0.48
		nPD:	0.21
		N. weight:	0.4
		Sc. PD:	0.023
		Sc. rank:	129.0

,

NP			
Pos.	422	obs:	exp:
ttt	F	1	0.39
ttc	F	0	0.61
cgt	R	0	39.09
cgc	R	0	46.49
cga	R	2	91.24
cgg	R	0	76.21
aaa	K	0	0.59
aag	K	1	0.41
aga	R	909	417.80
agg	R	4	244.20
---	--	------	------
mPD		0.024	0.97
		nPD:	0.02
		N. weight:	0.91
		Sc. PD:	-0.11
		Sc. rank:	-261.7

,

NP			
Pos.	423	obs:	exp:
tct	S	0	0.64
tcc	S	0	0.52
tca	S	3	1.01
tcg	S	1	0.23
act	T	0	0.52
acc	T	0	0.41
aca	T	2	0.92
acg	T	0	0.16
agt	S	0	0.82
agc	S	0	0.78
gtt	V	0	0.21
gtc	V	0	0.20
gta	V	1	0.21
gtg	V	0	0.39
gct	A	1	232.70
gcc	A	0	170.50
gca	A	838	424.50
gcg	A	69	80.31
ggt	G	0	0.27
ggc	G	0	0.25
gga	G	2	0.92
ggg	G	0	0.55
---	--	------	------
mPD		0.16	0.70
		nPD:	0.23
		N. weight:	0.75
		Sc. PD:	0.063
		Sc. rank:	263.4

,

NP			
Pos.	424	obs:	exp:
act	T	2	237.50
acc	T	915	186.70
aca	T	0	421.30
acg	T	0	71.43
---	--	------	------
mPD		0.0044	0.67
		nPD:	0.01
		N. weight:	1.9
		Sc. PD:	-0.25
		Sc. rank:	-1001.2

,

NP			
Pos.	425	obs:	exp:
att	I	857	334.60
atc	I	59	221.80
ata	I	1	360.60
---	--	------	------
mPD		0.12	0.65
		nPD:	0.19
		N. weight:	0.96
		Sc. PD:	0.038
		Sc. rank:	285.0

,

NP			
Pos.	426	obs:	exp:
att	I	0	0.73
atc	I	0	0.48
ata	I	2	0.79
atg	M	915	915.00
---	--	------	------
mPD		0.0044	0.0044
		nPD:	1.
		N. weight:	0.0025
		Sc. PD:	0.002
		Sc. rank:	1.6

,

NP			
Pos.	427	obs:	exp:
gct	A	2	235.00
gcc	A	0	172.20
gca	A	860	428.70
gcg	A	55	81.11
---	--	------	------
mPD		0.12	0.67
		nPD:	0.17
		N. weight:	0.76
		Sc. PD:	0.02
		Sc. rank:	208.8

,

NP			
Pos.	428	obs:	exp:
act	T	0	1.55
acc	T	0	1.22
aca	T	6	2.76
acg	T	0	0.47
gct	A	37	233.20
gcc	A	0	170.90
gca	A	840	425.50
gcg	A	33	80.49
gaa	E	1	0.60
gag	E	0	0.40
---	--	------	------
mPD		0.16	0.69
		nPD:	0.23
		N. weight:	0.64
		Sc. PD:	0.052
		Sc. rank:	223.3

,

NP

Pos. 429		obs:	exp:
ttt	F	869	361.90
ttc	F	48	555.10
mPD		0.099	0.48
		nPD:	0.21
		N. weight:	0.86
		Sc. PD:	0.05
		Sc. rank:	279.4

NP

Pos. 430		obs:	exp:
att	I	0	0.36
atc	I	0	0.24
ata	I	1	0.39
act	T	2	235.50
acc	T	3	185.10
aca	T	877	417.60
acg	T	27	70.81
aaa	K	1	0.59
aag	K	0	0.41
gct	A	0	1.54
gcc	A	0	1.13
gca	A	6	2.81
gcg	A	0	0.53
mPD		0.085	0.69
		nPD:	0.12
		N. weight:	0.82
		Sc. PD:	-0.018
		Sc. rank:	111.8

NP

Pos. 431		obs:	exp:
cgt	R	0	0.04
cgc	R	0	0.05
cga	R	1	0.10
cgg	R	0	0.08
aga	R	0	0.46
agg	R	0	0.27
gtt	V	2	0.41
gtc	V	0	0.40
gta	V	0	0.41
gtg	V	0	0.77
ggt	G	20	122.60
ggc	G	0	116.10
gga	G	830	422.60
ggg	G	64	252.70
mPD		0.18	0.68
		nPD:	0.26
		N. weight:	0.59
		Sc. PD:	0.066
		Sc. rank:	233.9

NP

Pos. 432		obs:	exp:
aat	N	867	466.50
aac	N	50	450.50
mPD		0.10	0.50
		nPD:	0.21
		N. weight:	0.57
		Sc. PD:	0.033
		Sc. rank:	182.4

NP

Pos. 433		obs:	exp:
act	T	603	208.30
acc	T	4	163.70
aca	T	195	369.40
acg	T	2	62.63
aat	N	94	47.82
aac	N	0	46.18
gct	A	18	4.87
gcc	A	0	3.57
gca	A	1	8.88
gcg	A	0	1.68
mPD		0.57	0.91
		nPD:	0.63
		N. weight:	0.78
		Sc. PD:	0.35
		Sc. rank:	480.1

NP

Pos. 434		obs:	exp:
gaa	E	72	551.60
gag	E	845	365.40
mPD		0.14	0.48
		nPD:	0.3
		N. weight:	0.75
		Sc. PD:	0.11
		Sc. rank:	319.5

NP

Pos. 435		obs:	exp:
ggt	G	739	123.00
ggc	G	93	116.50
gga	G	0	424.00
ggg	G	85	253.50
mPD		0.33	0.68
		nPD:	0.49
		N. weight:	1.6
		Sc. PD:	0.53
		Sc. rank:	946.5

NP

Pos. 436		obs:	exp:
cgt	R	0	39.17
cgc	R	0	46.59
cga	R	0	91.44
cgg	R	0	76.38
aga	R	912	418.70
agg	R	5	244.70
mPD		0.011	0.96
		nPD:	0.01
		N. weight:	0.92
		Sc. PD:	-0.12
		Sc. rank:	-413.1

NP

Pos. 437		obs:	exp:
atg	M	1	1.00
act	T	45	237.30
acc	T	1	186.50
aca	T	56	420.80
acg	T	814	71.35
mPD		0.21	0.68
		nPD:	0.3
		N. weight:	2.4
		Sc. PD:	0.36
		Sc. rank:	1036.0

NP

Pos. 438		obs:	exp:
tct	S	786	146.80
tcc	S	69	118.70
tca	S	62	231.40
tcg	S	0	53.36
agt	S	0	187.90
agc	S	0	178.80
mPD		0.26	1.7
		nPD:	0.15
		N. weight:	1.6
		Sc. PD:	0.013
		Sc. rank:	360.3

NP

Pos. 439		obs:	exp:
tat	Y	0	0.53
tac	Y	1	0.47
gat	D	7	494.50
gac	D	909	421.50
mPD		0.017	0.50
		nPD:	0.03
		N. weight:	0.89
		Sc. PD:	-0.094
		Sc. rank:	-195.0

NP

Pos. 440		obs:	exp:
atg	M	917	917.00
mPD		0	0
		nPD:	1.
		N. weight:	0.
		Sc. PD:	0
		Sc. rank:	0

NP

Pos. 441		obs:	exp:
cgt	R	0	39.17
cgc	R	0	46.59
cga	R	0	91.44
cgg	R	0	76.38
aga	R	73	418.70
agg	R	844	244.70
mPD		0.15	0.96
		nPD:	0.15
		N. weight:	1.2
		Sc. PD:	0.0072
		Sc. rank:	264.3

NP

Pos. 442		obs:	exp:
tct	S	0	0.16
tcc	S	0	0.13
tca	S	0	0.25
tcg	S	0	0.06
act	T	872	237.00
acc	T	22	186.30
aca	T	21	420.40
acg	T	0	71.28
agt	S	1	0.20
agc	S	0	0.20
gct	A	1	0.26
gcc	A	0	0.19
gca	A	0	0.47
gcg	A	0	0.09
mPD		0.095	0.68
		nPD:	0.14
		N. weight:	1.4
		Sc. PD:	-0.008
		Sc. rank:	237.2

NP

Pos. 443		obs:	exp:
gaa	E	784	551.60
gag	E	133	365.40
mPD		0.25	0.48
		nPD:	0.52
		N. weight:	0.19
		Sc. PD:	0.068
		Sc. rank:	113.4

NP

Pos. 444		obs:	exp:
att	I	5	334.60
atc	I	911	221.80
ata	I	1	360.60
mPD		0.013	0.65
		nPD:	0.02
		N. weight:	1.7
		Sc. PD:	-0.2
		Sc. rank:	-566.8

NP

Pos. 445		obs:	exp:
att	I	0	334.60
atc	I	1	221.80
ata	I	916	360.60
mPD		0.0022	0.65
		nPD:	0.
		N. weight:	1.1
		Sc. PD:	-0.15
		Sc. rank:	-632.7

NP

Pos. 446		obs:	exp:
cgt	R	0	38.28
cgc	R	0	45.52
cga	R	0	89.34
cgg	R	0	74.63
aaa	K	3	12.30
aag	K	18	8.70
aga	R	645	409.10
agg	R	251	239.10
mPD		0.46	1.0
		nPD:	0.46
		N. weight:	0.42
		Sc. PD:	0.13
		Sc. rank:	238.5

NP

Pos. 447		obs:	exp:
atg	M	914	914.00
act	T	0	0.78
acc	T	0	0.61
aca	T	0	1.38
acg	T	3	0.23
mPD		0.0065	0.013
		nPD:	0.52
		N. weight:	0.01
		Sc. PD:	0.0036
		Sc. rank:	6.0

NP

Pos. 448		obs:	exp:
atg	M	917	917.00
mPD		0	0
		nPD:	1.
		N. weight:	0.
		Sc. PD:	0
		Sc. rank:	0

NP

Pos. 449		obs:	exp:
gaa	E	909	551.60
gag	E	8	365.40
mPD		0.017	0.48
		nPD:	0.04
		N. weight:	0.56
		Sc. PD:	-0.059
		Sc. rank:	-115.4

NP

Pos. 450		obs:	exp:
tct	S	0	138.60
tcc	S	0	112.10
tca	S	0	218.60
tcg	S	0	50.40
aat	N	48	25.44
aac	N	2	24.56
agt	S	865	177.50
agc	S	1	168.90
ggt	G	1	0.13
ggc	G	0	0.13
gga	G	0	0.46
ggg	G	0	0.28
mPD		0.11	1.7
		nPD:	0.07
		N. weight:	1.9
		Sc. PD:	-0.14
		Sc. rank:	-70.4

NP

Pos. 451		obs:	exp:
gct	A	4	235.00
gcc	A	887	172.20
gca	A	26	428.70
gcg	A	0	81.11
mPD		0.064	0.67
		nPD:	0.09
		N. weight:	1.8
		Sc. PD:	-0.089
		Sc. rank:	55.6

NP

Pos. 452		obs:	exp:
cgt	R	0	38.23
cgc	R	0	45.47
cga	R	0	89.24
cgg	R	0	74.54
aaa	K	22	12.89
aag	K	0	9.11
aga	R	893	408.60
agg	R	2	238.90
mPD		0.051	1.0
		nPD:	0.05
		N. weight:	0.93
		Sc. PD:	-0.084
		Sc. rank:	-100.0

NP

Pos. 453		obs:	exp:
cct	P	45	231.90
ccc	P	0	162.60
cca	P	858	372.60
ccg	P	9	144.90
caa	Q	1	0.51
cag	Q	0	0.49
act	T	0	1.04
acc	T	0	0.81
aca	T	4	1.84
acg	T	0	0.31
mPD		0.12	0.72
		nPD:	0.17
		N. weight:	0.83
		Sc. PD:	0.019
		Sc. rank:	216.7

NP			
Pos.	454	obs:	exp:
gaa	E	888	551.60
gag	E	29	365.40
---	--	------	------
mPD		0.061	0.48
		nPD:	0.13
		N. weight:	0.47
		Sc. PD:	-0.008
		Sc. rank:	65.6

,

NP			
Pos.	455	obs:	exp:
gat	D	915	494.50
gac	D	1	421.50
gaa	E	1	0.60
gag	E	0	0.40
---	--	------	------
mPD		0.0044	0.50
		nPD:	0.01
		N. weight:	0.74
		Sc. PD:	-0.097
		Sc. rank:	-370.0

,

NP			
Pos.	456	obs:	exp:
atg	M	2	2.00
gtt	V	0	187.60
gtc	V	0	182.10
gta	V	0	188.20
gtg	V	909	351.00
gct	A	0	1.54
gcc	A	0	1.13
gca	A	0	2.81
gcg	A	6	0.53
---	--	------	------
mPD		0.017	0.74
		nPD:	0.02
		N. weight:	1.2
		Sc. PD:	-0.14
		Sc. rank:	-343.4

,

NP			
Pos.	457	obs:	exp:
tct	S	84	146.80
tcc	S	6	118.70
tca	S	623	231.40
tcg	S	204	53.36
agt	S	0	187.90
agc	S	0	178.80
---	--	------	------
mPD		0.48	1.7
		nPD:	0.29
		N. weight:	1.1
		Sc. PD:	0.15
		Sc. rank:	461.9

,

NP			
Pos.	458	obs:	exp:
ttt	F	0	361.90
ttc	F	917	555.10
---	--	------	------
mPD		0	0.48
		nPD:	0.
		N. weight:	0.61
		Sc. PD:	-0.085
		Sc. rank:	-396.6

,

NP			
Pos.	459	obs:	exp:
cat	H	1	0.57
cac	H	0	0.43
caa	Q	4	470.50
cag	Q	912	445.50
---	--	------	------
mPD		0.011	0.50
		nPD:	0.02
		N. weight:	0.84
		Sc. PD:	-0.1
		Sc. rank:	-274.0

,

NP			
Pos.	460	obs:	exp:
ggt	G	6	123.00
ggc	G	0	116.50
gga	G	11	424.00
ggg	G	900	253.50
---	--	------	------
mPD		0.037	0.68
		nPD:	0.05
		N. weight:	1.4
		Sc. PD:	-0.13
		Sc. rank:	-127.9

,

NP			
Pos.	461	obs:	exp:
cgt	R	0	39.17
cgc	R	0	46.59
cga	R	0	91.44
cgg	R	917	76.38
aga	R	0	418.70
agg	R	0	244.70
---	--	------	------
mPD		0	0.96
		nPD:	0.
		N. weight:	3.
		Sc. PD:	-0.42
		Sc. rank:	-1963.9

,

NP			
Pos.	462	obs:	exp:
ggt	G	0	123.00
ggc	G	0	116.50
gga	G	915	424.00
ggg	G	2	253.50
---	--	------	------
mPD		0.0044	0.68
		nPD:	0.01
		N. weight:	0.92
		Sc. PD:	-0.12
		Sc. rank:	-488.4

,

NP			
Pos.	463	obs:	exp:
gtt	V	0	189.10
gtc	V	913	183.50
gta	V	2	189.70
gtg	V	1	353.80
gct	A	0	0.26
gcc	A	1	0.19
gca	A	0	0.47
gcg	A	0	0.09
---	--	------	------
mPD		0.0087	0.73
		nPD:	0.01
		N. weight:	1.9
		Sc. PD:	-0.25
		Sc. rank:	-846.8

,

NP			
Pos.	464	obs:	exp:
ttt	F	2	361.90
ttc	F	915	555.10
---	--	------	------
mPD		0.0044	0.48
		nPD:	0.01
		N. weight:	0.59
		Sc. PD:	-0.077
		Sc. rank:	-292.9

,

NP			
Pos.	465	obs:	exp:
gaa	E	0	551.60
gag	E	917	365.40
---	--	------	------
mPD		0	0.48
		nPD:	0.
		N. weight:	1.1
		Sc. PD:	-0.16
		Sc. rank:	-727.0

,

NP			
Pos.	466	obs:	exp:
ttt	F	1	1.97
ttc	F	4	3.03
tta	L	1	80.07
ttg	L	0	170.00
ctt	L	2	154.40
ctc	L	836	152.60
cta	L	72	151.90
ctg	L	0	202.00
gtt	V	0	0.21
gtc	V	1	0.20
gta	V	0	0.21
gtg	V	0	0.39
---	--	------	------
mPD		0.17	1.1
		nPD:	0.15
		N. weight:	1.8
		Sc. PD:	0.0076
		Sc. rank:	383.4

,

NP			
Pos.	467	obs:	exp:
tct	S	1	146.80
tcc	S	0	118.70
tca	S	0	231.40
tcg	S	916	53.36
agt	S	0	187.90
agc	S	0	178.80
---	--	------	------
mPD		0.0022	1.7
		nPD:	0.
		N. weight:	3.5
		Sc. PD:	-0.47
		Sc. rank:	-2052.9

,

NP			
Pos.	468	obs:	exp:
gat	D	0	495.00
gac	D	917	422.00
---	--	------	------
mPD		0	0.50
		nPD:	0.
		N. weight:	0.94
		Sc. PD:	-0.13
		Sc. rank:	-613.3

,

NP			
Pos.	469	obs:	exp:
caa	Q	1	0.51
cag	Q	0	0.49
gaa	E	916	551.00
gag	E	0	365.00
---	--	------	------
mPD		0.0022	0.48
		nPD:	0.
		N. weight:	0.62
		Sc. PD:	-0.083
		Sc. rank:	-333.9

,

NP			
Pos.	470	obs:	exp:
cgt	R	0	0.09
cgc	R	0	0.10
cga	R	0	0.20
cgg	R	0	0.17
aaa	K	0	536.00
aag	K	915	379.00
aga	R	0	0.91
agg	R	2	0.53
---	--	------	------
mPD		0.0044	0.49
		nPD:	0.01
		N. weight:	1.1
		Sc. PD:	-0.14
		Sc. rank:	-532.9

,

NP			
Pos.	471	obs:	exp:
gct	A	0	235.00
gcc	A	0	172.20
gca	A	916	428.70
gcg	A	1	81.11
---	--	------	------
mPD		0.0022	0.67
		nPD:	0.
		N. weight:	0.92
		Sc. PD:	-0.12
		Sc. rank:	-523.9

,

NP			
Pos.	472	obs:	exp:
act	T	0	236.80
acc	T	1	186.10
aca	T	1	419.90
acg	T	912	71.20
gct	A	0	0.77
gcc	A	0	0.56
gca	A	0	1.40
gcg	A	3	0.27
---	--	------	------
mPD		0.011	0.68
		nPD:	0.02
		N. weight:	3.1
		Sc. PD:	-0.38
		Sc. rank:	-1149.5

,

NP			
Pos.	473	obs:	exp:
tct	S	0	0.48
tcc	S	0	0.39
tca	S	0	0.76
tcg	S	0	0.17
act	T	0	0.78
acc	T	3	0.61
aca	T	0	1.38
acg	T	0	0.23
aat	N	0	463.50
aac	N	911	447.50
agt	S	0	0.61
agc	S	3	0.59
---	--	------	------
mPD		0.013	0.52
		nPD:	0.03
		N. weight:	0.87
		Sc. PD:	-0.1
		Sc. rank:	-246.2

,

NP			
Pos.	474	obs:	exp:
cct	P	0	233.20
ccc	P	0	163.50
cca	P	0	374.60
ccg	P	917	145.70
---	--	------	------
mPD		0	0.71
		nPD:	0.
		N. weight:	2.2
		Sc. PD:	-0.31
		Sc. rank:	-1453.6

,

NP			
Pos.	475	obs:	exp:
att	I	0	334.60
atc	I	917	221.80
ata	I	0	360.60
---	--	------	------
mPD		0	0.65
		nPD:	0.
		N. weight:	1.7
		Sc. PD:	-0.24
		Sc. rank:	-1121.5

,

NP			
Pos.	476	obs:	exp:
gtt	V	1	189.30
gtc	V	0	183.70
gta	V	0	189.90
gtg	V	916	354.10
---	--	------	------
mPD		0.0022	0.73
		nPD:	0.
		N. weight:	1.1
		Sc. PD:	-0.16
		Sc. rank:	-665.5

,

NP			
Pos.	477	obs:	exp:
cct	P	912	232.90
ccc	P	4	163.30
cca	P	0	374.20
ccg	P	0	145.50
---	-	1	1.00
---	--	------	------
mPD		0.0087	0.71
		nPD:	0.01
		N. weight:	1.6
		Sc. PD:	-0.21
		Sc. rank:	-705.9

,

NP			
Pos.	478	obs:	exp:
tct	S	6	146.60
tcc	S	909	118.60
tca	S	1	231.20
tcg	S	0	53.31
agt	S	0	187.70
agc	S	0	178.60
---	-	1	1.00
---	--	------	------
mPD		0.015	1.7
		nPD:	0.01
		N. weight:	2.4
		Sc. PD:	-0.32
		Sc. rank:	-1150.8

,

NP

Pos.	479	obs:	exp:
ttt	F	915	361.90
ttc	F	2	555.10
---	--	------	------
mPD		0.0044	0.48
		nPD:	0.01
		N. weight:	1.1
		Sc. PD:	-0.14
		Sc. rank:	-548.2

,

NP

Pos.	480	obs:	exp:
tta	L	0	0.09
ttg	L	0	0.19
ctt	L	0	0.17
ctc	L	1	0.17
cta	L	0	0.17
ctg	L	0	0.22
aat	N	0	1.53
aac	N	3	1.47
gat	D	2	492.30
gac	D	910	419.70
gaa	E	1	0.60
gag	E	0	0.40
---	--	------	------
mPD		0.017	0.51
		nPD:	0.03
		N. weight:	0.93
		Sc. PD:	-0.099
		Sc. rank:	-205.2

,

NP

Pos.	481	obs:	exp:
tct	S	1	0.16
tcc	S	0	0.13
tca	S	0	0.25
tcg	S	0	0.06
aat	N	1	0.51
aac	N	0	0.49
agt	S	0	0.20
agc	S	0	0.20
---	-	915	915.00
---	--	------	------
mPD		4.8×10^{-6}	7.9×10^{-6}
		nPD:	0.6
		N. weight:	0.
		Sc. PD:	0
		Sc. rank:	0

,

NP

Pos.	482	obs:	exp:
tat	Y	0	0.53
tac	Y	1	0.47
gaa	E	1	0.60
gag	E	0	0.40
---	-	915	915.00
---	--	------	------
mPD		4.8×10^{-6}	5.9×10^{-6}
		nPD:	0.8
		N. weight:	0.
		Sc. PD:	0
		Sc. rank:	0

,

NP

Pos.	483	obs:	exp:
att	I	0	0.36
atc	I	0	0.24
ata	I	1	0.39
atg	M	916	916.00
---	--	------	------
mPD		0.0022	0.0022
		nPD:	1.
		N. weight:	0.0012
		Sc. PD:	0.001
		Sc. rank:	0.8

,

NP

Pos.	484	obs:	exp:
tct	S	0	26.73
tcc	S	0	21.62
tca	S	0	42.15
tcg	S	0	9.72
aat	N	748	381.60
aac	N	2	368.40
agt	S	145	34.22
agc	S	22	32.57
---	--	------	------
mPD		0.35	1.1
		nPD:	0.33
		N. weight:	0.93
		Sc. PD:	0.16
		Sc. rank:	422.4

,

NP

Pos.	485	obs:	exp:
aat	N	899	465.50
aac	N	16	449.50
aaa	K	1	0.59
aag	K	0	0.41
gaa	E	0	0.60
gag	E	1	0.40
---	--	------	------
mPD		0.041	0.50
		nPD:	0.08
		N. weight:	0.72
		Sc. PD:	-0.045
		Sc. rank:	-14.8

,

NP

Pos.	486	obs:	exp:
aaa	K	0	0.59
aag	K	1	0.41
gaa	E	863	551.00
gag	E	53	365.00
---	--	------	------
mPD		0.11	0.48
		nPD:	0.23
		N. weight:	0.38
		Sc. PD:	0.032
		Sc. rank:	134.2

,

NP

Pos.	487	obs:	exp:
gtt	V	0	0.21
gtc	V	0	0.20
gta	V	1	0.21
gtg	V	0	0.39
ggt	G	3	122.80
ggc	G	1	116.40
gga	G	890	423.50
ggg	G	22	253.30
---	--	------	------
mPD		0.058	0.68
		nPD:	0.08
		N. weight:	0.79
		Sc. PD:	-0.046
		Sc. rank:	-2.0

,

NP

Pos.	488	obs:	exp:
tct	S	917	146.80
tcc	S	0	118.70
tca	S	0	231.40
tcg	S	0	53.36
agt	S	0	187.90
agc	S	0	178.80
---	--	------	------
mPD		0	1.7
		nPD:	0.
		N. weight:	2.2
		Sc. PD:	-0.31
		Sc. rank:	-1447.6

,

NP

Pos.	489	obs:	exp:
tat	Y	916	486.20
tac	Y	1	430.80
---	--	------	------
mPD		0.0022	0.50
		nPD:	0.
		N. weight:	0.76
		Sc. PD:	-0.1
		Sc. rank:	-416.9

,

NP

Pos.	490	obs:	exp:
ttt	F	0	360.70
ttc	F	914	553.30
tta	L	1	0.09
ttg	L	0	0.19
tct	S	0	0.16
tcc	S	1	0.13
tca	S	0	0.25
tcg	S	0	0.06
ctt	L	0	0.17
ctc	L	0	0.17
cta	L	0	0.17
ctg	L	0	0.22
agt	S	0	0.20
agc	S	0	0.20
---	-	1	1.00
---	--	------	------
mPD		0.0044	0.48
		nPD:	0.01
		N. weight:	0.61
		Sc. PD:	-0.08
		Sc. rank:	-304.1

,

NP

Pos.	491	obs:	exp:
ttt	F	1	361.50
ttc	F	915	554.50
tct	S	1	0.16
tcc	S	0	0.13
tca	S	0	0.25
tcg	S	0	0.06
agt	S	0	0.20
agc	S	0	0.20
---	--	------	------
mPD		0.0065	0.48
		nPD:	0.01
		N. weight:	0.6
		Sc. PD:	-0.076
		Sc. rank:	-250.7

,

NP

Pos.	492	obs:	exp:
cgt	R	0	0.04
cgc	R	0	0.05
cga	R	0	0.10
cgg	R	1	0.08
aga	R	0	0.46
agg	R	0	0.27
ggt	G	0	122.80
ggc	G	0	116.40
gga	G	916	423.50
ggg	G	0	253.30
---	--	------	------
mPD		0.0044	0.68
		nPD:	0.01
		N. weight:	0.94
		Sc. PD:	-0.12
		Sc. rank:	-500.2

,

NP

Pos.	493	obs:	exp:
cgt	R	0	0.04
cgc	R	0	0.05
cga	R	0	0.10
cgg	R	0	0.08
aga	R	1	0.46
agg	R	0	0.27
gat	D	0	494.50
gac	D	916	421.50
---	--	------	------
mPD		0.0065	0.50
		nPD:	0.01
		N. weight:	0.94
		Sc. PD:	-0.12
		Sc. rank:	-401.5

,

NP

Pos.	494	obs:	exp:
tct	S	0	0.80
tcc	S	0	0.65
tca	S	0	1.26
tcg	S	0	0.29
caa	Q	1	0.51
cag	Q	0	0.49
aat	N	908	463.00
aac	N	2	447.00
agt	S	5	1.02
agc	S	0	0.98
gat	D	1	0.54
gac	D	0	0.46
---	--	------	------
mPD		0.022	0.52
		nPD:	0.04
		N. weight:	0.81
		Sc. PD:	-0.081
		Sc. rank:	-137.4

,

NP

Pos.	495	obs:	exp:
act	T	0	0.26
acc	T	0	0.20
aca	T	1	0.46
acg	T	0	0.08
gct	A	2	234.50
gcc	A	1	171.80
gca	A	912	427.80
gcg	A	0	80.93
gaa	E	1	0.60
gag	E	0	0.40
---	--	------	------
mPD		0.011	0.68
		nPD:	0.02
		N. weight:	0.9
		Sc. PD:	-0.11
		Sc. rank:	-329.6

,

NP

Pos.	496	obs:	exp:
gaa	E	12	551.00
gag	E	904	365.00
ggt	G	0	0.13
ggc	G	0	0.13
gga	G	0	0.46
ggg	G	1	0.28
---	--	------	------
mPD		0.028	0.48
		nPD:	0.06
		N. weight:	1.
		Sc. PD:	-0.086
		Sc. rank:	-73.7

,

NP			
Pos.	497	obs:	exp:
gat	D	0	1.08
gac	D	2	0.92
gaa	E	63	550.40
gag	E	852	364.60
---	--	------	------
mPD		0.13	0.48
		nPD:	0.27
		N. weight:	0.78
		Sc. PD:	0.096
		Sc. rank:	315.2

,

NP			
Pos.	498	obs:	exp:
ttt	F	3	1.18
ttc	F	0	1.82
tat	Y	913	484.60
tac	Y	1	429.40
---	--	------	------
mPD		0.0087	0.50
		nPD:	0.02
		N. weight:	0.76
		Sc. PD:	-0.094
		Sc. rank:	-272.2

,

NP			
Pos.	499	obs:	exp:
gat	D	3	492.90
gac	D	910	420.10
gaa	E	3	2.41
gag	E	1	1.59
---	--	------	------
mPD		0.015	0.50
		nPD:	0.03
		N. weight:	0.92
		Sc. PD:	-0.1
		Sc. rank:	-214.2

,

NP			
Pos.	500	obs:	exp:
tct	S	0	2.40
tcc	S	0	1.94
tca	S	0	3.79
tcg	S	0	0.87
tgt	C	1	0.41
tgc	C	0	0.59
caa	Q	1	0.51
cag	Q	0	0.49
att	I	1	0.36
atc	I	0	0.24
ata	I	0	0.39
aat	N	896	456.90
aac	N	2	441.10
aaa	K	1	0.59
aag	K	0	0.41
agt	S	15	3.07
agc	S	0	2.93
---	--	------	------
mPD		0.050	0.57
		nPD:	0.09
		N. weight:	0.82
		Sc. PD:	-0.046
		Sc. rank:	1.2

,

NP			
Pos.	501	obs:	exp:
taa	.	172	394.40
tag	.	2	231.90
tga	.	743	290.70
---	--	------	------
mPD		0.31	0.81
		nPD:	0.38
		N. weight:	0.73
		Sc. PD:	0.17
		Sc. rank:	387.0

}

Amino acid level conservation per codon

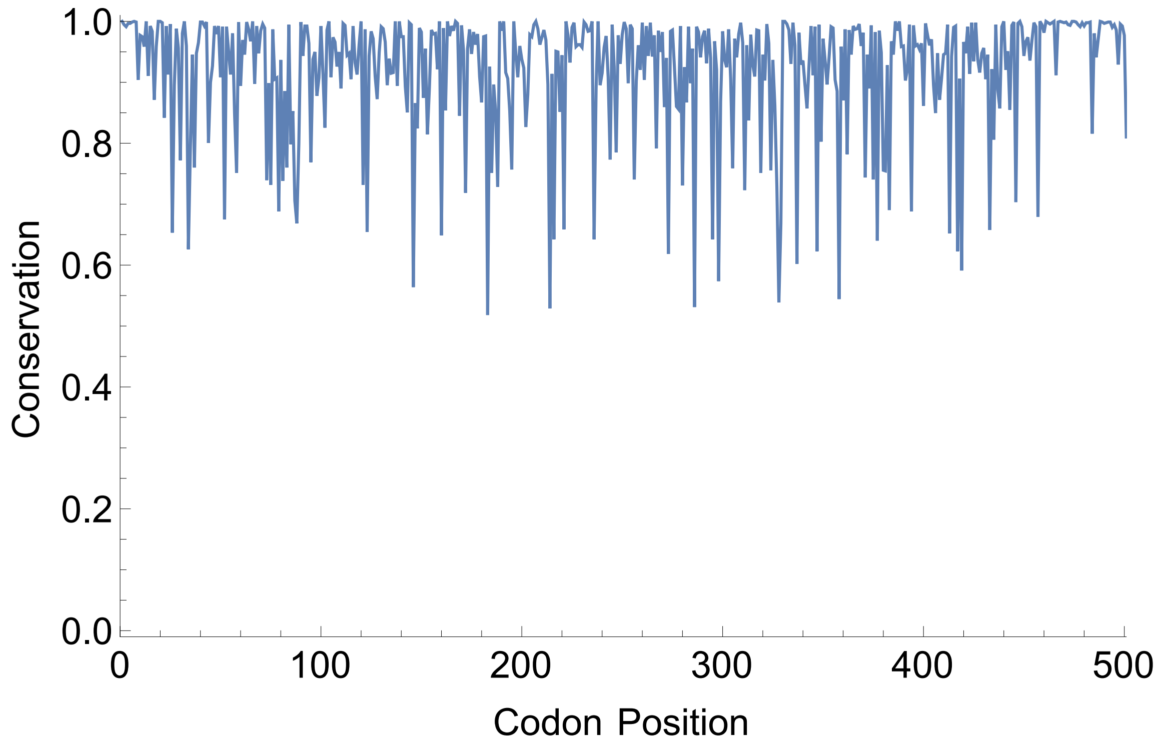

Conservation analysis plots

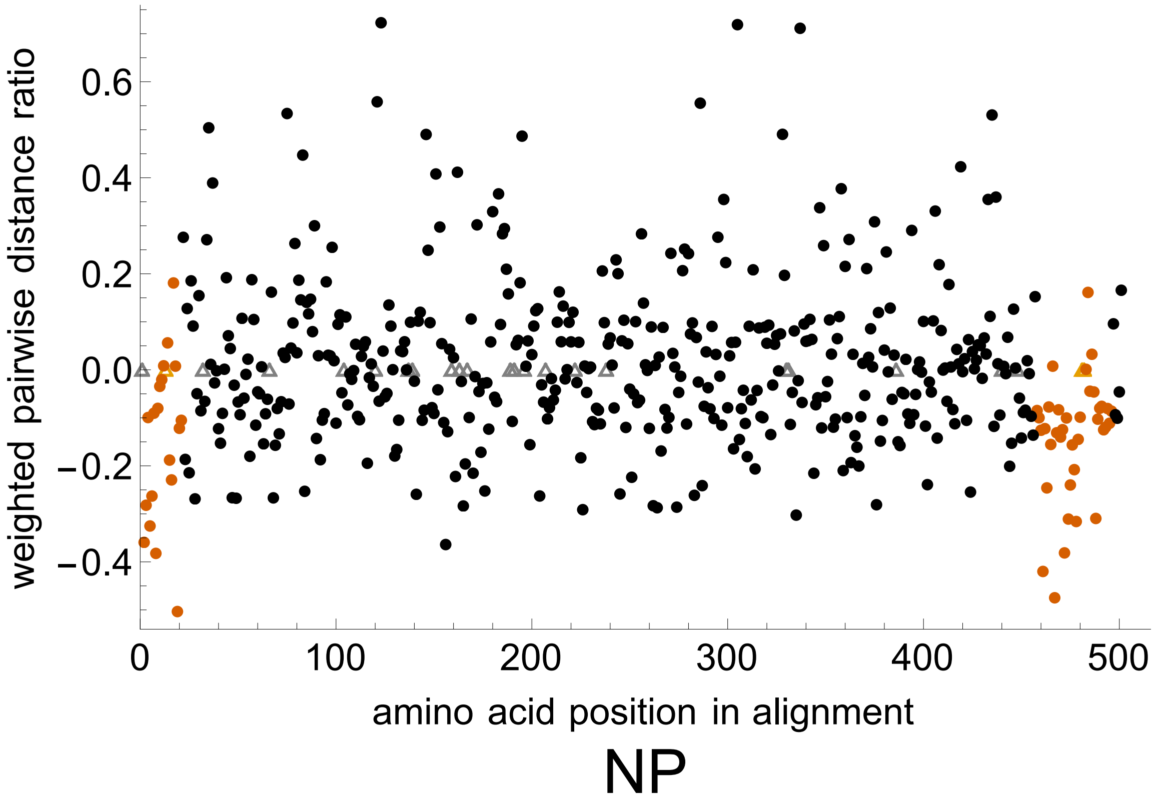

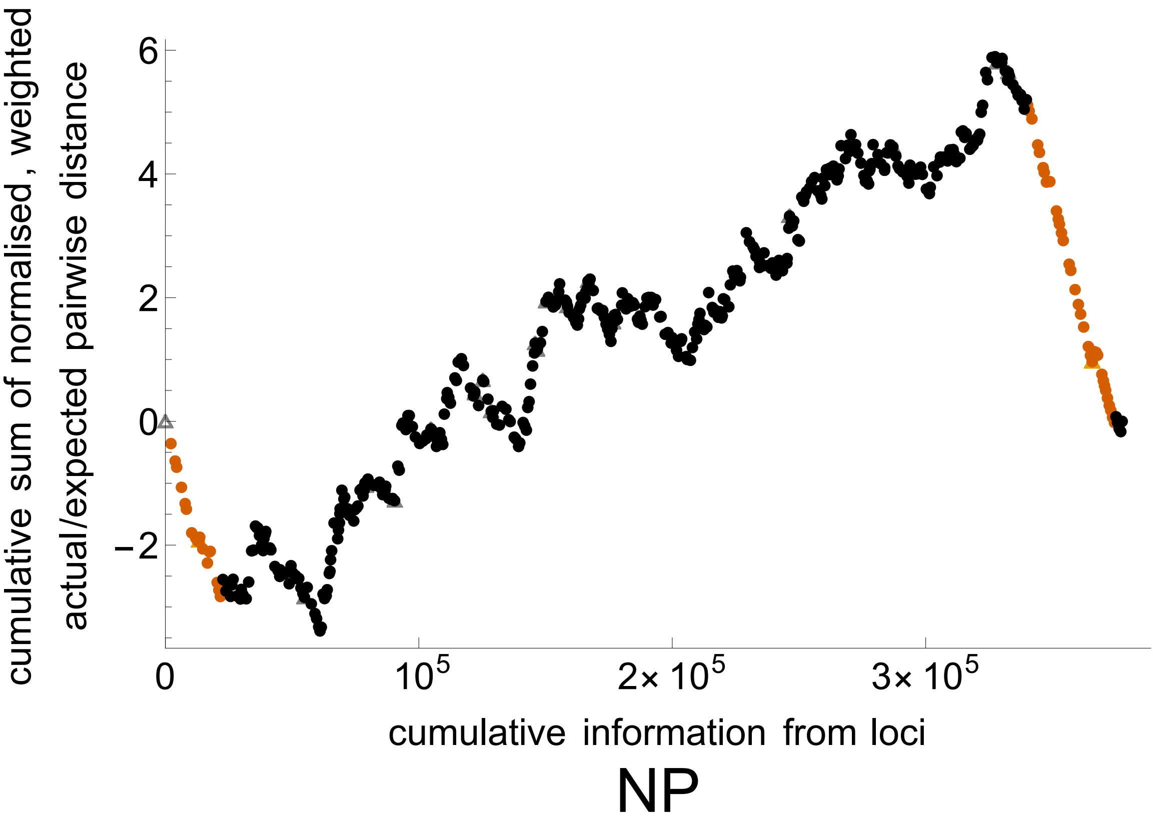

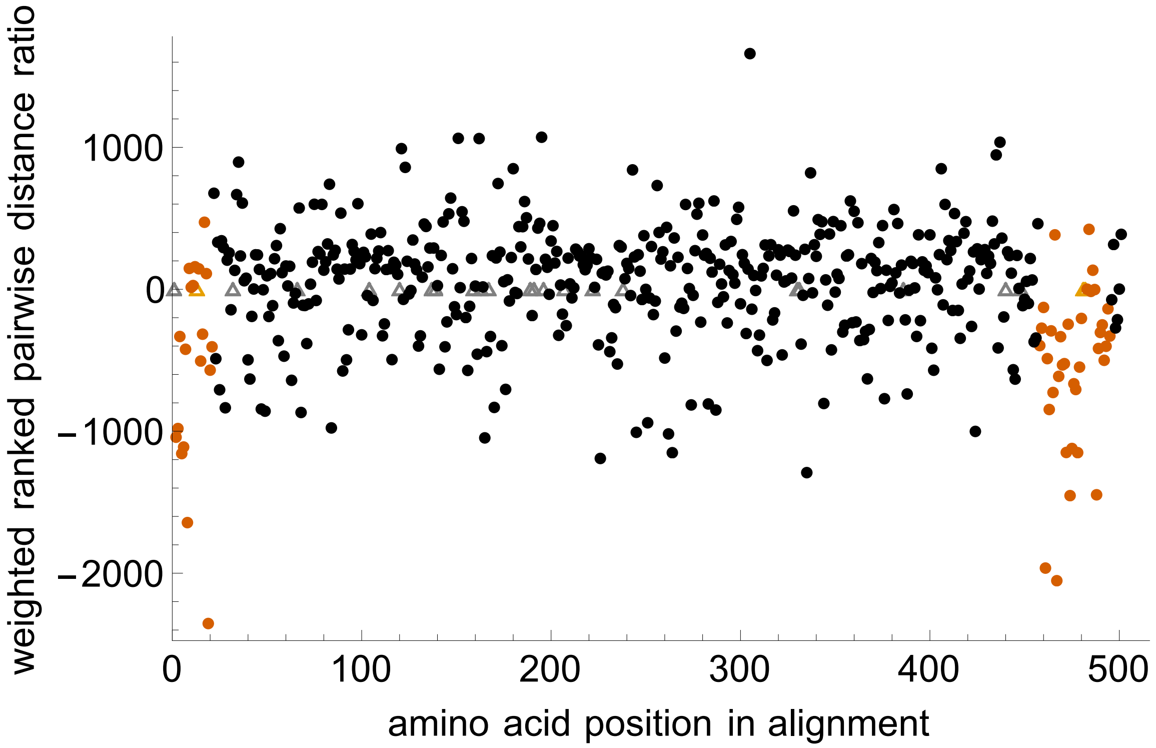
weighted ranked pairwise distance ratio
1000
0
−1000
−2000
0
100
200
300
400
500
amino acid position in alignment

NP

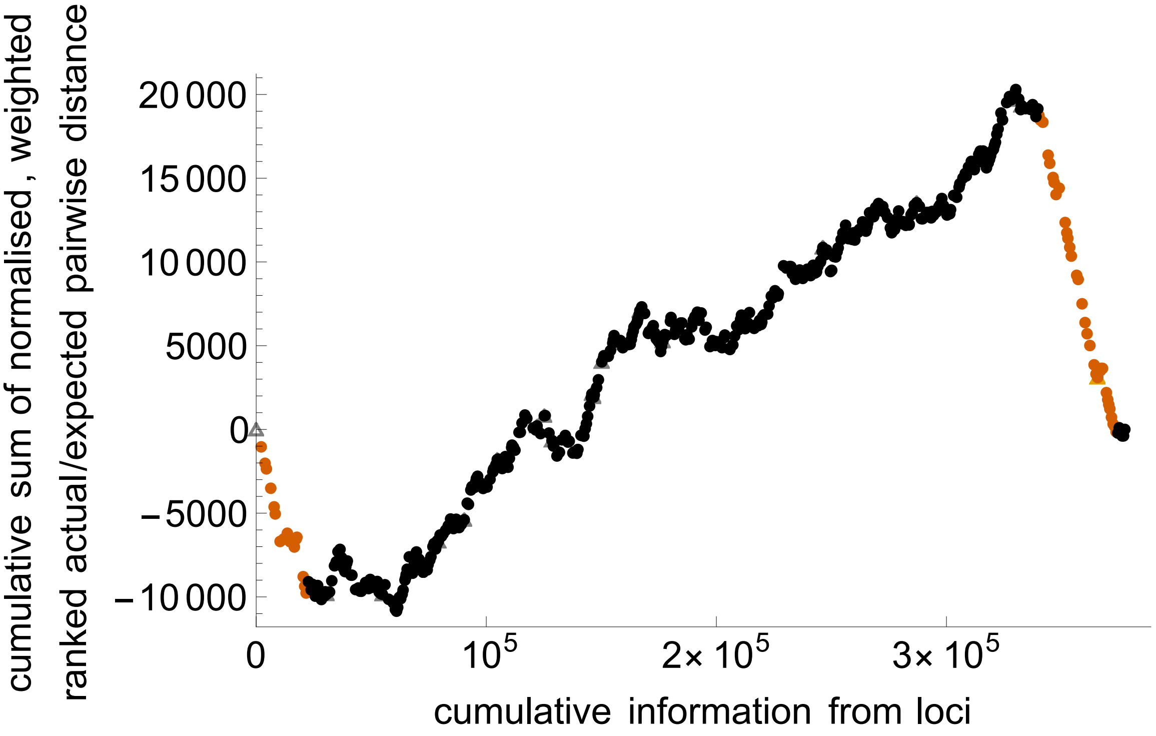
cumulative sum of normalised, weighted ranked actual/expected pairwise distance
20 000
15 000
10 000
5000
0
−5000
−10 000
0
10^5
2×10^5
3×10^5
cumulative information from loci

NP

NA

Gene length histogram

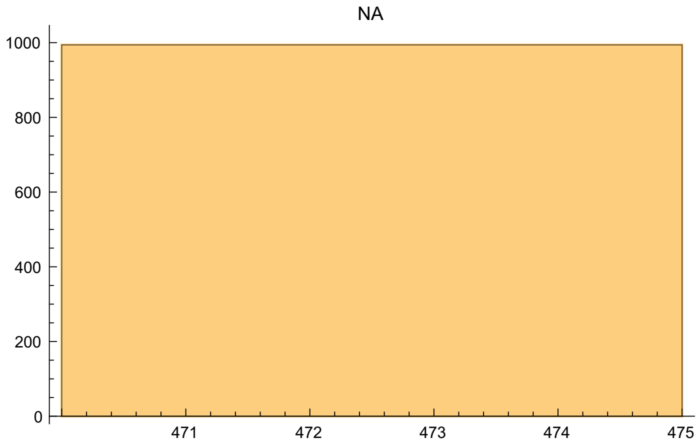

Information vs. nPD

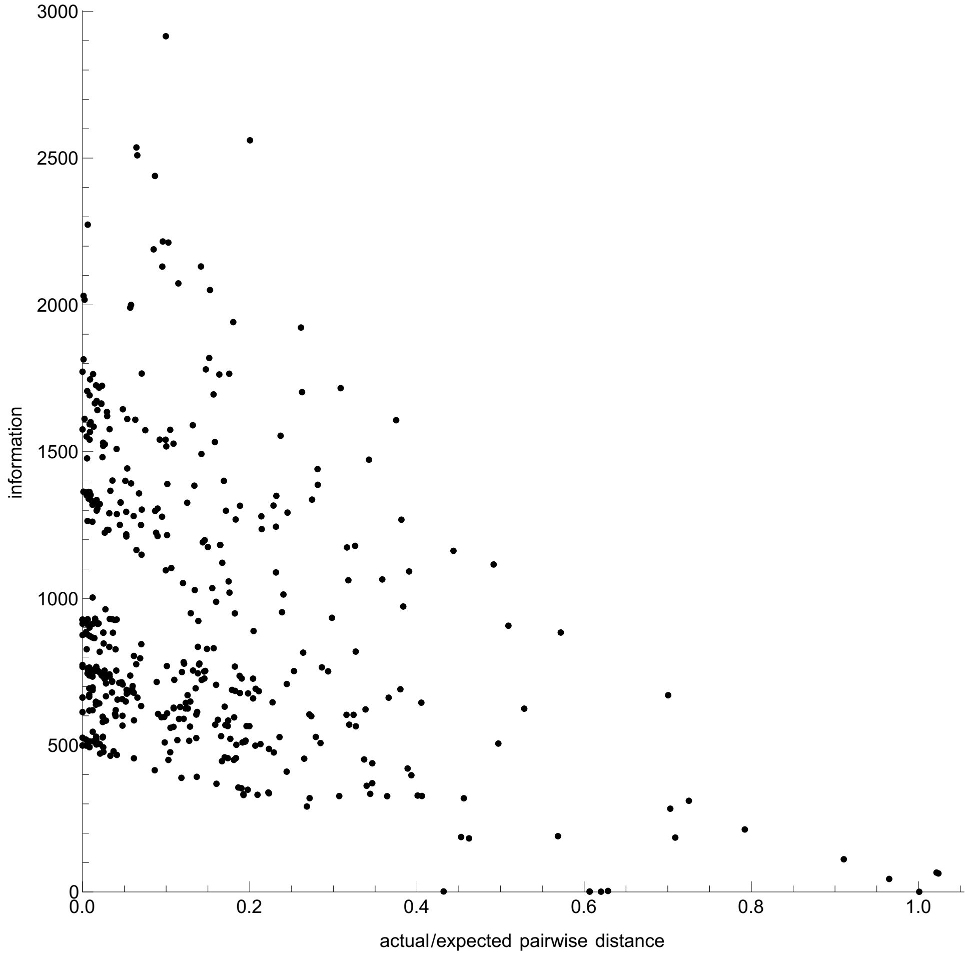

NA

Example sequences highlighted by regions found in analyses to be conserved

Interesting points (by weighted raw PD) highlighted for gene NA:

ATGAATCCAAATCAGAAGATTCTATGCACTTCTGCCACTGCTATTGTAATAGGCACAATTGCAGTACTCATAGGAA⋱
TAGCAAACCTAGGATTGAACATAGGACTACATCTGAAACCAAACTGCAACTGCTCACACTCACAACCTGAAGCAA⋱
CCAATGCAAGCCAAACAATAATAAACAACTACTATAACGAAACAAACATCACCCAAATAAGCAATACCAACATCC⋱
AAATGGAGGAGAAAGCAAATAGAGAATTCAATAACTTGACCAAAGGACTCTGCACTATAAATTCATGGCACATCT⋱
ATGGGAAAGACAATGCAGTAAGAATTGGGGAGAACTCAGATGTTCTAGTAACGAGAGAACCCTATGTCTCCTGTG⋱
ATCCAGATGAGTGCAGG
TTCTATGCTCTCAGCCAAGGAACAACGATTAGAGGGAAACACTCAAATGGAACAA
TACACGATAGATCCCAGTACCGTGCCCTGATAAGCTGGCCACTGTCATCACC
ACCCACAGTATACAACAGCAGAGTAGAATGCATTGGTTGGTCAAGTACTAGTTGTCATGATGGCAGAGC⋱
CAGGATGTCAATATGTGTATCAGGCCCAAACAACAATGCGTCTGCAGTAATCTGGTACAATAGAAGGCCT⋱
GTTACAGAAATCAATACATGGGCCCGAAACATACTACGAACACAAGAATCTGAATGCGTATGCCACAACG⋱
GTGTATGCCCGGTAGTATTCACAGATGGGTCTGCCACTGGACCTGCAGAAACAAGAGTGTACTATTTCAA⋱
GGAAGGGAAAATACTAAAATGGGAACCTCTAACTGGAACTGCTAAACACATTGAAGAATGCTCATGCTAT⋱
GGGGTGCAAGCAGGCATTACTTGCACATGCAGGGATAATTGGCAGGGTTCAAATAGACCAGTAATTCAAA⋱
TAGATCCAATGGCAATGACACATACTAGTCAGTATATATGTAGCCCCGTTCTCACAGATAATCCCCGGCC⋱
GAATGACCCAGCTGTAGGTAAGTGTAATGACCCTTATCCAGGGAATAACAACAAAGGAGTCAAAGGATTT⋱
TCATACCTGGATGGAGGTAATACTTGGCTAGGAAGAACAATAAGCACAGCTTCTAGATCCGGGTATGAGA⋱
TGCTAAAGGTGCCAAATGCATTGACCGATGATAGATCAAAACCCACCCAAGGTCAAACAATCGTGTTGAA⋱
CACTGACTGGAGTGGCTACAGTGGGTCCTTCGTGGACTATTGGGCCGAGGGGGAGTGCTACCGAGCGTGT⋱
TTTTATGTGGAGTTAATACGAGGAAGGCCCAAGGAGGACAAAGTATGGTGG
ACCAGTAATAGCATAGTATCAATGTGTTCCAGCACAGAATTCCTTGGACAATGGA
ACTGGCCTGATGGGGCTAAAATAGAGTACTTCCTCTAA

Interesting points (by weighted ranked PD) highlighted for gene NA:

ATGAATCCAAATCAGAAGATTCTATGCACTTCTGCCACTGCTATTGTAATAGGCACAATTGCAGTACTCATAGGAATA⋱
GCAAACCTAGGATTGAACATAGGACTACATCTGAAACCAAACTGCAACTGCTCACACTCACAACCTGAAGCAACCAA⋱
TGCAAGCCAAACAATAATAAACAACTACTATAACGAAACAAACATCACCCAAATAAGCAATACCAACATCCAAATGG⋱
AGGAGAAAGCAAATAGAGAATTCAATAACTTGACCAAAGGACTCTGCACTATAAATTCATGGCACATCTATGGGAAA⋱
GACAATGCAGTAAGAATTGGGGAGAACTCAGATGTTCTAGTAACGAGAGAACCCTATGTCTCCTGTGATCCAGATGA⋱
GTGCAGG
TTCTATGCTCTCAGCCAAGGAACAACGATTAGAGGGAAACACTCAAATGGAACAA
TACACGATAGATCCCAGTACCGTGCCCTGATAAGCTGGCCACTGTCATCACC
ACCCACAGTATACAACAGCAGAGTAGAATGCATTGGTTGGTCAAGTACTAGTTGTCATGATGGCAGAGC⋱
CAGGATGTCAATATGTGTATCAGGCCCAAACAACAATGCGTCTGCAGTAATCTGGTACAATAGAAGGCCT⋱
GTTACAGAAATCAATACATGGGCCCGAAACATACTACGAACACAAGAATCTGAATGCGTATGCCACAACG⋱
GTGTATGCCCGGTAGTATTCACAGATGGGTCTGCCACTGGACCTGCAGAAACAAGAGTGTACTATTTCAA⋱
GGAAGGGAAAATACTAAAATGGGAACCTCTAACTGGAACTGCTAAACACATTGAAGAATGCTCATGCTAT⋱
GGGGTGCAAGCAGGCATTACTTGCACATGCAGGGATAATTGGCAGGGTTCAAATAGACCAGTAATTCAAA⋱
TAGATCCAATGGCAATGACACATACTAGTCAGTATATATGTAGCCCCGTTCTCACAGATAATCCCCGGCC⋱
GAATGACCCAGCTGTAGGTAAGTGTAATGACCCTTATCCAGGGAATAACAACAAAGGAGTCAAAGGATTT⋱
TCATACCTGGATGGAGGTAATACTTGGCTAGGAAGAACAATAAGCACAGCTTCTAGATCCGGGTATGAGA⋱
TGCTAAAGGTGCCAAATGCATTGACCGATGATAGATCAAAACCCACCCAAGGTCAAACAATCGTGTTGAA⋱
CACTGACTGGAGTGGCTACAGTGGGTCCTTCGTGGACTATTGGGCCGAGGGGGAGTGCTACCGAGCGTGT⋱

TTTTATGTGGAGTTAATACGAGGAAGGCCCAAGGAGGACAAAGTATGGTGG

ACCAGTAATAGCATAGTATCAATGTGTTCCAGCACAGAATTCCTTGGACAATGGA

ACTGGCCTGATGGGGCTAAAATAGAGTACTTCCTCTAA

Per codon conservation report

NA

Pos.	1	obs:	exp:
atg	M	994	994.00
---	--	------	------
mPD		0	0
		nPD:	1.
		N. weight:	0.
		Sc. PD:	0
		Sc. rank:	0

NA

Pos.	2	obs:	exp:
cat	H	0	0.57
cac	H	1	0.43
aat	N	993	505.20
aac	N	0	487.80
---	--	------	------
mPD		0.0040	0.50
		nPD:	0.01
		N. weight:	0.75
		Sc. PD:	-0.07
		Sc. rank:	-452.0

NA

Pos.	3	obs:	exp:
tta	L	0	0.18
ttg	L	0	0.37
ctt	L	0	0.34
ctc	L	0	0.34
cta	L	2	0.33
ctg	L	0	0.44
cct	P	0	250.50
ccc	P	0	175.60
cca	P	984	402.40
ccg	P	1	156.50
caa	Q	5	2.57
cag	Q	0	2.43
act	T	0	0.26
acc	T	0	0.20
aca	T	1	0.46
acg	T	0	0.08
---	-	1	1.00
---	--	------	------
mPD		0.018	0.73
		nPD:	0.02
		N. weight:	0.98
		Sc. PD:	-0.077
		Sc. rank:	-276.5

NA

Pos.	4	obs:	exp:
aat	N	881	505.20
aac	N	112	487.80
---	-	1	1.00
---	--	------	------
mPD		0.20	0.50
		nPD:	0.4
		N. weight:	0.37
		Sc. PD:	0.1
		Sc. rank:	271.7

NA

Pos.	5	obs:	exp:
caa	Q	3	510.00
cag	Q	990	483.00
---	-	1	1.00
---	--	------	------
mPD		0.0060	0.50
		nPD:	0.01
		N. weight:	0.78
		Sc. PD:	-0.07
		Sc. rank:	-375.1

NA

Pos.	6	obs:	exp:
aaa	K	0	581.70
aag	K	993	411.30
---	-	1	1.00
---	--	------	------
mPD		0	0.48
		nPD:	0.
		N. weight:	0.98
		Sc. PD:	-0.099
		Sc. rank:	-756.1

NA

Pos.	7	obs:	exp:
tct	S	0	0.32
tcc	S	0	0.26
tca	S	0	0.50
tcg	S	0	0.12
att	I	989	360.90
atc	I	0	239.20
ata	I	0	388.90
act	T	2	0.52
acc	T	0	0.41
aca	T	0	0.92
acg	T	0	0.16
agt	S	2	0.41
agc	S	0	0.39
---	-	1	1.00
---	--	------	------
mPD		0.0080	0.66
		nPD:	0.01
		N. weight:	1.1
		Sc. PD:	-0.1
		Sc. rank:	-535.8

NA

Pos.	8	obs:	exp:
ttt	F	0	0.39
ttc	F	1	0.61
tta	L	1	87.01
ttg	L	0	184.70
ctt	L	0	167.80
ctc	L	0	165.90
cta	L	988	165.10
ctg	L	1	219.60
cct	P	0	0.25
ccc	P	0	0.18
cca	P	1	0.41
ccg	P	0	0.16
att	I	0	0.36
atc	I	0	0.24
ata	I	1	0.39
gtt	V	0	0.21
gtc	V	0	0.20
gta	V	1	0.21
gtg	V	0	0.39
---	--	------	------
mPD		0.014	1.1
		nPD:	0.01
		N. weight:	2.
		Sc. PD:	-0.18
		Sc. rank:	-910.0

NA

Pos.	9	obs:	exp:
tat	Y	0	1.06
tac	Y	2	0.94
tgt	C	0	406.40
tgc	C	990	583.60
ggt	G	0	0.27
ggc	G	2	0.25
gga	G	0	0.92
ggg	G	0	0.55
---	--	------	------
mPD		0.0080	0.49
		nPD:	0.02
		N. weight:	0.59
		Sc. PD:	-0.051
		Sc. rank:	-244.3

NA

Pos.	10	obs:	exp:
tct	S	0	0.48
tcc	S	0	0.39
tca	S	0	0.76
tcg	S	0	0.17
att	I	1	0.36
atc	I	0	0.24
ata	I	0	0.39
act	T	979	256.20
acc	T	9	201.40
aca	T	1	454.40
acg	T	0	77.04
agt	S	3	0.61
agc	S	0	0.59
---	-	1	1.00
---	--	------	------
mPD		0.028	0.68
		nPD:	0.04
		N. weight:	1.4
		Sc. PD:	-0.089
		Sc. rank:	-198.0

NA

Pos.	11	obs:	exp:
tta	L	1	0.09
ttg	L	0	0.19
tct	S	44	158.80
tcc	S	0	128.40
tca	S	948	250.30
tcg	S	0	57.73
ctt	L	0	0.17
ctc	L	0	0.17
cta	L	0	0.17
ctg	L	0	0.22
agt	S	0	203.30
agc	S	0	193.40
---	-	1	1.00
---	--	------	------
mPD		0.087	1.7
		nPD:	0.05
		N. weight:	1.3
		Sc. PD:	-0.069
		Sc. rank:	-104.9

NA

Pos.	12	obs:	exp:
tct	S	0	0.16
tcc	S	1	0.13
tca	S	0	0.25
tcg	S	0	0.06
agt	S	0	0.20
agc	S	0	0.20
gtt	V	0	1.03
gtc	V	5	1.00
gta	V	0	1.04
gtg	V	0	1.93
gct	A	4	252.90
gcc	A	983	185.30
gca	A	0	461.50
gcg	A	0	87.30
---	-	1	1.00
---	--	------	------
mPD		0.020	0.69
		nPD:	0.03
		N. weight:	1.8
		Sc. PD:	-0.13
		Sc. rank:	-429.9

NA

Pos.	13	obs:	exp:
cct	P	1	0.25
ccc	P	0	0.18
cca	P	0	0.41
ccg	P	0	0.16
att	I	4	1.46
atc	I	0	0.97
ata	I	0	1.57
act	T	978	255.70
acc	T	9	201.00
aca	T	0	453.40
acg	T	0	76.88
gct	A	0	0.51
gcc	A	2	0.38
gca	A	0	0.94
gcg	A	0	0.18
---	--	------	------
mPD		0.036	0.69
		nPD:	0.05
		N. weight:	1.4
		Sc. PD:	-0.074
		Sc. rank:	-125.1

NA

Pos.	14	obs:	exp:
act	T	12	3.11
acc	T	0	2.44
aca	T	0	5.51
acg	T	0	0.93
gct	A	909	251.60
gcc	A	65	184.40
gca	A	8	459.10
gcg	A	0	86.86
---	--	------	------
mPD		0.16	0.70
		nPD:	0.23
		N. weight:	1.2
		Sc. PD:	0.15
		Sc. rank:	702.7

NA

Pos.	15	obs:	exp:
att	I	47	362.00
atc	I	944	239.90
ata	I	1	390.10
act	T	0	0.52
acc	T	2	0.41
aca	T	0	0.92
acg	T	0	0.16
---	--	------	------
mPD		0.096	0.66
		nPD:	0.15
		N. weight:	1.3
		Sc. PD:	0.052
		Sc. rank:	449.2

NA			
Pos.	16	obs:	exp:
cgt	R	1	0.04
cgc	R	0	0.05
cga	R	0	0.10
cgg	R	0	0.08
att	I	0	183.50
atc	I	0	121.70
ata	I	503	197.80
act	T	0	96.11
acc	T	0	75.55
aca	T	370	170.40
acg	T	1	28.90
aga	R	0	0.46
agg	R	0	0.27
gtt	V	0	15.07
gtc	V	0	14.62
gta	V	73	15.11
gtg	V	0	28.19
gct	A	0	11.79
gcc	A	0	8.64
gca	A	46	21.51
gcg	A	0	4.07
---	--	------	------
mPD		0.71	1.4
		nPD:	0.51
		N. weight:	1.
		Sc. PD:	0.39
		Sc. rank:	770.4

,

NA			
Pos.	17	obs:	exp:
att	I	1	361.20
atc	I	1	239.50
ata	I	988	389.30
atg	M	1	1.00
act	T	0	0.78
acc	T	0	0.61
aca	T	3	1.38
acg	T	0	0.23
---	--	------	------
mPD		0.012	0.66
		nPD:	0.02
		N. weight:	1.
		Sc. PD:	-0.086
		Sc. rank:	-364.9

,

NA			
Pos.	18	obs:	exp:
tct	S	0	1.92
tcc	S	0	1.55
tca	S	0	3.03
tcg	S	0	0.70
tgt	C	0	0.82
tgc	C	2	1.18
agt	S	0	2.46
agc	S	12	2.34
ggt	G	6	131.40
ggc	G	973	124.50
gga	G	0	453.10
ggg	G	1	271.00
---	--	------	------
mPD		0.042	0.72
		nPD:	0.06
		N. weight:	2.2
		Sc. PD:	-0.1
		Sc. rank:	-92.8

,

NA			
Pos.	19	obs:	exp:
act	T	0	19.95
acc	T	3	15.68
aca	T	32	35.38
acg	T	42	6.00
gct	A	0	235.00
gcc	A	0	172.20
gca	A	915	428.70
gcg	A	2	81.11
---	--	------	------
mPD		0.23	0.82
		nPD:	0.29
		N. weight:	0.85
		Sc. PD:	0.15
		Sc. rank:	560.4

,

NA			
Pos.	20	obs:	exp:
tct	S	0	0.16
tcc	S	0	0.13
tca	S	0	0.25
tcg	S	0	0.06
att	I	50	360.90
atc	I	937	239.20
ata	I	2	388.90
act	T	0	0.78
acc	T	3	0.61
aca	T	0	1.38
acg	T	0	0.23
agt	S	0	0.20
agc	S	1	0.20
gtt	V	0	0.21
gtc	V	1	0.20
gta	V	0	0.21
gtg	V	0	0.39
---	--	------	------
mPD		0.11	0.67
		nPD:	0.16
		N. weight:	1.3
		Sc. PD:	0.074
		Sc. rank:	538.4

,

NA			
Pos.	21	obs:	exp:
cct	P	0	0.51
ccc	P	0	0.36
cca	P	2	0.82
ccg	P	0	0.32
act	T	0	32.64
acc	T	0	25.66
aca	T	126	57.89
acg	T	0	9.82
gct	A	0	221.90
gcc	A	1	162.60
gca	A	865	404.90
gcg	A	0	76.60
---	--	------	------
mPD		0.23	0.90
		nPD:	0.25
		N. weight:	0.84
		Sc. PD:	0.12
		Sc. rank:	512.1

,

NA			
Pos.	22	obs:	exp:
att	I	0	8.39
atc	I	0	5.56
ata	I	23	9.04
act	T	0	0.26
acc	T	0	0.20
aca	T	1	0.46
acg	T	0	0.08
gtt	V	0	193.80
gtc	V	0	188.10
gta	V	934	194.40
gtg	V	5	362.60
gct	A	0	7.94
gcc	A	0	5.82
gca	A	31	14.49
gcg	A	0	2.74
---	--	------	------
mPD		0.12	0.84
		nPD:	0.14
		N. weight:	1.7
		Sc. PD:	0.058
		Sc. rank:	531.3

,

NA			
Pos.	23	obs:	exp:
ttt	F	0	0.39
ttc	F	1	0.61
tta	L	0	87.27
ttg	L	0	185.30
ctt	L	3	168.30
ctc	L	985	166.40
cta	L	5	165.60
ctg	L	0	220.20
---	--	------	------
mPD		0.018	1.1
		nPD:	0.02
		N. weight:	1.9
		Sc. PD:	-0.17
		Sc. rank:	-800.9

,

NA			
Pos.	24	obs:	exp:
tta	L	0	0.09
ttg	L	0	0.19
ctt	L	0	0.17
ctc	L	0	0.17
cta	L	1	0.17
ctg	L	0	0.22
att	I	908	362.00
atc	I	1	239.90
ata	I	83	390.10
act	T	1	0.26
acc	T	0	0.20
aca	T	0	0.46
acg	T	0	0.08
---	--	------	------
mPD		0.16	0.66
		nPD:	0.24
		N. weight:	0.79
		Sc. PD:	0.1
		Sc. rank:	476.2

,

NA			
Pos.	25	obs:	exp:
tta	L	0	0.09
ttg	L	1	0.19
tgg	W	1	1.00
ctt	L	0	0.17
ctc	L	0	0.17
cta	L	0	0.17
ctg	L	0	0.22
ggt	G	0	133.00
ggc	G	0	126.00
gga	G	981	458.70
ggg	G	11	274.30
---	--	------	------
mPD		0.032	0.68
		nPD:	0.05
		N. weight:	0.8
		Sc. PD:	-0.045
		Sc. rank:	-91.0

,

NA			
Pos.	26	obs:	exp:
att	I	0	358.70
atc	I	0	237.80
ata	I	983	386.60
atg	M	1	1.00
act	T	0	1.81
acc	T	0	1.43
aca	T	7	3.22
acg	T	0	0.55
gtt	V	0	0.41
gtc	V	0	0.40
gta	V	2	0.41
gtg	V	0	0.77
gat	D	1	0.54
gac	D	0	0.46
---	--	------	------
mPD		0.026	0.68
		nPD:	0.04
		N. weight:	1.
		Sc. PD:	-0.067
		Sc. rank:	-169.6

,

NA			
Pos.	27	obs:	exp:
tct	S	0	0.32
tcc	S	0	0.26
tca	S	1	0.50
tcg	S	0	0.12
act	T	0	2.33
acc	T	0	1.83
aca	T	9	4.13
acg	T	0	0.70
agt	S	0	0.41
agc	S	1	0.39
gtt	V	0	0.41
gtc	V	0	0.40
gta	V	2	0.41
gtg	V	0	0.77
gct	A	0	251.40
gcc	A	0	184.20
gca	A	976	458.70
gcg	A	5	86.77
---	--	------	------
mPD		0.040	0.70
		nPD:	0.06
		N. weight:	0.82
		Sc. PD:	-0.039
		Sc. rank:	-40.2

,

NA			
Pos.	28	obs:	exp:
aat	N	21	505.20
aac	N	972	487.80
gat	D	1	0.54
gac	D	0	0.46
---	--	------	------
mPD		0.045	0.50
		nPD:	0.09
		N. weight:	0.68
		Sc. PD:	-0.01
		Sc. rank:	66.1

,

NA			
Pos.	29	obs:	exp:
tta	L	3	77.60
ttg	L	3	164.80
ctt	L	0	149.60
ctc	L	0	147.90
cta	L	807	147.20
ctg	L	70	195.80
att	I	0	39.77
atc	I	0	26.36
ata	I	109	42.86
atg	M	2	2.00
---	--	------	------
mPD		0.35	1.2
		nPD:	0.28
		N. weight:	1.5
		Sc. PD:	0.26
		Sc. rank:	1008.8

,

NA			
Pos.	30	obs:	exp:
gtt	V	0	0.62
gtc	V	0	0.60
gta	V	3	0.62
gtg	V	0	1.16
ggt	G	0	132.90
ggc	G	0	125.90
gga	G	953	458.20
ggg	G	38	274.00
---	--	------	------
mPD		0.080	0.68
		nPD:	0.12
		N. weight:	0.7
		Sc. PD:	0.0073
		Sc. rank:	156.9

,

NA

Pos.	31	obs:	exp:
tta	L	10	87.36
ttg	L	893	185.50
ctt	L	0	168.40
ctc	L	0	166.50
cta	L	2	165.70
ctg	L	89	220.40
---	--	------	------
mPD		0.19	1.1
		nPD:	0.17
		N. weight:	1.4
		Sc. PD:	0.091
		Sc. rank:	632.1

,

NA

Pos.	32	obs:	exp:
tct	S	0	0.48
tcc	S	0	0.39
tca	S	0	0.76
tcg	S	0	0.17
aat	N	15	504.20
aac	N	976	486.80
agt	S	0	0.61
agc	S	3	0.59
---	--	------	------
mPD		0.036	0.51
		nPD:	0.07
		N. weight:	0.71
		Sc. PD:	-0.024
		Sc. rank:	22.2

,

NA

Pos.	33	obs:	exp:
att	I	0	362.70
atc	I	0	240.40
ata	I	994	390.90
---	--	------	------
mPD		0	0.65
		nPD:	0.
		N. weight:	1.
		Sc. PD:	-0.11
		Sc. rank:	-801.5

,

NA

Pos.	34	obs:	exp:
cgt	R	0	0.13
cgc	R	0	0.15
cga	R	0	0.30
cgg	R	0	0.25
aga	R	3	1.37
agg	R	0	0.80
gaa	E	1	0.60
gag	E	0	0.40
ggt	G	0	132.80
ggc	G	1	125.80
gga	G	979	457.70
ggg	G	10	273.70
---	--	------	------
mPD		0.030	0.68
		nPD:	0.04
		N. weight:	0.79
		Sc. PD:	-0.047
		Sc. rank:	-104.2

,

NA

Pos.	35	obs:	exp:
tta	L	1	87.36
ttg	L	1	185.50
ctt	L	0	168.40
ctc	L	0	166.50
cta	L	49	165.70
ctg	L	943	220.40
---	--	------	------
mPD		0.10	1.1
		nPD:	0.09
		N. weight:	1.5
		Sc. PD:	-0.023
		Sc. rank:	132.5

,

NA

Pos.	36	obs:	exp:
tta	L	0	0.09
ttg	L	0	0.19
tat	Y	1	0.53
tac	Y	0	0.47
ctt	L	1	0.17
ctc	L	0	0.17
cta	L	0	0.17
ctg	L	0	0.22
cat	H	991	569.90
cac	H	1	422.10
---	--	------	------
mPD		0.0060	0.49
		nPD:	0.01
		N. weight:	0.61
		Sc. PD:	-0.055
		Sc. rank:	-288.6

,

NA

Pos.	37	obs:	exp:
tta	L	97	87.36
ttg	L	0	185.50
ctt	L	0	168.40
ctc	L	0	166.50
cta	L	855	165.70
ctg	L	42	220.40
---	--	------	------
mPD		0.26	1.1
		nPD:	0.23
		N. weight:	1.5
		Sc. PD:	0.18
		Sc. rank:	878.6

,

NA

Pos.	38	obs:	exp:
cgt	R	0	0.21
cgc	R	0	0.25
cga	R	0	0.50
cgg	R	0	0.42
aaa	K	988	579.40
aag	K	1	409.60
aga	R	5	2.28
agg	R	0	1.33
---	--	------	------
mPD		0.012	0.50
		nPD:	0.02
		N. weight:	0.59
		Sc. PD:	-0.046
		Sc. rank:	-175.6

,

NA

Pos.	39	obs:	exp:
tta	L	0	0.35
ttg	L	4	0.75
tct	S	0	19.53
tcc	S	0	15.79
tca	S	0	30.79
tcg	S	122	7.10
ctt	L	0	0.68
ctc	L	0	0.67
cta	L	0	0.67
ctg	L	0	0.89
cct	P	2	220.70
ccc	P	0	154.80
cca	P	46	354.60
ccg	P	820	137.90
agt	S	0	25.00
agc	S	0	23.79
---	--	------	------
mPD		0.32	1.0
		nPD:	0.31
		N. weight:	1.9
		Sc. PD:	0.37
		Sc. rank:	1283.5

,

NA

Pos.	40	obs:	exp:
tct	S	0	6.56
tcc	S	0	5.31
tca	S	0	10.35
tcg	S	0	2.39
act	T	0	0.26
acc	T	1	0.20
aca	T	0	0.46
acg	T	0	0.08
aat	N	1	20.35
aac	N	39	19.65
aaa	K	1	0.59
aag	K	0	0.41
agt	S	0	8.40
agc	S	41	8.00
gat	D	0	1.62
gac	D	3	1.38
ggt	G	4	121.80
ggc	G	901	115.40
gga	G	3	419.80
ggg	G	0	251.10
---	--	------	------
mPD		0.26	0.99
		nPD:	0.26
		N. weight:	2.1
		Sc. PD:	0.32
		Sc. rank:	1323.8

,

NA

Pos.	41	obs:	exp:
ttt	F	0	0.39
ttc	F	1	0.61
tgt	C	2	407.70
tgc	C	991	585.30
---	--	------	------
mPD		0.0060	0.49
		nPD:	0.01
		N. weight:	0.57
		Sc. PD:	-0.051
		Sc. rank:	-268.3

,

NA

Pos.	42	obs:	exp:
tct	S	0	0.16
tcc	S	0	0.13
tca	S	0	0.25
tcg	S	0	0.06
aat	N	898	504.20
aac	N	93	486.80
agt	S	1	0.20
agc	S	0	0.20
gat	D	2	1.08
gac	D	0	0.92
---	--	------	------
mPD		0.18	0.51
		nPD:	0.35
		N. weight:	0.41
		Sc. PD:	0.095
		Sc. rank:	292.4

,

NA

Pos.	43	obs:	exp:
tgt	C	1	408.10
tgc	C	993	585.90
---	--	------	------
mPD		0.0020	0.48
		nPD:	0.
		N. weight:	0.58
		Sc. PD:	-0.056
		Sc. rank:	-395.0

,

NA

Pos.	44	obs:	exp:
tta	L	2	0.18
ttg	L	0	0.37
tct	S	0	151.00
tcc	S	7	122.10
tca	S	935	238.00
tcg	S	1	54.88
ctt	L	0	0.34
ctc	L	0	0.34
cta	L	0	0.33
ctg	L	0	0.44
cct	P	0	2.03
ccc	P	0	1.43
cca	P	8	3.27
ccg	P	0	1.27
att	I	0	0.73
atc	I	0	0.48
ata	I	2	0.79
act	T	0	10.10
acc	T	0	7.94
aca	T	39	17.92
acg	T	0	3.04
agt	S	0	193.20
agc	S	0	183.90
---	--	------	------
mPD		0.12	1.7
		nPD:	0.07
		N. weight:	1.5
		Sc. PD:	-0.049
		Sc. rank:	72.7

,

NA

Pos.	45	obs:	exp:
tct	S	0	0.16
tcc	S	0	0.13
tca	S	0	0.25
tcg	S	0	0.06
tgt	C	0	0.82
tgc	C	2	1.18
cat	H	0	454.40
cac	H	791	336.60
caa	Q	5	2.57
cag	Q	0	2.43
cgt	R	0	6.92
cgc	R	161	8.23
cga	R	1	16.15
cgg	R	0	13.49
aat	N	3	16.79
aac	N	30	16.21
agt	S	1	0.20
agc	S	0	0.20
aga	R	0	73.97
agg	R	0	43.24
---	--	------	------
mPD		0.37	1.2
		nPD:	0.32
		N. weight:	1.3
		Sc. PD:	0.26
		Sc. rank:	886.2

,

NA			
Pos.	46	obs:	exp:
tta	L	2	0.18
ttg	L	0	0.37
tct	S	1	158.30
tcc	S	0	128.00
tca	S	988	249.60
tcg	S	0	57.55
ctt	L	0	0.34
ctc	L	0	0.34
cta	L	0	0.33
ctg	L	0	0.44
cct	P	0	0.76
ccc	P	0	0.53
cca	P	3	1.23
ccg	P	0	0.48
agt	S	0	202.70
agc	S	0	192.90
---	--	------	------
mPD		0.012	1.7
		nPD:	0.01
		N. weight:	1.5
		Sc. PD:	-0.14
		Sc. rank:	-928.0

,

NA			
Pos.	47	obs:	exp:
cct	P	0	0.25
ccc	P	0	0.18
cca	P	1	0.41
ccg	P	0	0.16
caa	Q	983	506.50
cag	Q	3	479.50
cgt	R	0	0.30
cgc	R	0	0.36
cga	R	7	0.70
cgg	R	0	0.58
aga	R	0	3.20
agg	R	0	1.87
---	--	------	------
mPD		0.022	0.53
		nPD:	0.04
		N. weight:	0.73
		Sc. PD:	-0.045
		Sc. rank:	-97.8

,

NA			
Pos.	48	obs:	exp:
tct	S	37	5.92
tcc	S	0	4.79
tca	S	0	9.34
tcg	S	0	2.15
cct	P	941	241.80
ccc	P	10	169.60
cca	P	0	388.50
ccg	P	0	151.10
cat	H	3	1.72
cac	H	0	1.28
act	T	3	0.78
acc	T	0	0.61
aca	T	0	1.38
acg	T	0	0.23
agt	S	0	7.58
agc	S	0	7.22
---	--	------	------
mPD		0.10	0.83
		nPD:	0.13
		N. weight:	1.5
		Sc. PD:	0.027
		Sc. rank:	367.7

,

NA			
Pos.	49	obs:	exp:
aaa	K	2	1.17
aag	K	0	0.83
gat	D	1	0.54
gac	D	0	0.46
gaa	E	937	565.40
gag	E	3	374.60
ggt	G	0	6.84
ggc	G	0	6.48
gga	G	51	23.58
ggg	G	0	14.10
---	--	------	------
mPD		0.11	0.60
		nPD:	0.18
		N. weight:	0.56
		Sc. PD:	0.042
		Sc. rank:	273.3

,

NA			
Pos.	50	obs:	exp:
tct	S	0	0.16
tcc	S	0	0.13
tca	S	1	0.25
tcg	S	0	0.06
att	I	0	11.68
atc	I	0	7.74
ata	I	32	12.58
act	T	0	243.20
acc	T	0	191.20
aca	T	939	431.40
acg	T	0	73.15
agt	S	0	0.20
agc	S	0	0.20
gct	A	0	5.38
gcc	A	0	3.94
gca	A	21	9.82
gcg	A	0	1.86
gaa	E	1	0.60
gag	E	0	0.40
---	--	------	------
mPD		0.11	0.78
		nPD:	0.14
		N. weight:	0.87
		Sc. PD:	0.028
		Sc. rank:	265.5

,

NA			
Pos.	51	obs:	exp:
tct	S	0	4.64
tcc	S	0	3.75
tca	S	0	7.32
tcg	S	0	1.69
act	T	1	248.20
acc	T	957	195.10
aca	T	0	440.10
acg	T	0	74.63
agt	S	0	5.94
agc	S	29	5.66
gtt	V	0	0.21
gtc	V	0	0.20
gta	V	1	0.21
gtg	V	0	0.39
gct	A	1	1.54
gcc	A	5	1.13
gca	A	0	2.81
gcg	A	0	0.53
---	--	------	------
mPD		0.079	0.75
		nPD:	0.1
		N. weight:	1.8
		Sc. PD:	-0.0017
		Sc. rank:	327.3

,

NA			
Pos.	52	obs:	exp:
aat	N	50	505.70
aac	N	944	488.30
---	--	------	------
mPD		0.096	0.50
		nPD:	0.19
		N. weight:	0.57
		Sc. PD:	0.047
		Sc. rank:	287.5

,

NA			
Pos.	53	obs:	exp:
tct	S	0	0.16
tcc	S	0	0.13
tca	S	1	0.25
tcg	S	0	0.06
att	I	0	1.09
atc	I	0	0.73
ata	I	3	1.18
act	T	0	244.00
acc	T	0	191.80
aca	T	937	432.80
acg	T	5	73.38
agt	S	0	0.20
agc	S	0	0.20
gtt	V	0	0.21
gtc	V	0	0.20
gta	V	0	0.21
gtg	V	1	0.39
gct	A	0	12.04
gcc	A	0	8.82
gca	A	47	21.97
gcg	A	0	4.16
---	--	------	------
mPD		0.11	0.78
		nPD:	0.15
		N. weight:	0.84
		Sc. PD:	0.033
		Sc. rank:	284.6

,

NA			
Pos.	54	obs:	exp:
tct	S	0	158.80
tcc	S	0	128.40
tca	S	0	250.30
tcg	S	0	57.73
aat	N	0	1.02
aac	N	2	0.98
agt	S	5	203.30
agc	S	987	193.40
---	--	------	------
mPD		0.014	1.7
		nPD:	0.01
		N. weight:	1.8
		Sc. PD:	-0.17
		Sc. rank:	-962.0

,

NA			
Pos.	55	obs:	exp:
caa	Q	994	510.60
cag	Q	0	483.40
---	--	------	------
mPD		0	0.50
		nPD:	0.
		N. weight:	0.74
		Sc. PD:	-0.075
		Sc. rank:	-572.1

,

NA			
Pos.	56	obs:	exp:
att	I	0	1.82
atc	I	0	1.21
ata	I	5	1.97
act	T	1	256.20
acc	T	0	201.40
aca	T	985	454.40
acg	T	3	77.04
---	--	------	------
mPD		0.018	0.68
		nPD:	0.03
		N. weight:	0.84
		Sc. PD:	-0.064
		Sc. rank:	-223.0

,

NA			
Pos.	57	obs:	exp:
att	I	0	342.60
atc	I	0	227.10
ata	I	939	369.30
act	T	0	12.95
acc	T	0	10.18
aca	T	50	22.97
acg	T	0	3.89
gtt	V	0	1.03
gtc	V	0	1.00
gta	V	5	1.04
gtg	V	0	1.93
---	--	------	------
mPD		0.11	0.76
		nPD:	0.14
		N. weight:	1.
		Sc. PD:	0.032
		Sc. rank:	309.2

,

NA			
Pos.	58	obs:	exp:
att	I	0	358.70
atc	I	0	237.80
ata	I	983	386.60
act	T	0	1.55
acc	T	0	1.22
aca	T	6	2.76
acg	T	0	0.47
gtt	V	0	1.03
gtc	V	0	1.00
gta	V	5	1.04
gtg	V	0	1.93
---	--	------	------
mPD		0.022	0.68
		nPD:	0.03
		N. weight:	1.
		Sc. PD:	-0.073
		Sc. rank:	-202.8

,

NA			
Pos.	59	obs:	exp:
act	T	0	0.26
acc	T	1	0.20
aca	T	0	0.46
acg	T	0	0.08
aat	N	2	505.20
aac	N	991	487.80
---	--	------	------
mPD		0.0060	0.50
		nPD:	0.01
		N. weight:	0.77
		Sc. PD:	-0.07
		Sc. rank:	-379.4

,

NA			
Pos.	60	obs:	exp:
aat	N	2	505.70
aac	N	992	488.30
---	--	------	------
mPD		0.0040	0.50
		nPD:	0.01
		N. weight:	0.77
		Sc. PD:	-0.073
		Sc. rank:	-462.5

,

NA			
Pos.	61	obs:	exp:
tat	Y	947	527.00
tac	Y	47	467.00
---	--	------	------
mPD		0.090	0.50
		nPD:	0.18
		N. weight:	0.5
		Sc. PD:	0.036
		Sc. rank:	235.6

,

NA			
Pos.	62	obs:	exp:
tat	Y	946	525.40
tac	Y	45	465.60
tgt	C	1	0.41
tgc	C	0	0.59
cat	H	1	0.57
cac	H	0	0.43
gat	D	1	0.54
gac	D	0	0.46
---	--	------	------
mPD		0.093	0.50
		nPD:	0.18
		N. weight:	0.51
		Sc. PD:	0.038
		Sc. rank:	247.6

,

NA			
Pos.	63	obs:	exp:
tct	S	0	1.44
tcc	S	0	1.16
tca	S	0	2.27
tcg	S	0	0.52
aat	N	936	499.60
aac	N	46	482.40
aaa	K	0	1.17
aag	K	2	0.83
agt	S	9	1.84
agc	S	0	1.76
---	-	1	1.00
---	--	------	------
mPD		0.11	0.53
		nPD:	0.21
		N. weight:	0.56
		Sc. PD:	0.054
		Sc. rank:	300.0

,

NA			
Pos.	64	obs:	exp:
gaa	E	986	596.10
gag	E	5	394.90
ggt	G	0	0.13
ggc	G	0	0.13
gga	G	1	0.46
ggg	G	0	0.28
---	-	2	2.00
---	--	------	------
mPD		0.012	0.48
		nPD:	0.03
		N. weight:	0.53
		Sc. PD:	-0.041
		Sc. rank:	-144.2

,

NA			
Pos.	65	obs:	exp:
act	T	0	254.40
acc	T	0	200.00
aca	T	978	451.10
acg	T	4	76.49
gct	A	0	2.56
gcc	A	0	1.88
gca	A	10	4.68
gcg	A	0	0.88
---	-	2	2.00
---	--	------	------
mPD		0.028	0.69
		nPD:	0.04
		N. weight:	0.84
		Sc. PD:	-0.053
		Sc. rank:	-127.5

,

NA			
Pos.	66	obs:	exp:
tct	S	0	0.48
tcc	S	0	0.39
tca	S	0	0.76
tcg	S	0	0.17
aat	N	26	502.60
aac	N	962	485.40
agt	S	0	0.61
agc	S	3	0.59
gat	D	0	0.54
gac	D	1	0.46
---	-	2	2.00
---	--	------	------
mPD		0.059	0.51
		nPD:	0.12
		N. weight:	0.66
		Sc. PD:	0.006
		Sc. rank:	145.2

,

NA			
Pos.	67	obs:	exp:
att	I	1	361.20
atc	I	983	239.50
ata	I	6	389.30
act	T	0	0.78
acc	T	3	0.61
aca	T	0	1.38
acg	T	0	0.23
gtt	V	0	0.21
gtc	V	1	0.20
gta	V	0	0.21
gtg	V	0	0.39
---	--	------	------
mPD		0.022	0.66
		nPD:	0.03
		N. weight:	1.5
		Sc. PD:	-0.11
		Sc. rank:	-290.4

,

NA			
Pos.	68	obs:	exp:
act	T	14	21.24
acc	T	68	16.70
aca	T	0	37.67
acg	T	0	6.39
---	-	912	912.00
---	--	------	------
mPD		0.0019	0.0046
		nPD:	0.42
		N. weight:	0.
		Sc. PD:	0
		Sc. rank:	0

,

NA			
Pos.	69	obs:	exp:
caa	Q	82	42.12
cag	Q	0	39.88
---	-	912	912.00
---	--	------	------
mPD		0	0.0034
		nPD:	0.
		N. weight:	0.
		Sc. PD:	0
		Sc. rank:	0

,

NA			
Pos.	70	obs:	exp:
att	I	0	29.92
atc	I	0	19.83
ata	I	82	32.25
---	-	912	912.00
---	--	------	------
mPD		0	0.0044
		nPD:	0.
		N. weight:	0.
		Sc. PD:	0
		Sc. rank:	0

,

NA			
Pos.	71	obs:	exp:
tct	S	0	13.13
tcc	S	0	10.61
tca	S	0	20.69
tcg	S	0	4.77
agt	S	40	16.80
agc	S	42	15.99
---	-	912	912.00
---	--	------	------
mPD		0.0034	0.011
		nPD:	0.3
		N. weight:	0.
		Sc. PD:	0
		Sc. rank:	0

,

NA			
Pos.	72	obs:	exp:
aat	N	26	41.72
aac	N	56	40.28
---	-	912	912.00
---	--	------	------
mPD		0.0030	0.0034
		nPD:	0.87
		N. weight:	0.
		Sc. PD:	0
		Sc. rank:	0

,

NA			
Pos.	73	obs:	exp:
act	T	1	257.50
acc	T	985	202.40
aca	T	5	456.70
acg	T	3	77.43
---	--	------	------
mPD		0.018	0.67
		nPD:	0.03
		N. weight:	1.7
		Sc. PD:	-0.13
		Sc. rank:	-429.3

,

NA			
Pos.	74	obs:	exp:
cat	H	0	0.57
cac	H	1	0.43
aat	N	9	504.20
aac	N	982	486.80
aaa	K	2	1.17
aag	K	0	0.83
---	--	------	------
mPD		0.024	0.50
		nPD:	0.05
		N. weight:	0.73
		Sc. PD:	-0.041
		Sc. rank:	-81.9

,

NA			
Pos.	75	obs:	exp:
att	I	14	362.70
atc	I	979	240.40
ata	I	1	390.90
---	--	------	------
mPD		0.030	0.65
		nPD:	0.05
		N. weight:	1.5
		Sc. PD:	-0.086
		Sc. rank:	-172.8

,

NA			
Pos.	76	obs:	exp:
tta	L	0	0.09
ttg	L	0	0.19
ctt	L	0	0.17
ctc	L	0	0.17
cta	L	1	0.17
ctg	L	0	0.22
cat	H	0	0.57
cac	H	1	0.43
caa	Q	984	509.50
cag	Q	8	482.50
---	--	------	------
mPD		0.020	0.50
		nPD:	0.04
		N. weight:	0.69
		Sc. PD:	-0.044
		Sc. rank:	-106.6

,

NA			
Pos.	77	obs:	exp:
tta	L	0	0.09
ttg	L	1	0.19
ctt	L	0	0.17
ctc	L	0	0.17
cta	L	0	0.17
ctg	L	0	0.22
cgt	R	0	0.17
cgc	R	0	0.20
cga	R	0	0.40
cgg	R	0	0.33
att	I	0	38.31
atc	I	0	25.40
ata	I	105	41.29
atg	M	879	879.00
act	T	0	0.26
acc	T	0	0.20
aca	T	0	0.46
acg	T	1	0.08
aga	R	0	1.83
agg	R	4	1.07
gtt	V	0	0.83
gtc	V	0	0.80
gta	V	0	0.83
gtg	V	4	1.54
---	--	------	------
mPD		0.21	0.23
		nPD:	0.91
		N. weight:	0.12
		Sc. PD:	0.096
		Sc. rank:	96.1

,

NA			
Pos.	78	obs:	exp:
cgt	R	0	0.17
cgc	R	0	0.20
cga	R	0	0.40
cgg	R	0	0.33
aaa	K	2	1.17
aag	K	0	0.83
aga	R	4	1.83
agg	R	0	1.07
gaa	E	845	529.90
gag	E	36	351.10
ggt	G	0	14.35
ggc	G	0	13.59
gga	G	106	49.47
ggg	G	1	29.58
---	--	------	------
mPD		0.28	0.72
		nPD:	0.39
		N. weight:	0.44
		Sc. PD:	0.12
		Sc. rank:	328.4

,

NA			
Pos.	79	obs:	exp:
aaa	K	8	9.37
aag	K	8	6.63
gaa	E	423	587.10
gag	E	553	388.90
ggt	G	0	0.27
ggc	G	0	0.25
gga	G	2	0.92
ggg	G	0	0.55
---	--	------	------
mPD		0.53	0.52
		nPD:	1.02
		N. weight:	0.07
		Sc. PD:	0.062
		Sc. rank:	54.5

,

NA			
Pos.	80	obs:	exp:
tct	S	0	0.48
tcc	S	0	0.39
tca	S	0	0.76
tcg	S	0	0.17
cgt	R	0	39.43
cgc	R	0	46.90
cga	R	0	92.03
cgg	R	0	76.88
aaa	K	49	31.63
aag	K	5	22.37
agt	S	2	0.61
agc	S	1	0.59
aga	R	918	421.40
agg	R	5	246.30
ggt	G	0	1.88
ggc	G	0	1.78
gga	G	14	6.47
ggg	G	0	3.87
---	--	------	------
mPD		0.16	1.1
		nPD:	0.15
		N. weight:	0.81
		Sc. PD:	0.031
		Sc. rank:	269.0

,

NA			
Pos.	81	obs:	exp:
tct	S	0	0.16
tcc	S	0	0.13
tca	S	1	0.25
tcg	S	0	0.06
act	T	0	237.00
acc	T	0	186.30
aca	T	915	420.40
acg	T	0	71.28
aaa	K	4	2.34
aag	K	0	1.66
agt	S	0	0.20
agc	S	0	0.20
gct	A	1	18.96
gcc	A	0	13.89
gca	A	73	34.60
gcg	A	0	6.55
---	--	------	------
mPD		0.15	0.82
		nPD:	0.18
		N. weight:	0.86
		Sc. PD:	0.063
		Sc. rank:	409.4

,

NA			
Pos.	82	obs:	exp:
tct	S	0	151.10
tcc	S	0	122.20
tca	S	0	238.20
tcg	S	0	54.93
aat	N	40	24.42
aac	N	8	23.58
aaa	K	1	1.17
aag	K	1	0.83
agt	S	5	193.40
agc	S	939	184.10
---	--	------	------
mPD		0.19	1.7
		nPD:	0.11
		N. weight:	1.7
		Sc. PD:	0.0048
		Sc. rank:	340.4

,

NA			
Pos.	83	obs:	exp:
cgt	R	0	32.17
cgc	R	0	38.26
cga	R	0	75.08
cgg	R	0	62.72
aat	N	1	0.51
aac	N	0	0.49
aaa	K	2	140.60
aag	K	238	99.41
aga	R	55	343.80
agg	R	698	201.00
---	--	------	------
mPD		0.48	1.2
		nPD:	0.38
		N. weight:	1.1
		Sc. PD:	0.29
		Sc. rank:	795.7

,

NA			
Pos.	84	obs:	exp:
tct	S	0	1.12
tcc	S	0	0.91
tca	S	0	1.77
tcg	S	0	0.41
tat	Y	2	1.06
tac	Y	0	0.94
aat	N	902	458.90
aac	N	0	443.10
agt	S	7	1.43
agc	S	0	1.37
gat	D	20	11.34
gac	D	1	9.66
gaa	E	44	26.47
gag	E	0	17.53
ggt	G	18	2.41
ggc	G	0	2.29
gga	G	0	8.32
ggg	G	0	4.98
---	--	------	------
mPD		0.29	0.77
		nPD:	0.38
		N. weight:	0.77
		Sc. PD:	0.2
		Sc. rank:	558.8

,

NA			
Pos.	85	obs:	exp:
ttt	F	0	392.30
ttc	F	994	601.70
---	--	------	------
mPD		0	0.48
		nPD:	0.
		N. weight:	0.56
		Sc. PD:	-0.057
		Sc. rank:	-431.1

,

NA

Pos.	86	obs:	exp:
aat	N	959	505.70
aac	N	35	488.30
---	--	------	------
mPD		0.068	0.50
		nPD:	0.14
		N. weight:	0.58
		Sc. PD:	0.017
		Sc. rank:	166.7

,

NA

Pos.	87	obs:	exp:
aat	N	7	505.70
aac	N	987	488.30
---	--	------	------
mPD		0.014	0.50
		nPD:	0.03
		N. weight:	0.74
		Sc. PD:	-0.056
		Sc. rank:	-183.2

,

NA

Pos.	88	obs:	exp:
tta	L	890	87.36
ttg	L	59	185.50
ctt	L	0	168.40
ctc	L	0	166.50
cta	L	44	165.70
ctg	L	1	220.40
---	--	------	------
mPD		0.20	1.1
		nPD:	0.18
		N. weight:	2.2
		Sc. PD:	0.15
		Sc. rank:	1013.2

,

NA

Pos.	89	obs:	exp:
act	T	935	257.50
acc	T	50	202.40
aca	T	0	456.70
acg	T	9	77.43
---	--	------	------
mPD		0.11	0.67
		nPD:	0.17
		N. weight:	1.2
		Sc. PD:	0.073
		Sc. rank:	525.7

,

NA

Pos.	90	obs:	exp:
cgt	R	0	0.04
cgc	R	0	0.05
cga	R	0	0.10
cgg	R	0	0.08
aat	N	0	0.51
aac	N	1	0.49
aaa	K	990	581.10
aag	K	2	410.90
aga	R	1	0.46
agg	R	0	0.27
---	--	------	------
mPD		0.0080	0.49
		nPD:	0.02
		N. weight:	0.58
		Sc. PD:	-0.05
		Sc. rank:	-238.9

,

NA

Pos.	91	obs:	exp:
tgg	W	1	1.00
cgt	R	0	0.98
cgc	R	0	1.17
cga	R	0	2.29
cgg	R	0	1.92
aga	R	0	10.50
agg	R	23	6.14
gaa	E	1	0.60
gag	E	0	0.40
ggt	G	0	129.90
ggc	G	0	123.10
gga	G	90	448.00
ggg	G	879	267.90
---	--	------	------
mPD		0.22	0.72
		nPD:	0.3
		N. weight:	1.
		Sc. PD:	0.19
		Sc. rank:	690.6

,

NA

Pos.	92	obs:	exp:
tta	L	0	87.36
ttg	L	0	185.50
ctt	L	5	168.40
ctc	L	989	166.50
cta	L	0	165.70
ctg	L	0	220.40
---	--	------	------
mPD		0.010	1.1
		nPD:	0.01
		N. weight:	1.9
		Sc. PD:	-0.18
		Sc. rank:	-1025.2

,

NA

Pos.	93	obs:	exp:
tgt	C	949	408.10
tgc	C	45	585.90
---	--	------	------
mPD		0.087	0.48
		nPD:	0.18
		N. weight:	0.77
		Sc. PD:	0.054
		Sc. rank:	353.7

,

NA

Pos.	94	obs:	exp:
act	T	988	257.50
acc	T	5	202.40
aca	T	1	456.70
acg	T	0	77.43
---	--	------	------
mPD		0.012	0.67
		nPD:	0.02
		N. weight:	1.5
		Sc. PD:	-0.12
		Sc. rank:	-534.2

,

NA

Pos.	95	obs:	exp:
att	I	0	362.70
atc	I	0	240.40
ata	I	994	390.90
---	--	------	------
mPD		0	0.65
		nPD:	0.
		N. weight:	1.
		Sc. PD:	-0.11
		Sc. rank:	-801.5

,

NA

Pos.	96	obs:	exp:
aat	N	989	505.70
aac	N	5	488.30
---	--	------	------
mPD		0.010	0.50
		nPD:	0.02
		N. weight:	0.72
		Sc. PD:	-0.059
		Sc. rank:	-246.1

,

NA

Pos.	97	obs:	exp:
tta	L	1	0.09
ttg	L	0	0.19
tct	S	0	159.00
tcc	S	0	128.50
tca	S	967	250.60
tcg	S	26	57.79
ctt	L	0	0.17
ctc	L	0	0.17
cta	L	0	0.17
ctg	L	0	0.22
agt	S	0	203.50
agc	S	0	193.60
---	--	------	------
mPD		0.053	1.7
		nPD:	0.03
		N. weight:	1.4
		Sc. PD:	-0.1
		Sc. rank:	-306.8

,

NA

Pos.	98	obs:	exp:
tgg	W	994	994.00
---	--	------	------
mPD		0	0
		nPD:	1.
		N. weight:	0.
		Sc. PD:	0
		Sc. rank:	0

,

NA

Pos.	99	obs:	exp:
cat	H	5	571.10
cac	H	989	422.90
---	--	------	------
mPD		0.010	0.49
		nPD:	0.02
		N. weight:	0.91
		Sc. PD:	-0.075
		Sc. rank:	-310.7

,

NA

Pos.	100	obs:	exp:
att	I	0	362.30
atc	I	40	240.20
ata	I	953	390.50
gtt	V	0	0.21
gtc	V	0	0.20
gta	V	1	0.21
gtg	V	0	0.39
---	--	------	------
mPD		0.079	0.66
		nPD:	0.12
		N. weight:	0.87
		Sc. PD:	0.013
		Sc. rank:	204.4

,

NA

Pos.	101	obs:	exp:
ttt	F	1	0.39
ttc	F	0	0.61
tat	Y	963	524.40
tac	Y	26	464.60
cat	H	2	2.30
cac	H	2	1.70
---	--	------	------
mPD		0.065	0.51
		nPD:	0.13
		N. weight:	0.57
		Sc. PD:	0.012
		Sc. rank:	146.6

,

NA

Pos.	102	obs:	exp:
ggt	G	12	133.30
ggc	G	0	126.30
gga	G	10	459.60
ggg	G	972	274.80
---	--	------	------
mPD		0.044	0.68
		nPD:	0.06
		N. weight:	1.3
		Sc. PD:	-0.052
		Sc. rank:	6.4

,

NA

Pos.	103	obs:	exp:
aaa	K	989	582.30
aag	K	5	411.70
---	--	------	------
mPD		0.010	0.49
		nPD:	0.02
		N. weight:	0.56
		Sc. PD:	-0.046
		Sc. rank:	-190.2

,

NA

Pos.	104	obs:	exp:
gat	D	3	536.60
gac	D	991	457.40
---	--	------	------
mPD		0.0060	0.50
		nPD:	0.01
		N. weight:	0.84
		Sc. PD:	-0.075
		Sc. rank:	-399.5

,

NA

Pos.	105	obs:	exp:
cat	H	1	0.57
cac	H	0	0.43
aat	N	880	505.20
aac	N	113	487.80
---	--	------	------
mPD		0.20	0.50
		nPD:	0.41
		N. weight:	0.36
		Sc. PD:	0.1
		Sc. rank:	271.8

,

NA

Pos.	106	obs:	exp:
gct	A	1	254.70
gcc	A	1	186.60
gca	A	990	464.70
gcg	A	2	87.92
---	--	------	------
mPD		0.0080	0.67
		nPD:	0.01
		N. weight:	0.82
		Sc. PD:	-0.074
		Sc. rank:	-403.1

,

NA

Pos.	107	obs:	exp:
tta	L	16	1.41
ttg	L	0	2.99
ctt	L	0	2.71
ctc	L	0	2.68
cta	L	0	2.67
ctg	L	0	3.55
att	I	0	5.47
atc	I	1	3.63
ata	I	14	5.90
gtt	V	0	198.80
gtc	V	0	192.90
gta	V	955	199.40
gtg	V	8	371.90
---	--	------	------
mPD		0.079	0.79
		nPD:	0.1
		N. weight:	1.7
		Sc. PD:	-0.0093
		Sc. rank:	264.4

,

NA

Pos.	108	obs:	exp:
cgt	R	0	42.33
cgc	R	0	50.35
cga	R	0	98.81
cgg	R	0	82.54
aaa	K	3	1.76
aag	K	0	1.24
aga	R	983	452.50
agg	R	8	264.50
---	--	------	------
mPD		0.022	0.97
		nPD:	0.02
		N. weight:	0.82
		Sc. PD:	-0.066
		Sc. rank:	-261.2

,

NA

Pos.	109	obs:	exp:
tta	L	0	0.18
ttg	L	0	0.37
ctt	L	2	0.34
ctc	L	0	0.34
cta	L	0	0.33
ctg	L	0	0.44
att	I	985	362.00
atc	I	5	239.90
ata	I	2	390.10
---	--	------	------
mPD		0.018	0.66
		nPD:	0.03
		N. weight:	1.1
		Sc. PD:	-0.081
		Sc. rank:	-267.3

,

NA

Pos.	110	obs:	exp:
gaa	E	1	0.60
gag	E	0	0.40
ggt	G	0	133.20
ggc	G	0	126.20
gga	G	946	459.10
ggg	G	47	274.60
---	--	------	------
mPD		0.092	0.68
		nPD:	0.14
		N. weight:	0.67
		Sc. PD:	0.02
		Sc. rank:	193.7

,

NA			
Pos.	111	obs:	exp:
aaa	K	0	0.59
aag	K	1	0.41
gaa	E	424	597.30
gag	E	569	395.70
---	--	------	------
mPD		0.49	0.48
		nPD:	1.02
		N. weight:	0.073
		Sc. PD:	0.065
		Sc. rank:	57.0

NA			
Pos.	112	obs:	exp:
tct	S	0	146.50
tcc	S	0	118.40
tca	S	0	230.90
tcg	S	0	53.25
aat	N	3	38.16
aac	N	72	36.84
agt	S	0	187.50
agc	S	915	178.40
gat	D	0	2.16
gac	D	4	1.84
---	--	------	------
mPD		0.16	1.7
		nPD:	0.09
		N. weight:	1.7
		Sc. PD:	-0.022
		Sc. rank:	177.6

NA			
Pos.	113	obs:	exp:
tct	S	2	159.10
tcc	S	0	128.70
tca	S	75	250.90
tcg	S	917	57.84
agt	S	0	203.70
agc	S	0	193.80
---	--	------	------
mPD		0.14	1.7
		nPD:	0.09
		N. weight:	2.7
		Sc. PD:	-0.05
		Sc. rank:	202.1

NA			
Pos.	114	obs:	exp:
gat	D	984	532.30
gac	D	2	453.70
gaa	E	8	4.81
gag	E	0	3.19
---	--	------	------
mPD		0.020	0.50
		nPD:	0.04
		N. weight:	0.67
		Sc. PD:	-0.043
		Sc. rank:	-104.7

NA			
Pos.	115	obs:	exp:
att	I	5	1.82
atc	I	0	1.21
ata	I	0	1.97
gtt	V	979	204.20
gtc	V	9	198.10
gta	V	0	204.80
gtg	V	1	381.90
---	--	------	------
mPD		0.030	0.74
		nPD:	0.04
		N. weight:	1.7
		Sc. PD:	-0.11
		Sc. rank:	-245.8

NA			
Pos.	116	obs:	exp:
tta	L	947	87.36
ttg	L	1	185.50
ctt	L	0	168.40
ctc	L	0	166.50
cta	L	44	165.70
ctg	L	2	220.40
---	--	------	------
mPD		0.094	1.1
		nPD:	0.09
		N. weight:	2.4
		Sc. PD:	-0.049
		Sc. rank:	155.8

NA			
Pos.	117	obs:	exp:
att	I	0	0.73
atc	I	2	0.48
ata	I	0	0.79
gtt	V	37	204.00
gtc	V	904	197.90
gta	V	44	204.60
gtg	V	3	381.60
gct	A	0	1.02
gcc	A	4	0.75
gca	A	0	1.87
gcg	A	0	0.35
---	--	------	------
mPD		0.17	0.74
		nPD:	0.23
		N. weight:	1.4
		Sc. PD:	0.17
		Sc. rank:	797.8

NA			
Pos.	118	obs:	exp:
act	T	18	257.50
acc	T	0	202.40
aca	T	930	456.70
acg	T	46	77.43
---	--	------	------
mPD		0.12	0.67
		nPD:	0.18
		N. weight:	0.66
		Sc. PD:	0.048
		Sc. rank:	313.0

NA			
Pos.	119	obs:	exp:
cgt	R	0	42.42
cgc	R	0	50.45
cga	R	0	99.01
cgg	R	0	82.71
act	T	0	0.26
acc	T	0	0.20
aca	T	1	0.46
acg	T	0	0.08
aga	R	990	453.40
agg	R	3	265.00
---	--	------	------
mPD		0.0080	0.97
		nPD:	0.01
		N. weight:	0.85
		Sc. PD:	-0.08
		Sc. rank:	-497.7

NA			
Pos.	120	obs:	exp:
tat	Y	0	1.06
tac	Y	2	0.94
gaa	E	956	596.70
gag	E	36	395.30
---	--	------	------
mPD		0.078	0.49
		nPD:	0.16
		N. weight:	0.41
		Sc. PD:	0.021
		Sc. rank:	162.5

NA			
Pos.	121	obs:	exp:
cct	P	1	252.70
ccc	P	991	177.20
cca	P	2	406.10
ccg	P	0	157.90
---	--	------	------
mPD		0.0060	0.71
		nPD:	0.01
		N. weight:	1.9
		Sc. PD:	-0.18
		Sc. rank:	-1032.7

NA			
Pos.	122	obs:	exp:
tat	Y	988	527.00
tac	Y	6	467.00
---	--	------	------
mPD		0.012	0.50
		nPD:	0.02
		N. weight:	0.66
		Sc. PD:	-0.052
		Sc. rank:	-201.7

NA			
Pos.	123	obs:	exp:
gtt	V	867	205.20
gtc	V	45	199.10
gta	V	0	205.80
gtg	V	82	383.90
---	--	------	------
mPD		0.23	0.73
		nPD:	0.32
		N. weight:	1.2
		Sc. PD:	0.24
		Sc. rank:	806.4

NA			
Pos.	124	obs:	exp:
tct	S	0	159.10
tcc	S	45	128.70
tca	S	949	250.90
tcg	S	0	57.84
agt	S	0	203.70
agc	S	0	193.80
---	--	------	------
mPD		0.087	1.7
		nPD:	0.05
		N. weight:	1.4
		Sc. PD:	-0.07
		Sc. rank:	-111.6

NA			
Pos.	125	obs:	exp:
tgt	C	50	408.10
tgc	C	944	585.90
---	--	------	------
mPD		0.096	0.48
		nPD:	0.2
		N. weight:	0.39
		Sc. PD:	0.034
		Sc. rank:	200.1

NA			
Pos.	126	obs:	exp:
aat	N	0	13.74
aac	N	27	13.26
gat	D	49	501.50
gac	D	880	427.50
ggt	G	0	5.10
ggc	G	38	4.83
gga	G	0	17.57
ggg	G	0	10.51
---	--	------	------
mPD		0.22	0.65
		nPD:	0.34
		N. weight:	0.69
		Sc. PD:	0.15
		Sc. rank:	484.5

NA			
Pos.	127	obs:	exp:
cct	P	0	252.70
ccc	P	0	177.20
cca	P	957	406.10
ccg	P	37	157.90
---	--	------	------
mPD		0.072	0.71
		nPD:	0.1
		N. weight:	0.86
		Sc. PD:	-0.0042
		Sc. rank:	136.5

NA			
Pos.	128	obs:	exp:
tat	Y	1	0.53
tac	Y	0	0.47
cat	H	2	1.15
cac	H	0	0.85
aat	N	6	3.05
aac	N	0	2.95
gat	D	984	531.70
gac	D	1	453.30
---	--	------	------
mPD		0.020	0.51
		nPD:	0.04
		N. weight:	0.68
		Sc. PD:	-0.043
		Sc. rank:	-109.5

NA			
Pos.	129	obs:	exp:
gaa	E	925	596.70
gag	E	67	395.30
ggt	G	0	0.27
ggc	G	0	0.25
gga	G	2	0.92
ggg	G	0	0.55
---	--	------	------
mPD		0.13	0.48
		nPD:	0.27
		N. weight:	0.32
		Sc. PD:	0.051
		Sc. rank:	204.3

NA			
Pos.	130	obs:	exp:
tgt	C	1	408.10
tgc	C	993	585.90
---	--	------	------
mPD		0.0020	0.48
		nPD:	0.
		N. weight:	0.58
		Sc. PD:	-0.056
		Sc. rank:	-395.0

NA			
Pos.	131	obs:	exp:
cgt	R	0	36.57
cgc	R	0	43.49
cga	R	1	85.35
cgg	R	0	71.30
act	T	0	0.52
acc	T	0	0.41
aca	T	0	0.92
acg	T	2	0.16
aaa	K	0	79.67
aag	K	136	56.33
aga	R	2	390.80
agg	R	853	228.50
---	--	------	------
mPD		0.25	1.2
		nPD:	0.21
		N. weight:	1.4
		Sc. PD:	0.14
		Sc. rank:	767.9

NA			
Pos.	132	obs:	exp:
ttt	F	7	391.10
ttc	F	984	599.90
tct	S	0	0.48
tcc	S	3	0.39
tca	S	0	0.76
tcg	S	0	0.17
agt	S	0	0.61
agc	S	0	0.59
---	--	------	------
mPD		0.020	0.49
		nPD:	0.04
		N. weight:	0.52
		Sc. PD:	-0.032
		Sc. rank:	-70.9

NA			
Pos.	133	obs:	exp:
tat	Y	992	527.00
tac	Y	2	467.00
---	--	------	------
mPD		0.0040	0.50
		nPD:	0.01
		N. weight:	0.69
		Sc. PD:	-0.065
		Sc. rank:	-410.4

NA			
Pos.	134	obs:	exp:
gct	A	988	254.70
gcc	A	6	186.60
gca	A	0	464.70
gcg	A	0	87.92
---	--	------	------
mPD		0.012	0.67
		nPD:	0.02
		N. weight:	1.5
		Sc. PD:	-0.12
		Sc. rank:	-532.7

NA			
Pos.	135	obs:	exp:
tta	L	1	87.36
ttg	L	0	185.50
ctt	L	8	168.40
ctc	L	984	166.50
cta	L	1	165.70
ctg	L	0	220.40
---	--	------	------
mPD		0.022	1.1
		nPD:	0.02
		N. weight:	1.9
		Sc. PD:	-0.16
		Sc. rank:	-668.1

NA			
Pos.	136	obs:	exp:
tct	S	0	158.50
tcc	S	0	128.10
tca	S	0	249.80
tcg	S	0	57.61
act	T	0	0.78
acc	T	3	0.61
aca	T	0	1.38
acg	T	0	0.23
agt	S	7	202.90
agc	S	983	193.10
ggt	G	0	0.13
ggc	G	1	0.13
gga	G	0	0.46
ggg	G	0	0.28
---	--	------	------
mPD		0.022	1.7
		nPD:	0.01
		N. weight:	1.8
		Sc. PD:	-0.16
		Sc. rank:	-807.4

NA			
Pos.	137	obs:	exp:
caa	Q	982	510.60
cag	Q	12	483.40
---	--	------	------
mPD		0.024	0.50
		nPD:	0.05
		N. weight:	0.67
		Sc. PD:	-0.037
		Sc. rank:	-73.4

NA			
Pos.	138	obs:	exp:
ggt	G	0	133.30
ggc	G	0	126.30
gga	G	976	459.60
ggg	G	18	274.80
---	--	------	------
mPD		0.036	0.68
		nPD:	0.05
		N. weight:	0.77
		Sc. PD:	-0.039
		Sc. rank:	-57.7

NA			
Pos.	139	obs:	exp:
act	T	1	257.50
acc	T	0	202.40
aca	T	991	456.70
acg	T	2	77.43
---	--	------	------
mPD		0.0060	0.67
		nPD:	0.01
		N. weight:	0.84
		Sc. PD:	-0.079
		Sc. rank:	-450.0

NA			
Pos.	140	obs:	exp:
att	I	0	0.36
atc	I	0	0.24
ata	I	1	0.39
act	T	1	257.20
acc	T	0	202.20
aca	T	950	456.20
acg	T	42	77.35
---	--	------	------
mPD		0.085	0.68
		nPD:	0.13
		N. weight:	0.75
		Sc. PD:	0.014
		Sc. rank:	187.7

NA			
Pos.	141	obs:	exp:
att	I	47	362.70
atc	I	945	240.40
ata	I	2	390.90
---	--	------	------
mPD		0.094	0.65
		nPD:	0.14
		N. weight:	1.3
		Sc. PD:	0.048
		Sc. rank:	433.5

NA			
Pos.	142	obs:	exp:
cgt	R	0	42.21
cgc	R	0	50.20
cga	R	1	98.51
cgg	R	0	82.29
att	I	0	0.36
atc	I	0	0.24
ata	I	1	0.39
act	T	0	0.52
acc	T	0	0.41
aca	T	2	0.92
acg	T	0	0.16
aaa	K	3	1.76
aag	K	0	1.24
aga	R	986	451.10
agg	R	1	263.70
---	--	------	------
mPD		0.016	0.97
		nPD:	0.02
		N. weight:	0.85
		Sc. PD:	-0.073
		Sc. rank:	-348.6

NA			
Pos.	143	obs:	exp:
tgg	W	2	2.00
ggt	G	0	133.00
ggc	G	0	126.00
gga	G	2	458.70
ggg	G	990	274.30
---	--	------	------
mPD		0.0080	0.68
		nPD:	0.01
		N. weight:	1.4
		Sc. PD:	-0.13
		Sc. rank:	-707.4

NA			
Pos.	144	obs:	exp:
cgt	R	0	0.04
cgc	R	0	0.05
cga	R	0	0.10
cgg	R	0	0.08
aaa	K	990	581.70
aag	K	3	411.30
aga	R	1	0.46
agg	R	0	0.27
---	--	------	------
mPD		0.0080	0.49
		nPD:	0.02
		N. weight:	0.57
		Sc. PD:	-0.049
		Sc. rank:	-235.2

NA			
Pos.	145	obs:	exp:
tta	L	0	0.18
ttg	L	0	0.37
ctt	L	0	0.34
ctc	L	2	0.34
cta	L	0	0.33
ctg	L	0	0.44
cat	H	14	569.90
cac	H	978	422.10
---	--	------	------
mPD		0.032	0.50
		nPD:	0.06
		N. weight:	0.86
		Sc. PD:	-0.035
		Sc. rank:	-7.5

NA			
Pos.	146	obs:	exp:
tct	S	1	159.10
tcc	S	0	128.70
tca	S	988	250.90
tcg	S	5	57.84
agt	S	0	203.70
agc	S	0	193.80
---	--	------	------
mPD		0.012	1.7
		nPD:	0.01
		N. weight:	1.5
		Sc. PD:	-0.14
		Sc. rank:	-904.9

NA			
Pos.	147	obs:	exp:
act	T	0	0.26
acc	T	1	0.20
aca	T	0	0.46
acg	T	0	0.08
aat	N	58	505.20
aac	N	935	487.80
---	--	------	------
mPD		0.11	0.50
		nPD:	0.22
		N. weight:	0.54
		Sc. PD:	0.061
		Sc. rank:	304.4

NA			
Pos.	148	obs:	exp:
cgt	R	0	0.04
cgc	R	0	0.05
cga	R	0	0.10
cgg	R	0	0.08
aga	R	1	0.46
agg	R	0	0.27
gaa	E	1	0.60
gag	E	0	0.40
ggt	G	33	133.00
ggc	G	0	126.00
gga	G	951	458.70
ggg	G	8	274.30
---	--	------	------
mPD		0.084	0.68
		nPD:	0.12
		N. weight:	0.7
		Sc. PD:	0.012
		Sc. rank:	168.3

NA			
Pos.	149	obs:	exp:
act	T	0	257.50
acc	T	0	202.40
aca	T	984	456.70
acg	T	10	77.43
---	--	------	------
mPD		0.020	0.67
		nPD:	0.03
		N. weight:	0.82
		Sc. PD:	-0.06
		Sc. rank:	-182.9

NA			
Pos.	150	obs:	exp:
tta	L	1	0.09
ttg	L	0	0.19
ctt	L	0	0.17
ctc	L	0	0.17
cta	L	0	0.17
ctg	L	0	0.22
cgt	R	0	0.13
cgc	R	0	0.15
cga	R	0	0.30
cgg	R	0	0.25
att	I	0	358.30
atc	I	0	237.50
ata	I	982	386.20
atg	M	2	2.00
act	T	0	0.52
acc	T	0	0.41
aca	T	2	0.92
acg	T	0	0.16
aga	R	3	1.37
agg	R	0	0.80
gtt	V	0	0.83
gtc	V	0	0.80
gta	V	4	0.83
gtg	V	0	1.54
---	--	------	------
mPD		0.024	0.68
		nPD:	0.04
		N. weight:	1.
		Sc. PD:	-0.07
		Sc. rank:	-186.8

NA			
Pos.	151	obs:	exp:
cat	H	5	568.20
cac	H	984	420.80
caa	Q	1	0.51
cag	Q	0	0.49
cgt	R	0	0.17
cgc	R	4	0.20
cga	R	0	0.40
cgg	R	0	0.33
aga	R	0	1.83
agg	R	0	1.07
---	--	------	------
mPD		0.020	0.51
		nPD:	0.04
		N. weight:	0.92
		Sc. PD:	-0.059
		Sc. rank:	-146.4

NA			
Pos.	152	obs:	exp:
gat	D	987	535.50
gac	D	5	456.50
gaa	E	2	1.20
gag	E	0	0.80
---	--	------	------
mPD		0.014	0.50
		nPD:	0.03
		N. weight:	0.65
		Sc. PD:	-0.049
		Sc. rank:	-157.3

NA			
Pos.	153	obs:	exp:
cgt	R	0	42.42
cgc	R	1	50.45
cga	R	0	99.01
cgg	R	0	82.71
aaa	K	0	0.59
aag	K	1	0.41
aga	R	47	453.40
agg	R	945	265.00
---	--	------	------
mPD		0.096	0.97
		nPD:	0.1
		N. weight:	1.2
		Sc. PD:	-0.0074
		Sc. rank:	171.0

NA			
Pos.	154	obs:	exp:
tct	S	1	159.10
tcc	S	992	128.70
tca	S	1	250.90
tcg	S	0	57.84
agt	S	0	203.70
agc	S	0	193.80
---	--	------	------
mPD		0.0040	1.7
		nPD:	0.
		N. weight:	2.2
		Sc. PD:	-0.22
		Sc. rank:	-1559.1

NA			
Pos.	155	obs:	exp:
caa	Q	13	510.60
cag	Q	981	483.40
---	--	------	------
mPD		0.026	0.50
		nPD:	0.05
		N. weight:	0.72
		Sc. PD:	-0.038
		Sc. rank:	-66.1

NA

Pos.	156	obs:	exp:
ttt	F	2	0.79
ttc	F	0	1.21
tat	Y	929	525.40
tac	Y	62	465.60
cat	H	1	0.57
cac	H	0	0.43
---	--	------	------
mPD		0.12	0.50
		nPD:	0.24
		N. weight:	0.46
		Sc. PD:	0.061
		Sc. rank:	274.3

NA

Pos.	157	obs:	exp:
tta	L	0	0.18
ttg	L	0	0.37
ctt	L	0	0.34
ctc	L	2	0.34
cta	L	0	0.33
ctg	L	0	0.44
cct	P	0	0.51
ccc	P	2	0.36
cca	P	0	0.82
ccg	P	0	0.32
cgt	R	67	42.29
cgc	R	889	50.30
cga	R	34	98.71
cgg	R	0	82.46
aga	R	0	452.00
agg	R	0	264.20
---	--	------	------
mPD		0.20	0.98
		nPD:	0.2
		N. weight:	2.9
		Sc. PD:	0.26
		Sc. rank:	1512.2

NA

Pos.	158	obs:	exp:
tct	S	0	0.16
tcc	S	1	0.13
tca	S	0	0.25
tcg	S	0	0.06
cct	P	0	0.25
ccc	P	1	0.18
cca	P	0	0.41
ccg	P	0	0.16
act	T	0	0.26
acc	T	1	0.20
aca	T	0	0.46
acg	T	0	0.08
agt	S	0	0.20
agc	S	0	0.20
gct	A	7	253.90
gcc	A	984	186.10
gca	A	0	463.30
gcg	A	0	87.65
---	--	------	------
mPD		0.020	0.68
		nPD:	0.03
		N. weight:	1.8
		Sc. PD:	-0.13
		Sc. rank:	-407.2

NA

Pos.	159	obs:	exp:
tta	L	0	87.36
ttg	L	1	185.50
ctt	L	1	168.40
ctc	L	0	166.50
cta	L	1	165.70
ctg	L	991	220.40
---	--	------	------
mPD		0.0060	1.1
		nPD:	0.01
		N. weight:	1.6
		Sc. PD:	-0.16
		Sc. rank:	-1081.5

NA

Pos.	160	obs:	exp:
att	I	0	362.00
atc	I	0	239.90
ata	I	992	390.10
gtt	V	0	0.41
gtc	V	0	0.40
gta	V	2	0.41
gtg	V	0	0.77
---	--	------	------
mPD		0.0040	0.66
		nPD:	0.01
		N. weight:	1.
		Sc. PD:	-0.099
		Sc. rank:	-653.4

NA

Pos.	161	obs:	exp:
tct	S	0	159.10
tcc	S	0	128.70
tca	S	0	250.90
tcg	S	0	57.84
agt	S	2	203.70
agc	S	992	193.80
---	--	------	------
mPD		0.0040	1.7
		nPD:	0.
		N. weight:	1.8
		Sc. PD:	-0.18
		Sc. rank:	-1258.8

NA

Pos.	162	obs:	exp:
tgg	W	994	994.00
---	--	------	------
mPD		0	0
		nPD:	1.
		N. weight:	0.
		Sc. PD:	0
		Sc. rank:	0

NA

Pos.	163	obs:	exp:
cct	P	1	252.70
ccc	P	0	177.20
cca	P	985	406.10
ccg	P	8	157.90
---	--	------	------
mPD		0.018	0.71
		nPD:	0.03
		N. weight:	0.94
		Sc. PD:	-0.073
		Sc. rank:	-252.9

NA

Pos.	164	obs:	exp:
tta	L	11	87.10
ttg	L	0	184.90
ctt	L	0	167.90
ctc	L	0	166.00
cta	L	937	165.20
ctg	L	43	219.80
caa	Q	1	0.51
cag	Q	0	0.49
att	I	0	0.73
atc	I	0	0.48
ata	I	2	0.79
---	--	------	------
mPD		0.11	1.1
		nPD:	0.1
		N. weight:	1.7
		Sc. PD:	-0.011
		Sc. rank:	233.5

NA

Pos.	165	obs:	exp:
tct	S	0	159.10
tcc	S	0	128.70
tca	S	993	250.90
tcg	S	1	57.84
agt	S	0	203.70
agc	S	0	193.80
---	--	------	------
mPD		0.0020	1.7
		nPD:	0.
		N. weight:	1.5
		Sc. PD:	-0.15
		Sc. rank:	-1090.6

NA

Pos.	166	obs:	exp:
tct	S	0	159.00
tcc	S	0	128.50
tca	S	992	250.60
tcg	S	1	57.79
act	T	0	0.26
acc	T	0	0.20
aca	T	1	0.46
acg	T	0	0.08
agt	S	0	203.50
agc	S	0	193.60
---	--	------	------
mPD		0.0040	1.7
		nPD:	0.
		N. weight:	1.5
		Sc. PD:	-0.15
		Sc. rank:	-1045.3

NA

Pos.	167	obs:	exp:
cct	P	7	252.70
ccc	P	1	177.20
cca	P	51	406.10
ccg	P	935	157.90
---	--	------	------
mPD		0.11	0.71
		nPD:	0.16
		N. weight:	1.7
		Sc. PD:	0.086
		Sc. rank:	659.3

NA

Pos.	168	obs:	exp:
tta	L	0	0.09
ttg	L	0	0.19
ctt	L	0	0.17
ctc	L	1	0.17
cta	L	0	0.17
ctg	L	0	0.22
cct	P	0	252.50
ccc	P	992	177.10
cca	P	1	405.70
ccg	P	0	157.80
---	--	------	------
mPD		0.0040	0.71
		nPD:	0.01
		N. weight:	1.9
		Sc. PD:	-0.18
		Sc. rank:	-1237.4

NA

Pos.	169	obs:	exp:
cct	P	0	0.25
ccc	P	0	0.18
cca	P	1	0.41
ccg	P	0	0.16
act	T	1	257.20
acc	T	0	202.20
aca	T	989	456.20
acg	T	3	77.35
---	--	------	------
mPD		0.010	0.68
		nPD:	0.01
		N. weight:	0.84
		Sc. PD:	-0.073
		Sc. rank:	-368.6

NA

Pos.	170	obs:	exp:
atg	M	1	1.00
act	T	0	0.26
acc	T	0	0.20
aca	T	0	0.46
acg	T	1	0.08
gtt	V	0	204.80
gtc	V	0	198.70
gta	V	77	205.40
gtg	V	915	383.10
---	--	------	------
mPD		0.15	0.73
		nPD:	0.2
		N. weight:	0.81
		Sc. PD:	0.076
		Sc. rank:	431.3

NA

Pos.	171	obs:	exp:
tat	Y	27	450.20
tac	Y	822	398.80
cat	H	0	82.73
cac	H	144	61.27
aat	N	0	0.51
aac	N	1	0.49
---	--	------	------
mPD		0.30	0.75
		nPD:	0.41
		N. weight:	0.72
		Sc. PD:	0.21
		Sc. rank:	535.3

NA

Pos.	172	obs:	exp:
aat	N	33	505.70
aac	N	961	488.30
---	--	------	------
mPD		0.064	0.50
		nPD:	0.13
		N. weight:	0.63
		Sc. PD:	0.014
		Sc. rank:	161.7

NA

Pos.	173	obs:	exp:
tct	S	0	158.80
tcc	S	0	128.40
tca	S	0	250.30
tcg	S	0	57.73
agt	S	4	203.30
agc	S	988	193.40
gct	A	1	0.26
gcc	A	0	0.19
gca	A	0	0.47
gcg	A	0	0.09
ggt	G	0	0.13
ggc	G	1	0.13
gga	G	0	0.46
ggg	G	0	0.28
---	--	------	------
mPD		0.016	1.7
		nPD:	0.01
		N. weight:	1.8
		Sc. PD:	-0.16
		Sc. rank:	-920.4

NA

Pos.	174	obs:	exp:
cgt	R	0	42.42
cgc	R	0	50.45
cga	R	0	99.01
cgg	R	3	82.71
aga	R	11	453.40
agg	R	979	265.00
ggt	G	0	0.13
ggc	G	0	0.13
gga	G	0	0.46
ggg	G	1	0.28
---	--	------	------
mPD		0.030	0.96
		nPD:	0.03
		N. weight:	1.4
		Sc. PD:	-0.099
		Sc. rank:	-299.8

NA			
Pos.	175	obs:	exp:
att	I	0	13.50
atc	I	0	8.95
ata	I	37	14.55
gtt	V	3	197.60
gtc	V	0	191.70
gta	V	8	198.10
gtg	V	946	369.60
---	--	------	------
mPD		0.16	0.80
		nPD:	0.2
		N. weight:	0.99
		Sc. PD:	0.094
		Sc. rank:	533.2

,

NA			
Pos.	176	obs:	exp:
gaa	E	871	597.90
gag	E	123	396.10
---	--	------	------
mPD		0.22	0.48
		nPD:	0.45
		N. weight:	0.21
		Sc. PD:	0.069
		Sc. rank:	156.6

,

NA			
Pos.	177	obs:	exp:
tgt	C	6	408.10
tgc	C	988	585.90
---	--	------	------
mPD		0.012	0.48
		nPD:	0.02
		N. weight:	0.55
		Sc. PD:	-0.043
		Sc. rank:	-153.2

,

NA			
Pos.	178	obs:	exp:
att	I	856	362.70
atc	I	138	240.40
ata	I	0	390.90
---	--	------	------
mPD		0.24	0.65
		nPD:	0.37
		N. weight:	0.74
		Sc. PD:	0.18
		Sc. rank:	529.3

,

NA			
Pos.	179	obs:	exp:
ggt	G	53	133.30
ggc	G	1	126.30
gga	G	8	459.60
ggg	G	932	274.80
---	--	------	------
mPD		0.12	0.68
		nPD:	0.17
		N. weight:	1.2
		Sc. PD:	0.078
		Sc. rank:	526.4

,

NA			
Pos.	180	obs:	exp:
tgg	W	994	994.00
---	--	------	------
mPD		0	0
		nPD:	1.
		N. weight:	0.
		Sc. PD:	0
		Sc. rank:	0

,

NA			
Pos.	181	obs:	exp:
tct	S	8	159.10
tcc	S	1	128.70
tca	S	980	250.90
tcg	S	5	57.84
agt	S	0	203.70
agc	S	0	193.80
---	--	------	------
mPD		0.028	1.7
		nPD:	0.02
		N. weight:	1.4
		Sc. PD:	-0.12
		Sc. rank:	-580.9

,

NA			
Pos.	182	obs:	exp:
tct	S	0	159.10
tcc	S	0	128.70
tca	S	0	250.90
tcg	S	0	57.84
agt	S	850	203.70
agc	S	144	193.80
---	--	------	------
mPD		0.25	1.7
		nPD:	0.15
		N. weight:	1.3
		Sc. PD:	0.055
		Sc. rank:	462.3

,

NA			
Pos.	183	obs:	exp:
act	T	914	238.10
acc	T	5	187.10
aca	T	0	422.20
acg	T	0	71.59
gtt	V	0	0.21
gtc	V	1	0.20
gta	V	0	0.21
gtg	V	0	0.39
gct	A	74	18.96
gcc	A	0	13.89
gca	A	0	34.60
gcg	A	0	6.55
---	--	------	------
mPD		0.15	0.82
		nPD:	0.19
		N. weight:	1.5
		Sc. PD:	0.12
		Sc. rank:	733.8

,

NA			
Pos.	184	obs:	exp:
tct	S	0	158.50
tcc	S	0	128.10
tca	S	0	249.80
tcg	S	0	57.61
att	I	2	0.73
atc	I	0	0.48
ata	I	0	0.79
aat	N	2	1.02
aac	N	0	0.98
agt	S	944	202.90
agc	S	46	193.10
---	--	------	------
mPD		0.096	1.7
		nPD:	0.06
		N. weight:	1.6
		Sc. PD:	-0.071
		Sc. rank:	-56.6

,

NA			
Pos.	185	obs:	exp:
tgt	C	48	408.10
tgc	C	946	585.90
---	--	------	------
mPD		0.092	0.48
		nPD:	0.19
		N. weight:	0.39
		Sc. PD:	0.032
		Sc. rank:	197.6

,

NA			
Pos.	186	obs:	exp:
cct	P	1	0.25
ccc	P	0	0.18
cca	P	0	0.41
ccg	P	0	0.16
cat	H	988	569.90
cac	H	4	422.10
aat	N	1	0.51
aac	N	0	0.49
---	--	------	------
mPD		0.012	0.49
		nPD:	0.02
		N. weight:	0.59
		Sc. PD:	-0.046
		Sc. rank:	-173.9

,

NA			
Pos.	187	obs:	exp:
gat	D	994	536.60
gac	D	0	457.40
---	--	------	------
mPD		0	0.50
		nPD:	0.
		N. weight:	0.68
		Sc. PD:	-0.069
		Sc. rank:	-529.4

,

NA			
Pos.	188	obs:	exp:
ggt	G	143	133.30
ggc	G	847	126.30
gga	G	3	459.60
ggg	G	1	274.80
---	--	------	------
mPD		0.25	0.68
		nPD:	0.38
		N. weight:	1.8
		Sc. PD:	0.46
		Sc. rank:	1295.8

,

NA			
Pos.	189	obs:	exp:
cgt	R	0	2.14
cgc	R	0	2.54
cga	R	0	4.99
cgg	R	0	4.16
aaa	K	876	553.00
aag	K	68	391.00
aga	R	50	22.83
agg	R	0	13.34
---	--	------	------
mPD		0.22	0.61
		nPD:	0.36
		N. weight:	0.36
		Sc. PD:	0.09
		Sc. rank:	260.0

,

NA			
Pos.	190	obs:	exp:
ttt	F	0	0.39
ttc	F	1	0.61
tct	S	3	151.80
tcc	S	945	122.70
tca	S	0	239.20
tcg	S	0	55.17
agt	S	0	194.30
agc	S	0	184.90
gct	A	1	11.53
gcc	A	44	8.45
gca	A	0	21.04
gcg	A	0	3.98
---	--	------	------
mPD		0.097	1.7
		nPD:	0.06
		N. weight:	2.2
		Sc. PD:	-0.1
		Sc. rank:	-114.6

,

NA			
Pos.	191	obs:	exp:
cgt	R	0	42.46
cgc	R	0	50.50
cga	R	0	99.11
cgg	R	1	82.79
aga	R	147	453.80
agg	R	846	265.30
---	--	------	------
mPD		0.25	0.96
		nPD:	0.26
		N. weight:	0.91
		Sc. PD:	0.14
		Sc. rank:	569.8

,

NA			
Pos.	192	obs:	exp:
atg	M	994	994.00
---	--	------	------
mPD		0	0
		nPD:	1.
		N. weight:	0.
		Sc. PD:	0
		Sc. rank:	0

,

NA			
Pos.	193	obs:	exp:
tct	S	1	158.20
tcc	S	1	127.90
tca	S	986	249.30
tcg	S	0	57.50
act	T	0	1.30
acc	T	0	1.02
aca	T	5	2.30
acg	T	0	0.39
agt	S	0	202.50
agc	S	0	192.70
gct	A	0	0.26
gcc	A	0	0.19
gca	A	1	0.47
gcg	A	0	0.09
---	--	------	------
mPD		0.016	1.7
		nPD:	0.01
		N. weight:	1.5
		Sc. PD:	-0.14
		Sc. rank:	-786.6

,

NA			
Pos.	194	obs:	exp:
att	I	0	360.90
atc	I	0	239.20
ata	I	989	388.90
gtt	V	0	1.03
gtc	V	0	1.00
gta	V	5	1.04
gtg	V	0	1.93
---	--	------	------
mPD		0.010	0.67
		nPD:	0.02
		N. weight:	1.
		Sc. PD:	-0.09
		Sc. rank:	-451.9

,

NA			
Pos.	195	obs:	exp:
tgt	C	979	408.10
tgc	C	15	585.90
---	--	------	------
mPD		0.030	0.48
		nPD:	0.06
		N. weight:	0.9
		Sc. PD:	-0.038
		Sc. rank:	-19.6

,

NA			
Pos.	196	obs:	exp:
att	I	2	361.60
atc	I	6	239.70
ata	I	983	389.70
gtt	V	0	0.62
gtc	V	0	0.60
gta	V	2	0.62
gtg	V	1	1.16
---	--	------	------
mPD		0.024	0.66
		nPD:	0.04
		N. weight:	0.98
		Sc. PD:	-0.066
		Sc. rank:	-166.7

,

NA			
Pos.	197	obs:	exp:
tct	S	0	159.10
tcc	S	0	128.70
tca	S	993	250.90
tcg	S	1	57.84
agt	S	0	203.70
agc	S	0	193.80
---	--	------	------
mPD		0.0020	1.7
		nPD:	0.
		N. weight:	1.5
		Sc. PD:	-0.15
		Sc. rank:	-1090.6

,

NA			
Pos.	198	obs:	exp:
ggt	G	0	133.30
ggc	G	44	126.30
gga	G	937	459.60
ggg	G	13	274.80
---	--	------	------
mPD		0.11	0.68
		nPD:	0.16
		N. weight:	0.65
		Sc. PD:	0.035
		Sc. rank:	260.0

,

NA			
Pos.	199	obs:	exp:
cct	P	1	252.70
ccc	P	0	177.20
cca	P	913	406.10
ccg	P	80	157.90
---	--	------	------
mPD		0.15	0.71
		nPD:	0.21
		N. weight:	0.76
		Sc. PD:	0.077
		Sc. rank:	416.4

,

NA			
Pos.	200	obs:	exp:
tct	S	0	0.48
tcc	S	0	0.39
tca	S	0	0.76
tcg	S	0	0.17
aat	N	5	503.70
aac	N	985	486.30
agt	S	0	0.61
agc	S	3	0.59
gat	D	0	0.54
gac	D	1	0.46
---	--	------	------
mPD		0.018	0.51
		nPD:	0.04
		N. weight:	0.76
		Sc. PD:	-0.051
		Sc. rank:	-141.2

,

NA			
Pos.	201	obs:	exp:
aat	N	3	505.70
aac	N	991	488.30
---	--	------	------
mPD		0.0060	0.50
		nPD:	0.01
		N. weight:	0.77
		Sc. PD:	-0.069
		Sc. rank:	-372.1

,

NA			
Pos.	202	obs:	exp:
aat	N	990	505.70
aac	N	4	488.30
---	--	------	------
mPD		0.0080	0.50
		nPD:	0.02
		N. weight:	0.72
		Sc. PD:	-0.062
		Sc. rank:	-310.2

,

NA			
Pos.	203	obs:	exp:
gct	A	2	254.70
gcc	A	0	186.60
gca	A	951	464.70
gcg	A	41	87.92
---	--	------	------
mPD		0.083	0.67
		nPD:	0.12
		N. weight:	0.72
		Sc. PD:	0.012
		Sc. rank:	175.1

,

NA			
Pos.	204	obs:	exp:
tct	S	948	159.10
tcc	S	2	128.70
tca	S	44	250.90
tcg	S	0	57.84
agt	S	0	203.70
agc	S	0	193.80
---	--	------	------
mPD		0.089	1.7
		nPD:	0.05
		N. weight:	1.8
		Sc. PD:	-0.09
		Sc. rank:	-110.4

,

NA			
Pos.	205	obs:	exp:
gct	A	53	254.70
gcc	A	0	186.60
gca	A	938	464.70
gcg	A	3	87.92
---	--	------	------
mPD		0.11	0.67
		nPD:	0.16
		N. weight:	0.63
		Sc. PD:	0.032
		Sc. rank:	246.4

,

NA			
Pos.	206	obs:	exp:
tta	L	1	0.09
ttg	L	0	0.19
ctt	L	0	0.17
ctc	L	0	0.17
cta	L	0	0.17
ctg	L	0	0.22
att	I	0	1.09
atc	I	0	0.73
ata	I	3	1.18
gtt	V	2	204.40
gtc	V	0	198.30
gta	V	985	205.00
gtg	V	3	382.30
---	--	------	------
mPD		0.018	0.73
		nPD:	0.02
		N. weight:	1.7
		Sc. PD:	-0.13
		Sc. rank:	-494.0

,

NA			
Pos.	207	obs:	exp:
att	I	36	50.72
atc	I	5	33.62
ata	I	98	54.66
gtt	V	0	176.50
gtc	V	8	171.30
gta	V	709	177.00
gtg	V	138	330.20
---	--	------	------
mPD		0.56	0.98
		nPD:	0.57
		N. weight:	0.98
		Sc. PD:	0.44
		Sc. rank:	756.8

,

NA			
Pos.	208	obs:	exp:
tgg	W	994	994.00
---	--	------	------
mPD		0	0
		nPD:	1.
		N. weight:	0.
		Sc. PD:	0
		Sc. rank:	0

,

NA			
Pos.	209	obs:	exp:
tat	Y	7	527.00
tac	Y	987	467.00
---	--	------	------
mPD		0.014	0.50
		nPD:	0.03
		N. weight:	0.79
		Sc. PD:	-0.059
		Sc. rank:	-193.2

,

NA			
Pos.	210	obs:	exp:
aat	N	49	504.70
aac	N	943	487.30
aaa	K	2	1.17
aag	K	0	0.83
---	--	------	------
mPD		0.098	0.50
		nPD:	0.19
		N. weight:	0.57
		Sc. PD:	0.049
		Sc. rank:	291.5

,

NA			
Pos.	211	obs:	exp:
cgt	R	0	42.33
cgc	R	0	50.35
cga	R	2	98.81
cgg	R	0	82.54
aaa	K	3	1.76
aag	K	0	1.24
aga	R	982	452.50
agg	R	7	264.50
---	--	------	------
mPD		0.024	0.97
		nPD:	0.02
		N. weight:	0.82
		Sc. PD:	-0.064
		Sc. rank:	-225.5

,

NA			
Pos.	212	obs:	exp:
cgt	R	0	42.38
cgc	R	0	50.40
cga	R	0	98.91
cgg	R	2	82.62
aaa	K	0	1.17
aag	K	2	0.83
aga	R	92	452.90
agg	R	898	264.80
---	--	------	------
mPD		0.18	0.97
		nPD:	0.18
		N. weight:	1.1
		Sc. PD:	0.077
		Sc. rank:	503.0

,

NA			
Pos.	213	obs:	exp:
cct	P	991	252.70
ccc	P	3	177.20
cca	P	0	406.10
ccg	P	0	157.90
---	--	------	------
mPD		0.0060	0.71
		nPD:	0.01
		N. weight:	1.5
		Sc. PD:	-0.14
		Sc. rank:	-835.3

,

NA			
Pos.	214	obs:	exp:
att	I	43	15.69
atc	I	0	10.40
ata	I	0	16.91
gtt	V	923	196.10
gtc	V	3	190.30
gta	V	0	196.70
gtg	V	24	366.90
gct	A	1	0.26
gcc	A	0	0.19
gca	A	0	0.47
gcg	A	0	0.09
---	--	------	------
mPD		0.14	0.82
		nPD:	0.17
		N. weight:	1.6
		Sc. PD:	0.095
		Sc. rank:	664.7

,

NA			
Pos.	215	obs:	exp:
tct	S	0	0.16
tcc	S	0	0.13
tca	S	1	0.25
tcg	S	0	0.06
act	T	0	18.39
acc	T	0	14.46
aca	T	68	32.62
acg	T	3	5.53
agt	S	0	0.20
agc	S	0	0.20
gtt	V	0	0.41
gtc	V	0	0.40
gta	V	2	0.41
gtg	V	0	0.77
gct	A	0	235.70
gcc	A	0	172.70
gca	A	916	430.10
gcg	A	4	81.37
---	--	------	------
mPD		0.15	0.81
		nPD:	0.19
		N. weight:	0.82
		Sc. PD:	0.065
		Sc. rank:	404.6

,

NA			
Pos.	216	obs:	exp:
gaa	E	986	597.90
gag	E	8	396.10
---	--	------	------
mPD		0.016	0.48
		nPD:	0.03
		N. weight:	0.52
		Sc. PD:	-0.036
		Sc. rank:	-97.7

,

NA			
Pos.	217	obs:	exp:
att	I	942	361.60
atc	I	49	239.70
ata	I	0	389.70
atg	M	1	1.00
gtt	V	0	0.41
gtc	V	2	0.40
gta	V	0	0.41
gtg	V	0	0.77
---	--	------	------
mPD		0.10	0.66
		nPD:	0.16
		N. weight:	0.92
		Sc. PD:	0.045
		Sc. rank:	351.9

,

NA			
Pos.	218	obs:	exp:
aat	N	46	505.70
aac	N	948	488.30
---	--	------	------
mPD		0.088	0.50
		nPD:	0.18
		N. weight:	0.58
		Sc. PD:	0.04
		Sc. rank:	266.5

,

NA			
Pos.	219	obs:	exp:
act	T	0	257.50
acc	T	0	202.40
aca	T	990	456.70
acg	T	4	77.43
---	--	------	------
mPD		0.0080	0.67
		nPD:	0.01
		N. weight:	0.84
		Sc. PD:	-0.076
		Sc. rank:	-420.7

,

NA

Pos.	220	obs:	exp:
tgg	W	994	994.00
---	--	------	------
mPD		0	0
		nPD:	1.
		N. weight:	0.
		Sc. PD:	0
		Sc. rank:	0

NA

Pos.	221	obs:	exp:
tct	S	0	0.16
tcc	S	1	0.13
tca	S	0	0.25
tcg	S	0	0.06
agt	S	0	0.20
agc	S	0	0.20
gct	A	0	253.40
gcc	A	989	185.70
gca	A	0	462.40
gcg	A	0	87.47
ggt	G	0	0.54
ggc	G	4	0.51
gga	G	0	1.85
ggg	G	0	1.11
---	--	------	------
mPD		0.010	0.68
		nPD:	0.01
		N. weight:	1.9
		Sc. PD:	-0.16
		Sc. rank:	-819.5

NA

Pos.	222	obs:	exp:
caa	Q	34	17.46
cag	Q	0	16.54
cgt	R	3	41.01
cgc	R	1	48.77
cga	R	944	95.72
cgg	R	2	79.96
aga	R	9	438.30
agg	R	1	256.20
---	--	------	------
mPD		0.10	1.0
		nPD:	0.1
		N. weight:	2.4
		Sc. PD:	-0.024
		Sc. rank:	280.5

NA

Pos.	223	obs:	exp:
tct	S	0	0.16
tcc	S	0	0.13
tca	S	0	0.25
tcg	S	0	0.06
aat	N	24	505.20
aac	N	969	487.80
agt	S	0	0.20
agc	S	1	0.20
---	--	------	------
mPD		0.049	0.50
		nPD:	0.1
		N. weight:	0.66
		Sc. PD:	-0.0052
		Sc. rank:	85.3

NA

Pos.	224	obs:	exp:
att	I	1	362.70
atc	I	1	240.40
ata	I	992	390.90
---	--	------	------
mPD		0.0040	0.65
		nPD:	0.01
		N. weight:	1.
		Sc. PD:	-0.098
		Sc. rank:	-636.3

NA

Pos.	225	obs:	exp:
tta	L	8	87.36
ttg	L	0	185.50
ctt	L	0	168.40
ctc	L	0	166.50
cta	L	967	165.70
ctg	L	19	220.40
---	--	------	------
mPD		0.054	1.1
		nPD:	0.05
		N. weight:	1.8
		Sc. PD:	-0.1
		Sc. rank:	-181.4

NA

Pos.	226	obs:	exp:
cgt	R	0	42.46
cgc	R	0	50.50
cga	R	9	99.11
cgg	R	36	82.79
aga	R	945	453.80
agg	R	4	265.30
---	--	------	------
mPD		0.16	0.96
		nPD:	0.17
		N. weight:	0.7
		Sc. PD:	0.043
		Sc. rank:	302.3

NA

Pos.	227	obs:	exp:
act	T	0	257.50
acc	T	43	202.40
aca	T	946	456.70
acg	T	5	77.43
---	--	------	------
mPD		0.092	0.67
		nPD:	0.14
		N. weight:	0.68
		Sc. PD:	0.02
		Sc. rank:	199.1

NA

Pos.	228	obs:	exp:
cat	H	1	0.57
cac	H	0	0.43
caa	Q	55	510.00
cag	Q	938	483.00
---	--	------	------
mPD		0.11	0.50
		nPD:	0.21
		N. weight:	0.56
		Sc. PD:	0.058
		Sc. rank:	307.7

NA

Pos.	229	obs:	exp:
gaa	E	868	597.90
gag	E	126	396.10
---	--	------	------
mPD		0.22	0.48
		nPD:	0.46
		N. weight:	0.2
		Sc. PD:	0.07
		Sc. rank:	153.1

NA

Pos.	230	obs:	exp:
tct	S	993	159.10
tcc	S	1	128.70
tca	S	0	250.90
tcg	S	0	57.84
agt	S	0	203.70
agc	S	0	193.80
---	--	------	------
mPD		0.0020	1.7
		nPD:	0.
		N. weight:	2.
		Sc. PD:	-0.2
		Sc. rank:	-1451.6

NA

Pos.	231	obs:	exp:
gaa	E	992	597.90
gag	E	2	396.10
---	--	------	------
mPD		0.0040	0.48
		nPD:	0.01
		N. weight:	0.55
		Sc. PD:	-0.051
		Sc. rank:	-317.8

NA

Pos.	232	obs:	exp:
tgt	C	948	408.10
tgc	C	46	585.90
---	--	------	------
mPD		0.088	0.48
		nPD:	0.18
		N. weight:	0.76
		Sc. PD:	0.056
		Sc. rank:	367.1

NA

Pos.	233	obs:	exp:
tta	L	1	0.09
ttg	L	0	0.19
ctt	L	0	0.17
ctc	L	0	0.17
cta	L	0	0.17
ctg	L	0	0.22
gtt	V	1	205.00
gtc	V	0	198.90
gta	V	923	205.60
gtg	V	69	383.50
---	--	------	------
mPD		0.13	0.73
		nPD:	0.18
		N. weight:	1.4
		Sc. PD:	0.1
		Sc. rank:	686.8

NA

Pos.	234	obs:	exp:
tgt	C	34	408.10
tgc	C	960	585.90
---	--	------	------
mPD		0.066	0.48
		nPD:	0.14
		N. weight:	0.44
		Sc. PD:	0.013
		Sc. rank:	126.3

NA

Pos.	235	obs:	exp:
tat	Y	0	0.53
tac	Y	1	0.47
cat	H	2	567.60
cac	H	986	420.40
aat	N	0	2.54
aac	N	5	2.46
---	--	------	------
mPD		0.016	0.50
		nPD:	0.03
		N. weight:	0.93
		Sc. PD:	-0.066
		Sc. rank:	-195.7

NA

Pos.	236	obs:	exp:
aat	N	70	505.20
aac	N	923	487.80
gat	D	0	0.54
gac	D	1	0.46
---	--	------	------
mPD		0.13	0.50
		nPD:	0.27
		N. weight:	0.51
		Sc. PD:	0.077
		Sc. rank:	318.1

NA

Pos.	237	obs:	exp:
ggt	G	91	133.30
ggc	G	897	126.30
gga	G	6	459.60
ggg	G	0	274.80
---	--	------	------
mPD		0.18	0.68
		nPD:	0.26
		N. weight:	1.9
		Sc. PD:	0.29
		Sc. rank:	1184.2

NA

Pos.	238	obs:	exp:
att	I	0	68.23
atc	I	27	45.23
ata	I	160	73.54
gtt	V	0	166.60
gtc	V	14	161.70
gta	V	785	167.10
gtg	V	8	311.70
---	--	------	------
mPD		0.40	1.0
		nPD:	0.38
		N. weight:	1.4
		Sc. PD:	0.37
		Sc. rank:	1032.7

NA

Pos.	239	obs:	exp:
tgt	C	2	408.10
tgc	C	992	585.90
---	--	------	------
mPD		0.0040	0.48
		nPD:	0.01
		N. weight:	0.57
		Sc. PD:	-0.054
		Sc. rank:	-336.7

NA

Pos.	240	obs:	exp:
cct	P	3	252.50
ccc	P	1	177.10
cca	P	941	405.70
ccg	P	48	157.80
act	T	0	0.26
acc	T	0	0.20
aca	T	1	0.46
acg	T	0	0.08
---	--	------	------
mPD		0.10	0.71
		nPD:	0.14
		N. weight:	0.8
		Sc. PD:	0.028
		Sc. rank:	259.3

NA

Pos.	241	obs:	exp:
tct	S	0	0.16
tcc	S	0	0.13
tca	S	0	0.25
tcg	S	0	0.06
att	I	0	57.65
atc	I	0	38.22
ata	I	158	62.13
agt	S	1	0.20
agc	S	0	0.20
gtt	V	0	171.50
gtc	V	0	166.50
gta	V	828	172.10
gtg	V	3	320.90
gct	A	0	1.02
gcc	A	0	0.75
gca	A	4	1.87
gcg	A	0	0.35
---	--	------	------
mPD		0.29	1.0
		nPD:	0.28
		N. weight:	1.6
		Sc. PD:	0.27
		Sc. rank:	1039.6

NA

Pos.	242	obs:	exp:
att	I	1	0.36
atc	I	0	0.24
ata	I	0	0.39
gtt	V	10	205.00
gtc	V	0	198.90
gta	V	43	205.60
gtg	V	940	383.50
---	--	------	------
mPD		0.11	0.73
		nPD:	0.15
		N. weight:	0.84
		Sc. PD:	0.032
		Sc. rank:	275.7

NA

Pos.	243	obs:	exp:
ttt	F	5	392.30
ttc	F	989	601.70
---	--	------	------
mPD		0.010	0.48
		nPD:	0.02
		N. weight:	0.53
		Sc. PD:	-0.043
		Sc. rank:	-174.6

NA

Pos.	244	obs:	exp:
cat	H	0	0.57
cac	H	1	0.43
act	T	240	257.20
acc	T	677	202.20
aca	T	75	456.20
acg	T	1	77.35
---	--	------	------
mPD		0.48	0.68
		nPD:	0.7
		N. weight:	0.75
		Sc. PD:	0.43
		Sc. rank:	575.7

NA

Pos.	245	obs:	exp:
cgt	R	0	0.04
cgc	R	0	0.05
cga	R	1	0.10
cgg	R	0	0.08
aga	R	0	0.46
agg	R	0	0.27
gat	D	984	536.10
gac	D	9	456.90
mPD		0.024	0.50
		nPD:	0.05
		N. weight:	0.63
		Sc. PD:	-0.035
		Sc. rank:	-68.0

,

NA

Pos.	246	obs:	exp:
tgg	W	1	1.00
ggt	G	0	133.20
ggc	G	0	126.20
gga	G	230	459.10
ggg	G	763	274.60
mPD		0.36	0.68
		nPD:	0.53
		N. weight:	0.7
		Sc. PD:	0.28
		Sc. rank:	532.0

,

NA

Pos.	247	obs:	exp:
ttt	F	1	0.39
ttc	F	0	0.61
tct	S	573	92.36
tcc	S	3	74.69
tca	S	0	145.60
tcg	S	1	33.58
cct	P	367	105.50
ccc	P	47	74.00
cca	P	1	169.50
ccg	P	0	65.93
agt	S	0	118.20
agc	S	0	112.50
gct	A	0	0.26
gcc	A	1	0.19
gca	A	0	0.47
gcg	A	0	0.09
mPD		0.59	1.7
		nPD:	0.34
		N. weight:	1.6
		Sc. PD:	0.37
		Sc. rank:	1158.4

,

NA

Pos.	248	obs:	exp:
tgt	C	0	0.41
tgc	C	1	0.59
act	T	0	0.26
acc	T	1	0.20
aca	T	0	0.46
acg	T	0	0.08
gct	A	3	254.20
gcc	A	989	186.30
gca	A	0	463.80
gcg	A	0	87.74
mPD		0.012	0.68
		nPD:	0.02
		N. weight:	1.8
		Sc. PD:	-0.15
		Sc. rank:	-693.7

,

NA

Pos.	249	obs:	exp:
cat	H	0	0.57
cac	H	1	0.43
act	T	989	257.00
acc	T	0	202.00
aca	T	3	455.70
acg	T	0	77.27
gct	A	1	0.26
gcc	A	0	0.19
gca	A	0	0.47
gcg	A	0	0.09
mPD		0.014	0.68
		nPD:	0.02
		N. weight:	1.5
		Sc. PD:	-0.12
		Sc. rank:	-492.0

,

NA

Pos.	250	obs:	exp:
tgg	W	1	1.00
ggt	G	0	133.20
ggc	G	1	126.20
gga	G	983	459.10
ggg	G	9	274.60
mPD		0.024	0.68
		nPD:	0.04
		N. weight:	0.8
		Sc. PD:	-0.054
		Sc. rank:	-146.7

,

NA

Pos.	251	obs:	exp:
cct	P	989	252.50
ccc	P	0	177.10
cca	P	4	405.70
ccg	P	0	157.80
act	T	0	0.26
acc	T	1	0.20
aca	T	0	0.46
acg	T	0	0.08
mPD		0.012	0.71
		nPD:	0.02
		N. weight:	1.5
		Sc. PD:	-0.13
		Sc. rank:	-589.6

,

NA

Pos.	252	obs:	exp:
tgt	C	0	0.41
tgc	C	1	0.59
gct	A	0	254.40
gcc	A	3	186.50
gca	A	987	464.30
gcg	A	3	87.83
mPD		0.018	0.68
		nPD:	0.03
		N. weight:	0.81
		Sc. PD:	-0.062
		Sc. rank:	-208.8

,

NA

Pos.	253	obs:	exp:
cgt	R	0	0.04
cgc	R	0	0.05
cga	R	0	0.10
cgg	R	0	0.08
aat	N	0	21.88
aac	N	43	21.12
aga	R	1	0.46
agg	R	0	0.27
gat	D	0	485.30
gac	D	899	413.70
gaa	E	50	30.68
gag	E	1	20.32
mPD		0.19	0.63
		nPD:	0.29
		N. weight:	0.84
		Sc. PD:	0.15
		Sc. rank:	552.9

,

NA

Pos.	254	obs:	exp:
cat	H	0	0.57
cac	H	1	0.43
act	T	3	257.20
acc	T	0	202.20
aca	T	990	456.20
acg	T	0	77.35
mPD		0.012	0.68
		nPD:	0.02
		N. weight:	0.84
		Sc. PD:	-0.071
		Sc. rank:	-317.2

,

NA

Pos.	255	obs:	exp:
cgt	R	0	42.42
cgc	R	0	50.45
cga	R	1	99.01
cgg	R	0	82.71
aaa	K	0	0.59
aag	K	1	0.41
aga	R	988	453.40
agg	R	4	265.00
mPD		0.014	0.97
		nPD:	0.01
		N. weight:	0.84
		Sc. PD:	-0.073
		Sc. rank:	-375.3

,

NA

Pos.	256	obs:	exp:
att	I	0	345.20
atc	I	0	228.80
ata	I	946	372.00
aat	N	1	0.51
aac	N	0	0.49
gtt	V	0	9.70
gtc	V	0	9.42
gta	V	45	9.73
gtg	V	2	18.15
mPD		0.098	0.76
		nPD:	0.13
		N. weight:	1.1
		Sc. PD:	0.024
		Sc. rank:	278.7

,

NA

Pos.	257	obs:	exp:
ttt	F	0	0.39
ttc	F	1	0.61
tat	Y	11	526.00
tac	Y	981	466.00
att	I	0	0.36
atc	I	0	0.24
ata	I	1	0.39
mPD		0.030	0.50
		nPD:	0.06
		N. weight:	0.77
		Sc. PD:	-0.034
		Sc. rank:	-26.2

,

NA

Pos.	258	obs:	exp:
tta	L	0	0.09
ttg	L	0	0.19
tat	Y	967	526.50
tac	Y	26	466.50
ctt	L	0	0.17
ctc	L	0	0.17
cta	L	1	0.17
ctg	L	0	0.22
mPD		0.057	0.50
		nPD:	0.11
		N. weight:	0.58
		Sc. PD:	0.0042
		Sc. rank:	121.7

,

NA

Pos.	259	obs:	exp:
ttt	F	910	392.30
ttc	F	84	601.70
mPD		0.15	0.48
		nPD:	0.32
		N. weight:	0.67
		Sc. PD:	0.14
		Sc. rank:	461.2

,

NA

Pos.	260	obs:	exp:
tta	L	1	0.09
ttg	L	0	0.19
ctt	L	0	0.17
ctc	L	0	0.17
cta	L	0	0.17
ctg	L	0	0.22
caa	Q	1	0.51
cag	Q	0	0.49
cgt	R	0	0.17
cgc	R	0	0.20
cga	R	0	0.40
cgg	R	0	0.33
aaa	K	969	578.80
aag	K	19	409.20
aga	R	4	1.83
agg	R	0	1.07
mPD		0.052	0.50
		nPD:	0.1
		N. weight:	0.5
		Sc. PD:	-0.0015
		Sc. rank:	90.6

,

NA

Pos.	261	obs:	exp:
gaa	E	52	597.90
gag	E	942	396.10
mPD		0.099	0.48
		nPD:	0.21
		N. weight:	0.77
		Sc. PD:	0.075
		Sc. rank:	418.5

,

NA

Pos.	262	obs:	exp:
ggt	G	0	133.30
ggc	G	0	126.30
gga	G	37	459.60
ggg	G	957	274.80
mPD		0.072	0.68
		nPD:	0.11
		N. weight:	1.2
		Sc. PD:	0.00035
		Sc. rank:	239.1

,

NA

Pos.	263	obs:	exp:
cgt	R	0	0.94
cgc	R	0	1.12
cga	R	0	2.19
cgg	R	0	1.83
aaa	K	967	569.40
aag	K	5	402.60
aga	R	22	10.04
agg	R	0	5.87
mPD		0.053	0.54
		nPD:	0.1
		N. weight:	0.57
		Sc. PD:	-0.0041
		Sc. rank:	74.0

,

NA			
Pos.	264	obs:	exp:
att	I	0	361.20
atc	I	0	239.50
ata	I	990	389.30
act	T	0	0.26
acc	T	0	0.20
aca	T	1	0.46
acg	T	0	0.08
gtt	V	0	0.62
gtc	V	0	0.60
gta	V	2	0.62
gtg	V	1	1.16
---	--	------	------
mPD		0.010	0.66
		nPD:	0.02
		N. weight:	1.
		Sc. PD:	-0.09
		Sc. rank:	-446.9

,

NA			
Pos.	265	obs:	exp:
tta	L	53	87.36
ttg	L	890	185.50
ctt	L	0	168.40
ctc	L	0	166.50
cta	L	43	165.70
ctg	L	8	220.40
---	--	------	------
mPD		0.27	1.1
		nPD:	0.25
		N. weight:	1.4
		Sc. PD:	0.19
		Sc. rank:	875.6

,

NA			
Pos.	266	obs:	exp:
cgt	R	0	0.04
cgc	R	0	0.05
cga	R	0	0.10
cgg	R	0	0.08
aaa	K	937	581.70
aag	K	56	411.30
aga	R	1	0.46
agg	R	0	0.27
---	--	------	------
mPD		0.11	0.49
		nPD:	0.22
		N. weight:	0.38
		Sc. PD:	0.042
		Sc. rank:	210.9

,

NA			
Pos.	267	obs:	exp:
tgt	C	0	0.41
tgc	C	1	0.59
tgg	W	993	993.00
---	--	------	------
mPD		0.0020	0.0020
		nPD:	1.
		N. weight:	0.00059
		Sc. PD:	0.00051
		Sc. rank:	0.5

,

NA			
Pos.	268	obs:	exp:
gat	D	1	0.54
gac	D	0	0.46
gaa	E	81	597.30
gag	E	912	395.70
---	--	------	------
mPD		0.15	0.48
		nPD:	0.32
		N. weight:	0.67
		Sc. PD:	0.14
		Sc. rank:	452.7

,

NA			
Pos.	269	obs:	exp:
ttt	F	6	2.37
ttc	F	0	3.63
tct	S	936	150.30
tcc	S	3	121.50
tca	S	0	237.00
tcg	S	0	54.64
tat	Y	1	0.53
tac	Y	0	0.47
cct	P	41	11.19
ccc	P	3	7.85
cca	P	0	17.98
ccg	P	0	6.99
act	T	3	0.78
acc	T	0	0.61
aca	T	0	1.38
acg	T	0	0.23
agt	S	0	192.40
agc	S	0	183.10
gct	A	0	0.26
gcc	A	1	0.19
gca	A	0	0.47
gcg	A	0	0.09
---	--	------	------
mPD		0.12	1.7
		nPD:	0.07
		N. weight:	2.
		Sc. PD:	-0.066
		Sc. rank:	89.1

,

NA			
Pos.	270	obs:	exp:
tta	L	0	87.36
ttg	L	3	185.50
ctt	L	1	168.40
ctc	L	1	166.50
cta	L	45	165.70
ctg	L	944	220.40
---	--	------	------
mPD		0.096	1.1
		nPD:	0.09
		N. weight:	1.4
		Sc. PD:	-0.026
		Sc. rank:	114.5

,

NA			
Pos.	271	obs:	exp:
att	I	10	3.65
atc	I	0	2.42
ata	I	0	3.93
act	T	979	254.10
acc	T	1	199.80
aca	T	0	450.70
acg	T	1	76.42
gtt	V	1	0.21
gtc	V	0	0.20
gta	V	0	0.21
gtg	V	0	0.39
gct	A	2	0.51
gcc	A	0	0.38
gca	A	0	0.94
gcg	A	0	0.18
---	--	------	------
mPD		0.032	0.70
		nPD:	0.05
		N. weight:	1.5
		Sc. PD:	-0.086
		Sc. rank:	-180.2

,

NA			
Pos.	272	obs:	exp:
ggt	G	0	133.30
ggc	G	0	126.30
gga	G	956	459.60
ggg	G	38	274.80
---	--	------	------
mPD		0.074	0.68
		nPD:	0.11
		N. weight:	0.7
		Sc. PD:	0.002
		Sc. rank:	143.0

,

NA			
Pos.	273	obs:	exp:
act	T	988	257.00
acc	T	2	202.00
aca	T	2	455.70
acg	T	0	77.27
gct	A	2	0.51
gcc	A	0	0.38
gca	A	0	0.94
gcg	A	0	0.18
---	--	------	------
mPD		0.012	0.68
		nPD:	0.02
		N. weight:	1.5
		Sc. PD:	-0.12
		Sc. rank:	-544.9

,

NA			
Pos.	274	obs:	exp:
gct	A	959	254.70
gcc	A	35	186.60
gca	A	0	464.70
gcg	A	0	87.92
---	--	------	------
mPD		0.068	0.67
		nPD:	0.1
		N. weight:	1.4
		Sc. PD:	-0.0062
		Sc. rank:	221.6

,

NA			
Pos.	275	obs:	exp:
cgt	R	0	0.09
cgc	R	0	0.10
cga	R	0	0.20
cgg	R	0	0.17
aaa	K	46	581.10
aag	K	946	410.90
aga	R	0	0.91
agg	R	2	0.53
---	--	------	------
mPD		0.092	0.49
		nPD:	0.19
		N. weight:	0.75
		Sc. PD:	0.06
		Sc. rank:	374.4

,

NA			
Pos.	276	obs:	exp:
cat	H	923	571.10
cac	H	71	422.90
---	--	------	------
mPD		0.13	0.49
		nPD:	0.27
		N. weight:	0.36
		Sc. PD:	0.057
		Sc. rank:	225.5

,

NA			
Pos.	277	obs:	exp:
att	I	929	341.90
atc	I	1	226.60
ata	I	7	368.50
gtt	V	57	11.77
gtc	V	0	11.42
gta	V	0	11.80
gtg	V	0	22.01
---	--	------	------
mPD		0.12	0.78
		nPD:	0.16
		N. weight:	1.1
		Sc. PD:	0.057
		Sc. rank:	434.3

,

NA			
Pos.	278	obs:	exp:
gaa	E	973	597.90
gag	E	21	396.10
---	--	------	------
mPD		0.041	0.48
		nPD:	0.09
		N. weight:	0.46
		Sc. PD:	-0.0087
		Sc. rank:	30.2

,

NA			
Pos.	279	obs:	exp:
gaa	E	993	597.90
gag	E	1	396.10
---	--	------	------
mPD		0.0020	0.48
		nPD:	0.
		N. weight:	0.56
		Sc. PD:	-0.054
		Sc. rank:	-375.8

,

NA			
Pos.	280	obs:	exp:
tgt	C	15	408.10
tgc	C	979	585.90
---	--	------	------
mPD		0.030	0.48
		nPD:	0.06
		N. weight:	0.51
		Sc. PD:	-0.022
		Sc. rank:	-11.1

,

NA			
Pos.	281	obs:	exp:
tct	S	0	159.10
tcc	S	0	128.70
tca	S	987	250.90
tcg	S	7	57.84
agt	S	0	203.70
agc	S	0	193.80
---	--	------	------
mPD		0.014	1.7
		nPD:	0.01
		N. weight:	1.5
		Sc. PD:	-0.14
		Sc. rank:	-847.2

,

NA			
Pos.	282	obs:	exp:
tgt	C	908	408.10
tgc	C	86	585.90
---	--	------	------
mPD		0.16	0.48
		nPD:	0.33
		N. weight:	0.63
		Sc. PD:	0.13
		Sc. rank:	437.2

,

NA			
Pos.	283	obs:	exp:
tat	Y	45	527.00
tac	Y	949	467.00
---	--	------	------
mPD		0.087	0.50
		nPD:	0.17
		N. weight:	0.63
		Sc. PD:	0.041
		Sc. rank:	276.2

,

NA			
Pos.	284	obs:	exp:
ggt	G	0	133.30
ggc	G	0	126.30
gga	G	9	459.60
ggg	G	985	274.80
---	--	------	------
mPD		0.018	0.68
		nPD:	0.03
		N. weight:	1.4
		Sc. PD:	-0.1
		Sc. rank:	-355.9

NA			
Pos.	285	obs:	exp:
aaa	K	139	82.01
aag	K	1	57.99
gtt	V	0	8.26
gtc	V	0	8.01
gta	V	0	8.28
gtg	V	40	15.45
gat	D	0	1.08
gac	D	2	0.92
gaa	E	809	488.40
gag	E	3	323.60
---	--	------	------
mPD		0.41	0.82
		nPD:	0.5
		N. weight:	0.56
		Sc. PD:	0.21
		Sc. rank:	427.5

NA			
Pos.	286	obs:	exp:
tta	L	0	0.26
ttg	L	0	0.56
ctt	L	0	0.51
ctc	L	0	0.50
cta	L	3	0.50
ctg	L	0	0.67
caa	Q	29	24.65
cag	Q	19	23.35
cgt	R	0	40.24
cgc	R	0	47.86
cga	R	935	93.93
cgg	R	1	78.46
aga	R	6	430.10
agg	R	0	251.40
gaa	E	0	0.60
gag	E	1	0.40
---	--	------	------
mPD		0.15	1.1
		nPD:	0.14
		N. weight:	2.4
		Sc. PD:	0.082
		Sc. rank:	745.7

NA			
Pos.	287	obs:	exp:
tct	S	0	0.16
tcc	S	0	0.13
tca	S	1	0.25
tcg	S	0	0.06
act	T	0	247.10
acc	T	0	194.30
aca	T	948	438.30
acg	T	6	74.31
agt	S	0	0.20
agc	S	0	0.20
gct	A	0	9.99
gcc	A	0	7.32
gca	A	39	18.23
gcg	A	0	3.45
---	--	------	------
mPD		0.089	0.75
		nPD:	0.12
		N. weight:	0.83
		Sc. PD:	0.01
		Sc. rank:	189.9

NA			
Pos.	288	obs:	exp:
tct	S	0	0.48
tcc	S	0	0.39
tca	S	0	0.76
tcg	S	0	0.17
cgt	R	0	0.09
cgc	R	0	0.10
cga	R	1	0.20
cgg	R	0	0.17
act	T	0	0.26
acc	T	0	0.20
aca	T	1	0.46
acg	T	0	0.08
agt	S	3	0.61
agc	S	0	0.59
aga	R	0	0.91
agg	R	1	0.53
ggt	G	38	132.50
ggc	G	3	125.50
gga	G	530	456.80
ggg	G	417	273.20
---	--	------	------
mPD		0.55	0.69
		nPD:	0.79
		N. weight:	0.24
		Sc. PD:	0.16
		Sc. rank:	183.7

NA			
Pos.	289	obs:	exp:
att	I	947	362.70
atc	I	47	240.40
ata	I	0	390.90
---	--	------	------
mPD		0.090	0.65
		nPD:	0.14
		N. weight:	0.93
		Sc. PD:	0.029
		Sc. rank:	273.8

NA			
Pos.	290	obs:	exp:
act	T	47	257.50
acc	T	947	202.40
aca	T	0	456.70
acg	T	0	77.43
---	--	------	------
mPD		0.090	0.67
		nPD:	0.13
		N. weight:	1.5
		Sc. PD:	0.041
		Sc. rank:	427.5

NA			
Pos.	291	obs:	exp:
tgt	C	4	408.10
tgc	C	990	585.90
---	--	------	------
mPD		0.0080	0.48
		nPD:	0.02
		N. weight:	0.56
		Sc. PD:	-0.048
		Sc. rank:	-227.3

NA			
Pos.	292	obs:	exp:
act	T	3	257.20
acc	T	3	202.20
aca	T	949	456.20
acg	T	38	77.35
gct	A	0	0.26
gcc	A	0	0.19
gca	A	1	0.47
gcg	A	0	0.09
---	--	------	------
mPD		0.087	0.68
		nPD:	0.13
		N. weight:	0.72
		Sc. PD:	0.016
		Sc. rank:	187.9

NA			
Pos.	293	obs:	exp:
tgt	C	0	408.10
tgc	C	994	585.90
---	--	------	------
mPD		0	0.48
		nPD:	0.
		N. weight:	0.59
		Sc. PD:	-0.06
		Sc. rank:	-453.9

NA			
Pos.	294	obs:	exp:
cgt	R	0	42.42
cgc	R	0	50.45
cga	R	0	99.01
cgg	R	0	82.71
aaa	K	0	0.59
aag	K	1	0.41
aga	R	34	453.40
agg	R	959	265.00
---	--	------	------
mPD		0.068	0.97
		nPD:	0.07
		N. weight:	1.3
		Sc. PD:	-0.043
		Sc. rank:	49.6

NA			
Pos.	295	obs:	exp:
gat	D	51	536.60
gac	D	943	457.40
---	--	------	------
mPD		0.097	0.50
		nPD:	0.2
		N. weight:	0.63
		Sc. PD:	0.055
		Sc. rank:	324.1

NA			
Pos.	296	obs:	exp:
aat	N	990	505.70
aac	N	4	488.30
---	--	------	------
mPD		0.0080	0.50
		nPD:	0.02
		N. weight:	0.72
		Sc. PD:	-0.062
		Sc. rank:	-310.2

NA			
Pos.	297	obs:	exp:
tgg	W	994	994.00
---	--	------	------
mPD		0	0
		nPD:	1.
		N. weight:	0.
		Sc. PD:	0
		Sc. rank:	0

NA			
Pos.	298	obs:	exp:
tta	L	0	0.35
ttg	L	0	0.75
ctt	L	0	0.68
ctc	L	0	0.67
cta	L	0	0.67
ctg	L	4	0.89
caa	Q	8	506.50
cag	Q	978	479.50
cgt	R	0	0.09
cgc	R	0	0.10
cga	R	0	0.20
cgg	R	2	0.17
aaa	K	0	1.17
aag	K	2	0.83
aga	R	0	0.91
agg	R	0	0.53
---	--	------	------
mPD		0.032	0.52
		nPD:	0.06
		N. weight:	0.76
		Sc. PD:	-0.032
		Sc. rank:	-18.3

NA			
Pos.	299	obs:	exp:
ggt	G	47	133.30
ggc	G	932	126.30
gga	G	14	459.60
ggg	G	1	274.80
---	--	------	------
mPD		0.12	0.68
		nPD:	0.18
		N. weight:	2.
		Sc. PD:	0.13
		Sc. rank:	891.3

NA			
Pos.	300	obs:	exp:
tct	S	4	159.10
tcc	S	0	128.70
tca	S	956	250.90
tcg	S	34	57.84
agt	S	0	203.70
agc	S	0	193.80
---	--	------	------
mPD		0.074	1.7
		nPD:	0.04
		N. weight:	1.4
		Sc. PD:	-0.082
		Sc. rank:	-176.6

NA			
Pos.	301	obs:	exp:
aat	N	990	505.70
aac	N	4	488.30
---	--	------	------
mPD		0.0080	0.50
		nPD:	0.02
		N. weight:	0.72
		Sc. PD:	-0.062
		Sc. rank:	-310.2

NA			
Pos.	302	obs:	exp:
cgt	R	0	42.46
cgc	R	0	50.50
cga	R	0	99.11
cgg	R	0	82.79
aga	R	968	453.80
agg	R	26	265.30
---	--	------	------
mPD		0.051	0.96
		nPD:	0.05
		N. weight:	0.75
		Sc. PD:	-0.038
		Sc. rank:	-54.7

NA			
Pos.	303	obs:	exp:
cct	P	3	252.70
ccc	P	0	177.20
cca	P	991	406.10
ccg	P	0	157.90
---	--	------	------
mPD		0.0060	0.71
		nPD:	0.01
		N. weight:	0.97
		Sc. PD:	-0.091
		Sc. rank:	-542.4

NA			
Pos.	304	obs:	exp:
gtt	V	0	205.20
gtc	V	1	199.10
gta	V	73	205.80
gtg	V	920	383.90
---	--	------	------
mPD		0.14	0.73
		nPD:	0.19
		N. weight:	0.81
		Sc. PD:	0.066
		Sc. rank:	408.9

NA			
Pos.	305	obs:	exp:
att	I	879	324.40
atc	I	10	215.00
ata	I	0	349.60
gtt	V	105	21.68
gtc	V	0	21.03
gta	V	0	21.74
gtg	V	0	40.55
---	--	------	------
mPD		0.21	0.87
		nPD:	0.24
		N. weight:	1.1
		Sc. PD:	0.15
		Sc. rank:	676.6

NA			
Pos.	306	obs:	exp:
tta	L	0	0.09
ttg	L	0	0.19
ctt	L	0	0.17
ctc	L	0	0.17
cta	L	0	0.17
ctg	L	1	0.22
cat	H	1	0.57
cac	H	0	0.43
caa	Q	53	501.80
cag	Q	924	475.20
cgt	R	0	0.56
cgc	R	0	0.66
cga	R	11	1.30
cgg	R	2	1.08
aaa	K	0	1.17
aag	K	2	0.83
aga	R	0	5.94
agg	R	0	3.47
---	--	------	------
mPD		0.15	0.55
		nPD:	0.28
		N. weight:	0.59
		Sc. PD:	0.098
		Sc. rank:	377.9

NA			
Pos.	307	obs:	exp:
att	I	47	362.70
atc	I	0	240.40
ata	I	947	390.90
---	--	------	------
mPD		0.090	0.65
		nPD:	0.14
		N. weight:	0.83
		Sc. PD:	0.026
		Sc. rank:	244.2

NA			
Pos.	308	obs:	exp:
aat	N	0	1.02
aac	N	2	0.98
gat	D	106	535.50
gac	D	886	456.50
---	--	------	------
mPD		0.19	0.50
		nPD:	0.39
		N. weight:	0.47
		Sc. PD:	0.13
		Sc. rank:	344.8

NA			
Pos.	309	obs:	exp:
cct	P	0	252.70
ccc	P	0	177.20
cca	P	989	406.10
ccg	P	5	157.90
---	--	------	------
mPD		0.010	0.71
		nPD:	0.01
		N. weight:	0.96
		Sc. PD:	-0.085
		Sc. rank:	-437.4

NA			
Pos.	310	obs:	exp:
att	I	0	5.47
atc	I	0	3.63
ata	I	15	5.90
atg	M	4	4.00
gtt	V	0	201.30
gtc	V	0	195.30
gta	V	912	201.90
gtg	V	63	376.50
---	--	------	------
mPD		0.16	0.76
		nPD:	0.21
		N. weight:	1.4
		Sc. PD:	0.15
		Sc. rank:	788.5

NA			
Pos.	311	obs:	exp:
tct	S	1	1.60
tcc	S	0	1.29
tca	S	9	2.52
tcg	S	0	0.58
act	T	0	2.33
acc	T	0	1.83
aca	T	9	4.13
acg	T	0	0.70
agt	S	0	2.05
agc	S	0	1.95
gtt	V	0	0.62
gtc	V	0	0.60
gta	V	3	0.62
gtg	V	0	1.16
gct	A	0	246.80
gcc	A	2	180.80
gca	A	954	450.20
gcg	A	7	85.18
gaa	E	4	2.41
gag	E	0	1.59
ggt	G	0	0.67
ggc	G	0	0.64
gga	G	5	2.31
ggg	G	0	1.38
---	--	------	------
mPD		0.082	0.74
		nPD:	0.11
		N. weight:	0.8
		Sc. PD:	0.003
		Sc. rank:	168.6

NA			
Pos.	312	obs:	exp:
tta	L	0	0.18
ttg	L	2	0.37
ctt	L	0	0.34
ctc	L	0	0.34
cta	L	0	0.33
ctg	L	0	0.44
atg	M	992	992.00
---	--	------	------
mPD		0.0040	0.0064
		nPD:	0.63
		N. weight:	0.0037
		Sc. PD:	0.0019
		Sc. rank:	2.9

NA			
Pos.	313	obs:	exp:
tct	S	0	0.16
tcc	S	0	0.13
tca	S	1	0.25
tcg	S	0	0.06
att	I	0	0.36
atc	I	0	0.24
ata	I	1	0.39
act	T	0	256.50
acc	T	2	201.60
aca	T	983	454.80
acg	T	5	77.12
aaa	K	2	1.17
aag	K	0	0.83
agt	S	0	0.20
agc	S	0	0.20
---	--	------	------
mPD		0.022	0.68
		nPD:	0.03
		N. weight:	0.83
		Sc. PD:	-0.058
		Sc. rank:	-171.5

NA			
Pos.	314	obs:	exp:
cat	H	34	571.10
cac	H	960	422.90
---	--	------	------
mPD		0.066	0.49
		nPD:	0.14
		N. weight:	0.77
		Sc. PD:	0.022
		Sc. rank:	219.1

NA			
Pos.	315	obs:	exp:
act	T	990	257.50
acc	T	4	202.40
aca	T	0	456.70
acg	T	0	77.43
---	--	------	------
mPD		0.0080	0.67
		nPD:	0.01
		N. weight:	1.5
		Sc. PD:	-0.13
		Sc. rank:	-734.3

NA			
Pos.	316	obs:	exp:
tct	S	0	159.10
tcc	S	0	128.70
tca	S	0	250.90
tcg	S	0	57.84
agt	S	990	203.70
agc	S	4	193.80
---	--	------	------
mPD		0.0080	1.7
		nPD:	0.
		N. weight:	1.7
		Sc. PD:	-0.17
		Sc. rank:	-1159.2

NA			
Pos.	317	obs:	exp:
caa	Q	901	510.60
cag	Q	93	483.40
---	--	------	------
mPD		0.17	0.50
		nPD:	0.34
		N. weight:	0.4
		Sc. PD:	0.091
		Sc. rank:	282.3

NA			
Pos.	318	obs:	exp:
tat	Y	988	527.00
tac	Y	6	467.00
---	--	------	------
mPD		0.012	0.50
		nPD:	0.02
		N. weight:	0.66
		Sc. PD:	-0.052
		Sc. rank:	-201.7

NA			
Pos.	319	obs:	exp:
att	I	0	362.00
atc	I	0	239.90
ata	I	992	390.10
atg	M	1	1.00
act	T	0	0.26
acc	T	0	0.20
aca	T	1	0.46
acg	T	0	0.08
---	--	------	------
mPD		0.0040	0.66
		nPD:	0.01
		N. weight:	1.
		Sc. PD:	-0.099
		Sc. rank:	-648.2

NA			
Pos.	320	obs:	exp:
tgt	C	47	408.10
tgc	C	947	585.90
---	--	------	------
mPD		0.090	0.48
		nPD:	0.19
		N. weight:	0.4
		Sc. PD:	0.031
		Sc. rank:	194.5

NA			
Pos.	321	obs:	exp:
tct	S	0	159.10
tcc	S	0	128.70
tca	S	0	250.90
tcg	S	0	57.84
agt	S	935	203.70
agc	S	59	193.80
---	--	------	------
mPD		0.11	1.7
		nPD:	0.07
		N. weight:	1.5
		Sc. PD:	-0.056
		Sc. rank:	32.7

NA			
Pos.	322	obs:	exp:
cct	P	988	252.70
ccc	P	6	177.20
cca	P	0	406.10
ccg	P	0	157.90
---	--	------	------
mPD		0.012	0.71
		nPD:	0.02
		N. weight:	1.5
		Sc. PD:	-0.13
		Sc. rank:	-579.5

NA			
Pos.	323	obs:	exp:
gtt	V	858	205.20
gtc	V	136	199.10
gta	V	0	205.80
gtg	V	0	383.90
---	--	------	------
mPD		0.24	0.73
		nPD:	0.33
		N. weight:	1.3
		Sc. PD:	0.28
		Sc. rank:	906.7

NA			
Pos.	324	obs:	exp:
tta	L	0	87.36
ttg	L	0	185.50
ctt	L	951	168.40
ctc	L	37	166.50
cta	L	5	165.70
ctg	L	1	220.40
---	--	------	------
mPD		0.083	1.1
		nPD:	0.08
		N. weight:	1.8
		Sc. PD:	-0.052
		Sc. rank:	97.9

NA			
Pos.	325	obs:	exp:
act	T	1	257.50
acc	T	1	202.40
aca	T	992	456.70
acg	T	0	77.43
---	--	------	------
mPD		0.0040	0.67
		nPD:	0.01
		N. weight:	0.85
		Sc. PD:	-0.081
		Sc. rank:	-542.6

NA			
Pos.	326	obs:	exp:
gat	D	45	536.60
gac	D	949	457.40
---	--	------	------
mPD		0.087	0.50
		nPD:	0.17
		N. weight:	0.65
		Sc. PD:	0.043
		Sc. rank:	288.0

NA			
Pos.	327	obs:	exp:
tct	S	0	62.75
tcc	S	0	50.74
tca	S	0	98.93
tcg	S	0	22.81
act	T	159	41.19
acc	T	0	32.38
aca	T	0	73.05
acg	T	0	12.39
aat	N	435	225.40
aac	N	8	217.60
agt	S	391	80.33
agc	S	1	76.44
---	--	------	------
mPD		0.64	1.6
		nPD:	0.39
		N. weight:	1.2
		Sc. PD:	0.33
		Sc. rank:	900.2

NA			
Pos.	328	obs:	exp:
cct	P	7	252.70
ccc	P	986	177.20
cca	P	1	406.10
ccg	P	0	157.90
---	--	------	------
mPD		0.016	0.71
		nPD:	0.02
		N. weight:	1.9
		Sc. PD:	-0.15
		Sc. rank:	-605.0

NA			
Pos.	329	obs:	exp:
cgt	R	0	42.46
cgc	R	0	50.50
cga	R	936	99.11
cgg	R	56	82.79
aga	R	2	453.80
agg	R	0	265.30
---	--	------	------
mPD		0.11	0.96
		nPD:	0.11
		N. weight:	2.3
		Sc. PD:	0.019
		Sc. rank:	505.6

NA			
Pos.	330	obs:	exp:
cct	P	0	252.70
ccc	P	0	177.20
cca	P	49	406.10
ccg	P	945	157.90
---	--	------	------
mPD		0.094	0.71
		nPD:	0.13
		N. weight:	1.8
		Sc. PD:	0.044
		Sc. rank:	477.1

NA			
Pos.	331	obs:	exp:
tat	Y	1	0.53
tac	Y	0	0.47
aat	N	932	500.10
aac	N	51	482.90
gat	D	10	5.40
gac	D	0	4.60
---	--	------	------
mPD		0.12	0.52
		nPD:	0.23
		N. weight:	0.53
		Sc. PD:	0.063
		Sc. rank:	302.2

NA			
Pos.	332	obs:	exp:
gat	D	12	536.60
gac	D	982	457.40
---	--	------	------
mPD		0.024	0.50
		nPD:	0.05
		N. weight:	0.79
		Sc. PD:	-0.044
		Sc. rank:	-82.7

NA			
Pos.	333	obs:	exp:
cct	P	1	252.70
ccc	P	1	177.20
cca	P	992	406.10
ccg	P	0	157.90
---	--	------	------
mPD		0.0040	0.71
		nPD:	0.01
		N. weight:	0.98
		Sc. PD:	-0.094
		Sc. rank:	-633.0

NA			
Pos.	334	obs:	exp:
tct	S	0	6.88
tcc	S	0	5.57
tca	S	0	10.85
tcg	S	0	2.50
cat	H	1	0.57
cac	H	0	0.43
atg	M	1	1.00
act	T	4	2.07
acc	T	1	1.63
aca	T	3	3.68
acg	T	0	0.62
aat	N	737	457.90
aac	N	163	442.10
agt	S	43	8.81
agc	S	0	8.39
gct	A	5	9.74
gcc	A	0	7.14
gca	A	33	17.77
gcg	A	0	3.36
gat	D	2	1.62
gac	D	1	1.38
---	--	------	------
mPD		0.59	0.83
		nPD:	0.7
		N. weight:	0.32
		Sc. PD:	0.18
		Sc. rank:	244.0

NA			
Pos.	335	obs:	exp:
att	I	0	324.00
atc	I	0	214.80
ata	I	888	349.20
atg	M	24	24.00
gtt	V	0	16.93
gtc	V	0	16.43
gta	V	81	16.98
gtg	V	1	31.67
---	--	------	------
mPD		0.20	0.84
		nPD:	0.24
		N. weight:	1.1
		Sc. PD:	0.14
		Sc. rank:	632.3

NA			
Pos.	336	obs:	exp:
ggt	G	936	133.30
ggc	G	53	126.30
gga	G	0	459.60
ggg	G	5	274.80
---	--	------	------
mPD		0.11	0.68
		nPD:	0.16
		N. weight:	2.
		Sc. PD:	0.11
		Sc. rank:	794.3

NA			
Pos.	337	obs:	exp:
aat	N	4	2.03
aac	N	0	1.97
aaa	K	19	575.30
aag	K	963	406.70
gaa	E	0	4.81
gag	E	8	3.19
---	--	------	------
mPD		0.061	0.51
		nPD:	0.12
		N. weight:	0.87
		Sc. PD:	0.013
		Sc. rank:	207.4

NA			
Pos.	338	obs:	exp:
tgt	C	954	408.10
tgc	C	40	585.90
---	--	------	------
mPD		0.077	0.48
		nPD:	0.16
		N. weight:	0.79
		Sc. PD:	0.041
		Sc. rank:	307.2

NA			
Pos.	339	obs:	exp:
cat	H	1	0.57
cac	H	0	0.43
act	T	1	0.26
acc	T	0	0.20
aca	T	0	0.46
acg	T	0	0.08
aat	N	966	503.70
aac	N	24	486.30
aaa	K	2	1.17
aag	K	0	0.83
---	--	------	------
mPD		0.055	0.51
		nPD:	0.11
		N. weight:	0.63
		Sc. PD:	0.0018
		Sc. rank:	126.6

NA			
Pos.	340	obs:	exp:
gat	D	11	530.10
gac	D	971	451.90
gaa	E	1	4.81
gag	E	7	3.19
ggt	G	0	0.54
ggc	G	4	0.51
gga	G	0	1.85
ggg	G	0	1.11
---	--	------	------
mPD		0.046	0.52
		nPD:	0.09
		N. weight:	0.8
		Sc. PD:	-0.013
		Sc. rank:	68.8

NA			
Pos.	341	obs:	exp:
cct	P	962	252.70
ccc	P	28	177.20
cca	P	0	406.10
ccg	P	4	157.90
---	--	------	------
mPD		0.063	0.71
		nPD:	0.09
		N. weight:	1.4
		Sc. PD:	-0.023
		Sc. rank:	114.1

NA			
Pos.	342	obs:	exp:
tat	Y	949	527.00
tac	Y	45	467.00
---	--	------	------
mPD		0.087	0.50
		nPD:	0.17
		N. weight:	0.51
		Sc. PD:	0.033
		Sc. rank:	222.7

NA			
Pos.	343	obs:	exp:
cct	P	1	252.70
ccc	P	0	177.20
cca	P	990	406.10
ccg	P	3	157.90
---	--	------	------
mPD		0.0080	0.71
		nPD:	0.01
		N. weight:	0.97
		Sc. PD:	-0.088
		Sc. rank:	-490.9

NA			
Pos.	344	obs:	exp:
ggt	G	939	133.30
ggc	G	1	126.30
gga	G	40	459.60
ggg	G	14	274.80
---	--	------	------
mPD		0.11	0.68
		nPD:	0.16
		N. weight:	1.9
		Sc. PD:	0.092
		Sc. rank:	712.7

,

NA			
Pos.	345	obs:	exp:
tct	S	0	0.16
tcc	S	0	0.13
tca	S	0	0.25
tcg	S	0	0.06
att	I	15	5.47
atc	I	0	3.63
ata	I	0	5.90
aat	N	857	497.60
aac	N	121	480.40
agt	S	1	0.20
agc	S	0	0.20
---	--	------	------
mPD		0.25	0.54
		nPD:	0.46
		N. weight:	0.36
		Sc. PD:	0.12
		Sc. rank:	267.5

,

NA			
Pos.	346	obs:	exp:
tct	S	0	0.16
tcc	S	0	0.13
tca	S	0	0.25
tcg	S	0	0.06
att	I	1	0.36
atc	I	0	0.24
ata	I	0	0.39
aat	N	745	476.20
aac	N	191	459.80
aaa	K	1	0.59
aag	K	0	0.41
agt	S	1	0.20
agc	S	0	0.20
gat	D	44	29.15
gac	D	10	24.85
ggt	G	1	0.13
ggc	G	0	0.13
gga	G	0	0.46
ggg	G	0	0.28
---	--	------	------
mPD		0.44	0.61
		nPD:	0.71
		N. weight:	0.21
		Sc. PD:	0.12
		Sc. rank:	159.5

,

NA			
Pos.	347	obs:	exp:
aat	N	24	505.70
aac	N	970	488.30
---	--	------	------
mPD		0.047	0.50
		nPD:	0.09
		N. weight:	0.66
		Sc. PD:	-0.0073
		Sc. rank:	70.0

,

NA			
Pos.	348	obs:	exp:
tct	S	0	1.60
tcc	S	0	1.29
tca	S	0	2.52
tcg	S	0	0.58
aat	N	961	498.60
aac	N	19	481.40
aaa	K	2	1.17
aag	K	0	0.83
agt	S	10	2.05
agc	S	0	1.95
gat	D	2	1.08
gac	D	0	0.92
---	--	------	------
mPD		0.065	0.54
		nPD:	0.12
		N. weight:	0.66
		Sc. PD:	0.0095
		Sc. rank:	155.4

,

NA			
Pos.	349	obs:	exp:
gaa	E	1	0.60
gag	E	0	0.40
ggt	G	2	133.20
ggc	G	0	126.20
gga	G	956	459.10
ggg	G	35	274.60
---	--	------	------
mPD		0.074	0.68
		nPD:	0.11
		N. weight:	0.7
		Sc. PD:	0.0021
		Sc. rank:	144.0

,

NA			
Pos.	350	obs:	exp:
att	I	0	45.97
atc	I	126	30.48
ata	I	0	49.55
gtt	V	0	179.20
gtc	V	865	173.90
gta	V	2	179.70
gtg	V	1	335.20
---	--	------	------
mPD		0.23	0.96
		nPD:	0.24
		N. weight:	1.7
		Sc. PD:	0.22
		Sc. rank:	1025.1

,

NA			
Pos.	351	obs:	exp:
aaa	K	71	582.30
aag	K	923	411.70
---	--	------	------
mPD		0.13	0.49
		nPD:	0.27
		N. weight:	0.67
		Sc. PD:	0.11
		Sc. rank:	424.0

,

NA			
Pos.	352	obs:	exp:
ggt	G	0	133.30
ggc	G	1	126.30
gga	G	933	459.60
ggg	G	60	274.80
---	--	------	------
mPD		0.12	0.68
		nPD:	0.17
		N. weight:	0.63
		Sc. PD:	0.04
		Sc. rank:	273.6

,

NA			
Pos.	353	obs:	exp:
ttt	F	50	391.10
ttc	F	941	599.90
tta	L	3	0.26
ttg	L	0	0.56
ctt	L	0	0.51
ctc	L	0	0.50
cta	L	0	0.50
ctg	L	0	0.67
---	--	------	------
mPD		0.10	0.48
		nPD:	0.21
		N. weight:	0.37
		Sc. PD:	0.037
		Sc. rank:	200.6

,

NA			
Pos.	354	obs:	exp:
tct	S	0	159.10
tcc	S	0	128.70
tca	S	985	250.90
tcg	S	9	57.84
agt	S	0	203.70
agc	S	0	193.80
---	--	------	------
mPD		0.018	1.7
		nPD:	0.01
		N. weight:	1.5
		Sc. PD:	-0.14
		Sc. rank:	-759.0

,

NA			
Pos.	355	obs:	exp:
tat	Y	13	526.50
tac	Y	980	466.50
tgt	C	0	0.41
tgc	C	1	0.59
---	--	------	------
mPD		0.028	0.50
		nPD:	0.06
		N. weight:	0.76
		Sc. PD:	-0.037
		Sc. rank:	-44.8

,

NA			
Pos.	356	obs:	exp:
tta	L	1	87.36
ttg	L	3	185.50
ctt	L	0	168.40
ctc	L	0	166.50
cta	L	15	165.70
ctg	L	975	220.40
---	--	------	------
mPD		0.040	1.1
		nPD:	0.04
		N. weight:	1.6
		Sc. PD:	-0.11
		Sc. rank:	-269.7

,

NA			
Pos.	357	obs:	exp:
aat	N	11	5.60
aac	N	0	5.40
gat	D	978	530.70
gac	D	5	452.30
---	--	------	------
mPD		0.032	0.52
		nPD:	0.06
		N. weight:	0.65
		Sc. PD:	-0.028
		Sc. rank:	-11.3

,

NA			
Pos.	358	obs:	exp:
cgt	R	0	0.04
cgc	R	0	0.05
cga	R	0	0.10
cgg	R	0	0.08
aga	R	1	0.46
agg	R	0	0.27
ggt	G	0	133.20
ggc	G	0	126.20
gga	G	61	459.10
ggg	G	932	274.60
---	--	------	------
mPD		0.12	0.68
		nPD:	0.18
		N. weight:	1.1
		Sc. PD:	0.076
		Sc. rank:	519.0

NA			
Pos.	359	obs:	exp:
ttt	F	1	0.39
ttc	F	0	0.61
att	I	1	0.36
atc	I	0	0.24
ata	I	0	0.39
act	T	7	1.81
acc	T	0	1.43
aca	T	0	3.22
acg	T	0	0.55
aat	N	45	22.89
aac	N	0	22.11
gtt	V	17	6.40
gtc	V	14	6.21
gta	V	0	6.42
gtg	V	0	11.97
gct	A	730	197.80
gcc	A	42	145.00
gca	A	0	360.90
gcg	A	0	68.28
gat	D	93	50.75
gac	D	1	43.25
ggt	G	43	5.77
ggc	G	0	5.46
gga	G	0	19.88
ggg	G	0	11.89
---	--	------	------
mPD		0.57	1.2
		nPD:	0.49
		N. weight:	1.2
		Sc. PD:	0.46
		Sc. rank:	941.2

,

NA			
Pos.	360	obs:	exp:
aat	N	43	505.70
aac	N	951	488.30
---	--	------	------
mPD		0.083	0.50
		nPD:	0.17
		N. weight:	0.59
		Sc. PD:	0.034
		Sc. rank:	245.4

,

NA			
Pos.	361	obs:	exp:
act	T	963	257.50
acc	T	30	202.40
aca	T	0	456.70
acg	T	1	77.43
---	--	------	------
mPD		0.061	0.67
		nPD:	0.09
		N. weight:	1.4
		Sc. PD:	-0.021
		Sc. rank:	129.0

,

NA			
Pos.	362	obs:	exp:
tgg	W	994	994.00
---	--	------	------
mPD		0	0
		nPD:	1.
		N. weight:	0.
		Sc. PD:	0
		Sc. rank:	0

,

NA			
Pos.	363	obs:	exp:
tta	L	2	87.01
ttg	L	0	184.70
ctt	L	0	167.80
ctc	L	0	165.90
cta	L	981	165.10
ctg	L	7	219.60
att	I	0	1.09
atc	I	0	0.73
ata	I	3	1.18
gtt	V	0	0.21
gtc	V	0	0.20
gta	V	1	0.21
gtg	V	0	0.39
---	--	------	------
mPD		0.026	1.1
		nPD:	0.02
		N. weight:	1.9
		Sc. PD:	-0.15
		Sc. rank:	-597.8

,

NA			
Pos.	364	obs:	exp:
ggt	G	2	133.30
ggc	G	0	126.30
gga	G	53	459.60
ggg	G	939	274.80
---	--	------	------
mPD		0.10	0.68
		nPD:	0.16
		N. weight:	1.2
		Sc. PD:	0.055
		Sc. rank:	428.0

NA			
Pos.	365	obs:	exp:
cgt	R	0	42.46
cgc	R	0	50.50
cga	R	0	99.11
cgg	R	0	82.79
aga	R	69	453.80
agg	R	925	265.30
---	--	------	------
mPD		0.13	0.96
		nPD:	0.13
		N. weight:	1.1
		Sc. PD:	0.031
		Sc. rank:	321.9

NA			
Pos.	366	obs:	exp:
act	T	1	257.50
acc	T	0	202.40
aca	T	992	456.70
acg	T	1	77.43
---	--	------	------
mPD		0.0040	0.67
		nPD:	0.01
		N. weight:	0.85
		Sc. PD:	-0.081
		Sc. rank:	-543.3

NA			
Pos.	367	obs:	exp:
att	I	0	357.60
atc	I	0	237.00
ata	I	980	385.40
act	T	0	2.85
acc	T	0	2.24
aca	T	11	5.05
acg	T	0	0.86
gtt	V	0	0.62
gtc	V	0	0.60
gta	V	3	0.62
gtg	V	0	1.16
---	--	------	------
mPD		0.028	0.68
		nPD:	0.04
		N. weight:	1.
		Sc. PD:	-0.065
		Sc. rank:	-147.6

NA			
Pos.	368	obs:	exp:
tct	S	0	158.80
tcc	S	0	128.40
tca	S	0	250.30
tcg	S	0	57.73
aat	N	0	1.02
aac	N	2	0.98
agt	S	159	203.30
agc	S	833	193.40
---	--	------	------
mPD		0.27	1.7
		nPD:	0.16
		N. weight:	1.3
		Sc. PD:	0.074
		Sc. rank:	544.0

NA			
Pos.	369	obs:	exp:
att	I	0	0.36
atc	I	0	0.24
ata	I	1	0.39
act	T	1	257.00
acc	T	1	202.00
aca	T	983	455.70
acg	T	7	77.27
aaa	K	1	0.59
aag	K	0	0.41
---	--	------	------
mPD		0.022	0.68
		nPD:	0.03
		N. weight:	0.82
		Sc. PD:	-0.058
		Sc. rank:	-162.7

NA			
Pos.	370	obs:	exp:
tct	S	0	8.00
tcc	S	50	6.47
tca	S	0	12.62
tcg	S	0	2.91
act	T	1	8.03
acc	T	30	6.31
aca	T	0	14.24
acg	T	0	2.41
agt	S	0	10.25
agc	S	0	9.75
gtt	V	0	0.21
gtc	V	1	0.20
gta	V	0	0.21
gtg	V	0	0.39
gct	A	78	233.40
gcc	A	785	171.10
gca	A	48	425.90
gcg	A	0	80.58
gat	D	0	0.54
gac	D	1	0.46
---	--	------	------
mPD		0.39	0.87
		nPD:	0.44
		N. weight:	1.3
		Sc. PD:	0.42
		Sc. rank:	971.7

NA			
Pos.	371	obs:	exp:
tta	L	0	0.09
ttg	L	1	0.19
tct	S	11	159.00
tcc	S	30	128.50
tca	S	14	250.60
tcg	S	938	57.79
ctt	L	0	0.17
ctc	L	0	0.17
cta	L	0	0.17
ctg	L	0	0.22
agt	S	0	203.50
agc	S	0	193.60
---	--	------	------
mPD		0.11	1.7
		nPD:	0.07
		N. weight:	2.8
		Sc. PD:	-0.11
		Sc. rank:	39.7

NA			
Pos.	372	obs:	exp:
cgt	R	0	42.46
cgc	R	0	50.50
cga	R	1	99.11
cgg	R	1	82.79
aga	R	58	453.80
agg	R	934	265.30
---	--	------	------
mPD		0.12	0.96
		nPD:	0.12
		N. weight:	1.2
		Sc. PD:	0.016
		Sc. rank:	271.3

NA			
Pos.	373	obs:	exp:
tct	S	946	158.20
tcc	S	6	127.90
tca	S	33	249.30
tcg	S	3	57.50
agt	S	0	202.50
agc	S	0	192.70
gct	A	0	1.54
gcc	A	0	1.13
gca	A	6	2.81
gcg	A	0	0.53
---	--	------	------
mPD		0.10	1.7
		nPD:	0.06
		N. weight:	1.8
		Sc. PD:	-0.074
		Sc. rank:	-20.6

NA			
Pos.	374	obs:	exp:
ggt	G	0	133.30
ggc	G	0	126.30
gga	G	864	459.60
ggg	G	130	274.80
---	--	------	------
mPD		0.23	0.68
		nPD:	0.34
		N. weight:	0.5
		Sc. PD:	0.11
		Sc. rank:	350.5

NA			
Pos.	375	obs:	exp:
ttt	F	0	2.37
ttc	F	6	3.63
tat	Y	57	523.90
tac	Y	931	464.10
---	--	------	------
mPD		0.12	0.51
		nPD:	0.24
		N. weight:	0.59
		Sc. PD:	0.073
		Sc. rank:	344.6

NA			
Pos.	376	obs:	exp:
gaa	E	2	597.90
gag	E	992	396.10
---	--	------	------
mPD		0.0040	0.48
		nPD:	0.01
		N. weight:	1.
		Sc. PD:	-0.094
		Sc. rank:	-580.7

NA			
Pos.	377	obs:	exp:
atg	M	993	993.00
gtt	V	0	0.21
gtc	V	0	0.20
gta	V	0	0.21
gtg	V	1	0.39
---	--	------	------
mPD		0.0020	0.0032
		nPD:	0.62
		N. weight:	0.0011
		Sc. PD:	0.00052
		Sc. rank:	0.8

NA			
Pos.	378	obs:	exp:
tta	L	910	87.27
ttg	L	2	185.30
ctt	L	0	168.30
ctc	L	0	166.40
cta	L	75	165.60
ctg	L	6	220.20
cct	P	0	0.25
ccc	P	0	0.18
cca	P	1	0.41
ccg	P	0	0.16
---	--	------	------
mPD		0.17	1.1
		nPD:	0.15
		N. weight:	2.3
		Sc. PD:	0.1
		Sc. rank:	839.3

NA			
Pos.	379	obs:	exp:
aaa	K	937	582.30
aag	K	57	411.70
---	--	------	------
mPD		0.11	0.49
		nPD:	0.22
		N. weight:	0.37
		Sc. PD:	0.042
		Sc. rank:	210.3

NA			
Pos.	380	obs:	exp:
gtt	V	0	205.20
gtc	V	0	199.10
gta	V	26	205.80
gtg	V	968	383.90
---	--	------	------
mPD		0.051	0.73
		nPD:	0.07
		N. weight:	0.94
		Sc. PD:	-0.032
		Sc. rank:	32.5

NA			
Pos.	381	obs:	exp:
cct	P	0	252.70
ccc	P	0	177.20
cca	P	989	406.10
ccg	P	5	157.90
---	--	------	------
mPD		0.010	0.71
		nPD:	0.01
		N. weight:	0.96
		Sc. PD:	-0.085
		Sc. rank:	-437.4

NA			
Pos.	382	obs:	exp:
aat	N	992	505.20
aac	N	1	487.80
aaa	K	1	0.59
aag	K	0	0.41
---	--	------	------
mPD		0.0040	0.50
		nPD:	0.01
		N. weight:	0.74
		Sc. PD:	-0.07
		Sc. rank:	-445.3

NA			
Pos.	383	obs:	exp:
gct	A	0	254.70
gcc	A	1	186.60
gca	A	992	464.70
gcg	A	1	87.92
---	--	------	------
mPD		0.0040	0.67
		nPD:	0.01
		N. weight:	0.83
		Sc. PD:	-0.08
		Sc. rank:	-526.4

NA			
Pos.	384	obs:	exp:
tta	L	10	87.36
ttg	L	976	185.50
ctt	L	0	168.40
ctc	L	0	166.50
cta	L	0	165.70
ctg	L	8	220.40
---	--	------	------
mPD		0.036	1.1
		nPD:	0.03
		N. weight:	1.8
		Sc. PD:	-0.12
		Sc. rank:	-354.6

NA			
Pos.	385	obs:	exp:
act	T	0	257.50
acc	T	41	202.40
aca	T	950	456.70
acg	T	3	77.43
---	--	------	------
mPD		0.085	0.67
		nPD:	0.13
		N. weight:	0.7
		Sc. PD:	0.013
		Sc. rank:	176.6

NA			
Pos.	386	obs:	exp:
gat	D	951	536.60
gac	D	43	457.40
---	--	------	------
mPD		0.083	0.50
		nPD:	0.17
		N. weight:	0.5
		Sc. PD:	0.029
		Sc. rank:	206.7

NA			
Pos.	387	obs:	exp:
gat	D	988	536.60
gac	D	6	457.40
---	--	------	------
mPD		0.012	0.50
		nPD:	0.02
		N. weight:	0.64
		Sc. PD:	-0.051
		Sc. rank:	-191.7

NA			
Pos.	388	obs:	exp:
cgt	R	0	42.46
cgc	R	0	50.50
cga	R	0	99.11
cgg	R	0	82.79
aga	R	943	453.80
agg	R	51	265.30
---	--	------	------
mPD		0.097	0.96
		nPD:	0.1
		N. weight:	0.68
		Sc. PD:	-0.0031
		Sc. rank:	112.5

NA			
Pos.	389	obs:	exp:
tct	S	1	159.00
tcc	S	0	128.50
tca	S	990	250.60
tcg	S	2	57.79
agt	S	0	203.50
agc	S	0	193.60
gct	A	0	0.26
gcc	A	0	0.19
gca	A	1	0.47
gcg	A	0	0.09
---	--	------	------
mPD		0.0080	1.7
		nPD:	0.
		N. weight:	1.5
		Sc. PD:	-0.15
		Sc. rank:	-1002.2

,

NA			
Pos.	390	obs:	exp:
atg	M	1	1.00
aaa	K	51	581.70
aag	K	942	411.30
---	--	------	------
mPD		0.099	0.49
		nPD:	0.2
		N. weight:	0.73
		Sc. PD:	0.069
		Sc. rank:	393.1

,

NA			
Pos.	391	obs:	exp:
cct	P	1	252.70
ccc	P	988	177.20
cca	P	5	406.10
ccg	P	0	157.90
---	--	------	------
mPD		0.012	0.71
		nPD:	0.02
		N. weight:	1.9
		Sc. PD:	-0.16
		Sc. rank:	-718.2

,

NA			
Pos.	392	obs:	exp:
tct	S	0	0.32
tcc	S	0	0.26
tca	S	0	0.50
tcg	S	0	0.12
att	I	878	339.70
atc	I	53	225.20
ata	I	0	366.10
act	T	35	13.99
acc	T	19	11.00
aca	T	0	24.81
acg	T	0	4.21
agt	S	2	0.41
agc	S	0	0.39
gtt	V	7	1.45
gtc	V	0	1.40
gta	V	0	1.45
gtg	V	0	2.70
---	--	------	------
mPD		0.26	0.78
		nPD:	0.33
		N. weight:	0.91
		Sc. PD:	0.19
		Sc. rank:	632.5

,

NA			
Pos.	393	obs:	exp:
caa	Q	994	510.60
cag	Q	0	483.40
---	--	------	------
mPD		0	0.50
		nPD:	0.
		N. weight:	0.74
		Sc. PD:	-0.075
		Sc. rank:	-572.1

,

NA			
Pos.	394	obs:	exp:
tct	S	0	1.60
tcc	S	0	1.29
tca	S	0	2.52
tcg	S	0	0.58
agt	S	10	2.05
agc	S	0	1.95
gat	D	4	2.16
gac	D	0	1.84
ggt	G	938	131.40
ggc	G	42	124.50
gga	G	0	453.10
ggg	G	0	271.00
---	--	------	------
mPD		0.11	0.72
		nPD:	0.15
		N. weight:	2.
		Sc. PD:	0.089
		Sc. rank:	729.3

,

NA			
Pos.	395	obs:	exp:
caa	Q	51	510.60
cag	Q	943	483.40
---	--	------	------
mPD		0.097	0.50
		nPD:	0.2
		N. weight:	0.57
		Sc. PD:	0.049
		Sc. rank:	294.4

,

NA			
Pos.	396	obs:	exp:
act	T	0	257.50
acc	T	10	202.40
aca	T	982	456.70
acg	T	2	77.43
---	--	------	------
mPD		0.024	0.67
		nPD:	0.04
		N. weight:	0.8
		Sc. PD:	-0.054
		Sc. rank:	-142.2

,

NA			
Pos.	397	obs:	exp:
att	I	943	362.30
atc	I	50	240.20
ata	I	0	390.50
gtt	V	1	0.21
gtc	V	0	0.20
gta	V	0	0.21
gtg	V	0	0.39
---	--	------	------
mPD		0.098	0.66
		nPD:	0.15
		N. weight:	0.92
		Sc. PD:	0.038
		Sc. rank:	321.8

,

NA			
Pos.	398	obs:	exp:
tta	L	2	0.18
ttg	L	0	0.37
ctt	L	0	0.34
ctc	L	0	0.34
cta	L	0	0.33
ctg	L	0	0.44
gtt	V	0	204.80
gtc	V	1	198.70
gta	V	956	205.40
gtg	V	35	383.10
---	--	------	------
mPD		0.074	0.73
		nPD:	0.1
		N. weight:	1.5
		Sc. PD:	-0.0066
		Sc. rank:	264.8

,

NA			
Pos.	399	obs:	exp:
ttt	F	0	0.39
ttc	F	1	0.61
tta	L	931	84.46
ttg	L	11	179.30
tct	S	0	5.12
tcc	S	0	4.14
tca	S	32	8.08
tcg	S	0	1.86
ctt	L	0	162.90
ctc	L	0	161.00
cta	L	19	160.20
ctg	L	0	213.10
agt	S	0	6.56
agc	S	0	6.24
---	--	------	------
mPD		0.12	1.2
		nPD:	0.1
		N. weight:	2.5
		Sc. PD:	-0.0077
		Sc. rank:	441.5

,

NA			
Pos.	400	obs:	exp:
act	T	0	1.55
acc	T	6	1.22
aca	T	0	2.76
acg	T	0	0.47
aat	N	9	501.60
aac	N	977	484.40
aaa	K	2	1.17
aag	K	0	0.83
---	--	------	------
mPD		0.034	0.52
		nPD:	0.07
		N. weight:	0.74
		Sc. PD:	-0.028
		Sc. rank:	12.3

,

NA			
Pos.	401	obs:	exp:
tct	S	1	0.16
tcc	S	0	0.13
tca	S	0	0.25
tcg	S	0	0.06
act	T	137	35.49
acc	T	0	27.90
aca	T	0	62.94
acg	T	0	10.67
agt	S	0	0.20
agc	S	0	0.20
gct	A	849	218.10
gcc	A	2	159.80
gca	A	0	397.90
gcg	A	0	75.27
gat	D	5	2.70
gac	D	0	2.30
---	--	------	------
mPD		0.25	0.92
		nPD:	0.27
		N. weight:	1.5
		Sc. PD:	0.24
		Sc. rank:	953.8

,

NA			
Pos.	402	obs:	exp:
gat	D	51	536.10
gac	D	942	456.90
gaa	E	1	0.60
gag	E	0	0.40
---	--	------	------
mPD		0.099	0.50
		nPD:	0.2
		N. weight:	0.63
		Sc. PD:	0.057
		Sc. rank:	327.7

,

NA			
Pos.	403	obs:	exp:
tgg	W	993	993.00
cgt	R	0	0.04
cgc	R	0	0.05
cga	R	0	0.10
cgg	R	0	0.08
aga	R	0	0.46
agg	R	1	0.27
---	--	------	------
mPD		0.0020	0.0033
		nPD:	0.61
		N. weight:	0.0015
		Sc. PD:	0.00071
		Sc. rank:	1.1

NA			
Pos.	404	obs:	exp:
tct	S	0	159.10
tcc	S	0	128.70
tca	S	0	250.90
tcg	S	0	57.84
agt	S	974	203.70
agc	S	20	193.80
---	--	------	------
mPD		0.039	1.7
		nPD:	0.02
		N. weight:	1.7
		Sc. PD:	-0.13
		Sc. rank:	-504.5

,

NA			
Pos.	405	obs:	exp:
ggt	G	942	133.30
ggc	G	48	126.30
gga	G	3	459.60
ggg	G	1	274.80
---	--	------	------
mPD		0.10	0.68
		nPD:	0.15
		N. weight:	2.
		Sc. PD:	0.079
		Sc. rank:	685.9

,

NA			
Pos.	406	obs:	exp:
tat	Y	2	527.00
tac	Y	992	467.00
---	--	------	------
mPD		0.0040	0.50
		nPD:	0.01
		N. weight:	0.82
		Sc. PD:	-0.077
		Sc. rank:	-490.0

,

NA			
Pos.	407	obs:	exp:
tct	S	0	159.10
tcc	S	0	128.70
tca	S	0	250.90
tcg	S	0	57.84
agt	S	994	203.70
agc	S	0	193.80
---	--	------	------
mPD		0	1.7
		nPD:	0.
		N. weight:	1.8
		Sc. PD:	-0.18
		Sc. rank:	-1361.3

,

NA			
Pos.	408	obs:	exp:
ggt	G	0	133.30
ggc	G	0	126.30
gga	G	569	459.60
ggg	G	425	274.80
---	--	------	------
mPD		0.49	0.68
		nPD:	0.73
		N. weight:	0.35
		Sc. PD:	0.21
		Sc. rank:	267.7

,

NA			
Pos.	409	obs:	exp:
tct	S	743	159.10
tcc	S	251	128.70
tca	S	0	250.90
tcg	S	0	57.84
agt	S	0	203.70
agc	S	0	193.80
---	--	------	------
mPD		0.38	1.7
		nPD:	0.23
		N. weight:	1.5
		Sc. PD:	0.17
		Sc. rank:	834.6

,

NA			
Pos.	410	obs:	exp:
ttt	F	48	392.30
ttc	F	946	601.70
---	--	------	------
mPD		0.092	0.48
		nPD:	0.19
		N. weight:	0.37
		Sc. PD:	0.031
		Sc. rank:	187.1

,

NA			
Pos.	411	obs:	exp:
att	I	2	19.34
atc	I	0	12.82
ata	I	51	20.84
atg	M	939	939.00
gtt	V	0	0.41
gtc	V	0	0.40
gta	V	0	0.41
gtg	V	2	0.77
---	--	------	------
mPD		0.11	0.11
		nPD:	0.96
		N. weight:	0.049
		Sc. PD:	0.041
		Sc. rank:	38.3

,

NA			
Pos.	412	obs:	exp:
gat	D	1	536.10
gac	D	992	456.90
gaa	E	0	0.60
gag	E	1	0.40
---	--	------	------
mPD		0.0040	0.50
		nPD:	0.01
		N. weight:	0.85
		Sc. PD:	-0.08
		Sc. rank:	-503.4

,

NA			
Pos.	413	obs:	exp:
tct	S	1	0.16
tcc	S	0	0.13
tca	S	0	0.25
tcg	S	0	0.06
tat	Y	991	526.50
tac	Y	2	466.50
agt	S	0	0.20
agc	S	0	0.20
---	--	------	------
mPD		0.0060	0.50
		nPD:	0.01
		N. weight:	0.69
		Sc. PD:	-0.062
		Sc. rank:	-336.7

,

NA			
Pos.	414	obs:	exp:
tgg	W	994	994.00
---	--	------	------
mPD		0	0
		nPD:	1.
		N. weight:	0.
		Sc. PD:	0
		Sc. rank:	0

,

NA			
Pos.	415	obs:	exp:
gct	A	974	253.20
gcc	A	10	185.50
gca	A	1	461.90
gcg	A	3	87.39
ggt	G	5	0.80
ggc	G	0	0.76
gga	G	1	2.77
ggg	G	0	1.66
---	--	------	------
mPD		0.042	0.69
		nPD:	0.06
		N. weight:	1.4
		Sc. PD:	-0.061
		Sc. rank:	-38.1

,

NA			
Pos.	416	obs:	exp:
aaa	K	14	8.20
aag	K	0	5.80
gat	D	2	4.32
gac	D	6	3.68
gaa	E	908	578.10
gag	E	53	382.90
ggt	G	0	1.48
ggc	G	0	1.40
gga	G	11	5.09
ggg	G	0	3.04
---	--	------	------
mPD		0.17	0.54
		nPD:	0.31
		N. weight:	0.36
		Sc. PD:	0.07
		Sc. rank:	242.0

,

NA			
Pos.	417	obs:	exp:
cgt	R	0	0.09
cgc	R	0	0.10
cga	R	0	0.20
cgg	R	0	0.17
aga	R	0	0.91
agg	R	2	0.53
gaa	E	0	0.60
gag	E	1	0.40
ggt	G	1	132.90
ggc	G	0	125.90
gga	G	6	458.20
ggg	G	984	274.00
---	--	------	------
mPD		0.020	0.68
		nPD:	0.03
		N. weight:	1.4
		Sc. PD:	-0.1
		Sc. rank:	-318.0

,

NA			
Pos.	418	obs:	exp:
gat	D	12	513.40
gac	D	939	437.60
gaa	E	24	25.87
gag	E	19	17.13
---	--	------	------
mPD		0.11	0.54
		nPD:	0.2
		N. weight:	0.75
		Sc. PD:	0.067
		Sc. rank:	390.7

,

NA			
Pos.	419	obs:	exp:
tgt	C	9	408.10
tgc	C	985	585.90
---	--	------	------
mPD		0.018	0.48
		nPD:	0.04
		N. weight:	0.53
		Sc. PD:	-0.035
		Sc. rank:	-89.5

,

NA			
Pos.	420	obs:	exp:
tat	Y	950	527.00
tac	Y	44	467.00
---	--	------	------
mPD		0.085	0.50
		nPD:	0.17
		N. weight:	0.51
		Sc. PD:	0.031
		Sc. rank:	218.5

,

NA			
Pos.	421	obs:	exp:
cgt	R	0	42.46
cgc	R	0	50.50
cga	R	991	99.11
cgg	R	3	82.79
aga	R	0	453.80
agg	R	0	265.30
---	--	------	------
mPD		0.0060	0.96
		nPD:	0.01
		N. weight:	2.5
		Sc. PD:	-0.24
		Sc. rank:	-1560.6

,

NA			
Pos.	422	obs:	exp:
gct	A	24	254.40
gcc	A	0	186.50
gca	A	8	464.30
gcg	A	961	87.83
ggt	G	0	0.13
ggc	G	0	0.13
gga	G	0	0.46
ggg	G	1	0.28
---	--	------	------
mPD		0.065	0.67
		nPD:	0.1
		N. weight:	2.5
		Sc. PD:	-0.023
		Sc. rank:	311.8

,

NA			
Pos.	423	obs:	exp:
tgt	C	936	408.10
tgc	C	58	585.90
---	--	------	------
mPD		0.11	0.48
		nPD:	0.23
		N. weight:	0.72
		Sc. PD:	0.084
		Sc. rank:	406.0

,

NA			
Pos.	424	obs:	exp:
ttt	F	992	392.30
ttc	F	2	601.70
---	--	------	------
mPD		0.0040	0.48
		nPD:	0.01
		N. weight:	1.
		Sc. PD:	-0.095
		Sc. rank:	-580.9

,

NA			
Pos.	425	obs:	exp:
tat	Y	900	527.00
tac	Y	94	467.00
---	--	------	------
mPD		0.17	0.50
		nPD:	0.34
		N. weight:	0.37
		Sc. PD:	0.085
		Sc. rank:	263.3

,

NA			
Pos.	426	obs:	exp:
gtt	V	0	205.00
gtc	V	0	198.90
gta	V	6	205.60
gtg	V	987	383.50
gaa	E	0	0.60
gag	E	1	0.40
---	--	------	------
mPD		0.014	0.73
		nPD:	0.02
		N. weight:	1.
		Sc. PD:	-0.085
		Sc. rank:	-362.1

,

NA			
Pos.	427	obs:	exp:
caa	Q	0	0.51
cag	Q	1	0.49
gaa	E	0	597.30
gag	E	993	395.70
---	--	------	------
mPD		0.0020	0.48
		nPD:	0.
		N. weight:	1.
		Sc. PD:	-0.1
		Sc. rank:	-690.9

,

NA			
Pos.	428	obs:	exp:
tta	L	50	87.18
ttg	L	821	185.10
ctt	L	0	168.10
ctc	L	0	166.20
cta	L	50	165.40
ctg	L	71	220.00
atg	M	1	1.00
gtt	V	0	0.21
gtc	V	0	0.20
gta	V	0	0.21
gtg	V	1	0.39
---	--	------	------
mPD		0.40	1.1
		nPD:	0.36
		N. weight:	1.2
		Sc. PD:	0.29
		Sc. rank:	847.1

NA			
Pos.	429	obs:	exp:
att	I	44	362.30
atc	I	0	240.20
ata	I	949	390.50
gtt	V	0	0.21
gtc	V	0	0.20
gta	V	1	0.21
gtg	V	0	0.39
---	--	------	------
mPD		0.087	0.66
		nPD:	0.13
		N. weight:	0.84
		Sc. PD:	0.021
		Sc. rank:	228.7

,

NA			
Pos.	430	obs:	exp:
cgt	R	944	42.46
cgc	R	46	50.50
cga	R	4	99.11
cgg	R	0	82.79
aga	R	0	453.80
agg	R	0	265.30
---	--	------	------
mPD		0.096	0.96
		nPD:	0.1
		N. weight:	3.2
		Sc. PD:	-0.02
		Sc. rank:	489.7

,

NA			
Pos.	431	obs:	exp:
cgt	R	0	0.04
cgc	R	0	0.05
cga	R	1	0.10
cgg	R	0	0.08
aga	R	0	0.46
agg	R	0	0.27
ggt	G	3	133.20
ggc	G	0	126.20
gga	G	860	459.10
ggg	G	130	274.60
---	--	------	------
mPD		0.23	0.68
		nPD:	0.35
		N. weight:	0.49
		Sc. PD:	0.11
		Sc. rank:	346.8

,

NA			
Pos.	432	obs:	exp:
cgt	R	0	36.87
cgc	R	0	43.85
cga	R	0	86.05
cgg	R	0	71.88
aaa	K	128	74.98
aag	K	0	53.02
aga	R	821	394.00
agg	R	42	230.30
ggt	G	0	0.40
ggc	G	0	0.38
gga	G	3	1.39
ggg	G	0	0.83
---	--	------	------
mPD		0.31	1.1
		nPD:	0.27
		N. weight:	0.67
		Sc. PD:	0.11
		Sc. rank:	426.0

,

NA			
Pos.	433	obs:	exp:
cct	P	6	252.70
ccc	P	986	177.20
cca	P	1	406.10
ccg	P	1	157.90
---	--	------	------
mPD		0.016	0.71
		nPD:	0.02
		N. weight:	1.9
		Sc. PD:	-0.15
		Sc. rank:	-593.1

,

NA			
Pos.	434	obs:	exp:
cgt	R	0	0.04
cgc	R	0	0.05
cga	R	0	0.10
cgg	R	0	0.08
aaa	K	15	581.10
aag	K	977	410.90
aga	R	0	0.46
agg	R	1	0.27
gaa	E	0	0.60
gag	E	1	0.40
---	--	------	------
mPD		0.034	0.49
		nPD:	0.07
		N. weight:	0.89
		Sc. PD:	-0.031
		Sc. rank:	21.8

,

NA			
Pos.	435	obs:	exp:
gaa	E	5	597.30
gag	E	988	395.70
ggt	G	0	0.13
ggc	G	0	0.13
gga	G	0	0.46
ggg	G	1	0.28
---	--	------	------
mPD		0.012	0.48
		nPD:	0.02
		N. weight:	0.98
		Sc. PD:	-0.076
		Sc. rank:	-268.7

,

NA			
Pos.	436	obs:	exp:
gat	D	902	511.20
gac	D	45	435.80
ggt	G	47	6.30
ggc	G	0	5.97
gga	G	0	21.73
ggg	G	0	12.99
---	--	------	------
mPD		0.18	0.62
		nPD:	0.28
		N. weight:	0.57
		Sc. PD:	0.097
		Sc. rank:	370.0

,

NA			
Pos.	437	obs:	exp:
cgt	R	0	0.26
cgc	R	0	0.30
cga	R	0	0.60
cgg	R	0	0.50
aat	N	0	3.56
aac	N	7	3.44
aaa	K	967	573.50
aag	K	12	405.50
aga	R	6	2.74
agg	R	0	1.60
gaa	E	2	1.20
gag	E	0	0.80
---	--	------	------
mPD		0.054	0.51
		nPD:	0.1
		N. weight:	0.53
		Sc. PD:	-0.00051
		Sc. rank:	99.8

,

NA			
Pos.	438	obs:	exp:
gtt	V	0	205.20
gtc	V	0	199.10
gta	V	53	205.80
gtg	V	941	383.90
---	--	------	------
mPD		0.10	0.73
		nPD:	0.14
		N. weight:	0.86
		Sc. PD:	0.028
		Sc. rank:	261.9

,

NA			
Pos.	439	obs:	exp:
tta	L	0	0.09
ttg	L	1	0.19
tgg	W	993	993.00
ctt	L	0	0.17
ctc	L	0	0.17
cta	L	0	0.17
ctg	L	0	0.22
---	--	------	------
mPD		0.0020	0.0047
		nPD:	0.43
		N. weight:	0.0019
		Sc. PD:	0.00059
		Sc. rank:	1.4

,

NA			
Pos.	440	obs:	exp:
tgg	W	994	994.00
---	--	------	------
mPD		0	0
		nPD:	1.
		N. weight:	0.
		Sc. PD:	0
		Sc. rank:	0

,

NA			
Pos.	441	obs:	exp:
act	T	1	257.20
acc	T	991	202.20
aca	T	1	456.20
acg	T	0	77.35
gct	A	0	0.26
gcc	A	1	0.19
gca	A	0	0.47
gcg	A	0	0.09
---	--	------	------
mPD		0.0060	0.68
		nPD:	0.01
		N. weight:	1.7
		Sc. PD:	-0.16
		Sc. rank:	-936.2

,

NA			
Pos.	442	obs:	exp:
tct	S	0	159.00
tcc	S	0	128.50
tca	S	0	250.60
tcg	S	0	57.79
agt	S	45	203.50
agc	S	948	193.60
ggt	G	0	0.13
ggc	G	1	0.13
gga	G	0	0.46
ggg	G	0	0.28
---	--	------	------
mPD		0.089	1.7
		nPD:	0.05
		N. weight:	1.6
		Sc. PD:	-0.081
		Sc. rank:	-108.4

,

NA			
Pos.	443	obs:	exp:
tct	S	0	0.16
tcc	S	0	0.13
tca	S	0	0.25
tcg	S	0	0.06
aat	N	967	504.70
aac	N	25	487.30
aaa	K	1	0.59
aag	K	0	0.41
agt	S	1	0.20
agc	S	0	0.20
---	--	------	------
mPD		0.053	0.50
		nPD:	0.11
		N. weight:	0.62
		Sc. PD:	-0.00042
		Sc. rank:	118.6

,

NA			
Pos.	444	obs:	exp:
tct	S	0	159.00
tcc	S	0	128.50
tca	S	0	250.60
tcg	S	0	57.79
cgt	R	0	0.04
cgc	R	0	0.05
cga	R	0	0.10
cgg	R	0	0.08
agt	S	987	203.50
agc	S	6	193.60
aga	R	0	0.46
agg	R	1	0.27
---	--	------	------
mPD		0.014	1.7
		nPD:	0.01
		N. weight:	1.7
		Sc. PD:	-0.16
		Sc. rank:	-965.2

,

NA			
Pos.	445	obs:	exp:
att	I	1	362.00
atc	I	1	239.90
ata	I	990	390.10
atg	M	1	1.00
aaa	K	1	0.59
aag	K	0	0.41
---	--	------	------
mPD		0.0080	0.66
		nPD:	0.01
		N. weight:	1.
		Sc. PD:	-0.092
		Sc. rank:	-479.6

,

NA			
Pos.	446	obs:	exp:
att	I	0	0.36
atc	I	0	0.24
ata	I	1	0.39
gtt	V	0	204.80
gtc	V	0	198.70
gta	V	985	205.40
gtg	V	7	383.10
ggt	G	0	0.13
ggc	G	0	0.13
gga	G	1	0.46
ggg	G	0	0.28
---	--	------	------
mPD		0.018	0.73
		nPD:	0.02
		N. weight:	1.7
		Sc. PD:	-0.13
		Sc. rank:	-481.1

,

NA			
Pos.	447	obs:	exp:
tct	S	0	159.00
tcc	S	0	128.50
tca	S	55	250.60
tcg	S	938	57.79
act	T	0	0.26
acc	T	0	0.20
aca	T	0	0.46
acg	T	1	0.08
agt	S	0	203.50
agc	S	0	193.60
---	--	------	------
mPD		0.11	1.7
		nPD:	0.06
		N. weight:	2.8
		Sc. PD:	-0.11
		Sc. rank:	1.9

NA			
Pos.	448	obs:	exp:
atg	M	994	994.00
---	--	------	------
mPD		0	0
		nPD:	1.
		N. weight:	0.
		Sc. PD:	0
		Sc. rank:	0

,

NA			
Pos.	449	obs:	exp:
ttt	F	1	0.39
ttc	F	0	0.61
tgt	C	993	407.70
tgc	C	0	585.30
---	--	------	------
mPD		0.0020	0.49
		nPD:	0.
		N. weight:	0.99
		Sc. PD:	-0.096
		Sc. rank:	-675.8

,

NA			
Pos.	450	obs:	exp:
tct	S	0	159.00
tcc	S	993	128.50
tca	S	0	250.60
tcg	S	0	57.79
tgt	C	0	0.41
tgc	C	1	0.59
agt	S	0	203.50
agc	S	0	193.60
---	--	------	------
mPD		0.0020	1.7
		nPD:	0.
		N. weight:	2.3
		Sc. PD:	-0.23
		Sc. rank:	-1637.2

,

NA			
Pos.	451	obs:	exp:
tct	S	0	159.10
tcc	S	0	128.70
tca	S	0	250.90
tcg	S	0	57.84
agt	S	950	203.70
agc	S	44	193.80
---	--	------	------
mPD		0.085	1.7
		nPD:	0.05
		N. weight:	1.6
		Sc. PD:	-0.082
		Sc. rank:	-146.4

,

NA			
Pos.	452	obs:	exp:
act	T	0	257.50
acc	T	0	202.40
aca	T	994	456.70
acg	T	0	77.43
---	--	------	------
mPD		0	0.67
		nPD:	0.
		N. weight:	0.86
		Sc. PD:	-0.088
		Sc. rank:	-667.9

NA			
Pos.	453	obs:	exp:
gaa	E	965	597.90
gag	E	29	396.10
---	--	------	------
mPD		0.057	0.48
		nPD:	0.12
		N. weight:	0.43
		Sc. PD:	0.0052
		Sc. rank:	97.4

NA			
Pos.	454	obs:	exp:
ttt	F	0	392.30
ttc	F	994	601.70
---	--	------	------
mPD		0	0.48
		nPD:	0.
		N. weight:	0.56
		Sc. PD:	-0.057
		Sc. rank:	-431.1

NA			
Pos.	455	obs:	exp:
tta	L	0	87.27
ttg	L	0	185.30
ctt	L	43	168.30
ctc	L	1	166.40
cta	L	10	165.60
ctg	L	939	220.20
cgt	R	0	0.04
cgc	R	0	0.05
cga	R	0	0.10
cgg	R	1	0.08
aga	R	0	0.46
agg	R	0	0.27
---	--	------	------
mPD		0.11	1.1
		nPD:	0.1
		N. weight:	1.4
		Sc. PD:	-0.015
		Sc. rank:	157.1

NA			
Pos.	456	obs:	exp:
ggt	G	0	133.30
ggc	G	0	126.30
gga	G	994	459.60
ggg	G	0	274.80
---	--	------	------
mPD		0	0.68
		nPD:	0.
		N. weight:	0.85
		Sc. PD:	-0.087
		Sc. rank:	-662.5

NA			
Pos.	457	obs:	exp:
caa	Q	990	510.60
cag	Q	4	483.40
---	--	------	------
mPD		0.0080	0.50
		nPD:	0.02
		N. weight:	0.71
		Sc. PD:	-0.061
		Sc. rank:	-300.6

NA			
Pos.	458	obs:	exp:
tgg	W	993	993.00
cgt	R	0	0.04
cgc	R	0	0.05
cga	R	0	0.10
cgg	R	0	0.08
aga	R	0	0.46
agg	R	1	0.27
---	--	------	------
mPD		0.0020	0.0033
		nPD:	0.61
		N. weight:	0.0015
		Sc. PD:	0.00071
		Sc. rank:	1.1

NA			
Pos.	459	obs:	exp:
tct	S	0	0.16
tcc	S	0	0.13
tca	S	0	0.25
tcg	S	0	0.06
aat	N	1	498.10
aac	N	978	480.90
agt	S	0	0.20
agc	S	1	0.20
gat	D	0	7.56
gac	D	14	6.44
---	--	------	------
mPD		0.032	0.53
		nPD:	0.06
		N. weight:	0.78
		Sc. PD:	-0.035
		Sc. rank:	-24.5

NA			
Pos.	460	obs:	exp:
tgg	W	994	994.00
---	--	------	------
mPD		0	0
		nPD:	1.
		N. weight:	0.
		Sc. PD:	0
		Sc. rank:	0

NA			
Pos.	461	obs:	exp:
tta	L	0	0.09
ttg	L	0	0.19
tct	S	2	0.32
tcc	S	0	0.26
tca	S	0	0.50
tcg	S	0	0.12
ctt	L	1	0.17
ctc	L	0	0.17
cta	L	0	0.17
ctg	L	0	0.22
cct	P	991	252.00
ccc	P	0	176.70
cca	P	0	404.90
ccg	P	0	157.40
agt	S	0	0.41
agc	S	0	0.39
---	--	------	------
mPD		0.0060	0.72
		nPD:	0.01
		N. weight:	1.5
		Sc. PD:	-0.14
		Sc. rank:	-882.3

NA			
Pos.	462	obs:	exp:
gat	D	825	536.60
gac	D	169	457.40
---	--	------	------
mPD		0.28	0.50
		nPD:	0.57
		N. weight:	0.21
		Sc. PD:	0.094
		Sc. rank:	162.0

NA			
Pos.	463	obs:	exp:
ggt	G	0	133.30
ggc	G	0	126.30
gga	G	2	459.60
ggg	G	992	274.80
---	--	------	------
mPD		0.0040	0.68
		nPD:	0.01
		N. weight:	1.4
		Sc. PD:	-0.14
		Sc. rank:	-905.4

NA			
Pos.	464	obs:	exp:
gct	A	972	253.90
gcc	A	0	186.10
gca	A	19	463.30
gcg	A	0	87.65
gat	D	1	0.54
gac	D	0	0.46
gaa	E	0	0.60
gag	E	1	0.40
ggt	G	0	0.13
ggc	G	1	0.13
gga	G	0	0.46
ggg	G	0	0.28
---	--	------	------
mPD		0.048	0.68
		nPD:	0.07
		N. weight:	1.4
		Sc. PD:	-0.048
		Sc. rank:	40.6

NA			
Pos.	465	obs:	exp:
cgt	R	0	6.58
cgc	R	0	7.82
cga	R	0	15.36
cgg	R	0	12.83
aat	N	0	1.53
aac	N	3	1.47
aaa	K	835	489.10
aag	K	0	345.90
aga	R	154	70.31
agg	R	0	41.10
gaa	E	1	0.60
gag	E	0	0.40
---	-	1	1.00
---	--	------	------
mPD		0.27	0.85
		nPD:	0.32
		N. weight:	0.63
		Sc. PD:	0.13
		Sc. rank:	434.2

NA			
Pos.	466	obs:	exp:
att	I	0	362.30
atc	I	0	240.20
ata	I	993	390.50
---	-	1	1.00
---	--	------	------
mPD		0	0.65
		nPD:	0.
		N. weight:	1.
		Sc. PD:	-0.11
		Sc. rank:	-800.7

NA			
Pos.	467	obs:	exp:
gaa	E	0	597.30
gag	E	993	395.70
---	-	1	1.00
---	--	------	------
mPD		0	0.48
		nPD:	0.
		N. weight:	1.
		Sc. PD:	-0.1
		Sc. rank:	-789.4

NA			
Pos.	468	obs:	exp:
tat	Y	0	524.40
tac	Y	989	464.60
tgt	C	0	0.82
tgc	C	2	1.18
act	T	1	0.26
acc	T	0	0.20
aca	T	0	0.46
acg	T	0	0.08
---	-	2	2.00
---	--	------	------
mPD		0.010	0.50
		nPD:	0.02
		N. weight:	0.84
		Sc. PD:	-0.069
		Sc. rank:	-289.2

NA			
Pos.	469	obs:	exp:
ttt	F	47	391.10
ttc	F	944	599.90
tta	L	0	0.09
ttg	L	0	0.19
ctt	L	0	0.17
ctc	L	1	0.17
cta	L	0	0.17
ctg	L	0	0.22
---	-	2	2.00
---	--	------	------
mPD		0.092	0.48
		nPD:	0.19
		N. weight:	0.37
		Sc. PD:	0.031
		Sc. rank:	188.4

NA			
Pos.	470	obs:	exp:
tta	L	0	87.18
ttg	L	0	185.10
ctt	L	0	168.10
ctc	L	992	166.20
cta	L	0	165.40
ctg	L	0	220.00
---	-	2	2.00
---	--	------	------
mPD		0	1.1
		nPD:	0.
		N. weight:	2.
		Sc. PD:	-0.2
		Sc. rank:	-1531.1

NA			
Pos.	471	obs:	exp:
taa	.	992	427.50
tag	.	2	251.30
tga	.	0	315.20
---	--	------	------
mPD		0.0040	0.81
		nPD:	0.
		N. weight:	0.92
		Sc. PD:	-0.089
		Sc. rank:	-610.3

Amino acid level conservation per codon

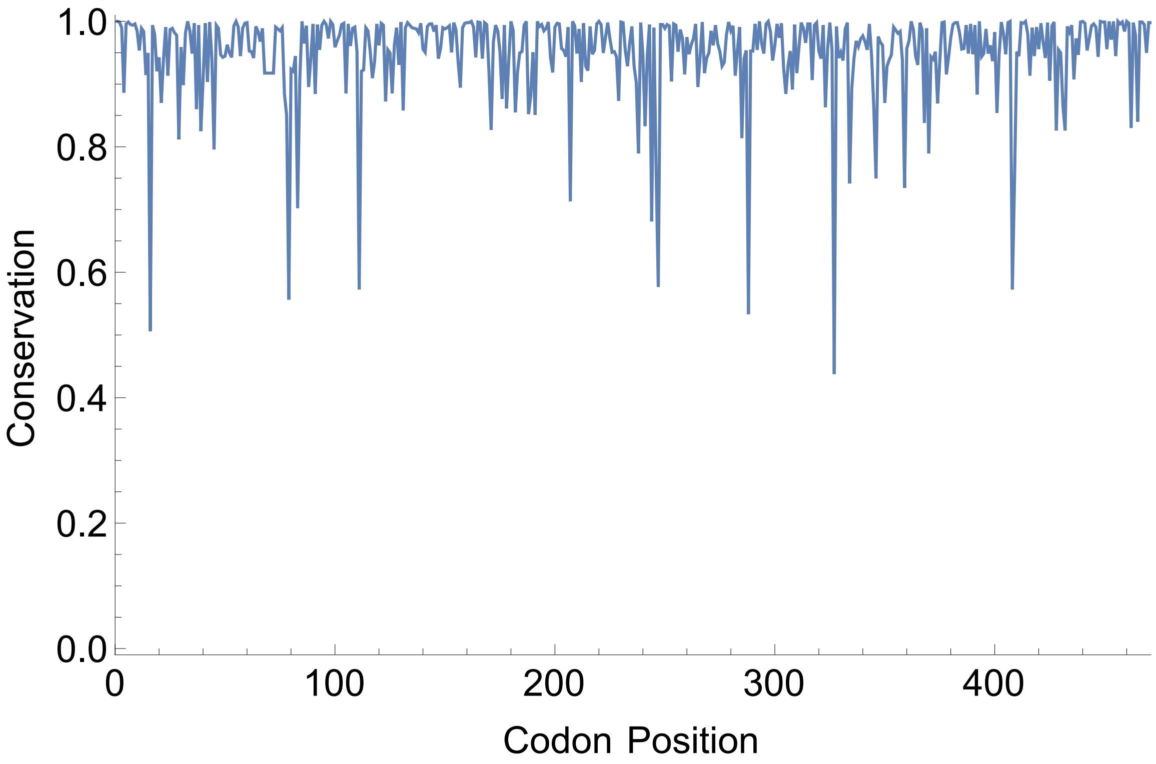

Conservation analysis plots

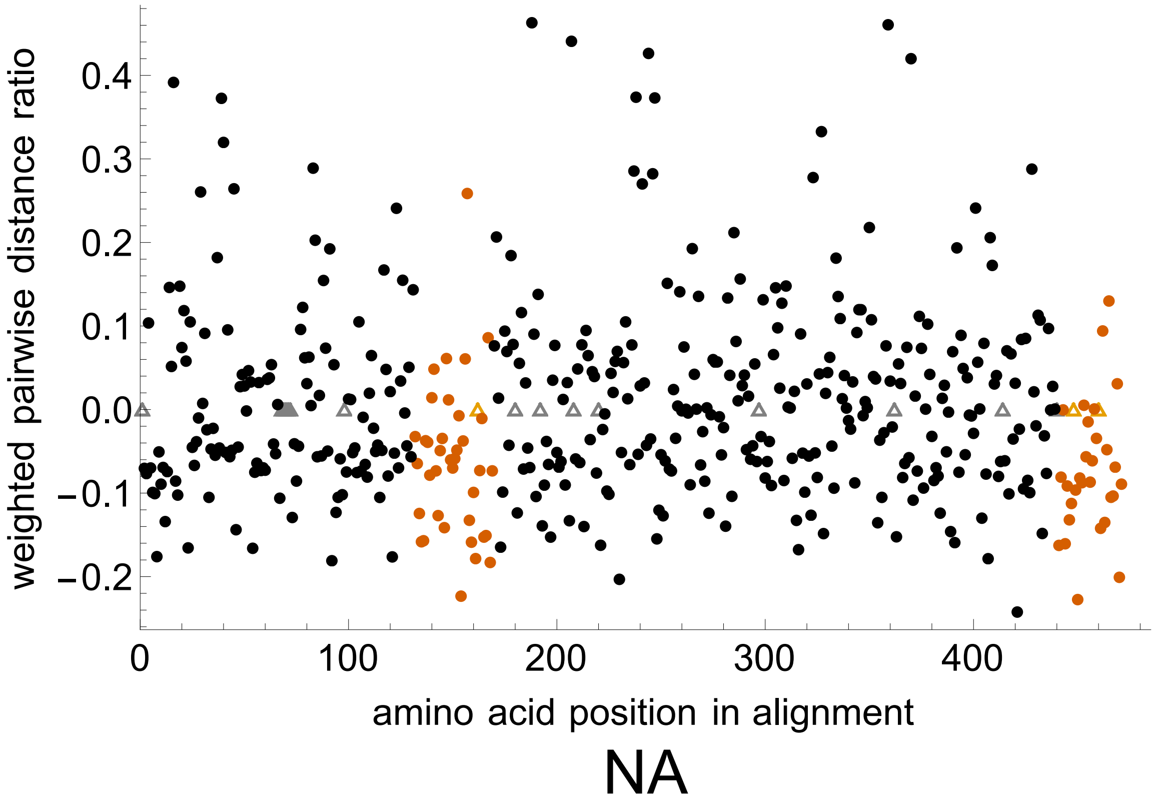

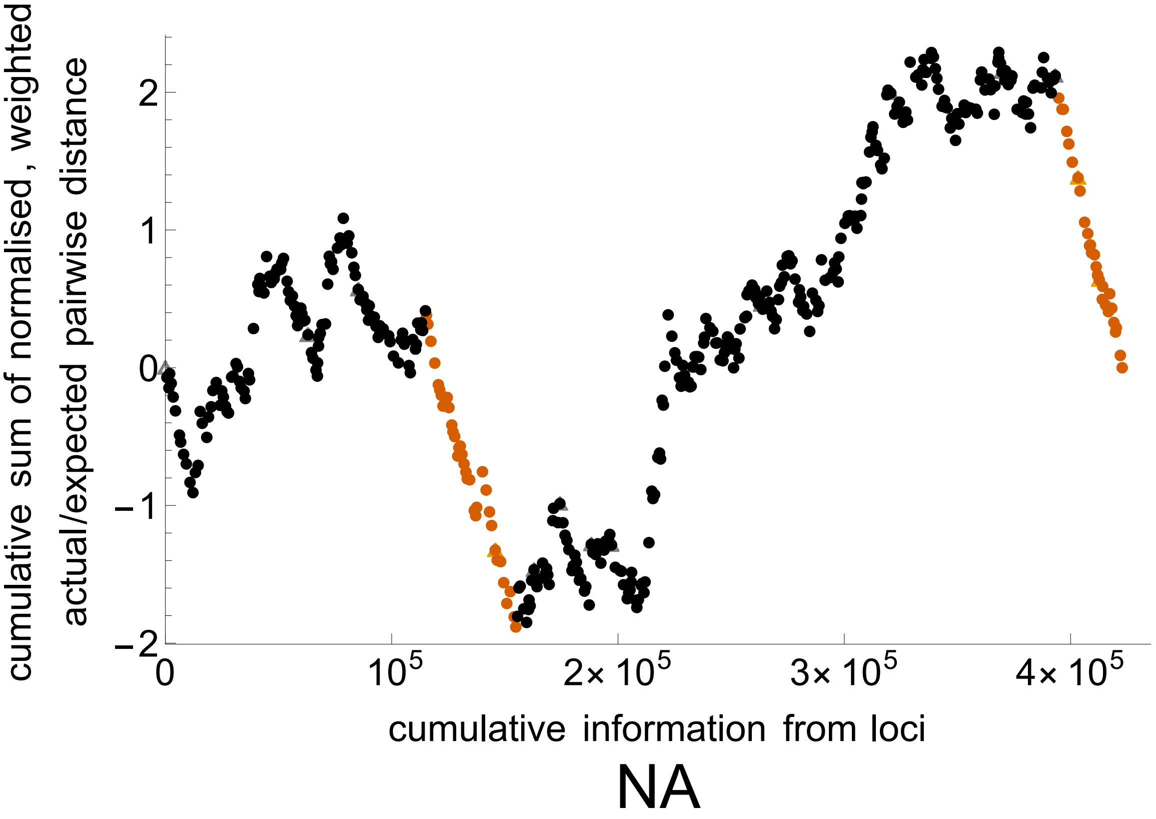

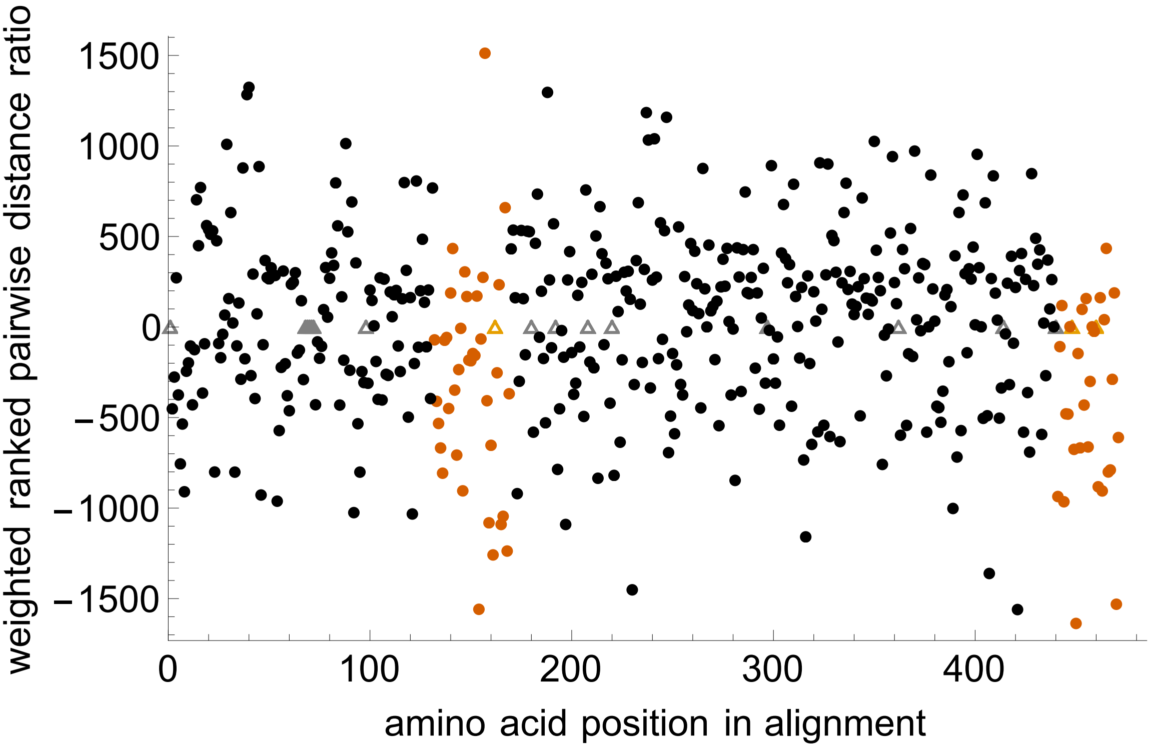
weighted ranked pairwise distance ratio
1500
1000
500
0
−500
−1000
−1500
0
100
200
300
400
amino acid position in alignment

NA

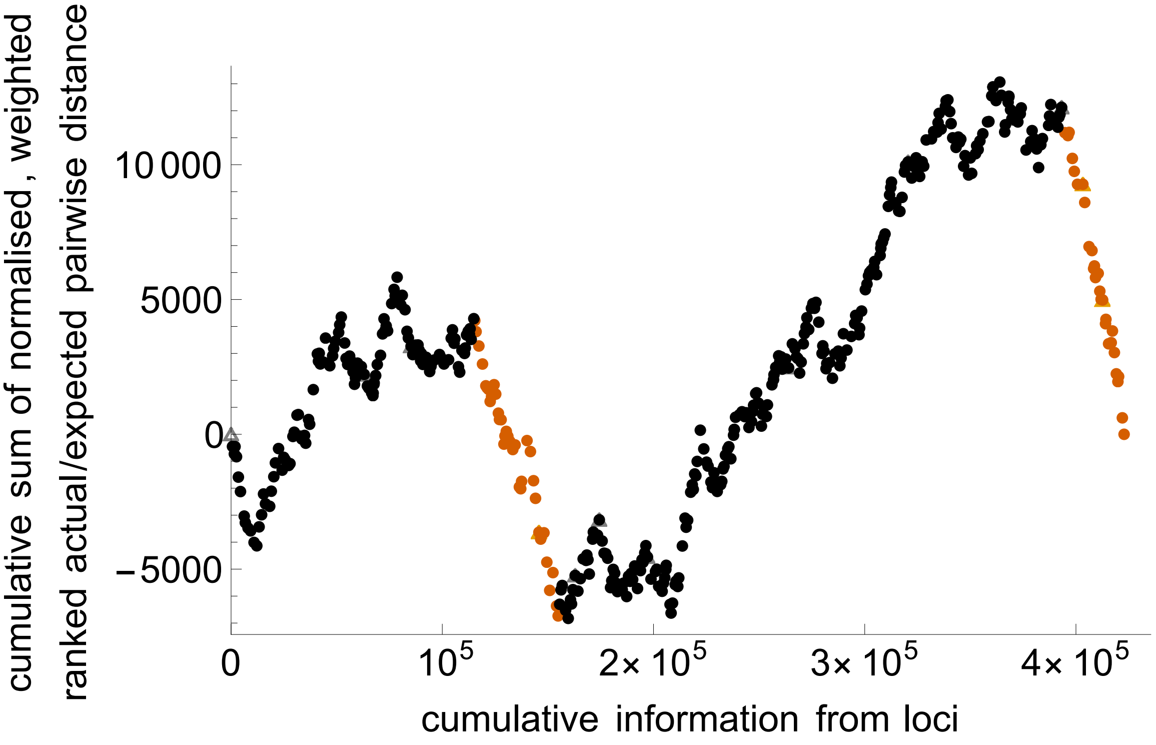
cumulative sum of normalised, weighted ranked actual/expected pairwise distance
10 000
5000
0
−5000
0
10^5
2×10^5
3×10^5
4×10^5
cumulative information from loci

NA

M1

Gene length histogram

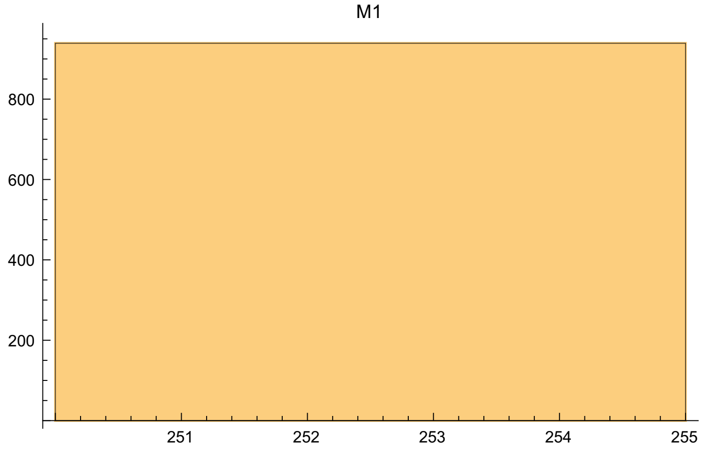

Information vs. nPD

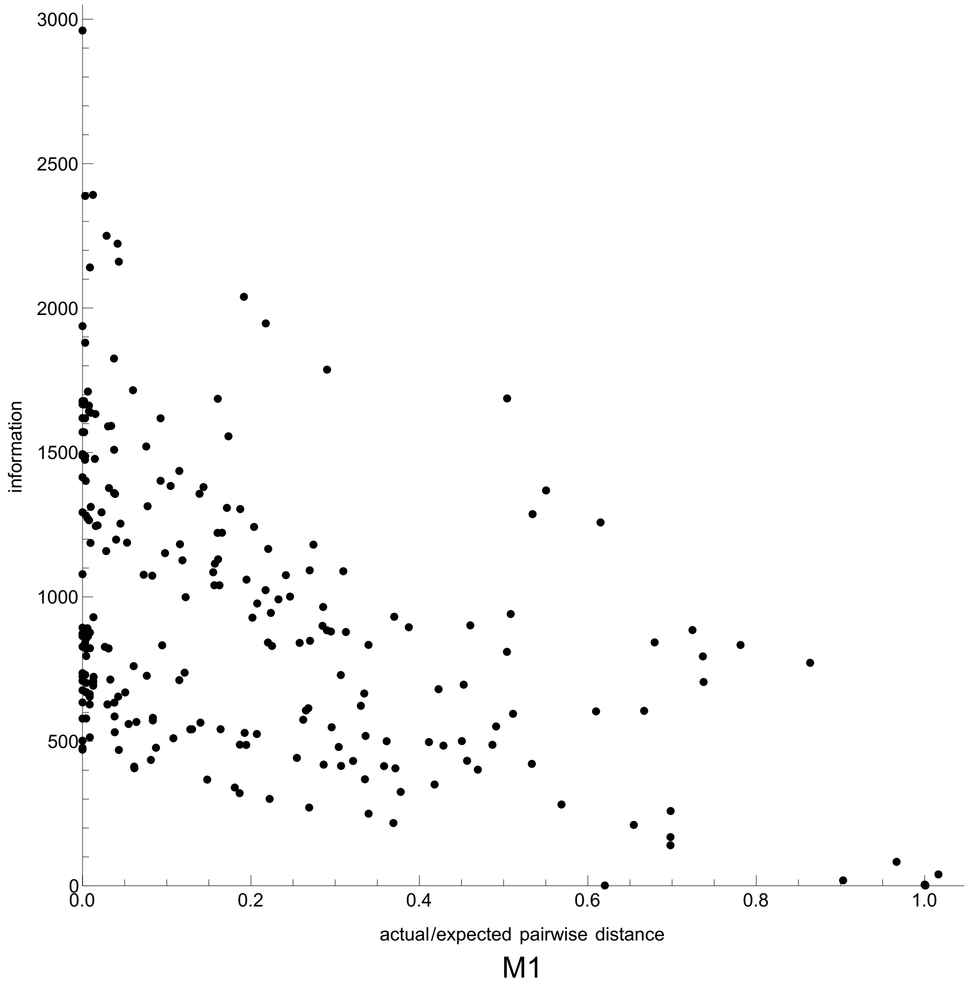

Example sequences highlighted by regions found in analyses to be conserved

Interesting points (by weighted raw PD) highlighted for gene M1:

ATG
AGTCTTCTAACCGAGGTCGAAACGTACGTTCTCTCTATCGTCCCGTCAGGCCCCC
TCAAAGCCGAGATCGCGCAGAGACTTGAAGATGTCTTTGCAGGGAAGAACAC
AGATCTCGAGGCTCTCATGGAATGGCTAAAGACAAGACCAATCCTGTCACCT
CTGACTAAGGGGATTTTGGGGTTTGTGTTCACGCTCACCGTGCCCAGTGAGC
GAGGACTGCAGCGTAGACGCTTTGTCCAAAATGCACTAAATGGAAATGGGGACCCAAACAACATG⋱
GACAGGGCAGTCAAATTATACAGGAAGCTGAAAAGAGAGATAACATTCCATGGAGCTAAGGAGGTT
GCACTTAGCTACTCAACCGGTGCACTTGCCAGTTGTATGGGTCTCATATACAACA⋱
GAATGGGAACAGTAACCACAGAAGTGGCTTTTGGCCTAGTGTGTGCCACTTGTGAGCAAATTGCTGACTC⋱
ACAGCACCGGTCCCACAGGCAGATGGTGACTACTACCAACCCACTAATCAGGCATGAGAACAGAATGGTG⋱
CTGGCCAGCACCACGGCTAAGGCCATGGAGCAGATGGCTGGATCAAGTGAACAGGCAGCAGAGGCCATGG⋱
AGGTTGCTAGTCAGGCCAGGCAGATGGTGCAGGCAATGAGGACAATTGGGACTCAT
CCTAGCTCCAGTGCCGGTCTGAAAGATGATCTTCTTGAAAATTTGCAGGCCTACC
AGAAACGGATGGGAGTGCAAATGCAGCGATTCAAGTGA

Interesting points (by weighted ranked PD) highlighted for gene M1:

ATGAGT
CTTCTAACCGAGGTCGAAACGTACGTTCTCTCTATCGTCCCGTCAGGCCCCCTCA
AAGCCGAGATCGCGCAGAGACTTGAAGATGTCTTTGCAGGGAAGAACACAGA
TCTCGAGGCTCTCATGGAATGGCTAAAGACAAGACCAATCCTGTCACCTCTG
ACTAAGGGGATTTTGGGGTTTGTGTTCACGCTCACCGTGCCCAGTGAGCGAG
GACTGCAGCGTAGACGCTTTGTCCAAAATGCACTAAATGGAAATGGGGACCCAAACAACATGGACA⋱
GGGCAGTCAAATTATACAGGAAGCTGAAAAGAGAGATAACATTCCATGGAGCTAAGGAGGTTGCACTTAG⋱
CTACTCAACCGGTGCACTTGCCAGTTGTATGGGTCTCATATACAACAGAATGGGAACAGTAACCACAGAA⋱
GTGGCTTTTGGCCTAGTGTGTGCCACTTGTGAGCAAATTGCTGACTCACAGCACCGGTCCCACAGGCAGA⋱
TGGTGACTACTACCAACCCACTAATCAGGCATGAGAACAGAATGGTGCTGGCCAGCACCACGGCTAAGGC⋱
CATGGAGCAGATGGCTGGATCAAGTGAACAGGCAGCAGAGGCCATGGAGGTTGCTAGTCAGGCCAGGCAG⋱
ATGGTGCAGGCAATGAGGACAATTGGGACTCATCCTAGCTCCAGTGCCGGTCTGAAAGATGATCTTCTTG⋱
AA**AATTTGCAG**GCCTACCAGAAACGGATGGGAGTGCAAATGCAGCGATTCAAGTGA

Per codon conservation report

M1			
Pos.	1	obs:	exp:
atg	M	939	939.00
---	--	------	------
mPD		0	0
		nPD:	1.
		N. weight:	0.
		Sc. PD:	0
		Sc. rank:	0

M1			
Pos.	2	obs:	exp:
tct	S	0	150.30
tcc	S	0	121.50
tca	S	0	237.00
tcg	S	0	54.64
agt	S	914	192.40
agc	S	25	183.10
---	--	------	------
mPD		0.052	1.7
		nPD:	0.03
		N. weight:	1.5
		Sc. PD:	-0.15
		Sc. rank:	-151.7

M1			
Pos.	3	obs:	exp:
tta	L	0	82.53
ttg	L	0	175.20
ctt	L	935	159.10
ctc	L	4	157.30
cta	L	0	156.60
ctg	L	0	208.30
---	--	------	------
mPD		0.0085	1.1
		nPD:	0.01
		N. weight:	1.8
		Sc. PD:	-0.22
		Sc. rank:	-648.6

M1			
Pos.	4	obs:	exp:
tta	L	3	82.53
ttg	L	0	175.20
ctt	L	1	159.10
ctc	L	0	157.30
cta	L	931	156.60
ctg	L	4	208.30
---	--	------	------
mPD		0.017	1.1
		nPD:	0.02
		N. weight:	1.8
		Sc. PD:	-0.21
		Sc. rank:	-353.4

M1			
Pos.	5	obs:	exp:
act	T	1	243.20
acc	T	938	191.20
aca	T	0	431.40
acg	T	0	73.15
---	--	------	------
mPD		0.0021	0.67
		nPD:	0.
		N. weight:	1.7
		Sc. PD:	-0.21
		Sc. rank:	-797.6

M1			
Pos.	6	obs:	exp:
gaa	E	0	564.80
gag	E	939	374.20
---	--	------	------
mPD		0	0.48
		nPD:	0.
		N. weight:	0.96
		Sc. PD:	-0.13
		Sc. rank:	-734.7

M1			
Pos.	7	obs:	exp:
gtt	V	3	193.80
gtc	V	934	188.10
gta	V	2	194.40
gtg	V	0	362.60
---	--	------	------
mPD		0.011	0.73
		nPD:	0.01
		N. weight:	1.6
		Sc. PD:	-0.19
		Sc. rank:	-338.3

M1			
Pos.	8	obs:	exp:
gaa	E	939	564.80
gag	E	0	374.20
---	--	------	------
mPD		0	0.48
		nPD:	0.
		N. weight:	0.53
		Sc. PD:	-0.069
		Sc. rank:	-405.8

M1			
Pos.	9	obs:	exp:
cgt	R	0	0.17
cgc	R	0	0.20
cga	R	0	0.40
cgg	R	0	0.33
act	T	0	242.20
acc	T	0	190.40
aca	T	0	429.60
acg	T	935	72.83
aga	R	0	1.83
agg	R	4	1.07
---	--	------	------
mPD		0.0085	0.69
		nPD:	0.01
		N. weight:	2.7
		Sc. PD:	-0.32
		Sc. rank:	-656.2

M1			
Pos.	10	obs:	exp:
tat	Y	1	497.90
tac	Y	938	441.10
---	--	------	------
mPD		0.0021	0.50
		nPD:	0.
		N. weight:	0.78
		Sc. PD:	-0.099
		Sc. rank:	-337.3

M1			
Pos.	11	obs:	exp:
gtt	V	938	193.80
gtc	V	1	188.10
gta	V	0	194.40
gtg	V	0	362.60
---	--	------	------
mPD		0.0021	0.73
		nPD:	0.
		N. weight:	1.6
		Sc. PD:	-0.21
		Sc. rank:	-883.2

M1			
Pos.	12	obs:	exp:
ttt	F	0	1.18
ttc	F	3	1.82
tta	L	0	82.17
ttg	L	0	174.50
ctt	L	0	158.40
ctc	L	898	156.60
cta	L	37	155.90
ctg	L	0	207.40
gtt	V	0	0.21
gtc	V	1	0.20
gta	V	0	0.21
gtg	V	0	0.39
---	--	------	------
mPD		0.084	1.1
		nPD:	0.08
		N. weight:	1.7
		Sc. PD:	-0.097
		Sc. rank:	145.9

M1			
Pos.	13	obs:	exp:
ttt	F	4	1.58
ttc	F	0	2.42
tct	S	934	149.70
tcc	S	0	121.00
tca	S	0	236.00
tcg	S	1	54.41
agt	S	0	191.60
agc	S	0	182.30
---	--	------	------
mPD		0.011	1.7
		nPD:	0.01
		N. weight:	1.9
		Sc. PD:	-0.24
		Sc. rank:	-708.4

M1			
Pos.	14	obs:	exp:
att	I	13	342.60
atc	I	925	227.10
ata	I	1	369.30
---	--	------	------
mPD		0.029	0.65
		nPD:	0.05
		N. weight:	1.4
		Sc. PD:	-0.12
		Sc. rank:	35.2

M1			
Pos.	15	obs:	exp:
att	I	793	307.60
atc	I	50	203.90
ata	I	0	331.50
act	T	1	0.26
acc	T	0	0.20
aca	T	0	0.46
acg	T	0	0.08
gtt	V	7	19.61
gtc	V	87	19.03
gta	V	1	19.67
gtg	V	0	36.69
---	--	------	------
mPD		0.44	0.86
		nPD:	0.5
		N. weight:	0.9
		Sc. PD:	0.32
		Sc. rank:	616.4

M1			
Pos.	16	obs:	exp:
cct	P	1	238.80
ccc	P	0	167.40
cca	P	839	383.60
ccg	P	99	149.20
---	--	------	------
mPD		0.19	0.71
		nPD:	0.27
		N. weight:	0.69
		Sc. PD:	0.088
		Sc. rank:	338.3

M1			
Pos.	17	obs:	exp:
tct	S	0	150.20
tcc	S	0	121.40
tca	S	936	236.70
tcg	S	2	54.59
act	T	0	0.26
acc	T	0	0.20
aca	T	1	0.46
acg	T	0	0.08
agt	S	0	192.20
agc	S	0	182.90
---	--	------	------
mPD		0.0064	1.7
		nPD:	0.
		N. weight:	1.4
		Sc. PD:	-0.18
		Sc. rank:	-642.3

M1			
Pos.	18	obs:	exp:
ggt	G	0	125.90
ggc	G	939	119.30
gga	G	0	434.20
ggg	G	0	259.60
---	--	------	------
mPD		0	0.68
		nPD:	0.
		N. weight:	2.2
		Sc. PD:	-0.28
		Sc. rank:	-1647.3

M1			
Pos.	19	obs:	exp:
cct	P	0	238.80
ccc	P	939	167.40
cca	P	0	383.60
ccg	P	0	149.20
---	--	------	------
mPD		0	0.71
		nPD:	0.
		N. weight:	1.8
		Sc. PD:	-0.23
		Sc. rank:	-1376.7

M1			
Pos.	20	obs:	exp:
tta	L	0	82.44
ttg	L	0	175.00
ctt	L	938	159.00
ctc	L	0	157.20
cta	L	0	156.40
ctg	L	0	208.00
cct	P	0	0.25
ccc	P	1	0.18
cca	P	0	0.41
ccg	P	0	0.16
---	--	------	------
mPD		0.0021	1.1
		nPD:	0.
		N. weight:	1.9
		Sc. PD:	-0.24
		Sc. rank:	-1070.8

M1			
Pos.	21	obs:	exp:
aaa	K	939	550.10
aag	K	0	388.90
---	--	------	------
mPD		0	0.49
		nPD:	0.
		N. weight:	0.56
		Sc. PD:	-0.073
		Sc. rank:	-427.0

M1			
Pos.	22	obs:	exp:
gct	A	0	240.60
gcc	A	939	176.30
gca	A	0	439.00
gcg	A	0	83.05
---	--	------	------
mPD		0	0.67
		nPD:	0.
		N. weight:	1.8
		Sc. PD:	-0.23
		Sc. rank:	-1335.4

M1			
Pos.	23	obs:	exp:
gaa	E	6	564.80
gag	E	933	374.20
---	--	------	------
mPD		0.013	0.48
		nPD:	0.03
		N. weight:	0.92
		Sc. PD:	-0.096
		Sc. rank:	-149.8

M1			
Pos.	24	obs:	exp:
tct	S	0	0.16
tcc	S	0	0.13
tca	S	0	0.25
tcg	S	0	0.06
att	I	3	342.30
atc	I	932	226.90
ata	I	3	368.90
agt	S	0	0.20
agc	S	1	0.20
---	--	------	------
mPD		0.015	0.66
		nPD:	0.02
		N. weight:	1.4
		Sc. PD:	-0.16
		Sc. rank:	-242.3

M1			
Pos.	25	obs:	exp:
gct	A	0	240.60
gcc	A	0	176.30
gca	A	274	439.00
gcg	A	665	83.05
---	--	------	------
mPD		0.41	0.67
		nPD:	0.62
		N. weight:	1.4
		Sc. PD:	0.65
		Sc. rank:	1038.7

M1			
Pos.	26	obs:	exp:
caa	Q	1	482.30
cag	Q	938	456.70
---	--	------	------
mPD		0.0021	0.50
		nPD:	0.
		N. weight:	0.75
		Sc. PD:	-0.094
		Sc. rank:	-332.2

,

M1			
Pos.	27	obs:	exp:
cgt	R	0	39.94
cgc	R	0	47.50
cga	R	0	93.23
cgg	R	0	77.88
aaa	K	4	2.34
aag	K	0	1.66
aga	R	933	426.90
agg	R	2	249.50
---	--	------	------
mPD		0.013	0.97
		nPD:	0.01
		N. weight:	0.81
		Sc. PD:	-0.095
		Sc. rank:	-173.7

,

M1			
Pos.	28	obs:	exp:
ttt	F	1	0.39
ttc	F	0	0.61
tta	L	0	82.44
ttg	L	0	175.00
ctt	L	938	159.00
ctc	L	0	157.20
cta	L	0	156.40
ctg	L	0	208.00
---	--	------	------
mPD		0.0021	1.1
		nPD:	0.
		N. weight:	1.9
		Sc. PD:	-0.24
		Sc. rank:	-1042.2

,

M1			
Pos.	29	obs:	exp:
gaa	E	109	564.80
gag	E	830	374.20
---	--	------	------
mPD		0.21	0.48
		nPD:	0.43
		N. weight:	0.54
		Sc. PD:	0.15
		Sc. rank:	351.6

,

M1			
Pos.	30	obs:	exp:
gat	D	938	506.40
gac	D	0	431.60
gaa	E	0	0.60
gag	E	1	0.40
---	--	------	------
mPD		0.0021	0.50
		nPD:	0.
		N. weight:	0.65
		Sc. PD:	-0.081
		Sc. rank:	-275.5

,

M1			
Pos.	31	obs:	exp:
gtt	V	797	193.80
gtc	V	55	188.10
gta	V	3	194.40
gtg	V	84	362.60
---	--	------	------
mPD		0.27	0.73
		nPD:	0.37
		N. weight:	1.
		Sc. PD:	0.24
		Sc. rank:	648.9

,

M1			
Pos.	32	obs:	exp:
ttt	F	939	370.60
ttc	F	0	568.40
---	--	------	------
mPD		0	0.48
		nPD:	0.
		N. weight:	0.97
		Sc. PD:	-0.13
		Sc. rank:	-742.4

,

M1			
Pos.	33	obs:	exp:
tct	S	0	1.60
tcc	S	0	1.29
tca	S	10	2.52
tcg	S	0	0.58
agt	S	0	2.05
agc	S	0	1.95
gct	A	1	238.00
gcc	A	0	174.40
gca	A	928	434.40
gcg	A	0	82.17
---	--	------	------
mPD		0.023	0.70
		nPD:	0.03
		N. weight:	0.8
		Sc. PD:	-0.078
		Sc. rank:	-74.7

,

M1			
Pos.	34	obs:	exp:
gaa	E	0	0.60
gag	E	1	0.40
ggt	G	0	125.80
ggc	G	0	119.20
gga	G	8	433.70
ggg	G	930	259.30
---	--	------	------
mPD		0.019	0.68
		nPD:	0.03
		N. weight:	1.3
		Sc. PD:	-0.13
		Sc. rank:	-199.6

,

M1			
Pos.	35	obs:	exp:
aaa	K	64	550.10
aag	K	875	388.90
---	--	------	------
mPD		0.13	0.49
		nPD:	0.26
		N. weight:	0.64
		Sc. PD:	0.078
		Sc. rank:	311.0

,

M1			
Pos.	36	obs:	exp:
tct	S	0	0.48
tcc	S	0	0.39
tca	S	0	0.76
tcg	S	0	0.17
aat	N	71	476.20
aac	N	865	459.80
agt	S	0	0.61
agc	S	3	0.59
---	--	------	------
mPD		0.15	0.51
		nPD:	0.29
		N. weight:	0.47
		Sc. PD:	0.068
		Sc. rank:	249.1

,

M1			
Pos.	37	obs:	exp:
act	T	19	25.90
acc	T	64	20.36
aca	T	17	45.94
acg	T	0	7.79
gtt	V	0	0.83
gtc	V	0	0.80
gta	V	4	0.83
gtg	V	0	1.54
gct	A	1	214.00
gcc	A	0	156.80
gca	A	832	390.40
gcg	A	2	73.85
---	--	------	------
mPD		0.37	0.87
		nPD:	0.42
		N. weight:	0.76
		Sc. PD:	0.21
		Sc. rank:	490.6

,

M1			
Pos.	38	obs:	exp:
gat	D	939	506.90
gac	D	0	432.10
---	--	------	------
mPD		0	0.50
		nPD:	0.
		N. weight:	0.65
		Sc. PD:	-0.084
		Sc. rank:	-492.2

,

M1			
Pos.	39	obs:	exp:
tta	L	0	82.53
ttg	L	0	175.20
ctt	L	60	159.10
ctc	L	747	157.30
cta	L	132	156.60
ctg	L	0	208.30
---	--	------	------
mPD		0.34	1.1
		nPD:	0.31
		N. weight:	1.2
		Sc. PD:	0.2
		Sc. rank:	703.4

,

M1			
Pos.	40	obs:	exp:
gaa	E	1	564.80
gag	E	938	374.20
---	--	------	------
mPD		0.0021	0.48
		nPD:	0.
		N. weight:	0.96
		Sc. PD:	-0.12
		Sc. rank:	-391.4

,

M1			
Pos.	41	obs:	exp:
tct	S	6	0.96
tcc	S	0	0.78
tca	S	0	1.51
tcg	S	0	0.35
agt	S	0	1.23
agc	S	0	1.17
gct	A	886	239.10
gcc	A	0	175.20
gca	A	8	436.20
gcg	A	39	82.52
---	--	------	------
mPD		0.11	0.69
		nPD:	0.16
		N. weight:	1.2
		Sc. PD:	0.026
		Sc. rank:	331.1

,

M1			
Pos.	42	obs:	exp:
ttt	F	0	0.39
ttc	F	1	0.61
tta	L	0	82.35
ttg	L	0	174.90
ctt	L	1	158.80
ctc	L	935	157.00
cta	L	1	156.20
ctg	L	0	207.80
att	I	0	0.36
atc	I	1	0.24
ata	I	0	0.39
---	--	------	------
mPD		0.0085	1.1
		nPD:	0.01
		N. weight:	1.9
		Sc. PD:	-0.23
		Sc. rank:	-635.2

,

M1			
Pos.	43	obs:	exp:
atg	M	939	939.00
---	--	------	------
mPD		0	0
		nPD:	1.
		N. weight:	0.
		Sc. PD:	0
		Sc. rank:	0

,

M1			
Pos.	44	obs:	exp:
gaa	E	104	564.80
gag	E	835	374.20
---	--	------	------
mPD		0.20	0.48
		nPD:	0.41
		N. weight:	0.56
		Sc. PD:	0.15
		Sc. rank:	354.7

,

M1			
Pos.	45	obs:	exp:
tgg	W	939	939.00
---	--	------	------
mPD		0	0
		nPD:	1.
		N. weight:	0.
		Sc. PD:	0
		Sc. rank:	0

,

M1			
Pos.	46	obs:	exp:
tta	L	2	8.53
ttg	L	0	18.10
ctt	L	0	16.44
ctc	L	0	16.25
cta	L	93	16.17
ctg	L	2	21.51
att	I	0	306.10
atc	I	0	202.90
ata	I	839	329.90
act	T	0	0.26
acc	T	0	0.20
aca	T	1	0.46
acg	T	0	0.08
gtt	V	0	0.41
gtc	V	0	0.40
gta	V	2	0.41
gtg	V	0	0.77
---	--	------	------
mPD		0.20	0.88
		nPD:	0.22
		N. weight:	1.1
		Sc. PD:	0.09
		Sc. rank:	469.6

,

M1			
Pos.	47	obs:	exp:
aaa	K	0	550.10
aag	K	939	388.90
---	--	------	------
mPD		0	0.49
		nPD:	0.
		N. weight:	0.92
		Sc. PD:	-0.12
		Sc. rank:	-703.7

,

M1			
Pos.	48	obs:	exp:
cgt	R	0	0.04
cgc	R	0	0.05
cga	R	0	0.10
cgg	R	0	0.08
act	T	0	243.00
acc	T	0	191.00
aca	T	938	430.90
acg	T	0	73.07
aga	R	1	0.46
agg	R	0	0.27
---	--	------	------
mPD		0.0021	0.68
		nPD:	0.
		N. weight:	0.82
		Sc. PD:	-0.1
		Sc. rank:	-413.0

,

M1			
Pos.	49	obs:	exp:
cgt	R	0	40.11
cgc	R	0	47.71
cga	R	0	93.63
cgg	R	0	78.21
aga	R	938	428.70
agg	R	1	250.60
---	--	------	------
mPD		0.0021	0.96
		nPD:	0.
		N. weight:	0.81
		Sc. PD:	-0.1
		Sc. rank:	-443.7

,

M1			
Pos.	50	obs:	exp:
cct	P	0	238.50
ccc	P	0	167.20
cca	P	938	383.20
ccg	P	0	149.00
gct	A	0	0.26
gcc	A	0	0.19
gca	A	1	0.47
gcg	A	0	0.09
---	--	------	------
mPD		0.0021	0.71
		nPD:	0.
		N. weight:	0.94
		Sc. PD:	-0.12
		Sc. rank:	-479.9

,

M1			
Pos.	51	obs:	exp:
att	I	2	342.60
atc	I	936	227.10
ata	I	1	369.30
---	--	------	------
mPD		0.0064	0.65
		nPD:	0.01
		N. weight:	1.5
		Sc. PD:	-0.18
		Sc. rank:	-383.8

,

M1			
Pos.	52	obs:	exp:
tta	L	0	82.53
ttg	L	93	175.20
ctt	L	0	159.10
ctc	L	0	157.30
cta	L	0	156.60
ctg	L	846	208.30
---	--	------	------
mPD		0.18	1.1
		nPD:	0.16
		N. weight:	1.3
		Sc. PD:	0.031
		Sc. rank:	370.4

,

M1			
Pos.	53	obs:	exp:
tct	S	0	150.30
tcc	S	0	121.50
tca	S	939	237.00
tcg	S	0	54.64
agt	S	0	192.40
agc	S	0	183.10
---	--	------	------
mPD		0	1.7
		nPD:	0.
		N. weight:	1.4
		Sc. PD:	-0.19
		Sc. rank:	-1099.4

,

M1			
Pos.	54	obs:	exp:
cct	P	937	238.80
ccc	P	0	167.40
cca	P	2	383.60
ccg	P	0	149.20
---	--	------	------
mPD		0.0043	0.71
		nPD:	0.01
		N. weight:	1.4
		Sc. PD:	-0.18
		Sc. rank:	-543.2

,

M1			
Pos.	55	obs:	exp:
tta	L	0	82.53
ttg	L	2	175.20
ctt	L	0	159.10
ctc	L	0	157.30
cta	L	0	156.60
ctg	L	937	208.30
---	--	------	------
mPD		0.0043	1.1
		nPD:	0.
		N. weight:	1.6
		Sc. PD:	-0.2
		Sc. rank:	-715.8

,

M1			
Pos.	56	obs:	exp:
tct	S	0	0.16
tcc	S	0	0.13
tca	S	0	0.25
tcg	S	0	0.06
act	T	934	243.00
acc	T	4	191.00
aca	T	0	430.90
acg	T	0	73.07
agt	S	1	0.20
agc	S	0	0.20
---	--	------	------
mPD		0.011	0.68
		nPD:	0.02
		N. weight:	1.4
		Sc. PD:	-0.16
		Sc. rank:	-257.6

,

M1			
Pos.	57	obs:	exp:
aaa	K	84	550.10
aag	K	855	388.90
---	--	------	------
mPD		0.16	0.49
		nPD:	0.34
		N. weight:	0.58
		Sc. PD:	0.11
		Sc. rank:	348.9

,

M1			
Pos.	58	obs:	exp:
ggt	G	0	125.90
ggc	G	0	119.30
gga	G	3	434.20
ggg	G	936	259.60
---	--	------	------
mPD		0.0064	0.68
		nPD:	0.01
		N. weight:	1.3
		Sc. PD:	-0.16
		Sc. rank:	-374.1

,

M1			
Pos.	59	obs:	exp:
tct	S	0	0.16
tcc	S	0	0.13
tca	S	0	0.25
tcg	S	0	0.06
att	I	935	342.30
atc	I	3	226.90
ata	I	0	368.90
agt	S	1	0.20
agc	S	0	0.20
---	--	------	------
mPD		0.0085	0.66
		nPD:	0.01
		N. weight:	1.
		Sc. PD:	-0.12
		Sc. rank:	-228.5

,

M1			
Pos.	60	obs:	exp:
tta	L	916	82.53
ttg	L	23	175.20
ctt	L	0	159.10
ctc	L	0	157.30
cta	L	0	156.60
ctg	L	0	208.30
---	--	------	------
mPD		0.048	1.1
		nPD:	0.04
		N. weight:	2.4
		Sc. PD:	-0.21
		Sc. rank:	32.1

,

M1			
Pos.	61	obs:	exp:
ggt	G	0	125.90
ggc	G	2	119.30
gga	G	93	434.20
ggg	G	844	259.60
---	--	------	------
mPD		0.18	0.68
		nPD:	0.27
		N. weight:	0.95
		Sc. PD:	0.12
		Sc. rank:	481.1

,

M1			
Pos.	62	obs:	exp:
ttt	F	911	370.60
ttc	F	28	568.40
---	--	------	------
mPD		0.058	0.48
		nPD:	0.12
		N. weight:	0.82
		Sc. PD:	-0.011
		Sc. rank:	168.3

,

M1			
Pos.	63	obs:	exp:
gtt	V	0	193.80
gtc	V	0	188.10
gta	V	3	194.40
gtg	V	936	362.60
---	--	------	------
mPD		0.0064	0.73
		nPD:	0.01
		N. weight:	0.98
		Sc. PD:	-0.12
		Sc. rank:	-304.1

,

M1			
Pos.	64	obs:	exp:
ttt	F	14	370.60
ttc	F	925	568.40
---	--	------	------
mPD		0.029	0.48
		nPD:	0.06
		N. weight:	0.45
		Sc. PD:	-0.032
		Sc. rank:	29.2

,

M1			
Pos.	65	obs:	exp:
act	T	0	243.20
acc	T	0	191.20
aca	T	1	431.40
acg	T	938	73.15
---	--	------	------
mPD		0.0021	0.67
		nPD:	0.
		N. weight:	2.7
		Sc. PD:	-0.34
		Sc. rank:	-1280.7

,

M1			
Pos.	66	obs:	exp:
tta	L	0	82.53
ttg	L	0	175.20
ctt	L	0	159.10
ctc	L	939	157.30
cta	L	0	156.60
ctg	L	0	208.30
---	--	------	------
mPD		0	1.1
		nPD:	0.
		N. weight:	1.9
		Sc. PD:	-0.24
		Sc. rank:	-1426.5

,

M1			
Pos.	67	obs:	exp:
act	T	0	243.20
acc	T	939	191.20
aca	T	0	431.40
acg	T	0	73.15
---	--	------	------
mPD		0	0.67
		nPD:	0.
		N. weight:	1.7
		Sc. PD:	-0.22
		Sc. rank:	-1270.7

,

M1			
Pos.	68	obs:	exp:
gtt	V	0	193.80
gtc	V	0	188.10
gta	V	0	194.40
gtg	V	939	362.60
---	--	------	------
mPD		0	0.73
		nPD:	0.
		N. weight:	1.
		Sc. PD:	-0.13
		Sc. rank:	-759.7

,

M1			
Pos.	69	obs:	exp:
cct	P	0	238.50
ccc	P	938	167.20
cca	P	0	383.20
ccg	P	0	149.00
cat	H	0	0.57
cac	H	1	0.43
---	--	------	------
mPD		0.0021	0.71
		nPD:	0.
		N. weight:	1.8
		Sc. PD:	-0.23
		Sc. rank:	-934.5

,

M1			
Pos.	70	obs:	exp:
tct	S	0	150.30
tcc	S	0	121.50
tca	S	0	237.00
tcg	S	0	54.64
agt	S	939	192.40
agc	S	0	183.10
---	--	------	------
mPD		0	1.7
		nPD:	0.
		N. weight:	1.7
		Sc. PD:	-0.22
		Sc. rank:	-1265.7

,

M1			
Pos.	71	obs:	exp:
gaa	E	1	564.80
gag	E	938	374.20
---	--	------	------
mPD		0.0021	0.48
		nPD:	0.
		N. weight:	0.96
		Sc. PD:	-0.12
		Sc. rank:	-391.4

M1			
Pos.	72	obs:	exp:
cgt	R	1	40.11
cgc	R	0	47.71
cga	R	935	93.63
cgg	R	3	78.21
aga	R	0	428.70
agg	R	0	250.60
---	--	------	------
mPD		0.0085	0.96
		nPD:	0.01
		N. weight:	2.4
		Sc. PD:	-0.29
		Sc. rank:	-694.2

M1			
Pos.	73	obs:	exp:
ggt	G	0	125.90
ggc	G	0	119.30
gga	G	939	434.20
ggg	G	0	259.60
---	--	------	------
mPD		0	0.68
		nPD:	0.
		N. weight:	0.81
		Sc. PD:	-0.11
		Sc. rank:	-615.9

M1			
Pos.	74	obs:	exp:
tta	L	0	82.53
ttg	L	0	175.20
ctt	L	0	159.10
ctc	L	0	157.30
cta	L	0	156.60
ctg	L	939	208.30
---	--	------	------
mPD		0	1.1
		nPD:	0.
		N. weight:	1.6
		Sc. PD:	-0.21
		Sc. rank:	-1202.5

M1			
Pos.	75	obs:	exp:
caa	Q	1	482.30
cag	Q	938	456.70
---	--	------	------
mPD		0.0021	0.50
		nPD:	0.
		N. weight:	0.75
		Sc. PD:	-0.094
		Sc. rank:	-332.2

M1			
Pos.	76	obs:	exp:
cgt	R	939	40.11
cgc	R	0	47.71
cga	R	0	93.63
cgg	R	0	78.21
aga	R	0	428.70
agg	R	0	250.60
---	--	------	------
mPD		0	0.96
		nPD:	0.
		N. weight:	3.3
		Sc. PD:	-0.43
		Sc. rank:	-2517.6

M1			
Pos.	77	obs:	exp:
cgt	R	0	40.11
cgc	R	0	47.71
cga	R	0	93.63
cgg	R	0	78.21
aga	R	939	428.70
agg	R	0	250.60
---	--	------	------
mPD		0	0.96
		nPD:	0.
		N. weight:	0.82
		Sc. PD:	-0.11
		Sc. rank:	-626.0

M1			
Pos.	78	obs:	exp:
cgt	R	190	40.11
cgc	R	96	47.71
cga	R	14	93.63
cgg	R	639	78.21
aga	R	0	428.70
agg	R	0	250.60
---	--	------	------
mPD		0.49	0.96
		nPD:	0.5
		N. weight:	1.9
		Sc. PD:	0.67
		Sc. rank:	1306.0

M1			
Pos.	79	obs:	exp:
ttt	F	938	370.60
ttc	F	1	568.40
---	--	------	------
mPD		0.0021	0.48
		nPD:	0.
		N. weight:	0.97
		Sc. PD:	-0.12
		Sc. rank:	-384.1

M1			
Pos.	80	obs:	exp:
gtt	V	105	193.80
gtc	V	834	188.10
gta	V	0	194.40
gtg	V	0	362.60
---	--	------	------
mPD		0.20	0.73
		nPD:	0.27
		N. weight:	1.3
		Sc. PD:	0.18
		Sc. rank:	680.7

M1			
Pos.	81	obs:	exp:
caa	Q	926	482.30
cag	Q	13	456.70
---	--	------	------
mPD		0.027	0.50
		nPD:	0.05
		N. weight:	0.63
		Sc. PD:	-0.048
		Sc. rank:	26.1

M1			
Pos.	82	obs:	exp:
tct	S	0	0.32
tcc	S	0	0.26
tca	S	0	0.50
tcg	S	0	0.12
act	T	0	3.37
acc	T	13	2.65
aca	T	0	5.97
acg	T	0	1.01
aat	N	188	470.10
aac	N	736	453.90
agt	S	2	0.41
agc	S	0	0.39
---	--	------	------
mPD		0.35	0.54
		nPD:	0.65
		N. weight:	0.24
		Sc. PD:	0.12
		Sc. rank:	174.3

M1			
Pos.	83	obs:	exp:
gct	A	8	240.60
gcc	A	905	176.30
gca	A	26	439.00
gcg	A	0	83.05
---	--	------	------
mPD		0.070	0.67
		nPD:	0.1
		N. weight:	1.5
		Sc. PD:	-0.045
		Sc. rank:	255.3

M1			
Pos.	84	obs:	exp:
tta	L	8	82.53
ttg	L	3	175.20
ctt	L	1	159.10
ctc	L	3	157.30
cta	L	866	156.60
ctg	L	12	208.30
---	--	------	------
mPD		0.15	1.1
		nPD:	0.14
		N. weight:	1.5
		Sc. PD:	0.0056
		Sc. rank:	345.2

M1			
Pos.	85	obs:	exp:
aat	N	930	477.70
aac	N	9	461.30
---	--	------	------
mPD		0.019	0.50
		nPD:	0.04
		N. weight:	0.65
		Sc. PD:	-0.061
		Sc. rank:	-27.7

M1			
Pos.	86	obs:	exp:
ggt	G	0	125.90
ggc	G	0	119.30
gga	G	27	434.20
ggg	G	912	259.60
---	--	------	------
mPD		0.056	0.68
		nPD:	0.08
		N. weight:	1.2
		Sc. PD:	-0.06
		Sc. rank:	132.1

M1			
Pos.	87	obs:	exp:
aat	N	937	477.20
aac	N	1	460.80
gat	D	1	0.54
gac	D	0	0.46
---	--	------	------
mPD		0.0043	0.50
		nPD:	0.01
		N. weight:	0.7
		Sc. PD:	-0.085
		Sc. rank:	-230.8

M1			
Pos.	88	obs:	exp:
ggt	G	0	125.90
ggc	G	0	119.30
gga	G	801	434.20
ggg	G	138	259.60
---	--	------	------
mPD		0.25	0.68
		nPD:	0.37
		N. weight:	0.45
		Sc. PD:	0.1
		Sc. rank:	284.6

M1			
Pos.	89	obs:	exp:
gat	D	51	506.90
gac	D	888	432.10
---	--	------	------
mPD		0.10	0.50
		nPD:	0.21
		N. weight:	0.59
		Sc. PD:	0.041
		Sc. rank:	232.2

M1			
Pos.	90	obs:	exp:
cct	P	12	238.80
ccc	P	0	167.40
cca	P	913	383.60
ccg	P	14	149.20
---	--	------	------
mPD		0.054	0.71
		nPD:	0.08
		N. weight:	0.81
		Sc. PD:	-0.046
		Sc. rank:	73.8

M1			
Pos.	91	obs:	exp:
aat	N	2	477.70
aac	N	937	461.30
---	--	------	------
mPD		0.0043	0.50
		nPD:	0.01
		N. weight:	0.73
		Sc. PD:	-0.089
		Sc. rank:	-237.3

M1			
Pos.	92	obs:	exp:
aat	N	7	477.70
aac	N	932	461.30
---	--	------	------
mPD		0.015	0.50
		nPD:	0.03
		N. weight:	0.7
		Sc. PD:	-0.071
		Sc. rank:	-94.3

M1			
Pos.	93	obs:	exp:
att	I	1	2.19
atc	I	0	1.45
ata	I	5	2.36
atg	M	933	933.00
---	--	------	------
mPD		0.013	0.013
		nPD:	1.
		N. weight:	0.0043
		Sc. PD:	0.0036
		Sc. rank:	3.4

M1			
Pos.	94	obs:	exp:
aat	N	0	0.51
aac	N	1	0.49
gat	D	11	506.40
gac	D	927	431.60
---	--	------	------
mPD		0.025	0.50
		nPD:	0.05
		N. weight:	0.75
		Sc. PD:	-0.061
		Sc. rank:	22.2

M1			
Pos.	95	obs:	exp:
cgt	R	0	4.87
cgc	R	0	5.79
cga	R	0	11.37
cgg	R	0	9.49
aaa	K	42	483.30
aag	K	783	341.70
aga	R	53	52.05
agg	R	61	30.43
---	--	------	------
mPD		0.40	0.77
		nPD:	0.51
		N. weight:	0.66
		Sc. PD:	0.24
		Sc. rank:	467.9

M1			
Pos.	96	obs:	exp:
gct	A	0	240.60
gcc	A	0	176.30
gca	A	669	439.00
gcg	A	270	83.05
---	--	------	------
mPD		0.41	0.67
		nPD:	0.61
		N. weight:	0.67
		Sc. PD:	0.31
		Sc. rank:	492.7

M1			
Pos.	97	obs:	exp:
att	I	0	0.73
atc	I	0	0.48
ata	I	2	0.79
gtt	V	757	193.40
gtc	V	97	187.70
gta	V	82	194.00
gtg	V	1	361.90
---	--	------	------
mPD		0.34	0.73
		nPD:	0.46
		N. weight:	1.
		Sc. PD:	0.31
		Sc. rank:	670.7

M1			
Pos.	98	obs:	exp:
aaa	K	904	550.10
aag	K	35	388.90
---	--	------	------
mPD		0.072	0.49
		nPD:	0.15
		N. weight:	0.41
		Sc. PD:	0.0051
		Sc. rank:	100.0

M1			
Pos.	99	obs:	exp:
tta	L	674	82.53
ttg	L	161	175.20
ctt	L	5	159.10
ctc	L	0	157.30
cta	L	13	156.60
ctg	L	86	208.30
---	--	------	------
mPD		0.59	1.1
		nPD:	0.53
		N. weight:	1.4
		Sc. PD:	0.55
		Sc. rank:	1027.8

M1			
Pos.	100	obs:	exp:
tat	Y	10	497.90
tac	Y	929	441.10
---	--	------	------
mPD		0.021	0.50
		nPD:	0.04
		N. weight:	0.73
		Sc. PD:	-0.065
		Sc. rank:	-1.1

M1			
Pos.	101	obs:	exp:
cgt	R	0	3.89
cgc	R	0	4.62
cga	R	0	9.07
cgg	R	0	7.58
aaa	K	2	496.80
aag	K	846	351.20
aga	R	3	41.55
agg	R	88	24.29
---	--	------	------
mPD		0.19	0.72
		nPD:	0.26
		N. weight:	0.94
		Sc. PD:	0.11
		Sc. rank:	451.3

,

M1			
Pos.	102	obs:	exp:
aaa	K	854	550.10
aag	K	85	388.90
---	--	------	------
mPD		0.16	0.49
		nPD:	0.34
		N. weight:	0.28
		Sc. PD:	0.055
		Sc. rank:	169.9

,

M1			
Pos.	103	obs:	exp:
tta	L	1	82.53
ttg	L	38	175.20
ctt	L	13	159.10
ctc	L	13	157.30
cta	L	22	156.60
ctg	L	852	208.30
---	--	------	------
mPD		0.18	1.1
		nPD:	0.16
		N. weight:	1.2
		Sc. PD:	0.031
		Sc. rank:	349.3

,

M1			
Pos.	104	obs:	exp:
aaa	K	73	550.10
aag	K	866	388.90
---	--	------	------
mPD		0.14	0.49
		nPD:	0.3
		N. weight:	0.61
		Sc. PD:	0.095
		Sc. rank:	343.1

,

M1			
Pos.	105	obs:	exp:
cgt	R	0	40.11
cgc	R	0	47.71
cga	R	0	93.63
cgg	R	0	78.21
aga	R	116	428.70
agg	R	823	250.60
---	--	------	------
mPD		0.22	0.96
		nPD:	0.22
		N. weight:	0.93
		Sc. PD:	0.08
		Sc. rank:	418.0

,

M1			
Pos.	106	obs:	exp:
gaa	E	895	564.80
gag	E	44	374.20
---	--	------	------
mPD		0.089	0.48
		nPD:	0.19
		N. weight:	0.36
		Sc. PD:	0.018
		Sc. rank:	120.6

,

M1			
Pos.	107	obs:	exp:
att	I	1	35.76
atc	I	1	23.70
ata	I	96	38.54
atg	M	841	841.00
---	--	------	------
mPD		0.19	0.19
		nPD:	0.97
		N. weight:	0.093
		Sc. PD:	0.074
		Sc. rank:	72.4

,

M1			
Pos.	108	obs:	exp:
act	T	3	243.20
acc	T	31	191.20
aca	T	893	431.40
acg	T	12	73.15
---	--	------	------
mPD		0.094	0.67
		nPD:	0.14
		N. weight:	0.63
		Sc. PD:	0.003
		Sc. rank:	146.1

,

M1			
Pos.	109	obs:	exp:
ttt	F	798	370.20
ttc	F	140	567.80
tta	L	0	0.09
ttg	L	0	0.19
ctt	L	1	0.17
ctc	L	0	0.17
cta	L	0	0.17
ctg	L	0	0.22
---	--	------	------
mPD		0.26	0.48
		nPD:	0.53
		N. weight:	0.47
		Sc. PD:	0.18
		Sc. rank:	333.0

,

M1			
Pos.	110	obs:	exp:
tat	Y	1	0.53
tac	Y	0	0.47
cat	H	937	538.90
cac	H	1	399.10
---	--	------	------
mPD		0.0043	0.49
		nPD:	0.01
		N. weight:	0.57
		Sc. PD:	-0.07
		Sc. rank:	-181.8

,

M1			
Pos.	111	obs:	exp:
ggt	G	1	125.90
ggc	G	0	119.30
gga	G	823	434.20
ggg	G	115	259.60
---	--	------	------
mPD		0.22	0.68
		nPD:	0.32
		N. weight:	0.48
		Sc. PD:	0.086
		Sc. rank:	283.5

,

M1			
Pos.	112	obs:	exp:
tct	S	0	0.16
tcc	S	0	0.13
tca	S	1	0.25
tcg	S	0	0.06
agt	S	0	0.20
agc	S	0	0.20
gct	A	33	240.40
gcc	A	18	176.10
gca	A	877	438.60
gcg	A	10	82.96
---	--	------	------
mPD		0.13	0.68
		nPD:	0.19
		N. weight:	0.55
		Sc. PD:	0.027
		Sc. rank:	185.6

,

M1			
Pos.	113	obs:	exp:
aaa	K	91	550.10
aag	K	848	388.90
---	--	------	------
mPD		0.18	0.49
		nPD:	0.36
		N. weight:	0.56
		Sc. PD:	0.12
		Sc. rank:	344.3

,

M1			
Pos.	114	obs:	exp:
gaa	E	874	564.80
gag	E	65	374.20
---	--	------	------
mPD		0.13	0.48
		nPD:	0.27
		N. weight:	0.3
		Sc. PD:	0.039
		Sc. rank:	149.6

,

M1			
Pos.	115	obs:	exp:
att	I	4	1.82
atc	I	0	1.21
ata	I	1	1.97
gtt	V	880	192.80
gtc	V	7	187.10
gta	V	1	193.40
gtg	V	46	360.70
---	--	------	------
mPD		0.12	0.74
		nPD:	0.17
		N. weight:	1.4
		Sc. PD:	0.04
		Sc. rank:	426.8

,

M1			
Pos.	116	obs:	exp:
tct	S	0	0.32
tcc	S	0	0.26
tca	S	2	0.50
tcg	S	0	0.12
agt	S	0	0.41
agc	S	0	0.39
gct	A	1	240.10
gcc	A	0	175.90
gca	A	935	438.10
gcg	A	1	82.88
---	--	------	------
mPD		0.0085	0.68
		nPD:	0.01
		N. weight:	0.79
		Sc. PD:	-0.093
		Sc. rank:	-189.3

,

M1			
Pos.	117	obs:	exp:
tta	L	0	82.53
ttg	L	0	175.20
ctt	L	11	159.10
ctc	L	923	157.30
cta	L	5	156.60
ctg	L	0	208.30
---	--	------	------
mPD		0.034	1.1
		nPD:	0.03
		N. weight:	1.8
		Sc. PD:	-0.18
		Sc. rank:	-212.0

,

M1			
Pos.	118	obs:	exp:
tct	S	0	150.30
tcc	S	0	121.50
tca	S	0	237.00
tcg	S	0	54.64
agt	S	908	192.40
agc	S	31	183.10
---	--	------	------
mPD		0.064	1.7
		nPD:	0.04
		N. weight:	1.5
		Sc. PD:	-0.14
		Sc. rank:	-44.6

,

M1			
Pos.	119	obs:	exp:
tat	Y	40	497.90
tac	Y	899	441.10
---	--	------	------
mPD		0.082	0.50
		nPD:	0.16
		N. weight:	0.61
		Sc. PD:	0.017
		Sc. rank:	184.2

,

M1			
Pos.	120	obs:	exp:
tct	S	1	150.20
tcc	S	1	121.40
tca	S	933	236.70
tcg	S	3	54.59
act	T	0	0.26
acc	T	0	0.20
aca	T	1	0.46
acg	T	0	0.08
agt	S	0	192.20
agc	S	0	182.90
---	--	------	------
mPD		0.013	1.7
		nPD:	0.01
		N. weight:	1.4
		Sc. PD:	-0.17
		Sc. rank:	-471.3

,

M1			
Pos.	121	obs:	exp:
act	T	887	243.00
acc	T	51	191.00
aca	T	0	430.90
acg	T	0	73.07
gct	A	1	0.26
gcc	A	0	0.19
gca	A	0	0.47
gcg	A	0	0.09
---	--	------	------
mPD		0.10	0.68
		nPD:	0.16
		N. weight:	1.2
		Sc. PD:	0.023
		Sc. rank:	304.4

,

M1			
Pos.	122	obs:	exp:
ggt	G	938	125.90
ggc	G	1	119.30
gga	G	0	434.20
ggg	G	0	259.60
---	--	------	------
mPD		0.0021	0.68
		nPD:	0.
		N. weight:	2.1
		Sc. PD:	-0.27
		Sc. rank:	-1036.0

,

M1			
Pos.	123	obs:	exp:
gct	A	1	240.60
gcc	A	1	176.30
gca	A	227	439.00
gcg	A	710	83.05
---	--	------	------
mPD		0.37	0.67
		nPD:	0.55
		N. weight:	1.5
		Sc. PD:	0.61
		Sc. rank:	1107.8

,

M1			
Pos.	124	obs:	exp:
tta	L	0	82.53
ttg	L	0	175.20
ctt	L	939	159.10
ctc	L	0	157.30
cta	L	0	156.60
ctg	L	0	208.30
---	--	------	------
mPD		0	1.1
		nPD:	0.
		N. weight:	1.9
		Sc. PD:	-0.24
		Sc. rank:	-1417.3

,

M1

Pos.	125	obs:	exp:
act	T	0	0.26
acc	T	1	0.20
aca	T	0	0.46
acg	T	0	0.08
gct	A	11	240.40
gcc	A	927	176.10
gca	A	0	438.60
gcg	A	0	82.96
mPD		0.025	0.67
		nPD:	0.04
		N. weight:	1.7
		Sc. PD:	-0.16
		Sc. rank:	-85.5

M1

Pos.	126	obs:	exp:
tct	S	0	149.70
tcc	S	0	121.00
tca	S	0	236.00
tcg	S	0	54.41
agt	S	60	191.60
agc	S	875	182.30
ggt	G	0	0.54
ggc	G	4	0.51
gga	G	0	1.85
ggg	G	0	1.11
mPD		0.13	1.7
		nPD:	0.08
		N. weight:	1.5
		Sc. PD:	-0.082
		Sc. rank:	146.6

M1

Pos.	127	obs:	exp:
tgt	C	43	385.50
tgc	C	896	553.50
mPD		0.087	0.48
		nPD:	0.18
		N. weight:	0.38
		Sc. PD:	0.017
		Sc. rank:	127.1

M1

Pos.	128	obs:	exp:
atg	M	939	939.00
mPD		0	0
		nPD:	1.
		N. weight:	0.
		Sc. PD:	0
		Sc. rank:	0

M1

Pos.	129	obs:	exp:
ggt	G	885	125.90
ggc	G	54	119.30
gga	G	0	434.20
ggg	G	0	259.60
mPD		0.11	0.68
		nPD:	0.16
		N. weight:	1.9
		Sc. PD:	0.046
		Sc. rank:	538.0

M1

Pos.	130	obs:	exp:
tta	L	0	82.35
ttg	L	0	174.90
ctt	L	13	158.80
ctc	L	921	157.00
cta	L	3	156.20
ctg	L	0	207.80
cct	P	0	0.25
ccc	P	1	0.18
cca	P	0	0.41
ccg	P	0	0.16
att	I	0	0.36
atc	I	1	0.24
ata	I	0	0.39
mPD		0.038	1.1
		nPD:	0.03
		N. weight:	1.8
		Sc. PD:	-0.17
		Sc. rank:	-147.3

M1

Pos.	131	obs:	exp:
att	I	0	342.60
atc	I	2	227.10
ata	I	937	369.30
mPD		0.0043	0.65
		nPD:	0.01
		N. weight:	0.97
		Sc. PD:	-0.12
		Sc. rank:	-352.2

M1

Pos.	132	obs:	exp:
tat	Y	0	497.90
tac	Y	939	441.10
mPD		0	0.50
		nPD:	0.
		N. weight:	0.79
		Sc. PD:	-0.1
		Sc. rank:	-603.2

M1

Pos.	133	obs:	exp:
aat	N	7	477.70
aac	N	932	461.30
mPD		0.015	0.50
		nPD:	0.03
		N. weight:	0.7
		Sc. PD:	-0.071
		Sc. rank:	-94.3

M1

Pos.	134	obs:	exp:
cgt	R	0	40.03
cgc	R	0	47.61
cga	R	0	93.43
cgg	R	0	78.04
aaa	K	0	1.17
aag	K	2	0.83
aga	R	295	427.80
agg	R	642	250.10
mPD		0.44	0.97
		nPD:	0.45
		N. weight:	0.56
		Sc. PD:	0.17
		Sc. rank:	364.9

M1

Pos.	135	obs:	exp:
atg	M	939	939.00
mPD		0	0
		nPD:	1.
		N. weight:	0.
		Sc. PD:	0
		Sc. rank:	0

M1

Pos.	136	obs:	exp:
ggt	G	0	125.90
ggc	G	0	119.30
gga	G	69	434.20
ggg	G	870	259.60
mPD		0.14	0.68
		nPD:	0.2
		N. weight:	1.
		Sc. PD:	0.066
		Sc. rank:	397.7

M1

Pos.	137	obs:	exp:
act	T	301	243.20
acc	T	0	191.20
aca	T	613	431.40
acg	T	25	73.15
mPD		0.47	0.67
		nPD:	0.7
		N. weight:	0.29
		Sc. PD:	0.16
		Sc. rank:	218.1

M1

Pos.	138	obs:	exp:
gtt	V	0	193.80
gtc	V	1	188.10
gta	V	558	194.40
gtg	V	380	362.60
mPD		0.48	0.73
		nPD:	0.67
		N. weight:	0.68
		Sc. PD:	0.35
		Sc. rank:	503.8

M1

Pos.	139	obs:	exp:
act	T	360	242.20
acc	T	560	190.40
aca	T	15	429.60
acg	T	0	72.83
gct	A	0	1.02
gcc	A	2	0.75
gca	A	2	1.87
gcg	A	0	0.35
mPD		0.50	0.68
		nPD:	0.74
		N. weight:	0.79
		Sc. PD:	0.46
		Sc. rank:	607.4

M1

Pos.	140	obs:	exp:
act	T	46	25.65
acc	T	1	20.16
aca	T	51	45.48
acg	T	1	7.71
gct	A	0	215.20
gcc	A	0	157.70
gca	A	839	392.70
gcg	A	1	74.30
mPD		0.29	0.86
		nPD:	0.33
		N. weight:	0.74
		Sc. PD:	0.14
		Sc. rank:	443.3

M1

Pos.	141	obs:	exp:
gaa	E	925	564.80
gag	E	14	374.20
mPD		0.029	0.48
		nPD:	0.06
		N. weight:	0.46
		Sc. PD:	-0.033
		Sc. rank:	28.3

M1

Pos.	142	obs:	exp:
gtt	V	0	20.85
gtc	V	0	20.23
gta	V	0	20.91
gtg	V	101	39.01
ggt	G	0	112.40
ggc	G	0	106.50
gga	G	1	387.50
ggg	G	837	231.70
mPD		0.19	0.88
		nPD:	0.22
		N. weight:	1.3
		Sc. PD:	0.11
		Sc. rank:	568.6

M1

Pos.	143	obs:	exp:
gct	A	869	240.60
gcc	A	1	176.30
gca	A	60	439.00
gcg	A	9	83.05
mPD		0.14	0.67
		nPD:	0.21
		N. weight:	1.1
		Sc. PD:	0.076
		Sc. rank:	439.3

M1

Pos.	144	obs:	exp:
ttt	F	99	40.65
ttc	F	4	62.35
tta	L	0	73.47
ttg	L	0	156.00
ctt	L	835	141.70
ctc	L	1	140.10
cta	L	0	139.40
ctg	L	0	185.40
mPD		0.21	1.2
		nPD:	0.17
		N. weight:	1.7
		Sc. PD:	0.064
		Sc. rank:	577.5

M1

Pos.	145	obs:	exp:
ggt	G	13	125.90
ggc	G	91	119.30
gga	G	832	434.20
ggg	G	3	259.60
mPD		0.21	0.68
		nPD:	0.3
		N. weight:	0.54
		Sc. PD:	0.087
		Sc. rank:	302.2

M1

Pos.	146	obs:	exp:
ttt	F	0	0.39
ttc	F	1	0.61
tta	L	20	82.44
ttg	L	337	175.00
ctt	L	0	159.00
ctc	L	0	157.20
cta	L	528	156.40
ctg	L	53	208.00
mPD		0.96	1.1
		nPD:	0.86
		N. weight:	0.86
		Sc. PD:	0.6
		Sc. rank:	674.0

M1

Pos.	147	obs:	exp:
tta	L	0	0.09
ttg	L	0	0.19
ctt	L	0	0.17
ctc	L	0	0.17
cta	L	1	0.17
ctg	L	0	0.22
gtt	V	1	193.60
gtc	V	0	187.90
gta	V	836	194.20
gtg	V	101	362.20
mPD		0.20	0.73
		nPD:	0.27
		N. weight:	1.2
		Sc. PD:	0.16
		Sc. rank:	612.5

M1

Pos.	148	obs:	exp:
tgt	C	925	385.50
tgc	C	14	553.50
mPD		0.029	0.48
		nPD:	0.06
		N. weight:	0.85
		Sc. PD:	-0.061
		Sc. rank:	49.8

M1

Pos.	149	obs:	exp:
gct	A	9	240.60
gcc	A	909	176.30
gca	A	21	439.00
gcg	A	0	83.05
mPD		0.062	0.67
		nPD:	0.09
		N. weight:	1.6
		Sc. PD:	-0.064
		Sc. rank:	222.5

M1			
Pos.	150	obs:	exp:
att	I	8	2.92
atc	I	0	1.93
ata	I	0	3.15
act	T	830	241.20
acc	T	53	189.60
aca	T	24	427.70
acg	T	24	72.52
---	--	------	------
mPD		0.22	0.69
		nPD:	0.31
		N. weight:	0.98
		Sc. PD:	0.17
		Sc. rank:	573.4

,

M1			
Pos.	151	obs:	exp:
tgt	C	937	385.50
tgc	C	2	553.50
---	--	------	------
mPD		0.0043	0.48
		nPD:	0.01
		N. weight:	0.92
		Sc. PD:	-0.11
		Sc. rank:	-280.1

,

M1			
Pos.	152	obs:	exp:
gaa	E	7	564.80
gag	E	932	374.20
---	--	------	------
mPD		0.015	0.48
		nPD:	0.03
		N. weight:	0.92
		Sc. PD:	-0.092
		Sc. rank:	-104.4

,

M1			
Pos.	153	obs:	exp:
caa	Q	20	482.30
cag	Q	919	456.70
---	--	------	------
mPD		0.042	0.50
		nPD:	0.08
		N. weight:	0.65
		Sc. PD:	-0.032
		Sc. rank:	76.7

,

M1			
Pos.	154	obs:	exp:
att	I	909	342.30
atc	I	29	226.90
ata	I	0	368.90
gtt	V	1	0.21
gtc	V	0	0.20
gta	V	0	0.21
gtg	V	0	0.39
---	--	------	------
mPD		0.062	0.66
		nPD:	0.09
		N. weight:	0.93
		Sc. PD:	-0.036
		Sc. rank:	137.4

,

M1			
Pos.	155	obs:	exp:
gct	A	854	240.60
gcc	A	80	176.30
gca	A	5	439.00
gcg	A	0	83.05
---	--	------	------
mPD		0.17	0.67
		nPD:	0.25
		N. weight:	1.1
		Sc. PD:	0.12
		Sc. rank:	527.5

,

M1			
Pos.	156	obs:	exp:
gat	D	158	505.80
gac	D	779	431.20
gaa	E	2	1.20
gag	E	0	0.80
---	--	------	------
mPD		0.28	0.50
		nPD:	0.57
		N. weight:	0.31
		Sc. PD:	0.13
		Sc. rank:	228.3

,

M1			
Pos.	157	obs:	exp:
tct	S	0	16.17
tcc	S	49	13.07
tca	S	52	25.49
tcg	S	0	5.88
agt	S	0	20.70
agc	S	0	19.70
gct	A	1	214.70
gcc	A	1	157.30
gca	A	836	391.80
gcg	A	0	74.12
---	--	------	------
mPD		0.30	0.96
		nPD:	0.31
		N. weight:	0.81
		Sc. PD:	0.13
		Sc. rank:	462.9

,

M1			
Pos.	158	obs:	exp:
caa	Q	728	482.30
cag	Q	211	456.70
---	--	------	------
mPD		0.35	0.50
		nPD:	0.7
		N. weight:	0.16
		Sc. PD:	0.085
		Sc. rank:	117.8

,

M1			
Pos.	159	obs:	exp:
cat	H	929	539.50
cac	H	10	399.50
---	--	------	------
mPD		0.021	0.49
		nPD:	0.04
		N. weight:	0.52
		Sc. PD:	-0.046
		Sc. rank:	8.7

,

M1			
Pos.	160	obs:	exp:
cgt	R	0	40.11
cgc	R	0	47.71
cga	R	5	93.63
cgg	R	926	78.21
aga	R	0	428.70
agg	R	8	250.60
---	--	------	------
mPD		0.028	0.96
		nPD:	0.03
		N. weight:	2.5
		Sc. PD:	-0.26
		Sc. rank:	-348.9

,

M1			
Pos.	161	obs:	exp:
tct	S	73	150.30
tcc	S	861	121.50
tca	S	5	237.00
tcg	S	0	54.64
agt	S	0	192.40
agc	S	0	183.10
---	--	------	------
mPD		0.15	1.7
		nPD:	0.09
		N. weight:	1.8
		Sc. PD:	-0.074
		Sc. rank:	239.5

,

M1			
Pos.	162	obs:	exp:
cat	H	1	539.50
cac	H	938	399.50
---	--	------	------
mPD		0.0021	0.49
		nPD:	0.
		N. weight:	0.89
		Sc. PD:	-0.11
		Sc. rank:	-373.5

,

M1			
Pos.	163	obs:	exp:
cgt	R	0	40.03
cgc	R	0	47.61
cga	R	0	93.43
cgg	R	0	78.04
aaa	K	0	1.17
aag	K	2	0.83
aga	R	111	427.80
agg	R	826	250.10
---	--	------	------
mPD		0.21	0.97
		nPD:	0.22
		N. weight:	0.94
		Sc. PD:	0.077
		Sc. rank:	403.1

,

M1			
Pos.	164	obs:	exp:
caa	Q	64	482.30
cag	Q	875	456.70
---	--	------	------
mPD		0.13	0.50
		nPD:	0.25
		N. weight:	0.49
		Sc. PD:	0.057
		Sc. rank:	234.7

,

M1			
Pos.	165	obs:	exp:
atg	M	938	938.00
gtt	V	0	0.21
gtc	V	0	0.20
gta	V	0	0.21
gtg	V	1	0.39
---	--	------	------
mPD		0.0021	0.0034
		nPD:	0.62
		N. weight:	0.0011
		Sc. PD:	0.0005
		Sc. rank:	0.8

,

M1			
Pos.	166	obs:	exp:
gtt	V	0	20.23
gtc	V	0	19.63
gta	V	26	20.29
gtg	V	72	37.85
gct	A	5	215.50
gcc	A	0	157.90
gca	A	392	393.20
gcg	A	444	74.38
---	--	------	------
mPD		0.69	0.88
		nPD:	0.78
		N. weight:	0.93
		Sc. PD:	0.58
		Sc. rank:	723.2

,

M1			
Pos.	167	obs:	exp:
act	T	859	238.10
acc	T	11	187.10
aca	T	49	422.20
acg	T	0	71.59
gct	A	18	5.12
gcc	A	1	3.76
gca	A	1	9.35
gcg	A	0	1.77
---	--	------	------
mPD		0.17	0.72
		nPD:	0.23
		N. weight:	1.1
		Sc. PD:	0.1
		Sc. rank:	506.7

,

M1			
Pos.	168	obs:	exp:
tct	S	0	1.76
tcc	S	0	1.42
tca	S	0	2.78
tcg	S	0	0.64
att	I	1	1.82
atc	I	2	1.21
ata	I	2	1.97
act	T	790	228.70
acc	T	46	179.80
aca	T	47	405.70
acg	T	0	68.78
agt	S	11	2.25
agc	S	0	2.15
gct	A	40	10.25
gcc	A	0	7.51
gca	A	0	18.70
gcg	A	0	3.54
---	--	------	------
mPD		0.31	0.79
		nPD:	0.39
		N. weight:	1.
		Sc. PD:	0.24
		Sc. rank:	634.7

,

M1			
Pos.	169	obs:	exp:
act	T	201	243.00
acc	T	734	191.00
aca	T	3	430.90
acg	T	0	73.07
aat	N	0	0.51
aac	N	1	0.49
---	--	------	------
mPD		0.34	0.68
		nPD:	0.51
		N. weight:	1.1
		Sc. PD:	0.38
		Sc. rank:	735.0

,

M1			
Pos.	170	obs:	exp:
aat	N	20	477.70
aac	N	919	461.30
---	--	------	------
mPD		0.042	0.50
		nPD:	0.08
		N. weight:	0.64
		Sc. PD:	-0.032
		Sc. rank:	73.0

,

M1			
Pos.	171	obs:	exp:
cct	P	0	238.80
ccc	P	1	167.40
cca	P	899	383.60
ccg	P	39	149.20
---	--	------	------
mPD		0.082	0.71
		nPD:	0.11
		N. weight:	0.79
		Sc. PD:	-0.015
		Sc. rank:	138.0

,

M1			
Pos.	172	obs:	exp:
tta	L	2	82.44
ttg	L	7	175.00
ctt	L	0	159.00
ctc	L	0	157.20
cta	L	883	156.40
ctg	L	46	208.00
att	I	0	0.36
atc	I	0	0.24
ata	I	1	0.39
---	--	------	------
mPD		0.13	1.1
		nPD:	0.11
		N. weight:	1.6
		Sc. PD:	-0.031
		Sc. rank:	294.1

,

M1			
Pos.	173	obs:	exp:
att	I	751	342.60
atc	I	187	227.10
ata	I	1	369.30
---	--	------	------
mPD		0.32	0.65
		nPD:	0.49
		N. weight:	0.62
		Sc. PD:	0.21
		Sc. rank:	416.4

,

M1			
Pos.	174	obs:	exp:
cgt	R	0	40.03
cgc	R	0	47.61
cga	R	0	93.43
cgg	R	0	78.04
aaa	K	1	1.17
aag	K	1	0.83
aga	R	56	427.80
agg	R	881	250.10
---	--	------	------
mPD		0.12	0.97
		nPD:	0.12
		N. weight:	1.1
		Sc. PD:	-0.014
		Sc. rank:	235.6

,

M1			
Pos.	175	obs:	exp:
cat	H	920	539.50
cac	H	19	399.50
---	--	------	------
mPD		0.040	0.49
		nPD:	0.08
		N. weight:	0.49
		Sc. PD:	-0.025
		Sc. rank:	50.1

M1			
Pos.	176	obs:	exp:
gaa	E	64	564.80
gag	E	875	374.20
---	--	------	------
mPD		0.13	0.48
		nPD:	0.27
		N. weight:	0.68
		Sc. PD:	0.085
		Sc. rank:	331.2

M1			
Pos.	177	obs:	exp:
aat	N	840	477.70
aac	N	99	461.30
---	--	------	------
mPD		0.19	0.50
		nPD:	0.38
		N. weight:	0.36
		Sc. PD:	0.085
		Sc. rank:	228.2

M1			
Pos.	178	obs:	exp:
cgt	R	0	40.11
cgc	R	0	47.71
cga	R	0	93.63
cgg	R	0	78.21
aga	R	876	428.70
agg	R	63	250.60
---	--	------	------
mPD		0.13	0.96
		nPD:	0.13
		N. weight:	0.61
		Sc. PD:	-0.0029
		Sc. rank:	132.2

M1			
Pos.	179	obs:	exp:
atg	M	939	939.00
---	--	------	------
mPD		0	0
		nPD:	1.
		N. weight:	0.
		Sc. PD:	0
		Sc. rank:	0

M1			
Pos.	180	obs:	exp:
gtt	V	2	193.80
gtc	V	0	188.10
gta	V	881	194.40
gtg	V	56	362.60
---	--	------	------
mPD		0.12	0.73
		nPD:	0.16
		N. weight:	1.4
		Sc. PD:	0.033
		Sc. rank:	374.1

M1			
Pos.	181	obs:	exp:
tta	L	2	82.26
ttg	L	1	174.70
ctt	L	1	158.60
ctc	L	0	156.80
cta	L	859	156.00
ctg	L	73	207.60
att	I	0	1.09
atc	I	0	0.73
ata	I	3	1.18
---	--	------	------
mPD		0.16	1.1
		nPD:	0.14
		N. weight:	1.5
		Sc. PD:	0.013
		Sc. rank:	371.7

M1			
Pos.	182	obs:	exp:
gct	A	58	240.60
gcc	A	876	176.30
gca	A	5	439.00
gcg	A	0	83.05
---	--	------	------
mPD		0.13	0.67
		nPD:	0.19
		N. weight:	1.5
		Sc. PD:	0.073
		Sc. rank:	508.5

M1			
Pos.	183	obs:	exp:
tct	S	0	150.30
tcc	S	0	121.50
tca	S	0	237.00
tcg	S	0	54.64
agt	S	395	192.40
agc	S	544	183.10
---	--	------	------
mPD		0.49	1.7
		nPD:	0.29
		N. weight:	0.98
		Sc. PD:	0.15
		Sc. rank:	546.6

M1			
Pos.	184	obs:	exp:
act	T	922	243.20
acc	T	16	191.20
aca	T	0	431.40
acg	T	1	73.15
---	--	------	------
mPD		0.036	0.67
		nPD:	0.05
		N. weight:	1.3
		Sc. PD:	-0.1
		Sc. rank:	50.2

M1			
Pos.	185	obs:	exp:
atg	M	2	2.00
act	T	5	242.70
acc	T	0	190.80
aca	T	58	430.50
acg	T	874	72.99
---	--	------	------
mPD		0.13	0.68
		nPD:	0.19
		N. weight:	2.3
		Sc. PD:	0.12
		Sc. rank:	826.8

M1			
Pos.	186	obs:	exp:
gct	A	907	240.60
gcc	A	32	176.30
gca	A	0	439.00
gcg	A	0	83.05
---	--	------	------
mPD		0.066	0.67
		nPD:	0.1
		N. weight:	1.3
		Sc. PD:	-0.046
		Sc. rank:	200.2

M1			
Pos.	187	obs:	exp:
aaa	K	119	550.10
aag	K	820	388.90
---	--	------	------
mPD		0.22	0.49
		nPD:	0.46
		N. weight:	0.48
		Sc. PD:	0.15
		Sc. rank:	318.9

M1			
Pos.	188	obs:	exp:
gct	A	873	240.60
gcc	A	65	176.30
gca	A	0	439.00
gcg	A	1	83.05
---	--	------	------
mPD		0.13	0.67
		nPD:	0.19
		N. weight:	1.2
		Sc. PD:	0.068
		Sc. rank:	446.6

M1			
Pos.	189	obs:	exp:
atg	M	939	939.00
---	--	------	------
mPD		0	0
		nPD:	1.
		N. weight:	0.
		Sc. PD:	0
		Sc. rank:	0

M1			
Pos.	190	obs:	exp:
gaa	E	7	564.80
gag	E	932	374.20
---	--	------	------
mPD		0.015	0.48
		nPD:	0.03
		N. weight:	0.92
		Sc. PD:	-0.092
		Sc. rank:	-104.4

M1			
Pos.	191	obs:	exp:
caa	Q	48	482.30
cag	Q	891	456.70
---	--	------	------
mPD		0.097	0.50
		nPD:	0.19
		N. weight:	0.54
		Sc. PD:	0.031
		Sc. rank:	201.5

M1			
Pos.	192	obs:	exp:
att	I	0	0.36
atc	I	0	0.24
ata	I	1	0.39
atg	M	938	938.00
---	--	------	------
mPD		0.0021	0.0021
		nPD:	1.
		N. weight:	0.001
		Sc. PD:	0.00087
		Sc. rank:	0.8

M1			
Pos.	193	obs:	exp:
gct	A	887	240.60
gcc	A	1	176.30
gca	A	47	439.00
gcg	A	4	83.05
---	--	------	------
mPD		0.11	0.67
		nPD:	0.16
		N. weight:	1.2
		Sc. PD:	0.024
		Sc. rank:	300.0

M1			
Pos.	194	obs:	exp:
ggt	G	0	125.90
ggc	G	0	119.30
gga	G	807	434.20
ggg	G	132	259.60
---	--	------	------
mPD		0.24	0.68
		nPD:	0.36
		N. weight:	0.46
		Sc. PD:	0.099
		Sc. rank:	283.4

M1			
Pos.	195	obs:	exp:
tct	S	1	150.30
tcc	S	0	121.50
tca	S	907	237.00
tcg	S	31	54.64
agt	S	0	192.40
agc	S	0	183.10
---	--	------	------
mPD		0.066	1.7
		nPD:	0.04
		N. weight:	1.3
		Sc. PD:	-0.12
		Sc. rank:	-28.4

M1			
Pos.	196	obs:	exp:
tct	S	0	149.80
tcc	S	0	121.20
tca	S	0	236.20
tcg	S	0	54.47
att	I	1	0.36
atc	I	0	0.24
ata	I	0	0.39
aat	N	2	1.02
aac	N	0	0.98
agt	S	839	191.80
agc	S	97	182.50
---	--	------	------
mPD		0.19	1.7
		nPD:	0.12
		N. weight:	1.3
		Sc. PD:	-0.025
		Sc. rank:	252.8

M1			
Pos.	197	obs:	exp:
gaa	E	642	564.20
gag	E	296	373.80
ggt	G	0	0.13
ggc	G	0	0.13
gga	G	0	0.46
ggg	G	1	0.28
---	--	------	------
mPD		0.44	0.48
		nPD:	0.9
		N. weight:	0.021
		Sc. PD:	0.015
		Sc. rank:	16.3

M1			
Pos.	198	obs:	exp:
caa	Q	31	482.30
cag	Q	908	456.70
---	--	------	------
mPD		0.064	0.50
		nPD:	0.13
		N. weight:	0.6
		Sc. PD:	-0.0042
		Sc. rank:	129.9

M1			
Pos.	199	obs:	exp:
gct	A	2	240.60
gcc	A	0	176.30
gca	A	935	439.00
gcg	A	2	83.05
---	--	------	------
mPD		0.0085	0.67
		nPD:	0.01
		N. weight:	0.77
		Sc. PD:	-0.091
		Sc. rank:	-175.1

M1			
Pos.	200	obs:	exp:
gtt	V	0	0.21
gtc	V	0	0.20
gta	V	0	0.21
gtg	V	1	0.39
gct	A	0	240.40
gcc	A	0	176.10
gca	A	102	438.60
gcg	A	836	82.96
---	--	------	------
mPD		0.20	0.68
		nPD:	0.29
		N. weight:	2.
		Sc. PD:	0.3
		Sc. rank:	1097.6

M1			
Pos.	201	obs:	exp:
gat	D	1	0.54
gac	D	0	0.46
gaa	E	886	564.20
gag	E	52	373.80
---	--	------	------
mPD		0.11	0.48
		nPD:	0.22
		N. weight:	0.34
		Sc. PD:	0.028
		Sc. rank:	147.3

M1			
Pos.	202	obs:	exp:
gct	A	46	240.60
gcc	A	882	176.30
gca	A	11	439.00
gcg	A	0	83.05
---	--	------	------
mPD		0.12	0.67
		nPD:	0.17
		N. weight:	1.5
		Sc. PD:	0.051
		Sc. rank:	470.0

M1			
Pos.	203	obs:	exp:
att	I	4	2.92
atc	I	0	1.93
ata	I	4	3.15
atg	M	931	931.00
---	--	------	------
mPD		0.017	0.017
		nPD:	1.
		N. weight:	0.0039
		Sc. PD:	0.0033
		Sc. rank:	3.1

M1

Pos.	204	obs:	exp:
gaa	E	847	564.80
gag	E	92	374.20
mPD		0.18	0.48
		nPD:	0.37
		N. weight:	0.24
		Sc. PD:	0.055
		Sc. rank:	149.8

M1

Pos.	205	obs:	exp:
att	I	0	0.73
atc	I	2	0.48
ata	I	0	0.79
gtt	V	391	193.40
gtc	V	542	187.70
gta	V	4	194.00
gtg	V	0	361.90
mPD		0.50	0.73
		nPD:	0.68
		N. weight:	0.94
		Sc. PD:	0.49
		Sc. rank:	706.4

M1

Pos.	206	obs:	exp:
gct	A	96	240.60
gcc	A	4	176.30
gca	A	831	439.00
gcg	A	8	83.05
mPD		0.21	0.67
		nPD:	0.31
		N. weight:	0.46
		Sc. PD:	0.077
		Sc. rank:	264.6

M1

Pos.	207	obs:	exp:
tct	S	0	149.50
tcc	S	0	120.90
tca	S	0	235.70
tcg	S	0	54.35
aat	N	5	2.54
aac	N	0	2.46
agt	S	578	191.40
agc	S	356	182.10
mPD		0.48	1.7
		nPD:	0.29
		N. weight:	0.99
		Sc. PD:	0.15
		Sc. rank:	531.2

M1

Pos.	208	obs:	exp:
caa	Q	1	482.30
cag	Q	938	456.70
mPD		0.0021	0.50
		nPD:	0.
		N. weight:	0.75
		Sc. PD:	-0.094
		Sc. rank:	-332.2

M1

Pos.	209	obs:	exp:
gct	A	900	240.60
gcc	A	38	176.30
gca	A	1	439.00
gcg	A	0	83.05
mPD		0.080	0.67
		nPD:	0.12
		N. weight:	1.3
		Sc. PD:	-0.02
		Sc. rank:	250.6

M1

Pos.	210	obs:	exp:
cgt	R	0	40.11
cgc	R	0	47.71
cga	R	0	93.63
cgg	R	1	78.21
aga	R	33	428.70
agg	R	905	250.60
mPD		0.070	0.96
		nPD:	0.07
		N. weight:	1.2
		Sc. PD:	-0.072
		Sc. rank:	90.8

M1

Pos.	211	obs:	exp:
caa	Q	814	469.00
cag	Q	99	444.00
cgt	R	0	0.98
cgc	R	0	1.17
cga	R	21	2.29
cgg	R	1	1.92
aaa	K	3	1.76
aag	K	0	1.24
aga	R	1	10.50
agg	R	0	6.14
mPD		0.25	0.59
		nPD:	0.42
		N. weight:	0.39
		Sc. PD:	0.11
		Sc. rank:	250.9

M1

Pos.	212	obs:	exp:
atg	M	939	939.00
mPD		0	0
		nPD:	1.
		N. weight:	0.
		Sc. PD:	0
		Sc. rank:	0

M1

Pos.	213	obs:	exp:
gtt	V	0	193.80
gtc	V	0	188.10
gta	V	1	194.40
gtg	V	938	362.60
mPD		0.0021	0.73
		nPD:	0.
		N. weight:	0.99
		Sc. PD:	-0.13
		Sc. rank:	-531.3

M1

Pos.	214	obs:	exp:
caa	Q	2	482.30
cag	Q	937	456.70
mPD		0.0043	0.50
		nPD:	0.01
		N. weight:	0.74
		Sc. PD:	-0.09
		Sc. rank:	-237.3

M1

Pos.	215	obs:	exp:
gct	A	841	240.60
gcc	A	0	176.30
gca	A	69	439.00
gcg	A	29	83.05
mPD		0.19	0.67
		nPD:	0.28
		N. weight:	1.
		Sc. PD:	0.14
		Sc. rank:	524.9

M1

Pos.	216	obs:	exp:
atg	M	939	939.00
mPD		0	0
		nPD:	1.
		N. weight:	0.
		Sc. PD:	0
		Sc. rank:	0

M1

Pos.	217	obs:	exp:
cgt	R	0	40.11
cgc	R	0	47.71
cga	R	0	93.63
cgg	R	0	78.21
aga	R	749	428.70
agg	R	190	250.60
mPD		0.32	0.96
		nPD:	0.34
		N. weight:	0.41
		Sc. PD:	0.079
		Sc. rank:	246.6

M1

Pos.	218	obs:	exp:
act	T	5	243.00
acc	T	57	191.00
aca	T	874	430.90
acg	T	2	73.07
gct	A	0	0.26
gcc	A	0	0.19
gca	A	1	0.47
gcg	A	0	0.09
mPD		0.13	0.68
		nPD:	0.19
		N. weight:	0.59
		Sc. PD:	0.033
		Sc. rank:	216.7

M1

Pos.	219	obs:	exp:
att	I	99	37.22
atc	I	3	24.67
ata	I	0	40.11
gtt	V	263	172.80
gtc	V	569	167.70
gta	V	5	173.30
gtg	V	0	323.20
mPD		0.67	0.93
		nPD:	0.72
		N. weight:	0.99
		Sc. PD:	0.56
		Sc. rank:	752.1

M1

Pos.	220	obs:	exp:
ggt	G	89	125.90
ggc	G	0	119.30
gga	G	31	434.20
ggg	G	819	259.60
mPD		0.23	0.68
		nPD:	0.34
		N. weight:	0.93
		Sc. PD:	0.18
		Sc. rank:	566.3

M1

Pos.	221	obs:	exp:
act	T	838	243.20
acc	T	99	191.20
aca	T	2	431.40
acg	T	0	73.15
mPD		0.19	0.67
		nPD:	0.29
		N. weight:	1.1
		Sc. PD:	0.16
		Sc. rank:	570.1

M1

Pos.	222	obs:	exp:
cat	H	124	539.50
cac	H	815	399.50
mPD		0.23	0.49
		nPD:	0.47
		N. weight:	0.45
		Sc. PD:	0.14
		Sc. rank:	300.3

M1

Pos.	223	obs:	exp:
cct	P	933	238.80
ccc	P	1	167.40
cca	P	5	383.60
ccg	P	0	149.20
mPD		0.013	0.71
		nPD:	0.02
		N. weight:	1.4
		Sc. PD:	-0.16
		Sc. rank:	-246.2

M1

Pos.	224	obs:	exp:
tct	S	0	17.13
tcc	S	0	13.85
tca	S	0	27.00
tcg	S	0	6.23
act	T	18	4.66
acc	T	0	3.67
aca	T	0	8.27
acg	T	0	1.40
aat	N	91	414.10
aac	N	723	399.90
agt	S	1	21.93
agc	S	106	20.87
mPD		0.44	0.91
		nPD:	0.49
		N. weight:	0.54
		Sc. PD:	0.18
		Sc. rank:	366.3

M1

Pos.	225	obs:	exp:
tct	S	48	150.30
tcc	S	890	121.50
tca	S	1	237.00
tcg	S	0	54.64
agt	S	0	192.40
agc	S	0	183.10
mPD		0.099	1.7
		nPD:	0.06
		N. weight:	1.9
		Sc. PD:	-0.14
		Sc. rank:	102.4

M1

Pos.	226	obs:	exp:
tct	S	0	150.30
tcc	S	0	121.50
tca	S	0	237.00
tcg	S	0	54.64
agt	S	909	192.40
agc	S	30	183.10
mPD		0.062	1.7
		nPD:	0.04
		N. weight:	1.5
		Sc. PD:	-0.14
		Sc. rank:	-92.7

M1

Pos.	227	obs:	exp:
act	T	4	217.60
acc	T	1	171.10
aca	T	834	385.90
acg	T	1	65.43
gct	A	50	25.37
gcc	A	47	18.59
gca	A	2	46.29
gcg	A	0	8.76
mPD		0.39	0.86
		nPD:	0.45
		N. weight:	0.78
		Sc. PD:	0.24
		Sc. rank:	510.8

M1

Pos.	228	obs:	exp:
ggt	G	927	125.90
ggc	G	12	119.30
gga	G	0	434.20
ggg	G	0	259.60
mPD		0.025	0.68
		nPD:	0.04
		N. weight:	2.
		Sc. PD:	-0.19
		Sc. rank:	-143.4

M1

Pos.	229	obs:	exp:
tta	L	2	82.44
ttg	L	0	175.00
ctt	L	23	159.00
ctc	L	0	157.20
cta	L	821	156.40
ctg	L	92	208.00
att	I	0	0.36
atc	I	0	0.24
ata	I	1	0.39
---	--	------	------
mPD		0.23	1.1
		nPD:	0.2
		N. weight:	1.4
		Sc. PD:	0.091
		Sc. rank:	544.1

M1

Pos.	230	obs:	exp:
cgt	R	0	0.26
cgc	R	0	0.30
cga	R	0	0.60
cgg	R	0	0.50
aaa	K	427	546.50
aag	K	506	386.50
aga	R	6	2.74
agg	R	0	1.60
---	--	------	------
mPD		0.51	0.50
		nPD:	1.02
		N. weight:	0.044
		Sc. PD:	0.037
		Sc. rank:	34.4

M1

Pos.	231	obs:	exp:
gat	D	930	506.90
gac	D	9	432.10
---	--	------	------
mPD		0.019	0.50
		nPD:	0.04
		N. weight:	0.59
		Sc. PD:	-0.055
		Sc. rank:	-23.0

M1

Pos.	232	obs:	exp:
aat	N	14	7.12
aac	N	0	6.88
gat	D	923	499.40
gac	D	2	425.60
---	--	------	------
mPD		0.034	0.53
		nPD:	0.06
		N. weight:	0.63
		Sc. PD:	-0.043
		Sc. rank:	43.1

M1

Pos.	233	obs:	exp:
tta	L	0	82.53
ttg	L	0	175.20
ctt	L	934	159.10
ctc	L	5	157.30
cta	L	0	156.60
ctg	L	0	208.30
---	--	------	------
mPD		0.011	1.1
		nPD:	0.01
		N. weight:	1.8
		Sc. PD:	-0.22
		Sc. rank:	-495.6

M1

Pos.	234	obs:	exp:
tta	L	0	8.79
ttg	L	0	18.66
ctt	L	100	16.95
ctc	L	0	16.75
cta	L	0	16.67
ctg	L	0	22.18
att	I	839	306.10
atc	I	0	202.90
ata	I	0	329.90
---	--	------	------
mPD		0.19	0.88
		nPD:	0.22
		N. weight:	1.1
		Sc. PD:	0.09
		Sc. rank:	468.0

M1

Pos.	235	obs:	exp:
aaa	K	22	12.89
aag	K	0	9.11
gaa	E	917	551.60
gag	E	0	365.40
---	--	------	------
mPD		0.046	0.53
		nPD:	0.09
		N. weight:	0.53
		Sc. PD:	-0.025
		Sc. rank:	64.8

M1

Pos.	236	obs:	exp:
aat	N	939	477.70
aac	N	0	461.30
---	--	------	------
mPD		0	0.50
		nPD:	0.
		N. weight:	0.71
		Sc. PD:	-0.092
		Sc. rank:	-539.6

M1

Pos.	237	obs:	exp:
tta	L	1	82.53
ttg	L	938	175.20
ctt	L	0	159.10
ctc	L	0	157.30
cta	L	0	156.60
ctg	L	0	208.30
---	--	------	------
mPD		0.0021	1.1
		nPD:	0.
		N. weight:	1.8
		Sc. PD:	-0.22
		Sc. rank:	-963.4

M1

Pos.	238	obs:	exp:
caa	Q	0	482.30
cag	Q	939	456.70
---	--	------	------
mPD		0	0.50
		nPD:	0.
		N. weight:	0.76
		Sc. PD:	-0.098
		Sc. rank:	-575.5

M1

Pos.	239	obs:	exp:
gct	A	417	240.60
gcc	A	521	176.30
gca	A	0	439.00
gcg	A	1	83.05
---	--	------	------
mPD		0.50	0.67
		nPD:	0.74
		N. weight:	0.89
		Sc. PD:	0.51
		Sc. rank:	679.4

M1

Pos.	240	obs:	exp:
tat	Y	0	496.30
tac	Y	936	439.70
cat	H	0	1.72
cac	H	3	1.28
---	--	------	------
mPD		0.0064	0.50
		nPD:	0.01
		N. weight:	0.79
		Sc. PD:	-0.093
		Sc. rank:	-183.2

M1

Pos.	241	obs:	exp:
caa	Q	211	482.30
cag	Q	728	456.70
---	--	------	------
mPD		0.35	0.50
		nPD:	0.7
		N. weight:	0.19
		Sc. PD:	0.1
		Sc. rank:	141.5

M1

Pos.	242	obs:	exp:
aat	N	2	426.30
aac	N	836	411.70
aaa	K	97	59.17
aag	K	4	41.83
---	--	------	------
mPD		0.20	0.60
		nPD:	0.33
		N. weight:	0.7
		Sc. PD:	0.13
		Sc. rank:	411.7

M1

Pos.	243	obs:	exp:
cgt	R	0	40.11
cgc	R	0	47.71
cga	R	10	93.63
cgg	R	921	78.21
aga	R	1	428.70
agg	R	7	250.60
---	--	------	------
mPD		0.040	0.96
		nPD:	0.04
		N. weight:	2.5
		Sc. PD:	-0.22
		Sc. rank:	-14.8

M1

Pos.	244	obs:	exp:
att	I	1	0.36
atc	I	0	0.24
ata	I	0	0.39
atg	M	938	938.00
---	--	------	------
mPD		0.0021	0.0021
		nPD:	1.
		N. weight:	0.0011
		Sc. PD:	0.00094
		Sc. rank:	0.9

M1

Pos.	245	obs:	exp:
ggt	G	3	125.90
ggc	G	0	119.30
gga	G	935	434.20
ggg	G	1	259.60
---	--	------	------
mPD		0.0085	0.68
		nPD:	0.01
		N. weight:	0.78
		Sc. PD:	-0.093
		Sc. rank:	-185.3

M1

Pos.	246	obs:	exp:
atg	M	2	2.00
gtt	V	0	193.40
gtc	V	0	187.70
gta	V	0	194.00
gtg	V	937	361.90
---	--	------	------
mPD		0.0043	0.73
		nPD:	0.01
		N. weight:	1.
		Sc. PD:	-0.12
		Sc. rank:	-389.4

M1

Pos.	247	obs:	exp:
caa	Q	913	482.30
cag	Q	26	456.70
---	--	------	------
mPD		0.054	0.50
		nPD:	0.11
		N. weight:	0.57
		Sc. PD:	-0.015
		Sc. rank:	96.2

M1

Pos.	248	obs:	exp:
tta	L	2	72.42
ttg	L	12	153.80
ctt	L	18	139.60
ctc	L	0	138.10
cta	L	3	137.40
ctg	L	789	182.70
atg	M	115	115.00
---	--	------	------
mPD		0.29	1.2
		nPD:	0.24
		N. weight:	1.2
		Sc. PD:	0.12
		Sc. rank:	558.3

M1

Pos.	249	obs:	exp:
caa	Q	8	481.80
cag	Q	930	456.20
cgt	R	0	0.04
cgc	R	0	0.05
cga	R	0	0.10
cgg	R	1	0.08
aga	R	0	0.46
agg	R	0	0.27
---	--	------	------
mPD		0.019	0.50
		nPD:	0.04
		N. weight:	0.71
		Sc. PD:	-0.066
		Sc. rank:	-32.8

M1

Pos.	250	obs:	exp:
cgt	R	4	40.11
cgc	R	1	47.71
cga	R	102	93.63
cgg	R	829	78.21
aga	R	0	428.70
agg	R	3	250.60
---	--	------	------
mPD		0.21	0.96
		nPD:	0.22
		N. weight:	2.2
		Sc. PD:	0.17
		Sc. rank:	919.3

M1

Pos.	251	obs:	exp:
ttt	F	0	370.60
ttc	F	939	568.40
---	--	------	------
mPD		0	0.48
		nPD:	0.
		N. weight:	0.53
		Sc. PD:	-0.068
		Sc. rank:	-400.8

M1

Pos.	252	obs:	exp:
aaa	K	1	550.10
aag	K	938	388.90
---	--	------	------
mPD		0.0021	0.49
		nPD:	0.
		N. weight:	0.92
		Sc. PD:	-0.12
		Sc. rank:	-380.2

M1

Pos.	253	obs:	exp:
taa	.	0	403.80
tag	.	0	237.40
tga	.	939	297.70
---	--	------	------
mPD		0	0.81
		nPD:	0.
		N. weight:	1.2
		Sc. PD:	-0.16
		Sc. rank:	-917.1

}

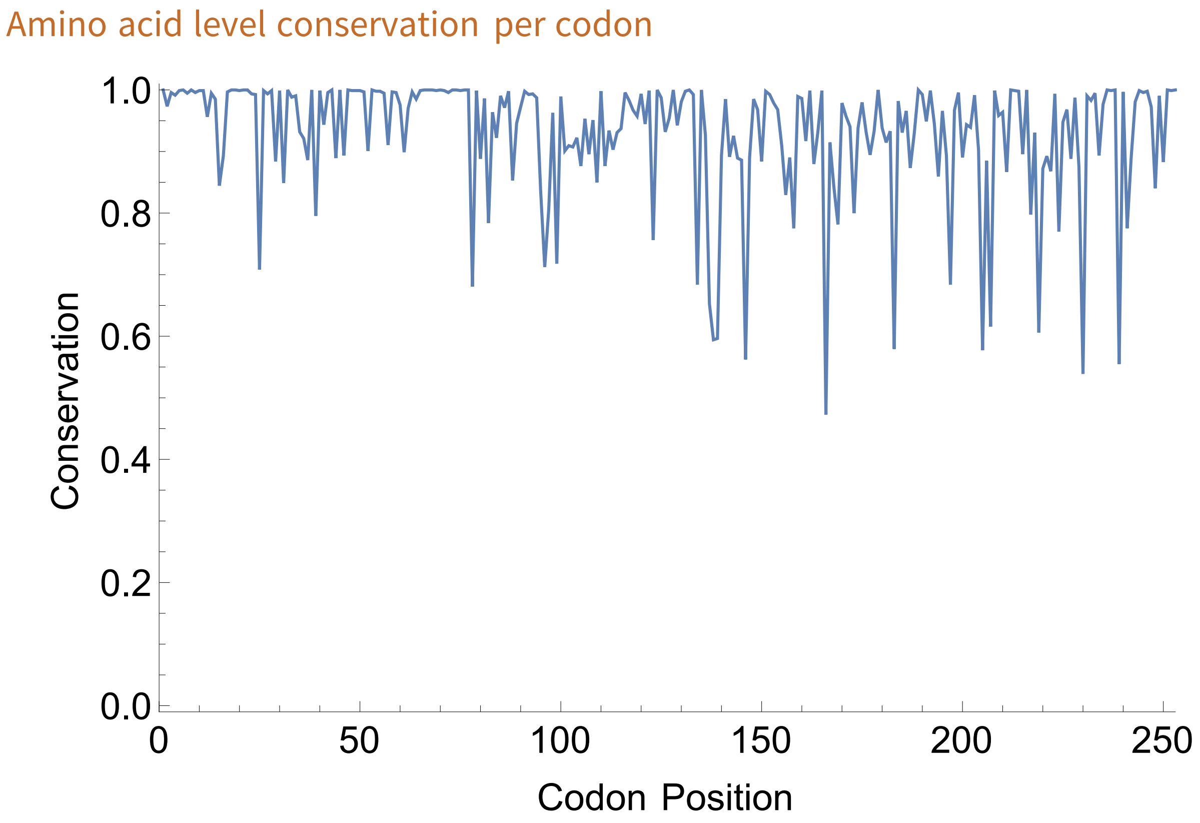
Amino acid level conservation per codon
Conservation
Codon Position
1.0
0.8
0.6
0.4
0.2
0.0
0
50
100
150
200
250

Conservation analysis plots

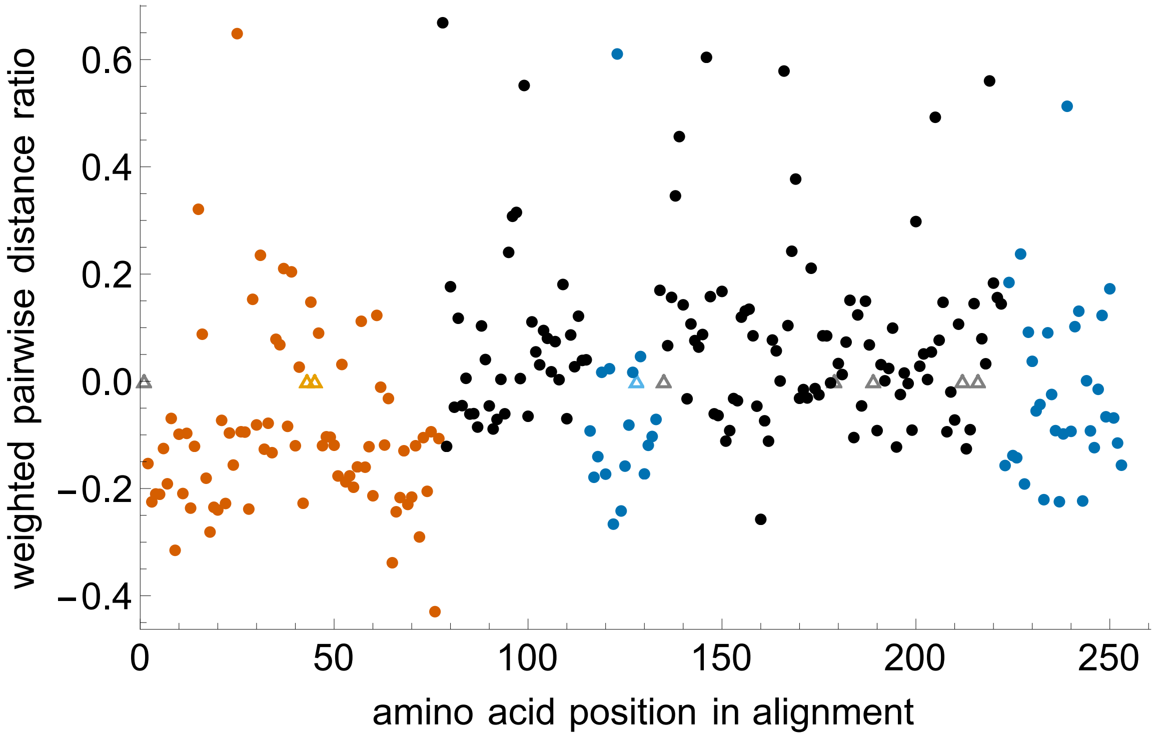

M1

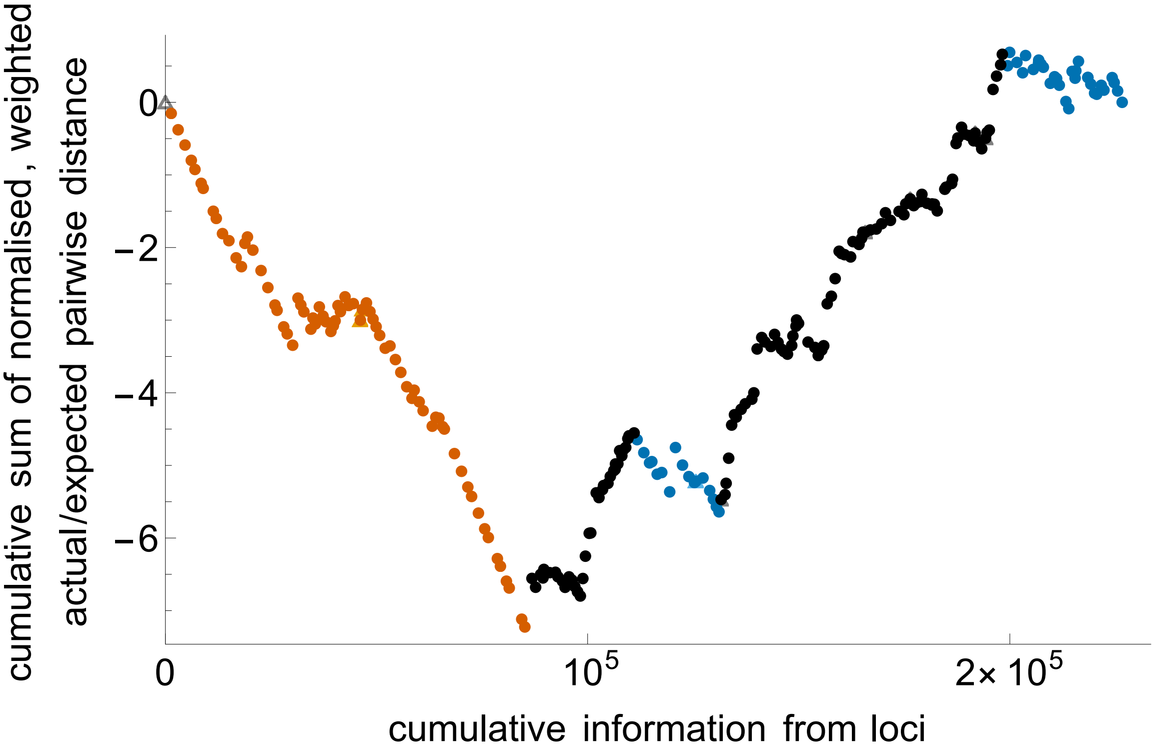

M1

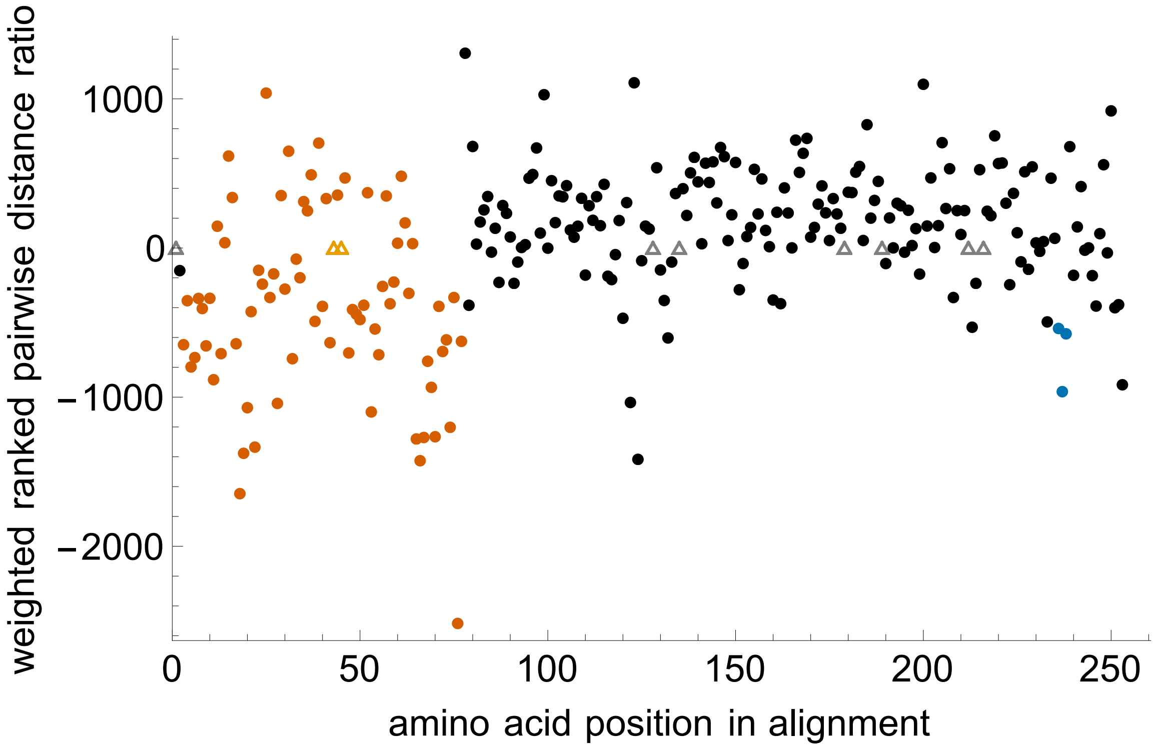
weighted ranked pairwise distance ratio
1000
0
−1000
−2000
0
50
100
150
200
250
amino acid position in alignment

M1

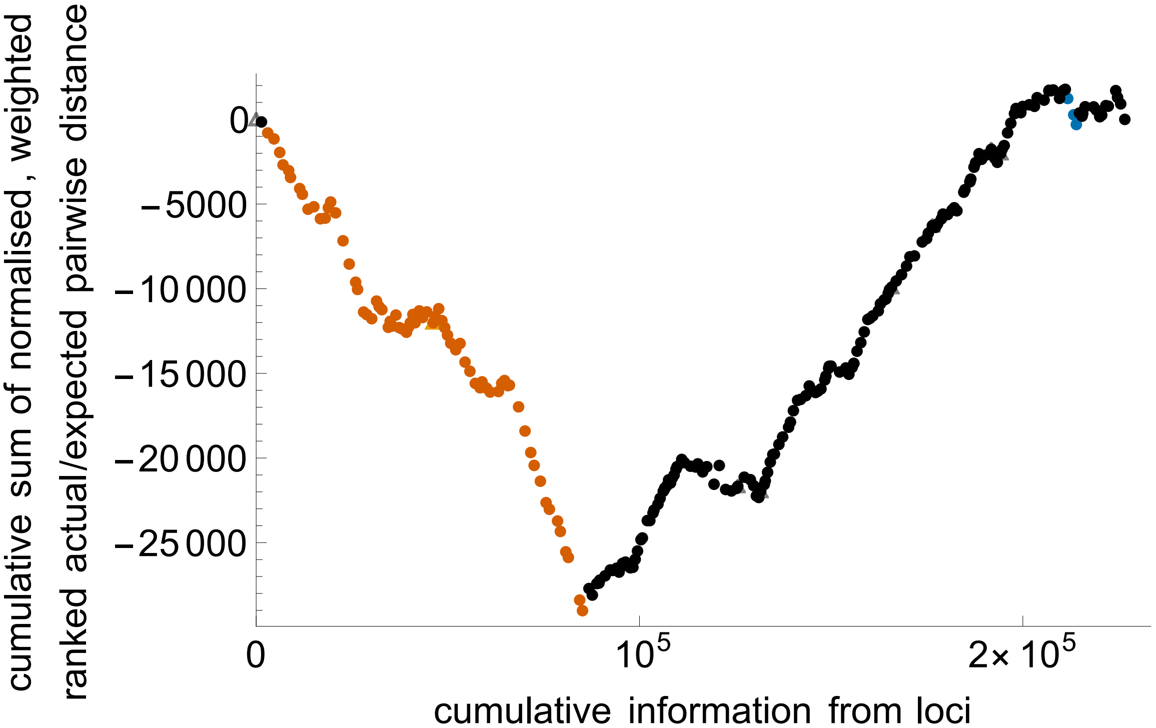
cumulative sum of normalised, weighted
ranked actual/expected pairwise distance
0
−5000
−10 000
−15 000
−20 000
−25 000
0
10^5
2×10^5
cumulative information from loci

M1

M2

Gene length histogram

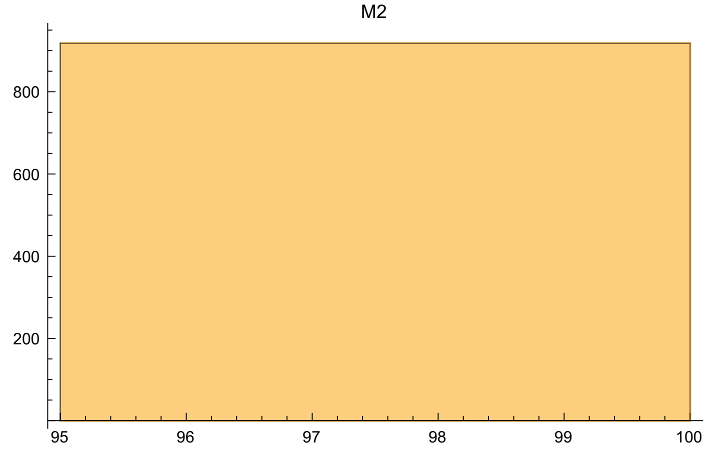

Information vs. nPD

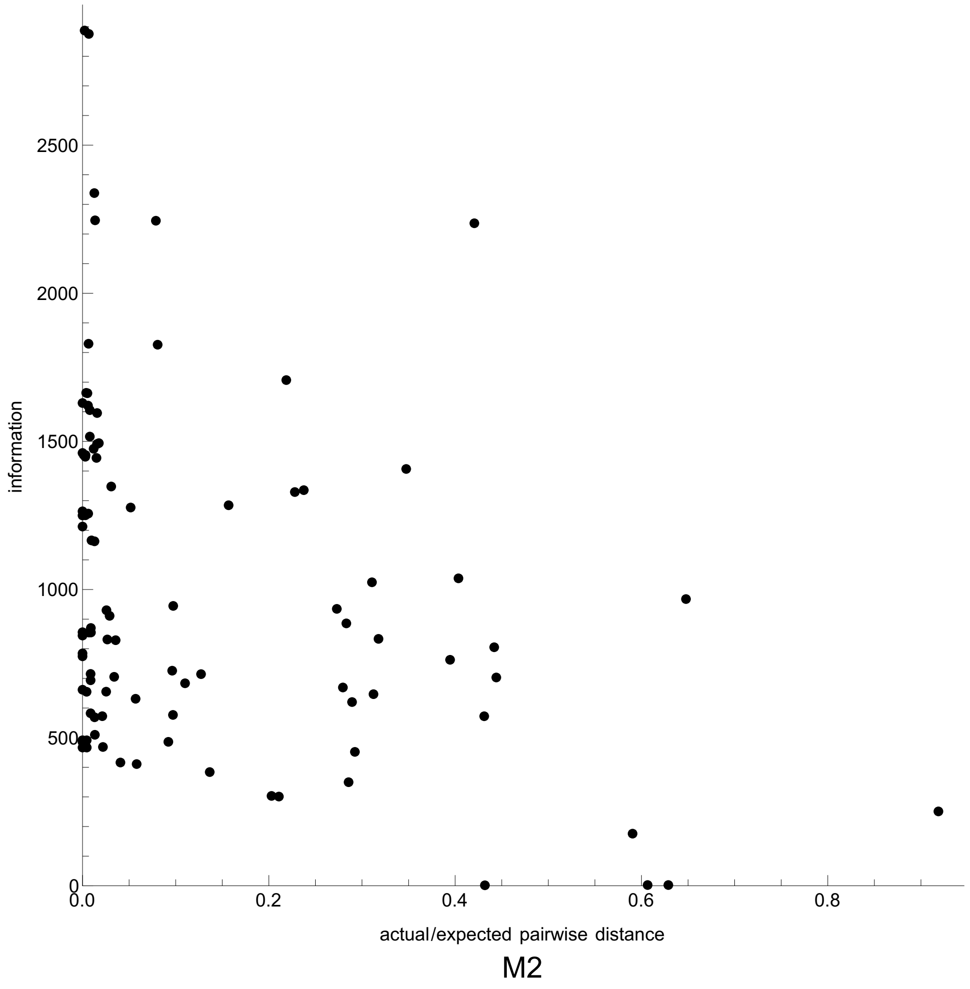

Example sequences highlighted by regions found in analyses to be conserved

Interesting points (by weighted raw PD) highlighted for gene M2:

ATG**AGTCTTCTAACCGAGGTCGAAACG**CCT
ACCAGAAACGGATGGGAGTGCAAATGCAGCGATTCAAGT
GATCCTCTCGTTATTGCCGCAAGTATC **ATTGGGATCTTGCAC**TTG
ATATTGTGGATTCTTGATCGTCTTTTCTTCAAATGCATTTATCGTCGCCTT
AAATACGGTTTGAAAAGAGGGCCTTCTACGGAAGGAGTGCCTGAGTCTATGAGGG
AAGAATACCGGCAGGAACAGCAGAGTGCTGTGGATGTTGACGATGGTCATTT
TGTCAACATAGAGCTGGAGTAA

Interesting points (by weighted ranked PD) highlighted for gene M2:

ATGAGTCTTCTAACCGAGGTCGAAACGCCTACCAGAAACGGATGGGAGTGCAAATGCAGCGATTCAAGTGATCCTCTC⋱
GTTATTGCCGCAAGTATCATTGGGATCTTGCACTTGATATTGTGGATTCTTGATCGTCTTTTCTTCAAATGCATTTA⋱
TCGTCGCCTTAAATACGGTTTGAAAAGAGGGCCTTCTACGGAAGGAGTGCCTGAGTCTATGAGGGAAGAATACCGGC⋱
AGGAACAGCAGAGTGCTGTGGATGTTGACGATGGTCATTTTGTCAACATAGAGCTGGAGTAA

Per codon conservation report

M2			
Pos.	1	obs:	exp:
atg	M	918	918.00
---	--	------	------
mPD		0	0
		nPD:	1.
		N. weight:	0.
		Sc. PD:	0
		Sc. rank:	0

M2			
Pos.	2	obs:	exp:
tct	S	0	146.90
tcc	S	0	118.80
tca	S	0	231.70
tcg	S	0	53.42
agt	S	894	188.10
agc	S	24	179.00
---	--	------	------
mPD		0.051	1.7
		nPD:	0.03
		N. weight:	1.4
		Sc. PD:	-0.08
		Sc. rank:	341.7

M2			
Pos.	3	obs:	exp:
tta	L	0	80.68
ttg	L	0	171.30
ctt	L	914	155.60
ctc	L	4	153.80
cta	L	0	153.00
ctg	L	0	203.60
---	--	------	------
mPD		0.0087	1.1
		nPD:	0.01
		N. weight:	1.6
		Sc. PD:	-0.13
		Sc. rank:	-352.0

M2			
Pos.	4	obs:	exp:
tta	L	3	80.68
ttg	L	0	171.30
ctt	L	1	155.60
ctc	L	0	153.80
cta	L	910	153.00
ctg	L	4	203.60
---	--	------	------
mPD		0.017	1.1
		nPD:	0.02
		N. weight:	1.6
		Sc. PD:	-0.12
		Sc. rank:	199.2

M2			
Pos.	5	obs:	exp:
act	T	1	237.80
acc	T	917	186.90
aca	T	0	421.70
acg	T	0	71.51
---	--	------	------
mPD		0.0022	0.67
		nPD:	0.
		N. weight:	1.5
		Sc. PD:	-0.13
		Sc. rank:	-706.3

M2			
Pos.	6	obs:	exp:
gaa	E	0	552.20
gag	E	918	365.80
---	--	------	------
mPD		0	0.48
		nPD:	0.
		N. weight:	0.85
		Sc. PD:	-0.076
		Sc. rank:	-715.8

M2			
Pos.	7	obs:	exp:
gtt	V	3	189.50
gtc	V	913	183.90
gta	V	2	190.10
gtg	V	0	354.50
---	--	------	------
mPD		0.011	0.73
		nPD:	0.01
		N. weight:	1.5
		Sc. PD:	-0.11
		Sc. rank:	100.7

M2			
Pos.	8	obs:	exp:
gaa	E	918	552.20
gag	E	0	365.80
---	--	------	------
mPD		0	0.48
		nPD:	0.
		N. weight:	0.47
		Sc. PD:	-0.042
		Sc. rank:	-395.4

M2			
Pos.	9	obs:	exp:
cgt	R	0	0.17
cgc	R	0	0.20
cga	R	0	0.40
cgg	R	0	0.33
act	T	0	236.80
acc	T	0	186.10
aca	T	0	419.90
acg	T	914	71.20
aga	R	0	1.83
agg	R	4	1.07
---	--	------	------
mPD		0.0087	0.69
		nPD:	0.01
		N. weight:	2.4
		Sc. PD:	-0.18
		Sc. rank:	-84.6

M2			
Pos.	10	obs:	exp:
tta	L	0	35.51
ttg	L	0	75.39
ctt	L	404	68.46
ctc	L	0	67.69
cta	L	0	67.35
ctg	L	0	89.60
cct	P	510	130.40
ccc	P	3	91.47
cca	P	0	209.60
ccg	P	0	81.50
cgt	R	1	0.04
cgc	R	0	0.05
cga	R	0	0.10
cgg	R	0	0.08
aga	R	0	0.46
agg	R	0	0.27
---	--	------	------
mPD		0.50	1.4
		nPD:	0.35
		N. weight:	1.4
		Sc. PD:	0.36
		Sc. rank:	1033.2

M2			
Pos.	11	obs:	exp:
act	T	0	237.80
acc	T	918	186.90
aca	T	0	421.70
acg	T	0	71.51
---	--	------	------
mPD		0	0.67
		nPD:	0.
		N. weight:	1.5
		Sc. PD:	-0.13
		Sc. rank:	-1238.1

M2			
Pos.	12	obs:	exp:
cgt	R	0	30.59
cgc	R	0	36.38
cga	R	0	71.39
cgg	R	0	59.63
aaa	K	202	118.30
aag	K	0	83.67
aga	R	716	326.90
agg	R	0	191.10
---	--	------	------
mPD		0.34	1.2
		nPD:	0.28
		N. weight:	0.68
		Sc. PD:	0.13
		Sc. rank:	417.2

M2			
Pos.	13	obs:	exp:
tct	S	0	0.64
tcc	S	0	0.52
tca	S	0	1.01
tcg	S	0	0.23
att	I	0	0.73
atc	I	2	0.48
ata	I	0	0.79
act	T	0	212.40
acc	T	813	167.00
aca	T	7	376.70
acg	T	0	63.88
aat	N	0	46.30
aac	N	91	44.70
aaa	K	1	0.59
aag	K	0	0.41
agt	S	0	0.82
agc	S	4	0.78
---	--	------	------
mPD		0.21	0.88
		nPD:	0.24
		N. weight:	1.3
		Sc. PD:	0.2
		Sc. rank:	794.2

M2			
Pos.	14	obs:	exp:
gaa	E	11	6.62
gag	E	0	4.38
ggt	G	0	121.60
ggc	G	0	115.20
gga	G	907	419.40
ggg	G	0	250.80
---	--	------	------
mPD		0.024	0.70
		nPD:	0.03
		N. weight:	0.71
		Sc. PD:	-0.039
		Sc. rank:	187.7

M2			
Pos.	15	obs:	exp:
tta	L	0	0.09
ttg	L	1	0.19
tgg	W	917	917.00
ctt	L	0	0.17
ctc	L	0	0.17
cta	L	0	0.17
ctg	L	0	0.22
---	--	------	------
mPD		0.0022	0.0050
		nPD:	0.43
		N. weight:	0.0017
		Sc. PD:	0.00057
		Sc. rank:	1.4

M2			
Pos.	16	obs:	exp:
gtt	V	0	0.62
gtc	V	0	0.60
gta	V	0	0.62
gtg	V	3	1.16
gaa	E	2	549.80
gag	E	912	364.20
ggt	G	0	0.13
ggc	G	0	0.13
gga	G	0	0.46
ggg	G	1	0.28
---	--	------	------
mPD		0.013	0.49
		nPD:	0.03
		N. weight:	0.84
		Sc. PD:	-0.053
		Sc. rank:	177.2

M2			
Pos.	17	obs:	exp:
tgt	C	0	376.90
tgc	C	918	541.10
---	--	------	------
mPD		0	0.48
		nPD:	0.
		N. weight:	0.49
		Sc. PD:	-0.043
		Sc. rank:	-411.2

M2			
Pos.	18	obs:	exp:
tct	S	0	0.32
tcc	S	0	0.26
tca	S	0	0.50
tcg	S	0	0.12
cgt	R	0	1.03
cgc	R	0	1.22
cga	R	0	2.39
cgg	R	0	2.00
aat	N	14	410.60
aac	N	793	396.40
aaa	K	85	49.79
aag	K	0	35.21
agt	S	0	0.41
agc	S	2	0.39
aga	R	24	10.96
agg	R	0	6.41
---	--	------	------
mPD		0.29	0.67
		nPD:	0.43
		N. weight:	0.58
		Sc. PD:	0.19
		Sc. rank:	467.3

M2

Pos.	19	obs:	exp:
ttt	F	0	7.10
ttc	F	18	10.90
tat	Y	0	2.65
tac	Y	5	2.35
tgt	C	0	367.40
tgc	C	895	527.60
---	--	------	------
mPD		0.049	0.53
		nPD:	0.09
		N. weight:	0.49
		Sc. PD:	0.00089
		Sc. rank:	199.7

M2

Pos.	20	obs:	exp:
tct	S	0	145.00
tcc	S	0	117.30
tca	S	0	228.60
tcg	S	0	52.72
cgt	R	0	0.13
cgc	R	0	0.15
cga	R	0	0.30
cgg	R	0	0.25
aat	N	0	4.07
aac	N	8	3.93
agt	S	0	185.70
agc	S	906	176.70
aga	R	3	1.37
agg	R	0	0.80
ggt	G	0	0.13
ggc	G	1	0.13
gga	G	0	0.46
ggg	G	0	0.28
---	--	------	------
mPD		0.026	1.7
		nPD:	0.02
		N. weight:	1.5
		Sc. PD:	-0.11
		Sc. rank:	143.6

M2

Pos.	21	obs:	exp:
gtt	V	4	0.83
gtc	V	0	0.80
gta	V	0	0.83
gtg	V	0	1.54
gct	A	1	0.26
gcc	A	0	0.19
gca	A	0	0.47
gcg	A	0	0.09
gat	D	97	52.37
gac	D	0	44.63
ggt	G	816	109.40
ggc	G	0	103.70
gga	G	0	377.30
ggg	G	0	225.60
---	--	------	------
mPD		0.20	0.91
		nPD:	0.22
		N. weight:	1.7
		Sc. PD:	0.22
		Sc. rank:	933.9

M2

Pos.	22	obs:	exp:
tct	S	0	146.90
tcc	S	0	118.80
tca	S	918	231.70
tcg	S	0	53.42
agt	S	0	188.10
agc	S	0	179.00
---	--	------	------
mPD		0	1.7
		nPD:	0.
		N. weight:	1.3
		Sc. PD:	-0.11
		Sc. rank:	-1071.2

M2

Pos.	23	obs:	exp:
tct	S	0	146.80
tcc	S	0	118.70
tca	S	0	231.40
tcg	S	0	53.36
aat	N	1	0.51
aac	N	0	0.49
agt	S	917	187.90
agc	S	0	178.80
---	--	------	------
mPD		0.0022	1.7
		nPD:	0.
		N. weight:	1.5
		Sc. PD:	-0.13
		Sc. rank:	-889.3

M2

Pos.	24	obs:	exp:
gat	D	431	359.50
gac	D	235	306.50
gaa	E	0	151.60
gag	E	252	100.40
---	--	------	------
mPD		0.64	0.70
		nPD:	0.92
		N. weight:	0.25
		Sc. PD:	0.21
		Sc. rank:	218.0

M2

Pos.	25	obs:	exp:
tta	L	0	0.26
ttg	L	0	0.56
ctt	L	2	0.51
ctc	L	1	0.50
cta	L	0	0.50
ctg	L	0	0.67
cct	P	765	232.70
ccc	P	6	163.10
cca	P	144	373.80
ccg	P	0	145.40
---	--	------	------
mPD		0.28	0.72
		nPD:	0.39
		N. weight:	0.77
		Sc. PD:	0.23
		Sc. rank:	570.5

M2

Pos.	26	obs:	exp:
ttt	F	0	2.76
ttc	F	7	4.24
tta	L	0	79.10
ttg	L	0	168.00
ctt	L	11	152.50
ctc	L	636	150.80
cta	L	252	150.00
ctg	L	1	199.60
att	I	0	3.65
atc	I	10	2.42
ata	I	0	3.93
gtt	V	0	0.21
gtc	V	0	0.20
gta	V	1	0.21
gtg	V	0	0.39
---	--	------	------
mPD		0.46	1.1
		nPD:	0.4
		N. weight:	1.
		Sc. PD:	0.32
		Sc. rank:	795.4

M2

Pos.	27	obs:	exp:
tct	S	0	0.16
tcc	S	0	0.13
tca	S	0	0.25
tcg	S	0	0.06
att	I	17	6.57
atc	I	1	4.35
ata	I	0	7.08
agt	S	1	0.20
agc	S	0	0.20
gtt	V	566	169.30
gtc	V	254	164.30
gta	V	0	169.80
gtg	V	0	316.70
gct	A	2	0.51
gcc	A	0	0.38
gca	A	0	0.94
gcg	A	0	0.18
ggt	G	76	10.33
ggc	G	1	9.78
gga	G	0	35.60
ggg	G	0	21.29
---	--	------	------
mPD		0.60	0.93
		nPD:	0.65
		N. weight:	0.98
		Sc. PD:	0.54
		Sc. rank:	836.2

M2

Pos.	28	obs:	exp:
att	I	94	34.30
atc	I	0	22.74
ata	I	0	36.97
gtt	V	812	170.10
gtc	V	3	165.10
gta	V	9	170.60
gtg	V	0	318.20
---	--	------	------
mPD		0.21	0.92
		nPD:	0.23
		N. weight:	1.3
		Sc. PD:	0.18
		Sc. rank:	758.7

M2

Pos.	29	obs:	exp:
gct	A	0	235.20
gcc	A	89	172.40
gca	A	818	429.20
gcg	A	11	81.20
---	--	------	------
mPD		0.20	0.67
		nPD:	0.29
		N. weight:	0.46
		Sc. PD:	0.091
		Sc. rank:	300.3

M2

Pos.	30	obs:	exp:
tct	S	1	0.16
tcc	S	0	0.13
tca	S	0	0.25
tcg	S	0	0.06
agt	S	0	0.20
agc	S	0	0.20
gct	A	820	235.00
gcc	A	0	172.20
gca	A	97	428.70
gcg	A	0	81.11
---	--	------	------
mPD		0.19	0.68
		nPD:	0.28
		N. weight:	0.89
		Sc. PD:	0.17
		Sc. rank:	566.1

M2

Pos.	31	obs:	exp:
tct	S	0	14.57
tcc	S	0	11.78
tca	S	0	22.97
tcg	S	0	5.30
aat	N	5	420.70
aac	N	822	406.30
agt	S	91	18.65
agc	S	0	17.75
---	--	------	------
mPD		0.37	0.82
		nPD:	0.44
		N. weight:	0.71
		Sc. PD:	0.25
		Sc. rank:	593.1

M2

Pos.	32	obs:	exp:
att	I	814	334.20
atc	I	101	221.60
ata	I	1	360.20
gtt	V	0	0.41
gtc	V	2	0.40
gta	V	0	0.41
gtg	V	0	0.77
---	--	------	------
mPD		0.21	0.66
		nPD:	0.31
		N. weight:	0.65
		Sc. PD:	0.14
		Sc. rank:	449.1

M2

Pos.	33	obs:	exp:
att	I	884	322.50
atc	I	0	213.80
ata	I	0	347.60
act	T	1	0.26
acc	T	0	0.20
aca	T	0	0.46
acg	T	0	0.08
gtt	V	33	6.81
gtc	V	0	6.61
gta	V	0	6.83
gtg	V	0	12.74
---	--	------	------
mPD		0.072	0.74
		nPD:	0.1
		N. weight:	0.95
		Sc. PD:	0.0066
		Sc. rank:	426.2

M2

Pos.	34	obs:	exp:
gaa	E	0	0.60
gag	E	1	0.40
ggt	G	0	123.00
ggc	G	0	116.50
gga	G	2	424.00
ggg	G	915	253.50
---	--	------	------
mPD		0.0065	0.68
		nPD:	0.01
		N. weight:	1.2
		Sc. PD:	-0.093
		Sc. rank:	-121.5

M2

Pos.	35	obs:	exp:
tct	S	0	0.96
tcc	S	0	0.78
tca	S	0	1.51
tcg	S	0	0.35
att	I	0	332.40
atc	I	86	220.30
ata	I	825	358.30
atg	M	1	1.00
agt	S	0	1.23
agc	S	6	1.17
---	--	------	------
mPD		0.20	0.68
		nPD:	0.29
		N. weight:	0.63
		Sc. PD:	0.12
		Sc. rank:	407.1

M2

Pos.	36	obs:	exp:
tta	L	0	80.68
ttg	L	914	171.30
ctt	L	0	155.60
ctc	L	0	153.80
cta	L	0	153.00
ctg	L	4	203.60
---	--	------	------
mPD		0.0087	1.1
		nPD:	0.01
		N. weight:	1.5
		Sc. PD:	-0.12
		Sc. rank:	-332.4

M2

Pos.	37	obs:	exp:
cat	H	0	527.40
cac	H	918	390.60
---	--	------	------
mPD		0	0.49
		nPD:	0.
		N. weight:	0.79
		Sc. PD:	-0.07
		Sc. rank:	-664.8

M2

Pos.	38	obs:	exp:
tta	L	0	80.68
ttg	L	715	171.30
ctt	L	0	155.60
ctc	L	0	153.80
cta	L	0	153.00
ctg	L	203	203.60
---	--	------	------
mPD		0.34	1.1
		nPD:	0.31
		N. weight:	1.
		Sc. PD:	0.22
		Sc. rank:	699.5

M2

Pos.	39	obs:	exp:
att	I	0	334.20
atc	I	0	221.60
ata	I	916	360.20
atg	M	2	2.00
---	--	------	------
mPD		0.0044	0.66
		nPD:	0.01
		N. weight:	0.86
		Sc. PD:	-0.071
		Sc. rank:	-255.1

,

M2

Pos.	40	obs:	exp:
tta	L	0	80.59
ttg	L	909	171.10
ctt	L	0	155.40
ctc	L	0	153.60
cta	L	0	152.90
ctg	L	8	203.40
atg	M	1	1.00
---	--	------	------
mPD		0.019	1.1
		nPD:	0.02
		N. weight:	1.5
		Sc. PD:	-0.11
		Sc. rank:	226.3

,

M2

Pos.	41	obs:	exp:
tgg	W	917	917.00
cgt	R	0	0.04
cgc	R	0	0.05
cga	R	0	0.10
cgg	R	1	0.08
aga	R	0	0.46
agg	R	0	0.27
---	--	------	------
mPD		0.0022	0.0036
		nPD:	0.61
		N. weight:	0.0025
		Sc. PD:	0.0013
		Sc. rank:	2.1

,

M2

Pos.	42	obs:	exp:
tct	S	0	0.64
tcc	S	0	0.52
tca	S	0	1.01
tcg	S	0	0.23
att	I	909	332.80
atc	I	3	220.60
ata	I	0	358.60
act	T	2	0.52
acc	T	0	0.41
aca	T	0	0.92
acg	T	0	0.16
agt	S	4	0.82
agc	S	0	0.78
---	--	------	------
mPD		0.020	0.67
		nPD:	0.03
		N. weight:	0.92
		Sc. PD:	-0.055
		Sc. rank:	209.1

,

M2

Pos.	43	obs:	exp:
ttt	F	1	0.39
ttc	F	0	0.61
tta	L	0	80.50
ttg	L	0	170.90
ctt	L	915	155.20
ctc	L	0	153.50
cta	L	0	152.70
ctg	L	1	203.10
att	I	1	0.36
atc	I	0	0.24
ata	I	0	0.39
---	--	------	------
mPD		0.0065	1.1
		nPD:	0.01
		N. weight:	1.6
		Sc. PD:	-0.14
		Sc. rank:	-597.7

,

M2

Pos.	44	obs:	exp:
aat	N	1	0.51
aac	N	0	0.49
gat	D	915	494.00
gac	D	0	421.00
ggt	G	2	0.27
ggc	G	0	0.25
gga	G	0	0.92
ggg	G	0	0.55
---	--	------	------
mPD		0.0065	0.50
		nPD:	0.01
		N. weight:	0.57
		Sc. PD:	-0.044
		Sc. rank:	-3.0

,

M2

Pos.	45	obs:	exp:
cgt	R	915	39.22
cgc	R	3	46.64
cga	R	0	91.53
cgg	R	0	76.46
aga	R	0	419.10
agg	R	0	245.00
---	--	------	------
mPD		0.0065	0.96
		nPD:	0.01
		N. weight:	2.9
		Sc. PD:	-0.24
		Sc. rank:	-710.5

,

M2

Pos.	46	obs:	exp:
tta	L	0	80.68
ttg	L	0	171.30
ctt	L	918	155.60
ctc	L	0	153.80
cta	L	0	153.00
ctg	L	0	203.60
---	--	------	------
mPD		0	1.1
		nPD:	0.
		N. weight:	1.6
		Sc. PD:	-0.15
		Sc. rank:	-1381.0

,

M2

Pos.	47	obs:	exp:
ttt	F	9	362.30
ttc	F	909	555.70
---	--	------	------
mPD		0.019	0.48
		nPD:	0.04
		N. weight:	0.42
		Sc. PD:	-0.02
		Sc. rank:	120.0

,

M2

Pos.	48	obs:	exp:
ttt	F	0	360.30
ttc	F	913	552.70
tct	S	0	0.80
tcc	S	5	0.65
tca	S	0	1.26
tcg	S	0	0.29
agt	S	0	1.02
agc	S	0	0.98
---	--	------	------
mPD		0.011	0.49
		nPD:	0.02
		N. weight:	0.47
		Sc. PD:	-0.032
		Sc. rank:	79.7

,

M2

Pos.	49	obs:	exp:
aaa	K	918	537.80
aag	K	0	380.20
---	--	------	------
mPD		0	0.49
		nPD:	0.
		N. weight:	0.5
		Sc. PD:	-0.044
		Sc. rank:	-416.0

,

M2

Pos.	50	obs:	exp:
tat	Y	0	3.71
tac	Y	7	3.29
tgt	C	25	374.00
tgc	C	886	537.00
---	--	------	------
mPD		0.068	0.50
		nPD:	0.14
		N. weight:	0.39
		Sc. PD:	0.018
		Sc. rank:	185.4

,

M2

Pos.	51	obs:	exp:
att	I	910	332.00
atc	I	0	220.10
ata	I	0	357.90
gtt	V	8	1.65
gtc	V	0	1.60
gta	V	0	1.66
gtg	V	0	3.09
---	--	------	------
mPD		0.017	0.67
		nPD:	0.03
		N. weight:	0.94
		Sc. PD:	-0.06
		Sc. rank:	184.5

,

M2

Pos.	52	obs:	exp:
tat	Y	916	485.70
tac	Y	0	430.30
cat	H	2	1.15
cac	H	0	0.85
---	--	------	------
mPD		0.0044	0.50
		nPD:	0.01
		N. weight:	0.59
		Sc. PD:	-0.047
		Sc. rank:	-97.4

,

M2

Pos.	53	obs:	exp:
cgt	R	917	39.22
cgc	R	0	46.64
cga	R	1	91.53
cgg	R	0	76.46
aga	R	0	419.10
agg	R	0	245.00
---	--	------	------
mPD		0.0022	0.96
		nPD:	0.
		N. weight:	2.9
		Sc. PD:	-0.25
		Sc. rank:	-1616.5

,

M2

Pos.	54	obs:	exp:
tta	L	0	0.09
ttg	L	0	0.19
tgt	C	1	0.41
tgc	C	0	0.59
ctt	L	0	0.17
ctc	L	1	0.17
cta	L	0	0.17
ctg	L	0	0.22
cat	H	0	0.57
cac	H	1	0.43
cgt	R	255	39.09
cgc	R	660	46.49
cga	R	0	91.24
cgg	R	0	76.21
aga	R	0	417.80
agg	R	0	244.20
---	--	------	------
mPD		0.41	0.97
		nPD:	0.42
		N. weight:	2.3
		Sc. PD:	0.73
		Sc. rank:	1803.3

,

M2

Pos.	55	obs:	exp:
ttt	F	822	324.40
ttc	F	0	497.60
tta	L	0	8.44
ttg	L	0	17.91
ctt	L	96	16.27
ctc	L	0	16.08
cta	L	0	16.01
ctg	L	0	21.29
---	--	------	------
mPD		0.19	0.69
		nPD:	0.27
		N. weight:	0.94
		Sc. PD:	0.17
		Sc. rank:	571.4

,

M2

Pos.	56	obs:	exp:
caa	Q	1	0.51
cag	Q	0	0.49
aaa	K	917	537.20
aag	K	0	379.80
---	--	------	------
mPD		0.0022	0.49
		nPD:	0.
		N. weight:	0.5
		Sc. PD:	-0.042
		Sc. rank:	-213.9

,

M2

Pos.	57	obs:	exp:
tat	Y	0	485.70
tac	Y	916	430.30
cat	H	0	1.15
cac	H	2	0.85
---	--	------	------
mPD		0.0044	0.50
		nPD:	0.01
		N. weight:	0.7
		Sc. PD:	-0.056
		Sc. rank:	-116.0

,

M2

Pos.	58	obs:	exp:
ggt	G	916	123.10
ggc	G	1	116.60
gga	G	1	424.40
ggg	G	0	253.80
---	--	------	------
mPD		0.0044	0.68
		nPD:	0.01
		N. weight:	1.8
		Sc. PD:	-0.15
		Sc. rank:	-573.9

,

M2			
Pos.	59	obs:	exp:
ttt	F	1	0.39
ttc	F	0	0.61
tta	L	73	80.50
ttg	L	832	170.90
ctt	L	0	155.20
ctc	L	0	153.50
cta	L	0	152.70
ctg	L	11	203.10
gtt	V	0	0.21
gtc	V	0	0.20
gta	V	0	0.21
gtg	V	1	0.39
mPD		0.17	1.1
		nPD:	0.16
		N. weight:	1.3
		Sc. PD:	0.085
		Sc. rank:	649.8

M2			
Pos.	60	obs:	exp:
aaa	K	918	537.80
aag	K	0	380.20
mPD		0	0.49
		nPD:	0.
		N. weight:	0.5
		Sc. PD:	-0.044
		Sc. rank:	-416.0

M2			
Pos.	61	obs:	exp:
cgt	R	0	37.17
cgc	R	0	44.20
cga	R	0	86.75
cgg	R	0	72.46
att	I	0	15.32
atc	I	0	10.16
ata	I	42	16.52
aaa	K	0	0.59
aag	K	1	0.41
aga	R	870	397.20
agg	R	0	232.20
ggt	G	0	0.67
ggc	G	0	0.64
gga	G	5	2.31
ggg	G	0	1.38
mPD		0.10	1.1
		nPD:	0.1
		N. weight:	0.73
		Sc. PD:	0.0043
		Sc. rank:	307.8

M2			
Pos.	62	obs:	exp:
cgt	R	0	0.04
cgc	R	0	0.05
cga	R	0	0.10
cgg	R	0	0.08
aga	R	0	0.46
agg	R	1	0.27
ggt	G	1	123.00
ggc	G	2	116.50
gga	G	0	424.00
ggg	G	914	253.50
mPD		0.0087	0.68
		nPD:	0.01
		N. weight:	1.2
		Sc. PD:	-0.089
		Sc. rank:	-17.9

M2			
Pos.	63	obs:	exp:
cct	P	917	233.20
ccc	P	0	163.50
cca	P	0	374.60
ccg	P	0	145.70
gat	D	1	0.54
gac	D	0	0.46
mPD		0.0044	0.72
		nPD:	0.01
		N. weight:	1.3
		Sc. PD:	-0.11
		Sc. rank:	-435.1

M2			
Pos.	64	obs:	exp:
tct	S	914	146.80
tcc	S	3	118.70
tca	S	0	231.40
tcg	S	0	53.36
tat	Y	1	0.53
tac	Y	0	0.47
agt	S	0	187.90
agc	S	0	178.80
mPD		0.0087	1.7
		nPD:	0.01
		N. weight:	1.7
		Sc. PD:	-0.14
		Sc. rank:	-661.2

M2			
Pos.	65	obs:	exp:
atg	M	1	1.00
act	T	0	233.70
acc	T	0	183.70
aca	T	10	414.40
acg	T	892	70.26
aaa	K	0	8.79
aag	K	15	6.21
mPD		0.056	0.71
		nPD:	0.08
		N. weight:	2.3
		Sc. PD:	-0.026
		Sc. rank:	830.1

M2			
Pos.	66	obs:	exp:
aaa	K	1	0.59
aag	K	0	0.41
gaa	E	917	551.60
gag	E	0	365.40
mPD		0.0022	0.48
		nPD:	0.
		N. weight:	0.47
		Sc. PD:	-0.04
		Sc. rank:	-199.4

M2			
Pos.	67	obs:	exp:
ggt	G	0	123.10
ggc	G	0	116.60
gga	G	900	424.40
ggg	G	18	253.80
mPD		0.038	0.68
		nPD:	0.06
		N. weight:	0.64
		Sc. PD:	-0.021
		Sc. rank:	203.5

M2			
Pos.	68	obs:	exp:
tta	L	0	0.09
ttg	L	1	0.19
ctt	L	0	0.17
ctc	L	0	0.17
cta	L	0	0.17
ctg	L	0	0.22
atg	M	729	729.00
gtt	V	0	38.81
gtc	V	0	37.66
gta	V	1	38.92
gtg	V	187	72.60
mPD		0.33	0.56
		nPD:	0.59
		N. weight:	0.18
		Sc. PD:	0.087
		Sc. rank:	149.0

M2			
Pos.	69	obs:	exp:
cct	P	917	233.40
ccc	P	1	163.70
cca	P	0	375.00
ccg	P	0	145.80
mPD		0.0022	0.71
		nPD:	0.
		N. weight:	1.3
		Sc. PD:	-0.11
		Sc. rank:	-640.0

M2			
Pos.	70	obs:	exp:
gat	D	1	0.54
gac	D	0	0.46
gaa	E	27	551.00
gag	E	889	365.00
ggt	G	0	0.13
ggc	G	0	0.13
gga	G	0	0.46
ggg	G	1	0.28
mPD		0.061	0.48
		nPD:	0.13
		N. weight:	0.72
		Sc. PD:	0.026
		Sc. rank:	340.1

M2			
Pos.	71	obs:	exp:
tct	S	915	146.90
tcc	S	3	118.80
tca	S	0	231.70
tcg	S	0	53.42
agt	S	0	188.10
agc	S	0	179.00
mPD		0.0065	1.7
		nPD:	0.
		N. weight:	1.7
		Sc. PD:	-0.14
		Sc. rank:	-758.9

M2			
Pos.	72	obs:	exp:
tta	L	0	0.18
ttg	L	1	0.37
ctt	L	0	0.34
ctc	L	0	0.34
cta	L	0	0.33
ctg	L	1	0.44
atg	M	916	916.00
mPD		0.0044	0.0069
		nPD:	0.63
		N. weight:	0.0025
		Sc. PD:	0.0013
		Sc. rank:	2.1

M2			
Pos.	73	obs:	exp:
cgt	R	0	39.22
cgc	R	0	46.64
cga	R	0	91.53
cgg	R	0	76.46
aga	R	0	419.10
agg	R	918	245.00
mPD		0	0.96
		nPD:	0.
		N. weight:	1.2
		Sc. PD:	-0.11
		Sc. rank:	-1027.6

M2			
Pos.	74	obs:	exp:
gtt	V	0	0.21
gtc	V	0	0.20
gta	V	1	0.21
gtg	V	0	0.39
gaa	E	905	551.60
gag	E	12	365.40
mPD		0.028	0.48
		nPD:	0.06
		N. weight:	0.41
		Sc. PD:	-0.013
		Sc. rank:	135.5

M2			
Pos.	75	obs:	exp:
gaa	E	871	552.20
gag	E	47	365.80
mPD		0.097	0.48
		nPD:	0.2
		N. weight:	0.31
		Sc. PD:	0.034
		Sc. rank:	155.1

M2			
Pos.	76	obs:	exp:
ttt	F	2	0.79
ttc	F	0	1.21
tat	Y	847	485.70
tac	Y	69	430.30
mPD		0.14	0.50
		nPD:	0.29
		N. weight:	0.35
		Sc. PD:	0.068
		Sc. rank:	225.6

M2			
Pos.	77	obs:	exp:
cgt	R	0	39.22
cgc	R	0	46.64
cga	R	6	91.53
cgg	R	912	76.46
aga	R	0	419.10
agg	R	0	245.00
mPD		0.013	0.96
		nPD:	0.01
		N. weight:	2.3
		Sc. PD:	-0.17
		Sc. rank:	98.7

M2			
Pos.	78	obs:	exp:
caa	Q	0	471.50
cag	Q	918	446.50
mPD		0	0.50
		nPD:	0.
		N. weight:	0.67
		Sc. PD:	-0.059
		Sc. rank:	-560.8

M2			
Pos.	79	obs:	exp:
gat	D	0	0.54
gac	D	1	0.46
gaa	E	869	551.60
gag	E	48	365.40
mPD		0.10	0.48
		nPD:	0.21
		N. weight:	0.3
		Sc. PD:	0.036
		Sc. rank:	155.6

M2			
Pos.	80	obs:	exp:
caa	Q	4	457.70
cag	Q	887	433.30
cgt	R	0	1.11
cgc	R	0	1.32
cga	R	0	2.59
cgg	R	26	2.17
aaa	K	0	0.59
aag	K	1	0.41
aga	R	0	11.87
agg	R	0	6.94
mPD		0.066	0.60
		nPD:	0.11
		N. weight:	0.69
		Sc. PD:	0.013
		Sc. rank:	316.7

M2			
Pos.	81	obs:	exp:
caa	Q	1	471.50
cag	Q	917	446.50
mPD		0.0022	0.50
		nPD:	0.
		N. weight:	0.66
		Sc. PD:	-0.056
		Sc. rank:	-291.0

M2			
Pos.	82	obs:	exp:
tct	S	0	36.66
tcc	S	0	29.64
tca	S	0	57.79
tcg	S	0	13.33
aat	N	681	346.50
aac	N	0	334.50
aaa	K	4	2.34
aag	K	0	1.66
agt	S	229	46.92
agc	S	0	44.66
ggt	G	4	0.54
ggc	G	0	0.51
gga	G	0	1.85
ggg	G	0	1.11
mPD		0.40	1.2
		nPD:	0.32
		N. weight:	0.84
		Sc. PD:	0.19
		Sc. rank:	591.1

M2			
Pos.	83	obs:	exp:
gct	A	918	235.20
gcc	A	0	172.40
gca	A	0	429.20
gcg	A	0	81.20
mPD		0	0.67
		nPD:	0.
		N. weight:	1.3
		Sc. PD:	-0.11
		Sc. rank:	-1059.3

M2

Pos.	84	obs:	exp:
gtt	V	0	189.10
gtc	V	0	183.50
gta	V	1	189.70
gtg	V	915	353.80
gct	A	0	0.51
gcc	A	0	0.38
gca	A	0	0.94
gcg	A	2	0.18
---	--	------	------
mPD		0.0065	0.73
		nPD:	0.01
		N. weight:	0.88
		Sc. PD:	-0.07
		Sc. rank:	-122.1

,

M2

Pos.	85	obs:	exp:
gtt	V	1	0.21
gtc	V	0	0.20
gta	V	0	0.21
gtg	V	0	0.39
gat	D	913	492.90
gac	D	0	420.10
ggt	G	4	0.54
ggc	G	0	0.51
gga	G	0	1.85
ggg	G	0	1.11
---	--	------	------
mPD		0.011	0.51
		nPD:	0.02
		N. weight:	0.58
		Sc. PD:	-0.039
		Sc. rank:	92.6

,

M2

Pos.	86	obs:	exp:
gtt	V	917	189.30
gtc	V	0	183.70
gta	V	0	189.90
gtg	V	0	354.10
gct	A	1	0.26
gcc	A	0	0.19
gca	A	0	0.47
gcg	A	0	0.09
---	--	------	------
mPD		0.0022	0.73
		nPD:	0.
		N. weight:	1.5
		Sc. PD:	-0.13
		Sc. rank:	-773.5

,

M2

Pos.	87	obs:	exp:
gat	D	0	494.50
gac	D	916	421.50
ggt	G	0	0.27
ggc	G	2	0.25
gga	G	0	0.92
ggg	G	0	0.55
---	--	------	------
mPD		0.0044	0.50
		nPD:	0.01
		N. weight:	0.72
		Sc. PD:	-0.058
		Sc. rank:	-127.7

,

M2

Pos.	88	obs:	exp:
tat	Y	8	4.24
tac	Y	0	3.76
aat	N	6	3.05
aac	N	0	2.95
gct	A	2	0.77
gcc	A	0	0.56
gca	A	1	1.40
gcg	A	0	0.27
gat	D	894	482.60
gac	D	0	411.40
ggt	G	7	0.94
ggc	G	0	0.89
gga	G	0	3.24
ggg	G	0	1.94
---	--	------	------
mPD		0.054	0.56
		nPD:	0.1
		N. weight:	0.58
		Sc. PD:	0.0039
		Sc. rank:	250.5

,

M2

Pos.	89	obs:	exp:
tct	S	0	3.36
tcc	S	0	2.72
tca	S	0	5.30
tcg	S	0	1.22
tgt	C	1	0.41
tgc	C	0	0.59
tgg	W	1	1.00
agt	S	21	4.30
agc	S	0	4.10
gtt	V	1	0.21
gtc	V	0	0.20
gta	V	0	0.21
gtg	V	0	0.39
gat	D	4	2.16
gac	D	0	1.84
ggt	G	890	119.40
ggc	G	0	113.10
gga	G	0	411.50
ggg	G	0	246.10
---	--	------	------
mPD		0.062	0.77
		nPD:	0.08
		N. weight:	1.8
		Sc. PD:	-0.017
		Sc. rank:	734.9

,

M2

Pos.	90	obs:	exp:
tct	S	0	0.16
tcc	S	0	0.13
tca	S	1	0.25
tcg	S	0	0.06
cat	H	917	526.80
cac	H	0	390.20
agt	S	0	0.20
agc	S	0	0.20
---	--	------	------
mPD		0.0065	0.49
		nPD:	0.01
		N. weight:	0.51
		Sc. PD:	-0.039
		Sc. rank:	2.0

,

M2

Pos.	91	obs:	exp:
ttt	F	916	361.50
ttc	F	0	554.50
tta	L	0	0.18
ttg	L	2	0.37
ctt	L	0	0.34
ctc	L	0	0.34
cta	L	0	0.33
ctg	L	0	0.44
---	--	------	------
mPD		0.0044	0.48
		nPD:	0.01
		N. weight:	0.86
		Sc. PD:	-0.069
		Sc. rank:	-106.9

,

M2

Pos.	92	obs:	exp:
tgt	C	1	0.41
tgc	C	0	0.59
att	I	0	0.36
atc	I	1	0.24
ata	I	0	0.39
gtt	V	0	189.10
gtc	V	916	183.50
gta	V	0	189.70
gtg	V	0	353.80
---	--	------	------
mPD		0.0087	0.73
		nPD:	0.01
		N. weight:	1.5
		Sc. PD:	-0.11
		Sc. rank:	-114.9

,

M2

Pos.	93	obs:	exp:
tct	S	0	0.16
tcc	S	0	0.13
tca	S	0	0.25
tcg	S	0	0.06
caa	Q	1	0.51
cag	Q	0	0.49
act	T	0	0.52
acc	T	2	0.41
aca	T	0	0.92
acg	T	0	0.16
aat	N	0	464.50
aac	N	913	448.50
agt	S	0	0.20
agc	S	1	0.20
gat	D	0	0.54
gac	D	1	0.46
---	--	------	------
mPD		0.013	0.51
		nPD:	0.03
		N. weight:	0.66
		Sc. PD:	-0.042
		Sc. rank:	119.1

,

M2

Pos.	94	obs:	exp:
att	I	0	334.60
atc	I	0	221.80
ata	I	917	360.60
---	-	1	1.00
---	--	------	------
mPD		0	0.65
		nPD:	0.
		N. weight:	0.86
		Sc. PD:	-0.077
		Sc. rank:	-725.3

,

M2

Pos.	95	obs:	exp:
cat	H	1	0.57
cac	H	0	0.43
aaa	K	1	1.76
aag	K	2	1.24
gaa	E	2	549.80
gag	E	912	364.20
---	--	------	------
mPD		0.017	0.49
		nPD:	0.04
		N. weight:	0.84
		Sc. PD:	-0.045
		Sc. rank:	232.9

,

M2

Pos.	96	obs:	exp:
tta	L	0	80.59
ttg	L	1	171.10
ctt	L	0	155.40
ctc	L	0	153.60
cta	L	23	152.90
ctg	L	893	203.40
cgt	R	0	0.04
cgc	R	0	0.05
cga	R	0	0.10
cgg	R	0	0.08
aga	R	1	0.46
agg	R	0	0.27
---	--	------	------
mPD		0.058	1.1
		nPD:	0.05
		N. weight:	1.3
		Sc. PD:	-0.049
		Sc. rank:	397.2

,

M2

Pos.	97	obs:	exp:
aaa	K	2	406.50
aag	K	692	287.50
gct	A	1	0.26
gcc	A	0	0.19
gca	A	0	0.47
gcg	A	0	0.09
gaa	E	0	134.10
gag	E	223	88.86
---	--	------	------
mPD		0.38	0.86
		nPD:	0.44
		N. weight:	0.81
		Sc. PD:	0.28
		Sc. rank:	669.1

,

M2

Pos.	98	obs:	exp:
taa	.	918	394.80
tag	.	0	232.10
tga	.	0	291.10
---	--	------	------
mPD		0	0.81
		nPD:	0.
		N. weight:	0.78
		Sc. PD:	-0.069
		Sc. rank:	-656.5

}

Amino acid level conservation per codon

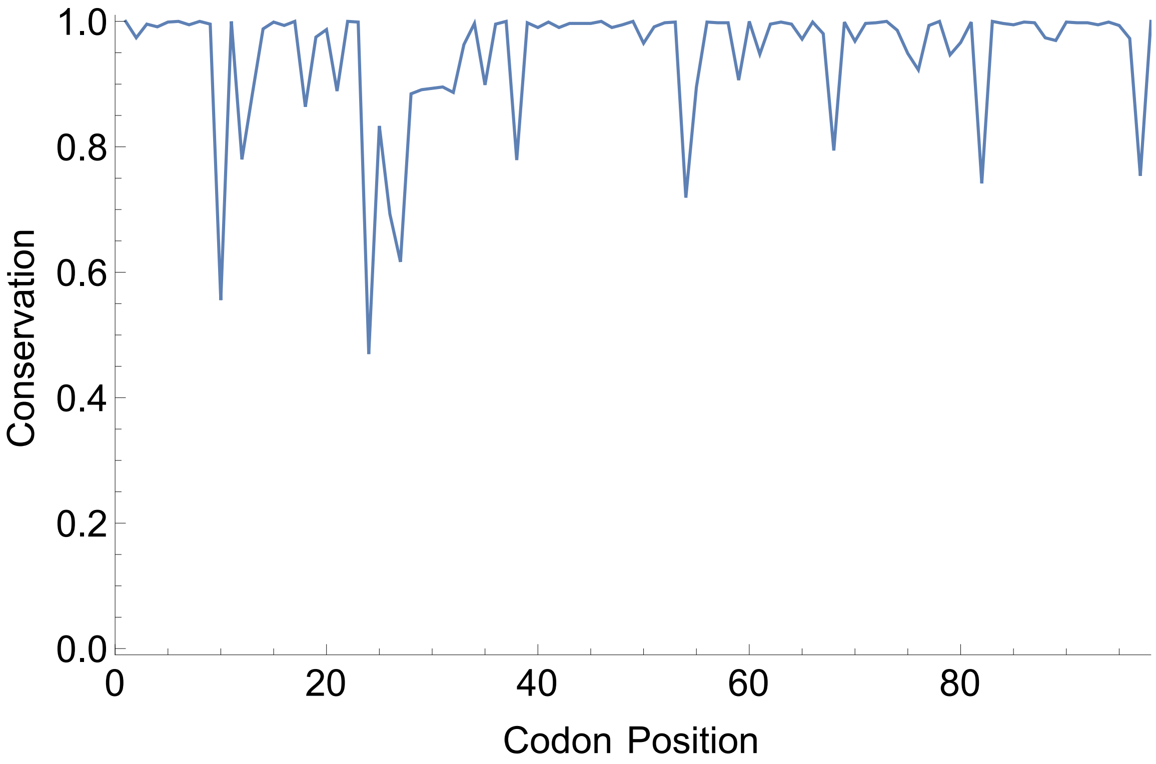

Conservation analysis plots

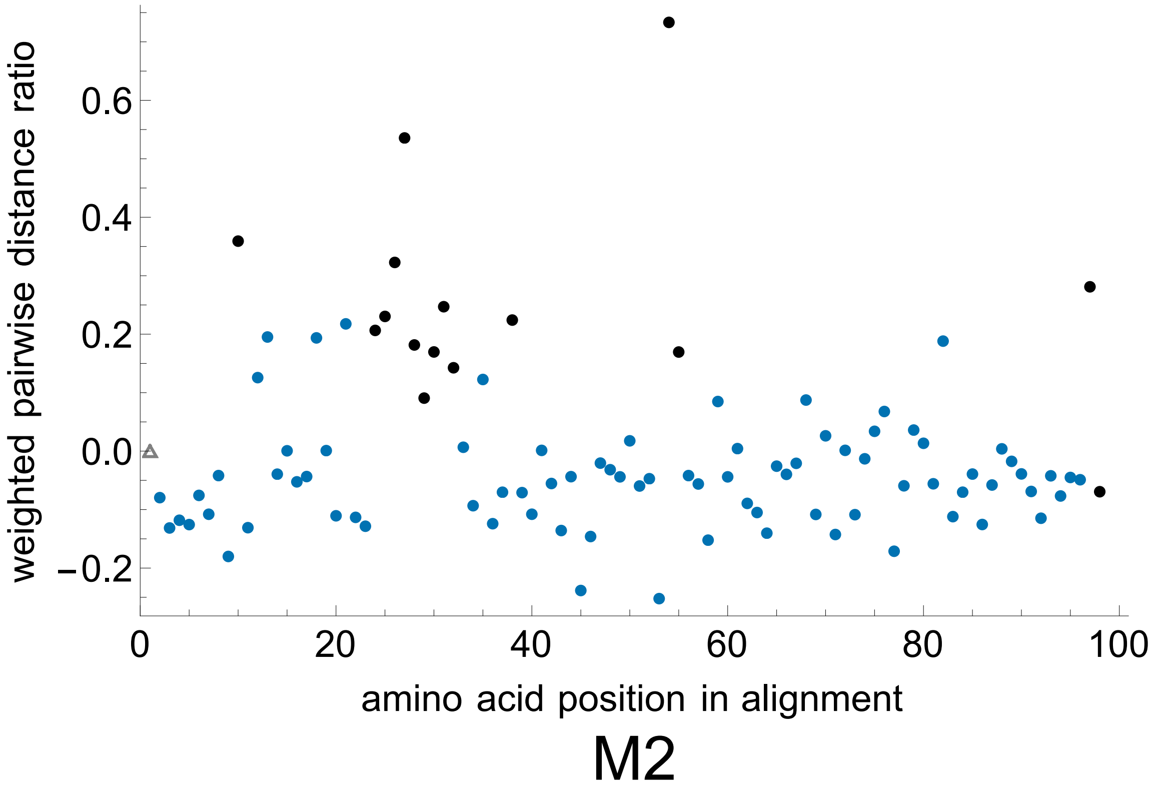

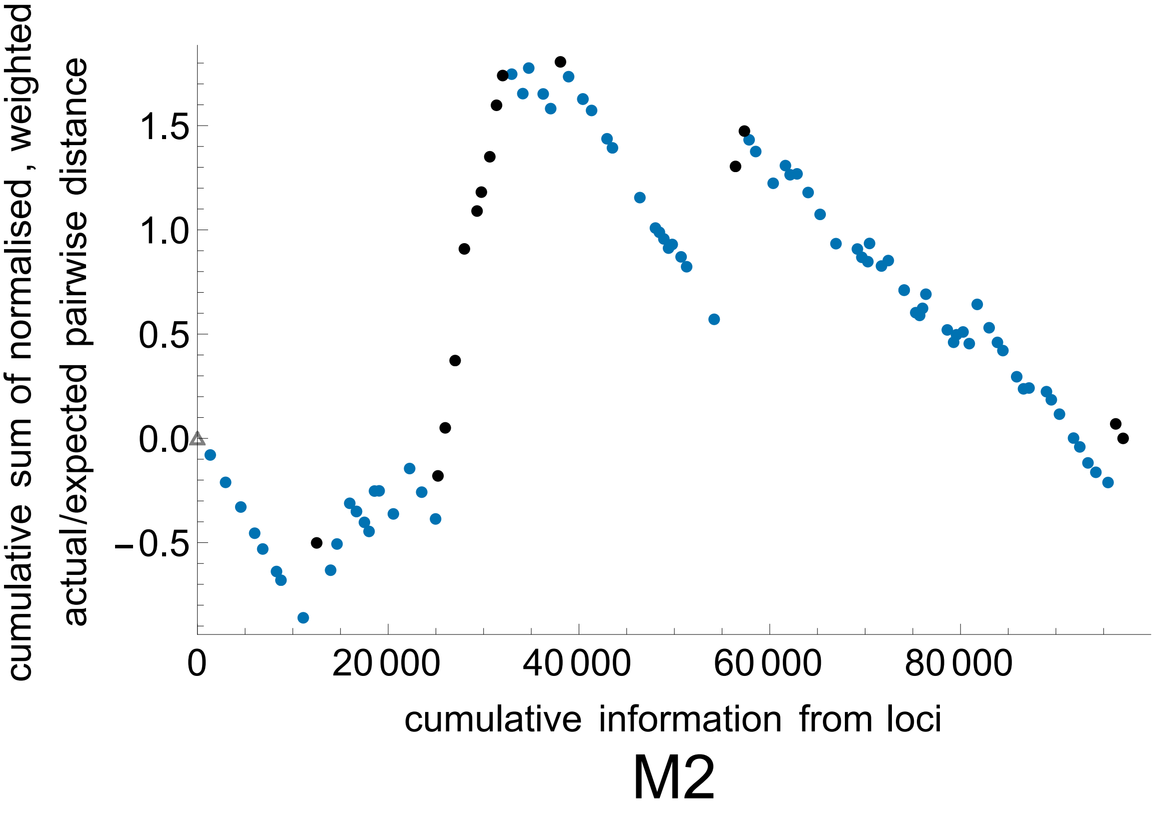

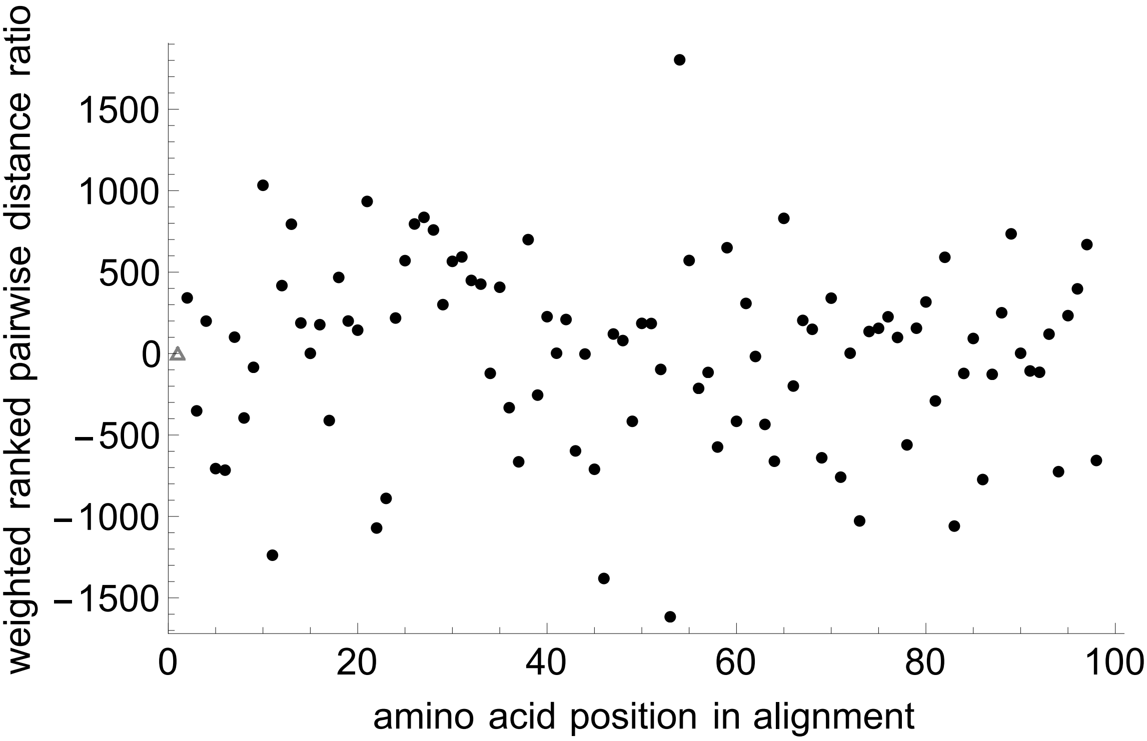
weighted ranked pairwise distance ratio
1500
1000
500
0
−500
−1000
−1500
0
20
40
60
80
100
amino acid position in alignment

M2

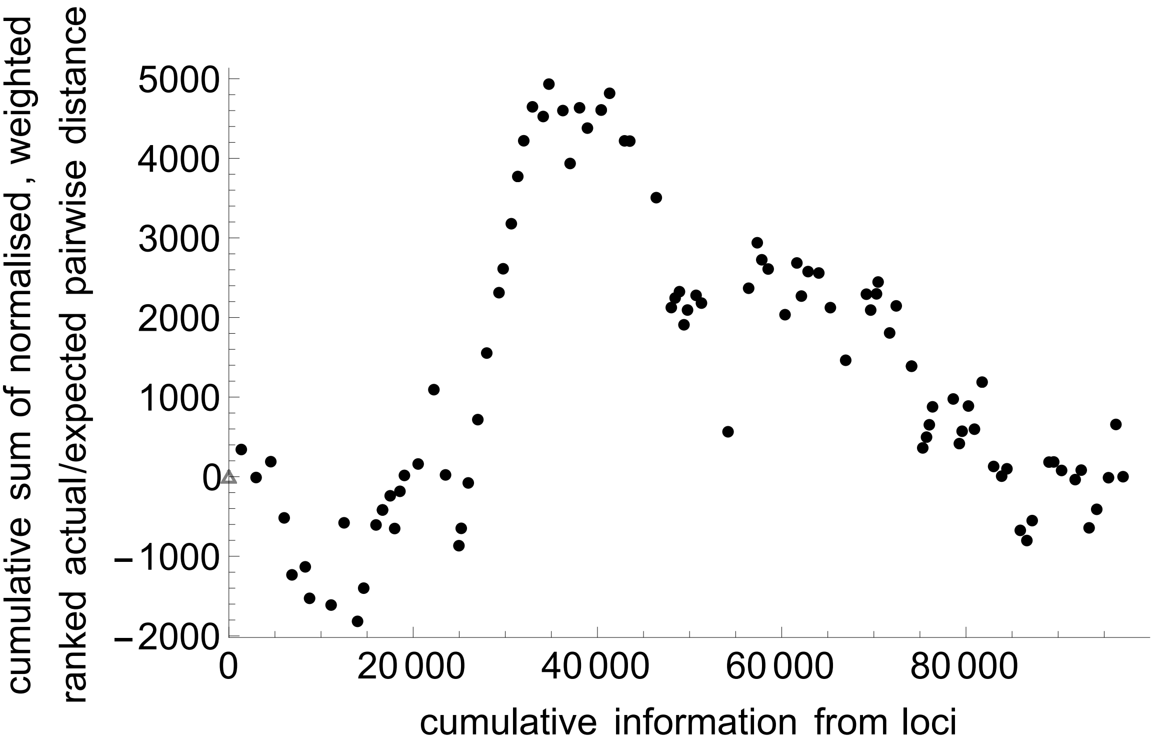
cumulative sum of normalised, weighted
ranked actual/expected pairwise distance
5000
4000
3000
2000
1000
0
−1000
−2000
0
20 000
40 000
60 000
80 000
cumulative information from loci

M2

NS1

Gene length histogram

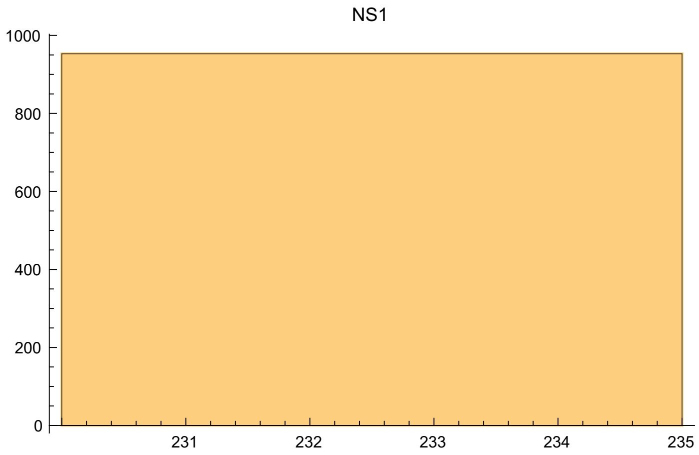

Information vs. nPD

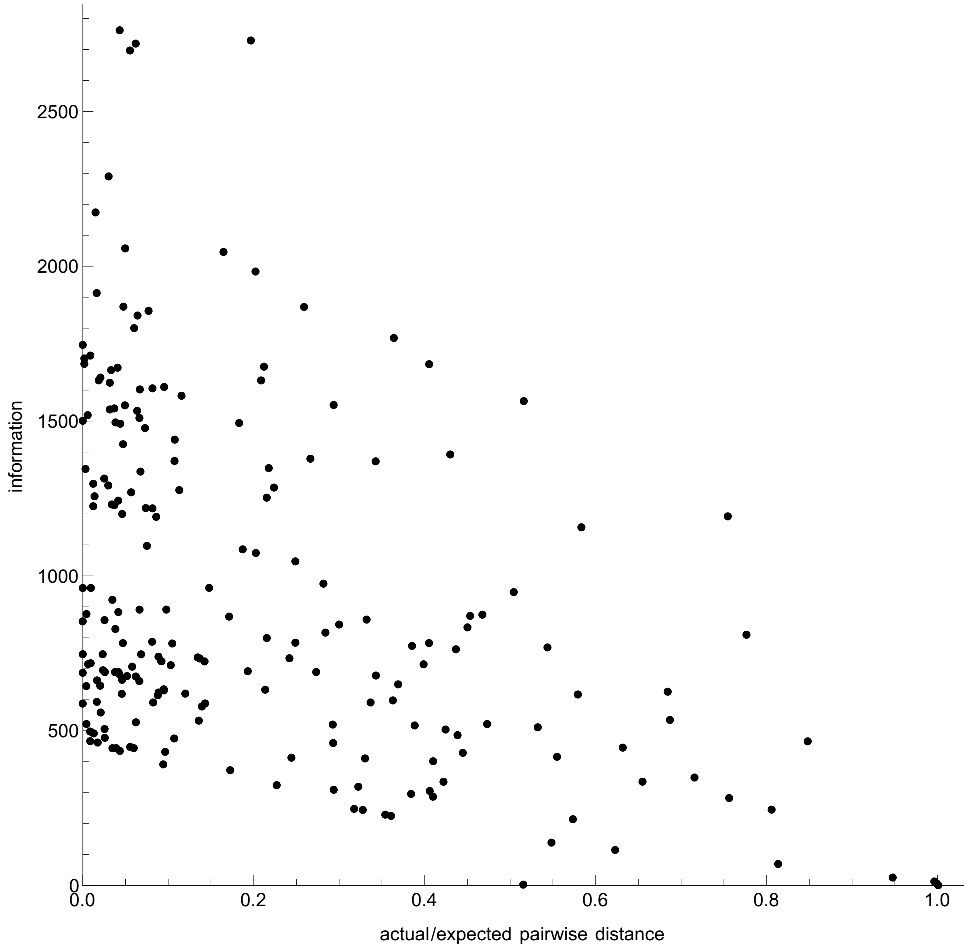

NS1

Example sequences highlighted by regions found in analyses to be conserved

Interesting points (by weighted raw PD) highlighted for gene NS1:

ATG
GATTCCAACACTGTGTCAAGCTTTCAGGTAGACTGCTTTCTTTGGCATGTCCGCA
AACGATTTGCAGACCAAGAACTGGGTGATGCCCCATTCCTTGACCGGCTTCG
CCGAGATCAGAAGTCCCTGAGAGGAAGAGGCAGCACTCTTGGTCTGGACATCGAG⋱
ACAGCCACTCGTGCGGGAAAGCAGATAGTGGAGCGGATTCTGGAGGAGGAATCTGATGAAGCACTTAAAA⋱
TGACTATTGCTTCAGTGCCAGCTTCACGCTACCTAACTGACATGACTCTTGAAGAAATGTCAAGGGACTG⋱
GTTCATGCTCATGCCCAAACAGAAAGTGGCAGGTTCCCTCTGCATCAGAATGGACCAGGCAATAATGGAT⋱
AAAAACATCATATTGAAGGCAAACTTCAGTGTGATTTTTGACCGATTGGAAACCCTAATACTGCTTAGAG⋱
CTTTCACAGAAGAAGGAGCAATTGTGGGAGAAATCTCACCGTTA
CCTTCTCTTCCAGGACATACTGATGAGGATGTCAAAAATGCAATTGGGGTCCTCA
TCGGAGGACTTGAATGGAATGATAACACAGTTCGAGTCTCTGAAACTCTACA
GAGATTCGCTTGGAGAAGCAGTAATGAGGATGGGAGACCTCCACTC --------------------
-------------------CCTCCAAAGCAGAAACGGAAAATGGCGAGAACAATTGAGTCAGAAGTT ------------
---------TGA

Interesting points (by weighted ranked PD) highlighted for gene NS1:

ATGGAT
TCCAACACTGTGTCAAGCTTTCAGGTAGACTGCTTTCTTTGGCATGTCCGCAAA
CGATTTGCAGACCAAGAACTGGGTGATGCCCCATTCCTTGAC
CGGCTTCGCCGAGATCAGAAGTCCCTGAGAGGAAGAGGCAGCACTCTTGGTCTGGA⋱
CATCGAGACAGCCACTCGTGCGGGAAAGCAGATAGTGGAGCGGATTCTGGAGGAGGAATCTGATGAAGCA⋱
CTTAAAATGACTATTGCTTCAGTGCCAGCTTCACGCTACCTAACTGACATGACTCTTGAAGAAATGTCAA⋱
GGGACTGGTTCATGCTCATGCCCAAACAGAAAGTGGCAGGTTCCCTCTGCATCAGAATGGACCAGGCAAT⋱
AATGGATAAAAACATCATATTGAAGGCAAACTTCAGTGTGATTTTTGACCGATTGGAAACCCTAATACTG⋱
CTTAGAGCTTTCACAGAAGAAGGAGCAATTGTGGGAGAAATCTCACCGTTACCT
TCTCTTCCAGGACATACTGATGAGGATGTCAAAAATGCAATTGGGGTCCTCATCG
GAGGACTTGAATGGAATGATAACACAGTTCGAGTCTCTGAAACTCTACAGAG
ATTCGCTTGGAGAAGCAGTAATGAGGATGGGAGACCTCCACTC --------------------------
-------------CCTCCAAAGCAGAAACGGAAAATGGCGAGAACAATTGAGTCAGAAGTT ------------------
---TGA

Per codon conservation report

NS1

Pos. 1		obs:	exp:
atg	M	953	953.00
---	--	------	------
mPD		0	0
		nPD:	1.
		N. weight:	0.
		Sc. PD:	0
		Sc. rank:	0

NS1

Pos. 2		obs:	exp:
aat	N	0	0.51
aac	N	1	0.49
gat	D	939	513.40
gac	D	12	437.60
gaa	E	1	0.60
gag	E	0	0.40
---	--	------	------
mPD		0.031	0.50
		nPD:	0.06
		N. weight:	0.67
		Sc. PD:	-0.05
		Sc. rank:	-46.8

NS1

Pos. 3		obs:	exp:
ttt	F	0	3.55
ttc	F	9	5.45
tct	S	0	151.00
tcc	S	940	122.10
tca	S	3	238.00
tcg	S	0	54.88
cct	P	0	0.25
ccc	P	1	0.18
cca	P	0	0.41
ccg	P	0	0.16
agt	S	0	193.20
agc	S	0	183.90
---	--	------	------
mPD		0.027	1.7
		nPD:	0.02
		N. weight:	2.4
		Sc. PD:	-0.27
		Sc. rank:	-1183.7

NS1

Pos. 4		obs:	exp:
aat	N	587	484.80
aac	N	366	468.20
---	--	------	------
mPD		0.47	0.50
		nPD:	0.95
		N. weight:	0.033
		Sc. PD:	0.022
		Sc. rank:	22.2

NS1

Pos. 5		obs:	exp:
act	T	941	246.90
acc	T	0	194.10
aca	T	3	437.80
acg	T	9	74.24
---	--	------	------
mPD		0.025	0.67
		nPD:	0.04
		N. weight:	1.6
		Sc. PD:	-0.15
		Sc. rank:	-489.2

NS1

Pos. 6		obs:	exp:
att	I	0	3.65
atc	I	0	2.42
ata	I	10	3.93
gtt	V	0	194.50
gtc	V	0	188.70
gta	V	3	195.00
gtg	V	939	363.80
gct	A	0	0.26
gcc	A	0	0.19
gca	A	0	0.47
gcg	A	1	0.09
---	--	------	------
mPD		0.050	0.75
		nPD:	0.07
		N. weight:	1.1
		Sc. PD:	-0.08
		Sc. rank:	-29.0

NS1

Pos. 7		obs:	exp:
tct	S	0	151.00
tcc	S	0	122.10
tca	S	943	238.00
tcg	S	0	54.88
act	T	0	2.59
acc	T	10	2.04
aca	T	0	4.59
acg	T	0	0.78
agt	S	0	193.20
agc	S	0	183.90
---	--	------	------
mPD		0.042	1.7
		nPD:	0.03
		N. weight:	1.7
		Sc. PD:	-0.18
		Sc. rank:	-710.4

NS1

Pos. 8		obs:	exp:
tct	S	0	152.40
tcc	S	0	123.20
tca	S	0	240.30
tcg	S	10	55.40
cgt	R	0	0.04
cgc	R	0	0.05
cga	R	0	0.10
cgg	R	0	0.08
agt	S	4	195.10
agc	S	938	185.60
aga	R	1	0.46
agg	R	0	0.27
---	--	------	------
mPD		0.073	1.7
		nPD:	0.04
		N. weight:	1.9
		Sc. PD:	-0.17
		Sc. rank:	-420.4

NS1

Pos. 9		obs:	exp:
ttt	F	147	375.30
ttc	F	804	575.70
tta	L	1	0.09
ttg	L	0	0.19
tct	S	0	0.16
tcc	S	1	0.13
tca	S	0	0.25
tcg	S	0	0.06
ctt	L	0	0.17
ctc	L	0	0.17
cta	L	0	0.17
ctg	L	0	0.22
agt	S	0	0.20
agc	S	0	0.20
---	--	------	------
mPD		0.27	0.48
		nPD:	0.55
		N. weight:	0.18
		Sc. PD:	0.059
		Sc. rank:	109.7

NS1

Pos. 10		obs:	exp:
caa	Q	0	489.50
cag	Q	953	463.50
---	--	------	------
mPD		0	0.50
		nPD:	0.
		N. weight:	0.87
		Sc. PD:	-0.11
		Sc. rank:	-591.6

NS1

Pos. 11		obs:	exp:
gtt	V	0	196.70
gtc	V	0	190.90
gta	V	953	197.30
gtg	V	0	368.00
---	--	------	------
mPD		0	0.73
		nPD:	0.
		N. weight:	1.9
		Sc. PD:	-0.24
		Sc. rank:	-1292.5

NS1

Pos. 12		obs:	exp:
gat	D	22	513.90
gac	D	930	438.10
gaa	E	1	0.60
gag	E	0	0.40
---	--	------	------
mPD		0.047	0.50
		nPD:	0.09
		N. weight:	0.8
		Sc. PD:	-0.038
		Sc. rank:	85.5

NS1

Pos. 13		obs:	exp:
tgt	C	176	391.20
tgc	C	777	561.80
---	--	------	------
mPD		0.30	0.48
		nPD:	0.62
		N. weight:	0.15
		Sc. PD:	0.058
		Sc. rank:	93.4

NS1

Pos. 14		obs:	exp:
ttt	F	943	372.20
ttc	F	0	570.80
tat	Y	10	5.30
tac	Y	0	4.70
---	--	------	------
mPD		0.021	0.50
		nPD:	0.04
		N. weight:	1.1
		Sc. PD:	-0.1
		Sc. rank:	-295.7

NS1

Pos. 15		obs:	exp:
tta	L	0	83.76
ttg	L	0	177.80
ctt	L	943	161.50
ctc	L	0	159.70
cta	L	5	158.90
ctg	L	5	211.40
---	--	------	------
mPD		0.021	1.1
		nPD:	0.02
		N. weight:	2.1
		Sc. PD:	-0.23
		Sc. rank:	-961.5

NS1

Pos. 16		obs:	exp:
tct	S	0	0.16
tcc	S	0	0.13
tca	S	0	0.25
tcg	S	1	0.06
tgg	W	952	952.00
agt	S	0	0.20
agc	S	0	0.20
---	--	------	------
mPD		0.0021	0.0041
		nPD:	0.52
		N. weight:	0.0036
		Sc. PD:	0.0011
		Sc. rank:	2.2

NS1

Pos. 17		obs:	exp:
tat	Y	0	0.53
tac	Y	1	0.47
cat	H	948	546.90
cac	H	4	405.10
---	--	------	------
mPD		0.013	0.49
		nPD:	0.03
		N. weight:	0.64
		Sc. PD:	-0.067
		Sc. rank:	-267.1

NS1

Pos. 18		obs:	exp:
tta	L	0	0.09
ttg	L	0	0.19
ctt	L	0	0.17
ctc	L	1	0.17
cta	L	0	0.17
ctg	L	0	0.22
att	I	0	4.01
atc	I	1	2.66
ata	I	10	4.33
gtt	V	2	194.30
gtc	V	939	188.50
gta	V	0	194.80
gtg	V	0	363.40
---	--	------	------
mPD		0.050	0.75
		nPD:	0.07
		N. weight:	1.9
		Sc. PD:	-0.14
		Sc. rank:	-60.9

NS1

Pos. 19		obs:	exp:
cgt	R	0	40.71
cgc	R	943	48.42
cga	R	0	95.02
cgg	R	0	79.37
aga	R	4	435.10
agg	R	6	254.40
---	--	------	------
mPD		0.042	0.96
		nPD:	0.04
		N. weight:	3.5
		Sc. PD:	-0.32
		Sc. rank:	-825.2

NS1

Pos. 20		obs:	exp:
aaa	K	940	558.30
aag	K	13	394.70
---	--	------	------
mPD		0.027	0.49
		nPD:	0.06
		N. weight:	0.57
		Sc. PD:	-0.045
		Sc. rank:	-71.5

NS1			
Pos.	21	obs:	exp:
tta	L	0	0.88
ttg	L	0	1.87
ctt	L	0	1.69
ctc	L	0	1.68
cta	L	1	1.67
ctg	L	9	2.22
caa	Q	3	2.57
cag	Q	2	2.43
cgt	R	1	40.07
cgc	R	0	47.66
cga	R	711	93.53
cgg	R	224	78.13
aga	R	2	428.30
agg	R	0	250.30
---	--	------	------
mPD		0.41	1.0
		nPD:	0.41
		N. weight:	2.1
		Sc. PD:	0.46
		Sc. rank:	1157.7

NS1			
Pos.	22	obs:	exp:
ttt	F	939	370.60
ttc	F	0	568.40
tta	L	0	1.23
ttg	L	0	2.61
ctt	L	4	2.37
ctc	L	10	2.35
cta	L	0	2.33
ctg	L	0	3.10
---	--	------	------
mPD		0.050	0.51
		nPD:	0.1
		N. weight:	1.1
		Sc. PD:	-0.05
		Sc. rank:	147.9

NS1			
Pos.	23	obs:	exp:
tct	S	0	1.60
tcc	S	0	1.29
tca	S	0	2.52
tcg	S	0	0.58
agt	S	1	2.05
agc	S	9	1.95
gct	A	1	241.60
gcc	A	0	177.10
gca	A	942	440.90
gcg	A	0	83.41
---	--	------	------
mPD		0.064	0.70
		nPD:	0.09
		N. weight:	0.92
		Sc. PD:	-0.046
		Sc. rank:	86.7

NS1			
Pos.	24	obs:	exp:
atg	M	10	10.00
aat	N	0	0.51
aac	N	1	0.49
gat	D	0	505.30
gac	D	936	430.70
gaa	E	1	2.41
gag	E	3	1.59
ggt	G	0	0.27
ggc	G	2	0.25
gga	G	0	0.92
ggg	G	0	0.55
---	--	------	------
mPD		0.077	0.56
		nPD:	0.14
		N. weight:	0.93
		Sc. PD:	-0.011
		Sc. rank:	187.4

NS1			
Pos.	25	obs:	exp:
cct	P	0	0.25
ccc	P	0	0.18
cca	P	1	0.41
ccg	P	0	0.16
caa	Q	940	483.90
cag	Q	2	458.10
cgt	R	0	0.43
cgc	R	0	0.51
cga	R	0	1.00
cgg	R	0	0.83
aga	R	10	4.57
agg	R	0	2.67
---	--	------	------
mPD		0.048	0.54
		nPD:	0.09
		N. weight:	0.79
		Sc. PD:	-0.041
		Sc. rank:	70.6

NS1			
Pos.	26	obs:	exp:
gat	D	1	6.48
gac	D	11	5.52
gaa	E	930	566.00
gag	E	11	375.00
---	--	------	------
mPD		0.047	0.49
		nPD:	0.1
		N. weight:	0.55
		Sc. PD:	-0.025
		Sc. rank:	68.3

NS1			
Pos.	27	obs:	exp:
tta	L	0	29.09
ttg	L	0	61.77
ctt	L	0	56.09
ctc	L	0	55.45
cta	L	0	55.18
ctg	L	331	73.41
cgt	R	0	0.04
cgc	R	0	0.05
cga	R	0	0.10
cgg	R	0	0.08
atg	M	490	490.00
act	T	0	0.26
acc	T	0	0.20
aca	T	0	0.46
acg	T	1	0.08
aaa	K	0	76.15
aag	K	130	53.85
aga	R	0	0.46
agg	R	1	0.27
---	--	------	------
mPD		0.69	1.2
		nPD:	0.58
		N. weight:	0.78
		Sc. PD:	0.28
		Sc. rank:	495.2

NS1			
Pos.	28	obs:	exp:
tgt	C	10	4.11
tgc	C	0	5.89
ggt	G	937	126.50
ggc	G	6	119.80
gga	G	0	436.00
ggg	G	0	260.70
---	--	------	------
mPD		0.033	0.70
		nPD:	0.05
		N. weight:	2.4
		Sc. PD:	-0.21
		Sc. rank:	-420.0

NS1			
Pos.	29	obs:	exp:
gat	D	948	514.50
gac	D	5	438.50
---	--	------	------
mPD		0.010	0.50
		nPD:	0.02
		N. weight:	0.71
		Sc. PD:	-0.077
		Sc. rank:	-315.5

NS1			
Pos.	30	obs:	exp:
gct	A	18	243.40
gcc	A	929	178.40
gca	A	3	444.20
gcg	A	0	84.03
gat	D	0	1.62
gac	D	3	1.38
---	--	------	------
mPD		0.050	0.68
		nPD:	0.07
		N. weight:	1.9
		Sc. PD:	-0.12
		Sc. rank:	18.5

NS1			
Pos.	31	obs:	exp:
cct	P	0	242.30
ccc	P	14	169.90
cca	P	921	389.30
ccg	P	18	151.40
---	--	------	------
mPD		0.066	0.71
		nPD:	0.09
		N. weight:	0.92
		Sc. PD:	-0.045
		Sc. rank:	91.2

NS1			
Pos.	32	obs:	exp:
ttt	F	802	375.30
ttc	F	149	575.70
tta	L	2	0.18
ttg	L	0	0.37
ctt	L	0	0.34
ctc	L	0	0.34
cta	L	0	0.33
ctg	L	0	0.44
---	--	------	------
mPD		0.27	0.48
		nPD:	0.55
		N. weight:	0.53
		Sc. PD:	0.18
		Sc. rank:	330.7

NS1			
Pos.	33	obs:	exp:
tta	L	1	82.53
ttg	L	0	175.20
ctt	L	84	159.10
ctc	L	11	157.30
cta	L	843	156.60
ctg	L	0	208.30
att	I	0	1.46
atc	I	0	0.97
ata	I	4	1.57
gat	D	10	5.40
gac	D	0	4.60
---	--	------	------
mPD		0.25	1.2
		nPD:	0.22
		N. weight:	1.7
		Sc. PD:	0.096
		Sc. rank:	609.7

NS1			
Pos.	34	obs:	exp:
gat	D	23	514.50
gac	D	930	438.50
---	--	------	------
mPD		0.047	0.50
		nPD:	0.09
		N. weight:	0.8
		Sc. PD:	-0.037
		Sc. rank:	88.5

NS1			
Pos.	35	obs:	exp:
cgt	R	0	40.71
cgc	R	0	48.42
cga	R	1	95.02
cgg	R	942	79.37
aga	R	3	435.10
agg	R	7	254.40
---	--	------	------
mPD		0.029	0.96
		nPD:	0.03
		N. weight:	2.9
		Sc. PD:	-0.29
		Sc. rank:	-1115.6

NS1			
Pos.	36	obs:	exp:
tta	L	0	83.32
ttg	L	0	176.90
ctt	L	936	160.70
ctc	L	10	158.80
cta	L	0	158.10
ctg	L	2	210.20
gtt	V	5	1.03
gtc	V	0	1.00
gta	V	0	1.04
gtg	V	0	1.93
---	--	------	------
mPD		0.035	1.1
		nPD:	0.03
		N. weight:	2.1
		Sc. PD:	-0.21
		Sc. rank:	-768.0

NS1			
Pos.	37	obs:	exp:
tgt	C	0	1.64
tgc	C	4	2.36
cgt	R	6	40.54
cgc	R	933	48.22
cga	R	1	94.63
cgg	R	0	79.04
aga	R	3	433.30
agg	R	6	253.30
---	--	------	------
mPD		0.060	0.97
		nPD:	0.06
		N. weight:	3.4
		Sc. PD:	-0.26
		Sc. rank:	-269.8

NS1			
Pos.	38	obs:	exp:
cgt	R	4	40.71
cgc	R	0	48.42
cga	R	930	95.02
cgg	R	7	79.37
aga	R	12	435.10
agg	R	0	254.40
---	--	------	------
mPD		0.048	0.96
		nPD:	0.05
		N. weight:	2.6
		Sc. PD:	-0.22
		Sc. rank:	-397.4

NS1			
Pos.	39	obs:	exp:
gat	D	927	514.50
gac	D	26	438.50
---	--	------	------
mPD		0.053	0.50
		nPD:	0.11
		N. weight:	0.6
		Sc. PD:	-0.022
		Sc. rank:	87.0

NS1			
Pos.	40	obs:	exp:
caa	Q	20	489.50
cag	Q	933	463.50
---	--	------	------
mPD		0.041	0.50
		nPD:	0.08
		N. weight:	0.75
		Sc. PD:	-0.043
		Sc. rank:	50.6

NS1			
Pos.	41	obs:	exp:
cgt	R	0	0.04
cgc	R	0	0.05
cga	R	0	0.10
cgg	R	0	0.08
aaa	K	20	557.70
aag	K	932	394.30
aga	R	0	0.46
agg	R	1	0.27
---	--	------	------
mPD		0.043	0.49
		nPD:	0.09
		N. weight:	0.94
		Sc. PD:	-0.049
		Sc. rank:	79.7

NS1			
Pos.	42	obs:	exp:
tct	S	28	151.00
tcc	S	914	122.10
tca	S	0	238.00
tcg	S	1	54.88
agt	S	0	193.20
agc	S	0	183.90
gct	A	0	2.56
gcc	A	0	1.88
gca	A	10	4.68
gcg	A	0	0.88
---	--	------	------
mPD		0.10	1.7
		nPD:	0.06
		N. weight:	2.3
		Sc. PD:	-0.17
		Sc. rank:	-221.3

NS1			
Pos.	43	obs:	exp:
tta	L	22	83.76
ttg	L	2	177.80
ctt	L	1	161.50
ctc	L	0	159.70
cta	L	101	158.90
ctg	L	827	211.40
---	--	------	------
mPD		0.28	1.1
		nPD:	0.25
		N. weight:	1.3
		Sc. PD:	0.11
		Sc. rank:	508.3

NS1			
Pos.	44	obs:	exp:
cgt	R	0	39.09
cgc	R	0	46.49
cga	R	0	91.24
cgg	R	0	76.21
aaa	K	28	22.26
aag	K	10	15.74
aga	R	777	417.80
agg	R	138	244.20
---	--	------	------
mPD		0.34	1.0
		nPD:	0.33
		N. weight:	0.52
		Sc. PD:	0.079
		Sc. rank:	240.2

NS1			
Pos.	45	obs:	exp:
cgt	R	0	0.13
cgc	R	0	0.15
cga	R	0	0.30
cgg	R	0	0.25
aga	R	3	1.37
agg	R	0	0.80
ggt	G	0	127.40
ggc	G	0	120.70
gga	G	939	439.20
ggg	G	11	262.70
---	--	------	------
mPD		0.029	0.68
		nPD:	0.04
		N. weight:	0.86
		Sc. PD:	-0.079
		Sc. rank:	-220.5

NS1			
Pos.	46	obs:	exp:
cgt	R	0	40.71
cgc	R	0	48.42
cga	R	2	95.02
cgg	R	0	79.37
aga	R	941	435.10
agg	R	10	254.40
---	--	------	------
mPD		0.025	0.96
		nPD:	0.03
		N. weight:	0.87
		Sc. PD:	-0.092
		Sc. rank:	-357.2

NS1			
Pos.	47	obs:	exp:
tct	S	0	135.60
tcc	S	0	109.60
tca	S	0	213.80
tcg	S	0	49.29
agt	S	2	173.60
agc	S	845	165.20
ggt	G	2	14.21
ggc	G	104	13.47
gga	G	0	49.01
ggg	G	0	29.31
---	--	------	------
mPD		0.21	1.8
		nPD:	0.12
		N. weight:	2.
		Sc. PD:	-0.059
		Sc. rank:	368.0

NS1			
Pos.	48	obs:	exp:
tct	S	0	148.70
tcc	S	0	120.30
tca	S	0	234.50
tcg	S	0	54.06
att	I	0	7.66
atc	I	21	5.08
ata	I	0	8.26
aat	N	0	1.53
aac	N	3	1.47
agt	S	2	190.40
agc	S	927	181.20
---	--	------	------
mPD		0.053	1.7
		nPD:	0.03
		N. weight:	1.9
		Sc. PD:	-0.19
		Sc. rank:	-706.6

NS1			
Pos.	49	obs:	exp:
act	T	938	246.90
acc	T	5	194.10
aca	T	10	437.80
acg	T	0	74.24
---	--	------	------
mPD		0.031	0.67
		nPD:	0.05
		N. weight:	1.5
		Sc. PD:	-0.13
		Sc. rank:	-312.3

NS1			
Pos.	50	obs:	exp:
tta	L	0	83.76
ttg	L	0	177.80
ctt	L	952	161.50
ctc	L	1	159.70
cta	L	0	158.90
ctg	L	0	211.40
---	--	------	------
mPD		0.0021	1.1
		nPD:	0.
		N. weight:	2.1
		Sc. PD:	-0.27
		Sc. rank:	-1311.9

NS1			
Pos.	51	obs:	exp:
tct	S	0	1.60
tcc	S	0	1.29
tca	S	0	2.52
tcg	S	0	0.58
cgt	R	0	0.04
cgc	R	0	0.05
cga	R	0	0.10
cgg	R	0	0.08
agt	S	10	2.05
agc	S	0	1.95
aga	R	1	0.46
agg	R	0	0.27
ggt	G	932	126.30
ggc	G	1	119.70
gga	G	8	435.50
ggg	G	1	260.50
---	--	------	------
mPD		0.046	0.71
		nPD:	0.06
		N. weight:	2.3
		Sc. PD:	-0.17
		Sc. rank:	-109.1

NS1			
Pos.	52	obs:	exp:
tta	L	4	83.67
ttg	L	7	177.70
ctt	L	123	161.30
ctc	L	11	159.50
cta	L	106	158.70
ctg	L	701	211.10
gtt	V	0	0.21
gtc	V	0	0.20
gta	V	1	0.21
gtg	V	0	0.39
---	--	------	------
mPD		0.44	1.1
		nPD:	0.4
		N. weight:	0.9
		Sc. PD:	0.19
		Sc. rank:	475.9

NS1			
Pos.	53	obs:	exp:
aat	N	0	0.51
aac	N	1	0.49
gat	D	9	513.90
gac	D	943	438.10
---	--	------	------
mPD		0.021	0.50
		nPD:	0.04
		N. weight:	0.87
		Sc. PD:	-0.08
		Sc. rank:	-226.7

NS1			
Pos.	54	obs:	exp:
tta	L	0	0.88
ttg	L	0	1.87
ctt	L	0	1.69
ctc	L	0	1.68
cta	L	1	1.67
ctg	L	9	2.22
att	I	1	344.10
atc	I	941	228.10
ata	I	1	370.80
---	--	------	------
mPD		0.046	0.68
		nPD:	0.07
		N. weight:	1.7
		Sc. PD:	-0.12
		Sc. rank:	-9.2

NS1			
Pos.	55	obs:	exp:
cgt	R	0	36.14
cgc	R	0	42.98
cga	R	3	84.36
cgg	R	0	70.46
aaa	K	6	3.51
aag	K	0	2.49
aga	R	842	386.30
agg	R	1	225.80
gaa	E	67	60.75
gag	E	34	40.25
---	--	------	------
mPD		0.47	1.3
		nPD:	0.37
		N. weight:	0.82
		Sc. PD:	0.15
		Sc. rank:	419.7

NS1			
Pos.	56	obs:	exp:
att	I	1	0.36
atc	I	0	0.24
ata	I	0	0.39
act	T	489	213.70
acc	T	206	168.00
aca	T	101	379.00
acg	T	29	64.27
aat	N	5	2.54
aac	N	0	2.46
gtt	V	0	2.06
gtc	V	0	2.00
gta	V	0	2.07
gtg	V	10	3.86
gct	A	112	28.70
gcc	A	0	21.03
gca	A	0	52.37
gcg	A	0	9.91
---	--	------	------
mPD		0.79	0.93
		nPD:	0.85
		N. weight:	0.59
		Sc. PD:	0.35
		Sc. rank:	400.7

NS1			
Pos.	57	obs:	exp:
gct	A	61	244.20
gcc	A	657	178.90
gca	A	235	445.60
gcg	A	0	84.29
---	--	------	------
mPD		0.46	0.67
		nPD:	0.68
		N. weight:	0.79
		Sc. PD:	0.36
		Sc. rank:	517.1

NS1			
Pos.	58	obs:	exp:
act	T	342	246.40
acc	T	5	193.70
aca	T	32	436.90
acg	T	572	74.08
gct	A	0	0.51
gcc	A	0	0.38
gca	A	0	0.94
gcg	A	2	0.18
---	--	------	------
mPD		0.51	0.68
		nPD:	0.75
		N. weight:	1.5
		Sc. PD:	0.77
		Sc. rank:	1006.5

NS1			
Pos.	59	obs:	exp:
tct	S	0	2.56
tcc	S	0	2.07
tca	S	0	4.04
tcg	S	0	0.93
tgt	C	0	2.05
tgc	C	5	2.95
cat	H	37	21.26
cac	H	0	15.74
cgt	R	866	37.76
cgc	R	9	44.91
cga	R	7	88.14
cgg	R	2	73.63
atg	M	10	10.00
agt	S	16	3.28
agc	S	0	3.12
aga	R	0	403.60
agg	R	0	235.90
ggt	G	1	0.13
ggc	G	0	0.13
gga	G	0	0.46
ggg	G	0	0.28
---	--	------	------
mPD		0.23	1.2
		nPD:	0.2
		N. weight:	3.5
		Sc. PD:	0.13
		Sc. rank:	986.6

NS1			
Pos.	60	obs:	exp:
act	T	2	0.52
acc	T	0	0.41
aca	T	0	0.92
acg	T	0	0.16
aaa	K	1	0.59
aag	K	0	0.41
gtt	V	1	0.41
gtc	V	0	0.40
gta	V	1	0.41
gtg	V	0	0.77
gct	A	42	24.86
gcc	A	0	18.21
gca	A	9	45.35
gcg	A	46	8.58
gaa	E	845	510.10
gag	E	3	337.90
ggt	G	0	0.40
ggc	G	0	0.38
gga	G	3	1.39
ggg	G	0	0.83
---	--	------	------
mPD		0.38	0.72
		nPD:	0.53
		N. weight:	0.65
		Sc. PD:	0.21
		Sc. rank:	400.6

NS1			
Pos.	61	obs:	exp:
ggt	G	0	127.80
ggc	G	3	121.10
gga	G	938	440.60
ggg	G	12	263.50
---	--	------	------
mPD		0.031	0.68
		nPD:	0.05
		N. weight:	0.84
		Sc. PD:	-0.074
		Sc. rank:	-179.8

,

NS1			
Pos.	62	obs:	exp:
caa	Q	0	0.51
cag	Q	1	0.49
aaa	K	10	557.70
aag	K	942	394.30
---	--	------	------
mPD		0.023	0.49
		nPD:	0.05
		N. weight:	0.99
		Sc. PD:	-0.087
		Sc. rank:	-198.4

,

NS1			
Pos.	63	obs:	exp:
tta	L	0	0.53
ttg	L	0	1.12
ctt	L	0	1.02
ctc	L	0	1.01
cta	L	5	1.00
ctg	L	1	1.33
cat	H	818	478.00
cac	H	14	354.00
caa	Q	2	48.28
cag	Q	92	45.72
cgt	R	2	0.09
cgc	R	0	0.10
cga	R	0	0.20
cgg	R	0	0.17
aat	N	9	4.58
aac	N	0	4.42
aaa	K	0	5.86
aag	K	10	4.14
aga	R	0	0.91
agg	R	0	0.53
---	--	------	------
mPD		0.29	0.66
		nPD:	0.44
		N. weight:	0.61
		Sc. PD:	0.15
		Sc. rank:	352.9

,

NS1			
Pos.	64	obs:	exp:
att	I	4	347.40
atc	I	7	230.30
ata	I	941	374.40
atg	M	1	1.00
---	--	------	------
mPD		0.025	0.65
		nPD:	0.04
		N. weight:	1.
		Sc. PD:	-0.099
		Sc. rank:	-318.9

,

NS1			
Pos.	65	obs:	exp:
atg	M	13	13.00
gtt	V	12	194.00
gtc	V	0	188.30
gta	V	13	194.60
gtg	V	915	363.00
---	--	------	------
mPD		0.078	0.75
		nPD:	0.1
		N. weight:	0.99
		Sc. PD:	-0.038
		Sc. rank:	139.9

,

NS1			
Pos.	66	obs:	exp:
aaa	K	0	0.59
aag	K	1	0.41
gaa	E	17	572.10
gag	E	934	378.90
ggt	G	0	0.13
ggc	G	0	0.13
gga	G	0	0.46
ggg	G	1	0.28
---	--	------	------
mPD		0.039	0.48
		nPD:	0.08
		N. weight:	1.
		Sc. PD:	-0.058
		Sc. rank:	43.9

,

NS1			
Pos.	67	obs:	exp:
caa	Q	0	1.54
cag	Q	3	1.46
cgt	R	0	40.11
cgc	R	0	47.71
cga	R	231	93.63
cgg	R	663	78.21
aaa	K	0	0.59
aag	K	1	0.41
aga	R	0	428.70
agg	R	45	250.60
gat	D	1	4.86
gac	D	8	4.14
ggt	G	0	0.13
ggc	G	1	0.13
gga	G	0	0.46
ggg	G	0	0.28
---	--	------	------
mPD		0.52	1.0
		nPD:	0.52
		N. weight:	2.
		Sc. PD:	0.61
		Sc. rank:	1219.3

,

NS1			
Pos.	68	obs:	exp:
att	I	942	346.30
atc	I	5	229.50
ata	I	2	373.20
gtt	V	4	0.83
gtc	V	0	0.80
gta	V	0	0.83
gtg	V	0	1.54
---	--	------	------
mPD		0.023	0.66
		nPD:	0.03
		N. weight:	1.2
		Sc. PD:	-0.11
		Sc. rank:	-393.2

,

NS1			
Pos.	69	obs:	exp:
tta	L	610	83.76
ttg	L	233	177.80
ctt	L	0	161.50
ctc	L	0	159.70
cta	L	28	158.90
ctg	L	82	211.40
---	--	------	------
mPD		0.65	1.1
		nPD:	0.58
		N. weight:	1.5
		Sc. PD:	0.53
		Sc. rank:	940.4

,

NS1			
Pos.	70	obs:	exp:
aaa	K	7	8.79
aag	K	8	6.21
gat	D	1	0.54
gac	D	0	0.46
gaa	E	235	562.40
gag	E	700	372.60
ggt	G	0	0.27
ggc	G	0	0.25
gga	G	0	0.92
ggg	G	2	0.55
---	--	------	------
mPD		0.42	0.52
		nPD:	0.81
		N. weight:	0.31
		Sc. PD:	0.17
		Sc. rank:	209.8

,

NS1			
Pos.	71	obs:	exp:
tct	S	0	1.60
tcc	S	0	1.29
tca	S	0	2.52
tcg	S	0	0.58
aaa	K	9	5.27
aag	K	0	3.73
agt	S	10	2.05
agc	S	0	1.95
gat	D	1	4.32
gac	D	7	3.68
gaa	E	907	557.00
gag	E	19	369.00
---	--	------	------
mPD		0.14	0.56
		nPD:	0.24
		N. weight:	0.52
		Sc. PD:	0.041
		Sc. rank:	196.7

,

NS1			
Pos.	72	obs:	exp:
aaa	K	0	0.59
aag	K	1	0.41
gaa	E	185	572.70
gag	E	767	379.30
---	--	------	------
mPD		0.32	0.48
		nPD:	0.65
		N. weight:	0.42
		Sc. PD:	0.18
		Sc. rank:	275.1

,

NS1			
Pos.	73	obs:	exp:
tta	L	1	0.09
ttg	L	0	0.19
tct	S	47	149.50
tcc	S	47	120.90
tca	S	833	235.70
tcg	S	7	54.35
ctt	L	0	0.17
ctc	L	0	0.17
cta	L	0	0.17
ctg	L	0	0.22
cct	P	3	2.03
ccc	P	0	1.43
cca	P	5	3.27
ccg	P	0	1.27
act	T	0	2.59
acc	T	0	2.04
aca	T	7	4.59
acg	T	3	0.78
agt	S	0	191.40
agc	S	0	182.10
---	--	------	------
mPD		0.25	1.7
		nPD:	0.15
		N. weight:	1.2
		Sc. PD:	-0.0028
		Sc. rank:	269.6

,

NS1			
Pos.	74	obs:	exp:
aat	N	0	0.51
aac	N	1	0.49
gat	D	686	513.90
gac	D	266	438.10
---	--	------	------
mPD		0.41	0.50
		nPD:	0.81
		N. weight:	0.088
		Sc. PD:	0.049
		Sc. rank:	59.7

,

NS1			
Pos.	75	obs:	exp:
gaa	E	862	573.30
gag	E	91	379.70
---	--	------	------
mPD		0.17	0.48
		nPD:	0.36
		N. weight:	0.28
		Sc. PD:	0.05
		Sc. rank:	139.3

,

NS1			
Pos.	76	obs:	exp:
tct	S	0	0.32
tcc	S	0	0.26
tca	S	2	0.50
tcg	S	0	0.12
act	T	0	0.52
acc	T	0	0.41
aca	T	2	0.92
acg	T	0	0.16
aat	N	8	5.09
aac	N	2	4.91
agt	S	0	0.41
agc	S	0	0.39
gct	A	0	240.60
gcc	A	0	176.30
gca	A	937	439.00
gcg	A	2	83.05
---	--	------	------
mPD		0.075	0.73
		nPD:	0.1
		N. weight:	0.9
		Sc. PD:	-0.036
		Sc. rank:	122.5

,

NS1			
Pos.	77	obs:	exp:
ttt	F	839	333.10
ttc	F	5	510.90
tta	L	0	9.58
ttg	L	1	20.34
ctt	L	54	18.47
ctc	L	54	18.26
cta	L	0	18.17
ctg	L	0	24.17
---	--	------	------
mPD		0.32	0.70
		nPD:	0.45
		N. weight:	1.1
		Sc. PD:	0.28
		Sc. rank:	648.9

,

NS1			
Pos.	78	obs:	exp:
aaa	K	949	557.70
aag	K	3	394.30
---	-	1	1.00
---	--	------	------
mPD		0.0063	0.48
		nPD:	0.01
		N. weight:	0.62
		Sc. PD:	-0.072
		Sc. rank:	-326.9

,

NS1			
Pos.	79	obs:	exp:
att	I	3	4.38
atc	I	0	2.90
ata	I	9	4.72
atg	M	940	940.00
---	-	1	1.00
---	--	------	------
mPD		0.025	0.025
		nPD:	1.
		N. weight:	0.0076
		Sc. PD:	0.0055
		Sc. rank:	5.2

,

NS1			
Pos.	80	obs:	exp:
tct	S	0	93.48
tcc	S	0	75.59
tca	S	0	147.40
tcg	S	0	33.99
act	T	327	85.49
acc	T	2	67.20
aca	T	1	151.60
acg	T	0	25.71
aat	N	27	13.74
aac	N	0	13.26
agt	S	582	119.70
agc	S	2	113.90
gct	A	1	2.56
gcc	A	6	1.88
gca	A	3	4.68
gcg	A	0	0.88
ggt	G	1	0.13
ggc	G	0	0.13
gga	G	0	0.46
ggg	G	0	0.28
---	-	1	1.00
---	--	------	------
mPD		0.55	1.6
		nPD:	0.34
		N. weight:	1.7
		Sc. PD:	0.28
		Sc. rank:	835.3

,

NS1			
Pos.	81	obs:	exp:
tta	L	0	0.09
ttg	L	0	0.19
ctt	L	1	0.17
ctc	L	0	0.17
cta	L	0	0.17
ctg	L	0	0.22
att	I	950	346.60
atc	I	0	229.80
ata	I	0	373.60
atg	M	1	1.00
gtt	V	1	0.21
gtc	V	0	0.20
gta	V	0	0.21
gtg	V	0	0.39
---	--	------	------
mPD		0.0063	0.66
		nPD:	0.01
		N. weight:	1.2
		Sc. PD:	-0.14
		Sc. rank:	-664.7

,

NS1			
Pos.	82	obs:	exp:
cct	P	1	0.25
ccc	P	0	0.18
cca	P	0	0.41
ccg	P	0	0.16
act	T	0	0.78
acc	T	3	0.61
aca	T	0	1.38
acg	T	0	0.23
gtt	V	0	0.21
gtc	V	1	0.20
gta	V	0	0.21
gtg	V	0	0.39
gct	A	887	242.90
gcc	A	56	178.00
gca	A	5	443.20
gcg	A	0	83.85
---	--	------	------
mPD		0.14	0.68
		nPD:	0.2
		N. weight:	1.4
		Sc. PD:	0.059
		Sc. rank:	416.9

,

NS1			
Pos.	83	obs:	exp:
tct	S	58	152.60
tcc	S	5	123.40
tca	S	890	240.50
tcg	S	0	55.46
agt	S	0	195.30
agc	S	0	185.80
---	--	------	------
mPD		0.12	1.7
		nPD:	0.08
		N. weight:	1.4
		Sc. PD:	-0.088
		Sc. rank:	35.9

,

NS1			
Pos.	84	obs:	exp:
tct	S	0	1.44
tcc	S	0	1.16
tca	S	0	2.27
tcg	S	0	0.52
att	I	0	0.73
atc	I	0	0.48
ata	I	2	0.79
atg	M	13	13.00
act	T	0	0.26
acc	T	0	0.20
aca	T	1	0.46
acg	T	0	0.08
aat	N	0	0.51
aac	N	1	0.49
agt	S	4	1.84
agc	S	5	1.76
gtt	V	1	191.20
gtc	V	3	185.50
gta	V	41	191.70
gtg	V	881	357.60
gct	A	0	0.26
gcc	A	0	0.19
gca	A	0	0.47
gcg	A	1	0.09
---	--	------	------
mPD		0.19	0.80
		nPD:	0.24
		N. weight:	0.93
		Sc. PD:	0.071
		Sc. rank:	347.1

,

NS1			
Pos.	85	obs:	exp:
tct	S	1	0.48
tcc	S	0	0.39
tca	S	2	0.76
tcg	S	0	0.17
cct	P	55	241.60
ccc	P	3	169.40
cca	P	845	388.10
ccg	P	47	150.90
agt	S	0	0.61
agc	S	0	0.59
---	--	------	------
mPD		0.21	0.72
		nPD:	0.29
		N. weight:	0.66
		Sc. PD:	0.079
		Sc. rank:	284.3

,

NS1			
Pos.	86	obs:	exp:
tct	S	19	3.04
tcc	S	0	2.46
tca	S	0	4.79
tcg	S	0	1.11
act	T	62	16.32
acc	T	1	12.83
aca	T	0	28.94
acg	T	0	4.91
agt	S	0	3.89
agc	S	0	3.71
gct	A	861	223.20
gcc	A	10	163.50
gca	A	0	407.20
gcg	A	0	77.04
---	--	------	------
mPD		0.18	0.85
		nPD:	0.22
		N. weight:	1.6
		Sc. PD:	0.086
		Sc. rank:	551.8

,

NS1			
Pos.	87	obs:	exp:
tta	L	0	0.18
ttg	L	0	0.37
tct	S	0	23.21
tcc	S	0	18.77
tca	S	143	36.59
tcg	S	2	8.44
ctt	L	0	0.34
ctc	L	0	0.34
cta	L	2	0.33
ctg	L	0	0.44
cct	P	10	204.90
ccc	P	1	143.70
cca	P	783	329.30
ccg	P	12	128.10
agt	S	0	29.71
agc	S	0	28.28
---	--	------	------
mPD		0.31	1.1
		nPD:	0.28
		N. weight:	1.
		Sc. PD:	0.12
		Sc. rank:	443.0

,

NS1			
Pos.	88	obs:	exp:
tgt	C	0	0.41
tgc	C	1	0.59
cat	H	0	1.15
cac	H	2	0.85
cgt	R	2	40.58
cgc	R	927	48.27
cga	R	12	94.73
cgg	R	9	79.12
aga	R	0	433.80
agg	R	0	253.50
---	--	------	------
mPD		0.054	0.97
		nPD:	0.06
		N. weight:	3.4
		Sc. PD:	-0.27
		Sc. rank:	-441.5

,

NS1			
Pos.	89	obs:	exp:
tat	Y	851	505.30
tac	Y	102	447.70
---	--	------	------
mPD		0.19	0.50
		nPD:	0.38
		N. weight:	0.37
		Sc. PD:	0.074
		Sc. rank:	191.9

,

NS1			
Pos.	90	obs:	exp:
tta	L	3	79.54
ttg	L	0	168.90
ctt	L	0	153.40
ctc	L	0	151.60
cta	L	876	150.90
ctg	L	26	200.70
att	I	1	10.22
atc	I	9	6.77
ata	I	18	11.01
atg	M	20	20.00
---	--	------	------
mPD		0.21	1.2
		nPD:	0.18
		N. weight:	1.9
		Sc. PD:	0.052
		Sc. rank:	481.2

,

NS1

Pos.	91	obs:	exp:
act	T	925	244.00
acc	T	2	191.80
aca	T	13	432.80
acg	T	2	73.38
gtt	V	1	0.21
gtc	V	0	0.20
gta	V	0	0.21
gtg	V	0	0.39
gct	A	10	2.56
gcc	A	0	1.88
gca	A	0	4.68
gcg	A	0	0.88
---	--	------	------
mPD		0.060	0.70
		nPD:	0.09
		N. weight:	1.5
		Sc. PD:	-0.082
		Sc. rank:	114.3

,

NS1

Pos.	92	obs:	exp:
tat	Y	0	0.53
tac	Y	1	0.47
gat	D	12	512.90
gac	D	938	437.10
gaa	E	2	1.20
gag	E	0	0.80
---	--	------	------
mPD		0.031	0.50
		nPD:	0.06
		N. weight:	0.85
		Sc. PD:	-0.064
		Sc. rank:	-63.0

,

NS1

Pos.	93	obs:	exp:
atg	M	953	953.00
---	--	------	------
mPD		0	0
		nPD:	1.
		N. weight:	0.
		Sc. PD:	0
		Sc. rank:	0

,

NS1

Pos.	94	obs:	exp:
tct	S	1	1.60
tcc	S	0	1.29
tca	S	0	2.52
tcg	S	0	0.58
cgt	R	0	0.04
cgc	R	0	0.05
cga	R	0	0.10
cgg	R	0	0.08
act	T	940	244.00
acc	T	2	191.80
aca	T	0	432.80
acg	T	0	73.38
agt	S	4	2.05
agc	S	5	1.95
aga	R	1	0.46
agg	R	0	0.27
---	--	------	------
mPD		0.040	0.70
		nPD:	0.06
		N. weight:	1.6
		Sc. PD:	-0.13
		Sc. rank:	-188.8

,

NS1

Pos.	95	obs:	exp:
tta	L	0	80.59
ttg	L	0	171.10
ctt	L	910	155.40
ctc	L	3	153.60
cta	L	0	152.90
ctg	L	4	203.40
cct	P	1	0.25
ccc	P	0	0.18
cca	P	0	0.41
ccg	P	0	0.16
cat	H	1	0.57
cac	H	0	0.43
att	I	24	10.58
atc	I	0	7.01
ata	I	5	11.40
gtt	V	0	0.83
gtc	V	0	0.80
gta	V	4	0.83
gtg	V	0	1.54
gct	A	0	0.26
gcc	A	0	0.19
gca	A	1	0.47
gcg	A	0	0.09
---	--	------	------
mPD		0.11	1.2
		nPD:	0.1
		N. weight:	2.
		Sc. PD:	-0.095
		Sc. rank:	248.7

,

NS1

Pos.	96	obs:	exp:
gat	D	0	0.54
gac	D	1	0.46
gaa	E	944	572.70
gag	E	8	379.30
---	--	------	------
mPD		0.019	0.48
		nPD:	0.04
		N. weight:	0.56
		Sc. PD:	-0.053
		Sc. rank:	-163.3

,

NS1

Pos.	97	obs:	exp:
gaa	E	871	572.70
gag	E	81	379.30
ggt	G	0	0.13
ggc	G	0	0.13
gga	G	1	0.46
ggg	G	0	0.28
---	--	------	------
mPD		0.16	0.48
		nPD:	0.33
		N. weight:	0.31
		Sc. PD:	0.046
		Sc. rank:	141.9

,

NS1

Pos.	98	obs:	exp:
att	I	0	5.11
atc	I	0	3.39
ata	I	14	5.51
atg	M	939	939.00
---	--	------	------
mPD		0.029	0.029
		nPD:	1.
		N. weight:	0.017
		Sc. PD:	0.012
		Sc. rank:	11.2

,

NS1

Pos.	99	obs:	exp:
tct	S	0	152.60
tcc	S	0	123.40
tca	S	940	240.50
tcg	S	3	55.46
agt	S	2	195.30
agc	S	8	185.80
---	--	------	------
mPD		0.069	1.7
		nPD:	0.04
		N. weight:	1.6
		Sc. PD:	-0.14
		Sc. rank:	-425.8

,

NS1

Pos.	100	obs:	exp:
cgt	R	0	40.71
cgc	R	0	48.42
cga	R	6	95.02
cgg	R	1	79.37
aga	R	888	435.10
agg	R	58	254.40
---	--	------	------
mPD		0.13	0.96
		nPD:	0.14
		N. weight:	0.67
		Sc. PD:	-0.0084
		Sc. rank:	132.7

,

NS1

Pos.	101	obs:	exp:
aat	N	1	0.51
aac	N	0	0.49
gat	D	842	508.50
gac	D	100	433.50
gaa	E	10	6.02
gag	E	0	3.98
---	--	------	------
mPD		0.21	0.51
		nPD:	0.41
		N. weight:	0.36
		Sc. PD:	0.079
		Sc. rank:	198.7

,

NS1

Pos.	102	obs:	exp:
tgg	W	953	953.00
---	--	------	------
mPD		0	0
		nPD:	1.
		N. weight:	0.
		Sc. PD:	0
		Sc. rank:	0

,

NS1

Pos.	103	obs:	exp:
ttt	F	0	39.86
ttc	F	101	61.14
tta	L	794	74.00
ttg	L	43	157.10
tat	Y	6	5.30
tac	Y	4	4.70
ctt	L	0	142.70
ctc	L	0	141.10
cta	L	5	140.40
ctg	L	0	186.70
---	--	------	------
mPD		0.31	1.2
		nPD:	0.26
		N. weight:	2.4
		Sc. PD:	0.22
		Sc. rank:	950.3

,

NS1

Pos.	104	obs:	exp:
att	I	0	0.36
atc	I	0	0.24
ata	I	1	0.39
atg	M	952	952.00
---	--	------	------
mPD		0.0021	0.0021
		nPD:	1.
		N. weight:	0.0012
		Sc. PD:	0.00085
		Sc. rank:	0.8

,

NS1

Pos.	105	obs:	exp:
tta	L	0	83.76
ttg	L	1	177.80
ctt	L	1	161.50
ctc	L	943	159.70
cta	L	1	158.90
ctg	L	7	211.40
---	--	------	------
mPD		0.023	1.1
		nPD:	0.02
		N. weight:	2.1
		Sc. PD:	-0.23
		Sc. rank:	-934.1

,

NS1

Pos.	106	obs:	exp:
att	I	615	305.80
atc	I	0	202.70
ata	I	223	329.50
atg	M	111	111.00
aat	N	4	2.03
aac	N	0	1.97
---	--	------	------
mPD		0.52	0.72
		nPD:	0.72
		N. weight:	0.44
		Sc. PD:	0.21
		Sc. rank:	290.9

,

NS1

Pos.	107	obs:	exp:
cct	P	13	242.10
ccc	P	936	169.70
cca	P	3	388.90
ccg	P	0	151.30
gct	A	0	0.26
gcc	A	1	0.19
gca	A	0	0.47
gcg	A	0	0.09
---	--	------	------
mPD		0.035	0.71
		nPD:	0.05
		N. weight:	2.
		Sc. PD:	-0.17
		Sc. rank:	-327.4

,

NS1

Pos.	108	obs:	exp:
cgt	R	0	0.51
cgc	R	0	0.61
cga	R	0	1.20
cgg	R	0	1.00
aaa	K	881	551.20
aag	K	60	389.80
aga	R	7	5.48
agg	R	5	3.20
---	--	------	------
mPD		0.15	0.52
		nPD:	0.29
		N. weight:	0.39
		Sc. PD:	0.047
		Sc. rank:	175.3

,

NS1

Pos.	109	obs:	exp:
caa	Q	114	489.50
cag	Q	839	463.50
---	--	------	------
mPD		0.21	0.50
		nPD:	0.42
		N. weight:	0.42
		Sc. PD:	0.097
		Sc. rank:	234.2

,

NS1

Pos.	110	obs:	exp:
aaa	K	947	558.30
aag	K	6	394.70
---	--	------	------
mPD		0.013	0.49
		nPD:	0.03
		N. weight:	0.6
		Sc. PD:	-0.063
		Sc. rank:	-250.2

,

NS1			
Pos.	111	obs:	exp:
att	I	1	214.20
atc	I	0	142.00
ata	I	586	230.80
atg	M	26	26.00
act	T	0	0.26
acc	T	0	0.20
aca	T	1	0.46
acg	T	0	0.08
gtt	V	1	69.98
gtc	V	0	67.91
gta	V	15	70.19
gtg	V	323	130.90
---	--	------	------
mPD		0.93	1.2
		nPD:	0.78
		N. weight:	1.
		Sc. PD:	0.54
		Sc. rank:	691.4

NS1			
Pos.	112	obs:	exp:
att	I	0	2.19
atc	I	0	1.45
ata	I	6	2.36
act	T	7	217.60
acc	T	0	171.10
aca	T	831	385.90
acg	T	2	65.43
gtt	V	0	0.41
gtc	V	0	0.40
gta	V	0	0.41
gtg	V	2	0.77
gct	A	2	26.91
gcc	A	0	19.72
gca	A	103	49.09
gcg	A	0	9.29
---	--	------	------
mPD		0.24	0.89
		nPD:	0.27
		N. weight:	0.87
		Sc. PD:	0.09
		Sc. rank:	363.1

NS1			
Pos.	113	obs:	exp:
cgt	R	0	0.04
cgc	R	0	0.05
cga	R	0	0.10
cgg	R	0	0.08
aga	R	0	0.46
agg	R	1	0.27
gaa	E	0	1.20
gag	E	2	0.80
ggt	G	70	127.40
ggc	G	37	120.70
gga	G	8	439.20
ggg	G	835	262.70
---	--	------	------
mPD		0.23	0.68
		nPD:	0.33
		N. weight:	1.1
		Sc. PD:	0.17
		Sc. rank:	509.0

NS1			
Pos.	114	obs:	exp:
tct	S	2	148.20
tcc	S	914	119.90
tca	S	8	233.70
tcg	S	2	53.89
cct	P	5	4.32
ccc	P	12	3.03
cca	P	0	6.95
ccg	P	0	2.70
agt	S	0	189.70
agc	S	0	180.60
ggt	G	9	1.34
ggc	G	1	1.27
gga	G	0	4.62
ggg	G	0	2.76
---	--	------	------
mPD		0.13	1.7
		nPD:	0.08
		N. weight:	2.3
		Sc. PD:	-0.15
		Sc. rank:	90.8

NS1			
Pos.	115	obs:	exp:
tta	L	8	83.76
ttg	L	0	177.80
ctt	L	301	161.50
ctc	L	44	159.70
cta	L	592	158.90
ctg	L	8	211.40
---	--	------	------
mPD		0.52	1.1
		nPD:	0.47
		N. weight:	1.1
		Sc. PD:	0.3
		Sc. rank:	658.5

NS1			
Pos.	116	obs:	exp:
ttt	F	0	2.37
ttc	F	6	3.63
tgt	C	52	384.70
tgc	C	885	552.30
atg	M	10	10.00
---	--	------	------
mPD		0.18	0.55
		nPD:	0.32
		N. weight:	0.4
		Sc. PD:	0.058
		Sc. rank:	184.9

NS1			
Pos.	117	obs:	exp:
att	I	840	344.10
atc	I	103	228.10
ata	I	0	370.80
gtt	V	0	2.06
gtc	V	0	2.00
gta	V	5	2.07
gtg	V	5	3.86
---	--	------	------
mPD		0.23	0.68
		nPD:	0.34
		N. weight:	0.86
		Sc. PD:	0.14
		Sc. rank:	417.5

NS1			
Pos.	118	obs:	exp:
cgt	R	0	39.81
cgc	R	0	47.35
cga	R	0	92.93
cgg	R	0	77.63
att	I	0	0.36
atc	I	0	0.24
ata	I	1	0.39
aaa	K	20	11.72
aag	K	0	8.28
aga	R	925	425.50
agg	R	7	248.70
---	--	------	------
mPD		0.058	1.0
		nPD:	0.06
		N. weight:	0.89
		Sc. PD:	-0.07
		Sc. rank:	-100.7

NS1			
Pos.	119	obs:	exp:
atg	M	953	953.00
---	--	------	------
mPD		0	0
		nPD:	1.
		N. weight:	0.
		Sc. PD:	0
		Sc. rank:	0

NS1			
Pos.	120	obs:	exp:
gat	D	9	514.50
gac	D	944	438.50
---	--	------	------
mPD		0.019	0.50
		nPD:	0.04
		N. weight:	0.87
		Sc. PD:	-0.083
		Sc. rank:	-270.3

NS1			
Pos.	121	obs:	exp:
caa	Q	844	489.50
cag	Q	109	463.50
---	--	------	------
mPD		0.20	0.50
		nPD:	0.41
		N. weight:	0.39
		Sc. PD:	0.083
		Sc. rank:	210.7

NS1			
Pos.	122	obs:	exp:
tct	S	0	0.16
tcc	S	0	0.13
tca	S	1	0.25
tcg	S	0	0.06
act	T	0	0.26
acc	T	0	0.20
aca	T	1	0.46
acg	T	0	0.08
agt	S	0	0.20
agc	S	0	0.20
gct	A	1	243.70
gcc	A	9	178.60
gca	A	905	444.60
gcg	A	36	84.11
---	--	------	------
mPD		0.097	0.68
		nPD:	0.14
		N. weight:	0.74
		Sc. PD:	-0.0047
		Sc. rank:	160.0

NS1			
Pos.	123	obs:	exp:
att	I	40	341.20
atc	I	8	226.20
ata	I	887	367.70
act	T	0	3.11
acc	T	0	2.44
aca	T	12	5.51
acg	T	0	0.93
gtt	V	0	1.24
gtc	V	0	1.20
gta	V	6	1.24
gtg	V	0	2.32
---	--	------	------
mPD		0.13	0.69
		nPD:	0.19
		N. weight:	0.88
		Sc. PD:	0.031
		Sc. rank:	233.7

NS1			
Pos.	124	obs:	exp:
tta	L	0	0.09
ttg	L	1	0.19
ctt	L	0	0.17
ctc	L	0	0.17
cta	L	0	0.17
ctg	L	0	0.22
atg	M	119	119.00
gtt	V	0	171.80
gtc	V	0	166.70
gta	V	2	172.30
gtg	V	830	321.30
gct	A	0	0.26
gcc	A	0	0.19
gca	A	0	0.47
gcg	A	1	0.09
---	--	------	------
mPD		0.23	0.91
		nPD:	0.25
		N. weight:	0.99
		Sc. PD:	0.082
		Sc. rank:	386.1

NS1			
Pos.	125	obs:	exp:
cat	H	0	0.57
cac	H	1	0.43
aat	N	0	1.02
aac	N	2	0.98
gat	D	105	510.70
gac	D	841	435.30
ggt	G	0	0.54
ggc	G	4	0.51
gga	G	0	1.85
ggg	G	0	1.11
---	--	------	------
mPD		0.21	0.51
		nPD:	0.41
		N. weight:	0.51
		Sc. PD:	0.11
		Sc. rank:	279.5

NS1			
Pos.	126	obs:	exp:
cgt	R	0	0.04
cgc	R	0	0.05
cga	R	0	0.10
cgg	R	0	0.08
aaa	K	897	557.10
aag	K	54	393.90
aga	R	1	0.46
agg	R	0	0.27
gaa	E	1	0.60
gag	E	0	0.40
---	--	------	------
mPD		0.11	0.49
		nPD:	0.23
		N. weight:	0.41
		Sc. PD:	0.026
		Sc. rank:	151.2

NS1			
Pos.	127	obs:	exp:
tct	S	0	0.16
tcc	S	0	0.13
tca	S	0	0.25
tcg	S	0	0.06
cgt	R	0	0.38
cgc	R	0	0.46
cga	R	0	0.90
cgg	R	0	0.75
act	T	0	54.14
acc	T	209	42.56
aca	T	0	96.02
acg	T	0	16.28
aat	N	22	372.90
aac	N	711	360.10
aaa	K	1	0.59
aag	K	0	0.41
agt	S	0	0.20
agc	S	1	0.20
aga	R	6	4.11
agg	R	3	2.40
---	--	------	------
mPD		0.42	0.97
		nPD:	0.44
		N. weight:	0.97
		Sc. PD:	0.23
		Sc. rank:	551.1

NS1			
Pos.	128	obs:	exp:
att	I	20	347.40
atc	I	927	230.30
ata	I	5	374.40
act	T	0	0.26
acc	T	1	0.20
aca	T	0	0.46
acg	T	0	0.08
---	--	------	------
mPD		0.053	0.66
		nPD:	0.08
		N. weight:	1.5
		Sc. PD:	-0.09
		Sc. rank:	80.9

NS1			
Pos.	129	obs:	exp:
att	I	1	28.10
atc	I	6	18.62
ata	I	70	30.28
atg	M	11	11.00
act	T	1	224.10
acc	T	40	176.10
aca	T	824	397.40
acg	T	0	67.38
---	--	------	------
mPD		0.29	0.85
		nPD:	0.34
		N. weight:	0.75
		Sc. PD:	0.12
		Sc. rank:	353.4

NS1			
Pos.	130	obs:	exp:
tta	L	4	83.76
ttg	L	904	177.80
ctt	L	1	161.50
ctc	L	9	159.70
cta	L	0	158.90
ctg	L	35	211.40
---	--	------	------
mPD		0.12	1.1
		nPD:	0.11
		N. weight:	1.7
		Sc. PD:	-0.063
		Sc. rank:	267.5

NS1

Pos.	131	obs:	exp:
aaa	K	939	558.30
aag	K	14	394.70
---	--	------	------
mPD		0.029	0.49
		nPD:	0.06
		N. weight:	0.56
		Sc. PD:	-0.043
		Sc. rank:	-61.5

,

NS1

Pos.	132	obs:	exp:
gct	A	0	244.20
gcc	A	2	178.90
gca	A	913	445.60
gcg	A	38	84.29
---	--	------	------
mPD		0.081	0.67
		nPD:	0.12
		N. weight:	0.78
		Sc. PD:	-0.02
		Sc. rank:	147.5

,

NS1

Pos.	133	obs:	exp:
aat	N	788	484.80
aac	N	165	468.20
---	--	------	------
mPD		0.29	0.50
		nPD:	0.57
		N. weight:	0.27
		Sc. PD:	0.096
		Sc. rank:	170.6

,

NS1

Pos.	134	obs:	exp:
ttt	F	2	376.10
ttc	F	951	576.90
---	--	------	------
mPD		0.0042	0.48
		nPD:	0.01
		N. weight:	0.59
		Sc. PD:	-0.07
		Sc. rank:	-336.2

,

NS1

Pos.	135	obs:	exp:
tct	S	8	150.50
tcc	S	0	121.70
tca	S	2	237.20
tcg	S	0	54.70
cgt	R	0	0.04
cgc	R	0	0.05
cga	R	0	0.10
cgg	R	0	0.08
aat	N	12	6.10
aac	N	0	5.90
agt	S	927	192.60
agc	S	3	183.30
aga	R	0	0.46
agg	R	1	0.27
---	--	------	------
mPD		0.079	1.7
		nPD:	0.05
		N. weight:	1.8
		Sc. PD:	-0.16
		Sc. rank:	-343.5

,

NS1

Pos.	136	obs:	exp:
att	I	0	0.73
atc	I	0	0.48
ata	I	2	0.79
atg	M	1	1.00
gtt	V	18	196.10
gtc	V	4	190.30
gta	V	130	196.70
gtg	V	798	366.90
---	--	------	------
mPD		0.28	0.73
		nPD:	0.39
		N. weight:	0.65
		Sc. PD:	0.13
		Sc. rank:	341.1

,

NS1

Pos.	137	obs:	exp:
tta	L	0	0.88
ttg	L	1	1.87
ctt	L	0	1.69
ctc	L	0	1.68
cta	L	9	1.67
ctg	L	0	2.22
att	I	890	341.90
atc	I	46	226.60
ata	I	1	368.50
act	T	1	0.26
acc	T	0	0.20
aca	T	0	0.46
acg	T	0	0.08
gtt	V	4	1.03
gtc	V	1	1.00
gta	V	0	1.04
gtg	V	0	1.93
---	--	------	------
mPD		0.15	0.69
		nPD:	0.22
		N. weight:	1.
		Sc. PD:	0.055
		Sc. rank:	343.3

,

NS1

Pos.	138	obs:	exp:
ttt	F	87	375.70
ttc	F	865	576.30
tta	L	0	0.09
ttg	L	0	0.19
ctt	L	1	0.17
ctc	L	0	0.17
cta	L	0	0.17
ctg	L	0	0.22
---	--	------	------
mPD		0.17	0.48
		nPD:	0.35
		N. weight:	0.29
		Sc. PD:	0.05
		Sc. rank:	141.4

,

NS1

Pos.	139	obs:	exp:
aat	N	836	431.40
aac	N	12	416.60
gat	D	50	53.98
gac	D	50	46.02
ggt	G	1	0.67
ggc	G	3	0.64
gga	G	1	2.31
ggg	G	0	1.38
---	--	------	------
mPD		0.34	0.71
		nPD:	0.47
		N. weight:	0.66
		Sc. PD:	0.18
		Sc. rank:	395.0

,

NS1

Pos.	140	obs:	exp:
caa	Q	9	6.68
cag	Q	4	6.32
cgt	R	0	40.16
cgc	R	0	47.76
cga	R	53	93.73
cgg	R	861	78.29
aga	R	0	429.20
agg	R	26	250.90
---	--	------	------
mPD		0.20	1.0
		nPD:	0.2
		N. weight:	2.5
		Sc. PD:	0.11
		Sc. rank:	751.2

,

NS1

Pos.	141	obs:	exp:
ttt	F	3	1.18
ttc	F	0	1.82
tta	L	7	80.68
ttg	L	30	171.30
ctt	L	565	155.60
ctc	L	7	153.80
cta	L	11	153.00
ctg	L	298	203.60
att	I	30	10.95
atc	I	0	7.26
ata	I	0	11.80
atg	M	1	1.00
gtt	V	1	0.21
gtc	V	0	0.20
gta	V	0	0.21
gtg	V	0	0.39
---	--	------	------
mPD		0.63	1.2
		nPD:	0.54
		N. weight:	0.97
		Sc. PD:	0.32
		Sc. rank:	607.9

,

NS1

Pos.	142	obs:	exp:
gaa	E	874	573.30
gag	E	79	379.70
---	--	------	------
mPD		0.15	0.48
		nPD:	0.32
		N. weight:	0.31
		Sc. PD:	0.044
		Sc. rank:	142.8

,

NS1

Pos.	143	obs:	exp:
tct	S	0	1.76
tcc	S	11	1.42
tca	S	0	2.78
tcg	S	0	0.64
act	T	29	31.09
acc	T	44	24.44
aca	T	40	55.13
acg	T	7	9.35
agt	S	0	2.25
agc	S	0	2.15
gct	A	2	210.60
gcc	A	819	154.30
gca	A	1	384.30
gcg	A	0	72.70
---	--	------	------
mPD		0.40	0.92
		nPD:	0.43
		N. weight:	1.8
		Sc. PD:	0.41
		Sc. rank:	996.4

,

NS1

Pos.	144	obs:	exp:
tta	L	4	83.67
ttg	L	5	177.70
ctt	L	5	161.30
ctc	L	1	159.50
cta	L	357	158.70
ctg	L	580	211.10
att	I	0	0.36
atc	I	0	0.24
ata	I	1	0.39
---	--	------	------
mPD		0.50	1.1
		nPD:	0.45
		N. weight:	1.1
		Sc. PD:	0.27
		Sc. rank:	615.0

,

NS1

Pos.	145	obs:	exp:
att	I	1	329.10
atc	I	2	218.20
ata	I	899	354.70
act	T	0	6.48
acc	T	0	5.09
aca	T	25	11.49
acg	T	0	1.95
gtt	V	0	5.37
gtc	V	10	5.21
gta	V	16	5.38
gtg	V	0	10.04
---	--	------	------
mPD		0.13	0.77
		nPD:	0.17
		N. weight:	1.1
		Sc. PD:	0.019
		Sc. rank:	265.6

,

NS1

Pos.	146	obs:	exp:
tta	L	3	82.88
ttg	L	0	176.00
tct	S	7	1.60
tcc	S	0	1.29
tca	S	3	2.52
tcg	S	0	0.58
ctt	L	0	159.80
ctc	L	0	158.00
cta	L	925	157.20
ctg	L	15	209.10
agt	S	0	2.05
agc	S	0	1.95
---	--	------	------
mPD		0.093	1.1
		nPD:	0.08
		N. weight:	2.
		Sc. PD:	-0.12
		Sc. rank:	129.2

,

NS1

Pos.	147	obs:	exp:
tta	L	0	83.67
ttg	L	0	177.70
ctt	L	893	161.30
ctc	L	47	159.50
cta	L	1	158.70
ctg	L	11	211.10
att	I	1	0.36
atc	I	0	0.24
ata	I	0	0.39
---	--	------	------
mPD		0.12	1.1
		nPD:	0.11
		N. weight:	1.8
		Sc. PD:	-0.066
		Sc. rank:	299.1

,

NS1

Pos.	148	obs:	exp:
cgt	R	0	40.71
cgc	R	0	48.42
cga	R	0	95.02
cgg	R	0	79.37
aga	R	942	435.10
agg	R	11	254.40
---	--	------	------
mPD		0.023	0.96
		nPD:	0.02
		N. weight:	0.88
		Sc. PD:	-0.094
		Sc. rank:	-383.7

,

NS1

Pos.	149	obs:	exp:
gct	A	942	244.20
gcc	A	2	178.90
gca	A	7	445.60
gcg	A	2	84.29
---	--	------	------
mPD		0.023	0.67
		nPD:	0.03
		N. weight:	1.6
		Sc. PD:	-0.15
		Sc. rank:	-534.5

,

NS1

Pos.	150	obs:	exp:
ttt	F	844	376.10
ttc	F	109	576.90
---	--	------	------
mPD		0.20	0.48
		nPD:	0.42
		N. weight:	0.64
		Sc. PD:	0.15
		Sc. rank:	354.4

,

NS1			
Pos.	151	obs:	exp:
cct	P	0	0.25
ccc	P	0	0.18
cca	P	0	0.41
ccg	P	1	0.16
act	T	44	246.40
acc	T	1	193.70
aca	T	84	436.90
acg	T	822	74.08
aaa	K	0	0.59
aag	K	1	0.41
---	--	------	------
mPD		0.25	0.68
		nPD:	0.36
		N. weight:	2.2
		Sc. PD:	0.4
		Sc. rank:	1132.2

NS1			
Pos.	152	obs:	exp:
aaa	K	2	1.17
aag	K	0	0.83
gat	D	233	130.60
gac	D	9	111.40
gaa	E	656	400.00
gag	E	9	265.00
ggt	G	0	5.90
ggc	G	0	5.59
gga	G	44	20.34
ggg	G	0	12.17
---	--	------	------
mPD		0.49	0.78
		nPD:	0.63
		N. weight:	0.56
		Sc. PD:	0.23
		Sc. rank:	364.1

NS1			
Pos.	153	obs:	exp:
gat	D	11	6.48
gac	D	1	5.52
gaa	E	912	565.40
gag	E	28	374.60
ggt	G	0	0.13
ggc	G	0	0.13
gga	G	0	0.46
ggg	G	1	0.28
---	--	------	------
mPD		0.085	0.49
		nPD:	0.17
		N. weight:	0.47
		Sc. PD:	0.0087
		Sc. rank:	115.1

NS1			
Pos.	154	obs:	exp:
gaa	E	1	0.60
gag	E	0	0.40
ggt	G	7	127.70
ggc	G	1	121.00
gga	G	924	440.20
ggg	G	20	263.20
---	--	------	------
mPD		0.060	0.68
		nPD:	0.09
		N. weight:	0.78
		Sc. PD:	-0.041
		Sc. rank:	62.3

NS1			
Pos.	155	obs:	exp:
act	T	0	2.07
acc	T	0	1.63
aca	T	8	3.68
acg	T	0	0.62
gtt	V	0	0.21
gtc	V	0	0.20
gta	V	1	0.21
gtg	V	0	0.39
gct	A	7	241.90
gcc	A	5	177.30
gca	A	931	441.40
gcg	A	1	83.49
---	--	------	------
mPD		0.046	0.69
		nPD:	0.07
		N. weight:	0.84
		Sc. PD:	-0.059
		Sc. rank:	-35.3

NS1			
Pos.	156	obs:	exp:
tta	L	0	0.09
ttg	L	0	0.19
ctt	L	1	0.17
ctc	L	0	0.17
cta	L	0	0.17
ctg	L	0	0.22
att	I	540	343.30
atc	I	3	227.60
ata	I	398	370.10
gtt	V	10	2.27
gtc	V	0	2.20
gta	V	1	2.28
gtg	V	0	4.25
---	--	------	------
mPD		0.52	0.68
		nPD:	0.76
		N. weight:	0.36
		Sc. PD:	0.18
		Sc. rank:	239.1

NS1			
Pos.	157	obs:	exp:
att	I	0	0.36
atc	I	0	0.24
ata	I	1	0.39
gtt	V	8	196.30
gtc	V	42	190.50
gta	V	604	196.90
gtg	V	297	367.30
gct	A	0	0.26
gcc	A	0	0.19
gca	A	1	0.47
gcg	A	0	0.09
---	--	------	------
mPD		0.50	0.73
		nPD:	0.69
		N. weight:	0.68
		Sc. PD:	0.31
		Sc. rank:	444.3

NS1			
Pos.	158	obs:	exp:
gct	A	10	2.56
gcc	A	0	1.88
gca	A	0	4.68
gcg	A	0	0.88
ggt	G	3	126.50
ggc	G	889	119.80
gga	G	50	436.00
ggg	G	1	260.70
---	--	------	------
mPD		0.15	0.70
		nPD:	0.21
		N. weight:	2.1
		Sc. PD:	0.11
		Sc. rank:	698.8

NS1			
Pos.	159	obs:	exp:
gaa	E	945	573.30
gag	E	8	379.70
---	--	------	------
mPD		0.017	0.48
		nPD:	0.03
		N. weight:	0.56
		Sc. PD:	-0.055
		Sc. rank:	-187.3

NS1			
Pos.	160	obs:	exp:
att	I	55	347.40
atc	I	891	230.30
ata	I	6	374.40
gtt	V	0	0.21
gtc	V	1	0.20
gta	V	0	0.21
gtg	V	0	0.39
---	--	------	------
mPD		0.12	0.66
		nPD:	0.19
		N. weight:	1.4
		Sc. PD:	0.042
		Sc. rank:	360.1

NS1			
Pos.	161	obs:	exp:
tct	S	10	152.60
tcc	S	1	123.40
tca	S	942	240.50
tcg	S	0	55.46
agt	S	0	195.30
agc	S	0	185.80
---	--	------	------
mPD		0.023	1.7
		nPD:	0.01
		N. weight:	1.6
		Sc. PD:	-0.18
		Sc. rank:	-822.5

NS1			
Pos.	162	obs:	exp:
cct	P	0	242.10
ccc	P	10	169.70
cca	P	875	388.90
ccg	P	67	151.30
act	T	0	0.26
acc	T	0	0.20
aca	T	1	0.46
acg	T	0	0.08
---	--	------	------
mPD		0.15	0.71
		nPD:	0.21
		N. weight:	0.8
		Sc. PD:	0.042
		Sc. rank:	267.3

NS1			
Pos.	163	obs:	exp:
tta	L	896	82.88
ttg	L	21	176.00
ctt	L	0	159.80
ctc	L	0	158.00
cta	L	1	157.20
ctg	L	25	209.10
att	I	10	3.65
atc	I	0	2.42
ata	I	0	3.93
---	--	------	------
mPD		0.19	1.1
		nPD:	0.16
		N. weight:	2.6
		Sc. PD:	0.031
		Sc. rank:	610.4

NS1			
Pos.	164	obs:	exp:
tta	L	0	0.09
ttg	L	0	0.19
tct	S	4	0.64
tcc	S	0	0.52
tca	S	0	1.01
tcg	S	0	0.23
ctt	L	1	0.17
ctc	L	0	0.17
cta	L	0	0.17
ctg	L	0	0.22
cct	P	927	240.80
ccc	P	20	168.90
cca	P	0	386.90
ccg	P	0	150.50
cat	H	1	0.57
cac	H	0	0.43
agt	S	0	0.82
agc	S	0	0.78
---	--	------	------
mPD		0.054	0.73
		nPD:	0.07
		N. weight:	1.5
		Sc. PD:	-0.1
		Sc. rank:	28.2

NS1			
Pos.	165	obs:	exp:
tct	S	946	152.40
tcc	S	6	123.20
tca	S	0	240.30
tcg	S	0	55.40
tat	Y	1	0.53
tac	Y	0	0.47
agt	S	0	195.10
agc	S	0	185.60
---	--	------	------
mPD		0.015	1.7
		nPD:	0.01
		N. weight:	2.2
		Sc. PD:	-0.26
		Sc. rank:	-1208.7

NS1			
Pos.	166	obs:	exp:
ttt	F	2	0.79
ttc	F	0	1.21
tta	L	0	82.70
ttg	L	0	175.60
ctt	L	941	159.50
ctc	L	0	157.70
cta	L	0	156.90
ctg	L	0	208.70
atg	M	10	10.00
---	--	------	------
mPD		0.046	1.1
		nPD:	0.04
		N. weight:	2.1
		Sc. PD:	-0.2
		Sc. rank:	-591.0

NS1			
Pos.	167	obs:	exp:
cct	P	0	242.30
ccc	P	0	169.90
cca	P	953	389.30
ccg	P	0	151.40
---	--	------	------
mPD		0	0.71
		nPD:	0.
		N. weight:	1.1
		Sc. PD:	-0.14
		Sc. rank:	-734.7

NS1			
Pos.	168	obs:	exp:
ggt	G	0	127.80
ggc	G	0	121.10
gga	G	950	440.60
ggg	G	3	263.50
---	--	------	------
mPD		0.0063	0.68
		nPD:	0.01
		N. weight:	0.91
		Sc. PD:	-0.11
		Sc. rank:	-502.5

NS1			
Pos.	169	obs:	exp:
cat	H	952	547.50
cac	H	1	405.50
---	--	------	------
mPD		0.0021	0.49
		nPD:	0.
		N. weight:	0.66
		Sc. PD:	-0.081
		Sc. rank:	-394.8

NS1			
Pos.	170	obs:	exp:
tct	S	10	1.60
tcc	S	0	1.29
tca	S	0	2.52
tcg	S	0	0.58
act	T	943	244.30
acc	T	0	192.00
aca	T	0	433.20
acg	T	0	73.46
agt	S	0	2.05
agc	S	0	1.95
---	--	------	------
mPD		0.021	0.70
		nPD:	0.03
		N. weight:	1.6
		Sc. PD:	-0.17
		Sc. rank:	-655.2

NS1

Pos.	171	obs:	exp:
act	T	0	2.59
acc	T	0	2.04
aca	T	10	4.59
acg	T	0	0.78
aat	N	1	0.51
aac	N	0	0.49
gat	D	93	506.90
gac	D	846	432.10
ggt	G	0	0.40
ggc	G	3	0.38
gga	G	0	1.39
ggg	G	0	0.83
---	--	------	------
mPD		0.25	0.55
		nPD:	0.44
		N. weight:	0.54
		Sc. PD:	0.13
		Sc. rank:	313.0

NS1

Pos.	172	obs:	exp:
caa	Q	0	3.60
cag	Q	7	3.40
aaa	K	0	493.20
aag	K	842	348.80
gaa	E	0	62.56
gag	E	104	41.44
---	--	------	------
mPD		0.21	0.69
		nPD:	0.3
		N. weight:	1.1
		Sc. PD:	0.13
		Sc. rank:	484.2

NS1

Pos.	173	obs:	exp:
gat	D	953	514.50
gac	D	0	438.50
---	--	------	------
mPD		0	0.50
		nPD:	0.
		N. weight:	0.74
		Sc. PD:	-0.094
		Sc. rank:	-506.0

NS1

Pos.	174	obs:	exp:
gtt	V	2	196.70
gtc	V	951	190.90
gta	V	0	197.30
gtg	V	0	368.00
---	--	------	------
mPD		0.0042	0.73
		nPD:	0.01
		N. weight:	1.9
		Sc. PD:	-0.23
		Sc. rank:	-1117.8

NS1

Pos.	175	obs:	exp:
aaa	K	951	558.30
aag	K	2	394.70
---	--	------	------
mPD		0.0042	0.49
		nPD:	0.01
		N. weight:	0.63
		Sc. PD:	-0.075
		Sc. rank:	-360.7

NS1

Pos.	176	obs:	exp:
att	I	4	1.46
atc	I	0	0.97
ata	I	0	1.57
aat	N	948	482.30
aac	N	0	465.70
gat	D	1	0.54
gac	D	0	0.46
---	--	------	------
mPD		0.010	0.51
		nPD:	0.02
		N. weight:	0.82
		Sc. PD:	-0.09
		Sc. rank:	-376.7

NS1

Pos.	177	obs:	exp:
gct	A	0	244.20
gcc	A	0	178.90
gca	A	951	445.60
gcg	A	2	84.29
---	--	------	------
mPD		0.0042	0.67
		nPD:	0.01
		N. weight:	0.9
		Sc. PD:	-0.11
		Sc. rank:	-521.5

NS1

Pos.	178	obs:	exp:
att	I	953	347.70
atc	I	0	230.50
ata	I	0	374.80
---	--	------	------
mPD		0	0.65
		nPD:	0.
		N. weight:	1.2
		Sc. PD:	-0.15
		Sc. rank:	-827.5

NS1

Pos.	179	obs:	exp:
gaa	E	21	503.50
gag	E	816	333.50
ggt	G	0	15.56
ggc	G	1	14.74
gga	G	10	53.63
ggg	G	105	32.07
---	--	------	------
mPD		0.28	0.72
		nPD:	0.39
		N. weight:	0.98
		Sc. PD:	0.19
		Sc. rank:	507.1

NS1

Pos.	180	obs:	exp:
att	I	0	297.70
atc	I	816	197.40
ata	I	0	320.90
act	T	0	1.55
acc	T	6	1.22
aca	T	0	2.76
acg	T	0	0.47
gtt	V	0	27.04
gtc	V	131	26.24
gta	V	0	27.12
gtg	V	0	50.59
---	--	------	------
mPD		0.25	0.94
		nPD:	0.27
		N. weight:	1.7
		Sc. PD:	0.17
		Sc. rank:	717.7

NS1

Pos.	181	obs:	exp:
tta	L	0	83.67
ttg	L	0	177.70
ctt	L	0	161.30
ctc	L	952	159.50
cta	L	0	158.70
ctg	L	0	211.10
att	I	0	0.36
atc	I	1	0.24
ata	I	0	0.39
---	--	------	------
mPD		0.0021	1.1
		nPD:	0.
		N. weight:	2.2
		Sc. PD:	-0.27
		Sc. rank:	-1350.5

NS1

Pos.	182	obs:	exp:
att	I	1	347.70
atc	I	952	230.50
ata	I	0	374.80
---	--	------	------
mPD		0.0021	0.65
		nPD:	0.
		N. weight:	1.7
		Sc. PD:	-0.21
		Sc. rank:	-1031.8

NS1

Pos.	183	obs:	exp:
cgt	R	0	0.09
cgc	R	0	0.10
cga	R	0	0.20
cgg	R	0	0.17
aga	R	2	0.91
agg	R	0	0.53
gaa	E	2	1.20
gag	E	0	0.80
ggt	G	10	127.30
ggc	G	0	120.60
gga	G	936	438.80
ggg	G	3	262.40
---	--	------	------
mPD		0.035	0.68
		nPD:	0.05
		N. weight:	0.86
		Sc. PD:	-0.071
		Sc. rank:	-126.7

NS1

Pos.	184	obs:	exp:
ggt	G	0	127.80
ggc	G	0	121.10
gga	G	847	440.60
ggg	G	106	263.50
---	--	------	------
mPD		0.20	0.68
		nPD:	0.29
		N. weight:	0.58
		Sc. PD:	0.07
		Sc. rank:	253.5

NS1

Pos.	185	obs:	exp:
ttt	F	848	334.70
ttc	F	0	513.30
tta	L	0	9.23
ttg	L	0	19.59
ctt	L	105	17.79
ctc	L	0	17.59
cta	L	0	17.51
ctg	L	0	23.29
---	--	------	------
mPD		0.20	0.70
		nPD:	0.28
		N. weight:	1.2
		Sc. PD:	0.14
		Sc. rank:	521.7

NS1

Pos.	186	obs:	exp:
gaa	E	949	573.30
gag	E	4	379.70
---	--	------	------
mPD		0.0084	0.48
		nPD:	0.02
		N. weight:	0.58
		Sc. PD:	-0.066
		Sc. rank:	-278.7

NS1

Pos.	187	obs:	exp:
tgg	W	953	953.00
---	--	------	------
mPD		0	0
		nPD:	1.
		N. weight:	0.
		Sc. PD:	0
		Sc. rank:	0

NS1

Pos.	188	obs:	exp:
aat	N	952	484.30
aac	N	0	467.70
gat	D	1	0.54
gac	D	0	0.46
---	--	------	------
mPD		0.0021	0.50
		nPD:	0.
		N. weight:	0.82
		Sc. PD:	-0.1
		Sc. rank:	-490.3

NS1

Pos.	189	obs:	exp:
gat	D	949	512.30
gac	D	0	436.70
ggt	G	4	0.54
ggc	G	0	0.51
gga	G	0	1.85
ggg	G	0	1.11
---	--	------	------
mPD		0.0084	0.51
		nPD:	0.02
		N. weight:	0.75
		Sc. PD:	-0.085
		Sc. rank:	-363.8

NS1

Pos.	190	obs:	exp:
aat	N	11	484.80
aac	N	942	468.20
---	--	------	------
mPD		0.023	0.50
		nPD:	0.05
		N. weight:	0.78
		Sc. PD:	-0.069
		Sc. rank:	-171.2

NS1

Pos.	191	obs:	exp:
tct	S	0	1.60
tcc	S	0	1.29
tca	S	10	2.52
tcg	S	0	0.58
act	T	0	233.40
acc	T	0	183.50
aca	T	898	413.90
acg	T	3	70.19
aaa	K	42	24.60
aag	K	0	17.40
agt	S	0	2.05
agc	S	0	1.95
---	--	------	------
mPD		0.11	0.78
		nPD:	0.14
		N. weight:	0.92
		Sc. PD:	-0.0063
		Sc. rank:	193.2

NS1

Pos.	192	obs:	exp:
att	I	14	5.11
atc	I	0	3.39
ata	I	0	5.51
gtt	V	939	193.80
gtc	V	0	188.10
gta	V	0	194.40
gtg	V	0	362.60
---	--	------	------
mPD		0.029	0.76
		nPD:	0.04
		N. weight:	1.9
		Sc. PD:	-0.18
		Sc. rank:	-556.2

NS1

Pos.	193	obs:	exp:
caa	Q	5	2.57
cag	Q	0	2.43
cgt	R	0	40.50
cgc	R	0	48.17
cga	R	946	94.53
cgg	R	1	78.96
aga	R	1	432.80
agg	R	0	253.00
---	--	------	------
mPD		0.015	0.98
		nPD:	0.01
		N. weight:	2.8
		Sc. PD:	-0.31
		Sc. rank:	-1381.1

NS1

Pos.	194	obs:	exp:
att	I	0	0.73
atc	I	2	0.48
ata	I	0	0.79
gtt	V	1	194.30
gtc	V	940	188.50
gta	V	0	194.80
gtg	V	0	363.40
gct	A	0	2.56
gcc	A	0	1.88
gca	A	0	4.68
gcg	A	10	0.88
---	--	------	------
mPD		0.048	0.75
		nPD:	0.06
		N. weight:	1.9
		Sc. PD:	-0.14
		Sc. rank:	-115.5

NS1

Pos.	195	obs:	exp:
tct	S	953	152.60
tcc	S	0	123.40
tca	S	0	240.50
tcg	S	0	55.46
agt	S	0	195.30
agc	S	0	185.80
---	--	------	------
mPD		0	1.7
		nPD:	0.
		N. weight:	2.2
		Sc. PD:	-0.28
		Sc. rank:	-1503.7

NS1			
Pos.	196	obs:	exp:
gaa	E	931	573.30
gag	E	22	379.70
---	--	------	------
mPD		0.045	0.48
		nPD:	0.09
		N. weight:	0.5
		Sc. PD:	-0.024
		Sc. rank:	50.6

NS1			
Pos.	197	obs:	exp:
att	I	1	0.36
atc	I	0	0.24
ata	I	0	0.39
act	T	911	236.00
acc	T	0	185.50
aca	T	0	418.50
acg	T	0	70.96
aat	N	15	7.63
aac	N	0	7.37
gct	A	26	6.66
gcc	A	0	4.88
gca	A	0	12.16
gcg	A	0	2.30
---	--	------	------
mPD		0.086	0.76
		nPD:	0.11
		N. weight:	1.6
		Sc. PD:	-0.051
		Sc. rank:	279.5

NS1			
Pos.	198	obs:	exp:
tta	L	24	82.61
ttg	L	0	175.40
ctt	L	0	159.30
ctc	L	0	157.50
cta	L	916	156.70
ctg	L	0	208.50
att	I	0	4.74
atc	I	0	3.14
ata	I	13	5.11
---	--	------	------
mPD		0.075	1.1
		nPD:	0.07
		N. weight:	2.
		Sc. PD:	-0.14
		Sc. rank:	-29.7

NS1			
Pos.	199	obs:	exp:
caa	Q	4	489.50
cag	Q	949	463.50
---	--	------	------
mPD		0.0084	0.50
		nPD:	0.02
		N. weight:	0.84
		Sc. PD:	-0.095
		Sc. rank:	-402.5

NS1			
Pos.	200	obs:	exp:
cgt	R	0	40.71
cgc	R	0	48.42
cga	R	0	95.02
cgg	R	0	79.37
aga	R	953	435.10
agg	R	0	254.40
---	--	------	------
mPD		0	0.96
		nPD:	0.
		N. weight:	0.95
		Sc. PD:	-0.12
		Sc. rank:	-643.5

NS1			
Pos.	201	obs:	exp:
ttt	F	9	375.70
ttc	F	943	576.30
tta	L	0	0.09
ttg	L	0	0.19
ctt	L	0	0.17
ctc	L	1	0.17
cta	L	0	0.17
ctg	L	0	0.22
---	--	------	------
mPD		0.021	0.48
		nPD:	0.04
		N. weight:	0.55
		Sc. PD:	-0.05
		Sc. rank:	-128.2

NS1			
Pos.	202	obs:	exp:
act	T	4	1.04
acc	T	0	0.81
aca	T	0	1.84
acg	T	0	0.31
gct	A	949	243.20
gcc	A	0	178.20
gca	A	0	443.70
gcg	A	0	83.94
---	--	------	------
mPD		0.0084	0.68
		nPD:	0.01
		N. weight:	1.6
		Sc. PD:	-0.19
		Sc. rank:	-868.8

NS1			
Pos.	203	obs:	exp:
tgg	W	953	953.00
---	--	------	------
mPD		0	0
		nPD:	1.
		N. weight:	0.
		Sc. PD:	0
		Sc. rank:	0

NS1			
Pos.	204	obs:	exp:
cgt	R	0	40.24
cgc	R	0	47.86
cga	R	0	93.93
cgg	R	0	78.46
aga	R	942	430.10
agg	R	0	251.40
ggt	G	0	1.48
ggc	G	0	1.40
gga	G	11	5.09
ggg	G	0	3.04
---	--	------	------
mPD		0.023	0.98
		nPD:	0.02
		N. weight:	0.95
		Sc. PD:	-0.1
		Sc. rank:	-416.8

NS1			
Pos.	205	obs:	exp:
tct	S	0	148.20
tcc	S	0	119.90
tca	S	0	233.70
tcg	S	0	53.89
tgt	C	0	0.41
tgc	C	1	0.59
cgt	R	0	0.30
cgc	R	0	0.36
cga	R	0	0.70
cgg	R	0	0.58
att	I	0	2.55
atc	I	7	1.69
ata	I	0	2.75
aat	N	0	4.58
aac	N	9	4.42
agt	S	0	189.70
agc	S	926	180.60
aga	R	7	3.20
agg	R	0	1.87
gtt	V	0	0.62
gtc	V	3	0.60
gta	V	0	0.62
gtg	V	0	1.16
---	--	------	------
mPD		0.062	1.7
		nPD:	0.04
		N. weight:	2.
		Sc. PD:	-0.19
		Sc. rank:	-630.1

NS1			
Pos.	206	obs:	exp:
tct	S	0	136.90
tcc	S	0	110.70
tca	S	0	215.80
tcg	S	0	49.76
tgt	C	8	4.11
tgc	C	2	5.89
cgt	R	10	0.43
cgc	R	0	0.51
cga	R	0	1.00
cgg	R	0	0.83
att	I	0	12.04
atc	I	33	7.98
ata	I	0	12.98
act	T	0	0.26
acc	T	1	0.20
aca	T	0	0.46
acg	T	0	0.08
aat	N	0	0.51
aac	N	1	0.49
agt	S	87	175.20
agc	S	768	166.70
aga	R	0	4.57
agg	R	0	2.67
ggt	G	1	5.77
ggc	G	42	5.46
gga	G	0	19.88
ggg	G	0	11.89
---	--	------	------
mPD		0.39	1.8
		nPD:	0.22
		N. weight:	1.6
		Sc. PD:	0.1
		Sc. rank:	595.7

NS1			
Pos.	207	obs:	exp:
cat	H	1	0.57
cac	H	0	0.43
aat	N	160	81.40
aac	N	0	78.60
gtt	V	1	0.21
gtc	V	0	0.20
gta	V	0	0.21
gtg	V	0	0.39
gat	D	791	427.00
gac	D	0	364.00
---	--	------	------
mPD		0.28	0.78
		nPD:	0.36
		N. weight:	0.76
		Sc. PD:	0.14
		Sc. rank:	373.6

NS1			
Pos.	208	obs:	exp:
aaa	K	0	0.59
aag	K	1	0.41
gaa	E	0	572.70
gag	E	952	379.30
---	--	------	------
mPD		0.0021	0.48
		nPD:	0.
		N. weight:	1.1
		Sc. PD:	-0.14
		Sc. rank:	-656.8

NS1			
Pos.	209	obs:	exp:
tct	S	0	0.16
tcc	S	0	0.13
tca	S	0	0.25
tcg	S	0	0.06
aat	N	24	12.21
aac	N	0	11.79
agt	S	1	0.20
agc	S	0	0.20
gct	A	1	0.26
gcc	A	0	0.19
gca	A	0	0.47
gcg	A	0	0.09
gat	D	915	496.10
gac	D	4	422.90
ggt	G	8	1.07
ggc	G	0	1.02
gga	G	0	3.70
ggg	G	0	2.21
---	--	------	------
mPD		0.080	0.58
		nPD:	0.14
		N. weight:	0.73
		Sc. PD:	-0.0069
		Sc. rank:	150.7

NS1			
Pos.	210	obs:	exp:
cgt	R	0	0.13
cgc	R	0	0.15
cga	R	0	0.30
cgg	R	0	0.25
aga	R	0	1.37
agg	R	3	0.80
gaa	E	0	0.60
gag	E	1	0.40
ggt	G	0	127.30
ggc	G	0	120.60
gga	G	0	438.80
ggg	G	949	262.40
---	--	------	------
mPD		0.0084	0.68
		nPD:	0.01
		N. weight:	1.6
		Sc. PD:	-0.18
		Sc. rank:	-834.0

NS1			
Pos.	211	obs:	exp:
cgt	R	0	39.26
cgc	R	0	46.69
cga	R	0	91.63
cgg	R	0	76.54
aga	R	919	419.60
agg	R	0	245.30
ggt	G	0	4.56
ggc	G	0	4.32
gga	G	34	15.72
ggg	G	0	9.40
---	--	------	------
mPD		0.069	1.0
		nPD:	0.07
		N. weight:	0.95
		Sc. PD:	-0.066
		Sc. rank:	-0.3

,

NS1			
Pos.	212	obs:	exp:
ttt	F	3	1.18
ttc	F	0	1.82
tta	L	0	0.18
ttg	L	0	0.37
tct	S	541	86.60
tcc	S	0	70.03
tca	S	0	136.50
tcg	S	0	31.48
tat	Y	4	2.12
tac	Y	0	1.88
ctt	L	2	0.34
ctc	L	0	0.34
cta	L	0	0.33
ctg	L	0	0.44
cct	P	403	102.50
ccc	P	0	71.86
cca	P	0	164.60
ccg	P	0	64.03
agt	S	0	110.90
agc	S	0	105.50
---	--	------	------
mPD		0.51	1.7
		nPD:	0.29
		N. weight:	2.
		Sc. PD:	0.24
		Sc. rank:	875.9

,

NS1			
Pos.	213	obs:	exp:
tta	L	0	0.09
ttg	L	0	0.19
tct	S	0	1.12
tcc	S	0	0.91
tca	S	7	1.77
tcg	S	0	0.41
ctt	L	0	0.17
ctc	L	0	0.17
cta	L	1	0.17
ctg	L	0	0.22
cct	P	0	240.00
ccc	P	0	168.30
cca	P	944	385.70
ccg	P	0	150.00
act	T	0	0.26
acc	T	0	0.20
aca	T	1	0.46
acg	T	0	0.08
agt	S	0	1.43
agc	S	0	1.37
---	--	------	------
mPD		0.019	0.74
		nPD:	0.03
		N. weight:	1.1
		Sc. PD:	-0.11
		Sc. rank:	-456.9

,

NS1			
Pos.	214	obs:	exp:
ttt	F	0	5.92
ttc	F	15	9.08
tta	L	0	82.44
ttg	L	0	175.00
ctt	L	3	159.00
ctc	L	935	157.20
cta	L	0	156.40
ctg	L	0	208.00
---	--	------	------
mPD		0.037	1.1
		nPD:	0.03
		N. weight:	2.1
		Sc. PD:	-0.21
		Sc. rank:	-740.7

,

NS1			
Pos.	215	obs:	exp:
cct	P	1	0.25
ccc	P	0	0.18
cca	P	0	0.41
ccg	P	0	0.16
---	-	952	952.00
---	--	------	------
mPD		0	7.8×10^{-7}
		nPD:	0.
		N. weight:	0.
		Sc. PD:	0
		Sc. rank:	0

,

NS1			
Pos.	216	obs:	exp:
cct	P	0	0.25
ccc	P	0	0.18
cca	P	1	0.41
ccg	P	0	0.16
---	-	952	952.00
---	--	------	------
mPD		0	7.8×10^{-7}
		nPD:	0.
		N. weight:	0.
		Sc. PD:	0
		Sc. rank:	0

,

NS1			
Pos.	217	obs:	exp:
aat	N	1	0.51
aac	N	0	0.49
---	-	952	952.00
---	--	------	------
mPD		0	5.5×10^{-7}
		nPD:	0.
		N. weight:	0.
		Sc. PD:	0
		Sc. rank:	0

,

NS1			
Pos.	218	obs:	exp:
caa	Q	0	0.51
cag	Q	1	0.49
---	-	952	952.00
---	--	------	------
mPD		0	5.5×10^{-7}
		nPD:	0.
		N. weight:	0.
		Sc. PD:	0
		Sc. rank:	0

,

NS1			
Pos.	219	obs:	exp:
aaa	K	1	0.59
aag	K	0	0.41
---	-	952	952.00
---	--	------	------
mPD		0	5.3×10^{-7}
		nPD:	0.
		N. weight:	0.
		Sc. PD:	0
		Sc. rank:	0

,

NS1			
Pos.	220	obs:	exp:
cgt	R	0	0.04
cgc	R	0	0.05
cga	R	0	0.10
cgg	R	1	0.08
aga	R	0	0.46
agg	R	0	0.27
---	-	952	952.00
---	--	------	------
mPD		0	1.1×10^{-6}
		nPD:	0.
		N. weight:	0.
		Sc. PD:	0
		Sc. rank:	0

,

NS1			
Pos.	221	obs:	exp:
aaa	K	1	0.59
aag	K	0	0.41
---	-	952	952.00
---	--	------	------
mPD		0	5.3×10^{-7}
		nPD:	0.
		N. weight:	0.
		Sc. PD:	0
		Sc. rank:	0

,

NS1			
Pos.	222	obs:	exp:
tta	L	0	0.09
ttg	L	0	0.19
ctt	L	0	0.17
ctc	L	0	0.17
cta	L	0	0.17
ctg	L	1	0.22
---	-	952	952.00
---	--	------	------
mPD		0	1.2×10^{-6}
		nPD:	0.
		N. weight:	0.
		Sc. PD:	0
		Sc. rank:	0

,

NS1			
Pos.	223	obs:	exp:
gct	A	0	0.26
gcc	A	0	0.19
gca	A	0	0.47
gcg	A	1	0.09
---	-	952	952.00
---	--	------	------
mPD		0	7.4×10^{-7}
		nPD:	0.
		N. weight:	0.
		Sc. PD:	0
		Sc. rank:	0

,

NS1			
Pos.	224	obs:	exp:
cgt	R	0	0.04
cgc	R	0	0.05
cga	R	0	0.10
cgg	R	0	0.08
aga	R	1	0.46
agg	R	0	0.27
---	-	952	952.00
---	--	------	------
mPD		0	1.1×10^{-6}
		nPD:	0.
		N. weight:	0.
		Sc. PD:	0
		Sc. rank:	0

,

NS1			
Pos.	225	obs:	exp:
gct	A	0	0.26
gcc	A	0	0.19
gca	A	1	0.47
gcg	A	0	0.09
---	-	952	952.00
---	--	------	------
mPD		0	7.4×10^{-7}
		nPD:	0.
		N. weight:	0.
		Sc. PD:	0
		Sc. rank:	0

,

NS1			
Pos.	226	obs:	exp:
att	I	1	0.36
atc	I	0	0.24
ata	I	0	0.39
---	-	952	952.00
---	--	------	------
mPD		0	7.2×10^{-7}
		nPD:	0.
		N. weight:	0.
		Sc. PD:	0
		Sc. rank:	0

,

NS1			
Pos.	227	obs:	exp:
gaa	E	0	0.60
gag	E	1	0.40
---	-	952	952.00
---	--	------	------
mPD		0	5.3×10^{-7}
		nPD:	0.
		N. weight:	0.
		Sc. PD:	0
		Sc. rank:	0

,

NS1			
Pos.	228	obs:	exp:
tct	S	721	115.60
tcc	S	0	93.46
tca	S	1	182.20
tcg	S	0	42.02
tat	Y	1	0.53
tac	Y	0	0.47
cct	P	230	58.48
ccc	P	0	41.01
cca	P	0	93.97
ccg	P	0	36.54
agt	S	0	147.90
agc	S	0	140.80
---	--	------	------
mPD		0.37	1.8
		nPD:	0.21
		N. weight:	2.1
		Sc. PD:	0.1
		Sc. rank:	656.5

,

NS1			
Pos.	229	obs:	exp:
tct	S	0	0.80
tcc	S	0	0.65
tca	S	5	1.26
tcg	S	0	0.29
cct	P	0	85.44
ccc	P	0	59.91
cca	P	336	137.30
ccg	P	0	53.38
act	T	0	157.80
acc	T	0	124.00
aca	T	609	279.80
acg	T	0	47.44
aaa	K	1	0.59
aag	K	0	0.41
agt	S	0	1.02
agc	S	0	0.98
gct	A	0	0.26
gcc	A	0	0.19
gca	A	1	0.47
gcg	A	0	0.09
gaa	E	1	0.60
gag	E	0	0.40
---	--	------	------
mPD		0.47	1.2
		nPD:	0.41
		N. weight:	0.99
		Sc. PD:	0.21
		Sc. rank:	527.0

,

NS1			
Pos.	230	obs:	exp:
aat	N	3	1.53
aac	N	0	1.47
aaa	K	20	552.40
aag	K	923	390.60
gtt	V	1	0.21
gtc	V	0	0.20
gta	V	0	0.21
gtg	V	0	0.39
gaa	E	1	3.61
gag	E	5	2.39
---	--	------	------
mPD		0.068	0.51
		nPD:	0.13
		N. weight:	0.93
		Sc. PD:	-0.013
		Sc. rank:	180.1

,

NS1			
Pos.	231	obs:	exp:
tat	Y	1	0.53
tac	Y	0	0.47
caa	Q	0	55.47
cag	Q	108	52.53
---	-	844	844.00
---	--	------	------
mPD		0.00048	0.0069
		nPD:	0.07
		N. weight:	0.
		Sc. PD:	0
		Sc. rank:	0

,

NS1			
Pos.	232	obs:	exp:
aaa	K	108	63.27
aag	K	0	44.73
gaa	E	1	0.60
gag	E	0	0.40
---	-	844	844.00
---	--	------	------
mPD		0.00024	0.0066
		nPD:	0.04
		N. weight:	0.
		Sc. PD:	0
		Sc. rank:	0

,

NS1			
Pos.	233	obs:	exp:
cgt	R	0	4.66
cgc	R	11	5.54
cga	R	0	10.87
cgg	R	98	9.08
aga	R	0	49.77
agg	R	0	29.09
---	-	844	844.00
---	--	------	------
mPD		0.0024	0.013
		nPD:	0.19
		N. weight:	0.
		Sc. PD:	0
		Sc. rank:	0

,

NS1			
Pos.	234	obs:	exp:
tat	Y	0	5.30
tac	Y	10	4.70
tgt	C	0	0.41
tgc	C	1	0.59
aaa	K	97	56.82
aag	K	0	40.18
gaa	E	1	0.60
gag	E	0	0.40
---	-	844	844.00
---	--	------	------
mPD		0.0052	0.010
		nPD:	0.5
		N. weight:	0.
		Sc. PD:	0
		Sc. rank:	0

,

NS1			
Pos.	235	obs:	exp:
tta	L	0	0.18
ttg	L	0	0.37
ctt	L	0	0.34
ctc	L	0	0.34
cta	L	0	0.33
ctg	L	2	0.44
atg	M	107	107.00
---	-	844	844.00
---	--	------	------
mPD		0.00047	0.00075
		nPD:	0.62
		N. weight:	0.
		Sc. PD:	0
		Sc. rank:	0

,

NS1			
Pos.	236	obs:	exp:
gct	A	0	27.42
gcc	A	0	20.09
gca	A	0	50.03
gcg	A	107	9.46
gaa	E	0	1.20
gag	E	2	0.80
---	-	844	844.00
---	--	------	------
mPD		0.00047	0.0093
		nPD:	0.05
		N. weight:	0.
		Sc. PD:	0
		Sc. rank:	0

,

NS1			
Pos.	237	obs:	exp:
cgt	R	0	4.53
cgc	R	0	5.39
cga	R	0	10.57
cgg	R	0	8.83
aaa	K	1	0.59
aag	K	0	0.41
aga	R	105	48.40
agg	R	1	28.29
ggt	G	0	0.27
ggc	G	0	0.25
gga	G	2	0.92
ggg	G	0	0.55
---	-	844	844.00
---	--	------	------
mPD		0.00095	0.013
		nPD:	0.07
		N. weight:	0.
		Sc. PD:	0
		Sc. rank:	0

,

NS1			
Pos.	238	obs:	exp:
cgt	R	0	0.47
cgc	R	0	0.56
cga	R	8	1.10
cgg	R	0	0.92
act	T	0	24.61
acc	T	0	19.35
aca	T	95	43.64
acg	T	0	7.40
aga	R	3	5.02
agg	R	0	2.94
gct	A	0	0.77
gcc	A	0	0.56
gca	A	3	1.40
gcg	A	0	0.27
---	-	844	844.00
---	--	------	------
mPD		0.0048	0.013
		nPD:	0.38
		N. weight:	0.
		Sc. PD:	0
		Sc. rank:	0

,

NS1			
Pos.	239	obs:	exp:
att	I	93	33.93
atc	I	0	22.49
ata	I	0	36.57
gtt	V	16	3.30
gtc	V	0	3.21
gta	V	0	3.31
gtg	V	0	6.18
---	-	844	844.00
---	--	------	------
mPD		0.0033	0.012
		nPD:	0.27
		N. weight:	0.
		Sc. PD:	0
		Sc. rank:	0

,

NS1			
Pos.	240	obs:	exp:
aaa	K	0	1.17
aag	K	2	0.83
gaa	E	0	64.36
gag	E	107	42.64
---	-	844	844.00
---	--	------	------
mPD		0.00047	0.0067
		nPD:	0.07
		N. weight:	0.
		Sc. PD:	0
		Sc. rank:	0

,

NS1			
Pos.	241	obs:	exp:
tct	S	0	17.13
tcc	S	0	13.85
tca	S	107	27.00
tcg	S	0	6.23
cct	P	0	0.51
ccc	P	0	0.36
cca	P	2	0.82
ccg	P	0	0.32
agt	S	0	21.93
agc	S	0	20.87
---	-	844	844.00
---	--	------	------
mPD		0.00047	0.022
		nPD:	0.02
		N. weight:	0.
		Sc. PD:	0
		Sc. rank:	0

,

NS1			
Pos.	242	obs:	exp:
gaa	E	109	65.57
gag	E	0	43.43
---	-	844	844.00
---	--	------	------
mPD		0	0.0063
		nPD:	0.
		N. weight:	0.
		Sc. PD:	0
		Sc. rank:	0

,

NS1			
Pos.	243	obs:	exp:
gtt	V	106	21.88
gtc	V	0	21.23
gta	V	0	21.95
gtg	V	0	40.94
---	-	847	847.00
---	--	------	------
mPD		0	0.0090
		nPD:	0.
		N. weight:	0.
		Sc. PD:	0
		Sc. rank:	0

,

NS1			
Pos.	244	obs:	exp:
cgt	R	0	0.09
cgc	R	0	0.10
cga	R	2	0.20
cgg	R	0	0.17
aga	R	0	0.91
agg	R	0	0.53
---	-	951	951.00
---	--	------	------
mPD		0	4.2×10^{-6}
		nPD:	0.
		N. weight:	0.
		Sc. PD:	0
		Sc. rank:	0

,

NS1			
Pos.	245	obs:	exp:
cgt	R	0	0.09
cgc	R	0	0.10
cga	R	0	0.20
cgg	R	0	0.17
aga	R	2	0.91
agg	R	0	0.53
---	-	951	951.00
---	--	------	------
mPD		0	4.2×10^{-6}
		nPD:	0.
		N. weight:	0.
		Sc. PD:	0
		Sc. rank:	0

,

NS1			
Pos.	246	obs:	exp:
aat	N	2	1.02
aac	N	0	0.98
---	-	951	951.00
---	--	------	------
mPD		0	2.2×10^{-6}
		nPD:	0.
		N. weight:	0.
		Sc. PD:	0
		Sc. rank:	0

,

NS1			
Pos.	247	obs:	exp:
aaa	K	0	1.17
aag	K	2	0.83
---	-	951	951.00
---	--	------	------
mPD		0	2.1×10^{-6}
		nPD:	0.
		N. weight:	0.
		Sc. PD:	0
		Sc. rank:	0

NS1			
Pos.	248	obs:	exp:
atg	M	1	1.00
gtt	V	0	0.21
gtc	V	0	0.20
gta	V	0	0.21
gtg	V	1	0.39
---	-	951	951.00
---	--	------	------
mPD		2.2×10^{-6}	4.4×10^{-6}
		nPD:	0.51
		N. weight:	0.
		Sc. PD:	0
		Sc. rank:	0

NS1			
Pos.	249	obs:	exp:
gtt	V	2	0.41
gtc	V	0	0.40
gta	V	0	0.41
gtg	V	0	0.77
---	-	951	951.00
---	--	------	------
mPD		0	3.2×10^{-6}
		nPD:	0.
		N. weight:	0.
		Sc. PD:	0
		Sc. rank:	0

NS1			
Pos.	250	obs:	exp:
gtt	V	3	0.62
gtc	V	0	0.60
gta	V	0	0.62
gtg	V	0	1.16
gat	D	2	1.08
gac	D	0	0.92
---	-	948	948.00
---	--	------	------
mPD		0.000013	0.000033
		nPD:	0.4
		N. weight:	0.
		Sc. PD:	0
		Sc. rank:	0

NS1			
Pos.	251	obs:	exp:
taa	.	0	409.90
tag	.	843	241.00
tga	.	110	302.20
---	--	------	------
mPD		0.41	0.81
		nPD:	0.5
		N. weight:	1.2
		Sc. PD:	0.36
		Sc. rank:	725.7

Amino acid level conservation per codon

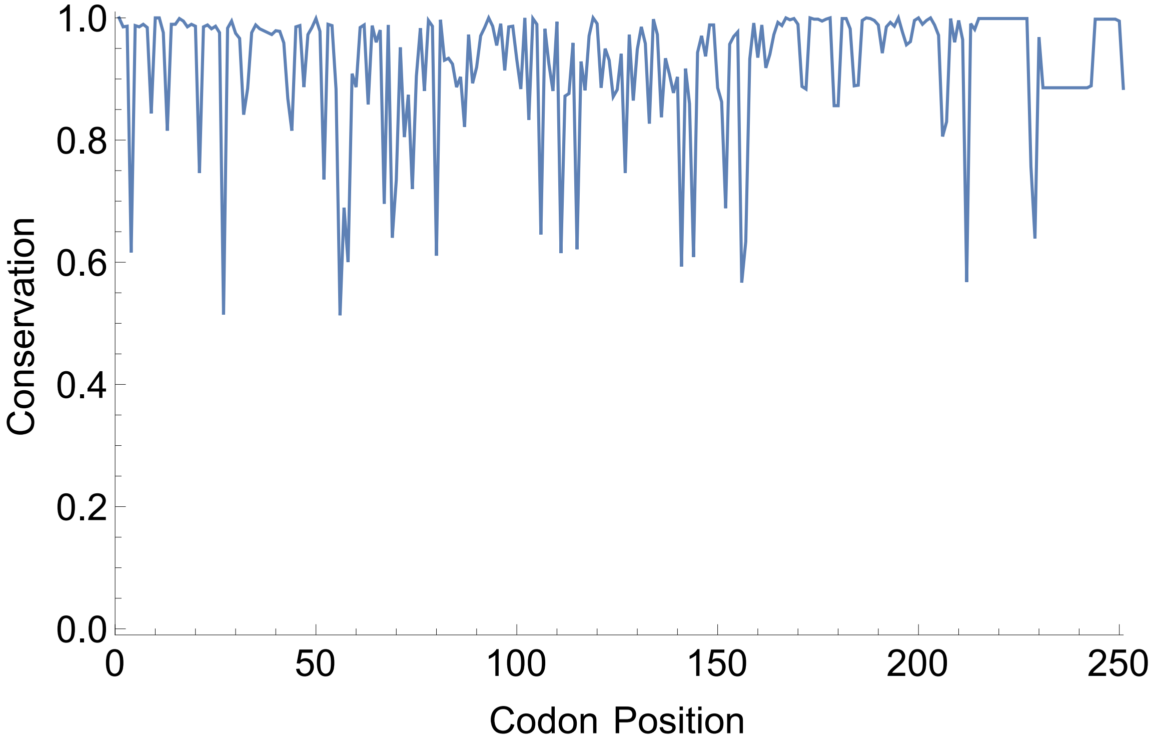

Conservation analysis plots

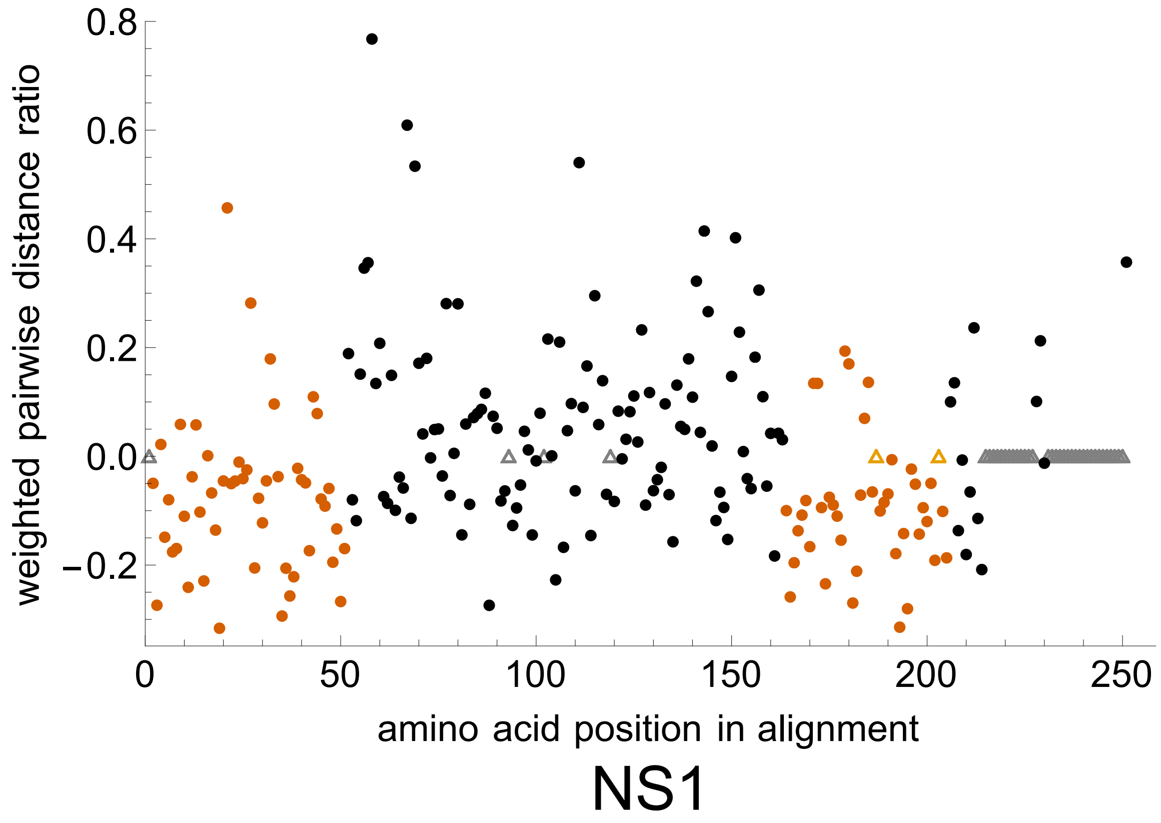

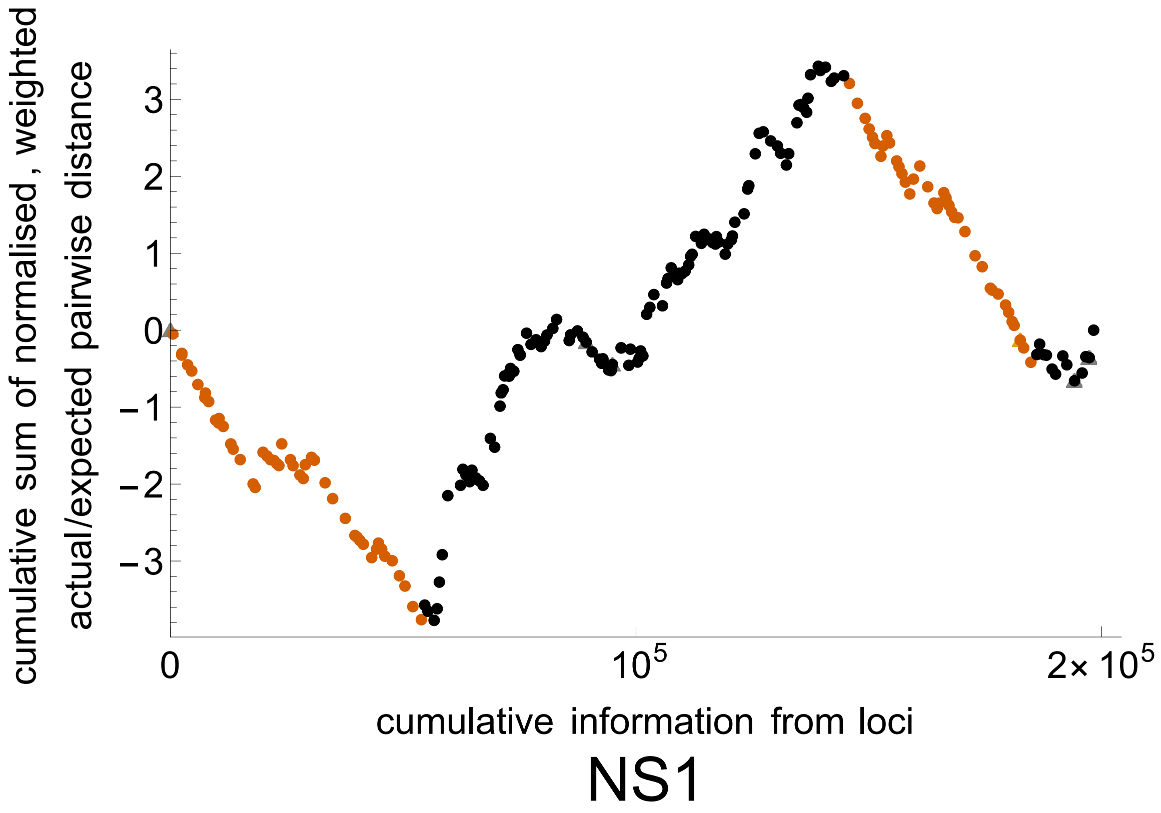

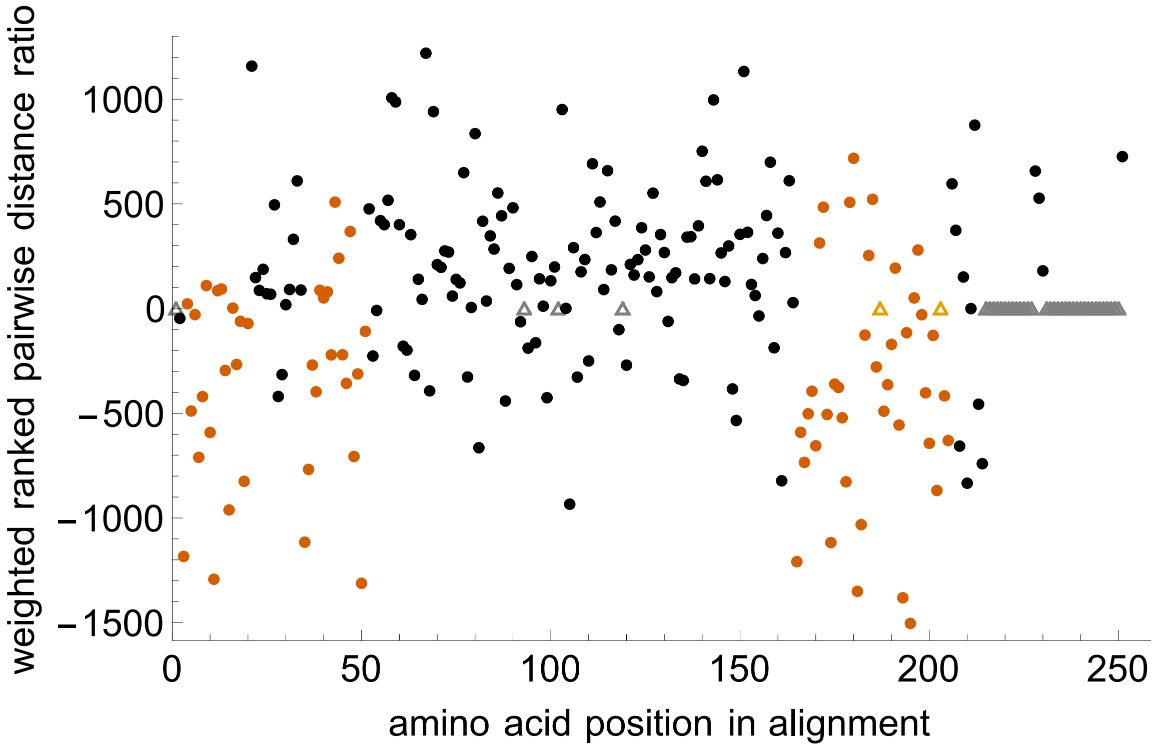
weighted ranked pairwise distance ratio
1000
500
0
−500
−1000
−1500
0
50
100
150
200
250
amino acid position in alignment

NS1

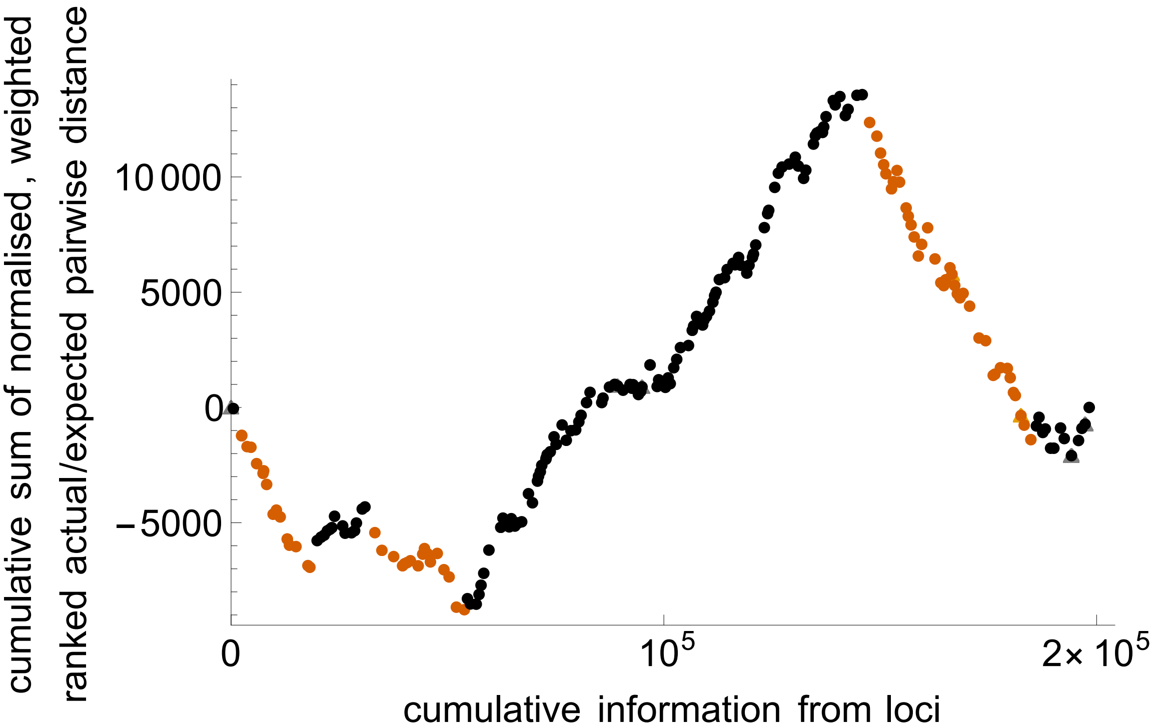
cumulative sum of normalised, weighted
ranked actual/expected pairwise distance
10 000
5000
0
−5000
0
10^5
2×10^5
cumulative information from loci

NS1

NS2

Gene length histogram

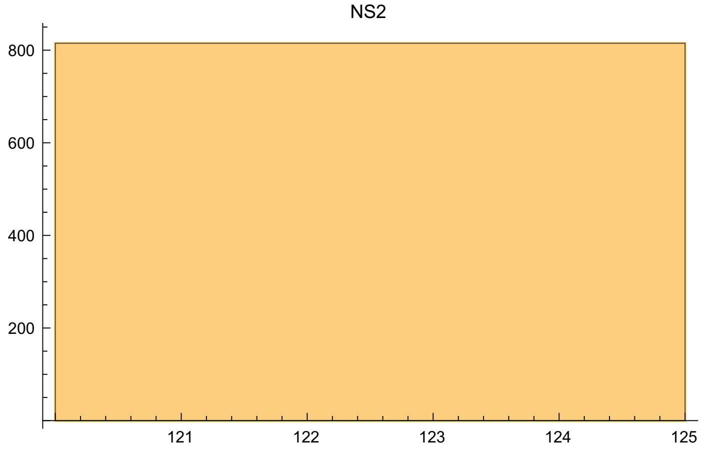

Information vs. nPD

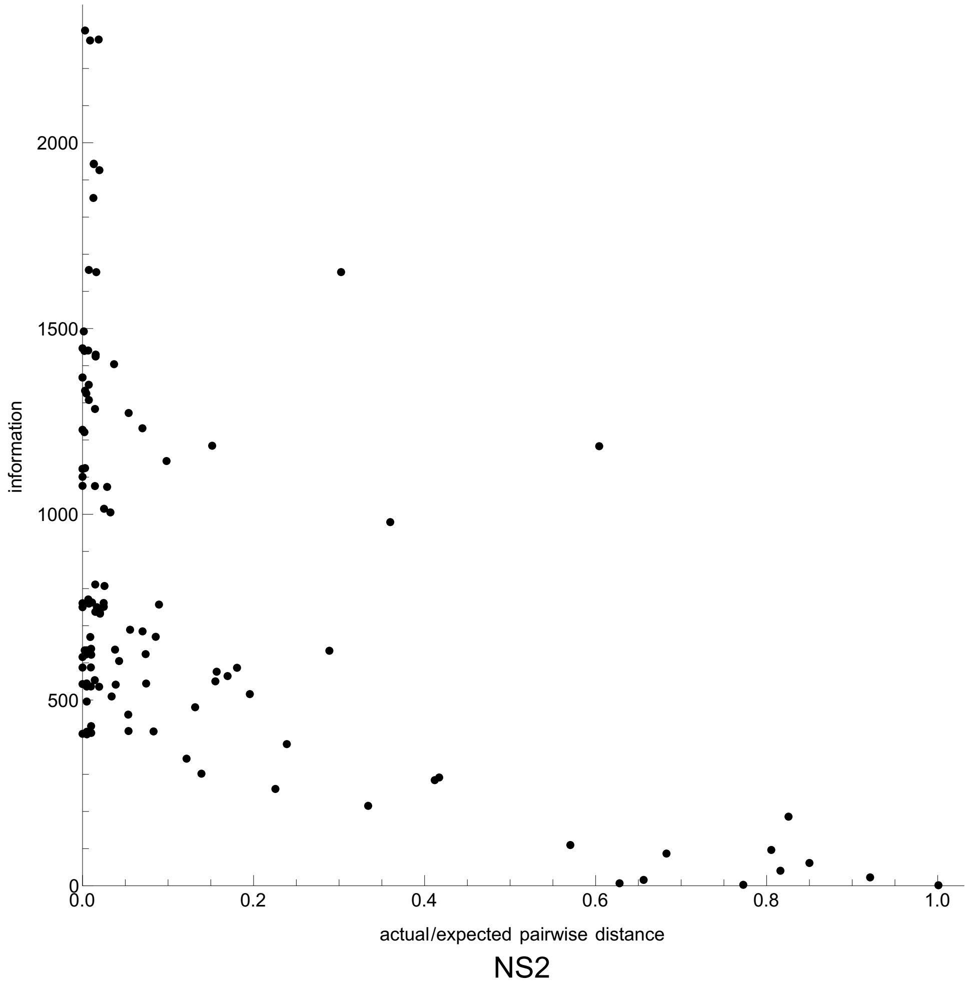

Example sequences highlighted by regions found in analyses to be conserved

Interesting points (by weighted raw PD) highlighted for gene NS2:

ATGGATTCCAAT
ACTGTGTCAAGCTTCCAGGACATACTGACAAGGATGTCAAAAATGCAATTGAGAT
CCTCATCGGAGGATTTGAATGGAATGATAACACAGTTCGAGTCTCTGAAACT
CTACAGAGATTCGCTTGGAGAAGCAGCGATGAGGATGGGAGATCTCCACTCTCTACAAAGTAG⋱
AAACGGGAAATGGAGAGAACAGTTAAGCCAGAAGTTCGAAGAAATAAGATGGTTGATTGAAGAAGTACGA⋱
CATAGATTAAAAATTACGGAGAATAGCTTTGAG
CAAATAACTTTTATGCAAGCCTTACAACTATTGCTTGAAGTGGAGCAAGAGATAA
GAACTTTCTCGTTTCAGCTTATTTAA

Interesting points (by weighted ranked PD) highlighted for gene NS2:

ATGGATTCCAAT
ACTGTGTCAAGCTTCCAGGACATACTGACAAGGATGTCAAAAATGCAATTGAGAT
CCTCATCGGAGGATTTGAATGGAATGATAACACAGTTCGAGTCTCTGAAACTCTACAGAGATTCGCTT⋱
GGAGAAGCAGCGATGAGGATGGGAGATCTCCACTCTCTACAAAGTAGAAACGGGAAATGGAGAGAACAGT⋱
TAAGCCAGAAGTTCGAAGAAATAAGATGGTTGATTGAAGAAGTACGACATAGATTAAAAATTACGGAGAA⋱
TAGCTTTGAGCAAATAACTTTTATGCAAGCCTTACAACTATTGCTTGAAGTGGAGCAAGAGATAAGAACT⋱
TTCTCGTTTCAGCTTATTTAA

Per codon conservation report

NS2			
Pos.	1	obs:	exp:
atg	M	815	815.00
---	--	------	------
mPD		0	0
		nPD:	1.
		N. weight:	0.
		Sc. PD:	0
		Sc. rank:	0

NS2			
Pos.	2	obs:	exp:
aat	N	0	0.51
aac	N	1	0.49
gat	D	805	438.90
gac	D	8	374.10
gaa	E	1	0.60
gag	E	0	0.40
---	--	------	------
mPD		0.027	0.50
		nPD:	0.05
		N. weight:	0.59
		Sc. PD:	0.00098
		Sc. rank:	234.6

NS2			
Pos.	3	obs:	exp:
ttt	F	0	3.55
ttc	F	9	5.45
tct	S	0	128.70
tcc	S	804	104.10
tca	S	0	202.90
tcg	S	0	46.79
cct	P	0	0.51
ccc	P	2	0.36
cca	P	0	0.82
ccg	P	0	0.32
agt	S	0	164.70
agc	S	0	156.80
---	--	------	------
mPD		0.027	1.7
		nPD:	0.02
		N. weight:	2.1
		Sc. PD:	-0.071
		Sc. rank:	347.3

NS2			
Pos.	4	obs:	exp:
aat	N	566	414.60
aac	N	249	400.40
---	--	------	------
mPD		0.42	0.50
		nPD:	0.85
		N. weight:	0.078
		Sc. PD:	0.06
		Sc. rank:	53.5

NS2			
Pos.	5	obs:	exp:
act	T	815	211.10
acc	T	0	166.00
aca	T	0	374.40
acg	T	0	63.49
---	--	------	------
mPD		0	0.67
		nPD:	0.
		N. weight:	1.4
		Sc. PD:	-0.069
		Sc. rank:	-927.1

NS2			
Pos.	6	obs:	exp:
gtt	V	0	168.00
gtc	V	0	163.10
gta	V	1	168.50
gtg	V	813	314.40
gct	A	0	0.26
gcc	A	0	0.19
gca	A	0	0.47
gcg	A	1	0.09
---	--	------	------
mPD		0.0049	0.73
		nPD:	0.01
		N. weight:	0.98
		Sc. PD:	-0.042
		Sc. rank:	-220.9

NS2			
Pos.	7	obs:	exp:
tct	S	0	130.50
tcc	S	0	105.50
tca	S	815	205.70
tcg	S	0	47.43
agt	S	0	167.00
agc	S	0	158.90
---	--	------	------
mPD		0	1.7
		nPD:	0.
		N. weight:	1.4
		Sc. PD:	-0.07
		Sc. rank:	-945.1

NS2			
Pos.	8	obs:	exp:
tct	S	0	130.30
tcc	S	0	105.40
tca	S	0	205.40
tcg	S	0	47.37
cgt	R	0	0.04
cgc	R	0	0.05
cga	R	0	0.10
cgg	R	0	0.08
agt	S	4	166.80
agc	S	810	158.70
aga	R	1	0.46
agg	R	0	0.27
---	--	------	------
mPD		0.012	1.7
		nPD:	0.01
		N. weight:	1.7
		Sc. PD:	-0.07
		Sc. rank:	-272.8

NS2			
Pos.	9	obs:	exp:
ttt	F	45	320.90
ttc	F	768	492.10
tta	L	1	0.09
ttg	L	0	0.19
tct	S	0	0.16
tcc	S	1	0.13
tca	S	0	0.25
tcg	S	0	0.06
ctt	L	0	0.17
ctc	L	0	0.17
cta	L	0	0.17
ctg	L	0	0.22
agt	S	0	0.20
agc	S	0	0.20
---	--	------	------
mPD		0.11	0.48
		nPD:	0.23
		N. weight:	0.33
		Sc. PD:	0.055
		Sc. rank:	197.8

NS2			
Pos.	10	obs:	exp:
caa	Q	0	418.60
cag	Q	815	396.40
---	--	------	------
mPD		0	0.50
		nPD:	0.
		N. weight:	0.75
		Sc. PD:	-0.037
		Sc. rank:	-494.8

NS2			
Pos.	11	obs:	exp:
gat	D	0	439.40
gac	D	814	374.60
ggt	G	0	0.13
ggc	G	1	0.13
gga	G	0	0.46
ggg	G	0	0.28
---	--	------	------
mPD		0.0025	0.50
		nPD:	0.
		N. weight:	0.81
		Sc. PD:	-0.036
		Sc. rank:	-237.7

NS2			
Pos.	12	obs:	exp:
att	I	0	297.40
atc	I	0	197.10
ata	I	815	320.50
---	--	------	------
mPD		0	0.65
		nPD:	0.
		N. weight:	0.97
		Sc. PD:	-0.048
		Sc. rank:	-640.6

NS2			
Pos.	13	obs:	exp:
tta	L	0	71.63
ttg	L	0	152.10
ctt	L	0	138.10
ctc	L	0	136.50
cta	L	1	135.90
ctg	L	814	180.80
---	--	------	------
mPD		0.0025	1.1
		nPD:	0.
		N. weight:	1.6
		Sc. PD:	-0.073
		Sc. rank:	-689.2

NS2			
Pos.	14	obs:	exp:
atg	M	1	1.00
act	T	0	210.10
acc	T	6	165.10
aca	T	805	372.60
acg	T	0	63.17
gct	A	0	0.77
gcc	A	0	0.56
gca	A	2	1.40
gcg	A	1	0.27
---	--	------	------
mPD		0.029	0.68
		nPD:	0.04
		N. weight:	0.77
		Sc. PD:	-0.0065
		Sc. rank:	303.0

NS2			
Pos.	15	obs:	exp:
cgt	R	0	34.82
cgc	R	0	41.41
cga	R	0	81.26
cgg	R	0	67.88
aga	R	0	372.10
agg	R	815	217.50
---	--	------	------
mPD		0	0.96
		nPD:	0.
		N. weight:	1.4
		Sc. PD:	-0.068
		Sc. rank:	-906.6

NS2			
Pos.	16	obs:	exp:
atg	M	815	815.00
---	--	------	------
mPD		0	0
		nPD:	1.
		N. weight:	0.
		Sc. PD:	0
		Sc. rank:	0

NS2			
Pos.	17	obs:	exp:
tta	L	2	0.18
ttg	L	0	0.37
tct	S	0	130.10
tcc	S	0	105.20
tca	S	813	205.20
tcg	S	0	47.31
ctt	L	0	0.34
ctc	L	0	0.34
cta	L	0	0.33
ctg	L	0	0.44
agt	S	0	166.60
agc	S	0	158.50
---	--	------	------
mPD		0.0049	1.7
		nPD:	0.
		N. weight:	1.4
		Sc. PD:	-0.067
		Sc. rank:	-571.8

NS2			
Pos.	18	obs:	exp:
cgt	R	0	0.04
cgc	R	0	0.05
cga	R	0	0.10
cgg	R	0	0.08
aaa	K	813	476.80
aag	K	1	337.20
aga	R	1	0.46
agg	R	0	0.27
---	--	------	------
mPD		0.0049	0.49
		nPD:	0.01
		N. weight:	0.55
		Sc. PD:	-0.022
		Sc. rank:	-42.9

NS2			
Pos.	19	obs:	exp:
tta	L	0	0.35
ttg	L	4	0.75
ctt	L	0	0.68
ctc	L	0	0.67
cta	L	0	0.67
ctg	L	0	0.89
atg	M	811	811.00
---	--	------	------
mPD		0.0098	0.016
		nPD:	0.63
		N. weight:	0.0086
		Sc. PD:	0.0047
		Sc. rank:	5.8

NS2			
Pos.	20	obs:	exp:
caa	Q	814	418.10
cag	Q	0	395.90
cgt	R	0	0.04
cgc	R	0	0.05
cga	R	1	0.10
cgg	R	0	0.08
aga	R	0	0.46
agg	R	0	0.27
---	--	------	------
mPD		0.0025	0.50
		nPD:	0.
		N. weight:	0.69
		Sc. PD:	-0.031
		Sc. rank:	-210.5

NS2

Pos.	21	obs:	exp:
tta	L	0	71.63
ttg	L	815	152.10
ctt	L	0	138.10
ctc	L	0	136.50
cta	L	0	135.90
ctg	L	0	180.80
---	--	------	------
mPD		0	1.1
		nPD:	0.
		N. weight:	1.7
		Sc. PD:	-0.086
		Sc. rank:	-1152.2

NS2

Pos.	22	obs:	exp:
cgt	R	0	33.45
cgc	R	0	39.78
cga	R	0	78.07
cgg	R	0	65.22
aaa	K	17	12.30
aag	K	4	8.70
aga	R	763	357.50
agg	R	20	209.00
gct	A	0	0.26
gcc	A	0	0.19
gca	A	0	0.47
gcg	A	1	0.09
ggt	G	0	1.34
ggc	G	0	1.27
gga	G	1	4.62
ggg	G	9	2.76
---	--	------	------
mPD		0.16	1.0
		nPD:	0.16
		N. weight:	0.7
		Sc. PD:	0.069
		Sc. rank:	392.8

NS2

Pos.	23	obs:	exp:
tct	S	0	129.80
tcc	S	810	105.00
tca	S	1	204.70
tcg	S	0	47.20
cct	P	0	1.02
ccc	P	4	0.71
cca	P	0	1.63
ccg	P	0	0.64
agt	S	0	166.20
agc	S	0	158.20
---	--	------	------
mPD		0.012	1.7
		nPD:	0.01
		N. weight:	2.1
		Sc. PD:	-0.089
		Sc. rank:	-385.0

NS2

Pos.	24	obs:	exp:
tct	S	0	130.50
tcc	S	0	105.50
tca	S	815	205.70
tcg	S	0	47.43
agt	S	0	167.00
agc	S	0	158.90
---	--	------	------
mPD		0	1.7
		nPD:	0.
		N. weight:	1.4
		Sc. PD:	-0.07
		Sc. rank:	-945.1

NS2

Pos.	25	obs:	exp:
tct	S	0	130.50
tcc	S	0	105.50
tca	S	2	205.70
tcg	S	813	47.43
agt	S	0	167.00
agc	S	0	158.90
---	--	------	------
mPD		0.0049	1.7
		nPD:	0.
		N. weight:	2.9
		Sc. PD:	-0.14
		Sc. rank:	-1075.0

NS2

Pos.	26	obs:	exp:
aaa	K	0	1.17
aag	K	2	0.83
gaa	E	0	487.20
gag	E	810	322.80
ggt	G	0	0.40
ggc	G	0	0.38
gga	G	0	1.39
ggg	G	3	0.83
---	--	------	------
mPD		0.012	0.49
		nPD:	0.02
		N. weight:	0.96
		Sc. PD:	-0.024
		Sc. rank:	272.9

NS2

Pos.	27	obs:	exp:
gat	D	706	382.80
gac	D	3	326.20
ggt	G	106	14.21
ggc	G	0	13.47
gga	G	0	49.01
ggg	G	0	29.31
---	--	------	------
mPD		0.23	0.81
		nPD:	0.29
		N. weight:	0.81
		Sc. PD:	0.18
		Sc. rank:	491.8

NS2

Pos.	28	obs:	exp:
tta	L	0	71.63
ttg	L	815	152.10
ctt	L	0	138.10
ctc	L	0	136.50
cta	L	0	135.90
ctg	L	0	180.80
---	--	------	------
mPD		0	1.1
		nPD:	0.
		N. weight:	1.7
		Sc. PD:	-0.086
		Sc. rank:	-1152.2

NS2

Pos.	29	obs:	exp:
tct	S	0	0.48
tcc	S	0	0.39
tca	S	0	0.76
tcg	S	0	0.17
aat	N	812	413.10
aac	N	0	398.90
agt	S	3	0.61
agc	S	0	0.59
---	--	------	------
mPD		0.0073	0.51
		nPD:	0.01
		N. weight:	0.71
		Sc. PD:	-0.025
		Sc. rank:	32.1

NS2

Pos.	30	obs:	exp:
ggt	G	0	109.30
ggc	G	0	103.50
gga	G	814	376.80
ggg	G	1	225.30
---	--	------	------
mPD		0.0025	0.68
		nPD:	0.
		N. weight:	0.79
		Sc. PD:	-0.036
		Sc. rank:	-268.7

NS2

Pos.	31	obs:	exp:
atg	M	815	815.00
---	--	------	------
mPD		0	0
		nPD:	1.
		N. weight:	0.
		Sc. PD:	0
		Sc. rank:	0

NS2

Pos.	32	obs:	exp:
att	I	0	296.30
atc	I	0	196.40
ata	I	812	319.30
gtt	V	0	0.62
gtc	V	0	0.60
gta	V	3	0.62
gtg	V	0	1.16
---	--	------	------
mPD		0.0073	0.66
		nPD:	0.01
		N. weight:	0.97
		Sc. PD:	-0.038
		Sc. rank:	-42.5

NS2

Pos.	33	obs:	exp:
att	I	0	4.01
atc	I	0	2.66
ata	I	11	4.33
act	T	0	208.30
acc	T	0	163.70
aca	T	804	369.40
acg	T	0	62.63
---	--	------	------
mPD		0.027	0.70
		nPD:	0.04
		N. weight:	0.81
		Sc. PD:	-0.011
		Sc. rank:	305.2

NS2

Pos.	34	obs:	exp:
caa	Q	2	395.50
cag	Q	768	374.50
cgt	R	0	0.13
cgc	R	0	0.15
cga	R	0	0.30
cgg	R	3	0.25
aaa	K	0	24.60
aag	K	42	17.40
aga	R	0	1.37
agg	R	0	0.80
---	--	------	------
mPD		0.11	0.61
		nPD:	0.18
		N. weight:	0.75
		Sc. PD:	0.092
		Sc. rank:	437.1

NS2

Pos.	35	obs:	exp:
ttt	F	0	321.60
ttc	F	815	493.40
---	--	------	------
mPD		0	0.48
		nPD:	0.
		N. weight:	0.52
		Sc. PD:	-0.026
		Sc. rank:	-344.5

NS2

Pos.	36	obs:	exp:
aaa	K	0	0.59
aag	K	1	0.41
gaa	E	2	489.00
gag	E	811	324.00
ggt	G	0	0.13
ggc	G	0	0.13
gga	G	0	0.46
ggg	G	1	0.28
---	--	------	------
mPD		0.0098	0.48
		nPD:	0.02
		N. weight:	0.94
		Sc. PD:	-0.028
		Sc. rank:	237.9

NS2

Pos.	37	obs:	exp:
ttt	F	1	0.39
ttc	F	0	0.61
tct	S	814	130.30
tcc	S	0	105.40
tca	S	0	205.40
tcg	S	0	47.37
agt	S	0	166.80
agc	S	0	158.70
---	--	------	------
mPD		0.0025	1.7
		nPD:	0.
		N. weight:	1.9
		Sc. PD:	-0.091
		Sc. rank:	-878.2

NS2

Pos.	38	obs:	exp:
tta	L	0	71.63
ttg	L	0	152.10
ctt	L	0	138.10
ctc	L	0	136.50
cta	L	0	135.90
ctg	L	815	180.80
---	--	------	------
mPD		0	1.1
		nPD:	0.
		N. weight:	1.6
		Sc. PD:	-0.077
		Sc. rank:	-1033.7

NS2

Pos.	39	obs:	exp:
cgt	R	0	0.09
cgc	R	0	0.10
cga	R	0	0.20
cgg	R	0	0.17
aaa	K	790	476.30
aag	K	23	336.70
aga	R	2	0.91
agg	R	0	0.53
---	--	------	------
mPD		0.060	0.49
		nPD:	0.12
		N. weight:	0.44
		Sc. PD:	0.029
		Sc. rank:	228.6

NS2

Pos.	40	obs:	exp:
tta	L	1	71.45
ttg	L	0	151.70
ctt	L	1	137.80
ctc	L	809	136.20
cta	L	2	135.50
ctg	L	0	180.30
att	I	0	0.73
atc	I	2	0.48
ata	I	0	0.79
---	--	------	------
mPD		0.017	1.1
		nPD:	0.02
		N. weight:	1.8
		Sc. PD:	-0.063
		Sc. rank:	257.9

NS2			
Pos.	41	obs:	exp:
tat	Y	0	432.10
tac	Y	815	382.90
---	--	------	------
mPD		0	0.50
		nPD:	0.
		N. weight:	0.78
		Sc. PD:	-0.039
		Sc. rank:	-518.5

NS2			
Pos.	42	obs:	exp:
cgt	R	0	34.64
cgc	R	0	41.20
cga	R	0	80.87
cgg	R	0	67.55
aaa	K	4	2.34
aag	K	0	1.66
aga	R	811	370.30
agg	R	0	216.50
---	--	------	------
mPD		0.0098	0.97
		nPD:	0.01
		N. weight:	0.81
		Sc. PD:	-0.032
		Sc. rank:	-56.3

NS2			
Pos.	43	obs:	exp:
gat	D	814	440.00
gac	D	1	375.00
---	--	------	------
mPD		0.0025	0.50
		nPD:	0.
		N. weight:	0.63
		Sc. PD:	-0.028
		Sc. rank:	-176.7

NS2			
Pos.	44	obs:	exp:
tta	L	0	0.79
ttg	L	9	1.68
tct	S	0	129.00
tcc	S	0	104.30
tca	S	4	203.40
tcg	S	802	46.90
ctt	L	0	1.53
ctc	L	0	1.51
cta	L	0	1.50
ctg	L	0	2.00
agt	S	0	165.20
agc	S	0	157.20
---	--	------	------
mPD		0.032	1.7
		nPD:	0.02
		N. weight:	2.9
		Sc. PD:	-0.091
		Sc. rank:	603.7

NS2			
Pos.	45	obs:	exp:
tta	L	0	71.63
ttg	L	0	152.10
ctt	L	815	138.10
ctc	L	0	136.50
cta	L	0	135.90
ctg	L	0	180.80
---	--	------	------
mPD		0	1.1
		nPD:	0.
		N. weight:	1.8
		Sc. PD:	-0.091
		Sc. rank:	-1218.4

NS2			
Pos.	46	obs:	exp:
ggt	G	0	109.30
ggc	G	0	103.50
gga	G	814	376.80
ggg	G	1	225.30
---	--	------	------
mPD		0.0025	0.68
		nPD:	0.
		N. weight:	0.79
		Sc. PD:	-0.036
		Sc. rank:	-268.7

NS2			
Pos.	47	obs:	exp:
gat	D	1	0.54
gac	D	0	0.46
gaa	E	814	489.60
gag	E	0	324.40
---	--	------	------
mPD		0.0025	0.48
		nPD:	0.01
		N. weight:	0.53
		Sc. PD:	-0.023
		Sc. rank:	-141.5

NS2			
Pos.	48	obs:	exp:
act	T	0	2.33
acc	T	0	1.83
aca	T	9	4.13
acg	T	0	0.70
gct	A	2	204.70
gcc	A	0	150.00
gca	A	756	373.60
gcg	A	41	70.67
gaa	E	7	4.21
gag	E	0	2.79
---	--	------	------
mPD		0.14	0.71
		nPD:	0.2
		N. weight:	0.66
		Sc. PD:	0.09
		Sc. rank:	389.2

NS2			
Pos.	49	obs:	exp:
tct	S	0	5.28
tcc	S	0	4.27
tca	S	0	8.33
tcg	S	33	1.92
act	T	0	0.26
acc	T	0	0.20
aca	T	0	0.46
acg	T	1	0.08
agt	S	0	6.76
agc	S	0	6.44
gtt	V	0	0.41
gtc	V	0	0.40
gta	V	2	0.41
gtg	V	0	0.77
gct	A	0	199.60
gcc	A	0	146.30
gca	A	67	364.20
gcg	A	712	68.90
---	--	------	------
mPD		0.24	0.79
		nPD:	0.3
		N. weight:	2.1
		Sc. PD:	0.5
		Sc. rank:	1333.4

NS2			
Pos.	50	obs:	exp:
tta	L	0	0.09
ttg	L	1	0.19
ctt	L	0	0.17
ctc	L	0	0.17
cta	L	0	0.17
ctg	L	0	0.22
att	I	0	0.36
atc	I	0	0.24
ata	I	1	0.39
atg	M	813	813.00
---	--	------	------
mPD		0.0049	0.0064
		nPD:	0.77
		N. weight:	0.0033
		Sc. PD:	0.0023
		Sc. rank:	2.3

NS2			
Pos.	51	obs:	exp:
cgt	R	0	34.82
cgc	R	0	41.41
cga	R	0	81.26
cgg	R	0	67.88
aga	R	13	372.10
agg	R	802	217.50
---	--	------	------
mPD		0.031	0.96
		nPD:	0.03
		N. weight:	1.3
		Sc. PD:	-0.023
		Sc. rank:	436.2

NS2			
Pos.	52	obs:	exp:
tta	L	0	0.09
ttg	L	0	0.19
ctt	L	0	0.17
ctc	L	0	0.17
cta	L	0	0.17
ctg	L	1	0.22
att	I	0	1.09
atc	I	0	0.73
ata	I	3	1.18
atg	M	804	804.00
act	T	0	0.78
acc	T	0	0.61
aca	T	0	1.38
acg	T	3	0.23
gtt	V	0	0.83
gtc	V	0	0.80
gta	V	0	0.83
gtg	V	4	1.54
---	--	------	------
mPD		0.027	0.041
		nPD:	0.66
		N. weight:	0.02
		Sc. PD:	0.012
		Sc. rank:	13.6

NS2			
Pos.	53	obs:	exp:
ggt	G	0	109.30
ggc	G	0	103.50
gga	G	794	376.80
ggg	G	21	225.30
---	--	------	------
mPD		0.050	0.68
		nPD:	0.07
		N. weight:	0.69
		Sc. PD:	0.015
		Sc. rank:	331.0

NS2			
Pos.	54	obs:	exp:
gat	D	527	440.00
gac	D	288	375.00
---	--	------	------
mPD		0.46	0.50
		nPD:	0.92
		N. weight:	0.029
		Sc. PD:	0.024
		Sc. rank:	19.7

NS2			
Pos.	55	obs:	exp:
ttt	F	0	1.58
ttc	F	4	2.42
tta	L	0	70.84
ttg	L	0	150.40
ctt	L	7	136.60
ctc	L	798	135.00
cta	L	1	134.40
ctg	L	0	178.80
att	I	0	1.82
atc	I	5	1.21
ata	I	0	1.97
---	--	------	------
mPD		0.041	1.1
		nPD:	0.04
		N. weight:	1.8
		Sc. PD:	-0.025
		Sc. rank:	658.0

NS2			
Pos.	56	obs:	exp:
tat	Y	0	0.53
tac	Y	1	0.47
cat	H	14	467.60
cac	H	800	346.40
---	--	------	------
mPD		0.036	0.49
		nPD:	0.07
		N. weight:	0.79
		Sc. PD:	0.017
		Sc. rank:	373.0

NS2			
Pos.	57	obs:	exp:
ttt	F	2	0.79
ttc	F	0	1.21
tct	S	696	130.10
tcc	S	117	105.20
tca	S	0	205.20
tcg	S	0	47.31
agt	S	0	166.60
agc	S	0	158.50
---	--	------	------
mPD		0.25	1.7
		nPD:	0.15
		N. weight:	1.5
		Sc. PD:	0.14
		Sc. rank:	833.6

NS2			
Pos.	58	obs:	exp:
tta	L	0	71.54
ttg	L	0	151.90
ctt	L	0	137.90
ctc	L	221	136.40
cta	L	592	135.70
ctg	L	1	180.50
att	I	0	0.36
atc	I	0	0.24
ata	I	1	0.39
---	--	------	------
mPD		0.40	1.1
		nPD:	0.36
		N. weight:	1.2
		Sc. PD:	0.37
		Sc. rank:	811.3

NS2			
Pos.	59	obs:	exp:
caa	Q	808	418.10
cag	Q	6	395.90
aaa	K	1	0.59
aag	K	0	0.41
---	--	------	------
mPD		0.017	0.50
		nPD:	0.03
		N. weight:	0.65
		Sc. PD:	-0.011
		Sc. rank:	225.8

NS2			
Pos.	60	obs:	exp:
tct	S	0	128.90
tcc	S	0	104.20
tca	S	0	203.20
tcg	S	0	46.85
att	I	2	0.73
atc	I	0	0.48
ata	I	0	0.79
aat	N	8	4.07
aac	N	0	3.93
agt	S	805	165.00
agc	S	0	157.00
---	--	------	------
mPD		0.024	1.7
		nPD:	0.01
		N. weight:	1.6
		Sc. PD:	-0.058
		Sc. rank:	129.3

NS2

Pos.	61	obs:	exp:
cgt	R	0	34.82
cgc	R	0	41.41
cga	R	0	81.26
cgg	R	0	67.88
aga	R	593	372.10
agg	R	222	217.50
---	--	------	------
mPD		0.40	0.96
		nPD:	0.41
		N. weight:	0.36
		Sc. PD:	0.12
		Sc. rank:	237.1

NS2

Pos.	62	obs:	exp:
att	I	0	0.36
atc	I	1	0.24
ata	I	0	0.39
aat	N	7	414.10
aac	N	807	399.90
---	--	------	------
mPD		0.020	0.50
		nPD:	0.04
		N. weight:	0.69
		Sc. PD:	-0.0085
		Sc. rank:	265.2

NS2

Pos.	63	obs:	exp:
tgg	W	1	1.00
gaa	E	0	0.60
gag	E	1	0.40
ggt	G	0	109.00
ggc	G	0	103.30
gga	G	5	375.90
ggg	G	808	224.80
---	--	------	------
mPD		0.017	0.68
		nPD:	0.03
		N. weight:	1.3
		Sc. PD:	-0.033
		Sc. rank:	387.5

NS2

Pos.	64	obs:	exp:
act	T	0	1.04
acc	T	0	0.81
aca	T	4	1.84
acg	T	0	0.31
aat	N	0	0.51
aac	N	1	0.49
aaa	K	640	472.70
aag	K	167	334.30
gaa	E	3	1.80
gag	E	0	1.20
---	--	------	------
mPD		0.35	0.51
		nPD:	0.68
		N. weight:	0.11
		Sc. PD:	0.066
		Sc. rank:	75.0

NS2

Pos.	65	obs:	exp:
tgg	W	815	815.00
---	--	------	------
mPD		0	0
		nPD:	1.
		N. weight:	0.
		Sc. PD:	0
		Sc. rank:	0

NS2

Pos.	66	obs:	exp:
cgt	R	0	34.82
cgc	R	0	41.41
cga	R	1	81.26
cgg	R	0	67.88
aga	R	814	372.10
agg	R	0	217.50
---	--	------	------
mPD		0.0025	0.96
		nPD:	0.
		N. weight:	0.81
		Sc. PD:	-0.038
		Sc. rank:	-335.2

NS2

Pos.	67	obs:	exp:
aaa	K	15	8.79
aag	K	0	6.21
gaa	E	797	479.40
gag	E	0	317.60
ggt	G	0	0.40
ggc	G	0	0.38
gga	G	3	1.39
ggg	G	0	0.83
---	--	------	------
mPD		0.044	0.52
		nPD:	0.08
		N. weight:	0.53
		Sc. PD:	0.016
		Sc. rank:	255.7

NS2

Pos.	68	obs:	exp:
caa	Q	227	418.60
cag	Q	588	396.40
---	--	------	------
mPD		0.40	0.50
		nPD:	0.81
		N. weight:	0.12
		Sc. PD:	0.088
		Sc. rank:	83.5

NS2

Pos.	69	obs:	exp:
tta	L	806	71.63
ttg	L	1	152.10
ctt	L	0	138.10
ctc	L	0	136.50
cta	L	8	135.90
ctg	L	0	180.80
---	--	------	------
mPD		0.022	1.1
		nPD:	0.02
		N. weight:	2.5
		Sc. PD:	-0.075
		Sc. rank:	596.3

NS2

Pos.	70	obs:	exp:
tct	S	0	130.30
tcc	S	0	105.40
tca	S	0	205.40
tcg	S	0	47.37
agt	S	0	166.80
agc	S	814	158.70
ggt	G	1	0.13
ggc	G	0	0.13
gga	G	0	0.46
ggg	G	0	0.28
---	--	------	------
mPD		0.0049	1.7
		nPD:	0.
		N. weight:	1.7
		Sc. PD:	-0.079
		Sc. rank:	-590.2

NS2

Pos.	71	obs:	exp:
tta	L	0	0.09
ttg	L	0	0.19
ctt	L	0	0.17
ctc	L	0	0.17
cta	L	0	0.17
ctg	L	1	0.22
caa	Q	24	417.10
cag	Q	788	394.90
aaa	K	1	1.17
aag	K	1	0.83
---	--	------	------
mPD		0.067	0.51
		nPD:	0.13
		N. weight:	0.61
		Sc. PD:	0.047
		Sc. rank:	325.3

NS2

Pos.	72	obs:	exp:
aaa	K	35	477.40
aag	K	780	337.60
---	--	------	------
mPD		0.082	0.49
		nPD:	0.17
		N. weight:	0.72
		Sc. PD:	0.081
		Sc. rank:	414.5

NS2

Pos.	73	obs:	exp:
ttt	F	28	321.60
ttc	F	787	493.40
---	--	------	------
mPD		0.066	0.48
		nPD:	0.14
		N. weight:	0.38
		Sc. PD:	0.032
		Sc. rank:	205.9

NS2

Pos.	74	obs:	exp:
gat	D	0	1.62
gac	D	3	1.38
gaa	E	744	487.80
gag	E	67	323.20
ggt	G	0	0.13
ggc	G	0	0.13
gga	G	0	0.46
ggg	G	1	0.28
---	--	------	------
mPD		0.16	0.49
		nPD:	0.33
		N. weight:	0.27
		Sc. PD:	0.074
		Sc. rank:	174.5

NS2

Pos.	75	obs:	exp:
gaa	E	682	490.20
gag	E	133	324.80
---	--	------	------
mPD		0.27	0.48
		nPD:	0.57
		N. weight:	0.14
		Sc. PD:	0.069
		Sc. rank:	92.3

NS2

Pos.	76	obs:	exp:
tta	L	1	0.09
ttg	L	0	0.19
ctt	L	0	0.17
ctc	L	0	0.17
cta	L	0	0.17
ctg	L	0	0.22
att	I	0	296.60
atc	I	0	196.60
ata	I	813	319.70
act	T	0	0.26
acc	T	0	0.20
aca	T	1	0.46
acg	T	0	0.08
---	--	------	------
mPD		0.0049	0.66
		nPD:	0.01
		N. weight:	0.97
		Sc. PD:	-0.041
		Sc. rank:	-148.5

NS2

Pos.	77	obs:	exp:
cgt	R	0	34.77
cgc	R	0	41.36
cga	R	1	81.16
cgg	R	0	67.80
aaa	K	1	0.59
aag	K	0	0.41
aga	R	811	371.70
agg	R	2	217.30
---	--	------	------
mPD		0.0098	0.97
		nPD:	0.01
		N. weight:	0.79
		Sc. PD:	-0.031
		Sc. rank:	-43.2

NS2

Pos.	78	obs:	exp:
tgg	W	815	815.00
---	--	------	------
mPD		0	0
		nPD:	1.
		N. weight:	0.
		Sc. PD:	0
		Sc. rank:	0

NS2

Pos.	79	obs:	exp:
tta	L	22	71.63
ttg	L	791	152.10
ctt	L	1	138.10
ctc	L	0	136.50
cta	L	0	135.90
ctg	L	1	180.80
---	--	------	------
mPD		0.060	1.1
		nPD:	0.05
		N. weight:	1.6
		Sc. PD:	0.0035
		Sc. rank:	686.9

NS2

Pos.	80	obs:	exp:
att	I	811	296.60
atc	I	2	196.60
ata	I	0	319.70
gtt	V	2	0.41
gtc	V	0	0.40
gta	V	0	0.41
gtg	V	0	0.77
---	--	------	------
mPD		0.0098	0.66
		nPD:	0.01
		N. weight:	1.
		Sc. PD:	-0.036
		Sc. rank:	93.6

NS2

Pos.	81	obs:	exp:
gaa	E	814	490.20
gag	E	1	324.80
---	--	------	------
mPD		0.0025	0.48
		nPD:	0.01
		N. weight:	0.52
		Sc. PD:	-0.023
		Sc. rank:	-136.3

NS2

Pos.	82	obs:	exp:
aaa	K	1	0.59
aag	K	0	0.41
gaa	E	814	489.60
gag	E	0	324.40
---	--	------	------
mPD		0.0025	0.48
		nPD:	0.01
		N. weight:	0.53
		Sc. PD:	-0.023
		Sc. rank:	-144.5

NS2

Pos.	83	obs:	exp:
att	I	0	0.73
atc	I	0	0.48
ata	I	2	0.79
gtt	V	17	167.60
gtc	V	0	162.70
gta	V	785	168.10
gtg	V	10	313.60
gct	A	0	0.26
gcc	A	0	0.19
gca	A	1	0.47
gcg	A	0	0.09
---	--	------	------
mPD		0.072	0.73
		nPD:	0.1
		N. weight:	1.5
		Sc. PD:	0.065
		Sc. rank:	756.9

NS2

Pos.	84	obs:	exp:
cgt	R	0	34.77
cgc	R	0	41.36
cga	R	810	81.16
cgg	R	3	67.80
aga	R	1	371.70
agg	R	0	217.30
ggt	G	0	0.13
ggc	G	0	0.13
gga	G	1	0.46
ggg	G	0	0.28
---	--	------	------
mPD		0.012	0.97
		nPD:	0.01
		N. weight:	2.4
		Sc. PD:	-0.088
		Sc. rank:	-41.1

NS2

Pos.	85	obs:	exp:
cct	P	1	0.25
ccc	P	0	0.18
cca	P	0	0.41
ccg	P	0	0.16
cat	H	804	465.90
cac	H	7	345.10
aat	N	1	0.51
aac	N	0	0.49
gat	D	2	1.08
gac	D	0	0.92
---	--	------	------
mPD		0.027	0.50
		nPD:	0.05
		N. weight:	0.53
		Sc. PD:	0.0011
		Sc. rank:	215.2

NS2			
Pos.	86	obs:	exp:
cgt	R	0	34.56
cgc	R	0	41.10
cga	R	0	80.67
cgg	R	0	67.38
aaa	K	0	3.51
aag	K	6	2.49
aga	R	597	369.40
agg	R	212	215.90
mPD		0.41	0.98
		nPD:	0.42
		N. weight:	0.37
		Sc. PD:	0.13
		Sc. rank:	244.6

NS2			
Pos.	87	obs:	exp:
tta	L	809	71.63
ttg	L	1	152.10
ctt	L	0	138.10
ctc	L	0	136.50
cta	L	5	135.90
ctg	L	0	180.80
mPD		0.015	1.1
		nPD:	0.01
		N. weight:	2.5
		Sc. PD:	-0.091
		Sc. rank:	93.4

NS2			
Pos.	88	obs:	exp:
cgt	R	0	0.04
cgc	R	0	0.05
cga	R	0	0.10
cgg	R	0	0.08
aaa	K	592	476.30
aag	K	221	336.70
aga	R	1	0.46
agg	R	0	0.27
gaa	E	1	0.60
gag	E	0	0.40
mPD		0.40	0.49
		nPD:	0.82
		N. weight:	0.052
		Sc. PD:	0.038
		Sc. rank:	35.1

NS2			
Pos.	89	obs:	exp:
ttt	F	3	1.18
ttc	F	0	1.82
att	I	791	294.10
atc	I	15	195.00
ata	I	0	317.00
act	T	1	0.52
acc	T	0	0.41
aca	T	1	0.92
acg	T	0	0.16
gtt	V	4	0.83
gtc	V	0	0.80
gta	V	0	0.83
gtg	V	0	1.54
mPD		0.061	0.68
		nPD:	0.09
		N. weight:	0.96
		Sc. PD:	0.035
		Sc. rank:	485.4

NS2			
Pos.	90	obs:	exp:
act	T	0	211.10
acc	T	1	166.00
aca	T	230	374.40
acg	T	584	63.49
mPD		0.41	0.67
		nPD:	0.6
		N. weight:	1.5
		Sc. PD:	0.79
		Sc. rank:	1020.6

NS2			
Pos.	91	obs:	exp:
gat	D	0	0.54
gac	D	1	0.46
gaa	E	12	489.00
gag	E	801	324.00
ggt	G	0	0.13
ggc	G	0	0.13
gga	G	0	0.46
ggg	G	1	0.28
mPD		0.034	0.48
		nPD:	0.07
		N. weight:	0.87
		Sc. PD:	0.015
		Sc. rank:	401.9

NS2			
Pos.	92	obs:	exp:
tct	S	0	6.08
tcc	S	0	4.92
tca	S	0	9.59
tcg	S	0	2.21
act	T	5	1.30
acc	T	0	1.02
aca	T	0	2.30
acg	T	0	0.39
aat	N	770	392.80
aac	N	2	379.20
agt	S	38	7.79
agc	S	0	7.41
mPD		0.11	0.67
		nPD:	0.16
		N. weight:	0.73
		Sc. PD:	0.074
		Sc. rank:	417.2

NS2			
Pos.	93	obs:	exp:
tct	S	0	130.10
tcc	S	0	105.20
tca	S	0	205.20
tcg	S	0	47.31
aat	N	0	0.51
aac	N	1	0.49
agt	S	1	166.60
agc	S	812	158.50
ggt	G	0	0.13
ggc	G	1	0.13
gga	G	0	0.46
ggg	G	0	0.28
mPD		0.0074	1.7
		nPD:	0.
		N. weight:	1.7
		Sc. PD:	-0.076
		Sc. rank:	-525.2

NS2			
Pos.	94	obs:	exp:
ttt	F	811	321.60
ttc	F	4	493.40
mPD		0.0098	0.48
		nPD:	0.02
		N. weight:	0.93
		Sc. PD:	-0.028
		Sc. rank:	246.2

NS2			
Pos.	95	obs:	exp:
gaa	E	221	490.20
gag	E	594	324.80
mPD		0.40	0.48
		nPD:	0.83
		N. weight:	0.24
		Sc. PD:	0.17
		Sc. rank:	161.9

NS2			
Pos.	96	obs:	exp:
caa	Q	814	418.60
cag	Q	1	396.40
mPD		0.0025	0.50
		nPD:	0.
		N. weight:	0.68
		Sc. PD:	-0.03
		Sc. rank:	-195.9

NS2			
Pos.	97	obs:	exp:
att	I	4	297.40
atc	I	0	197.10
ata	I	811	320.50
mPD		0.0098	0.65
		nPD:	0.01
		N. weight:	0.94
		Sc. PD:	-0.033
		Sc. rank:	94.9

NS2			
Pos.	98	obs:	exp:
act	T	811	211.10
acc	T	3	166.00
aca	T	1	374.40
acg	T	0	63.49
mPD		0.0098	0.67
		nPD:	0.01
		N. weight:	1.4
		Sc. PD:	-0.049
		Sc. rank:	83.4

NS2			
Pos.	99	obs:	exp:
ttt	F	798	321.60
ttc	F	17	493.40
mPD		0.041	0.48
		nPD:	0.09
		N. weight:	0.85
		Sc. PD:	0.028
		Sc. rank:	420.6

NS2			
Pos.	100	obs:	exp:
att	I	0	0.36
atc	I	1	0.24
ata	I	0	0.39
atg	M	814	814.00
mPD		0.0025	0.0025
		nPD:	1.
		N. weight:	0.0018
		Sc. PD:	0.0016
		Sc. rank:	1.2

NS2			
Pos.	101	obs:	exp:
caa	Q	815	418.60
cag	Q	0	396.40
mPD		0	0.50
		nPD:	0.
		N. weight:	0.69
		Sc. PD:	-0.034
		Sc. rank:	-457.3

NS2			
Pos.	102	obs:	exp:
gct	A	0	208.80
gcc	A	813	153.00
gca	A	2	381.10
gcg	A	0	72.09
mPD		0.0049	0.67
		nPD:	0.01
		N. weight:	1.7
		Sc. PD:	-0.073
		Sc. rank:	-353.7

NS2			
Pos.	103	obs:	exp:
tta	L	809	71.63
ttg	L	6	152.10
ctt	L	0	138.10
ctc	L	0	136.50
cta	L	0	135.90
ctg	L	0	180.80
mPD		0.015	1.1
		nPD:	0.01
		N. weight:	2.5
		Sc. PD:	-0.091
		Sc. rank:	25.1

NS2			
Pos.	104	obs:	exp:
cat	H	0	0.57
cac	H	1	0.43
caa	Q	813	418.10
cag	Q	1	395.90
mPD		0.0049	0.50
		nPD:	0.01
		N. weight:	0.68
		Sc. PD:	-0.027
		Sc. rank:	-57.7

NS2			
Pos.	105	obs:	exp:
tta	L	0	71.63
ttg	L	0	152.10
ctt	L	0	138.10
ctc	L	0	136.50
cta	L	812	135.90
ctg	L	3	180.80
mPD		0.0073	1.1
		nPD:	0.01
		N. weight:	1.8
		Sc. PD:	-0.079
		Sc. rank:	-433.1

NS2			
Pos.	106	obs:	exp:
tta	L	0	71.54
ttg	L	782	151.90
tct	S	0	0.16
tcc	S	0	0.13
tca	S	0	0.25
tcg	S	1	0.06
ctt	L	0	137.90
ctc	L	0	136.40
cta	L	0	135.70
ctg	L	32	180.50
agt	S	0	0.20
agc	S	0	0.20
mPD		0.078	1.1
		nPD:	0.07
		N. weight:	1.6
		Sc. PD:	0.027
		Sc. rank:	707.5

NS2			
Pos.	107	obs:	exp:
tta	L	0	71.63
ttg	L	0	152.10
ctt	L	814	138.10
ctc	L	0	136.50
cta	L	0	135.90
ctg	L	1	180.80
mPD		0.0025	1.1
		nPD:	0.
		N. weight:	1.8
		Sc. PD:	-0.087
		Sc. rank:	-812.7

NS2			
Pos.	108	obs:	exp:
gaa	E	814	490.20
gag	E	1	324.80
mPD		0.0025	0.48
		nPD:	0.01
		N. weight:	0.52
		Sc. PD:	-0.023
		Sc. rank:	-136.3

NS2			
Pos.	109	obs:	exp:
gtt	V	0	168.20
gtc	V	0	163.30
gta	V	5	168.70
gtg	V	810	314.70
mPD		0.012	0.73
		nPD:	0.02
		N. weight:	0.96
		Sc. PD:	-0.032
		Sc. rank:	167.8

NS2			
Pos.	110	obs:	exp:
gaa	E	11	490.20
gag	E	804	324.80
---	--	------	------
mPD		0.027	0.48
		nPD:	0.06
		N. weight:	0.88
		Sc. PD:	0.0033
		Sc. rank:	380.6

NS2			
Pos.	111	obs:	exp:
caa	Q	812	417.60
cag	Q	1	395.40
aaa	K	1	1.17
aag	K	1	0.83
---	--	------	------
mPD		0.0098	0.50
		nPD:	0.02
		N. weight:	0.68
		Sc. PD:	-0.021
		Sc. rank:	147.2

NS2			
Pos.	112	obs:	exp:
gaa	E	0	490.20
gag	E	815	324.80
---	--	------	------
mPD		0	0.48
		nPD:	0.
		N. weight:	0.96
		Sc. PD:	-0.047
		Sc. rank:	-631.6

NS2			
Pos.	113	obs:	exp:
att	I	0	296.60
atc	I	0	196.60
ata	I	813	319.70
atg	M	1	1.00
act	T	0	0.26
acc	T	0	0.20
aca	T	1	0.46
acg	T	0	0.08
---	--	------	------
mPD		0.0049	0.66
		nPD:	0.01
		N. weight:	0.97
		Sc. PD:	-0.041
		Sc. rank:	-137.6

NS2			
Pos.	114	obs:	exp:
cgt	R	0	34.82
cgc	R	0	41.41
cga	R	0	81.26
cgg	R	0	67.88
aga	R	707	372.10
agg	R	108	217.50
---	--	------	------
mPD		0.23	0.96
		nPD:	0.24
		N. weight:	0.49
		Sc. PD:	0.087
		Sc. rank:	292.1

NS2			
Pos.	115	obs:	exp:
cct	P	1	0.25
ccc	P	0	0.18
cca	P	0	0.41
ccg	P	0	0.16
act	T	808	210.60
acc	T	5	165.60
aca	T	0	373.50
acg	T	0	63.33
gct	A	0	0.26
gcc	A	1	0.19
gca	A	0	0.47
gcg	A	0	0.09
---	--	------	------
mPD		0.020	0.68
		nPD:	0.03
		N. weight:	1.4
		Sc. PD:	-0.03
		Sc. rank:	446.5

NS2			
Pos.	116	obs:	exp:
ttt	F	0	320.90
ttc	F	813	492.10
tta	L	0	0.09
ttg	L	0	0.19
ctt	L	0	0.17
ctc	L	1	0.17
cta	L	0	0.17
ctg	L	0	0.22
gtt	V	0	0.21
gtc	V	1	0.20
gta	V	0	0.21
gtg	V	0	0.39
---	--	------	------
mPD		0.0049	0.48
		nPD:	0.01
		N. weight:	0.52
		Sc. PD:	-0.021
		Sc. rank:	-33.2

NS2			
Pos.	117	obs:	exp:
tct	S	0	130.50
tcc	S	0	105.50
tca	S	6	205.70
tcg	S	809	47.43
agt	S	0	167.00
agc	S	0	158.90
---	--	------	------
mPD		0.015	1.7
		nPD:	0.01
		N. weight:	2.9
		Sc. PD:	-0.12
		Sc. rank:	-318.4

NS2			
Pos.	118	obs:	exp:
ttt	F	810	319.70
ttc	F	0	490.30
tct	S	1	0.16
tcc	S	0	0.13
tca	S	0	0.25
tcg	S	0	0.06
agt	S	0	0.20
agc	S	0	0.20
gtt	V	4	0.83
gtc	V	0	0.80
gta	V	0	0.83
gtg	V	0	1.54
---	--	------	------
mPD		0.012	0.49
		nPD:	0.02
		N. weight:	0.97
		Sc. PD:	-0.025
		Sc. rank:	266.3

NS2			
Pos.	119	obs:	exp:
cat	H	0	0.57
cac	H	1	0.43
caa	Q	0	417.60
cag	Q	813	395.40
gaa	E	0	0.60
gag	E	1	0.40
---	--	------	------
mPD		0.0049	0.50
		nPD:	0.01
		N. weight:	0.75
		Sc. PD:	-0.03
		Sc. rank:	-68.9

NS2			
Pos.	120	obs:	exp:
tta	L	0	71.36
ttg	L	0	151.50
ctt	L	808	137.60
ctc	L	4	136.00
cta	L	0	135.40
ctg	L	0	180.10
cat	H	1	0.57
cac	H	0	0.43
cgt	R	2	0.09
cgc	R	0	0.10
cga	R	0	0.20
cgg	R	0	0.17
aga	R	0	0.91
agg	R	0	0.53
---	--	------	------
mPD		0.017	1.1
		nPD:	0.02
		N. weight:	1.8
		Sc. PD:	-0.063
		Sc. rank:	220.1

NS2			
Pos.	121	obs:	exp:
att	I	808	295.50
atc	I	1	195.90
ata	I	1	318.50
aat	N	5	2.54
aac	N	0	2.46
---	--	------	------
mPD		0.017	0.67
		nPD:	0.03
		N. weight:	1.
		Sc. PD:	-0.025
		Sc. rank:	319.9

NS2			
Pos.	122	obs:	exp:
taa	.	812	350.50
tag	.	2	206.10
tga	.	1	258.40
---	--	------	------
mPD		0.0074	0.81
		nPD:	0.01
		N. weight:	0.85
		Sc. PD:	-0.035
		Sc. rank:	-85.6

Amino acid level conservation per codon

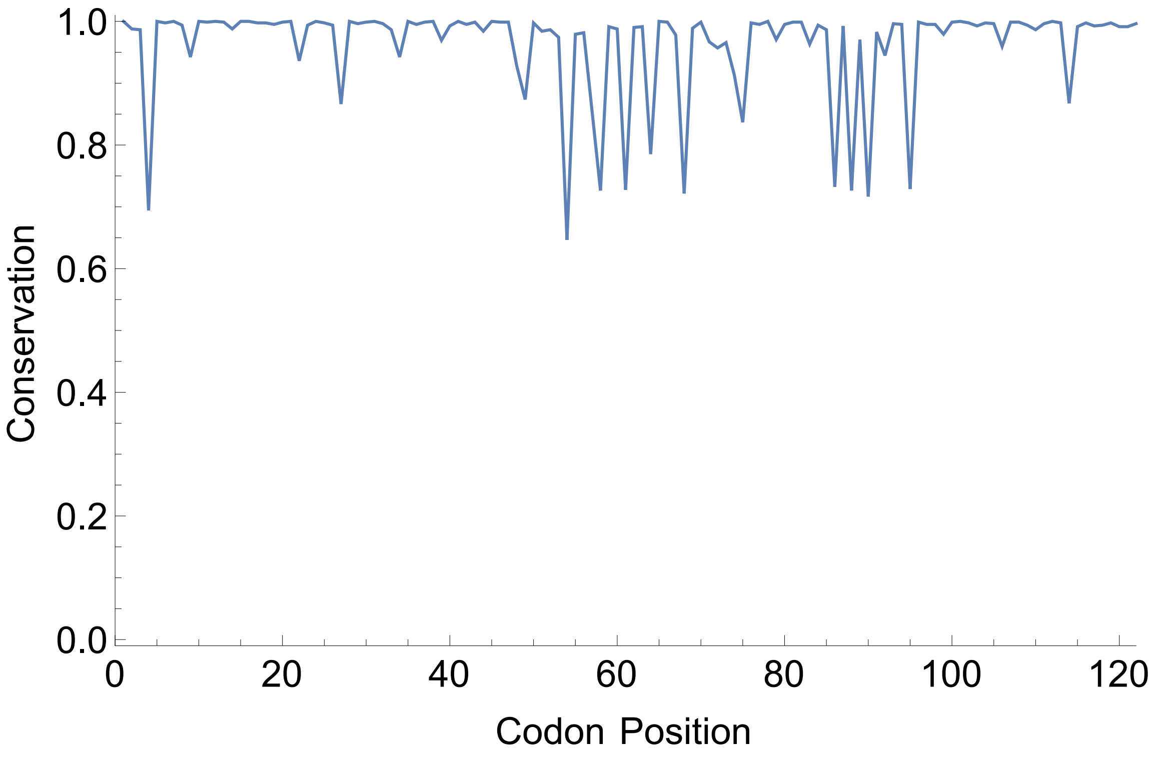

Conservation analysis plots

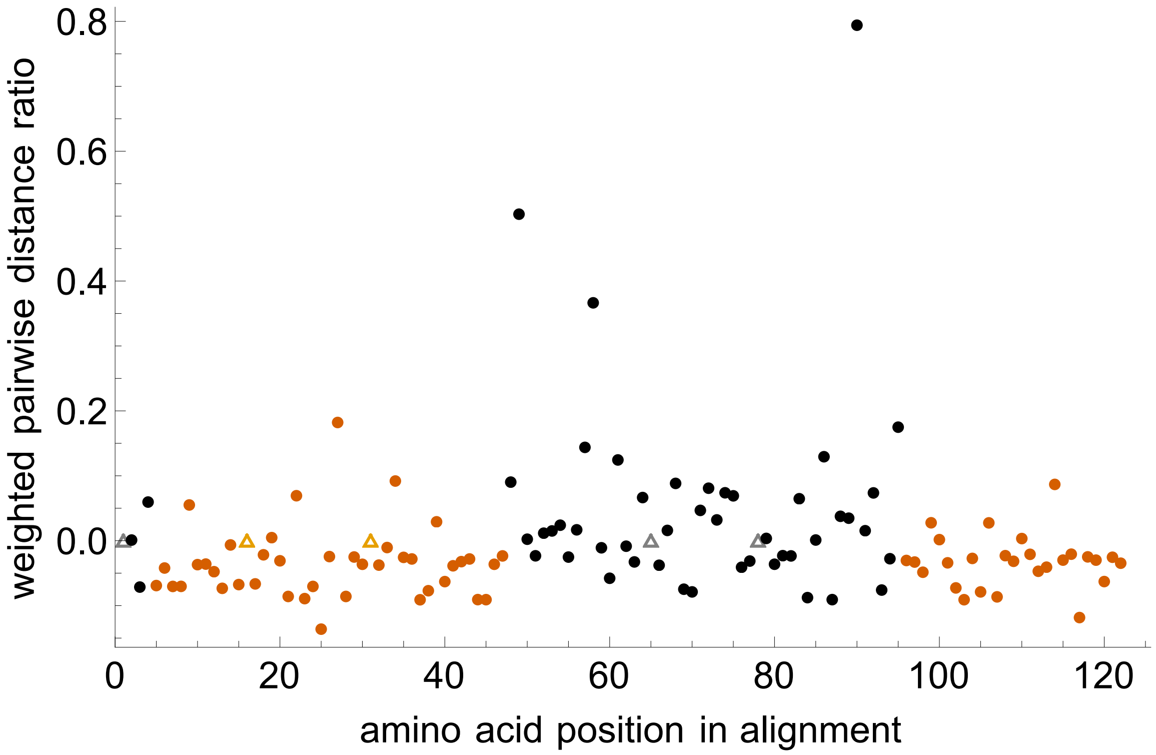

NS2

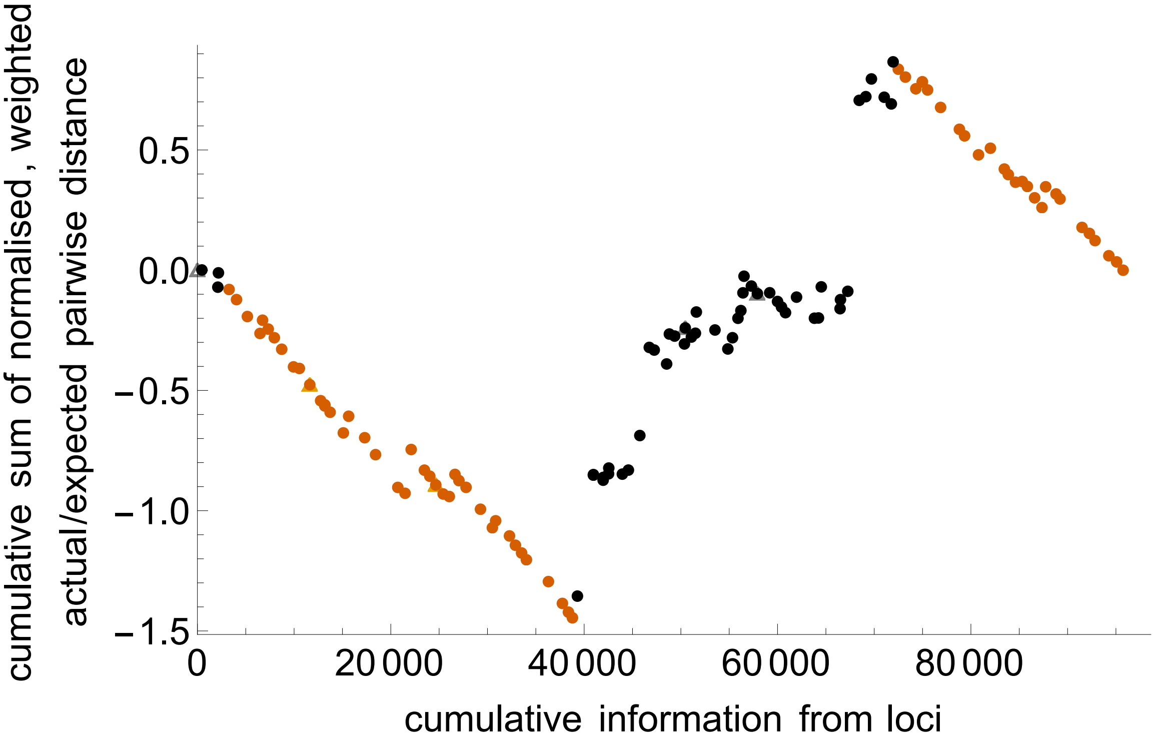

NS2

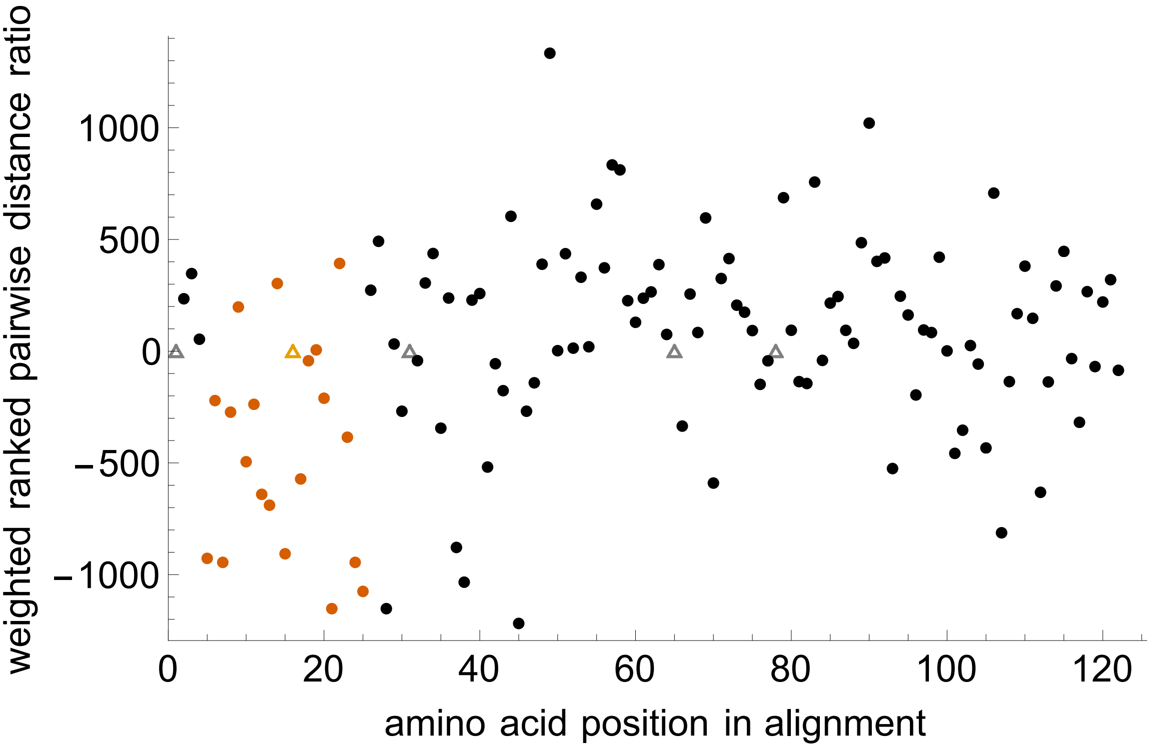
weighted ranked pairwise distance ratio
1000
500
0
−500
−1000
0
20
40
60
80
100
120
amino acid position in alignment

NS2

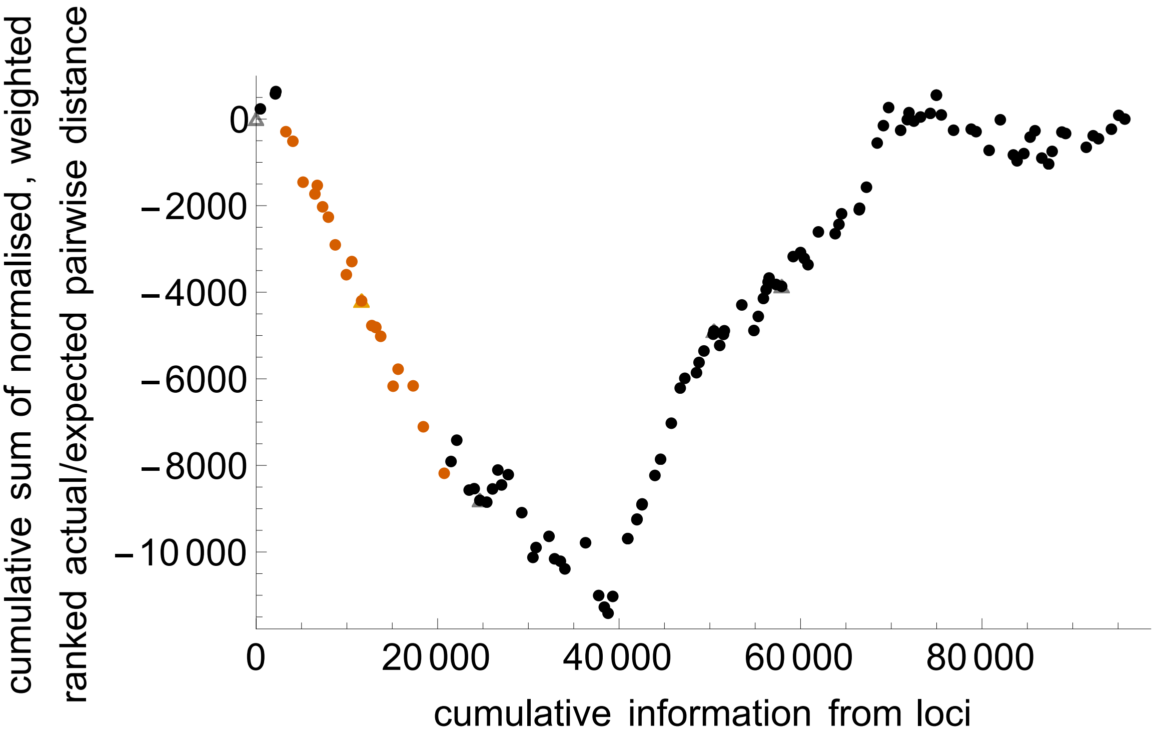
cumulative sum of normalised, weighted ranked actual/expected pairwise distance
0
−2000
−4000
−6000
−8000
−10 000
0
20 000
40 000
60 000
80 000
cumulative information from loci

NS2
