# Supplementary figures and images for: Locations and structures of influenza A virus packaging-associated signals and other functional elements via an in silico pipeline for predicting constrained features in RNA viruses (part 1 of 6)
Source: PLoS Comput Biol. 2024 Apr 22;20(4):e1012009. doi: 10.1371/journal.pcbi.1012009 (PMC11034665; doi:10.1371/journal.pcbi.1012009)

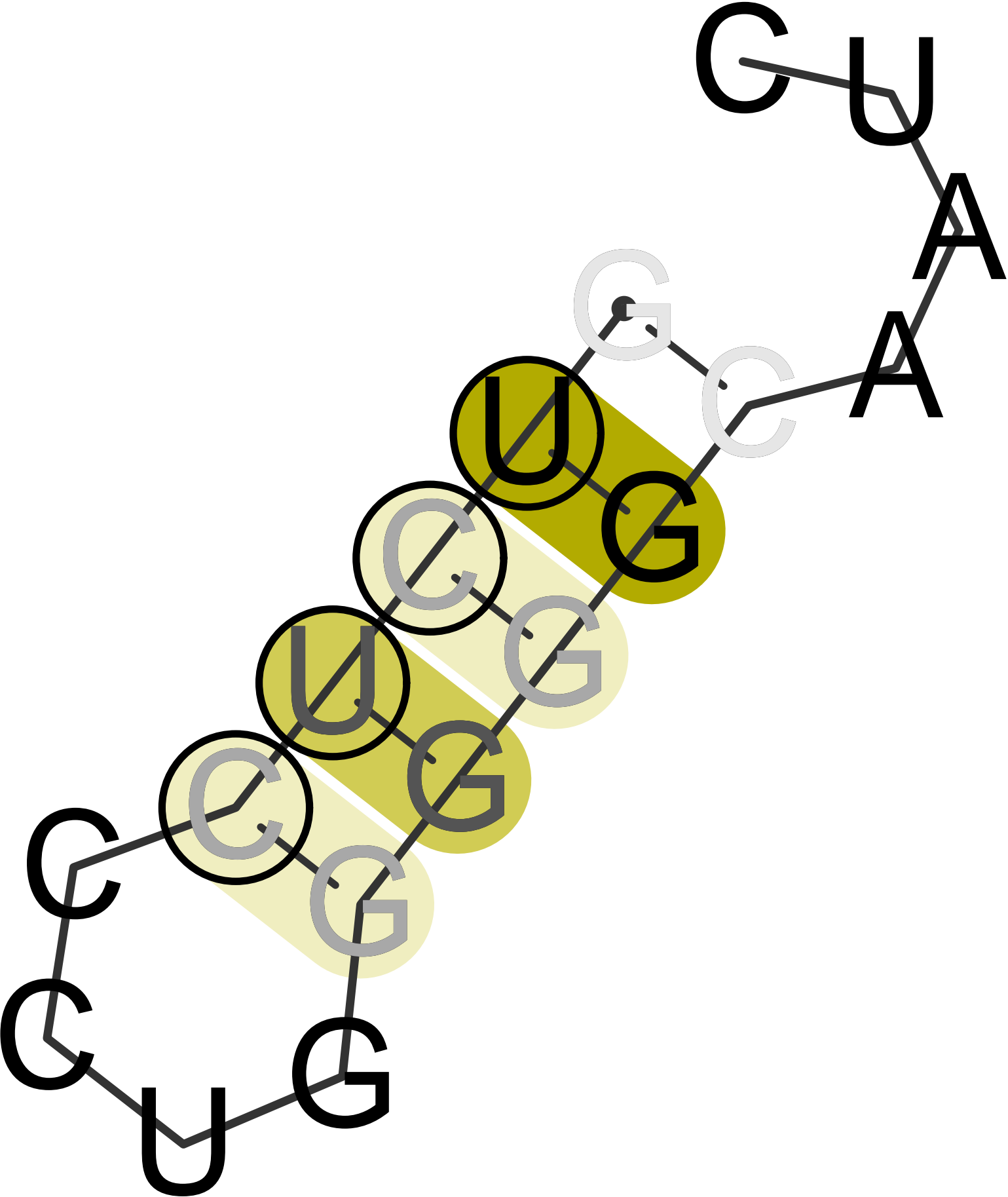

Supplement: S1 Fig — Filename convention within the zip file is as follows: HA/NA type, then host type, then whether the region was identified through analysis of raw (unranked) data, ranked data, or both, then the gene name, then the nucleotide location within the analysed alignment of the gene name, then the nucleotide location within the reference sequences (corresponding to locations listed in S2 Table and S10 Table), then a note if the analysis was performed using only one example of each distinct sequence, then a note if the fold uses the reverse complement of the cRNA (i.e. the vRNA), rather than the cRNA. All folds have been generated using alignments with loci where the consensus nucleotide is a gap removed. Base pairs are highlighted in deep/mid/light red when all/all but one/all but two sequences are capable of forming the pairs shown. Base pairs are highlighted in deep/mid/light yellow when all/all but one/all but two sequences are capable of forming the pair shown or one other pair (including GU pairs). Base pairs are highlighted in deep/mid/light green when all/all but one/all but two sequences are capable of forming the pair shown or one of two other pairs (including GU pairs). RNAalifold was used with input options disallowing lonely pairs, allowing G-quadruplexes, and with the ribosum scoring matrix enabled. (ZIP) [file pcbi.1012009.s122.zip › H1N1-human-ranked-HA-alignment-1642-1659-refseq-1633-1650-representative-sequences-only_alirna_nogap.pdf]

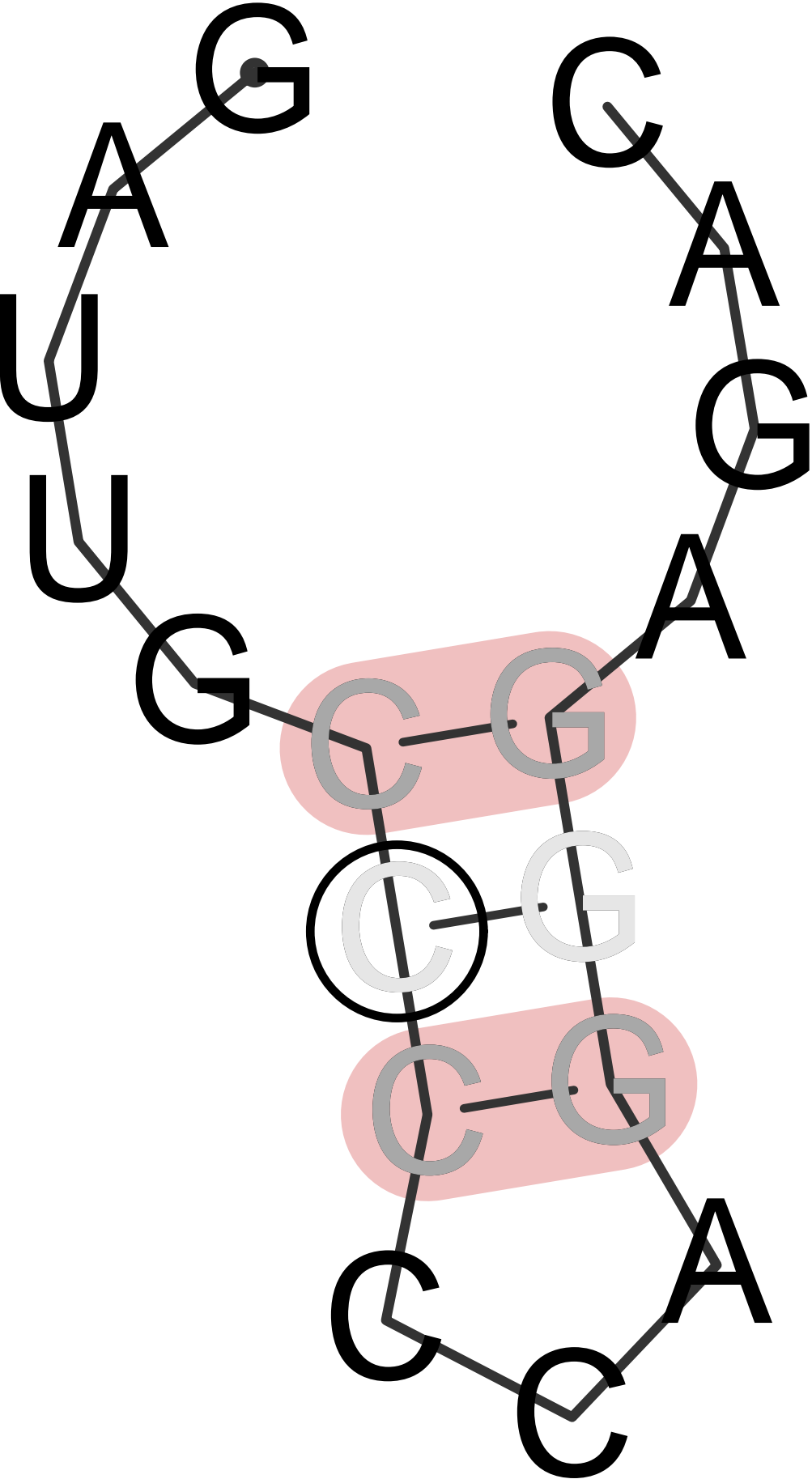

Supplement: S1 Fig — Filename convention within the zip file is as follows: HA/NA type, then host type, then whether the region was identified through analysis of raw (unranked) data, ranked data, or both, then the gene name, then the nucleotide location within the analysed alignment of the gene name, then the nucleotide location within the reference sequences (corresponding to locations listed in S2 Table and S10 Table), then a note if the analysis was performed using only one example of each distinct sequence, then a note if the fold uses the reverse complement of the cRNA (i.e. the vRNA), rather than the cRNA. All folds have been generated using alignments with loci where the consensus nucleotide is a gap removed. Base pairs are highlighted in deep/mid/light red when all/all but one/all but two sequences are capable of forming the pairs shown. Base pairs are highlighted in deep/mid/light yellow when all/all but one/all but two sequences are capable of forming the pair shown or one other pair (including GU pairs). Base pairs are highlighted in deep/mid/light green when all/all but one/all but two sequences are capable of forming the pair shown or one of two other pairs (including GU pairs). RNAalifold was used with input options disallowing lonely pairs, allowing G-quadruplexes, and with the ribosum scoring matrix enabled. (ZIP) [file pcbi.1012009.s122.zip › H1N1-human-ranked-HA-alignment-1642-1659-refseq-1633-1650-representative-sequences-only_revcomp_alirna_nogap.pdf]

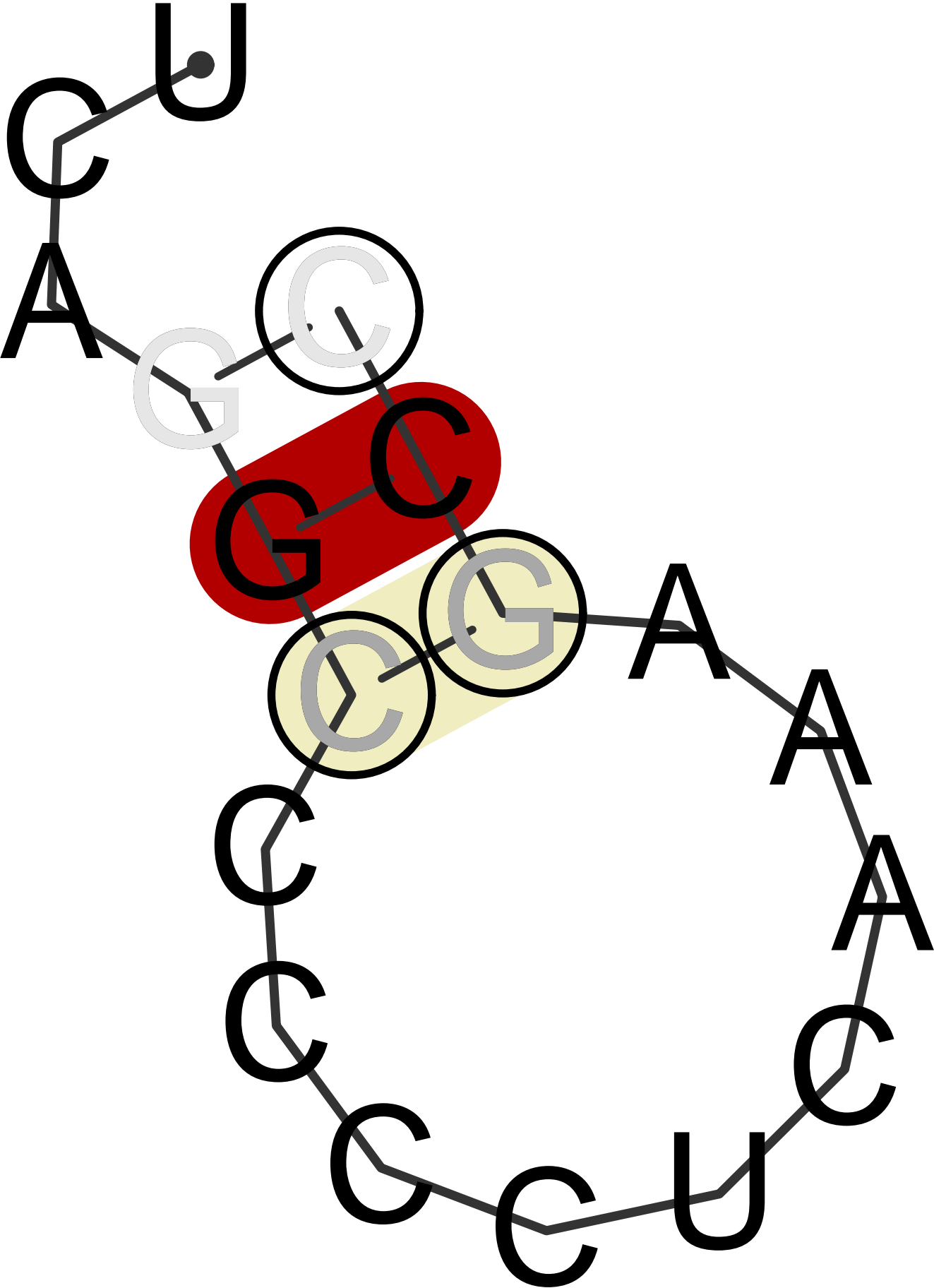

Supplement: S1 Fig — Filename convention within the zip file is as follows: HA/NA type, then host type, then whether the region was identified through analysis of raw (unranked) data, ranked data, or both, then the gene name, then the nucleotide location within the analysed alignment of the gene name, then the nucleotide location within the reference sequences (corresponding to locations listed in S2 Table and S10 Table), then a note if the analysis was performed using only one example of each distinct sequence, then a note if the fold uses the reverse complement of the cRNA (i.e. the vRNA), rather than the cRNA. All folds have been generated using alignments with loci where the consensus nucleotide is a gap removed. Base pairs are highlighted in deep/mid/light red when all/all but one/all but two sequences are capable of forming the pairs shown. Base pairs are highlighted in deep/mid/light yellow when all/all but one/all but two sequences are capable of forming the pair shown or one other pair (including GU pairs). Base pairs are highlighted in deep/mid/light green when all/all but one/all but two sequences are capable of forming the pair shown or one of two other pairs (including GU pairs). RNAalifold was used with input options disallowing lonely pairs, allowing G-quadruplexes, and with the ribosum scoring matrix enabled. (ZIP) [file pcbi.1012009.s122.zip › H1N1-human-ranked-M1-alignment-49-66-refseq-49-66-representative-sequences-only_alirna_nogap.pdf]

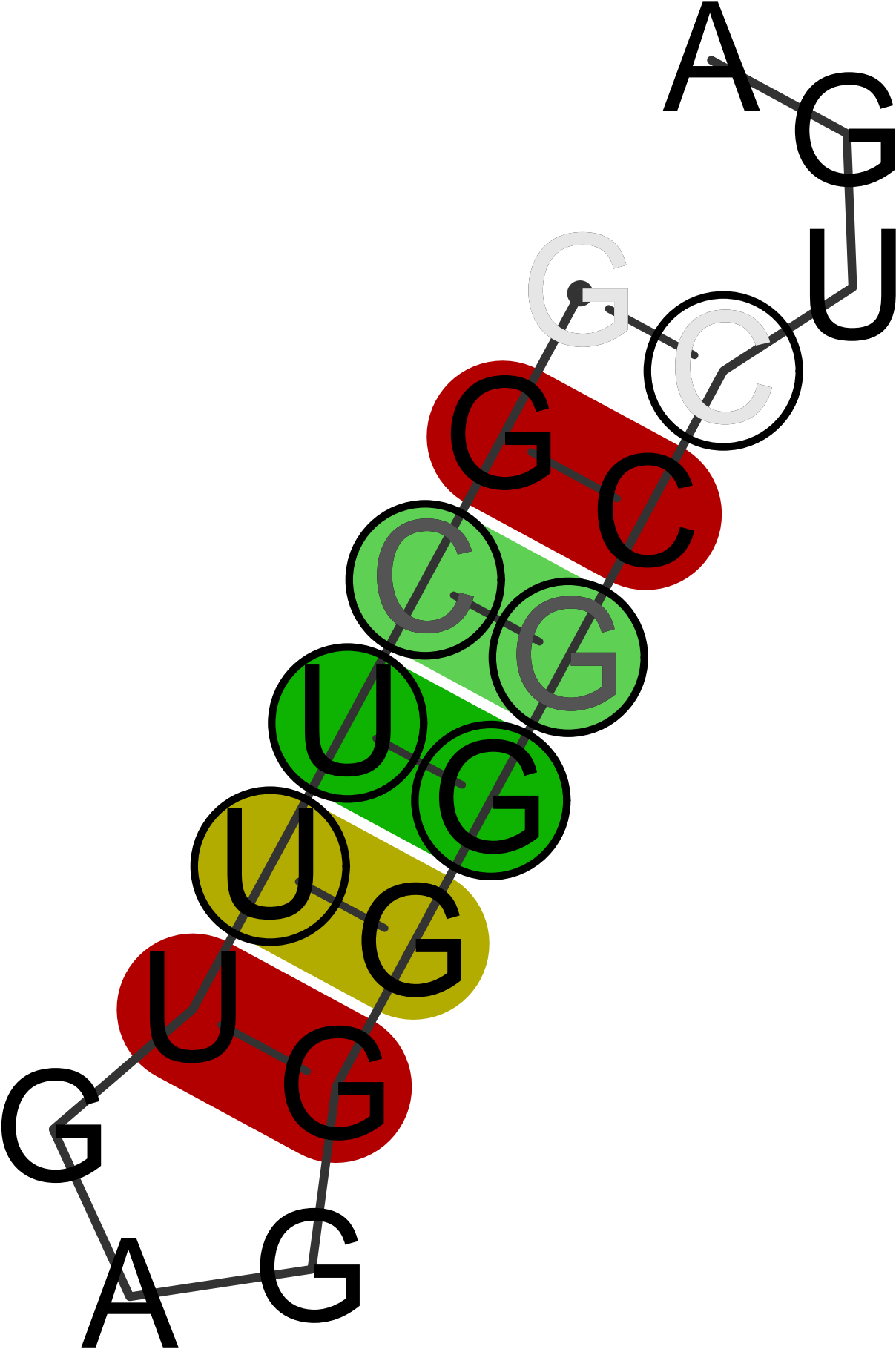

Supplement: S1 Fig — Filename convention within the zip file is as follows: HA/NA type, then host type, then whether the region was identified through analysis of raw (unranked) data, ranked data, or both, then the gene name, then the nucleotide location within the analysed alignment of the gene name, then the nucleotide location within the reference sequences (corresponding to locations listed in S2 Table and S10 Table), then a note if the analysis was performed using only one example of each distinct sequence, then a note if the fold uses the reverse complement of the cRNA (i.e. the vRNA), rather than the cRNA. All folds have been generated using alignments with loci where the consensus nucleotide is a gap removed. Base pairs are highlighted in deep/mid/light red when all/all but one/all but two sequences are capable of forming the pairs shown. Base pairs are highlighted in deep/mid/light yellow when all/all but one/all but two sequences are capable of forming the pair shown or one other pair (including GU pairs). Base pairs are highlighted in deep/mid/light green when all/all but one/all but two sequences are capable of forming the pair shown or one of two other pairs (including GU pairs). RNAalifold was used with input options disallowing lonely pairs, allowing G-quadruplexes, and with the ribosum scoring matrix enabled. (ZIP) [file pcbi.1012009.s122.zip › H1N1-human-ranked-M1-alignment-49-66-refseq-49-66-representative-sequences-only_revcomp_alirna_nogap.pdf]

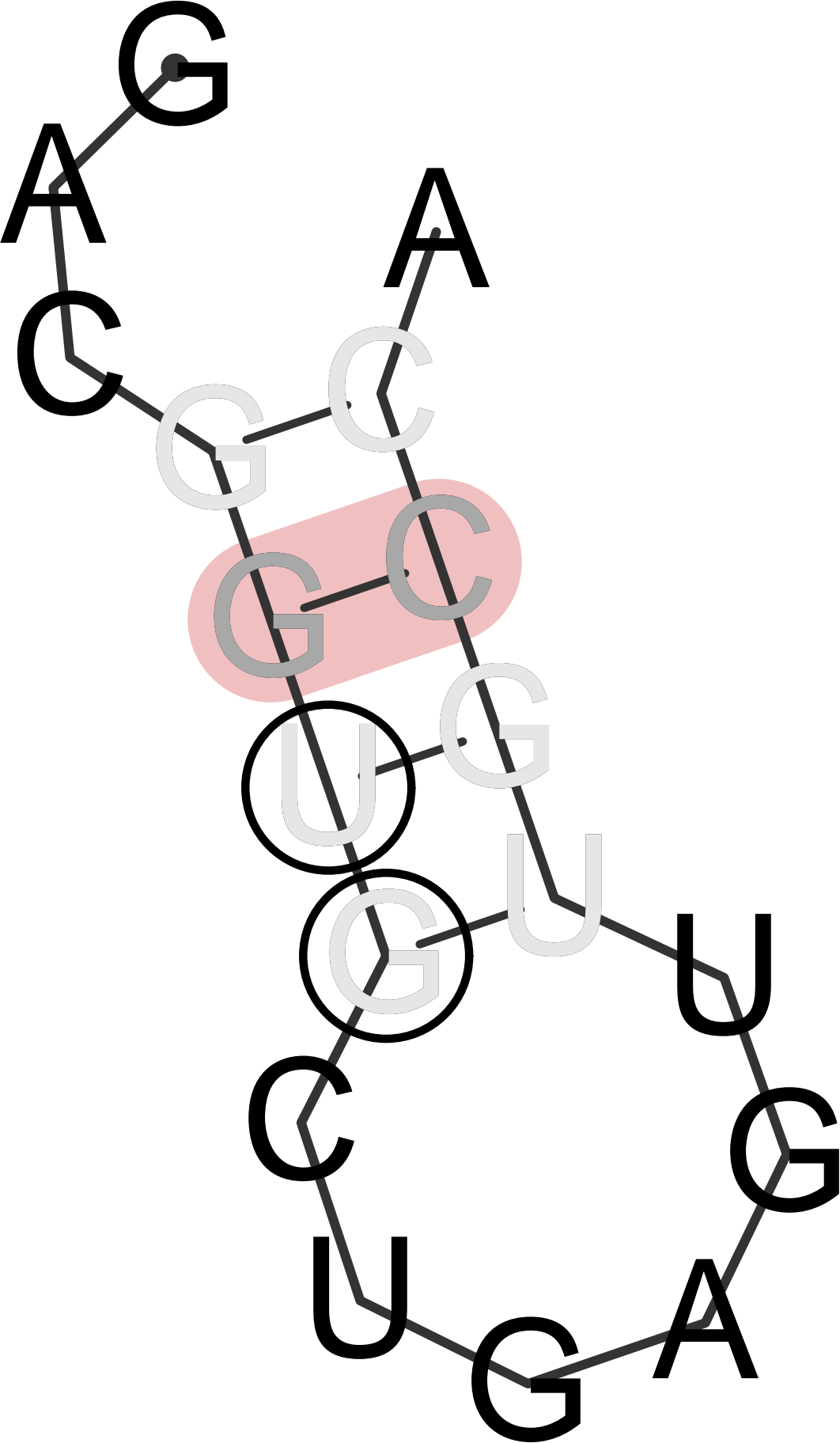

Supplement: S1 Fig — Filename convention within the zip file is as follows: HA/NA type, then host type, then whether the region was identified through analysis of raw (unranked) data, ranked data, or both, then the gene name, then the nucleotide location within the analysed alignment of the gene name, then the nucleotide location within the reference sequences (corresponding to locations listed in S2 Table and S10 Table), then a note if the analysis was performed using only one example of each distinct sequence, then a note if the fold uses the reverse complement of the cRNA (i.e. the vRNA), rather than the cRNA. All folds have been generated using alignments with loci where the consensus nucleotide is a gap removed. Base pairs are highlighted in deep/mid/light red when all/all but one/all but two sequences are capable of forming the pairs shown. Base pairs are highlighted in deep/mid/light yellow when all/all but one/all but two sequences are capable of forming the pair shown or one other pair (including GU pairs). Base pairs are highlighted in deep/mid/light green when all/all but one/all but two sequences are capable of forming the pair shown or one of two other pairs (including GU pairs). RNAalifold was used with input options disallowing lonely pairs, allowing G-quadruplexes, and with the ribosum scoring matrix enabled. (ZIP) [file pcbi.1012009.s122.zip › H1N1-human-ranked-NA-alignment-1390-1413-refseq-1375-1392-representative-sequences-only_alirna_nogap.pdf]

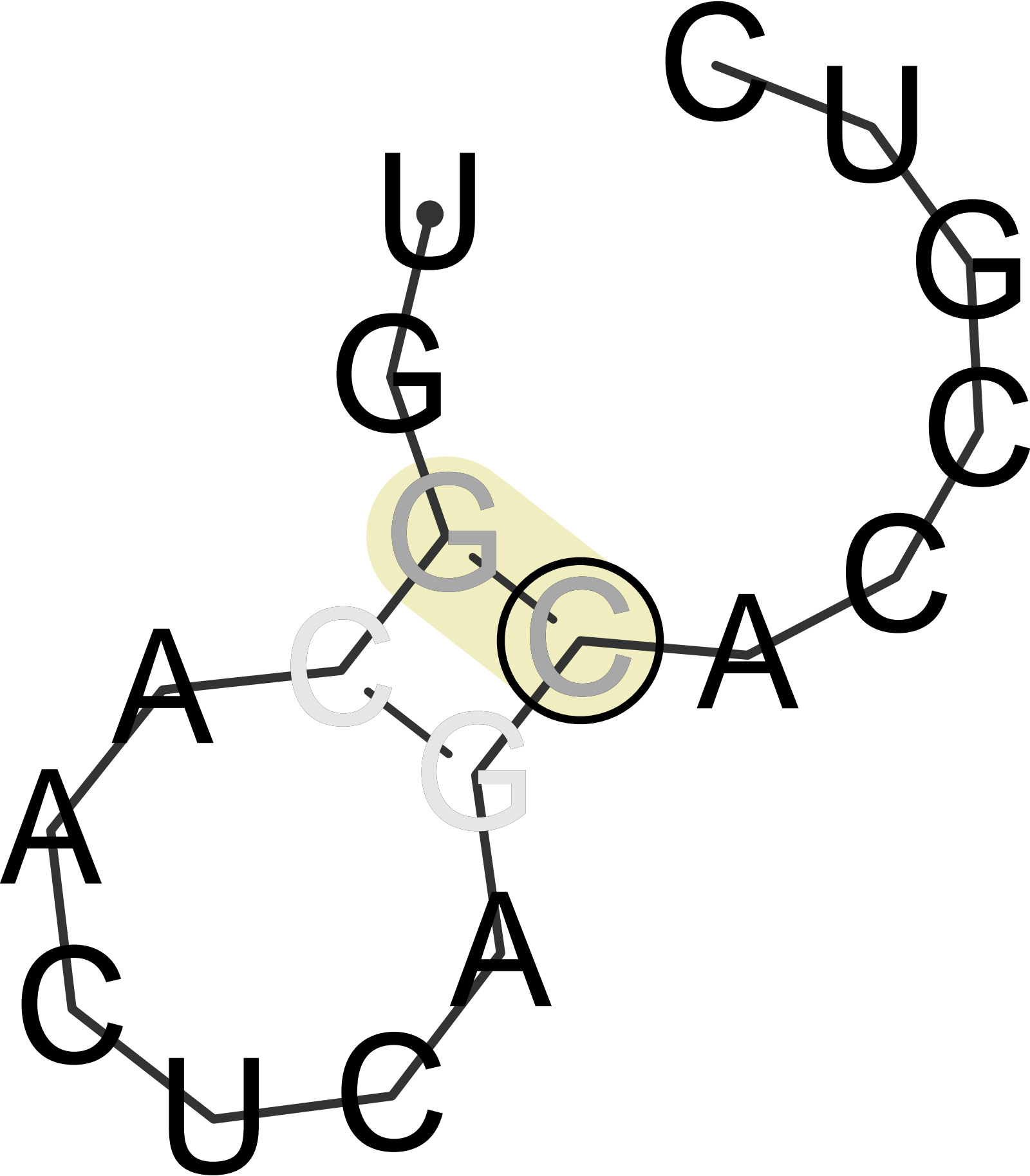

Supplement: S1 Fig — Filename convention within the zip file is as follows: HA/NA type, then host type, then whether the region was identified through analysis of raw (unranked) data, ranked data, or both, then the gene name, then the nucleotide location within the analysed alignment of the gene name, then the nucleotide location within the reference sequences (corresponding to locations listed in S2 Table and S10 Table), then a note if the analysis was performed using only one example of each distinct sequence, then a note if the fold uses the reverse complement of the cRNA (i.e. the vRNA), rather than the cRNA. All folds have been generated using alignments with loci where the consensus nucleotide is a gap removed. Base pairs are highlighted in deep/mid/light red when all/all but one/all but two sequences are capable of forming the pairs shown. Base pairs are highlighted in deep/mid/light yellow when all/all but one/all but two sequences are capable of forming the pair shown or one other pair (including GU pairs). Base pairs are highlighted in deep/mid/light green when all/all but one/all but two sequences are capable of forming the pair shown or one of two other pairs (including GU pairs). RNAalifold was used with input options disallowing lonely pairs, allowing G-quadruplexes, and with the ribosum scoring matrix enabled. (ZIP) [file pcbi.1012009.s122.zip › H1N1-human-ranked-NA-alignment-1390-1413-refseq-1375-1392-representative-sequences-only_revcomp_alirna_nogap.pdf]

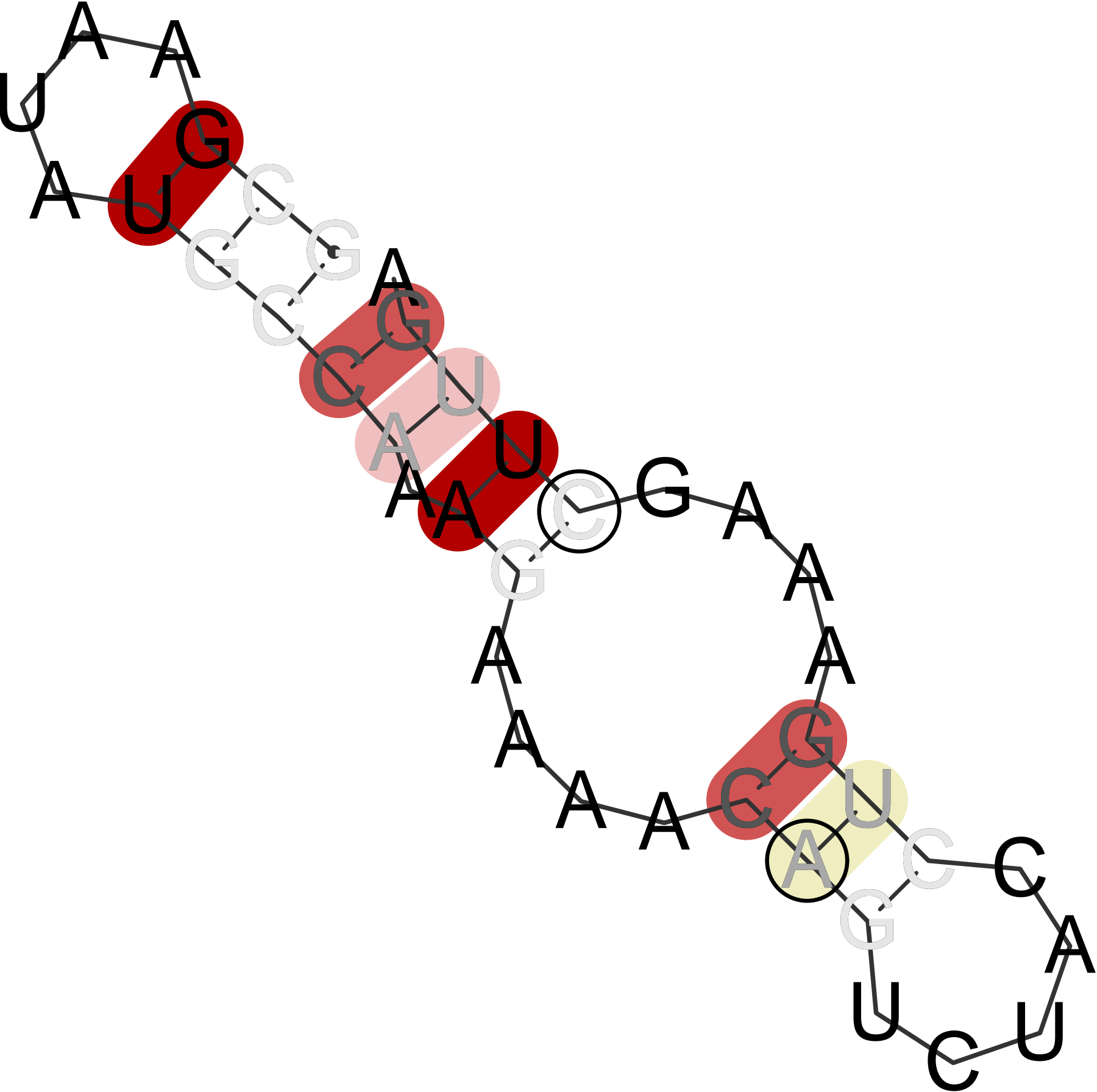

Supplement: S1 Fig — Filename convention within the zip file is as follows: HA/NA type, then host type, then whether the region was identified through analysis of raw (unranked) data, ranked data, or both, then the gene name, then the nucleotide location within the analysed alignment of the gene name, then the nucleotide location within the reference sequences (corresponding to locations listed in S2 Table and S10 Table), then a note if the analysis was performed using only one example of each distinct sequence, then a note if the fold uses the reverse complement of the cRNA (i.e. the vRNA), rather than the cRNA. All folds have been generated using alignments with loci where the consensus nucleotide is a gap removed. Base pairs are highlighted in deep/mid/light red when all/all but one/all but two sequences are capable of forming the pairs shown. Base pairs are highlighted in deep/mid/light yellow when all/all but one/all but two sequences are capable of forming the pair shown or one other pair (including GU pairs). Base pairs are highlighted in deep/mid/light green when all/all but one/all but two sequences are capable of forming the pair shown or one of two other pairs (including GU pairs). RNAalifold was used with input options disallowing lonely pairs, allowing G-quadruplexes, and with the ribosum scoring matrix enabled. (ZIP) [file pcbi.1012009.s122.zip › H1N1-human-ranked-NS1-alignment-37-75-refseq-19-57-representative-sequences-only_revcomp_alirna_nogap.pdf]

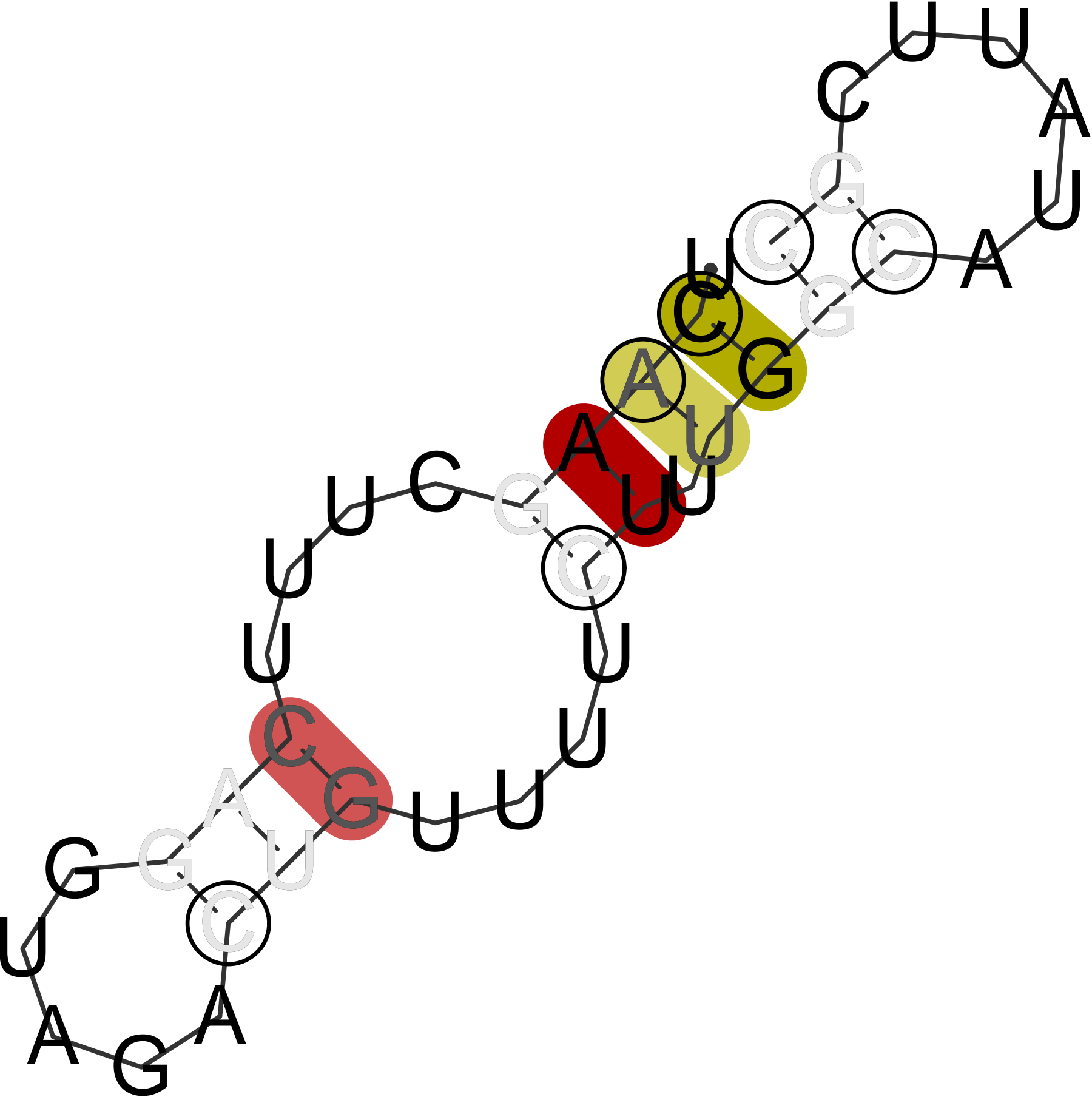

Supplement: S1 Fig — Filename convention within the zip file is as follows: HA/NA type, then host type, then whether the region was identified through analysis of raw (unranked) data, ranked data, or both, then the gene name, then the nucleotide location within the analysed alignment of the gene name, then the nucleotide location within the reference sequences (corresponding to locations listed in S2 Table and S10 Table), then a note if the analysis was performed using only one example of each distinct sequence, then a note if the fold uses the reverse complement of the cRNA (i.e. the vRNA), rather than the cRNA. All folds have been generated using alignments with loci where the consensus nucleotide is a gap removed. Base pairs are highlighted in deep/mid/light red when all/all but one/all but two sequences are capable of forming the pairs shown. Base pairs are highlighted in deep/mid/light yellow when all/all but one/all but two sequences are capable of forming the pair shown or one other pair (including GU pairs). Base pairs are highlighted in deep/mid/light green when all/all but one/all but two sequences are capable of forming the pair shown or one of two other pairs (including GU pairs). RNAalifold was used with input options disallowing lonely pairs, allowing G-quadruplexes, and with the ribosum scoring matrix enabled. (ZIP) [file pcbi.1012009.s122.zip › H1N1-human-ranked-NS1-alignment-37-75-refseq-19-57_alirna_nogap.pdf]

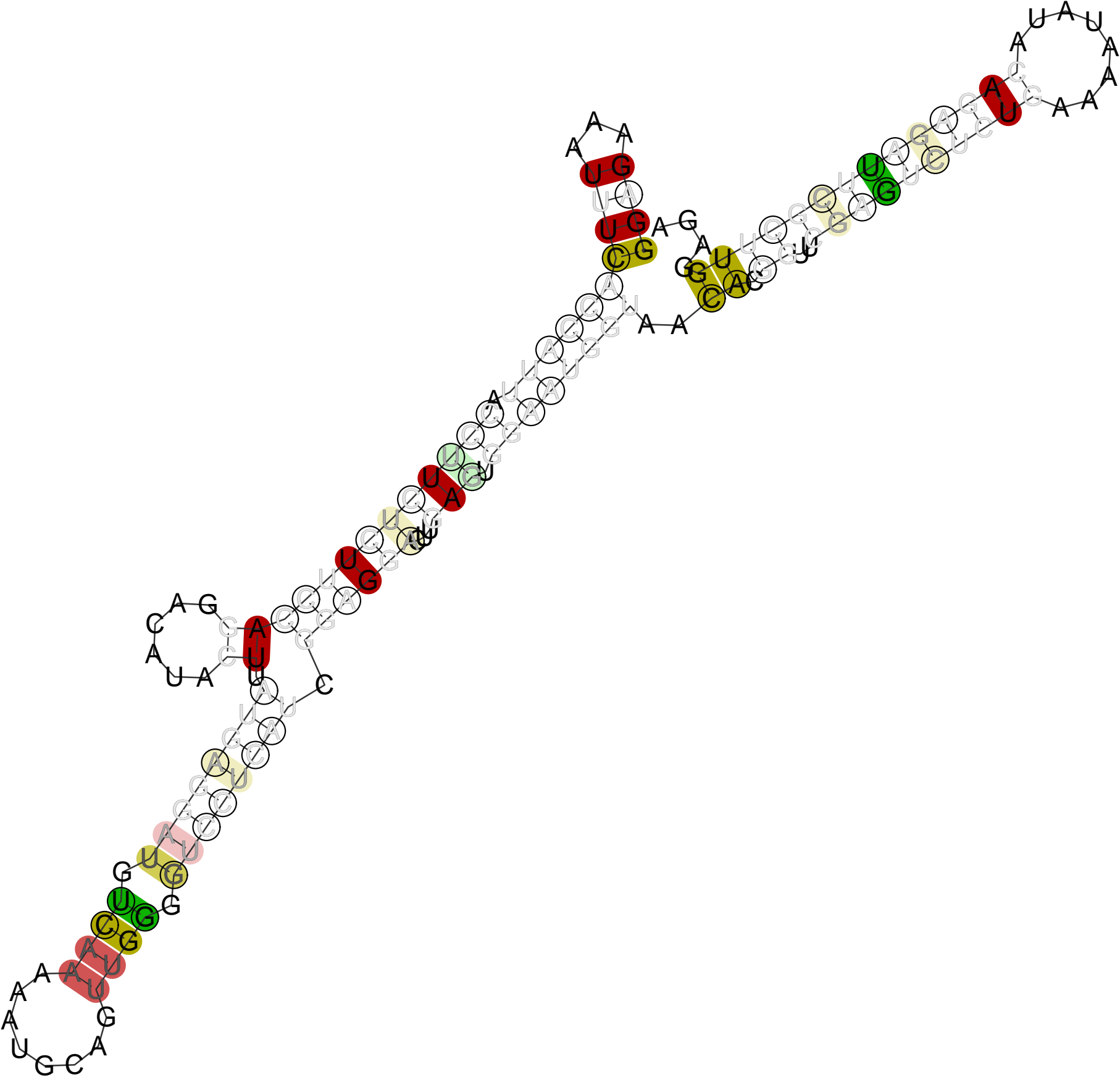

Supplement: S1 Fig — Filename convention within the zip file is as follows: HA/NA type, then host type, then whether the region was identified through analysis of raw (unranked) data, ranked data, or both, then the gene name, then the nucleotide location within the analysed alignment of the gene name, then the nucleotide location within the reference sequences (corresponding to locations listed in S2 Table and S10 Table), then a note if the analysis was performed using only one example of each distinct sequence, then a note if the fold uses the reverse complement of the cRNA (i.e. the vRNA), rather than the cRNA. All folds have been generated using alignments with loci where the consensus nucleotide is a gap removed. Base pairs are highlighted in deep/mid/light red when all/all but one/all but two sequences are capable of forming the pairs shown. Base pairs are highlighted in deep/mid/light yellow when all/all but one/all but two sequences are capable of forming the pair shown or one other pair (including GU pairs). Base pairs are highlighted in deep/mid/light green when all/all but one/all but two sequences are capable of forming the pair shown or one of two other pairs (including GU pairs). RNAalifold was used with input options disallowing lonely pairs, allowing G-quadruplexes, and with the ribosum scoring matrix enabled. (ZIP) [file pcbi.1012009.s122.zip › H1N1-human-ranked-NS1-alignment-490-630-refseq-472-612-representative-sequences-only_alirna_nogap.pdf]

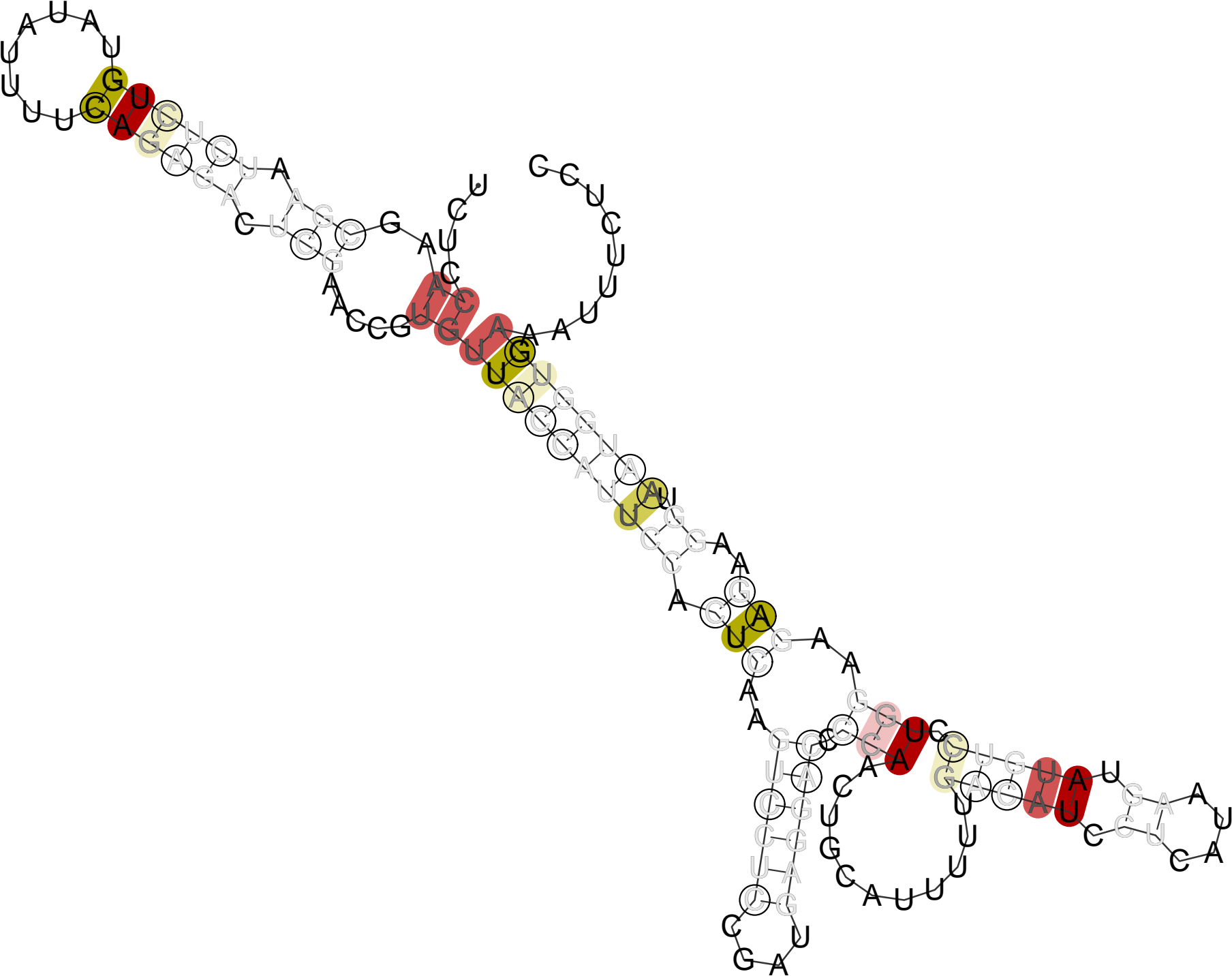

Supplement: S1 Fig — Filename convention within the zip file is as follows: HA/NA type, then host type, then whether the region was identified through analysis of raw (unranked) data, ranked data, or both, then the gene name, then the nucleotide location within the analysed alignment of the gene name, then the nucleotide location within the reference sequences (corresponding to locations listed in S2 Table and S10 Table), then a note if the analysis was performed using only one example of each distinct sequence, then a note if the fold uses the reverse complement of the cRNA (i.e. the vRNA), rather than the cRNA. All folds have been generated using alignments with loci where the consensus nucleotide is a gap removed. Base pairs are highlighted in deep/mid/light red when all/all but one/all but two sequences are capable of forming the pairs shown. Base pairs are highlighted in deep/mid/light yellow when all/all but one/all but two sequences are capable of forming the pair shown or one other pair (including GU pairs). Base pairs are highlighted in deep/mid/light green when all/all but one/all but two sequences are capable of forming the pair shown or one of two other pairs (including GU pairs). RNAalifold was used with input options disallowing lonely pairs, allowing G-quadruplexes, and with the ribosum scoring matrix enabled. (ZIP) [file pcbi.1012009.s122.zip › H1N1-human-ranked-NS1-alignment-490-630-refseq-472-612-representative-sequences-only_revcomp_alirna_nogap.pdf]

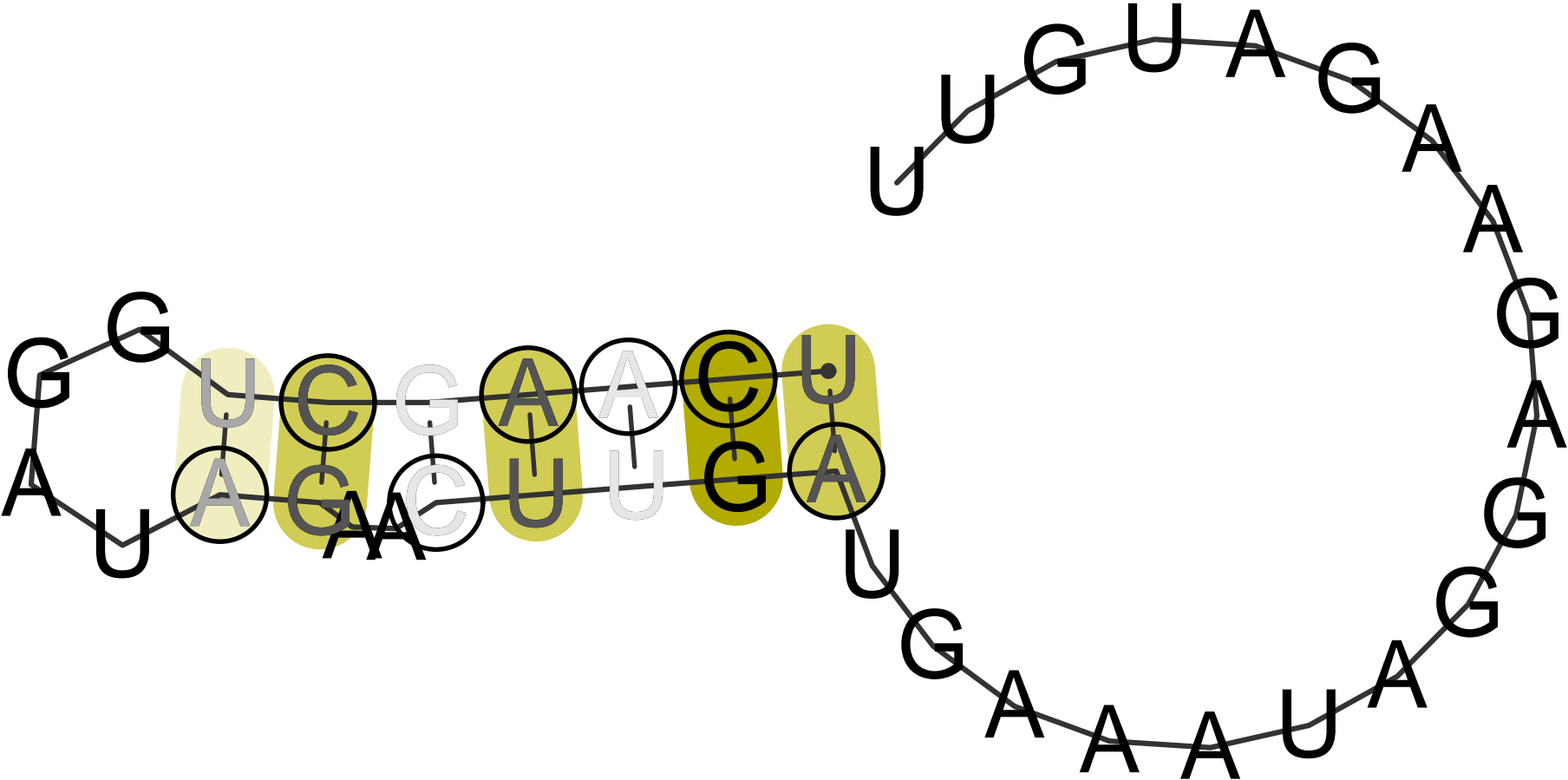

Supplement: S1 Fig — Filename convention within the zip file is as follows: HA/NA type, then host type, then whether the region was identified through analysis of raw (unranked) data, ranked data, or both, then the gene name, then the nucleotide location within the analysed alignment of the gene name, then the nucleotide location within the reference sequences (corresponding to locations listed in S2 Table and S10 Table), then a note if the analysis was performed using only one example of each distinct sequence, then a note if the fold uses the reverse complement of the cRNA (i.e. the vRNA), rather than the cRNA. All folds have been generated using alignments with loci where the consensus nucleotide is a gap removed. Base pairs are highlighted in deep/mid/light red when all/all but one/all but two sequences are capable of forming the pairs shown. Base pairs are highlighted in deep/mid/light yellow when all/all but one/all but two sequences are capable of forming the pair shown or one other pair (including GU pairs). Base pairs are highlighted in deep/mid/light green when all/all but one/all but two sequences are capable of forming the pair shown or one of two other pairs (including GU pairs). RNAalifold was used with input options disallowing lonely pairs, allowing G-quadruplexes, and with the ribosum scoring matrix enabled. (ZIP) [file pcbi.1012009.s122.zip › H1N1-human-ranked-PA-alignment-1258-1296-refseq-1258-1296-representative-sequences-only_alirna_nogap.pdf]

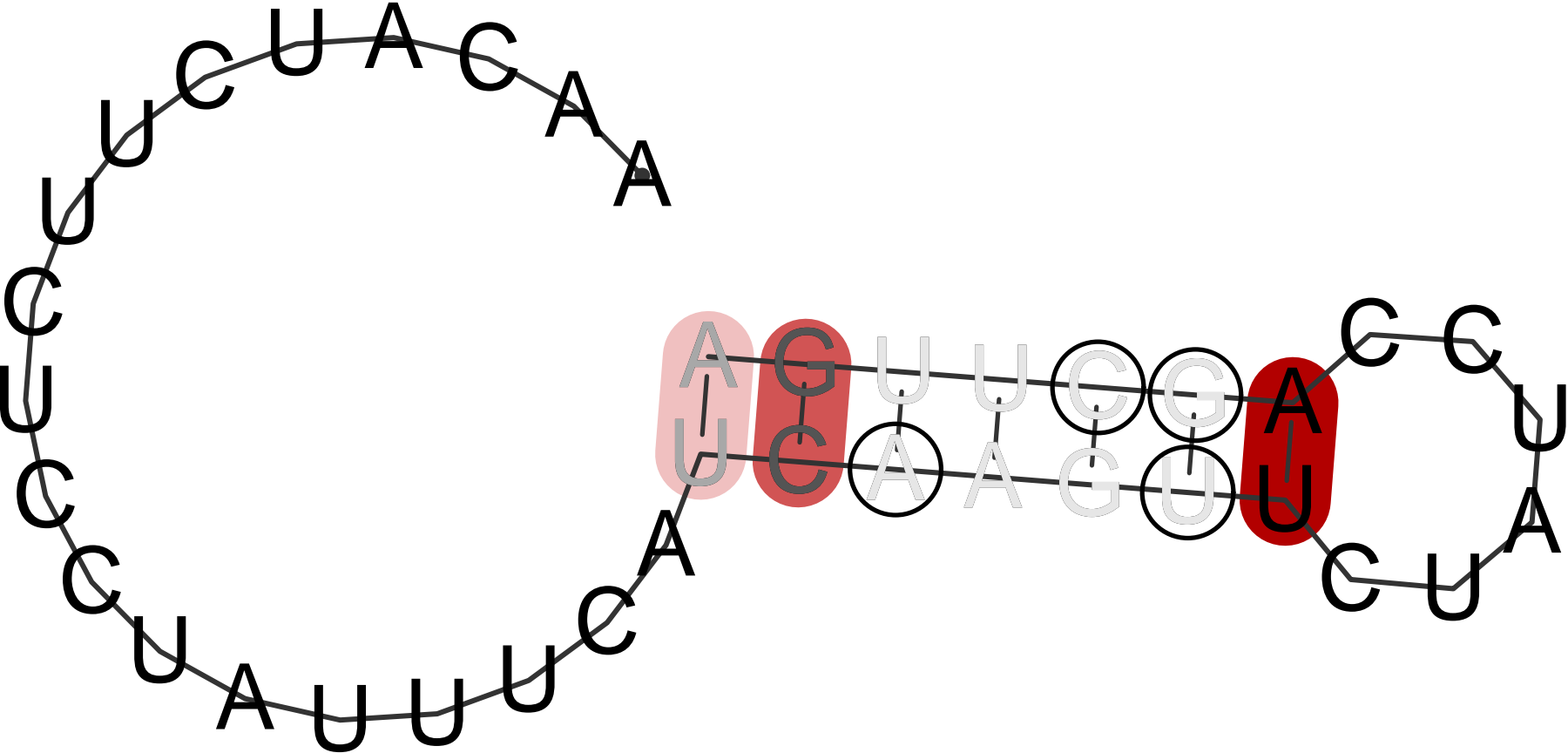

Supplement: S1 Fig — Filename convention within the zip file is as follows: HA/NA type, then host type, then whether the region was identified through analysis of raw (unranked) data, ranked data, or both, then the gene name, then the nucleotide location within the analysed alignment of the gene name, then the nucleotide location within the reference sequences (corresponding to locations listed in S2 Table and S10 Table), then a note if the analysis was performed using only one example of each distinct sequence, then a note if the fold uses the reverse complement of the cRNA (i.e. the vRNA), rather than the cRNA. All folds have been generated using alignments with loci where the consensus nucleotide is a gap removed. Base pairs are highlighted in deep/mid/light red when all/all but one/all but two sequences are capable of forming the pairs shown. Base pairs are highlighted in deep/mid/light yellow when all/all but one/all but two sequences are capable of forming the pair shown or one other pair (including GU pairs). Base pairs are highlighted in deep/mid/light green when all/all but one/all but two sequences are capable of forming the pair shown or one of two other pairs (including GU pairs). RNAalifold was used with input options disallowing lonely pairs, allowing G-quadruplexes, and with the ribosum scoring matrix enabled. (ZIP) [file pcbi.1012009.s122.zip › H1N1-human-ranked-PA-alignment-1258-1296-refseq-1258-1296-representative-sequences-only_revcomp_alirna_nogap.pdf]

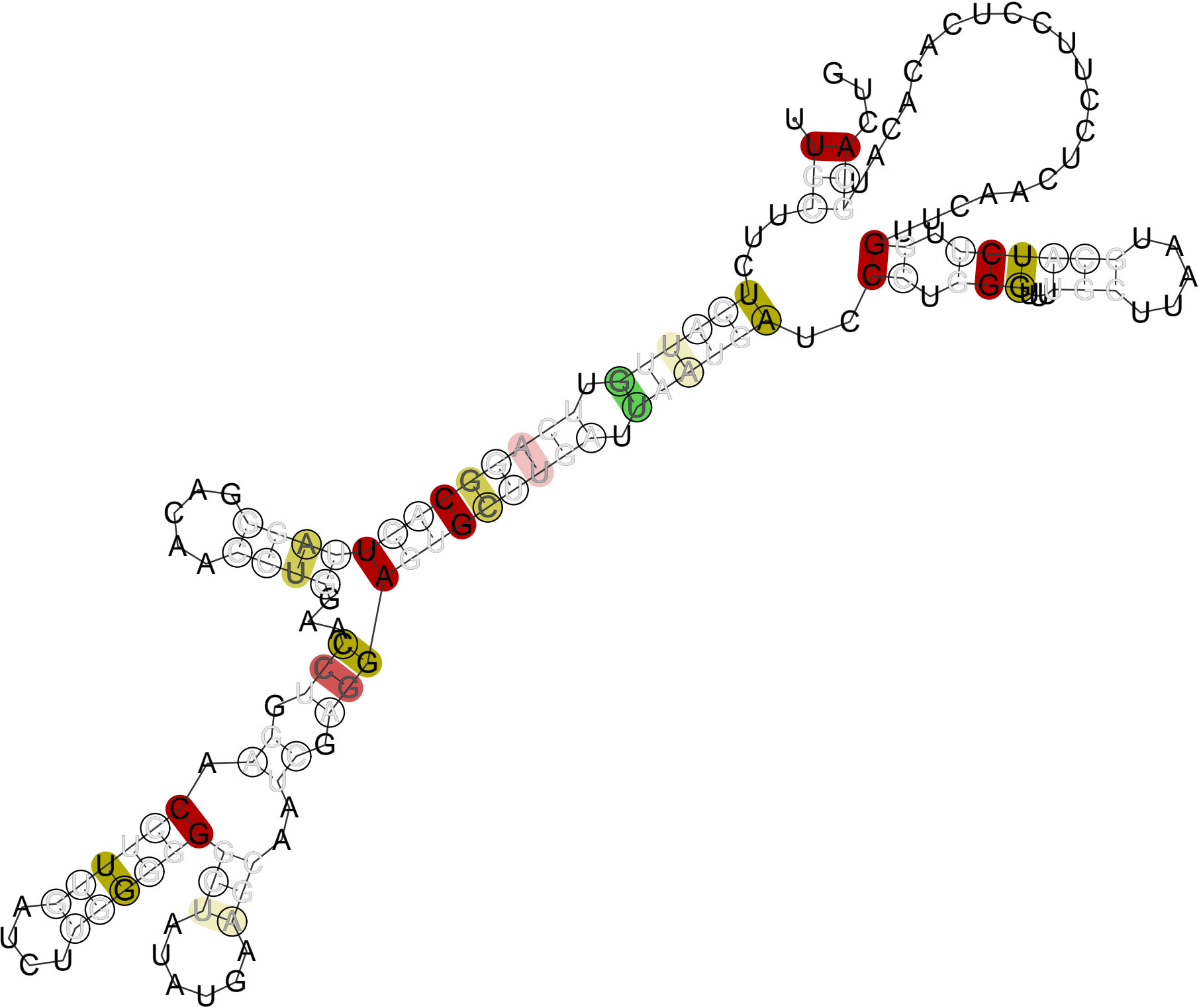

Supplement: S1 Fig — Filename convention within the zip file is as follows: HA/NA type, then host type, then whether the region was identified through analysis of raw (unranked) data, ranked data, or both, then the gene name, then the nucleotide location within the analysed alignment of the gene name, then the nucleotide location within the reference sequences (corresponding to locations listed in S2 Table and S10 Table), then a note if the analysis was performed using only one example of each distinct sequence, then a note if the fold uses the reverse complement of the cRNA (i.e. the vRNA), rather than the cRNA. All folds have been generated using alignments with loci where the consensus nucleotide is a gap removed. Base pairs are highlighted in deep/mid/light red when all/all but one/all but two sequences are capable of forming the pairs shown. Base pairs are highlighted in deep/mid/light yellow when all/all but one/all but two sequences are capable of forming the pair shown or one other pair (including GU pairs). Base pairs are highlighted in deep/mid/light green when all/all but one/all but two sequences are capable of forming the pair shown or one of two other pairs (including GU pairs). RNAalifold was used with input options disallowing lonely pairs, allowing G-quadruplexes, and with the ribosum scoring matrix enabled. (ZIP) [file pcbi.1012009.s122.zip › H1N1-human-ranked-PA-alignment-1993-2145-refseq-1993-2145-representative-sequences-only_alirna_nogap.pdf]

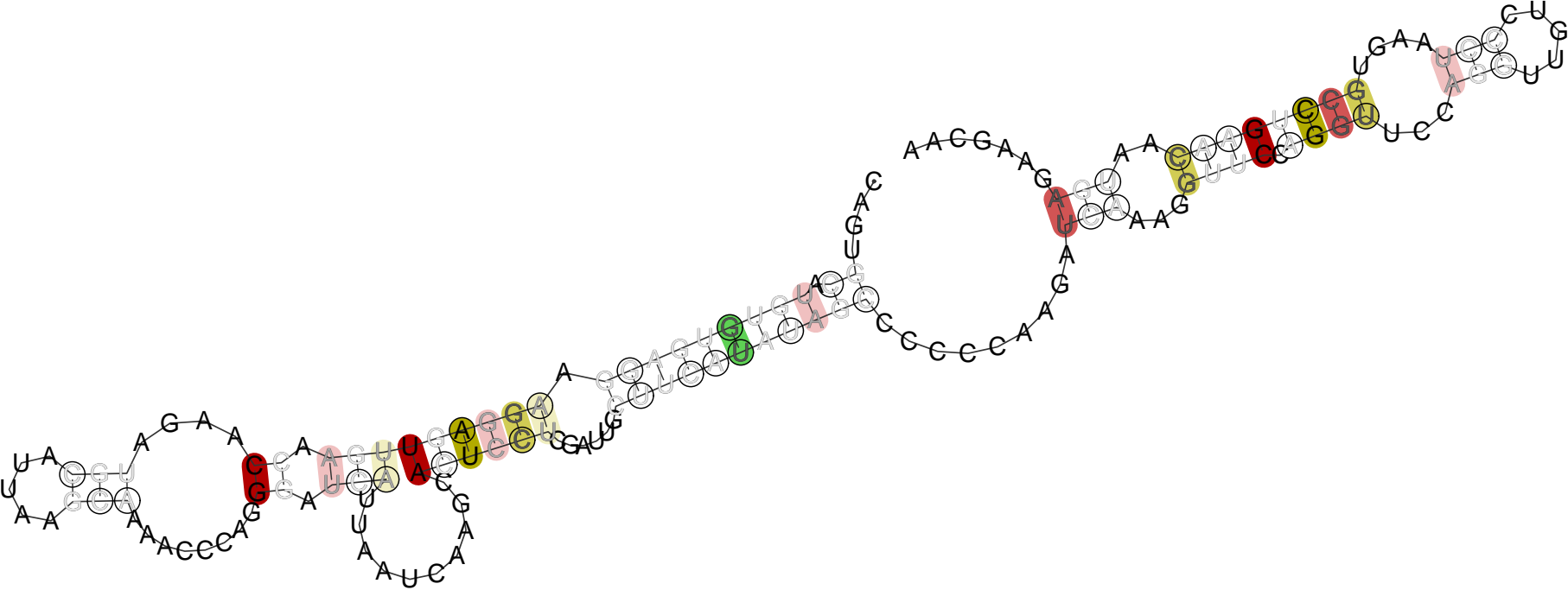

Supplement: S1 Fig — Filename convention within the zip file is as follows: HA/NA type, then host type, then whether the region was identified through analysis of raw (unranked) data, ranked data, or both, then the gene name, then the nucleotide location within the analysed alignment of the gene name, then the nucleotide location within the reference sequences (corresponding to locations listed in S2 Table and S10 Table), then a note if the analysis was performed using only one example of each distinct sequence, then a note if the fold uses the reverse complement of the cRNA (i.e. the vRNA), rather than the cRNA. All folds have been generated using alignments with loci where the consensus nucleotide is a gap removed. Base pairs are highlighted in deep/mid/light red when all/all but one/all but two sequences are capable of forming the pairs shown. Base pairs are highlighted in deep/mid/light yellow when all/all but one/all but two sequences are capable of forming the pair shown or one other pair (including GU pairs). Base pairs are highlighted in deep/mid/light green when all/all but one/all but two sequences are capable of forming the pair shown or one of two other pairs (including GU pairs). RNAalifold was used with input options disallowing lonely pairs, allowing G-quadruplexes, and with the ribosum scoring matrix enabled. (ZIP) [file pcbi.1012009.s122.zip › H1N1-human-ranked-PA-alignment-1993-2145-refseq-1993-2145-representative-sequences-only_revcomp_alirna_nogap.pdf]

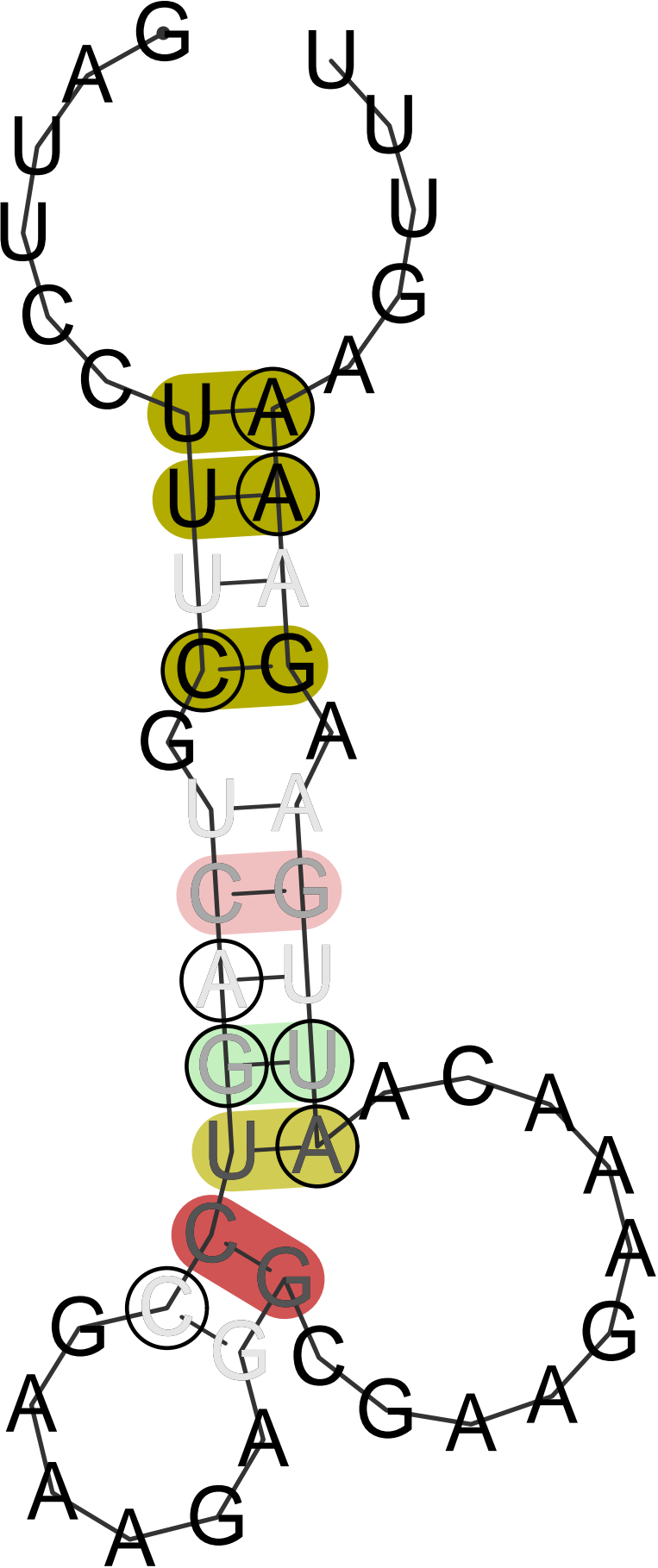

Supplement: S1 Fig — Filename convention within the zip file is as follows: HA/NA type, then host type, then whether the region was identified through analysis of raw (unranked) data, ranked data, or both, then the gene name, then the nucleotide location within the analysed alignment of the gene name, then the nucleotide location within the reference sequences (corresponding to locations listed in S2 Table and S10 Table), then a note if the analysis was performed using only one example of each distinct sequence, then a note if the fold uses the reverse complement of the cRNA (i.e. the vRNA), rather than the cRNA. All folds have been generated using alignments with loci where the consensus nucleotide is a gap removed. Base pairs are highlighted in deep/mid/light red when all/all but one/all but two sequences are capable of forming the pairs shown. Base pairs are highlighted in deep/mid/light yellow when all/all but one/all but two sequences are capable of forming the pair shown or one other pair (including GU pairs). Base pairs are highlighted in deep/mid/light green when all/all but one/all but two sequences are capable of forming the pair shown or one of two other pairs (including GU pairs). RNAalifold was used with input options disallowing lonely pairs, allowing G-quadruplexes, and with the ribosum scoring matrix enabled. (ZIP) [file pcbi.1012009.s122.zip › H1N1-human-ranked-PA-alignment-565-615-refseq-565-615-representative-sequences-only_alirna_nogap.pdf]

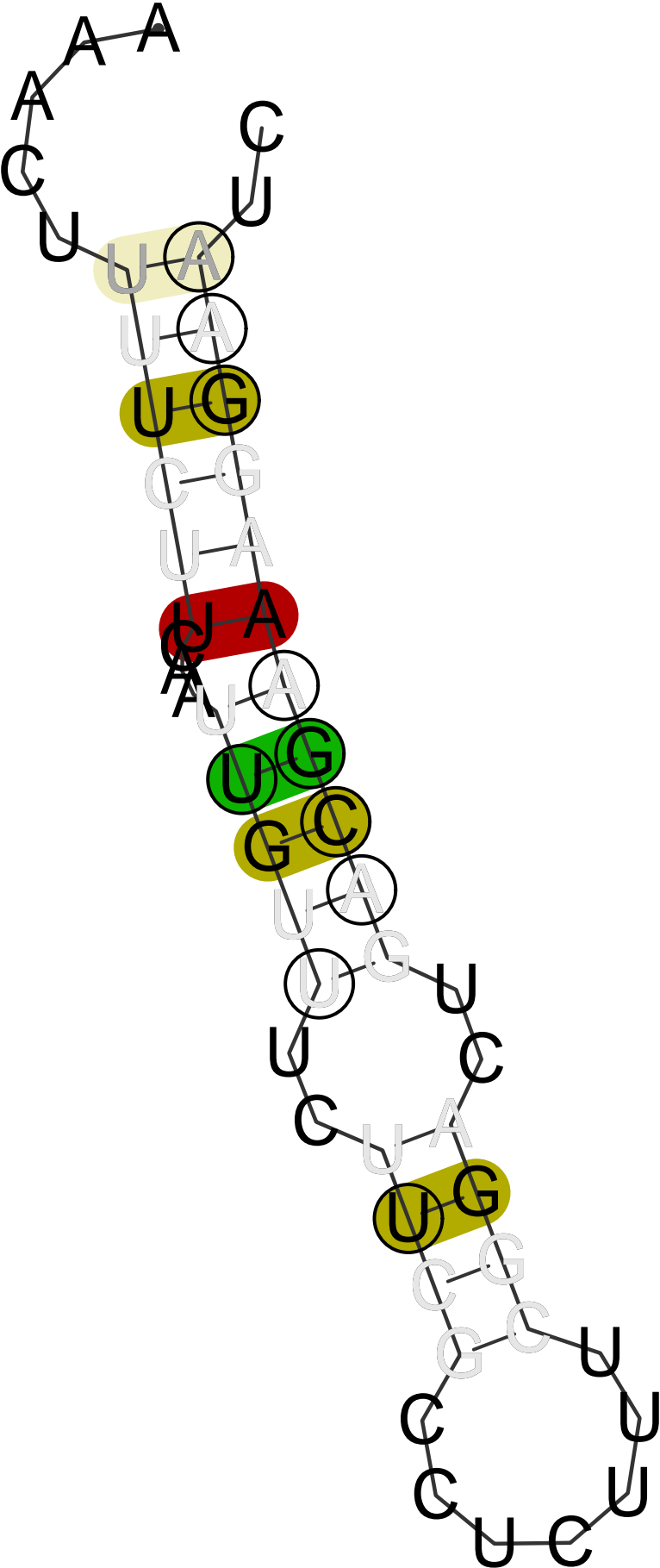

Supplement: S1 Fig — Filename convention within the zip file is as follows: HA/NA type, then host type, then whether the region was identified through analysis of raw (unranked) data, ranked data, or both, then the gene name, then the nucleotide location within the analysed alignment of the gene name, then the nucleotide location within the reference sequences (corresponding to locations listed in S2 Table and S10 Table), then a note if the analysis was performed using only one example of each distinct sequence, then a note if the fold uses the reverse complement of the cRNA (i.e. the vRNA), rather than the cRNA. All folds have been generated using alignments with loci where the consensus nucleotide is a gap removed. Base pairs are highlighted in deep/mid/light red when all/all but one/all but two sequences are capable of forming the pairs shown. Base pairs are highlighted in deep/mid/light yellow when all/all but one/all but two sequences are capable of forming the pair shown or one other pair (including GU pairs). Base pairs are highlighted in deep/mid/light green when all/all but one/all but two sequences are capable of forming the pair shown or one of two other pairs (including GU pairs). RNAalifold was used with input options disallowing lonely pairs, allowing G-quadruplexes, and with the ribosum scoring matrix enabled. (ZIP) [file pcbi.1012009.s122.zip › H1N1-human-ranked-PA-alignment-565-615-refseq-565-615-representative-sequences-only_revcomp_alirna_nogap.pdf]

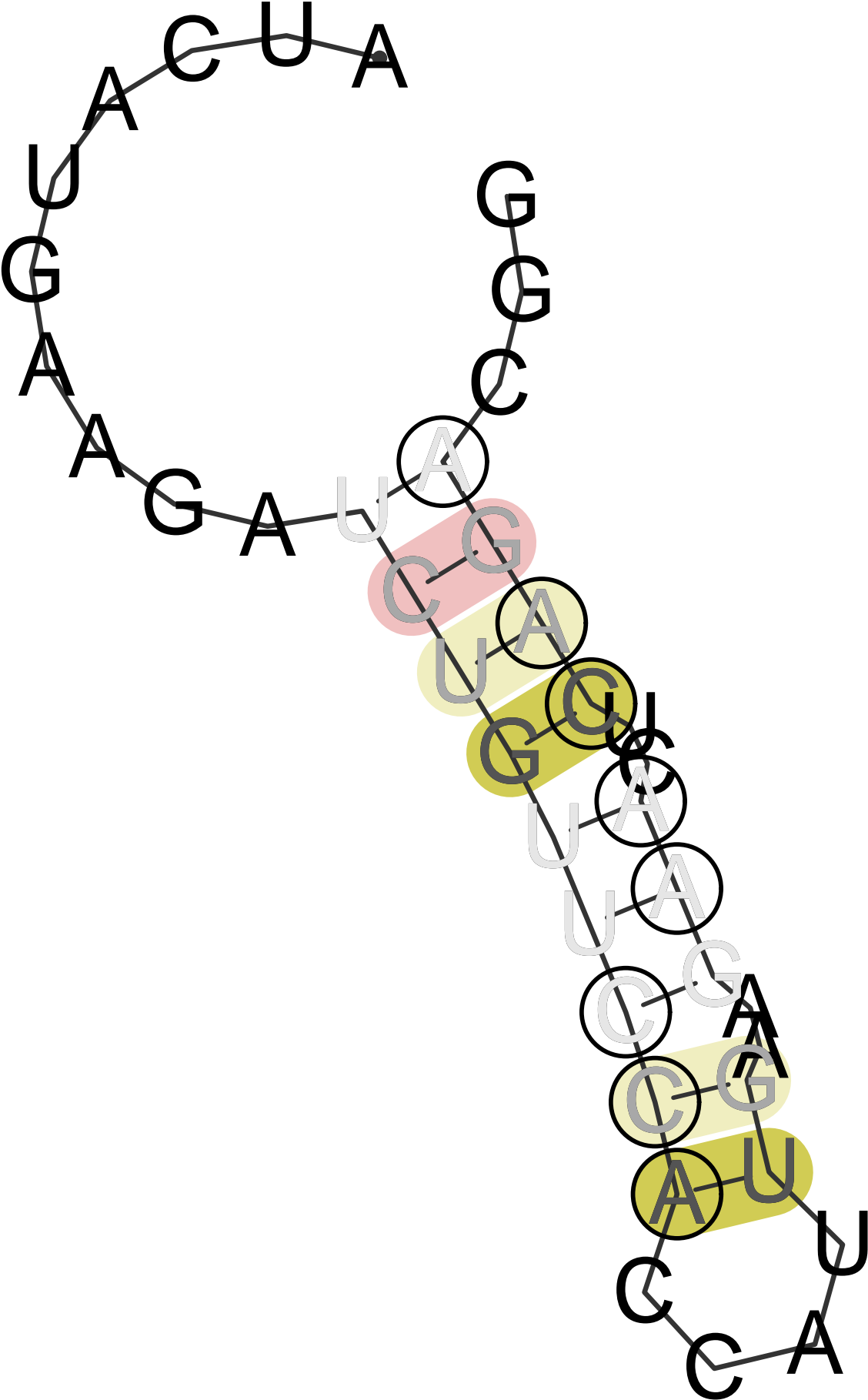

Supplement: S1 Fig — Filename convention within the zip file is as follows: HA/NA type, then host type, then whether the region was identified through analysis of raw (unranked) data, ranked data, or both, then the gene name, then the nucleotide location within the analysed alignment of the gene name, then the nucleotide location within the reference sequences (corresponding to locations listed in S2 Table and S10 Table), then a note if the analysis was performed using only one example of each distinct sequence, then a note if the fold uses the reverse complement of the cRNA (i.e. the vRNA), rather than the cRNA. All folds have been generated using alignments with loci where the consensus nucleotide is a gap removed. Base pairs are highlighted in deep/mid/light red when all/all but one/all but two sequences are capable of forming the pairs shown. Base pairs are highlighted in deep/mid/light yellow when all/all but one/all but two sequences are capable of forming the pair shown or one other pair (including GU pairs). Base pairs are highlighted in deep/mid/light green when all/all but one/all but two sequences are capable of forming the pair shown or one of two other pairs (including GU pairs). RNAalifold was used with input options disallowing lonely pairs, allowing G-quadruplexes, and with the ribosum scoring matrix enabled. (ZIP) [file pcbi.1012009.s122.zip › H1N1-human-ranked-PB1-alignment-2230-2268-refseq-2227-2265-representative-sequences-only_alirna_nogap.pdf]

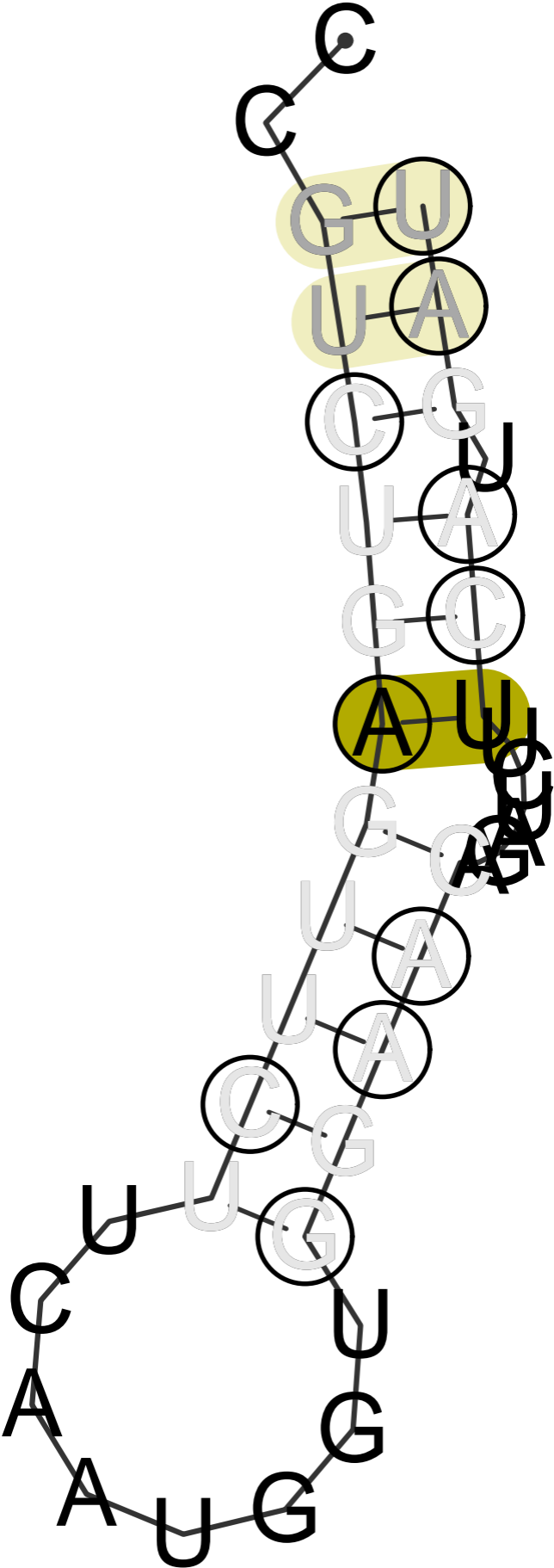

Supplement: S1 Fig — Filename convention within the zip file is as follows: HA/NA type, then host type, then whether the region was identified through analysis of raw (unranked) data, ranked data, or both, then the gene name, then the nucleotide location within the analysed alignment of the gene name, then the nucleotide location within the reference sequences (corresponding to locations listed in S2 Table and S10 Table), then a note if the analysis was performed using only one example of each distinct sequence, then a note if the fold uses the reverse complement of the cRNA (i.e. the vRNA), rather than the cRNA. All folds have been generated using alignments with loci where the consensus nucleotide is a gap removed. Base pairs are highlighted in deep/mid/light red when all/all but one/all but two sequences are capable of forming the pairs shown. Base pairs are highlighted in deep/mid/light yellow when all/all but one/all but two sequences are capable of forming the pair shown or one other pair (including GU pairs). Base pairs are highlighted in deep/mid/light green when all/all but one/all but two sequences are capable of forming the pair shown or one of two other pairs (including GU pairs). RNAalifold was used with input options disallowing lonely pairs, allowing G-quadruplexes, and with the ribosum scoring matrix enabled. (ZIP) [file pcbi.1012009.s122.zip › H1N1-human-ranked-PB1-alignment-2230-2268-refseq-2227-2265_revcomp_alirna_nogap.pdf]

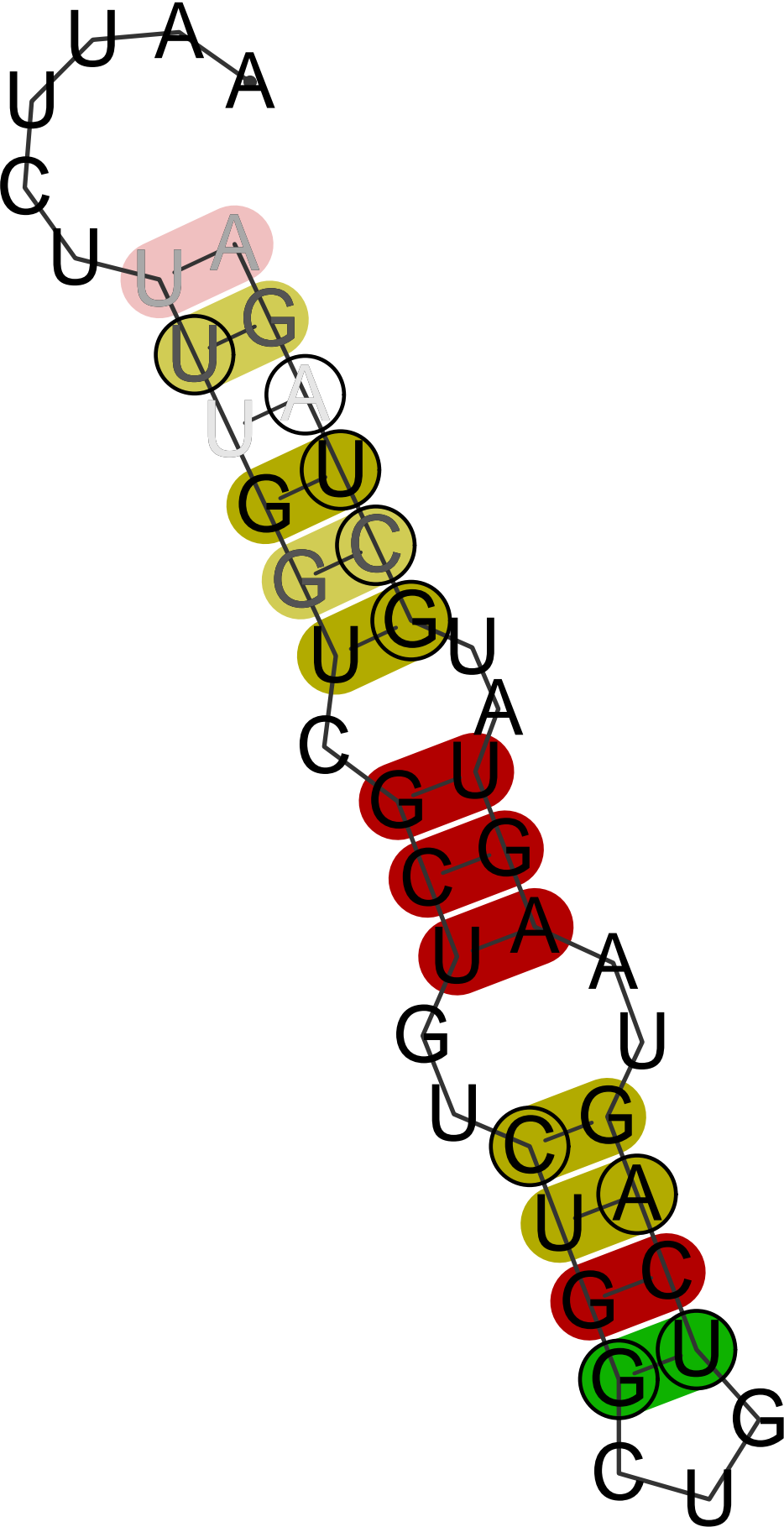

Supplement: S1 Fig — Filename convention within the zip file is as follows: HA/NA type, then host type, then whether the region was identified through analysis of raw (unranked) data, ranked data, or both, then the gene name, then the nucleotide location within the analysed alignment of the gene name, then the nucleotide location within the reference sequences (corresponding to locations listed in S2 Table and S10 Table), then a note if the analysis was performed using only one example of each distinct sequence, then a note if the fold uses the reverse complement of the cRNA (i.e. the vRNA), rather than the cRNA. All folds have been generated using alignments with loci where the consensus nucleotide is a gap removed. Base pairs are highlighted in deep/mid/light red when all/all but one/all but two sequences are capable of forming the pairs shown. Base pairs are highlighted in deep/mid/light yellow when all/all but one/all but two sequences are capable of forming the pair shown or one other pair (including GU pairs). Base pairs are highlighted in deep/mid/light green when all/all but one/all but two sequences are capable of forming the pair shown or one of two other pairs (including GU pairs). RNAalifold was used with input options disallowing lonely pairs, allowing G-quadruplexes, and with the ribosum scoring matrix enabled. (ZIP) [file pcbi.1012009.s122.zip › H1N1-human-ranked-PB2-alignment-2221-2262-refseq-2221-2262-representative-sequences-only_revcomp_alirna_nogap.pdf]

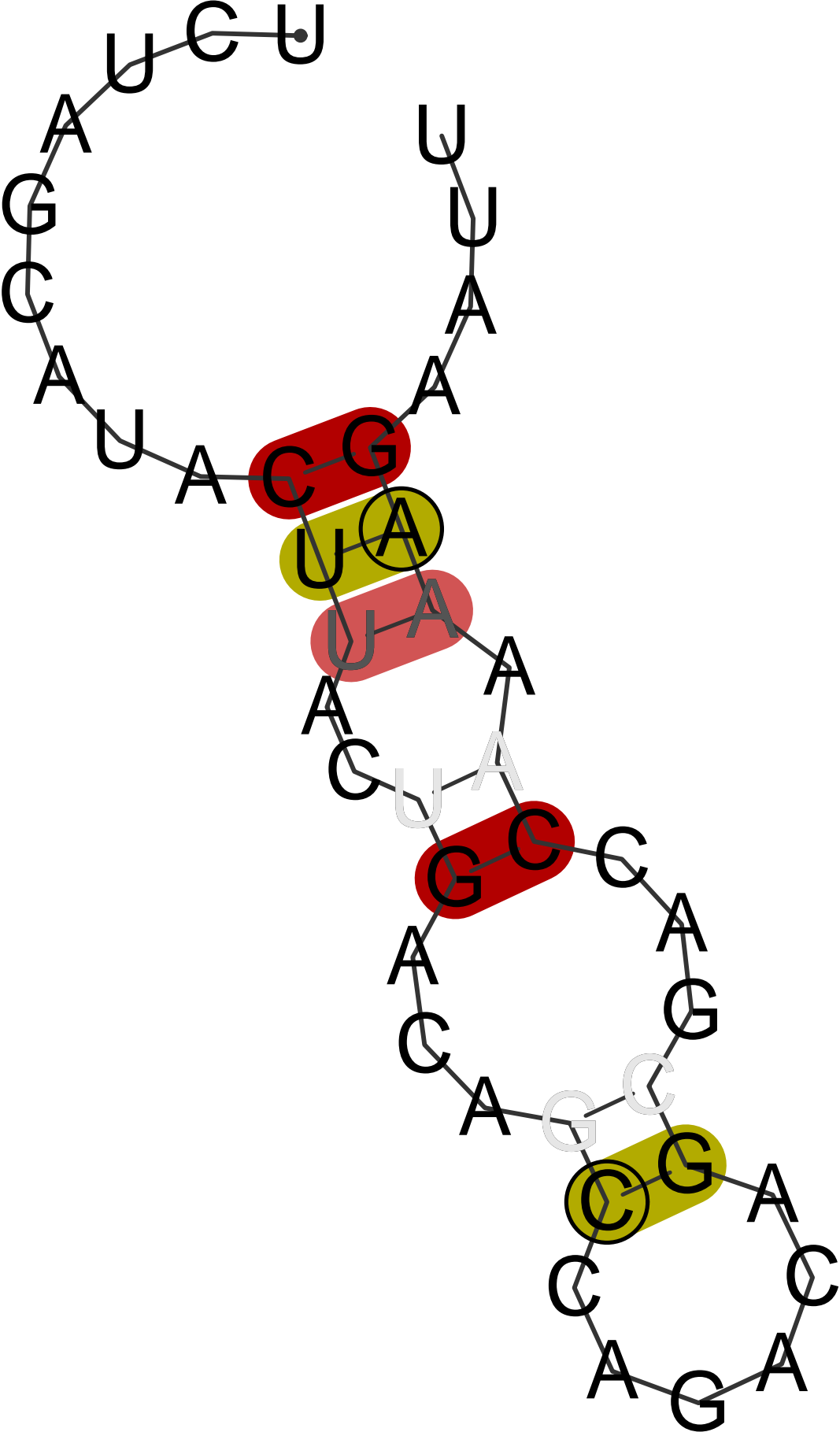

Supplement: S1 Fig — Filename convention within the zip file is as follows: HA/NA type, then host type, then whether the region was identified through analysis of raw (unranked) data, ranked data, or both, then the gene name, then the nucleotide location within the analysed alignment of the gene name, then the nucleotide location within the reference sequences (corresponding to locations listed in S2 Table and S10 Table), then a note if the analysis was performed using only one example of each distinct sequence, then a note if the fold uses the reverse complement of the cRNA (i.e. the vRNA), rather than the cRNA. All folds have been generated using alignments with loci where the consensus nucleotide is a gap removed. Base pairs are highlighted in deep/mid/light red when all/all but one/all but two sequences are capable of forming the pairs shown. Base pairs are highlighted in deep/mid/light yellow when all/all but one/all but two sequences are capable of forming the pair shown or one other pair (including GU pairs). Base pairs are highlighted in deep/mid/light green when all/all but one/all but two sequences are capable of forming the pair shown or one of two other pairs (including GU pairs). RNAalifold was used with input options disallowing lonely pairs, allowing G-quadruplexes, and with the ribosum scoring matrix enabled. (ZIP) [file pcbi.1012009.s122.zip › H1N1-human-ranked-PB2-alignment-2221-2262-refseq-2221-2262_alirna_nogap.pdf]

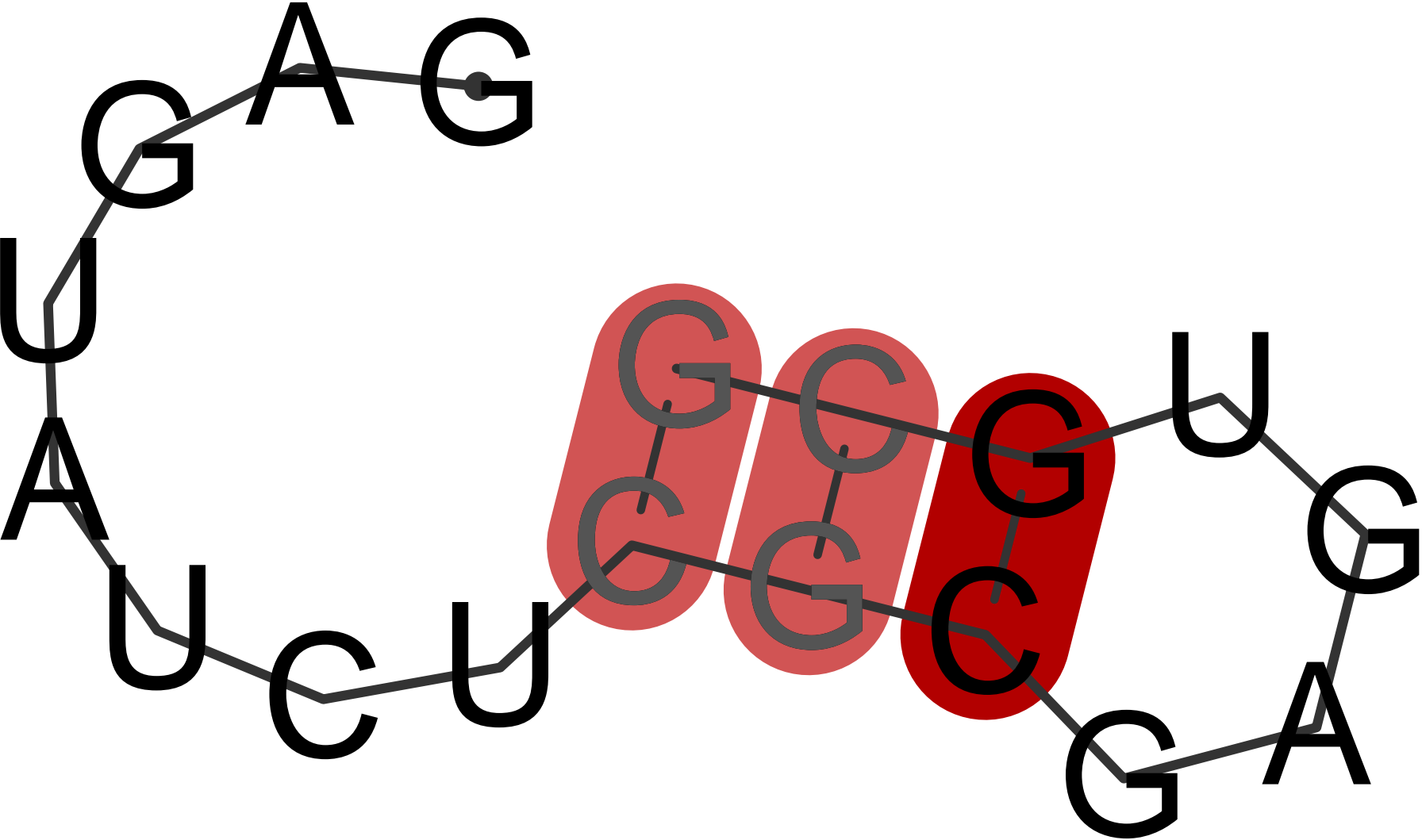

Supplement: S1 Fig — Filename convention within the zip file is as follows: HA/NA type, then host type, then whether the region was identified through analysis of raw (unranked) data, ranked data, or both, then the gene name, then the nucleotide location within the analysed alignment of the gene name, then the nucleotide location within the reference sequences (corresponding to locations listed in S2 Table and S10 Table), then a note if the analysis was performed using only one example of each distinct sequence, then a note if the fold uses the reverse complement of the cRNA (i.e. the vRNA), rather than the cRNA. All folds have been generated using alignments with loci where the consensus nucleotide is a gap removed. Base pairs are highlighted in deep/mid/light red when all/all but one/all but two sequences are capable of forming the pairs shown. Base pairs are highlighted in deep/mid/light yellow when all/all but one/all but two sequences are capable of forming the pair shown or one other pair (including GU pairs). Base pairs are highlighted in deep/mid/light green when all/all but one/all but two sequences are capable of forming the pair shown or one of two other pairs (including GU pairs). RNAalifold was used with input options disallowing lonely pairs, allowing G-quadruplexes, and with the ribosum scoring matrix enabled. (ZIP) [file pcbi.1012009.s122.zip › H1N1-human-ranked-PB2-alignment-43-60-refseq-43-60-representative-sequences-only_revcomp_alirna_nogap.pdf]

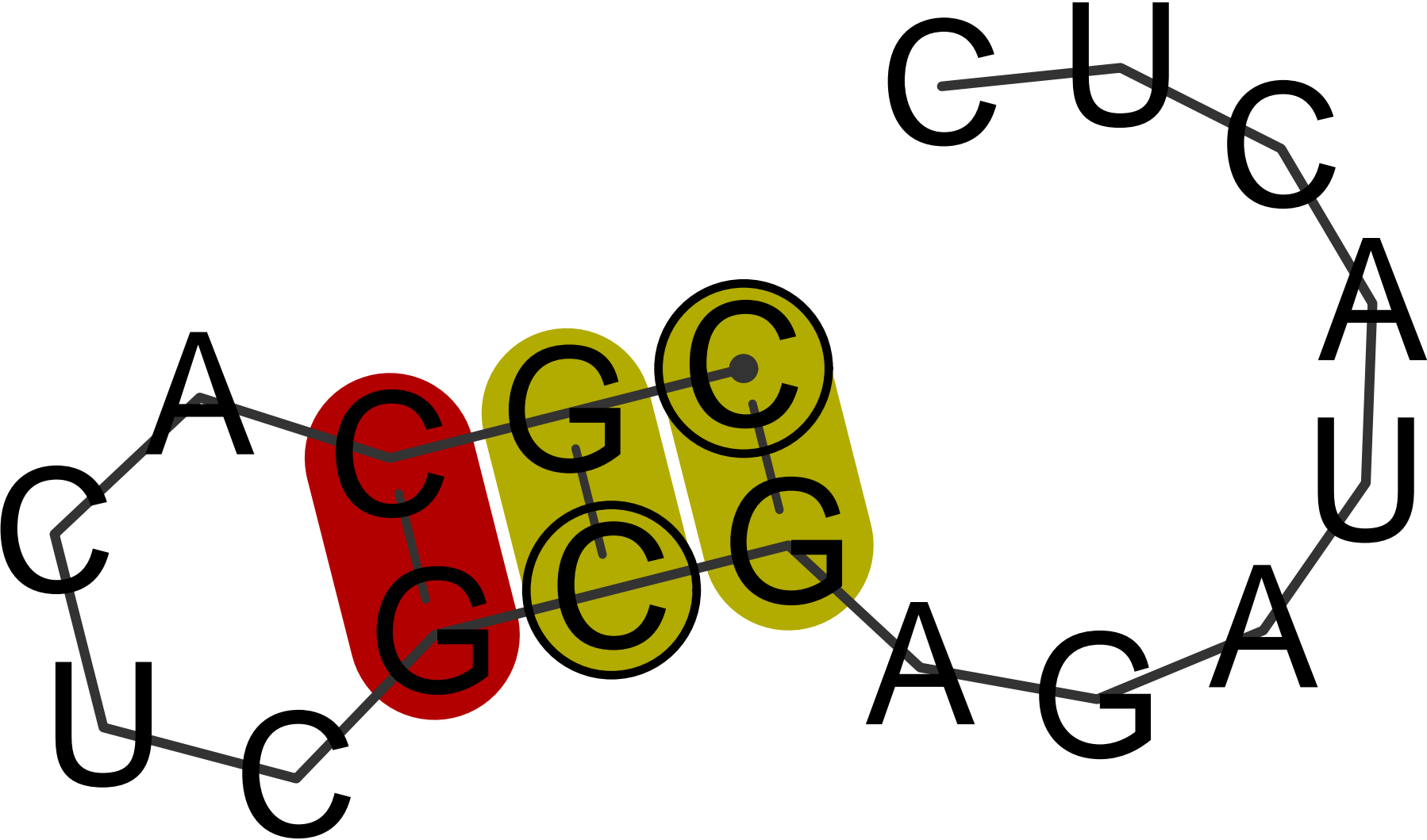

Supplement: S1 Fig — Filename convention within the zip file is as follows: HA/NA type, then host type, then whether the region was identified through analysis of raw (unranked) data, ranked data, or both, then the gene name, then the nucleotide location within the analysed alignment of the gene name, then the nucleotide location within the reference sequences (corresponding to locations listed in S2 Table and S10 Table), then a note if the analysis was performed using only one example of each distinct sequence, then a note if the fold uses the reverse complement of the cRNA (i.e. the vRNA), rather than the cRNA. All folds have been generated using alignments with loci where the consensus nucleotide is a gap removed. Base pairs are highlighted in deep/mid/light red when all/all but one/all but two sequences are capable of forming the pairs shown. Base pairs are highlighted in deep/mid/light yellow when all/all but one/all but two sequences are capable of forming the pair shown or one other pair (including GU pairs). Base pairs are highlighted in deep/mid/light green when all/all but one/all but two sequences are capable of forming the pair shown or one of two other pairs (including GU pairs). RNAalifold was used with input options disallowing lonely pairs, allowing G-quadruplexes, and with the ribosum scoring matrix enabled. (ZIP) [file pcbi.1012009.s122.zip › H1N1-human-ranked-PB2-alignment-43-60-refseq-43-60_alirna_nogap.pdf]

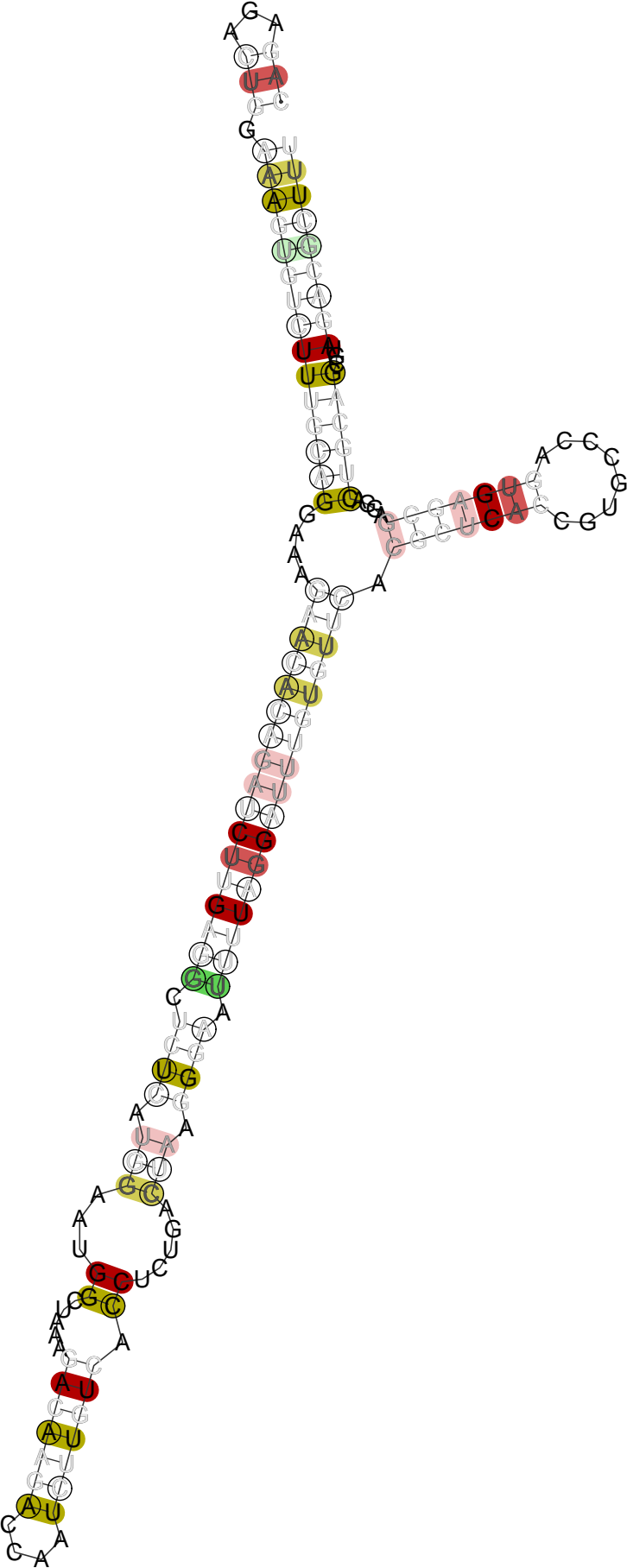

Supplement: S1 Fig — Filename convention within the zip file is as follows: HA/NA type, then host type, then whether the region was identified through analysis of raw (unranked) data, ranked data, or both, then the gene name, then the nucleotide location within the analysed alignment of the gene name, then the nucleotide location within the reference sequences (corresponding to locations listed in S2 Table and S10 Table), then a note if the analysis was performed using only one example of each distinct sequence, then a note if the fold uses the reverse complement of the cRNA (i.e. the vRNA), rather than the cRNA. All folds have been generated using alignments with loci where the consensus nucleotide is a gap removed. Base pairs are highlighted in deep/mid/light red when all/all but one/all but two sequences are capable of forming the pairs shown. Base pairs are highlighted in deep/mid/light yellow when all/all but one/all but two sequences are capable of forming the pair shown or one other pair (including GU pairs). Base pairs are highlighted in deep/mid/light green when all/all but one/all but two sequences are capable of forming the pair shown or one of two other pairs (including GU pairs). RNAalifold was used with input options disallowing lonely pairs, allowing G-quadruplexes, and with the ribosum scoring matrix enabled. (ZIP) [file pcbi.1012009.s122.zip › H1N1-human-raw-M1-alignment-76-237-refseq-76-237-representative-sequences-only_alirna_nogap.pdf]

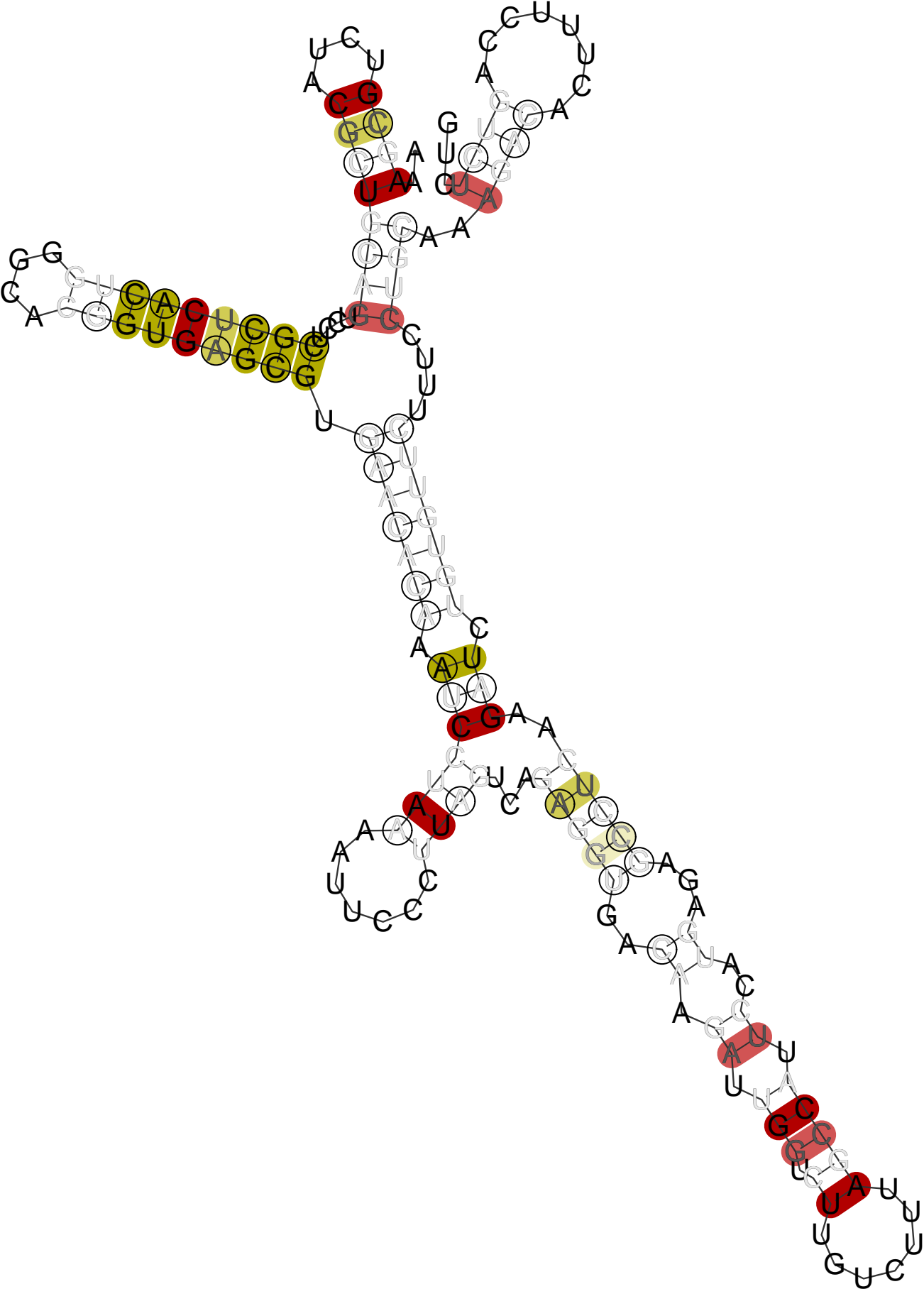

Supplement: S1 Fig — Filename convention within the zip file is as follows: HA/NA type, then host type, then whether the region was identified through analysis of raw (unranked) data, ranked data, or both, then the gene name, then the nucleotide location within the analysed alignment of the gene name, then the nucleotide location within the reference sequences (corresponding to locations listed in S2 Table and S10 Table), then a note if the analysis was performed using only one example of each distinct sequence, then a note if the fold uses the reverse complement of the cRNA (i.e. the vRNA), rather than the cRNA. All folds have been generated using alignments with loci where the consensus nucleotide is a gap removed. Base pairs are highlighted in deep/mid/light red when all/all but one/all but two sequences are capable of forming the pairs shown. Base pairs are highlighted in deep/mid/light yellow when all/all but one/all but two sequences are capable of forming the pair shown or one other pair (including GU pairs). Base pairs are highlighted in deep/mid/light green when all/all but one/all but two sequences are capable of forming the pair shown or one of two other pairs (including GU pairs). RNAalifold was used with input options disallowing lonely pairs, allowing G-quadruplexes, and with the ribosum scoring matrix enabled. (ZIP) [file pcbi.1012009.s122.zip › H1N1-human-raw-M1-alignment-76-237-refseq-76-237-representative-sequences-only_revcomp_alirna_nogap.pdf]

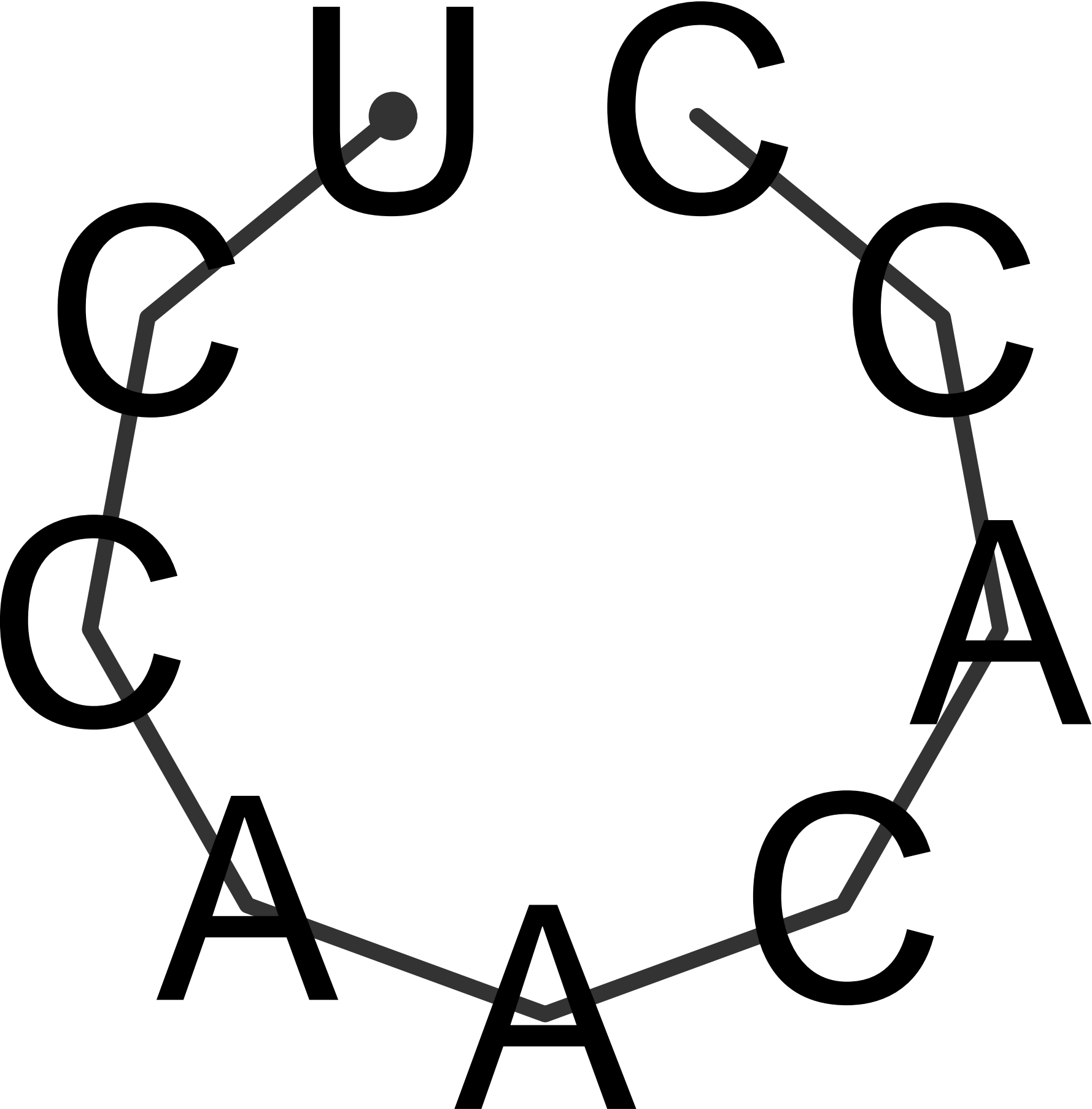

Supplement: S1 Fig — Filename convention within the zip file is as follows: HA/NA type, then host type, then whether the region was identified through analysis of raw (unranked) data, ranked data, or both, then the gene name, then the nucleotide location within the analysed alignment of the gene name, then the nucleotide location within the reference sequences (corresponding to locations listed in S2 Table and S10 Table), then a note if the analysis was performed using only one example of each distinct sequence, then a note if the fold uses the reverse complement of the cRNA (i.e. the vRNA), rather than the cRNA. All folds have been generated using alignments with loci where the consensus nucleotide is a gap removed. Base pairs are highlighted in deep/mid/light red when all/all but one/all but two sequences are capable of forming the pairs shown. Base pairs are highlighted in deep/mid/light yellow when all/all but one/all but two sequences are capable of forming the pair shown or one other pair (including GU pairs). Base pairs are highlighted in deep/mid/light green when all/all but one/all but two sequences are capable of forming the pair shown or one of two other pairs (including GU pairs). RNAalifold was used with input options disallowing lonely pairs, allowing G-quadruplexes, and with the ribosum scoring matrix enabled. (ZIP) [file pcbi.1012009.s122.zip › H1N1-human-raw-NS2-alignment-10-18-refseq-7-15_alirna_nogap.pdf]

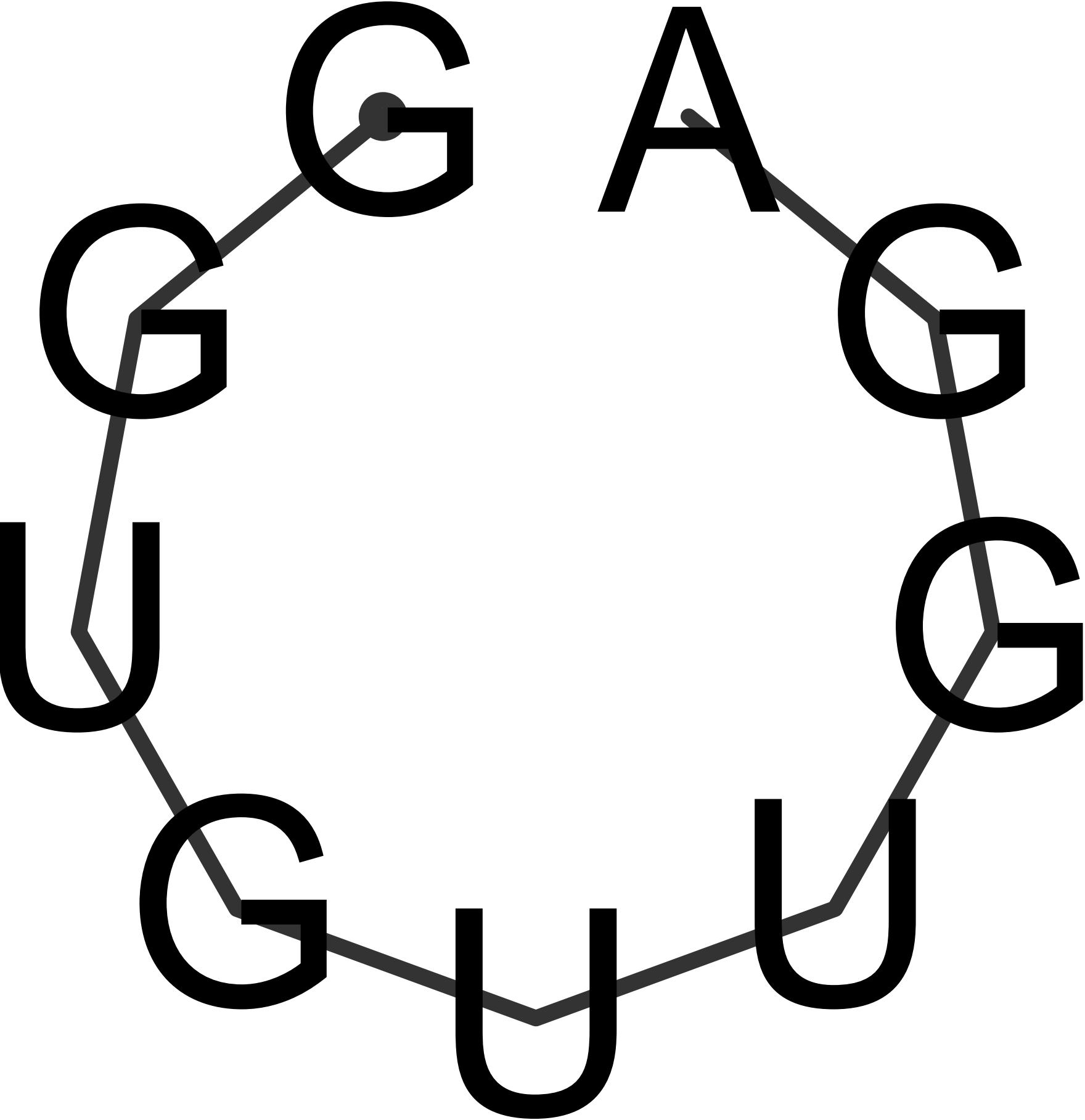

Supplement: S1 Fig — Filename convention within the zip file is as follows: HA/NA type, then host type, then whether the region was identified through analysis of raw (unranked) data, ranked data, or both, then the gene name, then the nucleotide location within the analysed alignment of the gene name, then the nucleotide location within the reference sequences (corresponding to locations listed in S2 Table and S10 Table), then a note if the analysis was performed using only one example of each distinct sequence, then a note if the fold uses the reverse complement of the cRNA (i.e. the vRNA), rather than the cRNA. All folds have been generated using alignments with loci where the consensus nucleotide is a gap removed. Base pairs are highlighted in deep/mid/light red when all/all but one/all but two sequences are capable of forming the pairs shown. Base pairs are highlighted in deep/mid/light yellow when all/all but one/all but two sequences are capable of forming the pair shown or one other pair (including GU pairs). Base pairs are highlighted in deep/mid/light green when all/all but one/all but two sequences are capable of forming the pair shown or one of two other pairs (including GU pairs). RNAalifold was used with input options disallowing lonely pairs, allowing G-quadruplexes, and with the ribosum scoring matrix enabled. (ZIP) [file pcbi.1012009.s122.zip › H1N1-human-raw-NS2-alignment-10-18-refseq-7-15_revcomp_alirna_nogap.pdf]

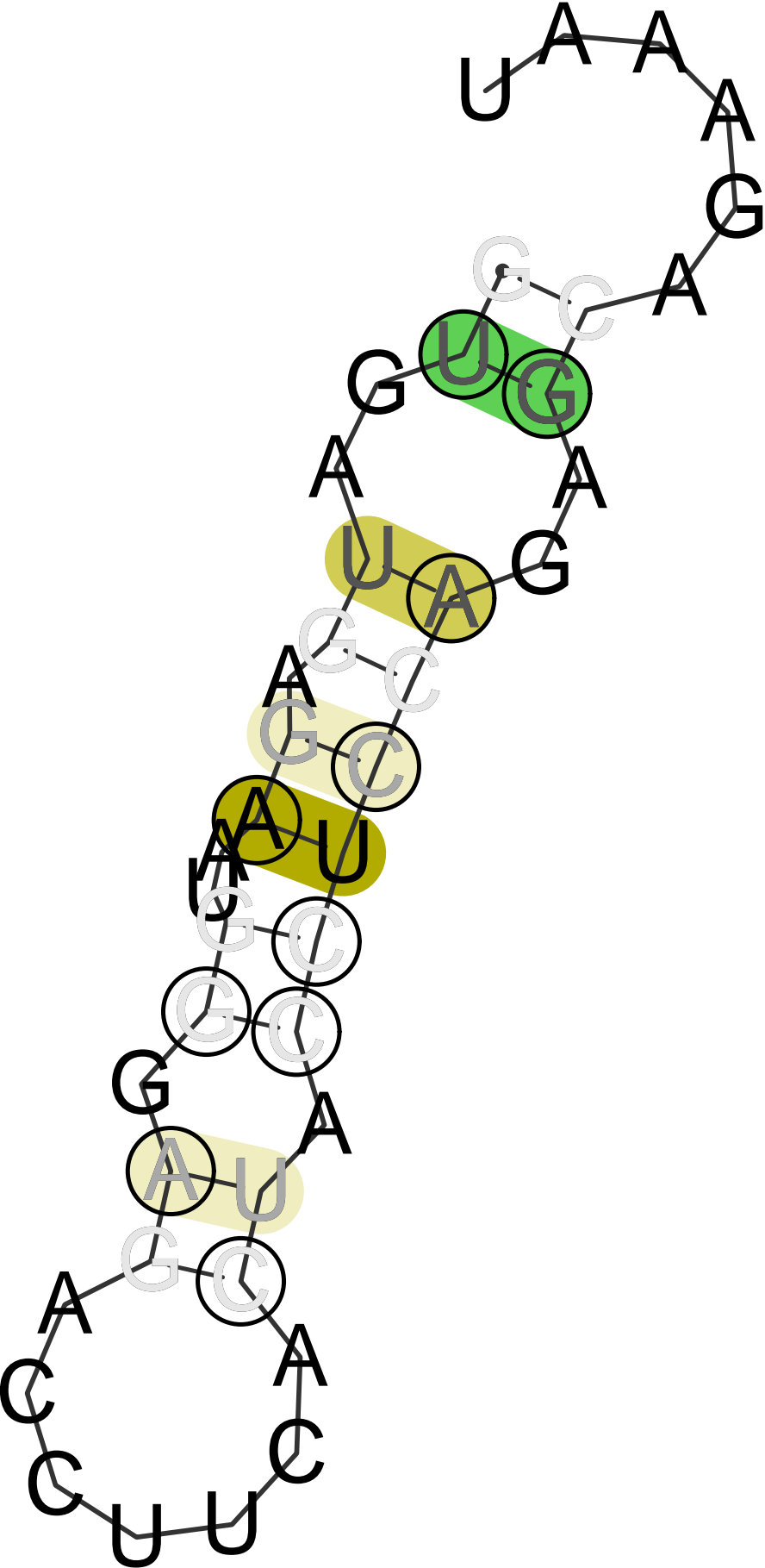

Supplement: S1 Fig — Filename convention within the zip file is as follows: HA/NA type, then host type, then whether the region was identified through analysis of raw (unranked) data, ranked data, or both, then the gene name, then the nucleotide location within the analysed alignment of the gene name, then the nucleotide location within the reference sequences (corresponding to locations listed in S2 Table and S10 Table), then a note if the analysis was performed using only one example of each distinct sequence, then a note if the fold uses the reverse complement of the cRNA (i.e. the vRNA), rather than the cRNA. All folds have been generated using alignments with loci where the consensus nucleotide is a gap removed. Base pairs are highlighted in deep/mid/light red when all/all but one/all but two sequences are capable of forming the pairs shown. Base pairs are highlighted in deep/mid/light yellow when all/all but one/all but two sequences are capable of forming the pair shown or one other pair (including GU pairs). Base pairs are highlighted in deep/mid/light green when all/all but one/all but two sequences are capable of forming the pair shown or one of two other pairs (including GU pairs). RNAalifold was used with input options disallowing lonely pairs, allowing G-quadruplexes, and with the ribosum scoring matrix enabled. (ZIP) [file pcbi.1012009.s122.zip › H1N1-human-raw-NS2-alignment-160-201-refseq-617-658-representative-sequences-only_alirna_nogap.pdf]

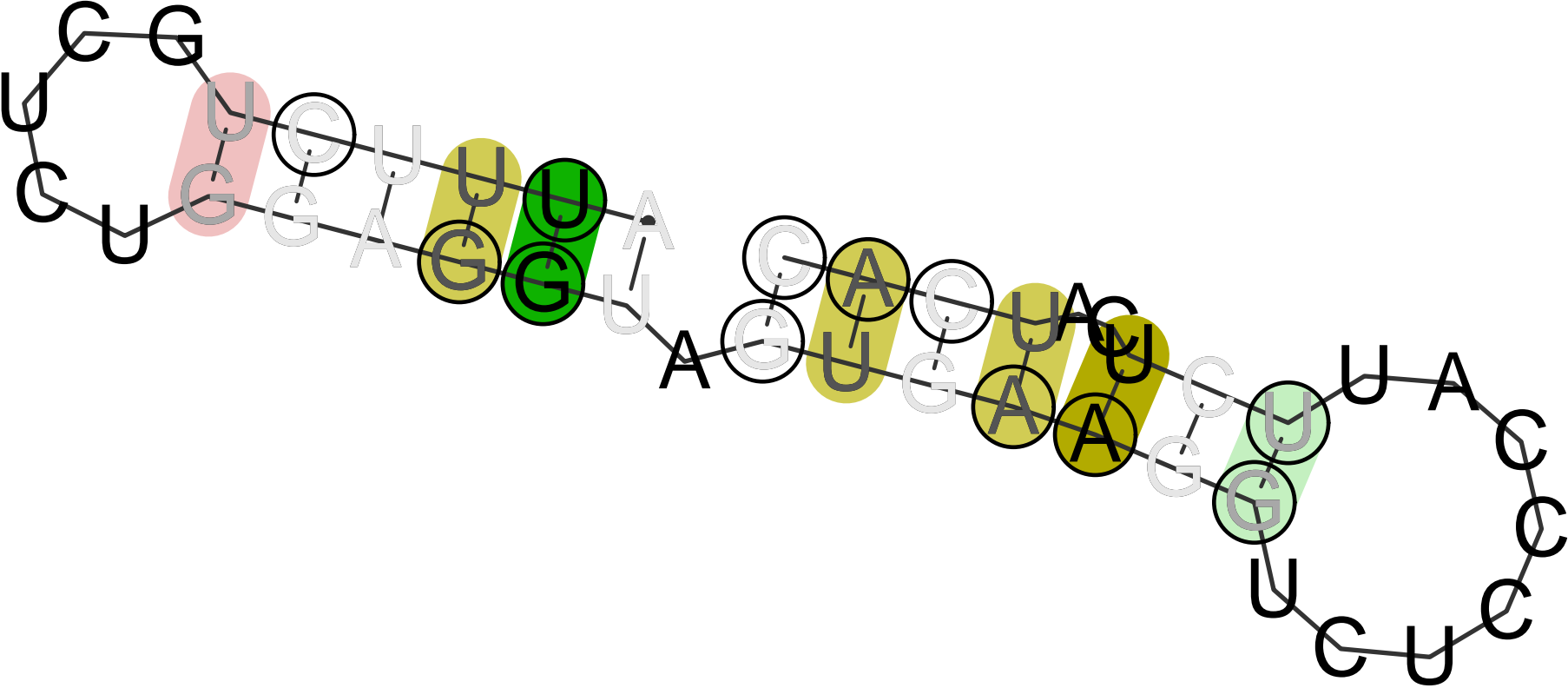

Supplement: S1 Fig — Filename convention within the zip file is as follows: HA/NA type, then host type, then whether the region was identified through analysis of raw (unranked) data, ranked data, or both, then the gene name, then the nucleotide location within the analysed alignment of the gene name, then the nucleotide location within the reference sequences (corresponding to locations listed in S2 Table and S10 Table), then a note if the analysis was performed using only one example of each distinct sequence, then a note if the fold uses the reverse complement of the cRNA (i.e. the vRNA), rather than the cRNA. All folds have been generated using alignments with loci where the consensus nucleotide is a gap removed. Base pairs are highlighted in deep/mid/light red when all/all but one/all but two sequences are capable of forming the pairs shown. Base pairs are highlighted in deep/mid/light yellow when all/all but one/all but two sequences are capable of forming the pair shown or one other pair (including GU pairs). Base pairs are highlighted in deep/mid/light green when all/all but one/all but two sequences are capable of forming the pair shown or one of two other pairs (including GU pairs). RNAalifold was used with input options disallowing lonely pairs, allowing G-quadruplexes, and with the ribosum scoring matrix enabled. (ZIP) [file pcbi.1012009.s122.zip › H1N1-human-raw-NS2-alignment-160-201-refseq-617-658-representative-sequences-only_revcomp_alirna_nogap.pdf]

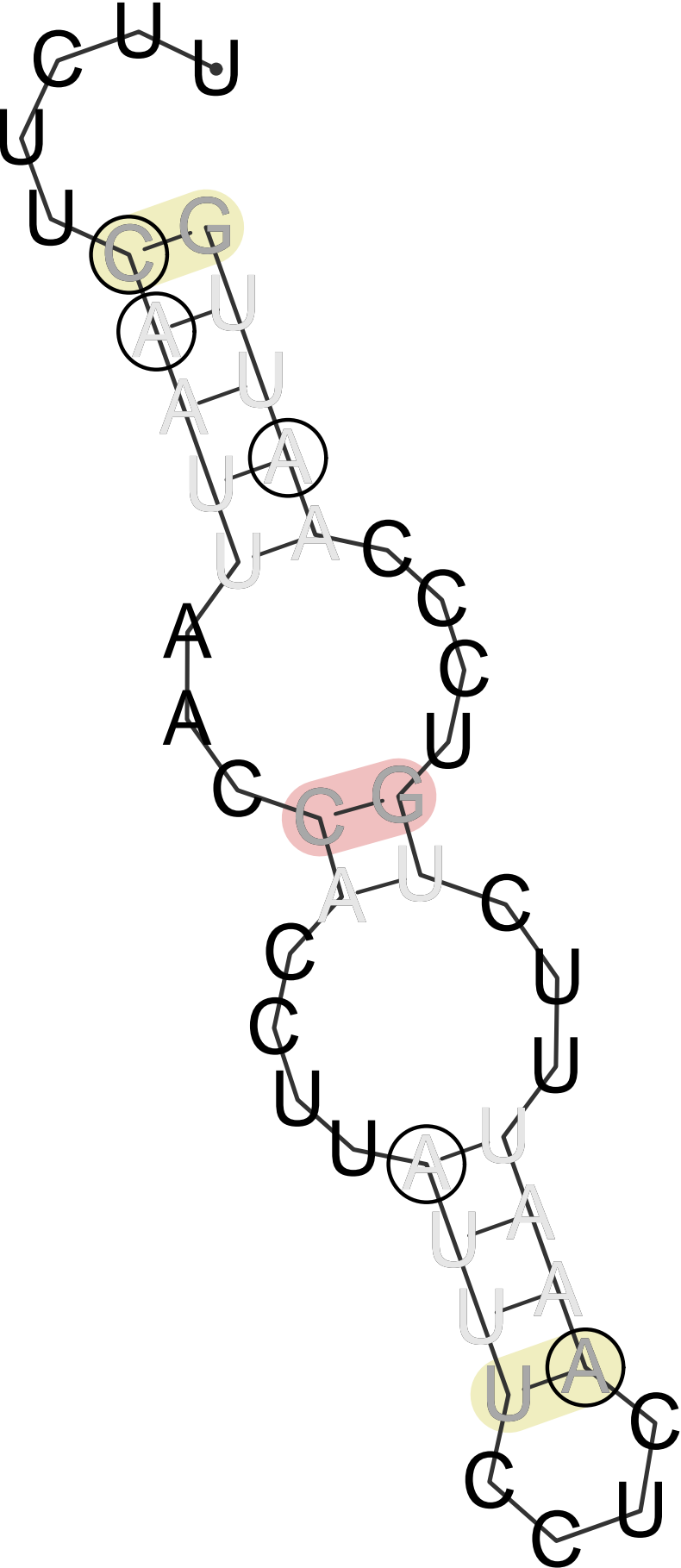

Supplement: S1 Fig — Filename convention within the zip file is as follows: HA/NA type, then host type, then whether the region was identified through analysis of raw (unranked) data, ranked data, or both, then the gene name, then the nucleotide location within the analysed alignment of the gene name, then the nucleotide location within the reference sequences (corresponding to locations listed in S2 Table and S10 Table), then a note if the analysis was performed using only one example of each distinct sequence, then a note if the fold uses the reverse complement of the cRNA (i.e. the vRNA), rather than the cRNA. All folds have been generated using alignments with loci where the consensus nucleotide is a gap removed. Base pairs are highlighted in deep/mid/light red when all/all but one/all but two sequences are capable of forming the pairs shown. Base pairs are highlighted in deep/mid/light yellow when all/all but one/all but two sequences are capable of forming the pair shown or one other pair (including GU pairs). Base pairs are highlighted in deep/mid/light green when all/all but one/all but two sequences are capable of forming the pair shown or one of two other pairs (including GU pairs). RNAalifold was used with input options disallowing lonely pairs, allowing G-quadruplexes, and with the ribosum scoring matrix enabled. (ZIP) [file pcbi.1012009.s122.zip › H1N1-human-raw-NS2-alignment-217-261-refseq-674-718-representative-sequences-only_revcomp_alirna_nogap.pdf]

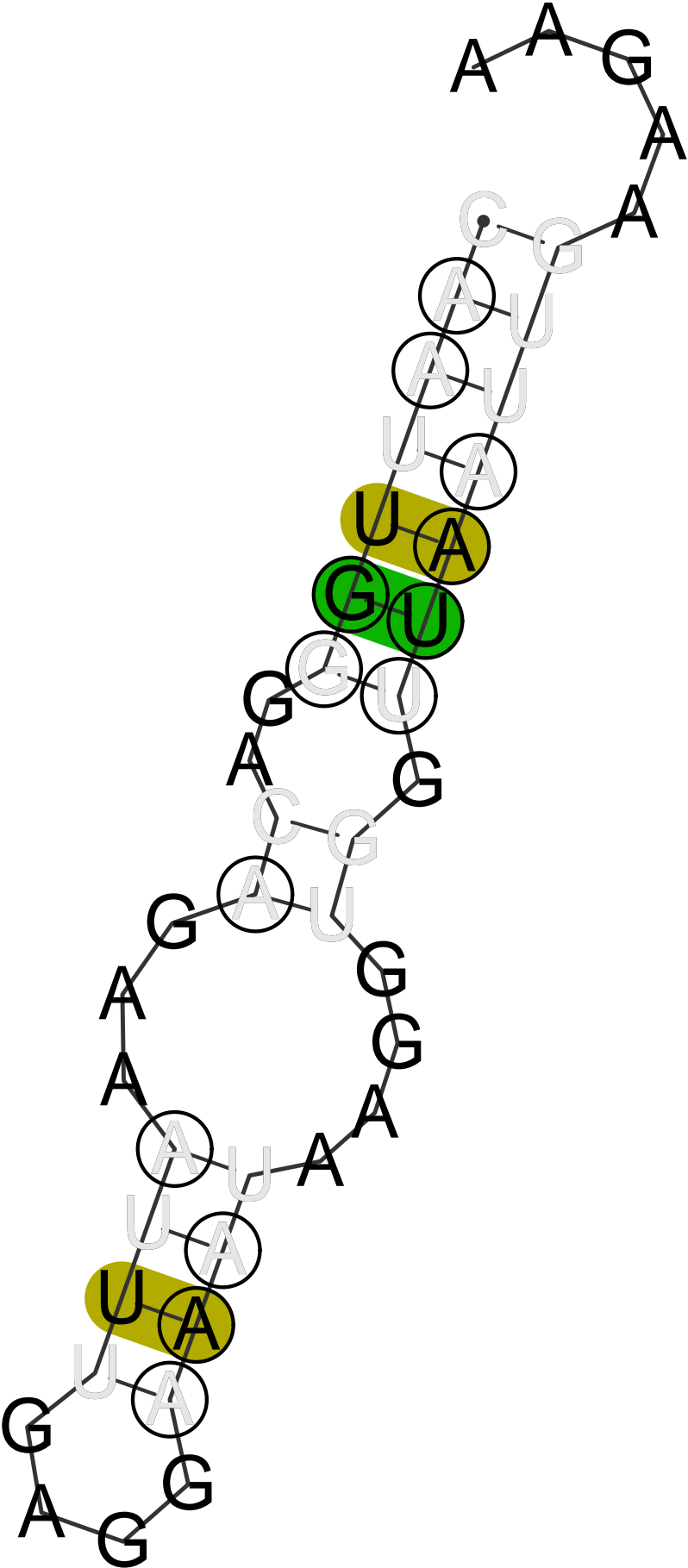

Supplement: S1 Fig — Filename convention within the zip file is as follows: HA/NA type, then host type, then whether the region was identified through analysis of raw (unranked) data, ranked data, or both, then the gene name, then the nucleotide location within the analysed alignment of the gene name, then the nucleotide location within the reference sequences (corresponding to locations listed in S2 Table and S10 Table), then a note if the analysis was performed using only one example of each distinct sequence, then a note if the fold uses the reverse complement of the cRNA (i.e. the vRNA), rather than the cRNA. All folds have been generated using alignments with loci where the consensus nucleotide is a gap removed. Base pairs are highlighted in deep/mid/light red when all/all but one/all but two sequences are capable of forming the pairs shown. Base pairs are highlighted in deep/mid/light yellow when all/all but one/all but two sequences are capable of forming the pair shown or one other pair (including GU pairs). Base pairs are highlighted in deep/mid/light green when all/all but one/all but two sequences are capable of forming the pair shown or one of two other pairs (including GU pairs). RNAalifold was used with input options disallowing lonely pairs, allowing G-quadruplexes, and with the ribosum scoring matrix enabled. (ZIP) [file pcbi.1012009.s122.zip › H1N1-human-raw-NS2-alignment-217-261-refseq-674-718_alirna_nogap.pdf]

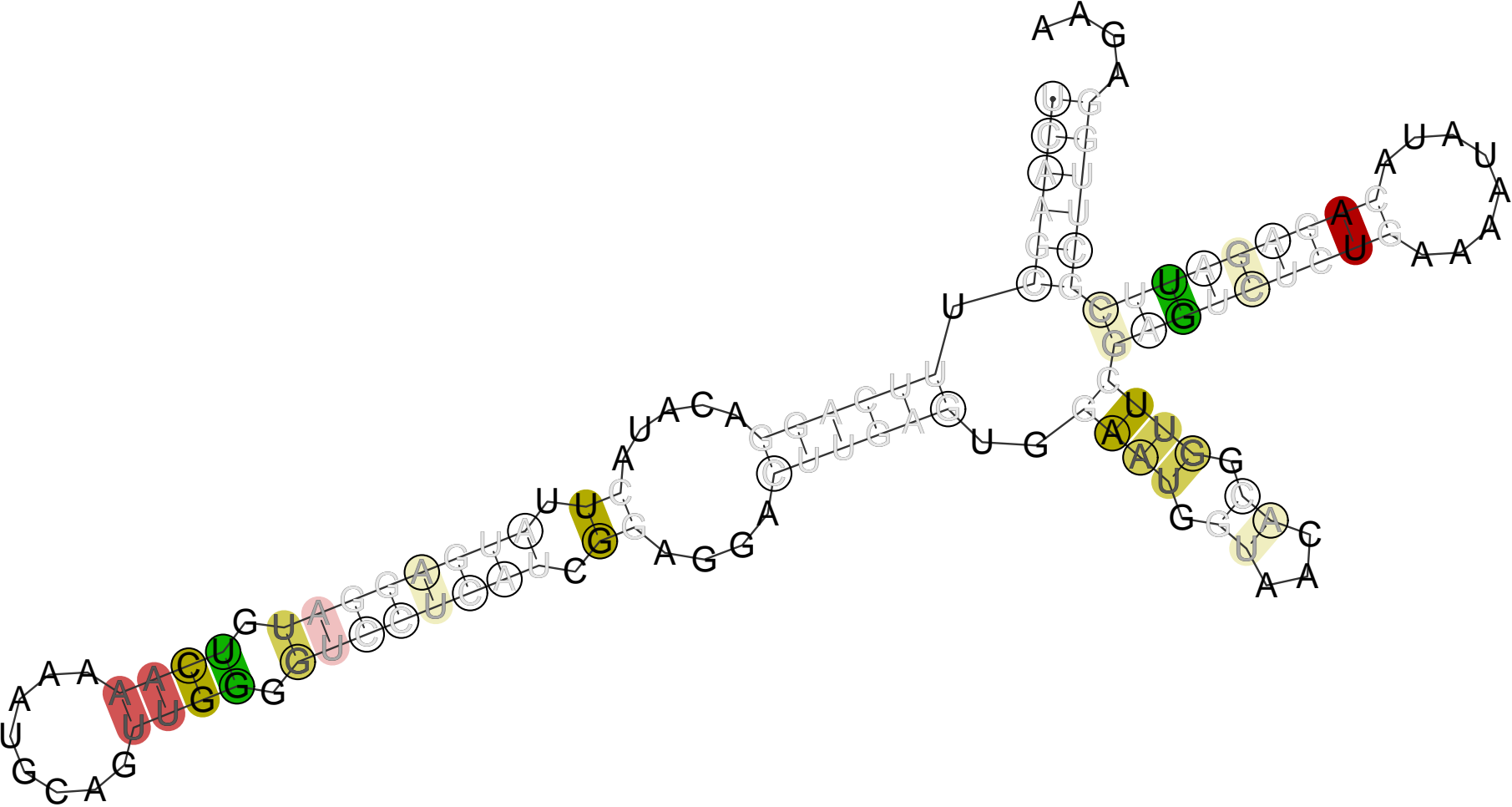

Supplement: S1 Fig — Filename convention within the zip file is as follows: HA/NA type, then host type, then whether the region was identified through analysis of raw (unranked) data, ranked data, or both, then the gene name, then the nucleotide location within the analysed alignment of the gene name, then the nucleotide location within the reference sequences (corresponding to locations listed in S2 Table and S10 Table), then a note if the analysis was performed using only one example of each distinct sequence, then a note if the fold uses the reverse complement of the cRNA (i.e. the vRNA), rather than the cRNA. All folds have been generated using alignments with loci where the consensus nucleotide is a gap removed. Base pairs are highlighted in deep/mid/light red when all/all but one/all but two sequences are capable of forming the pairs shown. Base pairs are highlighted in deep/mid/light yellow when all/all but one/all but two sequences are capable of forming the pair shown or one other pair (including GU pairs). Base pairs are highlighted in deep/mid/light green when all/all but one/all but two sequences are capable of forming the pair shown or one of two other pairs (including GU pairs). RNAalifold was used with input options disallowing lonely pairs, allowing G-quadruplexes, and with the ribosum scoring matrix enabled. (ZIP) [file pcbi.1012009.s122.zip › H1N1-human-raw-NS2-alignment-22-156-refseq-19-30--503-613-representative-sequences-only_alirna_nogap.pdf]

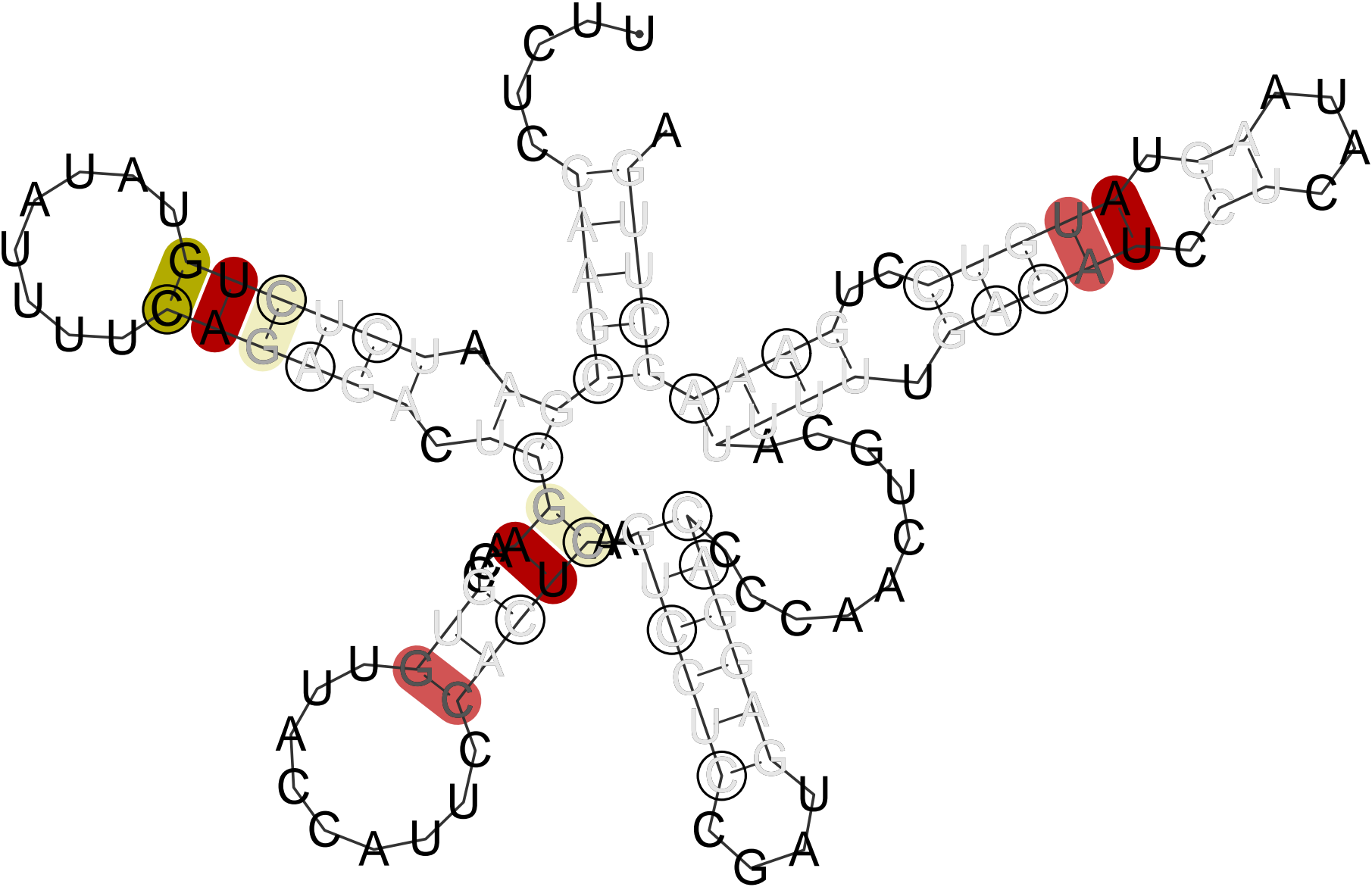

Supplement: S1 Fig — Filename convention within the zip file is as follows: HA/NA type, then host type, then whether the region was identified through analysis of raw (unranked) data, ranked data, or both, then the gene name, then the nucleotide location within the analysed alignment of the gene name, then the nucleotide location within the reference sequences (corresponding to locations listed in S2 Table and S10 Table), then a note if the analysis was performed using only one example of each distinct sequence, then a note if the fold uses the reverse complement of the cRNA (i.e. the vRNA), rather than the cRNA. All folds have been generated using alignments with loci where the consensus nucleotide is a gap removed. Base pairs are highlighted in deep/mid/light red when all/all but one/all but two sequences are capable of forming the pairs shown. Base pairs are highlighted in deep/mid/light yellow when all/all but one/all but two sequences are capable of forming the pair shown or one other pair (including GU pairs). Base pairs are highlighted in deep/mid/light green when all/all but one/all but two sequences are capable of forming the pair shown or one of two other pairs (including GU pairs). RNAalifold was used with input options disallowing lonely pairs, allowing G-quadruplexes, and with the ribosum scoring matrix enabled. (ZIP) [file pcbi.1012009.s122.zip › H1N1-human-raw-NS2-alignment-22-156-refseq-19-30--503-613-representative-sequences-only_revcomp_alirna_nogap.pdf]

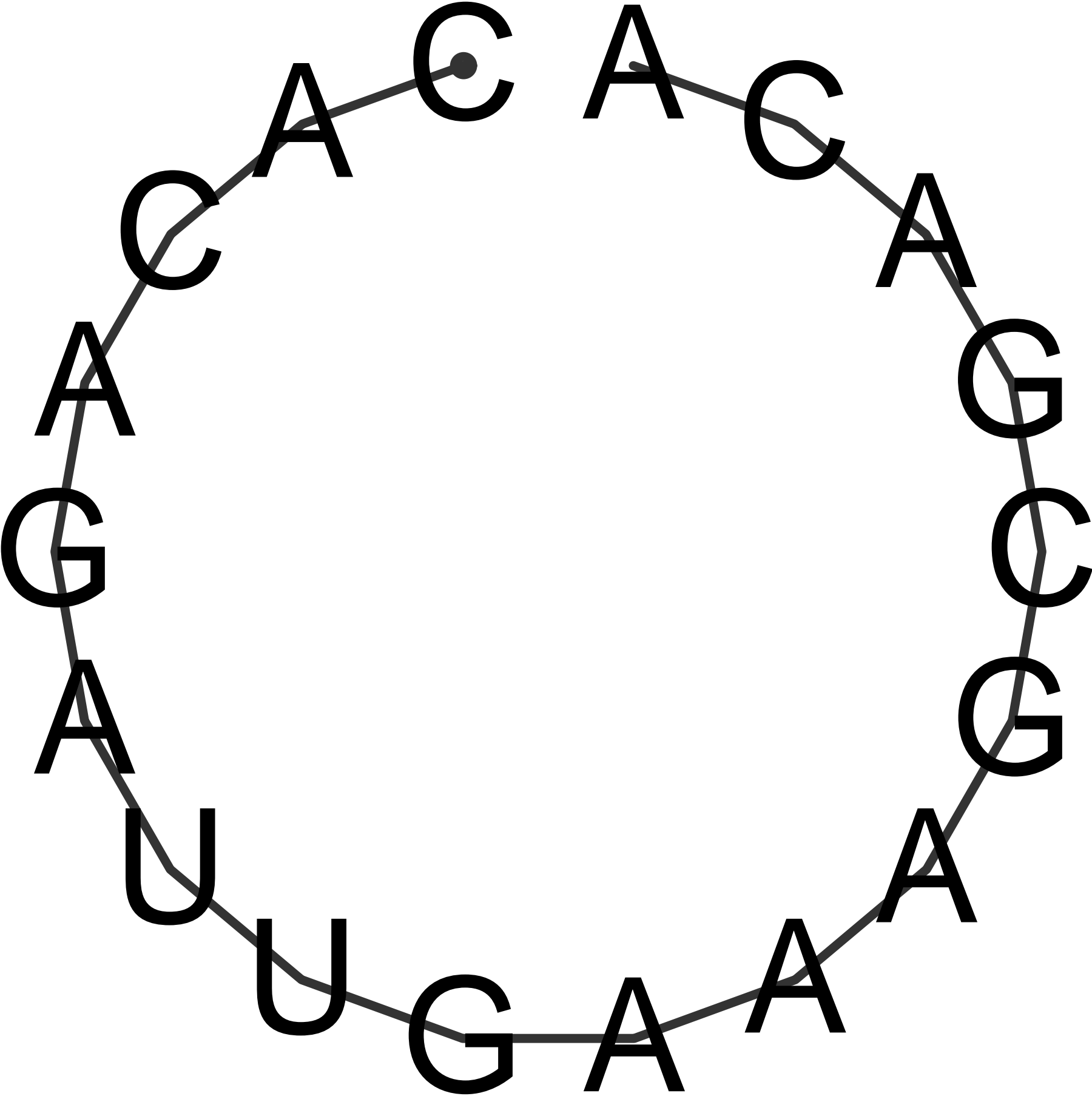

Supplement: S1 Fig — Filename convention within the zip file is as follows: HA/NA type, then host type, then whether the region was identified through analysis of raw (unranked) data, ranked data, or both, then the gene name, then the nucleotide location within the analysed alignment of the gene name, then the nucleotide location within the reference sequences (corresponding to locations listed in S2 Table and S10 Table), then a note if the analysis was performed using only one example of each distinct sequence, then a note if the fold uses the reverse complement of the cRNA (i.e. the vRNA), rather than the cRNA. All folds have been generated using alignments with loci where the consensus nucleotide is a gap removed. Base pairs are highlighted in deep/mid/light red when all/all but one/all but two sequences are capable of forming the pairs shown. Base pairs are highlighted in deep/mid/light yellow when all/all but one/all but two sequences are capable of forming the pair shown or one other pair (including GU pairs). Base pairs are highlighted in deep/mid/light green when all/all but one/all but two sequences are capable of forming the pair shown or one of two other pairs (including GU pairs). RNAalifold was used with input options disallowing lonely pairs, allowing G-quadruplexes, and with the ribosum scoring matrix enabled. (ZIP) [file pcbi.1012009.s122.zip › H1N1-human-raw-NS2-alignment-304-321-refseq-725-742-representative-sequences-only_alirna_nogap.pdf]

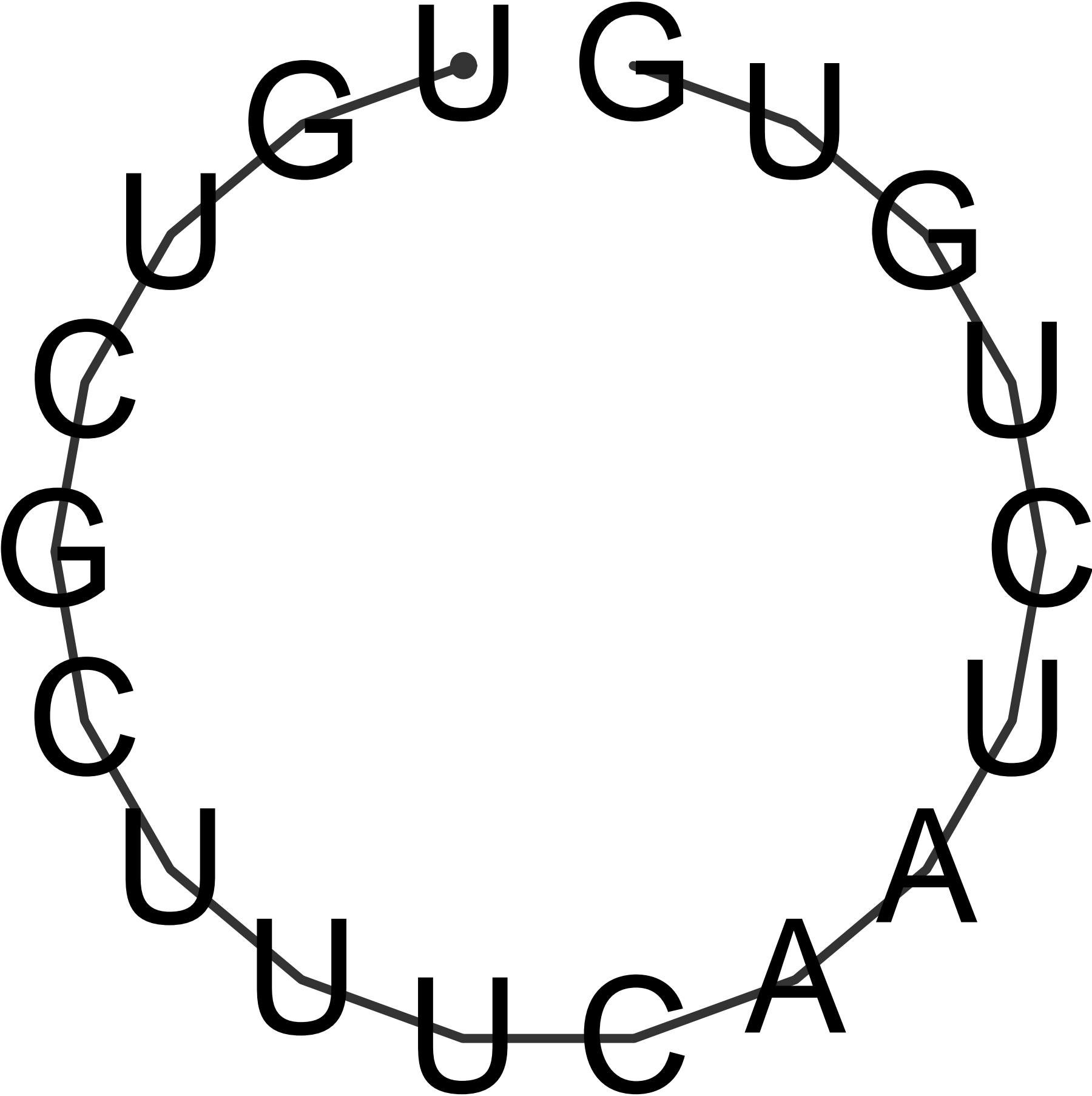

Supplement: S1 Fig — Filename convention within the zip file is as follows: HA/NA type, then host type, then whether the region was identified through analysis of raw (unranked) data, ranked data, or both, then the gene name, then the nucleotide location within the analysed alignment of the gene name, then the nucleotide location within the reference sequences (corresponding to locations listed in S2 Table and S10 Table), then a note if the analysis was performed using only one example of each distinct sequence, then a note if the fold uses the reverse complement of the cRNA (i.e. the vRNA), rather than the cRNA. All folds have been generated using alignments with loci where the consensus nucleotide is a gap removed. Base pairs are highlighted in deep/mid/light red when all/all but one/all but two sequences are capable of forming the pairs shown. Base pairs are highlighted in deep/mid/light yellow when all/all but one/all but two sequences are capable of forming the pair shown or one other pair (including GU pairs). Base pairs are highlighted in deep/mid/light green when all/all but one/all but two sequences are capable of forming the pair shown or one of two other pairs (including GU pairs). RNAalifold was used with input options disallowing lonely pairs, allowing G-quadruplexes, and with the ribosum scoring matrix enabled. (ZIP) [file pcbi.1012009.s122.zip › H1N1-human-raw-NS2-alignment-304-321-refseq-725-742-representative-sequences-only_revcomp_alirna_nogap.pdf]

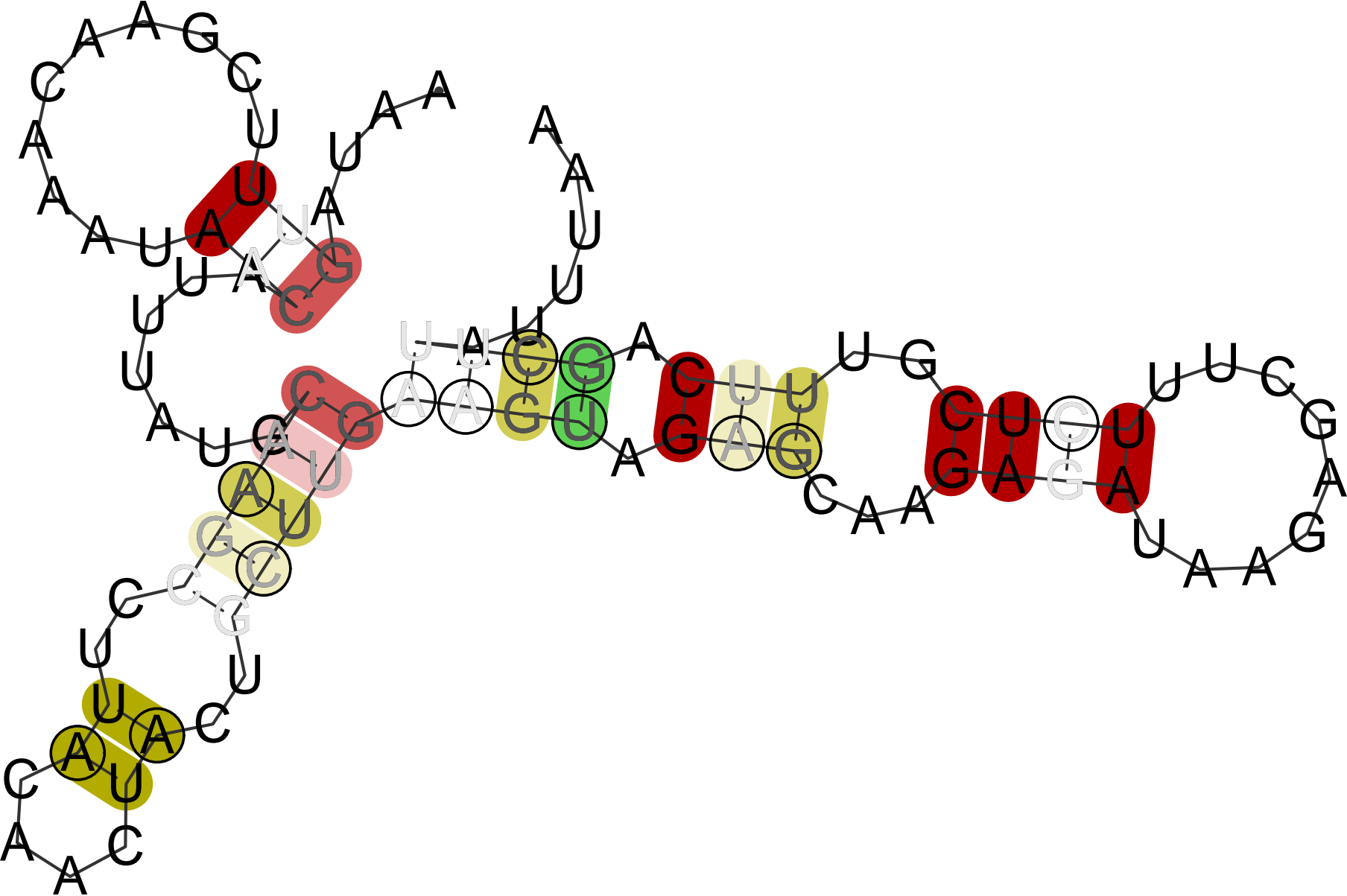

Supplement: S1 Fig — Filename convention within the zip file is as follows: HA/NA type, then host type, then whether the region was identified through analysis of raw (unranked) data, ranked data, or both, then the gene name, then the nucleotide location within the analysed alignment of the gene name, then the nucleotide location within the reference sequences (corresponding to locations listed in S2 Table and S10 Table), then a note if the analysis was performed using only one example of each distinct sequence, then a note if the fold uses the reverse complement of the cRNA (i.e. the vRNA), rather than the cRNA. All folds have been generated using alignments with loci where the consensus nucleotide is a gap removed. Base pairs are highlighted in deep/mid/light red when all/all but one/all but two sequences are capable of forming the pairs shown. Base pairs are highlighted in deep/mid/light yellow when all/all but one/all but two sequences are capable of forming the pair shown or one other pair (including GU pairs). Base pairs are highlighted in deep/mid/light green when all/all but one/all but two sequences are capable of forming the pair shown or one of two other pairs (including GU pairs). RNAalifold was used with input options disallowing lonely pairs, allowing G-quadruplexes, and with the ribosum scoring matrix enabled. (ZIP) [file pcbi.1012009.s122.zip › H1N1-human-raw-NS2-alignment-325-423-refseq-746-838-representative-sequences-only_alirna_nogap.pdf]

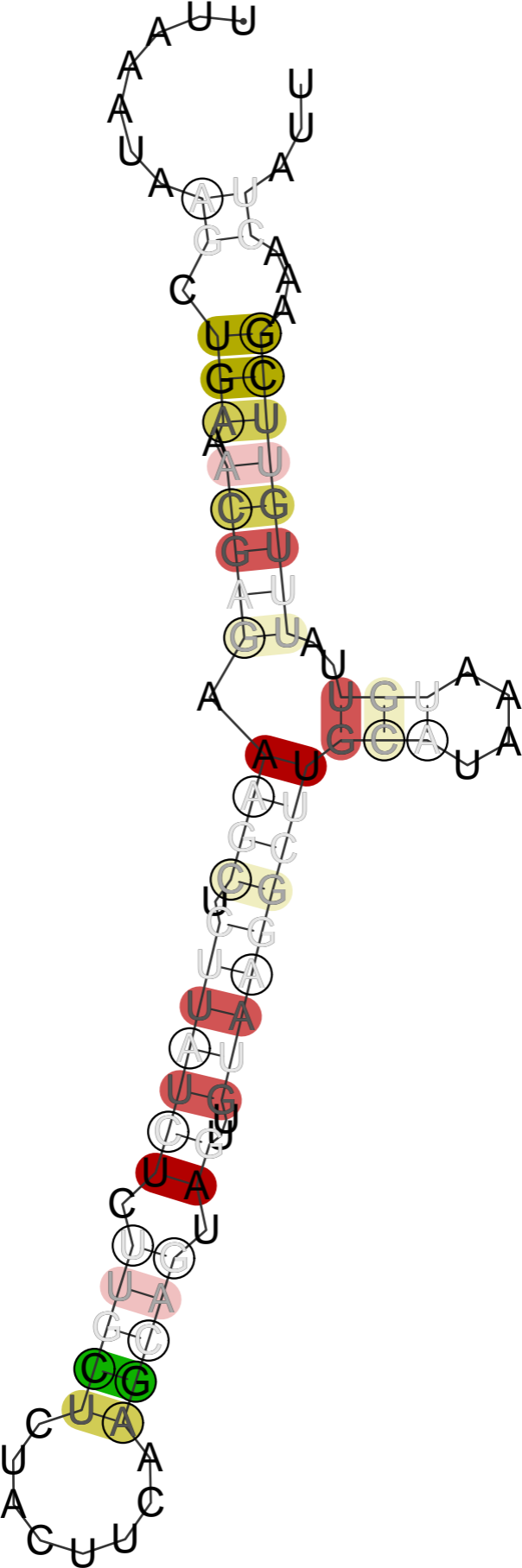

Supplement: S1 Fig — Filename convention within the zip file is as follows: HA/NA type, then host type, then whether the region was identified through analysis of raw (unranked) data, ranked data, or both, then the gene name, then the nucleotide location within the analysed alignment of the gene name, then the nucleotide location within the reference sequences (corresponding to locations listed in S2 Table and S10 Table), then a note if the analysis was performed using only one example of each distinct sequence, then a note if the fold uses the reverse complement of the cRNA (i.e. the vRNA), rather than the cRNA. All folds have been generated using alignments with loci where the consensus nucleotide is a gap removed. Base pairs are highlighted in deep/mid/light red when all/all but one/all but two sequences are capable of forming the pairs shown. Base pairs are highlighted in deep/mid/light yellow when all/all but one/all but two sequences are capable of forming the pair shown or one other pair (including GU pairs). Base pairs are highlighted in deep/mid/light green when all/all but one/all but two sequences are capable of forming the pair shown or one of two other pairs (including GU pairs). RNAalifold was used with input options disallowing lonely pairs, allowing G-quadruplexes, and with the ribosum scoring matrix enabled. (ZIP) [file pcbi.1012009.s122.zip › H1N1-human-raw-NS2-alignment-325-423-refseq-746-838-representative-sequences-only_revcomp_alirna_nogap.pdf]

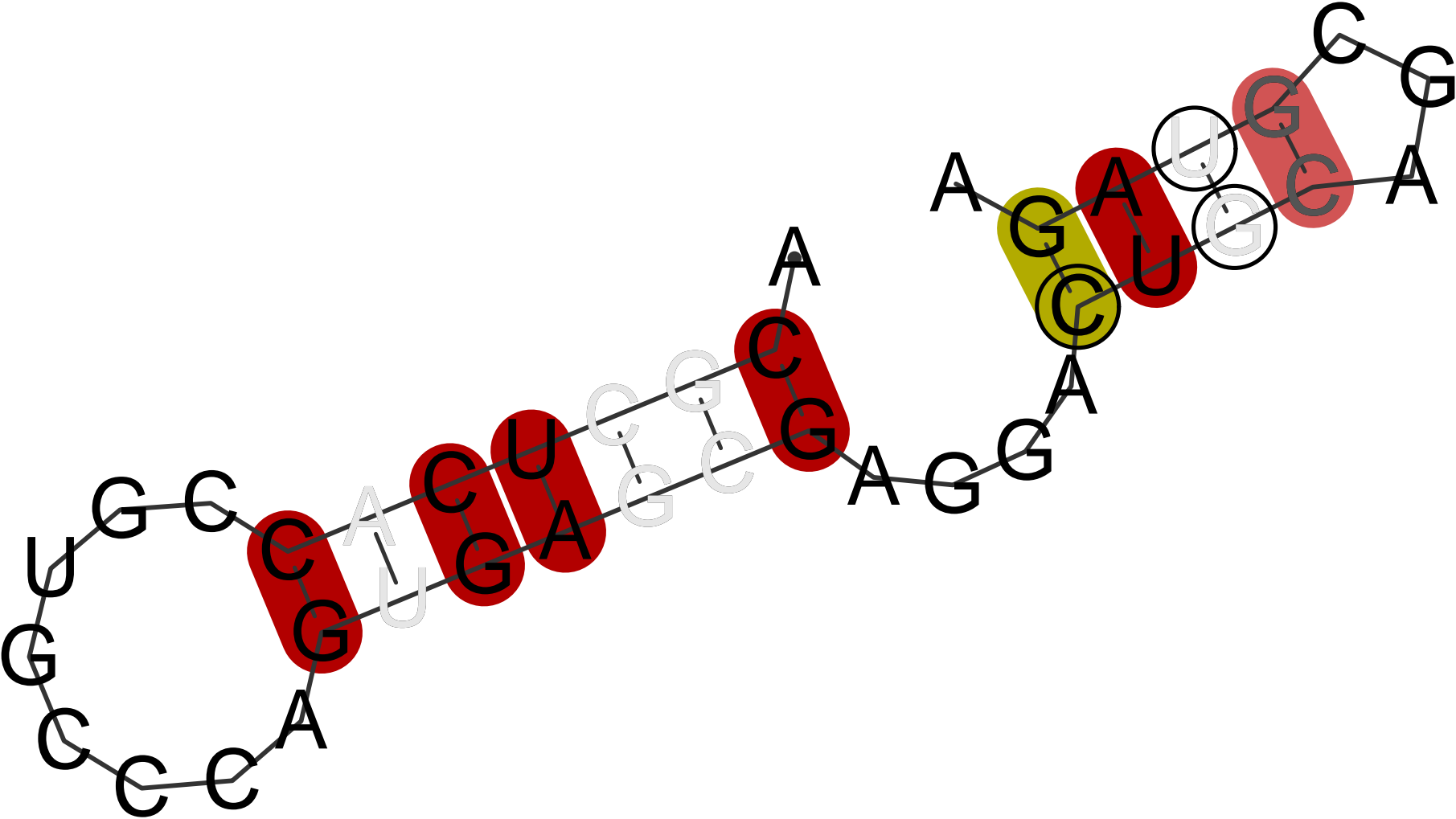

Supplement: S2 Fig — See the caption for S1 Fig for a description of the filename convention (save that the corresponding nucleotide locations in reference sequences are listed in S3 and S11 Tables), and an explanation of the RNAalifold options used and output. (ZIP) [file pcbi.1012009.s123.zip › H1N2-swine-ranked-M1-alignment-196-234-refseq-193-231_alirna_nogap.pdf]

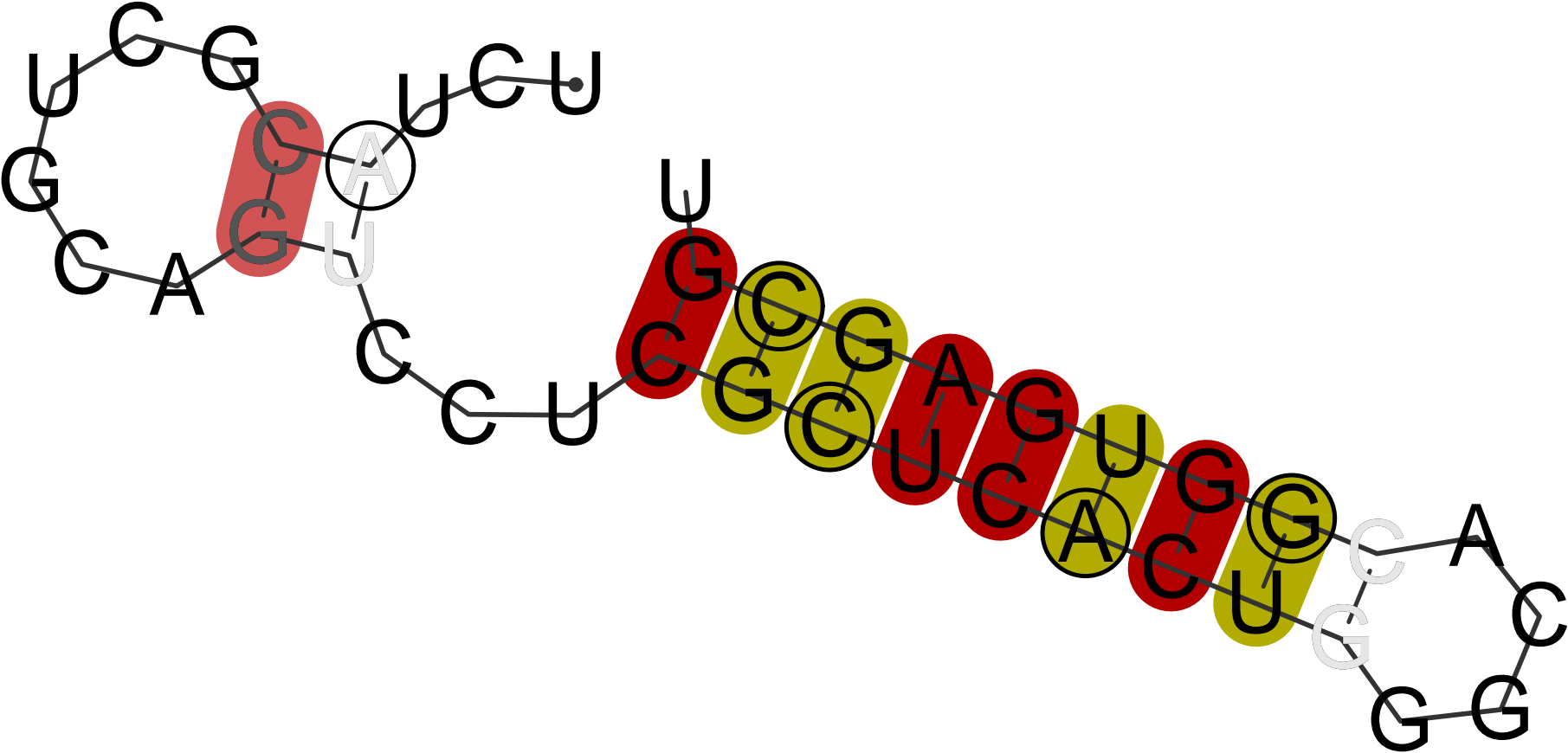

Supplement: S2 Fig — See the caption for S1 Fig for a description of the filename convention (save that the corresponding nucleotide locations in reference sequences are listed in S3 and S11 Tables), and an explanation of the RNAalifold options used and output. (ZIP) [file pcbi.1012009.s123.zip › H1N2-swine-ranked-M1-alignment-196-234-refseq-193-231_revcomp_alirna_nogap.pdf]

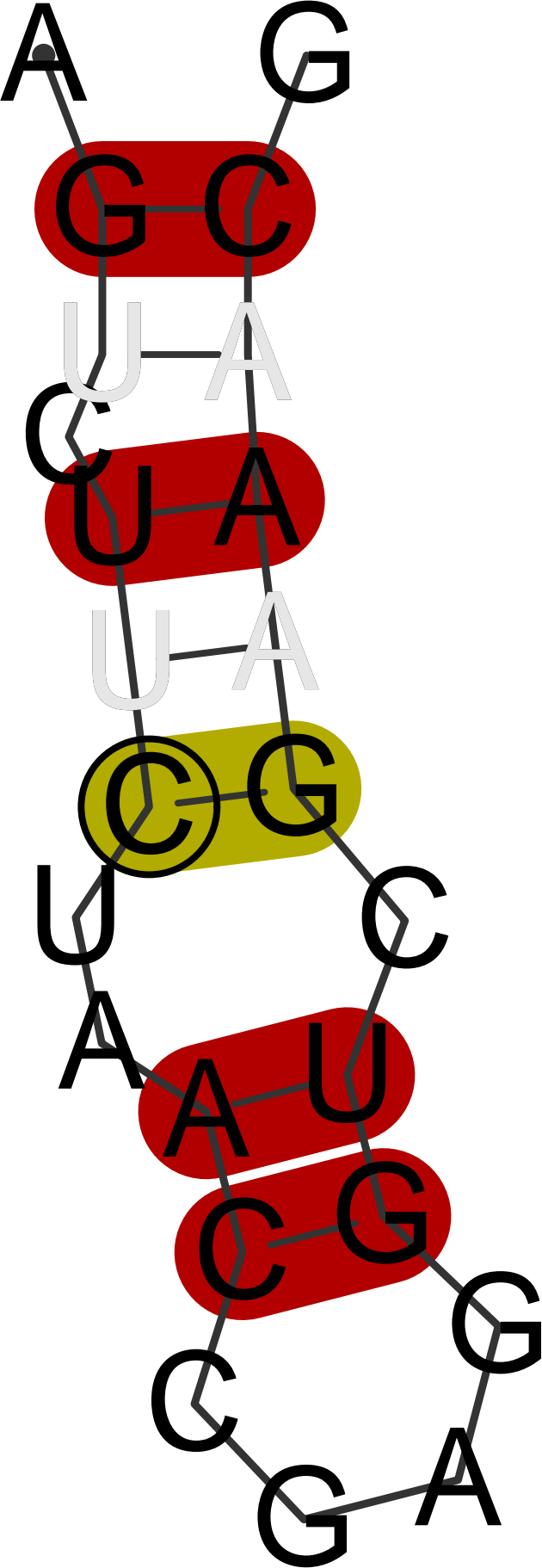

Supplement: S2 Fig — See the caption for S1 Fig for a description of the filename convention (save that the corresponding nucleotide locations in reference sequences are listed in S3 and S11 Tables), and an explanation of the RNAalifold options used and output. (ZIP) [file pcbi.1012009.s123.zip › H1N2-swine-ranked-M2-alignment-7-30-refseq-4-27_alirna_nogap.pdf]

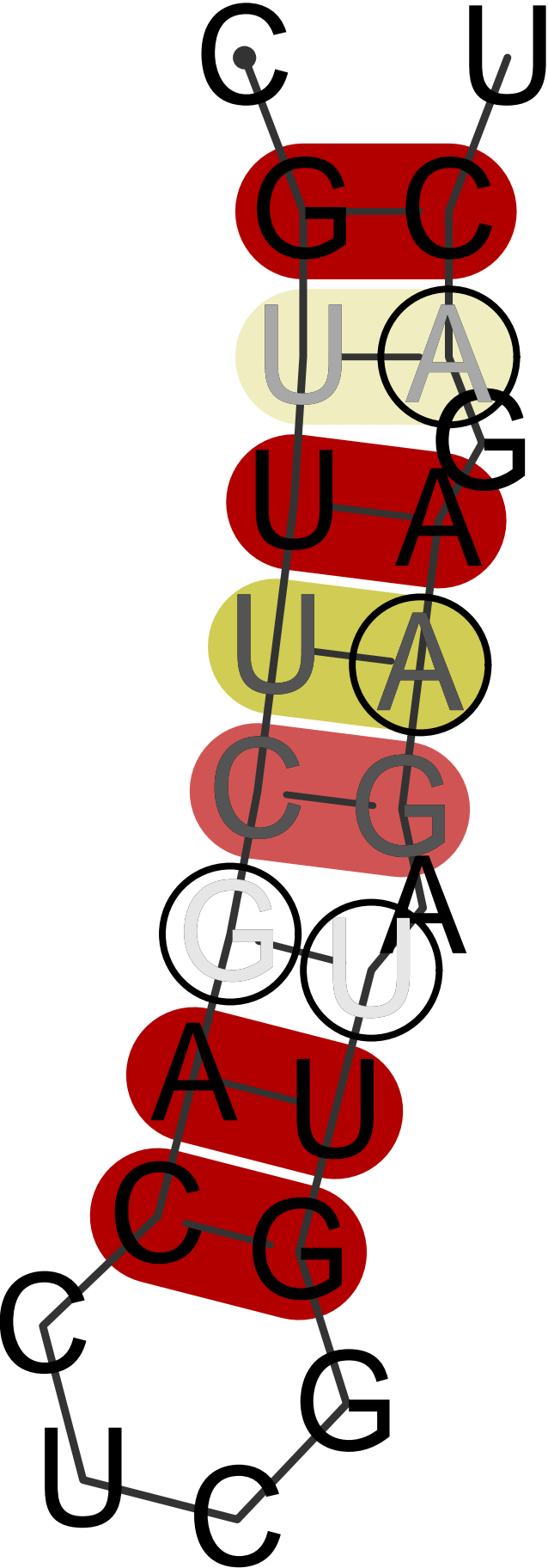

Supplement: S2 Fig — See the caption for S1 Fig for a description of the filename convention (save that the corresponding nucleotide locations in reference sequences are listed in S3 and S11 Tables), and an explanation of the RNAalifold options used and output. (ZIP) [file pcbi.1012009.s123.zip › H1N2-swine-ranked-M2-alignment-7-30-refseq-4-27_revcomp_alirna_nogap.pdf]

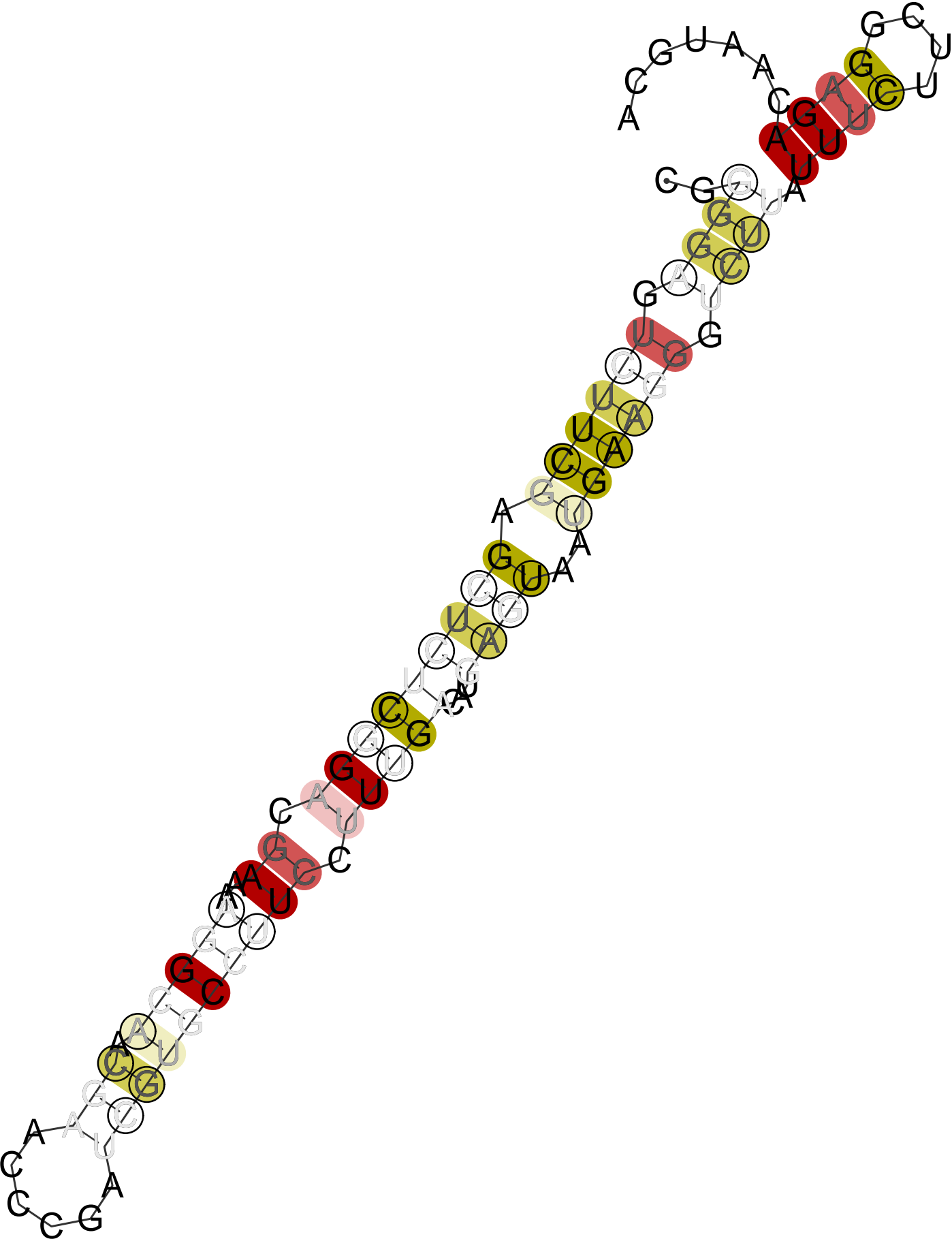

Supplement: S2 Fig — See the caption for S1 Fig for a description of the filename convention (save that the corresponding nucleotide locations in reference sequences are listed in S3 and S11 Tables), and an explanation of the RNAalifold options used and output. (ZIP) [file pcbi.1012009.s123.zip › H1N2-swine-ranked-NP-alignment-1399-1497-refseq-1381-1479_alirna_nogap.pdf]

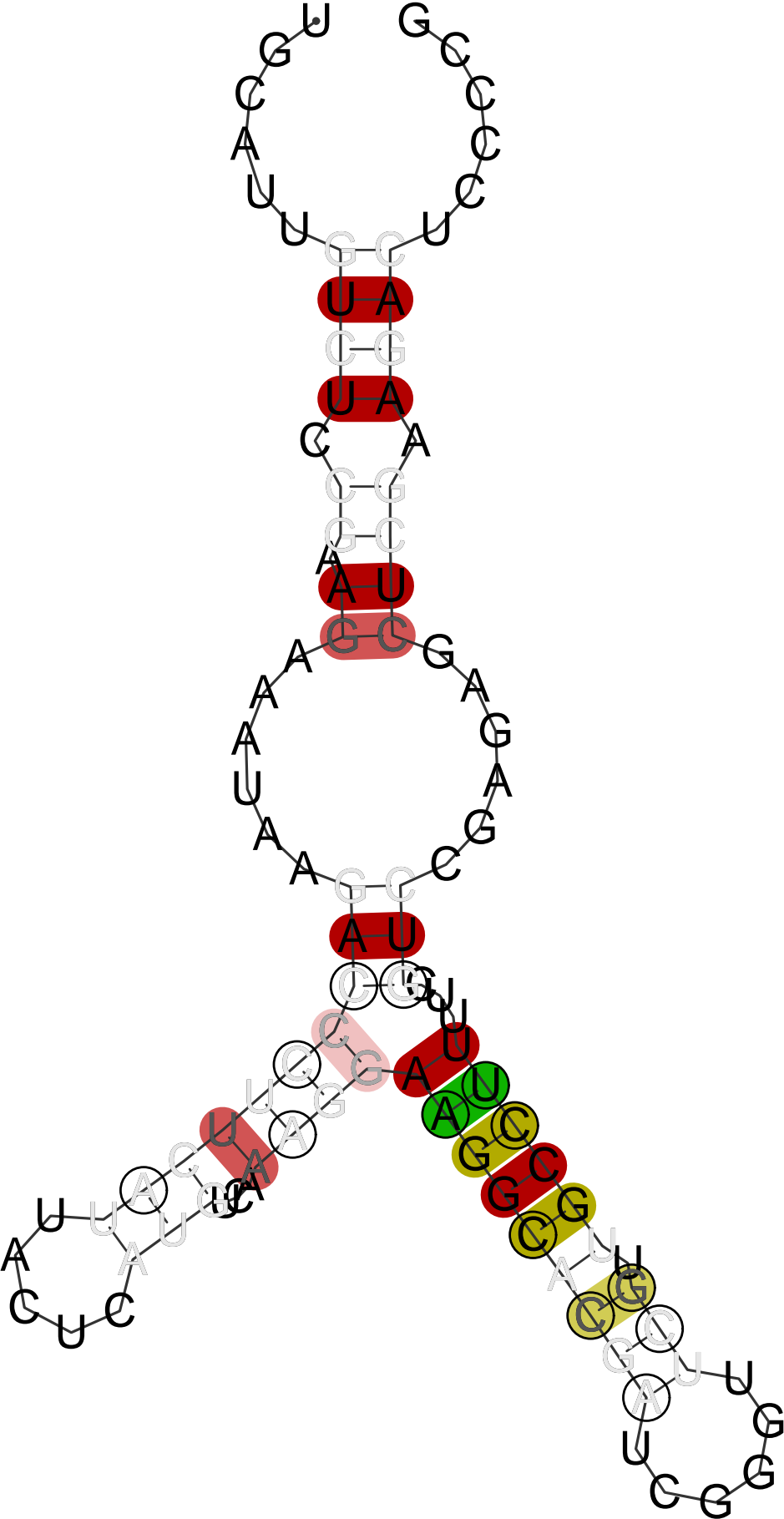

Supplement: S2 Fig — See the caption for S1 Fig for a description of the filename convention (save that the corresponding nucleotide locations in reference sequences are listed in S3 and S11 Tables), and an explanation of the RNAalifold options used and output. (ZIP) [file pcbi.1012009.s123.zip › H1N2-swine-ranked-NP-alignment-1399-1497-refseq-1381-1479_revcomp_alirna_nogap.pdf]

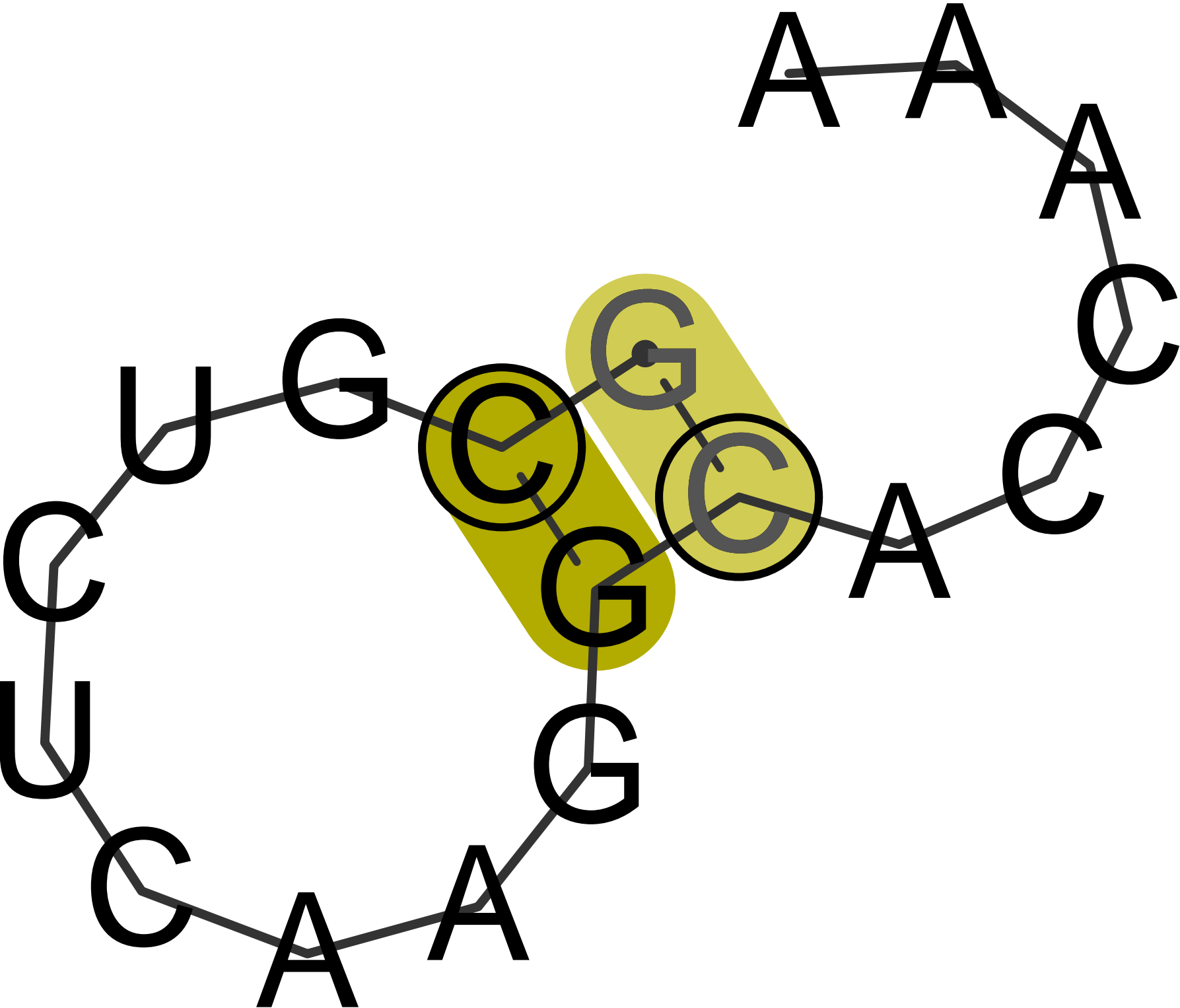

Supplement: S2 Fig — See the caption for S1 Fig for a description of the filename convention (save that the corresponding nucleotide locations in reference sequences are listed in S3 and S11 Tables), and an explanation of the RNAalifold options used and output. (ZIP) [file pcbi.1012009.s123.zip › H1N2-swine-ranked-NP-alignment-22-39-refseq-4-21_alirna_nogap.pdf]

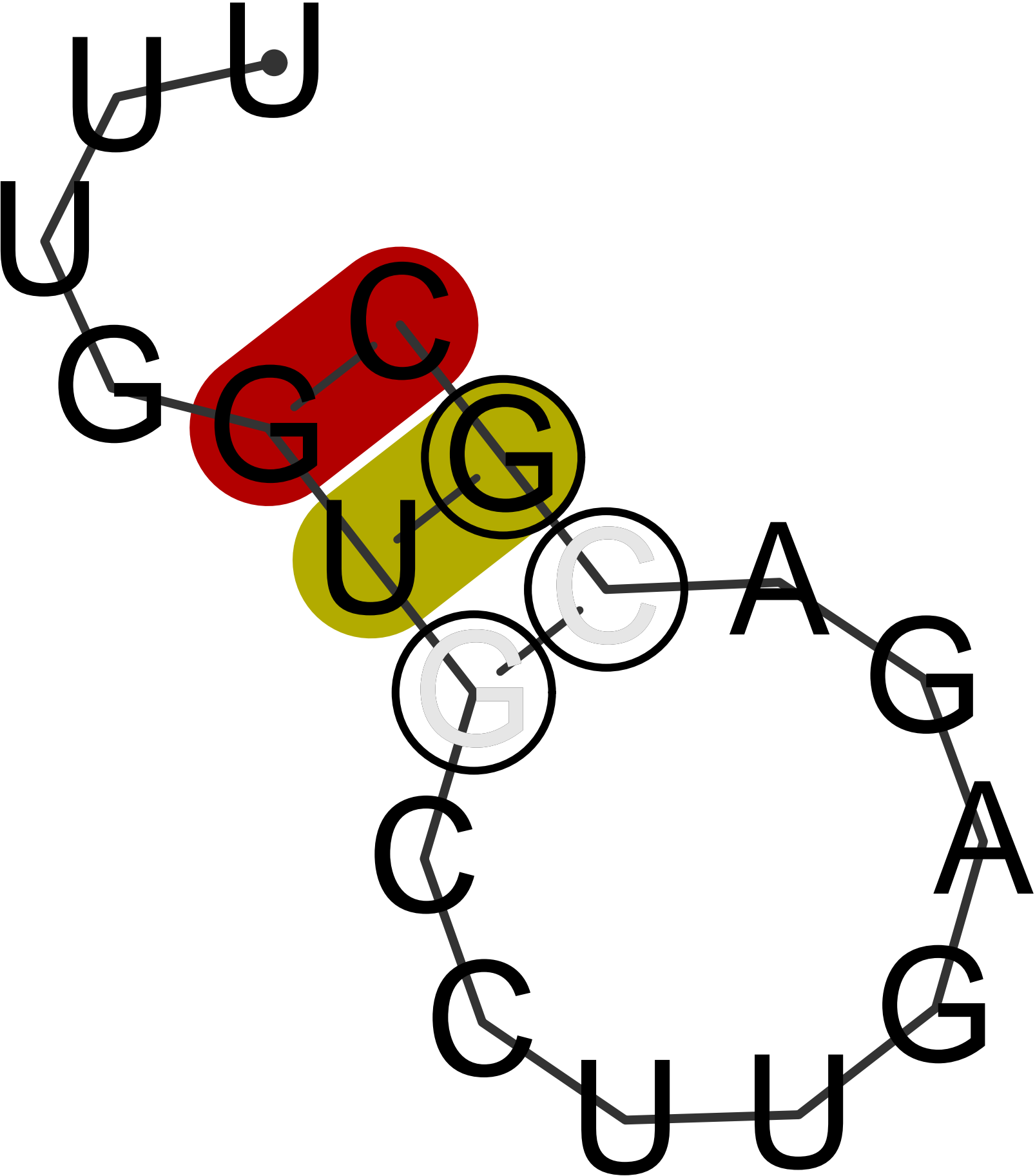

Supplement: S2 Fig — See the caption for S1 Fig for a description of the filename convention (save that the corresponding nucleotide locations in reference sequences are listed in S3 and S11 Tables), and an explanation of the RNAalifold options used and output. (ZIP) [file pcbi.1012009.s123.zip › H1N2-swine-ranked-NP-alignment-22-39-refseq-4-21_revcomp_alirna_nogap.pdf]

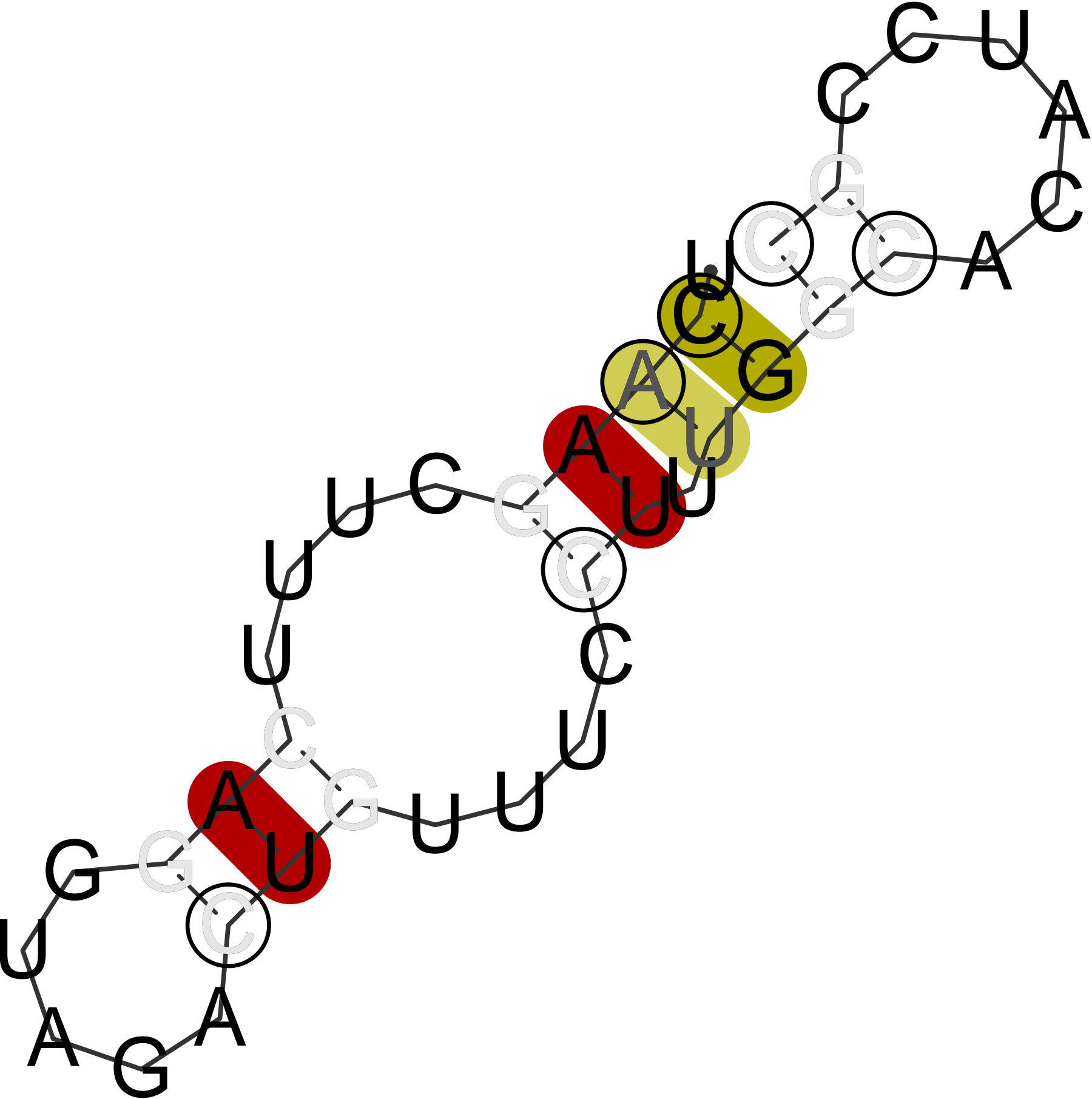

Supplement: S2 Fig — See the caption for S1 Fig for a description of the filename convention (save that the corresponding nucleotide locations in reference sequences are listed in S3 and S11 Tables), and an explanation of the RNAalifold options used and output. (ZIP) [file pcbi.1012009.s123.zip › H1N2-swine-ranked-NS1-alignment-22-60-refseq-19-57_alirna_nogap.pdf]

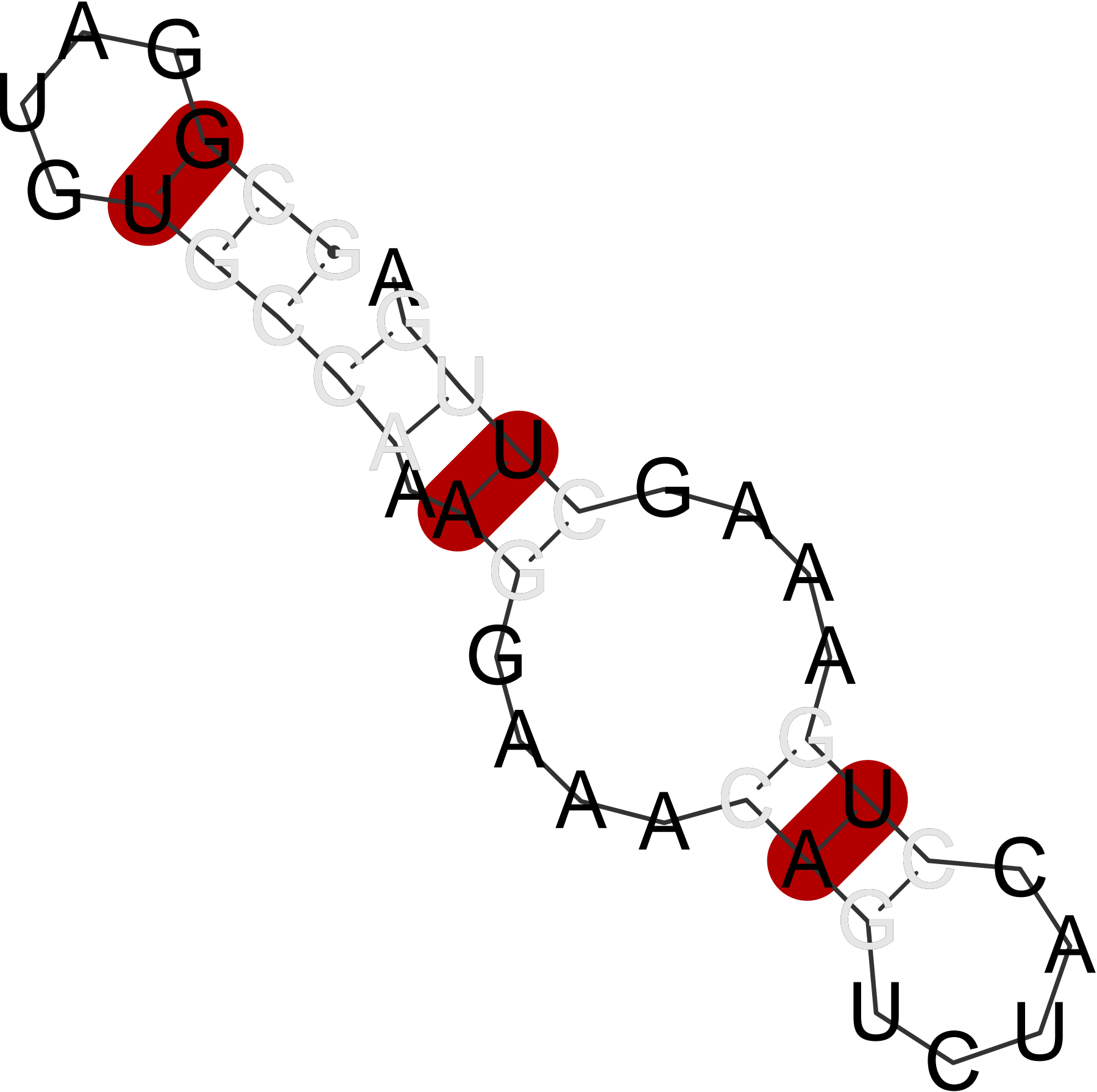

Supplement: S2 Fig — See the caption for S1 Fig for a description of the filename convention (save that the corresponding nucleotide locations in reference sequences are listed in S3 and S11 Tables), and an explanation of the RNAalifold options used and output. (ZIP) [file pcbi.1012009.s123.zip › H1N2-swine-ranked-NS1-alignment-22-60-refseq-19-57_revcomp_alirna_nogap.pdf]

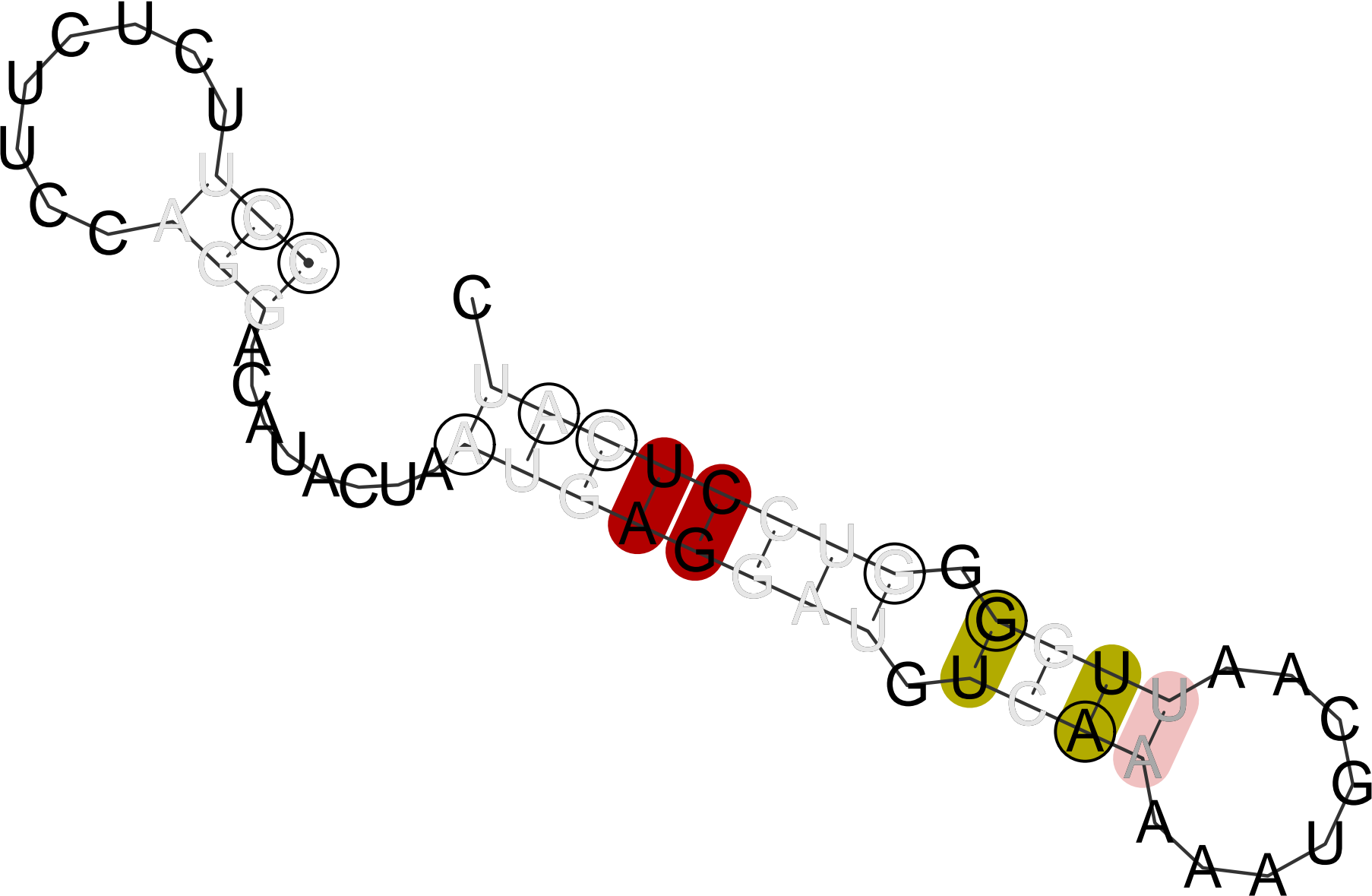

Supplement: S2 Fig — See the caption for S1 Fig for a description of the filename convention (save that the corresponding nucleotide locations in reference sequences are listed in S3 and S11 Tables), and an explanation of the RNAalifold options used and output. (ZIP) [file pcbi.1012009.s123.zip › H1N2-swine-ranked-NS1-alignment-493-549-refseq-490-546_alirna_nogap.pdf]

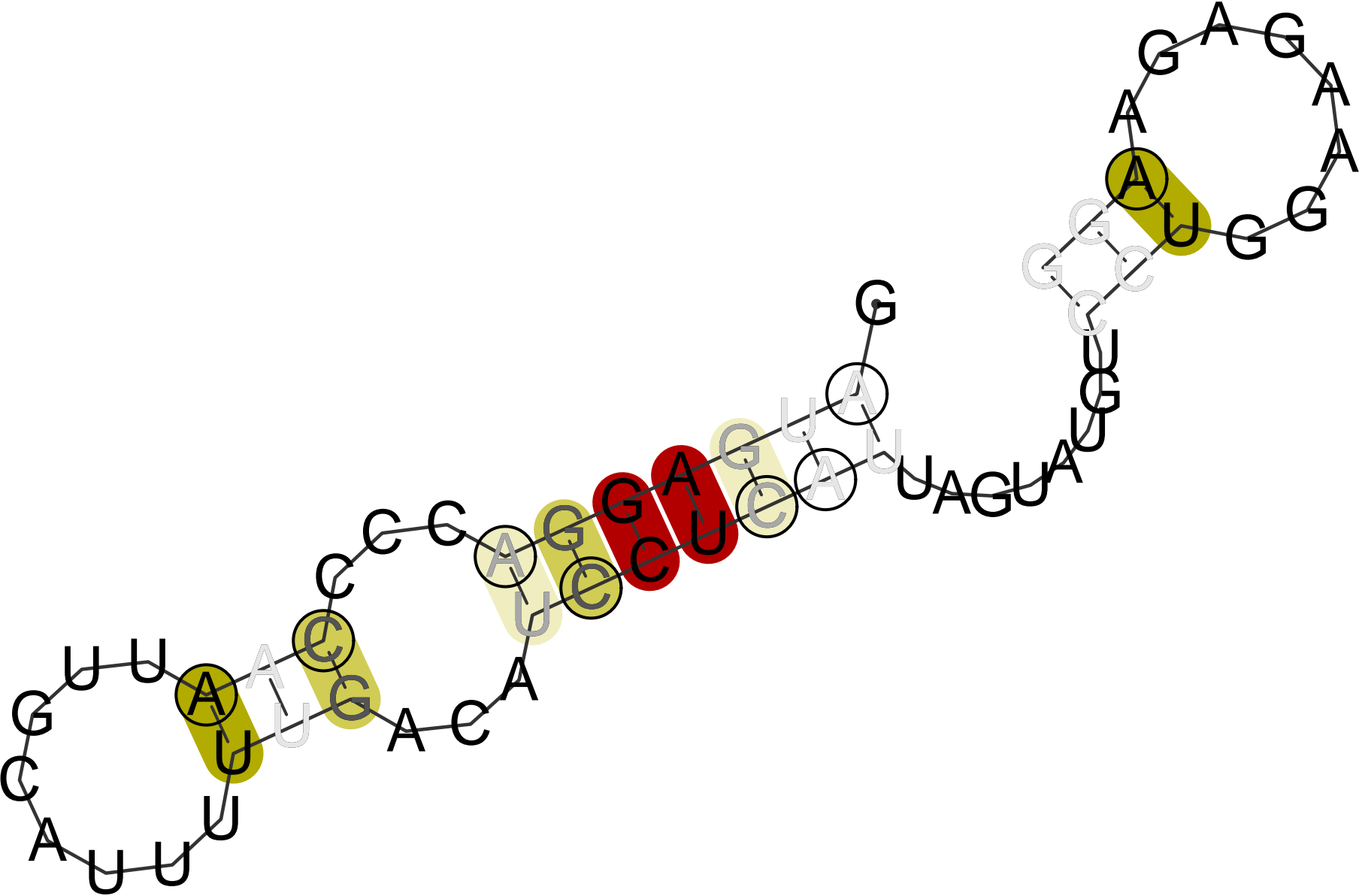

Supplement: S2 Fig — See the caption for S1 Fig for a description of the filename convention (save that the corresponding nucleotide locations in reference sequences are listed in S3 and S11 Tables), and an explanation of the RNAalifold options used and output. (ZIP) [file pcbi.1012009.s123.zip › H1N2-swine-ranked-NS1-alignment-493-549-refseq-490-546_revcomp_alirna_nogap.pdf]

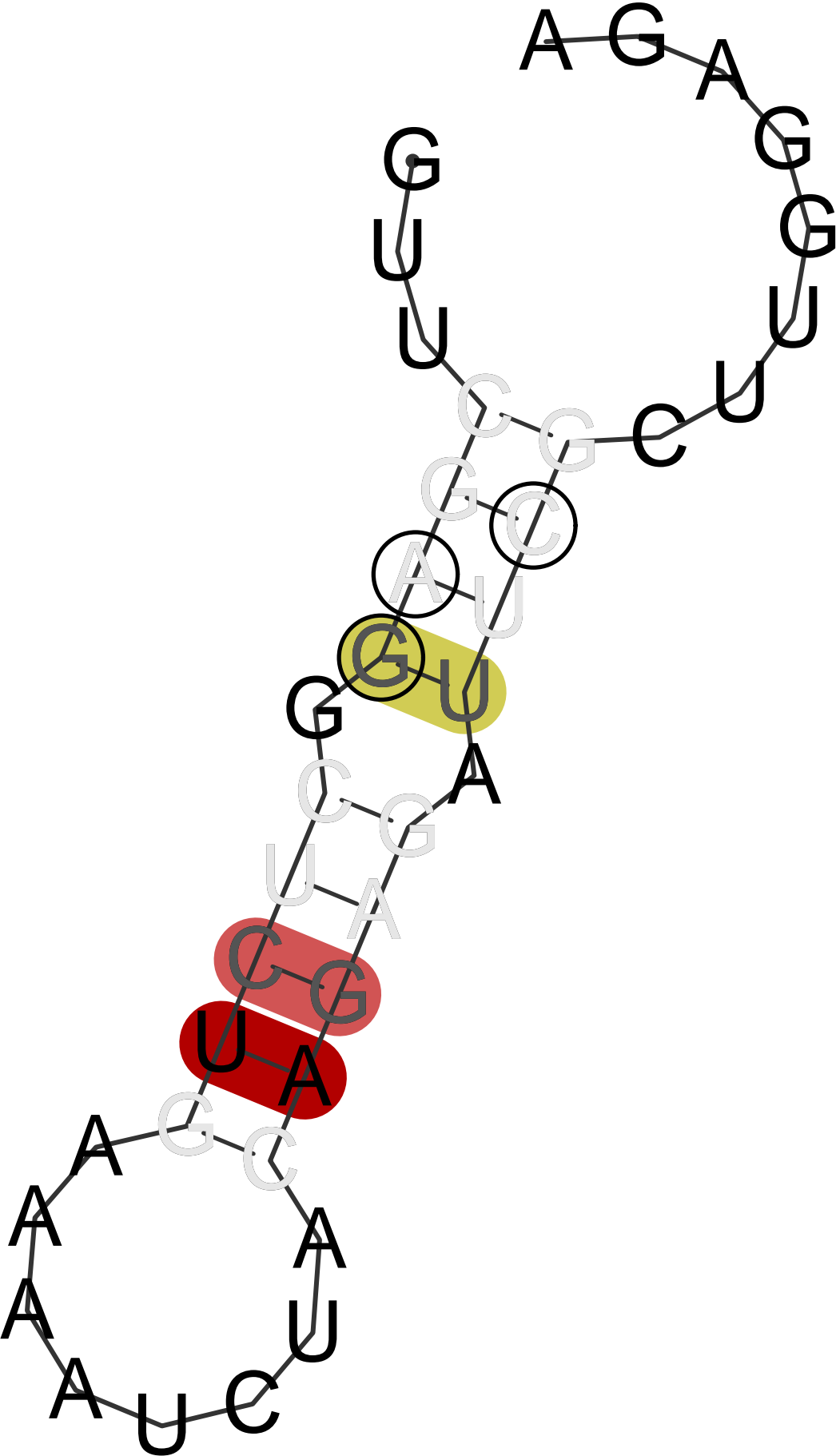

Supplement: S2 Fig — See the caption for S1 Fig for a description of the filename convention (save that the corresponding nucleotide locations in reference sequences are listed in S3 and S11 Tables), and an explanation of the RNAalifold options used and output. (ZIP) [file pcbi.1012009.s123.zip › H1N2-swine-ranked-NS1-alignment-577-615-refseq-574-612_alirna_nogap.pdf]

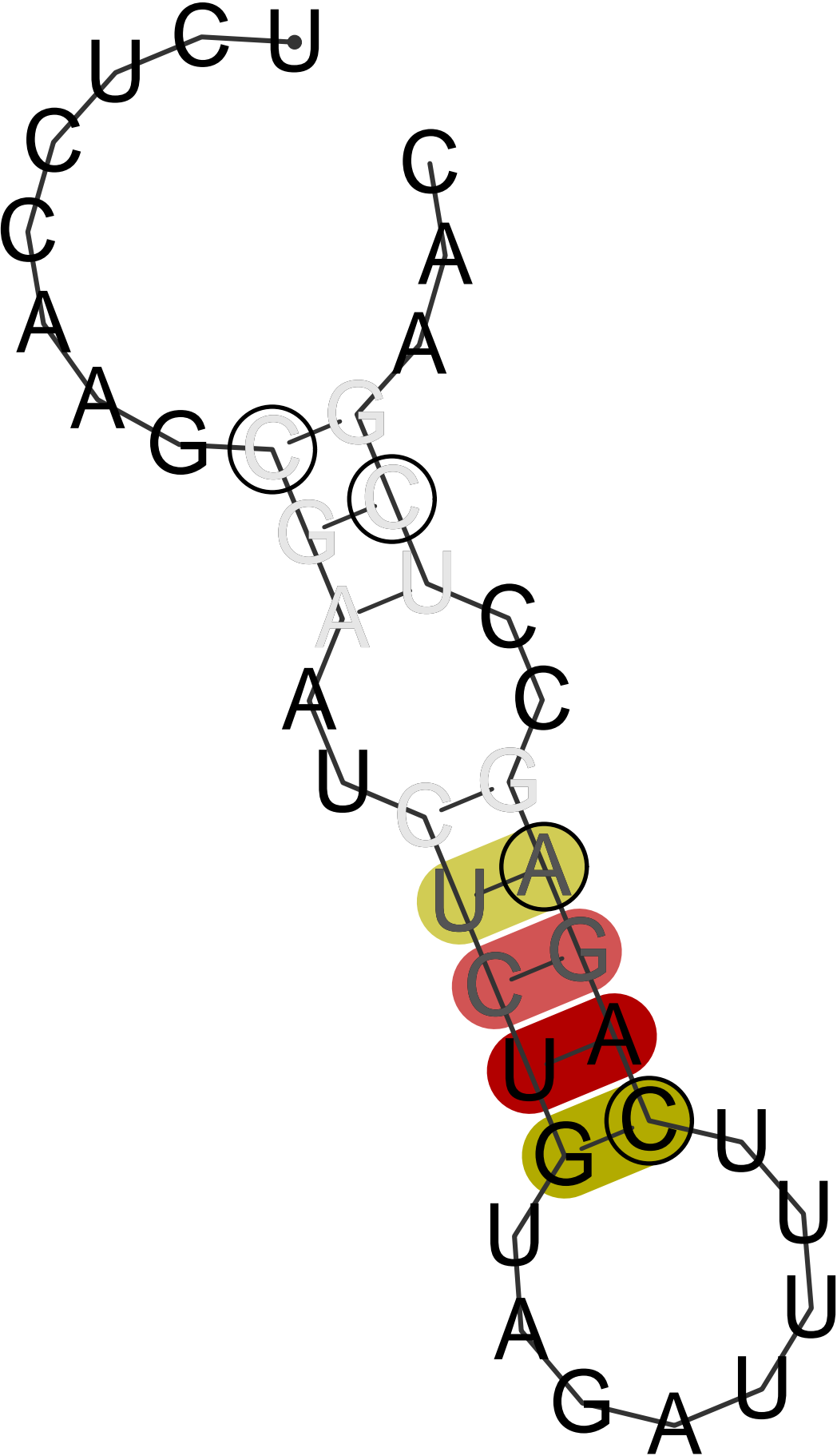

Supplement: S2 Fig — See the caption for S1 Fig for a description of the filename convention (save that the corresponding nucleotide locations in reference sequences are listed in S3 and S11 Tables), and an explanation of the RNAalifold options used and output. (ZIP) [file pcbi.1012009.s123.zip › H1N2-swine-ranked-NS1-alignment-577-615-refseq-574-612_revcomp_alirna_nogap.pdf]

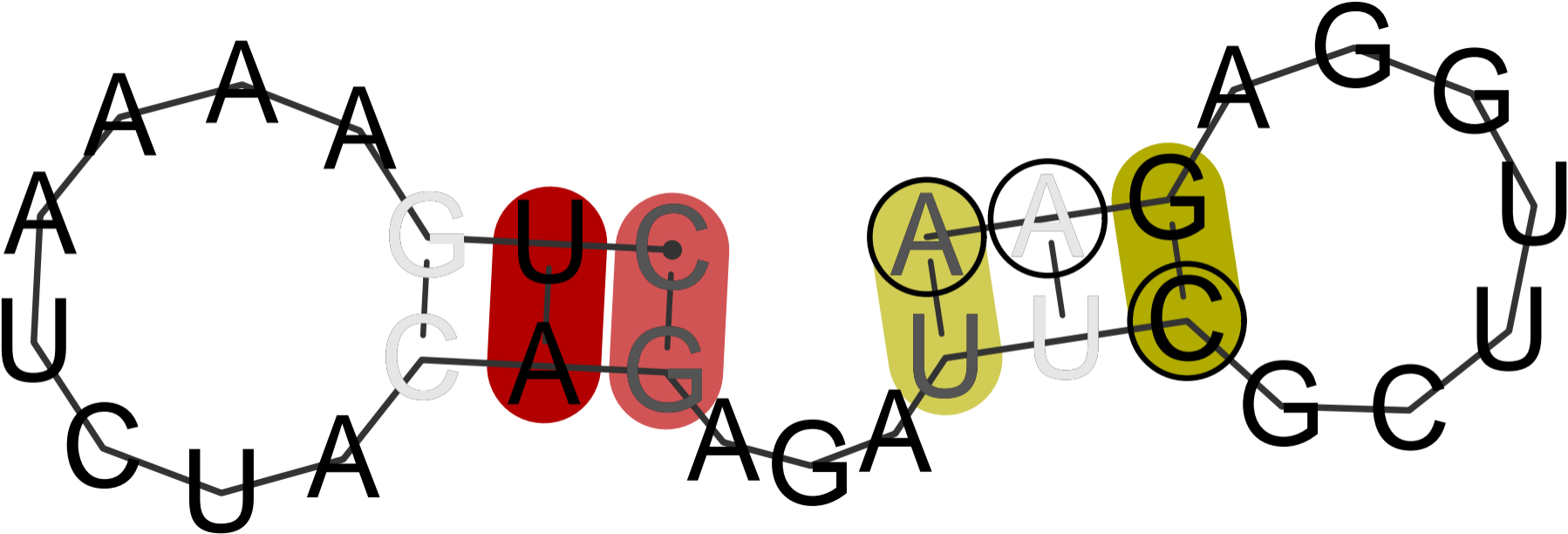

Supplement: S2 Fig — See the caption for S1 Fig for a description of the filename convention (save that the corresponding nucleotide locations in reference sequences are listed in S3 and S11 Tables), and an explanation of the RNAalifold options used and output. (ZIP) [file pcbi.1012009.s123.zip › H1N2-swine-ranked-NS2-alignment-115-144-refseq-584-613_alirna_nogap.pdf]

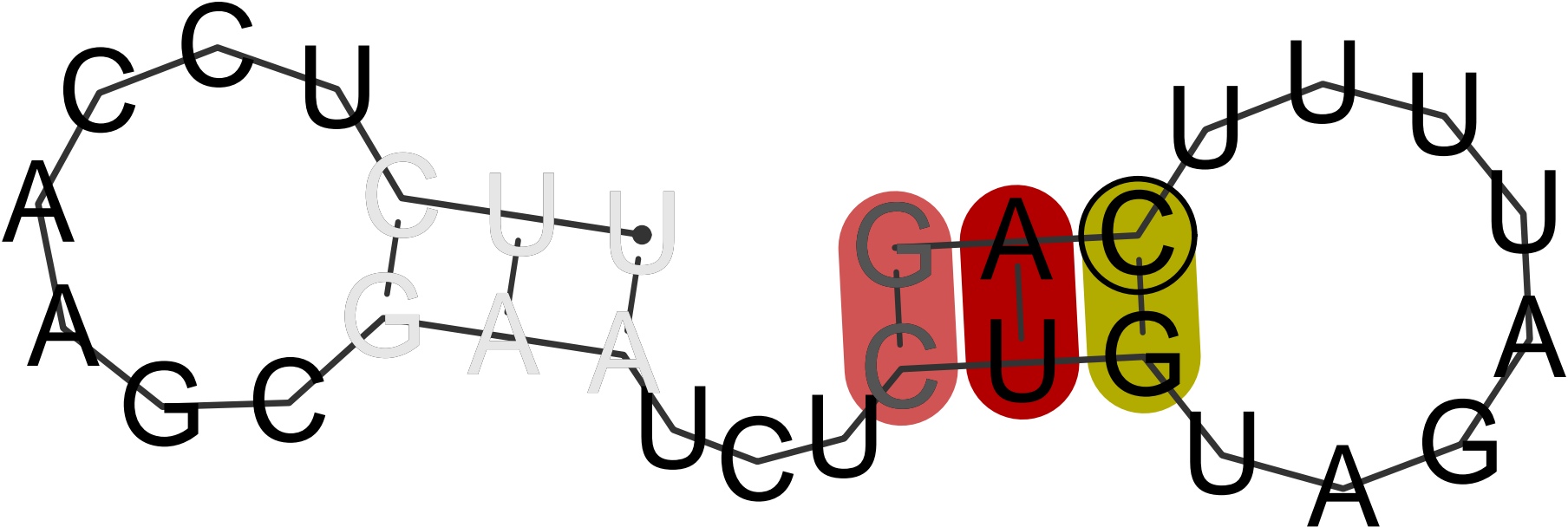

Supplement: S2 Fig — See the caption for S1 Fig for a description of the filename convention (save that the corresponding nucleotide locations in reference sequences are listed in S3 and S11 Tables), and an explanation of the RNAalifold options used and output. (ZIP) [file pcbi.1012009.s123.zip › H1N2-swine-ranked-NS2-alignment-115-144-refseq-584-613_revcomp_alirna_nogap.pdf]

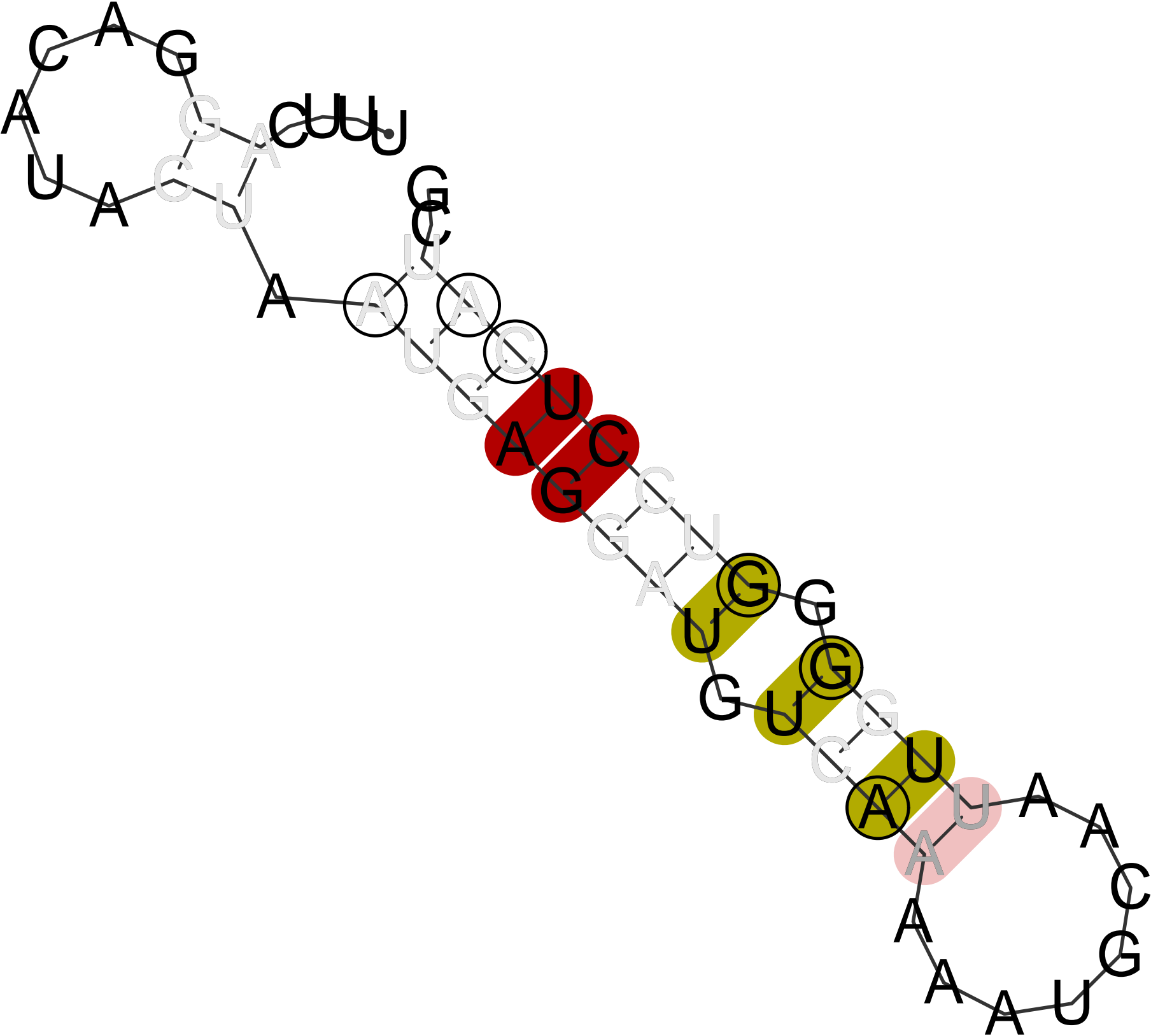

Supplement: S2 Fig — See the caption for S1 Fig for a description of the filename convention (save that the corresponding nucleotide locations in reference sequences are listed in S3 and S11 Tables), and an explanation of the RNAalifold options used and output. (ZIP) [file pcbi.1012009.s123.zip › H1N2-swine-ranked-NS2-alignment-28-78-refseq-25-547_alirna_nogap.pdf]

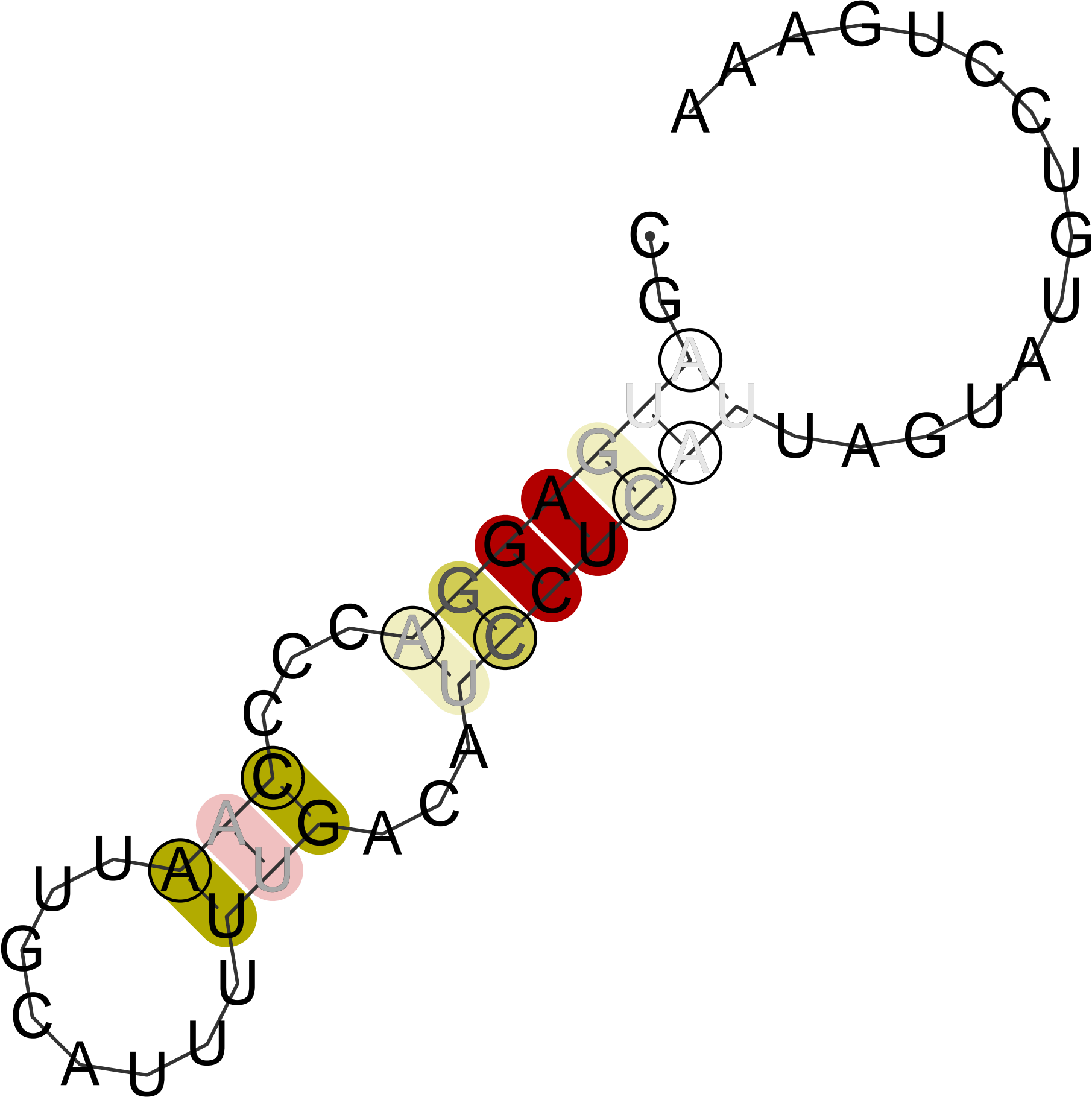

Supplement: S2 Fig — See the caption for S1 Fig for a description of the filename convention (save that the corresponding nucleotide locations in reference sequences are listed in S3 and S11 Tables), and an explanation of the RNAalifold options used and output. (ZIP) [file pcbi.1012009.s123.zip › H1N2-swine-ranked-NS2-alignment-28-78-refseq-25-547_revcomp_alirna_nogap.pdf]

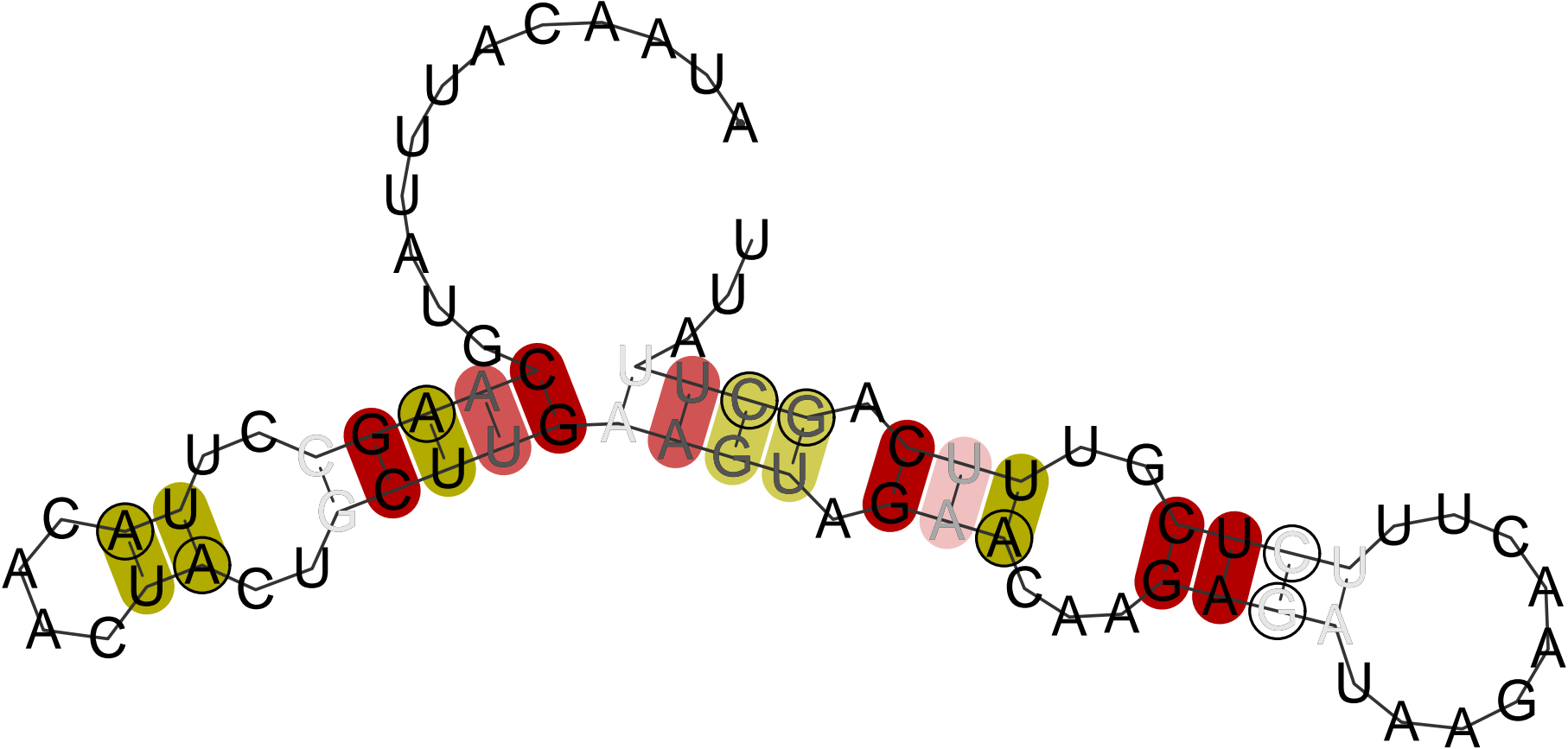

Supplement: S2 Fig — See the caption for S1 Fig for a description of the filename convention (save that the corresponding nucleotide locations in reference sequences are listed in S3 and S11 Tables), and an explanation of the RNAalifold options used and output. (ZIP) [file pcbi.1012009.s123.zip › H1N2-swine-ranked-NS2-alignment-292-366-refseq-761-835_alirna_nogap.pdf]

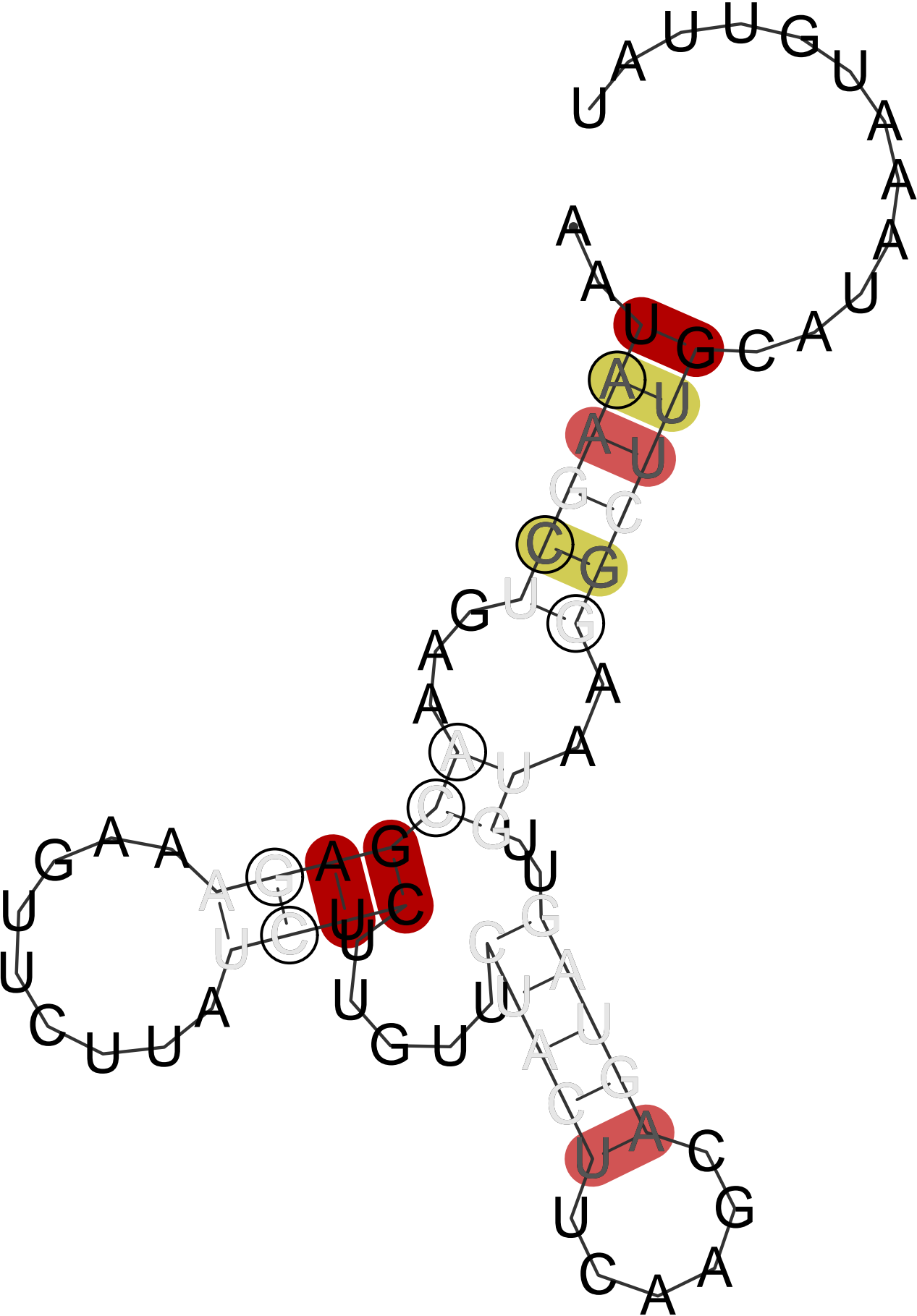

Supplement: S2 Fig — See the caption for S1 Fig for a description of the filename convention (save that the corresponding nucleotide locations in reference sequences are listed in S3 and S11 Tables), and an explanation of the RNAalifold options used and output. (ZIP) [file pcbi.1012009.s123.zip › H1N2-swine-ranked-NS2-alignment-292-366-refseq-761-835_revcomp_alirna_nogap.pdf]

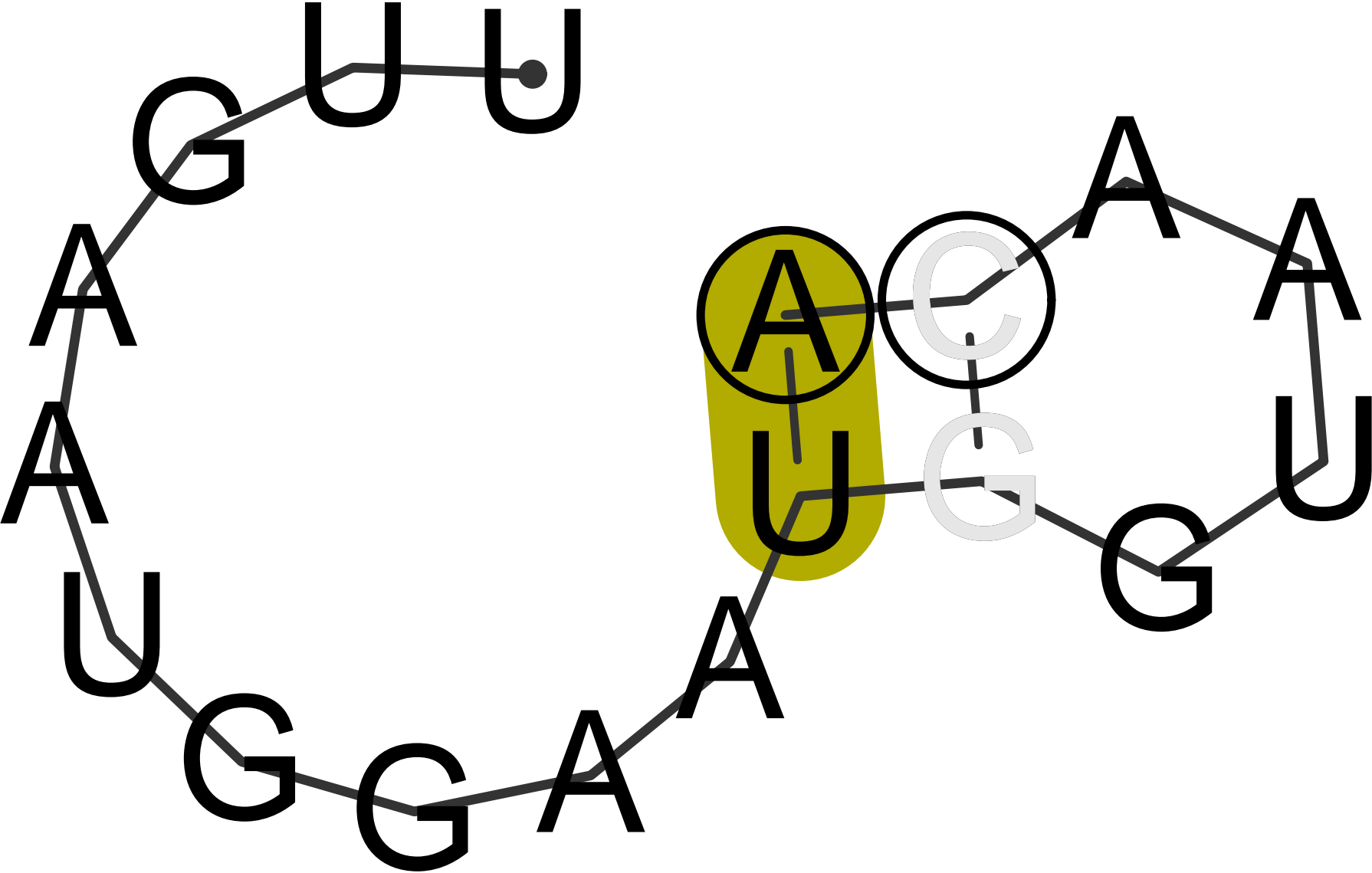

Supplement: S2 Fig — See the caption for S1 Fig for a description of the filename convention (save that the corresponding nucleotide locations in reference sequences are listed in S3 and S11 Tables), and an explanation of the RNAalifold options used and output. (ZIP) [file pcbi.1012009.s123.zip › H1N2-swine-ranked-NS2-alignment-85-102-refseq-554-571_alirna_nogap.pdf]

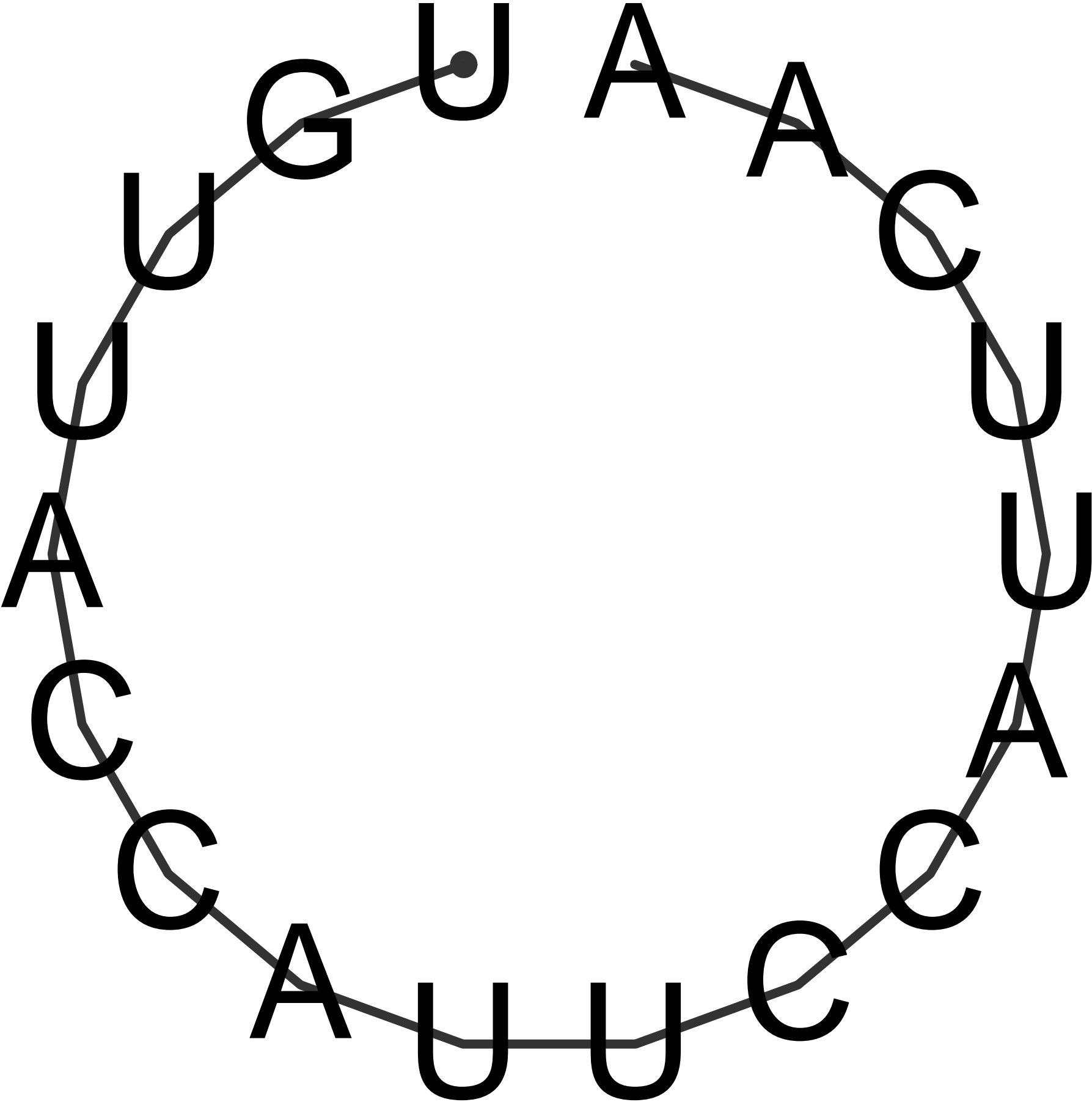

Supplement: S2 Fig — See the caption for S1 Fig for a description of the filename convention (save that the corresponding nucleotide locations in reference sequences are listed in S3 and S11 Tables), and an explanation of the RNAalifold options used and output. (ZIP) [file pcbi.1012009.s123.zip › H1N2-swine-ranked-NS2-alignment-85-102-refseq-554-571_revcomp_alirna_nogap.pdf]

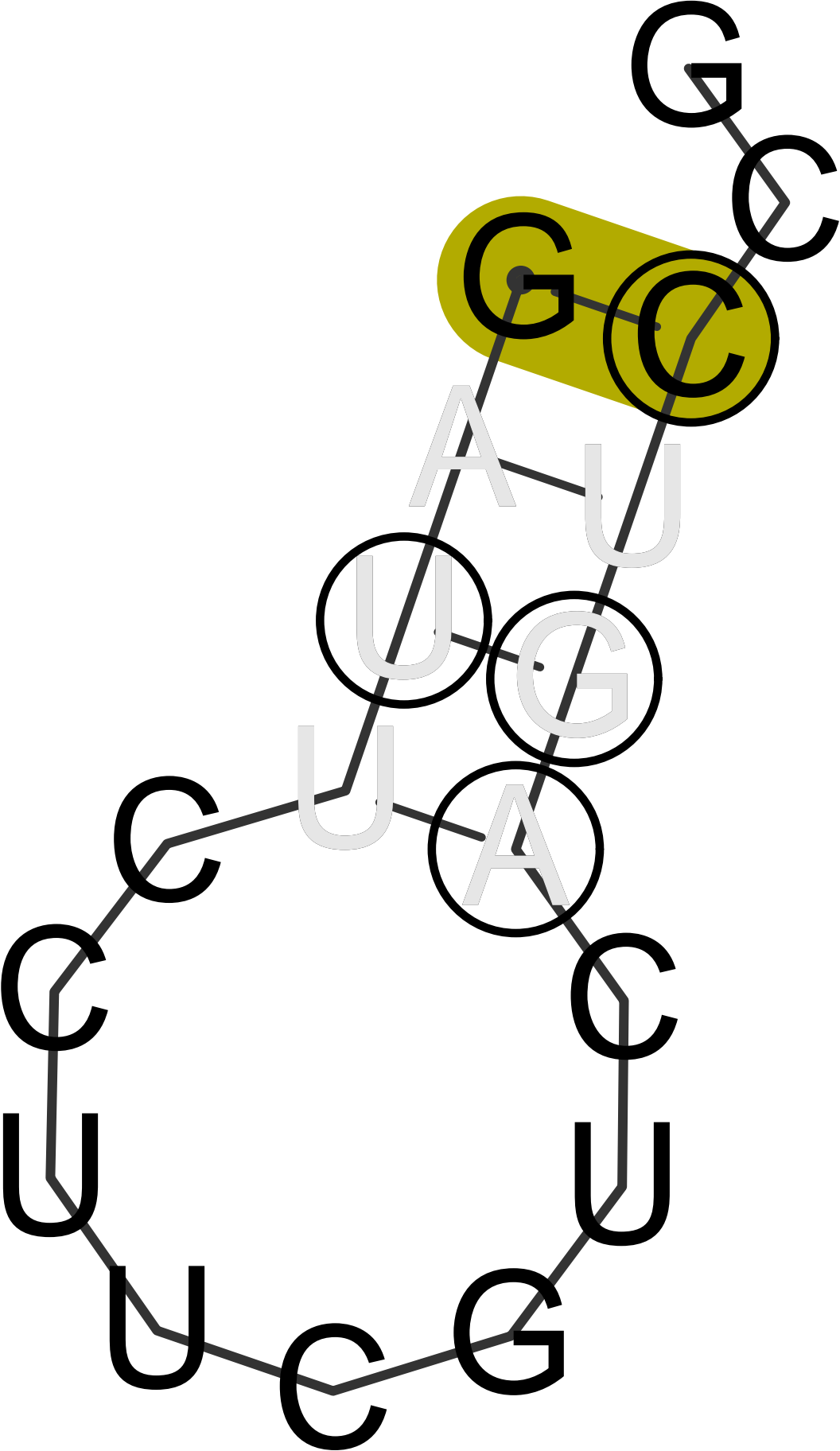

Supplement: S2 Fig — See the caption for S1 Fig for a description of the filename convention (save that the corresponding nucleotide locations in reference sequences are listed in S3 and S11 Tables), and an explanation of the RNAalifold options used and output. (ZIP) [file pcbi.1012009.s123.zip › H1N2-swine-ranked-PA-X-alignment-565-582-refseq-565-583_alirna_nogap.pdf]

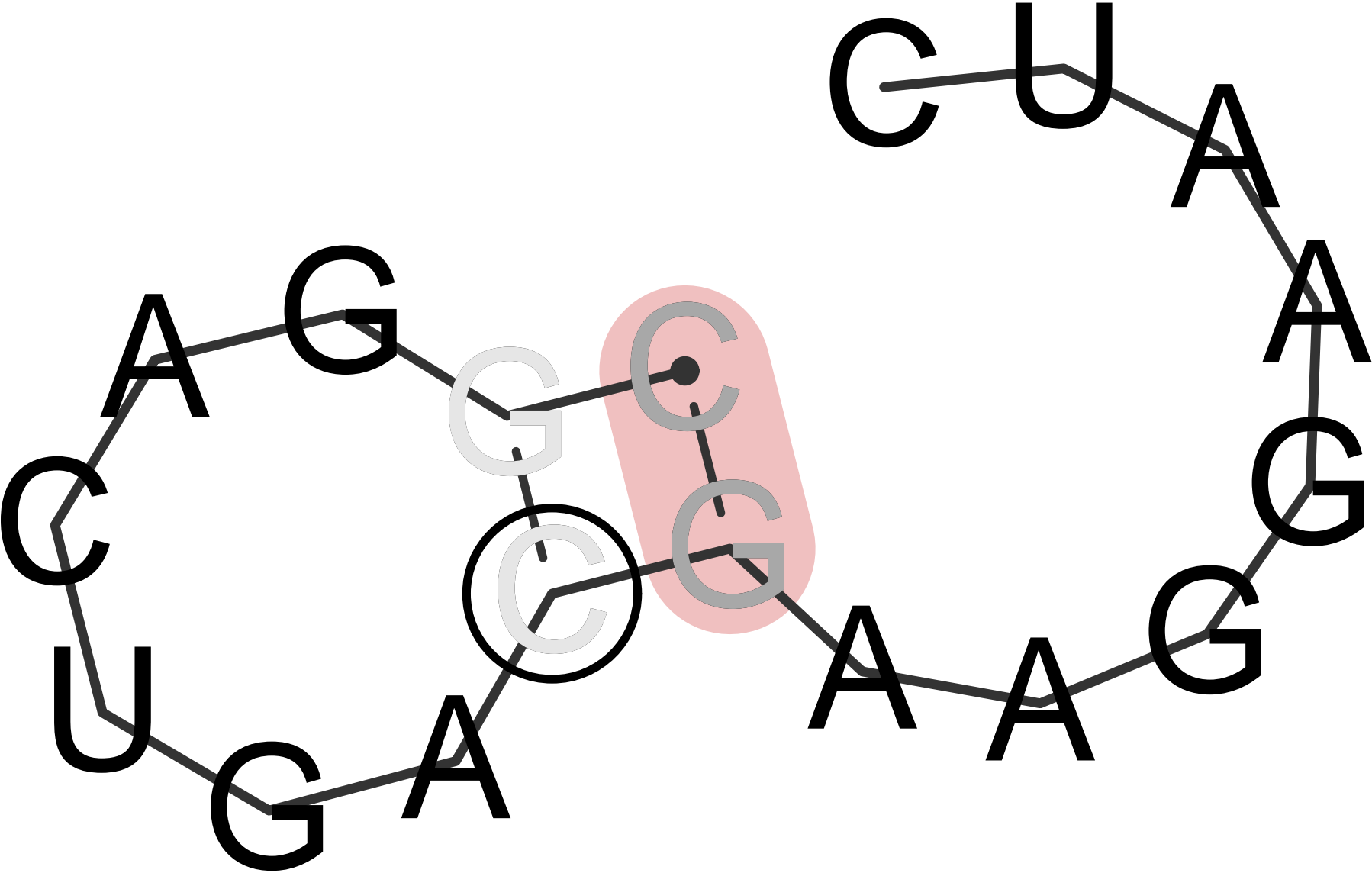

Supplement: S2 Fig — See the caption for S1 Fig for a description of the filename convention (save that the corresponding nucleotide locations in reference sequences are listed in S3 and S11 Tables), and an explanation of the RNAalifold options used and output. (ZIP) [file pcbi.1012009.s123.zip › H1N2-swine-ranked-PA-X-alignment-565-582-refseq-565-583_revcomp_alirna_nogap.pdf]

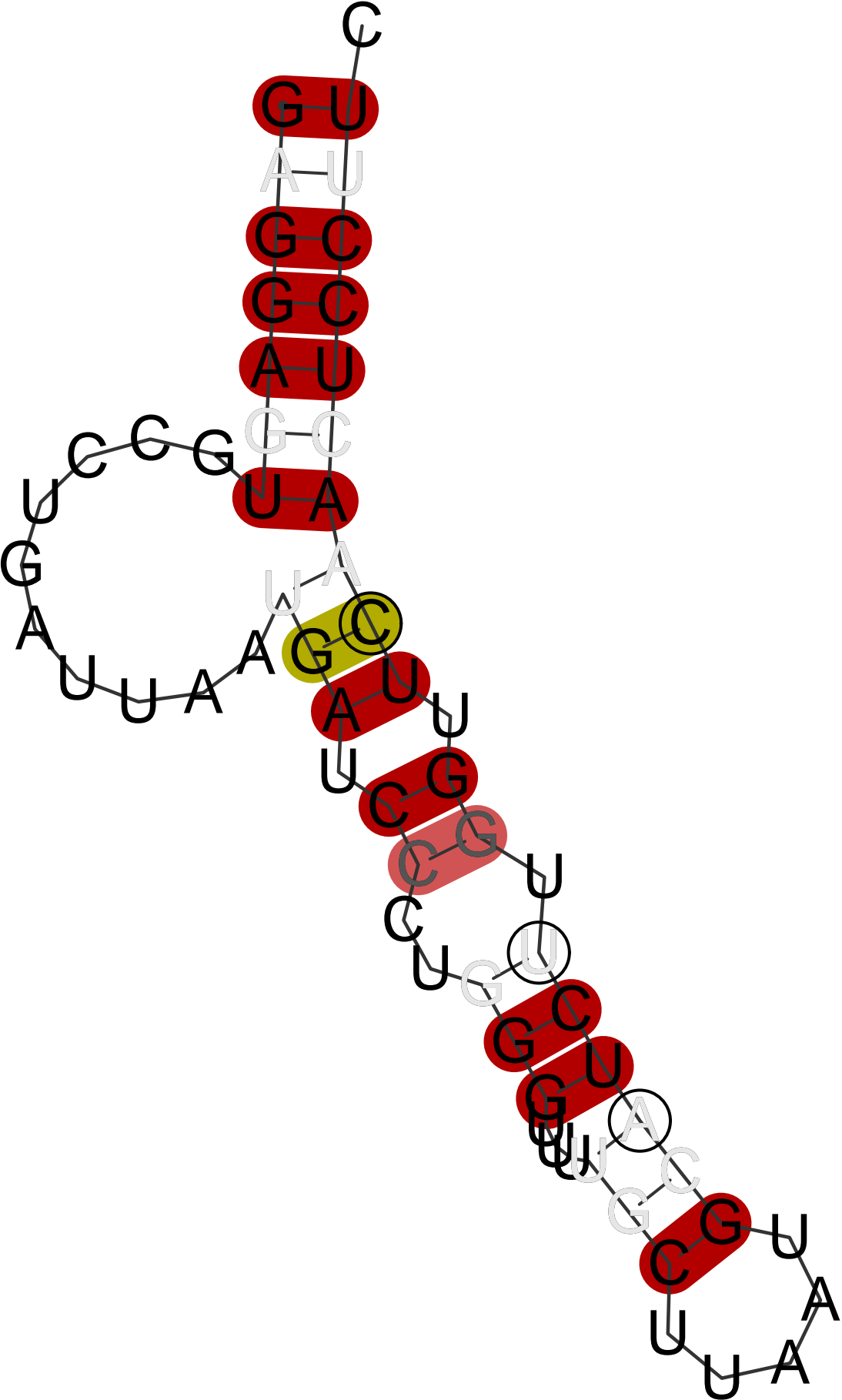

Supplement: S2 Fig — See the caption for S1 Fig for a description of the filename convention (save that the corresponding nucleotide locations in reference sequences are listed in S3 and S11 Tables), and an explanation of the RNAalifold options used and output. (ZIP) [file pcbi.1012009.s123.zip › H1N2-swine-ranked-PA-alignment-2071-2130-refseq-2071-2130_alirna_nogap.pdf]

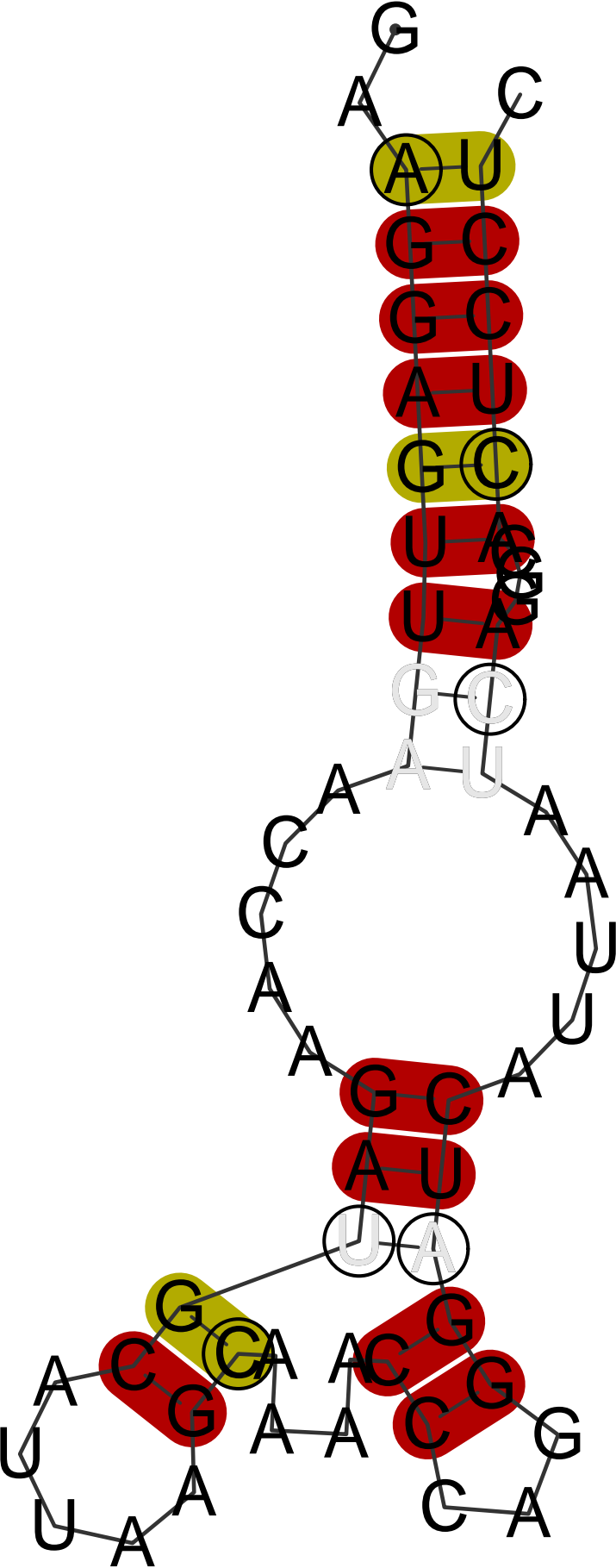

Supplement: S2 Fig — See the caption for S1 Fig for a description of the filename convention (save that the corresponding nucleotide locations in reference sequences are listed in S3 and S11 Tables), and an explanation of the RNAalifold options used and output. (ZIP) [file pcbi.1012009.s123.zip › H1N2-swine-ranked-PA-alignment-2071-2130-refseq-2071-2130_revcomp_alirna_nogap.pdf]

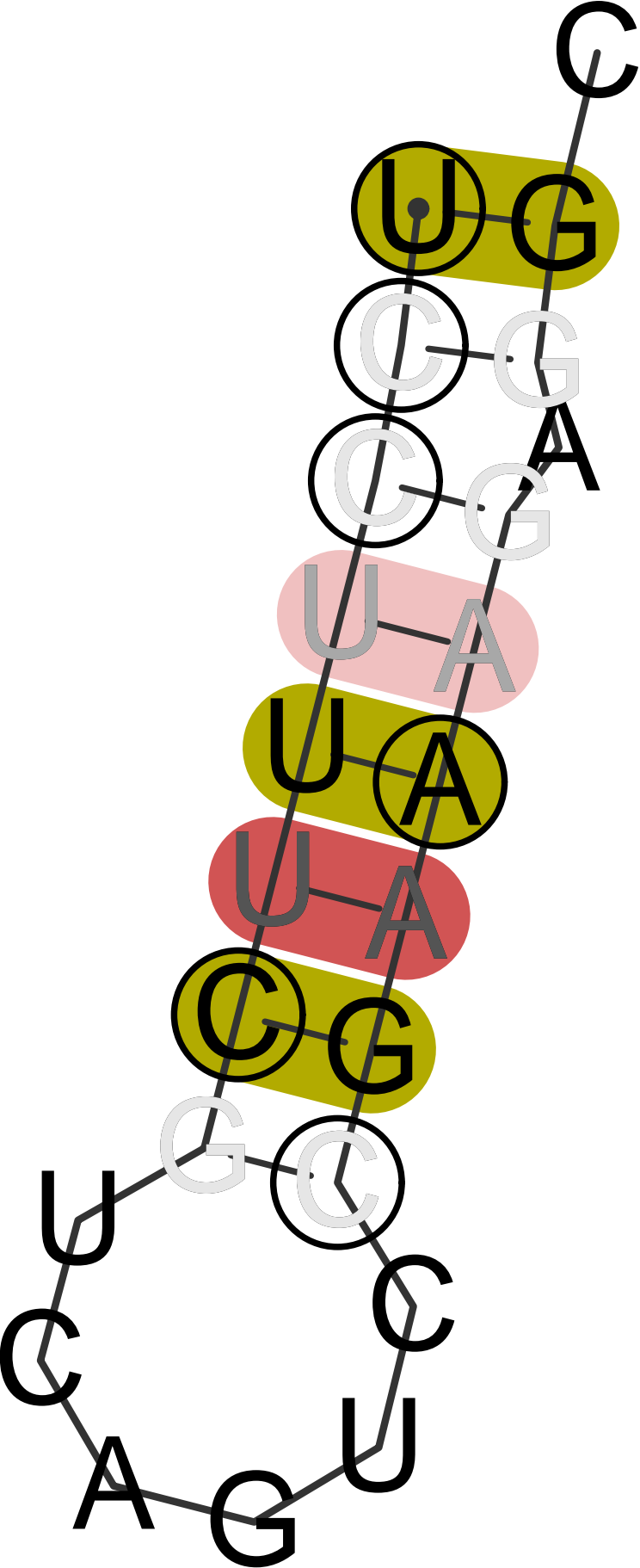

Supplement: S2 Fig — See the caption for S1 Fig for a description of the filename convention (save that the corresponding nucleotide locations in reference sequences are listed in S3 and S11 Tables), and an explanation of the RNAalifold options used and output. (ZIP) [file pcbi.1012009.s123.zip › H1N2-swine-ranked-PA-alignment-568-591-refseq-568-591_alirna_nogap.pdf]

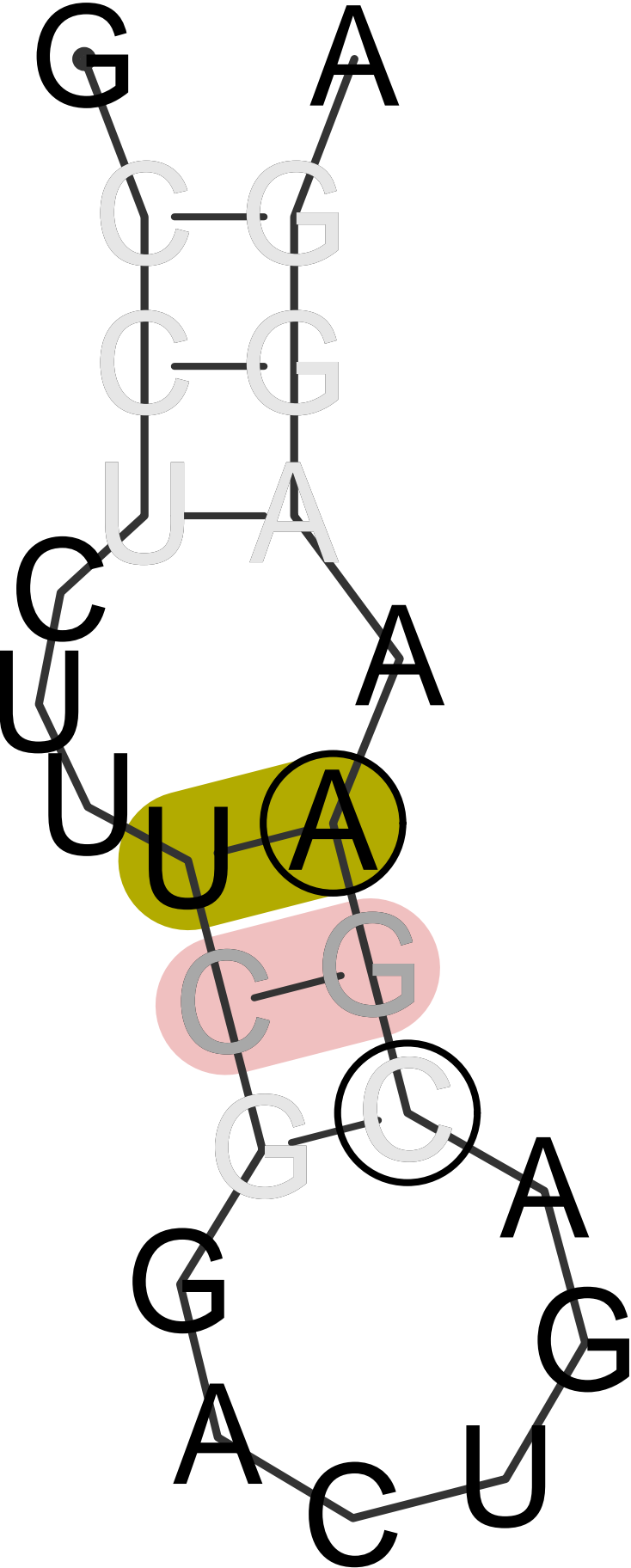

Supplement: S2 Fig — See the caption for S1 Fig for a description of the filename convention (save that the corresponding nucleotide locations in reference sequences are listed in S3 and S11 Tables), and an explanation of the RNAalifold options used and output. (ZIP) [file pcbi.1012009.s123.zip › H1N2-swine-ranked-PA-alignment-568-591-refseq-568-591_revcomp_alirna_nogap.pdf]

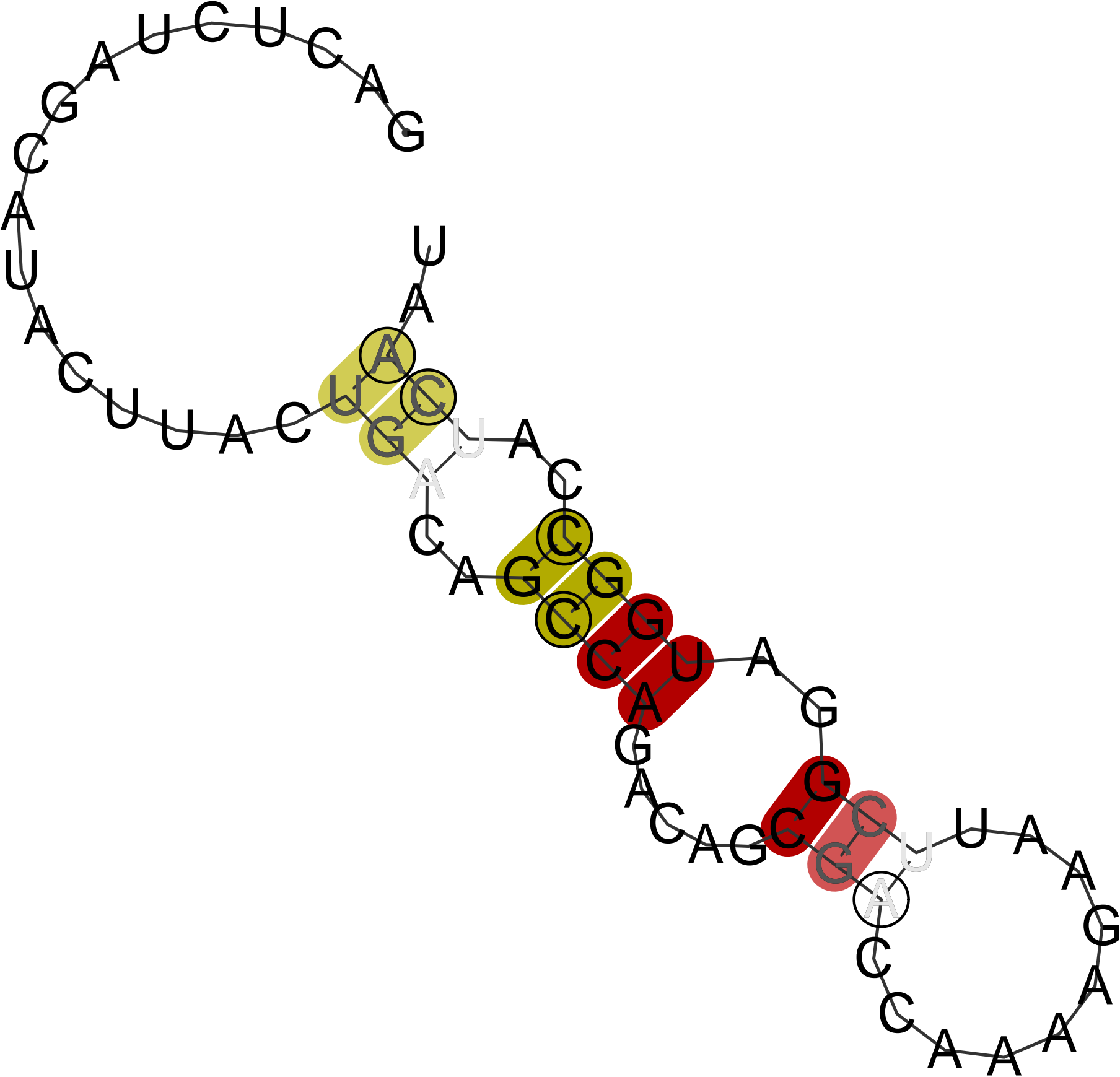

Supplement: S2 Fig — See the caption for S1 Fig for a description of the filename convention (save that the corresponding nucleotide locations in reference sequences are listed in S3 and S11 Tables), and an explanation of the RNAalifold options used and output. (ZIP) [file pcbi.1012009.s123.zip › H1N2-swine-ranked-PB2-alignment-2632-2895-refseq-2218-2277_alirna_nogap.pdf]

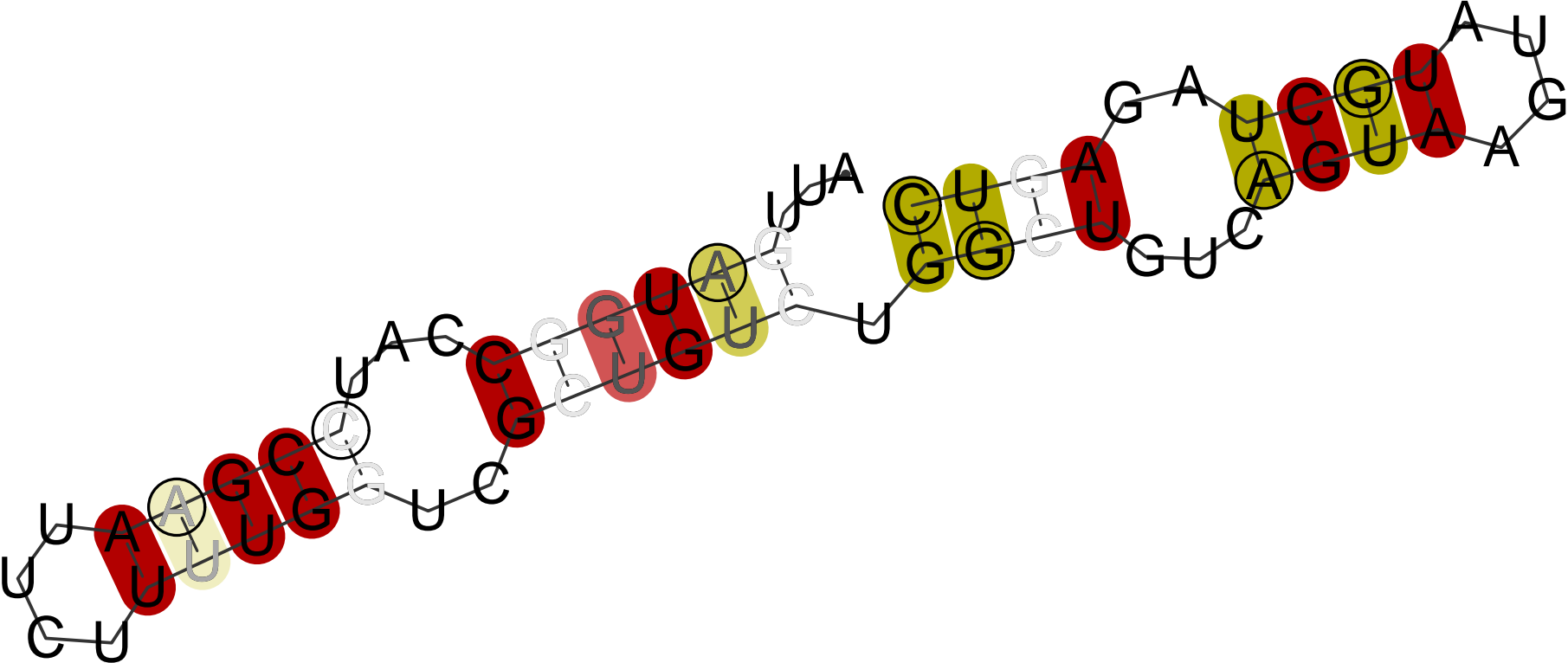

Supplement: S2 Fig — See the caption for S1 Fig for a description of the filename convention (save that the corresponding nucleotide locations in reference sequences are listed in S3 and S11 Tables), and an explanation of the RNAalifold options used and output. (ZIP) [file pcbi.1012009.s123.zip › H1N2-swine-ranked-PB2-alignment-2632-2895-refseq-2218-2277_revcomp_alirna_nogap.pdf]

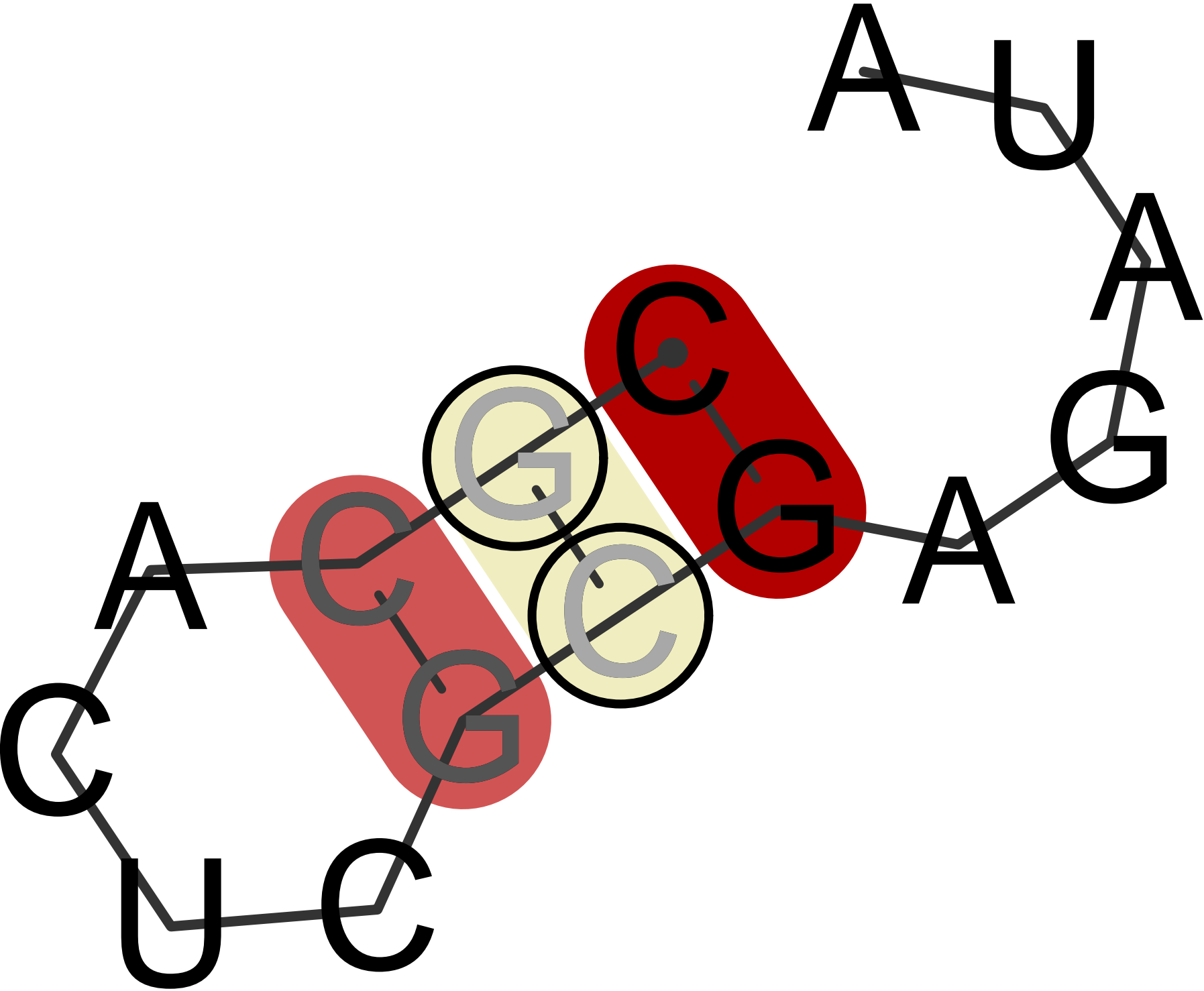

Supplement: S2 Fig — See the caption for S1 Fig for a description of the filename convention (save that the corresponding nucleotide locations in reference sequences are listed in S3 and S11 Tables), and an explanation of the RNAalifold options used and output. (ZIP) [file pcbi.1012009.s123.zip › H1N2-swine-ranked-PB2-alignment-43-57-refseq-43-57_alirna_nogap.pdf]

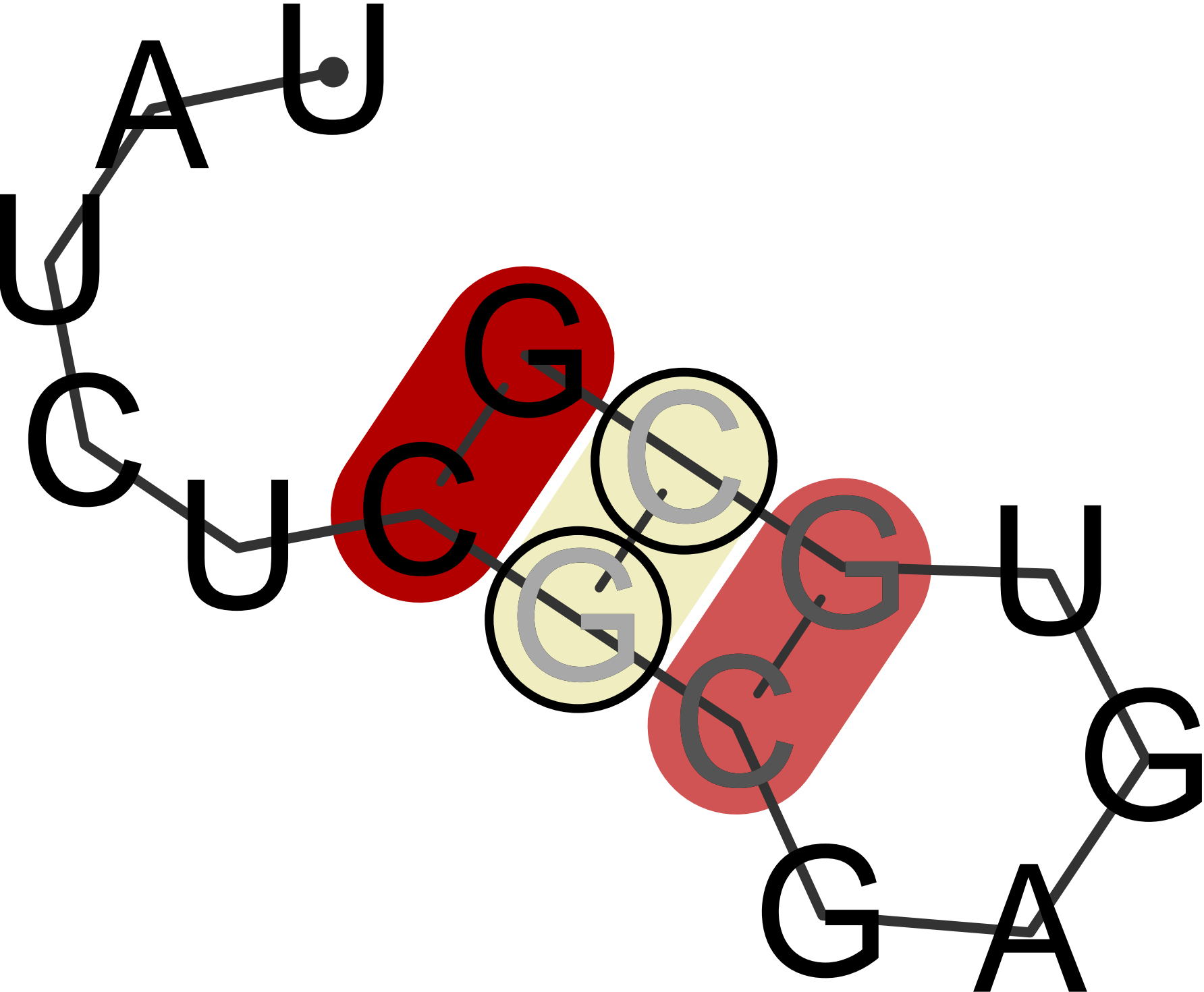

Supplement: S2 Fig — See the caption for S1 Fig for a description of the filename convention (save that the corresponding nucleotide locations in reference sequences are listed in S3 and S11 Tables), and an explanation of the RNAalifold options used and output. (ZIP) [file pcbi.1012009.s123.zip › H1N2-swine-ranked-PB2-alignment-43-57-refseq-43-57_revcomp_alirna_nogap.pdf]

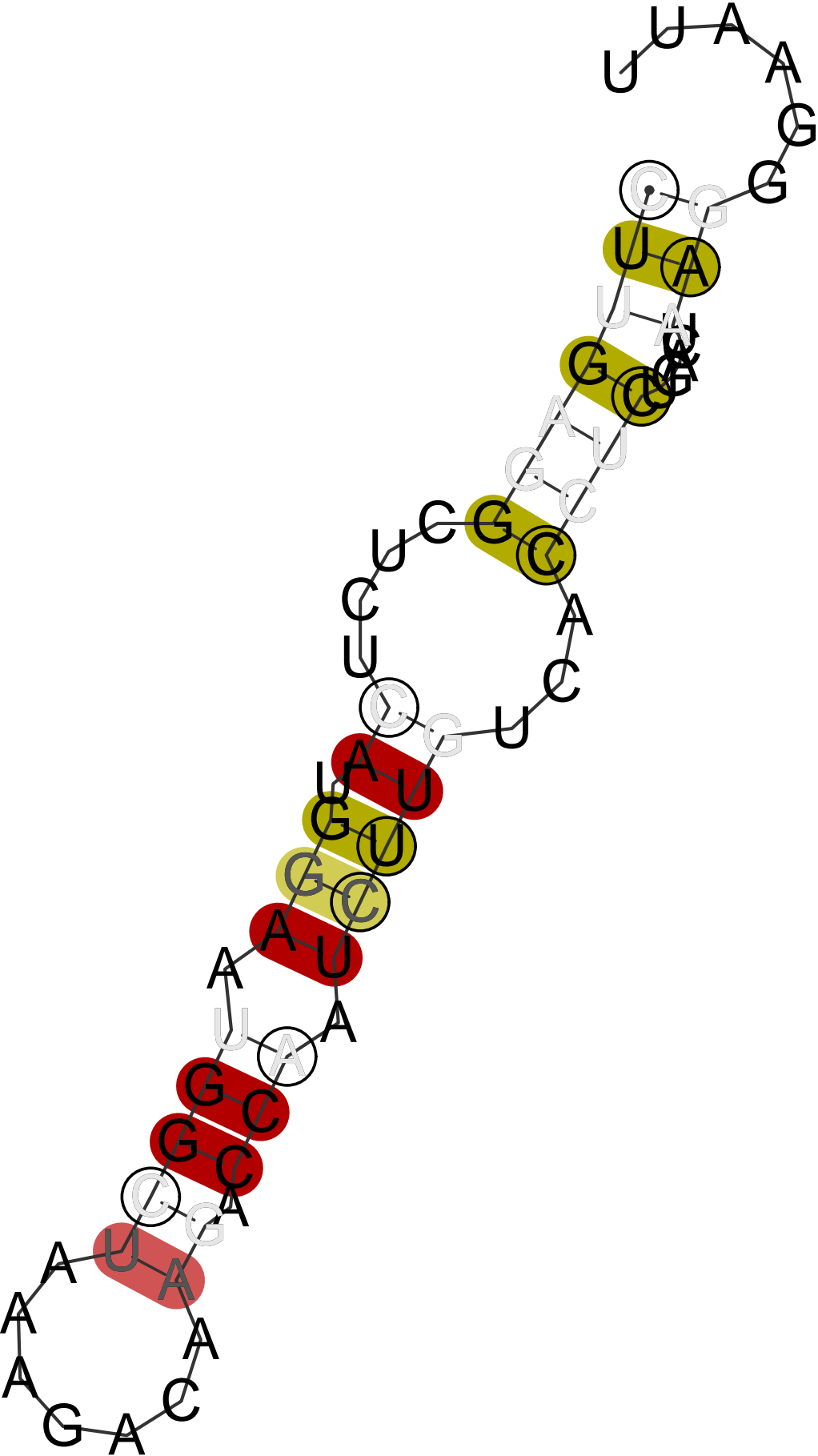

Supplement: S2 Fig — See the caption for S1 Fig for a description of the filename convention (save that the corresponding nucleotide locations in reference sequences are listed in S3 and S11 Tables), and an explanation of the RNAalifold options used and output. (ZIP) [file pcbi.1012009.s123.zip › H1N2-swine-raw-M1-alignment-118-180-refseq-115-177_alirna_nogap.pdf]

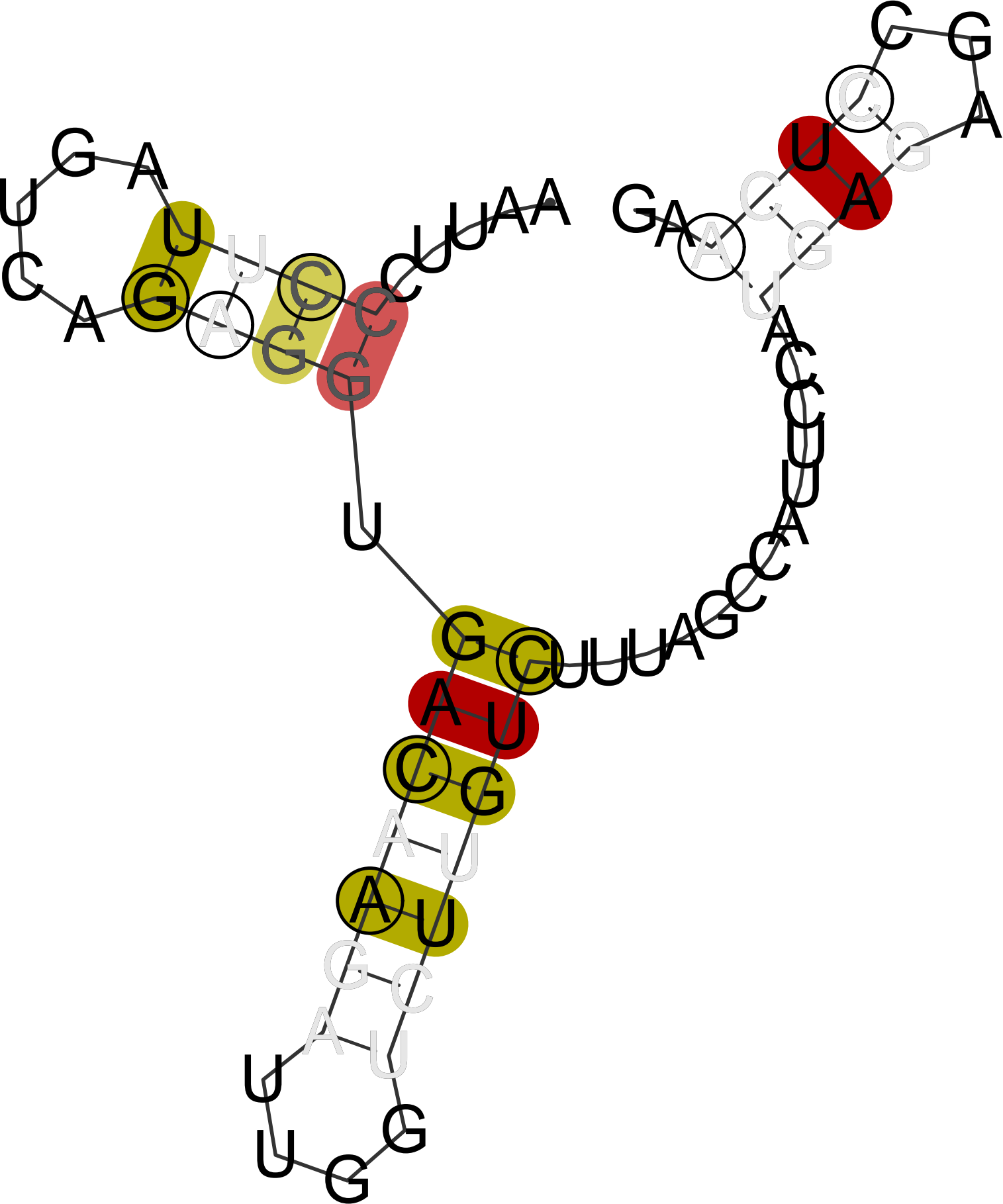

Supplement: S2 Fig — See the caption for S1 Fig for a description of the filename convention (save that the corresponding nucleotide locations in reference sequences are listed in S3 and S11 Tables), and an explanation of the RNAalifold options used and output. (ZIP) [file pcbi.1012009.s123.zip › H1N2-swine-raw-M1-alignment-118-180-refseq-115-177_revcomp_alirna_nogap.pdf]

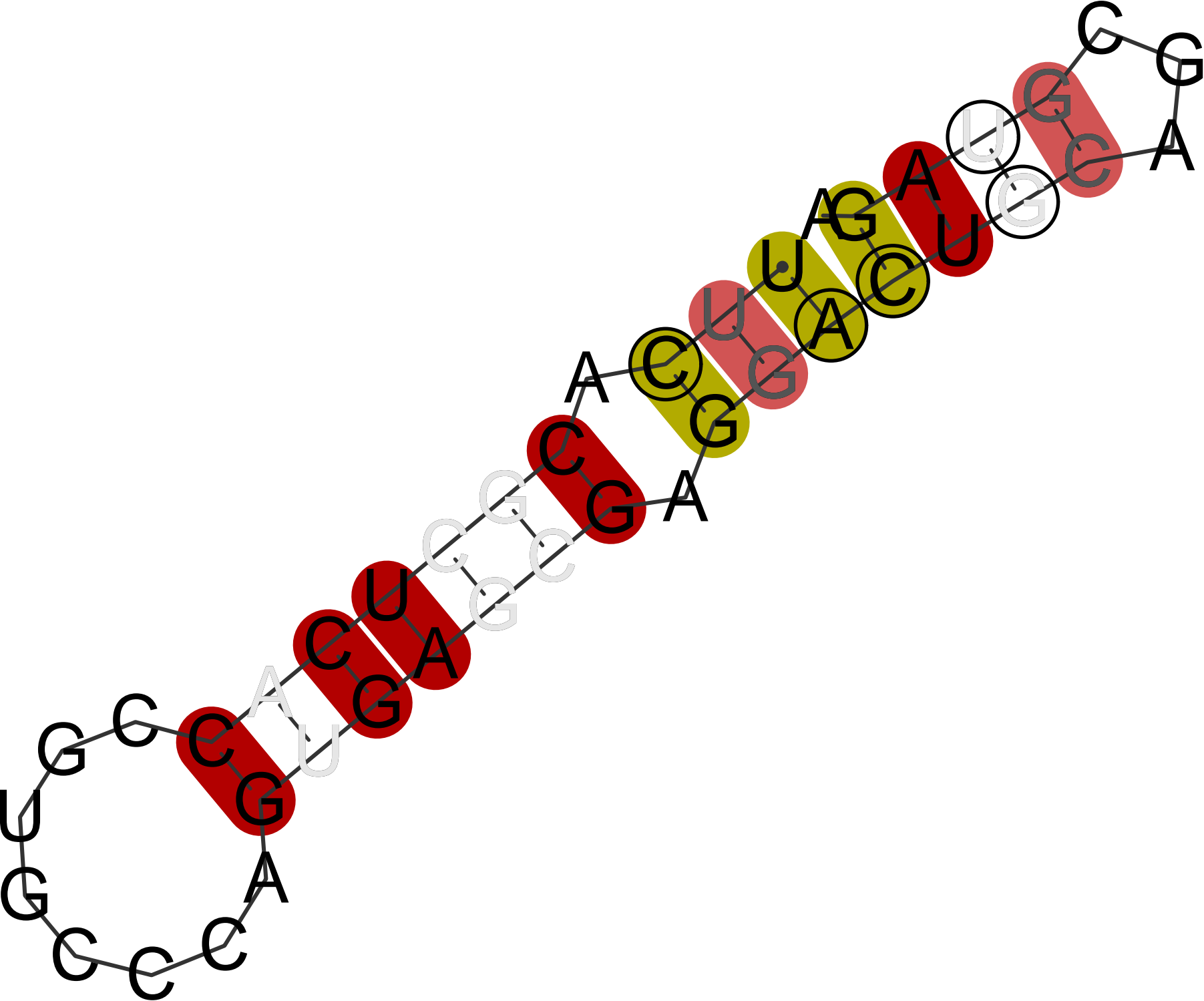

Supplement: S2 Fig — See the caption for S1 Fig for a description of the filename convention (save that the corresponding nucleotide locations in reference sequences are listed in S3 and S11 Tables), and an explanation of the RNAalifold options used and output. (ZIP) [file pcbi.1012009.s123.zip › H1N2-swine-raw-M1-alignment-193-234-refseq-190-231_alirna_nogap.pdf]

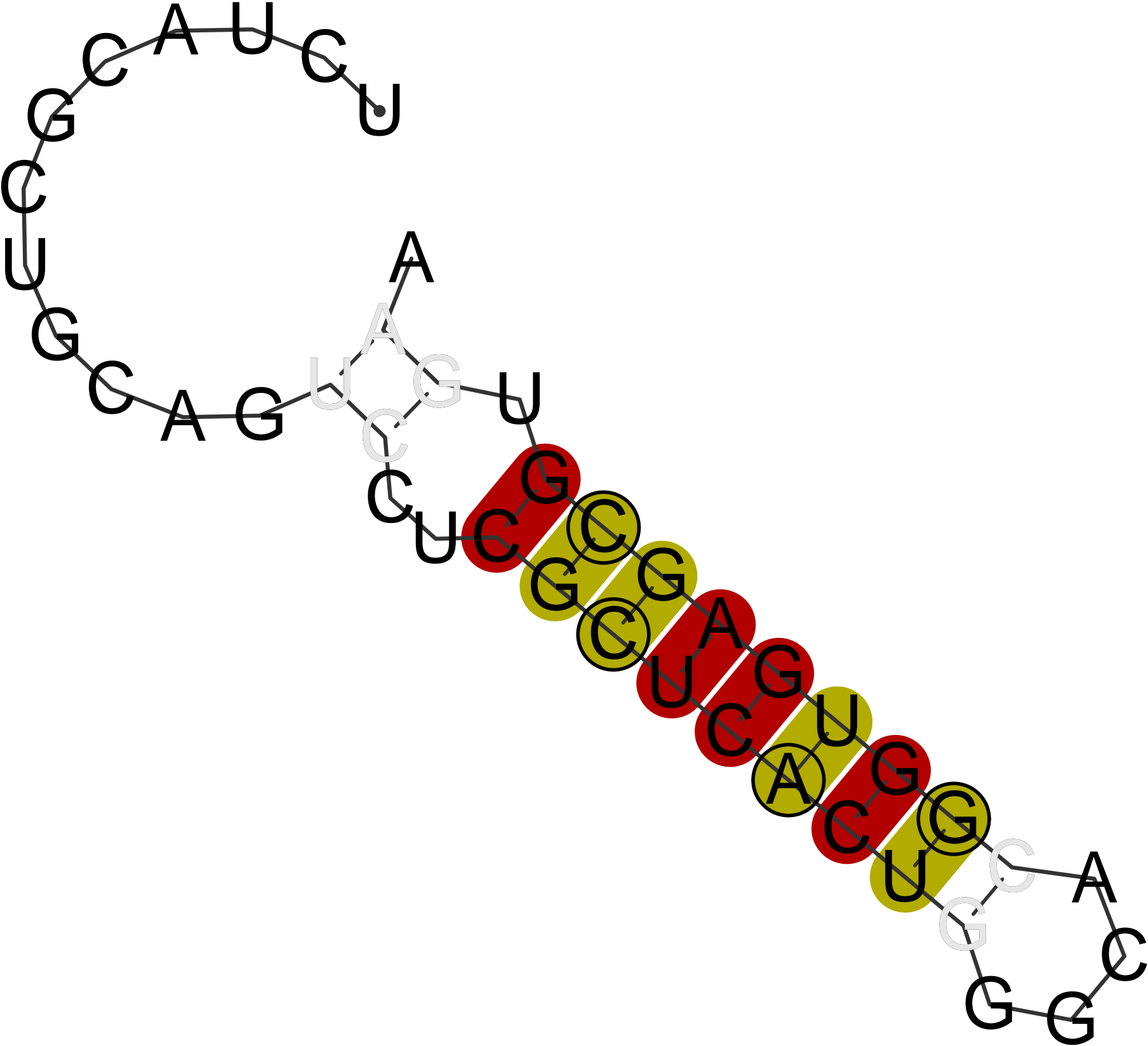

Supplement: S2 Fig — See the caption for S1 Fig for a description of the filename convention (save that the corresponding nucleotide locations in reference sequences are listed in S3 and S11 Tables), and an explanation of the RNAalifold options used and output. (ZIP) [file pcbi.1012009.s123.zip › H1N2-swine-raw-M1-alignment-193-234-refseq-190-231_revcomp_alirna_nogap.pdf]

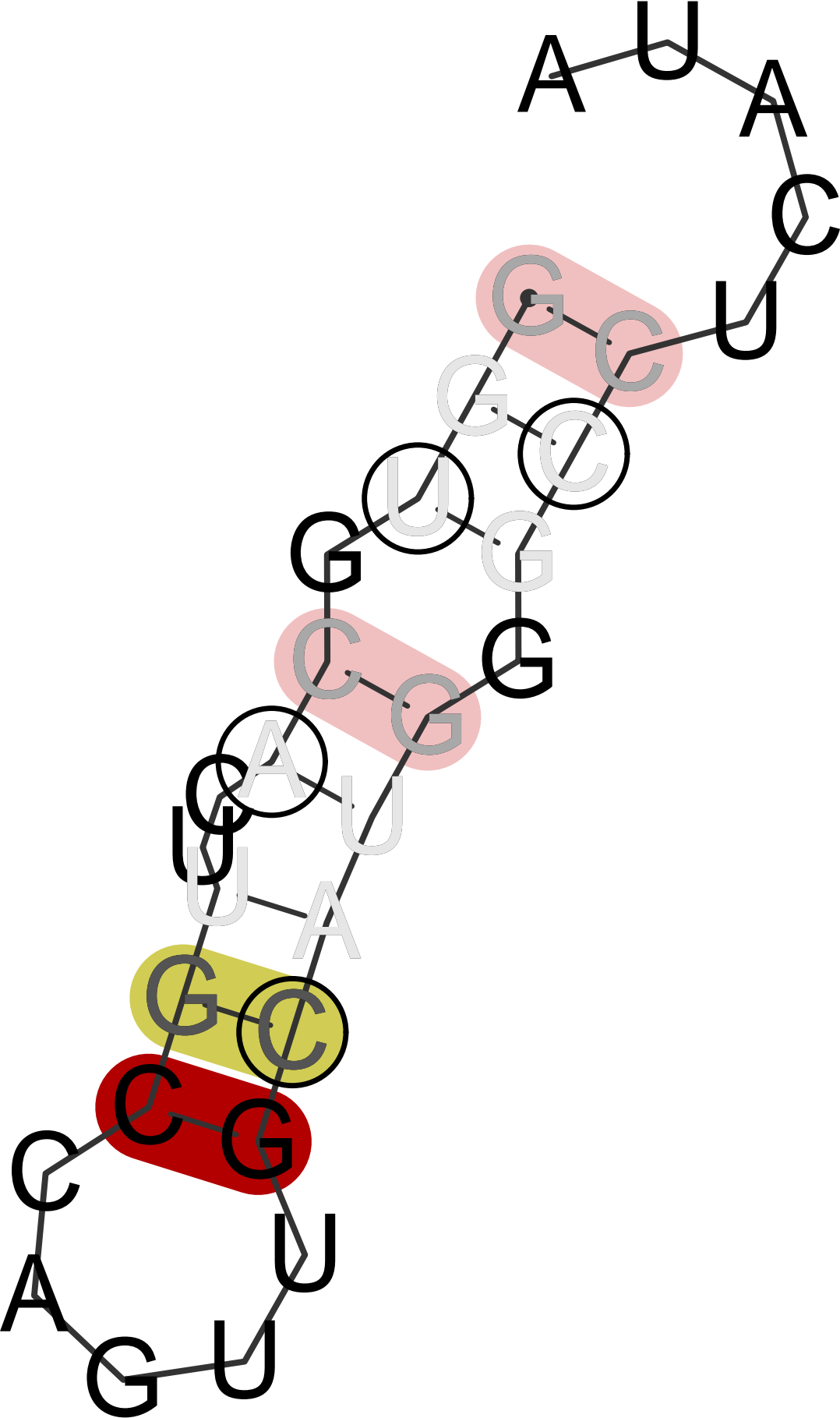

Supplement: S2 Fig — See the caption for S1 Fig for a description of the filename convention (save that the corresponding nucleotide locations in reference sequences are listed in S3 and S11 Tables), and an explanation of the RNAalifold options used and output. (ZIP) [file pcbi.1012009.s123.zip › H1N2-swine-raw-M1-alignment-367-396-refseq-364-393_alirna_nogap.pdf]

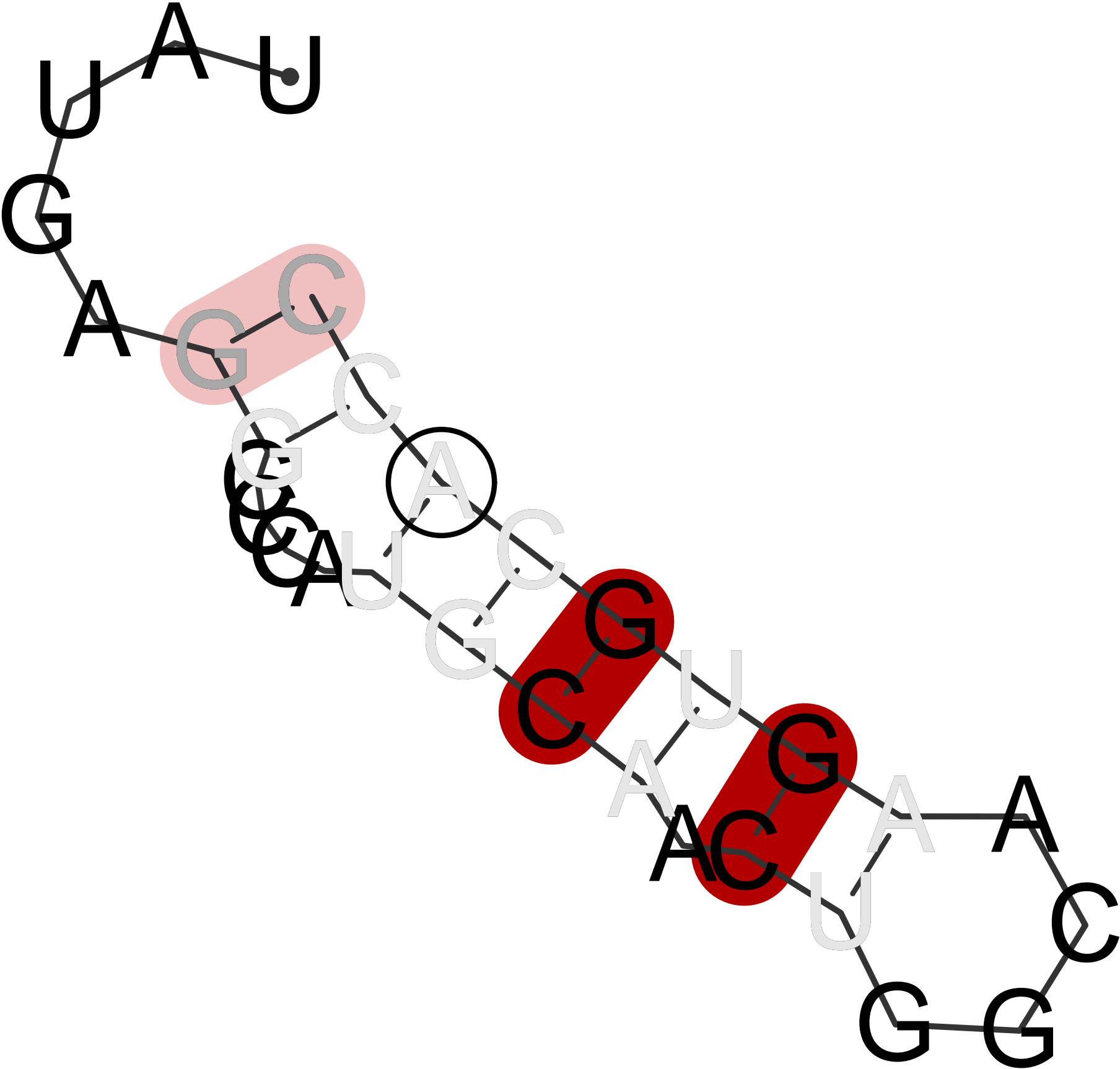

Supplement: S2 Fig — See the caption for S1 Fig for a description of the filename convention (save that the corresponding nucleotide locations in reference sequences are listed in S3 and S11 Tables), and an explanation of the RNAalifold options used and output. (ZIP) [file pcbi.1012009.s123.zip › H1N2-swine-raw-M1-alignment-367-396-refseq-364-393_revcomp_alirna_nogap.pdf]

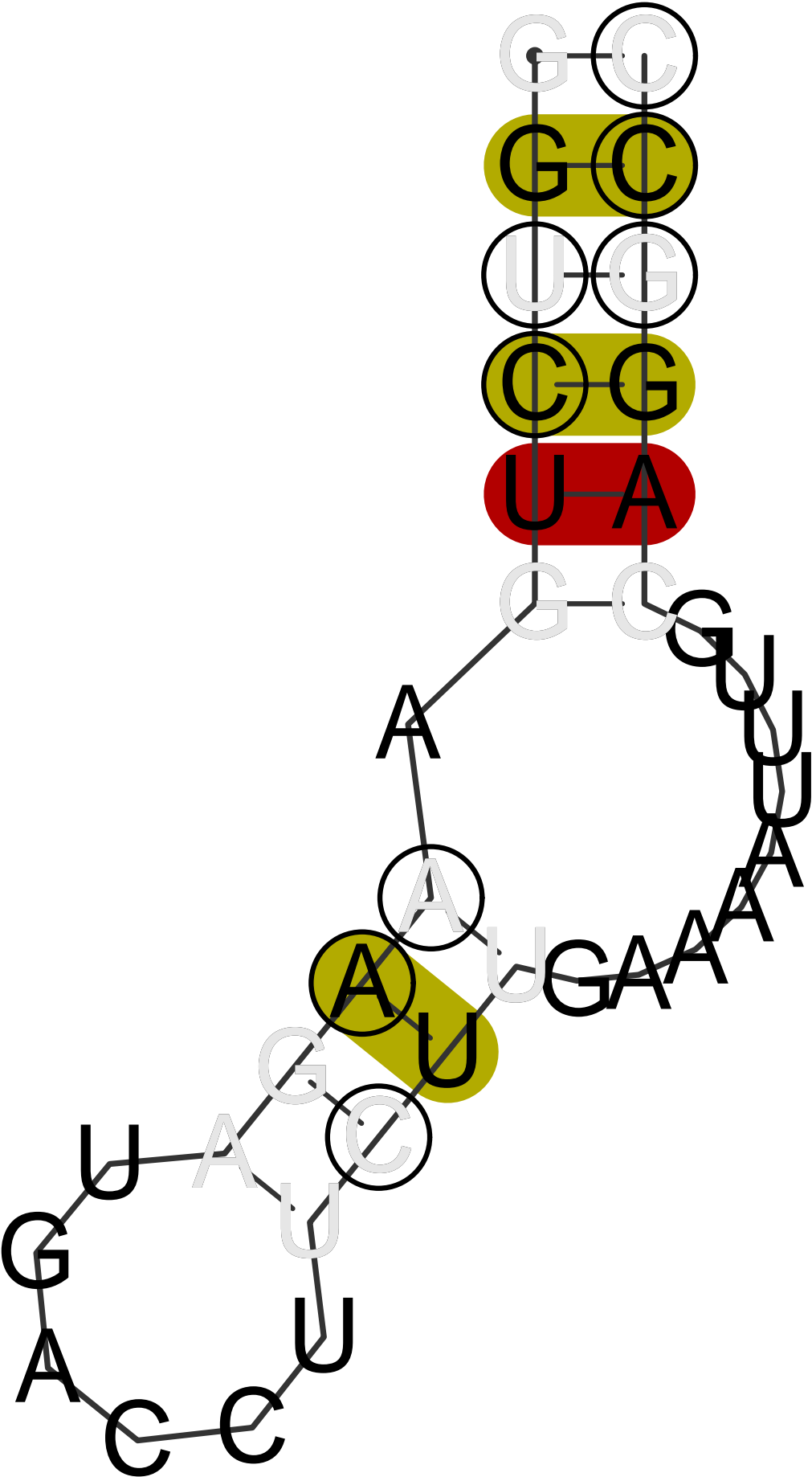

Supplement: S2 Fig — See the caption for S1 Fig for a description of the filename convention (save that the corresponding nucleotide locations in reference sequences are listed in S3 and S11 Tables), and an explanation of the RNAalifold options used and output. (ZIP) [file pcbi.1012009.s123.zip › H1N2-swine-raw-M1-alignment-685-720-refseq-682-717_alirna_nogap.pdf]

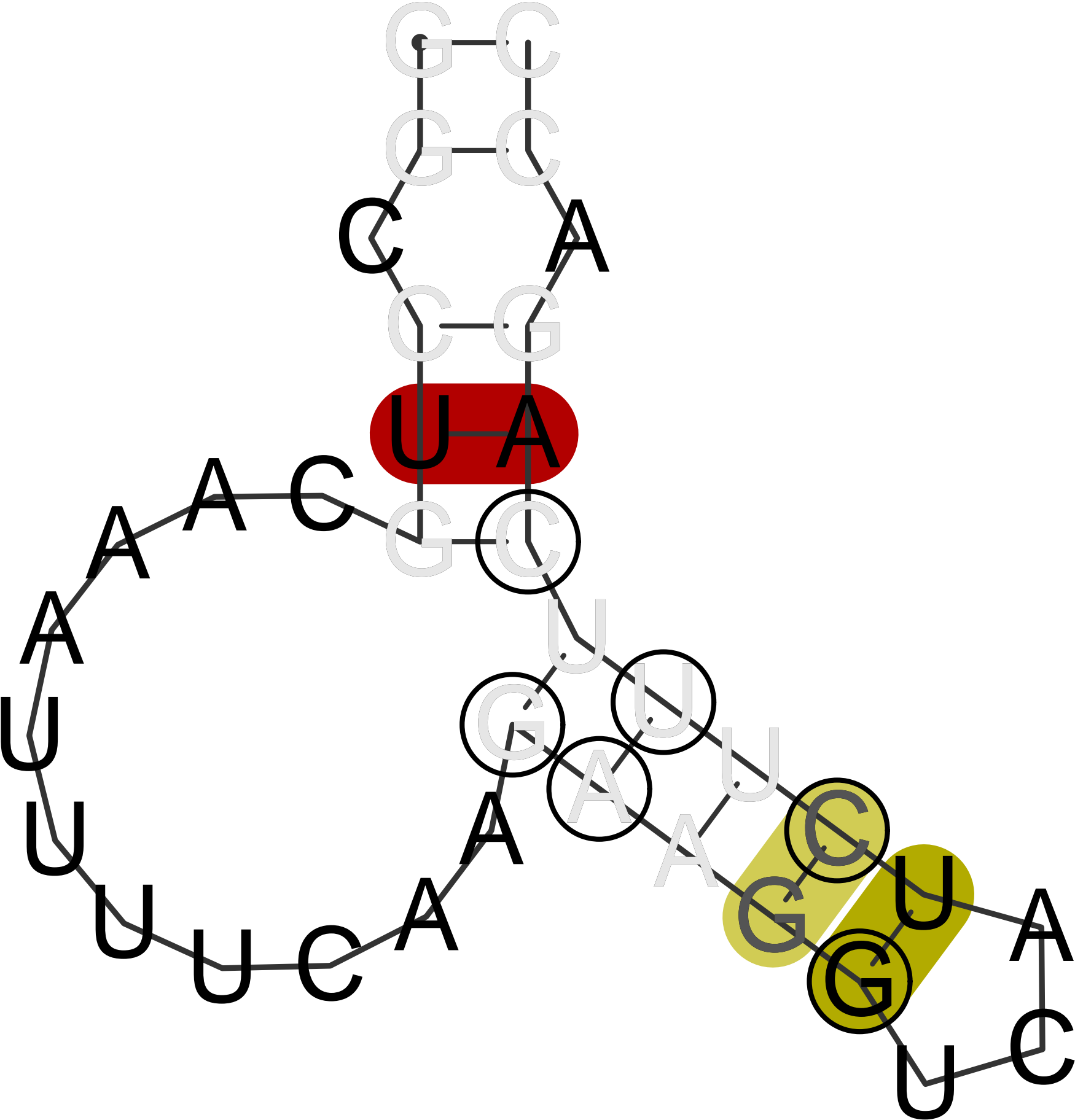

Supplement: S2 Fig — See the caption for S1 Fig for a description of the filename convention (save that the corresponding nucleotide locations in reference sequences are listed in S3 and S11 Tables), and an explanation of the RNAalifold options used and output. (ZIP) [file pcbi.1012009.s123.zip › H1N2-swine-raw-M1-alignment-685-720-refseq-682-717_revcomp_alirna_nogap.pdf]

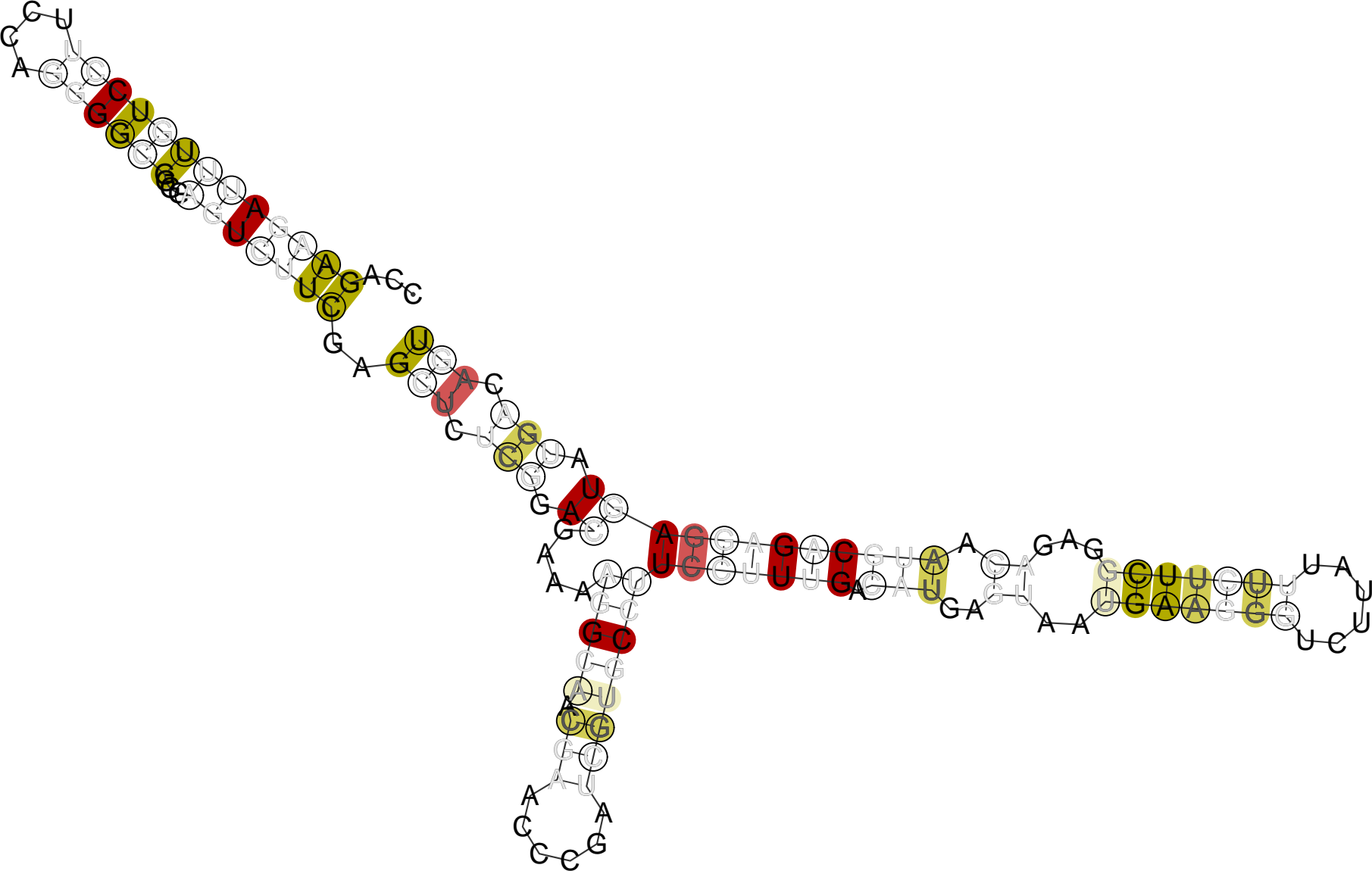

Supplement: S2 Fig — See the caption for S1 Fig for a description of the filename convention (save that the corresponding nucleotide locations in reference sequences are listed in S3 and S11 Tables), and an explanation of the RNAalifold options used and output. (ZIP) [file pcbi.1012009.s123.zip › H1N2-swine-raw-NP-alignment-1375-1512-refseq-1357-1494_alirna_nogap.pdf]

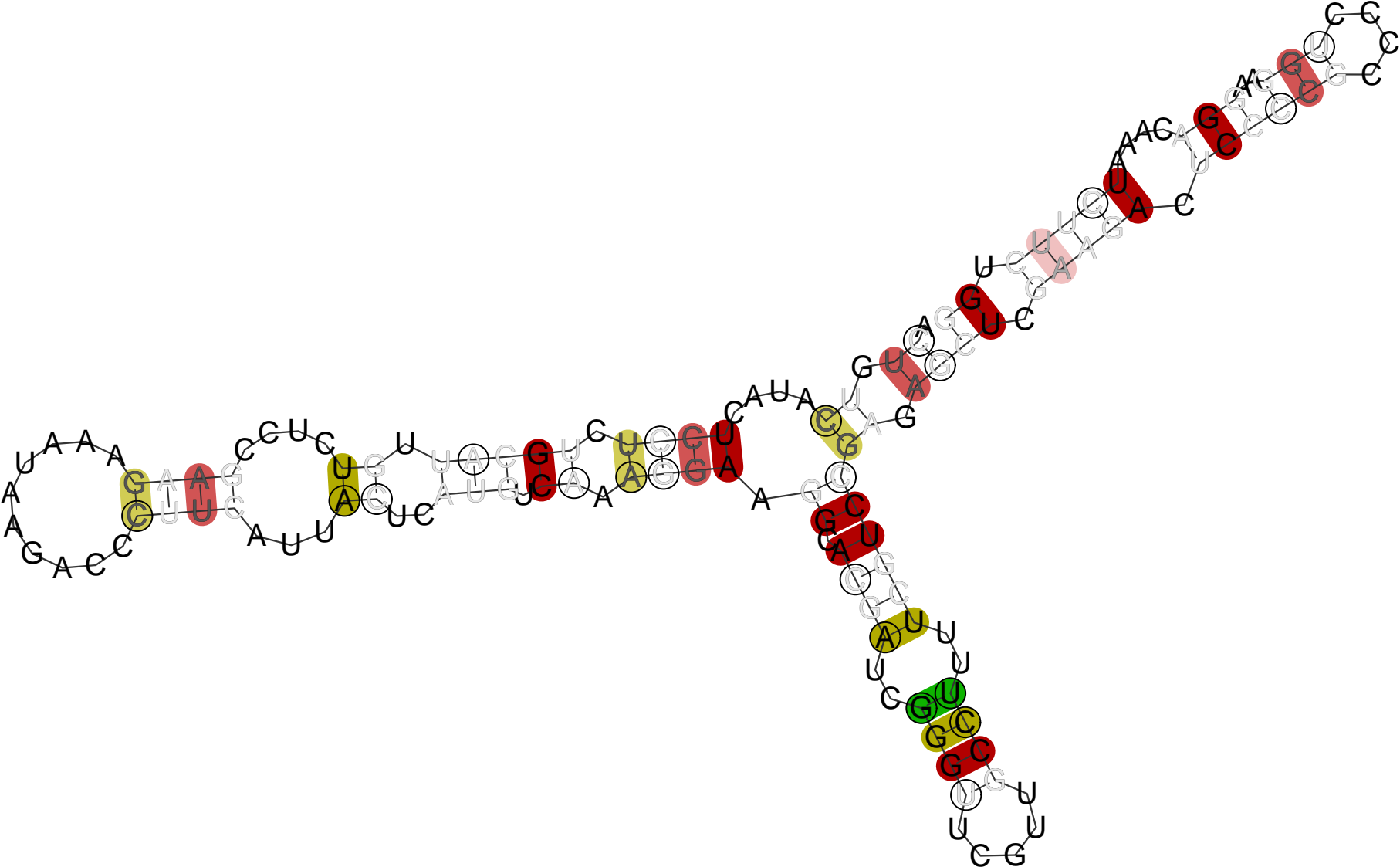

Supplement: S2 Fig — See the caption for S1 Fig for a description of the filename convention (save that the corresponding nucleotide locations in reference sequences are listed in S3 and S11 Tables), and an explanation of the RNAalifold options used and output. (ZIP) [file pcbi.1012009.s123.zip › H1N2-swine-raw-NP-alignment-1375-1512-refseq-1357-1494_revcomp_alirna_nogap.pdf]

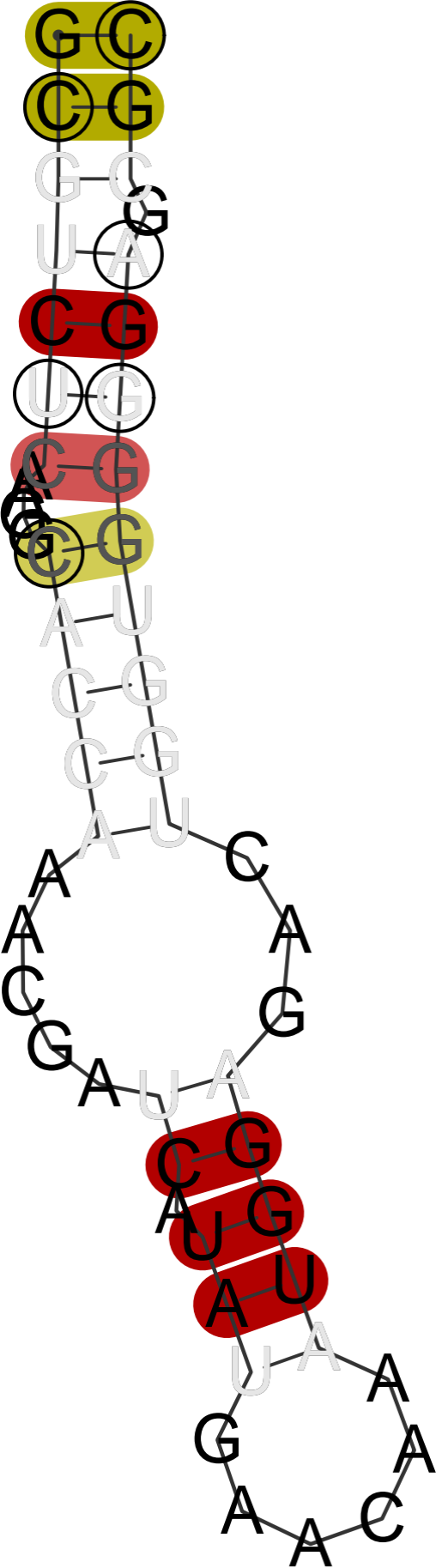

Supplement: S2 Fig — See the caption for S1 Fig for a description of the filename convention (save that the corresponding nucleotide locations in reference sequences are listed in S3 and S11 Tables), and an explanation of the RNAalifold options used and output. (ZIP) [file pcbi.1012009.s123.zip › H1N2-swine-raw-NP-alignment-22-75-refseq-4-57_alirna_nogap.pdf]

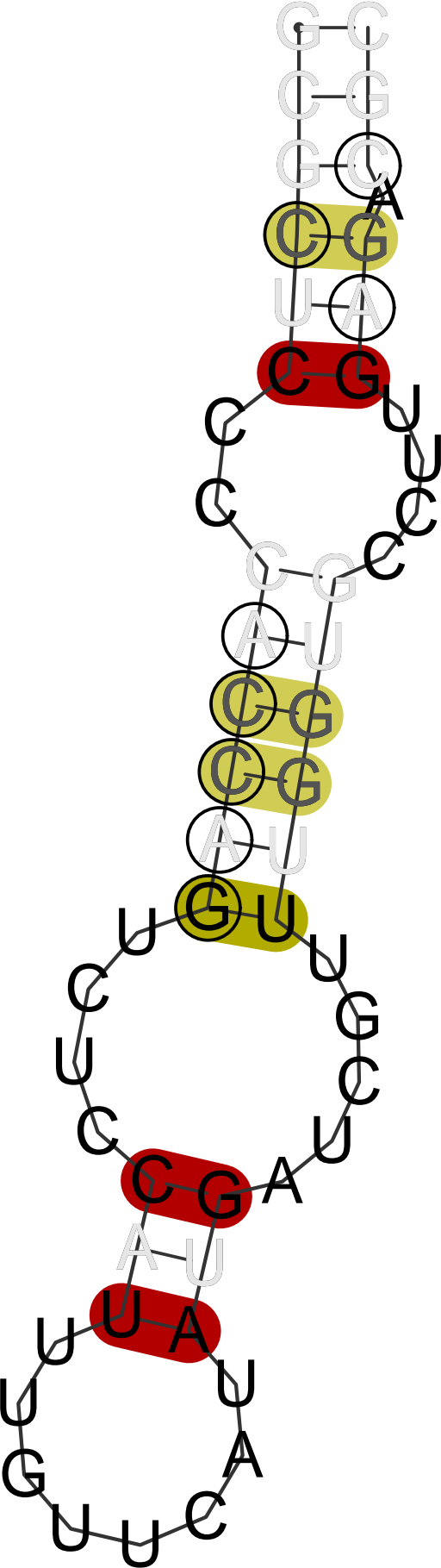

Supplement: S2 Fig — See the caption for S1 Fig for a description of the filename convention (save that the corresponding nucleotide locations in reference sequences are listed in S3 and S11 Tables), and an explanation of the RNAalifold options used and output. (ZIP) [file pcbi.1012009.s123.zip › H1N2-swine-raw-NP-alignment-22-75-refseq-4-57_revcomp_alirna_nogap.pdf]

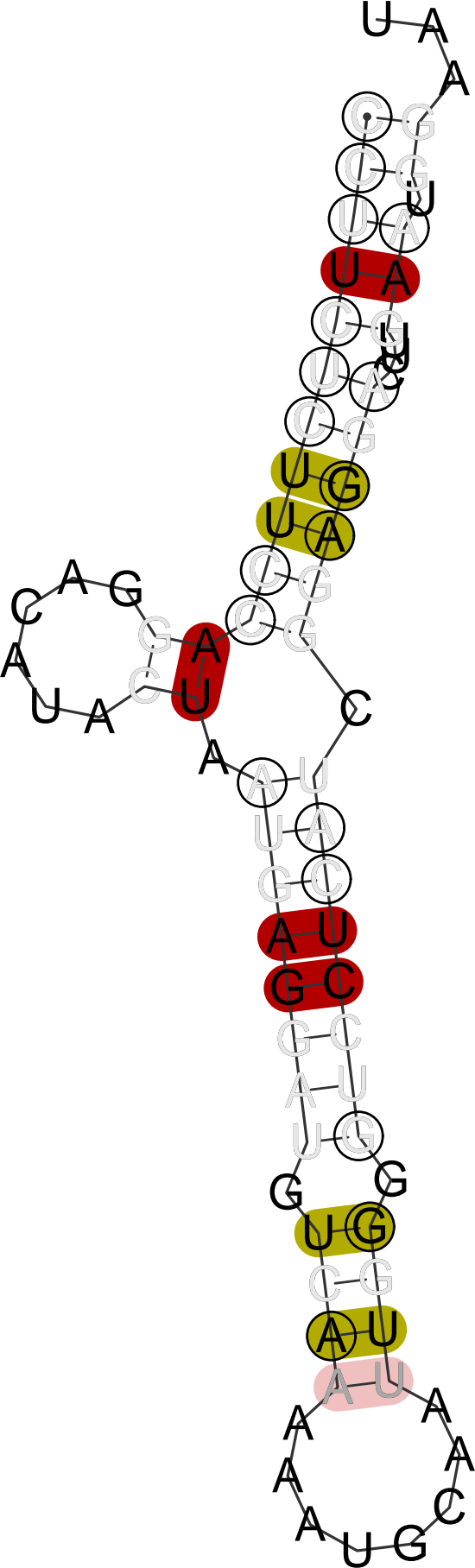

Supplement: S2 Fig — See the caption for S1 Fig for a description of the filename convention (save that the corresponding nucleotide locations in reference sequences are listed in S3 and S11 Tables), and an explanation of the RNAalifold options used and output. (ZIP) [file pcbi.1012009.s123.zip › H1N2-swine-raw-NS1-alignment-493-567-refseq-490-564_alirna_nogap.pdf]

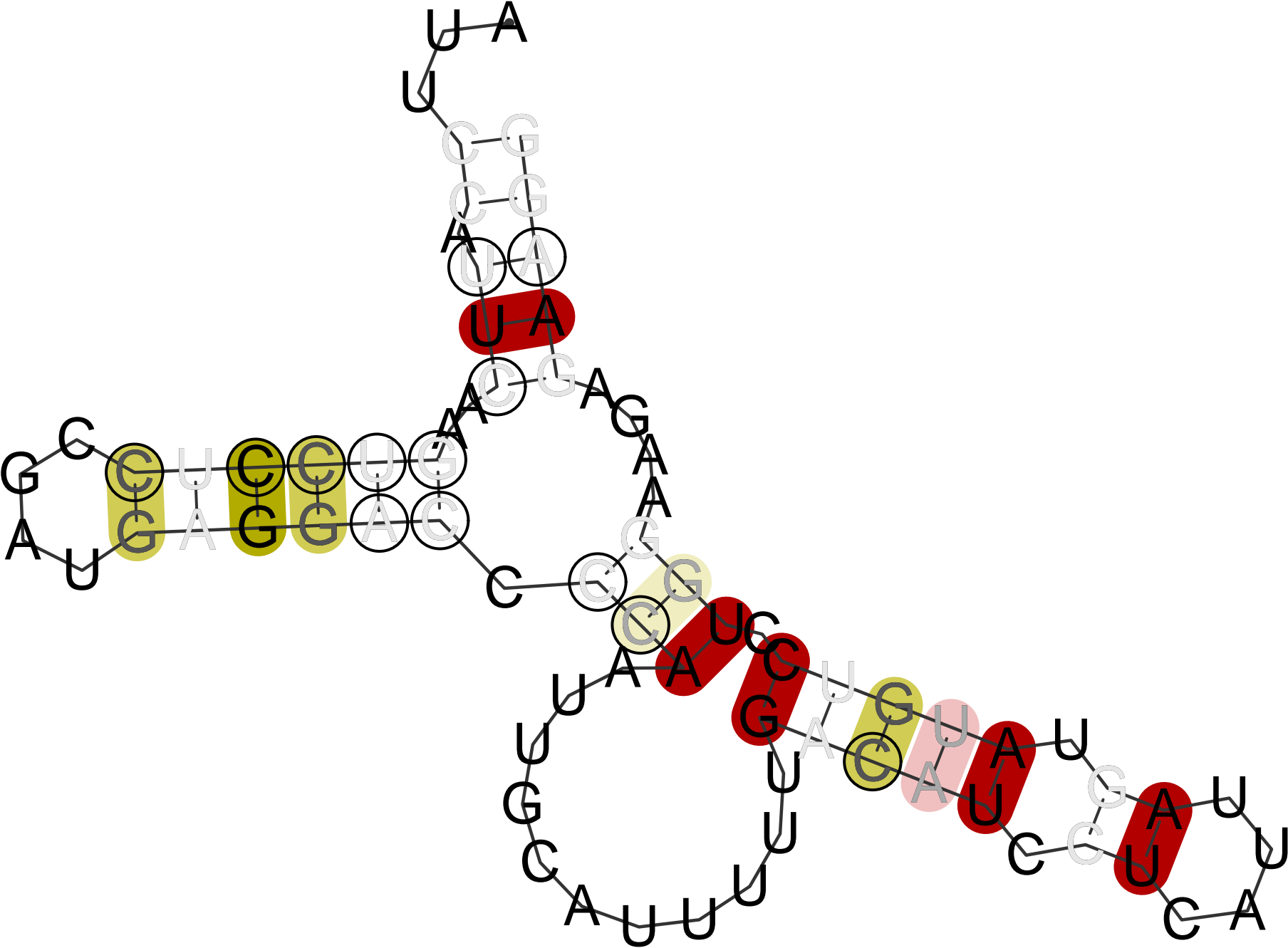

Supplement: S2 Fig — See the caption for S1 Fig for a description of the filename convention (save that the corresponding nucleotide locations in reference sequences are listed in S3 and S11 Tables), and an explanation of the RNAalifold options used and output. (ZIP) [file pcbi.1012009.s123.zip › H1N2-swine-raw-NS1-alignment-493-567-refseq-490-564_revcomp_alirna_nogap.pdf]

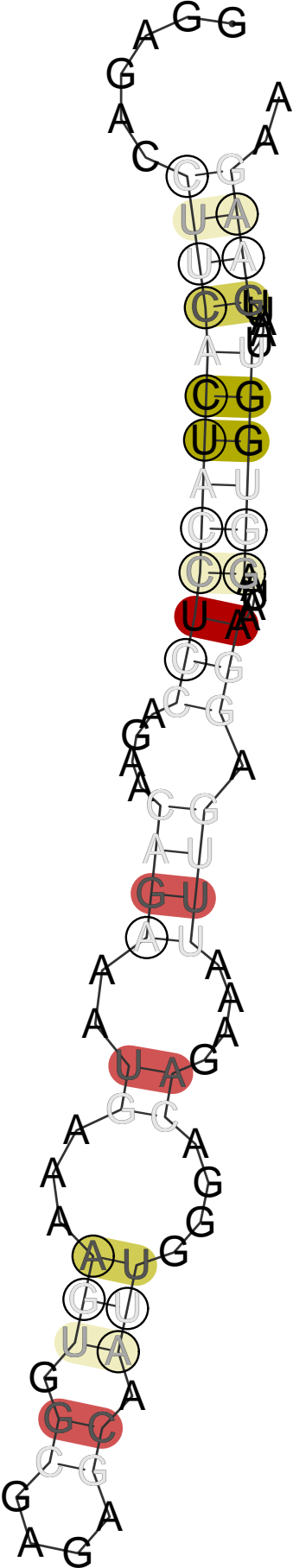

Supplement: S2 Fig — See the caption for S1 Fig for a description of the filename convention (save that the corresponding nucleotide locations in reference sequences are listed in S3 and S11 Tables), and an explanation of the RNAalifold options used and output. (ZIP) [file pcbi.1012009.s123.zip › H1N2-swine-raw-NS2-alignment-160-249-refseq-623-718_alirna_nogap.pdf]

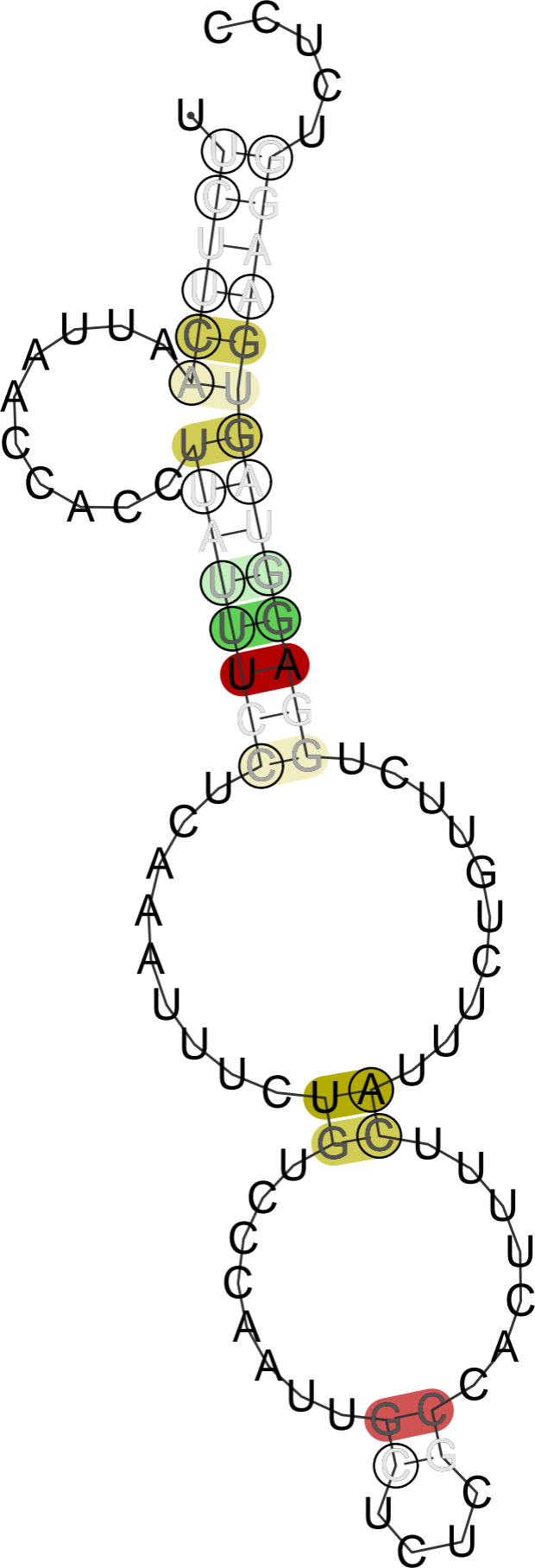

Supplement: S2 Fig — See the caption for S1 Fig for a description of the filename convention (save that the corresponding nucleotide locations in reference sequences are listed in S3 and S11 Tables), and an explanation of the RNAalifold options used and output. (ZIP) [file pcbi.1012009.s123.zip › H1N2-swine-raw-NS2-alignment-160-249-refseq-623-718_revcomp_alirna_nogap.pdf]

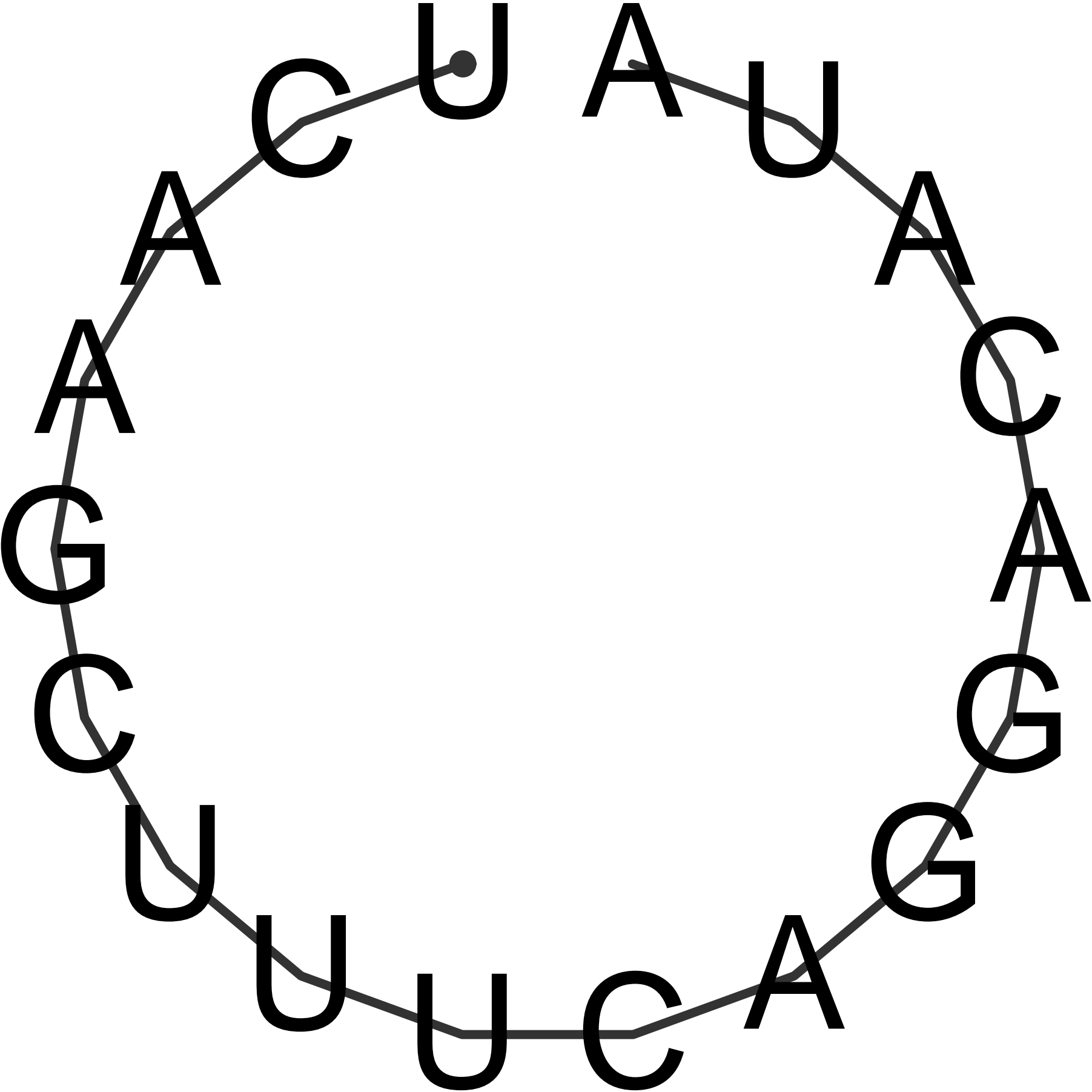

Supplement: S2 Fig — See the caption for S1 Fig for a description of the filename convention (save that the corresponding nucleotide locations in reference sequences are listed in S3 and S11 Tables), and an explanation of the RNAalifold options used and output. (ZIP) [file pcbi.1012009.s123.zip › H1N2-swine-raw-NS2-alignment-22-39-refseq-19-508_alirna_nogap.pdf]

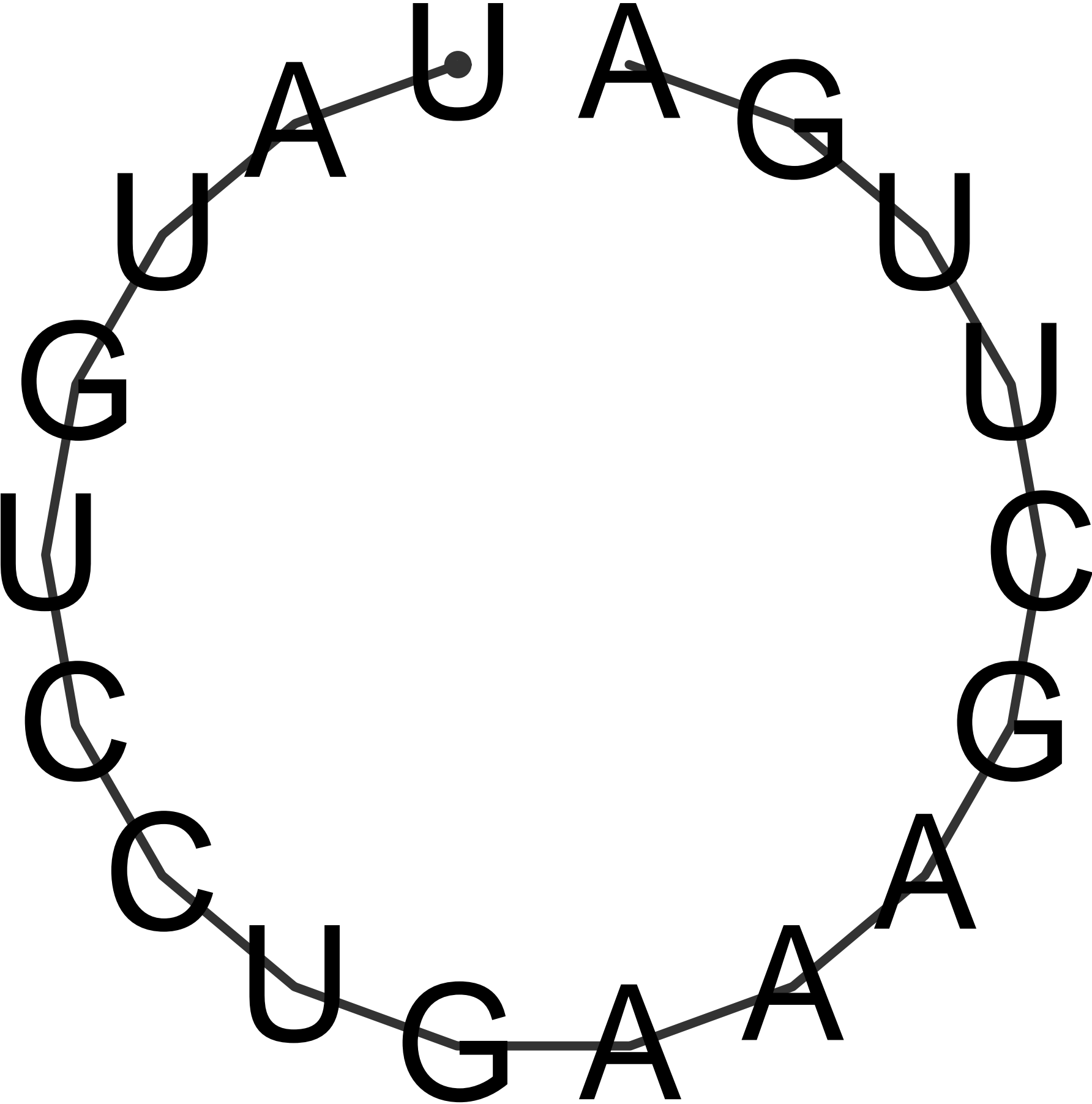

Supplement: S2 Fig — See the caption for S1 Fig for a description of the filename convention (save that the corresponding nucleotide locations in reference sequences are listed in S3 and S11 Tables), and an explanation of the RNAalifold options used and output. (ZIP) [file pcbi.1012009.s123.zip › H1N2-swine-raw-NS2-alignment-22-39-refseq-19-508_revcomp_alirna_nogap.pdf]

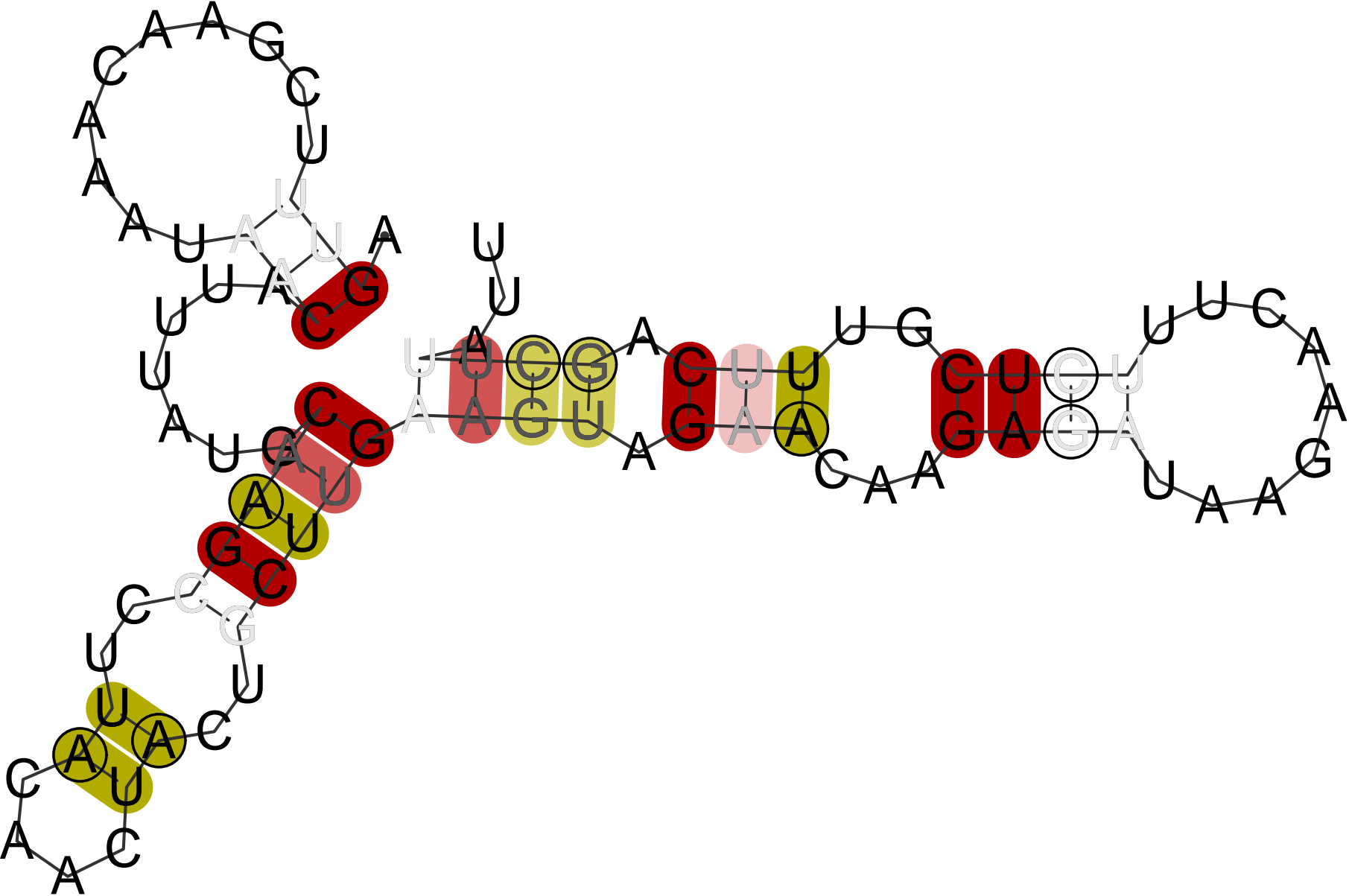

Supplement: S2 Fig — See the caption for S1 Fig for a description of the filename convention (save that the corresponding nucleotide locations in reference sequences are listed in S3 and S11 Tables), and an explanation of the RNAalifold options used and output. (ZIP) [file pcbi.1012009.s123.zip › H1N2-swine-raw-NS2-alignment-280-366-refseq-749-835_alirna_nogap.pdf]

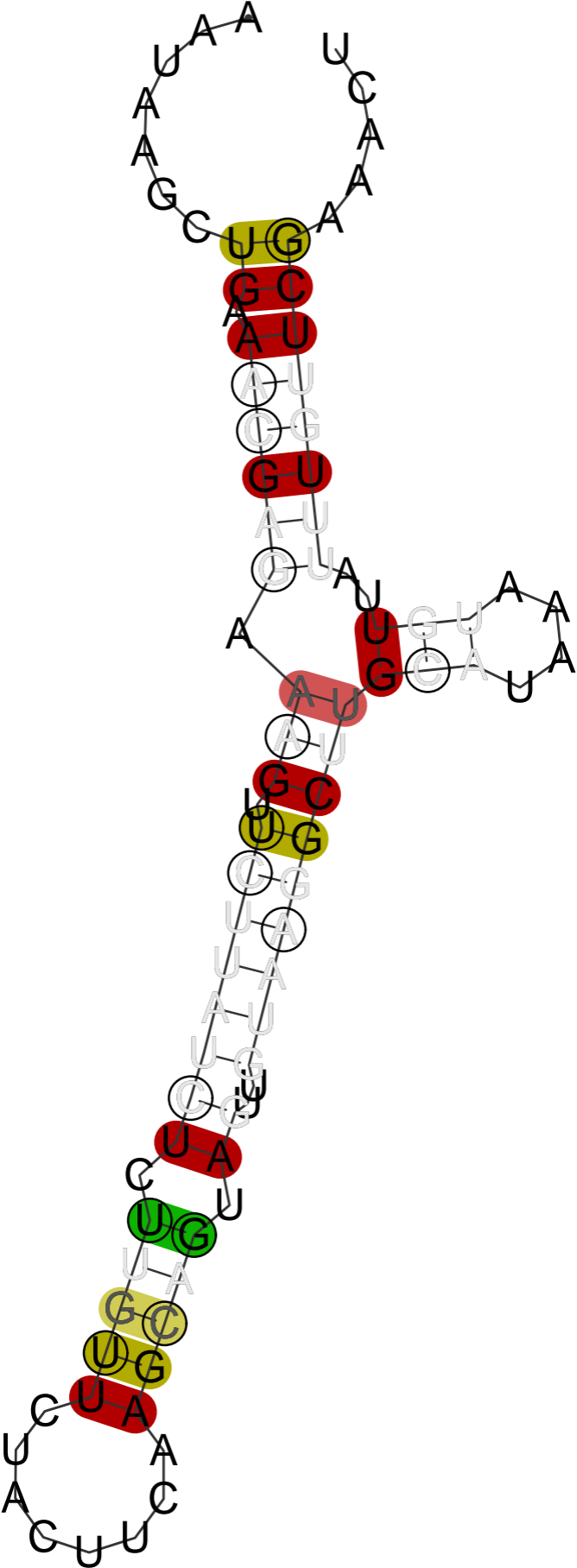

Supplement: S2 Fig — See the caption for S1 Fig for a description of the filename convention (save that the corresponding nucleotide locations in reference sequences are listed in S3 and S11 Tables), and an explanation of the RNAalifold options used and output. (ZIP) [file pcbi.1012009.s123.zip › H1N2-swine-raw-NS2-alignment-280-366-refseq-749-835_revcomp_alirna_nogap.pdf]

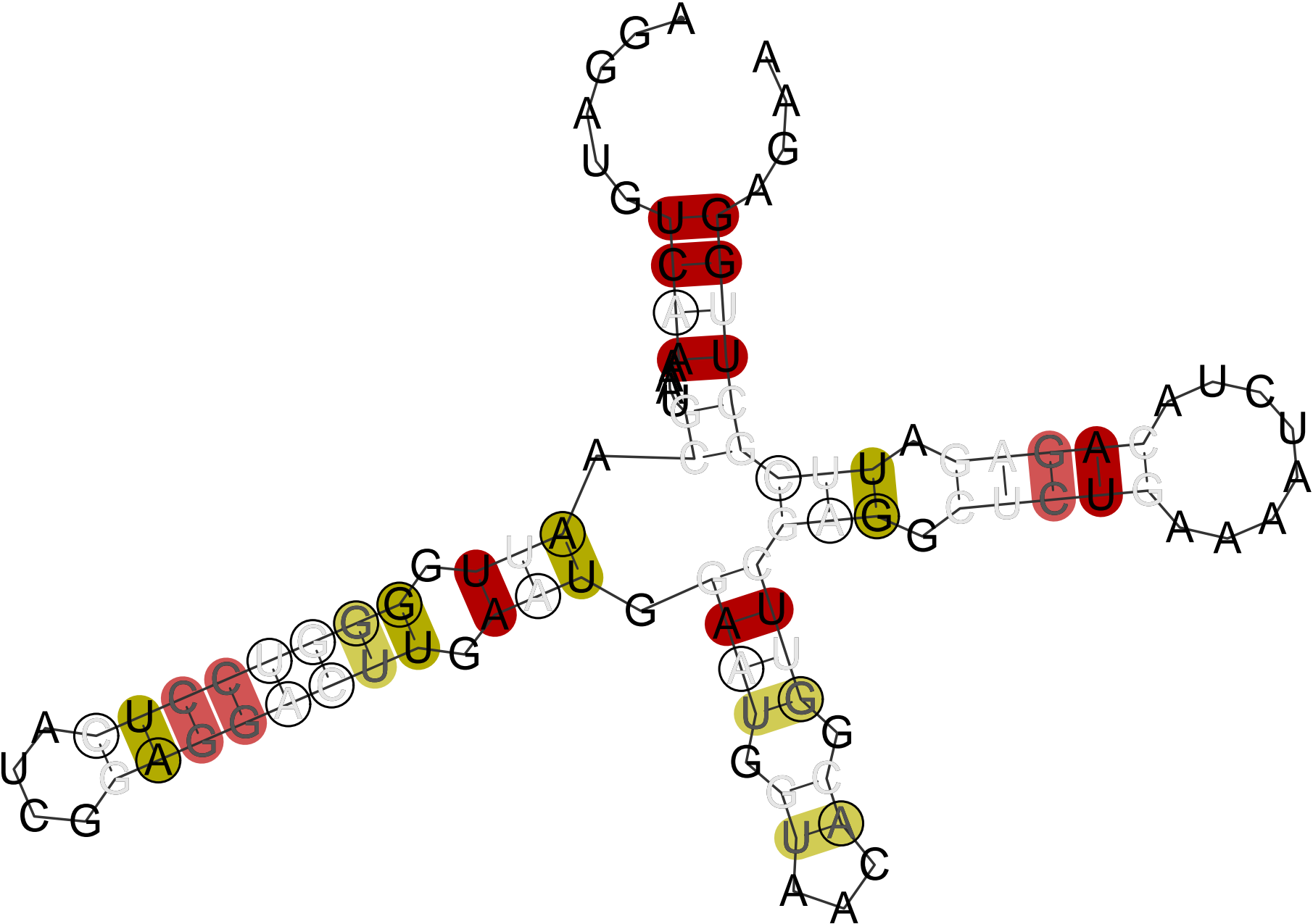

Supplement: S2 Fig — See the caption for S1 Fig for a description of the filename convention (save that the corresponding nucleotide locations in reference sequences are listed in S3 and S11 Tables), and an explanation of the RNAalifold options used and output. (ZIP) [file pcbi.1012009.s123.zip › H1N2-swine-raw-NS2-alignment-46-144-refseq-515-613_alirna_nogap.pdf]

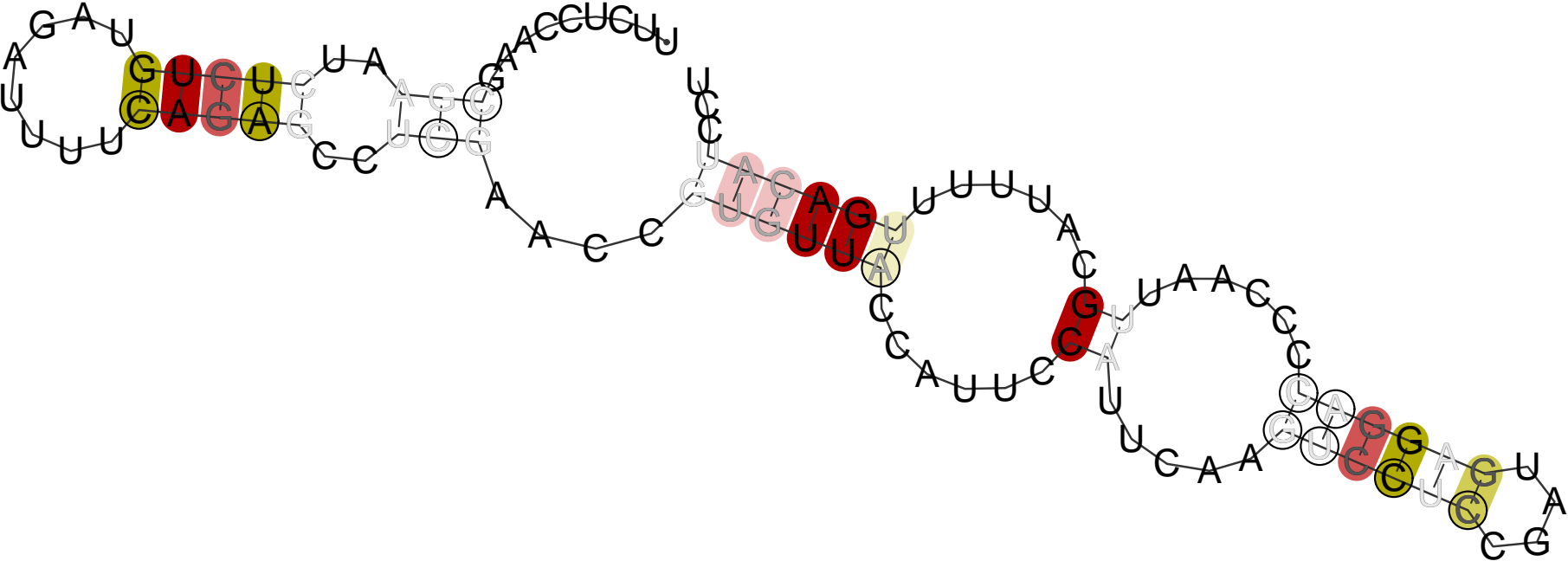

Supplement: S2 Fig — See the caption for S1 Fig for a description of the filename convention (save that the corresponding nucleotide locations in reference sequences are listed in S3 and S11 Tables), and an explanation of the RNAalifold options used and output. (ZIP) [file pcbi.1012009.s123.zip › H1N2-swine-raw-NS2-alignment-46-144-refseq-515-613_revcomp_alirna_nogap.pdf]

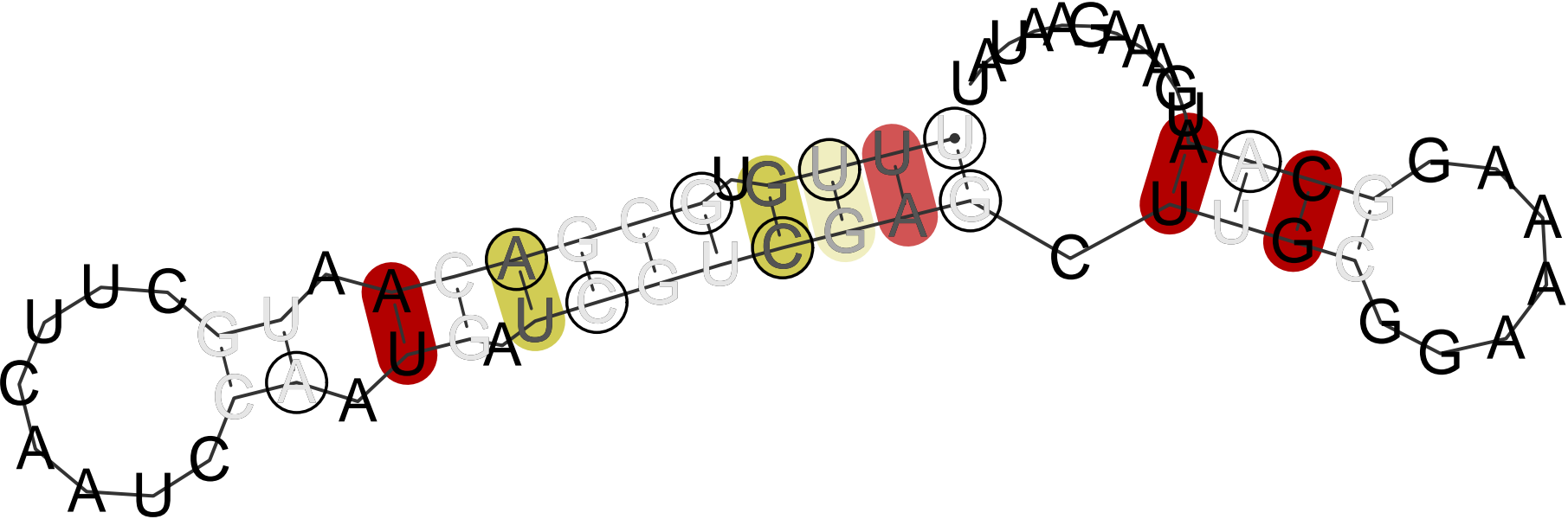

Supplement: S2 Fig — See the caption for S1 Fig for a description of the filename convention (save that the corresponding nucleotide locations in reference sequences are listed in S3 and S11 Tables), and an explanation of the RNAalifold options used and output. (ZIP) [file pcbi.1012009.s123.zip › H1N2-swine-raw-PA-X-alignment-10-72-refseq-10-72_alirna_nogap.pdf]

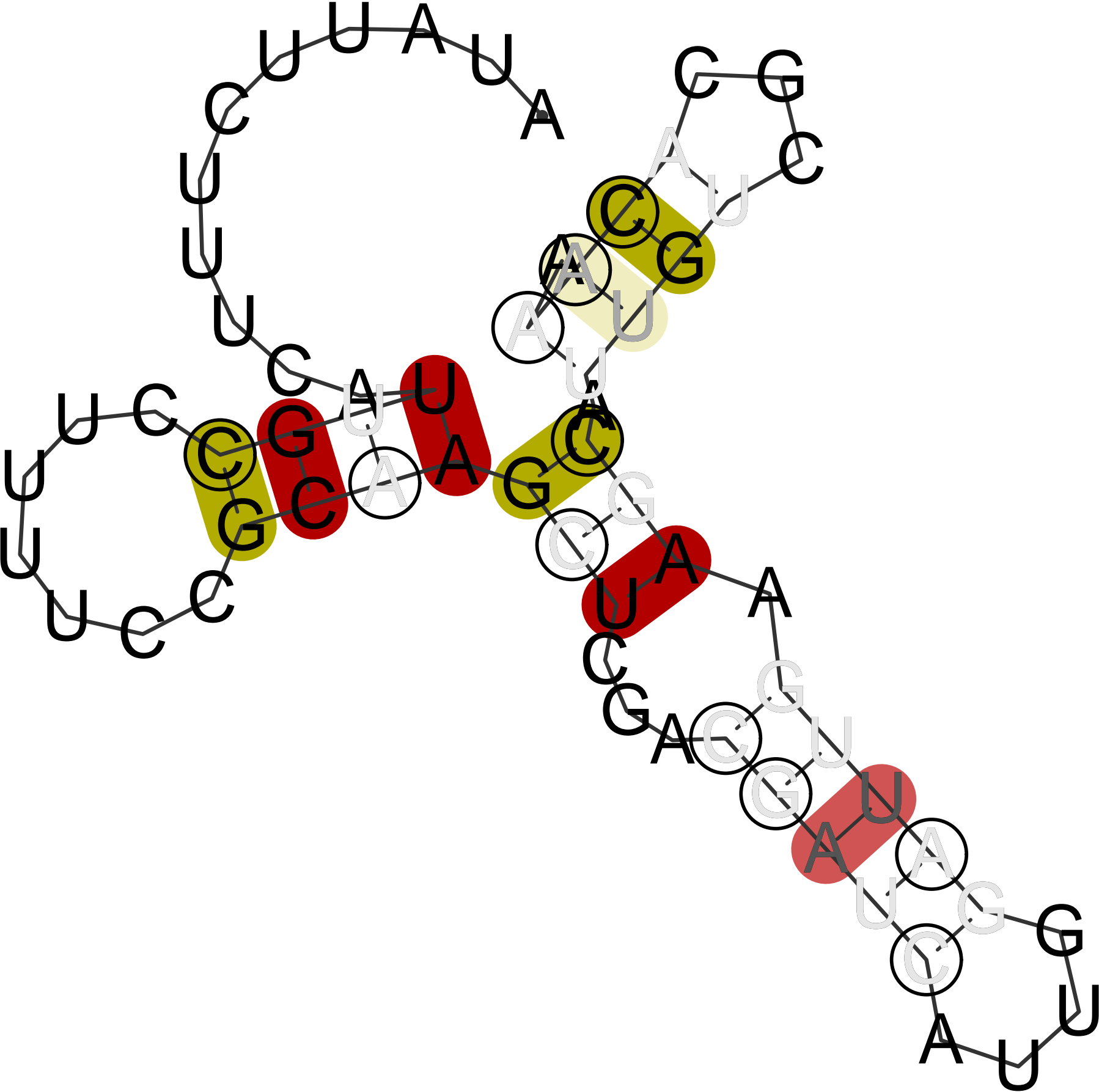

Supplement: S2 Fig — See the caption for S1 Fig for a description of the filename convention (save that the corresponding nucleotide locations in reference sequences are listed in S3 and S11 Tables), and an explanation of the RNAalifold options used and output. (ZIP) [file pcbi.1012009.s123.zip › H1N2-swine-raw-PA-X-alignment-10-72-refseq-10-72_revcomp_alirna_nogap.pdf]

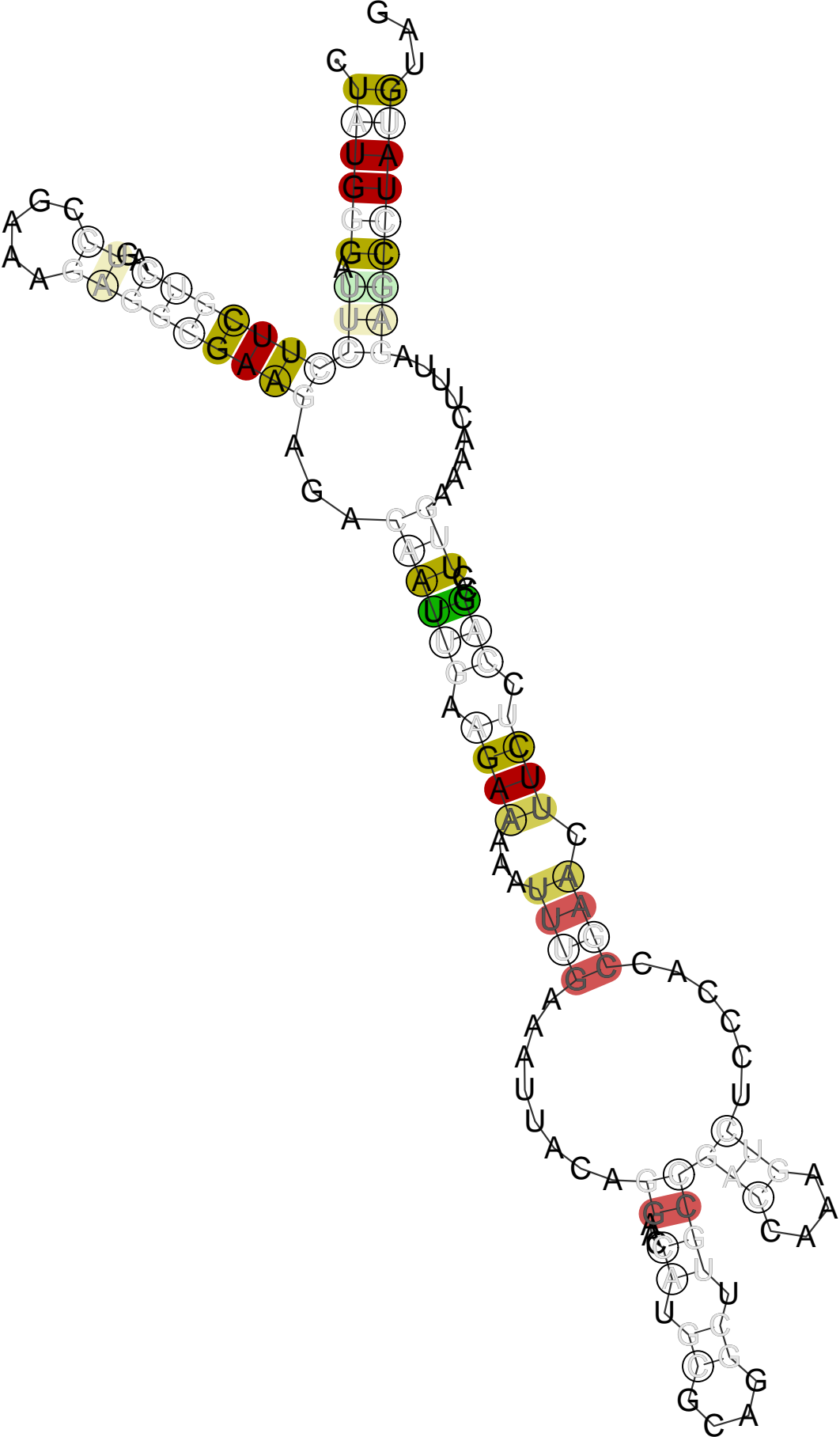

Supplement: S2 Fig — See the caption for S1 Fig for a description of the filename convention (save that the corresponding nucleotide locations in reference sequences are listed in S3 and S11 Tables), and an explanation of the RNAalifold options used and output. (ZIP) [file pcbi.1012009.s123.zip › H1N2-swine-raw-PA-X-alignment-559-699-refseq-559-700_alirna_nogap.pdf]

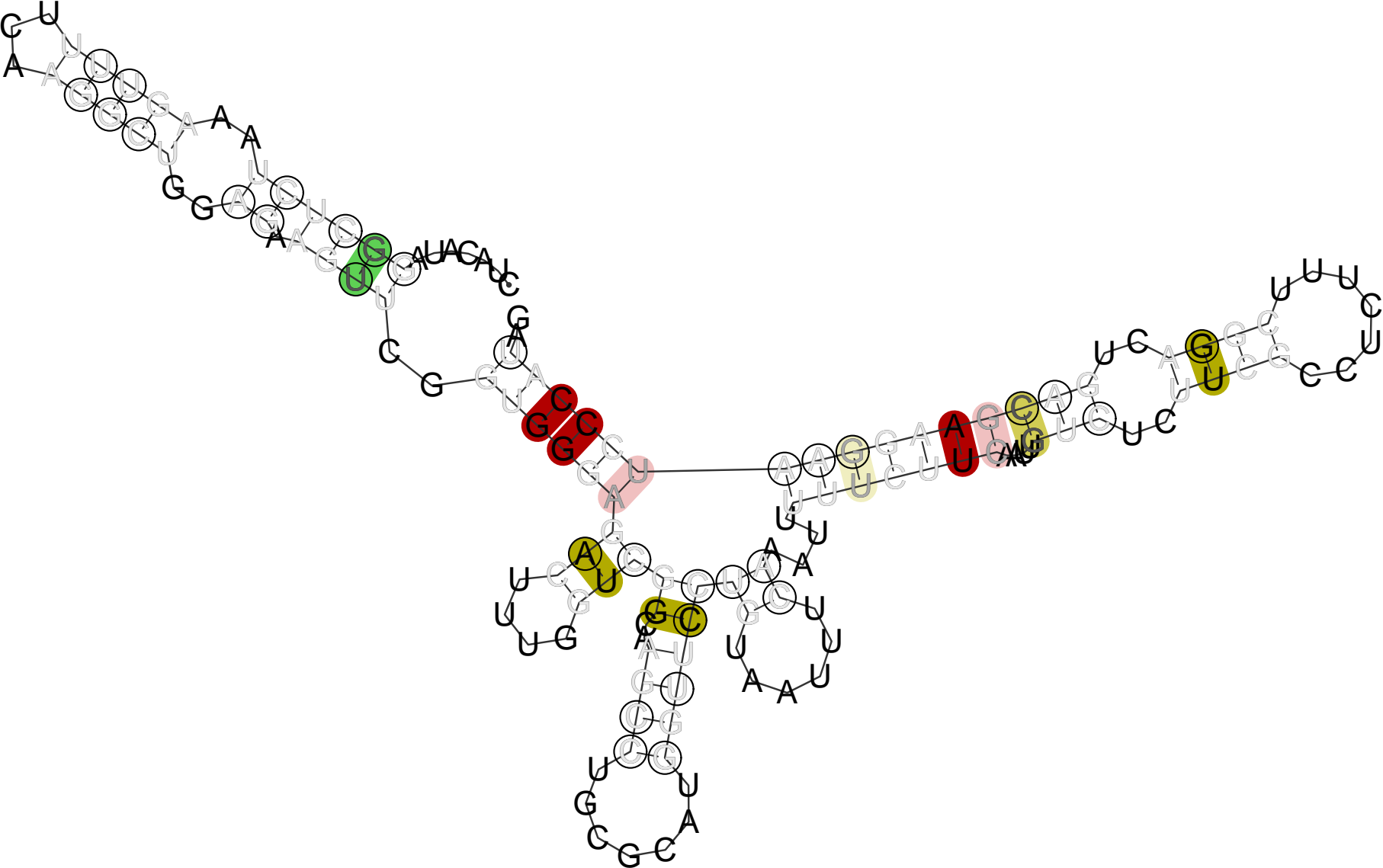

Supplement: S2 Fig — See the caption for S1 Fig for a description of the filename convention (save that the corresponding nucleotide locations in reference sequences are listed in S3 and S11 Tables), and an explanation of the RNAalifold options used and output. (ZIP) [file pcbi.1012009.s123.zip › H1N2-swine-raw-PA-X-alignment-559-699-refseq-559-700_revcomp_alirna_nogap.pdf]

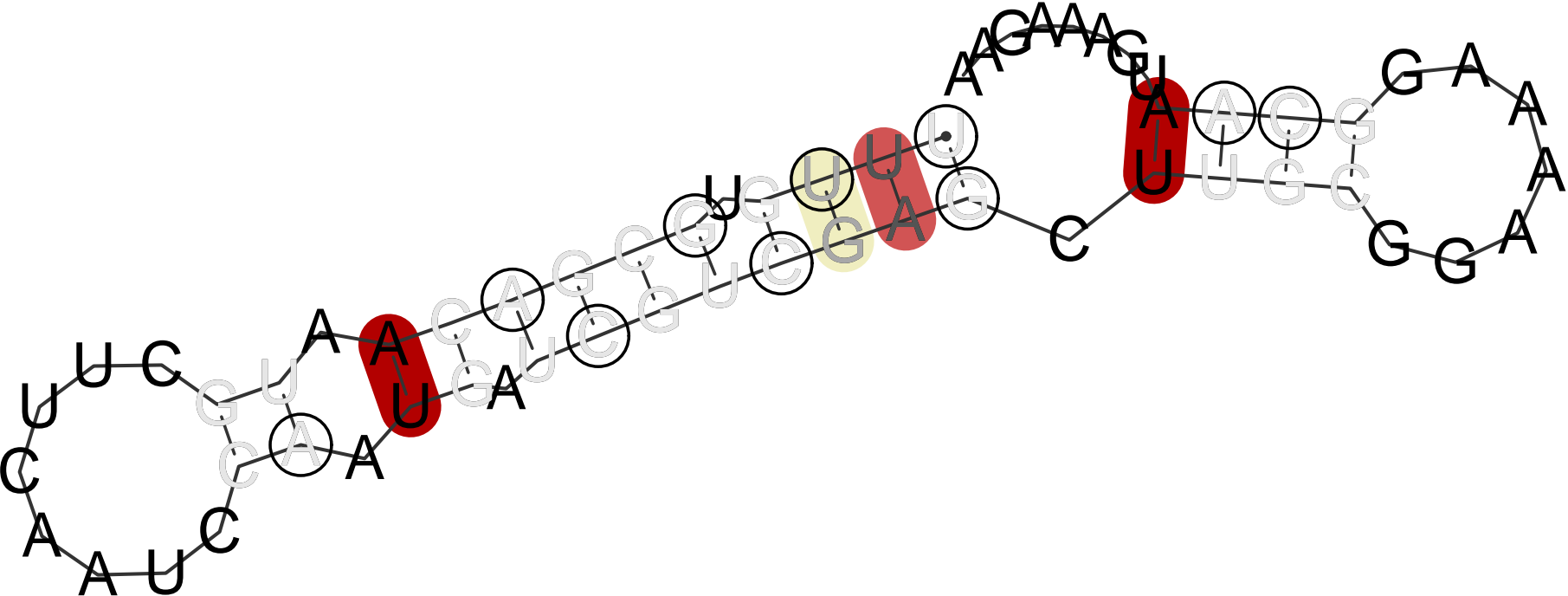

Supplement: S2 Fig — See the caption for S1 Fig for a description of the filename convention (save that the corresponding nucleotide locations in reference sequences are listed in S3 and S11 Tables), and an explanation of the RNAalifold options used and output. (ZIP) [file pcbi.1012009.s123.zip › H1N2-swine-raw-PA-alignment-10-69-refseq-10-69_alirna_nogap.pdf]

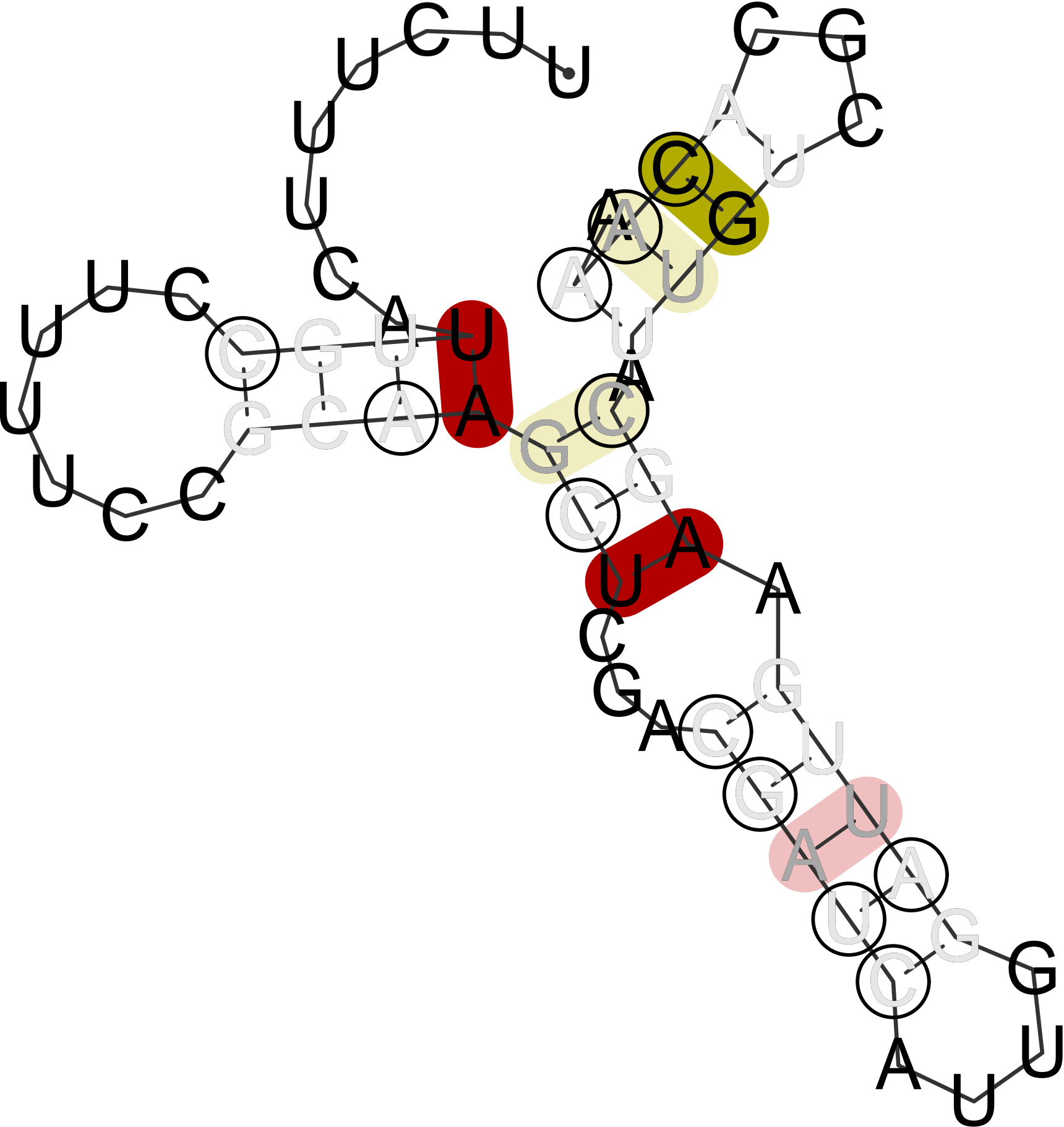

Supplement: S2 Fig — See the caption for S1 Fig for a description of the filename convention (save that the corresponding nucleotide locations in reference sequences are listed in S3 and S11 Tables), and an explanation of the RNAalifold options used and output. (ZIP) [file pcbi.1012009.s123.zip › H1N2-swine-raw-PA-alignment-10-69-refseq-10-69_revcomp_alirna_nogap.pdf]

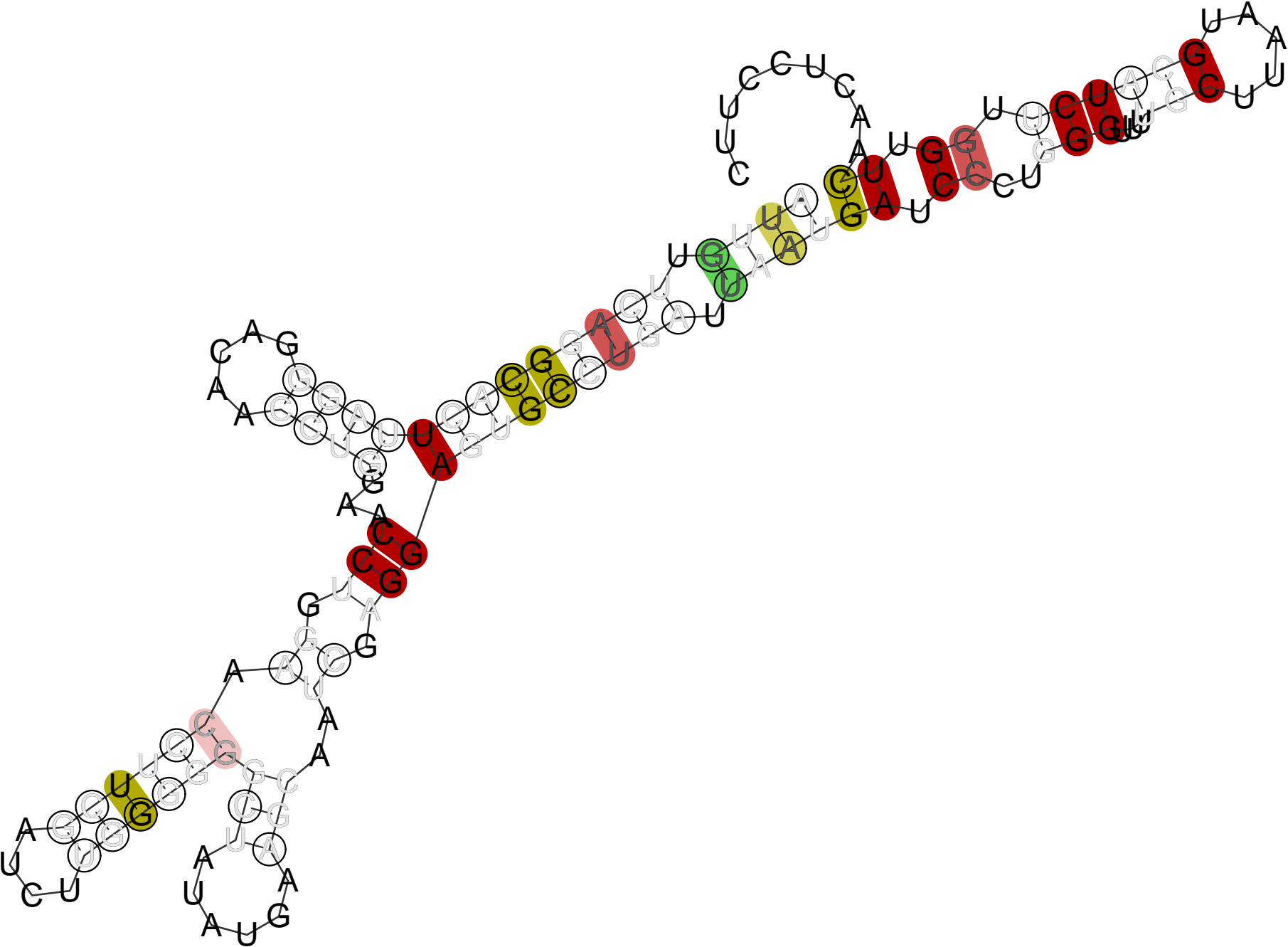

Supplement: S2 Fig — See the caption for S1 Fig for a description of the filename convention (save that the corresponding nucleotide locations in reference sequences are listed in S3 and S11 Tables), and an explanation of the RNAalifold options used and output. (ZIP) [file pcbi.1012009.s123.zip › H1N2-swine-raw-PA-alignment-2002-2130-refseq-2002-2130_alirna_nogap.pdf]

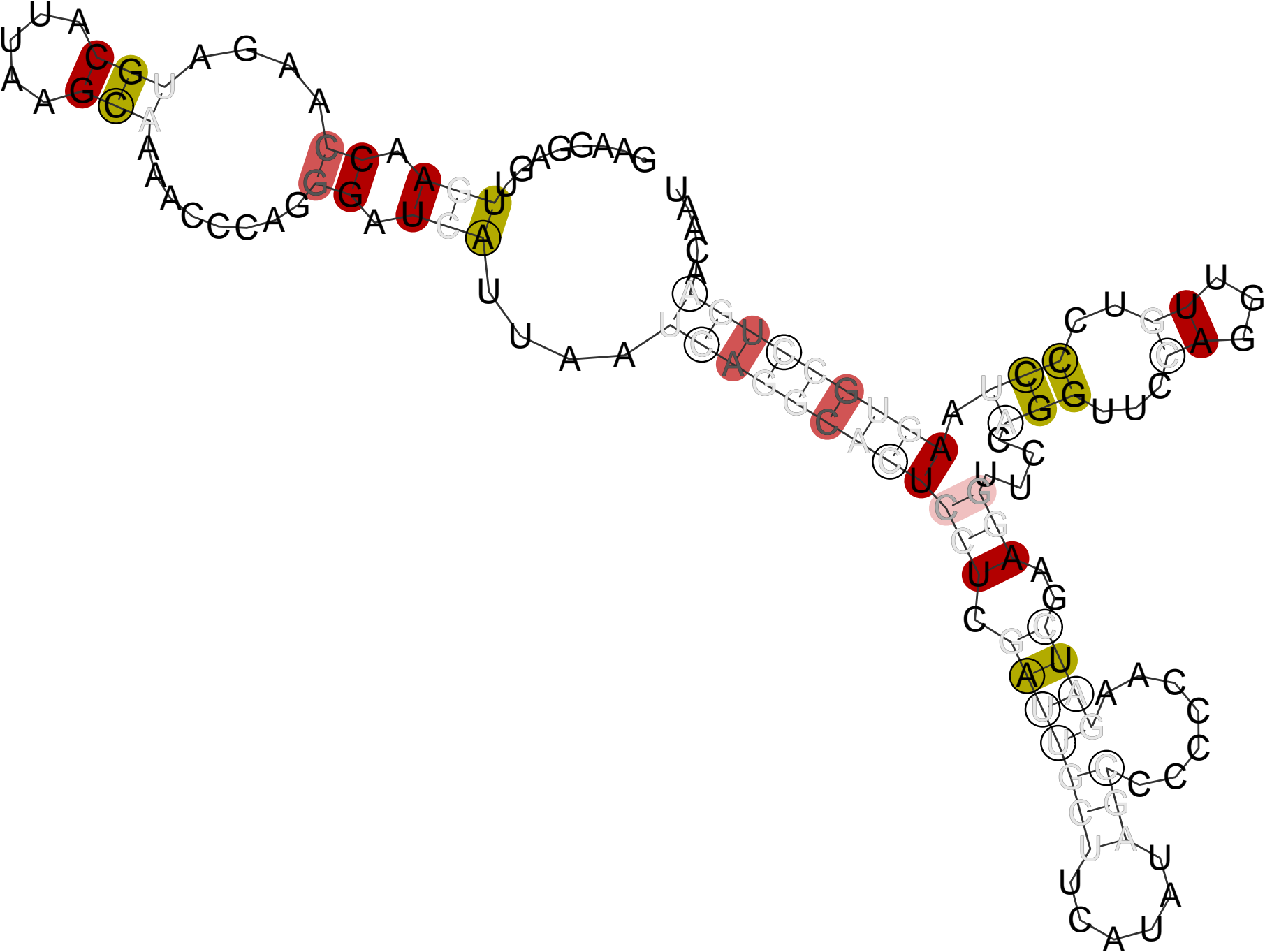

Supplement: S2 Fig — See the caption for S1 Fig for a description of the filename convention (save that the corresponding nucleotide locations in reference sequences are listed in S3 and S11 Tables), and an explanation of the RNAalifold options used and output. (ZIP) [file pcbi.1012009.s123.zip › H1N2-swine-raw-PA-alignment-2002-2130-refseq-2002-2130_revcomp_alirna_nogap.pdf]

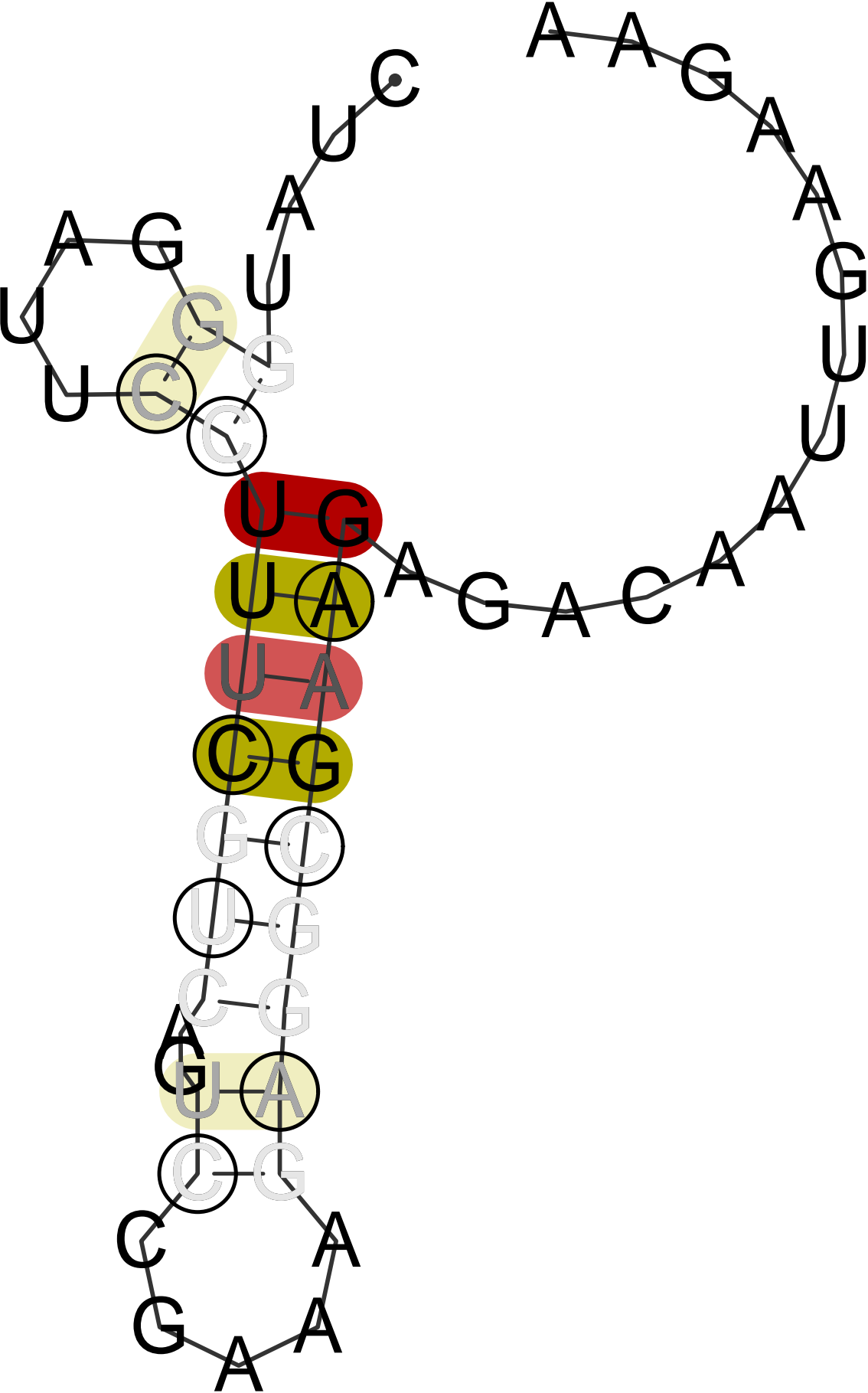

Supplement: S2 Fig — See the caption for S1 Fig for a description of the filename convention (save that the corresponding nucleotide locations in reference sequences are listed in S3 and S11 Tables), and an explanation of the RNAalifold options used and output. (ZIP) [file pcbi.1012009.s123.zip › H1N2-swine-raw-PA-alignment-559-609-refseq-559-609_alirna_nogap.pdf]

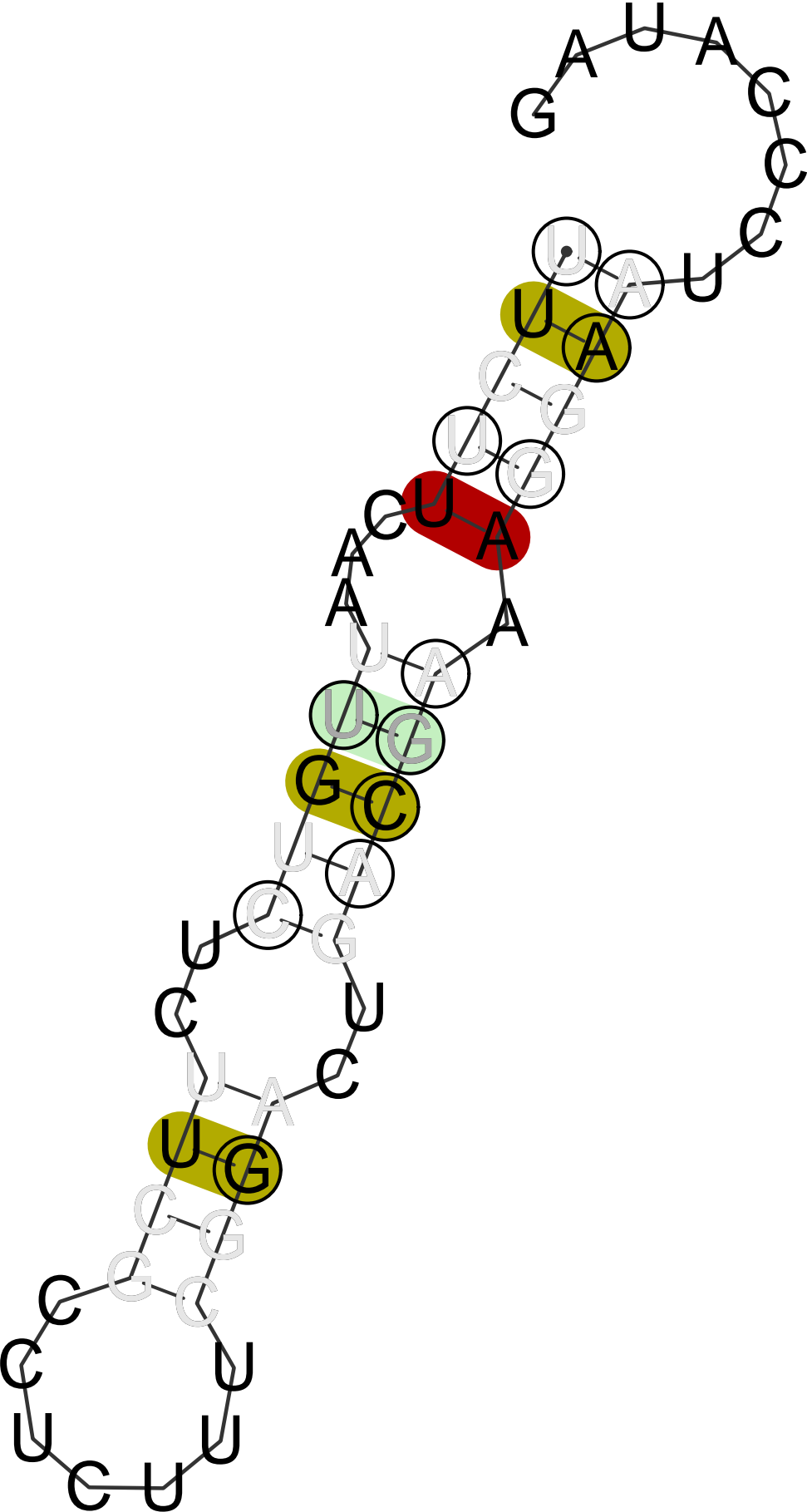

Supplement: S2 Fig — See the caption for S1 Fig for a description of the filename convention (save that the corresponding nucleotide locations in reference sequences are listed in S3 and S11 Tables), and an explanation of the RNAalifold options used and output. (ZIP) [file pcbi.1012009.s123.zip › H1N2-swine-raw-PA-alignment-559-609-refseq-559-609_revcomp_alirna_nogap.pdf]
