# Supplementary figures and images for: Locations and structures of influenza A virus packaging-associated signals and other functional elements via an in silico pipeline for predicting constrained features in RNA viruses (part 2 of 6)
Source: PLoS Comput Biol. 2024 Apr 22;20(4):e1012009. doi: 10.1371/journal.pcbi.1012009 (PMC11034665; doi:10.1371/journal.pcbi.1012009)

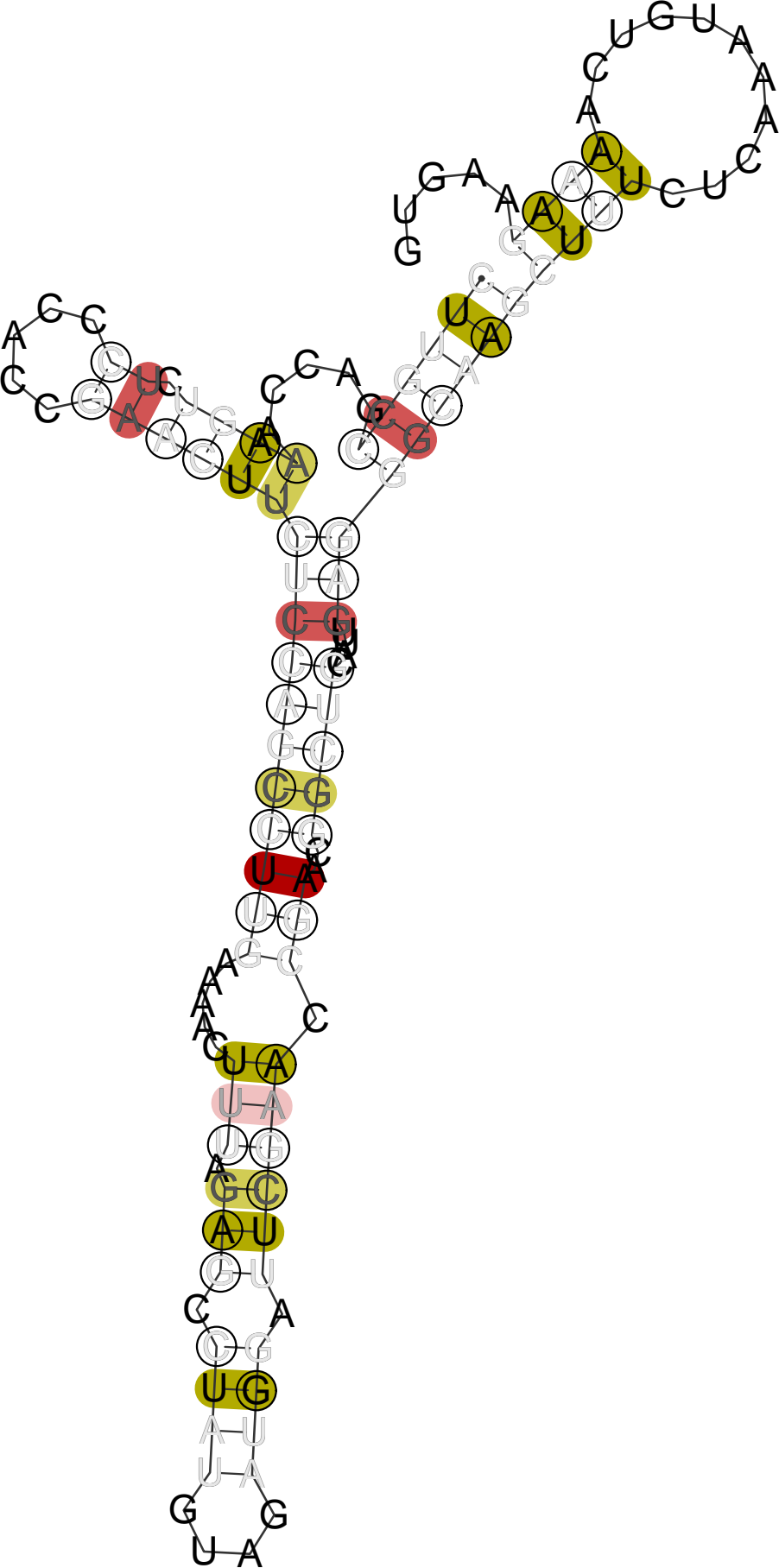

Supplement: S2 Fig — See the caption for S1 Fig for a description of the filename convention (save that the corresponding nucleotide locations in reference sequences are listed in S3 and S11 Tables), and an explanation of the RNAalifold options used and output. (ZIP) [file pcbi.1012009.s123.zip › H1N2-swine-raw-PA-alignment-640-759-refseq-640-759_alirna_nogap.pdf]

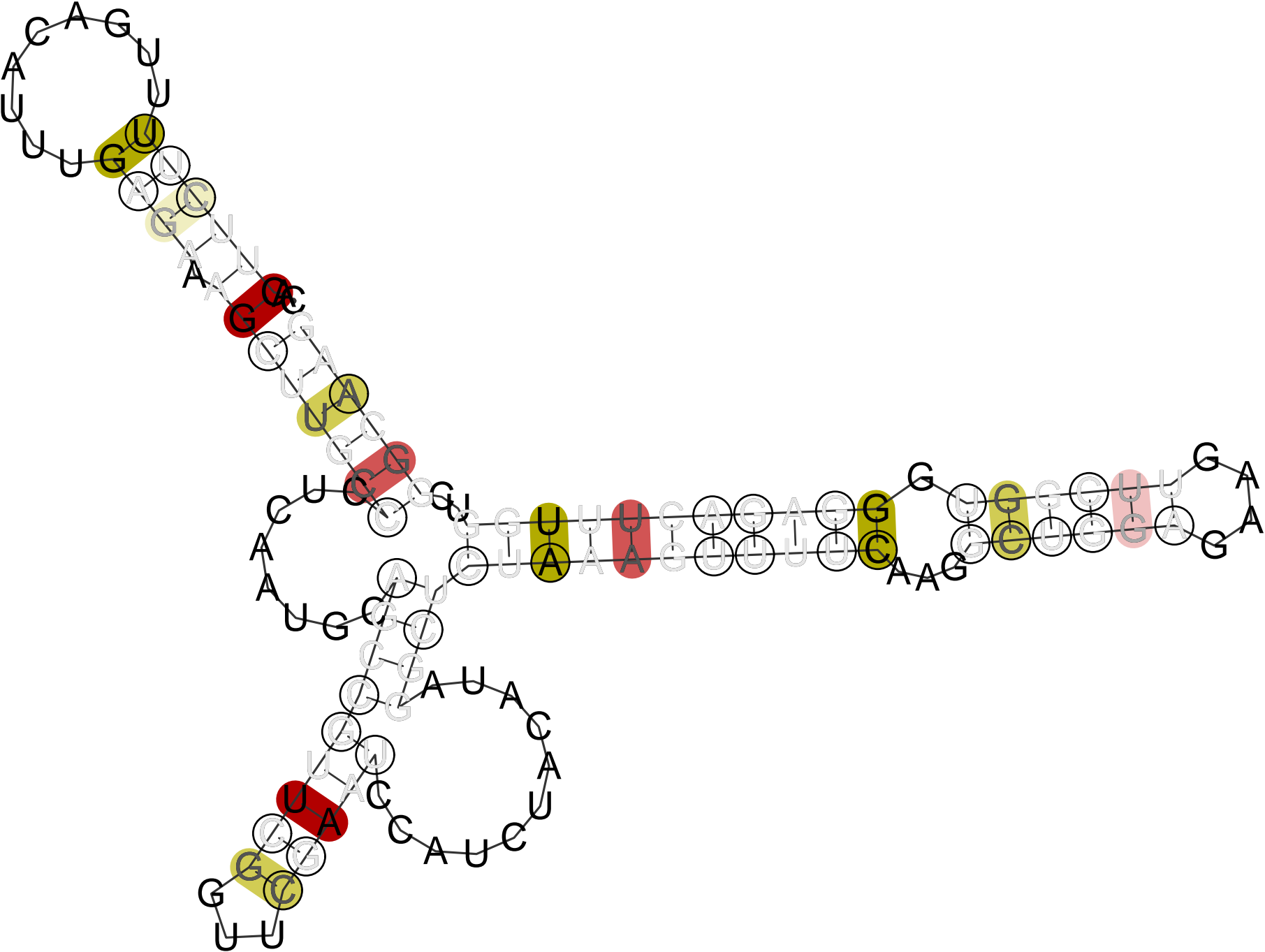

Supplement: S2 Fig — See the caption for S1 Fig for a description of the filename convention (save that the corresponding nucleotide locations in reference sequences are listed in S3 and S11 Tables), and an explanation of the RNAalifold options used and output. (ZIP) [file pcbi.1012009.s123.zip › H1N2-swine-raw-PA-alignment-640-759-refseq-640-759_revcomp_alirna_nogap.pdf]

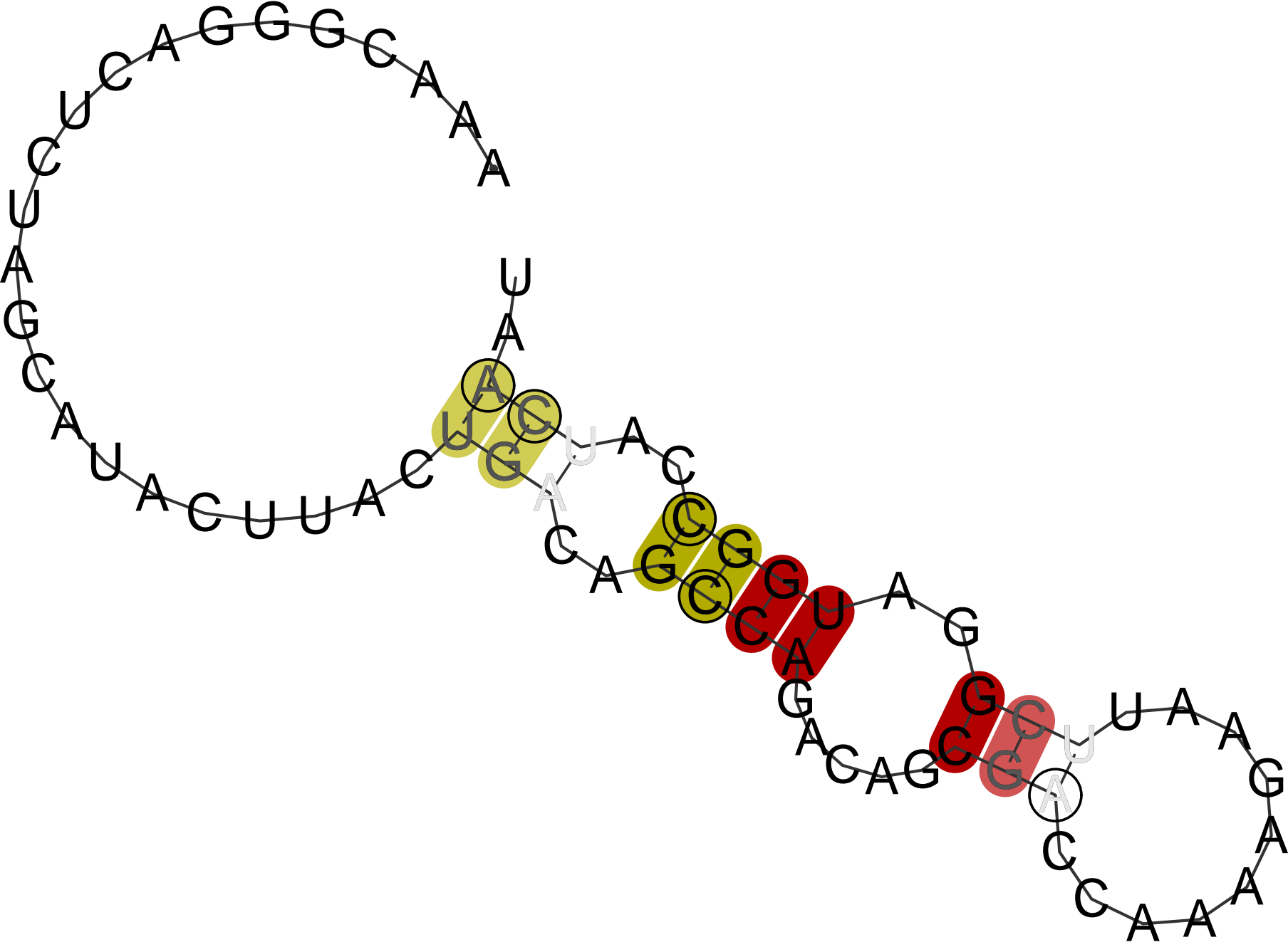

Supplement: S2 Fig — See the caption for S1 Fig for a description of the filename convention (save that the corresponding nucleotide locations in reference sequences are listed in S3 and S11 Tables), and an explanation of the RNAalifold options used and output. (ZIP) [file pcbi.1012009.s123.zip › H1N2-swine-raw-PB2-alignment-2626-2895-refseq-2212-2277_alirna_nogap.pdf]

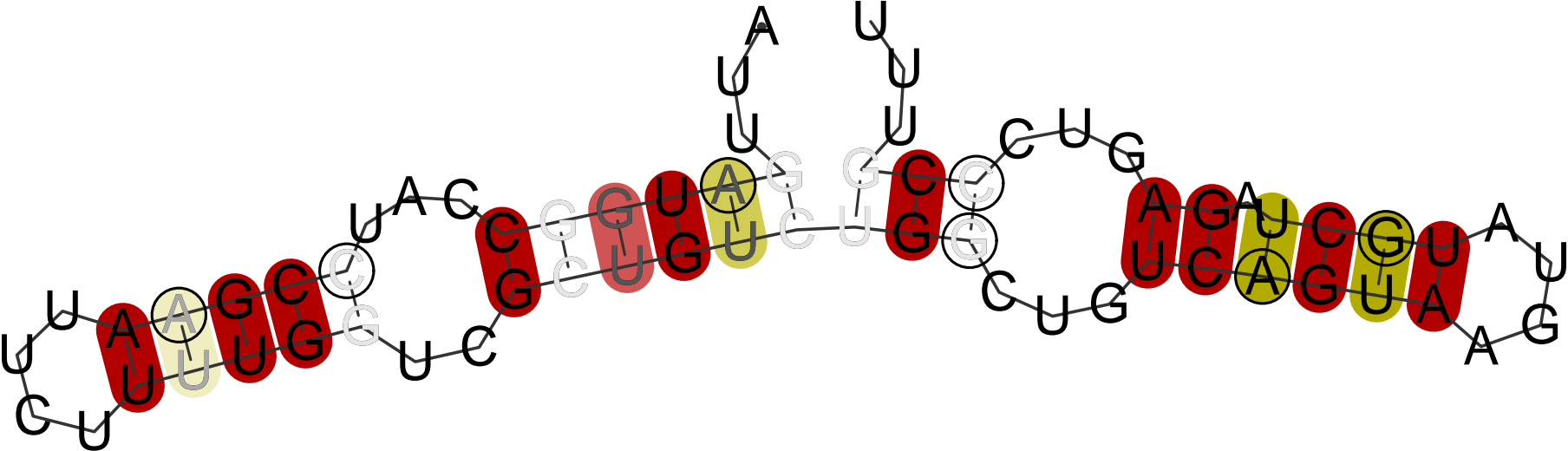

Supplement: S2 Fig — See the caption for S1 Fig for a description of the filename convention (save that the corresponding nucleotide locations in reference sequences are listed in S3 and S11 Tables), and an explanation of the RNAalifold options used and output. (ZIP) [file pcbi.1012009.s123.zip › H1N2-swine-raw-PB2-alignment-2626-2895-refseq-2212-2277_revcomp_alirna_nogap.pdf]

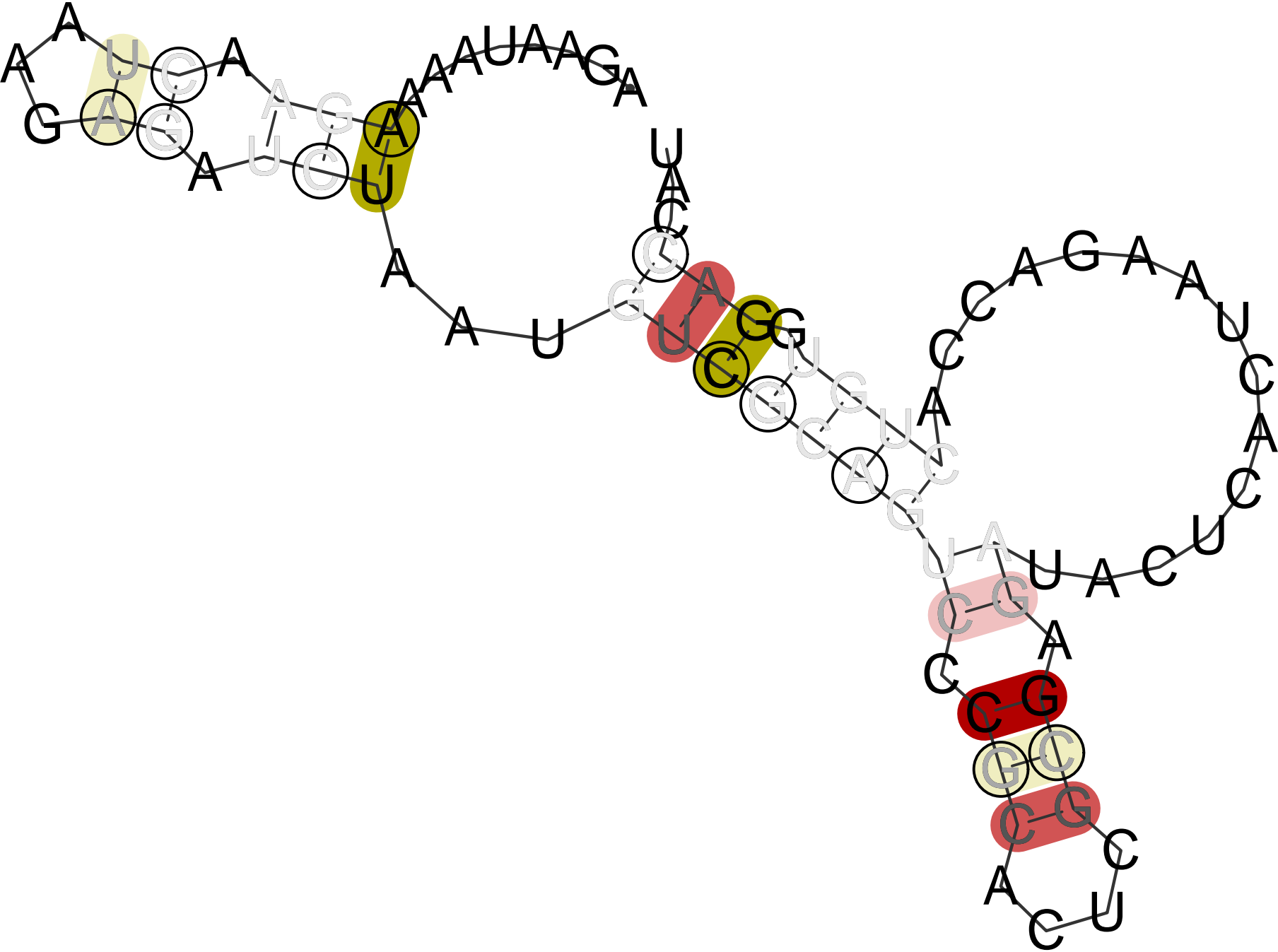

Supplement: S2 Fig — See the caption for S1 Fig for a description of the filename convention (save that the corresponding nucleotide locations in reference sequences are listed in S3 and S11 Tables), and an explanation of the RNAalifold options used and output. (ZIP) [file pcbi.1012009.s123.zip › H1N2-swine-raw-PB2-alignment-7-81-refseq-7-81_alirna_nogap.pdf]

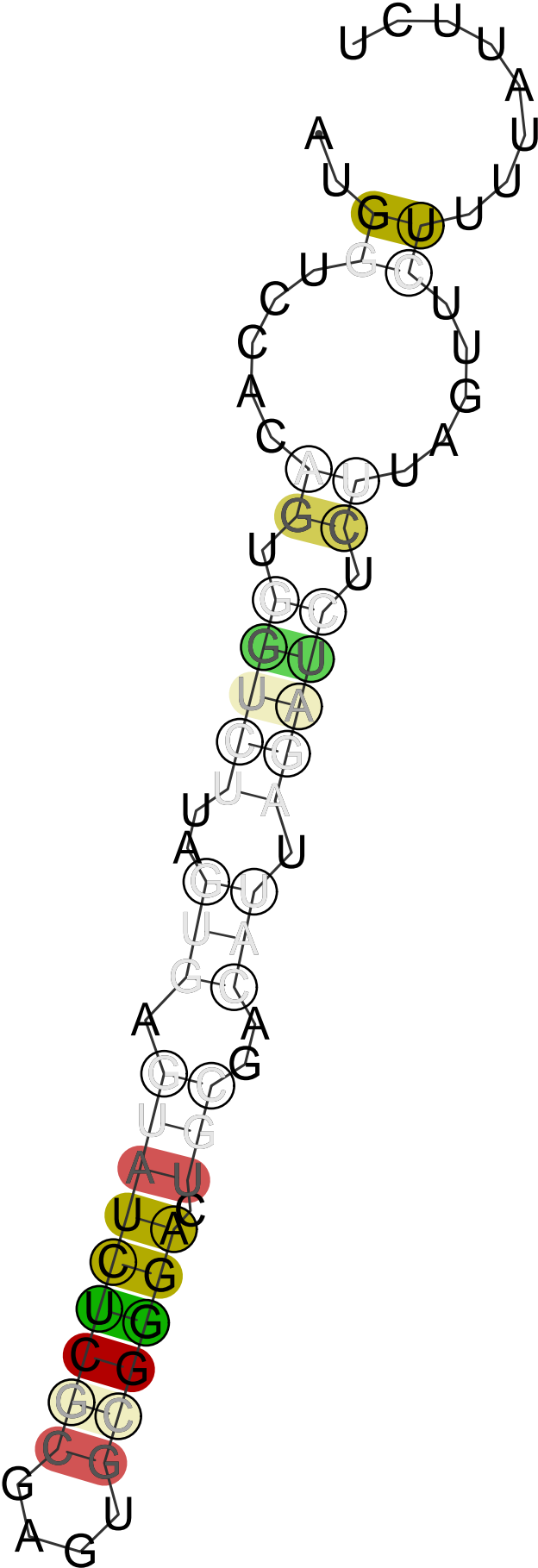

Supplement: S2 Fig — See the caption for S1 Fig for a description of the filename convention (save that the corresponding nucleotide locations in reference sequences are listed in S3 and S11 Tables), and an explanation of the RNAalifold options used and output. (ZIP) [file pcbi.1012009.s123.zip › H1N2-swine-raw-PB2-alignment-7-81-refseq-7-81_revcomp_alirna_nogap.pdf]

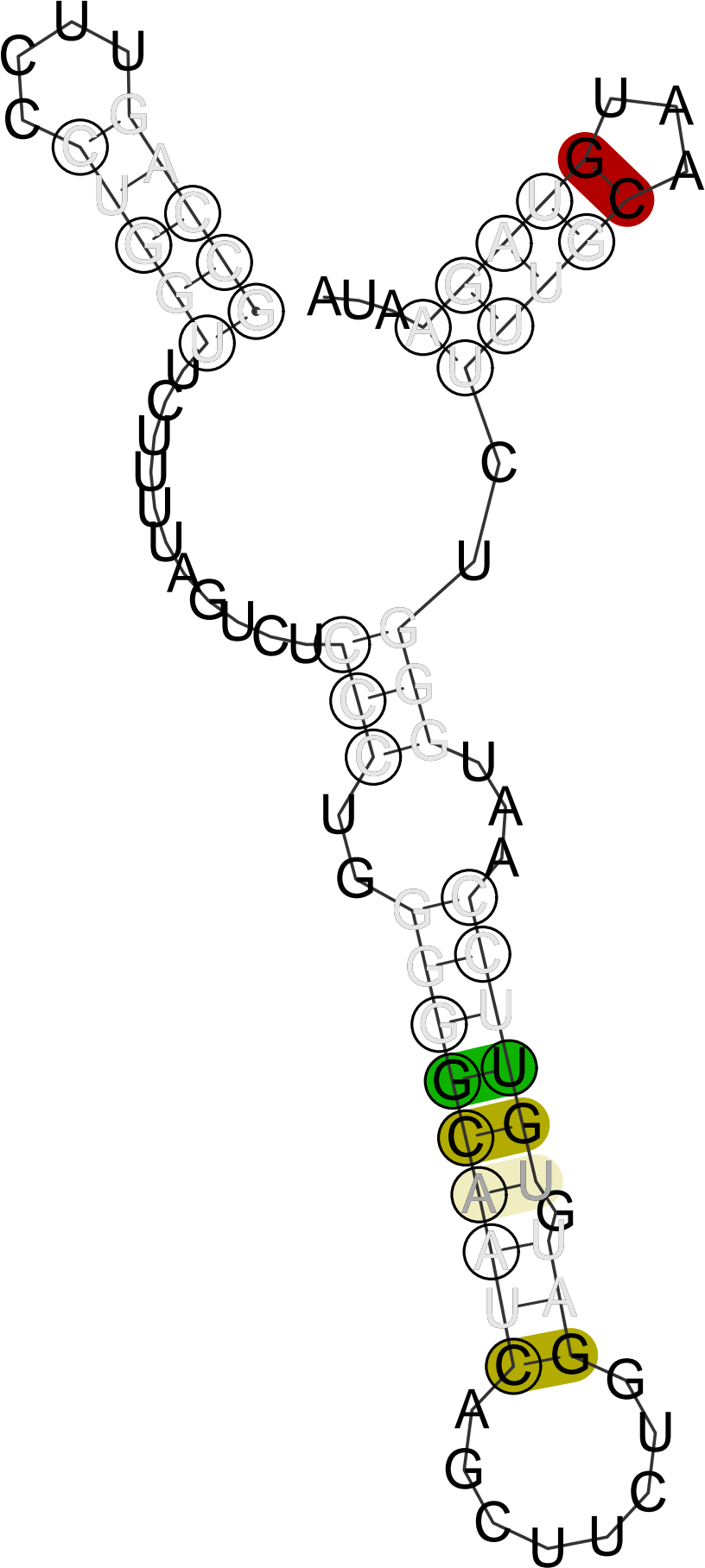

Supplement: S2 Fig — See the caption for S1 Fig for a description of the filename convention (save that the corresponding nucleotide locations in reference sequences are listed in S3 and S11 Tables), and an explanation of the RNAalifold options used and output. (ZIP) [file pcbi.1012009.s123.zip › H1N2-swine-raw-ranked-HA-alignment-1690-1770-refseq-1612-1692_alirna_nogap.pdf]

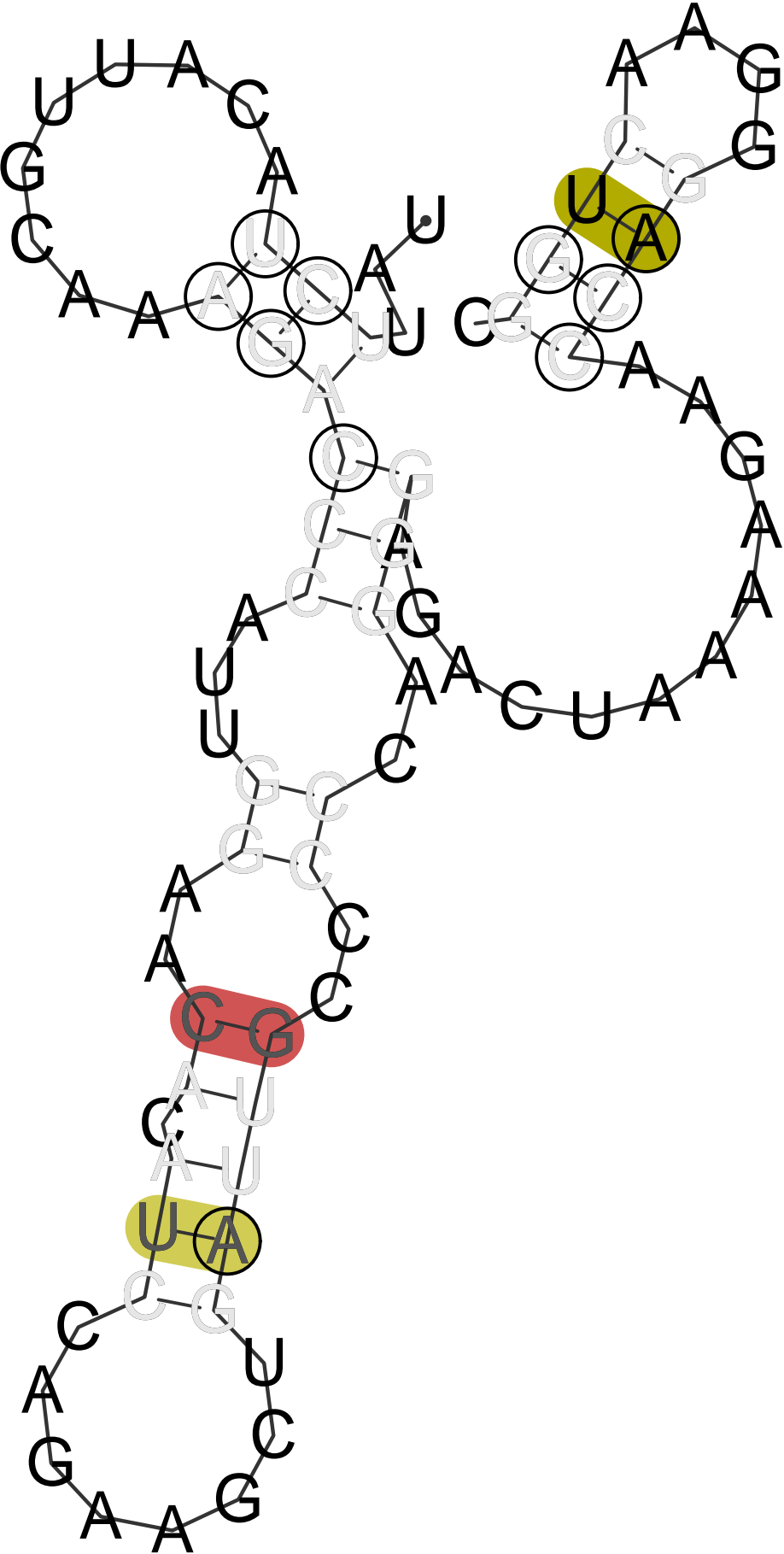

Supplement: S2 Fig — See the caption for S1 Fig for a description of the filename convention (save that the corresponding nucleotide locations in reference sequences are listed in S3 and S11 Tables), and an explanation of the RNAalifold options used and output. (ZIP) [file pcbi.1012009.s123.zip › H1N2-swine-raw-ranked-HA-alignment-1690-1770-refseq-1612-1692_revcomp_alirna_nogap.pdf]

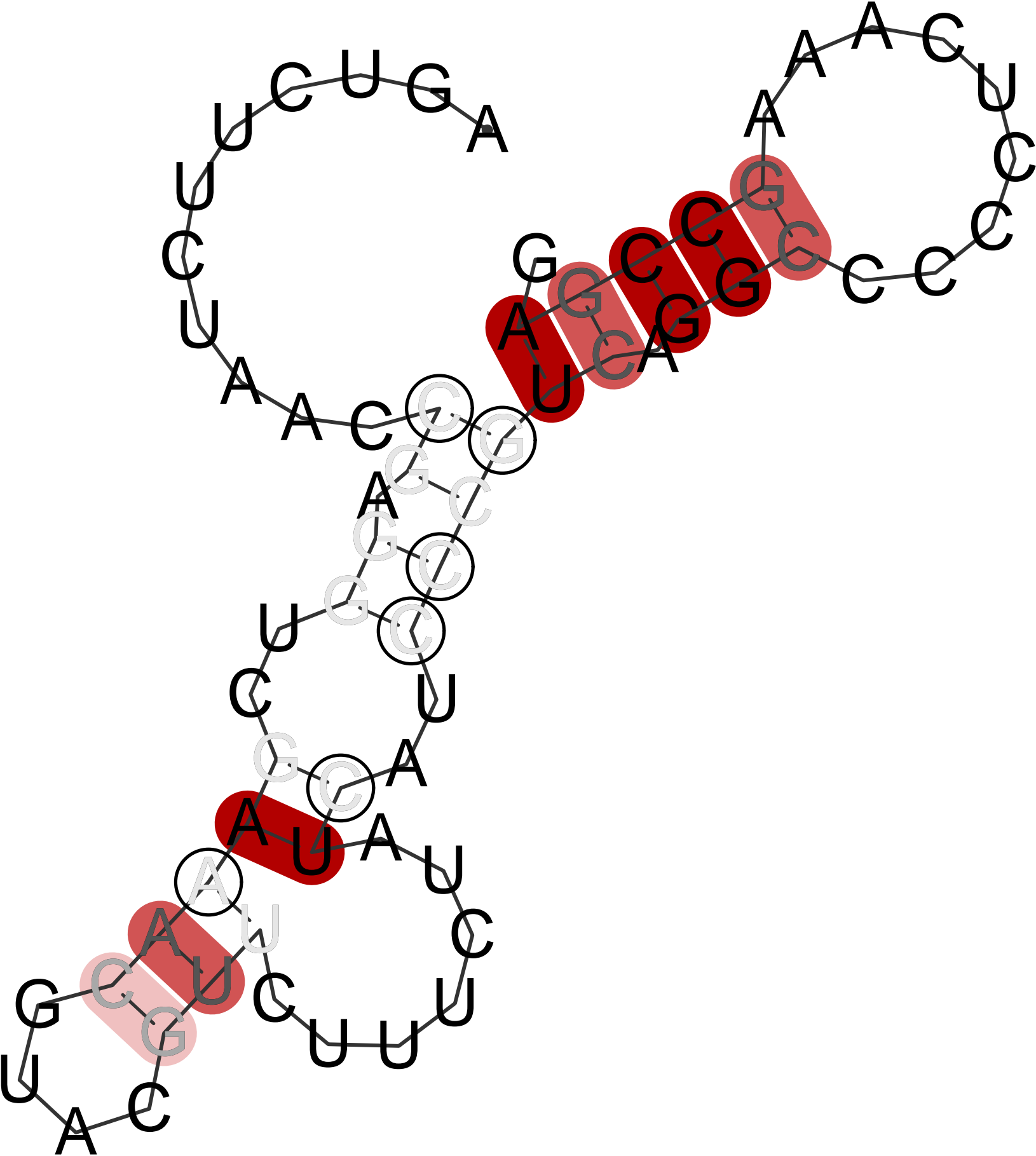

Supplement: S2 Fig — See the caption for S1 Fig for a description of the filename convention (save that the corresponding nucleotide locations in reference sequences are listed in S3 and S11 Tables), and an explanation of the RNAalifold options used and output. (ZIP) [file pcbi.1012009.s123.zip › H1N2-swine-raw-ranked-M1-alignment-7-72-refseq-4-69_alirna_nogap.pdf]

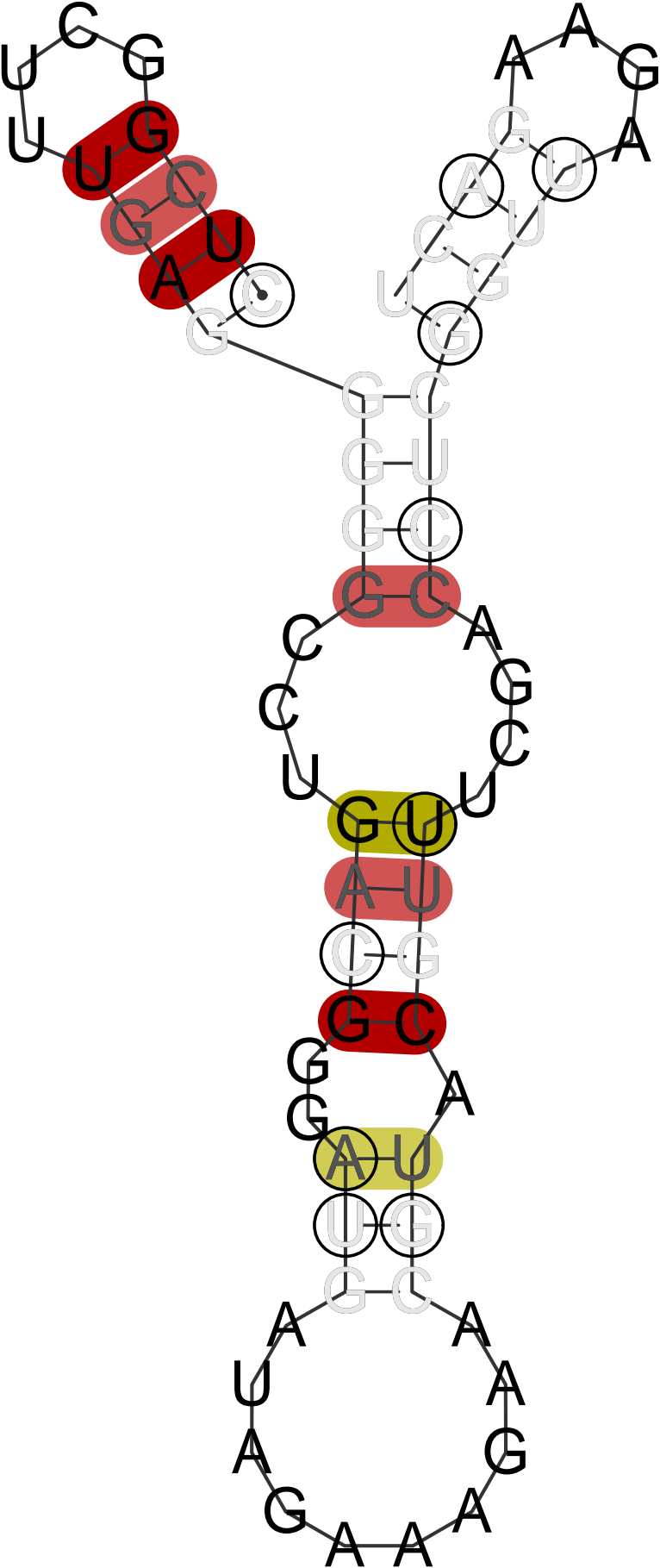

Supplement: S2 Fig — See the caption for S1 Fig for a description of the filename convention (save that the corresponding nucleotide locations in reference sequences are listed in S3 and S11 Tables), and an explanation of the RNAalifold options used and output. (ZIP) [file pcbi.1012009.s123.zip › H1N2-swine-raw-ranked-M1-alignment-7-72-refseq-4-69_revcomp_alirna_nogap.pdf]

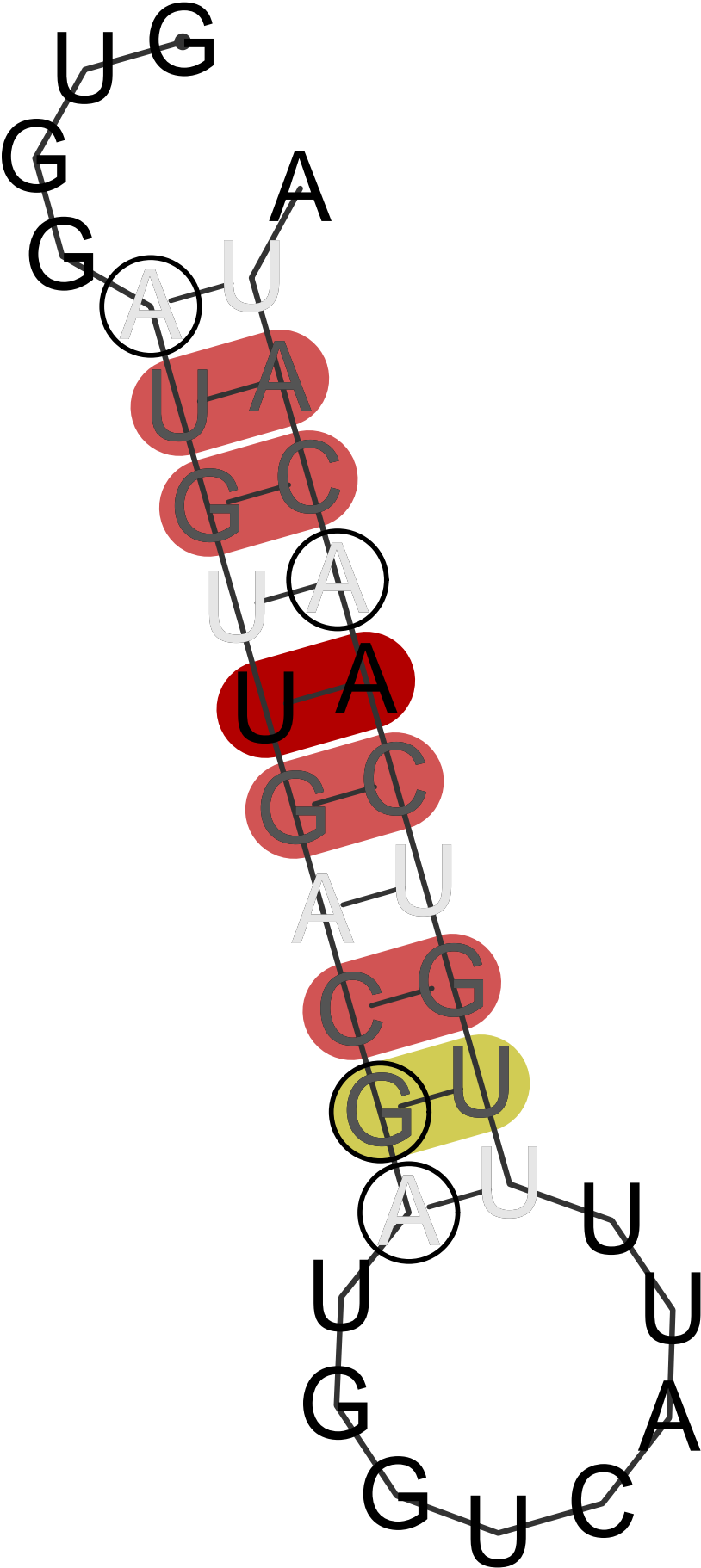

Supplement: S2 Fig — See the caption for S1 Fig for a description of the filename convention (save that the corresponding nucleotide locations in reference sequences are listed in S3 and S11 Tables), and an explanation of the RNAalifold options used and output. (ZIP) [file pcbi.1012009.s123.zip › H1N2-swine-raw-ranked-M2-alignment-253-285-refseq-938-970_alirna_nogap.pdf]

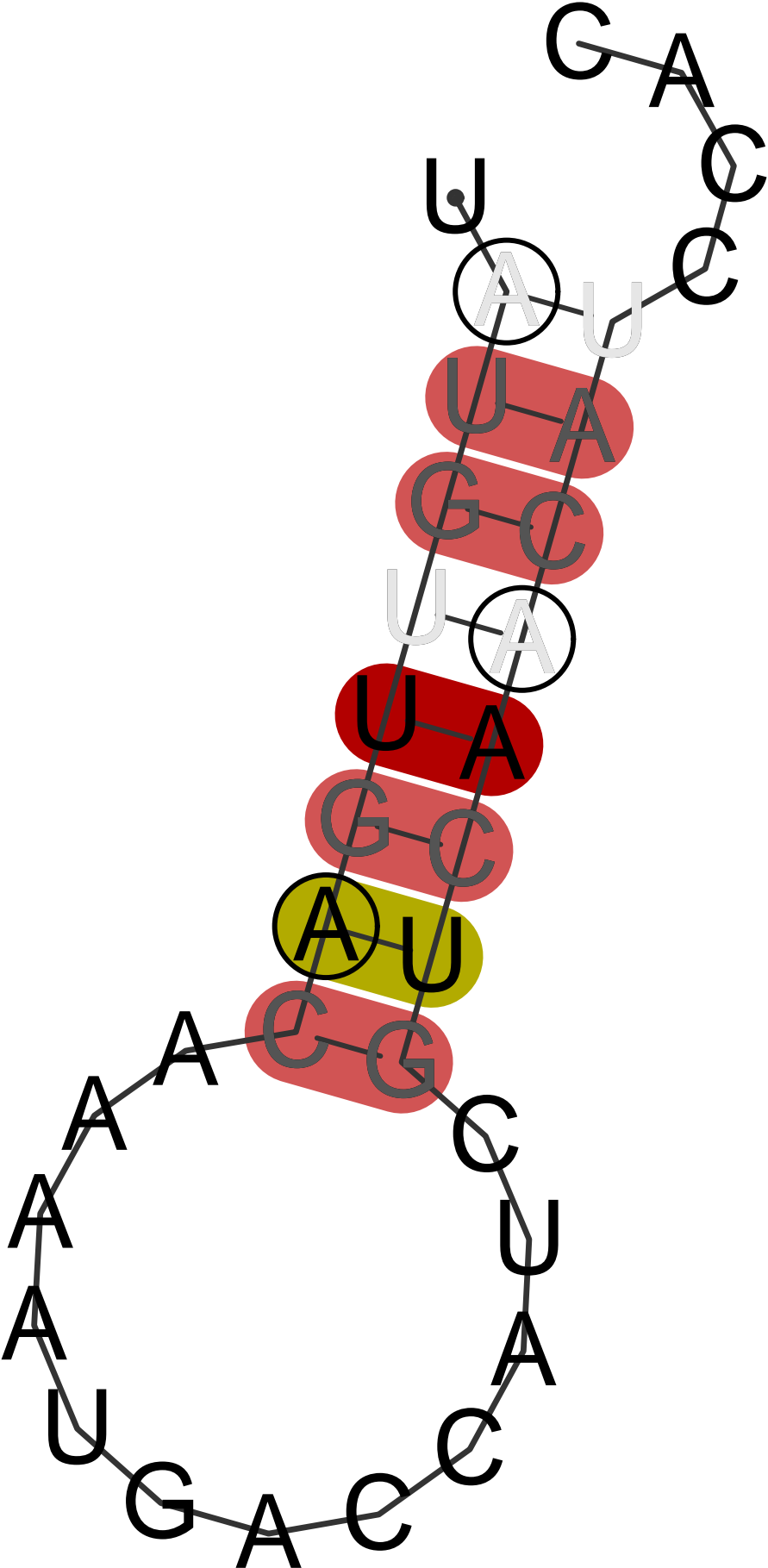

Supplement: S2 Fig — See the caption for S1 Fig for a description of the filename convention (save that the corresponding nucleotide locations in reference sequences are listed in S3 and S11 Tables), and an explanation of the RNAalifold options used and output. (ZIP) [file pcbi.1012009.s123.zip › H1N2-swine-raw-ranked-M2-alignment-253-285-refseq-938-970_revcomp_alirna_nogap.pdf]

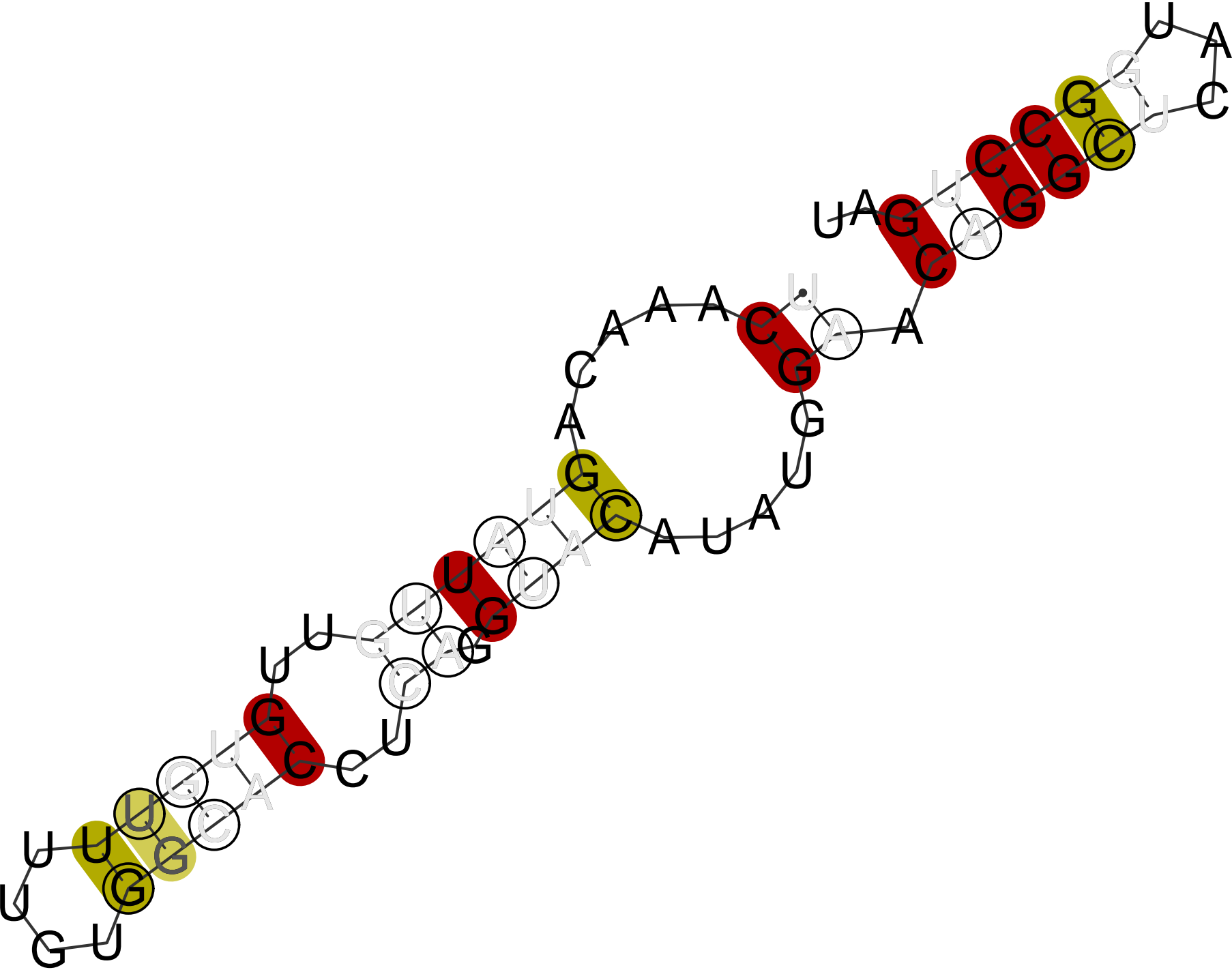

Supplement: S2 Fig — See the caption for S1 Fig for a description of the filename convention (save that the corresponding nucleotide locations in reference sequences are listed in S3 and S11 Tables), and an explanation of the RNAalifold options used and output. (ZIP) [file pcbi.1012009.s123.zip › H1N2-swine-raw-ranked-NA-alignment-1411-1473-refseq-1318-1380_alirna_nogap.pdf]

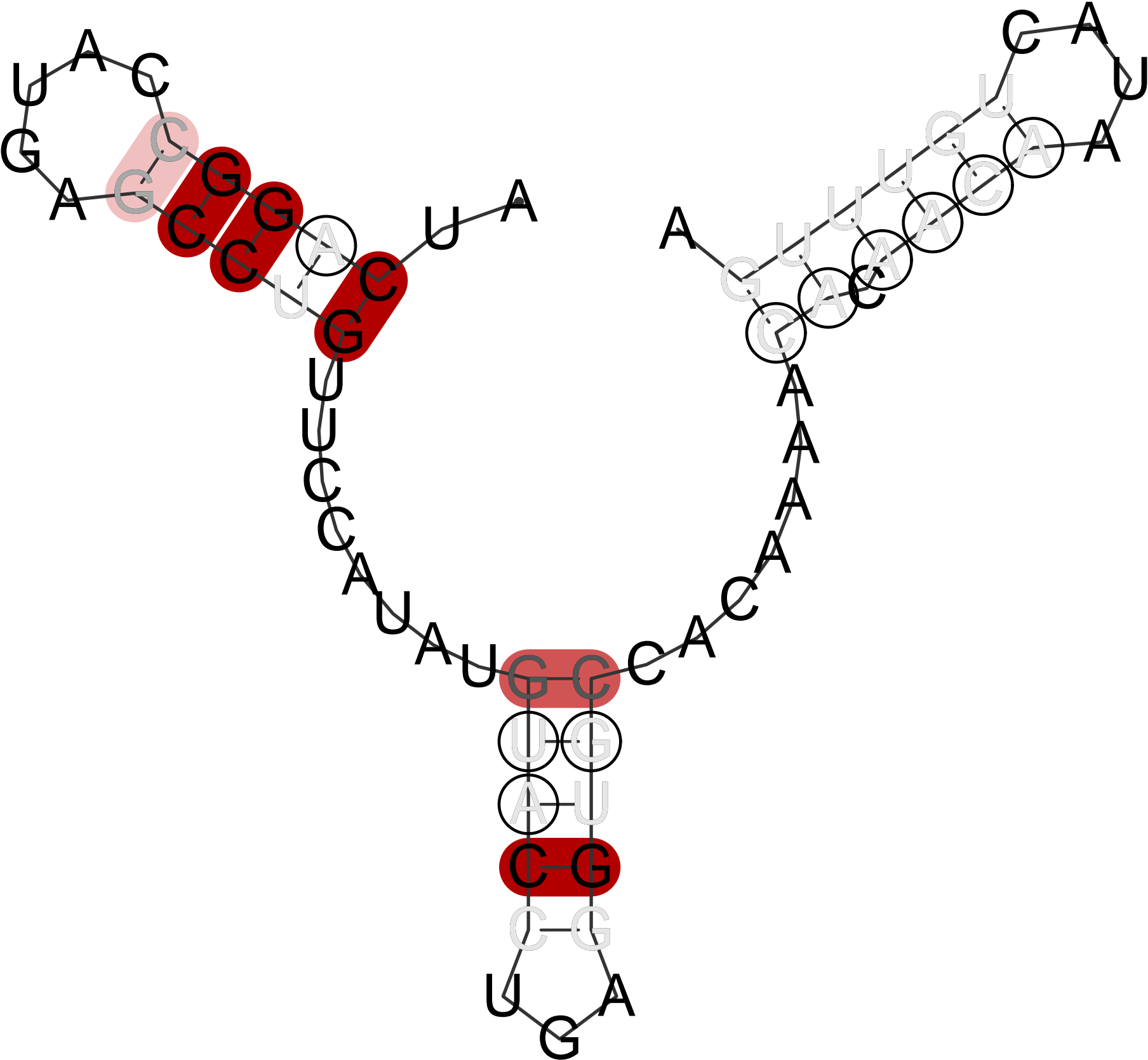

Supplement: S2 Fig — See the caption for S1 Fig for a description of the filename convention (save that the corresponding nucleotide locations in reference sequences are listed in S3 and S11 Tables), and an explanation of the RNAalifold options used and output. (ZIP) [file pcbi.1012009.s123.zip › H1N2-swine-raw-ranked-NA-alignment-1411-1473-refseq-1318-1380_revcomp_alirna_nogap.pdf]

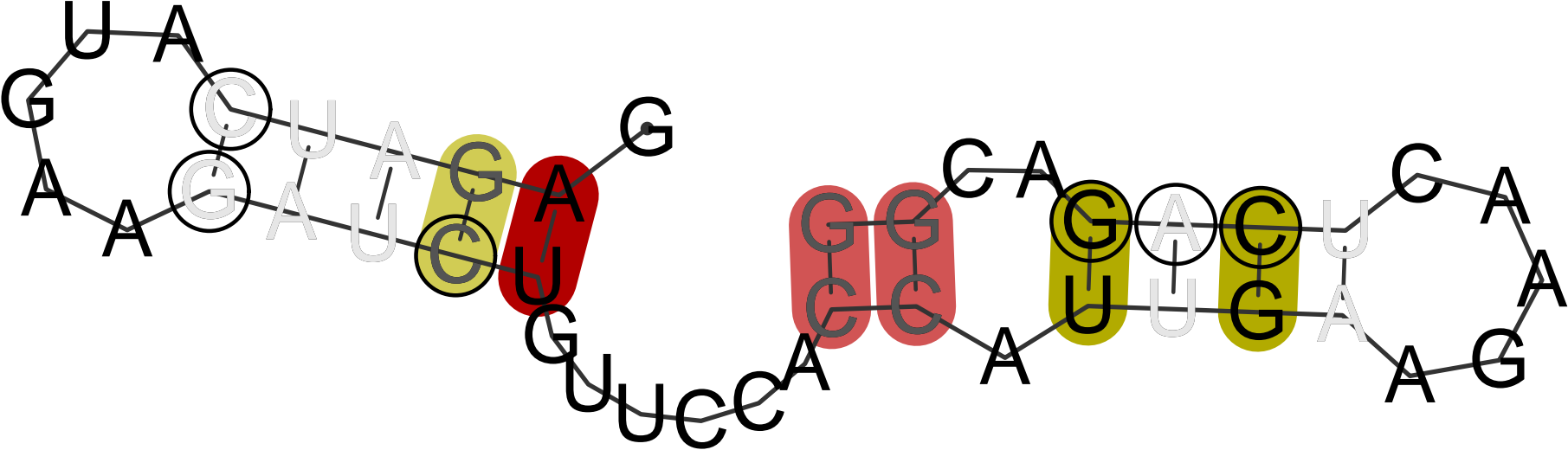

Supplement: S2 Fig — See the caption for S1 Fig for a description of the filename convention (save that the corresponding nucleotide locations in reference sequences are listed in S3 and S11 Tables), and an explanation of the RNAalifold options used and output. (ZIP) [file pcbi.1012009.s123.zip › H1N2-swine-raw-ranked-PB1-alignment-2224-2265-refseq-2224-2265_alirna_nogap.pdf]

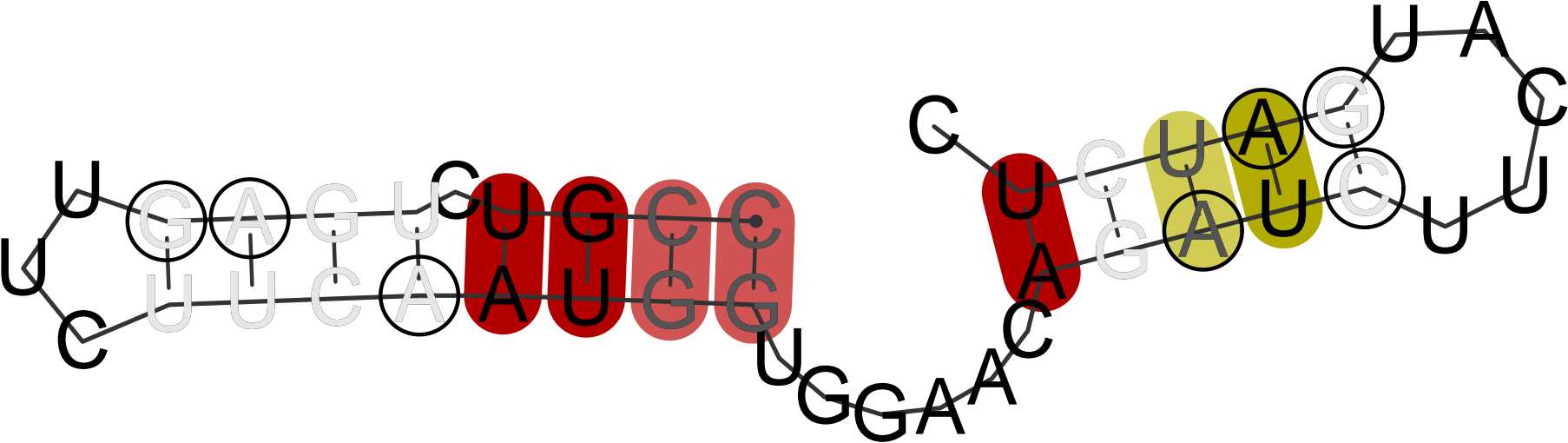

Supplement: S2 Fig — See the caption for S1 Fig for a description of the filename convention (save that the corresponding nucleotide locations in reference sequences are listed in S3 and S11 Tables), and an explanation of the RNAalifold options used and output. (ZIP) [file pcbi.1012009.s123.zip › H1N2-swine-raw-ranked-PB1-alignment-2224-2265-refseq-2224-2265_revcomp_alirna_nogap.pdf]

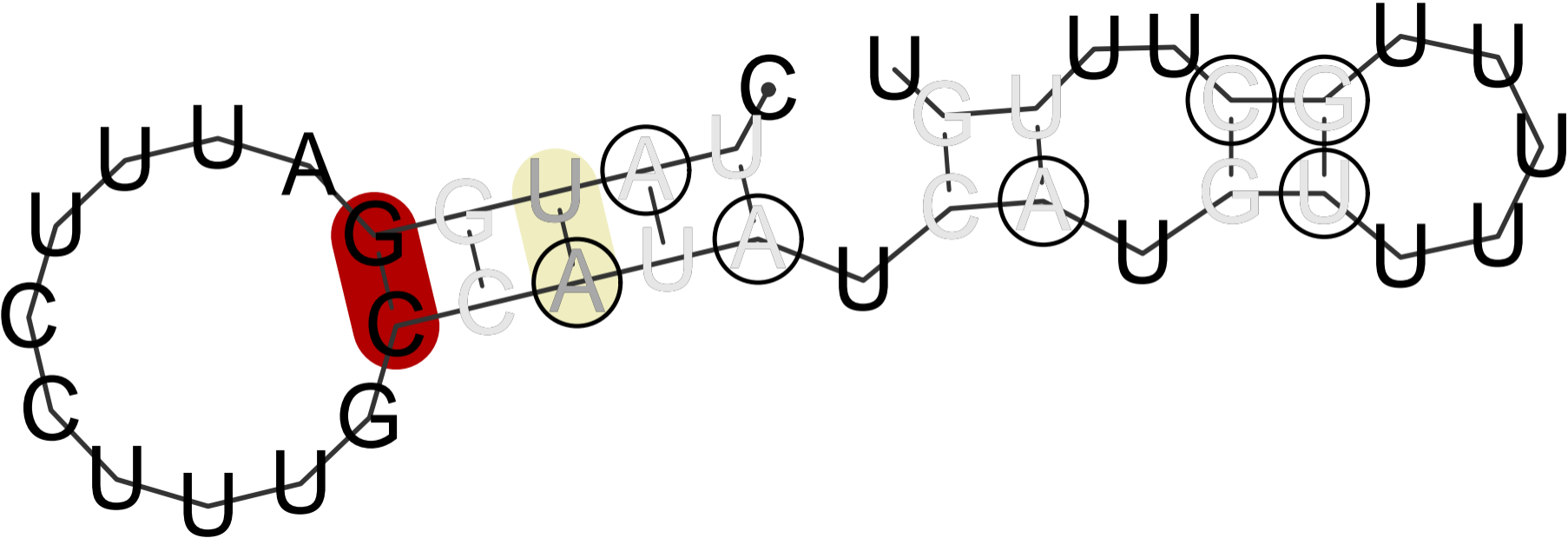

Supplement: S3 Fig — See the caption for S1 Fig for a description of the filename convention (save that the corresponding nucleotide locations in reference sequences are listed in S4 and S12 Tables), and an explanation of the RNAalifold options used and output. (ZIP) [file pcbi.1012009.s124.zip › H3N2-human-ranked-HA-alignment-1642-1680-refseq-1623-1661-representative-sequences-only_alirna_nogap.pdf]

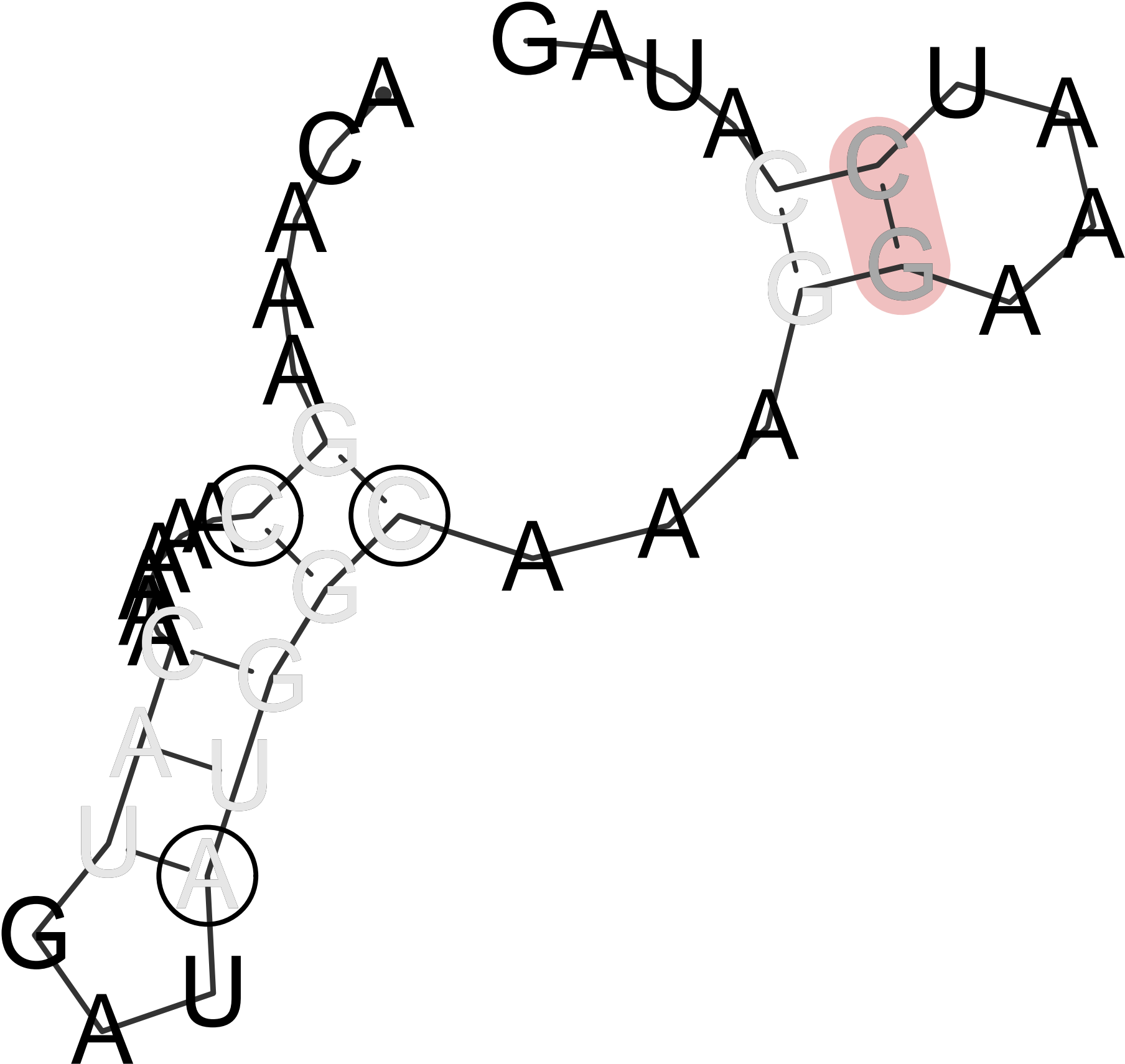

Supplement: S3 Fig — See the caption for S1 Fig for a description of the filename convention (save that the corresponding nucleotide locations in reference sequences are listed in S4 and S12 Tables), and an explanation of the RNAalifold options used and output. (ZIP) [file pcbi.1012009.s124.zip › H3N2-human-ranked-HA-alignment-1642-1680-refseq-1623-1661-representative-sequences-only_revcomp_alirna_nogap.pdf]

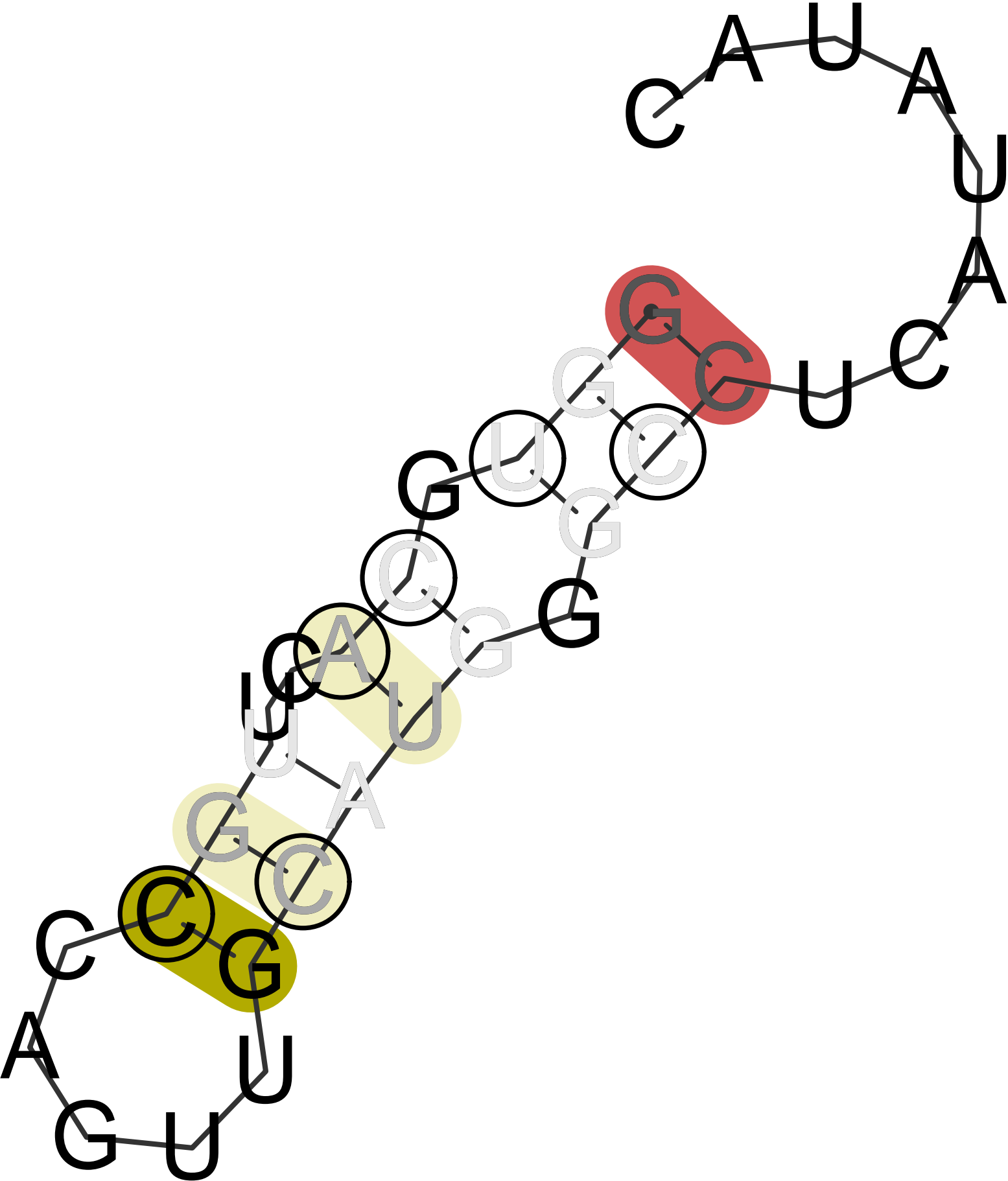

Supplement: S3 Fig — See the caption for S1 Fig for a description of the filename convention (save that the corresponding nucleotide locations in reference sequences are listed in S4 and S12 Tables), and an explanation of the RNAalifold options used and output. (ZIP) [file pcbi.1012009.s124.zip › H3N2-human-ranked-M1-alignment-364-396-refseq-389-421-representative-sequences-only_alirna_nogap.pdf]

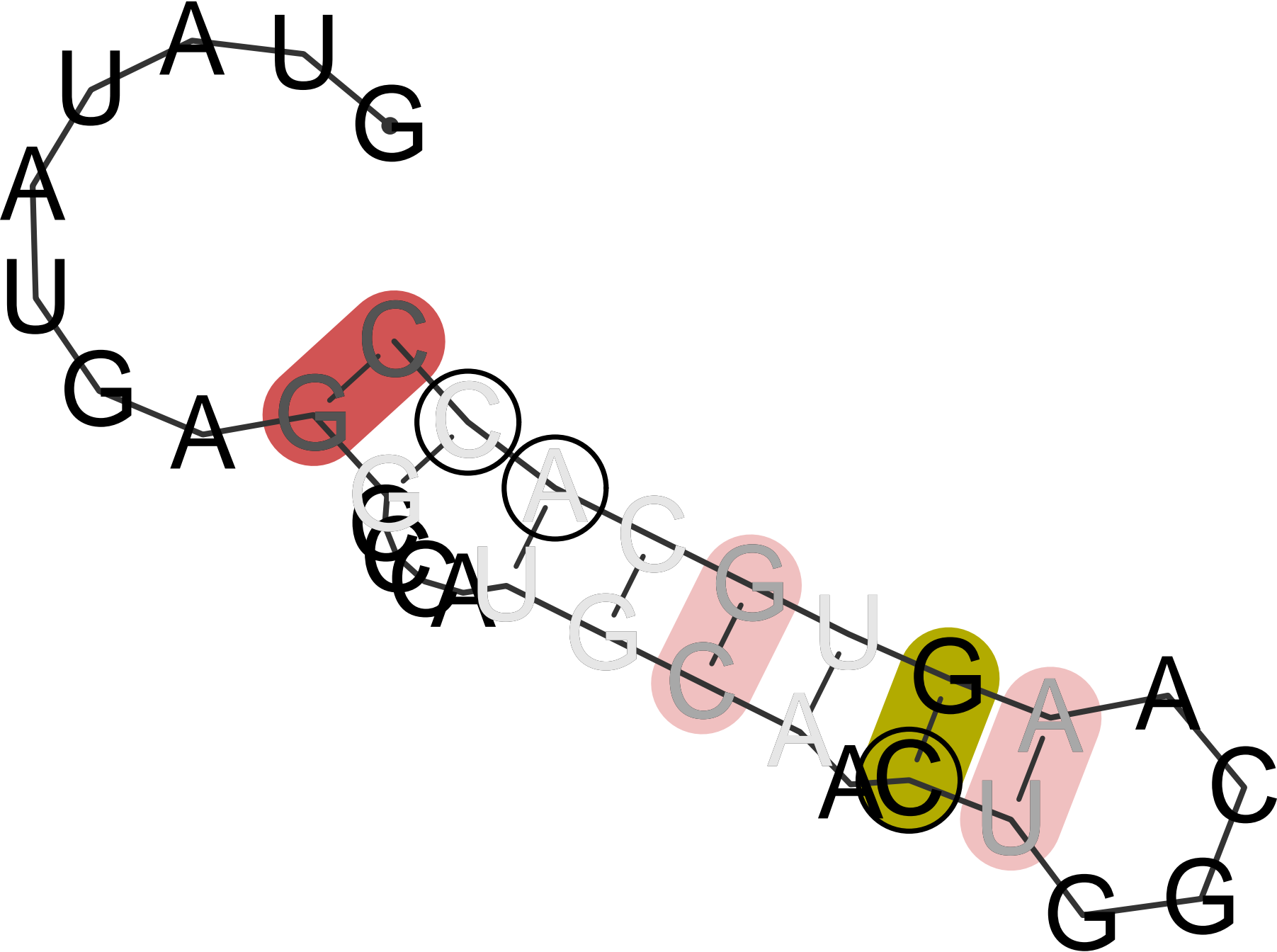

Supplement: S3 Fig — See the caption for S1 Fig for a description of the filename convention (save that the corresponding nucleotide locations in reference sequences are listed in S4 and S12 Tables), and an explanation of the RNAalifold options used and output. (ZIP) [file pcbi.1012009.s124.zip › H3N2-human-ranked-M1-alignment-364-396-refseq-389-421-representative-sequences-only_revcomp_alirna_nogap.pdf]

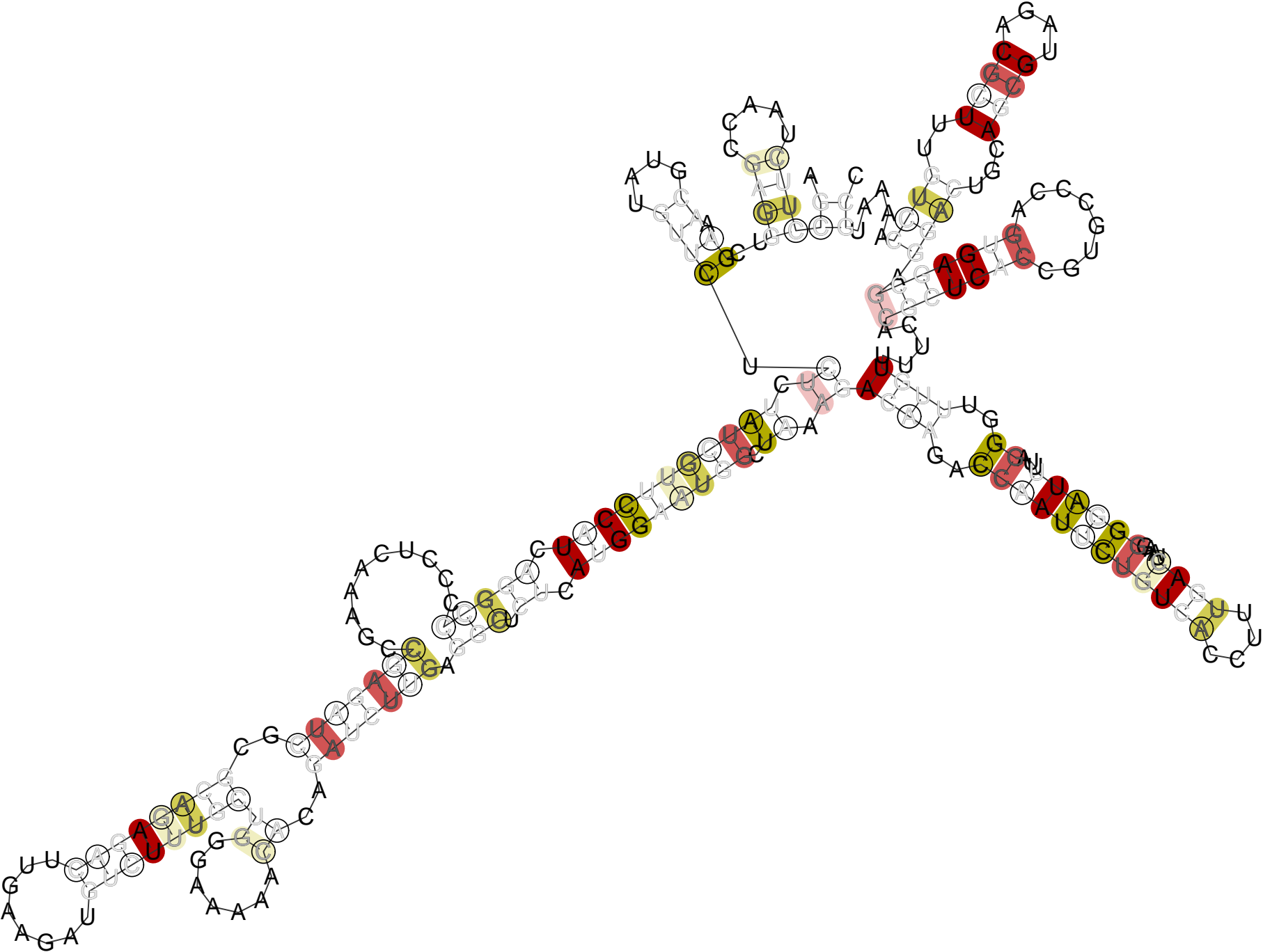

Supplement: S3 Fig — See the caption for S1 Fig for a description of the filename convention (save that the corresponding nucleotide locations in reference sequences are listed in S4 and S12 Tables), and an explanation of the RNAalifold options used and output. (ZIP) [file pcbi.1012009.s124.zip › H3N2-human-ranked-M1-alignment-4-249-refseq-29-274-representative-sequences-only_alirna_nogap.pdf]

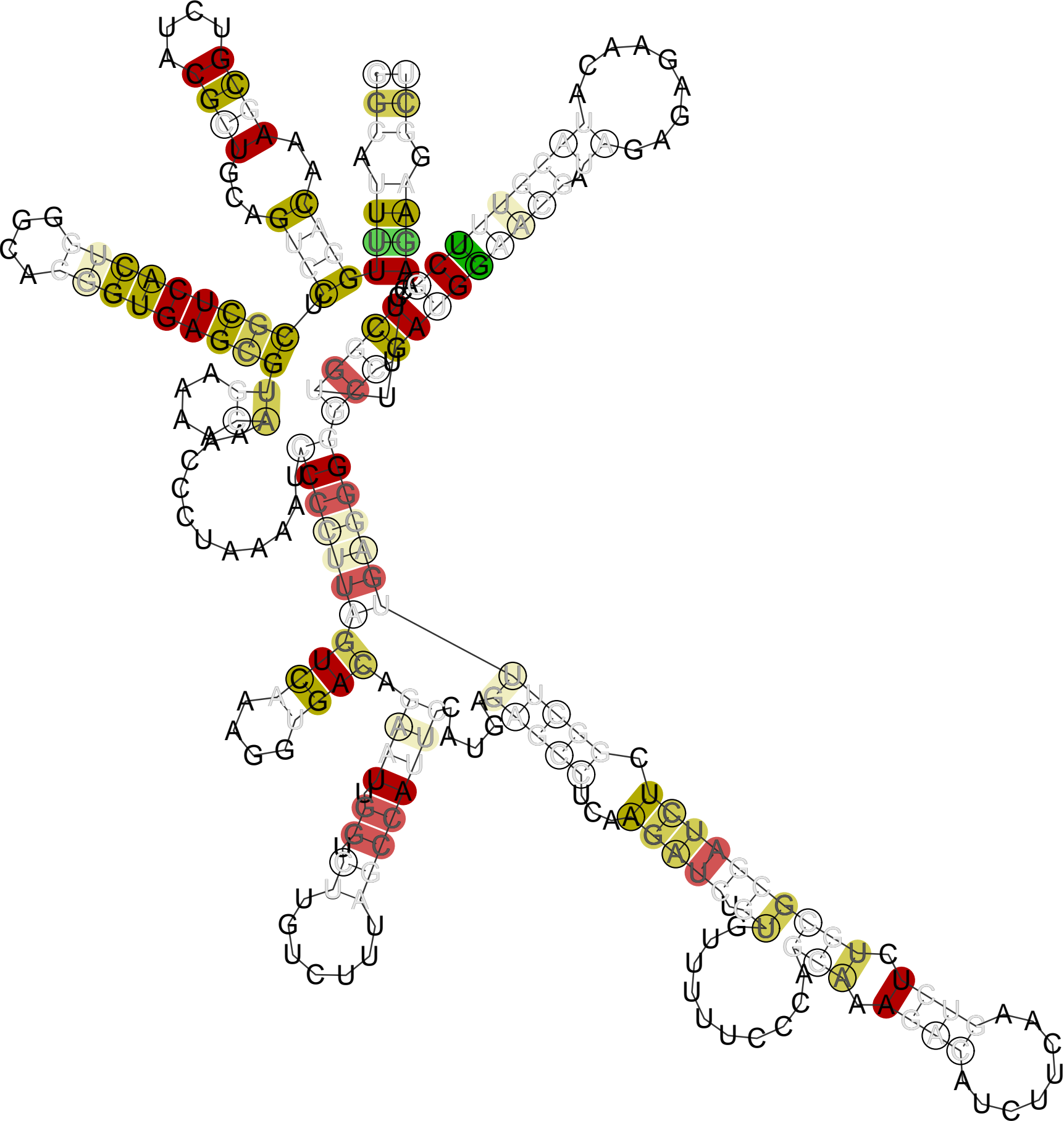

Supplement: S3 Fig — See the caption for S1 Fig for a description of the filename convention (save that the corresponding nucleotide locations in reference sequences are listed in S4 and S12 Tables), and an explanation of the RNAalifold options used and output. (ZIP) [file pcbi.1012009.s124.zip › H3N2-human-ranked-M1-alignment-4-249-refseq-29-274-representative-sequences-only_revcomp_alirna_nogap.pdf]

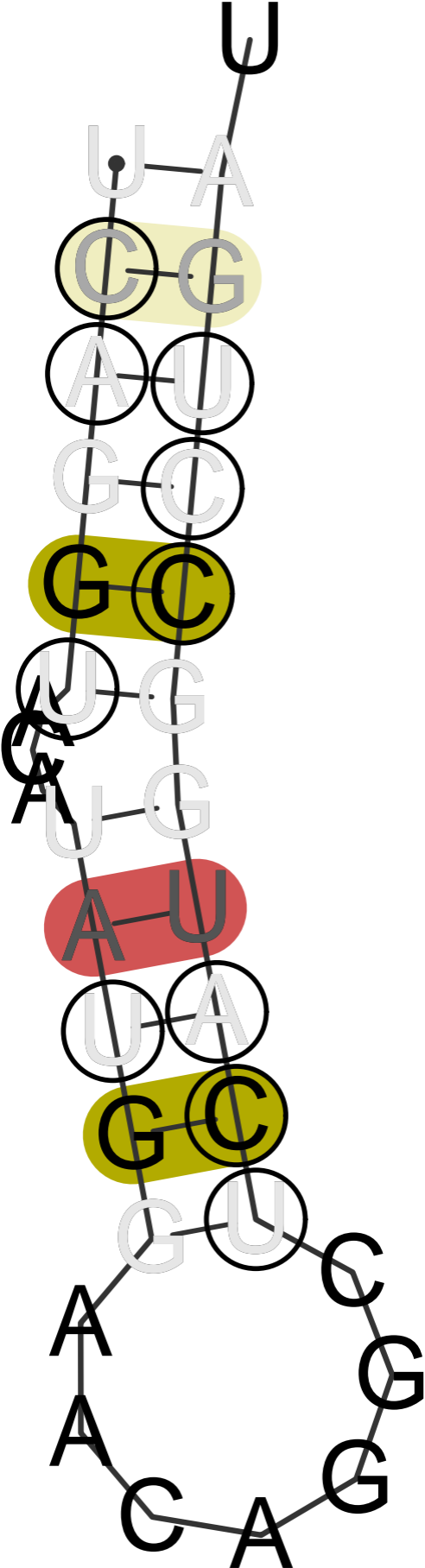

Supplement: S3 Fig — See the caption for S1 Fig for a description of the filename convention (save that the corresponding nucleotide locations in reference sequences are listed in S4 and S12 Tables), and an explanation of the RNAalifold options used and output. (ZIP) [file pcbi.1012009.s124.zip › H3N2-human-ranked-NA-alignment-1510-1560-refseq-1367-1399-representative-sequences-only_alirna_nogap.pdf]

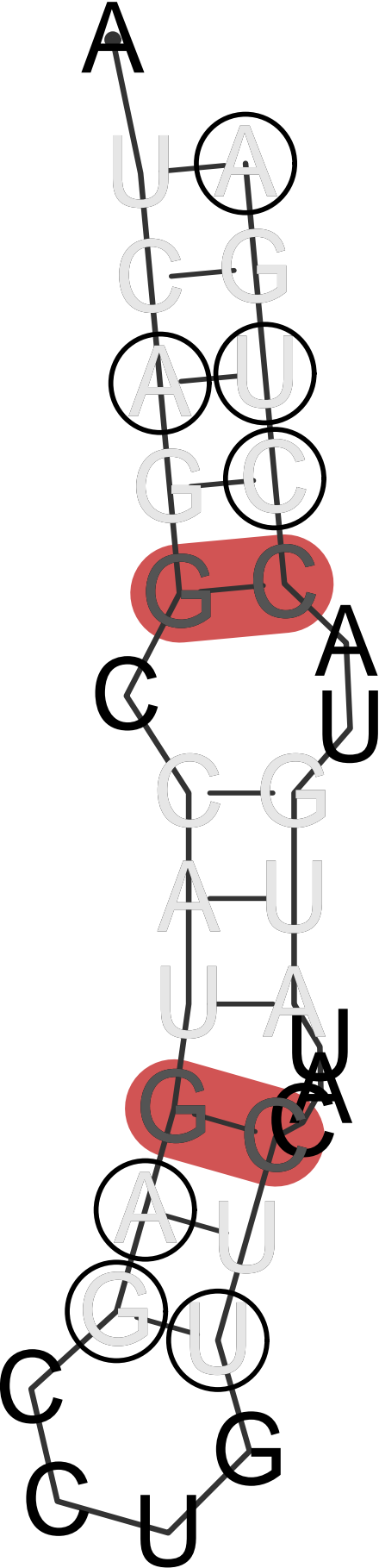

Supplement: S3 Fig — See the caption for S1 Fig for a description of the filename convention (save that the corresponding nucleotide locations in reference sequences are listed in S4 and S12 Tables), and an explanation of the RNAalifold options used and output. (ZIP) [file pcbi.1012009.s124.zip › H3N2-human-ranked-NA-alignment-1510-1560-refseq-1367-1399-representative-sequences-only_revcomp_alirna_nogap.pdf]

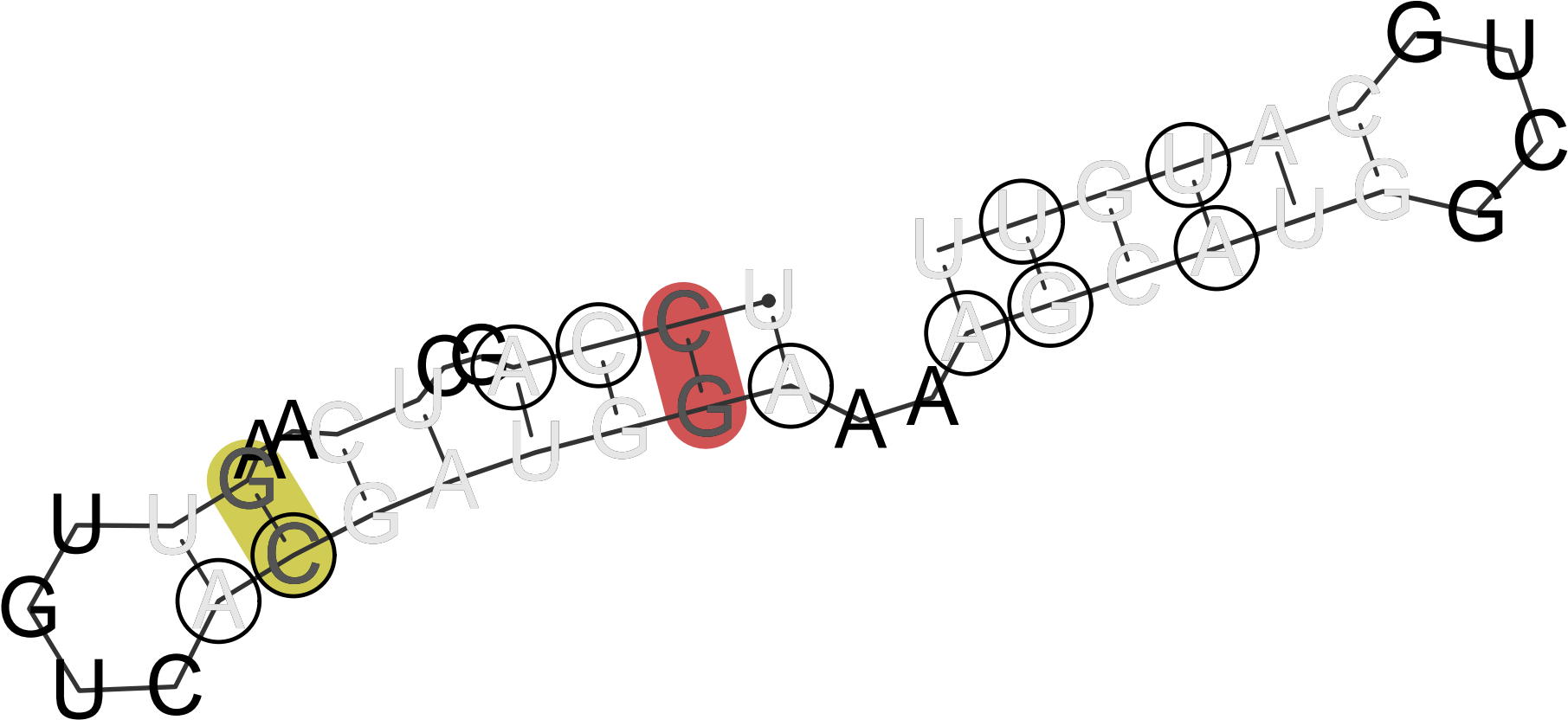

Supplement: S3 Fig — See the caption for S1 Fig for a description of the filename convention (save that the corresponding nucleotide locations in reference sequences are listed in S4 and S12 Tables), and an explanation of the RNAalifold options used and output. (ZIP) [file pcbi.1012009.s124.zip › H3N2-human-ranked-NA-alignment-658-714-refseq-554-595-representative-sequences-only_alirna_nogap.pdf]

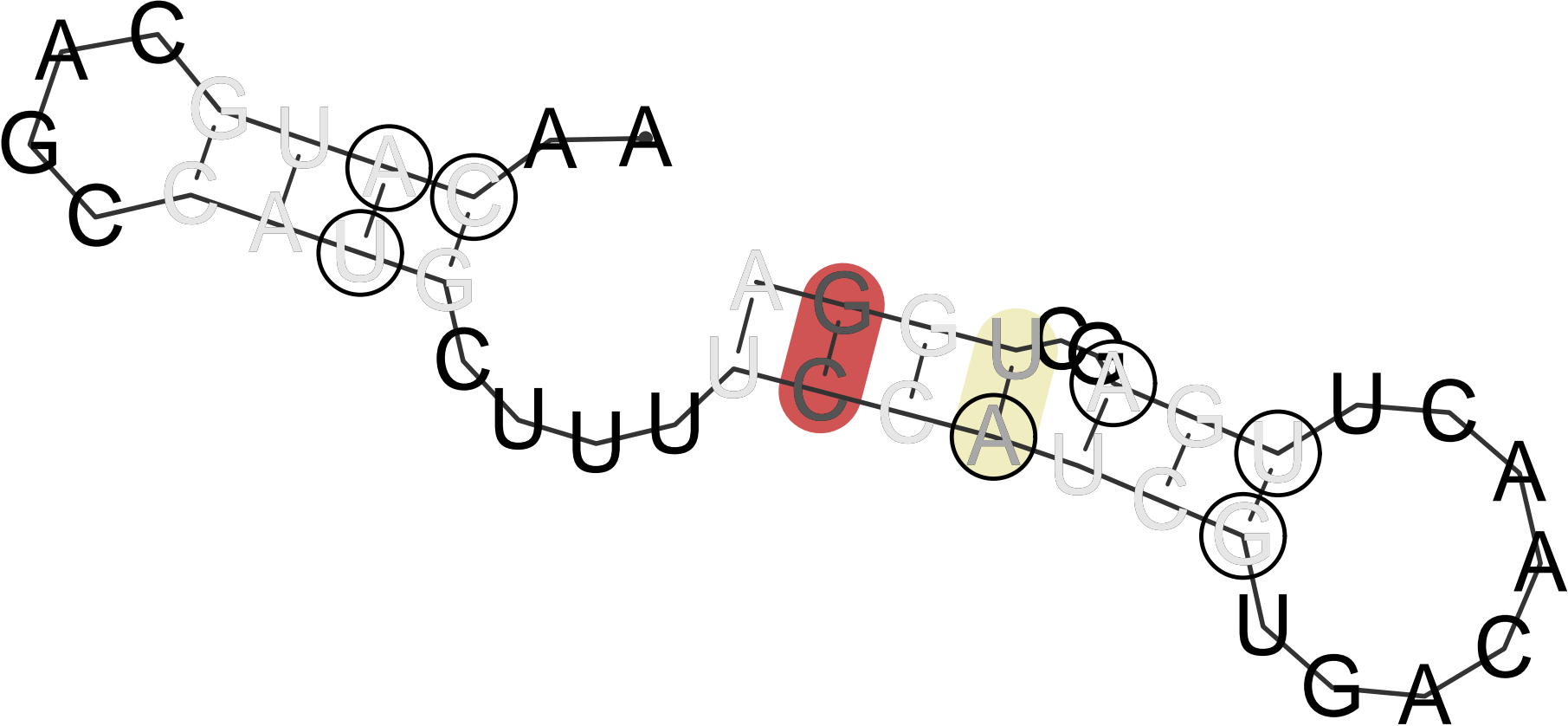

Supplement: S3 Fig — See the caption for S1 Fig for a description of the filename convention (save that the corresponding nucleotide locations in reference sequences are listed in S4 and S12 Tables), and an explanation of the RNAalifold options used and output. (ZIP) [file pcbi.1012009.s124.zip › H3N2-human-ranked-NA-alignment-658-714-refseq-554-595-representative-sequences-only_revcomp_alirna_nogap.pdf]

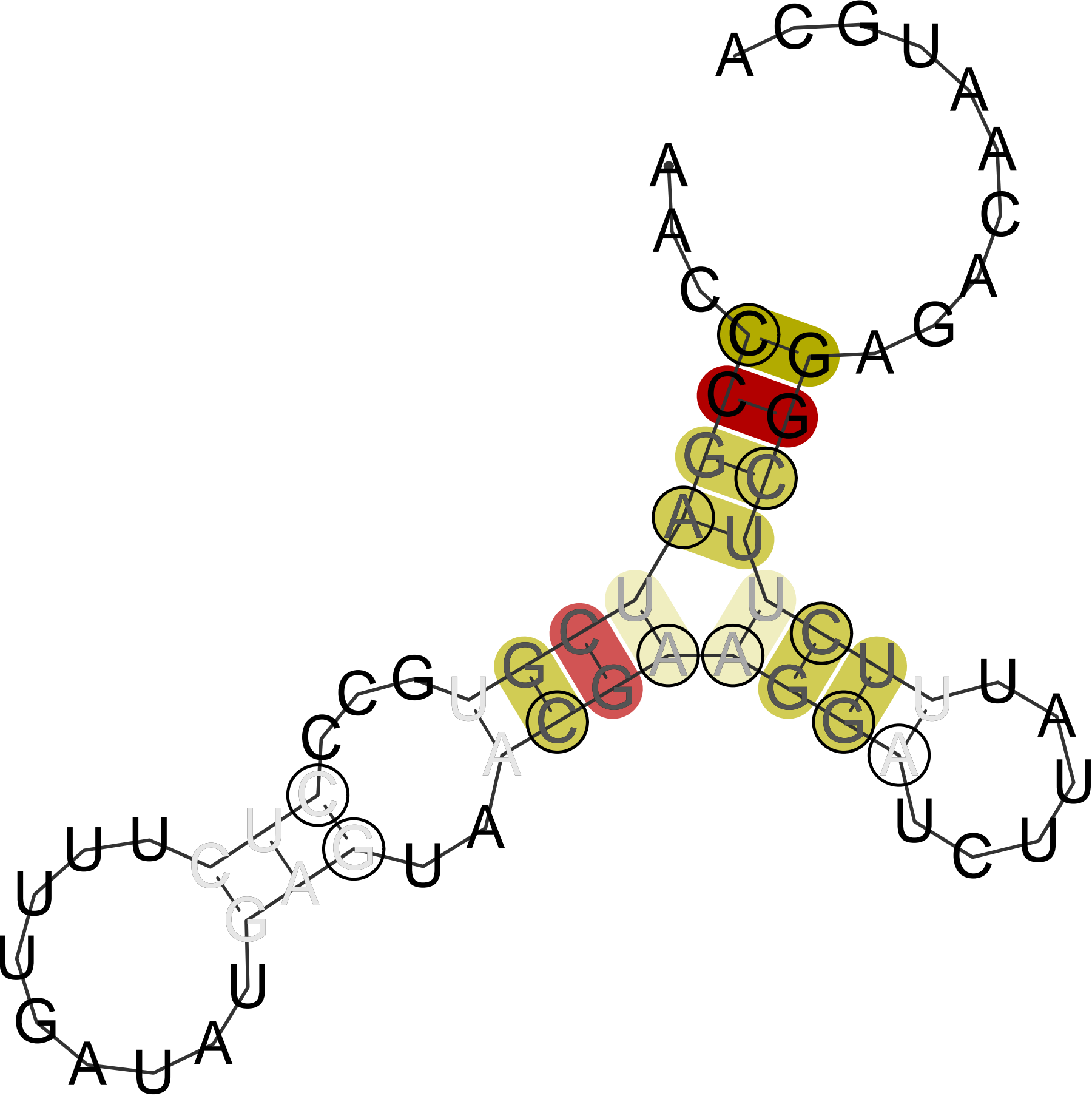

Supplement: S3 Fig — See the caption for S1 Fig for a description of the filename convention (save that the corresponding nucleotide locations in reference sequences are listed in S4 and S12 Tables), and an explanation of the RNAalifold options used and output. (ZIP) [file pcbi.1012009.s124.zip › H3N2-human-ranked-NP-alignment-1420-1482-refseq-1462-1524-representative-sequences-only_alirna_nogap.pdf]

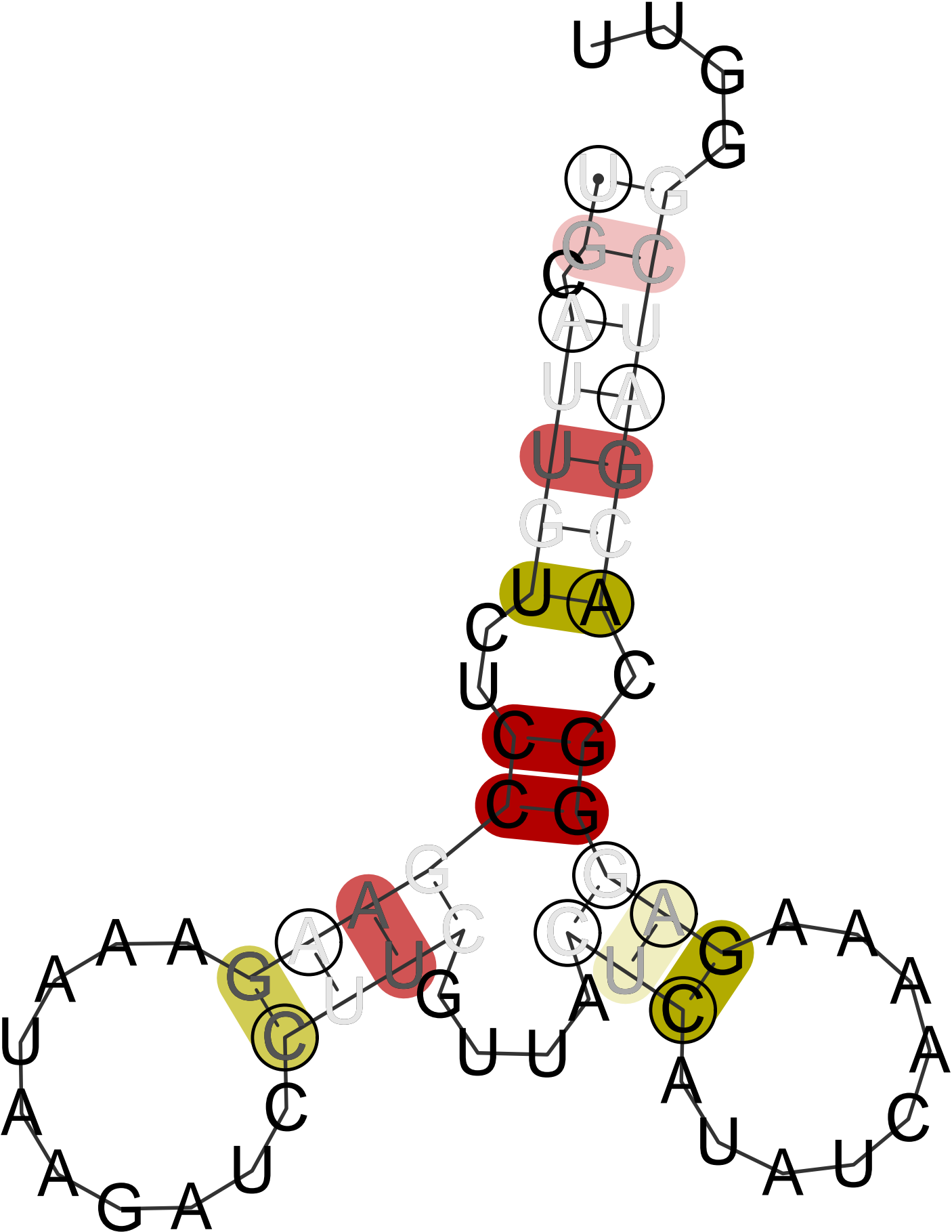

Supplement: S3 Fig — See the caption for S1 Fig for a description of the filename convention (save that the corresponding nucleotide locations in reference sequences are listed in S4 and S12 Tables), and an explanation of the RNAalifold options used and output. (ZIP) [file pcbi.1012009.s124.zip › H3N2-human-ranked-NP-alignment-1420-1482-refseq-1462-1524-representative-sequences-only_revcomp_alirna_nogap.pdf]

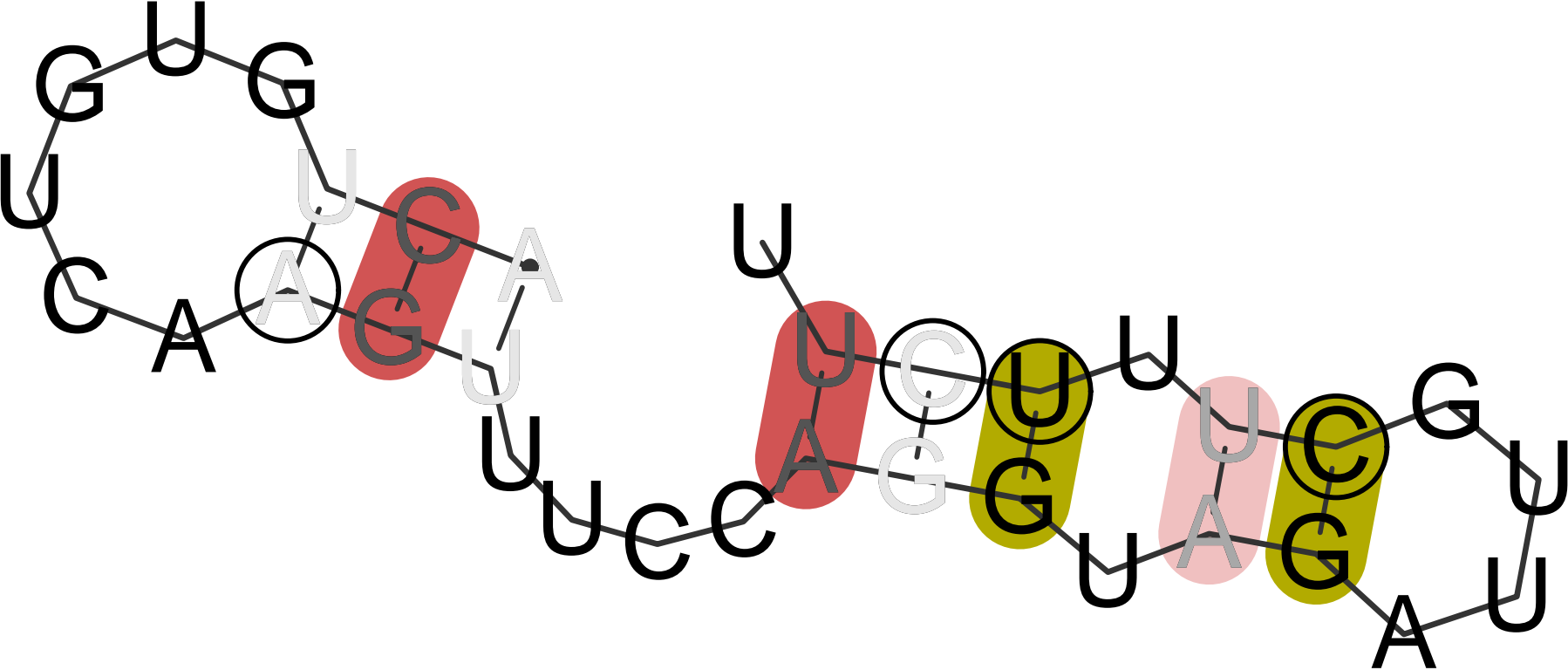

Supplement: S3 Fig — See the caption for S1 Fig for a description of the filename convention (save that the corresponding nucleotide locations in reference sequences are listed in S4 and S12 Tables), and an explanation of the RNAalifold options used and output. (ZIP) [file pcbi.1012009.s124.zip › H3N2-human-ranked-NS1-alignment-16-48-refseq-39-71-representative-sequences-only_alirna_nogap.pdf]

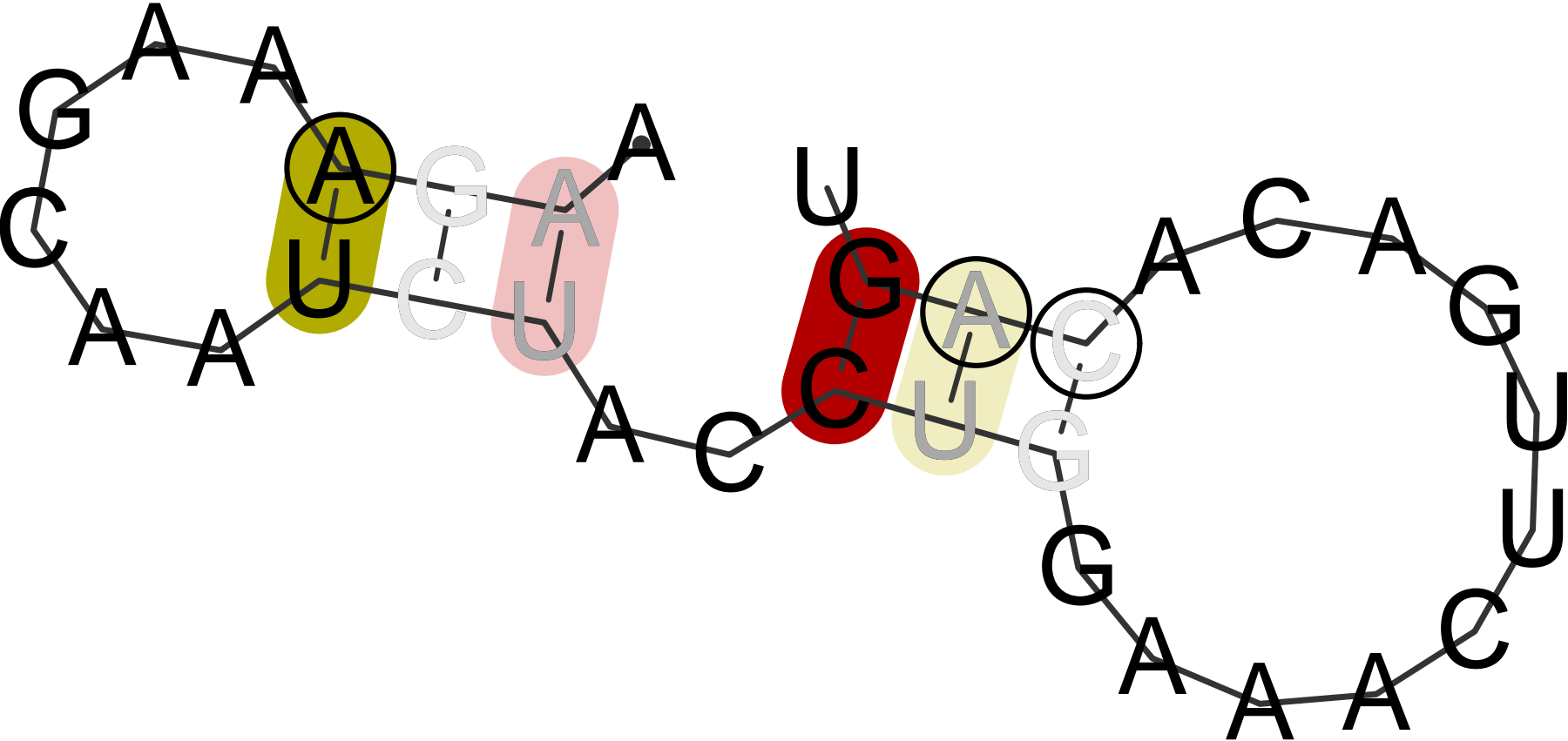

Supplement: S3 Fig — See the caption for S1 Fig for a description of the filename convention (save that the corresponding nucleotide locations in reference sequences are listed in S4 and S12 Tables), and an explanation of the RNAalifold options used and output. (ZIP) [file pcbi.1012009.s124.zip › H3N2-human-ranked-NS1-alignment-16-48-refseq-39-71-representative-sequences-only_revcomp_alirna_nogap.pdf]

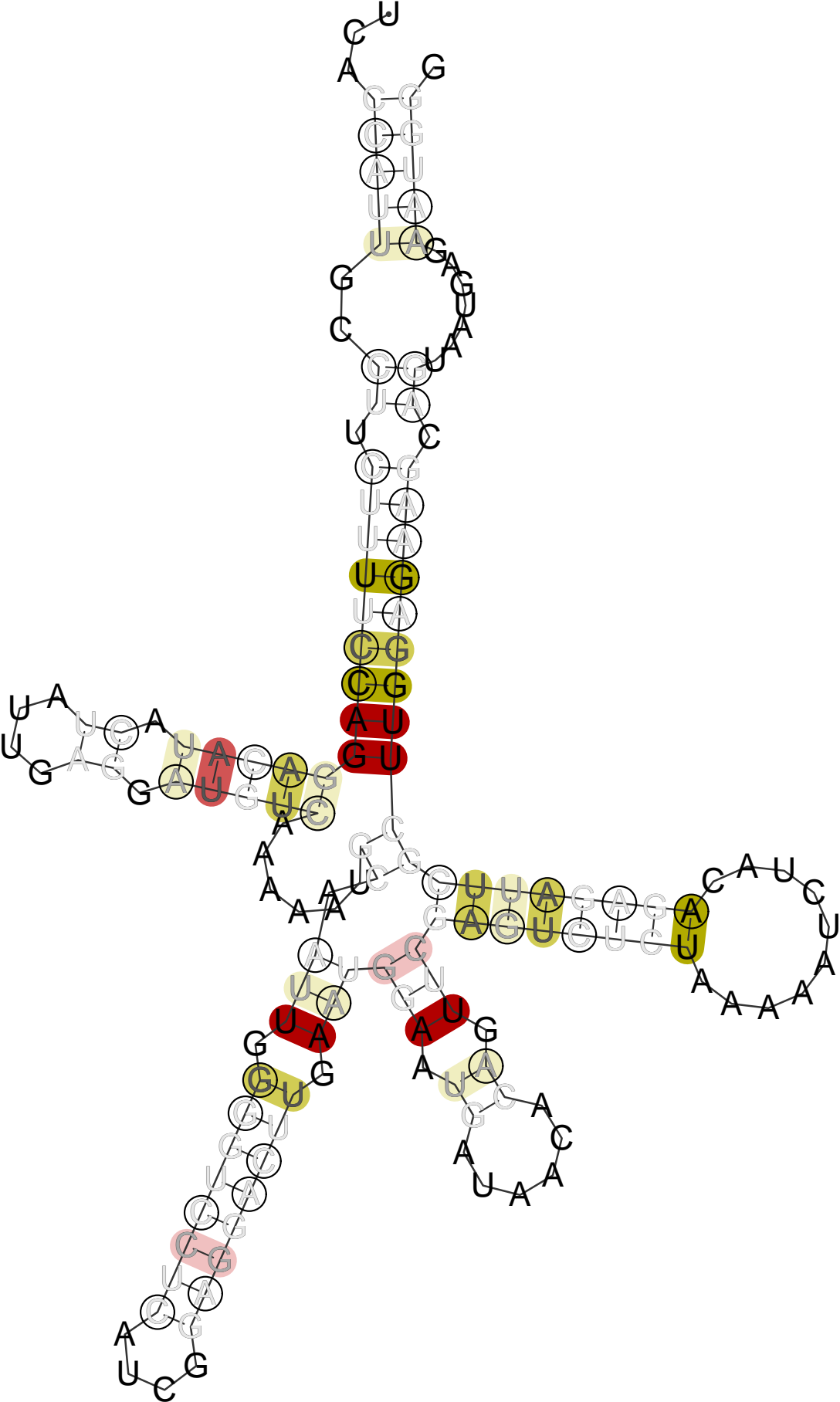

Supplement: S3 Fig — See the caption for S1 Fig for a description of the filename convention (save that the corresponding nucleotide locations in reference sequences are listed in S4 and S12 Tables), and an explanation of the RNAalifold options used and output. (ZIP) [file pcbi.1012009.s124.zip › H3N2-human-ranked-NS1-alignment-490-639-refseq-507-656-representative-sequences-only_alirna_nogap.pdf]

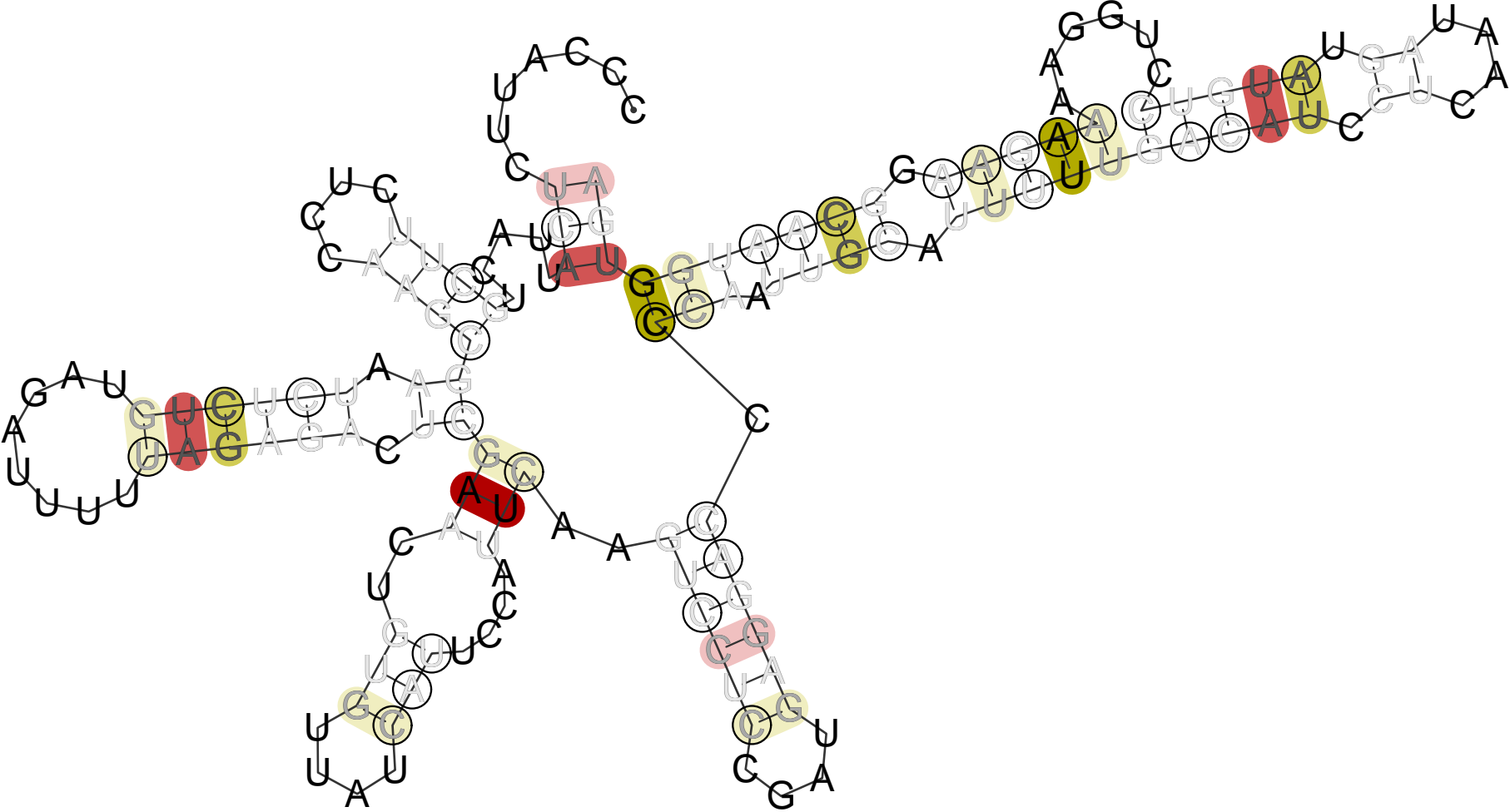

Supplement: S3 Fig — See the caption for S1 Fig for a description of the filename convention (save that the corresponding nucleotide locations in reference sequences are listed in S4 and S12 Tables), and an explanation of the RNAalifold options used and output. (ZIP) [file pcbi.1012009.s124.zip › H3N2-human-ranked-NS1-alignment-490-639-refseq-507-656-representative-sequences-only_revcomp_alirna_nogap.pdf]

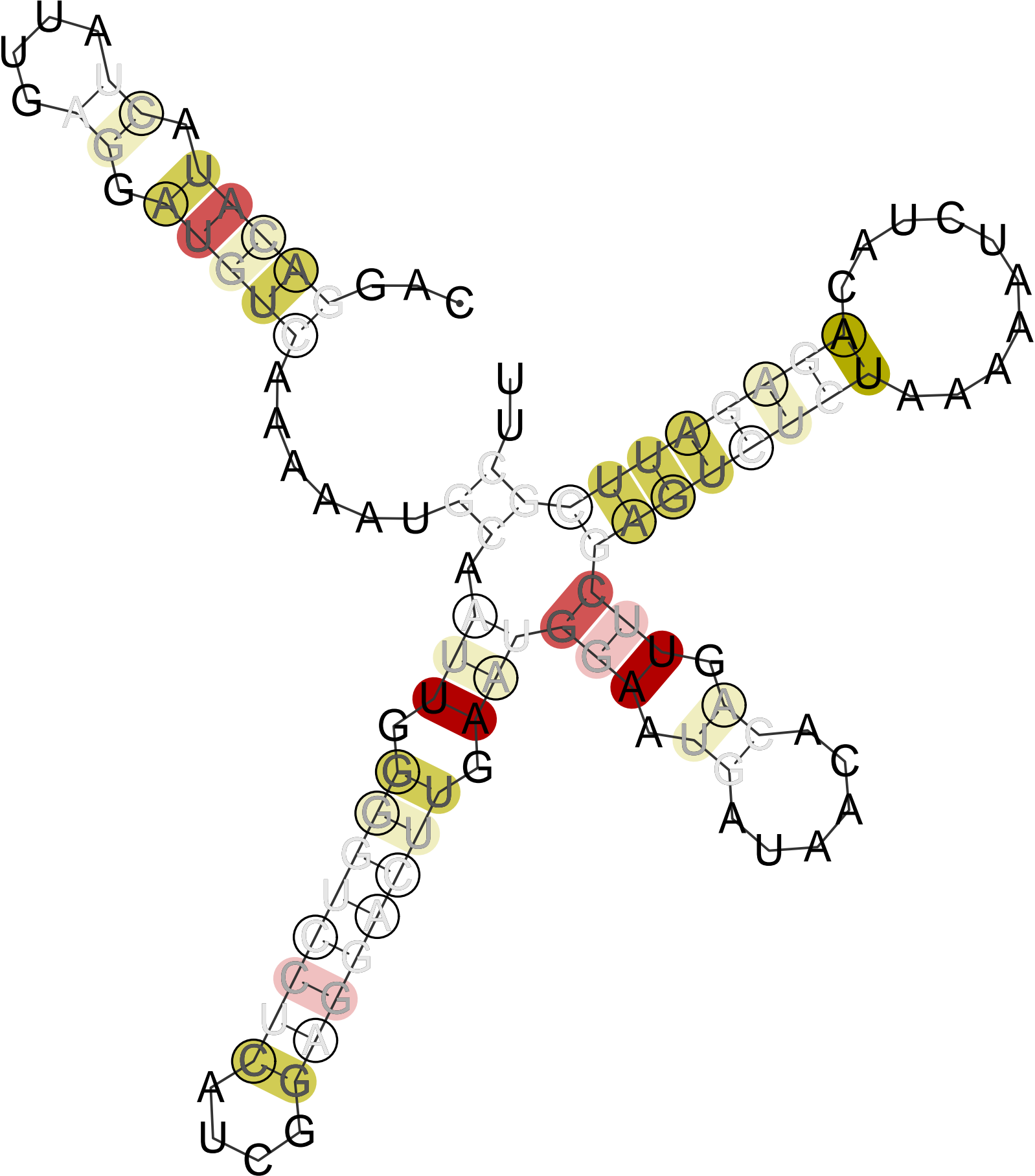

Supplement: S3 Fig — See the caption for S1 Fig for a description of the filename convention (save that the corresponding nucleotide locations in reference sequences are listed in S4 and S12 Tables), and an explanation of the RNAalifold options used and output. (ZIP) [file pcbi.1012009.s124.zip › H3N2-human-ranked-NS2-alignment-31-138-refseq-54-56-529-633-representative-sequences-only_alirna_nogap.pdf]

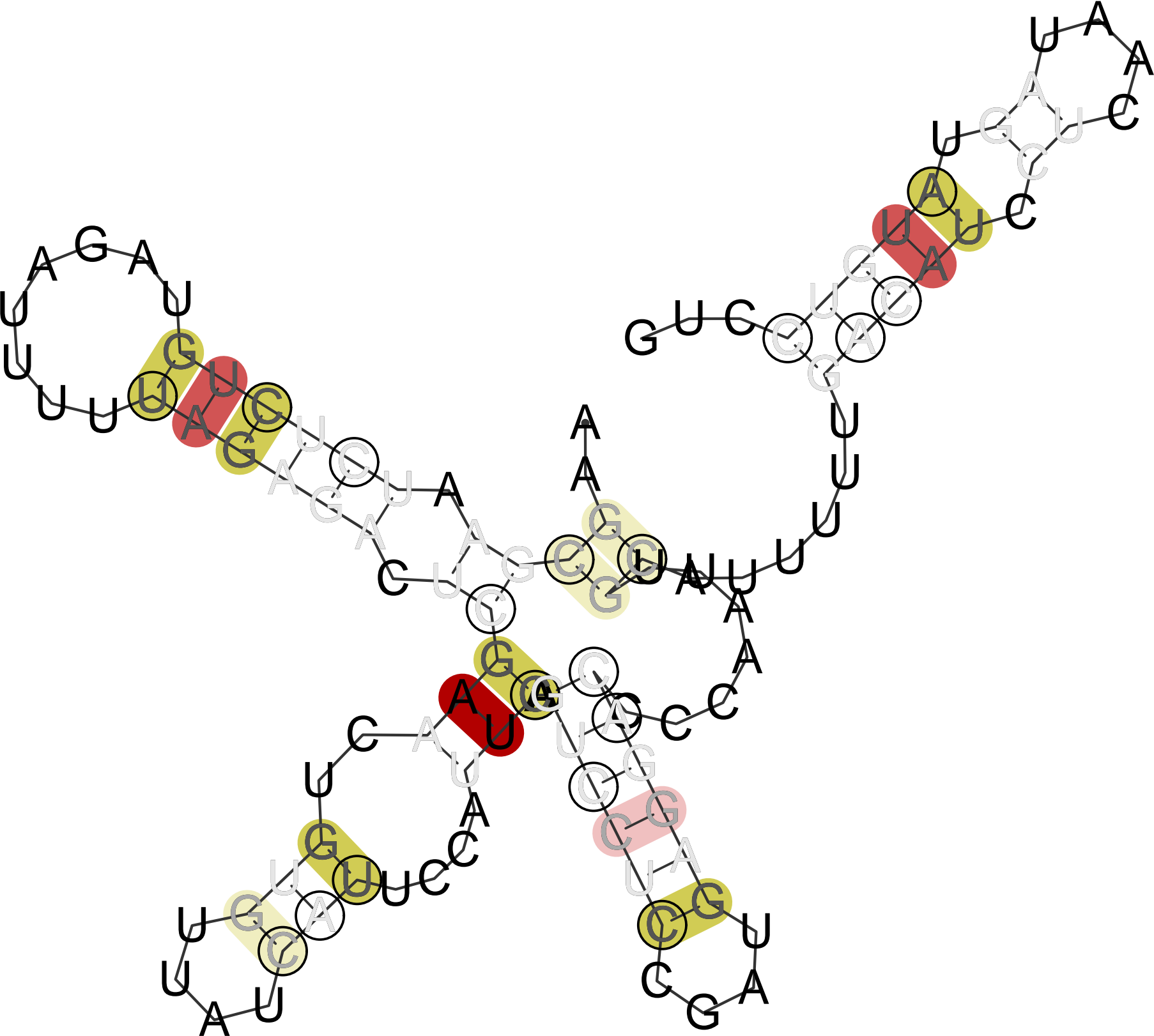

Supplement: S3 Fig — See the caption for S1 Fig for a description of the filename convention (save that the corresponding nucleotide locations in reference sequences are listed in S4 and S12 Tables), and an explanation of the RNAalifold options used and output. (ZIP) [file pcbi.1012009.s124.zip › H3N2-human-ranked-NS2-alignment-31-138-refseq-54-56-529-633-representative-sequences-only_revcomp_alirna_nogap.pdf]

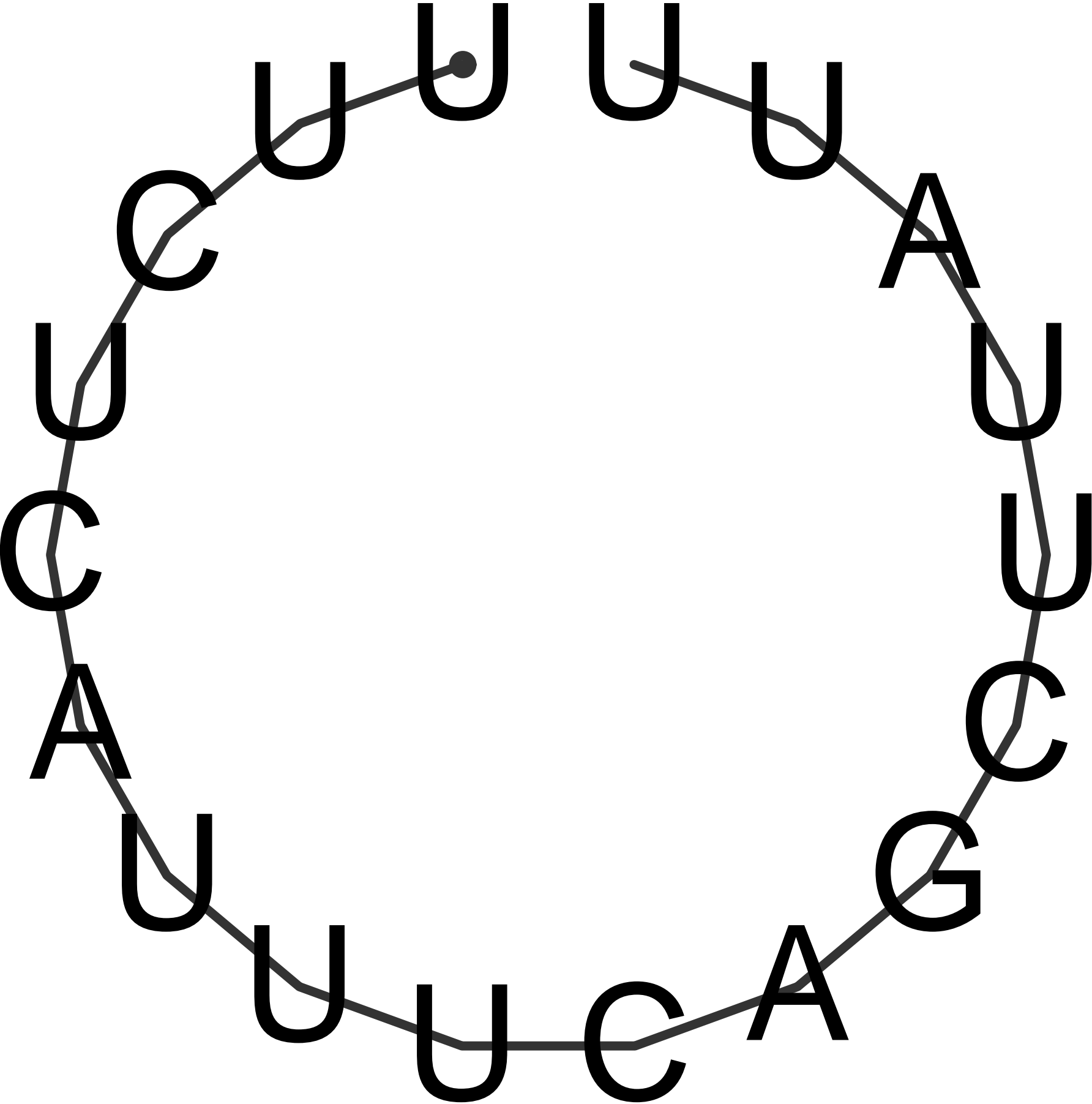

Supplement: S3 Fig — See the caption for S1 Fig for a description of the filename convention (save that the corresponding nucleotide locations in reference sequences are listed in S4 and S12 Tables), and an explanation of the RNAalifold options used and output. (ZIP) [file pcbi.1012009.s124.zip › H3N2-human-ranked-NS2-alignment-349-366-refseq-844-861-representative-sequences-only_alirna_nogap.pdf]

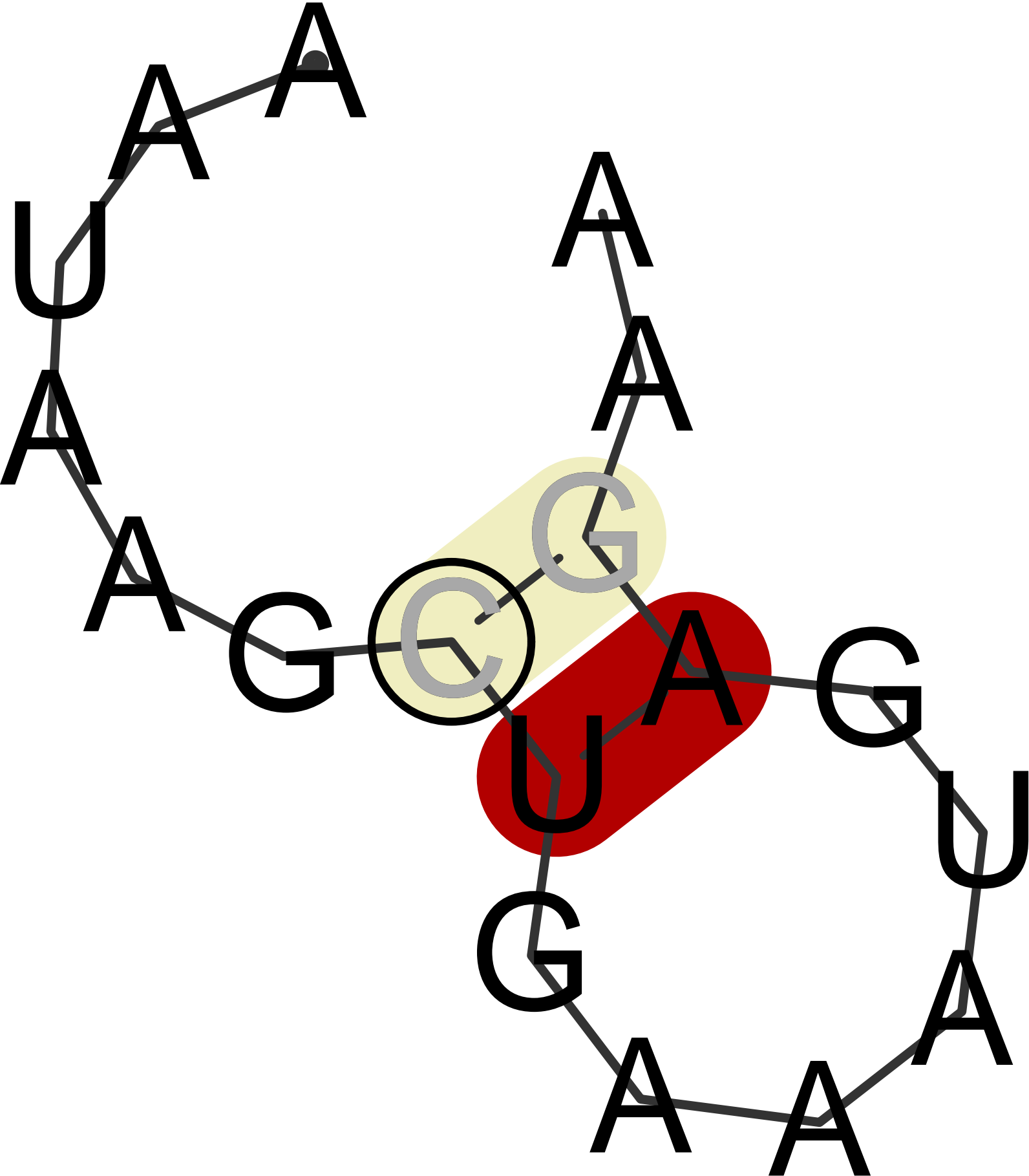

Supplement: S3 Fig — See the caption for S1 Fig for a description of the filename convention (save that the corresponding nucleotide locations in reference sequences are listed in S4 and S12 Tables), and an explanation of the RNAalifold options used and output. (ZIP) [file pcbi.1012009.s124.zip › H3N2-human-ranked-NS2-alignment-349-366-refseq-844-861-representative-sequences-only_revcomp_alirna_nogap.pdf]

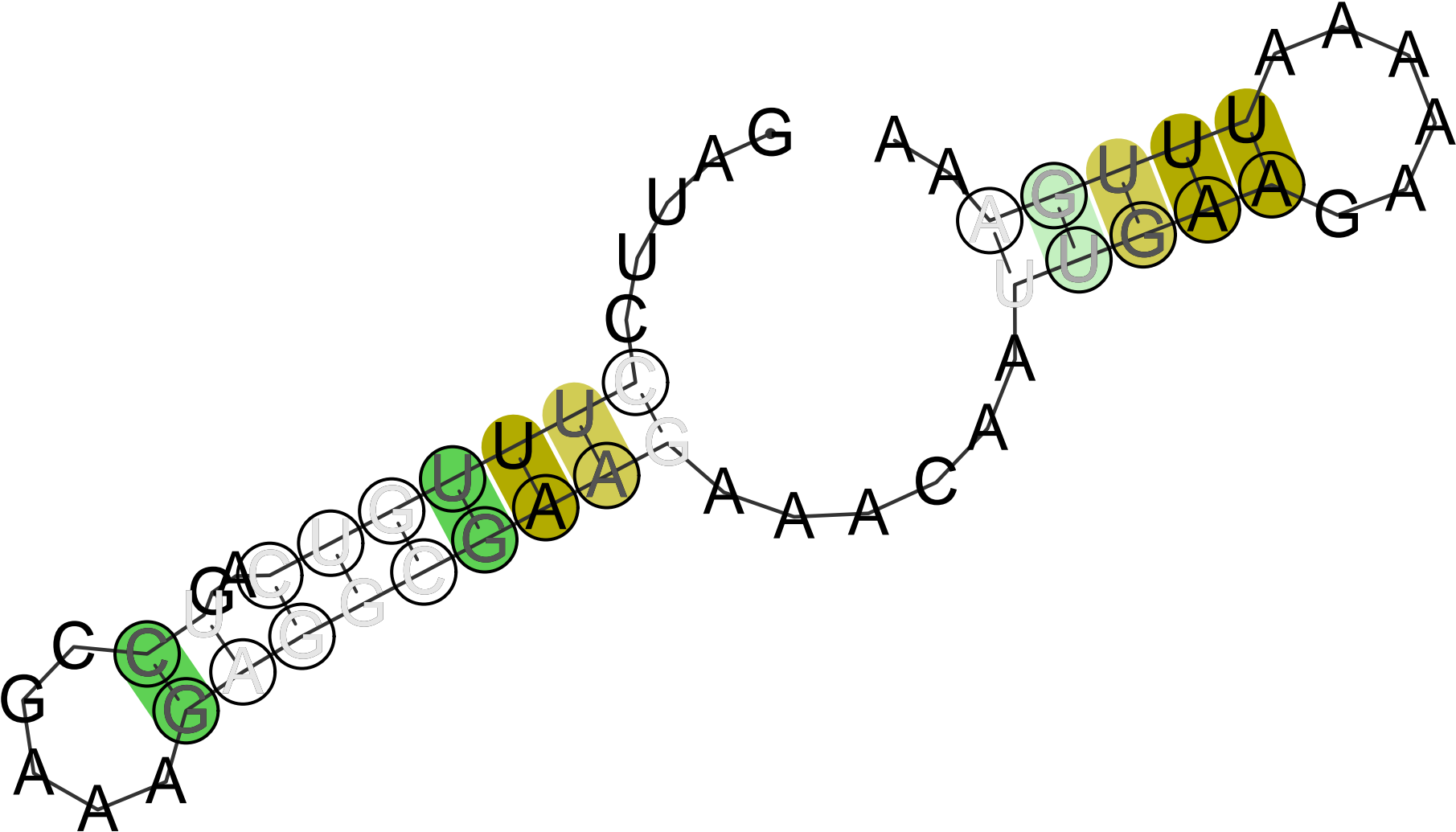

Supplement: S3 Fig — See the caption for S1 Fig for a description of the filename convention (save that the corresponding nucleotide locations in reference sequences are listed in S4 and S12 Tables), and an explanation of the RNAalifold options used and output. (ZIP) [file pcbi.1012009.s124.zip › H3N2-human-ranked-PA-X-alignment-571-624-refseq-589-643-representative-sequences-only_alirna_nogap.pdf]

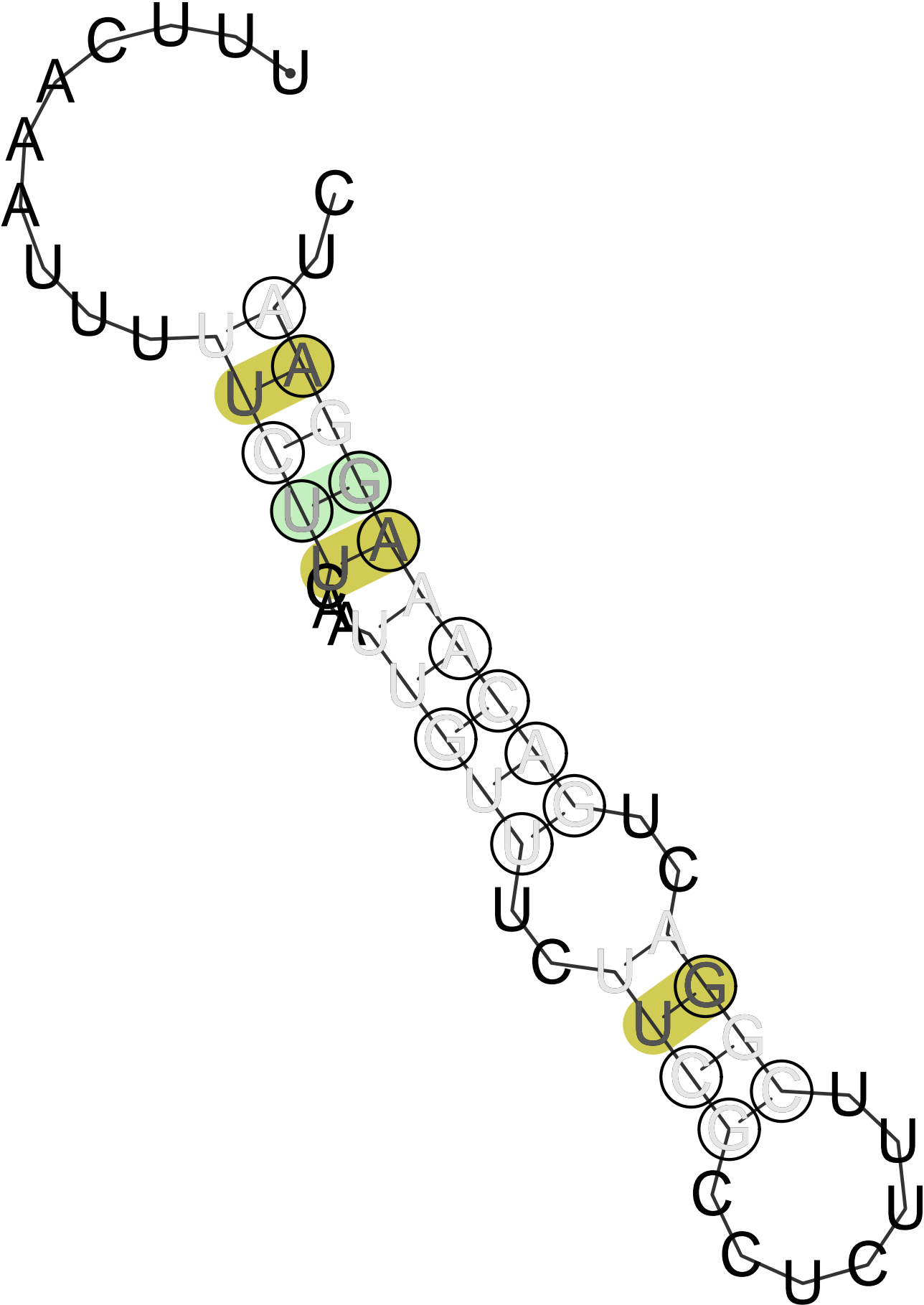

Supplement: S3 Fig — See the caption for S1 Fig for a description of the filename convention (save that the corresponding nucleotide locations in reference sequences are listed in S4 and S12 Tables), and an explanation of the RNAalifold options used and output. (ZIP) [file pcbi.1012009.s124.zip › H3N2-human-ranked-PA-X-alignment-571-624-refseq-589-643-representative-sequences-only_revcomp_alirna_nogap.pdf]

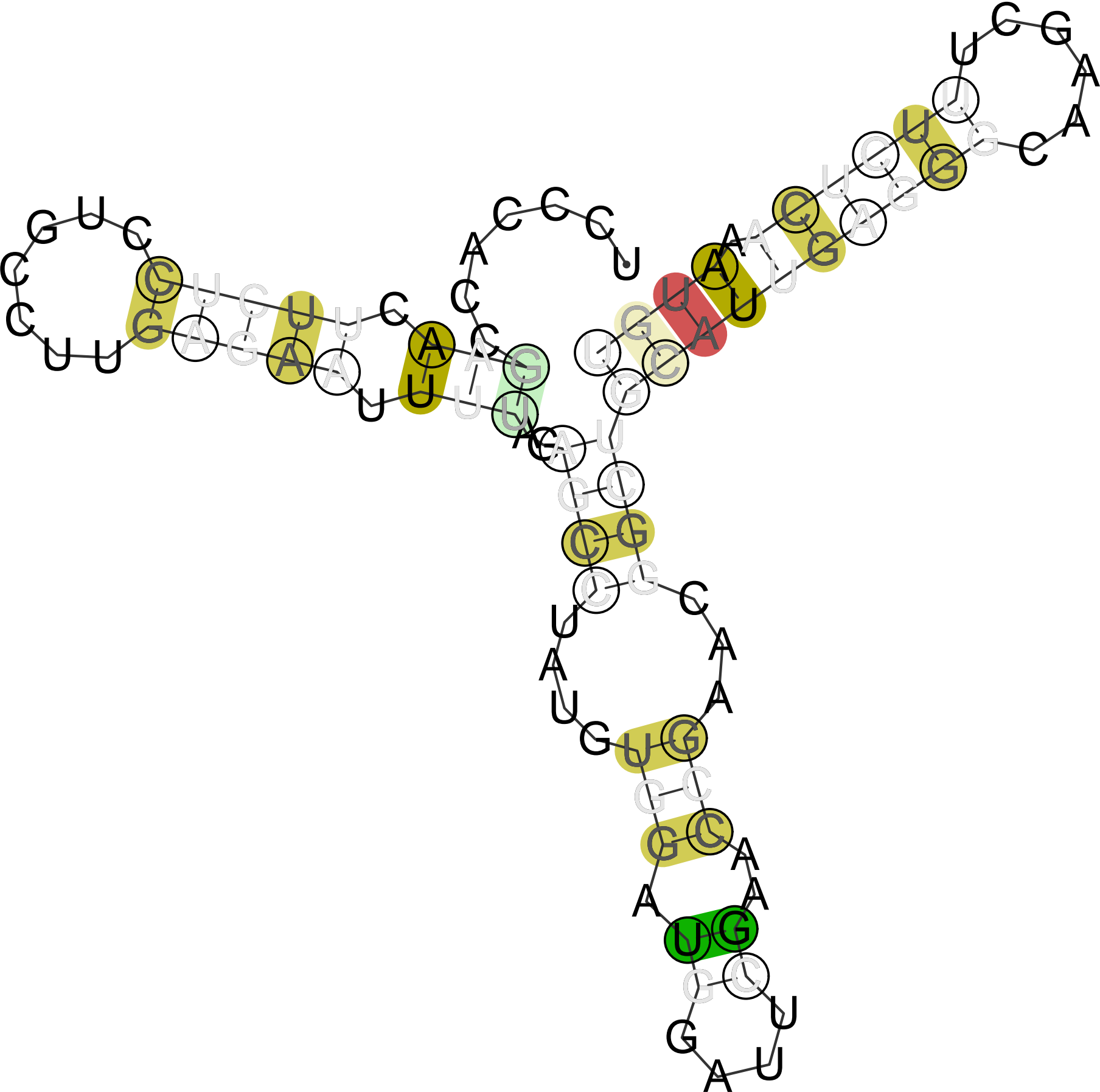

Supplement: S3 Fig — See the caption for S1 Fig for a description of the filename convention (save that the corresponding nucleotide locations in reference sequences are listed in S4 and S12 Tables), and an explanation of the RNAalifold options used and output. (ZIP) [file pcbi.1012009.s124.zip › H3N2-human-ranked-PA-X-alignment-661-753-refseq-680-772-representative-sequences-only_alirna_nogap.pdf]

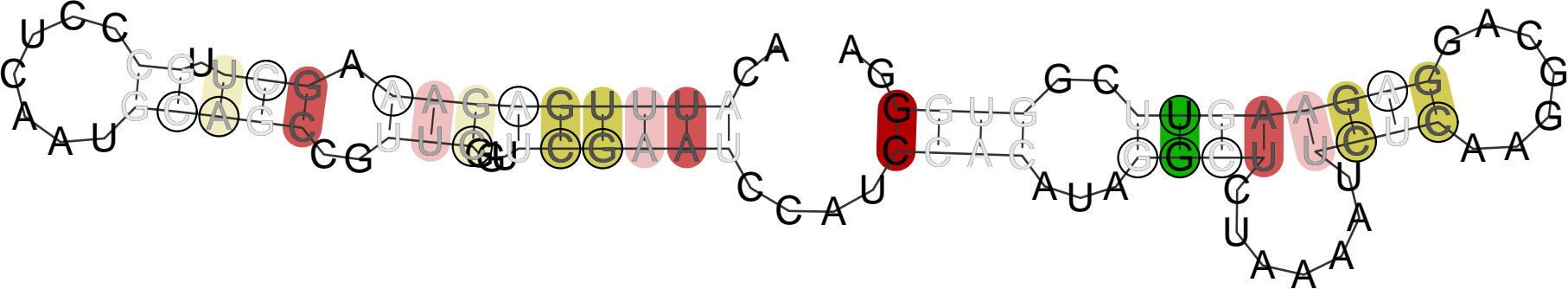

Supplement: S3 Fig — See the caption for S1 Fig for a description of the filename convention (save that the corresponding nucleotide locations in reference sequences are listed in S4 and S12 Tables), and an explanation of the RNAalifold options used and output. (ZIP) [file pcbi.1012009.s124.zip › H3N2-human-ranked-PA-X-alignment-661-753-refseq-680-772-representative-sequences-only_revcomp_alirna_nogap.pdf]

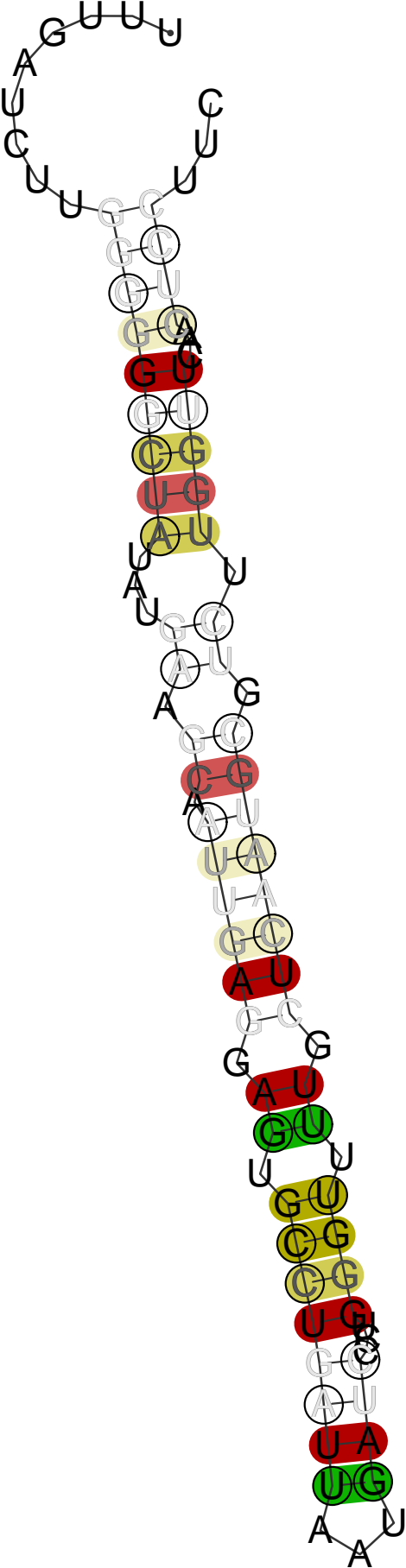

Supplement: S3 Fig — See the caption for S1 Fig for a description of the filename convention (save that the corresponding nucleotide locations in reference sequences are listed in S4 and S12 Tables), and an explanation of the RNAalifold options used and output. (ZIP) [file pcbi.1012009.s124.zip › H3N2-human-ranked-PA-alignment-2077-2166-refseq-2065-2154-representative-sequences-only_alirna_nogap.pdf]

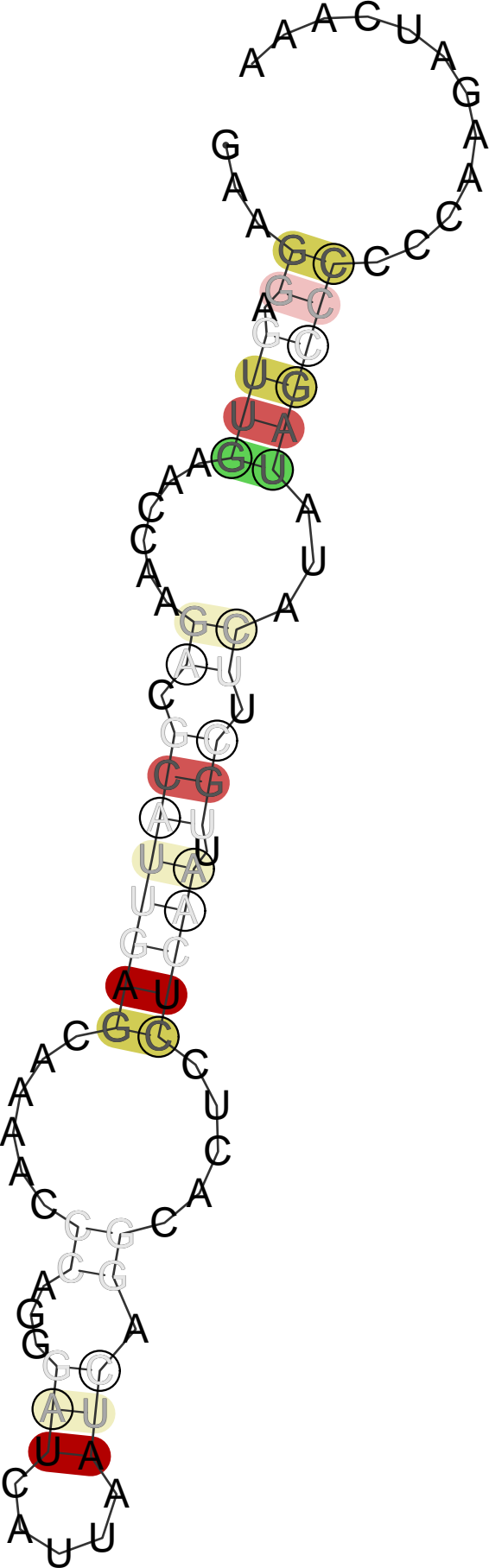

Supplement: S3 Fig — See the caption for S1 Fig for a description of the filename convention (save that the corresponding nucleotide locations in reference sequences are listed in S4 and S12 Tables), and an explanation of the RNAalifold options used and output. (ZIP) [file pcbi.1012009.s124.zip › H3N2-human-ranked-PA-alignment-2077-2166-refseq-2065-2154-representative-sequences-only_revcomp_alirna_nogap.pdf]

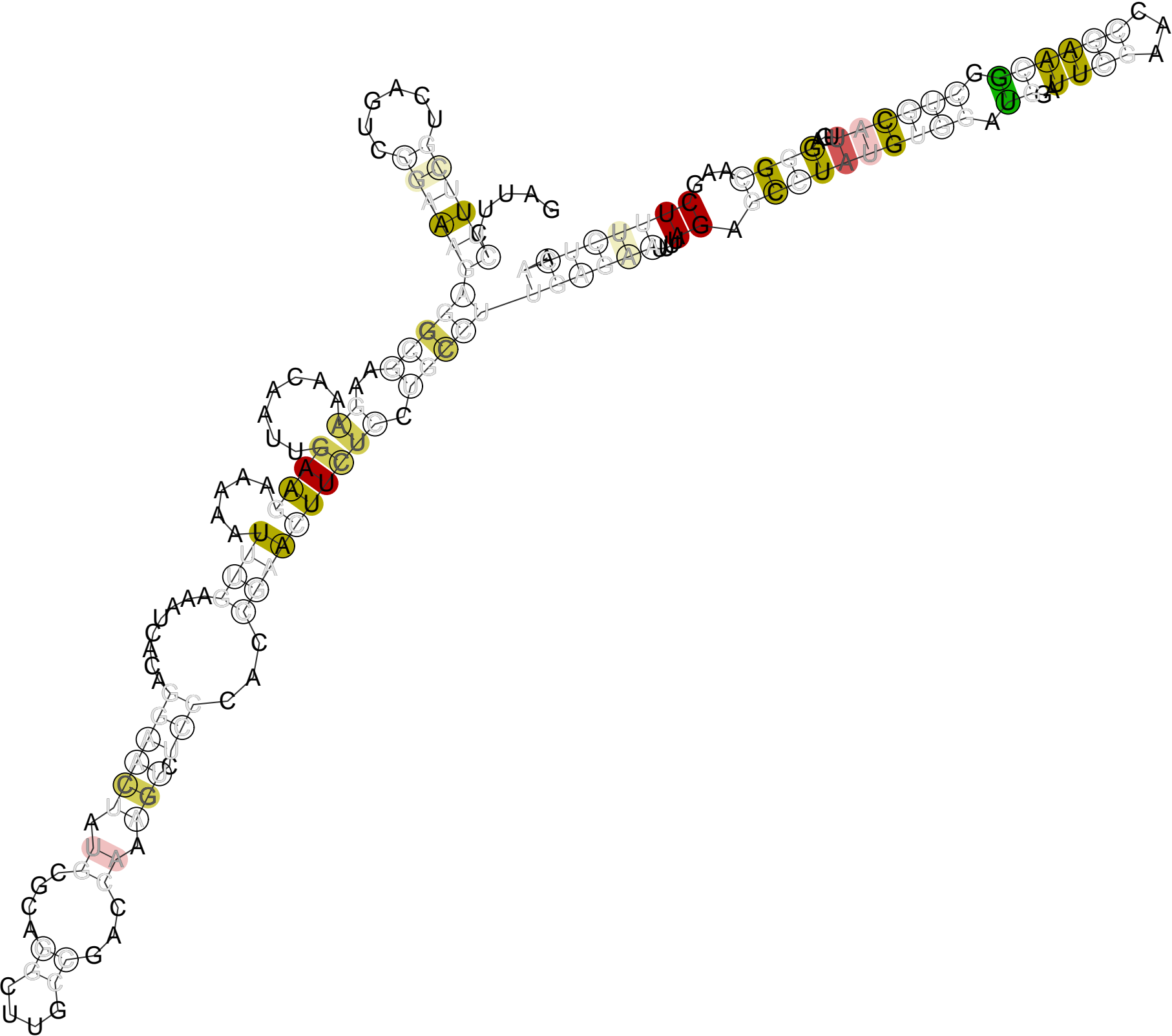

Supplement: S3 Fig — See the caption for S1 Fig for a description of the filename convention (save that the corresponding nucleotide locations in reference sequences are listed in S4 and S12 Tables), and an explanation of the RNAalifold options used and output. (ZIP) [file pcbi.1012009.s124.zip › H3N2-human-ranked-PA-alignment-598-777-refseq-589-768-representative-sequences-only_alirna_nogap.pdf]

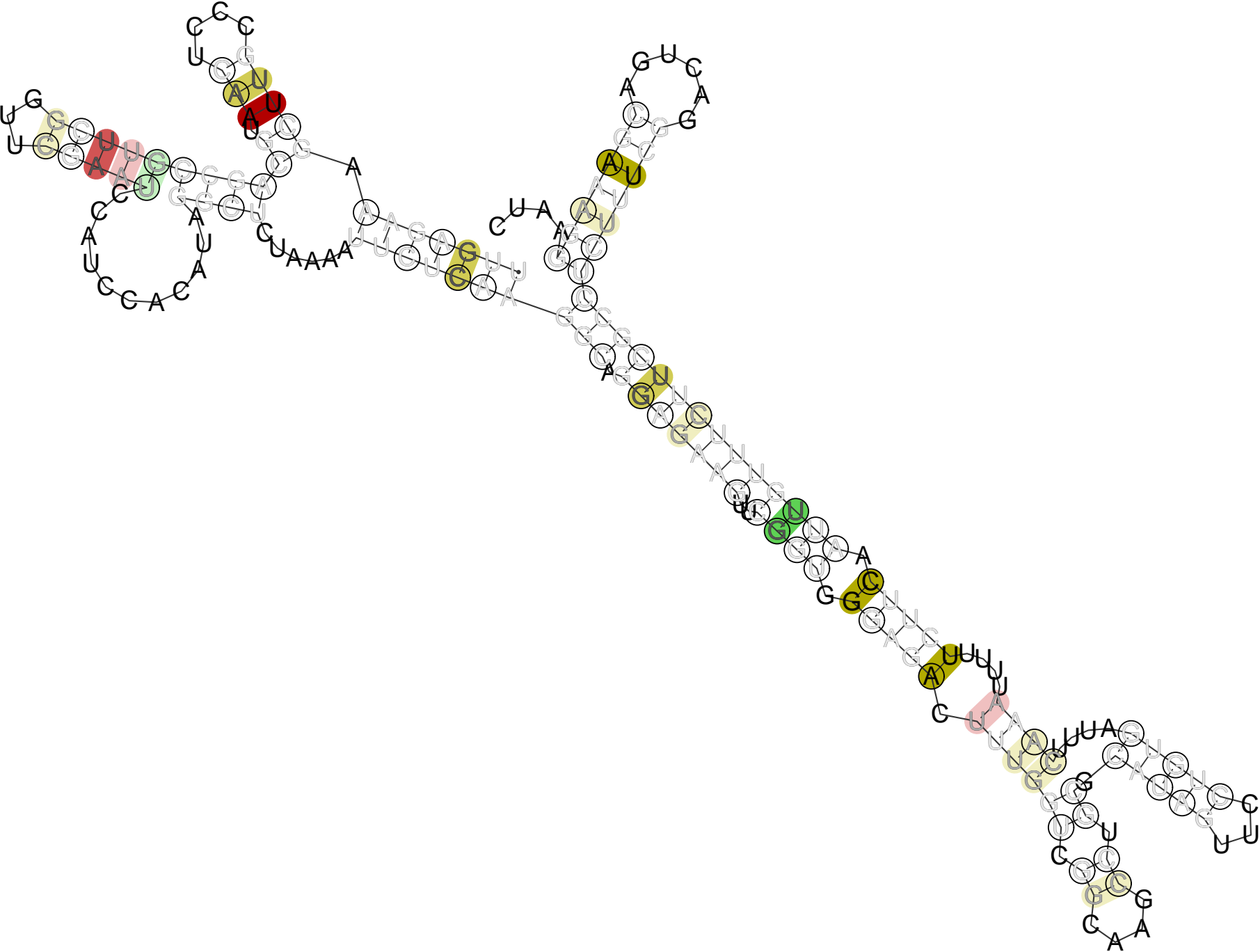

Supplement: S3 Fig — See the caption for S1 Fig for a description of the filename convention (save that the corresponding nucleotide locations in reference sequences are listed in S4 and S12 Tables), and an explanation of the RNAalifold options used and output. (ZIP) [file pcbi.1012009.s124.zip › H3N2-human-ranked-PA-alignment-598-777-refseq-589-768-representative-sequences-only_revcomp_alirna_nogap.pdf]

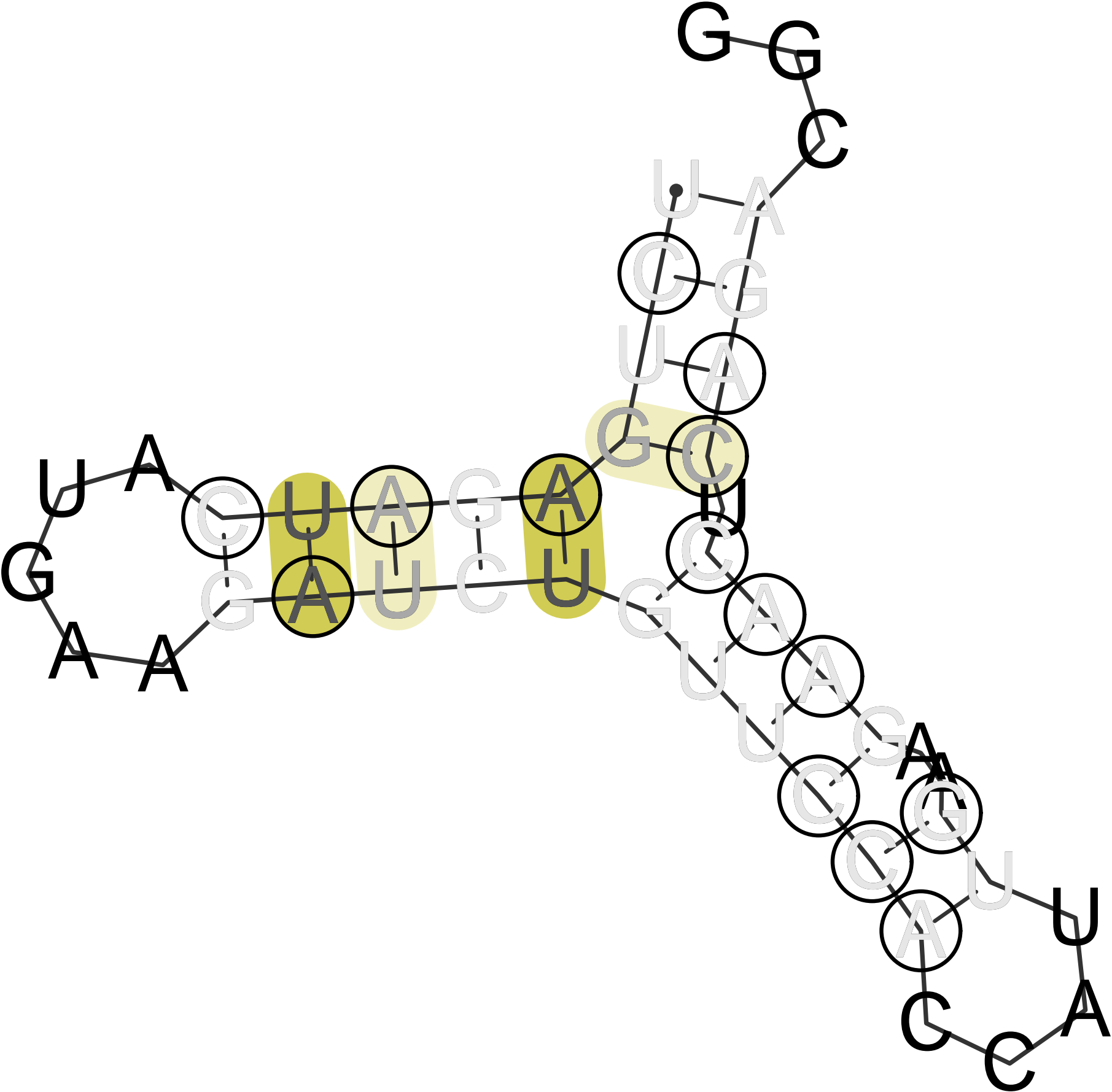

Supplement: S3 Fig — See the caption for S1 Fig for a description of the filename convention (save that the corresponding nucleotide locations in reference sequences are listed in S4 and S12 Tables), and an explanation of the RNAalifold options used and output. (ZIP) [file pcbi.1012009.s124.zip › H3N2-human-ranked-PB1-alignment-2227-2271-refseq-2245-2289-representative-sequences-only_alirna_nogap.pdf]

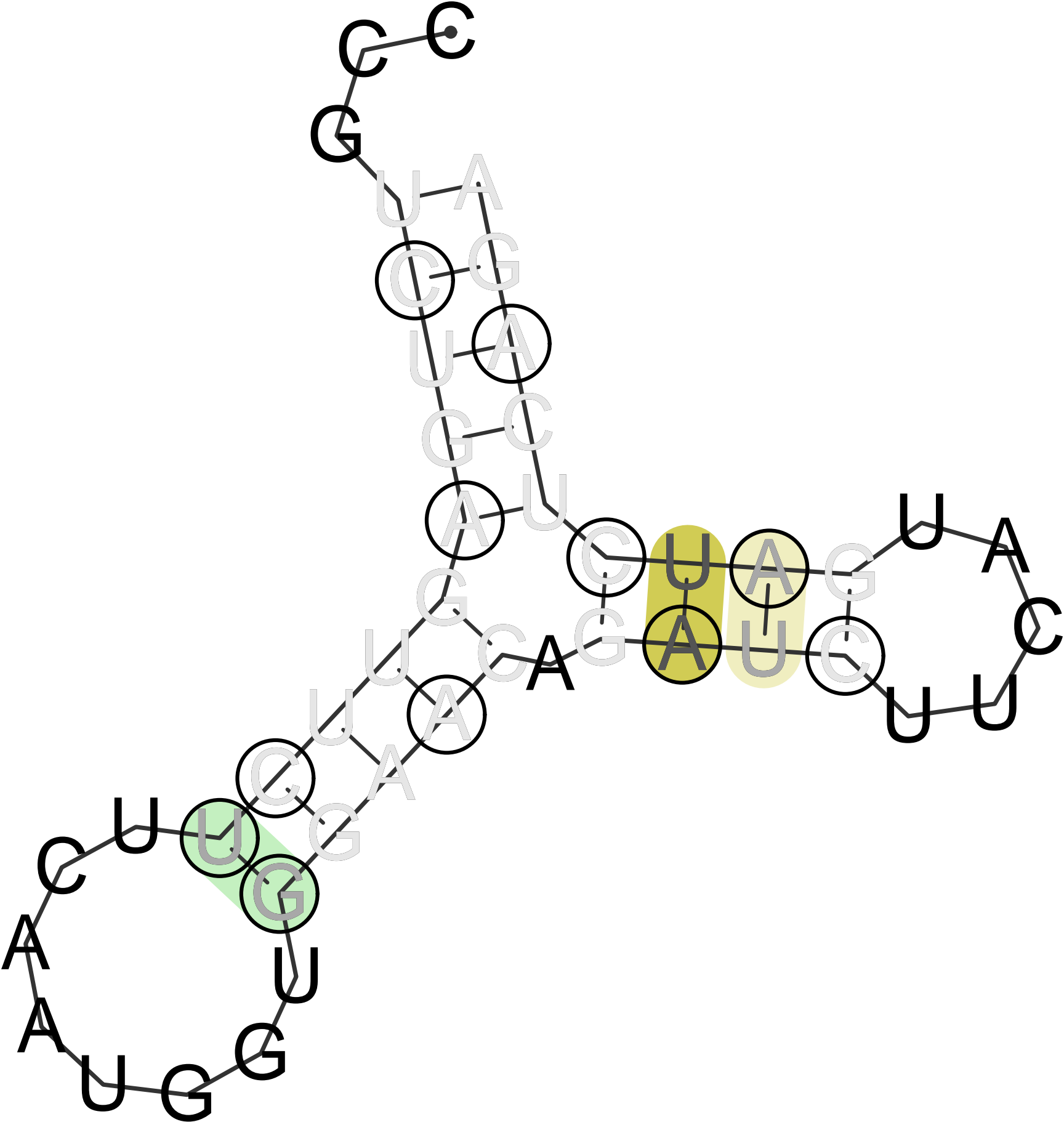

Supplement: S3 Fig — See the caption for S1 Fig for a description of the filename convention (save that the corresponding nucleotide locations in reference sequences are listed in S4 and S12 Tables), and an explanation of the RNAalifold options used and output. (ZIP) [file pcbi.1012009.s124.zip › H3N2-human-ranked-PB1-alignment-2227-2271-refseq-2245-2289-representative-sequences-only_revcomp_alirna_nogap.pdf]

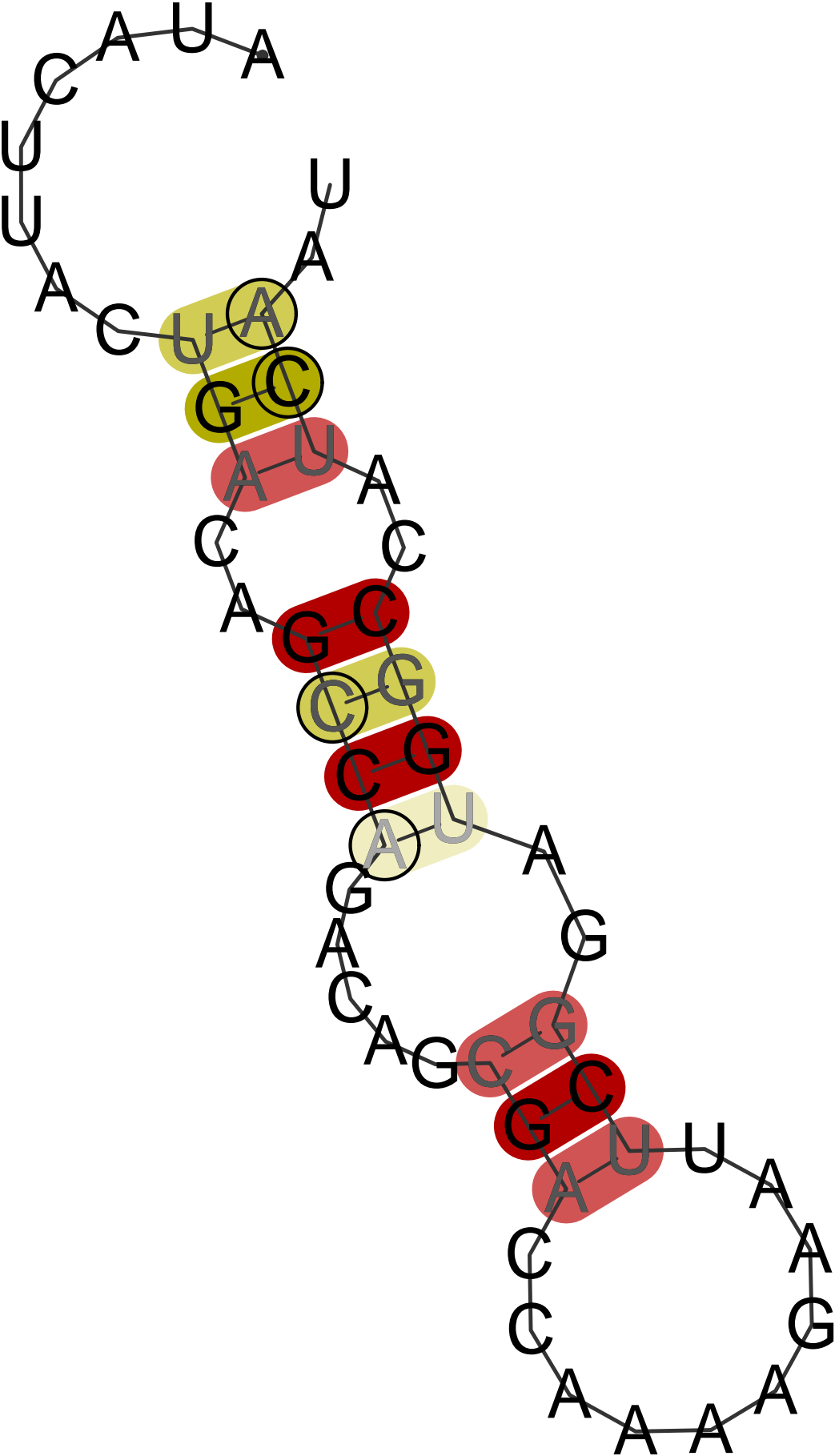

Supplement: S3 Fig — See the caption for S1 Fig for a description of the filename convention (save that the corresponding nucleotide locations in reference sequences are listed in S4 and S12 Tables), and an explanation of the RNAalifold options used and output. (ZIP) [file pcbi.1012009.s124.zip › H3N2-human-ranked-PB2-alignment-2239-2289-refseq-2254-2304-representative-sequences-only_alirna_nogap.pdf]

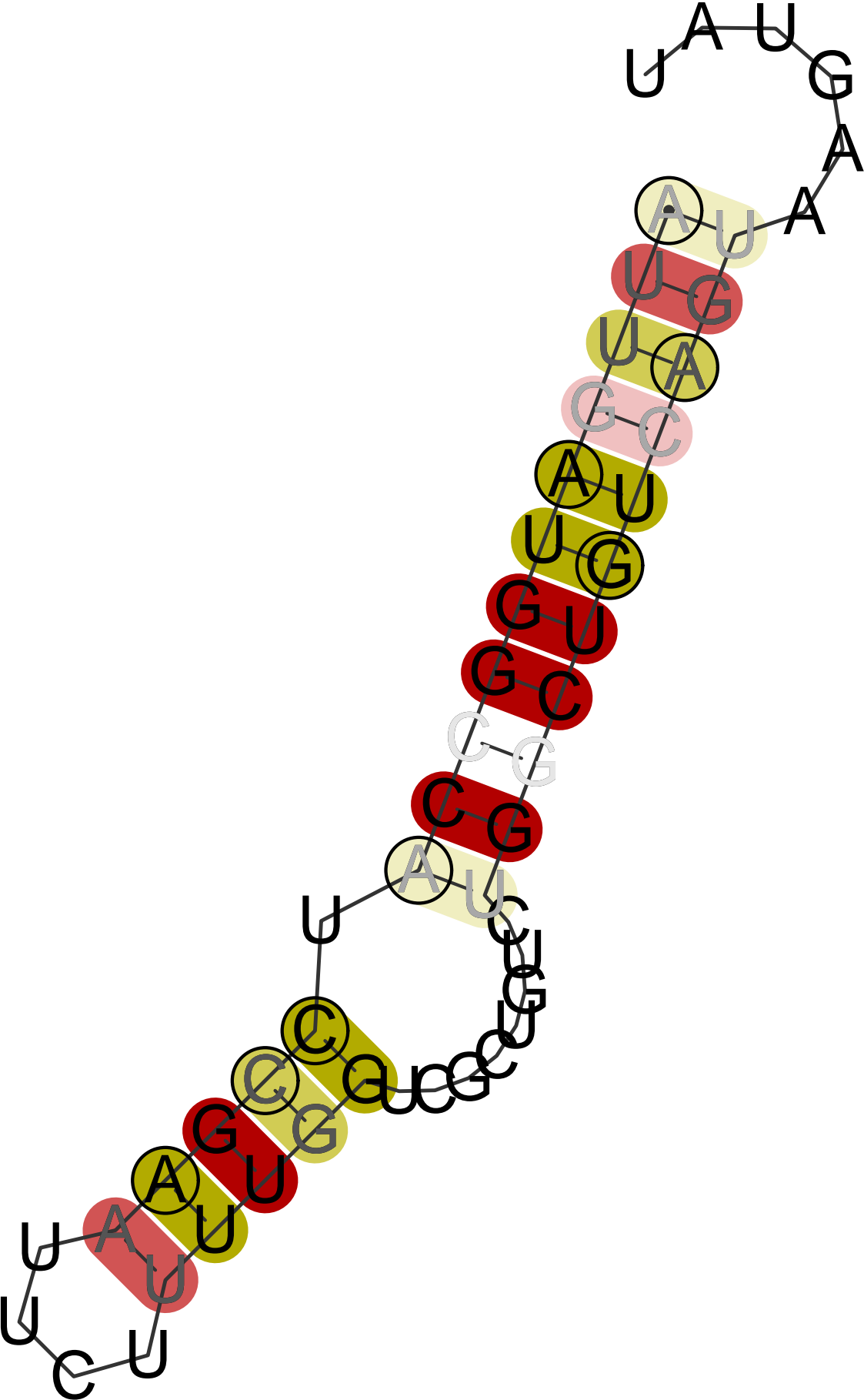

Supplement: S3 Fig — See the caption for S1 Fig for a description of the filename convention (save that the corresponding nucleotide locations in reference sequences are listed in S4 and S12 Tables), and an explanation of the RNAalifold options used and output. (ZIP) [file pcbi.1012009.s124.zip › H3N2-human-ranked-PB2-alignment-2239-2289-refseq-2254-2304-representative-sequences-only_revcomp_alirna_nogap.pdf]

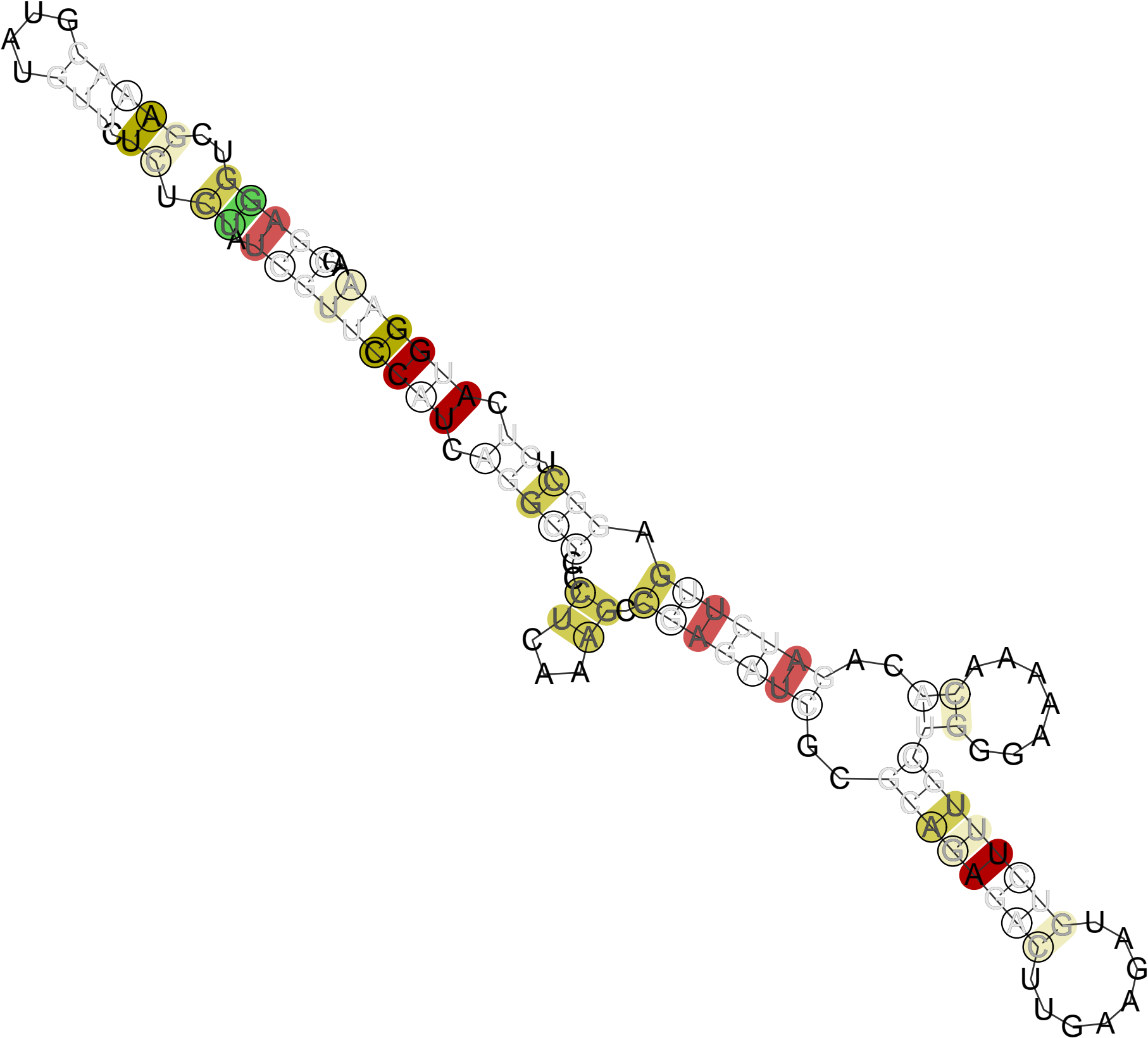

Supplement: S3 Fig — See the caption for S1 Fig for a description of the filename convention (save that the corresponding nucleotide locations in reference sequences are listed in S4 and S12 Tables), and an explanation of the RNAalifold options used and output. (ZIP) [file pcbi.1012009.s124.zip › H3N2-human-raw-M1-alignment-13-132-refseq-38-157-representative-sequences-only_alirna_nogap.pdf]

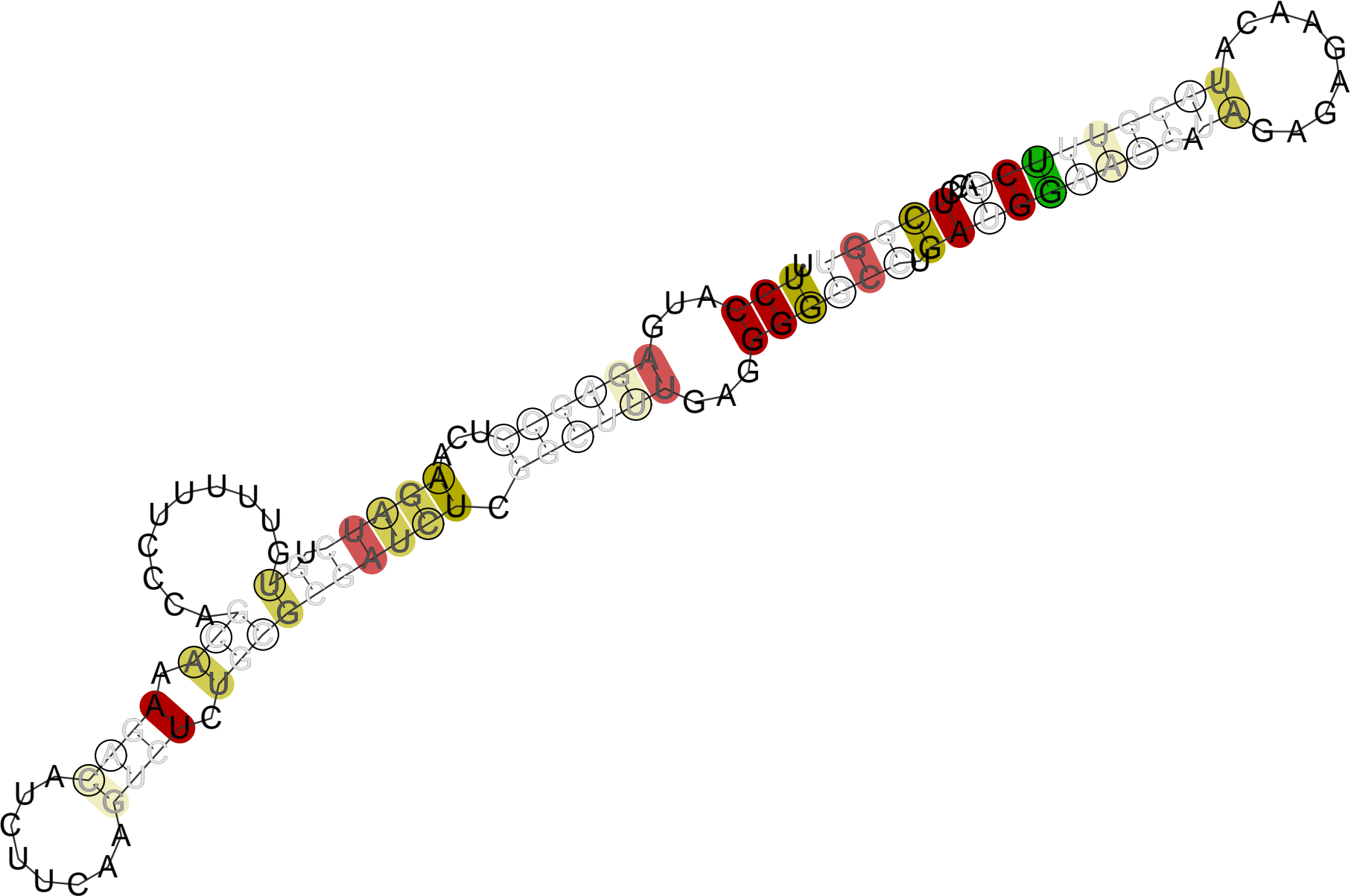

Supplement: S3 Fig — See the caption for S1 Fig for a description of the filename convention (save that the corresponding nucleotide locations in reference sequences are listed in S4 and S12 Tables), and an explanation of the RNAalifold options used and output. (ZIP) [file pcbi.1012009.s124.zip › H3N2-human-raw-M1-alignment-13-132-refseq-38-157-representative-sequences-only_revcomp_alirna_nogap.pdf]

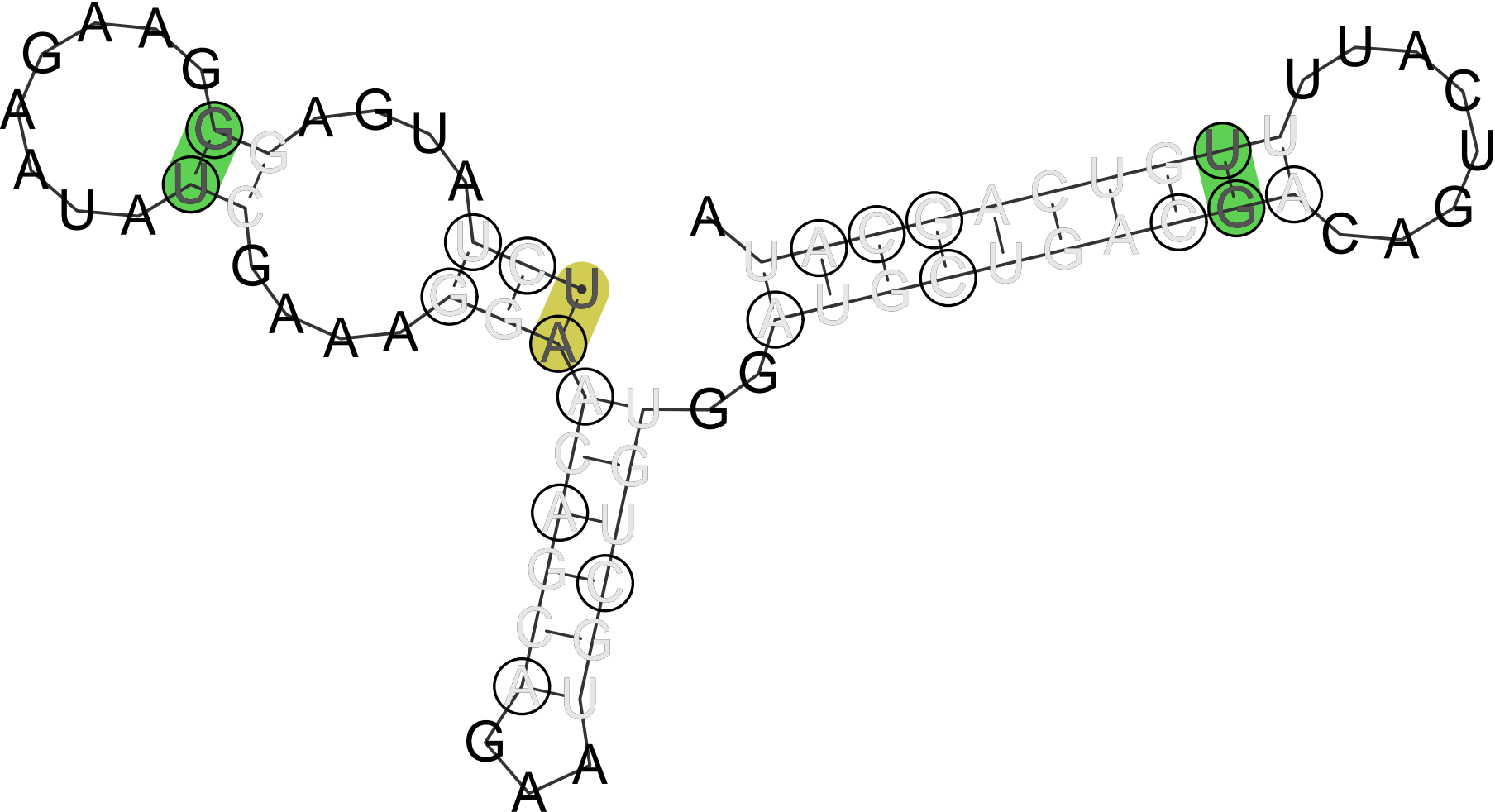

Supplement: S3 Fig — See the caption for S1 Fig for a description of the filename convention (save that the corresponding nucleotide locations in reference sequences are listed in S4 and S12 Tables), and an explanation of the RNAalifold options used and output. (ZIP) [file pcbi.1012009.s124.zip › H3N2-human-raw-M2-alignment-211-282-refseq-924-995-representative-sequences-only_alirna_nogap.pdf]

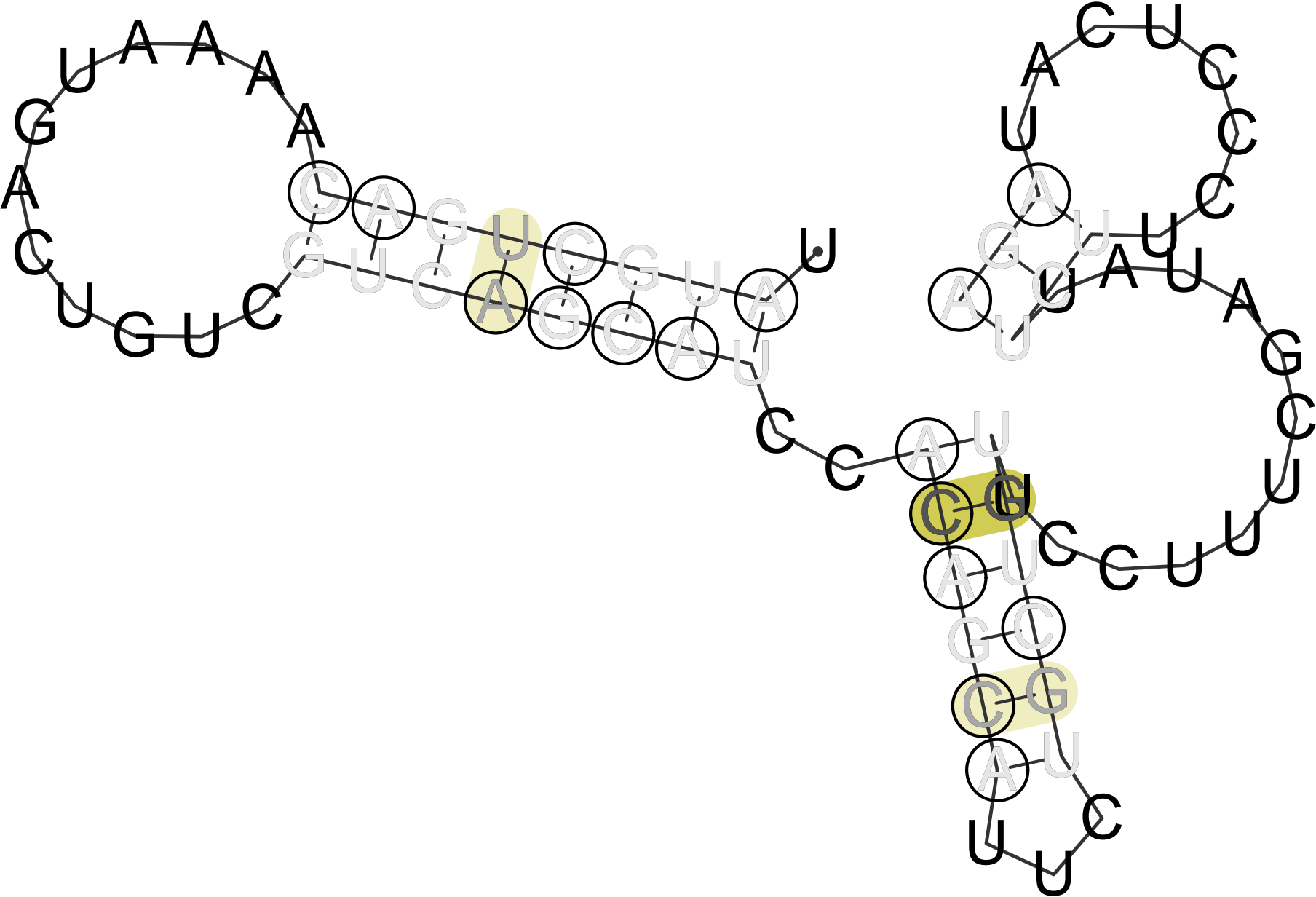

Supplement: S3 Fig — See the caption for S1 Fig for a description of the filename convention (save that the corresponding nucleotide locations in reference sequences are listed in S4 and S12 Tables), and an explanation of the RNAalifold options used and output. (ZIP) [file pcbi.1012009.s124.zip › H3N2-human-raw-M2-alignment-211-282-refseq-924-995-representative-sequences-only_revcomp_alirna_nogap.pdf]

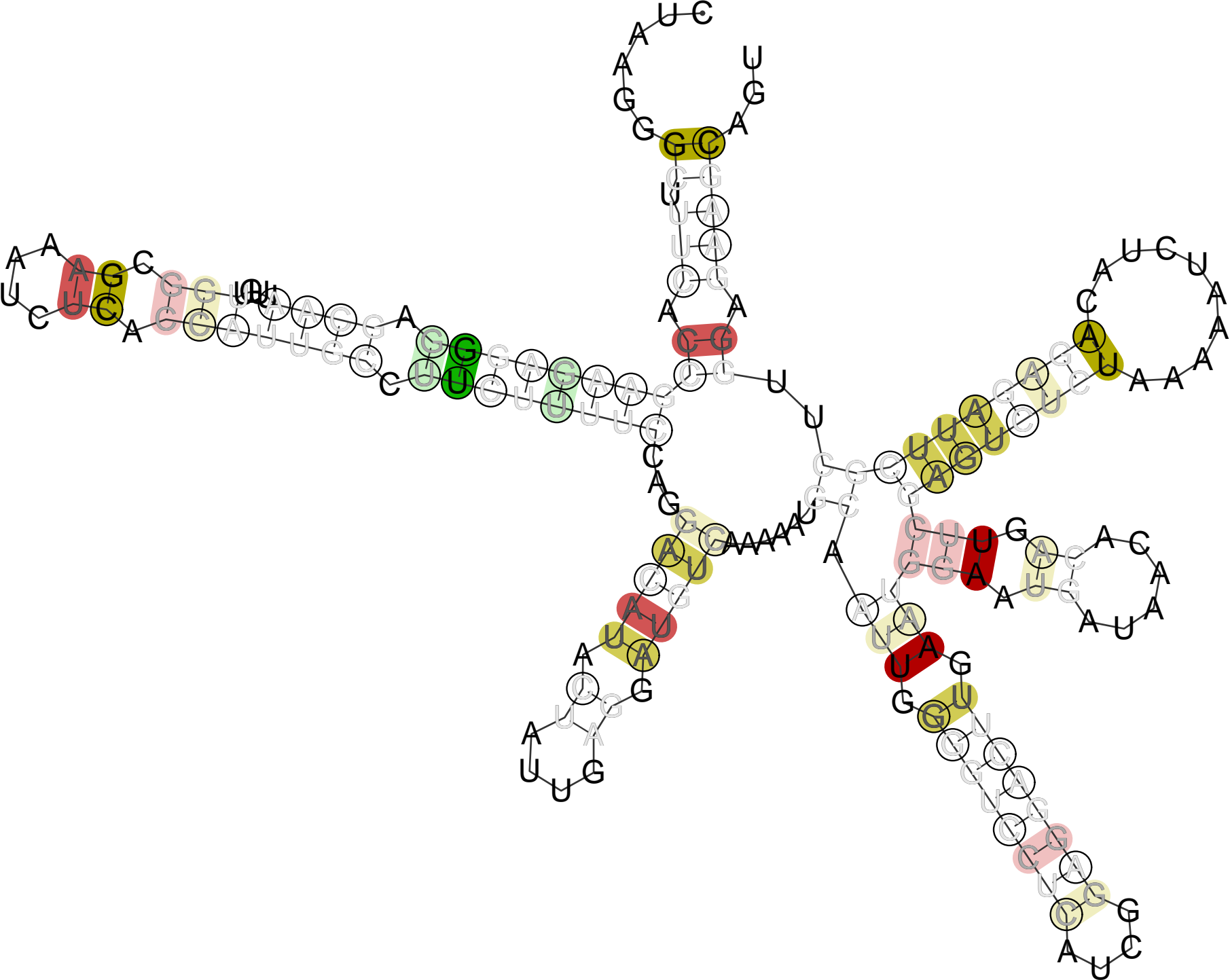

Supplement: S3 Fig — See the caption for S1 Fig for a description of the filename convention (save that the corresponding nucleotide locations in reference sequences are listed in S4 and S12 Tables), and an explanation of the RNAalifold options used and output. (ZIP) [file pcbi.1012009.s124.zip › H3N2-human-raw-NS1-alignment-448-627-refseq-465-644-representative-sequences-only_alirna_nogap.pdf]

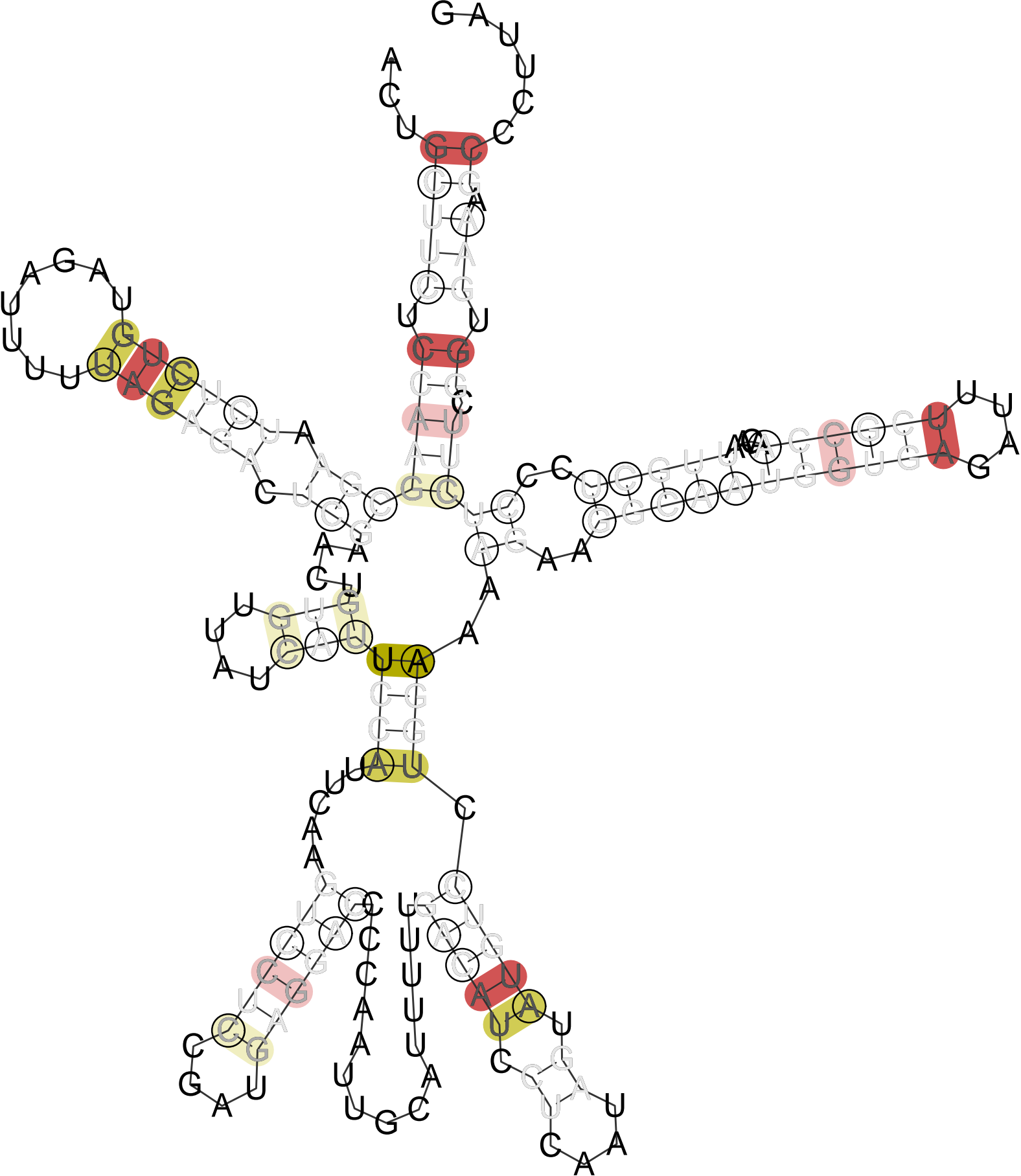

Supplement: S3 Fig — See the caption for S1 Fig for a description of the filename convention (save that the corresponding nucleotide locations in reference sequences are listed in S4 and S12 Tables), and an explanation of the RNAalifold options used and output. (ZIP) [file pcbi.1012009.s124.zip › H3N2-human-raw-NS1-alignment-448-627-refseq-465-644-representative-sequences-only_revcomp_alirna_nogap.pdf]

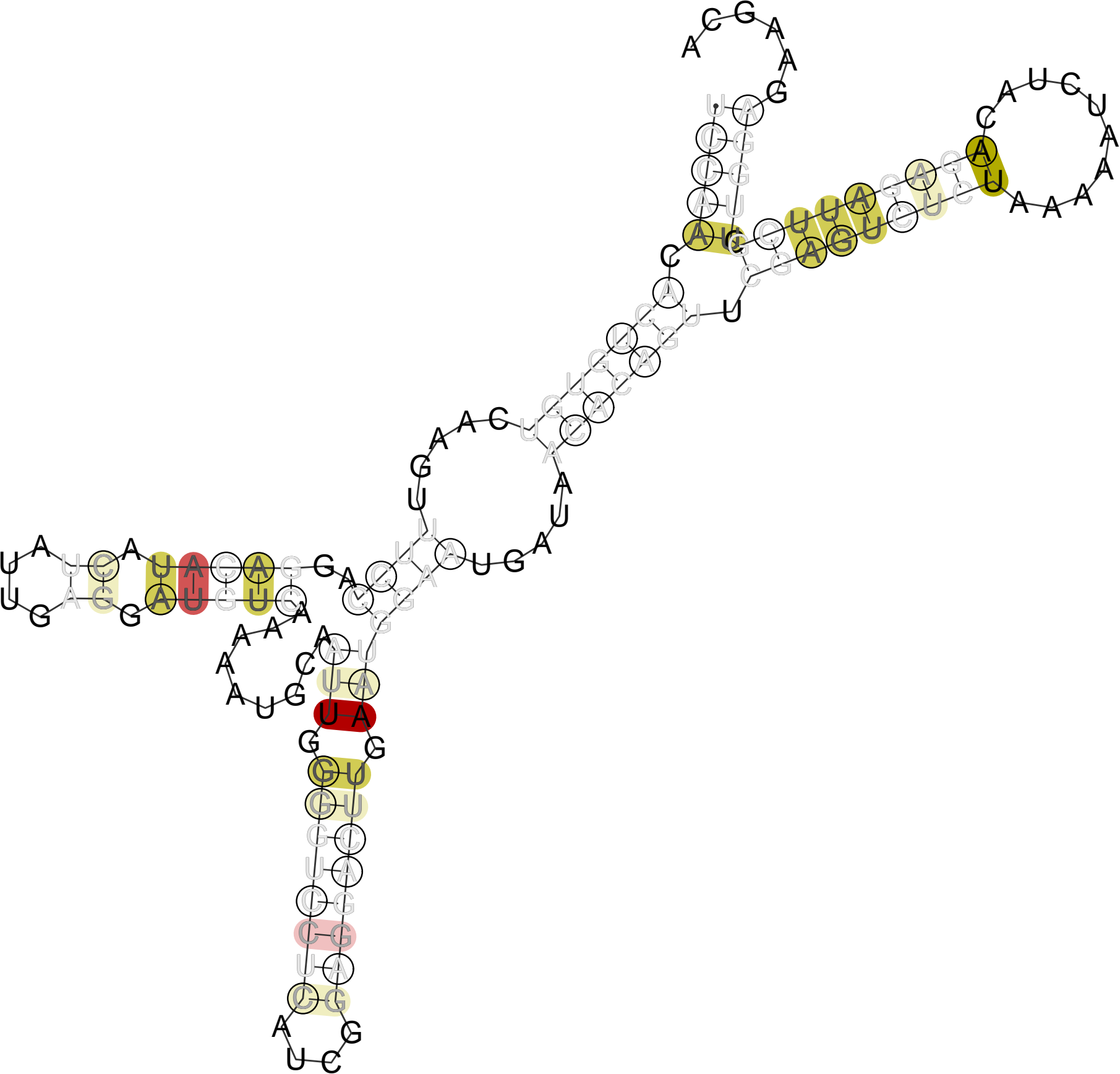

Supplement: S3 Fig — See the caption for S1 Fig for a description of the filename convention (save that the corresponding nucleotide locations in reference sequences are listed in S4 and S12 Tables), and an explanation of the RNAalifold options used and output. (ZIP) [file pcbi.1012009.s124.zip › H3N2-human-raw-NS2-alignment-10-147-refseq-33-56-529-642-representative-sequences-only_alirna_nogap.pdf]

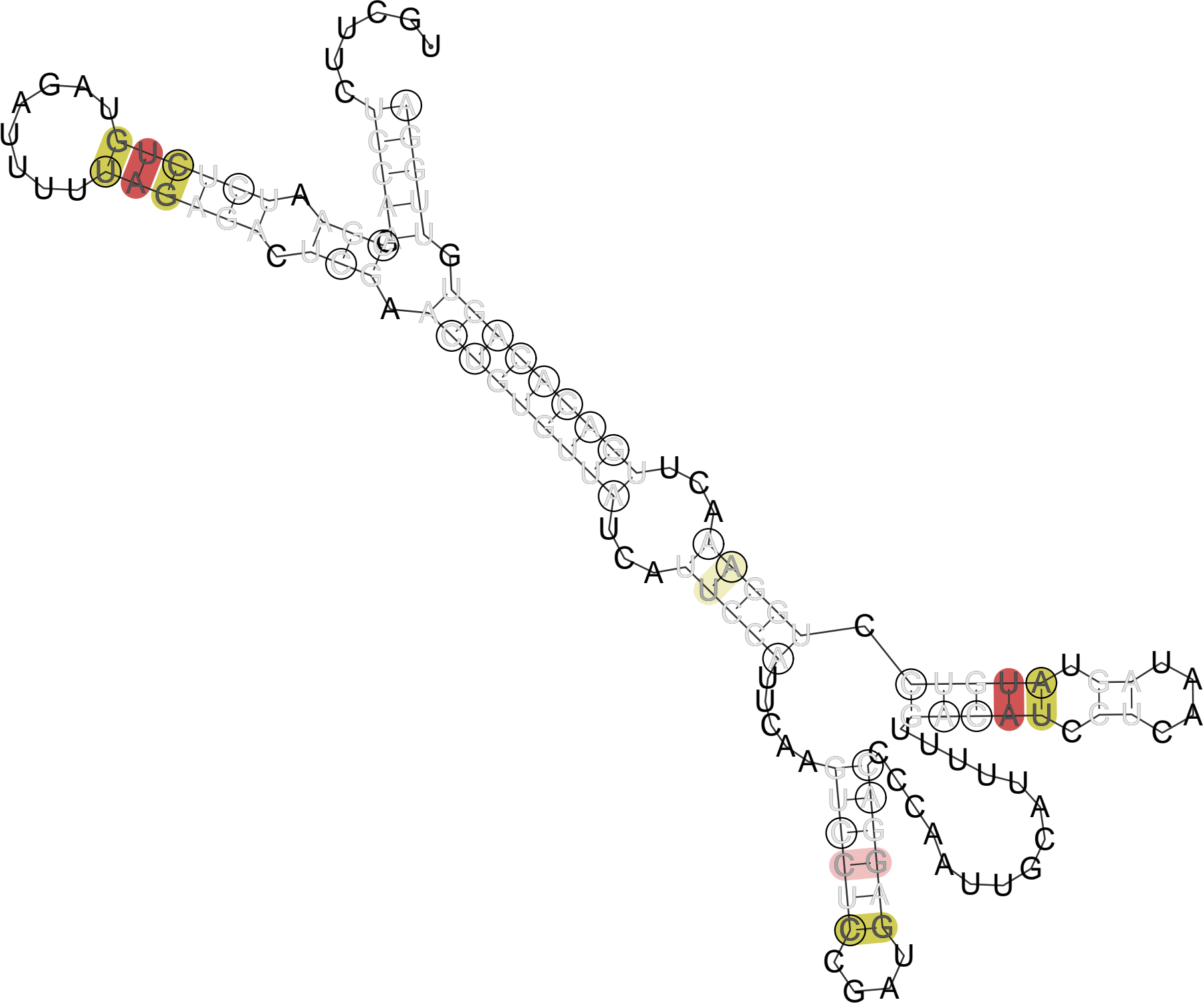

Supplement: S3 Fig — See the caption for S1 Fig for a description of the filename convention (save that the corresponding nucleotide locations in reference sequences are listed in S4 and S12 Tables), and an explanation of the RNAalifold options used and output. (ZIP) [file pcbi.1012009.s124.zip › H3N2-human-raw-NS2-alignment-10-147-refseq-33-56-529-642-representative-sequences-only_revcomp_alirna_nogap.pdf]

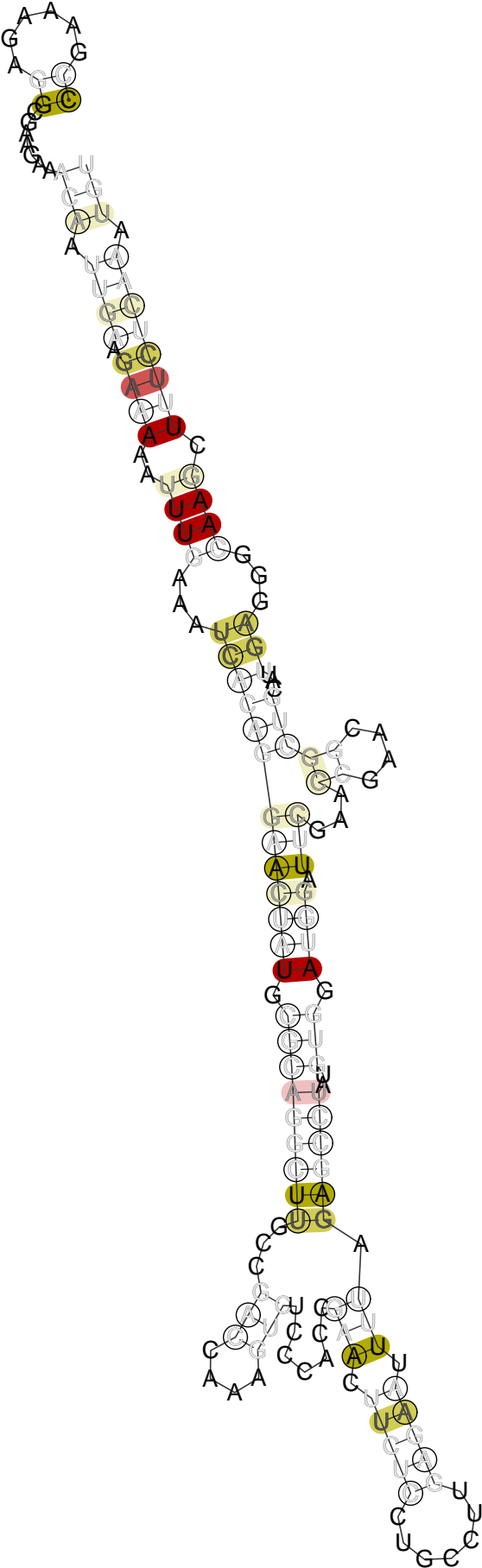

Supplement: S3 Fig — See the caption for S1 Fig for a description of the filename convention (save that the corresponding nucleotide locations in reference sequences are listed in S4 and S12 Tables), and an explanation of the RNAalifold options used and output. (ZIP) [file pcbi.1012009.s124.zip › H3N2-human-raw-PA-X-alignment-586-743-refseq-605-772-representative-sequences-only_alirna_nogap.pdf]

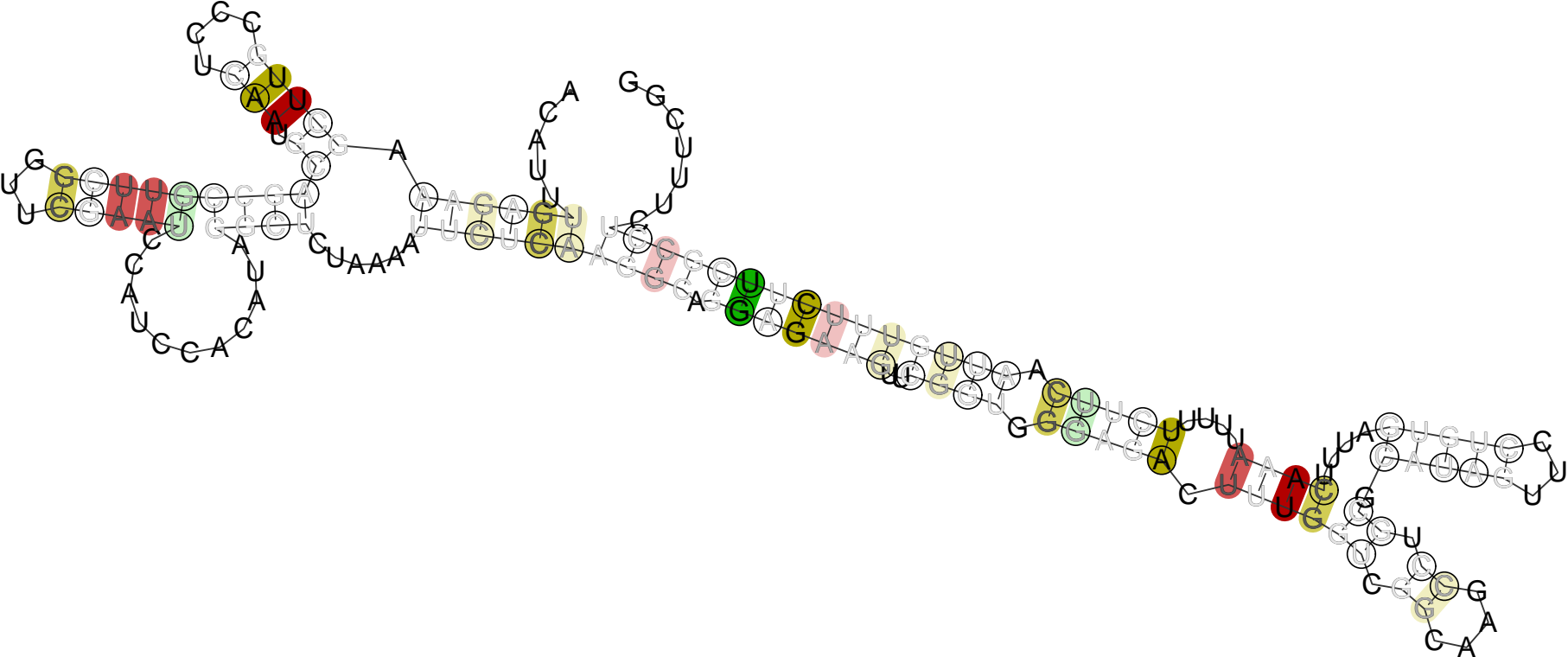

Supplement: S3 Fig — See the caption for S1 Fig for a description of the filename convention (save that the corresponding nucleotide locations in reference sequences are listed in S4 and S12 Tables), and an explanation of the RNAalifold options used and output. (ZIP) [file pcbi.1012009.s124.zip › H3N2-human-raw-PA-X-alignment-586-743-refseq-605-772-representative-sequences-only_revcomp_alirna_nogap.pdf]

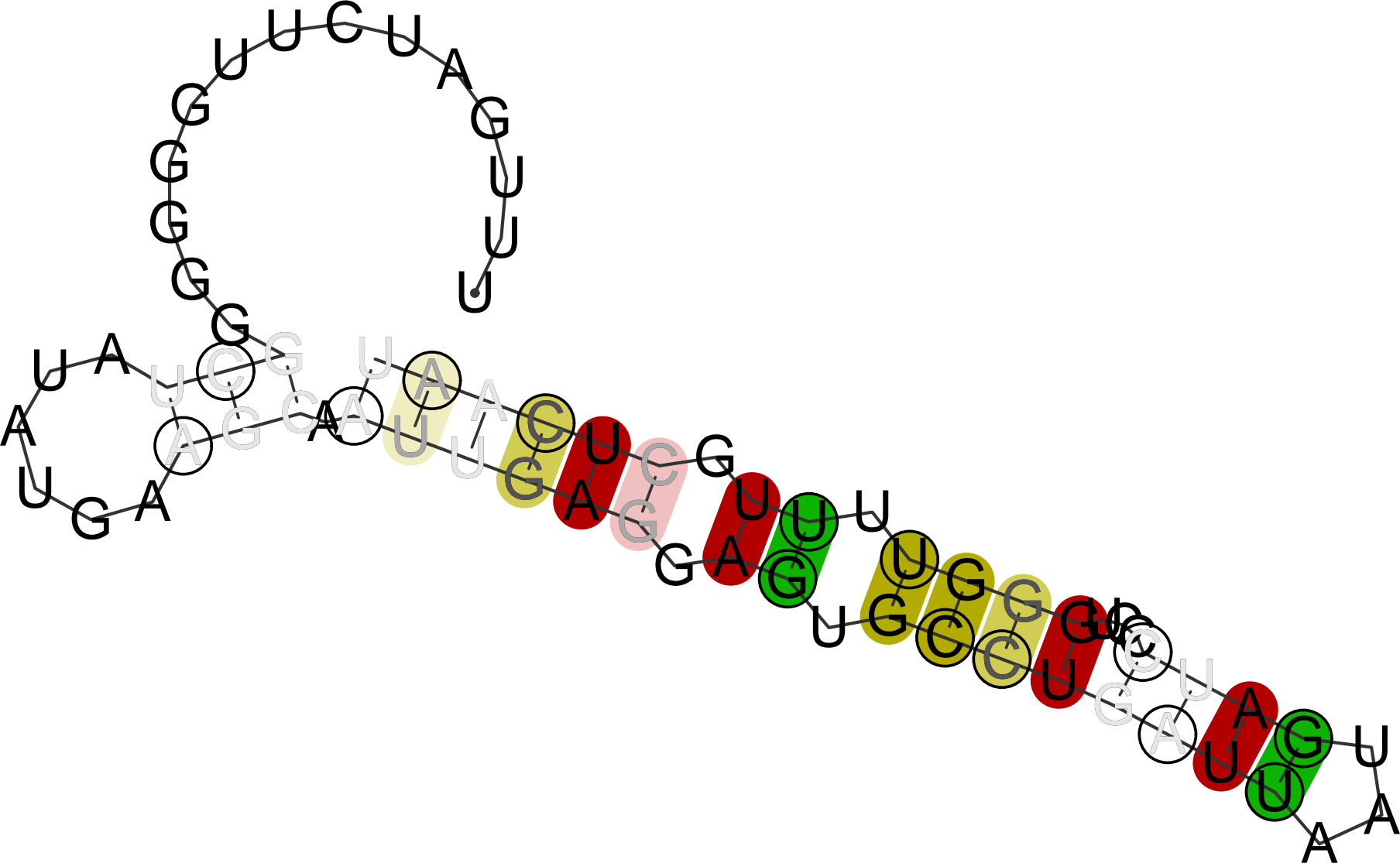

Supplement: S3 Fig — See the caption for S1 Fig for a description of the filename convention (save that the corresponding nucleotide locations in reference sequences are listed in S4 and S12 Tables), and an explanation of the RNAalifold options used and output. (ZIP) [file pcbi.1012009.s124.zip › H3N2-human-raw-PA-alignment-2077-2145-refseq-2065-2133-representative-sequences-only_alirna_nogap.pdf]

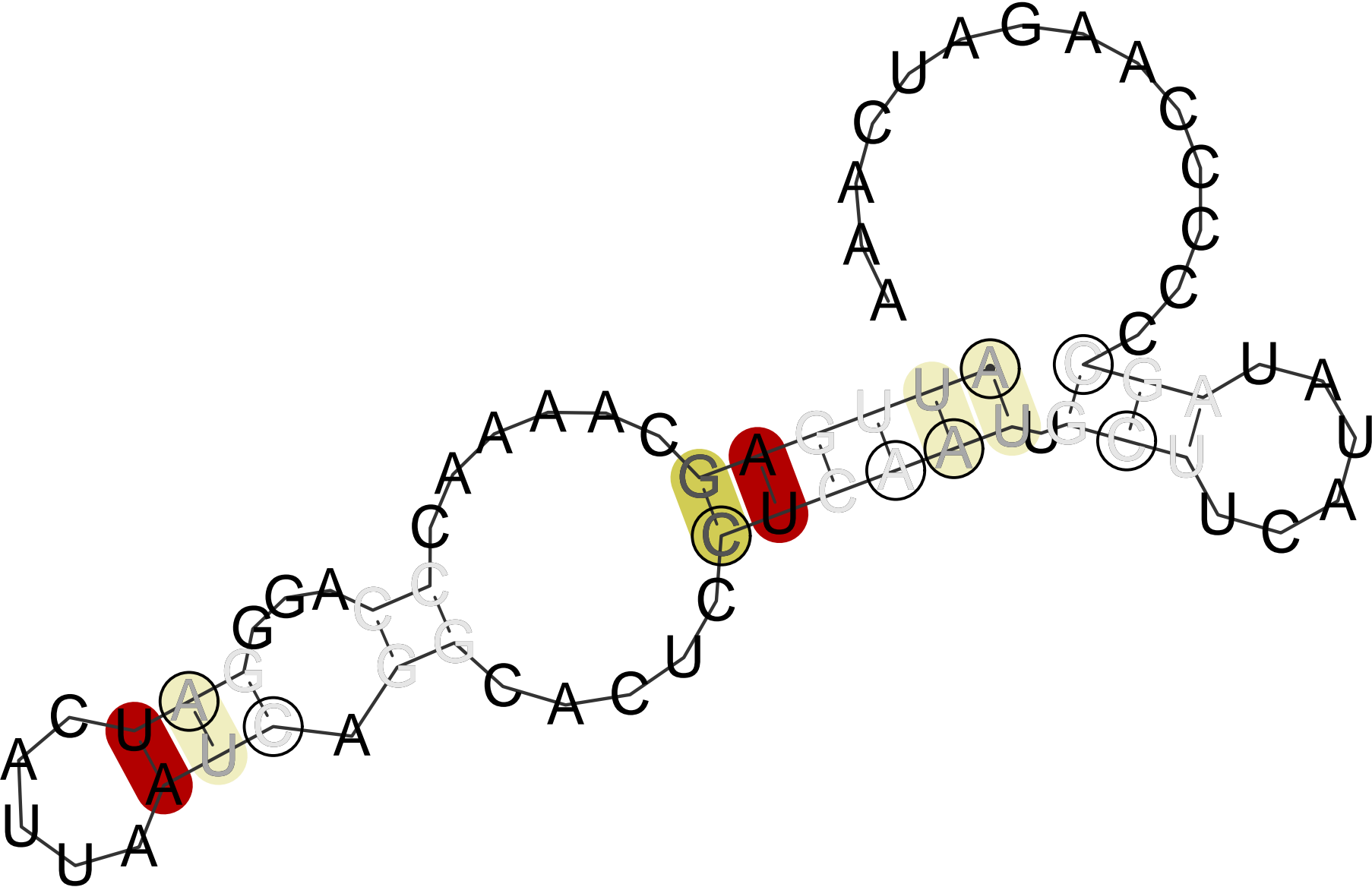

Supplement: S3 Fig — See the caption for S1 Fig for a description of the filename convention (save that the corresponding nucleotide locations in reference sequences are listed in S4 and S12 Tables), and an explanation of the RNAalifold options used and output. (ZIP) [file pcbi.1012009.s124.zip › H3N2-human-raw-PA-alignment-2077-2145-refseq-2065-2133-representative-sequences-only_revcomp_alirna_nogap.pdf]

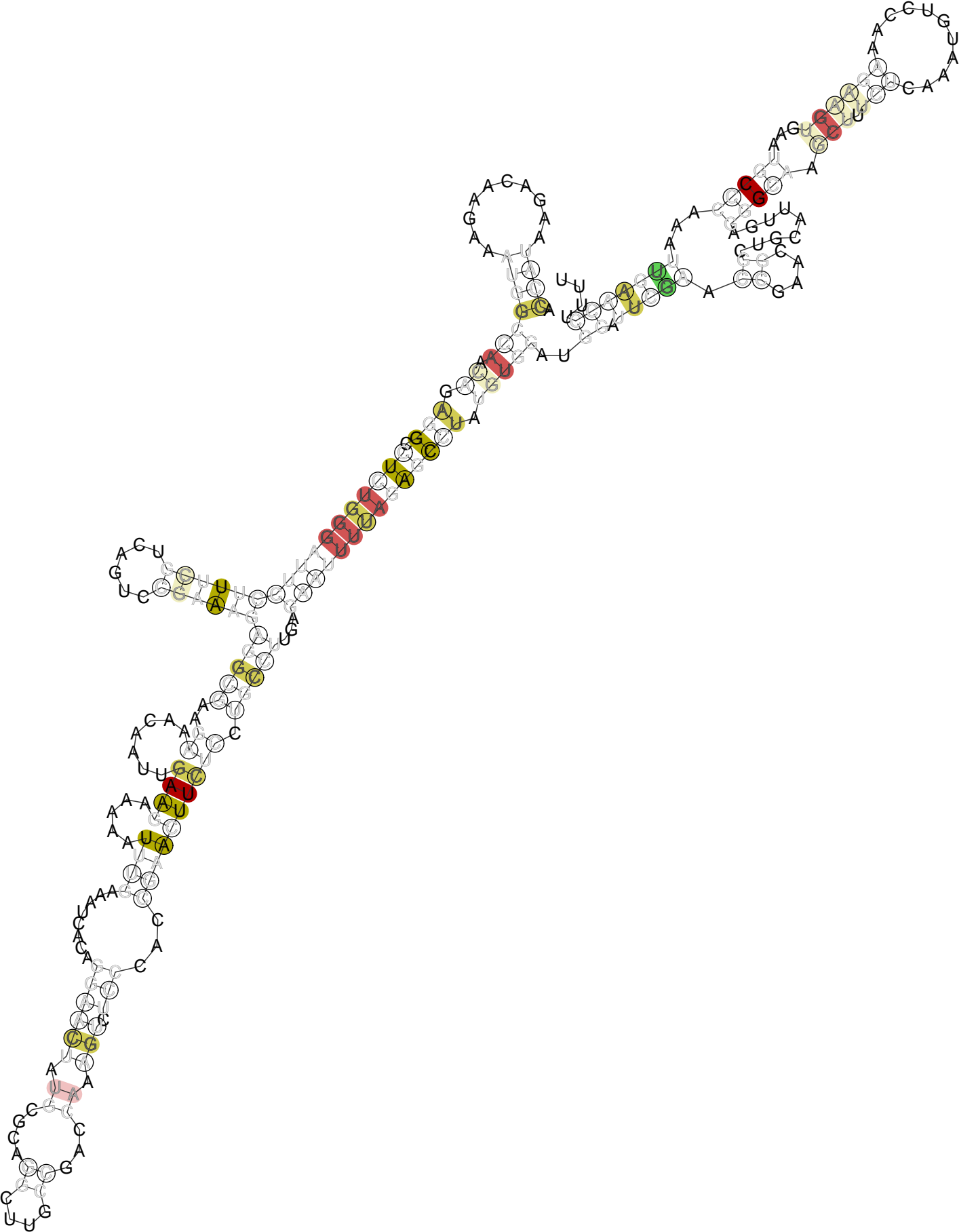

Supplement: S3 Fig — See the caption for S1 Fig for a description of the filename convention (save that the corresponding nucleotide locations in reference sequences are listed in S4 and S12 Tables), and an explanation of the RNAalifold options used and output. (ZIP) [file pcbi.1012009.s124.zip › H3N2-human-raw-PA-alignment-562-813-refseq-553-804-representative-sequences-only_alirna_nogap.pdf]

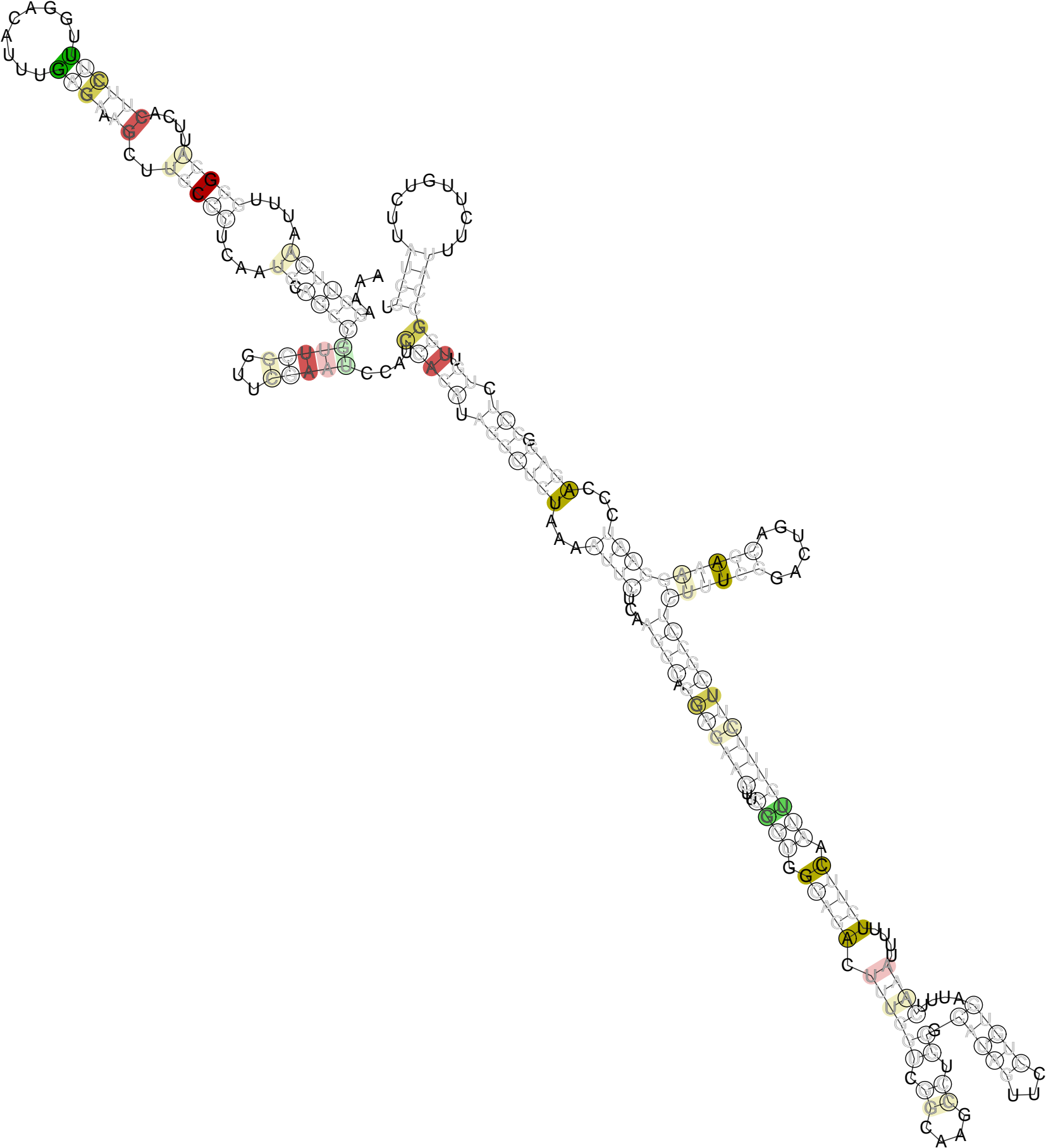

Supplement: S3 Fig — See the caption for S1 Fig for a description of the filename convention (save that the corresponding nucleotide locations in reference sequences are listed in S4 and S12 Tables), and an explanation of the RNAalifold options used and output. (ZIP) [file pcbi.1012009.s124.zip › H3N2-human-raw-PA-alignment-562-813-refseq-553-804-representative-sequences-only_revcomp_alirna_nogap.pdf]

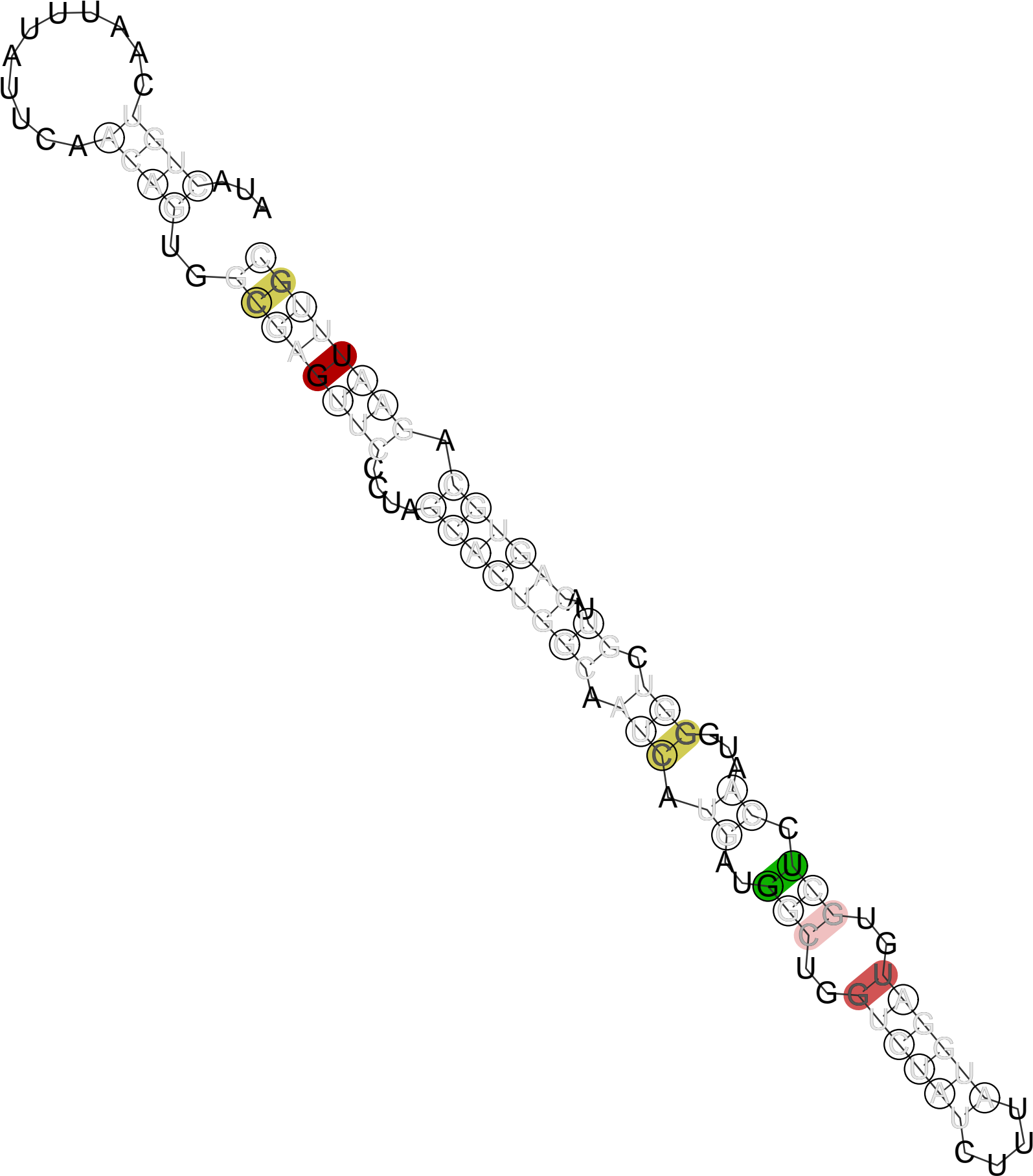

Supplement: S4 Fig — See the caption for S1 Fig for a description of the filename convention (save that the corresponding nucleotide locations in reference sequences are listed in S5 and S13 Tables), and an explanation of the RNAalifold options used and output (save that for these avian-origin viruses the folding temperature was set to 41°C). (ZIP) [file pcbi.1012009.s125.zip › H5N1-avian-ranked-HA-alignment-1627-1737-refseq-1615-1722-41C_alirna_nogap.pdf]

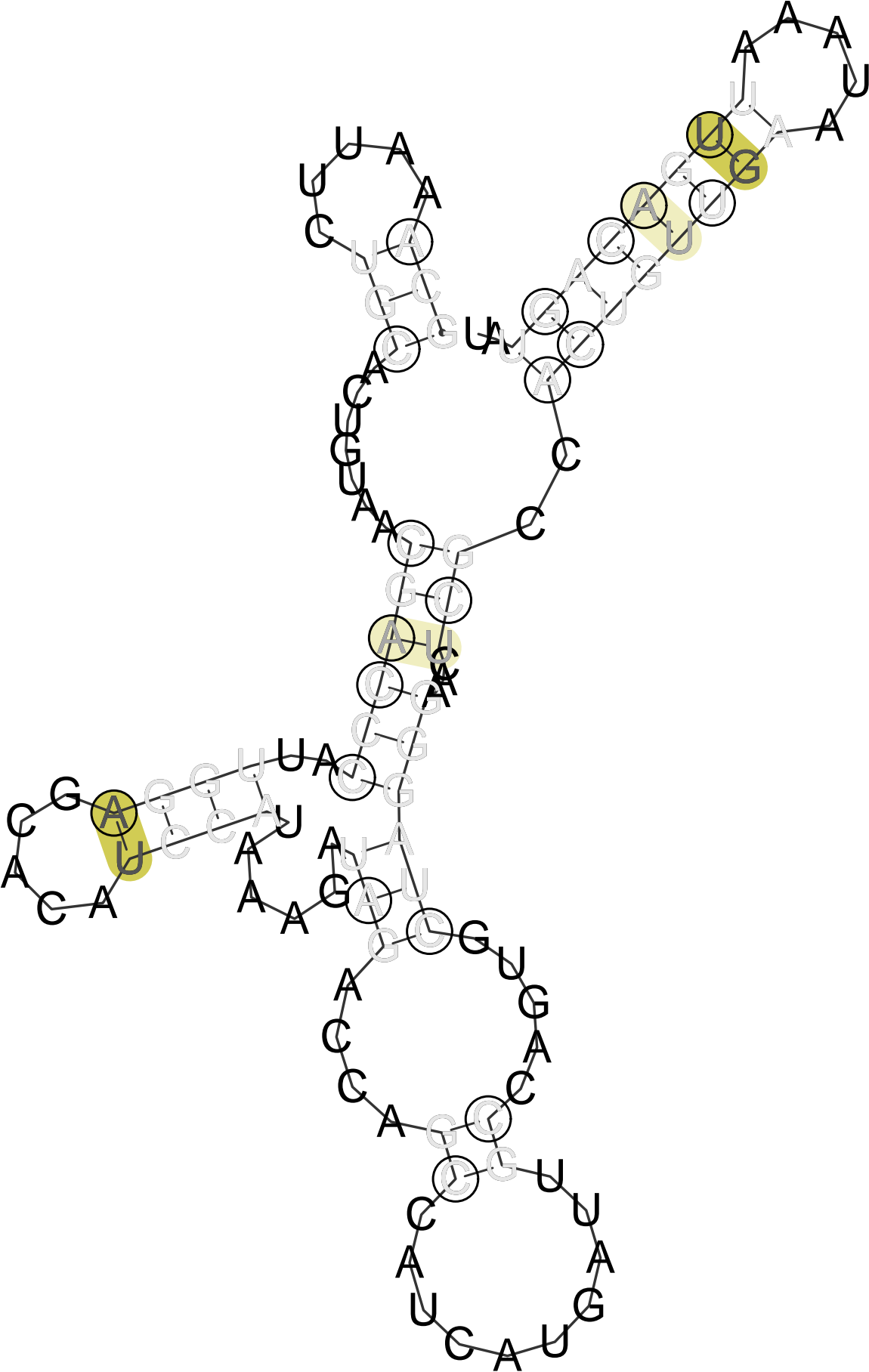

Supplement: S4 Fig — See the caption for S1 Fig for a description of the filename convention (save that the corresponding nucleotide locations in reference sequences are listed in S5 and S13 Tables), and an explanation of the RNAalifold options used and output (save that for these avian-origin viruses the folding temperature was set to 41°C). (ZIP) [file pcbi.1012009.s125.zip › H5N1-avian-ranked-HA-alignment-1627-1737-refseq-1615-1722-41C_revcomp_alirna_nogap.pdf]

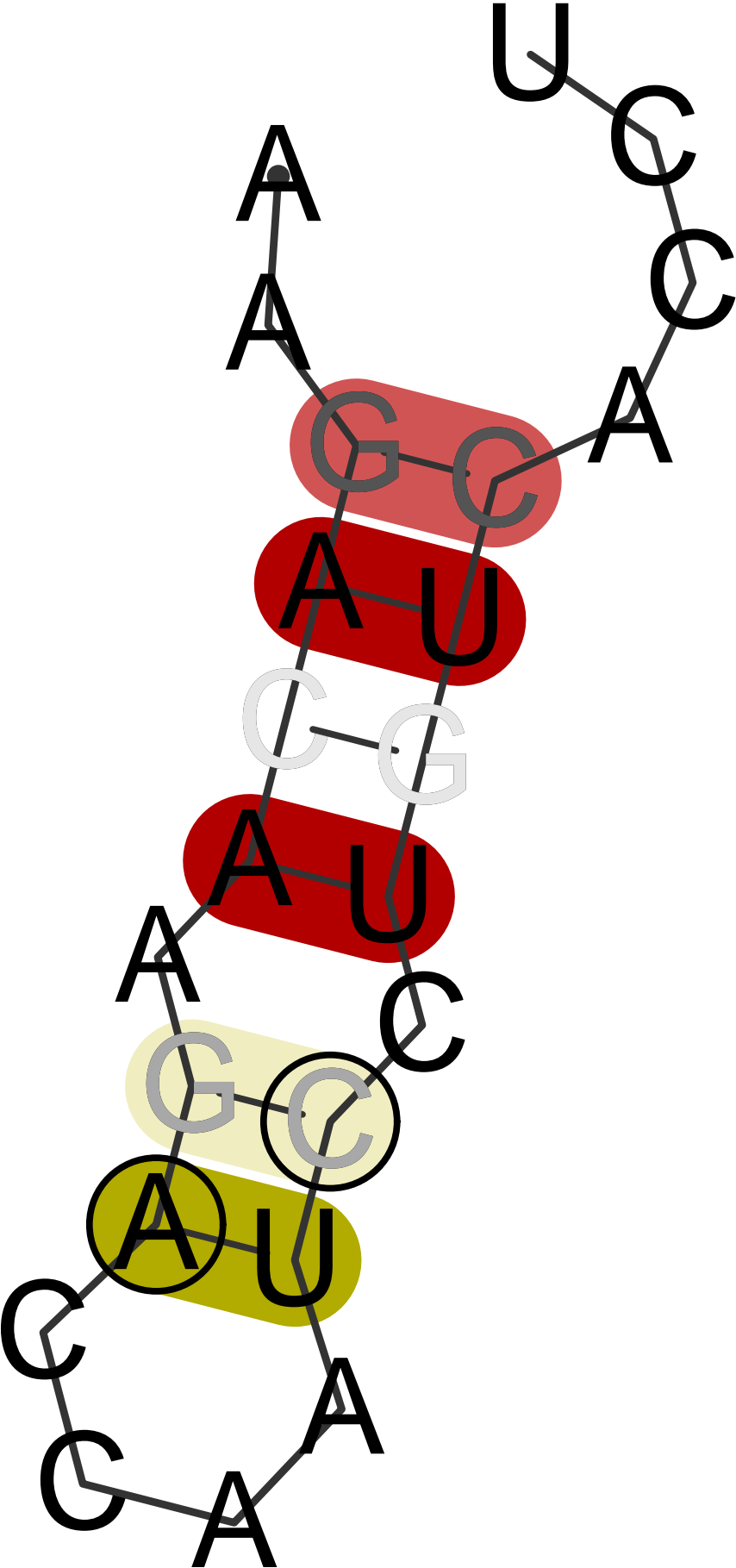

Supplement: S4 Fig — See the caption for S1 Fig for a description of the filename convention (save that the corresponding nucleotide locations in reference sequences are listed in S5 and S13 Tables), and an explanation of the RNAalifold options used and output (save that for these avian-origin viruses the folding temperature was set to 41°C). (ZIP) [file pcbi.1012009.s125.zip › H5N1-avian-ranked-M1-alignment-139-162-refseq-164-187-41C_alirna_nogap.pdf]

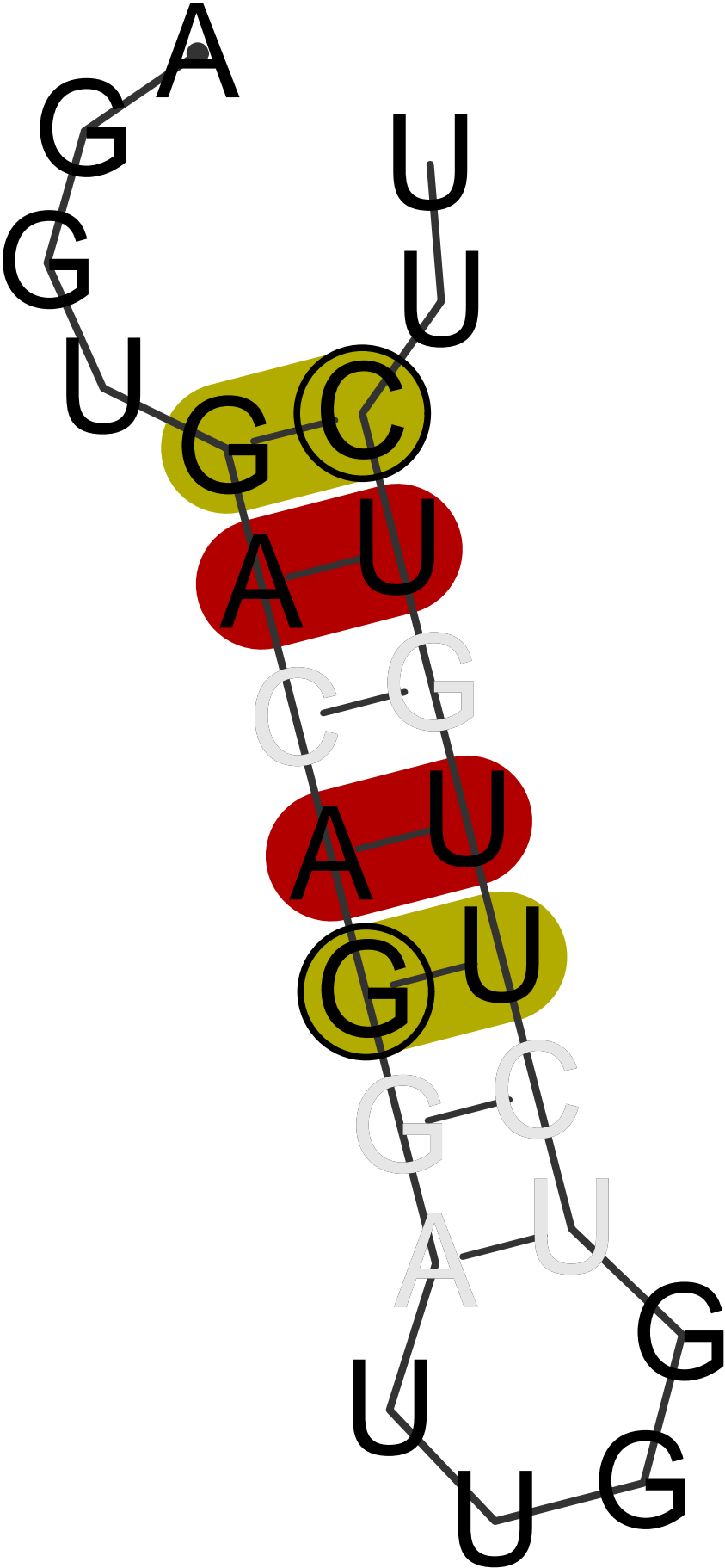

Supplement: S4 Fig — See the caption for S1 Fig for a description of the filename convention (save that the corresponding nucleotide locations in reference sequences are listed in S5 and S13 Tables), and an explanation of the RNAalifold options used and output (save that for these avian-origin viruses the folding temperature was set to 41°C). (ZIP) [file pcbi.1012009.s125.zip › H5N1-avian-ranked-M1-alignment-139-162-refseq-164-187-41C_revcomp_alirna_nogap.pdf]

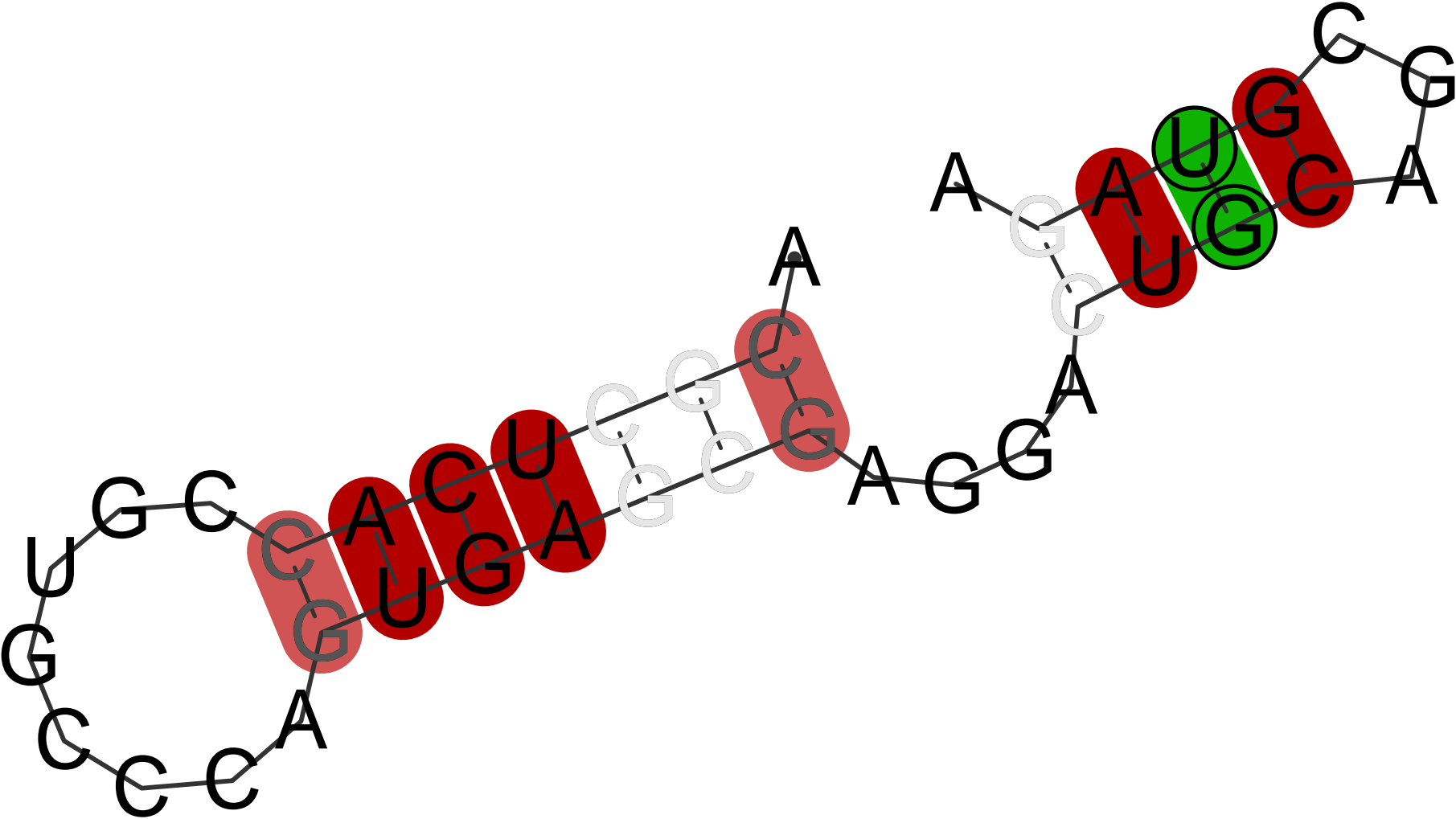

Supplement: S4 Fig — See the caption for S1 Fig for a description of the filename convention (save that the corresponding nucleotide locations in reference sequences are listed in S5 and S13 Tables), and an explanation of the RNAalifold options used and output (save that for these avian-origin viruses the folding temperature was set to 41°C). (ZIP) [file pcbi.1012009.s125.zip › H5N1-avian-ranked-M1-alignment-193-231-refseq-218-256-41C_alirna_nogap.pdf]

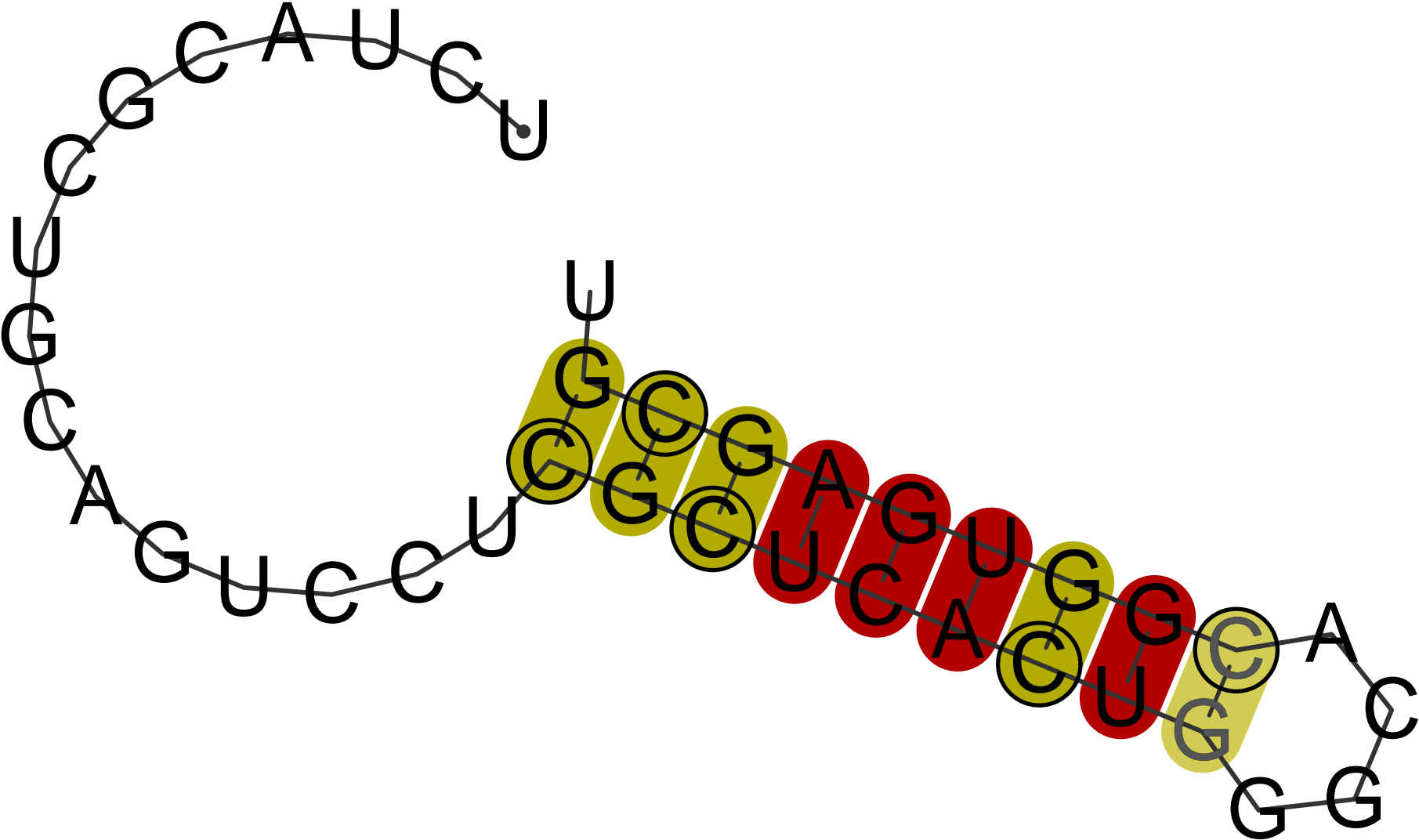

Supplement: S4 Fig — See the caption for S1 Fig for a description of the filename convention (save that the corresponding nucleotide locations in reference sequences are listed in S5 and S13 Tables), and an explanation of the RNAalifold options used and output (save that for these avian-origin viruses the folding temperature was set to 41°C). (ZIP) [file pcbi.1012009.s125.zip › H5N1-avian-ranked-M1-alignment-193-231-refseq-218-256-41C_revcomp_alirna_nogap.pdf]

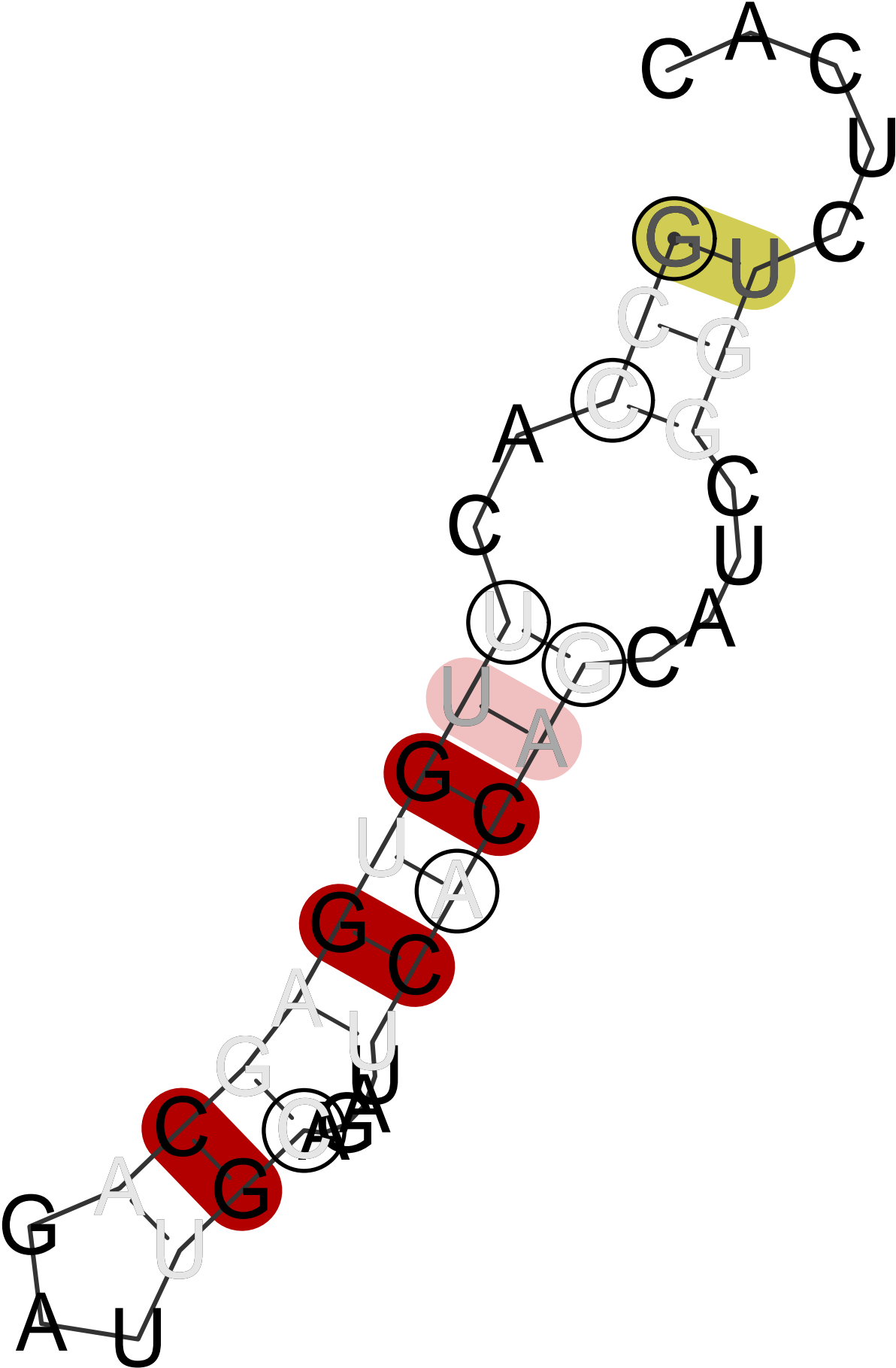

Supplement: S4 Fig — See the caption for S1 Fig for a description of the filename convention (save that the corresponding nucleotide locations in reference sequences are listed in S5 and S13 Tables), and an explanation of the RNAalifold options used and output (save that for these avian-origin viruses the folding temperature was set to 41°C). (ZIP) [file pcbi.1012009.s125.zip › H5N1-avian-ranked-M1-alignment-445-486-refseq-470-511-41C_alirna_nogap.pdf]

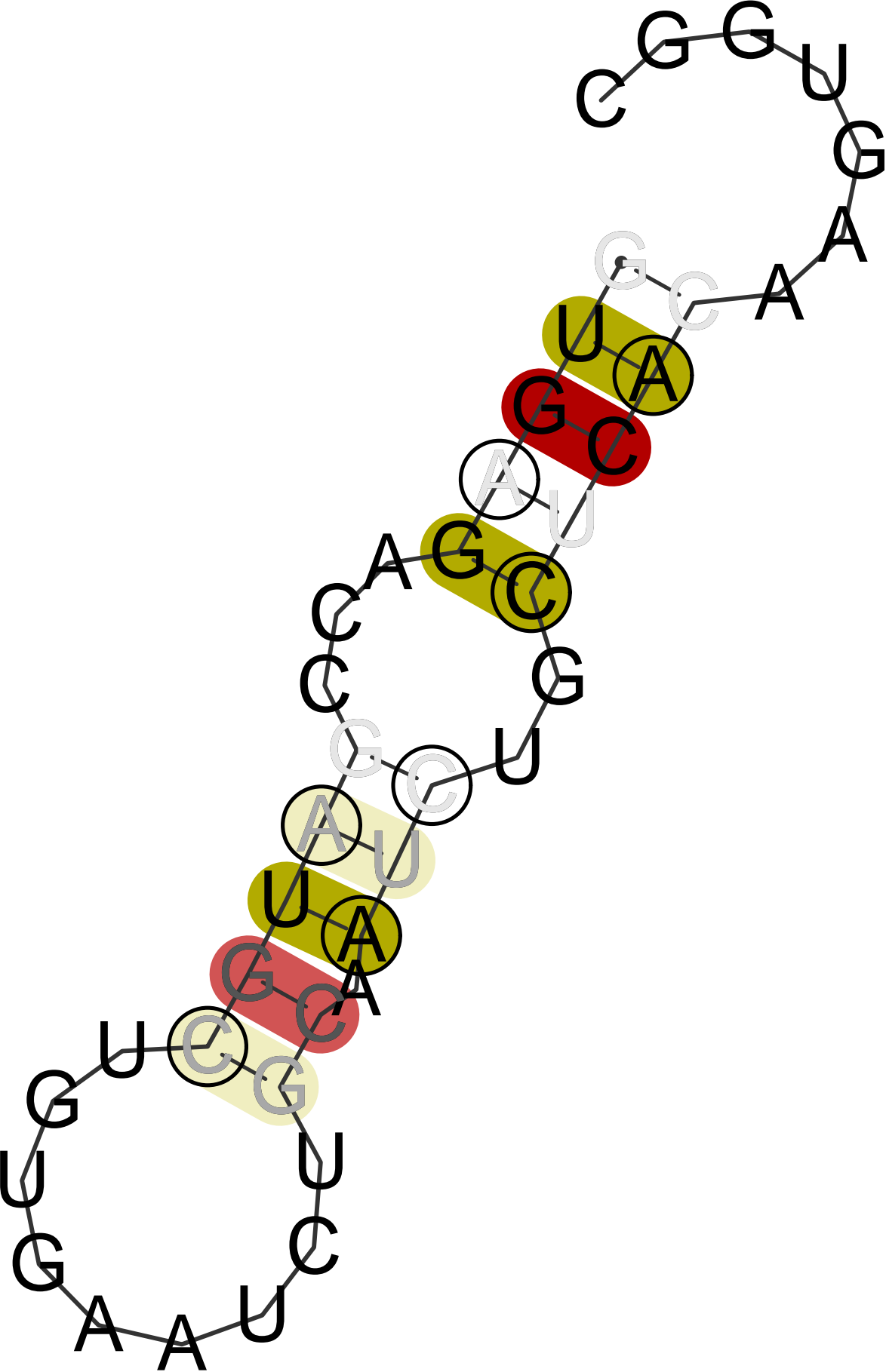

Supplement: S4 Fig — See the caption for S1 Fig for a description of the filename convention (save that the corresponding nucleotide locations in reference sequences are listed in S5 and S13 Tables), and an explanation of the RNAalifold options used and output (save that for these avian-origin viruses the folding temperature was set to 41°C). (ZIP) [file pcbi.1012009.s125.zip › H5N1-avian-ranked-M1-alignment-445-486-refseq-470-511-41C_revcomp_alirna_nogap.pdf]

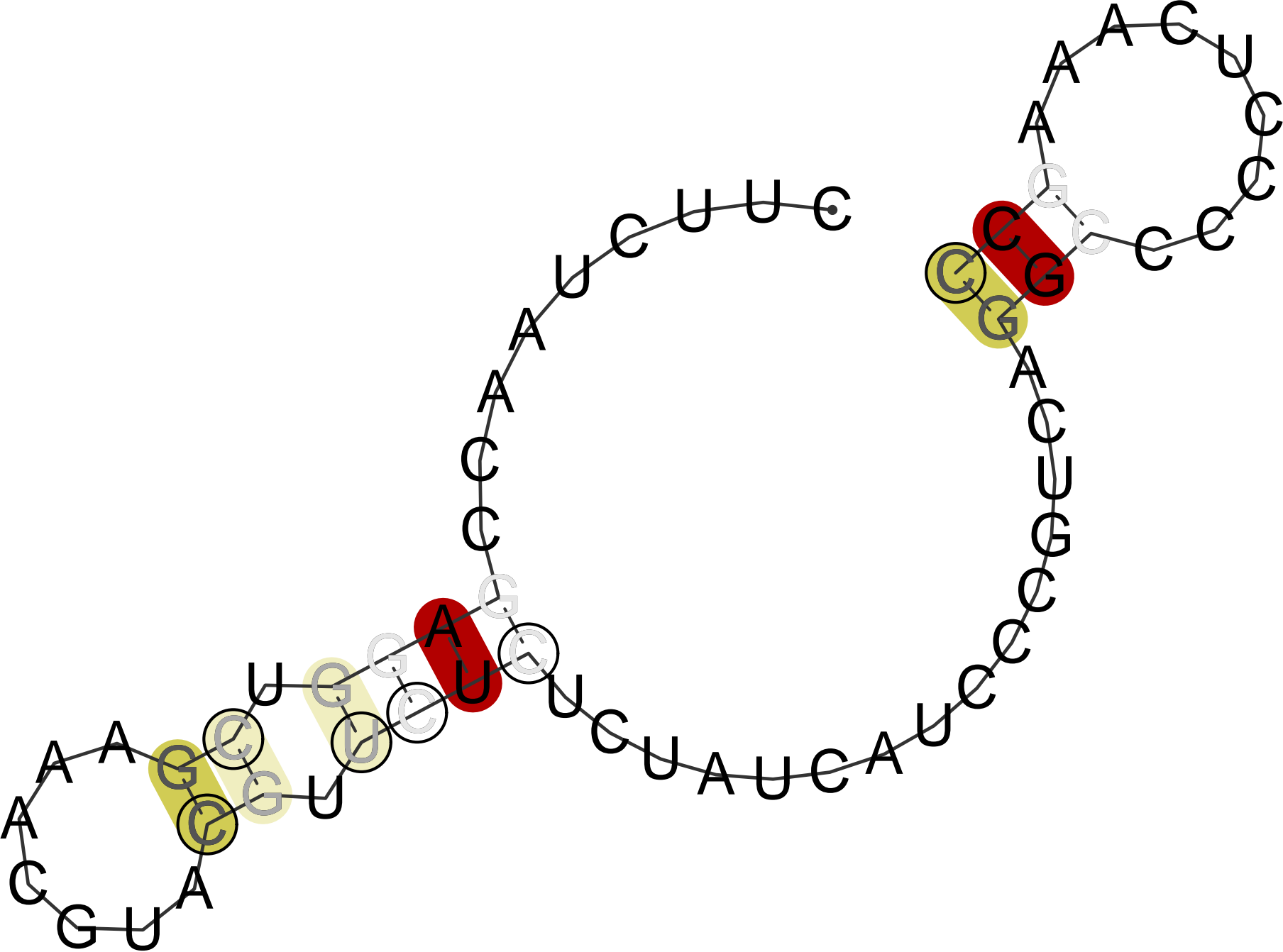

Supplement: S4 Fig — See the caption for S1 Fig for a description of the filename convention (save that the corresponding nucleotide locations in reference sequences are listed in S5 and S13 Tables), and an explanation of the RNAalifold options used and output (save that for these avian-origin viruses the folding temperature was set to 41°C). (ZIP) [file pcbi.1012009.s125.zip › H5N1-avian-ranked-M1-alignment-7-66-refseq-32-91-41C_alirna_nogap.pdf]

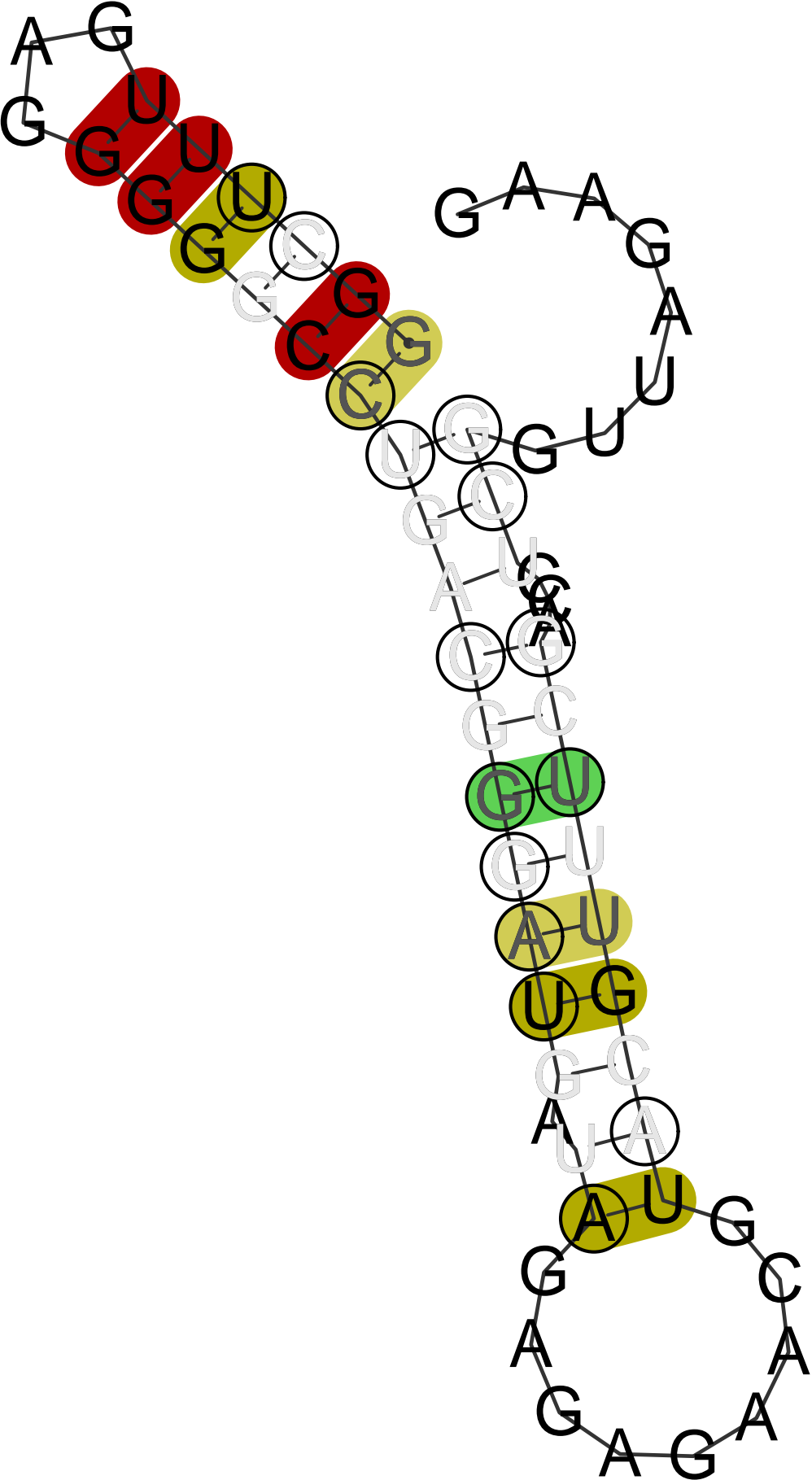

Supplement: S4 Fig — See the caption for S1 Fig for a description of the filename convention (save that the corresponding nucleotide locations in reference sequences are listed in S5 and S13 Tables), and an explanation of the RNAalifold options used and output (save that for these avian-origin viruses the folding temperature was set to 41°C). (ZIP) [file pcbi.1012009.s125.zip › H5N1-avian-ranked-M1-alignment-7-66-refseq-32-91-41C_revcomp_alirna_nogap.pdf]

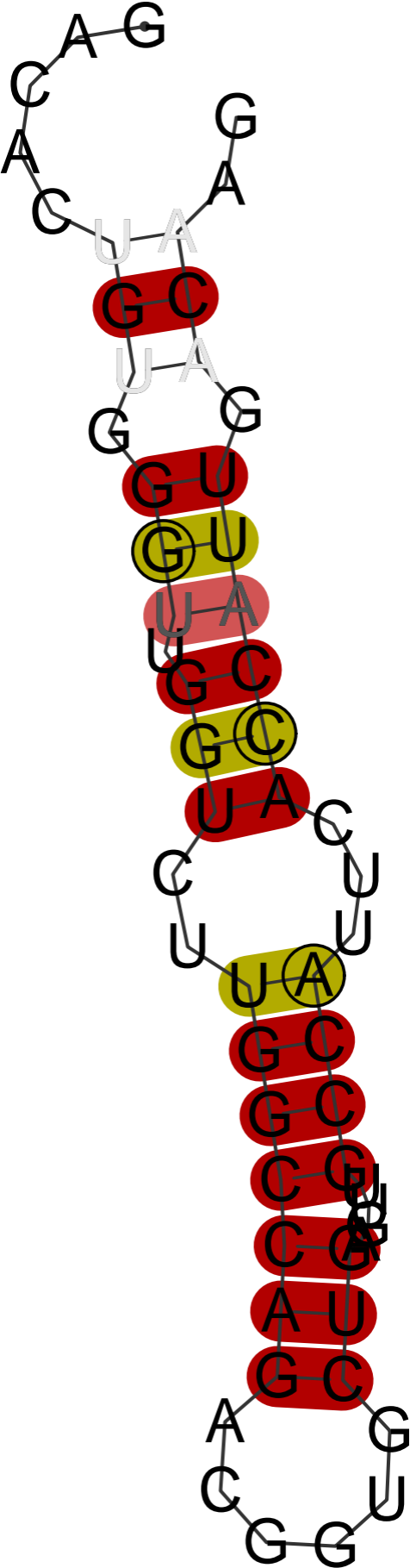

Supplement: S4 Fig — See the caption for S1 Fig for a description of the filename convention (save that the corresponding nucleotide locations in reference sequences are listed in S5 and S13 Tables), and an explanation of the RNAalifold options used and output (save that for these avian-origin viruses the folding temperature was set to 41°C). (ZIP) [file pcbi.1012009.s125.zip › H5N1-avian-ranked-NA-alignment-1354-1410-refseq-1371-1427-41C_alirna_nogap.pdf]

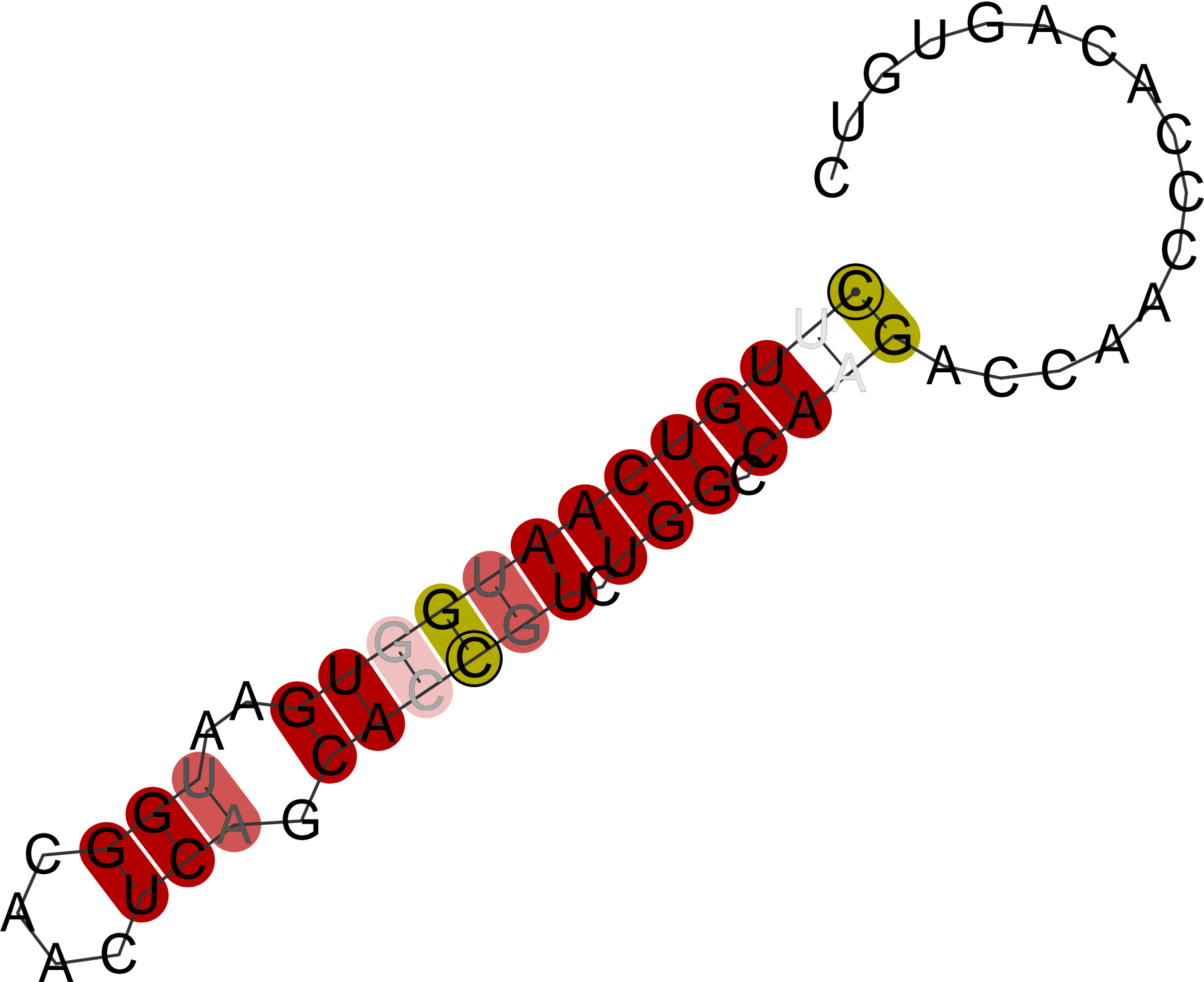

Supplement: S4 Fig — See the caption for S1 Fig for a description of the filename convention (save that the corresponding nucleotide locations in reference sequences are listed in S5 and S13 Tables), and an explanation of the RNAalifold options used and output (save that for these avian-origin viruses the folding temperature was set to 41°C). (ZIP) [file pcbi.1012009.s125.zip › H5N1-avian-ranked-NA-alignment-1354-1410-refseq-1371-1427-41C_revcomp_alirna_nogap.pdf]

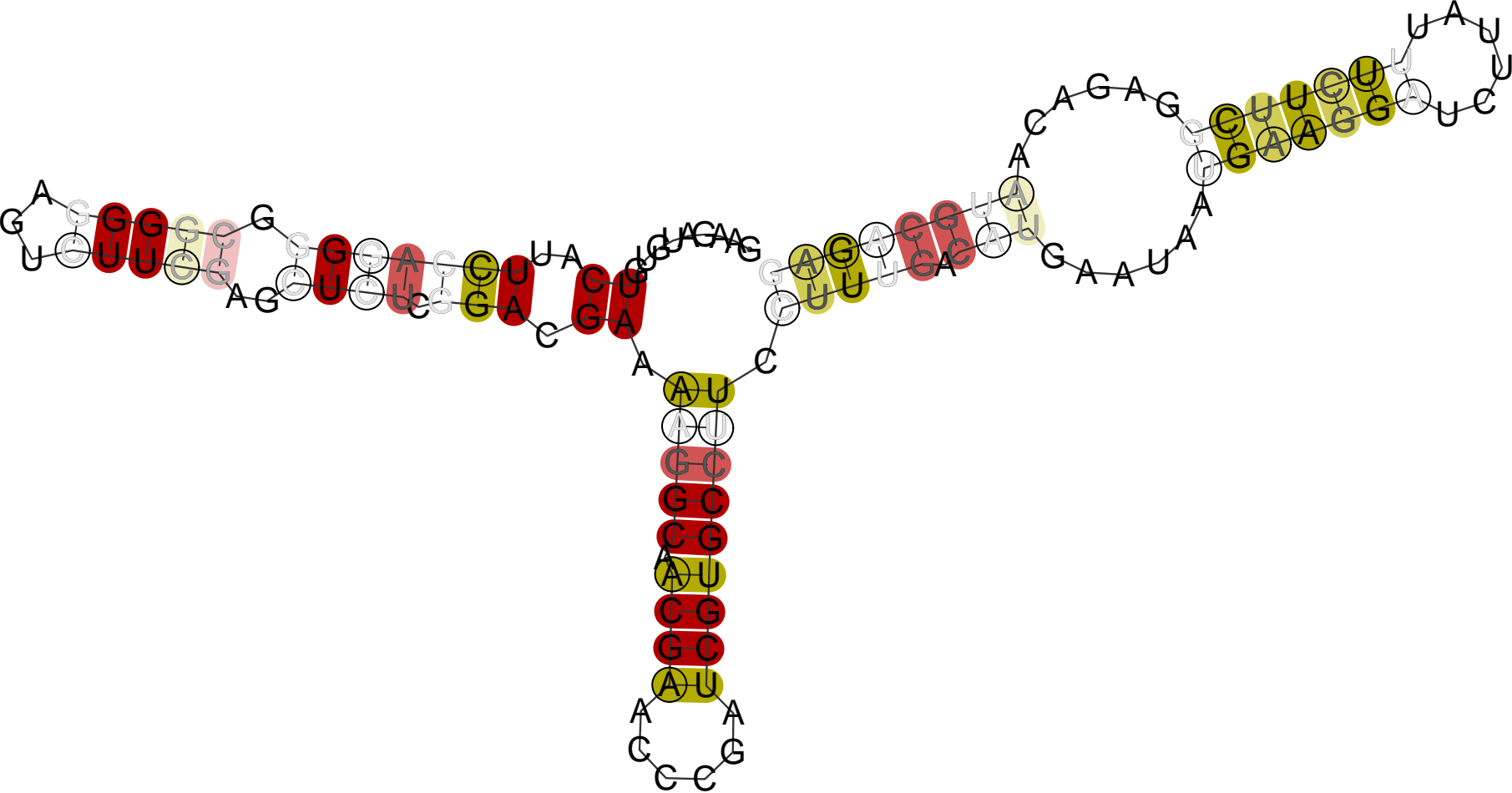

Supplement: S4 Fig — See the caption for S1 Fig for a description of the filename convention (save that the corresponding nucleotide locations in reference sequences are listed in S5 and S13 Tables), and an explanation of the RNAalifold options used and output (save that for these avian-origin viruses the folding temperature was set to 41°C). (ZIP) [file pcbi.1012009.s125.zip › H5N1-avian-ranked-NP-alignment-1360-1482-refseq-1405-1527-41C_alirna_nogap.pdf]

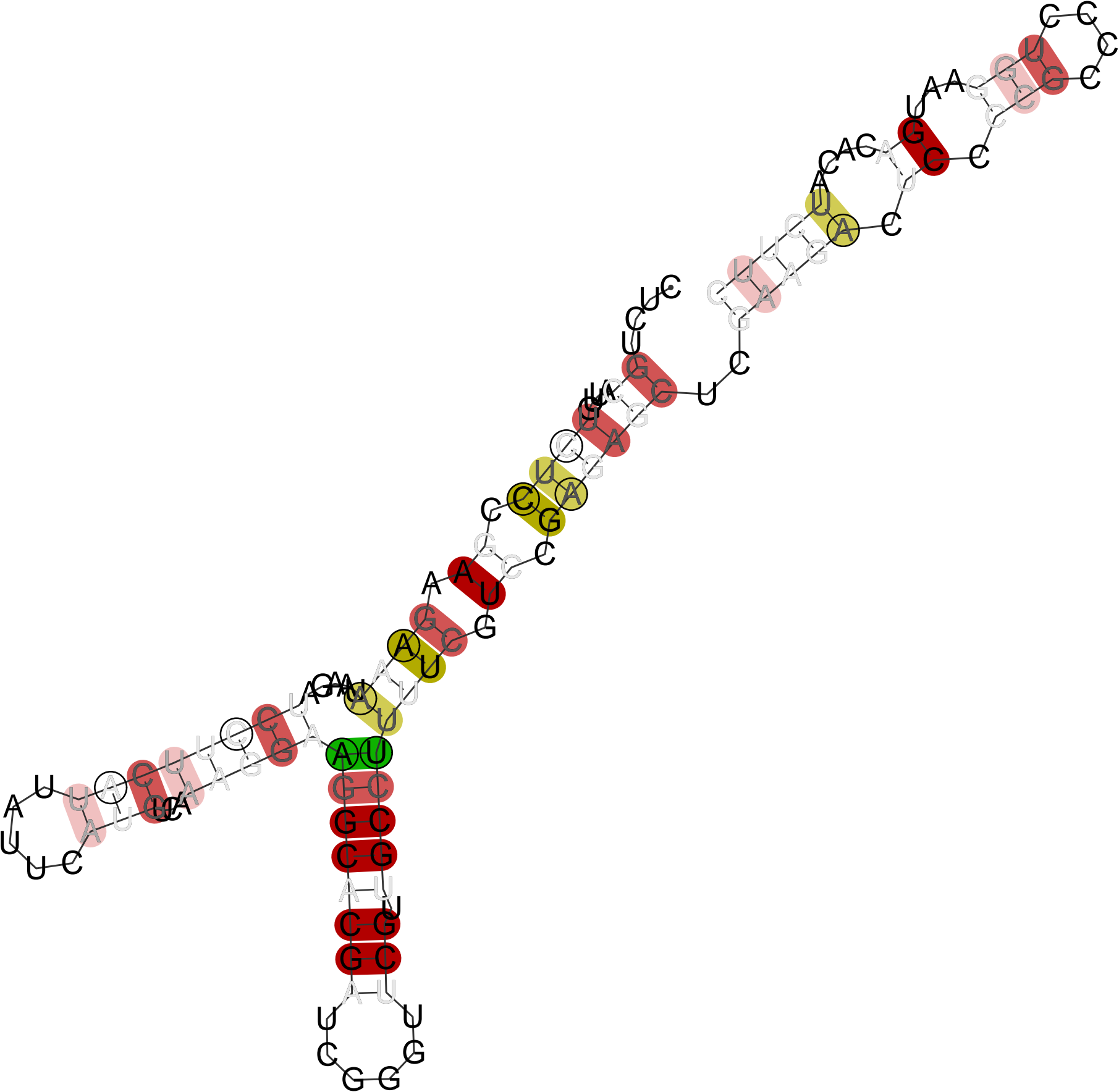

Supplement: S4 Fig — See the caption for S1 Fig for a description of the filename convention (save that the corresponding nucleotide locations in reference sequences are listed in S5 and S13 Tables), and an explanation of the RNAalifold options used and output (save that for these avian-origin viruses the folding temperature was set to 41°C). (ZIP) [file pcbi.1012009.s125.zip › H5N1-avian-ranked-NP-alignment-1360-1482-refseq-1405-1527-41C_revcomp_alirna_nogap.pdf]

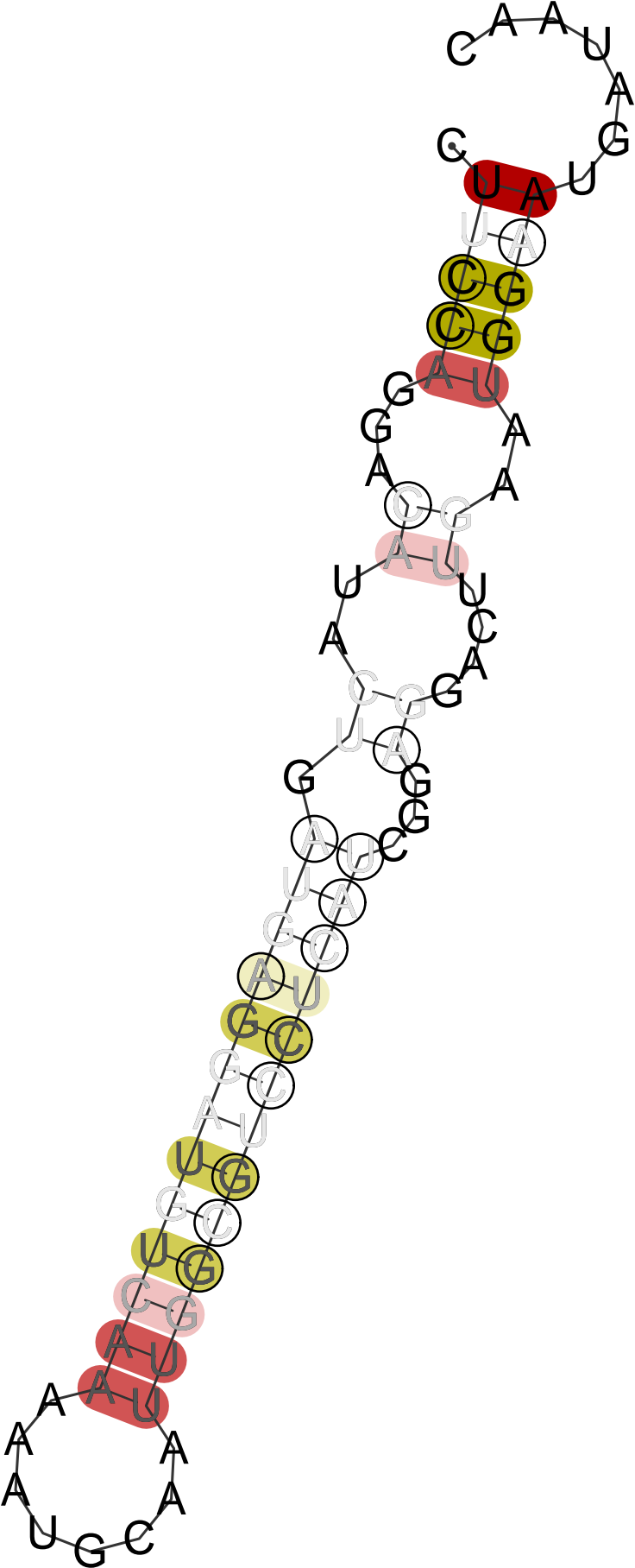

Supplement: S4 Fig — See the caption for S1 Fig for a description of the filename convention (save that the corresponding nucleotide locations in reference sequences are listed in S5 and S13 Tables), and an explanation of the RNAalifold options used and output (save that for these avian-origin viruses the folding temperature was set to 41°C). (ZIP) [file pcbi.1012009.s125.zip › H5N1-avian-ranked-NS1-alignment-529-606-refseq-510-584-41C_alirna_nogap.pdf]

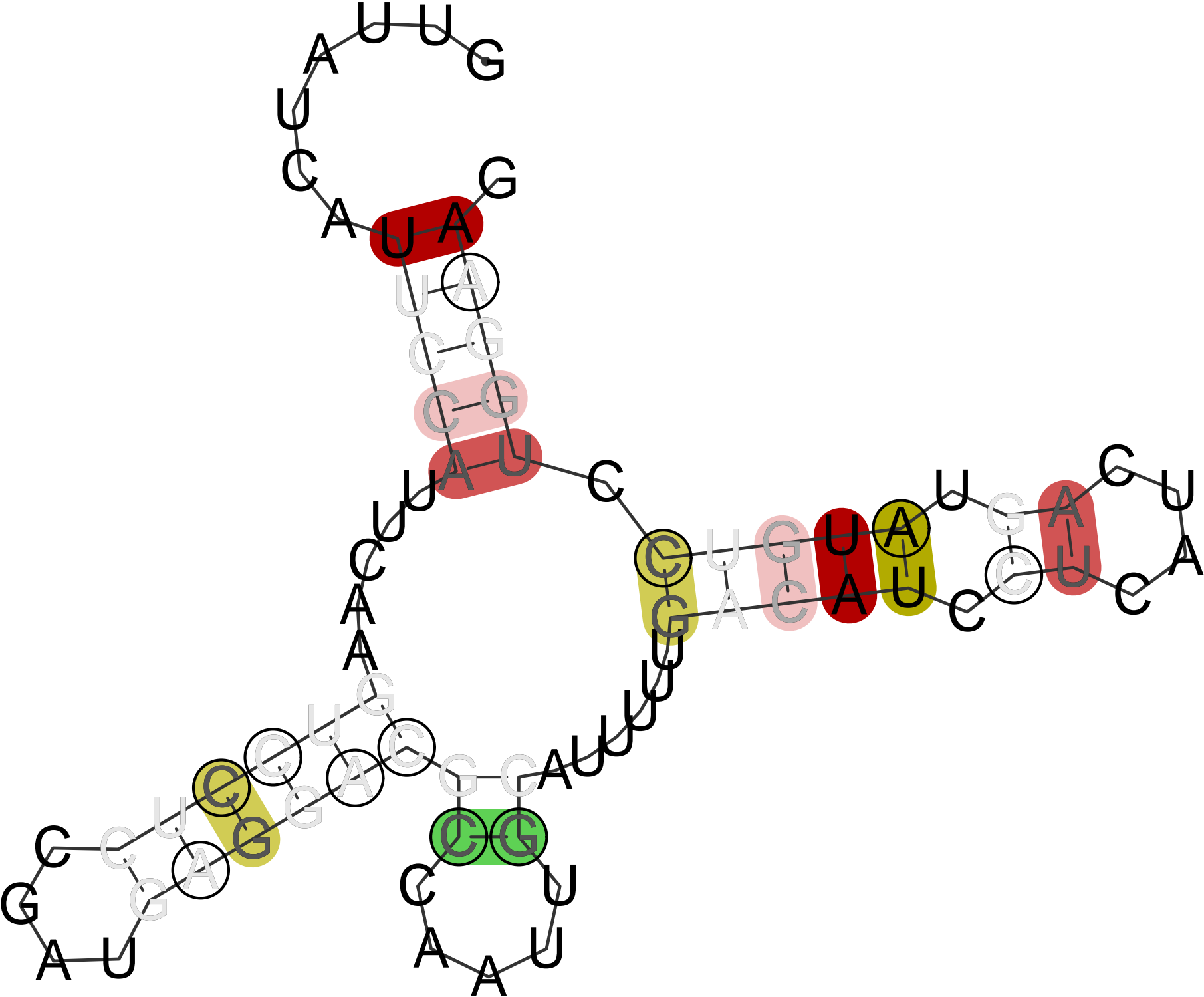

Supplement: S4 Fig — See the caption for S1 Fig for a description of the filename convention (save that the corresponding nucleotide locations in reference sequences are listed in S5 and S13 Tables), and an explanation of the RNAalifold options used and output (save that for these avian-origin viruses the folding temperature was set to 41°C). (ZIP) [file pcbi.1012009.s125.zip › H5N1-avian-ranked-NS1-alignment-529-606-refseq-510-584-41C_revcomp_alirna_nogap.pdf]

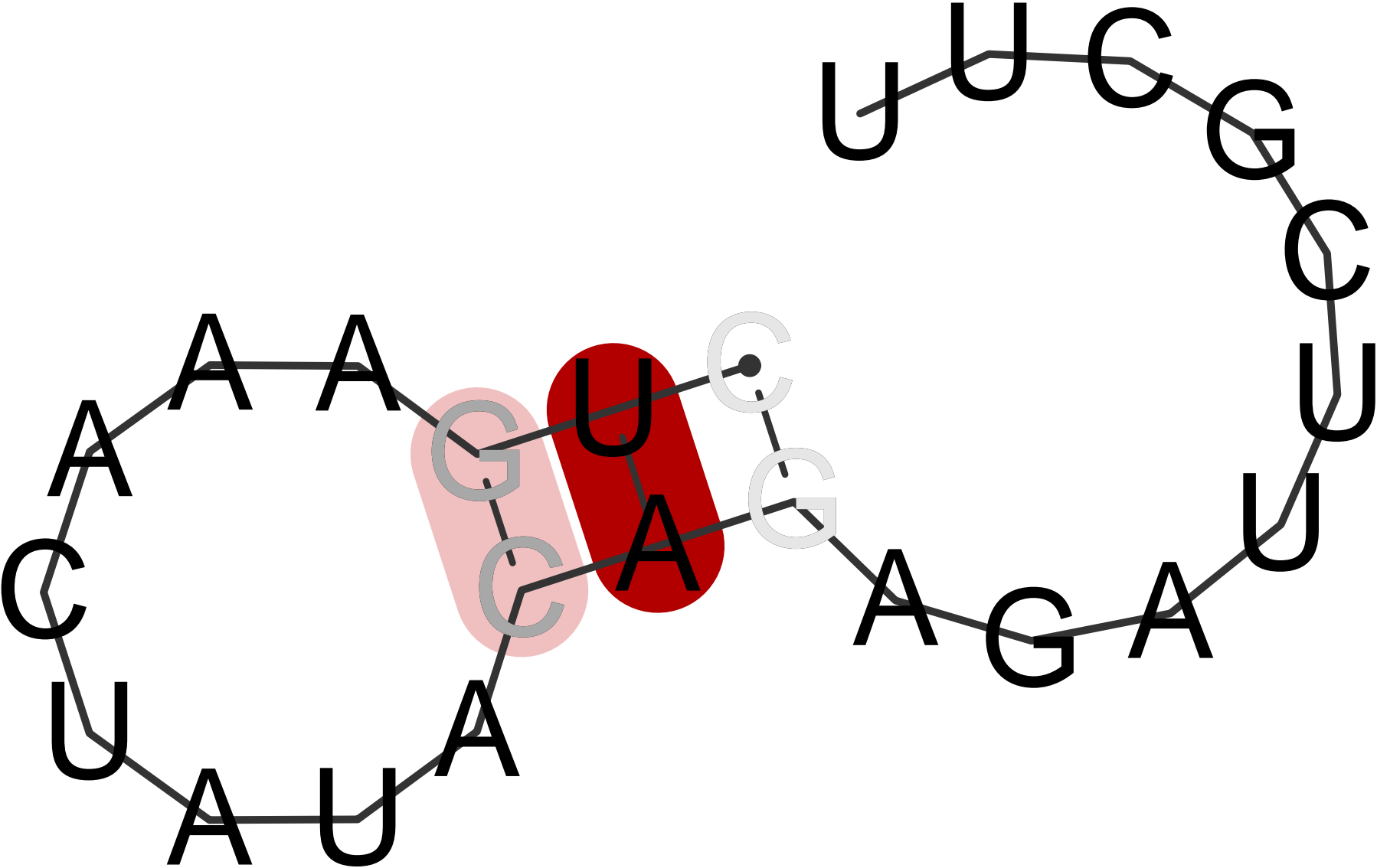

Supplement: S4 Fig — See the caption for S1 Fig for a description of the filename convention (save that the corresponding nucleotide locations in reference sequences are listed in S5 and S13 Tables), and an explanation of the RNAalifold options used and output (save that for these avian-origin viruses the folding temperature was set to 41°C). (ZIP) [file pcbi.1012009.s125.zip › H5N1-avian-ranked-NS2-alignment-121-144-refseq-598-621-41C_alirna_nogap.pdf]

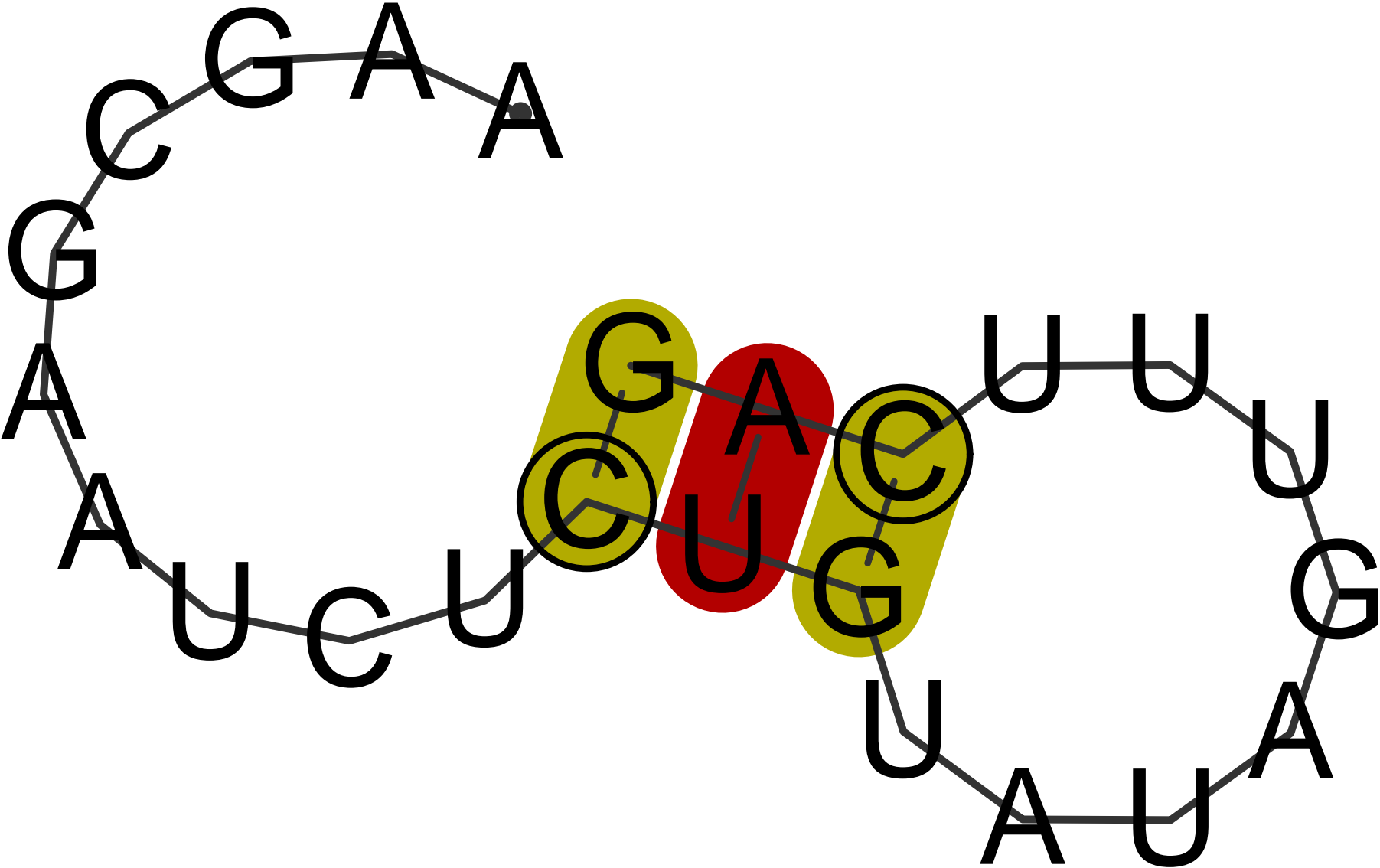

Supplement: S4 Fig — See the caption for S1 Fig for a description of the filename convention (save that the corresponding nucleotide locations in reference sequences are listed in S5 and S13 Tables), and an explanation of the RNAalifold options used and output (save that for these avian-origin viruses the folding temperature was set to 41°C). (ZIP) [file pcbi.1012009.s125.zip › H5N1-avian-ranked-NS2-alignment-121-144-refseq-598-621-41C_revcomp_alirna_nogap.pdf]

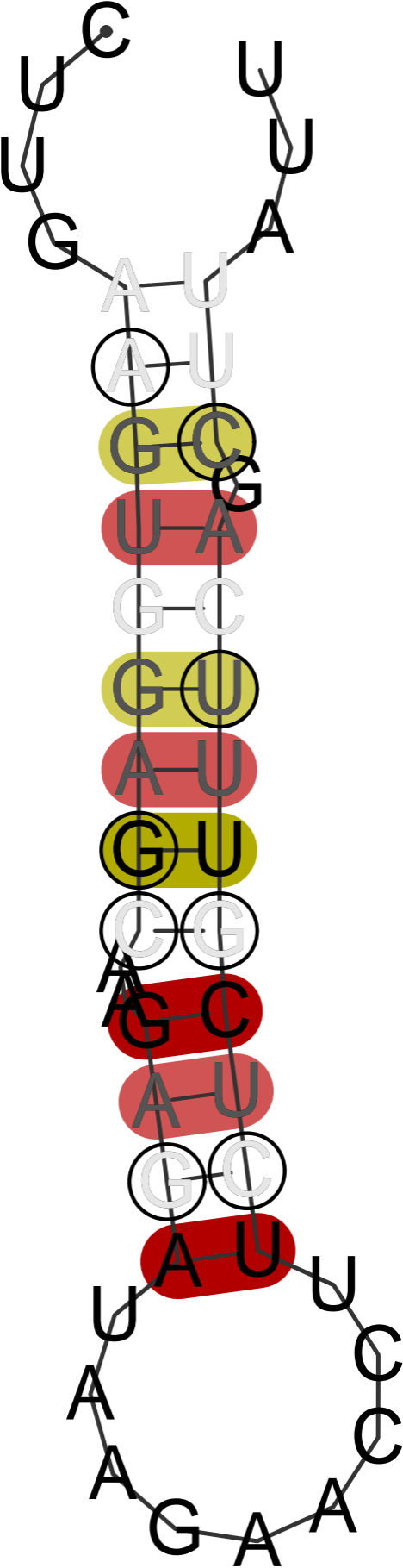

Supplement: S4 Fig — See the caption for S1 Fig for a description of the filename convention (save that the corresponding nucleotide locations in reference sequences are listed in S5 and S13 Tables), and an explanation of the RNAalifold options used and output (save that for these avian-origin viruses the folding temperature was set to 41°C). (ZIP) [file pcbi.1012009.s125.zip › H5N1-avian-ranked-NS2-alignment-328-372-refseq-805-849-41C_alirna_nogap.pdf]

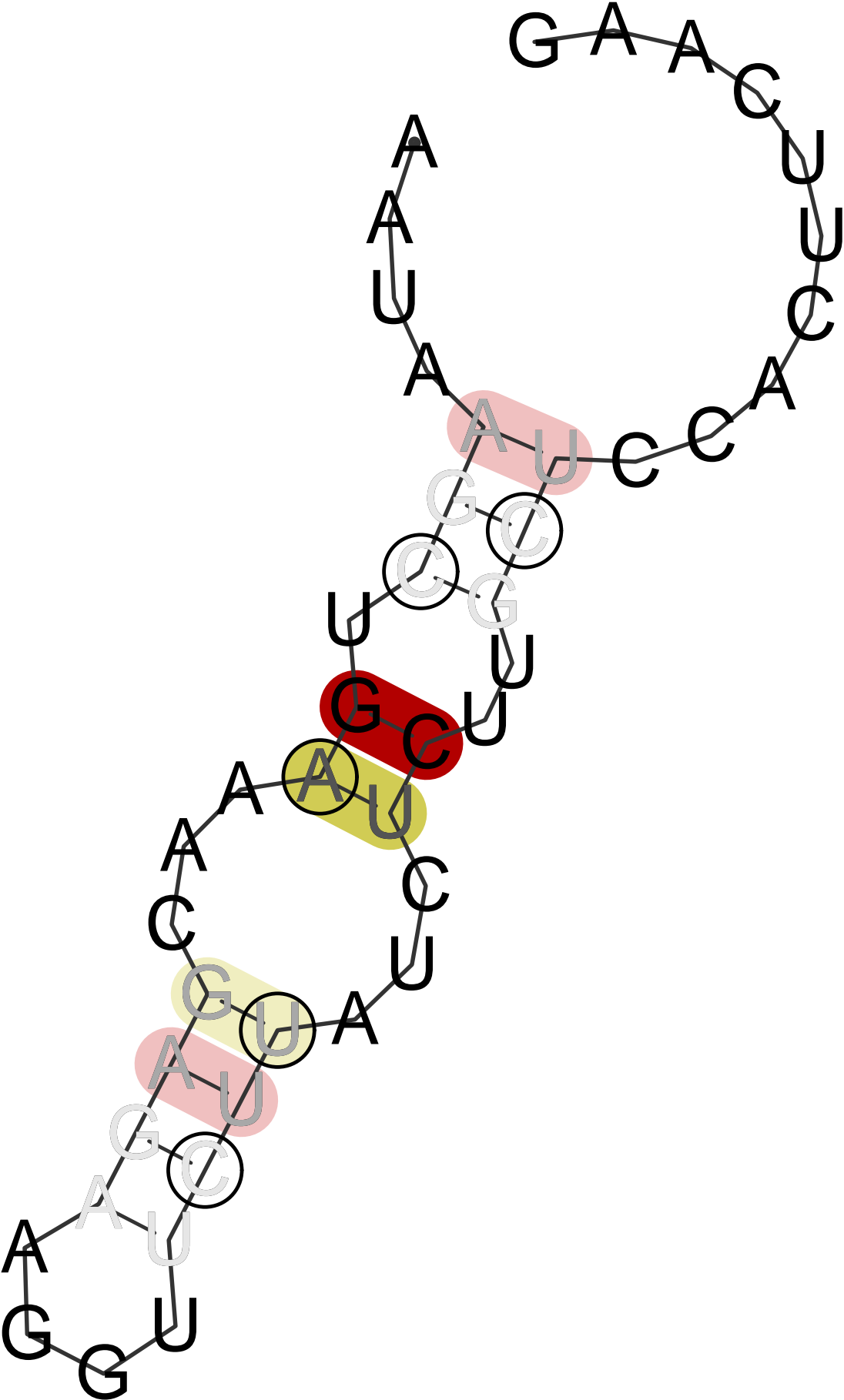

Supplement: S4 Fig — See the caption for S1 Fig for a description of the filename convention (save that the corresponding nucleotide locations in reference sequences are listed in S5 and S13 Tables), and an explanation of the RNAalifold options used and output (save that for these avian-origin viruses the folding temperature was set to 41°C). (ZIP) [file pcbi.1012009.s125.zip › H5N1-avian-ranked-NS2-alignment-328-372-refseq-805-849-41C_revcomp_alirna_nogap.pdf]

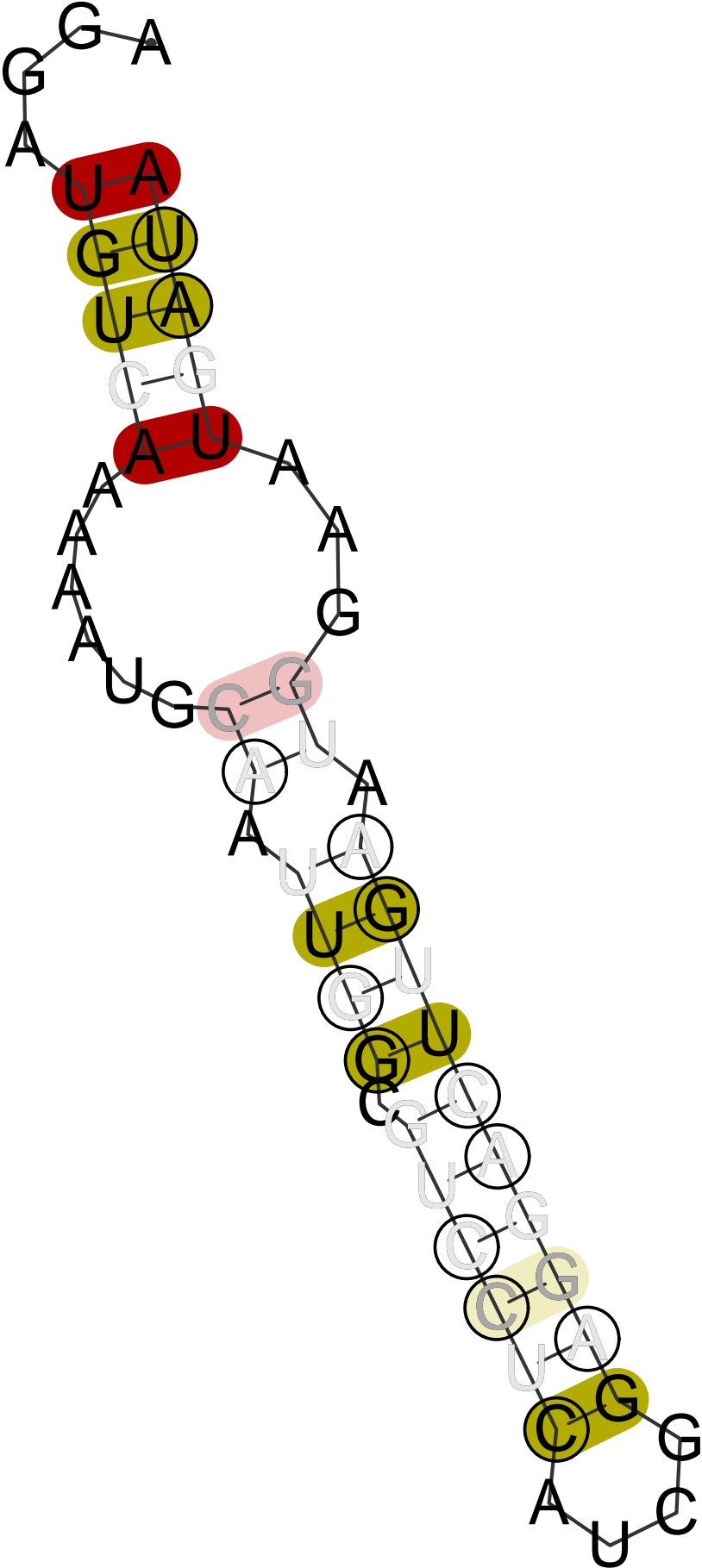

Supplement: S4 Fig — See the caption for S1 Fig for a description of the filename convention (save that the corresponding nucleotide locations in reference sequences are listed in S5 and S13 Tables), and an explanation of the RNAalifold options used and output (save that for these avian-origin viruses the folding temperature was set to 41°C). (ZIP) [file pcbi.1012009.s125.zip › H5N1-avian-ranked-NS2-alignment-49-105-refseq-529-582-41C_alirna_nogap.pdf]

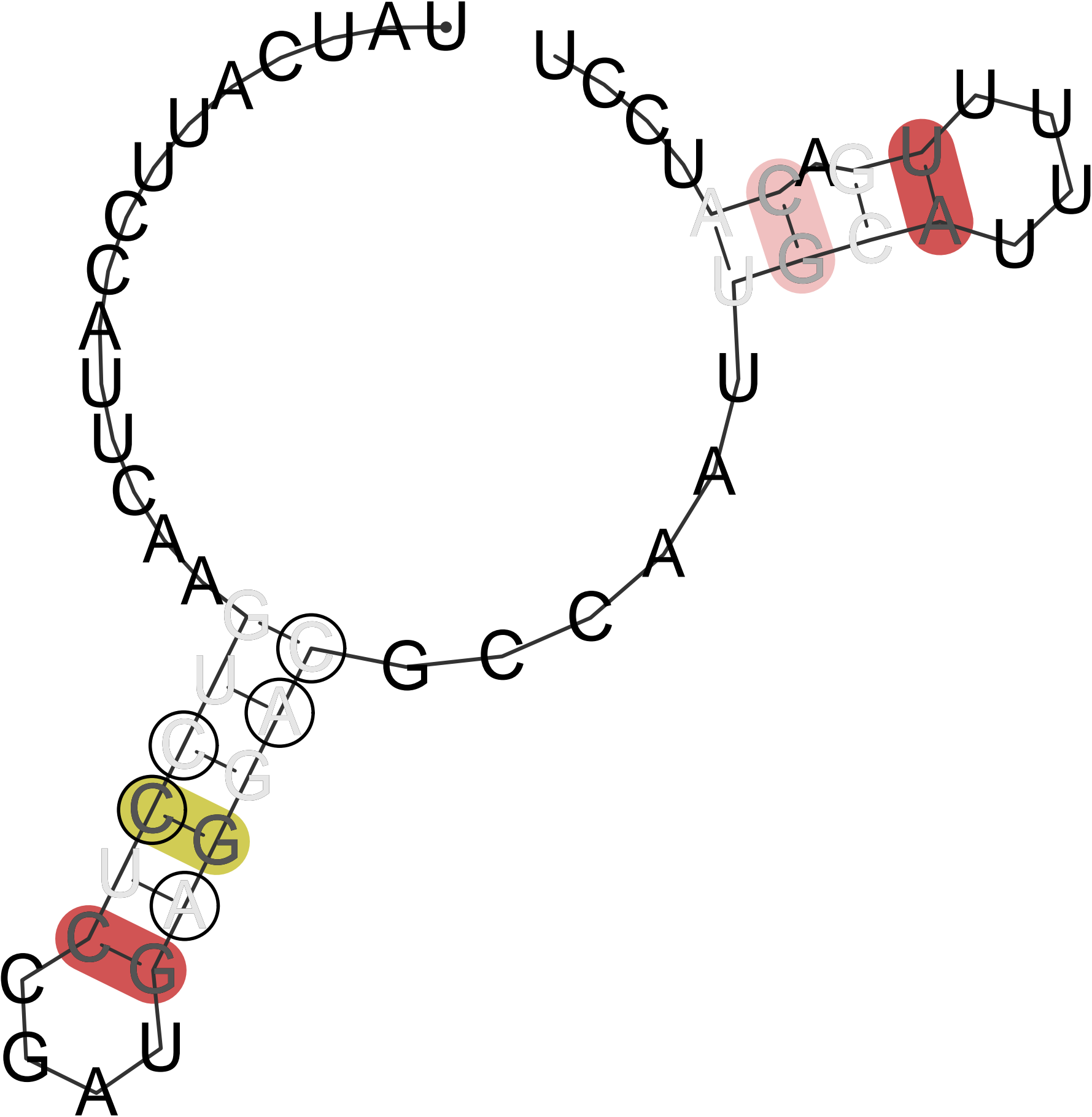

Supplement: S4 Fig — See the caption for S1 Fig for a description of the filename convention (save that the corresponding nucleotide locations in reference sequences are listed in S5 and S13 Tables), and an explanation of the RNAalifold options used and output (save that for these avian-origin viruses the folding temperature was set to 41°C). (ZIP) [file pcbi.1012009.s125.zip › H5N1-avian-ranked-NS2-alignment-49-105-refseq-529-582-41C_revcomp_alirna_nogap.pdf]

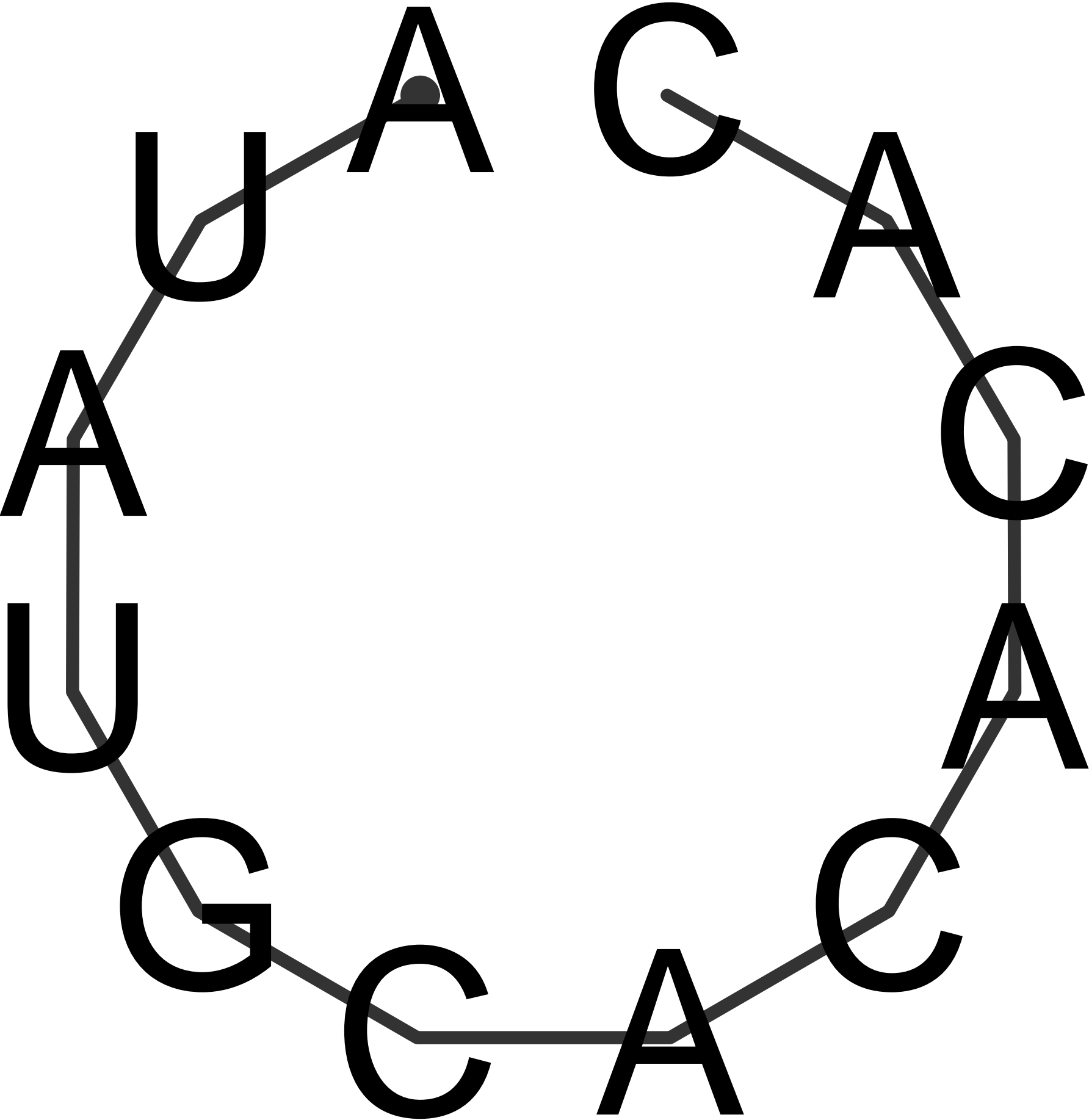

Supplement: S4 Fig — See the caption for S1 Fig for a description of the filename convention (save that the corresponding nucleotide locations in reference sequences are listed in S5 and S13 Tables), and an explanation of the RNAalifold options used and output (save that for these avian-origin viruses the folding temperature was set to 41°C). (ZIP) [file pcbi.1012009.s125.zip › H5N1-avian-ranked-PA-X-alignment-112-123-refseq-136-147-41C_alirna_nogap.pdf]

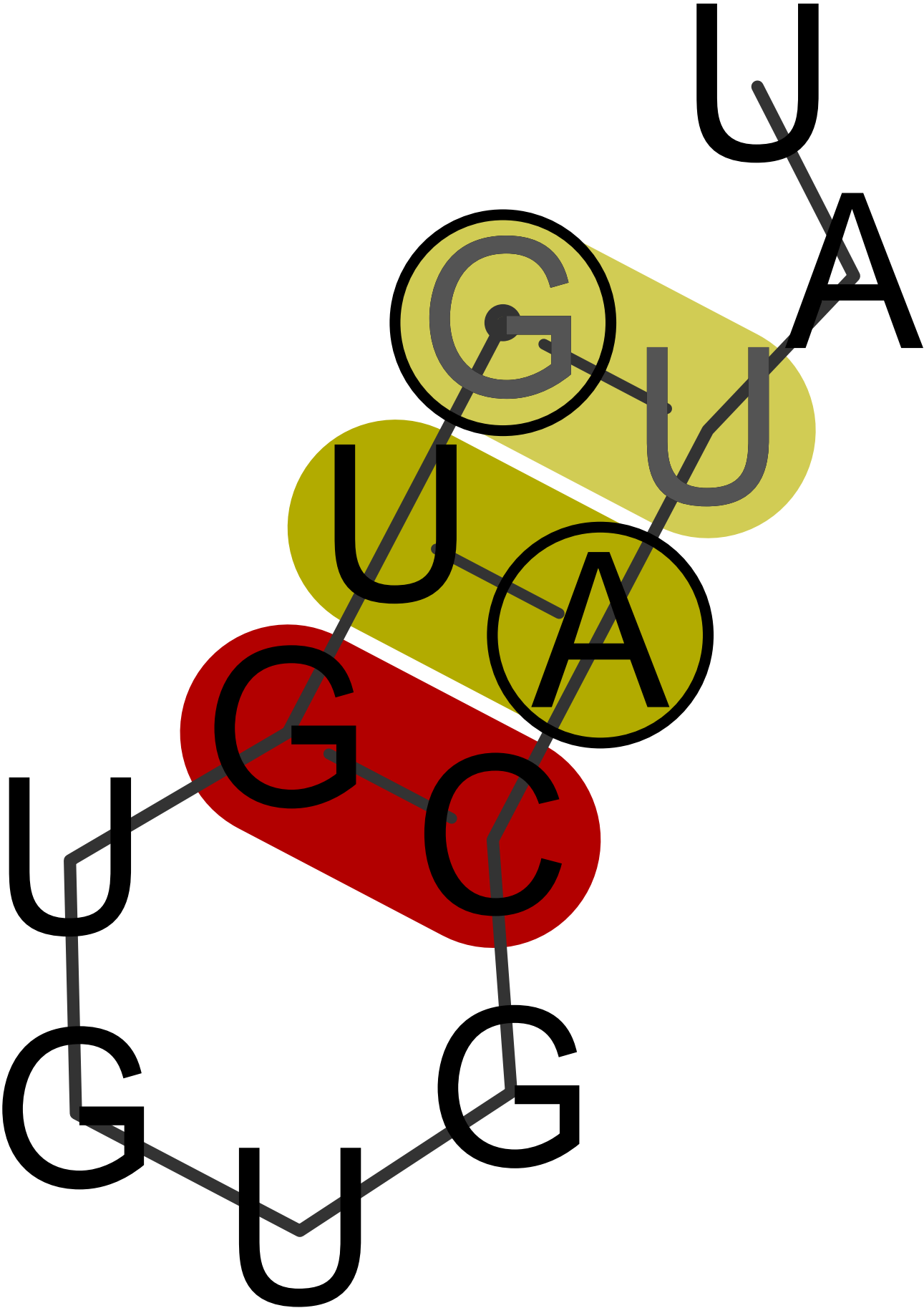

Supplement: S4 Fig — See the caption for S1 Fig for a description of the filename convention (save that the corresponding nucleotide locations in reference sequences are listed in S5 and S13 Tables), and an explanation of the RNAalifold options used and output (save that for these avian-origin viruses the folding temperature was set to 41°C). (ZIP) [file pcbi.1012009.s125.zip › H5N1-avian-ranked-PA-X-alignment-112-123-refseq-136-147-41C_revcomp_alirna_nogap.pdf]

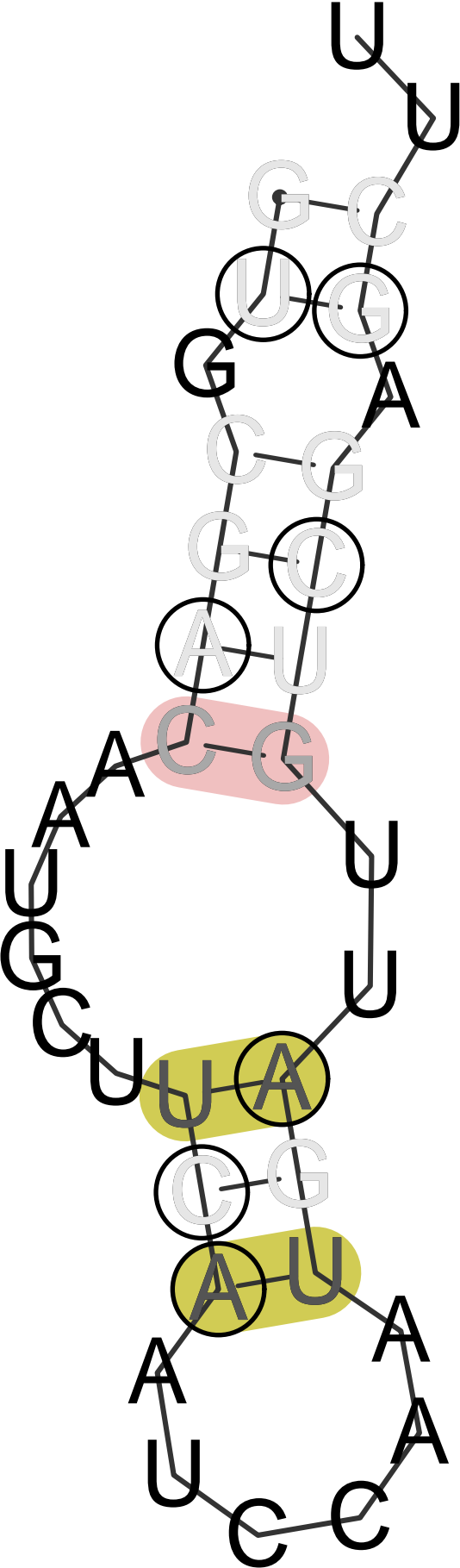

Supplement: S4 Fig — See the caption for S1 Fig for a description of the filename convention (save that the corresponding nucleotide locations in reference sequences are listed in S5 and S13 Tables), and an explanation of the RNAalifold options used and output (save that for these avian-origin viruses the folding temperature was set to 41°C). (ZIP) [file pcbi.1012009.s125.zip › H5N1-avian-ranked-PA-X-alignment-13-48-refseq-37-72-41C_alirna_nogap.pdf]

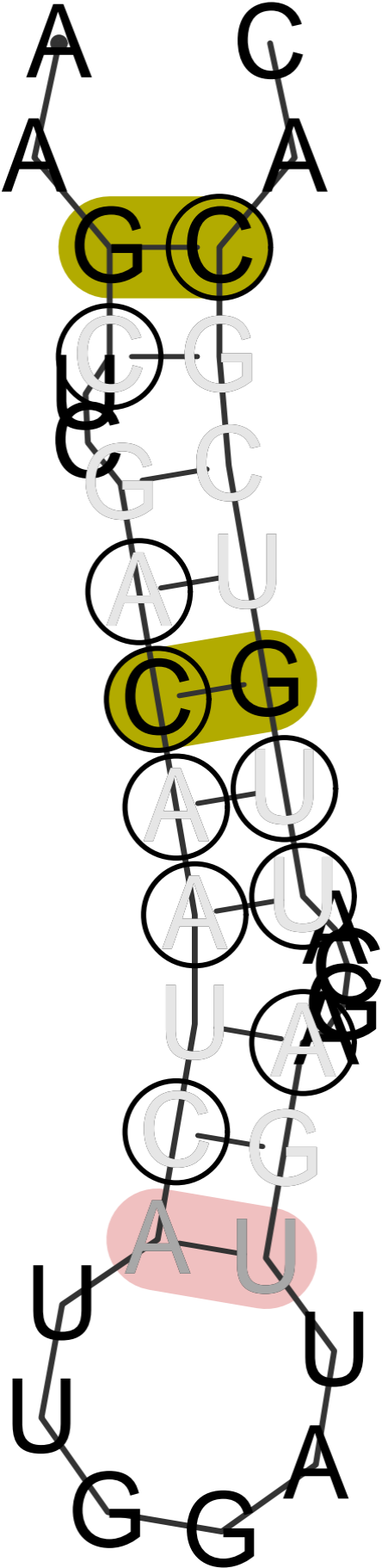

Supplement: S4 Fig — See the caption for S1 Fig for a description of the filename convention (save that the corresponding nucleotide locations in reference sequences are listed in S5 and S13 Tables), and an explanation of the RNAalifold options used and output (save that for these avian-origin viruses the folding temperature was set to 41°C). (ZIP) [file pcbi.1012009.s125.zip › H5N1-avian-ranked-PA-X-alignment-13-48-refseq-37-72-41C_revcomp_alirna_nogap.pdf]

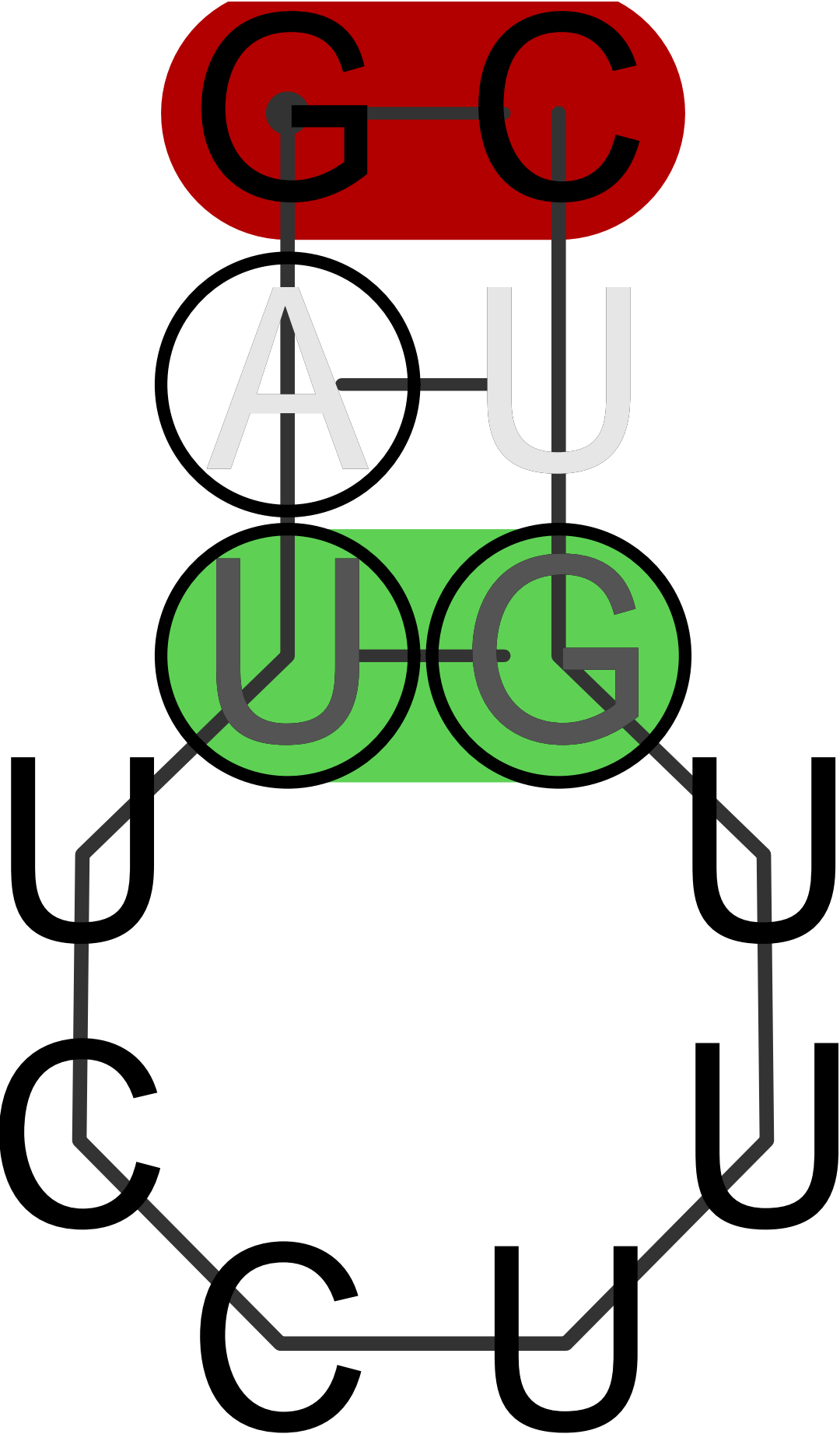

Supplement: S4 Fig — See the caption for S1 Fig for a description of the filename convention (save that the corresponding nucleotide locations in reference sequences are listed in S5 and S13 Tables), and an explanation of the RNAalifold options used and output (save that for these avian-origin viruses the folding temperature was set to 41°C). (ZIP) [file pcbi.1012009.s125.zip › H5N1-avian-ranked-PA-X-alignment-565-576-refseq-589-601-41C_alirna_nogap.pdf]

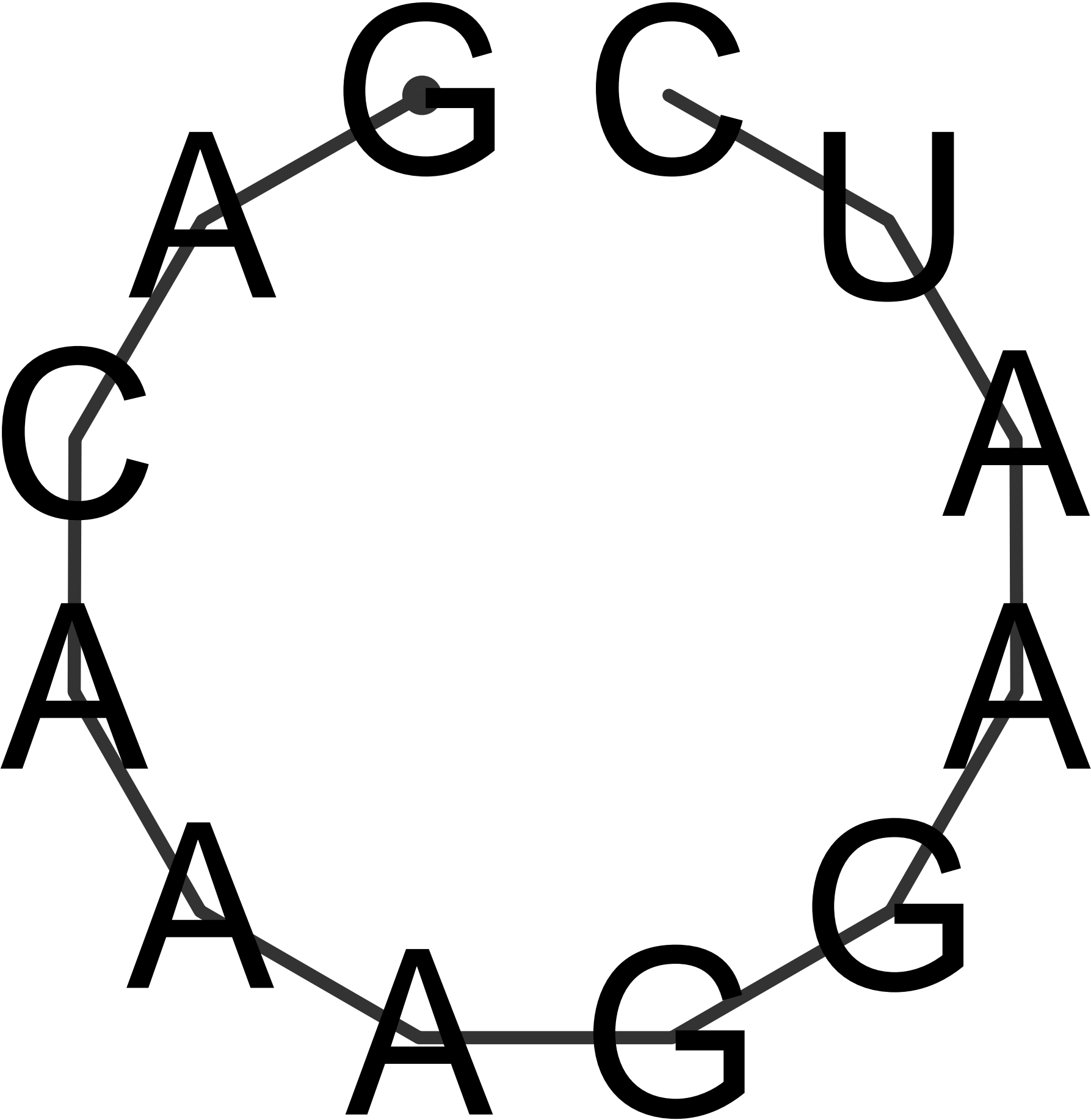

Supplement: S4 Fig — See the caption for S1 Fig for a description of the filename convention (save that the corresponding nucleotide locations in reference sequences are listed in S5 and S13 Tables), and an explanation of the RNAalifold options used and output (save that for these avian-origin viruses the folding temperature was set to 41°C). (ZIP) [file pcbi.1012009.s125.zip › H5N1-avian-ranked-PA-X-alignment-565-576-refseq-589-601-41C_revcomp_alirna_nogap.pdf]

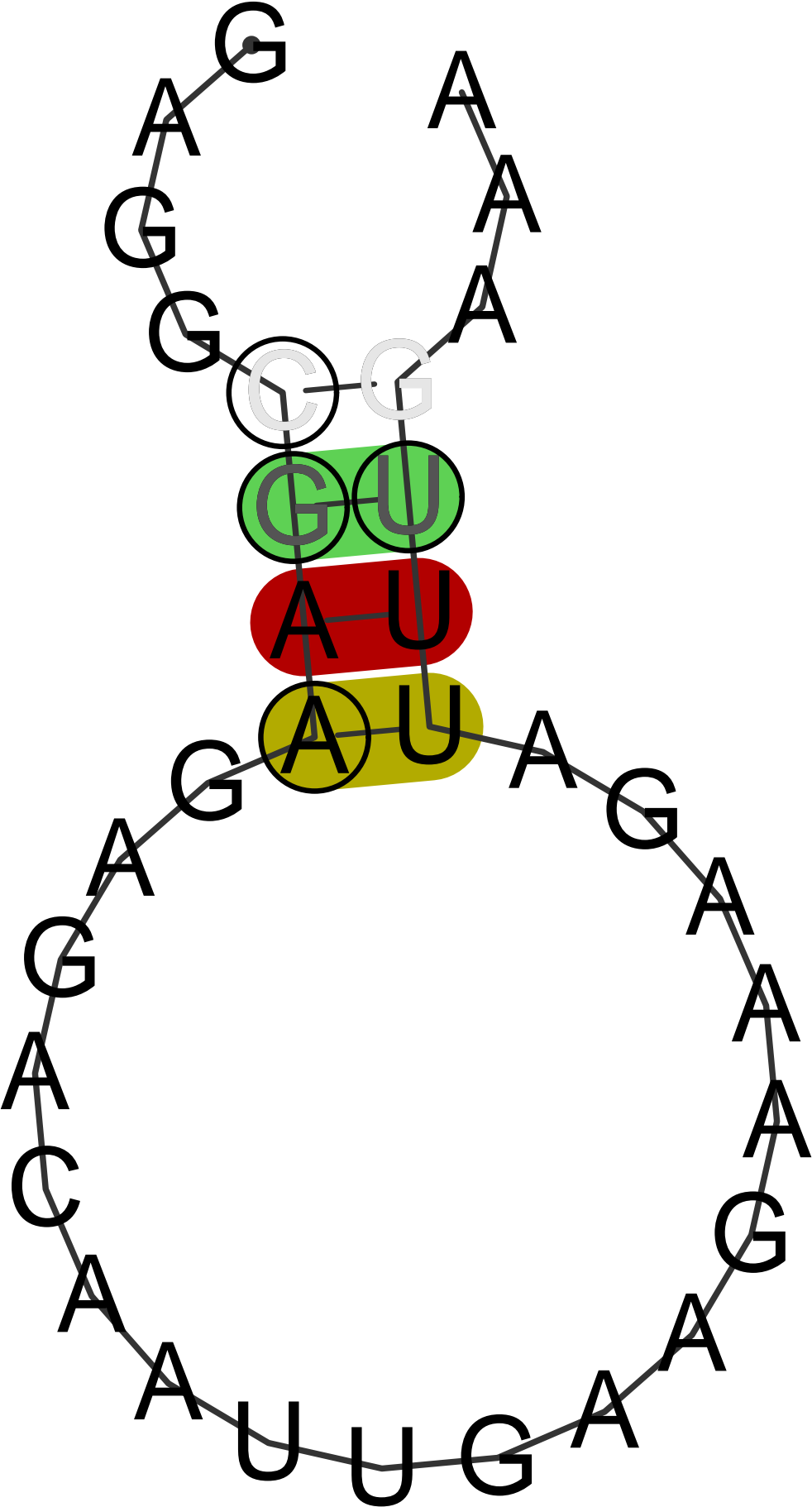

Supplement: S4 Fig — See the caption for S1 Fig for a description of the filename convention (save that the corresponding nucleotide locations in reference sequences are listed in S5 and S13 Tables), and an explanation of the RNAalifold options used and output (save that for these avian-origin viruses the folding temperature was set to 41°C). (ZIP) [file pcbi.1012009.s125.zip › H5N1-avian-ranked-PA-X-alignment-586-618-refseq-611-643-41C_alirna_nogap.pdf]

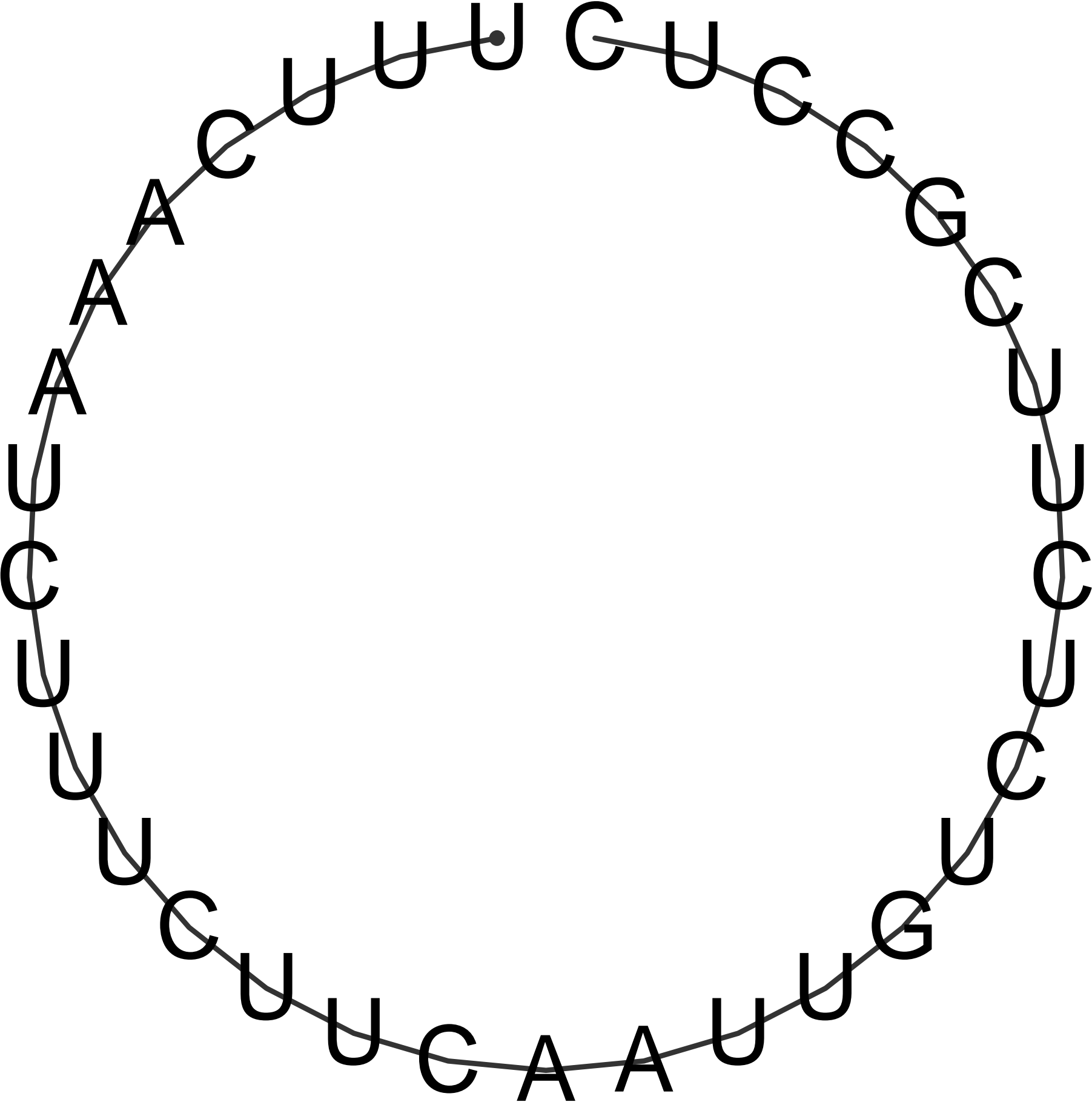

Supplement: S4 Fig — See the caption for S1 Fig for a description of the filename convention (save that the corresponding nucleotide locations in reference sequences are listed in S5 and S13 Tables), and an explanation of the RNAalifold options used and output (save that for these avian-origin viruses the folding temperature was set to 41°C). (ZIP) [file pcbi.1012009.s125.zip › H5N1-avian-ranked-PA-X-alignment-586-618-refseq-611-643-41C_revcomp_alirna_nogap.pdf]

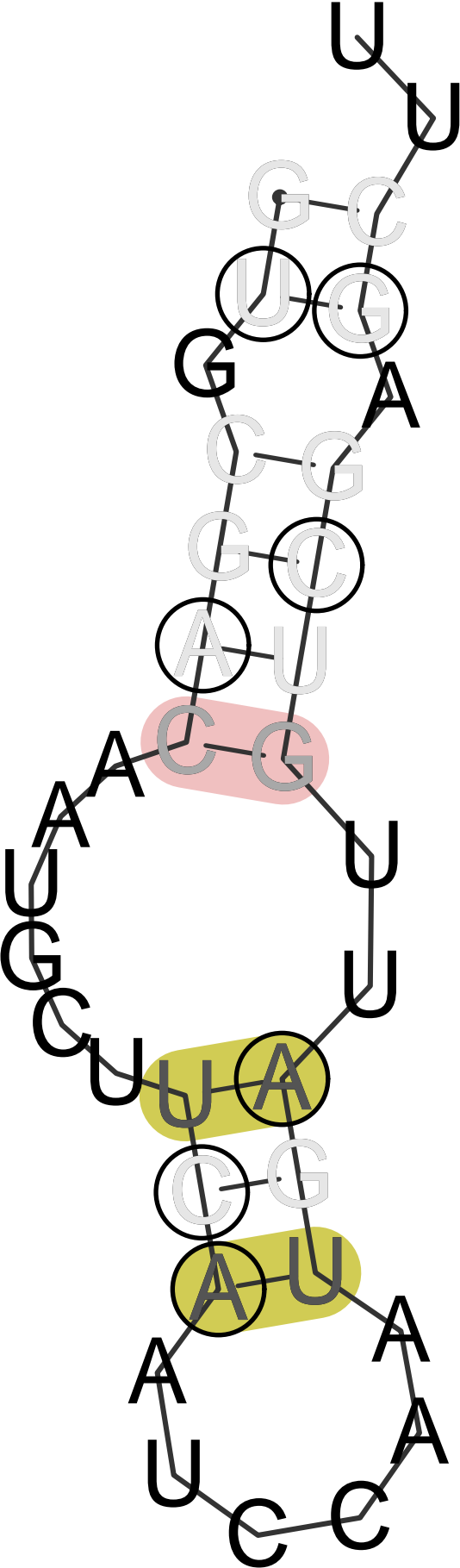

Supplement: S4 Fig — See the caption for S1 Fig for a description of the filename convention (save that the corresponding nucleotide locations in reference sequences are listed in S5 and S13 Tables), and an explanation of the RNAalifold options used and output (save that for these avian-origin viruses the folding temperature was set to 41°C). (ZIP) [file pcbi.1012009.s125.zip › H5N1-avian-ranked-PA-alignment-13-48-refseq-37-72-41C_alirna_nogap.pdf]

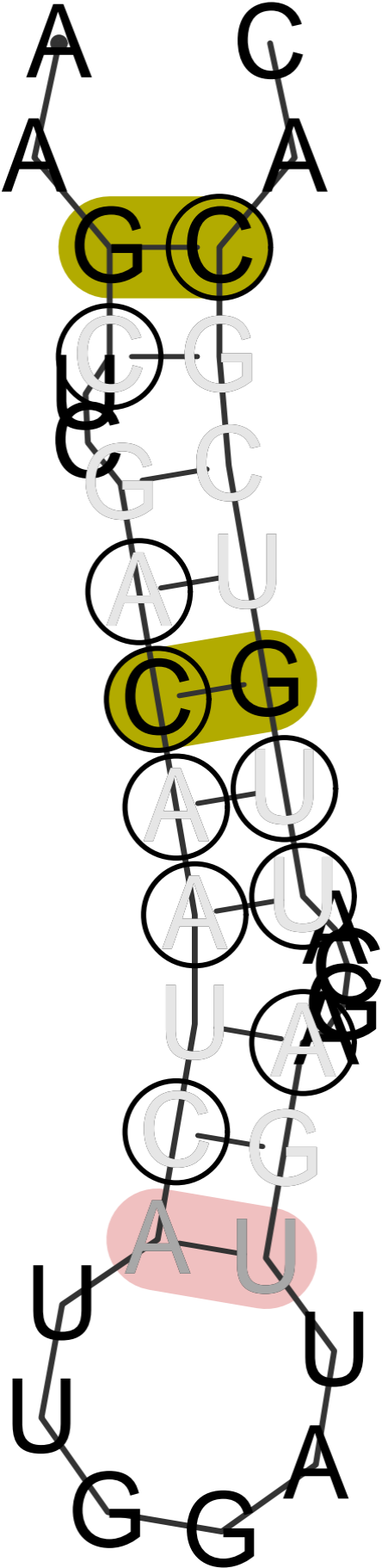

Supplement: S4 Fig — See the caption for S1 Fig for a description of the filename convention (save that the corresponding nucleotide locations in reference sequences are listed in S5 and S13 Tables), and an explanation of the RNAalifold options used and output (save that for these avian-origin viruses the folding temperature was set to 41°C). (ZIP) [file pcbi.1012009.s125.zip › H5N1-avian-ranked-PA-alignment-13-48-refseq-37-72-41C_revcomp_alirna_nogap.pdf]

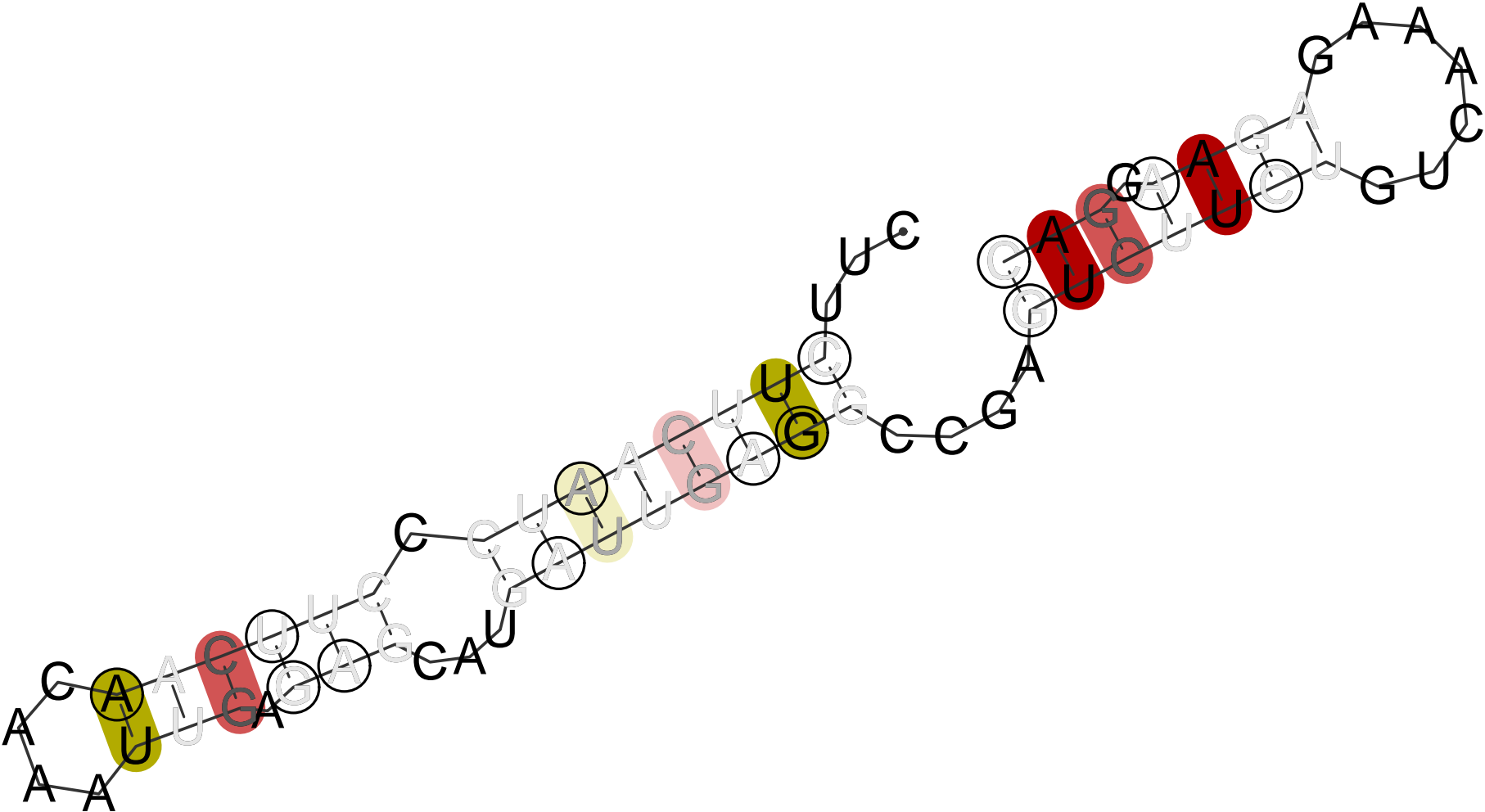

Supplement: S4 Fig — See the caption for S1 Fig for a description of the filename convention (save that the corresponding nucleotide locations in reference sequences are listed in S5 and S13 Tables), and an explanation of the RNAalifold options used and output (save that for these avian-origin viruses the folding temperature was set to 41°C). (ZIP) [file pcbi.1012009.s125.zip › H5N1-avian-ranked-PA-alignment-1753-1818-refseq-1777-1842-41C_alirna_nogap.pdf]

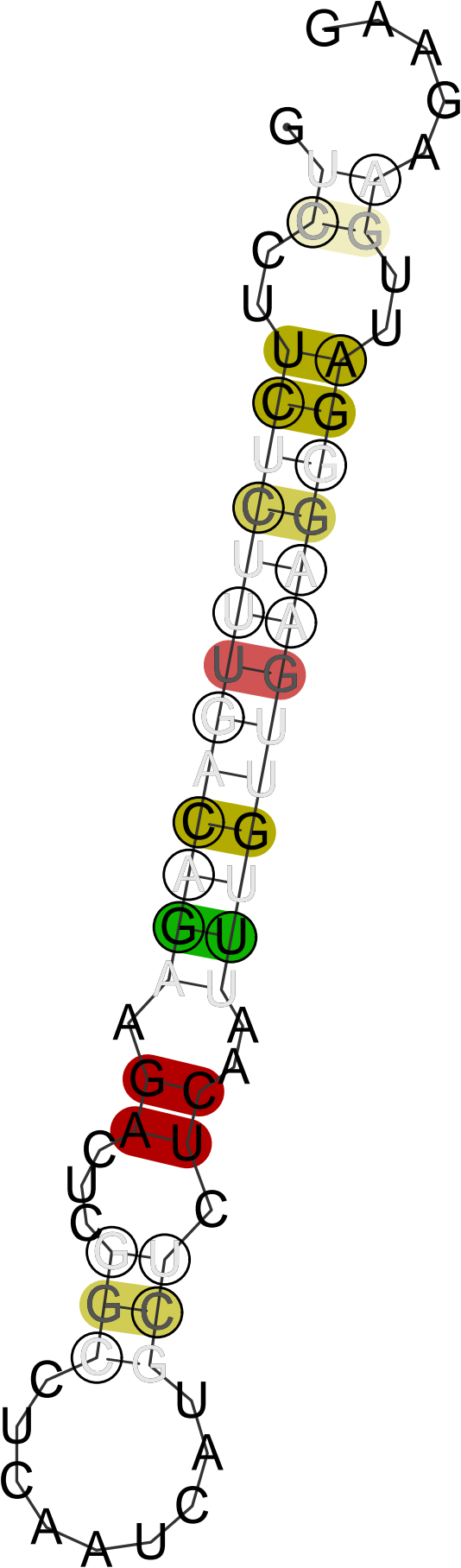

Supplement: S4 Fig — See the caption for S1 Fig for a description of the filename convention (save that the corresponding nucleotide locations in reference sequences are listed in S5 and S13 Tables), and an explanation of the RNAalifold options used and output (save that for these avian-origin viruses the folding temperature was set to 41°C). (ZIP) [file pcbi.1012009.s125.zip › H5N1-avian-ranked-PA-alignment-1753-1818-refseq-1777-1842-41C_revcomp_alirna_nogap.pdf]

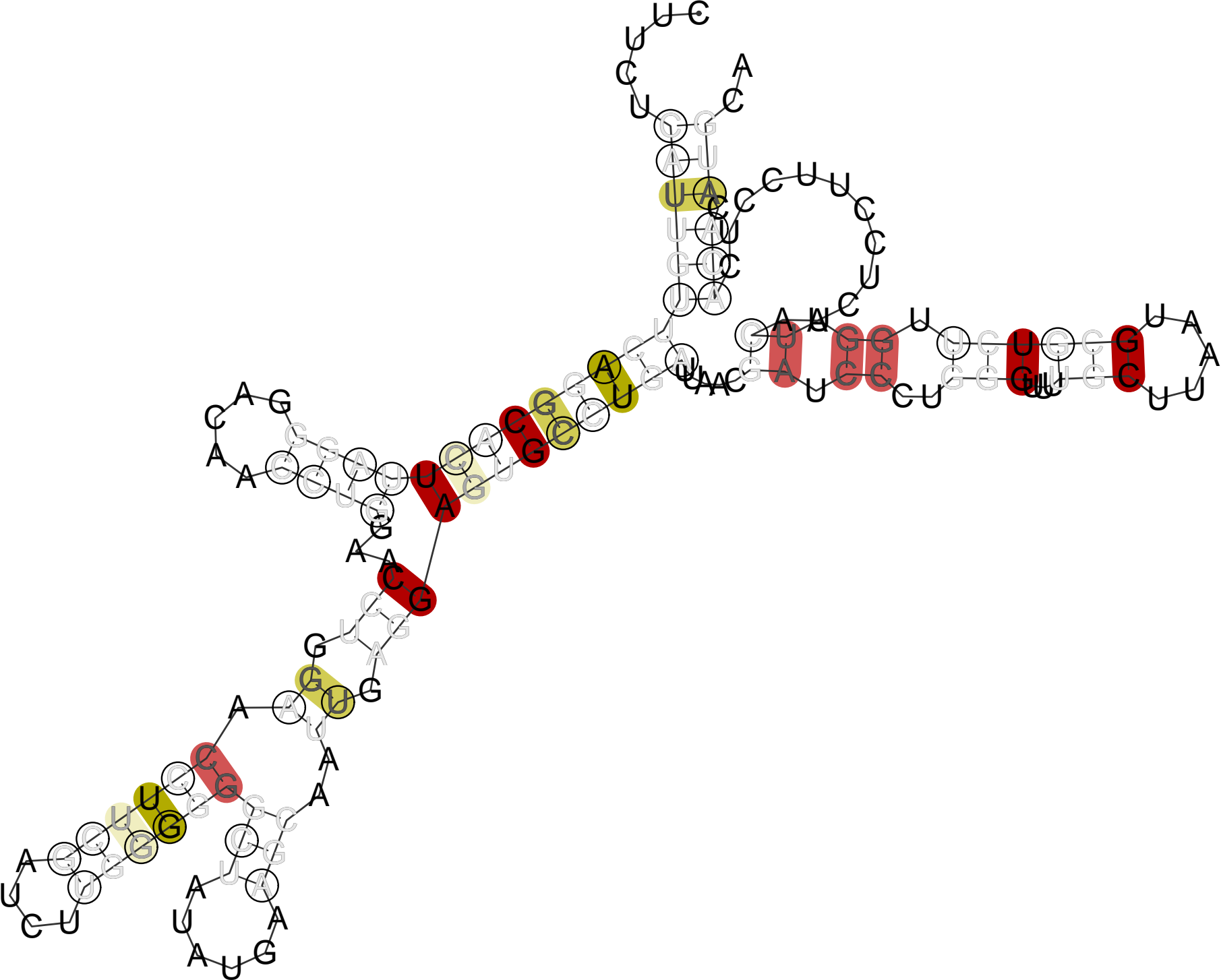

Supplement: S4 Fig — See the caption for S1 Fig for a description of the filename convention (save that the corresponding nucleotide locations in reference sequences are listed in S5 and S13 Tables), and an explanation of the RNAalifold options used and output (save that for these avian-origin viruses the folding temperature was set to 41°C). (ZIP) [file pcbi.1012009.s125.zip › H5N1-avian-ranked-PA-alignment-1996-2142-refseq-2020-2166-41C_alirna_nogap.pdf]

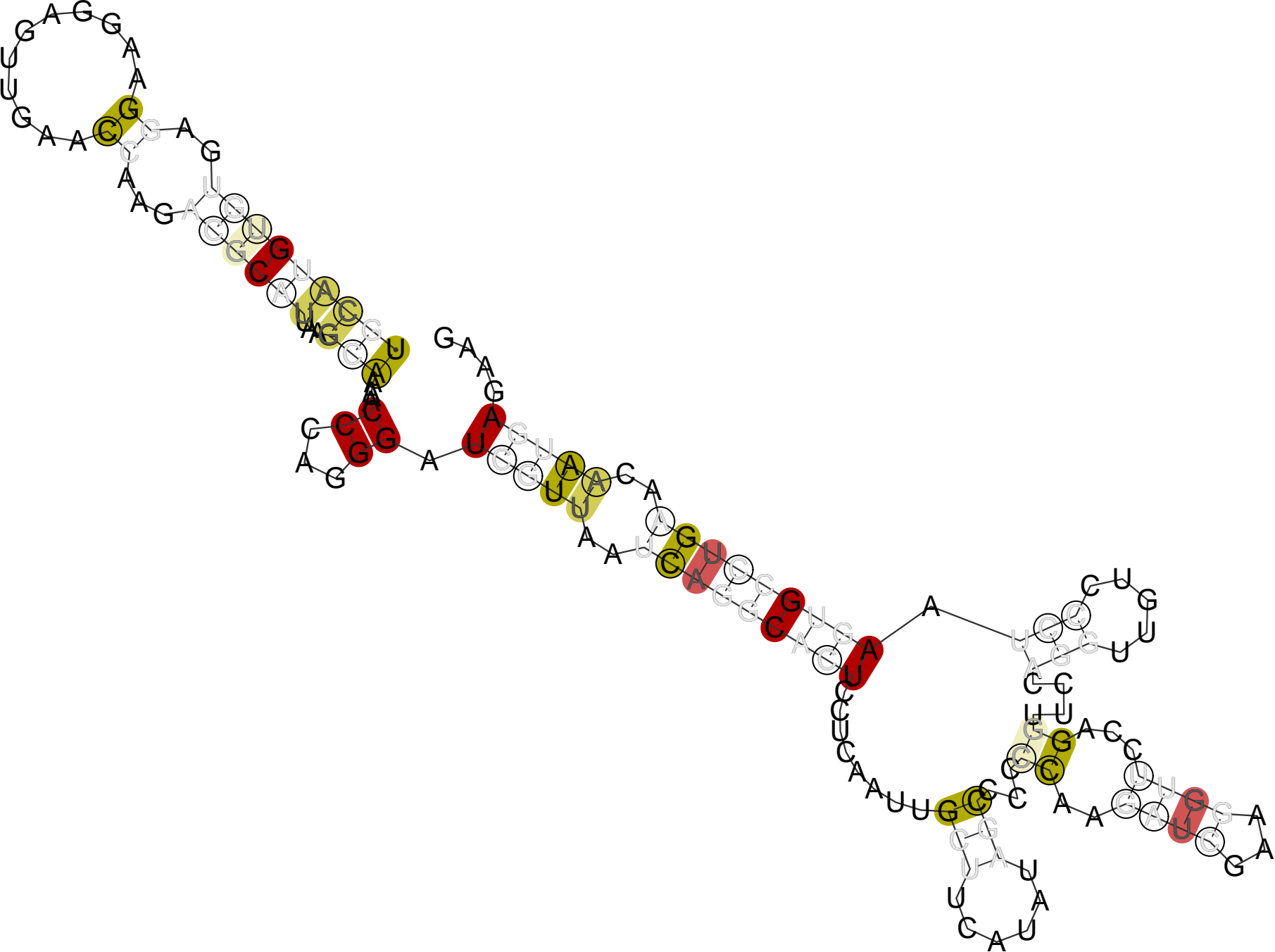

Supplement: S4 Fig — See the caption for S1 Fig for a description of the filename convention (save that the corresponding nucleotide locations in reference sequences are listed in S5 and S13 Tables), and an explanation of the RNAalifold options used and output (save that for these avian-origin viruses the folding temperature was set to 41°C). (ZIP) [file pcbi.1012009.s125.zip › H5N1-avian-ranked-PA-alignment-1996-2142-refseq-2020-2166-41C_revcomp_alirna_nogap.pdf]

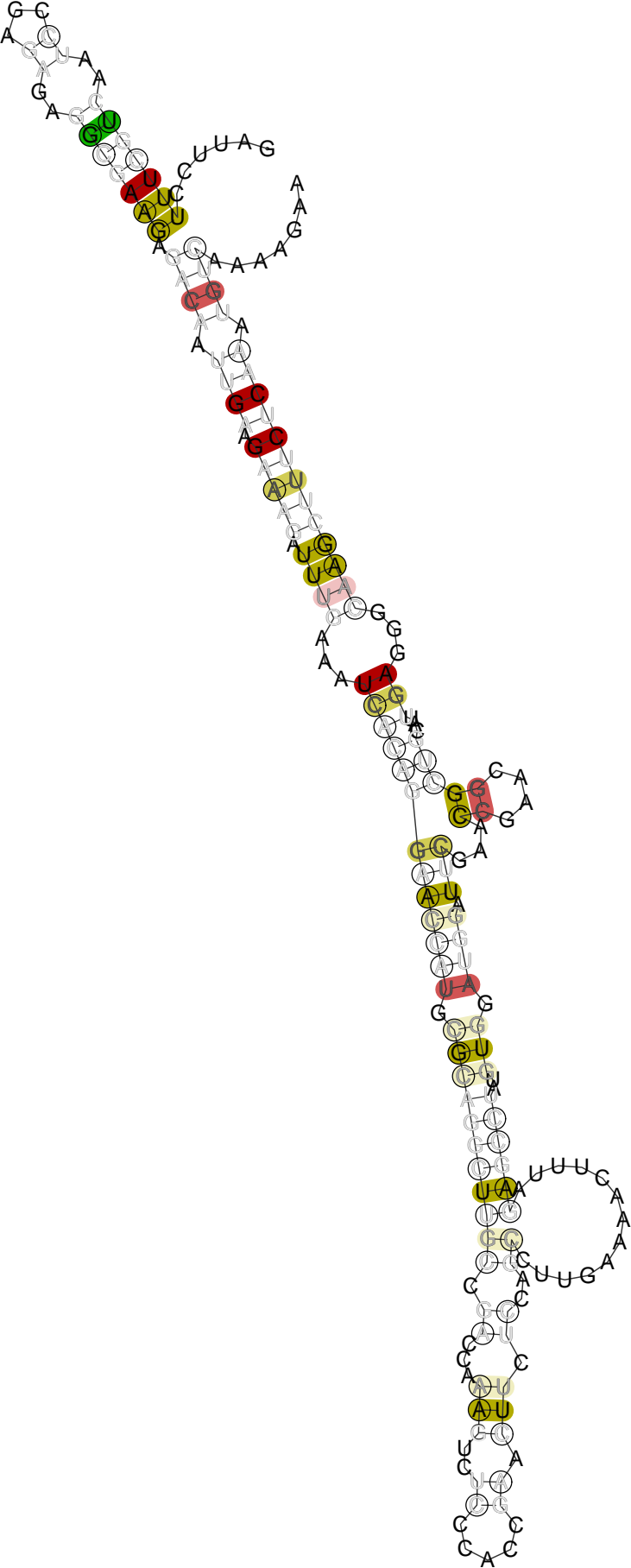

Supplement: S4 Fig — See the caption for S1 Fig for a description of the filename convention (save that the corresponding nucleotide locations in reference sequences are listed in S5 and S13 Tables), and an explanation of the RNAalifold options used and output (save that for these avian-origin viruses the folding temperature was set to 41°C). (ZIP) [file pcbi.1012009.s125.zip › H5N1-avian-ranked-PA-alignment-565-756-refseq-589-780-41C_alirna_nogap.pdf]

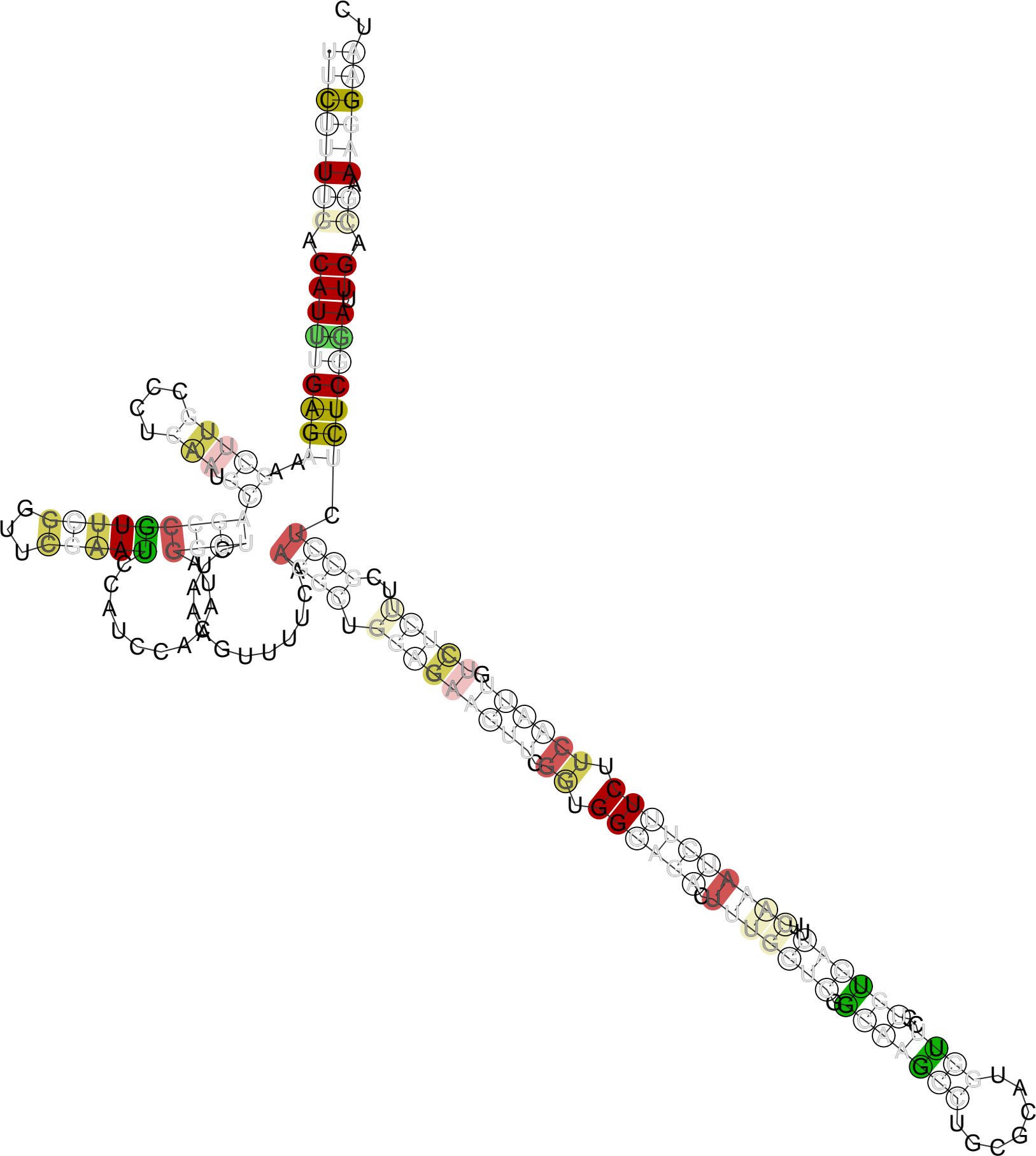

Supplement: S4 Fig — See the caption for S1 Fig for a description of the filename convention (save that the corresponding nucleotide locations in reference sequences are listed in S5 and S13 Tables), and an explanation of the RNAalifold options used and output (save that for these avian-origin viruses the folding temperature was set to 41°C). (ZIP) [file pcbi.1012009.s125.zip › H5N1-avian-ranked-PA-alignment-565-756-refseq-589-780-41C_revcomp_alirna_nogap.pdf]
