# Supplementary figures and images for: Locations and structures of influenza A virus packaging-associated signals and other functional elements via an in silico pipeline for predicting constrained features in RNA viruses (part 5 of 6)
Source: PLoS Comput Biol. 2024 Apr 22;20(4):e1012009. doi: 10.1371/journal.pcbi.1012009 (PMC11034665; doi:10.1371/journal.pcbi.1012009)

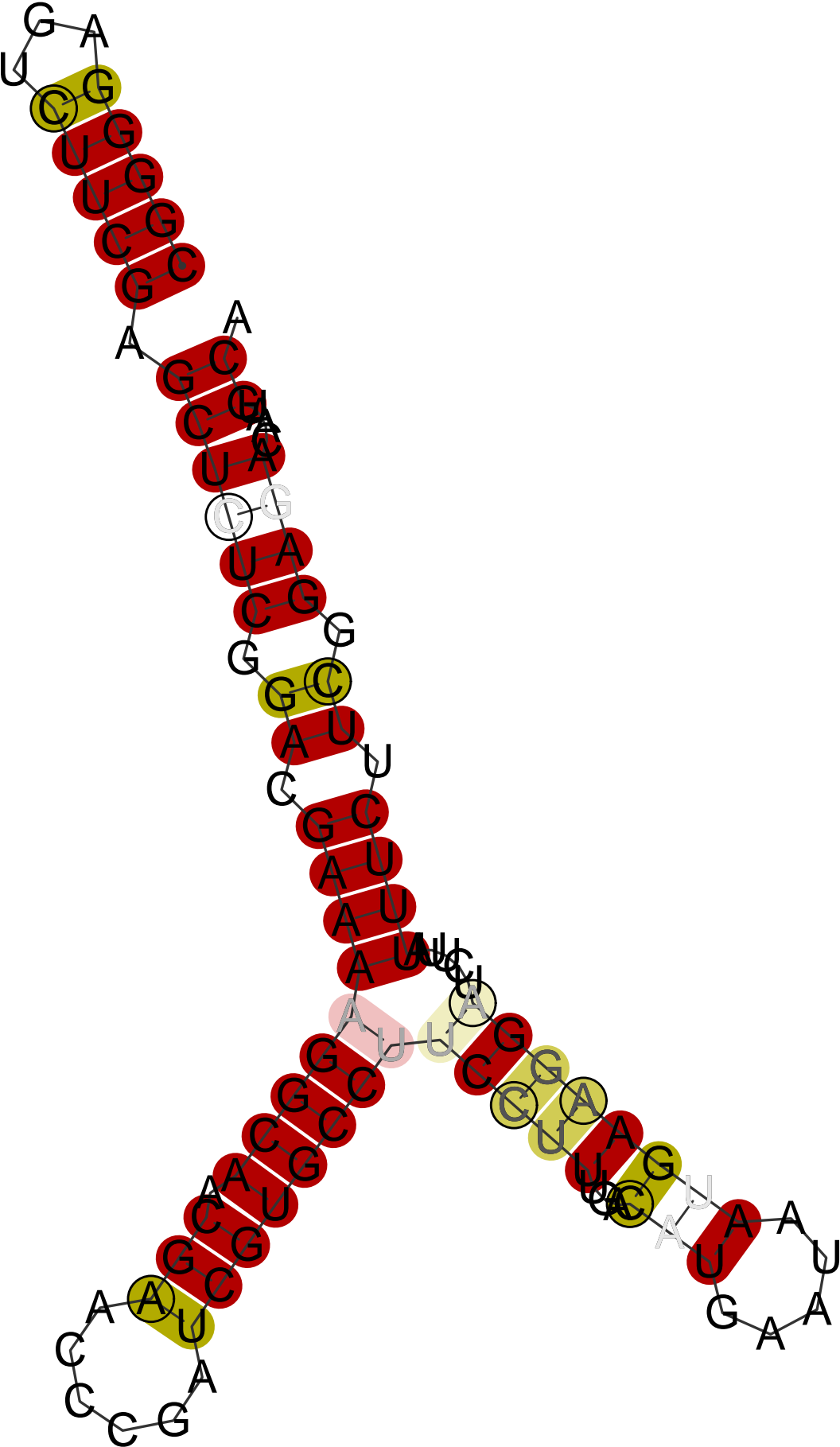

Supplement: S7 Fig — See the caption for S1 Fig for a description of the filename convention (save that the corresponding nucleotide locations in reference sequences are listed in S8 and S16 Tables), and an explanation of the RNAalifold options used and output. (ZIP) [file pcbi.1012009.s128.zip › H7N9-human-ranked-NP-alignment-1381-1479-refseq-1381-1479_alirna_nogap.pdf]

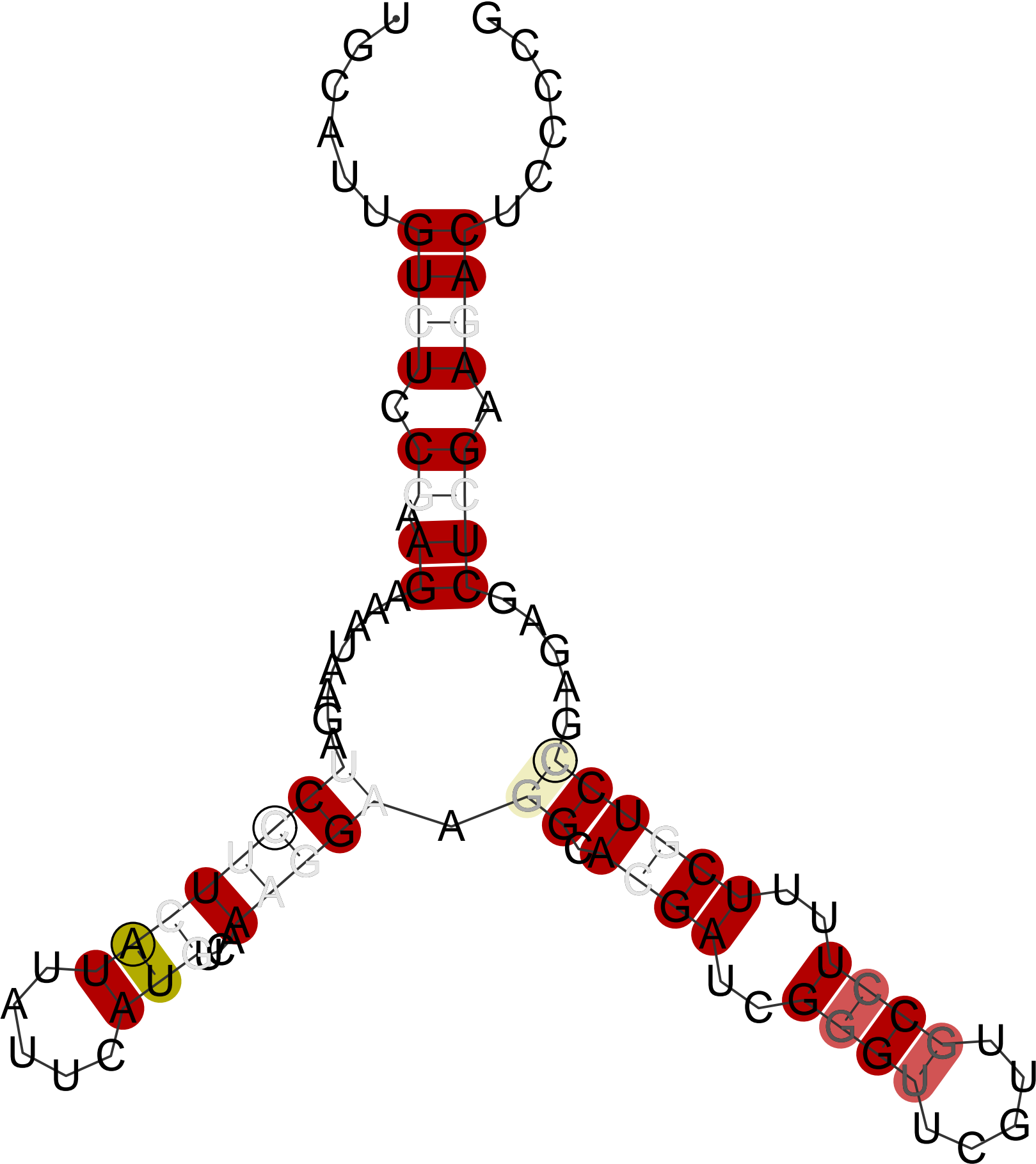

Supplement: S7 Fig — See the caption for S1 Fig for a description of the filename convention (save that the corresponding nucleotide locations in reference sequences are listed in S8 and S16 Tables), and an explanation of the RNAalifold options used and output. (ZIP) [file pcbi.1012009.s128.zip › H7N9-human-ranked-NP-alignment-1381-1479-refseq-1381-1479_revcomp_alirna_nogap.pdf]

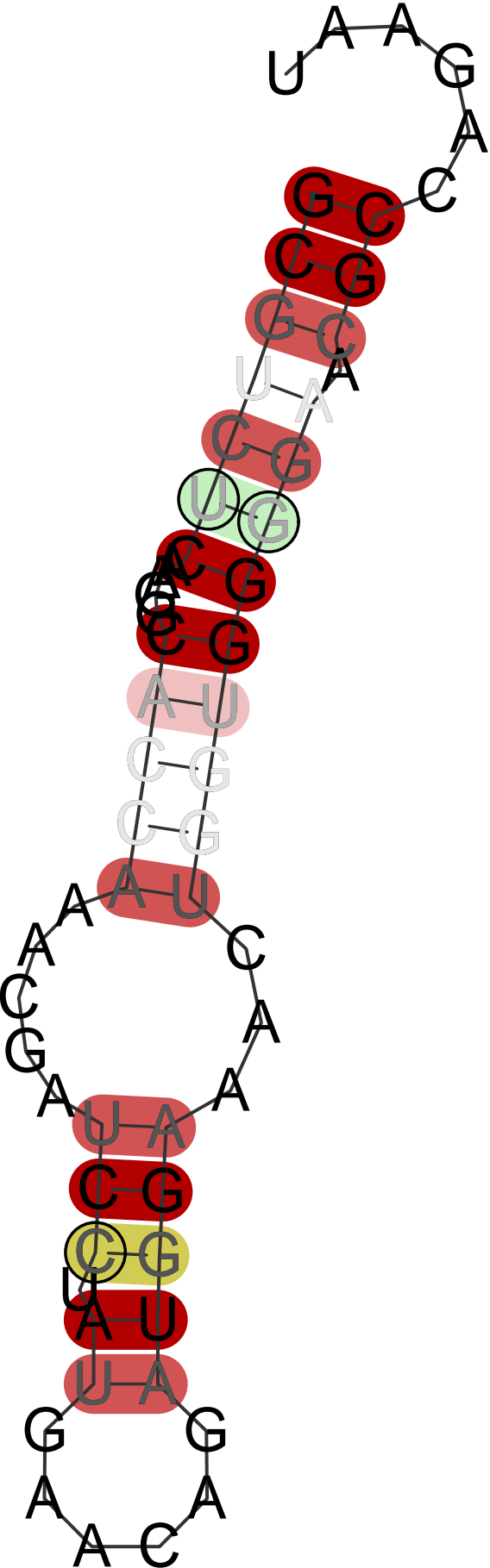

Supplement: S7 Fig — See the caption for S1 Fig for a description of the filename convention (save that the corresponding nucleotide locations in reference sequences are listed in S8 and S16 Tables), and an explanation of the RNAalifold options used and output. (ZIP) [file pcbi.1012009.s128.zip › H7N9-human-ranked-NP-alignment-4-63-refseq-4-63_alirna_nogap.pdf]

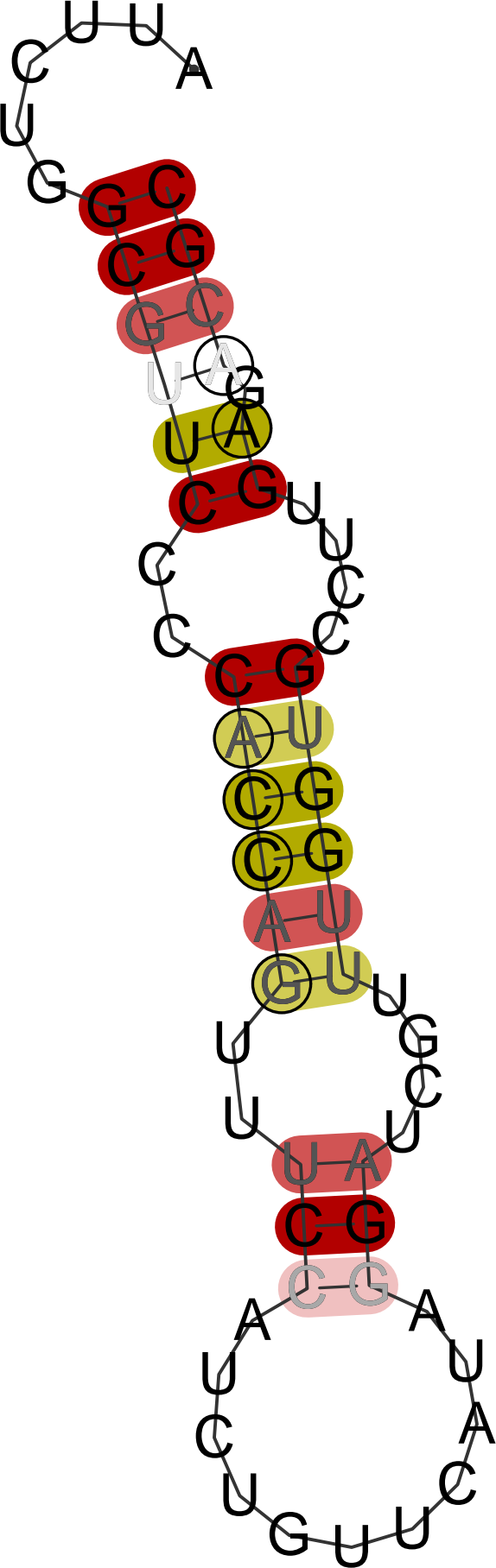

Supplement: S7 Fig — See the caption for S1 Fig for a description of the filename convention (save that the corresponding nucleotide locations in reference sequences are listed in S8 and S16 Tables), and an explanation of the RNAalifold options used and output. (ZIP) [file pcbi.1012009.s128.zip › H7N9-human-ranked-NP-alignment-4-63-refseq-4-63_revcomp_alirna_nogap.pdf]

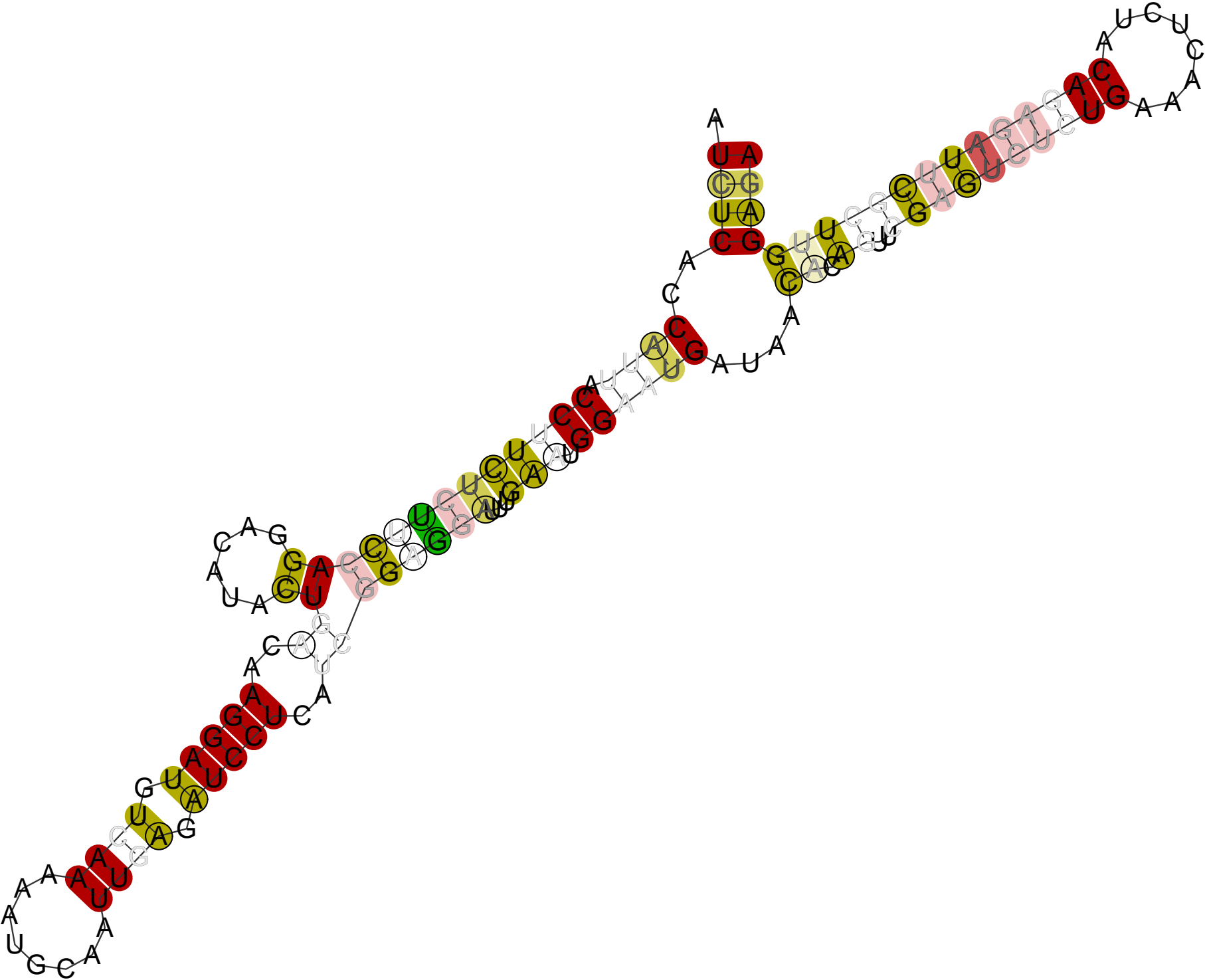

Supplement: S7 Fig — See the caption for S1 Fig for a description of the filename convention (save that the corresponding nucleotide locations in reference sequences are listed in S8 and S16 Tables), and an explanation of the RNAalifold options used and output. (ZIP) [file pcbi.1012009.s128.zip › H7N9-human-ranked-NS1-alignment-481-615-refseq-478-612_alirna_nogap.pdf]

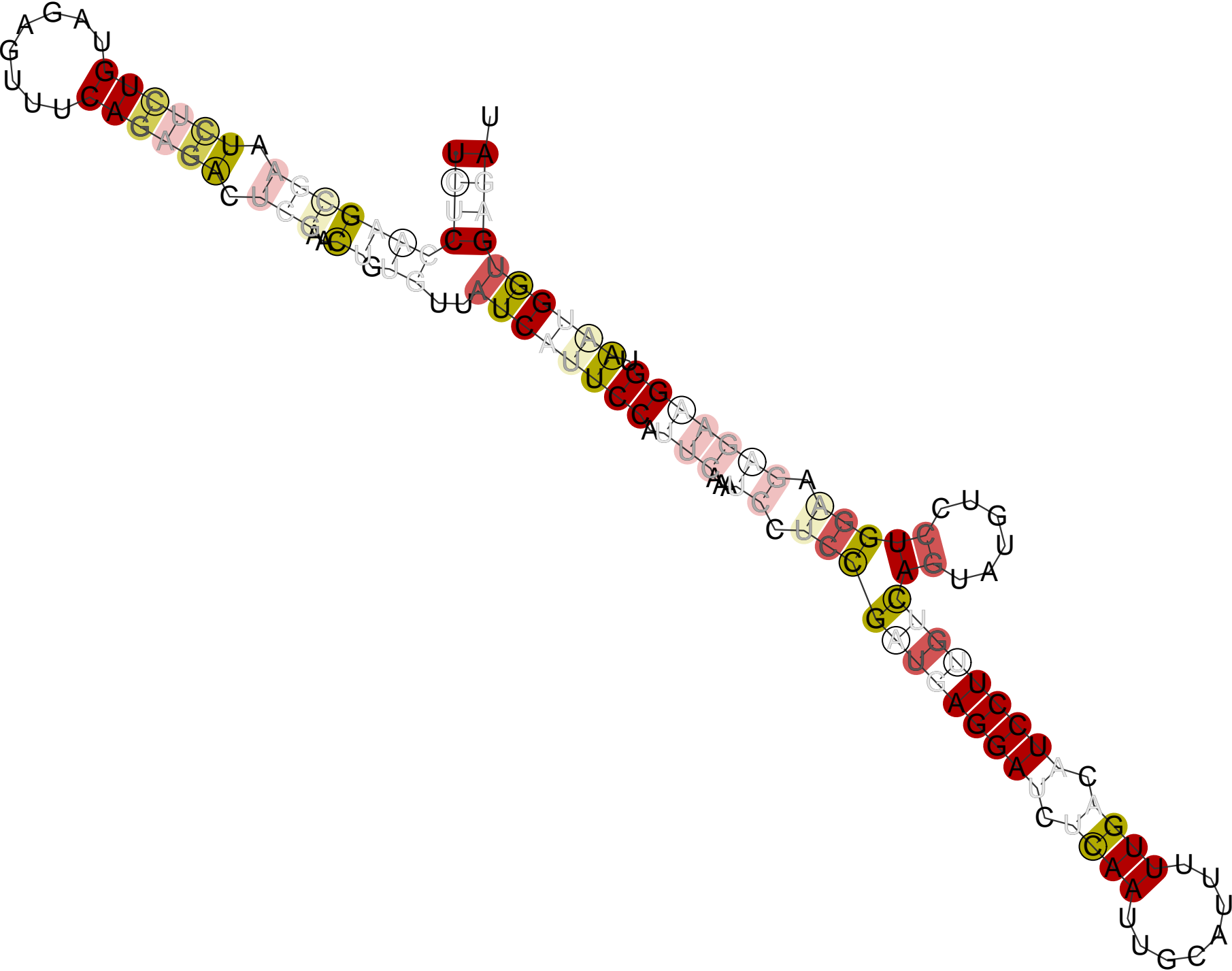

Supplement: S7 Fig — See the caption for S1 Fig for a description of the filename convention (save that the corresponding nucleotide locations in reference sequences are listed in S8 and S16 Tables), and an explanation of the RNAalifold options used and output. (ZIP) [file pcbi.1012009.s128.zip › H7N9-human-ranked-NS1-alignment-481-615-refseq-478-612_revcomp_alirna_nogap.pdf]

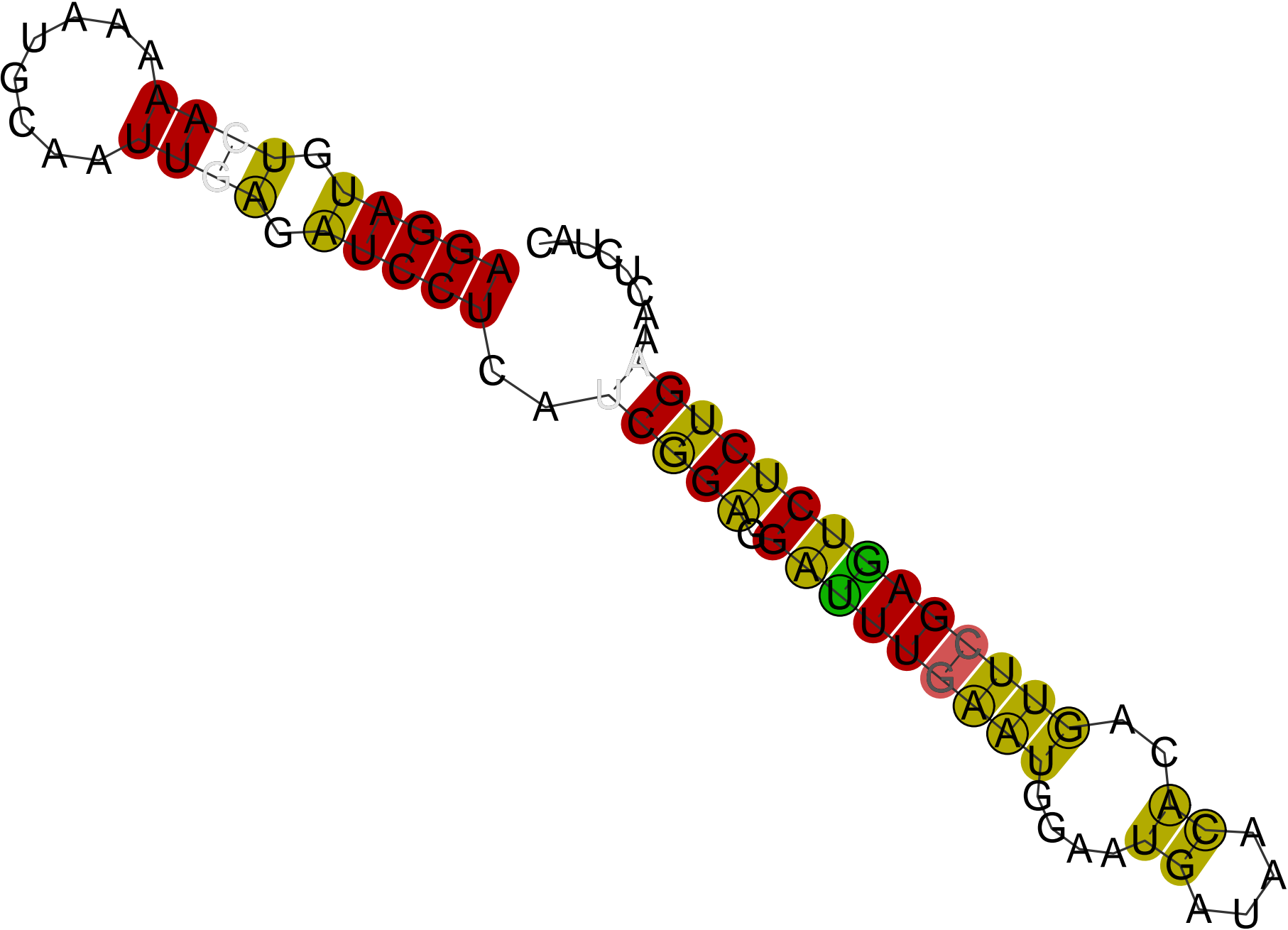

Supplement: S7 Fig — See the caption for S1 Fig for a description of the filename convention (save that the corresponding nucleotide locations in reference sequences are listed in S8 and S16 Tables), and an explanation of the RNAalifold options used and output. (ZIP) [file pcbi.1012009.s128.zip › H7N9-human-ranked-NS2-alignment-43-123-refseq-515-595_alirna_nogap.pdf]

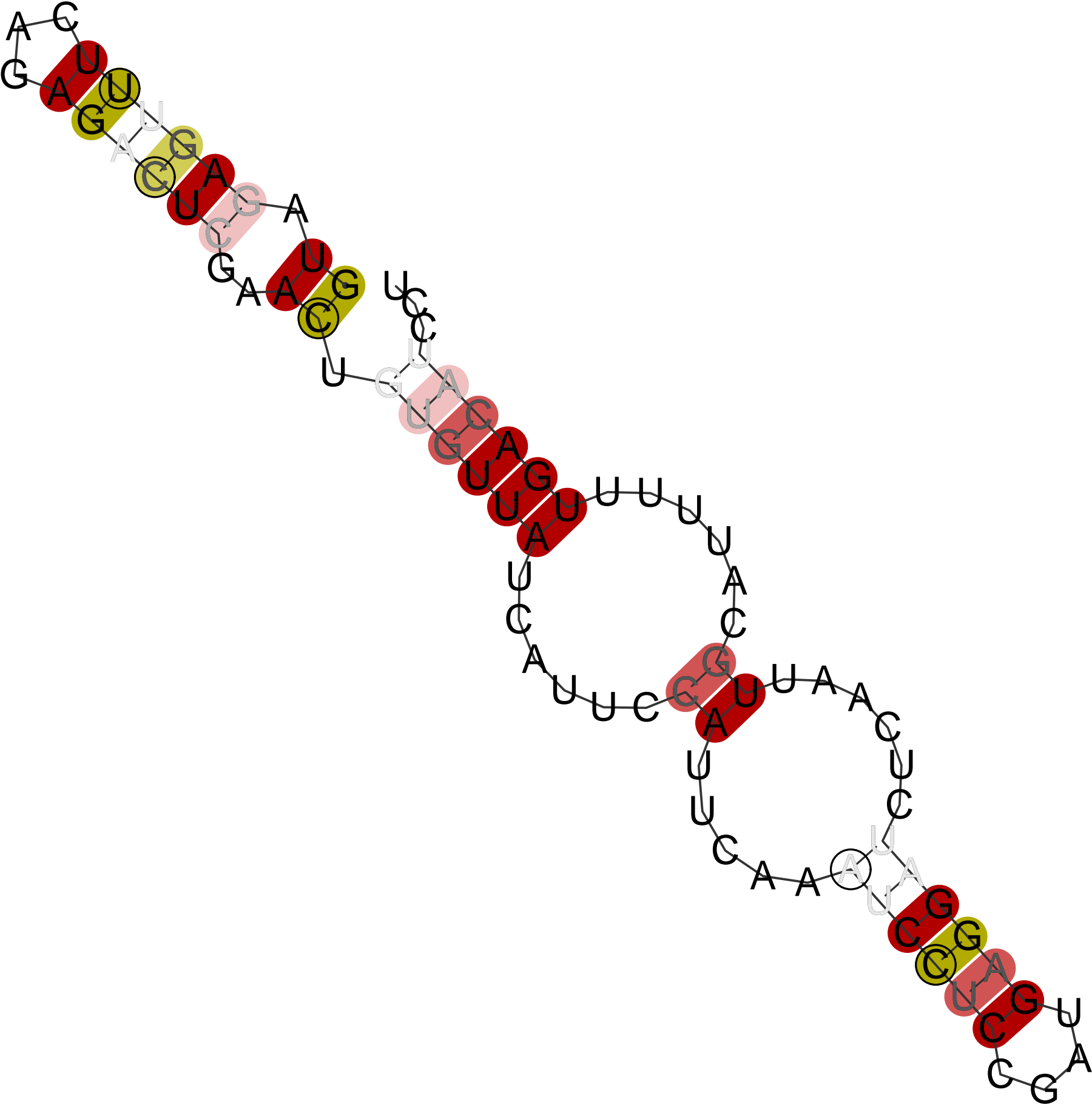

Supplement: S7 Fig — See the caption for S1 Fig for a description of the filename convention (save that the corresponding nucleotide locations in reference sequences are listed in S8 and S16 Tables), and an explanation of the RNAalifold options used and output. (ZIP) [file pcbi.1012009.s128.zip › H7N9-human-ranked-NS2-alignment-43-123-refseq-515-595_revcomp_alirna_nogap.pdf]

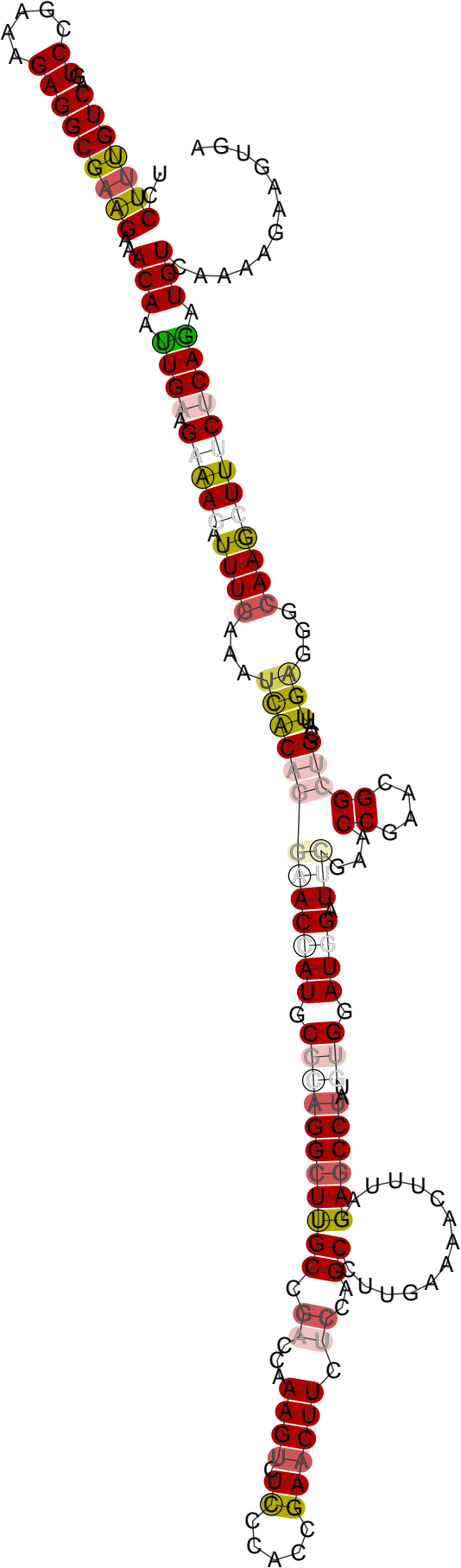

Supplement: S7 Fig — See the caption for S1 Fig for a description of the filename convention (save that the corresponding nucleotide locations in reference sequences are listed in S8 and S16 Tables), and an explanation of the RNAalifold options used and output. (ZIP) [file pcbi.1012009.s128.zip › H7N9-human-ranked-PA-X-alignment-568-759-refseq-568-570-572-760_alirna_nogap.pdf]

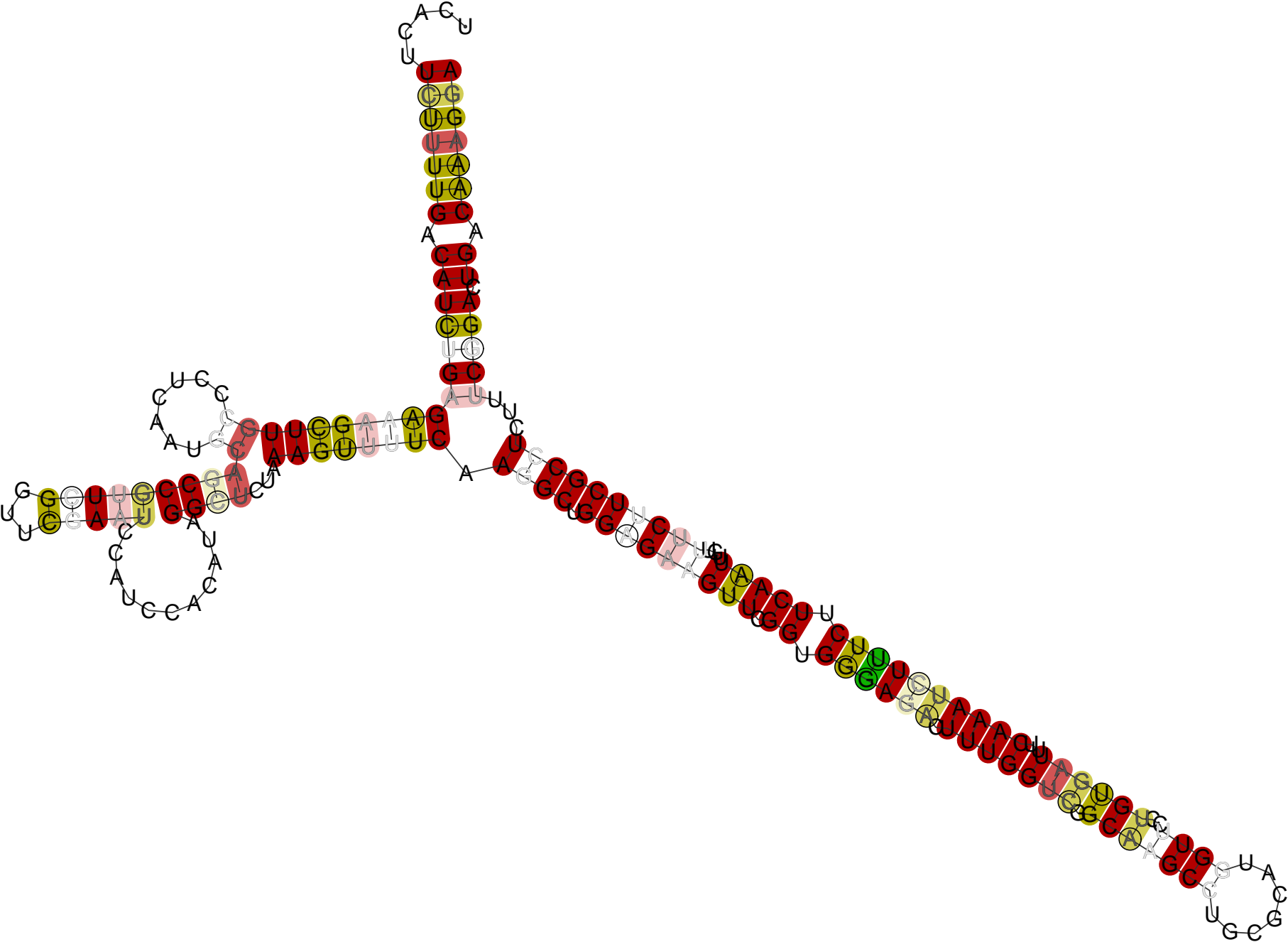

Supplement: S7 Fig — See the caption for S1 Fig for a description of the filename convention (save that the corresponding nucleotide locations in reference sequences are listed in S8 and S16 Tables), and an explanation of the RNAalifold options used and output. (ZIP) [file pcbi.1012009.s128.zip › H7N9-human-ranked-PA-X-alignment-568-759-refseq-568-570-572-760_revcomp_alirna_nogap.pdf]

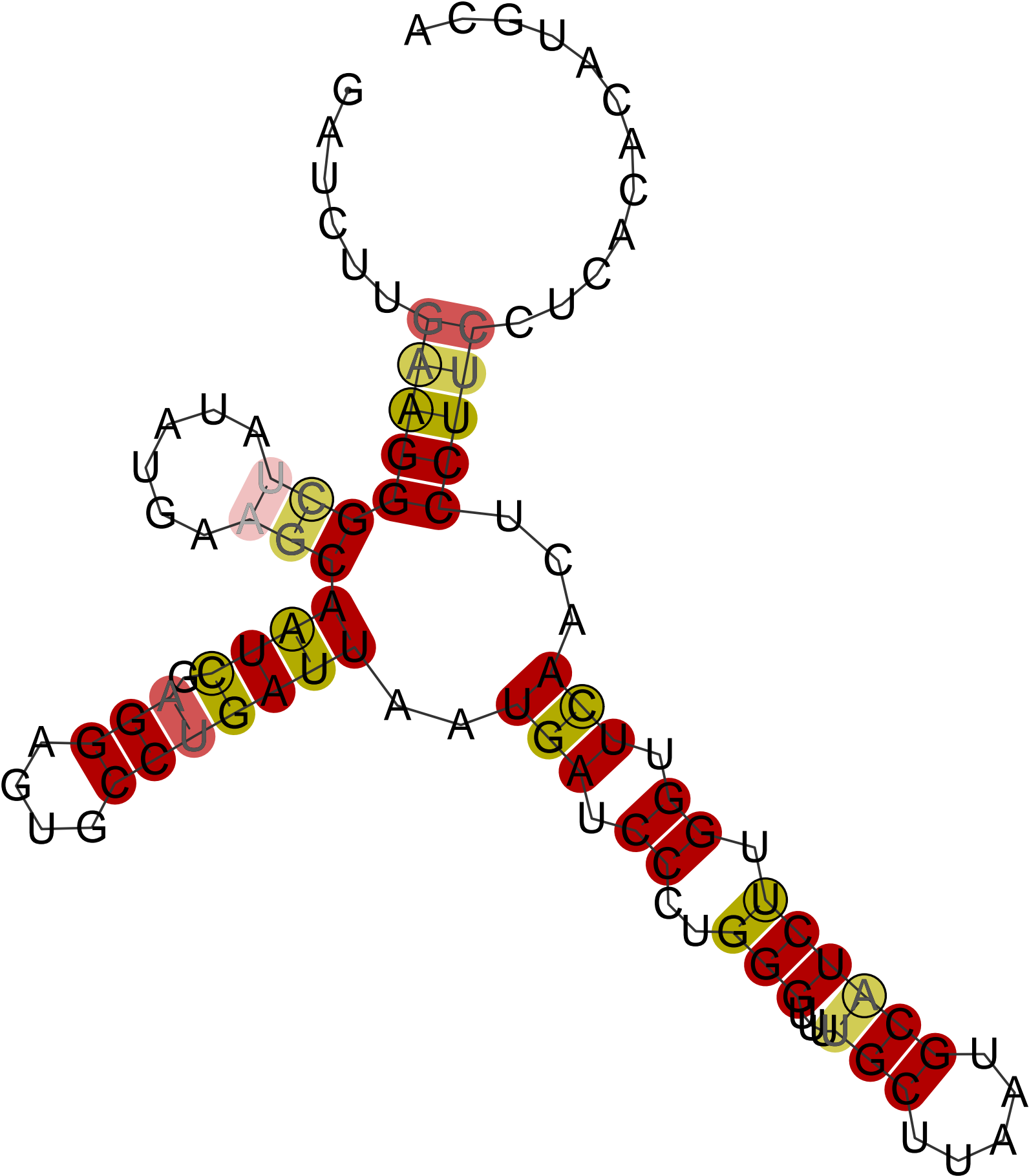

Supplement: S7 Fig — See the caption for S1 Fig for a description of the filename convention (save that the corresponding nucleotide locations in reference sequences are listed in S8 and S16 Tables), and an explanation of the RNAalifold options used and output. (ZIP) [file pcbi.1012009.s128.zip › H7N9-human-ranked-PA-alignment-2044-2142-refseq-2044-2142_alirna_nogap.pdf]

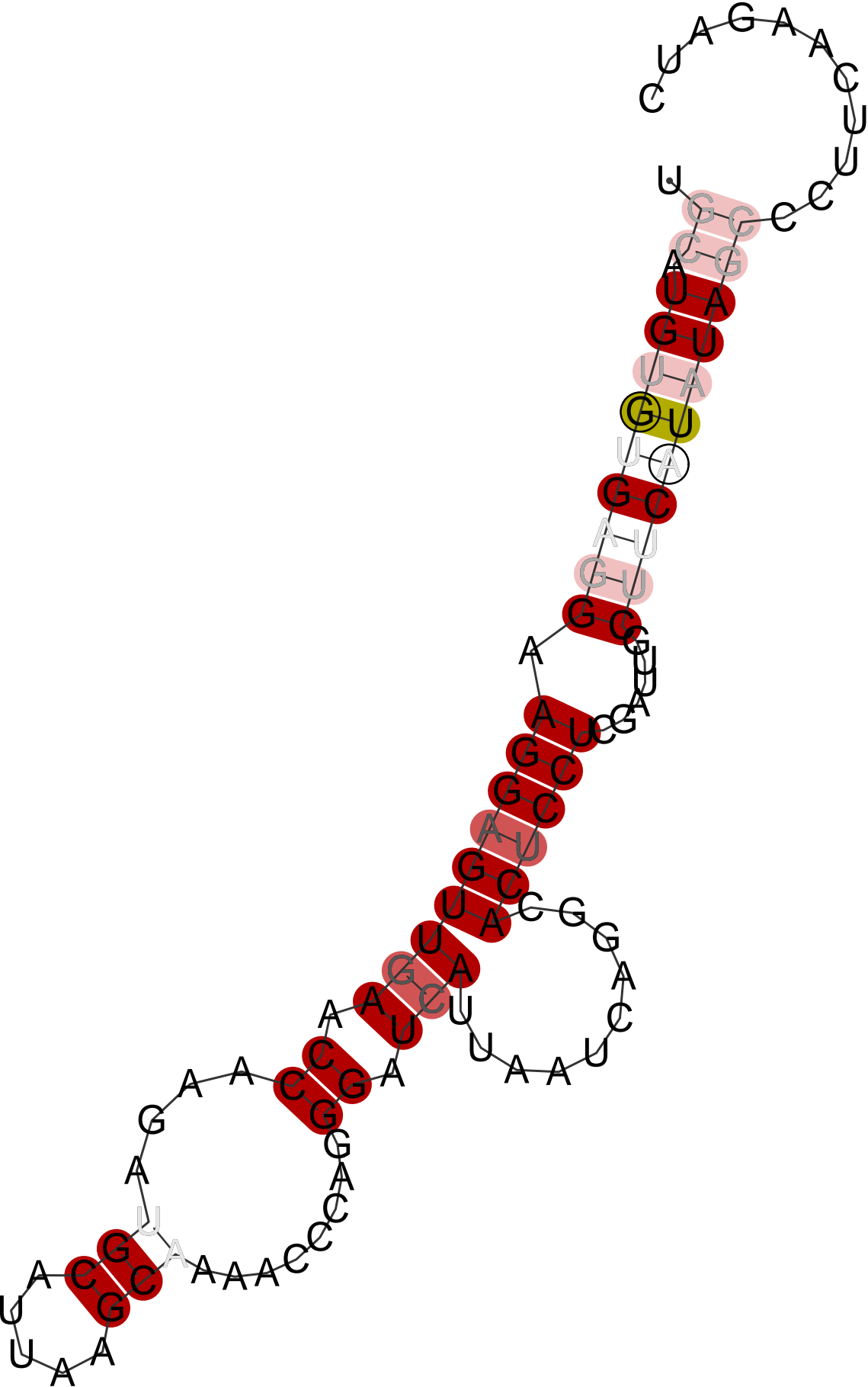

Supplement: S7 Fig — See the caption for S1 Fig for a description of the filename convention (save that the corresponding nucleotide locations in reference sequences are listed in S8 and S16 Tables), and an explanation of the RNAalifold options used and output. (ZIP) [file pcbi.1012009.s128.zip › H7N9-human-ranked-PA-alignment-2044-2142-refseq-2044-2142_revcomp_alirna_nogap.pdf]

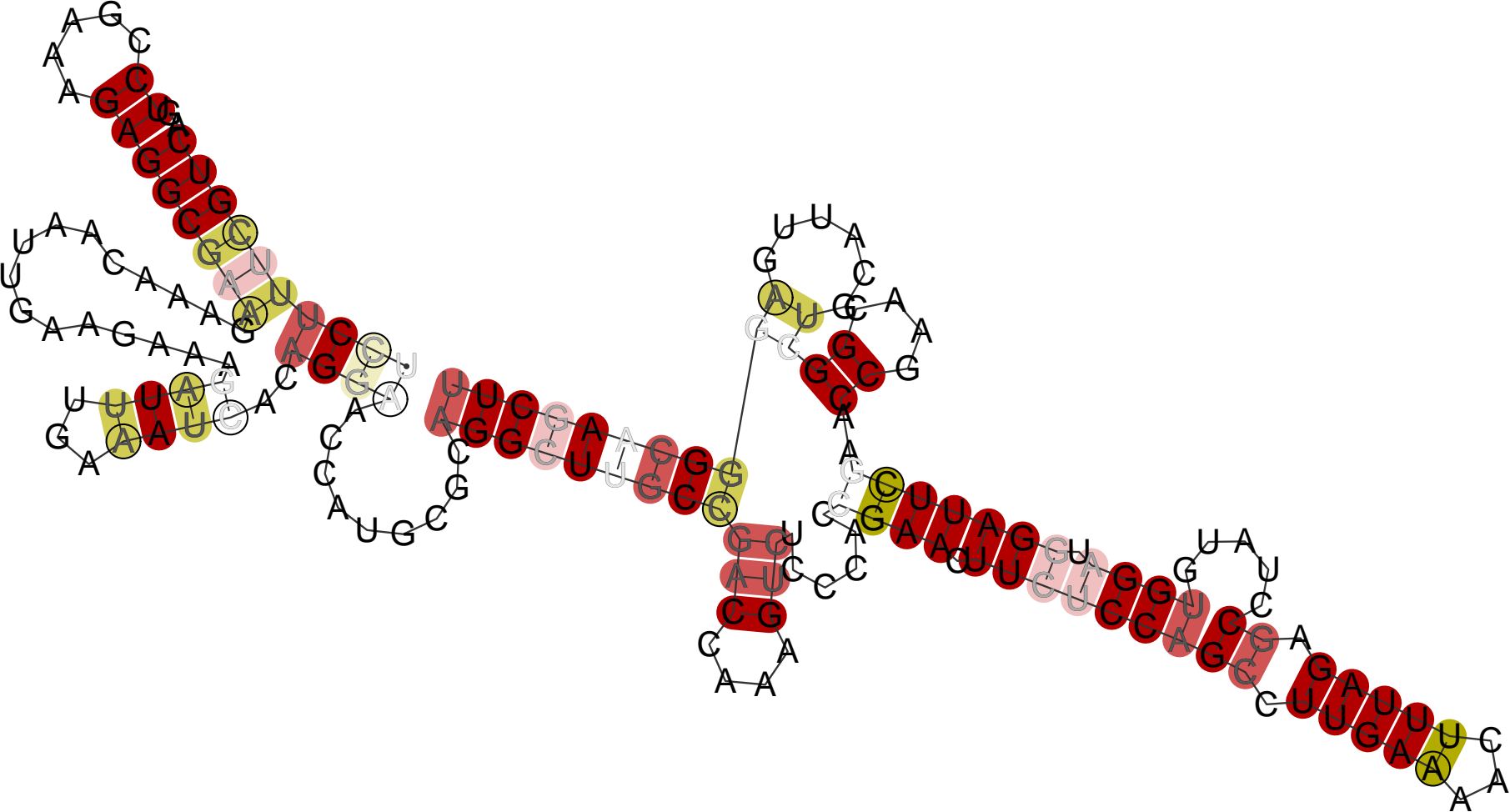

Supplement: S7 Fig — See the caption for S1 Fig for a description of the filename convention (save that the corresponding nucleotide locations in reference sequences are listed in S8 and S16 Tables), and an explanation of the RNAalifold options used and output. (ZIP) [file pcbi.1012009.s128.zip › H7N9-human-ranked-PA-alignment-568-738-refseq-568-738_alirna_nogap.pdf]

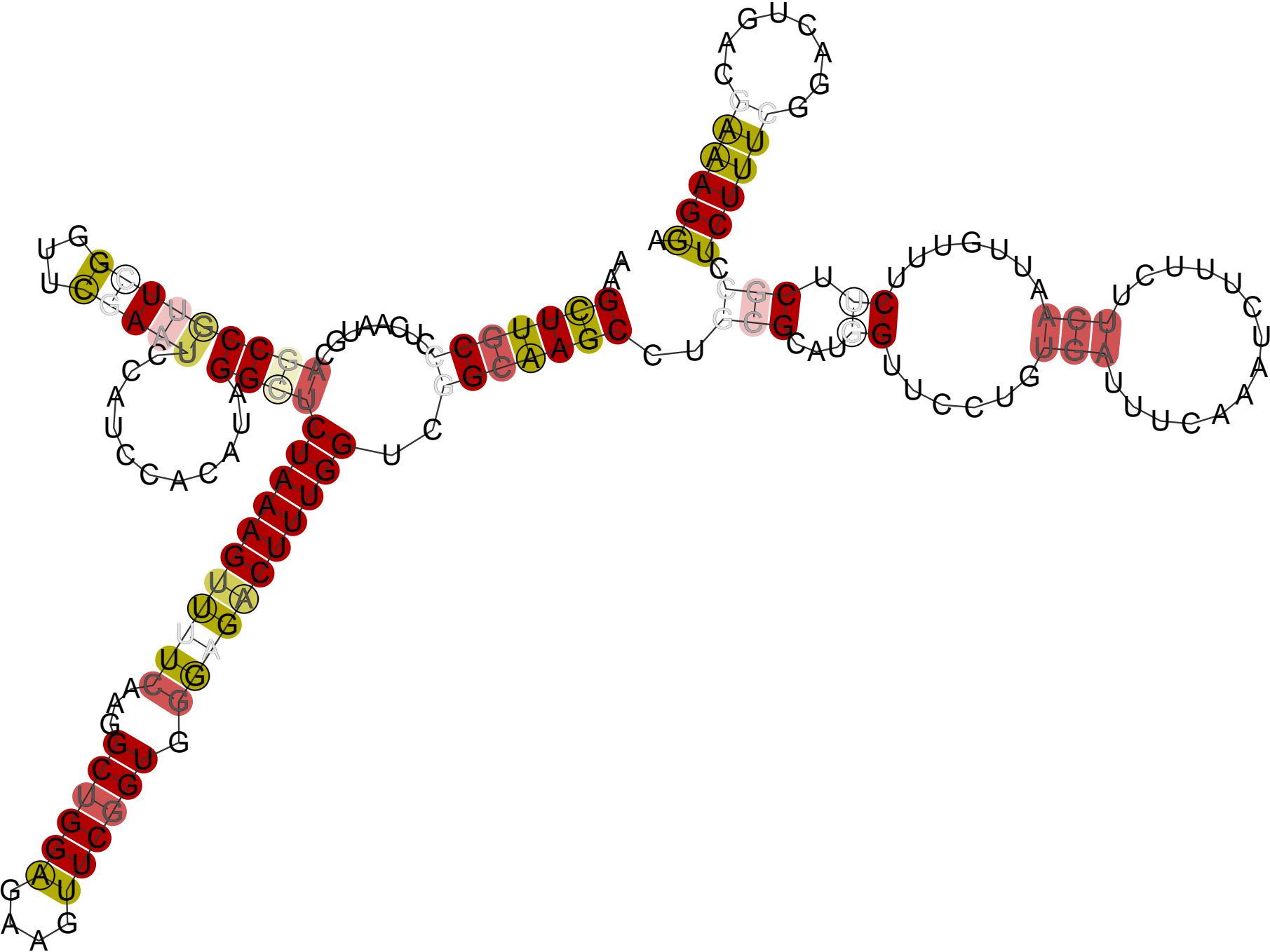

Supplement: S7 Fig — See the caption for S1 Fig for a description of the filename convention (save that the corresponding nucleotide locations in reference sequences are listed in S8 and S16 Tables), and an explanation of the RNAalifold options used and output. (ZIP) [file pcbi.1012009.s128.zip › H7N9-human-ranked-PA-alignment-568-738-refseq-568-738_revcomp_alirna_nogap.pdf]

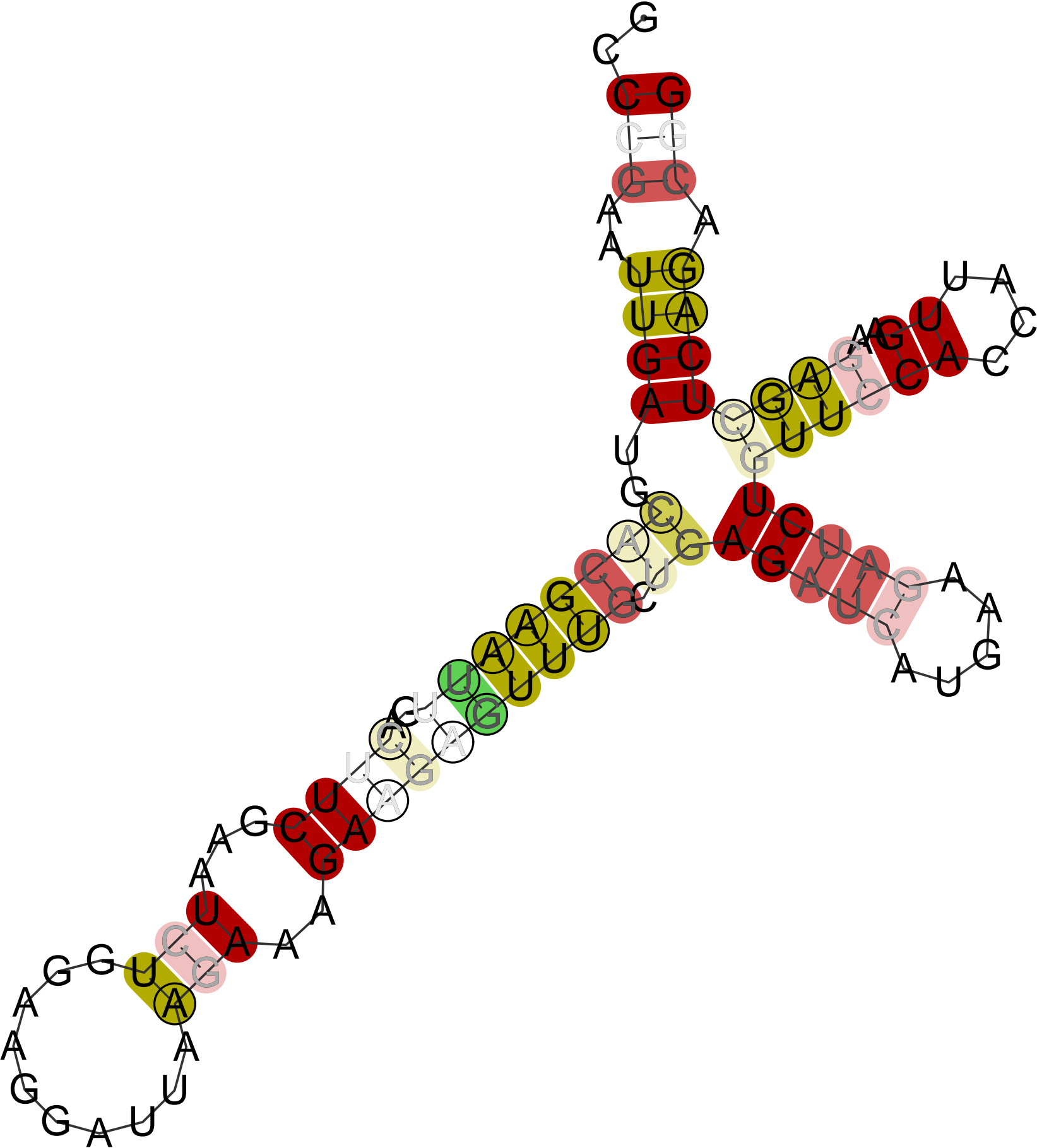

Supplement: S7 Fig — See the caption for S1 Fig for a description of the filename convention (save that the corresponding nucleotide locations in reference sequences are listed in S8 and S16 Tables), and an explanation of the RNAalifold options used and output. (ZIP) [file pcbi.1012009.s128.zip › H7N9-human-ranked-PB1-alignment-2164-2265-refseq-2164-2265_alirna_nogap.pdf]

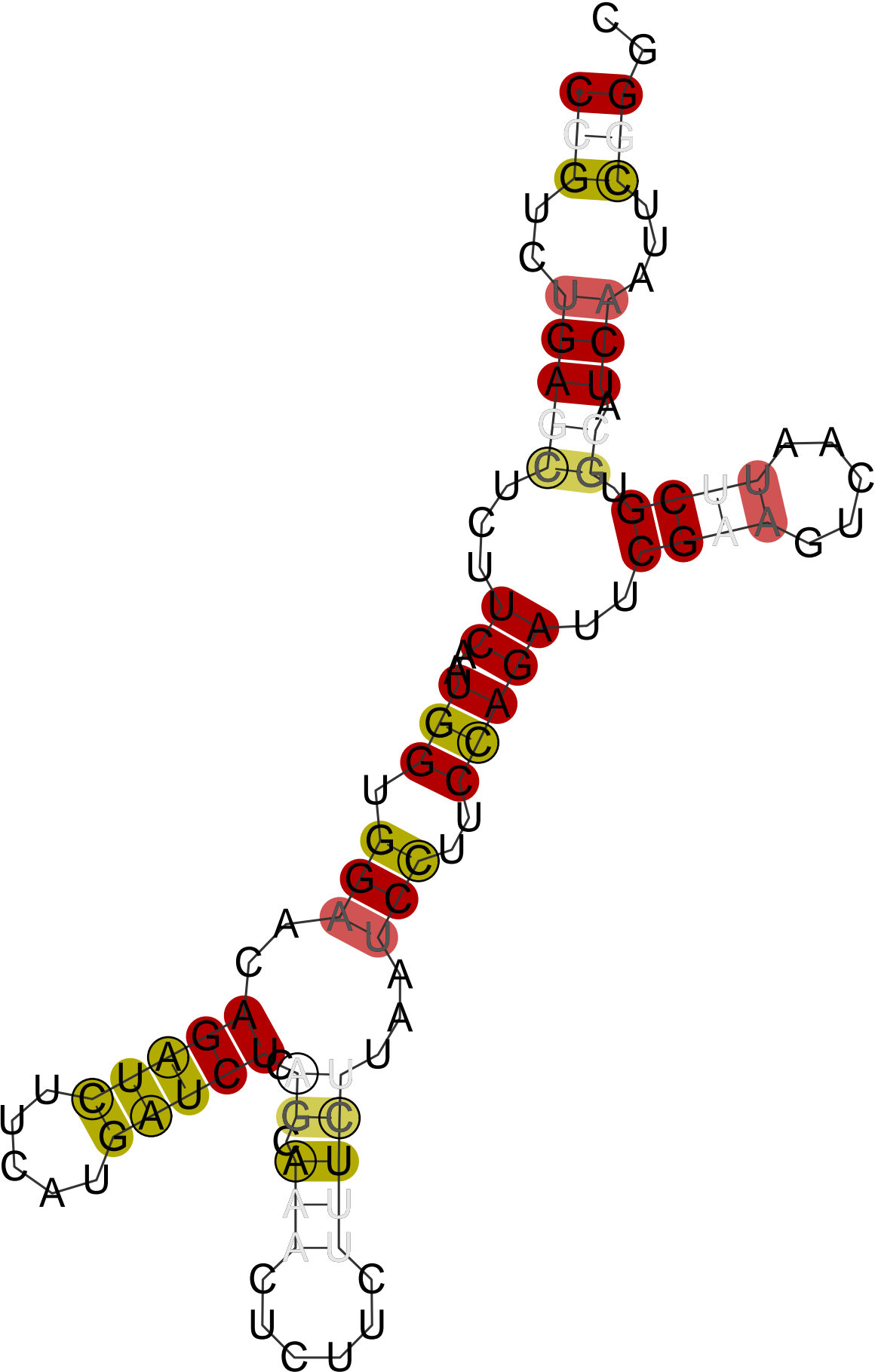

Supplement: S7 Fig — See the caption for S1 Fig for a description of the filename convention (save that the corresponding nucleotide locations in reference sequences are listed in S8 and S16 Tables), and an explanation of the RNAalifold options used and output. (ZIP) [file pcbi.1012009.s128.zip › H7N9-human-ranked-PB1-alignment-2164-2265-refseq-2164-2265_revcomp_alirna_nogap.pdf]

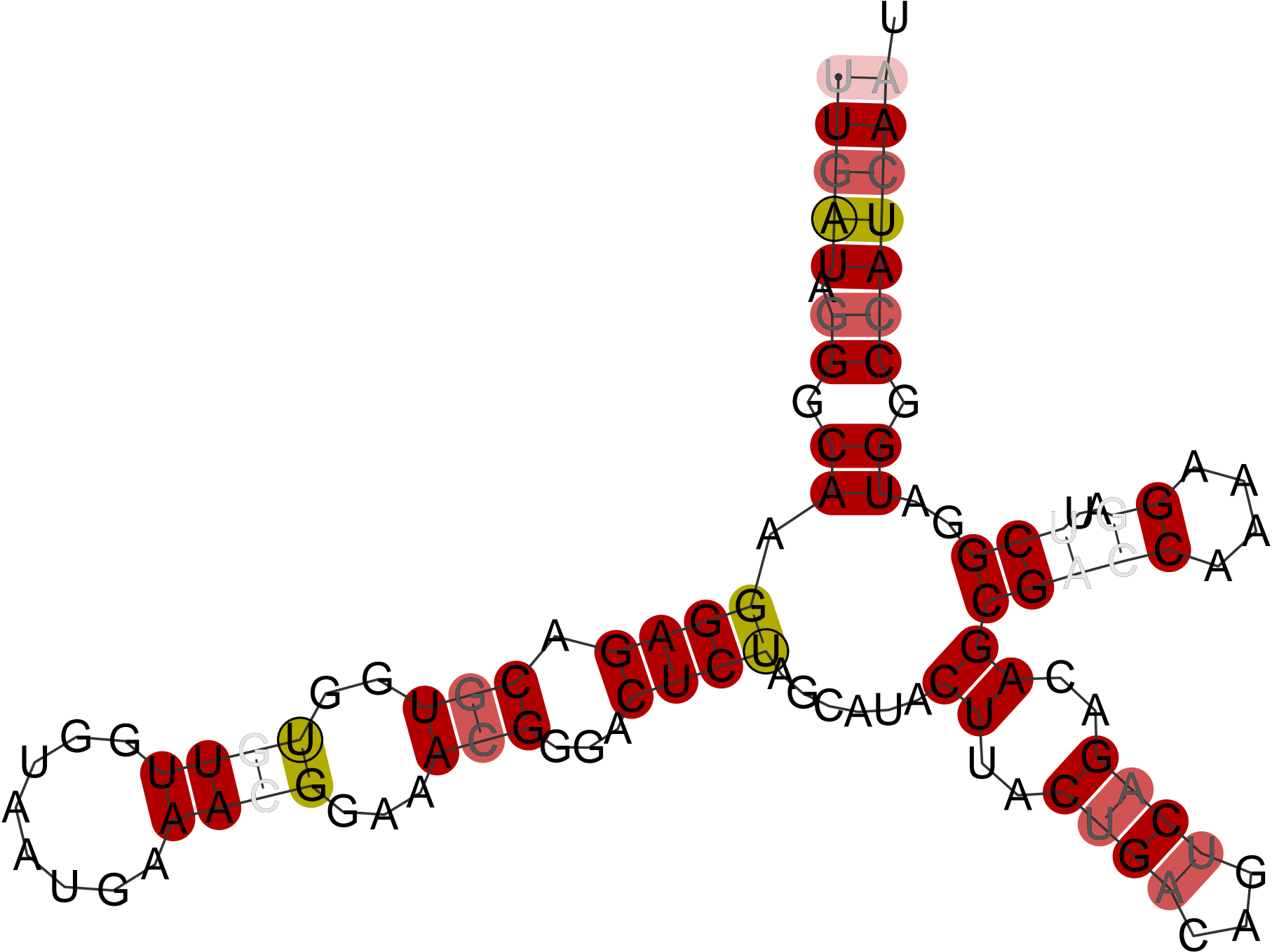

Supplement: S7 Fig — See the caption for S1 Fig for a description of the filename convention (save that the corresponding nucleotide locations in reference sequences are listed in S8 and S16 Tables), and an explanation of the RNAalifold options used and output. (ZIP) [file pcbi.1012009.s128.zip › H7N9-human-ranked-PB2-alignment-2173-2277-refseq-2173-2277_alirna_nogap.pdf]

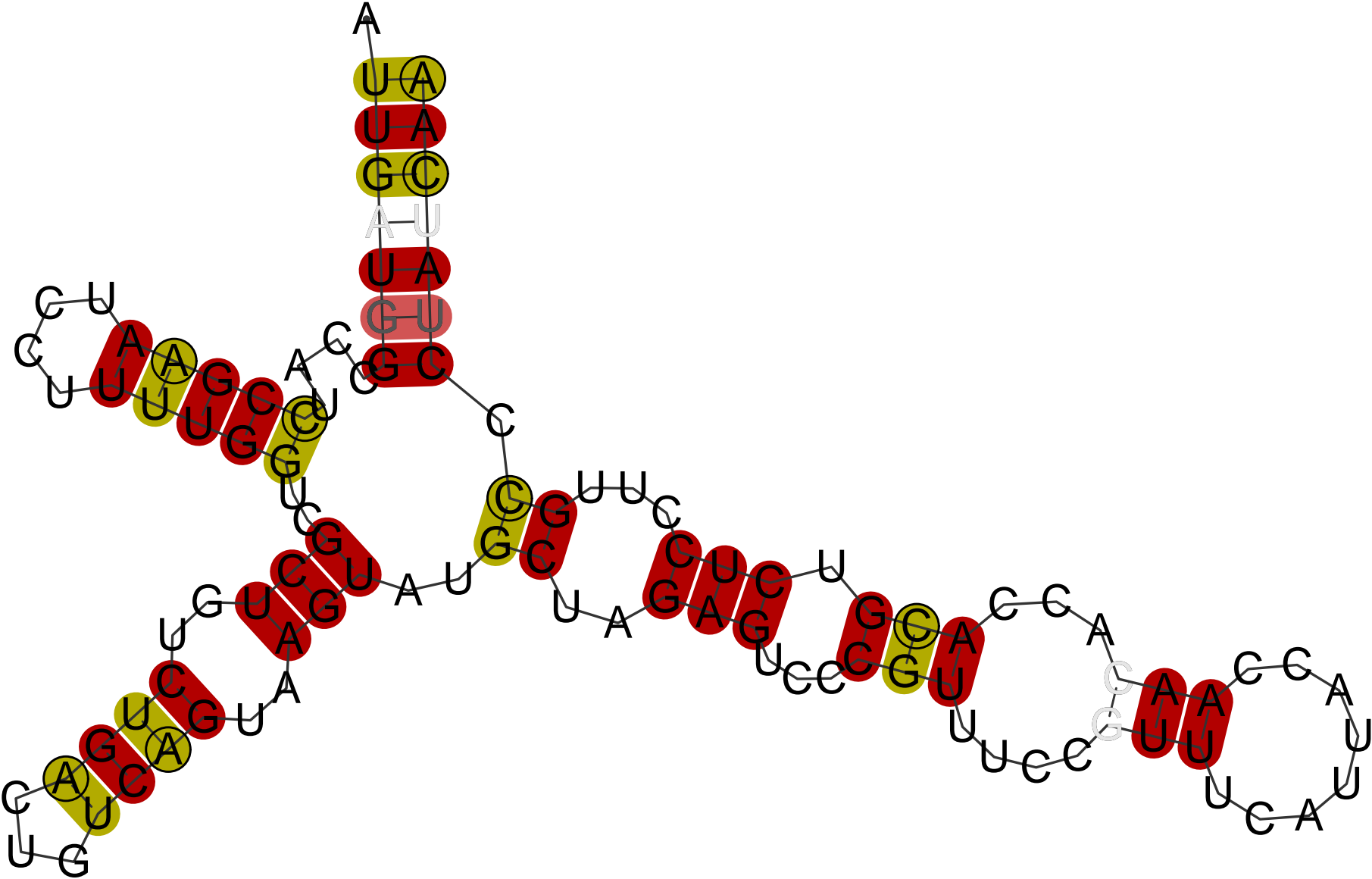

Supplement: S7 Fig — See the caption for S1 Fig for a description of the filename convention (save that the corresponding nucleotide locations in reference sequences are listed in S8 and S16 Tables), and an explanation of the RNAalifold options used and output. (ZIP) [file pcbi.1012009.s128.zip › H7N9-human-ranked-PB2-alignment-2173-2277-refseq-2173-2277_revcomp_alirna_nogap.pdf]

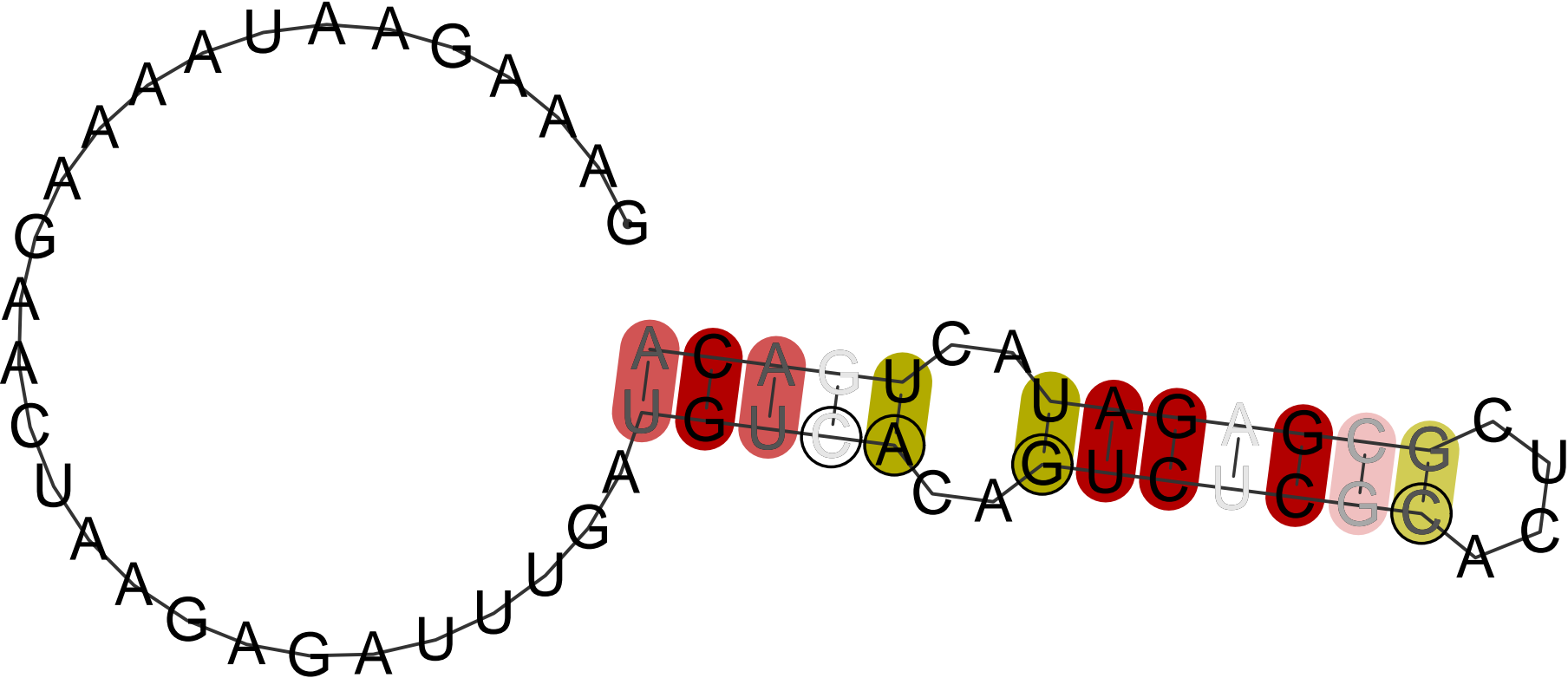

Supplement: S7 Fig — See the caption for S1 Fig for a description of the filename convention (save that the corresponding nucleotide locations in reference sequences are listed in S8 and S16 Tables), and an explanation of the RNAalifold options used and output. (ZIP) [file pcbi.1012009.s128.zip › H7N9-human-ranked-PB2-alignment-4-63-refseq-4-63_alirna_nogap.pdf]

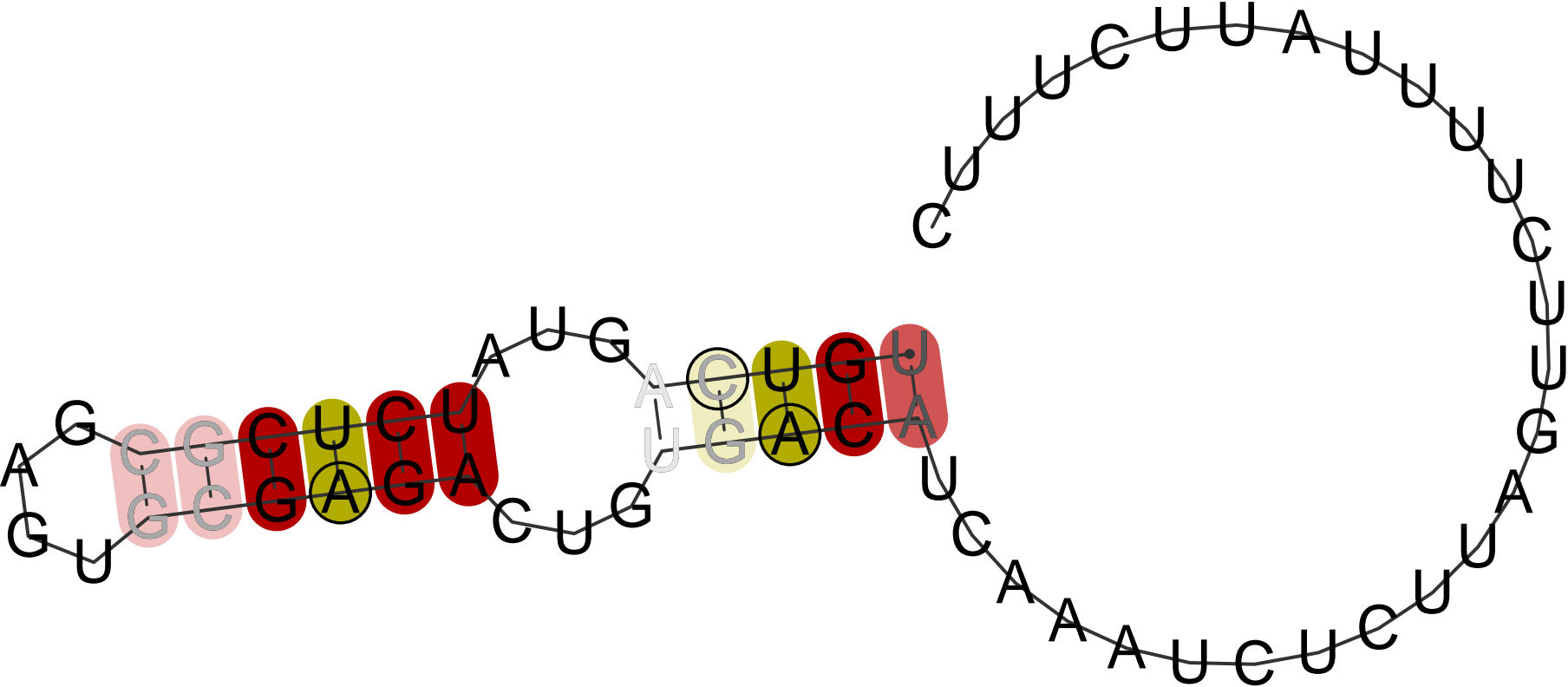

Supplement: S7 Fig — See the caption for S1 Fig for a description of the filename convention (save that the corresponding nucleotide locations in reference sequences are listed in S8 and S16 Tables), and an explanation of the RNAalifold options used and output. (ZIP) [file pcbi.1012009.s128.zip › H7N9-human-ranked-PB2-alignment-4-63-refseq-4-63_revcomp_alirna_nogap.pdf]

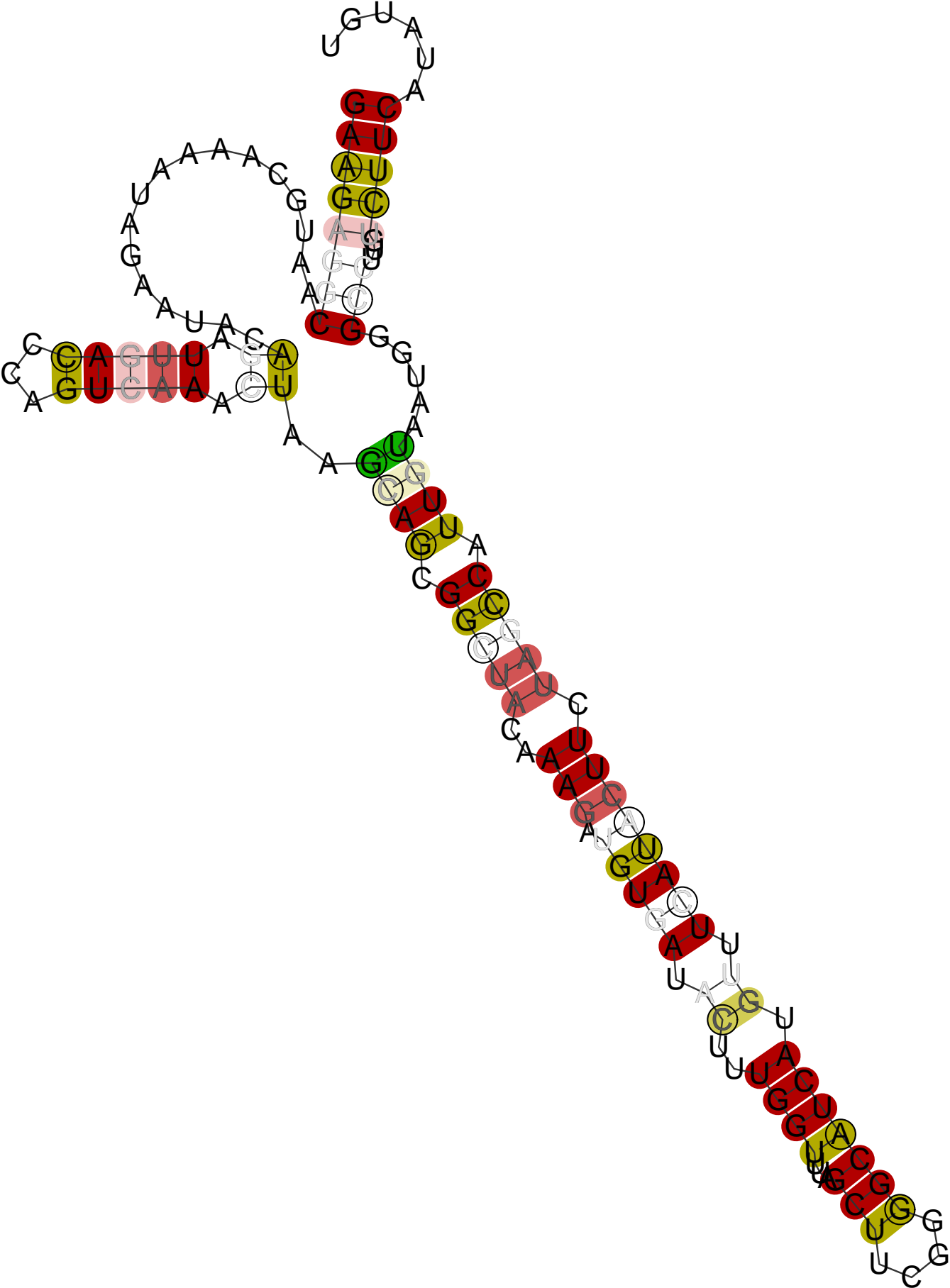

Supplement: S7 Fig — See the caption for S1 Fig for a description of the filename convention (save that the corresponding nucleotide locations in reference sequences are listed in S8 and S16 Tables), and an explanation of the RNAalifold options used and output. (ZIP) [file pcbi.1012009.s128.zip › H7N9-human-raw-HA-alignment-1519-1656-refseq-1507-1644_alirna_nogap.pdf]

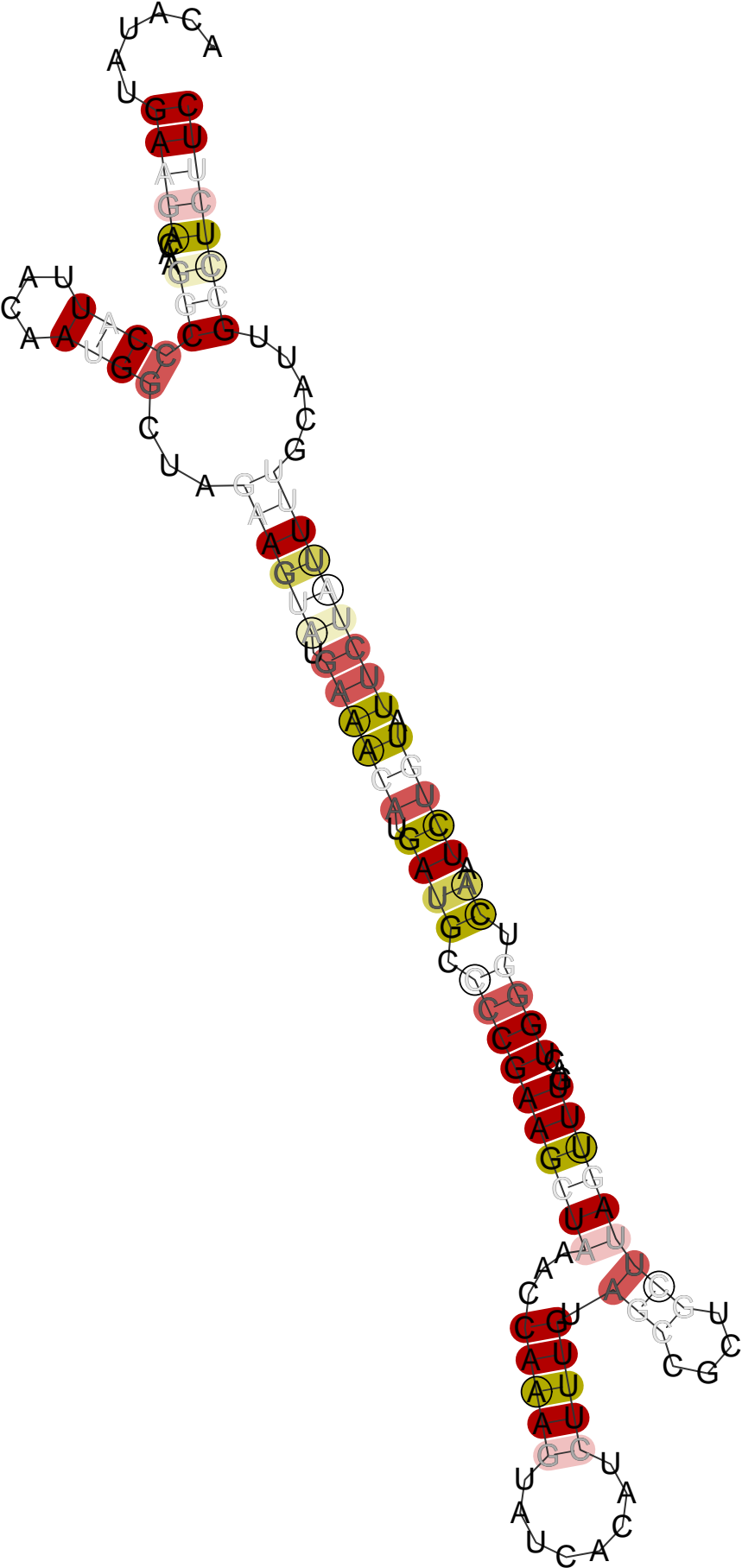

Supplement: S7 Fig — See the caption for S1 Fig for a description of the filename convention (save that the corresponding nucleotide locations in reference sequences are listed in S8 and S16 Tables), and an explanation of the RNAalifold options used and output. (ZIP) [file pcbi.1012009.s128.zip › H7N9-human-raw-HA-alignment-1519-1656-refseq-1507-1644_revcomp_alirna_nogap.pdf]

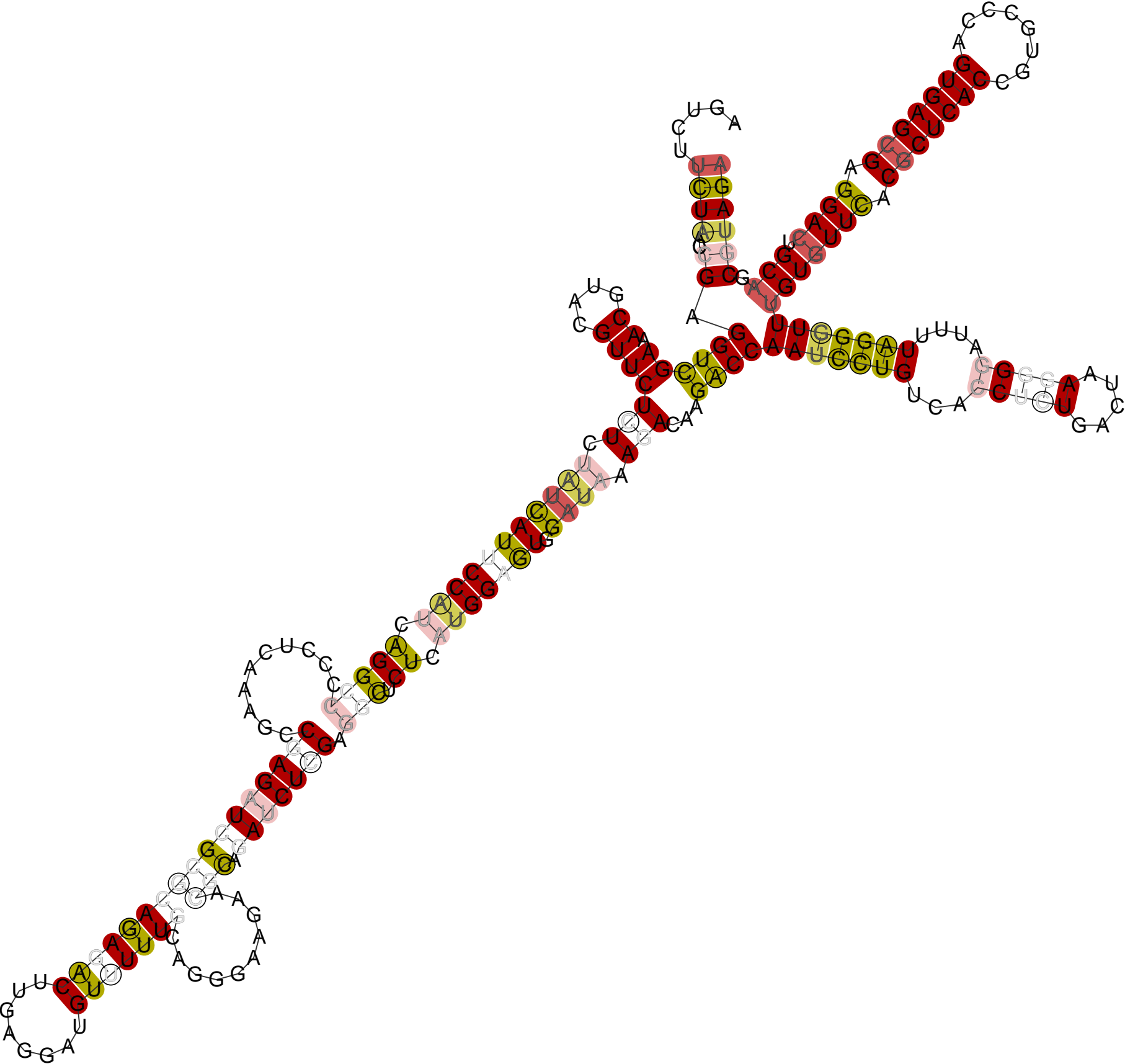

Supplement: S7 Fig — See the caption for S1 Fig for a description of the filename convention (save that the corresponding nucleotide locations in reference sequences are listed in S8 and S16 Tables), and an explanation of the RNAalifold options used and output. (ZIP) [file pcbi.1012009.s128.zip › H7N9-human-raw-M1-alignment-4-231-refseq-4-231_alirna_nogap.pdf]

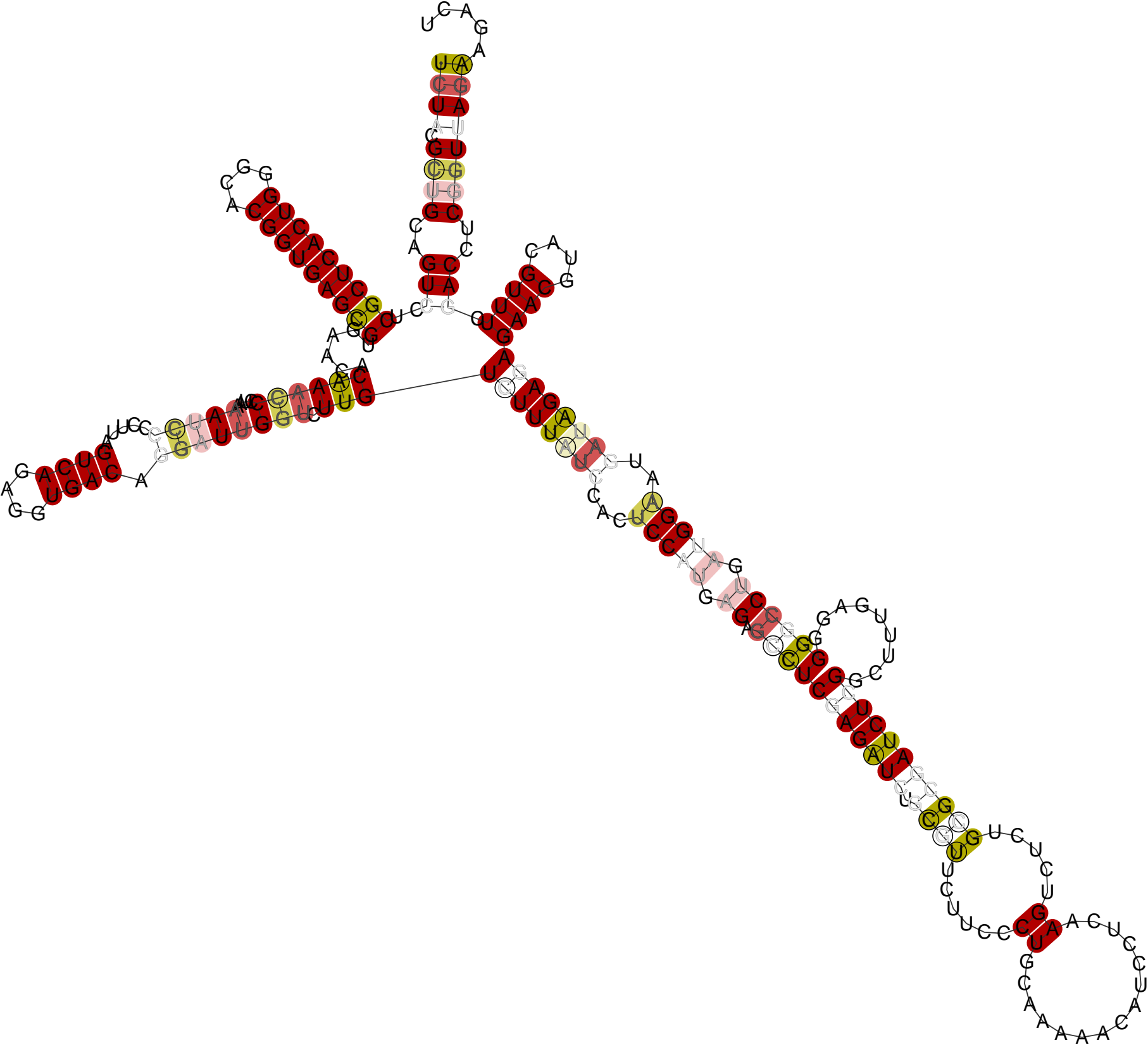

Supplement: S7 Fig — See the caption for S1 Fig for a description of the filename convention (save that the corresponding nucleotide locations in reference sequences are listed in S8 and S16 Tables), and an explanation of the RNAalifold options used and output. (ZIP) [file pcbi.1012009.s128.zip › H7N9-human-raw-M1-alignment-4-231-refseq-4-231_revcomp_alirna_nogap.pdf]

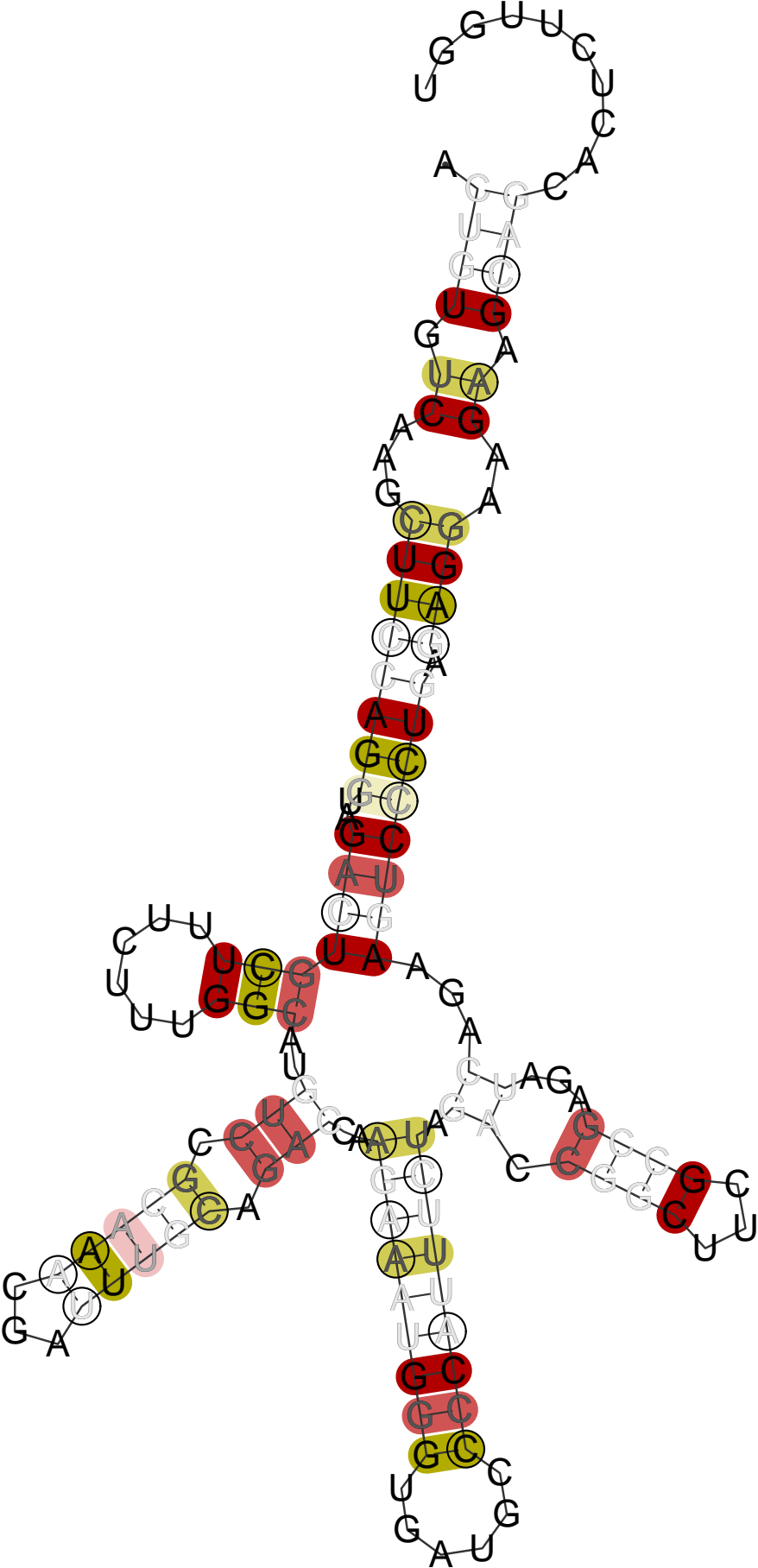

Supplement: S7 Fig — See the caption for S1 Fig for a description of the filename convention (save that the corresponding nucleotide locations in reference sequences are listed in S8 and S16 Tables), and an explanation of the RNAalifold options used and output. (ZIP) [file pcbi.1012009.s128.zip › H7N9-human-raw-NS1-alignment-13-153-refseq-13-153_alirna_nogap.pdf]

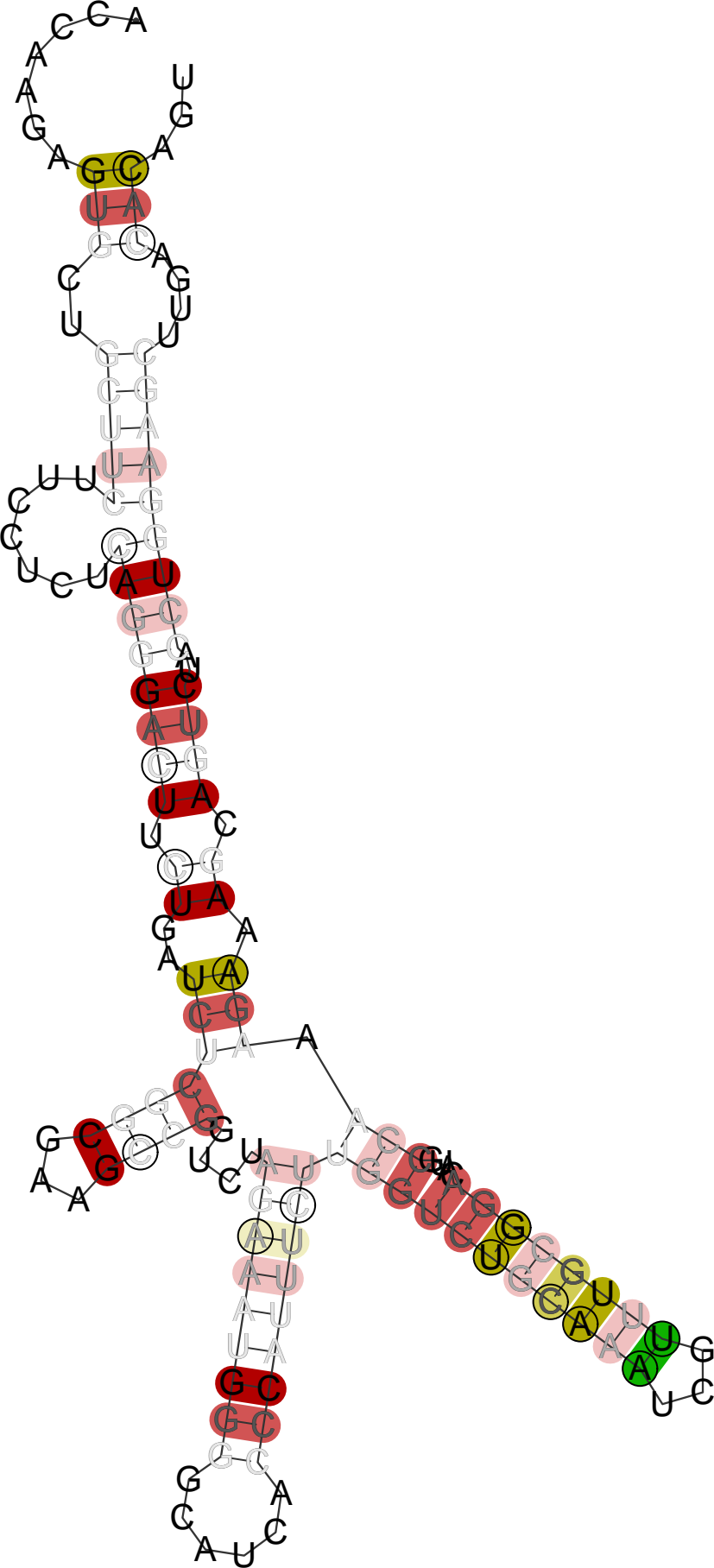

Supplement: S7 Fig — See the caption for S1 Fig for a description of the filename convention (save that the corresponding nucleotide locations in reference sequences are listed in S8 and S16 Tables), and an explanation of the RNAalifold options used and output. (ZIP) [file pcbi.1012009.s128.zip › H7N9-human-raw-NS1-alignment-13-153-refseq-13-153_revcomp_alirna_nogap.pdf]

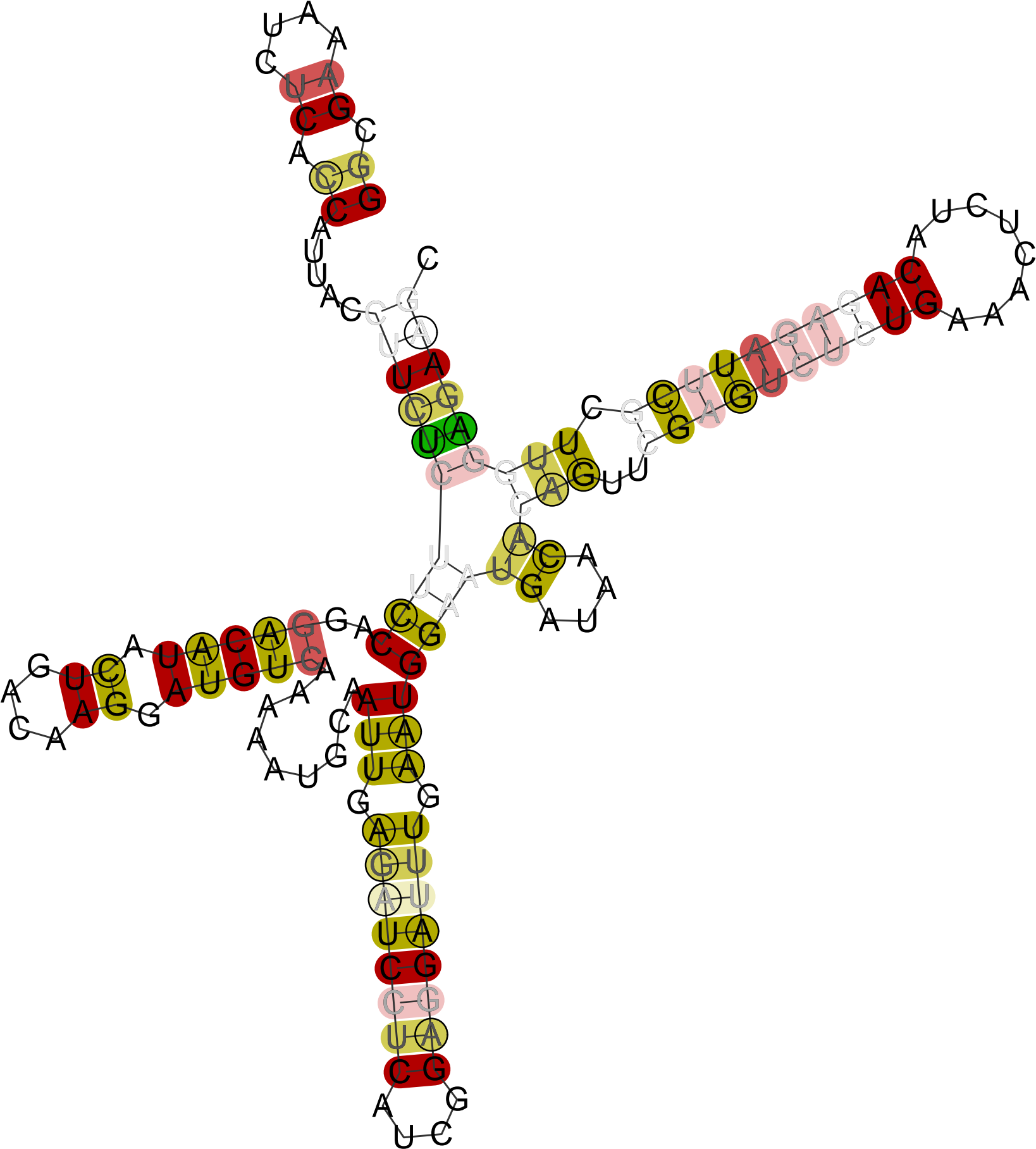

Supplement: S7 Fig — See the caption for S1 Fig for a description of the filename convention (save that the corresponding nucleotide locations in reference sequences are listed in S8 and S16 Tables), and an explanation of the RNAalifold options used and output. (ZIP) [file pcbi.1012009.s128.zip › H7N9-human-raw-NS1-alignment-475-618-refseq-472-615_alirna_nogap.pdf]

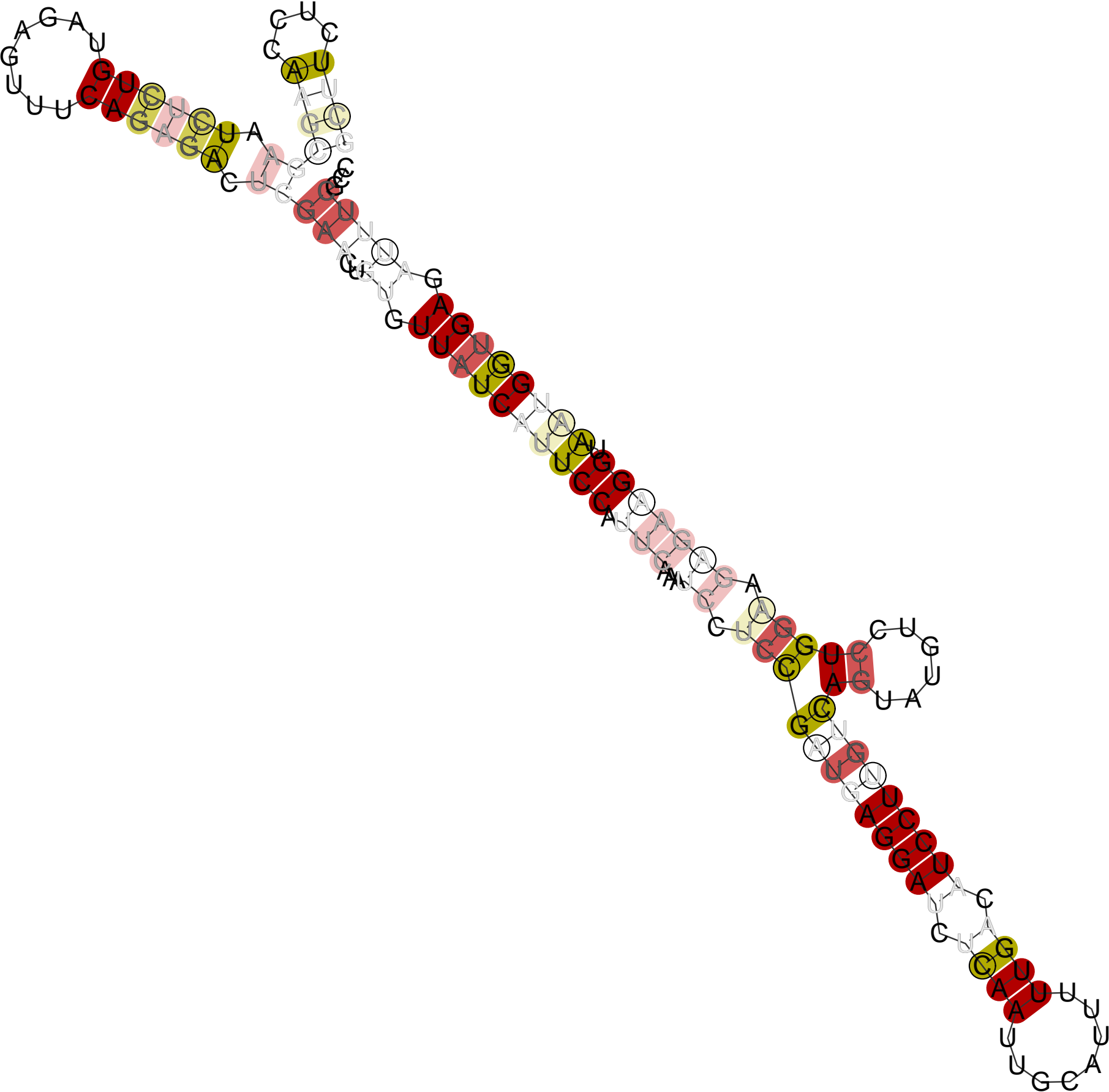

Supplement: S7 Fig — See the caption for S1 Fig for a description of the filename convention (save that the corresponding nucleotide locations in reference sequences are listed in S8 and S16 Tables), and an explanation of the RNAalifold options used and output. (ZIP) [file pcbi.1012009.s128.zip › H7N9-human-raw-NS1-alignment-475-618-refseq-472-615_revcomp_alirna_nogap.pdf]

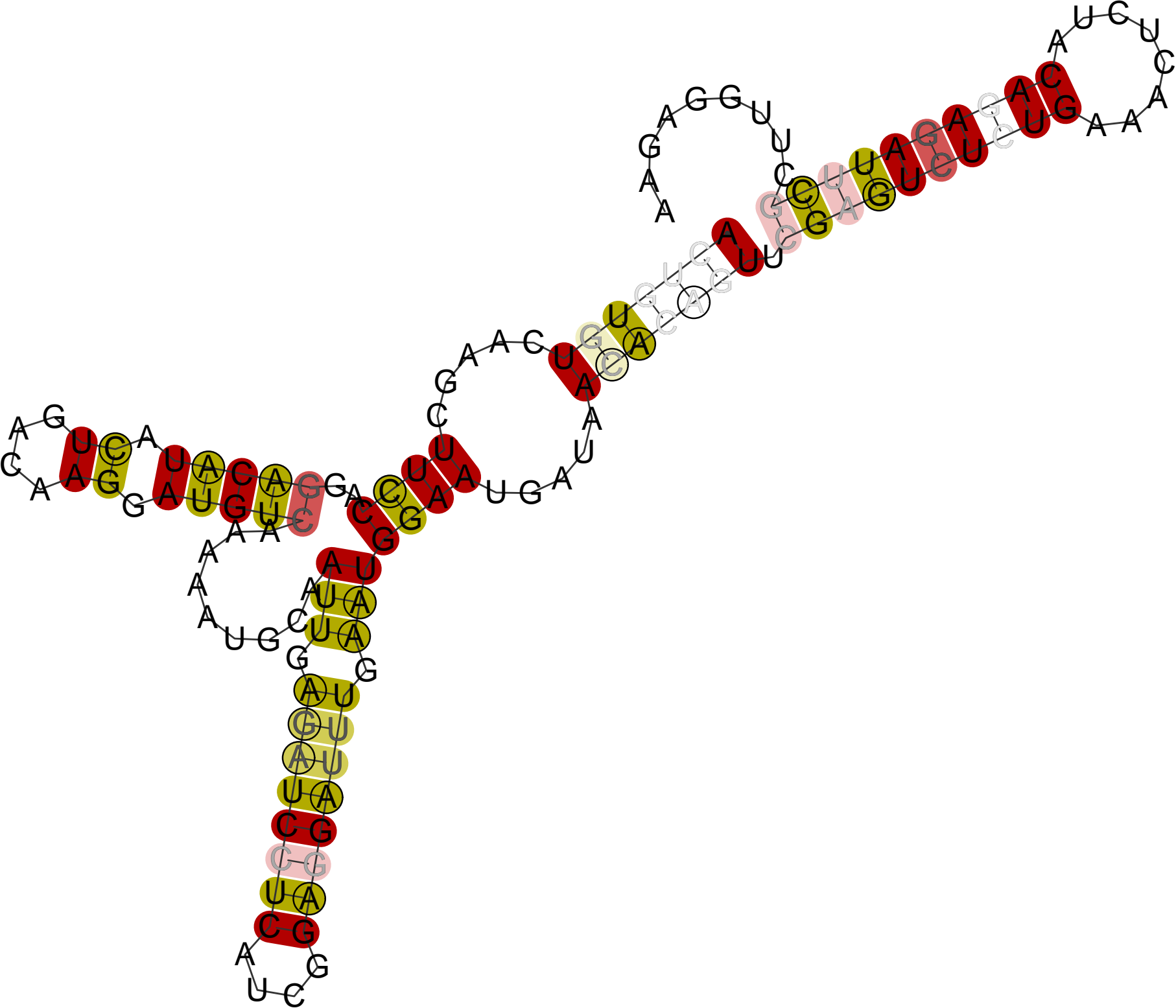

Supplement: S7 Fig — See the caption for S1 Fig for a description of the filename convention (save that the corresponding nucleotide locations in reference sequences are listed in S8 and S16 Tables), and an explanation of the RNAalifold options used and output. (ZIP) [file pcbi.1012009.s128.zip › H7N9-human-raw-NS2-alignment-13-141-refseq-13-30-503-613_alirna_nogap.pdf]

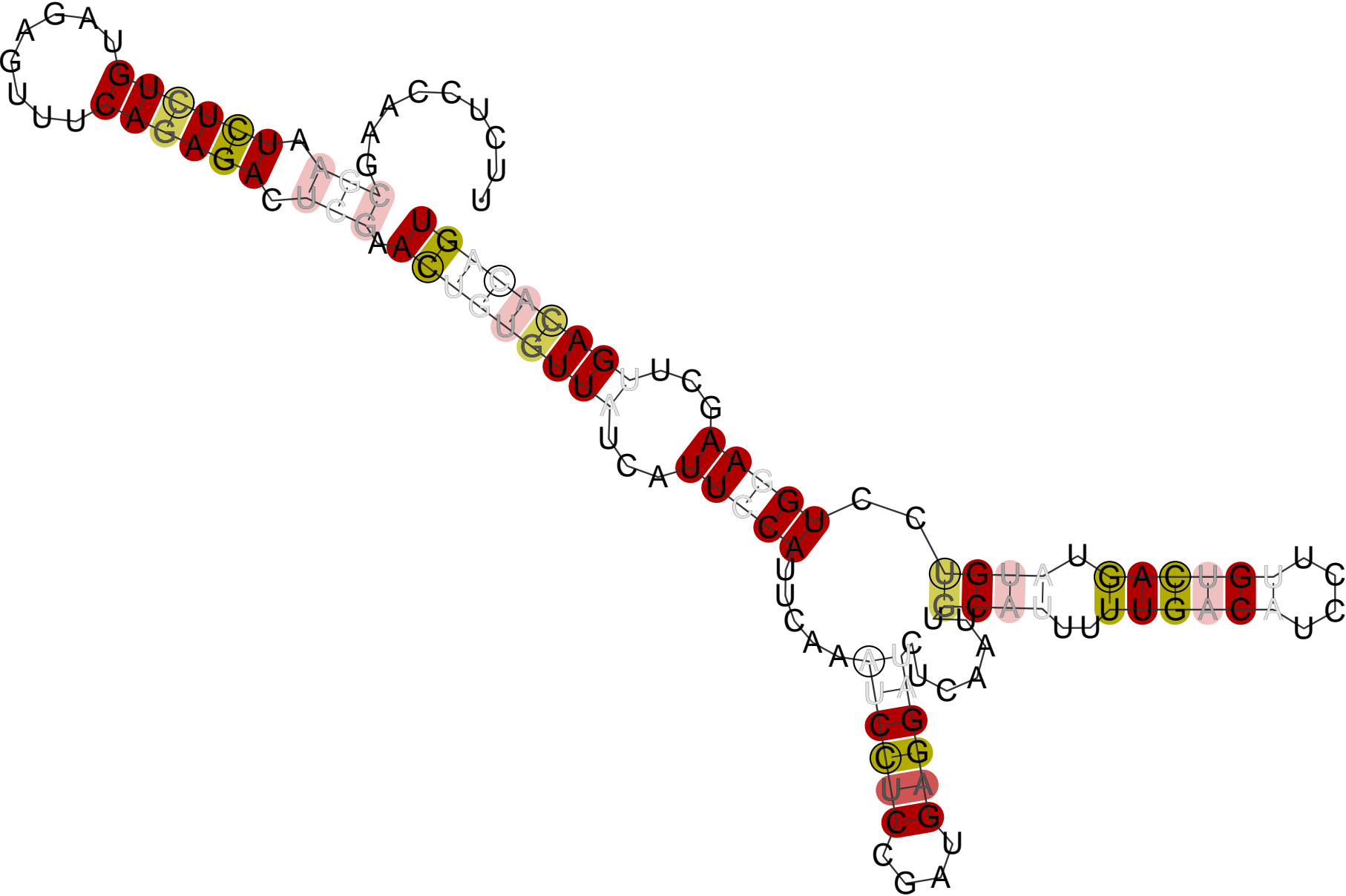

Supplement: S7 Fig — See the caption for S1 Fig for a description of the filename convention (save that the corresponding nucleotide locations in reference sequences are listed in S8 and S16 Tables), and an explanation of the RNAalifold options used and output. (ZIP) [file pcbi.1012009.s128.zip › H7N9-human-raw-NS2-alignment-13-141-refseq-13-30-503-613_revcomp_alirna_nogap.pdf]

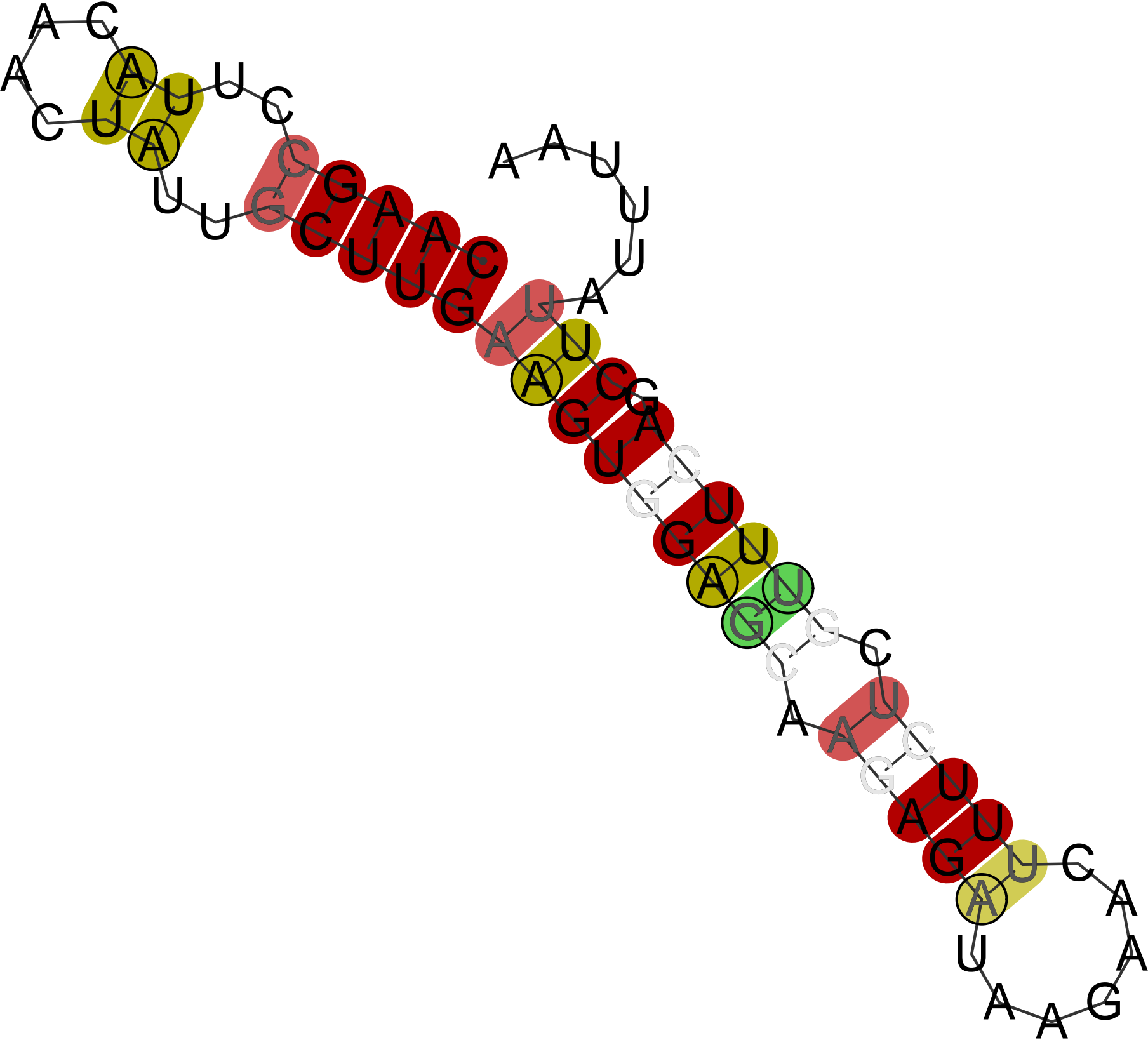

Supplement: S7 Fig — See the caption for S1 Fig for a description of the filename convention (save that the corresponding nucleotide locations in reference sequences are listed in S8 and S16 Tables), and an explanation of the RNAalifold options used and output. (ZIP) [file pcbi.1012009.s128.zip › H7N9-human-raw-NS2-alignment-301-366-refseq-773-838_alirna_nogap.pdf]

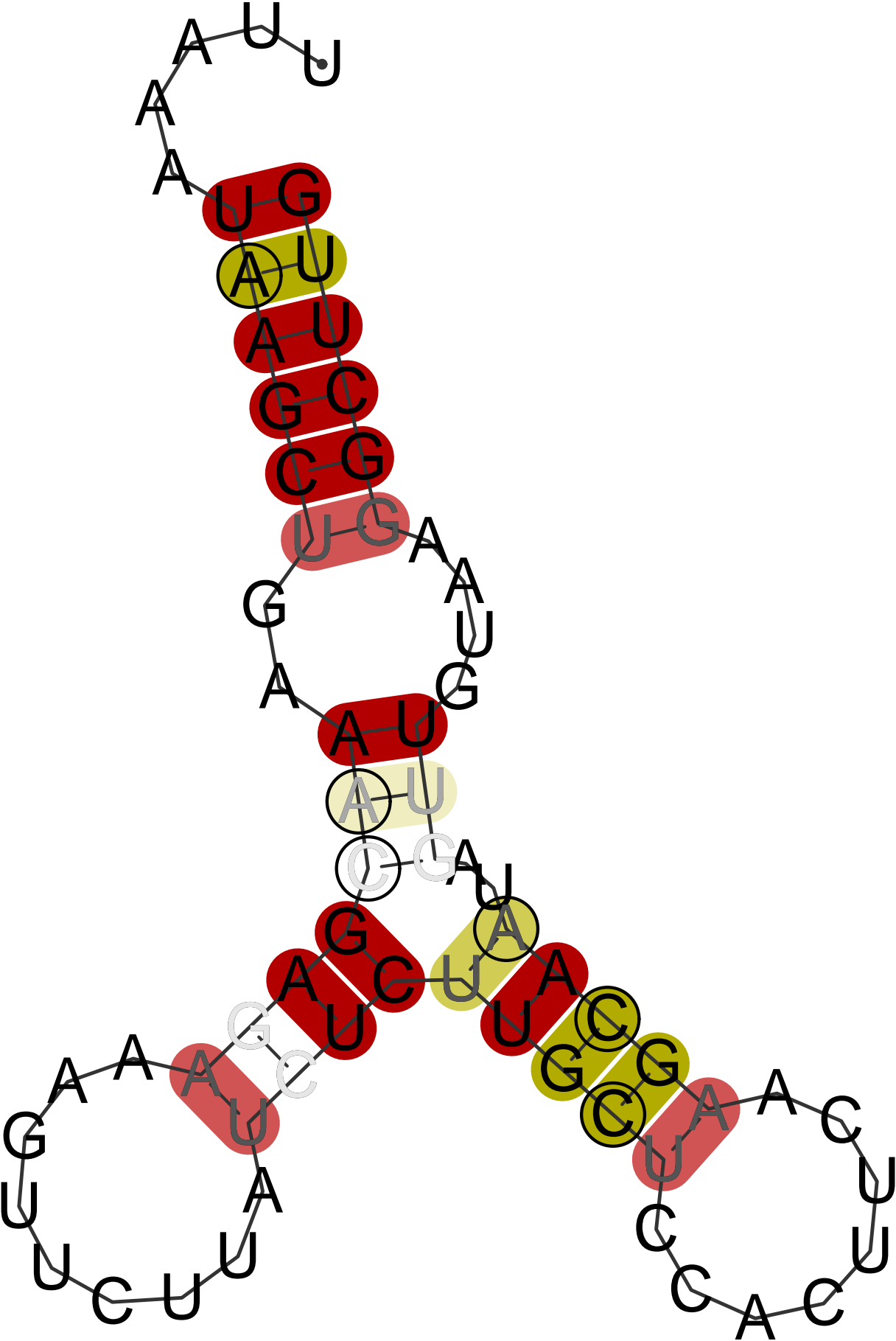

Supplement: S7 Fig — See the caption for S1 Fig for a description of the filename convention (save that the corresponding nucleotide locations in reference sequences are listed in S8 and S16 Tables), and an explanation of the RNAalifold options used and output. (ZIP) [file pcbi.1012009.s128.zip › H7N9-human-raw-NS2-alignment-301-366-refseq-773-838_revcomp_alirna_nogap.pdf]

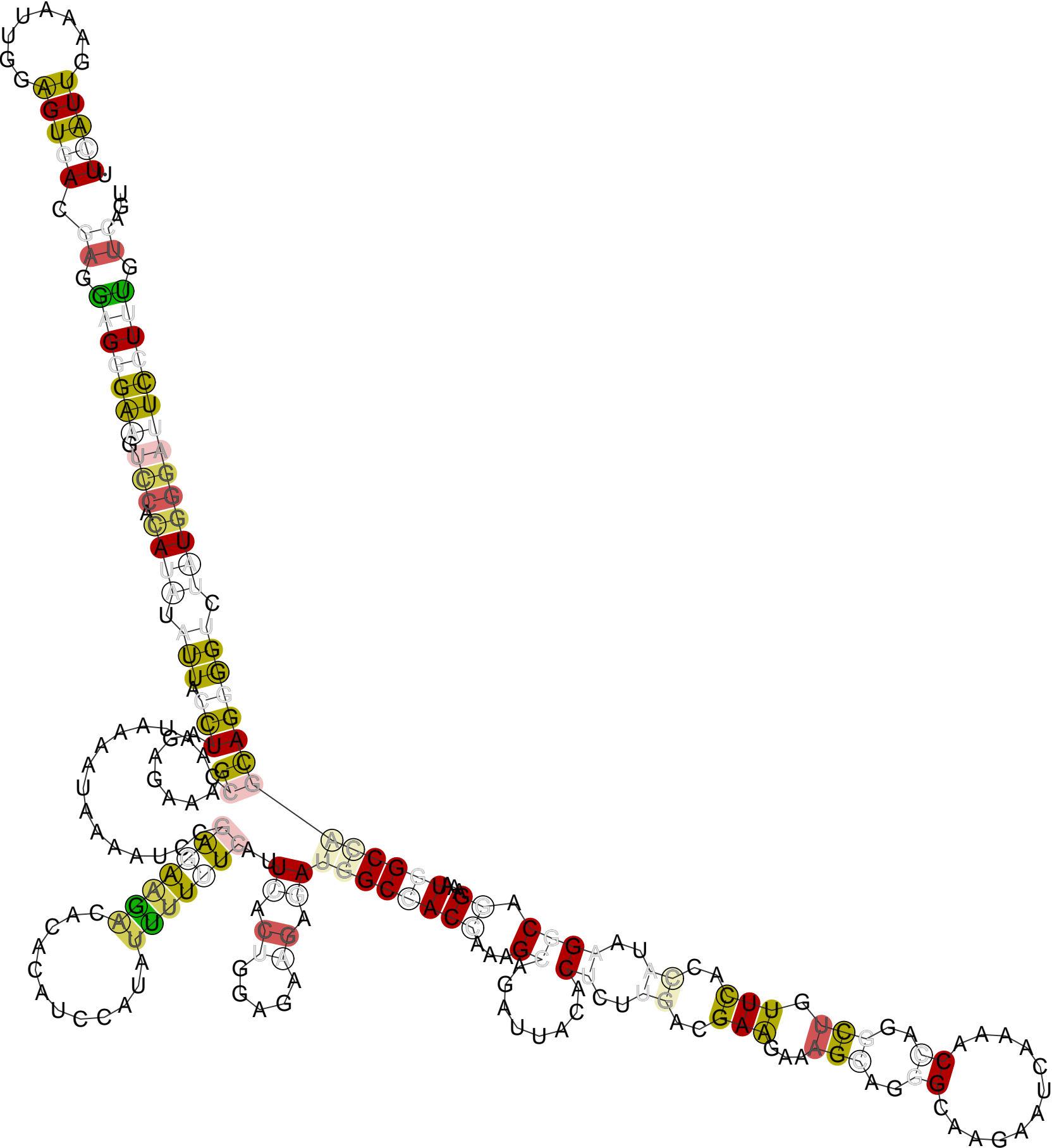

Supplement: S7 Fig — See the caption for S1 Fig for a description of the filename convention (save that the corresponding nucleotide locations in reference sequences are listed in S8 and S16 Tables), and an explanation of the RNAalifold options used and output. (ZIP) [file pcbi.1012009.s128.zip › H7N9-human-raw-PA-X-alignment-349-579-refseq-349-570-572-580_alirna_nogap.pdf]

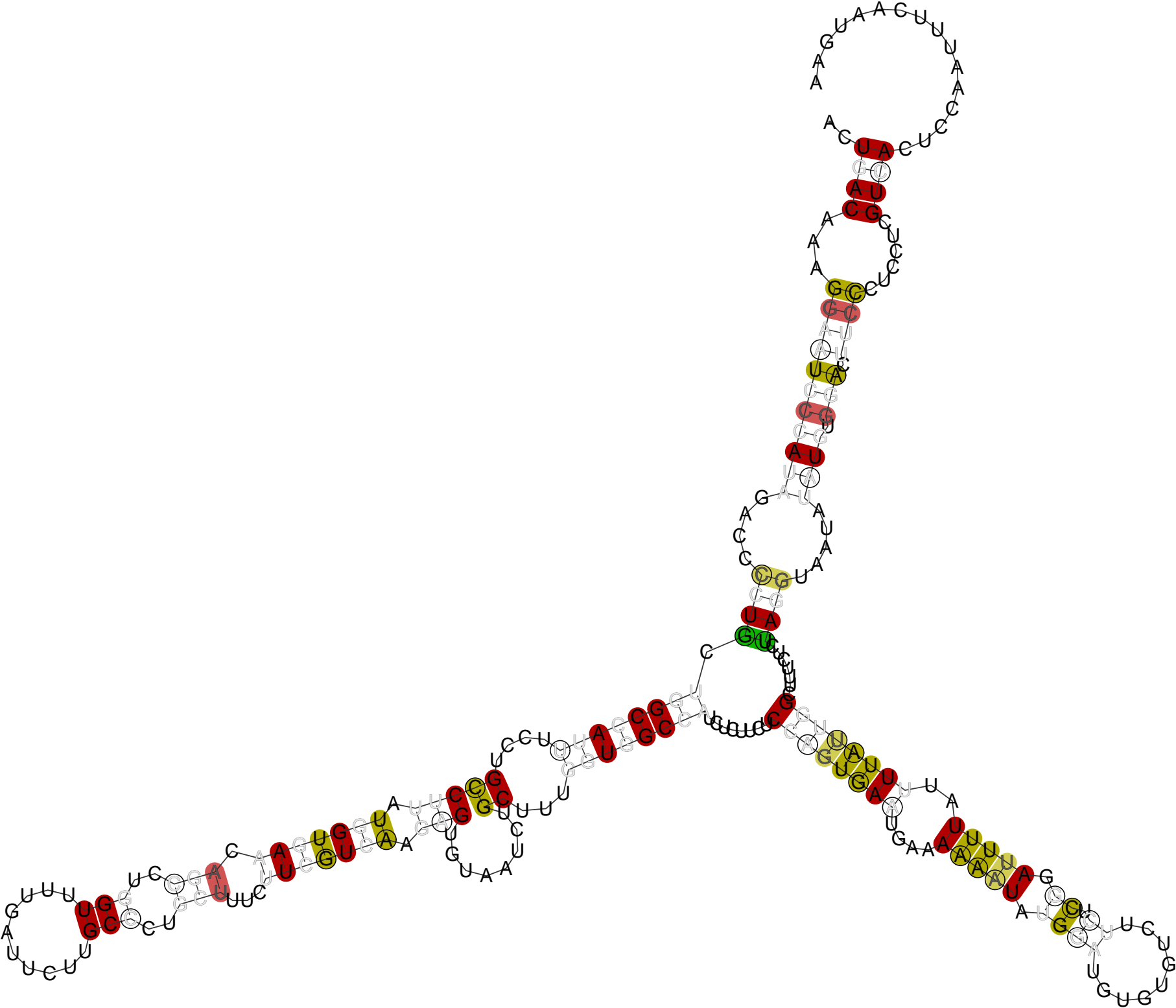

Supplement: S7 Fig — See the caption for S1 Fig for a description of the filename convention (save that the corresponding nucleotide locations in reference sequences are listed in S8 and S16 Tables), and an explanation of the RNAalifold options used and output. (ZIP) [file pcbi.1012009.s128.zip › H7N9-human-raw-PA-X-alignment-349-579-refseq-349-570-572-580_revcomp_alirna_nogap.pdf]

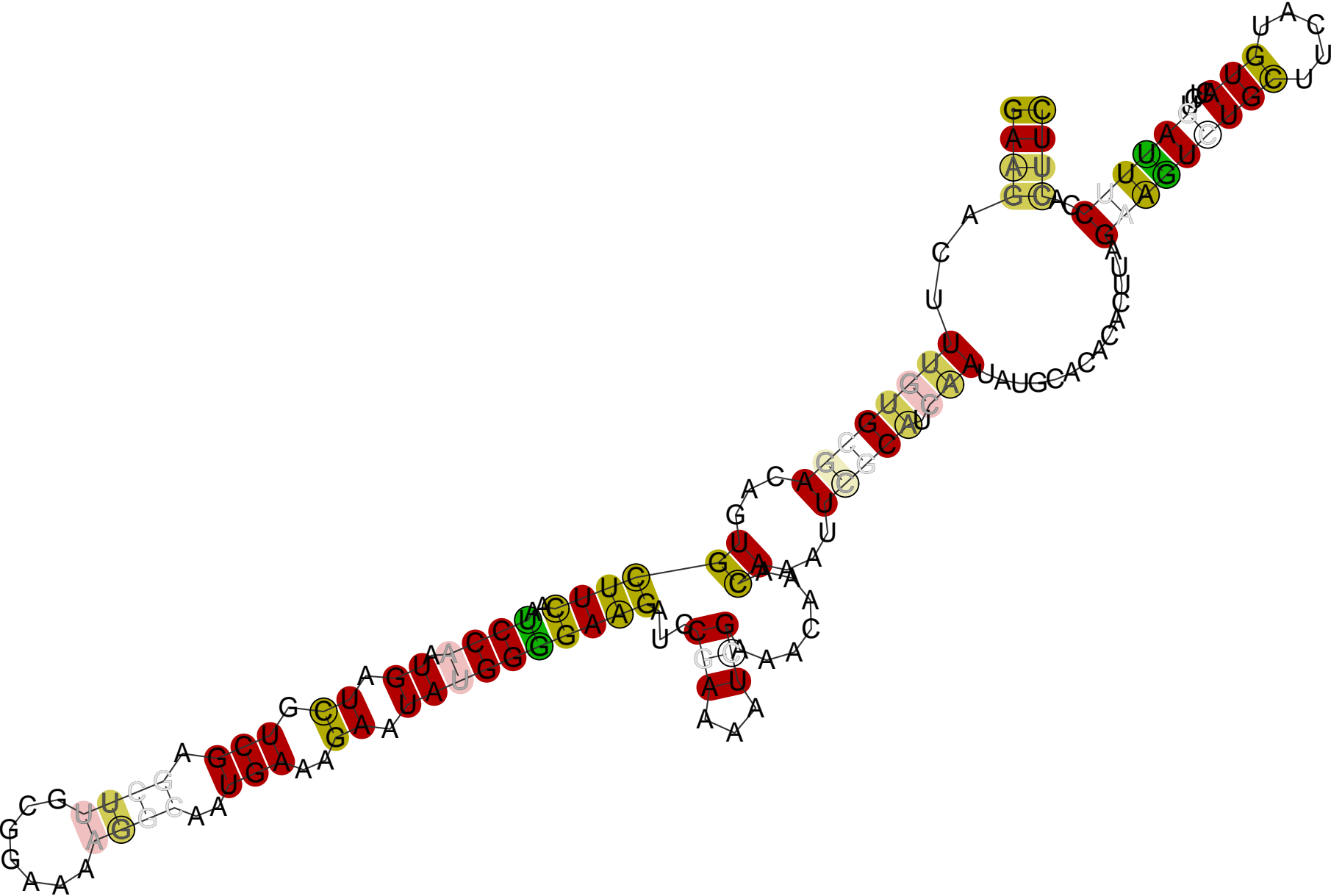

Supplement: S7 Fig — See the caption for S1 Fig for a description of the filename convention (save that the corresponding nucleotide locations in reference sequences are listed in S8 and S16 Tables), and an explanation of the RNAalifold options used and output. (ZIP) [file pcbi.1012009.s128.zip › H7N9-human-raw-PA-X-alignment-4-159-refseq-4-159_alirna_nogap.pdf]

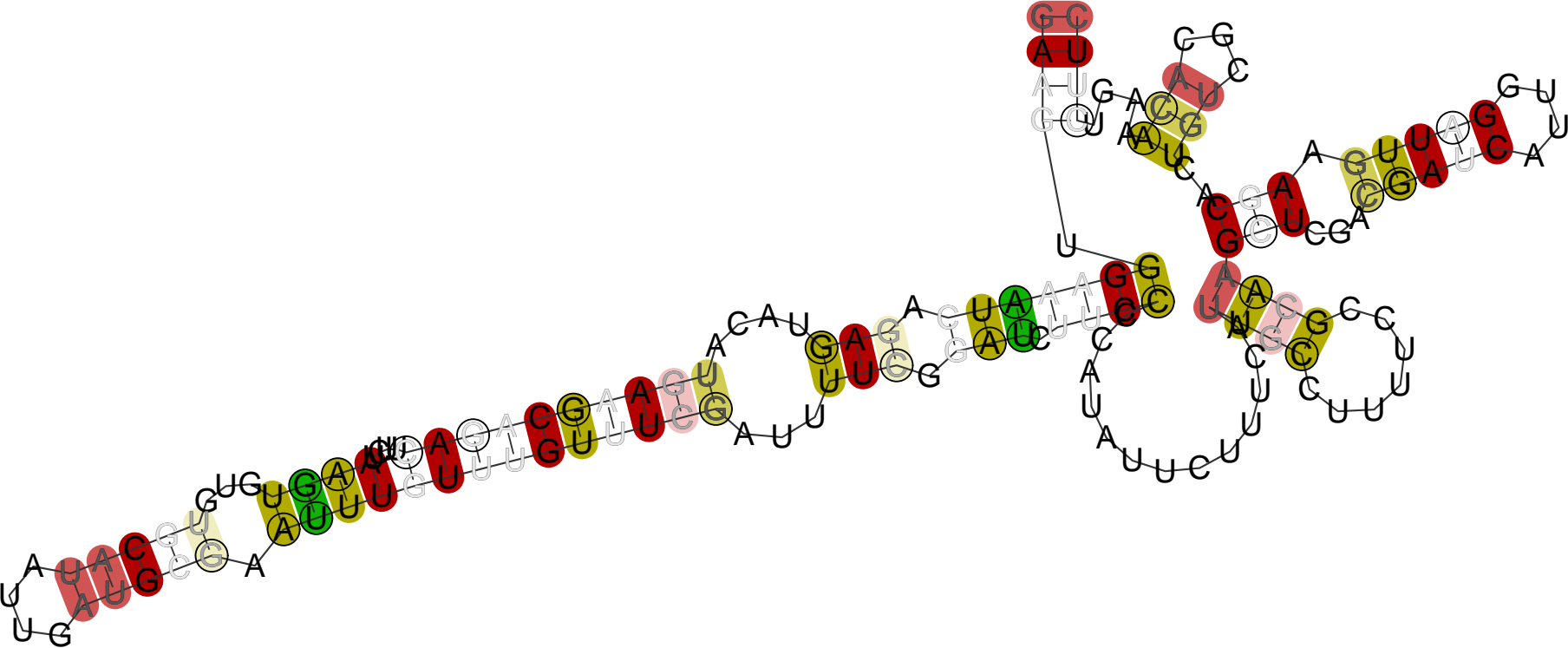

Supplement: S7 Fig — See the caption for S1 Fig for a description of the filename convention (save that the corresponding nucleotide locations in reference sequences are listed in S8 and S16 Tables), and an explanation of the RNAalifold options used and output. (ZIP) [file pcbi.1012009.s128.zip › H7N9-human-raw-PA-X-alignment-4-159-refseq-4-159_revcomp_alirna_nogap.pdf]

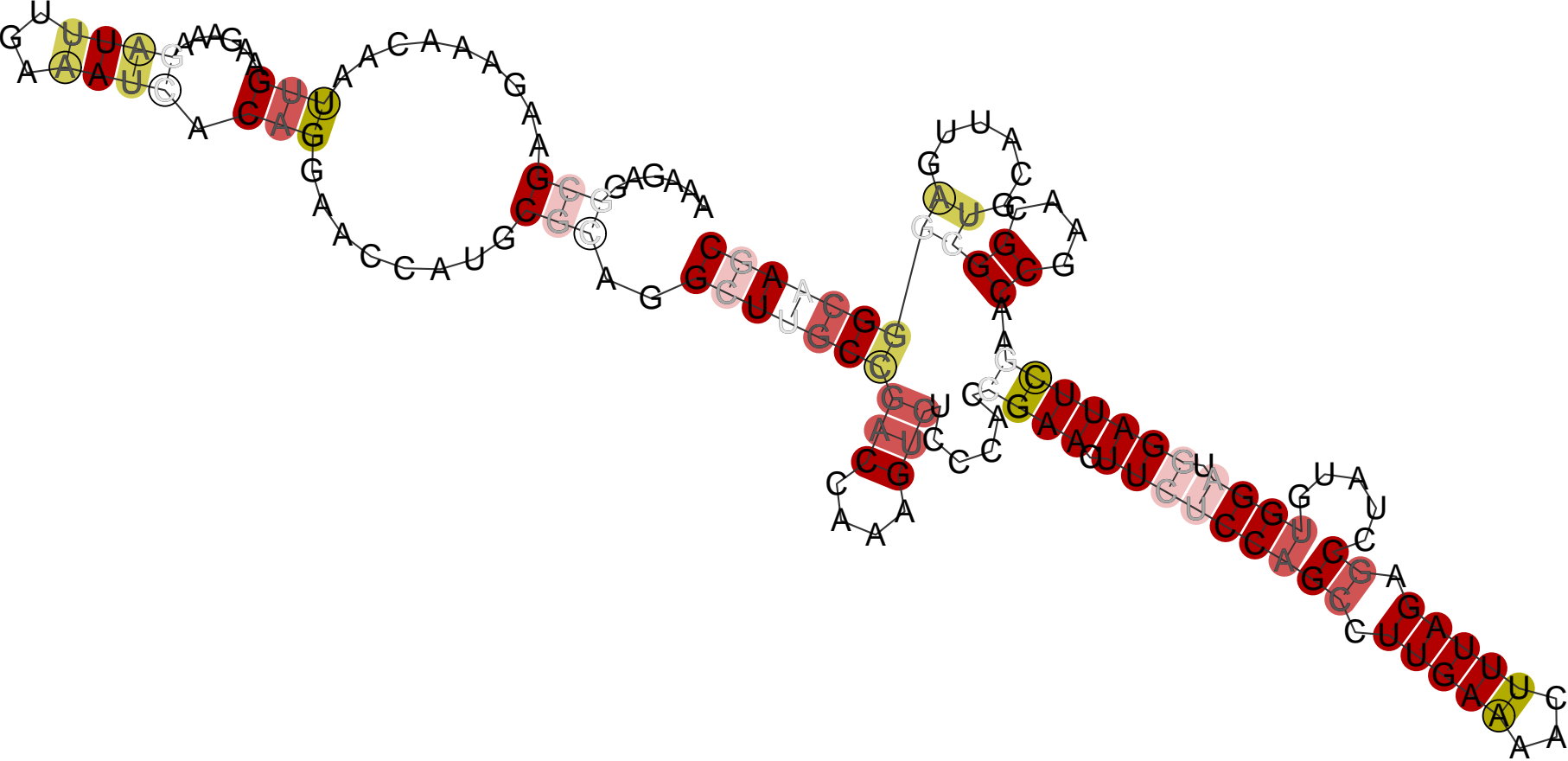

Supplement: S7 Fig — See the caption for S1 Fig for a description of the filename convention (save that the corresponding nucleotide locations in reference sequences are listed in S8 and S16 Tables), and an explanation of the RNAalifold options used and output. (ZIP) [file pcbi.1012009.s128.zip › H7N9-human-raw-PA-X-alignment-583-735-refseq-584-736_alirna_nogap.pdf]

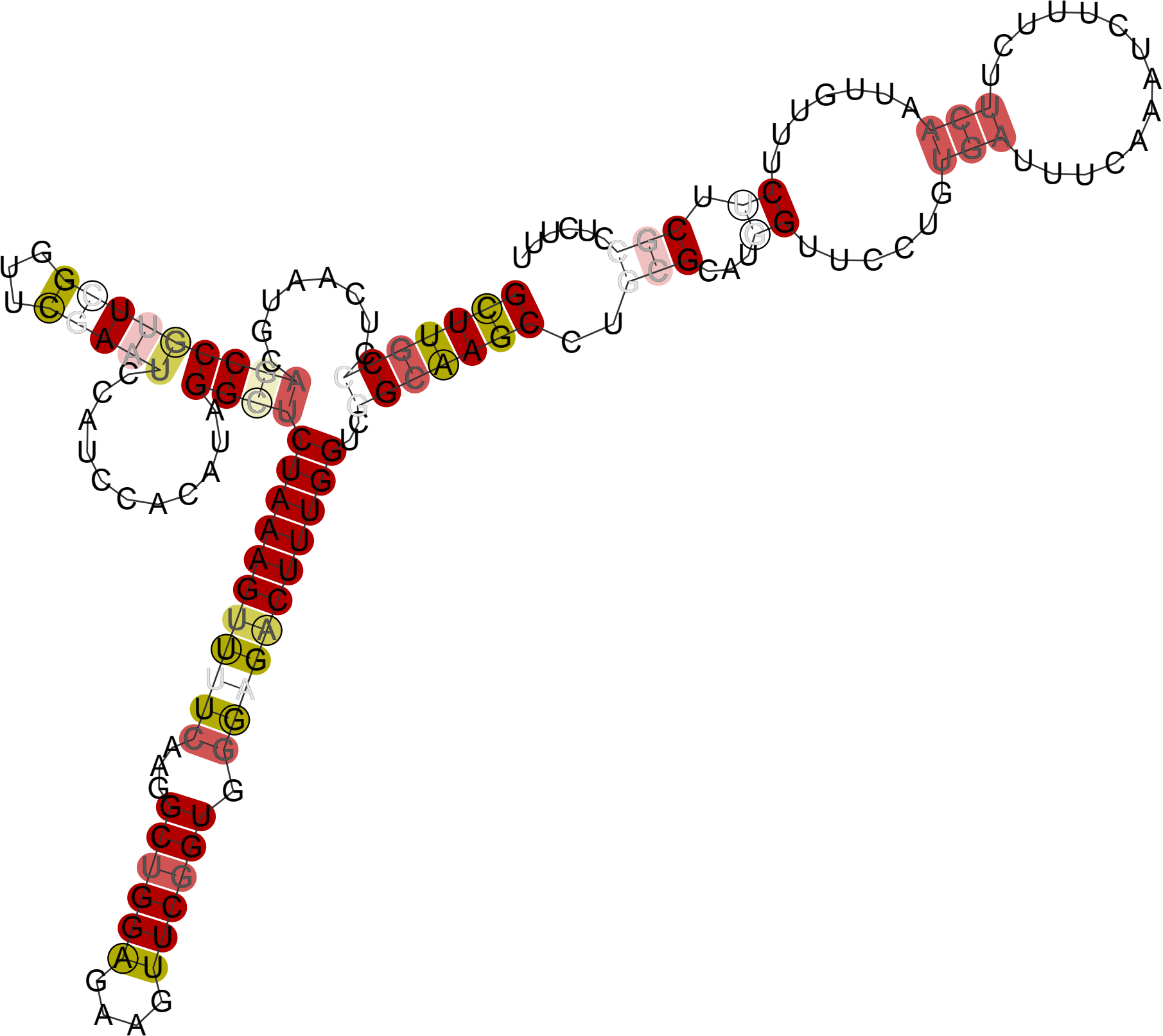

Supplement: S7 Fig — See the caption for S1 Fig for a description of the filename convention (save that the corresponding nucleotide locations in reference sequences are listed in S8 and S16 Tables), and an explanation of the RNAalifold options used and output. (ZIP) [file pcbi.1012009.s128.zip › H7N9-human-raw-PA-X-alignment-583-735-refseq-584-736_revcomp_alirna_nogap.pdf]

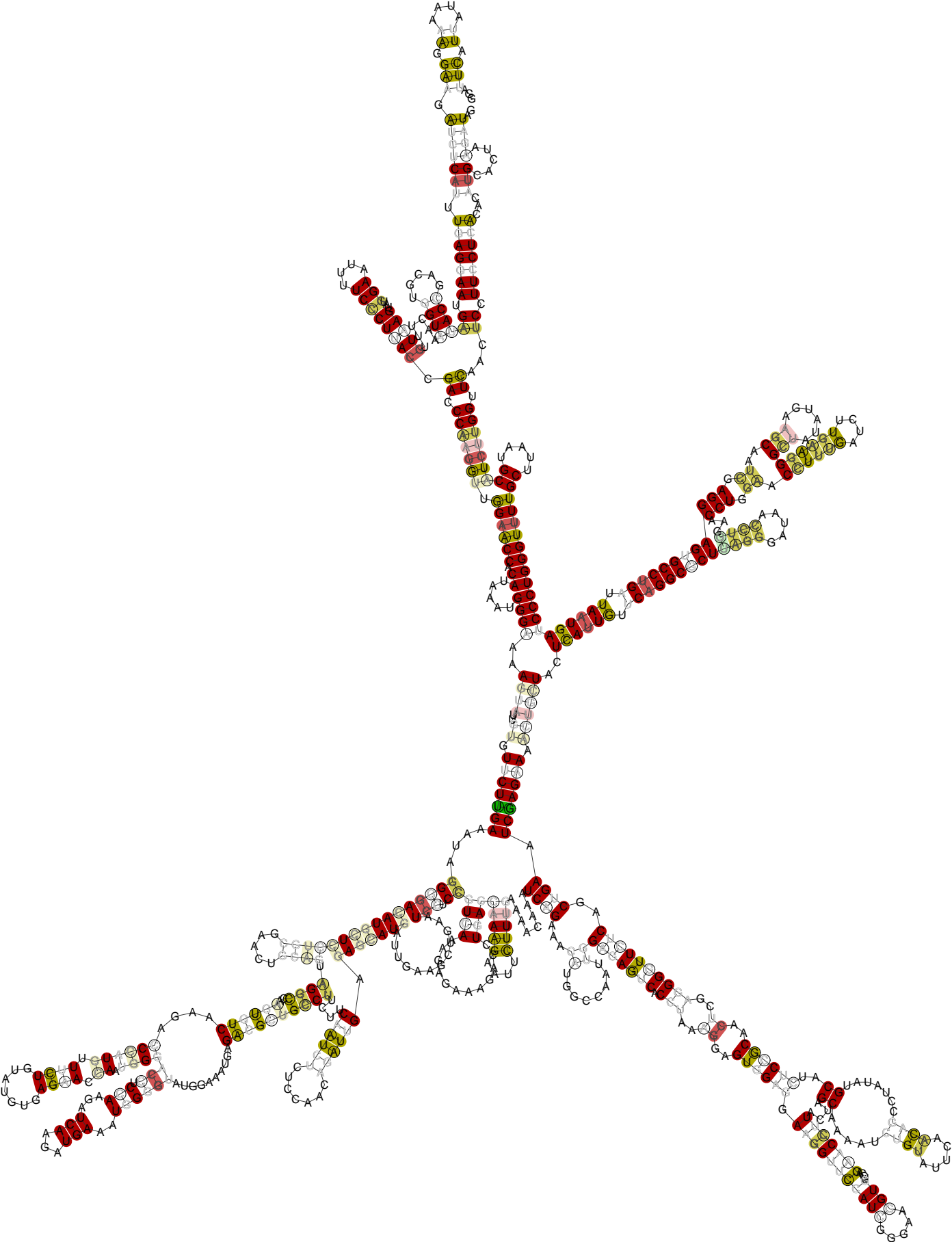

Supplement: S7 Fig — See the caption for S1 Fig for a description of the filename convention (save that the corresponding nucleotide locations in reference sequences are listed in S8 and S16 Tables), and an explanation of the RNAalifold options used and output. (ZIP) [file pcbi.1012009.s128.zip › H7N9-human-raw-PA-alignment-1504-2151-refseq-1504-2151_alirna_nogap.pdf]

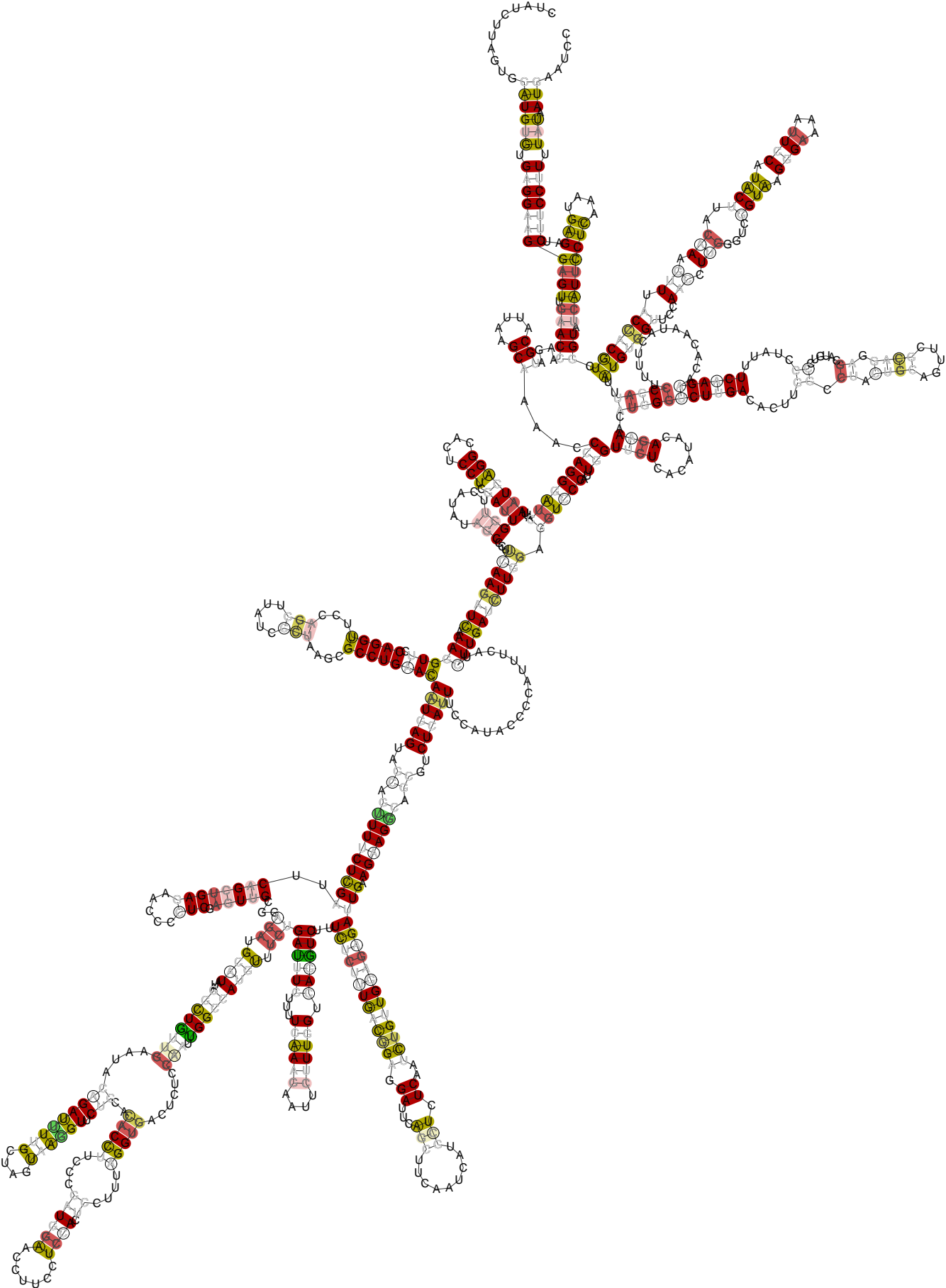

Supplement: S7 Fig — See the caption for S1 Fig for a description of the filename convention (save that the corresponding nucleotide locations in reference sequences are listed in S8 and S16 Tables), and an explanation of the RNAalifold options used and output. (ZIP) [file pcbi.1012009.s128.zip › H7N9-human-raw-PA-alignment-1504-2151-refseq-1504-2151_revcomp_alirna_nogap.pdf]

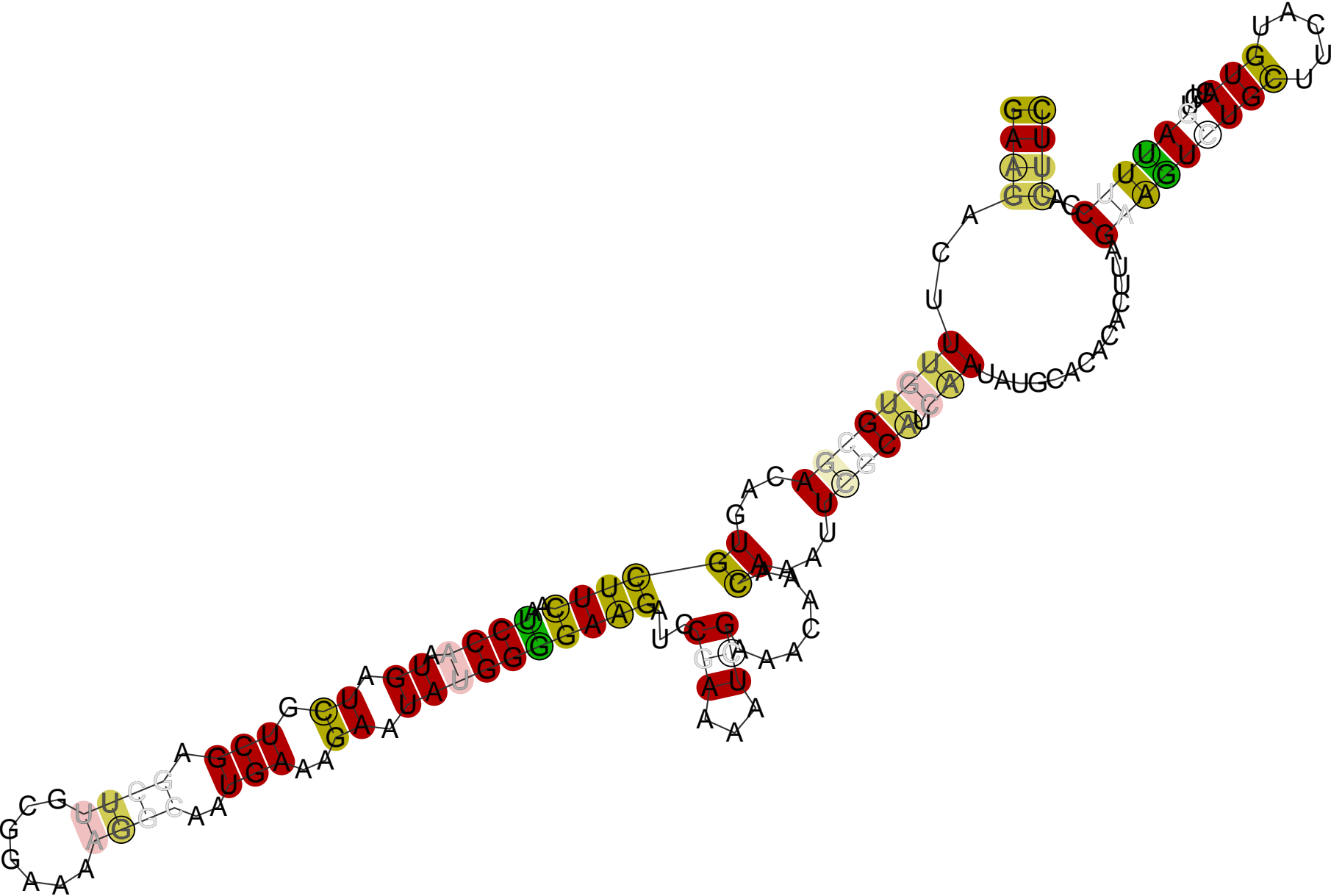

Supplement: S7 Fig — See the caption for S1 Fig for a description of the filename convention (save that the corresponding nucleotide locations in reference sequences are listed in S8 and S16 Tables), and an explanation of the RNAalifold options used and output. (ZIP) [file pcbi.1012009.s128.zip › H7N9-human-raw-PA-alignment-4-159-refseq-4-159_alirna_nogap.pdf]

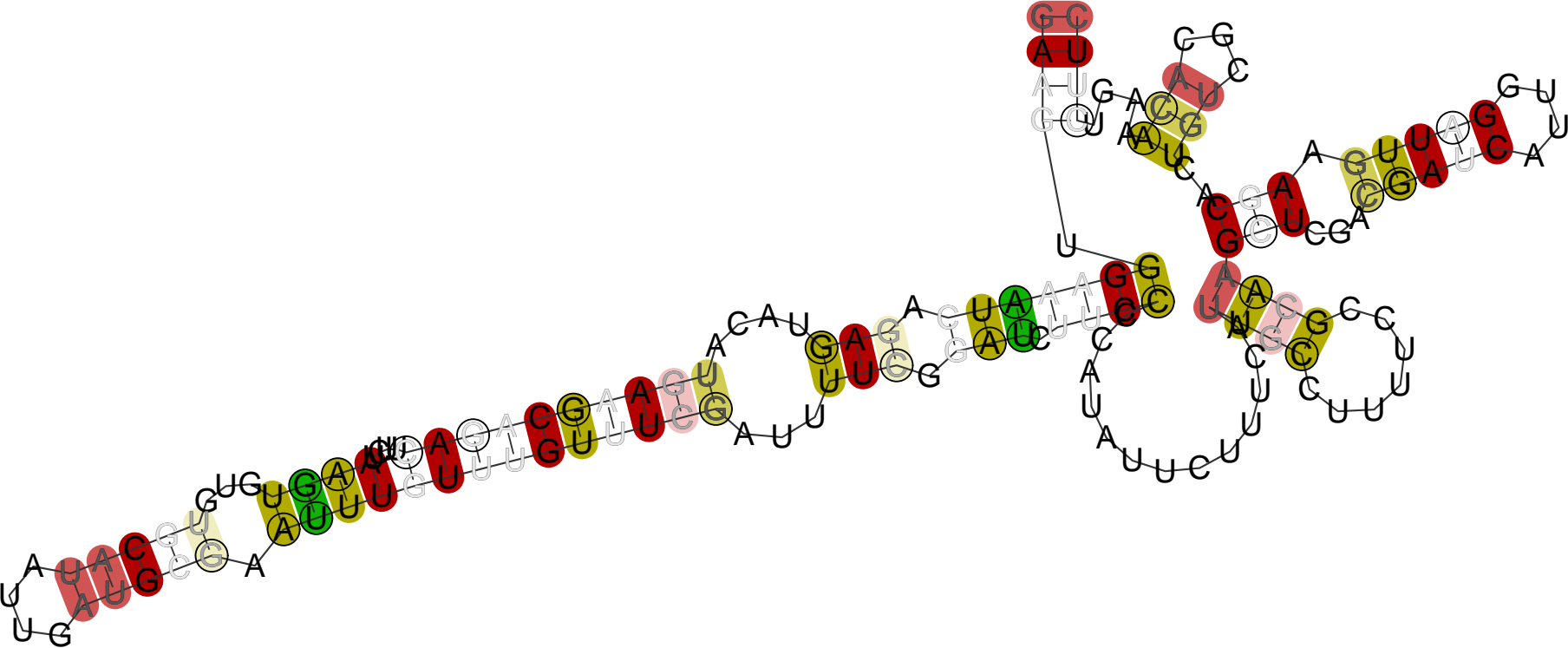

Supplement: S7 Fig — See the caption for S1 Fig for a description of the filename convention (save that the corresponding nucleotide locations in reference sequences are listed in S8 and S16 Tables), and an explanation of the RNAalifold options used and output. (ZIP) [file pcbi.1012009.s128.zip › H7N9-human-raw-PA-alignment-4-159-refseq-4-159_revcomp_alirna_nogap.pdf]

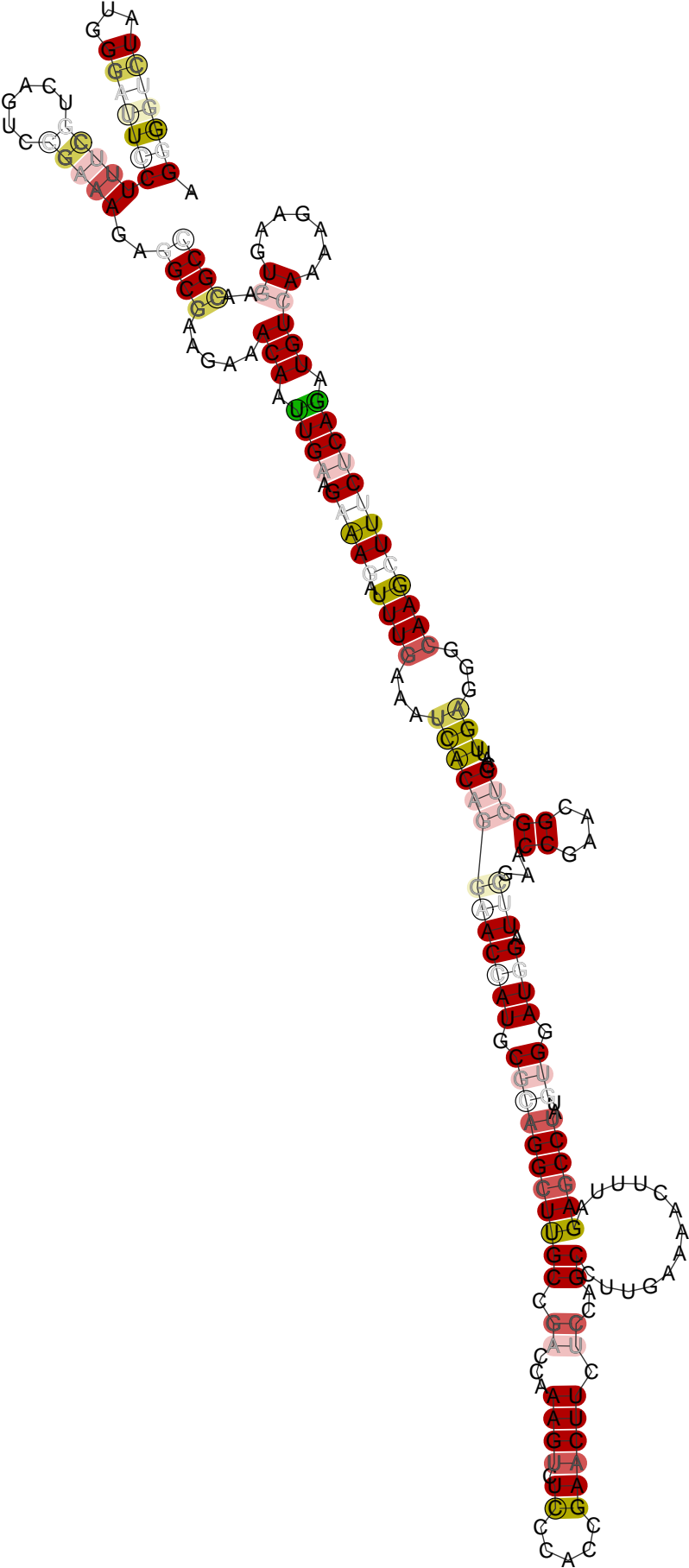

Supplement: S7 Fig — See the caption for S1 Fig for a description of the filename convention (save that the corresponding nucleotide locations in reference sequences are listed in S8 and S16 Tables), and an explanation of the RNAalifold options used and output. (ZIP) [file pcbi.1012009.s128.zip › H7N9-human-raw-PA-alignment-553-765-refseq-553-765_alirna_nogap.pdf]

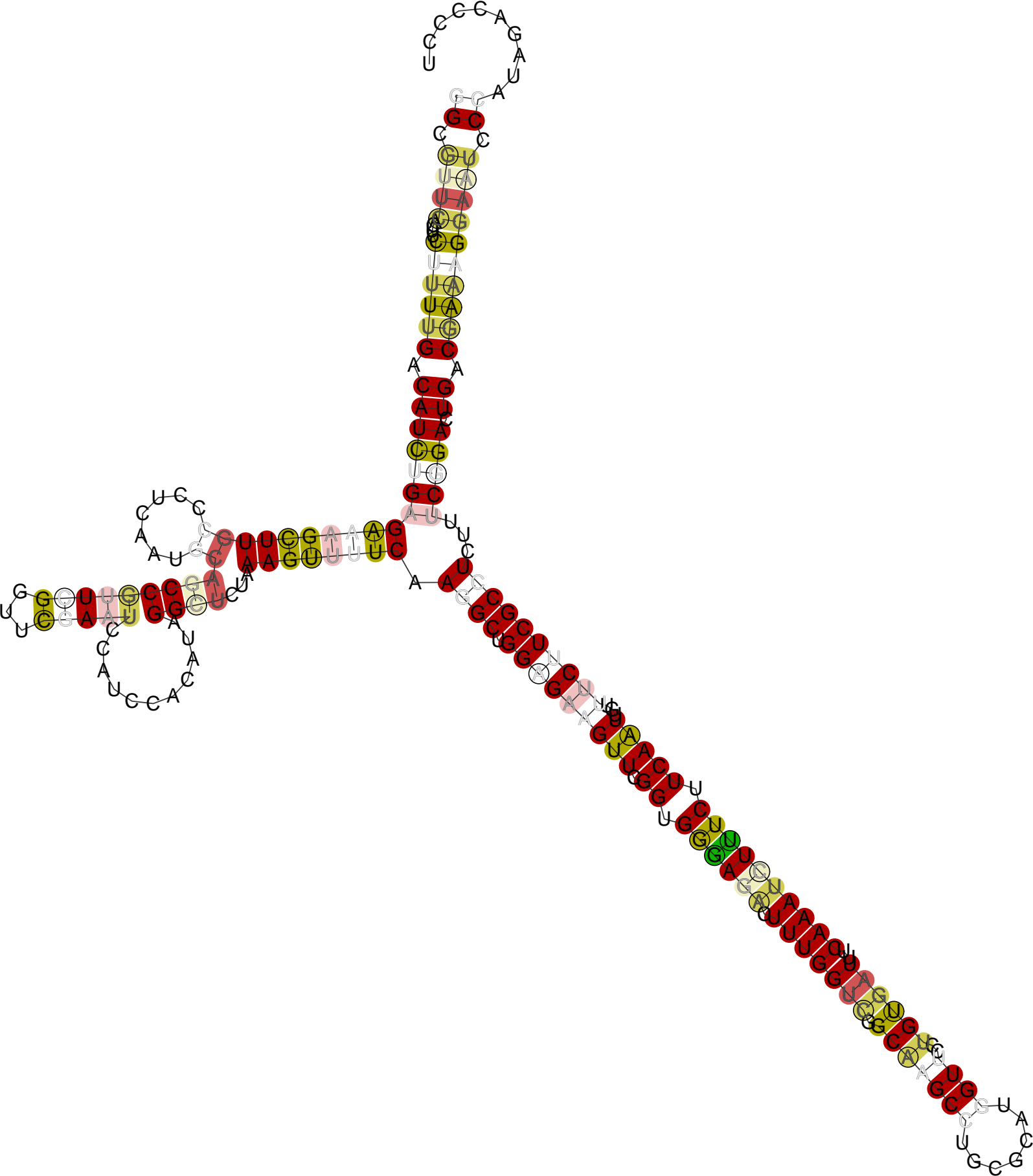

Supplement: S7 Fig — See the caption for S1 Fig for a description of the filename convention (save that the corresponding nucleotide locations in reference sequences are listed in S8 and S16 Tables), and an explanation of the RNAalifold options used and output. (ZIP) [file pcbi.1012009.s128.zip › H7N9-human-raw-PA-alignment-553-765-refseq-553-765_revcomp_alirna_nogap.pdf]

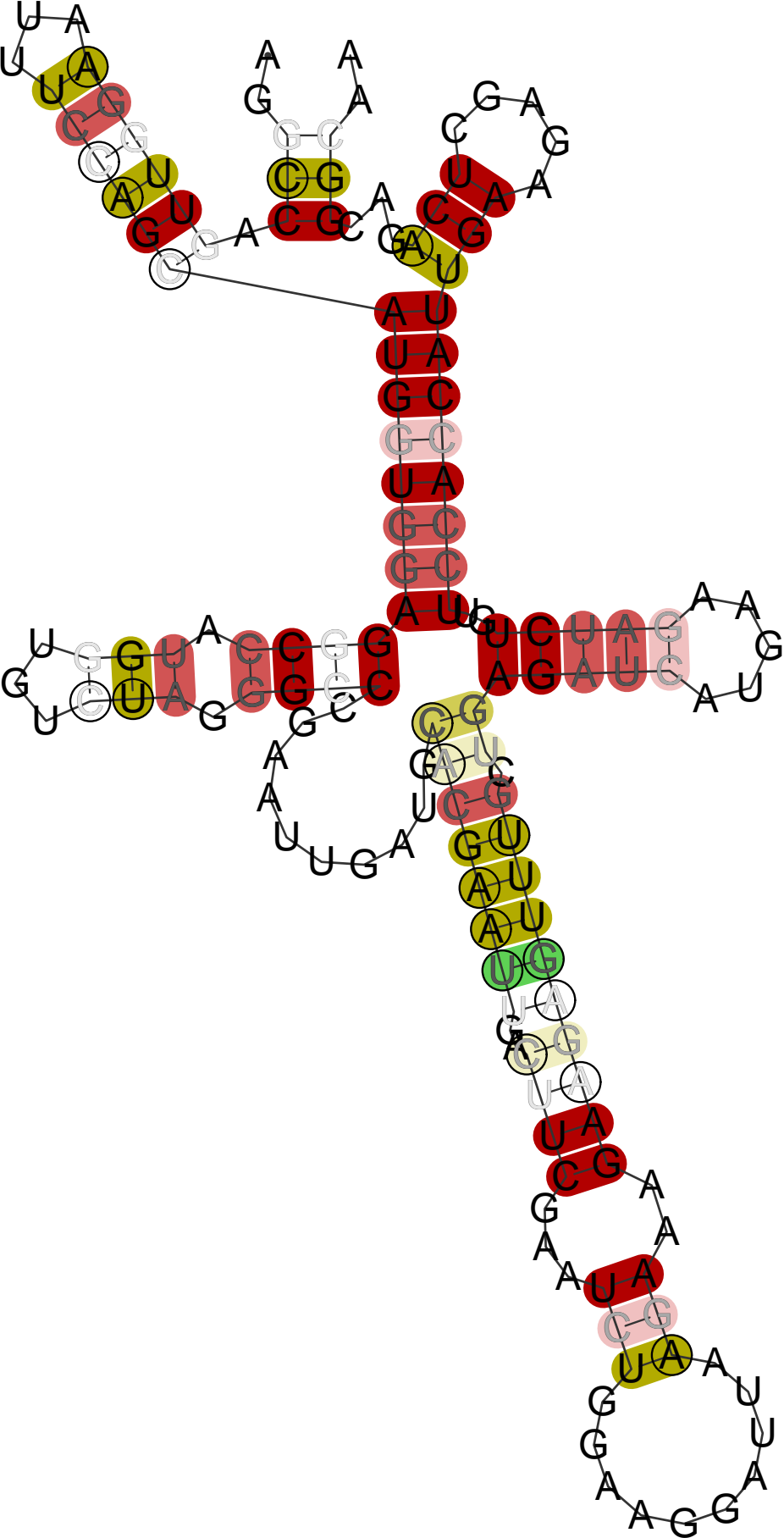

Supplement: S7 Fig — See the caption for S1 Fig for a description of the filename convention (save that the corresponding nucleotide locations in reference sequences are listed in S8 and S16 Tables), and an explanation of the RNAalifold options used and output. (ZIP) [file pcbi.1012009.s128.zip › H7N9-human-raw-PB1-alignment-2119-2268-refseq-2119-2268_alirna_nogap.pdf]

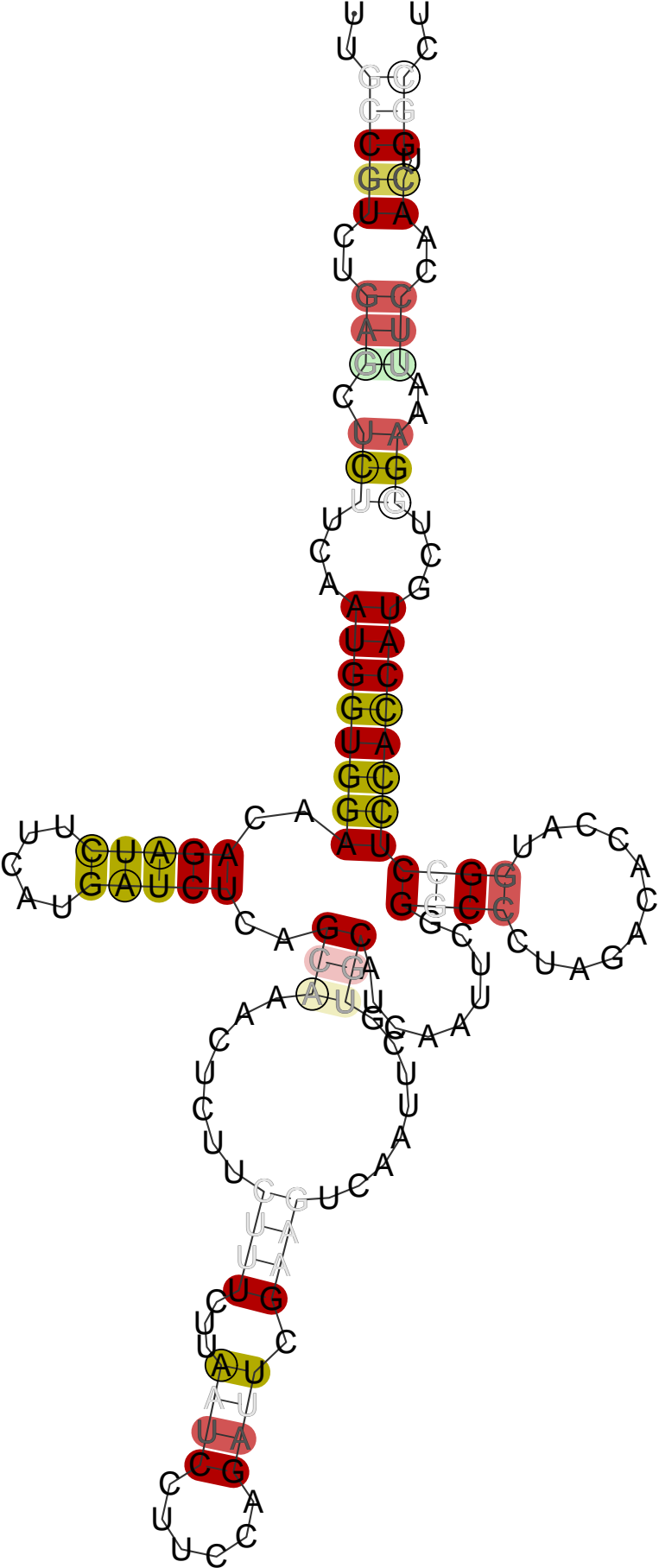

Supplement: S7 Fig — See the caption for S1 Fig for a description of the filename convention (save that the corresponding nucleotide locations in reference sequences are listed in S8 and S16 Tables), and an explanation of the RNAalifold options used and output. (ZIP) [file pcbi.1012009.s128.zip › H7N9-human-raw-PB1-alignment-2119-2268-refseq-2119-2268_revcomp_alirna_nogap.pdf]

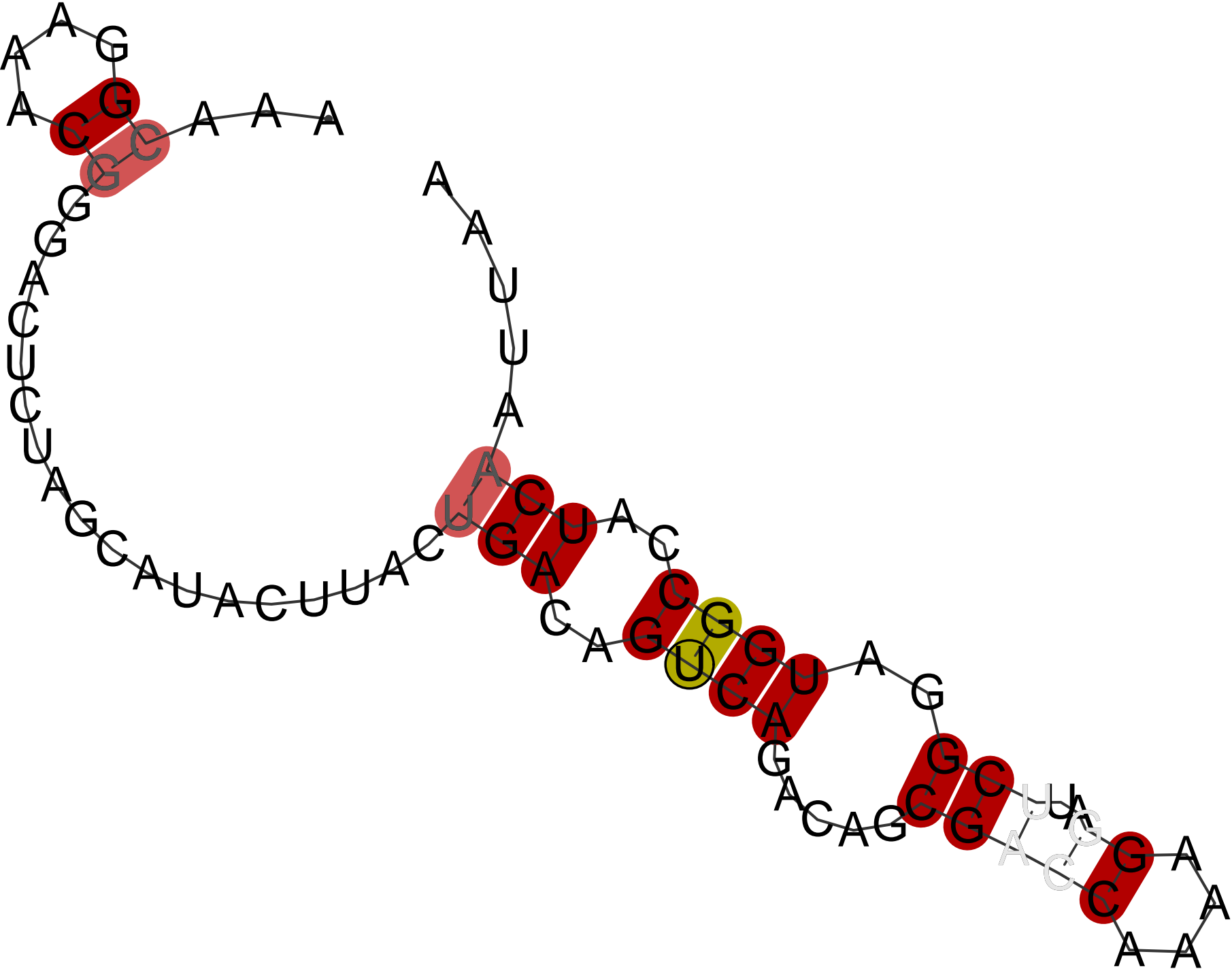

Supplement: S7 Fig — See the caption for S1 Fig for a description of the filename convention (save that the corresponding nucleotide locations in reference sequences are listed in S8 and S16 Tables), and an explanation of the RNAalifold options used and output. (ZIP) [file pcbi.1012009.s128.zip › H7N9-human-raw-PB2-alignment-2206-2280-refseq-2206-2280_alirna_nogap.pdf]

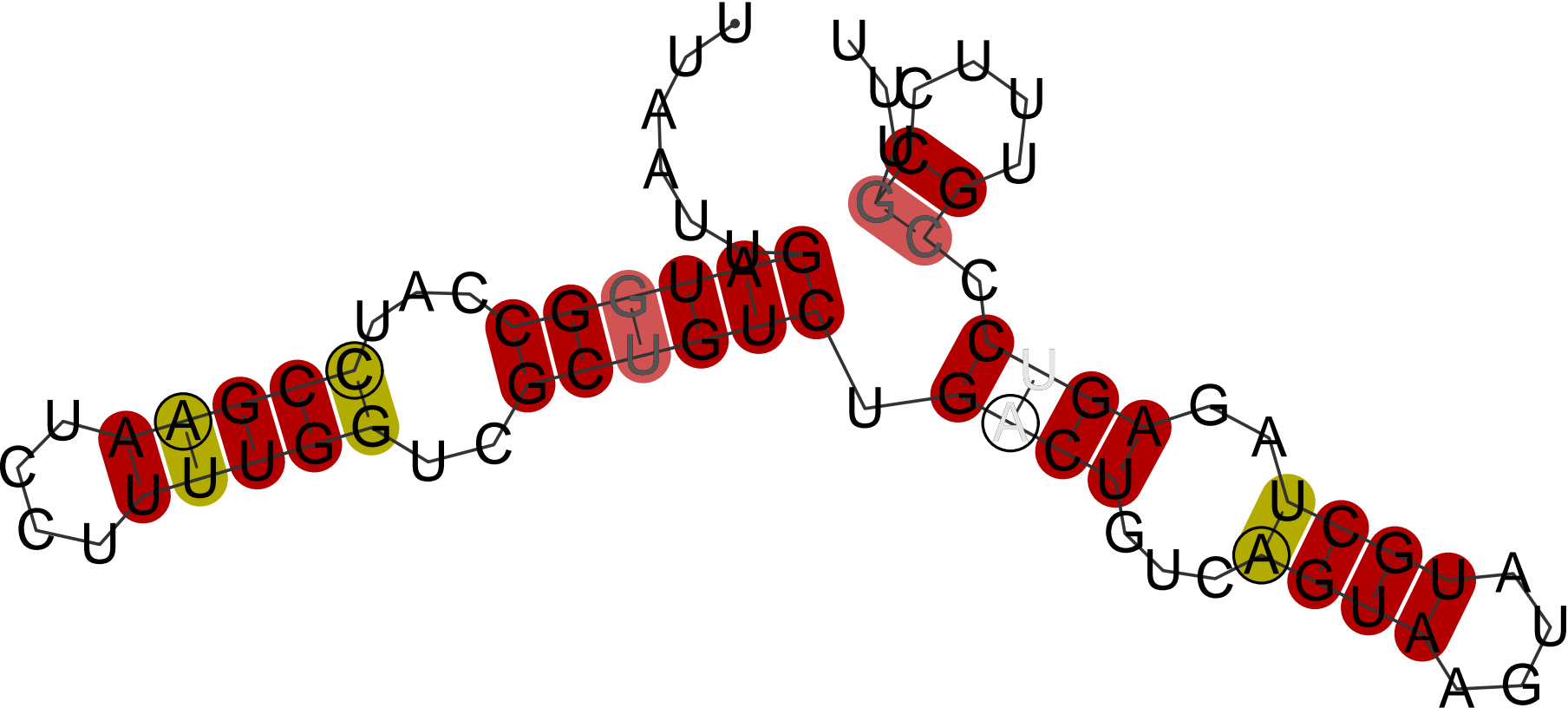

Supplement: S7 Fig — See the caption for S1 Fig for a description of the filename convention (save that the corresponding nucleotide locations in reference sequences are listed in S8 and S16 Tables), and an explanation of the RNAalifold options used and output. (ZIP) [file pcbi.1012009.s128.zip › H7N9-human-raw-PB2-alignment-2206-2280-refseq-2206-2280_revcomp_alirna_nogap.pdf]

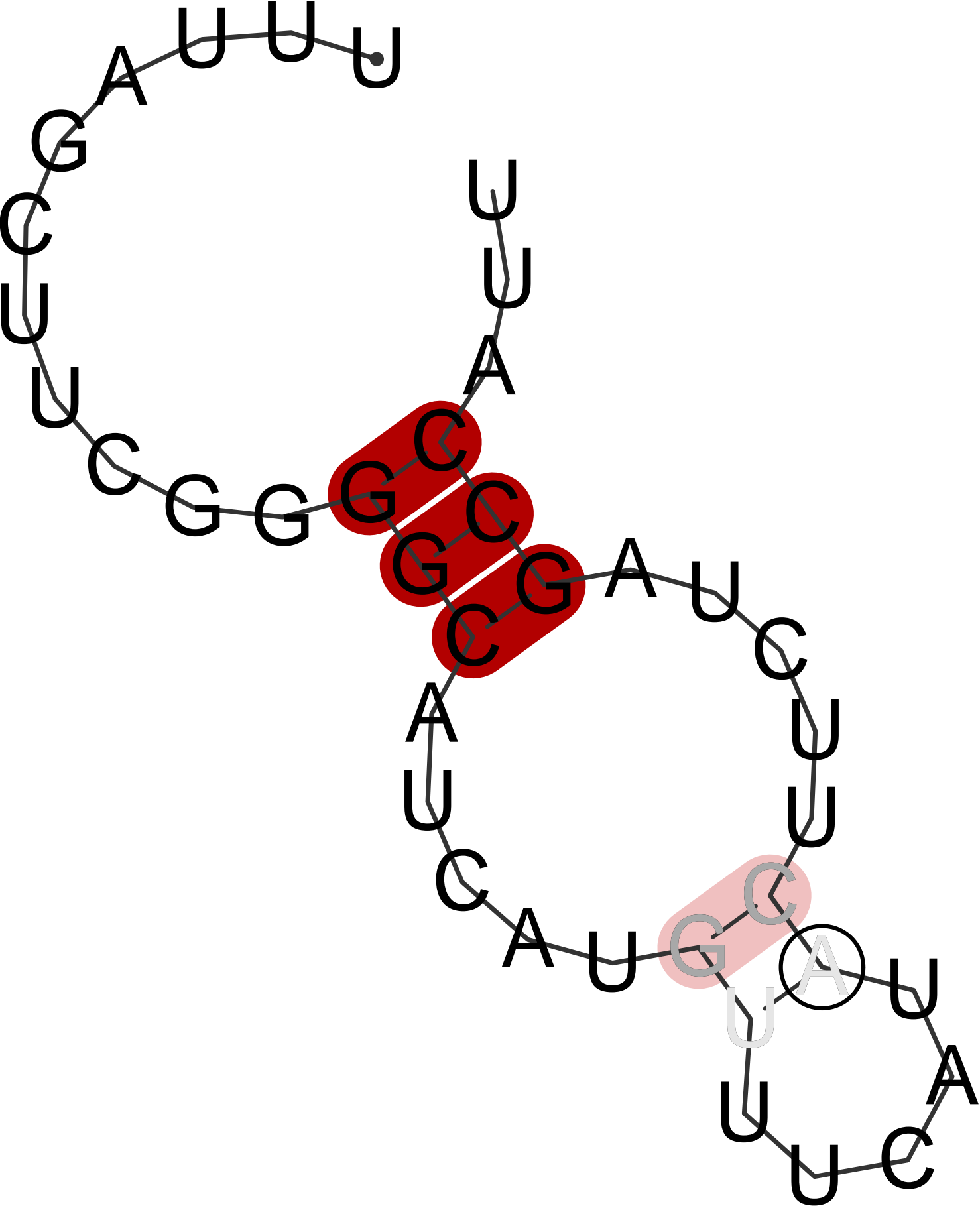

Supplement: S8 Fig — See the caption for S1 Fig for a description of the filename convention (save that the corresponding nucleotide locations in reference sequences are listed in S9 and S17 Tables), and an explanation of the RNAalifold options used and output (save that for these avian-origin viruses the folding temperature was set to 41°C). As some of the constrained regions predicted in the analysis of the M2 gene using raw (unranked) codon variability values are very close together, alignments of longer regions containing more than one conserved region are also included. (ZIP) [file pcbi.1012009.s129.zip › H7N9-avian-ranked-HA-alignment-1618-1656-refseq-1582-1620-41C_alirna_nogap.pdf]

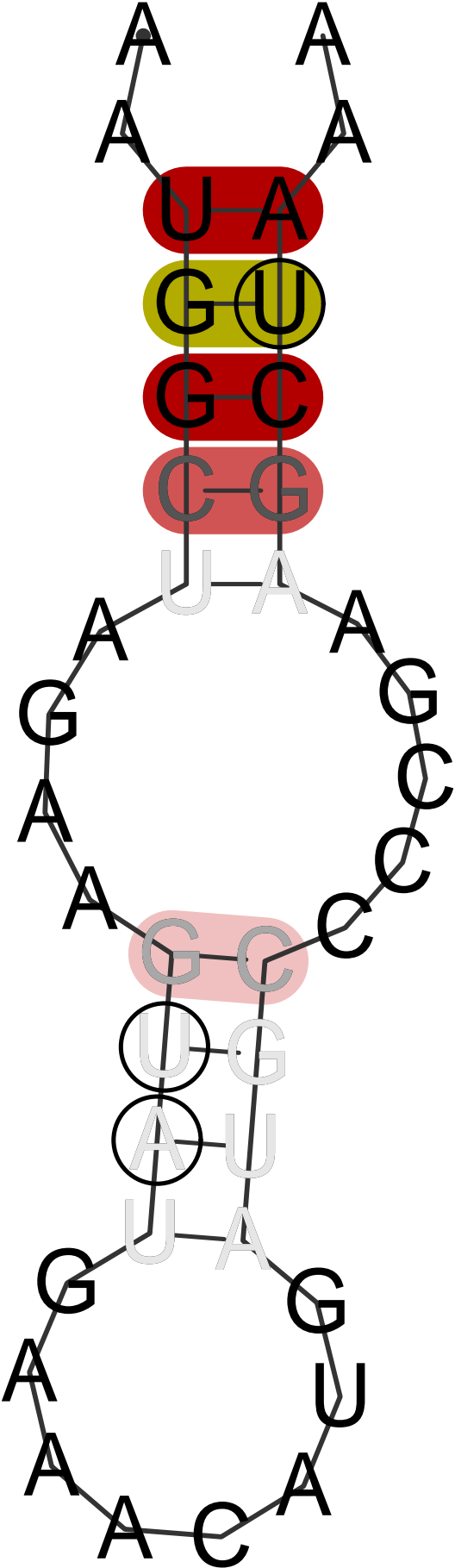

Supplement: S8 Fig — See the caption for S1 Fig for a description of the filename convention (save that the corresponding nucleotide locations in reference sequences are listed in S9 and S17 Tables), and an explanation of the RNAalifold options used and output (save that for these avian-origin viruses the folding temperature was set to 41°C). As some of the constrained regions predicted in the analysis of the M2 gene using raw (unranked) codon variability values are very close together, alignments of longer regions containing more than one conserved region are also included. (ZIP) [file pcbi.1012009.s129.zip › H7N9-avian-ranked-HA-alignment-1618-1656-refseq-1582-1620-41C_revcomp_alirna_nogap.pdf]

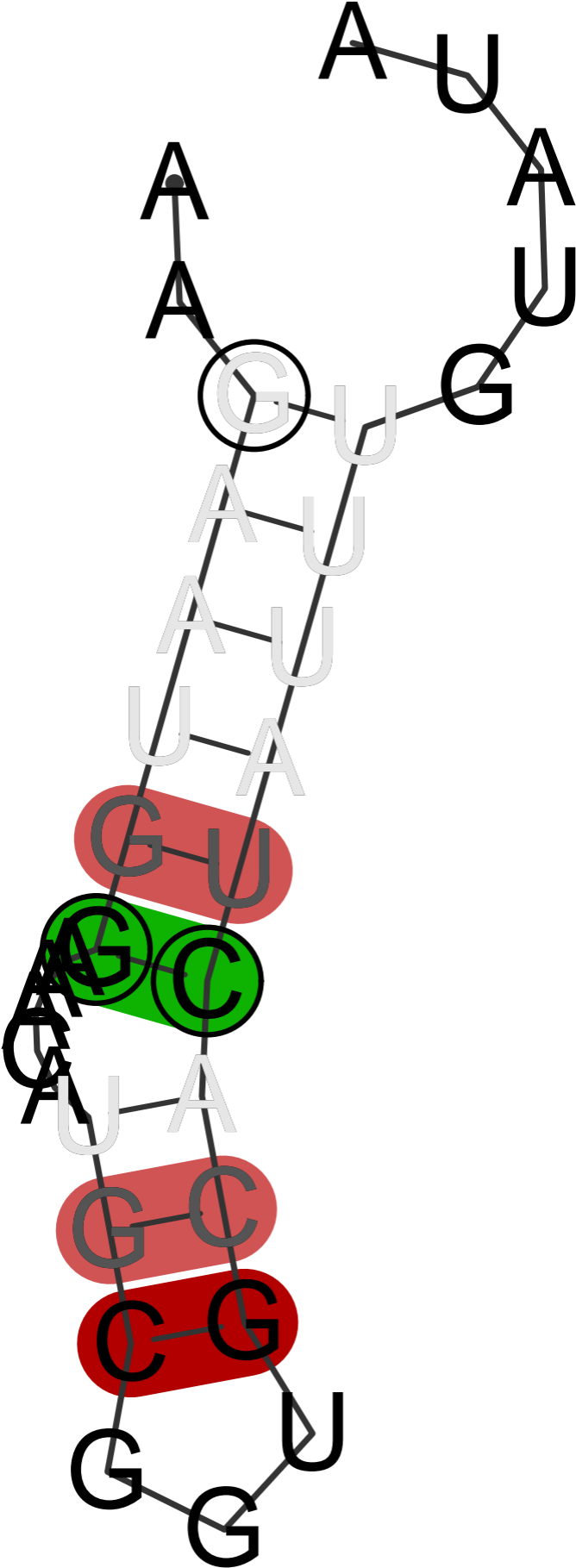

Supplement: S8 Fig — See the caption for S1 Fig for a description of the filename convention (save that the corresponding nucleotide locations in reference sequences are listed in S9 and S17 Tables), and an explanation of the RNAalifold options used and output (save that for these avian-origin viruses the folding temperature was set to 41°C). As some of the constrained regions predicted in the analysis of the M2 gene using raw (unranked) codon variability values are very close together, alignments of longer regions containing more than one conserved region are also included. (ZIP) [file pcbi.1012009.s129.zip › H7N9-avian-ranked-HA-alignment-1684-1716-refseq-1648-1680-41C_alirna_nogap.pdf]

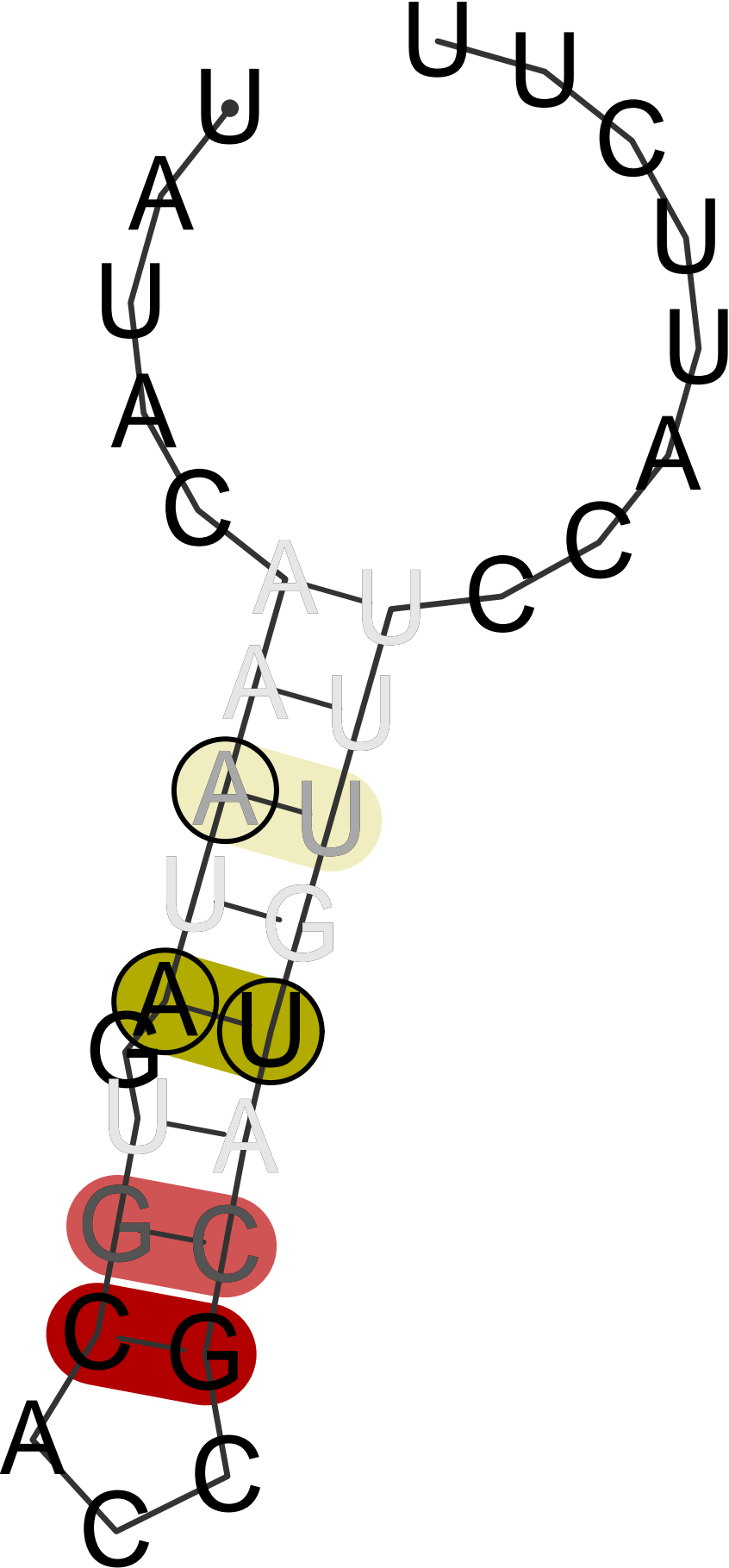

Supplement: S8 Fig — See the caption for S1 Fig for a description of the filename convention (save that the corresponding nucleotide locations in reference sequences are listed in S9 and S17 Tables), and an explanation of the RNAalifold options used and output (save that for these avian-origin viruses the folding temperature was set to 41°C). As some of the constrained regions predicted in the analysis of the M2 gene using raw (unranked) codon variability values are very close together, alignments of longer regions containing more than one conserved region are also included. (ZIP) [file pcbi.1012009.s129.zip › H7N9-avian-ranked-HA-alignment-1684-1716-refseq-1648-1680-41C_revcomp_alirna_nogap.pdf]

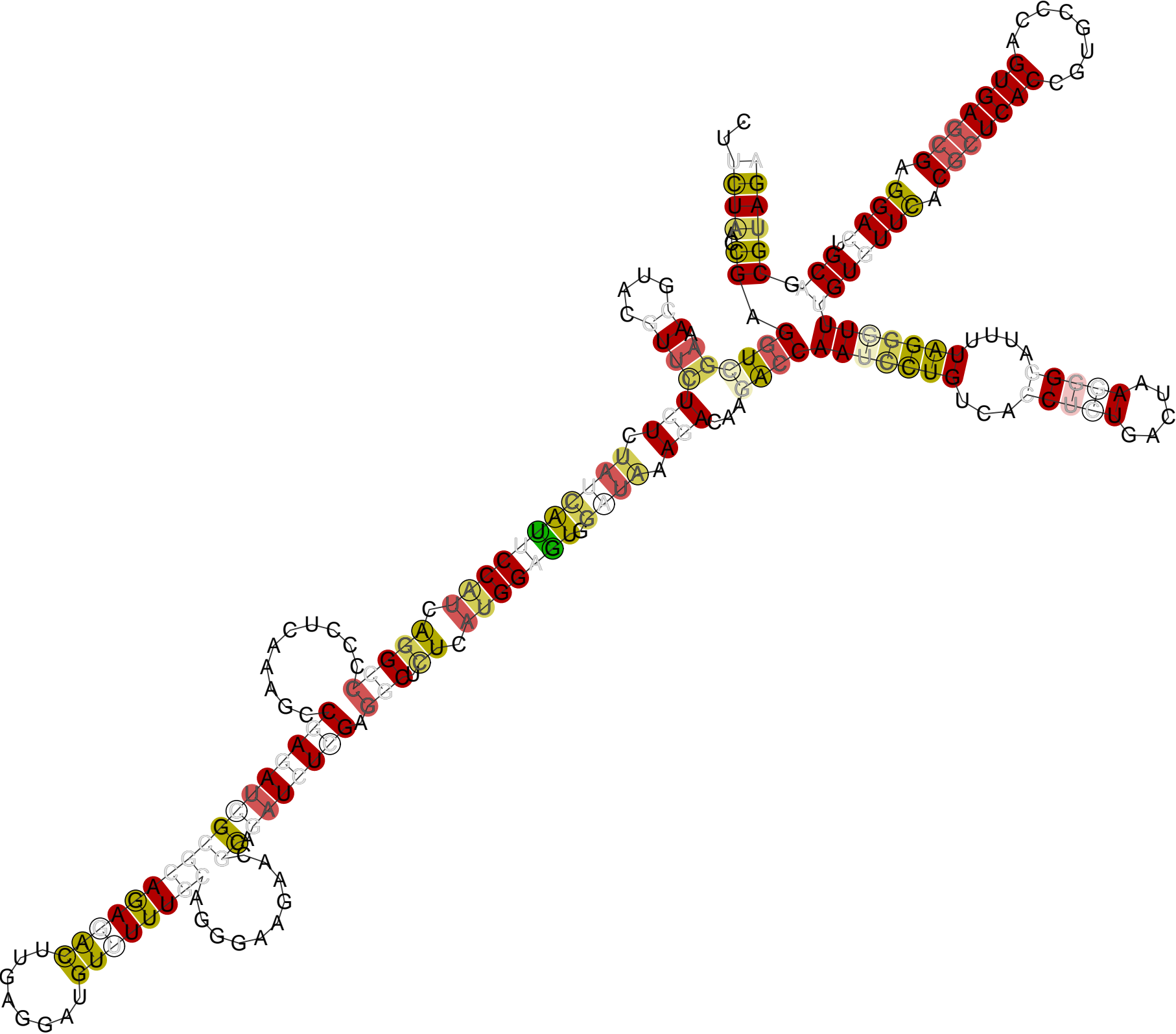

Supplement: S8 Fig — See the caption for S1 Fig for a description of the filename convention (save that the corresponding nucleotide locations in reference sequences are listed in S9 and S17 Tables), and an explanation of the RNAalifold options used and output (save that for these avian-origin viruses the folding temperature was set to 41°C). As some of the constrained regions predicted in the analysis of the M2 gene using raw (unranked) codon variability values are very close together, alignments of longer regions containing more than one conserved region are also included. (ZIP) [file pcbi.1012009.s129.zip › H7N9-avian-ranked-M1-alignment-7-231-refseq-7-231-41C_alirna_nogap.pdf]

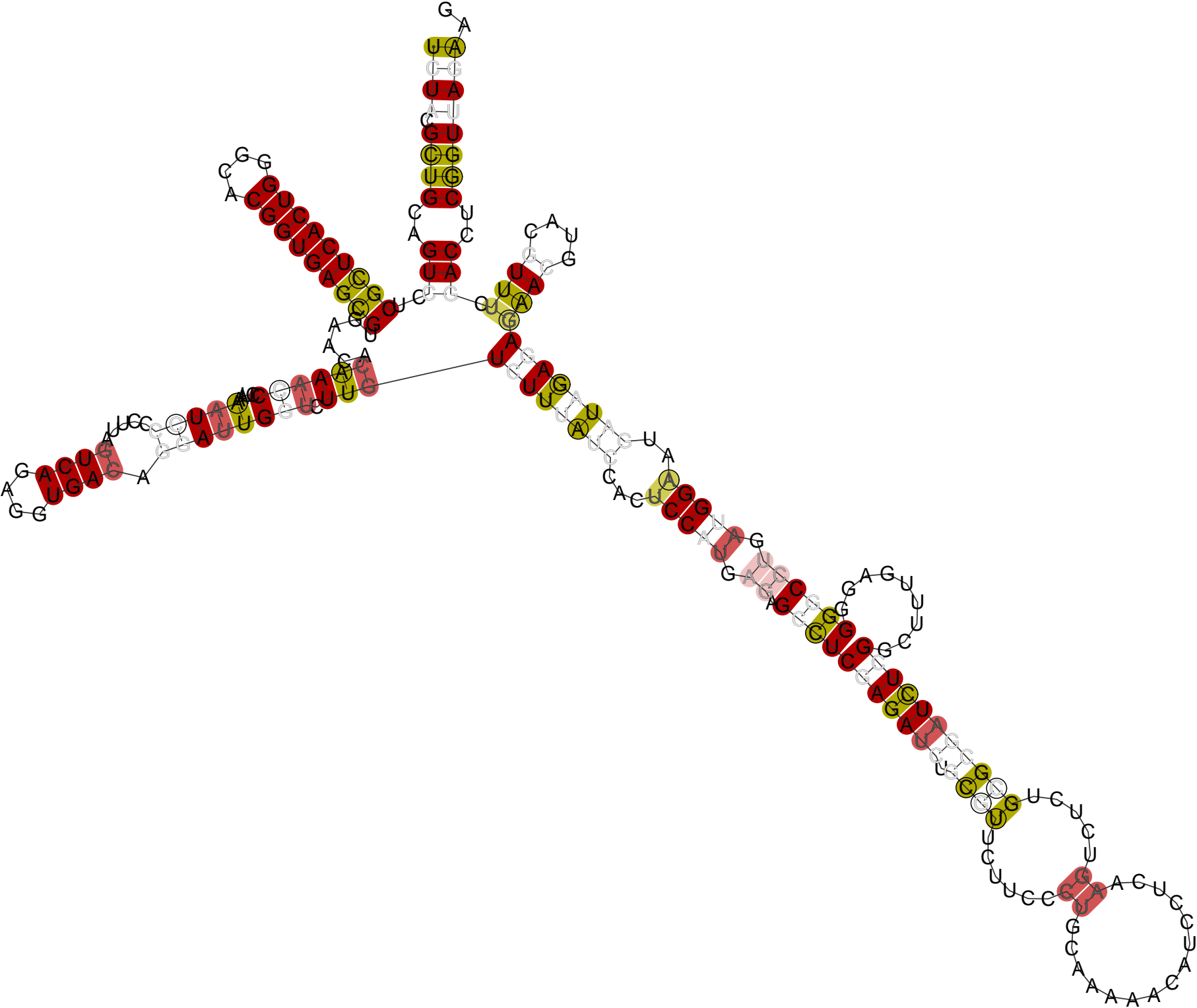

Supplement: S8 Fig — See the caption for S1 Fig for a description of the filename convention (save that the corresponding nucleotide locations in reference sequences are listed in S9 and S17 Tables), and an explanation of the RNAalifold options used and output (save that for these avian-origin viruses the folding temperature was set to 41°C). As some of the constrained regions predicted in the analysis of the M2 gene using raw (unranked) codon variability values are very close together, alignments of longer regions containing more than one conserved region are also included. (ZIP) [file pcbi.1012009.s129.zip › H7N9-avian-ranked-M1-alignment-7-231-refseq-7-231-41C_revcomp_alirna_nogap.pdf]

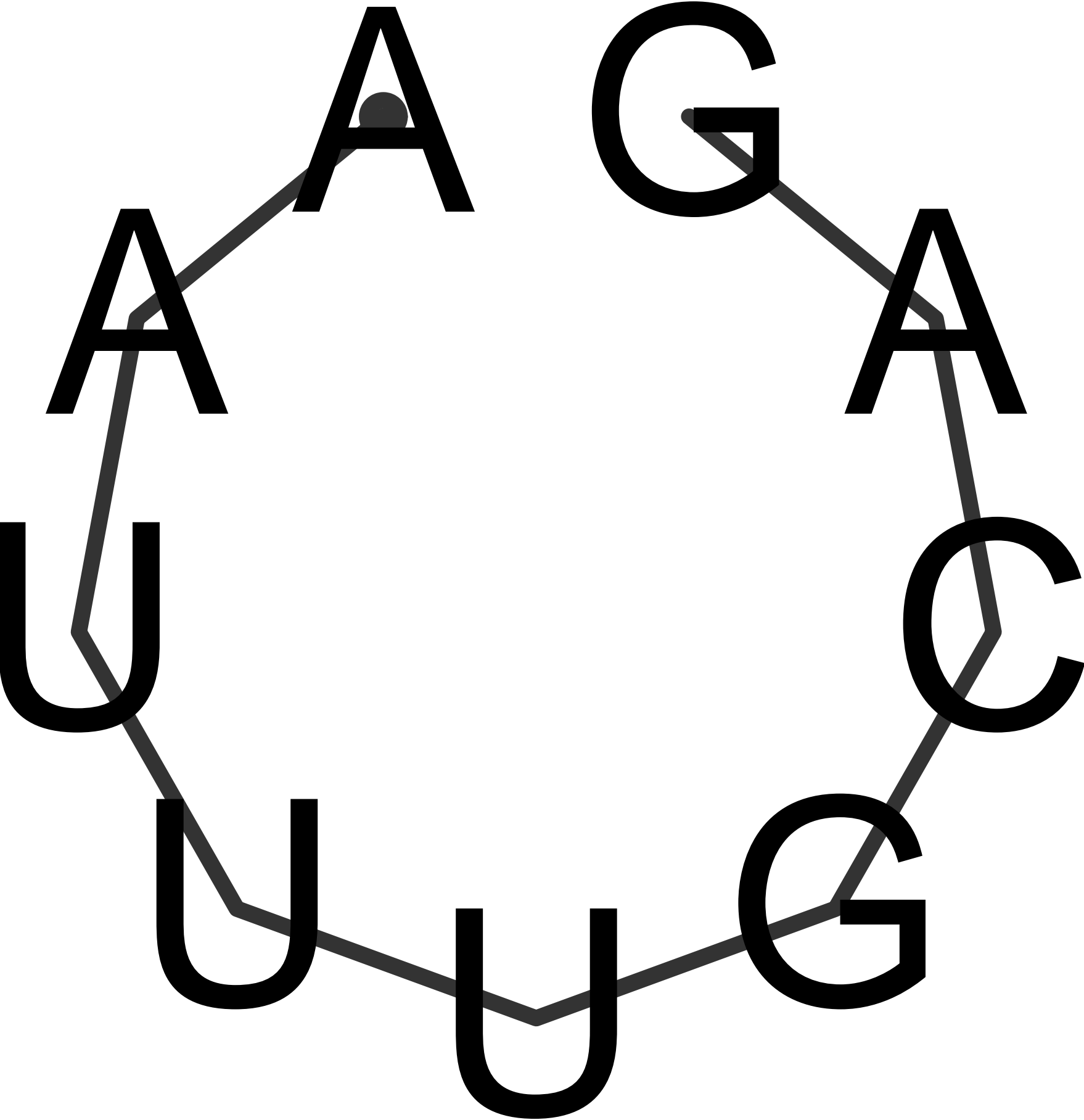

Supplement: S8 Fig — See the caption for S1 Fig for a description of the filename convention (save that the corresponding nucleotide locations in reference sequences are listed in S9 and S17 Tables), and an explanation of the RNAalifold options used and output (save that for these avian-origin viruses the folding temperature was set to 41°C). As some of the constrained regions predicted in the analysis of the M2 gene using raw (unranked) codon variability values are very close together, alignments of longer regions containing more than one conserved region are also included. (ZIP) [file pcbi.1012009.s129.zip › H7N9-avian-ranked-M1-alignment-706-714-refseq-706-714-41C_alirna_nogap.pdf]

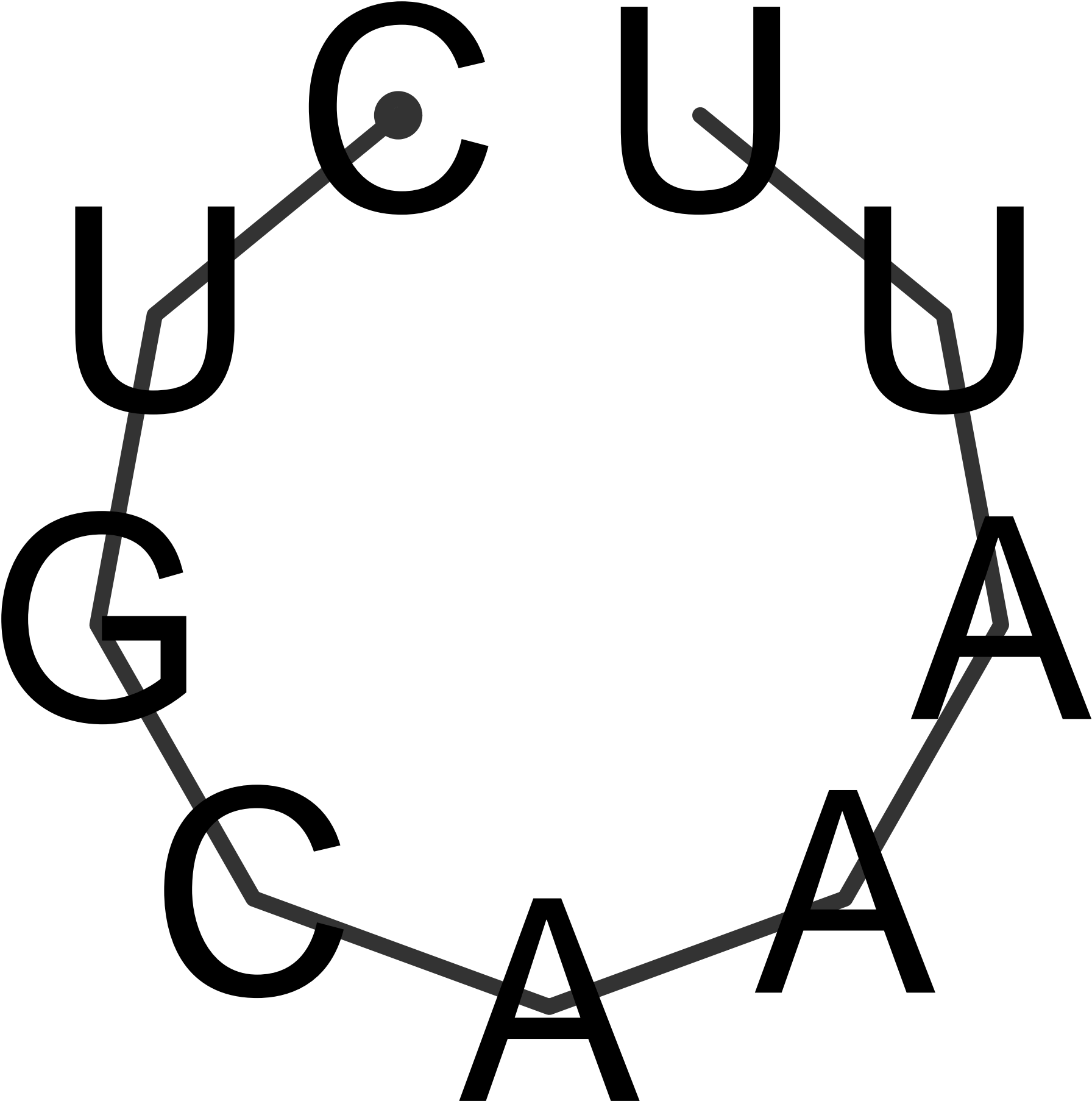

Supplement: S8 Fig — See the caption for S1 Fig for a description of the filename convention (save that the corresponding nucleotide locations in reference sequences are listed in S9 and S17 Tables), and an explanation of the RNAalifold options used and output (save that for these avian-origin viruses the folding temperature was set to 41°C). As some of the constrained regions predicted in the analysis of the M2 gene using raw (unranked) codon variability values are very close together, alignments of longer regions containing more than one conserved region are also included. (ZIP) [file pcbi.1012009.s129.zip › H7N9-avian-ranked-M1-alignment-706-714-refseq-706-714-41C_revcomp_alirna_nogap.pdf]

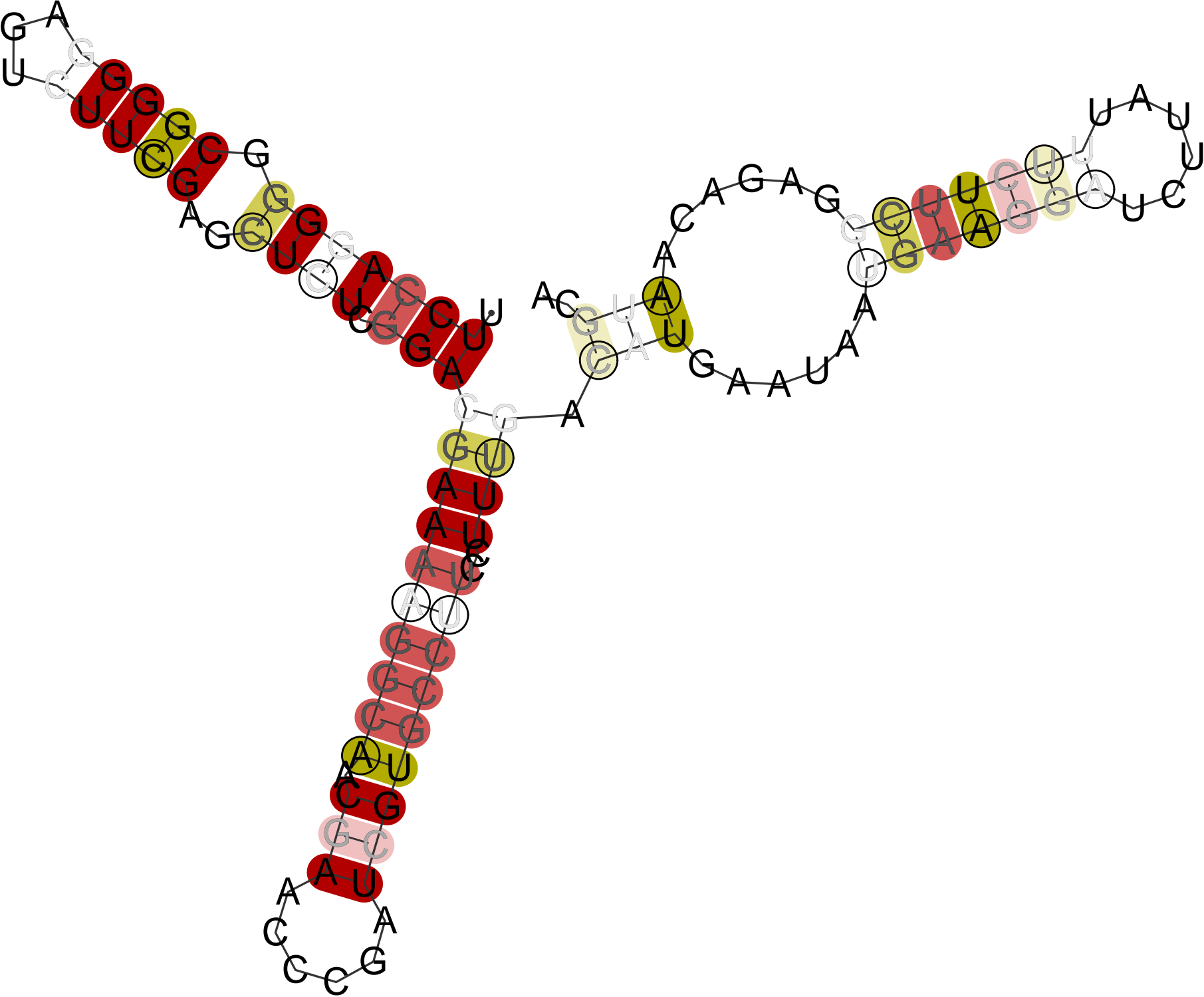

Supplement: S8 Fig — See the caption for S1 Fig for a description of the filename convention (save that the corresponding nucleotide locations in reference sequences are listed in S9 and S17 Tables), and an explanation of the RNAalifold options used and output (save that for these avian-origin viruses the folding temperature was set to 41°C). As some of the constrained regions predicted in the analysis of the M2 gene using raw (unranked) codon variability values are very close together, alignments of longer regions containing more than one conserved region are also included. (ZIP) [file pcbi.1012009.s129.zip › H7N9-avian-ranked-NP-alignment-1372-1485-refseq-1372-1479-41C_alirna_nogap.pdf]

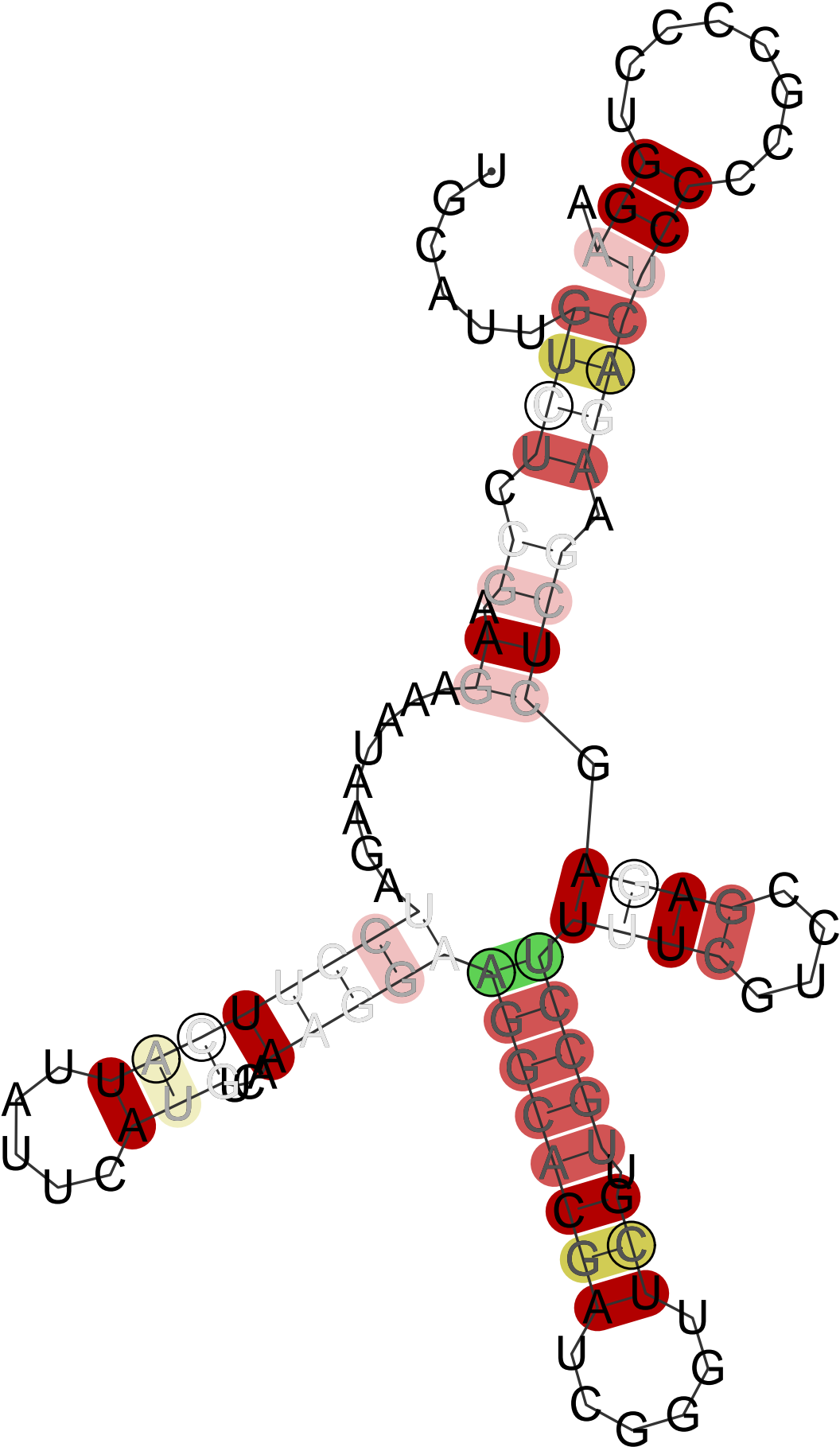

Supplement: S8 Fig — See the caption for S1 Fig for a description of the filename convention (save that the corresponding nucleotide locations in reference sequences are listed in S9 and S17 Tables), and an explanation of the RNAalifold options used and output (save that for these avian-origin viruses the folding temperature was set to 41°C). As some of the constrained regions predicted in the analysis of the M2 gene using raw (unranked) codon variability values are very close together, alignments of longer regions containing more than one conserved region are also included. (ZIP) [file pcbi.1012009.s129.zip › H7N9-avian-ranked-NP-alignment-1372-1485-refseq-1372-1479-41C_revcomp_alirna_nogap.pdf]

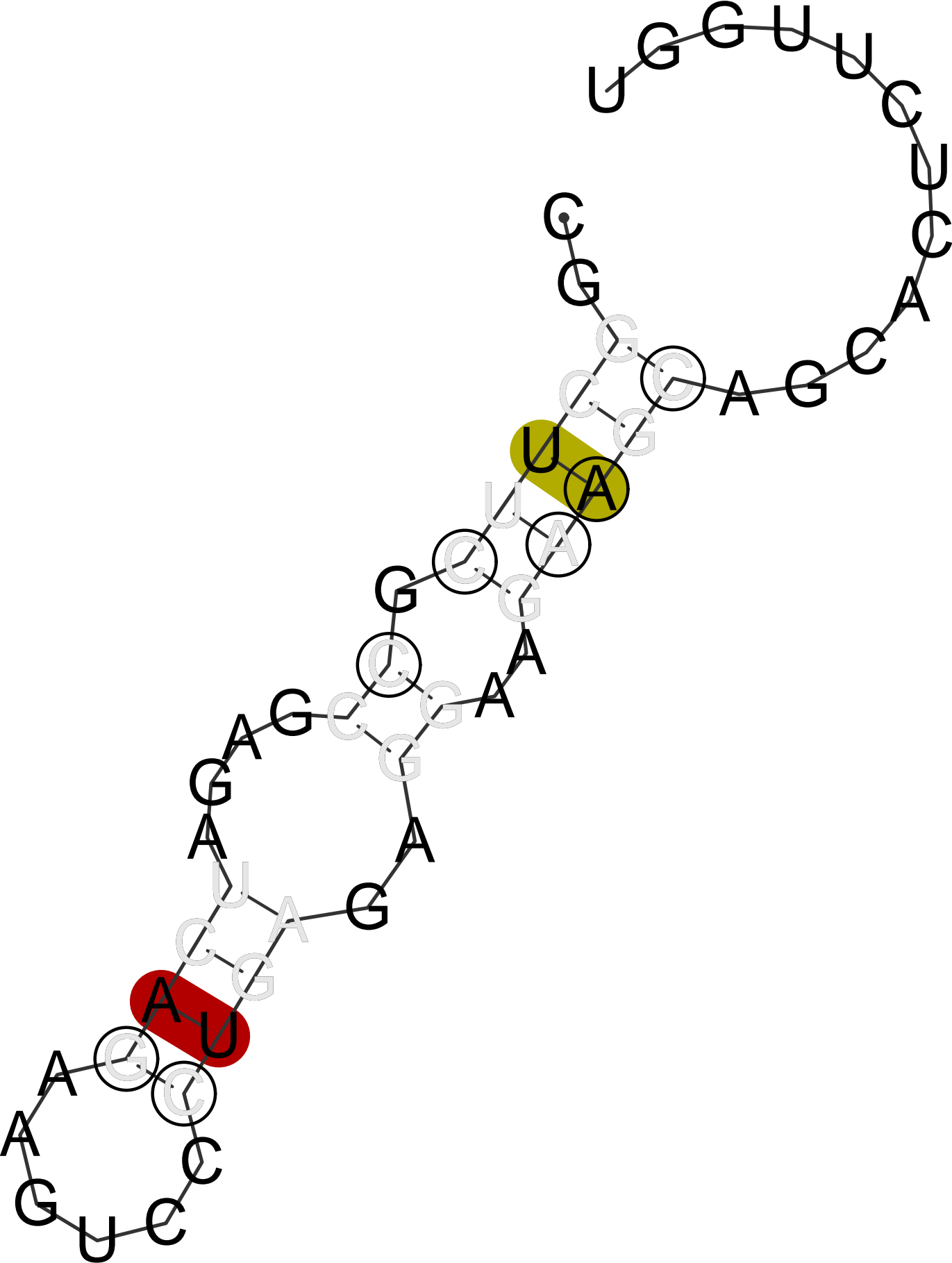

Supplement: S8 Fig — See the caption for S1 Fig for a description of the filename convention (save that the corresponding nucleotide locations in reference sequences are listed in S9 and S17 Tables), and an explanation of the RNAalifold options used and output (save that for these avian-origin viruses the folding temperature was set to 41°C). As some of the constrained regions predicted in the analysis of the M2 gene using raw (unranked) codon variability values are very close together, alignments of longer regions containing more than one conserved region are also included. (ZIP) [file pcbi.1012009.s129.zip › H7N9-avian-ranked-NS1-alignment-103-153-refseq-103-153-41C_alirna_nogap.pdf]

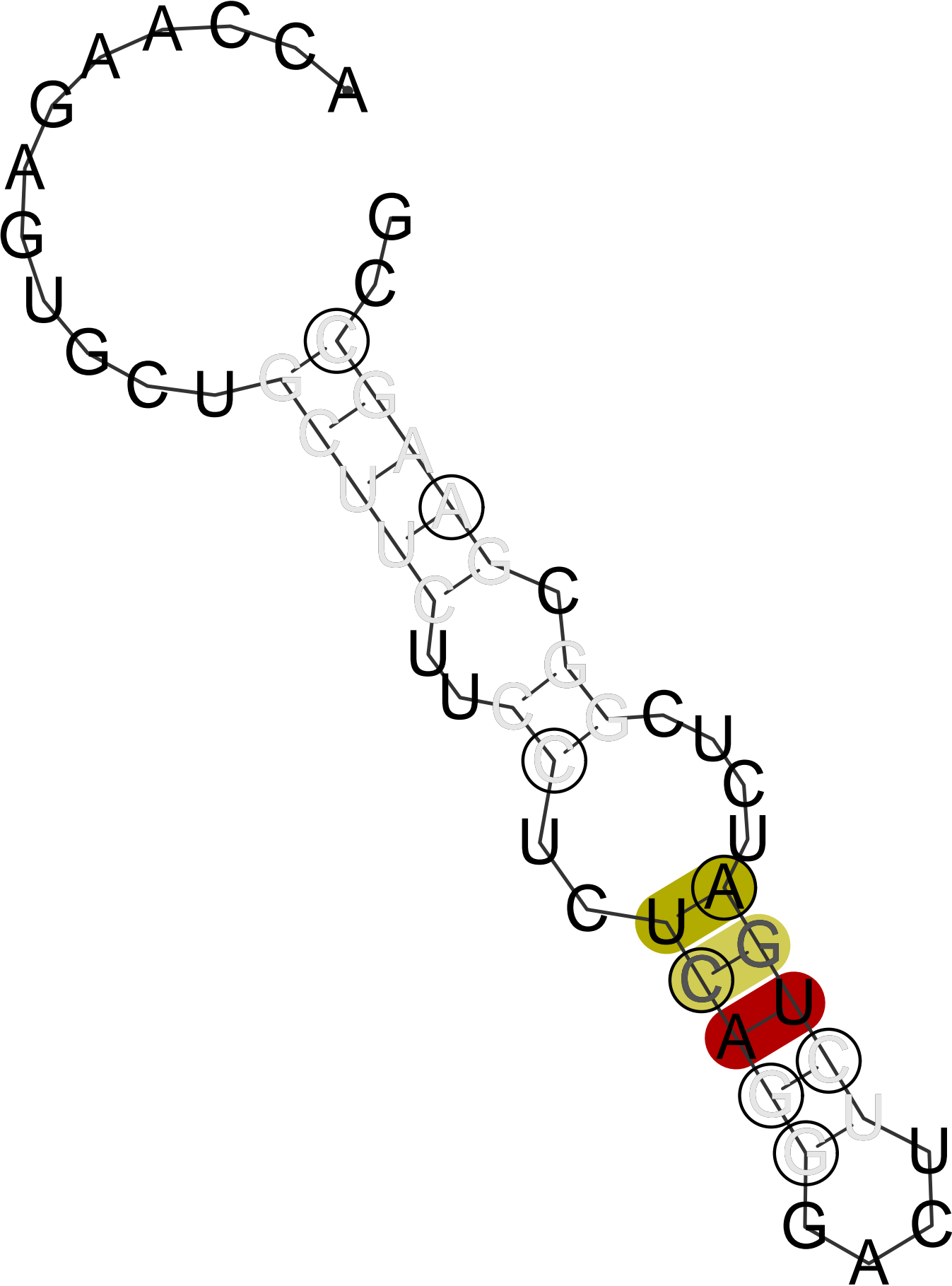

Supplement: S8 Fig — See the caption for S1 Fig for a description of the filename convention (save that the corresponding nucleotide locations in reference sequences are listed in S9 and S17 Tables), and an explanation of the RNAalifold options used and output (save that for these avian-origin viruses the folding temperature was set to 41°C). As some of the constrained regions predicted in the analysis of the M2 gene using raw (unranked) codon variability values are very close together, alignments of longer regions containing more than one conserved region are also included. (ZIP) [file pcbi.1012009.s129.zip › H7N9-avian-ranked-NS1-alignment-103-153-refseq-103-153-41C_revcomp_alirna_nogap.pdf]

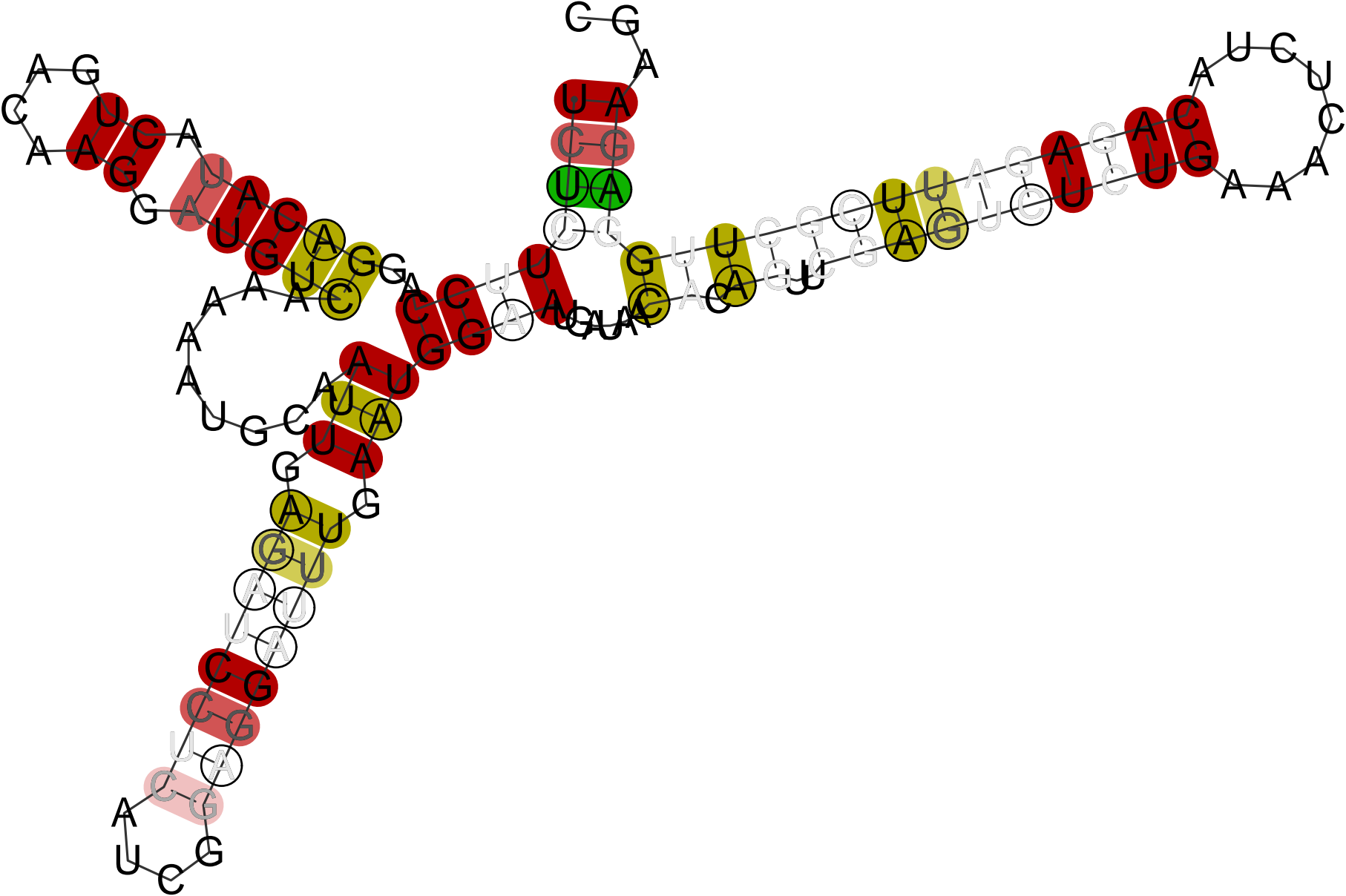

Supplement: S8 Fig — See the caption for S1 Fig for a description of the filename convention (save that the corresponding nucleotide locations in reference sequences are listed in S9 and S17 Tables), and an explanation of the RNAalifold options used and output (save that for these avian-origin viruses the folding temperature was set to 41°C). As some of the constrained regions predicted in the analysis of the M2 gene using raw (unranked) codon variability values are very close together, alignments of longer regions containing more than one conserved region are also included. (ZIP) [file pcbi.1012009.s129.zip › H7N9-avian-ranked-NS1-alignment-493-615-refseq-493-615-41C_alirna_nogap.pdf]

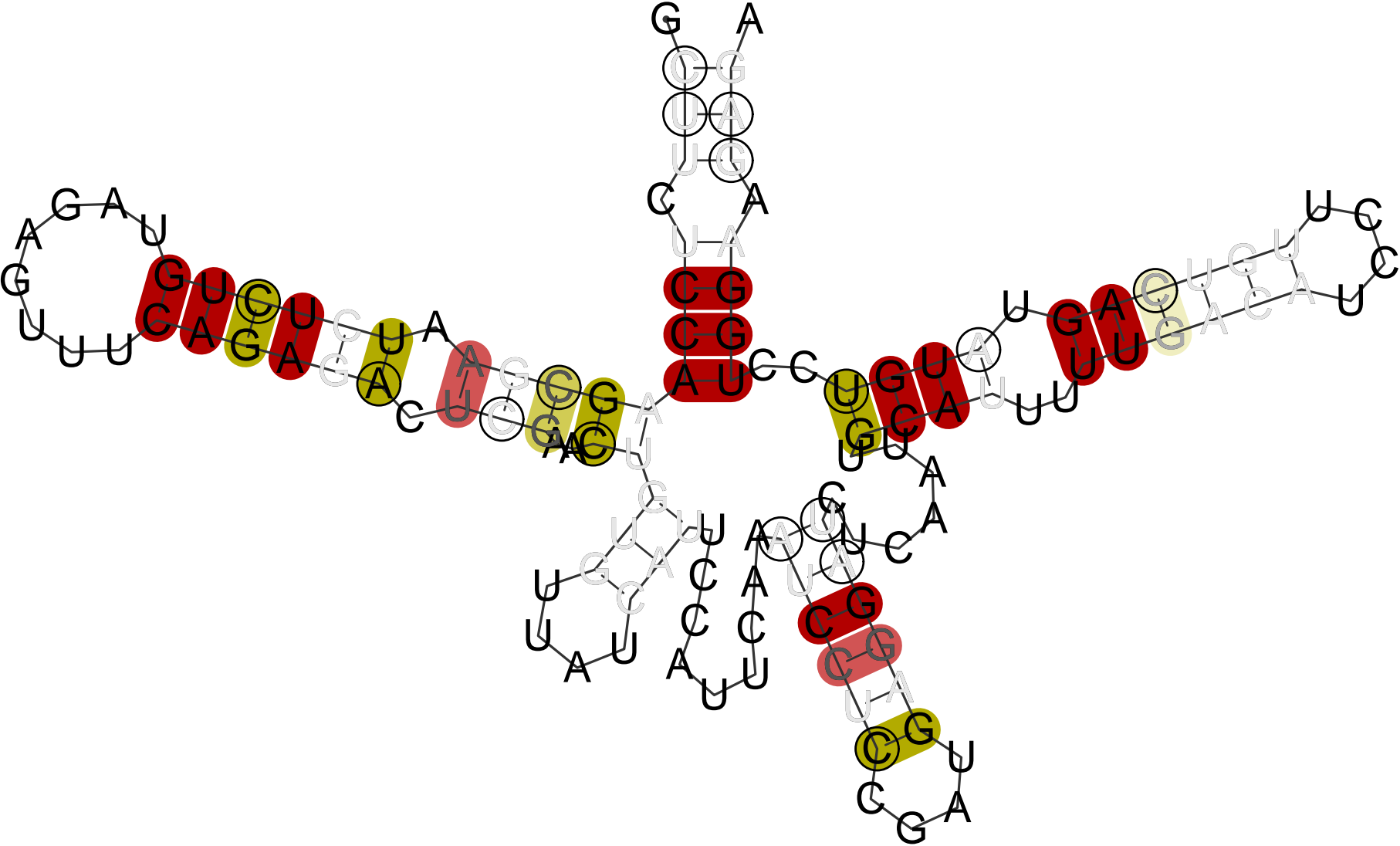

Supplement: S8 Fig — See the caption for S1 Fig for a description of the filename convention (save that the corresponding nucleotide locations in reference sequences are listed in S9 and S17 Tables), and an explanation of the RNAalifold options used and output (save that for these avian-origin viruses the folding temperature was set to 41°C). As some of the constrained regions predicted in the analysis of the M2 gene using raw (unranked) codon variability values are very close together, alignments of longer regions containing more than one conserved region are also included. (ZIP) [file pcbi.1012009.s129.zip › H7N9-avian-ranked-NS1-alignment-493-615-refseq-493-615-41C_revcomp_alirna_nogap.pdf]

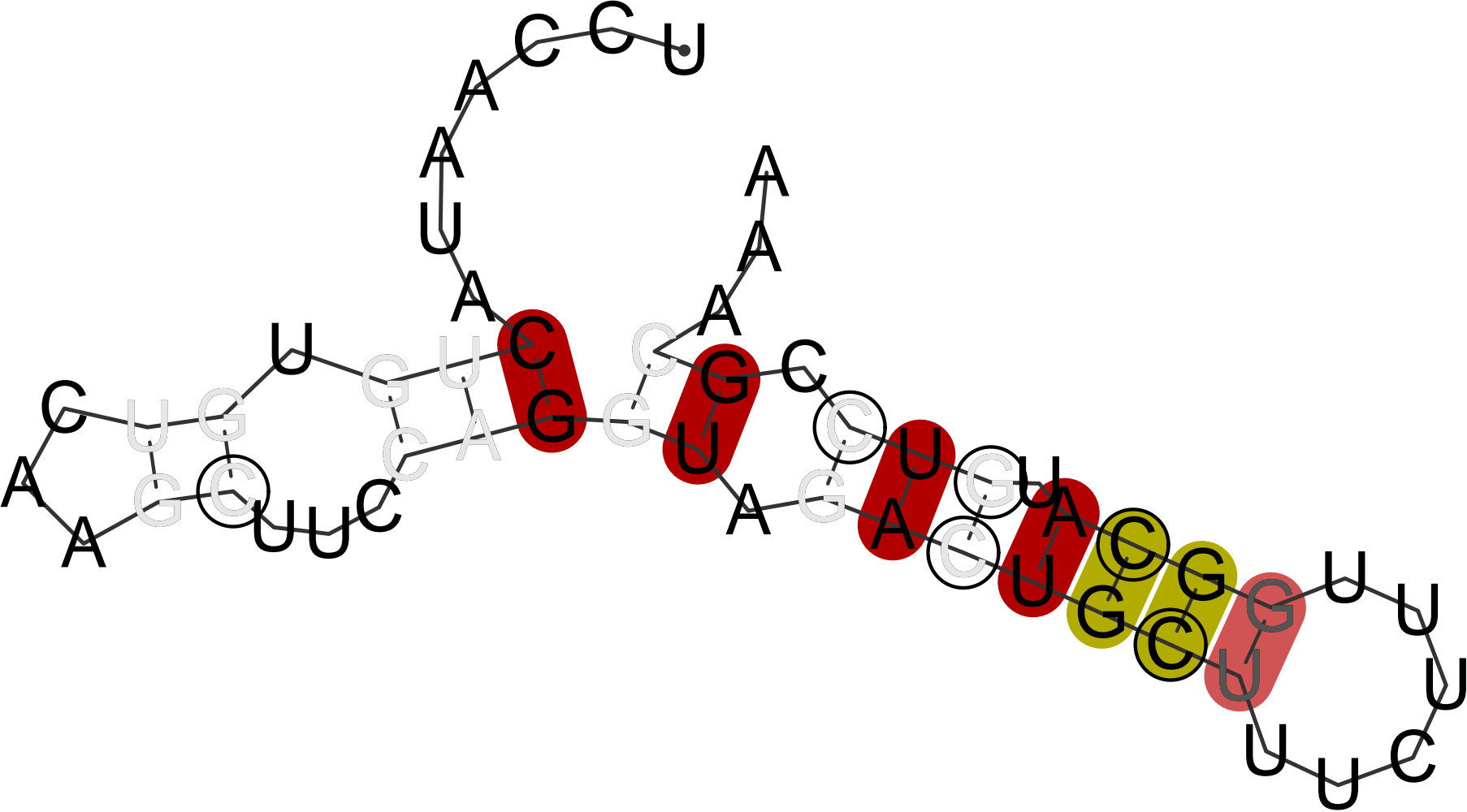

Supplement: S8 Fig — See the caption for S1 Fig for a description of the filename convention (save that the corresponding nucleotide locations in reference sequences are listed in S9 and S17 Tables), and an explanation of the RNAalifold options used and output (save that for these avian-origin viruses the folding temperature was set to 41°C). As some of the constrained regions predicted in the analysis of the M2 gene using raw (unranked) codon variability values are very close together, alignments of longer regions containing more than one conserved region are also included. (ZIP) [file pcbi.1012009.s129.zip › H7N9-avian-ranked-NS1-alignment-7-60-refseq-7-60-41C_alirna_nogap.pdf]

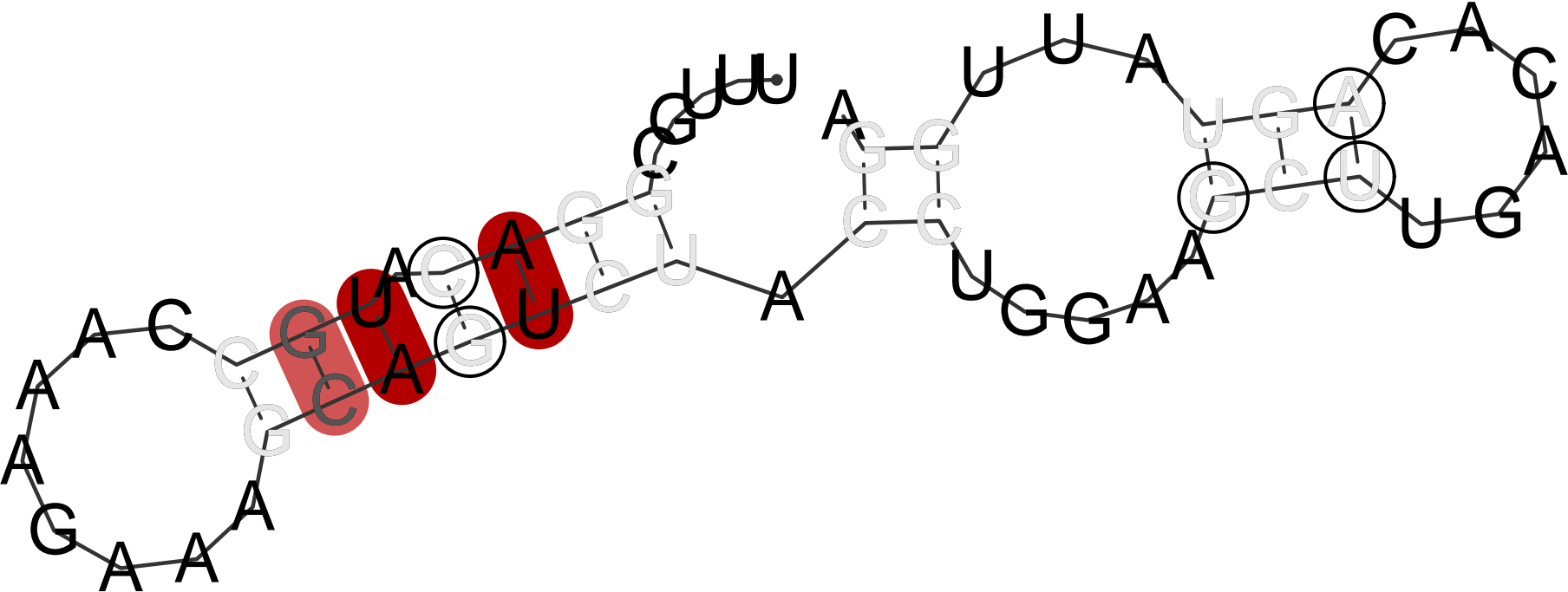

Supplement: S8 Fig — See the caption for S1 Fig for a description of the filename convention (save that the corresponding nucleotide locations in reference sequences are listed in S9 and S17 Tables), and an explanation of the RNAalifold options used and output (save that for these avian-origin viruses the folding temperature was set to 41°C). As some of the constrained regions predicted in the analysis of the M2 gene using raw (unranked) codon variability values are very close together, alignments of longer regions containing more than one conserved region are also included. (ZIP) [file pcbi.1012009.s129.zip › H7N9-avian-ranked-NS1-alignment-7-60-refseq-7-60-41C_revcomp_alirna_nogap.pdf]

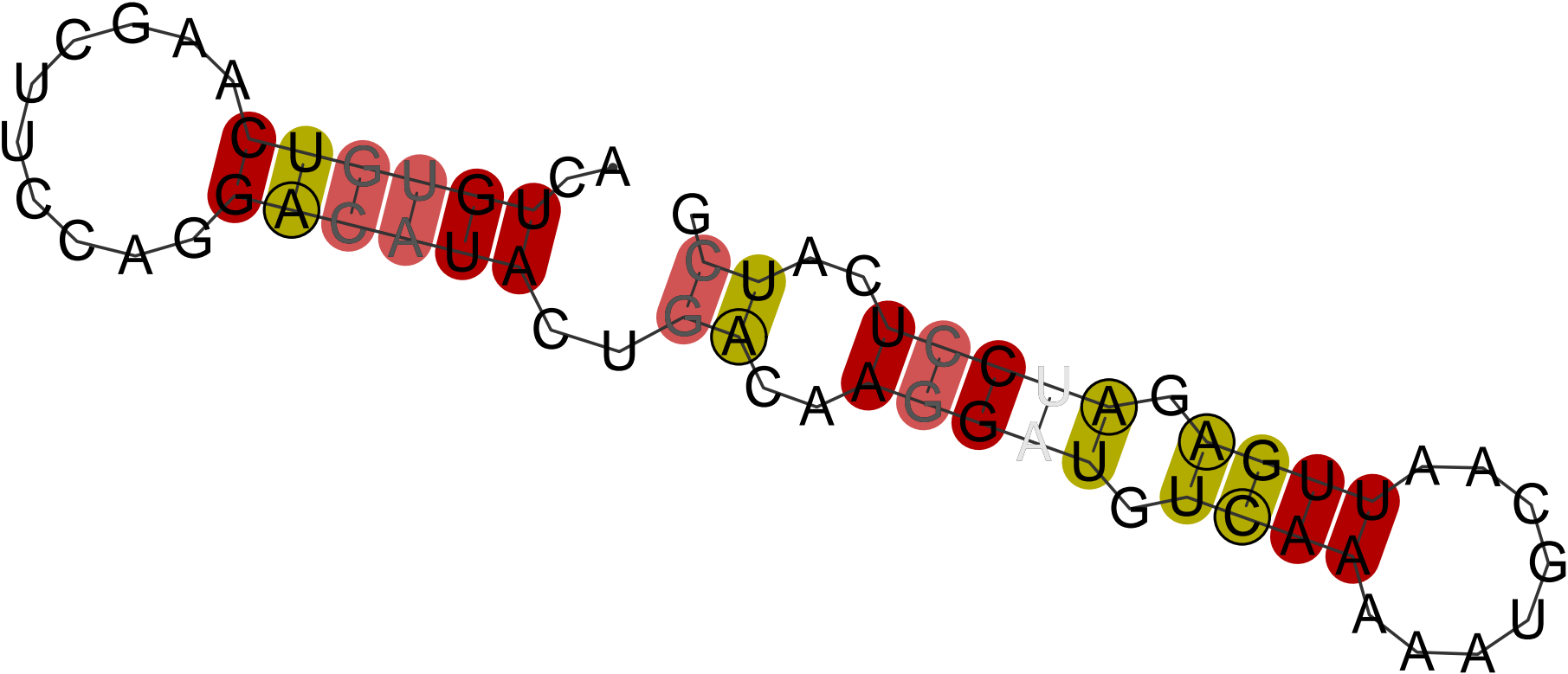

Supplement: S8 Fig — See the caption for S1 Fig for a description of the filename convention (save that the corresponding nucleotide locations in reference sequences are listed in S9 and S17 Tables), and an explanation of the RNAalifold options used and output (save that for these avian-origin viruses the folding temperature was set to 41°C). As some of the constrained regions predicted in the analysis of the M2 gene using raw (unranked) codon variability values are very close together, alignments of longer regions containing more than one conserved region are also included. (ZIP) [file pcbi.1012009.s129.zip › H7N9-avian-ranked-NS2-alignment-13-75-refseq-13-30-503-547-41C_alirna_nogap.pdf]

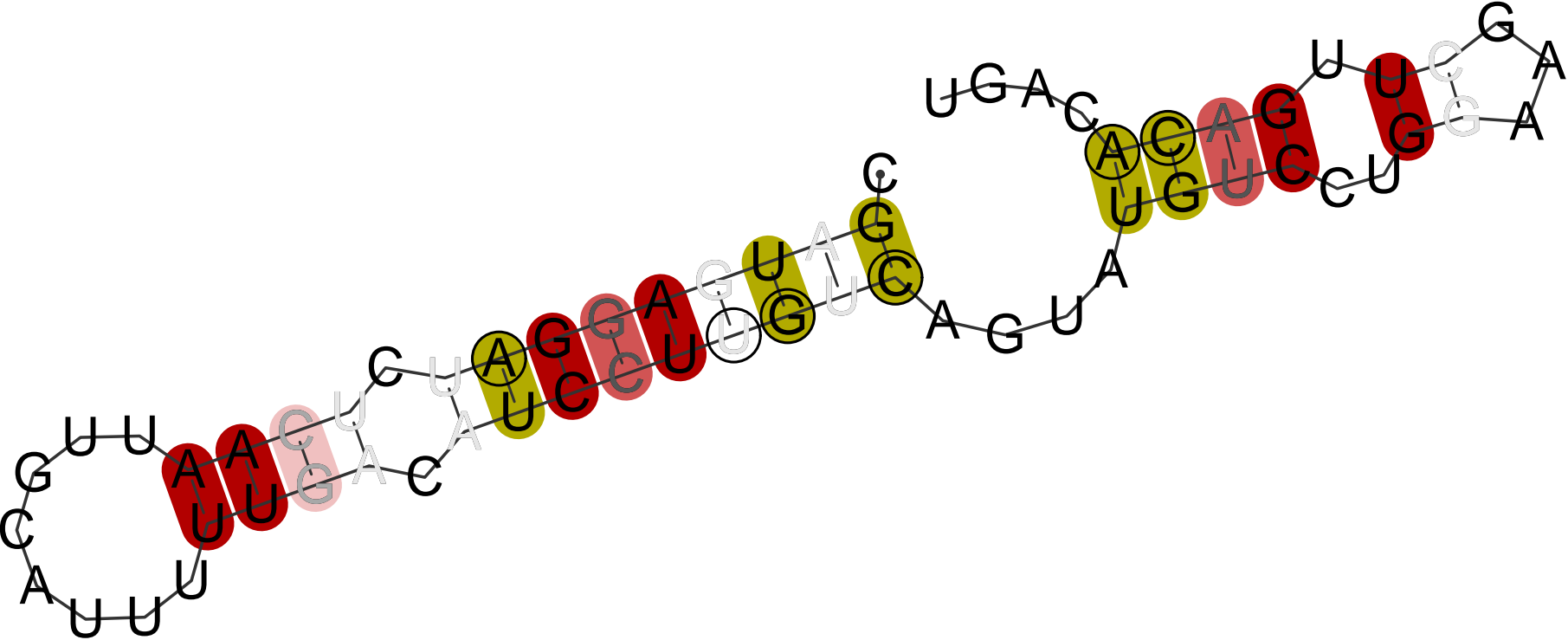

Supplement: S8 Fig — See the caption for S1 Fig for a description of the filename convention (save that the corresponding nucleotide locations in reference sequences are listed in S9 and S17 Tables), and an explanation of the RNAalifold options used and output (save that for these avian-origin viruses the folding temperature was set to 41°C). As some of the constrained regions predicted in the analysis of the M2 gene using raw (unranked) codon variability values are very close together, alignments of longer regions containing more than one conserved region are also included. (ZIP) [file pcbi.1012009.s129.zip › H7N9-avian-ranked-NS2-alignment-13-75-refseq-13-30-503-547-41C_revcomp_alirna_nogap.pdf]

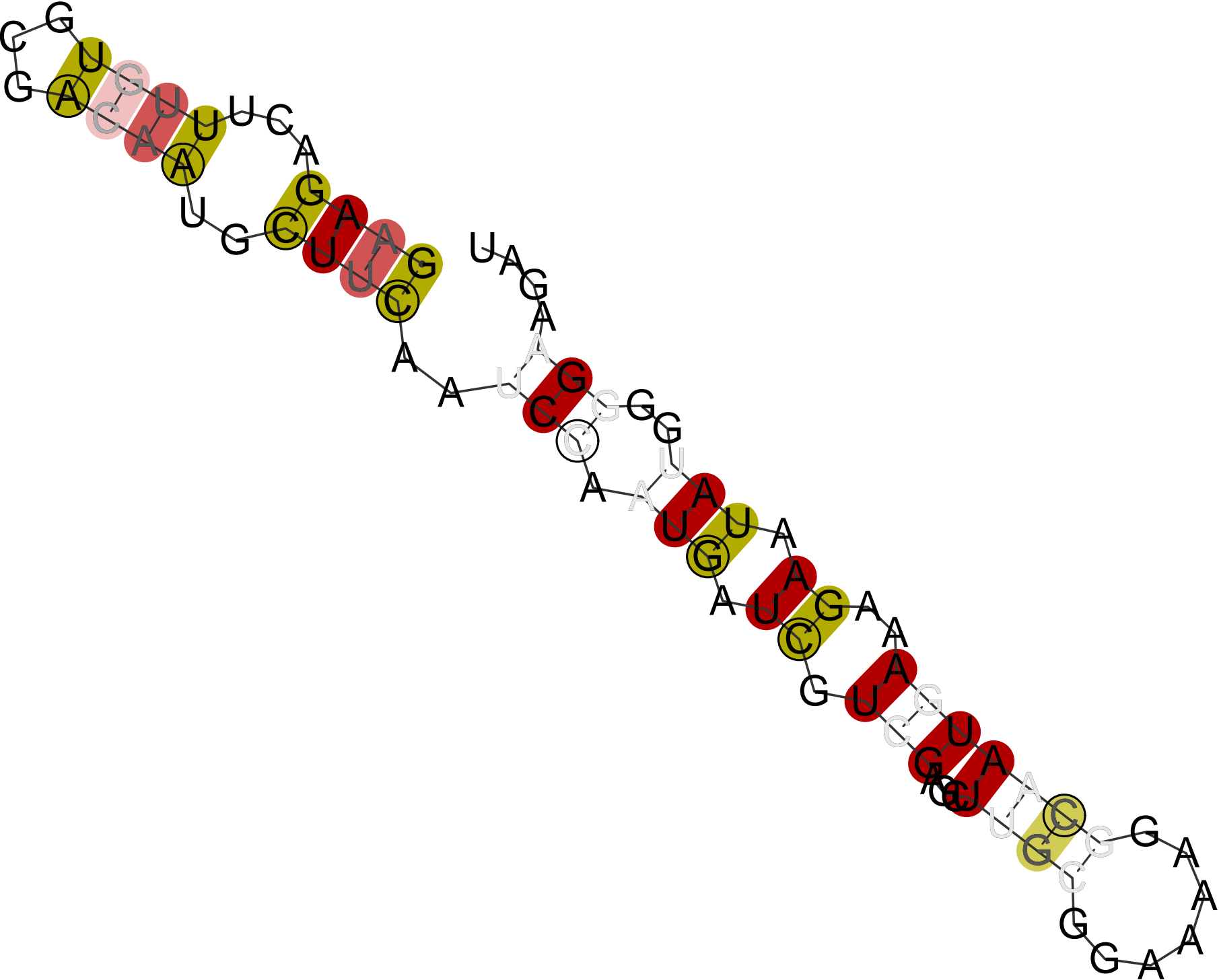

Supplement: S8 Fig — See the caption for S1 Fig for a description of the filename convention (save that the corresponding nucleotide locations in reference sequences are listed in S9 and S17 Tables), and an explanation of the RNAalifold options used and output (save that for these avian-origin viruses the folding temperature was set to 41°C). As some of the constrained regions predicted in the analysis of the M2 gene using raw (unranked) codon variability values are very close together, alignments of longer regions containing more than one conserved region are also included. (ZIP) [file pcbi.1012009.s129.zip › H7N9-avian-ranked-PA-X-alignment-4-81-refseq-4-81-41C_alirna_nogap.pdf]

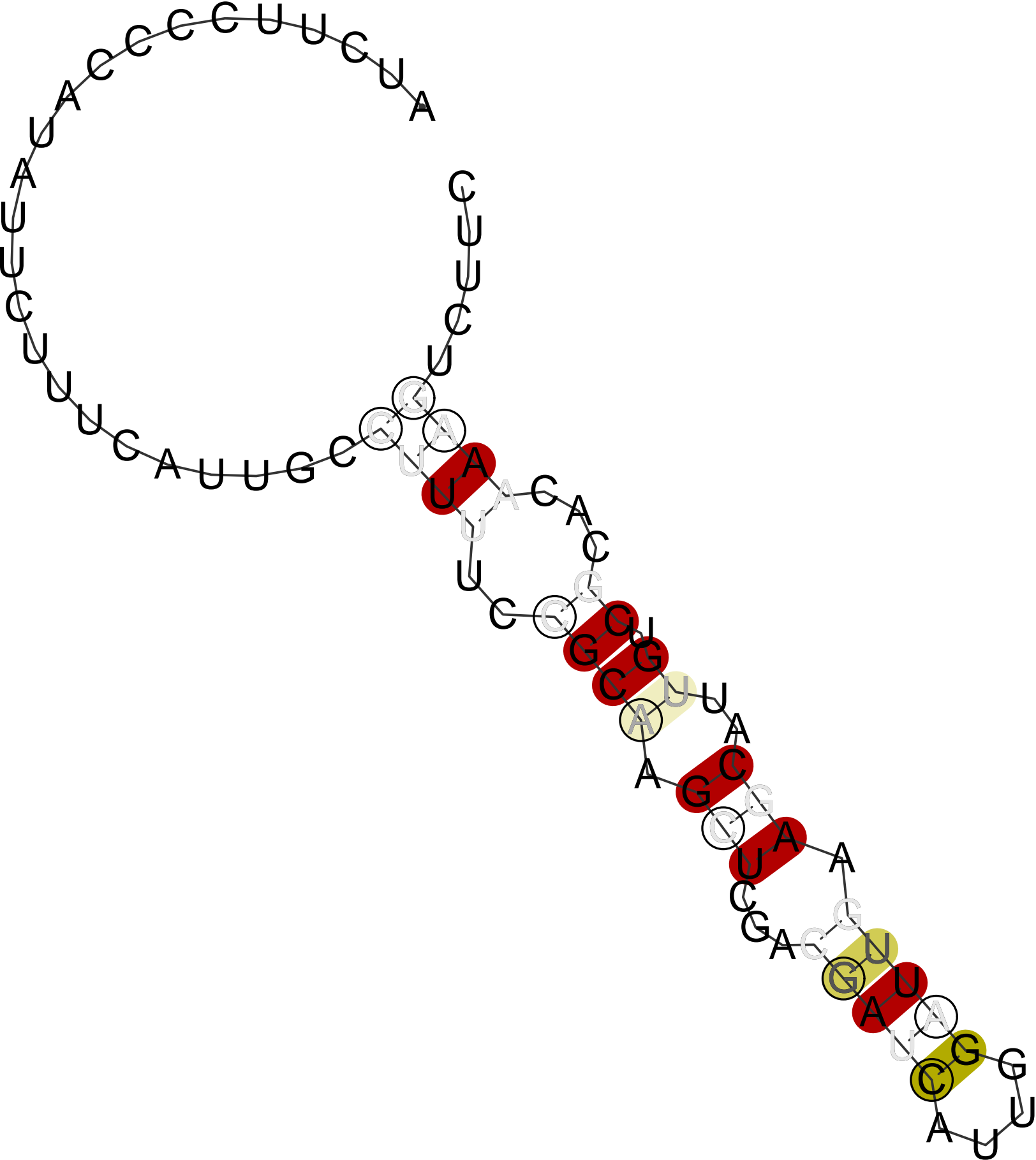

Supplement: S8 Fig — See the caption for S1 Fig for a description of the filename convention (save that the corresponding nucleotide locations in reference sequences are listed in S9 and S17 Tables), and an explanation of the RNAalifold options used and output (save that for these avian-origin viruses the folding temperature was set to 41°C). As some of the constrained regions predicted in the analysis of the M2 gene using raw (unranked) codon variability values are very close together, alignments of longer regions containing more than one conserved region are also included. (ZIP) [file pcbi.1012009.s129.zip › H7N9-avian-ranked-PA-X-alignment-4-81-refseq-4-81-41C_revcomp_alirna_nogap.pdf]

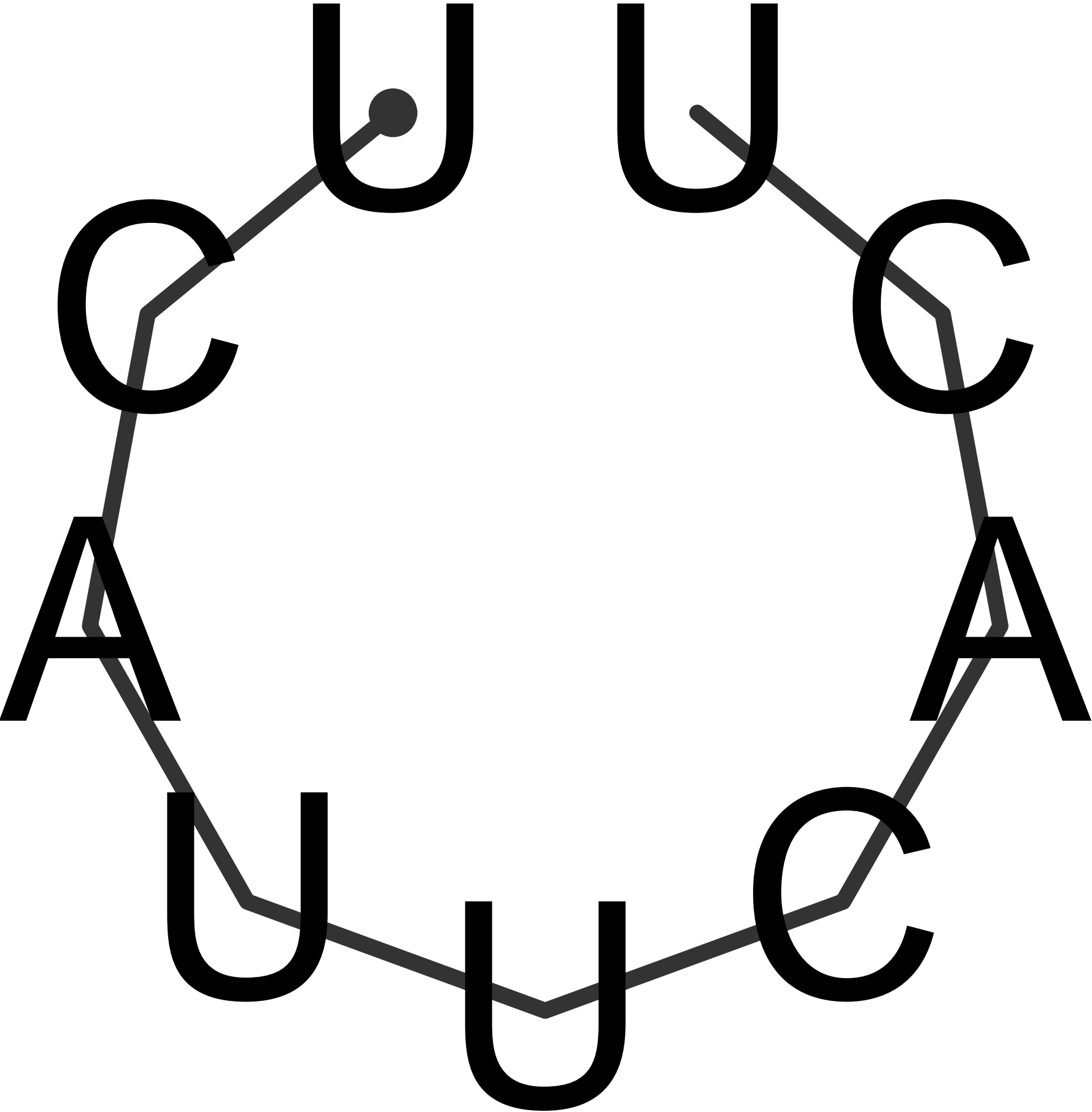

Supplement: S8 Fig — See the caption for S1 Fig for a description of the filename convention (save that the corresponding nucleotide locations in reference sequences are listed in S9 and S17 Tables), and an explanation of the RNAalifold options used and output (save that for these avian-origin viruses the folding temperature was set to 41°C). As some of the constrained regions predicted in the analysis of the M2 gene using raw (unranked) codon variability values are very close together, alignments of longer regions containing more than one conserved region are also included. (ZIP) [file pcbi.1012009.s129.zip › H7N9-avian-ranked-PA-X-alignment-445-453-refsseq-445-453-41C_alirna_nogap.pdf]

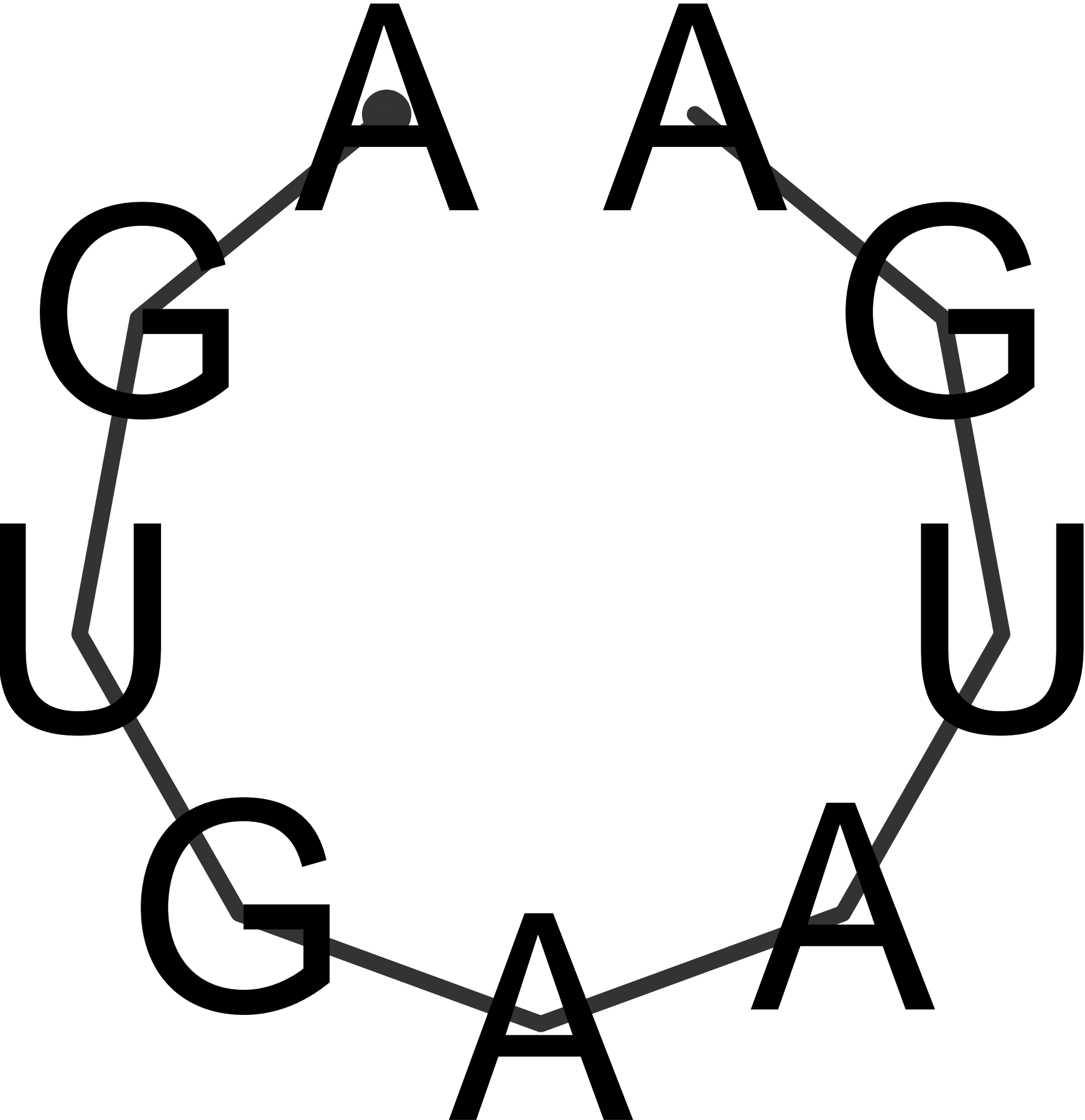

Supplement: S8 Fig — See the caption for S1 Fig for a description of the filename convention (save that the corresponding nucleotide locations in reference sequences are listed in S9 and S17 Tables), and an explanation of the RNAalifold options used and output (save that for these avian-origin viruses the folding temperature was set to 41°C). As some of the constrained regions predicted in the analysis of the M2 gene using raw (unranked) codon variability values are very close together, alignments of longer regions containing more than one conserved region are also included. (ZIP) [file pcbi.1012009.s129.zip › H7N9-avian-ranked-PA-X-alignment-445-453-refsseq-445-453-41C_revcomp_alirna_nogap.pdf]

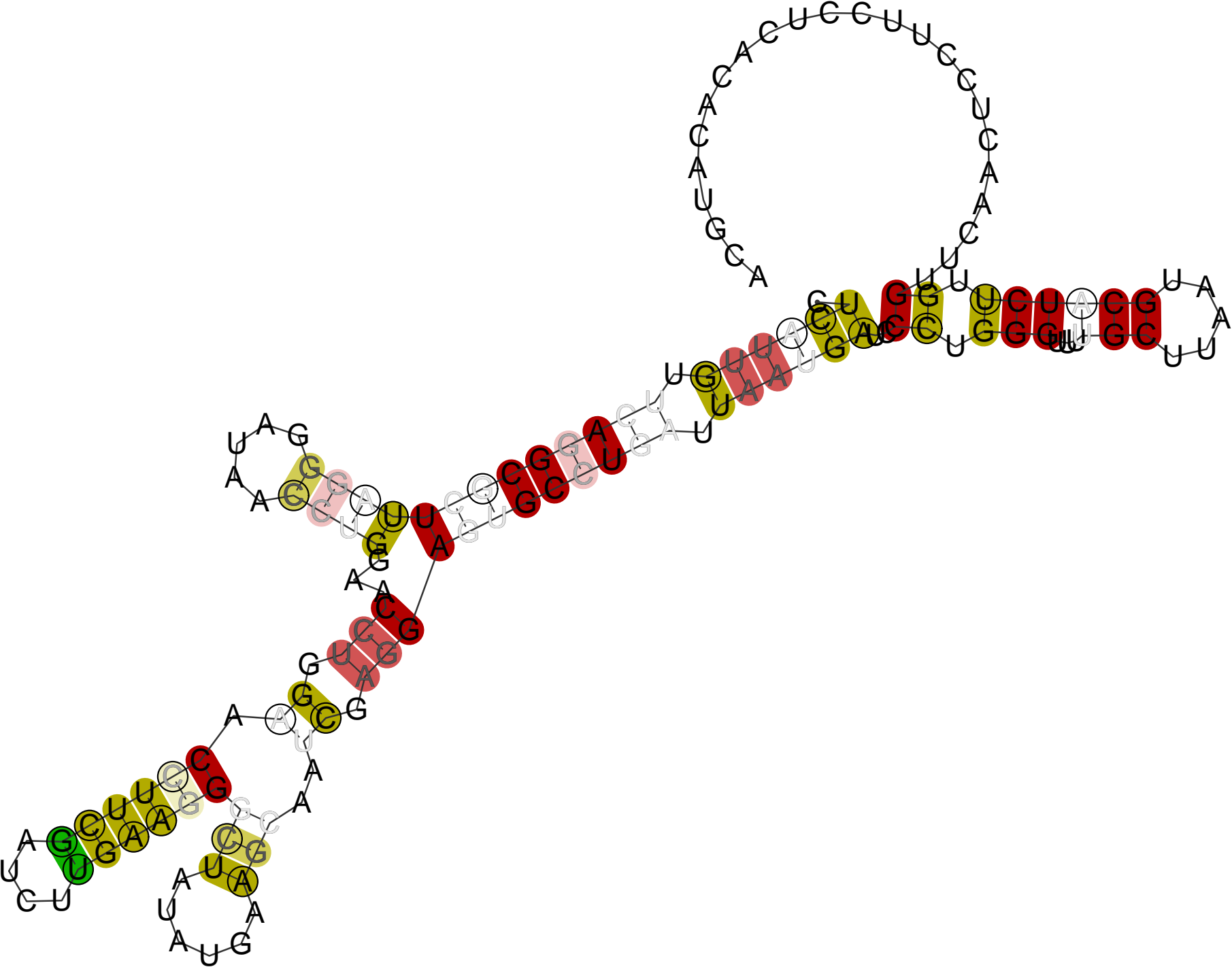

Supplement: S8 Fig — See the caption for S1 Fig for a description of the filename convention (save that the corresponding nucleotide locations in reference sequences are listed in S9 and S17 Tables), and an explanation of the RNAalifold options used and output (save that for these avian-origin viruses the folding temperature was set to 41°C). As some of the constrained regions predicted in the analysis of the M2 gene using raw (unranked) codon variability values are very close together, alignments of longer regions containing more than one conserved region are also included. (ZIP) [file pcbi.1012009.s129.zip › H7N9-avian-ranked-PA-alignment-1999-2142-refseq-1999-2142-41C_alirna_nogap.pdf]

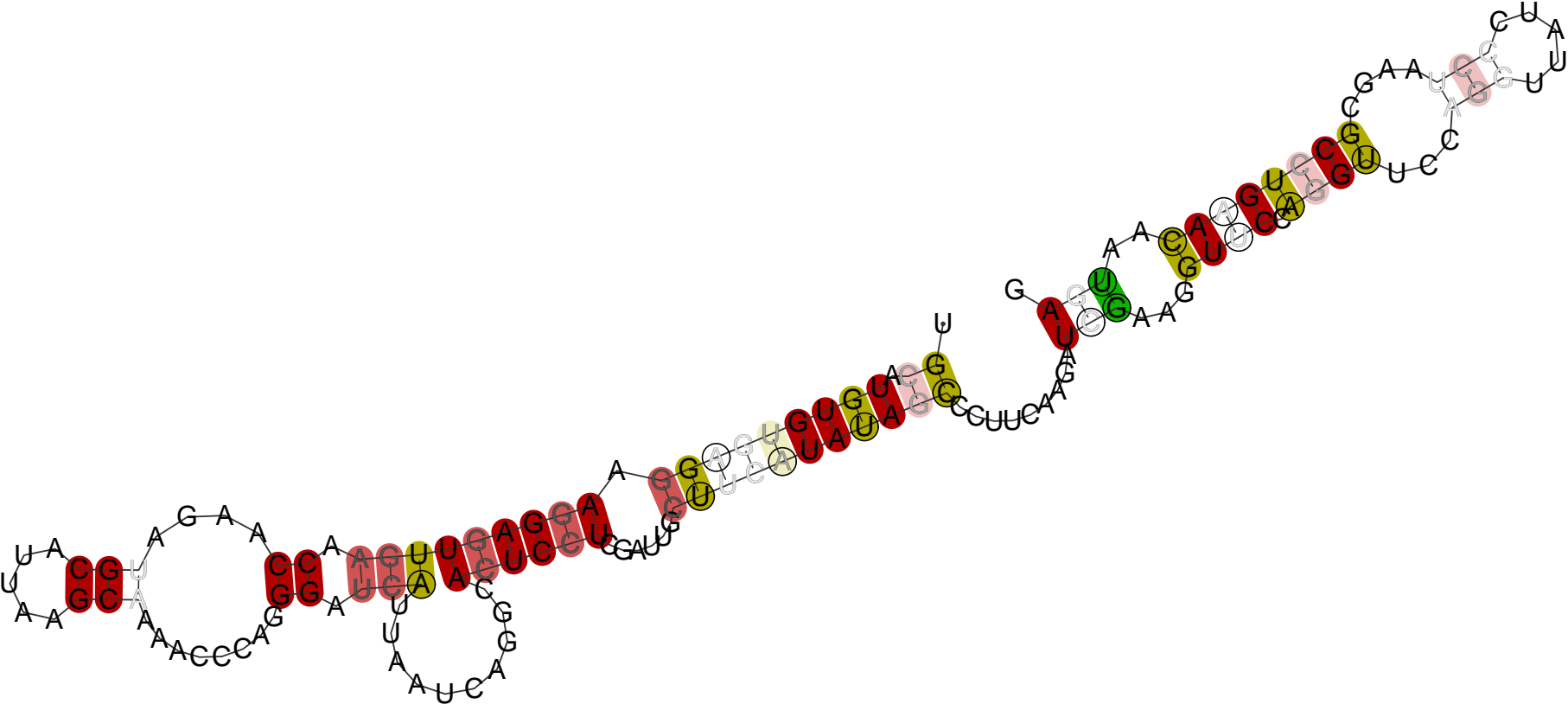

Supplement: S8 Fig — See the caption for S1 Fig for a description of the filename convention (save that the corresponding nucleotide locations in reference sequences are listed in S9 and S17 Tables), and an explanation of the RNAalifold options used and output (save that for these avian-origin viruses the folding temperature was set to 41°C). As some of the constrained regions predicted in the analysis of the M2 gene using raw (unranked) codon variability values are very close together, alignments of longer regions containing more than one conserved region are also included. (ZIP) [file pcbi.1012009.s129.zip › H7N9-avian-ranked-PA-alignment-1999-2142-refseq-1999-2142-41C_revcomp_alirna_nogap.pdf]

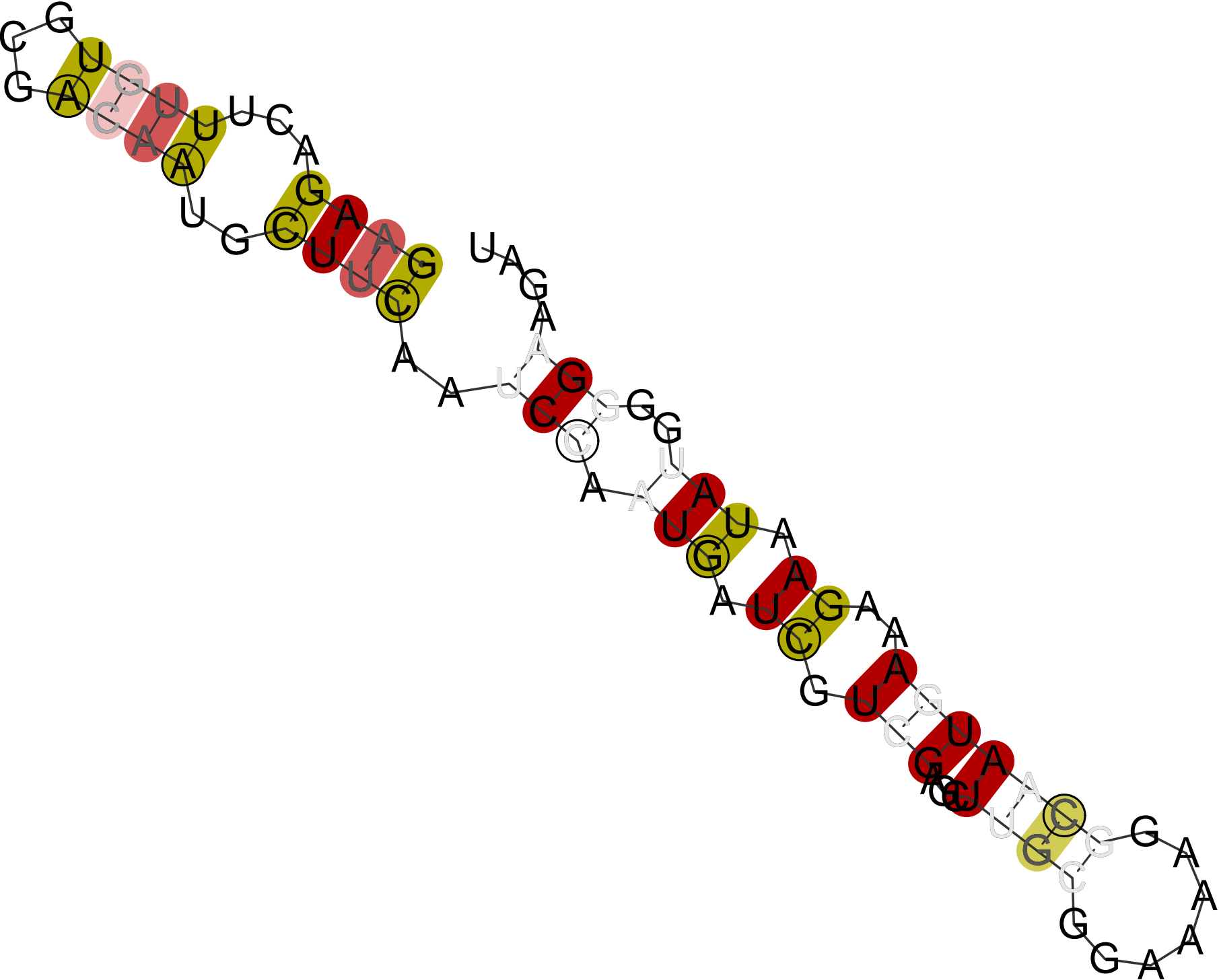

Supplement: S8 Fig — See the caption for S1 Fig for a description of the filename convention (save that the corresponding nucleotide locations in reference sequences are listed in S9 and S17 Tables), and an explanation of the RNAalifold options used and output (save that for these avian-origin viruses the folding temperature was set to 41°C). As some of the constrained regions predicted in the analysis of the M2 gene using raw (unranked) codon variability values are very close together, alignments of longer regions containing more than one conserved region are also included. (ZIP) [file pcbi.1012009.s129.zip › H7N9-avian-ranked-PA-alignment-4-81-refseq-4-81-41C_alirna_nogap.pdf]

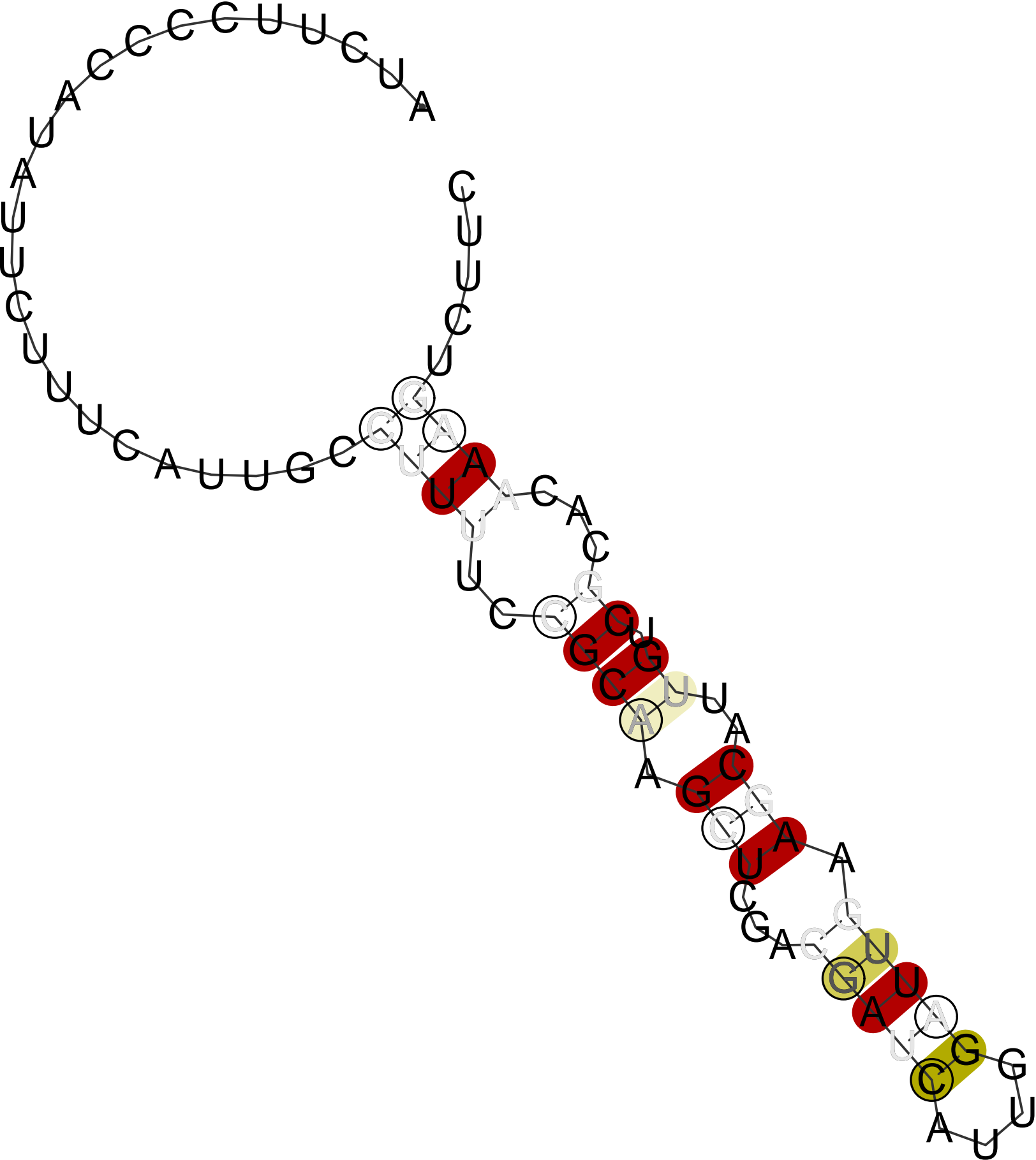

Supplement: S8 Fig — See the caption for S1 Fig for a description of the filename convention (save that the corresponding nucleotide locations in reference sequences are listed in S9 and S17 Tables), and an explanation of the RNAalifold options used and output (save that for these avian-origin viruses the folding temperature was set to 41°C). As some of the constrained regions predicted in the analysis of the M2 gene using raw (unranked) codon variability values are very close together, alignments of longer regions containing more than one conserved region are also included. (ZIP) [file pcbi.1012009.s129.zip › H7N9-avian-ranked-PA-alignment-4-81-refseq-4-81-41C_revcomp_alirna_nogap.pdf]

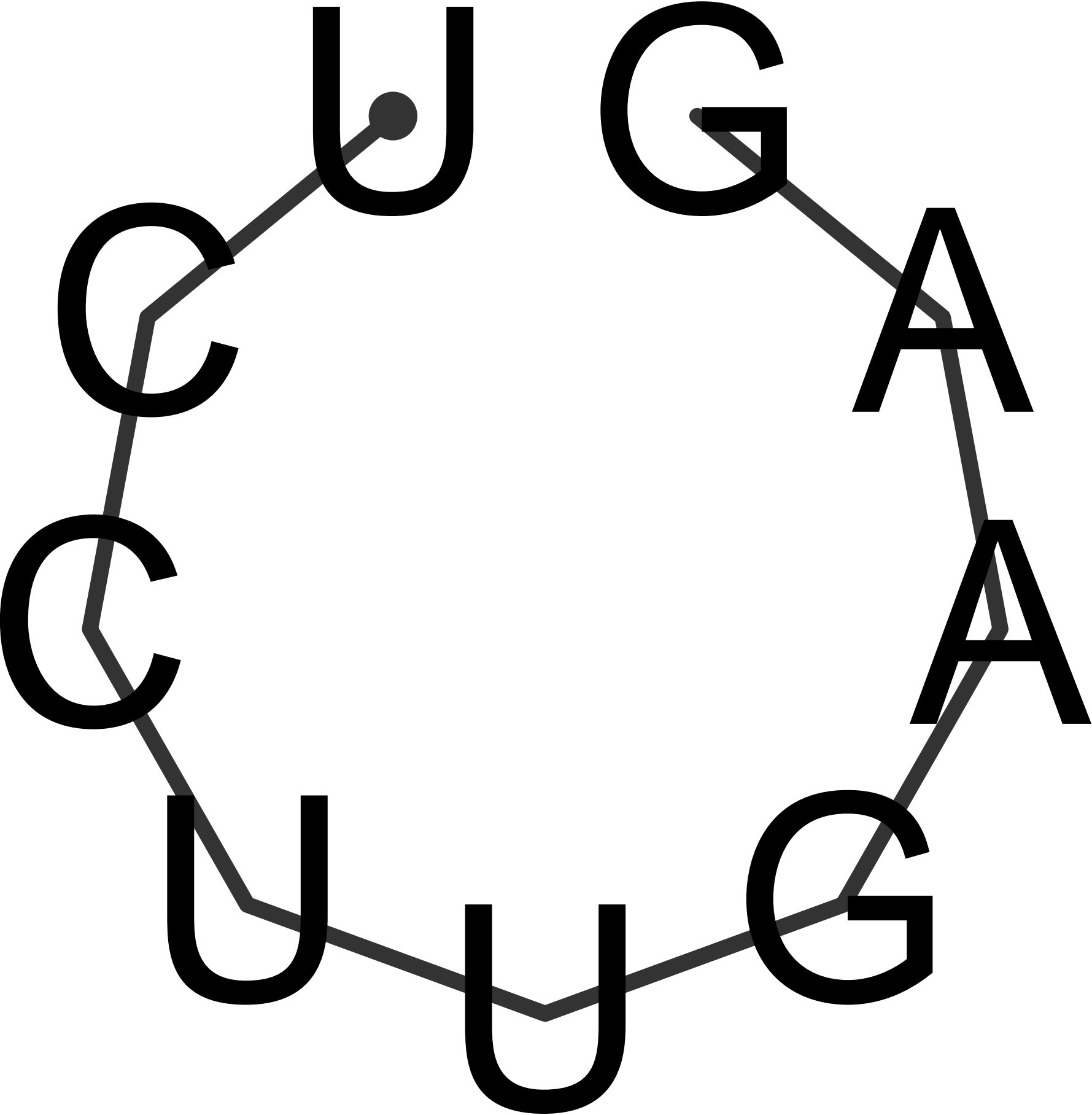

Supplement: S8 Fig — See the caption for S1 Fig for a description of the filename convention (save that the corresponding nucleotide locations in reference sequences are listed in S9 and S17 Tables), and an explanation of the RNAalifold options used and output (save that for these avian-origin viruses the folding temperature was set to 41°C). As some of the constrained regions predicted in the analysis of the M2 gene using raw (unranked) codon variability values are very close together, alignments of longer regions containing more than one conserved region are also included. (ZIP) [file pcbi.1012009.s129.zip › H7N9-avian-ranked-PB1-F2-alignment-187-195-refseq-281-289-41C_alirna_nogap.pdf]

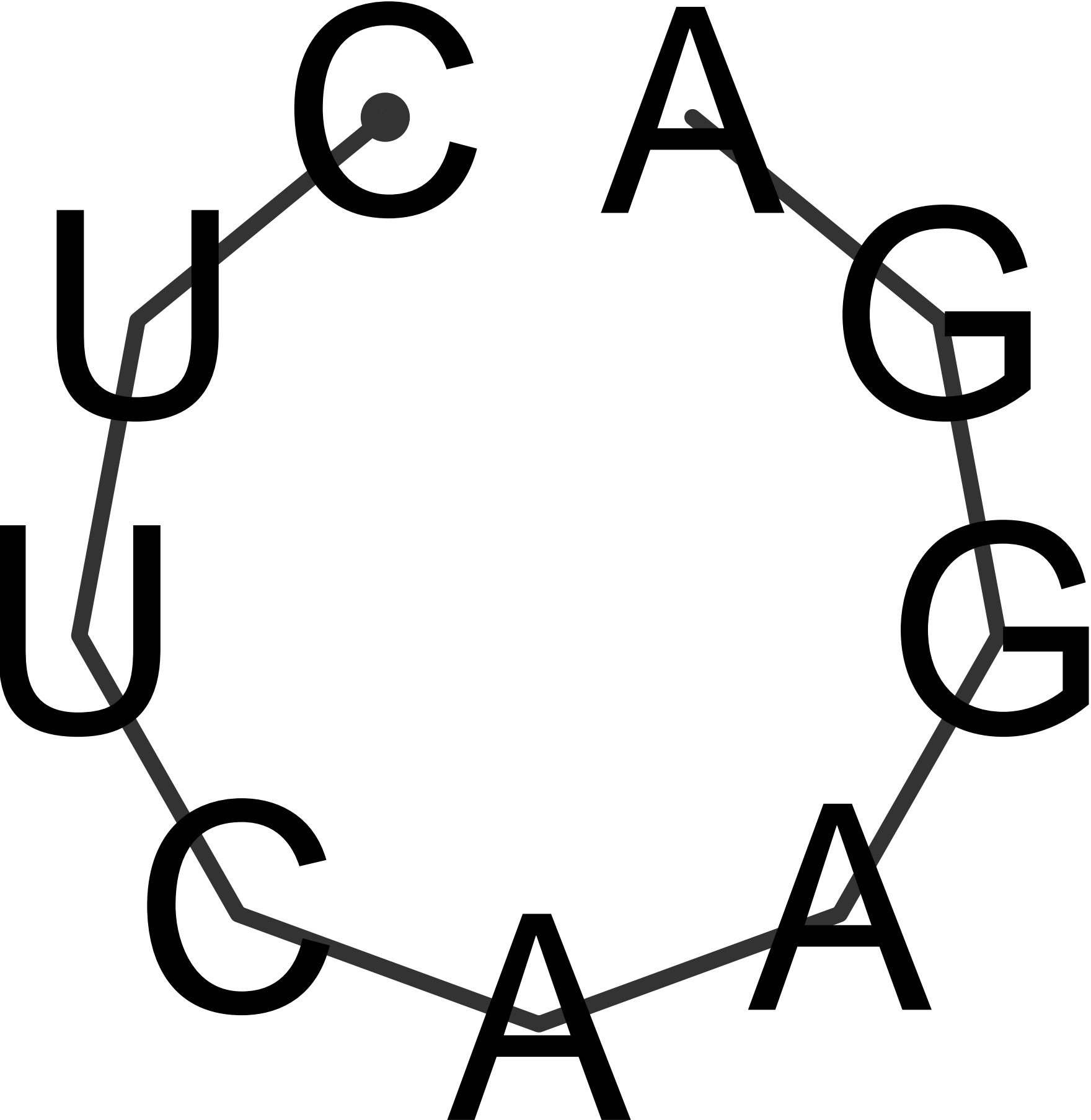

Supplement: S8 Fig — See the caption for S1 Fig for a description of the filename convention (save that the corresponding nucleotide locations in reference sequences are listed in S9 and S17 Tables), and an explanation of the RNAalifold options used and output (save that for these avian-origin viruses the folding temperature was set to 41°C). As some of the constrained regions predicted in the analysis of the M2 gene using raw (unranked) codon variability values are very close together, alignments of longer regions containing more than one conserved region are also included. (ZIP) [file pcbi.1012009.s129.zip › H7N9-avian-ranked-PB1-F2-alignment-187-195-refseq-281-289-41C_revcomp_alirna_nogap.pdf]

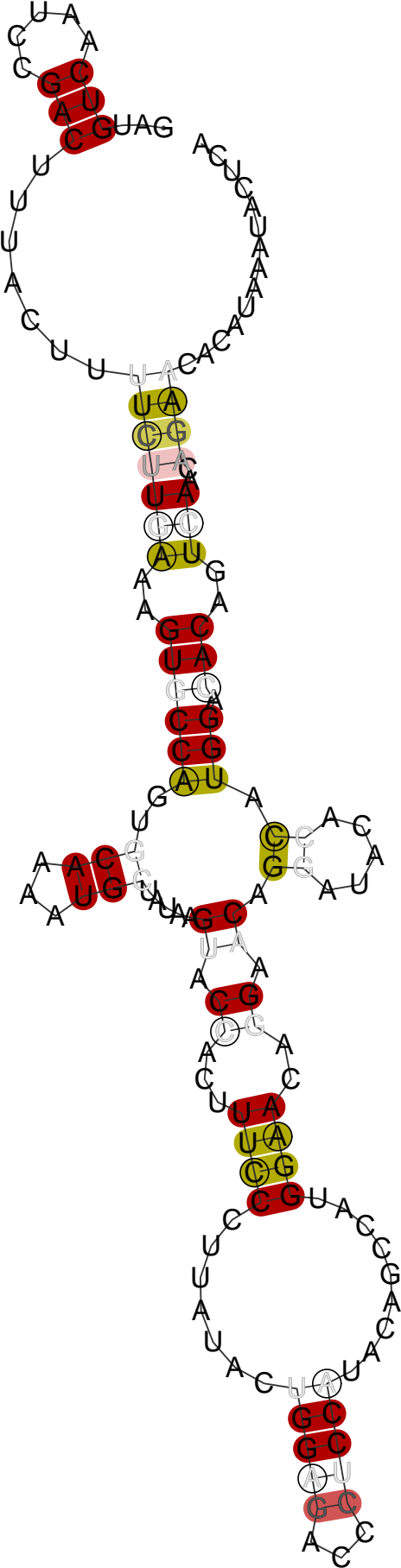

Supplement: S8 Fig — See the caption for S1 Fig for a description of the filename convention (save that the corresponding nucleotide locations in reference sequences are listed in S9 and S17 Tables), and an explanation of the RNAalifold options used and output (save that for these avian-origin viruses the folding temperature was set to 41°C). As some of the constrained regions predicted in the analysis of the M2 gene using raw (unranked) codon variability values are very close together, alignments of longer regions containing more than one conserved region are also included. (ZIP) [file pcbi.1012009.s129.zip › H7N9-avian-ranked-PB1-alignment-4-150-refseq-4-150-41C_alirna_nogap.pdf]

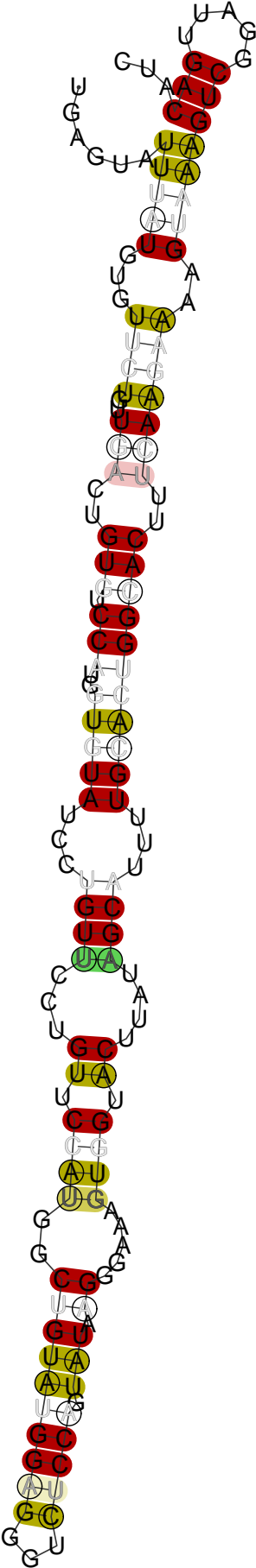

Supplement: S8 Fig — See the caption for S1 Fig for a description of the filename convention (save that the corresponding nucleotide locations in reference sequences are listed in S9 and S17 Tables), and an explanation of the RNAalifold options used and output (save that for these avian-origin viruses the folding temperature was set to 41°C). As some of the constrained regions predicted in the analysis of the M2 gene using raw (unranked) codon variability values are very close together, alignments of longer regions containing more than one conserved region are also included. (ZIP) [file pcbi.1012009.s129.zip › H7N9-avian-ranked-PB1-alignment-4-150-refseq-4-150-41C_revcomp_alirna_nogap.pdf]

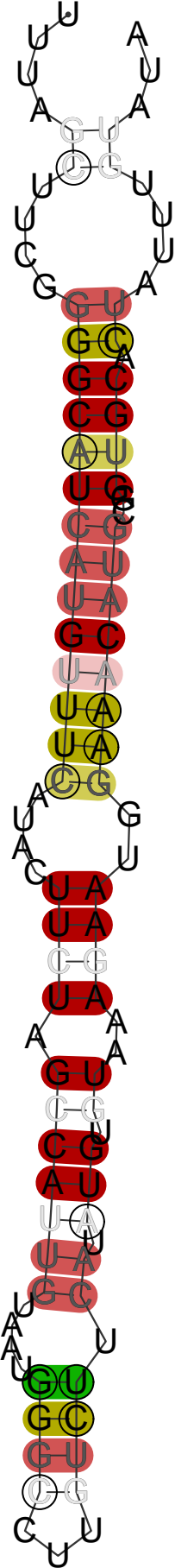

Supplement: S8 Fig — See the caption for S1 Fig for a description of the filename convention (save that the corresponding nucleotide locations in reference sequences are listed in S9 and S17 Tables), and an explanation of the RNAalifold options used and output (save that for these avian-origin viruses the folding temperature was set to 41°C). As some of the constrained regions predicted in the analysis of the M2 gene using raw (unranked) codon variability values are very close together, alignments of longer regions containing more than one conserved region are also included. (ZIP) [file pcbi.1012009.s129.zip › H7N9-avian-raw-HA-alignment-1618-1716-refseq-1582-1680-41C_alirna_nogap.pdf]

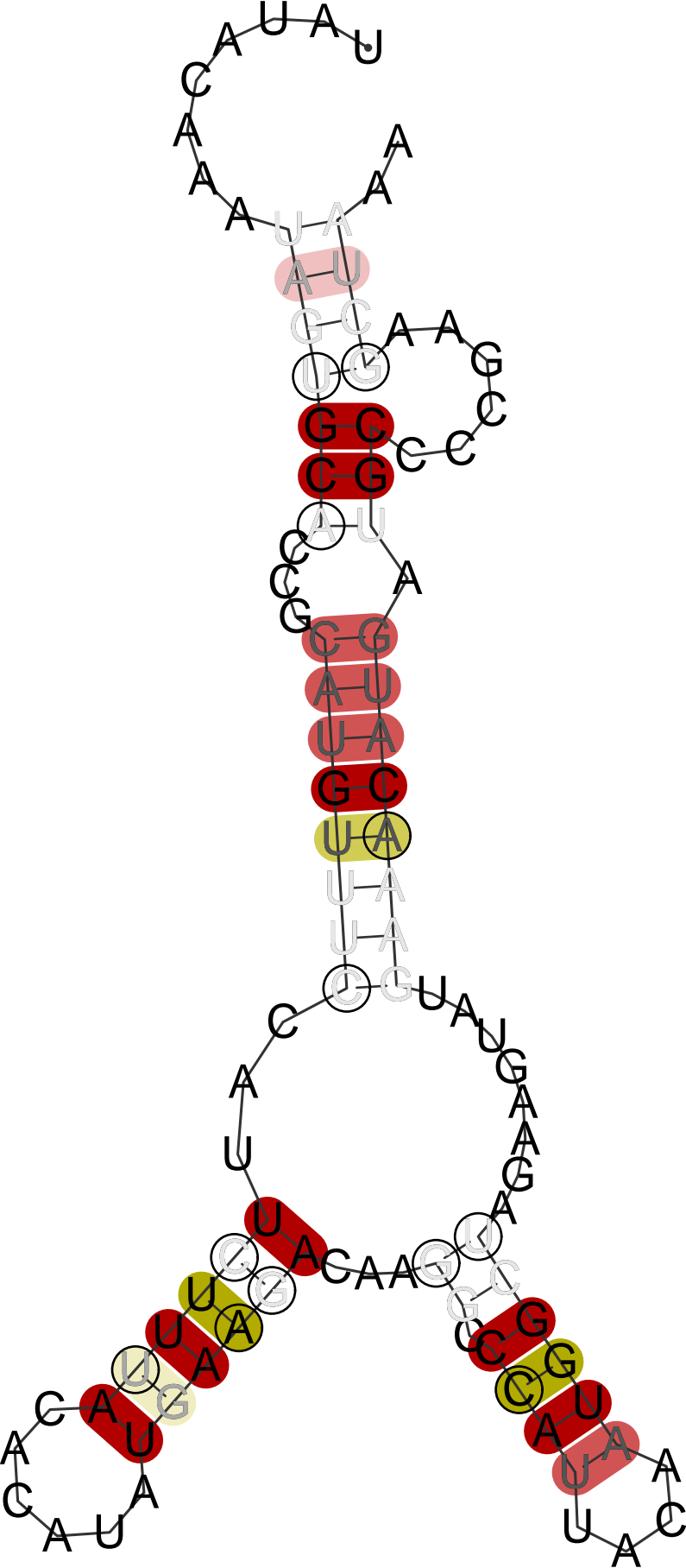

Supplement: S8 Fig — See the caption for S1 Fig for a description of the filename convention (save that the corresponding nucleotide locations in reference sequences are listed in S9 and S17 Tables), and an explanation of the RNAalifold options used and output (save that for these avian-origin viruses the folding temperature was set to 41°C). As some of the constrained regions predicted in the analysis of the M2 gene using raw (unranked) codon variability values are very close together, alignments of longer regions containing more than one conserved region are also included. (ZIP) [file pcbi.1012009.s129.zip › H7N9-avian-raw-HA-alignment-1618-1716-refseq-1582-1680-41C_revcomp_alirna_nogap.pdf]

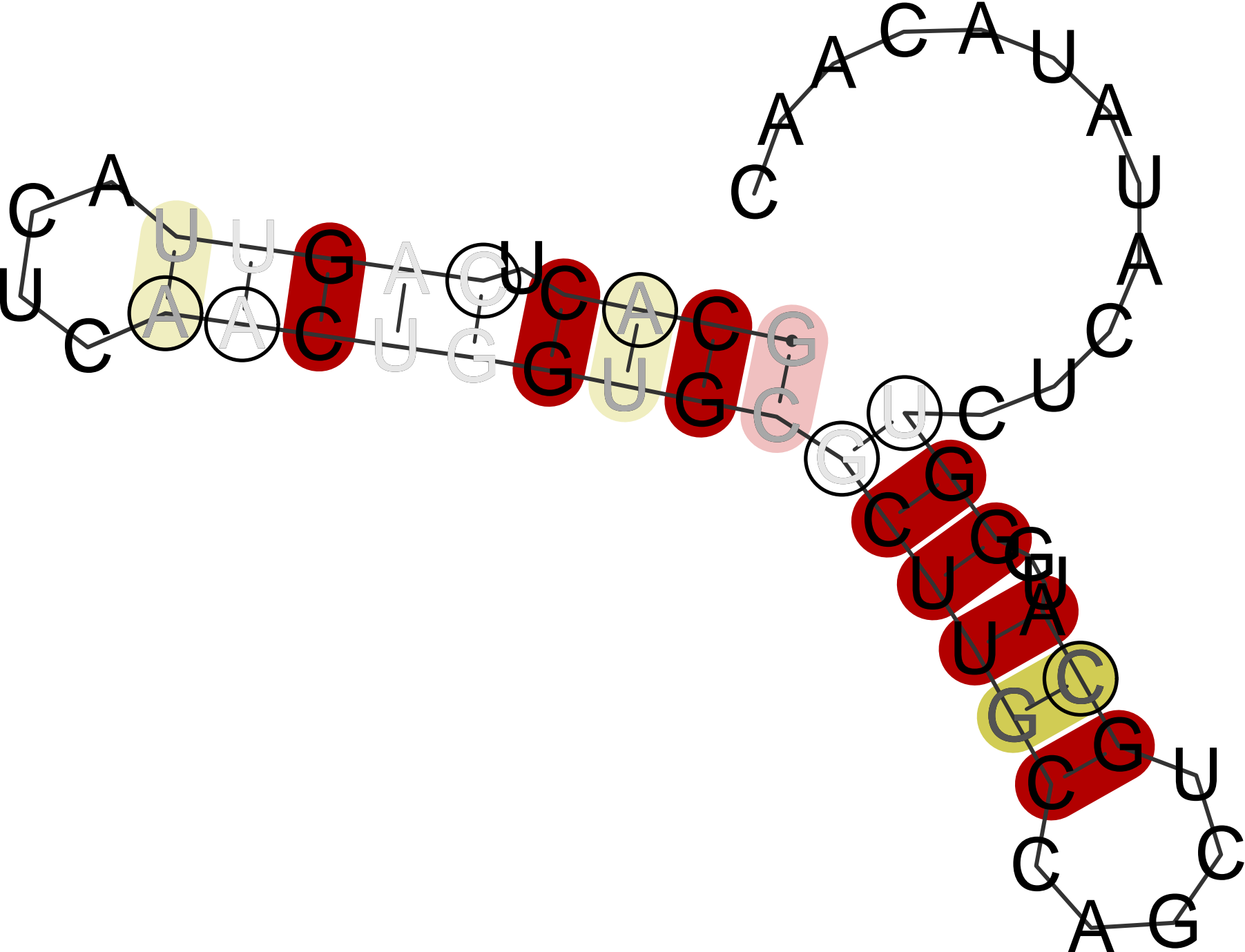

Supplement: S8 Fig — See the caption for S1 Fig for a description of the filename convention (save that the corresponding nucleotide locations in reference sequences are listed in S9 and S17 Tables), and an explanation of the RNAalifold options used and output (save that for these avian-origin viruses the folding temperature was set to 41°C). As some of the constrained regions predicted in the analysis of the M2 gene using raw (unranked) codon variability values are very close together, alignments of longer regions containing more than one conserved region are also included. (ZIP) [file pcbi.1012009.s129.zip › H7N9-avian-raw-M1-alignment-346-399-refseq-346-399-41C_alirna_nogap.pdf]

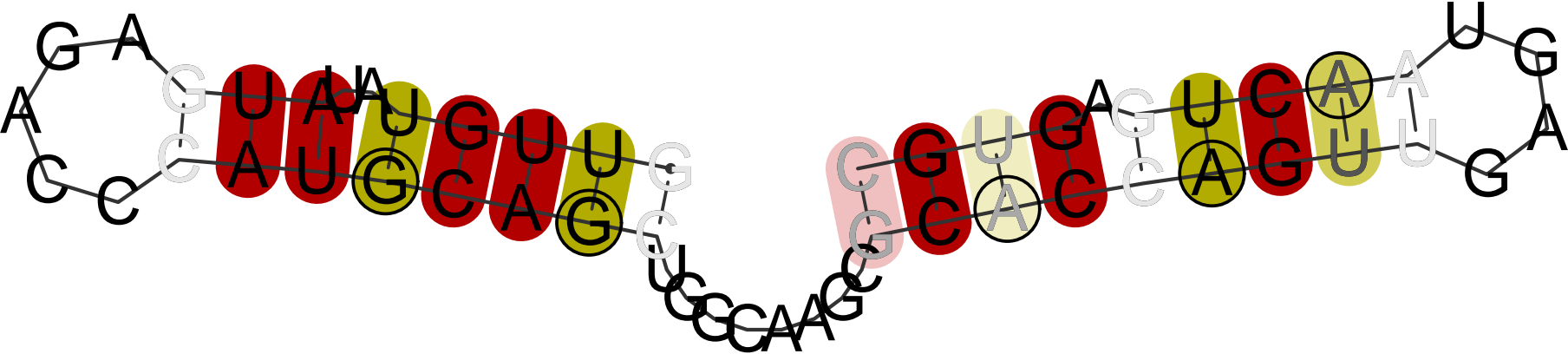

Supplement: S8 Fig — See the caption for S1 Fig for a description of the filename convention (save that the corresponding nucleotide locations in reference sequences are listed in S9 and S17 Tables), and an explanation of the RNAalifold options used and output (save that for these avian-origin viruses the folding temperature was set to 41°C). As some of the constrained regions predicted in the analysis of the M2 gene using raw (unranked) codon variability values are very close together, alignments of longer regions containing more than one conserved region are also included. (ZIP) [file pcbi.1012009.s129.zip › H7N9-avian-raw-M1-alignment-346-399-refseq-346-399-41C_revcomp_alirna_nogap.pdf]

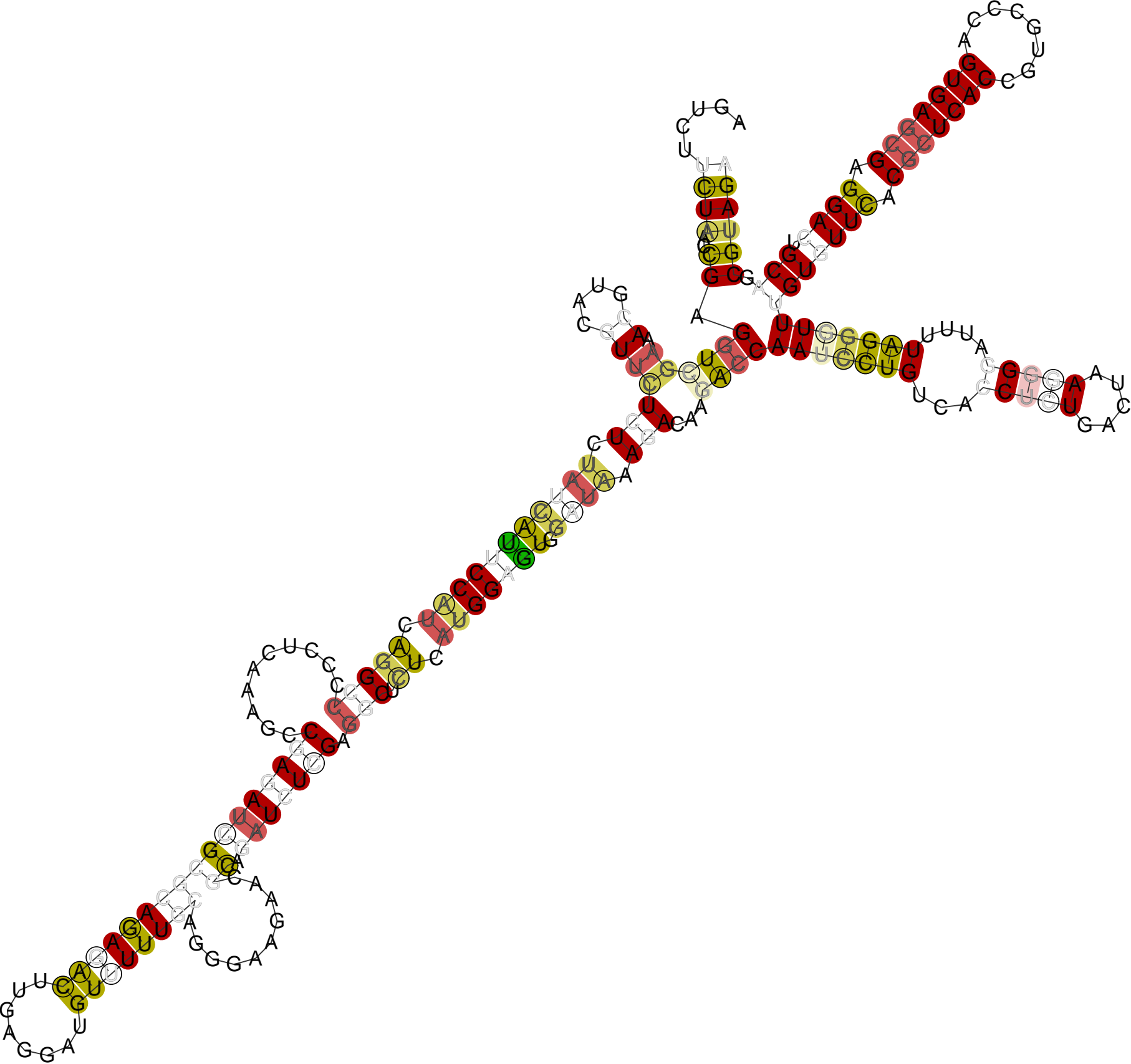

Supplement: S8 Fig — See the caption for S1 Fig for a description of the filename convention (save that the corresponding nucleotide locations in reference sequences are listed in S9 and S17 Tables), and an explanation of the RNAalifold options used and output (save that for these avian-origin viruses the folding temperature was set to 41°C). As some of the constrained regions predicted in the analysis of the M2 gene using raw (unranked) codon variability values are very close together, alignments of longer regions containing more than one conserved region are also included. (ZIP) [file pcbi.1012009.s129.zip › H7N9-avian-raw-M1-alignment-4-231-refseq-4-231-41C_alirna_nogap.pdf]

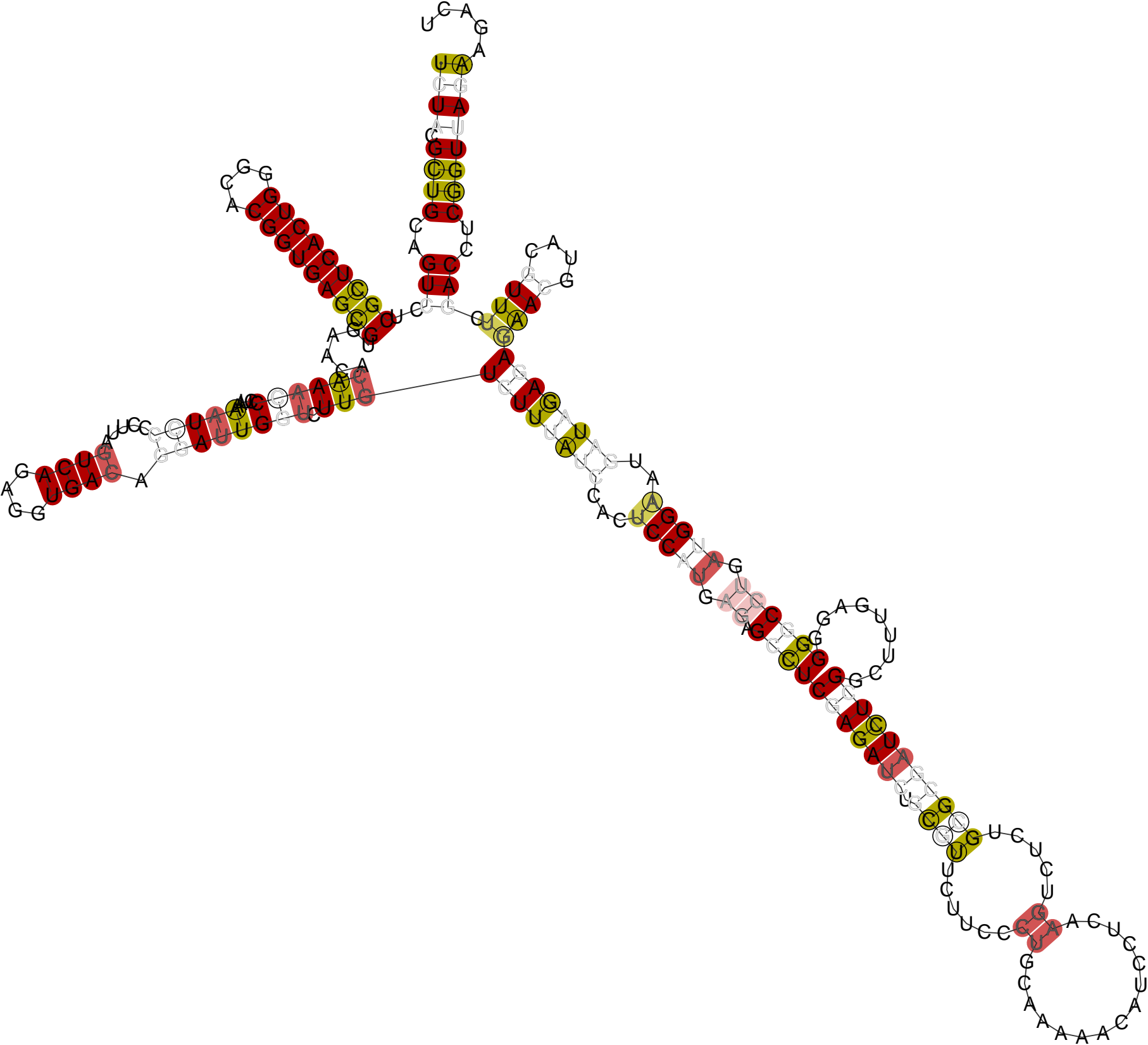

Supplement: S8 Fig — See the caption for S1 Fig for a description of the filename convention (save that the corresponding nucleotide locations in reference sequences are listed in S9 and S17 Tables), and an explanation of the RNAalifold options used and output (save that for these avian-origin viruses the folding temperature was set to 41°C). As some of the constrained regions predicted in the analysis of the M2 gene using raw (unranked) codon variability values are very close together, alignments of longer regions containing more than one conserved region are also included. (ZIP) [file pcbi.1012009.s129.zip › H7N9-avian-raw-M1-alignment-4-231-refseq-4-231-41C_revcomp_alirna_nogap.pdf]

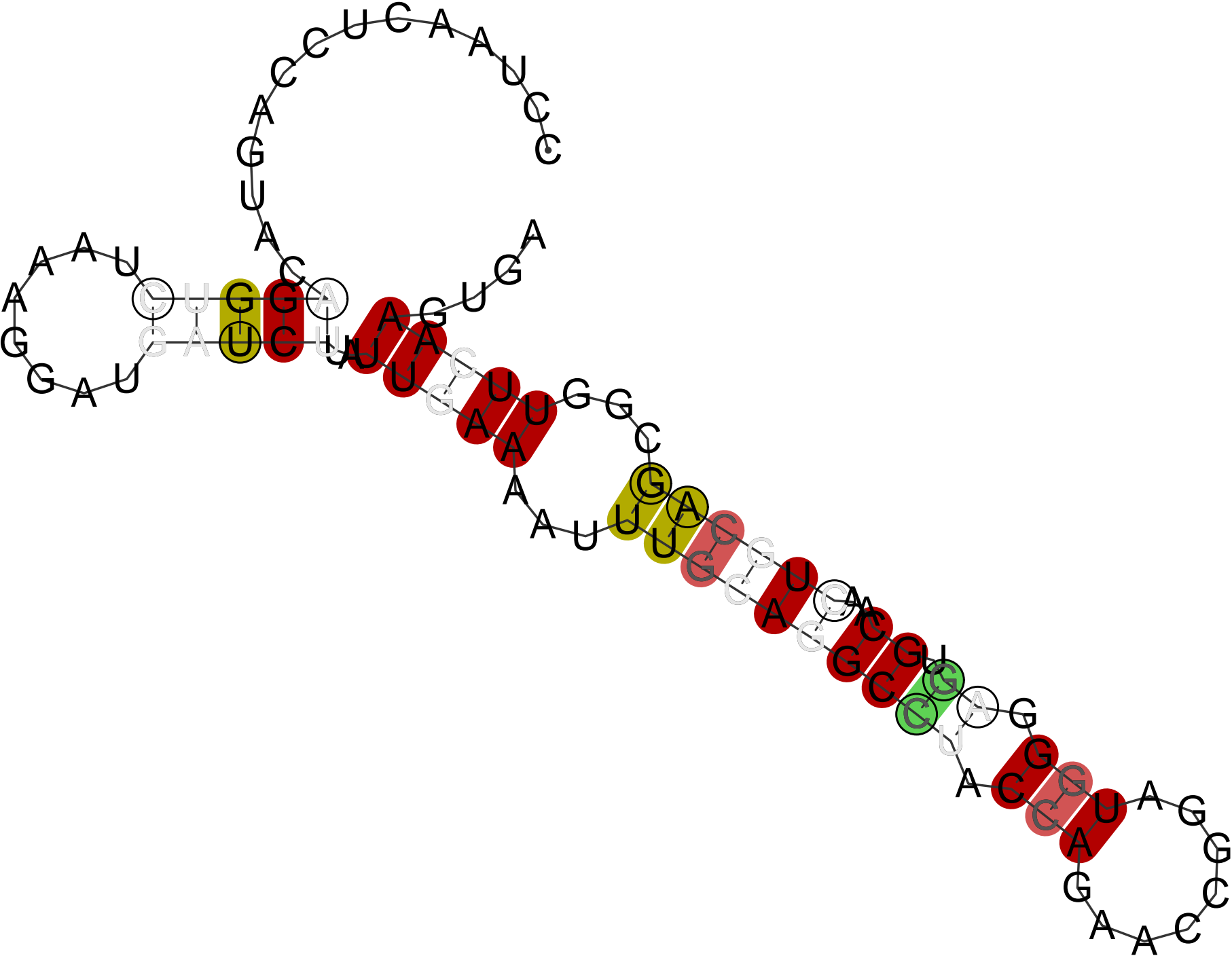

Supplement: S8 Fig — See the caption for S1 Fig for a description of the filename convention (save that the corresponding nucleotide locations in reference sequences are listed in S9 and S17 Tables), and an explanation of the RNAalifold options used and output (save that for these avian-origin viruses the folding temperature was set to 41°C). As some of the constrained regions predicted in the analysis of the M2 gene using raw (unranked) codon variability values are very close together, alignments of longer regions containing more than one conserved region are also included. (ZIP) [file pcbi.1012009.s129.zip › H7N9-avian-raw-M1-alignment-667-759-refseq-667-759-41C_alirna_nogap.pdf]

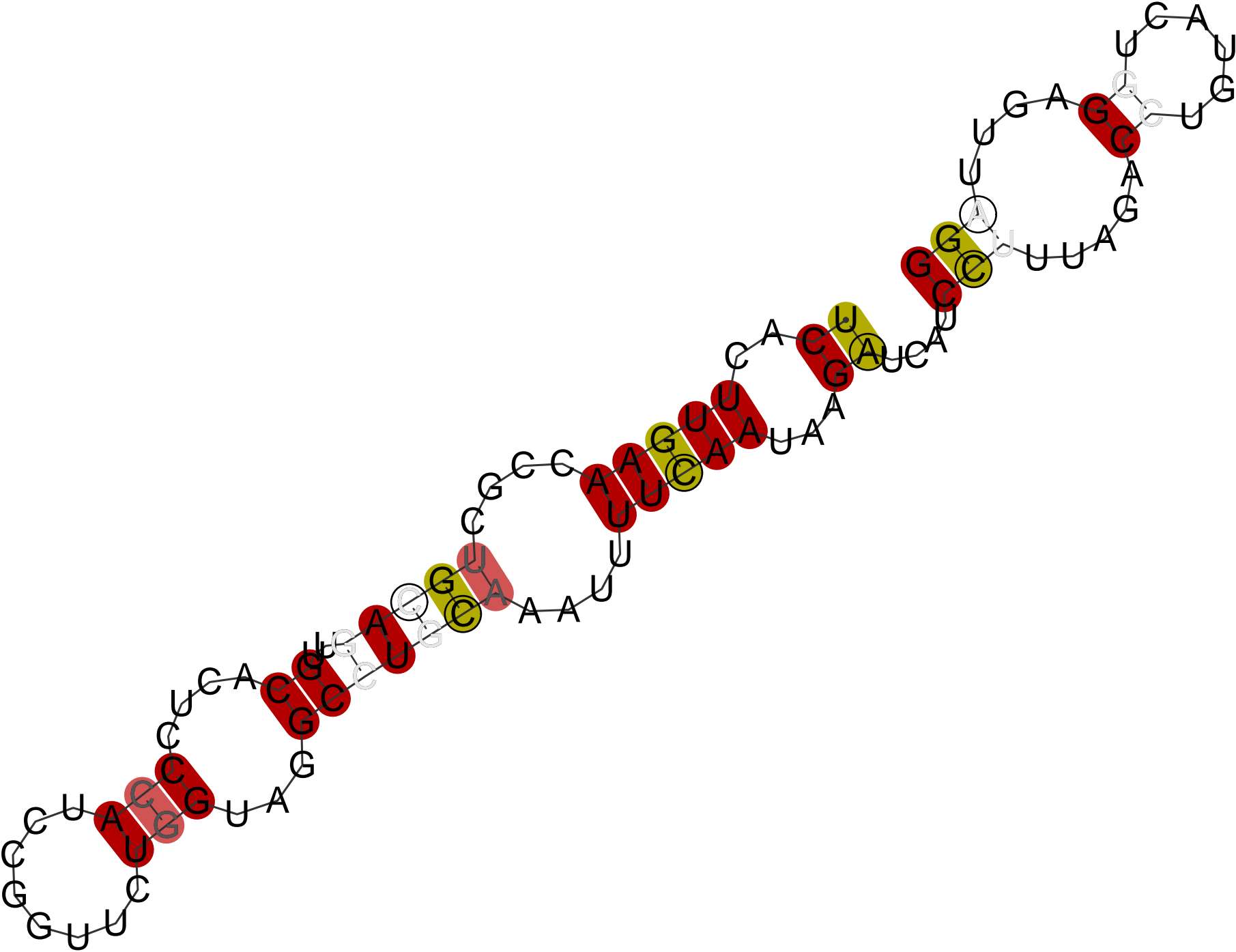

Supplement: S8 Fig — See the caption for S1 Fig for a description of the filename convention (save that the corresponding nucleotide locations in reference sequences are listed in S9 and S17 Tables), and an explanation of the RNAalifold options used and output (save that for these avian-origin viruses the folding temperature was set to 41°C). As some of the constrained regions predicted in the analysis of the M2 gene using raw (unranked) codon variability values are very close together, alignments of longer regions containing more than one conserved region are also included. (ZIP) [file pcbi.1012009.s129.zip › H7N9-avian-raw-M1-alignment-667-759-refseq-667-759-41C_revcomp_alirna_nogap.pdf]

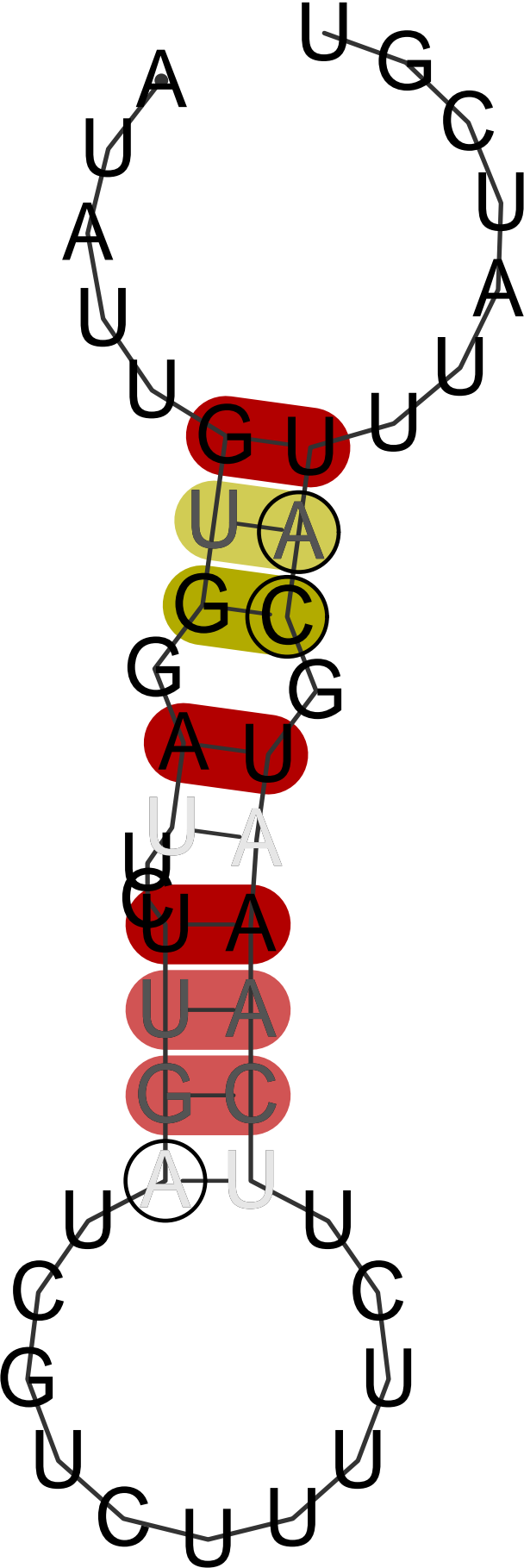

Supplement: S8 Fig — See the caption for S1 Fig for a description of the filename convention (save that the corresponding nucleotide locations in reference sequences are listed in S9 and S17 Tables), and an explanation of the RNAalifold options used and output (save that for these avian-origin viruses the folding temperature was set to 41°C). As some of the constrained regions predicted in the analysis of the M2 gene using raw (unranked) codon variability values are very close together, alignments of longer regions containing more than one conserved region are also included. (ZIP) [file pcbi.1012009.s129.zip › H7N9-avian-raw-M2-alignment-115-159-refseq-803-847-41C_alirna_nogap.pdf]

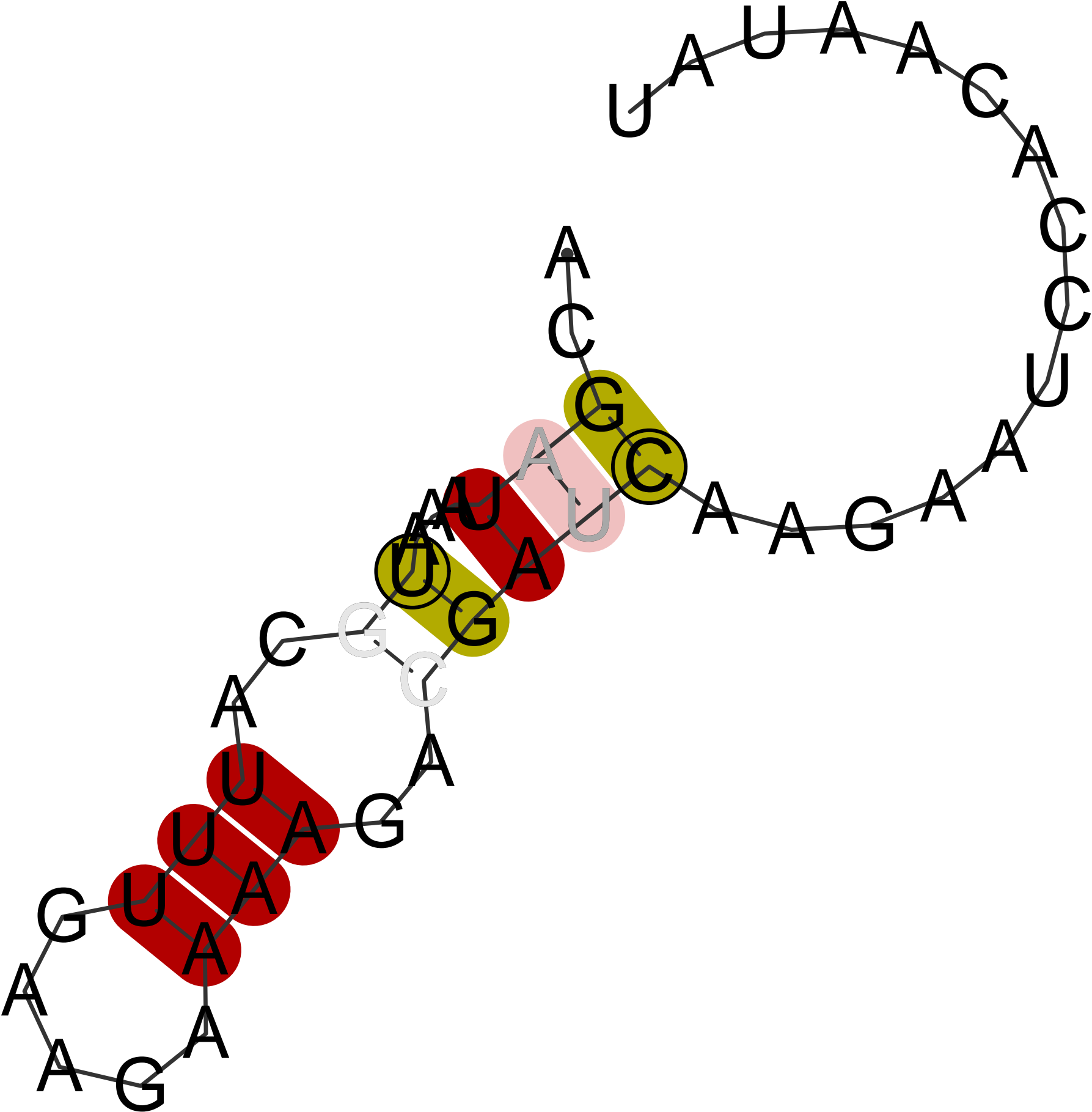

Supplement: S8 Fig — See the caption for S1 Fig for a description of the filename convention (save that the corresponding nucleotide locations in reference sequences are listed in S9 and S17 Tables), and an explanation of the RNAalifold options used and output (save that for these avian-origin viruses the folding temperature was set to 41°C). As some of the constrained regions predicted in the analysis of the M2 gene using raw (unranked) codon variability values are very close together, alignments of longer regions containing more than one conserved region are also included. (ZIP) [file pcbi.1012009.s129.zip › H7N9-avian-raw-M2-alignment-115-159-refseq-803-847-41C_revcomp_alirna_nogap.pdf]

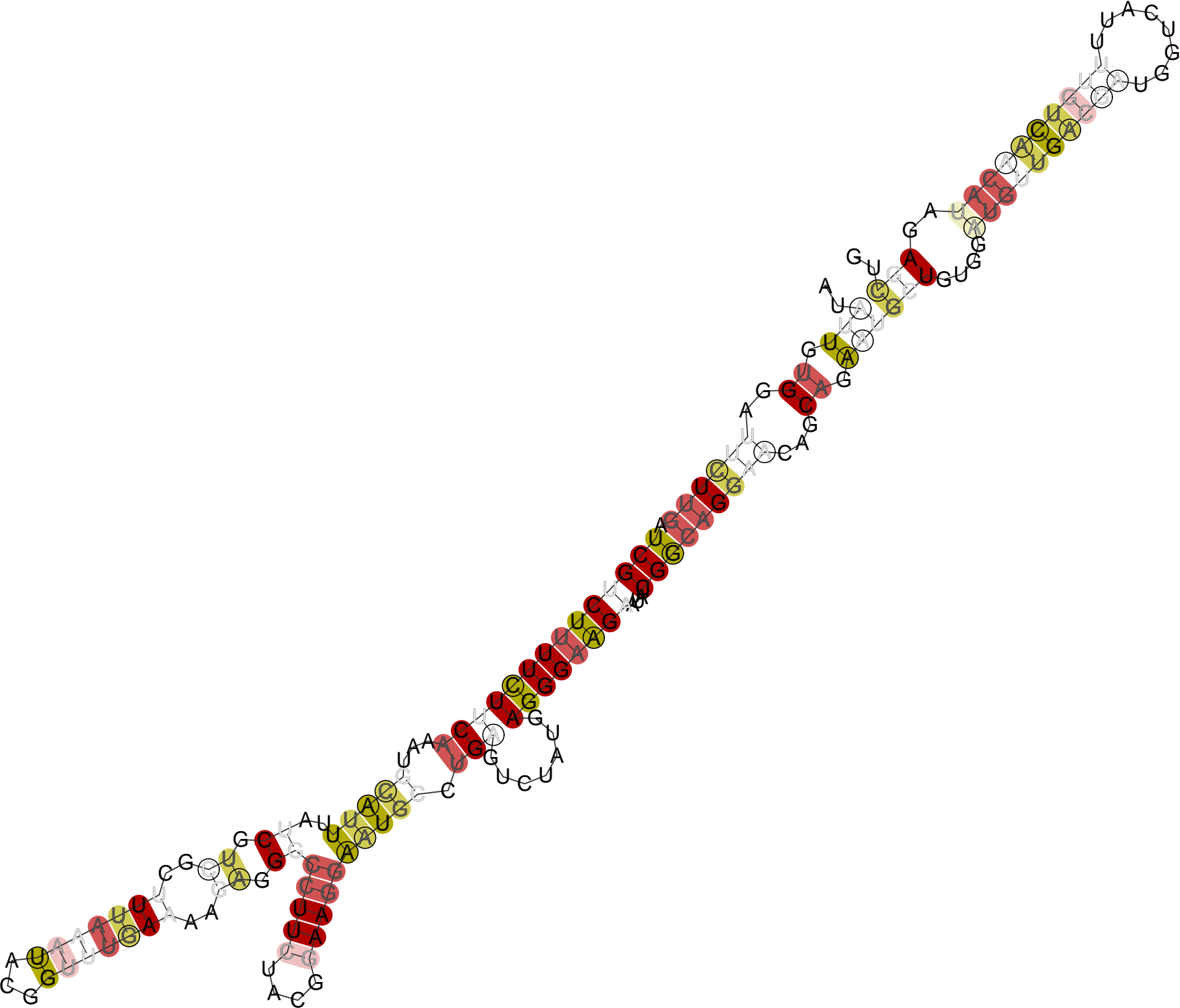

Supplement: S8 Fig — See the caption for S1 Fig for a description of the filename convention (save that the corresponding nucleotide locations in reference sequences are listed in S9 and S17 Tables), and an explanation of the RNAalifold options used and output (save that for these avian-origin viruses the folding temperature was set to 41°C). As some of the constrained regions predicted in the analysis of the M2 gene using raw (unranked) codon variability values are very close together, alignments of longer regions containing more than one conserved region are also included. (ZIP) [file pcbi.1012009.s129.zip › H7N9-avian-raw-M2-alignment-115-288-refseq-803-976-41C_alirna_nogap.pdf]

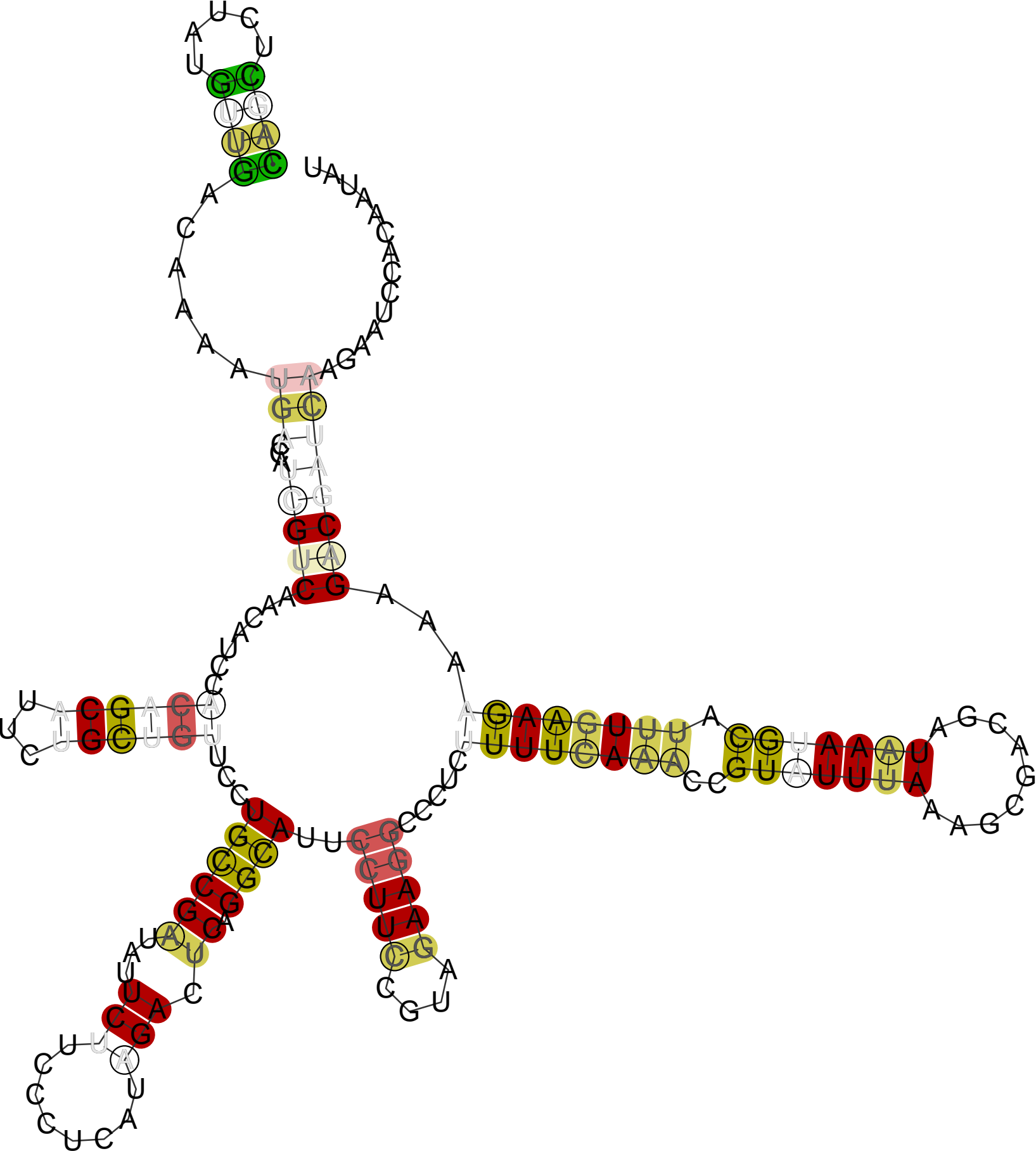

Supplement: S8 Fig — See the caption for S1 Fig for a description of the filename convention (save that the corresponding nucleotide locations in reference sequences are listed in S9 and S17 Tables), and an explanation of the RNAalifold options used and output (save that for these avian-origin viruses the folding temperature was set to 41°C). As some of the constrained regions predicted in the analysis of the M2 gene using raw (unranked) codon variability values are very close together, alignments of longer regions containing more than one conserved region are also included. (ZIP) [file pcbi.1012009.s129.zip › H7N9-avian-raw-M2-alignment-115-288-refseq-803-976-41C_revcomp_alirna_nogap.pdf]

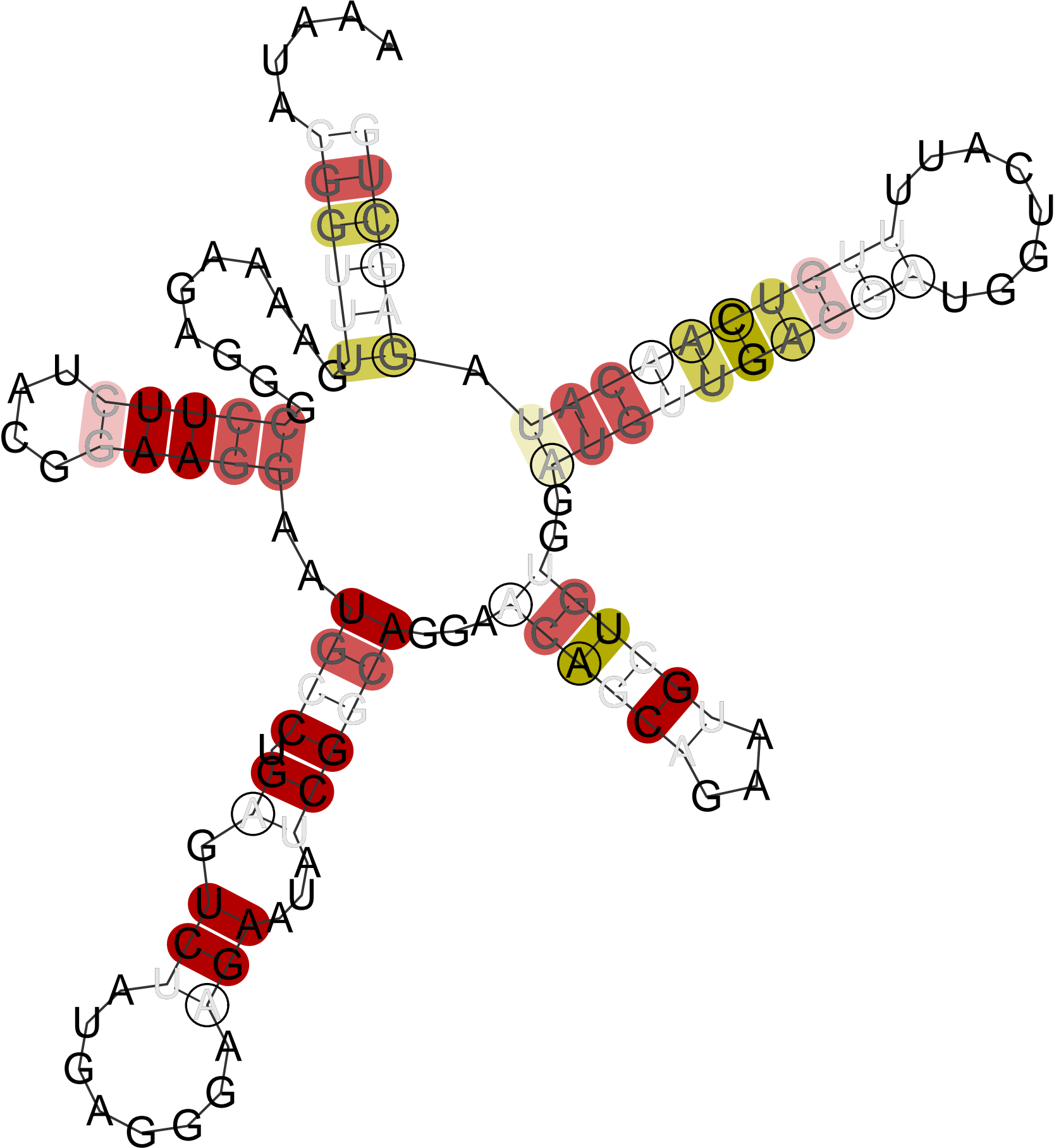

Supplement: S8 Fig — See the caption for S1 Fig for a description of the filename convention (save that the corresponding nucleotide locations in reference sequences are listed in S9 and S17 Tables), and an explanation of the RNAalifold options used and output (save that for these avian-origin viruses the folding temperature was set to 41°C). As some of the constrained regions predicted in the analysis of the M2 gene using raw (unranked) codon variability values are very close together, alignments of longer regions containing more than one conserved region are also included. (ZIP) [file pcbi.1012009.s129.zip › H7N9-avian-raw-M2-alignment-166-288-refseq-854-976-41C_alirna_nogap.pdf]

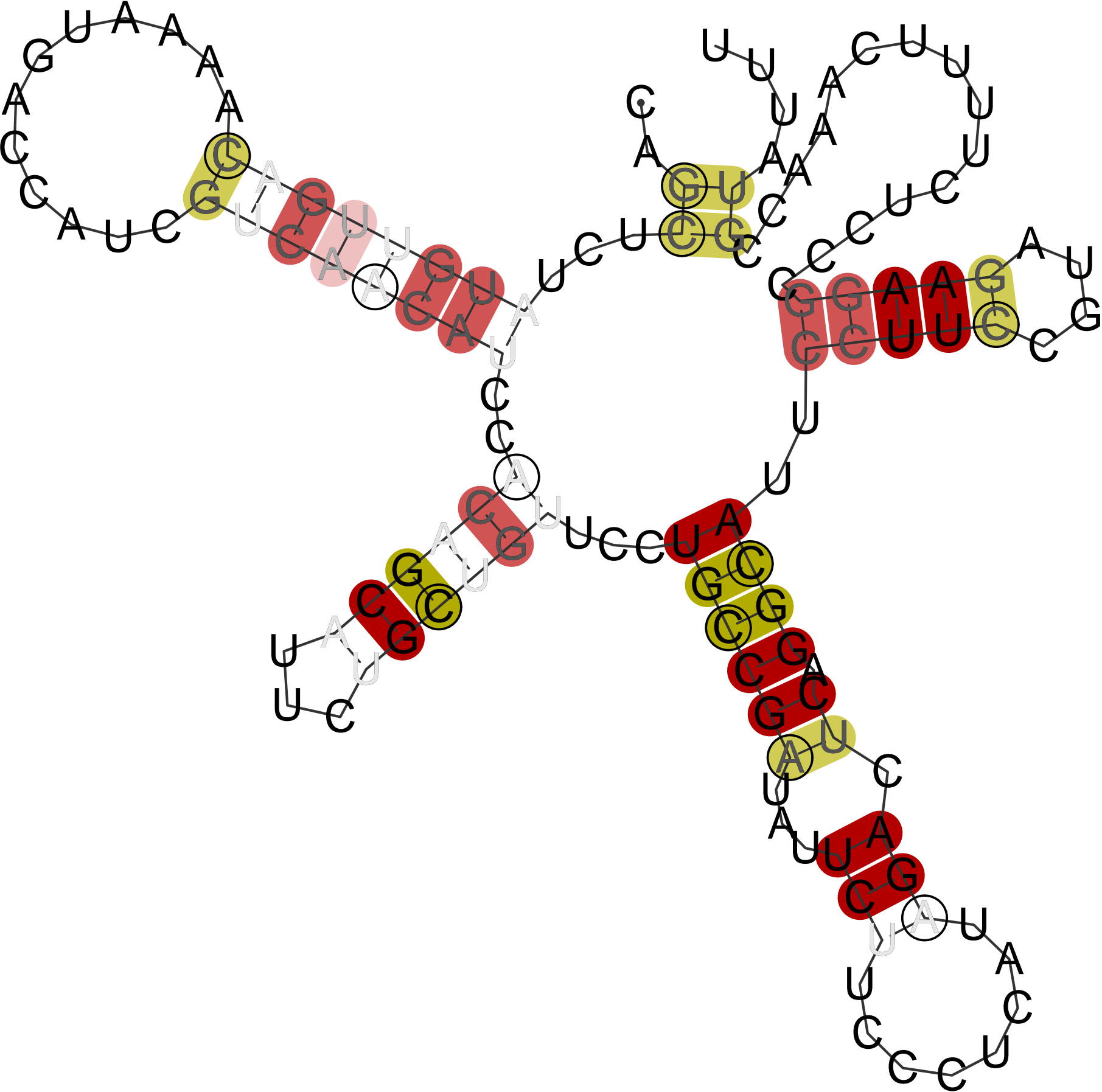

Supplement: S8 Fig — See the caption for S1 Fig for a description of the filename convention (save that the corresponding nucleotide locations in reference sequences are listed in S9 and S17 Tables), and an explanation of the RNAalifold options used and output (save that for these avian-origin viruses the folding temperature was set to 41°C). As some of the constrained regions predicted in the analysis of the M2 gene using raw (unranked) codon variability values are very close together, alignments of longer regions containing more than one conserved region are also included. (ZIP) [file pcbi.1012009.s129.zip › H7N9-avian-raw-M2-alignment-166-288-refseq-854-976-41C_revcomp_alirna_nogap.pdf]

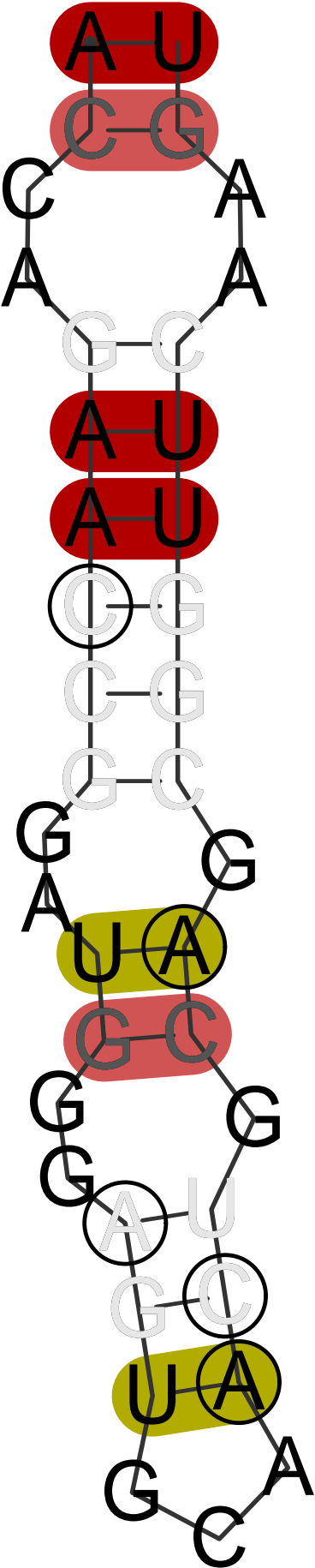

Supplement: S8 Fig — See the caption for S1 Fig for a description of the filename convention (save that the corresponding nucleotide locations in reference sequences are listed in S9 and S17 Tables), and an explanation of the RNAalifold options used and output (save that for these avian-origin viruses the folding temperature was set to 41°C). As some of the constrained regions predicted in the analysis of the M2 gene using raw (unranked) codon variability values are very close together, alignments of longer regions containing more than one conserved region are also included. (ZIP) [file pcbi.1012009.s129.zip › H7N9-avian-raw-M2-alignment-31-69-refseq-719-757-41C_alirna_nogap.pdf]

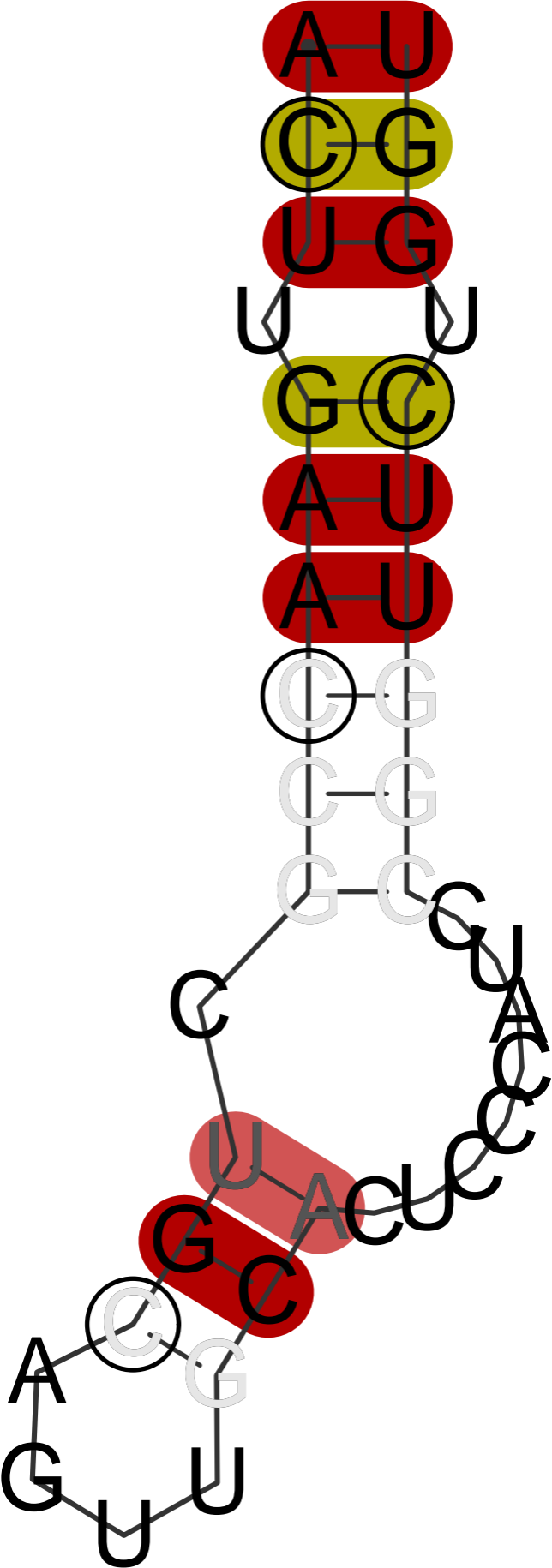

Supplement: S8 Fig — See the caption for S1 Fig for a description of the filename convention (save that the corresponding nucleotide locations in reference sequences are listed in S9 and S17 Tables), and an explanation of the RNAalifold options used and output (save that for these avian-origin viruses the folding temperature was set to 41°C). As some of the constrained regions predicted in the analysis of the M2 gene using raw (unranked) codon variability values are very close together, alignments of longer regions containing more than one conserved region are also included. (ZIP) [file pcbi.1012009.s129.zip › H7N9-avian-raw-M2-alignment-31-69-refseq-719-757-41C_revcomp_alirna_nogap.pdf]

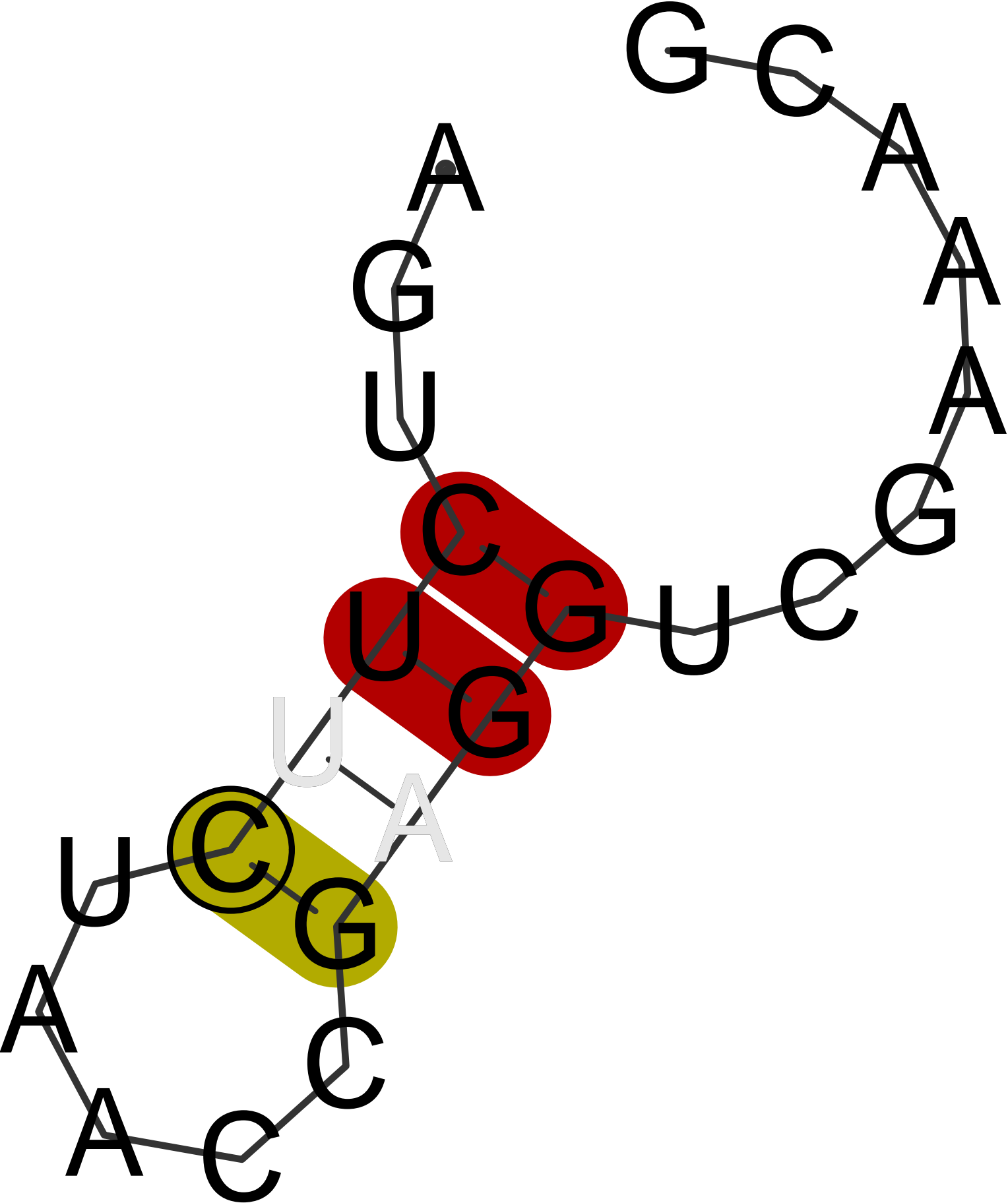

Supplement: S8 Fig — See the caption for S1 Fig for a description of the filename convention (save that the corresponding nucleotide locations in reference sequences are listed in S9 and S17 Tables), and an explanation of the RNAalifold options used and output (save that for these avian-origin viruses the folding temperature was set to 41°C). As some of the constrained regions predicted in the analysis of the M2 gene using raw (unranked) codon variability values are very close together, alignments of longer regions containing more than one conserved region are also included. (ZIP) [file pcbi.1012009.s129.zip › H7N9-avian-raw-M2-alignment-4-27-refseq-4-26-715-41C_alirna_nogap.pdf]

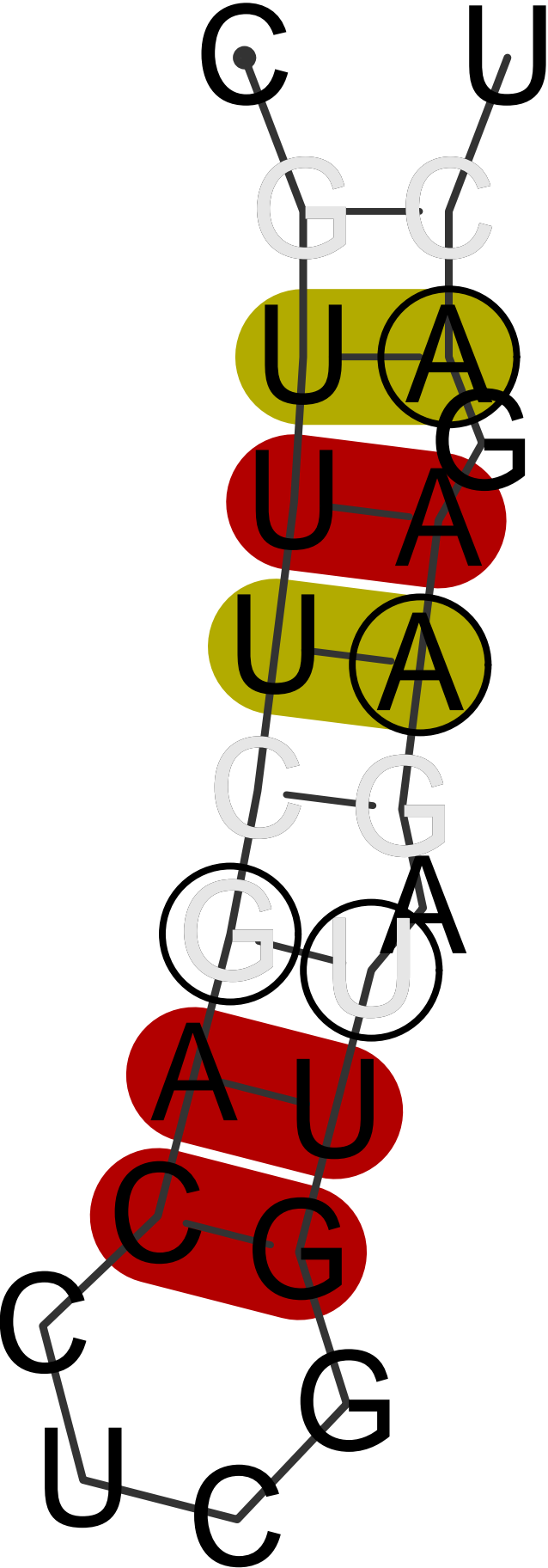

Supplement: S8 Fig — See the caption for S1 Fig for a description of the filename convention (save that the corresponding nucleotide locations in reference sequences are listed in S9 and S17 Tables), and an explanation of the RNAalifold options used and output (save that for these avian-origin viruses the folding temperature was set to 41°C). As some of the constrained regions predicted in the analysis of the M2 gene using raw (unranked) codon variability values are very close together, alignments of longer regions containing more than one conserved region are also included. (ZIP) [file pcbi.1012009.s129.zip › H7N9-avian-raw-M2-alignment-4-27-refseq-4-26-715-41C_revcomp_alirna_nogap.pdf]

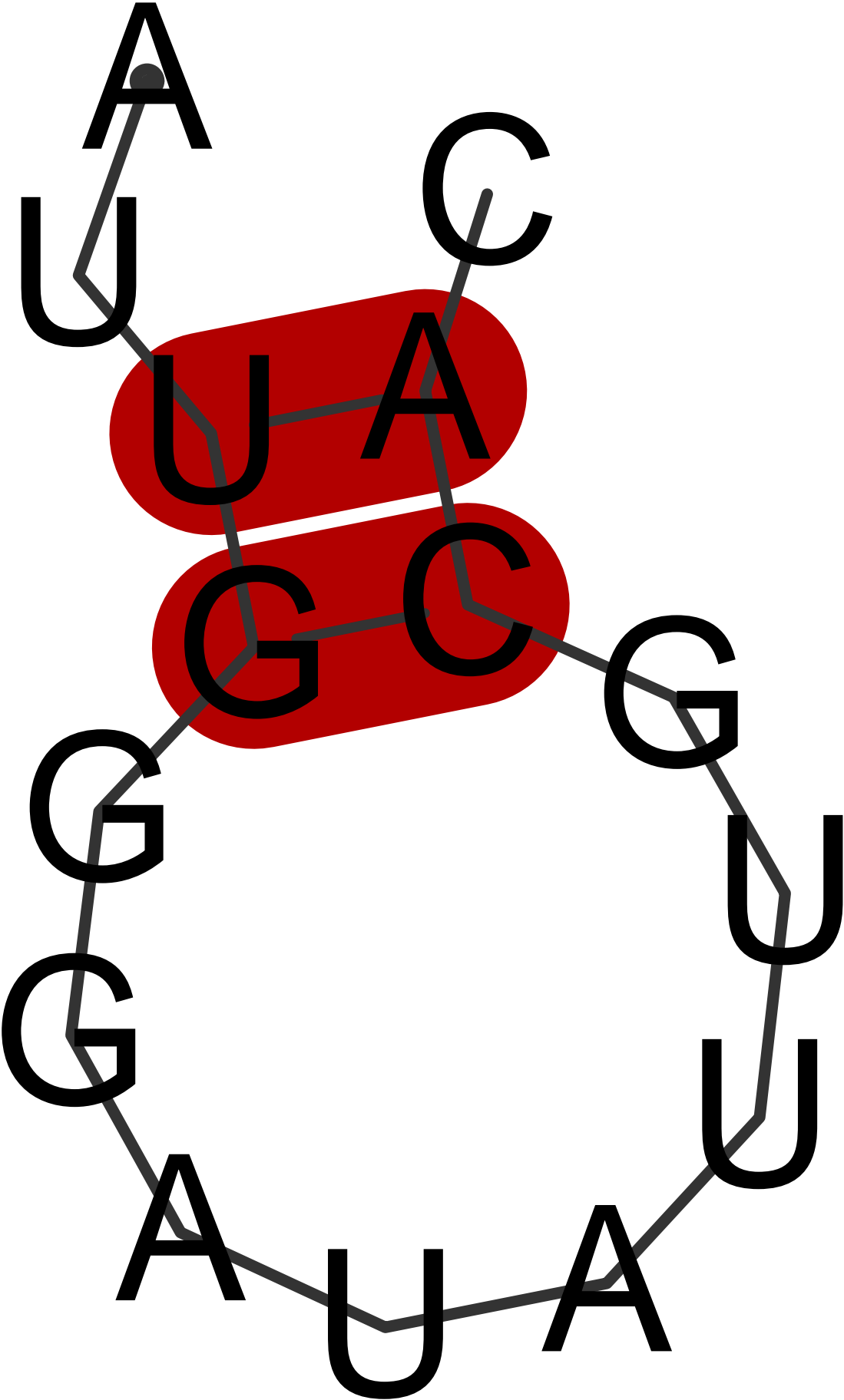

Supplement: S8 Fig — See the caption for S1 Fig for a description of the filename convention (save that the corresponding nucleotide locations in reference sequences are listed in S9 and S17 Tables), and an explanation of the RNAalifold options used and output (save that for these avian-origin viruses the folding temperature was set to 41°C). As some of the constrained regions predicted in the analysis of the M2 gene using raw (unranked) codon variability values are very close together, alignments of longer regions containing more than one conserved region are also included. (ZIP) [file pcbi.1012009.s129.zip › H7N9-avian-raw-M2-alignment-97-111-refseq-785-799-41C_alirna_nogap.pdf]

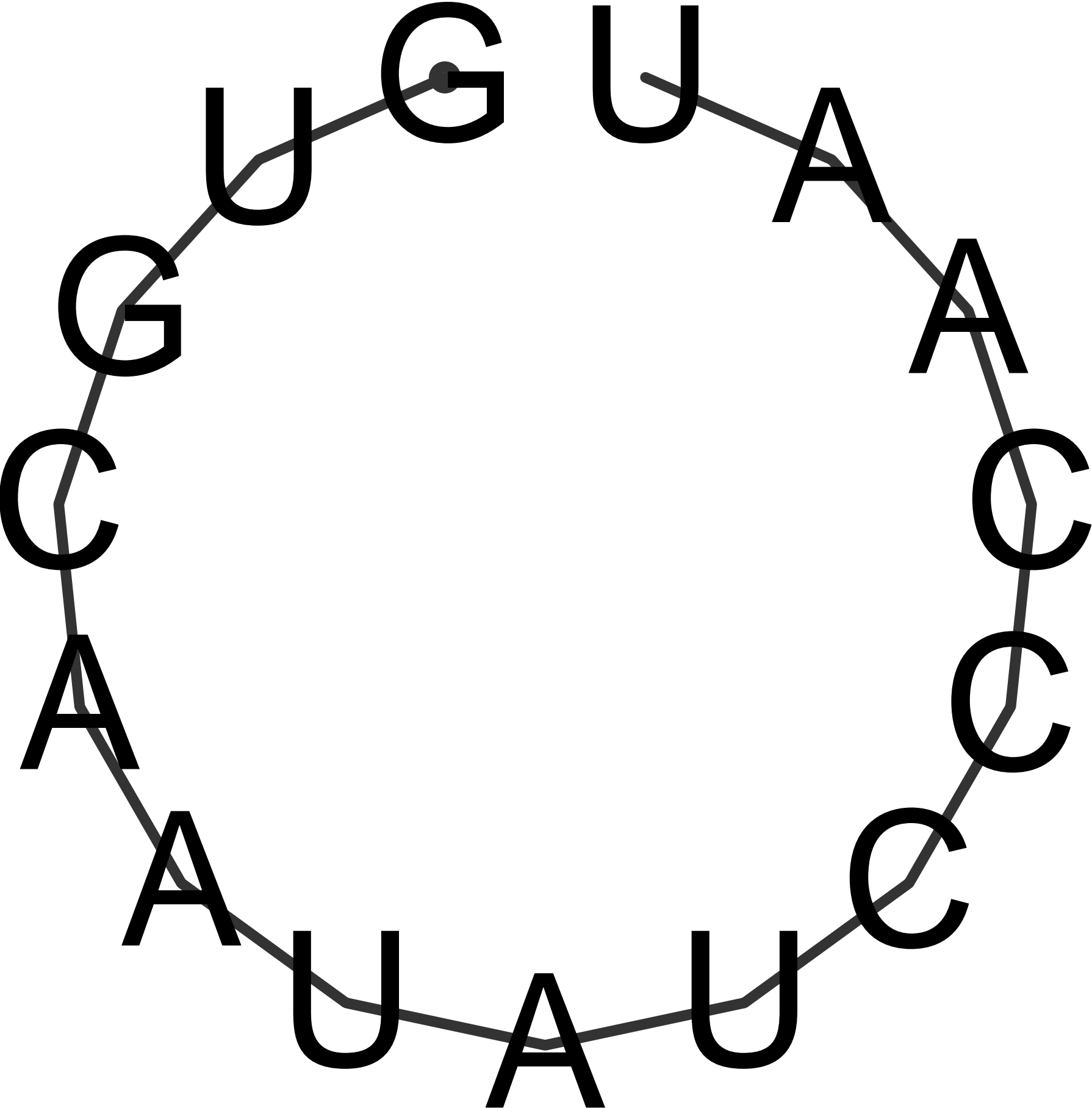

Supplement: S8 Fig — See the caption for S1 Fig for a description of the filename convention (save that the corresponding nucleotide locations in reference sequences are listed in S9 and S17 Tables), and an explanation of the RNAalifold options used and output (save that for these avian-origin viruses the folding temperature was set to 41°C). As some of the constrained regions predicted in the analysis of the M2 gene using raw (unranked) codon variability values are very close together, alignments of longer regions containing more than one conserved region are also included. (ZIP) [file pcbi.1012009.s129.zip › H7N9-avian-raw-M2-alignment-97-111-refseq-785-799-41C_revcomp_alirna_nogap.pdf]

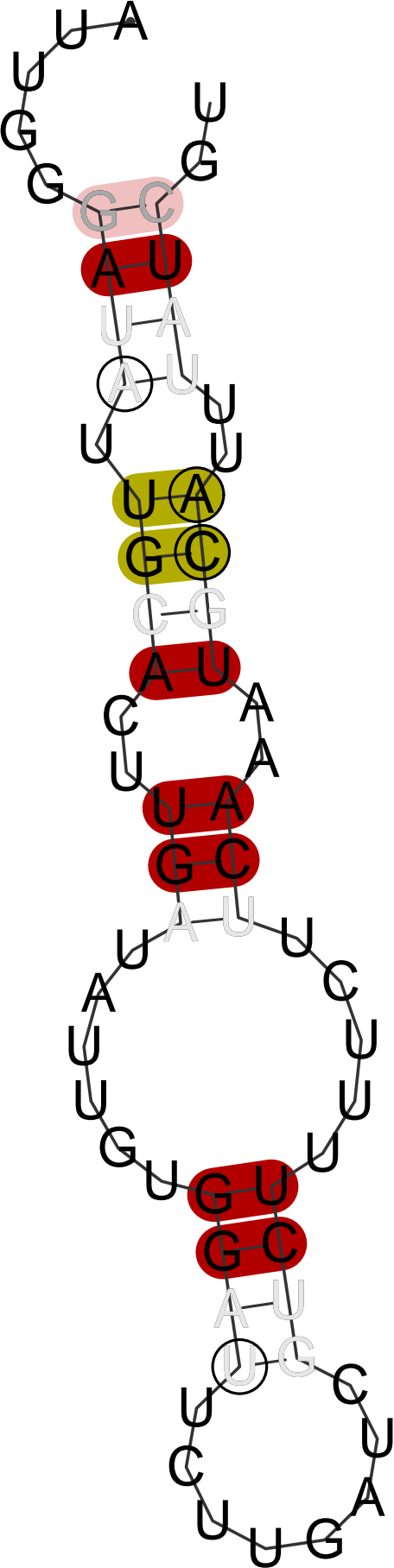

Supplement: S8 Fig — See the caption for S1 Fig for a description of the filename convention (save that the corresponding nucleotide locations in reference sequences are listed in S9 and S17 Tables), and an explanation of the RNAalifold options used and output (save that for these avian-origin viruses the folding temperature was set to 41°C). As some of the constrained regions predicted in the analysis of the M2 gene using raw (unranked) codon variability values are very close together, alignments of longer regions containing more than one conserved region are also included. (ZIP) [file pcbi.1012009.s129.zip › H7N9-avian-raw-M2-alignment-97-159-refseq-785-847-41C_alirna_nogap.pdf]

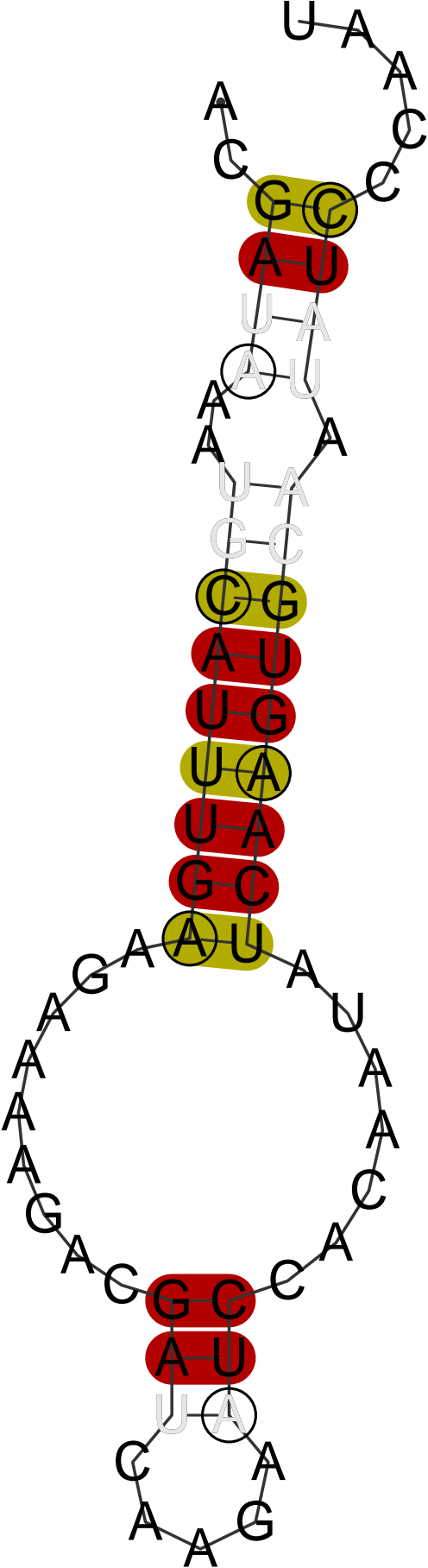

Supplement: S8 Fig — See the caption for S1 Fig for a description of the filename convention (save that the corresponding nucleotide locations in reference sequences are listed in S9 and S17 Tables), and an explanation of the RNAalifold options used and output (save that for these avian-origin viruses the folding temperature was set to 41°C). As some of the constrained regions predicted in the analysis of the M2 gene using raw (unranked) codon variability values are very close together, alignments of longer regions containing more than one conserved region are also included. (ZIP) [file pcbi.1012009.s129.zip › H7N9-avian-raw-M2-alignment-97-159-refseq-785-847-41C_revcomp_alirna_nogap.pdf]
