# Supplementary material for: Locations and structures of influenza A virus packaging-associated signals and other functional elements via an in silico pipeline for predicting constrained features in RNA viruses
Source: PLoS Comput Biol. 2024 Apr 22;20(4):e1012009. doi: 10.1371/journal.pcbi.1012009 (PMC11034665; doi:10.1371/journal.pcbi.1012009)
Supplement: S1 Code — The notebook contains, for each gene analysed: A histogram of amino acid lengths of analysed sequences;A plot of Shannon information versus the score (raw codon variability values) at each codon in the gene;An example sequence from the gene with nucleotide loci found to be constrained highlighted (in orange if found to be constrained without needing to adjust for potentially interfering regions, in blue if only found after adjusting for potentially interfering regions);A codon-level report of nucleotide usage (with observed and expected values for usage, mean distance, normalised ratio, weighted normalised ratio, weighted normalised ratio after all ratios across the gene are scaled to a mean of zero and variance of one, and ranking of the codon after weighting and scaling, with the scaled values highlighted in orange or blue if in constrained regions, similarly to the example sequence highlighting);A plot showing the proportion of sequences at each codon where the observed amino acid produced is the most common one;Plots showing the weighted constraint scores for each codon for the raw (unranked) and ranked analyses, plus plots of cumulative sums of these scores (again with codons in constrained regions highlighted orange or blue, similarly to the example sequence highlighting, and with codons with zero weight plotted with open triangles and with paler colours). The zip file contains a copy of the notebook in Mathematica format and in pdf. (ZIP) [file S1_code.pdf › S1_code.pdf]

H1N1pdm09 human hosts

PB2

Gene length histogram

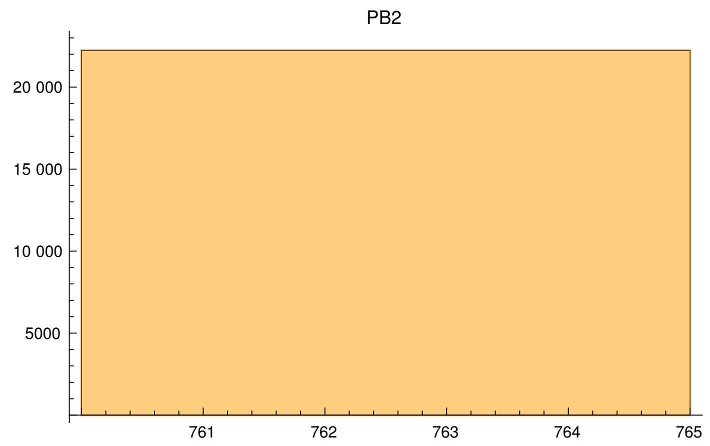

Information vs. nPD

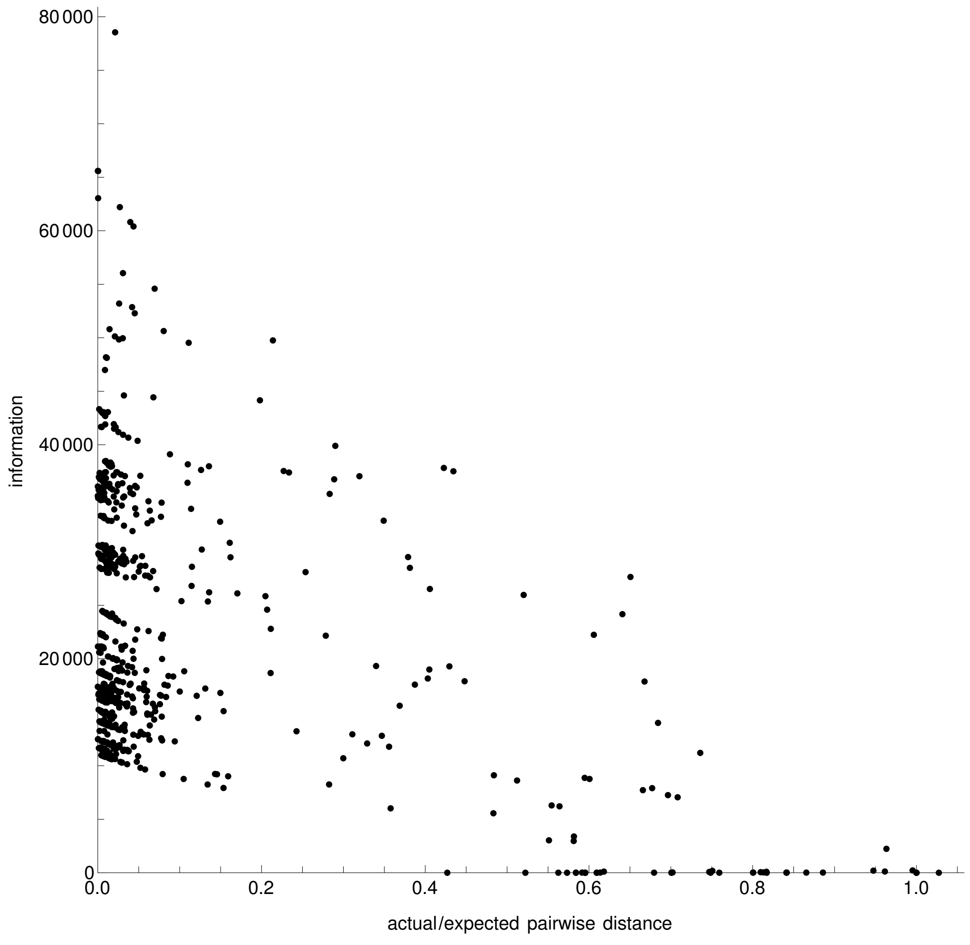

Example sequences highlighted by regions found in analyses to be conserved

Interesting points (by weighted raw PD) highlighted for gene PB2:

ATGGAGAGAATAAAAGAGCTGAGAGATCTAATGTCGCAGTCCCGCACTCGCGAGATACTCACTAAGACCACTGTGG⋱

ACCATATGGCCATCATCAAAAAGTACACATCGGGAAGGCAAGAGAAGAACCCCGCGCTCAGAATGAAGTGGATGA⋱

TGGCAATGAAATACCCAATTACGGCAGACAAGAGAATAATGGACATAATCCCAGAGAGGAATGAACAAGGACAAA⋱

CCCTCTGGAGCAAAATAAACGATGCTGGATCAGACCGAGTGATGGTATCACCTCTGGCAGTAACATGGTGGAATA⋱

GGAATGGTCCAACAACAAGTACAGTTCATTACCCTAAGGTATATAAAACTTATTTCGAAAAGGTCGAAAGGTTGA⋱

AACATGGTACCTTCGGCCCTGTCCACTTCAGAAATCAAGTTAAAATAAGGAGGAGAGTTGATACAAACCCTGGCC⋱

ATGCAGATCTCAGTGCCAAGGAGGCACAGGATGTGATTATGGAAGTTGTTTTCCCAAATGAAGTGGGGGCAAGAA⋱

TACTGACATCAGAGTCACAGCTGGCAATAACAAAAGAGAAGAAAGAAGAGCTCCAGAATTGTAAAATTGCTCCCT⋱

TGATGGTGGCGTACATGCTAGAAAGAGAATTGGTCCGTAAAACAAGGTTTCTCCCGGTAGCCGGTGGGACAAGCA⋱

GTGTTTATATTGAGGTGTTGCACTTGACCCAAGGGACTTGCTGGGAGCAGATGTACACTCCAGGAGGAGAAGTGA⋱

GAAATGATGATGTTGACCAAAGCTTGATTATCGCTGCTAGAAACATAGTAAGAAGAGCAGCAGTGTCAGCAGACC⋱

CATTAGCATCTCTCTTGGAAATGTGCCACAGCACACAGATTGGAGGTGTGAAGATGGTGGACATCCTTAAACAGA⋱

ATCCAACTGAGGAGCAAGCCGTAGACATATGCAAGGCAGCAATAGGGTTGAGGATCAGCTCATCTTTCAGTTTTG⋱

GTGGGTTCACTTTCAAAAGGACAAGCGGATCATCAGTCAAGAAAGAAGAAGAAATGCTAACGGGCAACCTCCAAA⋱

CACTGAAATTAAGAGTACATGAAGGGTATGAAGAATTCACAATGGTTGGGAGAAGAGCAACAGCTATTCTCAGAA⋱

AGGCAACCAGGAGATTGATCCAATTAATAGTAAGCGGGAGAGACGAGCAGTCAATAGCTGAAGCAATAATTGTGG⋱

CCATGGTATTCTCACAAGAGGATTGCATGATCAAAGCAGTTAGGGGCGATCTGAACTTTGTCAATAGGGCAAACC⋱

AGAGACTGAATCCCATGCACCAACTCTTGAGGCATTTCCAAAAAGATGCAAAAGTGCTTTTCCAGAACTGGGGAA⋱

TTGAAACCATCGACAATGTGATGGGAATGATCGGAATACTGCCCGACATGACCCCAAGTACGGAGATGTCACTGA⋱

GAGGAATAAGAGTCAGCAAGATGGGAGTAGATGAATACTCCAGCACGGAGAGAGTGGTAGTGAGTATTGACCGAT⋱

TTTTGAGGGTTAGAGATCAAAGAGGAAACGTACTGTTGTCTCCCGAAGAAGTTAGTGAAACGCAAGGAACTGAGA⋱

AGTTAACAATAACTTATTCGTCATCAATGATGTGGGAGATCAACGGCCCTGAGTCAGTGCTAGTCAACACTTATC⋱

AATGGATAATCAGAAACTGGGAAATTGTGAAAATTCAATGGTCACAAGACCCCACAATGTTATACAACAAAATGG⋱

AATTTGAACCATTTCAGTCTCTTGTTCCTAAGGCAACCAGAAGCCGGTACAGTGGATTCGTAAGGACACTGTTCC⋱

AGCAAATGAGGGATGTGCTTGGGACATTTGACACTGTTCAAATAATAAAACTTCTCCCCTTTGCTGCTGCTCCAC⋱

CAGAACAGAGCAGGATGCAATTTTCTTCATTGACTGTGAATGTGAGAGGATCAGGGTTAAGGATACTGGTAAGAG⋱

GCAATTCTCCAGTATTCAATTACAACAAGGCAACCAAACGACTTACGATTCTTGGAAAGGATGCAGGTGCATTGA⋱

CTGAAGATCCAGATGAAGGCACATCTGGGGTGGAGTCTGCTGTCCTGAGAGGATTCCTCATTTTAGGCAAAGAAG⋱

ACAAGAGATATGGCCCAGCATTAAGCATCAATGAACTGAGCAATCTTGCAAAAGGAGAGAAAGCTAATGTGCTAA⋱

TTGGGCAGGGGGACATAGTGTTAGTAATGAAACGAAAACGGGACTCTAGCATACTTACTGACAGCCAGACAGCGA⋱

CCAAAAGAATTCGGATGGCCATCAATTAG

Interesting points (by weighted ranked PD) highlighted for gene PB2:

ATGGAGAGAATAAAAGAGCTGAGAGATCTAATGTCGCAGTCC

CGCACTCGCGAGATACTCACTAAGACCACTGTGGACCATATGGCCATCATCAAAAAGTACACATCGGGA⋱

AGGCAAGAGAAGAACCCCGCGCTCAGAATGAAGTGGATGATGGCAATGAAATACCCAATTACGGCAGACA⋱

AGAGAATAATGGACATAATCCCAGAGAGGAATGAACAAGGACAAACCCTCTGGAGCAAAATAAACGATGC⋱

TGGATCAGACCGAGTGATGGTATCACCTCTGGCAGTAACATGGTGGAATAGGAATGGTCCAACAACAAGT⋱

ACAGTTCATTACCCTAAGGTATATAAAACTTATTTCGAAAAGGTCGAAAGGTTGAAACATGGTACCTTCG⋱

GCCCTGTCCACTTCAGAAATCAAGTTAAAATAAGGAGGAGAGTTGATACAAACCCTGGCCATGCAGATCT⋱

CAGTGCCAAGGAGGCACAGGATGTGATTATGGAAGTTGTTTTCCCAAATGAAGTGGGGGCAAGAATACTG⋱

ACATCAGAGTCACAGCTGGCAATAACAAAAGAGAAGAAAGAAGAGCTCCAGAATTGTAAAATTGCTCCCT⋱

TGATGGTGGCGTACATGCTAGAAAGAGAATTGGTCCGTAAAACAAGGTTTCTCCCGGTAGCCGGTGGGAC⋱

AAGCAGTGTTTATATTGAGGTGTTGCACTTGACCCAAGGGACTTGCTGGGAGCAGATGTACACTCCAGGA⋱
GGAGAAGTGAGAAATGATGATGTTGACCAAAGCTTGATTATCGCTGCTAGAAACATAGTAAGAAGAGCAG⋱
CAGTGTCAGCAGACCCATTAGCATCTCTCTTGGAAATGTGCCACAGCACACAGATTGGAGGTGTGAAGAT⋱
GGTGGACATCCTTAAACAGAATCCAACTGAGGAGCAAGCCGTAGACATATGCAAGGCAGCAATAGGGTTG⋱
AGGATCAGCTCATCTTTCAGTTTTGGTGGGTTCACTTTCAAAAGGACAAGCGGATCATCAGTCAAGAAAG⋱
AAGAAGAAATGCTAACGGGCAACCTCCAAACACTGAAATTAAGAGTACATGAAGGGTATGAAGAATTCAC⋱
AATGGTTGGGAGAAGAGCAACAGCTATTCTCAGAAAGGCAACCAGGAGATTGATCCAATTAATAGTAAGC⋱
GGGAGAGACGAGCAGTCAATAGCTGAAGCAATAATTGTGGCCATGGTATTCTCACAAGAGGATTGCATGA⋱
TCAAAGCAGTTAGGGGCGATCTGAACTTTGTCAATAGGGCAAACCAGAGACTGAATCCCATGCACCAACT⋱
CTTGAGGCATTTCCAAAAAGATGCAAAAGTGCTTTTCCAGAACTGGGGAATTGAAACCATCGACAATGTG⋱
ATGGGAATGATCGGAATACTGCCCGACATGACCCCAAGTACGGAGATGTCACTGAGAGGAATAAGAGTCA⋱
GCAAGATGGGAGTAGATGAATACTCCAGCACGGAGAGAGTGGTAGTGAGTATTGACCGATTTTTGAGGGT⋱
TAGAGATCAAAGAGGAAACGTACTGTTGTCTCCCGAAGAAGTTAGTGAAACGCAAGGAACTGAGAAGTTA⋱
ACAATAACTTATTCGTCATCAATGATGTGGGAGATCAACGGCCCTGAGTCAGTGCTAGTCAACACTTATC⋱
AATGGATAATCAGAAACTGGGAAATTGTGAAAATTCAATGGTCACAAGACCCCACAATGTTATACAACAA⋱
AATGGAATTTGAACCATTTCAGTCTCTTGTTCCTAAGGCAACCAGAAGCCGGTACAGTGGATTCGTAAGG⋱
ACACTGTTCCAGCAAATGAGGGATGTGCTTGGGACATTTGACACTGTTCAAATAATAAAACTTCTCCCCT⋱
TTGCTGCTGCTCCACCAGAACAGAGCAGGATGCAATTTTCTTCATTGACTGTGAATGTGAGAGGATCAGG⋱
GTTAAGGATACTGGTAAGAGGCAATTCTCCAGTATTCAATTACAACAAGGCAACCAAACGACTTACGATT⋱
CTTGGAAAGGATGCAGGTGCATTGACTGAAGATCCAGATGAAGGCACATCTGGGGTGGAGTCTGCTGTCC⋱
TGAGAGGATTCCTCATTTTAGGCAAAGAAGACAAGAGATATGGCCCAGCATTAAGCATCAATGAACTGAG⋱
CAATCTTGCAAAAGGAGAGAAAGCTAATGTGCTAATTGGGCAGGGGGACATAGTGTTAGTAATGAAACGA⋱
AAACGGGAC
TCTAGCATACTTACTGACAGCCAGACAGCGACCAAAAGAATTCGGATGGCCATCAATTAG

Per codon conservation report

PB2

Pos.	1	obs:	exp:
atg	M	22232	22232.00
---	--	------	------
mPD		0	0
		nPD:	1.
		N. weight:	0.
		Sc. PD:	0
		Sc. rank:	0

PB2

Pos.	2	obs:	exp:
aaa	K	0	0.57
aag	K	1	0.43
gat	D	2	1.05
gac	D	0	0.95
gaa	E	66	13460.00
gag	E	22163	8769.00
---	--	------	------
mPD		0.0062	0.48
		nPD:	0.01
		N. weight:	0.92
		Sc. PD:	-0.032
		Sc. rank:	-1463.1

PB2

Pos.	3	obs:	exp:
cgt	R	0	606.80
cgc	R	0	1162.00
cga	R	0	2177.00
cgg	R	0	1626.00
aaa	K	8	4.56
aag	K	0	3.44
aga	R	22094	11156.00
agg	R	128	5494.00
gaa	E	0	1.21
gag	E	2	0.79
---	--	------	------
mPD		0.013	0.91
		nPD:	0.01
		N. weight:	0.66
		Sc. PD:	-0.022
		Sc. rank:	-434.3

PB2

Pos.	4	obs:	exp:
att	I	0	8083.00
atc	I	0	5543.00
ata	I	22204	8579.00
atg	M	1	1.00
act	T	0	1.01
acc	T	0	0.82
aca	T	4	1.83
acg	T	0	0.34
gtt	V	0	5.08
gtc	V	0	4.18
gta	V	23	6.19
gtg	V	0	7.55
---	--	------	------
mPD		0.0025	0.66
		nPD:	0.
		N. weight:	0.96
		Sc. PD:	-0.042
		Sc. rank:	-13275.0

PB2

Pos.	5	obs:	exp:
cgt	R	0	0.57
cgc	R	0	1.10
cga	R	0	2.06
cgg	R	0	1.54
aaa	K	21325	12670.00
aag	K	886	9541.00
aga	R	21	10.54
agg	R	0	5.19
---	--	------	------
mPD		0.078	0.49
		nPD:	0.16
		N. weight:	0.41
		Sc. PD:	0.045
		Sc. rank:	6679.8

PB2

Pos.	6	obs:	exp:
aaa	K	1	5.70
aag	K	9	4.30
gtt	V	0	0.22
gtc	V	0	0.18
gta	V	1	0.27
gtg	V	0	0.33
gct	A	0	0.26
gcc	A	0	0.21
gca	A	0	0.47
gcg	A	1	0.06
gat	D	4	2.11
gac	D	0	1.89
gaa	E	4670	13449.00
gag	E	17541	8762.00
ggt	G	0	0.76
ggc	G	0	0.74
gga	G	4	2.22
ggg	G	1	1.28
---	--	------	------
mPD		0.33	0.48
		nPD:	0.7
		N. weight:	0.33
		Sc. PD:	0.21
		Sc. rank:	6256.0

PB2

Pos.	7	obs:	exp:
tta	L	1	2444.00
ttg	L	451	4185.00
ctt	L	9	4381.00
ctc	L	0	3140.00
cta	L	124	4280.00
ctg	L	21646	3801.00
atg	M	1	1.00
---	--	------	------
mPD		0.052	1.1
		nPD:	0.05
		N. weight:	1.6
		Sc. PD:	-0.0032
		Sc. rank:	18531.0

PB2

Pos.	8	obs:	exp:
cgt	R	0	606.90
cgc	R	0	1163.00
cga	R	0	2177.00
cgg	R	11	1627.00
att	I	0	0.36
atc	I	0	0.25
ata	I	1	0.39
aaa	K	2	1.14
aag	K	0	0.86
aga	R	22204	11160.00
agg	R	14	5495.00
---	--	------	------
mPD		0.0035	0.91
		nPD:	0.
		N. weight:	0.69
		Sc. PD:	-0.03
		Sc. rank:	-9481.5

PB2

Pos.	9	obs:	exp:
tat	Y	2	1.04
tac	Y	0	0.96
aat	N	98	58.55
aac	N	1	40.45
gat	D	21790	11654.00
gac	D	329	10465.00
gaa	E	0	1.21
gag	E	2	0.79
ggt	G	10	1.52
ggc	G	0	1.48
gga	G	0	4.45
ggg	G	0	2.55
---	--	------	------
mPD		0.039	0.51
		nPD:	0.08
		N. weight:	0.57
		Sc. PD:	0.017
		Sc. rank:	8091.3

PB2

Pos.	10	obs:	exp:
tta	L	20	2444.00
ttg	L	2	4185.00
ctt	L	2	4380.00
ctc	L	1	3140.00
cta	L	22153	4279.00
ctg	L	51	3801.00
caa	Q	1	1.06
cag	Q	1	0.94
att	I	0	0.36
atc	I	0	0.25
ata	I	1	0.39
---	--	------	------
mPD		0.0074	1.1
		nPD:	0.01
		N. weight:	1.6
		Sc. PD:	-0.067
		Sc. rank:	-15109.0

PB2

Pos.	11	obs:	exp:
att	I	0	0.73
atc	I	0	0.50
ata	I	2	0.77
atg	M	22228	22228.00
gtt	V	0	0.44
gtc	V	0	0.36
gta	V	0	0.54
gtg	V	2	0.66
---	--	------	------
mPD		0.00036	0.00048
		nPD:	0.75
		N. weight:	0.00019
		Sc. PD:	0.00013
		Sc. rank:	3.6

PB2

Pos.	12	obs:	exp:
tta	L	0	1.87
ttg	L	17	3.20
tct	S	8	4087.00
tcc	S	2	2573.00
tca	S	794	5781.00
tcg	S	21407	1182.00
tgg	W	2	2.00
ctt	L	0	3.35
ctc	L	0	2.40
cta	L	0	3.27
ctg	L	0	2.91
agt	S	0	4176.00
agc	S	0	4412.00
gct	A	0	0.53
gcc	A	0	0.41
gca	A	0	0.94
gcg	A	2	0.12
---	--	------	------
mPD		0.072	1.6
		nPD:	0.04
		N. weight:	2.7
		Sc. PD:	-0.011
		Sc. rank:	29823.0

PB2

Pos.	13	obs:	exp:
tta	L	0	0.22
ttg	L	0	0.38
ctt	L	0	0.39
ctc	L	0	0.28
cta	L	0	0.39
ctg	L	2	0.34
cat	H	5	2.94
cac	H	0	2.06
caa	Q	118	11724.00
cag	Q	22100	10494.00
cgt	R	0	0.05
cgc	R	0	0.10
cga	R	1	0.20
cgg	R	1	0.15
aaa	K	0	2.85
aag	K	5	2.15
aga	R	0	1.00
agg	R	0	0.49
---	--	------	------
mPD		0.012	0.50
		nPD:	0.02
		N. weight:	0.72
		Sc. PD:	-0.017
		Sc. rank:	3991.9

PB2

Pos.	14	obs:	exp:
ttt	F	0	1.72
ttc	F	4	2.28
tct	S	142	4088.00
tcc	S	22071	2573.00
tca	S	0	5781.00
tcg	S	0	1182.00
cct	P	0	1.38
ccc	P	5	0.93
cca	P	0	2.13
ccg	P	0	0.57
act	T	0	2.52
acc	T	10	2.05
aca	T	0	4.57
acg	T	0	0.85
agt	S	0	4177.00
agc	S	0	4412.00
---	--	------	------
mPD		0.014	1.6
		nPD:	0.01
		N. weight:	2.1
		Sc. PD:	-0.082
		Sc. rank:	-13655.0

PB2

Pos.	15	obs:	exp:
tgt	C	0	0.45
tgc	C	1	0.55
cgt	R	0	607.00
cgc	R	22231	1163.00
cga	R	0	2177.00
cgg	R	0	1627.00
aga	R	0	11161.00
agg	R	0	5496.00
---	--	------	------
mPD		0.000090	0.91
		nPD:	0.
		N. weight:	3.
		Sc. PD:	-0.14
		Sc. rank:	-55848.0

PB2

Pos.	16	obs :	exp :
tct	S	0	0.18
tcc	S	0	0.12
tca	S	0	0.26
tcg	S	0	0.05
att	I	2	0.73
atc	I	0	0.50
ata	I	0	0.77
act	T	22143	5604.00
acc	T	79	4559.00
aca	T	7	10169.00
acg	T	0	1897.00
agt	S	1	0.19
agc	S	0	0.20
---	--	------	------
mPD		0.0080	0.68
		nPD :	0.01
		N. weight :	1.4
		Sc. PD :	-0.049
		Sc. rank :	-3411.4

,

PB2

Pos.	17	obs :	exp :
tct	S	0	0.18
tcc	S	0	0.12
tca	S	0	0.26
tcg	S	0	0.05
tgt	C	0	0.90
tgc	C	2	1.10
cgt	R	1	606.90
cgc	R	22228	1163.00
cga	R	0	2177.00
cgg	R	0	1627.00
agt	S	0	0.19
agc	S	1	0.20
aga	R	0	11160.00
agg	R	0	5495.00
---	--	------	------
mPD		0.00036	0.91
		nPD :	0.
		N. weight :	3.
		Sc. PD :	-0.14
		Sc. rank :	-55078.0

,

PB2

Pos.	18	obs :	exp :
gaa	E	9	13461.00
gag	E	22223	8771.00
---	--	------	------
mPD		0.00081	0.48
		nPD :	0.
		N. weight :	0.94
		Sc. PD :	-0.043
		Sc. rank :	-16015.0

,

PB2

Pos.	19	obs :	exp :
att	I	0	8089.00
atc	I	1	5547.00
ata	I	22220	8585.00
atg	M	1	1.00
act	T	0	1.01
acc	T	0	0.82
aca	T	4	1.83
acg	T	0	0.34
gtt	V	0	1.33
gtc	V	0	1.09
gta	V	6	1.61
gtg	V	0	1.97
---	--	------	------
mPD		0.0011	0.66
		nPD :	0.
		N. weight :	0.96
		Sc. PD :	-0.044
		Sc. rank :	-16477.0

,

PB2

Pos.	20	obs :	exp :
ttt	F	0	0.43
ttc	F	1	0.57
tta	L	0	2443.00
ttg	L	0	4184.00
ctt	L	26	4380.00
ctc	L	22178	3139.00
cta	L	9	4279.00
ctg	L	13	3800.00
cct	P	0	0.28
ccc	P	1	0.19
cca	P	0	0.43
ccg	P	0	0.11
att	I	0	1.46
atc	I	4	1.00
ata	I	0	1.55
---	--	------	------
mPD		0.0049	1.1
		nPD :	0.
		N. weight :	2.
		Sc. PD :	-0.084
		Sc. rank :	-25358.0

,

PB2

Pos.	21	obs :	exp :
att	I	15	5.46
atc	I	0	3.74
ata	I	0	5.80
act	T	21918	5592.00
acc	T	245	4550.00
aca	T	15	10148.00
acg	T	4	1893.00
aat	N	8	4.73
aac	N	0	3.27
gct	A	10	7.15
gcc	A	17	5.59
gca	A	0	12.68
gcg	A	0	1.58
---	--	------	------
mPD		0.029	0.68
		nPD :	0.04
		N. weight :	1.3
		Sc. PD :	-0.0057
		Sc. rank :	14172.0

,

PB2

Pos.	22	obs :	exp :
caa	Q	0	0.53
cag	Q	1	0.47
cgt	R	0	0.22
cgc	R	0	0.42
cga	R	0	0.78
cgg	R	0	0.59
aaa	K	158	12675.00
aag	K	22062	9545.00
aga	R	0	4.02
agg	R	8	1.98
gaa	E	0	1.82
gag	E	3	1.18
---	--	------	------
mPD		0.015	0.49
		nPD :	0.03
		N. weight :	0.81
		Sc. PD :	-0.013
		Sc. rank :	6512.8

,

PB2

Pos.	23	obs :	exp :
att	I	0	2.55
atc	I	7	1.75
ata	I	0	2.70
act	T	9	5603.00
acc	T	22207	4559.00
aca	T	9	10167.00
acg	T	0	1896.00
---	--	------	------
mPD		0.0022	0.68
		nPD :	0.
		N. weight :	1.6
		Sc. PD :	-0.07
		Sc. rank :	-22934.0

,

PB2

Pos.	24	obs :	exp :
att	I	1	0.36
atc	I	0	0.25
ata	I	0	0.39
act	T	22145	5604.00
acc	T	48	4560.00
aca	T	32	10170.00
acg	T	5	1897.00
aat	N	1	0.59
aac	N	0	0.41
---	--	------	------
mPD		0.0078	0.68
		nPD :	0.01
		N. weight :	1.4
		Sc. PD :	-0.049
		Sc. rank :	-3960.0

,

PB2

Pos.	25	obs :	exp :
gtt	V	31	4915.00
gtc	V	0	4040.00
gta	V	26	5979.00
gtg	V	22174	7298.00
gct	A	0	0.26
gcc	A	0	0.21
gca	A	0	0.47
gcg	A	1	0.06
---	--	------	------
mPD		0.0052	0.74
		nPD :	0.01
		N. weight :	1.1
		Sc. PD :	-0.045
		Sc. rank :	-9407.5

,

PB2

Pos.	26	obs :	exp :
gat	D	83	11714.00
gac	D	22149	10518.00
---	--	------	------
mPD		0.0074	0.50
		nPD :	0.01
		N. weight :	0.73
		Sc. PD :	-0.024
		Sc. rank :	155.3

,

PB2

Pos.	27	obs :	exp :
cat	H	22170	13074.00
cac	H	62	9158.00
---	--	------	------
mPD		0.0056	0.48
		nPD :	0.01
		N. weight :	0.52
		Sc. PD :	-0.019
		Sc. rank :	-1589.5

,

PB2

Pos.	28	obs :	exp :
att	I	0	0.36
atc	I	0	0.25
ata	I	1	0.39
atg	M	22231	22231.00
---	--	------	------
mPD		0.000090	0.000090
		nPD :	1.
		N. weight :	0.000043
		Sc. PD :	0.000041
		Sc. rank :	0.8

,

PB2

Pos.	29	obs :	exp :
tct	S	0	0.55
tcc	S	3	0.35
tca	S	0	0.78
tcg	S	0	0.16
act	T	0	0.25
acc	T	1	0.21
aca	T	0	0.46
acg	T	0	0.09
agt	S	0	0.56
agc	S	0	0.60
gct	A	18	5885.00
gcc	A	22205	4601.00
gca	A	4	10440.00
gcg	A	1	1302.00
---	--	------	------
mPD		0.0024	0.66
		nPD :	0.
		N. weight :	1.6
		Sc. PD :	-0.069
		Sc. rank :	-22061.0

,

PB2

Pos.	30	obs :	exp :
att	I	235	8090.00
atc	I	13620	5547.00
ata	I	8368	8586.00
atg	M	3	3.00
gtt	V	0	1.33
gtc	V	3	1.09
gta	V	3	1.61
gtg	V	0	1.97
---	--	------	------
mPD		0.48	0.66
		nPD :	0.74
		N. weight :	0.51
		Sc. PD :	0.35
		Sc. rank :	9679.4

,

PB2

Pos.	31	obs :	exp :
tta	L	0	0.11
ttg	L	0	0.19
ctt	L	0	0.20
ctc	L	1	0.14
cta	L	0	0.19
ctg	L	0	0.17
att	I	62	8092.00
atc	I	22131	5549.00
ata	I	37	8589.00
gtt	V	0	0.22
gtc	V	1	0.18
gta	V	0	0.27
gtg	V	0	0.33
---	--	------	------
mPD		0.0091	0.66
		nPD :	0.01
		N. weight :	1.4
		Sc. PD :	-0.046
		Sc. rank :	-1065.8

,

PB2

Pos.	32	obs :	exp :
cgt	R	0	0.11
cgc	R	0	0.21
cga	R	0	0.39
cgg	R	0	0.29
aaa	K	22205	12680.00
aag	K	23	9548.00
aga	R	4	2.01
agg	R	0	0.99
---	--	------	------
mPD		0.0024	0.49
		nPD :	0.
		N. weight :	0.56
		Sc. PD :	-0.024
		Sc. rank :	-6489.5

,

PB2

Pos.	33	obs :	exp :
cgt	R	0	1.77
cgc	R	0	3.40
cga	R	0	6.37
cgg	R	0	4.76
aat	N	1	0.59
aac	N	0	0.41
aaa	K	814	12644.00
aag	K	21352	9522.00
aga	R	0	32.63
agg	R	65	16.07
---	--	------	------
mPD		0.076	0.50
		nPD :	0.15
		N. weight :	0.69
		Sc. PD :	0.072
		Sc. rank :	11177.0

,

PB2

Pos.	34	obs :	exp :
tat	Y	10	11551.00
tac	Y	22222	10681.00
---	--	------	------
mPD		0.00090	0.50
		nPD :	0.
		N. weight :	0.74
		Sc. PD :	-0.033
		Sc. rank :	-12478.0

,

PB2

Pos.	35	obs :	exp :
act	T	2	5604.00
acc	T	1	4560.00
aca	T	22220	10171.00
acg	T	9	1897.00
---	--	------	------
mPD		0.0011	0.68
		nPD :	0.
		N. weight :	0.79
		Sc. PD :	-0.036
		Sc. rank :	-13561.0

,

PB2			
Pos.	36	obs:	exp:
tta	L	0	0.11
ttg	L	1	0.19
tct	S	20	4090.00
tcc	S	0	2574.00
tca	S	8493	5784.00
tcg	S	13713	1183.00
ctt	L	0	0.20
ctc	L	0	0.14
cta	L	0	0.19
ctg	L	0	0.17
act	T	0	1.01
acc	T	0	0.82
aca	T	2	1.83
acg	T	2	0.34
agt	S	0	4179.00
agc	S	0	4415.00
---	-	1	1.00
---	--	------	------
mPD		0.47	1.6
		nPD:	0.29
		N. weight:	1.7
		Sc. PD:	0.4
		Sc. rank:	29113.0

,

PB2			
Pos.	37	obs:	exp:
gct	A	0	21.97
gcc	A	0	17.18
gca	A	83	38.98
gcg	A	0	4.86
gaa	E	4	2.42
gag	E	0	1.58
ggt	G	4	3358.00
ggc	G	1	3280.00
gga	G	22093	9851.00
ggg	G	47	5657.00
---	--	------	------
mPD		0.012	0.70
		nPD:	0.02
		N. weight:	0.8
		Sc. PD:	-0.024
		Sc. rank:	1570.0

,

PB2			
Pos.	38	obs:	exp:
cgt	R	0	606.80
cgc	R	0	1162.00
cga	R	0	2177.00
cgg	R	0	1626.00
aaa	K	0	5.13
aag	K	9	3.87
aga	R	197	11157.00
agg	R	22026	5494.00
---	--	------	------
mPD		0.018	0.91
		nPD:	0.02
		N. weight:	1.4
		Sc. PD:	-0.037
		Sc. rank:	4577.7

,

PB2			
Pos.	39	obs:	exp:
caa	Q	22159	11730.00
cag	Q	71	10500.00
cgt	R	0	0.03
cgc	R	0	0.05
cga	R	1	0.10
cgg	R	0	0.07
aaa	K	1	0.57
aag	K	0	0.43
aga	R	0	0.50
agg	R	0	0.25
---	--	------	------
mPD		0.0065	0.50
		nPD:	0.01
		N. weight:	0.62
		Sc. PD:	-0.021
		Sc. rank:	-820.6

,

PB2			
Pos.	40	obs:	exp:
gct	A	0	0.26
gcc	A	0	0.21
gca	A	0	0.47
gcg	A	1	0.06
gaa	E	99	13461.00
gag	E	22132	8770.00
---	--	------	------
mPD		0.0090	0.48
		nPD:	0.02
		N. weight:	0.91
		Sc. PD:	-0.026
		Sc. rank:	2151.6

,

PB2			
Pos.	41	obs:	exp:
cgt	R	0	0.05
cgc	R	0	0.10
cga	R	0	0.20
cgg	R	0	0.15
aat	N	1	0.59
aac	N	0	0.41
aaa	K	140	12680.00
aag	K	22089	9549.00
aga	R	0	1.00
agg	R	2	0.49
---	--	------	------
mPD		0.013	0.49
		nPD:	0.03
		N. weight:	0.81
		Sc. PD:	-0.017
		Sc. rank:	5115.7

,

PB2			
Pos.	42	obs:	exp:
act	T	0	0.25
acc	T	1	0.21
aca	T	0	0.46
acg	T	0	0.09
aat	N	126	13148.00
aac	N	22105	9083.00
---	--	------	------
mPD		0.011	0.48
		nPD:	0.02
		N. weight:	0.87
		Sc. PD:	-0.021
		Sc. rank:	4612.1

,

PB2			
Pos.	43	obs:	exp:
tct	S	0	0.18
tcc	S	1	0.12
tca	S	0	0.26
tcg	S	0	0.05
cct	P	431	6125.00
ccc	P	21744	4123.00
cca	P	45	9466.00
ccg	P	11	2517.00
agt	S	0	0.19
agc	S	0	0.20
---	--	------	------
mPD		0.043	0.70
		nPD:	0.06
		N. weight:	1.6
		Sc. PD:	0.022
		Sc. rank:	20650.0

,

PB2			
Pos.	44	obs:	exp:
tct	S	0	3.86
tcc	S	0	2.43
tca	S	18	5.47
tcg	S	3	1.12
act	T	0	6.05
acc	T	0	4.92
aca	T	2	10.98
acg	T	22	2.05
agt	S	0	3.95
agc	S	0	4.17
gct	A	10	5874.00
gcc	A	2	4593.00
gca	A	14893	10420.00
gcg	A	7282	1300.00
---	--	------	------
mPD		0.45	0.67
		nPD:	0.67
		N. weight:	0.81
		Sc. PD:	0.5
		Sc. rank:	15355.0

,

PB2			
Pos.	45	obs:	exp:
ttt	F	0	0.43
ttc	F	1	0.57
tta	L	0	2444.00
ttg	L	0	4185.00
ctt	L	66	4381.00
ctc	L	22157	3140.00
cta	L	8	4280.00
ctg	L	0	3801.00
---	--	------	------
mPD		0.0067	1.1
		nPD:	0.01
		N. weight:	2.
		Sc. PD:	-0.081
		Sc. rank:	-19849.0

,

PB2			
Pos.	46	obs:	exp:
cgt	R	0	607.00
cgc	R	0	1163.00
cga	R	3	2177.00
cgg	R	0	1627.00
att	I	0	0.36
atc	I	0	0.25
ata	I	1	0.39
aga	R	21407	11161.00
agg	R	821	5496.00
---	--	------	------
mPD		0.071	0.91
		nPD:	0.08
		N. weight:	0.56
		Sc. PD:	0.017
		Sc. rank:	8114.9

,

PB2			
Pos.	47	obs:	exp:
att	I	0	2.91
atc	I	0	2.00
ata	I	8	3.09
atg	M	22221	22221.00
act	T	0	0.76
acc	T	0	0.62
aca	T	0	1.37
acg	T	3	0.26
---	--	------	------
mPD		0.00099	0.0012
		nPD:	0.8
		N. weight:	0.00068
		Sc. PD:	0.00051
		Sc. rank:	13.0

,

PB2			
Pos.	48	obs:	exp:
cgt	R	0	0.11
cgc	R	0	0.21
cga	R	0	0.39
cgg	R	0	0.29
aaa	K	122	12679.00
aag	K	22105	9548.00
aga	R	0	2.01
agg	R	4	0.99
gaa	E	1	0.61
gag	E	0	0.39
---	--	------	------
mPD		0.011	0.49
		nPD:	0.02
		N. weight:	0.82
		Sc. PD:	-0.02
		Sc. rank:	4279.6

,

PB2			
Pos.	49	obs:	exp:
tgg	W	22230	22230.00
cgt	R	0	0.03
cgc	R	0	0.05
cga	R	0	0.10
cgg	R	0	0.07
aga	R	0	0.50
agg	R	1	0.25
ggt	G	0	0.15
ggc	G	0	0.15
gga	G	0	0.44
ggg	G	1	0.26
---	--	------	------
mPD		0.00018	0.00031
		nPD:	0.58
		N. weight:	0.00013
		Sc. PD:	0.000067
		Sc. rank:	2.3

,

PB2			
Pos.	50	obs:	exp:
att	I	0	4.00
atc	I	0	2.75
ata	I	11	4.25
atg	M	22217	22217.00
act	T	0	0.25
acc	T	0	0.21
aca	T	0	0.46
acg	T	1	0.09
aaa	K	0	0.57
aag	K	1	0.43
gtt	V	0	0.44
gtc	V	0	0.36
gta	V	0	0.54
gtg	V	2	0.66
---	--	------	------
mPD		0.0013	0.0016
		nPD:	0.84
		N. weight:	0.00073
		Sc. PD:	0.00057
		Sc. rank:	13.8

,

PB2			
Pos.	51	obs:	exp:
att	I	0	0.73
atc	I	0	0.50
ata	I	2	0.77
atg	M	22229	22229.00
gtt	V	0	0.22
gtc	V	0	0.18
gta	V	0	0.27
gtg	V	1	0.33
---	--	------	------
mPD		0.00027	0.00033
		nPD:	0.82
		N. weight:	0.00014
		Sc. PD:	0.0001
		Sc. rank:	2.6

,

PB2			
Pos.	52	obs:	exp:
act	T	0	0.25
acc	T	0	0.21
aca	T	1	0.46
acg	T	0	0.09
gct	A	0	5886.00
gcc	A	3	4602.00
gca	A	22224	10441.00
gcg	A	4	1302.00
---	--	------	------
mPD		0.00072	0.66
		nPD:	0.
		N. weight:	0.76
		Sc. PD:	-0.035
		Sc. rank:	-13706.0

,

PB2			
Pos.	53	obs:	exp:
tta	L	0	0.11
ttg	L	0	0.19
ctt	L	0	0.20
ctc	L	0	0.14
cta	L	0	0.19
ctg	L	1	0.17
atg	M	22230	22230.00
act	T	0	0.25
acc	T	0	0.21
aca	T	0	0.46
acg	T	1	0.09
---	--	------	------
mPD		0.00018	0.00032
		nPD:	0.56
		N. weight:	0.00019
		Sc. PD:	0.000098
		Sc. rank:	3.6

,

PB2			
Pos.	54	obs:	exp:
cgt	R	0	93.79
cgc	R	0	179.70
cga	R	0	336.50
cgg	R	0	251.40
act	T	0	0.25
acc	T	0	0.21
aca	T	1	0.46
acg	T	0	0.09
aaa	K	18671	10722.00
aag	K	125	8074.00
aga	R	3411	1724.00
agg	R	24	849.20
---	--	------	------
mPD		0.27	0.83
		nPD:	0.33
		N. weight:	0.55
		Sc. PD:	0.15
		Sc. rank:	9707.4

,

PB2			
Pos.	55	obs:	exp:
tat	Y	140	11548.00
tac	Y	22086	10678.00
tgt	C	0	0.45
tgc	C	1	0.55
cat	H	0	2.94
cac	H	5	2.06
---	--	------	------
mPD		0.013	0.50
		nPD:	0.03
		N. weight:	0.7
		Sc. PD:	-0.015
		Sc. rank:	4522.9

,

PB2			
Pos.	56	obs:	exp:
tct	S	0	0.37
tcc	S	0	0.23
tca	S	2	0.52
tcg	S	0	0.11
cct	P	1	6124.00
ccc	P	9	4123.00
cca	P	22190	9465.00
ccg	P	30	2517.00
agt	S	0	0.38
agc	S	0	0.40
---	--	------	------
mPD		0.0038	0.70
		nPD:	0.01
		N. weight:	0.85
		Sc. PD:	-0.036
		Sc. rank:	-9264.7

,

PB2			
Pos.	57	obs:	exp:
tta	L	0	0.44
ttg	L	0	0.75
ctt	L	4	0.79
ctc	L	0	0.56
cta	L	0	0.77
ctg	L	0	0.68
att	I	22074	8091.00
atc	I	151	5548.00
ata	I	1	8587.00
gtt	V	2	0.44
gtc	V	0	0.36
gta	V	0	0.54
gtg	V	0	0.66
---	--	------	------
mPD		0.014	0.66
		nPD:	0.02
		N. weight:	0.98
		Sc. PD:	-0.025
		Sc. rank:	4141.1

,

PB2			
Pos.	58	obs:	exp:
tct	S	0	0.18
tcc	S	0	0.12
tca	S	0	0.26
tcg	S	1	0.05
act	T	50	5601.00
acc	T	3	4558.00
aca	T	3773	10165.00
acg	T	18394	1896.00
aaa	K	0	0.57
aag	K	1	0.43
agt	S	0	0.19
agc	S	0	0.20
gct	A	0	2.65
gcc	A	0	2.07
gca	A	2	4.70
gcg	A	8	0.59
---	--	------	------
mPD		0.29	0.68
		nPD:	0.42
		N. weight:	1.7
		Sc. PD:	0.64
		Sc. rank:	31474.0

,

PB2			
Pos.	59	obs:	exp:
gct	A	44	5884.00
gcc	A	4	4600.00
gca	A	22097	10437.00
gcg	A	78	1302.00
ggt	G	0	1.36
ggc	G	0	1.33
gga	G	9	4.00
ggg	G	0	2.30
---	--	------	------
mPD		0.012	0.66
		nPD:	0.02
		N. weight:	0.73
		Sc. PD:	-0.021
		Sc. rank:	1573.6

,

PB2			
Pos.	60	obs:	exp:
tct	S	0	0.18
tcc	S	0	0.12
tca	S	0	0.26
tcg	S	0	0.05
aat	N	0	55.60
aac	N	94	38.40
agt	S	1	0.19
agc	S	0	0.20
gct	A	0	0.26
gcc	A	1	0.21
gca	A	0	0.47
gcg	A	0	0.06
gat	D	32	11658.00
gac	D	22093	10467.00
gaa	E	9	5.45
gag	E	0	3.55
ggt	G	0	0.30
ggc	G	2	0.30
gga	G	0	0.89
ggg	G	0	0.51
---	--	------	------
mPD		0.013	0.51
		nPD:	0.02
		N. weight:	0.75
		Sc. PD:	-0.017
		Sc. rank:	4351.9

,

PB2			
Pos.	61	obs:	exp:
cgt	R	0	0.44
cgc	R	0	0.84
cga	R	0	1.57
cgg	R	0	1.17
aat	N	4	2.37
aac	N	0	1.63
aaa	K	58	12669.00
aag	K	22151	9540.00
aga	R	0	8.03
agg	R	16	3.96
gaa	E	0	0.61
gag	E	1	0.39
ggt	G	0	0.30
ggc	G	0	0.30
gga	G	0	0.89
ggg	G	2	0.51
---	--	------	------
mPD		0.0075	0.49
		nPD:	0.02
		N. weight:	0.83
		Sc. PD:	-0.027
		Sc. rank:	267.1

,

PB2			
Pos.	62	obs:	exp:
cgt	R	0	604.90
cgc	R	0	1159.00
cga	R	0	2170.00
cgg	R	0	1621.00
att	I	0	0.73
atc	I	0	0.50
ata	I	2	0.77
aaa	K	63	35.94
aag	K	0	27.06
aga	R	22029	11123.00
agg	R	126	5477.00
ggt	G	0	1.82
ggc	G	0	1.78
gga	G	12	5.34
ggg	G	0	3.07
---	--	------	------
mPD		0.018	0.92
		nPD:	0.02
		N. weight:	0.66
		Sc. PD:	-0.018
		Sc. rank:	2026.9

,

PB2			
Pos.	63	obs:	exp:
tta	L	0	0.22
ttg	L	0	0.38
ctt	L	0	0.39
ctc	L	0	0.28
cta	L	2	0.39
ctg	L	0	0.34
att	I	0	8082.00
atc	I	1	5542.00
ata	I	22200	8577.00
atg	M	4	4.00
act	T	0	2.02
acc	T	0	1.64
aca	T	8	3.66
acg	T	0	0.68
gtt	V	0	3.76
gtc	V	0	3.09
gta	V	17	4.57
gtg	V	0	5.58
---	--	------	------
mPD		0.0029	0.66
		nPD:	0.
		N. weight:	0.96
		Sc. PD:	-0.041
		Sc. rank:	-12336.0

,

PB2			
Pos.	64	obs:	exp:
tta	L	0	0.11
ttg	L	0	0.19
ctt	L	0	0.20
ctc	L	0	0.14
cta	L	0	0.19
ctg	L	1	0.17
cgt	R	0	0.14
cgc	R	0	0.26
cga	R	0	0.49
cgg	R	0	0.37
att	I	10	25.12
atc	I	1	17.22
ata	I	58	26.66
atg	M	22119	22119.00
act	T	0	7.06
acc	T	0	5.74
aca	T	10	12.81
acg	T	18	2.39
aga	R	0	2.51
agg	R	5	1.24
gtt	V	0	2.21
gtc	V	0	1.82
gta	V	0	2.69
gtg	V	10	3.28
---	--	------	------
mPD		0.011	0.014
		nPD:	0.82
		N. weight:	0.0042
		Sc. PD:	0.0032
		Sc. rank:	79.2

,

PB2			
Pos.	65	obs:	exp:
tat	Y	0	0.52
tac	Y	1	0.48
aat	N	0	402.20
aac	N	680	277.80
gtt	V	0	0.22
gtc	V	1	0.18
gta	V	0	0.27
gtg	V	0	0.33
gct	A	0	0.26
gcc	A	1	0.21
gca	A	0	0.47
gcg	A	0	0.06
gat	D	32	11321.00
gac	D	21454	10165.00
gaa	E	25	15.14
gag	E	0	9.86
ggt	G	0	5.76
ggc	G	38	5.63
gga	G	0	16.90
ggg	G	0	9.71
---	--	------	------
mPD		0.068	0.56
		nPD:	0.12
		N. weight:	0.75
		Sc. PD:	0.055
		Sc. rank:	11743.0

,

PB2			
Pos.	66	obs:	exp:
tta	L	0	0.11
ttg	L	0	0.19
tct	S	0	0.18
tcc	S	0	0.12
tca	S	0	0.26
tcg	S	0	0.05
ctt	L	0	0.20
ctc	L	0	0.14
cta	L	1	0.19
ctg	L	0	0.17
att	I	2	6707.00
atc	I	1	4599.00
ata	I	18421	7118.00
atg	M	3685	3685.00
act	T	0	28.49
acc	T	0	23.18
aca	T	109	51.69
acg	T	4	9.64
agt	S	0	0.19
agc	S	1	0.20
gtt	V	0	1.77
gtc	V	0	1.45
gta	V	6	2.15
gtg	V	2	2.63
---	--	------	------
mPD		0.29	0.74
		nPD:	0.39
		N. weight:	0.8
		Sc. PD:	0.27
		Sc. rank:	14442.0

,

PB2			
Pos.	67	obs:	exp:
ttt	F	0	0.43
ttc	F	1	0.57
att	I	3717	8084.00
atc	I	18475	5543.00
ata	I	15	8580.00
atg	M	1	1.00
act	T	0	0.25
acc	T	1	0.21
aca	T	0	0.46
acg	T	0	0.09
gtt	V	15	4.86
gtc	V	7	4.00
gta	V	0	5.92
gtg	V	0	7.22
---	--	------	------
mPD		0.28	0.66
		nPD:	0.43
		N. weight:	0.88
		Sc. PD:	0.33
		Sc. rank:	16085.0

,

PB2			
Pos.	68	obs:	exp:
cct	P	7	6125.00
ccc	P	2	4123.00
cca	P	22201	9466.00
ccg	P	22	2517.00
---	--	------	------
mPD		0.0028	0.70
		nPD:	0.
		N. weight:	0.85
		Sc. PD:	-0.037
		Sc. rank:	-11493.0

,

PB2			
Pos.	69	obs:	exp:
aaa	K	0	1.14
aag	K	2	0.86
gtt	V	0	0.22
gtc	V	0	0.18
gta	V	0	0.27
gtg	V	1	0.33
gct	A	0	0.26
gcc	A	0	0.21
gca	A	0	0.47
gcg	A	1	0.06
gat	D	3	1.58
gac	D	0	1.42
gaa	E	90	13455.00
gag	E	22132	8767.00
ggt	G	0	0.45
ggc	G	0	0.44
gga	G	0	1.33
ggg	G	3	0.77
---	--	------	------
mPD		0.0090	0.48
		nPD:	0.02
		N. weight:	0.91
		Sc. PD:	-0.026
		Sc. rank:	2115.8

,

PB2			
Pos.	70	obs:	exp:
tct	S	0	0.18
tcc	S	0	0.12
tca	S	0	0.26
tcg	S	0	0.05
cgt	R	0	606.60
cgc	R	0	1162.00
cga	R	0	2176.00
cgg	R	0	1626.00
atg	M	2	2.00
aaa	K	0	5.70
aag	K	10	4.30
agt	S	1	0.19
agc	S	0	0.20
aga	R	86	11154.00
agg	R	22132	5493.00
ggt	G	0	0.15
ggc	G	0	0.15
gga	G	0	0.44
ggg	G	1	0.26
---	--	------	------
mPD		0.0090	0.91
		nPD:	0.01
		N. weight:	1.4
		Sc. PD:	-0.052
		Sc. rank:	-6494.2

,

PB2			
Pos.	71	obs:	exp:
tct	S	0	0.18
tcc	S	0	0.12
tca	S	0	0.26
tcg	S	0	0.05
aat	N	22181	13148.00
aac	N	49	9082.00
agt	S	1	0.19
agc	S	0	0.20
gat	D	1	0.53
gac	D	0	0.47
---	--	------	------
mPD		0.0046	0.48
		nPD:	0.01
		N. weight:	0.52
		Sc. PD:	-0.02
		Sc. rank:	-2765.5

,

PB2			
Pos.	72	obs:	exp:
caa	Q	1	0.53
cag	Q	0	0.47
aaa	K	1	0.57
aag	K	0	0.43
gat	D	0	0.53
gac	D	1	0.47
gaa	E	22074	13458.00
gag	E	152	8768.00
ggt	G	0	0.45
ggc	G	0	0.44
gga	G	3	1.33
ggg	G	0	0.77
---	--	------	------
mPD		0.014	0.48
		nPD:	0.03
		N. weight:	0.47
		Sc. PD:	-0.0084
		Sc. rank:	3439.7

,

PB2			
Pos.	73	obs:	exp:
tta	L	0	0.11
ttg	L	0	0.19
ctt	L	0	0.20
ctc	L	0	0.14
cta	L	1	0.19
ctg	L	0	0.17
cat	H	11	7.65
cac	H	2	5.35
caa	Q	22183	11723.00
cag	Q	33	10493.00
aaa	K	2	1.14
aag	K	0	0.86
---	--	------	------
mPD		0.0044	0.50
		nPD:	0.01
		N. weight:	0.63
		Sc. PD:	-0.024
		Sc. rank:	-3976.9

,

PB2			
Pos.	74	obs:	exp:
cgt	R	0	0.22
cgc	R	0	0.42
cga	R	0	0.78
cgg	R	0	0.59
aga	R	8	4.02
agg	R	0	1.98
gaa	E	15	9.08
gag	E	0	5.92
ggt	G	0	3368.00
ggc	G	0	3289.00
gga	G	22082	9879.00
ggg	G	127	5673.00
---	--	------	------
mPD		0.013	0.69
		nPD:	0.02
		N. weight:	0.79
		Sc. PD:	-0.022
		Sc. rank:	2161.5

,

PB2			
Pos.	75	obs:	exp:
tta	L	0	0.11
ttg	L	0	0.19
ctt	L	0	0.20
ctc	L	0	0.14
cta	L	1	0.19
ctg	L	0	0.17
cat	H	1	1.76
cac	H	2	1.24
caa	Q	22187	11729.00
cag	Q	40	10498.00
aaa	K	1	0.57
aag	K	0	0.43
---	--	------	------
mPD		0.0040	0.50
		nPD:	0.01
		N. weight:	0.63
		Sc. PD:	-0.025
		Sc. rank:	-4451.7

,

PB2			
Pos.	76	obs:	exp:
tct	S	0	0.55
tcc	S	1	0.35
tca	S	0	0.78
tcg	S	0	0.16
cct	P	0	1.10
ccc	P	4	0.74
cca	P	0	1.70
ccg	P	0	0.45
att	I	0	10.19
atc	I	28	6.99
ata	I	0	10.82
act	T	406	5584.00
acc	T	21728	4544.00
aca	T	18	10134.00
acg	T	0	1890.00
aat	N	4	7.10
aac	N	8	4.90
agt	S	1	0.56
agc	S	1	0.60
gct	A	0	8.74
gcc	A	33	6.83
gca	A	0	15.50
gcg	A	0	1.93
---	--	------	------
mPD		0.045	0.69
		nPD:	0.07
		N. weight:	1.5
		Sc. PD:	0.027
		Sc. rank:	20126.0

,

PB2			
Pos.	77	obs:	exp:
ttt	F	0	0.86
ttc	F	2	1.14
tta	L	1	2443.00
ttg	L	0	4185.00
ctt	L	46	4380.00
ctc	L	22125	3140.00
cta	L	52	4279.00
ctg	L	3	3800.00
att	I	0	1.09
atc	I	3	0.75
ata	I	0	1.16
---	--	------	------
mPD		0.0097	1.1
		nPD:	0.01
		N. weight:	1.9
		Sc. PD:	-0.075
		Sc. rank:	-12878.0

,

PB2			
Pos.	78	obs:	exp:
tgg	W	22231	22231.00
cgt	R	0	0.03
cgc	R	0	0.05
cga	R	0	0.10
cgg	R	1	0.07
aga	R	0	0.50
agg	R	0	0.25
---	--	------	------
mPD		0.000090	0.00015
		nPD:	0.6
		N. weight:	0.00012
		Sc. PD:	0.000065
		Sc. rank:	2.2

,

PB2			
Pos.	79	obs:	exp:
tct	S	0	4087.00
tcc	S	0	2572.00
tca	S	0	5780.00
tcg	S	0	1182.00
att	I	0	0.73
atc	I	2	0.50
ata	I	0	0.77
aat	N	0	8.87
aac	N	15	6.13
agt	S	33	4176.00
agc	S	22175	4411.00
ggt	G	0	1.06
ggc	G	7	1.04
gga	G	0	3.11
ggg	G	0	1.79
---	--	------	------
mPD		0.0051	1.6
		nPD:	0.
		N. weight:	1.6
		Sc. PD:	-0.072
		Sc. rank:	-24128.0

,

PB2			
Pos.	80	obs:	exp:
caa	Q	1	0.53
cag	Q	0	0.47
cgt	R	0	0.41
cgc	R	0	0.78
cga	R	0	1.47
cgg	R	0	1.10
aaa	K	22192	12672.00
aag	K	23	9543.00
aga	R	15	7.53
agg	R	0	3.71
gaa	E	1	0.61
gag	E	0	0.39
---	--	------	------
mPD		0.0036	0.49
		nPD:	0.01
		N. weight:	0.56
		Sc. PD:	-0.022
		Sc. rank:	-4513.0

,

PB2			
Pos.	81	obs:	exp:
att	I	0	2428.00
atc	I	0	1665.00
ata	I	6671	2577.00
atg	M	734	734.00
act	T	0	3734.00
acc	T	0	3038.00
aca	T	14805	6776.00
acg	T	6	1264.00
gtt	V	0	0.88
gtc	V	0	0.73
gta	V	4	1.08
gtg	V	0	1.31
gct	A	0	3.18
gcc	A	0	2.48
gca	A	12	5.64
gcg	A	0	0.70
---	--	------	------
mPD		0.51	1.1
		nPD:	0.45
		N. weight:	0.81
		Sc. PD:	0.32
		Sc. rank:	15030.0

,

PB2			
Pos.	82	obs:	exp:
tct	S	0	14.91
tcc	S	0	9.38
tca	S	0	21.08
tcg	S	0	4.31
act	T	0	1.01
acc	T	4	0.82
aca	T	0	1.83
acg	T	0	0.34
aat	N	87	13085.00
aac	N	22037	9039.00
aaa	K	18	10.27
aag	K	0	7.73
agt	S	10	15.23
agc	S	71	16.09
gat	D	0	2.63
gac	D	5	2.37
---	--	------	------
mPD		0.018	0.50
		nPD:	0.04
		N. weight:	0.88
		Sc. PD:	-0.0093
		Sc. rank:	8371.5

,

PB2			
Pos.	83	obs:	exp:
aat	N	1	0.59
aac	N	0	0.41
gat	D	22217	11713.00
gac	D	14	10518.00
---	--	------	------
mPD		0.0013	0.50
		nPD:	0.
		N. weight:	0.64
		Sc. PD:	-0.029
		Sc. rank:	-9899.0

,

PB2			
Pos.	84	obs:	exp:
act	T	2	0.50
acc	T	0	0.41
aca	T	0	0.91
acg	T	0	0.17
gtt	V	1	0.22
gtc	V	0	0.18
gta	V	0	0.27
gtg	V	0	0.33
gct	A	22200	5885.00
gcc	A	21	4601.00
gca	A	4	10440.00
gcg	A	3	1302.00
ggt	G	0	0.15
ggc	G	0	0.15
gga	G	1	0.44
ggg	G	0	0.26
---	--	------	------
mPD		0.0030	0.66
		nPD:	0.
		N. weight:	1.3
		Sc. PD:	-0.057
		Sc. rank:	-16837.0

,

PB2			
Pos.	85	obs:	exp:
gtt	V	0	0.22
gtc	V	0	0.18
gta	V	1	0.27
gtg	V	0	0.33
gaa	E	1	0.61
gag	E	0	0.39
ggt	G	2	3371.00
ggc	G	0	3293.00
gga	G	22013	9888.00
ggg	G	215	5678.00
---	--	------	------
mPD		0.020	0.69
		nPD:	0.03
		N. weight:	0.77
		Sc. PD:	-0.015
		Sc. rank:	5352.1

,

PB2			
Pos.	86	obs:	exp:
tct	S	2	4091.00
tcc	S	11	2575.00
tca	S	22207	5785.00
tcg	S	10	1183.00
act	T	0	0.50
acc	T	0	0.41
aca	T	2	0.91
acg	T	0	0.17
agt	S	0	4180.00
agc	S	0	4416.00
---	--	------	------
mPD		0.0022	1.6
		nPD:	0.
		N. weight:	1.4
		Sc. PD:	-0.062
		Sc. rank:	-23751.0

,

PB2			
Pos.	87	obs:	exp:
aat	N	0	18.93
aac	N	32	13.07
gct	A	0	0.26
gcc	A	1	0.21
gca	A	0	0.47
gcg	A	0	0.06
gat	D	142	11690.00
gac	D	22045	10497.00
gaa	E	7	4.24
gag	E	0	2.76
ggt	G	0	0.76
ggc	G	5	0.74
gga	G	0	2.22
ggg	G	0	1.28
---	--	------	------
mPD		0.017	0.50
		nPD:	0.03
		N. weight:	0.72
		Sc. PD:	-0.01
		Sc. rank:	6438.3

,

PB2			
Pos.	88	obs:	exp:
cgt	R	6	607.00
cgc	R	1	1163.00
cga	R	22020	2178.00
cgg	R	84	1627.00
aga	R	121	11161.00
agg	R	0	5496.00
---	--	------	------
mPD		0.019	0.91
		nPD:	0.02
		N. weight:	2.3
		Sc. PD:	-0.06
		Sc. rank:	8447.9

,

PB2			
Pos.	89	obs:	exp:
tta	L	0	0.77
ttg	L	6	1.32
ctt	L	0	1.38
ctc	L	0	0.99
cta	L	0	1.35
ctg	L	1	1.20
att	I	0	0.36
atc	I	0	0.25
ata	I	1	0.39
atg	M	65	65.00
gtt	V	5	4899.00
gtc	V	1	4026.00
gta	V	63	5959.00
gtg	V	22088	7273.00
gct	A	0	0.26
gcc	A	0	0.21
gca	A	0	0.47
gcg	A	1	0.06
ggt	G	0	0.15
ggc	G	0	0.15
gga	G	0	0.44
ggg	G	1	0.26
---	--	------	------
mPD		0.013	0.74
		nPD:	0.02
		N. weight:	1.1
		Sc. PD:	-0.033
		Sc. rank:	1926.3

,

PB2			
Pos.	90	obs:	exp:
tta	L	0	0.33
ttg	L	2	0.56
ctt	L	0	0.59
ctc	L	0	0.42
cta	L	0	0.58
ctg	L	1	0.51
att	I	2	4.37
atc	I	0	3.00
ata	I	10	4.64
atg	M	22200	22200.00
act	T	0	0.25
acc	T	0	0.21
aca	T	0	0.46
acg	T	1	0.09
gtt	V	0	3.54
gtc	V	0	2.91
gta	V	0	4.30
gtg	V	16	5.25
---	--	------	------
mPD		0.0029	0.0041
		nPD:	0.7
		N. weight:	0.0014
		Sc. PD:	0.00093
		Sc. rank:	27.3

,

PB2			
Pos.	91	obs:	exp:
att	I	0	7.28
atc	I	0	4.99
ata	I	20	7.73
gtt	V	4	4910.00
gtc	V	5	4035.00
gta	V	22129	5973.00
gtg	V	70	7290.00
gct	A	0	1.06
gcc	A	0	0.83
gca	A	4	1.88
gcg	A	0	0.23
---	--	------	------
mPD		0.0092	0.74
		nPD:	0.01
		N. weight:	1.3
		Sc. PD:	-0.045
		Sc. rank:	-2456.7

,

PB2			
Pos.	92	obs:	exp:
tct	S	0	4091.00
tcc	S	2	2575.00
tca	S	22220	5786.00
tcg	S	9	1183.00
cct	P	0	0.28
ccc	P	0	0.19
cca	P	1	0.43
ccg	P	0	0.11
agt	S	0	4180.00
agc	S	0	4416.00
---	--	------	------
mPD		0.0011	1.6
		nPD:	0.
		N. weight:	1.4
		Sc. PD:	-0.063
		Sc. rank:	-24670.0

,

PB2			
Pos.	93	obs:	exp:
tta	L	0	0.44
ttg	L	0	0.75
tct	S	17	3.13
tcc	S	0	1.97
tca	S	0	4.42
tcg	S	0	0.90
ctt	L	4	0.79
ctc	L	0	0.56
cta	L	0	0.77
ctg	L	0	0.68
cct	P	22172	6119.00
ccc	P	34	4119.00
cca	P	3	9457.00
ccg	P	1	2515.00
act	T	1	0.25
acc	T	0	0.21
aca	T	0	0.46
acg	T	0	0.09
agt	S	0	3.20
agc	S	0	3.38
---	--	------	------
mPD		0.0054	0.70
		nPD:	0.01
		N. weight:	1.3
		Sc. PD:	-0.051
		Sc. rank:	-9681.6

,

PB2			
Pos.	94	obs:	exp:
tta	L	0	2443.00
ttg	L	109	4184.00
ctt	L	50	4379.00
ctc	L	0	3139.00
cta	L	119	4278.00
ctg	L	21943	3799.00
caa	Q	0	0.53
cag	Q	1	0.47
atg	M	10	10.00
---	--	------	------
mPD		0.026	1.1
		nPD:	0.02
		N. weight:	1.7
		Sc. PD:	-0.042
		Sc. rank:	7894.0

,

PB2			
Pos.	95	obs:	exp:
tct	S	0	0.18
tcc	S	1	0.12
tca	S	0	0.26
tcg	S	0	0.05
act	T	0	0.25
acc	T	1	0.21
aca	T	0	0.46
acg	T	0	0.09
agt	S	0	0.19
agc	S	0	0.20
gct	A	94	5885.00
gcc	A	14604	4602.00
gca	A	7528	10441.00
gcg	A	4	1302.00
---	--	------	------
mPD		0.45	0.66
		nPD:	0.68
		N. weight:	0.64
		Sc. PD:	0.4
		Sc. rank:	12065.0

,

PB2			
Pos.	96	obs:	exp:
att	I	0	40.77
atc	I	0	27.96
ata	I	112	43.27
gtt	V	1	4890.00
gtc	V	1	4019.00
gta	V	22072	5949.00
gtg	V	46	7261.00
---	--	------	------
mPD		0.014	0.75
		nPD:	0.02
		N. weight:	1.3
		Sc. PD:	-0.037
		Sc. rank:	3401.8

,

PB2			
Pos.	97	obs:	exp:
cgt	R	0	0.03
cgc	R	0	0.05
cga	R	0	0.10
cgg	R	0	0.07
att	I	0	0.36
atc	I	0	0.25
ata	I	1	0.39
act	T	0	5604.00
acc	T	1	4560.00
aca	T	22208	10170.00
acg	T	21	1897.00
aga	R	1	0.50
agg	R	0	0.25
---	--	------	------
mPD		0.0022	0.68
		nPD:	0.
		N. weight:	0.78
		Sc. PD:	-0.035
		Sc. rank:	-11510.0

,

PB2			
Pos.	98	obs:	exp:
tgg	W	22231	22231.00
ggt	G	0	0.15
ggc	G	0	0.15
gga	G	0	0.44
ggg	G	1	0.26
---	--	------	------
mPD		0.000090	0.00016
		nPD:	0.57
		N. weight:	0.000062
		Sc. PD:	0.000032
		Sc. rank:	1.2

,

PB2			
Pos.	99	obs:	exp:
tgg	W	22231	22231.00
cgt	R	0	0.03
cgc	R	0	0.05
cga	R	0	0.10
cgg	R	0	0.07
aga	R	0	0.50
agg	R	1	0.25
---	--	------	------
mPD		0.000090	0.00015
		nPD:	0.6
		N. weight:	0.000063
		Sc. PD:	0.000035
		Sc. rank:	1.2

,

PB2			
Pos.	100	obs:	exp:
cat	H	1	0.59
cac	H	0	0.41
aat	N	22209	13147.00
aac	N	20	9082.00
aaa	K	1	0.57
aag	K	0	0.43
gat	D	1	0.53
gac	D	0	0.47
---	--	------	------
mPD		0.0021	0.48
		nPD:	0.
		N. weight:	0.52
		Sc. PD:	-0.023
		Sc. rank:	-6765.6

,

PB2

Pos.	101	obs:	exp:
cgt	R	0	606.90
cgc	R	0	1163.00
cga	R	0	2177.00
cgg	R	0	1627.00
aga	R	630	11159.00
agg	R	21597	5495.00
ggt	G	0	0.76
ggc	G	0	0.74
gga	G	1	2.22
ggg	G	4	1.28
---	--	------	------
mPD		0.056	0.91
		nPD:	0.06
		N. weight:	1.3
		Sc. PD:	0.017
		Sc. rank:	16373.0

,

PB2

Pos.	102	obs:	exp:
tct	S	0	11.59
tcc	S	0	7.30
tca	S	0	16.40
tcg	S	0	3.35
tat	Y	2	1.04
tac	Y	0	0.96
cat	H	2	1.18
cac	H	0	0.82
act	T	2	0.50
acc	T	0	0.41
aca	T	0	0.91
acg	T	0	0.17
aat	N	22124	13098.00
aac	N	22	9048.00
aaa	K	10	6.85
aag	K	2	5.15
agt	S	63	11.85
agc	S	0	12.51
gat	D	5	2.63
gac	D	0	2.37
---	--	------	------
mPD		0.0097	0.49
		nPD:	0.02
		N. weight:	0.53
		Sc. PD:	-0.015
		Sc. rank:	1568.3

,

PB2

Pos.	103	obs:	exp:
gct	A	0	0.53
gcc	A	2	0.41
gca	A	0	0.94
gcg	A	0	0.12
ggt	G	7495	3371.00
ggc	G	14681	3293.00
gga	G	37	9888.00
ggg	G	17	5678.00
---	--	------	------
mPD		0.45	0.69
		nPD:	0.65
		N. weight:	1.3
		Sc. PD:	0.75
		Sc. rank:	23677.0

,

PB2

Pos.	104	obs:	exp:
tta	L	0	0.99
ttg	L	0	1.69
tct	S	0	1.10
tcc	S	0	0.69
tca	S	6	1.56
tcg	S	0	0.32
ctt	L	0	1.77
ctc	L	0	1.27
cta	L	8	1.73
ctg	L	1	1.54
cct	P	3	6120.00
ccc	P	10	4120.00
cca	P	22191	9458.00
ccg	P	9	2515.00
caa	Q	1	0.53
cag	Q	0	0.47
act	T	0	0.50
acc	T	0	0.41
aca	T	2	0.91
acg	T	0	0.17
agt	S	0	1.13
agc	S	0	1.19
gct	A	0	0.26
gcc	A	0	0.21
gca	A	1	0.47
gcg	A	0	0.06
---	--	------	------
mPD		0.0038	0.70
		nPD:	0.01
		N. weight:	0.85
		Sc. PD:	-0.036
		Sc. rank:	-9352.1

,

PB2

Pos.	105	obs:	exp:
cct	P	0	0.83
ccc	P	0	0.56
cca	P	3	1.28
ccg	P	0	0.34
cgt	R	0	0.03
cgc	R	0	0.05
cga	R	0	0.10
cgg	R	0	0.07
att	I	0	2.18
atc	I	0	1.50
ata	I	6	2.32
act	T	0	5591.00
acc	T	0	4549.00
aca	T	22166	10146.00
acg	T	13	1892.00
aaa	K	13	7.42
aag	K	0	5.58
aga	R	1	0.50
agg	R	0	0.25
gtt	V	0	2.21
gtc	V	0	1.82
gta	V	1	2.69
gtg	V	9	3.28
gct	A	0	5.30
gcc	A	0	4.14
gca	A	20	9.39
gcg	A	0	1.17
---	--	------	------
mPD		0.0076	0.68
		nPD:	0.01
		N. weight:	0.79
		Sc. PD:	-0.028
		Sc. rank:	-2558.5

,

PB2

Pos.	106	obs:	exp:
tct	S	0	2.76
tcc	S	0	1.74
tca	S	15	3.90
tcg	S	0	0.80
act	T	2	5595.00
acc	T	0	4552.00
aca	T	22186	10153.00
acg	T	6	1894.00
agt	S	0	2.82
agc	S	0	2.98
gct	A	0	6.09
gcc	A	0	4.76
gca	A	23	10.80
gcg	A	0	1.35
---	--	------	------
mPD		0.0041	0.68
		nPD:	0.01
		N. weight:	0.79
		Sc. PD:	-0.032
		Sc. rank:	-7825.1

,

PB2

Pos.	107	obs:	exp:
tct	S	0	3974.00
tcc	S	0	2501.00
tca	S	0	5620.00
tcg	S	0	1149.00
tgt	C	3	1.35
tgc	C	0	1.65
cgt	R	0	0.03
cgc	R	0	0.05
cga	R	0	0.10
cgg	R	0	0.07
act	T	3	0.76
acc	T	0	0.62
aca	T	0	1.37
acg	T	0	0.26
aat	N	614	363.10
aac	N	0	250.90
agt	S	21414	4061.00
agc	S	182	4290.00
aga	R	0	0.50
agg	R	1	0.25
gat	D	2	1.05
gac	D	0	0.95
ggt	G	13	1.97
ggc	G	0	1.93
gga	G	0	5.78
ggg	G	0	3.32
---	--	------	------
mPD		0.072	1.7
		nPD:	0.04
		N. weight:	1.6
		Sc. PD:	-0.0072
		Sc. rank:	17041.0

,

PB2

Pos.	108	obs:	exp:
tct	S	0	0.18
tcc	S	0	0.12
tca	S	1	0.26
tcg	S	0	0.05
att	I	0	1.09
atc	I	0	0.75
ata	I	3	1.16
act	T	6	5590.00
acc	T	5	4549.00
aca	T	22149	10145.00
acg	T	16	1892.00
agt	S	0	0.19
agc	S	0	0.20
gct	A	0	13.77
gcc	A	0	10.76
gca	A	52	24.42
gcg	A	0	3.05
---	--	------	------
mPD		0.0075	0.68
		nPD:	0.01
		N. weight:	0.78
		Sc. PD:	-0.028
		Sc. rank:	-2861.8

,

PB2

Pos.	109	obs:	exp:
ttt	F	9	3.86
ttc	F	0	5.14
att	I	19	6.92
atc	I	0	4.74
ata	I	0	7.34
gtt	V	22093	4908.00
gtc	V	103	4034.00
gta	V	1	5970.00
gtg	V	1	7287.00
gct	A	5	1.32
gcc	A	0	1.03
gca	A	0	2.35
gcg	A	0	0.29
gat	D	1	0.53
gac	D	0	0.47
---	--	------	------
mPD		0.012	0.74
		nPD:	0.02
		N. weight:	1.5
		Sc. PD:	-0.046
		Sc. rank:	2111.6

,

PB2

Pos.	110	obs:	exp:
tta	L	0	0.11
ttg	L	0	0.19
tat	Y	1	0.52
tac	Y	0	0.48
ctt	L	1	0.20
ctc	L	0	0.14
cta	L	0	0.19
ctg	L	0	0.17
cat	H	22202	13071.00
cac	H	25	9156.00
aat	N	3	1.77
aac	N	0	1.23
---	--	------	------
mPD		0.0027	0.48
		nPD:	0.01
		N. weight:	0.53
		Sc. PD:	-0.022
		Sc. rank:	-5585.5

,

PB2

Pos.	111	obs:	exp:
tat	Y	29	11551.00
tac	Y	22202	10680.00
tgt	C	0	0.45
tgc	C	1	0.55
---	--	------	------
mPD		0.0027	0.50
		nPD:	0.01
		N. weight:	0.73
		Sc. PD:	-0.031
		Sc. rank:	-8043.6

,

PB2

Pos.	112	obs:	exp:
tta	L	0	0.88
ttg	L	0	1.51
tct	S	15	2.76
tcc	S	0	1.74
tca	S	0	3.90
tcg	S	0	0.80
ctt	L	8	1.58
ctc	L	0	1.13
cta	L	0	1.54
ctg	L	0	1.37
cct	P	22149	6115.00
ccc	P	28	4117.00
cca	P	15	9451.00
ccg	P	4	2513.00
cat	H	11	6.47
cac	H	0	4.53
act	T	1	0.25
acc	T	0	0.21
aca	T	0	0.46
acg	T	0	0.09
aat	N	1	0.59
aac	N	0	0.41
agt	S	0	2.82
agc	S	0	2.98
---	--	------	------
mPD		0.0075	0.70
		nPD:	0.01
		N. weight:	1.3
		Sc. PD:	-0.047
		Sc. rank:	-4862.2

,

PB2

Pos.	113	obs:	exp:
aat	N	4	2.37
aac	N	0	1.63
aaa	K	307	12680.00
aag	K	21921	9548.00
---	--	------	------
mPD		0.028	0.49
		nPD:	0.06
		N. weight:	0.78
		Sc. PD:	0.0067
		Sc. rank:	9646.7

,

PB2

Pos.	114	obs:	exp:
att	I	0	4.37
atc	I	0	3.00
ata	I	12	4.64
gtt	V	38	4911.00
gtc	V	1	4036.00
gta	V	21948	5974.00
gtg	V	226	7292.00
gct	A	0	1.85
gcc	A	0	1.45
gca	A	7	3.29
gcg	A	0	0.41
---	--	------	------
mPD		0.025	0.74
		nPD:	0.03
		N. weight:	1.3
		Sc. PD:	-0.017
		Sc. rank:	11488.0

,

PB2

Pos.	115	obs:	exp:
tat	Y	22111	11551.00
tac	Y	120	10680.00
aat	N	1	0.59
aac	N	0	0.41
---	--	------	------
mPD		0.011	0.50
		nPD:	0.02
		N. weight:	0.63
		Sc. PD:	-0.016
		Sc. rank:	2667.8

,

PB2

Pos.	116	obs:	exp:
cgt	R	0	0.25
cgc	R	0	0.47
cga	R	0	0.88
cgg	R	0	0.66
att	I	0	0.36
atc	I	0	0.25
ata	I	1	0.39
aat	N	1	1.18
aac	N	1	0.82
aaa	K	22166	12675.00
aag	K	54	9545.00
aga	R	9	4.52
agg	R	0	2.22
---	--	------	------
mPD		0.0059	0.49
		nPD:	0.01
		N. weight:	0.55
		Sc. PD:	-0.019
		Sc. rank:	-1343.9

,

PB2

Pos.	117	obs:	exp:
cct	P	8	2.20
ccc	P	0	1.48
cca	P	0	3.41
ccg	P	0	0.91
att	I	10	3.64
atc	I	0	2.50
ata	I	0	3.86
act	T	22185	5599.00
acc	T	21	4556.00
aca	T	1	10161.00
acg	T	3	1895.00
aat	N	3	1.77
aac	N	0	1.23
gct	A	1	0.26
gcc	A	0	0.21
gca	A	0	0.47
gcg	A	0	0.06
---	--	------	------
mPD		0.0042	0.68
		nPD:	0.01
		N. weight:	1.4
		Sc. PD:	-0.057
		Sc. rank:	-13469.0

,

PB2

Pos.	118	obs:	exp:
ttt	F	3	1.29
ttc	F	0	1.71
tat	Y	22190	11549.00
tac	Y	37	10678.00
cat	H	1	1.18
cac	H	1	0.82
---	--	------	------
mPD		0.0039	0.50
		nPD:	0.01
		N. weight:	0.65
		Sc. PD:	-0.026
		Sc. rank:	-4850.8

,

PB2

Pos.	119	obs:	exp:
ttt	F	88	9540.00
ttc	F	22143	12691.00
tgt	C	0	0.45
tgc	C	1	0.55
---	--	------	------
mPD		0.0080	0.49
		nPD:	0.02
		N. weight:	0.54
		Sc. PD:	-0.017
		Sc. rank:	486.3

,

PB2

Pos.	120	obs:	exp:
aaa	K	8	4.56
aag	K	0	3.44
gat	D	0	6.85
gac	D	13	6.15
gaa	E	22199	13445.00
gag	E	6	8760.00
ggt	G	0	0.91
ggc	G	0	0.89
gga	G	6	2.67
ggg	G	0	1.53
---	--	------	------
mPD		0.0030	0.48
		nPD:	0.01
		N. weight:	0.5
		Sc. PD:	-0.021
		Sc. rank:	-4976.7

,

PB2			
Pos.	121	obs:	exp:
cgt	R	0	0.25
cgc	R	0	0.47
cga	R	0	0.88
cgg	R	0	0.66
aaa	K	325	12677.00
aag	K	21898	9546.00
aga	R	0	4.52
agg	R	9	2.22
---	--	------	------
mPD		0.030	0.49
		nPD:	0.06
		N. weight:	0.77
		Sc. PD:	0.0098
		Sc. rank:	9872.1

,

PB2			
Pos.	122	obs:	exp:
ttt	F	0	0.43
ttc	F	1	0.57
tta	L	0	0.11
ttg	L	0	0.19
ctt	L	0	0.20
ctc	L	1	0.14
cta	L	0	0.19
ctg	L	0	0.17
att	I	0	15.29
atc	I	41	10.48
ata	I	1	16.23
act	T	0	0.25
acc	T	1	0.21
aca	T	0	0.46
acg	T	0	0.09
gtt	V	77	4904.00
gtc	V	22074	4031.00
gta	V	30	5965.00
gtg	V	0	7281.00
gct	A	0	1.32
gcc	A	5	1.03
gca	A	0	2.35
gcg	A	0	0.29
ggt	G	0	0.15
ggc	G	1	0.15
gga	G	0	0.44
ggg	G	0	0.26
---	--	------	------
mPD		0.014	0.74
		nPD:	0.02
		N. weight:	1.7
		Sc. PD:	-0.047
		Sc. rank:	4574.1

,

PB2			
Pos.	123	obs:	exp:
caa	Q	1	0.53
cag	Q	0	0.47
aaa	K	8	4.56
aag	K	0	3.44
gat	D	1	1.05
gac	D	1	0.95
gaa	E	22180	13449.00
gag	E	32	8763.00
ggt	G	0	1.36
ggc	G	0	1.33
gga	G	9	4.00
ggg	G	0	2.30
---	--	------	------
mPD		0.0047	0.48
		nPD:	0.01
		N. weight:	0.5
		Sc. PD:	-0.019
		Sc. rank:	-2514.5

,

PB2			
Pos.	124	obs:	exp:
cgt	R	0	607.00
cgc	R	0	1163.00
cga	R	0	2177.00
cgg	R	13	1627.00
aaa	K	0	0.57
aag	K	1	0.43
aga	R	152	11161.00
agg	R	22066	5496.00
---	--	------	------
mPD		0.015	0.91
		nPD:	0.02
		N. weight:	1.4
		Sc. PD:	-0.042
		Sc. rank:	1360.7

,

PB2			
Pos.	125	obs:	exp:
ttt	F	1	0.43
ttc	F	0	0.57
tta	L	277	2444.00
ttg	L	21938	4185.00
ctt	L	1	4381.00
ctc	L	0	3140.00
cta	L	0	4280.00
ctg	L	15	3801.00
---	--	------	------
mPD		0.026	1.1
		nPD:	0.02
		N. weight:	1.6
		Sc. PD:	-0.039
		Sc. rank:	7980.4

,

PB2			
Pos.	126	obs:	exp:
cgt	R	0	0.19
cgc	R	0	0.37
cga	R	0	0.69
cgg	R	0	0.51
aat	N	0	1.77
aac	N	3	1.23
aaa	K	22145	12676.00
aag	K	76	9545.00
aga	R	7	3.51
agg	R	0	1.73
gaa	E	1	0.61
gag	E	0	0.39
---	--	------	------
mPD		0.0078	0.49
		nPD:	0.02
		N. weight:	0.55
		Sc. PD:	-0.017
		Sc. rank:	418.2

,

PB2			
Pos.	127	obs:	exp:
tat	Y	48	24.94
tac	Y	0	23.06
cat	H	22107	13020.00
cac	H	33	9120.00
caa	Q	6	5.28
cag	Q	4	4.72
cgt	R	15	0.41
cgc	R	0	0.78
cga	R	0	1.47
cgg	R	0	1.10
aat	N	19	11.24
aac	N	0	7.76
aga	R	0	7.53
agg	R	0	3.71
---	--	------	------
mPD		0.011	0.49
		nPD:	0.02
		N. weight:	0.53
		Sc. PD:	-0.013
		Sc. rank:	2504.8

,

PB2			
Pos.	128	obs:	exp:
tct	S	0	0.18
tcc	S	0	0.12
tca	S	0	0.26
tcg	S	0	0.05
tgt	C	1	0.45
tgc	C	0	0.55
agt	S	1	0.19
agc	S	0	0.20
ggt	G	21352	3371.00
ggc	G	863	3293.00
gga	G	15	9888.00
ggg	G	0	5678.00
---	--	------	------
mPD		0.076	0.69
		nPD:	0.11
		N. weight:	1.7
		Sc. PD:	0.11
		Sc. rank:	26491.0

,

PB2			
Pos.	129	obs:	exp:
tct	S	0	0.92
tcc	S	5	0.58
tca	S	0	1.30
tcg	S	0	0.27
att	I	1	21.11
atc	I	57	14.48
ata	I	0	22.41
act	T	252	5587.00
acc	T	21875	4546.00
aca	T	35	10139.00
acg	T	0	1891.00
aat	N	3	2.96
aac	N	2	2.04
agt	S	0	0.94
agc	S	0	0.99
gct	A	0	0.53
gcc	A	2	0.41
gca	A	0	0.94
gcg	A	0	0.12
---	--	------	------
mPD		0.032	0.68
		nPD:	0.05
		N. weight:	1.5
		Sc. PD:	-0.00082
		Sc. rank:	17574.0

,

PB2			
Pos.	130	obs:	exp:
ttt	F	203	9540.00
ttc	F	22029	12692.00
---	--	------	------
mPD		0.018	0.49
		nPD:	0.04
		N. weight:	0.52
		Sc. PD:	-0.0054
		Sc. rank:	4937.5

,

PB2			
Pos.	131	obs:	exp:
ggt	G	195	3371.00
ggc	G	21942	3293.00
gga	G	3	9889.00
ggg	G	92	5679.00
---	--	------	------
mPD		0.026	0.69
		nPD:	0.04
		N. weight:	1.8
		Sc. PD:	-0.019
		Sc. rank:	17879.0

,

PB2			
Pos.	132	obs:	exp:
tta	L	0	0.11
ttg	L	0	0.19
ctt	L	1	0.20
ctc	L	0	0.14
cta	L	0	0.19
ctg	L	0	0.17
cct	P	22215	6125.00
ccc	P	4	4123.00
cca	P	12	9466.00
ccg	P	0	2517.00
---	--	------	------
mPD		0.0015	0.70
		nPD:	0.
		N. weight:	1.3
		Sc. PD:	-0.058
		Sc. rank:	-20734.0

,

PB2			
Pos.	133	obs:	exp:
att	I	0	3.64
atc	I	9	2.50
ata	I	1	3.86
gtt	V	118	4913.00
gtc	V	21908	4038.00
gta	V	196	5976.00
gtg	V	0	7295.00
---	--	------	------
mPD		0.029	0.74
		nPD:	0.04
		N. weight:	1.6
		Sc. PD:	-0.014
		Sc. rank:	16050.0

,

PB2			
Pos.	134	obs:	exp:
tat	Y	0	2.08
tac	Y	4	1.92
cat	H	89	13039.00
cac	H	22083	9133.00
caa	Q	44	23.22
cag	Q	0	20.78
cgt	R	0	0.33
cgc	R	12	0.63
cga	R	0	1.18
cgg	R	0	0.88
aga	R	0	6.02
agg	R	0	2.97
---	--	------	------
mPD		0.013	0.49
		nPD:	0.03
		N. weight:	0.87
		Sc. PD:	-0.017
		Sc. rank:	5873.3

,

PB2			
Pos.	135	obs:	exp:
ttt	F	122	9540.00
ttc	F	22110	12692.00
---	--	------	------
mPD		0.011	0.49
		nPD:	0.02
		N. weight:	0.53
		Sc. PD:	-0.013
		Sc. rank:	2417.1

,

PB2			
Pos.	136	obs:	exp:
cgt	R	0	606.90
cgc	R	0	1163.00
cga	R	1	2177.00
cgg	R	0	1627.00
aaa	K	6	3.42
aag	K	0	2.58
aga	R	22183	11158.00
agg	R	42	5495.00
---	--	------	------
mPD		0.0044	0.91
		nPD:	0.
		N. weight:	0.68
		Sc. PD:	-0.029
		Sc. rank:	-8060.3

,

PB2			
Pos.	137	obs:	exp:
tct	S	0	3.13
tcc	S	0	1.97
tca	S	0	4.42
tcg	S	0	0.90
act	T	2	0.50
acc	T	0	0.41
aca	T	0	0.91
acg	T	0	0.17
aat	N	22111	13138.00
aac	N	102	9075.00
agt	S	17	3.20
agc	S	0	3.38
---	--	------	------
mPD		0.011	0.49
		nPD:	0.02
		N. weight:	0.5
		Sc. PD:	-0.013
		Sc. rank:	2294.6

,

PB2			
Pos.	138	obs:	exp:
caa	Q	22217	11731.00
cag	Q	14	10500.00
cgt	R	0	0.03
cgc	R	0	0.05
cga	R	1	0.10
cgg	R	0	0.07
aga	R	0	0.50
agg	R	0	0.25
---	--	------	------
mPD		0.0013	0.50
		nPD:	0.
		N. weight:	0.64
		Sc. PD:	-0.029
		Sc. rank:	-9856.9

,

PB2			
Pos.	139	obs:	exp:
att	I	42	15.29
atc	I	0	10.48
ata	I	0	16.23
gtt	V	21878	4906.00
gtc	V	308	4032.00
gta	V	1	5968.00
gtg	V	3	7284.00
---	--	------	------
mPD		0.031	0.74
		nPD:	0.04
		N. weight:	1.5
		Sc. PD:	-0.0074
		Sc. rank:	15088.0

,

PB2			
Pos.	140	obs:	exp:
cgt	R	0	0.03
cgc	R	0	0.05
cga	R	0	0.10
cgg	R	0	0.07
aaa	K	22199	12681.00
aag	K	32	9550.00
aga	R	1	0.50
agg	R	0	0.25
---	--	------	------
mPD		0.0030	0.49
		nPD:	0.01
		N. weight:	0.56
		Sc. PD:	-0.023
		Sc. rank:	-5552.8

,

PB2			
Pos.	141	obs:	exp:
att	I	0	8088.00
atc	I	0	5546.00
ata	I	22218	8584.00
atg	M	6	6.00
act	T	0	1.01
acc	T	0	0.82
aca	T	4	1.83
acg	T	0	0.34
gtt	V	0	0.88
gtc	V	0	0.73
gta	V	4	1.08
gtg	V	0	1.31
---	--	------	------
mPD		0.0013	0.66
		nPD:	0.
		N. weight:	0.96
		Sc. PD:	-0.044
		Sc. rank:	-15924.0

,

PB2			
Pos.	142	obs:	exp:
cgt	R	0	607.00
cgc	R	4	1163.00
cga	R	6	2178.00
cgg	R	5	1627.00
aga	R	189	11161.00
agg	R	22028	5496.00
---	--	------	------
mPD		0.019	0.91
		nPD:	0.02
		N. weight:	1.4
		Sc. PD:	-0.036
		Sc. rank:	5102.2

,

PB2			
Pos.	143	obs:	exp:
cgt	R	0	606.70
cgc	R	0	1162.00
cga	R	0	2176.00
cgg	R	1	1626.00
aaa	K	0	6.85
aag	K	12	5.15
aga	R	113	11155.00
agg	R	22106	5493.00
---	--	------	------
mPD		0.011	0.91
		nPD:	0.01
		N. weight:	1.4
		Sc. PD:	-0.048
		Sc. rank:	-2687.4

,

PB2			
Pos.	144	obs:	exp:
cgt	R	0	606.70
cgc	R	0	1162.00
cga	R	0	2176.00
cgg	R	0	1626.00
act	T	0	0.25
acc	T	0	0.21
aca	T	1	0.46
acg	T	0	0.09
aaa	K	11	6.27
aag	K	0	4.73
aga	R	22172	11155.00
agg	R	48	5493.00
---	--	------	------
mPD		0.0054	0.91
		nPD:	0.01
		N. weight:	0.68
		Sc. PD:	-0.028
		Sc. rank:	-6911.2

,

PB2			
Pos.	145	obs:	exp:
tct	S	0	1.10
tcc	S	0	0.69
tca	S	0	1.56
tcg	S	0	0.32
att	I	2	0.73
atc	I	0	0.50
ata	I	0	0.77
agt	S	6	1.13
agc	S	0	1.19
gtt	V	22189	4913.00
gtc	V	10	4038.00
gta	V	24	5977.00
gtg	V	1	7295.00
---	--	------	------
mPD		0.0044	0.74
		nPD:	0.01
		N. weight:	1.5
		Sc. PD:	-0.062
		Sc. rank:	-15122.0

,

PB2			
Pos.	146	obs:	exp:
tat	Y	2	1.04
tac	Y	0	0.96
gtt	V	4	0.88
gtc	V	0	0.73
gta	V	0	1.08
gtg	V	0	1.31
gct	A	1	0.26
gcc	A	0	0.21
gca	A	0	0.47
gcg	A	0	0.06
gat	D	22109	11708.00
gac	D	111	10512.00
gaa	E	2	1.82
gag	E	1	1.18
ggt	G	2	0.30
ggc	G	0	0.30
gga	G	0	0.89
ggg	G	0	0.51
---	--	------	------
mPD		0.011	0.50
		nPD:	0.02
		N. weight:	0.62
		Sc. PD:	-0.016
		Sc. rank:	2751.7

,

PB2			
Pos.	147	obs:	exp:
att	I	0	16.38
atc	I	0	11.23
ata	I	45	17.39
act	T	1	5593.00
acc	T	3	4550.00
aca	T	22105	10149.00
acg	T	76	1893.00
gct	A	0	0.53
gcc	A	0	0.41
gca	A	2	0.94
gcg	A	0	0.12
---	--	------	------
mPD		0.011	0.68
		nPD:	0.02
		N. weight:	0.77
		Sc. PD:	-0.024
		Sc. rank:	885.2

,

PB2			
Pos.	148	obs:	exp:
tat	Y	0	0.52
tac	Y	1	0.48
act	T	0	1.51
acc	T	6	1.23
aca	T	0	2.74
acg	T	0	0.51
aat	N	462	13145.00
aac	N	21763	9080.00
---	--	------	------
mPD		0.041	0.48
		nPD:	0.09
		N. weight:	0.79
		Sc. PD:	0.03
		Sc. rank:	11717.0

,

PB2			
Pos.	149	obs:	exp:
cct	P	22143	6125.00
ccc	P	36	4123.00
cca	P	10	9466.00
ccg	P	43	2517.00
---	--	------	------
mPD		0.0080	0.70
		nPD:	0.01
		N. weight:	1.3
		Sc. PD:	-0.046
		Sc. rank:	-3943.9

,

PB2			
Pos.	150	obs:	exp:
tct	S	0	0.18
tcc	S	0	0.12
tca	S	0	0.26
tcg	S	0	0.05
agt	S	0	0.19
agc	S	1	0.20
gtt	V	0	0.44
gtc	V	2	0.36
gta	V	0	0.54
gtg	V	0	0.66
ggt	G	140	3371.00
ggc	G	22078	3292.00
gga	G	7	9888.00
ggg	G	4	5678.00
---	--	------	------
mPD		0.014	0.69
		nPD:	0.02
		N. weight:	1.9
		Sc. PD:	-0.052
		Sc. rank:	5928.3

,

PB2			
Pos.	151	obs:	exp:
tat	Y	2	1.04
tac	Y	0	0.96
cat	H	22215	13072.00
cac	H	13	9156.00
aat	N	2	1.18
aac	N	0	0.82
---	--	------	------
mPD		0.0015	0.48
		nPD:	0.
		N. weight:	0.53
		Sc. PD:	-0.023
		Sc. rank:	-7871.9

,

PB2			
Pos.	152	obs:	exp:
tct	S	0	1.29
tcc	S	0	0.81
tca	S	7	1.82
tcg	S	0	0.37
act	T	0	5.29
acc	T	0	4.31
aca	T	21	9.61
acg	T	0	1.79
agt	S	0	1.32
agc	S	0	1.39
gtt	V	0	0.66
gtc	V	0	0.55
gta	V	3	0.81
gtg	V	0	0.98
gct	A	28	5878.00
gcc	A	9	4595.00
gca	A	22146	10427.00
gcg	A	17	1301.00
gaa	E	1	0.61
gag	E	0	0.39
---	--	------	------
mPD		0.0077	0.67
		nPD:	0.01
		N. weight:	0.75
		Sc. PD:	-0.027
		Sc. rank:	-2141.6

,

PB2			
Pos.	153	obs:	exp:
aat	N	3	1.77
aac	N	0	1.23
gat	D	22047	11708.00
gac	D	174	10513.00
gaa	E	3	1.82
gag	E	0	1.18
ggt	G	5	0.76
ggc	G	0	0.74
gga	G	0	2.22
ggg	G	0	1.28
---	--	------	------
mPD		0.017	0.50
		nPD:	0.03
		N. weight:	0.6
		Sc. PD:	-0.0087
		Sc. rank:	5336.4

,

PB2			
Pos.	154	obs:	exp:
ttt	F	0	2.57
ttc	F	6	3.43
tta	L	1	2432.00
ttg	L	0	4166.00
ctt	L	34	4360.00
ctc	L	22077	3125.00
cta	L	14	4260.00
ctg	L	0	3783.00
cct	P	1	0.28
ccc	P	0	0.19
cca	P	0	0.43
ccg	P	0	0.11
att	I	0	36.04
atc	I	98	24.71
ata	I	1	38.25
---	--	------	------
mPD		0.014	1.1
		nPD:	0.01
		N. weight:	2.
		Sc. PD:	-0.068
		Sc. rank:	-4047.5

,

PB2			
Pos.	155	obs:	exp:
tct	S	0	4074.00
tcc	S	0	2564.00
tca	S	0	5762.00
tcg	S	0	1178.00
tgt	C	1	0.45
tgc	C	0	0.55
cgt	R	0	0.05
cgc	R	0	0.10
cga	R	0	0.20
cgg	R	0	0.15
act	T	6	1.51
acc	T	0	1.23
aca	T	0	2.74
acg	T	0	0.51
aat	N	82	48.50
aac	N	0	33.50
agt	S	22134	4163.00
agc	S	4	4397.00
aga	R	2	1.00
agg	R	0	0.49
ggt	G	3	0.45
ggc	G	0	0.44
gga	G	0	1.33
ggg	G	0	0.77
---	--	------	------
mPD		0.0088	1.6
		nPD:	0.01
		N. weight:	1.7
		Sc. PD:	-0.071
		Sc. rank:	-18582.0

,

PB2			
Pos.	156	obs:	exp:
tct	S	0	1.66
tcc	S	9	1.04
tca	S	0	2.34
tcg	S	0	0.48
att	I	0	0.73
atc	I	2	0.50
ata	I	0	0.77
act	T	0	5.29
acc	T	21	4.31
aca	T	0	9.61
acg	T	0	1.79
agt	S	0	1.69
agc	S	0	1.79
gtt	V	0	4.86
gtc	V	22	4.00
gta	V	0	5.92
gtg	V	0	7.22
gct	A	17	5871.00
gcc	A	22143	4591.00
gca	A	17	10416.00
gcg	A	0	1299.00
gat	D	0	0.53
gac	D	1	0.47
---	--	------	------
mPD		0.0082	0.67
		nPD:	0.01
		N. weight:	1.6
		Sc. PD:	-0.055
		Sc. rank:	-3726.2

,

PB2			
Pos.	157	obs:	exp:
caa	Q	2	6.86
cag	Q	11	6.14
cgt	R	0	0.30
cgc	R	0	0.58
cga	R	0	1.08
cgg	R	0	0.81
att	I	0	0.36
atc	I	0	0.25
ata	I	1	0.39
act	T	0	0.76
acc	T	0	0.62
aca	T	1	1.37
acg	T	2	0.26
aat	N	0	1.18
aac	N	2	0.82
aaa	K	1380	12665.00
aag	K	20822	9537.00
aga	R	3	5.52
agg	R	8	2.72
---	--	------	------
mPD		0.12	0.49
		nPD:	0.24
		N. weight:	0.6
		Sc. PD:	0.12
		Sc. rank:	10291.0

,

PB2			
Pos.	158	obs:	exp:
gaa	E	824	13460.00
gag	E	21406	8770.00
ggt	G	0	0.30
ggc	G	0	0.30
gga	G	0	0.89
ggg	G	2	0.51
---	--	------	------
mPD		0.072	0.48
		nPD:	0.15
		N. weight:	0.76
		Sc. PD:	0.078
		Sc. rank:	12396.0

,

PB2			
Pos.	159	obs:	exp:
act	T	0	0.76
acc	T	0	0.62
aca	T	3	1.37
acg	T	0	0.26
gtt	V	0	0.22
gtc	V	0	0.18
gta	V	1	0.27
gtg	V	0	0.33
gct	A	0	5885.00
gcc	A	2	4601.00
gca	A	22218	10440.00
gcg	A	8	1302.00
---	--	------	------
mPD		0.0013	0.66
		nPD:	0.
		N. weight:	0.76
		Sc. PD:	-0.034
		Sc. rank:	-12641.0

,

PB2			
Pos.	160	obs:	exp:
cat	H	1	0.59
cac	H	0	0.41
caa	Q	105	11729.00
cag	Q	22122	10498.00
cgt	R	0	0.11
cgc	R	0	0.21
cga	R	0	0.39
cgg	R	4	0.29
aga	R	0	2.01
agg	R	0	0.99
---	--	------	------
mPD		0.0099	0.50
		nPD:	0.02
		N. weight:	0.73
		Sc. PD:	-0.02
		Sc. rank:	2193.6

,

PB2

Pos.	161	obs:	exp:
aat	N	8	4.73
aac	N	0	3.27
gat	D	22209	11709.00
gac	D	13	10513.00
gaa	E	0	0.61
gag	E	1	0.39
ggt	G	1	0.15
ggc	G	0	0.15
gga	G	0	0.44
ggg	G	0	0.26
---	--	------	------
mPD		0.0021	0.50
		nPD:	0.
		N. weight:	0.64
		Sc. PD:	-0.028
		Sc. rank:	-8511.3

,

PB2

Pos.	162	obs:	exp:
atg	M	3	3.00
gtt	V	602	4914.00
gtc	V	0	4039.00
gta	V	52	5978.00
gtg	V	21573	7296.00
gct	A	0	0.53
gcc	A	0	0.41
gca	A	0	0.94
gcg	A	2	0.12
---	--	------	------
mPD		0.058	0.74
		nPD:	0.08
		N. weight:	0.99
		Sc. PD:	0.03
		Sc. rank:	14254.0

,

PB2

Pos.	163	obs:	exp:
att	I	21986	8092.00
atc	I	242	5549.00
ata	I	1	8588.00
gtt	V	3	0.66
gtc	V	0	0.55
gta	V	0	0.81
gtg	V	0	0.98
---	--	------	------
mPD		0.022	0.66
		nPD:	0.03
		N. weight:	0.96
		Sc. PD:	-0.014
		Sc. rank:	8704.5

,

PB2

Pos.	164	obs:	exp:
att	I	0	1.09
atc	I	0	0.75
ata	I	3	1.16
atg	M	22229	22229.00
---	--	------	------
mPD		0.00027	0.00027
		nPD:	1.
		N. weight:	0.00013
		Sc. PD:	0.00012
		Sc. rank:	2.5

,

PB2

Pos.	165	obs:	exp:
gaa	E	21803	13461.00
gag	E	429	8771.00
---	--	------	------
mPD		0.038	0.48
		nPD:	0.08
		N. weight:	0.42
		Sc. PD:	0.013
		Sc. rank:	6059.9

,

PB2

Pos.	166	obs:	exp:
att	I	8	3.28
atc	I	1	2.25
ata	I	0	3.48
gtt	V	22164	4913.00
gtc	V	51	4038.00
gta	V	7	5977.00
gtg	V	1	7295.00
---	--	------	------
mPD		0.0062	0.74
		nPD:	0.01
		N. weight:	1.5
		Sc. PD:	-0.059
		Sc. rank:	-10276.0

,

PB2

Pos.	167	obs:	exp:
ttt	F	1	0.43
ttc	F	0	0.57
att	I	3	1.09
atc	I	0	0.75
ata	I	0	1.16
gtt	V	22203	4914.00
gtc	V	25	4039.00
gta	V	0	5978.00
gtg	V	0	7297.00
---	--	------	------
mPD		0.0026	0.74
		nPD:	0.
		N. weight:	1.5
		Sc. PD:	-0.066
		Sc. rank:	-21411.0

,

PB2

Pos.	168	obs:	exp:
ttt	F	101	9538.00
ttc	F	22125	12688.00
tat	Y	0	3.12
tac	Y	6	2.88
---	--	------	------
mPD		0.0096	0.49
		nPD:	0.02
		N. weight:	0.54
		Sc. PD:	-0.015
		Sc. rank:	1538.7

,

PB2

Pos.	169	obs:	exp:
cct	P	2	6125.00
ccc	P	11	4123.00
cca	P	22211	9466.00
ccg	P	8	2517.00
---	--	------	------
mPD		0.0019	0.70
		nPD:	0.
		N. weight:	0.85
		Sc. PD:	-0.038
		Sc. rank:	-13120.0

,

PB2

Pos.	170	obs:	exp:
aat	N	22225	13147.00
aac	N	3	9081.00
aaa	K	1	0.57
aag	K	0	0.43
gat	D	3	1.58
gac	D	0	1.42
---	--	------	------
mPD		0.00063	0.48
		nPD:	0.
		N. weight:	0.53
		Sc. PD:	-0.024
		Sc. rank:	-9415.3

,

PB2

Pos.	171	obs:	exp:
gaa	E	22209	13461.00
gag	E	23	8771.00
---	--	------	------
mPD		0.0021	0.48
		nPD:	0.
		N. weight:	0.5
		Sc. PD:	-0.021
		Sc. rank:	-6435.6

,

PB2

Pos.	172	obs:	exp:
atg	M	3	3.00
gtt	V	30	4914.00
gtc	V	0	4039.00
gta	V	74	5978.00
gtg	V	22125	7297.00
---	--	------	------
mPD		0.0096	0.74
		nPD:	0.01
		N. weight:	1.1
		Sc. PD:	-0.038
		Sc. rank:	-1535.6

,

PB2

Pos.	173	obs:	exp:
ggt	G	9	3371.00
ggc	G	5	3293.00
gga	G	186	9889.00
ggg	G	22032	5679.00
---	--	------	------
mPD		0.018	0.69
		nPD:	0.03
		N. weight:	1.3
		Sc. PD:	-0.029
		Sc. rank:	8098.8

,

PB2

Pos.	174	obs:	exp:
cct	P	0	0.28
ccc	P	0	0.19
cca	P	1	0.43
ccg	P	0	0.11
gct	A	6	5886.00
gcc	A	10	4602.00
gca	A	22204	10441.00
gcg	A	11	1302.00
---	--	------	------
mPD		0.0025	0.66
		nPD:	0.
		N. weight:	0.75
		Sc. PD:	-0.033
		Sc. rank:	-10459.0

,

PB2

Pos.	175	obs:	exp:
tct	S	0	0.18
tcc	S	0	0.12
tca	S	0	0.26
tcg	S	0	0.05
cgt	R	0	605.20
cgc	R	0	1159.00
cga	R	1	2171.00
cgg	R	0	1622.00
att	I	0	0.73
atc	I	0	0.50
ata	I	2	0.77
aaa	K	63	35.94
aag	K	0	27.06
agt	S	1	0.19
agc	S	0	0.20
aga	R	22086	11128.00
agg	R	79	5480.00
---	--	------	------
mPD		0.013	0.91
		nPD:	0.01
		N. weight:	0.67
		Sc. PD:	-0.022
		Sc. rank:	-269.6

,

PB2

Pos.	176	obs:	exp:
tta	L	1	0.99
ttg	L	0	1.69
ctt	L	0	1.77
ctc	L	0	1.27
cta	L	8	1.73
ctg	L	0	1.54
att	I	1	8083.00
atc	I	2	5543.00
ata	I	22202	8579.00
atg	M	4	4.00
act	T	0	1.01
acc	T	0	0.82
aca	T	4	1.83
acg	T	0	0.34
gtt	V	0	2.21
gtc	V	0	1.82
gta	V	10	2.69
gtg	V	0	3.28
---	--	------	------
mPD		0.0027	0.66
		nPD:	0.
		N. weight:	0.96
		Sc. PD:	-0.041
		Sc. rank:	-12857.0

,

PB2

Pos.	177	obs:	exp:
tta	L	1	2444.00
ttg	L	47	4185.00
ctt	L	62	4381.00
ctc	L	1	3140.00
cta	L	59	4280.00
ctg	L	22061	3801.00
atg	M	1	1.00
---	--	------	------
mPD		0.015	1.1
		nPD:	0.01
		N. weight:	1.7
		Sc. PD:	-0.058
		Sc. rank:	-1677.0

,

PB2

Pos.	178	obs:	exp:
tct	S	0	0.74
tcc	S	0	0.46
tca	S	4	1.04
tcg	S	0	0.21
att	I	0	1.09
atc	I	0	0.75
ata	I	3	1.16
act	T	5	5599.00
acc	T	6	4555.00
aca	T	22169	10160.00
acg	T	29	1895.00
agt	S	0	0.75
agc	S	0	0.79
gct	A	0	4.24
gcc	A	0	3.31
gca	A	16	7.51
gcg	A	0	0.94
---	--	------	------
mPD		0.0057	0.68
		nPD:	0.01
		N. weight:	0.78
		Sc. PD:	-0.03
		Sc. rank:	-5371.9

,

PB2

Pos.	179	obs:	exp:
tta	L	2	0.22
ttg	L	0	0.38
tct	S	2	4090.00
tcc	S	2	2574.00
tca	S	22205	5784.00
tcg	S	17	1183.00
ctt	L	0	0.39
ctc	L	0	0.28
cta	L	0	0.39
ctg	L	0	0.34
act	T	0	0.25
acc	T	0	0.21
aca	T	1	0.46
acg	T	0	0.09
agt	S	0	4179.00
agc	S	0	4415.00
gct	A	0	0.79
gcc	A	0	0.62
gca	A	3	1.41
gcg	A	0	0.18
---	--	------	------
mPD		0.0024	1.6
		nPD:	0.
		N. weight:	1.4
		Sc. PD:	-0.062
		Sc. rank:	-23622.0

,

PB2

Pos.	180	obs:	exp:
aaa	K	0	0.57
aag	K	1	0.43
gat	D	11	5.80
gac	D	0	5.20
gaa	E	108	13452.00
gag	E	22109	8765.00
ggt	G	0	0.45
ggc	G	0	0.44
gga	G	0	1.33
ggg	G	3	0.77
---	--	------	------
mPD		0.011	0.48
		nPD:	0.02
		N. weight:	0.91
		Sc. PD:	-0.022
		Sc. rank:	4391.7

,

PB2

Pos.	181	obs:	exp:
tct	S	2	4091.00
tcc	S	0	2575.00
tca	S	22195	5785.00
tcg	S	33	1183.00
cct	P	0	0.28
ccc	P	0	0.19
cca	P	1	0.43
ccg	P	0	0.11
act	T	0	0.25
acc	T	0	0.21
aca	T	1	0.46
acg	T	0	0.09
agt	S	0	4180.00
agc	S	0	4416.00
---	--	------	------
mPD		0.0033	1.6
		nPD:	0.
		N. weight:	1.3
		Sc. PD:	-0.061
		Sc. rank:	-22250.0

,

PB2

Pos.	182	obs:	exp:
cat	H	1	0.59
cac	H	0	0.41
caa	Q	339	11731.00
cag	Q	21892	10500.00
---	--	------	------
mPD		0.030	0.50
		nPD:	0.06
		N. weight:	0.68
		Sc. PD:	0.0087
		Sc. rank:	8665.0

,

PB2

Pos.	183	obs:	exp:
tta	L	0	2436.00
ttg	L	24	4171.00
ctt	L	11	4366.00
ctc	L	0	3130.00
cta	L	104	4266.00
ctg	L	22018	3788.00
caa	Q	0	0.53
cag	Q	1	0.47
cgt	R	0	0.05
cgc	R	0	0.10
cga	R	0	0.20
cgg	R	2	0.15
atg	M	72	72.00
aga	R	0	1.00
agg	R	0	0.49
---	--	------	------
mPD		0.019	1.1
		nPD:	0.02
		N. weight:	1.7
		Sc. PD:	-0.053
		Sc. rank:	2205.8

,

PB2

Pos.	184	obs:	exp:
tct	S	0	0.55
tcc	S	0	0.35
tca	S	3	0.78
tcg	S	0	0.16
act	T	0	137.40
acc	T	0	111.80
aca	T	544	249.30
acg	T	1	46.50
agt	S	0	0.56
agc	S	0	0.60
gtt	V	0	2.65
gtc	V	0	2.18
gta	V	12	3.23
gtg	V	0	3.94
gct	A	2	5732.00
gcc	A	1	4482.00
gca	A	21616	10169.00
gcg	A	32	1268.00
gaa	E	21	12.72
gag	E	0	8.28
---	--	------	------
mPD		0.054	0.71
		nPD:	0.08
		N. weight:	0.75
		Sc. PD:	0.021
		Sc. rank:	10578.0

,

PB2

Pos.	185	obs:	exp:
tta	L	0	0.22
ttg	L	0	0.38
ctt	L	0	0.39
ctc	L	0	0.28
cta	L	2	0.39
ctg	L	0	0.34
att	I	2	8083.00
atc	I	2	5542.00
ata	I	22199	8578.00
atg	M	6	6.00
act	T	0	1.01
acc	T	0	0.82
aca	T	4	1.83
acg	T	0	0.34
gtt	V	0	3.76
gtc	V	0	3.09
gta	V	17	4.57
gtg	V	0	5.58
---	--	------	------
mPD		0.0030	0.66
		nPD:	0.
		N. weight:	0.96
		Sc. PD:	-0.041
		Sc. rank:	-12042.0

,

PB2

Pos.	186	obs:	exp:
att	I	0	0.73
atc	I	0	0.50
ata	I	2	0.77
act	T	9	5604.00
acc	T	5	4560.00
aca	T	22209	10170.00
acg	T	7	1897.00
---	--	------	------
mPD		0.0021	0.68
		nPD:	0.
		N. weight:	0.78
		Sc. PD:	-0.035
		Sc. rank:	-11710.0

,

PB2

Pos.	187	obs:	exp:
cgt	R	0	0.33
cgc	R	0	0.63
cga	R	0	1.18
cgg	R	0	0.88
act	T	0	0.25
acc	T	0	0.21
aca	T	1	0.46
acg	T	0	0.09
aat	N	0	1.18
aac	N	2	0.82
aaa	K	22033	12672.00
aag	K	182	9543.00
aga	R	12	6.02
agg	R	0	2.97
gaa	E	2	1.21
gag	E	0	0.79
---	--	------	------
mPD		0.018	0.49
		nPD:	0.04
		N. weight:	0.52
		Sc. PD:	-0.0059
		Sc. rank:	4895.5

,

PB2

Pos.	188	obs:	exp:
aat	N	1	0.59
aac	N	0	0.41
aaa	K	0	13.69
aag	K	24	10.31
gtt	V	0	0.22
gtc	V	0	0.18
gta	V	0	0.27
gtg	V	1	0.33
gct	A	0	0.26
gcc	A	0	0.21
gca	A	0	0.47
gcg	A	1	0.06
gat	D	10	5.27
gac	D	0	4.73
gaa	E	427	13436.00
gag	E	21763	8754.00
ggt	G	0	0.76
ggc	G	0	0.74
gga	G	0	2.22
ggg	G	5	1.28
---	--	------	------
mPD		0.042	0.48
		nPD:	0.09
		N. weight:	0.84
		Sc. PD:	0.032
		Sc. rank:	12354.0

,

PB2			
Pos.	189	obs:	exp:
cgt	R	0	0.03
cgc	R	0	0.05
cga	R	0	0.10
cgg	R	0	0.07
aat	N	0	0.59
aac	N	1	0.41
aaa	K	79	12681.00
aag	K	22151	9549.00
aga	R	0	0.50
agg	R	1	0.25
---	--	------	------
mPD		0.0073	0.49
		nPD:	0.01
		N. weight:	0.83
		Sc. PD:	-0.027
		Sc. rank:	145.7

,

PB2			
Pos.	190	obs:	exp:
cgt	R	0	0.19
cgc	R	0	0.37
cga	R	0	0.69
cgg	R	0	0.51
act	T	0	0.25
acc	T	0	0.21
aca	T	1	0.46
acg	T	0	0.09
aaa	K	22186	12677.00
aag	K	38	9547.00
aga	R	7	3.51
agg	R	0	1.73
---	--	------	------
mPD		0.0041	0.49
		nPD:	0.01
		N. weight:	0.55
		Sc. PD:	-0.022
		Sc. rank:	-3773.8

,

PB2			
Pos.	191	obs:	exp:
aaa	K	219	124.90
aag	K	0	94.08
gtt	V	0	0.22
gtc	V	0	0.18
gta	V	1	0.27
gtg	V	0	0.33
gat	D	1	2.63
gac	D	4	2.37
gaa	E	21957	13320.00
gag	E	41	8678.00
ggt	G	0	1.36
ggc	G	0	1.33
gga	G	9	4.00
ggg	G	0	2.30
---	--	------	------
mPD		0.025	0.50
		nPD:	0.05
		N. weight:	0.49
		Sc. PD:	0.00082
		Sc. rank:	5851.8

,

PB2			
Pos.	192	obs:	exp:
aaa	K	0	1.14
aag	K	2	0.86
gat	D	7	3.69
gac	D	0	3.31
gaa	E	308	13454.00
gag	E	21912	8766.00
ggt	G	0	0.45
ggc	G	0	0.44
gga	G	0	1.33
ggg	G	3	0.77
---	--	------	------
mPD		0.028	0.48
		nPD:	0.06
		N. weight:	0.86
		Sc. PD:	0.01
		Sc. rank:	10921.0

,

PB2			
Pos.	193	obs:	exp:
ttt	F	0	0.86
ttc	F	2	1.14
tta	L	0	2444.00
ttg	L	0	4185.00
ctt	L	49	4380.00
ctc	L	22158	3140.00
cta	L	21	4279.00
ctg	L	0	3800.00
att	I	0	0.73
atc	I	2	0.50
ata	I	0	0.77
---	--	------	------
mPD		0.0066	1.1
		nPD:	0.01
		N. weight:	2.
		Sc. PD:	-0.081
		Sc. rank:	-20357.0

,

PB2			
Pos.	194	obs:	exp:
tta	L	0	0.55
ttg	L	0	0.94
ctt	L	0	0.99
ctc	L	0	0.71
cta	L	0	0.96
ctg	L	5	0.85
cat	H	11	6.47
cac	H	0	4.53
caa	Q	48	11700.00
cag	Q	22125	10473.00
cgt	R	0	0.38
cgc	R	0	0.73
cga	R	10	1.37
cgg	R	4	1.02
aaa	K	0	16.54
aag	K	29	12.46
aga	R	0	7.03
agg	R	0	3.46
---	--	------	------
mPD		0.011	0.50
		nPD:	0.02
		N. weight:	0.74
		Sc. PD:	-0.02
		Sc. rank:	2671.0

,

PB2			
Pos.	195	obs:	exp:
tct	S	0	2.58
tcc	S	0	1.62
tca	S	0	3.64
tcg	S	0	0.75
tat	Y	5	2.60
tac	Y	0	2.40
act	T	1	0.25
acc	T	0	0.21
aca	T	0	0.46
acg	T	0	0.09
aat	N	18739	11110.00
aac	N	45	7674.00
aaa	K	1	0.57
aag	K	0	0.43
agt	S	14	2.63
agc	S	0	2.78
gat	D	3423	1805.00
gac	D	2	1620.00
ggt	G	2	0.30
ggc	G	0	0.30
gga	G	0	0.89
ggg	G	0	0.51
---	--	------	------
mPD		0.27	0.75
		nPD:	0.36
		N. weight:	0.54
		Sc. PD:	0.16
		Sc. rank:	9557.4

,

PB2			
Pos.	196	obs:	exp:
ttt	F	1	0.43
ttc	F	0	0.57
tct	S	0	0.37
tcc	S	0	0.23
tca	S	0	0.52
tcg	S	0	0.11
tat	Y	1	0.52
tac	Y	0	0.48
tgt	C	22134	10033.00
tgc	C	94	12195.00
agt	S	0	0.38
agc	S	2	0.40
---	--	------	------
mPD		0.0090	0.50
		nPD:	0.02
		N. weight:	0.78
		Sc. PD:	-0.023
		Sc. rank:	1610.9

,

PB2			
Pos.	197	obs:	exp:
cgt	R	0	0.30
cgc	R	0	0.58
cga	R	0	1.08
cgg	R	0	0.81
act	T	0	0.25
acc	T	0	0.21
aca	T	1	0.46
acg	T	0	0.09
aat	N	0	1.77
aac	N	3	1.23
aaa	K	22169	12673.00
aag	K	47	9543.00
aga	R	10	5.52
agg	R	1	2.72
gaa	E	1	0.61
gag	E	0	0.39
---	--	------	------
mPD		0.0057	0.49
		nPD:	0.01
		N. weight:	0.55
		Sc. PD:	-0.02
		Sc. rank:	-1511.5

,

PB2			
Pos.	198	obs:	exp:
att	I	22160	8090.00
atc	I	45	5548.00
ata	I	19	8586.00
atg	M	1	1.00
act	T	1	0.25
acc	T	0	0.21
aca	T	0	0.46
acg	T	0	0.09
gtt	V	6	1.33
gtc	V	0	1.09
gta	V	0	1.61
gtg	V	0	1.97
---	--	------	------
mPD		0.0065	0.66
		nPD:	0.01
		N. weight:	1.
		Sc. PD:	-0.037
		Sc. rank:	-4765.6

,

PB2			
Pos.	199	obs:	exp:
tct	S	14	2.58
tcc	S	0	1.62
tca	S	0	3.64
tcg	S	0	0.75
att	I	1	0.36
atc	I	0	0.25
ata	I	0	0.39
act	T	8	2.02
acc	T	0	1.64
aca	T	0	3.66
acg	T	0	0.68
agt	S	0	2.63
agc	S	0	2.78
gct	A	22146	5879.00
gcc	A	51	4597.00
gca	A	8	10429.00
gcg	A	1	1301.00
gat	D	3	1.58
gac	D	0	1.42
---	--	------	------
mPD		0.0078	0.67
		nPD:	0.01
		N. weight:	1.3
		Sc. PD:	-0.047
		Sc. rank:	-3481.5

,

PB2			
Pos.	200	obs:	exp:
cct	P	48	6125.00
ccc	P	22158	4123.00
cca	P	26	9466.00
ccg	P	0	2517.00
---	--	------	------
mPD		0.0066	0.70
		nPD:	0.01
		N. weight:	1.7
		Sc. PD:	-0.063
		Sc. rank:	-8844.9

,

PB2			
Pos.	201	obs:	exp:
ttt	F	1	0.43
ttc	F	0	0.57
tta	L	82	2444.00
ttg	L	22105	4185.00
ctt	L	0	4381.00
ctc	L	0	3140.00
cta	L	0	4280.00
ctg	L	44	3801.00
---	--	------	------
mPD		0.011	1.1
		nPD:	0.01
		N. weight:	1.6
		Sc. PD:	-0.062
		Sc. rank:	-6972.1

,

PB2

Pos.	202	obs:	exp:
tta	L	0	0.11
ttg	L	1	0.19
ctt	L	0	0.20
ctc	L	0	0.14
cta	L	0	0.19
ctg	L	0	0.17
att	I	2	4.37
atc	I	0	3.00
ata	I	10	4.64
atg	M	22214	22214.00
act	T	0	0.50
acc	T	0	0.41
aca	T	0	0.91
acg	T	2	0.17
gtt	V	0	0.66
gtc	V	0	0.55
gta	V	0	0.81
gtg	V	3	0.98
---	--	------	------
mPD		0.0016	0.0020
		nPD:	0.8
		N. weight:	0.00078
		Sc. PD:	0.00059
		Sc. rank:	14.9

,

PB2

Pos.	203	obs:	exp:
atg	M	2	2.00
gtt	V	20	4915.00
gtc	V	9	4039.00
gta	V	158	5979.00
gtg	V	22043	7297.00
---	--	------	------
mPD		0.017	0.74
		nPD:	0.02
		N. weight:	1.1
		Sc. PD:	-0.026
		Sc. rank:	5154.3

,

PB2

Pos.	204	obs:	exp:
gct	A	29	5886.00
gcc	A	1	4602.00
gca	A	266	10442.00
gcg	A	21936	1302.00
---	--	------	------
mPD		0.026	0.66
		nPD:	0.04
		N. weight:	2.8
		Sc. PD:	-0.022
		Sc. rank:	27640.0

,

PB2

Pos.	205	obs:	exp:
tat	Y	18	11549.00
tac	Y	22210	10679.00
cat	H	0	2.35
cac	H	4	1.65
---	--	------	------
mPD		0.0020	0.50
		nPD:	0.
		N. weight:	0.73
		Sc. PD:	-0.032
		Sc. rank:	-9926.6

,

PB2

Pos.	206	obs:	exp:
atg	M	22231	22231.00
act	T	0	0.25
acc	T	0	0.21
aca	T	0	0.46
acg	T	1	0.09
---	--	------	------
mPD		0.000090	0.00017
		nPD:	0.52
		N. weight:	0.00011
		Sc. PD:	0.000053
		Sc. rank:	2.1

,

PB2

Pos.	207	obs:	exp:
tta	L	84	2444.00
ttg	L	1	4185.00
ctt	L	5	4381.00
ctc	L	1	3140.00
cta	L	21985	4280.00
ctg	L	155	3801.00
gtt	V	0	0.22
gtc	V	0	0.18
gta	V	1	0.27
gtg	V	0	0.33
---	--	------	------
mPD		0.022	1.1
		nPD:	0.02
		N. weight:	1.6
		Sc. PD:	-0.045
		Sc. rank:	4522.0

,

PB2

Pos.	208	obs:	exp:
gat	D	0	0.53
gac	D	1	0.47
gaa	E	22201	13461.00
gag	E	30	8770.00
---	--	------	------
mPD		0.0028	0.48
		nPD:	0.01
		N. weight:	0.5
		Sc. PD:	-0.021
		Sc. rank:	-5170.4

,

PB2

Pos.	209	obs:	exp:
cgt	R	0	607.00
cgc	R	0	1163.00
cga	R	2	2177.00
cgg	R	0	1627.00
aga	R	22190	11161.00
agg	R	39	5496.00
ggt	G	0	0.15
ggc	G	0	0.15
gga	G	1	0.44
ggg	G	0	0.26
---	--	------	------
mPD		0.0038	0.91
		nPD:	0.
		N. weight:	0.68
		Sc. PD:	-0.029
		Sc. rank:	-9034.6

,

PB2

Pos.	210	obs:	exp:
gaa	E	22141	13461.00
gag	E	90	8770.00
ggt	G	0	0.15
ggc	G	0	0.15
gga	G	1	0.44
ggg	G	0	0.26
---	--	------	------
mPD		0.0082	0.48
		nPD:	0.02
		N. weight:	0.48
		Sc. PD:	-0.015
		Sc. rank:	708.0

,

PB2

Pos.	211	obs:	exp:
ttt	F	1	0.43
ttc	F	0	0.57
tta	L	138	2444.00
ttg	L	22013	4185.00
ctt	L	9	4380.00
ctc	L	0	3140.00
cta	L	1	4279.00
ctg	L	68	3801.00
atg	M	2	2.00
---	--	------	------
mPD		0.020	1.1
		nPD:	0.02
		N. weight:	1.6
		Sc. PD:	-0.048
		Sc. rank:	3318.2

,

PB2

Pos.	212	obs:	exp:
att	I	0	6.55
atc	I	18	4.49
ata	I	0	6.95
gtt	V	50	4911.00
gtc	V	22151	4036.00
gta	V	2	5974.00
gtg	V	9	7291.00
gct	A	0	0.53
gcc	A	2	0.41
gca	A	0	0.94
gcg	A	0	0.12
---	--	------	------
mPD		0.0073	0.74
		nPD:	0.01
		N. weight:	1.7
		Sc. PD:	-0.064
		Sc. rank:	-8191.6

,

PB2

Pos.	213	obs:	exp:
cgt	R	22016	607.00
cgc	R	174	1163.00
cga	R	28	2178.00
cgg	R	14	1627.00
aga	R	0	11161.00
agg	R	0	5496.00
---	--	------	------
mPD		0.019	0.91
		nPD:	0.02
		N. weight:	3.6
		Sc. PD:	-0.093
		Sc. rank:	14446.0

,

PB2

Pos.	214	obs:	exp:
cgt	R	0	0.38
cgc	R	0	0.73
cga	R	0	1.37
cgg	R	0	1.02
aaa	K	22174	12674.00
aag	K	44	9544.00
aga	R	14	7.03
agg	R	0	3.46
---	--	------	------
mPD		0.0052	0.49
		nPD:	0.01
		N. weight:	0.55
		Sc. PD:	-0.02
		Sc. rank:	-2128.4

,

PB2

Pos.	215	obs:	exp:
cct	P	0	0.28
ccc	P	0	0.19
cca	P	1	0.43
ccg	P	0	0.11
act	T	4	5603.00
acc	T	1	4559.00
aca	T	22213	10168.00
acg	T	9	1896.00
aaa	K	1	0.57
aag	K	0	0.43
gct	A	0	0.79
gcc	A	0	0.62
gca	A	3	1.41
gcg	A	0	0.18
---	--	------	------
mPD		0.0017	0.68
		nPD:	0.
		N. weight:	0.78
		Sc. PD:	-0.035
		Sc. rank:	-12230.0

,

PB2

Pos.	216	obs:	exp:
cgt	R	0	606.80
cgc	R	0	1163.00
cga	R	0	2177.00
cgg	R	2	1627.00
aaa	K	0	3.99
aag	K	7	3.01
aga	R	121	11158.00
agg	R	22102	5494.00
---	--	------	------
mPD		0.012	0.91
		nPD:	0.01
		N. weight:	1.4
		Sc. PD:	-0.047
		Sc. rank:	-2251.3

,

PB2

Pos.	217	obs:	exp:
ttt	F	22173	9540.00
ttc	F	59	12692.00
---	--	------	------
mPD		0.0053	0.49
		nPD:	0.01
		N. weight:	0.84
		Sc. PD:	-0.03
		Sc. rank:	-3093.1

,

PB2			
Pos.	218	obs:	exp:
ttt	F	0	0.86
ttc	F	2	1.14
tta	L	0	2444.00
ttg	L	0	4185.00
ctt	L	613	4380.00
ctc	L	21599	3140.00
cta	L	16	4279.00
ctg	L	0	3800.00
att	I	0	0.73
atc	I	2	0.50
ata	I	0	0.77
---	--	------	------
mPD		0.055	1.1
		nPD:	0.05
		N. weight:	1.8
		Sc. PD:	0.0019
		Sc. rank:	21644.0

,

PB2			
Pos.	219	obs:	exp:
cct	P	60	6125.00
ccc	P	2	4123.00
cca	P	7330	9466.00
ccg	P	14840	2517.00
---	--	------	------
mPD		0.45	0.70
		nPD:	0.64
		N. weight:	1.1
		Sc. PD:	0.65
		Sc. rank:	20632.0

,

PB2			
Pos.	220	obs:	exp:
att	I	0	18.57
atc	I	0	12.73
ata	I	51	19.70
gtt	V	11	4904.00
gtc	V	8	4031.00
gta	V	22066	5965.00
gtg	V	96	7281.00
---	--	------	------
mPD		0.015	0.74
		nPD:	0.02
		N. weight:	1.3
		Sc. PD:	-0.035
		Sc. rank:	4173.2

,

PB2			
Pos.	221	obs:	exp:
tct	S	0	13.25
tcc	S	72	8.34
tca	S	0	18.74
tcg	S	0	3.83
act	T	0	28.99
acc	T	115	23.59
aca	T	0	52.61
acg	T	0	9.81
agt	S	0	13.54
agc	S	0	14.30
gtt	V	0	1.99
gtc	V	9	1.64
gta	V	0	2.42
gtg	V	0	2.95
gct	A	138	5834.00
gcc	A	21883	4561.00
gca	A	12	10350.00
gcg	A	3	1291.00
---	--	------	------
mPD		0.031	0.68
		nPD:	0.05
		N. weight:	1.5
		Sc. PD:	-0.0029
		Sc. rank:	17575.0

,

PB2			
Pos.	222	obs:	exp:
ggt	G	19109	3371.00
ggc	G	3111	3293.00
gga	G	3	9889.00
ggg	G	9	5679.00
---	--	------	------
mPD		0.24	0.69
		nPD:	0.35
		N. weight:	1.5
		Sc. PD:	0.45
		Sc. rank:	26655.0

,

PB2			
Pos.	223	obs:	exp:
ggt	G	17	3371.00
ggc	G	0	3293.00
gga	G	3703	9889.00
ggg	G	18512	5679.00
---	--	------	------
mPD		0.28	0.69
		nPD:	0.4
		N. weight:	0.82
		Sc. PD:	0.29
		Sc. rank:	14946.0

,

PB2			
Pos.	224	obs:	exp:
att	I	0	4.73
atc	I	0	3.25
ata	I	13	5.02
act	T	47	5600.00
acc	T	29	4556.00
aca	T	22121	10162.00
acg	T	16	1895.00
gct	A	0	1.59
gcc	A	0	1.24
gca	A	6	2.82
gcg	A	0	0.35
---	--	------	------
mPD		0.010	0.68
		nPD:	0.01
		N. weight:	0.76
		Sc. PD:	-0.025
		Sc. rank:	5.2

,

PB2			
Pos.	225	obs:	exp:
tct	S	0	1361.00
tcc	S	0	856.40
tca	S	0	1924.00
tcg	S	0	393.50
agt	S	3	1390.00
agc	S	7391	1469.00
ggt	G	44	2250.00
ggc	G	14787	2198.00
gga	G	6	6600.00
ggg	G	1	3790.00
---	--	------	------
mPD		0.45	1.5
		nPD:	0.29
		N. weight:	1.8
		Sc. PD:	0.44
		Sc. rank:	31752.0

,

PB2			
Pos.	226	obs:	exp:
tct	S	0	4091.00
tcc	S	0	2575.00
tca	S	0	5785.00
tcg	S	0	1183.00
act	T	1	0.25
acc	T	0	0.21
aca	T	0	0.46
acg	T	0	0.09
aat	N	1	0.59
aac	N	0	0.41
agt	S	22208	4180.00
agc	S	21	4415.00
ggt	G	1	0.15
ggc	G	0	0.15
gga	G	0	0.44
ggg	G	0	0.26
---	--	------	------
mPD		0.0022	1.6
		nPD:	0.
		N. weight:	1.7
		Sc. PD:	-0.077
		Sc. rank:	-29751.0

,

PB2			
Pos.	227	obs:	exp:
att	I	132	51.33
atc	I	0	35.20
ata	I	9	54.48
gtt	V	22047	4882.00
gtc	V	33	4013.00
gta	V	0	5939.00
gtg	V	3	7249.00
gct	A	8	2.12
gcc	A	0	1.66
gca	A	0	3.76
gcg	A	0	0.47
---	--	------	------
mPD		0.017	0.75
		nPD:	0.02
		N. weight:	1.5
		Sc. PD:	-0.037
		Sc. rank:	7676.9

,

PB2			
Pos.	228	obs:	exp:
tat	Y	22154	11551.00
tac	Y	78	10681.00
---	--	------	------
mPD		0.0070	0.50
		nPD:	0.01
		N. weight:	0.64
		Sc. PD:	-0.021
		Sc. rank:	-396.7

,

PB2			
Pos.	229	obs:	exp:
att	I	22196	8090.00
atc	I	22	5548.00
ata	I	6	8586.00
gtt	V	8	1.77
gtc	V	0	1.45
gta	V	0	2.15
gtg	V	0	2.63
---	--	------	------
mPD		0.0032	0.66
		nPD:	0.
		N. weight:	1.
		Sc. PD:	-0.043
		Sc. rank:	-11761.0

,

PB2			
Pos.	230	obs:	exp:
aaa	K	1	0.57
aag	K	0	0.43
gaa	E	3810	13461.00
gag	E	18421	8770.00
---	--	------	------
mPD		0.28	0.48
		nPD:	0.59
		N. weight:	0.4
		Sc. PD:	0.22
		Sc. rank:	7519.1

,

PB2			
Pos.	231	obs:	exp:
atg	M	3	3.00
gtt	V	22	4914.00
gtc	V	45	4039.00
gta	V	54	5978.00
gtg	V	22106	7296.00
gct	A	0	0.53
gcc	A	0	0.41
gca	A	0	0.94
gcg	A	2	0.12
---	--	------	------
mPD		0.011	0.74
		nPD:	0.02
		N. weight:	1.1
		Sc. PD:	-0.035
		Sc. rank:	496.5

,

PB2			
Pos.	232	obs:	exp:
tta	L	785	2444.00
ttg	L	21405	4186.00
ctt	L	0	4381.00
ctc	L	0	3140.00
cta	L	6	4280.00
ctg	L	36	3801.00
---	--	------	------
mPD		0.072	1.1
		nPD:	0.06
		N. weight:	1.5
		Sc. PD:	0.024
		Sc. rank:	20417.0

,

PB2			
Pos.	233	obs:	exp:
cat	H	553	13074.00
cac	H	21679	9158.00
---	--	------	------
mPD		0.049	0.48
		nPD:	0.1
		N. weight:	0.77
		Sc. PD:	0.04
		Sc. rank:	11531.0

,

PB2			
Pos.	234	obs:	exp:
ttt	F	6	2.57
ttc	F	0	3.43
tta	L	7827	2443.00
ttg	L	14337	4184.00
ctt	L	0	4379.00
ctc	L	0	3139.00
cta	L	3	4279.00
ctg	L	57	3800.00
atg	M	2	2.00
---	--	------	------
mPD		0.46	1.1
		nPD:	0.41
		N. weight:	1.2
		Sc. PD:	0.43
		Sc. rank:	21961.0

,

PB2			
Pos.	235	obs:	exp:
act	T	41	5604.00
acc	T	22185	4560.00
aca	T	5	10170.00
acg	T	0	1897.00
gct	A	0	0.26
gcc	A	1	0.21
gca	A	0	0.47
gcg	A	0	0.06
---	--	------	------
mPD		0.0042	0.68
		nPD:	0.01
		N. weight:	1.6
		Sc. PD:	-0.065
		Sc. rank:	-15172.0

,

PB2

Pos.	236	obs:	exp:
cat	H	0	1.18
cac	H	2	0.82
caa	Q	22162	11730.00
cag	Q	67	10499.00
aaa	K	1	0.57
aag	K	0	0.43
---	--	------	------
mPD		0.0063	0.50
		nPD:	0.01
		N. weight:	0.63
		Sc. PD:	-0.022
		Sc. rank:	-1142.1

,

PB2

Pos.	237	obs:	exp:
ggt	G	7	3371.00
ggc	G	0	3293.00
gga	G	227	9889.00
ggg	G	21998	5679.00
---	--	------	------
mPD		0.021	0.69
		nPD:	0.03
		N. weight:	1.3
		Sc. PD:	-0.023
		Sc. rank:	10015.0

,

PB2

Pos.	238	obs:	exp:
tct	S	0	0.18
tcc	S	0	0.12
tca	S	1	0.26
tcg	S	0	0.05
atg	M	7	7.00
act	T	260	5602.00
acc	T	0	4558.00
aca	T	350	10166.00
acg	T	21613	1896.00
agt	S	0	0.19
agc	S	0	0.20
gct	A	0	0.26
gcc	A	0	0.21
gca	A	0	0.47
gcg	A	1	0.06
---	--	------	------
mPD		0.055	0.68
		nPD:	0.08
		N. weight:	2.3
		Sc. PD:	0.075
		Sc. rank:	33647.0

,

PB2

Pos.	239	obs:	exp:
tct	S	0	0.18
tcc	S	0	0.12
tca	S	0	0.26
tcg	S	0	0.05
tgt	C	64	10034.00
tgc	C	22167	12197.00
agt	S	0	0.19
agc	S	1	0.20
---	--	------	------
mPD		0.0058	0.50
		nPD:	0.01
		N. weight:	0.59
		Sc. PD:	-0.021
		Sc. rank:	-1449.2

,

PB2

Pos.	240	obs:	exp:
tct	S	0	0.18
tcc	S	1	0.12
tca	S	0	0.26
tcg	S	0	0.05
tgg	W	22231	22231.00
agt	S	0	0.19
agc	S	0	0.20
---	--	------	------
mPD		0.00018	0.00018
		nPD:	1.03
		N. weight:	0.000098
		Sc. PD:	0.000095
		Sc. rank:	1.9

,

PB2

Pos.	241	obs:	exp:
gtt	V	0	0.22
gtc	V	0	0.18
gta	V	0	0.27
gtg	V	1	0.33
gaa	E	64	13460.00
gag	E	22166	8770.00
ggt	G	0	0.15
ggc	G	0	0.15
gga	G	0	0.44
ggg	G	1	0.26
---	--	------	------
mPD		0.0059	0.48
		nPD:	0.01
		N. weight:	0.92
		Sc. PD:	-0.032
		Sc. rank:	-1858.4

,

PB2

Pos.	242	obs:	exp:
caa	Q	364	11731.00
cag	Q	21867	10500.00
cgt	R	0	0.03
cgc	R	0	0.05
cga	R	1	0.10
cgg	R	0	0.07
aga	R	0	0.50
agg	R	0	0.25
---	--	------	------
mPD		0.032	0.50
		nPD:	0.06
		N. weight:	0.67
		Sc. PD:	0.012
		Sc. rank:	8983.6

,

PB2

Pos.	243	obs:	exp:
tta	L	0	6.60
ttg	L	59	11.30
ctt	L	0	11.82
ctc	L	0	8.47
cta	L	0	11.55
ctg	L	1	10.26
cgt	R	0	0.03
cgc	R	0	0.05
cga	R	0	0.10
cgg	R	0	0.07
att	I	1	1.46
atc	I	0	1.00
ata	I	3	1.55
atg	M	22163	22163.00
act	T	0	0.50
acc	T	0	0.41
aca	T	0	0.91
acg	T	2	0.17
aga	R	0	0.50
agg	R	1	0.25
gtt	V	0	0.44
gtc	V	0	0.36
gta	V	0	0.54
gtg	V	2	0.66
---	--	------	------
mPD		0.0062	0.010
		nPD:	0.62
		N. weight:	0.0049
		Sc. PD:	0.0028
		Sc. rank:	91.3

,

PB2

Pos.	244	obs:	exp:
tat	Y	52	11551.00
tac	Y	22180	10681.00
---	--	------	------
mPD		0.0047	0.50
		nPD:	0.01
		N. weight:	0.72
		Sc. PD:	-0.027
		Sc. rank:	-4012.0

,

PB2

Pos.	245	obs:	exp:
att	I	1	0.36
atc	I	0	0.25
ata	I	0	0.39
act	T	21829	5603.00
acc	T	393	4559.00
aca	T	6	10169.00
acg	T	0	1897.00
aat	N	2	1.18
aac	N	0	0.82
gct	A	1	0.26
gcc	A	0	0.21
gca	A	0	0.47
gcg	A	0	0.06
---	--	------	------
mPD		0.036	0.68
		nPD:	0.05
		N. weight:	1.3
		Sc. PD:	0.0064
		Sc. rank:	15933.0

,

PB2

Pos.	246	obs:	exp:
cct	P	9	6125.00
ccc	P	15	4123.00
cca	P	22134	9466.00
ccg	P	73	2517.00
caa	Q	1	0.53
cag	Q	0	0.47
---	--	------	------
mPD		0.0088	0.70
		nPD:	0.01
		N. weight:	0.84
		Sc. PD:	-0.029
		Sc. rank:	-1491.9

,

PB2

Pos.	247	obs:	exp:
ggt	G	13	3371.00
ggc	G	0	3293.00
gga	G	21246	9889.00
ggg	G	973	5679.00
---	--	------	------
mPD		0.085	0.69
		nPD:	0.12
		N. weight:	0.66
		Sc. PD:	0.049
		Sc. rank:	10293.0

,

PB2

Pos.	248	obs:	exp:
cgt	R	0	0.03
cgc	R	0	0.05
cga	R	0	0.10
cgg	R	0	0.07
aga	R	1	0.50
agg	R	0	0.25
ggt	G	3	3371.00
ggc	G	0	3293.00
gga	G	22174	9889.00
ggg	G	54	5679.00
---	--	------	------
mPD		0.0052	0.69
		nPD:	0.01
		N. weight:	0.8
		Sc. PD:	-0.032
		Sc. rank:	-6373.6

,

PB2

Pos.	249	obs:	exp:
aaa	K	17	9.70
aag	K	0	7.30
gct	A	0	0.26
gcc	A	0	0.21
gca	A	1	0.47
gcg	A	0	0.06
gat	D	0	1.58
gac	D	3	1.42
gaa	E	22154	13432.00
gag	E	29	8751.00
ggt	G	1	4.25
ggc	G	0	4.15
gga	G	27	12.45
ggg	G	0	7.15
---	--	------	------
mPD		0.0071	0.48
		nPD:	0.01
		N. weight :	0.5
		Sc. PD:	-0.016
		Sc. rank :	34.5

PB2

Pos.	250	obs:	exp:
atg	M	2	2.00
gtt	V	11	4915.00
gtc	V	0	4039.00
gta	V	102	5979.00
gtg	V	22117	7297.00
---	--	------	------
mPD		0.010	0.74
		nPD:	0.01
		N. weight :	1.1
		Sc. PD:	-0.036
		Sc. rank :	-750.5

PB2

Pos.	251	obs:	exp:
cgt	R	0	598.10
cgc	R	0	1146.00
cga	R	1	2146.00
cgg	R	0	1603.00
att	I	0	1.82
atc	I	0	1.25
ata	I	5	1.93
aaa	K	302	172.30
aag	K	0	129.70
aga	R	21563	10997.00
agg	R	341	5415.00
ggt	G	0	3.03
ggc	G	0	2.96
gga	G	20	8.90
ggg	G	0	5.11
---	--	------	------
mPD		0.059	0.93
		nPD:	0.06
		N. weight :	0.63
		Sc. PD:	0.0099
		Sc. rank :	8255.8

PB2

Pos.	252	obs:	exp:
tct	S	0	0.18
tcc	S	0	0.12
tca	S	0	0.26
tcg	S	0	0.05
act	T	1	0.25
acc	T	0	0.21
aca	T	0	0.46
acg	T	0	0.09
aat	N	22200	13148.00
aac	N	30	9082.00
agt	S	1	0.19
agc	S	0	0.20
---	--	------	------
mPD		0.0029	0.48
		nPD:	0.01
		N. weight :	0.52
		Sc. PD:	-0.022
		Sc. rank :	-5250.1

PB2

Pos.	253	obs:	exp:
aat	N	8	4.73
aac	N	0	3.27
gat	D	22159	11709.00
gac	D	64	10514.00
gaa	E	0	0.61
gag	E	1	0.39
---	--	------	------
mPD		0.0066	0.50
		nPD:	0.01
		N. weight :	0.63
		Sc. PD:	-0.021
		Sc. rank :	-844.5

PB2

Pos.	254	obs:	exp:
tat	Y	1	0.52
tac	Y	0	0.48
gat	D	22188	11713.00
gac	D	42	10517.00
gaa	E	1	0.61
gag	E	0	0.39
---	--	------	------
mPD		0.0040	0.50
		nPD:	0.01
		N. weight :	0.63
		Sc. PD:	-0.025
		Sc. rank :	-4550.7

PB2

Pos.	255	obs:	exp:
ttt	F	6	2.57
ttc	F	0	3.43
tta	L	0	0.11
ttg	L	0	0.19
ctt	L	1	0.20
ctc	L	0	0.14
cta	L	0	0.19
ctg	L	0	0.17
att	I	97	35.67
atc	I	1	24.46
ata	I	0	37.86
gtt	V	21966	4892.00
gtc	V	51	4020.00
gta	V	106	5950.00
gtg	V	2	7263.00
gct	A	2	0.53
gcc	A	0	0.41
gca	A	0	0.94
gcg	A	0	0.12
---	--	------	------
mPD		0.024	0.75
		nPD:	0.03
		N. weight :	1.5
		Sc. PD:	-0.023
		Sc. rank :	12598.0

PB2

Pos.	256	obs:	exp:
tat	Y	0	0.52
tac	Y	1	0.48
aat	N	0	2.37
aac	N	4	1.63
gat	D	188	11709.00
gac	D	22035	10514.00
ggt	G	2	0.61
ggc	G	2	0.59
gga	G	0	1.78
ggg	G	0	1.02
---	--	------	------
mPD		0.018	0.50
		nPD:	0.04
		N. weight :	0.71
		Sc. PD:	-0.0084
		Sc. rank :	6539.3

PB2

Pos.	257	obs:	exp:
cat	H	1	0.59
cac	H	0	0.41
caa	Q	22204	11727.00
cag	Q	21	10498.00
cgt	R	0	0.08
cgc	R	0	0.16
cga	R	3	0.29
cgg	R	0	0.22
aaa	K	3	1.71
aag	K	0	1.29
aga	R	0	1.51
agg	R	0	0.74
---	--	------	------
mPD		0.0025	0.50
		nPD:	0.01
		N. weight :	0.64
		Sc. PD:	-0.027
		Sc. rank :	-7362.3

PB2

Pos.	258	obs:	exp:
tct	S	0	4091.00
tcc	S	0	2575.00
tca	S	0	5786.00
tcg	S	0	1183.00
agt	S	4813	4180.00
agc	S	17418	4416.00
ggt	G	1	0.15
ggc	G	0	0.15
gga	G	0	0.44
ggg	G	0	0.26
---	--	------	------
mPD		0.34	1.6
		nPD:	0.21
		N. weight :	1.1
		Sc. PD:	0.18
		Sc. rank :	18678.0

PB2

Pos.	259	obs:	exp:
tta	L	71	2444.00
ttg	L	22121	4185.00
ctt	L	0	4381.00
ctc	L	0	3140.00
cta	L	9	4280.00
ctg	L	30	3801.00
gtt	V	0	0.22
gtc	V	0	0.18
gta	V	0	0.27
gtg	V	1	0.33
---	--	------	------
mPD		0.011	1.1
		nPD:	0.01
		N. weight :	1.7
		Sc. PD:	-0.063
		Sc. rank :	-8908.7

PB2

Pos.	260	obs:	exp:
att	I	21644	8091.00
atc	I	578	5548.00
ata	I	3	8587.00
act	T	1	0.25
acc	T	0	0.21
aca	T	0	0.46
acg	T	0	0.09
gtt	V	6	1.33
gtc	V	0	1.09
gta	V	0	1.61
gtg	V	0	1.97
---	--	------	------
mPD		0.052	0.66
		nPD:	0.08
		N. weight :	0.91
		Sc. PD:	0.028
		Sc. rank :	13085.0

PB2

Pos.	261	obs:	exp:
att	I	799	8083.00
atc	I	21364	5543.00
ata	I	41	8579.00
act	T	0	3.28
acc	T	13	2.67
aca	T	0	5.95
acg	T	0	1.11
gtt	V	1	3.32
gtc	V	14	2.73
gta	V	0	4.03
gtg	V	0	4.92
---	--	------	------
mPD		0.075	0.66
		nPD:	0.11
		N. weight :	1.2
		Sc. PD:	0.081
		Sc. rank :	18911.0

PB2

Pos.	262	obs:	exp:
tct	S	2	0.37
tcc	S	0	0.23
tca	S	0	0.52
tcg	S	0	0.11
act	T	3	0.76
acc	T	0	0.62
aca	T	0	1.37
acg	T	0	0.26
agt	S	0	0.38
agc	S	0	0.40
gct	A	22201	5885.00
gcc	A	15	4601.00
gca	A	1	10439.00
gcg	A	10	1302.00
---	--	------	------
mPD		0.0028	0.66
		nPD:	0.
		N. weight :	1.3
		Sc. PD:	-0.057
		Sc. rank :	-17480.0

PB2

Pos.	263	obs:	exp:
tct	S	6	1.10
tcc	S	0	0.69
tca	S	0	1.56
tcg	S	0	0.32
act	T	3	0.76
acc	T	0	0.62
aca	T	0	1.37
acg	T	0	0.26
agt	S	0	1.13
agc	S	0	1.19
gct	A	22132	5884.00
gcc	A	54	4600.00
gca	A	12	10437.00
gcg	A	25	1302.00
---	--	------	------
mPD		0.0090	0.66
		nPD:	0.01
		N. weight :	1.3
		Sc. PD:	-0.044
		Sc. rank :	-1504.3

PB2

Pos.	264	obs:	exp:
cgt	R	0	607.00
cgc	R	0	1163.00
cga	R	2	2178.00
cgg	R	0	1627.00
aga	R	22052	11161.00
agg	R	178	5496.00
---	--	------	------
mPD		0.016	0.91
		nPD:	0.02
		N. weight :	0.65
		Sc. PD:	-0.019
		Sc. rank :	1224.1

PB2

Pos.	265	obs:	exp:
tct	S	0	76.19
tcc	S	0	47.95
tca	S	0	107.70
tcg	S	0	22.03
cat	H	0	4.70
cac	H	8	3.30
act	T	0	0.25
acc	T	1	0.21
aca	T	0	0.46
acg	T	0	0.09
aat	N	232	12899.00
aac	N	21577	8910.00
agt	S	0	77.85
agc	S	414	82.24
---	--	------	------
mPD		0.058	0.55
		nPD:	0.11
		N. weight :	0.86
		Sc. PD:	0.049
		Sc. rank :	12924.0

PB2

Pos.	266	obs:	exp:
att	I	1	8087.00
atc	I	0	5545.00
ata	I	22214	8583.00
atg	M	5	5.00
aaa	K	1	0.57
aag	K	0	0.43
gtt	V	0	2.43
gtc	V	0	2.00
gta	V	11	2.96
gtg	V	0	3.61
---	--	------	------
mPD		0.0016	0.66
		nPD:	0.
		N. weight:	0.96
		Sc. PD:	-0.043
		Sc. rank:	-15072.0

PB2

Pos.	267	obs:	exp:
att	I	0	8.01
atc	I	0	5.49
ata	I	22	8.50
gtt	V	1	4910.00
gtc	V	1	4036.00
gta	V	22110	5973.00
gtg	V	98	7291.00
---	--	------	------
mPD		0.011	0.74
		nPD:	0.01
		N. weight:	1.3
		Sc. PD:	-0.042
		Sc. rank:	174.0

PB2

Pos.	268	obs:	exp:
cgt	R	0	607.00
cgc	R	0	1163.00
cga	R	5	2178.00
cgg	R	0	1627.00
aga	R	22222	11161.00
agg	R	5	5496.00
---	--	------	------
mPD		0.00090	0.91
		nPD:	0.
		N. weight:	0.69
		Sc. PD:	-0.032
		Sc. rank:	-12500.0

PB2

Pos.	269	obs:	exp:
cgt	R	0	607.00
cgc	R	0	1163.00
cga	R	7	2178.00
cgg	R	0	1627.00
aga	R	22150	11161.00
agg	R	75	5496.00
---	--	------	------
mPD		0.0074	0.91
		nPD:	0.01
		N. weight:	0.67
		Sc. PD:	-0.026
		Sc. rank:	-4759.7

PB2

Pos.	270	obs:	exp:
tct	S	0	71.40
tcc	S	0	44.94
tca	S	388	101.00
tcg	S	0	20.65
act	T	0	0.50
acc	T	0	0.41
aca	T	2	0.91
acg	T	0	0.17
agt	S	0	72.96
agc	S	0	77.07
gct	A	67	5783.00
gcc	A	10	4521.00
gca	A	21752	10258.00
gcg	A	13	1280.00
---	--	------	------
mPD		0.043	0.71
		nPD:	0.06
		N. weight:	0.75
		Sc. PD:	0.009
		Sc. rank:	9526.4

PB2

Pos.	271	obs:	exp:
tct	S	0	7.91
tcc	S	0	4.98
tca	S	43	11.19
tcg	S	0	2.29
act	T	0	1.01
acc	T	0	0.82
aca	T	4	1.83
acg	T	0	0.34
agt	S	0	8.09
agc	S	0	8.54
gct	A	10	5874.00
gcc	A	4	4592.00
gca	A	22163	10420.00
gcg	A	8	1300.00
---	--	------	------
mPD		0.0062	0.67
		nPD:	0.01
		N. weight:	0.76
		Sc. PD:	-0.029
		Sc. rank:	-4243.2

PB2

Pos.	272	obs:	exp:
gtt	V	5	4915.00
gtc	V	1	4040.00
gta	V	80	5979.00
gtg	V	22146	7298.00
---	--	------	------
mPD		0.0077	0.74
		nPD:	0.01
		N. weight:	1.1
		Sc. PD:	-0.041
		Sc. rank:	-4306.2

PB2

Pos.	273	obs:	exp:
tct	S	47	4091.00
tcc	S	5	2575.00
tca	S	22172	5786.00
tcg	S	8	1183.00
agt	S	0	4180.00
agc	S	0	4416.00
---	--	------	------
mPD		0.0054	1.6
		nPD:	0.
		N. weight:	1.3
		Sc. PD:	-0.059
		Sc. rank:	-19604.0

PB2

Pos.	274	obs:	exp:
tct	S	0	2.21
tcc	S	0	1.39
tca	S	12	3.12
tcg	S	0	0.64
act	T	0	59.74
acc	T	1	48.61
aca	T	236	108.40
acg	T	0	20.22
agt	S	0	2.26
agc	S	0	2.38
gtt	V	0	0.22
gtc	V	0	0.18
gta	V	1	0.27
gtg	V	0	0.33
gct	A	62	5820.00
gcc	A	5	4550.00
gca	A	21903	10324.00
gcg	A	12	1288.00
---	--	------	------
mPD		0.029	0.69
		nPD:	0.04
		N. weight:	0.74
		Sc. PD:	-0.0034
		Sc. rank:	7767.3

PB2

Pos.	275	obs:	exp:
gat	D	127	11713.00
gac	D	22103	10517.00
gaa	E	2	1.21
gag	E	0	0.79
---	--	------	------
mPD		0.012	0.50
		nPD:	0.02
		N. weight:	0.72
		Sc. PD:	-0.017
		Sc. rank:	3738.1

PB2

Pos.	276	obs:	exp:
tct	S	0	0.92
tcc	S	0	0.58
tca	S	5	1.30
tcg	S	0	0.27
cct	P	2	6123.00
ccc	P	6	4123.00
cca	P	22150	9464.00
ccg	P	69	2517.00
agt	S	0	0.94
agc	S	0	0.99
---	--	------	------
mPD		0.0074	0.70
		nPD:	0.01
		N. weight:	0.84
		Sc. PD:	-0.031
		Sc. rank:	-3264.0

PB2

Pos.	277	obs:	exp:
tta	L	22100	2444.00
ttg	L	100	4186.00
ctt	L	2	4381.00
ctc	L	6	3140.00
cta	L	24	4280.00
ctg	L	0	3801.00
---	--	------	------
mPD		0.013	1.1
		nPD:	0.01
		N. weight:	2.2
		Sc. PD:	-0.079
		Sc. rank:	-7685.6

PB2

Pos.	278	obs:	exp:
tct	S	0	0.74
tcc	S	0	0.46
tca	S	4	1.04
tcg	S	0	0.21
act	T	0	0.25
acc	T	0	0.21
aca	T	1	0.46
acg	T	0	0.09
agt	S	0	0.75
agc	S	0	0.79
gtt	V	0	0.22
gtc	V	0	0.18
gta	V	1	0.27
gtg	V	0	0.33
gct	A	1	5884.00
gcc	A	7	4601.00
gca	A	22174	10438.00
gcg	A	43	1302.00
gaa	E	1	0.61
gag	E	0	0.39
---	--	------	------
mPD		0.0052	0.66
		nPD:	0.01
		N. weight:	0.75
		Sc. PD:	-0.03
		Sc. rank:	-5557.7

PB2

Pos.	279	obs:	exp:
ttt	F	1	0.43
ttc	F	0	0.57
tct	S	22117	4090.00
tcc	S	104	2574.00
tca	S	6	5785.00
tcg	S	0	1183.00
tat	Y	2	1.04
tac	Y	0	0.96
agt	S	0	4179.00
agc	S	0	4415.00
gct	A	2	0.53
gcc	A	0	0.41
gca	A	0	0.94
gcg	A	0	0.12
---	--	------	------
mPD		0.010	1.6
		nPD:	0.01
		N. weight:	1.7
		Sc. PD:	-0.069
		Sc. rank:	-15943.0

PB2

Pos.	280	obs:	exp:
tta	L	9	2444.00
ttg	L	0	4185.00
ctt	L	96	4381.00
ctc	L	21991	3140.00
cta	L	135	4280.00
ctg	L	0	3801.00
att	I	0	0.36
atc	I	1	0.25
ata	I	0	0.39
---	--	------	------
mPD		0.022	1.1
		nPD:	0.02
		N. weight:	1.9
		Sc. PD:	-0.053
		Sc. rank:	5626.1

PB2

Pos.	281	obs:	exp:
tta	L	31	2444.00
ttg	L	22163	4185.00
tct	S	0	0.18
tcc	S	0	0.12
tca	S	0	0.26
tcg	S	1	0.05
ctt	L	0	4381.00
ctc	L	0	3140.00
cta	L	0	4280.00
ctg	L	36	3801.00
agt	S	0	0.19
agc	S	0	0.20
gtt	V	0	0.22
gtc	V	0	0.18
gta	V	0	0.27
gtg	V	1	0.33
---	--	------	------
mPD		0.0062	1.1
		nPD:	0.01
		N. weight:	1.7
		Sc. PD:	-0.07
		Sc. rank:	-17869.0

PB2

Pos.	282	obs:	exp:
aaa	K	1	0.57
aag	K	0	0.43
gat	D	4	2.63
gac	D	1	2.37
gaa	E	21660	13457.00
gag	E	565	8768.00
ggt	G	0	0.15
ggc	G	0	0.15
gga	G	1	0.44
ggg	G	0	0.26
---	--	------	------
mPD		0.050	0.48
		nPD:	0.1
		N. weight:	0.4
		Sc. PD:	0.023
		Sc. rank:	5999.4

PB2

Pos.	283	obs:	exp:
tta	L	0	0.33
ttg	L	1	0.56
ctt	L	0	0.59
ctc	L	0	0.42
cta	L	0	0.58
ctg	L	2	0.51
att	I	0	0.73
atc	I	0	0.50
ata	I	2	0.77
atg	M	22221	22221.00
act	T	0	1.01
acc	T	0	0.82
aca	T	0	1.83
acg	T	4	0.34
gtt	V	0	0.44
gtc	V	0	0.36
gta	V	0	0.54
gtg	V	2	0.66
---	--	------	------
mPD		0.00099	0.0016
		nPD:	0.61
		N. weight:	0.00082
		Sc. PD:	0.00046
		Sc. rank:	15.4

PB2

Pos.	284	obs:	exp:
tgt	C	172	10034.00
tgc	C	22059	12197.00
cgt	R	0	0.03
cgc	R	1	0.05
cga	R	0	0.10
cgg	R	0	0.07
aga	R	0	0.50
agg	R	0	0.25
---	--	------	------
mPD		0.015	0.50
		nPD:	0.03
		N. weight:	0.56
		Sc. PD:	-0.0091
		Sc. rank:	4678.5

PB2

Pos.	285	obs:	exp:
tat	Y	0	3.12
tac	Y	6	2.88
cat	H	100	13069.00
cac	H	22122	9153.00
caa	Q	3	1.58
cag	Q	0	1.42
aat	N	0	0.59
aac	N	1	0.41
---	--	------	------
mPD		0.0099	0.49
		nPD:	0.02
		N. weight:	0.87
		Sc. PD:	-0.023
		Sc. rank:	2963.0

PB2

Pos.	286	obs:	exp:
tct	S	0	4089.00
tcc	S	0	2573.00
tca	S	0	5782.00
tcg	S	0	1182.00
aat	N	0	2.37
aac	N	4	1.63
agt	S	30	4178.00
agc	S	22187	4413.00
ggt	G	0	1.67
ggc	G	11	1.63
gga	G	0	4.89
ggg	G	0	2.81
---	--	------	------
mPD		0.0040	1.6
		nPD:	0.
		N. weight:	1.6
		Sc. PD:	-0.073
		Sc. rank:	-25337.0

PB2

Pos.	287	obs:	exp:
tct	S	0	0.55
tcc	S	0	0.35
tca	S	3	0.78
tcg	S	0	0.16
act	T	4	5604.00
acc	T	2	4559.00
aca	T	22184	10169.00
acg	T	39	1897.00
agt	S	0	0.56
agc	S	0	0.60
---	--	------	------
mPD		0.0043	0.68
		nPD:	0.01
		N. weight:	0.78
		Sc. PD:	-0.032
		Sc. rank:	-7291.6

PB2

Pos.	288	obs:	exp:
tta	L	0	0.66
ttg	L	0	1.13
ctt	L	0	1.18
ctc	L	0	0.85
cta	L	0	1.16
ctg	L	6	1.03
cct	P	0	0.28
ccc	P	0	0.19
cca	P	0	0.43
ccg	P	1	0.11
cat	H	47	27.64
cac	H	0	19.36
caa	Q	140	11698.00
cag	Q	22029	10471.00
cgt	R	0	0.19
cgc	R	0	0.37
cga	R	0	0.69
cgg	R	7	0.51
aaa	K	0	1.14
aag	K	2	0.86
aga	R	0	3.51
agg	R	0	1.73
---	--	------	------
mPD		0.018	0.50
		nPD:	0.04
		N. weight:	0.72
		Sc. PD:	-0.0082
		Sc. rank:	6728.5

PB2

Pos.	289	obs:	exp:
att	I	22187	8085.00
atc	I	18	5544.00
ata	I	5	8581.00
gtt	V	22	4.86
gtc	V	0	4.00
gta	V	0	5.92
gtg	V	0	7.22
---	--	------	------
mPD		0.0040	0.66
		nPD:	0.01
		N. weight:	1.
		Sc. PD:	-0.042
		Sc. rank:	-10014.0

PB2

Pos.	290	obs:	exp:
cgt	R	0	0.08
cgc	R	0	0.16
cga	R	0	0.29
cgg	R	0	0.22
aga	R	3	1.51
agg	R	0	0.74
ggt	G	14	3371.00
ggc	G	12	3292.00
gga	G	22061	9888.00
ggg	G	142	5678.00
---	--	------	------
mPD		0.015	0.69
		nPD:	0.02
		N. weight:	0.77
		Sc. PD:	-0.02
		Sc. rank:	3487.4

PB2

Pos.	291	obs:	exp:
tct	S	0	2.58
tcc	S	0	1.62
tca	S	0	3.64
tcg	S	0	0.75
agt	S	14	2.63
agc	S	0	2.78
gat	D	2	1.05
gac	D	0	0.95
ggt	G	18753	3369.00
ggc	G	12	3290.00
gga	G	3446	9882.00
ggg	G	5	5675.00
---	--	------	------
mPD		0.26	0.69
		nPD:	0.38
		N. weight:	1.3
		Sc. PD:	0.43
		Sc. rank:	23355.0

PB2

Pos.	292	obs:	exp:
tta	L	0	0.55
ttg	L	2	0.94
ctt	L	0	0.99
ctc	L	0	0.71
cta	L	0	0.96
ctg	L	3	0.85
att	I	0	4.37
atc	I	0	3.00
ata	I	12	4.64
atg	M	56	56.00
act	T	0	2.77
acc	T	0	2.26
aca	T	11	5.03
acg	T	0	0.94
gtt	V	35	4893.00
gtc	V	0	4021.00
gta	V	3437	5952.00
gtg	V	18658	7264.00
gct	A	0	3.44
gcc	A	0	2.69
gca	A	1	6.11
gcg	A	12	0.76
gaa	E	2	1.21
gag	E	0	0.79
ggt	G	0	0.45
ggc	G	0	0.44
gga	G	0	1.33
ggg	G	3	0.77
---	--	------	------
mPD		0.28	0.75
		nPD:	0.37
		N. weight:	0.71
		Sc. PD:	0.23
		Sc. rank:	12701.0

PB2

Pos.	293	obs:	exp:
cgt	R	0	93.73
cgc	R	0	179.60
cga	R	0	336.30
cgg	R	3	251.20
act	T	0	0.76
acc	T	0	0.62
aca	T	0	1.37
acg	T	3	0.26
aat	N	8	4.73
aac	N	0	3.27
aaa	K	222	10710.00
aag	K	18553	8065.00
aga	R	11	1723.00
agg	R	3419	848.70
gaa	E	0	7.27
gag	E	12	4.73
ggt	G	0	0.15
ggc	G	0	0.15
gga	G	0	0.44
ggg	G	1	0.26
---	--	------	------
mPD		0.28	0.84
		nPD:	0.34
		N. weight:	0.88
		Sc. PD:	0.26
		Sc. rank:	15562.0

PB2

Pos.	294	obs:	exp:
tta	L	0	0.11
ttg	L	0	0.19
ctt	L	0	0.20
ctc	L	0	0.14
cta	L	0	0.19
ctg	L	1	0.17
att	I	3	88.46
atc	I	0	60.66
ata	I	240	93.88
atg	M	21987	21987.00
gtt	V	0	0.22
gtc	V	0	0.18
gta	V	1	0.27
gtg	V	0	0.33
---	--	------	------
mPD		0.022	0.022
		nPD:	1.
		N. weight:	0.01
		Sc. PD:	0.0094
		Sc. rank:	189.9

PB2			
Pos .	295	obs :	exp :
tta	L	0	0.22
ttg	L	1	0.38
ctt	L	0	0.39
ctc	L	0	0.28
cta	L	0	0.39
ctg	L	1	0.34
att	I	0	0.36
atc	I	0	0.25
ata	I	1	0.39
atg	M	63	63.00
gtt	V	3	4899.00
gtc	V	1	4027.00
gta	V	444	5959.00
gtg	V	21711	7274.00
gct	A	0	1.85
gcc	A	0	1.45
gca	A	0	3.29
gcg	A	7	0.41
---	--	------	------
mPD		0.046	0.74
		nPD :	0.06
		N. weight :	1.
		Sc. PD :	0.015
		Sc. rank :	13487.0

,

PB2			
Pos .	296	obs :	exp :
tat	Y	0	2.08
tac	Y	4	1.92
aat	N	0	96.40
aac	N	163	66.60
gat	D	210	11612.00
gac	D	21829	10427.00
gaa	E	19	11.50
gag	E	0	7.50
ggt	G	0	1.06
ggc	G	7	1.04
gga	G	0	3.11
ggg	G	0	1.79
---	--	------	------
mPD		0.036	0.52
		nPD :	0.07
		N. weight :	0.7
		Sc. PD :	0.016
		Sc. rank :	9725.1

,

PB2			
Pos .	297	obs :	exp :
ttt	F	0	0.43
ttc	F	1	0.57
tta	L	0	0.11
ttg	L	0	0.19
ctt	L	0	0.20
ctc	L	1	0.14
cta	L	0	0.19
ctg	L	0	0.17
att	I	167	8079.00
atc	I	22002	5540.00
ata	I	25	8575.00
act	T	0	7.31
acc	T	29	5.95
aca	T	0	13.27
acg	T	0	2.47
gtt	V	0	1.55
gtc	V	7	1.27
gta	V	0	1.88
gtg	V	0	2.30
---	--	------	------
mPD		0.021	0.66
		nPD :	0.03
		N. weight :	1.3
		Sc. PD :	-0.022
		Sc. rank :	11156.0

,

PB2			
Pos .	298	obs :	exp :
ttt	F	0	0.43
ttc	F	1	0.57
tta	L	0	2443.00
ttg	L	0	4184.00
ctt	L	21857	4379.00
ctc	L	357	3139.00
cta	L	3	4278.00
ctg	L	6	3800.00
att	I	7	2.91
atc	I	1	2.00
ata	I	0	3.09
---	--	------	------
mPD		0.033	1.1
		nPD :	0.03
		N. weight :	1.6
		Sc. PD :	-0.028
		Sc. rank :	11371.0

,

PB2			
Pos .	299	obs :	exp :
cgt	R	0	116.70
cgc	R	0	223.50
cga	R	0	418.50
cgg	R	0	312.70
aaa	K	17929	10244.00
aag	K	30	7715.00
aga	R	4271	2145.00
agg	R	2	1056.00
---	--	------	------
mPD		0.31	0.90
		nPD :	0.35
		N. weight :	0.58
		Sc. PD :	0.17
		Sc. rank :	10326.0

,

PB2			
Pos .	300	obs :	exp :
tta	L	0	0.11
ttg	L	0	0.19
ctt	L	0	0.20
ctc	L	0	0.14
cta	L	0	0.19
ctg	L	1	0.17
cat	H	8	4.70
cac	H	0	3.30
caa	Q	67	11724.00
cag	Q	22151	10494.00
cgt	R	0	0.05
cgc	R	0	0.10
cga	R	0	0.20
cgg	R	2	0.15
aaa	K	0	1.71
aag	K	3	1.29
aga	R	0	1.00
agg	R	0	0.49
---	--	------	------
mPD		0.0073	0.50
		nPD :	0.01
		N. weight :	0.74
		Sc. PD :	-0.024
		Sc. rank :	-79.5

,

PB2			
Pos .	301	obs :	exp :
tct	S	0	0.18
tcc	S	0	0.12
tca	S	0	0.26
tcg	S	0	0.05
act	T	1	0.25
acc	T	0	0.21
aca	T	0	0.46
acg	T	0	0.09
aat	N	21973	13148.00
aac	N	257	9082.00
agt	S	1	0.19
agc	S	0	0.20
---	--	------	------
mPD		0.023	0.48
		nPD :	0.05
		N. weight :	0.47
		Sc. PD :	0.000042
		Sc. rank :	5494.8

,

PB2			
Pos .	302	obs :	exp :
tta	L	0	0.11
ttg	L	0	0.19
tct	S	0	0.74
tcc	S	0	0.46
tca	S	4	1.04
tcg	S	0	0.21
ctt	L	0	0.20
ctc	L	0	0.14
cta	L	1	0.19
ctg	L	0	0.17
cct	P	1	6123.00
ccc	P	81	4122.00
cca	P	22119	9464.00
ccg	P	25	2517.00
caa	Q	1	0.53
cag	Q	0	0.47
agt	S	0	0.75
agc	S	0	0.79
---	--	------	------
mPD		0.010	0.70
		nPD :	0.01
		N. weight :	0.83
		Sc. PD :	-0.027
		Sc. rank :	-120.8

,

PB2			
Pos .	303	obs :	exp :
tct	S	1	0.37
tcc	S	0	0.23
tca	S	0	0.52
tcg	S	0	0.11
att	I	8	2.91
atc	I	0	2.00
ata	I	0	3.09
act	T	22177	5600.00
acc	T	18	4557.00
aca	T	12	10163.00
acg	T	9	1896.00
agt	S	1	0.38
agc	S	0	0.40
gct	A	6	1.59
gcc	A	0	1.24
gca	A	0	2.82
gcg	A	0	0.35
---	--	------	------
mPD		0.0049	0.68
		nPD :	0.01
		N. weight :	1.4
		Sc. PD :	-0.055
		Sc. rank :	-11153.0

,

PB2			
Pos .	304	obs :	exp :
gtt	V	0	0.66
gtc	V	0	0.55
gta	V	1	0.81
gtg	V	2	0.98
gct	A	0	0.26
gcc	A	0	0.21
gca	A	0	0.47
gcg	A	1	0.06
gat	D	1	0.53
gac	D	0	0.47
gaa	E	715	13458.00
gag	E	21512	8769.00
---	--	------	------
mPD		0.063	0.48
		nPD :	0.13
		N. weight :	0.78
		Sc. PD :	0.065
		Sc. rank :	12403.0

,

PB2			
Pos .	305	obs :	exp :
aaa	K	0	0.57
aag	K	1	0.43
gaa	E	4410	13460.00
gag	E	17820	8770.00
ggt	G	0	0.15
ggc	G	0	0.15
gga	G	0	0.44
ggg	G	1	0.26
---	--	------	------
mPD		0.32	0.48
		nPD :	0.67
		N. weight :	0.35
		Sc. PD :	0.22
		Sc. rank :	6619.2

,

PB2			
Pos .	306	obs :	exp :
caa	Q	22212	11731.00
cag	Q	20	10501.00
---	--	------	------
mPD		0.0018	0.50
		nPD :	0.
		N. weight :	0.64
		Sc. PD :	-0.028
		Sc. rank :	-8956.2

,

PB2			
Pos .	307	obs :	exp :
tct	S	0	1.47
tcc	S	8	0.93
tca	S	0	2.08
tcg	S	0	0.43
agt	S	0	1.50
agc	S	0	1.59
gct	A	438	5884.00
gcc	A	21775	4600.00
gca	A	11	10438.00
gcg	A	0	1302.00
---	--	------	------
mPD		0.040	0.66
		nPD :	0.06
		N. weight :	1.5
		Sc. PD :	0.019
		Sc. rank :	19171.0

,

PB2

Pos.	308	obs:	exp:
tta	L	1	0.11
ttg	L	0	0.19
ctt	L	0	0.20
ctc	L	0	0.14
cta	L	0	0.19
ctg	L	0	0.17
att	I	0	27.30
atc	I	0	18.72
ata	I	75	28.98
gtt	V	10	4898.00
gtc	V	2	4026.00
gta	V	21625	5958.00
gtg	V	518	7273.00
gct	A	0	0.26
gcc	A	0	0.21
gca	A	1	0.47
gcg	A	0	0.06
---	--	------	------
mPD		0.053	0.75
		nPD:	0.07
		N. weight:	1.2
		Sc. PD:	0.029
		Sc. rank:	16790.0

,

PB2

Pos.	309	obs:	exp:
cat	H	0	0.59
cac	H	1	0.41
aat	N	0	0.59
aac	N	1	0.41
gat	D	31	11711.00
gac	D	22196	10516.00
gaa	E	3	1.82
gag	E	0	1.18
---	--	------	------
mPD		0.0032	0.50
		nPD:	0.01
		N. weight:	0.75
		Sc. PD:	-0.03
		Sc. rank:	-6841.5

,

PB2

Pos.	310	obs:	exp:
att	I	2	8088.00
atc	I	19	5546.00
ata	I	22198	8584.00
atg	M	10	10.00
act	T	0	0.50
acc	T	0	0.41
aca	T	2	0.91
acg	T	0	0.17
gtt	V	0	0.22
gtc	V	0	0.18
gta	V	1	0.27
gtg	V	0	0.33
---	--	------	------
mPD		0.0031	0.66
		nPD:	0.
		N. weight:	0.95
		Sc. PD:	-0.041
		Sc. rank:	-11652.0

,

PB2

Pos.	311	obs:	exp:
tgt	C	11	10034.00
tgc	C	22220	12197.00
ggt	G	0	0.15
ggc	G	1	0.15
gga	G	0	0.44
ggg	G	0	0.26
---	--	------	------
mPD		0.0011	0.50
		nPD:	0.
		N. weight:	0.6
		Sc. PD:	-0.027
		Sc. rank:	-9819.0

,

PB2

Pos.	312	obs:	exp:
caa	Q	0	0.53
cag	Q	1	0.47
cgt	R	0	0.11
cgc	R	0	0.21
cga	R	0	0.39
cgg	R	0	0.29
aaa	K	209	12679.00
aag	K	22018	9548.00
aga	R	0	2.01
agg	R	4	0.99
---	--	------	------
mPD		0.019	0.49
		nPD:	0.04
		N. weight:	0.8
		Sc. PD:	-0.0069
		Sc. rank:	7768.0

,

PB2

Pos.	313	obs:	exp:
gct	A	426	5886.00
gcc	A	5	4602.00
gca	A	21777	10442.00
gcg	A	24	1302.00
---	--	------	------
mPD		0.040	0.66
		nPD:	0.06
		N. weight:	0.67
		Sc. PD:	0.0087
		Sc. rank:	8617.5

,

PB2

Pos.	314	obs:	exp:
tct	S	0	2.39
tcc	S	0	1.51
tca	S	13	3.38
tcg	S	0	0.69
act	T	0	0.25
acc	T	0	0.21
aca	T	1	0.46
acg	T	0	0.09
agt	S	0	2.44
agc	S	0	2.58
gct	A	3	5882.00
gcc	A	3	4599.00
gca	A	22158	10435.00
gcg	A	54	1302.00
---	--	------	------
mPD		0.0066	0.66
		nPD:	0.01
		N. weight:	0.75
		Sc. PD:	-0.028
		Sc. rank:	-3310.4

,

PB2

Pos.	315	obs:	exp:
tta	L	3	0.66
ttg	L	0	1.13
ctt	L	0	1.18
ctc	L	0	0.85
cta	L	3	1.16
ctg	L	0	1.03
att	I	5	8077.00
atc	I	1	5539.00
ata	I	22182	8572.00
atg	M	23	23.00
act	T	0	1.01
acc	T	0	0.82
aca	T	4	1.83
acg	T	0	0.34
gtt	V	0	2.43
gtc	V	0	2.00
gta	V	11	2.96
gtg	V	0	3.61
---	--	------	------
mPD		0.0045	0.66
		nPD:	0.01
		N. weight:	0.96
		Sc. PD:	-0.039
		Sc. rank:	-8566.6

,

PB2

Pos.	316	obs:	exp:
ggt	G	12	3371.00
ggc	G	4	3293.00
gga	G	486	9889.00
ggg	G	21730	5679.00
---	--	------	------
mPD		0.044	0.69
		nPD:	0.06
		N. weight:	1.3
		Sc. PD:	0.02
		Sc. rank:	16736.0

,

PB2

Pos.	317	obs:	exp:
ttt	F	1	0.43
ttc	F	0	0.57
tta	L	48	2443.00
ttg	L	22135	4184.00
tct	S	0	0.18
tcc	S	0	0.12
tca	S	0	0.26
tcg	S	1	0.05
ctt	L	0	4380.00
ctc	L	0	3139.00
cta	L	0	4279.00
ctg	L	42	3800.00
atg	M	4	4.00
agt	S	0	0.19
agc	S	0	0.20
gtt	V	0	0.22
gtc	V	0	0.18
gta	V	0	0.27
gtg	V	1	0.33
---	--	------	------
mPD		0.0087	1.1
		nPD:	0.01
		N. weight:	1.7
		Sc. PD:	-0.066
		Sc. rank:	-12774.0

,

PB2

Pos.	318	obs:	exp:
cgt	R	0	606.80
cgc	R	0	1163.00
cga	R	0	2177.00
cgg	R	1	1627.00
act	T	0	0.25
acc	T	0	0.21
aca	T	0	0.46
acg	T	1	0.09
aaa	K	0	3.42
aag	K	6	2.58
aga	R	104	11158.00
agg	R	22120	5494.00
---	--	------	------
mPD		0.010	0.91
		nPD:	0.01
		N. weight:	1.4
		Sc. PD:	-0.05
		Sc. rank:	-4748.3

,

PB2

Pos.	319	obs:	exp:
att	I	3116	8083.00
atc	I	18784	5543.00
ata	I	304	8579.00
gtt	V	1	6.19
gtc	V	27	5.09
gta	V	0	7.53
gtg	V	0	9.19
---	--	------	------
mPD		0.27	0.66
		nPD:	0.4
		N. weight:	0.86
		Sc. PD:	0.31
		Sc. rank:	15675.0

,

PB2

Pos.	320	obs:	exp:
tct	S	0	4091.00
tcc	S	0	2575.00
tca	S	0	5785.00
tcg	S	0	1183.00
aat	N	0	1.77
aac	N	3	1.23
agt	S	40	4180.00
agc	S	22189	4415.00
---	--	------	------
mPD		0.0039	1.6
		nPD:	0.
		N. weight:	1.6
		Sc. PD:	-0.073
		Sc. rank:	-25500.0

,

PB2

Pos.	321	obs:	exp:
tct	S	7	4091.00
tcc	S	9	2575.00
tca	S	22208	5786.00
tcg	S	7	1183.00
cct	P	0	0.28
ccc	P	0	0.19
cca	P	1	0.43
ccg	P	0	0.11
agt	S	0	4180.00
agc	S	0	4416.00
---	--	------	------
mPD		0.0022	1.6
		nPD:	0.
		N. weight:	1.4
		Sc. PD:	-0.062
		Sc. rank:	-23836.0

,

PB2

Pos.	322	obs:	exp:
ttt	F	2	0.86
ttc	F	0	1.14
tct	S	22118	4091.00
tcc	S	101	2575.00
tca	S	4	5785.00
tcg	S	7	1183.00
agt	S	0	4180.00
agc	S	0	4416.00
---	--	------	------
mPD		0.010	1.6
		nPD:	0.01
		N. weight:	1.7
		Sc. PD:	-0.069
		Sc. rank:	-16209.0

,

PB2

Pos.	323	obs:	exp:
ttt	F	807	9539.00
ttc	F	21422	12690.00
tta	L	0	0.22
ttg	L	2	0.38
tct	S	0	0.18
tcc	S	1	0.12
tca	S	0	0.26
tcg	S	0	0.05
ctt	L	0	0.39
ctc	L	0	0.28
cta	L	0	0.39
ctg	L	0	0.34
agt	S	0	0.19
agc	S	0	0.20
---	--	------	------
mPD		0.070	0.49
		nPD:	0.14
		N. weight:	0.42
		Sc. PD:	0.04
		Sc. rank:	6754.1

,

PB2

Pos.	324	obs:	exp:
tct	S	0	4091.00
tcc	S	0	2575.00
tca	S	0	5785.00
tcg	S	0	1183.00
tgt	C	1	0.45
tgc	C	0	0.55
cgt	R	0	0.03
cgc	R	0	0.05
cga	R	0	0.10
cgg	R	0	0.07
agt	S	22181	4180.00
agc	S	49	4416.00
aga	R	0	0.50
agg	R	1	0.25
---	--	------	------
mPD		0.0046	1.6
		nPD:	0.
		N. weight:	1.7
		Sc. PD:	-0.074
		Sc. rank:	-25514.0

,

PB2

Pos.	325	obs:	exp:
ttt	F	22204	9540.00
ttc	F	27	12691.00
tat	Y	1	0.52
tac	Y	0	0.48
---	--	------	------
mPD		0.0025	0.49
		nPD:	0.01
		N. weight:	0.84
		Sc. PD:	-0.036
		Sc. rank:	-9704.5

,

PB2

Pos.	326	obs:	exp:
ggt	G	22202	3371.00
ggc	G	16	3293.00
gga	G	12	9889.00
ggg	G	2	5679.00
---	--	------	------
mPD		0.0027	0.69
		nPD:	0.
		N. weight:	1.9
		Sc. PD:	-0.082
		Sc. rank:	-25832.0

,

PB2

Pos.	327	obs:	exp:
cgt	R	0	0.05
cgc	R	0	0.10
cga	R	0	0.20
cgg	R	0	0.15
aga	R	0	1.00
agg	R	2	0.49
gaa	E	0	5.45
gag	E	9	3.55
ggt	G	79	3369.00
ggc	G	11	3291.00
gga	G	352	9884.00
ggg	G	21779	5676.00
---	--	------	------
mPD		0.040	0.69
		nPD:	0.06
		N. weight:	1.3
		Sc. PD:	0.013
		Sc. rank:	15843.0

,

PB2

Pos.	328	obs:	exp:
ttt	F	27	9535.00
ttc	F	22193	12685.00
tta	L	1	0.22
ttg	L	0	0.38
tat	Y	0	5.20
tac	Y	10	4.80
ctt	L	0	0.39
ctc	L	1	0.28
cta	L	0	0.39
ctg	L	0	0.34
---	--	------	------
mPD		0.0035	0.49
		nPD:	0.01
		N. weight:	0.56
		Sc. PD:	-0.022
		Sc. rank:	-4709.5

,

PB2

Pos.	329	obs:	exp:
att	I	16	5.82
atc	I	0	3.99
ata	I	0	6.18
act	T	22157	5600.00
acc	T	43	4556.00
aca	T	12	10162.00
acg	T	2	1895.00
aat	N	2	1.18
aac	N	0	0.82
---	--	------	------
mPD		0.0067	0.68
		nPD:	0.01
		N. weight:	1.4
		Sc. PD:	-0.051
		Sc. rank:	-6349.4

,

PB2

Pos.	330	obs:	exp:
ttt	F	107	9540.00
ttc	F	22123	12690.00
tta	L	1	0.22
ttg	L	0	0.38
ctt	L	0	0.39
ctc	L	1	0.28
cta	L	0	0.39
ctg	L	0	0.34
---	--	------	------
mPD		0.0098	0.49
		nPD:	0.02
		N. weight:	0.54
		Sc. PD:	-0.015
		Sc. rank:	1703.2

,

PB2

Pos.	331	obs:	exp:
aaa	K	22209	12682.00
aag	K	23	9550.00
---	--	------	------
mPD		0.0021	0.49
		nPD:	0.
		N. weight:	0.56
		Sc. PD:	-0.024
		Sc. rank:	-7294.4

,

PB2

Pos.	332	obs:	exp:
cgt	R	0	606.40
cgc	R	0	1162.00
cga	R	0	2175.00
cgg	R	1	1625.00
atg	M	2	2.00
aaa	K	0	10.27
aag	K	18	7.73
aga	R	337	11150.00
agg	R	21872	5491.00
ggt	G	0	0.30
ggc	G	0	0.30
gga	G	0	0.89
ggg	G	2	0.51
---	--	------	------
mPD		0.032	0.91
		nPD:	0.04
		N. weight:	1.3
		Sc. PD:	-0.016
		Sc. rank:	12183.0

,

PB2

Pos.	333	obs:	exp:
att	I	0	2.18
atc	I	0	1.50
ata	I	6	2.32
act	T	2	5602.00
acc	T	1	4558.00
aca	T	22195	10167.00
acg	T	26	1896.00
gct	A	0	0.53
gcc	A	0	0.41
gca	A	2	0.94
gcg	A	0	0.12
---	--	------	------
mPD		0.0033	0.68
		nPD:	0.
		N. weight:	0.78
		Sc. PD:	-0.033
		Sc. rank:	-9132.3

,

PB2

Pos.	334	obs:	exp:
tct	S	0	4090.00
tcc	S	0	2574.00
tca	S	0	5785.00
tcg	S	0	1183.00
aat	N	0	1.77
aac	N	3	1.23
agt	S	78	4179.00
agc	S	22149	4415.00
ggt	G	0	0.30
ggc	G	2	0.30
gga	G	0	0.89
ggg	G	0	0.51
---	--	------	------
mPD		0.0074	1.6
		nPD:	0.
		N. weight:	1.6
		Sc. PD:	-0.069
		Sc. rank:	-20079.0

,

PB2

Pos.	335	obs:	exp:
gaa	E	1	0.61
gag	E	0	0.39
ggt	G	102	3371.00
ggc	G	1	3293.00
gga	G	22044	9889.00
ggg	G	84	5679.00
---	--	------	------
mPD		0.017	0.69
		nPD:	0.02
		N. weight:	0.77
		Sc. PD:	-0.018
		Sc. rank:	4278.7

,

PB2

Pos.	336	obs:	exp:
tct	S	17	4090.00
tcc	S	2	2574.00
tca	S	22190	5785.00
tcg	S	18	1183.00
cct	P	0	0.28
ccc	P	0	0.19
cca	P	1	0.43
ccg	P	0	0.11
act	T	0	1.01
acc	T	0	0.82
aca	T	4	1.83
acg	T	0	0.34
agt	S	0	4179.00
agc	S	0	4415.00
---	--	------	------
mPD		0.0038	1.6
		nPD:	0.
		N. weight:	1.3
		Sc. PD:	-0.061
		Sc. rank:	-21379.0

,

PB2

Pos.	337	obs:	exp:
tct	S	9	4090.00
tcc	S	0	2574.00
tca	S	22198	5784.00
tcg	S	19	1183.00
cct	P	0	0.28
ccc	P	0	0.19
cca	P	1	0.43
ccg	P	0	0.11
act	T	0	1.01
acc	T	0	0.82
aca	T	4	1.83
acg	T	0	0.34
agt	S	0	4179.00
agc	S	0	4415.00
gct	A	0	0.26
gcc	A	0	0.21
gca	A	1	0.47
gcg	A	0	0.06
---	--	------	------
mPD		0.0031	1.6
		nPD:	0.
		N. weight:	1.4
		Sc. PD:	-0.061
		Sc. rank:	-22564.0

,

PB2

Pos.	338	obs:	exp:
ttt	F	0	2.15
ttc	F	5	2.85
tta	L	0	0.11
ttg	L	0	0.19
ctt	L	0	0.20
ctc	L	1	0.14
cta	L	0	0.19
ctg	L	0	0.17
att	I	0	297.00
atc	I	816	203.70
ata	I	0	315.30
act	T	0	0.25
acc	T	1	0.21
aca	T	0	0.46
acg	T	0	0.09
gtt	V	33	4730.00
gtc	V	21211	3888.00
gta	V	150	5754.00
gtg	V	0	7023.00
gct	A	0	3.97
gcc	A	15	3.10
gca	A	0	7.04
gcg	A	0	0.88
---	--	------	------
mPD		0.089	0.81
		nPD:	0.11
		N. weight:	1.7
		Sc. PD:	0.1
		Sc. rank:	25150.0

,

PB2

Pos.	339	obs:	exp:
cgt	R	0	0.74
cgc	R	0	1.41
cga	R	0	2.64
cgg	R	0	1.98
atg	M	2	2.00
act	T	0	3.53
acc	T	0	2.87
aca	T	1	6.40
acg	T	13	1.19
aat	N	4	2.37
aac	N	0	1.63
aaa	K	265	12655.00
aag	K	21920	9530.00
aga	R	0	13.55
agg	R	27	6.67
---	--	------	------
mPD		0.028	0.50
		nPD:	0.06
		N. weight:	0.79
		Sc. PD:	0.0068
		Sc. rank:	9743.8

,

PB2

Pos.	340	obs:	exp:
caa	Q	1	0.53
cag	Q	0	0.47
cgt	R	0	8.82
cgc	R	0	16.90
cga	R	0	31.64
cgg	R	0	23.64
att	I	0	0.36
atc	I	0	0.25
ata	I	1	0.39
act	T	0	2.02
acc	T	0	1.64
aca	T	8	3.66
acg	T	0	0.68
aat	N	183	109.40
aac	N	2	75.58
aaa	K	21663	12375.00
aag	K	32	9320.00
aga	R	323	162.20
agg	R	0	79.85
gaa	E	19	11.50
gag	E	0	7.50
---	--	------	------
mPD		0.051	0.54
		nPD:	0.09
		N. weight:	0.56
		Sc. PD:	0.026
		Sc. rank:	8337.2

,

PB2

Pos.	341	obs:	exp:
aaa	K	1	0.57
aag	K	0	0.43
gat	D	2	4.22
gac	D	6	3.78
gaa	E	21952	13456.00
gag	E	271	8767.00
---	--	------	------
mPD		0.025	0.48
		nPD:	0.05
		N. weight:	0.45
		Sc. PD:	0.002
		Sc. rank:	5405.6

,

PB2

Pos.	342	obs:	exp:
aaa	K	1	0.57
aag	K	0	0.43
gat	D	0	1.05
gac	D	2	0.95
gaa	E	21495	13460.00
gag	E	734	8769.00
---	--	------	------
mPD		0.064	0.48
		nPD:	0.13
		N. weight:	0.37
		Sc. PD:	0.032
		Sc. rank:	5948.7

,

PB2

Pos.	343	obs:	exp:
gtt	V	0	0.22
gtc	V	0	0.18
gta	V	1	0.27
gtg	V	0	0.33
gat	D	0	1.05
gac	D	2	0.95
gaa	E	22180	13459.00
gag	E	48	8769.00
ggt	G	0	0.15
ggc	G	0	0.15
gga	G	1	0.44
ggg	G	0	0.26
---	--	------	------
mPD		0.0047	0.48
		nPD:	0.01
		N. weight:	0.49
		Sc. PD:	-0.018
		Sc. rank:	-2480.3

,

PB2

Pos.	344	obs:	exp:
tta	L	0	1.21
ttg	L	3	2.07
ctt	L	0	2.17
ctc	L	0	1.55
cta	L	0	2.12
ctg	L	8	1.88
att	I	4	5.10
atc	I	0	3.49
ata	I	10	5.41
atg	M	19120	19120.00
gtt	V	10	682.50
gtc	V	0	560.90
gta	V	1	830.20
gtg	V	3076	1013.00
---	--	------	------
mPD		0.24	0.42
		nPD:	0.58
		N. weight:	0.15
		Sc. PD:	0.082
		Sc. rank:	2871.6

,

PB2

Pos.	345	obs:	exp:
tta	L	26	2444.00
ttg	L	0	4185.00
ctt	L	10	4380.00
ctc	L	2	3140.00
cta	L	22143	4279.00
ctg	L	47	3800.00
att	I	0	1.46
atc	I	0	1.00
ata	I	4	1.55
---	--	------	------
mPD		0.0080	1.1
		nPD:	0.01
		N. weight:	1.6
		Sc. PD:	-0.066
		Sc. rank:	-14125.0

,

PB2

Pos.	346	obs:	exp:
cct	P	0	0.28
ccc	P	0	0.19
cca	P	0	0.43
ccg	P	1	0.11
act	T	30	5604.00
acc	T	0	4560.00
aca	T	314	10170.00
acg	T	21887	1897.00
---	--	------	------
mPD		0.031	0.68
		nPD:	0.05
		N. weight:	2.4
		Sc. PD:	-0.0057
		Sc. rank:	26502.0

,

PB2

Pos.	347	obs:	exp:
ggt	G	37	3371.00
ggc	G	22162	3293.00
gga	G	32	9889.00
ggg	G	1	5679.00
---	--	------	------
mPD		0.0063	0.69
		nPD:	0.01
		N. weight:	1.9
		Sc. PD:	-0.073
		Sc. rank:	-11328.0

,

PB2			
Pos.	348	obs:	exp:
tct	S	0	0.92
tcc	S	0	0.58
tca	S	0	1.30
tcg	S	0	0.27
aat	N	442	13146.00
aac	N	21785	9081.00
agt	S	0	0.94
agc	S	5	0.99
---	--	------	------
mPD		0.039	0.48
		nPD:	0.08
		N. weight:	0.8
		Sc. PD:	0.027
		Sc. rank:	11712.0

PB2			
Pos.	349	obs:	exp:
tta	L	0	2444.00
ttg	L	0	4185.00
ctt	L	56	4381.00
ctc	L	22165	3140.00
cta	L	10	4280.00
ctg	L	0	3801.00
att	I	0	0.36
atc	I	1	0.25
ata	I	0	0.39
---	--	------	------
mPD		0.0060	1.1
		nPD:	0.01
		N. weight:	2.
		Sc. PD:	-0.082
		Sc. rank:	-21833.0

PB2			
Pos.	350	obs:	exp:
caa	Q	21877	11731.00
cag	Q	355	10501.00
---	--	------	------
mPD		0.031	0.50
		nPD:	0.06
		N. weight:	0.56
		Sc. PD:	0.0087
		Sc. rank:	7440.8

PB2			
Pos.	351	obs:	exp:
cct	P	0	0.55
ccc	P	0	0.37
cca	P	2	0.85
ccg	P	0	0.23
act	T	7	5600.00
acc	T	1	4557.00
aca	T	22149	10163.00
acg	T	58	1895.00
gct	A	0	3.97
gcc	A	0	3.10
gca	A	15	7.04
gcg	A	0	0.88
---	--	------	------
mPD		0.0075	0.68
		nPD:	0.01
		N. weight:	0.77
		Sc. PD:	-0.028
		Sc. rank:	-2802.6

PB2			
Pos.	352	obs:	exp:
tta	L	0	2439.00
ttg	L	104	4178.00
ctt	L	9	4373.00
ctc	L	0	3134.00
cta	L	66	4272.00
ctg	L	22011	3794.00
atg	M	42	42.00
---	--	------	------
mPD		0.020	1.1
		nPD:	0.02
		N. weight:	1.7
		Sc. PD:	-0.052
		Sc. rank:	2925.4

PB2			
Pos.	353	obs:	exp:
cgt	R	0	0.76
cgc	R	0	1.46
cga	R	0	2.74
cgg	R	0	2.05
act	T	0	1.26
acc	T	0	1.03
aca	T	5	2.29
acg	T	0	0.43
aat	N	1	0.59
aac	N	0	0.41
aaa	K	22096	12662.00
aag	K	102	9536.00
aga	R	28	14.06
agg	R	0	6.92
---	--	------	------
mPD		0.012	0.49
		nPD:	0.02
		N. weight:	0.54
		Sc. PD:	-0.012
		Sc. rank:	3052.6

PB2			
Pos.	354	obs:	exp:
tta	L	18959	2100.00
ttg	L	94	3596.00
ctt	L	0	3764.00
ctc	L	0	2698.00
cta	L	47	3677.00
ctg	L	0	3266.00
att	I	0	1139.00
atc	I	0	780.80
ata	I	3128	1209.00
atg	M	1	1.00
gtt	V	0	0.66
gtc	V	0	0.55
gta	V	3	0.81
gtg	V	0	0.98
---	--	------	------
mPD		0.25	1.3
		nPD:	0.2
		N. weight:	2.
		Sc. PD:	0.3
		Sc. rank:	33311.0

PB2			
Pos.	355	obs:	exp:
tct	S	0	0.18
tcc	S	0	0.12
tca	S	0	0.26
tcg	S	0	0.05
cgt	R	0	606.50
cgc	R	0	1162.00
cga	R	3	2176.00
cgg	R	0	1626.00
att	I	0	1.82
atc	I	0	1.25
ata	I	5	1.93
act	T	0	0.25
acc	T	0	0.21
aca	T	1	0.46
acg	T	0	0.09
aaa	K	6	3.42
aag	K	0	2.58
agt	S	1	0.19
agc	S	0	0.20
aga	R	22168	11151.00
agg	R	41	5491.00
ggt	G	0	1.06
ggc	G	0	1.04
gga	G	7	3.11
ggg	G	0	1.79
---	--	------	------
mPD		0.0058	0.91
		nPD:	0.01
		N. weight:	0.68
		Sc. PD:	-0.028
		Sc. rank:	-6460.3

PB2			
Pos.	356	obs:	exp:
att	I	0	24.39
atc	I	0	16.72
ata	I	67	25.89
gtt	V	1	4900.00
gtc	V	1	4027.00
gta	V	22108	5961.00
gtg	V	54	7276.00
gct	A	0	0.26
gcc	A	0	0.21
gca	A	1	0.47
gcg	A	0	0.06
---	--	------	------
mPD		0.011	0.74
		nPD:	0.01
		N. weight:	1.3
		Sc. PD:	-0.042
		Sc. rank:	363.4

PB2			
Pos.	357	obs:	exp:
tat	Y	4	2.08
tac	Y	0	1.92
cat	H	22206	13072.00
cac	H	22	9156.00
---	--	------	------
mPD		0.0023	0.48
		nPD:	0.
		N. weight:	0.53
		Sc. PD:	-0.022
		Sc. rank:	-6256.7

PB2			
Pos.	358	obs:	exp:
aaa	K	3	1.71
aag	K	0	1.29
gaa	E	22161	13459.00
gag	E	67	8769.00
ggt	G	0	0.15
ggc	G	0	0.15
gga	G	1	0.44
ggg	G	0	0.26
---	--	------	------
mPD		0.0064	0.48
		nPD:	0.01
		N. weight:	0.49
		Sc. PD:	-0.017
		Sc. rank:	-628.7

PB2			
Pos.	359	obs:	exp:
ggt	G	10	3371.00
ggc	G	1	3293.00
gga	G	1076	9889.00
ggg	G	21145	5679.00
---	--	------	------
mPD		0.093	0.69
		nPD:	0.13
		N. weight:	1.2
		Sc. PD:	0.1
		Sc. rank:	18352.0

PB2			
Pos.	360	obs:	exp:
ttt	F	1	0.43
ttc	F	0	0.57
tat	Y	22065	11550.00
tac	Y	165	10680.00
tgt	C	1	0.45
tgc	C	0	0.55
---	--	------	------
mPD		0.015	0.50
		nPD:	0.03
		N. weight:	0.62
		Sc. PD:	-0.011
		Sc. rank:	4621.5

PB2			
Pos.	361	obs:	exp:
gaa	E	22183	13461.00
gag	E	49	8771.00
---	--	------	------
mPD		0.0044	0.48
		nPD:	0.01
		N. weight:	0.49
		Sc. PD:	-0.019
		Sc. rank:	-2776.5

PB2			
Pos.	362	obs:	exp:
gaa	E	22148	13461.00
gag	E	84	8771.00
---	--	------	------
mPD		0.0075	0.48
		nPD:	0.02
		N. weight:	0.48
		Sc. PD:	-0.015
		Sc. rank:	315.9

PB2			
Pos.	363	obs:	exp:
ttt	F	22	9536.00
ttc	F	22200	12686.00
tta	L	4	0.88
ttg	L	0	1.51
tct	S	0	0.37
tcc	S	2	0.23
tca	S	0	0.52
tcg	S	0	0.11
ctt	L	0	1.58
ctc	L	4	1.13
cta	L	0	1.54
ctg	L	0	1.37
agt	S	0	0.38
agc	S	0	0.40
---	--	------	------
mPD		0.0029	0.49
		nPD:	0.01
		N. weight:	0.56
		Sc. PD:	-0.023
		Sc. rank:	-5740.4

PB2			
Pos.	364	obs:	exp:
tct	S	0	0.18
tcc	S	0	0.12
tca	S	1	0.26
tcg	S	0	0.05
act	T	1	5603.00
acc	T	19	4559.00
aca	T	22193	10168.00
acg	T	14	1896.00
agt	S	0	0.19
agc	S	0	0.20
gct	A	0	1.06
gcc	A	0	0.83
gca	A	4	1.88
gcg	A	0	0.23
---	--	------	------
mPD		0.0035	0.68
		nPD:	0.01
		N. weight:	0.78
		Sc. PD:	-0.033
		Sc. rank:	-8920.3

PB2

Pos.	365	obs:	exp:
tta	L	0	0.22
ttg	L	2	0.38
ctt	L	0	0.39
ctc	L	0	0.28
cta	L	0	0.39
ctg	L	0	0.34
att	I	5	5.46
atc	I	0	3.74
ata	I	10	5.80
atg	M	22211	22211.00
gtt	V	0	0.88
gtc	V	0	0.73
gta	V	0	1.08
gtg	V	4	1.31
---	--	------	------
mPD		0.0019	0.0022
		nPD:	0.84
		N. weight:	0.00065
		Sc. PD:	0.00051
		Sc. rank:	12.4

,

PB2

Pos.	366	obs:	exp:
att	I	8	2.91
atc	I	0	2.00
ata	I	0	3.09
gtt	V	22171	4913.00
gtc	V	37	4038.00
gta	V	1	5976.00
gtg	V	12	7294.00
gct	A	3	0.79
gcc	A	0	0.62
gca	A	0	1.41
gcg	A	0	0.18
---	--	------	------
mPD		0.0055	0.74
		nPD:	0.01
		N. weight:	1.5
		Sc. PD:	-0.06
		Sc. rank:	-12046.0

,

PB2

Pos.	367	obs:	exp:
ggt	G	5	3371.00
ggc	G	1	3293.00
gga	G	100	9889.00
ggg	G	22126	5679.00
---	--	------	------
mPD		0.0095	0.69
		nPD:	0.01
		N. weight:	1.3
		Sc. PD:	-0.045
		Sc. rank:	-1139.5

,

PB2

Pos.	368	obs:	exp:
cgt	R	0	595.30
cgc	R	0	1140.00
cga	R	0	2135.00
cgg	R	0	1595.00
att	I	0	0.73
atc	I	0	0.50
ata	I	2	0.77
aaa	K	405	240.70
aag	K	17	181.30
aga	R	21776	10945.00
agg	R	25	5390.00
ggt	G	0	1.06
ggc	G	0	1.04
gga	G	7	3.11
ggg	G	0	1.79
---	--	------	------
mPD		0.042	0.94
		nPD:	0.04
		N. weight:	0.68
		Sc. PD:	-0.0021
		Sc. rank:	7536.0

,

PB2

Pos.	369	obs:	exp:
tct	S	0	0.74
tcc	S	0	0.46
tca	S	0	1.04
tcg	S	0	0.21
cgt	R	0	605.40
cgc	R	0	1160.00
cga	R	1	2172.00
cgg	R	0	1623.00
aaa	K	47	27.38
aag	K	1	20.62
agt	S	3	0.75
agc	S	1	0.79
aga	R	22048	11131.00
agg	R	123	5481.00
ggt	G	0	1.21
ggc	G	0	1.18
gga	G	8	3.56
ggg	G	0	2.04
---	--	------	------
mPD		0.017	0.91
		nPD:	0.02
		N. weight:	0.66
		Sc. PD:	-0.019
		Sc. rank:	1403.6

,

PB2

Pos.	370	obs:	exp:
tct	S	0	0.18
tcc	S	0	0.12
tca	S	1	0.26
tcg	S	0	0.05
act	T	0	1.01
acc	T	0	0.82
aca	T	4	1.83
acg	T	0	0.34
agt	S	0	0.19
agc	S	0	0.20
gtt	V	0	0.44
gtc	V	0	0.36
gta	V	2	0.54
gtg	V	0	0.66
gct	A	6	5884.00
gcc	A	0	4601.00
gca	A	22218	10438.00
gcg	A	1	1302.00
---	--	------	------
mPD		0.0013	0.66
		nPD:	0.
		N. weight:	0.76
		Sc. PD:	-0.035
		Sc. rank:	-12681.0

,

PB2

Pos.	371	obs:	exp:
att	I	0	0.36
atc	I	0	0.25
ata	I	1	0.39
act	T	515	5604.00
acc	T	1	4560.00
aca	T	21691	10170.00
acg	T	24	1897.00
---	--	------	------
mPD		0.048	0.68
		nPD:	0.07
		N. weight:	0.69
		Sc. PD:	0.015
		Sc. rank:	9518.5

,

PB2

Pos.	372	obs:	exp:
tct	S	1	0.18
tcc	S	0	0.12
tca	S	0	0.26
tcg	S	0	0.05
act	T	3	0.76
acc	T	0	0.62
aca	T	0	1.37
acg	T	0	0.26
agt	S	0	0.19
agc	S	0	0.20
gct	A	22157	5885.00
gcc	A	66	4601.00
gca	A	3	10440.00
gcg	A	2	1302.00
---	--	------	------
mPD		0.0067	0.66
		nPD:	0.01
		N. weight:	1.3
		Sc. PD:	-0.049
		Sc. rank:	-5492.8

,

PB2

Pos.	373	obs:	exp:
att	I	21990	8088.00
atc	I	203	5546.00
ata	I	26	8584.00
atg	M	1	1.00
gtt	V	12	2.65
gtc	V	0	2.18
gta	V	0	3.23
gtg	V	0	3.94
---	--	------	------
mPD		0.022	0.66
		nPD:	0.03
		N. weight:	0.96
		Sc. PD:	-0.014
		Sc. rank:	8503.8

,

PB2

Pos.	374	obs:	exp:
ttt	F	0	15.02
ttc	F	35	19.98
tta	L	0	2440.00
ttg	L	0	4179.00
ctt	L	22	4374.00
ctc	L	22143	3135.00
cta	L	30	4273.00
ctg	L	0	3795.00
cct	P	0	0.28
ccc	P	1	0.19
cca	P	0	0.43
ccg	P	0	0.11
att	I	0	0.36
atc	I	1	0.25
ata	I	0	0.39
---	--	------	------
mPD		0.0080	1.1
		nPD:	0.01
		N. weight:	2.
		Sc. PD:	-0.079
		Sc. rank:	-17047.0

,

PB2

Pos.	375	obs:	exp:
cgt	R	0	606.80
cgc	R	0	1163.00
cga	R	1	2177.00
cgg	R	0	1627.00
aaa	K	5	2.85
aag	K	0	2.15
aga	R	21895	11158.00
agg	R	329	5494.00
ggt	G	0	0.30
ggc	G	0	0.30
gga	G	2	0.89
ggg	G	0	0.51
---	--	------	------
mPD		0.030	0.91
		nPD:	0.03
		N. weight:	0.63
		Sc. PD:	-0.0092
		Sc. rank:	5520.4

,

PB2

Pos.	376	obs:	exp:
cgt	R	0	0.03
cgc	R	0	0.05
cga	R	0	0.10
cgg	R	0	0.07
aaa	K	429	12681.00
aag	K	21802	9550.00
aga	R	0	0.50
agg	R	1	0.25
---	--	------	------
mPD		0.038	0.49
		nPD:	0.08
		N. weight:	0.75
		Sc. PD:	0.022
		Sc. rank:	10681.0

,

PB2

Pos.	377	obs:	exp:
tct	S	0	0.18
tcc	S	0	0.12
tca	S	1	0.26
tcg	S	0	0.05
cct	P	0	0.28
ccc	P	0	0.19
cca	P	1	0.43
ccg	P	0	0.11
act	T	0	0.25
acc	T	0	0.21
aca	T	1	0.46
acg	T	0	0.09
agt	S	0	0.19
agc	S	0	0.20
gtt	V	0	0.22
gtc	V	0	0.18
gta	V	1	0.27
gtg	V	0	0.33
gct	A	4	5884.00
gcc	A	16	4601.00
gca	A	22201	10439.00
gcg	A	5	1302.00
ggt	G	0	0.30
ggc	G	0	0.30
gga	G	2	0.89
ggg	G	0	0.51
---	--	------	------
mPD		0.0028	0.66
		nPD:	0.
		N. weight:	0.75
		Sc. PD:	-0.033
		Sc. rank:	-9948.3

PB2

Pos.	378	obs:	exp:
act	T	15	5604.00
acc	T	22191	4560.00
aca	T	26	10171.00
acg	T	0	1897.00
---	--	------	------
mPD		0.0037	0.68
		nPD:	0.01
		N. weight:	1.6
		Sc. PD:	-0.066
		Sc. rank:	-17136.0

PB2

Pos.	379	obs:	exp:
tct	S	0	0.92
tcc	S	0	0.58
tca	S	0	1.30
tcg	S	0	0.27
cgt	R	0	606.40
cgc	R	0	1162.00
cga	R	0	2175.00
cgg	R	6	1625.00
atg	M	2	2.00
aaa	K	0	9.70
aag	K	17	7.30
agt	S	5	0.94
agc	S	0	0.99
aga	R	102	11149.00
agg	R	22100	5490.00
---	--	------	------
mPD		0.012	0.91
		nPD:	0.01
		N. weight:	1.4
		Sc. PD:	-0.047
		Sc. rank:	-2008.0

PB2

Pos.	380	obs:	exp:
cgt	R	0	607.00
cgc	R	0	1163.00
cga	R	3	2177.00
cgg	R	0	1627.00
aaa	K	1	0.57
aag	K	0	0.43
aga	R	21597	11160.00
agg	R	630	5496.00
gat	D	1	0.53
gac	D	0	0.47
---	--	------	------
mPD		0.056	0.91
		nPD:	0.06
		N. weight:	0.59
		Sc. PD:	0.008
		Sc. rank:	7614.6

PB2

Pos.	381	obs:	exp:
ttt	F	1	0.43
ttc	F	0	0.57
tta	L	35	2444.00
ttg	L	22038	4185.00
ctt	L	0	4380.00
ctc	L	0	3140.00
cta	L	0	4279.00
ctg	L	155	3800.00
atg	M	3	3.00
---	--	------	------
mPD		0.017	1.1
		nPD:	0.02
		N. weight:	1.6
		Sc. PD:	-0.052
		Sc. rank:	656.8

PB2

Pos.	382	obs:	exp:
tta	L	0	0.11
ttg	L	0	0.19
ctt	L	0	0.20
ctc	L	1	0.14
cta	L	0	0.19
ctg	L	0	0.17
att	I	65	8075.00
atc	I	22114	5537.00
ata	I	4	8570.00
gtt	V	9	10.61
gtc	V	39	8.72
gta	V	0	12.91
gtg	V	0	15.76
---	--	------	------
mPD		0.011	0.66
		nPD:	0.02
		N. weight:	1.4
		Sc. PD:	-0.042
		Sc. rank:	2162.9

PB2

Pos.	383	obs:	exp:
cat	H	1	1.18
cac	H	1	0.82
caa	Q	19118	11730.00
cag	Q	3112	10500.00
---	--	------	------
mPD		0.24	0.50
		nPD:	0.48
		N. weight:	0.25
		Sc. PD:	0.11
		Sc. rank:	4671.3

PB2

Pos.	384	obs:	exp:
ttt	F	7	3.43
ttc	F	1	4.57
tta	L	18744	2443.00
ttg	L	3456	4184.00
ctt	L	1	4379.00
ctc	L	8	3139.00
cta	L	12	4278.00
ctg	L	2	3800.00
gtt	V	0	0.22
gtc	V	0	0.18
gta	V	1	0.27
gtg	V	0	0.33
---	--	------	------
mPD		0.27	1.1
		nPD:	0.23
		N. weight:	1.7
		Sc. PD:	0.31
		Sc. rank:	29059.0

PB2

Pos.	385	obs:	exp:
att	I	11	8090.00
atc	I	4	5548.00
ata	I	22209	8586.00
atg	M	1	1.00
gtt	V	0	1.55
gtc	V	0	1.27
gta	V	7	1.88
gtg	V	0	2.30
---	--	------	------
mPD		0.0021	0.66
		nPD:	0.
		N. weight:	0.95
		Sc. PD:	-0.042
		Sc. rank:	-14167.0

PB2

Pos.	386	obs:	exp:
tta	L	1	0.11
ttg	L	0	0.19
ctt	L	0	0.20
ctc	L	0	0.14
cta	L	0	0.19
ctg	L	0	0.17
att	I	0	5.82
atc	I	0	3.99
ata	I	16	6.18
gtt	V	5	4908.00
gtc	V	0	4034.00
gta	V	22135	5971.00
gtg	V	61	7288.00
gct	A	0	3.71
gcc	A	0	2.90
gca	A	14	6.58
gcg	A	0	0.82
---	--	------	------
mPD		0.0087	0.74
		nPD:	0.01
		N. weight:	1.3
		Sc. PD:	-0.046
		Sc. rank:	-3347.0

PB2

Pos.	387	obs:	exp:
tct	S	0	4089.00
tcc	S	0	2574.00
tca	S	0	5783.00
tcg	S	0	1183.00
aat	N	0	6.51
aac	N	11	4.49
agt	S	132	4178.00
agc	S	22088	4414.00
ggt	G	0	0.15
ggc	G	1	0.15
gga	G	0	0.44
ggg	G	0	0.26
---	--	------	------
mPD		0.013	1.6
		nPD:	0.01
		N. weight:	1.6
		Sc. PD:	-0.063
		Sc. rank:	-11690.0

PB2

Pos.	388	obs:	exp:
ggt	G	0	3371.00
ggc	G	0	3293.00
gga	G	250	9889.00
ggg	G	21982	5679.00
---	--	------	------
mPD		0.022	0.69
		nPD:	0.03
		N. weight:	1.3
		Sc. PD:	-0.02
		Sc. rank:	11285.0

PB2

Pos.	389	obs:	exp:
cgt	R	0	603.50
cgc	R	0	1156.00
cga	R	3	2165.00
cgg	R	0	1618.00
att	I	0	0.73
atc	I	0	0.50
ata	I	2	0.77
aat	N	0	0.59
aac	N	1	0.41
aaa	K	124	70.73
aag	K	0	53.27
aga	R	22053	11096.00
agg	R	47	5464.00
ggt	G	0	0.30
ggc	G	0	0.30
gga	G	2	0.89
ggg	G	0	0.51
---	--	------	------
mPD		0.016	0.92
		nPD:	0.02
		N. weight:	0.68
		Sc. PD:	-0.02
		Sc. rank:	1214.4

,

PB2

Pos.	390	obs:	exp:
aat	N	0	15.38
aac	N	26	10.62
gat	D	121	11697.00
gac	D	22078	10502.00
gaa	E	6	3.63
gag	E	0	2.37
ggt	G	0	0.15
ggc	G	1	0.15
gga	G	0	0.44
ggg	G	0	0.26
---	--	------	------
mPD		0.014	0.50
		nPD:	0.03
		N. weight:	0.72
		Sc. PD:	-0.014
		Sc. rank:	4952.1

,

PB2

Pos.	391	obs:	exp:
gtt	V	0	0.22
gtc	V	0	0.18
gta	V	0	0.27
gtg	V	1	0.33
gat	D	138	72.71
gac	D	0	65.29
gaa	E	93	13374.00
gag	E	21995	8714.00
ggt	G	0	0.76
ggc	G	0	0.74
gga	G	0	2.22
ggg	G	5	1.28
---	--	------	------
mPD		0.021	0.48
		nPD:	0.04
		N. weight:	0.91
		Sc. PD:	-0.0035
		Sc. rank:	9917.3

,

PB2

Pos.	392	obs:	exp:
cct	P	0	0.55
ccc	P	0	0.37
cca	P	0	0.85
ccg	P	2	0.23
cat	H	11	6.47
cac	H	0	4.53
caa	Q	68	11724.00
cag	Q	22151	10495.00
---	--	------	------
mPD		0.0073	0.50
		nPD:	0.01
		N. weight:	0.74
		Sc. PD:	-0.024
		Sc. rank:	-52.3

,

PB2

Pos.	393	obs:	exp:
tct	S	7	4091.00
tcc	S	5	2575.00
tca	S	22211	5786.00
tcg	S	9	1183.00
agt	S	0	4180.00
agc	S	0	4416.00
---	--	------	------
mPD		0.0019	1.6
		nPD:	0.
		N. weight:	1.4
		Sc. PD:	-0.062
		Sc. rank:	-24156.0

,

PB2

Pos.	394	obs:	exp:
att	I	8069	8073.00
atc	I	3	5536.00
ata	I	14105	8568.00
atg	M	1	1.00
act	T	2	1.51
acc	T	0	1.23
aca	T	4	2.74
acg	T	0	0.51
aat	N	1	0.59
aac	N	0	0.41
gtt	V	32	10.39
gtc	V	0	8.54
gta	V	15	12.64
gtg	V	0	15.43
---	--	------	------
mPD		0.47	0.66
		nPD:	0.71
		N. weight:	0.32
		Sc. PD:	0.21
		Sc. rank:	6088.3

,

PB2

Pos.	395	obs:	exp:
tct	S	4	0.74
tcc	S	0	0.46
tca	S	0	1.04
tcg	S	0	0.21
act	T	8	2.02
acc	T	0	1.64
aca	T	0	3.66
acg	T	0	0.68
agt	S	0	0.75
agc	S	0	0.79
gtt	V	1	0.22
gtc	V	0	0.18
gta	V	0	0.27
gtg	V	0	0.33
gct	A	22171	5883.00
gcc	A	38	4599.00
gca	A	10	10435.00
gcg	A	0	1302.00
---	--	------	------
mPD		0.0055	0.66
		nPD:	0.01
		N. weight:	1.3
		Sc. PD:	-0.052
		Sc. rank:	-9206.6

,

PB2

Pos.	396	obs:	exp:
gat	D	1	1.58
gac	D	2	1.42
gaa	E	18761	13455.00
gag	E	3461	8767.00
ggt	G	0	1.06
ggc	G	0	1.04
gga	G	7	3.11
ggg	G	0	1.79
---	--	------	------
mPD		0.26	0.48
		nPD:	0.55
		N. weight:	0.14
		Sc. PD:	0.069
		Sc. rank:	2561.4

,

PB2

Pos.	397	obs:	exp:
tct	S	0	0.18
tcc	S	0	0.12
tca	S	1	0.26
tcg	S	0	0.05
act	T	0	4.03
acc	T	0	3.28
aca	T	16	7.32
acg	T	0	1.37
agt	S	0	0.19
agc	S	0	0.20
gtt	V	1	5.31
gtc	V	0	4.36
gta	V	19	6.45
gtg	V	4	7.88
gct	A	22	5875.00
gcc	A	1	4593.00
gca	A	22146	10422.00
gcg	A	22	1300.00
---	--	------	------
mPD		0.0082	0.67
		nPD:	0.01
		N. weight:	0.75
		Sc. PD:	-0.026
		Sc. rank:	-1687.5

,

PB2

Pos.	398	obs:	exp:
tta	L	8	0.99
ttg	L	0	1.69
ctt	L	0	1.77
ctc	L	0	1.27
cta	L	1	1.73
ctg	L	0	1.54
att	I	3	8020.00
atc	I	14	5500.00
ata	I	22015	8512.00
atg	M	174	174.00
act	T	0	1.01
acc	T	0	0.82
aca	T	4	1.83
acg	T	0	0.34
gtt	V	0	2.87
gtc	V	0	2.36
gta	V	13	3.50
gtg	V	0	4.27
---	--	------	------
mPD		0.019	0.66
		nPD:	0.03
		N. weight:	0.95
		Sc. PD:	-0.017
		Sc. rank:	6773.2

,

PB2

Pos.	399	obs:	exp:
att	I	21918	8093.00
atc	I	242	5550.00
ata	I	72	8589.00
---	--	------	------
mPD		0.028	0.66
		nPD:	0.04
		N. weight:	0.94
		Sc. PD:	-0.0047
		Sc. rank:	9844.3

,

PB2

Pos.	400	obs:	exp:
gtt	V	12	4912.00
gtc	V	0	4037.00
gta	V	125	5975.00
gtg	V	22079	7293.00
gct	A	0	3.97
gcc	A	0	3.10
gca	A	0	7.04
gcg	A	15	0.88
ggt	G	0	0.15
ggc	G	0	0.15
gga	G	0	0.44
ggg	G	1	0.26
---	--	------	------
mPD		0.014	0.74
		nPD:	0.02
		N. weight:	1.1
		Sc. PD:	-0.031
		Sc. rank:	2446.4

,

PB2			
Pos.	401	obs:	exp:
tct	S	0	0.37
tcc	S	2	0.23
tca	S	0	0.52
tcg	S	0	0.11
agt	S	0	0.38
agc	S	0	0.40
gct	A	42	5885.00
gcc	A	22083	4602.00
gca	A	105	10441.00
gcg	A	0	1302.00
---	--	------	------
mPD		0.013	0.66
		nPD:	0.02
		N. weight:	1.5
		Sc. PD:	-0.042
		Sc. rank:	5111.1

PB2			
Pos.	402	obs:	exp:
tta	L	0	0.33
ttg	L	0	0.56
ctt	L	0	0.59
ctc	L	0	0.42
cta	L	0	0.58
ctg	L	3	0.51
att	I	3	6.55
atc	I	0	4.49
ata	I	15	6.95
atg	M	22210	22210.00
act	T	0	0.25
acc	T	0	0.21
aca	T	0	0.46
acg	T	1	0.09
---	--	------	------
mPD		0.0020	0.0022
		nPD:	0.89
		N. weight:	0.00083
		Sc. PD:	0.00069
		Sc. rank:	15.9

PB2			
Pos.	403	obs:	exp:
att	I	0	2.55
atc	I	0	1.75
ata	I	7	2.70
gtt	V	1816	4914.00
gtc	V	6	4039.00
gta	V	20343	5977.00
gtg	V	60	7296.00
---	--	------	------
mPD		0.16	0.74
		nPD:	0.21
		N. weight:	1.
		Sc. PD:	0.17
		Sc. rank:	17415.0

PB2			
Pos.	404	obs:	exp:
ttt	F	105	9540.00
ttc	F	22127	12692.00
---	--	------	------
mPD		0.0094	0.49
		nPD:	0.02
		N. weight:	0.54
		Sc. PD:	-0.015
		Sc. rank:	1411.7

PB2			
Pos.	405	obs:	exp:
tct	S	2	4090.00
tcc	S	2	2574.00
tca	S	22136	5784.00
tcg	S	85	1183.00
cct	P	0	0.28
ccc	P	0	0.19
cca	P	1	0.43
ccg	P	0	0.11
act	T	0	1.51
acc	T	0	1.23
aca	T	6	2.74
acg	T	0	0.51
agt	S	0	4179.00
agc	S	0	4415.00
---	--	------	------
mPD		0.0086	1.6
		nPD:	0.01
		N. weight:	1.3
		Sc. PD:	-0.056
		Sc. rank:	-15086.0

PB2			
Pos.	406	obs:	exp:
caa	Q	22138	11731.00
cag	Q	93	10500.00
gaa	E	1	0.61
gag	E	0	0.39
---	--	------	------
mPD		0.0084	0.50
		nPD:	0.02
		N. weight:	0.62
		Sc. PD:	-0.019
		Sc. rank:	894.1

PB2			
Pos.	407	obs:	exp:
aaa	K	0	0.57
aag	K	1	0.43
gaa	E	161	13461.00
gag	E	22070	8770.00
---	--	------	------
mPD		0.014	0.48
		nPD:	0.03
		N. weight:	0.89
		Sc. PD:	-0.015
		Sc. rank:	6854.0

PB2			
Pos.	408	obs:	exp:
aat	N	4	2.37
aac	N	0	1.63
gat	D	22136	11710.00
gac	D	89	10515.00
gaa	E	0	1.21
gag	E	2	0.79
ggt	G	1	0.15
ggc	G	0	0.15
gga	G	0	0.44
ggg	G	0	0.26
---	--	------	------
mPD		0.0086	0.50
		nPD:	0.02
		N. weight:	0.62
		Sc. PD:	-0.019
		Sc. rank:	932.1

PB2			
Pos.	409	obs:	exp:
tct	S	0	1.29
tcc	S	0	0.81
tca	S	0	1.82
tcg	S	0	0.37
tat	Y	0	0.52
tac	Y	1	0.48
tgt	C	18	10024.00
tgc	C	22190	12184.00
cgt	R	0	0.41
cgc	R	14	0.78
cga	R	1	1.47
cgg	R	0	1.10
agt	S	0	1.32
agc	S	7	1.39
aga	R	0	7.53
agg	R	0	3.71
ggt	G	0	0.15
ggc	G	1	0.15
gga	G	0	0.44
ggg	G	0	0.26
---	--	------	------
mPD		0.0039	0.50
		nPD:	0.01
		N. weight:	0.6
		Sc. PD:	-0.024
		Sc. rank:	-4479.1

PB2			
Pos.	410	obs:	exp:
atg	M	22219	22219.00
act	T	0	0.25
acc	T	0	0.21
aca	T	0	0.46
acg	T	1	0.09
gtt	V	0	2.65
gtc	V	0	2.18
gta	V	0	3.23
gtg	V	12	3.94
---	--	------	------
mPD		0.0012	0.0020
		nPD:	0.59
		N. weight:	0.00072
		Sc. PD:	0.00039
		Sc. rank:	13.4

PB2			
Pos.	411	obs:	exp:
tta	L	0	0.22
ttg	L	0	0.38
ctt	L	0	0.39
ctc	L	2	0.28
cta	L	0	0.39
ctg	L	0	0.34
att	I	406	8083.00
atc	I	21730	5543.00
ata	I	68	8579.00
act	T	0	0.50
acc	T	2	0.41
aca	T	0	0.91
acg	T	0	0.17
aaa	K	1	0.57
aag	K	0	0.43
gtt	V	1	5.08
gtc	V	22	4.18
gta	V	0	6.19
gtg	V	0	7.55
---	--	------	------
mPD		0.045	0.66
		nPD:	0.07
		N. weight:	1.3
		Sc. PD:	0.026
		Sc. rank:	17359.0

PB2			
Pos.	412	obs:	exp:
cgt	R	0	0.25
cgc	R	0	0.47
cga	R	0	0.88
cgg	R	0	0.66
aat	N	0	1.18
aac	N	2	0.82
aaa	K	14076	12676.00
aag	K	8145	9545.00
aga	R	3	4.52
agg	R	6	2.22
---	--	------	------
mPD		0.47	0.49
		nPD:	0.95
		N. weight:	0.0089
		Sc. PD:	0.008
		Sc. rank:	169.2

PB2			
Pos.	413	obs:	exp:
tct	S	0	1.10
tcc	S	0	0.69
tca	S	6	1.56
tcg	S	0	0.32
act	T	0	0.25
acc	T	0	0.21
aca	T	1	0.46
acg	T	0	0.09
agt	S	0	1.13
agc	S	0	1.19
gct	A	26	5884.00
gcc	A	0	4601.00
gca	A	22193	10438.00
gcg	A	6	1302.00
---	--	------	------
mPD		0.0035	0.66
		nPD:	0.01
		N. weight:	0.75
		Sc. PD:	-0.032
		Sc. rank:	-8386.4

PB2			
Pos.	414	obs:	exp:
att	I	32	11.65
atc	I	0	7.99
ata	I	0	12.36
gtt	V	22174	4908.00
gtc	V	22	4034.00
gta	V	0	5970.00
gtg	V	3	7287.00
gct	A	1	0.26
gcc	A	0	0.21
gca	A	0	0.47
gcg	A	0	0.06
---	--	------	------
mPD		0.0052	0.74
		nPD:	0.01
		N. weight:	1.5
		Sc. PD:	-0.061
		Sc. rank:	-12963.0

PB2			
Pos.	415	obs:	exp:
cgt	R	0	607.00
cgc	R	0	1163.00
cga	R	0	2178.00
cgg	R	1	1627.00
aga	R	211	11161.00
agg	R	22020	5496.00
---	--	------	------
mPD		0.019	0.91
		nPD:	0.02
		N. weight:	1.4
		Sc. PD:	-0.036
		Sc. rank:	4804.4

PB2			
Pos.	416	obs:	exp:
tct	S	0	1.84
tcc	S	0	1.16
tca	S	0	2.60
tcg	S	0	0.53
agt	S	0	1.88
agc	S	10	1.99
ggt	G	144	3370.00
ggc	G	21992	3291.00
gga	G	86	9885.00
ggg	G	0	5676.00
---	--	------	------
mPD		0.021	0.69
		nPD:	0.03
		N. weight:	1.9
		Sc. PD:	-0.031
		Sc. rank:	14893.0

PB2			
Pos.	417	obs:	exp:
aat	N	2	1.18
aac	N	0	0.82
gat	D	21955	11713.00
gac	D	275	10517.00
---	--	------	------
mPD		0.025	0.50
		nPD:	0.05
		N. weight:	0.58
		Sc. PD:	0.001
		Sc. rank:	6892.5

PB2			
Pos.	418	obs:	exp:
tta	L	1	2443.00
ttg	L	31	4185.00
ctt	L	19	4380.00
ctc	L	0	3140.00
cta	L	85	4279.00
ctg	L	22091	3800.00
atg	M	5	5.00
---	--	------	------
mPD		0.013	1.1
		nPD:	0.01
		N. weight:	1.7
		Sc. PD:	-0.063
		Sc. rank:	-5732.3

PB2			
Pos.	419	obs:	exp:
aat	N	196	13149.00
aac	N	22036	9083.00
---	--	------	------
mPD		0.017	0.48
		nPD:	0.04
		N. weight:	0.85
		Sc. PD:	-0.0096
		Sc. rank:	8043.3

PB2			
Pos.	420	obs:	exp:
ttt	F	22214	9540.00
ttc	F	18	12692.00
---	--	------	------
mPD		0.0016	0.49
		nPD:	0.
		N. weight:	0.85
		Sc. PD:	-0.037
		Sc. rank:	-12275.0

PB2			
Pos.	421	obs:	exp:
att	I	0	1.09
atc	I	3	0.75
ata	I	0	1.16
gtt	V	55	4914.00
gtc	V	22162	4039.00
gta	V	12	5978.00
gtg	V	0	7297.00
---	--	------	------
mPD		0.0063	0.74
		nPD:	0.01
		N. weight:	1.7
		Sc. PD:	-0.066
		Sc. rank:	-11127.0

PB2			
Pos.	422	obs:	exp:
tct	S	0	0.18
tcc	S	0	0.12
tca	S	0	0.26
tcg	S	0	0.05
cat	H	1	0.59
cac	H	0	0.41
act	T	2	0.50
acc	T	0	0.41
aca	T	0	0.91
acg	T	0	0.17
aat	N	22194	13147.00
aac	N	34	9081.00
agt	S	1	0.19
agc	S	0	0.20
---	--	------	------
mPD		0.0034	0.48
		nPD:	0.01
		N. weight:	0.52
		Sc. PD:	-0.021
		Sc. rank:	-4405.6

PB2			
Pos.	423	obs:	exp:
cgt	R	0	607.00
cgc	R	0	1163.00
cga	R	0	2177.00
cgg	R	1	1627.00
aaa	K	0	0.57
aag	K	1	0.43
aga	R	67	11161.00
agg	R	22163	5496.00
---	--	------	------
mPD		0.0062	0.91
		nPD:	0.01
		N. weight:	1.4
		Sc. PD:	-0.056
		Sc. rank:	-12490.0

PB2			
Pos.	424	obs:	exp:
gct	A	3	5886.00
gcc	A	1	4602.00
gca	A	22219	10442.00
gcg	A	9	1302.00
---	--	------	------
mPD		0.0012	0.66
		nPD:	0.
		N. weight:	0.76
		Sc. PD:	-0.035
		Sc. rank:	-12886.0

PB2			
Pos.	425	obs:	exp:
act	T	0	0.25
acc	T	1	0.21
aca	T	0	0.46
acg	T	0	0.09
aat	N	160	13148.00
aac	N	22071	9083.00
---	--	------	------
mPD		0.014	0.48
		nPD:	0.03
		N. weight:	0.86
		Sc. PD:	-0.015
		Sc. rank:	6395.1

PB2			
Pos.	426	obs:	exp:
caa	Q	60	11731.00
cag	Q	22172	10501.00
---	--	------	------
mPD		0.0054	0.50
		nPD:	0.01
		N. weight:	0.74
		Sc. PD:	-0.027
		Sc. rank:	-2766.0

PB2			
Pos.	427	obs:	exp:
caa	Q	1	0.53
cag	Q	0	0.47
cgt	R	3	607.00
cgc	R	0	1163.00
cga	R	3382	2177.00
cgg	R	55	1627.00
aaa	K	1	0.57
aag	K	0	0.43
aga	R	18725	11160.00
agg	R	65	5496.00
---	--	------	------
mPD		0.27	0.91
		nPD:	0.3
		N. weight:	0.49
		Sc. PD:	0.12
		Sc. rank:	8529.3

PB2			
Pos.	428	obs:	exp:
tta	L	0	2444.00
ttg	L	46	4186.00
ctt	L	9	4381.00
ctc	L	0	3140.00
cta	L	57	4280.00
ctg	L	22120	3801.00
---	--	------	------
mPD		0.010	1.1
		nPD:	0.01
		N. weight:	1.7
		Sc. PD:	-0.067
		Sc. rank:	-10741.0

PB2			
Pos.	429	obs:	exp:
aat	N	18474	13149.00
aac	N	3758	9083.00
---	--	------	------
mPD		0.28	0.48
		nPD:	0.58
		N. weight:	0.13
		Sc. PD:	0.072
		Sc. rank:	2514.6

PB2			
Pos.	430	obs:	exp:
tct	S	0	0.18
tcc	S	1	0.12
tca	S	0	0.26
tcg	S	0	0.05
cct	P	251	6125.00
ccc	P	21919	4123.00
cca	P	61	9466.00
ccg	P	0	2517.00
agt	S	0	0.19
agc	S	0	0.20
---	--	------	------
mPD		0.028	0.70
		nPD:	0.04
		N. weight:	1.6
		Sc. PD:	-0.012
		Sc. rank:	16274.0

PB2			
Pos.	431	obs:	exp:
tta	L	0	0.11
ttg	L	0	0.19
ctt	L	0	0.20
ctc	L	0	0.14
cta	L	0	0.19
ctg	L	1	0.17
atg	M	22231	22231.00
---	--	------	------
mPD		0.000090	0.00015
		nPD:	0.61
		N. weight:	0.00008
		Sc. PD:	0.000045
		Sc. rank:	1.5

PB2			
Pos.	432	obs:	exp:
tat	Y	0	2.08
tac	Y	4	1.92
cat	H	134	13072.00
cac	H	22094	9156.00
---	--	------	------
mPD		0.012	0.48
		nPD:	0.03
		N. weight:	0.86
		Sc. PD:	-0.019
		Sc. rank:	5199.6

PB2			
Pos.	433	obs:	exp:
caa	Q	21989	11731.00
cag	Q	243	10501.00
---	--	------	------
mPD		0.022	0.50
		nPD:	0.04
		N. weight:	0.59
		Sc. PD:	-0.0024
		Sc. rank:	6290.3

PB2			
Pos.	434	obs:	exp:
ttt	F	0	0.43
ttc	F	1	0.57
tta	L	0	2444.00
ttg	L	0	4185.00
ctt	L	39	4381.00
ctc	L	22116	3140.00
cta	L	76	4280.00
ctg	L	0	3801.00
---	--	------	------
mPD		0.010	1.1
		nPD:	0.01
		N. weight:	1.9
		Sc. PD:	-0.074
		Sc. rank:	-11197.0

PB2			
Pos.	435	obs:	exp:
ttt	F	1	0.43
ttc	F	0	0.57
tta	L	38	2444.00
ttg	L	22158	4185.00
ctt	L	0	4381.00
ctc	L	0	3140.00
cta	L	0	4280.00
ctg	L	35	3801.00
---	--	------	------
mPD		0.0066	1.1
		nPD:	0.01
		N. weight:	1.7
		Sc. PD:	-0.069
		Sc. rank:	-17121.0

PB2			
Pos.	436	obs:	exp:
cgt	R	0	607.00
cgc	R	0	1163.00
cga	R	0	2178.00
cgg	R	2	1627.00
aga	R	54	11161.00
agg	R	22176	5496.00
---	--	------	------
mPD		0.0050	0.91
		nPD:	0.01
		N. weight:	1.4
		Sc. PD:	-0.058
		Sc. rank:	-14763.0

PB2

Pos.	437	obs:	exp:
cat	H	22200	13074.00
cac	H	31	9157.00
caa	Q	1	0.53
cag	Q	0	0.47
---	--	------	------
mPD		0.0029	0.48
		nPD:	0.01
		N. weight:	0.53
		Sc. PD:	-0.022
		Sc. rank:	-5317.7

,

PB2

Pos.	438	obs:	exp:
ttt	F	69	9540.00
ttc	F	22161	12690.00
tat	Y	0	1.04
tac	Y	2	0.96
---	--	------	------
mPD		0.0064	0.49
		nPD:	0.01
		N. weight:	0.55
		Sc. PD:	-0.019
		Sc. rank:	-751.8

,

PB2

Pos.	439	obs:	exp:
cat	H	1	1.76
cac	H	2	1.24
caa	Q	22082	11730.00
cag	Q	147	10499.00
---	--	------	------
mPD		0.013	0.50
		nPD:	0.03
		N. weight:	0.61
		Sc. PD:	-0.012
		Sc. rank:	3986.9

,

PB2

Pos.	440	obs:	exp:
cgt	R	0	0.14
cgc	R	0	0.26
cga	R	0	0.49
cgg	R	0	0.37
aaa	K	22103	12679.00
aag	K	124	9548.00
aga	R	5	2.51
agg	R	0	1.24
---	--	------	------
mPD		0.012	0.49
		nPD:	0.02
		N. weight:	0.53
		Sc. PD:	-0.013
		Sc. rank:	2855.1

,

PB2

Pos.	441	obs:	exp:
cat	H	2	1.18
cac	H	0	0.82
aat	N	22	13.01
aac	N	0	8.99
gat	D	22193	11698.00
gac	D	8	10503.00
gaa	E	2	2.42
gag	E	2	1.58
ggt	G	3	0.45
ggc	G	0	0.44
gga	G	0	1.33
ggg	G	0	0.77
---	--	------	------
mPD		0.0035	0.50
		nPD:	0.01
		N. weight:	0.64
		Sc. PD:	-0.026
		Sc. rank:	-5698.7

,

PB2

Pos.	442	obs:	exp:
act	T	0	0.50
acc	T	0	0.41
aca	T	2	0.91
acg	T	0	0.17
gtt	V	0	0.22
gtc	V	0	0.18
gta	V	1	0.27
gtg	V	0	0.33
gct	A	3	5885.00
gcc	A	7	4601.00
gca	A	22108	10440.00
gcg	A	110	1302.00
ggt	G	0	0.15
ggc	G	0	0.15
gga	G	1	0.44
ggg	G	0	0.26
---	--	------	------
mPD		0.011	0.66
		nPD:	0.02
		N. weight:	0.74
		Sc. PD:	-0.023
		Sc. rank:	874.8

,

PB2

Pos.	443	obs:	exp:
caa	Q	3	1.58
cag	Q	0	1.42
cgt	R	0	0.03
cgc	R	0	0.05
cga	R	0	0.10
cgg	R	0	0.07
aaa	K	20073	12680.00
aag	K	2155	9548.00
aga	R	1	0.50
agg	R	0	0.25
---	--	------	------
mPD		0.18	0.49
		nPD:	0.36
		N. weight:	0.27
		Sc. PD:	0.084
		Sc. rank:	4888.5

,

PB2

Pos.	444	obs:	exp:
tta	L	0	0.22
ttg	L	1	0.38
ctt	L	0	0.39
ctc	L	0	0.28
cta	L	0	0.39
ctg	L	1	0.34
att	I	0	0.73
atc	I	0	0.50
ata	I	2	0.77
atg	M	13	13.00
gtt	V	10	4911.00
gtc	V	0	4037.00
gta	V	147	5974.00
gtg	V	22057	7292.00
gct	A	0	0.26
gcc	A	0	0.21
gca	A	0	0.47
gcg	A	1	0.06
---	--	------	------
mPD		0.016	0.74
		nPD:	0.02
		N. weight:	1.1
		Sc. PD:	-0.028
		Sc. rank:	4510.2

,

PB2

Pos.	445	obs:	exp:
tta	L	0	2444.00
ttg	L	0	4186.00
ctt	L	22192	4381.00
ctc	L	35	3140.00
cta	L	0	4280.00
ctg	L	5	3801.00
---	--	------	------
mPD		0.0036	1.1
		nPD:	0.
		N. weight:	1.6
		Sc. PD:	-0.072
		Sc. rank:	-23968.0

,

PB2

Pos.	446	obs:	exp:
ttt	F	205	9539.00
ttc	F	22024	12690.00
tta	L	2	0.33
ttg	L	0	0.56
ctt	L	0	0.59
ctc	L	1	0.42
cta	L	0	0.58
ctg	L	0	0.51
---	--	------	------
mPD		0.019	0.49
		nPD:	0.04
		N. weight:	0.52
		Sc. PD:	-0.005
		Sc. rank:	5009.8

,

PB2

Pos.	447	obs:	exp:
tta	L	0	0.44
ttg	L	0	0.75
ctt	L	0	0.79
ctc	L	0	0.56
cta	L	0	0.77
ctg	L	4	0.68
cat	H	23	13.53
cac	H	0	9.47
caa	Q	162	11710.00
cag	Q	22029	10481.00
cgt	R	0	0.30
cgc	R	0	0.58
cga	R	0	1.08
cgg	R	11	0.81
aaa	K	0	1.71
aag	K	3	1.29
aga	R	0	5.52
agg	R	0	2.72
---	--	------	------
mPD		0.018	0.50
		nPD:	0.04
		N. weight:	0.71
		Sc. PD:	-0.0081
		Sc. rank:	6729.1

,

PB2

Pos.	448	obs:	exp:
tct	S	0	0.74
tcc	S	0	0.46
tca	S	0	1.04
tcg	S	0	0.21
cat	H	0	0.59
cac	H	1	0.41
aat	N	116	13142.00
aac	N	22105	9079.00
aaa	K	4	2.28
aag	K	0	1.72
agt	S	0	0.75
agc	S	4	0.79
gat	D	0	1.05
gac	D	2	0.95
---	--	------	------
mPD		0.011	0.48
		nPD:	0.02
		N. weight:	0.87
		Sc. PD:	-0.021
		Sc. rank:	4587.9

,

PB2			
Pos.	449	obs:	exp:
tgg	W	22232	22232.00
---	--	------	------
mPD		0	0
		nPD:	1.
		N. weight:	0.
		Sc. PD:	0
		Sc. rank:	0

PB2			
Pos.	450	obs:	exp:
gaa	E	2	1.21
gag	E	0	0.79
ggt	G	0	3371.00
ggc	G	1	3293.00
gga	G	22098	9888.00
ggg	G	131	5678.00
---	--	------	------
mPD		0.012	0.69
		nPD:	0.02
		N. weight:	0.78
		Sc. PD:	-0.024
		Sc. rank:	1262.1

PB2			
Pos.	451	obs:	exp:
ttt	F	2	0.86
ttc	F	0	1.14
tta	L	0	0.66
ttg	L	0	1.13
ctt	L	6	1.18
ctc	L	0	0.85
cta	L	0	1.16
ctg	L	0	1.03
att	I	21633	7913.00
atc	I	26	5426.00
ata	I	79	8399.00
atg	M	2	2.00
act	T	68	17.14
acc	T	0	13.95
aca	T	0	31.11
acg	T	0	5.80
aat	N	1	0.59
aac	N	0	0.41
gtt	V	401	91.53
gtc	V	13	75.23
gta	V	0	111.30
gtg	V	0	135.90
gct	A	1	0.26
gcc	A	0	0.21
gca	A	0	0.47
gcg	A	0	0.06
---	--	------	------
mPD		0.054	0.70
		nPD:	0.08
		N. weight:	1.
		Sc. PD:	0.029
		Sc. rank:	14089.0

PB2			
Pos.	452	obs:	exp:
gat	D	0	0.53
gac	D	1	0.47
gaa	E	22160	13460.00
gag	E	70	8770.00
ggt	G	0	0.15
ggc	G	0	0.15
gga	G	1	0.44
ggg	G	0	0.26
---	--	------	------
mPD		0.0065	0.48
		nPD:	0.01
		N. weight:	0.49
		Sc. PD:	-0.016
		Sc. rank:	-545.4

PB2			
Pos.	453	obs:	exp:
ttt	F	0	1.29
ttc	F	3	1.71
tta	L	0	0.11
ttg	L	0	0.19
tct	S	4	769.40
tcc	S	4176	484.30
tca	S	0	1088.00
tcg	S	0	222.50
tat	Y	0	0.52
tac	Y	1	0.48
ctt	L	0	0.20
ctc	L	1	0.14
cta	L	0	0.19
ctg	L	0	0.17
cct	P	0	67.22
ccc	P	244	45.26
cca	P	0	103.90
ccg	P	0	27.63
cat	H	0	5.88
cac	H	10	4.12
att	I	0	8.74
atc	I	24	5.99
ata	I	0	9.27
act	T	61	4468.00
acc	T	17653	3635.00
aca	T	9	8108.00
acg	T	0	1512.00
aat	N	0	14.79
aac	N	25	10.21
agt	S	0	786.20
agc	S	1	830.50
gct	A	0	5.30
gcc	A	20	4.14
gca	A	0	9.39
gcg	A	0	1.17
---	--	------	------
mPD		0.34	1.1
		nPD:	0.32
		N. weight:	1.7
		Sc. PD:	0.46
		Sc. rank:	29728.0

PB2			
Pos.	454	obs:	exp:
att	I	204	8090.00
atc	I	21983	5547.00
ata	I	36	8586.00
act	T	0	1.01
acc	T	4	0.82
aca	T	0	1.83
acg	T	0	0.34
gtt	V	0	1.11
gtc	V	5	0.91
gta	V	0	1.34
gtg	V	0	1.64
---	--	------	------
mPD		0.022	0.66
		nPD:	0.03
		N. weight:	1.3
		Sc. PD:	-0.018
		Sc. rank:	12127.0

PB2			
Pos.	455	obs:	exp:
gat	D	25	11713.00
gac	D	22205	10517.00
gaa	E	0	1.21
gag	E	2	0.79
---	--	------	------
mPD		0.0024	0.50
		nPD:	0.
		N. weight:	0.75
		Sc. PD:	-0.032
		Sc. rank:	-8786.5

PB2			
Pos.	456	obs:	exp:
tct	S	0	34.05
tcc	S	0	21.43
tca	S	0	48.15
tcg	S	0	9.85
tat	Y	6	3.12
tac	Y	0	2.88
cat	H	27	15.88
cac	H	0	11.12
aat	N	21978	13007.00
aac	N	14	8985.00
agt	S	185	34.79
agc	S	0	36.75
gat	D	22	11.59
gac	D	0	10.41
---	--	------	------
mPD		0.023	0.52
		nPD:	0.04
		N. weight:	0.54
		Sc. PD:	-0.002
		Sc. rank:	5855.8

PB2			
Pos.	457	obs:	exp:
att	I	0	0.73
atc	I	0	0.50
ata	I	2	0.77
atg	M	9	9.00
gtt	V	4	4913.00
gtc	V	0	4038.00
gta	V	1876	5976.00
gtg	V	20341	7294.00
---	--	------	------
mPD		0.16	0.74
		nPD:	0.21
		N. weight:	0.85
		Sc. PD:	0.14
		Sc. rank:	14216.0

PB2			
Pos.	458	obs:	exp:
tta	L	0	0.44
ttg	L	3	0.75
ctt	L	0	0.79
ctc	L	0	0.56
cta	L	0	0.77
ctg	L	1	0.68
att	I	0	0.73
atc	I	0	0.50
ata	I	2	0.77
atg	M	22226	22226.00
---	--	------	------
mPD		0.00054	0.00077
		nPD:	0.7
		N. weight:	0.00033
		Sc. PD:	0.00022
		Sc. rank:	6.3

PB2			
Pos.	459	obs:	exp:
gaa	E	1	0.61
gag	E	0	0.39
ggt	G	2	3371.00
ggc	G	1	3293.00
gga	G	21953	9889.00
ggg	G	275	5679.00
---	--	------	------
mPD		0.025	0.69
		nPD:	0.04
		N. weight:	0.76
		Sc. PD:	-0.0088
		Sc. rank:	7037.9

PB2			
Pos.	460	obs:	exp:
att	I	1	1.09
atc	I	0	0.75
ata	I	2	1.16
atg	M	22227	22227.00
act	T	0	0.25
acc	T	0	0.21
aca	T	0	0.46
acg	T	1	0.09
gtt	V	0	0.22
gtc	V	0	0.18
gta	V	0	0.27
gtg	V	1	0.33
---	--	------	------
mPD		0.00045	0.00059
		nPD:	0.76
		N. weight:	0.00024
		Sc. PD:	0.00017
		Sc. rank:	4.7

PB2

Pos. 461		obs:	exp:
tta	L	0	0.22
ttg	L	0	0.38
ctt	L	0	0.39
ctc	L	2	0.28
cta	L	0	0.39
ctg	L	0	0.34
att	I	105	8018.00
atc	I	21843	5498.00
ata	I	78	8510.00
act	T	0	2.02
acc	T	8	1.64
aca	T	0	3.66
acg	T	0	0.68
gtt	V	10	43.33
gtc	V	176	35.62
gta	V	10	52.71
gtg	V	0	64.34
---	--	------	------
mPD		0.037	0.68
		nPD:	0.05
		N. weight:	1.3
		Sc. PD:	0.0086
		Sc. rank:	16525.0

,

PB2

Pos. 462		obs:	exp:
ggt	G	1	3371.00
ggc	G	0	3293.00
gga	G	21702	9889.00
ggg	G	529	5679.00
---	--	------	------
mPD		0.047	0.69
		nPD:	0.07
		N. weight:	0.72
		Sc. PD:	0.014
		Sc. rank:	9680.2

,

PB2

Pos. 463		obs:	exp:
tta	L	6	1.32
ttg	L	0	2.26
ctt	L	0	2.36
ctc	L	0	1.69
cta	L	6	2.31
ctg	L	0	2.05
att	I	4	8017.00
atc	I	0	5497.00
ata	I	22019	8509.00
atg	M	31	31.00
act	T	0	0.25
acc	T	0	0.21
aca	T	1	0.46
acg	T	0	0.09
gtt	V	0	36.48
gtc	V	1	29.98
gta	V	162	44.38
gtg	V	2	54.16
---	--	------	------
mPD		0.019	0.67
		nPD:	0.03
		N. weight:	0.96
		Sc. PD:	-0.018
		Sc. rank:	6816.4

,

PB2

Pos. 464		obs:	exp:
tta	L	11	2440.00
ttg	L	185	4179.00
ctt	L	18	4374.00
ctc	L	0	3136.00
cta	L	105	4274.00
ctg	L	21880	3795.00
caa	Q	0	1.06
cag	Q	2	0.94
atg	M	31	31.00
---	--	------	------
mPD		0.032	1.1
		nPD:	0.03
		N. weight:	1.7
		Sc. PD:	-0.032
		Sc. rank:	11927.0

,

PB2

Pos. 465		obs:	exp:
tct	S	0	4.97
tcc	S	27	3.13
tca	S	0	7.03
tcg	S	0	1.44
cct	P	116	6117.00
ccc	P	22038	4118.00
cca	P	47	9454.00
ccg	P	1	2514.00
cgt	R	0	0.03
cgc	R	1	0.05
cga	R	0	0.10
cgg	R	0	0.07
agt	S	0	5.08
agc	S	0	5.36
aga	R	0	0.50
agg	R	0	0.25
gct	A	0	0.53
gcc	A	2	0.41
gca	A	0	0.94
gcg	A	0	0.12
---	--	------	------
mPD		0.017	0.70
		nPD:	0.02
		N. weight:	1.7
		Sc. PD:	-0.037
		Sc. rank:	9568.9

,

PB2

Pos. 466		obs:	exp:
cat	H	0	0.59
cac	H	1	0.41
aat	N	0	3.55
aac	N	6	2.45
gat	D	227	11706.00
gac	D	21990	10511.00
ggt	G	0	1.21
ggc	G	8	1.18
gga	G	0	3.56
ggg	G	0	2.04
---	--	------	------
mPD		0.022	0.50
		nPD:	0.04
		N. weight:	0.7
		Sc. PD:	-0.0031
		Sc. rank:	7421.9

,

PB2

Pos. 467		obs:	exp:
tta	L	0	0.55
ttg	L	5	0.94
ctt	L	0	0.99
ctc	L	0	0.71
cta	L	0	0.96
ctg	L	0	0.85
att	I	4	4.37
atc	I	0	3.00
ata	I	8	4.64
atg	M	22215	22215.00
---	--	------	------
mPD		0.0015	0.0018
		nPD:	0.84
		N. weight:	0.00063
		Sc. PD:	0.00049
		Sc. rank:	11.9

,

PB2

Pos. 468		obs:	exp:
att	I	0	0.36
atc	I	1	0.25
ata	I	0	0.39
act	T	82	5601.00
acc	T	22131	4557.00
aca	T	6	10165.00
acg	T	0	1896.00
gct	A	0	3.18
gcc	A	12	2.48
gca	A	0	5.64
gcg	A	0	0.70
---	--	------	------
mPD		0.0091	0.68
		nPD:	0.01
		N. weight:	1.6
		Sc. PD:	-0.053
		Sc. rank:	-1903.5

,

PB2

Pos. 469		obs:	exp:
cct	P	23	6125.00
ccc	P	2	4123.00
cca	P	22195	9466.00
ccg	P	11	2517.00
caa	Q	1	0.53
cag	Q	0	0.47
---	--	------	------
mPD		0.0033	0.70
		nPD:	0.
		N. weight:	0.85
		Sc. PD:	-0.036
		Sc. rank:	-10306.0

,

PB2

Pos. 470		obs:	exp:
tct	S	0	4089.00
tcc	S	0	2574.00
tca	S	0	5783.00
tcg	S	0	1183.00
tgt	C	0	0.45
tgc	C	1	0.55
aat	N	1	5.91
aac	N	9	4.09
agt	S	2330	4178.00
agc	S	19891	4414.00
---	--	------	------
mPD		0.19	1.6
		nPD:	0.11
		N. weight:	1.3
		Sc. PD:	0.087
		Sc. rank:	20257.0

,

PB2

Pos. 471		obs:	exp:
tct	S	0	0.55
tcc	S	0	0.35
tca	S	0	0.78
tcg	S	3	0.16
cct	P	0	0.28
ccc	P	0	0.19
cca	P	0	0.43
ccg	P	1	0.11
cgt	R	0	0.03
cgc	R	0	0.05
cga	R	0	0.10
cgg	R	0	0.07
atg	M	67	67.00
act	T	27	5578.00
acc	T	0	4539.00
aca	T	193	10123.00
acg	T	21909	1888.00
aaa	K	0	11.98
aag	K	21	9.02
agt	S	0	0.56
agc	S	0	0.60
aga	R	0	0.50
agg	R	1	0.25
gct	A	0	2.65
gcc	A	0	2.07
gca	A	0	4.70
gcg	A	10	0.59
---	--	------	------
mPD		0.029	0.69
		nPD:	0.04
		N. weight:	2.4
		Sc. PD:	-0.013
		Sc. rank:	24801.0

,

PB2

Pos. 472		obs:	exp:
caa	Q	0	0.53
cag	Q	1	0.47
aaa	K	0	0.57
aag	K	1	0.43
gat	D	2	1.58
gac	D	1	1.42
gaa	E	118	13456.00
gag	E	22105	8767.00
ggt	G	0	0.61
ggc	G	0	0.59
gga	G	0	1.78
ggg	G	4	1.02
---	--	------	------
mPD		0.011	0.48
		nPD:	0.02
		N. weight:	0.9
		Sc. PD:	-0.021
		Sc. rank:	4951.4

,

PB2

Pos. 473		obs:	exp:
tta	L	0	0.44
ttg	L	1	0.75
ctt	L	0	0.79
ctc	L	0	0.56
cta	L	0	0.77
ctg	L	3	0.68
cgt	R	0	0.05
cgc	R	0	0.10
cga	R	0	0.20
cgg	R	0	0.15
att	I	6	36.40
atc	I	3	24.96
ata	I	91	38.64
atg	M	22039	22039.00
act	T	0	1.76
acc	T	0	1.44
aca	T	0	3.20
acg	T	7	0.60
aaa	K	0	1.71
aag	K	3	1.29
aga	R	0	1.00
agg	R	2	0.49
gtt	V	0	17.02
gtc	V	0	13.99
gta	V	0	20.71
gtg	V	77	25.28
---	--	------	------
mPD		0.017	0.023
		nPD:	0.75
		N. weight:	0.0081
		Sc. PD:	0.0057
		Sc. rank:	153.8

,

PB2			
Pos.	474	obs:	exp:
tct	S	4	4091.00
tcc	S	6	2575.00
tca	S	17488	5785.00
tcg	S	4732	1183.00
act	T	0	0.50
acc	T	0	0.41
aca	T	2	0.91
acg	T	0	0.17
agt	S	0	4180.00
agc	S	0	4416.00
---	--	------	------
mPD		0.34	1.6
		nPD:	0.2
		N. weight:	1.2
		Sc. PD:	0.18
		Sc. rank:	19570.0

,

PB2			
Pos.	475	obs:	exp:
tta	L	0	2434.00
ttg	L	35	4168.00
ctt	L	7	4363.00
ctc	L	0	3127.00
cta	L	65	4263.00
ctg	L	22034	3786.00
att	I	0	0.36
atc	I	0	0.25
ata	I	1	0.39
atg	M	90	90.00
---	--	------	------
mPD		0.018	1.1
		nPD:	0.02
		N. weight:	1.7
		Sc. PD:	-0.055
		Sc. rank:	1004.6

,

PB2			
Pos.	476	obs:	exp:
cgt	R	0	606.90
cgc	R	0	1163.00
cga	R	0	2177.00
cgg	R	0	1627.00
aaa	K	6	3.42
aag	K	0	2.58
aga	R	22202	11158.00
agg	R	24	5495.00
---	--	------	------
mPD		0.0027	0.91
		nPD:	0.
		N. weight:	0.69
		Sc. PD:	-0.03
		Sc. rank:	-10423.0

,

PB2			
Pos.	477	obs:	exp:
cgt	R	0	0.30
cgc	R	0	0.58
cga	R	0	1.08
cgg	R	0	0.81
aaa	K	1	0.57
aag	K	0	0.43
aga	R	11	5.52
agg	R	0	2.72
ggt	G	5	3369.00
ggc	G	0	3291.00
gga	G	17508	9884.00
ggg	G	4707	5676.00
---	--	------	------
mPD		0.34	0.69
		nPD:	0.48
		N. weight:	0.41
		Sc. PD:	0.18
		Sc. rank:	7664.3

,

PB2			
Pos.	478	obs:	exp:
att	I	3	8080.00
atc	I	0	5541.00
ata	I	22194	8576.00
aaa	K	1	0.57
aag	K	0	0.43
gtt	V	0	7.52
gtc	V	0	6.18
gta	V	34	9.14
gtg	V	0	11.16
---	--	------	------
mPD		0.0034	0.66
		nPD:	0.01
		N. weight:	0.96
		Sc. PD:	-0.04
		Sc. rank:	-10942.0

,

PB2			
Pos.	479	obs:	exp:
cgt	R	0	606.80
cgc	R	0	1162.00
cga	R	2	2177.00
cgg	R	0	1626.00
aaa	K	10	5.70
aag	K	0	4.30
aga	R	22140	11156.00
agg	R	80	5494.00
---	--	------	------
mPD		0.0083	0.91
		nPD:	0.01
		N. weight:	0.67
		Sc. PD:	-0.026
		Sc. rank:	-4074.0

,

PB2			
Pos.	480	obs:	exp:
tta	L	0	0.22
ttg	L	0	0.38
ctt	L	0	0.39
ctc	L	2	0.28
cta	L	0	0.39
ctg	L	0	0.34
att	I	0	59.34
atc	I	163	40.69
ata	I	0	62.98
gtt	V	42	4878.00
gtc	V	21953	4009.00
gta	V	67	5933.00
gtg	V	0	7242.00
gct	A	0	1.32
gcc	A	5	1.03
gca	A	0	2.35
gcg	A	0	0.29
---	--	------	------
mPD		0.025	0.75
		nPD:	0.03
		N. weight:	1.7
		Sc. PD:	-0.024
		Sc. rank:	15067.0

,

PB2			
Pos.	481	obs:	exp:
tct	S	0	4091.00
tcc	S	0	2575.00
tca	S	0	5785.00
tcg	S	0	1183.00
tgt	C	0	0.45
tgc	C	1	0.55
aat	N	0	0.59
aac	N	1	0.41
agt	S	17	4180.00
agc	S	22212	4415.00
ggt	G	0	0.15
ggc	G	1	0.15
gga	G	0	0.44
ggg	G	0	0.26
---	--	------	------
mPD		0.0018	1.6
		nPD:	0.
		N. weight:	1.6
		Sc. PD:	-0.075
		Sc. rank:	-29163.0

,

PB2			
Pos.	482	obs:	exp:
cgt	R	0	0.46
cgc	R	0	0.89
cga	R	0	1.67
cgg	R	0	1.24
act	T	0	0.25
acc	T	0	0.21
aca	T	0	0.46
acg	T	1	0.09
aat	N	7	4.73
aac	N	1	3.27
aaa	K	3246	12666.00
aag	K	18959	9539.00
aga	R	9	8.53
agg	R	8	4.20
gaa	E	0	0.61
gag	E	1	0.39
---	--	------	------
mPD		0.25	0.49
		nPD:	0.51
		N. weight:	0.39
		Sc. PD:	0.18
		Sc. rank:	7260.2

,

PB2			
Pos.	483	obs:	exp:
att	I	1	9.10
atc	I	0	6.24
ata	I	24	9.66
atg	M	22194	22194.00
gtt	V	0	2.87
gtc	V	0	2.36
gta	V	0	3.50
gtg	V	13	4.27
---	--	------	------
mPD		0.0034	0.0042
		nPD:	0.81
		N. weight:	0.0016
		Sc. PD:	0.0012
		Sc. rank:	30.3

,

PB2			
Pos.	484	obs:	exp:
ggt	G	18	3371.00
ggc	G	0	3293.00
gga	G	21768	9889.00
ggg	G	446	5679.00
---	--	------	------
mPD		0.041	0.69
		nPD:	0.06
		N. weight:	0.72
		Sc. PD:	0.0083
		Sc. rank:	9173.8

,

PB2			
Pos.	485	obs:	exp:
att	I	0	0.73
atc	I	0	0.50
ata	I	2	0.77
gtt	V	123	4915.00
gtc	V	6	4039.00
gta	V	22041	5979.00
gtg	V	60	7297.00
---	--	------	------
mPD		0.017	0.74
		nPD:	0.02
		N. weight:	1.3
		Sc. PD:	-0.031
		Sc. rank:	6556.2

,

PB2			
Pos.	486	obs:	exp:
gat	D	22221	11714.00
gac	D	11	10518.00
---	--	------	------
mPD		0.00099	0.50
		nPD:	0.
		N. weight:	0.64
		Sc. PD:	-0.029
		Sc. rank:	-10642.0

,

PB2			
Pos.	487	obs:	exp:
gat	D	1	0.53
gac	D	0	0.47
gaa	E	22199	13457.00
gag	E	25	8767.00
ggt	G	0	1.06
ggc	G	0	1.04
gga	G	7	3.11
ggg	G	0	1.79
---	--	------	------
mPD		0.0030	0.48
		nPD:	0.01
		N. weight:	0.5
		Sc. PD:	-0.02
		Sc. rank:	-4904.3

,

PB2			
Pos.	488	obs:	exp:
ttt	F	0	0.43
ttc	F	1	0.57
tat	Y	56	11547.00
tac	Y	22168	10677.00
tgt	C	0	0.45
tgc	C	1	0.55
cat	H	0	3.53
cac	H	6	2.47
---	--	------	------
mPD		0.0057	0.50
		nPD:	0.01
		N. weight:	0.72
		Sc. PD:	-0.026
		Sc. rank:	-2191.4

,

PB2			
Pos.	489	obs:	exp:
ttt	F	0	3.86
ttc	F	9	5.14
tct	S	464	4089.00
tcc	S	21637	2574.00
tca	S	119	5783.00
tcg	S	0	1183.00
tat	Y	0	0.52
tac	Y	1	0.48
cct	P	0	0.55
ccc	P	2	0.37
cca	P	0	0.85
ccg	P	0	0.23
agt	S	0	4178.00
agc	S	0	4414.00
---	--	------	------
mPD		0.052	1.6
		nPD:	0.03
		N. weight:	2.
		Sc. PD:	-0.031
		Sc. rank:	17175.0

,

PB2

Pos.	490	obs:	exp:
tct	S	0	4087.00
tcc	S	0	2573.00
tca	S	0	5781.00
tcg	S	0	1182.00
tgt	C	0	0.45
tgc	C	1	0.55
cgt	R	0	0.27
cgc	R	4	0.52
cga	R	0	0.98
cgg	R	0	0.73
att	I	0	0.36
atc	I	1	0.25
ata	I	0	0.39
act	T	0	0.50
acc	T	2	0.41
aca	T	0	0.91
acg	T	0	0.17
aat	N	0	2.37
aac	N	4	1.63
agt	S	54	4176.00
agc	S	22157	4412.00
aga	R	6	5.02
agg	R	0	2.47
ggt	G	0	0.45
ggc	G	3	0.44
gga	G	0	1.33
ggg	G	0	0.77
---	--	------	------
mPD		0.0067	1.6
		nPD:	0.
		N. weight:	1.6
		Sc. PD:	-0.07
		Sc. rank:	-21513.0

,

PB2

Pos.	491	obs:	exp:
tct	S	0	0.18
tcc	S	0	0.12
tca	S	0	0.26
tcg	S	1	0.05
atg	M	1	1.00
act	T	39	5604.00
acc	T	0	4560.00
aca	T	157	10170.00
acg	T	22034	1897.00
agt	S	0	0.19
agc	S	0	0.20
---	--	------	------
mPD		0.018	0.68
		nPD:	0.03
		N. weight:	2.4
		Sc. PD:	-0.052
		Sc. rank:	15496.0

,

PB2

Pos.	492	obs:	exp:
aaa	K	0	0.57
aag	K	1	0.43
gat	D	1	0.53
gac	D	0	0.47
gaa	E	14	13459.00
gag	E	22214	8769.00
ggt	G	0	0.30
ggc	G	0	0.30
gga	G	0	0.89
ggg	G	2	0.51
---	--	------	------
mPD		0.0016	0.48
		nPD:	0.
		N. weight:	0.93
		Sc. PD:	-0.041
		Sc. rank:	-13344.0

,

PB2

Pos.	493	obs:	exp:
cgt	R	0	606.90
cgc	R	0	1163.00
cga	R	0	2177.00
cgg	R	0	1627.00
aaa	K	3	1.71
aag	K	0	1.29
aga	R	22161	11160.00
agg	R	68	5495.00
---	--	------	------
mPD		0.0064	0.91
		nPD:	0.01
		N. weight:	0.68
		Sc. PD:	-0.027
		Sc. rank:	-5970.3

,

PB2

Pos.	494	obs:	exp:
atg	M	3	3.00
gtt	V	2	4914.00
gtc	V	0	4039.00
gta	V	40	5978.00
gtg	V	22186	7297.00
gct	A	0	0.26
gcc	A	0	0.21
gca	A	0	0.47
gcg	A	1	0.06
---	--	------	------
mPD		0.0041	0.74
		nPD:	0.01
		N. weight:	1.1
		Sc. PD:	-0.046
		Sc. rank:	-11697.0

,

PB2

Pos.	495	obs:	exp:
tta	L	3	0.33
ttg	L	0	0.56
ctt	L	0	0.59
ctc	L	0	0.42
cta	L	0	0.58
ctg	L	0	0.51
att	I	0	31.31
atc	I	0	21.47
ata	I	86	33.23
gtt	V	4	4891.00
gtc	V	7	4020.00
gta	V	21860	5950.00
gtg	V	253	7262.00
gct	A	0	4.77
gcc	A	0	3.73
gca	A	18	8.45
gcg	A	0	1.05
ggt	G	0	0.15
ggc	G	0	0.15
gga	G	1	0.44
ggg	G	0	0.26
---	--	------	------
mPD		0.033	0.75
		nPD:	0.04
		N. weight:	1.3
		Sc. PD:	-0.004
		Sc. rank:	13813.0

,

PB2

Pos.	496	obs:	exp:
gtt	V	24	4915.00
gtc	V	3	4039.00
gta	V	372	5979.00
gtg	V	21831	7297.00
gct	A	0	0.26
gcc	A	0	0.21
gca	A	1	0.47
gcg	A	0	0.06
gaa	E	0	0.61
gag	E	1	0.39
---	--	------	------
mPD		0.036	0.74
		nPD:	0.05
		N. weight:	1.
		Sc. PD:	0.00064
		Sc. rank:	12102.0

,

PB2

Pos.	497	obs:	exp:
tct	S	0	4078.00
tcc	S	0	2567.00
tca	S	0	5767.00
tcg	S	0	1179.00
tgt	C	1	0.45
tgc	C	0	0.55
cgt	R	1	0.03
cgc	R	0	0.05
cga	R	0	0.10
cgg	R	0	0.07
act	T	5	1.26
acc	T	0	1.03
aca	T	0	2.29
acg	T	0	0.43
aat	N	60	35.49
aac	N	0	24.51
agt	S	22055	4167.00
agc	S	105	4402.00
aga	R	0	0.50
agg	R	0	0.25
ggt	G	5	0.76
ggc	G	0	0.74
gga	G	0	2.22
ggg	G	0	1.28
---	--	------	------
mPD		0.016	1.6
		nPD:	0.01
		N. weight:	1.7
		Sc. PD:	-0.062
		Sc. rank:	-8596.6

,

PB2

Pos.	498	obs:	exp:
att	I	22184	8092.00
atc	I	40	5549.00
ata	I	4	8588.00
gtt	V	4	0.88
gtc	V	0	0.73
gta	V	0	1.08
gtg	V	0	1.31
---	--	------	------
mPD		0.0043	0.66
		nPD:	0.01
		N. weight:	1.
		Sc. PD:	-0.041
		Sc. rank:	-9151.8

,

PB2

Pos.	499	obs:	exp:
aat	N	0	0.59
aac	N	1	0.41
gat	D	121	11713.00
gac	D	22110	10518.00
---	--	------	------
mPD		0.011	0.50
		nPD:	0.02
		N. weight:	0.72
		Sc. PD:	-0.018
		Sc. rank:	3159.2

,

PB2

Pos.	500	obs:	exp:
caa	Q	14	7.39
cag	Q	0	6.61
cgt	R	6	606.60
cgc	R	2	1162.00
cga	R	21969	2176.00
cgg	R	73	1626.00
aga	R	168	11154.00
agg	R	0	5493.00
---	--	------	------
mPD		0.024	0.91
		nPD:	0.03
		N. weight:	2.3
		Sc. PD:	-0.049
		Sc. rank:	14151.0

,

PB2

Pos.	501	obs:	exp:
ttt	F	21768	9540.00
ttc	F	463	12691.00
tta	L	0	0.11
ttg	L	0	0.19
ctt	L	1	0.20
ctc	L	0	0.14
cta	L	0	0.19
ctg	L	0	0.17
---	--	------	------
mPD		0.041	0.49
		nPD:	0.08
		N. weight:	0.75
		Sc. PD:	0.027
		Sc. rank:	10977.0

,

PB2

Pos.	502	obs:	exp:
tta	L	3576	2444.00
ttg	L	18441	4186.00
ctt	L	0	4381.00
ctc	L	0	3140.00
cta	L	0	4280.00
ctg	L	215	3801.00
---	--	------	------
mPD		0.29	1.1
		nPD:	0.25
		N. weight:	1.3
		Sc. PD:	0.26
		Sc. rank:	21948.0

,

PB2			
Pos.	503	obs:	exp:
cgt	R	0	607.00
cgc	R	0	1163.00
cga	R	0	2178.00
cgg	R	17	1627.00
aga	R	178	11161.00
agg	R	22037	5496.00
---	--	------	------
mPD		0.017	0.91
		nPD:	0.02
		N. weight:	1.4
		Sc. PD:	-0.038
		Sc. rank:	3430.3

,

PB2			
Pos.	504	obs:	exp:
att	I	8	2.91
atc	I	0	2.00
ata	I	0	3.09
gtt	V	22127	4913.00
gtc	V	89	4038.00
gta	V	1	5977.00
gtg	V	7	7295.00
---	--	------	------
mPD		0.0094	0.74
		nPD:	0.01
		N. weight:	1.5
		Sc. PD:	-0.052
		Sc. rank:	-2555.5

,

PB2			
Pos.	505	obs:	exp:
tct	S	0	0.92
tcc	S	0	0.58
tca	S	0	1.30
tcg	S	0	0.27
cgt	R	0	605.60
cgc	R	0	1160.00
cga	R	15	2172.00
cgg	R	2	1623.00
aaa	K	48	27.95
aag	K	1	21.05
agt	S	5	0.94
agc	S	0	0.99
aga	R	22139	11134.00
agg	R	22	5483.00
---	--	------	------
mPD		0.0086	0.91
		nPD:	0.01
		N. weight:	0.68
		Sc. PD:	-0.026
		Sc. rank:	-3740.3

,

PB2			
Pos.	506	obs:	exp:
cat	H	1	0.59
cac	H	0	0.41
aat	N	4	2.37
aac	N	0	1.63
gat	D	22126	11711.00
gac	D	101	10516.00
---	--	------	------
mPD		0.0095	0.50
		nPD:	0.02
		N. weight:	0.62
		Sc. PD:	-0.018
		Sc. rank:	1525.4

,

PB2			
Pos.	507	obs:	exp:
tta	L	0	0.11
ttg	L	0	0.19
ctt	L	0	0.20
ctc	L	0	0.14
cta	L	1	0.19
ctg	L	0	0.17
cct	P	0	0.28
ccc	P	0	0.19
cca	P	1	0.43
ccg	P	0	0.11
cat	H	3	4.70
cac	H	5	3.30
caa	Q	22203	11725.00
cag	Q	17	10495.00
cgt	R	0	0.03
cgc	R	0	0.05
cga	R	1	0.10
cgg	R	0	0.07
aaa	K	1	0.57
aag	K	0	0.43
aga	R	0	0.50
agg	R	0	0.25
---	--	------	------
mPD		0.0026	0.50
		nPD:	0.01
		N. weight:	0.64
		Sc. PD:	-0.027
		Sc. rank:	-7270.8

,

PB2			
Pos.	508	obs:	exp:
tta	L	0	0.11
ttg	L	0	0.19
ctt	L	0	0.20
ctc	L	0	0.14
cta	L	1	0.19
ctg	L	0	0.17
cgt	R	10	606.50
cgc	R	0	1162.00
cga	R	12	2176.00
cgg	R	0	1626.00
att	I	0	1.82
atc	I	0	1.25
ata	I	5	1.93
aaa	K	9	5.70
aag	K	1	4.30
aga	R	22078	11152.00
agg	R	114	5492.00
ggt	G	0	0.30
ggc	G	0	0.30
gga	G	2	0.89
ggg	G	0	0.51
---	--	------	------
mPD		0.015	0.91
		nPD:	0.02
		N. weight:	0.66
		Sc. PD:	-0.021
		Sc. rank:	682.8

,

PB2			
Pos.	509	obs:	exp:
cgt	R	0	0.05
cgc	R	0	0.10
cga	R	0	0.20
cgg	R	0	0.15
aga	R	0	1.00
agg	R	2	0.49
gaa	E	6	4.24
gag	E	1	2.76
ggt	G	3	3370.00
ggc	G	0	3291.00
gga	G	13916	9885.00
ggg	G	8304	5677.00
---	--	------	------
mPD		0.47	0.69
		nPD:	0.68
		N. weight:	0.36
		Sc. PD:	0.22
		Sc. rank:	6801.0

,

PB2			
Pos.	510	obs:	exp:
tct	S	0	0.74
tcc	S	0	0.46
tca	S	0	1.04
tcg	S	0	0.21
act	T	0	0.76
acc	T	3	0.62
aca	T	0	1.37
acg	T	0	0.26
aat	N	123	13132.00
aac	N	22081	9072.00
aaa	K	2	1.14
aag	K	0	0.86
agt	S	0	0.75
agc	S	4	0.79
gat	D	0	10.01
gac	D	19	8.99
---	--	------	------
mPD		0.014	0.49
		nPD:	0.03
		N. weight:	0.87
		Sc. PD:	-0.017
		Sc. rank:	6013.0

,

PB2			
Pos.	511	obs:	exp:
att	I	0	170.70
atc	I	0	117.10
ata	I	469	181.20
gtt	V	2	4811.00
gtc	V	2	3955.00
gta	V	21718	5853.00
gtg	V	41	7144.00
---	--	------	------
mPD		0.045	0.78
		nPD:	0.06
		N. weight:	1.3
		Sc. PD:	0.014
		Sc. rank:	16449.0

,

PB2			
Pos.	512	obs:	exp:
tta	L	250	2443.00
ttg	L	0	4185.00
ctt	L	1	4380.00
ctc	L	2	3140.00
cta	L	20526	4279.00
ctg	L	1448	3800.00
att	I	0	1.82
atc	I	0	1.25
ata	I	5	1.93
---	--	------	------
mPD		0.14	1.1
		nPD:	0.13
		N. weight:	1.4
		Sc. PD:	0.11
		Sc. rank:	21708.0

,

PB2			
Pos.	513	obs:	exp:
tta	L	382	2444.00
ttg	L	21817	4185.00
tct	S	0	0.18
tcc	S	0	0.12
tca	S	0	0.26
tcg	S	1	0.05
ctt	L	0	4381.00
ctc	L	0	3140.00
cta	L	0	4280.00
ctg	L	32	3801.00
agt	S	0	0.19
agc	S	0	0.20
---	--	------	------
mPD		0.037	1.1
		nPD:	0.03
		N. weight:	1.6
		Sc. PD:	-0.024
		Sc. rank:	13941.0

,

PB2			
Pos.	514	obs:	exp:
tct	S	22144	4090.00
tcc	S	80	2574.00
tca	S	2	5785.00
tcg	S	1	1183.00
act	T	5	1.26
acc	T	0	1.03
aca	T	0	2.29
acg	T	0	0.43
agt	S	0	4179.00
agc	S	0	4415.00
---	--	------	------
mPD		0.0079	1.6
		nPD:	0.
		N. weight:	1.7
		Sc. PD:	-0.072
		Sc. rank:	-20132.0

,

PB2			
Pos.	515	obs:	exp:
tct	S	0	0.55
tcc	S	3	0.35
tca	S	0	0.78
tcg	S	0	0.16
cct	P	155	6123.00
ccc	P	22040	4123.00
cca	P	32	9464.00
ccg	P	0	2517.00
act	T	0	0.50
acc	T	2	0.41
aca	T	0	0.91
acg	T	0	0.17
agt	S	0	0.56
agc	S	0	0.60
---	--	------	------
mPD		0.017	0.70
		nPD:	0.02
		N. weight:	1.6
		Sc. PD:	-0.037
		Sc. rank:	9271.0

,

PB2			
Pos.	516	obs:	exp:
gat	D	1	0.53
gac	D	0	0.47
gaa	E	22201	13461.00
gag	E	30	8770.00
---	--	------	------
mPD		0.0028	0.48
		nPD:	0.01
		N. weight:	0.5
		Sc. PD:	-0.021
		Sc. rank:	-5170.3

,

PB2			
Pos.	517	obs:	exp:
aaa	K	2	1.14
aag	K	0	0.86
gaa	E	21384	13460.00
gag	E	846	8770.00
---	--	------	------
mPD		0.073	0.48
		nPD:	0.15
		N. weight:	0.36
		Sc. PD:	0.038
		Sc. rank:	5852.2

,

PB2			
Pos.	518	obs:	exp:
att	I	0	17.11
atc	I	47	11.73
ata	I	0	18.16
gtt	V	1254	4905.00
gtc	V	20928	4031.00
gta	V	2	5966.00
gtg	V	1	7283.00
---	--	------	------
mPD		0.11	0.74
		nPD:	0.15
		N. weight:	1.5
		Sc. PD:	0.15
		Sc. rank:	24139.0

,

PB2			
Pos.	519	obs:	exp:
tct	S	0	4091.00
tcc	S	0	2575.00
tca	S	0	5786.00
tcg	S	0	1183.00
aat	N	1	0.59
aac	N	0	0.41
agt	S	22199	4180.00
agc	S	32	4416.00
---	--	------	------
mPD		0.0030	1.6
		nPD:	0.
		N. weight:	1.7
		Sc. PD:	-0.076
		Sc. rank:	-28267.0

,

PB2			
Pos.	520	obs:	exp:
gaa	E	22191	13461.00
gag	E	41	8771.00
---	--	------	------
mPD		0.0037	0.48
		nPD:	0.01
		N. weight:	0.49
		Sc. PD:	-0.02
		Sc. rank:	-3740.3

,

PB2			
Pos.	521	obs:	exp:
atg	M	3	3.00
act	T	20	5603.00
acc	T	0	4559.00
aca	T	844	10168.00
acg	T	21363	1896.00
aaa	K	1	0.57
aag	K	0	0.43
gct	A	0	0.26
gcc	A	0	0.21
gca	A	0	0.47
gcg	A	1	0.06
---	--	------	------
mPD		0.075	0.68
		nPD:	0.11
		N. weight:	2.3
		Sc. PD:	0.14
		Sc. rank:	34628.0

,

PB2			
Pos.	522	obs:	exp:
cat	H	1	1.18
cac	H	1	0.82
caa	Q	22183	11730.00
cag	Q	47	10500.00
---	--	------	------
mPD		0.0044	0.50
		nPD:	0.01
		N. weight:	0.63
		Sc. PD:	-0.024
		Sc. rank:	-3933.2

,

PB2			
Pos.	523	obs:	exp:
ggt	G	0	3371.00
ggc	G	0	3293.00
gga	G	22167	9889.00
ggg	G	65	5679.00
---	--	------	------
mPD		0.0058	0.69
		nPD:	0.01
		N. weight:	0.8
		Sc. PD:	-0.031
		Sc. rank:	-5406.9

,

PB2			
Pos.	524	obs:	exp:
tct	S	1	0.18
tcc	S	0	0.12
tca	S	0	0.26
tcg	S	0	0.05
att	I	34	12.38
atc	I	0	8.49
ata	I	0	13.14
act	T	21878	5554.00
acc	T	104	4519.00
aca	T	38	10079.00
acg	T	11	1880.00
aat	N	141	83.98
aac	N	1	58.02
agt	S	0	0.19
agc	S	0	0.20
gct	A	23	6.35
gcc	A	0	4.97
gca	A	1	11.27
gcg	A	0	1.41
---	--	------	------
mPD		0.032	0.70
		nPD:	0.05
		N. weight:	1.3
		Sc. PD:	-0.0025
		Sc. rank:	15301.0

,

PB2			
Pos.	525	obs:	exp:
gaa	E	123	13461.00
gag	E	22109	8771.00
---	--	------	------
mPD		0.011	0.48
		nPD:	0.02
		N. weight:	0.9
		Sc. PD:	-0.022
		Sc. rank:	4528.4

,

PB2			
Pos.	526	obs:	exp:
cgt	R	0	3.36
cgc	R	0	6.43
cga	R	0	12.05
cgg	R	0	9.00
aaa	K	186	12612.00
aag	K	21923	9497.00
aga	R	11	61.75
agg	R	112	30.41
---	--	------	------
mPD		0.029	0.50
		nPD:	0.06
		N. weight:	0.8
		Sc. PD:	0.0073
		Sc. rank:	10021.0

,

PB2			
Pos.	527	obs:	exp:
tta	L	949	2444.00
ttg	L	21215	4185.00
ctt	L	0	4381.00
ctc	L	0	3140.00
cta	L	1	4280.00
ctg	L	66	3801.00
gtt	V	0	0.22
gtc	V	0	0.18
gta	V	0	0.27
gtg	V	1	0.33
---	--	------	------
mPD		0.088	1.1
		nPD:	0.08
		N. weight:	1.5
		Sc. PD:	0.044
		Sc. rank:	21296.0

,

PB2			
Pos.	528	obs:	exp:
act	T	3	5604.00
acc	T	60	4560.00
aca	T	22110	10170.00
acg	T	58	1897.00
gct	A	0	0.26
gcc	A	0	0.21
gca	A	1	0.47
gcg	A	0	0.06
---	--	------	------
mPD		0.011	0.68
		nPD:	0.02
		N. weight:	0.76
		Sc. PD:	-0.024
		Sc. rank:	660.7

,

PB2			
Pos.	529	obs:	exp:
att	I	2	8089.00
atc	I	0	5547.00
ata	I	22220	8586.00
act	T	0	0.25
acc	T	0	0.21
aca	T	1	0.46
acg	T	0	0.09
gtt	V	0	1.99
gtc	V	0	1.64
gta	V	9	2.42
gtg	V	0	2.95
---	--	------	------
mPD		0.0011	0.66
		nPD:	0.
		N. weight:	0.96
		Sc. PD:	-0.044
		Sc. rank:	-16518.0

,

PB2			
Pos.	530	obs:	exp:
tct	S	0	0.18
tcc	S	0	0.12
tca	S	0	0.26
tcg	S	0	0.05
att	I	1	0.36
atc	I	0	0.25
ata	I	0	0.39
act	T	22205	5603.00
acc	T	22	4559.00
aca	T	0	10169.00
acg	T	1	1897.00
agt	S	1	0.19
agc	S	0	0.20
gct	A	2	0.53
gcc	A	0	0.41
gca	A	0	0.94
gcg	A	0	0.12
---	--	------	------
mPD		0.0024	0.68
		nPD:	0.
		N. weight:	1.4
		Sc. PD:	-0.06
		Sc. rank:	-19451.0

,

PB2			
Pos.	531	obs:	exp:
tat	Y	21922	11551.00
tac	Y	310	10681.00
---	--	------	------
mPD		0.028	0.50
		nPD:	0.06
		N. weight:	0.59
		Sc. PD:	0.0044
		Sc. rank:	7250.9

,

PB2			
Pos.	532	obs:	exp:
tta	L	0	8.68
ttg	L	79	14.87
tct	S	90	4075.00
tcc	S	0	2565.00
tca	S	324	5764.00
tcg	S	21732	1179.00
tgg	W	4	4.00
ctt	L	0	15.57
ctc	L	0	11.16
cta	L	0	15.21
ctg	L	0	13.51
agt	S	0	4164.00
agc	S	0	4399.00
gct	A	0	0.79
gcc	A	0	0.62
gca	A	0	1.41
gcg	A	3	0.18
---	--	------	------
mPD		0.044	1.6
		nPD:	0.03
		N. weight:	2.8
		Sc. PD:	-0.058
		Sc. rank:	18982.0

,

PB2			
Pos.	533	obs:	exp:
tct	S	4	4090.00
tcc	S	2	2574.00
tca	S	22190	5785.00
tcg	S	31	1183.00
act	T	0	1.01
acc	T	0	0.82
aca	T	4	1.83
acg	T	0	0.34
agt	S	0	4179.00
agc	S	0	4415.00
gct	A	0	0.26
gcc	A	0	0.21
gca	A	1	0.47
gcg	A	0	0.06
---	--	------	------
mPD		0.0038	1.6
		nPD:	0.
		N. weight:	1.3
		Sc. PD:	-0.061
		Sc. rank:	-21490.0

,

PB2			
Pos.	534	obs:	exp:
tct	S	2	4091.00
tcc	S	34	2575.00
tca	S	22171	5785.00
tcg	S	22	1183.00
cct	P	0	0.28
ccc	P	0	0.19
cca	P	1	0.43
ccg	P	0	0.11
agt	S	0	4180.00
agc	S	0	4415.00
gct	A	0	0.53
gcc	A	0	0.41
gca	A	2	0.94
gcg	A	0	0.12
---	--	------	------
mPD		0.0055	1.6
		nPD:	0.
		N. weight:	1.3
		Sc. PD:	-0.059
		Sc. rank:	-19239.0

,

PB2			
Pos.	535	obs:	exp:
tta	L	0	0.55
ttg	L	1	0.94
ctt	L	0	0.99
ctc	L	0	0.71
cta	L	0	0.96
ctg	L	4	0.85
atg	M	22227	22227.00
---	--	------	------
mPD		0.00045	0.00074
		nPD:	0.61
		N. weight:	0.00032
		Sc. PD:	0.00018
		Sc. rank:	6.1

,

PB2			
Pos.	536	obs:	exp:
tta	L	0	0.22
ttg	L	0	0.38
ctt	L	0	0.39
ctc	L	0	0.28
cta	L	0	0.39
ctg	L	2	0.34
att	I	0	0.36
atc	I	0	0.25
ata	I	1	0.39
atg	M	22229	22229.00
---	--	------	------
mPD		0.00027	0.00039
		nPD:	0.7
		N. weight:	0.0002
		Sc. PD:	0.00013
		Sc. rank:	3.9

,

PB2			
Pos.	537	obs:	exp:
tgg	W	22231	22231.00
cgt	R	0	0.03
cgc	R	0	0.05
cga	R	0	0.10
cgg	R	1	0.07
aga	R	0	0.50
agg	R	0	0.25
---	--	------	------
mPD		0.000090	0.00015
		nPD:	0.6
		N. weight:	0.00012
		Sc. PD:	0.000065
		Sc. rank:	2.2

,

PB2			
Pos.	538	obs:	exp:
gaa	E	3860	13461.00
gag	E	18372	8771.00
---	--	------	------
mPD		0.29	0.48
		nPD:	0.6
		N. weight:	0.4
		Sc. PD:	0.22
		Sc. rank:	7442.6

,

PB2			
Pos.	539	obs:	exp:
ttt	F	0	1.29
ttc	F	3	1.71
att	I	177	8087.00
atc	I	21852	5545.00
ata	I	185	8582.00
act	T	0	2.52
acc	T	10	2.05
aca	T	0	4.57
acg	T	0	0.85
gtt	V	0	1.11
gtc	V	5	0.91
gta	V	0	1.34
gtg	V	0	1.64
---	--	------	------
mPD		0.034	0.66
		nPD:	0.05
		N. weight:	1.3
		Sc. PD:	0.005
		Sc. rank:	15631.0

,

PB2			
Pos.	540	obs:	exp:
tct	S	0	0.37
tcc	S	0	0.23
tca	S	0	0.52
tcg	S	0	0.11
act	T	0	0.25
acc	T	1	0.21
aca	T	0	0.46
acg	T	0	0.09
aat	N	8203	13147.00
aac	N	14026	9082.00
agt	S	1	0.38
agc	S	1	0.40
---	--	------	------
mPD		0.47	0.48
		nPD:	0.96
		N. weight:	0.1
		Sc. PD:	0.092
		Sc. rank:	1932.9

,

PB2			
Pos.	541	obs:	exp:
ggt	G	1087	3371.00
ggc	G	21134	3293.00
gga	G	9	9889.00
ggg	G	2	5679.00
---	--	------	------
mPD		0.094	0.69
		nPD:	0.14
		N. weight:	1.7
		Sc. PD:	0.15
		Sc. rank:	27649.0

,

PB2			
Pos.	542	obs:	exp:
cct	P	22198	6125.00
ccc	P	23	4123.00
cca	P	8	9466.00
ccg	P	3	2517.00
---	--	------	------
mPD		0.0031	0.70
		nPD:	0.
		N. weight:	1.3
		Sc. PD:	-0.055
		Sc. rank:	-16423.0

,

PB2			
Pos.	543	obs:	exp:
gaa	E	97	13461.00
gag	E	22134	8770.00
ggt	G	0	0.15
ggc	G	0	0.15
gga	G	0	0.44
ggg	G	1	0.26
---	--	------	------
mPD		0.0088	0.48
		nPD:	0.02
		N. weight:	0.91
		Sc. PD:	-0.026
		Sc. rank:	1994.8

,

PB2			
Pos.	544	obs:	exp:
tct	S	6	4091.00
tcc	S	0	2575.00
tca	S	22193	5786.00
tcg	S	32	1183.00
cct	P	0	0.28
ccc	P	0	0.19
cca	P	1	0.43
ccg	P	0	0.11
agt	S	0	4180.00
agc	S	0	4416.00
---	--	------	------
mPD		0.0035	1.6
		nPD:	0.
		N. weight:	1.3
		Sc. PD:	-0.061
		Sc. rank:	-22029.0

,

PB2			
Pos.	545	obs:	exp:
tta	L	0	0.11
ttg	L	0	0.19
ctt	L	0	0.20
ctc	L	0	0.14
cta	L	0	0.19
ctg	L	1	0.17
att	I	2	0.73
atc	I	0	0.50
ata	I	0	0.77
atg	M	1	1.00
gtt	V	170	4914.00
gtc	V	0	4039.00
gta	V	86	5978.00
gtg	V	21972	7297.00
---	--	------	------
mPD		0.023	0.74
		nPD:	0.03
		N. weight:	1.1
		Sc. PD:	-0.017
		Sc. rank:	8860.9

,

PB2			
Pos.	546	obs:	exp:
tta	L	28	2444.00
ttg	L	12	4185.00
ctt	L	0	4381.00
ctc	L	28	3140.00
cta	L	22147	4280.00
ctg	L	15	3801.00
att	I	0	0.73
atc	I	0	0.50
ata	I	2	0.77
---	--	------	------
mPD		0.0087	1.1
		nPD:	0.01
		N. weight:	1.6
		Sc. PD:	-0.065
		Sc. rank:	-12465.0

,

PB2			
Pos.	547	obs:	exp:
tta	L	0	0.11
ttg	L	0	0.19
ctt	L	0	0.20
ctc	L	1	0.14
cta	L	0	0.19
ctg	L	0	0.17
att	I	0	126.70
atc	I	347	86.87
ata	I	1	134.50
gtt	V	83	4838.00
gtc	V	21783	3976.00
gta	V	15	5885.00
gtg	V	1	7183.00
gct	A	0	0.26
gcc	A	1	0.21
gca	A	0	0.47
gcg	A	0	0.06
---	--	------	------
mPD		0.040	0.77
		nPD:	0.05
		N. weight:	1.7
		Sc. PD:	0.0073
		Sc. rank:	20408.0

PB2			
Pos.	548	obs:	exp:
tct	S	0	0.18
tcc	S	0	0.12
tca	S	0	0.26
tcg	S	0	0.05
aat	N	32	13148.00
aac	N	22198	9082.00
agt	S	0	0.19
agc	S	1	0.20
gat	D	0	0.53
gac	D	1	0.47
---	--	------	------
mPD		0.0031	0.48
		nPD:	0.01
		N. weight:	0.89
		Sc. PD:	-0.037
		Sc. rank:	-8405.2

PB2			
Pos.	549	obs:	exp:
act	T	22158	5604.00
acc	T	70	4560.00
aca	T	3	10171.00
acg	T	1	1897.00
---	--	------	------
mPD		0.0066	0.68
		nPD:	0.01
		N. weight:	1.4
		Sc. PD:	-0.051
		Sc. rank:	-6710.9

PB2			
Pos.	550	obs:	exp:
tat	Y	21916	11551.00
tac	Y	316	10681.00
---	--	------	------
mPD		0.028	0.50
		nPD:	0.06
		N. weight:	0.59
		Sc. PD:	0.005
		Sc. rank:	7254.2

PB2			
Pos.	551	obs:	exp:
cat	H	0	0.59
cac	H	1	0.41
caa	Q	22185	11730.00
cag	Q	44	10499.00
aaa	K	1	0.57
aag	K	0	0.43
gaa	E	1	0.61
gag	E	0	0.39
---	--	------	------
mPD		0.0042	0.50
		nPD:	0.01
		N. weight:	0.63
		Sc. PD:	-0.025
		Sc. rank:	-4249.8

PB2			
Pos.	552	obs:	exp:
tta	L	0	0.11
ttg	L	1	0.19
tgg	W	22231	22231.00
ctt	L	0	0.20
ctc	L	0	0.14
cta	L	0	0.19
ctg	L	0	0.17
---	--	------	------
mPD		0.000090	0.00021
		nPD:	0.43
		N. weight:	0.000076
		Sc. PD:	0.000029
		Sc. rank:	1.4

PB2			
Pos.	553	obs:	exp:
tta	L	3	0.33
ttg	L	0	0.56
ctt	L	0	0.59
ctc	L	0	0.42
cta	L	0	0.58
ctg	L	0	0.51
att	I	3	8082.00
atc	I	19	5542.00
ata	I	22179	8577.00
atg	M	2	2.00
gtt	V	0	5.75
gtc	V	0	4.72
gta	V	26	6.99
gtg	V	0	8.53
---	--	------	------
mPD		0.0048	0.66
		nPD:	0.01
		N. weight:	0.95
		Sc. PD:	-0.038
		Sc. rank:	-7901.8

PB2			
Pos.	554	obs:	exp:
att	I	994	8086.00
atc	I	21192	5545.00
ata	I	27	8582.00
act	T	0	3.53
acc	T	14	2.87
aca	T	0	6.40
acg	T	0	1.19
gtt	V	0	1.11
gtc	V	5	0.91
gta	V	0	1.34
gtg	V	0	1.64
---	--	------	------
mPD		0.089	0.66
		nPD:	0.14
		N. weight:	1.2
		Sc. PD:	0.1
		Sc. rank:	19148.0

PB2			
Pos.	555	obs:	exp:
cgt	R	0	606.50
cgc	R	0	1162.00
cga	R	5	2176.00
cgg	R	0	1626.00
act	T	0	0.25
acc	T	0	0.21
aca	T	0	0.46
acg	T	1	0.09
aaa	K	18	10.84
aag	K	1	8.16
aga	R	18871	11151.00
agg	R	3336	5491.00
---	--	------	------
mPD		0.26	0.91
		nPD:	0.28
		N. weight:	0.37
		Sc. PD:	0.088
		Sc. rank:	6471.4

PB2			
Pos.	556	obs:	exp:
tct	S	0	4.42
tcc	S	0	2.78
tca	S	0	6.25
tcg	S	0	1.28
tat	Y	0	0.52
tac	Y	1	0.48
cat	H	0	1.18
cac	H	2	0.82
aat	N	111	13129.00
aac	N	22088	9070.00
aaa	K	6	3.42
aag	K	0	2.58
agt	S	0	4.51
agc	S	24	4.77
---	--	------	------
mPD		0.013	0.49
		nPD:	0.03
		N. weight:	0.87
		Sc. PD:	-0.018
		Sc. rank:	5655.3

PB2			
Pos.	557	obs:	exp:
tgg	W	22232	22232.00
---	--	------	------
mPD		0	0
		nPD:	1.
		N. weight:	0.
		Sc. PD:	0
		Sc. rank:	0

PB2			
Pos.	558	obs:	exp:
gaa	E	22075	13461.00
gag	E	157	8771.00
---	--	------	------
mPD		0.014	0.48
		nPD:	0.03
		N. weight:	0.47
		Sc. PD:	-0.0085
		Sc. rank:	3420.7

PB2			
Pos.	559	obs:	exp:
ttt	F	451	193.50
ttc	F	0	257.50
tta	L	0	0.22
ttg	L	0	0.38
tct	S	0	1.10
tcc	S	0	0.69
tca	S	0	1.56
tcg	S	0	0.32
ctt	L	2	0.39
ctc	L	0	0.28
cta	L	0	0.39
ctg	L	0	0.34
att	I	21604	7873.00
atc	I	12	5399.00
ata	I	12	8356.00
atg	M	5	5.00
act	T	54	13.61
acc	T	0	11.08
aca	T	0	24.70
acg	T	0	4.61
aat	N	9	5.32
aac	N	0	3.68
agt	S	4	1.13
agc	S	2	1.19
gtt	V	66	14.59
gtc	V	0	11.99
gta	V	0	17.75
gtg	V	0	21.67
gct	A	11	2.91
gcc	A	0	2.28
gca	A	0	5.17
gcg	A	0	0.64
---	--	------	------
mPD		0.057	0.71
		nPD:	0.08
		N. weight:	1.
		Sc. PD:	0.032
		Sc. rank:	14667.0

PB2

Pos.	560	obs:	exp:
tta	L	0	0.88
ttg	L	8	1.51
ctt	L	0	1.58
ctc	L	0	1.13
cta	L	0	1.54
ctg	L	0	1.37
atg	M	10	10.00
gtt	V	7	4910.00
gtc	V	25	4036.00
gta	V	31	5973.00
gtg	V	22147	7291.00
gct	A	0	1.06
gcc	A	0	0.83
gca	A	0	1.88
gcg	A	4	0.23
---	--	------	------
mPD		0.0076	0.74
		nPD:	0.01
		N. weight:	1.1
		Sc. PD:	-0.041
		Sc. rank:	-4458.2

,

PB2

Pos.	561	obs:	exp:
aat	N	2	1.18
aac	N	0	0.82
aaa	K	22054	12681.00
aag	K	176	9549.00
---	--	------	------
mPD		0.016	0.49
		nPD:	0.03
		N. weight:	0.52
		Sc. PD:	-0.0079
		Sc. rank:	4574.8

,

PB2

Pos.	562	obs:	exp:
tta	L	0	0.33
ttg	L	0	0.56
tgt	C	1	0.45
tgc	C	0	0.55
ctt	L	3	0.59
ctc	L	0	0.42
cta	L	0	0.58
ctg	L	0	0.51
att	I	21892	7992.00
atc	I	60	5480.00
ata	I	3	8482.00
atg	M	264	264.00
act	T	0	0.25
acc	T	1	0.21
aca	T	0	0.46
acg	T	0	0.09
gtt	V	8	1.77
gtc	V	0	1.45
gta	V	0	2.15
gtg	V	0	2.63
---	--	------	------
mPD		0.030	0.67
		nPD:	0.05
		N. weight:	0.99
		Sc. PD:	-0.0018
		Sc. rank:	11359.0

,

PB2

Pos.	563	obs:	exp:
caa	Q	22168	11726.00
cag	Q	55	10497.00
cgt	R	0	0.11
cgc	R	0	0.21
cga	R	4	0.39
cgg	R	0	0.29
aaa	K	5	2.85
aag	K	0	2.15
aga	R	0	2.01
agg	R	0	0.99
---	--	------	------
mPD		0.0057	0.50
		nPD:	0.01
		N. weight:	0.63
		Sc. PD:	-0.023
		Sc. rank:	-1869.8

,

PB2

Pos.	564	obs:	exp:
tgg	W	22232	22232.00
---	--	------	------
mPD		0	0
		nPD:	1.
		N. weight:	0.
		Sc. PD:	0
		Sc. rank:	0

,

PB2

Pos.	565	obs:	exp:
tct	S	20	4091.00
tcc	S	1	2575.00
tca	S	22145	5785.00
tcg	S	62	1183.00
act	T	0	1.01
acc	T	0	0.82
aca	T	4	1.83
acg	T	0	0.34
agt	S	0	4180.00
agc	S	0	4415.00
---	--	------	------
mPD		0.0078	1.6
		nPD:	0.
		N. weight:	1.3
		Sc. PD:	-0.057
		Sc. rank:	-16267.0

,

PB2

Pos.	566	obs:	exp:
caa	Q	22203	11731.00
cag	Q	29	10501.00
---	--	------	------
mPD		0.0026	0.50
		nPD:	0.01
		N. weight:	0.64
		Sc. PD:	-0.027
		Sc. rank:	-7208.8

,

PB2

Pos.	567	obs:	exp:
tat	Y	0	1.04
tac	Y	2	0.96
aat	N	13	30.75
aac	N	39	21.25
gct	A	0	0.79
gcc	A	3	0.62
gca	A	0	1.41
gcg	A	0	0.18
gat	D	3709	11682.00
gac	D	18462	10489.00
gaa	E	4	2.42
gag	E	0	1.58
---	--	------	------
mPD		0.28	0.50
		nPD:	0.56
		N. weight:	0.28
		Sc. PD:	0.14
		Sc. rank:	5256.1

,

PB2

Pos.	568	obs:	exp:
cct	P	24	6125.00
ccc	P	22176	4123.00
cca	P	31	9466.00
ccg	P	0	2517.00
cat	H	0	0.59
cac	H	1	0.41
---	--	------	------
mPD		0.0050	0.70
		nPD:	0.01
		N. weight:	1.7
		Sc. PD:	-0.067
		Sc. rank:	-14015.0

,

PB2

Pos.	569	obs:	exp:
act	T	4	5601.00
acc	T	42	4558.00
aca	T	22159	10165.00
acg	T	15	1896.00
gct	A	0	3.18
gcc	A	0	2.48
gca	A	12	5.64
gcg	A	0	0.70
---	--	------	------
mPD		0.0066	0.68
		nPD:	0.01
		N. weight:	0.77
		Sc. PD:	-0.029
		Sc. rank:	-3979.9

,

PB2

Pos.	570	obs:	exp:
tta	L	0	0.22
ttg	L	1	0.38
ctt	L	0	0.39
ctc	L	0	0.28
cta	L	1	0.39
ctg	L	0	0.34
cgt	R	0	0.05
cgc	R	0	0.10
cga	R	0	0.20
cgg	R	0	0.15
att	I	6	14.56
atc	I	0	9.98
ata	I	34	15.45
atg	M	22172	22172.00
act	T	0	1.51
acc	T	0	1.23
aca	T	0	2.74
acg	T	6	0.51
aga	R	0	1.00
agg	R	2	0.49
gtt	V	0	2.21
gtc	V	0	1.82
gta	V	0	2.69
gtg	V	10	3.28
---	--	------	------
mPD		0.0055	0.0068
		nPD:	0.81
		N. weight:	0.0025
		Sc. PD:	0.0019
		Sc. rank:	47.1

,

PB2

Pos.	571	obs:	exp:
ttt	F	1	0.43
ttc	F	0	0.57
tta	L	22105	2444.00
ttg	L	78	4185.00
tct	S	0	0.18
tcc	S	0	0.12
tca	S	1	0.26
tcg	S	0	0.05
ctt	L	0	4380.00
ctc	L	0	3140.00
cta	L	45	4279.00
ctg	L	0	3800.00
att	I	0	0.36
atc	I	0	0.25
ata	I	1	0.39
agt	S	0	0.19
agc	S	0	0.20
gtt	V	0	0.22
gtc	V	0	0.18
gta	V	1	0.27
gtg	V	0	0.33
---	--	------	------
mPD		0.011	1.1
		nPD:	0.01
		N. weight:	2.2
		Sc. PD:	-0.082
		Sc. rank:	-9429.6

,

PB2			
Pos.	572	obs:	exp:
tat	Y	240	11551.00
tac	Y	21991	10680.00
aat	N	0	0.59
aac	N	1	0.41
---	--	------	------
mPD		0.021	0.50
		nPD:	0.04
		N. weight:	0.68
		Sc. PD:	-0.0031
		Sc. rank:	7147.8

PB2			
Pos.	573	obs:	exp:
tct	S	0	0.18
tcc	S	0	0.12
tca	S	0	0.26
tcg	S	0	0.05
act	T	0	0.25
acc	T	1	0.21
aca	T	0	0.46
acg	T	0	0.09
aat	N	1818	13148.00
aac	N	20412	9082.00
agt	S	0	0.19
agc	S	1	0.20
---	--	------	------
mPD		0.15	0.48
		nPD:	0.31
		N. weight:	0.59
		Sc. PD:	0.15
		Sc. rank:	10330.0

PB2			
Pos.	574	obs:	exp:
cgt	R	0	0.52
cgc	R	0	0.99
cga	R	0	1.86
cgg	R	0	1.39
aaa	K	22087	12671.00
aag	K	126	9542.00
aga	R	18	9.54
agg	R	1	4.70
---	--	------	------
mPD		0.013	0.49
		nPD:	0.03
		N. weight:	0.53
		Sc. PD:	-0.011
		Sc. rank:	3491.2

PB2			
Pos.	575	obs:	exp:
tta	L	0	2.42
ttg	L	22	4.14
ctt	L	0	4.34
ctc	L	0	3.11
cta	L	0	4.24
ctg	L	0	3.76
att	I	3	12.38
atc	I	0	8.49
ata	I	31	13.14
atg	M	22162	22162.00
act	T	0	0.25
acc	T	0	0.21
aca	T	0	0.46
acg	T	1	0.09
gtt	V	0	2.87
gtc	V	0	2.36
gta	V	0	3.50
gtg	V	13	4.27
---	--	------	------
mPD		0.0063	0.0084
		nPD:	0.75
		N. weight:	0.0035
		Sc. PD:	0.0024
		Sc. rank:	67.0

PB2			
Pos.	576	obs:	exp:
gtt	V	0	0.44
gtc	V	0	0.36
gta	V	2	0.54
gtg	V	0	0.66
gaa	E	22040	13460.00
gag	E	190	8770.00
---	--	------	------
mPD		0.017	0.48
		nPD:	0.04
		N. weight:	0.46
		Sc. PD:	-0.0054
		Sc. rank:	4297.3

PB2			
Pos.	577	obs:	exp:
ttt	F	22218	9540.00
ttc	F	13	12691.00
tta	L	0	0.11
ttg	L	0	0.19
ctt	L	1	0.20
ctc	L	0	0.14
cta	L	0	0.19
ctg	L	0	0.17
---	--	------	------
mPD		0.0013	0.49
		nPD:	0.
		N. weight:	0.85
		Sc. PD:	-0.038
		Sc. rank:	-13197.0

PB2			
Pos.	578	obs:	exp:
gaa	E	22085	13458.00
gag	E	142	8769.00
ggt	G	0	0.76
ggc	G	0	0.74
gga	G	4	2.22
ggg	G	1	1.28
---	--	------	------
mPD		0.013	0.48
		nPD:	0.03
		N. weight:	0.47
		Sc. PD:	-0.0093
		Sc. rank:	3241.2

PB2			
Pos.	579	obs:	exp:
tct	S	0	0.18
tcc	S	0	0.12
tca	S	1	0.26
tcg	S	0	0.05
cct	P	5	6125.00
ccc	P	0	4123.00
cca	P	22122	9466.00
ccg	P	104	2517.00
agt	S	0	0.19
agc	S	0	0.20
---	--	------	------
mPD		0.0099	0.70
		nPD:	0.01
		N. weight:	0.84
		Sc. PD:	-0.028
		Sc. rank:	-485.0

PB2			
Pos.	580	obs:	exp:
ttt	F	22189	9540.00
ttc	F	43	12692.00
---	--	------	------
mPD		0.0039	0.49
		nPD:	0.01
		N. weight:	0.84
		Sc. PD:	-0.033
		Sc. rank:	-6117.0

PB2			
Pos.	581	obs:	exp:
cat	H	1	0.59
cac	H	0	0.41
caa	Q	67	11731.00
cag	Q	22164	10500.00
---	--	------	------
mPD		0.0061	0.50
		nPD:	0.01
		N. weight:	0.74
		Sc. PD:	-0.026
		Sc. rank:	-1712.4

PB2			
Pos.	582	obs:	exp:
ttt	F	1	0.43
ttc	F	0	0.57
tct	S	22183	4091.00
tcc	S	40	2575.00
tca	S	5	5785.00
tcg	S	2	1183.00
act	T	1	0.25
acc	T	0	0.21
aca	T	0	0.46
acg	T	0	0.09
agt	S	0	4180.00
agc	S	0	4416.00
---	--	------	------
mPD		0.0044	1.6
		nPD:	0.
		N. weight:	1.7
		Sc. PD:	-0.076
		Sc. rank:	-26179.0

PB2			
Pos.	583	obs:	exp:
tta	L	11	2443.00
ttg	L	0	4184.00
ctt	L	22182	4380.00
ctc	L	11	3139.00
cta	L	21	4279.00
ctg	L	1	3800.00
att	I	6	2.18
atc	I	0	1.50
ata	I	0	2.32
---	--	------	------
mPD		0.0055	1.1
		nPD:	0.
		N. weight:	1.6
		Sc. PD:	-0.069
		Sc. rank:	-19265.0

PB2			
Pos.	584	obs:	exp:
att	I	1	13.11
atc	I	35	8.99
ata	I	0	13.91
gtt	V	7452	4907.00
gtc	V	14740	4033.00
gta	V	4	5969.00
gtg	V	0	7286.00
---	--	------	------
mPD		0.45	0.74
		nPD:	0.61
		N. weight:	1.
		Sc. PD:	0.56
		Sc. rank:	18929.0

PB2			
Pos.	585	obs:	exp:
tct	S	1	0.18
tcc	S	0	0.12
tca	S	0	0.26
tcg	S	0	0.05
cct	P	22127	6124.00
ccc	P	97	4123.00
cca	P	3	9465.00
ccg	P	2	2517.00
cat	H	1	0.59
cac	H	0	0.41
cgt	R	1	0.03
cgc	R	0	0.05
cga	R	0	0.10
cgg	R	0	0.07
agt	S	0	0.19
agc	S	0	0.20
aga	R	0	0.50
agg	R	0	0.25
---	--	------	------
mPD		0.0094	0.70
		nPD:	0.01
		N. weight:	1.3
		Sc. PD:	-0.043
		Sc. rank:	-1345.6

PB2			
Pos.	586	obs:	exp:
caa	Q	0	0.53
cag	Q	1	0.47
cgt	R	0	0.87
cgc	R	0	1.67
cga	R	0	3.13
cgg	R	0	2.34
act	T	0	0.25
acc	T	0	0.21
aca	T	0	0.46
acg	T	1	0.09
aaa	K	188	12662.00
aag	K	22010	9536.00
aga	R	0	16.07
agg	R	32	7.91
---	--	------	------
mPD		0.020	0.49
		nPD:	0.04
		N. weight:	0.8
		Sc. PD:	-0.0059
		Sc. rank:	8128.5

PB2			
Pos.	587	obs:	exp:
gct	A	9	5886.00
gcc	A	14	4602.00
gca	A	22196	10442.00
gcg	A	13	1302.00
---	--	------	------
mPD		0.0032	0.66
		nPD:	0.
		N. weight:	0.75
		Sc. PD:	-0.032
		Sc. rank:	-8811.9

PB2			
Pos.	588	obs:	exp:
tct	S	0	0.55
tcc	S	1	0.35
tca	S	0	0.78
tcg	S	0	0.16
att	I	1	160.90
atc	I	441	110.30
ata	I	0	170.80
act	T	58	5461.00
acc	T	21592	4443.00
aca	T	12	9910.00
acg	T	0	1848.00
aat	N	0	11.83
aac	N	20	8.17
agt	S	0	0.56
agc	S	2	0.60
gct	A	0	27.80
gcc	A	105	21.73
gca	A	0	49.31
gcg	A	0	6.15
---	--	------	------
mPD		0.057	0.73
		nPD:	0.08
		N. weight:	1.6
		Sc. PD:	0.047
		Sc. rank:	22448.0

PB2			
Pos.	589	obs:	exp:
cgt	R	0	606.80
cgc	R	0	1163.00
cga	R	5	2177.00
cgg	R	0	1627.00
att	I	0	0.36
atc	I	0	0.25
ata	I	1	0.39
aaa	K	6	3.42
aag	K	0	2.58
aga	R	22191	11158.00
agg	R	29	5494.00
---	--	------	------
mPD		0.0037	0.91
		nPD:	0.
		N. weight:	0.69
		Sc. PD:	-0.03
		Sc. rank:	-9216.1

,

PB2			
Pos.	590	obs:	exp:
tct	S	0	4055.00
tcc	S	0	2552.00
tca	S	0	5735.00
tcg	S	0	1173.00
cgt	R	0	0.03
cgc	R	1	0.05
cga	R	0	0.10
cgg	R	0	0.07
aat	N	0	83.98
aac	N	142	58.02
agt	S	223	4144.00
agc	S	21813	4377.00
aga	R	0	0.50
agg	R	0	0.25
ggt	G	1	8.04
ggc	G	52	7.85
gga	G	0	23.58
ggg	G	0	13.54
---	--	------	------
mPD		0.037	1.7
		nPD:	0.02
		N. weight:	1.6
		Sc. PD:	-0.039
		Sc. rank:	7421.5

,

PB2			
Pos.	591	obs:	exp:
tgg	W	1	1.00
cct	P	0	0.28
ccc	P	0	0.19
cca	P	0	0.43
ccg	P	1	0.11
caa	Q	12	16.89
cag	Q	20	15.11
cgt	R	13	606.10
cgc	R	1	1161.00
cga	R	624	2174.00
cgg	R	21520	1625.00
aga	R	0	11144.00
agg	R	40	5488.00
---	--	------	------
mPD		0.063	0.91
		nPD:	0.07
		N. weight:	2.5
		Sc. PD:	0.054
		Sc. rank:	34246.0

,

PB2			
Pos.	592	obs:	exp:
tat	Y	51	11551.00
tac	Y	22181	10681.00
---	--	------	------
mPD		0.0046	0.50
		nPD:	0.01
		N. weight:	0.72
		Sc. PD:	-0.028
		Sc. rank:	-4103.9

,

PB2			
Pos.	593	obs:	exp:
tct	S	0	4091.00
tcc	S	0	2575.00
tca	S	0	5786.00
tcg	S	0	1183.00
agt	S	22192	4180.00
agc	S	40	4416.00
---	--	------	------
mPD		0.0036	1.6
		nPD:	0.
		N. weight:	1.7
		Sc. PD:	-0.075
		Sc. rank:	-27161.0

,

PB2			
Pos.	594	obs:	exp:
ggt	G	0	3371.00
ggc	G	4	3293.00
gga	G	21905	9889.00
ggg	G	323	5679.00
---	--	------	------
mPD		0.029	0.69
		nPD:	0.04
		N. weight:	0.75
		Sc. PD:	-0.0042
		Sc. rank:	7638.5

,

PB2			
Pos.	595	obs:	exp:
ttt	F	111	9540.00
ttc	F	22120	12691.00
tta	L	0	0.11
ttg	L	1	0.19
ctt	L	0	0.20
ctc	L	0	0.14
cta	L	0	0.19
ctg	L	0	0.17
---	--	------	------
mPD		0.010	0.49
		nPD:	0.02
		N. weight:	0.54
		Sc. PD:	-0.014
		Sc. rank:	1850.3

,

PB2			
Pos.	596	obs:	exp:
tta	L	1	0.11
ttg	L	0	0.19
ctt	L	0	0.20
ctc	L	0	0.14
cta	L	0	0.19
ctg	L	0	0.17
att	I	0	3.28
atc	I	0	2.25
ata	I	9	3.48
gtt	V	0	4913.00
gtc	V	13	4038.00
gta	V	22136	5976.00
gtg	V	71	7294.00
gct	A	0	0.53
gcc	A	0	0.41
gca	A	2	0.94
gcg	A	0	0.12
---	--	------	------
mPD		0.0086	0.74
		nPD:	0.01
		N. weight:	1.3
		Sc. PD:	-0.046
		Sc. rank:	-3590.9

,

PB2			
Pos.	597	obs:	exp:
cgt	R	0	607.00
cgc	R	0	1163.00
cga	R	0	2178.00
cgg	R	3	1627.00
aga	R	277	11161.00
agg	R	21952	5496.00
---	--	------	------
mPD		0.025	0.91
		nPD:	0.03
		N. weight:	1.3
		Sc. PD:	-0.027
		Sc. rank:	9106.9

,

PB2			
Pos.	598	obs:	exp:
tct	S	0	0.18
tcc	S	0	0.12
tca	S	1	0.26
tcg	S	0	0.05
att	I	0	0.73
atc	I	0	0.50
ata	I	2	0.77
act	T	13	5601.00
acc	T	2	4558.00
aca	T	22144	10165.00
acg	T	61	1896.00
agt	S	0	0.19
agc	S	0	0.20
gct	A	0	2.38
gcc	A	0	1.86
gca	A	9	4.23
gcg	A	0	0.53
---	--	------	------
mPD		0.0079	0.68
		nPD:	0.01
		N. weight:	0.77
		Sc. PD:	-0.027
		Sc. rank:	-2178.8

,

PB2			
Pos.	599	obs:	exp:
tta	L	8	2444.00
ttg	L	75	4186.00
ctt	L	15	4381.00
ctc	L	0	3140.00
cta	L	197	4280.00
ctg	L	21937	3801.00
---	--	------	------
mPD		0.027	1.1
		nPD:	0.02
		N. weight:	1.7
		Sc. PD:	-0.04
		Sc. rank:	9236.8

,

PB2			
Pos.	600	obs:	exp:
ttt	F	200	9540.00
ttc	F	22031	12691.00
gtt	V	0	0.22
gtc	V	1	0.18
gta	V	0	0.27
gtg	V	0	0.33
---	--	------	------
mPD		0.018	0.49
		nPD:	0.04
		N. weight:	0.52
		Sc. PD:	-0.0056
		Sc. rank:	4907.5

,

PB2			
Pos.	601	obs:	exp:
caa	Q	68	11731.00
cag	Q	22163	10500.00
---	-	1	1.00
---	--	------	------
mPD		0.0061	0.50
		nPD:	0.01
		N. weight:	0.74
		Sc. PD:	-0.026
		Sc. rank:	-1684.5

,

PB2			
Pos.	602	obs:	exp:
caa	Q	22169	11731.00
cag	Q	63	10501.00
---	--	------	------
mPD		0.0057	0.50
		nPD:	0.01
		N. weight:	0.63
		Sc. PD:	-0.023
		Sc. rank:	-1978.9

,

PB2			
Pos.	603	obs:	exp:
att	I	0	0.36
atc	I	0	0.25
ata	I	1	0.39
atg	M	22231	22231.00
---	--	------	------
mPD		0.000090	0.000090
		nPD:	1.
		N. weight:	0.000043
		Sc. PD:	0.000041
		Sc. rank:	0.8

,

PB2			
Pos.	604	obs:	exp:
cgt	R	2	607.00
cgc	R	0	1163.00
cga	R	18	2177.00
cgg	R	8243	1627.00
aga	R	48	11161.00
agg	R	13920	5496.00
ggt	G	0	0.15
ggc	G	0	0.15
gga	G	0	0.44
ggg	G	1	0.26
---	--	------	------
mPD		0.47	0.91
		nPD:	0.52
		N. weight:	1.2
		Sc. PD:	0.55
		Sc. rank:	21934.0

,

PB2

Pos.	605	obs:	exp:
gat	D	22207	11714.00
gac	D	25	10518.00
---	--	------	------
mPD		0.0022	0.50
		nPD:	0.
		N. weight:	0.64
		Sc. PD:	-0.027
		Sc. rank:	-8050.0

,

PB2

Pos.	606	obs:	exp:
tta	L	0	0.11
ttg	L	1	0.19
ctt	L	0	0.20
ctc	L	0	0.14
cta	L	0	0.19
ctg	L	0	0.17
att	I	2	0.73
atc	I	0	0.50
ata	I	0	0.77
gtt	V	15	4914.00
gtc	V	1	4039.00
gta	V	123	5978.00
gtg	V	22089	7297.00
gct	A	0	0.26
gcc	A	0	0.21
gca	A	0	0.47
gcg	A	1	0.06
---	--	------	------
mPD		0.013	0.74
		nPD:	0.02
		N. weight:	1.1
		Sc. PD:	-0.032
		Sc. rank:	1997.1

,

PB2

Pos.	607	obs:	exp:
ttt	F	5	2.15
ttc	F	0	2.85
tta	L	0	2440.00
ttg	L	0	4179.00
ctt	L	22173	4374.00
ctc	L	23	3135.00
cta	L	1	4273.00
ctg	L	0	3795.00
att	I	30	10.92
atc	I	0	7.49
ata	I	0	11.59
---	--	------	------
mPD		0.0053	1.1
		nPD:	0.
		N. weight:	1.6
		Sc. PD:	-0.07
		Sc. rank:	-20028.0

,

PB2

Pos.	608	obs:	exp:
ggt	G	10	3371.00
ggc	G	0	3293.00
gga	G	217	9889.00
ggg	G	22005	5679.00
---	--	------	------
mPD		0.020	0.69
		nPD:	0.03
		N. weight:	1.3
		Sc. PD:	-0.024
		Sc. rank:	9481.5

,

PB2

Pos.	609	obs:	exp:
act	T	3	5604.00
acc	T	1	4560.00
aca	T	22209	10171.00
acg	T	19	1897.00
---	--	------	------
mPD		0.0021	0.68
		nPD:	0.
		N. weight:	0.78
		Sc. PD:	-0.035
		Sc. rank:	-11747.0

,

PB2

Pos.	610	obs:	exp:
ttt	F	22197	9540.00
ttc	F	33	12690.00
tta	L	0	0.22
ttg	L	0	0.38
ctt	L	2	0.39
ctc	L	0	0.28
cta	L	0	0.39
ctg	L	0	0.34
---	--	------	------
mPD		0.0031	0.49
		nPD:	0.01
		N. weight:	0.84
		Sc. PD:	-0.034
		Sc. rank:	-7839.6

,

PB2

Pos.	611	obs:	exp:
aat	N	0	2.96
aac	N	5	2.04
gat	D	42	11709.00
gac	D	22180	10513.00
gaa	E	5	3.03
gag	E	0	1.97
---	--	------	------
mPD		0.0047	0.50
		nPD:	0.01
		N. weight:	0.74
		Sc. PD:	-0.028
		Sc. rank:	-4085.5

,

PB2

Pos.	612	obs:	exp:
tct	S	1	0.18
tcc	S	0	0.12
tca	S	0	0.26
tcg	S	0	0.05
att	I	5	1.82
atc	I	0	1.25
ata	I	0	1.93
act	T	22158	5602.00
acc	T	10	4558.00
aca	T	40	10166.00
acg	T	13	1896.00
agt	S	0	0.19
agc	S	0	0.20
gct	A	5	1.32
gcc	A	0	1.03
gca	A	0	2.35
gcg	A	0	0.29
---	--	------	------
mPD		0.0066	0.68
		nPD:	0.01
		N. weight:	1.4
		Sc. PD:	-0.051
		Sc. rank:	-6805.6

,

PB2

Pos.	613	obs:	exp:
att	I	5	31.67
atc	I	82	21.72
ata	I	0	33.61
gtt	V	861	4891.00
gtc	V	21255	4020.00
gta	V	5	5949.00
gtg	V	0	7262.00
gct	A	0	6.35
gcc	A	24	4.97
gca	A	0	11.27
gcg	A	0	1.41
---	--	------	------
mPD		0.085	0.75
		nPD:	0.11
		N. weight:	1.5
		Sc. PD:	0.1
		Sc. rank:	23899.0

,

PB2

Pos.	614	obs:	exp:
caa	Q	22202	11731.00
cag	Q	30	10501.00
---	--	------	------
mPD		0.0027	0.50
		nPD:	0.01
		N. weight:	0.64
		Sc. PD:	-0.027
		Sc. rank:	-6979.3

,

PB2

Pos.	615	obs:	exp:
tta	L	0	0.11
ttg	L	0	0.19
ctt	L	0	0.20
ctc	L	0	0.14
cta	L	1	0.19
ctg	L	0	0.17
att	I	2	8086.00
atc	I	0	5545.00
ata	I	22210	8582.00
atg	M	1	1.00
act	T	0	1.51
acc	T	0	1.23
aca	T	6	2.74
acg	T	0	0.51
gtt	V	0	2.65
gtc	V	0	2.18
gta	V	12	3.23
gtg	V	0	3.94
---	--	------	------
mPD		0.0020	0.66
		nPD:	0.
		N. weight:	0.96
		Sc. PD:	-0.042
		Sc. rank:	-14489.0

,

PB2

Pos.	616	obs:	exp:
att	I	1	8091.00
atc	I	1	5548.00
ata	I	22223	8587.00
atg	M	1	1.00
aaa	K	1	0.57
aag	K	0	0.43
gtt	V	0	1.11
gtc	V	0	0.91
gta	V	5	1.34
gtg	V	0	1.64
---	--	------	------
mPD		0.00081	0.66
		nPD:	0.
		N. weight:	0.96
		Sc. PD:	-0.044
		Sc. rank:	-17098.0

,

PB2

Pos.	617	obs:	exp:
aaa	K	22193	12682.00
aag	K	39	9550.00
---	--	------	------
mPD		0.0035	0.49
		nPD:	0.01
		N. weight:	0.55
		Sc. PD:	-0.022
		Sc. rank:	-4670.4

,

PB2

Pos.	618	obs:	exp:
tta	L	0	2444.00
ttg	L	0	4186.00
ctt	L	22131	4381.00
ctc	L	100	3140.00
cta	L	1	4280.00
ctg	L	0	3801.00
---	--	------	------
mPD		0.0090	1.1
		nPD:	0.01
		N. weight:	1.6
		Sc. PD:	-0.063
		Sc. rank:	-11452.0

,

PB2

Pos.	619	obs:	exp:
tta	L	0	2444.00
ttg	L	0	4185.00
ctt	L	19	4381.00
ctc	L	22120	3140.00
cta	L	92	4280.00
ctg	L	0	3801.00
att	I	0	0.36
atc	I	1	0.25
ata	I	0	0.39
---	--	------	------
mPD		0.010	1.1
		nPD:	0.01
		N. weight:	1.9
		Sc. PD:	-0.075
		Sc. rank:	-12230.0

,

PB2

Pos.	620	obs:	exp:
cct	P	63	6125.00
ccc	P	22155	4123.00
cca	P	14	9466.00
ccg	P	0	2517.00
---	--	------	------
mPD		0.0069	0.70
		nPD:	0.01
		N. weight:	1.7
		Sc. PD:	-0.063
		Sc. rank:	-7464.6

,

PB2			
Pos.	621	obs:	exp:
ttt	F	22224	9540.00
ttc	F	8	12692.00
---	--	------	------
mPD		0.00072	0.49
		nPD:	0.
		N. weight:	0.85
		Sc. PD:	-0.039
		Sc. rank:	-14920.0

PB2			
Pos.	622	obs:	exp:
tct	S	1	0.18
tcc	S	0	0.12
tca	S	0	0.26
tcg	S	0	0.05
act	T	4	1.01
acc	T	0	0.82
aca	T	0	1.83
acg	T	0	0.34
agt	S	0	0.19
agc	S	0	0.20
gct	A	22155	5884.00
gcc	A	11	4601.00
gca	A	59	10439.00
gcg	A	1	1302.00
ggt	G	1	0.15
ggc	G	0	0.15
gga	G	0	0.44
ggg	G	0	0.26
---	--	------	------
mPD		0.0069	0.66
		nPD:	0.01
		N. weight:	1.3
		Sc. PD:	-0.049
		Sc. rank:	-5234.2

PB2			
Pos.	623	obs:	exp:
tct	S	3	0.55
tcc	S	0	0.35
tca	S	0	0.78
tcg	S	0	0.16
act	T	1	0.25
acc	T	0	0.21
aca	T	0	0.46
acg	T	0	0.09
agt	S	0	0.56
agc	S	0	0.60
gct	A	22074	5885.00
gcc	A	141	4601.00
gca	A	11	10440.00
gcg	A	2	1302.00
---	--	------	------
mPD		0.014	0.66
		nPD:	0.02
		N. weight:	1.3
		Sc. PD:	-0.034
		Sc. rank:	5347.6

PB2			
Pos.	624	obs:	exp:
tct	S	7	1.47
tcc	S	1	0.93
tca	S	0	2.08
tcg	S	0	0.43
agt	S	0	1.50
agc	S	0	1.59
gtt	V	0	0.44
gtc	V	2	0.36
gta	V	0	0.54
gtg	V	0	0.66
gct	A	22139	5883.00
gcc	A	65	4600.00
gca	A	14	10437.00
gcg	A	4	1302.00
---	--	------	------
mPD		0.0086	0.66
		nPD:	0.01
		N. weight:	1.3
		Sc. PD:	-0.045
		Sc. rank:	-2011.1

PB2			
Pos.	625	obs:	exp:
tta	L	0	0.11
ttg	L	0	0.19
ctt	L	0	0.20
ctc	L	0	0.14
cta	L	1	0.19
ctg	L	0	0.17
cct	P	5	6125.00
ccc	P	3	4123.00
cca	P	22203	9466.00
ccg	P	20	2517.00
---	--	------	------
mPD		0.0026	0.70
		nPD:	0.
		N. weight:	0.85
		Sc. PD:	-0.037
		Sc. rank:	-11863.0

PB2			
Pos.	626	obs:	exp:
cct	P	21	6124.00
ccc	P	3	4123.00
cca	P	22165	9465.00
ccg	P	41	2517.00
act	T	0	0.25
acc	T	0	0.21
aca	T	1	0.46
acg	T	0	0.09
---	-	1	1.00
---	--	------	------
mPD		0.0059	0.70
		nPD:	0.01
		N. weight:	0.84
		Sc. PD:	-0.033
		Sc. rank:	-5483.1

PB2			
Pos.	627	obs:	exp:
aaa	K	3	7.99
aag	K	11	6.01
gtt	V	0	0.44
gtc	V	0	0.36
gta	V	2	0.54
gtg	V	0	0.66
gaa	E	22129	13451.00
gag	E	86	8764.00
---	-	1	1.00
---	--	------	------
mPD		0.010	0.48
		nPD:	0.02
		N. weight:	0.48
		Sc. PD:	-0.013
		Sc. rank:	1868.5

PB2			
Pos.	628	obs:	exp:
tta	L	0	0.88
ttg	L	1	1.51
ctt	L	0	1.58
ctc	L	0	1.13
cta	L	0	1.54
ctg	L	7	1.37
cat	H	8	4.70
cac	H	0	3.30
caa	Q	56	11721.00
cag	Q	22156	10491.00
cgt	R	0	0.05
cgc	R	0	0.10
cga	R	0	0.20
cgg	R	2	0.15
aaa	K	0	0.57
aag	K	1	0.43
aga	R	0	1.00
agg	R	0	0.49
gaa	E	0	0.61
gag	E	1	0.39
---	--	------	------
mPD		0.0069	0.50
		nPD:	0.01
		N. weight:	0.74
		Sc. PD:	-0.025
		Sc. rank:	-548.9

PB2			
Pos.	629	obs:	exp:
tct	S	0	4087.00
tcc	S	0	2573.00
tca	S	0	5780.00
tcg	S	0	1182.00
aat	N	0	11.83
aac	N	20	8.17
agt	S	3708	4176.00
agc	S	18502	4412.00
ggt	G	0	0.30
ggc	G	2	0.30
gga	G	0	0.89
ggg	G	0	0.51
---	--	------	------
mPD		0.28	1.6
		nPD:	0.17
		N. weight:	1.2
		Sc. PD:	0.14
		Sc. rank:	19578.0

PB2			
Pos.	630	obs:	exp:
cgt	R	0	606.50
cgc	R	0	1162.00
cga	R	0	2176.00
cgg	R	2	1626.00
act	T	0	0.25
acc	T	0	0.21
aca	T	0	0.46
acg	T	1	0.09
aaa	K	0	9.13
aag	K	16	6.87
aga	R	173	11152.00
agg	R	22039	5492.00
ggt	G	0	0.15
ggc	G	0	0.15
gga	G	0	0.44
ggg	G	1	0.26
---	--	------	------
mPD		0.017	0.91
		nPD:	0.02
		N. weight:	1.4
		Sc. PD:	-0.039
		Sc. rank:	3309.6

PB2			
Pos.	631	obs:	exp:
tta	L	0	0.33
ttg	L	3	0.56
ctt	L	0	0.59
ctc	L	0	0.42
cta	L	0	0.58
ctg	L	0	0.51
att	I	5	5.82
atc	I	0	3.99
ata	I	11	6.18
atg	M	22211	22211.00
gtt	V	0	0.44
gtc	V	0	0.36
gta	V	0	0.54
gtg	V	2	0.66
---	--	------	------
mPD		0.0019	0.0022
		nPD:	0.87
		N. weight:	0.00065
		Sc. PD:	0.00053
		Sc. rank:	12.4

PB2			
Pos.	632	obs:	exp:
caa	Q	22092	11731.00
cag	Q	140	10501.00
---	--	------	------
mPD		0.013	0.50
		nPD:	0.03
		N. weight:	0.61
		Sc. PD:	-0.014
		Sc. rank:	3607.2

PB2			
Pos.	633	obs:	exp:
ttt	F	21953	9540.00
ttc	F	279	12692.00
---	--	------	------
mPD		0.025	0.49
		nPD:	0.05
		N. weight:	0.78
		Sc. PD:	0.0024
		Sc. rank:	9362.9

PB2			
Pos.	634	obs:	exp:
tct	S	18725	4091.00
tcc	S	3494	2575.00
tca	S	6	5786.00
tcg	S	6	1183.00
tat	Y	1	0.52
tac	Y	0	0.48
agt	S	0	4180.00
agc	S	0	4416.00
---	--	------	------
mPD		0.27	1.6
		nPD:	0.16
		N. weight:	1.3
		Sc. PD:	0.15
		Sc. rank:	22028.0

PB2			
Pos.	635	obs:	exp:
tct	S	11	4091.00
tcc	S	1	2575.00
tca	S	22192	5786.00
tcg	S	28	1183.00
agt	S	0	4180.00
agc	S	0	4416.00
---	--	------	------
mPD		0.0036	1.6
		nPD:	0.
		N. weight:	1.3
		Sc. PD:	-0.061
		Sc. rank:	-21650.0

PB2			
Pos.	636	obs:	exp:
ttt	F	10	4.29
ttc	F	0	5.71
tta	L	245	2443.00
ttg	L	21836	4184.00
ctt	L	0	4379.00
ctc	L	0	3139.00
cta	L	0	4278.00
ctg	L	141	3799.00
---	--	------	------
mPD		0.035	1.1
		nPD:	0.03
		N. weight:	1.6
		Sc. PD:	-0.026
		Sc. rank:	12952.0

PB2			
Pos.	637	obs:	exp:
tct	S	1	0.18
tcc	S	0	0.12
tca	S	0	0.26
tcg	S	0	0.05
att	I	6	2.18
atc	I	0	1.50
ata	I	0	2.32
act	T	22172	5596.00
acc	T	19	4553.00
aca	T	6	10155.00
acg	T	1	1894.00
agt	S	0	0.19
agc	S	0	0.20
gct	A	27	7.15
gcc	A	0	5.59
gca	A	0	12.68
gcg	A	0	1.58
---	--	------	------
mPD		0.0054	0.68
		nPD:	0.01
		N. weight:	1.4
		Sc. PD:	-0.054
		Sc. rank:	-9969.3

,

PB2			
Pos.	638	obs:	exp:
tta	L	0	0.33
ttg	L	3	0.56
ctt	L	0	0.59
ctc	L	0	0.42
cta	L	0	0.58
ctg	L	0	0.51
gtt	V	4	4914.00
gtc	V	0	4039.00
gta	V	38	5978.00
gtg	V	22187	7297.00
---	--	------	------
mPD		0.0040	0.74
		nPD:	0.01
		N. weight:	1.1
		Sc. PD:	-0.046
		Sc. rank:	-11865.0

,

PB2			
Pos.	639	obs:	exp:
tct	S	0	0.74
tcc	S	0	0.46
tca	S	0	1.04
tcg	S	0	0.21
act	T	1	0.25
acc	T	0	0.21
aca	T	0	0.46
acg	T	0	0.09
aat	N	22196	13146.00
aac	N	31	9081.00
agt	S	4	0.75
agc	S	0	0.79
---	--	------	------
mPD		0.0032	0.48
		nPD:	0.01
		N. weight:	0.52
		Sc. PD:	-0.021
		Sc. rank:	-4720.5

,

PB2			
Pos.	640	obs:	exp:
atg	M	4	4.00
gtt	V	27	4914.00
gtc	V	0	4039.00
gta	V	150	5978.00
gtg	V	22051	7297.00
---	--	------	------
mPD		0.016	0.74
		nPD:	0.02
		N. weight:	1.1
		Sc. PD:	-0.027
		Sc. rank:	4773.6

,

PB2			
Pos.	641	obs:	exp:
cgt	R	0	607.00
cgc	R	0	1163.00
cga	R	0	2178.00
cgg	R	0	1627.00
aga	R	22185	11161.00
agg	R	47	5496.00
---	--	------	------
mPD		0.0042	0.91
		nPD:	0.
		N. weight:	0.68
		Sc. PD:	-0.029
		Sc. rank:	-8433.3

,

PB2			
Pos.	642	obs:	exp:
ggt	G	84	3371.00
ggc	G	0	3293.00
gga	G	22072	9889.00
ggg	G	76	5679.00
---	--	------	------
mPD		0.014	0.69
		nPD:	0.02
		N. weight:	0.78
		Sc. PD:	-0.021
		Sc. rank:	2709.8

,

PB2			
Pos.	643	obs:	exp:
tta	L	4	0.44
ttg	L	0	0.75
tct	S	10	4074.00
tcc	S	1	2564.00
tca	S	22105	5761.00
tcg	S	21	1178.00
ctt	L	0	0.79
ctc	L	0	0.56
cta	L	0	0.77
ctg	L	0	0.68
act	T	0	22.69
acc	T	0	18.46
aca	T	90	41.17
acg	T	0	7.68
agt	S	0	4163.00
agc	S	0	4397.00
gct	A	0	0.26
gcc	A	0	0.21
gca	A	1	0.47
gcg	A	0	0.06
---	--	------	------
mPD		0.011	1.6
		nPD:	0.01
		N. weight:	1.3
		Sc. PD:	-0.054
		Sc. rank:	-11965.0

,

PB2			
Pos.	644	obs:	exp:
ggt	G	4	3371.00
ggc	G	1	3293.00
gga	G	189	9889.00
ggg	G	22038	5679.00
---	--	------	------
mPD		0.017	0.69
		nPD:	0.03
		N. weight:	1.3
		Sc. PD:	-0.03
		Sc. rank:	7805.4

,

PB2			
Pos.	645	obs:	exp:
tta	L	17780	2442.00
ttg	L	4413	4183.00
tct	S	0	0.18
tcc	S	0	0.12
tca	S	0	0.26
tcg	S	1	0.05
ctt	L	0	4378.00
ctc	L	0	3138.00
cta	L	16	4277.00
ctg	L	8	3799.00
atg	M	12	12.00
agt	S	0	0.19
agc	S	0	0.20
gtt	V	0	0.44
gtc	V	0	0.36
gta	V	1	0.54
gtg	V	1	0.66
---	--	------	------
mPD		0.32	1.1
		nPD:	0.28
		N. weight:	1.6
		Sc. PD:	0.38
		Sc. rank:	27897.0

,

PB2			
Pos.	646	obs:	exp:
cgt	R	0	607.00
cgc	R	0	1163.00
cga	R	0	2178.00
cgg	R	70	1627.00
aga	R	144	11161.00
agg	R	22018	5496.00
---	--	------	------
mPD		0.019	0.91
		nPD:	0.02
		N. weight:	1.3
		Sc. PD:	-0.036
		Sc. rank:	5191.0

,

PB2			
Pos.	647	obs:	exp:
att	I	15	8091.00
atc	I	2	5548.00
ata	I	22208	8587.00
atg	M	3	3.00
gtt	V	0	0.88
gtc	V	0	0.73
gta	V	4	1.08
gtg	V	0	1.31
---	--	------	------
mPD		0.0022	0.66
		nPD:	0.
		N. weight:	0.95
		Sc. PD:	-0.042
		Sc. rank:	-13905.0

,

PB2			
Pos.	648	obs:	exp:
tta	L	0	2441.00
ttg	L	56	4181.00
ctt	L	13	4376.00
ctc	L	0	3137.00
cta	L	33	4275.00
ctg	L	22106	3797.00
cct	P	0	0.83
ccc	P	0	0.56
cca	P	0	1.28
ccg	P	3	0.34
caa	Q	0	2.64
cag	Q	5	2.36
atg	M	16	16.00
---	--	------	------
mPD		0.011	1.1
		nPD:	0.01
		N. weight:	1.7
		Sc. PD:	-0.065
		Sc. rank:	-7936.6

,

PB2

Pos.	649	obs:	exp:
tta	L	0	0.11
ttg	L	0	0.19
ctt	L	0	0.20
ctc	L	0	0.14
cta	L	1	0.19
ctg	L	0	0.17
att	I	0	35.31
atc	I	0	24.21
ata	I	97	37.48
gtt	V	1	4893.00
gtc	V	7	4022.00
gta	V	22111	5953.00
gtg	V	15	7266.00
---	--	------	------
mPD		0.011	0.75
		nPD:	0.01
		N. weight:	1.3
		Sc. PD:	-0.043
		Sc. rank:	-287.1

,

PB2

Pos.	650	obs:	exp:
cgt	R	0	607.00
cgc	R	0	1163.00
cga	R	4	2178.00
cgg	R	0	1627.00
aga	R	22167	11161.00
agg	R	61	5496.00
---	--	------	------
mPD		0.0058	0.91
		nPD:	0.01
		N. weight:	0.68
		Sc. PD:	-0.028
		Sc. rank:	-6330.3

,

PB2

Pos.	651	obs:	exp:
ggt	G	150	3371.00
ggc	G	22037	3293.00
gga	G	45	9889.00
ggg	G	0	5679.00
---	--	------	------
mPD		0.017	0.69
		nPD:	0.03
		N. weight:	1.9
		Sc. PD:	-0.042
		Sc. rank:	11278.0

,

PB2

Pos.	652	obs:	exp:
tct	S	0	1.66
tcc	S	0	1.04
tca	S	0	2.34
tcg	S	0	0.48
att	I	1	0.36
atc	I	0	0.25
ata	I	0	0.39
aat	N	22184	13143.00
aac	N	38	9079.00
agt	S	9	1.69
agc	S	0	1.79
---	--	------	------
mPD		0.0043	0.48
		nPD:	0.01
		N. weight:	0.52
		Sc. PD:	-0.02
		Sc. rank:	-3177.3

,

PB2

Pos.	653	obs:	exp:
tct	S	22169	4091.00
tcc	S	55	2575.00
tca	S	6	5786.00
tcg	S	2	1183.00
agt	S	0	4180.00
agc	S	0	4416.00
---	--	------	------
mPD		0.0057	1.6
		nPD:	0.
		N. weight:	1.7
		Sc. PD:	-0.074
		Sc. rank:	-24003.0

,

PB2

Pos.	654	obs:	exp:
tta	L	0	0.11
ttg	L	0	0.19
tct	S	0	0.74
tcc	S	0	0.46
tca	S	4	1.04
tcg	S	0	0.21
ctt	L	0	0.20
ctc	L	1	0.14
cta	L	0	0.19
ctg	L	0	0.17
cct	P	17	6122.00
ccc	P	4	4121.00
cca	P	22164	9462.00
ccg	P	36	2516.00
caa	Q	6	3.17
cag	Q	0	2.83
agt	S	0	0.75
agc	S	0	0.79
---	--	------	------
mPD		0.0062	0.70
		nPD:	0.01
		N. weight:	0.85
		Sc. PD:	-0.032
		Sc. rank:	-5138.5

,

PB2

Pos.	655	obs:	exp:
tta	L	2	0.22
ttg	L	0	0.38
ctt	L	0	0.39
ctc	L	0	0.28
cta	L	0	0.39
ctg	L	0	0.34
att	I	0	1.82
atc	I	0	1.25
ata	I	5	1.93
gtt	V	7	4908.00
gtc	V	3	4034.00
gta	V	21359	5971.00
gtg	V	832	7288.00
gct	A	0	6.35
gcc	A	0	4.97
gca	A	24	11.27
gcg	A	0	1.41
---	--	------	------
mPD		0.076	0.74
		nPD:	0.1
		N. weight:	1.2
		Sc. PD:	0.063
		Sc. rank:	17349.0

,

PB2

Pos.	656	obs:	exp:
ttt	F	205	9540.00
ttc	F	22027	12692.00
---	--	------	------
mPD		0.018	0.49
		nPD:	0.04
		N. weight:	0.52
		Sc. PD:	-0.0053
		Sc. rank:	4947.0

,

PB2

Pos.	657	obs:	exp:
aat	N	22182	13148.00
aac	N	49	9083.00
aaa	K	1	0.57
aag	K	0	0.43
---	--	------	------
mPD		0.0045	0.48
		nPD:	0.01
		N. weight:	0.52
		Sc. PD:	-0.02
		Sc. rank:	-2876.8

,

PB2

Pos.	658	obs:	exp:
tat	Y	388	11551.00
tac	Y	21843	10680.00
cat	H	0	0.59
cac	H	1	0.41
---	--	------	------
mPD		0.034	0.50
		nPD:	0.07
		N. weight:	0.65
		Sc. PD:	0.014
		Sc. rank:	8901.3

,

PB2

Pos.	659	obs:	exp:
tct	S	0	21.35
tcc	S	0	13.44
tca	S	0	30.19
tcg	S	0	6.17
tat	Y	0	0.52
tac	Y	1	0.48
att	I	0	0.36
atc	I	1	0.25
ata	I	0	0.39
aat	N	117	13079.00
aac	N	21997	9035.00
agt	S	0	21.81
agc	S	116	23.04
---	--	------	------
mPD		0.021	0.50
		nPD:	0.04
		N. weight:	0.87
		Sc. PD:	-0.0049
		Sc. rank:	8876.8

,

PB2

Pos.	660	obs:	exp:
cgt	R	0	12.53
cgc	R	0	24.01
cga	R	0	44.96
cgg	R	0	33.59
aaa	K	105	12420.00
aag	K	21668	9353.00
aga	R	1	230.40
agg	R	458	113.50
---	--	------	------
mPD		0.050	0.54
		nPD:	0.09
		N. weight:	0.83
		Sc. PD:	0.037
		Sc. rank:	12431.0

,

PB2

Pos.	661	obs:	exp:
act	T	0	12.86
acc	T	11	10.46
aca	T	40	23.33
acg	T	0	4.35
gtt	V	0	0.66
gtc	V	0	0.55
gta	V	3	0.81
gtg	V	0	0.98
gct	A	7	5870.00
gcc	A	1	4590.00
gca	A	22156	10414.00
gcg	A	9	1299.00
gaa	E	3	1.82
gag	E	0	1.18
ggt	G	0	0.30
ggc	G	0	0.30
gga	G	2	0.89
ggg	G	0	0.51
---	--	------	------
mPD		0.0078	0.67
		nPD:	0.01
		N. weight:	0.76
		Sc. PD:	-0.027
		Sc. rank:	-2042.4

,

PB2

Pos.	662	obs:	exp:
tct	S	0	3.31
tcc	S	16	2.08
tca	S	0	4.68
tcg	S	0	0.96
att	I	0	1.09
atc	I	3	0.75
ata	I	0	1.16
act	T	34	5598.00
acc	T	22163	4555.00
aca	T	10	10159.00
acg	T	0	1895.00
aat	N	0	2.37
aac	N	4	1.63
agt	S	0	3.38
agc	S	2	3.58
---	--	------	------
mPD		0.0062	0.68
		nPD:	0.01
		N. weight:	1.6
		Sc. PD:	-0.061
		Sc. rank:	-9300.5

,

PB2

Pos.	663	obs:	exp:
cgt	R	0	0.41
cgc	R	0	0.78
cga	R	0	1.47
cgg	R	0	1.10
act	T	0	0.25
acc	T	0	0.21
aca	T	1	0.46
acg	T	0	0.09
aat	N	0	1.18
aac	N	2	0.82
aaa	K	22169	12672.00
aag	K	45	9542.00
aga	R	15	7.53
agg	R	0	3.71
---	--	------	------
mPD		0.0057	0.49
		nPD:	0.01
		N. weight:	0.55
		Sc. PD:	-0.02
		Sc. rank:	-1661.9

,

PB2

Pos.	664	obs:	exp:
cgt	R	0	607.00
cgc	R	0	1163.00
cga	R	21938	2178.00
cgg	R	250	1627.00
aga	R	26	11161.00
agg	R	18	5496.00
---	--	------	------
mPD		0.028	0.91
		nPD:	0.03
		N. weight:	2.3
		Sc. PD:	-0.038
		Sc. rank:	17675.0

,

PB2

Pos.	665	obs:	exp:
ttt	F	2	0.86
ttc	F	0	1.14
tta	L	0	2443.00
ttg	L	0	4183.00
ctt	L	22136	4379.00
ctc	L	64	3139.00
cta	L	15	4278.00
ctg	L	5	3799.00
cat	H	1	0.59
cac	H	0	0.41
att	I	9	3.28
atc	I	0	2.25
ata	I	0	3.48
---	--	------	------
mPD		0.0086	1.1
		nPD:	0.01
		N. weight:	1.6
		Sc. PD:	-0.064
		Sc. rank:	-12564.0

,

PB2

Pos.	666	obs:	exp:
tct	S	0	0.18
tcc	S	0	0.12
tca	S	0	0.26
tcg	S	1	0.05
att	I	0	0.73
atc	I	0	0.50
ata	I	2	0.77
atg	M	88	88.00
act	T	16	5580.00
acc	T	0	4540.00
aca	T	3896	10127.00
acg	T	18224	1889.00
agt	S	0	0.19
agc	S	0	0.20
gct	A	0	1.32
gcc	A	0	1.03
gca	A	0	2.35
gcg	A	5	0.29
---	--	------	------
mPD		0.30	0.69
		nPD:	0.43
		N. weight:	1.7
		Sc. PD:	0.66
		Sc. rank:	31435.0

,

PB2

Pos.	667	obs:	exp:
att	I	7460	2726.00
atc	I	28	1869.00
ata	I	0	2893.00
gtt	V	14725	3260.00
gtc	V	18	2679.00
gta	V	1	3965.00
gtg	V	0	4840.00
---	--	------	------
mPD		0.45	1.2
		nPD:	0.38
		N. weight:	1.3
		Sc. PD:	0.44
		Sc. rank:	24094.0

,

PB2

Pos.	668	obs:	exp:
ttt	F	1	0.43
ttc	F	0	0.57
tta	L	0	2442.00
ttg	L	0	4183.00
ctt	L	22196	4378.00
ctc	L	14	3138.00
cta	L	7	4277.00
ctg	L	0	3799.00
att	I	14	5.10
atc	I	0	3.49
ata	I	0	5.41
---	--	------	------
mPD		0.0032	1.1
		nPD:	0.
		N. weight:	1.6
		Sc. PD:	-0.073
		Sc. rank:	-24775.0

,

PB2

Pos.	669	obs:	exp:
ggt	G	1	3371.00
ggc	G	3	3293.00
gga	G	22119	9889.00
ggg	G	109	5679.00
---	--	------	------
mPD		0.010	0.69
		nPD:	0.01
		N. weight:	0.79
		Sc. PD:	-0.026
		Sc. rank:	-24.8

,

PB2

Pos.	670	obs:	exp:
cgt	R	0	0.52
cgc	R	0	0.99
cga	R	0	1.86
cgg	R	0	1.39
aaa	K	199	12671.00
aag	K	22014	9542.00
aga	R	0	9.54
agg	R	19	4.70
---	--	------	------
mPD		0.019	0.49
		nPD:	0.04
		N. weight:	0.8
		Sc. PD:	-0.0064
		Sc. rank:	7891.3

,

PB2

Pos.	671	obs:	exp:
aat	N	20	11.83
aac	N	0	8.17
gtt	V	1	0.22
gtc	V	0	0.18
gta	V	0	0.27
gtg	V	0	0.33
gat	D	22190	11702.00
gac	D	19	10507.00
gaa	E	2	1.21
gag	E	0	0.79
---	--	------	------
mPD		0.0038	0.50
		nPD:	0.01
		N. weight:	0.64
		Sc. PD:	-0.025
		Sc. rank:	-5036.9

,

PB2

Pos.	672	obs:	exp:
act	T	0	0.25
acc	T	0	0.21
aca	T	1	0.46
acg	T	0	0.09
gct	A	6	5886.00
gcc	A	12	4602.00
gca	A	22201	10441.00
gcg	A	12	1302.00
---	--	------	------
mPD		0.0028	0.66
		nPD:	0.
		N. weight:	0.75
		Sc. PD:	-0.032
		Sc. rank:	-9850.8

,

PB2

Pos.	673	obs:	exp:
cgt	R	2	0.05
cgc	R	0	0.10
cga	R	0	0.20
cgg	R	0	0.15
aga	R	0	1.00
agg	R	0	0.49
ggt	G	22195	3371.00
ggc	G	26	3293.00
gga	G	7	9888.00
ggg	G	2	5678.00
---	--	------	------
mPD		0.0033	0.69
		nPD:	0.
		N. weight:	1.9
		Sc. PD:	-0.08
		Sc. rank:	-22755.0

,

PB2

Pos.	674	obs:	exp:
tct	S	0	1.10
tcc	S	0	0.69
tca	S	6	1.56
tcg	S	0	0.32
act	T	11	6.30
acc	T	0	5.13
aca	T	14	11.44
acg	T	0	2.13
agt	S	0	1.13
agc	S	0	1.19
gtt	V	0	1.77
gtc	V	0	1.45
gta	V	8	2.15
gtg	V	0	2.63
gct	A	34	5873.00
gcc	A	1	4592.00
gca	A	22134	10419.00
gcg	A	15	1300.00
gaa	E	7	4.24
gag	E	0	2.76
ggt	G	0	0.30
ggc	G	0	0.30
gga	G	2	0.89
ggg	G	0	0.51
---	--	------	------
mPD		0.0098	0.67
		nPD:	0.01
		N. weight:	0.75
		Sc. PD:	-0.024
		Sc. rank:	33.2

,

PB2

Pos.	675	obs:	exp:
tta	L	107	2444.00
ttg	L	22076	4185.00
ctt	L	0	4381.00
ctc	L	0	3140.00
cta	L	1	4280.00
ctg	L	47	3801.00
atg	M	1	1.00
---	--	------	------
mPD		0.014	1.1
		nPD:	0.01
		N. weight:	1.6
		Sc. PD:	-0.057
		Sc. rank:	-3561.5

,

PB2

Pos.	676	obs:	exp:
tct	S	0	0.55
tcc	S	0	0.35
tca	S	0	0.78
tcg	S	0	0.16
att	I	60	21.84
atc	I	0	14.98
ata	I	0	23.18
act	T	21991	5554.00
acc	T	20	4519.00
aca	T	13	10080.00
acg	T	9	1880.00
aat	N	105	62.10
aac	N	0	42.90
agt	S	3	0.56
agc	S	0	0.60
gct	A	31	8.21
gcc	A	0	6.42
gca	A	0	14.56
gcg	A	0	1.82
---	--	------	------
mPD		0.022	0.70
		nPD:	0.03
		N. weight:	1.4
		Sc. PD:	-0.023
		Sc. rank:	11243.0

,

PB2			
Pos.	677	obs:	exp:
aaa	K	17	9.70
aag	K	0	7.30
gct	A	0	2.12
gcc	A	0	1.66
gca	A	8	3.76
gcg	A	0	0.47
gat	D	0	1.58
gac	D	3	1.42
gaa	E	22148	13425.00
gag	E	23	8746.00
ggt	G	0	5.00
ggc	G	0	4.89
gga	G	33	14.68
ggg	G	0	8.43
---	--	------	------
mPD		0.0075	0.48
		nPD:	0.02
		N. weight:	0.5
		Sc. PD:	-0.016
		Sc. rank:	300.8

,

PB2			
Pos.	678	obs:	exp:
aat	N	4	2.37
aac	N	0	1.63
gat	D	22151	11704.00
gac	D	62	10509.00
gaa	E	6	5.45
gag	E	3	3.55
ggt	G	6	0.91
ggc	G	0	0.89
gga	G	0	2.67
ggg	G	0	1.53
---	--	------	------
mPD		0.0073	0.50
		nPD:	0.01
		N. weight:	0.63
		Sc. PD:	-0.021
		Sc. rank:	-117.3

,

PB2			
Pos.	679	obs:	exp:
tta	L	0	0.22
ttg	L	0	0.38
tct	S	0	2.39
tcc	S	0	1.51
tca	S	13	3.38
tcg	S	0	0.69
ctt	L	0	0.39
ctc	L	0	0.28
cta	L	2	0.39
ctg	L	0	0.34
cct	P	2	6120.00
ccc	P	4	4120.00
cca	P	22193	9459.00
ccg	P	15	2515.00
caa	Q	2	1.06
cag	Q	0	0.94
act	T	0	0.25
acc	T	0	0.21
aca	T	1	0.46
acg	T	0	0.09
agt	S	0	2.44
agc	S	0	2.58
---	--	------	------
mPD		0.0035	0.70
		nPD:	0.01
		N. weight:	0.86
		Sc. PD:	-0.036
		Sc. rank:	-9893.5

,

PB2			
Pos.	680	obs:	exp:
tat	Y	2	1.04
tac	Y	0	0.96
aat	N	11	6.51
aac	N	0	4.49
gat	D	22162	11695.00
gac	D	33	10500.00
gaa	E	14	10.90
gag	E	4	7.10
ggt	G	6	0.91
ggc	G	0	0.89
gga	G	0	2.67
ggg	G	0	1.53
---	--	------	------
mPD		0.0063	0.50
		nPD:	0.01
		N. weight:	0.64
		Sc. PD:	-0.022
		Sc. rank:	-1181.4

,

PB2			
Pos.	681	obs:	exp:
gaa	E	22199	13461.00
gag	E	33	8771.00
---	--	------	------
mPD		0.0030	0.48
		nPD:	0.01
		N. weight:	0.5
		Sc. PD:	-0.02
		Sc. rank:	-4868.7

,

PB2			
Pos.	682	obs:	exp:
tct	S	0	9.75
tcc	S	0	6.14
tca	S	0	13.79
tcg	S	0	2.82
tgt	C	0	1.35
tgc	C	3	1.65
cgt	R	0	0.08
cgc	R	3	0.16
cga	R	0	0.29
cgg	R	0	0.22
agt	S	0	9.97
agc	S	53	10.53
aga	R	0	1.51
agg	R	0	0.74
gat	D	0	1.05
gac	D	2	0.95
ggt	G	70	3362.00
ggc	G	22061	3284.00
gga	G	40	9862.00
ggg	G	0	5663.00
---	--	------	------
mPD		0.015	0.70
		nPD:	0.02
		N. weight:	1.9
		Sc. PD:	-0.048
		Sc. rank:	8215.4

,

PB2			
Pos.	683	obs:	exp:
tct	S	0	0.37
tcc	S	0	0.23
tca	S	2	0.52
tcg	S	0	0.11
cgt	R	0	0.11
cgc	R	0	0.21
cga	R	0	0.39
cgg	R	0	0.29
att	I	0	1.46
atc	I	0	1.00
ata	I	4	1.55
act	T	3	5538.00
acc	T	265	4506.00
aca	T	21633	10049.00
acg	T	66	1874.00
aaa	K	242	138.00
aag	K	0	104.00
agt	S	0	0.38
agc	S	0	0.40
aga	R	4	2.01
agg	R	0	0.99
gct	A	0	3.44
gcc	A	0	2.69
gca	A	13	6.11
gcg	A	0	0.76
---	--	------	------
mPD		0.053	0.70
		nPD:	0.08
		N. weight:	0.72
		Sc. PD:	0.02
		Sc. rank:	10003.0

,

PB2			
Pos.	684	obs:	exp:
ttt	F	1	0.43
ttc	F	0	0.57
tct	S	21971	4089.00
tcc	S	28	2574.00
tca	S	26	5783.00
tcg	S	195	1183.00
tat	Y	1	0.52
tac	Y	0	0.48
tgt	C	6	2.71
tgc	C	0	3.29
cct	P	2	0.55
ccc	P	0	0.37
cca	P	0	0.85
ccg	P	0	0.23
agt	S	0	4178.00
agc	S	0	4414.00
gct	A	2	0.53
gcc	A	0	0.41
gca	A	0	0.94
gcg	A	0	0.12
---	--	------	------
mPD		0.023	1.6
		nPD:	0.01
		N. weight:	1.7
		Sc. PD:	-0.055
		Sc. rank:	-820.3

,

PB2			
Pos.	685	obs:	exp:
gtt	V	0	0.22
gtc	V	0	0.18
gta	V	0	0.27
gtg	V	1	0.33
gaa	E	0	3.03
gag	E	5	1.97
ggt	G	12	3370.00
ggc	G	1	3292.00
gga	G	212	9887.00
ggg	G	22001	5677.00
---	--	------	------
mPD		0.021	0.69
		nPD:	0.03
		N. weight:	1.3
		Sc. PD:	-0.023
		Sc. rank:	9732.5

,

PB2			
Pos.	686	obs:	exp:
atg	M	3	3.00
gtt	V	5	4914.00
gtc	V	0	4039.00
gta	V	101	5977.00
gtg	V	22119	7296.00
ggt	G	0	0.61
ggc	G	0	0.59
gga	G	0	1.78
ggg	G	4	1.02
---	--	------	------
mPD		0.010	0.74
		nPD:	0.01
		N. weight:	1.1
		Sc. PD:	-0.037
		Sc. rank:	-1001.6

,

PB2			
Pos.	687	obs:	exp:
gaa	E	122	13461.00
gag	E	22109	8770.00
ggt	G	0	0.15
ggc	G	0	0.15
gga	G	0	0.44
ggg	G	1	0.26
---	--	------	------
mPD		0.011	0.48
		nPD:	0.02
		N. weight:	0.9
		Sc. PD:	-0.022
		Sc. rank:	4488.9

,

PB2			
Pos.	688	obs:	exp:
tct	S	22148	4091.00
tcc	S	21	2575.00
tca	S	58	5786.00
tcg	S	5	1183.00
agt	S	0	4180.00
agc	S	0	4416.00
---	--	------	------
mPD		0.0075	1.6
		nPD:	0.
		N. weight:	1.7
		Sc. PD:	-0.072
		Sc. rank:	-20842.0

,

PB2

Pos.	689	obs:	exp:
gct	A	22157	5886.00
gcc	A	43	4602.00
gca	A	28	10442.00
gcg	A	4	1302.00
---	--	------	------
mPD		0.0067	0.66
		nPD:	0.01
		N. weight:	1.3
		Sc. PD:	-0.049
		Sc. rank:	-5390.5

,

PB2

Pos.	690	obs:	exp:
gtt	V	218	4915.00
gtc	V	21983	4040.00
gta	V	31	5979.00
gtg	V	0	7298.00
---	--	------	------
mPD		0.022	0.74
		nPD:	0.03
		N. weight:	1.7
		Sc. PD:	-0.029
		Sc. rank:	12509.0

,

PB2

Pos.	691	obs:	exp:
tta	L	2	2444.00
ttg	L	86	4185.00
ctt	L	9	4380.00
ctc	L	1	3140.00
cta	L	98	4279.00
ctg	L	22033	3801.00
atg	M	3	3.00
---	--	------	------
mPD		0.018	1.1
		nPD:	0.02
		N. weight:	1.7
		Sc. PD:	-0.054
		Sc. rank:	1275.6

,

PB2

Pos.	692	obs:	exp:
cgt	R	0	606.80
cgc	R	0	1163.00
cga	R	0	2177.00
cgg	R	3	1627.00
act	T	0	0.76
acc	T	0	0.62
aca	T	3	1.37
acg	T	0	0.26
aaa	K	3	1.71
aag	K	0	1.29
aga	R	21692	11158.00
agg	R	530	5494.00
ggt	G	0	0.15
ggc	G	0	0.15
gga	G	1	0.44
ggg	G	0	0.26
---	--	------	------
mPD		0.048	0.91
		nPD:	0.05
		N. weight:	0.6
		Sc. PD:	0.0029
		Sc. rank:	7277.9

,

PB2

Pos.	693	obs:	exp:
ggt	G	4	3371.00
ggc	G	2	3293.00
gga	G	22104	9889.00
ggg	G	122	5679.00
---	--	------	------
mPD		0.011	0.69
		nPD:	0.02
		N. weight:	0.78
		Sc. PD:	-0.024
		Sc. rank:	870.1

,

PB2

Pos.	694	obs:	exp:
ttt	F	8441	9540.00
ttc	F	13791	12692.00
---	--	------	------
mPD		0.47	0.49
		nPD:	0.96
		N. weight:	0.0053
		Sc. PD:	0.0048
		Sc. rank:	101.5

,

PB2

Pos.	695	obs:	exp:
tta	L	0	2444.00
ttg	L	0	4186.00
ctt	L	18	4381.00
ctc	L	22210	3140.00
cta	L	4	4280.00
ctg	L	0	3801.00
---	--	------	------
mPD		0.0020	1.1
		nPD:	0.
		N. weight:	2.
		Sc. PD:	-0.09
		Sc. rank:	-33492.0

,

PB2

Pos.	696	obs:	exp:
ttt	F	1	0.43
ttc	F	0	0.57
att	I	22200	8092.00
atc	I	30	5549.00
ata	I	0	8589.00
gtt	V	1	0.22
gtc	V	0	0.18
gta	V	0	0.27
gtg	V	0	0.33
---	--	------	------
mPD		0.0029	0.66
		nPD:	0.
		N. weight:	1.
		Sc. PD:	-0.043
		Sc. rank:	-12936.0

,

PB2

Pos.	697	obs:	exp:
ttt	F	1	0.43
ttc	F	0	0.57
tta	L	18872	2443.00
ttg	L	3290	4183.00
ctt	L	0	4379.00
ctc	L	0	3139.00
cta	L	52	4278.00
ctg	L	6	3799.00
att	I	0	4.00
atc	I	0	2.75
ata	I	11	4.25
---	--	------	------
mPD		0.26	1.1
		nPD:	0.23
		N. weight:	1.7
		Sc. PD:	0.3
		Sc. rank:	29022.0

,

PB2

Pos.	698	obs:	exp:
tct	S	0	1.84
tcc	S	0	1.16
tca	S	0	2.60
tcg	S	0	0.53
agt	S	1	1.88
agc	S	9	1.99
gct	A	0	0.26
gcc	A	1	0.21
gca	A	0	0.47
gcg	A	0	0.06
gat	D	0	0.53
gac	D	1	0.47
ggt	G	625	3369.00
ggc	G	21534	3291.00
gga	G	61	9884.00
ggg	G	0	5676.00
---	--	------	------
mPD		0.061	0.69
		nPD:	0.09
		N. weight:	1.8
		Sc. PD:	0.072
		Sc. rank:	26433.0

,

PB2

Pos.	699	obs:	exp:
cgt	R	0	1.58
cgc	R	0	3.03
cga	R	0	5.68
cgg	R	0	4.24
aaa	K	22129	12649.00
aag	K	45	9525.00
aga	R	58	29.12
agg	R	0	14.34
---	--	------	------
mPD		0.0092	0.50
		nPD:	0.02
		N. weight:	0.55
		Sc. PD:	-0.016
		Sc. rank:	1259.3

,

PB2

Pos.	700	obs:	exp:
aaa	K	1	0.57
aag	K	0	0.43
gat	D	0	0.53
gac	D	1	0.47
gaa	E	22191	13460.00
gag	E	38	8769.00
ggt	G	0	0.15
ggc	G	0	0.15
gga	G	1	0.44
ggg	G	0	0.26
---	--	------	------
mPD		0.0037	0.48
		nPD:	0.01
		N. weight:	0.49
		Sc. PD:	-0.02
		Sc. rank:	-3758.8

,

PB2

Pos.	701	obs:	exp:
aat	N	0	5.32
aac	N	9	3.68
gat	D	9	11707.00
gac	D	22209	10511.00
gaa	E	1	1.82
gag	E	2	1.18
ggt	G	0	0.30
ggc	G	2	0.30
gga	G	0	0.89
ggg	G	0	0.51
---	--	------	------
mPD		0.0021	0.50
		nPD:	0.
		N. weight:	0.75
		Sc. PD:	-0.032
		Sc. rank:	-9994.9

,

PB2

Pos.	702	obs:	exp:
cgt	R	0	5.95
cgc	R	0	11.40
cga	R	0	21.35
cgg	R	0	15.95
atg	M	1	1.00
aat	N	10	6.51
aac	N	1	4.49
aaa	K	21	12551.00
aag	K	21981	9451.00
aga	R	10	109.40
agg	R	208	53.89
---	--	------	------
mPD		0.023	0.52
		nPD:	0.05
		N. weight:	0.85
		Sc. PD:	-0.002
		Sc. rank:	9505.5

,

PB2

Pos.	703	obs:	exp:
cgt	R	0	606.80
cgc	R	0	1162.00
cga	R	6	2177.00
cgg	R	0	1626.00
att	I	0	0.36
atc	I	0	0.25
ata	I	1	0.39
aaa	K	7	3.99
aag	K	0	3.01
aga	R	22065	11156.00
agg	R	151	5494.00
ggt	G	0	0.15
ggc	G	0	0.15
gga	G	1	0.44
ggg	G	0	0.26
---	-	1	1.00
---	--	------	------
mPD		0.015	0.91
		nPD:	0.02
		N. weight:	0.66
		Sc. PD:	-0.02
		Sc. rank:	612.7

,

PB2

Pos.	704	obs:	exp:
tat	Y	22052	11547.00
tac	Y	171	10676.00
tgt	C	2	0.90
tgc	C	0	1.10
cat	H	5	3.53
cac	H	1	2.47
---	-	1	1.00
---	--	------	------
mPD		0.016	0.50
		nPD:	0.03
		N. weight:	0.62
		Sc. PD:	-0.0094
		Sc. rank:	5328.6

,

PB2

Pos.	705	obs:	exp:
ggt	G	825	3371.00
ggc	G	21223	3293.00
gga	G	40	9889.00
ggg	G	144	5679.00
---	--	------	------
mPD		0.087	0.69
		nPD:	0.13
		N. weight:	1.7
		Sc. PD:	0.13
		Sc. rank:	26935.0

,

PB2

Pos.	706	obs:	exp:
cct	P	4	6125.00
ccc	P	3	4123.00
cca	P	22202	9466.00
ccg	P	22	2517.00
cgt	R	0	0.03
cgc	R	0	0.05
cga	R	1	0.10
cgg	R	0	0.07
aga	R	0	0.50
agg	R	0	0.25
---	--	------	------
mPD		0.0027	0.70
		nPD:	0.
		N. weight:	0.85
		Sc. PD:	-0.037
		Sc. rank:	-11722.0

,

PB2

Pos.	707	obs:	exp:
tct	S	0	1.29
tcc	S	0	0.81
tca	S	7	1.82
tcg	S	0	0.37
cct	P	0	0.28
ccc	P	0	0.19
cca	P	1	0.43
ccg	P	0	0.11
act	T	0	1.26
acc	T	0	1.03
aca	T	5	2.29
acg	T	0	0.43
agt	S	0	1.32
agc	S	0	1.39
gtt	V	0	0.22
gtc	V	0	0.18
gta	V	1	0.27
gtg	V	0	0.33
gct	A	4	5882.00
gcc	A	3	4599.00
gca	A	22184	10435.00
gcg	A	27	1302.00
---	--	------	------
mPD		0.0043	0.66
		nPD:	0.01
		N. weight:	0.75
		Sc. PD:	-0.031
		Sc. rank:	-6893.5

,

PB2

Pos.	708	obs:	exp:
ttt	F	5	2.15
ttc	F	0	2.85
tta	L	21350	2443.00
ttg	L	212	4184.00
tct	S	0	0.37
tcc	S	0	0.23
tca	S	2	0.52
tcg	S	0	0.11
ctt	L	0	4379.00
ctc	L	0	3139.00
cta	L	661	4278.00
ctg	L	0	3800.00
att	I	0	0.36
atc	I	0	0.25
ata	I	1	0.39
agt	S	0	0.38
agc	S	0	0.40
gtt	V	0	0.22
gtc	V	0	0.18
gta	V	1	0.27
gtg	V	0	0.33
---	--	------	------
mPD		0.077	1.1
		nPD:	0.07
		N. weight:	2.
		Sc. PD:	0.041
		Sc. rank:	27560.0

,

PB2

Pos.	709	obs:	exp:
tct	S	0	4089.00
tcc	S	0	2574.00
tca	S	0	5783.00
tcg	S	0	1183.00
tgt	C	0	0.45
tgc	C	1	0.55
cgt	R	0	0.03
cgc	R	0	0.05
cga	R	0	0.10
cgg	R	0	0.07
aat	N	0	3.55
aac	N	6	2.45
agt	S	26	4178.00
agc	S	22194	4414.00
aga	R	1	0.50
agg	R	0	0.25
ggt	G	0	0.61
ggc	G	4	0.59
gga	G	0	1.78
ggg	G	0	1.02
---	--	------	------
mPD		0.0034	1.6
		nPD:	0.
		N. weight:	1.6
		Sc. PD:	-0.073
		Sc. rank:	-26623.0

,

PB2

Pos.	710	obs:	exp:
att	I	24	8092.00
atc	I	22165	5549.00
ata	I	41	8589.00
aat	N	0	1.18
aac	N	2	0.82
---	--	------	------
mPD		0.0060	0.66
		nPD:	0.01
		N. weight:	1.4
		Sc. PD:	-0.053
		Sc. rank:	-7864.3

,

PB2

Pos.	711	obs:	exp:
tct	S	0	4.42
tcc	S	0	2.78
tca	S	0	6.25
tcg	S	0	1.28
aat	N	22188	13132.00
aac	N	15	9071.00
agt	S	24	4.51
agc	S	0	4.77
gat	D	5	2.63
gac	D	0	2.37
---	--	------	------
mPD		0.0040	0.49
		nPD:	0.01
		N. weight:	0.53
		Sc. PD:	-0.021
		Sc. rank:	-3689.9

,

PB2

Pos.	712	obs:	exp:
gaa	E	21921	13461.00
gag	E	311	8771.00
---	--	------	------
mPD		0.028	0.48
		nPD:	0.06
		N. weight:	0.44
		Sc. PD:	0.0044
		Sc. rank:	5479.5

,

PB2

Pos.	713	obs:	exp:
tta	L	0	2443.00
ttg	L	371	4184.00
ctt	L	2	4380.00
ctc	L	0	3139.00
cta	L	231	4279.00
ctg	L	21621	3800.00
atg	M	7	7.00
---	--	------	------
mPD		0.054	1.1
		nPD:	0.05
		N. weight:	1.6
		Sc. PD:	3.8×10^{-6}
		Sc. rank:	19034.0

,

PB2

Pos.	714	obs:	exp:
tct	S	0	4086.00
tcc	S	0	2572.00
tca	S	0	5779.00
tcg	S	0	1182.00
tgt	C	0	0.90
tgc	C	2	1.10
cgt	R	0	0.11
cgc	R	0	0.21
cga	R	0	0.39
cgg	R	0	0.29
att	I	0	1.09
atc	I	3	0.75
ata	I	0	1.16
act	T	0	0.25
acc	T	1	0.21
aca	T	0	0.46
acg	T	0	0.09
aat	N	0	5.32
aac	N	9	3.68
agt	S	44	4175.00
agc	S	22160	4411.00
aga	R	4	2.01
agg	R	0	0.99
ggt	G	0	1.36
ggc	G	9	1.33
gga	G	0	4.00
ggg	G	0	2.30
---	--	------	------
mPD		0.0065	1.6
		nPD:	0.
		N. weight:	1.6
		Sc. PD:	-0.07
		Sc. rank:	-21942.0

,

PB2

Pos.	715	obs:	exp:
tct	S	0	16.56
tcc	S	0	10.42
tca	S	0	23.42
tcg	S	0	4.79
cat	H	3	1.76
cac	H	0	1.24
att	I	1	0.36
atc	I	0	0.25
ata	I	0	0.39
act	T	2	0.50
acc	T	0	0.41
aca	T	0	0.91
acg	T	0	0.17
aat	N	22063	13086.00
aac	N	62	9039.00
aaa	K	0	3.99
aag	K	7	3.01
agt	S	90	16.92
agc	S	0	17.88
ggt	G	4	0.61
ggc	G	0	0.59
gga	G	0	1.78
ggg	G	0	1.02
---	--	------	------
mPD		0.016	0.50
		nPD:	0.03
		N. weight:	0.52
		Sc. PD:	-0.0085
		Sc. rank:	4255.1

,

PB2

Pos.	716	obs:	exp:
ttt	F	1	0.43
ttc	F	0	0.57
tta	L	0	2444.00
ttg	L	0	4185.00
ctt	L	22209	4381.00
ctc	L	5	3140.00
cta	L	3	4280.00
ctg	L	13	3801.00
att	I	1	0.36
atc	I	0	0.25
ata	I	0	0.39
---	--	------	------
mPD		0.0021	1.1
		nPD:	0.
		N. weight:	1.6
		Sc. PD:	-0.074
		Sc. rank:	-27383.0

,

PB2			
Pos.	717	obs:	exp:
tct	S	0	1.29
tcc	S	0	0.81
tca	S	7	1.82
tcg	S	0	0.37
act	T	0	15.13
acc	T	0	12.31
aca	T	60	27.45
acg	T	0	5.12
agt	S	0	1.32
agc	S	0	1.39
gtt	V	2	0.88
gtc	V	0	0.73
gta	V	2	1.08
gtg	V	0	1.31
gct	A	508	5867.00
gcc	A	5	4587.00
gca	A	21640	10408.00
gcg	A	8	1298.00
---	--	------	------
mPD		0.052	0.67
		nPD:	0.08
		N. weight:	0.66
		Sc. PD:	0.02
		Sc. rank:	9523.1

,

PB2			
Pos.	718	obs:	exp:
cgt	R	0	1.50
cgc	R	0	2.88
cga	R	0	5.39
cgg	R	0	4.03
aaa	K	22086	12650.00
aag	K	90	9526.00
aga	R	54	27.61
agg	R	1	13.60
gaa	E	1	0.61
gag	E	0	0.39
---	--	------	------
mPD		0.013	0.50
		nPD:	0.03
		N. weight:	0.54
		Sc. PD:	-0.011
		Sc. rank:	3531.7

,

PB2			
Pos.	719	obs:	exp:
cgt	R	0	0.05
cgc	R	0	0.10
cga	R	0	0.20
cgg	R	0	0.15
aga	R	2	1.00
agg	R	0	0.49
gaa	E	1	0.61
gag	E	0	0.39
ggt	G	0	3371.00
ggc	G	2	3292.00
gga	G	22176	9888.00
ggg	G	51	5678.00
---	--	------	------
mPD		0.0050	0.69
		nPD:	0.01
		N. weight:	0.8
		Sc. PD:	-0.032
		Sc. rank:	-6553.1

,

PB2			
Pos.	720	obs:	exp:
gaa	E	94	13461.00
gag	E	22137	8770.00
ggt	G	0	0.15
ggc	G	0	0.15
gga	G	0	0.44
ggg	G	1	0.26
---	--	------	------
mPD		0.0085	0.48
		nPD:	0.02
		N. weight:	0.91
		Sc. PD:	-0.027
		Sc. rank:	1745.0

,

PB2			
Pos.	721	obs:	exp:
cgt	R	0	0.05
cgc	R	0	0.10
cga	R	0	0.20
cgg	R	0	0.15
aaa	K	21409	12681.00
aag	K	821	9549.00
aga	R	1	1.00
agg	R	1	0.49
---	--	------	------
mPD		0.071	0.49
		nPD:	0.15
		N. weight:	0.42
		Sc. PD:	0.041
		Sc. rank:	6741.1

,

PB2			
Pos.	722	obs:	exp:
tct	S	5	0.92
tcc	S	0	0.58
tca	S	0	1.30
tcg	S	0	0.27
act	T	12	3.03
acc	T	0	2.46
aca	T	0	5.49
acg	T	0	1.02
agt	S	0	0.94
agc	S	0	0.99
gtt	V	6	1.33
gtc	V	0	1.09
gta	V	0	1.61
gtg	V	0	1.97
gct	A	22113	5880.00
gcc	A	89	4597.00
gca	A	5	10431.00
gcg	A	2	1301.00
---	--	------	------
mPD		0.011	0.67
		nPD:	0.02
		N. weight:	1.3
		Sc. PD:	-0.041
		Sc. rank:	1095.0

,

PB2			
Pos.	723	obs:	exp:
aat	N	22155	13149.00
aac	N	77	9083.00
---	--	------	------
mPD		0.0069	0.48
		nPD:	0.01
		N. weight:	0.51
		Sc. PD:	-0.017
		Sc. rank:	-239.7

,

PB2			
Pos.	724	obs:	exp:
atg	M	1	1.00
gtt	V	9	4914.00
gtc	V	0	4039.00
gta	V	50	5978.00
gtg	V	22170	7297.00
gct	A	0	0.53
gcc	A	0	0.41
gca	A	0	0.94
gcg	A	2	0.12
---	--	------	------
mPD		0.0056	0.74
		nPD:	0.01
		N. weight:	1.1
		Sc. PD:	-0.044
		Sc. rank:	-8738.0

,

PB2			
Pos.	725	obs:	exp:
tta	L	35	2444.00
ttg	L	1	4185.00
ctt	L	0	4381.00
ctc	L	0	3140.00
cta	L	22140	4280.00
ctg	L	55	3801.00
---	-	1	1.00
---	--	------	------
mPD		0.0083	1.1
		nPD:	0.01
		N. weight:	1.6
		Sc. PD:	-0.066
		Sc. rank:	-13424.0

,

PB2			
Pos.	726	obs:	exp:
att	I	22208	8093.00
atc	I	22	5550.00
ata	I	2	8589.00
---	--	------	------
mPD		0.0022	0.66
		nPD:	0.
		N. weight:	1.
		Sc. PD:	-0.045
		Sc. rank:	-14702.0

,

PB2			
Pos.	727	obs:	exp:
gct	A	0	0.53
gcc	A	0	0.41
gca	A	0	0.94
gcg	A	2	0.12
ggt	G	4	3371.00
ggc	G	3	3293.00
gga	G	121	9888.00
ggg	G	22102	5678.00
---	--	------	------
mPD		0.012	0.69
		nPD:	0.02
		N. weight:	1.3
		Sc. PD:	-0.041
		Sc. rank:	1792.0

,

PB2			
Pos.	728	obs:	exp:
caa	Q	3681	11731.00
cag	Q	18551	10501.00
---	--	------	------
mPD		0.28	0.50
		nPD:	0.55
		N. weight:	0.29
		Sc. PD:	0.14
		Sc. rank:	5323.9

,

PB2			
Pos.	729	obs:	exp:
ggt	G	4	3371.00
ggc	G	0	3293.00
gga	G	387	9889.00
ggg	G	21841	5679.00
---	--	------	------
mPD		0.035	0.69
		nPD:	0.05
		N. weight:	1.3
		Sc. PD:	0.0031
		Sc. rank:	15241.0

,

PB2			
Pos.	730	obs:	exp:
gat	D	13	11714.00
gac	D	22219	10518.00
---	--	------	------
mPD		0.0012	0.50
		nPD:	0.
		N. weight:	0.75
		Sc. PD:	-0.034
		Sc. rank:	-11887.0

,

PB2			
Pos.	731	obs:	exp:
tta	L	1	0.11
ttg	L	0	0.19
ctt	L	0	0.20
ctc	L	0	0.14
cta	L	0	0.19
ctg	L	0	0.17
att	I	6	6845.00
atc	I	4	4694.00
ata	I	18793	7265.00
atg	M	7	7.00
act	T	0	2.02
acc	T	0	1.64
aca	T	8	3.66
acg	T	0	0.68
gtt	V	0	754.60
gtc	V	0	620.20
gta	V	3392	917.90
gtg	V	21	1120.00
---	--	------	------
mPD		0.26	0.95
		nPD:	0.28
		N. weight:	1.
		Sc. PD:	0.23
		Sc. rank:	17357.0

,

PB2			
Pos.	732	obs:	exp:
atg	M	1	1.00
gtt	V	117	4915.00
gtc	V	0	4039.00
gta	V	86	5979.00
gtg	V	22027	7297.00
gaa	E	0	0.61
gag	E	1	0.39
---	--	------	------
mPD		0.018	0.74
		nPD:	0.02
		N. weight:	1.1
		Sc. PD:	-0.024
		Sc. rank:	6113.1

,

PB2			
Pos.	733	obs:	exp:
tta	L	2223	2444.00
ttg	L	19969	4185.00
tct	S	0	0.37
tcc	S	0	0.23
tca	S	0	0.52
tcg	S	2	0.11
ctt	L	0	4380.00
ctc	L	0	3140.00
cta	L	1	4279.00
ctg	L	35	3800.00
atg	M	1	1.00
agt	S	0	0.38
agc	S	0	0.40
gtt	V	0	0.22
gtc	V	0	0.18
gta	V	0	0.27
gtg	V	1	0.33
---	--	------	------
mPD		0.18	1.1
		nPD:	0.16
		N. weight:	1.4
		Sc. PD:	0.16
		Sc. rank:	22946.0

PB2			
Pos.	734	obs:	exp:
att	I	0	6.92
atc	I	0	4.74
ata	I	19	7.34
gtt	V	4	4910.00
gtc	V	1	4035.00
gta	V	22139	5973.00
gtg	V	64	7290.00
gct	A	0	1.32
gcc	A	0	1.03
gca	A	5	2.35
gcg	A	0	0.29
---	--	------	------
mPD		0.0083	0.74
		nPD:	0.01
		N. weight:	1.3
		Sc. PD:	-0.047
		Sc. rank:	-4158.4

PB2			
Pos.	735	obs:	exp:
tta	L	0	0.55
ttg	L	3	0.94
ctt	L	0	0.99
ctc	L	0	0.71
cta	L	0	0.96
ctg	L	2	0.85
att	I	0	0.73
atc	I	0	0.50
ata	I	2	0.77
atg	M	22224	22224.00
aaa	K	0	0.57
aag	K	1	0.43
---	--	------	------
mPD		0.00072	0.0011
		nPD:	0.68
		N. weight:	0.00041
		Sc. PD:	0.00026
		Sc. rank:	7.7

PB2			
Pos.	736	obs:	exp:
cgt	R	0	0.03
cgc	R	0	0.05
cga	R	0	0.10
cgg	R	0	0.07
aaa	K	22231	12681.00
aag	K	0	9550.00
aga	R	1	0.50
agg	R	0	0.25
---	--	------	------
mPD		0.000090	0.49
		nPD:	0.
		N. weight:	0.57
		Sc. PD:	-0.027
		Sc. rank:	-10582.0

PB2			
Pos.	737	obs:	exp:
tta	L	0	0.11
ttg	L	0	0.19
ctt	L	0	0.20
ctc	L	0	0.14
cta	L	1	0.19
ctg	L	0	0.17
caa	Q	2	1.06
cag	Q	0	0.94
cgt	R	5	606.90
cgc	R	1	1163.00
cga	R	22086	2177.00
cgg	R	136	1627.00
aga	R	1	11160.00
agg	R	0	5495.00
---	--	------	------
mPD		0.013	0.91
		nPD:	0.01
		N. weight:	2.3
		Sc. PD:	-0.076
		Sc. rank:	-655.9

PB2			
Pos.	738	obs:	exp:
cgt	R	0	0.05
cgc	R	0	0.10
cga	R	0	0.20
cgg	R	0	0.15
aaa	K	22146	12681.00
aag	K	84	9549.00
aga	R	2	1.00
agg	R	0	0.49
---	--	------	------
mPD		0.0077	0.49
		nPD:	0.02
		N. weight:	0.54
		Sc. PD:	-0.017
		Sc. rank:	341.7

PB2			
Pos.	739	obs:	exp:
cct	P	0	0.28
ccc	P	0	0.19
cca	P	0	0.43
ccg	P	1	0.11
cgt	R	1	607.00
cgc	R	0	1163.00
cga	R	153	2177.00
cgg	R	21921	1627.00
aga	R	2	11161.00
agg	R	154	5496.00
---	--	------	------
mPD		0.028	0.91
		nPD:	0.03
		N. weight:	2.5
		Sc. PD:	-0.043
		Sc. rank:	20153.0

PB2			
Pos.	740	obs:	exp:
aat	N	0	7.69
aac	N	13	5.31
gat	D	17	11681.00
gac	D	22152	10488.00
gaa	E	49	30.28
gag	E	1	19.72
---	--	------	------
mPD		0.0072	0.50
		nPD:	0.01
		N. weight:	0.75
		Sc. PD:	-0.025
		Sc. rank:	-324.9

PB2			
Pos.	741	obs:	exp:
tct	S	22192	4091.00
tcc	S	34	2575.00
tca	S	2	5785.00
tcg	S	1	1183.00
tgt	C	1	0.45
tgc	C	0	0.55
act	T	2	0.50
acc	T	0	0.41
aca	T	0	0.91
acg	T	0	0.17
agt	S	0	4180.00
agc	S	0	4415.00
---	--	------	------
mPD		0.0036	1.6
		nPD:	0.
		N. weight:	1.7
		Sc. PD:	-0.077
		Sc. rank:	-27388.0

PB2			
Pos.	742	obs:	exp:
tct	S	0	4091.00
tcc	S	0	2575.00
tca	S	0	5785.00
tcg	S	0	1183.00
act	T	0	0.25
acc	T	1	0.21
aca	T	0	0.46
acg	T	0	0.09
aat	N	0	0.59
aac	N	1	0.41
agt	S	26	4180.00
agc	S	22203	4415.00
ggt	G	0	0.15
ggc	G	1	0.15
gga	G	0	0.44
ggg	G	0	0.26
---	--	------	------
mPD		0.0026	1.6
		nPD:	0.
		N. weight:	1.6
		Sc. PD:	-0.074
		Sc. rank:	-28081.0

PB2			
Pos.	743	obs:	exp:
att	I	0	8093.00
atc	I	0	5549.00
ata	I	22231	8589.00
gtt	V	0	0.22
gtc	V	0	0.18
gta	V	1	0.27
gtg	V	0	0.33
---	--	------	------
mPD		0.000090	0.66
		nPD:	0.
		N. weight:	0.96
		Sc. PD:	-0.045
		Sc. rank:	-17955.0

PB2			
Pos.	744	obs:	exp:
tta	L	0	2444.00
ttg	L	0	4186.00
ctt	L	22231	4381.00
ctc	L	1	3140.00
cta	L	0	4280.00
ctg	L	0	3801.00
---	--	------	------
mPD		0.000090	1.1
		nPD:	0.
		N. weight:	1.6
		Sc. PD:	-0.077
		Sc. rank:	-30982.0

PB2			
Pos.	745	obs:	exp:
act	T	22226	5604.00
acc	T	6	4560.00
aca	T	0	10171.00
acg	T	0	1897.00
---	--	------	------
mPD		0.00054	0.68
		nPD:	0.
		N. weight:	1.4
		Sc. PD:	-0.065
		Sc. rank:	-25130.0

PB2			
Pos.	746	obs:	exp:
gat	D	90	11713.00
gac	D	22141	10518.00
ggt	G	0	0.15
ggc	G	1	0.15
gga	G	0	0.44
ggg	G	0	0.26
---	--	------	------
mPD		0.0082	0.50
		nPD:	0.02
		N. weight:	0.73
		Sc. PD:	-0.023
		Sc. rank:	779.6

PB2			
Pos.	747	obs:	exp:
tct	S	0	4091.00
tcc	S	0	2575.00
tca	S	0	5785.00
tcg	S	0	1183.00
act	T	0	0.50
acc	T	2	0.41
aca	T	0	0.91
acg	T	0	0.17
aat	N	0	0.59
aac	N	1	0.41
agt	S	25	4180.00
agc	S	22204	4415.00
---	--	------	------
mPD		0.0025	1.6
		nPD:	0.
		N. weight:	1.6
		Sc. PD:	-0.074
		Sc. rank:	-28218.0

PB2			
Pos.	748	obs:	exp:
caa	Q	4	11731.00
cag	Q	22228	10501.00
---	--	------	------
mPD		0.00036	0.50
		nPD:	0.
		N. weight:	0.76
		Sc. PD:	-0.035
		Sc. rank:	-13725.0

PB2			
Pos.	749	obs:	exp:
act	T	0	5604.00
acc	T	0	4560.00
aca	T	22232	10171.00
acg	T	0	1897.00
---	--	------	------
mPD		0	0.68
		nPD:	0.
		N. weight:	0.79
		Sc. PD:	-0.037
		Sc. rank:	-15033.0

PB2			
Pos.	750	obs:	exp:
gct	A	2	5886.00
gcc	A	1	4602.00
gca	A	0	10442.00
gcg	A	22229	1302.00
---	--	------	------
mPD		0.00027	0.66
		nPD:	0.
		N. weight:	2.9
		Sc. PD:	-0.13
		Sc. rank:	-52308.0

PB2			
Pos.	751	obs:	exp:
act	T	0	5604.00
acc	T	22232	4560.00
aca	T	0	10171.00
acg	T	0	1897.00
---	--	------	------
mPD		0	0.68
		nPD:	0.
		N. weight:	1.6
		Sc. PD:	-0.076
		Sc. rank:	-30453.0

PB2			
Pos.	752	obs:	exp:
cgt	R	0	0.05
cgc	R	0	0.10
cga	R	0	0.20
cgg	R	0	0.15
aaa	K	22230	12681.00
aag	K	0	9549.00
aga	R	2	1.00
agg	R	0	0.49
---	--	------	------
mPD		0.00018	0.49
		nPD:	0.
		N. weight:	0.57
		Sc. PD:	-0.027
		Sc. rank:	-10566.0

PB2			
Pos.	753	obs:	exp:
cgt	R	0	607.00
cgc	R	0	1163.00
cga	R	0	2177.00
cgg	R	0	1627.00
aga	R	22076	11161.00
agg	R	155	5496.00
ggt	G	0	0.15
ggc	G	0	0.15
gga	G	1	0.44
ggg	G	0	0.26
---	--	------	------
mPD		0.014	0.91
		nPD:	0.02
		N. weight:	0.66
		Sc. PD:	-0.021
		Sc. rank:	322.7

PB2			
Pos.	754	obs:	exp:
ttt	F	5	2.15
ttc	F	0	2.85
tta	L	0	0.66
ttg	L	0	1.13
ctt	L	6	1.18
ctc	L	0	0.85
cta	L	0	1.16
ctg	L	0	1.03
att	I	22210	8088.00
atc	I	9	5546.00
ata	I	0	8584.00
gtt	V	2	0.44
gtc	V	0	0.36
gta	V	0	0.54
gtg	V	0	0.66
---	--	------	------
mPD		0.0020	0.66
		nPD:	0.
		N. weight:	1.
		Sc. PD:	-0.045
		Sc. rank:	-15307.0

PB2			
Pos.	755	obs:	exp:
tta	L	0	0.22
ttg	L	0	0.38
tgg	W	1	1.00
ctt	L	0	0.39
ctc	L	0	0.28
cta	L	0	0.39
ctg	L	2	0.34
cgt	R	1	606.90
cgc	R	1	1163.00
cga	R	2422	2177.00
cgg	R	19804	1627.00
aga	R	0	11159.00
agg	R	0	5495.00
ggt	G	0	0.15
ggc	G	0	0.15
gga	G	0	0.44
ggg	G	1	0.26
---	--	------	------
mPD		0.19	0.91
		nPD:	0.21
		N. weight:	2.3
		Sc. PD:	0.37
		Sc. rank:	38263.0

PB2			
Pos.	756	obs:	exp:
atg	M	22226	22226.00
act	T	0	0.25
acc	T	0	0.21
aca	T	0	0.46
acg	T	1	0.09
gtt	V	0	1.11
gtc	V	0	0.91
gta	V	0	1.34
gtg	V	5	1.64
---	--	------	------
mPD		0.00054	0.00092
		nPD:	0.58
		N. weight:	0.00036
		Sc. PD:	0.00019
		Sc. rank:	6.8

PB2			
Pos.	757	obs:	exp:
gtt	V	0	0.44
gtc	V	2	0.36
gta	V	0	0.54
gtg	V	0	0.66
gct	A	0	5885.00
gcc	A	22229	4601.00
gca	A	0	10440.00
gcg	A	0	1302.00
gat	D	0	0.53
gac	D	1	0.47
---	--	------	------
mPD		0.00027	0.66
		nPD:	0.
		N. weight:	1.6
		Sc. PD:	-0.075
		Sc. rank:	-29281.0

PB2			
Pos.	758	obs:	exp:
tta	L	0	0.11
ttg	L	0	0.19
ctt	L	0	0.20
ctc	L	1	0.14
cta	L	0	0.19
ctg	L	0	0.17
att	I	33	8080.00
atc	I	22161	5540.00
ata	I	1	8575.00
act	T	0	7.56
acc	T	30	6.15
aca	T	0	13.72
acg	T	0	2.56
aat	N	0	2.96
aac	N	5	2.04
gtt	V	0	0.22
gtc	V	1	0.18
gta	V	0	0.27
gtg	V	0	0.33
---	--	------	------
mPD		0.0064	0.66
		nPD:	0.01
		N. weight:	1.4
		Sc. PD:	-0.052
		Sc. rank:	-7070.8

PB2			
Pos.	759	obs:	exp:
tat	Y	1	0.52
tac	Y	0	0.48
aat	N	22208	13139.00
aac	N	8	9077.00
aaa	K	10	6.85
aag	K	2	5.15
gat	D	3	1.58
gac	D	0	1.42
---	--	------	------
mPD		0.0022	0.48
		nPD:	0.
		N. weight:	0.53
		Sc. PD:	-0.023
		Sc. rank:	-6699.8

PB2			
Pos.	760	obs:	exp:
taa	.	96	10098.00
tag	.	22135	6056.00
tga	.	1	6079.00
---	--	------	------
mPD		0.0088	0.79
		nPD:	0.01
		N. weight:	1.3
		Sc. PD:	-0.047
		Sc. rank:	-4339.1

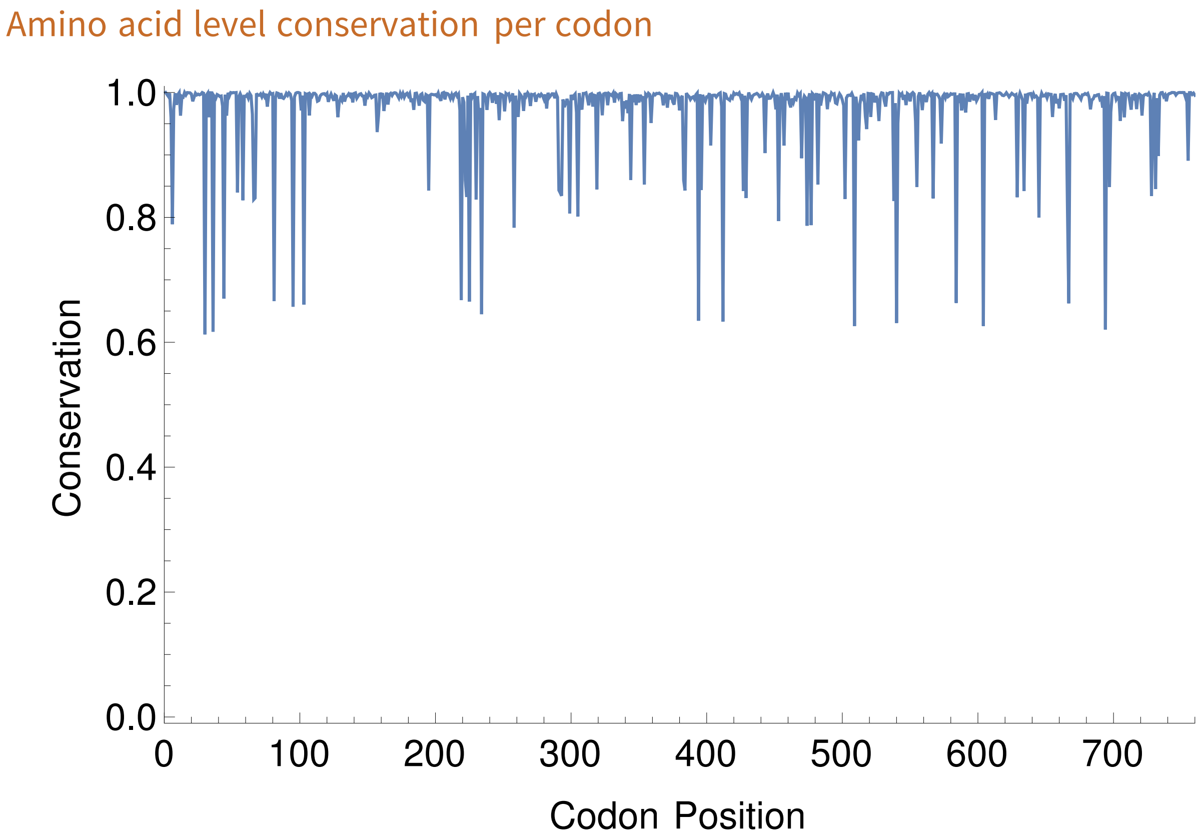
Amino acid level conservation per codon
Conservation
Codon Position
1.0
0.8
0.6
0.4
0.2
0.0
0
100
200
300
400
500
600
700

Conservation analysis plots

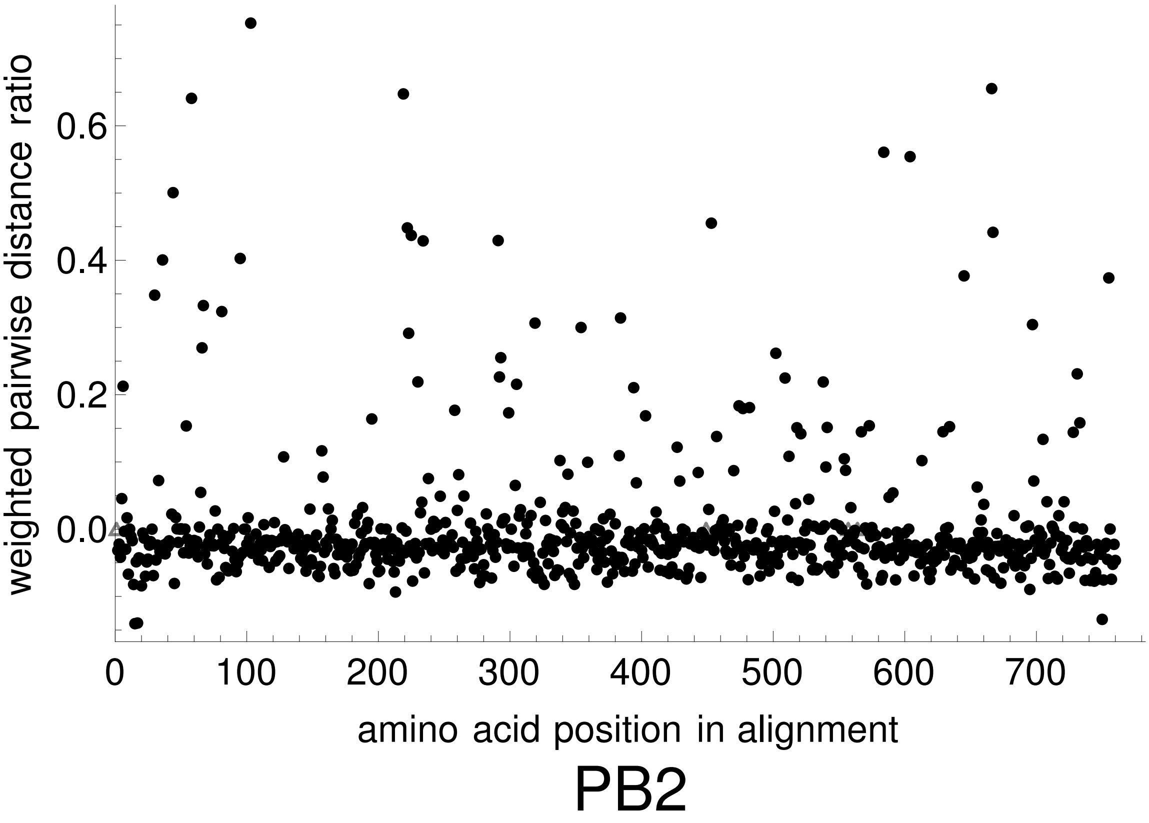

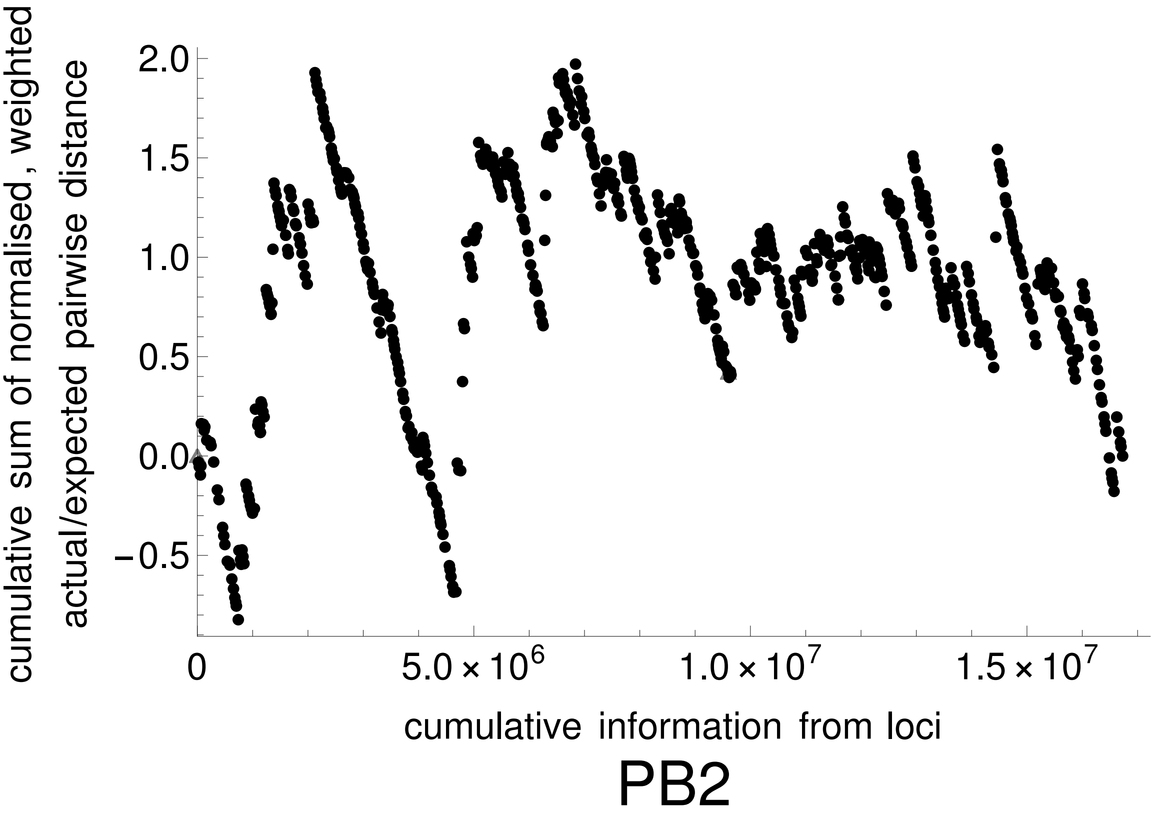

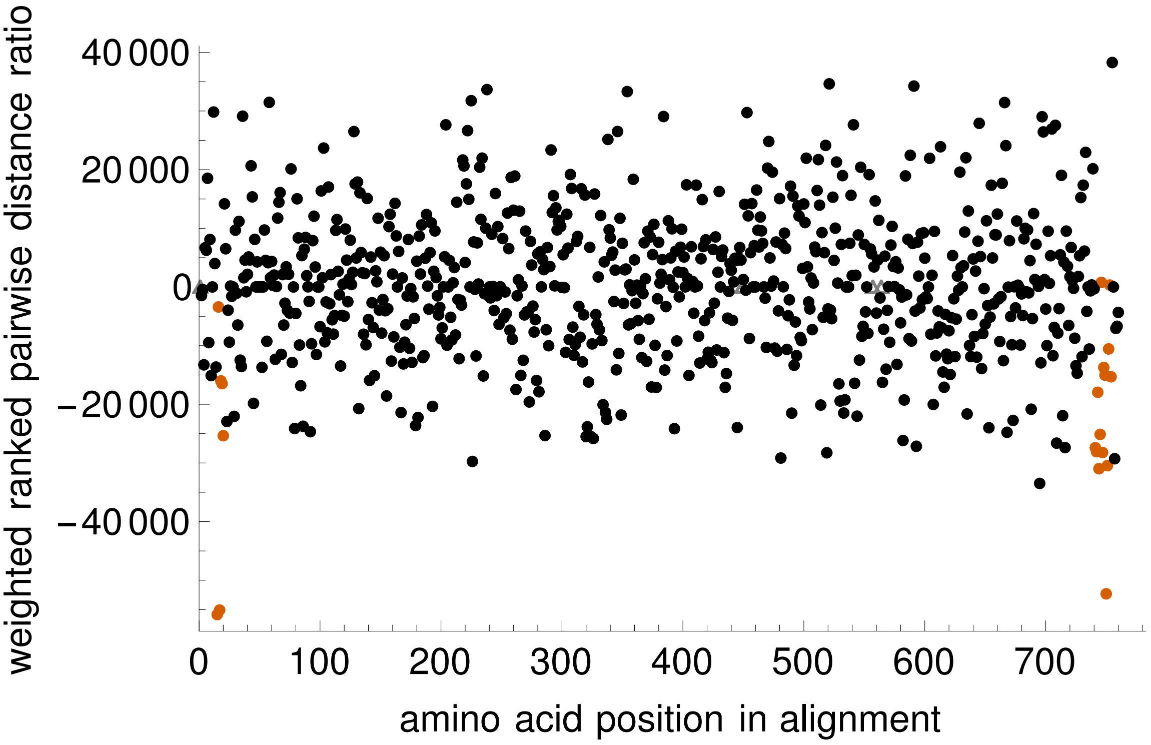
weighted ranked pairwise distance ratio
40 000
20 000
0
−20 000
−40 000
0
100
200
300
400
500
600
700
amino acid position in alignment

PB2

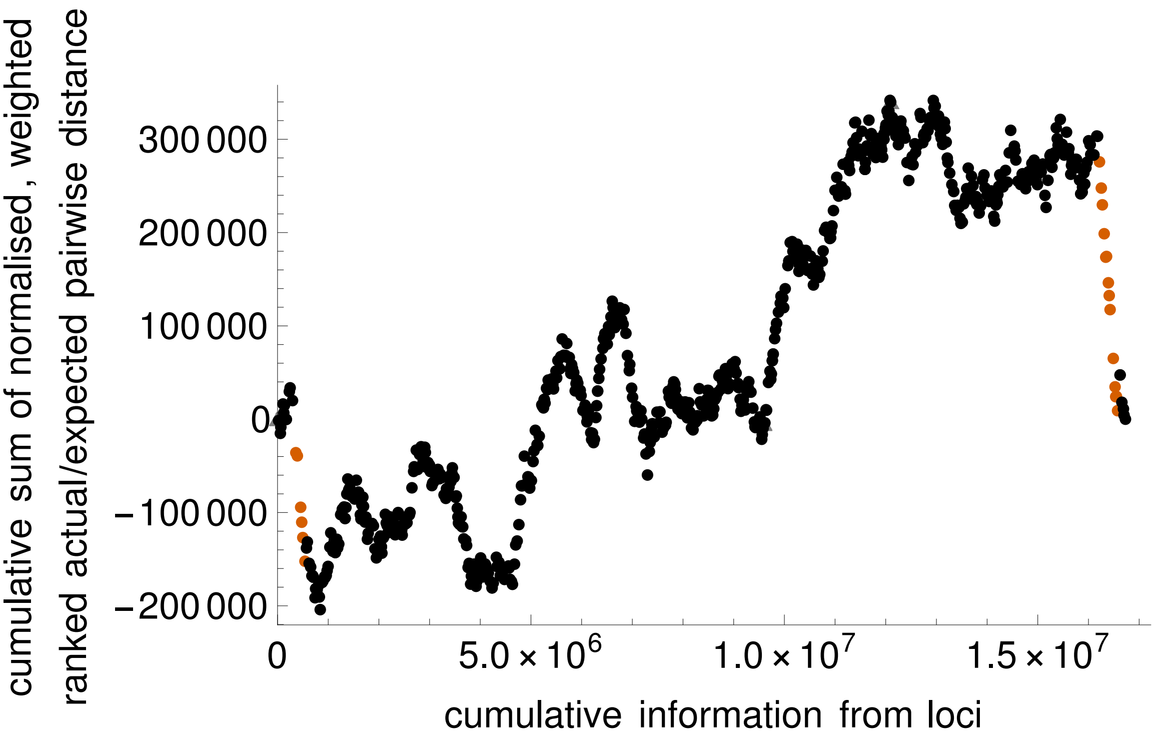
cumulative sum of normalised, weighted ranked actual/expected pairwise distance
300 000
200 000
100 000
0
−100 000
−200 000
0
5.0 × 10^6
1.0 × 10^7
1.5 × 10^7
cumulative information from loci

PB2

PB1

Gene length histogram

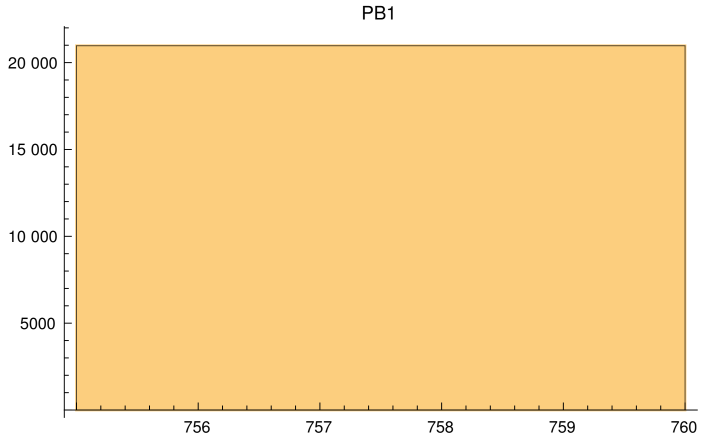

Information vs. nPD

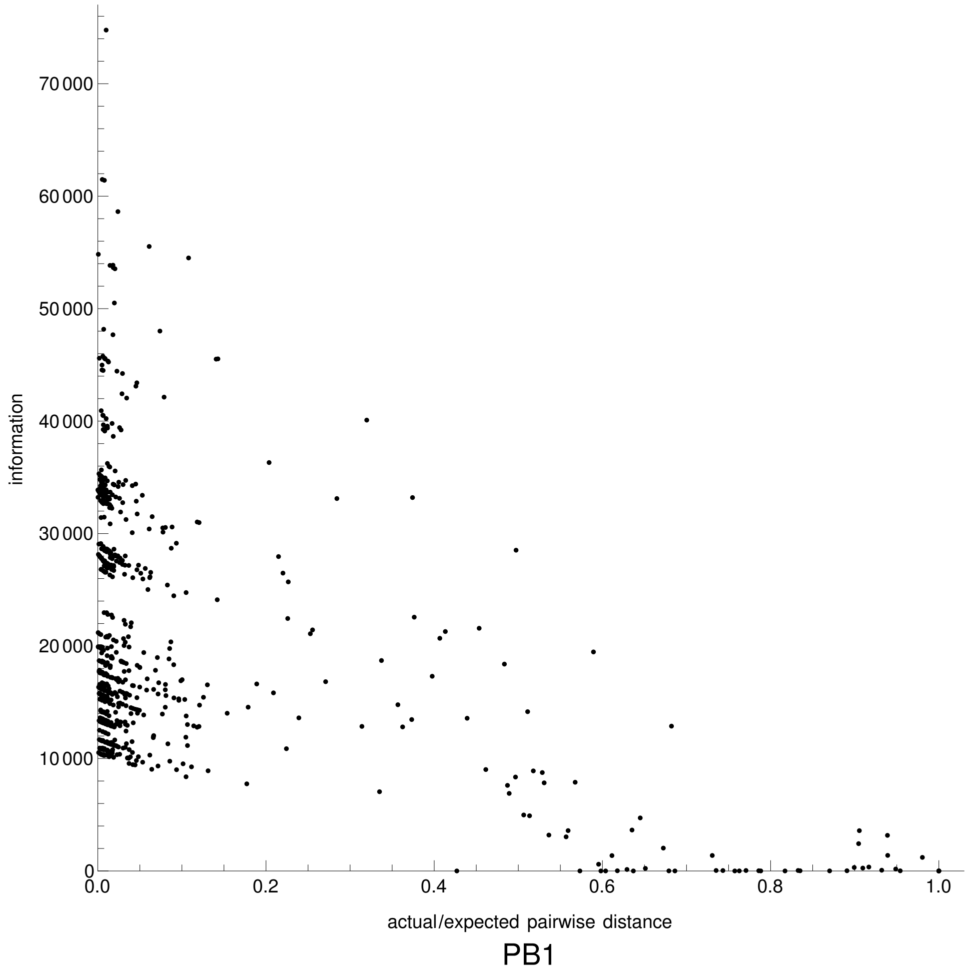

Example sequences highlighted by regions found in analyses to be conserved

```
Interesting points (by weighted raw PD) highlighted for gene PB1:
ATGGATGTCAATCCGACTCTACTTTTCCTAAAAATTCCAGCACAAAATGCCATAAGCACCACATTCCCTTATACTG⋱
    GAGATCCTCCATACAGCCATGGAACAGGAACAGGATACACCATGGACACAGTAAACAGAACACACCAATACTCAG⋱
    AAAAGGGGAAGTGGACAACCAACACAGAGACTGGTGCACCCCAGCTCAACCCGATTGACGGACCACTACCTGAAG⋱
    ATAATGAACCAAGTGGGTATGCACAAACAGACTGTGTTCTAGAGGCTATGGCTTTCCTTGAAGAATCCCATCCAG⋱
    GAATATTTGAAAATTCATGCCTTGAAACAATGGAAGTTGTTCAACAAACAAGGGTAGATAAACTGACTCAAGGTC⋱
    GCCAGACTTATGATTGGACATTAAACAGAAATCAACCGGCAGCAACTGCATTGGCCAACACCATAGAAGTTTTCA⋱
    GATCGAATGACCTAACAGCTAACGAGTCAGGAAGGCTAATAGATTTCTTAAAGGATGTGATGGAATCAATGAACA⋱
    AAGAGGAAATAGAGATAACAACCCACTTTCAAAGAAAAAGGAGAGTAAGAGACAACATGACCAAGAAGATGATCA⋱
    CGCAAAGAACAATAGGGAAGAAAAAACAGAGACTGAATAAGAGAGGCTATCTAATAAGAGCACTGACATTAAATA⋱
    CGATGACCAAAGATGCAGAGAGAGGCAAGTTAAAAAGAAGGGCTATCGCAACACCTGGGATGCAGATTAGAGGTT⋱
    TCGTATACTTTGTTGAAACTTTAGCTAGGAGCATTTGCGAAAAGCTTGAACAGTCTGGGCTCCCAGTAGGGGGCA⋱
    ATGAAAAGAAGGCCAAATTGGCAAATGTTGTGAGAAAGATGATGACTAATTCACAAGATACAGAGATTTCTTTCA⋱
    CAATCACTGGGGACAACACTAAATGGAATGAGAATCAAAATCCTCGAATGTTCCTGGCGATGATTACATATATCA⋱
    CCAGAAATCAACCCGAGTGGTTTAGAAACATCCTGAGCATGGCACCCATAATGTTCTCAAATAAAATGGCAAGAC⋱
    TAGGGAAAGGGTACATGTTCGAGAGTAAAAGAATGAAGATTCGAACACAAATACCAGCAGAAATGCTAGCAAGCA⋱
    TTGATCTAAAGTATTTCAATGAATCAACAAGGAAGAAAATTGAGAAGATAAGGCCTCTTTTAATGGATGGCACAG⋱
    CATCACTGAGTCCTGGGATGATGATGGGCATGTTCAACATGCTAAGTACTGTCTTGGGAGTCTCGATACTGAATC⋱
    TTGGACAAAAGAAATACACCAAGACAACATACTGGTGGGATGGGCTCCAATCATCCGACGATTTTGCTCTCATAG⋱
    TGAATGCACCAAACCATGAAGGAATACAAGCAGGAGTGGACAGATTCTACAGGACCTGCAAATTAGTGGGAATCA⋱
    ACATGAGCAAAAAGAAGTCCTATATAAATAGGACAGGGACATTTGAATTCACAAGCTTTTTTTATCGCTATGGAT⋱
    TTGTGGCTAATTTTAGCATGGAGCTACCCAGCTTTGGAGTGTCTGGAGTAAATGAATCAGCTGACATGAGTATTG⋱
    GAGTAACAGTGATAAAGAACAACATGATAAACAATGACCTTGGACCTGCAACGGCTCAGATGGCTCTTCAATTGT⋱
    TCATCAAAGACTACAGATACACATATAGGTGTCATAGGGGAGACACACAAATTCAGACGAGAAGATCATTTGAGT⋱
    TGAAGAAGCTATGGGATCAAACCCAATCAAAGGTAGGGCTATTAGTATCAGATGGAGGACCAAACTTATACAACA⋱
    TACGGAATCTTCACATTCCT ---GAAGTCTGCTTAAAATGGGAGCTAATGGATGATGATTATCGGGGAAGACTTTG⋱
    TAATCCCCTGAATCCCTTTGTGAGTCATAAGGAGATTGATTCTGTAAACAATGCTGTGGTACTGCCAGCCCATGG⋱
    CCCAGCCAAAAGCATGGAATATGATGCCGTCGCTACTACACATTCCTGGATTCCCAAGAGGAATCGTTCTATTCT⋱
    CAACACAAGCCAAAGGGGAATTCTTGAGGATGAACAGATGTACCAGAAGTGTTGCAATTTATTCGAGAAATTTTT⋱
    CCCTAGCAGTTCATATAGGAGACCGGTTGGAATTTCTAGCATGGTGGAGGCCATGGTGTCTAGGGCCCGGATTGA⋱
    TGCCAGAGTCGACTTTGAGTCTGGACGGATTAAGAAAGAAGAGTTCTCTGAGATCATGAAGATCTGTTCCACCAT⋱
    TGAAGAACTCAGACGGCAAAAATAA
```

```
Interesting points (by weighted ranked PD) highlighted for gene PB1:
ATGGATGTCAATCCGACTCTACTTTTCCTAAAAATTCCAGCACAAAATGCCATAAGCACCACATTCCCTTATACTGGA⋱
    GATCCTCCATACAGCCATGGAACAGGAACAGGATACACCATGGACACAGTAAACAGAACACACCAATACTCAGAAAA⋱
    GGGGAAGTGGACAACCAACACAGAGACTGGTGCACCCCAGCTCAACCCGATTGACGGACCACTACCTGAAGATAATG⋱
    AACCAAGTGGGTATGCACAAACAGACTGTGTTCTAGAGGCTATGGCTTTCCTTGAAGAATCCCATCCAGGAATATTT⋱
    GAAAATTCATGCCTTGAAACAATGGAAGTTGTTCAACAAACAAGGGTAGATAAACTGACTCAAGGTCGCCAGACTTA⋱
    TGATTGGACATTAAACAGAAATCAACCGGCAGCAACTGCATTGGCCAACACCATAGAAGTTTTCAGATCGAATGACC⋱
    TAACAGCTAACGAGTCAGGAAGGCTAATAGATTTCTTAAAGGATGTGATGGAATCAATGAACAAAGAGGAAATAGAG⋱
    ATAACAACCCACTTTCAAAGAAAAAGGAGAGTAAGAGACAACATGACCAAGAAGATGATCACGCAAAGAACAATAGG⋱
    GAAGAAAAAACAGAGACTGAATAAGAGAGGCTATCTAATAAGAGCACTGACATTAAATACGATGACCAAAGATGCAG⋱
    AGAGAGGCAAGTTAAAAAGAAGGGCTATCGCAACACCTGGGATGCAGATTAGAGGTTTCGTATACTTTGTTGAAACT⋱
```

TTAGCTAGGAGCATTTGCGAAAAGCTTGAACAGTCTGGGCTCCCAGTAGGGGGCAATGAAAAGAAGGCCAAATTGGC⋱

AAATGTTGTGAGAAAGATGATGACTAATTCACAAGATACAGAGATTTCTTTCACAATCACTGGGGACAACACTAAAT⋱

GGAATGAGAATCAAAATCCTCGAATGTTCCTGGCGATGATTACATATATCACCAGAAATCAACCCGAGTGGTTTAGA⋱

AACATCCTGAGCATGGCACCCATAATGTTCTCAAATAAAATGGCAAGACTAGGGAAAGGGTACATGTTCGAGAGTAA⋱

AAGAATGAAGATTCGAACACAAATACCAGCAGAAATGCTAGCAAGCATTGATCTAAAGTATTTCAATGAATCAACAA⋱

GGAAGAAAATTGAGAAGATAAGGCCTCTTTTAATGGATGGCACAGCATCACTGAGTCCTGGGATGATGATGGGCATG⋱

TTCAACATGCTAAGTACTGTCTTGGGAGTCTCGATACTGAATCTTGGACAAAAGAAATACACCAAGACAACATACTG⋱

GTGGGATGGGCTCCAATCATCCGACGATTTTGCTCTCATAGTGAATGCACCAAACCATGAAGGAATACAAGCAGGAG⋱

TGGACAGATTCTACAGGACCTGCAAATTAGTGGGAATCAACATGAGCAAAAAGAAGTCCTATATAAATAGGACAGGG⋱

ACATTTGAATTCACAAGCTTTTTTTATCGCTATGGATTTGTGGCTAATTTTAGCATGGAGCTACCCAGCTTTGGAGT⋱

GTCTGGAGTAAATGAATCAGCTGACATGAGTATTGGAGTAACAGTGATAAAGAACAACATGATAAACAATGACCTTG⋱

GACCTGCAACGGCTCAGATGGCTCTTCAATTGTTCATCAAAGACTACAGATACACATATAGGTGTCATAGGGGAGAC⋱

ACACAAATTCAGACGAGAAGATCATTTGAGTTGAAGAAGCTATGGGATCAAACCCAATCAAAGGTAGGGCTATTAGT⋱

ATCAGATGGAGGACCAAACTTATACAACATACGGAATCTTCACATTCCT ---GAAGTCTGCTTAAAATGGGAGCTAAT

GGATGATGATTATCGGGGAAGACTTTGTAATCCCCTGAATCCCTTTGTGAGTCATAAGGAGATTGATTCTGTAAA⋱

CAATGCTGTGGTACTGCCAGCCCATGGCCCAGCCAAAAGCATGGAATATGATGCCGTCGCTACTACACATTCCTG⋱

GATTCCCAAGAGGAATCGTTCTATTCTCAACACAAGCCAAAGGGGAATTCTTGAGGATGAACAGATGTACCAGAA⋱

GTGTTGCAATTTATTCGAGAAATTTTTCCCTAGCAGTTCATATAGGAGACCGGTTGGAATTTCTAGCATGGTGGA⋱

GGCCATGGTGTCTAGGGCCCGGATTGATGCCAGAGTCGACTTTGAGTCTGGACGGATTAAGAAAGAAGAGTTCTC⋱

TGAG**ATCATGAAGATCTGTTCCACCATTGAAGAACTCAGACGG**CAAAAATAA

Per codon conservation report

PB1			
Pos.	1	obs:	exp:
atg	M	20972	20972.00
---	--	------	------
mPD		0	0
		nPD:	1.
		N. weight:	0.
		Sc. PD:	0
		Sc. rank:	0

PB1			
Pos.	2	obs:	exp:
aat	N	1	0.59
aac	N	0	0.41
gat	D	20957	11049.00
gac	D	13	9921.00
ggt	G	1	0.15
ggc	G	0	0.15
gga	G	0	0.44
ggg	G	0	0.26
---	--	------	------
mPD		0.0014	0.50
		nPD:	0.
		N. weight:	0.67
		Sc. PD:	-0.024
		Sc. rank:	-9588.2

PB1			
Pos.	3	obs:	exp:
att	I	0	8.01
atc	I	22	5.49
ata	I	0	8.50
gtt	V	10	4632.00
gtc	V	20939	3807.00
gta	V	1	5634.00
gtg	V	0	6877.00
---	--	------	------
mPD		0.0031	0.74
		nPD:	0.
		N. weight:	1.8
		Sc. PD:	-0.063
		Sc. rank:	-20740.0

PB1			
Pos.	4	obs:	exp:
att	I	1	0.36
atc	I	0	0.25
ata	I	0	0.39
aat	N	20885	12401.00
aac	N	83	8567.00
aaa	K	3	1.71
aag	K	0	1.29
---	--	------	------
mPD		0.0083	0.48
		nPD:	0.02
		N. weight:	0.53
		Sc. PD:	-0.012
		Sc. rank:	1852.0

PB1			
Pos.	5	obs:	exp:
tta	L	0	0.11
ttg	L	0	0.19
tct	S	0	0.37
tcc	S	0	0.23
tca	S	0	0.52
tcg	S	2	0.11
ctt	L	0	0.20
ctc	L	0	0.14
cta	L	0	0.19
ctg	L	1	0.17
cct	P	1	5777.00
ccc	P	1	3889.00
cca	P	7	8928.00
ccg	P	20959	2374.00
agt	S	0	0.38
agc	S	0	0.40
gct	A	0	0.26
gcc	A	0	0.21
gca	A	0	0.47
gcg	A	1	0.06
---	--	------	------
mPD		0.0012	0.70
		nPD:	0.
		N. weight:	2.3
		Sc. PD:	-0.086
		Sc. rank:	-36041.0

PB1			
Pos.	6	obs:	exp:
tct	S	1	0.18
tcc	S	0	0.12
tca	S	0	0.26
tcg	S	0	0.05
att	I	15	5.46
atc	I	0	3.74
ata	I	0	5.80
act	T	20736	5266.00
acc	T	151	4284.00
aca	T	1	9556.00
acg	T	0	1782.00
aat	N	11	6.51
aac	N	0	4.49
agt	S	0	0.19
agc	S	0	0.20
gct	A	57	15.09
gcc	A	0	11.80
gca	A	0	26.77
gcg	A	0	3.34
---	--	------	------
mPD		0.022	0.69
		nPD:	0.03
		N. weight:	1.4
		Sc. PD:	-0.01
		Sc. rank:	12798.0

PB1			
Pos.	7	obs:	exp:
tta	L	761	2304.00
ttg	L	0	3946.00
ctt	L	1	4130.00
ctc	L	0	2960.00
cta	L	20171	4035.00
ctg	L	25	3583.00
att	I	0	5.10
atc	I	0	3.49
ata	I	14	5.41
---	--	------	------
mPD		0.074	1.1
		nPD:	0.06
		N. weight:	1.6
		Sc. PD:	0.039
		Sc. rank:	20121.0

PB1			
Pos.	8	obs:	exp:
ttt	F	2	0.86
ttc	F	0	1.14
tta	L	0	2305.00
ttg	L	2	3947.00
ctt	L	20913	4131.00
ctc	L	36	2961.00
cta	L	0	4036.00
ctg	L	12	3584.00
cct	P	1	0.28
ccc	P	0	0.19
cca	P	0	0.43
ccg	P	0	0.11
att	I	6	2.18
atc	I	0	1.50
ata	I	0	2.32
---	--	------	------
mPD		0.0058	1.1
		nPD:	0.01
		N. weight:	1.7
		Sc. PD:	-0.058
		Sc. rank:	-16890.0

PB1			
Pos.	9	obs:	exp:
ttt	F	105	8996.00
ttc	F	20859	11968.00
tta	L	1	0.44
ttg	L	1	0.75
tct	S	0	0.55
tcc	S	3	0.35
tca	S	0	0.78
tcg	S	0	0.16
ctt	L	0	0.79
ctc	L	2	0.56
cta	L	0	0.77
ctg	L	0	0.68
att	I	0	0.36
atc	I	1	0.25
ata	I	0	0.39
agt	S	0	0.56
agc	S	0	0.60
---	--	------	------
mPD		0.011	0.49
		nPD:	0.02
		N. weight:	0.56
		Sc. PD:	-0.0099
		Sc. rank:	3313.6

PB1			
Pos.	10	obs:	exp:
tta	L	257	2304.00
ttg	L	2	3945.00
ctt	L	8	4129.00
ctc	L	0	2960.00
cta	L	20617	4034.00
ctg	L	71	3583.00
cct	P	0	0.28
ccc	P	0	0.19
cca	P	1	0.43
ccg	P	0	0.11
caa	Q	3	1.58
cag	Q	0	1.42
att	I	0	4.37
atc	I	0	3.00
ata	I	12	4.64
atg	M	1	1.00
---	--	------	------
mPD		0.034	1.1
		nPD:	0.03
		N. weight:	1.7
		Sc. PD:	-0.017
		Sc. rank:	13800.0

PB1			
Pos.	11	obs:	exp:
cgt	R	0	0.05
cgc	R	0	0.10
cga	R	0	0.20
cgg	R	0	0.15
act	T	0	0.25
acc	T	0	0.21
aca	T	1	0.46
acg	T	0	0.09
aat	N	1	0.59
aac	N	0	0.41
aaa	K	20870	11961.00
aag	K	98	9007.00
aga	R	2	1.00
agg	R	0	0.49
---	--	------	------
mPD		0.0097	0.49
		nPD:	0.02
		N. weight:	0.57
		Sc. PD:	-0.011
		Sc. rank:	2953.2

PB1			
Pos.	12	obs:	exp:
tta	L	0	1.65
ttg	L	0	2.82
ctt	L	15	2.96
ctc	L	0	2.12
cta	L	0	2.89
ctg	L	0	2.56
att	I	20760	7590.00
atc	I	80	5205.00
ata	I	10	8055.00
act	T	22	5.55
acc	T	0	4.51
aca	T	0	10.06
acg	T	0	1.88
aat	N	3	1.77
aac	N	0	1.23
gtt	V	79	18.13
gtc	V	0	14.90
gta	V	0	22.05
gtg	V	3	26.92
---	--	------	------
mPD		0.020	0.67
		nPD:	0.03
		N. weight:	1.
		Sc. PD:	-0.0095
		Sc. rank:	8902.9

PB1			
Pos.	13	obs:	exp:
tct	S	0	1.29
tcc	S	0	0.81
tca	S	7	1.82
tcg	S	0	0.37
cct	P	2	5776.00
ccc	P	1	3888.00
cca	P	20958	8927.00
ccg	P	4	2374.00
agt	S	0	1.32
agc	S	0	1.39
---	--	------	------
mPD		0.0013	0.70
		nPD:	0.
		N. weight:	0.9
		Sc. PD:	-0.034
		Sc. rank:	-14016.0

PB1			
Pos.	14	obs:	exp:
act	T	0	3.03
acc	T	0	2.46
aca	T	6	5.49
acg	T	6	1.02
gtt	V	0	5.53
gtc	V	0	4.54
gta	V	12	6.72
gtg	V	13	8.21
gct	A	3	5543.00
gcc	A	0	4333.00
gca	A	17952	9832.00
gcg	A	2980	1226.00
---	--	------	------
mPD		0.25	0.67
		nPD:	0.37
		N. weight:	0.68
		Sc. PD:	0.22
		Sc. rank:	11085.0

PB1			
Pos.	15	obs:	exp:
caa	Q	20959	11066.00
cag	Q	13	9906.00
---	--	------	------
mPD		0.0012	0.50
		nPD:	0.
		N. weight:	0.67
		Sc. PD:	-0.025
		Sc. rank:	-9856.6

PB1			
Pos.	16	obs:	exp:
tct	S	0	2.76
tcc	S	0	1.74
tca	S	0	3.90
tcg	S	0	0.80
aat	N	20914	12394.00
aac	N	41	8561.00
aaa	K	2	1.14
aag	K	0	0.86
agt	S	15	2.82
agc	S	0	2.98
---	--	------	------
mPD		0.0055	0.49
		nPD:	0.01
		N. weight:	0.54
		Sc. PD:	-0.015
		Sc. rank:	-415.5

,

PB1			
Pos.	17	obs:	exp:
gct	A	60	5552.00
gcc	A	20874	4341.00
gca	A	38	9850.00
gcg	A	0	1229.00
---	--	------	------
mPD		0.0093	0.66
		nPD:	0.01
		N. weight:	1.6
		Sc. PD:	-0.041
		Sc. rank:	2470.0

,

PB1			
Pos.	18	obs:	exp:
att	I	0	7633.00
atc	I	2	5234.00
ata	I	20967	8101.00
act	T	0	0.25
acc	T	0	0.21
aca	T	1	0.46
acg	T	0	0.09
gtt	V	0	0.44
gtc	V	0	0.36
gta	V	2	0.54
gtg	V	0	0.66
---	--	------	------
mPD		0.00048	0.66
		nPD:	0.
		N. weight:	1.
		Sc. PD:	-0.039
		Sc. rank:	-16799.0

,

PB1			
Pos.	19	obs:	exp:
tct	S	0	3859.00
tcc	S	0	2429.00
tca	S	0	5458.00
tcg	S	0	1116.00
aat	N	0	1.18
aac	N	2	0.82
agt	S	18	3943.00
agc	S	20952	4165.00
---	--	------	------
mPD		0.0019	1.6
		nPD:	0.
		N. weight:	1.7
		Sc. PD:	-0.065
		Sc. rank:	-27934.0

,

PB1			
Pos.	20	obs:	exp:
att	I	0	7.64
atc	I	21	5.24
ata	I	0	8.11
act	T	18	5281.00
acc	T	20903	4297.00
aca	T	29	9584.00
acg	T	0	1788.00
gct	A	0	0.26
gcc	A	1	0.21
gca	A	0	0.47
gcg	A	0	0.06
---	--	------	------
mPD		0.0066	0.68
		nPD:	0.01
		N. weight:	1.7
		Sc. PD:	-0.049
		Sc. rank:	-4423.5

,

PB1			
Pos.	21	obs:	exp:
act	T	6	5287.00
acc	T	30	4302.00
aca	T	20883	9594.00
acg	T	53	1789.00
---	--	------	------
mPD		0.0085	0.68
		nPD:	0.01
		N. weight:	0.8
		Sc. PD:	-0.021
		Sc. rank:	513.5

,

PB1			
Pos.	22	obs:	exp:
ttt	F	83	8999.00
ttc	F	20887	11971.00
tta	L	0	0.11
ttg	L	0	0.19
ctt	L	0	0.20
ctc	L	1	0.14
cta	L	0	0.19
ctg	L	0	0.17
att	I	0	0.36
atc	I	1	0.25
ata	I	0	0.39
---	--	------	------
mPD		0.0081	0.49
		nPD:	0.02
		N. weight:	0.57
		Sc. PD:	-0.013
		Sc. rank:	1803.4

,

PB1			
Pos.	23	obs:	exp:
tta	L	0	0.11
ttg	L	0	0.19
ctt	L	1	0.20
ctc	L	0	0.14
cta	L	0	0.19
ctg	L	0	0.17
cct	P	20942	5777.00
ccc	P	25	3890.00
cca	P	1	8929.00
ccg	P	3	2375.00
---	--	------	------
mPD		0.0029	0.70
		nPD:	0.
		N. weight:	1.4
		Sc. PD:	-0.047
		Sc. rank:	-16286.0

,

PB1			
Pos.	24	obs:	exp:
tat	Y	20858	10897.00
tac	Y	114	10075.00
---	--	------	------
mPD		0.011	0.50
		nPD:	0.02
		N. weight:	0.66
		Sc. PD:	-0.012
		Sc. rank:	3861.2

,

PB1			
Pos.	25	obs:	exp:
tct	S	0	0.18
tcc	S	0	0.12
tca	S	0	0.26
tcg	S	1	0.05
act	T	20941	5287.00
acc	T	13	4301.00
aca	T	15	9594.00
acg	T	2	1789.00
agt	S	0	0.19
agc	S	0	0.20
---	--	------	------
mPD		0.0030	0.68
		nPD:	0.
		N. weight:	1.4
		Sc. PD:	-0.05
		Sc. rank:	-16165.0

,

PB1			
Pos.	26	obs:	exp:
cgt	R	0	0.03
cgc	R	0	0.05
cga	R	0	0.10
cgg	R	0	0.07
aga	R	1	0.50
agg	R	0	0.25
ggt	G	2	3180.00
ggc	G	0	3106.00
gga	G	20916	9328.00
ggg	G	53	5357.00
---	--	------	------
mPD		0.0053	0.69
		nPD:	0.01
		N. weight:	0.84
		Sc. PD:	-0.026
		Sc. rank:	-4594.9

,

PB1			
Pos.	27	obs:	exp:
aat	N	1	0.59
aac	N	0	0.41
gat	D	20896	11050.00
gac	D	75	9921.00
---	--	------	------
mPD		0.0072	0.50
		nPD:	0.01
		N. weight:	0.65
		Sc. PD:	-0.016
		Sc. rank:	1205.6

,

PB1			
Pos.	28	obs:	exp:
cct	P	20937	5778.00
ccc	P	34	3890.00
cca	P	1	8930.00
ccg	P	0	2375.00
---	--	------	------
mPD		0.0033	0.70
		nPD:	0.
		N. weight:	1.4
		Sc. PD:	-0.046
		Sc. rank:	-14387.0

,

PB1			
Pos.	29	obs:	exp:
cct	P	3	5778.00
ccc	P	2	3890.00
cca	P	20960	8930.00
ccg	P	7	2375.00
---	--	------	------
mPD		0.0011	0.70
		nPD:	0.
		N. weight:	0.9
		Sc. PD:	-0.034
		Sc. rank:	-14366.0

,

PB1			
Pos.	30	obs:	exp:
tat	Y	279	10892.00
tac	Y	20683	10070.00
tgt	C	0	0.45
tgc	C	1	0.55
cat	H	1	5.29
cac	H	8	3.71
---	--	------	------
mPD		0.027	0.50
		nPD:	0.05
		N. weight:	0.7
		Sc. PD:	0.01
		Sc. rank:	8347.9

,

PB1			
Pos.	31	obs:	exp:
tct	S	0	3859.00
tcc	S	0	2429.00
tca	S	0	5458.00
tcg	S	0	1116.00
agt	S	14	3943.00
agc	S	20957	4166.00
ggt	G	0	0.15
ggc	G	1	0.15
gga	G	0	0.44
ggg	G	0	0.26
---	--	------	------
mPD		0.0014	1.6
		nPD:	0.
		N. weight:	1.7
		Sc. PD:	-0.065
		Sc. rank:	-28350.0

,

PB1			
Pos.	32	obs:	exp:
cat	H	20943	12333.00
cac	H	29	8639.00
---	--	------	------
mPD		0.0028	0.48
		nPD:	0.01
		N. weight:	0.55
		Sc. PD:	-0.018
		Sc. rank:	-4886.8

,

PB1			
Pos.	33	obs:	exp:
ggt	G	3	3180.00
ggc	G	2	3106.00
gga	G	20629	9329.00
ggg	G	338	5357.00
---	--	------	------
mPD		0.032	0.69
		nPD:	0.05
		N. weight:	0.78
		Sc. PD:	0.0051
		Sc. rank:	8705.3

,

PB1			
Pos.	34	obs:	exp:
act	T	2	5286.00
acc	T	0	4301.00
aca	T	20950	9593.00
acg	T	18	1789.00
gct	A	0	0.53
gcc	A	0	0.41
gca	A	2	0.94
gcg	A	0	0.12
---	--	------	------
mPD		0.0021	0.68
		nPD:	0.
		N. weight:	0.82
		Sc. PD:	-0.03
		Sc. rank:	-11455.0

,

PB1			
Pos.	35	obs:	exp:
ggt	G	1	3180.00
ggc	G	0	3106.00
gga	G	20907	9329.00
ggg	G	64	5357.00
---	--	------	------
mPD		0.0062	0.69
		nPD:	0.01
		N. weight:	0.84
		Sc. PD:	-0.025
		Sc. rank:	-2716.5

,

PB1

Pos.	36	obs:	exp:
att	I	0	0.36
atc	I	0	0.25
ata	I	1	0.39
act	T	19	5287.00
acc	T	6	4301.00
aca	T	20938	9594.00
acg	T	8	1789.00
mPD		0.0032	0.68
		nPD:	0.
		N. weight:	0.82
		Sc. PD:	-0.028
		Sc. rank:	-8727.6

PB1

Pos.	37	obs:	exp:
ggt	G	2	3180.00
ggc	G	2	3106.00
gga	G	20909	9329.00
ggg	G	59	5357.00
mPD		0.0060	0.69
		nPD:	0.01
		N. weight:	0.84
		Sc. PD:	-0.026
		Sc. rank:	-3340.7

PB1

Pos.	38	obs:	exp:
tat	Y	8	10886.00
tac	Y	20943	10065.00
tgt	C	0	0.90
tgc	C	2	1.10
cat	H	0	11.17
cac	H	19	7.83
mPD		0.0028	0.50
		nPD:	0.01
		N. weight:	0.77
		Sc. PD:	-0.026
		Sc. rank:	-6889.4

PB1

Pos.	39	obs:	exp:
att	I	0	0.36
atc	I	1	0.25
ata	I	0	0.39
act	T	68	5285.00
acc	T	20886	4300.00
aca	T	12	9591.00
acg	T	0	1789.00
gct	A	0	1.32
gcc	A	5	1.03
gca	A	0	2.35
gcg	A	0	0.29
mPD		0.0082	0.68
		nPD:	0.01
		N. weight:	1.6
		Sc. PD:	-0.045
		Sc. rank:	570.9

PB1

Pos.	40	obs:	exp:
tta	L	0	1.43
ttg	L	3	2.45
ctt	L	0	2.56
ctc	L	0	1.84
cta	L	0	2.50
ctg	L	10	2.22
att	I	8	14.20
atc	I	0	9.74
ata	I	31	15.07
atg	M	20916	20916.00
act	T	0	0.25
acc	T	0	0.21
aca	T	0	0.46
acg	T	1	0.09
gtt	V	0	0.66
gtc	V	0	0.55
gta	V	0	0.81
gtg	V	3	0.98
mPD		0.0053	0.0064
		nPD:	0.83
		N. weight:	0.0021
		Sc. PD:	0.0017
		Sc. rank:	36.6

PB1

Pos.	41	obs:	exp:
gat	D	636	11050.00
gac	D	20335	9921.00
gaa	E	1	0.61
gag	E	0	0.39
mPD		0.059	0.50
		nPD:	0.12
		N. weight:	0.65
		Sc. PD:	0.05
		Sc. rank:	9506.6

PB1

Pos.	42	obs:	exp:
tct	S	0	0.18
tcc	S	0	0.12
tca	S	1	0.26
tcg	S	0	0.05
act	T	6	5287.00
acc	T	3	4301.00
aca	T	20949	9594.00
acg	T	13	1789.00
agt	S	0	0.19
agc	S	0	0.20
mPD		0.0022	0.68
		nPD:	0.
		N. weight:	0.82
		Sc. PD:	-0.029
		Sc. rank:	-11311.0

PB1

Pos.	43	obs:	exp:
tta	L	1	0.11
ttg	L	0	0.19
ctt	L	0	0.20
ctc	L	0	0.14
cta	L	0	0.19
ctg	L	0	0.17
att	I	0	0.73
atc	I	0	0.50
ata	I	2	0.77
gtt	V	2	4636.00
gtc	V	16	3810.00
gta	V	20890	5639.00
gtg	V	60	6883.00
ggt	G	0	0.15
ggc	G	0	0.15
gga	G	1	0.44
ggg	G	0	0.26
mPD		0.0078	0.74
		nPD:	0.01
		N. weight:	1.4
		Sc. PD:	-0.039
		Sc. rank:	-2101.7

PB1

Pos.	44	obs:	exp:
tct	S	0	1.29
tcc	S	0	0.81
tca	S	0	1.82
tcg	S	0	0.37
tat	Y	0	0.52
tac	Y	1	0.48
act	T	0	1.01
acc	T	4	0.82
aca	T	0	1.83
acg	T	0	0.34
aat	N	13	12396.00
aac	N	20946	8563.00
agt	S	0	1.32
agc	S	7	1.39
gat	D	0	0.53
gac	D	1	0.47
mPD		0.0025	0.49
		nPD:	0.01
		N. weight:	0.94
		Sc. PD:	-0.032
		Sc. rank:	-9243.4

PB1

Pos.	45	obs:	exp:
cgt	R	0	572.60
cgc	R	0	1097.00
cga	R	7	2054.00
cgg	R	0	1535.00
aga	R	20932	10529.00
agg	R	33	5185.00
mPD		0.0038	0.91
		nPD:	0.
		N. weight:	0.72
		Sc. PD:	-0.025
		Sc. rank:	-8452.7

PB1

Pos.	46	obs:	exp:
act	T	1	5287.00
acc	T	5	4302.00
aca	T	20949	9594.00
acg	T	17	1789.00
mPD		0.0022	0.68
		nPD:	0.
		N. weight:	0.82
		Sc. PD:	-0.029
		Sc. rank:	-11343.0

PB1

Pos.	47	obs:	exp:
cat	H	658	12333.00
cac	H	20313	8638.00
aat	N	0	0.59
aac	N	1	0.41
mPD		0.061	0.48
		nPD:	0.13
		N. weight:	0.78
		Sc. PD:	0.066
		Sc. rank:	11675.0

PB1

Pos.	48	obs:	exp:
tta	L	0	0.11
ttg	L	0	0.19
ctt	L	0	0.20
ctc	L	0	0.14
cta	L	1	0.19
ctg	L	0	0.17
cat	H	0	0.59
cac	H	1	0.41
caa	Q	20941	11062.00
cag	Q	22	9901.00
cgt	R	0	0.05
cgc	R	0	0.10
cga	R	2	0.20
cgg	R	0	0.15
aaa	K	5	2.85
aag	K	0	2.15
aga	R	0	1.00
agg	R	0	0.49
mPD		0.0030	0.50
		nPD:	0.01
		N. weight:	0.67
		Sc. PD:	-0.022
		Sc. rank:	-5578.5

PB1

Pos.	49	obs:	exp:
tat	Y	74	10896.00
tac	Y	20897	10075.00
tgt	C	1	0.45
tgc	C	0	0.55
mPD		0.0072	0.50
		nPD:	0.01
		N. weight:	0.75
		Sc. PD:	-0.019
		Sc. rank:	1363.0

PB1

Pos.	50	obs:	exp:
tct	S	11	3859.00
tcc	S	10	2429.00
tca	S	20931	5457.00
tcg	S	17	1116.00
agt	S	0	3943.00
agc	S	0	4165.00
gct	A	0	0.79
gcc	A	0	0.62
gca	A	3	1.41
gcg	A	0	0.18
mPD		0.0039	1.6
		nPD:	0.
		N. weight:	1.4
		Sc. PD:	-0.052
		Sc. rank:	-20980.0

PB1

Pos.	51	obs:	exp:
aaa	K	4	2.28
aag	K	0	1.72
gtt	V	0	0.22
gtc	V	0	0.18
gta	V	1	0.27
gtg	V	0	0.33
gct	A	0	0.26
gcc	A	0	0.21
gca	A	1	0.47
gcg	A	0	0.06
gat	D	0	0.53
gac	D	1	0.47
gaa	E	20747	12691.00
gag	E	213	8269.00
ggt	G	0	0.76
ggc	G	0	0.74
gga	G	5	2.22
ggg	G	0	1.28
---	--	------	------
mPD		0.021	0.48
		nPD:	0.04
		N. weight:	0.48
		Sc. PD:	0.0021
		Sc. rank:	5102.0

,

PB1

Pos.	52	obs:	exp:
cgt	R	0	1.34
cgc	R	0	2.56
cga	R	0	4.80
cgg	R	0	3.59
atg	M	1	1.00
aat	N	4	2.37
aac	N	0	1.63
aaa	K	113	11932.00
aag	K	20804	8985.00
aga	R	4	24.60
agg	R	45	12.11
gaa	E	0	0.61
gag	E	1	0.39
---	--	------	------
mPD		0.016	0.50
		nPD:	0.03
		N. weight:	0.86
		Sc. PD:	-0.0059
		Sc. rank:	7894.8

,

PB1

Pos.	53	obs:	exp:
cgt	R	0	0.03
cgc	R	0	0.05
cga	R	0	0.10
cgg	R	0	0.07
aga	R	0	0.50
agg	R	1	0.25
ggt	G	3	3180.00
ggc	G	0	3106.00
gga	G	3449	9328.00
ggg	G	17519	5357.00
---	--	------	------
mPD		0.28	0.69
		nPD:	0.4
		N. weight:	0.87
		Sc. PD:	0.31
		Sc. rank:	14401.0

,

PB1

Pos.	54	obs:	exp:
cgt	R	0	3.11
cgc	R	0	5.96
cga	R	0	11.17
cgg	R	0	8.34
atg	M	3	3.00
act	T	0	0.50
acc	T	0	0.41
aca	T	0	0.91
acg	T	2	0.17
aat	N	4	2.96
aac	N	1	2.04
aaa	K	299	11892.00
aag	K	20548	8955.00
aga	R	6	57.23
agg	R	108	28.18
gaa	E	0	0.61
gag	E	1	0.39
---	--	------	------
mPD		0.041	0.50
		nPD:	0.08
		N. weight:	0.81
		Sc. PD:	0.032
		Sc. rank:	10893.0

,

PB1

Pos.	55	obs:	exp:
tgg	W	20972	20972.00
---	--	------	------
mPD		0	0
		nPD:	1.
		N. weight:	0.
		Sc. PD:	0
		Sc. rank:	0

,

PB1

Pos.	56	obs:	exp:
att	I	0	0.73
atc	I	0	0.50
ata	I	2	0.77
atg	M	9	9.00
act	T	3	5278.00
acc	T	0	4295.00
aca	T	17974	9579.00
acg	T	2962	1787.00
aaa	K	4	2.85
aag	K	1	2.15
gct	A	0	4.24
gcc	A	0	3.31
gca	A	12	7.51
gcg	A	4	0.94
---	-	1	1.00
---	--	------	------
mPD		0.25	0.68
		nPD:	0.36
		N. weight:	0.65
		Sc. PD:	0.2
		Sc. rank:	10526.0

,

PB1

Pos.	57	obs:	exp:
att	I	0	12.74
atc	I	1	8.74
ata	I	34	13.52
act	T	0	5275.00
acc	T	716	4292.00
aca	T	20196	9572.00
acg	T	12	1785.00
aaa	K	8	4.56
aag	K	0	3.44
gct	A	0	1.32
gcc	A	0	1.03
gca	A	5	2.35
gcg	A	0	0.29
---	--	------	------
mPD		0.072	0.68
		nPD:	0.11
		N. weight:	0.7
		Sc. PD:	0.045
		Sc. rank:	10069.0

,

PB1

Pos.	58	obs:	exp:
act	T	0	0.25
acc	T	1	0.21
aca	T	0	0.46
acg	T	0	0.09
aat	N	948	12403.00
aac	N	20023	8568.00
---	--	------	------
mPD		0.086	0.48
		nPD:	0.18
		N. weight:	0.74
		Sc. PD:	0.1
		Sc. rank:	11306.0

,

PB1

Pos.	59	obs:	exp:
tct	S	0	0.55
tcc	S	0	0.35
tca	S	3	0.78
tcg	S	0	0.16
cct	P	0	0.83
ccc	P	0	0.56
cca	P	3	1.28
ccg	P	0	0.34
act	T	6	5285.00
acc	T	2	4300.00
aca	T	20884	9591.00
acg	T	73	1789.00
agt	S	0	0.56
agc	S	0	0.60
gct	A	0	0.26
gcc	A	0	0.21
gca	A	1	0.47
gcg	A	0	0.06
---	--	------	------
mPD		0.0084	0.68
		nPD:	0.01
		N. weight:	0.81
		Sc. PD:	-0.022
		Sc. rank:	436.3

,

PB1

Pos.	60	obs:	exp:
aaa	K	0	0.57
aag	K	1	0.43
gtt	V	0	0.44
gtc	V	0	0.36
gta	V	0	0.54
gtg	V	2	0.66
gaa	E	1710	12696.00
gag	E	19258	8272.00
ggt	G	0	0.15
ggc	G	0	0.15
gga	G	0	0.44
ggg	G	1	0.26
---	--	------	------
mPD		0.15	0.48
		nPD:	0.31
		N. weight:	0.65
		Sc. PD:	0.17
		Sc. rank:	10425.0

,

PB1

Pos.	61	obs:	exp:
att	I	1	0.36
atc	I	0	0.25
ata	I	0	0.39
act	T	20916	5287.00
acc	T	44	4301.00
aca	T	7	9594.00
acg	T	4	1789.00
---	--	------	------
mPD		0.0053	0.68
		nPD:	0.01
		N. weight:	1.4
		Sc. PD:	-0.045
		Sc. rank:	-7115.4

,

PB1

Pos.	62	obs:	exp:
tct	S	0	0.92
tcc	S	0	0.58
tca	S	0	1.30
tcg	S	0	0.27
agt	S	5	0.94
agc	S	0	0.99
gat	D	8	4.22
gac	D	0	3.78
ggt	G	20925	3178.00
ggc	G	6	3104.00
gga	G	8	9323.00
ggg	G	20	5354.00
---	--	------	------
mPD		0.0045	0.69
		nPD:	0.01
		N. weight:	2.
		Sc. PD:	-0.065
		Sc. rank:	-14710.0

,

PB1

Pos.	63	obs:	exp:
tct	S	0	0.18
tcc	S	0	0.12
tca	S	1	0.26
tcg	S	0	0.05
act	T	0	0.76
acc	T	0	0.62
aca	T	3	1.37
acg	T	0	0.26
agt	S	0	0.19
agc	S	0	0.20
gtt	V	0	0.88
gtc	V	0	0.73
gta	V	4	1.08
gtg	V	0	1.31
gct	A	10	5550.00
gcc	A	4	4339.00
gca	A	20895	9846.00
gcg	A	54	1228.00
gaa	E	1	0.61
gag	E	0	0.39
---	--	------	------
mPD		0.0073	0.66
		nPD:	0.01
		N. weight:	0.78
		Sc. PD:	-0.022
		Sc. rank:	-948.1

,

PB1

Pos.	64	obs:	exp:
tta	L	0	1.98
ttg	L	0	3.39
tct	S	0	7.73
tcc	S	40	4.86
tca	S	0	10.93
tcg	S	0	2.24
ctt	L	0	3.55
ctc	L	18	2.54
cta	L	0	3.47
ctg	L	0	3.08
cct	P	88	5758.00
ccc	P	20791	3876.00
cca	P	19	8899.00
ccg	P	1	2366.00
cat	H	0	3.53
cac	H	6	2.47
act	T	0	0.76
acc	T	3	0.62
aca	T	0	1.37
acg	T	0	0.26
agt	S	2	7.90
agc	S	0	8.34
gct	A	0	1.06
gcc	A	4	0.83
gca	A	0	1.88
gcg	A	0	0.23
---	--	------	------
mPD		0.018	0.70
		nPD:	0.02
		N. weight:	1.7
		Sc. PD:	-0.025
		Sc. rank:	12293.0

,

PB1

Pos.	65	obs:	exp:
caa	Q	62	11066.00
cag	Q	20909	9905.00
cgt	R	0	0.03
cgc	R	0	0.05
cga	R	0	0.10
cgg	R	1	0.07
aga	R	0	0.50
agg	R	0	0.25
---	--	------	------
mPD		0.0060	0.50
		nPD:	0.01
		N. weight:	0.77
		Sc. PD:	-0.021
		Sc. rank:	159.8

,

PB1

Pos.	66	obs:	exp:
tta	L	0	2305.00
ttg	L	0	3948.00
ctt	L	65	4132.00
ctc	L	20895	2962.00
cta	L	11	4037.00
ctg	L	0	3586.00
att	I	0	0.36
atc	I	1	0.25
ata	I	0	0.39
---	--	------	------
mPD		0.0073	1.1
		nPD:	0.01
		N. weight:	2.
		Sc. PD:	-0.067
		Sc. rank:	-15358.0

,

PB1

Pos.	67	obs:	exp:
aat	N	302	12404.00
aac	N	20670	8568.00
---	--	------	------
mPD		0.028	0.48
		nPD:	0.06
		N. weight:	0.86
		Sc. PD:	0.016
		Sc. rank:	10437.0

,

PB1

Pos.	68	obs:	exp:
cct	P	21	5778.00
ccc	P	0	3890.00
cca	P	146	8930.00
ccg	P	20805	2375.00
---	--	------	------
mPD		0.016	0.70
		nPD:	0.02
		N. weight:	2.2
		Sc. PD:	-0.038
		Sc. rank:	13963.0

,

PB1

Pos.	69	obs:	exp:
att	I	20347	7524.00
atc	I	316	5159.00
ata	I	6	7986.00
act	T	3	0.76
acc	T	0	0.62
aca	T	0	1.37
acg	T	0	0.26
gtt	V	300	66.33
gtc	V	0	54.51
gta	V	0	80.68
gtg	V	0	98.48
---	--	------	------
mPD		0.059	0.69
		nPD:	0.09
		N. weight:	1.
		Sc. PD:	0.045
		Sc. rank:	13690.0

,

PB1

Pos.	70	obs:	exp:
tat	Y	0	0.52
tac	Y	1	0.48
aat	N	0	0.59
aac	N	1	0.41
gat	D	4220	11049.00
gac	D	16750	9921.00
---	--	------	------
mPD		0.32	0.50
		nPD:	0.64
		N. weight:	0.24
		Sc. PD:	0.14
		Sc. rank:	4061.5

,

PB1

Pos.	71	obs:	exp:
ggt	G	1	3180.00
ggc	G	2	3106.00
gga	G	20882	9329.00
ggg	G	87	5357.00
---	--	------	------
mPD		0.0085	0.69
		nPD:	0.01
		N. weight:	0.83
		Sc. PD:	-0.022
		Sc. rank:	480.5

,

PB1

Pos.	72	obs:	exp:
tct	S	0	0.18
tcc	S	0	0.12
tca	S	1	0.26
tcg	S	0	0.05
cct	P	9	5777.00
ccc	P	3	3890.00
cca	P	20929	8929.00
ccg	P	30	2375.00
agt	S	0	0.19
agc	S	0	0.20
---	--	------	------
mPD		0.0041	0.70
		nPD:	0.01
		N. weight:	0.89
		Sc. PD:	-0.03
		Sc. rank:	-7478.0

,

PB1

Pos.	73	obs:	exp:
tta	L	244	2305.00
ttg	L	0	3948.00
ctt	L	0	4132.00
ctc	L	2	2962.00
cta	L	20714	4037.00
ctg	L	11	3586.00
att	I	0	0.36
atc	I	0	0.25
ata	I	1	0.39
---	--	------	------
mPD		0.024	1.1
		nPD:	0.02
		N. weight:	1.7
		Sc. PD:	-0.03
		Sc. rank:	9693.2

,

PB1

Pos.	74	obs:	exp:
cct	P	18931	5777.00
ccc	P	2031	3890.00
cca	P	8	8929.00
ccg	P	1	2375.00
gct	A	1	0.26
gcc	A	0	0.21
gca	A	0	0.47
gcg	A	0	0.06
---	--	------	------
mPD		0.18	0.70
		nPD:	0.25
		N. weight:	1.1
		Sc. PD:	0.22
		Sc. rank:	16902.0

,

PB1

Pos.	75	obs:	exp:
aaa	K	6	5.70
aag	K	4	4.30
gtt	V	0	0.22
gtc	V	0	0.18
gta	V	1	0.27
gtg	V	0	0.33
gat	D	1	1.05
gac	D	1	0.95
gaa	E	16240	12685.00
gag	E	4710	8265.00
ggt	G	0	1.36
ggc	G	0	1.33
gga	G	8	4.00
ggg	G	1	2.30
---	--	------	------
mPD		0.35	0.48
		nPD:	0.73
		N. weight:	0.07
		Sc. PD:	0.047
		Sc. rank:	1188.5

,

PB1

Pos.	76	obs:	exp:
aat	N	9	5.32
aac	N	0	3.68
gat	D	20880	11040.00
gac	D	73	9913.00
ggt	G	9	1.36
ggc	G	0	1.33
gga	G	0	4.00
ggg	G	0	2.30
---	-	1	1.00
---	--	------	------
mPD		0.0087	0.50
		nPD:	0.02
		N. weight:	0.66
		Sc. PD:	-0.014
		Sc. rank:	2402.5

,

PB1

Pos.	77	obs:	exp:
tct	S	0	0.18
tcc	S	0	0.12
tca	S	0	0.26
tcg	S	0	0.05
aat	N	20901	12399.00
aac	N	63	8565.00
agt	S	1	0.19
agc	S	0	0.20
gat	D	6	3.16
gac	D	0	2.84
---	-	1	1.00
---	--	------	------
mPD		0.0067	0.48
		nPD:	0.01
		N. weight:	0.54
		Sc. PD:	-0.014
		Sc. rank:	693.3

,

PB1

Pos.	78	obs:	exp:
aaa	K	4	2.28
aag	K	0	1.72
gtt	V	0	0.44
gtc	V	0	0.36
gta	V	2	0.54
gtg	V	0	0.66
gat	D	0	2.63
gac	D	5	2.37
gaa	E	20904	12692.00
gag	E	57	8269.00
---	--	------	------
mPD		0.0065	0.48
		nPD:	0.01
		N. weight:	0.51
		Sc. PD:	-0.013
		Sc. rank:	604.4

,

PB1

Pos.	79	obs:	exp:
cct	P	4	5777.00
ccc	P	4	3890.00
cca	P	20936	8929.00
ccg	P	27	2375.00
act	T	0	0.25
acc	T	0	0.21
aca	T	1	0.46
acg	T	0	0.09
---	--	------	------
mPD		0.0034	0.70
		nPD:	0.
		N. weight:	0.89
		Sc. PD:	-0.031
		Sc. rank:	-9289.2

,

PB1

Pos.	80	obs:	exp:
tct	S	0	3858.00
tcc	S	0	2428.00
tca	S	0	5455.00
tcg	S	0	1116.00
tgt	C	1	0.45
tgc	C	0	0.55
att	I	8	2.91
atc	I	0	2.00
ata	I	0	3.09
aat	N	1	0.59
aac	N	0	0.41
agt	S	20931	3942.00
agc	S	31	4164.00
---	--	------	------
mPD		0.0039	1.6
		nPD:	0.
		N. weight:	1.8
		Sc. PD:	-0.065
		Sc. rank:	-26234.0

,

PB1

Pos.	81	obs:	exp:
ggt	G	9	3180.00
ggc	G	3	3106.00
gga	G	150	9329.00
ggg	G	20810	5357.00
---	--	------	------
mPD		0.015	0.69
		nPD:	0.02
		N. weight:	1.4
		Sc. PD:	-0.024
		Sc. rank:	8461.5

,

PB1

Pos.	82	obs:	exp:
tat	Y	20951	10896.00
tac	Y	20	10075.00
cat	H	1	0.59
cac	H	0	0.41
---	--	------	------
mPD		0.0020	0.50
		nPD:	0.
		N. weight:	0.69
		Sc. PD:	-0.024
		Sc. rank:	-8515.4

,

PB1

Pos.	83	obs:	exp:
tct	S	0	1.29
tcc	S	0	0.81
tca	S	7	1.82
tcg	S	0	0.37
agt	S	0	1.32
agc	S	0	1.39
gct	A	1	5550.00
gcc	A	2	4339.00
gca	A	20951	9846.00
gcg	A	10	1228.00
ggt	G	0	0.15
ggc	G	0	0.15
gga	G	1	0.44
ggg	G	0	0.26
---	--	------	------
mPD		0.0020	0.66
		nPD:	0.
		N. weight:	0.8
		Sc. PD:	-0.029
		Sc. rank:	-11127.0

,

PB1

Pos.	84	obs:	exp:
caa	Q	20527	11066.00
cag	Q	445	9906.00
---	--	------	------
mPD		0.042	0.50
		nPD:	0.08
		N. weight:	0.57
		Sc. PD:	0.024
		Sc. rank:	7774.8

,

PB1

Pos.	85	obs:	exp:
act	T	3	5287.00
acc	T	0	4302.00
aca	T	20941	9594.00
acg	T	28	1789.00
---	--	------	------
mPD		0.0030	0.68
		nPD:	0.
		N. weight:	0.82
		Sc. PD:	-0.028
		Sc. rank:	-9361.0

,

PB1			
Pos.	86	obs :	exp :
gat	D	206	11049.00
gac	D	20764	9921.00
gaa	E	2	1.21
gag	E	0	0.79
---	--	------	------
mPD		0.020	0.50
		nPD :	0.04
		N. weight :	0.73
		Sc. PD :	-0.00033
		Sc. rank :	7474.0

,

PB1			
Pos.	87	obs :	exp :
tgt	C	20961	9466.00
tgc	C	11	11506.00
---	--	------	------
mPD		0.0010	0.50
		nPD :	0.
		N. weight :	0.84
		Sc. PD :	-0.031
		Sc. rank :	-12790.0

,

PB1			
Pos.	88	obs :	exp :
gtt	V	20273	4637.00
gtc	V	694	3811.00
gta	V	4	5640.00
gtg	V	1	6884.00
---	--	------	------
mPD		0.064	0.74
		nPD :	0.09
		N. weight :	1.5
		Sc. PD :	0.067
		Sc. rank :	20057.0

,

PB1			
Pos.	89	obs :	exp :
tta	L	27	2305.00
ttg	L	0	3948.00
ctt	L	1	4132.00
ctc	L	1	2962.00
cta	L	20890	4037.00
ctg	L	51	3585.00
cct	P	0	0.28
ccc	P	0	0.19
cca	P	1	0.43
ccg	P	0	0.11
att	I	0	0.36
atc	I	0	0.25
ata	I	1	0.39
---	--	------	------
mPD		0.0078	1.1
		nPD :	0.01
		N. weight :	1.7
		Sc. PD :	-0.056
		Sc. rank :	-11524.0

,

PB1			
Pos.	90	obs :	exp :
gaa	E	80	12699.00
gag	E	20892	8273.00
---	--	------	------
mPD		0.0076	0.48
		nPD :	0.02
		N. weight :	0.96
		Sc. PD :	-0.022
		Sc. rank :	2713.6

,

PB1			
Pos.	91	obs :	exp :
tct	S	1	0.18
tcc	S	0	0.12
tca	S	0	0.26
tcg	S	0	0.05
act	T	3	0.76
acc	T	0	0.62
aca	T	0	1.37
acg	T	0	0.26
agt	S	0	0.19
agc	S	0	0.20
gct	A	20883	5551.00
gcc	A	55	4340.00
gca	A	19	9848.00
gcg	A	11	1228.00
---	--	------	------
mPD		0.0085	0.66
		nPD :	0.01
		N. weight :	1.4
		Sc. PD :	-0.037
		Sc. rank :	1110.2

,

PB1			
Pos.	92	obs :	exp :
att	I	0	4.73
atc	I	0	3.25
ata	I	13	5.02
atg	M	20958	20958.00
gtt	V	0	0.22
gtc	V	0	0.18
gta	V	0	0.27
gtg	V	1	0.33
---	--	------	------
mPD		0.0013	0.0014
		nPD :	0.95
		N. weight :	0.00068
		Sc. PD :	0.00061
		Sc. rank :	11.6

,

PB1			
Pos.	93	obs :	exp :
act	T	4	1.01
acc	T	0	0.82
aca	T	0	1.83
acg	T	0	0.34
gtt	V	8	1.77
gtc	V	0	1.45
gta	V	0	2.15
gtg	V	0	2.63
gct	A	20926	5549.00
gcc	A	26	4339.00
gca	A	1	9844.00
gcg	A	7	1228.00
---	--	------	------
mPD		0.0044	0.66
		nPD :	0.01
		N. weight :	1.4
		Sc. PD :	-0.045
		Sc. rank :	-10030.0

,

PB1			
Pos.	94	obs :	exp :
ttt	F	3380	8959.00
ttc	F	17496	11917.00
tta	L	7	9.67
ttg	L	0	16.57
tct	S	0	0.92
tcc	S	5	0.58
tca	S	0	1.30
tcg	S	0	0.27
tat	Y	0	0.52
tac	Y	1	0.48
tgt	C	0	0.45
tgc	C	1	0.55
ctt	L	1	17.34
ctc	L	80	12.43
cta	L	0	16.94
ctg	L	0	15.05
agt	S	0	0.94
agc	S	0	0.99
gtt	V	0	0.22
gtc	V	1	0.18
gta	V	0	0.27
gtg	V	0	0.33
---	--	------	------
mPD		0.28	0.50
		nPD :	0.56
		N. weight :	0.18
		Sc. PD :	0.092
		Sc. rank :	3073.9

,

PB1			
Pos.	95	obs :	exp :
tta	L	0	2306.00
ttg	L	0	3948.00
ctt	L	20948	4133.00
ctc	L	13	2962.00
cta	L	10	4037.00
ctg	L	1	3586.00
---	--	------	------
mPD		0.0023	1.1
		nPD :	0.
		N. weight :	1.7
		Sc. PD :	-0.063
		Sc. rank :	-26348.0

,

PB1			
Pos.	96	obs :	exp :
aaa	K	2	1.14
aag	K	0	0.86
gct	A	0	0.26
gcc	A	0	0.21
gca	A	1	0.47
gcg	A	0	0.06
gat	D	5	3.69
gac	D	2	3.31
gaa	E	20936	12691.00
gag	E	23	8268.00
ggt	G	0	0.45
ggc	G	0	0.44
gga	G	3	1.33
ggg	G	0	0.77
---	--	------	------
mPD		0.0034	0.48
		nPD :	0.01
		N. weight :	0.52
		Sc. PD :	-0.017
		Sc. rank :	-3382.2

,

PB1			
Pos.	97	obs :	exp :
aaa	K	22	12.55
aag	K	0	9.45
gat	D	3	5.27
gac	D	7	4.73
gaa	E	20895	12677.00
gag	E	42	8260.00
ggt	G	0	0.45
ggc	G	0	0.44
gga	G	3	1.33
ggg	G	0	0.77
---	--	------	------
mPD		0.0073	0.48
		nPD :	0.02
		N. weight :	0.52
		Sc. PD :	-0.012
		Sc. rank :	1279.7

,

PB1			
Pos.	98	obs :	exp :
ttt	F	0	0.43
ttc	F	1	0.57
tct	S	95	3858.00
tcc	S	20862	2428.00
tca	S	5	5455.00
tcg	S	0	1116.00
act	T	0	2.27
acc	T	9	1.85
aca	T	0	4.12
acg	T	0	0.77
agt	S	0	3942.00
agc	S	0	4164.00
---	--	------	------
mPD		0.010	1.6
		nPD :	0.01
		N. weight :	2.2
		Sc. PD :	-0.074
		Sc. rank :	-17236.0

,

PB1			
Pos.	99	obs :	exp :
cat	H	16676	12333.00
cac	H	4296	8639.00
---	--	------	------
mPD		0.33	0.48
		nPD :	0.67
		N. weight :	0.1
		Sc. PD :	0.064
		Sc. rank :	1752.0

,

PB1			
Pos.	100	obs :	exp :
tta	L	0	0.33
ttg	L	0	0.56
ctt	L	0	0.59
ctc	L	0	0.42
cta	L	3	0.58
ctg	L	0	0.51
cct	P	6	5777.00
ccc	P	3	3889.00
cca	P	20797	8929.00
ccg	P	163	2374.00
---	--	------	------
mPD		0.017	0.70
		nPD :	0.02
		N. weight :	0.86
		Sc. PD :	-0.014
		Sc. rank :	5538.0

,

PB1			
Pos.	101	obs :	exp :
cgt	R	0	0.08
cgc	R	0	0.16
cga	R	0	0.29
cgg	R	0	0.22
aga	R	3	1.51
agg	R	0	0.74
gaa	E	2	1.21
gag	E	0	0.79
ggt	G	14	3179.00
ggc	G	2	3105.00
gga	G	20907	9327.00
ggg	G	44	5356.00
---	--	------	------
mPD		0.0062	0.69
		nPD :	0.01
		N. weight :	0.84
		Sc. PD :	-0.025
		Sc. rank :	-2653.1

,

PB1			
Pos.	102	obs :	exp :
tta	L	0	0.22
ttg	L	0	0.38
ctt	L	0	0.39
ctc	L	0	0.28
cta	L	2	0.39
ctg	L	0	0.34
att	I	3	7629.00
atc	I	13	5232.00
ata	I	20942	8097.00
atg	M	10	10.00
gtt	V	0	0.44
gtc	V	0	0.36
gta	V	2	0.54
gtg	V	0	0.66
---	--	------	------
mPD		0.0029	0.66
		nPD :	0.
		N. weight :	1.
		Sc. PD :	-0.035
		Sc. rank :	-11475.0

,

PB1			
Pos.	103	obs :	exp :
ttt	F	20944	8998.00
ttc	F	23	11969.00
tat	Y	5	2.60
tac	Y	0	2.40
---	--	------	------
mPD		0.0027	0.49
		nPD :	0.01
		N. weight :	0.89
		Sc. PD :	-0.03
		Sc. rank :	-8020.0

,

PB1			
Pos.	104	obs :	exp :
aaa	K	0	0.57
aag	K	1	0.43
gtt	V	0	0.44
gtc	V	0	0.36
gta	V	0	0.54
gtg	V	2	0.66
gat	D	2	1.05
gac	D	0	0.95
gaa	E	6634	12693.00
gag	E	14329	8270.00
ggt	G	0	0.61
ggc	G	0	0.59
gga	G	1	1.78
ggg	G	3	1.02
---	--	------	------
mPD		0.43	0.48
		nPD :	0.91
		N. weight :	0.18
		Sc. PD :	0.15
		Sc. rank :	3095.7

,

PB1

Pos.	105	obs:	exp:
tct	S	0	2.58
tcc	S	0	1.62
tca	S	0	3.64
tcg	S	0	0.75
cat	H	1	0.59
cac	H	0	0.41
act	T	5	1.26
acc	T	0	1.03
aca	T	0	2.29
acg	T	0	0.43
aat	N	20884	12390.00
aac	N	65	8559.00
agt	S	14	2.63
agc	S	0	2.78
gat	D	3	1.58
gac	D	0	1.42
---	--	------	------
mPD		0.0084	0.49
		nPD:	0.02
		N. weight:	0.54
		Sc. PD:	-0.012
		Sc. rank:	1932.1

,

PB1

Pos.	106	obs:	exp:
tct	S	2	3858.00
tcc	S	19	2428.00
tca	S	20891	5456.00
tcg	S	52	1116.00
cct	P	0	0.28
ccc	P	0	0.19
cca	P	1	0.43
ccg	P	0	0.11
agt	S	0	3942.00
agc	S	0	4164.00
gct	A	0	1.85
gcc	A	0	1.45
gca	A	7	3.29
gcg	A	0	0.41
---	--	------	------
mPD		0.0077	1.6
		nPD:	0.
		N. weight:	1.4
		Sc. PD:	-0.048
		Sc. rank:	-15230.0

,

PB1

Pos.	107	obs:	exp:
tgt	C	49	9465.00
tgc	C	20922	11506.00
cgt	R	0	0.03
cgc	R	1	0.05
cga	R	0	0.10
cgg	R	0	0.07
aga	R	0	0.50
agg	R	0	0.25
---	--	------	------
mPD		0.0048	0.50
		nPD:	0.01
		N. weight:	0.62
		Sc. PD:	-0.018
		Sc. rank:	-1724.0

,

PB1

Pos.	108	obs:	exp:
ttt	F	20	8.58
ttc	F	0	11.42
tta	L	0	2302.00
ttg	L	0	3943.00
ctt	L	20909	4127.00
ctc	L	32	2958.00
cta	L	0	4032.00
ctg	L	0	3580.00
cat	H	1	0.59
cac	H	0	0.41
cgt	R	1	0.03
cgc	R	0	0.05
cga	R	0	0.10
cgg	R	0	0.07
att	I	9	3.28
atc	I	0	2.25
ata	I	0	3.48
aga	R	0	0.50
agg	R	0	0.25
---	--	------	------
mPD		0.0060	1.1
		nPD:	0.01
		N. weight:	1.7
		Sc. PD:	-0.058
		Sc. rank:	-16231.0

,

PB1

Pos.	109	obs:	exp:
gaa	E	20954	12699.00
gag	E	18	8273.00
---	--	------	------
mPD		0.0017	0.48
		nPD:	0.
		N. weight:	0.52
		Sc. PD:	-0.019
		Sc. rank:	-6929.2

,

PB1

Pos.	110	obs:	exp:
att	I	0	1.09
atc	I	0	0.75
ata	I	3	1.16
act	T	0	5284.00
acc	T	1	4300.00
aca	T	20952	9590.00
acg	T	9	1789.00
gct	A	0	1.85
gcc	A	0	1.45
gca	A	7	3.29
gcg	A	0	0.41
---	--	------	------
mPD		0.0019	0.68
		nPD:	0.
		N. weight:	0.83
		Sc. PD:	-0.03
		Sc. rank:	-11768.0

,

PB1

Pos.	111	obs:	exp:
tta	L	0	0.77
ttg	L	2	1.32
ctt	L	0	1.38
ctc	L	0	0.99
cta	L	0	1.35
ctg	L	5	1.20
att	I	7	29.12
atc	I	0	19.97
ata	I	73	30.91
atg	M	20882	20882.00
gtt	V	0	0.66
gtc	V	0	0.55
gta	V	0	0.81
gtg	V	3	0.98
---	--	------	------
mPD		0.0086	0.0092
		nPD:	0.93
		N. weight:	0.0034
		Sc. PD:	0.003
		Sc. rank:	57.9

,

PB1

Pos.	112	obs:	exp:
gat	D	2	3.16
gac	D	4	2.84
gaa	E	20931	12694.00
gag	E	34	8271.00
ggt	G	0	0.15
ggc	G	0	0.15
gga	G	1	0.44
ggg	G	0	0.26
---	--	------	------
mPD		0.0039	0.48
		nPD:	0.01
		N. weight:	0.52
		Sc. PD:	-0.016
		Sc. rank:	-2416.1

,

PB1

Pos.	113	obs:	exp:
ttt	F	12	5.15
ttc	F	0	6.85
tta	L	0	0.11
ttg	L	0	0.19
ctt	L	1	0.20
ctc	L	0	0.14
cta	L	0	0.19
ctg	L	0	0.17
att	I	210	76.45
atc	I	0	52.42
ata	I	0	81.13
gtt	V	20221	4573.00
gtc	V	392	3759.00
gta	V	72	5563.00
gtg	V	1	6790.00
gct	A	52	16.68
gcc	A	11	13.04
gca	A	0	29.59
gcg	A	0	3.69
---	--	------	------
mPD		0.072	0.77
		nPD:	0.09
		N. weight:	1.5
		Sc. PD:	0.077
		Sc. rank:	20764.0

,

PB1

Pos.	114	obs:	exp:
att	I	20	7.28
atc	I	0	4.99
ata	I	0	7.73
gtt	V	20650	4632.00
gtc	V	290	3807.00
gta	V	7	5634.00
gtg	V	3	6877.00
gct	A	2	0.53
gcc	A	0	0.41
gca	A	0	0.94
gcg	A	0	0.12
---	--	------	------
mPD		0.030	0.74
		nPD:	0.04
		N. weight:	1.5
		Sc. PD:	0.0016
		Sc. rank:	15753.0

,

PB1

Pos.	115	obs:	exp:
caa	Q	20951	11066.00
cag	Q	21	9906.00
---	--	------	------
mPD		0.0020	0.50
		nPD:	0.
		N. weight:	0.67
		Sc. PD:	-0.023
		Sc. rank:	-8288.3

,

PB1

Pos.	116	obs:	exp:
caa	Q	20937	11065.00
cag	Q	32	9904.00
aaa	K	2	1.14
aag	K	0	0.86
gaa	E	1	0.61
gag	E	0	0.39
---	--	------	------
mPD		0.0033	0.50
		nPD:	0.01
		N. weight:	0.67
		Sc. PD:	-0.022
		Sc. rank:	-4629.2

,

PB1

Pos.	117	obs:	exp:
act	T	1	5286.00
acc	T	0	4301.00
aca	T	20947	9593.00
acg	T	22	1789.00
gct	A	0	0.53
gcc	A	0	0.41
gca	A	2	0.94
gcg	A	0	0.12
---	--	------	------
mPD		0.0024	0.68
		nPD:	0.
		N. weight:	0.82
		Sc. PD:	-0.029
		Sc. rank:	-10940.0

,

PB1

Pos.	118	obs:	exp:
cgt	R	0	572.60
cgc	R	0	1097.00
cga	R	0	2054.00
cgg	R	2	1535.00
aaa	K	0	0.57
aag	K	1	0.43
aga	R	280	10528.00
agg	R	20689	5184.00
---	--	------	------
mPD		0.027	0.91
		nPD:	0.03
		N. weight:	1.4
		Sc. PD:	-0.014
		Sc. rank:	11255.0

,

PB1

Pos.	119	obs:	exp:
tta	L	1	0.11
ttg	L	0	0.19
ctt	L	0	0.20
ctc	L	0	0.14
cta	L	0	0.19
ctg	L	0	0.17
att	I	0	10.19
atc	I	0	6.99
ata	I	28	10.82
gtt	V	69	4630.00
gtc	V	2	3806.00
gta	V	20824	5632.00
gtg	V	48	6875.00
---	--	------	------
mPD		0.014	0.74
		nPD:	0.02
		N. weight:	1.4
		Sc. PD:	-0.028
		Sc. rank:	6430.3

,

PB1

Pos.	120	obs:	exp:
gat	D	20881	11050.00
gac	D	91	9922.00
---	--	------	------
mPD		0.0086	0.50
		nPD:	0.02
		N. weight:	0.65
		Sc. PD:	-0.014
		Sc. rank:	2429.8

,

PB1

Pos.	121	obs:	exp:
caa	Q	1	0.53
cag	Q	0	0.47
cgt	R	0	0.11
cgc	R	0	0.21
cga	R	0	0.39
cgg	R	0	0.29
aaa	K	20816	11960.00
aag	K	150	9006.00
aga	R	4	2.01
agg	R	0	0.99
gaa	E	1	0.61
gag	E	0	0.39
---	--	------	------
mPD		0.015	0.49
		nPD:	0.03
		N. weight:	0.55
		Sc. PD:	-0.0053
		Sc. rank:	4683.5

,

PB1

Pos.	122	obs:	exp:
tta	L	7	2305.00
ttg	L	15	3948.00
ctt	L	4	4132.00
ctc	L	1	2962.00
cta	L	2946	4037.00
ctg	L	17995	3585.00
atg	M	4	4.00
---	--	------	------
mPD		0.24	1.1
		nPD:	0.21
		N. weight:	1.4
		Sc. PD:	0.24
		Sc. rank:	22012.0

,

PB1

Pos.	123	obs:	exp:
att	I	1	0.36
atc	I	0	0.25
ata	I	0	0.39
act	T	20886	5281.00
acc	T	58	4297.00
aca	T	5	9584.00
acg	T	1	1788.00
gct	A	21	5.56
gcc	A	0	4.35
gca	A	0	9.86
gcg	A	0	1.23
---	--	------	------
mPD		0.0082	0.68
		nPD:	0.01
		N. weight:	1.4
		Sc. PD:	-0.039
		Sc. rank:	390.5

,

PB1

Pos.	124	obs:	exp:
caa	Q	20890	11065.00
cag	Q	79	9904.00
aaa	K	3	1.71
aag	K	0	1.29
---	--	------	------
mPD		0.0078	0.50
		nPD:	0.02
		N. weight:	0.65
		Sc. PD:	-0.015
		Sc. rank:	1760.8

,

PB1

Pos.	125	obs:	exp:
ggt	G	20912	3180.00
ggc	G	51	3106.00
gga	G	8	9329.00
ggg	G	1	5357.00
---	--	------	------
mPD		0.0057	0.69
		nPD:	0.01
		N. weight:	2.
		Sc. PD:	-0.061
		Sc. rank:	-8942.9

,

PB1

Pos.	126	obs:	exp:
tta	L	0	0.11
ttg	L	0	0.19
tgt	C	0	0.45
tgc	C	1	0.55
ctt	L	0	0.20
ctc	L	1	0.14
cta	L	0	0.19
ctg	L	0	0.17
cat	H	0	5.29
cac	H	9	3.71
cgt	R	48	572.30
cgc	R	20898	1096.00
cga	R	12	2053.00
cgg	R	2	1534.00
aga	R	1	10523.00
agg	R	0	5182.00
---	--	------	------
mPD		0.0071	0.91
		nPD:	0.01
		N. weight:	3.1
		Sc. PD:	-0.097
		Sc. rank:	-15613.0

,

PB1

Pos.	127	obs:	exp:
caa	Q	43	11066.00
cag	Q	20929	9906.00
---	--	------	------
mPD		0.0041	0.50
		nPD:	0.01
		N. weight:	0.78
		Sc. PD:	-0.024
		Sc. rank:	-3594.7

,

PB1

Pos.	128	obs:	exp:
act	T	20909	5287.00
acc	T	57	4302.00
aca	T	5	9594.00
acg	T	1	1789.00
---	--	------	------
mPD		0.0060	0.68
		nPD:	0.01
		N. weight:	1.4
		Sc. PD:	-0.044
		Sc. rank:	-4889.8

,

PB1

Pos.	129	obs:	exp:
tat	Y	20398	10896.00
tac	Y	573	10075.00
tgt	C	1	0.45
tgc	C	0	0.55
---	--	------	------
mPD		0.053	0.50
		nPD:	0.11
		N. weight:	0.56
		Sc. PD:	0.037
		Sc. rank:	8179.1

,

PB1

Pos.	130	obs:	exp:
gat	D	20795	11050.00
gac	D	177	9922.00
---	--	------	------
mPD		0.017	0.50
		nPD:	0.03
		N. weight:	0.63
		Sc. PD:	-0.0039
		Sc. rank:	5908.1

,

PB1

Pos.	131	obs:	exp:
tgg	W	20972	20972.00
---	--	------	------
mPD		0	0
		nPD:	1.
		N. weight:	0.
		Sc. PD:	0
		Sc. rank:	0

,

PB1

Pos.	132	obs:	exp:
act	T	3	5287.00
acc	T	12	4302.00
aca	T	20951	9594.00
acg	T	6	1789.00
---	--	------	------
mPD		0.0020	0.68
		nPD:	0.
		N. weight:	0.82
		Sc. PD:	-0.03
		Sc. rank:	-11561.0

,

PB1

Pos.	133	obs:	exp:
tta	L	20869	2305.00
ttg	L	36	3947.00
ctt	L	0	4132.00
ctc	L	0	2962.00
cta	L	61	4037.00
ctg	L	1	3585.00
att	I	0	0.73
atc	I	0	0.50
ata	I	2	0.77
gtt	V	0	0.66
gtc	V	0	0.55
gta	V	3	0.81
gtg	V	0	0.98
---	--	------	------
mPD		0.0099	1.1
		nPD:	0.01
		N. weight:	2.3
		Sc. PD:	-0.07
		Sc. rank:	-8937.3

,

PB1

Pos.	134	obs:	exp:
tct	S	0	0.18
tcc	S	0	0.12
tca	S	0	0.26
tcg	S	0	0.05
cat	H	0	1.76
cac	H	3	1.24
aat	N	32	12388.00
aac	N	20914	8558.00
agt	S	0	0.19
agc	S	1	0.20
gat	D	0	11.59
gac	D	22	10.41
---	--	------	------
mPD		0.0055	0.49
		nPD:	0.01
		N. weight:	0.94
		Sc. PD:	-0.026
		Sc. rank:	-676.1

PB1

Pos.	135	obs:	exp:
cgt	R	0	572.10
cgc	R	0	1096.00
cga	R	1	2052.00
cgg	R	0	1533.00
aaa	K	19	10.84
aag	K	0	8.16
aga	R	20907	10519.00
agg	R	45	5180.00
---	--	------	------
mPD		0.0062	0.91
		nPD:	0.01
		N. weight:	0.72
		Sc. PD:	-0.023
		Sc. rank:	-4885.7

PB1

Pos.	136	obs:	exp:
tat	Y	1	0.52
tac	Y	0	0.48
aat	N	20737	12403.00
aac	N	234	8568.00
---	--	------	------
mPD		0.022	0.48
		nPD:	0.05
		N. weight:	0.5
		Sc. PD:	0.0029
		Sc. rank:	5465.3

PB1

Pos.	137	obs:	exp:
caa	Q	20939	11066.00
cag	Q	33	9906.00
---	--	------	------
mPD		0.0031	0.50
		nPD:	0.01
		N. weight:	0.67
		Sc. PD:	-0.022
		Sc. rank:	-5167.2

PB1

Pos.	138	obs:	exp:
cct	P	26	5777.00
ccc	P	0	3889.00
cca	P	562	8929.00
ccg	P	20381	2374.00
caa	Q	0	0.53
cag	Q	1	0.47
cgt	R	0	0.03
cgc	R	0	0.05
cga	R	0	0.10
cgg	R	1	0.07
act	T	0	0.25
acc	T	0	0.21
aca	T	0	0.46
acg	T	1	0.09
aga	R	0	0.50
agg	R	0	0.25
---	--	------	------
mPD		0.055	0.70
		nPD:	0.08
		N. weight:	2.1
		Sc. PD:	0.081
		Sc. rank:	28343.0

PB1

Pos.	139	obs:	exp:
tct	S	0	0.18
tcc	S	0	0.12
tca	S	1	0.26
tcg	S	0	0.05
agt	S	0	0.19
agc	S	0	0.20
gct	A	7	5552.00
gcc	A	4	4341.00
gca	A	20946	9849.00
gcg	A	14	1229.00
---	--	------	------
mPD		0.0025	0.66
		nPD:	0.
		N. weight:	0.79
		Sc. PD:	-0.028
		Sc. rank:	-10145.0

PB1

Pos.	140	obs:	exp:
gct	A	15	5552.00
gcc	A	1	4341.00
gca	A	20947	9850.00
gcg	A	9	1229.00
---	--	------	------
mPD		0.0024	0.66
		nPD:	0.
		N. weight:	0.79
		Sc. PD:	-0.028
		Sc. rank:	-10425.0

PB1

Pos.	141	obs:	exp:
act	T	20937	5286.00
acc	T	12	4301.00
aca	T	13	9592.00
acg	T	5	1789.00
aat	N	4	2.37
aac	N	0	1.63
gct	A	1	0.26
gcc	A	0	0.21
gca	A	0	0.47
gcg	A	0	0.06
---	--	------	------
mPD		0.0033	0.68
		nPD:	0.
		N. weight:	1.4
		Sc. PD:	-0.05
		Sc. rank:	-15183.0

PB1

Pos.	142	obs:	exp:
gtt	V	0	0.22
gtc	V	0	0.18
gta	V	1	0.27
gtg	V	0	0.33
gct	A	3	5552.00
gcc	A	2	4341.00
gca	A	20943	9849.00
gcg	A	23	1229.00
---	--	------	------
mPD		0.0028	0.66
		nPD:	0.
		N. weight:	0.79
		Sc. PD:	-0.028
		Sc. rank:	-9490.6

PB1

Pos.	143	obs:	exp:
tta	L	35	2306.00
ttg	L	20866	3948.00
ctt	L	0	4133.00
ctc	L	0	2962.00
cta	L	0	4037.00
ctg	L	71	3586.00
---	--	------	------
mPD		0.010	1.1
		nPD:	0.01
		N. weight:	1.7
		Sc. PD:	-0.053
		Sc. rank:	-5760.0

PB1

Pos.	144	obs:	exp:
tct	S	0	0.37
tcc	S	2	0.23
tca	S	0	0.52
tcg	S	0	0.11
act	T	0	0.25
acc	T	1	0.21
aca	T	0	0.46
acg	T	0	0.09
agt	S	0	0.38
agc	S	0	0.40
gtt	V	0	0.22
gtc	V	1	0.18
gta	V	0	0.27
gtg	V	0	0.33
gct	A	21	5551.00
gcc	A	20937	4340.00
gca	A	10	9848.00
gcg	A	0	1228.00
---	--	------	------
mPD		0.0033	0.66
		nPD:	0.01
		N. weight:	1.7
		Sc. PD:	-0.056
		Sc. rank:	-16834.0

PB1

Pos.	145	obs:	exp:
aat	N	16	12403.00
aac	N	20955	8568.00
gat	D	0	0.53
gac	D	1	0.47
---	--	------	------
mPD		0.0016	0.48
		nPD:	0.
		N. weight:	0.94
		Sc. PD:	-0.034
		Sc. rank:	-12675.0

PB1

Pos.	146	obs:	exp:
act	T	35	5287.00
acc	T	20919	4302.00
aca	T	18	9594.00
acg	T	0	1789.00
---	--	------	------
mPD		0.0050	0.68
		nPD:	0.01
		N. weight:	1.7
		Sc. PD:	-0.053
		Sc. rank:	-10261.0

PB1

Pos.	147	obs:	exp:
att	I	42	7634.00
atc	I	3	5235.00
ata	I	20925	8102.00
gtt	V	0	0.44
gtc	V	0	0.36
gta	V	2	0.54
gtg	V	0	0.66
---	--	------	------
mPD		0.0045	0.66
		nPD:	0.01
		N. weight:	0.99
		Sc. PD:	-0.032
		Sc. rank:	-6728.3

PB1

Pos.	148	obs:	exp:
caa	Q	1	0.53
cag	Q	0	0.47
gaa	E	20939	12698.00
gag	E	32	8273.00
---	--	------	------
mPD		0.0031	0.48
		nPD:	0.01
		N. weight:	0.52
		Sc. PD:	-0.017
		Sc. rank:	-3811.9

PB1

Pos.	149	obs:	exp:
att	I	10	5.46
atc	I	5	3.74
ata	I	0	5.80
gtt	V	16526	4633.00
gtc	V	4415	3808.00
gta	V	10	5636.00
gtg	V	6	6879.00
---	--	------	------
mPD		0.34	0.74
		nPD:	0.45
		N. weight:	1.1
		Sc. PD:	0.44
		Sc. rank:	18141.0

PB1			
Pos.	150	obs:	exp:
ttt	F	7706	8999.00
ttc	F	13265	11972.00
tta	L	1	0.11
ttg	L	0	0.19
ctt	L	0	0.20
ctc	L	0	0.14
cta	L	0	0.19
ctg	L	0	0.17
---	--	------	------
mPD		0.46	0.49
		nPD:	0.95
		N. weight:	0.0087
		Sc. PD:	0.0078
		Sc. rank:	149.1

,

PB1			
Pos.	151	obs:	exp:
cgt	R	1	572.30
cgc	R	0	1096.00
cga	R	269	2053.00
cgg	R	0	1534.00
aaa	K	11	6.27
aag	K	0	4.73
aga	R	20661	10523.00
agg	R	29	5182.00
ggt	G	0	0.15
ggc	G	1	0.15
gga	G	0	0.44
ggg	G	0	0.26
---	--	------	------
mPD		0.030	0.91
		nPD:	0.03
		N. weight:	0.67
		Sc. PD:	-0.0049
		Sc. rank:	5981.0

,

PB1			
Pos.	152	obs:	exp:
tta	L	0	5.17
ttg	L	46	8.85
tct	S	19	3850.00
tcc	S	0	2423.00
tca	S	1033	5445.00
tcg	S	19868	1113.00
ctt	L	0	9.26
ctc	L	0	6.64
cta	L	1	9.05
ctg	L	0	8.04
act	T	0	0.76
acc	T	0	0.62
aca	T	0	1.37
acg	T	3	0.26
agt	S	0	3934.00
agc	S	0	4155.00
gct	A	0	0.53
gcc	A	0	0.41
gca	A	0	0.94
gcg	A	2	0.12
---	--	------	------
mPD		0.10	1.6
		nPD:	0.06
		N. weight:	2.8
		Sc. PD:	0.058
		Sc. rank:	34634.0

,

PB1			
Pos.	153	obs:	exp:
tct	S	0	0.18
tcc	S	0	0.12
tca	S	0	0.26
tcg	S	0	0.05
aat	N	20952	12403.00
aac	N	19	8568.00
agt	S	1	0.19
agc	S	0	0.20
---	--	------	------
mPD		0.0019	0.48
		nPD:	0.
		N. weight:	0.55
		Sc. PD:	-0.019
		Sc. rank:	-6850.2

,

PB1			
Pos.	154	obs:	exp:
tct	S	0	0.18
tcc	S	0	0.12
tca	S	0	0.26
tcg	S	0	0.05
tat	Y	0	3.64
tac	Y	7	3.36
act	T	0	0.50
acc	T	2	0.41
aca	T	0	0.91
acg	T	0	0.17
aat	N	0	194.60
aac	N	329	134.40
agt	S	0	0.19
agc	S	1	0.20
gat	D	73	9218.00
gac	D	17422	8277.00
gaa	E	3	2.42
gag	E	1	1.58
ggt	G	16	475.20
ggc	G	3105	464.20
gga	G	13	1394.00
ggg	G	0	800.50
---	--	------	------
mPD		0.30	0.88
		nPD:	0.34
		N. weight:	0.95
		Sc. PD:	0.28
		Sc. rank:	15317.0

,

PB1			
Pos.	155	obs:	exp:
tta	L	35	2305.00
ttg	L	0	3947.00
ctt	L	1	4131.00
ctc	L	1	2961.00
cta	L	20900	4036.00
ctg	L	27	3584.00
att	I	0	2.91
atc	I	0	2.00
ata	I	8	3.09
---	--	------	------
mPD		0.0069	1.1
		nPD:	0.01
		N. weight:	1.7
		Sc. PD:	-0.057
		Sc. rank:	-13887.0

,

PB1			
Pos.	156	obs:	exp:
cgt	R	0	0.08
cgc	R	0	0.16
cga	R	0	0.29
cgg	R	0	0.22
att	I	0	4.73
atc	I	0	3.25
ata	I	13	5.02
act	T	2	5280.00
acc	T	1	4296.00
aca	T	20920	9581.00
acg	T	21	1787.00
aaa	K	9	5.13
aag	K	0	3.87
aga	R	3	1.51
agg	R	0	0.74
gct	A	0	0.79
gcc	A	0	0.62
gca	A	3	1.41
gcg	A	0	0.18
---	--	------	------
mPD		0.0050	0.68
		nPD:	0.01
		N. weight:	0.82
		Sc. PD:	-0.026
		Sc. rank:	-5191.6

,

PB1			
Pos.	157	obs:	exp:
tct	S	4	0.74
tcc	S	0	0.46
tca	S	0	1.04
tcg	S	0	0.21
act	T	14	3.53
acc	T	0	2.87
aca	T	0	6.40
acg	T	0	1.19
agt	S	0	0.75
agc	S	0	0.79
gtt	V	3	0.66
gtc	V	0	0.55
gta	V	0	0.81
gtg	V	0	0.98
gct	A	20840	5546.00
gcc	A	90	4336.00
gca	A	10	9839.00
gcg	A	9	1227.00
gat	D	2	1.05
gac	D	0	0.95
---	--	------	------
mPD		0.013	0.67
		nPD:	0.02
		N. weight:	1.4
		Sc. PD:	-0.028
		Sc. rank:	6453.2

,

PB1			
Pos.	158	obs:	exp:
tct	S	0	2.39
tcc	S	0	1.51
tca	S	0	3.38
tcg	S	0	0.69
aat	N	3149	12395.00
aac	N	17809	8563.00
aaa	K	0	0.57
aag	K	1	0.43
agt	S	1	2.44
agc	S	12	2.58
---	--	------	------
mPD		0.26	0.49
		nPD:	0.53
		N. weight:	0.44
		Sc. PD:	0.21
		Sc. rank:	7477.8

,

PB1			
Pos.	159	obs:	exp:
aaa	K	0	3.99
aag	K	7	3.01
gat	D	1	0.53
gac	D	0	0.47
gaa	E	136	12693.00
gag	E	20827	8270.00
ggt	G	0	0.15
ggc	G	0	0.15
gga	G	0	0.44
ggg	G	1	0.26
---	--	------	------
mPD		0.014	0.48
		nPD:	0.03
		N. weight:	0.94
		Sc. PD:	-0.01
		Sc. rank:	7410.2

,

PB1			
Pos.	160	obs:	exp:
tct	S	30	3859.00
tcc	S	0	2429.00
tca	S	20936	5458.00
tcg	S	5	1116.00
act	T	0	0.25
acc	T	0	0.21
aca	T	1	0.46
acg	T	0	0.09
agt	S	0	3943.00
agc	S	0	4166.00
---	--	------	------
mPD		0.0034	1.6
		nPD:	0.
		N. weight:	1.4
		Sc. PD:	-0.052
		Sc. rank:	-21625.0

,

PB1			
Pos.	161	obs:	exp:
ggt	G	2	3180.00
ggc	G	10	3106.00
gga	G	20910	9329.00
ggg	G	50	5357.00
---	--	------	------
mPD		0.0059	0.69
		nPD:	0.01
		N. weight:	0.84
		Sc. PD:	-0.026
		Sc. rank:	-3499.2

,

PB1			
Pos.	162	obs:	exp:
cgt	R	0	572.60
cgc	R	0	1097.00
cga	R	0	2054.00
cgg	R	0	1535.00
aga	R	148	10529.00
agg	R	20824	5185.00
---	--	------	------
mPD		0.014	0.91
		nPD:	0.02
		N. weight:	1.4
		Sc. PD:	-0.034
		Sc. rank:	3684.4

,

PB1

Pos.	163	obs:	exp:
tta	L	75	2306.00
ttg	L	0	3948.00
ctt	L	2	4133.00
ctc	L	0	2962.00
cta	L	20860	4037.00
ctg	L	35	3586.00
---	--	------	------
mPD		0.011	1.1
		nPD:	0.01
		N. weight:	1.7
		Sc. PD:	-0.051
		Sc. rank:	-4955.1

PB1

Pos.	164	obs:	exp:
tta	L	1	0.22
ttg	L	0	0.38
ctt	L	0	0.39
ctc	L	0	0.28
cta	L	1	0.39
ctg	L	0	0.34
att	I	0	7626.00
atc	I	1	5230.00
ata	I	20949	8094.00
gtt	V	0	4.42
gtc	V	0	3.63
gta	V	20	5.38
gtg	V	0	6.57
---	--	------	------
mPD		0.0022	0.66
		nPD:	0.
		N. weight:	1.
		Sc. PD:	-0.036
		Sc. rank:	-13652.0

PB1

Pos.	165	obs:	exp:
tat	Y	1	0.52
tac	Y	0	0.48
gat	D	20921	11050.00
gac	D	50	9921.00
---	--	------	------
mPD		0.0049	0.50
		nPD:	0.01
		N. weight:	0.66
		Sc. PD:	-0.02
		Sc. rank:	-1704.3

PB1

Pos.	166	obs:	exp:
ttt	F	53	8997.00
ttc	F	20913	11969.00
tta	L	3	0.55
ttg	L	0	0.94
tct	S	0	0.18
tcc	S	1	0.12
tca	S	0	0.26
tcg	S	0	0.05
ctt	L	0	0.99
ctc	L	2	0.71
cta	L	0	0.96
ctg	L	0	0.85
agt	S	0	0.19
agc	S	0	0.20
---	--	------	------
mPD		0.0056	0.49
		nPD:	0.01
		N. weight:	0.58
		Sc. PD:	-0.016
		Sc. rank:	-266.1

PB1

Pos.	167	obs:	exp:
tta	L	20839	2306.00
ttg	L	102	3948.00
ctt	L	1	4133.00
ctc	L	10	2962.00
cta	L	20	4037.00
ctg	L	0	3586.00
---	--	------	------
mPD		0.014	1.1
		nPD:	0.01
		N. weight:	2.3
		Sc. PD:	-0.062
		Sc. rank:	393.4

PB1

Pos.	168	obs:	exp:
cgt	R	0	0.11
cgc	R	0	0.21
cga	R	0	0.39
cgg	R	0	0.29
aaa	K	210	11961.00
aag	K	20758	9007.00
aga	R	0	2.01
agg	R	4	0.99
---	--	------	------
mPD		0.020	0.49
		nPD:	0.04
		N. weight:	0.83
		Sc. PD:	0.0011
		Sc. rank:	8752.9

PB1

Pos.	169	obs:	exp:
gat	D	20939	11050.00
gac	D	33	9922.00
---	--	------	------
mPD		0.0031	0.50
		nPD:	0.01
		N. weight:	0.67
		Sc. PD:	-0.022
		Sc. rank:	-5199.2

PB1

Pos.	170	obs:	exp:
att	I	0	0.36
atc	I	0	0.25
ata	I	1	0.39
gtt	V	7	4636.00
gtc	V	0	3811.00
gta	V	4255	5640.00
gtg	V	16709	6884.00
---	--	------	------
mPD		0.32	0.74
		nPD:	0.44
		N. weight:	0.69
		Sc. PD:	0.27
		Sc. rank:	11382.0

PB1

Pos.	171	obs:	exp:
tta	L	0	0.33
ttg	L	2	0.56
ctt	L	0	0.59
ctc	L	0	0.42
cta	L	0	0.58
ctg	L	1	0.51
att	I	3	8.01
atc	I	0	5.49
ata	I	19	8.50
atg	M	20925	20925.00
act	T	0	0.25
acc	T	0	0.21
aca	T	0	0.46
acg	T	1	0.09
gtt	V	0	4.64
gtc	V	0	3.82
gta	V	0	5.65
gtg	V	21	6.89
---	--	------	------
mPD		0.0045	0.0061
		nPD:	0.74
		N. weight:	0.0022
		Sc. PD:	0.0015
		Sc. rank:	37.6

PB1

Pos.	172	obs:	exp:
aaa	K	12	6.85
aag	K	0	5.15
gat	D	0	2.63
gac	D	5	2.37
gaa	E	20877	12682.00
gag	E	67	8262.00
ggt	G	0	1.67
ggc	G	0	1.63
gga	G	11	4.89
ggg	G	0	2.81
---	--	------	------
mPD		0.0090	0.48
		nPD:	0.02
		N. weight:	0.51
		Sc. PD:	-0.011
		Sc. rank:	2369.4

PB1

Pos.	173	obs:	exp:
tct	S	94	3859.00
tcc	S	0	2429.00
tca	S	20864	5458.00
tcg	S	12	1116.00
cct	P	0	0.28
ccc	P	0	0.19
cca	P	1	0.43
ccg	P	0	0.11
agt	S	0	3943.00
agc	S	0	4165.00
gct	A	0	0.26
gcc	A	0	0.21
gca	A	1	0.47
gcg	A	0	0.06
---	--	------	------
mPD		0.010	1.6
		nPD:	0.01
		N. weight:	1.4
		Sc. PD:	-0.046
		Sc. rank:	-10908.0

PB1

Pos.	174	obs:	exp:
tta	L	0	0.22
ttg	L	2	0.38
ctt	L	0	0.39
ctc	L	0	0.28
cta	L	0	0.39
ctg	L	0	0.34
att	I	0	1.46
atc	I	0	1.00
ata	I	4	1.55
atg	M	20447	20447.00
act	T	0	2.52
acc	T	0	2.05
aca	T	0	4.57
acg	T	10	0.85
gtt	V	0	112.50
gtc	V	0	92.49
gta	V	0	136.90
gtg	V	509	167.10
---	--	------	------
mPD		0.049	0.082
		nPD:	0.6
		N. weight:	0.03
		Sc. PD:	0.016
		Sc. rank:	514.8

PB1

Pos.	175	obs:	exp:
tct	S	0	2.58
tcc	S	0	1.62
tca	S	0	3.64
tcg	S	0	0.75
act	T	0	0.25
acc	T	1	0.21
aca	T	0	0.46
acg	T	0	0.09
aat	N	43	12136.00
aac	N	20477	8384.00
aaa	K	2	1.14
aag	K	0	0.86
agt	S	0	2.63
agc	S	14	2.78
gat	D	13	229.20
gac	D	422	205.80
---	--	------	------
mPD		0.048	0.53
		nPD:	0.09
		N. weight:	0.93
		Sc. PD:	0.046
		Sc. rank:	12922.0

PB1

Pos.	176	obs:	exp:
caa	Q	1	0.53
cag	Q	0	0.47
cgt	R	0	0.82
cgc	R	0	1.57
cga	R	0	2.94
cgg	R	0	2.20
aaa	K	20888	11945.00
aag	K	53	8996.00
aga	R	30	15.06
agg	R	0	7.42
---	--	------	------
mPD		0.0080	0.49
		nPD:	0.02
		N. weight:	0.58
		Sc. PD:	-0.013
		Sc. rank:	1686.9

PB1

Pos.	177	obs:	exp:
aaa	K	1	7.42
aag	K	12	5.58
gct	A	0	0.26
gcc	A	0	0.21
gca	A	0	0.47
gcg	A	1	0.06
gat	D	19	10.01
gac	D	0	8.99
gaa	E	201	12677.00
gag	E	20736	8260.00
ggt	G	0	0.30
ggc	G	1	0.30
gga	G	0	0.89
ggg	G	1	0.51
---	--	------	------
mPD		0.022	0.48
		nPD:	0.05
		N. weight:	0.92
		Sc. PD:	0.0063
		Sc. rank:	10492.0

PB1

Pos.	178	obs:	exp:
aaa	K	14	7.99
aag	K	0	6.01
gat	D	3	1.58
gac	D	0	1.42
gaa	E	20901	12683.00
gag	E	45	8263.00
ggt	G	0	1.36
ggc	G	0	1.33
gga	G	9	4.00
ggg	G	0	2.30
---	--	------	------
mPD		0.0068	0.48
		nPD:	0.01
		N. weight:	0.52
		Sc. PD:	-0.013
		Sc. rank:	794.0

PB1

Pos.	179	obs:	exp:
tta	L	0	0.11
ttg	L	0	0.19
ctt	L	0	0.20
ctc	L	0	0.14
cta	L	1	0.19
ctg	L	0	0.17
att	I	10	7609.00
atc	I	0	5218.00
ata	I	20892	8076.00
atg	M	61	61.00
gtt	V	0	1.77
gtc	V	0	1.45
gta	V	8	2.15
gtg	V	0	2.63
---	--	------	------
mPD		0.0076	0.66
		nPD:	0.01
		N. weight:	1.
		Sc. PD:	-0.028
		Sc. rank:	-326.7

PB1

Pos.	180	obs:	exp:
aaa	K	0	2.28
aag	K	4	1.72
gat	D	5	2.63
gac	D	0	2.37
gaa	E	129	12693.00
gag	E	20833	8269.00
ggt	G	0	0.15
ggc	G	0	0.15
gga	G	0	0.44
ggg	G	1	0.26
---	--	------	------
mPD		0.013	0.48
		nPD:	0.03
		N. weight:	0.94
		Sc. PD:	-0.011
		Sc. rank:	7260.6

PB1

Pos.	181	obs:	exp:
att	I	0	7630.00
atc	I	4	5232.00
ata	I	20955	8098.00
atg	M	4	4.00
gtt	V	0	1.99
gtc	V	1	1.64
gta	V	8	2.42
gtg	V	0	2.95
---	--	------	------
mPD		0.0017	0.66
		nPD:	0.
		N. weight:	1.
		Sc. PD:	-0.037
		Sc. rank:	-14681.0

PB1

Pos.	182	obs:	exp:
cct	P	0	0.28
ccc	P	0	0.19
cca	P	1	0.43
ccg	P	0	0.11
att	I	0	10.19
atc	I	0	6.99
ata	I	28	10.82
act	T	1	5279.00
acc	T	1	4296.00
aca	T	20826	9581.00
acg	T	115	1787.00
---	--	------	------
mPD		0.014	0.68
		nPD:	0.02
		N. weight:	0.8
		Sc. PD:	-0.015
		Sc. rank:	4293.6

PB1

Pos.	183	obs:	exp:
att	I	0	1.09
atc	I	3	0.75
ata	I	0	1.16
act	T	25	5286.00
acc	T	20861	4301.00
aca	T	82	9592.00
acg	T	0	1789.00
gct	A	0	0.26
gcc	A	1	0.21
gca	A	0	0.47
gcg	A	0	0.06
---	--	------	------
mPD		0.011	0.68
		nPD:	0.02
		N. weight:	1.6
		Sc. PD:	-0.039
		Sc. rank:	4378.6

PB1

Pos.	184	obs:	exp:
tat	Y	0	0.52
tac	Y	1	0.48
cat	H	74	12333.00
cac	H	20897	8638.00
---	--	------	------
mPD		0.0071	0.48
		nPD:	0.01
		N. weight:	0.91
		Sc. PD:	-0.022
		Sc. rank:	1954.4

PB1

Pos.	185	obs:	exp:
ttt	F	20852	9000.00
ttc	F	120	11972.00
---	--	------	------
mPD		0.011	0.49
		nPD:	0.02
		N. weight:	0.86
		Sc. PD:	-0.014
		Sc. rank:	5433.6

PB1

Pos.	186	obs:	exp:
caa	Q	20947	11066.00
cag	Q	25	9906.00
---	--	------	------
mPD		0.0024	0.50
		nPD:	0.
		N. weight:	0.67
		Sc. PD:	-0.023
		Sc. rank:	-7164.4

PB1

Pos.	187	obs:	exp:
cgt	R	0	572.60
cgc	R	0	1097.00
cga	R	0	2054.00
cgg	R	0	1535.00
aaa	K	1	0.57
aag	K	0	0.43
aga	R	20715	10528.00
agg	R	256	5184.00
---	--	------	------
mPD		0.024	0.91
		nPD:	0.03
		N. weight:	0.67
		Sc. PD:	-0.0087
		Sc. rank:	5057.3

PB1

Pos.	188	obs:	exp:
aaa	K	20383	11963.00
aag	K	589	9009.00
---	--	------	------
mPD		0.055	0.49
		nPD:	0.11
		N. weight:	0.47
		Sc. PD:	0.033
		Sc. rank:	6859.0

PB1

Pos.	189	obs:	exp:
cgt	R	0	572.40
cgc	R	0	1097.00
cga	R	0	2053.00
cgg	R	0	1534.00
aaa	K	0	2.85
aag	K	5	2.15
aga	R	95	10525.00
agg	R	20870	5183.00
ggt	G	0	0.30
ggc	G	0	0.30
gga	G	0	0.89
ggg	G	2	0.51
---	--	------	------
mPD		0.0097	0.91
		nPD:	0.01
		N. weight:	1.4
		Sc. PD:	-0.041
		Sc. rank:	-2136.0

PB1

Pos.	190	obs:	exp:
cgt	R	0	572.30
cgc	R	0	1096.00
cga	R	0	2053.00
cgg	R	0	1534.00
aaa	K	13	7.42
aag	K	0	5.58
aga	R	20874	10522.00
agg	R	85	5181.00
---	--	------	------
mPD		0.0093	0.91
		nPD:	0.01
		N. weight:	0.71
		Sc. PD:	-0.02
		Sc. rank:	-1384.0

PB1

Pos.	191	obs:	exp:
tta	L	6	0.66
ttg	L	0	1.13
ctt	L	0	1.18
ctc	L	0	0.85
cta	L	0	1.16
ctg	L	0	1.03
att	I	0	28.03
atc	I	0	19.22
ata	I	77	29.75
gtt	V	6	4618.00
gtc	V	1	3795.00
gta	V	20846	5617.00
gtg	V	34	6856.00
gct	A	0	0.53
gcc	A	0	0.41
gca	A	2	0.94
gcg	A	0	0.12
---	--	------	------
mPD		0.012	0.75
		nPD:	0.02
		N. weight:	1.4
		Sc. PD:	-0.032
		Sc. rank:	3980.3

PB1

Pos.	192	obs:	exp:
cgt	R	0	572.30
cgc	R	0	1097.00
cga	R	3	2053.00
cgg	R	0	1534.00
att	I	0	0.36
atc	I	0	0.25
ata	I	1	0.39
aaa	K	9	5.13
aag	K	0	3.87
aga	R	20844	10524.00
agg	R	115	5182.00
---	--	------	------
mPD		0.012	0.91
		nPD:	0.01
		N. weight:	0.7
		Sc. PD:	-0.018
		Sc. rank:	779.1

PB1

Pos.	193	obs:	exp:
aat	N	0	0.59
aac	N	1	0.41
gat	D	245	11050.00
gac	D	20726	9921.00
---	--	------	------
mPD		0.023	0.50
		nPD:	0.05
		N. weight:	0.72
		Sc. PD:	0.0047
		Sc. rank:	8076.4

PB1

Pos.	194	obs:	exp:
tct	S	0	5.15
tcc	S	0	3.24
tca	S	0	7.29
tcg	S	0	1.49
tat	Y	0	0.52
tac	Y	1	0.48
act	T	0	0.25
acc	T	1	0.21
aca	T	0	0.46
acg	T	0	0.09
aat	N	157	12384.00
aac	N	20781	8554.00
aaa	K	4	2.28
aag	K	0	1.72
agt	S	0	5.26
agc	S	28	5.56
---	--	------	------
mPD		0.018	0.49
		nPD:	0.04
		N. weight:	0.9
		Sc. PD:	-0.0025
		Sc. rank:	8958.7

PB1

Pos.	195	obs:	exp:
tta	L	0	0.88
ttg	L	5	1.51
ctt	L	0	1.58
ctc	L	0	1.13
cta	L	0	1.54
ctg	L	3	1.37
cgt	R	0	0.25
cgc	R	0	0.47
cga	R	0	0.88
cgg	R	0	0.66
att	I	5	8.37
atc	I	0	5.74
ata	I	18	8.89
atg	M	20882	20882.00
act	T	1	9.58
acc	T	0	7.79
aca	T	0	17.38
acg	T	37	3.24
aga	R	0	4.52
agg	R	9	2.22
gtt	V	0	2.65
gtc	V	0	2.18
gta	V	1	3.23
gtg	V	11	3.94
---	--	------	------
mPD		0.0088	0.014
		nPD:	0.63
		N. weight:	0.0068
		Sc. PD:	0.0039
		Sc. rank:	115.1

PB1

Pos.	196	obs:	exp:
tct	S	0	0.18
tcc	S	1	0.12
tca	S	0	0.26
tcg	S	0	0.05
act	T	38	5287.00
acc	T	20898	4301.00
aca	T	35	9594.00
acg	T	0	1789.00
agt	S	0	0.19
agc	S	0	0.20
---	--	------	------
mPD		0.0070	0.68
		nPD:	0.01
		N. weight:	1.7
		Sc. PD:	-0.048
		Sc. rank:	-2966.1

,

PB1

Pos.	197	obs:	exp:
cgt	R	0	0.44
cgc	R	0	0.84
cga	R	0	1.57
cgg	R	0	1.17
aat	N	2	1.77
aac	N	1	1.23
aaa	K	115	11952.00
aag	K	20838	9001.00
aga	R	0	8.03
agg	R	16	3.96
---	--	------	------
mPD		0.013	0.49
		nPD:	0.03
		N. weight:	0.86
		Sc. PD:	-0.012
		Sc. rank:	6163.5

,

PB1

Pos.	198	obs:	exp:
cgt	R	0	0.30
cgc	R	0	0.58
cga	R	0	1.08
cgg	R	0	0.81
aat	N	1	0.59
aac	N	0	0.41
aaa	K	74	11956.00
aag	K	20886	9004.00
aga	R	0	5.52
agg	R	11	2.72
---	--	------	------
mPD		0.0082	0.49
		nPD:	0.02
		N. weight:	0.87
		Sc. PD:	-0.02
		Sc. rank:	2795.6

,

PB1

Pos.	199	obs:	exp:
att	I	1	2.55
atc	I	0	1.75
ata	I	6	2.70
atg	M	20963	20963.00
gtt	V	0	0.44
gtc	V	0	0.36
gta	V	0	0.54
gtg	V	2	0.66
---	--	------	------
mPD		0.00086	0.00099
		nPD:	0.87
		N. weight:	0.00035
		Sc. PD:	0.00029
		Sc. rank:	6.0

,

PB1

Pos.	200	obs:	exp:
ttt	F	0	0.43
ttc	F	1	0.57
att	I	3	2449.00
atc	I	6702	1679.00
ata	I	23	2599.00
act	T	0	0.25
acc	T	1	0.21
aca	T	0	0.46
acg	T	0	0.09
gtt	V	1	3149.00
gtc	V	14211	2588.00
gta	V	29	3830.00
gtg	V	1	4675.00
---	--	------	------
mPD		0.44	1.2
		nPD:	0.37
		N. weight:	1.7
		Sc. PD:	0.55
		Sc. rank:	27463.0

,

PB1

Pos.	201	obs:	exp:
atg	M	4	4.00
act	T	164	5286.00
acc	T	1	4301.00
aca	T	367	9592.00
acg	T	20436	1789.00
---	--	------	------
mPD		0.050	0.68
		nPD:	0.07
		N. weight:	2.4
		Sc. PD:	0.081
		Sc. rank:	31648.0

,

PB1

Pos.	202	obs:	exp:
tta	L	0	0.22
ttg	L	0	0.38
ctt	L	0	0.39
ctc	L	0	0.28
cta	L	2	0.39
ctg	L	0	0.34
cat	H	1	1.18
cac	H	1	0.82
caa	Q	20935	11064.00
cag	Q	32	9903.00
aaa	K	1	0.57
aag	K	0	0.43
---	--	------	------
mPD		0.0035	0.50
		nPD:	0.01
		N. weight:	0.67
		Sc. PD:	-0.021
		Sc. rank:	-4344.9

,

PB1

Pos.	203	obs:	exp:
cgt	R	0	572.60
cgc	R	0	1097.00
cga	R	3	2054.00
cgg	R	0	1535.00
aaa	K	0	0.57
aag	K	1	0.43
aga	R	20924	10528.00
agg	R	44	5184.00
---	--	------	------
mPD		0.0047	0.91
		nPD:	0.01
		N. weight:	0.71
		Sc. PD:	-0.024
		Sc. rank:	-6958.7

,

PB1

Pos.	204	obs:	exp:
att	I	0	1.09
atc	I	0	0.75
ata	I	3	1.16
act	T	9	5286.00
acc	T	0	4301.00
aca	T	20958	9593.00
acg	T	2	1789.00
---	--	------	------
mPD		0.0013	0.68
		nPD:	0.
		N. weight:	0.82
		Sc. PD:	-0.031
		Sc. rank:	-12755.0

,

PB1

Pos.	205	obs:	exp:
tta	L	0	0.22
ttg	L	0	0.38
ctt	L	0	0.39
ctc	L	0	0.28
cta	L	2	0.39
ctg	L	0	0.34
att	I	3	7627.00
atc	I	1	5230.00
ata	I	20949	8095.00
atg	M	6	6.00
act	T	0	0.25
acc	T	0	0.21
aca	T	1	0.46
acg	T	0	0.09
gtt	V	0	2.21
gtc	V	0	1.82
gta	V	10	2.69
gtg	V	0	3.28
---	--	------	------
mPD		0.0022	0.66
		nPD:	0.
		N. weight:	1.
		Sc. PD:	-0.036
		Sc. rank:	-13580.0

,

PB1

Pos.	206	obs:	exp:
cgt	R	0	0.08
cgc	R	0	0.16
cga	R	0	0.29
cgg	R	0	0.22
aga	R	0	1.51
agg	R	3	0.74
ggt	G	21	3180.00
ggc	G	4	3106.00
gga	G	590	9327.00
ggg	G	20354	5356.00
---	--	------	------
mPD		0.057	0.69
		nPD:	0.08
		N. weight:	1.3
		Sc. PD:	0.054
		Sc. rank:	17451.0

,

PB1

Pos.	207	obs:	exp:
cgt	R	0	0.11
cgc	R	0	0.21
cga	R	0	0.39
cgg	R	0	0.29
aaa	K	1	11959.00
aag	K	20964	9006.00
aga	R	0	2.01
agg	R	4	0.99
gaa	E	0	1.82
gag	E	3	1.18
---	--	------	------
mPD		0.00076	0.49
		nPD:	0.
		N. weight:	0.9
		Sc. PD:	-0.034
		Sc. rank:	-14395.0

,

PB1

Pos.	208	obs:	exp:
caa	Q	4	2.11
cag	Q	0	1.89
cgt	R	0	0.11
cgc	R	0	0.21
cga	R	0	0.39
cgg	R	0	0.29
aaa	K	20522	11958.00
aag	K	442	9006.00
aga	R	4	2.01
agg	R	0	0.99
---	--	------	------
mPD		0.042	0.49
		nPD:	0.09
		N. weight:	0.49
		Sc. PD:	0.022
		Sc. rank:	6766.4

,

PB1

Pos.	209	obs:	exp:
cgt	R	0	0.33
cgc	R	0	0.63
cga	R	0	1.18
cgg	R	0	0.88
aat	N	0	0.59
aac	N	1	0.41
aaa	K	20778	11956.00
aag	K	181	9003.00
aga	R	12	6.02
agg	R	0	2.97
---	--	------	------
mPD		0.018	0.49
		nPD:	0.04
		N. weight:	0.55
		Sc. PD:	-0.0013
		Sc. rank:	5476.5

,

PB1

Pos.	210	obs:	exp:
tta	L	0	2.86
ttg	L	0	4.90
ctt	L	0	5.12
ctc	L	0	3.67
cta	L	1	5.01
ctg	L	25	4.45
cat	H	6	3.53
cac	H	0	2.47
caa	Q	2962	11048.00
cag	Q	17976	9890.00
cgt	R	0	0.03
cgc	R	0	0.05
cga	R	1	0.10
cgg	R	0	0.07
aaa	K	0	0.57
aag	K	1	0.43
aga	R	0	0.50
agg	R	0	0.25
---	--	------	------
mPD		0.25	0.50
		nPD:	0.49
		N. weight:	0.35
		Sc. PD:	0.15
		Sc. rank:	5830.4

,

PB1

Pos.	211	obs:	exp:
tct	S	0	0.37
tcc	S	0	0.23
tca	S	0	0.52
tcg	S	0	0.11
cgt	R	0	562.20
cgc	R	0	1077.00
cga	R	0	2017.00
cgg	R	0	1507.00
att	I	0	0.36
atc	I	0	0.25
ata	I	1	0.39
aaa	K	362	212.20
aag	K	10	159.80
agt	S	0	0.38
agc	S	2	0.40
aga	R	19922	10337.00
agg	R	669	5090.00
gtt	V	0	0.22
gtc	V	0	0.18
gta	V	1	0.27
gtg	V	0	0.33
ggt	G	0	0.76
ggc	G	0	0.74
gga	G	5	2.22
ggg	G	0	1.28
---	--	------	------
mPD		0.098	0.94
		nPD:	0.1
		N. weight:	0.6
		Sc. PD:	0.038
		Sc. rank:	8625.6

,

PB1

Pos.	212	obs:	exp:
tta	L	1	2304.00
ttg	L	170	3945.00
ctt	L	18	4129.00
ctc	L	0	2960.00
cta	L	41	4034.00
ctg	L	20725	3583.00
atg	M	8	8.00
gtt	V	0	1.99
gtc	V	0	1.64
gta	V	0	2.42
gtg	V	9	2.95
---	--	------	------
mPD		0.023	1.1
		nPD:	0.02
		N. weight:	1.8
		Sc. PD:	-0.034
		Sc. rank:	10103.0

,

PB1

Pos.	213	obs:	exp:
tct	S	0	10.31
tcc	S	0	6.49
tca	S	0	14.57
tcg	S	0	2.98
aat	N	20723	12356.00
aac	N	169	8536.00
aaa	K	0	0.57
aag	K	1	0.43
agt	S	56	10.53
agc	S	0	11.12
gat	D	20	12.12
gac	D	3	10.88
---	--	------	------
mPD		0.024	0.50
		nPD:	0.05
		N. weight:	0.51
		Sc. PD:	0.0042
		Sc. rank:	5874.1

,

PB1

Pos.	214	obs:	exp:
cgt	R	0	0.41
cgc	R	0	0.78
cga	R	0	1.47
cgg	R	0	1.10
aaa	K	95	11955.00
aag	K	20862	9002.00
aga	R	0	7.53
agg	R	15	3.71
---	--	------	------
mPD		0.010	0.49
		nPD:	0.02
		N. weight:	0.86
		Sc. PD:	-0.016
		Sc. rank:	4918.3

,

PB1

Pos.	215	obs:	exp:
cgt	R	0	568.40
cgc	R	0	1089.00
cga	R	2	2039.00
cgg	R	0	1523.00
aaa	K	153	87.85
aag	K	1	66.15
aga	R	20767	10451.00
agg	R	48	5146.00
ggt	G	0	0.15
ggc	G	0	0.15
gga	G	1	0.44
ggg	G	0	0.26
---	--	------	------
mPD		0.020	0.92
		nPD:	0.02
		N. weight:	0.71
		Sc. PD:	-0.013
		Sc. rank:	4030.6

,

PB1

Pos.	216	obs:	exp:
tct	S	0	11.41
tcc	S	0	7.18
tca	S	0	16.14
tcg	S	0	3.30
tgt	C	0	1.81
tgc	C	4	2.19
agt	S	4	11.66
agc	S	58	12.32
gtt	V	0	0.22
gtc	V	1	0.18
gta	V	0	0.27
gtg	V	0	0.33
gct	A	0	0.26
gcc	A	1	0.21
gca	A	0	0.47
gcg	A	0	0.06
gat	D	0	28.98
gac	D	55	26.02
ggt	G	24	3161.00
ggc	G	20783	3088.00
gga	G	42	9274.00
ggg	G	0	5326.00
---	--	------	------
mPD		0.018	0.71
		nPD:	0.03
		N. weight:	2.
		Sc. PD:	-0.027
		Sc. rank:	14484.0

,

PB1

Pos.	217	obs:	exp:
tat	Y	20919	10896.00
tac	Y	51	10074.00
tgt	C	1	0.45
tgc	C	0	0.55
gat	D	1	0.53
gac	D	0	0.47
---	--	------	------
mPD		0.0050	0.50
		nPD:	0.01
		N. weight:	0.68
		Sc. PD:	-0.02
		Sc. rank:	-1348.0

,

PB1

Pos.	218	obs:	exp:
tta	L	62	2303.00
ttg	L	0	3944.00
ctt	L	6	4128.00
ctc	L	9	2959.00
cta	L	20853	4033.00
ctg	L	17	3581.00
att	I	0	9.10
atc	I	0	6.24
ata	I	25	9.66
---	--	------	------
mPD		0.011	1.1
		nPD:	0.01
		N. weight:	1.7
		Sc. PD:	-0.05
		Sc. rank:	-4005.5

,

PB1

Pos.	219	obs:	exp:
tta	L	0	0.22
ttg	L	0	0.38
ctt	L	0	0.39
ctc	L	0	0.28
cta	L	2	0.39
ctg	L	0	0.34
att	I	4	7630.00
atc	I	0	5232.00
ata	I	20957	8098.00
atg	M	2	2.00
act	T	0	0.25
acc	T	0	0.21
aca	T	1	0.46
acg	T	0	0.09
gtt	V	0	1.33
gtc	V	0	1.09
gta	V	6	1.61
gtg	V	0	1.97
---	--	------	------
mPD		0.0014	0.66
		nPD:	0.
		N. weight:	1.
		Sc. PD:	-0.037
		Sc. rank:	-15198.0

,

PB1			
Pos.	220	obs:	exp:
cgt	R	0	572.60
cgc	R	0	1097.00
cga	R	2	2054.00
cgg	R	0	1535.00
aaa	K	1	0.57
aag	K	0	0.43
aga	R	20939	10528.00
agg	R	30	5184.00
---	--	------	------
mPD		0.0031	0.91
		nPD:	0.
		N. weight:	0.72
		Sc. PD:	-0.026
		Sc. rank:	-9632.4

,

PB1			
Pos.	221	obs:	exp:
tct	S	0	0.18
tcc	S	0	0.12
tca	S	1	0.26
tcg	S	0	0.05
act	T	1	0.50
acc	T	0	0.41
aca	T	1	0.91
acg	T	0	0.17
agt	S	0	0.19
agc	S	0	0.20
gct	A	26	5552.00
gcc	A	10	4341.00
gca	A	20919	9848.00
gcg	A	14	1228.00
---	--	------	------
mPD		0.0051	0.66
		nPD:	0.01
		N. weight:	0.78
		Sc. PD:	-0.025
		Sc. rank:	-4113.1

,

PB1			
Pos.	222	obs:	exp:
tta	L	4	2305.00
ttg	L	51	3948.00
ctt	L	360	4132.00
ctc	L	0	2962.00
cta	L	78	4037.00
ctg	L	20478	3586.00
caa	Q	0	0.53
cag	Q	1	0.47
---	--	------	------
mPD		0.047	1.1
		nPD:	0.04
		N. weight:	1.7
		Sc. PD:	0.002
		Sc. rank:	18077.0

,

PB1			
Pos.	223	obs:	exp:
act	T	7	5287.00
acc	T	5	4301.00
aca	T	20934	9594.00
acg	T	25	1789.00
gct	A	0	0.26
gcc	A	0	0.21
gca	A	1	0.47
gcg	A	0	0.06
---	--	------	------
mPD		0.0036	0.68
		nPD:	0.01
		N. weight:	0.82
		Sc. PD:	-0.028
		Sc. rank:	-7455.7

,

PB1			
Pos.	224	obs:	exp:
tta	L	20425	2306.00
ttg	L	345	3948.00
ctt	L	0	4133.00
ctc	L	0	2962.00
cta	L	200	4037.00
ctg	L	2	3586.00
---	--	------	------
mPD		0.052	1.1
		nPD:	0.05
		N. weight:	2.2
		Sc. PD:	0.012
		Sc. rank:	23779.0

,

PB1			
Pos.	225	obs:	exp:
aat	N	20792	12404.00
aac	N	180	8568.00
---	--	------	------
mPD		0.017	0.48
		nPD:	0.04
		N. weight:	0.51
		Sc. PD:	-0.0023
		Sc. rank:	4954.5

,

PB1			
Pos.	226	obs:	exp:
tct	S	0	0.18
tcc	S	0	0.12
tca	S	0	0.26
tcg	S	1	0.05
cct	P	0	0.28
ccc	P	0	0.19
cca	P	0	0.43
ccg	P	1	0.11
act	T	59	5286.00
acc	T	0	4301.00
aca	T	989	9593.00
acg	T	19922	1789.00
agt	S	0	0.19
agc	S	0	0.20
---	--	------	------
mPD		0.095	0.68
		nPD:	0.14
		N. weight:	2.3
		Sc. PD:	0.23
		Sc. rank:	34773.0

,

PB1			
Pos.	227	obs:	exp:
atg	M	20972	20972.00
---	--	------	------
mPD		0	0
		nPD:	1.
		N. weight:	0.
		Sc. PD:	0
		Sc. rank:	0

,

PB1			
Pos.	228	obs:	exp:
act	T	11	5287.00
acc	T	20406	4301.00
aca	T	554	9594.00
acg	T	0	1789.00
gct	A	0	0.26
gcc	A	1	0.21
gca	A	0	0.47
gcg	A	0	0.06
---	--	------	------
mPD		0.053	0.68
		nPD:	0.08
		N. weight:	1.5
		Sc. PD:	0.056
		Sc. rank:	20122.0

,

PB1			
Pos.	229	obs:	exp:
att	I	0	0.36
atc	I	0	0.25
ata	I	1	0.39
aaa	K	20942	11962.00
aag	K	29	9009.00
---	--	------	------
mPD		0.0029	0.49
		nPD:	0.01
		N. weight:	0.58
		Sc. PD:	-0.019
		Sc. rank:	-5021.2

,

PB1			
Pos.	230	obs:	exp:
aat	N	1	0.59
aac	N	0	0.41
gat	D	20913	11050.00
gac	D	58	9921.00
---	--	------	------
mPD		0.0056	0.50
		nPD:	0.01
		N. weight:	0.66
		Sc. PD:	-0.018
		Sc. rank:	-623.1

,

PB1			
Pos.	231	obs:	exp:
gct	A	298	5552.00
gcc	A	0	4341.00
gca	A	20658	9848.00
gcg	A	13	1228.00
gaa	E	1	0.61
gag	E	0	0.39
ggt	G	0	0.30
ggc	G	0	0.30
gga	G	1	0.89
ggg	G	1	0.51
---	--	------	------
mPD		0.030	0.66
		nPD:	0.04
		N. weight:	0.73
		Sc. PD:	0.0034
		Sc. rank:	7804.1

,

PB1			
Pos.	232	obs:	exp:
gaa	E	171	12699.00
gag	E	20801	8273.00
---	--	------	------
mPD		0.016	0.48
		nPD:	0.03
		N. weight:	0.93
		Sc. PD:	-0.0055
		Sc. rank:	8830.9

,

PB1			
Pos.	233	obs:	exp:
cgt	R	0	572.60
cgc	R	0	1097.00
cga	R	2	2054.00
cgg	R	0	1535.00
aga	R	20923	10529.00
agg	R	47	5185.00
---	--	------	------
mPD		0.0047	0.91
		nPD:	0.01
		N. weight:	0.71
		Sc. PD:	-0.024
		Sc. rank:	-6977.3

,

PB1			
Pos.	234	obs:	exp:
tct	S	0	0.37
tcc	S	0	0.23
tca	S	0	0.52
tcg	S	0	0.11
agt	S	0	0.38
agc	S	2	0.40
ggt	G	26	3180.00
ggc	G	20924	3106.00
gga	G	19	9328.00
ggg	G	1	5357.00
---	--	------	------
mPD		0.0046	0.69
		nPD:	0.01
		N. weight:	2.
		Sc. PD:	-0.065
		Sc. rank:	-14151.0

,

PB1			
Pos.	235	obs:	exp:
caa	Q	0	0.53
cag	Q	1	0.47
aat	N	1	0.59
aac	N	0	0.41
aaa	K	152	11962.00
aag	K	20818	9008.00
---	--	------	------
mPD		0.015	0.49
		nPD:	0.03
		N. weight:	0.85
		Sc. PD:	-0.0084
		Sc. rank:	7103.3

,

PB1			
Pos.	236	obs:	exp:
tta	L	20875	2305.00
ttg	L	42	3948.00
ctt	L	0	4132.00
ctc	L	0	2962.00
cta	L	53	4037.00
ctg	L	0	3585.00
att	I	0	0.36
atc	I	0	0.25
ata	I	1	0.39
gtt	V	0	0.22
gtc	V	0	0.18
gta	V	1	0.27
gtg	V	0	0.33
---	--	------	------
mPD		0.0092	1.1
		nPD:	0.01
		N. weight:	2.3
		Sc. PD:	-0.072
		Sc. rank:	-10761.0

,

PB1			
Pos.	237	obs:	exp:
cgt	R	0	0.16
cgc	R	0	0.31
cga	R	0	0.59
cgg	R	0	0.44
att	I	0	0.36
atc	I	0	0.25
ata	I	1	0.39
aaa	K	20905	11959.00
aag	K	60	9006.00
aga	R	6	3.01
agg	R	0	1.48
---	--	------	------
mPD		0.0064	0.49
		nPD:	0.01
		N. weight:	0.58
		Sc. PD:	-0.015
		Sc. rank:	533.7

,

PB1			
Pos.	238	obs:	exp:
cgt	R	0	572.60
cgc	R	1	1097.00
cga	R	20	2054.00
cgg	R	0	1535.00
aga	R	20930	10529.00
agg	R	21	5185.00
---	--	------	------
mPD		0.0041	0.91
		nPD:	0.
		N. weight:	0.72
		Sc. PD:	-0.025
		Sc. rank:	-7973.0

,

PB1			
Pos.	239	obs:	exp:
cgt	R	0	572.60
cgc	R	0	1097.00
cga	R	0	2054.00
cgg	R	0	1535.00
aga	R	206	10529.00
agg	R	20766	5185.00
---	--	------	------
mPD		0.019	0.91
		nPD:	0.02
		N. weight:	1.4
		Sc. PD:	-0.026
		Sc. rank:	8254.6

,

PB1			
Pos.	240	obs:	exp:
cct	P	1	0.28
ccc	P	0	0.19
cca	P	0	0.43
ccg	P	0	0.11
gct	A	20920	5552.00
gcc	A	40	4341.00
gca	A	6	9849.00
gcg	A	5	1229.00
---	--	------	------
mPD		0.0049	0.66
		nPD:	0.01
		N. weight:	1.4
		Sc. PD:	-0.044
		Sc. rank:	-8514.6

,

PB1			
Pos.	241	obs:	exp:
att	I	109	7634.00
atc	I	19049	5235.00
ata	I	1814	8103.00
---	--	------	------
mPD		0.17	0.66
		nPD:	0.26
		N. weight:	1.1
		Sc. PD:	0.23
		Sc. rank:	17228.0

,

PB1			
Pos.	242	obs:	exp:
act	T	0	0.25
acc	T	0	0.21
aca	T	1	0.46
acg	T	0	0.09
gct	A	0	5552.00
gcc	A	1	4341.00
gca	A	20961	9849.00
gcg	A	9	1229.00
---	--	------	------
mPD		0.0010	0.66
		nPD:	0.
		N. weight:	0.8
		Sc. PD:	-0.03
		Sc. rank:	-12763.0

,

PB1			
Pos.	243	obs:	exp:
act	T	1	5287.00
acc	T	2	4302.00
aca	T	20965	9594.00
acg	T	4	1789.00
---	--	------	------
mPD		0.00067	0.68
		nPD:	0.
		N. weight:	0.83
		Sc. PD:	-0.031
		Sc. rank:	-13647.0

,

PB1			
Pos.	244	obs:	exp:
tct	S	1	0.18
tcc	S	0	0.12
tca	S	0	0.26
tcg	S	0	0.05
cct	P	20844	5777.00
ccc	P	43	3890.00
cca	P	78	8929.00
ccg	P	6	2375.00
agt	S	0	0.19
agc	S	0	0.20
---	--	------	------
mPD		0.012	0.70
		nPD:	0.02
		N. weight:	1.3
		Sc. PD:	-0.029
		Sc. rank:	5154.1

,

PB1			
Pos.	245	obs:	exp:
ggt	G	3	3180.00
ggc	G	0	3106.00
gga	G	187	9329.00
ggg	G	20782	5357.00
---	--	------	------
mPD		0.018	0.69
		nPD:	0.03
		N. weight:	1.4
		Sc. PD:	-0.019
		Sc. rank:	10164.0

,

PB1			
Pos.	246	obs:	exp:
atg	M	20970	20970.00
gtt	V	0	0.44
gtc	V	0	0.36
gta	V	0	0.54
gtg	V	2	0.66
---	--	------	------
mPD		0.00019	0.00032
		nPD:	0.6
		N. weight:	0.00011
		Sc. PD:	0.000062
		Sc. rank:	1.9

,

PB1			
Pos.	247	obs:	exp:
cat	H	2	1.18
cac	H	0	0.82
caa	Q	612	11065.00
cag	Q	20358	9905.00
---	--	------	------
mPD		0.057	0.50
		nPD:	0.11
		N. weight:	0.65
		Sc. PD:	0.047
		Sc. rank:	9575.6

,

PB1			
Pos.	248	obs:	exp:
att	I	20714	7632.00
atc	I	248	5233.00
ata	I	3	8100.00
atg	M	1	1.00
act	T	3	1.01
acc	T	1	0.82
aca	T	0	1.83
acg	T	0	0.34
gtt	V	2	0.44
gtc	V	0	0.36
gta	V	0	0.54
gtg	V	0	0.66
---	--	------	------
mPD		0.024	0.66
		nPD:	0.04
		N. weight:	1.
		Sc. PD:	-0.0026
		Sc. rank:	10084.0

,

PB1			
Pos.	249	obs:	exp:
cgt	R	0	572.60
cgc	R	0	1097.00
cga	R	0	2054.00
cgg	R	0	1535.00
att	I	0	0.36
atc	I	1	0.25
ata	I	0	0.39
aga	R	20934	10528.00
agg	R	37	5184.00
---	--	------	------
mPD		0.0037	0.91
		nPD:	0.
		N. weight:	0.72
		Sc. PD:	-0.025
		Sc. rank:	-8842.5

,

PB1			
Pos.	250	obs:	exp:
tct	S	0	0.18
tcc	S	0	0.12
tca	S	0	0.26
tcg	S	0	0.05
tgt	C	1	0.45
tgc	C	0	0.55
agt	S	1	0.19
agc	S	0	0.20
ggt	G	20838	3180.00
ggc	G	80	3106.00
gga	G	1	9328.00
ggg	G	51	5357.00
---	--	------	------
mPD		0.013	0.69
		nPD:	0.02
		N. weight:	2.
		Sc. PD:	-0.041
		Sc. rank:	8857.4

,

PB1			
Pos.	251	obs:	exp:
ttt	F	117	8998.00
ttc	F	20852	11971.00
tta	L	0	0.22
ttg	L	1	0.38
tat	Y	0	0.52
tac	Y	1	0.48
ctt	L	0	0.39
ctc	L	1	0.28
cta	L	0	0.39
ctg	L	0	0.34
---	--	------	------
mPD		0.011	0.49
		nPD:	0.02
		N. weight:	0.56
		Sc. PD:	-0.0091
		Sc. rank:	3523.0

,

PB1			
Pos.	252	obs:	exp:
att	I	0	0.36
atc	I	0	0.25
ata	I	1	0.39
gtt	V	0	4636.00
gtc	V	2	3811.00
gta	V	20913	5640.00
gtg	V	56	6884.00
---	--	------	------
mPD		0.0056	0.74
		nPD:	0.01
		N. weight:	1.4
		Sc. PD:	-0.043
		Sc. rank:	-7878.7

,

PB1			
Pos.	253	obs:	exp:
ttt	F	0	1.29
ttc	F	3	1.71
tat	Y	28	10891.00
tac	Y	20933	10070.00
tgt	C	0	0.45
tgc	C	1	0.55
cat	H	0	3.53
cac	H	6	2.47
gat	D	0	0.53
gac	D	1	0.47
---	--	------	------
mPD		0.0037	0.50
		nPD:	0.01
		N. weight:	0.77
		Sc. PD:	-0.024
		Sc. rank:	-4800.1

,

PB1			
Pos.	254	obs:	exp:
ttt	F	20924	8998.00
ttc	F	43	11969.00
tta	L	0	0.44
ttg	L	0	0.75
ctt	L	4	0.79
ctc	L	0	0.56
cta	L	0	0.77
ctg	L	0	0.68
gtt	V	1	0.22
gtc	V	0	0.18
gta	V	0	0.27
gtg	V	0	0.33
---	--	------	------
mPD		0.0046	0.49
		nPD:	0.01
		N. weight:	0.88
		Sc. PD:	-0.026
		Sc. rank:	-2620.6

,

PB1			
Pos.	255	obs:	exp:
gtt	V	20942	4636.00
gtc	V	13	3810.00
gta	V	15	5640.00
gtg	V	0	6884.00
gct	A	2	0.53
gcc	A	0	0.41
gca	A	0	0.94
gcg	A	0	0.12
---	--	------	------
mPD		0.0029	0.74
		nPD:	0.
		N. weight:	1.6
		Sc. PD:	-0.056
		Sc. rank:	-19999.0

,

PB1			
Pos.	256	obs:	exp:
gat	D	0	0.53
gac	D	1	0.47
gaa	E	20954	12698.00
gag	E	17	8273.00
---	--	------	------
mPD		0.0017	0.48
		nPD:	0.
		N. weight:	0.53
		Sc. PD:	-0.019
		Sc. rank:	-6946.2

,

PB1			
Pos.	257	obs:	exp:
tct	S	0	0.37
tcc	S	0	0.23
tca	S	0	0.52
tcg	S	0	0.11
cat	H	1	0.59
cac	H	0	0.41
att	I	7	2.55
atc	I	0	1.75
ata	I	0	2.70
act	T	20834	5261.00
acc	T	30	4281.00
aca	T	4	9547.00
acg	T	2	1781.00
aat	N	7	4.14
aac	N	0	2.86
agt	S	2	0.38
agc	S	0	0.40
gct	A	85	22.50
gcc	A	0	17.59
gca	A	0	39.92
gcg	A	0	4.98
---	--	------	------
mPD		0.013	0.69
		nPD:	0.02
		N. weight:	1.4
		Sc. PD:	-0.029
		Sc. rank:	7106.0

,

PB1			
Pos.	258	obs:	exp:
tta	L	20881	2306.00
ttg	L	65	3948.00
ctt	L	0	4133.00
ctc	L	0	2962.00
cta	L	25	4037.00
ctg	L	1	3586.00
---	--	------	------
mPD		0.0088	1.1
		nPD:	0.01
		N. weight:	2.3
		Sc. PD:	-0.073
		Sc. rank:	-12688.0

,

PB1			
Pos.	259	obs:	exp:
gct	A	20916	5552.00
gcc	A	33	4341.00
gca	A	15	9850.00
gcg	A	8	1229.00
---	--	------	------
mPD		0.0053	0.66
		nPD:	0.01
		N. weight:	1.4
		Sc. PD:	-0.043
		Sc. rank:	-6624.6

,

PB1			
Pos.	260	obs:	exp:
cgt	R	0	572.30
cgc	R	0	1097.00
cga	R	0	2053.00
cgg	R	2	1534.00
aaa	K	0	4.56
aag	K	8	3.44
aga	R	100	10524.00
agg	R	20860	5182.00
ggt	G	0	0.30
ggc	G	0	0.30
gga	G	0	0.89
ggg	G	2	0.51
---	--	------	------
mPD		0.011	0.91
		nPD:	0.01
		N. weight:	1.4
		Sc. PD:	-0.04
		Sc. rank:	-223.1

,

PB1			
Pos.	261	obs:	exp:
tct	S	0	3844.00
tcc	S	0	2419.00
tca	S	0	5436.00
tcg	S	0	1112.00
tgt	C	0	0.45
tgc	C	1	0.55
cgt	R	0	0.08
cgc	R	0	0.16
cga	R	0	0.29
cgg	R	0	0.22
att	I	0	0.73
atc	I	2	0.50
ata	I	0	0.77
act	T	0	0.50
acc	T	2	0.41
aca	T	0	0.91
acg	T	0	0.17
aat	N	0	43.18
aac	N	73	29.82
agt	S	15	3927.00
agc	S	20871	4149.00
aga	R	2	1.51
agg	R	1	0.74
ggt	G	0	0.76
ggc	G	5	0.74
gga	G	0	2.22
ggg	G	0	1.28
---	--	------	------
mPD		0.0096	1.6
		nPD:	0.01
		N. weight:	1.7
		Sc. PD:	-0.057
		Sc. rank:	-14510.0

,

PB1			
Pos.	262	obs:	exp:
att	I	20907	7631.00
atc	I	45	5233.00
ata	I	12	8099.00
gtt	V	8	1.77
gtc	V	0	1.45
gta	V	0	2.15
gtg	V	0	2.63
---	--	------	------
mPD		0.0062	0.66
		nPD:	0.01
		N. weight:	1.1
		Sc. PD:	-0.031
		Sc. rank:	-2996.0

,

PB1			
Pos.	263	obs:	exp:
tct	S	0	0.18
tcc	S	0	0.12
tca	S	0	0.26
tcg	S	0	0.05
tgt	C	63	9465.00
tgc	C	20908	11506.00
agt	S	0	0.19
agc	S	1	0.20
---	--	------	------
mPD		0.0061	0.50
		nPD:	0.01
		N. weight:	0.62
		Sc. PD:	-0.017
		Sc. rank:	310.2

,

PB1			
Pos.	264	obs:	exp:
aaa	K	2	1.14
aag	K	0	0.86
gaa	E	20901	12674.00
gag	E	31	8258.00
ggt	G	0	5.76
ggc	G	0	5.63
gga	G	38	16.90
ggg	G	0	9.71
---	--	------	------
mPD		0.0068	0.48
		nPD:	0.01
		N. weight:	0.52
		Sc. PD:	-0.013
		Sc. rank:	750.4

,

PB1			
Pos.	265	obs:	exp:
cgt	R	0	0.22
cgc	R	0	0.42
cga	R	0	0.78
cgg	R	0	0.59
aaa	K	82	11958.00
aag	K	20881	9005.00
aga	R	0	4.02
agg	R	8	1.98
gaa	E	0	0.61
gag	E	1	0.39
---	--	------	------
mPD		0.0086	0.49
		nPD:	0.02
		N. weight:	0.87
		Sc. PD:	-0.019
		Sc. rank:	3461.8

,

PB1

Pos.	266	obs:	exp:
ttt	F	1	0.43
ttc	F	0	0.57
tta	L	0	2305.00
ttg	L	0	3948.00
ctt	L	20954	4132.00
ctc	L	14	2962.00
cta	L	0	4037.00
ctg	L	1	3585.00
att	I	2	0.73
atc	I	0	0.50
ata	I	0	0.77
---	--	------	------
mPD		0.0017	1.1
		nPD:	0.
		N. weight:	1.7
		Sc. PD:	-0.064
		Sc. rank:	-27706.0

,

PB1

Pos.	267	obs:	exp:
aaa	K	2	1.14
aag	K	0	0.86
gaa	E	20958	12697.00
gag	E	12	8273.00
---	--	------	------
mPD		0.0013	0.48
		nPD:	0.
		N. weight:	0.53
		Sc. PD:	-0.019
		Sc. rank:	-7534.1

,

PB1

Pos.	268	obs:	exp:
caa	Q	132	11062.00
cag	Q	20832	9902.00
cgt	R	0	0.19
cgc	R	0	0.37
cga	R	0	0.69
cgg	R	7	0.51
aaa	K	0	0.57
aag	K	1	0.43
aga	R	0	3.51
agg	R	0	1.73
---	--	------	------
mPD		0.013	0.50
		nPD:	0.03
		N. weight:	0.75
		Sc. PD:	-0.0098
		Sc. rank:	5674.8

,

PB1

Pos.	269	obs:	exp:
tct	S	20948	3859.00
tcc	S	9	2429.00
tca	S	3	5458.00
tcg	S	11	1116.00
agt	S	0	3943.00
agc	S	0	4166.00
gct	A	1	0.26
gcc	A	0	0.21
gca	A	0	0.47
gcg	A	0	0.06
---	--	------	------
mPD		0.0023	1.6
		nPD:	0.
		N. weight:	1.8
		Sc. PD:	-0.067
		Sc. rank:	-28967.0

,

PB1

Pos.	270	obs:	exp:
ggt	G	0	3180.00
ggc	G	0	3106.00
gga	G	461	9329.00
ggg	G	20511	5357.00
---	--	------	------
mPD		0.043	0.69
		nPD:	0.06
		N. weight:	1.3
		Sc. PD:	0.029
		Sc. rank:	16501.0

,

PB1

Pos.	271	obs:	exp:
tta	L	0	2305.00
ttg	L	1	3948.00
ctt	L	54	4132.00
ctc	L	20902	2962.00
cta	L	14	4037.00
ctg	L	0	3586.00
att	I	0	0.36
atc	I	1	0.25
ata	I	0	0.39
---	--	------	------
mPD		0.0068	1.1
		nPD:	0.01
		N. weight:	2.
		Sc. PD:	-0.068
		Sc. rank:	-16882.0

,

PB1

Pos.	272	obs:	exp:
cct	P	17	5778.00
ccc	P	7	3890.00
cca	P	20919	8930.00
ccg	P	29	2375.00
---	--	------	------
mPD		0.0050	0.70
		nPD:	0.01
		N. weight:	0.89
		Sc. PD:	-0.028
		Sc. rank:	-5639.3

,

PB1

Pos.	273	obs:	exp:
att	I	0	1.46
atc	I	0	1.00
ata	I	4	1.55
gtt	V	15	4636.00
gtc	V	0	3810.00
gta	V	20881	5639.00
gtg	V	72	6883.00
---	--	------	------
mPD		0.0086	0.74
		nPD:	0.01
		N. weight:	1.4
		Sc. PD:	-0.037
		Sc. rank:	-126.6

,

PB1

Pos.	274	obs:	exp:
ggt	G	18	3180.00
ggc	G	0	3106.00
gga	G	773	9329.00
ggg	G	20181	5357.00
---	--	------	------
mPD		0.073	0.69
		nPD:	0.1
		N. weight:	1.3
		Sc. PD:	0.08
		Sc. rank:	18047.0

,

PB1

Pos.	275	obs:	exp:
tct	S	0	0.18
tcc	S	0	0.12
tca	S	0	0.26
tcg	S	0	0.05
agt	S	0	0.19
agc	S	1	0.20
ggt	G	25	3180.00
ggc	G	20888	3106.00
gga	G	52	9328.00
ggg	G	6	5357.00
---	--	------	------
mPD		0.0080	0.69
		nPD:	0.01
		N. weight:	2.
		Sc. PD:	-0.055
		Sc. rank:	-739.6

,

PB1

Pos.	276	obs:	exp:
aat	N	20959	12404.00
aac	N	13	8568.00
---	--	------	------
mPD		0.0012	0.48
		nPD:	0.
		N. weight:	0.55
		Sc. PD:	-0.02
		Sc. rank:	-8072.4

,

PB1

Pos.	277	obs:	exp:
gaa	E	20949	12699.00
gag	E	23	8273.00
---	--	------	------
mPD		0.0022	0.48
		nPD:	0.
		N. weight:	0.52
		Sc. PD:	-0.018
		Sc. rank:	-5790.0

,

PB1

Pos.	278	obs:	exp:
aaa	K	37	11963.00
aag	K	20935	9009.00
---	--	------	------
mPD		0.0035	0.49
		nPD:	0.01
		N. weight:	0.88
		Sc. PD:	-0.028
		Sc. rank:	-5641.8

,

PB1

Pos.	279	obs:	exp:
caa	Q	0	4.22
cag	Q	8	3.78
aat	N	0	0.59
aac	N	1	0.41
aaa	K	106	11958.00
aag	K	20857	9005.00
---	--	------	------
mPD		0.011	0.49
		nPD:	0.02
		N. weight:	0.86
		Sc. PD:	-0.015
		Sc. rank:	5262.4

,

PB1

Pos.	280	obs:	exp:
gct	A	33	5552.00
gcc	A	20926	4341.00
gca	A	13	9850.00
gcg	A	0	1229.00
---	--	------	------
mPD		0.0044	0.66
		nPD:	0.01
		N. weight:	1.7
		Sc. PD:	-0.054
		Sc. rank:	-11528.0

,

PB1

Pos.	281	obs:	exp:
aaa	K	20944	11963.00
aag	K	28	9009.00
---	--	------	------
mPD		0.0027	0.49
		nPD:	0.01
		N. weight:	0.58
		Sc. PD:	-0.02
		Sc. rank:	-5269.7

,

PB1

Pos.	282	obs:	exp:
tta	L	15	2305.00
ttg	L	17899	3948.00
ctt	L	0	4132.00
ctc	L	0	2962.00
cta	L	2	4037.00
ctg	L	3055	3586.00
cct	P	0	0.28
ccc	P	0	0.19
cca	P	0	0.43
ccg	P	1	0.11
---	--	------	------
mPD		0.25	1.1
		nPD:	0.22
		N. weight:	1.3
		Sc. PD:	0.24
		Sc. rank:	20935.0

,

PB1

Pos.	283	obs:	exp:
tct	S	0	0.37
tcc	S	0	0.23
tca	S	2	0.52
tcg	S	0	0.11
agt	S	0	0.38
agc	S	0	0.40
gtt	V	0	0.22
gtc	V	0	0.18
gta	V	1	0.27
gtg	V	0	0.33
gct	A	1	5551.00
gcc	A	0	4340.00
gca	A	20932	9848.00
gcg	A	35	1228.00
gaa	E	1	0.61
gag	E	0	0.39
---	--	------	------
mPD		0.0038	0.66
		nPD:	0.01
		N. weight:	0.79
		Sc. PD:	-0.026
		Sc. rank:	-6927.0

,

PB1

Pos.	284	obs:	exp:
aat	N	20945	12404.00
aac	N	27	8568.00
---	--	------	------
mPD		0.0026	0.48
		nPD:	0.01
		N. weight:	0.55
		Sc. PD:	-0.018
		Sc. rank:	-5174.9

,

PB1

Pos.	285	obs:	exp:
gtt	V	20858	4637.00
gtc	V	9	3811.00
gta	V	94	5640.00
gtg	V	11	6884.00
---	--	------	------
mPD		0.011	0.74
		nPD:	0.01
		N. weight:	1.6
		Sc. PD:	-0.038
		Sc. rank:	3270.6

,

PB1

Pos.	286	obs:	exp:
tta	L	0	0.11
ttg	L	1	0.19
ctt	L	0	0.20
ctc	L	0	0.14
cta	L	0	0.19
ctg	L	0	0.17
atg	M	1	1.00
gtt	V	185	4636.00
gtc	V	1	3810.00
gta	V	67	5639.00
gtg	V	20716	6883.00
gct	A	0	0.26
gcc	A	0	0.21
gca	A	0	0.47
gcg	A	1	0.06
---	--	------	------
mPD		0.024	0.74
		nPD:	0.03
		N. weight:	1.1
		Sc. PD:	-0.0077
		Sc. rank:	10106.0

,

PB1

Pos.	287	obs:	exp:
cgt	R	0	572.50
cgc	R	0	1097.00
cga	R	4	2054.00
cgg	R	0	1535.00
act	T	0	0.25
acc	T	0	0.21
aca	T	1	0.46
acg	T	0	0.09
aaa	K	1	0.57
aag	K	0	0.43
aga	R	20917	10527.00
agg	R	48	5184.00
ggt	G	0	0.15
ggc	G	0	0.15
gga	G	1	0.44
ggg	G	0	0.26
---	--	------	------
mPD		0.0052	0.91
		nPD:	0.01
		N. weight:	0.71
		Sc. PD:	-0.024
		Sc. rank:	-6222.9

,

PB1

Pos.	288	obs:	exp:
aaa	K	375	11963.00
aag	K	20597	9009.00
---	--	------	------
mPD		0.035	0.49
		nPD:	0.07
		N. weight:	0.8
		Sc. PD:	0.025
		Sc. rank:	10257.0

,

PB1

Pos.	289	obs:	exp:
atg	M	20970	20970.00
aaa	K	0	1.14
aag	K	2	0.86
---	--	------	------
mPD		0.00019	0.00030
		nPD:	0.64
		N. weight:	0.000085
		Sc. PD:	0.00005
		Sc. rank:	1.5

,

PB1

Pos.	290	obs:	exp:
att	I	2	1.09
atc	I	0	0.75
ata	I	1	1.16
atg	M	20967	20967.00
gtt	V	0	0.44
gtc	V	0	0.36
gta	V	0	0.54
gtg	V	2	0.66
---	--	------	------
mPD		0.00048	0.00060
		nPD:	0.79
		N. weight:	0.00021
		Sc. PD:	0.00015
		Sc. rank:	3.5

,

PB1

Pos.	291	obs:	exp:
tct	S	0	0.18
tcc	S	0	0.12
tca	S	0	0.26
tcg	S	0	0.05
att	I	2	0.73
atc	I	0	0.50
ata	I	0	0.77
act	T	20833	5284.00
acc	T	74	4299.00
aca	T	36	9588.00
acg	T	16	1788.00
aat	N	1	0.59
aac	N	0	0.41
agt	S	1	0.19
agc	S	0	0.20
gct	A	9	2.38
gcc	A	0	1.86
gca	A	0	4.23
gcg	A	0	0.53
---	--	------	------
mPD		0.013	0.68
		nPD:	0.02
		N. weight:	1.4
		Sc. PD:	-0.028
		Sc. rank:	7051.6

,

PB1

Pos.	292	obs:	exp:
tct	S	0	2.58
tcc	S	0	1.62
tca	S	0	3.64
tcg	S	0	0.75
att	I	1	0.36
atc	I	0	0.25
ata	I	0	0.39
act	T	1	0.25
acc	T	0	0.21
aca	T	0	0.46
acg	T	0	0.09
aat	N	20891	12393.00
aac	N	63	8561.00
aaa	K	1	0.57
aag	K	0	0.43
agt	S	14	2.63
agc	S	0	2.78
gat	D	1	0.53
gac	D	0	0.47
---	--	------	------
mPD		0.0077	0.49
		nPD:	0.02
		N. weight:	0.54
		Sc. PD:	-0.013
		Sc. rank:	1498.9

,

PB1

Pos.	293	obs:	exp:
tta	L	2	0.22
ttg	L	0	0.38
tct	S	13	3859.00
tcc	S	4	2429.00
tca	S	20942	5458.00
tcg	S	11	1116.00
ctt	L	0	0.39
ctc	L	0	0.28
cta	L	0	0.39
ctg	L	0	0.34
agt	S	0	3943.00
agc	S	0	4165.00
---	--	------	------
mPD		0.0029	1.6
		nPD:	0.
		N. weight:	1.4
		Sc. PD:	-0.053
		Sc. rank:	-22404.0

,

PB1

Pos.	294	obs:	exp:
cat	H	0	1.18
cac	H	2	0.82
caa	Q	20938	11064.00
cag	Q	30	9904.00
aaa	K	2	1.14
aag	K	0	0.86
---	--	------	------
mPD		0.0032	0.50
		nPD:	0.01
		N. weight:	0.67
		Sc. PD:	-0.022
		Sc. rank:	-4896.6

,

PB1

Pos.	295	obs:	exp:
gat	D	17864	11050.00
gac	D	3108	9922.00
---	--	------	------
mPD		0.25	0.50
		nPD:	0.51
		N. weight:	0.25
		Sc. PD:	0.11
		Sc. rank:	4232.8

,

PB1

Pos.	296	obs:	exp:
att	I	0	1.09
atc	I	0	0.75
ata	I	3	1.16
act	T	143	5286.00
acc	T	12	4301.00
aca	T	20805	9592.00
acg	T	8	1789.00
gct	A	0	0.26
gcc	A	0	0.21
gca	A	1	0.47
gcg	A	0	0.06
---	--	------	------
mPD		0.016	0.68
		nPD:	0.02
		N. weight:	0.78
		Sc. PD:	-0.013
		Sc. rank:	4986.5

,

PB1

Pos.	297	obs:	exp:
gat	D	2	1.05
gac	D	0	0.95
gaa	E	153	12697.00
gag	E	20817	8273.00
---	--	------	------
mPD		0.015	0.48
		nPD:	0.03
		N. weight:	0.94
		Sc. PD:	-0.0084
		Sc. rank:	8028.4

,

PB1

Pos.	298	obs:	exp:
tta	L	0	1.65
ttg	L	0	2.82
ctt	L	15	2.96
ctc	L	0	2.12
cta	L	0	2.89
ctg	L	0	2.56
att	I	20373	7617.00
atc	I	549	5223.00
ata	I	2	8084.00
atg	M	8	8.00
act	T	3	1.26
acc	T	2	1.03
aca	T	0	2.29
acg	T	0	0.43
gtt	V	20	4.42
gtc	V	0	3.63
gta	V	0	5.38
gtg	V	0	6.57
---	--	------	------
mPD		0.056	0.66
		nPD:	0.08
		N. weight:	0.95
		Sc. PD:	0.042
		Sc. rank:	13008.0

,

PB1

Pos.	299	obs:	exp:
ttt	F	1	0.43
ttc	F	0	0.57
tct	S	20921	3859.00
tcc	S	43	2429.00
tca	S	2	5458.00
tcg	S	4	1116.00
act	T	1	0.25
acc	T	0	0.21
aca	T	0	0.46
acg	T	0	0.09
agt	S	0	3943.00
agc	S	0	4165.00
---	--	------	------
mPD		0.0049	1.6
		nPD:	0.
		N. weight:	1.8
		Sc. PD:	-0.064
		Sc. rank:	-24884.0

,

PB1

Pos.	300	obs:	exp:
ttt	F	19	9000.00
ttc	F	20953	11972.00
---	--	------	------
mPD		0.0018	0.49
		nPD:	0.
		N. weight:	0.59
		Sc. PD:	-0.021
		Sc. rank:	-7536.8

,

PB1

Pos.	301	obs:	exp:
act	T	5	5287.00
acc	T	5	4302.00
aca	T	20862	9594.00
acg	T	100	1789.00
---	--	------	------
mPD		0.010	0.68
		nPD:	0.02
		N. weight:	0.8
		Sc. PD:	-0.019
		Sc. rank:	2010.5

,

PB1

Pos.	302	obs:	exp:
tta	L	0	0.11
ttg	L	0	0.19
ctt	L	0	0.20
ctc	L	1	0.14
cta	L	0	0.19
ctg	L	0	0.17
att	I	20	7631.00
atc	I	20878	5233.00
ata	I	66	8099.00
gtt	V	0	1.55
gtc	V	7	1.27
gta	V	0	1.88
gtg	V	0	2.30
---	--	------	------
mPD		0.0089	0.66
		nPD:	0.01
		N. weight:	1.4
		Sc. PD:	-0.037
		Sc. rank:	1825.0

,

PB1

Pos.	303	obs:	exp:
tct	S	1	0.18
tcc	S	0	0.12
tca	S	0	0.26
tcg	S	0	0.05
act	T	20867	5287.00
acc	T	74	4301.00
aca	T	29	9594.00
acg	T	1	1789.00
agt	S	0	0.19
agc	S	0	0.20
---	--	------	------
mPD		0.010	0.68
		nPD:	0.01
		N. weight:	1.4
		Sc. PD:	-0.035
		Sc. rank:	3137.0

,

PB1

Pos.	304	obs:	exp:
ggt	G	2	3180.00
ggc	G	0	3106.00
gga	G	235	9329.00
ggg	G	20735	5357.00
---	--	------	------
mPD		0.022	0.69
		nPD:	0.03
		N. weight:	1.4
		Sc. PD:	-0.01
		Sc. rank:	12229.0

,

PB1

Pos.	305	obs:	exp:
gat	D	221	11050.00
gac	D	20751	9922.00
---	--	------	------
mPD		0.021	0.50
		nPD:	0.04
		N. weight:	0.73
		Sc. PD:	0.0014
		Sc. rank:	7752.8

,

PB1

Pos.	306	obs:	exp:
tct	S	0	0.18
tcc	S	0	0.12
tca	S	0	0.26
tcg	S	0	0.05
aat	N	415	12403.00
aac	N	20556	8568.00
agt	S	0	0.19
agc	S	1	0.20
---	--	------	------
mPD		0.039	0.48
		nPD:	0.08
		N. weight:	0.84
		Sc. PD:	0.033
		Sc. rank:	11305.0

,

PB1

Pos.	307	obs:	exp:
cct	P	1	0.28
ccc	P	0	0.19
cca	P	0	0.43
ccg	P	0	0.11
act	T	20897	5286.00
acc	T	59	4301.00
aca	T	13	9593.00
acg	T	1	1789.00
gct	A	1	0.26
gcc	A	0	0.21
gca	A	0	0.47
gcg	A	0	0.06
---	--	------	------
mPD		0.0071	0.68
		nPD:	0.01
		N. weight:	1.4
		Sc. PD:	-0.041
		Sc. rank:	-2374.6

,

PB1

Pos.	308	obs:	exp:
aaa	K	13826	11963.00
aag	K	7146	9009.00
---	--	------	------
mPD		0.45	0.49
		nPD:	0.92
		N. weight:	0.018
		Sc. PD:	0.015
		Sc. rank:	302.9

,

PB1

Pos.	309	obs:	exp:
tgg	W	20972	20972.00
---	--	------	------
mPD		0	0
		nPD:	1.
		N. weight:	0.
		Sc. PD:	0
		Sc. rank:	0

,

PB1

Pos.	310	obs:	exp:
aat	N	20961	12404.00
aac	N	11	8568.00
---	--	------	------
mPD		0.0010	0.48
		nPD:	0.
		N. weight:	0.55
		Sc. PD:	-0.02
		Sc. rank:	-8366.1

,

PB1

Pos.	311	obs:	exp:
gaa	E	14419	12699.00
gag	E	6553	8273.00
---	--	------	------
mPD		0.43	0.48
		nPD:	0.9
		N. weight:	0.016
		Sc. PD:	0.013
		Sc. rank:	267.1

,

PB1

Pos.	312	obs:	exp:
tct	S	0	0.18
tcc	S	0	0.12
tca	S	0	0.26
tcg	S	0	0.05
aat	N	20784	12403.00
aac	N	187	8568.00
agt	S	1	0.19
agc	S	0	0.20
---	--	------	------
mPD		0.018	0.48
		nPD:	0.04
		N. weight:	0.51
		Sc. PD:	-0.0015
		Sc. rank:	4987.3

,

PB1			
Pos.	313	obs:	exp:
caa	Q	20911	11066.00
cag	Q	61	9906.00
---	--	------	------
mPD		0.0058	0.50
		nPD:	0.01
		N. weight:	0.66
		Sc. PD:	-0.018
		Sc. rank:	-194.9

,

PB1			
Pos.	314	obs:	exp:
tct	S	0	0.37
tcc	S	0	0.23
tca	S	0	0.52
tcg	S	0	0.11
aat	N	20698	12402.00
aac	N	271	8567.00
aaa	K	1	0.57
aag	K	0	0.43
agt	S	2	0.38
agc	S	0	0.40
---	--	------	------
mPD		0.026	0.48
		nPD:	0.05
		N. weight:	0.49
		Sc. PD:	0.0065
		Sc. rank:	5776.1

,

PB1			
Pos.	315	obs:	exp:
tct	S	1	0.18
tcc	S	0	0.12
tca	S	0	0.26
tcg	S	0	0.05
cct	P	20528	5777.00
ccc	P	435	3890.00
cca	P	7	8929.00
ccg	P	1	2375.00
agt	S	0	0.19
agc	S	0	0.20
---	--	------	------
mPD		0.041	0.70
		nPD:	0.06
		N. weight:	1.3
		Sc. PD:	0.024
		Sc. rank:	15359.0

,

PB1			
Pos.	316	obs:	exp:
cgt	R	1	572.60
cgc	R	2	1097.00
cga	R	20905	2054.00
cgg	R	25	1535.00
aga	R	39	10529.00
agg	R	0	5185.00
---	--	------	------
mPD		0.0064	0.91
		nPD:	0.01
		N. weight:	2.4
		Sc. PD:	-0.078
		Sc. rank:	-15959.0

,

PB1			
Pos.	317	obs:	exp:
att	I	5	7.28
atc	I	0	4.99
ata	I	15	7.73
atg	M	20936	20936.00
act	T	0	1.76
acc	T	0	1.44
aca	T	0	3.20
acg	T	7	0.60
gtt	V	0	1.99
gtc	V	0	1.64
gta	V	0	2.42
gtg	V	9	2.95
---	--	------	------
mPD		0.0034	0.0046
		nPD:	0.74
		N. weight:	0.0019
		Sc. PD:	0.0013
		Sc. rank:	31.9

,

PB1			
Pos.	318	obs:	exp:
ttt	F	99	9000.00
ttc	F	20873	11972.00
---	--	------	------
mPD		0.0094	0.49
		nPD:	0.02
		N. weight:	0.56
		Sc. PD:	-0.011
		Sc. rank:	2734.6

,

PB1			
Pos.	319	obs:	exp:
tta	L	4	2305.00
ttg	L	63	3948.00
ctt	L	21	4132.00
ctc	L	0	2962.00
cta	L	1448	4037.00
ctg	L	19435	3586.00
atg	M	1	1.00
---	--	------	------
mPD		0.14	1.1
		nPD:	0.12
		N. weight:	1.6
		Sc. PD:	0.12
		Sc. rank:	23307.0

,

PB1			
Pos.	320	obs:	exp:
gct	A	9	5552.00
gcc	A	1	4341.00
gca	A	769	9850.00
gcg	A	20193	1229.00
---	--	------	------
mPD		0.072	0.66
		nPD:	0.11
		N. weight:	2.8
		Sc. PD:	0.18
		Sc. rank:	40321.0

,

PB1			
Pos.	321	obs:	exp:
att	I	0	0.36
atc	I	0	0.25
ata	I	1	0.39
atg	M	20971	20971.00
---	--	------	------
mPD		0.000095	0.000095
		nPD:	1.
		N. weight:	0.000048
		Sc. PD:	0.000045
		Sc. rank:	0.8

,

PB1			
Pos.	322	obs:	exp:
att	I	20898	7626.00
atc	I	51	5229.00
ata	I	0	8094.00
gtt	V	22	5.08
gtc	V	1	4.18
gta	V	0	6.19
gtg	V	0	7.55
---	--	------	------
mPD		0.0071	0.66
		nPD:	0.01
		N. weight:	1.1
		Sc. PD:	-0.03
		Sc. rank:	-1387.0

,

PB1			
Pos.	323	obs:	exp:
tct	S	0	0.74
tcc	S	0	0.46
tca	S	4	1.04
tcg	S	0	0.21
act	T	11	5285.00
acc	T	23	4300.00
aca	T	20910	9591.00
acg	T	22	1789.00
aaa	K	1	0.57
aag	K	0	0.43
agt	S	0	0.75
agc	S	0	0.79
gct	A	0	0.26
gcc	A	0	0.21
gca	A	1	0.47
gcg	A	0	0.06
---	--	------	------
mPD		0.0059	0.68
		nPD:	0.01
		N. weight:	0.81
		Sc. PD:	-0.025
		Sc. rank:	-3084.4

,

PB1			
Pos.	324	obs:	exp:
tat	Y	20833	10897.00
tac	Y	139	10075.00
---	--	------	------
mPD		0.013	0.50
		nPD:	0.03
		N. weight:	0.65
		Sc. PD:	-0.0086
		Sc. rank:	4842.4

,

PB1			
Pos.	325	obs:	exp:
att	I	41	7634.00
atc	I	20914	5235.00
ata	I	16	8102.00
gtt	V	0	0.22
gtc	V	1	0.18
gta	V	0	0.27
gtg	V	0	0.33
---	--	------	------
mPD		0.0055	0.66
		nPD:	0.01
		N. weight:	1.4
		Sc. PD:	-0.045
		Sc. rank:	-6218.1

,

PB1			
Pos.	326	obs:	exp:
tct	S	0	0.37
tcc	S	2	0.23
tca	S	0	0.52
tcg	S	0	0.11
act	T	31	5286.00
acc	T	20778	4301.00
aca	T	161	9593.00
acg	T	0	1789.00
agt	S	0	0.38
agc	S	0	0.40
---	--	------	------
mPD		0.018	0.68
		nPD:	0.03
		N. weight:	1.6
		Sc. PD:	-0.02
		Sc. rank:	12296.0

,

PB1			
Pos.	327	obs:	exp:
cgt	R	0	571.00
cgc	R	0	1094.00
cga	R	0	2048.00
cgg	R	0	1530.00
aaa	K	50	34.80
aag	K	11	26.20
aga	R	20875	10498.00
agg	R	36	5170.00
---	--	------	------
mPD		0.010	0.91
		nPD:	0.01
		N. weight:	0.72
		Sc. PD:	-0.02
		Sc. rank:	-698.7

,

PB1			
Pos.	328	obs:	exp:
tct	S	0	2.76
tcc	S	0	1.74
tca	S	0	3.90
tcg	S	0	0.80
cat	H	38	22.35
cac	H	0	15.65
act	T	1	0.25
acc	T	0	0.21
aca	T	0	0.46
acg	T	0	0.09
aat	N	20757	12325.00
aac	N	82	8514.00
aaa	K	20	11.41
aag	K	0	8.59
agt	S	15	2.82
agc	S	0	2.98
gat	D	59	31.09
gac	D	0	27.91
---	--	------	------
mPD		0.020	0.50
		nPD:	0.04
		N. weight:	0.53
		Sc. PD:	0.00069
		Sc. rank:	5572.9

,

PB1			
Pos.	329	obs:	exp:
caa	Q	20946	11066.00
cag	Q	26	9906.00
---	--	------	------
mPD		0.0025	0.50
		nPD:	0.
		N. weight:	0.67
		Sc. PD:	-0.023
		Sc. rank:	-6926.1

,

PB1

Pos.	330	obs:	exp:
tct	S	0	0.18
tcc	S	1	0.12
tca	S	0	0.26
tcg	S	0	0.05
cct	P	103	5777.00
ccc	P	20838	3890.00
cca	P	28	8929.00
ccg	P	2	2375.00
agt	S	0	0.19
agc	S	0	0.20
---	--	------	------
mPD		0.013	0.70
		nPD:	0.02
		N. weight:	1.7
		Sc. PD:	-0.037
		Sc. rank:	7517.2

,

PB1

Pos.	331	obs:	exp:
aaa	K	0	3.99
aag	K	7	3.01
gtt	V	0	0.22
gtc	V	0	0.18
gta	V	0	0.27
gtg	V	1	0.33
gat	D	46	25.29
gac	D	2	22.71
gaa	E	289	12661.00
gag	E	20621	8249.00
ggt	G	0	0.91
ggc	G	0	0.89
gga	G	0	2.67
ggg	G	6	1.53
---	--	------	------
mPD		0.033	0.48
		nPD:	0.07
		N. weight:	0.9
		Sc. PD:	0.025
		Sc. rank:	11539.0

,

PB1

Pos.	332	obs:	exp:
tgg	W	20971	20971.00
ggt	G	0	0.15
ggc	G	0	0.15
gga	G	0	0.44
ggg	G	1	0.26
---	--	------	------
mPD		0.000095	0.00017
		nPD:	0.57
		N. weight:	0.000069
		Sc. PD:	0.000036
		Sc. rank:	1.2

,

PB1

Pos.	333	obs:	exp:
ttt	F	17998	8998.00
ttc	F	2969	11969.00
tta	L	0	0.44
ttg	L	0	0.75
tct	S	0	0.18
tcc	S	1	0.12
tca	S	0	0.26
tcg	S	0	0.05
ctt	L	4	0.79
ctc	L	0	0.56
cta	L	0	0.77
ctg	L	0	0.68
agt	S	0	0.19
agc	S	0	0.20
---	--	------	------
mPD		0.24	0.49
		nPD:	0.5
		N. weight:	0.42
		Sc. PD:	0.19
		Sc. rank:	7069.7

,

PB1

Pos.	334	obs:	exp:
cgt	R	0	572.60
cgc	R	0	1097.00
cga	R	9	2054.00
cgg	R	0	1535.00
aga	R	20935	10529.00
agg	R	28	5185.00
---	--	------	------
mPD		0.0035	0.91
		nPD:	0.
		N. weight:	0.72
		Sc. PD:	-0.025
		Sc. rank:	-9013.5

,

PB1

Pos.	335	obs:	exp:
aat	N	8	12404.00
aac	N	20964	8568.00
---	--	------	------
mPD		0.00076	0.48
		nPD:	0.
		N. weight:	0.95
		Sc. PD:	-0.035
		Sc. rank:	-15157.0

,

PB1

Pos.	336	obs:	exp:
att	I	234	7625.00
atc	I	20634	5229.00
ata	I	79	8093.00
act	T	0	2.27
acc	T	9	1.85
aca	T	0	4.12
acg	T	0	0.77
aat	N	0	0.59
aac	N	1	0.41
gtt	V	0	3.32
gtc	V	15	2.73
gta	V	0	4.03
gtg	V	0	4.92
---	--	------	------
mPD		0.032	0.66
		nPD:	0.05
		N. weight:	1.4
		Sc. PD:	0.012
		Sc. rank:	15810.0

,

PB1

Pos.	337	obs:	exp:
tta	L	0	2305.00
ttg	L	87	3948.00
ctt	L	25	4132.00
ctc	L	1	2962.00
cta	L	285	4037.00
ctg	L	20571	3585.00
cct	P	0	0.28
ccc	P	0	0.19
cca	P	0	0.43
ccg	P	1	0.11
atg	M	2	2.00
---	--	------	------
mPD		0.038	1.1
		nPD:	0.03
		N. weight:	1.8
		Sc. PD:	-0.011
		Sc. rank:	16320.0

,

PB1

Pos.	338	obs:	exp:
tct	S	0	3859.00
tcc	S	0	2429.00
tca	S	0	5458.00
tcg	S	0	1116.00
act	T	0	0.25
acc	T	1	0.21
aca	T	0	0.46
acg	T	0	0.09
agt	S	112	3943.00
agc	S	20858	4165.00
ggt	G	0	0.15
ggc	G	1	0.15
gga	G	0	0.44
ggg	G	0	0.26
---	--	------	------
mPD		0.011	1.6
		nPD:	0.01
		N. weight:	1.7
		Sc. PD:	-0.055
		Sc. rank:	-12165.0

,

PB1

Pos.	339	obs:	exp:
tta	L	0	0.11
ttg	L	0	0.19
ctt	L	0	0.20
ctc	L	0	0.14
cta	L	0	0.19
ctg	L	1	0.17
att	I	4	12.74
atc	I	11	8.74
ata	I	20	13.52
atg	M	20930	20930.00
act	T	0	0.50
acc	T	0	0.41
aca	T	0	0.91
acg	T	2	0.17
gtt	V	0	0.88
gtc	V	0	0.73
gta	V	0	1.08
gtg	V	4	1.31
---	--	------	------
mPD		0.0040	0.0045
		nPD:	0.89
		N. weight:	0.001
		Sc. PD:	0.00086
		Sc. rank:	17.6

,

PB1

Pos.	340	obs:	exp:
gct	A	1	5552.00
gcc	A	1	4341.00
gca	A	20911	9850.00
gcg	A	59	1229.00
---	--	------	------
mPD		0.0058	0.66
		nPD:	0.01
		N. weight:	0.79
		Sc. PD:	-0.024
		Sc. rank:	-2882.1

,

PB1

Pos.	341	obs:	exp:
cct	P	28	5778.00
ccc	P	20889	3890.00
cca	P	55	8930.00
ccg	P	0	2375.00
---	--	------	------
mPD		0.0079	0.70
		nPD:	0.01
		N. weight:	1.8
		Sc. PD:	-0.049
		Sc. rank:	-1383.3

,

PB1

Pos.	342	obs:	exp:
att	I	0	7594.00
atc	I	4	5208.00
ata	I	20858	8060.00
gtt	V	0	24.32
gtc	V	0	19.99
gta	V	110	29.58
gtg	V	0	36.11
---	--	------	------
mPD		0.011	0.67
		nPD:	0.02
		N. weight:	1.
		Sc. PD:	-0.023
		Sc. rank:	2991.4

,

PB1

Pos.	343	obs:	exp:
att	I	0	0.36
atc	I	0	0.25
ata	I	1	0.39
atg	M	20970	20970.00
act	T	0	0.25
acc	T	0	0.21
aca	T	0	0.46
acg	T	1	0.09
---	--	------	------
mPD		0.00019	0.00028
		nPD:	0.69
		N. weight:	0.00017
		Sc. PD:	0.00011
		Sc. rank:	2.9

,

PB1

Pos.	344	obs:	exp:
ttt	F	20	8999.00
ttc	F	20951	11972.00
tta	L	0	0.11
ttg	L	0	0.19
ctt	L	0	0.20
ctc	L	1	0.14
cta	L	0	0.19
ctg	L	0	0.17
---	--	------	------
mPD		0.0020	0.49
		nPD:	0.
		N. weight:	0.59
		Sc. PD:	-0.021
		Sc. rank:	-7207.5

,

PB1

Pos.	345	obs:	exp:
tct	S	1	3859.00
tcc	S	1	2429.00
tca	S	20939	5458.00
tcg	S	31	1116.00
agt	S	0	3943.00
agc	S	0	4166.00
---	--	------	------
mPD		0.0031	1.6
		nPD:	0.
		N. weight:	1.4
		Sc. PD:	-0.053
		Sc. rank:	-22067.0

,

PB1			
Pos.	346	obs:	exp:
tct	S	0	0.18
tcc	S	0	0.12
tca	S	0	0.26
tcg	S	0	0.05
aat	N	17611	12403.00
aac	N	3360	8568.00
agt	S	1	0.19
agc	S	0	0.20
---	--	------	------
mPD		0.27	0.48
		nPD:	0.56
		N. weight:	0.15
		Sc. PD:	0.078
		Sc. rank:	2598.7

PB1			
Pos.	347	obs:	exp:
aaa	K	20953	11963.00
aag	K	19	9009.00
---	--	------	------
mPD		0.0018	0.49
		nPD:	0.
		N. weight:	0.59
		Sc. PD:	-0.021
		Sc. rank:	-7586.4

PB1			
Pos.	348	obs:	exp:
tta	L	0	0.44
ttg	L	4	0.75
ctt	L	0	0.79
ctc	L	0	0.56
cta	L	0	0.77
ctg	L	0	0.68
att	I	0	0.36
atc	I	0	0.25
ata	I	1	0.39
atg	M	20957	20957.00
gtt	V	0	2.21
gtc	V	0	1.82
gta	V	0	2.69
gtg	V	10	3.28
---	--	------	------
mPD		0.0014	0.0023
		nPD:	0.62
		N. weight:	0.00095
		Sc. PD:	0.00054
		Sc. rank:	16.1

PB1			
Pos.	349	obs:	exp:
act	T	0	0.25
acc	T	0	0.21
aca	T	1	0.46
acg	T	0	0.09
gct	A	3	5552.00
gcc	A	4	4341.00
gca	A	20895	9849.00
gcg	A	69	1229.00
---	--	------	------
mPD		0.0073	0.66
		nPD:	0.01
		N. weight:	0.78
		Sc. PD:	-0.022
		Sc. rank:	-920.1

PB1			
Pos.	350	obs:	exp:
cgt	R	0	572.00
cgc	R	0	1096.00
cga	R	7	2052.00
cgg	R	0	1533.00
aaa	K	22	12.55
aag	K	0	9.45
aga	R	20318	10518.00
agg	R	625	5179.00
---	--	------	------
mPD		0.061	0.91
		nPD:	0.07
		N. weight:	0.61
		Sc. PD:	0.016
		Sc. rank:	7758.4

PB1			
Pos.	351	obs:	exp:
tta	L	18	2304.00
ttg	L	1	3947.00
ctt	L	6	4131.00
ctc	L	0	2961.00
cta	L	19968	4036.00
ctg	L	969	3584.00
att	I	0	3.28
atc	I	0	2.25
ata	I	9	3.48
atg	M	1	1.00
---	--	------	------
mPD		0.092	1.1
		nPD:	0.08
		N. weight:	1.5
		Sc. PD:	0.061
		Sc. rank:	20763.0

PB1			
Pos.	352	obs:	exp:
ggt	G	4	3180.00
ggc	G	0	3106.00
gga	G	15345	9329.00
ggg	G	5623	5357.00
---	--	------	------
mPD		0.39	0.69
		nPD:	0.57
		N. weight:	0.4
		Sc. PD:	0.21
		Sc. rank:	6765.0

PB1			
Pos.	353	obs:	exp:
cgt	R	0	4.78
cgc	R	0	9.15
cga	R	0	17.14
cgg	R	0	12.81
aat	N	0	1.18
aac	N	2	0.82
aaa	K	20752	11862.00
aag	K	43	8933.00
aga	R	175	87.86
agg	R	0	43.26
---	--	------	------
mPD		0.021	0.51
		nPD:	0.04
		N. weight:	0.58
		Sc. PD:	0.00051
		Sc. rank:	5983.7

PB1			
Pos.	354	obs:	exp:
ggt	G	27	3180.00
ggc	G	0	3106.00
gga	G	188	9329.00
ggg	G	20757	5357.00
---	--	------	------
mPD		0.020	0.69
		nPD:	0.03
		N. weight:	1.4
		Sc. PD:	-0.014
		Sc. rank:	11336.0

PB1			
Pos.	355	obs:	exp:
ttt	F	0	1.29
ttc	F	3	1.71
tat	Y	14	10894.00
tac	Y	20953	10073.00
tgt	C	0	0.45
tgc	C	1	0.55
gat	D	0	0.53
gac	D	1	0.47
---	--	------	------
mPD		0.0018	0.50
		nPD:	0.
		N. weight:	0.77
		Sc. PD:	-0.027
		Sc. rank:	-10074.0

PB1			
Pos.	356	obs:	exp:
tta	L	0	0.11
ttg	L	0	0.19
ctt	L	0	0.20
ctc	L	0	0.14
cta	L	0	0.19
ctg	L	1	0.17
att	I	0	0.36
atc	I	0	0.25
ata	I	1	0.39
atg	M	20970	20970.00
---	--	------	------
mPD		0.00019	0.00025
		nPD:	0.76
		N. weight:	0.00014
		Sc. PD:	0.000097
		Sc. rank:	2.3

PB1			
Pos.	357	obs:	exp:
ttt	F	64	9000.00
ttc	F	20908	11972.00
---	--	------	------
mPD		0.0061	0.49
		nPD:	0.01
		N. weight:	0.57
		Sc. PD:	-0.015
		Sc. rank:	346.2

PB1			
Pos.	358	obs:	exp:
aaa	K	0	1.14
aag	K	2	0.86
gtt	V	0	0.22
gtc	V	0	0.18
gta	V	0	0.27
gtg	V	1	0.33
gct	A	0	0.26
gcc	A	0	0.21
gca	A	0	0.47
gcg	A	1	0.06
gat	D	2	1.05
gac	D	0	0.95
gaa	E	49	12694.00
gag	E	20916	8271.00
ggt	G	0	0.15
ggc	G	0	0.15
gga	G	0	0.44
ggg	G	1	0.26
---	--	------	------
mPD		0.0053	0.48
		nPD:	0.01
		N. weight:	0.97
		Sc. PD:	-0.027
		Sc. rank:	-974.5

PB1			
Pos.	359	obs:	exp:
tct	S	0	3859.00
tcc	S	0	2429.00
tca	S	0	5458.00
tcg	S	0	1116.00
agt	S	20669	3943.00
agc	S	303	4166.00
---	--	------	------
mPD		0.028	1.6
		nPD:	0.02
		N. weight:	1.7
		Sc. PD:	-0.037
		Sc. rank:	6490.6

PB1			
Pos.	360	obs:	exp:
caa	Q	1	0.53
cag	Q	0	0.47
cgt	R	0	0.16
cgc	R	0	0.31
cga	R	0	0.59
cgg	R	0	0.44
aat	N	0	1.18
aac	N	2	0.82
aaa	K	20916	11955.00
aag	K	41	9002.00
aga	R	6	3.01
agg	R	0	1.48
gaa	E	6	3.63
gag	E	0	2.37
---	--	------	------
mPD		0.0053	0.49
		nPD:	0.01
		N. weight:	0.58
		Sc. PD:	-0.016
		Sc. rank:	-748.7

PB1			
Pos.	361	obs:	exp:
tct	S	0	0.37
tcc	S	0	0.23
tca	S	0	0.52
tcg	S	0	0.11
cgt	R	0	563.80
cgc	R	0	1080.00
cga	R	29	2022.00
cgg	R	0	1511.00
att	I	0	0.73
atc	I	0	0.50
ata	I	2	0.77
aaa	K	308	177.40
aag	K	3	133.60
agt	S	1	0.38
agc	S	1	0.40
aga	R	20583	10366.00
agg	R	36	5105.00
ggt	G	0	1.36
ggc	G	0	1.33
gga	G	9	4.00
ggg	G	0	2.30
---	--	------	------
mPD		0.037	0.94
		nPD:	0.04
		N. weight:	0.71
		Sc. PD:	-0.00026
		Sc. rank:	7225.9

PB1

Pos .	362	obs :	exp :
tta	L	0	0.33
ttg	L	1	0.56
ctt	L	0	0.59
ctc	L	0	0.42
cta	L	0	0.58
ctg	L	2	0.51
att	I	0	0.73
atc	I	0	0.50
ata	I	2	0.77
atg	M	20966	20966.00
act	T	0	0.25
acc	T	0	0.21
aca	T	0	0.46
acg	T	1	0.09
---	--	------	------
mPD		0.00057	0.00084
		nPD :	0.68
		N. weight :	0.00043
		Sc. PD :	0.00027
		Sc. rank :	7.3

,

PB1

Pos .	363	obs :	exp :
cgt	R	0	0.49
cgc	R	0	0.94
cga	R	0	1.76
cgg	R	0	1.32
aaa	K	624	11953.00
aag	K	20330	9001.00
aga	R	1	9.04
agg	R	17	4.45
---	--	------	------
mPD		0.060	0.49
		nPD :	0.12
		N. weight :	0.75
		Sc. PD :	0.059
		Sc. rank :	11120.0

,

PB1

Pos .	364	obs :	exp :
tta	L	0	1.65
ttg	L	0	2.82
ctt	L	1	2.96
ctc	L	13	2.12
cta	L	0	2.89
ctg	L	1	2.56
att	I	20727	7569.00
atc	I	53	5190.00
ata	I	13	8033.00
act	T	1	0.25
acc	T	0	0.21
aca	T	0	0.46
acg	T	0	0.09
gtt	V	163	36.04
gtc	V	0	29.62
gta	V	0	43.84
gtg	V	0	53.51
---	--	------	------
mPD		0.025	0.67
		nPD :	0.04
		N. weight :	1.1
		Sc. PD :	-0.0036
		Sc. rank :	10344.0

,

PB1

Pos .	365	obs :	exp :
cgt	R	2	572.60
cgc	R	4	1097.00
cga	R	20799	2054.00
cgg	R	153	1535.00
aga	R	14	10529.00
agg	R	0	5185.00
---	--	------	------
mPD		0.016	0.91
		nPD :	0.02
		N. weight :	2.4
		Sc. PD :	-0.052
		Sc. rank :	9935.4

,

PB1

Pos .	366	obs :	exp :
act	T	1	5286.00
acc	T	0	4301.00
aca	T	20815	9592.00
acg	T	152	1789.00
gct	A	0	1.06
gcc	A	0	0.83
gca	A	4	1.88
gcg	A	0	0.23
---	--	------	------
mPD		0.015	0.68
		nPD :	0.02
		N. weight :	0.8
		Sc. PD :	-0.014
		Sc. rank :	4751.3

,

PB1

Pos .	367	obs :	exp :
caa	Q	20950	11066.00
cag	Q	21	9905.00
cgt	R	0	0.03
cgc	R	0	0.05
cga	R	1	0.10
cgg	R	0	0.07
aga	R	0	0.50
agg	R	0	0.25
---	--	------	------
mPD		0.0021	0.50
		nPD :	0.
		N. weight :	0.67
		Sc. PD :	-0.023
		Sc. rank :	-7875.6

,

PB1

Pos .	368	obs :	exp :
tta	L	1	0.11
ttg	L	0	0.19
ctt	L	0	0.20
ctc	L	0	0.14
cta	L	0	0.19
ctg	L	0	0.17
att	I	18	7629.00
atc	I	0	5231.00
ata	I	20938	8096.00
gtt	V	0	3.32
gtc	V	0	2.73
gta	V	15	4.03
gtg	V	0	4.92
---	--	------	------
mPD		0.0032	0.66
		nPD :	0.
		N. weight :	1.
		Sc. PD :	-0.034
		Sc. rank :	-10455.0

,

PB1

Pos .	369	obs :	exp :
tct	S	0	1.84
tcc	S	0	1.16
tca	S	10	2.60
tcg	S	0	0.53
cct	P	28	5774.00
ccc	P	23	3887.00
cca	P	20891	8924.00
ccg	P	16	2373.00
caa	Q	3	1.58
cag	Q	0	1.42
agt	S	0	1.88
agc	S	0	1.99
gct	A	0	0.26
gcc	A	0	0.21
gca	A	1	0.47
gcg	A	0	0.06
---	--	------	------
mPD		0.0077	0.70
		nPD :	0.01
		N. weight :	0.88
		Sc. PD :	-0.025
		Sc. rank :	-947.2

,

PB1

Pos .	370	obs :	exp :
tct	S	0	0.74
tcc	S	0	0.46
tca	S	4	1.04
tcg	S	0	0.21
act	T	0	4.03
acc	T	0	3.28
aca	T	16	7.32
acg	T	0	1.37
agt	S	0	0.75
agc	S	0	0.79
gtt	V	0	1.11
gtc	V	0	0.91
gta	V	5	1.34
gtg	V	0	1.64
gct	A	157	5546.00
gcc	A	0	4336.00
gca	A	20426	9838.00
gcg	A	364	1227.00
---	--	------	------
mPD		0.051	0.67
		nPD :	0.08
		N. weight :	0.71
		Sc. PD :	0.025
		Sc. rank :	9220.1

,

PB1

Pos .	371	obs :	exp :
aaa	K	4	2.28
aag	K	0	1.72
gaa	E	20784	12695.00
gag	E	182	8271.00
ggt	G	0	0.30
ggc	G	0	0.30
gga	G	2	0.89
ggg	G	0	0.51
---	--	------	------
mPD		0.018	0.48
		nPD :	0.04
		N. weight :	0.48
		Sc. PD :	-0.0013
		Sc. rank :	4818.1

,

PB1

Pos .	372	obs :	exp :
tta	L	0	0.11
ttg	L	1	0.19
ctt	L	0	0.20
ctc	L	0	0.14
cta	L	0	0.19
ctg	L	0	0.17
cgt	R	0	0.03
cgc	R	0	0.05
cga	R	0	0.10
cgg	R	0	0.07
att	I	1	8.01
atc	I	0	5.49
ata	I	21	8.50
atg	M	20937	20937.00
act	T	0	1.51
acc	T	0	1.23
aca	T	0	2.74
acg	T	6	0.51
aga	R	0	0.50
agg	R	1	0.25
gtt	V	0	1.11
gtc	V	0	0.91
gta	V	0	1.34
gtg	V	5	1.64
---	--	------	------
mPD		0.0033	0.0043
		nPD :	0.77
		N. weight :	0.0021
		Sc. PD :	0.0015
		Sc. rank :	35.6

,

PB1

Pos .	373	obs :	exp :
tta	L	53	2304.00
ttg	L	0	3947.00
ctt	L	2	4131.00
ctc	L	1	2961.00
cta	L	20846	4036.00
ctg	L	60	3584.00
att	I	0	3.28
atc	I	0	2.25
ata	I	9	3.48
gtt	V	0	0.22
gtc	V	0	0.18
gta	V	1	0.27
gtg	V	0	0.33
---	--	------	------
mPD		0.012	1.1
		nPD :	0.01
		N. weight :	1.7
		Sc. PD :	-0.049
		Sc. rank :	-2936.4

,

PB1

Pos.	374	obs:	exp:
tct	S	0	2.76
tcc	S	0	1.74
tca	S	15	3.90
tcg	S	0	0.80
att	I	0	1.46
atc	I	0	1.00
ata	I	4	1.55
act	T	0	29.24
acc	T	0	23.79
aca	T	116	53.07
acg	T	0	9.90
agt	S	0	2.82
agc	S	0	2.98
gtt	V	0	18.79
gtc	V	0	15.45
gta	V	85	22.86
gtg	V	0	27.90
gct	A	3	5493.00
gcc	A	2	4295.00
gca	A	20731	9745.00
gcg	A	13	1216.00
gaa	E	3	1.82
gag	E	0	1.18
---	--	------	------
mPD		0.023	0.69
		nPD:	0.03
		N. weight:	0.8
		Sc. PD:	-0.0046
		Sc. rank:	7586.5

,

PB1

Pos.	375	obs:	exp:
tct	S	0	3843.00
tcc	S	0	2419.00
tca	S	0	5435.00
tcg	S	0	1111.00
cgt	R	0	0.05
cgc	R	1	0.10
cga	R	0	0.20
cgg	R	0	0.15
att	I	0	2.18
atc	I	6	1.50
ata	I	0	2.32
aat	N	0	41.40
aac	N	70	28.60
agt	S	84	3927.00
agc	S	20800	4148.00
aga	R	1	1.00
agg	R	0	0.49
ggt	G	0	1.52
ggc	G	10	1.48
gga	G	0	4.45
ggg	G	0	2.55
---	--	------	------
mPD		0.016	1.6
		nPD:	0.01
		N. weight:	1.7
		Sc. PD:	-0.049
		Sc. rank:	-3521.7

,

PB1

Pos.	376	obs:	exp:
att	I	20947	7633.00
atc	I	16	5234.00
ata	I	6	8101.00
gtt	V	3	0.66
gtc	V	0	0.55
gta	V	0	0.81
gtg	V	0	0.98
---	--	------	------
mPD		0.0024	0.66
		nPD:	0.
		N. weight:	1.1
		Sc. PD:	-0.038
		Sc. rank:	-13825.0

,

PB1

Pos.	377	obs:	exp:
aat	N	5	2.96
aac	N	0	2.04
gat	D	17810	11045.00
gac	D	3153	9918.00
gaa	E	1	0.61
gag	E	0	0.39
ggt	G	2	0.45
ggc	G	1	0.44
gga	G	0	1.33
ggg	G	0	0.77
---	--	------	------
mPD		0.26	0.50
		nPD:	0.51
		N. weight:	0.25
		Sc. PD:	0.12
		Sc. rank:	4184.1

,

PB1

Pos.	378	obs:	exp:
tta	L	41	2306.00
ttg	L	16	3948.00
ctt	L	2	4133.00
ctc	L	0	2962.00
cta	L	13209	4037.00
ctg	L	7704	3586.00
---	--	------	------
mPD		0.47	1.1
		nPD:	0.41
		N. weight:	1.1
		Sc. PD:	0.39
		Sc. rank:	17812.0

,

PB1

Pos.	379	obs:	exp:
cgt	R	0	0.38
cgc	R	0	0.73
cga	R	0	1.37
cgg	R	0	1.02
aat	N	2	1.18
aac	N	0	0.82
aaa	K	459	11954.00
aag	K	20497	9002.00
aga	R	2	7.03
agg	R	12	3.46
---	--	------	------
mPD		0.045	0.49
		nPD:	0.09
		N. weight:	0.78
		Sc. PD:	0.039
		Sc. rank:	10913.0

,

PB1

Pos.	380	obs:	exp:
ttt	F	1	0.43
ttc	F	0	0.57
tat	Y	16827	10895.00
tac	Y	4142	10074.00
cat	H	2	1.18
cac	H	0	0.82
---	--	------	------
mPD		0.32	0.50
		nPD:	0.64
		N. weight:	0.18
		Sc. PD:	0.11
		Sc. rank:	3131.5

,

PB1

Pos.	381	obs:	exp:
ttt	F	9	9000.00
ttc	F	20963	11972.00
---	--	------	------
mPD		0.00086	0.49
		nPD:	0.
		N. weight:	0.59
		Sc. PD:	-0.022
		Sc. rank:	-9296.3

,

PB1

Pos.	382	obs:	exp:
tct	S	0	1.10
tcc	S	0	0.69
tca	S	0	1.56
tcg	S	0	0.32
tat	Y	1	0.52
tac	Y	0	0.48
aat	N	20952	12400.00
aac	N	13	8565.00
agt	S	6	1.13
agc	S	0	1.19
---	--	------	------
mPD		0.0019	0.48
		nPD:	0.
		N. weight:	0.55
		Sc. PD:	-0.019
		Sc. rank:	-6893.2

,

PB1

Pos.	383	obs:	exp:
aaa	K	1	0.57
aag	K	0	0.43
gat	D	2	6.32
gac	D	10	5.68
gaa	E	20916	12685.00
gag	E	33	8264.00
ggt	G	0	1.52
ggc	G	0	1.48
gga	G	10	4.45
ggg	G	0	2.55
---	--	------	------
mPD		0.0053	0.48
		nPD:	0.01
		N. weight:	0.52
		Sc. PD:	-0.015
		Sc. rank:	-546.7

,

PB1

Pos.	384	obs:	exp:
tta	L	8	0.88
ttg	L	0	1.51
tct	S	1	3842.00
tcc	S	0	2418.00
tca	S	20011	5433.00
tcg	S	864	1111.00
ctt	L	0	1.58
ctc	L	0	1.13
cta	L	0	1.54
ctg	L	0	1.37
cct	P	0	23.42
ccc	P	0	15.77
cca	P	85	36.19
ccg	P	0	9.62
act	T	0	0.25
acc	T	0	0.21
aca	T	1	0.46
acg	T	0	0.09
agt	S	0	3925.00
agc	S	0	4147.00
gct	A	0	0.53
gcc	A	0	0.41
gca	A	2	0.94
gcg	A	0	0.12
---	--	------	------
mPD		0.088	1.6
		nPD:	0.05
		N. weight:	1.3
		Sc. PD:	0.018
		Sc. rank:	15581.0

,

PB1

Pos.	385	obs:	exp:
act	T	4	5287.00
acc	T	1	4302.00
aca	T	20917	9594.00
acg	T	50	1789.00
---	--	------	------
mPD		0.0052	0.68
		nPD:	0.01
		N. weight:	0.81
		Sc. PD:	-0.026
		Sc. rank:	-4323.5

,

PB1

Pos.	386	obs:	exp:
caa	Q	0	0.53
cag	Q	1	0.47
cgt	R	0	179.60
cgc	R	0	344.10
cga	R	0	644.40
cgg	R	1	481.50
atg	M	1	1.00
act	T	0	0.50
acc	T	0	0.41
aca	T	0	0.91
acg	T	2	0.17
aaa	K	81	8208.00
aag	K	14308	6181.00
aga	R	19	3303.00
agg	R	6559	1626.00
---	--	------	------
mPD		0.44	1.1
		nPD:	0.41
		N. weight:	1.
		Sc. PD:	0.38
		Sc. rank:	17257.0

,

PB1			
Pos.	387	obs:	exp:
caa	Q	0	1.58
cag	Q	3	1.42
cgt	R	0	1.06
cgc	R	0	2.04
cga	R	0	3.82
cgg	R	0	2.85
atg	M	2	2.00
act	T	0	1.51
acc	T	0	1.23
aca	T	0	2.74
acg	T	6	0.51
aaa	K	290	11929.00
aag	K	20623	8984.00
aga	R	0	19.58
agg	R	39	9.64
gaa	E	0	4.84
gag	E	8	3.16
ggt	G	0	0.15
ggc	G	0	0.15
gga	G	0	0.44
ggg	G	1	0.26
---	--	------	------
mPD		0.033	0.50
		nPD:	0.07
		N. weight:	0.82
		Sc. PD:	0.021
		Sc. rank:	10385.0

,

PB1			
Pos.	388	obs:	exp:
cgt	R	0	0.76
cgc	R	0	1.46
cga	R	0	2.74
cgg	R	0	2.05
aaa	K	20435	11947.00
aag	K	509	8997.00
aga	R	28	14.06
agg	R	0	6.92
---	--	------	------
mPD		0.050	0.49
		nPD:	0.1
		N. weight:	0.48
		Sc. PD:	0.029
		Sc. rank:	6881.6

,

PB1			
Pos.	389	obs:	exp:
att	I	20960	7634.00
atc	I	8	5235.00
ata	I	3	8102.00
gtt	V	1	0.22
gtc	V	0	0.18
gta	V	0	0.27
gtg	V	0	0.33
---	--	------	------
mPD		0.0011	0.66
		nPD:	0.
		N. weight:	1.1
		Sc. PD:	-0.04
		Sc. rank:	-16818.0

,

PB1			
Pos.	390	obs:	exp:
aaa	K	0	2.85
aag	K	5	2.15
gtt	V	0	1.11
gtc	V	0	0.91
gta	V	0	1.34
gtg	V	5	1.64
gat	D	4	2.11
gac	D	0	1.89
gaa	E	237	12689.00
gag	E	20719	8267.00
ggt	G	0	0.30
ggc	G	0	0.30
gga	G	0	0.89
ggg	G	2	0.51
---	--	------	------
mPD		0.024	0.48
		nPD:	0.05
		N. weight:	0.92
		Sc. PD:	0.0089
		Sc. rank:	10598.0

,

PB1			
Pos.	391	obs:	exp:
caa	Q	0	0.53
cag	Q	1	0.47
cgt	R	0	0.60
cgc	R	0	1.15
cga	R	0	2.15
cgg	R	0	1.61
aat	N	3	1.77
aac	N	0	1.23
aaa	K	8431	11948.00
aag	K	12515	8998.00
aga	R	10	11.04
agg	R	12	5.44
---	--	------	------
mPD		0.48	0.49
		nPD:	0.98
		N. weight:	0.061
		Sc. PD:	0.056
		Sc. rank:	1043.7

,

PB1			
Pos.	392	obs:	exp:
tta	L	2	0.22
ttg	L	0	0.38
ctt	L	0	0.39
ctc	L	0	0.28
cta	L	0	0.39
ctg	L	0	0.34
att	I	1	7629.00
atc	I	2	5231.00
ata	I	20953	8096.00
atg	M	3	3.00
act	T	0	0.25
acc	T	0	0.21
aca	T	1	0.46
acg	T	0	0.09
gtt	V	0	2.21
gtc	V	0	1.82
gta	V	10	2.69
gtg	V	0	3.28
---	--	------	------
mPD		0.0018	0.66
		nPD:	0.
		N. weight:	1.
		Sc. PD:	-0.037
		Sc. rank:	-14416.0

,

PB1			
Pos.	393	obs:	exp:
cgt	R	0	572.10
cgc	R	0	1096.00
cga	R	1	2052.00
cgg	R	6	1533.00
act	T	0	0.25
acc	T	0	0.21
aca	T	0	0.46
acg	T	1	0.09
aaa	K	0	9.70
aag	K	17	7.30
aga	R	229	10520.00
agg	R	20718	5180.00
---	--	------	------
mPD		0.024	0.91
		nPD:	0.03
		N. weight:	1.4
		Sc. PD:	-0.018
		Sc. rank:	10526.0

,

PB1			
Pos.	394	obs:	exp:
tct	S	3	0.55
tcc	S	0	0.35
tca	S	0	0.78
tcg	S	0	0.16
cct	P	20901	5776.00
ccc	P	34	3889.00
cca	P	9	8928.00
ccg	P	23	2374.00
cat	H	1	0.59
cac	H	0	0.41
agt	S	0	0.56
agc	S	0	0.60
gct	A	1	0.26
gcc	A	0	0.21
gca	A	0	0.47
gcg	A	0	0.06
---	--	------	------
mPD		0.0068	0.70
		nPD:	0.01
		N. weight:	1.3
		Sc. PD:	-0.04
		Sc. rank:	-3497.5

,

PB1			
Pos.	395	obs:	exp:
ttt	F	1	0.43
ttc	F	0	0.57
tta	L	1	2305.00
ttg	L	0	3948.00
ctt	L	20923	4132.00
ctc	L	43	2962.00
cta	L	0	4037.00
ctg	L	3	3585.00
att	I	1	0.36
atc	I	0	0.25
ata	I	0	0.39
---	--	------	------
mPD		0.0048	1.1
		nPD:	0.
		N. weight:	1.7
		Sc. PD:	-0.06
		Sc. rank:	-20161.0

,

PB1			
Pos.	396	obs:	exp:
tta	L	18182	2305.00
ttg	L	31	3948.00
ctt	L	0	4132.00
ctc	L	1	2962.00
cta	L	2751	4037.00
ctg	L	5	3585.00
att	I	0	0.73
atc	I	0	0.50
ata	I	2	0.77
---	--	------	------
mPD		0.23	1.1
		nPD:	0.2
		N. weight:	1.8
		Sc. PD:	0.29
		Sc. rank:	28412.0

,

PB1			
Pos.	397	obs:	exp:
tta	L	0	0.11
ttg	L	1	0.19
ctt	L	0	0.20
ctc	L	0	0.14
cta	L	0	0.19
ctg	L	0	0.17
cgt	R	0	0.14
cgc	R	0	0.26
cga	R	0	0.49
cgg	R	0	0.37
att	I	3	1211.00
atc	I	0	830.20
ata	I	3323	1285.00
atg	M	17627	17627.00
act	T	0	1.26
acc	T	0	1.03
aca	T	0	2.29
acg	T	5	0.43
aaa	K	1	3.99
aag	K	6	3.01
aga	R	0	2.51
agg	R	5	1.24
gtt	V	0	0.22
gtc	V	0	0.18
gta	V	0	0.27
gtg	V	1	0.33
---	--	------	------
mPD		0.27	0.29
		nPD:	0.94
		N. weight:	0.16
		Sc. PD:	0.14
		Sc. rank:	2735.9

,

PB1			
Pos.	398	obs:	exp:
aat	N	45	26.61
aac	N	0	18.39
gat	D	20837	11011.00
gac	D	60	9886.00
gaa	E	7	7.27
gag	E	5	4.73
ggt	G	18	2.73
ggc	G	0	2.67
gga	G	0	8.01
ggg	G	0	4.60
---	--	------	------
mPD		0.013	0.51
		nPD:	0.03
		N. weight:	0.66
		Sc. PD:	-0.0093
		Sc. rank:	4659.0

,

PB1

Pos.	399	obs:	exp:
tct	S	0	0.92
tcc	S	0	0.58
tca	S	0	1.30
tcg	S	0	0.27
agt	S	0	0.94
agc	S	5	0.99
gat	D	0	9.48
gac	D	18	8.52
ggt	G	37	3177.00
ggc	G	20889	3103.00
gga	G	22	9319.00
ggg	G	1	5351.00
---	--	------	------
mPD		0.0079	0.69
		nPD:	0.01
		N. weight:	2.
		Sc. PD:	-0.056
		Sc. rank:	-1263.5

,

PB1

Pos.	400	obs:	exp:
tct	S	0	0.55
tcc	S	0	0.35
tca	S	3	0.78
tcg	S	0	0.16
act	T	72	5284.00
acc	T	21	4300.00
aca	T	20849	9590.00
acg	T	20	1789.00
aaa	K	1	0.57
aag	K	0	0.43
agt	S	0	0.56
agc	S	0	0.60
gct	A	0	1.59
gcc	A	0	1.24
gca	A	6	2.82
gcg	A	0	0.35
---	--	------	------
mPD		0.012	0.68
		nPD:	0.02
		N. weight:	0.79
		Sc. PD:	-0.018
		Sc. rank:	2883.9

,

PB1

Pos.	401	obs:	exp:
gtt	V	0	0.22
gtc	V	0	0.18
gta	V	1	0.27
gtg	V	0	0.33
gct	A	19	5552.00
gcc	A	0	4341.00
gca	A	20944	9849.00
gcg	A	8	1229.00
---	--	------	------
mPD		0.0027	0.66
		nPD:	0.
		N. weight:	0.79
		Sc. PD:	-0.028
		Sc. rank:	-9769.9

,

PB1

Pos.	402	obs:	exp:
tct	S	2	3859.00
tcc	S	2	2429.00
tca	S	20960	5458.00
tcg	S	7	1116.00
cct	P	0	0.28
ccc	P	0	0.19
cca	P	1	0.43
ccg	P	0	0.11
agt	S	0	3943.00
agc	S	0	4166.00
---	--	------	------
mPD		0.0011	1.6
		nPD:	0.
		N. weight:	1.4
		Sc. PD:	-0.055
		Sc. rank:	-23791.0

,

PB1

Pos.	403	obs:	exp:
tta	L	0	2306.00
ttg	L	76	3948.00
ctt	L	3	4133.00
ctc	L	0	2962.00
cta	L	80	4037.00
ctg	L	20813	3586.00
---	--	------	------
mPD		0.015	1.1
		nPD:	0.01
		N. weight:	1.8
		Sc. PD:	-0.047
		Sc. rank:	1973.9

,

PB1

Pos.	404	obs:	exp:
tct	S	0	3859.00
tcc	S	0	2429.00
tca	S	0	5458.00
tcg	S	0	1116.00
agt	S	20871	3943.00
agc	S	101	4166.00
---	--	------	------
mPD		0.0096	1.6
		nPD:	0.01
		N. weight:	1.7
		Sc. PD:	-0.058
		Sc. rank:	-14668.0

,

PB1

Pos.	405	obs:	exp:
cct	P	20923	5778.00
ccc	P	34	3890.00
cca	P	8	8930.00
ccg	P	7	2375.00
---	--	------	------
mPD		0.0047	0.70
		nPD:	0.01
		N. weight:	1.3
		Sc. PD:	-0.044
		Sc. rank:	-9250.6

,

PB1

Pos.	406	obs:	exp:
ggt	G	21	3180.00
ggc	G	0	3106.00
gga	G	356	9329.00
ggg	G	20595	5357.00
---	--	------	------
mPD		0.035	0.69
		nPD:	0.05
		N. weight:	1.3
		Sc. PD:	0.015
		Sc. rank:	15676.0

,

PB1

Pos.	407	obs:	exp:
atg	M	20972	20972.00
---	--	------	------
mPD		0	0
		nPD:	1.
		N. weight:	0.
		Sc. PD:	0
		Sc. rank:	0

,

PB1

Pos.	408	obs:	exp:
atg	M	20972	20972.00
---	--	------	------
mPD		0	0
		nPD:	1.
		N. weight:	0.
		Sc. PD:	0
		Sc. rank:	0

,

PB1

Pos.	409	obs:	exp:
tta	L	0	0.11
ttg	L	1	0.19
ctt	L	0	0.20
ctc	L	0	0.14
cta	L	0	0.19
ctg	L	0	0.17
atg	M	20970	20970.00
gtt	V	0	0.22
gtc	V	0	0.18
gta	V	0	0.27
gtg	V	1	0.33
---	--	------	------
mPD		0.00019	0.00032
		nPD:	0.6
		N. weight:	0.00014
		Sc. PD:	0.000078
		Sc. rank:	2.4

,

PB1

Pos.	410	obs:	exp:
ggt	G	24	3180.00
ggc	G	20897	3106.00
gga	G	51	9329.00
ggg	G	0	5357.00
---	--	------	------
mPD		0.0071	0.69
		nPD:	0.01
		N. weight:	2.
		Sc. PD:	-0.058
		Sc. rank:	-3732.2

,

PB1

Pos.	411	obs:	exp:
atg	M	20972	20972.00
---	--	------	------
mPD		0	0
		nPD:	1.
		N. weight:	0.
		Sc. PD:	0
		Sc. rank:	0

,

PB1

Pos.	412	obs:	exp:
ttt	F	45	9000.00
ttc	F	20927	11972.00
---	--	------	------
mPD		0.0043	0.49
		nPD:	0.01
		N. weight:	0.58
		Sc. PD:	-0.018
		Sc. rank:	-2142.7

,

PB1

Pos.	413	obs:	exp:
aat	N	51	12404.00
aac	N	20921	8568.00
---	--	------	------
mPD		0.0049	0.48
		nPD:	0.01
		N. weight:	0.93
		Sc. PD:	-0.027
		Sc. rank:	-1906.3

,

PB1

Pos.	414	obs:	exp:
atg	M	20972	20972.00
---	--	------	------
mPD		0	0
		nPD:	1.
		N. weight:	0.
		Sc. PD:	0
		Sc. rank:	0

,

PB1

Pos.	415	obs:	exp:
tta	L	13	2306.00
ttg	L	1	3948.00
ctt	L	1	4133.00
ctc	L	7	2962.00
cta	L	20934	4037.00
ctg	L	16	3586.00
---	--	------	------
mPD		0.0037	1.1
		nPD:	0.
		N. weight:	1.7
		Sc. PD:	-0.062
		Sc. rank:	-23652.0

,

PB1

Pos.	416	obs:	exp:
tct	S	0	3859.00
tcc	S	0	2429.00
tca	S	0	5458.00
tcg	S	0	1116.00
agt	S	20896	3943.00
agc	S	76	4166.00
---	--	------	------
mPD		0.0072	1.6
		nPD:	0.
		N. weight:	1.7
		Sc. PD:	-0.061
		Sc. rank:	-19745.0

,

PB1

Pos.	417	obs:	exp:
act	T	2298	5287.00
acc	T	1	4302.00
aca	T	257	9594.00
acg	T	18416	1789.00
---	--	------	------
mPD		0.22	0.68
		nPD:	0.32
		N. weight:	2.
		Sc. PD:	0.56
		Sc. rank:	32698.0

,

PB1

Pos.	418	obs:	exp:
att	I	0	1.46
atc	I	4	1.00
ata	I	0	1.55
gtt	V	61	4636.00
gtc	V	20555	3810.00
gta	V	350	5639.00
gtg	V	2	6883.00
---	--	------	------
mPD		0.039	0.74
		nPD:	0.05
		N. weight:	1.7
		Sc. PD:	0.022
		Sc. rank:	19910.0

,

PB1

Pos.	419	obs:	exp:
tta	L	55	2305.00
ttg	L	20871	3948.00
ctt	L	0	4132.00
ctc	L	0	2962.00
cta	L	1	4037.00
ctg	L	44	3586.00
atg	M	1	1.00
---	--	------	------
mPD		0.0097	1.1
		nPD:	0.01
		N. weight:	1.7
		Sc. PD:	-0.053
		Sc. rank:	-7316.8

,

PB1

Pos.	420	obs:	exp:
ggt	G	6	3180.00
ggc	G	2	3106.00
gga	G	20925	9329.00
ggg	G	39	5357.00
---	--	------	------
mPD		0.0045	0.69
		nPD:	0.01
		N. weight:	0.84
		Sc. PD:	-0.028
		Sc. rank:	-6213.7

,

PB1

Pos.	421	obs:	exp:
gtt	V	262	4636.00
gtc	V	20023	3811.00
gta	V	686	5640.00
gtg	V	0	6884.00
gct	A	0	0.26
gcc	A	1	0.21
gca	A	0	0.47
gcg	A	0	0.06
mPD		0.087	0.74
		nPD:	0.12
		N. weight:	1.6
		Sc. PD:	0.12
		Sc. rank:	23190.0

PB1

Pos.	422	obs:	exp:
tct	S	256	3859.00
tcc	S	1	2429.00
tca	S	159	5458.00
tcg	S	20554	1116.00
agt	S	0	3943.00
agc	S	0	4165.00
gct	A	0	0.53
gcc	A	0	0.41
gca	A	0	0.94
gcg	A	2	0.12
mPD		0.039	1.6
		nPD:	0.02
		N. weight:	3.
		Sc. PD:	-0.046
		Sc. rank:	19692.0

PB1

Pos.	423	obs:	exp:
att	I	6	7623.00
atc	I	0	5227.00
ata	I	20934	8090.00
atg	M	3	3.00
act	T	0	0.25
acc	T	0	0.21
aca	T	1	0.46
acg	T	0	0.09
gtt	V	0	6.19
gtc	V	0	5.09
gta	V	28	7.53
gtg	V	0	9.19
mPD		0.0036	0.66
		nPD:	0.01
		N. weight:	1.
		Sc. PD:	-0.034
		Sc. rank:	-8990.8

PB1

Pos.	424	obs:	exp:
tta	L	0	2305.00
ttg	L	73	3948.00
ctt	L	4	4132.00
ctc	L	0	2962.00
cta	L	90	4037.00
ctg	L	20801	3585.00
atg	M	4	4.00
mPD		0.016	1.1
		nPD:	0.01
		N. weight:	1.8
		Sc. PD:	-0.045
		Sc. rank:	3072.6

PB1

Pos.	425	obs:	exp:
aat	N	20914	12404.00
aac	N	58	8568.00
mPD		0.0055	0.48
		nPD:	0.01
		N. weight:	0.54
		Sc. PD:	-0.015
		Sc. rank:	-306.6

PB1

Pos.	426	obs:	exp:
ttt	F	1	0.86
ttc	F	1	1.14
tta	L	0	2303.00
ttg	L	0	3945.00
ctt	L	20007	4129.00
ctc	L	943	2959.00
cta	L	2	4034.00
ctg	L	0	3582.00
att	I	18	6.55
atc	I	0	4.49
ata	I	0	6.95
mPD		0.088	1.1
		nPD:	0.08
		N. weight:	1.5
		Sc. PD:	0.056
		Sc. rank:	20266.0

PB1

Pos.	427	obs:	exp:
ggt	G	0	3180.00
ggc	G	1	3106.00
gga	G	20716	9329.00
ggg	G	255	5357.00
mPD		0.024	0.69
		nPD:	0.03
		N. weight:	0.8
		Sc. PD:	-0.0039
		Sc. rank:	7693.9

PB1

Pos.	428	obs:	exp:
caa	Q	20957	11066.00
cag	Q	14	9905.00
aaa	K	1	0.57
aag	K	0	0.43
mPD		0.0014	0.50
		nPD:	0.
		N. weight:	0.67
		Sc. PD:	-0.024
		Sc. rank:	-9539.7

PB1

Pos.	429	obs:	exp:
cgt	R	0	0.49
cgc	R	0	0.94
cga	R	0	1.76
cgg	R	0	1.32
aaa	K	6	11952.00
aag	K	20947	9001.00
aga	R	0	9.04
agg	R	18	4.45
gaa	E	0	0.61
gag	E	1	0.39
mPD		0.0024	0.49
		nPD:	0.
		N. weight:	0.89
		Sc. PD:	-0.031
		Sc. rank:	-9427.6

PB1

Pos.	430	obs:	exp:
cgt	R	0	2.68
cgc	R	0	5.13
cga	R	0	9.60
cgg	R	0	7.17
aat	N	0	0.59
aac	N	1	0.41
aaa	K	20792	11903.00
aag	K	74	8963.00
aga	R	98	49.20
agg	R	0	24.23
gaa	E	7	4.24
gag	E	0	2.76
mPD		0.017	0.50
		nPD:	0.03
		N. weight:	0.57
		Sc. PD:	-0.0033
		Sc. rank:	5454.3

PB1

Pos.	431	obs:	exp:
tat	Y	47	10886.00
tac	Y	20904	10065.00
tgt	C	0	0.45
tgc	C	1	0.55
cat	H	0	11.76
cac	H	20	8.24
mPD		0.0065	0.50
		nPD:	0.01
		N. weight:	0.76
		Sc. PD:	-0.02
		Sc. rank:	685.6

PB1

Pos.	432	obs:	exp:
att	I	0	0.36
atc	I	1	0.25
ata	I	0	0.39
act	T	22	5286.00
acc	T	20931	4301.00
aca	T	17	9593.00
acg	T	0	1789.00
gct	A	0	0.26
gcc	A	1	0.21
gca	A	0	0.47
gcg	A	0	0.06
mPD		0.0039	0.68
		nPD:	0.01
		N. weight:	1.7
		Sc. PD:	-0.055
		Sc. rank:	-14376.0

PB1

Pos.	433	obs:	exp:
cgt	R	0	0.38
cgc	R	0	0.73
cga	R	0	1.37
cgg	R	0	1.02
aat	N	1	0.59
aac	N	0	0.41
aaa	K	410	11955.00
aag	K	20547	9002.00
aga	R	1	7.03
agg	R	13	3.46
mPD		0.040	0.49
		nPD:	0.08
		N. weight:	0.79
		Sc. PD:	0.032
		Sc. rank:	10658.0

PB1

Pos.	434	obs:	exp:
act	T	1	5287.00
acc	T	0	4302.00
aca	T	20910	9594.00
acg	T	61	1789.00
mPD		0.0059	0.68
		nPD:	0.01
		N. weight:	0.81
		Sc. PD:	-0.025
		Sc. rank:	-3124.6

PB1

Pos.	435	obs:	exp:
cct	P	0	0.28
ccc	P	0	0.19
cca	P	1	0.43
ccg	P	0	0.11
att	I	0	1006.00
atc	I	0	689.70
ata	I	2763	1067.00
atg	M	1	1.00
act	T	1	4557.00
acc	T	2	3708.00
aca	T	18062	8270.00
acg	T	12	1542.00
gtt	V	0	28.30
gtc	V	0	23.26
gta	V	128	34.42
gtg	V	0	42.02
gct	A	0	0.53
gcc	A	0	0.41
gca	A	2	0.94
gcg	A	0	0.12
mPD		0.25	0.93
		nPD:	0.27
		N. weight:	0.85
		Sc. PD:	0.19
		Sc. rank:	13560.0

PB1

Pos.	436	obs:	exp:
tat	Y	126	10895.00
tac	Y	20842	10073.00
tgt	C	0	0.45
tgc	C	1	0.55
cat	H	0	1.76
cac	H	3	1.24
mPD		0.012	0.50
		nPD:	0.02
		N. weight:	0.74
		Sc. PD:	-0.011
		Sc. rank:	5050.9

PB1

Pos.	437	obs:	exp:
tta	L	0	0.11
ttg	L	1	0.19
tgg	W	20971	20971.00
ctt	L	0	0.20
ctc	L	0	0.14
cta	L	0	0.19
ctg	L	0	0.17
mPD		0.000095	0.00022
		nPD:	0.43
		N. weight:	0.000084
		Sc. PD:	0.000032
		Sc. rank:	1.4

PB1			
Pos.	438	obs:	exp:
tgg	W	20972	20972.00
---	--	------	------
mPD		0	0
		nPD:	1.
		N. weight:	0.
		Sc. PD:	0
		Sc. rank:	0

,

PB1			
Pos.	439	obs:	exp:
aat	N	6	3.55
aac	N	0	2.45
gat	D	20945	11047.00
gac	D	21	9919.00
---	--	------	------
mPD		0.0026	0.50
		nPD:	0.01
		N. weight:	0.67
		Sc. PD:	-0.023
		Sc. rank:	-6466.2

,

PB1			
Pos.	440	obs:	exp:
ggt	G	5	3180.00
ggc	G	2	3106.00
gga	G	118	9329.00
ggg	G	20847	5357.00
---	--	------	------
mPD		0.012	0.69
		nPD:	0.02
		N. weight:	1.4
		Sc. PD:	-0.031
		Sc. rank:	5017.4

,

PB1			
Pos.	441	obs:	exp:
tta	L	0	2305.00
ttg	L	0	3948.00
ctt	L	332	4132.00
ctc	L	20636	2962.00
cta	L	2	4037.00
ctg	L	0	3585.00
att	I	0	0.73
atc	I	2	0.50
ata	I	0	0.77
---	--	------	------
mPD		0.032	1.1
		nPD:	0.03
		N. weight:	2.
		Sc. PD:	-0.024
		Sc. rank:	15429.0

,

PB1			
Pos.	442	obs:	exp:
caa	Q	20958	11066.00
cag	Q	14	9906.00
---	--	------	------
mPD		0.0013	0.50
		nPD:	0.
		N. weight:	0.67
		Sc. PD:	-0.024
		Sc. rank:	-9695.0

,

PB1			
Pos.	443	obs:	exp:
tct	S	2	3859.00
tcc	S	15	2429.00
tca	S	20890	5458.00
tcg	S	65	1116.00
agt	S	0	3943.00
agc	S	0	4166.00
---	--	------	------
mPD		0.0078	1.6
		nPD:	0.
		N. weight:	1.4
		Sc. PD:	-0.048
		Sc. rank:	-15092.0

,

PB1			
Pos.	444	obs:	exp:
tct	S	575	3859.00
tcc	S	20365	2429.00
tca	S	32	5458.00
tcg	S	0	1116.00
agt	S	0	3943.00
agc	S	0	4166.00
---	--	------	------
mPD		0.056	1.6
		nPD:	0.03
		N. weight:	2.1
		Sc. PD:	-0.012
		Sc. rank:	20468.0

,

PB1			
Pos.	445	obs:	exp:
aat	N	0	0.59
aac	N	1	0.41
gat	D	129	11050.00
gac	D	20842	9921.00
---	--	------	------
mPD		0.012	0.50
		nPD:	0.02
		N. weight:	0.75
		Sc. PD:	-0.011
		Sc. rank:	5210.0

,

PB1			
Pos.	446	obs:	exp:
gat	D	20921	11050.00
gac	D	51	9922.00
---	--	------	------
mPD		0.0049	0.50
		nPD:	0.01
		N. weight:	0.66
		Sc. PD:	-0.02
		Sc. rank:	-1683.8

,

PB1			
Pos.	447	obs:	exp:
ttt	F	20957	9000.00
ttc	F	15	11972.00
---	--	------	------
mPD		0.0014	0.49
		nPD:	0.
		N. weight:	0.89
		Sc. PD:	-0.032
		Sc. rank:	-12605.0

,

PB1			
Pos.	448	obs:	exp:
tct	S	1	0.18
tcc	S	0	0.12
tca	S	0	0.26
tcg	S	0	0.05
act	T	1	0.25
acc	T	0	0.21
aca	T	0	0.46
acg	T	0	0.09
agt	S	0	0.19
agc	S	0	0.20
gtt	V	2	0.44
gtc	V	0	0.36
gta	V	0	0.54
gtg	V	0	0.66
gct	A	20895	5551.00
gcc	A	45	4340.00
gca	A	6	9848.00
gcg	A	22	1228.00
---	--	------	------
mPD		0.0073	0.66
		nPD:	0.01
		N. weight:	1.4
		Sc. PD:	-0.039
		Sc. rank:	-1541.1

,

PB1			
Pos.	449	obs:	exp:
ttt	F	0	0.43
ttc	F	1	0.57
tta	L	0	2305.00
ttg	L	1	3948.00
ctt	L	191	4132.00
ctc	L	20768	2962.00
cta	L	11	4037.00
ctg	L	0	3586.00
---	--	------	------
mPD		0.019	1.1
		nPD:	0.02
		N. weight:	2.
		Sc. PD:	-0.045
		Sc. rank:	6878.9

,

PB1			
Pos.	450	obs:	exp:
att	I	1	7634.00
atc	I	4	5235.00
ata	I	20966	8102.00
gtt	V	0	0.22
gtc	V	0	0.18
gta	V	1	0.27
gtg	V	0	0.33
---	--	------	------
mPD		0.00057	0.66
		nPD:	0.
		N. weight:	1.
		Sc. PD:	-0.038
		Sc. rank:	-16654.0

,

PB1			
Pos.	451	obs:	exp:
tta	L	0	0.22
ttg	L	2	0.38
ctt	L	0	0.39
ctc	L	0	0.28
cta	L	0	0.39
ctg	L	0	0.34
att	I	0	7.28
atc	I	0	4.99
ata	I	20	7.73
atg	M	15	15.00
gtt	V	7	4628.00
gtc	V	0	3804.00
gta	V	181	5630.00
gtg	V	20747	6872.00
---	--	------	------
mPD		0.023	0.74
		nPD:	0.03
		N. weight:	1.1
		Sc. PD:	-0.0095
		Sc. rank:	9758.9

,

PB1			
Pos.	452	obs:	exp:
act	T	1	0.25
acc	T	0	0.21
aca	T	0	0.46
acg	T	0	0.09
aat	N	20935	12403.00
aac	N	36	8568.00
---	--	------	------
mPD		0.0035	0.48
		nPD:	0.01
		N. weight:	0.54
		Sc. PD:	-0.017
		Sc. rank:	-3427.2

,

PB1			
Pos.	453	obs:	exp:
tct	S	0	0.18
tcc	S	0	0.12
tca	S	1	0.26
tcg	S	0	0.05
agt	S	0	0.19
agc	S	0	0.20
gct	A	2	5552.00
gcc	A	2	4341.00
gca	A	20958	9849.00
gcg	A	9	1229.00
---	--	------	------
mPD		0.0013	0.66
		nPD:	0.
		N. weight:	0.8
		Sc. PD:	-0.03
		Sc. rank:	-12229.0

,

PB1			
Pos.	454	obs:	exp:
tta	L	0	0.11
ttg	L	0	0.19
tct	S	0	27.97
tcc	S	0	17.61
tca	S	152	39.56
tcg	S	0	8.09
ctt	L	0	0.20
ctc	L	0	0.14
cta	L	1	0.19
ctg	L	0	0.17
cct	P	1	5710.00
ccc	P	6	3844.00
cca	P	20415	8825.00
ccg	P	304	2347.00
caa	Q	12	6.33
cag	Q	0	5.67
act	T	0	20.42
acc	T	0	16.61
aca	T	81	37.06
acg	T	0	6.91
agt	S	0	28.58
agc	S	0	30.19
---	--	------	------
mPD		0.052	0.72
		nPD:	0.07
		N. weight:	0.85
		Sc. PD:	0.027
		Sc. rank:	10941.0

,

PB1			
Pos.	455	obs:	exp:
tct	S	0	6.81
tcc	S	0	4.29
tca	S	0	9.63
tcg	S	0	1.97
act	T	0	0.76
acc	T	3	0.62
aca	T	0	1.37
acg	T	0	0.26
aat	N	1246	12369.00
aac	N	19667	8544.00
agt	S	0	6.96
agc	S	37	7.35
gat	D	2	10.01
gac	D	17	8.99
---	--	------	------
mPD		0.12	0.49
		nPD:	0.24
		N. weight:	0.69
		Sc. PD:	0.13
		Sc. rank:	10875.0

,

PB1			
Pos.	456	obs:	exp:
tta	L	0	0.33
ttg	L	0	0.56
tat	Y	29	15.07
tac	Y	0	13.93
ctt	L	3	0.59
ctc	L	0	0.42
cta	L	0	0.58
ctg	L	0	0.51
cat	H	20026	12307.00
cac	H	901	8620.00
caa	Q	4	2.64
cag	Q	1	2.36
aat	N	7	4.73
aac	N	1	3.27
---	--	------	------
mPD		0.087	0.49
		nPD:	0.18
		N. weight:	0.39
		Sc. PD:	0.053
		Sc. rank:	6001.0

,

PB1			
Pos.	457	obs:	exp:
aaa	K	9	5.13
aag	K	0	3.87
gtt	V	0	20.78
gtc	V	0	17.08
gta	V	92	25.28
gtg	V	2	30.86
gct	A	0	0.79
gcc	A	0	0.62
gca	A	3	1.41
gcg	A	0	0.18
gat	D	2	15.81
gac	D	28	14.19
gaa	E	17747	12610.00
gag	E	3078	8215.00
ggt	G	0	1.67
ggc	G	0	1.63
gga	G	10	4.89
ggg	G	1	2.81
---	--	------	------
mPD		0.26	0.49
		nPD:	0.54
		N. weight:	0.16
		Sc. PD:	0.078
		Sc. rank:	2733.0

,

PB1			
Pos.	458	obs:	exp:
gaa	E	5	3.03
gag	E	0	1.97
ggt	G	8	3179.00
ggc	G	0	3105.00
gga	G	20779	9327.00
ggg	G	180	5356.00
---	--	------	------
mPD		0.018	0.69
		nPD:	0.03
		N. weight:	0.81
		Sc. PD:	-0.011
		Sc. rank:	5976.1

,

PB1			
Pos.	459	obs:	exp:
att	I	4	7629.00
atc	I	917	5231.00
ata	I	20035	8096.00
atg	M	1	1.00
gtt	V	0	2.87
gtc	V	0	2.36
gta	V	13	3.50
gtg	V	0	4.27
gct	A	0	0.53
gcc	A	0	0.41
gca	A	2	0.94
gcg	A	0	0.12
---	--	------	------
mPD		0.086	0.66
		nPD:	0.13
		N. weight:	0.84
		Sc. PD:	0.074
		Sc. rank:	12541.0

,

PB1			
Pos.	460	obs:	exp:
cat	H	1	0.59
cac	H	0	0.41
caa	Q	20942	11063.00
cag	Q	24	9903.00
cgt	R	0	0.03
cgc	R	0	0.05
cga	R	1	0.10
cgg	R	0	0.07
aaa	K	4	2.28
aag	K	0	1.72
aga	R	0	0.50
agg	R	0	0.25
---	--	------	------
mPD		0.0029	0.50
		nPD:	0.01
		N. weight:	0.67
		Sc. PD:	-0.022
		Sc. rank:	-5895.5

,

PB1			
Pos.	461	obs:	exp:
tct	S	0	0.92
tcc	S	0	0.58
tca	S	5	1.30
tcg	S	0	0.27
act	T	0	1.26
acc	T	0	1.03
aca	T	5	2.29
acg	T	0	0.43
agt	S	0	0.94
agc	S	0	0.99
gtt	V	0	0.22
gtc	V	0	0.18
gta	V	1	0.27
gtg	V	0	0.33
gct	A	27	5549.00
gcc	A	0	4339.00
gca	A	20917	9844.00
gcg	A	16	1228.00
gaa	E	1	0.61
gag	E	0	0.39
---	--	------	------
mPD		0.0052	0.66
		nPD:	0.01
		N. weight:	0.79
		Sc. PD:	-0.025
		Sc. rank:	-3859.4

,

PB1			
Pos.	462	obs:	exp:
ggt	G	2	3180.00
ggc	G	1	3106.00
gga	G	20889	9329.00
ggg	G	80	5357.00
---	--	------	------
mPD		0.0079	0.69
		nPD:	0.01
		N. weight:	0.83
		Sc. PD:	-0.023
		Sc. rank:	-495.2

,

PB1			
Pos.	463	obs:	exp:
atg	M	1	1.00
gtt	V	45	4636.00
gtc	V	0	3811.00
gta	V	260	5640.00
gtg	V	20666	6884.00
---	--	------	------
mPD		0.029	0.74
		nPD:	0.04
		N. weight:	1.1
		Sc. PD:	-0.00087
		Sc. rank:	11142.0

,

PB1			
Pos.	464	obs:	exp:
aat	N	4	75.70
aac	N	124	52.30
gat	D	44	10982.00
gac	D	20799	9861.00
gaa	E	1	0.61
gag	E	0	0.39
---	--	------	------
mPD		0.017	0.51
		nPD:	0.03
		N. weight:	0.78
		Sc. PD:	-0.0053
		Sc. rank:	7112.8

,

PB1			
Pos.	465	obs:	exp:
cgt	R	0	572.10
cgc	R	0	1096.00
cga	R	20	2052.00
cgg	R	0	1533.00
aaa	K	19	10.84
aag	K	0	8.16
aga	R	20654	10519.00
agg	R	279	5180.00
---	--	------	------
mPD		0.030	0.91
		nPD:	0.03
		N. weight:	0.66
		Sc. PD:	-0.0045
		Sc. rank:	6077.8

,

PB1			
Pos.	466	obs:	exp:
ttt	F	695	8999.00
ttc	F	20275	11971.00
tat	Y	0	1.04
tac	Y	2	0.96
---	--	------	------
mPD		0.064	0.49
		nPD:	0.13
		N. weight:	0.45
		Sc. PD:	0.04
		Sc. rank:	6759.6

,

PB1

Pos.	467	obs:	exp:
tat	Y	45	10897.00
tac	Y	20927	10075.00
---	--	------	------
mPD		0.0043	0.50
		nPD:	0.01
		N. weight:	0.76
		Sc. PD:	-0.023
		Sc. rank:	-3128.2

PB1

Pos.	468	obs:	exp:
cgt	R	0	572.60
cgc	R	0	1097.00
cga	R	1	2054.00
cgg	R	1	1535.00
aaa	K	0	0.57
aag	K	1	0.43
aga	R	57	10528.00
agg	R	20912	5184.00
---	--	------	------
mPD		0.0058	0.91
		nPD:	0.01
		N. weight:	1.5
		Sc. PD:	-0.048
		Sc. rank:	-11081.0

PB1

Pos.	469	obs:	exp:
tct	S	0	8.83
tcc	S	2	5.56
tca	S	0	12.49
tcg	S	0	2.55
att	I	0	8.74
atc	I	24	5.99
ata	I	0	9.27
act	T	229	5264.00
acc	T	20632	4283.00
aca	T	19	9552.00
acg	T	0	1782.00
agt	S	0	9.03
agc	S	46	9.53
gct	A	0	5.30
gcc	A	20	4.14
gca	A	0	9.39
gcg	A	0	1.17
---	--	------	------
mPD		0.032	0.69
		nPD:	0.05
		N. weight:	1.6
		Sc. PD:	0.011
		Sc. rank:	18149.0

PB1

Pos.	470	obs:	exp:
tct	S	0	0.18
tcc	S	0	0.12
tca	S	0	0.26
tcg	S	0	0.05
tgt	C	9	9465.00
tgc	C	20962	11506.00
agt	S	0	0.19
agc	S	1	0.20
---	--	------	------
mPD		0.00095	0.50
		nPD:	0.
		N. weight:	0.63
		Sc. PD:	-0.023
		Sc. rank:	-9809.1

PB1

Pos.	471	obs:	exp:
cgt	R	0	0.03
cgc	R	0	0.05
cga	R	0	0.10
cgg	R	0	0.07
aaa	K	6956	11962.00
aag	K	14015	9009.00
aga	R	0	0.50
agg	R	1	0.25
---	--	------	------
mPD		0.44	0.49
		nPD:	0.9
		N. weight:	0.12
		Sc. PD:	0.1
		Sc. rank:	2096.9

PB1

Pos.	472	obs:	exp:
tta	L	20869	2306.00
ttg	L	50	3948.00
ctt	L	0	4133.00
ctc	L	0	2962.00
cta	L	51	4037.00
ctg	L	2	3586.00
---	--	------	------
mPD		0.010	1.1
		nPD:	0.01
		N. weight:	2.3
		Sc. PD:	-0.07
		Sc. rank:	-8198.0

PB1

Pos.	473	obs:	exp:
tta	L	0	0.11
ttg	L	0	0.19
ctt	L	0	0.20
ctc	L	1	0.14
cta	L	0	0.19
ctg	L	0	0.17
atg	M	41	41.00
gtt	V	11	4627.00
gtc	V	0	3803.00
gta	V	73	5629.00
gtg	V	20846	6871.00
---	--	------	------
mPD		0.012	0.74
		nPD:	0.02
		N. weight:	1.2
		Sc. PD:	-0.027
		Sc. rank:	3550.7

PB1

Pos.	474	obs:	exp:
ggt	G	1	3180.00
ggc	G	0	3106.00
gga	G	20935	9329.00
ggg	G	36	5357.00
---	--	------	------
mPD		0.0035	0.69
		nPD:	0.01
		N. weight:	0.85
		Sc. PD:	-0.029
		Sc. rank:	-8456.1

PB1

Pos.	475	obs:	exp:
att	I	33	7633.00
atc	I	20807	5234.00
ata	I	128	8101.00
gtt	V	0	0.88
gtc	V	4	0.73
gta	V	0	1.08
gtg	V	0	1.31
---	--	------	------
mPD		0.016	0.66
		nPD:	0.02
		N. weight:	1.4
		Sc. PD:	-0.022
		Sc. rank:	9165.9

PB1

Pos.	476	obs:	exp:
tct	S	0	0.18
tcc	S	0	0.12
tca	S	0	0.26
tcg	S	0	0.05
aat	N	55	12403.00
aac	N	20916	8568.00
agt	S	0	0.19
agc	S	1	0.20
---	--	------	------
mPD		0.0053	0.48
		nPD:	0.01
		N. weight:	0.93
		Sc. PD:	-0.026
		Sc. rank:	-1158.1

PB1

Pos.	477	obs:	exp:
att	I	0	0.36
atc	I	0	0.25
ata	I	1	0.39
atg	M	20971	20971.00
---	--	------	------
mPD		0.000095	0.000095
		nPD:	1.
		N. weight:	0.000048
		Sc. PD:	0.000045
		Sc. rank:	0.8

PB1

Pos.	478	obs:	exp:
tct	S	0	3859.00
tcc	S	0	2429.00
tca	S	0	5458.00
tcg	S	0	1116.00
agt	S	54	3943.00
agc	S	20918	4166.00
---	--	------	------
mPD		0.0051	1.6
		nPD:	0.
		N. weight:	1.7
		Sc. PD:	-0.061
		Sc. rank:	-23475.0

PB1

Pos.	479	obs:	exp:
aaa	K	20898	11963.00
aag	K	74	9009.00
---	--	------	------
mPD		0.0070	0.49
		nPD:	0.01
		N. weight:	0.57
		Sc. PD:	-0.014
		Sc. rank:	1016.1

PB1

Pos.	480	obs:	exp:
cgt	R	0	4.40
cgc	R	0	8.42
cga	R	0	15.77
cgg	R	0	11.78
aaa	K	7	11871.00
aag	K	20804	8940.00
aga	R	1	80.83
agg	R	160	39.80
---	--	------	------
mPD		0.016	0.51
		nPD:	0.03
		N. weight:	0.9
		Sc. PD:	-0.0074
		Sc. rank:	7805.7

PB1

Pos.	481	obs:	exp:
cgt	R	0	0.03
cgc	R	0	0.05
cga	R	0	0.10
cgg	R	0	0.07
aaa	K	822	11962.00
aag	K	20149	9009.00
aga	R	0	0.50
agg	R	1	0.25
---	--	------	------
mPD		0.075	0.49
		nPD:	0.15
		N. weight:	0.71
		Sc. PD:	0.079
		Sc. rank:	10850.0

PB1

Pos.	482	obs:	exp:
tct	S	462	3859.00
tcc	S	20466	2429.00
tca	S	44	5458.00
tcg	S	0	1116.00
agt	S	0	3943.00
agc	S	0	4166.00
---	--	------	------
mPD		0.047	1.6
		nPD:	0.03
		N. weight:	2.1
		Sc. PD:	-0.023
		Sc. rank:	17084.0

PB1			
Pos.	483	obs:	exp:
tat	Y	20926	10897.00
tac	Y	46	10075.00
---	--	------	------
mPD		0.0044	0.50
		nPD:	0.01
		N. weight:	0.68
		Sc. PD:	-0.021
		Sc. rank:	-2394.1

,

PB1			
Pos.	484	obs:	exp:
tta	L	1	0.22
ttg	L	0	0.38
ctt	L	0	0.39
ctc	L	0	0.28
cta	L	1	0.39
ctg	L	0	0.34
att	I	0	7632.00
atc	I	0	5233.00
ata	I	20965	8100.00
atg	M	3	3.00
gtt	V	0	0.44
gtc	V	0	0.36
gta	V	2	0.54
gtg	V	0	0.66
---	--	------	------
mPD		0.00067	0.66
		nPD:	0.
		N. weight:	1.
		Sc. PD:	-0.038
		Sc. rank:	-16583.0

,

PB1			
Pos.	485	obs:	exp:
aat	N	20893	12404.00
aac	N	79	8568.00
---	--	------	------
mPD		0.0075	0.48
		nPD:	0.02
		N. weight:	0.53
		Sc. PD:	-0.013
		Sc. rank:	1382.0

,

PB1			
Pos.	486	obs:	exp:
cgt	R	0	1.37
cgc	R	0	2.62
cga	R	0	4.90
cgg	R	0	3.66
aaa	K	499	11935.00
aag	K	20423	8987.00
aga	R	1	25.10
agg	R	49	12.36
---	--	------	------
mPD		0.051	0.50
		nPD:	0.1
		N. weight:	0.77
		Sc. PD:	0.048
		Sc. rank:	11038.0

,

PB1			
Pos.	487	obs:	exp:
act	T	55	5287.00
acc	T	16	4302.00
aca	T	20880	9594.00
acg	T	21	1789.00
---	--	------	------
mPD		0.0087	0.68
		nPD:	0.01
		N. weight:	0.8
		Sc. PD:	-0.021
		Sc. rank:	693.2

,

PB1			
Pos.	488	obs:	exp:
ggt	G	38	3180.00
ggc	G	1	3106.00
gga	G	418	9329.00
ggg	G	20515	5357.00
---	--	------	------
mPD		0.043	0.69
		nPD:	0.06
		N. weight:	1.3
		Sc. PD:	0.028
		Sc. rank:	16344.0

,

PB1			
Pos.	489	obs:	exp:
act	T	8	5287.00
acc	T	2	4302.00
aca	T	20925	9594.00
acg	T	37	1789.00
---	--	------	------
mPD		0.0045	0.68
		nPD:	0.01
		N. weight:	0.81
		Sc. PD:	-0.027
		Sc. rank:	-5820.8

,

PB1			
Pos.	490	obs:	exp:
ttt	F	20929	9000.00
ttc	F	43	11972.00
---	--	------	------
mPD		0.0041	0.49
		nPD:	0.01
		N. weight:	0.88
		Sc. PD:	-0.027
		Sc. rank:	-3837.7

,

PB1			
Pos.	491	obs:	exp:
gaa	E	20952	12699.00
gag	E	20	8273.00
---	--	------	------
mPD		0.0019	0.48
		nPD:	0.
		N. weight:	0.52
		Sc. PD:	-0.018
		Sc. rank:	-6523.1

,

PB1			
Pos.	492	obs:	exp:
ttt	F	25	8999.00
ttc	F	20946	11972.00
tta	L	0	0.11
ttg	L	0	0.19
ctt	L	0	0.20
ctc	L	1	0.14
cta	L	0	0.19
ctg	L	0	0.17
---	--	------	------
mPD		0.0025	0.49
		nPD:	0.01
		N. weight:	0.58
		Sc. PD:	-0.02
		Sc. rank:	-5869.9

,

PB1			
Pos.	493	obs:	exp:
act	T	28	5287.00
acc	T	0	4302.00
aca	T	20915	9594.00
acg	T	29	1789.00
---	--	------	------
mPD		0.0054	0.68
		nPD:	0.01
		N. weight:	0.81
		Sc. PD:	-0.025
		Sc. rank:	-3921.5

,

PB1			
Pos.	494	obs:	exp:
tct	S	0	3859.00
tcc	S	0	2429.00
tca	S	0	5458.00
tcg	S	0	1116.00
agt	S	2	3943.00
agc	S	20970	4166.00
---	--	------	------
mPD		0.00019	1.6
		nPD:	0.
		N. weight:	1.7
		Sc. PD:	-0.067
		Sc. rank:	-29253.0

,

PB1			
Pos.	495	obs:	exp:
ttt	F	20953	8999.00
ttc	F	18	11972.00
tct	S	1	0.18
tcc	S	0	0.12
tca	S	0	0.26
tcg	S	0	0.05
agt	S	0	0.19
agc	S	0	0.20
---	--	------	------
mPD		0.0018	0.49
		nPD:	0.
		N. weight:	0.89
		Sc. PD:	-0.031
		Sc. rank:	-11443.0

,

PB1			
Pos.	496	obs:	exp:
ttt	F	20961	9000.00
ttc	F	11	11972.00
---	--	------	------
mPD		0.0010	0.49
		nPD:	0.
		N. weight:	0.89
		Sc. PD:	-0.033
		Sc. rank:	-13570.0

,

PB1			
Pos.	497	obs:	exp:
tat	Y	20926	10897.00
tac	Y	46	10075.00
---	--	------	------
mPD		0.0044	0.50
		nPD:	0.01
		N. weight:	0.68
		Sc. PD:	-0.021
		Sc. rank:	-2394.1

,

PB1			
Pos.	498	obs:	exp:
cgt	R	20	572.60
cgc	R	20921	1097.00
cga	R	31	2054.00
cgg	R	0	1535.00
aga	R	0	10529.00
agg	R	0	5185.00
---	--	------	------
mPD		0.0049	0.91
		nPD:	0.01
		N. weight:	3.1
		Sc. PD:	-0.11
		Sc. rank:	-28741.0

,

PB1			
Pos.	499	obs:	exp:
ttt	F	1	0.43
ttc	F	0	0.57
tat	Y	20941	10896.00
tac	Y	29	10074.00
tgt	C	1	0.45
tgc	C	0	0.55
---	--	------	------
mPD		0.0030	0.50
		nPD:	0.01
		N. weight:	0.68
		Sc. PD:	-0.023
		Sc. rank:	-5714.0

,

PB1			
Pos.	500	obs:	exp:
ggt	G	5	3180.00
ggc	G	1	3106.00
gga	G	20758	9329.00
ggg	G	208	5357.00
---	--	------	------
mPD		0.020	0.69
		nPD:	0.03
		N. weight:	0.8
		Sc. PD:	-0.0084
		Sc. rank:	6428.3

,

PB1			
Pos.	501	obs:	exp:
ttt	F	20961	9000.00
ttc	F	11	11972.00
---	--	------	------
mPD		0.0010	0.49
		nPD:	0.
		N. weight:	0.89
		Sc. PD:	-0.033
		Sc. rank:	-13570.0

,

PB1			
Pos.	502	obs:	exp:
tta	L	0	0.22
ttg	L	2	0.38
ctt	L	0	0.39
ctc	L	0	0.28
cta	L	0	0.39
ctg	L	0	0.34
atg	M	1	1.00
gtt	V	18	4636.00
gtc	V	1	3810.00
gta	V	35	5639.00
gtg	V	20914	6883.00
gct	A	0	0.26
gcc	A	0	0.21
gca	A	0	0.47
gcg	A	1	0.06
---	--	------	------
mPD		0.0055	0.74
		nPD:	0.01
		N. weight:	1.2
		Sc. PD:	-0.037
		Sc. rank:	-7051.2

,

PB1

Pos.	503	obs:	exp:
gct	A	20919	5552.00
gcc	A	51	4341.00
gca	A	1	9850.00
gcg	A	1	1229.00
---	--	------	------
mPD		0.0050	0.66
		nPD:	0.01
		N. weight:	1.4
		Sc. PD:	-0.044
		Sc. rank:	-7902.5

PB1

Pos.	504	obs:	exp:
aat	N	20855	12403.00
aac	N	116	8568.00
gat	D	1	0.53
gac	D	0	0.47
---	--	------	------
mPD		0.011	0.48
		nPD:	0.02
		N. weight:	0.52
		Sc. PD:	-0.0086
		Sc. rank:	3277.6

PB1

Pos.	505	obs:	exp:
ttt	F	20912	9000.00
ttc	F	60	11972.00
---	--	------	------
mPD		0.0057	0.49
		nPD:	0.01
		N. weight:	0.88
		Sc. PD:	-0.024
		Sc. rank:	-225.1

PB1

Pos.	506	obs:	exp:
tct	S	0	3859.00
tcc	S	0	2429.00
tca	S	0	5457.00
tcg	S	0	1116.00
agt	S	38	3943.00
agc	S	20930	4165.00
ggt	G	0	0.61
ggc	G	4	0.59
gga	G	0	1.78
ggg	G	0	1.02
---	--	------	------
mPD		0.0040	1.6
		nPD:	0.
		N. weight:	1.7
		Sc. PD:	-0.062
		Sc. rank:	-25104.0

PB1

Pos.	507	obs:	exp:
att	I	0	3.64
atc	I	0	2.50
ata	I	10	3.86
atg	M	20957	20957.00
act	T	0	0.76
acc	T	0	0.62
aca	T	0	1.37
acg	T	3	0.26
gtt	V	0	0.44
gtc	V	0	0.36
gta	V	0	0.54
gtg	V	2	0.66
---	--	------	------
mPD		0.0014	0.0018
		nPD:	0.79
		N. weight:	0.00097
		Sc. PD:	0.00071
		Sc. rank:	16.5

PB1

Pos.	508	obs:	exp:
gat	D	1	0.53
gac	D	0	0.47
gaa	E	86	12698.00
gag	E	20885	8273.00
---	--	------	------
mPD		0.0083	0.48
		nPD:	0.02
		N. weight:	0.96
		Sc. PD:	-0.021
		Sc. rank:	3544.3

PB1

Pos.	509	obs:	exp:
tta	L	11	2305.00
ttg	L	0	3948.00
ctt	L	2	4132.00
ctc	L	1	2962.00
cta	L	20916	4037.00
ctg	L	40	3585.00
att	I	0	0.73
atc	I	0	0.50
ata	I	2	0.77
---	--	------	------
mPD		0.0053	1.1
		nPD:	0.
		N. weight:	1.7
		Sc. PD:	-0.059
		Sc. rank:	-18824.0

PB1

Pos.	510	obs:	exp:
tct	S	0	0.55
tcc	S	3	0.35
tca	S	0	0.78
tcg	S	0	0.16
cct	P	152	5777.00
ccc	P	20794	3889.00
cca	P	23	8929.00
ccg	P	0	2374.00
agt	S	0	0.56
agc	S	0	0.60
---	--	------	------
mPD		0.017	0.70
		nPD:	0.02
		N. weight:	1.7
		Sc. PD:	-0.026
		Sc. rank:	11726.0

PB1

Pos.	511	obs:	exp:
tct	S	0	3858.00
tcc	S	0	2428.00
tca	S	0	5457.00
tcg	S	0	1116.00
aat	N	0	1.77
aac	N	3	1.23
agt	S	588	3942.00
agc	S	20378	4165.00
ggt	G	0	0.45
ggc	G	3	0.44
gga	G	0	1.33
ggg	G	0	0.77
---	--	------	------
mPD		0.055	1.6
		nPD:	0.03
		N. weight:	1.6
		Sc. PD:	-0.0097
		Sc. rank:	14798.0

PB1

Pos.	512	obs:	exp:
ttt	F	20752	8999.00
ttc	F	218	11971.00
tta	L	0	0.22
ttg	L	1	0.38
ctt	L	1	0.39
ctc	L	0	0.28
cta	L	0	0.39
ctg	L	0	0.34
---	--	------	------
mPD		0.021	0.49
		nPD:	0.04
		N. weight:	0.83
		Sc. PD:	0.0021
		Sc. rank:	8893.9

PB1

Pos.	513	obs:	exp:
gaa	E	1	0.61
gag	E	0	0.39
ggt	G	16	3180.00
ggc	G	2	3106.00
gga	G	20740	9328.00
ggg	G	213	5357.00
---	--	------	------
mPD		0.022	0.69
		nPD:	0.03
		N. weight:	0.8
		Sc. PD:	-0.0064
		Sc. rank:	6977.4

PB1

Pos.	514	obs:	exp:
atg	M	3	3.00
gtt	V	5	4636.00
gtc	V	7	3810.00
gta	V	121	5639.00
gtg	V	20835	6883.00
gct	A	0	0.26
gcc	A	0	0.21
gca	A	0	0.47
gcg	A	1	0.06
---	--	------	------
mPD		0.013	0.74
		nPD:	0.02
		N. weight:	1.1
		Sc. PD:	-0.025
		Sc. rank:	4500.9

PB1

Pos.	515	obs:	exp:
tct	S	20930	3859.00
tcc	S	16	2429.00
tca	S	16	5457.00
tcg	S	6	1116.00
cct	P	3	0.83
ccc	P	0	0.56
cca	P	0	1.28
ccg	P	0	0.34
act	T	1	0.25
acc	T	0	0.21
aca	T	0	0.46
acg	T	0	0.09
agt	S	0	3943.00
agc	S	0	4165.00
---	--	------	------
mPD		0.0040	1.6
		nPD:	0.
		N. weight:	1.8
		Sc. PD:	-0.065
		Sc. rank:	-26135.0

PB1

Pos.	516	obs:	exp:
ggt	G	9	3180.00
ggc	G	1	3106.00
gga	G	20694	9329.00
ggg	G	268	5357.00
---	--	------	------
mPD		0.026	0.69
		nPD:	0.04
		N. weight:	0.79
		Sc. PD:	-0.0016
		Sc. rank:	7962.2

PB1

Pos.	517	obs:	exp:
tta	L	1	0.11
ttg	L	0	0.19
ctt	L	0	0.20
ctc	L	0	0.14
cta	L	0	0.19
ctg	L	0	0.17
att	I	0	19.66
atc	I	0	13.48
ata	I	54	20.86
gtt	V	2	4624.00
gtc	V	2	3800.00
gta	V	20722	5624.00
gtg	V	187	6865.00
gct	A	0	0.53
gcc	A	0	0.41
gca	A	2	0.94
gcg	A	0	0.12
ggt	G	0	0.30
ggc	G	0	0.30
gga	G	2	0.89
ggg	G	0	0.51
---	--	------	------
mPD		0.024	0.74
		nPD:	0.03
		N. weight:	1.3
		Sc. PD:	-0.01
		Sc. rank:	11736.0

PB1

Pos.	518	obs:	exp:
aat	N	20948	12404.00
aac	N	24	8568.00
---	--	------	------
mPD		0.0023	0.48
		nPD:	0.
		N. weight:	0.55
		Sc. PD:	-0.019
		Sc. rank:	-5925.9

PB1			
Pos.	519	obs:	exp:
gaa	E	20946	12699.00
gag	E	26	8273.00
---	--	------	------
mPD		0.0025	0.48
		nPD:	0.01
		N. weight:	0.52
		Sc. PD:	-0.018
		Sc. rank:	-5021.7

,

PB1			
Pos.	520	obs:	exp:
tct	S	23	3859.00
tcc	S	1	2429.00
tca	S	20899	5458.00
tcg	S	49	1116.00
agt	S	0	3943.00
agc	S	0	4166.00
---	--	------	------
mPD		0.0069	1.6
		nPD:	0.
		N. weight:	1.4
		Sc. PD:	-0.049
		Sc. rank:	-16387.0

,

PB1			
Pos.	521	obs:	exp:
tct	S	1	0.18
tcc	S	0	0.12
tca	S	0	0.26
tcg	S	0	0.05
act	T	3	0.76
acc	T	0	0.62
aca	T	0	1.37
acg	T	0	0.26
agt	S	0	0.19
agc	S	0	0.20
gct	A	20914	5551.00
gcc	A	30	4340.00
gca	A	21	9848.00
gcg	A	3	1228.00
---	--	------	------
mPD		0.0055	0.66
		nPD:	0.01
		N. weight:	1.4
		Sc. PD:	-0.043
		Sc. rank:	-6102.6

,

PB1			
Pos.	522	obs:	exp:
gat	D	249	11049.00
gac	D	20721	9921.00
ggt	G	0	0.30
ggc	G	2	0.30
gga	G	0	0.89
ggg	G	0	0.51
---	--	------	------
mPD		0.024	0.50
		nPD:	0.05
		N. weight:	0.72
		Sc. PD:	0.0054
		Sc. rank:	8266.5

,

PB1			
Pos.	523	obs:	exp:
att	I	0	0.36
atc	I	0	0.25
ata	I	1	0.39
atg	M	20971	20971.00
---	--	------	------
mPD		0.000095	0.000095
		nPD:	1.
		N. weight:	0.000048
		Sc. PD:	0.000045
		Sc. rank:	0.8

,

PB1			
Pos.	524	obs:	exp:
tct	S	0	3858.00
tcc	S	0	2428.00
tca	S	0	5456.00
tcg	S	0	1116.00
att	I	1	0.36
atc	I	0	0.25
ata	I	0	0.39
aat	N	3	1.77
aac	N	0	1.23
agt	S	20715	3942.00
agc	S	248	4164.00
ggt	G	5	0.76
ggc	G	0	0.74
gga	G	0	2.22
ggg	G	0	1.28
---	--	------	------
mPD		0.024	1.6
		nPD:	0.01
		N. weight:	1.7
		Sc. PD:	-0.042
		Sc. rank:	3880.5

,

PB1			
Pos.	525	obs:	exp:
att	I	20889	7625.00
atc	I	20	5228.00
ata	I	36	8092.00
atg	M	2	2.00
gtt	V	25	5.53
gtc	V	0	4.54
gta	V	0	6.72
gtg	V	0	8.21
---	--	------	------
mPD		0.0079	0.66
		nPD:	0.01
		N. weight:	1.1
		Sc. PD:	-0.029
		Sc. rank:	71.7

,

PB1			
Pos.	526	obs:	exp:
ggt	G	4	3180.00
ggc	G	1	3106.00
gga	G	20907	9329.00
ggg	G	60	5357.00
---	--	------	------
mPD		0.0062	0.69
		nPD:	0.01
		N. weight:	0.84
		Sc. PD:	-0.025
		Sc. rank:	-2683.0

,

PB1			
Pos.	527	obs:	exp:
att	I	1	26.94
atc	I	0	18.47
ata	I	73	28.59
atg	M	2	2.00
act	T	0	0.50
acc	T	0	0.41
aca	T	2	0.91
acg	T	0	0.17
gtt	V	184	4619.00
gtc	V	2	3796.00
gta	V	20649	5618.00
gtg	V	56	6858.00
gct	A	0	0.79
gcc	A	0	0.62
gca	A	3	1.41
gcg	A	0	0.18
---	--	------	------
mPD		0.031	0.75
		nPD:	0.04
		N. weight:	1.3
		Sc. PD:	0.0023
		Sc. rank:	13919.0

,

PB1			
Pos.	528	obs:	exp:
act	T	7	5287.00
acc	T	3	4302.00
aca	T	20949	9594.00
acg	T	13	1789.00
---	--	------	------
mPD		0.0022	0.68
		nPD:	0.
		N. weight:	0.82
		Sc. PD:	-0.029
		Sc. rank:	-11275.0

,

PB1			
Pos.	529	obs:	exp:
gtt	V	10	4637.00
gtc	V	7	3811.00
gta	V	678	5640.00
gtg	V	20277	6884.00
---	--	------	------
mPD		0.064	0.74
		nPD:	0.09
		N. weight:	1.
		Sc. PD:	0.047
		Sc. rank:	14170.0

,

PB1			
Pos.	530	obs:	exp:
att	I	0	7634.00
atc	I	1	5235.00
ata	I	20969	8102.00
gtt	V	0	0.44
gtc	V	0	0.36
gta	V	2	0.54
gtg	V	0	0.66
---	--	------	------
mPD		0.00029	0.66
		nPD:	0.
		N. weight:	1.
		Sc. PD:	-0.039
		Sc. rank:	-17043.0

,

PB1			
Pos.	531	obs:	exp:
cgt	R	0	0.16
cgc	R	0	0.31
cga	R	0	0.59
cgg	R	0	0.44
aaa	K	297	11960.00
aag	K	20669	9006.00
aga	R	0	3.01
agg	R	6	1.48
---	--	------	------
mPD		0.028	0.49
		nPD:	0.06
		N. weight:	0.81
		Sc. PD:	0.015
		Sc. rank:	9794.2

,

PB1			
Pos.	532	obs:	exp:
tct	S	0	0.18
tcc	S	0	0.12
tca	S	0	0.26
tcg	S	0	0.05
act	T	0	0.76
acc	T	3	0.62
aca	T	0	1.37
acg	T	0	0.26
aat	N	42	12401.00
aac	N	20925	8566.00
agt	S	0	0.19
agc	S	1	0.20
gat	D	0	0.53
gac	D	1	0.47
---	--	------	------
mPD		0.0045	0.48
		nPD:	0.01
		N. weight:	0.93
		Sc. PD:	-0.028
		Sc. rank:	-2812.9

,

PB1			
Pos.	533	obs:	exp:
tct	S	0	2.21
tcc	S	0	1.39
tca	S	0	3.12
tcg	S	0	0.64
aat	N	23	12397.00
aac	N	20937	8563.00
agt	S	0	2.26
agc	S	12	2.38
---	--	------	------
mPD		0.0033	0.49
		nPD:	0.01
		N. weight:	0.94
		Sc. PD:	-0.03
		Sc. rank:	-6263.9

,

PB1			
Pos.	534	obs:	exp:
att	I	4	1.46
atc	I	0	1.00
ata	I	0	1.55
atg	M	20968	20968.00
---	--	------	------
mPD		0.00038	0.00038
		nPD:	1.
		N. weight:	0.0002
		Sc. PD:	0.00019
		Sc. rank:	3.5

,

PB1			
Pos.	535	obs:	exp:
tta	L	1	0.11
ttg	L	0	0.19
ctt	L	0	0.20
ctc	L	0	0.14
cta	L	0	0.19
ctg	L	0	0.17
att	I	2	7623.00
atc	I	0	5228.00
ata	I	20940	8091.00
atg	M	9	9.00
act	T	0	0.50
acc	T	0	0.41
aca	T	2	0.91
acg	T	0	0.17
gtt	V	0	3.98
gtc	V	0	3.27
gta	V	18	4.84
gtg	V	0	5.91
---	--	------	------
mPD		0.0030	0.66
		nPD:	0.
		N. weight:	1.
		Sc. PD:	-0.035
		Sc. rank:	-11060.0

,

PB1			
Pos.	536	obs:	exp:
aat	N	27	12404.00
aac	N	20945	8568.00
---	--	------	------
mPD		0.0026	0.48
		nPD:	0.01
		N. weight:	0.94
		Sc. PD:	-0.032
		Sc. rank:	-8875.6

,

PB1			
Pos.	537	obs:	exp:
tct	S	0	3.50
tcc	S	0	2.20
tca	S	0	4.94
tcg	S	0	1.01
aat	N	20932	12392.00
aac	N	21	8561.00
agt	S	19	3.57
agc	S	0	3.77
---	--	------	------
mPD		0.0038	0.49
		nPD:	0.01
		N. weight:	0.55
		Sc. PD:	-0.017
		Sc. rank:	-2752.4

,

PB1			
Pos.	538	obs:	exp:
gat	D	571	11049.00
gac	D	20399	9921.00
gaa	E	1	0.61
gag	E	0	0.39
ggt	G	0	0.15
ggc	G	1	0.15
gga	G	0	0.44
ggg	G	0	0.26
---	--	------	------
mPD		0.053	0.50
		nPD:	0.11
		N. weight:	0.66
		Sc. PD:	0.043
		Sc. rank:	9531.8

,

PB1			
Pos.	539	obs:	exp:
tta	L	0	2305.00
ttg	L	0	3948.00
ctt	L	20804	4132.00
ctc	L	140	2962.00
cta	L	25	4037.00
ctg	L	2	3586.00
att	I	1	0.36
atc	I	0	0.25
ata	I	0	0.39
---	--	------	------
mPD		0.016	1.1
		nPD:	0.01
		N. weight:	1.7
		Sc. PD:	-0.042
		Sc. rank:	2365.7

,

PB1			
Pos.	540	obs:	exp:
ggt	G	4	3180.00
ggc	G	2	3106.00
gga	G	20920	9329.00
ggg	G	46	5357.00
---	--	------	------
mPD		0.0049	0.69
		nPD:	0.01
		N. weight:	0.84
		Sc. PD:	-0.027
		Sc. rank:	-5455.8

,

PB1			
Pos.	541	obs:	exp:
cct	P	20909	5778.00
ccc	P	16	3890.00
cca	P	46	8930.00
ccg	P	1	2375.00
---	--	------	------
mPD		0.0060	0.70
		nPD:	0.01
		N. weight:	1.3
		Sc. PD:	-0.041
		Sc. rank:	-5438.5

,

PB1			
Pos.	542	obs:	exp:
gct	A	16	5552.00
gcc	A	5	4341.00
gca	A	20940	9850.00
gcg	A	11	1229.00
---	--	------	------
mPD		0.0030	0.66
		nPD:	0.
		N. weight:	0.79
		Sc. PD:	-0.027
		Sc. rank:	-8706.5

,

PB1			
Pos.	543	obs:	exp:
atg	M	1	1.00
act	T	17	5286.00
acc	T	0	4301.00
aca	T	1050	9593.00
acg	T	19903	1789.00
aaa	K	0	0.57
aag	K	1	0.43
---	--	------	------
mPD		0.097	0.68
		nPD:	0.14
		N. weight:	2.3
		Sc. PD:	0.23
		Sc. rank:	35156.0

,

PB1			
Pos.	544	obs:	exp:
tct	S	1	0.18
tcc	S	0	0.12
tca	S	0	0.26
tcg	S	0	0.05
agt	S	0	0.19
agc	S	0	0.20
gct	A	16768	5552.00
gcc	A	4202	4341.00
gca	A	1	9849.00
gcg	A	0	1229.00
---	--	------	------
mPD		0.32	0.66
		nPD:	0.48
		N. weight:	0.93
		Sc. PD:	0.4
		Sc. rank:	15522.0

,

PB1			
Pos.	545	obs:	exp:
caa	Q	177	11066.00
cag	Q	20795	9906.00
---	--	------	------
mPD		0.017	0.50
		nPD:	0.03
		N. weight:	0.74
		Sc. PD:	-0.0046
		Sc. rank:	7003.6

,

PB1			
Pos.	546	obs:	exp:
att	I	0	0.36
atc	I	0	0.25
ata	I	1	0.39
atg	M	20971	20971.00
---	--	------	------
mPD		0.000095	0.000095
		nPD:	1.
		N. weight:	0.000048
		Sc. PD:	0.000045
		Sc. rank:	0.8

,

PB1			
Pos.	547	obs:	exp:
gct	A	20839	5552.00
gcc	A	123	4341.00
gca	A	8	9849.00
gcg	A	1	1229.00
ggt	G	1	0.15
ggc	G	0	0.15
gga	G	0	0.44
ggg	G	0	0.26
---	--	------	------
mPD		0.013	0.66
		nPD:	0.02
		N. weight:	1.4
		Sc. PD:	-0.028
		Sc. rank:	6598.3

,

PB1			
Pos.	548	obs:	exp:
tta	L	0	2305.00
ttg	L	0	3948.00
ctt	L	20880	4132.00
ctc	L	80	2962.00
cta	L	6	4037.00
ctg	L	3	3585.00
att	I	3	1.09
atc	I	0	0.75
ata	I	0	1.16
---	--	------	------
mPD		0.0087	1.1
		nPD:	0.01
		N. weight:	1.7
		Sc. PD:	-0.053
		Sc. rank:	-9490.1

,

PB1			
Pos.	549	obs:	exp:
caa	Q	20925	11066.00
cag	Q	47	9906.00
---	--	------	------
mPD		0.0045	0.50
		nPD:	0.01
		N. weight:	0.66
		Sc. PD:	-0.02
		Sc. rank:	-2073.0

,

PB1			
Pos.	550	obs:	exp:
ttt	F	1	0.43
ttc	F	0	0.57
tta	L	806	2305.00
ttg	L	19882	3948.00
ctt	L	0	4132.00
ctc	L	0	2962.00
cta	L	0	4037.00
ctg	L	282	3585.00
atg	M	1	1.00
---	--	------	------
mPD		0.10	1.1
		nPD:	0.09
		N. weight:	1.5
		Sc. PD:	0.073
		Sc. rank:	21486.0

,

PB1			
Pos.	551	obs:	exp:
ttt	F	26	8999.00
ttc	F	20944	11971.00
tta	L	0	0.22
ttg	L	0	0.38
ctt	L	0	0.39
ctc	L	2	0.28
cta	L	0	0.39
ctg	L	0	0.34
---	--	------	------
mPD		0.0027	0.49
		nPD:	0.01
		N. weight:	0.58
		Sc. PD:	-0.02
		Sc. rank:	-5254.0

,

PB1

Pos.	552	obs:	exp:
tta	L	0	0.11
ttg	L	0	0.19
ctt	L	0	0.20
ctc	L	1	0.14
cta	L	0	0.19
ctg	L	0	0.17
att	I	17	7630.00
atc	I	19283	5232.00
ata	I	1659	8098.00
act	T	0	0.76
acc	T	3	0.62
aca	T	0	1.37
acg	T	0	0.26
gtt	V	0	1.99
gtc	V	9	1.64
gta	V	0	2.42
gtg	V	0	2.95
---	--	------	------
mPD		0.15	0.66
		nPD:	0.23
		N. weight:	1.1
		Sc. PD:	0.21
		Sc. rank:	17831.0

PB1

Pos.	553	obs:	exp:
caa	Q	1	0.53
cag	Q	0	0.47
aaa	K	20920	11962.00
aag	K	51	9009.00
---	--	------	------
mPD		0.0049	0.49
		nPD:	0.01
		N. weight:	0.58
		Sc. PD:	-0.017
		Sc. rank:	-1169.4

PB1

Pos.	554	obs:	exp:
tct	S	0	0.18
tcc	S	0	0.12
tca	S	0	0.26
tcg	S	0	0.05
aat	N	0	0.59
aac	N	1	0.41
agt	S	0	0.19
agc	S	1	0.20
gat	D	257	11048.00
gac	D	20711	9920.00
gaa	E	2	1.21
gag	E	0	0.79
---	--	------	------
mPD		0.025	0.50
		nPD:	0.05
		N. weight:	0.72
		Sc. PD:	0.0068
		Sc. rank:	8329.8

PB1

Pos.	555	obs:	exp:
tat	Y	96	10896.00
tac	Y	20875	10075.00
cat	H	0	0.59
cac	H	1	0.41
---	--	------	------
mPD		0.0092	0.50
		nPD:	0.02
		N. weight:	0.75
		Sc. PD:	-0.016
		Sc. rank:	3407.5

PB1

Pos.	556	obs:	exp:
cgt	R	0	572.60
cgc	R	0	1097.00
cga	R	5	2054.00
cgg	R	0	1535.00
aga	R	20926	10529.00
agg	R	41	5185.00
---	--	------	------
mPD		0.0044	0.91
		nPD:	0.
		N. weight:	0.72
		Sc. PD:	-0.025
		Sc. rank:	-7575.7

PB1

Pos.	557	obs:	exp:
tat	Y	12	10897.00
tac	Y	20960	10075.00
---	--	------	------
mPD		0.0011	0.50
		nPD:	0.
		N. weight:	0.77
		Sc. PD:	-0.028
		Sc. rank:	-11574.0

PB1

Pos.	558	obs:	exp:
act	T	2	5287.00
acc	T	6	4301.00
aca	T	20941	9594.00
acg	T	22	1789.00
gct	A	0	0.26
gcc	A	0	0.21
gca	A	1	0.47
gcg	A	0	0.06
---	--	------	------
mPD		0.0030	0.68
		nPD:	0.
		N. weight:	0.82
		Sc. PD:	-0.028
		Sc. rank:	-9325.0

PB1

Pos.	559	obs:	exp:
tat	Y	20889	10897.00
tac	Y	83	10075.00
---	--	------	------
mPD		0.0079	0.50
		nPD:	0.02
		N. weight:	0.67
		Sc. PD:	-0.016
		Sc. rank:	1821.8

PB1

Pos.	560	obs:	exp:
cgt	R	0	572.60
cgc	R	0	1097.00
cga	R	4	2054.00
cgg	R	11	1535.00
aga	R	434	10529.00
agg	R	20523	5185.00
---	--	------	------
mPD		0.042	0.91
		nPD:	0.05
		N. weight:	1.4
		Sc. PD:	0.0089
		Sc. rank:	15213.0

PB1

Pos.	561	obs:	exp:
tgt	C	13255	9466.00
tgc	C	7717	11506.00
---	--	------	------
mPD		0.47	0.50
		nPD:	0.94
		N. weight:	0.07
		Sc. PD:	0.062
		Sc. rank:	1197.2

PB1

Pos.	562	obs:	exp:
cct	P	1	0.28
ccc	P	0	0.19
cca	P	0	0.43
ccg	P	0	0.11
cat	H	20485	12332.00
cac	H	485	8638.00
caa	Q	1	0.53
cag	Q	0	0.47
---	--	------	------
mPD		0.045	0.48
		nPD:	0.09
		N. weight:	0.46
		Sc. PD:	0.024
		Sc. rank:	6425.6

PB1

Pos.	563	obs:	exp:
cgt	R	0	568.80
cgc	R	0	1090.00
cga	R	1	2041.00
cgg	R	2	1525.00
aaa	K	1	77.01
aag	K	134	57.99
aga	R	410	10459.00
agg	R	20420	5150.00
ggt	G	0	0.61
ggc	G	0	0.59
gga	G	0	1.78
ggg	G	4	1.02
---	--	------	------
mPD		0.052	0.92
		nPD:	0.06
		N. weight:	1.4
		Sc. PD:	0.022
		Sc. rank:	16330.0

PB1

Pos.	564	obs:	exp:
ggt	G	9	3180.00
ggc	G	1	3106.00
gga	G	20373	9329.00
ggg	G	589	5357.00
---	--	------	------
mPD		0.056	0.69
		nPD:	0.08
		N. weight:	0.74
		Sc. PD:	0.029
		Sc. rank:	9818.5

PB1

Pos.	565	obs:	exp:
aat	N	0	2.96
aac	N	5	2.04
gat	D	1237	11044.00
gac	D	19724	9917.00
gaa	E	2	1.21
gag	E	0	0.79
ggt	G	0	0.61
ggc	G	4	0.59
gga	G	0	1.78
ggg	G	0	1.02
---	--	------	------
mPD		0.11	0.50
		nPD:	0.22
		N. weight:	0.55
		Sc. PD:	0.099
		Sc. rank:	8607.6

PB1

Pos.	566	obs:	exp:
tct	S	0	0.37
tcc	S	0	0.23
tca	S	2	0.52
tcg	S	0	0.11
att	I	0	1.82
atc	I	0	1.25
ata	I	5	1.93
act	T	0	5277.00
acc	T	0	4294.00
aca	T	20928	9577.00
acg	T	7	1786.00
aaa	K	4	2.28
aag	K	0	1.72
agt	S	0	0.38
agc	S	0	0.40
gct	A	0	6.88
gcc	A	0	5.38
gca	A	26	12.21
gcg	A	0	1.52
---	--	------	------
mPD		0.0042	0.68
		nPD:	0.01
		N. weight:	0.83
		Sc. PD:	-0.027
		Sc. rank:	-6518.5

PB1

Pos.	567	obs:	exp:
tta	L	0	0.88
ttg	L	0	1.51
ctt	L	0	1.58
ctc	L	0	1.13
cta	L	8	1.54
ctg	L	0	1.37
cct	P	0	0.28
ccc	P	0	0.19
cca	P	1	0.43
ccg	P	0	0.11
cat	H	0	1.18
cac	H	2	0.82
caa	Q	20927	11055.00
cag	Q	23	9895.00
cgt	R	0	0.11
cgc	R	0	0.21
cga	R	4	0.39
cgg	R	0	0.29
aaa	K	7	3.99
aag	K	0	3.01
aga	R	0	2.01
agg	R	0	0.99
---	--	------	------
mPD		0.0043	0.50
		nPD:	0.01
		N. weight:	0.67
		Sc. PD:	-0.021
		Sc. rank:	-2776.1

PB1

Pos.	568	obs:	exp:
tta	L	0	0.11
ttg	L	0	0.19
ctt	L	1	0.20
ctc	L	0	0.14
cta	L	0	0.19
ctg	L	0	0.17
att	I	20590	7629.00
atc	I	353	5232.00
ata	I	15	8097.00
act	T	3	1.01
acc	T	1	0.82
aca	T	0	1.83
acg	T	0	0.34
gtt	V	9	1.99
gtc	V	0	1.64
gta	V	0	2.42
gtg	V	0	2.95
---	--	------	------
mPD		0.036	0.66
		nPD:	0.05
		N. weight:	0.98
		Sc. PD:	0.014
		Sc. rank:	11730.0

,

PB1

Pos.	569	obs:	exp:
tta	L	0	0.66
ttg	L	0	1.13
ctt	L	0	1.18
ctc	L	0	0.85
cta	L	0	1.16
ctg	L	6	1.03
cat	H	3	4.70
cac	H	5	3.30
caa	Q	630	11055.00
cag	Q	20321	9896.00
cgt	R	0	0.16
cgc	R	0	0.31
cga	R	0	0.59
cgg	R	6	0.44
aaa	K	0	0.57
aag	K	1	0.43
aga	R	0	3.01
agg	R	0	1.48
---	--	------	------
mPD		0.060	0.50
		nPD:	0.12
		N. weight:	0.65
		Sc. PD:	0.051
		Sc. rank:	9622.2

,

PB1

Pos.	570	obs:	exp:
act	T	16	5287.00
acc	T	1	4301.00
aca	T	123	9594.00
acg	T	20831	1789.00
gct	A	0	0.26
gcc	A	0	0.21
gca	A	0	0.47
gcg	A	1	0.06
---	--	------	------
mPD		0.013	0.68
		nPD:	0.02
		N. weight:	2.6
		Sc. PD:	-0.05
		Sc. rank:	13275.0

,

PB1

Pos.	571	obs:	exp:
cgt	R	0	572.30
cgc	R	0	1096.00
cga	R	1	2053.00
cgg	R	0	1534.00
aaa	K	10	5.70
aag	K	0	4.30
aga	R	20898	10523.00
agg	R	61	5182.00
ggt	G	0	0.30
ggc	G	0	0.30
gga	G	2	0.89
ggg	G	0	0.51
---	--	------	------
mPD		0.0070	0.91
		nPD:	0.01
		N. weight:	0.71
		Sc. PD:	-0.022
		Sc. rank:	-3757.6

,

PB1

Pos.	572	obs:	exp:
cgt	R	0	572.60
cgc	R	0	1097.00
cga	R	2	2054.00
cgg	R	0	1535.00
aaa	K	1	0.57
aag	K	0	0.43
aga	R	20924	10528.00
agg	R	45	5184.00
---	--	------	------
mPD		0.0046	0.91
		nPD:	0.01
		N. weight:	0.72
		Sc. PD:	-0.024
		Sc. rank:	-7321.1

,

PB1

Pos.	573	obs:	exp:
tta	L	1	0.11
ttg	L	0	0.19
tct	S	235	3856.00
tcc	S	6	2427.00
tca	S	20686	5453.00
tcg	S	27	1115.00
ctt	L	0	0.20
ctc	L	0	0.14
cta	L	0	0.19
ctg	L	0	0.17
act	T	0	4.29
acc	T	0	3.49
aca	T	17	7.78
acg	T	0	1.45
agt	S	0	3940.00
agc	S	0	4162.00
---	--	------	------
mPD		0.027	1.6
		nPD:	0.02
		N. weight:	1.4
		Sc. PD:	-0.031
		Sc. rank:	4265.1

,

PB1

Pos.	574	obs:	exp:
ttt	F	20923	8990.00
ttc	F	26	11959.00
tta	L	0	0.88
ttg	L	0	1.51
tct	S	12	2.21
tcc	S	0	1.39
tca	S	0	3.12
tcg	S	0	0.64
ctt	L	8	1.58
ctc	L	0	1.13
cta	L	0	1.54
ctg	L	0	1.37
agt	S	0	2.26
agc	S	0	2.38
gtt	V	3	0.66
gtc	V	0	0.55
gta	V	0	0.81
gtg	V	0	0.98
---	--	------	------
mPD		0.0047	0.49
		nPD:	0.01
		N. weight:	0.89
		Sc. PD:	-0.026
		Sc. rank:	-2493.8

,

PB1

Pos.	575	obs:	exp:
gaa	E	142	12699.00
gag	E	20830	8273.00
---	--	------	------
mPD		0.013	0.48
		nPD:	0.03
		N. weight:	0.94
		Sc. PD:	-0.011
		Sc. rank:	7357.5

,

PB1

Pos.	576	obs:	exp:
tta	L	4175	2303.00
ttg	L	15585	3945.00
ctt	L	0	4129.00
ctc	L	0	2959.00
cta	L	7	4034.00
ctg	L	1185	3582.00
att	I	0	4.73
atc	I	0	3.25
ata	I	13	5.02
atg	M	4	4.00
gtt	V	0	0.66
gtc	V	0	0.55
gta	V	0	0.81
gtg	V	3	0.98
---	--	------	------
mPD		0.43	1.1
		nPD:	0.38
		N. weight:	1.1
		Sc. PD:	0.38
		Sc. rank:	18725.0

,

PB1

Pos.	577	obs:	exp:
cgt	R	0	0.52
cgc	R	0	0.99
cga	R	0	1.86
cgg	R	0	1.39
atg	M	1	1.00
act	T	0	0.25
acc	T	0	0.21
aca	T	0	0.46
acg	T	1	0.09
aat	N	9	5.32
aac	N	0	3.68
aaa	K	90	11944.00
aag	K	20848	8994.00
aga	R	0	9.54
agg	R	19	4.70
gaa	E	0	2.42
gag	E	4	1.58
---	--	------	------
mPD		0.012	0.49
		nPD:	0.02
		N. weight:	0.87
		Sc. PD:	-0.014
		Sc. rank:	5636.7

,

PB1

Pos.	578	obs:	exp:
caa	Q	0	1.58
cag	Q	3	1.42
cgt	R	0	0.25
cgc	R	0	0.47
cga	R	0	0.88
cgg	R	0	0.66
aat	N	4	2.37
aac	N	0	1.63
aaa	K	239	11951.00
aag	K	20712	9000.00
aga	R	0	4.52
agg	R	9	2.22
gaa	E	0	3.03
gag	E	5	1.97
---	--	------	------
mPD		0.025	0.49
		nPD:	0.05
		N. weight:	0.83
		Sc. PD:	0.0081
		Sc. rank:	9600.1

,

PB1

Pos.	579	obs:	exp:
tta	L	34	2305.00
ttg	L	3	3948.00
ctt	L	24	4132.00
ctc	L	0	2962.00
cta	L	17807	4037.00
ctg	L	3100	3585.00
att	I	0	1.09
atc	I	0	0.75
ata	I	3	1.16
atg	M	1	1.00
---	--	------	------
mPD		0.26	1.1
		nPD:	0.23
		N. weight:	1.3
		Sc. PD:	0.24
		Sc. rank:	20493.0

,

PB1

Pos.	580	obs:	exp:
tgg	W	20972	20972.00
---	--	------	------
mPD		0	0
		nPD:	1.
		N. weight:	0.
		Sc. PD:	0
		Sc. rank:	0

,

PB1

Pos.	581	obs:	exp:
aat	N	43	25.43
aac	N	0	17.57
gat	D	20618	11017.00
gac	D	292	9893.00
gaa	E	3	2.42
gag	E	1	1.58
ggt	G	15	2.27
ggc	G	0	2.22
gga	G	0	6.67
ggg	G	0	3.83
---	--	------	------
mPD		0.033	0.50
		nPD:	0.07
		N. weight:	0.6
		Sc. PD:	0.016
		Sc. rank:	7663.3

,

PB1

Pos.	582	obs:	exp:
tta	L	0	0.11
ttg	L	0	0.19
ctt	L	0	0.20
ctc	L	0	0.14
cta	L	1	0.19
ctg	L	0	0.17
cat	H	1	0.59
cac	H	0	0.41
caa	Q	20945	11062.00
cag	Q	19	9902.00
cgt	R	0	0.03
cgc	R	0	0.05
cga	R	1	0.10
cgg	R	0	0.07
aaa	K	5	2.85
aag	K	0	2.15
aga	R	0	0.50
agg	R	0	0.25
---	--	------	------
mPD		0.0026	0.50
		nPD:	0.01
		N. weight:	0.67
		Sc. PD:	-0.023
		Sc. rank:	-6479.7

,

PB1

Pos.	583	obs:	exp:
tct	S	0	0.37
tcc	S	2	0.23
tca	S	0	0.52
tcg	S	0	0.11
act	T	51	5286.00
acc	T	20917	4301.00
aca	T	1	9593.00
acg	T	0	1789.00
agt	S	0	0.38
agc	S	0	0.40
gct	A	0	0.26
gcc	A	1	0.21
gca	A	0	0.47
gcg	A	0	0.06
---	--	------	------
mPD		0.0052	0.68
		nPD:	0.01
		N. weight:	1.7
		Sc. PD:	-0.052
		Sc. rank:	-8886.2

,

PB1

Pos.	584	obs:	exp:
cat	H	0	1.18
cac	H	2	0.82
caa	Q	20909	11043.00
cag	Q	18	9884.00
cgt	R	0	0.85
cgc	R	1	1.62
cga	R	30	3.04
cgg	R	0	2.27
aaa	K	12	6.85
aag	K	0	5.15
aga	R	0	15.56
agg	R	0	7.66
---	--	------	------
mPD		0.0061	0.50
		nPD:	0.01
		N. weight:	0.67
		Sc. PD:	-0.018
		Sc. rank:	304.2

,

PB1

Pos.	585	obs:	exp:
tct	S	5	3859.00
tcc	S	1	2429.00
tca	S	20829	5458.00
tcg	S	137	1116.00
agt	S	0	3943.00
agc	S	0	4166.00
---	--	------	------
mPD		0.014	1.6
		nPD:	0.01
		N. weight:	1.4
		Sc. PD:	-0.043
		Sc. rank:	-6215.9

,

PB1

Pos.	586	obs:	exp:
caa	Q	0	0.53
cag	Q	1	0.47
cgt	R	0	0.66
cgc	R	0	1.26
cga	R	0	2.35
cgg	R	0	1.76
act	T	0	0.25
acc	T	0	0.21
aca	T	1	0.46
acg	T	0	0.09
aaa	K	482	11948.00
aag	K	20464	8998.00
aga	R	0	12.05
agg	R	24	5.93
---	--	------	------
mPD		0.047	0.49
		nPD:	0.1
		N. weight:	0.77
		Sc. PD:	0.043
		Sc. rank:	10949.0

,

PB1

Pos.	587	obs:	exp:
att	I	0	19.29
atc	I	0	13.23
ata	I	53	20.48
act	T	3	3.28
acc	T	0	2.67
aca	T	10	5.95
acg	T	0	1.11
gtt	V	2	4582.00
gtc	V	0	3766.00
gta	V	20692	5573.00
gtg	V	29	6803.00
gct	A	1	33.62
gcc	A	0	26.29
gca	A	126	59.65
gcg	A	0	7.44
ggt	G	0	8.49
ggc	G	0	8.29
gga	G	56	24.91
ggg	G	0	14.30
---	--	------	------
mPD		0.028	0.76
		nPD:	0.04
		N. weight:	1.4
		Sc. PD:	-0.004
		Sc. rank:	13608.0

,

PB1

Pos.	588	obs:	exp:
ggt	G	0	3180.00
ggc	G	9	3106.00
gga	G	99	9329.00
ggg	G	20864	5357.00
---	--	------	------
mPD		0.010	0.69
		nPD:	0.01
		N. weight:	1.4
		Sc. PD:	-0.035
		Sc. rank:	3299.9

,

PB1

Pos.	589	obs:	exp:
tta	L	36	2306.00
ttg	L	0	3948.00
ctt	L	2	4133.00
ctc	L	2	2962.00
cta	L	20836	4037.00
ctg	L	96	3586.00
---	--	------	------
mPD		0.013	1.1
		nPD:	0.01
		N. weight:	1.7
		Sc. PD:	-0.048
		Sc. rank:	-1477.6

,

PB1

Pos.	590	obs:	exp:
tta	L	20617	2306.00
ttg	L	333	3948.00
ctt	L	0	4133.00
ctc	L	0	2962.00
cta	L	21	4037.00
ctg	L	1	3586.00
---	--	------	------
mPD		0.033	1.1
		nPD:	0.03
		N. weight:	2.2
		Sc. PD:	-0.023
		Sc. rank:	18257.0

,

PB1

Pos.	591	obs:	exp:
tta	L	1	0.11
ttg	L	0	0.19
ctt	L	0	0.20
ctc	L	0	0.14
cta	L	0	0.19
ctg	L	0	0.17
att	I	0	10.92
atc	I	0	7.49
ata	I	30	11.59
gtt	V	8	4630.00
gtc	V	8	3805.00
gta	V	20880	5632.00
gtg	V	45	6874.00
---	--	------	------
mPD		0.0088	0.74
		nPD:	0.01
		N. weight:	1.4
		Sc. PD:	-0.038
		Sc. rank:	-42.7

,

PB1

Pos.	592	obs:	exp:
tct	S	15	3859.00
tcc	S	0	2429.00
tca	S	20925	5457.00
tcg	S	28	1116.00
agt	S	0	3943.00
agc	S	0	4165.00
gct	A	0	1.06
gcc	A	0	0.83
gca	A	4	1.88
gcg	A	0	0.23
---	--	------	------
mPD		0.0045	1.6
		nPD:	0.
		N. weight:	1.4
		Sc. PD:	-0.051
		Sc. rank:	-20289.0

,

PB1

Pos.	593	obs:	exp:
gat	D	20942	11050.00
gac	D	29	9921.00
ggt	G	1	0.15
ggc	G	0	0.15
gga	G	0	0.44
ggg	G	0	0.26
---	--	------	------
mPD		0.0029	0.50
		nPD:	0.01
		N. weight:	0.67
		Sc. PD:	-0.022
		Sc. rank:	-5874.4

,

PB1

Pos.	594	obs:	exp:
ggt	G	4	3180.00
ggc	G	1	3106.00
gga	G	20908	9329.00
ggg	G	59	5357.00
---	--	------	------
mPD		0.0061	0.69
		nPD:	0.01
		N. weight:	0.84
		Sc. PD:	-0.025
		Sc. rank:	-2902.4

,

PB1

Pos.	595	obs:	exp:
ggt	G	1	3180.00
ggc	G	7	3106.00
gga	G	20887	9329.00
ggg	G	77	5357.00
---	--	------	------
mPD		0.0081	0.69
		nPD:	0.01
		N. weight:	0.83
		Sc. PD:	-0.023
		Sc. rank:	-182.8

,

PB1			
Pos.	596	obs:	exp:
tta	L	0	0.22
ttg	L	0	0.38
tct	S	0	0.18
tcc	S	0	0.12
tca	S	1	0.26
tcg	S	0	0.05
ctt	L	0	0.39
ctc	L	0	0.28
cta	L	2	0.39
ctg	L	0	0.34
cct	P	2	5777.00
ccc	P	12	3889.00
cca	P	20942	8929.00
ccg	P	13	2374.00
agt	S	0	0.19
agc	S	0	0.20
---	--	------	------
mPD		0.0029	0.70
		nPD:	0.
		N. weight:	0.9
		Sc. PD:	-0.031
		Sc. rank:	-10809.0

,

PB1			
Pos.	597	obs:	exp:
aat	N	76	12404.00
aac	N	20896	8568.00
---	--	------	------
mPD		0.0072	0.48
		nPD:	0.01
		N. weight:	0.92
		Sc. PD:	-0.022
		Sc. rank:	2198.6

,

PB1			
Pos.	598	obs:	exp:
tta	L	20900	2305.00
ttg	L	56	3948.00
tct	S	0	0.18
tcc	S	0	0.12
tca	S	1	0.26
tcg	S	0	0.05
ctt	L	0	4132.00
ctc	L	0	2962.00
cta	L	13	4037.00
ctg	L	0	3585.00
att	I	0	0.36
atc	I	0	0.25
ata	I	1	0.39
agt	S	0	0.19
agc	S	0	0.20
gtt	V	0	0.22
gtc	V	0	0.18
gta	V	1	0.27
gtg	V	0	0.33
---	--	------	------
mPD		0.0069	1.1
		nPD:	0.01
		N. weight:	2.3
		Sc. PD:	-0.077
		Sc. rank:	-18825.0

,

PB1			
Pos.	599	obs:	exp:
tat	Y	26	10896.00
tac	Y	20945	10075.00
cat	H	0	0.59
cac	H	1	0.41
---	--	------	------
mPD		0.0026	0.50
		nPD:	0.01
		N. weight:	0.77
		Sc. PD:	-0.026
		Sc. rank:	-7438.0

,

PB1			
Pos.	600	obs:	exp:
aat	N	3075	12404.00
aac	N	17897	8568.00
---	--	------	------
mPD		0.25	0.48
		nPD:	0.52
		N. weight:	0.45
		Sc. PD:	0.21
		Sc. rank:	7594.9

,

PB1			
Pos.	601	obs:	exp:
att	I	4	7632.00
atc	I	17	5234.00
ata	I	20945	8100.00
atg	M	1	1.00
gtt	V	0	1.11
gtc	V	0	0.91
gta	V	5	1.34
gtg	V	0	1.64
---	--	------	------
mPD		0.0026	0.66
		nPD:	0.
		N. weight:	1.
		Sc. PD:	-0.035
		Sc. rank:	-12512.0

,

PB1			
Pos.	602	obs:	exp:
cct	P	0	0.28
ccc	P	0	0.19
cca	P	0	0.43
ccg	P	1	0.11
cgt	R	5	572.60
cgc	R	0	1097.00
cga	R	92	2054.00
cgg	R	20835	1535.00
aga	R	2	10528.00
agg	R	37	5184.00
---	--	------	------
mPD		0.013	0.91
		nPD:	0.01
		N. weight:	2.7
		Sc. PD:	-0.067
		Sc. rank:	5346.6

,

PB1			
Pos.	603	obs:	exp:
tct	S	0	0.18
tcc	S	0	0.12
tca	S	0	0.26
tcg	S	0	0.05
act	T	1	0.25
acc	T	0	0.21
aca	T	0	0.46
acg	T	0	0.09
aat	N	20910	12402.00
aac	N	60	8568.00
agt	S	1	0.19
agc	S	0	0.20
---	--	------	------
mPD		0.0059	0.48
		nPD:	0.01
		N. weight:	0.54
		Sc. PD:	-0.015
		Sc. rank:	255.8

,

PB1			
Pos.	604	obs:	exp:
ttt	F	7	3.00
ttc	F	0	4.00
tta	L	0	2304.00
ttg	L	0	3946.00
ctt	L	20813	4130.00
ctc	L	146	2961.00
cta	L	1	4035.00
ctg	L	0	3584.00
att	I	5	1.82
atc	I	0	1.25
ata	I	0	1.93
---	--	------	------
mPD		0.015	1.1
		nPD:	0.01
		N. weight:	1.7
		Sc. PD:	-0.044
		Sc. rank:	1685.3

,

PB1			
Pos.	605	obs:	exp:
cat	H	45	12332.00
cac	H	20925	8638.00
caa	Q	1	0.53
cag	Q	0	0.47
aat	N	0	0.59
aac	N	1	0.41
---	--	------	------
mPD		0.0045	0.48
		nPD:	0.01
		N. weight:	0.92
		Sc. PD:	-0.028
		Sc. rank:	-2822.4

,

PB1			
Pos.	606	obs:	exp:
tta	L	0	0.88
ttg	L	0	1.51
ctt	L	8	1.58
ctc	L	0	1.13
cta	L	0	1.54
ctg	L	0	1.37
att	I	20820	7630.00
atc	I	139	5232.00
ata	I	0	8098.00
act	T	1	0.25
acc	T	0	0.21
aca	T	0	0.46
acg	T	0	0.09
gtt	V	4	0.88
gtc	V	0	0.73
gta	V	0	1.08
gtg	V	0	1.31
---	--	------	------
mPD		0.014	0.66
		nPD:	0.02
		N. weight:	1.
		Sc. PD:	-0.018
		Sc. rank:	6123.2

,

PB1			
Pos.	607	obs:	exp:
tct	S	1	0.18
tcc	S	0	0.12
tca	S	0	0.26
tcg	S	0	0.05
cct	P	20944	5777.00
ccc	P	9	3890.00
cca	P	16	8929.00
ccg	P	2	2375.00
agt	S	0	0.19
agc	S	0	0.20
---	--	------	------
mPD		0.0027	0.70
		nPD:	0.
		N. weight:	1.4
		Sc. PD:	-0.048
		Sc. rank:	-17161.0

,

PB1			
Pos.	608	obs:	exp:
ggt	G	1	0.15
ggc	G	0	0.15
gga	G	0	0.44
ggg	G	0	0.26
---	-	20971	20971.00
---	--	------	------
mPD		0	1.6×10^{-9}
		nPD:	0.
		N. weight:	0.
		Sc. PD:	0
		Sc. rank:	0

,

PB1			
Pos.	609	obs:	exp:
cat	H	1	0.59
cac	H	0	0.41
gaa	E	20947	12697.00
gag	E	23	8273.00
ggt	G	1	0.15
ggc	G	0	0.15
gga	G	0	0.44
ggg	G	0	0.26
---	--	------	------
mPD		0.0026	0.48
		nPD:	0.01
		N. weight:	0.52
		Sc. PD:	-0.018
		Sc. rank:	-4751.3

,

PB1			
Pos.	610	obs:	exp:
tct	S	0	0.18
tcc	S	0	0.12
tca	S	0	0.26
tcg	S	0	0.05
cat	H	1	0.59
cac	H	0	0.41
att	I	0	1.09
atc	I	3	0.75
ata	I	0	1.16
agt	S	0	0.19
agc	S	1	0.20
gtt	V	45	4634.00
gtc	V	20740	3809.00
gta	V	175	5637.00
gtg	V	0	6880.00
gct	A	0	1.85
gcc	A	7	1.45
gca	A	0	3.29
gcg	A	0	0.41
---	--	------	------
mPD		0.022	0.74
		nPD:	0.03
		N. weight:	1.7
		Sc. PD:	-0.017
		Sc. rank:	14680.0

,

PB1			
Pos.	611	obs:	exp:
tct	S	0	0.74
tcc	S	0	0.46
tca	S	0	1.04
tcg	S	0	0.21
tgt	C	41	9464.00
tgc	C	20927	11504.00
agt	S	0	0.75
agc	S	4	0.79
---	--	------	------
mPD		0.0043	0.50
		nPD:	0.01
		N. weight:	0.62
		Sc. PD:	-0.019
		Sc. rank:	-2502.7

,

PB1			
Pos.	612	obs:	exp:
ttt	F	0	0.43
ttc	F	1	0.57
tta	L	20819	2305.00
ttg	L	118	3948.00
tct	S	0	0.18
tcc	S	0	0.12
tca	S	1	0.26
tcg	S	0	0.05
ctt	L	0	4132.00
ctc	L	0	2962.00
cta	L	32	4037.00
ctg	L	0	3585.00
att	I	0	0.36
atc	I	0	0.25
ata	I	1	0.39
agt	S	0	0.19
agc	S	0	0.20
---	--	------	------
mPD		0.015	1.1
		nPD:	0.01
		N. weight:	2.3
		Sc. PD:	-0.061
		Sc. rank:	1700.2

,

PB1			
Pos.	613	obs:	exp:
aat	N	0	0.59
aac	N	1	0.41
aaa	K	20906	11962.00
aag	K	65	9009.00
---	--	------	------
mPD		0.0063	0.49
		nPD:	0.01
		N. weight:	0.57
		Sc. PD:	-0.015
		Sc. rank:	477.1

,

PB1			
Pos.	614	obs:	exp:
tgg	W	20972	20972.00
---	--	------	------
mPD		0	0
		nPD:	1.
		N. weight:	0.
		Sc. PD:	0
		Sc. rank:	0

,

PB1			
Pos.	615	obs:	exp:
aaa	K	0	2.85
aag	K	5	2.15
gat	D	11	6.85
gac	D	2	6.15
gaa	E	59	12685.00
gag	E	20890	8264.00
ggt	G	0	0.76
ggc	G	0	0.74
gga	G	0	2.22
ggg	G	5	1.28
---	--	------	------
mPD		0.0078	0.48
		nPD:	0.02
		N. weight:	0.96
		Sc. PD:	-0.022
		Sc. rank:	2925.4

,

PB1			
Pos.	616	obs:	exp:
tta	L	40	2305.00
ttg	L	1	3948.00
ctt	L	1	4132.00
ctc	L	0	2962.00
cta	L	20900	4037.00
ctg	L	29	3586.00
gtt	V	0	0.22
gtc	V	0	0.18
gta	V	1	0.27
gtg	V	0	0.33
---	--	------	------
mPD		0.0069	1.1
		nPD:	0.01
		N. weight:	1.7
		Sc. PD:	-0.057
		Sc. rank:	-13692.0

,

PB1			
Pos.	617	obs:	exp:
atg	M	20972	20972.00
---	--	------	------
mPD		0	0
		nPD:	1.
		N. weight:	0.
		Sc. PD:	0
		Sc. rank:	0

,

PB1			
Pos.	618	obs:	exp:
gat	D	20964	11050.00
gac	D	8	9922.00
---	--	------	------
mPD		0.00076	0.50
		nPD:	0.
		N. weight:	0.68
		Sc. PD:	-0.025
		Sc. rank:	-10892.0

,

PB1			
Pos.	619	obs:	exp:
aat	N	28	16.56
aac	N	0	11.44
gtt	V	3	0.66
gtc	V	0	0.55
gta	V	0	0.81
gtg	V	0	0.98
gct	A	3	0.79
gcc	A	0	0.62
gca	A	0	1.41
gcg	A	0	0.18
gat	D	20850	10993.00
gac	D	13	9870.00
gaa	E	16	18.77
gag	E	15	12.23
ggt	G	44	6.67
ggc	G	0	6.52
gga	G	0	19.57
ggg	G	0	11.24
---	--	------	------
mPD		0.012	0.51
		nPD:	0.02
		N. weight:	0.68
		Sc. PD:	-0.011
		Sc. rank:	4221.6

,

PB1			
Pos.	620	obs:	exp:
tat	Y	2	1.04
tac	Y	0	0.96
aat	N	48	28.39
aac	N	0	19.61
gct	A	1	0.26
gcc	A	0	0.21
gca	A	0	0.47
gcg	A	0	0.06
gat	D	20743	10961.00
gac	D	59	9841.00
gaa	E	2	2.42
gag	E	2	1.58
ggt	G	114	17.44
ggc	G	1	17.03
gga	G	0	51.15
ggg	G	0	29.38
---	--	------	------
mPD		0.022	0.52
		nPD:	0.04
		N. weight:	0.67
		Sc. PD:	0.0015
		Sc. rank:	7088.3

,

PB1			
Pos.	621	obs:	exp:
tat	Y	20943	10897.00
tac	Y	29	10075.00
---	--	------	------
mPD		0.0028	0.50
		nPD:	0.01
		N. weight:	0.68
		Sc. PD:	-0.023
		Sc. rank:	-6061.9

,

PB1			
Pos.	622	obs:	exp:
caa	Q	0	10.55
cag	Q	20	9.45
cgt	R	3	572.10
cgc	R	0	1096.00
cga	R	100	2052.00
cgg	R	20777	1533.00
aga	R	1	10519.00
agg	R	71	5180.00
---	--	------	------
mPD		0.019	0.91
		nPD:	0.02
		N. weight:	2.7
		Sc. PD:	-0.052
		Sc. rank:	14817.0

,

PB1			
Pos.	623	obs:	exp:
cgt	R	0	0.05
cgc	R	0	0.10
cga	R	0	0.20
cgg	R	0	0.15
aga	R	2	1.00
agg	R	0	0.49
gaa	E	3	1.82
gag	E	0	1.18
ggt	G	6	3179.00
ggc	G	2	3105.00
gga	G	20716	9327.00
ggg	G	243	5356.00
---	--	------	------
mPD		0.024	0.69
		nPD:	0.03
		N. weight:	0.8
		Sc. PD:	-0.0039
		Sc. rank:	7717.8

,

PB1			
Pos.	624	obs:	exp:
cgt	R	1	572.60
cgc	R	0	1097.00
cga	R	2	2054.00
cgg	R	0	1535.00
aga	R	20633	10529.00
agg	R	336	5185.00
---	--	------	------
mPD		0.032	0.91
		nPD:	0.04
		N. weight:	0.65
		Sc. PD:	-0.0031
		Sc. rank:	6369.0

,

PB1			
Pos.	625	obs:	exp:
tta	L	1	2305.00
ttg	L	0	3948.00
ctt	L	20924	4132.00
ctc	L	25	2962.00
cta	L	3	4037.00
ctg	L	15	3585.00
att	I	4	1.46
atc	I	0	1.00
ata	I	0	1.55
---	--	------	------
mPD		0.0047	1.1
		nPD:	0.
		N. weight:	1.7
		Sc. PD:	-0.06
		Sc. rank:	-20650.0

,

PB1			
Pos.	626	obs:	exp:
tat	Y	1	0.52
tac	Y	0	0.48
tgt	C	20902	9465.00
tgc	C	69	11506.00
---	--	------	------
mPD		0.0067	0.50
		nPD:	0.01
		N. weight:	0.82
		Sc. PD:	-0.021
		Sc. rank:	945.5

,

PB1			
Pos.	627	obs:	exp:
tct	S	0	1.29
tcc	S	0	0.81
tca	S	0	1.82
tcg	S	0	0.37
cat	H	1	0.59
cac	H	0	0.41
aat	N	20602	12399.00
aac	N	362	8565.00
agt	S	7	1.32
agc	S	0	1.39
---	--	------	------
mPD		0.035	0.48
		nPD:	0.07
		N. weight:	0.47
		Sc. PD:	0.015
		Sc. rank:	6065.4

,

PB1			
Pos.	628	obs:	exp:
tct	S	0	1.10
tcc	S	6	0.69
tca	S	0	1.56
tcg	S	0	0.32
cct	P	48	5776.00
ccc	P	20828	3889.00
cca	P	90	8927.00
ccg	P	0	2374.00
agt	S	0	1.13
agc	S	0	1.19
---	--	------	------
mPD		0.014	0.70
		nPD:	0.02
		N. weight:	1.7
		Sc. PD:	-0.034
		Sc. rank:	8823.4

,

PB1			
Pos.	629	obs:	exp:
tta	L	1	2302.00
ttg	L	40	3943.00
ctt	L	4	4127.00
ctc	L	0	2958.00
cta	L	61	4032.00
ctg	L	20836	3581.00
caa	Q	0	4.75
cag	Q	9	4.25
atg	M	21	21.00
---	--	------	------
mPD		0.013	1.1
		nPD:	0.01
		N. weight:	1.8
		Sc. PD:	-0.051
		Sc. rank:	-892.7

,

PB1			
Pos.	630	obs:	exp:
aat	N	20791	12404.00
aac	N	181	8568.00
---	--	------	------
mPD		0.017	0.48
		nPD:	0.04
		N. weight:	0.51
		Sc. PD:	-0.0022
		Sc. rank:	4964.0

,

PB1			
Pos.	631	obs:	exp:
tta	L	0	0.11
ttg	L	0	0.19
ctt	L	0	0.20
ctc	L	1	0.14
cta	L	0	0.19
ctg	L	0	0.17
cct	P	27	5777.00
ccc	P	20933	3890.00
cca	P	10	8929.00
ccg	P	1	2375.00
---	--	------	------
mPD		0.0037	0.70
		nPD:	0.01
		N. weight:	1.8
		Sc. PD:	-0.06
		Sc. rank:	-16227.0

,

PB1			
Pos.	632	obs:	exp:
ttt	F	20954	8999.00
ttc	F	17	11972.00
tta	L	0	0.11
ttg	L	1	0.19
ctt	L	0	0.20
ctc	L	0	0.14
cta	L	0	0.19
ctg	L	0	0.17
---	--	------	------
mPD		0.0017	0.49
		nPD:	0.
		N. weight:	0.89
		Sc. PD:	-0.032
		Sc. rank:	-11877.0

,

PB1			
Pos.	633	obs:	exp:
att	I	0	0.36
atc	I	1	0.25
ata	I	0	0.39
atg	M	3	3.00
gtt	V	11	4635.00
gtc	V	2712	3810.00
gta	V	453	5638.00
gtg	V	17789	6882.00
gct	A	0	0.79
gcc	A	1	0.62
gca	A	0	1.41
gcg	A	2	0.18
---	--	------	------
mPD		0.26	0.74
		nPD:	0.36
		N. weight:	0.75
		Sc. PD:	0.23
		Sc. rank:	12125.0

,

PB1			
Pos.	634	obs:	exp:
tct	S	0	3844.00
tcc	S	0	2420.00
tca	S	0	5437.00
tcg	S	0	1112.00
act	T	0	0.50
acc	T	2	0.41
aca	T	0	0.91
acg	T	0	0.17
aat	N	45	26.61
aac	N	0	18.39
agt	S	20522	3928.00
agc	S	368	4150.00
ggt	G	35	5.31
ggc	G	0	5.18
gga	G	0	15.57
ggg	G	0	8.94
---	--	------	------
mPD		0.042	1.6
		nPD:	0.03
		N. weight:	1.7
		Sc. PD:	-0.023
		Sc. rank:	11959.0

,

PB1			
Pos.	635	obs:	exp:
tat	Y	0	0.52
tac	Y	1	0.48
cat	H	20774	12329.00
cac	H	190	8635.00
cgt	R	7	0.19
cgc	R	0	0.37
cga	R	0	0.69
cgg	R	0	0.51
aga	R	0	3.51
agg	R	0	1.73
---	--	------	------
mPD		0.019	0.49
		nPD:	0.04
		N. weight:	0.51
		Sc. PD:	-0.00057
		Sc. rank:	5178.9

,

PB1

Pos.	636	obs:	exp:
cgt	R	0	0.38
cgc	R	0	0.73
cga	R	0	1.37
cgg	R	0	1.02
aaa	K	3212	11955.00
aag	K	17746	9003.00
aga	R	4	7.03
agg	R	10	3.46
---	--	------	------
mPD		0.26	0.49
		nPD:	0.53
		N. weight:	0.4
		Sc. PD:	0.19
		Sc. rank:	6703.0

,

PB1

Pos.	637	obs:	exp:
aaa	K	0	0.57
aag	K	1	0.43
gat	D	2	1.05
gac	D	0	0.95
gaa	E	241	12691.00
gag	E	20718	8268.00
ggt	G	0	1.52
ggc	G	0	1.48
gga	G	0	4.45
ggg	G	10	2.55
---	--	------	------
mPD		0.024	0.48
		nPD:	0.05
		N. weight:	0.91
		Sc. PD:	0.0091
		Sc. rank:	10660.0

,

PB1

Pos.	638	obs:	exp:
ttt	F	2	0.86
ttc	F	0	1.14
att	I	20876	7614.00
atc	I	39	5221.00
ata	I	1	8081.00
act	T	2	0.50
acc	T	0	0.41
aca	T	0	0.91
acg	T	0	0.17
gtt	V	52	11.50
gtc	V	0	9.45
gta	V	0	13.98
gtg	V	0	17.07
---	--	------	------
mPD		0.0091	0.66
		nPD:	0.01
		N. weight:	1.1
		Sc. PD:	-0.027
		Sc. rank:	1418.6

,

PB1

Pos.	639	obs:	exp:
aat	N	11	6.51
aac	N	0	4.49
gat	D	20620	11029.00
gac	D	311	9902.00
gaa	E	19	13.32
gag	E	3	8.68
ggt	G	8	1.21
ggc	G	0	1.18
gga	G	0	3.56
ggg	G	0	2.04
---	--	------	------
mPD		0.033	0.50
		nPD:	0.07
		N. weight:	0.6
		Sc. PD:	0.015
		Sc. rank:	7588.5

,

PB1

Pos.	640	obs:	exp:
ttt	F	7	3.00
ttc	F	0	4.00
tct	S	20899	3857.00
tcc	S	50	2428.00
tca	S	8	5455.00
tcg	S	2	1115.00
tat	Y	5	2.60
tac	Y	0	2.40
cct	P	1	0.28
ccc	P	0	0.19
cca	P	0	0.43
ccg	P	0	0.11
agt	S	0	3941.00
agc	S	0	4163.00
---	--	------	------
mPD		0.0069	1.6
		nPD:	0.
		N. weight:	1.8
		Sc. PD:	-0.062
		Sc. rank:	-20543.0

,

PB1

Pos.	641	obs:	exp:
tta	L	3	0.33
ttg	L	0	0.56
ctt	L	0	0.59
ctc	L	0	0.42
cta	L	0	0.58
ctg	L	0	0.51
att	I	0	14.56
atc	I	0	9.98
ata	I	40	15.45
gtt	V	0	4626.00
gtc	V	5	3802.00
gta	V	20858	5628.00
gtg	V	62	6869.00
gct	A	0	1.06
gcc	A	0	0.83
gca	A	4	1.88
gcg	A	0	0.23
---	--	------	------
mPD		0.011	0.74
		nPD:	0.01
		N. weight:	1.4
		Sc. PD:	-0.034
		Sc. rank:	2770.6

,

PB1

Pos.	642	obs:	exp:
aat	N	45	12404.00
aac	N	20927	8568.00
---	--	------	------
mPD		0.0043	0.48
		nPD:	0.01
		N. weight:	0.93
		Sc. PD:	-0.028
		Sc. rank:	-3055.2

,

PB1

Pos.	643	obs:	exp:
tct	S	0	26.13
tcc	S	0	16.45
tca	S	0	36.96
tcg	S	0	7.56
cat	H	1	0.59
cac	H	0	0.41
att	I	2	0.73
atc	I	0	0.50
ata	I	0	0.77
aat	N	20804	12317.00
aac	N	22	8509.00
agt	S	135	26.70
agc	S	7	28.21
gat	D	1	0.53
gac	D	0	0.47
---	--	------	------
mPD		0.017	0.51
		nPD:	0.03
		N. weight:	0.56
		Sc. PD:	-0.0039
		Sc. rank:	5035.0

,

PB1

Pos.	644	obs:	exp:
cct	P	1	0.28
ccc	P	0	0.19
cca	P	0	0.43
ccg	P	0	0.11
act	T	87	22.18
acc	T	1	18.05
aca	T	0	40.26
acg	T	0	7.51
gtt	V	2	0.44
gtc	V	0	0.36
gta	V	0	0.54
gtg	V	0	0.66
gct	A	19930	5528.00
gcc	A	926	4322.00
gca	A	20	9807.00
gcg	A	4	1223.00
ggt	G	1	0.15
ggc	G	0	0.15
gga	G	0	0.44
ggg	G	0	0.26
---	--	------	------
mPD		0.095	0.67
		nPD:	0.14
		N. weight:	1.2
		Sc. PD:	0.12
		Sc. rank:	18492.0

,

PB1

Pos.	645	obs:	exp:
tta	L	0	0.11
ttg	L	1	0.19
ctt	L	0	0.20
ctc	L	0	0.14
cta	L	0	0.19
ctg	L	0	0.17
atg	M	10	10.00
gtt	V	7	4634.00
gtc	V	2	3809.00
gta	V	72	5637.00
gtg	V	20880	6881.00
---	--	------	------
mPD		0.0087	0.74
		nPD:	0.01
		N. weight:	1.2
		Sc. PD:	-0.032
		Sc. rank:	24.0

,

PB1

Pos.	646	obs:	exp:
tta	L	1	0.11
ttg	L	0	0.19
ctt	L	0	0.20
ctc	L	0	0.14
cta	L	0	0.19
ctg	L	0	0.17
att	I	0	17.11
atc	I	0	11.73
ata	I	47	18.16
atg	M	12	12.00
gtt	V	4	4622.00
gtc	V	1	3799.00
gta	V	20258	5622.00
gtg	V	642	6862.00
gct	A	0	1.85
gcc	A	0	1.45
gca	A	7	3.29
gcg	A	0	0.41
---	--	------	------
mPD		0.067	0.74
		nPD:	0.09
		N. weight:	1.2
		Sc. PD:	0.061
		Sc. rank:	17309.0

,

PB1

Pos.	647	obs:	exp:
tta	L	1	92.56
ttg	L	35	158.50
ctt	L	0	165.90
ctc	L	0	118.90
cta	L	0	162.10
ctg	L	806	144.00
att	I	5	11.65
atc	I	16	7.99
ata	I	11	12.36
atg	M	20086	20086.00
act	T	0	2.02
acc	T	0	1.64
aca	T	0	3.66
acg	T	8	0.68
gtt	V	0	0.88
gtc	V	0	0.73
gta	V	0	1.08
gtg	V	4	1.31
---	--	------	------
mPD		0.081	0.13
		nPD:	0.61
		N. weight:	0.069
		Sc. PD:	0.039
		Sc. rank:	1176.3

,

PB1			
Pos.	648	obs:	exp:
tct	S	0	0.18
tcc	S	0	0.12
tca	S	1	0.26
tcg	S	0	0.05
cct	P	4	5776.00
ccc	P	25	3889.00
cca	P	20933	8928.00
ccg	P	5	2374.00
caa	Q	4	2.11
cag	Q	0	1.89
agt	S	0	0.19
agc	S	0	0.20
---	--	------	------
mPD		0.0037	0.70
		nPD:	0.01
		N. weight:	0.89
		Sc. PD:	-0.03
		Sc. rank:	-8381.2

,

PB1			
Pos.	649	obs:	exp:
tct	S	0	1.84
tcc	S	10	1.16
tca	S	0	2.60
tcg	S	0	0.53
act	T	0	5.04
acc	T	20	4.10
aca	T	0	9.15
acg	T	0	1.71
agt	S	0	1.88
agc	S	0	1.99
gtt	V	0	1.77
gtc	V	8	1.45
gta	V	0	2.15
gtg	V	0	2.63
gct	A	42	5542.00
gcc	A	20873	4333.00
gca	A	6	9832.00
gcg	A	13	1226.00
---	--	------	------
mPD		0.0094	0.67
		nPD:	0.01
		N. weight:	1.6
		Sc. PD:	-0.041
		Sc. rank:	2652.0

,

PB1			
Pos.	650	obs:	exp:
tat	Y	1	0.52
tac	Y	0	0.48
cat	H	20889	12331.00
cac	H	79	8637.00
caa	Q	0	1.06
cag	Q	2	0.94
aat	N	1	0.59
aac	N	0	0.41
---	--	------	------
mPD		0.0079	0.48
		nPD:	0.02
		N. weight:	0.54
		Sc. PD:	-0.012
		Sc. rank:	1609.4

,

PB1			
Pos.	651	obs:	exp:
ggt	G	4549	3180.00
ggc	G	16364	3106.00
gga	G	57	9329.00
ggg	G	2	5357.00
---	--	------	------
mPD		0.34	0.69
		nPD:	0.5
		N. weight:	1.4
		Sc. PD:	0.65
		Sc. rank:	24237.0

,

PB1			
Pos.	652	obs:	exp:
cct	P	83	5778.00
ccc	P	59	3890.00
cca	P	20791	8930.00
ccg	P	39	2375.00
---	--	------	------
mPD		0.017	0.70
		nPD:	0.02
		N. weight:	0.85
		Sc. PD:	-0.013
		Sc. rank:	5852.3

,

PB1			
Pos.	653	obs:	exp:
tct	S	0	8.28
tcc	S	45	5.21
tca	S	0	11.71
tcg	S	0	2.39
att	I	0	0.73
atc	I	2	0.50
ata	I	0	0.77
act	T	0	27.23
acc	T	108	22.15
aca	T	0	49.41
acg	T	0	9.21
agt	S	0	8.46
agc	S	0	8.94
gtt	V	1	34.27
gtc	V	154	28.17
gta	V	0	41.69
gtg	V	0	50.88
gct	A	19	5470.00
gcc	A	20639	4277.00
gca	A	4	9704.00
gcg	A	0	1210.00
---	--	------	------
mPD		0.032	0.70
		nPD:	0.05
		N. weight:	1.7
		Sc. PD:	0.0096
		Sc. rank:	18262.0

,

PB1			
Pos.	654	obs:	exp:
cgt	R	0	0.19
cgc	R	0	0.37
cga	R	0	0.69
cgg	R	0	0.51
aat	N	0	0.59
aac	N	1	0.41
aaa	K	20941	11958.00
aag	K	23	9006.00
aga	R	7	3.51
agg	R	0	1.73
---	--	------	------
mPD		0.0030	0.49
		nPD:	0.01
		N. weight:	0.59
		Sc. PD:	-0.019
		Sc. rank:	-4711.1

,

PB1			
Pos.	655	obs:	exp:
tct	S	0	3842.00
tcc	S	0	2418.00
tca	S	0	5434.00
tcg	S	0	1111.00
tgt	C	0	1.81
tgc	C	4	2.19
cgt	R	0	0.05
cgc	R	0	0.10
cga	R	0	0.20
cgg	R	0	0.15
att	I	0	8.01
atc	I	22	5.49
ata	I	0	8.50
act	T	0	0.25
acc	T	1	0.21
aca	T	0	0.46
acg	T	0	0.09
aat	N	1	35.49
aac	N	59	24.51
agt	S	116	3926.00
agc	S	20764	4148.00
aga	R	2	1.00
agg	R	0	0.49
ggt	G	0	0.45
ggc	G	3	0.44
gga	G	0	1.33
ggg	G	0	0.77
---	--	------	------
mPD		0.020	1.6
		nPD:	0.01
		N. weight:	1.7
		Sc. PD:	-0.046
		Sc. rank:	705.9

,

PB1			
Pos.	656	obs:	exp:
tta	L	0	11.65
ttg	L	1	19.96
ctt	L	0	20.89
ctc	L	0	14.97
cta	L	0	20.41
ctg	L	105	18.12
att	I	1	9.83
atc	I	0	6.74
ata	I	26	10.43
atg	M	20824	20824.00
act	T	0	1.26
acc	T	0	1.03
aca	T	0	2.29
acg	T	5	0.43
gtt	V	0	2.21
gtc	V	0	1.82
gta	V	0	2.69
gtg	V	10	3.28
---	--	------	------
mPD		0.014	0.022
		nPD:	0.65
		N. weight:	0.012
		Sc. PD:	0.0069
		Sc. rank:	196.6

,

PB1			
Pos.	657	obs:	exp:
gat	D	2	1.58
gac	D	1	1.42
gaa	E	20761	12696.00
gag	E	206	8271.00
ggt	G	0	0.30
ggc	G	0	0.30
gga	G	2	0.89
ggg	G	0	0.51
---	--	------	------
mPD		0.020	0.48
		nPD:	0.04
		N. weight:	0.48
		Sc. PD:	0.00086
		Sc. rank:	5057.7

,

PB1			
Pos.	658	obs:	exp:
tat	Y	20958	10897.00
tac	Y	14	10075.00
---	--	------	------
mPD		0.0013	0.50
		nPD:	0.
		N. weight:	0.69
		Sc. PD:	-0.025
		Sc. rank:	-9951.6

,

PB1			
Pos.	659	obs:	exp:
gat	D	20944	11047.00
gac	D	22	9919.00
gaa	E	1	3.03
gag	E	4	1.97
ggt	G	1	0.15
ggc	G	0	0.15
gga	G	0	0.44
ggg	G	0	0.26
---	--	------	------
mPD		0.0027	0.50
		nPD:	0.01
		N. weight:	0.67
		Sc. PD:	-0.023
		Sc. rank:	-6104.4

,

PB1			
Pos.	660	obs:	exp:
tct	S	0	0.37
tcc	S	2	0.23
tca	S	0	0.52
tcg	S	0	0.11
agt	S	0	0.38
agc	S	0	0.40
gct	A	111	5552.00
gcc	A	20540	4341.00
gca	A	319	9849.00
gcg	A	0	1229.00
---	--	------	------
mPD		0.041	0.66
		nPD:	0.06
		N. weight:	1.5
		Sc. PD:	0.032
		Sc. rank:	18771.0

PB1			
Pos.	661	obs:	exp:
att	I	19	8.01
atc	I	3	5.49
ata	I	0	8.50
gtt	V	14330	4632.00
gtc	V	6553	3807.00
gta	V	66	5634.00
gtg	V	0	6877.00
gct	A	1	0.26
gcc	A	0	0.21
gca	A	0	0.47
gcg	A	0	0.06
---	--	------	------
mPD		0.44	0.74
		nPD:	0.59
		N. weight:	0.98
		Sc. PD:	0.53
		Sc. rank:	16744.0

PB1			
Pos.	662	obs:	exp:
tct	S	2	0.37
tcc	S	0	0.23
tca	S	0	0.52
tcg	S	0	0.11
act	T	5	2.27
acc	T	0	1.85
aca	T	4	4.12
acg	T	0	0.77
agt	S	0	0.38
agc	S	0	0.40
gtt	V	1	0.22
gtc	V	0	0.18
gta	V	0	0.27
gtg	V	0	0.33
gct	A	16464	5548.00
gcc	A	39	4337.00
gca	A	4440	9841.00
gcg	A	11	1228.00
ggt	G	0	0.91
ggc	G	0	0.89
gga	G	6	2.67
ggg	G	0	1.53
---	--	------	------
mPD		0.34	0.66
		nPD:	0.51
		N. weight:	0.72
		Sc. PD:	0.33
		Sc. rank:	12067.0

PB1			
Pos.	663	obs:	exp:
tct	S	1	0.18
tcc	S	0	0.12
tca	S	0	0.26
tcg	S	0	0.05
act	T	20799	5286.00
acc	T	165	4301.00
aca	T	2	9592.00
acg	T	2	1789.00
aat	N	3	1.77
aac	N	0	1.23
agt	S	0	0.19
agc	S	0	0.20
---	--	------	------
mPD		0.016	0.68
		nPD:	0.02
		N. weight:	1.4
		Sc. PD:	-0.022
		Sc. rank:	9472.0

PB1			
Pos.	664	obs:	exp:
tct	S	0	0.18
tcc	S	0	0.12
tca	S	1	0.26
tcg	S	0	0.05
act	T	2	5285.00
acc	T	1	4300.00
aca	T	20951	9591.00
acg	T	12	1789.00
agt	S	0	0.19
agc	S	0	0.20
gct	A	0	1.32
gcc	A	0	1.03
gca	A	5	2.35
gcg	A	0	0.29
---	--	------	------
mPD		0.0020	0.68
		nPD:	0.
		N. weight:	0.82
		Sc. PD:	-0.03
		Sc. rank:	-11629.0

PB1			
Pos.	665	obs:	exp:
cat	H	20839	12333.00
cac	H	133	8639.00
---	--	------	------
mPD		0.013	0.48
		nPD:	0.03
		N. weight:	0.52
		Sc. PD:	-0.0071
		Sc. rank:	3858.5

PB1			
Pos.	666	obs:	exp:
tct	S	52	3859.00
tcc	S	20886	2429.00
tca	S	34	5458.00
tcg	S	0	1116.00
agt	S	0	3943.00
agc	S	0	4166.00
---	--	------	------
mPD		0.0082	1.6
		nPD:	0.
		N. weight:	2.3
		Sc. PD:	-0.077
		Sc. rank:	-23123.0

PB1			
Pos.	667	obs:	exp:
tgg	W	20972	20972.00
---	--	------	------
mPD		0	0
		nPD:	1.
		N. weight:	0.
		Sc. PD:	0
		Sc. rank:	0

PB1			
Pos.	668	obs:	exp:
ttt	F	4	1.72
ttc	F	0	2.28
tct	S	0	0.74
tcc	S	0	0.46
tca	S	0	1.04
tcg	S	0	0.21
att	I	20745	7609.00
atc	I	154	5217.00
ata	I	2	8075.00
atg	M	7	7.00
act	T	24	6.05
acc	T	0	4.92
aca	T	0	10.98
acg	T	0	2.05
agt	S	4	0.75
agc	S	0	0.79
gtt	V	31	7.07
gtc	V	1	5.81
gta	V	0	8.61
gtg	V	0	10.50
---	--	------	------
mPD		0.022	0.66
		nPD:	0.03
		N. weight:	1.
		Sc. PD:	-0.0073
		Sc. rank:	9228.2

PB1			
Pos.	669	obs:	exp:
tct	S	0	2.21
tcc	S	12	1.39
tca	S	0	3.12
tcg	S	0	0.64
cct	P	42	5774.00
ccc	P	20908	3887.00
cca	P	8	8924.00
ccg	P	0	2373.00
act	T	0	0.50
acc	T	2	0.41
aca	T	0	0.91
acg	T	0	0.17
agt	S	0	2.26
agc	S	0	2.38
---	--	------	------
mPD		0.0061	0.70
		nPD:	0.01
		N. weight:	1.8
		Sc. PD:	-0.054
		Sc. rank:	-6585.6

PB1			
Pos.	670	obs:	exp:
cgt	R	0	0.22
cgc	R	0	0.42
cga	R	0	0.78
cgg	R	0	0.59
act	T	0	0.25
acc	T	0	0.21
aca	T	0	0.46
acg	T	1	0.09
aat	N	0	0.59
aac	N	1	0.41
aaa	K	119	11957.00
aag	K	20843	9005.00
aga	R	0	4.02
agg	R	8	1.98
---	--	------	------
mPD		0.012	0.49
		nPD:	0.02
		N. weight:	0.86
		Sc. PD:	-0.013
		Sc. rank:	5964.0

PB1			
Pos.	671	obs:	exp:
cgt	R	0	572.60
cgc	R	0	1097.00
cga	R	0	2054.00
cgg	R	2	1535.00
aaa	K	0	0.57
aag	K	1	0.43
aga	R	188	10528.00
agg	R	20781	5184.00
---	--	------	------
mPD		0.018	0.91
		nPD:	0.02
		N. weight:	1.4
		Sc. PD:	-0.028
		Sc. rank:	7509.8

PB1

Pos.	672	obs:	exp:
aat	N	20885	12404.00
aac	N	87	8568.00
---	--	------	------
mPD		0.0083	0.48
		nPD:	0.02
		N. weight:	0.53
		Sc. PD:	-0.012
		Sc. rank:	1860.9

PB1

Pos.	673	obs:	exp:
tta	L	0	0.11
ttg	L	0	0.19
ctt	L	1	0.20
ctc	L	0	0.14
cta	L	0	0.19
ctg	L	0	0.17
cgt	R	20877	572.60
cgc	R	47	1097.00
cga	R	1	2054.00
cgg	R	46	1535.00
aga	R	0	10528.00
agg	R	0	5184.00
---	--	------	------
mPD		0.0090	0.91
		nPD:	0.01
		N. weight:	3.8
		Sc. PD:	-0.11
		Sc. rank:	-8192.7

PB1

Pos.	674	obs:	exp:
tct	S	20895	3859.00
tcc	S	72	2429.00
tca	S	2	5458.00
tcg	S	2	1116.00
cct	P	1	0.28
ccc	P	0	0.19
cca	P	0	0.43
ccg	P	0	0.11
agt	S	0	3943.00
agc	S	0	4166.00
---	--	------	------
mPD		0.0073	1.6
		nPD:	0.
		N. weight:	1.8
		Sc. PD:	-0.061
		Sc. rank:	-19875.0

PB1

Pos.	675	obs:	exp:
ttt	F	1	0.43
ttc	F	0	0.57
att	I	19581	7631.00
atc	I	1381	5233.00
ata	I	0	8099.00
gtt	V	7	1.99
gtc	V	2	1.64
gta	V	0	2.42
gtg	V	0	2.95
---	--	------	------
mPD		0.12	0.66
		nPD:	0.19
		N. weight:	0.84
		Sc. PD:	0.12
		Sc. rank:	12940.0

PB1

Pos.	676	obs:	exp:
ttt	F	0	0.43
ttc	F	1	0.57
tta	L	0	2305.00
ttg	L	1	3948.00
ctt	L	35	4132.00
ctc	L	20854	2962.00
cta	L	79	4037.00
ctg	L	0	3585.00
att	I	0	0.73
atc	I	2	0.50
ata	I	0	0.77
---	--	------	------
mPD		0.011	1.1
		nPD:	0.01
		N. weight:	2.
		Sc. PD:	-0.06
		Sc. rank:	-4911.6

PB1

Pos.	677	obs:	exp:
aat	N	49	12404.00
aac	N	20923	8568.00
---	--	------	------
mPD		0.0047	0.48
		nPD:	0.01
		N. weight:	0.93
		Sc. PD:	-0.028
		Sc. rank:	-2550.2

PB1

Pos.	678	obs:	exp:
act	T	5	5287.00
acc	T	0	4301.00
aca	T	20953	9594.00
acg	T	13	1789.00
aaa	K	1	0.57
aag	K	0	0.43
---	--	------	------
mPD		0.0018	0.68
		nPD:	0.
		N. weight:	0.82
		Sc. PD:	-0.03
		Sc. rank:	-11918.0

PB1

Pos.	679	obs:	exp:
tct	S	0	3856.00
tcc	S	0	2427.00
tca	S	0	5453.00
tcg	S	0	1115.00
tgt	C	0	0.45
tgc	C	1	0.55
cgt	R	0	0.05
cgc	R	1	0.10
cga	R	0	0.20
cgg	R	0	0.15
att	I	0	0.73
atc	I	2	0.50
ata	I	0	0.77
act	T	0	0.50
acc	T	2	0.41
aca	T	0	0.91
acg	T	0	0.17
aat	N	0	7.69
aac	N	13	5.31
agt	S	164	3939.00
agc	S	20787	4162.00
aga	R	1	1.00
agg	R	0	0.49
ggt	G	0	0.15
ggc	G	1	0.15
gga	G	0	0.44
ggg	G	0	0.26
---	--	------	------
mPD		0.018	1.6
		nPD:	0.01
		N. weight:	1.7
		Sc. PD:	-0.048
		Sc. rank:	-2332.1

PB1

Pos.	680	obs:	exp:
cat	H	2	1.18
cac	H	0	0.82
caa	Q	20937	11065.00
cag	Q	33	9905.00
---	--	------	------
mPD		0.0033	0.50
		nPD:	0.01
		N. weight:	0.67
		Sc. PD:	-0.022
		Sc. rank:	-4607.0

PB1

Pos.	681	obs:	exp:
cgt	R	0	572.00
cgc	R	0	1096.00
cga	R	0	2052.00
cgg	R	375	1533.00
aaa	K	1	13.12
aag	K	22	9.88
aga	R	211	10517.00
agg	R	20363	5179.00
---	--	------	------
mPD		0.057	0.91
		nPD:	0.06
		N. weight:	1.3
		Sc. PD:	0.03
		Sc. rank:	16832.0

PB1

Pos.	682	obs:	exp:
ggt	G	5	3180.00
ggc	G	0	3106.00
gga	G	20916	9329.00
ggg	G	51	5357.00
---	--	------	------
mPD		0.0053	0.69
		nPD:	0.01
		N. weight:	0.84
		Sc. PD:	-0.026
		Sc. rank:	-4559.8

PB1

Pos.	683	obs:	exp:
att	I	19430	7631.00
atc	I	1420	5233.00
ata	I	114	8099.00
aat	N	1	0.59
aac	N	0	0.41
gtt	V	6	1.55
gtc	V	1	1.27
gta	V	0	1.88
gtg	V	0	2.30
---	--	------	------
mPD		0.14	0.66
		nPD:	0.21
		N. weight:	0.8
		Sc. PD:	0.13
		Sc. rank:	12419.0

PB1

Pos.	684	obs:	exp:
tta	L	0	2305.00
ttg	L	0	3948.00
ctt	L	20945	4132.00
ctc	L	9	2962.00
cta	L	5	4037.00
ctg	L	9	3585.00
cat	H	1	0.59
cac	H	0	0.41
att	I	3	1.09
atc	I	0	0.75
ata	I	0	1.16
---	--	------	------
mPD		0.0026	1.1
		nPD:	0.
		N. weight:	1.7
		Sc. PD:	-0.063
		Sc. rank:	-25721.0

PB1

Pos.	685	obs:	exp:
aaa	K	0	1.71
aag	K	3	1.29
gtt	V	0	0.22
gtc	V	0	0.18
gta	V	0	0.27
gtg	V	1	0.33
gat	D	1	0.53
gac	D	0	0.47
gaa	E	81	12696.00
gag	E	20886	8271.00
---	--	------	------
mPD		0.0082	0.48
		nPD:	0.02
		N. weight:	0.96
		Sc. PD:	-0.021
		Sc. rank:	3316.2

PB1

Pos.	686	obs:	exp:
gat	D	20950	11050.00
gac	D	21	9921.00
gaa	E	0	0.61
gag	E	1	0.39
---	--	------	------
mPD		0.0021	0.50
		nPD:	0.
		N. weight:	0.67
		Sc. PD:	-0.023
		Sc. rank:	-7872.6

PB1

Pos.	687	obs:	exp:
gaa	E	20936	12699.00
gag	E	36	8273.00
---	--	------	------
mPD		0.0034	0.48
		nPD:	0.01
		N. weight:	0.52
		Sc. PD:	-0.017
		Sc. rank:	-3344.2

PB1			
Pos.	688	obs:	exp:
tta	L	0	1.32
ttg	L	0	2.26
ctt	L	0	2.36
ctc	L	0	1.69
cta	L	0	2.31
ctg	L	12	2.05
cat	H	450	265.20
cac	H	1	185.80
caa	Q	70	10819.00
cag	Q	20433	9684.00
cgt	R	0	0.08
cgc	R	0	0.16
cga	R	0	0.29
cgg	R	3	0.22
aaa	K	0	1.71
aag	K	3	1.29
aga	R	0	1.51
agg	R	0	0.74
---	--	------	------
mPD		0.050	0.52
		nPD:	0.1
		N. weight:	0.77
		Sc. PD:	0.042
		Sc. rank:	10880.0

,

PB1			
Pos.	689	obs:	exp:
att	I	0	4.37
atc	I	0	3.00
ata	I	12	4.64
atg	M	20955	20955.00
gtt	V	0	1.11
gtc	V	0	0.91
gta	V	0	1.34
gtg	V	5	1.64
---	--	------	------
mPD		0.0016	0.0019
		nPD:	0.84
		N. weight:	0.00086
		Sc. PD:	0.00067
		Sc. rank:	14.7

,

PB1			
Pos.	690	obs:	exp:
tat	Y	245	10894.00
tac	Y	20722	10073.00
cat	H	0	2.35
cac	H	4	1.65
gat	D	0	0.53
gac	D	1	0.47
---	--	------	------
mPD		0.024	0.50
		nPD:	0.05
		N. weight:	0.71
		Sc. PD:	0.0051
		Sc. rank:	8071.4

,

PB1			
Pos.	691	obs:	exp:
cat	H	1	0.59
cac	H	0	0.41
caa	Q	79	11064.00
cag	Q	20889	9904.00
cgt	R	0	0.03
cgc	R	0	0.05
cga	R	0	0.10
cgg	R	1	0.07
aaa	K	0	1.14
aag	K	2	0.86
aga	R	0	0.50
agg	R	0	0.25
---	--	------	------
mPD		0.0079	0.50
		nPD:	0.02
		N. weight:	0.77
		Sc. PD:	-0.018
		Sc. rank:	2125.7

,

PB1			
Pos.	692	obs:	exp:
cgt	R	0	0.11
cgc	R	0	0.21
cga	R	0	0.39
cgg	R	0	0.29
aat	N	1	0.59
aac	N	0	0.41
aaa	K	73	11960.00
aag	K	20894	9007.00
aga	R	0	2.01
agg	R	4	0.99
---	--	------	------
mPD		0.0074	0.49
		nPD:	0.02
		N. weight:	0.87
		Sc. PD:	-0.021
		Sc. rank:	2109.2

,

PB1			
Pos.	693	obs:	exp:
tct	S	1	0.18
tcc	S	0	0.12
tca	S	0	0.26
tcg	S	0	0.05
tgt	C	18029	9465.00
tgc	C	2942	11506.00
agt	S	0	0.19
agc	S	0	0.20
---	--	------	------
mPD		0.24	0.50
		nPD:	0.49
		N. weight:	0.38
		Sc. PD:	0.17
		Sc. rank:	6428.9

,

PB1			
Pos.	694	obs:	exp:
tat	Y	0	1.04
tac	Y	2	0.96
tgt	C	27	9465.00
tgc	C	20942	11504.00
cgt	R	0	0.03
cgc	R	1	0.05
cga	R	0	0.10
cgg	R	0	0.07
aga	R	0	0.50
agg	R	0	0.25
---	--	------	------
mPD		0.0029	0.50
		nPD:	0.01
		N. weight:	0.63
		Sc. PD:	-0.021
		Sc. rank:	-5397.8

,

PB1			
Pos.	695	obs:	exp:
tct	S	0	5.70
tcc	S	0	3.59
tca	S	0	8.07
tcg	S	0	1.65
tat	Y	8	4.16
tac	Y	0	3.84
cat	H	5	2.94
cac	H	0	2.06
att	I	2	0.73
atc	I	0	0.50
ata	I	0	0.77
act	T	4	1.01
acc	T	0	0.82
aca	T	0	1.83
acg	T	0	0.34
aat	N	20881	12373.00
aac	N	40	8548.00
aaa	K	0	0.57
aag	K	1	0.43
agt	S	31	5.83
agc	S	0	6.16
---	--	------	------
mPD		0.0087	0.49
		nPD:	0.02
		N. weight:	0.54
		Sc. PD:	-0.012
		Sc. rank:	2187.6

,

PB1			
Pos.	696	obs:	exp:
tta	L	16749	2305.00
ttg	L	56	3947.00
tct	S	0	0.92
tcc	S	0	0.58
tca	S	5	1.30
tcg	S	0	0.27
ctt	L	1	4131.00
ctc	L	0	2961.00
cta	L	4159	4036.00
ctg	L	0	3585.00
att	I	0	0.36
atc	I	0	0.25
ata	I	1	0.39
agt	S	0	0.94
agc	S	0	0.99
gtt	V	0	0.22
gtc	V	0	0.18
gta	V	1	0.27
gtg	V	0	0.33
---	--	------	------
mPD		0.32	1.1
		nPD:	0.28
		N. weight:	1.7
		Sc. PD:	0.4
		Sc. rank:	26807.0

,

PB1			
Pos.	697	obs:	exp:
ttt	F	127	9000.00
ttc	F	20845	11972.00
---	--	------	------
mPD		0.012	0.49
		nPD:	0.02
		N. weight:	0.56
		Sc. PD:	-0.0083
		Sc. rank:	3794.7

,

PB1			
Pos.	698	obs:	exp:
aaa	K	0	1.71
aag	K	3	1.29
gat	D	3	1.58
gac	D	0	1.42
gaa	E	78	12694.00
gag	E	20887	8271.00
ggt	G	0	0.15
ggc	G	0	0.15
gga	G	0	0.44
ggg	G	1	0.26
---	--	------	------
mPD		0.0081	0.48
		nPD:	0.02
		N. weight:	0.96
		Sc. PD:	-0.022
		Sc. rank:	3190.9

,

PB1			
Pos.	699	obs:	exp:
cgt	R	0	0.60
cgc	R	0	1.15
cga	R	0	2.15
cgg	R	0	1.61
aat	N	8	5.91
aac	N	2	4.09
aaa	K	20882	11943.00
aag	K	55	8994.00
aga	R	22	11.04
agg	R	0	5.44
gaa	E	3	1.82
gag	E	0	1.18
---	--	------	------
mPD		0.0086	0.49
		nPD:	0.02
		N. weight:	0.58
		Sc. PD:	-0.013
		Sc. rank:	2169.8

,

PB1			
Pos.	700	obs:	exp:
ttt	F	20933	9000.00
ttc	F	39	11972.00
---	--	------	------
mPD		0.0037	0.49
		nPD:	0.01
		N. weight:	0.88
		Sc. PD:	-0.028
		Sc. rank:	-5188.4

,

PB1			
Pos.	701	obs:	exp:
ttt	F	77	8999.00
ttc	F	20894	11972.00
tta	L	0	0.11
ttg	L	0	0.19
ctt	L	0	0.20
ctc	L	1	0.14
cta	L	0	0.19
ctg	L	0	0.17
---	--	------	------
mPD		0.0074	0.49
		nPD:	0.02
		N. weight:	0.57
		Sc. PD:	-0.014
		Sc. rank:	1395.8

,

PB1			
Pos.	702	obs:	exp:
tct	S	2	0.37
tcc	S	0	0.23
tca	S	0	0.52
tcg	S	0	0.11
cct	P	20867	5777.00
ccc	P	33	3889.00
cca	P	66	8928.00
ccg	P	2	2374.00
cat	H	2	1.18
cac	H	0	0.82
agt	S	0	0.38
agc	S	0	0.40
---	--	------	------
mPD		0.010	0.70
		nPD:	0.01
		N. weight:	1.3
		Sc. PD:	-0.033
		Sc. rank:	2331.0

,

PB1			
Pos.	703	obs:	exp:
tct	S	0	3859.00
tcc	S	0	2429.00
tca	S	0	5458.00
tcg	S	0	1116.00
agt	S	70	3943.00
agc	S	20900	4165.00
ggt	G	0	0.30
ggc	G	2	0.30
gga	G	0	0.89
ggg	G	0	0.51
---	--	------	------
mPD		0.0068	1.6
		nPD:	0.
		N. weight:	1.7
		Sc. PD:	-0.059
		Sc. rank:	-20113.0

,

PB1			
Pos.	704	obs:	exp:
tct	S	0	3858.00
tcc	S	0	2428.00
tca	S	0	5456.00
tcg	S	0	1116.00
tgt	C	3	1.35
tgc	C	0	1.65
aat	N	5	2.96
aac	N	0	2.04
agt	S	20837	3942.00
agc	S	127	4164.00
---	--	------	------
mPD		0.013	1.6
		nPD:	0.01
		N. weight:	1.7
		Sc. PD:	-0.054
		Sc. rank:	-8952.3

,

PB1			
Pos.	705	obs:	exp:
tct	S	9	3859.00
tcc	S	2	2429.00
tca	S	20926	5457.00
tcg	S	31	1116.00
cct	P	0	0.28
ccc	P	0	0.19
cca	P	1	0.43
ccg	P	0	0.11
agt	S	0	3943.00
agc	S	0	4165.00
gct	A	0	0.79
gcc	A	0	0.62
gca	A	3	1.41
gcg	A	0	0.18
---	--	------	------
mPD		0.0044	1.6
		nPD:	0.
		N. weight:	1.4
		Sc. PD:	-0.051
		Sc. rank:	-20526.0

,

PB1			
Pos.	706	obs:	exp:
ttt	F	2	0.86
ttc	F	0	1.14
tat	Y	20940	10896.00
tac	Y	30	10074.00
---	--	------	------
mPD		0.0030	0.50
		nPD:	0.01
		N. weight:	0.68
		Sc. PD:	-0.023
		Sc. rank:	-5411.0

,

PB1			
Pos.	707	obs:	exp:
cgt	R	0	572.30
cgc	R	0	1097.00
cga	R	0	2053.00
cgg	R	0	1534.00
atg	M	1	1.00
aaa	K	0	5.13
aag	K	9	3.87
aga	R	188	10524.00
agg	R	20774	5182.00
---	--	------	------
mPD		0.019	0.91
		nPD:	0.02
		N. weight:	1.4
		Sc. PD:	-0.027
		Sc. rank:	7866.4

,

PB1			
Pos.	708	obs:	exp:
cgt	R	0	572.40
cgc	R	0	1097.00
cga	R	6	2053.00
cgg	R	0	1534.00
att	I	0	0.36
atc	I	0	0.25
ata	I	1	0.39
act	T	0	1.26
acc	T	0	1.03
aca	T	5	2.29
acg	T	0	0.43
aaa	K	2	1.14
aag	K	0	0.86
aga	R	20707	10524.00
agg	R	250	5182.00
ggt	G	0	0.15
ggc	G	0	0.15
gga	G	1	0.44
ggg	G	0	0.26
---	--	------	------
mPD		0.025	0.91
		nPD:	0.03
		N. weight:	0.67
		Sc. PD:	-0.0081
		Sc. rank:	5118.5

,

PB1			
Pos.	709	obs:	exp:
tct	S	0	0.37
tcc	S	0	0.23
tca	S	0	0.52
tcg	S	2	0.11
cct	P	25	5777.00
ccc	P	1	3889.00
cca	P	316	8929.00
ccg	P	20628	2375.00
agt	S	0	0.38
agc	S	0	0.40
---	--	------	------
mPD		0.032	0.70
		nPD:	0.05
		N. weight:	2.2
		Sc. PD:	0.014
		Sc. rank:	24376.0

,

PB1			
Pos.	710	obs:	exp:
att	I	33	12.01
atc	I	0	8.24
ata	I	0	12.75
gtt	V	20913	4628.00
gtc	V	10	3804.00
gta	V	3	5629.00
gtg	V	6	6871.00
gct	A	1	0.26
gcc	A	0	0.21
gca	A	0	0.47
gcg	A	0	0.06
gat	D	6	3.16
gac	D	0	2.84
---	--	------	------
mPD		0.0056	0.74
		nPD:	0.01
		N. weight:	1.6
		Sc. PD:	-0.05
		Sc. rank:	-9418.3

,

PB1			
Pos.	711	obs:	exp:
ggt	G	1	3180.00
ggc	G	0	3106.00
gga	G	20900	9329.00
ggg	G	71	5357.00
---	--	------	------
mPD		0.0068	0.69
		nPD:	0.01
		N. weight:	0.84
		Sc. PD:	-0.025
		Sc. rank:	-2097.3

,

PB1			
Pos.	712	obs:	exp:
tta	L	0	3.08
ttg	L	0	5.27
ctt	L	28	5.52
ctc	L	0	3.95
cta	L	0	5.39
ctg	L	0	4.79
att	I	20843	7623.00
atc	I	42	5227.00
ata	I	55	8090.00
atg	M	1	1.00
gtt	V	3	0.66
gtc	V	0	0.55
gta	V	0	0.81
gtg	V	0	0.98
---	--	------	------
mPD		0.012	0.66
		nPD:	0.02
		N. weight:	1.
		Sc. PD:	-0.022
		Sc. rank:	4792.3

,

PB1			
Pos.	713	obs:	exp:
tct	S	20842	3858.00
tcc	S	122	2429.00
tca	S	3	5457.00
tcg	S	0	1116.00
tgt	C	5	2.26
tgc	C	0	2.74
agt	S	0	3943.00
agc	S	0	4165.00
---	--	------	------
mPD		0.012	1.6
		nPD:	0.01
		N. weight:	1.8
		Sc. PD:	-0.056
		Sc. rank:	-10536.0

,

PB1			
Pos.	714	obs:	exp:
tct	S	0	3859.00
tcc	S	0	2429.00
tca	S	0	5458.00
tcg	S	0	1116.00
cgt	R	0	0.05
cgc	R	0	0.10
cga	R	0	0.20
cgg	R	0	0.15
agt	S	27	3943.00
agc	S	20943	4165.00
aga	R	2	1.00
agg	R	0	0.49
---	--	------	------
mPD		0.0028	1.6
		nPD:	0.
		N. weight:	1.7
		Sc. PD:	-0.064
		Sc. rank:	-27046.0

,

PB1			
Pos.	715	obs:	exp:
tta	L	0	0.11
ttg	L	1	0.19
ctt	L	0	0.20
ctc	L	0	0.14
cta	L	0	0.19
ctg	L	0	0.17
att	I	0	1.09
atc	I	0	0.75
ata	I	3	1.16
atg	M	20967	20967.00
act	T	0	0.25
acc	T	0	0.21
aca	T	0	0.46
acg	T	1	0.09
---	--	------	------
mPD		0.00048	0.00063
		nPD:	0.76
		N. weight:	0.00035
		Sc. PD:	0.00025
		Sc. rank:	6.0

,

PB1

Pos.	716	obs:	exp:
atg	M	14	14.00
gtt	V	13	4631.00
gtc	V	0	3806.00
gta	V	43	5633.00
gtg	V	20890	6876.00
gct	A	0	2.65
gcc	A	0	2.07
gca	A	0	4.70
gcg	A	10	0.59
ggt	G	0	0.30
ggc	G	0	0.30
gga	G	0	0.89
ggg	G	2	0.51
---	--	------	------
mPD		0.0078	0.74
		nPD:	0.01
		N. weight:	1.2
		Sc. PD:	-0.033
		Sc. rank:	-1860.3

,

PB1

Pos.	717	obs:	exp:
caa	Q	0	0.53
cag	Q	1	0.47
gct	A	0	0.26
gcc	A	0	0.21
gca	A	0	0.47
gcg	A	1	0.06
gat	D	7	3.69
gac	D	0	3.31
gaa	E	14	12693.00
gag	E	20949	8270.00
---	--	------	------
mPD		0.0022	0.48
		nPD:	0.
		N. weight:	0.98
		Sc. PD:	-0.034
		Sc. rank:	-10864.0

,

PB1

Pos.	718	obs:	exp:
tct	S	0	0.18
tcc	S	1	0.12
tca	S	0	0.26
tcg	S	0	0.05
agt	S	0	0.19
agc	S	0	0.20
gct	A	74	5550.00
gcc	A	20855	4339.00
gca	A	34	9846.00
gcg	A	0	1228.00
ggt	G	0	1.21
ggc	G	8	1.18
gga	G	0	3.56
ggg	G	0	2.04
---	--	------	------
mPD		0.011	0.66
		nPD:	0.02
		N. weight:	1.6
		Sc. PD:	-0.037
		Sc. rank:	5356.7

,

PB1

Pos.	719	obs:	exp:
att	I	1	0.73
atc	I	0	0.50
ata	I	1	0.77
atg	M	20969	20969.00
gtt	V	0	0.22
gtc	V	0	0.18
gta	V	0	0.27
gtg	V	1	0.33
---	--	------	------
mPD		0.00029	0.00035
		nPD:	0.82
		N. weight:	0.00012
		Sc. PD:	0.000092
		Sc. rank:	2.1

,

PB1

Pos.	720	obs:	exp:
tta	L	0	0.44
ttg	L	4	0.75
ctt	L	0	0.79
ctc	L	0	0.56
cta	L	0	0.77
ctg	L	0	0.68
att	I	0	6.19
atc	I	0	4.24
ata	I	17	6.57
atg	M	52	52.00
gtt	V	21	4620.00
gtc	V	1	3797.00
gta	V	198	5620.00
gtg	V	20675	6859.00
gct	A	0	1.06
gcc	A	0	0.83
gca	A	0	1.88
gcg	A	4	0.23
---	--	------	------
mPD		0.030	0.75
		nPD:	0.04
		N. weight:	1.1
		Sc. PD:	4.4×10^{-6}
		Sc. rank:	11452.0

,

PB1

Pos.	721	obs:	exp:
ttt	F	1	0.43
ttc	F	0	0.57
tct	S	20907	3857.00
tcc	S	45	2428.00
tca	S	4	5455.00
tcg	S	3	1115.00
tat	Y	1	0.52
tac	Y	0	0.48
agt	S	0	3941.00
agc	S	0	4163.00
gct	A	11	2.91
gcc	A	0	2.28
gca	A	0	5.17
gcg	A	0	0.64
---	--	------	------
mPD		0.0062	1.6
		nPD:	0.
		N. weight:	1.8
		Sc. PD:	-0.063
		Sc. rank:	-22596.0

,

PB1

Pos.	722	obs:	exp:
cgt	R	0	572.40
cgc	R	0	1097.00
cga	R	0	2053.00
cgg	R	0	1534.00
aaa	K	0	4.56
aag	K	8	3.44
aga	R	26	10525.00
agg	R	20938	5183.00
---	--	------	------
mPD		0.0032	0.91
		nPD:	0.
		N. weight:	1.5
		Sc. PD:	-0.052
		Sc. rank:	-19475.0

,

PB1

Pos.	723	obs:	exp:
act	T	0	0.76
acc	T	3	0.62
aca	T	0	1.37
acg	T	0	0.26
gct	A	16	5549.00
gcc	A	20919	4338.00
gca	A	23	9843.00
gcg	A	0	1228.00
ggt	G	0	1.67
ggc	G	11	1.63
gga	G	0	4.89
ggg	G	0	2.81
---	--	------	------
mPD		0.0050	0.66
		nPD:	0.01
		N. weight:	1.7
		Sc. PD:	-0.052
		Sc. rank:	-9601.7

,

PB1

Pos.	724	obs:	exp:
tta	L	0	0.22
ttg	L	0	0.38
tgg	W	3	3.00
ctt	L	0	0.39
ctc	L	0	0.28
cta	L	0	0.39
ctg	L	2	0.34
caa	Q	2	19.52
cag	Q	35	17.48
cgt	R	0	571.40
cgc	R	0	1095.00
cga	R	111	2050.00
cgg	R	20800	1532.00
aga	R	0	10507.00
agg	R	18	5174.00
ggt	G	0	0.15
ggc	G	0	0.15
gga	G	0	0.44
ggg	G	1	0.26
---	--	------	------
mPD		0.017	0.92
		nPD:	0.02
		N. weight:	2.7
		Sc. PD:	-0.058
		Sc. rank:	11559.0

,

PB1

Pos.	725	obs:	exp:
att	I	20755	7634.00
atc	I	207	5235.00
ata	I	8	8102.00
gtt	V	2	0.44
gtc	V	0	0.36
gta	V	0	0.54
gtg	V	0	0.66
---	--	------	------
mPD		0.020	0.66
		nPD:	0.03
		N. weight:	1.
		Sc. PD:	-0.0085
		Sc. rank:	8726.5

,

PB1

Pos.	726	obs:	exp:
gat	D	20869	11050.00
gac	D	103	9922.00
---	--	------	------
mPD		0.0098	0.50
		nPD:	0.02
		N. weight:	0.65
		Sc. PD:	-0.013
		Sc. rank:	3239.6

,

PB1

Pos.	727	obs:	exp:
tct	S	0	0.18
tcc	S	1	0.12
tca	S	0	0.26
tcg	S	0	0.05
act	T	0	1.26
acc	T	5	1.03
aca	T	0	2.29
acg	T	0	0.43
agt	S	0	0.19
agc	S	0	0.20
gtt	V	0	1.11
gtc	V	5	0.91
gta	V	0	1.34
gtg	V	0	1.64
gct	A	8	5550.00
gcc	A	20949	4339.00
gca	A	4	9845.00
gcg	A	0	1228.00
---	--	------	------
mPD		0.0022	0.66
		nPD:	0.
		N. weight:	1.7
		Sc. PD:	-0.06
		Sc. rank:	-22612.0

,

PB1			
Pos.	728	obs:	exp:
cgt	R	0	572.60
cgc	R	0	1097.00
cga	R	9	2054.00
cgg	R	5	1535.00
aga	R	17053	10529.00
agg	R	3905	5185.00
---	--	------	------
mPD		0.30	0.91
		nPD:	0.33
		N. weight:	0.36
		Sc. PD:	0.1
		Sc. rank:	5753.8

,

PB1			
Pos.	729	obs:	exp:
ttt	F	0	0.43
ttc	F	1	0.57
att	I	19	48.78
atc	I	115	33.45
ata	I	0	51.77
gtt	V	168	4606.00
gtc	V	20633	3786.00
gta	V	31	5603.00
gtg	V	1	6839.00
gct	A	0	1.06
gcc	A	4	0.83
gca	A	0	1.88
gcg	A	0	0.23
---	--	------	------
mPD		0.034	0.75
		nPD:	0.05
		N. weight:	1.7
		Sc. PD:	0.0089
		Sc. rank:	18806.0

,

PB1			
Pos.	730	obs:	exp:
aat	N	0	2.96
aac	N	5	2.04
gat	D	143	11044.00
gac	D	20817	9916.00
gaa	E	4	2.42
gag	E	0	1.58
ggt	G	0	0.45
ggc	G	3	0.44
gga	G	0	1.33
ggg	G	0	0.77
---	--	------	------
mPD		0.015	0.50
		nPD:	0.03
		N. weight:	0.75
		Sc. PD:	-0.0077
		Sc. rank:	6193.9

,

PB1			
Pos.	731	obs:	exp:
ttt	F	18253	8980.00
ttc	F	2673	11946.00
tta	L	0	0.77
ttg	L	1	1.32
tct	S	8	6.81
tcc	S	29	4.29
tca	S	0	9.63
tcg	S	0	1.97
tat	Y	0	0.52
tac	Y	1	0.48
ctt	L	5	1.38
ctc	L	1	0.99
cta	L	0	1.35
ctg	L	0	1.20
agt	S	0	6.96
agc	S	0	7.35
gtt	V	1	0.22
gtc	V	0	0.18
gta	V	0	0.27
gtg	V	0	0.33
---	--	------	------
mPD		0.23	0.50
		nPD:	0.46
		N. weight:	0.46
		Sc. PD:	0.19
		Sc. rank:	7591.0

,

PB1			
Pos.	732	obs:	exp:
gat	D	2	1.05
gac	D	0	0.95
gaa	E	505	12697.00
gag	E	20464	8272.00
---	-	1	1.00
---	--	------	------
mPD		0.047	0.48
		nPD:	0.1
		N. weight:	0.85
		Sc. PD:	0.049
		Sc. rank:	12161.0

,

PB1			
Pos.	733	obs:	exp:
tct	S	20910	3859.00
tcc	S	42	2429.00
tca	S	8	5458.00
tcg	S	11	1116.00
agt	S	1	3943.00
agc	S	0	4166.00
---	--	------	------
mPD		0.0060	1.6
		nPD:	0.
		N. weight:	1.8
		Sc. PD:	-0.063
		Sc. rank:	-22919.0

,

PB1			
Pos.	734	obs:	exp:
cat	H	1	0.59
cac	H	0	0.41
cgt	R	0	0.14
cgc	R	0	0.26
cga	R	0	0.49
cgg	R	0	0.37
aga	R	5	2.51
agg	R	0	1.24
gaa	E	17	10.29
gag	E	0	6.71
ggt	G	5	3177.00
ggc	G	0	3103.00
gga	G	20896	9319.00
ggg	G	48	5351.00
---	--	------	------
mPD		0.0074	0.69
		nPD:	0.01
		N. weight:	0.84
		Sc. PD:	-0.024
		Sc. rank:	-1145.7

,

PB1			
Pos.	735	obs:	exp:
tta	L	0	0.22
ttg	L	0	0.38
tgg	W	3	3.00
ctt	L	0	0.39
ctc	L	0	0.28
cta	L	0	0.39
ctg	L	2	0.34
caa	Q	0	2.64
cag	Q	5	2.36
cgt	R	6	572.30
cgc	R	0	1097.00
cga	R	111	2053.00
cgg	R	20797	1534.00
aga	R	1	10524.00
agg	R	47	5182.00
---	--	------	------
mPD		0.017	0.91
		nPD:	0.02
		N. weight:	2.7
		Sc. PD:	-0.057
		Sc. rank:	12057.0

,

PB1			
Pos.	736	obs:	exp:
tct	S	0	0.18
tcc	S	0	0.12
tca	S	0	0.26
tcg	S	0	0.05
att	I	6966	7620.00
atc	I	13929	5225.00
ata	I	38	8088.00
act	T	3	3.28
acc	T	10	2.67
aca	T	0	5.95
acg	T	0	1.11
agt	S	0	0.19
agc	S	1	0.20
gtt	V	9	5.53
gtc	V	16	4.54
gta	V	0	6.72
gtg	V	0	8.21
---	--	------	------
mPD		0.45	0.66
		nPD:	0.68
		N. weight:	0.65
		Sc. PD:	0.41
		Sc. rank:	11109.0

,

PB1			
Pos.	737	obs:	exp:
cgt	R	0	0.38
cgc	R	0	0.73
cga	R	0	1.37
cgg	R	0	1.02
act	T	0	0.50
acc	T	0	0.41
aca	T	0	0.91
acg	T	2	0.17
aat	N	5	2.96
aac	N	0	2.04
aaa	K	131	11830.00
aag	K	20607	8908.00
aga	R	0	7.03
agg	R	14	3.46
gaa	E	0	6.66
gag	E	11	4.34
ggt	G	0	30.63
ggc	G	0	29.92
gga	G	0	89.85
ggg	G	202	51.60
---	--	------	------
mPD		0.054	0.53
		nPD:	0.1
		N. weight:	0.86
		Sc. PD:	0.051
		Sc. rank:	12263.0

,

PB1			
Pos.	738	obs:	exp:
cgt	R	0	0.41
cgc	R	0	0.78
cga	R	0	1.47
cgg	R	0	1.10
att	I	0	0.36
atc	I	0	0.25
ata	I	1	0.39
aat	N	0	0.59
aac	N	1	0.41
aaa	K	20651	11949.00
aag	K	297	8999.00
aga	R	14	7.53
agg	R	1	3.71
gaa	E	3	1.82
gag	E	0	1.18
ggt	G	0	0.45
ggc	G	0	0.44
gga	G	3	1.33
ggg	G	0	0.77
---	-	1	1.00
---	--	------	------
mPD		0.030	0.49
		nPD:	0.06
		N. weight:	0.52
		Sc. PD:	0.011
		Sc. rank:	6468.9

,

PB1			
Pos.	739	obs:	exp:
aaa	K	2	1.14
aag	K	0	0.86
gct	A	0	0.26
gcc	A	0	0.21
gca	A	1	0.47
gcg	A	0	0.06
gat	D	2	4.22
gac	D	6	3.78
gaa	E	20645	12689.00
gag	E	311	8267.00
ggt	G	0	0.76
ggc	G	0	0.74
gga	G	5	2.22
ggg	G	0	1.28
---	--	------	------
mPD		0.031	0.48
		nPD:	0.06
		N. weight:	0.46
		Sc. PD:	0.011
		Sc. rank:	5741.0

,

PB1			
Pos.	740	obs:	exp:
cgt	R	0	0.03
cgc	R	0	0.05
cga	R	0	0.10
cgg	R	0	0.07
aaa	K	0	3.99
aag	K	7	3.01
aga	R	0	0.50
agg	R	1	0.25
gtt	V	0	0.66
gtc	V	0	0.55
gta	V	0	0.81
gtg	V	3	0.98
gct	A	0	0.53
gcc	A	0	0.41
gca	A	0	0.94
gcg	A	2	0.12
gat	D	289	152.30
gac	D	0	136.70
gaa	E	63	12512.00
gag	E	20601	8152.00
ggt	G	0	0.91
ggc	G	0	0.89
gga	G	0	2.67
ggg	G	6	1.53
---	--	------	------
mPD		0.035	0.49
		nPD:	0.07
		N. weight:	0.96
		Sc. PD:	0.029
		Sc. rank:	12315.0

PB1			
Pos.	741	obs:	exp:
ttt	F	57	8994.00
ttc	F	20902	11965.00
tta	L	4	1.43
ttg	L	0	2.45
ctt	L	0	2.56
ctc	L	9	1.84
cta	L	0	2.50
ctg	L	0	2.22
---	--	------	------
mPD		0.0067	0.49
		nPD:	0.01
		N. weight:	0.58
		Sc. PD:	-0.015
		Sc. rank:	693.0

PB1			
Pos.	742	obs:	exp:
ttt	F	4	1.72
ttc	F	0	2.28
tct	S	20884	3857.00
tcc	S	26	2427.00
tca	S	45	5454.00
tcg	S	2	1115.00
tat	Y	7	3.64
tac	Y	0	3.36
act	T	2	0.50
acc	T	0	0.41
aca	T	0	0.91
acg	T	0	0.17
agt	S	0	3941.00
agc	S	0	4163.00
gct	A	2	0.53
gcc	A	0	0.41
gca	A	0	0.94
gcg	A	0	0.12
---	--	------	------
mPD		0.0084	1.6
		nPD:	0.01
		N. weight:	1.8
		Sc. PD:	-0.06
		Sc. rank:	-17381.0

PB1			
Pos.	743	obs:	exp:
gat	D	105	55.32
gac	D	0	49.68
gaa	E	4	12633.00
gag	E	20860	8231.00
ggt	G	0	0.45
ggc	G	0	0.44
gga	G	0	1.33
ggg	G	3	0.77
---	--	------	------
mPD		0.011	0.48
		nPD:	0.02
		N. weight:	0.98
		Sc. PD:	-0.017
		Sc. rank:	5908.0

PB1			
Pos.	744	obs:	exp:
att	I	2	7633.00
atc	I	20963	5234.00
ata	I	2	8101.00
act	T	0	1.01
acc	T	4	0.82
aca	T	0	1.83
acg	T	0	0.34
gtt	V	0	0.22
gtc	V	1	0.18
gta	V	0	0.27
gtg	V	0	0.33
---	--	------	------
mPD		0.00086	0.66
		nPD:	0.
		N. weight:	1.5
		Sc. PD:	-0.055
		Sc. rank:	-23957.0

PB1			
Pos.	745	obs:	exp:
tta	L	0	2.09
ttg	L	19	3.58
ctt	L	0	3.74
ctc	L	0	2.68
cta	L	0	3.66
ctg	L	0	3.25
att	I	2	80.45
atc	I	0	55.17
ata	I	219	85.38
atg	M	20715	20715.00
act	T	0	1.51
acc	T	0	1.23
aca	T	0	2.74
acg	T	6	0.51
aaa	K	0	0.57
aag	K	1	0.43
gtt	V	0	2.21
gtc	V	0	1.82
gta	V	0	2.69
gtg	V	10	3.28
---	--	------	------
mPD		0.024	0.027
		nPD:	0.91
		N. weight:	0.013
		Sc. PD:	0.011
		Sc. rank:	223.4

PB1			
Pos.	746	obs:	exp:
cgt	R	0	0.33
cgc	R	0	0.63
cga	R	0	1.18
cgg	R	0	0.88
act	T	0	0.25
acc	T	0	0.21
aca	T	0	0.46
acg	T	1	0.09
aaa	K	14	11956.00
aag	K	20945	9003.00
aga	R	0	6.02
agg	R	12	2.97
---	--	------	------
mPD		0.0026	0.49
		nPD:	0.01
		N. weight:	0.89
		Sc. PD:	-0.03
		Sc. rank:	-8521.0

PB1			
Pos.	747	obs:	exp:
att	I	0	7625.00
atc	I	20947	5229.00
ata	I	0	8093.00
act	T	0	5.80
acc	T	23	4.72
aca	T	0	10.52
acg	T	0	1.96
gtt	V	0	0.44
gtc	V	2	0.36
gta	V	0	0.54
gtg	V	0	0.66
---	--	------	------
mPD		0.0024	0.66
		nPD:	0.
		N. weight:	1.5
		Sc. PD:	-0.052
		Sc. rank:	-19270.0

PB1			
Pos.	748	obs:	exp:
tgt	C	20804	9466.00
tgc	C	168	11506.00
---	--	------	------
mPD		0.016	0.50
		nPD:	0.03
		N. weight:	0.79
		Sc. PD:	-0.006
		Sc. rank:	7001.5

PB1			
Pos.	749	obs:	exp:
ttt	F	0	22.31
ttc	F	52	29.69
tta	L	0	0.77
ttg	L	0	1.32
tct	S	5	3847.00
tcc	S	20892	2421.00
tca	S	9	5441.00
tcg	S	0	1113.00
tat	Y	0	1.56
tac	Y	3	1.44
ctt	L	0	1.38
ctc	L	7	0.99
cta	L	0	1.35
ctg	L	0	1.20
act	T	0	0.76
acc	T	3	0.62
aca	T	0	1.37
acg	T	0	0.26
agt	S	0	3931.00
agc	S	0	4153.00
gct	A	0	0.26
gcc	A	1	0.21
gca	A	0	0.47
gcg	A	0	0.06
---	--	------	------
mPD		0.0083	1.6
		nPD:	0.01
		N. weight:	2.3
		Sc. PD:	-0.077
		Sc. rank:	-22874.0

PB1			
Pos.	750	obs:	exp:
tct	S	0	0.18
tcc	S	1	0.12
tca	S	0	0.26
tcg	S	0	0.05
act	T	0	5286.00
acc	T	20970	4301.00
aca	T	0	9593.00
acg	T	0	1789.00
agt	S	0	0.19
agc	S	0	0.20
gct	A	0	0.26
gcc	A	1	0.21
gca	A	0	0.47
gcg	A	0	0.06
---	--	------	------
mPD		0.00019	0.68
		nPD:	0.
		N. weight:	1.7
		Sc. PD:	-0.065
		Sc. rank:	-28562.0

PB1			
Pos.	751	obs:	exp:
ttt	F	1	0.43
ttc	F	0	0.57
att	I	20969	7633.00
atc	I	0	5234.00
ata	I	0	8101.00
act	T	2	0.50
acc	T	0	0.41
aca	T	0	0.91
acg	T	0	0.17
---	--	------	------
mPD		0.00029	0.66
		nPD:	0.
		N. weight:	1.1
		Sc. PD:	-0.041
		Sc. rank:	-18167.0

PB1

Pos.	752	obs:	exp:
aaa	K	3	1.71
aag	K	0	1.29
gaa	E	20967	12696.00
gag	E	0	8271.00
ggt	G	0	0.30
ggc	G	0	0.30
gga	G	2	0.89
ggg	G	0	0.51
---	--	------	------
mPD		0.00048	0.48
		nPD:	0.
		N. weight:	0.53
		Sc. PD:	-0.02
		Sc. rank:	-8773.3

PB1

Pos.	753	obs:	exp:
cgt	R	0	0.03
cgc	R	0	0.05
cga	R	0	0.10
cgg	R	0	0.07
aaa	K	5	2.85
aag	K	0	2.15
aga	R	1	0.50
agg	R	0	0.25
gct	A	0	0.26
gcc	A	0	0.21
gca	A	1	0.47
gcg	A	0	0.06
gat	D	1	4.22
gac	D	7	3.78
gaa	E	20431	12676.00
gag	E	503	8258.00
ggt	G	0	3.49
ggc	G	0	3.41
gga	G	23	10.23
ggg	G	0	5.88
---	--	------	------
mPD		0.051	0.48
		nPD:	0.1
		N. weight:	0.42
		Sc. PD:	0.027
		Sc. rank:	6082.9

PB1

Pos.	754	obs:	exp:
ttt	F	0	3.00
ttc	F	7	4.00
tta	L	0	2301.00
ttg	L	0	3941.00
ctt	L	7	4125.00
ctc	L	20925	2957.00
cta	L	3	4030.00
ctg	L	0	3579.00
cct	P	0	0.28
ccc	P	1	0.19
cca	P	0	0.43
ccg	P	0	0.11
att	I	0	10.19
atc	I	28	6.99
ata	I	0	10.82
act	T	1	0.25
acc	T	0	0.21
aca	T	0	0.46
acg	T	0	0.09
---	--	------	------
mPD		0.0047	1.1
		nPD:	0.
		N. weight:	2.1
		Sc. PD:	-0.072
		Sc. rank:	-25221.0

PB1

Pos.	755	obs:	exp:
tct	S	0	0.18
tcc	S	0	0.12
tca	S	0	0.26
tcg	S	0	0.05
caa	Q	0	0.53
cag	Q	1	0.47
cgt	R	0	572.40
cgc	R	0	1097.00
cga	R	22	2053.00
cgg	R	0	1534.00
aaa	K	5	2.85
aag	K	0	2.15
agt	S	1	0.19
agc	S	0	0.20
aga	R	20941	10524.00
agg	R	0	5182.00
ggt	G	0	0.30
ggc	G	0	0.30
gga	G	2	0.89
ggg	G	0	0.51
---	--	------	------
mPD		0.0031	0.91
		nPD:	0.
		N. weight:	0.72
		Sc. PD:	-0.026
		Sc. rank:	-9713.4

PB1

Pos.	756	obs:	exp:
caa	Q	0	0.53
cag	Q	1	0.47
cgt	R	0	572.50
cgc	R	0	1097.00
cga	R	0	2054.00
cgg	R	20969	1535.00
act	T	0	0.25
acc	T	0	0.21
aca	T	0	0.46
acg	T	1	0.09
aaa	K	1	0.57
aag	K	0	0.43
aga	R	0	10527.00
agg	R	0	5184.00
---	--	------	------
mPD		0.00057	0.91
		nPD:	0.
		N. weight:	2.8
		Sc. PD:	-0.11
		Sc. rank:	-46531.0

PB1

Pos.	757	obs:	exp:
tta	L	0	0.44
ttg	L	0	0.75
ctt	L	0	0.79
ctc	L	0	0.56
cta	L	4	0.77
ctg	L	0	0.68
cat	H	2	4.12
cac	H	5	2.88
caa	Q	20929	11053.00
cag	Q	18	9894.00
cgt	R	0	0.03
cgc	R	0	0.05
cga	R	1	0.10
cgg	R	0	0.07
aaa	K	10	5.70
aag	K	0	4.30
aga	R	0	0.50
agg	R	0	0.25
gct	A	0	0.26
gcc	A	0	0.21
gca	A	1	0.47
gcg	A	0	0.06
---	-	2	2.00
---	--	------	------
mPD		0.0040	0.50
		nPD:	0.01
		N. weight:	0.67
		Sc. PD:	-0.021
		Sc. rank:	-3268.7

PB1

Pos.	758	obs:	exp:
caa	Q	5	2.64
cag	Q	0	2.36
cgt	R	0	1.99
cgc	R	0	3.82
cga	R	0	7.15
cgg	R	0	5.34
att	I	0	0.36
atc	I	0	0.25
ata	I	1	0.39
atg	M	1	1.00
aat	N	6	4.14
aac	N	1	2.86
aaa	K	20865	11912.00
aag	K	17	8970.00
aga	R	73	36.65
agg	R	0	18.05
gaa	E	1	0.61
gag	E	0	0.39
---	-	2	2.00
---	--	------	------
mPD		0.010	0.50
		nPD:	0.02
		N. weight:	0.59
		Sc. PD:	-0.011
		Sc. rank:	3130.2

PB1

Pos.	759	obs:	exp:
taa	.	20896	9525.00
tag	.	37	5712.00
tga	.	39	5734.00
---	--	------	------
mPD		0.0072	0.79
		nPD:	0.01
		N. weight:	0.81
		Sc. PD:	-0.024
		Sc. rank:	-2512.2

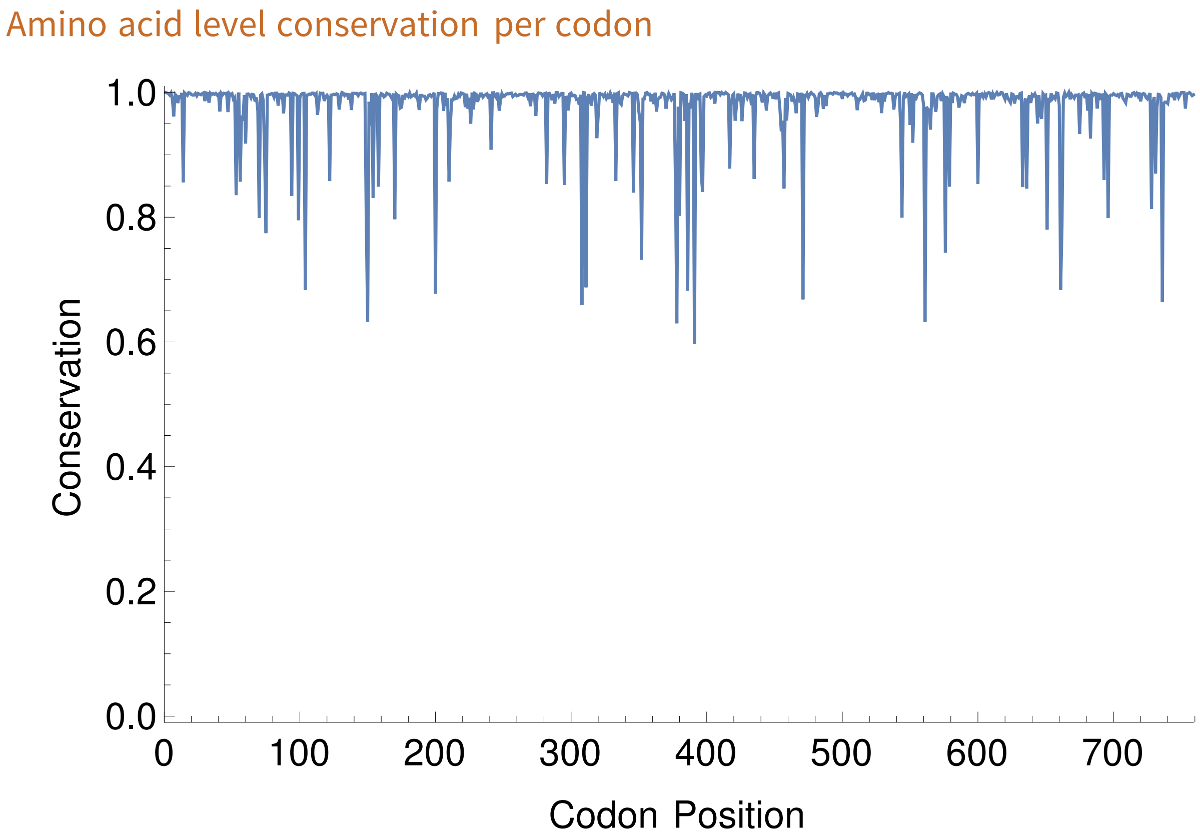

Amino acid level conservation per codon
Conservation
Codon Position
1.0
0.8
0.6
0.4
0.2
0.0
0
100
200
300
400
500
600
700

Conservation analysis plots

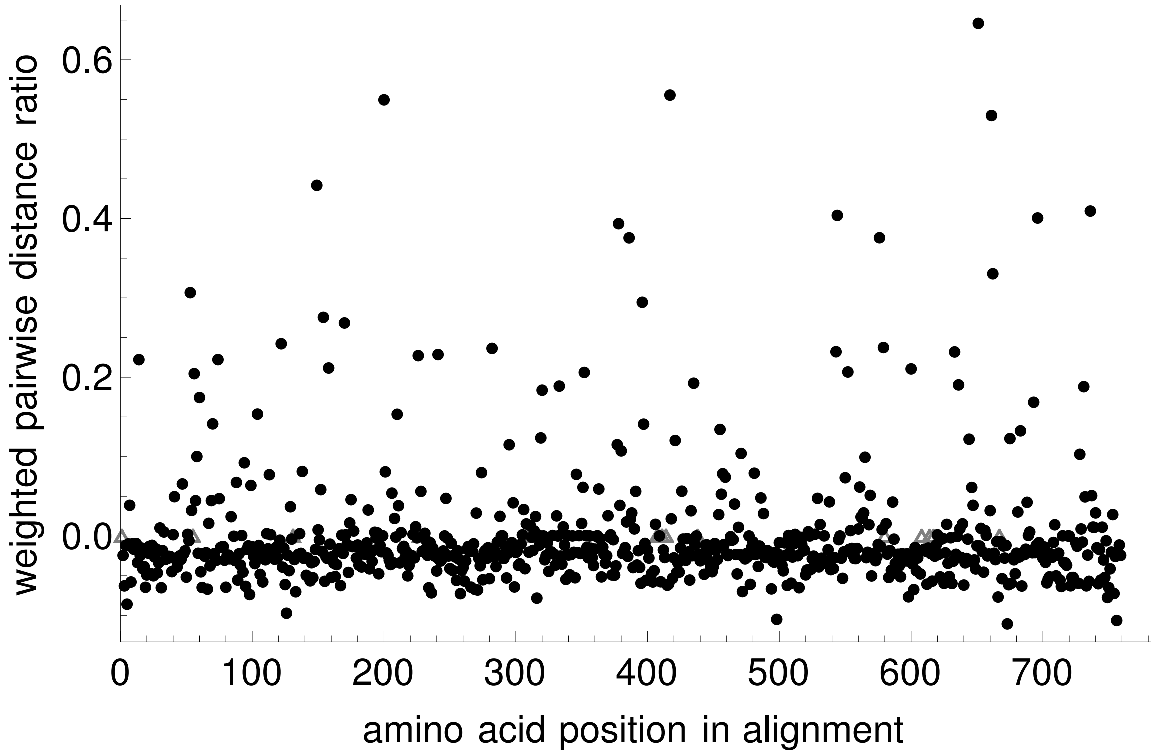

PB1

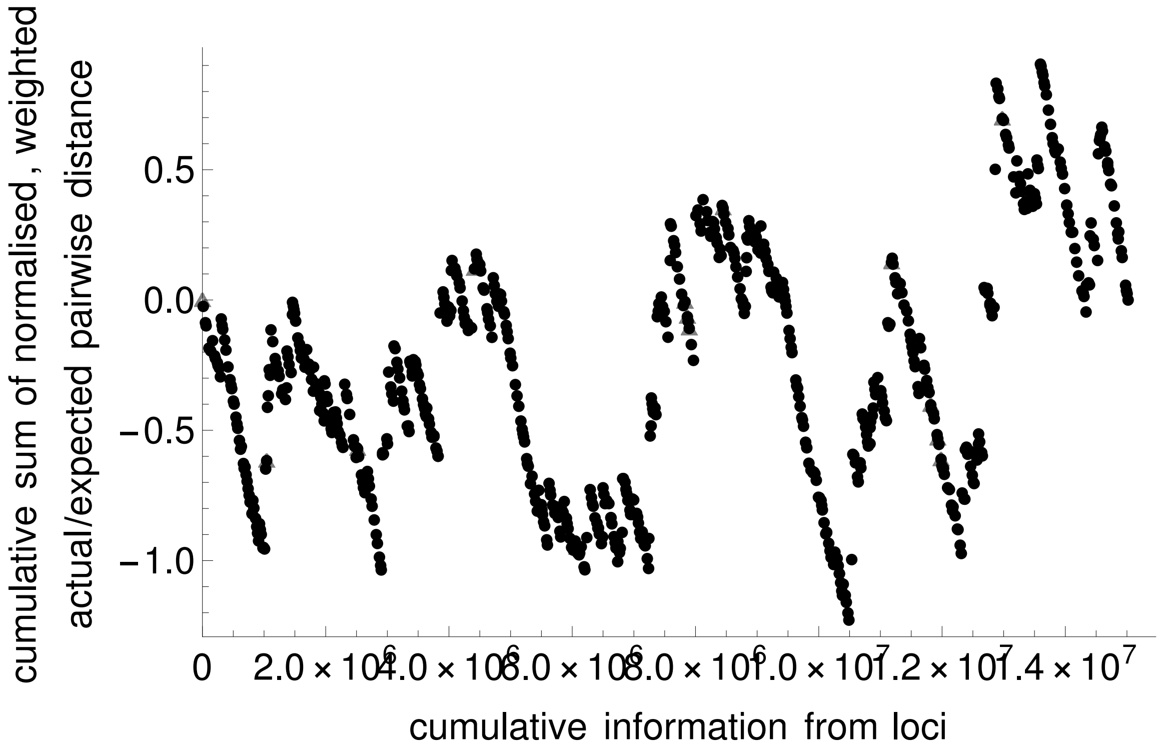

PB1

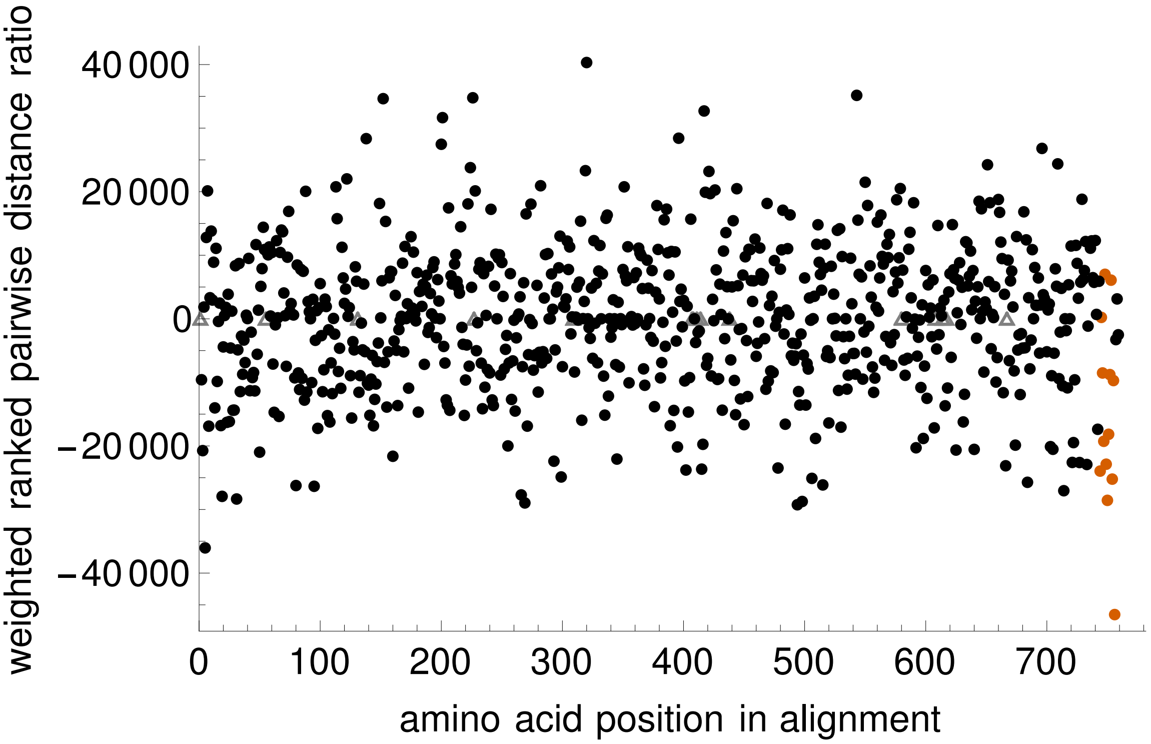
weighted ranked pairwise distance ratio
40 000
20 000
0
−20 000
−40 000
0
100
200
300
400
500
600
700
amino acid position in alignment

PB1

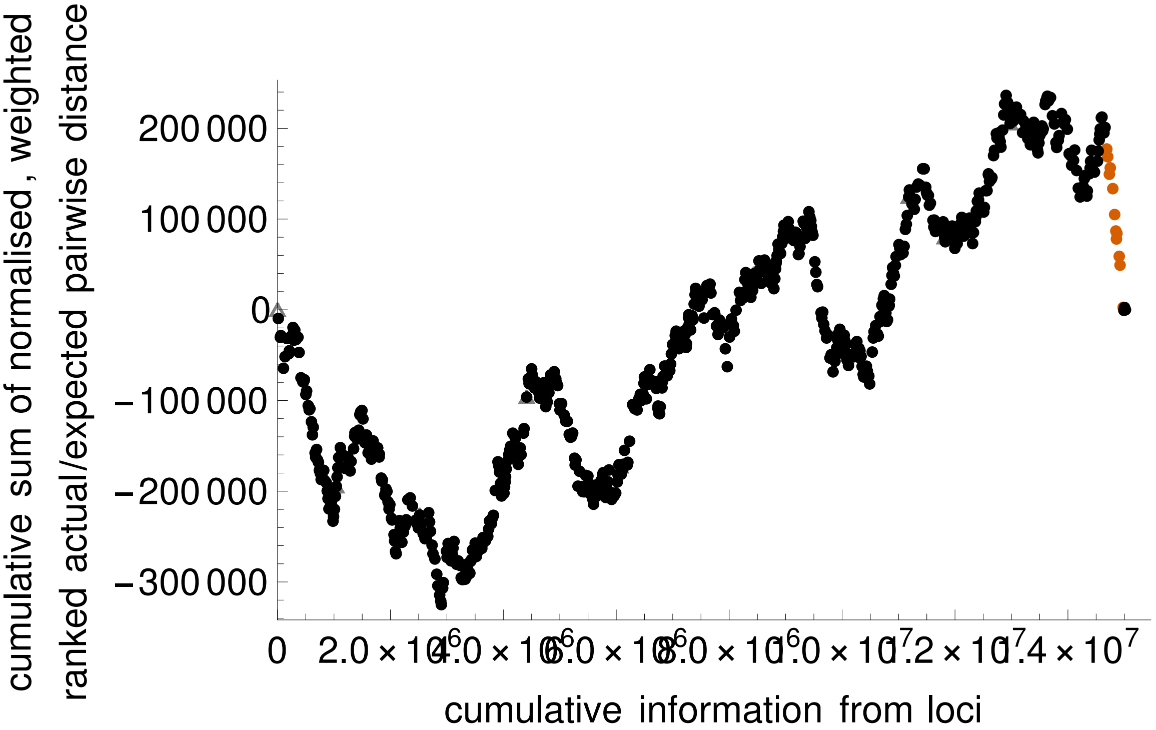
cumulative sum of normalised, weighted ranked actual/expected pairwise distance
200 000
100 000
0
−100 000
−200 000
−300 000
0
2.0 × 10^6
4.0 × 10^6
6.0 × 10^6
8.0 × 10^6
1.0 × 10^7
1.2 × 10^7
1.4 × 10^7
cumulative information from loci

PB1

PA

Gene length histogram

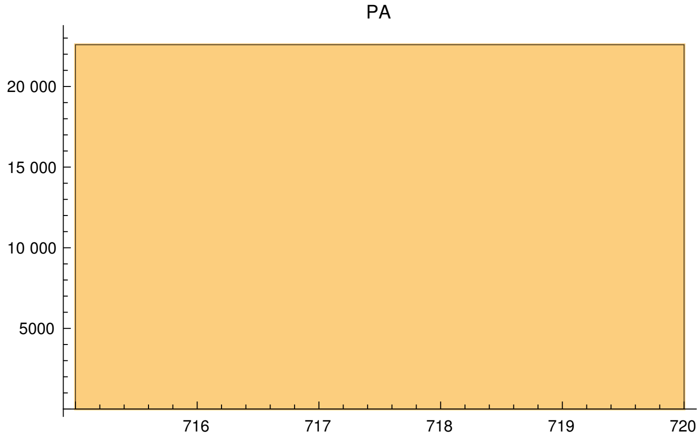

Information vs. nPD

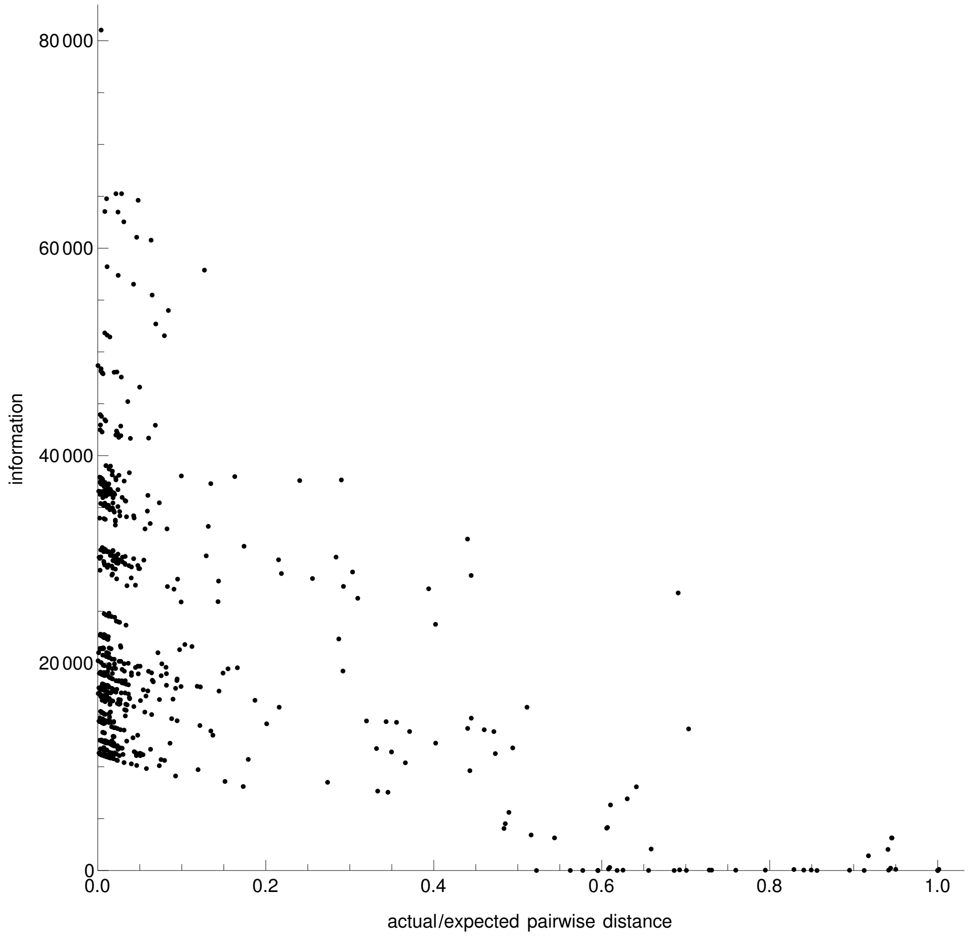

PA

Example sequences highlighted by regions found in analyses to be conserved

Interesting points (by weighted raw PD) highlighted for gene PA:

ATGGAAGACTTTGTGCGACAATGCTTCAATCCAATGATCGTCGAGCTTGCGGAAAAGGCAATGAAAGAATATGGGG⋱
AAGACCCCAAAATTGAAACTAATAAGTTTGCTGCAATTTGCACACATTTGGAAGTTTGTTTCATGTATTCGGATT⋱
TCCATTTCATTGATGAACGGGGTGAATCAATAATTGTAGAATCTGGTGACCCAAATGCACTATTGAAGCACCGAT⋱
TTGAGATAATTGAAGGAAGAGACCGAATCATGGCCTGGACAGTGGTGAACAGTATATGTAACACAACAGGGATAG⋱
AGAAGCCTAAATTTCTTCCTGATTTGTATGATTACAAAGAGAACCGGTTCATTGAAATTGGAGTAACACGGAGAG⋱
AAGTCCACATATATTACCTAGAGAAAGCCAACAAAATAAAATCTGAGAAGACACACATTCACATCTTTTCATTCA⋱
CTGGAGAGGAGATGGCCACCAAAGCGGACTACACCCTTGACGAAGAAAGCAGAGCAAGAATCAAAACTAGGCTTT⋱
TCACTATAAGACAAGAAATGGCCAGTAGGAGTCTATGGGATTCCTTTCGTCAGTCCGAAAGAGGCGAAGAAACAA⋱
TTGAAGAAAAGTTTGAAATTACAGGAACTATGCGCAAGCTTGCCGACCAAAGTCTCCCACCAAACTTCTCCAGCC⋱
TTGAAAACTTCAGAGCCTATGTAGATGGATTCGAGCCGAACGGCTGCATTGAGGGCAAGCTTTCCCAAATGTCAA⋱
AAGAAGTGAACGCCAAAATTGAACCATTCTTGAGGACGACACCACGCCCCCTCAGATTACCTGATGGACCTCTTT⋱
GCCATCAGCGGTCAAAGTTCCTGCTGATGGATGCTCTGAAATTAAGCATTGAAGACCCGAATCACGAGGGGGAAG⋱
GAATACCACTATATGATGCAATCAAATGCATGAAGACATTCTTTGGCTGGAAAGAGCCTAAAATAGTCAAACCAC⋱
ATGAGAAAGGCGTAAATCCCAATTACCTCATGGCTTGGAAGCAGGTGCTAGCAGAGCTACAGGACATTGAAAATG⋱
AAGAGAAAATCCCAAGGACAAAGAACATGAAGAAAACAAGCCAATTGAAGTGGGCACTCGGTGAAAATATGGCAC⋱
CTGAAAAAGTGGACTTTGATGACTGTAAAGATGTTGGAGACCTTAAACAGTATGACAGTGATGAGCCAGAGCCCA⋱
GATCTCTAGCAAGCTGGGTCCAAAATGAATTCAATAAGGCATGTGAATTGACTGATTCAAGCTGGATAGAACTTG⋱
ATGAAATAGGAGAAGATGTTGCCCCGATTGAACATATAGCAAGCATGAGGAGGAACTATTTTACAGCAGAAGTGT⋱
CCCACTGCAGGGCTACTGAATACATAATGAAGGGAGTGTACATAAATACGGCCTTGCTTAATGCATCCTGTGCAG⋱
CCATGGATGACTTTCAGCTGATTCCAATGATAAGCAAATGTAGGACCAAAGAAGGAAGACGGAAAACAAACCTGT⋱
ATGGGTTCATTATAAAAGGAAGGTCTCATTTGAGAAATGATACTGATGTAGTGAACTTTGTAAGTATGGAGTTCT⋱
CACTCACTGATCCAAGACTGGAACCACACAAATGGGAAAAATACTGTGTTCTTGAAATAGGAGACATGCTATTGA⋱
GGACTGCGATAGGTCAAGTGTCGAGGCCCATGTTCCTATATGTGAGAACCAATGGAACCTCCAAGATCAAGATGA⋱
AATGGGGCATGGAAATGAGGCGCTGCCTTCTTCAATCTCTTCAACAGATTGAGAGCATGATTGAAGCCGAGTCTT⋱
CTGTCAAAGAGAAAGACATGACCAAGGAATTCTTTGAAAACAAATCGGAAACATGGCCAATCGGAGAGTCACCCA⋱
GGGGAGTGGAGGAAGGCTCTATTGGAAAAGTGTGCAGAACCTTACTGGCAAAATCTGTGTTCAACAGTCTATATG⋱
CTTCTCCACAACTTGAGGGGTTTTCGGCTGAATCAAGAAAATTGCTTCTCATTGTTCAGGCACTTAGGGACAACC⋱
TGGAACCTGGAACCTTTGATCTTGGGGGGCTATATGAAGCAATCGAGGAGTGCTTGATTAATGATCCCTGGGTTT⋱
TGCTTAATGCATCTTGGTTCAACTCCTTCCTCACACATGCACTGAAGTAG

Interesting points (by weighted ranked PD) highlighted for gene PA:

ATGGAAGACTTTGTGCGACAATGCTTCAATCCAATGATCGTCGAGCTTGCGGAAAAGGCAATGAAAGAATATGGGGAA⋱
GACCCCAAAATTGAAACTAATAAGTTTGCTGCAATTTGCACACATTTGGAAGTTTGTTTCATGTATTCGGATTTCCA⋱
TTTCATTGATGAACGGGGTGAATCAATAATTGTAGAATCTGGTGACCCAAATGCACTATTGAAGCACCGATTTGAGA⋱
TAATTGAAGGAAGAGACCGAATCATGGCCTGGACAGTGGTGAACAGTATATGTAACACAACAGGGATAGAGAAGCCT⋱
AAATTTCTTCCTGATTTGTATGATTACAAAGAGAACCGGTTCATTGAAATTGGAGTAACACGGAGAGAAGTCCACAT⋱
ATATTACCTAGAGAAAGCCAACAAAATAAAATCTGAGAAGACACACATTCACATCTTTTCATTCACTGGAGAGGAGA⋱
TGGCCACCAAAGCGGACTACACCCTTGACGAAGAAAGCAGAGCAAGAATCAAAACTAGGCTTTTCACTATAAGACAA⋱
GAAATGGCCAGTAGGAGTCTATGG
GATTCCTTTCGTCAGTCCGAAAGAGGCGAAGAAACAATTGAAGAAAAGTTTGAAAT⋱
TACAGGAACTATGCGCAAGCTTGCCGACCAAAGTCTCCCACCAAACTTCTCCAGCCTTGAAAACTTCAGA⋱
GCCTATGTAGATGGATTCGAGCCGAACGGCTGCATTGAGGGCAAGCTTTCCCAAATGTCAAAAGAAGTGA⋱
ACGCCAAAATTGAACCATTCTTGAGGACGACACCACGCCCCCTCAGATTACCTGATGGACCTCTTTGCCA⋱

TCAGCGGTCAAAGTTCCTGCTGATGGATGCTCTGAAATTAAGCATTGAAGACCCGAATCACGAGGGGGAA∵.
GGAATACCACTATATGATGCAATCAAATGCATGAAGACATTCTTTGGCTGGAAAGAGCCTAAAATAGTCA∵.
AACCACATGAGAAAGGCGTAAATCCCAATTACCTCATGGCTTGGAAGCAGGTGCTAGCAGAGCTACAGGA∵.
CATTGAAAATGAAGAGAAAATCCCAAGGACAAAGAACATGAAGAAAACAAGCCAATTGAAGTGGGCACTC∵.
GGTGAAAATATGGCACCTGAAAAAGTGGACTTTGATGACTGTAAAGATGTTGGAGACCTTAAACAGTATG∵.
ACAGTGATGAGCCAGAGCCCAGATCTCTAGCAAGCTGGGTCCAAAATGAATTCAATAAGGCATGTGAATT∵.
GACTGAT
TCAAGCTGGATAGAACTTGATGAAATAGGAGAAGATGTTGCCCCGATTGAACATATAGCAA∵.
GCATGAGGAGGAACTATTTTACAGCAGAAGTGTCCCACTGCAGGGCTACTGAATACATAATGAAGGGAGT∵.
GTACATAAATACGGCCTTGCTTAATGCATCCTGTGCAGCCATGGATGACTTTCAGCTGATTCCAATGATA∵.
AGCAAATGTAGGACCAAAGAAGGAAGACGGAAAACAAACCTGTATGGGTTCATTATAAAAGGAAGGTCTC∵.
ATTTGAGAAATGATACTGATGTAGTGAACTTTGTAAGTATGGAGTTCTCACTCACTGATCCAAGACTGGA∵.
ACCACACAAATGGGAAAAATACTGTGTTCTTGAAATAGGAGACATGCTATTGAGGACTGCGATAGGTCAA∵.
GTGTCGAGGCCCATGTTCCTATATGTGAGAACCAATGGAACCTCCAAGATCAAGATGAAATGGGGCATGG∵.
AAATGAGGCGCTGCCTTCTTCAATCTCTTCAACAGATTGAGAGCATGATTGAAGCCGAGTCTTCTGTCAA∵.
AGAGAAAGACATGACCAAGGAATTCTTTGAAAACAAATCGGAAACATGGCCAATCGGAGAGTCACCCAGG∵.
GGAGTGGAGGAAGGCTCTATTGGAAAAGTGTGCAGAACCTTACTGGCAAAATCTGTGTTCAACAGTCTAT∵.
ATGCTTCTCCACAACTTGAGGGGTTTTCGGCTGAATCAAGAAAA
TTGCTTCTCATTGTTCAGGCACTTAGGGACAACCTGGAACCTGGAACCTTTGATC
TTGGGGGGCTATATGAAGCAATCGAGGAGTGCTTGATTAATGATCCCTGGGT
TTTGCTTAATGCATCTTGGTTCAACTCCTTCCTCACACATGCACTGAAGTAG

Per codon conservation report

PA

Pos.	1	obs:	exp:
atg	M	22589	22589.00
mPD		0	0
		nPD:	1.
		N. weight:	0.
		Sc. PD:	0
		Sc. rank:	0

PA

Pos.	2	obs:	exp:
aaa	K	5	2.85
aag	K	0	2.15
gaa	E	22565	13673.00
gag	E	16	8908.00
ggt	G	0	0.45
ggc	G	0	0.44
gga	G	3	1.33
ggg	G	0	0.77
mPD		0.0021	0.48
		nPD:	0.
		N. weight:	0.52
		Sc. PD:	-0.02
		Sc. rank:	-7170.4

PA

Pos.	3	obs:	exp:
tat	Y	0	0.52
tac	Y	1	0.48
aat	N	0	5.32
aac	N	9	3.68
gat	D	79	11862.00
gac	D	22434	10651.00
gaa	E	5	3.03
gag	E	0	1.97
ggt	G	0	9.25
ggc	G	61	9.03
gga	G	0	27.13
ggg	G	0	15.58
mPD		0.014	0.51
		nPD:	0.03
		N. weight:	0.76
		Sc. PD:	-0.012
		Sc. rank:	5665.4

PA

Pos.	4	obs:	exp:
ttt	F	22567	9691.00
ttc	F	15	12891.00
tta	L	1	0.66
ttg	L	2	1.13
tct	S	1	0.18
tcc	S	0	0.12
tca	S	0	0.26
tcg	S	0	0.05
ctt	L	3	1.18
ctc	L	0	0.85
cta	L	0	1.16
ctg	L	0	1.03
agt	S	0	0.19
agc	S	0	0.20
mPD		0.0019	0.49
		nPD:	0.
		N. weight:	0.88
		Sc. PD:	-0.034
		Sc. rank:	-12926.0

PA

Pos.	5	obs:	exp:
tta	L	0	0.44
ttg	L	0	0.75
ctt	L	0	0.79
ctc	L	0	0.56
cta	L	0	0.77
ctg	L	4	0.68
atg	M	3	3.00
gtt	V	230	4992.00
gtc	V	3	4103.00
gta	V	42	6073.00
gtg	V	22306	7413.00
gct	A	0	0.26
gcc	A	0	0.21
gca	A	0	0.47
gcg	A	1	0.06
mPD		0.025	0.74
		nPD:	0.03
		N. weight:	1.1
		Sc. PD:	-0.01
		Sc. rank:	10593.0

PA

Pos.	6	obs:	exp:
caa	Q	2	1.06
cag	Q	0	0.94
cgt	R	1	616.70
cgc	R	9	1182.00
cga	R	22441	2212.00
cgg	R	93	1653.00
aga	R	43	11339.00
agg	R	0	5584.00
mPD		0.013	0.91
		nPD:	0.01
		N. weight:	2.4
		Sc. PD:	-0.067
		Sc. rank:	-778.2

PA

Pos.	7	obs:	exp:
caa	Q	22550	11918.00
cag	Q	37	10669.00
cgt	R	0	0.05
cgc	R	0	0.10
cga	R	2	0.20
cgg	R	0	0.15
aga	R	0	1.00
agg	R	0	0.49
mPD		0.0034	0.50
		nPD:	0.01
		N. weight:	0.65
		Sc. PD:	-0.023
		Sc. rank:	-6652.7

PA

Pos.	8	obs:	exp:
tct	S	0	128.50
tcc	S	0	80.85
tca	S	0	181.70
tcg	S	0	37.15
tat	Y	0	4.16
tac	Y	8	3.84
tgt	C	43	9864.00
tgc	C	21810	11989.00
att	I	0	0.36
atc	I	1	0.25
ata	I	0	0.39
aat	N	0	17.15
aac	N	29	11.85
agt	S	0	131.20
agc	S	698	138.60
mPD		0.070	0.57
		nPD:	0.12
		N. weight:	0.65
		Sc. PD:	0.05
		Sc. rank:	10154.0

PA

Pos.	9	obs:	exp:
ttt	F	25	9693.00
ttc	F	22563	12895.00
tat	Y	0	0.52
tac	Y	1	0.48
mPD		0.0023	0.49
		nPD:	0.
		N. weight:	0.58
		Sc. PD:	-0.022
		Sc. rank:	-7701.1

PA

Pos.	10	obs:	exp:
tct	S	0	0.18
tcc	S	0	0.12
tca	S	0	0.26
tcg	S	0	0.05
att	I	1	0.36
atc	I	0	0.25
ata	I	0	0.39
aat	N	22511	13359.00
aac	N	76	9228.00
agt	S	1	0.19
agc	S	0	0.20
mPD		0.0069	0.48
		nPD:	0.01
		N. weight:	0.53
		Sc. PD:	-0.015
		Sc. rank:	-305.6

PA

Pos.	11	obs:	exp:
cct	P	1	6223.00
ccc	P	2	4190.00
cca	P	22553	9618.00
ccg	P	33	2558.00
mPD		0.0032	0.70
		nPD:	0.
		N. weight:	0.88
		Sc. PD:	-0.033
		Sc. rank:	-11985.0

PA

Pos.	12	obs:	exp:
att	I	0	2.18
atc	I	0	1.50
ata	I	6	2.32
atg	M	22582	22582.00
gtt	V	0	0.22
gtc	V	0	0.18
gta	V	0	0.27
gtg	V	1	0.33
mPD		0.00062	0.00068
		nPD:	0.91
		N. weight:	0.00031
		Sc. PD:	0.00027
		Sc. rank:	5.9

PA

Pos.	13	obs:	exp:
att	I	199	7892.00
atc	I	21470	5411.00
ata	I	10	8376.00
act	T	0	2.02
acc	T	8	1.64
aca	T	0	3.66
acg	T	0	0.68
gtt	V	1	199.40
gtc	V	901	163.90
gta	V	0	242.60
gtg	V	0	296.10
mPD		0.096	0.74
		nPD:	0.13
		N. weight:	1.4
		Sc. PD:	0.12
		Sc. rank:	22393.0

PA

Pos.	14	obs:	exp:
att	I	0	116.90
atc	I	321	80.13
ata	I	0	124.00
gtt	V	6	4923.00
gtc	V	22259	4046.00
gta	V	1	5988.00
gtg	V	0	7309.00
gct	A	0	0.53
gcc	A	2	0.41
gca	A	0	0.94
gcg	A	0	0.12
mPD		0.029	0.77
		nPD:	0.04
		N. weight:	1.8
		Sc. PD:	-0.0092
		Sc. rank:	18493.0

PA

Pos.	15	obs:	exp:
aaa	K	0	0.57
aag	K	1	0.43
gat	D	3	2.11
gac	D	1	1.89
gaa	E	65	13675.00
gag	E	22519	8909.00
mPD		0.0062	0.48
		nPD:	0.01
		N. weight:	0.95
		Sc. PD:	-0.028
		Sc. rank:	-1624.5

PA			
Pos.	16	obs:	exp:
tta	L	0	2483.00
ttg	L	0	4253.00
ctt	L	22575	4451.00
ctc	L	5	3191.00
cta	L	8	4349.00
ctg	L	1	3862.00
---	--	------	------
mPD		0.0012	1.1
		nPD:	0.
		N. weight:	1.7
		Sc. PD:	-0.069
		Sc. rank:	-31135.0

,

PA			
Pos.	17	obs:	exp:
act	T	0	0.50
acc	T	0	0.41
aca	T	1	0.91
acg	T	1	0.17
gct	A	6	5980.00
gcc	A	0	4675.00
gca	A	54	10608.00
gcg	A	22527	1323.00
---	--	------	------
mPD		0.0056	0.66
		nPD:	0.01
		N. weight:	2.9
		Sc. PD:	-0.1
		Sc. rank:	-21193.0

,

PA			
Pos.	18	obs:	exp:
aaa	K	1	0.57
aag	K	0	0.43
gat	D	1	0.53
gac	D	0	0.47
gaa	E	22558	13672.00
gag	E	22	8908.00
ggt	G	0	1.06
ggc	G	0	1.04
gga	G	7	3.11
ggg	G	0	1.79
---	--	------	------
mPD		0.0027	0.48
		nPD:	0.01
		N. weight:	0.52
		Sc. PD:	-0.019
		Sc. rank:	-5906.1

,

PA			
Pos.	19	obs:	exp:
cgt	R	0	0.11
cgc	R	0	0.21
cga	R	0	0.39
cgg	R	0	0.29
aat	N	1	0.59
aac	N	0	0.41
aaa	K	169	12882.00
aag	K	22414	9701.00
aga	R	0	2.01
agg	R	4	0.99
gaa	E	0	0.61
gag	E	1	0.39
---	--	------	------
mPD		0.015	0.49
		nPD:	0.03
		N. weight:	0.83
		Sc. PD:	-0.0094
		Sc. rank:	7392.5

,

PA			
Pos.	20	obs:	exp:
tct	S	0	1.29
tcc	S	0	0.81
tca	S	7	1.82
tcg	S	0	0.37
act	T	0	16.39
acc	T	0	13.33
aca	T	65	29.74
acg	T	0	5.55
agt	S	0	1.32
agc	S	0	1.39
gtt	V	0	0.66
gtc	V	0	0.55
gta	V	3	0.81
gtg	V	0	0.98
gct	A	0	5960.00
gcc	A	0	4660.00
gca	A	22505	10573.00
gcg	A	6	1319.00
gaa	E	2	1.21
gag	E	0	0.79
---	-	1	1.00
---	--	------	------
mPD		0.0073	0.67
		nPD:	0.01
		N. weight:	0.79
		Sc. PD:	-0.025
		Sc. rank:	-2855.7

,

PA			
Pos.	21	obs:	exp:
tta	L	0	0.11
ttg	L	0	0.19
ctt	L	0	0.20
ctc	L	0	0.14
cta	L	0	0.19
ctg	L	1	0.17
atg	M	22585	22585.00
act	T	0	0.25
acc	T	0	0.21
aca	T	0	0.46
acg	T	1	0.09
---	-	2	2.00
---	--	------	------
mPD		0.00018	0.00031
		nPD:	0.56
		N. weight:	0.0002
		Sc. PD:	0.0001
		Sc. rank:	3.6

,

PA			
Pos.	22	obs:	exp:
cgt	R	0	0.38
cgc	R	0	0.73
cga	R	0	1.37
cgg	R	0	1.02
aaa	K	22534	12877.00
aag	K	41	9698.00
aga	R	14	7.03
agg	R	0	3.46
---	--	------	------
mPD		0.0049	0.49
		nPD:	0.01
		N. weight:	0.57
		Sc. PD:	-0.019
		Sc. rank:	-2861.8

,

PA			
Pos.	23	obs:	exp:
aaa	K	3	1.71
aag	K	0	1.29
gaa	E	22547	13670.00
gag	E	30	8907.00
ggt	G	0	1.36
ggc	G	0	1.33
gga	G	9	4.00
ggg	G	0	2.30
---	--	------	------
mPD		0.0037	0.48
		nPD:	0.01
		N. weight:	0.51
		Sc. PD:	-0.018
		Sc. rank:	-4317.7

,

PA			
Pos.	24	obs:	exp:
ttt	F	5	2.15
ttc	F	0	2.85
tat	Y	22482	11720.00
tac	Y	74	10836.00
tgt	C	5	2.26
tgc	C	0	2.74
cat	H	23	13.53
cac	H	0	9.47
---	--	------	------
mPD		0.0095	0.50
		nPD:	0.02
		N. weight:	0.66
		Sc. PD:	-0.016
		Sc. rank:	1963.8

,

PA			
Pos.	25	obs:	exp:
gaa	E	0	0.61
gag	E	1	0.39
ggt	G	0	3425.00
ggc	G	0	3346.00
gga	G	141	10048.00
ggg	G	22447	5770.00
---	--	------	------
mPD		0.012	0.69
		nPD:	0.02
		N. weight:	1.4
		Sc. PD:	-0.034
		Sc. rank:	3259.2

,

PA			
Pos.	26	obs:	exp:
gaa	E	22462	13677.00
gag	E	126	8911.00
ggt	G	0	0.15
ggc	G	0	0.15
gga	G	1	0.44
ggg	G	0	0.26
---	--	------	------
mPD		0.011	0.48
		nPD:	0.02
		N. weight:	0.49
		Sc. PD:	-0.0094
		Sc. rank:	2756.9

,

PA			
Pos.	27	obs:	exp:
tat	Y	0	1.56
tac	Y	3	1.44
aat	N	9	34.30
aac	N	49	23.70
gtt	V	0	0.22
gtc	V	1	0.18
gta	V	0	0.27
gtg	V	0	0.33
gat	D	7950	11852.00
gac	D	14543	10641.00
gaa	E	33	19.98
gag	E	0	13.02
ggt	G	1	0.15
ggc	G	0	0.15
gga	G	0	0.44
ggg	G	0	0.26
---	--	------	------
mPD		0.46	0.51
		nPD:	0.92
		N. weight:	0.066
		Sc. PD:	0.057
		Sc. rank:	1226.8

,

PA			
Pos.	28	obs:	exp:
tta	L	0	1.10
ttg	L	0	1.88
tct	S	0	2.39
tcc	S	10	1.51
tca	S	0	3.38
tcg	S	3	0.69
ctt	L	0	1.97
ctc	L	0	1.41
cta	L	0	1.93
ctg	L	10	1.71
cct	P	41	6216.00
ccc	P	18314	4185.00
cca	P	87	9607.00
ccg	P	4121	2555.00
cat	H	0	1.76
cac	H	3	1.24
agt	S	0	2.44
agc	S	0	2.58
---	--	------	------
mPD		0.31	0.70
		nPD:	0.44
		N. weight:	1.3
		Sc. PD:	0.52
		Sc. rank:	23998.0

,

PA			
Pos.	29	obs:	exp:
cgt	R	0	0.05
cgc	R	0	0.10
cga	R	0	0.20
cgg	R	0	0.15
att	I	0	0.36
atc	I	0	0.25
ata	I	1	0.39
aat	N	0	0.59
aac	N	1	0.41
aaa	K	22561	12883.00
aag	K	24	9702.00
aga	R	2	1.00
agg	R	0	0.49
---	--	------	------
mPD		0.0025	0.49
		nPD:	0.01
		N. weight:	0.58
		Sc. PD:	-0.021
		Sc. rank:	-7450.5

,

PA			
Pos.	30	obs:	exp:
tta	L	0	0.11
ttg	L	0	0.19
tct	S	0	0.18
tcc	S	0	0.12
tca	S	0	0.26
tcg	S	0	0.05
ctt	L	1	0.20
ctc	L	0	0.14
cta	L	0	0.19
ctg	L	0	0.17
att	I	7840	8154.00
atc	I	14537	5591.00
ata	I	23	8654.00
act	T	3	12.10
acc	T	45	9.85
aca	T	0	21.96
acg	T	0	4.10
aat	N	10	8.28
aac	N	4	5.72
agt	S	1	0.19
agc	S	0	0.20
gtt	V	93	27.64
gtc	V	32	22.71
gta	V	0	33.62
gtg	V	0	41.03
---	--	------	------
mPD		0.47	0.67
		nPD:	0.7
		N. weight:	0.63
		Sc. PD:	0.41
		Sc. rank:	11780.0

,

PA

Pos.	31	obs:	exp:
aaa	K	4	2.28
aag	K	0	1.72
gtt	V	0	0.22
gtc	V	0	0.18
gta	V	1	0.27
gtg	V	0	0.33
gct	A	0	0.53
gcc	A	0	0.41
gca	A	2	0.94
gcg	A	0	0.12
gaa	E	22508	13673.00
gag	E	73	8908.00
ggt	G	0	0.15
ggc	G	0	0.15
gga	G	1	0.44
ggg	G	0	0.26
---	--	------	------
mPD		0.0072	0.48
		nPD:	0.01
		N. weight:	0.5
		Sc. PD:	-0.014
		Sc. rank:	7.9

,

PA

Pos.	32	obs:	exp:
tta	L	0	0.22
ttg	L	0	0.38
tct	S	0	0.18
tcc	S	0	0.12
tca	S	0	0.26
tcg	S	0	0.05
ctt	L	2	0.39
ctc	L	0	0.28
cta	L	0	0.39
ctg	L	0	0.34
att	I	28	10.19
atc	I	0	6.99
ata	I	0	10.82
act	T	22431	5685.00
acc	T	105	4625.00
aca	T	12	10316.00
acg	T	2	1924.00
aat	N	7	4.14
aac	N	0	2.86
agt	S	1	0.19
agc	S	0	0.20
gct	A	1	0.26
gcc	A	0	0.21
gca	A	0	0.47
gcg	A	0	0.06
---	--	------	------
mPD		0.014	0.68
		nPD:	0.02
		N. weight:	1.4
		Sc. PD:	-0.031
		Sc. rank:	5547.6

,

PA

Pos.	33	obs:	exp:
tat	Y	1	0.52
tac	Y	0	0.48
aat	N	19536	13359.00
aac	N	3051	9228.00
---	-	1	1.00
---	--	------	------
mPD		0.23	0.48
		nPD:	0.48
		N. weight:	0.19
		Sc. PD:	0.081
		Sc. rank:	3445.7

,

PA

Pos.	34	obs:	exp:
cgt	R	0	0.03
cgc	R	0	0.05
cga	R	0	0.10
cgg	R	0	0.07
aaa	K	1173	12885.00
aag	K	21415	9703.00
aga	R	0	0.50
agg	R	1	0.25
---	--	------	------
mPD		0.099	0.49
		nPD:	0.2
		N. weight:	0.65
		Sc. PD:	0.1
		Sc. rank:	11000.0

,

PA

Pos.	35	obs:	exp:
ttt	F	22560	9688.00
ttc	F	16	12888.00
tta	L	3	1.21
ttg	L	0	2.07
tat	Y	1	0.52
tac	Y	0	0.48
ctt	L	8	2.17
ctc	L	0	1.55
cta	L	0	2.12
ctg	L	0	1.88
gtt	V	1	0.22
gtc	V	0	0.18
gta	V	0	0.27
gtg	V	0	0.33
---	--	------	------
mPD		0.0026	0.49
		nPD:	0.01
		N. weight:	0.88
		Sc. PD:	-0.032
		Sc. rank:	-10757.0

,

PA

Pos.	36	obs:	exp:
act	T	1	0.25
acc	T	0	0.21
aca	T	0	0.46
acg	T	0	0.09
gtt	V	2	0.44
gtc	V	0	0.36
gta	V	0	0.54
gtg	V	0	0.66
gct	A	22531	5980.00
gcc	A	22	4675.00
gca	A	31	10608.00
gcg	A	2	1323.00
---	--	------	------
mPD		0.0051	0.66
		nPD:	0.01
		N. weight:	1.4
		Sc. PD:	-0.047
		Sc. rank:	-11522.0

,

PA

Pos.	37	obs:	exp:
tct	S	0	0.37
tcc	S	0	0.23
tca	S	2	0.52
tcg	S	0	0.11
act	T	0	0.25
acc	T	0	0.21
aca	T	1	0.46
acg	T	0	0.09
agt	S	0	0.38
agc	S	0	0.40
gct	A	0	5980.00
gcc	A	0	4675.00
gca	A	22585	10608.00
gcg	A	1	1323.00
---	--	------	------
mPD		0.00035	0.66
		nPD:	0.
		N. weight:	0.79
		Sc. PD:	-0.033
		Sc. rank:	-14639.0

,

PA

Pos.	38	obs:	exp:
tta	L	0	0.22
ttg	L	0	0.38
tct	S	0	0.18
tcc	S	0	0.12
tca	S	0	0.26
tcg	S	0	0.05
ctt	L	2	0.39
ctc	L	0	0.28
cta	L	0	0.39
ctg	L	0	0.34
att	I	18267	8217.00
atc	I	65	5634.00
ata	I	4240	8721.00
atg	M	1	1.00
act	T	1	0.25
acc	T	0	0.21
aca	T	0	0.46
acg	T	0	0.09
agt	S	1	0.19
agc	S	0	0.20
gtt	V	10	2.65
gtc	V	0	2.18
gta	V	2	3.23
gtg	V	0	3.94
---	--	------	------
mPD		0.31	0.66
		nPD:	0.47
		N. weight:	0.52
		Sc. PD:	0.22
		Sc. rank:	9577.3

,

PA

Pos.	39	obs:	exp:
tgt	C	33	10196.00
tgc	C	22556	12393.00
---	--	------	------
mPD		0.0029	0.50
		nPD:	0.01
		N. weight:	0.62
		Sc. PD:	-0.022
		Sc. rank:	-6988.4

,

PA

Pos.	40	obs:	exp:
tct	S	0	0.18
tcc	S	0	0.12
tca	S	1	0.26
tcg	S	0	0.05
att	I	0	1.09
atc	I	0	0.75
ata	I	3	1.16
act	T	12	5692.00
acc	T	2	4631.00
aca	T	22533	10330.00
acg	T	33	1927.00
agt	S	0	0.19
agc	S	0	0.20
gct	A	0	1.32
gcc	A	0	1.03
gca	A	5	2.35
gcg	A	0	0.29
---	--	------	------
mPD		0.0050	0.68
		nPD:	0.01
		N. weight:	0.8
		Sc. PD:	-0.028
		Sc. rank:	-7287.1

,

PA

Pos.	41	obs:	exp:
cat	H	22530	13284.00
cac	H	58	9304.00
caa	Q	0	0.53
cag	Q	1	0.47
---	--	------	------
mPD		0.0052	0.48
		nPD:	0.01
		N. weight:	0.54
		Sc. PD:	-0.017
		Sc. rank:	-2132.9

,

PA

Pos.	42	obs:	exp:
ttt	F	2	0.86
ttc	F	0	1.14
tta	L	129	2483.00
ttg	L	22432	4252.00
ctt	L	0	4451.00
ctc	L	0	3190.00
cta	L	0	4348.00
ctg	L	26	3862.00
---	--	------	------
mPD		0.014	1.1
		nPD:	0.01
		N. weight:	1.7
		Sc. PD:	-0.051
		Sc. rank:	-4083.4

,

PA

Pos.	43	obs:	exp:
gaa	E	22077	13677.00
gag	E	511	8911.00
---	-	1	1.00
---	--	------	------
mPD		0.044	0.48
		nPD:	0.09
		N. weight:	0.42
		Sc. PD:	0.021
		Sc. rank:	6330.2

,

PA

Pos.	44	obs:	exp:
att	I	63	22.93
atc	I	0	15.73
ata	I	0	24.34
gtt	V	22414	4979.00
gtc	V	74	4093.00
gta	V	1	6057.00
gtg	V	34	7393.00
gct	A	2	0.79
gcc	A	1	0.62
gca	A	0	1.41
gcg	A	0	0.18
---	--	------	------
mPD		0.016	0.74
		nPD:	0.02
		N. weight:	1.5
		Sc. PD:	-0.033
		Sc. rank:	6212.5

,

PA

Pos.	45	obs:	exp:
tgt	C	22468	10196.00
tgc	C	121	12393.00
---	--	------	------
mPD		0.011	0.50
		nPD:	0.02
		N. weight:	0.79
		Sc. PD:	-0.017
		Sc. rank:	3616.2

,

PA

Pos.	46	obs:	exp:
ttt	F	421	9692.00
ttc	F	22164	12893.00
tta	L	0	0.33
ttg	L	0	0.56
tat	Y	0	0.52
tac	Y	1	0.48
ctt	L	0	0.59
ctc	L	3	0.42
cta	L	0	0.58
ctg	L	0	0.51
---	--	------	------
mPD		0.037	0.49
		nPD:	0.08
		N. weight:	0.49
		Sc. PD:	0.016
		Sc. rank:	7011.3

,

PA

Pos.	47	obs:	exp:
atg	M	22588	22588.00
act	T	0	0.25
acc	T	0	0.21
aca	T	0	0.46
acg	T	1	0.09
---	--	------	------
mPD		0.000089	0.00017
		nPD:	0.52
		N. weight:	0.00011
		Sc. PD:	0.000054
		Sc. rank:	2.1

,

PA

Pos.	48	obs:	exp:
tat	Y	22547	11737.00
tac	Y	42	10852.00
---	--	------	------
mPD		0.0037	0.50
		nPD:	0.01
		N. weight:	0.67
		Sc. PD:	-0.023
		Sc. rank:	-5957.1

,

PA

Pos.	49	obs:	exp:
tta	L	0	0.22
ttg	L	2	0.38
tct	S	45	4156.00
tcc	S	0	2616.00
tca	S	146	5878.00
tcg	S	22394	1202.00
ctt	L	0	0.39
ctc	L	0	0.28
cta	L	0	0.39
ctg	L	0	0.34
cct	P	0	0.28
ccc	P	0	0.19
cca	P	0	0.43
ccg	P	1	0.11
act	T	0	0.25
acc	T	0	0.21
aca	T	0	0.46
acg	T	1	0.09
agt	S	0	4247.00
agc	S	0	4486.00
---	--	------	------
mPD		0.017	1.6
		nPD:	0.01
		N. weight:	3.
		Sc. PD:	-0.095
		Sc. rank:	-13097.0

,

PA

Pos.	50	obs:	exp:
gat	D	22563	11902.00
gac	D	25	10686.00
ggt	G	1	0.15
ggc	G	0	0.15
gga	G	0	0.44
ggg	G	0	0.26
---	--	------	------
mPD		0.0023	0.50
		nPD:	0.
		N. weight:	0.66
		Sc. PD:	-0.025
		Sc. rank:	-8902.2

,

PA

Pos.	51	obs:	exp:
ttt	F	299	9692.00
ttc	F	22287	12894.00
tta	L	2	0.33
ttg	L	0	0.56
ctt	L	0	0.59
ctc	L	1	0.42
cta	L	0	0.58
ctg	L	0	0.51
---	--	------	------
mPD		0.026	0.49
		nPD:	0.05
		N. weight:	0.52
		Sc. PD:	0.0056
		Sc. rank:	6432.5

,

PA

Pos.	52	obs:	exp:
cat	H	22181	13283.00
cac	H	406	9304.00
caa	Q	1	0.53
cag	Q	0	0.47
aat	N	1	0.59
aac	N	0	0.41
---	--	------	------
mPD		0.035	0.48
		nPD:	0.07
		N. weight:	0.47
		Sc. PD:	0.014
		Sc. rank:	6553.0

,

PA

Pos.	53	obs:	exp:
ttt	F	52	9694.00
ttc	F	22537	12895.00
---	--	------	------
mPD		0.0046	0.49
		nPD:	0.01
		N. weight:	0.57
		Sc. PD:	-0.019
		Sc. rank:	-3451.2

,

PA

Pos.	54	obs:	exp:
tta	L	0	0.88
ttg	L	0	1.51
ctt	L	7	1.58
ctc	L	1	1.13
cta	L	0	1.54
ctg	L	0	1.37
att	I	18406	8216.00
atc	I	4158	5634.00
ata	I	6	8720.00
gtt	V	10	2.43
gtc	V	1	2.00
gta	V	0	2.96
gtg	V	0	3.61
---	--	------	------
mPD		0.30	0.66
		nPD:	0.46
		N. weight:	0.63
		Sc. PD:	0.26
		Sc. rank:	11492.0

,

PA

Pos.	55	obs:	exp:
aat	N	51	41.99
aac	N	20	29.01
gat	D	18386	11864.00
gac	D	4131	10653.00
gaa	E	1	0.61
gag	E	0	0.39
---	--	------	------
mPD		0.31	0.50
		nPD:	0.61
		N. weight:	0.19
		Sc. PD:	0.11
		Sc. rank:	3552.7

,

PA

Pos.	56	obs:	exp:
caa	Q	1	0.53
cag	Q	0	0.47
gct	A	0	0.79
gcc	A	0	0.62
gca	A	3	1.41
gcg	A	0	0.18
gat	D	0	0.53
gac	D	1	0.47
gaa	E	22272	13674.00
gag	E	311	8909.00
ggt	G	0	0.15
ggc	G	0	0.15
gga	G	1	0.44
ggg	G	0	0.26
---	--	------	------
mPD		0.028	0.48
		nPD:	0.06
		N. weight:	0.45
		Sc. PD:	0.0067
		Sc. rank:	5796.8

,

PA

Pos.	57	obs:	exp:
tta	L	0	1.76
ttg	L	0	3.01
tgg	W	30	30.00
ctt	L	0	3.15
ctc	L	0	2.26
cta	L	0	3.08
ctg	L	16	2.74
caa	Q	7	59.63
cag	Q	106	53.37
cgt	R	4	612.40
cgc	R	0	1173.00
cga	R	195	2197.00
cgg	R	21912	1641.00
aaa	K	0	0.57
aag	K	1	0.43
aga	R	0	11260.00
agg	R	318	5545.00
---	--	------	------
mPD		0.060	0.93
		nPD:	0.06
		N. weight:	2.6
		Sc. PD:	0.055
		Sc. rank:	34849.0

,

PA

Pos.	58	obs:	exp:
tct	S	0	1.10
tcc	S	0	0.69
tca	S	0	1.56
tcg	S	0	0.32
agt	S	6	1.13
agc	S	0	1.19
gat	D	3	1.58
gac	D	0	1.42
ggt	G	22565	3424.00
ggc	G	10	3344.00
gga	G	0	10043.00
ggg	G	3	5767.00
---	-	2	2.00
---	--	------	------
mPD		0.0019	0.69
		nPD:	0.
		N. weight:	2.
		Sc. PD:	-0.077
		Sc. rank:	-32708.0

,

PA

Pos.	59	obs:	exp:
caa	Q	1	0.53
cag	Q	0	0.47
aaa	K	8	4.56
aag	K	0	3.44
gtt	V	0	1.11
gtc	V	0	0.91
gta	V	5	1.34
gtg	V	0	1.64
gat	D	2	4.22
gac	D	6	3.78
gaa	E	22489	13641.00
gag	E	40	8888.00
ggt	G	0	5.76
ggc	G	0	5.63
gga	G	37	16.90
ggg	G	1	9.71
---	--	------	------
mPD		0.0089	0.48
		nPD:	0.02
		N. weight:	0.51
		Sc. PD:	-0.012
		Sc. rank:	1370.7

,

PA

Pos.	60	obs:	exp:
tct	S	9	4148.00
tcc	S	0	2611.00
tca	S	22522	5866.00
tcg	S	10	1200.00
cct	P	0	12.67
ccc	P	0	8.53
cca	P	46	19.59
ccg	P	0	5.21
agt	S	0	4238.00
agc	S	0	4477.00
gct	A	0	0.53
gcc	A	0	0.41
gca	A	2	0.94
gcg	A	0	0.12
---	--	------	------
mPD		0.0059	1.6
		nPD:	0.
		N. weight:	1.4
		Sc. PD:	-0.054
		Sc. rank:	-21464.0

,

PA

Pos.	61	obs:	exp:
tta	L	4	0.88
ttg	L	0	1.51
ctt	L	0	1.58
ctc	L	0	1.13
cta	L	4	1.54
ctg	L	0	1.37
cgt	R	0	0.03
cgc	R	0	0.05
cga	R	0	0.10
cgg	R	0	0.07
att	I	2	8023.00
atc	I	4	5501.00
ata	I	22033	8515.00
atg	M	468	468.00
act	T	0	3.28
acc	T	0	2.67
aca	T	13	5.95
acg	T	0	1.11
aga	R	1	0.50
agg	R	0	0.25
gtt	V	0	13.27
gtc	V	0	10.90
gta	V	58	16.14
gtg	V	2	19.70
---	--	------	------
mPD		0.049	0.68
		nPD:	0.07
		N. weight:	0.97
		Sc. PD:	0.027
		Sc. rank:	13591.0

,

PA

Pos.	62	obs:	exp:
tta	L	0	0.22
ttg	L	0	0.38
ctt	L	1	0.39
ctc	L	0	0.28
cta	L	0	0.39
ctg	L	1	0.34
att	I	22502	8205.00
atc	I	25	5626.00
ata	I	13	8708.00
act	T	9	2.27
acc	T	0	1.85
aca	T	0	4.12
acg	T	0	0.77
gtt	V	32	8.40
gtc	V	0	6.90
gta	V	6	10.22
gtg	V	0	12.47
---	--	------	------
mPD		0.0083	0.66
		nPD:	0.01
		N. weight:	1.
		Sc. PD:	-0.031
		Sc. rank:	-2091.3

,

PA

Pos.	63	obs:	exp:
ttt	F	1	0.43
ttc	F	0	0.57
tta	L	2069	227.60
ttg	L	1	389.70
tct	S	0	0.37
tcc	S	0	0.23
tca	S	2	0.52
tcg	S	0	0.11
ctt	L	0	407.90
ctc	L	0	292.40
cta	L	0	398.50
ctg	L	0	353.90
att	I	0	17.11
atc	I	0	11.73
ata	I	47	18.16
agt	S	0	0.38
agc	S	0	0.40
gtt	V	12	4516.00
gtc	V	17	3712.00
gta	V	20172	5494.00
gtg	V	226	6705.00
gct	A	0	10.06
gcc	A	0	7.87
gca	A	38	17.85
gcg	A	0	2.23
gaa	E	1	0.61
gag	E	0	0.39
ggt	G	0	0.45
ggc	G	0	0.44
gga	G	3	1.33
ggg	G	0	0.77
---	--	------	------
mPD		0.20	0.91
		nPD:	0.22
		N. weight:	1.4
		Sc. PD:	0.23
		Sc. rank:	23394.0

,

PA

Pos.	64	obs:	exp:
aaa	K	1	0.57
aag	K	0	0.43
gaa	E	22553	13677.00
gag	E	35	8911.00
---	--	------	------
mPD		0.0032	0.48
		nPD:	0.01
		N. weight:	0.51
		Sc. PD:	-0.018
		Sc. rank:	-5408.2

,

PA

Pos.	65	obs:	exp:
ttt	F	4	1.72
ttc	F	0	2.28
tta	L	0	0.66
ttg	L	0	1.13
tct	S	22146	4130.00
tcc	S	285	2599.00
tca	S	0	5840.00
tcg	S	10	1194.00
tat	Y	1	0.52
tac	Y	0	0.48
ctt	L	6	1.18
ctc	L	0	0.85
cta	L	0	1.16
ctg	L	0	1.03
cct	P	15	4.13
ccc	P	0	2.78
cca	P	0	6.39
ccg	P	0	1.70
act	T	122	30.75
acc	T	0	25.02
aca	T	0	55.81
acg	T	0	10.41
agt	S	0	4220.00
agc	S	0	4458.00
---	--	------	------
mPD		0.039	1.6
		nPD:	0.02
		N. weight:	1.7
		Sc. PD:	-0.032
		Sc. rank:	10064.0

,

PA

Pos.	66	obs:	exp:
tct	S	0	8.10
tcc	S	0	5.10
tca	S	0	11.45
tcg	S	0	2.34
cgt	R	1	0.03
cgc	R	0	0.05
cga	R	0	0.10
cgg	R	0	0.07
aat	N	3	1.77
aac	N	0	1.23
agt	S	44	8.27
agc	S	0	8.74
aga	R	0	0.50
agg	R	0	0.25
gct	A	2	0.53
gcc	A	0	0.41
gca	A	0	0.94
gcg	A	0	0.12
gat	D	398	212.90
gac	D	6	191.10
ggt	G	22085	3356.00
ggc	G	45	3278.00
gga	G	3	9846.00
ggg	G	2	5654.00
---	--	------	------
mPD		0.045	0.74
		nPD:	0.06
		N. weight:	1.9
		Sc. PD:	0.033
		Sc. rank:	25280.0

,

PA

Pos.	67	obs:	exp:
aat	N	0	1.18
aac	N	2	0.82
gat	D	128	11900.00
gac	D	22457	10685.00
ggt	G	0	0.30
ggc	G	2	0.30
gga	G	0	0.89
ggg	G	0	0.51
---	--	------	------
mPD		0.012	0.50
		nPD:	0.02
		N. weight:	0.74
		Sc. PD:	-0.014
		Sc. rank:	4166.3

,

PA

Pos.	68	obs:	exp:
tta	L	0	0.33
ttg	L	0	0.56
tct	S	0	6.81
tcc	S	0	4.29
tca	S	34	9.63
tcg	S	3	1.97
ctt	L	0	0.59
ctc	L	0	0.42
cta	L	3	0.58
ctg	L	0	0.51
cct	P	3	6212.00
ccc	P	1	4182.00
cca	P	19451	9601.00
ccg	P	3094	2553.00
agt	S	0	6.96
agc	S	0	7.35
---	--	------	------
mPD		0.24	0.70
		nPD:	0.34
		N. weight:	0.66
		Sc. PD:	0.2
		Sc. rank:	11782.0

,

PA

Pos.	69	obs:	exp:
aat	N	22544	13360.00
aac	N	45	9229.00
---	--	------	------
mPD		0.0040	0.48
		nPD:	0.01
		N. weight:	0.53
		Sc. PD:	-0.018
		Sc. rank:	-4165.1

,

PA

Pos.	70	obs:	exp:
tct	S	0	0.18
tcc	S	0	0.12
tca	S	1	0.26
tcg	S	0	0.05
att	I	0	0.36
atc	I	0	0.25
ata	I	1	0.39
act	T	0	1.51
acc	T	0	1.23
aca	T	6	2.74
acg	T	0	0.51
agt	S	0	0.19
agc	S	0	0.20
gtt	V	0	55.05
gtc	V	0	45.25
gta	V	247	66.97
gtg	V	2	81.74
gct	A	1	5913.00
gcc	A	14	4623.00
gca	A	22306	10489.00
gcg	A	11	1308.00
---	--	------	------
mPD		0.025	0.69
		nPD:	0.04
		N. weight:	0.79
		Sc. PD:	-0.0049
		Sc. rank:	8011.2

,

PA

Pos.	71	obs:	exp:
tta	L	142	2483.00
ttg	L	3	4252.00
ctt	L	0	4450.00
ctc	L	4	3190.00
cta	L	21768	4348.00
ctg	L	666	3861.00
cct	P	0	0.28
ccc	P	0	0.19
cca	P	1	0.43
ccg	P	0	0.11
att	I	0	1.82
atc	I	0	1.25
ata	I	5	1.93
---	--	------	------
mPD		0.071	1.1
		nPD:	0.06
		N. weight:	1.5
		Sc. PD:	0.03
		Sc. rank:	20410.0

,

PA

Pos.	72	obs:	exp:
tta	L	116	2483.00
ttg	L	22394	4252.00
tct	S	0	0.18
tcc	S	0	0.12
tca	S	0	0.26
tcg	S	1	0.05
ctt	L	0	4451.00
ctc	L	0	3190.00
cta	L	0	4348.00
ctg	L	77	3862.00
atg	M	1	1.00
agt	S	0	0.19
agc	S	0	0.20
---	--	------	------
mPD		0.017	1.1
		nPD:	0.02
		N. weight:	1.7
		Sc. PD:	-0.046
		Sc. rank:	366.2

,

PA

Pos.	73	obs:	exp:
cgt	R	0	0.08
cgc	R	0	0.16
cga	R	0	0.29
cgg	R	0	0.22
aaa	K	185	12884.00
aag	K	22401	9702.00
aga	R	0	1.51
agg	R	3	0.74
---	--	------	------
mPD		0.017	0.49
		nPD:	0.03
		N. weight:	0.83
		Sc. PD:	-0.0075
		Sc. rank:	8081.9

,

PA

Pos.	74	obs:	exp:
tat	Y	0	0.52
tac	Y	1	0.48
cat	H	64	13281.00
cac	H	22520	9303.00
caa	Q	1	0.53
cag	Q	0	0.47
cgt	R	0	0.05
cgc	R	2	0.10
cga	R	0	0.20
cgg	R	0	0.15
aat	N	0	0.59
aac	N	1	0.41
aga	R	0	1.00
agg	R	0	0.49
---	--	------	------
mPD		0.0061	0.49
		nPD:	0.01
		N. weight:	0.9
		Sc. PD:	-0.027
		Sc. rank:	-1892.8

,

PA

Pos.	75	obs:	exp:
cgt	R	2	616.80
cgc	R	0	1182.00
cga	R	22506	2213.00
cgg	R	47	1653.00
aga	R	32	11340.00
agg	R	2	5584.00
---	--	------	------
mPD		0.0075	0.91
		nPD:	0.01
		N. weight:	2.4
		Sc. PD:	-0.082
		Sc. rank:	-18378.0

,

PA

Pos.	76	obs:	exp:
ttt	F	22550	9693.00
ttc	F	38	12895.00
tct	S	1	0.18
tcc	S	0	0.12
tca	S	0	0.26
tcg	S	0	0.05
agt	S	0	0.19
agc	S	0	0.20
---	--	------	------
mPD		0.0034	0.49
		nPD:	0.01
		N. weight:	0.87
		Sc. PD:	-0.031
		Sc. rank:	-8554.5

,

PA

Pos.	77	obs:	exp:
gaa	E	520	13678.00
gag	E	22069	8911.00
---	--	------	------
mPD		0.045	0.48
		nPD:	0.09
		N. weight:	0.85
		Sc. PD:	0.043
		Sc. rank:	12795.0

,

PA

Pos.	78	obs:	exp:
tta	L	1	0.22
ttg	L	0	0.38
ctt	L	0	0.39
ctc	L	0	0.28
cta	L	1	0.39
ctg	L	0	0.34
att	I	16	8222.00
atc	I	2	5638.00
ata	I	22569	8727.00
---	--	------	------
mPD		0.0018	0.66
		nPD:	0.
		N. weight:	0.99
		Sc. PD:	-0.039
		Sc. rank:	-16758.0

,

PA

Pos.	79	obs:	exp:
tta	L	0	0.11
ttg	L	0	0.19
tct	S	0	0.18
tcc	S	0	0.12
tca	S	0	0.26
tcg	S	0	0.05
ctt	L	1	0.20
ctc	L	0	0.14
cta	L	0	0.19
ctg	L	0	0.17
att	I	22553	8220.00
atc	I	20	5637.00
ata	I	9	8725.00
agt	S	1	0.19
agc	S	0	0.20
gtt	V	5	1.11
gtc	V	0	0.91
gta	V	0	1.34
gtg	V	0	1.64
---	--	------	------
mPD		0.0032	0.66
		nPD:	0.
		N. weight:	1.
		Sc. PD:	-0.039
		Sc. rank:	-13725.0

,

PA

Pos.	80	obs:	exp:
gaa	E	22572	13678.00
gag	E	17	8911.00
---	--	------	------
mPD		0.0015	0.48
		nPD:	0.
		N. weight:	0.52
		Sc. PD:	-0.02
		Sc. rank:	-8404.0

,

PA

Pos.	81	obs:	exp:
ggt	G	4	3425.00
ggc	G	0	3346.00
gga	G	22512	10048.00
ggg	G	73	5770.00
---	--	------	------
mPD		0.0068	0.69
		nPD:	0.01
		N. weight:	0.82
		Sc. PD:	-0.027
		Sc. rank:	-4289.5

,

PA

Pos.	82	obs:	exp:
cgt	R	0	616.70
cgc	R	0	1182.00
cga	R	1	2212.00
cgg	R	0	1653.00
att	I	0	0.73
atc	I	0	0.50
ata	I	2	0.77
aga	R	22543	11339.00
agg	R	43	5584.00
---	--	------	------
mPD		0.0041	0.91
		nPD:	0.
		N. weight:	0.71
		Sc. PD:	-0.027
		Sc. rank:	-9745.9

,

PA

Pos.	83	obs:	exp:
gat	D	57	11902.00
gac	D	22531	10686.00
ggt	G	0	0.15
ggc	G	1	0.15
gga	G	0	0.44
ggg	G	0	0.26
---	--	------	------
mPD		0.0051	0.50
		nPD:	0.01
		N. weight:	0.76
		Sc. PD:	-0.024
		Sc. rank:	-3506.4

,

PA

Pos.	84	obs:	exp:
tta	L	0	0.11
ttg	L	0	0.19
ctt	L	0	0.20
ctc	L	0	0.14
cta	L	1	0.19
ctg	L	0	0.17
caa	Q	1	0.53
cag	Q	0	0.47
cgt	R	1	616.70
cgc	R	1	1182.00
cga	R	22480	2212.00
cgg	R	43	1653.00
aga	R	57	11339.00
agg	R	5	5584.00
---	--	------	------
mPD		0.010	0.91
		nPD:	0.01
		N. weight:	2.4
		Sc. PD:	-0.075
		Sc. rank:	-7841.2

,

PA

Pos.	85	obs:	exp:
tct	S	0	0.18
tcc	S	0	0.12
tca	S	0	0.26
tcg	S	0	0.05
att	I	37	8199.00
atc	I	22462	5622.00
ata	I	25	8702.00
act	T	3	6.55
acc	T	18	5.33
aca	T	5	11.89
acg	T	0	2.22
aat	N	0	0.59
aac	N	1	0.41
agt	S	0	0.19
agc	S	1	0.20
gtt	V	0	8.18
gtc	V	37	6.72
gta	V	0	9.95
gtg	V	0	12.15
---	--	------	------
mPD		0.012	0.66
		nPD:	0.02
		N. weight:	1.4
		Sc. PD:	-0.035
		Sc. rank:	3260.7

,

PA

Pos.	86	obs:	exp:
tta	L	0	0.11
ttg	L	1	0.19
ctt	L	0	0.20
ctc	L	0	0.14
cta	L	0	0.19
ctg	L	0	0.17
att	I	6	6.92
atc	I	0	4.74
ata	I	13	7.34
atg	M	22555	22555.00
act	T	0	0.76
acc	T	0	0.62
aca	T	0	1.37
acg	T	3	0.26
gtt	V	0	2.43
gtc	V	0	2.00
gta	V	0	2.96
gtg	V	11	3.61
---	--	------	------
mPD		0.0030	0.0040
		nPD:	0.76
		N. weight:	0.0014
		Sc. PD:	0.00096
		Sc. rank:	25.5

,

PA

Pos.	87	obs:	exp:
tct	S	0	0.18
tcc	S	1	0.12
tca	S	0	0.26
tcg	S	0	0.05
agt	S	0	0.19
agc	S	0	0.20
gct	A	2455	5980.00
gcc	A	19987	4675.00
gca	A	144	10608.00
gcg	A	0	1323.00
ggt	G	0	0.30
ggc	G	2	0.30
gga	G	0	0.89
ggg	G	0	0.51
---	--	------	------
mPD		0.21	0.66
		nPD:	0.31
		N. weight:	1.2
		Sc. PD:	0.32
		Sc. rank:	21404.0

,

PA

Pos.	88	obs:	exp:
tgg	W	22588	22588.00
cgt	R	0	0.03
cgc	R	0	0.05
cga	R	0	0.10
cgg	R	1	0.07
aga	R	0	0.50
agg	R	0	0.25
---	--	------	------
mPD		0.000089	0.00015
		nPD:	0.6
		N. weight:	0.00012
		Sc. PD:	0.000066
		Sc. rank:	2.2

,

PA

Pos.	89	obs:	exp:
act	T	6	5694.00
acc	T	3	4633.00
aca	T	22565	10333.00
acg	T	12	1927.00
gct	A	0	0.79
gcc	A	0	0.62
gca	A	3	1.41
gcg	A	0	0.18
---	--	------	------
mPD		0.0021	0.68
		nPD:	0.
		N. weight:	0.81
		Sc. PD:	-0.032
		Sc. rank:	-13156.0

,

PA

Pos.	90	obs:	exp:
tta	L	0	0.22
ttg	L	2	0.38
ctt	L	0	0.39
ctc	L	0	0.28
cta	L	0	0.39
ctg	L	0	0.34
atg	M	36	36.00
gtt	V	30	4985.00
gtc	V	0	4097.00
gta	V	65	6064.00
gtg	V	22452	7401.00
gct	A	0	0.79
gcc	A	0	0.62
gca	A	0	1.41
gcg	A	3	0.18
ggt	G	0	0.15
ggc	G	0	0.15
gga	G	0	0.44
ggg	G	1	0.26
---	--	------	------
mPD		0.012	0.74
		nPD:	0.02
		N. weight:	1.1
		Sc. PD:	-0.03
		Sc. rank:	1011.9

,

PA

Pos.	91	obs:	exp:
atg	M	4	4.00
gtt	V	7	4993.00
gtc	V	39	4104.00
gta	V	135	6074.00
gtg	V	22404	7414.00
---	--	------	------
mPD		0.016	0.74
		nPD:	0.02
		N. weight:	1.1
		Sc. PD:	-0.023
		Sc. rank:	5559.1

,

PA

Pos.	92	obs:	exp:
tct	S	0	0.18
tcc	S	0	0.12
tca	S	0	0.26
tcg	S	0	0.05
aat	N	21	13359.00
aac	N	22567	9229.00
agt	S	0	0.19
agc	S	1	0.20
---	--	------	------
mPD		0.0019	0.48
		nPD:	0.
		N. weight:	0.93
		Sc. PD:	-0.035
		Sc. rank:	-13389.0

,

PA

Pos.	93	obs:	exp:
tct	S	0	4157.00
tcc	S	0	2616.00
tca	S	0	5879.00
tcg	S	0	1202.00
agt	S	22545	4247.00
agc	S	44	4487.00
---	--	------	------
mPD		0.0039	1.6
		nPD:	0.
		N. weight:	1.7
		Sc. PD:	-0.069
		Sc. rank:	-30388.0

,

PA

Pos.	94	obs:	exp:
att	I	6	8220.00
atc	I	65	5637.00
ata	I	22511	8725.00
gtt	V	0	1.55
gtc	V	0	1.27
gta	V	7	1.88
gtg	V	0	2.30
---	--	------	------
mPD		0.0069	0.66
		nPD:	0.01
		N. weight:	0.97
		Sc. PD:	-0.031
		Sc. rank:	-4158.9

,

PA

Pos.	95	obs:	exp:
tgt	C	22301	10195.00
tgc	C	287	12393.00
cgt	R	1	0.03
cgc	R	0	0.05
cga	R	0	0.10
cgg	R	0	0.07
aga	R	0	0.50
agg	R	0	0.25
---	--	------	------
mPD		0.025	0.50
		nPD:	0.05
		N. weight:	0.76
		Sc. PD:	0.0059
		Sc. rank:	9396.4

,

PA

Pos.	96	obs:	exp:
tct	S	0	0.37
tcc	S	0	0.23
tca	S	0	0.52
tcg	S	0	0.11
cat	H	0	0.59
cac	H	1	0.41
aat	N	112	13358.00
aac	N	22473	9227.00
aaa	K	1	0.57
aag	K	0	0.43
agt	S	0	0.38
agc	S	2	0.40
---	--	------	------
mPD		0.010	0.48
		nPD:	0.02
		N. weight:	0.9
		Sc. PD:	-0.019
		Sc. rank:	3885.1

,

PA

Pos.	97	obs:	exp:
att	I	0	0.36
atc	I	0	0.25
ata	I	1	0.39
act	T	7	5693.00
acc	T	7	4632.00
aca	T	22561	10332.00
acg	T	9	1927.00
gct	A	0	1.06
gcc	A	0	0.83
gca	A	4	1.88
gcg	A	0	0.23
---	--	------	------
mPD		0.0025	0.68
		nPD:	0.
		N. weight:	0.81
		Sc. PD:	-0.031
		Sc. rank:	-12309.0

,

PA

Pos.	98	obs:	exp:
act	T	10	5692.00
acc	T	0	4631.00
aca	T	22471	10329.00
acg	T	97	1926.00
gct	A	0	2.91
gcc	A	0	2.28
gca	A	11	5.17
gcg	A	0	0.64
---	--	------	------
mPD		0.010	0.68
		nPD:	0.02
		N. weight:	0.79
		Sc. PD:	-0.021
		Sc. rank:	328.6

,

PA

Pos.	99	obs:	exp:
cgt	R	0	0.87
cgc	R	0	1.67
cga	R	0	3.13
cgg	R	0	2.34
aga	R	0	16.07
agg	R	32	7.91
gtt	V	0	0.22
gtc	V	0	0.18
gta	V	0	0.27
gtg	V	1	0.33
gaa	E	0	8.48
gag	E	14	5.52
ggt	G	0	3418.00
ggc	G	0	3339.00
gga	G	212	10027.00
ggg	G	22330	5758.00
---	--	------	------
mPD		0.023	0.70
		nPD:	0.03
		N. weight:	1.4
		Sc. PD:	-0.014
		Sc. rank:	12918.0

,

PA

Pos.	100	obs:	exp:
att	I	14	6942.00
atc	I	0	4760.00
ata	I	19057	7368.00
atg	M	146	146.00
act	T	0	2.52
acc	T	0	2.05
aca	T	9	4.57
acg	T	1	0.85
aaa	K	1	0.57
aag	K	0	0.43
gtt	V	8	741.30
gtc	V	1	609.30
gta	V	3336	901.80
gtg	V	8	1101.00
gct	A	5	1.85
gcc	A	0	1.45
gca	A	2	3.29
gcg	A	0	0.41
gat	D	1	0.53
gac	D	0	0.47
---	--	------	------
mPD		0.27	0.95
		nPD:	0.29
		N. weight:	1.
		Sc. PD:	0.25
		Sc. rank:	17859.0

,

PA

Pos.	101	obs:	exp:
cgt	R	0	0.03
cgc	R	0	0.05
cga	R	0	0.10
cgg	R	0	0.07
aaa	K	0	1.71
aag	K	3	1.29
aga	R	1	0.50
agg	R	0	0.25
gtt	V	0	0.22
gtc	V	0	0.18
gta	V	0	0.27
gtg	V	1	0.33
gat	D	61	32.67
gac	D	1	29.33
gaa	E	362	13626.00
gag	E	22142	8878.00
ggt	G	0	2.73
ggc	G	0	2.67
gga	G	4	8.01
ggg	G	14	4.60
---	--	------	------
mPD		0.039	0.48
		nPD:	0.08
		N. weight:	0.88
		Sc. PD:	0.034
		Sc. rank:	12735.0

,

PA

Pos.	102	obs:	exp:
aaa	K	122	12885.00
aag	K	22467	9704.00
---	--	------	------
mPD		0.011	0.49
		nPD:	0.02
		N. weight:	0.84
		Sc. PD:	-0.017
		Sc. rank:	4181.5

,

PA

Pos.	103	obs:	exp:
tta	L	0	0.11
ttg	L	0	0.19
ctt	L	1	0.20
ctc	L	0	0.14
cta	L	0	0.19
ctg	L	0	0.17
cct	P	21781	6223.00
ccc	P	794	4189.00
cca	P	6	9618.00
ccg	P	7	2558.00
---	--	------	------
mPD		0.069	0.70
		nPD:	0.1
		N. weight:	1.2
		Sc. PD:	0.066
		Sc. rank:	18450.0

,

PA

Pos.	104	obs:	exp:
cgt	R	0	0.11
cgc	R	0	0.21
cga	R	0	0.39
cgg	R	0	0.29
act	T	0	0.25
acc	T	0	0.21
aca	T	1	0.46
acg	T	0	0.09
aat	N	0	1.18
aac	N	2	0.82
aaa	K	22469	12881.00
aag	K	113	9701.00
aga	R	4	2.01
agg	R	0	0.99
---	--	------	------
mPD		0.011	0.49
		nPD:	0.02
		N. weight:	0.55
		Sc. PD:	-0.012
		Sc. rank:	2540.9

,

PA

Pos.	105	obs:	exp:
ttt	F	21439	9224.00
ttc	F	55	12270.00
tta	L	0	119.40
ttg	L	1	204.50
tgt	C	1	0.45
tgc	C	0	0.55
ctt	L	1085	214.00
ctc	L	0	153.40
cta	L	0	209.10
ctg	L	0	185.70
cat	H	8	4.70
cac	H	0	3.30
---	--	------	------
mPD		0.098	0.59
		nPD:	0.17
		N. weight:	0.9
		Sc. PD:	0.11
		Sc. rank:	15025.0

,

PA

Pos.	106	obs:	exp:
tta	L	2	2483.00
ttg	L	0	4252.00
ctt	L	22028	4451.00
ctc	L	548	3190.00
cta	L	6	4348.00
ctg	L	2	3862.00
att	I	3	1.09
atc	I	0	0.75
ata	I	0	1.16
---	--	------	------
mPD		0.049	1.1
		nPD:	0.04
		N. weight:	1.6
		Sc. PD:	-0.00022
		Sc. rank:	17628.0

,

PA

Pos.	107	obs:	exp:
cct	P	22568	6223.00
ccc	P	11	4190.00
cca	P	1	9618.00
ccg	P	9	2558.00
---	--	------	------
mPD		0.0019	0.70
		nPD:	0.
		N. weight:	1.3
		Sc. PD:	-0.053
		Sc. rank:	-22926.0

,

PA

Pos.	108	obs:	exp:
aaa	K	1	0.57
aag	K	0	0.43
gat	D	22558	11902.00
gac	D	30	10686.00
---	--	------	------
mPD		0.0028	0.50
		nPD:	0.01
		N. weight:	0.66
		Sc. PD:	-0.024
		Sc. rank:	-7637.6

,

PA			
Pos.	109	obs:	exp:
tta	L	372	2483.00
ttg	L	22164	4253.00
ctt	L	0	4451.00
ctc	L	0	3190.00
cta	L	0	4349.00
ctg	L	52	3862.00
atg	M	1	1.00
---	--	------	------
mPD		0.037	1.1
		nPD:	0.03
		N. weight:	1.6
		Sc. PD:	-0.017
		Sc. rank:	15303.0

,

PA			
Pos.	110	obs:	exp:
tat	Y	22541	11736.00
tac	Y	47	10852.00
cat	H	1	0.59
cac	H	0	0.41
---	--	------	------
mPD		0.0042	0.50
		nPD:	0.01
		N. weight:	0.67
		Sc. PD:	-0.023
		Sc. rank:	-4666.2

,

PA			
Pos.	111	obs:	exp:
gat	D	22436	11902.00
gac	D	153	10687.00
---	--	------	------
mPD		0.013	0.50
		nPD:	0.03
		N. weight:	0.63
		Sc. PD:	-0.0098
		Sc. rank:	4652.0

,

PA			
Pos.	112	obs:	exp:
tat	Y	192	11737.00
tac	Y	22397	10852.00
---	--	------	------
mPD		0.017	0.50
		nPD:	0.03
		N. weight:	0.71
		Sc. PD:	-0.0064
		Sc. rank:	6972.9

,

PA			
Pos.	113	obs:	exp:
caa	Q	2	1.06
cag	Q	0	0.94
cgt	R	0	0.33
cgc	R	0	0.63
cga	R	0	1.18
cgg	R	0	0.88
aaa	K	22535	12877.00
aag	K	40	9698.00
aga	R	12	6.02
agg	R	0	2.97
---	--	------	------
mPD		0.0048	0.49
		nPD:	0.01
		N. weight:	0.57
		Sc. PD:	-0.019
		Sc. rank:	-3084.1

,

PA			
Pos.	114	obs:	exp:
aaa	K	0	5.13
aag	K	9	3.87
gat	D	3	1.58
gac	D	0	1.42
gaa	E	93	13670.00
gag	E	22483	8906.00
ggt	G	0	0.15
ggc	G	0	0.15
gga	G	0	0.44
ggg	G	1	0.26
---	--	------	------
mPD		0.0094	0.48
		nPD:	0.02
		N. weight:	0.94
		Sc. PD:	-0.022
		Sc. rank:	3228.2

,

PA			
Pos.	115	obs:	exp:
tct	S	0	11.23
tcc	S	0	7.07
tca	S	0	15.88
tcg	S	0	3.25
cat	H	0	0.59
cac	H	1	0.41
aat	N	486	13314.00
aac	N	22025	9197.00
agt	S	0	11.47
agc	S	61	12.12
gat	D	0	8.43
gac	D	16	7.57
---	--	------	------
mPD		0.049	0.49
		nPD:	0.1
		N. weight:	0.82
		Sc. PD:	0.045
		Sc. rank:	12591.0

,

PA			
Pos.	116	obs:	exp:
cgt	R	141	616.80
cgc	R	1	1182.00
cga	R	258	2213.00
cgg	R	22152	1653.00
aga	R	6	11340.00
agg	R	31	5584.00
---	--	------	------
mPD		0.039	0.91
		nPD:	0.04
		N. weight:	2.6
		Sc. PD:	-0.0007
		Sc. rank:	28725.0

,

PA			
Pos.	117	obs:	exp:
ttt	F	13	9694.00
ttc	F	22576	12895.00
---	--	------	------
mPD		0.0012	0.49
		nPD:	0.
		N. weight:	0.58
		Sc. PD:	-0.023
		Sc. rank:	-10248.0

,

PA			
Pos.	118	obs:	exp:
tta	L	0	0.33
ttg	L	0	0.56
ctt	L	3	0.59
ctc	L	0	0.42
cta	L	0	0.58
ctg	L	0	0.51
att	I	22546	8218.00
atc	I	17	5635.00
ata	I	12	8722.00
gtt	V	11	2.43
gtc	V	0	2.00
gta	V	0	2.96
gtg	V	0	3.61
---	--	------	------
mPD		0.0038	0.66
		nPD:	0.01
		N. weight:	1.
		Sc. PD:	-0.038
		Sc. rank:	-11891.0

,

PA			
Pos.	119	obs:	exp:
gaa	E	22535	13678.00
gag	E	54	8911.00
---	--	------	------
mPD		0.0048	0.48
		nPD:	0.01
		N. weight:	0.51
		Sc. PD:	-0.016
		Sc. rank:	-2445.1

,

PA			
Pos.	120	obs:	exp:
att	I	22555	8219.00
atc	I	23	5636.00
ata	I	1	8723.00
gtt	V	10	2.21
gtc	V	0	1.82
gta	V	0	2.69
gtg	V	0	3.28
---	--	------	------
mPD		0.0030	0.66
		nPD:	0.
		N. weight:	1.
		Sc. PD:	-0.039
		Sc. rank:	-14188.0

,

PA			
Pos.	121	obs:	exp:
ggt	G	2	3425.00
ggc	G	0	3346.00
gga	G	22484	10048.00
ggg	G	103	5770.00
---	--	------	------
mPD		0.0093	0.69
		nPD:	0.01
		N. weight:	0.82
		Sc. PD:	-0.024
		Sc. rank:	-977.7

,

PA			
Pos.	122	obs:	exp:
att	I	0	12.74
atc	I	0	8.74
ata	I	35	13.52
gtt	V	0	4986.00
gtc	V	2	4098.00
gta	V	22493	6065.00
gtg	V	57	7403.00
gct	A	0	0.53
gcc	A	0	0.41
gca	A	2	0.94
gcg	A	0	0.12
---	--	------	------
mPD		0.0085	0.74
		nPD:	0.01
		N. weight:	1.3
		Sc. PD:	-0.042
		Sc. rank:	-3944.3

,

PA			
Pos.	123	obs:	exp:
act	T	4	5694.00
acc	T	14	4633.00
aca	T	22506	10334.00
acg	T	65	1927.00
---	--	------	------
mPD		0.0073	0.68
		nPD:	0.01
		N. weight:	0.79
		Sc. PD:	-0.025
		Sc. rank:	-2994.7

,

PA			
Pos.	124	obs:	exp:
cgt	R	3	616.80
cgc	R	0	1182.00
cga	R	72	2213.00
cgg	R	22478	1653.00
aga	R	3	11340.00
agg	R	33	5584.00
---	--	------	------
mPD		0.010	0.91
		nPD:	0.01
		N. weight:	2.7
		Sc. PD:	-0.084
		Sc. rank:	-9177.4

,

PA

Pos.	125	obs:	exp:
cgt	R	0	616.80
cgc	R	0	1182.00
cga	R	0	2213.00
cgg	R	0	1653.00
aga	R	18180	11340.00
agg	R	4409	5584.00
---	--	------	------
mPD		0.31	0.91
		nPD:	0.35
		N. weight:	0.35
		Sc. PD:	0.1
		Sc. rank:	6198.1

,

PA

Pos.	126	obs:	exp:
aaa	K	1	0.57
aag	K	0	0.43
gat	D	3	2.63
gac	D	2	2.37
gaa	E	22547	13669.00
gag	E	28	8906.00
ggt	G	0	1.21
ggc	G	0	1.18
gga	G	8	3.56
ggg	G	0	2.04
---	--	------	------
mPD		0.0037	0.48
		nPD:	0.01
		N. weight:	0.51
		Sc. PD:	-0.018
		Sc. rank:	-4308.0

,

PA

Pos.	127	obs:	exp:
att	I	4	81.91
atc	I	221	56.16
ata	I	0	86.93
gtt	V	2421	4934.00
gtc	V	19887	4055.00
gta	V	8	6002.00
gtg	V	0	7326.00
gct	A	1	12.71
gcc	A	47	9.94
gca	A	0	22.54
gcg	A	0	2.81
---	--	------	------
mPD		0.22	0.76
		nPD:	0.28
		N. weight:	1.4
		Sc. PD:	0.33
		Sc. rank:	24097.0

,

PA

Pos.	128	obs:	exp:
tat	Y	0	0.52
tac	Y	1	0.48
cct	P	0	0.28
ccc	P	1	0.19
cca	P	0	0.43
ccg	P	0	0.11
cat	H	37	13281.00
cac	H	22546	9302.00
caa	Q	2	1.06
cag	Q	0	0.94
aat	N	0	1.18
aac	N	2	0.82
---	--	------	------
mPD		0.0038	0.48
		nPD:	0.01
		N. weight:	0.91
		Sc. PD:	-0.031
		Sc. rank:	-7567.9

,

PA

Pos.	129	obs:	exp:
tta	L	29	3.41
ttg	L	0	5.84
ctt	L	0	6.11
ctc	L	0	4.38
cta	L	2	5.97
ctg	L	0	5.30
att	I	0	8187.00
atc	I	3	5614.00
ata	I	22487	8689.00
atg	M	37	37.00
act	T	0	5.55
acc	T	0	4.51
aca	T	22	10.06
acg	T	0	1.88
gtt	V	0	1.99
gtc	V	0	1.64
gta	V	9	2.42
gtg	V	0	2.95
---	--	------	------
mPD		0.0090	0.66
		nPD:	0.01
		N. weight:	0.99
		Sc. PD:	-0.029
		Sc. rank:	-883.3

,

PA

Pos.	130	obs:	exp:
tat	Y	22524	11736.00
tac	Y	63	10851.00
cat	H	1	0.59
cac	H	0	0.41
---	-	1	1.00
---	--	------	------
mPD		0.0057	0.50
		nPD:	0.01
		N. weight:	0.66
		Sc. PD:	-0.021
		Sc. rank:	-2073.9

,

PA

Pos.	131	obs:	exp:
tat	Y	82	11737.00
tac	Y	22507	10852.00
---	--	------	------
mPD		0.0072	0.50
		nPD:	0.01
		N. weight:	0.74
		Sc. PD:	-0.021
		Sc. rank:	-151.5

,

PA

Pos.	132	obs:	exp:
tta	L	40	2483.00
ttg	L	0	4252.00
ctt	L	10	4450.00
ctc	L	29	3190.00
cta	L	22458	4348.00
ctg	L	47	3861.00
cgt	R	0	0.03
cgc	R	0	0.05
cga	R	1	0.10
cgg	R	0	0.07
att	I	0	1.09
atc	I	0	0.75
ata	I	3	1.16
aga	R	0	0.50
agg	R	0	0.25
gtt	V	0	0.22
gtc	V	0	0.18
gta	V	1	0.27
gtg	V	0	0.33
---	--	------	------
mPD		0.012	1.1
		nPD:	0.01
		N. weight:	1.7
		Sc. PD:	-0.054
		Sc. rank:	-7897.1

,

PA

Pos.	133	obs:	exp:
aaa	K	1	3.99
aag	K	6	3.01
gtt	V	0	0.22
gtc	V	0	0.18
gta	V	0	0.27
gtg	V	1	0.33
gat	D	5	2.63
gac	D	0	2.37
gaa	E	317	13670.00
gag	E	22259	8906.00
---	--	------	------
mPD		0.029	0.48
		nPD:	0.06
		N. weight:	0.89
		Sc. PD:	0.015
		Sc. rank:	11557.0

,

PA

Pos.	134	obs:	exp:
aaa	K	22542	12884.00
aag	K	45	9703.00
gaa	E	2	1.21
gag	E	0	0.79
---	--	------	------
mPD		0.0042	0.49
		nPD:	0.01
		N. weight:	0.57
		Sc. PD:	-0.019
		Sc. rank:	-4032.1

,

PA

Pos.	135	obs:	exp:
tct	S	0	0.55
tcc	S	3	0.35
tca	S	0	0.78
tcg	S	0	0.16
act	T	0	0.50
acc	T	2	0.41
aca	T	0	0.91
acg	T	0	0.17
agt	S	0	0.56
agc	S	0	0.60
gct	A	67	5979.00
gcc	A	22505	4675.00
gca	A	12	10607.00
gcg	A	0	1323.00
---	--	------	------
mPD		0.0074	0.66
		nPD:	0.01
		N. weight:	1.6
		Sc. PD:	-0.05
		Sc. rank:	-5179.4

,

PA

Pos.	136	obs:	exp:
tct	S	0	0.37
tcc	S	0	0.23
tca	S	0	0.52
tcg	S	0	0.11
act	T	0	0.25
acc	T	1	0.21
aca	T	0	0.46
acg	T	0	0.09
aat	N	103	13358.00
aac	N	22482	9227.00
agt	S	0	0.38
agc	S	2	0.40
gat	D	0	0.53
gac	D	1	0.47
---	--	------	------
mPD		0.0094	0.48
		nPD:	0.02
		N. weight:	0.9
		Sc. PD:	-0.021
		Sc. rank:	3050.6

,

PA			
Pos.	137	obs:	exp:
cgt	R	0	0.05
cgc	R	0	0.10
cga	R	0	0.20
cgg	R	0	0.15
aaa	K	22528	12884.00
aag	K	58	9702.00
aga	R	2	1.00
agg	R	0	0.49
gaa	E	1	0.61
gag	E	0	0.39
---	--	------	------
mPD		0.0054	0.49
		nPD:	0.01
		N. weight:	0.57
		Sc. PD:	-0.018
		Sc. rank:	-2044.3

,

PA			
Pos.	138	obs:	exp:
att	I	6	8219.00
atc	I	1	5636.00
ata	I	22571	8723.00
atg	M	11	11.00
---	--	------	------
mPD		0.0016	0.66
		nPD:	0.
		N. weight:	0.99
		Sc. PD:	-0.039
		Sc. rank:	-17257.0

,

PA			
Pos.	139	obs:	exp:
cgt	R	0	0.05
cgc	R	0	0.10
cga	R	0	0.20
cgg	R	0	0.15
act	T	0	0.25
acc	T	0	0.21
aca	T	1	0.46
acg	T	0	0.09
aat	N	0	1.18
aac	N	2	0.82
aaa	K	22298	12883.00
aag	K	286	9701.00
aga	R	2	1.00
agg	R	0	0.49
---	--	------	------
mPD		0.025	0.49
		nPD:	0.05
		N. weight:	0.52
		Sc. PD:	0.0046
		Sc. rank:	6457.1

,

PA			
Pos.	140	obs:	exp:
ttt	F	5	2.15
ttc	F	0	2.85
tct	S	22527	4154.00
tcc	S	28	2614.00
tca	S	13	5874.00
tcg	S	3	1201.00
tat	Y	2	1.04
tac	Y	0	0.96
cct	P	3	0.83
ccc	P	0	0.56
cca	P	0	1.28
ccg	P	0	0.34
act	T	1	0.25
acc	T	0	0.21
aca	T	0	0.46
acg	T	0	0.09
agt	S	0	4244.00
agc	S	0	4483.00
gct	A	7	1.85
gcc	A	0	1.45
gca	A	0	3.29
gcg	A	0	0.41
---	--	------	------
mPD		0.0055	1.6
		nPD:	0.
		N. weight:	1.7
		Sc. PD:	-0.068
		Sc. rank:	-27613.0

,

PA			
Pos.	141	obs:	exp:
aaa	K	0	0.57
aag	K	1	0.43
gtt	V	0	0.22
gtc	V	0	0.18
gta	V	0	0.27
gtg	V	1	0.33
gat	D	6	3.16
gac	D	0	2.84
gaa	E	1221	13670.00
gag	E	21355	8906.00
ggt	G	0	0.76
ggc	G	0	0.74
gga	G	0	2.22
ggg	G	5	1.28
---	--	------	------
mPD		0.10	0.48
		nPD:	0.22
		N. weight:	0.73
		Sc. PD:	0.12
		Sc. rank:	12321.0

,

PA			
Pos.	142	obs:	exp:
cgt	R	0	0.35
cgc	R	0	0.68
cga	R	0	1.27
cgg	R	0	0.95
atg	M	2	2.00
act	T	0	3.03
acc	T	0	2.46
aca	T	0	5.49
acg	T	12	1.02
aat	N	3	5.32
aac	N	6	3.68
aaa	K	148	12751.00
aag	K	22206	9603.00
aga	R	0	6.53
agg	R	13	3.21
gat	D	1	0.53
gac	D	0	0.47
gaa	E	0	119.90
gag	E	198	78.11
---	--	------	------
mPD		0.034	0.51
		nPD:	0.07
		N. weight:	0.84
		Sc. PD:	0.019
		Sc. rank:	11540.0

,

PA			
Pos.	143	obs:	exp:
cct	P	0	0.28
ccc	P	0	0.19
cca	P	1	0.43
ccg	P	0	0.11
att	I	0	0.36
atc	I	0	0.25
ata	I	1	0.39
act	T	0	5693.00
acc	T	5	4632.00
aca	T	22565	10332.00
acg	T	14	1927.00
gct	A	0	0.79
gcc	A	0	0.62
gca	A	3	1.41
gcg	A	0	0.18
---	--	------	------
mPD		0.0021	0.68
		nPD:	0.
		N. weight:	0.81
		Sc. PD:	-0.032
		Sc. rank:	-13208.0

,

PA			
Pos.	144	obs:	exp:
tat	Y	0	0.52
tac	Y	1	0.48
cat	H	30	13281.00
cac	H	22554	9303.00
caa	Q	3	1.58
cag	Q	0	1.42
aat	N	0	0.59
aac	N	1	0.41
---	--	------	------
mPD		0.0031	0.48
		nPD:	0.01
		N. weight:	0.91
		Sc. PD:	-0.033
		Sc. rank:	-9879.2

,

PA			
Pos.	145	obs:	exp:
att	I	22388	8222.00
atc	I	167	5638.00
ata	I	31	8726.00
gtt	V	3	0.66
gtc	V	0	0.55
gta	V	0	0.81
gtg	V	0	0.98
---	--	------	------
mPD		0.018	0.66
		nPD:	0.03
		N. weight:	1.
		Sc. PD:	-0.016
		Sc. rank:	7393.6

,

PA			
Pos.	146	obs:	exp:
cat	H	72	13284.00
cac	H	22517	9305.00
---	--	------	------
mPD		0.0064	0.48
		nPD:	0.01
		N. weight:	0.9
		Sc. PD:	-0.026
		Sc. rank:	-1256.5

,

PA			
Pos.	147	obs:	exp:
tta	L	0	0.11
ttg	L	0	0.19
ctt	L	0	0.20
ctc	L	1	0.14
cta	L	0	0.19
ctg	L	0	0.17
att	I	57	8222.00
atc	I	22508	5638.00
ata	I	22	8727.00
gtt	V	0	0.22
gtc	V	1	0.18
gta	V	0	0.27
gtg	V	0	0.33
---	--	------	------
mPD		0.0072	0.66
		nPD:	0.01
		N. weight:	1.4
		Sc. PD:	-0.045
		Sc. rank:	-5250.7

,

PA			
Pos.	148	obs:	exp:
ttt	F	22561	9693.00
ttc	F	27	12895.00
tta	L	0	0.11
ttg	L	0	0.19
ctt	L	1	0.20
ctc	L	0	0.14
cta	L	0	0.19
ctg	L	0	0.17
---	--	------	------
mPD		0.0025	0.49
		nPD:	0.01
		N. weight:	0.87
		Sc. PD:	-0.032
		Sc. rank:	-11221.0

,

PA

Pos.	149	obs:	exp:
tct	S	14	4157.00
tcc	S	12	2616.00
tca	S	22546	5879.00
tcg	S	17	1202.00
agt	S	0	4247.00
agc	S	0	4487.00
---	--	------	------
mPD		0.0038	1.6
		nPD:	0.
		N. weight:	1.4
		Sc. PD:	-0.055
		Sc. rank:	-24601.0

,

PA

Pos.	150	obs:	exp:
ttt	F	253	9694.00
ttc	F	22336	12895.00
---	--	------	------
mPD		0.022	0.49
		nPD:	0.05
		N. weight:	0.52
		Sc. PD:	0.0012
		Sc. rank:	6001.3

,

PA

Pos.	151	obs:	exp:
att	I	14	5.10
atc	I	0	3.49
ata	I	0	5.41
act	T	22523	5689.00
acc	T	32	4629.00
aca	T	11	10325.00
acg	T	3	1926.00
aat	N	3	1.77
aac	N	0	1.23
gct	A	3	0.79
gcc	A	0	0.62
gca	A	0	1.41
gcg	A	0	0.18
---	--	------	------
mPD		0.0058	0.68
		nPD:	0.01
		N. weight:	1.4
		Sc. PD:	-0.048
		Sc. rank:	-9762.3

,

PA

Pos.	152	obs:	exp:
ggt	G	0	3425.00
ggc	G	0	3346.00
gga	G	22456	10048.00
ggg	G	133	5770.00
---	--	------	------
mPD		0.012	0.69
		nPD:	0.02
		N. weight:	0.81
		Sc. PD:	-0.021
		Sc. rank:	1022.4

,

PA

Pos.	153	obs:	exp:
gaa	E	350	13677.00
gag	E	22238	8911.00
ggt	G	0	0.15
ggc	G	0	0.15
gga	G	0	0.44
ggg	G	1	0.26
---	--	------	------
mPD		0.031	0.48
		nPD:	0.06
		N. weight:	0.88
		Sc. PD:	0.018
		Sc. rank:	11822.0

,

PA

Pos.	154	obs:	exp:
gaa	E	657	13677.00
gag	E	21931	8911.00
ggt	G	0	0.15
ggc	G	0	0.15
gga	G	0	0.44
ggg	G	1	0.26
---	--	------	------
mPD		0.057	0.48
		nPD:	0.12
		N. weight:	0.82
		Sc. PD:	0.061
		Sc. rank:	12854.0

,

PA

Pos.	155	obs:	exp:
tta	L	0	0.11
ttg	L	1	0.19
ctt	L	0	0.20
ctc	L	0	0.14
cta	L	0	0.19
ctg	L	0	0.17
att	I	0	0.36
atc	I	0	0.25
ata	I	1	0.39
atg	M	22585	22585.00
act	T	0	0.50
acc	T	0	0.41
aca	T	0	0.91
acg	T	2	0.17
---	--	------	------
mPD		0.00035	0.00057
		nPD:	0.62
		N. weight:	0.00035
		Sc. PD:	0.0002
		Sc. rank:	6.5

,

PA

Pos.	156	obs:	exp:
act	T	0	0.25
acc	T	1	0.21
aca	T	0	0.46
acg	T	0	0.09
gtt	V	0	0.66
gtc	V	3	0.55
gta	V	0	0.81
gtg	V	0	0.98
gct	A	28	5979.00
gcc	A	22537	4675.00
gca	A	20	10607.00
gcg	A	0	1323.00
---	--	------	------
mPD		0.0046	0.66
		nPD:	0.01
		N. weight:	1.6
		Sc. PD:	-0.057
		Sc. rank:	-16373.0

,

PA

Pos.	157	obs:	exp:
tct	S	0	0.18
tcc	S	1	0.12
tca	S	0	0.26
tcg	S	0	0.05
act	T	78	5694.00
acc	T	22493	4633.00
aca	T	15	10333.00
acg	T	0	1927.00
aat	N	0	0.59
aac	N	1	0.41
agt	S	0	0.19
agc	S	0	0.20
gct	A	0	0.26
gcc	A	1	0.21
gca	A	0	0.47
gcg	A	0	0.06
---	--	------	------
mPD		0.0085	0.68
		nPD:	0.01
		N. weight:	1.6
		Sc. PD:	-0.048
		Sc. rank:	-3471.5

,

PA

Pos.	158	obs:	exp:
caa	Q	3	1.58
cag	Q	0	1.42
cgt	R	0	1.58
cgc	R	0	3.03
cga	R	0	5.68
cgg	R	0	4.24
aat	N	1	2.37
aac	N	3	1.63
aaa	K	22499	12846.00
aag	K	21	9674.00
aga	R	53	29.12
agg	R	5	14.34
gaa	E	4	2.42
gag	E	0	1.58
---	--	------	------
mPD		0.0084	0.50
		nPD:	0.02
		N. weight:	0.58
		Sc. PD:	-0.015
		Sc. rank:	687.3

,

PA

Pos.	159	obs:	exp:
tct	S	0	2.21
tcc	S	0	1.39
tca	S	1	3.12
tcg	S	11	0.64
act	T	0	0.50
acc	T	0	0.41
aca	T	0	0.91
acg	T	2	0.17
agt	S	0	2.26
agc	S	0	2.38
gtt	V	0	0.22
gtc	V	0	0.18
gta	V	0	0.27
gtg	V	1	0.33
gct	A	15	5977.00
gcc	A	0	4673.00
gca	A	455	10602.00
gcg	A	22104	1322.00
---	--	------	------
mPD		0.042	0.66
		nPD:	0.06
		N. weight:	2.8
		Sc. PD:	0.057
		Sc. rank:	37566.0

,

PA

Pos.	160	obs:	exp:
aat	N	0	3.55
aac	N	6	2.45
gat	D	84	11893.00
gac	D	22488	10679.00
gaa	E	11	6.66
gag	E	0	4.34
---	--	------	------
mPD		0.0089	0.50
		nPD:	0.02
		N. weight:	0.75
		Sc. PD:	-0.019
		Sc. rank:	1553.4

,

PA

Pos.	161	obs:	exp:
ttt	F	0	2.15
ttc	F	5	2.85
tat	Y	18	11731.00
tac	Y	22560	10847.00
cat	H	0	3.53
cac	H	6	2.47
---	--	------	------
mPD		0.0026	0.50
		nPD:	0.01
		N. weight:	0.76
		Sc. PD:	-0.028
		Sc. rank:	-9462.9

,

PA

Pos.	162	obs:	exp:
tct	S	0	2.76
tcc	S	15	1.74
tca	S	0	3.90
tcg	S	0	0.80
att	I	0	5.82
atc	I	16	3.99
ata	I	0	6.18
act	T	2481	5686.00
acc	T	20067	4627.00
aca	T	8	10319.00
acg	T	0	1925.00
aat	N	0	0.59
aac	N	1	0.41
agt	S	0	2.82
agc	S	0	2.98
gct	A	0	0.26
gcc	A	1	0.21
gca	A	0	0.47
gcg	A	0	0.06
---	--	------	------
mPD		0.20	0.68
		nPD:	0.29
		N. weight:	1.3
		Sc. PD:	0.31
		Sc. rank:	22170.0

,

PA

Pos.	163	obs:	exp:
ttt	F	1	0.43
ttc	F	0	0.57
tta	L	0	2481.00
ttg	L	0	4249.00
ctt	L	22549	4448.00
ctc	L	14	3188.00
cta	L	6	4345.00
ctg	L	1	3859.00
att	I	18	6.55
atc	I	0	4.49
ata	I	0	6.95
---	--	------	------
mPD		0.0035	1.1
		nPD:	0.
		N. weight:	1.7
		Sc. PD:	-0.066
		Sc. rank:	-27620.0

,

PA

Pos.	164	obs:	exp:
aat	N	0	0.59
aac	N	1	0.41
gat	D	83	11901.00
gac	D	22504	10686.00
ggt	G	0	0.15
ggc	G	1	0.15
gga	G	0	0.44
ggg	G	0	0.26
---	--	------	------
mPD		0.0075	0.50
		nPD:	0.02
		N. weight:	0.76
		Sc. PD:	-0.021
		Sc. rank:	97.1

,

PA

Pos.	165	obs:	exp:
gct	A	0	0.26
gcc	A	0	0.21
gca	A	1	0.47
gcg	A	0	0.06
gat	D	1	1.58
gac	D	2	1.42
gaa	E	22536	13675.00
gag	E	48	8909.00
ggt	G	0	0.15
ggc	G	0	0.15
gga	G	1	0.44
ggg	G	0	0.26
---	--	------	------
mPD		0.0047	0.48
		nPD:	0.01
		N. weight:	0.51
		Sc. PD:	-0.017
		Sc. rank:	-2673.1

,

PA

Pos.	166	obs:	exp:
aaa	K	1	0.57
aag	K	0	0.43
gaa	E	7774	13677.00
gag	E	14814	8911.00
---	--	------	------
mPD		0.45	0.48
		nPD:	0.95
		N. weight:	0.15
		Sc. PD:	0.13
		Sc. rank:	2715.6

,

PA

Pos.	167	obs:	exp:
tct	S	0	4157.00
tcc	S	0	2616.00
tca	S	0	5879.00
tcg	S	0	1202.00
act	T	0	0.25
acc	T	1	0.21
aca	T	0	0.46
acg	T	0	0.09
agt	S	351	4247.00
agc	S	22237	4487.00
---	--	------	------
mPD		0.031	1.6
		nPD:	0.02
		N. weight:	1.6
		Sc. PD:	-0.038
		Sc. rank:	4530.5

,

PA

Pos.	168	obs:	exp:
cgt	R	0	616.70
cgc	R	0	1182.00
cga	R	4	2212.00
cgg	R	0	1653.00
act	T	0	0.25
acc	T	0	0.21
aca	T	0	0.46
acg	T	1	0.09
aga	R	19302	11340.00
agg	R	3282	5584.00
---	--	------	------
mPD		0.25	0.91
		nPD:	0.27
		N. weight:	0.39
		Sc. PD:	0.089
		Sc. rank:	6754.3

,

PA

Pos.	169	obs:	exp:
act	T	0	0.25
acc	T	0	0.21
aca	T	1	0.46
acg	T	0	0.09
gct	A	44	5980.00
gcc	A	1	4676.00
gca	A	22337	10609.00
gcg	A	206	1323.00
---	--	------	------
mPD		0.022	0.66
		nPD:	0.03
		N. weight:	0.74
		Sc. PD:	-0.0069
		Sc. rank:	7137.1

,

PA

Pos.	170	obs:	exp:
cgt	R	0	616.80
cgc	R	0	1182.00
cga	R	1	2213.00
cgg	R	0	1653.00
aga	R	22548	11340.00
agg	R	40	5584.00
---	--	------	------
mPD		0.0036	0.91
		nPD:	0.
		N. weight:	0.71
		Sc. PD:	-0.027
		Sc. rank:	-10250.0

,

PA

Pos.	171	obs:	exp:
att	I	37	8221.00
atc	I	22502	5637.00
ata	I	45	8725.00
gtt	V	0	1.11
gtc	V	5	0.91
gta	V	0	1.34
gtg	V	0	1.64
---	--	------	------
mPD		0.0077	0.66
		nPD:	0.01
		N. weight:	1.4
		Sc. PD:	-0.044
		Sc. rank:	-3839.4

,

PA

Pos.	172	obs:	exp:
caa	Q	1	0.53
cag	Q	0	0.47
act	T	0	0.25
acc	T	0	0.21
aca	T	1	0.46
acg	T	0	0.09
aaa	K	22564	12884.00
aag	K	23	9703.00
---	--	------	------
mPD		0.0022	0.49
		nPD:	0.
		N. weight:	0.58
		Sc. PD:	-0.022
		Sc. rank:	-7943.8

,

PA

Pos.	173	obs:	exp:
act	T	22536	5694.00
acc	T	46	4633.00
aca	T	4	10334.00
acg	T	3	1927.00
---	--	------	------
mPD		0.0047	0.68
		nPD:	0.01
		N. weight:	1.4
		Sc. PD:	-0.05
		Sc. rank:	-14476.0

,

PA

Pos.	174	obs:	exp:
cgt	R	0	616.70
cgc	R	0	1182.00
cga	R	0	2212.00
cgg	R	5	1653.00
aaa	K	0	0.57
aag	K	1	0.43
aga	R	227	11340.00
agg	R	22356	5584.00
---	--	------	------
mPD		0.020	0.91
		nPD:	0.02
		N. weight:	1.4
		Sc. PD:	-0.028
		Sc. rank:	7434.7

,

PA

Pos.	175	obs:	exp:
tta	L	0	2483.00
ttg	L	0	4253.00
ctt	L	22500	4451.00
ctc	L	76	3191.00
cta	L	1	4349.00
ctg	L	12	3862.00
---	--	------	------
mPD		0.0079	1.1
		nPD:	0.01
		N. weight:	1.7
		Sc. PD:	-0.059
		Sc. rank:	-17122.0

,

PA

Pos.	176	obs:	exp:
ttt	F	30	9690.00
ttc	F	22550	12890.00
tta	L	0	0.11
ttg	L	0	0.19
tat	Y	0	4.16
tac	Y	8	3.84
ctt	L	0	0.20
ctc	L	1	0.14
cta	L	0	0.19
ctg	L	0	0.17
---	--	------	------
mPD		0.0034	0.49
		nPD:	0.01
		N. weight:	0.57
		Sc. PD:	-0.02
		Sc. rank:	-5701.1

,

PA

Pos.	177	obs:	exp:
att	I	1	0.36
atc	I	0	0.25
ata	I	0	0.39
act	T	22203	5694.00
acc	T	240	4633.00
aca	T	4	10333.00
acg	T	141	1927.00
---	--	------	------
mPD		0.034	0.68
		nPD:	0.05
		N. weight:	1.3
		Sc. PD:	0.0092
		Sc. rank:	16321.0

,

PA

Pos.	178	obs:	exp:
tta	L	1	0.22
ttg	L	0	0.38
ctt	L	0	0.39
ctc	L	0	0.28
cta	L	1	0.39
ctg	L	0	0.34
att	I	0	8222.00
atc	I	92	5638.00
ata	I	22493	8726.00
gtt	V	0	0.44
gtc	V	0	0.36
gta	V	2	0.54
gtg	V	0	0.66
---	--	------	------
mPD		0.0085	0.66
		nPD:	0.01
		N. weight:	0.97
		Sc. PD:	-0.029
		Sc. rank:	-1703.4

,

PA

Pos.	179	obs:	exp:
cgt	R	0	616.70
cgc	R	0	1182.00
cga	R	4	2212.00
cgg	R	0	1653.00
aaa	K	1	0.57
aag	K	0	0.43
aga	R	22539	11340.00
agg	R	45	5584.00
---	--	------	------
mPD		0.0044	0.91
		nPD:	0.
		N. weight:	0.7
		Sc. PD:	-0.026
		Sc. rank:	-9231.6

,

PA

Pos.	180	obs:	exp:
cat	H	0	1.18
cac	H	2	0.82
caa	Q	22549	11917.00
cag	Q	36	10668.00
aaa	K	1	0.57
aag	K	0	0.43
gaa	E	1	0.61
gag	E	0	0.39
---	--	------	------
mPD		0.0035	0.50
		nPD:	0.01
		N. weight:	0.65
		Sc. PD:	-0.023
		Sc. rank:	-6344.1

,

PA

Pos.	181	obs:	exp:
cct	P	0	0.28
ccc	P	0	0.19
cca	P	1	0.43
ccg	P	0	0.11
gaa	E	22467	13676.00
gag	E	120	8911.00
ggt	G	0	0.15
ggc	G	0	0.15
gga	G	1	0.44
ggg	G	0	0.26
---	--	------	------
mPD		0.011	0.48
		nPD:	0.02
		N. weight:	0.49
		Sc. PD:	-0.0098
		Sc. rank:	2661.4

,

PA

Pos.	182	obs:	exp:
att	I	0	0.36
atc	I	0	0.25
ata	I	1	0.39
atg	M	22588	22588.00
---	--	------	------
mPD		0.000089	0.000089
		nPD:	1.
		N. weight:	0.000044
		Sc. PD:	0.000041
		Sc. rank:	0.8

,

PA

Pos.	183	obs:	exp:
cct	P	0	0.28
ccc	P	0	0.19
cca	P	1	0.43
ccg	P	0	0.11
act	T	0	0.50
acc	T	2	0.41
aca	T	0	0.91
acg	T	0	0.17
gtt	V	0	0.44
gtc	V	2	0.36
gta	V	0	0.54
gtg	V	0	0.66
gct	A	35	5979.00
gcc	A	22537	4675.00
gca	A	12	10607.00
gcg	A	0	1323.00
---	--	------	------
mPD		0.0047	0.66
		nPD:	0.01
		N. weight:	1.6
		Sc. PD:	-0.057
		Sc. rank:	-15860.0

,

PA

Pos.	184	obs:	exp:
tct	S	0	4132.00
tcc	S	0	2600.00
tca	S	0	5843.00
tcg	S	0	1195.00
tat	Y	1	0.52
tac	Y	0	0.48
cgt	R	0	0.03
cgc	R	0	0.05
cga	R	0	0.10
cgg	R	0	0.07
att	I	4	1.46
atc	I	0	1.00
ata	I	0	1.55
act	T	2	0.50
acc	T	0	0.41
aca	T	0	0.91
acg	T	0	0.17
aat	N	7	7.69
aac	N	6	5.31
agt	S	22364	4222.00
agc	S	87	4460.00
aga	R	0	0.50
agg	R	1	0.25
ggt	G	117	17.74
ggc	G	0	17.33
gga	G	0	52.04
ggg	G	0	29.89
---	--	------	------
mPD		0.020	1.6
		nPD:	0.01
		N. weight:	1.7
		Sc. PD:	-0.051
		Sc. rank:	-3820.8

,

PA

Pos.	185	obs:	exp:
cgt	R	0	616.40
cgc	R	0	1181.00
cga	R	0	2211.00
cgg	R	0	1652.00
aaa	K	0	7.42
aag	K	13	5.58
aga	R	374	11333.00
agg	R	22201	5581.00
ggt	G	0	0.15
ggc	G	0	0.15
gga	G	0	0.44
ggg	G	1	0.26
---	--	------	------
mPD		0.034	0.91
		nPD:	0.04
		N. weight:	1.4
		Sc. PD:	-0.0076
		Sc. rank:	13993.0

,

PA

Pos.	186	obs:	exp:
tct	S	0	4146.00
tcc	S	0	2609.00
tca	S	0	5863.00
tcg	S	0	1199.00
aat	N	44	26.02
aac	N	0	17.98
agt	S	22479	4236.00
agc	S	49	4475.00
ggt	G	11	2.58
ggc	G	6	2.52
gga	G	0	7.56
ggg	G	0	4.34
---	--	------	------
mPD		0.010	1.6
		nPD:	0.01
		N. weight:	1.7
		Sc. PD:	-0.062
		Sc. rank:	-18991.0

,

PA

Pos.	187	obs:	exp:
tta	L	925	2481.00
ttg	L	1	4249.00
ctt	L	0	4447.00
ctc	L	135	3188.00
cta	L	21489	4345.00
ctg	L	18	3859.00
att	I	0	7.64
atc	I	0	5.24
ata	I	21	8.11
---	--	------	------
mPD		0.094	1.1
		nPD:	0.08
		N. weight:	1.5
		Sc. PD:	0.059
		Sc. rank:	22286.0

,

PA

Pos.	188	obs:	exp:
tgg	W	22588	22588.00
cgt	R	0	0.03
cgc	R	0	0.05
cga	R	0	0.10
cgg	R	1	0.07
aga	R	0	0.50
agg	R	0	0.25
---	--	------	------
mPD		0.000089	0.00015
		nPD:	0.6
		N. weight:	0.00012
		Sc. PD:	0.000066
		Sc. rank:	2.2

,

PA

Pos.	189	obs:	exp:
aat	N	1	0.59
aac	N	0	0.41
gct	A	1	0.26
gcc	A	0	0.21
gca	A	0	0.47
gcg	A	0	0.06
gat	D	22545	11900.00
gac	D	40	10685.00
gaa	E	1	0.61
gag	E	0	0.39
ggt	G	1	0.15
ggc	G	0	0.15
gga	G	0	0.44
ggg	G	0	0.26
---	--	------	------
mPD		0.0039	0.50
		nPD:	0.01
		N. weight:	0.66
		Sc. PD:	-0.023
		Sc. rank:	-5468.1

,

PA

Pos.	190	obs:	exp:
ttt	F	0	1.72
ttc	F	4	2.28
tct	S	14	4156.00
tcc	S	22491	2616.00
tca	S	78	5877.00
tcg	S	0	1202.00
cct	P	0	0.28
ccc	P	1	0.19
cca	P	0	0.43
ccg	P	0	0.11
agt	S	0	4246.00
agc	S	0	4486.00
gct	A	0	0.26
gcc	A	1	0.21
gca	A	0	0.47
gcg	A	0	0.06
---	--	------	------
mPD		0.0086	1.6
		nPD:	0.01
		N. weight:	2.2
		Sc. PD:	-0.082
		Sc. rank:	-26680.0

,

PA

Pos.	191	obs:	exp:
ttt	F	22579	9694.00
ttc	F	10	12895.00
---	--	------	------
mPD		0.00089	0.49
		nPD:	0.
		N. weight:	0.88
		Sc. PD:	-0.036
		Sc. rank:	-15969.0

,

PA

Pos.	192	obs:	exp:
tgt	C	1	0.45
tgc	C	0	0.55
cat	H	6	3.53
cac	H	0	2.47
cgt	R	22548	616.60
cgc	R	31	1181.00
cga	R	1	2212.00
cgg	R	2	1653.00
aga	R	0	11337.00
agg	R	0	5583.00
---	--	------	------
mPD		0.0036	0.91
		nPD:	0.
		N. weight:	3.7
		Sc. PD:	-0.14
		Sc. rank:	-54438.0

,

PA

Pos.	193	obs:	exp:
tta	L	0	0.11
ttg	L	0	0.19
ctt	L	0	0.20
ctc	L	0	0.14
cta	L	0	0.19
ctg	L	1	0.17
cat	H	2	1.18
cac	H	0	0.82
caa	Q	206	11898.00
cag	Q	22343	10651.00
cgt	R	0	0.82
cgc	R	0	1.57
cga	R	0	2.94
cgg	R	30	2.20
aaa	K	0	3.99
aag	K	7	3.01
aga	R	0	15.06
agg	R	0	7.42
---	--	------	------
mPD		0.022	0.50
		nPD:	0.04
		N. weight:	0.73
		Sc. PD:	0.000023
		Sc. rank:	8175.0

,

PA

Pos.	194	obs:	exp:
tct	S	113	4157.00
tcc	S	22472	2616.00
tca	S	4	5879.00
tcg	S	0	1202.00
agt	S	0	4247.00
agc	S	0	4487.00
---	--	------	------
mPD		0.010	1.6
		nPD:	0.01
		N. weight:	2.2
		Sc. PD:	-0.08
		Sc. rank:	-24005.0

,

PA

Pos.	195	obs:	exp:
gaa	E	22567	13678.00
gag	E	22	8911.00
---	--	------	------
mPD		0.0019	0.48
		nPD:	0.
		N. weight:	0.52
		Sc. PD:	-0.02
		Sc. rank:	-7313.4

,

PA

Pos.	196	obs:	exp:
tct	S	0	0.74
tcc	S	0	0.46
tca	S	0	1.04
tcg	S	0	0.21
cgt	R	0	615.20
cgc	R	0	1179.00
cga	R	2	2207.00
cgg	R	0	1649.00
att	I	0	0.36
atc	I	0	0.25
ata	I	1	0.39
aaa	K	51	29.66
aag	K	1	22.34
agt	S	4	0.75
agc	S	0	0.79
aga	R	22455	11312.00
agg	R	75	5570.00
---	--	------	------
mPD		0.012	0.91
		nPD:	0.01
		N. weight:	0.7
		Sc. PD:	-0.02
		Sc. rank:	-1116.9

,

PA

Pos.	197	obs:	exp:
tct	S	0	0.55
tcc	S	0	0.35
tca	S	0	0.78
tcg	S	0	0.16
agt	S	0	0.56
agc	S	3	0.60
gat	D	0	0.53
gac	D	1	0.47
ggt	G	16	3425.00
ggc	G	22564	3345.00
gga	G	5	10046.00
ggg	G	0	5769.00
---	--	------	------
mPD		0.0022	0.69
		nPD:	0.
		N. weight:	2.
		Sc. PD:	-0.077
		Sc. rank:	-31752.0

,

PA

Pos.	198	obs:	exp:
gat	D	1	0.53
gac	D	0	0.47
gaa	E	22506	13676.00
gag	E	80	8910.00
ggt	G	0	0.30
ggc	G	0	0.30
gga	G	2	0.89
ggg	G	0	0.51
---	--	------	------
mPD		0.0073	0.48
		nPD:	0.02
		N. weight:	0.5
		Sc. PD:	-0.014
		Sc. rank:	187.3

,

PA

Pos.	199	obs:	exp:
aaa	K	0	0.57
aag	K	1	0.43
gat	D	4	2.63
gac	D	1	2.37
gaa	E	14722	13673.00
gag	E	7859	8908.00
ggt	G	0	0.30
ggc	G	0	0.30
gga	G	2	0.89
ggg	G	0	0.51
---	--	------	------
mPD		0.45	0.48
		nPD:	0.95
		N. weight:	0.0052
		Sc. PD:	0.0047
		Sc. rank:	97.6

,

PA

Pos.	200	obs:	exp:
act	T	0	5694.00
acc	T	0	4633.00
aca	T	22579	10333.00
acg	T	8	1927.00
gct	A	0	0.53
gcc	A	0	0.41
gca	A	2	0.94
gcg	A	0	0.12
---	--	------	------
mPD		0.00089	0.68
		nPD:	0.
		N. weight:	0.81
		Sc. PD:	-0.033
		Sc. rank:	-14959.0

,

PA			
Pos.	201	obs:	exp:
ttt	F	1	0.43
ttc	F	0	0.57
tct	S	0	0.18
tcc	S	0	0.12
tca	S	0	0.26
tcg	S	0	0.05
att	I	22520	8207.00
atc	I	22	5627.00
ata	I	2	8710.00
atg	M	1	1.00
act	T	12	3.03
acc	T	0	2.46
aca	T	0	5.49
acg	T	0	1.02
agt	S	1	0.19
agc	S	0	0.20
gtt	V	30	6.63
gtc	V	0	5.45
gta	V	0	8.07
gtg	V	0	9.85
---	--	------	------
mPD		0.0061	0.66
		nPD:	0.01
		N. weight:	1.
		Sc. PD:	-0.035
		Sc. rank:	-6461.2

,

PA			
Pos.	202	obs:	exp:
gaa	E	22419	13678.00
gag	E	170	8911.00
---	--	------	------
mPD		0.015	0.48
		nPD:	0.03
		N. weight:	0.48
		Sc. PD:	-0.0055
		Sc. rank:	4226.5

,

PA			
Pos.	203	obs:	exp:
gaa	E	22578	13678.00
gag	E	11	8911.00
---	--	------	------
mPD		0.00097	0.48
		nPD:	0.
		N. weight:	0.52
		Sc. PD:	-0.021
		Sc. rank:	-9319.5

,

PA			
Pos.	204	obs:	exp:
cgt	R	0	1.28
cgc	R	0	2.46
cga	R	0	4.60
cgg	R	0	3.44
aat	N	1	1.18
aac	N	1	0.82
aaa	K	4290	12857.00
aag	K	18249	9682.00
aga	R	4	23.60
agg	R	43	11.62
gaa	E	1	0.61
gag	E	0	0.39
---	--	------	------
mPD		0.31	0.50
		nPD:	0.63
		N. weight:	0.32
		Sc. PD:	0.18
		Sc. rank:	5928.7

,

PA			
Pos.	205	obs:	exp:
ttt	F	22568	9693.00
ttc	F	19	12894.00
tta	L	1	0.11
ttg	L	0	0.19
ctt	L	0	0.20
ctc	L	0	0.14
cta	L	0	0.19
ctg	L	0	0.17
gtt	V	1	0.22
gtc	V	0	0.18
gta	V	0	0.27
gtg	V	0	0.33
---	--	------	------
mPD		0.0019	0.49
		nPD:	0.
		N. weight:	0.88
		Sc. PD:	-0.034
		Sc. rank:	-13079.0

,

PA			
Pos.	206	obs:	exp:
aaa	K	0	1.14
aag	K	2	0.86
gtt	V	0	0.88
gtc	V	0	0.73
gta	V	1	1.08
gtg	V	3	1.31
gat	D	3	1.58
gac	D	0	1.42
gaa	E	7783	13669.00
gag	E	14792	8906.00
ggt	G	0	0.76
ggc	G	0	0.74
gga	G	2	2.22
ggg	G	3	1.28
---	--	------	------
mPD		0.45	0.48
		nPD:	0.95
		N. weight:	0.14
		Sc. PD:	0.13
		Sc. rank:	2711.1

,

PA			
Pos.	207	obs:	exp:
tta	L	0	0.11
ttg	L	0	0.19
ctt	L	1	0.20
ctc	L	0	0.14
cta	L	0	0.19
ctg	L	0	0.17
att	I	22511	8222.00
atc	I	72	5638.00
ata	I	2	8726.00
gtt	V	3	0.66
gtc	V	0	0.55
gta	V	0	0.81
gtg	V	0	0.98
---	--	------	------
mPD		0.0069	0.66
		nPD:	0.01
		N. weight:	1.
		Sc. PD:	-0.033
		Sc. rank:	-4367.6

,

PA			
Pos.	208	obs:	exp:
att	I	0	3.64
atc	I	0	2.50
ata	I	10	3.86
act	T	16	5684.00
acc	T	1	4625.00
aca	T	22523	10315.00
acg	T	7	1924.00
aaa	K	2	1.14
aag	K	0	0.86
gct	A	0	7.94
gcc	A	0	6.21
gca	A	30	14.09
gcg	A	0	1.76
---	--	------	------
mPD		0.0058	0.68
		nPD:	0.01
		N. weight:	0.81
		Sc. PD:	-0.027
		Sc. rank:	-5609.3

,

PA			
Pos.	209	obs:	exp:
cgt	R	0	0.19
cgc	R	0	0.37
cga	R	0	0.69
cgg	R	0	0.51
aga	R	7	3.51
agg	R	0	1.73
gaa	E	8	4.84
gag	E	0	3.16
ggt	G	1	3423.00
ggc	G	0	3343.00
gga	G	22501	10041.00
ggg	G	72	5766.00
---	--	------	------
mPD		0.0078	0.69
		nPD:	0.01
		N. weight:	0.82
		Sc. PD:	-0.026
		Sc. rank:	-2605.1

,

PA			
Pos.	210	obs:	exp:
cct	P	1	0.28
ccc	P	0	0.19
cca	P	0	0.43
ccg	P	0	0.11
att	I	2	0.73
atc	I	0	0.50
ata	I	0	0.77
act	T	22456	5694.00
acc	T	129	4633.00
aca	T	1	10333.00
acg	T	0	1927.00
---	--	------	------
mPD		0.012	0.68
		nPD:	0.02
		N. weight:	1.4
		Sc. PD:	-0.035
		Sc. rank:	2291.0

,

PA			
Pos.	211	obs:	exp:
tta	L	0	0.33
ttg	L	2	0.56
ctt	L	0	0.59
ctc	L	0	0.42
cta	L	0	0.58
ctg	L	1	0.51
att	I	5	6.55
atc	I	1	4.49
ata	I	12	6.95
atg	M	22556	22556.00
act	T	0	2.27
acc	T	0	1.85
aca	T	0	4.12
acg	T	9	0.77
gtt	V	0	0.66
gtc	V	0	0.55
gta	V	0	0.81
gtg	V	3	0.98
---	--	------	------
mPD		0.0029	0.0040
		nPD:	0.73
		N. weight:	0.0017
		Sc. PD:	0.0011
		Sc. rank:	30.8

,

PA			
Pos.	212	obs:	exp:
tta	L	0	1.32
ttg	L	0	2.26
tct	S	0	0.37
tcc	S	0	0.23
tca	S	0	0.52
tcg	S	0	0.11
tgt	C	0	4.06
tgc	C	9	4.94
ctt	L	0	2.36
ctc	L	12	1.69
cta	L	0	2.31
ctg	L	0	2.05
cat	H	0	18.23
cac	H	31	12.77
cgt	R	214	615.30
cgc	R	22293	1179.00
cga	R	28	2207.00
cgg	R	0	1649.00
agt	S	0	0.38
agc	S	2	0.40
aga	R	0	11313.00
agg	R	0	5571.00
---	--	------	------
mPD		0.026	0.92
		nPD:	0.03
		N. weight:	3.
		Sc. PD:	-0.043
		Sc. rank:	24563.0

,

PA

Pos.	213	obs:	exp:
cgt	R	0	29.05
cgc	R	0	55.66
cga	R	0	104.20
cgg	R	0	77.87
aat	N	21	12.42
aac	N	0	8.58
aaa	K	28	12267.00
aag	K	21476	9237.00
aga	R	0	534.20
agg	R	1064	263.00
---	--	------	------
mPD		0.094	0.61
		nPD:	0.16
		N. weight:	0.9
		Sc. PD:	0.099
		Sc. rank:	14822.0

,

PA

Pos.	214	obs:	exp:
ttt	F	9	3.86
ttc	F	0	5.14
tta	L	0	2482.00
ttg	L	0	4251.00
ctt	L	22563	4450.00
ctc	L	16	3189.00
cta	L	1	4347.00
ctg	L	0	3861.00
---	--	------	------
mPD		0.0023	1.1
		nPD:	0.
		N. weight:	1.7
		Sc. PD:	-0.068
		Sc. rank:	-30337.0

,

PA

Pos.	215	obs:	exp:
act	T	0	1.01
acc	T	4	0.82
aca	T	0	1.83
acg	T	0	0.34
gtt	V	0	0.22
gtc	V	1	0.18
gta	V	0	0.27
gtg	V	0	0.33
gct	A	81	5979.00
gcc	A	22441	4675.00
gca	A	61	10606.00
gcg	A	0	1323.00
ggt	G	0	0.15
ggc	G	1	0.15
gga	G	0	0.44
ggg	G	0	0.26
---	--	------	------
mPD		0.013	0.66
		nPD:	0.02
		N. weight:	1.6
		Sc. PD:	-0.036
		Sc. rank:	5726.5

,

PA

Pos.	216	obs:	exp:
aat	N	0	23.66
aac	N	40	16.34
gat	D	506	11881.00
gac	D	22042	10667.00
ggt	G	0	0.15
ggc	G	1	0.15
gga	G	0	0.44
ggg	G	0	0.26
---	--	------	------
mPD		0.047	0.50
		nPD:	0.09
		N. weight:	0.67
		Sc. PD:	0.034
		Sc. rank:	10112.0

,

PA

Pos.	217	obs:	exp:
caa	Q	22480	11919.00
cag	Q	108	10669.00
cgt	R	0	0.03
cgc	R	0	0.05
cga	R	1	0.10
cgg	R	0	0.07
aga	R	0	0.50
agg	R	0	0.25
---	--	------	------
mPD		0.0096	0.50
		nPD:	0.02
		N. weight:	0.64
		Sc. PD:	-0.015
		Sc. rank:	1953.5

,

PA

Pos.	218	obs:	exp:
tct	S	0	4157.00
tcc	S	0	2616.00
tca	S	0	5879.00
tcg	S	0	1202.00
agt	S	22514	4247.00
agc	S	74	4487.00
ggt	G	1	0.15
ggc	G	0	0.15
gga	G	0	0.44
ggg	G	0	0.26
---	--	------	------
mPD		0.0066	1.6
		nPD:	0.
		N. weight:	1.7
		Sc. PD:	-0.066
		Sc. rank:	-24524.0

,

PA

Pos.	219	obs:	exp:
tta	L	3	2483.00
ttg	L	0	4253.00
ctt	L	134	4451.00
ctc	L	22302	3191.00
cta	L	148	4349.00
ctg	L	2	3862.00
---	--	------	------
mPD		0.025	1.1
		nPD:	0.02
		N. weight:	2.
		Sc. PD:	-0.04
		Sc. rank:	10190.0

,

PA

Pos.	220	obs:	exp:
cct	P	90	6223.00
ccc	P	0	4190.00
cca	P	22476	9618.00
ccg	P	23	2558.00
---	--	------	------
mPD		0.010	0.70
		nPD:	0.01
		N. weight:	0.86
		Sc. PD:	-0.024
		Sc. rank:	-387.9

,

PA

Pos.	221	obs:	exp:
tta	L	0	0.11
ttg	L	0	0.19
tct	S	0	0.55
tcc	S	0	0.35
tca	S	3	0.78
tcg	S	0	0.16
ctt	L	0	0.20
ctc	L	0	0.14
cta	L	0	0.19
ctg	L	1	0.17
cct	P	6	6222.00
ccc	P	3	4189.00
cca	P	19710	9616.00
ccg	P	2865	2557.00
caa	Q	1	0.53
cag	Q	0	0.47
agt	S	0	0.56
agc	S	0	0.60
---	--	------	------
mPD		0.22	0.70
		nPD:	0.32
		N. weight:	0.67
		Sc. PD:	0.18
		Sc. rank:	11784.0

,

PA

Pos.	222	obs:	exp:
tct	S	0	0.74
tcc	S	0	0.46
tca	S	0	1.04
tcg	S	0	0.21
cat	H	0	4.12
cac	H	7	2.88
aat	N	159	13351.00
aac	N	22415	9223.00
aaa	K	3	1.71
aag	K	0	1.29
agt	S	0	0.75
agc	S	4	0.79
gat	D	0	0.53
gac	D	1	0.47
---	--	------	------
mPD		0.015	0.48
		nPD:	0.03
		N. weight:	0.89
		Sc. PD:	-0.0099
		Sc. rank:	8036.4

,

PA

Pos.	223	obs:	exp:
ttt	F	108	9694.00
ttc	F	22481	12895.00
---	--	------	------
mPD		0.0095	0.49
		nPD:	0.02
		N. weight:	0.55
		Sc. PD:	-0.013
		Sc. rank:	1786.0

,

PA

Pos.	224	obs:	exp:
ttt	F	0	1.29
ttc	F	3	1.71
tct	S	29	4150.00
tcc	S	22517	2612.00
tca	S	6	5869.00
tcg	S	0	1200.00
tat	Y	0	1.56
tac	Y	3	1.44
cct	P	0	7.99
ccc	P	29	5.38
cca	P	0	12.35
ccg	P	0	3.28
act	T	0	0.50
acc	T	2	0.41
aca	T	0	0.91
acg	T	0	0.17
agt	S	0	4241.00
agc	S	0	4480.00
---	--	------	------
mPD		0.0064	1.6
		nPD:	0.
		N. weight:	2.2
		Sc. PD:	-0.086
		Sc. rank:	-33121.0

,

PA

Pos.	225	obs:	exp:
tct	S	0	3686.00
tcc	S	0	2320.00
tca	S	0	5212.00
tcg	S	0	1066.00
tgt	C	5	1086.00
tgc	C	2400	1319.00
cgt	R	0	0.05
cgc	R	0	0.10
cga	R	0	0.20
cgg	R	0	0.15
att	I	0	0.36
atc	I	1	0.25
ata	I	0	0.39
aat	N	4	71.56
aac	N	117	49.44
agt	S	126	3766.00
agc	S	19901	3978.00
aga	R	1	1.00
agg	R	1	0.49
ggt	G	1	5.00
ggc	G	32	4.89
gga	G	0	14.68
ggg	G	0	8.43
---	--	------	------
mPD		0.22	1.6
		nPD:	0.13
		N. weight:	1.5
		Sc. PD:	0.13
		Sc. rank:	24613.0

,

PA

Pos.	226	obs:	exp:
ttt	F	30	12.87
ttc	F	0	17.13
tta	L	0	2458.00
ttg	L	0	4210.00
ctt	L	22343	4406.00
ctc	L	6	3158.00
cta	L	7	4305.00
ctg	L	3	3823.00
att	I	200	72.81
atc	I	0	49.92
ata	I	0	77.27
---	--	------	------
mPD		0.022	1.2
		nPD:	0.02
		N. weight:	1.7
		Sc. PD:	-0.04
		Sc. rank:	4897.5

,

PA

Pos.	227	obs:	exp:
aaa	K	12	6.85
aag	K	0	5.15
gtt	V	0	0.22
gtc	V	0	0.18
gta	V	1	0.27
gtg	V	0	0.33
gat	D	8	5.80
gac	D	3	5.20
gaa	E	22534	13661.00
gag	E	27	8900.00
ggt	G	0	0.61
ggc	G	0	0.59
gga	G	4	1.78
ggg	G	0	1.02
---	--	------	------
mPD		0.0049	0.48
		nPD:	0.01
		N. weight:	0.51
		Sc. PD:	-0.017
		Sc. rank:	-2466.7

,

PA

Pos.	228	obs:	exp:
tct	S	0	5.15
tcc	S	0	3.24
tca	S	0	7.29
tcg	S	0	1.49
tat	Y	0	0.52
tac	Y	1	0.48
cat	H	0	1.76
cac	H	3	1.24
act	T	0	3.28
acc	T	13	2.67
aca	T	0	5.95
acg	T	0	1.11
aat	N	213	13327.00
aac	N	22320	9206.00
aaa	K	10	5.70
aag	K	0	4.30
agt	S	0	5.26
agc	S	28	5.56
gat	D	0	0.53
gac	D	1	0.47
---	--	------	------
mPD		0.024	0.49
		nPD:	0.05
		N. weight:	0.88
		Sc. PD:	0.0046
		Sc. rank:	10509.0

,

PA

Pos.	229	obs:	exp:
ttt	F	3106	9691.00
ttc	F	19476	12891.00
tta	L	0	0.66
ttg	L	0	1.13
tat	Y	0	0.52
tac	Y	1	0.48
ctt	L	3	1.18
ctc	L	3	0.85
cta	L	0	1.16
ctg	L	0	1.03
---	--	------	------
mPD		0.24	0.49
		nPD:	0.49
		N. weight:	0.21
		Sc. PD:	0.091
		Sc. rank:	3840.4

,

PA

Pos.	230	obs:	exp:
cgt	R	0	616.70
cgc	R	0	1181.00
cga	R	20	2212.00
cgg	R	0	1653.00
aaa	K	3	1.71
aag	K	0	1.29
aga	R	22512	11339.00
agg	R	54	5584.00
---	--	------	------
mPD		0.0068	0.91
		nPD:	0.01
		N. weight:	0.7
		Sc. PD:	-0.024
		Sc. rank:	-6092.3

,

PA

Pos.	231	obs:	exp:
tct	S	0	0.92
tcc	S	5	0.58
tca	S	0	1.30
tcg	S	0	0.27
act	T	0	3.53
acc	T	14	2.87
aca	T	0	6.40
acg	T	0	1.19
agt	S	0	0.94
agc	S	0	0.99
gtt	V	0	3.76
gtc	V	17	3.09
gta	V	0	4.57
gtg	V	0	5.58
gct	A	17	5971.00
gcc	A	22509	4668.00
gca	A	26	10592.00
gcg	A	0	1321.00
ggt	G	0	0.15
ggc	G	1	0.15
gga	G	0	0.44
ggg	G	0	0.26
---	--	------	------
mPD		0.0071	0.67
		nPD:	0.01
		N. weight:	1.6
		Sc. PD:	-0.052
		Sc. rank:	-6581.4

,

PA

Pos.	232	obs:	exp:
ttt	F	3	1.29
ttc	F	0	1.71
tat	Y	22528	11734.00
tac	Y	56	10850.00
cat	H	2	1.18
cac	H	0	0.82
---	--	------	------
mPD		0.0054	0.50
		nPD:	0.01
		N. weight:	0.67
		Sc. PD:	-0.021
		Sc. rank:	-2563.1

,

PA

Pos.	233	obs:	exp:
att	I	0	29.85
atc	I	0	20.47
ata	I	82	31.68
gtt	V	17	4976.00
gtc	V	223	4090.00
gta	V	22251	6053.00
gtg	V	16	7388.00
---	--	------	------
mPD		0.030	0.75
		nPD:	0.04
		N. weight:	1.3
		Sc. PD:	-0.0039
		Sc. rank:	13874.0

,

PA

Pos.	234	obs:	exp:
tat	Y	2	1.04
tac	Y	0	0.96
aat	N	1	0.59
aac	N	0	0.41
gat	D	22557	11901.00
gac	D	29	10685.00
---	--	------	------
mPD		0.0028	0.50
		nPD:	0.01
		N. weight:	0.66
		Sc. PD:	-0.024
		Sc. rank:	-7663.7

,

PA

Pos.	235	obs:	exp:
cgt	R	0	0.52
cgc	R	0	0.99
cga	R	0	1.86
cgg	R	0	1.39
aga	R	19	9.54
agg	R	0	4.70
gaa	E	2	1.21
gag	E	0	0.79
ggt	G	1	3422.00
ggc	G	3	3343.00
gga	G	22494	10039.00
ggg	G	70	5765.00
---	--	------	------
mPD		0.0084	0.69
		nPD:	0.01
		N. weight:	0.82
		Sc. PD:	-0.025
		Sc. rank:	-2168.1

,

PA

Pos.	236	obs:	exp:
ttt	F	140	9693.00
ttc	F	22448	12895.00
tat	Y	0	0.52
tac	Y	1	0.48
---	--	------	------
mPD		0.012	0.49
		nPD:	0.03
		N. weight:	0.55
		Sc. PD:	-0.0094
		Sc. rank:	3748.2

,

PA

Pos.	237	obs:	exp:
aaa	K	0	11.98
aag	K	21	9.02
gtt	V	1	0.66
gtc	V	0	0.55
gta	V	0	0.81
gtg	V	2	0.98
gct	A	0	0.26
gcc	A	0	0.21
gca	A	0	0.47
gcg	A	1	0.06
gat	D	6	3.16
gac	D	0	2.84
gaa	E	163	13657.00
gag	E	22392	8898.00
ggt	G	0	0.45
ggc	G	0	0.44
gga	G	0	1.33
ggg	G	3	0.77
---	--	------	------
mPD		0.017	0.48
		nPD:	0.04
		N. weight:	0.92
		Sc. PD:	-0.006
		Sc. rank:	9366.9

,

PA			
Pos.	238	obs:	exp:
tct	S	0	0.37
tcc	S	0	0.23
tca	S	1	0.52
tcg	S	1	0.11
cct	P	27	6222.00
ccc	P	6	4189.00
cca	P	359	9616.00
ccg	P	22192	2557.00
caa	Q	0	0.53
cag	Q	1	0.47
agt	S	0	0.38
agc	S	0	0.40
gct	A	0	0.53
gcc	A	0	0.41
gca	A	0	0.94
gcg	A	2	0.12
---	--	------	------
mPD		0.035	0.70
		nPD:	0.05
		N. weight:	2.2
		Sc. PD:	0.015
		Sc. rank:	26369.0

,

PA			
Pos.	239	obs:	exp:
tct	S	0	0.92
tcc	S	0	0.58
tca	S	0	1.30
tcg	S	0	0.27
att	I	0	2.18
atc	I	6	1.50
ata	I	0	2.32
act	T	0	1.01
acc	T	4	0.82
aca	T	0	1.83
acg	T	0	0.34
aat	N	339	13347.00
aac	N	22228	9220.00
aaa	K	7	3.99
aag	K	0	3.01
agt	S	0	0.94
agc	S	5	0.99
---	--	------	------
mPD		0.032	0.49
		nPD:	0.06
		N. weight:	0.85
		Sc. PD:	0.018
		Sc. rank:	11561.0

,

PA			
Pos.	240	obs:	exp:
tct	S	0	1.47
tcc	S	0	0.93
tca	S	0	2.08
tcg	S	0	0.43
cgt	R	0	0.03
cgc	R	0	0.05
cga	R	0	0.10
cgg	R	0	0.07
agt	S	0	1.50
agc	S	8	1.59
aga	R	1	0.50
agg	R	0	0.25
ggt	G	110	3424.00
ggc	G	21489	3344.00
gga	G	974	10044.00
ggg	G	7	5768.00
---	--	------	------
mPD		0.093	0.69
		nPD:	0.13
		N. weight:	1.7
		Sc. PD:	0.16
		Sc. rank:	27825.0

,

PA			
Pos.	241	obs:	exp:
ttt	F	0	4.29
ttc	F	10	5.71
tct	S	0	0.37
tcc	S	1	0.23
tca	S	0	0.52
tcg	S	0	0.11
tat	Y	0	48.84
tac	Y	94	45.16
tgt	C	132	10147.00
tgc	C	22349	12334.00
cgt	R	0	0.05
cgc	R	2	0.10
cga	R	0	0.20
cgg	R	0	0.15
agt	S	0	0.38
agc	S	1	0.40
aga	R	0	1.00
agg	R	0	0.49
---	--	------	------
mPD		0.021	0.50
		nPD:	0.04
		N. weight:	0.59
		Sc. PD:	-0.00057
		Sc. rank:	6423.1

,

PA			
Pos.	242	obs:	exp:
tta	L	0	0.11
ttg	L	0	0.19
ctt	L	1	0.20
ctc	L	0	0.14
cta	L	0	0.19
ctg	L	0	0.17
att	I	22535	8222.00
atc	I	12	5638.00
ata	I	38	8726.00
gtt	V	3	0.66
gtc	V	0	0.55
gta	V	0	0.81
gtg	V	0	0.98
---	--	------	------
mPD		0.0048	0.66
		nPD:	0.01
		N. weight:	1.
		Sc. PD:	-0.036
		Sc. rank:	-9601.5

,

PA			
Pos.	243	obs:	exp:
aaa	K	0	1.14
aag	K	2	0.86
gat	D	4	2.11
gac	D	0	1.89
gaa	E	64	13671.00
gag	E	22514	8907.00
ggt	G	0	0.76
ggc	G	0	0.74
gga	G	0	2.22
ggg	G	5	1.28
---	--	------	------
mPD		0.0066	0.48
		nPD:	0.01
		N. weight:	0.95
		Sc. PD:	-0.027
		Sc. rank:	-636.9

,

PA			
Pos.	244	obs:	exp:
gct	A	0	3.71
gcc	A	14	2.90
gca	A	0	6.58
gcg	A	0	0.82
ggt	G	1337	3423.00
ggc	G	21233	3344.00
gga	G	5	10042.00
ggg	G	0	5767.00
---	--	------	------
mPD		0.11	0.69
		nPD:	0.16
		N. weight:	1.8
		Sc. PD:	0.21
		Sc. rank:	29108.0

,

PA			
Pos.	245	obs:	exp:
caa	Q	0	0.53
cag	Q	1	0.47
aat	N	1	0.59
aac	N	0	0.41
aaa	K	159	12884.00
aag	K	22428	9703.00
---	--	------	------
mPD		0.014	0.49
		nPD:	0.03
		N. weight:	0.84
		Sc. PD:	-0.011
		Sc. rank:	6886.1

,

PA			
Pos.	246	obs:	exp:
tta	L	0	2483.00
ttg	L	0	4253.00
ctt	L	22543	4451.00
ctc	L	33	3191.00
cta	L	11	4349.00
ctg	L	2	3862.00
---	--	------	------
mPD		0.0041	1.1
		nPD:	0.
		N. weight:	1.7
		Sc. PD:	-0.065
		Sc. rank:	-25919.0

,

PA			
Pos.	247	obs:	exp:
tct	S	87	4157.00
tcc	S	22491	2616.00
tca	S	10	5879.00
tcg	S	0	1202.00
cct	P	0	0.28
ccc	P	1	0.19
cca	P	0	0.43
ccg	P	0	0.11
agt	S	0	4247.00
agc	S	0	4487.00
---	--	------	------
mPD		0.0086	1.6
		nPD:	0.01
		N. weight:	2.2
		Sc. PD:	-0.082
		Sc. rank:	-26942.0

,

PA			
Pos.	248	obs:	exp:
tta	L	0	0.11
ttg	L	0	0.19
ctt	L	0	0.20
ctc	L	0	0.14
cta	L	1	0.19
ctg	L	0	0.17
caa	Q	22579	11919.00
cag	Q	9	10669.00
---	--	------	------
mPD		0.00089	0.50
		nPD:	0.
		N. weight:	0.66
		Sc. PD:	-0.027
		Sc. rank:	-12090.0

,

PA			
Pos.	249	obs:	exp:
tta	L	0	0.88
ttg	L	5	1.51
ctt	L	0	1.58
ctc	L	0	1.13
cta	L	0	1.54
ctg	L	3	1.37
att	I	2	1.46
atc	I	0	1.00
ata	I	2	1.55
atg	M	22577	22577.00
---	--	------	------
mPD		0.0011	0.0015
		nPD:	0.7
		N. weight:	0.00054
		Sc. PD:	0.00035
		Sc. rank:	10.1

,

PA			
Pos.	250	obs:	exp:
tta	L	7	0.77
ttg	L	0	1.32
tct	S	1	4154.00
tcc	S	24	2614.00
tca	S	22456	5874.00
tcg	S	91	1201.00
ctt	L	0	1.38
ctc	L	0	0.99
cta	L	0	1.35
ctg	L	0	1.20
cct	P	0	1.38
ccc	P	0	0.93
cca	P	5	2.13
ccg	P	0	0.57
act	T	0	1.26
acc	T	0	1.03
aca	T	5	2.29
acg	T	0	0.43
agt	S	0	4244.00
agc	S	0	4484.00
---	--	------	------
mPD		0.012	1.6
		nPD:	0.01
		N. weight:	1.4
		Sc. PD:	-0.048
		Sc. rank:	-13150.0

,

PA			
Pos.	251	obs:	exp:
cgt	R	0	0.22
cgc	R	0	0.42
cga	R	0	0.78
cgg	R	0	0.59
aaa	K	22485	12881.00
aag	K	96	9700.00
aga	R	8	4.02
agg	R	0	1.98
---	--	------	------
mPD		0.0092	0.49
		nPD:	0.02
		N. weight:	0.56
		Sc. PD:	-0.013
		Sc. rank:	1530.9

,

PA			
Pos.	252	obs:	exp:
aaa	K	213	121.50
aag	K	0	91.50
gtt	V	0	0.88
gtc	V	0	0.73
gta	V	4	1.08
gtg	V	0	1.31
gat	D	0	2.11
gac	D	4	1.89
gaa	E	22301	13528.00
gag	E	41	8814.00
ggt	G	0	3.94
ggc	G	0	3.85
gga	G	26	11.57
ggg	G	0	6.64
---	--	------	------
mPD		0.025	0.50
		nPD:	0.05
		N. weight:	0.51
		Sc. PD:	0.0039
		Sc. rank:	6334.5

,

PA			
Pos.	253	obs:	exp:
gtt	V	12	4994.00
gtc	V	2	4105.00
gta	V	204	6075.00
gtg	V	22371	7415.00
---	--	------	------
mPD		0.019	0.74
		nPD:	0.03
		N. weight:	1.1
		Sc. PD:	-0.018
		Sc. rank:	7835.5

,

PA

Pos.	254	obs:	exp:
tct	S	0	2.58
tcc	S	0	1.62
tca	S	0	3.64
tcg	S	0	0.75
act	T	0	0.50
acc	T	2	0.41
aca	T	0	0.91
acg	T	0	0.17
aat	N	190	13343.00
aac	N	22370	9217.00
aaa	K	4	2.28
aag	K	0	1.72
agt	S	3	2.63
agc	S	11	2.78
gat	D	0	4.74
gac	D	9	4.26
---	--	------	------
mPD		0.020	0.49
		nPD:	0.04
		N. weight:	0.88
		Sc. PD:	-0.0024
		Sc. rank:	9435.9

,

PA

Pos.	255	obs:	exp:
tct	S	0	0.37
tcc	S	2	0.23
tca	S	0	0.52
tcg	S	0	0.11
cct	P	0	0.28
ccc	P	1	0.19
cca	P	0	0.43
ccg	P	0	0.11
agt	S	0	0.38
agc	S	0	0.40
gtt	V	0	0.22
gtc	V	1	0.18
gta	V	0	0.27
gtg	V	0	0.33
gct	A	14	5979.00
gcc	A	22563	4675.00
gca	A	8	10607.00
gcg	A	0	1323.00
---	--	------	------
mPD		0.0023	0.66
		nPD:	0.
		N. weight:	1.6
		Sc. PD:	-0.063
		Sc. rank:	-25501.0

,

PA

Pos.	256	obs:	exp:
caa	Q	412	217.40
cag	Q	0	194.60
cgt	R	0	0.87
cgc	R	0	1.67
cga	R	1	3.13
cgg	R	0	2.34
att	I	0	0.36
atc	I	0	0.25
ata	I	1	0.39
act	T	0	0.25
acc	T	0	0.21
aca	T	1	0.46
acg	T	0	0.09
aat	N	0	1.77
aac	N	3	1.23
aaa	K	22067	12627.00
aag	K	69	9509.00
aga	R	31	16.07
agg	R	0	7.91
gaa	E	4	2.42
gag	E	0	1.58
---	--	------	------
mPD		0.046	0.53
		nPD:	0.09
		N. weight:	0.57
		Sc. PD:	0.024
		Sc. rank:	8431.3

,

PA

Pos.	257	obs:	exp:
ttt	F	3	1.29
ttc	F	0	1.71
att	I	22551	8219.00
atc	I	24	5636.00
ata	I	3	8723.00
act	T	1	0.25
acc	T	0	0.21
aca	T	0	0.46
acg	T	0	0.09
gtt	V	7	1.55
gtc	V	0	1.27
gta	V	0	1.88
gtg	V	0	2.30
---	--	------	------
mPD		0.0034	0.66
		nPD:	0.01
		N. weight:	1.
		Sc. PD:	-0.039
		Sc. rank:	-13084.0

,

PA

Pos.	258	obs:	exp:
aaa	K	7	3.99
aag	K	0	3.01
gtt	V	0	0.22
gtc	V	0	0.18
gta	V	1	0.27
gtg	V	0	0.33
gct	A	0	0.26
gcc	A	0	0.21
gca	A	1	0.47
gcg	A	0	0.06
gat	D	1	0.53
gac	D	0	0.47
gaa	E	22532	13671.00
gag	E	46	8907.00
ggt	G	0	0.15
ggc	G	0	0.15
gga	G	1	0.44
ggg	G	0	0.26
---	--	------	------
mPD		0.0050	0.48
		nPD:	0.01
		N. weight:	0.51
		Sc. PD:	-0.016
		Sc. rank:	-2140.8

,

PA

Pos.	259	obs:	exp:
tta	L	0	0.44
ttg	L	0	0.75
tct	S	0	2.94
tcc	S	0	1.85
tca	S	16	4.16
tcg	S	0	0.85
ctt	L	0	0.79
ctc	L	0	0.56
cta	L	4	0.77
ctg	L	0	0.68
cct	P	10	6214.00
ccc	P	1	4184.00
cca	P	22502	9604.00
ccg	P	43	2554.00
caa	Q	7	3.69
cag	Q	0	3.31
act	T	0	1.51
acc	T	0	1.23
aca	T	6	2.74
acg	T	0	0.51
agt	S	0	3.01
agc	S	0	3.18
---	--	------	------
mPD		0.0077	0.70
		nPD:	0.01
		N. weight:	0.88
		Sc. PD:	-0.027
		Sc. rank:	-3110.9

,

PA

Pos.	260	obs:	exp:
ttt	F	36	9692.00
ttc	F	22548	12892.00
tta	L	2	0.22
ttg	L	0	0.38
tct	S	0	0.37
tcc	S	2	0.23
tca	S	0	0.52
tcg	S	0	0.11
ctt	L	0	0.39
ctc	L	0	0.28
cta	L	0	0.39
ctg	L	0	0.34
agt	S	0	0.38
agc	S	0	0.40
gtt	V	0	0.22
gtc	V	1	0.18
gta	V	0	0.27
gtg	V	0	0.33
---	--	------	------
mPD		0.0036	0.49
		nPD:	0.01
		N. weight:	0.57
		Sc. PD:	-0.02
		Sc. rank:	-5149.5

,

PA

Pos.	261	obs:	exp:
ttt	F	8	3.43
ttc	F	0	4.57
tta	L	27	2478.00
ttg	L	22396	4244.00
tct	S	0	1.47
tcc	S	0	0.93
tca	S	0	2.08
tcg	S	8	0.43
ctt	L	0	4442.00
ctc	L	0	3184.00
cta	L	0	4340.00
ctg	L	118	3854.00
atg	M	31	31.00
agt	S	0	1.50
agc	S	0	1.59
gtt	V	0	0.22
gtc	V	0	0.18
gta	V	0	0.27
gtg	V	1	0.33
---	--	------	------
mPD		0.017	1.1
		nPD:	0.01
		N. weight:	1.7
		Sc. PD:	-0.047
		Sc. rank:	-67.9

,

PA

Pos.	262	obs:	exp:
tct	S	0	0.37
tcc	S	0	0.23
tca	S	0	0.52
tcg	S	0	0.11
tgg	W	2	2.00
cgt	R	0	614.30
cgc	R	0	1177.00
cga	R	0	2204.00
cgg	R	2	1647.00
atg	M	3	3.00
aaa	K	3	45.63
aag	K	77	34.37
agt	S	2	0.38
agc	S	0	0.40
aga	R	95	11296.00
agg	R	22403	5562.00
ggt	G	0	0.30
ggc	G	0	0.30
gga	G	0	0.89
ggg	G	2	0.51
---	--	------	------
mPD		0.017	0.92
		nPD:	0.02
		N. weight:	1.4
		Sc. PD:	-0.035
		Sc. rank:	3509.0

,

PA

Pos.	263	obs:	exp:
tct	S	0	2.76
tcc	S	0	1.74
tca	S	0	3.90
tcg	S	15	0.80
atg	M	41	41.00
act	T	30	5661.00
acc	T	0	4606.00
aca	T	375	10273.00
acg	T	22051	1916.00
aat	N	1	0.59
aac	N	0	0.41
aaa	K	1	9.13
aag	K	15	6.87
agt	S	0	2.82
agc	S	0	2.98
gtt	V	0	1.33
gtc	V	0	1.09
gta	V	0	1.61
gtg	V	6	1.97
gct	A	0	14.30
gcc	A	0	11.18
gca	A	0	25.36
gcg	A	54	3.16
---	--	------	------
mPD		0.048	0.69
		nPD:	0.07
		N. weight:	2.4
		Sc. PD:	0.063
		Sc. rank:	33985.0

,

PA

Pos.	264	obs:	exp:
tct	S	0	0.92
tcc	S	0	0.58
tca	S	5	1.30
tcg	S	0	0.27
cct	P	0	0.28
ccc	P	0	0.19
cca	P	1	0.43
ccg	P	0	0.11
att	I	0	0.73
atc	I	0	0.50
ata	I	2	0.77
act	T	1	5692.00
acc	T	3	4631.00
aca	T	22569	10330.00
acg	T	7	1927.00
agt	S	0	0.94
agc	S	0	0.99
gct	A	0	0.26
gcc	A	0	0.21
gca	A	1	0.47
gcg	A	0	0.06
---	--	------	------
mPD		0.0018	0.68
		nPD:	0.
		N. weight:	0.81
		Sc. PD:	-0.032
		Sc. rank:	-13980.0

,

PA

Pos.	265	obs:	exp:
tta	L	0	0.22
ttg	L	0	0.38
tct	S	0	0.37
tcc	S	0	0.23
tca	S	2	0.52
tcg	S	0	0.11
ctt	L	0	0.39
ctc	L	0	0.28
cta	L	2	0.39
ctg	L	0	0.34
cct	P	2	6222.00
ccc	P	75	4189.00
cca	P	22486	9616.00
ccg	P	21	2557.00
act	T	0	0.25
acc	T	0	0.21
aca	T	1	0.46
acg	T	0	0.09
agt	S	0	0.38
agc	S	0	0.40
---	--	------	------
mPD		0.0091	0.70
		nPD:	0.01
		N. weight:	0.86
		Sc. PD:	-0.025
		Sc. rank:	-1344.2

,

PA

Pos.	266	obs:	exp:
tta	L	0	0.66
ttg	L	0	1.13
tct	S	0	0.37
tcc	S	0	0.23
tca	S	0	0.52
tcg	S	0	0.11
tgt	C	0	4.96
tgc	C	11	6.04
ctt	L	0	1.18
ctc	L	6	0.85
cta	L	0	1.16
ctg	L	0	1.03
cat	H	0	95.86
cac	H	163	67.14
cgt	R	296	611.80
cgc	R	22080	1172.00
cga	R	11	2195.00
cgg	R	15	1640.00
agt	S	0	0.38
agc	S	2	0.40
aga	R	5	11249.00
agg	R	0	5539.00
---	--	------	------
mPD		0.045	0.94
		nPD:	0.05
		N. weight:	3.
		Sc. PD:	0.015
		Sc. rank:	35669.0

,

PA

Pos.	267	obs:	exp:
tta	L	0	1.21
ttg	L	0	2.07
tct	S	1	8.83
tcc	S	47	5.56
tca	S	0	12.49
tcg	S	0	2.55
ctt	L	0	2.17
ctc	L	11	1.55
cta	L	0	2.12
ctg	L	0	1.88
cct	P	2623	6204.00
ccc	P	19879	4177.00
cca	P	18	9589.00
ccg	P	0	2550.00
cat	H	1	0.59
cac	H	0	0.41
act	T	8	2.27
acc	T	1	1.85
aca	T	0	4.12
acg	T	0	0.77
agt	S	0	9.03
agc	S	0	9.53
---	--	------	------
mPD		0.21	0.70
		nPD:	0.3
		N. weight:	1.3
		Sc. PD:	0.34
		Sc. rank:	23386.0

,

PA

Pos.	268	obs:	exp:
ttt	F	0	31.76
ttc	F	74	42.24
tta	L	0	2472.00
ttg	L	0	4234.00
ctt	L	260	4432.00
ctc	L	22082	3177.00
cta	L	148	4330.00
ctg	L	0	3845.00
att	I	0	9.10
atc	I	25	6.24
ata	I	0	9.66
---	--	------	------
mPD		0.044	1.1
		nPD:	0.04
		N. weight:	1.9
		Sc. PD:	-0.0077
		Sc. rank:	20359.0

,

PA

Pos.	269	obs:	exp:
tct	S	0	0.18
tcc	S	0	0.12
tca	S	0	0.26
tcg	S	0	0.05
cgt	R	0	610.50
cgc	R	0	1170.00
cga	R	0	2190.00
cgg	R	0	1636.00
att	I	0	16.02
atc	I	0	10.98
ata	I	44	17.00
aaa	K	180	102.70
aag	K	0	77.32
agt	S	0	0.19
agc	S	1	0.20
aga	R	22243	11226.00
agg	R	118	5528.00
ggt	G	0	0.45
ggc	G	0	0.44
gga	G	3	1.33
ggg	G	0	0.77
---	--	------	------
mPD		0.030	0.93
		nPD:	0.03
		N. weight:	0.69
		Sc. PD:	-0.0068
		Sc. rank:	6550.0

,

PA

Pos.	270	obs:	exp:
ttt	F	4	3.43
ttc	F	4	4.57
tta	L	18969	2480.00
ttg	L	3516	4248.00
tct	S	0	3.50
tcc	S	0	2.20
tca	S	19	4.94
tcg	S	0	1.01
ctt	L	5	4446.00
ctc	L	0	3187.00
cta	L	35	4343.00
ctg	L	36	3857.00
att	I	0	0.36
atc	I	0	0.25
ata	I	1	0.39
agt	S	0	3.57
agc	S	0	3.77
---	--	------	------
mPD		0.27	1.1
		nPD:	0.24
		N. weight:	1.7
		Sc. PD:	0.34
		Sc. rank:	29701.0

,

PA

Pos.	271	obs:	exp:
tta	L	0	1.32
ttg	L	0	2.26
tct	S	8	1.47
tcc	S	0	0.93
tca	S	0	2.08
tcg	S	0	0.43
ctt	L	12	2.36
ctc	L	0	1.69
cta	L	0	2.31
ctg	L	0	2.05
cct	P	22456	6215.00
ccc	P	93	4184.00
cca	P	7	9605.00
ccg	P	2	2554.00
cat	H	6	3.53
cac	H	0	2.47
act	T	5	1.26
acc	T	0	1.03
aca	T	0	2.29
acg	T	0	0.43
agt	S	0	1.50
agc	S	0	1.59
---	--	------	------
mPD		0.012	0.70
		nPD:	0.02
		N. weight:	1.3
		Sc. PD:	-0.034
		Sc. rank:	1524.1

,

PA

Pos.	272	obs:	exp:
tct	S	0	1.29
tcc	S	0	0.81
tca	S	0	1.82
tcg	S	0	0.37
aat	N	28	16.56
aac	N	0	11.44
agt	S	7	1.32
agc	S	0	1.39
gtt	V	3	0.66
gtc	V	0	0.55
gta	V	0	0.81
gtg	V	0	0.98
gat	D	22487	11859.00
gac	D	21	10649.00
gaa	E	4	3.03
gag	E	1	1.97
ggt	G	38	5.76
ggc	G	0	5.63
gga	G	0	16.90
ggg	G	0	9.71
---	--	------	------
mPD		0.0096	0.51
		nPD:	0.02
		N. weight:	0.66
		Sc. PD:	-0.016
		Sc. rank:	1995.5

,

PA

Pos.	273	obs:	exp:
cgt	R	0	0.05
cgc	R	0	0.10
cga	R	0	0.20
cgg	R	0	0.15
aga	R	2	1.00
agg	R	0	0.49
gaa	E	2	1.21
gag	E	0	0.79
ggt	G	55	3425.00
ggc	G	8	3345.00
gga	G	19240	10046.00
ggg	G	3282	5769.00
---	--	------	------
mPD		0.25	0.69
		nPD:	0.37
		N. weight:	0.48
		Sc. PD:	0.15
		Sc. rank:	8577.3

,

PA

Pos.	274	obs:	exp:
tta	L	0	1.21
ttg	L	0	2.07
tct	S	27	4.97
tcc	S	0	3.13
tca	S	0	7.03
tcg	S	0	1.44
ctt	L	11	2.17
ctc	L	0	1.55
cta	L	0	2.12
ctg	L	0	1.88
cct	P	22450	6209.00
ccc	P	81	4180.00
cca	P	2	9596.00
ccg	P	3	2552.00
cat	H	7	4.12
cac	H	0	2.88
act	T	7	1.76
acc	T	0	1.44
aca	T	0	3.20
acg	T	0	0.60
agt	S	0	5.08
agc	S	0	5.36
gct	A	1	0.26
gcc	A	0	0.21
gca	A	0	0.47
gcg	A	0	0.06
---	--	------	------
mPD		0.012	0.70
		nPD:	0.02
		N. weight:	1.3
		Sc. PD:	-0.033
		Sc. rank:	2553.0

,

PA

Pos.	275	obs:	exp:
ttt	F	175	75.10
ttc	F	0	99.90
tta	L	0	2458.00
ttg	L	0	4209.00
ctt	L	22337	4405.00
ctc	L	19	3158.00
cta	L	0	4304.00
ctg	L	0	3822.00
cct	P	36	9.92
ccc	P	0	6.68
cca	P	0	15.33
ccg	P	0	4.08
cat	H	1	0.59
cac	H	0	0.41
cgt	R	2	0.05
cgc	R	0	0.10
cga	R	0	0.20
cgg	R	0	0.15
att	I	18	6.55
atc	I	0	4.49
ata	I	0	6.95
aga	R	0	1.00
agg	R	0	0.49
gtt	V	1	0.22
gtc	V	0	0.18
gta	V	0	0.27
gtg	V	0	0.33
---	--	------	------
mPD		0.022	1.1
		nPD:	0.02
		N. weight:	1.7
		Sc. PD:	-0.039
		Sc. rank:	5309.1

,

PA			
Pos.	276	obs:	exp:
tct	S	0	0.37
tcc	S	0	0.23
tca	S	0	0.52
tcg	S	0	0.11
tgt	C	101	10194.00
tgc	C	22484	12391.00
agt	S	0	0.38
agc	S	2	0.40
ggt	G	0	0.30
ggc	G	2	0.30
gga	G	0	0.89
ggg	G	0	0.51
mPD		0.0093	0.50
		nPD:	0.02
		N. weight:	0.6
		Sc. PD:	-0.014
		Sc. rank:	1620.9

,

PA			
Pos.	277	obs:	exp:
tta	L	0	0.22
ttg	L	0	0.38
tct	S	1	0.74
tcc	S	3	0.46
tca	S	0	1.04
tcg	S	0	0.21
tat	Y	41	21.82
tac	Y	1	20.18
ctt	L	2	0.39
ctc	L	0	0.28
cta	L	0	0.39
ctg	L	0	0.34
cct	P	5	1.38
ccc	P	0	0.93
cca	P	0	2.13
ccg	P	0	0.57
cat	H	22340	13240.00
cac	H	174	9274.00
caa	Q	1	1.58
cag	Q	2	1.42
cgt	R	10	0.27
cgc	R	0	0.52
cga	R	0	0.98
cgg	R	0	0.73
aat	N	9	5.32
aac	N	0	3.68
agt	S	0	0.75
agc	S	0	0.79
aga	R	0	5.02
agg	R	0	2.47
mPD		0.023	0.49
		nPD:	0.05
		N. weight:	0.51
		Sc. PD:	0.0015
		Sc. rank:	5871.7

,

PA			
Pos.	278	obs:	exp:
tta	L	0	0.11
ttg	L	0	0.19
ctt	L	0	0.20
ctc	L	0	0.14
cta	L	0	0.19
ctg	L	1	0.17
caa	Q	311	11915.00
cag	Q	22270	10666.00
cgt	R	0	0.05
cgc	R	0	0.10
cga	R	1	0.20
cgg	R	1	0.15
aaa	K	0	2.85
aag	K	5	2.15
aga	R	0	1.00
agg	R	0	0.49
mPD		0.028	0.50
		nPD:	0.06
		N. weight:	0.71
		Sc. PD:	0.0091
		Sc. rank:	8906.7

,

PA			
Pos.	279	obs:	exp:
tta	L	0	0.11
ttg	L	0	0.19
tgg	W	1	1.00
ctt	L	0	0.20
ctc	L	0	0.14
cta	L	0	0.19
ctg	L	1	0.17
caa	Q	0	2.64
cag	Q	5	2.36
cgt	R	2	616.60
cgc	R	1	1181.00
cga	R	140	2212.00
cgg	R	21704	1653.00
aaa	K	0	0.57
aag	K	1	0.43
aga	R	3	11336.00
agg	R	731	5582.00
mPD		0.077	0.91
		nPD:	0.08
		N. weight:	2.5
		Sc. PD:	0.1
		Sc. rank:	37022.0

,

PA			
Pos.	280	obs:	exp:
tct	S	1	4155.00
tcc	S	6	2615.00
tca	S	22545	5876.00
tcg	S	27	1202.00
cct	P	0	0.28
ccc	P	0	0.19
cca	P	1	0.43
ccg	P	0	0.11
act	T	0	2.02
acc	T	0	1.64
aca	T	8	3.66
acg	T	0	0.68
agt	S	0	4246.00
agc	S	0	4485.00
gct	A	0	0.26
gcc	A	0	0.21
gca	A	1	0.47
gcg	A	0	0.06
mPD		0.0039	1.6
		nPD:	0.
		N. weight:	1.4
		Sc. PD:	-0.056
		Sc. rank:	-24399.0

,

PA			
Pos.	281	obs:	exp:
cgt	R	0	0.03
cgc	R	0	0.05
cga	R	0	0.10
cgg	R	0	0.07
aaa	K	120	12885.00
aag	K	22468	9703.00
aga	R	0	0.50
agg	R	1	0.25
mPD		0.011	0.49
		nPD:	0.02
		N. weight:	0.85
		Sc. PD:	-0.018
		Sc. rank:	4146.8

,

PA			
Pos.	282	obs:	exp:
ttt	F	231	9689.00
ttc	F	22347	12889.00
tta	L	0	0.22
ttg	L	0	0.38
tct	S	0	1.10
tcc	S	6	0.69
tca	S	0	1.56
tcg	S	0	0.32
tat	Y	0	1.56
tac	Y	3	1.44
ctt	L	1	0.39
ctc	L	1	0.28
cta	L	0	0.39
ctg	L	0	0.34
agt	S	0	1.13
agc	S	0	1.19
mPD		0.021	0.49
		nPD:	0.04
		N. weight:	0.53
		Sc. PD:	0.00028
		Sc. rank:	5982.2

,

PA			
Pos.	283	obs:	exp:
tta	L	1	2483.00
ttg	L	46	4252.00
ctt	L	16	4451.00
ctc	L	6	3190.00
cta	L	102	4348.00
ctg	L	22416	3862.00
atg	M	1	1.00
gtt	V	0	0.22
gtc	V	0	0.18
gta	V	0	0.27
gtg	V	1	0.33
mPD		0.015	1.1
		nPD:	0.01
		N. weight:	1.8
		Sc. PD:	-0.052
		Sc. rank:	-1810.8

,

PA			
Pos.	284	obs:	exp:
tta	L	0	2472.00
ttg	L	37	4234.00
ctt	L	22	4432.00
ctc	L	0	3177.00
cta	L	38	4330.00
ctg	L	22393	3845.00
atg	M	99	99.00
mPD		0.017	1.1
		nPD:	0.02
		N. weight:	1.8
		Sc. PD:	-0.049
		Sc. rank:	560.1

,

PA			
Pos.	285	obs:	exp:
att	I	0	0.36
atc	I	0	0.25
ata	I	1	0.39
atg	M	22587	22587.00
act	T	0	0.25
acc	T	0	0.21
aca	T	0	0.46
acg	T	1	0.09
mPD		0.00018	0.00026
		nPD:	0.69
		N. weight:	0.00016
		Sc. PD:	0.0001
		Sc. rank:	2.9

,

PA			
Pos.	286	obs:	exp:
aat	N	13	7.69
aac	N	0	5.31
gat	D	22558	11894.00
gac	D	15	10679.00
ggt	G	3	0.45
ggc	G	0	0.44
gga	G	0	1.33
ggg	G	0	0.77
mPD		0.0027	0.50
		nPD:	0.01
		N. weight:	0.66
		Sc. PD:	-0.024
		Sc. rank:	-7833.0

,

PA			
Pos.	287	obs:	exp:
tct	S	11	2.02
tcc	S	0	1.27
tca	S	0	2.86
tcg	S	0	0.59
act	T	6	1.51
acc	T	0	1.23
aca	T	0	2.74
acg	T	0	0.51
agt	S	0	2.07
agc	S	0	2.18
gct	A	22463	5976.00
gcc	A	104	4672.00
gca	A	3	10601.00
gcg	A	1	1322.00
ggt	G	1	0.15
ggc	G	0	0.15
gga	G	0	0.44
ggg	G	0	0.26
mPD		0.011	0.67
		nPD:	0.02
		N. weight:	1.4
		Sc. PD:	-0.035
		Sc. rank:	1477.6

,

PA			
Pos.	288	obs:	exp:
tta	L	1	2483.00
ttg	L	57	4252.00
tct	S	0	0.18
tcc	S	0	0.12
tca	S	0	0.26
tcg	S	1	0.05
ctt	L	4	4451.00
ctc	L	0	3190.00
cta	L	157	4348.00
ctg	L	22367	3862.00
atg	M	2	2.00
agt	S	0	0.19
agc	S	0	0.20
---	--	------	------
mPD		0.020	1.1
		nPD:	0.02
		N. weight:	1.8
		Sc. PD:	-0.045
		Sc. rank:	3154.7

,

PA			
Pos.	289	obs:	exp:
aaa	K	22550	12885.00
aag	K	39	9704.00
---	--	------	------
mPD		0.0034	0.49
		nPD:	0.01
		N. weight:	0.57
		Sc. PD:	-0.02
		Sc. rank:	-5665.1

,

PA			
Pos.	290	obs:	exp:
tta	L	22229	2483.00
ttg	L	165	4253.00
ctt	L	3	4451.00
ctc	L	0	3191.00
cta	L	192	4349.00
ctg	L	0	3862.00
---	--	------	------
mPD		0.032	1.1
		nPD:	0.03
		N. weight:	2.2
		Sc. PD:	-0.032
		Sc. rank:	17561.0

,

PA			
Pos.	291	obs:	exp:
tct	S	0	4138.00
tcc	S	0	2604.00
tca	S	0	5852.00
tcg	S	0	1197.00
cgt	R	0	0.14
cgc	R	1	0.26
cga	R	0	0.49
cgg	R	0	0.37
att	I	0	1.82
atc	I	5	1.25
ata	I	0	1.93
act	T	4	1.01
acc	T	0	0.82
aca	T	0	1.83
acg	T	0	0.34
aat	N	1	27.80
aac	N	46	19.20
agt	S	2951	4228.00
agc	S	19533	4466.00
aga	R	4	2.51
agg	R	0	1.24
ggt	G	0	6.67
ggc	G	44	6.52
gga	G	0	19.57
ggg	G	0	11.24
---	--	------	------
mPD		0.24	1.6
		nPD:	0.14
		N. weight:	1.3
		Sc. PD:	0.13
		Sc. rank:	21066.0

,

PA			
Pos.	292	obs:	exp:
tta	L	0	0.22
ttg	L	0	0.38
ctt	L	2	0.39
ctc	L	0	0.28
cta	L	0	0.39
ctg	L	0	0.34
att	I	22543	8221.00
atc	I	37	5637.00
ata	I	3	8725.00
gtt	V	4	0.88
gtc	V	0	0.73
gta	V	0	1.08
gtg	V	0	1.31
---	--	------	------
mPD		0.0041	0.66
		nPD:	0.01
		N. weight:	1.
		Sc. PD:	-0.038
		Sc. rank:	-11558.0

,

PA			
Pos.	293	obs:	exp:
gat	D	1	0.53
gac	D	0	0.47
gaa	E	22562	13676.00
gag	E	25	8911.00
ggt	G	0	0.15
ggc	G	0	0.15
gga	G	1	0.44
ggg	G	0	0.26
---	--	------	------
mPD		0.0024	0.48
		nPD:	0.
		N. weight:	0.51
		Sc. PD:	-0.019
		Sc. rank:	-6674.7

,

PA			
Pos.	294	obs:	exp:
aat	N	0	18.33
aac	N	31	12.67
gat	D	58	11858.00
gac	D	22447	10647.00
gaa	E	52	32.09
gag	E	1	20.91
---	--	------	------
mPD		0.013	0.50
		nPD:	0.02
		N. weight:	0.76
		Sc. PD:	-0.013
		Sc. rank:	5010.3

,

PA			
Pos.	295	obs:	exp:
tta	L	0	0.11
ttg	L	0	0.19
ctt	L	0	0.20
ctc	L	0	0.14
cta	L	0	0.19
ctg	L	1	0.17
cct	P	15	6223.00
ccc	P	3	4189.00
cca	P	135	9617.00
ccg	P	22434	2558.00
---	-	1	1.00
---	--	------	------
mPD		0.014	0.70
		nPD:	0.02
		N. weight:	2.2
		Sc. PD:	-0.051
		Sc. rank:	7399.1

,

PA			
Pos.	296	obs:	exp:
tct	S	0	4132.00
tcc	S	0	2601.00
tca	S	0	5844.00
tcg	S	0	1195.00
cgt	R	2	0.08
cgc	R	0	0.16
cga	R	0	0.29
cgg	R	0	0.22
act	T	1	0.25
acc	T	0	0.21
aca	T	0	0.46
acg	T	0	0.09
aat	N	118	69.79
aac	N	0	48.21
agt	S	22211	4222.00
agc	S	244	4460.00
aga	R	1	1.51
agg	R	0	0.74
ggt	G	12	1.82
ggc	G	0	1.78
gga	G	0	5.34
ggg	G	0	3.07
---	--	------	------
mPD		0.033	1.6
		nPD:	0.02
		N. weight:	1.7
		Sc. PD:	-0.037
		Sc. rank:	6405.5

,

PA			
Pos.	297	obs:	exp:
tat	Y	0	11.43
tac	Y	22	10.57
cat	H	200	13271.00
cac	H	22367	9296.00
---	--	------	------
mPD		0.019	0.49
		nPD:	0.04
		N. weight:	0.87
		Sc. PD:	-0.0024
		Sc. rank:	9356.9

,

PA			
Pos.	298	obs:	exp:
aaa	K	0	9.13
aag	K	16	6.87
gat	D	5	2.63
gac	D	0	2.37
gaa	E	128	13665.00
gag	E	22440	8903.00
---	--	------	------
mPD		0.013	0.48
		nPD:	0.03
		N. weight:	0.93
		Sc. PD:	-0.014
		Sc. rank:	7156.2

,

PA			
Pos.	299	obs:	exp:
cgt	R	0	0.05
cgc	R	0	0.10
cga	R	0	0.20
cgg	R	0	0.15
aga	R	0	1.00
agg	R	2	0.49
gaa	E	0	0.61
gag	E	1	0.39
ggt	G	10	3425.00
ggc	G	1	3345.00
gga	G	176	10047.00
ggg	G	22399	5769.00
---	--	------	------
mPD		0.017	0.69
		nPD:	0.02
		N. weight:	1.4
		Sc. PD:	-0.025
		Sc. rank:	8562.9

,

PA			
Pos.	300	obs:	exp:
aaa	K	3	1.71
aag	K	0	1.29
gaa	E	19122	13675.00
gag	E	3463	8910.00
ggt	G	0	0.15
ggc	G	0	0.15
gga	G	1	0.44
ggg	G	0	0.26
---	--	------	------
mPD		0.26	0.48
		nPD:	0.54
		N. weight:	0.15
		Sc. PD:	0.072
		Sc. rank:	2689.1

,

PA

Pos.	301	obs:	exp:
gaa	E	1	0.61
gag	E	0	0.39
ggt	G	4	3425.00
ggc	G	0	3346.00
gga	G	22484	10048.00
ggg	G	100	5770.00
---	--	------	------
mPD		0.0093	0.69
		nPD:	0.01
		N. weight:	0.82
		Sc. PD:	-0.024
		Sc. rank:	-942.7

,

PA

Pos.	302	obs:	exp:
att	I	1	8221.00
atc	I	14	5637.00
ata	I	22568	8725.00
gtt	V	0	1.33
gtc	V	0	1.09
gta	V	6	1.61
gtg	V	0	1.97
---	--	------	------
mPD		0.0019	0.66
		nPD:	0.
		N. weight:	0.99
		Sc. PD:	-0.039
		Sc. rank:	-16397.0

,

PA

Pos.	303	obs:	exp:
cct	P	2	6223.00
ccc	P	1	4190.00
cca	P	22534	9618.00
ccg	P	52	2558.00
---	--	------	------
mPD		0.0049	0.70
		nPD:	0.01
		N. weight:	0.88
		Sc. PD:	-0.031
		Sc. rank:	-8710.7

,

PA

Pos.	304	obs:	exp:
tta	L	168	2482.00
ttg	L	0	4250.00
ctt	L	1	4448.00
ctc	L	1	3189.00
cta	L	22353	4346.00
ctg	L	51	3860.00
att	I	0	5.46
atc	I	0	3.74
ata	I	15	5.80
---	--	------	------
mPD		0.021	1.1
		nPD:	0.02
		N. weight:	1.7
		Sc. PD:	-0.04
		Sc. rank:	4237.5

,

PA

Pos.	305	obs:	exp:
ttt	F	3	1.29
ttc	F	0	1.71
tat	Y	22511	11716.00
tac	Y	38	10833.00
tgt	C	21	9.48
tgc	C	0	11.52
cat	H	16	9.41
cac	H	0	6.59
---	--	------	------
mPD		0.0069	0.50
		nPD:	0.01
		N. weight:	0.67
		Sc. PD:	-0.019
		Sc. rank:	-510.1

,

PA

Pos.	306	obs:	exp:
gat	D	22578	11902.00
gac	D	11	10687.00
---	--	------	------
mPD		0.00097	0.50
		nPD:	0.
		N. weight:	0.66
		Sc. PD:	-0.027
		Sc. rank:	-12024.0

,

PA

Pos.	307	obs:	exp:
tct	S	0	0.74
tcc	S	0	0.46
tca	S	4	1.04
tcg	S	0	0.21
act	T	0	0.25
acc	T	0	0.21
aca	T	1	0.46
acg	T	0	0.09
agt	S	0	0.75
agc	S	0	0.79
gct	A	1	5979.00
gcc	A	11	4675.00
gca	A	22566	10607.00
gcg	A	6	1323.00
---	--	------	------
mPD		0.0020	0.66
		nPD:	0.
		N. weight:	0.78
		Sc. PD:	-0.031
		Sc. rank:	-12885.0

,

PA

Pos.	308	obs:	exp:
tta	L	0	0.33
ttg	L	0	0.56
ctt	L	0	0.59
ctc	L	3	0.42
cta	L	0	0.58
ctg	L	0	0.51
att	I	93	8202.00
atc	I	22410	5624.00
ata	I	28	8705.00
act	T	0	3.03
acc	T	12	2.46
aca	T	0	5.49
acg	T	0	1.02
aat	N	0	1.18
aac	N	2	0.82
gtt	V	1	9.06
gtc	V	40	7.45
gta	V	0	11.03
gtg	V	0	13.46
---	--	------	------
mPD		0.016	0.66
		nPD:	0.02
		N. weight:	1.4
		Sc. PD:	-0.026
		Sc. rank:	8462.7

,

PA

Pos.	309	obs:	exp:
cgt	R	0	0.35
cgc	R	0	0.68
cga	R	0	1.27
cgg	R	0	0.95
aat	N	0	2.37
aac	N	4	1.63
aaa	K	22540	12876.00
aag	K	32	9696.00
aga	R	13	6.53
agg	R	0	3.21
---	--	------	------
mPD		0.0043	0.49
		nPD:	0.01
		N. weight:	0.58
		Sc. PD:	-0.019
		Sc. rank:	-3775.9

,

PA

Pos.	310	obs:	exp:
tct	S	0	0.37
tcc	S	1	0.23
tca	S	0	0.52
tcg	S	0	0.11
tgt	C	32	10195.00
tgc	C	22555	12392.00
agt	S	0	0.38
agc	S	1	0.40
---	--	------	------
mPD		0.0030	0.50
		nPD:	0.01
		N. weight:	0.62
		Sc. PD:	-0.022
		Sc. rank:	-6867.4

,

PA

Pos.	311	obs:	exp:
tta	L	0	2.42
ttg	L	16	4.14
ctt	L	0	4.34
ctc	L	0	3.11
cta	L	0	4.24
ctg	L	6	3.76
att	I	2	25.12
atc	I	0	17.22
ata	I	67	26.66
atg	M	22490	22490.00
act	T	0	1.01
acc	T	0	0.82
aca	T	0	1.83
acg	T	4	0.34
gtt	V	0	0.88
gtc	V	0	0.73
gta	V	0	1.08
gtg	V	4	1.31
---	--	------	------
mPD		0.0087	0.011
		nPD:	0.83
		N. weight:	0.0045
		Sc. PD:	0.0035
		Sc. rank:	84.9

,

PA

Pos.	312	obs:	exp:
caa	Q	0	0.53
cag	Q	1	0.47
cgt	R	0	0.14
cgc	R	0	0.26
cga	R	0	0.49
cgg	R	0	0.37
act	T	0	0.50
acc	T	0	0.41
aca	T	0	0.91
acg	T	2	0.17
aat	N	1	1.18
aac	N	1	0.82
aaa	K	322	12878.00
aag	K	22254	9698.00
aga	R	0	2.51
agg	R	5	1.24
gaa	E	0	1.82
gag	E	3	1.18
---	--	------	------
mPD		0.029	0.49
		nPD:	0.06
		N. weight:	0.8
		Sc. PD:	0.013
		Sc. rank:	10312.0

,

PA

Pos.	313	obs:	exp:
act	T	12	5694.00
acc	T	3	4633.00
aca	T	22568	10334.00
acg	T	6	1927.00
---	--	------	------
mPD		0.0019	0.68
		nPD:	0.
		N. weight:	0.81
		Sc. PD:	-0.032
		Sc. rank:	-13631.0

,

PA

Pos.	314	obs:	exp:
ttt	F	102	9694.00
ttc	F	22487	12895.00
---	--	------	------
mPD		0.0090	0.49
		nPD:	0.02
		N. weight:	0.56
		Sc. PD:	-0.013
		Sc. rank:	1436.1

,

PA

Pos.	315	obs:	exp:
ttt	F	22540	9686.00
ttc	F	31	12885.00
tta	L	2	1.32
ttg	L	6	2.26
tat	Y	4	2.08
tac	Y	0	1.92
ctt	L	4	2.36
ctc	L	0	1.69
cta	L	0	2.31
ctg	L	0	2.05
att	I	2	0.73
atc	I	0	0.50
ata	I	0	0.77
---	--	------	------
mPD		0.0043	0.49
		nPD:	0.01
		N. weight:	0.87
		Sc. PD:	-0.029
		Sc. rank:	-5681.5

,

PA

Pos.	316	obs:	exp:
gat	D	0	3.69
gac	D	7	3.31
ggt	G	43	3424.00
ggc	G	22392	3345.00
gga	G	143	10045.00
ggg	G	4	5768.00
---	--	------	------
mPD		0.017	0.69
		nPD:	0.03
		N. weight:	1.9
		Sc. PD:	-0.034
		Sc. rank:	12885.0

,

PA			
Pos.	317	obs:	exp:
tgg	W	22589	22589.00
---	--	------	------
mPD		0	0
		nPD:	1.
		N. weight:	0.
		Sc. PD:	0
		Sc. rank:	0

PA			
Pos.	318	obs:	exp:
cgt	R	0	0.03
cgc	R	0	0.05
cga	R	0	0.10
cgg	R	0	0.07
aaa	K	22540	12885.00
aag	K	48	9703.00
aga	R	1	0.50
agg	R	0	0.25
---	--	------	------
mPD		0.0043	0.49
		nPD:	0.01
		N. weight:	0.57
		Sc. PD:	-0.019
		Sc. rank:	-3702.7

PA			
Pos.	319	obs:	exp:
aaa	K	0	1.14
aag	K	2	0.86
gct	A	0	0.26
gcc	A	0	0.21
gca	A	0	0.47
gcg	A	1	0.06
gat	D	13	8.96
gac	D	4	8.04
gaa	E	776	13657.00
gag	E	21779	8898.00
ggt	G	0	2.12
ggc	G	0	2.07
gga	G	0	6.23
ggg	G	14	3.58
---	--	------	------
mPD		0.069	0.48
		nPD:	0.14
		N. weight:	0.8
		Sc. PD:	0.08
		Sc. rank:	13094.0

PA			
Pos.	320	obs:	exp:
tta	L	0	0.11
ttg	L	0	0.19
tct	S	31	6.07
tcc	S	2	3.82
tca	S	0	8.59
tcg	S	0	1.76
ctt	L	1	0.20
ctc	L	0	0.14
cta	L	0	0.19
ctg	L	0	0.17
cct	P	22232	6214.00
ccc	P	309	4183.00
cca	P	8	9603.00
ccg	P	5	2554.00
act	T	1	0.25
acc	T	0	0.21
aca	T	0	0.46
acg	T	0	0.09
agt	S	0	6.21
agc	S	0	6.55
---	--	------	------
mPD		0.031	0.70
		nPD:	0.04
		N. weight:	1.3
		Sc. PD:	0.0025
		Sc. rank:	14449.0

PA			
Pos.	321	obs:	exp:
tct	S	0	1.47
tcc	S	0	0.93
tca	S	0	2.08
tcg	S	0	0.43
tat	Y	5	2.60
tac	Y	0	2.40
tgt	C	1	0.45
tgc	C	0	0.55
cat	H	0	0.59
cac	H	1	0.41
caa	Q	1	0.53
cag	Q	0	0.47
cgt	R	0	0.08
cgc	R	0	0.16
cga	R	0	0.29
cgg	R	0	0.22
att	I	0	0.36
atc	I	1	0.25
ata	I	0	0.39
act	T	0	0.76
acc	T	3	0.62
aca	T	0	1.37
acg	T	0	0.26
aat	N	11	1673.00
aac	N	2817	1155.00
aaa	K	19715	11259.00
aag	K	22	8478.00
agt	S	0	1.50
agc	S	8	1.59
aga	R	3	1.51
agg	R	0	0.74
gtt	V	0	0.22
gtc	V	1	0.18
gta	V	0	0.27
gtg	V	0	0.33
---	--	------	------
mPD		0.22	0.60
		nPD:	0.37
		N. weight:	0.62
		Sc. PD:	0.2
		Sc. rank:	11087.0

PA			
Pos.	322	obs:	exp:
tta	L	1	0.55
ttg	L	0	0.94
ctt	L	0	0.99
ctc	L	0	0.71
cta	L	4	0.96
ctg	L	0	0.85
cgt	R	0	0.03
cgc	R	0	0.05
cga	R	0	0.10
cgg	R	0	0.07
att	I	3	7927.00
atc	I	1	5436.00
ata	I	21772	8413.00
atg	M	67	67.00
act	T	0	169.90
acc	T	0	138.20
aca	T	674	308.30
acg	T	0	57.51
aga	R	1	0.50
agg	R	0	0.25
gtt	V	0	14.59
gtc	V	0	11.99
gta	V	66	17.75
gtg	V	0	21.67
---	--	------	------
mPD		0.071	0.72
		nPD:	0.1
		N. weight:	0.98
		Sc. PD:	0.053
		Sc. rank:	15070.0

PA			
Pos.	323	obs:	exp:
att	I	0	26.57
atc	I	73	18.22
ata	I	0	28.20
gtt	V	32	4972.00
gtc	V	22444	4086.00
gta	V	12	6048.00
gtg	V	0	7382.00
gct	A	0	7.41
gcc	A	28	5.80
gca	A	0	13.15
gcg	A	0	1.64
---	--	------	------
mPD		0.013	0.75
		nPD:	0.02
		N. weight:	1.8
		Sc. PD:	-0.045
		Sc. rank:	2431.3

PA			
Pos.	324	obs:	exp:
cgt	R	0	0.05
cgc	R	0	0.10
cga	R	0	0.20
cgg	R	0	0.15
aaa	K	22565	12884.00
aag	K	22	9703.00
aga	R	2	1.00
agg	R	0	0.49
---	--	------	------
mPD		0.0021	0.49
		nPD:	0.
		N. weight:	0.58
		Sc. PD:	-0.022
		Sc. rank:	-8081.1

PA			
Pos.	325	obs:	exp:
tta	L	0	1.54
ttg	L	0	2.64
tct	S	0	9.20
tcc	S	0	5.79
tca	S	50	13.01
tcg	S	0	2.66
ctt	L	0	2.76
ctc	L	0	1.98
cta	L	14	2.70
ctg	L	0	2.39
cct	P	4	6203.00
ccc	P	0	4176.00
cca	P	22496	9586.00
ccg	P	14	2549.00
caa	Q	8	4.22
cag	Q	0	3.78
act	T	0	0.50
acc	T	0	0.41
aca	T	2	0.91
acg	T	0	0.17
agt	S	0	9.40
agc	S	0	9.93
gct	A	0	0.26
gcc	A	0	0.21
gca	A	1	0.47
gcg	A	0	0.06
---	--	------	------
mPD		0.0082	0.70
		nPD:	0.01
		N. weight:	0.89
		Sc. PD:	-0.027
		Sc. rank:	-2511.6

PA			
Pos.	326	obs:	exp:
cat	H	22478	13284.00
cac	H	110	9304.00
aat	N	1	0.59
aac	N	0	0.41
---	--	------	------
mPD		0.0098	0.48
		nPD:	0.02
		N. weight:	0.52
		Sc. PD:	-0.012
		Sc. rank:	2017.3

PA			
Pos.	327	obs:	exp:
aaa	K	0	12.55
aag	K	22	9.45
gtt	V	0	0.66
gtc	V	0	0.55
gta	V	0	0.81
gtg	V	3	0.98
gct	A	0	2.12
gcc	A	0	1.66
gca	A	0	3.76
gcg	A	8	0.47
gat	D	11	5.80
gac	D	0	5.20
gaa	E	221	13644.00
gag	E	22312	8889.00
ggt	G	0	1.82
ggc	G	0	1.78
gga	G	0	5.34
ggg	G	12	3.07
---	--	------	------
mPD		0.024	0.48
		nPD:	0.05
		N. weight:	0.91
		Sc. PD:	0.0068
		Sc. rank:	11236.0

PA			
Pos.	328	obs:	exp:
cgt	R	0	4.45
cgc	R	0	8.53
cga	R	2	15.97
cgg	R	0	11.93
aaa	K	22392	12790.00
aag	K	30	9632.00
aga	R	161	81.83
agg	R	0	40.30
gaa	E	4	2.42
gag	E	0	1.58
---	--	------	------
mPD		0.018	0.51
		nPD:	0.03
		N. weight:	0.58
		Sc. PD:	-0.0048
		Sc. rank:	5699.1

PA			
Pos.	329	obs:	exp:
ggt	G	117	3425.00
ggc	G	22420	3346.00
gga	G	52	10048.00
ggg	G	0	5770.00
---	--	------	------
mPD		0.015	0.69
		nPD:	0.02
		N. weight:	1.9
		Sc. PD:	-0.041
		Sc. rank:	8749.2

PA			
Pos.	330	obs:	exp:
att	I	0	1190.00
atc	I	1	816.30
ata	I	3269	1263.00
atg	M	5	5.00
act	T	0	0.50
acc	T	0	0.41
aca	T	2	0.91
acg	T	0	0.17
gtt	V	4	4265.00
gtc	V	2	3505.00
gta	V	19251	5188.00
gtg	V	33	6332.00
gct	A	0	5.82
gcc	A	0	4.55
gca	A	22	10.33
gcg	A	0	1.29
---	--	------	------
mPD		0.25	0.99
		nPD:	0.26
		N. weight:	1.3
		Sc. PD:	0.27
		Sc. rank:	22329.0

,

PA			
Pos.	331	obs:	exp:
act	T	1	0.25
acc	T	0	0.21
aca	T	0	0.46
acg	T	0	0.09
aat	N	22448	13359.00
aac	N	139	9228.00
gat	D	1	0.53
gac	D	0	0.47
---	--	------	------
mPD		0.012	0.48
		nPD:	0.03
		N. weight:	0.51
		Sc. PD:	-0.0087
		Sc. rank:	3596.2

,

PA			
Pos.	332	obs:	exp:
tta	L	0	0.33
ttg	L	0	0.56
tct	S	0	19.69
tcc	S	101	12.39
tca	S	6	27.85
tcg	S	0	5.69
ctt	L	0	0.59
ctc	L	3	0.42
cta	L	0	0.58
ctg	L	0	0.51
cct	P	50	6192.00
ccc	P	22421	4169.00
cca	P	5	9570.00
ccg	P	0	2545.00
act	T	0	0.76
acc	T	3	0.62
aca	T	0	1.37
acg	T	0	0.26
agt	S	0	20.12
agc	S	0	21.25
---	--	------	------
mPD		0.015	0.71
		nPD:	0.02
		N. weight:	1.7
		Sc. PD:	-0.036
		Sc. rank:	8403.0

,

PA			
Pos.	333	obs:	exp:
tct	S	0	0.18
tcc	S	0	0.12
tca	S	0	0.26
tcg	S	0	0.05
act	T	1	0.25
acc	T	0	0.21
aca	T	0	0.46
acg	T	0	0.09
aat	N	22559	13358.00
aac	N	27	9228.00
aaa	K	1	0.57
aag	K	0	0.43
agt	S	1	0.19
agc	S	0	0.20
---	--	------	------
mPD		0.0027	0.48
		nPD:	0.01
		N. weight:	0.54
		Sc. PD:	-0.02
		Sc. rank:	-6352.4

,

PA			
Pos.	334	obs:	exp:
tat	Y	179	11735.00
tac	Y	22407	10851.00
tgt	C	0	0.45
tgc	C	1	0.55
cat	H	0	1.18
cac	H	2	0.82
---	--	------	------
mPD		0.016	0.50
		nPD:	0.03
		N. weight:	0.72
		Sc. PD:	-0.0076
		Sc. rank:	6510.2

,

PA			
Pos.	335	obs:	exp:
ttt	F	0	73.81
ttc	F	172	98.19
tta	L	0	2462.00
ttg	L	0	4217.00
ctt	L	35	4413.00
ctc	L	22233	3164.00
cta	L	125	4312.00
ctg	L	4	3829.00
cct	P	0	0.55
ccc	P	2	0.37
cca	P	0	0.85
ccg	P	0	0.23
cgt	R	0	0.05
cgc	R	2	0.10
cga	R	0	0.20
cgg	R	0	0.15
att	I	0	5.82
atc	I	16	3.99
ata	I	0	6.18
aga	R	0	1.00
agg	R	0	0.49
---	--	------	------
mPD		0.031	1.1
		nPD:	0.03
		N. weight:	2.
		Sc. PD:	-0.03
		Sc. rank:	15108.0

,

PA			
Pos.	336	obs:	exp:
tta	L	0	1.21
ttg	L	1	2.07
ctt	L	0	2.17
ctc	L	0	1.55
cta	L	0	2.12
ctg	L	10	1.88
cgt	R	0	0.14
cgc	R	0	0.26
cga	R	0	0.49
cgg	R	0	0.37
att	I	0	6.92
atc	I	0	4.74
ata	I	19	7.34
atg	M	22542	22542.00
act	T	0	1.26
acc	T	0	1.03
aca	T	0	2.29
acg	T	5	0.43
aaa	K	0	0.57
aag	K	1	0.43
aga	R	0	2.51
agg	R	5	1.24
gtt	V	0	1.33
gtc	V	0	1.09
gta	V	0	1.61
gtg	V	6	1.97
---	--	------	------
mPD		0.0042	0.0060
		nPD:	0.69
		N. weight:	0.0029
		Sc. PD:	0.0018
		Sc. rank:	53.3

,

PA			
Pos.	337	obs:	exp:
tct	S	0	1.10
tcc	S	0	0.69
tca	S	6	1.56
tcg	S	0	0.32
act	T	322	81.68
acc	T	2	66.46
aca	T	0	148.20
acg	T	0	27.64
agt	S	0	1.13
agc	S	0	1.19
gtt	V	4	0.88
gtc	V	0	0.73
gta	V	0	1.08
gtg	V	0	1.31
gct	A	21842	5891.00
gcc	A	409	4606.00
gca	A	0	10451.00
gcg	A	0	1304.00
ggt	G	3	0.61
ggc	G	1	0.59
gga	G	0	1.78
ggg	G	0	1.02
---	--	------	------
mPD		0.066	0.69
		nPD:	0.1
		N. weight:	1.3
		Sc. PD:	0.067
		Sc. rank:	19815.0

,

PA			
Pos.	338	obs:	exp:
tgg	W	22589	22589.00
---	--	------	------
mPD		0	0
		nPD:	1.
		N. weight:	0.
		Sc. PD:	0
		Sc. rank:	0

,

PA			
Pos.	339	obs:	exp:
cgt	R	0	0.46
cgc	R	0	0.89
cga	R	0	1.67
cgg	R	0	1.24
aat	N	2	1.18
aac	N	0	0.82
aaa	K	120	12875.00
aag	K	22450	9695.00
aga	R	1	8.53
agg	R	16	4.20
---	--	------	------
mPD		0.012	0.49
		nPD:	0.03
		N. weight:	0.85
		Sc. PD:	-0.015
		Sc. rank:	5687.8

,

PA			
Pos.	340	obs:	exp:
tta	L	0	0.11
ttg	L	0	0.19
ctt	L	0	0.20
ctc	L	0	0.14
cta	L	0	0.19
ctg	L	1	0.17
cat	H	2	1.18
cac	H	0	0.82
caa	Q	83	11917.00
cag	Q	22502	10668.00
aaa	K	0	0.57
aag	K	1	0.43
---	--	------	------
mPD		0.0077	0.50
		nPD:	0.02
		N. weight:	0.76
		Sc. PD:	-0.02
		Sc. rank:	344.5

,

PA			
Pos.	341	obs:	exp:
tta	L	0	1.87
ttg	L	11	3.20
ctt	L	0	3.35
ctc	L	0	2.40
cta	L	0	3.27
ctg	L	6	2.91
att	I	1	0.36
atc	I	0	0.25
ata	I	0	0.39
atg	M	142	142.00
gtt	V	733	4957.00
gtc	V	1	4074.00
gta	V	75	6029.00
gtg	V	21610	7359.00
gct	A	0	2.65
gcc	A	0	2.07
gca	A	0	4.70
gcg	A	10	0.59
---	--	------	------
mPD		0.084	0.75
		nPD:	0.11
		N. weight:	1.
		Sc. PD:	0.068
		Sc. rank:	15586.0

,

PA			
Pos.	342	obs:	exp:
tta	L	118	2476.00
ttg	L	0	4241.00
ctt	L	2	4439.00
ctc	L	0	3182.00
cta	L	22380	4337.00
ctg	L	27	3852.00
caa	Q	55	29.02
cag	Q	0	25.98
att	I	0	2.55
atc	I	0	1.75
ata	I	7	2.70
---	--	------	------
mPD		0.018	1.1
		nPD:	0.02
		N. weight:	1.7
		Sc. PD:	-0.044
		Sc. rank:	1251.9

,

PA			
Pos.	343	obs:	exp:
tct	S	0	7.18
tcc	S	0	4.52
tca	S	39	10.15
tcg	S	0	2.08
act	T	0	87.47
acc	T	1	71.17
aca	T	346	158.70
acg	T	0	29.61
aaa	K	1	0.57
aag	K	0	0.43
agt	S	0	7.33
agc	S	0	7.75
gtt	V	0	2.65
gtc	V	0	2.18
gta	V	12	3.23
gtg	V	0	3.94
gct	A	20	5871.00
gcc	A	0	4590.00
gca	A	22131	10415.00
gcg	A	24	1299.00
gaa	E	15	9.08
gag	E	0	5.92
---	--	------	------
mPD		0.040	0.70
		nPD:	0.06
		N. weight:	0.78
		Sc. PD:	0.011
		Sc. rank:	9900.2

,

PA			
Pos.	344	obs:	exp:
aaa	K	0	0.57
aag	K	1	0.43
gat	D	1	0.53
gac	D	0	0.47
gaa	E	241	13675.00
gag	E	22344	8910.00
ggt	G	0	0.30
ggc	G	0	0.30
gga	G	0	0.89
ggg	G	2	0.51
---	--	------	------
mPD		0.021	0.48
		nPD:	0.04
		N. weight:	0.9
		Sc. PD:	0.0018
		Sc. rank:	10326.0

,

PA			
Pos.	345	obs:	exp:
tta	L	37	2481.00
ttg	L	6	4250.00
ctt	L	1	4448.00
ctc	L	5	3188.00
cta	L	22474	4346.00
ctg	L	49	3859.00
att	I	0	5.10
atc	I	0	3.49
ata	I	14	5.41
aaa	K	2	1.14
aag	K	0	0.86
gtt	V	0	0.22
gtc	V	0	0.18
gta	V	1	0.27
gtg	V	0	0.33
---	--	------	------
mPD		0.011	1.1
		nPD:	0.01
		N. weight:	1.7
		Sc. PD:	-0.055
		Sc. rank:	-9705.6

,

PA			
Pos.	346	obs:	exp:
tta	L	0	0.22
ttg	L	0	0.38
ctt	L	0	0.39
ctc	L	0	0.28
cta	L	0	0.39
ctg	L	2	0.34
cct	P	0	0.28
ccc	P	0	0.19
cca	P	0	0.43
ccg	P	1	0.11
cat	H	11	6.47
cac	H	0	4.53
caa	Q	125	11907.00
cag	Q	22440	10658.00
cgt	R	0	0.14
cgc	R	0	0.26
cga	R	0	0.49
cgg	R	5	0.37
aaa	K	0	2.85
aag	K	5	2.15
aga	R	0	2.51
agg	R	0	1.24
---	--	------	------
mPD		0.013	0.50
		nPD:	0.03
		N. weight:	0.75
		Sc. PD:	-0.012
		Sc. rank:	5386.1

,

PA			
Pos.	347	obs:	exp:
aat	N	0	7.10
aac	N	12	4.90
gct	A	0	0.26
gcc	A	1	0.21
gca	A	0	0.47
gcg	A	0	0.06
gat	D	47	11886.00
gac	D	22511	10672.00
gaa	E	2	1.21
gag	E	0	0.79
ggt	G	0	2.43
ggc	G	16	2.37
gga	G	0	7.12
ggg	G	0	4.09
---	--	------	------
mPD		0.0069	0.50
		nPD:	0.01
		N. weight:	0.77
		Sc. PD:	-0.022
		Sc. rank:	-552.2

,

PA			
Pos.	348	obs:	exp:
ttt	F	2	0.86
ttc	F	0	1.14
tct	S	0	1.10
tcc	S	0	0.69
tca	S	0	1.56
tcg	S	0	0.32
att	I	22533	8206.00
atc	I	9	5627.00
ata	I	1	8710.00
act	T	22	5.55
acc	T	0	4.51
aca	T	0	10.06
acg	T	0	1.88
aat	N	8	4.73
aac	N	0	3.27
agt	S	6	1.13
agc	S	0	1.19
gtt	V	8	1.77
gtc	V	0	1.45
gta	V	0	2.15
gtg	V	0	2.63
---	--	------	------
mPD		0.0050	0.66
		nPD:	0.01
		N. weight:	1.1
		Sc. PD:	-0.037
		Sc. rank:	-9122.1

,

PA			
Pos.	349	obs:	exp:
aaa	K	13	7.42
aag	K	0	5.58
gat	D	1	7.38
gac	D	13	6.62
gaa	E	21597	13595.00
gag	E	856	8858.00
ggt	G	0	16.53
ggc	G	0	16.14
gga	G	108	48.49
ggg	G	1	27.84
---	--	------	------
mPD		0.085	0.49
		nPD:	0.17
		N. weight:	0.37
		Sc. PD:	0.048
		Sc. rank:	6231.6

,

PA			
Pos.	350	obs:	exp:
tct	S	0	9.57
tcc	S	0	6.02
tca	S	0	13.53
tcg	S	0	2.77
cat	H	2	1.18
cac	H	0	0.82
att	I	26	9.46
atc	I	0	6.49
ata	I	0	10.05
act	T	6	1.76
acc	T	1	1.44
aca	T	0	3.20
acg	T	0	0.60
aat	N	22454	13301.00
aac	N	36	9189.00
aaa	K	4	3.99
aag	K	3	3.01
agt	S	52	9.78
agc	S	0	10.33
gat	D	4	2.11
gac	D	0	1.89
---	-	1	1.00
---	--	------	------
mPD		0.012	0.50
		nPD:	0.02
		N. weight:	0.54
		Sc. PD:	-0.01
		Sc. rank:	3257.5

,

PA			
Pos.	351	obs:	exp:
aaa	K	12	6.85
aag	K	0	5.15
gtt	V	0	0.44
gtc	V	0	0.36
gta	V	2	0.54
gtg	V	0	0.66
gct	A	0	0.26
gcc	A	0	0.21
gca	A	1	0.47
gcg	A	0	0.06
gat	D	2	4.74
gac	D	7	4.26
gaa	E	22513	13655.00
gag	E	38	8896.00
ggt	G	0	2.12
ggc	G	0	2.07
gga	G	14	6.23
ggg	G	0	3.58
---	--	------	------
mPD		0.0067	0.48
		nPD:	0.01
		N. weight:	0.51
		Sc. PD:	-0.015
		Sc. rank:	-329.6

,

PA			
Pos.	352	obs:	exp:
aaa	K	0	2.28
aag	K	4	1.72
gtt	V	0	0.22
gtc	V	0	0.18
gta	V	0	0.27
gtg	V	1	0.33
gct	A	0	0.26
gcc	A	0	0.21
gca	A	0	0.47
gcg	A	1	0.06
gat	D	32	16.86
gac	D	0	15.14
gaa	E	483	13653.00
gag	E	22066	8896.00
ggt	G	0	0.30
ggc	G	0	0.30
gga	G	0	0.89
ggg	G	2	0.51
---	--	------	------
mPD		0.045	0.48
		nPD:	0.09
		N. weight:	0.85
		Sc. PD:	0.043
		Sc. rank:	12968.0

,

PA			
Pos.	353	obs:	exp:
cgt	R	0	0.30
cgc	R	0	0.58
cga	R	0	1.08
cgg	R	0	0.81
act	T	0	0.25
acc	T	0	0.21
aca	T	0	0.46
acg	T	1	0.09
aat	N	3	1.77
aac	N	0	1.23
aaa	K	14369	12875.00
aag	K	8201	9695.00
aga	R	4	5.52
agg	R	7	2.72
gaa	E	4	2.42
gag	E	0	1.58
---	--	------	------
mPD		0.46	0.49
		nPD:	0.94
		N. weight:	0.01
		Sc. PD:	0.0091
		Sc. rank:	192.7

,

PA			
Pos.	354	obs :	exp :
ttt	F	0	0.43
ttc	F	1	0.57
tta	L	0	0.44
ttg	L	0	0.75
tct	S	0	0.18
tcc	S	0	0.12
tca	S	0	0.26
tcg	S	0	0.05
ctt	L	0	0.79
ctc	L	4	0.56
cta	L	0	0.77
ctg	L	0	0.68
att	I	235	8160.00
atc	I	22173	5595.00
ata	I	8	8660.00
act	T	0	9.08
acc	T	36	7.38
aca	T	0	16.47
acg	T	0	3.07
aat	N	0	1.77
aac	N	3	1.23
agt	S	0	0.19
agc	S	1	0.20
gtt	V	1	27.64
gtc	V	124	22.71
gta	V	0	33.62
gtg	V	0	41.03
gct	A	0	0.79
gcc	A	3	0.62
gca	A	0	1.41
gcg	A	0	0.18
---	--	------	------
mPD		0.037	0.67
		nPD :	0.05
		N. weight :	1.4
		Sc. PD :	0.016
		Sc. rank :	17400.0

,

PA			
Pos.	355	obs :	exp :
tta	L	0	0.11
ttg	L	0	0.19
tct	S	0	0.37
tcc	S	0	0.23
tca	S	2	0.52
tcg	S	0	0.11
ctt	L	0	0.20
ctc	L	0	0.14
cta	L	1	0.19
ctg	L	0	0.17
cct	P	14	6222.00
ccc	P	2	4189.00
cca	P	22564	9616.00
ccg	P	4	2557.00
caa	Q	2	1.06
cag	Q	0	0.94
agt	S	0	0.38
agc	S	0	0.40
---	--	------	------
mPD		0.0022	0.70
		nPD :	0.
		N. weight :	0.88
		Sc. PD :	-0.035
		Sc. rank :	-14231.0

,

PA			
Pos.	356	obs :	exp :
cgt	R	0	614.30
cgc	R	0	1177.00
cga	R	0	2204.00
cgg	R	6	1647.00
atg	M	1	1.00
aaa	K	4	50.77
aag	K	85	38.23
aga	R	413	11295.00
agg	R	22080	5562.00
---	--	------	------
mPD		0.045	0.92
		nPD :	0.05
		N. weight :	1.3
		Sc. PD :	0.0079
		Sc. rank :	16223.0

,

PA			
Pos.	357	obs :	exp :
tct	S	0	0.18
tcc	S	0	0.12
tca	S	1	0.26
tcg	S	0	0.05
att	I	0	6.19
atc	I	0	4.24
ata	I	17	6.57
act	T	30	5678.00
acc	T	21	4620.00
aca	T	22460	10305.00
acg	T	14	1922.00
aaa	K	4	2.28
aag	K	0	1.72
agt	S	0	0.19
agc	S	0	0.20
gct	A	0	11.12
gcc	A	0	8.69
gca	A	42	19.73
gcg	A	0	2.46
---	--	------	------
mPD		0.011	0.68
		nPD :	0.02
		N. weight :	0.8
		Sc. PD :	-0.02
		Sc. rank :	810.4

,

PA			
Pos.	358	obs :	exp :
cgt	R	0	0.11
cgc	R	0	0.21
cga	R	0	0.39
cgg	R	0	0.29
act	T	0	0.25
acc	T	0	0.21
aca	T	0	0.46
acg	T	1	0.09
aaa	K	298	12883.00
aag	K	22286	9701.00
aga	R	1	2.01
agg	R	3	0.99
---	--	------	------
mPD		0.027	0.49
		nPD :	0.05
		N. weight :	0.8
		Sc. PD :	0.009
		Sc. rank :	10071.0

,

PA			
Pos.	359	obs :	exp :
tct	S	0	1.84
tcc	S	0	1.16
tca	S	0	2.60
tcg	S	0	0.53
tat	Y	0	0.52
tac	Y	1	0.48
act	T	0	0.25
acc	T	1	0.21
aca	T	0	0.46
acg	T	0	0.09
aat	N	58	13350.00
aac	N	22514	9222.00
aaa	K	3	1.71
aag	K	0	1.29
agt	S	0	1.88
agc	S	10	1.99
gat	D	0	1.05
gac	D	2	0.95
---	--	------	------
mPD		0.0066	0.49
		nPD :	0.01
		N. weight :	0.91
		Sc. PD :	-0.026
		Sc. rank :	-727.9

,

PA			
Pos.	360	obs :	exp :
tta	L	0	0.44
ttg	L	3	0.75
ctt	L	0	0.79
ctc	L	0	0.56
cta	L	0	0.77
ctg	L	1	0.68
att	I	6	9.10
atc	I	0	6.24
ata	I	19	9.66
atg	M	22551	22551.00
act	T	0	1.26
acc	T	0	1.03
aca	T	0	2.29
acg	T	5	0.43
gtt	V	0	0.88
gtc	V	0	0.73
gta	V	0	1.08
gtg	V	4	1.31
---	--	------	------
mPD		0.0034	0.0042
		nPD :	0.79
		N. weight :	0.0016
		Sc. PD :	0.0012
		Sc. rank :	29.5

,

PA			
Pos.	361	obs :	exp :
cgt	R	0	25.64
cgc	R	0	49.12
cga	R	0	91.97
cgg	R	0	68.72
atg	M	1	1.00
aat	N	10	5.91
aac	N	0	4.09
aaa	K	76	12341.00
aag	K	21559	9294.00
aga	R	7	471.40
agg	R	932	232.10
gtt	V	0	0.22
gtc	V	0	0.18
gta	V	0	0.27
gtg	V	1	0.33
gaa	E	0	0.61
gag	E	1	0.39
ggt	G	0	0.30
ggc	G	0	0.30
gga	G	0	0.89
ggg	G	2	0.51
---	--	------	------
mPD		0.089	0.59
		nPD :	0.15
		N. weight :	0.88
		Sc. PD :	0.092
		Sc. rank :	14447.0

,

PA

Pos.	362	obs:	exp:
cgt	R	0	88.71
cgc	R	0	170.00
cga	R	0	318.20
cgg	R	0	237.80
aat	N	1	0.59
aac	N	0	0.41
aaa	K	19066	11029.00
aag	K	268	8305.00
aga	R	3249	1631.00
agg	R	0	803.20
gaa	E	4	2.42
gag	E	0	1.58
ggt	G	0	0.15
ggc	G	0	0.15
gga	G	1	0.44
ggg	G	0	0.26
---	--	------	------
mPD		0.27	0.81
		nPD:	0.33
		N. weight:	0.54
		Sc. PD:	0.15
		Sc. rank:	9626.6

,

PA

Pos.	363	obs:	exp:
tct	S	0	0.18
tcc	S	0	0.12
tca	S	1	0.26
tcg	S	0	0.05
att	I	0	0.73
atc	I	0	0.50
ata	I	2	0.77
act	T	2	5691.00
acc	T	0	4630.00
aca	T	22530	10327.00
acg	T	43	1926.00
aaa	K	2	1.14
aag	K	0	0.86
agt	S	0	0.19
agc	S	0	0.20
gct	A	0	2.38
gcc	A	0	1.86
gca	A	9	4.23
gcg	A	0	0.53
---	--	------	------
mPD		0.0052	0.68
		nPD:	0.01
		N. weight:	0.8
		Sc. PD:	-0.028
		Sc. rank:	-6837.6

,

PA

Pos.	364	obs:	exp:
tct	S	0	4112.00
tcc	S	0	2588.00
tca	S	0	5815.00
tcg	S	0	1189.00
cgt	R	0	0.05
cgc	R	0	0.10
cga	R	0	0.20
cgg	R	0	0.15
att	I	0	8.74
atc	I	24	5.99
ata	I	0	9.27
act	T	0	0.76
acc	T	3	0.62
aca	T	0	1.37
acg	T	0	0.26
aat	N	1	56.78
aac	N	95	39.22
agt	S	834	4202.00
agc	S	21511	4439.00
aga	R	2	1.00
agg	R	0	0.49
ggt	G	4	18.04
ggc	G	115	17.63
gga	G	0	52.93
ggg	G	0	30.40
---	--	------	------
mPD		0.093	1.7
		nPD:	0.06
		N. weight:	1.5
		Sc. PD:	0.02
		Sc. rank:	19326.0

,

PA

Pos.	365	obs:	exp:
tta	L	0	0.11
ttg	L	0	0.19
ctt	L	0	0.20
ctc	L	0	0.14
cta	L	1	0.19
ctg	L	0	0.17
cct	P	0	0.55
ccc	P	0	0.37
cca	P	2	0.85
ccg	P	0	0.23
cat	H	7	4.70
cac	H	1	3.30
caa	Q	22549	11913.00
cag	Q	28	10664.00
cgt	R	0	0.03
cgc	R	0	0.05
cga	R	1	0.10
cgg	R	0	0.07
aga	R	0	0.50
agg	R	0	0.25
---	--	------	------
mPD		0.0035	0.50
		nPD:	0.01
		N. weight:	0.66
		Sc. PD:	-0.023
		Sc. rank:	-6389.7

,

PA

Pos.	366	obs:	exp:
tta	L	64	2481.00
ttg	L	22483	4249.00
ctt	L	0	4447.00
ctc	L	0	3188.00
cta	L	2	4345.00
ctg	L	19	3859.00
atg	M	20	20.00
gtt	V	0	0.22
gtc	V	0	0.18
gta	V	0	0.27
gtg	V	1	0.33
---	--	------	------
mPD		0.0095	1.1
		nPD:	0.01
		N. weight:	1.7
		Sc. PD:	-0.058
		Sc. rank:	-12643.0

,

PA

Pos.	367	obs:	exp:
cgt	R	0	0.11
cgc	R	0	0.21
cga	R	0	0.39
cgg	R	0	0.29
aat	N	1	0.59
aac	N	0	0.41
aaa	K	197	12881.00
aag	K	22385	9701.00
aga	R	0	2.01
agg	R	4	0.99
gaa	E	0	1.21
gag	E	2	0.79
---	--	------	------
mPD		0.018	0.49
		nPD:	0.04
		N. weight:	0.83
		Sc. PD:	-0.0052
		Sc. rank:	8465.4

,

PA

Pos.	368	obs:	exp:
tgg	W	22589	22589.00
---	--	------	------
mPD		0	0
		nPD:	1.
		N. weight:	0.
		Sc. PD:	0
		Sc. rank:	0

,

PA

Pos.	369	obs:	exp:
tct	S	0	1.10
tcc	S	0	0.69
tca	S	6	1.56
tcg	S	0	0.32
act	T	0	6.81
acc	T	0	5.54
aca	T	27	12.35
acg	T	0	2.30
agt	S	0	1.13
agc	S	0	1.19
gtt	V	0	1.11
gtc	V	0	0.91
gta	V	5	1.34
gtg	V	0	1.64
gct	A	11	5970.00
gcc	A	10	4668.00
gca	A	22524	10591.00
gcg	A	6	1321.00
---	--	------	------
mPD		0.0057	0.67
		nPD:	0.01
		N. weight:	0.78
		Sc. PD:	-0.026
		Sc. rank:	-5323.1

,

PA

Pos.	370	obs:	exp:
ttt	F	1	0.43
ttc	F	0	0.57
tta	L	0	2483.00
ttg	L	0	4252.00
ctt	L	55	4451.00
ctc	L	22467	3190.00
cta	L	58	4348.00
ctg	L	5	3861.00
att	I	0	0.73
atc	I	2	0.50
ata	I	0	0.77
gtt	V	0	0.22
gtc	V	1	0.18
gta	V	0	0.27
gtg	V	0	0.33
---	--	------	------
mPD		0.011	1.1
		nPD:	0.01
		N. weight:	2.
		Sc. PD:	-0.066
		Sc. rank:	-11700.0

,

PA

Pos.	371	obs:	exp:
ggt	G	22549	3425.00
ggc	G	23	3346.00
gga	G	2	10048.00
ggg	G	15	5770.00
---	--	------	------
mPD		0.0035	0.69
		nPD:	0.01
		N. weight:	2.
		Sc. PD:	-0.073
		Sc. rank:	-24729.0

,

PA

Pos.	372	obs:	exp:
gaa	E	22561	13678.00
gag	E	28	8911.00
---	--	------	------
mPD		0.0025	0.48
		nPD:	0.01
		N. weight:	0.51
		Sc. PD:	-0.019
		Sc. rank:	-6346.1

,

PA

Pos.	373	obs:	exp:
tct	S	0	0.92
tcc	S	0	0.58
tca	S	0	1.30
tcg	S	0	0.27
act	T	1	0.25
acc	T	0	0.21
aca	T	0	0.46
acg	T	0	0.09
aat	N	22540	13356.00
aac	N	42	9226.00
agt	S	5	0.94
agc	S	0	0.99
gat	D	1	0.53
gac	D	0	0.47
---	--	------	------
mPD		0.0043	0.48
		nPD:	0.01
		N. weight:	0.53
		Sc. PD:	-0.018
		Sc. rank:	-3345.9

,

PA

Pos.	374	obs:	exp:
tta	L	0	0.22
ttg	L	0	0.38
ctt	L	0	0.39
ctc	L	0	0.28
cta	L	0	0.39
ctg	L	2	0.34
att	I	5	12.38
atc	I	0	8.49
ata	I	29	13.14
atg	M	22544	22544.00
act	T	0	1.26
acc	T	0	1.03
aca	T	0	2.29
acg	T	5	0.43
gtt	V	0	0.88
gtc	V	0	0.73
gta	V	0	1.08
gtg	V	4	1.31
---	--	------	------
mPD		0.0040	0.0047
		nPD:	0.84
		N. weight:	0.0019
		Sc. PD:	0.0015
		Sc. rank:	34.9

,

PA

Pos.	375	obs:	exp:
gtt	V	0	0.22
gtc	V	0	0.18
gta	V	1	0.27
gtg	V	0	0.33
gct	A	0	5980.00
gcc	A	62	4676.00
gca	A	22325	10609.00
gcg	A	201	1323.00
---	--	------	------
mPD		0.023	0.66
		nPD:	0.03
		N. weight:	0.74
		Sc. PD:	-0.0058
		Sc. rank:	7372.2

,

PA

Pos.	376	obs:	exp:
cct	P	7548	6223.00
ccc	P	3	4190.00
cca	P	15024	9618.00
ccg	P	14	2558.00
---	--	------	------
mPD		0.45	0.70
		nPD:	0.64
		N. weight:	0.37
		Sc. PD:	0.22
		Sc. rank:	6927.4

,

PA

Pos.	377	obs:	exp:
gaa	E	22554	13677.00
gag	E	34	8911.00
ggt	G	0	0.15
ggc	G	0	0.15
gga	G	1	0.44
ggg	G	0	0.26
---	--	------	------
mPD		0.0031	0.48
		nPD:	0.01
		N. weight:	0.51
		Sc. PD:	-0.018
		Sc. rank:	-5470.0

,

PA

Pos.	378	obs:	exp:
cgt	R	0	0.03
cgc	R	0	0.05
cga	R	0	0.10
cgg	R	0	0.07
aat	N	0	0.59
aac	N	1	0.41
aaa	K	22140	12884.00
aag	K	447	9703.00
aga	R	1	0.50
agg	R	0	0.25
---	--	------	------
mPD		0.039	0.49
		nPD:	0.08
		N. weight:	0.49
		Sc. PD:	0.018
		Sc. rank:	7076.8

,

PA

Pos.	379	obs:	exp:
tta	L	1	1.43
ttg	L	11	2.45
ctt	L	0	2.56
ctc	L	0	1.84
cta	L	1	2.50
ctg	L	0	2.22
att	I	0	8.01
atc	I	0	5.49
ata	I	22	8.50
atg	M	435	435.00
gtt	V	10	4890.00
gtc	V	2	4019.00
gta	V	4186	5948.00
gtg	V	17919	7260.00
gct	A	0	0.26
gcc	A	0	0.21
gca	A	1	0.47
gcg	A	0	0.06
ggt	G	0	0.15
ggc	G	0	0.15
gga	G	0	0.44
ggg	G	1	0.26
---	--	------	------
mPD		0.35	0.78
		nPD:	0.44
		N. weight:	0.68
		Sc. PD:	0.27
		Sc. rank:	12417.0

,

PA

Pos.	380	obs:	exp:
gat	D	58	11901.00
gac	D	22528	10685.00
gaa	E	3	1.82
gag	E	0	1.18
---	--	------	------
mPD		0.0054	0.50
		nPD:	0.01
		N. weight:	0.76
		Sc. PD:	-0.024
		Sc. rank:	-2905.3

,

PA

Pos.	381	obs:	exp:
ttt	F	22574	9693.00
ttc	F	13	12894.00
tta	L	0	0.22
ttg	L	0	0.38
ctt	L	2	0.39
ctc	L	0	0.28
cta	L	0	0.39
ctg	L	0	0.34
---	--	------	------
mPD		0.0013	0.49
		nPD:	0.
		N. weight:	0.88
		Sc. PD:	-0.035
		Sc. rank:	-14835.0

,

PA

Pos.	382	obs:	exp:
tat	Y	1	0.52
tac	Y	0	0.48
aat	N	4	2.37
aac	N	0	1.63
gat	D	22501	11867.00
gac	D	21	10655.00
gaa	E	11	37.54
gag	E	51	24.46
---	--	------	------
mPD		0.0078	0.50
		nPD:	0.02
		N. weight:	0.66
		Sc. PD:	-0.018
		Sc. rank:	323.3

,

PA

Pos.	383	obs:	exp:
aat	N	0	4.14
aac	N	7	2.86
gat	D	58	11895.00
gac	D	22517	10680.00
gaa	E	5	3.63
gag	E	1	2.37
ggt	G	0	0.15
ggc	G	1	0.15
gga	G	0	0.44
ggg	G	0	0.26
---	--	------	------
mPD		0.0064	0.50
		nPD:	0.01
		N. weight:	0.76
		Sc. PD:	-0.023
		Sc. rank:	-1381.3

,

PA

Pos.	384	obs:	exp:
tat	Y	2	1.04
tac	Y	0	0.96
tgt	C	18396	10195.00
tgc	C	4191	12392.00
---	--	------	------
mPD		0.30	0.50
		nPD:	0.61
		N. weight:	0.29
		Sc. PD:	0.16
		Sc. rank:	5414.5

,

PA

Pos.	385	obs:	exp:
caa	Q	2	1.06
cag	Q	0	0.94
cgt	R	0	0.60
cgc	R	0	1.15
cga	R	0	2.15
cgg	R	0	1.61
act	T	0	0.25
acc	T	0	0.21
aca	T	1	0.46
acg	T	0	0.09
aaa	K	22536	12869.00
aag	K	25	9692.00
aga	R	22	11.04
agg	R	0	5.44
gaa	E	3	1.82
gag	E	0	1.18
---	--	------	------
mPD		0.0047	0.49
		nPD:	0.01
		N. weight:	0.58
		Sc. PD:	-0.019
		Sc. rank:	-3434.1

,

PA

Pos.	386	obs:	exp:
tat	Y	1	0.52
tac	Y	0	0.48
aat	N	8	4.73
aac	N	0	3.27
gat	D	22557	11894.00
gac	D	16	10679.00
gaa	E	1	1.21
gag	E	1	0.79
ggt	G	5	0.76
ggc	G	0	0.74
gga	G	0	2.22
ggg	G	0	1.28
---	--	------	------
mPD		0.0028	0.50
		nPD:	0.01
		N. weight:	0.66
		Sc. PD:	-0.024
		Sc. rank:	-7736.6

,

PA

Pos.	387	obs:	exp:
ttt	F	19	8.15
ttc	F	0	10.85
att	I	120	45.50
atc	I	0	31.20
ata	I	5	48.29
gtt	V	22416	4962.00
gtc	V	22	4078.00
gta	V	5	6036.00
gtg	V	0	7367.00
gct	A	2	0.53
gcc	A	0	0.41
gca	A	0	0.94
gcg	A	0	0.12
---	--	------	------
mPD		0.016	0.75
		nPD:	0.02
		N. weight:	1.6
		Sc. PD:	-0.034
		Sc. rank:	6429.2

,

PA

Pos.	388	obs:	exp:
tct	S	0	0.92
tcc	S	0	0.58
tca	S	0	1.30
tcg	S	0	0.27
cgt	R	0	0.05
cgc	R	0	0.10
cga	R	0	0.20
cgg	R	0	0.15
agt	S	0	0.94
agc	S	5	0.99
aga	R	2	1.00
agg	R	0	0.49
gtt	V	0	1.77
gtc	V	0	1.45
gta	V	8	2.15
gtg	V	0	2.63
gaa	E	19	11.50
gag	E	0	7.50
ggt	G	25	3420.00
ggc	G	2	3341.00
gga	G	22337	10033.00
ggg	G	191	5761.00
---	--	------	------
mPD		0.023	0.70
		nPD:	0.03
		N. weight:	0.79
		Sc. PD:	-0.0081
		Sc. rank:	7280.0

,

PA

Pos.	389	obs:	exp:
gat	D	144	11902.00
gac	D	22445	10687.00
---	--	------	------
mPD		0.013	0.50
		nPD:	0.03
		N. weight:	0.74
		Sc. PD:	-0.013
		Sc. rank:	5183.9

,

PA

Pos.	390	obs:	exp:
tta	L	0	2483.00
ttg	L	5	4253.00
ctt	L	22282	4451.00
ctc	L	290	3191.00
cta	L	7	4349.00
ctg	L	5	3862.00
---	--	------	------
mPD		0.027	1.1
		nPD:	0.02
		N. weight:	1.6
		Sc. PD:	-0.03
		Sc. rank:	9472.4

,

PA

Pos.	391	obs:	exp:
caa	Q	5	2.64
cag	Q	0	2.36
cgt	R	0	0.16
cgc	R	0	0.31
cga	R	0	0.59
cgg	R	0	0.44
att	I	0	0.73
atc	I	0	0.50
ata	I	2	0.77
aat	N	1	0.59
aac	N	0	0.41
aaa	K	22519	12869.00
aag	K	42	9692.00
aga	R	6	3.01
agg	R	0	1.48
gaa	E	14	8.48
gag	E	0	5.52
---	--	------	------
mPD		0.0062	0.49
		nPD:	0.01
		N. weight:	0.57
		Sc. PD:	-0.017
		Sc. rank:	-1180.7

,

PA

Pos.	392	obs:	exp:
caa	Q	137	11920.00
cag	Q	22452	10669.00
---	--	------	------
mPD		0.012	0.50
		nPD:	0.02
		N. weight:	0.74
		Sc. PD:	-0.014
		Sc. rank:	4682.8

,

PA

Pos.	393	obs:	exp:
ttt	F	1	0.43
ttc	F	0	0.57
tat	Y	22575	11735.00
tac	Y	11	10851.00
tgt	C	1	0.45
tgc	C	0	0.55
cat	H	1	0.59
cac	H	0	0.41
---	--	------	------
mPD		0.0012	0.50
		nPD:	0.
		N. weight:	0.68
		Sc. PD:	-0.027
		Sc. rank:	-11823.0

,

PA

Pos.	394	obs:	exp:
tat	Y	0	0.52
tac	Y	1	0.48
cat	H	0	0.59
cac	H	1	0.41
aat	N	1	72.16
aac	N	121	49.84
gat	D	38	11807.00
gac	D	22371	10602.00
gaa	E	21	14.53
gag	E	3	9.47
ggt	G	0	4.85
ggc	G	32	4.74
gga	G	0	14.23
ggg	G	0	8.17
---	--	------	------
mPD		0.019	0.51
		nPD:	0.04
		N. weight:	0.77
		Sc. PD:	-0.004
		Sc. rank:	7962.9

,

PA

Pos.	395	obs:	exp:
tct	S	0	4151.00
tcc	S	0	2613.00
tca	S	0	5870.00
tcg	S	0	1200.00
tgt	C	3	1.35
tgc	C	0	1.65
act	T	1	0.25
acc	T	0	0.21
aca	T	0	0.46
acg	T	0	0.09
aat	N	26	15.38
aac	N	0	10.62
agt	S	22525	4241.00
agc	S	31	4480.00
ggt	G	3	0.45
ggc	G	0	0.44
gga	G	0	1.33
ggg	G	0	0.77
---	--	------	------
mPD		0.0057	1.6
		nPD:	0.
		N. weight:	1.7
		Sc. PD:	-0.067
		Sc. rank:	-27161.0

,

PA

Pos.	396	obs:	exp:
tat	Y	3	1.56
tac	Y	0	1.44
aat	N	29	17.15
aac	N	0	11.85
gat	D	22513	11877.00
gac	D	28	10664.00
gaa	E	8	6.06
gag	E	2	3.94
ggt	G	6	0.91
ggc	G	0	0.89
gga	G	0	2.67
ggg	G	0	1.53
---	--	------	------
mPD		0.0067	0.50
		nPD:	0.01
		N. weight:	0.66
		Sc. PD:	-0.019
		Sc. rank:	-816.7

,

PA

Pos.	397	obs:	exp:
aaa	K	2	3.42
aag	K	4	2.58
gtt	V	0	1.99
gtc	V	0	1.64
gta	V	1	2.42
gtg	V	8	2.95
gat	D	18	9.48
gac	D	0	8.52
gaa	E	1022	13655.00
gag	E	21529	8896.00
ggt	G	0	0.76
ggc	G	0	0.74
gga	G	0	2.22
ggg	G	5	1.28
---	--	------	------
mPD		0.090	0.48
		nPD:	0.19
		N. weight:	0.76
		Sc. PD:	0.11
		Sc. rank:	12733.0

,

PA			
Pos.	398	obs:	exp:
cct	P	440	6223.00
ccc	P	99	4189.00
cca	P	22000	9618.00
ccg	P	49	2558.00
act	T	0	0.25
acc	T	0	0.21
aca	T	1	0.46
acg	T	0	0.09
---	--	------	------
mPD		0.051	0.70
		nPD:	0.07
		N. weight:	0.76
		Sc. PD:	0.023
		Sc. rank:	10783.0

,

PA			
Pos.	399	obs:	exp:
aaa	K	0	42.21
aag	K	74	31.79
gtt	V	0	0.22
gtc	V	0	0.18
gta	V	0	0.27
gtg	V	1	0.33
gct	A	0	0.53
gcc	A	0	0.41
gca	A	0	0.94
gcg	A	2	0.12
gat	D	144	76.40
gac	D	1	68.60
gaa	E	229	13540.00
gag	E	22133	8822.00
ggt	G	0	0.76
ggc	G	0	0.74
gga	G	0	2.22
ggg	G	5	1.28
---	--	------	------
mPD		0.040	0.49
		nPD:	0.08
		N. weight:	0.9
		Sc. PD:	0.034
		Sc. rank:	13104.0

,

PA			
Pos.	400	obs:	exp:
tta	L	5	89.60
ttg	L	0	153.40
tct	S	0	11.04
tcc	S	60	6.95
tca	S	0	15.62
tcg	S	0	3.19
ctt	L	0	160.60
ctc	L	810	115.10
cta	L	0	156.90
ctg	L	0	139.30
cct	P	27	5979.00
ccc	P	21672	4025.00
cca	P	2	9240.00
ccg	P	0	2457.00
cat	H	0	0.59
cac	H	1	0.41
act	T	0	2.77
acc	T	11	2.26
aca	T	0	5.03
acg	T	0	0.94
agt	S	0	11.28
agc	S	0	11.92
gct	A	0	0.26
gcc	A	1	0.21
gca	A	0	0.47
gcg	A	0	0.06
---	--	------	------
mPD		0.079	0.80
		nPD:	0.1
		N. weight:	1.8
		Sc. PD:	0.098
		Sc. rank:	27271.0

,

PA			
Pos.	401	obs:	exp:
cgt	R	0	613.80
cgc	R	0	1176.00
cga	R	12	2202.00
cgg	R	0	1645.00
att	I	0	0.73
atc	I	0	0.50
ata	I	2	0.77
aaa	K	99	56.47
aag	K	0	42.53
aga	R	22436	11286.00
agg	R	33	5558.00
ggt	G	0	1.06
ggc	G	0	1.04
gga	G	7	3.11
ggg	G	0	1.79
---	--	------	------
mPD		0.014	0.92
		nPD:	0.01
		N. weight:	0.7
		Sc. PD:	-0.02
		Sc. rank:	-90.7

,

PA			
Pos.	402	obs:	exp:
ttt	F	6	2.57
ttc	F	0	3.43
tct	S	22501	4152.00
tcc	S	41	2613.00
tca	S	10	5871.00
tcg	S	8	1201.00
tat	Y	1	0.52
tac	Y	0	0.48
tgt	C	1	0.45
tgc	C	0	0.55
agt	S	0	4242.00
agc	S	0	4481.00
gct	A	21	5.56
gcc	A	0	4.35
gca	A	0	9.86
gcg	A	0	1.23
---	--	------	------
mPD		0.0078	1.6
		nPD:	0.
		N. weight:	1.7
		Sc. PD:	-0.066
		Sc. rank:	-23167.0

,

PA			
Pos.	403	obs:	exp:
tta	L	97	2482.00
ttg	L	0	4250.00
ctt	L	10	4449.00
ctc	L	4	3189.00
cta	L	22429	4346.00
ctg	L	35	3860.00
cct	P	0	1.10
ccc	P	0	0.74
cca	P	4	1.70
ccg	P	0	0.45
att	I	0	3.64
atc	I	0	2.50
ata	I	10	3.86
---	--	------	------
mPD		0.014	1.1
		nPD:	0.01
		N. weight:	1.7
		Sc. PD:	-0.05
		Sc. rank:	-3877.2

,

PA			
Pos.	404	obs:	exp:
tct	S	0	2.94
tcc	S	0	1.85
tca	S	16	4.16
tcg	S	0	0.85
cct	P	0	0.28
ccc	P	0	0.19
cca	P	1	0.43
ccg	P	0	0.11
act	T	0	1.76
acc	T	0	1.44
aca	T	7	3.20
acg	T	0	0.60
agt	S	0	3.01
agc	S	0	3.18
gct	A	7	5974.00
gcc	A	3	4671.00
gca	A	22533	10598.00
gcg	A	22	1322.00
---	--	------	------
mPD		0.0050	0.67
		nPD:	0.01
		N. weight:	0.78
		Sc. PD:	-0.027
		Sc. rank:	-6904.5

,

PA			
Pos.	405	obs:	exp:
tct	S	0	4153.00
tcc	S	0	2614.00
tca	S	0	5873.00
tcg	S	0	1201.00
cgt	R	0	0.03
cgc	R	0	0.05
cga	R	0	0.10
cgg	R	0	0.07
att	I	0	2.18
atc	I	6	1.50
ata	I	0	2.32
aat	N	0	7.69
aac	N	13	5.31
agt	S	22	4243.00
agc	S	22543	4482.00
aga	R	0	0.50
agg	R	1	0.25
gct	A	0	0.26
gcc	A	1	0.21
gca	A	0	0.47
gcg	A	0	0.06
ggt	G	0	0.45
ggc	G	3	0.44
gga	G	0	1.33
ggg	G	0	0.77
---	--	------	------
mPD		0.0042	1.6
		nPD:	0.
		N. weight:	1.7
		Sc. PD:	-0.067
		Sc. rank:	-29077.0

,

PA			
Pos.	406	obs:	exp:
tgg	W	22588	22588.00
cgt	R	0	0.03
cgc	R	0	0.05
cga	R	0	0.10
cgg	R	0	0.07
aga	R	0	0.50
agg	R	1	0.25
---	--	------	------
mPD		0.000089	0.00015
		nPD:	0.6
		N. weight:	0.000065
		Sc. PD:	0.000035
		Sc. rank:	1.2

,

PA			
Pos.	407	obs:	exp:
att	I	0	40.77
atc	I	107	27.96
ata	I	5	43.27
act	T	0	0.25
acc	T	1	0.21
aca	T	0	0.46
acg	T	0	0.09
gtt	V	125	4967.00
gtc	V	22328	4082.00
gta	V	4	6042.00
gtg	V	9	7375.00
gct	A	0	2.65
gcc	A	10	2.07
gca	A	0	4.70
gcg	A	0	0.59
---	--	------	------
mPD		0.024	0.75
		nPD:	0.03
		N. weight:	1.7
		Sc. PD:	-0.02
		Sc. rank:	15536.0

,

PA			
Pos.	408	obs:	exp:
caa	Q	22496	11919.00
cag	Q	92	10669.00
cgt	R	0	0.03
cgc	R	0	0.05
cga	R	1	0.10
cgg	R	0	0.07
aga	R	0	0.50
agg	R	0	0.25
---	--	------	------
mPD		0.0082	0.50
		nPD:	0.02
		N. weight:	0.64
		Sc. PD:	-0.017
		Sc. rank:	594.1

,

PA			
Pos.	409	obs:	exp:
tct	S	0	7.73
tcc	S	0	4.86
tca	S	0	10.93
tcg	S	0	2.24
att	I	1	0.36
atc	I	0	0.25
ata	I	0	0.39
aat	N	22422	13335.00
aac	N	124	9211.00
agt	S	42	7.90
agc	S	0	8.34
---	--	------	------
mPD		0.015	0.49
		nPD:	0.03
		N. weight:	0.52
		Sc. PD:	-0.0065
		Sc. rank:	4453.8

,

PA			
Pos.	410	obs:	exp:
gat	D	1	0.53
gac	D	0	0.47
gaa	E	22530	13677.00
gag	E	58	8911.00
---	--	------	------
mPD		0.0052	0.48
		nPD:	0.01
		N. weight:	0.51
		Sc. PD:	-0.016
		Sc. rank:	-1857.4

,

PA			
Pos.	411	obs:	exp:
ttt	F	49	9692.00
ttc	F	22535	12892.00
tta	L	0	0.22
ttg	L	0	0.38
tat	Y	0	1.04
tac	Y	2	0.96
ctt	L	0	0.39
ctc	L	2	0.28
cta	L	0	0.39
ctg	L	0	0.34
att	I	0	0.36
atc	I	1	0.25
ata	I	0	0.39
---	--	------	------
mPD		0.0048	0.49
		nPD:	0.01
		N. weight:	0.57
		Sc. PD:	-0.019
		Sc. rank:	-3050.2

,

PA			
Pos.	412	obs:	exp:
act	T	1	0.25
acc	T	0	0.21
aca	T	0	0.46
acg	T	0	0.09
aat	N	22544	13359.00
aac	N	44	9229.00
---	--	------	------
mPD		0.0040	0.48
		nPD:	0.01
		N. weight:	0.53
		Sc. PD:	-0.018
		Sc. rank:	-4182.4

,

PA			
Pos.	413	obs:	exp:
caa	Q	1	0.53
cag	Q	0	0.47
cgt	R	0	0.14
cgc	R	0	0.26
cga	R	0	0.49
cgg	R	0	0.37
aaa	K	90	12882.00
aag	K	22493	9701.00
aga	R	0	2.51
agg	R	5	1.24
---	--	------	------
mPD		0.0086	0.49
		nPD:	0.02
		N. weight:	0.85
		Sc. PD:	-0.021
		Sc. rank:	1591.7

,

PA			
Pos.	414	obs:	exp:
tct	S	0	0.18
tcc	S	0	0.12
tca	S	1	0.26
tcg	S	0	0.05
act	T	0	1.76
acc	T	0	1.44
aca	T	7	3.20
acg	T	0	0.60
agt	S	0	0.19
agc	S	0	0.20
gct	A	5	5978.00
gcc	A	45	4674.00
gca	A	22487	10605.00
gcg	A	44	1323.00
---	--	------	------
mPD		0.0090	0.66
		nPD:	0.01
		N. weight:	0.76
		Sc. PD:	-0.022
		Sc. rank:	-719.8

,

PA			
Pos.	415	obs:	exp:
tta	L	0	0.11
ttg	L	0	0.19
tgt	C	22561	10195.00
tgc	C	26	12392.00
ctt	L	1	0.20
ctc	L	0	0.14
cta	L	0	0.19
ctg	L	0	0.17
cgt	R	1	0.03
cgc	R	0	0.05
cga	R	0	0.10
cgg	R	0	0.07
aga	R	0	0.50
agg	R	0	0.25
---	--	------	------
mPD		0.0026	0.50
		nPD:	0.01
		N. weight:	0.82
		Sc. PD:	-0.03
		Sc. rank:	-10152.0

,

PA			
Pos.	416	obs:	exp:
gaa	E	22564	13678.00
gag	E	25	8911.00
---	--	------	------
mPD		0.0022	0.48
		nPD:	0.
		N. weight:	0.51
		Sc. PD:	-0.019
		Sc. rank:	-6935.2

,

PA			
Pos.	417	obs:	exp:
tta	L	358	2483.00
ttg	L	22160	4252.00
tct	S	0	0.37
tcc	S	0	0.23
tca	S	0	0.52
tcg	S	2	0.11
ctt	L	0	4451.00
ctc	L	0	3190.00
cta	L	6	4348.00
ctg	L	61	3861.00
agt	S	0	0.38
agc	S	0	0.40
gtt	V	0	0.44
gtc	V	0	0.36
gta	V	0	0.54
gtg	V	2	0.66
---	--	------	------
mPD		0.038	1.1
		nPD:	0.03
		N. weight:	1.6
		Sc. PD:	-0.015
		Sc. rank:	15791.0

,

PA			
Pos.	418	obs:	exp:
tct	S	0	0.18
tcc	S	0	0.12
tca	S	0	0.26
tcg	S	1	0.05
att	I	18	6.55
atc	I	0	4.49
ata	I	0	6.95
act	T	22470	5684.00
acc	T	25	4625.00
aca	T	3	10316.00
acg	T	51	1924.00
aat	N	3	1.77
aac	N	0	1.23
agt	S	0	0.19
agc	S	0	0.20
gct	A	18	4.77
gcc	A	0	3.73
gca	A	0	8.45
gcg	A	0	1.05
---	--	------	------
mPD		0.011	0.68
		nPD:	0.02
		N. weight:	1.4
		Sc. PD:	-0.038
		Sc. rank:	796.2

,

PA			
Pos.	419	obs:	exp:
tat	Y	1	0.52
tac	Y	0	0.48
aat	N	11	6.51
aac	N	0	4.49
gat	D	22411	11894.00
gac	D	162	10679.00
ggt	G	4	0.61
ggc	G	0	0.59
gga	G	0	1.78
ggg	G	0	1.02
---	--	------	------
mPD		0.016	0.50
		nPD:	0.03
		N. weight:	0.63
		Sc. PD:	-0.0071
		Sc. rank:	5517.4

,

PA			
Pos.	420	obs:	exp:
tta	L	2	0.22
ttg	L	0	0.38
tct	S	2	4156.00
tcc	S	11	2616.00
tca	S	22541	5878.00
tcg	S	32	1202.00
ctt	L	0	0.39
ctc	L	0	0.28
cta	L	0	0.39
ctg	L	0	0.34
agt	S	0	4247.00
agc	S	0	4486.00
gct	A	0	0.26
gcc	A	0	0.21
gca	A	1	0.47
gcg	A	0	0.06
---	--	------	------
mPD		0.0042	1.6
		nPD:	0.
		N. weight:	1.4
		Sc. PD:	-0.055
		Sc. rank:	-24004.0

,

PA			
Pos.	421	obs:	exp:
tct	S	0	4153.00
tcc	S	0	2614.00
tca	S	0	5874.00
tcg	S	0	1201.00
tgt	C	0	0.45
tgc	C	1	0.55
cgt	R	0	0.03
cgc	R	0	0.05
cga	R	0	0.10
cgg	R	0	0.07
att	I	0	2.55
atc	I	7	1.75
ata	I	0	2.70
act	T	0	0.25
acc	T	1	0.21
aca	T	0	0.46
acg	T	0	0.09
aat	N	0	0.59
aac	N	1	0.41
agt	S	86	4244.00
agc	S	22484	4483.00
aga	R	1	0.50
agg	R	0	0.25
gtt	V	1	1.11
gtc	V	4	0.91
gta	V	0	1.34
gtg	V	0	1.64
ggt	G	0	0.45
ggc	G	3	0.44
gga	G	0	1.33
ggg	G	0	0.77
---	--	------	------
mPD		0.0098	1.6
		nPD:	0.01
		N. weight:	1.7
		Sc. PD:	-0.06
		Sc. rank:	-18632.0

,

PA

Pos.	422	obs:	exp:
tgg	W	22589	22589.00
---	--	------	------
mPD		0	0
		nPD:	1.
		N. weight:	0.
		Sc. PD:	0
		Sc. rank:	0

,

PA

Pos.	423	obs:	exp:
tta	L	1	0.22
ttg	L	0	0.38
ctt	L	0	0.39
ctc	L	0	0.28
cta	L	1	0.39
ctg	L	0	0.34
att	I	1	8214.00
atc	I	6	5632.00
ata	I	22557	8718.00
atg	M	11	11.00
act	T	0	0.50
acc	T	0	0.41
aca	T	2	0.91
acg	T	0	0.17
gtt	V	0	2.21
gtc	V	0	1.82
gta	V	10	2.69
gtg	V	0	3.28
---	--	------	------
mPD		0.0028	0.66
		nPD:	0.
		N. weight:	0.99
		Sc. PD:	-0.038
		Sc. rank:	-13858.0

,

PA

Pos.	424	obs:	exp:
gat	D	0	1.05
gac	D	2	0.95
gaa	E	22544	13676.00
gag	E	43	8911.00
---	--	------	------
mPD		0.0040	0.48
		nPD:	0.01
		N. weight:	0.51
		Sc. PD:	-0.017
		Sc. rank:	-3878.6

,

PA

Pos.	425	obs:	exp:
ttt	F	35	15.02
ttc	F	0	19.98
tta	L	0	2479.00
ttg	L	0	4246.00
ctt	L	22537	4444.00
ctc	L	17	3186.00
cta	L	0	4342.00
ctg	L	0	3856.00
---	--	------	------
mPD		0.0046	1.1
		nPD:	0.
		N. weight:	1.7
		Sc. PD:	-0.064
		Sc. rank:	-24203.0

,

PA

Pos.	426	obs:	exp:
gat	D	22581	11902.00
gac	D	7	10686.00
ggt	G	1	0.15
ggc	G	0	0.15
gga	G	0	0.44
ggg	G	0	0.26
---	--	------	------
mPD		0.00071	0.50
		nPD:	0.
		N. weight:	0.67
		Sc. PD:	-0.027
		Sc. rank:	-12221.0

,

PA

Pos.	427	obs:	exp:
gaa	E	22561	13678.00
gag	E	28	8911.00
---	--	------	------
mPD		0.0025	0.48
		nPD:	0.01
		N. weight:	0.51
		Sc. PD:	-0.019
		Sc. rank:	-6346.1

,

PA

Pos.	428	obs:	exp:
tta	L	1	0.22
ttg	L	0	0.38
ctt	L	0	0.39
ctc	L	0	0.28
cta	L	1	0.39
ctg	L	0	0.34
att	I	12	8216.00
atc	I	0	5634.00
ata	I	22559	8720.00
atg	M	1	1.00
gtt	V	0	3.32
gtc	V	0	2.73
gta	V	15	4.03
gtg	V	0	4.92
---	--	------	------
mPD		0.0027	0.66
		nPD:	0.
		N. weight:	0.99
		Sc. PD:	-0.038
		Sc. rank:	-14030.0

,

PA

Pos.	429	obs:	exp:
ggt	G	5	3425.00
ggc	G	0	3346.00
gga	G	22514	10048.00
ggg	G	70	5770.00
---	--	------	------
mPD		0.0066	0.69
		nPD:	0.01
		N. weight:	0.82
		Sc. PD:	-0.027
		Sc. rank:	-4630.0

,

PA

Pos.	430	obs:	exp:
caa	Q	1	0.53
cag	Q	0	0.47
gat	D	2	3.69
gac	D	5	3.31
gaa	E	22552	13672.00
gag	E	27	8907.00
ggt	G	0	0.30
ggc	G	0	0.30
gga	G	2	0.89
ggg	G	0	0.51
---	--	------	------
mPD		0.0033	0.48
		nPD:	0.01
		N. weight:	0.51
		Sc. PD:	-0.018
		Sc. rank:	-5381.1

,

PA

Pos.	431	obs:	exp:
aat	N	4	2.37
aac	N	0	1.63
gat	D	22563	11900.00
gac	D	22	10685.00
---	--	------	------
mPD		0.0023	0.50
		nPD:	0.
		N. weight:	0.66
		Sc. PD:	-0.025
		Sc. rank:	-8936.6

,

PA

Pos.	432	obs:	exp:
ttt	F	1	0.43
ttc	F	0	0.57
tta	L	0	0.11
ttg	L	0	0.19
ctt	L	1	0.20
ctc	L	0	0.14
cta	L	0	0.19
ctg	L	0	0.17
att	I	40	14.56
atc	I	0	9.98
ata	I	0	15.45
gtt	V	22528	4984.00
gtc	V	3	4096.00
gta	V	10	6063.00
gtg	V	3	7400.00
gct	A	3	0.79
gcc	A	0	0.62
gca	A	0	1.41
gcg	A	0	0.18
---	--	------	------
mPD		0.0054	0.74
		nPD:	0.01
		N. weight:	1.6
		Sc. PD:	-0.055
		Sc. rank:	-14593.0

,

PA

Pos.	433	obs:	exp:
gct	A	71	5981.00
gcc	A	22481	4676.00
gca	A	37	10609.00
gcg	A	0	1323.00
---	--	------	------
mPD		0.0095	0.66
		nPD:	0.01
		N. weight:	1.6
		Sc. PD:	-0.045
		Sc. rank:	-391.2

,

PA

Pos.	434	obs:	exp:
tta	L	0	0.44
ttg	L	0	0.75
ctt	L	0	0.79
ctc	L	0	0.56
cta	L	0	0.77
ctg	L	4	0.68
cct	P	36	6222.00
ccc	P	0	4189.00
cca	P	2526	9617.00
ccg	P	20023	2557.00
---	--	------	------
mPD		0.20	0.70
		nPD:	0.29
		N. weight:	1.7
		Sc. PD:	0.42
		Sc. rank:	30283.0

PA

Pos.	435	obs:	exp:
tta	L	0	0.22
ttg	L	0	0.38
ctt	L	2	0.39
ctc	L	0	0.28
cta	L	0	0.39
ctg	L	0	0.34
att	I	22499	8221.00
atc	I	76	5637.00
ata	I	9	8725.00
gtt	V	3	0.66
gtc	V	0	0.55
gta	V	0	0.81
gtg	V	0	0.98
---	--	------	------
mPD		0.0079	0.66
		nPD:	0.01
		N. weight:	1.
		Sc. PD:	-0.031
		Sc. rank:	-2653.3

PA

Pos.	436	obs:	exp:
gaa	E	22560	13678.00
gag	E	29	8911.00
---	--	------	------
mPD		0.0026	0.48
		nPD:	0.01
		N. weight:	0.51
		Sc. PD:	-0.019
		Sc. rank:	-6088.7

PA

Pos.	437	obs:	exp:
tta	L	0	0.33
ttg	L	0	0.56
tat	Y	310	161.60
tac	Y	1	149.40
ctt	L	3	0.59
ctc	L	0	0.42
cta	L	0	0.58
ctg	L	0	0.51
cat	H	22222	13099.00
cac	H	51	9174.00
aat	N	2	1.18
aac	N	0	0.82
---	--	------	------
mPD		0.032	0.51
		nPD:	0.06
		N. weight:	0.54
		Sc. PD:	0.011
		Sc. rank:	7145.8

PA

Pos.	438	obs:	exp:
att	I	78	8178.00
atc	I	4376	5608.00
ata	I	18011	8679.00
atg	M	2	2.00
gtt	V	0	26.97
gtc	V	21	22.17
gta	V	101	32.81
gtg	V	0	40.05
---	--	------	------
mPD		0.33	0.67
		nPD:	0.49
		N. weight:	0.55
		Sc. PD:	0.24
		Sc. rank:	10078.0

PA

Pos.	439	obs:	exp:
tct	S	0	0.74
tcc	S	0	0.46
tca	S	4	1.04
tcg	S	0	0.21
agt	S	0	0.75
agc	S	0	0.79
gct	A	8	5979.00
gcc	A	5	4675.00
gca	A	22557	10607.00
gcg	A	15	1323.00
---	--	------	------
mPD		0.0028	0.66
		nPD:	0.
		N. weight:	0.78
		Sc. PD:	-0.03
		Sc. rank:	-10961.0

PA

Pos.	440	obs:	exp:
tct	S	0	4157.00
tcc	S	0	2616.00
tca	S	0	5879.00
tcg	S	0	1202.00
att	I	0	0.36
atc	I	1	0.25
ata	I	0	0.39
agt	S	236	4247.00
agc	S	22352	4487.00
---	--	------	------
mPD		0.021	1.6
		nPD:	0.01
		N. weight:	1.6
		Sc. PD:	-0.048
		Sc. rank:	-3013.1

PA

Pos.	441	obs:	exp:
cgt	R	0	0.63
cgc	R	0	1.20
cga	R	0	2.25
cgg	R	0	1.68
att	I	2	6.19
atc	I	0	4.24
ata	I	15	6.57
atg	M	22473	22473.00
act	T	0	3.28
acc	T	0	2.67
aca	T	0	5.95
acg	T	13	1.11
aaa	K	0	3.99
aag	K	7	3.01
aga	R	0	11.55
agg	R	23	5.69
gtt	V	0	12.38
gtc	V	0	10.18
gta	V	0	15.06
gtg	V	56	18.38
---	--	------	------
mPD		0.010	0.017
		nPD:	0.61
		N. weight:	0.0066
		Sc. PD:	0.0037
		Sc. rank:	123.1

PA

Pos.	442	obs:	exp:
cgt	R	0	616.70
cgc	R	0	1182.00
cga	R	0	2212.00
cgg	R	1	1653.00
aaa	K	0	0.57
aag	K	1	0.43
aga	R	262	11340.00
agg	R	22325	5584.00
---	--	------	------
mPD		0.023	0.91
		nPD:	0.03
		N. weight:	1.4
		Sc. PD:	-0.024
		Sc. rank:	9635.6

PA

Pos.	443	obs:	exp:
cgt	R	0	616.70
cgc	R	0	1182.00
cga	R	0	2212.00
cgg	R	0	1653.00
aga	R	202	11340.00
agg	R	22386	5584.00
ggt	G	0	0.15
ggc	G	0	0.15
gga	G	0	0.44
ggg	G	1	0.26
---	--	------	------
mPD		0.018	0.91
		nPD:	0.02
		N. weight:	1.4
		Sc. PD:	-0.032
		Sc. rank:	4900.1

PA

Pos.	444	obs:	exp:
tct	S	0	0.55
tcc	S	0	0.35
tca	S	0	0.78
tcg	S	0	0.16
aat	N	52	13346.00
aac	N	22514	9220.00
agt	S	0	0.56
agc	S	3	0.60
gat	D	0	10.54
gac	D	20	9.46
---	--	------	------
mPD		0.0066	0.49
		nPD:	0.01
		N. weight:	0.92
		Sc. PD:	-0.026
		Sc. rank:	-772.9

PA

Pos.	445	obs:	exp:
ttt	F	1	0.43
ttc	F	0	0.57
tat	Y	22551	11735.00
tac	Y	35	10851.00
cat	H	2	1.18
cac	H	0	0.82
---	--	------	------
mPD		0.0034	0.50
		nPD:	0.01
		N. weight:	0.67
		Sc. PD:	-0.024
		Sc. rank:	-7039.8

PA

Pos.	446	obs:	exp:
ttt	F	20452	9694.00
ttc	F	2137	12895.00
---	--	------	------
mPD		0.17	0.49
		nPD:	0.35
		N. weight:	0.53
		Sc. PD:	0.16
		Sc. rank:	9409.3

PA

Pos.	447	obs:	exp:
tct	S	0	0.18
tcc	S	0	0.12
tca	S	1	0.26
tcg	S	0	0.05
act	T	8	5692.00
acc	T	12	4632.00
aca	T	22552	10330.00
acg	T	9	1927.00
agt	S	0	0.19
agc	S	0	0.20
gct	A	0	1.85
gcc	A	0	1.45
gca	A	7	3.29
gcg	A	0	0.41
---	--	------	------
mPD		0.0033	0.68
		nPD:	0.
		N. weight:	0.81
		Sc. PD:	-0.03
		Sc. rank:	-10639.0

PA

Pos.	448	obs:	exp:
tct	S	0	0.55
tcc	S	0	0.35
tca	S	3	0.78
tcg	S	0	0.16
act	T	0	4.03
acc	T	0	3.28
aca	T	16	7.32
acg	T	0	1.37
agt	S	0	0.56
agc	S	0	0.60
gtt	V	0	0.44
gtc	V	0	0.36
gta	V	2	0.54
gtg	V	0	0.66
gct	A	8	5972.00
gcc	A	1	4669.00
gca	A	22509	10593.00
gcg	A	37	1321.00
gaa	E	13	7.87
gag	E	0	5.13
---	--	------	------
mPD		0.0071	0.67
		nPD:	0.01
		N. weight:	0.78
		Sc. PD:	-0.025
		Sc. rank:	-3108.1

PA

Pos.	449	obs:	exp:
gaa	E	22563	13678.00
gag	E	26	8911.00
---	--	------	------
mPD		0.0023	0.48
		nPD:	0.
		N. weight:	0.51
		Sc. PD:	-0.019
		Sc. rank:	-6817.3

PA

Pos.	450	obs:	exp:
cgt	R	0	0.03
cgc	R	0	0.05
cga	R	0	0.10
cgg	R	1	0.07
atg	M	3	3.00
aga	R	0	0.50
agg	R	0	0.25
gtt	V	33	4993.00
gtc	V	1	4104.00
gta	V	43	6074.00
gtg	V	22507	7413.00
gct	A	0	0.26
gcc	A	0	0.21
gca	A	0	0.47
gcg	A	1	0.06
---	--	------	------
mPD		0.0073	0.74
		nPD:	0.01
		N. weight:	1.1
		Sc. PD:	-0.037
		Sc. rank:	-5615.7

,

PA

Pos.	451	obs:	exp:
tta	L	0	0.22
ttg	L	0	0.38
tct	S	1331	4156.00
tcc	S	21242	2616.00
tca	S	10	5877.00
tcg	S	0	1202.00
ctt	L	0	0.39
ctc	L	2	0.28
cta	L	0	0.39
ctg	L	0	0.34
cct	P	0	0.28
ccc	P	1	0.19
cca	P	0	0.43
ccg	P	0	0.11
agt	S	0	4246.00
agc	S	0	4486.00
gct	A	0	0.79
gcc	A	3	0.62
gca	A	0	1.41
gcg	A	0	0.18
---	--	------	------
mPD		0.11	1.6
		nPD:	0.07
		N. weight:	2.
		Sc. PD:	0.05
		Sc. rank:	27433.0

,

PA

Pos.	452	obs:	exp:
tct	S	0	0.18
tcc	S	0	0.12
tca	S	0	0.26
tcg	S	0	0.05
tat	Y	1	3.12
tac	Y	5	2.88
cat	H	83	13279.00
cac	H	22497	9301.00
caa	Q	2	1.06
cag	Q	0	0.94
agt	S	1	0.19
agc	S	0	0.20
---	--	------	------
mPD		0.0084	0.49
		nPD:	0.02
		N. weight:	0.9
		Sc. PD:	-0.023
		Sc. rank:	1511.0

,

PA

Pos.	453	obs:	exp:
tct	S	0	0.37
tcc	S	1	0.23
tca	S	0	0.52
tcg	S	0	0.11
tgt	C	43	10195.00
tgc	C	22544	12392.00
agt	S	0	0.38
agc	S	1	0.40
---	--	------	------
mPD		0.0040	0.50
		nPD:	0.01
		N. weight:	0.61
		Sc. PD:	-0.021
		Sc. rank:	-4979.9

,

PA

Pos.	454	obs:	exp:
cgt	R	0	616.80
cgc	R	0	1182.00
cga	R	0	2213.00
cgg	R	5	1653.00
aga	R	50	11340.00
agg	R	22534	5584.00
---	--	------	------
mPD		0.0049	0.91
		nPD:	0.01
		N. weight:	1.4
		Sc. PD:	-0.053
		Sc. rank:	-17206.0

,

PA

Pos.	455	obs:	exp:
tct	S	2	0.37
tcc	S	0	0.23
tca	S	0	0.52
tcg	S	0	0.11
act	T	4	1.01
acc	T	0	0.82
aca	T	0	1.83
acg	T	0	0.34
agt	S	0	0.38
agc	S	0	0.40
gtt	V	2	0.44
gtc	V	0	0.36
gta	V	0	0.54
gtg	V	0	0.66
gct	A	22494	5978.00
gcc	A	78	4674.00
gca	A	2	10605.00
gcg	A	6	1323.00
ggt	G	1	0.15
ggc	G	0	0.15
gga	G	0	0.44
ggg	G	0	0.26
---	--	------	------
mPD		0.0084	0.66
		nPD:	0.01
		N. weight:	1.4
		Sc. PD:	-0.04
		Sc. rank:	-2636.3

,

PA

Pos.	456	obs:	exp:
act	T	22474	5694.00
acc	T	98	4633.00
aca	T	15	10333.00
acg	T	1	1927.00
aat	N	1	0.59
aac	N	0	0.41
---	--	------	------
mPD		0.010	0.68
		nPD:	0.01
		N. weight:	1.4
		Sc. PD:	-0.039
		Sc. rank:	125.0

,

PA

Pos.	457	obs:	exp:
gaa	E	22545	13678.00
gag	E	44	8911.00
---	--	------	------
mPD		0.0039	0.48
		nPD:	0.01
		N. weight:	0.51
		Sc. PD:	-0.017
		Sc. rank:	-4087.3

,

PA

Pos.	458	obs:	exp:
tat	Y	800	11736.00
tac	Y	21788	10852.00
cat	H	0	0.59
cac	H	1	0.41
---	--	------	------
mPD		0.068	0.50
		nPD:	0.14
		N. weight:	0.6
		Sc. PD:	0.056
		Sc. rank:	9768.9

,

PA

Pos.	459	obs:	exp:
tta	L	1	0.22
ttg	L	0	0.38
ctt	L	0	0.39
ctc	L	0	0.28
cta	L	1	0.39
ctg	L	0	0.34
att	I	0	8206.00
atc	I	0	5627.00
ata	I	22541	8709.00
atg	M	4	4.00
act	T	0	9.08
acc	T	0	7.38
aca	T	36	16.47
acg	T	0	3.07
gtt	V	0	1.33
gtc	V	0	1.09
gta	V	6	1.61
gtg	V	0	1.97
---	--	------	------
mPD		0.0042	0.66
		nPD:	0.01
		N. weight:	0.99
		Sc. PD:	-0.036
		Sc. rank:	-10665.0

,

PA

Pos.	460	obs:	exp:
tta	L	0	0.11
ttg	L	1	0.19
ctt	L	0	0.20
ctc	L	0	0.14
cta	L	0	0.19
ctg	L	0	0.17
att	I	2	14.20
atc	I	0	9.74
ata	I	37	15.07
atg	M	22546	22546.00
gtt	V	0	0.66
gtc	V	0	0.55
gta	V	0	0.81
gtg	V	3	0.98
---	--	------	------
mPD		0.0038	0.0040
		nPD:	0.94
		N. weight:	0.0016
		Sc. PD:	0.0015
		Sc. rank:	30.7

,

PA

Pos.	461	obs:	exp:
cgt	R	0	0.19
cgc	R	0	0.37
cga	R	0	0.69
cgg	R	0	0.51
aaa	K	130	12881.00
aag	K	22452	9701.00
aga	R	0	3.51
agg	R	7	1.73
---	--	------	------
mPD		0.012	0.49
		nPD:	0.02
		N. weight:	0.84
		Sc. PD:	-0.015
		Sc. rank:	5465.7

,

PA

Pos.	462	obs:	exp:
cgt	R	0	0.03
cgc	R	0	0.05
cga	R	0	0.10
cgg	R	0	0.07
aga	R	1	0.50
agg	R	0	0.25
ggt	G	6	3425.00
ggc	G	0	3346.00
gga	G	22507	10048.00
ggg	G	75	5770.00
---	--	------	------
mPD		0.0072	0.69
		nPD:	0.01
		N. weight:	0.82
		Sc. PD:	-0.026
		Sc. rank:	-3562.6

,

PA

Pos.	463	obs:	exp:
tta	L	0	0.11
ttg	L	1	0.19
ctt	L	0	0.20
ctc	L	0	0.14
cta	L	0	0.19
ctg	L	0	0.17
atg	M	6	6.00
gtt	V	14	4992.00
gtc	V	1	4103.00
gta	V	40	6073.00
gtg	V	22526	7413.00
gct	A	0	0.26
gcc	A	0	0.21
gca	A	0	0.47
gcg	A	1	0.06
---	--	------	------
mPD		0.0056	0.74
		nPD:	0.01
		N. weight:	1.1
		Sc. PD:	-0.04
		Sc. rank:	-9856.5

,

PA

Pos.	464	obs:	exp:
tat	Y	38	11731.00
tac	Y	22540	10847.00
tgt	C	0	0.45
tgc	C	1	0.55
cat	H	0	5.88
cac	H	10	4.12
---	--	------	------
mPD		0.0043	0.50
		nPD:	0.01
		N. weight:	0.75
		Sc. PD:	-0.025
		Sc. rank:	-5079.1

,

PA

Pos.	465	obs:	exp:
tta	L	0	1.87
ttg	L	0	3.20
ctt	L	0	3.35
ctc	L	0	2.40
cta	L	17	3.27
ctg	L	0	2.91
att	I	7	8183.00
atc	I	2	5611.00
ata	I	22470	8685.00
atg	M	36	36.00
act	T	0	11.34
acc	T	0	9.23
aca	T	45	20.59
acg	T	0	3.84
gtt	V	0	2.65
gtc	V	0	2.18
gta	V	12	3.23
gtg	V	0	3.94
---	--	------	------
mPD		0.011	0.66
		nPD:	0.02
		N. weight:	0.99
		Sc. PD:	-0.026
		Sc. rank:	607.3

,

PA

Pos.	466	obs:	exp:
tct	S	0	2.21
tcc	S	0	1.39
tca	S	0	3.12
tcg	S	0	0.64
act	T	1	0.25
acc	T	0	0.21
aca	T	0	0.46
acg	T	0	0.09
aat	N	21727	13351.00
aac	N	846	9222.00
aaa	K	3	1.71
aag	K	0	1.29
agt	S	12	2.26
agc	S	0	2.38
---	--	------	------
mPD		0.074	0.49
		nPD:	0.15
		N. weight:	0.4
		Sc. PD:	0.042
		Sc. rank:	6526.0

,

PA

Pos.	467	obs:	exp:
act	T	51	5694.00
acc	T	0	4633.00
aca	T	572	10334.00
acg	T	21966	1927.00
---	--	------	------
mPD		0.054	0.68
		nPD:	0.08
		N. weight:	2.4
		Sc. PD:	0.086
		Sc. rank:	34311.0

,

PA

Pos.	468	obs:	exp:
gct	A	74	5981.00
gcc	A	22391	4676.00
gca	A	123	10609.00
gcg	A	1	1323.00
---	--	------	------
mPD		0.017	0.66
		nPD:	0.03
		N. weight:	1.6
		Sc. PD:	-0.026
		Sc. rank:	11521.0

,

PA

Pos.	469	obs:	exp:
tta	L	63	2482.00
ttg	L	22486	4251.00
ctt	L	0	4449.00
ctc	L	3	3189.00
cta	L	0	4346.00
ctg	L	25	3860.00
atg	M	5	5.00
gtt	V	0	1.55
gtc	V	0	1.27
gta	V	0	1.88
gtg	V	7	2.30
---	--	------	------
mPD		0.0094	1.1
		nPD:	0.01
		N. weight:	1.7
		Sc. PD:	-0.058
		Sc. rank:	-13585.0

,

PA

Pos.	470	obs:	exp:
ttt	F	0	0.43
ttc	F	1	0.57
tta	L	0	2483.00
ttg	L	0	4252.00
ctt	L	7656	4450.00
ctc	L	14924	3190.00
cta	L	4	4348.00
ctg	L	0	3861.00
att	I	2	1.46
atc	I	2	1.00
ata	I	0	1.55
---	--	------	------
mPD		0.45	1.1
		nPD:	0.39
		N. weight:	1.3
		Sc. PD:	0.43
		Sc. rank:	22553.0

,

PA

Pos.	471	obs:	exp:
aat	N	22572	13360.00
aac	N	17	9229.00
---	--	------	------
mPD		0.0015	0.48
		nPD:	0.
		N. weight:	0.54
		Sc. PD:	-0.021
		Sc. rank:	-8861.9

,

PA

Pos.	472	obs:	exp:
tct	S	0	0.74
tcc	S	0	0.46
tca	S	4	1.04
tcg	S	0	0.21
agt	S	0	0.75
agc	S	0	0.79
gct	A	3	5979.00
gcc	A	6	4675.00
gca	A	22561	10607.00
gcg	A	15	1323.00
---	--	------	------
mPD		0.0025	0.66
		nPD:	0.
		N. weight:	0.78
		Sc. PD:	-0.03
		Sc. rank:	-11745.0

,

PA

Pos.	473	obs:	exp:
ttt	F	0	0.43
ttc	F	1	0.57
tct	S	650	4156.00
tcc	S	21908	2616.00
tca	S	28	5878.00
tcg	S	0	1202.00
agt	S	0	4247.00
agc	S	0	4486.00
gct	A	0	0.53
gcc	A	2	0.41
gca	A	0	0.94
gcg	A	0	0.12
---	--	------	------
mPD		0.059	1.6
		nPD:	0.04
		N. weight:	2.1
		Sc. PD:	-0.015
		Sc. rank:	21098.0

,

PA

Pos.	474	obs:	exp:
tct	S	0	0.18
tcc	S	0	0.12
tca	S	0	0.26
tcg	S	0	0.05
tgt	C	22563	10195.00
tgc	C	25	12393.00
agt	S	1	0.19
agc	S	0	0.20
---	--	------	------
mPD		0.0023	0.50
		nPD:	0.
		N. weight:	0.82
		Sc. PD:	-0.031
		Sc. rank:	-11018.0

,

PA

Pos.	475	obs:	exp:
tct	S	0	0.37
tcc	S	0	0.23
tca	S	2	0.52
tcg	S	0	0.11
act	T	0	2.27
acc	T	0	1.85
aca	T	9	4.12
acg	T	0	0.77
agt	S	0	0.38
agc	S	0	0.40
gtt	V	0	0.44
gtc	V	0	0.36
gta	V	2	0.54
gtg	V	0	0.66
gct	A	121	5977.00
gcc	A	2	4673.00
gca	A	22390	10603.00
gcg	A	63	1323.00
---	--	------	------
mPD		0.018	0.66
		nPD:	0.03
		N. weight:	0.74
		Sc. PD:	-0.012
		Sc. rank:	5445.2

,

PA

Pos.	476	obs:	exp:
tct	S	0	1.29
tcc	S	7	0.81
tca	S	0	1.82
tcg	S	0	0.37
act	T	0	1.51
acc	T	6	1.23
aca	T	0	2.74
acg	T	0	0.51
agt	S	0	1.32
agc	S	0	1.39
gtt	V	2	2.87
gtc	V	11	2.36
gta	V	0	3.50
gtg	V	0	4.27
gct	A	189	5974.00
gcc	A	22332	4670.00
gca	A	42	10597.00
gcg	A	0	1322.00
---	--	------	------
mPD		0.023	0.67
		nPD:	0.03
		N. weight:	1.6
		Sc. PD:	-0.013
		Sc. rank:	15531.0

,

PA

Pos.	477	obs:	exp:
att	I	0	0.36
atc	I	0	0.25
ata	I	1	0.39
atg	M	22588	22588.00
---	--	------	------
mPD		0.000089	0.000089
		nPD:	1.
		N. weight:	0.000044
		Sc. PD:	0.000041
		Sc. rank:	0.8

,

PA			
Pos.	478	obs:	exp:
aat	N	3	1.77
aac	N	0	1.23
gct	A	1	0.26
gcc	A	0	0.21
gca	A	0	0.47
gcg	A	0	0.06
gat	D	22560	11895.00
gac	D	16	10681.00
gaa	E	2	1.21
gag	E	0	0.79
ggt	G	7	1.06
ggc	G	0	1.04
gga	G	0	3.11
ggg	G	0	1.79
---	--	------	------
mPD		0.0026	0.50
		nPD:	0.01
		N. weight:	0.66
		Sc. PD:	-0.025
		Sc. rank:	-8252.3

,

PA			
Pos.	479	obs:	exp:
tat	Y	0	1.04
tac	Y	2	0.96
aat	N	1	3.55
aac	N	5	2.45
gat	D	452	11873.00
gac	D	22082	10661.00
gaa	E	43	26.04
gag	E	0	16.96
ggt	G	0	0.61
ggc	G	4	0.59
gga	G	0	1.78
ggg	G	0	1.02
---	--	------	------
mPD		0.044	0.50
		nPD:	0.09
		N. weight:	0.68
		Sc. PD:	0.03
		Sc. rank:	10082.0

,

PA			
Pos.	480	obs:	exp:
ttt	F	22500	9692.00
ttc	F	84	12892.00
tta	L	0	0.33
ttg	L	0	0.56
tct	S	1	0.18
tcc	S	0	0.12
tca	S	0	0.26
tcg	S	0	0.05
tat	Y	1	0.52
tac	Y	0	0.48
ctt	L	3	0.59
ctc	L	0	0.42
cta	L	0	0.58
ctg	L	0	0.51
agt	S	0	0.19
agc	S	0	0.20
---	--	------	------
mPD		0.0079	0.49
		nPD:	0.02
		N. weight:	0.86
		Sc. PD:	-0.023
		Sc. rank:	565.0

,

PA			
Pos.	481	obs:	exp:
caa	Q	785	11920.00
cag	Q	21804	10669.00
---	--	------	------
mPD		0.067	0.50
		nPD:	0.13
		N. weight:	0.62
		Sc. PD:	0.056
		Sc. rank:	10055.0

,

PA			
Pos.	482	obs:	exp:
tta	L	0	2482.00
ttg	L	86	4250.00
ctt	L	6	4449.00
ctc	L	1	3189.00
cta	L	116	4346.00
ctg	L	22366	3860.00
atg	M	14	14.00
---	--	------	------
mPD		0.020	1.1
		nPD:	0.02
		N. weight:	1.8
		Sc. PD:	-0.045
		Sc. rank:	2619.4

,

PA			
Pos.	483	obs:	exp:
tta	L	0	0.22
ttg	L	0	0.38
ctt	L	2	0.39
ctc	L	0	0.28
cta	L	0	0.39
ctg	L	0	0.34
att	I	18270	8216.00
atc	I	4291	5634.00
ata	I	10	8720.00
act	T	13	3.28
acc	T	0	2.67
aca	T	0	5.95
acg	T	0	1.11
gtt	V	3	0.66
gtc	V	0	0.55
gta	V	0	0.81
gtg	V	0	0.98
---	--	------	------
mPD		0.31	0.66
		nPD:	0.47
		N. weight:	0.62
		Sc. PD:	0.26
		Sc. rank:	11364.0

,

PA			
Pos.	484	obs:	exp:
tct	S	0	1.47
tcc	S	0	0.93
tca	S	8	2.08
tcg	S	0	0.43
cct	P	1	6221.00
ccc	P	7	4188.00
cca	P	22512	9615.00
ccg	P	61	2557.00
agt	S	0	1.50
agc	S	0	1.59
---	--	------	------
mPD		0.0068	0.70
		nPD:	0.01
		N. weight:	0.87
		Sc. PD:	-0.028
		Sc. rank:	-4630.6

,

PA			
Pos.	485	obs:	exp:
att	I	0	1.09
atc	I	0	0.75
ata	I	3	1.16
atg	M	22585	22585.00
gtt	V	0	0.22
gtc	V	0	0.18
gta	V	0	0.27
gtg	V	1	0.33
---	--	------	------
mPD		0.00035	0.00041
		nPD:	0.86
		N. weight:	0.00018
		Sc. PD:	0.00015
		Sc. rank:	3.4

,

PA			
Pos.	486	obs:	exp:
tta	L	5	0.66
ttg	L	0	1.13
ctt	L	0	1.18
ctc	L	0	0.85
cta	L	1	1.16
ctg	L	0	1.03
att	I	3	8206.00
atc	I	3	5627.00
ata	I	22537	8710.00
atg	M	14	14.00
act	T	0	0.76
acc	T	0	0.62
aca	T	3	1.37
acg	T	0	0.26
gtt	V	0	5.08
gtc	V	0	4.18
gta	V	23	6.19
gtg	V	0	7.55
---	--	------	------
mPD		0.0046	0.66
		nPD:	0.01
		N. weight:	0.99
		Sc. PD:	-0.035
		Sc. rank:	-9888.0

,

PA			
Pos.	487	obs:	exp:
tct	S	0	4149.00
tcc	S	0	2612.00
tca	S	0	5868.00
tcg	S	0	1200.00
tgt	C	0	0.45
tgc	C	1	0.55
act	T	0	0.25
acc	T	1	0.21
aca	T	0	0.46
acg	T	0	0.09
aat	N	0	20.70
aac	N	35	14.30
agt	S	18	4240.00
agc	S	22530	4479.00
ggt	G	0	0.61
ggc	G	4	0.59
gga	G	0	1.78
ggg	G	0	1.02
---	--	------	------
mPD		0.0052	1.6
		nPD:	0.
		N. weight:	1.7
		Sc. PD:	-0.066
		Sc. rank:	-27114.0

,

PA			
Pos.	488	obs:	exp:
cgt	R	0	0.05
cgc	R	0	0.10
cga	R	0	0.20
cgg	R	0	0.15
aat	N	0	0.59
aac	N	1	0.41
aaa	K	22309	12884.00
aag	K	277	9702.00
aga	R	2	1.00
agg	R	0	0.49
---	--	------	------
mPD		0.024	0.49
		nPD:	0.05
		N. weight:	0.52
		Sc. PD:	0.0036
		Sc. rank:	6390.4

,

PA			
Pos.	489	obs:	exp:
tct	S	0	0.74
tcc	S	0	0.46
tca	S	0	1.04
tcg	S	0	0.21
tgt	C	22544	10194.00
tgc	C	41	12391.00
agt	S	0	0.75
agc	S	4	0.79
---	--	------	------
mPD		0.0043	0.50
		nPD:	0.01
		N. weight:	0.82
		Sc. PD:	-0.027
		Sc. rank:	-5381.4

,

PA

Pos.	490	obs:	exp:
cgt	R	0	616.70
cgc	R	0	1181.00
cga	R	0	2212.00
cgg	R	0	1653.00
aaa	K	0	1.71
aag	K	3	1.29
aga	R	332	11339.00
agg	R	22254	5584.00
mPD		0.029	0.91
		nPD:	0.03
		N. weight:	1.4
		Sc. PD:	-0.014
		Sc. rank:	12527.0

,

PA

Pos.	491	obs:	exp:
att	I	0	5.82
atc	I	16	3.99
ata	I	0	6.18
act	T	29	5689.00
acc	T	22525	4629.00
aca	T	15	10325.00
acg	T	0	1926.00
aat	N	0	0.59
aac	N	1	0.41
gct	A	0	0.79
gcc	A	3	0.62
gca	A	0	1.41
gcg	A	0	0.18
mPD		0.0057	0.68
		nPD:	0.01
		N. weight:	1.6
		Sc. PD:	-0.056
		Sc. rank:	-12212.0

,

PA

Pos.	492	obs:	exp:
caa	Q	1	0.53
cag	Q	0	0.47
cgt	R	0	0.33
cgc	R	0	0.63
cga	R	0	1.18
cgg	R	0	0.88
aaa	K	22514	12877.00
aag	K	61	9698.00
aga	R	12	6.02
agg	R	0	2.97
gaa	E	1	0.61
gag	E	0	0.39
mPD		0.0066	0.49
		nPD:	0.01
		N. weight:	0.57
		Sc. PD:	-0.016
		Sc. rank:	-557.7

,

PA

Pos.	493	obs:	exp:
gct	A	0	0.53
gcc	A	0	0.41
gca	A	2	0.94
gcg	A	0	0.12
gat	D	1	0.53
gac	D	0	0.47
gaa	E	22540	13672.00
gag	E	40	8908.00
ggt	G	0	0.91
ggc	G	0	0.89
gga	G	6	2.67
ggg	G	0	1.53
mPD		0.0043	0.48
		nPD:	0.01
		N. weight:	0.51
		Sc. PD:	-0.017
		Sc. rank:	-3181.8

,

PA

Pos.	494	obs:	exp:
cgt	R	0	0.03
cgc	R	0	0.05
cga	R	0	0.10
cgg	R	0	0.07
aga	R	1	0.50
agg	R	0	0.25
gat	D	0	0.53
gac	D	1	0.47
gaa	E	8	4.84
gag	E	0	3.16
ggt	G	1	3424.00
ggc	G	54	3344.00
gga	G	22432	10044.00
ggg	G	92	5768.00
mPD		0.014	0.69
		nPD:	0.02
		N. weight:	0.8
		Sc. PD:	-0.018
		Sc. rank:	3007.7

,

PA

Pos.	495	obs:	exp:
cgt	R	0	616.60
cgc	R	0	1181.00
cga	R	3	2212.00
cgg	R	0	1653.00
att	I	0	0.73
atc	I	0	0.50
ata	I	2	0.77
act	T	0	0.25
acc	T	0	0.21
aca	T	1	0.46
acg	T	0	0.09
aaa	K	2	1.14
aag	K	0	0.86
aga	R	22520	11338.00
agg	R	61	5583.00
mPD		0.0061	0.91
		nPD:	0.01
		N. weight:	0.7
		Sc. PD:	-0.025
		Sc. rank:	-7371.4

,

PA

Pos.	496	obs:	exp:
tta	L	0	0.22
ttg	L	0	0.38
tgg	W	1	1.00
ctt	L	0	0.39
ctc	L	0	0.28
cta	L	0	0.39
ctg	L	2	0.34
cgt	R	0	616.70
cgc	R	0	1181.00
cga	R	157	2212.00
cgg	R	22338	1653.00
aga	R	0	11339.00
agg	R	91	5584.00
mPD		0.022	0.91
		nPD:	0.02
		N. weight:	2.6
		Sc. PD:	-0.048
		Sc. rank:	17062.0

,

PA

Pos.	497	obs:	exp:
cgt	R	0	0.60
cgc	R	0	1.15
cga	R	0	2.15
cgg	R	0	1.61
aaa	K	22459	12872.00
aag	K	107	9694.00
aga	R	22	11.04
agg	R	0	5.44
gaa	E	1	0.61
gag	E	0	0.39
mPD		0.011	0.49
		nPD:	0.02
		N. weight:	0.56
		Sc. PD:	-0.011
		Sc. rank:	3091.7

,

PA

Pos.	498	obs:	exp:
cct	P	0	0.28
ccc	P	0	0.19
cca	P	1	0.43
ccg	P	0	0.11
caa	Q	1	0.53
cag	Q	0	0.47
att	I	0	1.82
atc	I	0	1.25
ata	I	5	1.93
act	T	4	5693.00
acc	T	13	4632.00
aca	T	22558	10331.00
acg	T	7	1927.00
mPD		0.0028	0.68
		nPD:	0.
		N. weight:	0.81
		Sc. PD:	-0.031
		Sc. rank:	-11389.0

,

PA

Pos.	499	obs:	exp:
tct	S	0	0.74
tcc	S	0	0.46
tca	S	0	1.04
tcg	S	0	0.21
act	T	0	0.76
acc	T	3	0.62
aca	T	0	1.37
acg	T	0	0.26
aat	N	449	13356.00
aac	N	22133	9226.00
agt	S	0	0.75
agc	S	4	0.79
mPD		0.040	0.48
		nPD:	0.08
		N. weight:	0.82
		Sc. PD:	0.032
		Sc. rank:	12018.0

,

PA

Pos.	500	obs:	exp:
tta	L	5	2483.00
ttg	L	18	4252.00
ctt	L	2407	4451.00
ctc	L	0	3190.00
cta	L	65	4348.00
ctg	L	20090	3861.00
att	I	1	0.36
atc	I	0	0.25
ata	I	0	0.39
atg	M	3	3.00
mPD		0.20	1.1
		nPD:	0.17
		N. weight:	1.4
		Sc. PD:	0.19
		Sc. rank:	24164.0

,

PA

Pos.	501	obs:	exp:
tat	Y	22530	11734.00
tac	Y	54	10850.00
tgt	C	1	0.45
tgc	C	0	0.55
cat	H	4	2.35
cac	H	0	1.65
mPD		0.0052	0.50
		nPD:	0.01
		N. weight:	0.67
		Sc. PD:	-0.021
		Sc. rank:	-3019.2

,

PA

Pos.	502	obs:	exp:
ggt	G	14	3425.00
ggc	G	4	3346.00
gga	G	714	10048.00
ggg	G	21857	5770.00
mPD		0.063	0.69
		nPD:	0.09
		N. weight:	1.3
		Sc. PD:	0.059
		Sc. rank:	18813.0

,

PA

Pos.	503	obs:	exp:
ttt	F	11	9688.00
ttc	F	22564	12887.00
tta	L	5	1.32
ttg	L	0	2.26
tat	Y	0	0.52
tac	Y	1	0.48
ctt	L	0	2.36
ctc	L	7	1.69
cta	L	0	2.31
ctg	L	0	2.05
gtt	V	0	0.22
gtc	V	1	0.18
gta	V	0	0.27
gtg	V	0	0.33
mPD		0.0022	0.49
		nPD:	0.
		N. weight:	0.58
		Sc. PD:	-0.022
		Sc. rank:	-8010.8

,

PA

Pos.	504	obs:	exp:
ttt	F	3	1.29
ttc	F	0	1.71
tta	L	0	0.88
ttg	L	0	1.51
ctt	L	8	1.58
ctc	L	0	1.13
cta	L	0	1.54
ctg	L	0	1.37
att	I	22528	8212.00
atc	I	31	5631.00
ata	I	0	8716.00
act	T	4	1.26
acc	T	1	1.03
aca	T	0	2.29
acg	T	0	0.43
gtt	V	14	3.10
gtc	V	0	2.54
gta	V	0	3.77
gtg	V	0	4.60
mPD		0.0055	0.66
		nPD:	0.01
		N. weight:	1.
		Sc. PD:	-0.036
		Sc. rank:	-7887.3

,

PA

Pos.	505	obs:	exp:
tta	L	0	0.11
ttg	L	0	0.19
ctt	L	0	0.20
ctc	L	0	0.14
cta	L	1	0.19
ctg	L	0	0.17
att	I	702	7337.00
atc	I	7	5031.00
ata	I	19446	7787.00
atg	M	9	9.00
act	T	0	1.51
acc	T	0	1.23
aca	T	6	2.74
acg	T	0	0.51
gtt	V	7	534.60
gtc	V	1	439.40
gta	V	2409	650.30
gtg	V	1	793.70
mPD		0.25	0.87
		nPD:	0.29
		N. weight:	0.89
		Sc. PD:	0.22
		Sc. rank:	15506.0

,

PA			
Pos .	506	obs :	exp :
aaa	K	22437	12885.00
aag	K	152	9704.00
---	--	------	------
mPD		0.013	0.49
		nPD :	0.03
		N. weight :	0.55
		Sc . PD :	-0.0084
		Sc . rank :	4109.7

,

PA			
Pos .	507	obs :	exp :
gaa	E	1	0.61
gag	E	0	0.39
ggt	G	1	3425.00
ggc	G	5	3346.00
gga	G	22532	10048.00
ggg	G	50	5770.00
---	--	------	------
mPD		0.0050	0.69
		nPD :	0.01
		N. weight :	0.83
		Sc . PD :	-0.029
		Sc . rank :	-7629.4

,

PA			
Pos .	508	obs :	exp :
cgt	R	0	616.60
cgc	R	0	1181.00
cga	R	0	2212.00
cgg	R	2	1653.00
atg	M	1	1.00
aaa	K	0	2.85
aag	K	5	2.15
aga	R	297	11337.00
agg	R	22283	5583.00
ggt	G	0	0.15
ggc	G	0	0.15
gga	G	0	0.44
ggg	G	1	0.26
---	--	------	------
mPD		0.027	0.91
		nPD :	0.03
		N. weight :	1.4
		Sc . PD :	-0.018
		Sc . rank :	11704.0

,

PA			
Pos .	509	obs :	exp :
tct	S	22440	4157.00
tcc	S	139	2616.00
tca	S	9	5879.00
tcg	S	1	1202.00
agt	S	0	4247.00
agc	S	0	4487.00
---	--	------	------
mPD		0.013	1.6
		nPD :	0.01
		N. weight :	1.7
		Sc . PD :	-0.059
		Sc . rank :	-14088.0

,

PA			
Pos .	510	obs :	exp :
cat	H	22533	13284.00
cac	H	56	9305.00
---	--	------	------
mPD		0.0049	0.48
		nPD :	0.01
		N. weight :	0.54
		Sc . PD :	-0.017
		Sc . rank :	-2511.9

,

PA			
Pos .	511	obs :	exp :
tta	L	33	2483.00
ttg	L	22529	4253.00
tgg	W	1	1.00
ctt	L	0	4451.00
ctc	L	0	3190.00
cta	L	3	4349.00
ctg	L	23	3862.00
---	--	------	------
mPD		0.0056	1.1
		nPD :	0.
		N. weight :	1.7
		Sc . PD :	-0.064
		Sc . rank :	-22423.0

,

PA			
Pos .	512	obs :	exp :
cgt	R	0	616.70
cgc	R	0	1182.00
cga	R	1	2212.00
cgg	R	0	1653.00
aaa	K	1	0.57
aag	K	0	0.43
aga	R	22555	11340.00
agg	R	32	5584.00
---	--	------	------
mPD		0.0030	0.91
		nPD :	0.
		N. weight :	0.71
		Sc . PD :	-0.028
		Sc . rank :	-11274.0

,

PA			
Pos .	513	obs :	exp :
tct	S	0	0.92
tcc	S	0	0.58
tca	S	0	1.30
tcg	S	0	0.27
aat	N	22550	13356.00
aac	N	32	9226.00
agt	S	5	0.94
agc	S	0	0.99
gat	D	2	1.05
gac	D	0	0.95
---	--	------	------
mPD		0.0034	0.48
		nPD :	0.01
		N. weight :	0.54
		Sc . PD :	-0.019
		Sc . rank :	-5178.2

,

PA			
Pos .	514	obs :	exp :
tat	Y	1	0.52
tac	Y	0	0.48
gat	D	22472	11901.00
gac	D	115	10686.00
ggt	G	1	0.15
ggc	G	0	0.15
gga	G	0	0.44
ggg	G	0	0.26
---	--	------	------
mPD		0.010	0.50
		nPD :	0.02
		N. weight :	0.64
		Sc . PD :	-0.014
		Sc . rank :	2470.7

,

PA			
Pos .	515	obs :	exp :
tct	S	1	0.18
tcc	S	0	0.12
tca	S	0	0.26
tcg	S	0	0.05
cct	P	1	0.28
ccc	P	0	0.19
cca	P	0	0.43
ccg	P	0	0.11
act	T	22562	5694.00
acc	T	11	4633.00
aca	T	13	10333.00
acg	T	1	1927.00
agt	S	0	0.19
agc	S	0	0.20
---	--	------	------
mPD		0.0024	0.68
		nPD :	0.
		N. weight :	1.4
		Sc . PD :	-0.055
		Sc . rank :	-22171.0

,

PA			
Pos .	516	obs :	exp :
gat	D	22561	11902.00
gac	D	28	10687.00
---	--	------	------
mPD		0.0025	0.50
		nPD :	0.
		N. weight :	0.66
		Sc . PD :	-0.025
		Sc . rank :	-8555.5

,

PA			
Pos .	517	obs :	exp :
att	I	0	0.73
atc	I	0	0.50
ata	I	2	0.77
gtt	V	4	4994.00
gtc	V	2	4104.00
gta	V	16897	6075.00
gtg	V	5684	7414.00
---	--	------	------
mPD		0.38	0.74
		nPD :	0.51
		N. weight :	0.73
		Sc . PD :	0.34
		Sc . rank :	13446.0

,

PA			
Pos .	518	obs :	exp :
tta	L	0	0.11
ttg	L	1	0.19
ctt	L	0	0.20
ctc	L	0	0.14
cta	L	0	0.19
ctg	L	0	0.17
gtt	V	10	4992.00
gtc	V	0	4103.00
gta	V	92	6073.00
gtg	V	22479	7413.00
gct	A	0	1.85
gcc	A	0	1.45
gca	A	0	3.29
gcg	A	7	0.41
---	--	------	------
mPD		0.0097	0.74
		nPD :	0.01
		N. weight :	1.1
		Sc . PD :	-0.033
		Sc . rank :	-1510.6

,

PA			
Pos .	519	obs :	exp :
tct	S	0	1.29
tcc	S	0	0.81
tca	S	0	1.82
tcg	S	0	0.37
tat	Y	0	0.52
tac	Y	1	0.48
aat	N	348	13352.00
aac	N	22227	9223.00
agt	S	0	1.32
agc	S	7	1.39
gat	D	1	3.16
gac	D	5	2.84
---	--	------	------
mPD		0.032	0.49
		nPD :	0.07
		N. weight :	0.85
		Sc . PD :	0.019
		Sc . rank :	11570.0

,

PA			
Pos .	520	obs :	exp :
ttt	F	22438	9688.00
ttc	F	137	12887.00
tta	L	0	0.99
ttg	L	0	1.69
tat	Y	1	0.52
tac	Y	0	0.48
ctt	L	9	1.77
ctc	L	0	1.27
cta	L	0	1.73
ctg	L	0	1.54
att	I	1	0.36
atc	I	0	0.25
ata	I	0	0.39
gtt	V	3	0.66
gtc	V	0	0.55
gta	V	0	0.81
gtg	V	0	0.98
---	--	------	------
mPD		0.013	0.49
		nPD :	0.03
		N. weight :	0.84
		Sc . PD :	-0.013
		Sc . rank :	6322.8

,

PA			
Pos .	521	obs :	exp :
tta	L	7	0.77
ttg	L	0	1.32
ctt	L	0	1.38
ctc	L	0	0.99
cta	L	0	1.35
ctg	L	0	1.20
att	I	0	10.56
atc	I	0	7.24
ata	I	29	11.20
gtt	V	0	4976.00
gtc	V	3	4090.00
gta	V	22437	6053.00
gtg	V	67	7388.00
gct	A	0	12.18
gcc	A	0	9.52
gca	A	46	21.60
gcg	A	0	2.69
---	--	------	------
mPD		0.013	0.75
		nPD :	0.02
		N. weight :	1.3
		Sc . PD :	-0.033
		Sc . rank :	2979.4

,

PA

Pos.	522	obs:	exp:
tct	S	0	4136.00
tcc	S	0	2603.00
tca	S	0	5849.00
tcg	S	0	1196.00
tgt	C	4	1.81
tgc	C	0	2.19
att	I	3	1.09
atc	I	0	0.75
ata	I	0	1.16
act	T	1	0.25
acc	T	0	0.21
aca	T	0	0.46
acg	T	0	0.09
aat	N	7	4.14
aac	N	0	2.86
agt	S	22427	4226.00
agc	S	48	4464.00
ggt	G	99	15.01
ggc	G	0	14.66
gga	G	0	44.04
ggg	G	0	25.29
---	--	------	------
mPD		0.014	1.6
		nPD :	0.01
		N. weight :	1.7
		Sc. PD :	-0.058
		Sc. rank :	-11516.0

,

PA

Pos.	523	obs:	exp:
tta	L	0	0.22
ttg	L	0	0.38
ctt	L	0	0.39
ctc	L	0	0.28
cta	L	0	0.39
ctg	L	2	0.34
att	I	0	0.36
atc	I	0	0.25
ata	I	1	0.39
atg	M	22584	22584.00
gtt	V	0	0.44
gtc	V	0	0.36
gta	V	0	0.54
gtg	V	2	0.66
---	--	------	------
mPD		0.00044	0.00068
		nPD :	0.66
		N. weight :	0.00031
		Sc. PD :	0.00019
		Sc. rank :	5.8

,

PA

Pos.	524	obs:	exp:
gaa	E	171	13678.00
gag	E	22418	8911.00
---	--	------	------
mPD		0.015	0.48
		nPD :	0.03
		N. weight :	0.92
		Sc. PD :	-0.01
		Sc. rank :	8293.1

,

PA

Pos.	525	obs:	exp:
ttt	F	155	9691.00
ttc	F	22427	12891.00
tta	L	3	0.77
ttg	L	0	1.32
ctt	L	0	1.38
ctc	L	4	0.99
cta	L	0	1.35
ctg	L	0	1.20
---	--	------	------
mPD		0.014	0.49
		nPD :	0.03
		N. weight :	0.54
		Sc. PD :	-0.0074
		Sc. rank :	4574.7

,

PA

Pos.	526	obs:	exp:
tct	S	7	4157.00
tcc	S	9	2616.00
tca	S	22560	5879.00
tcg	S	12	1202.00
cct	P	0	0.28
ccc	P	0	0.19
cca	P	1	0.43
ccg	P	0	0.11
agt	S	0	4247.00
agc	S	0	4487.00
---	--	------	------
mPD		0.0026	1.6
		nPD :	0.
		N. weight :	1.4
		Sc. PD :	-0.057
		Sc. rank :	-25462.0

,

PA

Pos.	527	obs:	exp:
ttt	F	0	0.86
ttc	F	2	1.14
tta	L	0	2483.00
ttg	L	0	4252.00
ctt	L	26	4450.00
ctc	L	22480	3190.00
cta	L	78	4348.00
ctg	L	0	3861.00
att	I	0	1.09
atc	I	3	0.75
ata	I	0	1.16
---	--	------	------
mPD		0.0096	1.1
		nPD :	0.01
		N. weight :	2.
		Sc. PD :	-0.068
		Sc. rank :	-14224.0

,

PA

Pos.	528	obs:	exp:
tct	S	0	0.18
tcc	S	0	0.12
tca	S	0	0.26
tcg	S	0	0.05
att	I	27	9.83
atc	I	0	6.74
ata	I	0	10.43
act	T	22536	5686.00
acc	T	10	4627.00
aca	T	10	10319.00
acg	T	0	1925.00
aat	N	4	2.37
aac	N	0	1.63
agt	S	1	0.19
agc	S	0	0.20
gct	A	1	0.26
gcc	A	0	0.21
gca	A	0	0.47
gcg	A	0	0.06
---	--	------	------
mPD		0.0047	0.68
		nPD :	0.01
		N. weight :	1.4
		Sc. PD :	-0.051
		Sc. rank :	-14799.0

,

PA

Pos.	529	obs:	exp:
tat	Y	1	0.52
tac	Y	0	0.48
aat	N	10	7.10
aac	N	2	4.90
gct	A	1	0.26
gcc	A	0	0.21
gca	A	0	0.47
gcg	A	0	0.06
gat	D	19374	11894.00
gac	D	3200	10680.00
gaa	E	1	0.61
gag	E	0	0.39
---	--	------	------
mPD		0.24	0.50
		nPD :	0.49
		N. weight :	0.26
		Sc. PD :	0.11
		Sc. rank :	4771.1

,

PA

Pos.	530	obs:	exp:
tct	S	0	3.86
tcc	S	0	2.43
tca	S	21	5.47
tcg	S	0	1.12
cct	P	2	6217.00
ccc	P	0	4186.00
cca	P	19317	9609.00
ccg	P	3249	2555.00
agt	S	0	3.95
agc	S	0	4.17
---	--	------	------
mPD		0.25	0.70
		nPD :	0.36
		N. weight :	0.66
		Sc. PD :	0.2
		Sc. rank :	11780.0

,

PA

Pos.	531	obs:	exp:
cgt	R	0	613.60
cgc	R	0	1176.00
cga	R	1	2201.00
cgg	R	0	1645.00
att	I	0	4.00
atc	I	0	2.75
ata	I	11	4.25
aaa	K	100	57.04
aag	K	0	42.96
aga	R	22431	11282.00
agg	R	41	5556.00
ggt	G	0	0.76
ggc	G	0	0.74
gga	G	5	2.22
ggg	G	0	1.28
---	--	------	------
mPD		0.014	0.92
		nPD :	0.02
		N. weight :	0.71
		Sc. PD :	-0.019
		Sc. rank :	245.5

,

PA

Pos.	532	obs:	exp:
tta	L	2	2477.00
ttg	L	180	4242.00
ctt	L	50	4439.00
ctc	L	0	3182.00
cta	L	33	4337.00
ctg	L	22264	3852.00
cct	P	0	0.28
ccc	P	0	0.19
cca	P	0	0.43
ccg	P	1	0.11
caa	Q	0	0.53
cag	Q	1	0.47
atg	M	58	58.00
---	--	------	------
mPD		0.029	1.1
		nPD :	0.03
		N. weight :	1.8
		Sc. PD :	-0.031
		Sc. rank :	11997.0

,

PA

Pos.	533	obs:	exp:
gat	D	3	1.58
gac	D	0	1.42
gaa	E	18168	13673.00
gag	E	4414	8909.00
ggt	G	0	0.61
ggc	G	0	0.59
gga	G	3	1.78
ggg	G	1	1.02
---	--	------	------
mPD		0.32	0.48
		nPD :	0.66
		N. weight :	0.096
		Sc. PD :	0.058
		Sc. rank :	1779.8

,

PA			
Pos.	534	obs:	exp:
tta	L	0	3.19
ttg	L	0	5.46
tct	S	0	7.73
tcc	S	0	4.86
tca	S	42	10.93
tcg	S	0	2.24
ctt	L	0	5.71
ctc	L	0	4.10
cta	L	29	5.58
ctg	L	0	4.96
cct	P	16	6198.00
ccc	P	6	4172.00
cca	P	22450	9579.00
ccg	P	24	2547.00
caa	Q	10	5.28
cag	Q	0	4.72
act	T	0	3.03
acc	T	0	2.46
aca	T	12	5.49
acg	T	0	1.02
agt	S	0	7.90
agc	S	0	8.34
---	--	------	------
mPD		0.012	0.71
		nPD:	0.02
		N. weight:	0.88
		Sc. PD:	-0.022
		Sc. rank:	1597.4

,

PA			
Pos.	535	obs:	exp:
tta	L	0	0.33
ttg	L	0	0.56
tat	Y	0	104.40
tac	Y	201	96.56
ctt	L	0	0.59
ctc	L	3	0.42
cta	L	0	0.58
ctg	L	0	0.51
cct	P	0	0.83
ccc	P	3	0.56
cca	P	0	1.28
ccg	P	0	0.34
cat	H	41	13147.00
cac	H	22315	9209.00
caa	Q	6	3.17
cag	Q	0	2.83
cgt	R	0	0.05
cgc	R	2	0.10
cga	R	0	0.20
cgg	R	0	0.15
aat	N	0	10.65
aac	N	18	7.35
aga	R	0	1.00
agg	R	0	0.49
---	--	------	------
mPD		0.024	0.51
		nPD:	0.05
		N. weight:	0.91
		Sc. PD:	0.0043
		Sc. rank:	10669.0

,

PA			
Pos.	536	obs:	exp:
cgt	R	0	0.03
cgc	R	0	0.05
cga	R	0	0.10
cgg	R	0	0.07
aaa	K	22566	12885.00
aag	K	22	9703.00
aga	R	1	0.50
agg	R	0	0.25
---	--	------	------
mPD		0.0020	0.49
		nPD:	0.
		N. weight:	0.58
		Sc. PD:	-0.022
		Sc. rank:	-8176.3

,

PA			
Pos.	537	obs:	exp:
tgg	W	22589	22589.00
---	--	------	------
mPD		0	0
		nPD:	1.
		N. weight:	0.
		Sc. PD:	0
		Sc. rank:	0

,

PA			
Pos.	538	obs:	exp:
aat	N	1	0.59
aac	N	0	0.41
aaa	K	41	23.39
aag	K	0	17.61
gtt	V	0	2.87
gtc	V	0	2.36
gta	V	13	3.50
gtg	V	0	4.27
gat	D	121	64.28
gac	D	1	57.72
gaa	E	21902	13478.00
gag	E	358	8782.00
ggt	G	0	23.05
ggc	G	0	22.51
gga	G	151	67.61
ggg	G	1	38.83
---	--	------	------
mPD		0.060	0.50
		nPD:	0.12
		N. weight:	0.45
		Sc. PD:	0.034
		Sc. rank:	7047.5

,

PA			
Pos.	539	obs:	exp:
cgt	R	0	0.66
cgc	R	0	1.26
cga	R	0	2.35
cgg	R	0	1.76
aat	N	2	1.18
aac	N	0	0.82
aaa	K	22542	12871.00
aag	K	21	9692.00
aga	R	24	12.05
agg	R	0	5.93
---	--	------	------
mPD		0.0042	0.49
		nPD:	0.01
		N. weight:	0.58
		Sc. PD:	-0.02
		Sc. rank:	-4160.0

,

PA			
Pos.	540	obs:	exp:
ttt	F	0	1.29
ttc	F	3	1.71
tat	Y	40	11715.00
tac	Y	22506	10831.00
tgt	C	0	1.35
tgc	C	3	1.65
cat	H	0	21.76
cac	H	37	15.24
---	--	------	------
mPD		0.0073	0.50
		nPD:	0.01
		N. weight:	0.75
		Sc. PD:	-0.021
		Sc. rank:	-124.4

,

PA			
Pos.	541	obs:	exp:
tct	S	3	0.92
tcc	S	0	0.58
tca	S	0	1.30
tcg	S	0	0.27
tgt	C	22554	10193.00
tgc	C	30	12391.00
agt	S	2	0.94
agc	S	0	0.99
---	--	------	------
mPD		0.0031	0.50
		nPD:	0.01
		N. weight:	0.82
		Sc. PD:	-0.03
		Sc. rank:	-8986.1

,

PA			
Pos.	542	obs:	exp:
att	I	83	30.21
atc	I	0	20.72
ata	I	0	32.07
gtt	V	22411	4966.00
gtc	V	48	4082.00
gta	V	4	6041.00
gtg	V	1	7374.00
gct	A	42	11.12
gcc	A	0	8.69
gca	A	0	19.73
gcg	A	0	2.46
---	--	------	------
mPD		0.016	0.75
		nPD:	0.02
		N. weight:	1.6
		Sc. PD:	-0.033
		Sc. rank:	6642.0

,

PA			
Pos.	543	obs:	exp:
ttt	F	5	2.15
ttc	F	0	2.85
tta	L	0	2469.00
ttg	L	0	4229.00
ctt	L	22441	4426.00
ctc	L	8	3173.00
cta	L	14	4325.00
ctg	L	0	3841.00
att	I	118	42.96
atc	I	0	29.46
ata	I	0	45.59
gtt	V	3	0.66
gtc	V	0	0.55
gta	V	0	0.81
gtg	V	0	0.98
---	--	------	------
mPD		0.013	1.1
		nPD:	0.01
		N. weight:	1.7
		Sc. PD:	-0.052
		Sc. rank:	-5112.4

,

PA			
Pos.	544	obs:	exp:
aaa	K	1	0.57
aag	K	0	0.43
gtt	V	0	0.22
gtc	V	0	0.18
gta	V	1	0.27
gtg	V	0	0.33
gaa	E	22487	13672.00
gag	E	93	8908.00
ggt	G	0	1.06
ggc	G	0	1.04
gga	G	7	3.11
ggg	G	0	1.79
---	--	------	------
mPD		0.0090	0.48
		nPD:	0.02
		N. weight:	0.5
		Sc. PD:	-0.012
		Sc. rank:	1462.6

,

PA			
Pos.	545	obs:	exp:
att	I	2	8147.00
atc	I	0	5587.00
ata	I	22379	8647.00
atg	M	4	4.00
act	T	0	1.76
acc	T	0	1.44
aca	T	7	3.20
acg	T	0	0.60
gtt	V	0	43.55
gtc	V	0	35.80
gta	V	196	52.98
gtg	V	1	64.67
---	--	------	------
mPD		0.019	0.68
		nPD:	0.03
		N. weight:	0.99
		Sc. PD:	-0.015
		Sc. rank:	7689.4

,

PA			
Pos.	546	obs:	exp:
ggt	G	2	3425.00
ggc	G	10	3346.00
gga	G	22527	10048.00
ggg	G	50	5770.00
---	--	------	------
mPD		0.0055	0.69
		nPD:	0.01
		N. weight:	0.83
		Sc. PD:	-0.028
		Sc. rank:	-6816.4

,

PA			
Pos.	547	obs:	exp:
aat	N	0	6.51
aac	N	11	4.49
gat	D	41	11808.00
gac	D	22370	10603.00
gaa	E	165	99.91
gag	E	0	65.09
ggt	G	0	0.30
ggc	G	2	0.30
gga	G	0	0.89
ggg	G	0	0.51
---	--	------	------
mPD		0.019	0.51
		nPD:	0.04
		N. weight:	0.77
		Sc. PD:	-0.0037
		Sc. rank:	8019.6

,

PA			
Pos.	548	obs:	exp:
att	I	2	7.28
atc	I	1	4.99
ata	I	17	7.73
atg	M	22569	22569.00
---	--	------	------
mPD		0.0018	0.0018
		nPD:	1.
		N. weight:	0.00053
		Sc. PD:	0.0005
		Sc. rank:	9.9

,

PA			
Pos.	549	obs:	exp:
tta	L	19	2482.00
ttg	L	0	4251.00
ctt	L	1	4449.00
ctc	L	3223	3189.00
cta	L	19313	4347.00
ctg	L	23	3860.00
att	I	0	3.64
atc	I	2	2.50
ata	I	8	3.86
---	--	------	------
mPD		0.25	1.1
		nPD:	0.22
		N. weight:	1.3
		Sc. PD:	0.23
		Sc. rank:	22501.0

,

PA			
Pos.	550	obs:	exp:
tta	L	164	2483.00
ttg	L	22384	4252.00
ctt	L	0	4451.00
ctc	L	0	3190.00
cta	L	5	4348.00
ctg	L	33	3862.00
gtt	V	0	0.66
gtc	V	0	0.55
gta	V	0	0.81
gtg	V	3	0.98
---	--	------	------
mPD		0.018	1.1
		nPD:	0.02
		N. weight:	1.7
		Sc. PD:	-0.044
		Sc. rank:	1410.8

,

PA			
Pos.	551	obs:	exp:
cgt	R	0	616.30
cgc	R	0	1181.00
cga	R	0	2211.00
cgg	R	0	1652.00
aaa	K	2	10.27
aag	K	16	7.73
aga	R	399	11331.00
agg	R	22171	5580.00
ggt	G	0	0.15
ggc	G	0	0.15
gga	G	0	0.44
ggg	G	1	0.26
---	--	------	------
mPD		0.037	0.91
		nPD:	0.04
		N. weight:	1.4
		Sc. PD:	-0.0036
		Sc. rank:	14647.0

,

PA			
Pos.	552	obs:	exp:
tct	S	1	1.10
tcc	S	0	0.69
tca	S	0	1.56
tcg	S	0	0.32
att	I	14	5.10
atc	I	0	3.49
ata	I	0	5.41
act	T	22530	5683.00
acc	T	3	4624.00
aca	T	6	10312.00
acg	T	3	1923.00
aat	N	3	1.77
aac	N	0	1.23
agt	S	5	1.13
agc	S	0	1.19
gct	A	24	6.35
gcc	A	0	4.97
gca	A	0	11.27
gcg	A	0	1.41
---	--	------	------
mPD		0.0052	0.68
		nPD:	0.01
		N. weight:	1.4
		Sc. PD:	-0.05
		Sc. rank:	-12238.0

,

PA			
Pos.	553	obs:	exp:
tct	S	0	0.18
tcc	S	0	0.12
tca	S	0	0.26
tcg	S	1	0.05
act	T	0	0.25
acc	T	0	0.21
aca	T	0	0.46
acg	T	1	0.09
agt	S	0	0.19
agc	S	0	0.20
gtt	V	0	0.66
gtc	V	0	0.55
gta	V	0	0.81
gtg	V	3	0.98
gct	A	15	5979.00
gcc	A	5	4675.00
gca	A	968	10606.00
gcg	A	21595	1323.00
ggt	G	0	0.15
ggc	G	0	0.15
gga	G	0	0.44
ggg	G	1	0.26
---	--	------	------
mPD		0.084	0.66
		nPD:	0.13
		N. weight:	2.7
		Sc. PD:	0.22
		Sc. rank:	42528.0

,

PA			
Pos.	554	obs:	exp:
tta	L	1	0.11
ttg	L	0	0.19
ctt	L	0	0.20
ctc	L	0	0.14
cta	L	0	0.19
ctg	L	0	0.17
att	I	9	8210.00
atc	I	2	5629.00
ata	I	22541	8713.00
atg	M	19	19.00
act	T	0	0.25
acc	T	0	0.21
aca	T	1	0.46
acg	T	0	0.09
gtt	V	0	3.54
gtc	V	0	2.91
gta	V	16	4.30
gtg	V	0	5.25
---	--	------	------
mPD		0.0042	0.66
		nPD:	0.01
		N. weight:	0.99
		Sc. PD:	-0.035
		Sc. rank:	-10564.0

,

PA			
Pos.	555	obs:	exp:
tct	S	0	54.84
tcc	S	0	34.52
tca	S	0	77.56
tcg	S	0	15.86
agt	S	298	56.03
agc	S	0	59.19
gat	D	0	0.53
gac	D	1	0.47
ggt	G	18178	3380.00
ggc	G	4108	3301.00
gga	G	4	9915.00
ggg	G	0	5694.00
---	--	------	------
mPD		0.32	0.74
		nPD:	0.44
		N. weight:	1.5
		Sc. PD:	0.58
		Sc. rank:	26773.0

,

PA			
Pos.	556	obs:	exp:
tta	L	0	0.11
ttg	L	0	0.19
ctt	L	0	0.20
ctc	L	0	0.14
cta	L	1	0.19
ctg	L	0	0.17
cat	H	2	2.35
cac	H	2	1.65
caa	Q	22456	11915.00
cag	Q	125	10666.00
cgt	R	0	0.05
cgc	R	0	0.10
cga	R	2	0.20
cgg	R	0	0.15
aaa	K	1	0.57
aag	K	0	0.43
aga	R	0	1.00
agg	R	0	0.49
---	--	------	------
mPD		0.012	0.50
		nPD:	0.02
		N. weight:	0.63
		Sc. PD:	-0.012
		Sc. rank:	3576.8

,

PA			
Pos.	557	obs:	exp:
tta	L	0	0.55
ttg	L	5	0.94
ctt	L	0	0.99
ctc	L	0	0.71
cta	L	0	0.96
ctg	L	0	0.85
att	I	6	2.18
atc	I	0	1.50
ata	I	0	2.32
atg	M	49	49.00
gtt	V	3	4980.00
gtc	V	0	4093.00
gta	V	40	6058.00
gtg	V	22483	7394.00
gct	A	0	0.79
gcc	A	0	0.62
gca	A	0	1.41
gcg	A	3	0.18
---	--	------	------
mPD		0.0099	0.74
		nPD:	0.01
		N. weight:	1.1
		Sc. PD:	-0.033
		Sc. rank:	-1459.8

,

PA			
Pos.	558	obs:	exp:
tta	L	0	1.21
ttg	L	11	2.07
tct	S	34	4154.00
tcc	S	1	2615.00
tca	S	539	5875.00
tcg	S	22001	1201.00
ctt	L	0	2.17
ctc	L	0	1.55
cta	L	0	2.12
ctg	L	0	1.88
cct	P	0	0.83
ccc	P	0	0.56
cca	P	0	1.28
ccg	P	3	0.34
agt	S	0	4245.00
agc	S	0	4484.00
---	--	------	------
mPD		0.051	1.6
		nPD:	0.03
		N. weight:	2.9
		Sc. PD:	-0.034
		Sc. rank:	25323.0

,

PA			
Pos.	559	obs:	exp:
cgt	R	0	615.40
cgc	R	0	1179.00
cga	R	0	2207.00
cgg	R	1	1649.00
aaa	K	2	29.66
aag	K	50	22.34
aga	R	831	11314.00
agg	R	21705	5572.00
---	--	------	------
mPD		0.076	0.91
		nPD:	0.08
		N. weight:	1.3
		Sc. PD:	0.05
		Sc. rank:	18609.0

,

PA			
Pos.	560	obs:	exp:
tct	S	0	17.85
tcc	S	97	11.24
tca	S	0	25.24
tcg	S	0	5.16
cct	P	424	6188.00
ccc	P	21992	4166.00
cca	P	42	9565.00
ccg	P	5	2544.00
act	T	0	0.50
acc	T	2	0.41
aca	T	0	0.91
acg	T	0	0.17
agt	S	0	18.24
agc	S	0	19.27
gct	A	0	7.15
gcc	A	27	5.59
gca	A	0	12.68
gcg	A	0	1.58
---	--	------	------
mPD		0.052	0.71
		nPD:	0.07
		N. weight:	1.6
		Sc. PD:	0.049
		Sc. rank:	23128.0

,

PA			
Pos.	561	obs:	exp:
tta	L	0	2.75
ttg	L	9	4.71
ctt	L	0	4.93
ctc	L	0	3.53
cta	L	0	4.81
ctg	L	16	4.27
att	I	11	7.64
atc	I	0	5.24
ata	I	10	8.11
atg	M	22539	22539.00
gtt	V	0	0.88
gtc	V	0	0.73
gta	V	0	1.08
gtg	V	4	1.31
---	--	------	------
mPD		0.0044	0.0061
		nPD:	0.73
		N. weight:	0.0019
		Sc. PD:	0.0013
		Sc. rank:	35.4

,

PA			
Pos.	562	obs:	exp:
ttt	F	114	9691.00
ttc	F	22468	12891.00
tta	L	2	0.77
ttg	L	0	1.32
ctt	L	0	1.38
ctc	L	5	0.99
cta	L	0	1.35
ctg	L	0	1.20
---	--	------	------
mPD		0.011	0.49
		nPD:	0.02
		N. weight:	0.55
		Sc. PD:	-0.012
		Sc. rank:	2691.1

,

PA			
Pos.	563	obs:	exp:
tta	L	128	2483.00
ttg	L	8	4253.00
ctt	L	1	4451.00
ctc	L	2	3191.00
cta	L	22440	4349.00
ctg	L	10	3862.00
---	--	------	------
mPD		0.014	1.1
		nPD:	0.01
		N. weight:	1.7
		Sc. PD:	-0.051
		Sc. rank:	-4179.2

,

PA			
Pos.	564	obs:	exp:
ttt	F	7	3.00
ttc	F	0	4.00
tat	Y	22557	11732.00
tac	Y	22	10847.00
tgt	C	3	1.35
tgc	C	0	1.65
---	--	------	------
mPD		0.0028	0.50
		nPD:	0.01
		N. weight:	0.68
		Sc. PD:	-0.025
		Sc. rank:	-7905.8

,

PA			
Pos.	565	obs:	exp:
tta	L	0	0.44
ttg	L	4	0.75
ctt	L	0	0.79
ctc	L	0	0.56
cta	L	0	0.77
ctg	L	0	0.68
att	I	0	0.36
atc	I	0	0.25
ata	I	1	0.39
atg	M	45	45.00
gtt	V	23	4981.00
gtc	V	0	4094.00
gta	V	86	6059.00
gtg	V	22421	7396.00
gct	A	0	2.38
gcc	A	0	1.86
gca	A	0	4.23
gcg	A	9	0.53
---	--	------	------
mPD		0.015	0.74
		nPD:	0.02
		N. weight:	1.1
		Sc. PD:	-0.025
		Sc. rank:	4166.7

,

PA			
Pos.	566	obs:	exp:
cgt	R	0	616.70
cgc	R	0	1182.00
cga	R	0	2212.00
cgg	R	0	1653.00
aaa	K	1	0.57
aag	K	0	0.43
aga	R	22500	11340.00
agg	R	88	5584.00
---	--	------	------
mPD		0.0078	0.91
		nPD:	0.01
		N. weight:	0.7
		Sc. PD:	-0.023
		Sc. rank:	-4724.0

,

PA			
Pos.	567	obs:	exp:
cct	P	0	0.28
ccc	P	1	0.19
cca	P	0	0.43
ccg	P	0	0.11
act	T	22	5694.00
acc	T	22527	4633.00
aca	T	37	10333.00
acg	T	0	1927.00
gct	A	0	0.53
gcc	A	2	0.41
gca	A	0	0.94
gcg	A	0	0.12
---	--	------	------
mPD		0.0055	0.68
		nPD:	0.01
		N. weight:	1.6
		Sc. PD:	-0.056
		Sc. rank:	-13120.0

,

PA			
Pos.	568	obs:	exp:
aat	N	22567	13360.00
aac	N	22	9229.00
---	--	------	------
mPD		0.0019	0.48
		nPD:	0.
		N. weight:	0.54
		Sc. PD:	-0.021
		Sc. rank:	-7798.8

,

PA			
Pos.	569	obs:	exp:
ggt	G	1	3425.00
ggc	G	0	3346.00
gga	G	22531	10048.00
ggg	G	57	5770.00
---	--	------	------
mPD		0.0051	0.69
		nPD:	0.01
		N. weight:	0.83
		Sc. PD:	-0.029
		Sc. rank:	-7399.2

,

PA			
Pos.	570	obs:	exp:
tct	S	0	0.37
tcc	S	2	0.23
tca	S	0	0.52
tcg	S	0	0.11
att	I	0	3.64
atc	I	10	2.50
ata	I	0	3.86
act	T	140	5688.00
acc	T	22388	4628.00
aca	T	36	10322.00
acg	T	0	1925.00
aat	N	0	3.55
aac	N	6	2.45
agt	S	0	0.38
agc	S	0	0.40
gct	A	0	1.85
gcc	A	7	1.45
gca	A	0	3.29
gcg	A	0	0.41
---	--	------	------
mPD		0.018	0.68
		nPD:	0.03
		N. weight:	1.6
		Sc. PD:	-0.026
		Sc. rank:	11466.0

,

PA			
Pos.	571	obs:	exp:
ttt	F	0	0.43
ttc	F	1	0.57
tct	S	43	4157.00
tcc	S	22520	2616.00
tca	S	25	5879.00
tcg	S	0	1202.00
agt	S	0	4247.00
agc	S	0	4487.00
---	--	------	------
mPD		0.0061	1.6
		nPD:	0.
		N. weight:	2.2
		Sc. PD:	-0.086
		Sc. rank:	-33628.0

,

PA			
Pos.	572	obs:	exp:
aaa	K	507	12885.00
aag	K	22082	9704.00
---	--	------	------
mPD		0.044	0.49
		nPD:	0.09
		N. weight:	0.76
		Sc. PD:	0.035
		Sc. rank:	11408.0

,

PA			
Pos.	573	obs:	exp:
tta	L	0	0.11
ttg	L	0	0.19
ctt	L	0	0.20
ctc	L	1	0.14
cta	L	0	0.19
ctg	L	0	0.17
att	I	26	8217.00
atc	I	22521	5635.00
ata	I	26	8721.00
act	T	0	1.26
acc	T	5	1.03
aca	T	0	2.29
acg	T	0	0.43
gtt	V	1	2.21
gtc	V	9	1.82
gta	V	0	2.69
gtg	V	0	3.28
---	--	------	------
mPD		0.0061	0.66
		nPD:	0.01
		N. weight:	1.4
		Sc. PD:	-0.047
		Sc. rank:	-8712.2

,

PA			
Pos.	574	obs:	exp:
tct	S	0	0.18
tcc	S	0	0.12
tca	S	0	0.26
tcg	S	0	0.05
cgt	R	0	0.05
cgc	R	0	0.10
cga	R	0	0.20
cgg	R	0	0.15
aaa	K	158	12884.00
aag	K	22428	9702.00
agt	S	1	0.19
agc	S	0	0.20
aga	R	0	1.00
agg	R	2	0.49
---	--	------	------
mPD		0.014	0.49
		nPD:	0.03
		N. weight:	0.84
		Sc. PD:	-0.011
		Sc. rank:	7061.1

,

PA			
Pos.	575	obs:	exp:
tta	L	0	0.22
ttg	L	1	0.38
ctt	L	0	0.39
ctc	L	0	0.28
cta	L	0	0.39
ctg	L	1	0.34
atg	M	22586	22586.00
act	T	0	0.25
acc	T	0	0.21
aca	T	0	0.46
acg	T	1	0.09
---	--	------	------
mPD		0.00027	0.00046
		nPD:	0.58
		N. weight:	0.00024
		Sc. PD:	0.00013
		Sc. rank:	4.4

,

PA			
Pos.	576	obs:	exp:
cgt	R	0	0.22
cgc	R	0	0.42
cga	R	0	0.78
cgg	R	0	0.59
aat	N	0	0.59
aac	N	1	0.41
aaa	K	22506	12880.00
aag	K	74	9700.00
aga	R	8	4.02
agg	R	0	1.98
---	--	------	------
mPD		0.0073	0.49
		nPD:	0.01
		N. weight:	0.56
		Sc. PD:	-0.016
		Sc. rank:	-5.9

,

PA			
Pos.	577	obs:	exp:
tgg	W	22589	22589.00
---	--	------	------
mPD		0	0
		nPD:	1.
		N. weight:	0.
		Sc. PD:	0
		Sc. rank:	0

,

PA			
Pos.	578	obs:	exp:
tct	S	0	9.02
tcc	S	0	5.68
tca	S	0	12.75
tcg	S	0	2.61
tgt	C	0	0.90
tgc	C	2	1.10
agt	S	0	9.21
agc	S	49	9.73
gct	A	0	0.53
gcc	A	2	0.41
gca	A	0	0.94
gcg	A	0	0.12
ggt	G	39	3417.00
ggc	G	22371	3338.00
gga	G	126	10024.00
ggg	G	0	5757.00
---	--	------	------
mPD		0.019	0.70
		nPD:	0.03
		N. weight:	1.9
		Sc. PD:	-0.029
		Sc. rank:	15173.0

,

PA			
Pos.	579	obs:	exp:
tta	L	0	0.44
ttg	L	2	0.75
ctt	L	0	0.79
ctc	L	0	0.56
cta	L	0	0.77
ctg	L	2	0.68
att	I	7	11.65
atc	I	0	7.99
ata	I	25	12.36
atg	M	22550	22550.00
gtt	V	0	0.66
gtc	V	0	0.55
gta	V	0	0.81
gtg	V	3	0.98
---	--	------	------
mPD		0.0034	0.0039
		nPD:	0.89
		N. weight:	0.0011
		Sc. PD:	0.00094
		Sc. rank:	20.9

,

PA			
Pos.	580	obs:	exp:
gaa	E	22562	13678.00
gag	E	27	8911.00
---	--	------	------
mPD		0.0024	0.48
		nPD:	0.
		N. weight:	0.51
		Sc. PD:	-0.019
		Sc. rank:	-6653.0

,

PA			
Pos.	581	obs:	exp:
tta	L	0	17.26
ttg	L	2	29.56
ctt	L	0	30.94
ctc	L	0	22.18
cta	L	1	30.23
ctg	L	154	26.84
att	I	1	0.73
atc	I	0	0.50
ata	I	1	0.77
atg	M	22409	22409.00
act	T	0	2.27
acc	T	0	1.85
aca	T	0	4.12
acg	T	9	0.77
aaa	K	0	2.28
aag	K	4	1.72
gtt	V	0	1.77
gtc	V	0	1.45
gta	V	0	2.15
gtg	V	8	2.63
---	--	------	------
mPD		0.016	0.026
		nPD:	0.61
		N. weight:	0.014
		Sc. PD:	0.0077
		Sc. rank:	255.3

,

PA

Pos.	582	obs:	exp:
cgt	R	0	616.80
cgc	R	0	1182.00
cga	R	0	2213.00
cgg	R	0	1653.00
aga	R	74	11340.00
agg	R	22515	5584.00
---	--	------	------
mPD		0.0065	0.91
		nPD:	0.01
		N. weight:	1.4
		Sc. PD:	-0.05
		Sc. rank:	-13587.0

PA

Pos.	583	obs:	exp:
tta	L	0	0.11
ttg	L	0	0.19
tct	S	0	0.18
tcc	S	0	0.12
tca	S	0	0.26
tcg	S	0	0.05
ctt	L	0	0.20
ctc	L	1	0.14
cta	L	0	0.19
ctg	L	0	0.17
cat	H	0	5.29
cac	H	9	3.71
cgt	R	73	616.50
cgc	R	22365	1181.00
cga	R	140	2211.00
cgg	R	0	1652.00
agt	S	0	0.19
agc	S	1	0.20
aga	R	0	11335.00
agg	R	0	5582.00
---	--	------	------
mPD		0.020	0.91
		nPD:	0.02
		N. weight:	3.
		Sc. PD:	-0.063
		Sc. rank:	14285.0

PA

Pos.	584	obs:	exp:
tct	S	0	0.18
tcc	S	0	0.12
tca	S	0	0.26
tcg	S	0	0.05
tgt	C	101	10195.00
tgc	C	22487	12393.00
agt	S	0	0.19
agc	S	1	0.20
---	--	------	------
mPD		0.0090	0.50
		nPD:	0.02
		N. weight:	0.6
		Sc. PD:	-0.015
		Sc. rank:	1429.0

PA

Pos.	585	obs:	exp:
ttt	F	5	2.15
ttc	F	0	2.85
tta	L	0	2483.00
ttg	L	0	4252.00
ctt	L	22530	4450.00
ctc	L	50	3190.00
cta	L	3	4348.00
ctg	L	0	3861.00
att	I	1	0.36
atc	I	0	0.25
ata	I	0	0.39
---	--	------	------
mPD		0.0052	1.1
		nPD:	0.
		N. weight:	1.7
		Sc. PD:	-0.063
		Sc. rank:	-22920.0

PA

Pos.	586	obs:	exp:
tta	L	0	2483.00
ttg	L	0	4253.00
ctt	L	22355	4451.00
ctc	L	226	3191.00
cta	L	8	4349.00
ctg	L	0	3862.00
---	--	------	------
mPD		0.021	1.1
		nPD:	0.02
		N. weight:	1.6
		Sc. PD:	-0.04
		Sc. rank:	3507.6

PA

Pos.	587	obs:	exp:
caa	Q	18402	11920.00
cag	Q	4187	10669.00
---	--	------	------
mPD		0.30	0.50
		nPD:	0.61
		N. weight:	0.19
		Sc. PD:	0.1
		Sc. rank:	3490.0

PA

Pos.	588	obs:	exp:
tct	S	22537	4157.00
tcc	S	37	2616.00
tca	S	11	5878.00
tcg	S	2	1202.00
cct	P	1	0.28
ccc	P	0	0.19
cca	P	0	0.43
ccg	P	0	0.11
agt	S	0	4247.00
agc	S	0	4487.00
gct	A	1	0.26
gcc	A	0	0.21
gca	A	0	0.47
gcg	A	0	0.06
---	--	------	------
mPD		0.0046	1.6
		nPD:	0.
		N. weight:	1.7
		Sc. PD:	-0.069
		Sc. rank:	-29332.0

PA

Pos.	589	obs:	exp:
ttt	F	6	2.57
ttc	F	0	3.43
tta	L	0	2479.00
ttg	L	0	4245.00
ctt	L	22464	4443.00
ctc	L	63	3185.00
cta	L	16	4341.00
ctg	L	3	3855.00
att	I	37	13.47
atc	I	0	9.24
ata	I	0	14.30
---	--	------	------
mPD		0.011	1.1
		nPD:	0.01
		N. weight:	1.7
		Sc. PD:	-0.054
		Sc. rank:	-9182.8

PA

Pos.	590	obs:	exp:
caa	Q	10816	11920.00
cag	Q	11773	10669.00
---	--	------	------
mPD		0.50	0.50
		nPD:	1.
		N. weight:	0.0052
		Sc. PD:	0.0049
		Sc. rank:	97.8

PA

Pos.	591	obs:	exp:
tta	L	0	0.11
ttg	L	0	0.19
ctt	L	0	0.20
ctc	L	0	0.14
cta	L	0	0.19
ctg	L	1	0.17
caa	Q	366	11919.00
cag	Q	22222	10669.00
---	--	------	------
mPD		0.032	0.50
		nPD:	0.06
		N. weight:	0.69
		Sc. PD:	0.015
		Sc. rank:	9351.6

PA

Pos.	592	obs:	exp:
ttt	F	1	0.43
ttc	F	0	0.57
att	I	22014	8216.00
atc	I	188	5634.00
ata	I	367	8720.00
gtt	V	17	4.20
gtc	V	1	3.45
gta	V	1	5.11
gtg	V	0	6.24
---	--	------	------
mPD		0.050	0.66
		nPD:	0.08
		N. weight:	0.92
		Sc. PD:	0.03
		Sc. rank:	13146.0

PA

Pos.	593	obs:	exp:
gtt	V	0	0.22
gtc	V	0	0.18
gta	V	0	0.27
gtg	V	1	0.33
gaa	E	31	13677.00
gag	E	22557	8911.00
---	--	------	------
mPD		0.0028	0.48
		nPD:	0.01
		N. weight:	0.96
		Sc. PD:	-0.035
		Sc. rank:	-10846.0

PA

Pos.	594	obs:	exp:
tct	S	0	4154.00
tcc	S	0	2614.00
tca	S	0	5874.00
tcg	S	0	1201.00
att	I	0	0.36
atc	I	1	0.25
ata	I	0	0.39
aat	N	0	7.10
aac	N	12	4.90
agt	S	32	4244.00
agc	S	22539	4483.00
ggt	G	0	0.76
ggc	G	5	0.74
gga	G	0	2.22
ggg	G	0	1.28
---	--	------	------
mPD		0.0044	1.6
		nPD:	0.
		N. weight:	1.7
		Sc. PD:	-0.066
		Sc. rank:	-28570.0

PA

Pos.	595	obs:	exp:
tta	L	0	0.11
ttg	L	1	0.19
ctt	L	0	0.20
ctc	L	0	0.14
cta	L	0	0.19
ctg	L	0	0.17
att	I	1	13.11
atc	I	0	8.99
ata	I	35	13.91
atg	M	22542	22542.00
act	T	0	1.01
acc	T	0	0.82
aca	T	0	1.83
acg	T	4	0.34
gtt	V	0	1.33
gtc	V	0	1.09
gta	V	0	1.61
gtg	V	6	1.97
---	--	------	------
mPD		0.0042	0.0049
		nPD:	0.85
		N. weight:	0.0023
		Sc. PD:	0.0018
		Sc. rank:	42.2

PA

Pos.	596	obs:	exp:
att	I	22502	8213.00
atc	I	58	5632.00
ata	I	2	8717.00
act	T	5	1.26
acc	T	0	1.03
aca	T	0	2.29
acg	T	0	0.43
gtt	V	22	4.86
gtc	V	0	4.00
gta	V	0	5.92
gtg	V	0	7.22
---	--	------	------
mPD		0.0077	0.66
		nPD:	0.01
		N. weight:	1.
		Sc. PD:	-0.032
		Sc. rank:	-2881.2

PA

Pos.	597	obs:	exp:
gat	D	1	0.53
gac	D	0	0.47
gaa	E	19337	13676.00
gag	E	3250	8911.00
ggt	G	0	0.15
ggc	G	0	0.15
gga	G	1	0.44
ggg	G	0	0.26
---	--	------	------
mPD		0.25	0.48
		nPD:	0.52
		N. weight:	0.16
		Sc. PD:	0.074
		Sc. rank:	2927.5

PA			
Pos.	598	obs:	exp:
tct	S	0	2.21
tcc	S	12	1.39
tca	S	0	3.12
tcg	S	0	0.64
cct	P	0	0.28
ccc	P	1	0.19
cca	P	0	0.43
ccg	P	0	0.11
act	T	0	8.82
acc	T	35	7.18
aca	T	0	16.01
acg	T	0	2.99
agt	S	0	2.26
agc	S	0	2.38
gtt	V	0	3.76
gtc	V	17	3.09
gta	V	0	4.57
gtg	V	0	5.58
gct	A	239	5963.00
gcc	A	22259	4662.00
gca	A	26	10579.00
gcg	A	0	1320.00
---	--	------	------
mPD		0.029	0.67
		nPD:	0.04
		N. weight:	1.6
		Sc. PD:	0.00065
		Sc. rank:	17705.0

,

PA			
Pos.	599	obs:	exp:
aaa	K	0	1.14
aag	K	2	0.86
gaa	E	129	13672.00
gag	E	22451	8908.00
ggt	G	0	1.06
ggc	G	0	1.04
gga	G	0	3.11
ggg	G	7	1.79
---	--	------	------
mPD		0.012	0.48
		nPD:	0.03
		N. weight:	0.93
		Sc. PD:	-0.016
		Sc. rank:	6421.2

,

PA			
Pos.	600	obs:	exp:
tct	S	22411	4156.00
tcc	S	60	2616.00
tca	S	4	5878.00
tcg	S	111	1202.00
agt	S	0	4247.00
agc	S	0	4486.00
gct	A	3	0.79
gcc	A	0	0.62
gca	A	0	1.41
gcg	A	0	0.18
---	--	------	------
mPD		0.016	1.6
		nPD:	0.01
		N. weight:	1.7
		Sc. PD:	-0.056
		Sc. rank:	-9749.1

,

PA			
Pos.	601	obs:	exp:
tct	S	22495	4157.00
tcc	S	40	2616.00
tca	S	10	5879.00
tcg	S	43	1202.00
agt	S	0	4247.00
agc	S	0	4487.00
gct	A	1	0.26
gcc	A	0	0.21
gca	A	0	0.47
gcg	A	0	0.06
---	--	------	------
mPD		0.0083	1.6
		nPD:	0.01
		N. weight:	1.7
		Sc. PD:	-0.065
		Sc. rank:	-22180.0

,

PA			
Pos.	602	obs:	exp:
ttt	F	0	0.43
ttc	F	1	0.57
att	I	0	32.03
atc	I	88	21.97
ata	I	0	34.00
gtt	V	82	4974.00
gtc	V	22409	4088.00
gta	V	9	6051.00
gtg	V	0	7386.00
---	--	------	------
mPD		0.016	0.75
		nPD:	0.02
		N. weight:	1.7
		Sc. PD:	-0.037
		Sc. rank:	7711.4

,

PA			
Pos.	603	obs:	exp:
cgt	R	0	0.49
cgc	R	0	0.94
cga	R	0	1.76
cgg	R	0	1.32
att	I	0	0.36
atc	I	0	0.25
ata	I	1	0.39
aat	N	1	1.18
aac	N	1	0.82
aaa	K	22530	12873.00
aag	K	37	9694.00
aga	R	18	9.04
agg	R	0	4.45
gaa	E	1	0.61
gag	E	0	0.39
---	--	------	------
mPD		0.0052	0.49
		nPD:	0.01
		N. weight:	0.57
		Sc. PD:	-0.018
		Sc. rank:	-2369.8

,

PA			
Pos.	604	obs:	exp:
aaa	K	0	1.71
aag	K	3	1.29
gtt	V	0	0.22
gtc	V	0	0.18
gta	V	0	0.27
gtg	V	1	0.33
gaa	E	673	13673.00
gag	E	21909	8909.00
ggt	G	0	0.45
ggc	G	0	0.44
gga	G	0	1.33
ggg	G	3	0.77
---	--	------	------
mPD		0.058	0.48
		nPD:	0.12
		N. weight:	0.82
		Sc. PD:	0.064
		Sc. rank:	12891.0

,

PA			
Pos.	605	obs:	exp:
cgt	R	0	0.03
cgc	R	0	0.05
cga	R	0	0.10
cgg	R	0	0.07
aaa	K	22563	12885.00
aag	K	25	9703.00
aga	R	1	0.50
agg	R	0	0.25
---	--	------	------
mPD		0.0023	0.49
		nPD:	0.
		N. weight:	0.58
		Sc. PD:	-0.022
		Sc. rank:	-7729.1

,

PA			
Pos.	606	obs:	exp:
aat	N	0	1.77
aac	N	3	1.23
gat	D	18	11901.00
gac	D	22568	10685.00
---	--	------	------
mPD		0.0019	0.50
		nPD:	0.
		N. weight:	0.77
		Sc. PD:	-0.03
		Sc. rank:	-11675.0

,

PA			
Pos.	607	obs:	exp:
tta	L	0	1.76
ttg	L	16	3.01
ctt	L	0	3.15
ctc	L	0	2.26
cta	L	0	3.08
ctg	L	0	2.74
att	I	0	0.73
atc	I	0	0.50
ata	I	2	0.77
atg	M	22567	22567.00
act	T	0	0.25
acc	T	0	0.21
aca	T	0	0.46
acg	T	1	0.09
gtt	V	0	0.66
gtc	V	0	0.55
gta	V	0	0.81
gtg	V	3	0.98
---	--	------	------
mPD		0.0019	0.0031
		nPD:	0.63
		N. weight:	0.0016
		Sc. PD:	0.00091
		Sc. rank:	29.5

,

PA			
Pos.	608	obs:	exp:
tct	S	0	0.18
tcc	S	1	0.12
tca	S	0	0.26
tcg	S	0	0.05
act	T	125	5693.00
acc	T	22454	4632.00
aca	T	4	10331.00
acg	T	0	1927.00
agt	S	0	0.19
agc	S	0	0.20
gct	A	0	1.32
gcc	A	5	1.03
gca	A	0	2.35
gcg	A	0	0.29
---	--	------	------
mPD		0.012	0.68
		nPD:	0.02
		N. weight:	1.6
		Sc. PD:	-0.04
		Sc. rank:	3257.7

,

PA			
Pos.	609	obs:	exp:
cgt	R	0	1.39
cgc	R	0	2.67
cga	R	0	5.00
cgg	R	0	3.73
atg	M	1	1.00
aat	N	6	3.55
aac	N	0	2.45
aaa	K	126	12852.00
aag	K	22405	9679.00
aga	R	0	25.60
agg	R	51	12.61
---	--	------	------
mPD		0.016	0.50
		nPD:	0.03
		N. weight:	0.85
		Sc. PD:	-0.0085
		Sc. rank:	7951.2

,

PA

Pos.	610	obs:	exp:
aaa	K	3	1.71
aag	K	0	1.29
gtt	V	0	0.22
gtc	V	0	0.18
gta	V	1	0.27
gtg	V	0	0.33
gat	D	8	5.80
gac	D	3	5.20
gaa	E	22371	13666.00
gag	E	198	8903.00
ggt	G	0	0.76
ggc	G	0	0.74
gga	G	5	2.22
ggg	G	0	1.28
---	--	------	------
mPD		0.019	0.48
		nPD:	0.04
		N. weight:	0.47
		Sc. PD:	-0.0014
		Sc. rank:	5070.4

,

PA

Pos.	611	obs:	exp:
ttt	F	267	9687.00
ttc	F	22307	12887.00
tta	L	1	0.44
ttg	L	0	0.75
tct	S	0	0.55
tcc	S	3	0.35
tca	S	0	0.78
tcg	S	0	0.16
tat	Y	0	3.64
tac	Y	7	3.36
ctt	L	0	0.79
ctc	L	3	0.56
cta	L	0	0.77
ctg	L	0	0.68
att	I	0	0.36
atc	I	1	0.25
ata	I	0	0.39
agt	S	0	0.56
agc	S	0	0.60
---	--	------	------
mPD		0.025	0.49
		nPD:	0.05
		N. weight:	0.52
		Sc. PD:	0.0038
		Sc. rank:	6424.6

,

PA

Pos.	612	obs:	exp:
ttt	F	22543	9692.00
ttc	F	42	12893.00
tta	L	0	0.22
ttg	L	0	0.38
ctt	L	2	0.39
ctc	L	0	0.28
cta	L	0	0.39
ctg	L	0	0.34
gtt	V	2	0.44
gtc	V	0	0.36
gta	V	0	0.54
gtg	V	0	0.66
---	--	------	------
mPD		0.0041	0.49
		nPD:	0.01
		N. weight:	0.87
		Sc. PD:	-0.03
		Sc. rank:	-6625.8

,

PA

Pos.	613	obs:	exp:
aaa	K	7	3.99
aag	K	0	3.01
gtt	V	0	0.44
gtc	V	0	0.36
gta	V	2	0.54
gtg	V	0	0.66
gat	D	2	2.63
gac	D	3	2.37
gaa	E	22336	13667.00
gag	E	236	8905.00
ggt	G	0	0.45
ggc	G	0	0.44
gga	G	3	1.33
ggg	G	0	0.77
---	--	------	------
mPD		0.022	0.48
		nPD:	0.05
		N. weight:	0.47
		Sc. PD:	0.0016
		Sc. rank:	5447.6

,

PA

Pos.	614	obs:	exp:
tct	S	0	2.39
tcc	S	0	1.51
tca	S	0	3.38
tcg	S	0	0.69
cat	H	0	1.76
cac	H	3	1.24
act	T	0	9.08
acc	T	36	7.38
aca	T	0	16.47
acg	T	0	3.07
aat	N	45	13322.00
aac	N	22479	9202.00
aaa	K	2	1.14
aag	K	0	0.86
agt	S	0	2.44
agc	S	13	2.58
gat	D	0	5.80
gac	D	11	5.20
---	--	------	------
mPD		0.0097	0.49
		nPD:	0.02
		N. weight:	0.92
		Sc. PD:	-0.021
		Sc. rank:	3344.1

,

PA

Pos.	615	obs:	exp:
cgt	R	0	1.20
cgc	R	0	2.30
cga	R	0	4.31
cgg	R	0	3.22
aaa	K	22482	12860.00
aag	K	63	9685.00
aga	R	44	22.09
agg	R	0	10.88
---	--	------	------
mPD		0.0095	0.50
		nPD:	0.02
		N. weight:	0.57
		Sc. PD:	-0.013
		Sc. rank:	1722.0

,

PA

Pos.	616	obs:	exp:
tta	L	0	2.53
ttg	L	23	4.33
tct	S	3	4150.00
tcc	S	0	2612.00
tca	S	412	5869.00
tcg	S	22135	1200.00
ctt	L	0	4.53
ctc	L	0	3.25
cta	L	0	4.43
ctg	L	0	3.93
cct	P	0	2.75
ccc	P	0	1.85
cca	P	0	4.26
ccg	P	10	1.13
act	T	0	1.26
acc	T	0	1.03
aca	T	0	2.29
acg	T	5	0.43
agt	S	0	4240.00
agc	S	0	4479.00
gct	A	0	0.26
gcc	A	0	0.21
gca	A	0	0.47
gcg	A	1	0.06
---	--	------	------
mPD		0.040	1.6
		nPD:	0.02
		N. weight:	2.9
		Sc. PD:	-0.054
		Sc. rank:	18145.0

,

PA

Pos.	617	obs:	exp:
aaa	K	7	3.99
aag	K	0	3.01
gaa	E	22542	13671.00
gag	E	36	8907.00
ggt	G	0	0.61
ggc	G	0	0.59
gga	G	4	1.78
ggg	G	0	1.02
---	--	------	------
mPD		0.0042	0.48
		nPD:	0.01
		N. weight:	0.51
		Sc. PD:	-0.017
		Sc. rank:	-3398.2

,

PA

Pos.	618	obs:	exp:
atg	M	10	10.00
act	T	3	5658.00
acc	T	1	4604.00
aca	T	22422	10268.00
acg	T	20	1915.00
aaa	K	118	67.31
aag	K	0	50.69
gct	A	0	3.97
gcc	A	0	3.10
gca	A	15	7.04
gcg	A	0	0.88
---	--	------	------
mPD		0.016	0.69
		nPD:	0.02
		N. weight:	0.81
		Sc. PD:	-0.016
		Sc. rank:	4349.3

,

PA

Pos.	619	obs:	exp:
tgg	W	22589	22589.00
---	--	------	------
mPD		0	0
		nPD:	1.
		N. weight:	0.
		Sc. PD:	0
		Sc. rank:	0

,

PA

Pos.	620	obs:	exp:
tct	S	0	5.34
tcc	S	0	3.36
tca	S	29	7.55
tcg	S	0	1.54
cct	P	3	6215.00
ccc	P	8	4184.00
cca	P	22531	9606.00
ccg	P	17	2554.00
act	T	0	0.25
acc	T	0	0.21
aca	T	1	0.46
acg	T	0	0.09
agt	S	0	5.45
agc	S	0	5.76
---	--	------	------
mPD		0.0051	0.70
		nPD:	0.01
		N. weight:	0.88
		Sc. PD:	-0.031
		Sc. rank:	-7972.9

,

PA

Pos.	621	obs:	exp:
att	I	152	8194.00
atc	I	22343	5619.00
ata	I	15	8697.00
act	T	0	1.26
acc	T	5	1.03
aca	T	0	2.29
acg	T	0	0.43
gtt	V	0	16.36
gtc	V	74	13.45
gta	V	0	19.90
gtg	V	0	24.29
---	--	------	------
mPD		0.022	0.66
		nPD:	0.03
		N. weight:	1.4
		Sc. PD:	-0.014
		Sc. rank:	13094.0

,

PA			
Pos.	622	obs:	exp:
gtt	V	0	0.22
gtc	V	0	0.18
gta	V	1	0.27
gtg	V	0	0.33
ggt	G	140	3425.00
ggc	G	0	3346.00
gga	G	22369	10048.00
ggg	G	79	5770.00
---	--	------	------
mPD		0.019	0.69
		nPD:	0.03
		N. weight:	0.79
		Sc. PD:	-0.012
		Sc. rank:	6230.4

PA			
Pos.	623	obs:	exp:
aaa	K	1	1.14
aag	K	1	0.86
gat	D	1	1.58
gac	D	2	1.42
gaa	E	2423	13671.00
gag	E	20155	8907.00
ggt	G	0	0.91
ggc	G	0	0.89
gga	G	0	2.67
ggg	G	6	1.53
---	--	------	------
mPD		0.19	0.48
		nPD:	0.4
		N. weight:	0.57
		Sc. PD:	0.2
		Sc. rank:	10246.0

PA			
Pos.	624	obs:	exp:
tct	S	96	4157.00
tcc	S	39	2616.00
tca	S	22436	5879.00
tcg	S	17	1202.00
act	T	0	0.25
acc	T	0	0.21
aca	T	1	0.46
acg	T	0	0.09
agt	S	0	4247.00
agc	S	0	4487.00
---	--	------	------
mPD		0.013	1.6
		nPD:	0.01
		N. weight:	1.4
		Sc. PD:	-0.046
		Sc. rank:	-10692.0

PA			
Pos.	625	obs:	exp:
tct	S	0	0.37
tcc	S	2	0.23
tca	S	0	0.52
tcg	S	0	0.11
cct	P	53	6223.00
ccc	P	22486	4189.00
cca	P	48	9617.00
ccg	P	0	2558.00
agt	S	0	0.38
agc	S	0	0.40
---	--	------	------
mPD		0.0091	0.70
		nPD:	0.01
		N. weight:	1.7
		Sc. PD:	-0.051
		Sc. rank:	-2525.9

PA			
Pos.	626	obs:	exp:
tct	S	0	2.02
tcc	S	0	1.27
tca	S	0	2.86
tcg	S	0	0.59
tgg	W	4	4.00
cgt	R	0	610.40
cgc	R	0	1169.00
cga	R	0	2190.00
cgg	R	5	1636.00
aaa	K	9	118.10
aag	K	198	88.92
agt	S	11	2.07
agc	S	0	2.18
aga	R	205	11224.00
agg	R	22147	5527.00
ggt	G	0	1.52
ggc	G	0	1.48
gga	G	0	4.45
ggg	G	10	2.55
---	--	------	------
mPD		0.040	0.93
		nPD:	0.04
		N. weight:	1.4
		Sc. PD:	-0.00023
		Sc. rank:	15380.0

PA			
Pos.	627	obs:	exp:
cgt	R	0	0.14
cgc	R	0	0.26
cga	R	0	0.49
cgg	R	0	0.37
aga	R	5	2.51
agg	R	0	1.24
gaa	E	7	4.24
gag	E	0	2.76
ggt	G	4	3423.00
ggc	G	2	3344.00
gga	G	22506	10043.00
ggg	G	65	5767.00
---	--	------	------
mPD		0.0073	0.69
		nPD:	0.01
		N. weight:	0.82
		Sc. PD:	-0.026
		Sc. rank:	-3428.3

PA			
Pos.	628	obs:	exp:
tta	L	0	4.51
ttg	L	40	7.72
ctt	L	0	8.08
ctc	L	0	5.79
cta	L	0	7.89
ctg	L	1	7.01
att	I	0	3.28
atc	I	0	2.25
ata	I	9	3.48
atg	M	32	32.00
gtt	V	17	4974.00
gtc	V	0	4088.00
gta	V	784	6050.00
gtg	V	21696	7385.00
gct	A	0	2.38
gcc	A	0	1.86
gca	A	0	4.23
gcg	A	9	0.53
ggt	G	0	0.15
ggc	G	0	0.15
gga	G	0	0.44
ggg	G	1	0.26
---	--	------	------
mPD		0.077	0.75
		nPD:	0.1
		N. weight:	1.
		Sc. PD:	0.06
		Sc. rank:	15673.0

PA			
Pos.	629	obs:	exp:
gat	D	1	0.53
gac	D	0	0.47
gaa	E	412	13676.00
gag	E	22174	8910.00
ggt	G	0	0.30
ggc	G	0	0.30
gga	G	0	0.89
ggg	G	2	0.51
---	--	------	------
mPD		0.036	0.48
		nPD:	0.08
		N. weight:	0.87
		Sc. PD:	0.028
		Sc. rank:	12348.0

PA			
Pos.	630	obs:	exp:
gtt	V	0	0.22
gtc	V	0	0.18
gta	V	1	0.27
gtg	V	0	0.33
gat	D	3	3.69
gac	D	4	3.31
gaa	E	22581	13673.00
gag	E	0	8908.00
---	--	------	------
mPD		0.00071	0.48
		nPD:	0.
		N. weight:	0.52
		Sc. PD:	-0.021
		Sc. rank:	-9593.4

PA			
Pos.	631	obs:	exp:
tct	S	0	6.81
tcc	S	0	4.29
tca	S	0	9.63
tcg	S	0	1.97
tgt	C	0	2.71
tgc	C	6	3.29
agt	S	0	6.96
agc	S	37	7.35
ggt	G	113	3419.00
ggc	G	22403	3339.00
gga	G	30	10029.00
ggg	G	0	5759.00
---	--	------	------
mPD		0.016	0.70
		nPD:	0.02
		N. weight:	1.9
		Sc. PD:	-0.037
		Sc. rank:	11214.0

PA			
Pos.	632	obs:	exp:
ttt	F	14	6.87
ttc	F	2	9.13
tct	S	22042	4151.00
tcc	S	513	2613.00
tca	S	3	5871.00
tcg	S	0	1201.00
tat	Y	2	1.04
tac	Y	0	0.96
cct	P	9	2.75
ccc	P	1	1.85
cca	P	0	4.26
ccg	P	0	1.13
agt	S	0	4242.00
agc	S	0	4481.00
gct	A	3	0.79
gcc	A	0	0.62
gca	A	0	1.41
gcg	A	0	0.18
---	--	------	------
mPD		0.048	1.6
		nPD:	0.03
		N. weight:	1.7
		Sc. PD:	-0.023
		Sc. rank:	13909.0

PA			
Pos.	633	obs:	exp:
tta	L	0	0.22
ttg	L	0	0.38
ctt	L	2	0.39
ctc	L	0	0.28
cta	L	0	0.39
ctg	L	0	0.34
att	I	22569	8222.00
atc	I	12	5638.00
ata	I	6	8727.00
---	--	------	------
mPD		0.0018	0.66
		nPD:	0.
		N. weight:	1.
		Sc. PD:	-0.041
		Sc. rank:	-17754.0

PA			
Pos.	634	obs:	exp:
gtt	V	0	0.22
gtc	V	0	0.18
gta	V	1	0.27
gtg	V	0	0.33
gaa	E	0	0.61
gag	E	1	0.39
ggt	G	18	3425.00
ggc	G	0	3345.00
gga	G	18327	10047.00
ggg	G	4242	5770.00
---	--	------	------
mPD		0.31	0.69
		nPD:	0.44
		N. weight:	0.44
		Sc. PD:	0.18
		Sc. rank:	8090.6

,

PA			
Pos.	635	obs:	exp:
aaa	K	22495	12885.00
aag	K	94	9704.00
---	--	------	------
mPD		0.0083	0.49
		nPD:	0.02
		N. weight:	0.56
		Sc. PD:	-0.014
		Sc. rank:	680.8

,

PA			
Pos.	636	obs:	exp:
tta	L	0	0.11
ttg	L	1	0.19
ctt	L	0	0.20
ctc	L	0	0.14
cta	L	0	0.19
ctg	L	0	0.17
att	I	0	0.73
atc	I	0	0.50
ata	I	2	0.77
gtt	V	13	4993.00
gtc	V	15	4104.00
gta	V	173	6074.00
gtg	V	22384	7414.00
gct	A	0	0.26
gcc	A	0	0.21
gca	A	0	0.47
gcg	A	1	0.06
---	--	------	------
mPD		0.018	0.74
		nPD:	0.02
		N. weight:	1.1
		Sc. PD:	-0.02
		Sc. rank:	7240.4

,

PA			
Pos.	637	obs:	exp:
tgt	C	109	10196.00
tgc	C	22480	12393.00
---	--	------	------
mPD		0.0096	0.50
		nPD:	0.02
		N. weight:	0.6
		Sc. PD:	-0.014
		Sc. rank:	1899.8

,

PA			
Pos.	638	obs:	exp:
cgt	R	0	616.80
cgc	R	0	1182.00
cga	R	5	2213.00
cgg	R	1	1653.00
aga	R	18387	11340.00
agg	R	4196	5584.00
---	--	------	------
mPD		0.30	0.91
		nPD:	0.33
		N. weight:	0.35
		Sc. PD:	0.1
		Sc. rank:	6272.5

,

PA			
Pos.	639	obs:	exp:
cct	P	0	0.28
ccc	P	1	0.19
cca	P	0	0.43
ccg	P	0	0.11
att	I	0	0.73
atc	I	2	0.50
ata	I	0	0.77
act	T	41	5693.00
acc	T	22521	4632.00
aca	T	21	10331.00
acg	T	0	1927.00
gct	A	0	0.79
gcc	A	3	0.62
gca	A	0	1.41
gcg	A	0	0.18
---	--	------	------
mPD		0.0060	0.68
		nPD:	0.01
		N. weight:	1.6
		Sc. PD:	-0.055
		Sc. rank:	-10440.0

,

PA			
Pos.	640	obs:	exp:
ttt	F	0	0.43
ttc	F	1	0.57
tta	L	22295	2483.00
ttg	L	268	4253.00
ctt	L	0	4451.00
ctc	L	0	3190.00
cta	L	23	4349.00
ctg	L	2	3862.00
---	--	------	------
mPD		0.026	1.1
		nPD:	0.02
		N. weight:	2.2
		Sc. PD:	-0.044
		Sc. rank:	12280.0

,

PA			
Pos.	641	obs:	exp:
tta	L	0	2483.00
ttg	L	77	4252.00
ctt	L	4	4451.00
ctc	L	0	3190.00
cta	L	41	4348.00
ctg	L	22464	3862.00
atg	M	2	2.00
gtt	V	0	0.22
gtc	V	0	0.18
gta	V	0	0.27
gtg	V	1	0.33
---	--	------	------
mPD		0.011	1.1
		nPD:	0.01
		N. weight:	1.8
		Sc. PD:	-0.059
		Sc. rank:	-9760.5

,

PA			
Pos.	642	obs:	exp:
tct	S	0	0.37
tcc	S	0	0.23
tca	S	2	0.52
tcg	S	0	0.11
act	T	0	1.76
acc	T	0	1.44
aca	T	7	3.20
acg	T	0	0.60
agt	S	0	0.38
agc	S	0	0.40
gct	A	7	5978.00
gcc	A	0	4674.00
gca	A	22564	10605.00
gcg	A	9	1323.00
---	--	------	------
mPD		0.0022	0.66
		nPD:	0.
		N. weight:	0.78
		Sc. PD:	-0.03
		Sc. rank:	-12429.0

,

PA			
Pos.	643	obs:	exp:
cgt	R	0	0.11
cgc	R	0	0.21
cga	R	0	0.39
cgg	R	0	0.29
aaa	K	22548	12883.00
aag	K	37	9702.00
aga	R	4	2.01
agg	R	0	0.99
---	--	------	------
mPD		0.0036	0.49
		nPD:	0.01
		N. weight:	0.57
		Sc. PD:	-0.02
		Sc. rank:	-5168.4

,

PA			
Pos.	644	obs:	exp:
tct	S	22549	4157.00
tcc	S	18	2616.00
tca	S	19	5879.00
tcg	S	2	1202.00
tgt	C	1	0.45
tgc	C	0	0.55
agt	S	0	4247.00
agc	S	0	4487.00
---	--	------	------
mPD		0.0035	1.6
		nPD:	0.
		N. weight:	1.8
		Sc. PD:	-0.07
		Sc. rank:	-31271.0

,

PA			
Pos.	645	obs:	exp:
att	I	0	4.00
atc	I	0	2.75
ata	I	11	4.25
atg	M	1	1.00
gtt	V	331	4990.00
gtc	V	0	4102.00
gta	V	4150	6071.00
gtg	V	18091	7410.00
gct	A	0	1.32
gcc	A	0	1.03
gca	A	2	2.35
gcg	A	3	0.29
---	--	------	------
mPD		0.33	0.74
		nPD:	0.44
		N. weight:	0.63
		Sc. PD:	0.25
		Sc. rank:	11499.0

,

PA

Pos.	646	obs:	exp:
ttt	F	35	9684.00
ttc	F	22532	12883.00
tta	L	4	2.09
ttg	L	0	3.58
tct	S	0	0.18
tcc	S	0	0.12
tca	S	1	0.26
tcg	S	0	0.05
tgt	C	0	0.90
tgc	C	2	1.10
ctt	L	0	3.74
ctc	L	15	2.68
cta	L	0	3.66
ctg	L	0	3.25
agt	S	0	0.19
agc	S	0	0.20
---	--	------	------
mPD		0.0051	0.49
		nPD:	0.01
		N. weight:	0.57
		Sc. PD:	-0.018
		Sc. rank:	-2624.2

,

PA

Pos.	647	obs:	exp:
tct	S	0	4.05
tcc	S	0	2.55
tca	S	0	5.73
tcg	S	0	1.17
tat	Y	0	0.52
tac	Y	1	0.48
aat	N	51	13346.00
aac	N	22515	9220.00
agt	S	0	4.14
agc	S	22	4.37
---	--	------	------
mPD		0.0065	0.49
		nPD:	0.01
		N. weight:	0.92
		Sc. PD:	-0.027
		Sc. rank:	-1014.7

,

PA

Pos.	648	obs:	exp:
tct	S	0	4151.00
tcc	S	0	2613.00
tca	S	0	5871.00
tcg	S	0	1201.00
cgt	R	0	0.08
cgc	R	0	0.16
cga	R	0	0.29
cgg	R	0	0.22
aat	N	25	14.79
aac	N	0	10.21
agt	S	22450	4242.00
agc	S	109	4481.00
aga	R	0	1.51
agg	R	3	0.74
ggt	G	2	0.30
ggc	G	0	0.30
gga	G	0	0.89
ggg	G	0	0.51
---	--	------	------
mPD		0.012	1.6
		nPD:	0.01
		N. weight:	1.7
		Sc. PD:	-0.06
		Sc. rank:	-15000.0

,

PA

Pos.	649	obs:	exp:
tta	L	461	2454.00
ttg	L	1	4203.00
ctt	L	3	4399.00
ctc	L	5	3153.00
cta	L	21818	4298.00
ctg	L	37	3817.00
cct	P	0	1.10
ccc	P	0	0.74
cca	P	4	1.70
ccg	P	0	0.45
att	I	0	94.65
atc	I	15	64.90
ata	I	245	100.50
---	--	------	------
mPD		0.068	1.2
		nPD:	0.06
		N. weight:	1.6
		Sc. PD:	0.025
		Sc. rank:	20570.0

,

PA

Pos.	650	obs:	exp:
ttt	F	8	3.43
ttc	F	0	4.57
tct	S	1	0.18
tcc	S	0	0.12
tca	S	0	0.26
tcg	S	0	0.05
tat	Y	21531	11719.00
tac	Y	1024	10836.00
tgt	C	4	1.81
tgc	C	0	2.19
cat	H	13	7.65
cac	H	0	5.35
agt	S	0	0.19
agc	S	0	0.20
gtt	V	6	1.33
gtc	V	0	1.09
gta	V	0	1.61
gtg	V	0	1.97
gat	D	2	1.05
gac	D	0	0.95
---	--	------	------
mPD		0.090	0.50
		nPD:	0.18
		N. weight:	0.49
		Sc. PD:	0.066
		Sc. rank:	8293.8

,

PA

Pos.	651	obs:	exp:
act	T	4	1.51
acc	T	0	1.23
aca	T	0	2.74
acg	T	2	0.51
gct	A	14652	5979.00
gcc	A	18	4674.00
gca	A	55	10606.00
gcg	A	7857	1323.00
gat	D	1	0.53
gac	D	0	0.47
---	--	------	------
mPD		0.46	0.66
		nPD:	0.69
		N. weight:	1.2
		Sc. PD:	0.79
		Sc. rank:	23054.0

,

PA

Pos.	652	obs:	exp:
ttt	F	1	0.43
ttc	F	0	0.57
tct	S	22388	4156.00
tcc	S	185	2616.00
tca	S	9	5877.00
tcg	S	0	1202.00
act	T	5	1.26
acc	T	0	1.03
aca	T	0	2.29
acg	T	0	0.43
agt	S	0	4246.00
agc	S	0	4486.00
gct	A	1	0.26
gcc	A	0	0.21
gca	A	0	0.47
gcg	A	0	0.06
---	--	------	------
mPD		0.018	1.6
		nPD:	0.01
		N. weight:	1.7
		Sc. PD:	-0.054
		Sc. rank:	-6673.1

,

PA

Pos.	653	obs:	exp:
tta	L	0	0.22
ttg	L	0	0.38
tct	S	0	3.31
tcc	S	0	2.08
tca	S	18	4.68
tcg	S	0	0.96
ctt	L	0	0.39
ctc	L	0	0.28
cta	L	2	0.39
ctg	L	0	0.34
cct	P	0	6217.00
ccc	P	3	4185.00
cca	P	22499	9608.00
ccg	P	63	2555.00
act	T	0	1.01
acc	T	0	0.82
aca	T	4	1.83
acg	T	0	0.34
agt	S	0	3.38
agc	S	0	3.58
---	--	------	------
mPD		0.0080	0.70
		nPD:	0.01
		N. weight:	0.87
		Sc. PD:	-0.027
		Sc. rank:	-2615.6

,

PA

Pos.	654	obs:	exp:
cct	P	0	0.28
ccc	P	0	0.19
cca	P	1	0.43
ccg	P	0	0.11
caa	Q	22560	11918.00
cag	Q	27	10669.00
cgt	R	0	0.03
cgc	R	0	0.05
cga	R	1	0.10
cgg	R	0	0.07
aga	R	0	0.50
agg	R	0	0.25
---	--	------	------
mPD		0.0026	0.50
		nPD:	0.01
		N. weight:	0.66
		Sc. PD:	-0.024
		Sc. rank:	-8163.5

,

PA

Pos.	655	obs:	exp:
ttt	F	20	8.58
ttc	F	0	11.42
tta	L	0	2478.00
ttg	L	6	4244.00
ctt	L	22501	4442.00
ctc	L	18	3184.00
cta	L	14	4340.00
ctg	L	5	3854.00
att	I	25	9.10
atc	I	0	6.24
ata	I	0	9.66
---	--	------	------
mPD		0.0083	1.1
		nPD:	0.01
		N. weight:	1.7
		Sc. PD:	-0.059
		Sc. rank:	-15307.0

,

PA

Pos.	656	obs:	exp:
gaa	E	90	13678.00
gag	E	22499	8911.00
---	--	------	------
mPD		0.0079	0.48
		nPD:	0.02
		N. weight:	0.94
		Sc. PD:	-0.024
		Sc. rank:	920.5

,

PA

Pos.	657	obs:	exp:
ggt	G	29	3425.00
ggc	G	1	3346.00
gga	G	144	10048.00
ggg	G	22415	5770.00
---	--	------	------
mPD		0.015	0.69
		nPD:	0.02
		N. weight:	1.4
		Sc. PD:	-0.028
		Sc. rank:	6957.7

,

PA			
Pos.	658	obs:	exp:
ttt	F	22429	9681.00
ttc	F	130	12878.00
tta	L	0	3.19
ttg	L	2	5.46
tgt	C	1	0.45
tgc	C	0	0.55
ctt	L	27	5.71
ctc	L	0	4.10
cta	L	0	5.58
ctg	L	0	4.96
---	--	------	------
mPD		0.014	0.49
		nPD:	0.03
		N. weight:	0.84
		Sc. PD:	-0.012
		Sc. rank:	6923.1

,

PA			
Pos.	659	obs:	exp:
tta	L	0	0.22
ttg	L	2	0.38
tct	S	12	4156.00
tcc	S	0	2616.00
tca	S	875	5878.00
tcg	S	21697	1202.00
tgg	W	1	1.00
ctt	L	0	0.39
ctc	L	0	0.28
cta	L	0	0.39
ctg	L	0	0.34
act	T	0	0.25
acc	T	0	0.21
aca	T	0	0.46
acg	T	1	0.09
agt	S	0	4247.00
agc	S	0	4486.00
gct	A	0	0.26
gcc	A	0	0.21
gca	A	0	0.47
gcg	A	1	0.06
---	--	------	------
mPD		0.076	1.6
		nPD:	0.05
		N. weight:	2.8
		Sc. PD:	0.0095
		Sc. rank:	32769.0

,

PA			
Pos.	660	obs:	exp:
tct	S	5	0.92
tcc	S	0	0.58
tca	S	0	1.30
tcg	S	0	0.27
act	T	4	1.01
acc	T	0	0.82
aca	T	0	1.83
acg	T	0	0.34
agt	S	0	0.94
agc	S	0	0.99
gtt	V	3	0.66
gtc	V	0	0.55
gta	V	0	0.81
gtg	V	0	0.98
gct	A	22431	5977.00
gcc	A	97	4673.00
gca	A	45	10603.00
gcg	A	2	1323.00
ggt	G	2	0.30
ggc	G	0	0.30
gga	G	0	0.89
ggg	G	0	0.51
---	--	------	------
mPD		0.014	0.66
		nPD:	0.02
		N. weight:	1.3
		Sc. PD:	-0.029
		Sc. rank:	5624.8

,

PA			
Pos.	661	obs:	exp:
aaa	K	1	0.57
aag	K	0	0.43
gaa	E	22518	13677.00
gag	E	70	8911.00
---	--	------	------
mPD		0.0063	0.48
		nPD:	0.01
		N. weight:	0.5
		Sc. PD:	-0.015
		Sc. rank:	-724.6

,

PA			
Pos.	662	obs:	exp:
tct	S	15	4154.00
tcc	S	47	2615.00
tca	S	19537	5875.00
tcg	S	2975	1201.00
tgt	C	1	0.45
tgc	C	0	0.55
act	T	0	3.53
acc	T	0	2.87
aca	T	14	6.40
acg	T	0	1.19
agt	S	0	4245.00
agc	S	0	4484.00
---	--	------	------
mPD		0.23	1.6
		nPD:	0.14
		N. weight:	1.2
		Sc. PD:	0.12
		Sc. rank:	19489.0

,

PA			
Pos.	663	obs:	exp:
cgt	R	0	616.70
cgc	R	0	1182.00
cga	R	0	2212.00
cgg	R	0	1653.00
att	I	0	0.36
atc	I	0	0.25
ata	I	1	0.39
aga	R	22444	11340.00
agg	R	144	5584.00
---	--	------	------
mPD		0.013	0.91
		nPD:	0.01
		N. weight:	0.68
		Sc. PD:	-0.019
		Sc. rank:	-416.0

,

PA			
Pos.	664	obs:	exp:
caa	Q	1	0.53
cag	Q	0	0.47
cgt	R	0	0.35
cgc	R	0	0.68
cga	R	0	1.27
cgg	R	0	0.95
aaa	K	22523	12877.00
aag	K	52	9698.00
aga	R	13	6.53
agg	R	0	3.21
---	--	------	------
mPD		0.0058	0.49
		nPD:	0.01
		N. weight:	0.57
		Sc. PD:	-0.017
		Sc. rank:	-1524.1

,

PA			
Pos.	665	obs:	exp:
ttt	F	1	0.43
ttc	F	0	0.57
tta	L	69	2483.00
ttg	L	22468	4251.00
ctt	L	0	4450.00
ctc	L	0	3190.00
cta	L	0	4347.00
ctg	L	45	3861.00
atg	M	6	6.00
---	--	------	------
mPD		0.011	1.1
		nPD:	0.01
		N. weight:	1.7
		Sc. PD:	-0.056
		Sc. rank:	-10208.0

,

PA			
Pos.	666	obs:	exp:
ttt	F	0	0.43
ttc	F	1	0.57
tta	L	0	2481.00
ttg	L	0	4249.00
ctt	L	22525	4448.00
ctc	L	44	3188.00
cta	L	1	4345.00
ctg	L	1	3859.00
att	I	16	5.82
atc	I	0	3.99
ata	I	0	6.18
act	T	1	0.25
acc	T	0	0.21
aca	T	0	0.46
acg	T	0	0.09
---	--	------	------
mPD		0.0058	1.1
		nPD:	0.01
		N. weight:	1.7
		Sc. PD:	-0.062
		Sc. rank:	-21119.0

,

PA			
Pos.	667	obs:	exp:
tta	L	0	2483.00
ttg	L	0	4253.00
ctt	L	53	4451.00
ctc	L	22532	3191.00
cta	L	4	4349.00
ctg	L	0	3862.00
---	--	------	------
mPD		0.0050	1.1
		nPD:	0.
		N. weight:	2.
		Sc. PD:	-0.077
		Sc. rank:	-28059.0

,

PA			
Pos.	668	obs:	exp:
tta	L	0	0.11
ttg	L	0	0.19
ctt	L	1	0.20
ctc	L	0	0.14
cta	L	0	0.19
ctg	L	0	0.17
att	I	22561	8220.00
atc	I	19	5636.00
ata	I	0	8724.00
act	T	3	0.76
acc	T	0	0.62
aca	T	0	1.37
acg	T	0	0.26
gtt	V	5	1.11
gtc	V	0	0.91
gta	V	0	1.34
gtg	V	0	1.64
---	--	------	------
mPD		0.0025	0.66
		nPD:	0.
		N. weight:	1.
		Sc. PD:	-0.04
		Sc. rank:	-15702.0

,

PA			
Pos.	669	obs:	exp:
ttt	F	5	2.15
ttc	F	0	2.85
att	I	33	12.01
atc	I	0	8.24
ata	I	0	12.75
gtt	V	22514	4983.00
gtc	V	13	4096.00
gta	V	9	6062.00
gtg	V	3	7399.00
gct	A	9	2.38
gcc	A	0	1.86
gca	A	0	4.23
gcg	A	0	0.53
ggt	G	3	0.45
ggc	G	0	0.44
gga	G	0	1.33
ggg	G	0	0.77
---	--	------	------
mPD		0.0066	0.74
		nPD:	0.01
		N. weight:	1.6
		Sc. PD:	-0.052
		Sc. rank:	-9824.1

,

PA			
Pos.	670	obs:	exp:
caa	Q	32	11920.00
cag	Q	22557	10669.00
---	--	------	------
mPD		0.0028	0.50
		nPD:	0.01
		N. weight:	0.77
		Sc. PD:	-0.028
		Sc. rank:	-8919.4

PA			
Pos.	671	obs:	exp:
gct	A	15	5981.00
gcc	A	4	4676.00
gca	A	22512	10609.00
gcg	A	58	1323.00
---	--	------	------
mPD		0.0068	0.66
		nPD:	0.01
		N. weight:	0.77
		Sc. PD:	-0.025
		Sc. rank:	-3566.1

PA			
Pos.	672	obs:	exp:
ttt	F	20	8.58
ttc	F	0	11.42
tta	L	0	2481.00
ttg	L	0	4249.00
ctt	L	22542	4447.00
ctc	L	22	3188.00
cta	L	2	4345.00
ctg	L	2	3859.00
att	I	1	0.36
atc	I	0	0.25
ata	I	0	0.39
---	--	------	------
mPD		0.0042	1.1
		nPD:	0.
		N. weight:	1.7
		Sc. PD:	-0.065
		Sc. rank:	-25739.0

PA			
Pos.	673	obs:	exp:
tct	S	0	0.18
tcc	S	0	0.12
tca	S	0	0.26
tcg	S	0	0.05
cgt	R	0	616.30
cgc	R	0	1181.00
cga	R	0	2211.00
cgg	R	2	1652.00
atg	M	3	3.00
aaa	K	0	7.42
aag	K	13	5.58
agt	S	1	0.19
agc	S	0	0.20
aga	R	114	11331.00
agg	R	22455	5580.00
ggt	G	0	0.15
ggc	G	0	0.15
gga	G	0	0.44
ggg	G	1	0.26
---	--	------	------
mPD		0.012	0.91
		nPD:	0.01
		N. weight:	1.4
		Sc. PD:	-0.042
		Sc. rank:	-2326.5

PA			
Pos.	674	obs:	exp:
gat	D	50	11902.00
gac	D	22539	10687.00
---	--	------	------
mPD		0.0044	0.50
		nPD:	0.01
		N. weight:	0.76
		Sc. PD:	-0.026
		Sc. rank:	-4864.2

PA			
Pos.	675	obs:	exp:
tct	S	0	2.76
tcc	S	0	1.74
tca	S	0	3.90
tcg	S	0	0.80
tat	Y	0	0.52
tac	Y	1	0.48
cat	H	0	1.76
cac	H	3	1.24
act	T	0	0.25
acc	T	1	0.21
aca	T	0	0.46
acg	T	0	0.09
aat	N	129	13342.00
aac	N	22429	9216.00
aaa	K	7	3.99
aag	K	0	3.01
agt	S	0	2.82
agc	S	15	2.98
gat	D	0	2.11
gac	D	4	1.89
---	--	------	------
mPD		0.014	0.49
		nPD:	0.03
		N. weight:	0.9
		Sc. PD:	-0.012
		Sc. rank:	7419.0

PA			
Pos.	676	obs:	exp:
tta	L	0	2483.00
ttg	L	730	4252.00
ctt	L	7	4451.00
ctc	L	7	3190.00
cta	L	43	4348.00
ctg	L	21798	3861.00
atg	M	4	4.00
---	--	------	------
mPD		0.068	1.1
		nPD:	0.06
		N. weight:	1.7
		Sc. PD:	0.028
		Sc. rank:	21683.0

PA			
Pos.	677	obs:	exp:
aaa	K	8	4.56
aag	K	0	3.44
gat	D	0	0.53
gac	D	1	0.47
gaa	E	22536	13672.00
gag	E	44	8908.00
---	--	------	------
mPD		0.0047	0.48
		nPD:	0.01
		N. weight:	0.51
		Sc. PD:	-0.017
		Sc. rank:	-2692.4

PA			
Pos.	678	obs:	exp:
tct	S	2	0.37
tcc	S	0	0.23
tca	S	0	0.52
tcg	S	0	0.11
cct	P	22313	6223.00
ccc	P	25	4189.00
cca	P	247	9617.00
ccg	P	2	2558.00
agt	S	0	0.38
agc	S	0	0.40
---	--	------	------
mPD		0.024	0.70
		nPD:	0.03
		N. weight:	1.3
		Sc. PD:	-0.01
		Sc. rank:	12625.0

PA			
Pos.	679	obs:	exp:
ggt	G	7	3425.00
ggc	G	0	3346.00
gga	G	22546	10048.00
ggg	G	36	5770.00
---	--	------	------
mPD		0.0038	0.69
		nPD:	0.01
		N. weight:	0.83
		Sc. PD:	-0.031
		Sc. rank:	-9762.1

PA			
Pos.	680	obs:	exp:
act	T	73	5694.00
acc	T	22479	4633.00
aca	T	37	10334.00
acg	T	0	1927.00
---	--	------	------
mPD		0.0097	0.68
		nPD:	0.01
		N. weight:	1.6
		Sc. PD:	-0.045
		Sc. rank:	-802.4

PA			
Pos.	681	obs:	exp:
ttt	F	14451	9693.00
ttc	F	8137	12895.00
tta	L	1	0.11
ttg	L	0	0.19
ctt	L	0	0.20
ctc	L	0	0.14
cta	L	0	0.19
ctg	L	0	0.17
---	--	------	------
mPD		0.46	0.49
		nPD:	0.94
		N. weight:	0.094
		Sc. PD:	0.083
		Sc. rank:	1754.4

PA			
Pos.	682	obs:	exp:
tat	Y	2	1.04
tac	Y	0	0.96
aat	N	13	7.69
aac	N	0	5.31
gat	D	22540	11894.00
gac	D	33	10679.00
---	-	1	1.00
---	--	------	------
mPD		0.0042	0.50
		nPD:	0.01
		N. weight:	0.66
		Sc. PD:	-0.022
		Sc. rank:	-4615.2

PA			
Pos.	683	obs:	exp:
ttt	F	2	0.86
ttc	F	0	1.14
tta	L	0	2479.00
ttg	L	0	4245.00
ctt	L	22505	4443.00
ctc	L	10	3185.00
cta	L	34	4341.00
ctg	L	0	3855.00
att	I	38	13.83
atc	I	0	9.49
ata	I	0	14.68
---	--	------	------
mPD		0.0074	1.1
		nPD:	0.01
		N. weight:	1.7
		Sc. PD:	-0.06
		Sc. rank:	-17764.0

PA			
Pos.	684	obs:	exp:
tgg	W	7	7.00
cgt	R	0	2.92
cgc	R	0	5.60
cga	R	0	10.48
cgg	R	0	7.83
aga	R	0	53.72
agg	R	107	26.45
gaa	E	0	24.22
gag	E	40	15.78
ggt	G	9	3402.00
ggc	G	0	3323.00
gga	G	72	9980.00
ggg	G	22354	5731.00
---	--	------	------
mPD		0.021	0.70
		nPD:	0.03
		N. weight:	1.4
		Sc. PD:	-0.018
		Sc. rank:	11984.0

PA			
Pos.	685	obs:	exp:
cgt	R	0	0.05
cgc	R	0	0.10
cga	R	0	0.20
cgg	R	0	0.15
aga	R	0	1.00
agg	R	2	0.49
ggt	G	1	3425.00
ggc	G	5	3345.00
gga	G	137	10047.00
ggg	G	22444	5770.00
---	--	------	------
mPD		0.013	0.69
		nPD:	0.02
		N. weight:	1.4
		Sc. PD:	-0.033
		Sc. rank:	3661.1

PA

Pos.	686	obs:	exp:
tta	L	11	2483.00
ttg	L	0	4252.00
ctt	L	9	4451.00
ctc	L	1	3190.00
cta	L	22496	4348.00
ctg	L	69	3862.00
att	I	0	0.36
atc	I	0	0.25
ata	I	1	0.39
gtt	V	0	0.44
gtc	V	0	0.36
gta	V	2	0.54
gtg	V	0	0.66
---	--	------	------
mPD		0.0082	1.1
		nPD:	0.01
		N. weight:	1.7
		Sc. PD:	-0.059
		Sc. rank:	-15875.0

PA

Pos.	687	obs:	exp:
tat	Y	22567	11736.00
tac	Y	21	10852.00
tgt	C	1	0.45
tgc	C	0	0.55
---	--	------	------
mPD		0.0019	0.50
		nPD:	0.
		N. weight:	0.68
		Sc. PD:	-0.026
		Sc. rank:	-9989.4

PA

Pos.	688	obs:	exp:
aaa	K	5	2.85
aag	K	0	2.15
gtt	V	0	0.22
gtc	V	0	0.18
gta	V	1	0.27
gtg	V	0	0.33
gct	A	0	0.26
gcc	A	0	0.21
gca	A	1	0.47
gcg	A	0	0.06
gat	D	1	2.63
gac	D	4	2.37
gaa	E	22466	13614.00
gag	E	18	8870.00
ggt	G	0	14.10
ggc	G	0	13.77
gga	G	93	41.37
ggg	G	0	23.76
---	--	------	------
mPD		0.011	0.49
		nPD:	0.02
		N. weight:	0.52
		Sc. PD:	-0.011
		Sc. rank:	2646.9

PA

Pos.	689	obs:	exp:
tct	S	0	0.92
tcc	S	0	0.58
tca	S	5	1.30
tcg	S	0	0.27
agt	S	0	0.94
agc	S	0	0.99
gct	A	1	5979.00
gcc	A	0	4675.00
gca	A	22575	10607.00
gcg	A	8	1323.00
---	--	------	------
mPD		0.0012	0.66
		nPD:	0.
		N. weight:	0.79
		Sc. PD:	-0.032
		Sc. rank:	-14250.0

PA

Pos.	690	obs:	exp:
att	I	216	8206.00
atc	I	22231	5627.00
ata	I	94	8709.00
atg	M	7	7.00
act	T	0	1.01
acc	T	4	0.82
aca	T	0	1.83
acg	T	0	0.34
gtt	V	1	8.18
gtc	V	36	6.72
gta	V	0	9.95
gtg	V	0	12.15
---	--	------	------
mPD		0.031	0.66
		nPD:	0.05
		N. weight:	1.4
		Sc. PD:	0.0065
		Sc. rank:	16020.0

PA

Pos.	691	obs:	exp:
aaa	K	0	1.14
aag	K	2	0.86
gaa	E	1	13675.00
gag	E	22584	8910.00
ggt	G	0	0.30
ggc	G	0	0.30
gga	G	0	0.89
ggg	G	2	0.51
---	--	------	------
mPD		0.00044	0.48
		nPD:	0.
		N. weight:	0.97
		Sc. PD:	-0.04
		Sc. rank:	-17967.0

PA

Pos.	692	obs:	exp:
gat	D	5	2.63
gac	D	0	2.37
gaa	E	49	13675.00
gag	E	22535	8909.00
---	--	------	------
mPD		0.0048	0.48
		nPD:	0.01
		N. weight:	0.95
		Sc. PD:	-0.031
		Sc. rank:	-4660.8

PA

Pos.	693	obs:	exp:
tgt	C	38	10195.00
tgc	C	22550	12393.00
tgg	W	1	1.00
---	--	------	------
mPD		0.0034	0.50
		nPD:	0.01
		N. weight:	0.61
		Sc. PD:	-0.022
		Sc. rank:	-6164.5

PA

Pos.	694	obs:	exp:
tta	L	5	2483.00
ttg	L	14602	4252.00
tct	S	0	0.18
tcc	S	0	0.12
tca	S	0	0.26
tcg	S	1	0.05
ctt	L	2	4451.00
ctc	L	0	3190.00
cta	L	4	4348.00
ctg	L	7973	3862.00
cct	P	0	0.28
ccc	P	0	0.19
cca	P	0	0.43
ccg	P	1	0.11
atg	M	1	1.00
agt	S	0	0.19
agc	S	0	0.20
---	--	------	------
mPD		0.46	1.1
		nPD:	0.4
		N. weight:	1.1
		Sc. PD:	0.39
		Sc. rank:	19769.0

PA

Pos.	695	obs:	exp:
att	I	22565	8216.00
atc	I	6	5634.00
ata	I	0	8720.00
act	T	2	0.50
acc	T	0	0.41
aca	T	0	0.91
acg	T	0	0.17
gtt	V	16	3.54
gtc	V	0	2.91
gta	V	0	4.30
gtg	V	0	5.25
---	--	------	------
mPD		0.0021	0.66
		nPD:	0.
		N. weight:	1.1
		Sc. PD:	-0.041
		Sc. rank:	-16783.0

PA

Pos.	696	obs:	exp:
tct	S	0	0.92
tcc	S	0	0.58
tca	S	0	1.30
tcg	S	0	0.27
tat	Y	1	0.52
tac	Y	0	0.48
aat	N	22496	13356.00
aac	N	86	9226.00
aaa	K	0	0.57
aag	K	1	0.43
agt	S	5	0.94
agc	S	0	0.99
---	--	------	------
mPD		0.0082	0.48
		nPD:	0.02
		N. weight:	0.52
		Sc. PD:	-0.013
		Sc. rank:	674.6

PA

Pos.	697	obs:	exp:
aat	N	4	2.37
aac	N	0	1.63
gat	D	22555	11899.00
gac	D	28	10684.00
ggt	G	2	0.30
ggc	G	0	0.30
gga	G	0	0.89
ggg	G	0	0.51
---	--	------	------
mPD		0.0030	0.50
		nPD:	0.01
		N. weight:	0.66
		Sc. PD:	-0.024
		Sc. rank:	-7371.3

PA

Pos.	698	obs:	exp:
tct	S	0	0.37
tcc	S	2	0.23
tca	S	0	0.52
tcg	S	0	0.11
cct	P	23	6222.00
ccc	P	22544	4189.00
cca	P	19	9617.00
ccg	P	0	2557.00
act	T	0	0.25
acc	T	1	0.21
aca	T	0	0.46
acg	T	0	0.09
agt	S	0	0.38
agc	S	0	0.40
---	--	------	------
mPD		0.0040	0.70
		nPD:	0.01
		N. weight:	1.7
		Sc. PD:	-0.064
		Sc. rank:	-19979.0

PA

Pos.	699	obs:	exp:
tgg	W	22589	22589.00
---	--	------	------
mPD		0	0
		nPD:	1.
		N. weight:	0.
		Sc. PD:	0
		Sc. rank:	0

PA

Pos.	700	obs:	exp:
att	I	1	0.36
atc	I	0	0.25
ata	I	0	0.39
gtt	V	22571	4993.00
gtc	V	10	4104.00
gta	V	1	6074.00
gtg	V	2	7413.00
gct	A	4	1.06
gcc	A	0	0.83
gca	A	0	1.88
gcg	A	0	0.23
---	--	------	------
mPD		0.0016	0.74
		nPD:	0.
		N. weight:	1.6
		Sc. PD:	-0.063
		Sc. rank:	-27887.0

PA

Pos.	701	obs:	exp:
tta	L	13	2483.00
ttg	L	22365	4252.00
tct	S	0	0.37
tcc	S	0	0.23
tca	S	0	0.52
tcg	S	2	0.11
ctt	L	0	4451.00
ctc	L	0	3190.00
cta	L	0	4348.00
ctg	L	208	3862.00
agt	S	0	0.38
agc	S	0	0.40
gtt	V	0	0.22
gtc	V	0	0.18
gta	V	0	0.27
gtg	V	1	0.33
---	--	------	------
mPD		0.020	1.1
		nPD:	0.02
		N. weight:	1.7
		Sc. PD:	-0.042
		Sc. rank:	2629.0

PA

Pos.	702	obs:	exp:
tta	L	0	2483.00
ttg	L	0	4252.00
ctt	L	22520	4450.00
ctc	L	62	3190.00
cta	L	2	4348.00
ctg	L	0	3861.00
att	I	5	1.82
atc	I	0	1.25
ata	I	0	1.93
---	--	------	------
mPD		0.0061	1.1
		nPD:	0.01
		N. weight:	1.7
		Sc. PD:	-0.062
		Sc. rank:	-19891.0

,

PA

Pos.	703	obs:	exp:
tct	S	0	2.94
tcc	S	0	1.85
tca	S	0	4.16
tcg	S	0	0.85
att	I	1	0.36
atc	I	0	0.25
ata	I	0	0.39
act	T	2	0.50
acc	T	0	0.41
aca	T	0	0.91
acg	T	0	0.17
aat	N	22536	13349.00
aac	N	34	9221.00
agt	S	16	3.01
agc	S	0	3.18
---	--	------	------
mPD		0.0047	0.49
		nPD:	0.01
		N. weight:	0.54
		Sc. PD:	-0.018
		Sc. rank:	-3009.2

,

PA

Pos.	704	obs:	exp:
tct	S	0	0.37
tcc	S	0	0.23
tca	S	2	0.52
tcg	S	0	0.11
act	T	0	0.50
acc	T	0	0.41
aca	T	2	0.91
acg	T	0	0.17
agt	S	0	0.38
agc	S	0	0.40
gtt	V	0	0.66
gtc	V	0	0.55
gta	V	3	0.81
gtg	V	0	0.98
gct	A	25	5979.00
gcc	A	0	4674.00
gca	A	22514	10606.00
gcg	A	43	1323.00
---	--	------	------
mPD		0.0066	0.66
		nPD:	0.01
		N. weight:	0.77
		Sc. PD:	-0.025
		Sc. rank:	-3734.3

,

PA

Pos.	705	obs:	exp:
tct	S	22405	4157.00
tcc	S	181	2616.00
tca	S	3	5879.00
tcg	S	0	1202.00
agt	S	0	4247.00
agc	S	0	4487.00
---	--	------	------
mPD		0.016	1.6
		nPD:	0.01
		N. weight:	1.7
		Sc. PD:	-0.056
		Sc. rank:	-8648.0

,

PA

Pos.	706	obs:	exp:
tgg	W	22589	22589.00
---	--	------	------
mPD		0	0
		nPD:	1.
		N. weight:	0.
		Sc. PD:	0
		Sc. rank:	0

,

PA

Pos.	707	obs:	exp:
ttt	F	35	9692.00
ttc	F	22551	12894.00
tta	L	1	0.33
ttg	L	0	0.56
ctt	L	0	0.59
ctc	L	2	0.42
cta	L	0	0.58
ctg	L	0	0.51
---	--	------	------
mPD		0.0034	0.49
		nPD:	0.01
		N. weight:	0.57
		Sc. PD:	-0.02
		Sc. rank:	-5982.5

,

PA

Pos.	708	obs:	exp:
aat	N	0	13359.00
aac	N	22587	9228.00
aaa	K	1	0.57
aag	K	0	0.43
gat	D	0	0.53
gac	D	1	0.47
---	--	------	------
mPD		0.00018	0.48
		nPD:	0.
		N. weight:	0.93
		Sc. PD:	-0.039
		Sc. rank:	-17384.0

,

PA

Pos.	709	obs:	exp:
ttt	F	0	0.43
ttc	F	1	0.57
tct	S	1	4156.00
tcc	S	22585	2616.00
tca	S	0	5878.00
tcg	S	0	1202.00
tat	Y	0	0.52
tac	Y	1	0.48
cct	P	0	0.28
ccc	P	1	0.19
cca	P	0	0.43
ccg	P	0	0.11
agt	S	0	4247.00
agc	S	0	4486.00
---	--	------	------
mPD		0.00035	1.6
		nPD:	0.
		N. weight:	2.2
		Sc. PD:	-0.094
		Sc. rank:	-41963.0

,

PA

Pos.	710	obs:	exp:
ttt	F	1	9574.00
ttc	F	22309	12736.00
tta	L	0	30.56
ttg	L	0	52.34
tct	S	0	0.18
tcc	S	1	0.12
tca	S	0	0.26
tcg	S	0	0.05
ctt	L	0	54.78
ctc	L	278	39.27
cta	L	0	53.52
ctg	L	0	47.53
agt	S	0	0.19
agc	S	0	0.20
---	--	------	------
mPD		0.024	0.52
		nPD:	0.05
		N. weight:	0.6
		Sc. PD:	0.0028
		Sc. rank:	7034.6

,

PA

Pos.	711	obs:	exp:
ttt	F	0	3.00
ttc	F	7	4.00
tta	L	0	2482.00
ttg	L	0	4251.00
ctt	L	13	4449.00
ctc	L	22551	3189.00
cta	L	3	4347.00
ctg	L	12	3860.00
att	I	0	1.09
atc	I	3	0.75
ata	I	0	1.16
---	--	------	------
mPD		0.0034	1.1
		nPD:	0.
		N. weight:	2.
		Sc. PD:	-0.08
		Sc. rank:	-33520.0

,

PA

Pos.	712	obs:	exp:
att	I	0	0.73
atc	I	0	0.50
ata	I	2	0.77
act	T	9	5692.00
acc	T	0	4631.00
aca	T	22501	10330.00
acg	T	70	1927.00
aaa	K	1	0.57
aag	K	0	0.43
gct	A	0	1.59
gcc	A	0	1.24
gca	A	6	2.82
gcg	A	0	0.35
---	--	------	------
mPD		0.0078	0.68
		nPD:	0.01
		N. weight:	0.8
		Sc. PD:	-0.025
		Sc. rank:	-2293.5

,

PA

Pos.	713	obs:	exp:
cct	P	1	0.28
ccc	P	0	0.19
cca	P	0	0.43
ccg	P	0	0.11
cat	H	22550	13274.00
cac	H	22	9298.00
caa	Q	2	4.22
cag	Q	6	3.78
cgt	R	8	0.22
cgc	R	0	0.42
cga	R	0	0.78
cgg	R	0	0.59
aga	R	0	4.02
agg	R	0	1.98
---	--	------	------
mPD		0.0035	0.49
		nPD:	0.01
		N. weight:	0.55
		Sc. PD:	-0.019
		Sc. rank:	-5286.5

,

PA

Pos.	714	obs:	exp:
act	T	0	2.52
acc	T	0	2.05
aca	T	10	4.57
acg	T	0	0.85
gtt	V	0	1.11
gtc	V	0	0.91
gta	V	5	1.34
gtg	V	0	1.64
gct	A	0	5977.00
gcc	A	0	4673.00
gca	A	22574	10602.00
gcg	A	0	1322.00
---	--	------	------
mPD		0.0013	0.66
		nPD:	0.
		N. weight:	0.79
		Sc. PD:	-0.032
		Sc. rank:	-14242.0

,

PA

Pos.	715	obs:	exp:
tta	L	6	2483.00
ttg	L	62	4253.00
ctt	L	2	4451.00
ctc	L	3	3190.00
cta	L	47	4349.00
ctg	L	22468	3862.00
cct	P	0	0.28
ccc	P	0	0.19
cca	P	0	0.43
ccg	P	1	0.11
---	--	------	------
mPD		0.011	1.1
		nPD:	0.01
		N. weight:	1.8
		Sc. PD:	-0.059
		Sc. rank:	-9225.7

,

PA

Pos.	716	obs:	exp:
caa	Q	0	122.40
cag	Q	232	109.60
cgt	R	0	0.22
cgc	R	0	0.42
cga	R	0	0.78
cgg	R	0	0.59
aat	N	13	8.28
aac	N	1	5.72
aaa	K	263	12725.00
aag	K	22045	9583.00
aga	R	0	4.02
agg	R	8	1.98
gaa	E	0	16.35
gag	E	27	10.65
---	--	------	------
mPD		0.048	0.51
		nPD:	0.09
		N. weight:	0.81
		Sc. PD:	0.04
		Sc. rank:	12234.0

,

PA

Pos.	717	obs:	exp:
taa	.	203	10260.00
tag	.	22386	6153.00
tga	.	0	6177.00
---	--	------	------
mPD		0.018	0.79
		nPD:	0.02
		N. weight:	1.3
		Sc. PD:	-0.026
		Sc. rank:	6851.0

}

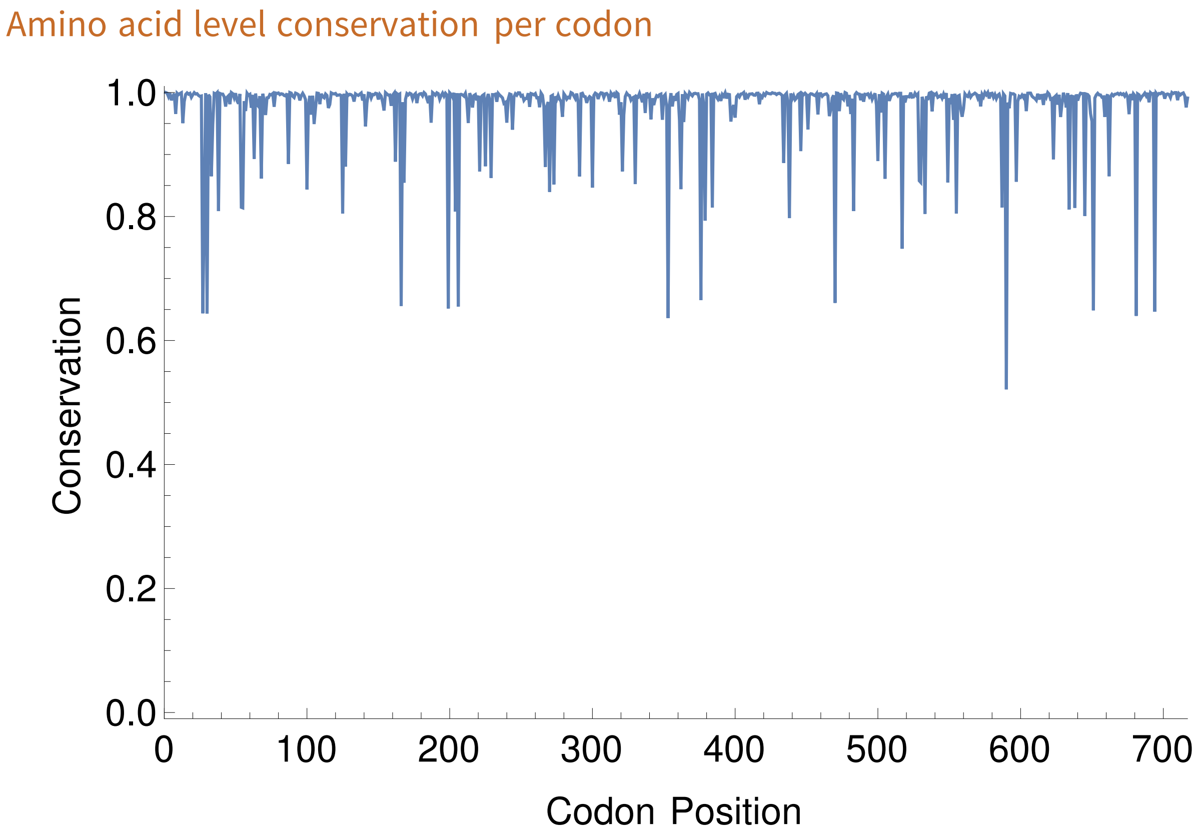
Amino acid level conservation per codon
Conservation
Codon Position
1.0
0.8
0.6
0.4
0.2
0.0
0
100
200
300
400
500
600
700

Conservation analysis plots

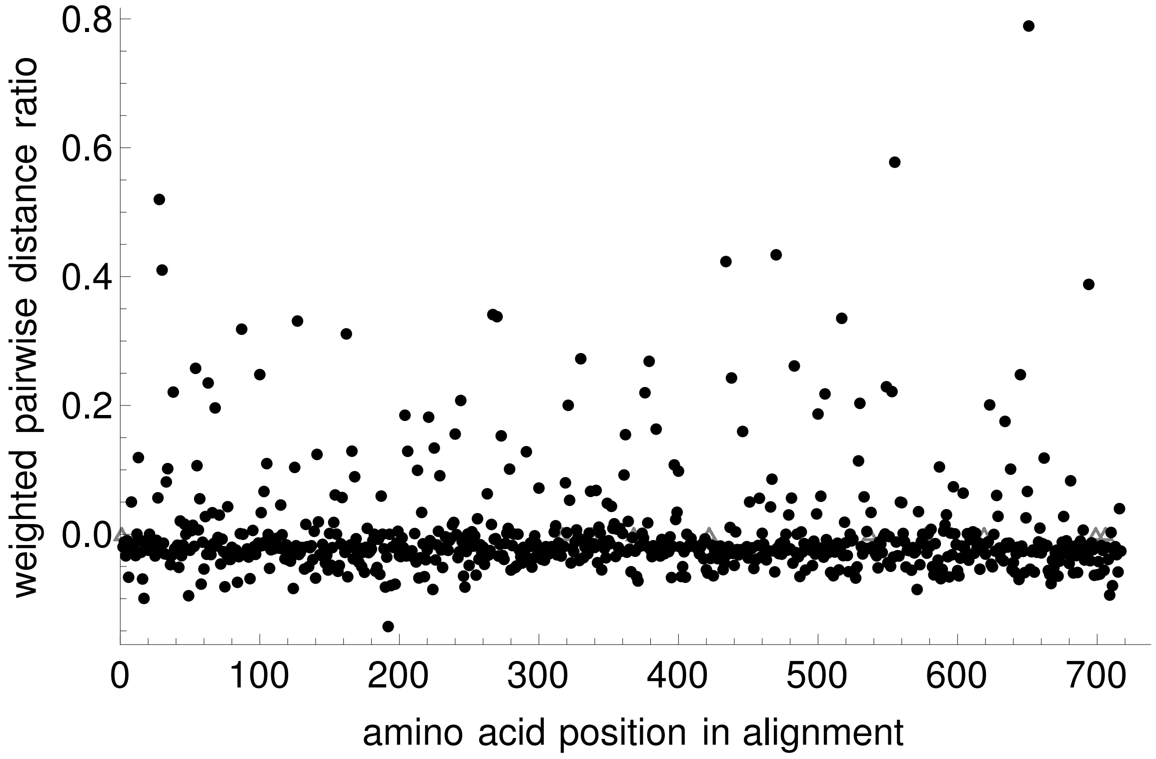

PA

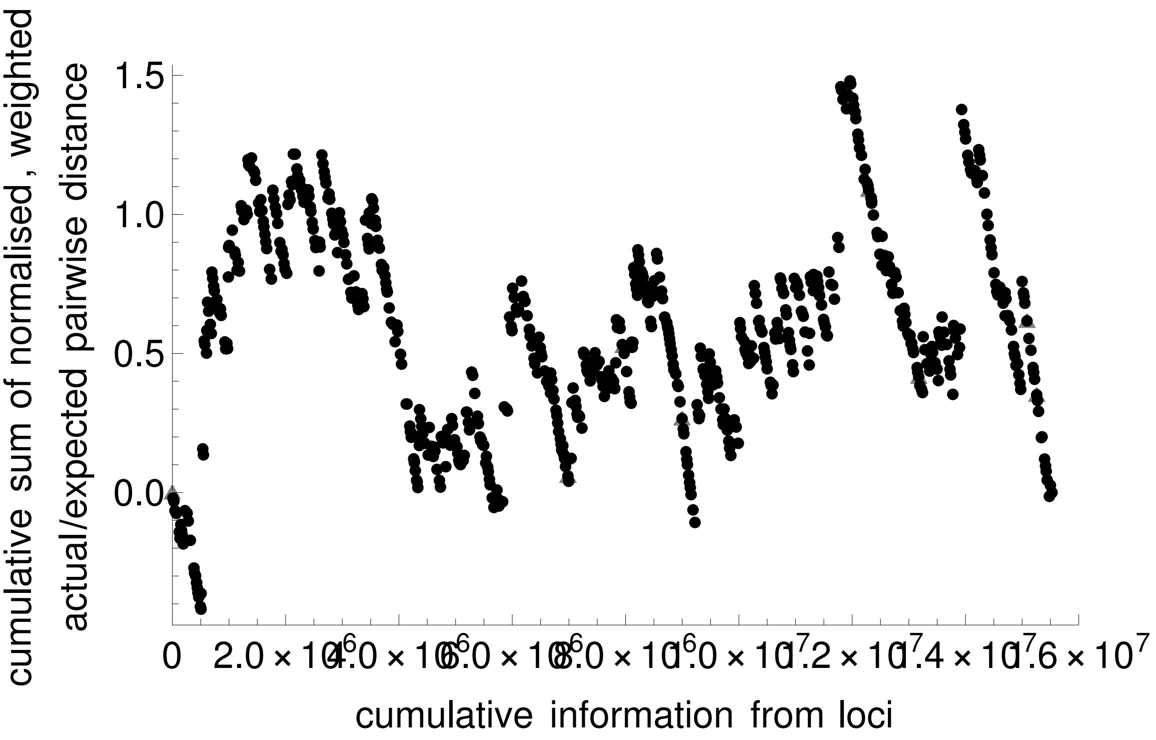

PA

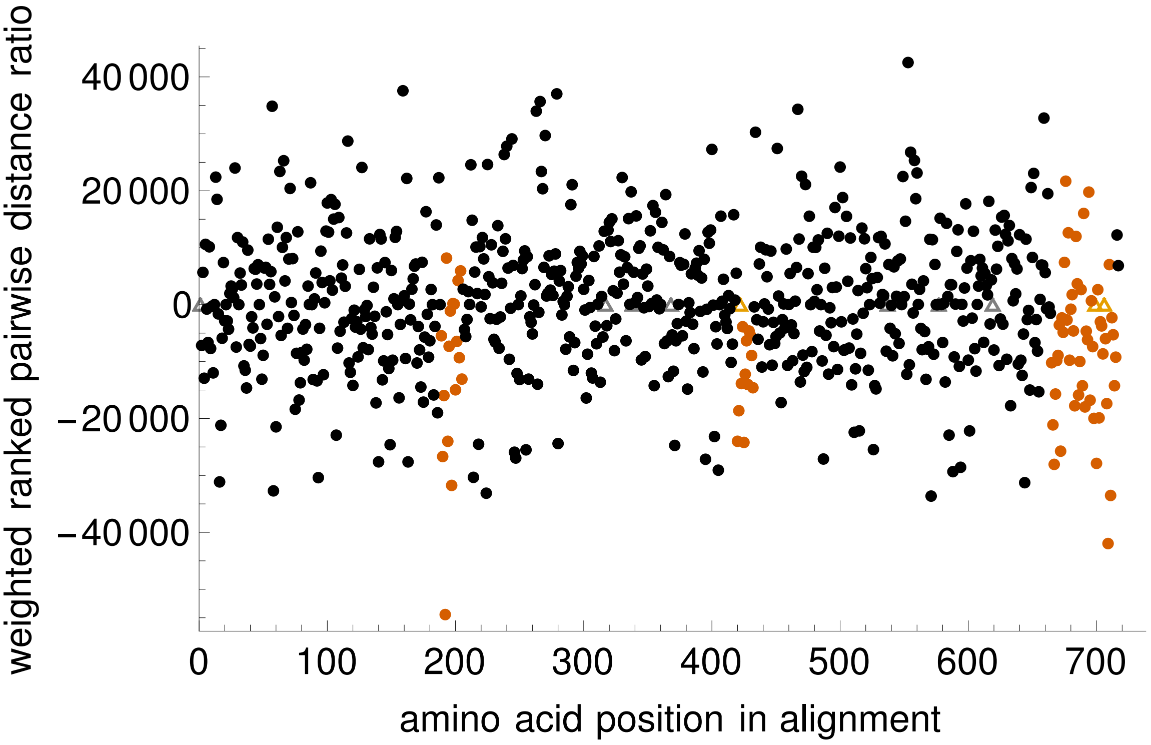
weighted ranked pairwise distance ratio
40 000
20 000
0
−20 000
−40 000
0
100
200
300
400
500
600
700
amino acid position in alignment

PA

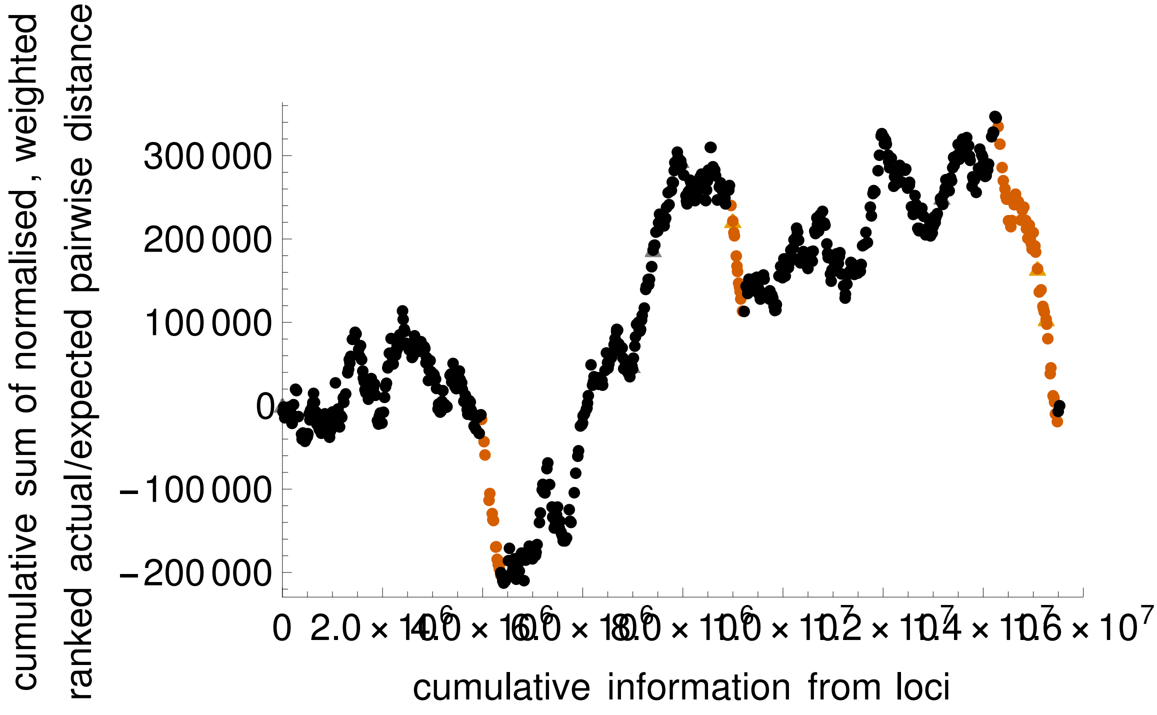
cumulative sum of normalised, weighted
ranked actual/expected pairwise distance
300 000
200 000
100 000
0
−100 000
−200 000
cumulative information from loci

PA

PA-X

Gene length histogram

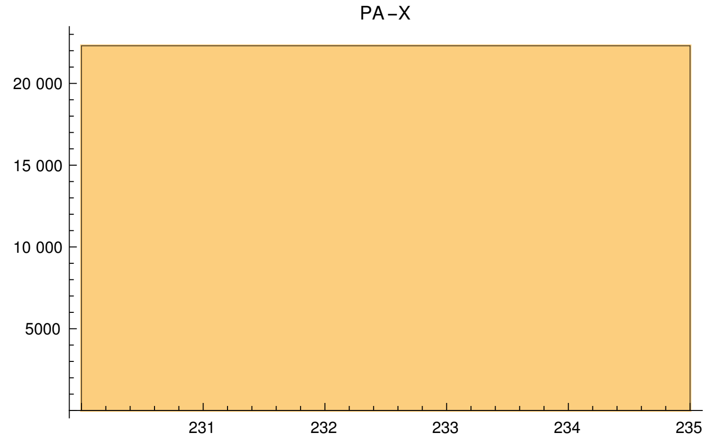

Information vs. nPD

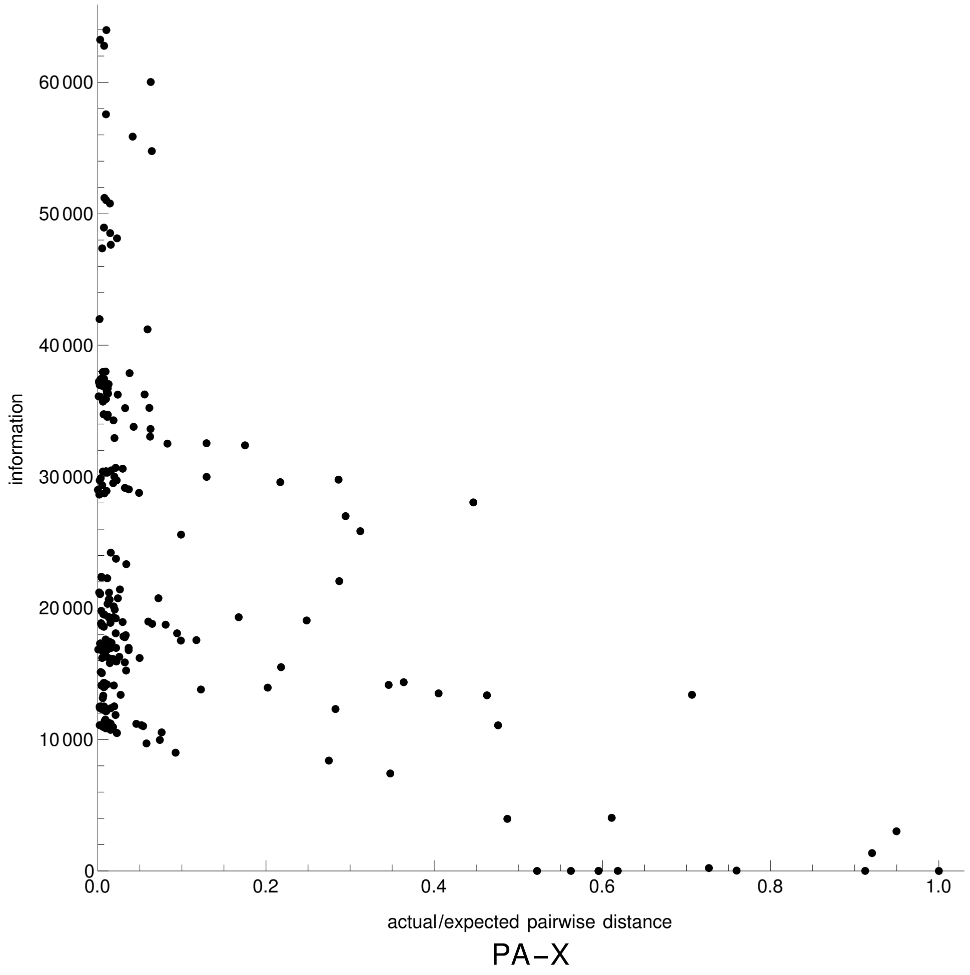

Example sequences highlighted by regions found in analyses to be conserved

```
Interesting points (by weighted raw PD) highlighted for gene PA-X:
ATGGAAGACTTTGTGCGACAATGCTTCAATCCAATGATCGTCGAGCTTGCGGAAAAGGCAATGAAAGAATATGGGGAA
    GACCCCAAAATTGAAACTAATAAGTTTGCTGCAATTTGCACACATTTGGAAGTTTGTTTCATGTATTCGGATTTCCA
    TTTCATTGATGAACGGGGTGAATCAATAATTGTAGAATCTGGTGACCCAAATGCACTATTGAAGCACCGATTTGAGA
    TAATTGAAGGAAGAGACCGAATCATGGCCTGGACAGTGGTGAACAGTATATGTAACACAACAGGGATAGAGAAGCCT
    AAATTTCTTCCTGATTTGTATGATTACAAAGAGAACCGGTTCATTGAAATTGGAGTAACACGGAGAGAAGTCCACAT
    ATATTACCTAGAGAAAGCCAACAAAATAAAATCTGAGAAGACACACATTCACATCTTTTCATTCACTGGAGAGGAGA
    TGGCCACCAAAGCGGACTACACCCTTGACGAAGAAAGCAGAGCAAGAATCAAAACTAGGCTTTTCACTATAAGACAA
    GAAATGGCCAGTAGGAGTCTATGGGATTCCTTCGTCAGTCCGAAAGAGGCGAAGAAACAATTGAAGAAAAGTTTGAA
    ATTACAGGAACTATGCGCAAGCTTGCCGACCAAAGTCTCCCACCAAACTTCTCCAGCCTTGAAAACTTCAGAGCCTA
    TGTAG
```

```
Interesting points (by weighted ranked PD) highlighted for gene PA-X:
ATGGAAGACTTTGTGCGACAATGCTTCAATCCAATGATCGTCGAGCTTGCGGAAAAGGCAATGAAAGAATATGGGGAA
    GACCCCAAAATTGAAACTAATAAGTTTGCTGCAATTTGCACACATTTGGAAGTTTGTTTCATGTATTCGGATTTCCA
    TTTCATTGATGAACGGGGTGAATCAATAATTGTAGAATCTGGTGACCCAAATGCACTATTGAAGCACCGATTTGAGA
    TAATTGAAGGAAGAGACCGAATCATGGCCTGGACAGTGGTGAACAGTATATGTAACACAACAGGGATAGAGAAGCCT
    AAATTTCTTCCTGATTTGTATGATTACAAAGAGAACCGGTTCATTGAAATTGGAGTAACACGGAGAGAAGTCCACAT
    ATATTACCTAGAGAAAGCCAACAAAATAAAATCTGAGAAGACACACATTCACATCTTTTCATTCACTGGAGAGGAGA
    TGGCCACCAAAGCGGACTACACCCTTGACGAAGAAAGCAGAGCAAGAATCAAAACTAGGCTTTTCACTATAAGACAA
    GAAATGGCCAGTAGGAGTCTATGGGATTCCTTCGTCAGTCCGAAAGAGGCGAAGAAACAATTGAAGAAAAGTTTGAA
    ATTACAGGAACTATGCGCAAGCTTGCCGACCAAAGTCTCCCACCAAACTTCTCCAGCCTTGAAAACTTCAGAGCCTA
    TGTAG
```

Per codon conservation report

PA-X

Pos.	1	obs:	exp:
atg	M	22301	22301.00
mPD		0	0
		nPD:	1.
		N. weight:	0.
		Sc. PD:	0
		Sc. rank:	0

PA-X

Pos.	2	obs:	exp:
aaa	K	5	2.85
aag	K	0	2.15
gaa	E	22278	13498.00
gag	E	15	8795.00
ggt	G	0	0.45
ggc	G	0	0.44
gga	G	3	1.33
ggg	G	0	0.77
mPD		0.0021	0.48
		nPD:	0.
		N. weight:	0.5
		Sc. PD:	-0.02
		Sc. rank:	-6419.3

PA-X

Pos.	3	obs:	exp:
tat	Y	0	0.52
tac	Y	1	0.48
aat	N	0	5.32
aac	N	9	3.68
gat	D	69	11710.00
gac	D	22156	10515.00
gaa	E	5	3.03
gag	E	0	1.97
ggt	G	0	9.25
ggc	G	61	9.03
gga	G	0	27.13
ggg	G	0	15.58
mPD		0.013	0.51
		nPD:	0.03
		N. weight:	0.74
		Sc. PD:	-0.013
		Sc. rank:	5773.1

PA-X

Pos.	4	obs:	exp:
ttt	F	22279	9567.00
ttc	F	15	12727.00
tta	L	1	0.66
ttg	L	2	1.13
tct	S	1	0.18
tcc	S	0	0.12
tca	S	0	0.26
tcg	S	0	0.05
ctt	L	3	1.18
ctc	L	0	0.85
cta	L	0	1.16
ctg	L	0	1.03
agt	S	0	0.19
agc	S	0	0.20
mPD		0.0020	0.49
		nPD:	0.
		N. weight:	0.85
		Sc. PD:	-0.033
		Sc. rank:	-11507.0

PA-X

Pos.	5	obs:	exp:
tta	L	0	0.44
ttg	L	0	0.75
ctt	L	0	0.79
ctc	L	0	0.56
cta	L	0	0.77
ctg	L	4	0.68
atg	M	3	3.00
gtt	V	229	4929.00
gtc	V	3	4051.00
gta	V	42	5996.00
gtg	V	22019	7318.00
gct	A	0	0.26
gcc	A	0	0.21
gca	A	0	0.47
gcg	A	1	0.06
mPD		0.025	0.74
		nPD:	0.03
		N. weight:	1.1
		Sc. PD:	-0.01
		Sc. rank:	10284.0

PA-X

Pos.	6	obs:	exp:
caa	Q	2	1.06
cag	Q	0	0.94
cgt	R	1	608.90
cgc	R	9	1166.00
cga	R	22154	2184.00
cgg	R	92	1632.00
aga	R	43	11195.00
agg	R	0	5513.00
mPD		0.013	0.91
		nPD:	0.01
		N. weight:	2.3
		Sc. PD:	-0.067
		Sc. rank:	4301.5

PA-X

Pos.	7	obs:	exp:
caa	Q	22262	11767.00
cag	Q	37	10532.00
cgt	R	0	0.05
cgc	R	0	0.10
cga	R	2	0.20
cgg	R	0	0.15
aga	R	0	1.00
agg	R	0	0.49
mPD		0.0035	0.50
		nPD:	0.01
		N. weight:	0.63
		Sc. PD:	-0.023
		Sc. rank:	-5687.1

PA-X

Pos.	8	obs:	exp:
tct	S	0	128.10
tcc	S	0	80.62
tca	S	0	181.10
tcg	S	0	37.04
tat	Y	0	4.16
tac	Y	8	3.84
tgt	C	43	9734.00
tgc	C	21524	11833.00
att	I	0	0.36
atc	I	1	0.25
ata	I	0	0.39
aat	N	0	17.15
aac	N	29	11.85
agt	S	0	130.90
agc	S	696	138.30
mPD		0.070	0.57
		nPD:	0.12
		N. weight:	0.62
		Sc. PD:	0.049
		Sc. rank:	9813.1

PA-X

Pos.	9	obs:	exp:
ttt	F	25	9570.00
ttc	F	22275	12730.00
tat	Y	0	0.52
tac	Y	1	0.48
mPD		0.0023	0.49
		nPD:	0.
		N. weight:	0.56
		Sc. PD:	-0.021
		Sc. rank:	-6856.5

PA-X

Pos.	10	obs:	exp:
tct	S	0	0.18
tcc	S	0	0.12
tca	S	0	0.26
tcg	S	0	0.05
att	I	1	0.36
atc	I	0	0.25
ata	I	0	0.39
aat	N	22231	13189.00
aac	N	68	9110.00
agt	S	1	0.19
agc	S	0	0.20
mPD		0.0063	0.48
		nPD:	0.01
		N. weight:	0.51
		Sc. PD:	-0.016
		Sc. rank:	327.3

PA-X

Pos.	11	obs:	exp:
cct	P	0	6144.00
ccc	P	2	4136.00
cca	P	22271	9496.00
ccg	P	28	2525.00
mPD		0.0027	0.70
		nPD:	0.
		N. weight:	0.85
		Sc. PD:	-0.034
		Sc. rank:	-11690.0

PA-X

Pos.	12	obs:	exp:
att	I	0	2.18
atc	I	0	1.50
ata	I	6	2.32
atg	M	22294	22294.00
gtt	V	0	0.22
gtc	V	0	0.18
gta	V	0	0.27
gtg	V	1	0.33
mPD		0.00063	0.00069
		nPD:	0.91
		N. weight:	0.00031
		Sc. PD:	0.00027
		Sc. rank:	5.9

PA-X

Pos.	13	obs:	exp:
att	I	191	7788.00
atc	I	21192	5340.00
ata	I	10	8265.00
act	T	0	2.02
acc	T	8	1.64
aca	T	0	3.66
acg	T	0	0.68
gtt	V	1	199.00
gtc	V	899	163.50
gta	V	0	242.00
gtg	V	0	295.40
mPD		0.096	0.74
		nPD:	0.13
		N. weight:	1.4
		Sc. PD:	0.12
		Sc. rank:	21940.0

PA-X

Pos.	14	obs:	exp:
att	I	0	116.50
atc	I	320	79.88
ata	I	0	123.60
gtt	V	5	4859.00
gtc	V	21973	3994.00
gta	V	1	5911.00
gtg	V	0	7215.00
gct	A	0	0.53
gcc	A	2	0.41
gca	A	0	0.94
gcg	A	0	0.12
mPD		0.029	0.77
		nPD:	0.04
		N. weight:	1.7
		Sc. PD:	-0.0099
		Sc. rank:	17968.0

PA-X

Pos.	15	obs:	exp:
aaa	K	0	0.57
aag	K	1	0.43
gat	D	3	2.11
gac	D	1	1.89
gaa	E	57	13500.00
gag	E	22239	8796.00
mPD		0.0055	0.48
		nPD:	0.01
		N. weight:	0.92
		Sc. PD:	-0.029
		Sc. rank:	-814.7

PA-X

Pos.	16	obs:	exp:
tta	L	0	2452.00
ttg	L	0	4199.00
ctt	L	22288	4395.00
ctc	L	4	3150.00
cta	L	8	4293.00
ctg	L	1	3813.00
---	--	------	------
mPD		0.0012	1.1
		nPD:	0.
		N. weight:	1.6
		Sc. PD:	-0.069
		Sc. rank:	-30437.0

,

PA-X

Pos.	17	obs:	exp:
act	T	0	0.50
acc	T	0	0.41
aca	T	1	0.91
acg	T	1	0.17
gct	A	6	5904.00
gcc	A	0	4616.00
gca	A	47	10473.00
gcg	A	22246	1306.00
---	--	------	------
mPD		0.0050	0.66
		nPD:	0.01
		N. weight:	2.8
		Sc. PD:	-0.1
		Sc. rank:	-19161.0

,

PA-X

Pos.	18	obs:	exp:
aaa	K	1	0.57
aag	K	0	0.43
gat	D	1	0.53
gac	D	0	0.47
gaa	E	22270	13498.00
gag	E	22	8794.00
ggt	G	0	1.06
ggc	G	0	1.04
gga	G	7	3.11
ggg	G	0	1.79
---	--	------	------
mPD		0.0028	0.48
		nPD:	0.01
		N. weight:	0.5
		Sc. PD:	-0.019
		Sc. rank:	-5394.7

,

PA-X

Pos.	19	obs:	exp:
cgt	R	0	0.11
cgc	R	0	0.21
cga	R	0	0.39
cgg	R	0	0.29
aat	N	1	0.59
aac	N	0	0.41
aaa	K	162	12718.00
aag	K	22133	9577.00
aga	R	0	2.01
agg	R	4	0.99
gaa	E	0	0.61
gag	E	1	0.39
---	--	------	------
mPD		0.015	0.49
		nPD:	0.03
		N. weight:	0.81
		Sc. PD:	-0.011
		Sc. rank:	6936.3

,

PA-X

Pos.	20	obs:	exp:
tct	S	0	1.29
tcc	S	0	0.81
tca	S	7	1.82
tcg	S	0	0.37
act	T	0	16.13
acc	T	0	13.13
aca	T	64	29.28
acg	T	0	5.46
agt	S	0	1.32
agc	S	0	1.39
gtt	V	0	0.66
gtc	V	0	0.55
gta	V	3	0.81
gtg	V	0	0.98
gct	A	0	5884.00
gcc	A	0	4600.00
gca	A	22218	10438.00
gcg	A	6	1302.00
gaa	E	2	1.21
gag	E	0	0.79
---	-	1	1.00
---	--	------	------
mPD		0.0073	0.67
		nPD:	0.01
		N. weight:	0.76
		Sc. PD:	-0.025
		Sc. rank:	-1205.9

,

PA-X

Pos.	21	obs:	exp:
tta	L	0	0.11
ttg	L	0	0.19
ctt	L	0	0.20
ctc	L	0	0.14
cta	L	0	0.19
ctg	L	1	0.17
atg	M	22297	22297.00
act	T	0	0.25
acc	T	0	0.21
aca	T	0	0.46
acg	T	1	0.09
---	-	2	2.00
---	--	------	------
mPD		0.00018	0.00032
		nPD:	0.56
		N. weight:	0.00019
		Sc. PD:	0.000098
		Sc. rank:	3.6

,

PA-X

Pos.	22	obs:	exp:
cgt	R	0	0.38
cgc	R	0	0.73
cga	R	0	1.37
cgg	R	0	1.02
aaa	K	22246	12713.00
aag	K	41	9574.00
aga	R	14	7.03
agg	R	0	3.46
---	--	------	------
mPD		0.0049	0.49
		nPD:	0.01
		N. weight:	0.55
		Sc. PD:	-0.018
		Sc. rank:	-1803.2

,

PA-X

Pos.	23	obs:	exp:
aaa	K	3	1.71
aag	K	0	1.29
gaa	E	22265	13496.00
gag	E	24	8793.00
ggt	G	0	1.36
ggc	G	0	1.33
gga	G	9	4.00
ggg	G	0	2.30
---	--	------	------
mPD		0.0032	0.48
		nPD:	0.01
		N. weight:	0.5
		Sc. PD:	-0.018
		Sc. rank:	-4529.0

,

PA-X

Pos.	24	obs:	exp:
ttt	F	5	2.15
ttc	F	0	2.85
tat	Y	22194	11570.00
tac	Y	74	10698.00
tgt	C	5	2.26
tgc	C	0	2.74
cat	H	23	13.53
cac	H	0	9.47
---	--	------	------
mPD		0.0096	0.50
		nPD:	0.02
		N. weight:	0.64
		Sc. PD:	-0.016
		Sc. rank:	3086.4

,

PA-X

Pos.	25	obs:	exp:
gaa	E	0	0.61
gag	E	1	0.39
ggt	G	0	3381.00
ggc	G	0	3303.00
gga	G	141	9919.00
ggg	G	22159	5696.00
---	--	------	------
mPD		0.013	0.69
		nPD:	0.02
		N. weight:	1.3
		Sc. PD:	-0.034
		Sc. rank:	5582.5

,

PA-X

Pos.	26	obs:	exp:
gaa	E	22180	13503.00
gag	E	120	8797.00
ggt	G	0	0.15
ggc	G	0	0.15
gga	G	1	0.44
ggg	G	0	0.26
---	--	------	------
mPD		0.011	0.48
		nPD:	0.02
		N. weight:	0.47
		Sc. PD:	-0.0099
		Sc. rank:	3254.9

,

PA-X

Pos.	27	obs:	exp:
tat	Y	0	1.56
tac	Y	3	1.44
aat	N	9	33.12
aac	N	47	22.88
gtt	V	0	0.22
gtc	V	1	0.18
gta	V	0	0.27
gtg	V	0	0.33
gat	D	7910	11701.00
gac	D	14298	10507.00
gaa	E	32	19.38
gag	E	0	12.62
ggt	G	1	0.15
ggc	G	0	0.15
gga	G	0	0.44
ggg	G	0	0.26
---	--	------	------
mPD		0.47	0.51
		nPD:	0.92
		N. weight:	0.061
		Sc. PD:	0.054
		Sc. rank:	1168.1

,

PA-X

Pos.	28	obs:	exp:
tta	L	0	0.44
ttg	L	0	0.75
tct	S	0	1.84
tcc	S	10	1.16
tca	S	0	2.60
tcg	S	0	0.53
ctt	L	0	0.79
ctc	L	0	0.56
cta	L	0	0.77
ctg	L	4	0.68
cct	P	41	6139.00
ccc	P	18050	4133.00
cca	P	86	9488.00
ccg	P	4107	2523.00
cat	H	0	1.76
cac	H	3	1.24
agt	S	0	1.88
agc	S	0	1.99
---	--	------	------
mPD		0.31	0.70
		nPD:	0.45
		N. weight:	1.3
		Sc. PD:	0.51
		Sc. rank:	23653.0

,

PA-X

Pos.	29	obs:	exp:
cgt	R	0	0.05
cgc	R	0	0.10
cga	R	0	0.20
cgg	R	0	0.15
att	I	0	0.36
atc	I	0	0.25
ata	I	1	0.39
aat	N	0	0.59
aac	N	1	0.41
aaa	K	22273	12719.00
aag	K	24	9578.00
aga	R	2	1.00
agg	R	0	0.49
---	--	------	------
mPD		0.0025	0.49
		nPD:	0.01
		N. weight:	0.56
		Sc. PD:	-0.021
		Sc. rank:	-6756.5

,

PA-X

Pos.	30	obs:	exp:
tta	L	0	0.11
ttg	L	0	0.19
tct	S	0	0.18
tcc	S	0	0.12
tca	S	0	0.26
tcg	S	0	0.05
ctt	L	1	0.20
ctc	L	0	0.14
cta	L	0	0.19
ctg	L	0	0.17
att	I	7818	8049.00
atc	I	14272	5520.00
ata	I	22	8543.00
act	T	3	12.10
acc	T	45	9.85
aca	T	0	21.96
acg	T	0	4.10
aat	N	10	8.28
aac	N	4	5.72
agt	S	1	0.19
agc	S	0	0.20
gtt	V	93	27.64
gtc	V	32	22.71
gta	V	0	33.62
gtg	V	0	41.03
---	--	------	------
mPD		0.48	0.67
		nPD:	0.71
		N. weight:	0.61
		Sc. PD:	0.4
		Sc. rank:	11513.0

,

PA-X

Pos.	31	obs:	exp:
aaa	K	4	2.28
aag	K	0	1.72
gtt	V	0	0.22
gtc	V	0	0.18
gta	V	1	0.27
gtg	V	0	0.33
gct	A	0	0.53
gcc	A	0	0.41
gca	A	2	0.94
gcg	A	0	0.12
gaa	E	22220	13498.00
gag	E	73	8795.00
ggt	G	0	0.15
ggc	G	0	0.15
gga	G	1	0.44
ggg	G	0	0.26
---	--	------	------
mPD		0.0072	0.48
		nPD:	0.02
		N. weight:	0.49
		Sc. PD:	-0.014
		Sc. rank:	1310.3

,

PA-X

Pos.	32	obs:	exp:
tta	L	0	0.22
ttg	L	0	0.38
tct	S	0	0.18
tcc	S	0	0.12
tca	S	0	0.26
tcg	S	0	0.05
ctt	L	2	0.39
ctc	L	0	0.28
cta	L	0	0.39
ctg	L	0	0.34
att	I	28	10.19
atc	I	0	6.99
ata	I	0	10.82
act	T	22153	5612.00
acc	T	95	4566.00
aca	T	12	10184.00
acg	T	2	1899.00
aat	N	7	4.14
aac	N	0	2.86
agt	S	1	0.19
agc	S	0	0.20
gct	A	1	0.26
gcc	A	0	0.21
gca	A	0	0.47
gcg	A	0	0.06
---	--	------	------
mPD		0.013	0.68
		nPD:	0.02
		N. weight:	1.4
		Sc. PD:	-0.032
		Sc. rank:	6989.5

,

PA-X

Pos.	33	obs:	exp:
tat	Y	1	0.52
tac	Y	0	0.48
aat	N	19262	13189.00
aac	N	3037	9110.00
---	-	1	1.00
---	--	------	------
mPD		0.24	0.48
		nPD:	0.49
		N. weight:	0.18
		Sc. PD:	0.079
		Sc. rank:	3382.8

,

PA-X

Pos.	34	obs:	exp:
cgt	R	0	0.03
cgc	R	0	0.05
cga	R	0	0.10
cgg	R	0	0.07
aaa	K	1164	12721.00
aag	K	21136	9579.00
aga	R	0	0.50
agg	R	1	0.25
---	--	------	------
mPD		0.099	0.49
		nPD:	0.2
		N. weight:	0.63
		Sc. PD:	0.099
		Sc. rank:	10515.0

,

PA-X

Pos.	35	obs:	exp:
ttt	F	22275	9564.00
ttc	F	13	12724.00
tta	L	3	1.21
ttg	L	0	2.07
tat	Y	1	0.52
tac	Y	0	0.48
ctt	L	8	2.17
ctc	L	0	1.55
cta	L	0	2.12
ctg	L	0	1.88
gtt	V	1	0.22
gtc	V	0	0.18
gta	V	0	0.27
gtg	V	0	0.33
---	--	------	------
mPD		0.0023	0.49
		nPD:	0.
		N. weight:	0.85
		Sc. PD:	-0.033
		Sc. rank:	-10521.0

,

PA-X

Pos.	36	obs:	exp:
act	T	1	0.25
acc	T	0	0.21
aca	T	0	0.46
acg	T	0	0.09
gtt	V	2	0.44
gtc	V	0	0.36
gta	V	0	0.54
gtg	V	0	0.66
gct	A	22262	5903.00
gcc	A	20	4616.00
gca	A	14	10473.00
gcg	A	2	1306.00
---	--	------	------
mPD		0.0035	0.66
		nPD:	0.01
		N. weight:	1.3
		Sc. PD:	-0.051
		Sc. rank:	-14602.0

,

PA-X

Pos.	37	obs:	exp:
tct	S	0	0.37
tcc	S	0	0.23
tca	S	2	0.52
tcg	S	0	0.11
act	T	0	0.25
acc	T	0	0.21
aca	T	1	0.46
acg	T	0	0.09
agt	S	0	0.38
agc	S	0	0.40
gct	A	0	5903.00
gcc	A	0	4616.00
gca	A	22297	10473.00
gcg	A	1	1306.00
---	--	------	------
mPD		0.00036	0.66
		nPD:	0.
		N. weight:	0.76
		Sc. PD:	-0.033
		Sc. rank:	-14405.0

,

PA-X

Pos.	38	obs:	exp:
tta	L	0	0.22
ttg	L	0	0.38
tct	S	0	0.18
tcc	S	0	0.12
tca	S	0	0.26
tcg	S	0	0.05
ctt	L	2	0.39
ctc	L	0	0.28
cta	L	0	0.39
ctg	L	0	0.34
att	I	18003	8112.00
atc	I	65	5563.00
ata	I	4216	8609.00
atg	M	1	1.00
act	T	1	0.25
acc	T	0	0.21
aca	T	0	0.46
acg	T	0	0.09
agt	S	1	0.19
agc	S	0	0.20
gtt	V	10	2.65
gtc	V	0	2.18
gta	V	2	3.23
gtg	V	0	3.94
---	--	------	------
mPD		0.31	0.66
		nPD:	0.48
		N. weight:	0.5
		Sc. PD:	0.22
		Sc. rank:	9437.5

,

PA-X

Pos.	39	obs:	exp:
tgt	C	33	10066.00
tgc	C	22268	12235.00
---	--	------	------
mPD		0.0030	0.50
		nPD:	0.01
		N. weight:	0.59
		Sc. PD:	-0.022
		Sc. rank:	-6300.1

,

PA-X

Pos.	40	obs:	exp:
tct	S	0	0.18
tcc	S	0	0.12
tca	S	1	0.26
tcg	S	0	0.05
att	I	0	1.09
atc	I	0	0.75
ata	I	3	1.16
act	T	5	5620.00
acc	T	2	4572.00
aca	T	22255	10198.00
acg	T	30	1902.00
agt	S	0	0.19
agc	S	0	0.20
gct	A	0	1.32
gcc	A	0	1.03
gca	A	5	2.35
gcg	A	0	0.29
---	--	------	------
mPD		0.0041	0.68
		nPD:	0.01
		N. weight:	0.78
		Sc. PD:	-0.029
		Sc. rank:	-7853.9

,

PA-X

Pos.	41	obs:	exp:
cat	H	22252	13114.00
cac	H	48	9186.00
caa	Q	0	0.53
cag	Q	1	0.47
---	--	------	------
mPD		0.0044	0.48
		nPD:	0.01
		N. weight:	0.52
		Sc. PD:	-0.018
		Sc. rank:	-2772.7

,

PA-X

Pos.	42	obs:	exp:
ttt	F	2	0.86
ttc	F	0	1.14
tta	L	128	2451.00
ttg	L	22145	4198.00
ctt	L	0	4394.00
ctc	L	0	3150.00
cta	L	0	4293.00
ctg	L	26	3813.00
---	--	------	------
mPD		0.014	1.1
		nPD:	0.01
		N. weight:	1.6
		Sc. PD:	-0.051
		Sc. rank:	228.6

,

PA-X

Pos.	43	obs:	exp:
gaa	E	21796	13503.00
gag	E	504	8797.00
---	-	1	1.00
---	--	------	------
mPD		0.044	0.48
		nPD:	0.09
		N. weight:	0.41
		Sc. PD:	0.02
		Sc. rank:	6116.9

,

PA-X

Pos.	44	obs:	exp:
att	I	62	22.57
atc	I	0	15.48
ata	I	0	23.95
gtt	V	22137	4916.00
gtc	V	71	4041.00
gta	V	1	5980.00
gtg	V	27	7299.00
gct	A	2	0.79
gcc	A	1	0.62
gca	A	0	1.41
gcg	A	0	0.18
---	--	------	------
mPD		0.015	0.74
		nPD:	0.02
		N. weight:	1.5
		Sc. PD:	-0.035
		Sc. rank:	8038.0

,

PA-X

Pos.	45	obs:	exp:
tgt	C	22180	10066.00
tgc	C	121	12235.00
---	--	------	------
mPD		0.011	0.50
		nPD:	0.02
		N. weight:	0.77
		Sc. PD:	-0.017
		Sc. rank:	4941.5

,

PA-X

Pos.	46	obs:	exp:
ttt	F	420	9569.00
ttc	F	21878	12729.00
tta	L	0	0.22
ttg	L	0	0.38
tat	Y	0	0.52
tac	Y	1	0.48
ctt	L	0	0.39
ctc	L	2	0.28
cta	L	0	0.39
ctg	L	0	0.34
---	--	------	------
mPD		0.037	0.49
		nPD:	0.08
		N. weight:	0.48
		Sc. PD:	0.015
		Sc. rank:	6953.6

PA-X

Pos.	47	obs:	exp:
atg	M	22300	22300.00
act	T	0	0.25
acc	T	0	0.21
aca	T	0	0.46
acg	T	1	0.09
---	--	------	------
mPD		0.000090	0.00017
		nPD:	0.52
		N. weight:	0.00011
		Sc. PD:	0.000053
		Sc. rank:	2.1

PA-X

Pos.	48	obs:	exp:
tat	Y	22261	11587.00
tac	Y	40	10714.00
---	--	------	------
mPD		0.0036	0.50
		nPD:	0.01
		N. weight:	0.65
		Sc. PD:	-0.023
		Sc. rank:	-5325.4

PA-X

Pos.	49	obs:	exp:
tta	L	0	0.22
ttg	L	2	0.38
tct	S	45	4103.00
tcc	S	0	2583.00
tca	S	139	5803.00
tcg	S	22113	1187.00
ctt	L	0	0.39
ctc	L	0	0.28
cta	L	0	0.39
ctg	L	0	0.34
cct	P	0	0.28
ccc	P	0	0.19
cca	P	0	0.43
ccg	P	1	0.11
act	T	0	0.25
acc	T	0	0.21
aca	T	0	0.46
acg	T	1	0.09
agt	S	0	4193.00
agc	S	0	4429.00
---	--	------	------
mPD		0.017	1.6
		nPD:	0.01
		N. weight:	2.9
		Sc. PD:	-0.096
		Sc. rank:	-8053.2

PA-X

Pos.	50	obs:	exp:
gat	D	22278	11750.00
gac	D	22	10550.00
ggt	G	1	0.15
ggc	G	0	0.15
gga	G	0	0.44
ggg	G	0	0.26
---	--	------	------
mPD		0.0021	0.50
		nPD:	0.
		N. weight:	0.64
		Sc. PD:	-0.025
		Sc. rank:	-8450.5

PA-X

Pos.	51	obs:	exp:
ttt	F	296	9569.00
ttc	F	22002	12729.00
tta	L	2	0.33
ttg	L	0	0.56
ctt	L	0	0.59
ctc	L	1	0.42
cta	L	0	0.58
ctg	L	0	0.51
---	--	------	------
mPD		0.026	0.49
		nPD:	0.05
		N. weight:	0.5
		Sc. PD:	0.0051
		Sc. rank:	5850.2

PA-X

Pos.	52	obs:	exp:
cat	H	21895	13114.00
cac	H	404	9185.00
caa	Q	1	0.53
cag	Q	0	0.47
aat	N	1	0.59
aac	N	0	0.41
---	--	------	------
mPD		0.036	0.48
		nPD:	0.07
		N. weight:	0.45
		Sc. PD:	0.013
		Sc. rank:	6537.0

PA-X

Pos.	53	obs:	exp:
ttt	F	51	9570.00
ttc	F	22250	12731.00
---	--	------	------
mPD		0.0046	0.49
		nPD:	0.01
		N. weight:	0.55
		Sc. PD:	-0.019
		Sc. rank:	-2650.5

PA-X

Pos.	54	obs:	exp:
tta	L	0	0.88
ttg	L	0	1.51
ctt	L	7	1.58
ctc	L	1	1.13
cta	L	0	1.54
ctg	L	0	1.37
att	I	18140	8111.00
atc	I	4136	5562.00
ata	I	6	8609.00
gtt	V	10	2.43
gtc	V	1	2.00
gta	V	0	2.96
gtg	V	0	3.61
---	--	------	------
mPD		0.30	0.66
		nPD:	0.46
		N. weight:	0.6
		Sc. PD:	0.25
		Sc. rank:	11333.0

PA-X

Pos.	55	obs:	exp:
aat	N	45	38.44
aac	N	20	26.56
gat	D	18116	11716.00
gac	D	4119	10519.00
gaa	E	1	0.61
gag	E	0	0.39
---	--	------	------
mPD		0.31	0.50
		nPD:	0.61
		N. weight:	0.18
		Sc. PD:	0.1
		Sc. rank:	3454.8

PA-X

Pos.	56	obs:	exp:
caa	Q	1	0.53
cag	Q	0	0.47
gct	A	0	0.79
gcc	A	0	0.62
gca	A	3	1.41
gcg	A	0	0.18
gat	D	0	0.53
gac	D	1	0.47
gaa	E	21988	13500.00
gag	E	307	8795.00
ggt	G	0	0.15
ggc	G	0	0.15
gga	G	1	0.44
ggg	G	0	0.26
---	--	------	------
mPD		0.028	0.48
		nPD:	0.06
		N. weight:	0.44
		Sc. PD:	0.0062
		Sc. rank:	5301.5

PA-X

Pos.	57	obs:	exp:
tta	L	0	1.65
ttg	L	0	2.82
tgg	W	30	30.00
ctt	L	0	2.96
ctc	L	0	2.12
cta	L	0	2.89
ctg	L	15	2.56
caa	Q	2	55.41
cag	Q	103	49.59
cgt	R	4	604.80
cgc	R	0	1159.00
cga	R	195	2170.00
cgg	R	21633	1621.00
aaa	K	0	0.57
aag	K	1	0.43
aga	R	0	11120.00
agg	R	318	5476.00
---	--	------	------
mPD		0.060	0.93
		nPD:	0.06
		N. weight:	2.5
		Sc. PD:	0.051
		Sc. rank:	35011.0

PA-X

Pos.	58	obs:	exp:
tct	S	0	0.92
tcc	S	0	0.58
tca	S	0	1.30
tcg	S	0	0.27
agt	S	5	0.94
agc	S	0	0.99
gat	D	3	1.58
gac	D	0	1.42
ggt	G	22283	3380.00
ggc	G	5	3302.00
gga	G	0	9915.00
ggg	G	3	5694.00
---	-	2	2.00
---	--	------	------
mPD		0.0014	0.69
		nPD:	0.
		N. weight:	1.9
		Sc. PD:	-0.078
		Sc. rank:	-32520.0

PA-X

Pos.	59	obs:	exp:
caa	Q	1	0.53
cag	Q	0	0.47
aaa	K	8	4.56
aag	K	0	3.44
gtt	V	0	1.11
gtc	V	0	0.91
gta	V	5	1.34
gtg	V	0	1.64
gat	D	2	4.22
gac	D	6	3.78
gaa	E	22204	13467.00
gag	E	37	8774.00
ggt	G	0	5.76
ggc	G	0	5.63
gga	G	37	16.90
ggg	G	1	9.71
---	--	------	------
mPD		0.0088	0.48
		nPD:	0.02
		N. weight:	0.49
		Sc. PD:	-0.013
		Sc. rank:	1962.3

PA-X

Pos.	60	obs:	exp:
tct	S	5	4095.00
tcc	S	0	2578.00
tca	S	22238	5791.00
tcg	S	10	1184.00
cct	P	0	12.67
ccc	P	0	8.53
cca	P	46	19.59
ccg	P	0	5.21
agt	S	0	4184.00
agc	S	0	4420.00
gct	A	0	0.53
gcc	A	0	0.41
gca	A	2	0.94
gcg	A	0	0.12
---	--	------	------
mPD		0.0056	1.6
		nPD:	0.
		N. weight:	1.4
		Sc. PD:	-0.054
		Sc. rank:	-19002.0

PA-X

Pos.	61	obs:	exp:
tta	L	4	0.88
ttg	L	0	1.51
ctt	L	0	1.58
ctc	L	0	1.13
cta	L	4	1.54
ctg	L	0	1.37
cgt	R	0	0.03
cgc	R	0	0.05
cga	R	0	0.10
cgg	R	0	0.07
att	I	2	7918.00
atc	I	1	5429.00
ata	I	21748	8404.00
atg	M	468	468.00
act	T	0	3.28
acc	T	0	2.67
aca	T	13	5.95
acg	T	0	1.11
aga	R	1	0.50
agg	R	0	0.25
gtt	V	0	13.27
gtc	V	0	10.90
gta	V	58	16.14
gtg	V	2	19.70
---	--	------	------
mPD		0.049	0.68
		nPD:	0.07
		N. weight:	0.94
		Sc. PD:	0.027
		Sc. rank:	13541.0

,

PA-X

Pos.	62	obs:	exp:
tta	L	0	0.22
ttg	L	0	0.38
ctt	L	1	0.39
ctc	L	0	0.28
cta	L	0	0.39
ctg	L	1	0.34
att	I	22220	8102.00
atc	I	25	5556.00
ata	I	12	8599.00
act	T	9	2.27
acc	T	0	1.85
aca	T	0	4.12
acg	T	0	0.77
gtt	V	32	7.30
gtc	V	0	6.00
gta	V	1	8.88
gtg	V	0	10.83
---	--	------	------
mPD		0.0074	0.66
		nPD:	0.01
		N. weight:	1.
		Sc. PD:	-0.032
		Sc. rank:	-1303.1

,

PA-X

Pos.	63	obs:	exp:
ttt	F	1	0.43
ttc	F	0	0.57
tta	L	2065	227.10
ttg	L	1	389.00
tct	S	0	0.37
tcc	S	0	0.23
tca	S	2	0.52
tcg	S	0	0.11
ctt	L	0	407.10
ctc	L	0	291.80
cta	L	0	397.70
ctg	L	0	353.20
att	I	0	17.11
atc	I	0	11.73
ata	I	47	18.16
agt	S	0	0.38
agc	S	0	0.40
gtt	V	12	4453.00
gtc	V	17	3660.00
gta	V	19888	5417.00
gtg	V	226	6612.00
gct	A	0	10.06
gcc	A	0	7.87
gca	A	38	17.85
gcg	A	0	2.23
gaa	E	1	0.61
gag	E	0	0.39
ggt	G	0	0.45
ggc	G	0	0.44
gga	G	3	1.33
ggg	G	0	0.77
---	--	------	------
mPD		0.20	0.92
		nPD:	0.22
		N. weight:	1.3
		Sc. PD:	0.23
		Sc. rank:	22593.0

,

PA-X

Pos.	64	obs:	exp:
aaa	K	1	0.57
aag	K	0	0.43
gaa	E	22268	13503.00
gag	E	32	8797.00
---	--	------	------
mPD		0.0030	0.48
		nPD:	0.01
		N. weight:	0.5
		Sc. PD:	-0.018
		Sc. rank:	-4720.7

,

PA-X

Pos.	65	obs:	exp:
ttt	F	4	1.72
ttc	F	0	2.28
tta	L	0	0.11
ttg	L	0	0.19
tct	S	21863	4078.00
tcc	S	285	2566.00
tca	S	0	5767.00
tcg	S	10	1179.00
tat	Y	1	0.52
tac	Y	0	0.48
ctt	L	1	0.20
ctc	L	0	0.14
cta	L	0	0.19
ctg	L	0	0.17
cct	P	15	4.13
ccc	P	0	2.78
cca	P	0	6.39
ccg	P	0	1.70
act	T	122	30.75
acc	T	0	25.02
aca	T	0	55.81
acg	T	0	10.41
agt	S	0	4166.00
agc	S	0	4401.00
---	--	------	------
mPD		0.039	1.6
		nPD:	0.02
		N. weight:	1.6
		Sc. PD:	-0.032
		Sc. rank:	12392.0

,

PA-X

Pos.	66	obs:	exp:
tct	S	0	7.55
tcc	S	0	4.75
tca	S	0	10.67
tcg	S	0	2.18
cgt	R	1	0.03
cgc	R	0	0.05
cga	R	0	0.10
cgg	R	0	0.07
aat	N	3	1.77
aac	N	0	1.23
agt	S	41	7.71
agc	S	0	8.14
aga	R	0	0.50
agg	R	0	0.25
gct	A	2	0.53
gcc	A	0	0.41
gca	A	0	0.94
gcg	A	0	0.12
gat	D	395	208.70
gac	D	1	187.30
ggt	G	21810	3314.00
ggc	G	45	3237.00
gga	G	1	9723.00
ggg	G	2	5583.00
---	--	------	------
mPD		0.044	0.74
		nPD:	0.06
		N. weight:	1.9
		Sc. PD:	0.029
		Sc. rank:	23080.0

,

PA-X

Pos.	67	obs:	exp:
aat	N	0	1.18
aac	N	2	0.82
gat	D	120	11748.00
gac	D	22177	10549.00
ggt	G	0	0.30
ggc	G	2	0.30
gga	G	0	0.89
ggg	G	0	0.51
---	--	------	------
mPD		0.011	0.50
		nPD:	0.02
		N. weight:	0.72
		Sc. PD:	-0.015
		Sc. rank:	4726.7

,

PA-X

Pos.	68	obs:	exp:
tta	L	0	0.33
ttg	L	0	0.56
tct	S	0	6.26
tcc	S	0	3.94
tca	S	31	8.85
tcg	S	3	1.81
ctt	L	0	0.59
ctc	L	0	0.42
cta	L	3	0.58
ctg	L	0	0.51
cct	P	3	6134.00
ccc	P	1	4129.00
cca	P	19176	9480.00
ccg	P	3084	2521.00
agt	S	0	6.39
agc	S	0	6.75
---	--	------	------
mPD		0.24	0.70
		nPD:	0.35
		N. weight:	0.64
		Sc. PD:	0.19
		Sc. rank:	11638.0

,

PA-X

Pos.	69	obs:	exp:
aat	N	22257	13190.00
aac	N	44	9111.00
---	--	------	------
mPD		0.0039	0.48
		nPD:	0.01
		N. weight:	0.52
		Sc. PD:	-0.018
		Sc. rank:	-2950.2

,

PA-X

Pos.	70	obs:	exp:
tct	S	0	0.18
tcc	S	0	0.12
tca	S	1	0.26
tcg	S	0	0.05
att	I	0	0.36
atc	I	0	0.25
ata	I	1	0.39
act	T	0	1.51
acc	T	0	1.23
aca	T	6	2.74
acg	T	0	0.51
agt	S	0	0.19
agc	S	0	0.20
gtt	V	0	54.61
gtc	V	0	44.88
gta	V	245	66.43
gtg	V	2	81.08
gct	A	1	5837.00
gcc	A	14	4563.00
gca	A	22020	10354.00
gcg	A	11	1292.00
---	--	------	------
mPD		0.025	0.69
		nPD:	0.04
		N. weight:	0.76
		Sc. PD:	-0.0052
		Sc. rank:	7497.0

,

PA-X

Pos.	71	obs:	exp:
tta	L	142	2451.00
ttg	L	0	4197.00
ctt	L	0	4393.00
ctc	L	4	3149.00
cta	L	21490	4292.00
ctg	L	659	3812.00
cct	P	0	0.28
ccc	P	0	0.19
cca	P	1	0.43
ccg	P	0	0.11
att	I	0	1.82
atc	I	0	1.25
ata	I	5	1.93
---	--	------	------
mPD		0.071	1.1
		nPD:	0.06
		N. weight:	1.5
		Sc. PD:	0.028
		Sc. rank:	19477.0

,

PA-X

Pos.	72	obs:	exp:
tta	L	63	2451.00
ttg	L	22162	4198.00
tct	S	0	0.18
tcc	S	0	0.12
tca	S	0	0.26
tcg	S	1	0.05
ctt	L	0	4394.00
ctc	L	0	3150.00
cta	L	0	4293.00
ctg	L	74	3813.00
atg	M	1	1.00
agt	S	0	0.19
agc	S	0	0.20
---	--	------	------
mPD		0.012	1.1
		nPD:	0.01
		N. weight:	1.6
		Sc. PD:	-0.053
		Sc. rank:	-2985.6

,

PA-X

Pos.	73	obs:	exp:
cgt	R	0	0.08
cgc	R	0	0.16
cga	R	0	0.29
cgg	R	0	0.22
aaa	K	174	12719.00
aag	K	22124	9579.00
aga	R	0	1.51
agg	R	3	0.74
---	--	------	------
mPD		0.016	0.49
		nPD:	0.03
		N. weight:	0.8
		Sc. PD:	-0.0092
		Sc. rank:	7018.3

,

PA-X

Pos.	74	obs:	exp:
tat	Y	0	0.52
tac	Y	1	0.48
cat	H	64	13112.00
cac	H	22232	9184.00
caa	Q	1	0.53
cag	Q	0	0.47
cgt	R	0	0.05
cgc	R	2	0.10
cga	R	0	0.20
cgg	R	0	0.15
aat	N	0	0.59
aac	N	1	0.41
aga	R	0	1.00
agg	R	0	0.49
---	--	------	------
mPD		0.0062	0.49
		nPD:	0.01
		N. weight:	0.87
		Sc. PD:	-0.027
		Sc. rank:	247.0

,

PA-X

Pos.	75	obs:	exp:
cgt	R	2	608.90
cgc	R	0	1167.00
cga	R	22223	2184.00
cgg	R	47	1632.00
aga	R	27	11196.00
agg	R	2	5513.00
---	--	------	------
mPD		0.0072	0.91
		nPD:	0.01
		N. weight:	2.3
		Sc. PD:	-0.082
		Sc. rank:	-14254.0

,

PA-X			
Pos.	76	obs:	exp:
ttt	F	22262	9570.00
ttc	F	38	12730.00
tct	S	1	0.18
tcc	S	0	0.12
tca	S	0	0.26
tcg	S	0	0.05
agt	S	0	0.19
agc	S	0	0.20
---	--	------	------
mPD		0.0035	0.49
		nPD:	0.01
		N. weight:	0.84
		Sc. PD:	-0.03
		Sc. rank:	-7218.3

,

PA-X			
Pos.	77	obs:	exp:
gaa	E	514	13503.00
gag	E	21787	8798.00
---	--	------	------
mPD		0.045	0.48
		nPD:	0.09
		N. weight:	0.82
		Sc. PD:	0.041
		Sc. rank:	12399.0

,

PA-X			
Pos.	78	obs:	exp:
tta	L	1	0.22
ttg	L	0	0.38
ctt	L	0	0.39
ctc	L	0	0.28
cta	L	1	0.39
ctg	L	0	0.34
att	I	16	8117.00
atc	I	2	5566.00
ata	I	22281	8615.00
---	--	------	------
mPD		0.0018	0.66
		nPD:	0.
		N. weight:	0.95
		Sc. PD:	-0.039
		Sc. rank:	-15062.0

,

PA-X			
Pos.	79	obs:	exp:
tta	L	0	0.11
ttg	L	0	0.19
tct	S	0	0.18
tcc	S	0	0.12
tca	S	0	0.26
tcg	S	0	0.05
ctt	L	1	0.20
ctc	L	0	0.14
cta	L	0	0.19
ctg	L	0	0.17
att	I	22270	8116.00
atc	I	15	5565.00
ata	I	9	8613.00
agt	S	1	0.19
agc	S	0	0.20
gtt	V	5	1.11
gtc	V	0	0.91
gta	V	0	1.34
gtg	V	0	1.64
---	--	------	------
mPD		0.0028	0.66
		nPD:	0.
		N. weight:	1.
		Sc. PD:	-0.04
		Sc. rank:	-13036.0

,

PA-X			
Pos.	80	obs:	exp:
gaa	E	22289	13503.00
gag	E	12	8798.00
---	--	------	------
mPD		0.0011	0.48
		nPD:	0.
		N. weight:	0.5
		Sc. PD:	-0.021
		Sc. rank:	-8505.0

,

PA-X			
Pos.	81	obs:	exp:
ggt	G	4	3382.00
ggc	G	0	3303.00
gga	G	22229	9920.00
ggg	G	68	5697.00
---	--	------	------
mPD		0.0064	0.69
		nPD:	0.01
		N. weight:	0.8
		Sc. PD:	-0.027
		Sc. rank:	-3911.3

,

PA-X			
Pos.	82	obs:	exp:
cgt	R	0	608.90
cgc	R	0	1166.00
cga	R	1	2184.00
cgg	R	0	1632.00
att	I	0	0.73
atc	I	0	0.50
ata	I	2	0.77
aga	R	22255	11195.00
agg	R	43	5513.00
---	--	------	------
mPD		0.0041	0.91
		nPD:	0.
		N. weight:	0.68
		Sc. PD:	-0.026
		Sc. rank:	-8665.8

,

PA-X			
Pos.	83	obs:	exp:
gat	D	57	11750.00
gac	D	22243	10550.00
ggt	G	0	0.15
ggc	G	1	0.15
gga	G	0	0.44
ggg	G	0	0.26
---	--	------	------
mPD		0.0052	0.50
		nPD:	0.01
		N. weight:	0.74
		Sc. PD:	-0.024
		Sc. rank:	-1536.8

,

PA-X			
Pos.	84	obs:	exp:
tta	L	0	0.11
ttg	L	0	0.19
ctt	L	0	0.20
ctc	L	0	0.14
cta	L	1	0.19
ctg	L	0	0.17
caa	Q	1	0.53
cag	Q	0	0.47
cgt	R	1	608.90
cgc	R	1	1166.00
cga	R	22201	2184.00
cgg	R	42	1632.00
aga	R	53	11195.00
agg	R	1	5513.00
---	--	------	------
mPD		0.0090	0.91
		nPD:	0.01
		N. weight:	2.3
		Sc. PD:	-0.077
		Sc. rank:	-7730.7

,

PA-X			
Pos.	85	obs:	exp:
tct	S	0	0.18
tcc	S	0	0.12
tca	S	0	0.26
tcg	S	0	0.05
att	I	37	8098.00
atc	I	22183	5553.00
ata	I	25	8594.00
act	T	0	4.54
acc	T	18	3.69
aca	T	0	8.23
acg	T	0	1.54
aat	N	0	0.59
aac	N	1	0.41
agt	S	0	0.19
agc	S	1	0.20
gtt	V	0	7.96
gtc	V	36	6.54
gta	V	0	9.68
gtg	V	0	11.82
---	--	------	------
mPD		0.011	0.66
		nPD:	0.02
		N. weight:	1.4
		Sc. PD:	-0.038
		Sc. rank:	5009.2

,

PA-X			
Pos.	86	obs:	exp:
tta	L	0	0.11
ttg	L	1	0.19
ctt	L	0	0.20
ctc	L	0	0.14
cta	L	0	0.19
ctg	L	0	0.17
att	I	6	6.92
atc	I	0	4.74
ata	I	13	7.34
atg	M	22267	22267.00
act	T	0	0.76
acc	T	0	0.62
aca	T	0	1.37
acg	T	3	0.26
gtt	V	0	2.43
gtc	V	0	2.00
gta	V	0	2.96
gtg	V	11	3.61
---	--	------	------
mPD		0.0030	0.0040
		nPD:	0.76
		N. weight:	0.0013
		Sc. PD:	0.00095
		Sc. rank:	25.4

,

PA-X			
Pos.	87	obs:	exp:
tct	S	0	0.18
tcc	S	1	0.12
tca	S	0	0.26
tcg	S	0	0.05
agt	S	0	0.19
agc	S	0	0.20
gct	A	2448	5903.00
gcc	A	19707	4616.00
gca	A	143	10473.00
gcg	A	0	1306.00
ggt	G	0	0.30
ggc	G	2	0.30
gga	G	0	0.89
ggg	G	0	0.51
---	--	------	------
mPD		0.21	0.66
		nPD:	0.31
		N. weight:	1.2
		Sc. PD:	0.31
		Sc. rank:	21133.0

,

PA-X			
Pos.	88	obs:	exp:
tgg	W	22300	22300.00
cgt	R	0	0.03
cgc	R	0	0.05
cga	R	0	0.10
cgg	R	1	0.07
aga	R	0	0.50
agg	R	0	0.25
---	--	------	------
mPD		0.000090	0.00015
		nPD:	0.6
		N. weight:	0.00012
		Sc. PD:	0.000065
		Sc. rank:	2.2

,

PA-X			
Pos.	89	obs:	exp:
act	T	6	5621.00
acc	T	3	4574.00
aca	T	22277	10201.00
acg	T	12	1903.00
gct	A	0	0.79
gcc	A	0	0.62
gca	A	3	1.41
gcg	A	0	0.18
---	--	------	------
mPD		0.0022	0.68
		nPD:	0.
		N. weight:	0.78
		Sc. PD:	-0.031
		Sc. rank:	-11454.0

,

PA-X			
Pos.	90	obs:	exp:
tta	L	0	0.22
ttg	L	2	0.38
ctt	L	0	0.39
ctc	L	0	0.28
cta	L	0	0.39
ctg	L	0	0.34
atg	M	36	36.00
gtt	V	30	4921.00
gtc	V	0	4045.00
gta	V	56	5986.00
gtg	V	22173	7307.00
gct	A	0	0.79
gcc	A	0	0.62
gca	A	0	1.41
gcg	A	3	0.18
ggt	G	0	0.15
ggc	G	0	0.15
gga	G	0	0.44
ggg	G	1	0.26
---	--	------	------
mPD		0.011	0.74
		nPD:	0.02
		N. weight:	1.1
		Sc. PD:	-0.031
		Sc. rank:	3345.0

,

PA-X			
Pos.	91	obs:	exp:
atg	M	4	4.00
gtt	V	7	4930.00
gtc	V	39	4052.00
gta	V	129	5997.00
gtg	V	22122	7319.00
---	--	------	------
mPD		0.016	0.74
		nPD:	0.02
		N. weight:	1.1
		Sc. PD:	-0.023
		Sc. rank:	6782.3

PA-X			
Pos.	92	obs:	exp:
tct	S	0	0.18
tcc	S	0	0.12
tca	S	0	0.26
tcg	S	0	0.05
aat	N	21	13189.00
aac	N	22279	9111.00
agt	S	0	0.19
agc	S	1	0.20
---	--	------	------
mPD		0.0020	0.48
		nPD:	0.
		N. weight:	0.89
		Sc. PD:	-0.035
		Sc. rank:	-11937.0

PA-X			
Pos.	93	obs:	exp:
tct	S	0	4104.00
tcc	S	0	2583.00
tca	S	0	5804.00
tcg	S	0	1187.00
agt	S	22258	4193.00
agc	S	43	4430.00
---	--	------	------
mPD		0.0038	1.6
		nPD:	0.
		N. weight:	1.7
		Sc. PD:	-0.068
		Sc. rank:	-27506.0

PA-X			
Pos.	94	obs:	exp:
att	I	5	8116.00
atc	I	9	5565.00
ata	I	22280	8613.00
gtt	V	0	1.55
gtc	V	0	1.27
gta	V	7	1.88
gtg	V	0	2.30
---	--	------	------
mPD		0.0019	0.66
		nPD:	0.
		N. weight:	0.95
		Sc. PD:	-0.039
		Sc. rank:	-14223.0

PA-X			
Pos.	95	obs:	exp:
tgt	C	22023	10065.00
tgc	C	277	12235.00
cgt	R	1	0.03
cgc	R	0	0.05
cga	R	0	0.10
cgg	R	0	0.07
aga	R	0	0.50
agg	R	0	0.25
---	--	------	------
mPD		0.025	0.50
		nPD:	0.05
		N. weight:	0.73
		Sc. PD:	0.0044
		Sc. rank:	8480.5

PA-X			
Pos.	96	obs:	exp:
tct	S	0	0.37
tcc	S	0	0.23
tca	S	0	0.52
tcg	S	0	0.11
cat	H	0	0.59
cac	H	1	0.41
aat	N	112	13187.00
aac	N	22185	9110.00
aaa	K	1	0.57
aag	K	0	0.43
agt	S	0	0.38
agc	S	2	0.40
---	--	------	------
mPD		0.010	0.48
		nPD:	0.02
		N. weight:	0.87
		Sc. PD:	-0.019
		Sc. rank:	5334.5

PA-X			
Pos.	97	obs:	exp:
att	I	0	0.36
atc	I	0	0.25
ata	I	1	0.39
act	T	2	5621.00
acc	T	7	4574.00
aca	T	22281	10201.00
acg	T	9	1903.00
gct	A	0	0.26
gcc	A	0	0.21
gca	A	1	0.47
gcg	A	0	0.06
---	--	------	------
mPD		0.0018	0.68
		nPD:	0.
		N. weight:	0.78
		Sc. PD:	-0.032
		Sc. rank:	-12706.0

PA-X			
Pos.	98	obs:	exp:
act	T	5	5619.00
acc	T	0	4572.00
aca	T	22188	10197.00
acg	T	97	1902.00
gct	A	0	2.91
gcc	A	0	2.28
gca	A	11	5.17
gcg	A	0	0.64
---	--	------	------
mPD		0.010	0.68
		nPD:	0.01
		N. weight:	0.77
		Sc. PD:	-0.022
		Sc. rank:	1807.4

PA-X			
Pos.	99	obs:	exp:
cgt	R	0	0.82
cgc	R	0	1.57
cga	R	0	2.94
cgg	R	0	2.20
aga	R	0	15.06
agg	R	30	7.42
gtt	V	0	0.22
gtc	V	0	0.18
gta	V	0	0.27
gtg	V	1	0.33
gaa	E	0	8.48
gag	E	14	5.52
ggt	G	0	3375.00
ggc	G	0	3296.00
gga	G	207	9900.00
ggg	G	22049	5685.00
---	--	------	------
mPD		0.022	0.70
		nPD:	0.03
		N. weight:	1.3
		Sc. PD:	-0.015
		Sc. rank:	11944.0

PA-X			
Pos.	100	obs:	exp:
att	I	14	6846.00
atc	I	0	4694.00
ata	I	18791	7265.00
atg	M	143	143.00
act	T	0	2.52
acc	T	0	2.05
aca	T	9	4.57
acg	T	1	0.85
aaa	K	1	0.57
aag	K	0	0.43
gtt	V	8	738.20
gtc	V	1	606.70
gta	V	3322	898.00
gtg	V	8	1096.00
gct	A	0	0.53
gcc	A	0	0.41
gca	A	2	0.94
gcg	A	0	0.12
gat	D	1	0.53
gac	D	0	0.47
---	--	------	------
mPD		0.27	0.95
		nPD:	0.29
		N. weight:	1.
		Sc. PD:	0.24
		Sc. rank:	17635.0

PA-X			
Pos.	101	obs:	exp:
cgt	R	0	0.03
cgc	R	0	0.05
cga	R	0	0.10
cgg	R	0	0.07
aaa	K	0	1.14
aag	K	2	0.86
aga	R	1	0.50
agg	R	0	0.25
gtt	V	0	0.22
gtc	V	0	0.18
gta	V	0	0.27
gtg	V	1	0.33
gat	D	60	32.14
gac	D	1	28.86
gaa	E	360	13455.00
gag	E	21862	8767.00
ggt	G	0	2.12
ggc	G	0	2.07
gga	G	0	6.23
ggg	G	14	3.58
---	--	------	------
mPD		0.039	0.48
		nPD:	0.08
		N. weight:	0.85
		Sc. PD:	0.031
		Sc. rank:	12473.0

PA-X			
Pos.	102	obs:	exp:
aaa	K	117	12721.00
aag	K	22184	9580.00
---	--	------	------
mPD		0.010	0.49
		nPD:	0.02
		N. weight:	0.82
		Sc. PD:	-0.018
		Sc. rank:	4902.1

PA-X			
Pos.	103	obs:	exp:
tta	L	0	0.11
ttg	L	0	0.19
ctt	L	1	0.20
ctc	L	0	0.14
cta	L	0	0.19
ctg	L	0	0.17
cct	P	21505	6144.00
ccc	P	787	4136.00
cca	P	6	9495.00
ccg	P	2	2525.00
---	--	------	------
mPD		0.069	0.70
		nPD:	0.1
		N. weight:	1.2
		Sc. PD:	0.064
		Sc. rank:	17918.0

PA-X			
Pos.	104	obs:	exp:
cgt	R	0	0.11
cgc	R	0	0.21
cga	R	0	0.39
cgg	R	0	0.29
act	T	0	0.25
acc	T	0	0.21
aca	T	1	0.46
acg	T	0	0.09
aat	N	0	1.18
aac	N	2	0.82
aaa	K	22185	12717.00
aag	K	109	9577.00
aga	R	4	2.01
agg	R	0	0.99
---	--	------	------
mPD		0.010	0.49
		nPD:	0.02
		N. weight:	0.54
		Sc. PD:	-0.012
		Sc. rank:	3022.4

PA-X			
Pos.	105	obs:	exp:
ttt	F	21154	9101.00
ttc	F	55	12108.00
tta	L	0	119.10
ttg	L	1	203.90
tgt	C	1	0.45
tgc	C	0	0.55
ctt	L	1082	213.40
ctc	L	0	153.00
cta	L	0	208.50
ctg	L	0	185.20
cat	H	8	4.70
cac	H	0	3.30
---	--	------	------
mPD		0.099	0.59
		nPD:	0.17
		N. weight:	0.87
		Sc. PD:	0.11
		Sc. rank:	14252.0

PA-X			
Pos.	106	obs:	exp:
tta	L	0	2451.00
ttg	L	0	4198.00
ctt	L	21746	4394.00
ctc	L	547	3150.00
cta	L	3	4293.00
ctg	L	2	3812.00
att	I	3	1.09
atc	I	0	0.75
ata	I	0	1.16
---	--	------	------
mPD		0.049	1.1
		nPD:	0.04
		N. weight:	1.5
		Sc. PD:	-0.0015
		Sc. rank:	17050.0

PA-X			
Pos.	107	obs:	exp:
cct	P	22287	6144.00
ccc	P	11	4136.00
cca	P	1	9496.00
ccg	P	2	2525.00
---	--	------	------
mPD		0.0013	0.70
		nPD:	0.
		N. weight:	1.3
		Sc. PD:	-0.054
		Sc. rank:	-22584.0

PA-X			
Pos.	108	obs:	exp:
aaa	K	1	0.57
aag	K	0	0.43
gat	D	22270	11750.00
gac	D	30	10550.00
---	--	------	------
mPD		0.0029	0.50
		nPD:	0.01
		N. weight:	0.64
		Sc. PD:	-0.024
		Sc. rank:	-6933.1

PA-X			
Pos.	109	obs:	exp:
tta	L	366	2452.00
ttg	L	21882	4198.00
ctt	L	0	4394.00
ctc	L	0	3150.00
cta	L	0	4293.00
ctg	L	52	3813.00
atg	M	1	1.00
---	--	------	------
mPD		0.037	1.1
		nPD:	0.03
		N. weight:	1.6
		Sc. PD:	-0.018
		Sc. rank:	14847.0

PA-X			
Pos.	110	obs:	exp:
tat	Y	22256	11587.00
tac	Y	44	10713.00
cat	H	1	0.59
cac	H	0	0.41
---	--	------	------
mPD		0.0040	0.50
		nPD:	0.01
		N. weight:	0.65
		Sc. PD:	-0.023
		Sc. rank:	-3748.3

PA-X			
Pos.	111	obs:	exp:
gat	D	22149	11750.00
gac	D	152	10551.00
---	--	------	------
mPD		0.014	0.50
		nPD:	0.03
		N. weight:	0.61
		Sc. PD:	-0.0099
		Sc. rank:	4905.3

PA-X			
Pos.	112	obs:	exp:
tat	Y	189	11587.00
tac	Y	22112	10714.00
---	--	------	------
mPD		0.017	0.50
		nPD:	0.03
		N. weight:	0.69
		Sc. PD:	-0.0068
		Sc. rank:	6599.1

PA-X			
Pos.	113	obs:	exp:
caa	Q	2	1.06
cag	Q	0	0.94
cgt	R	0	0.33
cgc	R	0	0.63
cga	R	0	1.18
cgg	R	0	0.88
aaa	K	22249	12713.00
aag	K	38	9574.00
aga	R	12	6.02
agg	R	0	2.97
---	--	------	------
mPD		0.0047	0.49
		nPD:	0.01
		N. weight:	0.55
		Sc. PD:	-0.019
		Sc. rank:	-2620.9

PA-X			
Pos.	114	obs:	exp:
aaa	K	0	5.13
aag	K	9	3.87
gat	D	2	1.05
gac	D	0	0.95
gaa	E	89	13496.00
gag	E	22200	8793.00
ggt	G	0	0.15
ggc	G	0	0.15
gga	G	0	0.44
ggg	G	1	0.26
---	--	------	------
mPD		0.0090	0.48
		nPD:	0.02
		N. weight:	0.91
		Sc. PD:	-0.022
		Sc. rank:	4306.3

PA-X			
Pos.	115	obs:	exp:
tct	S	0	11.23
tcc	S	0	7.07
tca	S	0	15.88
tcg	S	0	3.25
cat	H	0	0.59
cac	H	1	0.41
aat	N	477	13144.00
aac	N	21746	9079.00
agt	S	0	11.47
agc	S	61	12.12
gat	D	0	8.43
gac	D	16	7.57
---	--	------	------
mPD		0.049	0.49
		nPD:	0.1
		N. weight:	0.79
		Sc. PD:	0.043
		Sc. rank:	12123.0

PA-X			
Pos.	116	obs:	exp:
cgt	R	141	608.90
cgc	R	1	1167.00
cga	R	253	2184.00
cgg	R	21875	1632.00
aga	R	0	11196.00
agg	R	31	5513.00
---	--	------	------
mPD		0.038	0.91
		nPD:	0.04
		N. weight:	2.5
		Sc. PD:	-0.0054
		Sc. rank:	27555.0

PA-X			
Pos.	117	obs:	exp:
ttt	F	13	9570.00
ttc	F	22288	12731.00
---	--	------	------
mPD		0.0012	0.49
		nPD:	0.
		N. weight:	0.56
		Sc. PD:	-0.023
		Sc. rank:	-9175.9

PA-X			
Pos.	118	obs:	exp:
tta	L	0	0.33
ttg	L	0	0.56
ctt	L	3	0.59
ctc	L	0	0.42
cta	L	0	0.58
ctg	L	0	0.51
att	I	22267	8114.00
atc	I	11	5564.00
ata	I	12	8612.00
gtt	V	8	1.77
gtc	V	0	1.45
gta	V	0	2.15
gtg	V	0	2.63
---	--	------	------
mPD		0.0030	0.66
		nPD:	0.
		N. weight:	1.
		Sc. PD:	-0.039
		Sc. rank:	-12676.0

PA-X			
Pos.	119	obs:	exp:
gaa	E	22250	13503.00
gag	E	51	8798.00
---	--	------	------
mPD		0.0046	0.48
		nPD:	0.01
		N. weight:	0.49
		Sc. PD:	-0.017
		Sc. rank:	-2281.5

PA-X			
Pos.	120	obs:	exp:
att	I	22270	8115.00
atc	I	20	5564.00
ata	I	1	8612.00
gtt	V	10	2.21
gtc	V	0	1.82
gta	V	0	2.69
gtg	V	0	3.28
---	--	------	------
mPD		0.0028	0.66
		nPD:	0.
		N. weight:	1.
		Sc. PD:	-0.04
		Sc. rank:	-13225.0

PA-X			
Pos.	121	obs:	exp:
ggt	G	2	3382.00
ggc	G	0	3303.00
gga	G	22196	9920.00
ggg	G	103	5697.00
---	--	------	------
mPD		0.0094	0.69
		nPD:	0.01
		N. weight:	0.79
		Sc. PD:	-0.024
		Sc. rank:	966.9

PA-X			
Pos.	122	obs:	exp:
att	I	0	12.74
atc	I	0	8.74
ata	I	35	13.52
gtt	V	0	4922.00
gtc	V	2	4046.00
gta	V	22215	5988.00
gtg	V	47	7308.00
gct	A	0	0.53
gcc	A	0	0.41
gca	A	2	0.94
gcg	A	0	0.12
---	--	------	------
mPD		0.0077	0.74
		nPD:	0.01
		N. weight:	1.3
		Sc. PD:	-0.043
		Sc. rank:	-3240.6

PA-X			
Pos.	123	obs:	exp:
act	T	4	5622.00
acc	T	14	4574.00
aca	T	22218	10202.00
acg	T	65	1903.00
---	--	------	------
mPD		0.0074	0.68
		nPD:	0.01
		N. weight:	0.77
		Sc. PD:	-0.025
		Sc. rank:	-1120.0

PA-X			
Pos.	124	obs:	exp:
cgt	R	3	608.90
cgc	R	0	1167.00
cga	R	70	2184.00
cgg	R	22202	1632.00
aga	R	0	11196.00
agg	R	26	5513.00
---	--	------	------
mPD		0.0089	0.91
		nPD:	0.01
		N. weight:	2.6
		Sc. PD:	-0.088
		Sc. rank:	-10989.0

PA-X

Pos.	125	obs:	exp:
cgt	R	0	608.90
cgc	R	0	1167.00
cga	R	0	2184.00
cgg	R	0	1632.00
aga	R	17909	11196.00
agg	R	4392	5513.00
---	--	------	------
mPD		0.32	0.91
		nPD:	0.35
		N. weight:	0.34
		Sc. PD:	0.1
		Sc. rank:	6119.0

PA-X

Pos.	126	obs:	exp:
aaa	K	1	0.57
aag	K	0	0.43
gat	D	3	2.63
gac	D	2	2.37
gaa	E	22262	13495.00
gag	E	25	8792.00
ggt	G	0	1.21
ggc	G	0	1.18
gga	G	8	3.56
ggg	G	0	2.04
---	--	------	------
mPD		0.0035	0.48
		nPD:	0.01
		N. weight:	0.5
		Sc. PD:	-0.018
		Sc. rank:	-4002.9

PA-X

Pos.	127	obs:	exp:
att	I	4	81.54
atc	I	220	55.91
ata	I	0	86.54
gtt	V	2416	4870.00
gtc	V	19605	4003.00
gta	V	8	5925.00
gtg	V	0	7231.00
gct	A	1	12.71
gcc	A	47	9.94
gca	A	0	22.54
gcg	A	0	2.81
---	--	------	------
mPD		0.22	0.76
		nPD:	0.29
		N. weight:	1.3
		Sc. PD:	0.32
		Sc. rank:	23588.0

PA-X

Pos.	128	obs:	exp:
tat	Y	0	0.52
tac	Y	1	0.48
cct	P	0	0.28
ccc	P	1	0.19
cca	P	0	0.43
ccg	P	0	0.11
cat	H	37	13111.00
cac	H	22258	9184.00
caa	Q	2	1.06
cag	Q	0	0.94
aat	N	0	1.18
aac	N	2	0.82
---	--	------	------
mPD		0.0039	0.48
		nPD:	0.01
		N. weight:	0.88
		Sc. PD:	-0.031
		Sc. rank:	-5207.7

PA-X

Pos.	129	obs:	exp:
tta	L	29	3.41
ttg	L	0	5.84
ctt	L	0	6.11
ctc	L	0	4.38
cta	L	2	5.97
ctg	L	0	5.30
att	I	0	8083.00
atc	I	3	5543.00
ata	I	22202	8579.00
atg	M	37	37.00
act	T	0	4.79
acc	T	0	3.90
aca	T	19	8.69
acg	T	0	1.62
gtt	V	0	1.99
gtc	V	0	1.64
gta	V	9	2.42
gtg	V	0	2.95
---	--	------	------
mPD		0.0089	0.66
		nPD:	0.01
		N. weight:	0.96
		Sc. PD:	-0.029
		Sc. rank:	1049.1

PA-X

Pos.	130	obs:	exp:
tat	Y	22239	11586.00
tac	Y	60	10713.00
cat	H	1	0.59
cac	H	0	0.41
---	-	1	1.00
---	--	------	------
mPD		0.0055	0.50
		nPD:	0.01
		N. weight:	0.64
		Sc. PD:	-0.021
		Sc. rank:	-1097.8

PA-X

Pos.	131	obs:	exp:
tat	Y	80	11587.00
tac	Y	22221	10714.00
---	--	------	------
mPD		0.0071	0.50
		nPD:	0.01
		N. weight:	0.72
		Sc. PD:	-0.021
		Sc. rank:	1069.9

PA-X

Pos.	132	obs:	exp:
tta	L	40	2451.00
ttg	L	0	4198.00
ctt	L	4	4394.00
ctc	L	28	3149.00
cta	L	22177	4292.00
ctg	L	47	3812.00
cgt	R	0	0.03
cgc	R	0	0.05
cga	R	1	0.10
cgg	R	0	0.07
att	I	0	1.09
atc	I	0	0.75
ata	I	3	1.16
aga	R	0	0.50
agg	R	0	0.25
gtt	V	0	0.22
gtc	V	0	0.18
gta	V	1	0.27
gtg	V	0	0.33
---	--	------	------
mPD		0.011	1.1
		nPD:	0.01
		N. weight:	1.6
		Sc. PD:	-0.055
		Sc. rank:	-6421.1

PA-X

Pos.	133	obs:	exp:
aaa	K	1	3.99
aag	K	6	3.01
gtt	V	0	0.22
gtc	V	0	0.18
gta	V	0	0.27
gtg	V	1	0.33
gat	D	5	2.63
gac	D	0	2.37
gaa	E	311	13495.00
gag	E	21977	8793.00
---	--	------	------
mPD		0.029	0.48
		nPD:	0.06
		N. weight:	0.86
		Sc. PD:	0.014
		Sc. rank:	10748.0

PA-X

Pos.	134	obs:	exp:
aaa	K	22260	12720.00
aag	K	39	9579.00
gaa	E	2	1.21
gag	E	0	0.79
---	--	------	------
mPD		0.0037	0.49
		nPD:	0.01
		N. weight:	0.55
		Sc. PD:	-0.02
		Sc. rank:	-4150.0

PA-X

Pos.	135	obs:	exp:
tct	S	0	0.55
tcc	S	3	0.35
tca	S	0	0.78
tcg	S	0	0.16
act	T	0	0.50
acc	T	2	0.41
aca	T	0	0.91
acg	T	0	0.17
agt	S	0	0.56
agc	S	0	0.60
gct	A	67	5903.00
gcc	A	22217	4615.00
gca	A	12	10472.00
gcg	A	0	1306.00
---	--	------	------
mPD		0.0075	0.66
		nPD:	0.01
		N. weight:	1.6
		Sc. PD:	-0.05
		Sc. rank:	-1620.8

PA-X

Pos.	136	obs:	exp:
tct	S	0	0.37
tcc	S	0	0.23
tca	S	0	0.52
tcg	S	0	0.11
act	T	0	0.25
acc	T	1	0.21
aca	T	0	0.46
acg	T	0	0.09
aat	N	97	13187.00
aac	N	22200	9110.00
agt	S	0	0.38
agc	S	2	0.40
gat	D	0	0.53
gac	D	1	0.47
---	--	------	------
mPD		0.0090	0.48
		nPD:	0.02
		N. weight:	0.87
		Sc. PD:	-0.022
		Sc. rank:	3778.1

PA-X

Pos.	137	obs:	exp:
cgt	R	0	0.05
cgc	R	0	0.10
cga	R	0	0.20
cgg	R	0	0.15
aaa	K	22245	12719.00
aag	K	53	9579.00
aga	R	2	1.00
agg	R	0	0.49
gaa	E	1	0.61
gag	E	0	0.39
---	--	------	------
mPD		0.0050	0.49
		nPD:	0.01
		N. weight:	0.55
		Sc. PD:	-0.018
		Sc. rank:	-1481.5

PA-X

Pos.	138	obs:	exp:
att	I	0	8114.00
atc	I	1	5564.00
ata	I	22289	8612.00
atg	M	11	11.00
---	--	------	------
mPD		0.0011	0.66
		nPD:	0.
		N. weight:	0.96
		Sc. PD:	-0.04
		Sc. rank:	-16915.0

PA-X

Pos.	139	obs:	exp:
cgt	R	0	0.05
cgc	R	0	0.10
cga	R	0	0.20
cgg	R	0	0.15
act	T	0	0.25
acc	T	0	0.21
aca	T	1	0.46
acg	T	0	0.09
aat	N	0	1.18
aac	N	2	0.82
aaa	K	22013	12718.00
aag	K	283	9578.00
aga	R	2	1.00
agg	R	0	0.49
---	--	------	------
mPD		0.026	0.49
		nPD:	0.05
		N. weight:	0.5
		Sc. PD:	0.0042
		Sc. rank:	5844.6

PA-X

Pos.	140	obs:	exp:
ttt	F	5	2.15
ttc	F	0	2.85
tct	S	22242	4101.00
tcc	S	25	2581.00
tca	S	13	5799.00
tcg	S	3	1186.00
tat	Y	2	1.04
tac	Y	0	0.96
cct	P	3	0.83
ccc	P	0	0.56
cca	P	0	1.28
ccg	P	0	0.34
act	T	1	0.25
acc	T	0	0.21
aca	T	0	0.46
acg	T	0	0.09
agt	S	0	4190.00
agc	S	0	4426.00
gct	A	7	1.85
gcc	A	0	1.45
gca	A	0	3.29
gcg	A	0	0.41
---	--	------	------
mPD		0.0053	1.6
		nPD:	0.
		N. weight:	1.7
		Sc. PD:	-0.068
		Sc. rank:	-24327.0

PA-X

Pos.	141	obs:	exp:
aaa	K	0	0.57
aag	K	1	0.43
gtt	V	0	0.22
gtc	V	0	0.18
gta	V	0	0.27
gtg	V	1	0.33
gat	D	6	3.16
gac	D	0	2.84
gaa	E	1217	13495.00
gag	E	21071	8793.00
ggt	G	0	0.76
ggc	G	0	0.74
gga	G	0	2.22
ggg	G	5	1.28
---	--	------	------
mPD		0.10	0.48
		nPD:	0.22
		N. weight:	0.7
		Sc. PD:	0.12
		Sc. rank:	11923.0

PA-X

Pos.	142	obs:	exp:
cgt	R	0	0.33
cgc	R	0	0.63
cga	R	0	1.18
cgg	R	0	0.88
atg	M	2	2.00
act	T	0	3.03
acc	T	0	2.46
aca	T	0	5.49
acg	T	12	1.02
aat	N	3	1.77
aac	N	0	1.23
aaa	K	148	12701.00
aag	K	22117	9564.00
aga	R	0	6.02
agg	R	12	2.97
gaa	E	0	4.24
gag	E	7	2.76
---	--	------	------
mPD		0.016	0.49
		nPD:	0.03
		N. weight:	0.81
		Sc. PD:	-0.0084
		Sc. rank:	7672.1

PA-X

Pos.	143	obs:	exp:
cct	P	0	0.28
ccc	P	0	0.19
cca	P	1	0.43
ccg	P	0	0.11
att	I	0	0.36
atc	I	0	0.25
ata	I	1	0.39
act	T	0	5621.00
acc	T	5	4573.00
aca	T	22277	10200.00
acg	T	14	1902.00
gct	A	0	0.79
gcc	A	0	0.62
gca	A	3	1.41
gcg	A	0	0.18
---	--	------	------
mPD		0.0022	0.68
		nPD:	0.
		N. weight:	0.78
		Sc. PD:	-0.031
		Sc. rank:	-11570.0

PA-X

Pos.	144	obs:	exp:
tat	Y	0	0.52
tac	Y	1	0.48
cat	H	30	13112.00
cac	H	22266	9184.00
caa	Q	3	1.58
cag	Q	0	1.42
aat	N	0	0.59
aac	N	1	0.41
---	--	------	------
mPD		0.0031	0.48
		nPD:	0.01
		N. weight:	0.88
		Sc. PD:	-0.033
		Sc. rank:	-8194.4

PA-X

Pos.	145	obs:	exp:
att	I	22107	8117.00
atc	I	160	5566.00
ata	I	31	8615.00
gtt	V	3	0.66
gtc	V	0	0.55
gta	V	0	0.81
gtg	V	0	0.98
---	--	------	------
mPD		0.017	0.66
		nPD:	0.03
		N. weight:	0.97
		Sc. PD:	-0.017
		Sc. rank:	7744.3

PA-X

Pos.	146	obs:	exp:
cat	H	72	13115.00
cac	H	22229	9186.00
---	--	------	------
mPD		0.0064	0.48
		nPD:	0.01
		N. weight:	0.87
		Sc. PD:	-0.026
		Sc. rank:	817.6

PA-X

Pos.	147	obs:	exp:
tta	L	0	0.11
ttg	L	0	0.19
ctt	L	0	0.20
ctc	L	1	0.14
cta	L	0	0.19
ctg	L	0	0.17
att	I	48	8117.00
atc	I	22229	5566.00
ata	I	22	8615.00
gtt	V	0	0.22
gtc	V	1	0.18
gta	V	0	0.27
gtg	V	0	0.33
---	--	------	------
mPD		0.0064	0.66
		nPD:	0.01
		N. weight:	1.4
		Sc. PD:	-0.046
		Sc. rank:	-5128.1

PA-X

Pos.	148	obs:	exp:
ttt	F	22273	9570.00
ttc	F	27	12730.00
tta	L	0	0.11
ttg	L	0	0.19
ctt	L	1	0.20
ctc	L	0	0.14
cta	L	0	0.19
ctg	L	0	0.17
---	--	------	------
mPD		0.0025	0.49
		nPD:	0.01
		N. weight:	0.84
		Sc. PD:	-0.032
		Sc. rank:	-10096.0

PA-X

Pos.	149	obs:	exp:
tct	S	14	4104.00
tcc	S	12	2583.00
tca	S	22259	5804.00
tcg	S	16	1187.00
agt	S	0	4193.00
agc	S	0	4430.00
---	--	------	------
mPD		0.0038	1.6
		nPD:	0.
		N. weight:	1.3
		Sc. PD:	-0.055
		Sc. rank:	-22485.0

,

PA-X

Pos.	150	obs:	exp:
ttt	F	253	9570.00
ttc	F	22048	12731.00
---	--	------	------
mPD		0.022	0.49
		nPD:	0.05
		N. weight:	0.51
		Sc. PD:	0.0011
		Sc. rank:	5689.6

,

PA-X

Pos.	151	obs:	exp:
att	I	14	5.10
atc	I	0	3.49
ata	I	0	5.41
act	T	22235	5617.00
acc	T	32	4570.00
aca	T	11	10193.00
acg	T	3	1901.00
aat	N	3	1.77
aac	N	0	1.23
gct	A	3	0.79
gcc	A	0	0.62
gca	A	0	1.41
gcg	A	0	0.18
---	--	------	------
mPD		0.0059	0.68
		nPD:	0.01
		N. weight:	1.4
		Sc. PD:	-0.048
		Sc. rank:	-7539.5

,

PA-X

Pos.	152	obs:	exp:
ggt	G	0	3382.00
ggc	G	0	3303.00
gga	G	22174	9920.00
ggg	G	127	5697.00
---	--	------	------
mPD		0.011	0.69
		nPD:	0.02
		N. weight:	0.78
		Sc. PD:	-0.021
		Sc. rank:	2954.0

,

PA-X

Pos.	153	obs:	exp:
gaa	E	349	13503.00
gag	E	21951	8797.00
ggt	G	0	0.15
ggc	G	0	0.15
gga	G	0	0.44
ggg	G	1	0.26
---	--	------	------
mPD		0.031	0.48
		nPD:	0.06
		N. weight:	0.85
		Sc. PD:	0.018
		Sc. rank:	12138.0

,

PA-X

Pos.	154	obs:	exp:
gaa	E	642	13503.00
gag	E	21658	8797.00
ggt	G	0	0.15
ggc	G	0	0.15
gga	G	0	0.44
ggg	G	1	0.26
---	--	------	------
mPD		0.056	0.48
		nPD:	0.12
		N. weight:	0.79
		Sc. PD:	0.058
		Sc. rank:	12402.0

,

PA-X

Pos.	155	obs:	exp:
tta	L	0	0.11
ttg	L	1	0.19
ctt	L	0	0.20
ctc	L	0	0.14
cta	L	0	0.19
ctg	L	0	0.17
att	I	0	0.36
atc	I	0	0.25
ata	I	1	0.39
atg	M	22297	22297.00
act	T	0	0.50
acc	T	0	0.41
aca	T	0	0.91
acg	T	2	0.17
---	--	------	------
mPD		0.00036	0.00058
		nPD:	0.62
		N. weight:	0.00034
		Sc. PD:	0.00019
		Sc. rank:	6.4

,

PA-X

Pos.	156	obs:	exp:
act	T	0	0.25
acc	T	1	0.21
aca	T	0	0.46
acg	T	0	0.09
gtt	V	0	0.66
gtc	V	3	0.55
gta	V	0	0.81
gtg	V	0	0.98
gct	A	28	5903.00
gcc	A	22249	4615.00
gca	A	20	10472.00
gcg	A	0	1306.00
---	--	------	------
mPD		0.0047	0.66
		nPD:	0.01
		N. weight:	1.6
		Sc. PD:	-0.057
		Sc. rank:	-13711.0

,

PA-X

Pos.	157	obs:	exp:
tct	S	0	0.18
tcc	S	1	0.12
tca	S	0	0.26
tcg	S	0	0.05
act	T	78	5621.00
acc	T	22211	4574.00
aca	T	9	10201.00
acg	T	0	1903.00
aat	N	0	0.59
aac	N	1	0.41
agt	S	0	0.19
agc	S	0	0.20
gct	A	0	0.26
gcc	A	1	0.21
gca	A	0	0.47
gcg	A	0	0.06
---	--	------	------
mPD		0.0080	0.68
		nPD:	0.01
		N. weight:	1.6
		Sc. PD:	-0.049
		Sc. rank:	-205.1

,

PA-X

Pos.	158	obs:	exp:
caa	Q	3	1.58
cag	Q	0	1.42
cgt	R	0	1.47
cgc	R	0	2.82
cga	R	0	5.29
cgg	R	0	3.95
aat	N	1	2.37
aac	N	3	1.63
aaa	K	22217	12684.00
aag	K	19	9552.00
aga	R	53	27.11
agg	R	1	13.35
gaa	E	4	2.42
gag	E	0	1.58
---	--	------	------
mPD		0.0076	0.50
		nPD:	0.02
		N. weight:	0.56
		Sc. PD:	-0.016
		Sc. rank:	1608.8

,

PA-X

Pos.	159	obs:	exp:
tct	S	0	2.21
tcc	S	0	1.39
tca	S	1	3.12
tcg	S	11	0.64
act	T	0	0.50
acc	T	0	0.41
aca	T	0	0.91
acg	T	2	0.17
agt	S	0	2.26
agc	S	0	2.38
gtt	V	0	0.22
gtc	V	0	0.18
gta	V	0	0.27
gtg	V	1	0.33
gct	A	14	5900.00
gcc	A	0	4613.00
gca	A	446	10467.00
gcg	A	21826	1306.00
---	--	------	------
mPD		0.042	0.66
		nPD:	0.06
		N. weight:	2.7
		Sc. PD:	0.052
		Sc. rank:	37268.0

,

PA-X

Pos.	160	obs:	exp:
aat	N	0	3.55
aac	N	6	2.45
gat	D	84	11742.00
gac	D	22201	10543.00
gaa	E	10	6.06
gag	E	0	3.94
---	--	------	------
mPD		0.0089	0.50
		nPD:	0.02
		N. weight:	0.73
		Sc. PD:	-0.019
		Sc. rank:	2832.8

,

PA-X

Pos.	161	obs:	exp:
ttt	F	0	2.15
ttc	F	5	2.85
tat	Y	18	11582.00
tac	Y	22272	10708.00
cat	H	0	3.53
cac	H	6	2.47
---	--	------	------
mPD		0.0026	0.50
		nPD:	0.01
		N. weight:	0.73
		Sc. PD:	-0.028
		Sc. rank:	-8480.5

,

PA-X

Pos.	162	obs:	exp:
tct	S	0	2.76
tcc	S	15	1.74
tca	S	0	3.90
tcg	S	0	0.80
att	I	0	5.82
atc	I	16	3.99
ata	I	0	6.18
act	T	2470	5613.00
acc	T	19790	4567.00
aca	T	8	10187.00
acg	T	0	1900.00
aat	N	0	0.59
aac	N	1	0.41
agt	S	0	2.82
agc	S	0	2.98
gct	A	0	0.26
gcc	A	1	0.21
gca	A	0	0.47
gcg	A	0	0.06
---	--	------	------
mPD		0.20	0.68
		nPD:	0.29
		N. weight:	1.2
		Sc. PD:	0.3
		Sc. rank:	21834.0

,

PA-X

Pos.	163	obs:	exp:
ttt	F	1	0.43
ttc	F	0	0.57
tta	L	0	2450.00
ttg	L	0	4195.00
ctt	L	22267	4391.00
ctc	L	8	3147.00
cta	L	6	4290.00
ctg	L	1	3810.00
att	I	18	6.55
atc	I	0	4.49
ata	I	0	6.95
---	--	------	------
mPD		0.0030	1.1
		nPD:	0.
		N. weight:	1.6
		Sc. PD:	-0.066
		Sc. rank:	-26057.0

,

PA-X

Pos.	164	obs:	exp:
aat	N	0	0.59
aac	N	1	0.41
gat	D	81	11749.00
gac	D	22218	10550.00
ggt	G	0	0.15
ggc	G	1	0.15
gga	G	0	0.44
ggg	G	0	0.26
---	--	------	------
mPD		0.0074	0.50
		nPD:	0.01
		N. weight:	0.73
		Sc. PD:	-0.021
		Sc. rank:	1810.8

,

PA-X

Pos.	165	obs:	exp:
gct	A	0	0.26
gcc	A	0	0.21
gca	A	1	0.47
gcg	A	0	0.06
gat	D	1	1.58
gac	D	2	1.42
gaa	E	22253	13500.00
gag	E	43	8796.00
ggt	G	0	0.15
ggc	G	0	0.15
gga	G	1	0.44
ggg	G	0	0.26
---	--	------	------
mPD		0.0043	0.48
		nPD:	0.01
		N. weight:	0.49
		Sc. PD:	-0.017
		Sc. rank:	-2665.9

,

PA-X

Pos.	166	obs:	exp:
aaa	K	1	0.57
aag	K	0	0.43
gaa	E	7755	13503.00
gag	E	14545	8797.00
---	--	------	------
mPD		0.45	0.48
		nPD:	0.95
		N. weight:	0.14
		Sc. PD:	0.12
		Sc. rank:	2597.3

,

PA-X

Pos.	167	obs:	exp:
tct	S	0	4104.00
tcc	S	0	2583.00
tca	S	0	5804.00
tcg	S	0	1187.00
act	T	0	0.25
acc	T	1	0.21
aca	T	0	0.46
acg	T	0	0.09
agt	S	346	4193.00
agc	S	21954	4430.00
---	--	------	------
mPD		0.031	1.6
		nPD:	0.02
		N. weight:	1.6
		Sc. PD:	-0.038
		Sc. rank:	7103.4

,

PA-X

Pos.	168	obs:	exp:
cgt	R	0	608.90
cgc	R	0	1167.00
cga	R	1	2184.00
cgg	R	0	1632.00
act	T	0	0.25
acc	T	0	0.21
aca	T	0	0.46
acg	T	1	0.09
aga	R	19038	11195.00
agg	R	3261	5513.00
---	--	------	------
mPD		0.25	0.91
		nPD:	0.27
		N. weight:	0.38
		Sc. PD:	0.087
		Sc. rank:	6534.0

,

PA-X

Pos.	169	obs:	exp:
act	T	0	0.25
acc	T	0	0.21
aca	T	1	0.46
acg	T	0	0.09
gct	A	38	5904.00
gcc	A	1	4616.00
gca	A	22061	10474.00
gcg	A	200	1306.00
---	--	------	------
mPD		0.021	0.66
		nPD:	0.03
		N. weight:	0.72
		Sc. PD:	-0.0082
		Sc. rank:	6347.2

,

PA-X

Pos.	170	obs:	exp:
cgt	R	0	608.90
cgc	R	0	1167.00
cga	R	1	2184.00
cgg	R	0	1632.00
aga	R	22266	11196.00
agg	R	34	5513.00
---	--	------	------
mPD		0.0031	0.91
		nPD:	0.
		N. weight:	0.68
		Sc. PD:	-0.027
		Sc. rank:	-9544.8

,

PA-X

Pos.	171	obs:	exp:
att	I	37	8116.00
atc	I	22214	5566.00
ata	I	45	8614.00
gtt	V	0	1.11
gtc	V	5	0.91
gta	V	0	1.34
gtg	V	0	1.64
---	--	------	------
mPD		0.0078	0.66
		nPD:	0.01
		N. weight:	1.4
		Sc. PD:	-0.043
		Sc. rank:	-906.8

,

PA-X

Pos.	172	obs:	exp:
caa	Q	1	0.53
cag	Q	0	0.47
act	T	0	0.25
acc	T	0	0.21
aca	T	1	0.46
acg	T	0	0.09
aaa	K	22279	12720.00
aag	K	20	9579.00
---	--	------	------
mPD		0.0020	0.49
		nPD:	0.
		N. weight:	0.56
		Sc. PD:	-0.022
		Sc. rank:	-7541.7

,

PA-X

Pos.	173	obs:	exp:
act	T	22255	5622.00
acc	T	39	4574.00
aca	T	4	10202.00
acg	T	3	1903.00
---	--	------	------
mPD		0.0041	0.68
		nPD:	0.01
		N. weight:	1.4
		Sc. PD:	-0.051
		Sc. rank:	-13562.0

,

PA-X

Pos.	174	obs:	exp:
cgt	R	0	608.90
cgc	R	0	1167.00
cga	R	0	2184.00
cgg	R	5	1632.00
aaa	K	0	0.57
aag	K	1	0.43
aga	R	224	11195.00
agg	R	22071	5513.00
---	--	------	------
mPD		0.020	0.91
		nPD:	0.02
		N. weight:	1.3
		Sc. PD:	-0.028
		Sc. rank:	9110.0

,

PA-X

Pos.	175	obs:	exp:
tta	L	0	2452.00
ttg	L	0	4199.00
ctt	L	22223	4395.00
ctc	L	71	3150.00
cta	L	1	4293.00
ctg	L	6	3813.00
---	--	------	------
mPD		0.0070	1.1
		nPD:	0.01
		N. weight:	1.6
		Sc. PD:	-0.06
		Sc. rank:	-15507.0

,

PA-X

Pos.	176	obs:	exp:
ttt	F	24	9566.00
ttc	F	22268	12726.00
tta	L	0	0.11
ttg	L	0	0.19
tat	Y	0	4.16
tac	Y	8	3.84
ctt	L	0	0.20
ctc	L	1	0.14
cta	L	0	0.19
ctg	L	0	0.17
---	--	------	------
mPD		0.0030	0.49
		nPD:	0.01
		N. weight:	0.56
		Sc. PD:	-0.021
		Sc. rank:	-5854.9

,

PA-X			
Pos.	177	obs :	exp :
att	I	1	0.36
atc	I	0	0.25
ata	I	0	0.39
act	T	21925	5622.00
acc	T	230	4574.00
aca	T	4	10202.00
acg	T	141	1903.00
---	--	------	------
mPD		0.033	0.68
		nPD :	0.05
		N. weight :	1.3
		Sc. PD :	0.0071
		Sc. rank :	14899.0

,

PA-X			
Pos.	178	obs :	exp :
tta	L	1	0.22
ttg	L	0	0.38
ctt	L	0	0.39
ctc	L	0	0.28
cta	L	1	0.39
ctg	L	0	0.34
att	I	0	8117.00
atc	I	92	5566.00
ata	I	22205	8615.00
gtt	V	0	0.44
gtc	V	0	0.36
gta	V	2	0.54
gtg	V	0	0.66
---	--	------	------
mPD		0.0086	0.66
		nPD :	0.01
		N. weight :	0.93
		Sc. PD :	-0.028
		Sc. rank :	742.6

,

PA-X			
Pos.	179	obs :	exp :
cgt	R	0	608.90
cgc	R	0	1167.00
cga	R	4	2184.00
cgg	R	0	1632.00
aaa	K	1	0.57
aag	K	0	0.43
aga	R	22252	11195.00
agg	R	44	5513.00
---	--	------	------
mPD		0.0044	0.91
		nPD :	0.
		N. weight :	0.68
		Sc. PD :	-0.026
		Sc. rank :	-8303.4

,

PA-X			
Pos.	180	obs :	exp :
cat	H	0	1.18
cac	H	2	0.82
caa	Q	22261	11765.00
cag	Q	36	10532.00
aaa	K	1	0.57
aag	K	0	0.43
gaa	E	1	0.61
gag	E	0	0.39
---	--	------	------
mPD		0.0036	0.50
		nPD :	0.01
		N. weight :	0.63
		Sc. PD :	-0.023
		Sc. rank :	-5141.4

,

PA-X			
Pos.	181	obs :	exp :
cct	P	0	0.28
ccc	P	0	0.19
cca	P	1	0.43
ccg	P	0	0.11
gaa	E	22180	13503.00
gag	E	120	8797.00
---	--	------	------
mPD		0.011	0.48
		nPD :	0.02
		N. weight :	0.47
		Sc. PD :	-0.0098
		Sc. rank :	3291.9

,

PA-X			
Pos.	182	obs :	exp :
att	I	0	0.36
atc	I	0	0.25
ata	I	1	0.39
atg	M	22300	22300.00
---	--	------	------
mPD		0.000090	0.000090
		nPD :	1.
		N. weight :	0.000043
		Sc. PD :	0.000041
		Sc. rank :	0.8

,

PA-X			
Pos.	183	obs :	exp :
cct	P	0	0.28
ccc	P	0	0.19
cca	P	1	0.43
ccg	P	0	0.11
act	T	0	0.50
acc	T	2	0.41
aca	T	0	0.91
acg	T	0	0.17
gtt	V	0	0.44
gtc	V	2	0.36
gta	V	0	0.54
gtg	V	0	0.66
gct	A	35	5903.00
gcc	A	22249	4615.00
gca	A	12	10472.00
gcg	A	0	1306.00
---	--	------	------
mPD		0.0047	0.66
		nPD :	0.01
		N. weight :	1.6
		Sc. PD :	-0.057
		Sc. rank :	-13095.0

,

PA-X			
Pos.	184	obs :	exp :
tct	S	0	4080.00
tcc	S	0	2568.00
tca	S	0	5770.00
tcg	S	0	1180.00
tat	Y	1	0.52
tac	Y	0	0.48
cgt	R	0	0.03
cgc	R	0	0.05
cga	R	0	0.10
cgg	R	0	0.07
att	I	4	1.46
atc	I	0	1.00
ata	I	0	1.55
act	T	2	0.50
acc	T	0	0.41
aca	T	0	0.91
acg	T	0	0.17
aat	N	7	4.14
aac	N	0	2.86
agt	S	22083	4169.00
agc	S	86	4404.00
aga	R	0	0.50
agg	R	1	0.25
ggt	G	117	17.74
ggc	G	0	17.33
gga	G	0	52.04
ggg	G	0	29.89
---	--	------	------
mPD		0.020	1.6
		nPD :	0.01
		N. weight :	1.7
		Sc. PD :	-0.052
		Sc. rank :	-645.1

,

PA-X			
Pos.	185	obs :	exp :
cgt	R	0	608.50
cgc	R	0	1166.00
cga	R	0	2183.00
cgg	R	0	1631.00
aaa	K	0	7.42
aag	K	13	5.58
aga	R	367	11189.00
agg	R	21920	5510.00
ggt	G	0	0.15
ggc	G	0	0.15
gga	G	0	0.44
ggg	G	1	0.26
---	--	------	------
mPD		0.034	0.91
		nPD :	0.04
		N. weight :	1.3
		Sc. PD :	-0.0087
		Sc. rank :	13404.0

,

PA-X			
Pos.	186	obs :	exp :
tct	S	0	4094.00
tcc	S	0	2577.00
tca	S	0	5790.00
tcg	S	0	1184.00
aat	N	44	26.02
aac	N	0	17.98
agt	S	22206	4184.00
agc	S	43	4419.00
ggt	G	8	1.21
ggc	G	0	1.18
gga	G	0	3.56
ggg	G	0	2.04
---	--	------	------
mPD		0.0085	1.6
		nPD :	0.01
		N. weight :	1.7
		Sc. PD :	-0.064
		Sc. rank :	-19517.0

,

PA-X			
Pos.	187	obs :	exp :
tta	L	923	2449.00
ttg	L	1	4195.00
ctt	L	0	4390.00
ctc	L	129	3147.00
cta	L	21209	4289.00
ctg	L	18	3809.00
att	I	0	7.64
atc	I	0	5.24
ata	I	21	8.11
---	--	------	------
mPD		0.094	1.1
		nPD :	0.08
		N. weight :	1.5
		Sc. PD :	0.057
		Sc. rank :	22000.0

,

PA-X			
Pos.	188	obs :	exp :
tgg	W	22300	22300.00
cgt	R	0	0.03
cgc	R	0	0.05
cga	R	0	0.10
cgg	R	1	0.07
aga	R	0	0.50
agg	R	0	0.25
---	--	------	------
mPD		0.000090	0.00015
		nPD :	0.6
		N. weight :	0.00012
		Sc. PD :	0.000065
		Sc. rank :	2.2

,

PA-X			
Pos.	189	obs:	exp:
aat	N	1	0.59
aac	N	0	0.41
gct	A	1	0.26
gcc	A	0	0.21
gca	A	0	0.47
gcg	A	0	0.06
gat	D	22257	11748.00
gac	D	40	10549.00
gaa	E	1	0.61
gag	E	0	0.39
ggt	G	1	0.15
ggc	G	0	0.15
gga	G	0	0.44
ggg	G	0	0.26
---	--	------	------
mPD		0.0039	0.50
		nPD:	0.01
		N. weight:	0.63
		Sc. PD:	-0.022
		Sc. rank:	-3832.8

,

PA-X			
Pos.	190	obs:	exp:
ttt	F	0	1.29
ttc	F	3	1.71
tct	S	14	4103.00
tcc	S	22205	2583.00
tca	S	78	5803.00
tcg	S	0	1187.00
agt	S	0	4193.00
agc	S	0	4429.00
gct	A	0	0.26
gcc	A	1	0.21
gca	A	0	0.47
gcg	A	0	0.06
---	--	------	------
mPD		0.0086	1.6
		nPD:	0.01
		N. weight:	2.1
		Sc. PD:	-0.082
		Sc. rank:	-24037.0

,

PA-X			
Pos.	191	obs:	exp:
ttt	F	8	9566.00
ttc	F	22283	12725.00
tct	S	0	1.84
tcc	S	10	1.16
tca	S	0	2.60
tcg	S	0	0.53
agt	S	0	1.88
agc	S	0	1.99
---	--	------	------
mPD		0.0016	0.49
		nPD:	0.
		N. weight:	0.56
		Sc. PD:	-0.023
		Sc. rank:	-8042.2

,

PA-X			
Pos.	192	obs:	exp:
cgt	R	1	0.03
cgc	R	0	0.05
cga	R	0	0.10
cgg	R	0	0.07
att	I	0	2.18
atc	I	6	1.50
ata	I	0	2.32
aga	R	0	0.50
agg	R	0	0.25
gtt	V	0	4921.00
gtc	V	22253	4045.00
gta	V	7	5987.00
gtg	V	0	7307.00
gct	A	0	8.21
gcc	A	31	6.42
gca	A	0	14.56
gcg	A	0	1.82
gat	D	0	0.53
gac	D	1	0.47
ggt	G	0	0.30
ggc	G	2	0.30
gga	G	0	0.89
ggg	G	0	0.51
---	--	------	------
mPD		0.0045	0.74
		nPD:	0.01
		N. weight:	1.7
		Sc. PD:	-0.064
		Sc. rank:	-17548.0

,

PA-X			
Pos.	193	obs:	exp:
tct	S	0	4060.00
tcc	S	0	2555.00
tca	S	0	5742.00
tcg	S	0	1174.00
tgt	C	1	0.45
tgc	C	0	0.55
att	I	2	0.73
atc	I	0	0.50
ata	I	0	0.77
aat	N	206	121.80
aac	N	0	84.16
agt	S	22062	4148.00
agc	S	0	4382.00
ggt	G	30	4.55
ggc	G	0	4.44
gga	G	0	13.34
ggg	G	0	7.66
---	--	------	------
mPD		0.021	1.7
		nPD:	0.01
		N. weight:	1.7
		Sc. PD:	-0.051
		Sc. rank:	934.2

,

PA-X			
Pos.	194	obs:	exp:
tta	L	0	12.42
ttg	L	0	21.27
ctt	L	0	22.27
ctc	L	0	15.96
cta	L	0	21.75
ctg	L	113	19.32
cct	P	0	6112.00
ccc	P	0	4115.00
cca	P	0	9446.00
ccg	P	22184	2512.00
caa	Q	0	2.11
cag	Q	4	1.89
---	--	------	------
mPD		0.010	0.71
		nPD:	0.01
		N. weight:	2.2
		Sc. PD:	-0.063
		Sc. rank:	4901.4

,

PA-X			
Pos.	195	obs:	exp:
cgt	R	0	0.52
cgc	R	0	0.99
cga	R	0	1.86
cgg	R	0	1.39
aat	N	0	1.18
aac	N	2	0.82
aaa	K	22280	12709.00
aag	K	0	9571.00
aga	R	19	9.54
agg	R	0	4.70
---	--	------	------
mPD		0.0019	0.49
		nPD:	0.
		N. weight:	0.57
		Sc. PD:	-0.022
		Sc. rank:	-7846.3

,

PA-X			
Pos.	196	obs:	exp:
cgt	R	0	0.03
cgc	R	0	0.05
cga	R	0	0.10
cgg	R	0	0.07
aaa	K	0	26.81
aag	K	47	20.19
aga	R	0	0.50
agg	R	1	0.25
gtt	V	0	0.88
gtc	V	0	0.73
gta	V	0	1.08
gtg	V	4	1.31
gaa	E	3	13426.00
gag	E	22171	8748.00
ggt	G	0	11.37
ggc	G	0	11.11
gga	G	0	33.36
ggg	G	75	19.16
---	--	------	------
mPD		0.012	0.49
		nPD:	0.02
		N. weight:	0.94
		Sc. PD:	-0.018
		Sc. rank:	7237.7

,

PA-X			
Pos.	197	obs:	exp:
act	T	0	0.25
acc	T	0	0.21
aca	T	0	0.46
acg	T	1	0.09
gtt	V	0	3.54
gtc	V	0	2.91
gta	V	0	4.30
gtg	V	16	5.25
gct	A	0	5899.00
gcc	A	0	4612.00
gca	A	0	10464.00
gcg	A	22280	1305.00
gaa	E	0	2.42
gag	E	4	1.58
---	--	------	------
mPD		0.0019	0.67
		nPD:	0.
		N. weight:	2.9
		Sc. PD:	-0.12
		Sc. rank:	-43090.0

,

PA-X			
Pos.	198	obs:	exp:
cgt	R	0	2.18
cgc	R	0	4.18
cga	R	0	7.84
cgg	R	0	5.85
atg	M	1	1.00
aaa	K	1	12674.00
aag	K	22217	9544.00
aga	R	0	40.16
agg	R	80	19.78
gaa	E	0	1.21
gag	E	2	0.79
---	--	------	------
mPD		0.0075	0.50
		nPD:	0.02
		N. weight:	0.85
		Sc. PD:	-0.024
		Sc. rank:	2236.2

,

PA-X			
Pos.	199	obs:	exp:
cgt	R	0	213.80
cgc	R	0	409.60
cga	R	0	766.90
cgg	R	0	573.00
att	I	0	1.46
atc	I	0	1.00
ata	I	4	1.55
act	T	0	0.25
acc	T	0	0.21
aca	T	1	0.46
acg	T	0	0.09
aaa	K	14464	8251.00
aag	K	0	6213.00
aga	R	7829	3931.00
agg	R	1	1936.00
gaa	E	2	1.21
gag	E	0	0.79
---	--	------	------
mPD		0.46	1.1
		nPD:	0.41
		N. weight:	0.61
		Sc. PD:	0.22
		Sc. rank:	11271.0

,

PA-X			
Pos.	200	obs:	exp:
cat	H	1	0.59
cac	H	0	0.41
caa	Q	22265	11764.00
cag	Q	30	10531.00
cgt	R	0	0.14
cgc	R	0	0.26
cga	R	5	0.49
cgg	R	0	0.37
aga	R	0	2.51
agg	R	0	1.24
---	--	------	------
mPD		0.0032	0.50
		nPD:	0.01
		N. weight:	0.63
		Sc. PD:	-0.023
		Sc. rank:	-5980.3

,

PA-X			
Pos.	201	obs:	exp:
tta	L	0	2449.00
ttg	L	22266	4194.00
tct	S	0	4.05
tcc	S	0	2.55
tca	S	0	5.73
tcg	S	22	1.17
tgg	W	1	1.00
ctt	L	0	4390.00
ctc	L	0	3147.00
cta	L	0	4289.00
ctg	L	11	3809.00
agt	S	0	4.14
agc	S	0	4.37
gtt	V	0	0.22
gtc	V	0	0.18
gta	V	0	0.27
gtg	V	1	0.33
---	--	------	------
mPD		0.0031	1.1
		nPD:	0.
		N. weight:	1.7
		Sc. PD:	-0.068
		Sc. rank:	-26119.0

,

PA-X			
Pos.	202	obs:	exp:
cgt	R	0	4.61
cgc	R	0	8.84
cga	R	0	16.55
cgg	R	0	12.37
aaa	K	0	12625.00
aag	K	22132	9507.00
aga	R	0	84.84
agg	R	169	41.78
---	--	------	------
mPD		0.015	0.51
		nPD:	0.03
		N. weight:	0.86
		Sc. PD:	-0.012
		Sc. rank:	7053.0

,

PA-X			
Pos.	203	obs:	exp:
cgt	R	0	0.30
cgc	R	0	0.58
cga	R	0	1.08
cgg	R	0	0.81
aaa	K	22289	12715.00
aag	K	1	9575.00
aga	R	11	5.52
agg	R	0	2.72
---	--	------	------
mPD		0.0011	0.49
		nPD:	0.
		N. weight:	0.57
		Sc. PD:	-0.023
		Sc. rank:	-9638.2

,

PA-X			
Pos.	204	obs:	exp:
tct	S	0	3310.00
tcc	S	0	2083.00
tca	S	0	4680.00
tcg	S	0	957.10
cgt	R	0	0.03
cgc	R	0	0.05
cga	R	0	0.10
cgg	R	0	0.07
att	I	1	0.36
atc	I	0	0.25
ata	I	0	0.39
act	T	1	0.25
acc	T	0	0.21
aca	T	0	0.46
acg	T	0	0.09
aat	N	4267	2524.00
aac	N	0	1743.00
agt	S	17984	3382.00
agc	S	0	3572.00
aga	R	0	0.50
agg	R	1	0.25
gat	D	4	2.11
gac	D	0	1.89
ggt	G	43	6.52
ggc	G	0	6.37
gga	G	0	19.13
ggg	G	0	10.98
---	--	------	------
mPD		0.31	1.8
		nPD:	0.18
		N. weight:	1.5
		Sc. PD:	0.19
		Sc. rank:	24259.0

,

PA-X			
Pos.	205	obs:	exp:
tta	L	2	2450.00
ttg	L	22283	4196.00
tct	S	0	2.94
tcc	S	0	1.85
tca	S	0	4.16
tcg	S	16	0.85
ctt	L	0	4391.00
ctc	L	0	3148.00
cta	L	0	4290.00
ctg	L	0	3810.00
agt	S	0	3.01
agc	S	0	3.18
---	--	------	------
mPD		0.0016	1.1
		nPD:	0.
		N. weight:	1.7
		Sc. PD:	-0.071
		Sc. rank:	-30927.0

,

PA-X			
Pos.	206	obs:	exp:
cgt	R	0	396.80
cgc	R	0	760.20
cga	R	0	1423.00
cgg	R	0	1064.00
att	I	0	1.09
atc	I	0	0.75
ata	I	3	1.16
aat	N	0	0.59
aac	N	1	0.41
aaa	K	7759	4426.00
aag	K	0	3333.00
aga	R	14531	7296.00
agg	R	2	3593.00
gaa	E	2	1.21
gag	E	0	0.79
ggt	G	0	0.45
ggc	G	0	0.44
gga	G	3	1.33
ggg	G	0	0.77
---	--	------	------
mPD		0.45	1.3
		nPD:	0.36
		N. weight:	0.65
		Sc. PD:	0.21
		Sc. rank:	11913.0

,

PA-X			
Pos.	207	obs:	exp:
tta	L	22207	2444.00
ttg	L	29	4186.00
tct	S	0	11.96
tcc	S	0	7.53
tca	S	65	16.92
tcg	S	0	3.46
ctt	L	0	4382.00
ctc	L	0	3141.00
cta	L	0	4281.00
ctg	L	0	3802.00
agt	S	0	12.22
agc	S	0	12.91
---	--	------	------
mPD		0.0084	1.1
		nPD:	0.01
		N. weight:	2.2
		Sc. PD:	-0.08
		Sc. rank:	-16995.0

,

PA-X			
Pos.	208	obs:	exp:
tta	L	0	1.76
ttg	L	0	3.01
ctt	L	0	3.15
ctc	L	0	2.26
cta	L	0	3.08
ctg	L	16	2.74
cct	P	0	0.28
ccc	P	0	0.19
cca	P	0	0.43
ccg	P	1	0.11
caa	Q	7	11754.00
cag	Q	22268	10521.00
cgt	R	0	0.19
cgc	R	0	0.37
cga	R	0	0.69
cgg	R	7	0.51
aaa	K	0	1.14
aag	K	2	0.86
aga	R	0	3.51
agg	R	0	1.73
---	--	------	------
mPD		0.0030	0.50
		nPD:	0.01
		N. weight:	0.75
		Sc. PD:	-0.028
		Sc. rank:	-8073.6

,

PA-X			
Pos.	209	obs:	exp:
aaa	K	8	4.56
aag	K	0	3.44
gtt	V	0	0.22
gtc	V	0	0.18
gta	V	1	0.27
gtg	V	0	0.33
gat	D	0	0.53
gac	D	1	0.47
gaa	E	22218	13453.00
gag	E	0	8765.00
ggt	G	0	11.07
ggc	G	0	10.81
gga	G	73	32.47
ggg	G	0	18.65
---	--	------	------
mPD		0.0074	0.49
		nPD:	0.02
		N. weight:	0.51
		Sc. PD:	-0.014
		Sc. rank:	1410.9

,

PA-X			
Pos.	210	obs:	exp:
tta	L	2	2438.00
ttg	L	0	4175.00
ctt	L	2	4370.00
ctc	L	1	3132.00
cta	L	22167	4269.00
ctg	L	2	3791.00
cct	P	0	34.71
ccc	P	0	23.37
cca	P	126	53.65
ccg	P	0	14.27
caa	Q	1	0.53
cag	Q	0	0.47
---	--	------	------
mPD		0.012	1.1
		nPD:	0.01
		N. weight:	1.7
		Sc. PD:	-0.055
		Sc. rank:	-3650.2

,

PA-X			
Pos.	211	obs:	exp:
ttt	F	0	2.15
ttc	F	5	2.85
tct	S	0	0.18
tcc	S	1	0.12
tca	S	0	0.26
tcg	S	0	0.05
tat	Y	0	6.24
tac	Y	12	5.76
tgt	C	9	10054.00
tgc	C	22265	12220.00
cgt	R	0	0.25
cgc	R	9	0.47
cga	R	0	0.88
cgg	R	0	0.66
agt	S	0	0.19
agc	S	0	0.20
aga	R	0	4.52
agg	R	0	2.22
---	--	------	------
mPD		0.0032	0.50
		nPD:	0.01
		N. weight:	0.6
		Sc. PD:	-0.022
		Sc. rank:	-5532.2

,

PA-X			
Pos.	212	obs:	exp:
tct	S	0	2.21
tcc	S	0	1.39
tca	S	12	3.12
tcg	S	0	0.64
act	T	0	7.81
acc	T	0	6.36
aca	T	31	14.18
acg	T	0	2.65
agt	S	0	2.26
agc	S	0	2.38
gtt	V	0	47.31
gtc	V	0	38.89
gta	V	214	57.55
gtg	V	0	70.25
gct	A	0	5829.00
gcc	A	0	4557.00
gca	A	22016	10340.00
gcg	A	0	1290.00
gaa	E	28	16.95
gag	E	0	11.05
---	--	------	------
mPD		0.025	0.69
		nPD:	0.04
		N. weight:	0.77
		Sc. PD:	-0.0053
		Sc. rank:	7671.7

,

PA-X

Pos.	213	obs:	exp:
tct	S	0	3901.00
tcc	S	0	2456.00
tca	S	0	5517.00
tcg	S	0	1128.00
att	I	0	7.64
atc	I	21	5.24
ata	I	0	8.11
aat	N	0	16.56
aac	N	28	11.44
agt	S	9	3986.00
agc	S	21191	4211.00
ggt	G	0	159.50
ggc	G	1052	155.80
gga	G	0	468.00
ggg	G	0	268.70
---	--	------	------
mPD		0.095	1.7
		nPD:	0.06
		N. weight:	1.6
		Sc. PD:	0.02
		Sc. rank:	19686.0

,

PA-X

Pos.	214	obs:	exp:
tta	L	4	2450.00
ttg	L	22282	4196.00
tct	S	0	2.76
tcc	S	0	1.74
tca	S	0	3.90
tcg	S	15	0.80
ctt	L	0	4392.00
ctc	L	0	3148.00
cta	L	0	4290.00
ctg	L	0	3810.00
agt	S	0	2.82
agc	S	0	2.98
---	--	------	------
mPD		0.0017	1.1
		nPD:	0.
		N. weight:	1.7
		Sc. PD:	-0.07
		Sc. rank:	-30446.0

,

PA-X

Pos.	215	obs:	exp:
tta	L	0	8.90
ttg	L	0	15.25
tct	S	0	0.18
tcc	S	0	0.12
tca	S	0	0.26
tcg	S	1	0.05
ctt	L	0	15.96
ctc	L	0	11.44
cta	L	0	15.59
ctg	L	81	13.85
cct	P	0	6104.00
ccc	P	0	4110.00
cca	P	38	9434.00
ccg	P	22119	2509.00
caa	Q	0	32.19
cag	Q	61	28.81
agt	S	0	0.19
agc	S	0	0.20
gct	A	0	0.26
gcc	A	0	0.21
gca	A	0	0.47
gcg	A	1	0.06
---	--	------	------
mPD		0.016	0.71
		nPD:	0.02
		N. weight:	2.2
		Sc. PD:	-0.045
		Sc. rank:	15873.0

,

PA-X

Pos.	216	obs:	exp:
att	I	0	183.50
atc	I	504	125.80
ata	I	0	194.70
act	T	0	5495.00
acc	T	21796	4471.00
aca	T	0	9971.00
acg	T	0	1860.00
gct	A	0	0.26
gcc	A	1	0.21
gca	A	0	0.47
gcg	A	0	0.06
---	--	------	------
mPD		0.044	0.72
		nPD:	0.06
		N. weight:	1.6
		Sc. PD:	0.028
		Sc. rank:	20375.0

,

PA-X

Pos.	217	obs:	exp:
cgt	R	0	2.95
cgc	R	0	5.65
cga	R	0	10.58
cgg	R	0	7.90
aaa	K	22191	12659.00
aag	K	1	9533.00
aga	R	108	54.22
agg	R	0	26.70
gaa	E	1	0.61
gag	E	0	0.39
---	--	------	------
mPD		0.0098	0.50
		nPD:	0.02
		N. weight:	0.57
		Sc. PD:	-0.014
		Sc. rank:	2791.5

,

PA-X

Pos.	218	obs:	exp:
gtt	V	3	4914.00
gtc	V	22224	4039.00
gta	V	0	5978.00
gtg	V	0	7296.00
gct	A	0	19.59
gcc	A	74	15.32
gca	A	0	34.76
gcg	A	0	4.34
---	--	------	------
mPD		0.0069	0.74
		nPD:	0.01
		N. weight:	1.7
		Sc. PD:	-0.059
		Sc. rank:	-8662.1

,

PA-X

Pos.	219	obs:	exp:
ttt	F	0	57.50
ttc	F	134	76.50
tct	S	0	4051.00
tcc	S	22014	2550.00
tca	S	0	5729.00
tcg	S	0	1172.00
tat	Y	0	78.46
tac	Y	151	72.54
tgt	C	0	0.90
tgc	C	2	1.10
agt	S	0	4139.00
agc	S	0	4373.00
---	--	------	------
mPD		0.025	1.7
		nPD:	0.02
		N. weight:	2.2
		Sc. PD:	-0.06
		Sc. rank:	7342.9

,

PA-X

Pos.	220	obs:	exp:
tta	L	0	9.89
ttg	L	0	16.94
ctt	L	0	17.74
ctc	L	90	12.71
cta	L	0	17.33
ctg	L	0	15.39
cat	H	3	13050.00
cac	H	22188	9141.00
cgt	R	0	0.55
cgc	R	20	1.05
cga	R	0	1.96
cgg	R	0	1.46
aga	R	0	10.04
agg	R	0	4.94
---	--	------	------
mPD		0.010	0.50
		nPD:	0.02
		N. weight:	0.9
		Sc. PD:	-0.021
		Sc. rank:	4986.5

,

PA-X

Pos.	221	obs:	exp:
tta	L	0	0.55
ttg	L	0	0.94
ctt	L	0	0.99
ctc	L	0	0.71
cta	L	5	0.96
ctg	L	0	0.85
cct	P	0	0.83
ccc	P	0	0.56
cca	P	3	1.28
ccg	P	0	0.34
cat	H	0	4.12
cac	H	7	2.88
caa	Q	19431	10254.00
cag	Q	1	9178.00
cgt	R	0	77.90
cgc	R	0	149.20
cga	R	2853	279.40
cgg	R	0	208.80
aaa	K	1	0.57
aag	K	0	0.43
aga	R	0	1432.00
agg	R	0	705.30
---	--	------	------
mPD		0.22	0.90
		nPD:	0.25
		N. weight:	0.86
		Sc. PD:	0.18
		Sc. rank:	14778.0

,

PA-X

Pos.	222	obs:	exp:
att	I	158	57.52
atc	I	0	39.44
ata	I	0	61.04
act	T	22136	5580.00
acc	T	0	4540.00
aca	T	0	10127.00
acg	T	0	1889.00
aat	N	3	1.77
aac	N	0	1.23
gct	A	4	1.06
gcc	A	0	0.83
gca	A	0	1.88
gcg	A	0	0.23
---	--	------	------
mPD		0.015	0.69
		nPD:	0.02
		N. weight:	1.4
		Sc. PD:	-0.031
		Sc. rank:	8127.9

,

PA-X

Pos.	223	obs:	exp:
ttt	F	105	46.35
ttc	F	3	61.65
tct	S	22165	4084.00
tcc	S	26	2571.00
tca	S	2	5776.00
tcg	S	0	1181.00
agt	S	0	4173.00
agc	S	0	4408.00
---	--	------	------
mPD		0.012	1.6
		nPD:	0.01
		N. weight:	1.7
		Sc. PD:	-0.061
		Sc. rank:	-12216.0

,

PA-X

Pos.	224	obs:	exp:
tta	L	0	3.08
ttg	L	0	5.27
tct	S	1	0.55
tcc	S	0	0.35
tca	S	2	0.78
tcg	S	0	0.16
ctt	L	4	5.52
ctc	L	0	3.95
cta	L	24	5.39
ctg	L	0	4.79
cct	P	2387	6133.00
ccc	P	0	4129.00
cca	P	19842	9479.00
ccg	P	32	2521.00
cat	H	3	1.76
cac	H	0	1.24
caa	Q	3	1.58
cag	Q	0	1.42
act	T	0	0.76
acc	T	0	0.62
aca	T	3	1.37
acg	T	0	0.26
agt	S	0	0.56
agc	S	0	0.60
---	--	------	------
mPD		0.20	0.70
		nPD:	0.28
		N. weight:	0.56
		Sc. PD:	0.13
		Sc. rank:	9637.6

,

PA-X

Pos.	225	obs:	exp:
tct	S	0	0.18
tcc	S	1	0.12
tca	S	0	0.26
tcg	S	0	0.05
att	I	0	1.46
atc	I	4	1.00
ata	I	0	1.55
act	T	0	29.49
acc	T	117	24.00
aca	T	0	53.52
acg	T	0	9.98
agt	S	0	0.19
agc	S	0	0.20
gtt	V	0	28.52
gtc	V	129	23.44
gta	V	0	34.69
gtg	V	0	42.35
gct	A	30	5837.00
gcc	A	21821	4564.00
gca	A	197	10355.00
gcg	A	0	1292.00
gat	D	0	0.53
gac	D	1	0.47
ggt	G	0	0.15
ggc	G	1	0.15
gga	G	0	0.44
ggg	G	0	0.26
---	--	------	------
mPD		0.043	0.69
		nPD:	0.06
		N. weight:	1.5
		Sc. PD:	0.029
		Sc. rank:	20200.0

,

PA-X

Pos.	226	obs:	exp:
tta	L	11	2451.00
ttg	L	22281	4197.00
tct	S	0	1.10
tcc	S	0	0.69
tca	S	0	1.56
tcg	S	6	0.32
tgg	W	3	3.00
ctt	L	0	4393.00
ctc	L	0	3149.00
cta	L	0	4292.00
ctg	L	0	3811.00
agt	S	0	1.13
agc	S	0	1.19
---	--	------	------
mPD		0.0018	1.1
		nPD:	0.
		N. weight:	1.7
		Sc. PD:	-0.07
		Sc. rank:	-29927.0

,

PA-X

Pos.	227	obs:	exp:
cgt	R	0	0.57
cgc	R	0	1.10
cga	R	0	2.06
cgg	R	0	1.54
att	I	0	2.91
atc	I	0	2.00
ata	I	8	3.09
act	T	0	0.76
acc	T	0	0.62
aca	T	3	1.37
acg	T	0	0.26
aat	N	1	2.37
aac	N	3	1.63
aaa	K	22260	12698.00
aag	K	1	9563.00
aga	R	21	10.54
agg	R	0	5.19
gaa	E	4	2.42
gag	E	0	1.58
---	--	------	------
mPD		0.0037	0.49
		nPD:	0.01
		N. weight:	0.57
		Sc. PD:	-0.02
		Sc. rank:	-4292.6

,

PA-X

Pos.	228	obs:	exp:
cct	P	13	3.58
ccc	P	0	2.41
cca	P	0	5.54
ccg	P	0	1.47
att	I	175	63.70
atc	I	0	43.68
ata	I	0	67.61
act	T	22069	5565.00
acc	T	6	4528.00
aca	T	0	10099.00
acg	T	0	1884.00
aat	N	10	5.91
aac	N	0	4.09
gct	A	28	7.41
gcc	A	0	5.80
gca	A	0	13.15
gcg	A	0	1.64
---	--	------	------
mPD		0.021	0.70
		nPD:	0.03
		N. weight:	1.4
		Sc. PD:	-0.019
		Sc. rank:	11712.0

,

PA-X

Pos.	229	obs:	exp:
tta	L	3089	339.60
ttg	L	0	581.60
tct	S	0	3535.00
tcc	S	20	2225.00
tca	S	19191	5000.00
tcg	S	0	1022.00
ctt	L	0	608.70
ctc	L	0	436.30
cta	L	0	594.70
ctg	L	0	528.10
act	T	0	0.25
acc	T	0	0.21
aca	T	1	0.46
acg	T	0	0.09
agt	S	0	3612.00
agc	S	0	3816.00
---	--	------	------
mPD		0.24	1.9
		nPD:	0.13
		N. weight:	1.5
		Sc. PD:	0.13
		Sc. rank:	23486.0

,

PA-X

Pos.	230	obs:	exp:
aaa	K	0	1.71
aag	K	3	1.29
gat	D	5	2.63
gac	D	0	2.37
gaa	E	14	13466.00
gag	E	22225	8773.00
ggt	G	0	8.19
ggc	G	0	8.00
gga	G	0	24.02
ggg	G	54	13.79
---	--	------	------
mPD		0.0068	0.48
		nPD:	0.01
		N. weight:	0.93
		Sc. PD:	-0.027
		Sc. rank:	1286.8

,

PA-X

Pos.	231	obs:	exp:
tta	L	0	1.87
ttg	L	0	3.20
tct	S	15	2.76
tcc	S	0	1.74
tca	S	0	3.90
tcg	S	0	0.80
ctt	L	17	3.35
ctc	L	0	2.40
cta	L	0	3.27
ctg	L	0	2.91
cct	P	22240	6128.00
ccc	P	2	4125.00
cca	P	0	9471.00
ccg	P	0	2519.00
cat	H	26	15.29
cac	H	0	10.71
agt	S	0	2.82
agc	S	0	2.98
gct	A	1	0.26
gcc	A	0	0.21
gca	A	0	0.47
gcg	A	0	0.06
---	--	------	------
mPD		0.0055	0.70
		nPD:	0.01
		N. weight:	1.3
		Sc. PD:	-0.046
		Sc. rank:	-8489.9

,

PA-X

Pos.	232	obs:	exp:
tta	L	0	0.33
ttg	L	3	0.56
ctt	L	0	0.59
ctc	L	0	0.42
cta	L	0	0.58
ctg	L	0	0.51
att	I	0	29.85
atc	I	0	20.47
ata	I	82	31.68
atg	M	22160	22160.00
act	T	0	14.12
acc	T	0	11.49
aca	T	0	25.62
acg	T	56	4.78
---	--	------	------
mPD		0.013	0.017
		nPD:	0.73
		N. weight:	0.01
		Sc. PD:	0.0068
		Sc. rank:	189.7

,

PA-X

Pos.	233	obs:	exp:
taa	.	1	10129.00
tag	.	22300	6074.00
tga	.	0	6098.00
---	--	------	------
mPD		0.000090	0.79
		nPD:	0.
		N. weight:	1.3
		Sc. PD:	-0.057
		Sc. rank:	-24955.0

}

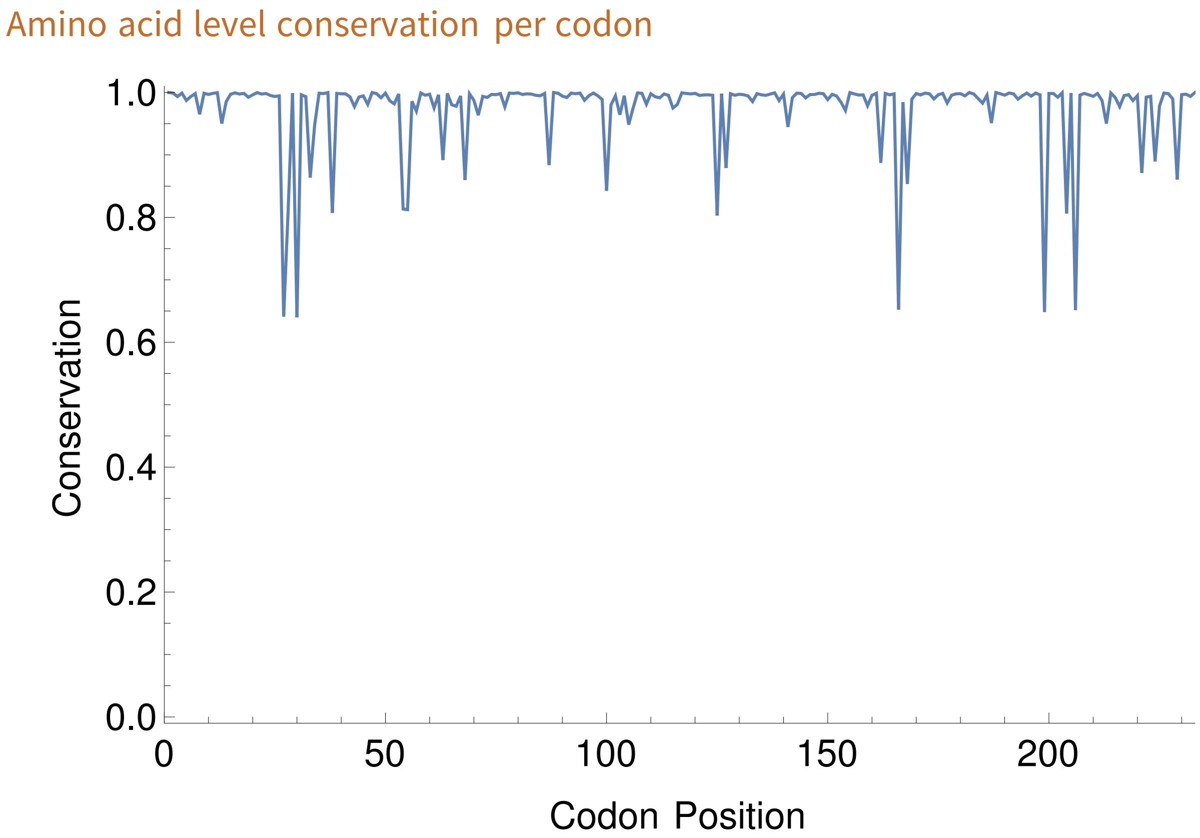
Amino acid level conservation per codon
1.0
0.8
0.6
0.4
0.2
0.0
Conservation
0
50
100
150
200
Codon Position

Conservation analysis plots

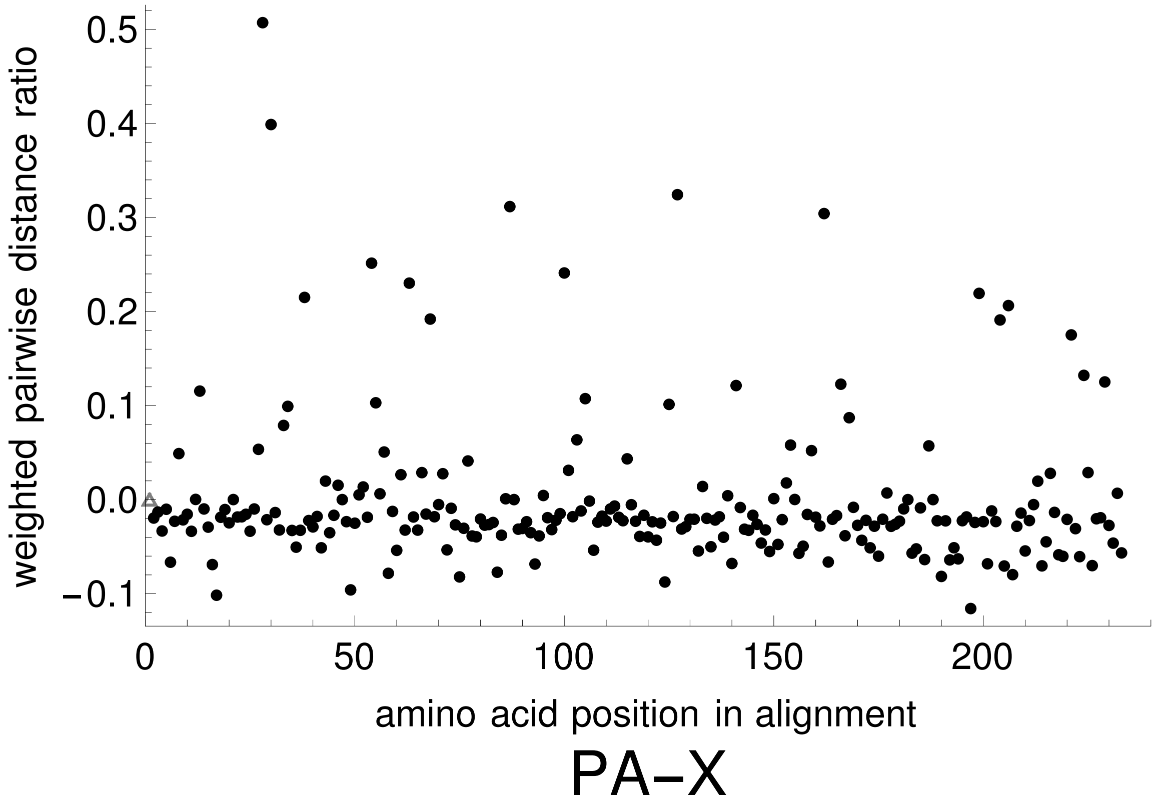

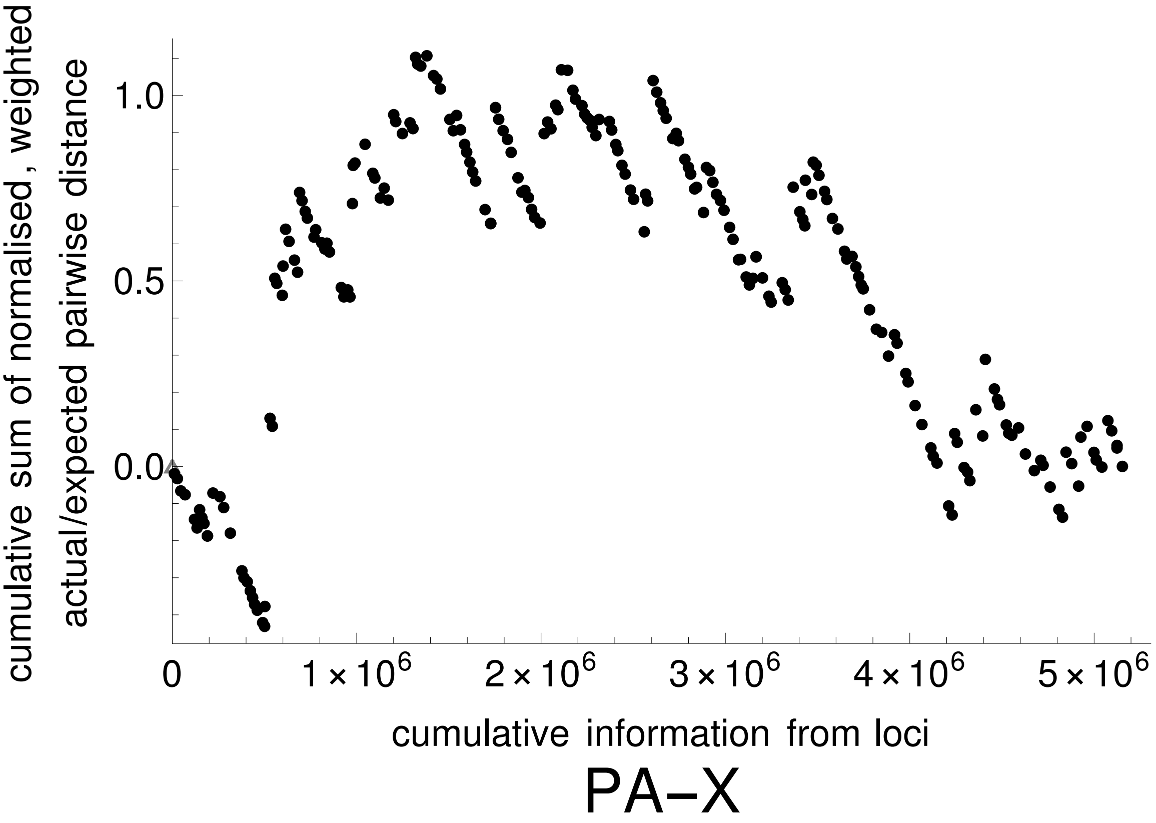

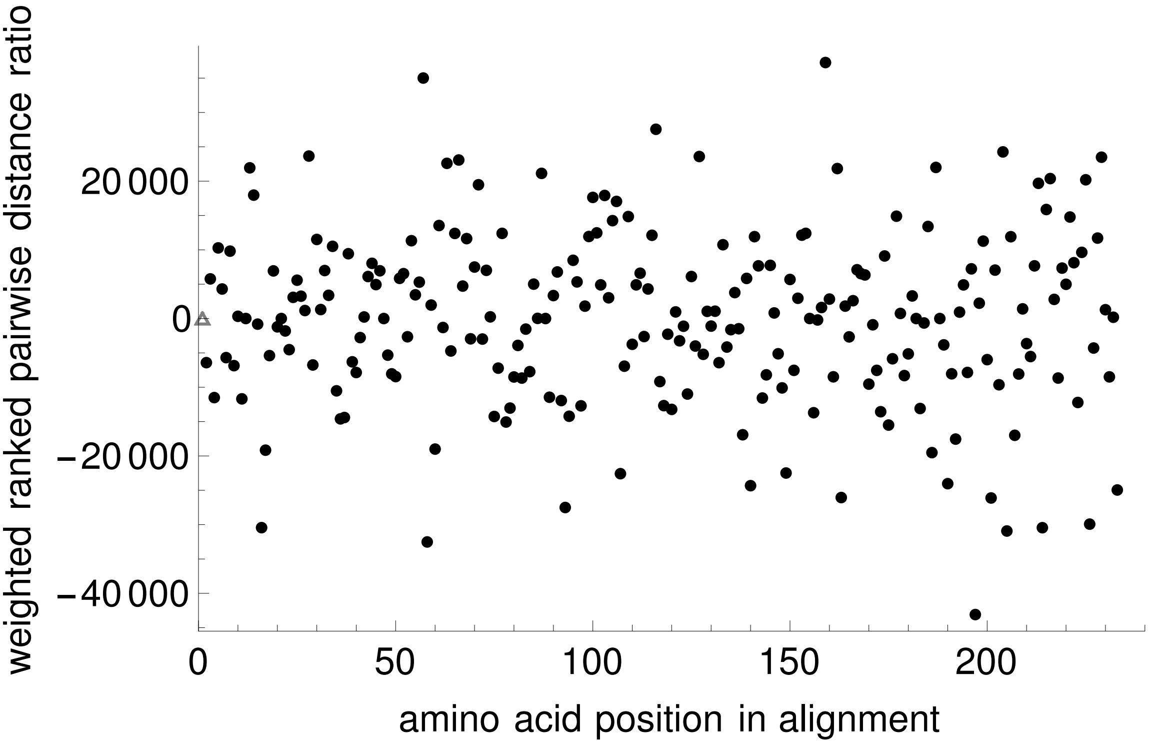
weighted ranked pairwise distance ratio
20 000
0
−20 000
−40 000
0
50
100
150
200
amino acid position in alignment

PA−X

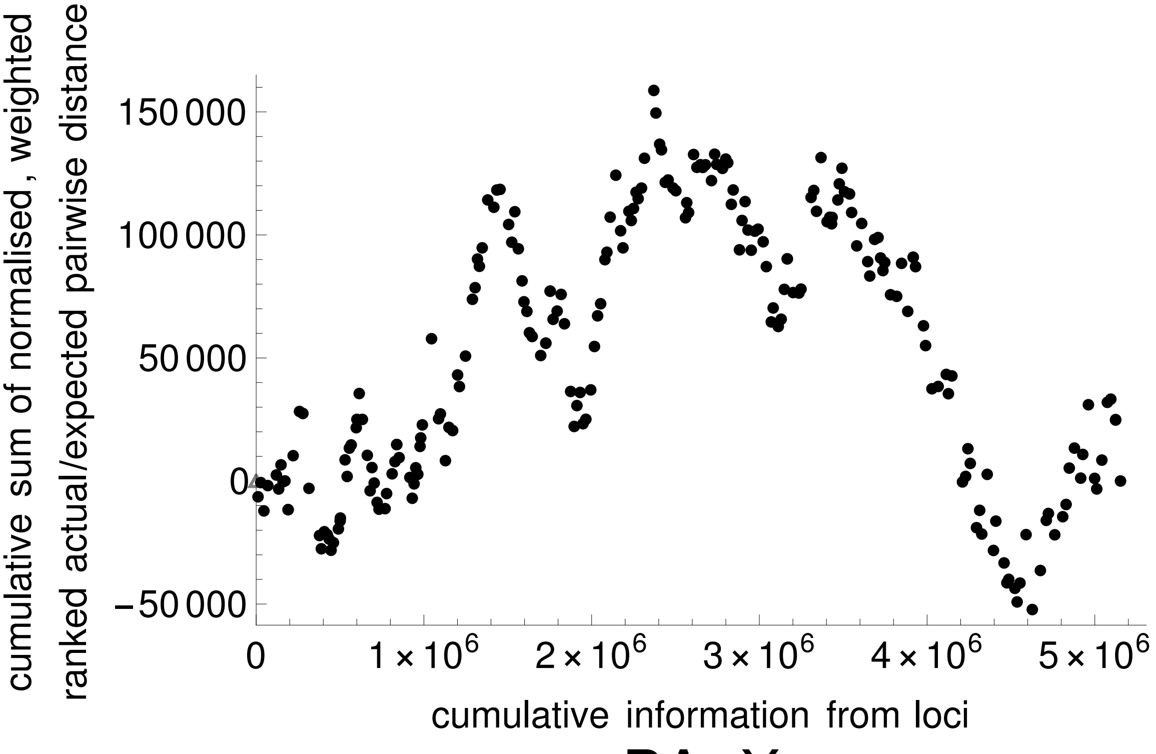
cumulative sum of normalised, weighted ranked actual/expected pairwise distance
150 000
100 000
50 000
0
−50 000
0
1 × 10^6
2 × 10^6
3 × 10^6
4 × 10^6
5 × 10^6
cumulative information from loci

PA−X

HA

Gene length histogram

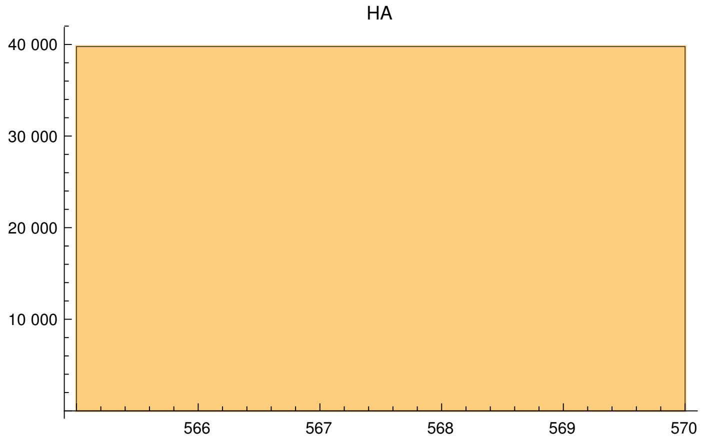

Information vs. nPD

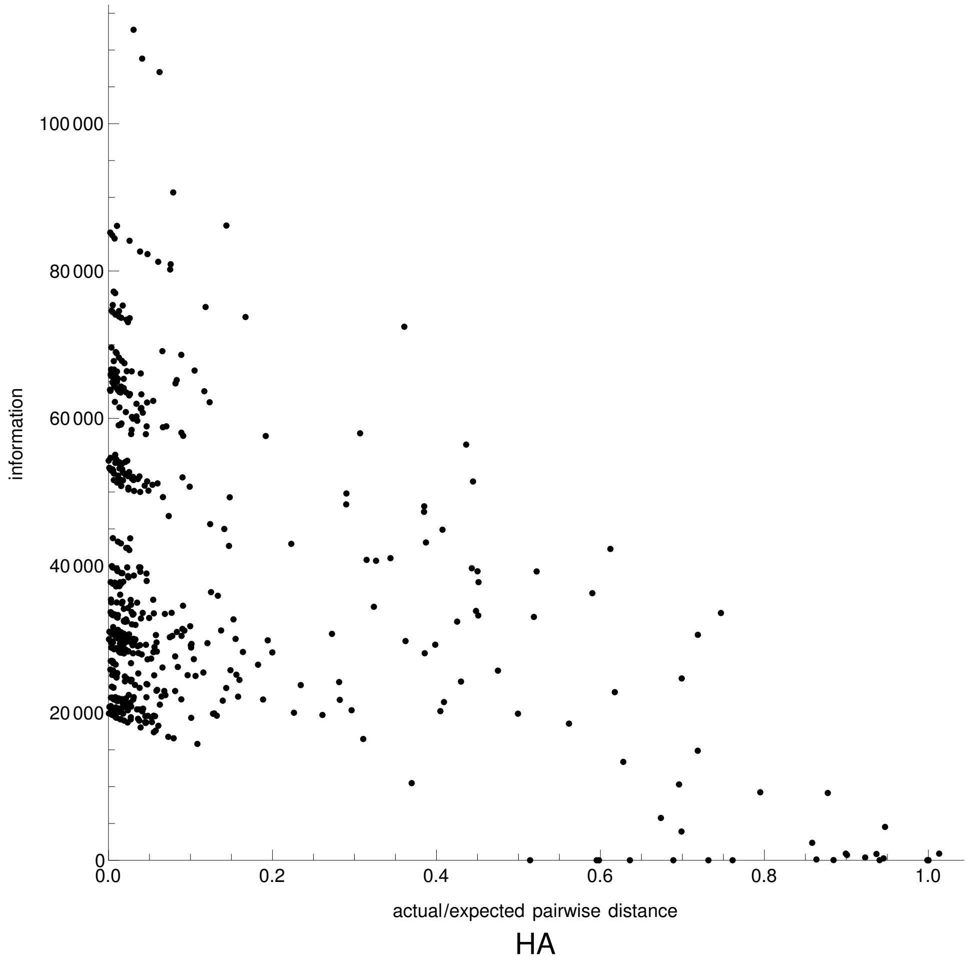

Example sequences highlighted by regions found in analyses to be conserved

Interesting points (by weighted raw PD) highlighted for gene HA:

ATGAAGGCAATACTAGTAGTTCTGCTGTATACATTTACAACCGCAAATGCAGACACATTATGTATAGGTTATCATG⋱
CGAACAATTCAACAGACACTGTAGACACAGTACTAGAAAAGAATGTAACAGTAACACACTCTGTCAATCTTCTGG⋱
AAGACAAGCATAACGGAAAACTATGCAAACTAAGAGGGGTAGCCCCATTGCATTTGGGTAAATGTAACATTGCTG⋱
GCTGGATCCTGGGAAATCCAGAGTGTGAATCACTCTCCACAGCAAGATCATGGTCCTACATTGTGGAAACATCTA⋱
ATTCAGACAATGGAACGTGTTACCCAGGAGATTTCATCAATTATGAGGAGCTAAGAGAGCAATTGAGCTCAGTGT⋱
CATCATTTGAAAGGTTTGAAATATTCCCCAAGACAAGTTCATGGCCTAATCATGACTCGGACAAAGGTGTAACGG⋱
CAGCATGTCCTCACGCTGGAGCAAAAAGCTTCTACAAAAACTTGATATGGCTGGTTAAAAAAGGAAATTCATACC⋱
CAAAGCTCAACCAAACCTACATTAATGATAAAGGGAAAGAAGTCCTCGTGCTGTGGGGCATTCACCATCCACCTA⋱
CTATTACTGCCCAAGAAAGTCTCTATCAGAATGCAGATGCATATGTTTTTGTGGGGACATCAAGATACAGCAAGA⋱
AGTTCAAGCCGGAAATAGCAACAAGACCCAAAGTGAGGGATCAAGAAGGGAGAATGAACTATTACTGGACACTAG⋱
TAGAACCGGGAGACAAAATAACATTCGAAGCAACTGGAAATCTAGTGGTACCGAGATATGCATTCACAATGGAAA⋱
GAGATGCTGGATCTGGTATTATCATTTCAGATACACCAGTCCACGATTGCAACACAACTTGTCAGACACCCGAGG⋱
GTGCTATAAAC ---------ACCAGCCTTCCATTTCAGAATGTACATCCGATTACAATTGGGAAATGTCCAAAGTAT⋱
GTAAAAAGCACAAAATTGAGACTGGCCACAGGATTGAGGAATGTCCCGTCTATTCAATCTAGAGGCCTATTCGGG⋱
GCCATTGCTGGCTTCATTGAAGGGGGGTGGACAGGGATGGTAGATGGATGGTACGGTTATCACCATCAAAATGAG⋱
CAGGGGTCAGGATATGCAGCCGATCTGAAGAGCACACAAAATGCCATTGATAAGATTACTAACAAAGTAAATTCT⋱
GTTATTGAAAAGATGAATACACAGTTCACAGCAGTGGGTAAAGAGTTCAACCACCTTGAAAAAAGAATAGAGAAT⋱
CTAAATAAAAAAGTTGATGATGGTTTCCTGGACATTTGGACTTACAATGCCGAACTGTTGGTTCTACTGGAAAAC⋱
GAAAGAACTTTGGACTATCACGATTCAAATGTGAAGAACTTGTATGAAAAAGTAAGAAACCAGTTAAAAAACAAT⋱
GCCAAGGAAATTGGAAACGGCTGCTTTGAATTTTACCACAAATGCGACAACACATGCATGGAAAGTGTCAAGAAT⋱
GGGACTTATGACTACCCAAAATACTCAGAGGAAGCAAAATTAAACAGAGAAAAAATAGATGGAGTAAAGCTGGAA⋱
TCAACAAGGATCTACCAGATTTTGGCGATCTATTCAACTGTTGCCAGTTCGTTGGTACTGGTAGTCTCCCTGGGG⋱
GCAATCAGCTTCTGGATGTGCTCTAAT ---GGG------TCT---CTACAGTGTAGAATATGTATTTAA

Interesting points (by weighted ranked PD) highlighted for gene HA:

ATGAAGGCAATACTAGTAGTTCTGCTGTATACATTTACAACCGCAAATGCAGACACATTATGTATAGGTTATCATGCG⋱
AACAATTCAACAGACACTGTAGACACAGTACTAGAAAAGAATGTAACAGTAACACACTCTGTCAATCTTCTGGAAGA⋱
CAAGCATAACGGAAAACTATGCAAACTAAGAGGGGTAGCCCCATTGCATTTGGGTAAATGTAACATTGCTGGCTGGA⋱
TCCTGGGAAATCCAGAGTGTGAATCACTCTCCACAGCAAGATCATGGTCCTACATTGTGGAAACATCTAATTCAGAC⋱
AATGGAACGTGTTACCCAGGAGATTTCATCAATTATGAGGAGCTAAGAGAGCAATTGAGCTCAGTGTCATCATTTGA⋱
AAGGTTTGAAATATTCCCCAAGACAAGTTCATGGCCTAATCATGACTCGGACAAAGGTGTAACGGCAGCATGTCCTC⋱
ACGCTGGAGCAAAAAGCTTCTACAAAAACTTGATATGGCTGGTTAAAAAAGGAAATTCATACCCAAAGCTCAACCAA⋱
ACCTACATTAATGATAAAGGGAAAGAAGTCCTCGTGCTGTGGGGCATTCACCATCCACCTACTATTACTGCCCAAGA⋱
AAGTCTCTATCAGAATGCAGATGCATATGTTTTTGTGGGGACATCAAGATACAGCAAGAAGTTCAAGCCGGAAATAG⋱
CAACAAGACCCAAAGTGAGGGATCAAGAAGGGAGAATGAACTATTACTGGACACTAGTAGAACCGGGAGACAAAATA⋱
ACATTCGAAGCAACTGGAAATCTAGTGGTACCGAGATATGCATTCACAATGGAAAGAGATGCTGGATCTGGTATTAT⋱
CATTTCAGATACACCAGTCCACGATTGCAACACAACTTGTCAGACACCCGAGGGTGCTATAAAC ---------ACCAG
CTTCCATTTCAGAATGTACATCCGATTACAATTGGGAAATGTCCAAAGTATGTAAAAAGCACAAAATTGAGACTG⋱
GCCACAGGATTGAGGAATGTCCCGTCTATTCAATCTAGAGGCCTATTCGGGGCCATTGCTGGCTTCATTGAAGGG⋱
GGGTGGACAGGGATGGTAGATGGATGGTACGGTTATCACCATCAAAATGAGCAGGGGTCAGGATATGCAGCCGAT⋱
CTGAAGAGCACACAAAATGCCATTGATAAGATTACTAACAAAGTAAATTCTGTTATTGAAAAGATGAATACACAG⋱
TTCACAGCAGTGGGTAAAGAGTTCAACCACCTTGAAAAAAGAATAGAGAATCTAAATAAAAAAGTTGATGATGGT⋱
TTCCTGGACATTTGGACTTACAATGCCGAACTGTTGGTTCTACTGGAAAACGAAAGAACTTTGGACTATCACGAT⋱

TCAAATGTGAAGAACTTGTATGAAAAAGTAAGAAACCAGTTAAAAAACAATGCCAAGGAAATTGGAAACGGCTGC∵.

TTTGAATTTTACCACAAATGCGACAACACATGCATGGAAAGTGTCAAGAATGGGACTTATGACTACCCAAAATAC∵.

TCAGAGGAAGCAAAATTAAACAGAGAAAAAATAGATGGAGTAAAGCTGGAATCAACAAGGATCTACCAGATTTTG∵.

GCGATCTATTCAACTGTTGCCAGTTCGTTGGTACTGGTA

GTCTCCCTGGGGGCAATCAGCTTCTGGATGTGCTCTAAT ---GGG------TCT---

CTACAGTGTAGAATATGTATTTAA

Per codon conservation report

{

HA

Pos.	1	obs:	exp:
atg	M	39770	39770.00
---	--	------	------
mPD		0	0
		nPD:	1.
		N. weight:	0.
		Sc. PD:	0
		Sc. rank:	0

,

HA

Pos.	2	obs:	exp:
caa	Q	0	0.53
cag	Q	1	0.47
cgt	R	0	0.05
cgc	R	0	0.10
cga	R	0	0.20
cgg	R	0	0.15
atg	M	1	1.00
act	T	0	0.25
acc	T	0	0.21
aca	T	0	0.46
acg	T	1	0.09
aat	N	14	8.28
aac	N	0	5.72
aaa	K	110	22522.00
aag	K	39373	16961.00
aga	R	0	1.00
agg	R	2	0.49
gaa	E	2	162.30
gag	E	266	105.70
---	--	------	------
mPD		0.020	0.50
		nPD:	0.04
		N. weight:	0.89
		Sc. PD:	-0.021
		Sc. rank:	10691.0

,

HA

Pos.	3	obs:	exp:
cct	P	0	0.28
ccc	P	0	0.19
cca	P	1	0.43
ccg	P	0	0.11
att	I	0	1.09
atc	I	0	0.75
ata	I	3	1.16
act	T	0	62.27
acc	T	0	50.66
aca	T	247	113.00
acg	T	0	21.07
gtt	V	0	192.30
gtc	V	0	158.10
gta	V	870	234.00
gtg	V	0	285.60
gct	A	6	10231.00
gcc	A	1	7999.00
gca	A	38634	18150.00
gcg	A	3	2264.00
gat	D	0	0.53
gac	D	1	0.47
gaa	E	3	1.82
gag	E	0	1.18
ggt	G	0	0.15
ggc	G	0	0.15
gga	G	1	0.44
ggg	G	0	0.26
---	--	------	------
mPD		0.056	0.72
		nPD:	0.08
		N. weight:	0.83
		Sc. PD:	0.011
		Sc. rank:	16522.0

,

HA

Pos.	4	obs:	exp:
tta	L	1	0.22
ttg	L	0	0.38
ctt	L	0	0.39
ctc	L	0	0.28
cta	L	1	0.39
ctg	L	0	0.34
cgt	R	0	0.03
cgc	R	0	0.05
cga	R	0	0.10
cgg	R	0	0.07
att	I	3	14410.00
atc	I	0	9881.00
ata	I	39583	15294.00
atg	M	19	19.00
act	T	0	35.80
acc	T	0	29.13
aca	T	142	64.96
acg	T	0	12.12
aaa	K	5	2.85
aag	K	0	2.15
aga	R	1	0.50
agg	R	0	0.25
gtt	V	0	3.32
gtc	V	0	2.73
gta	V	15	4.03
gtg	V	0	4.92
---	--	------	------
mPD		0.0094	0.66
		nPD:	0.01
		N. weight:	1.
		Sc. PD:	-0.05
		Sc. rank:	-7888.3

,

HA

Pos.	5	obs:	exp:
tta	L	40	4368.00
ttg	L	0	7481.00
ctt	L	14	7830.00
ctc	L	1	5613.00
cta	L	39658	7650.00
ctg	L	24	6794.00
cct	P	0	1.38
ccc	P	0	0.93
cca	P	5	2.13
ccg	P	0	0.57
caa	Q	2	1.06
cag	Q	0	0.94
cgt	R	0	0.03
cgc	R	0	0.05
cga	R	1	0.10
cgg	R	0	0.07
att	I	0	8.01
atc	I	0	5.49
ata	I	22	8.50
aga	R	0	0.50
agg	R	0	0.25
gtt	V	0	0.66
gtc	V	0	0.55
gta	V	3	0.81
gtg	V	0	0.98
---	--	------	------
mPD		0.0056	1.1
		nPD:	0.
		N. weight:	1.8
		Sc. PD:	-0.1
		Sc. rank:	-42400.0

,

HA

Pos.	6	obs:	exp:
tta	L	18	2.09
ttg	L	0	3.58
ctt	L	0	3.74
ctc	L	0	2.68
cta	L	1	3.66
ctg	L	0	3.25
att	I	0	53.88
atc	I	0	36.94
ata	I	148	57.18
gtt	V	4	8703.00
gtc	V	2	7153.00
gta	V	39335	10586.00
gtg	V	22	12921.00
gct	A	0	62.75
gcc	A	0	49.06
gca	A	237	111.30
gcg	A	0	13.88
ggt	G	0	0.45
ggc	G	0	0.44
gga	G	3	1.33
ggg	G	0	0.77
---	--	------	------
mPD		0.022	0.76
		nPD:	0.03
		N. weight:	1.4
		Sc. PD:	-0.048
		Sc. rank:	9507.2

,

HA

Pos.	7	obs:	exp:
ttt	F	11	4.72
ttc	F	0	6.28
att	I	76	27.67
atc	I	0	18.97
ata	I	0	29.36
gtt	V	39549	8769.00
gtc	V	94	7207.00
gta	V	10	10667.00
gtg	V	10	13020.00
gct	A	15	4.24
gcc	A	1	3.31
gca	A	0	7.51
gcg	A	0	0.94
gat	D	1	0.53
gac	D	0	0.47
ggt	G	3	0.45
ggc	G	0	0.44
gga	G	0	1.33
ggg	G	0	0.77
---	--	------	------
mPD		0.011	0.74
		nPD:	0.01
		N. weight:	1.6
		Sc. PD:	-0.077
		Sc. rank:	-10440.0

,

HA

Pos.	8	obs:	exp:
tta	L	0	4113.00
ttg	L	67	7044.00
ctt	L	15	7372.00
ctc	L	0	5284.00
cta	L	632	7203.00
ctg	L	36699	6397.00
att	I	0	1.82
atc	I	0	1.25
ata	I	5	1.93
atg	M	2351	2351.00
act	T	0	0.25
acc	T	0	0.21
aca	T	0	0.46
acg	T	1	0.09
---	--	------	------
mPD		0.15	1.2
		nPD:	0.12
		N. weight:	1.7
		Sc. PD:	0.099
		Sc. rank:	40543.0

,

HA

Pos.	9	obs:	exp:
tta	L	22	4367.00
ttg	L	22	7479.00
ctt	L	9	7828.00
ctc	L	60	5611.00
cta	L	19034	7648.00
ctg	L	20579	6792.00
cct	P	0	0.28
ccc	P	0	0.19
cca	P	0	0.43
ccg	P	1	0.11
cgt	R	0	0.03
cgc	R	0	0.05
cga	R	0	0.10
cgg	R	1	0.07
att	I	0	13.11
atc	I	0	8.99
ata	I	36	13.91
atg	M	5	5.00
aga	R	0	0.50
agg	R	0	0.25
gtt	V	0	0.22
gtc	V	0	0.18
gta	V	1	0.27
gtg	V	0	0.33
---	--	------	------
mPD		0.51	1.1
		nPD:	0.44
		N. weight:	1.1
		Sc. PD:	0.4
		Sc. rank:	32301.0

,

HA

Pos.	10	obs:	exp:
ttt	F	10	4.29
ttc	F	0	5.71
tat	Y	36296	20262.00
tac	Y	2701	18735.00
tgt	C	651	293.80
tgc	C	0	357.20
cat	H	63	60.57
cac	H	40	42.43
aat	N	0	5.32
aac	N	9	3.68
---	--	------	------
mPD		0.17	0.54
		nPD:	0.31
		N. weight:	0.45
		Sc. PD:	0.11
		Sc. rank:	12466.0

,

HA

Pos. 11		obs:	exp:
tct	S	0	0.18
tcc	S	0	0.12
tca	S	1	0.26
tcg	S	0	0.05
cct	P	0	0.83
ccc	P	0	0.56
cca	P	3	1.28
ccg	P	0	0.34
att	I	0	13.83
atc	I	0	9.49
ata	I	38	14.68
act	T	2	9981.00
acc	T	3	8121.00
aca	T	39568	18112.00
acg	T	19	3378.00
aaa	K	5	2.85
aag	K	0	2.15
agt	S	0	0.19
agc	S	0	0.20
gtt	V	0	1.55
gtc	V	0	1.27
gta	V	7	1.88
gtg	V	0	2.30
gct	A	0	32.83
gcc	A	0	25.67
gca	A	124	58.24
gcg	A	0	7.26
---	--	------	------
mPD		0.010	0.69
		nPD:	0.02
		N. weight:	0.84
		Sc. PD:	-0.04
		Sc. rank:	-5052.8

HA

Pos. 12		obs:	exp:
ttt	F	37882	16951.00
ttc	F	1619	22550.00
tta	L	12	21.22
ttg	L	0	36.34
tct	S	13	3.86
tcc	S	8	2.43
tca	S	0	5.47
tcg	S	0	1.12
tat	Y	11	5.72
tac	Y	0	5.28
tgt	C	11	4.96
tgc	C	0	6.04
ctt	L	180	38.03
ctc	L	1	27.26
cta	L	0	37.16
ctg	L	0	33.00
att	I	18	7.64
atc	I	3	5.24
ata	I	0	8.11
agt	S	0	3.95
agc	S	0	4.17
gtt	V	12	2.65
gtc	V	0	2.18
gta	V	0	3.23
gtg	V	0	3.94
---	--	------	------
mPD		0.092	0.50
		nPD:	0.18
		N. weight:	0.72
		Sc. PD:	0.084
		Sc. rank:	18979.0

HA

Pos. 13		obs:	exp:
tct	S	0	0.55
tcc	S	0	0.35
tca	S	3	0.78
tcg	S	0	0.16
cgt	R	0	0.03
cgc	R	0	0.05
cga	R	0	0.10
cgg	R	0	0.07
att	I	0	1.82
atc	I	0	1.25
ata	I	5	1.93
act	T	31	7010.00
acc	T	166	5704.00
aca	T	27547	12722.00
acg	T	65	2373.00
aaa	K	43	24.53
aag	K	0	18.47
agt	S	0	0.56
agc	S	0	0.60
aga	R	1	0.50
agg	R	0	0.25
gtt	V	0	3.98
gtc	V	0	3.27
gta	V	18	4.84
gtg	V	0	5.91
gct	A	3	3144.00
gcc	A	4	2458.00
gca	A	11865	5577.00
gcg	A	3	695.70
gaa	E	12	7.27
gag	E	0	4.73
ggt	G	0	0.61
ggc	G	0	0.59
gga	G	4	1.78
ggg	G	0	1.02
---	--	------	------
mPD		0.44	1.1
		nPD:	0.4
		N. weight:	0.8
		Sc. PD:	0.26
		Sc. rank:	23271.0

HA

Pos. 14		obs:	exp:
cct	P	0	2.48
ccc	P	9	1.67
cca	P	0	3.83
ccg	P	0	1.02
att	I	4	30.21
atc	I	79	20.72
ata	I	0	32.07
act	T	195	9944.00
acc	T	39218	8091.00
aca	T	31	18046.00
acg	T	2	3366.00
aat	N	0	7.10
aac	N	12	4.90
gct	A	0	58.25
gcc	A	220	45.54
gca	A	0	103.30
gcg	A	0	12.89
---	--	------	------
mPD		0.028	0.69
		nPD:	0.04
		N. weight:	1.7
		Sc. PD:	-0.039
		Sc. rank:	20306.0

HA

Pos. 15		obs:	exp:
tct	S	0	5.52
tcc	S	0	3.47
tca	S	30	7.81
tcg	S	0	1.60
cct	P	0	0.28
ccc	P	0	0.19
cca	P	1	0.43
ccg	P	0	0.11
act	T	0	66.30
acc	T	0	53.95
aca	T	263	120.30
acg	T	0	22.44
agt	S	0	5.64
agc	S	0	5.96
gtt	V	0	5.75
gtc	V	0	4.72
gta	V	26	6.99
gtg	V	0	8.53
gct	A	8	10445.00
gcc	A	20	8166.00
gca	A	39369	18528.00
gcg	A	53	2311.00
---	--	------	------
mPD		0.020	0.68
		nPD:	0.03
		N. weight:	0.8
		Sc. PD:	-0.027
		Sc. rank:	5571.2

HA

Pos. 16		obs:	exp:
tct	S	0	22.82
tcc	S	0	14.36
tca	S	0	32.27
tcg	S	0	6.60
tat	Y	4	2.08
tac	Y	0	1.92
cat	H	2	1.18
cac	H	0	0.82
act	T	35	9.08
acc	T	0	7.38
aca	T	1	16.47
acg	T	0	3.07
aat	N	39248	23393.00
aac	N	304	16159.00
aaa	K	14	9.13
aag	K	2	6.87
agt	S	124	23.32
agc	S	0	24.63
gat	D	35	18.44
gac	D	0	16.56
ggt	G	1	0.15
ggc	G	0	0.15
gga	G	0	0.44
ggg	G	0	0.26
---	--	------	------
mPD		0.026	0.50
		nPD:	0.05
		N. weight:	0.53
		Sc. PD:	-0.0059
		Sc. rank:	8205.9

HA

Pos. 17		obs:	exp:
tct	S	0	19.69
tcc	S	0	12.39
tca	S	107	27.85
tcg	S	0	5.69
cct	P	0	0.55
ccc	P	0	0.37
cca	P	2	0.85
ccg	P	0	0.23
act	T	0	8.07
acc	T	0	6.56
aca	T	32	14.64
acg	T	0	2.73
agt	S	0	20.12
agc	S	0	21.25
gct	A	35	10492.00
gcc	A	4	8203.00
gca	A	39573	18612.00
gcg	A	16	2322.00
ggt	G	0	0.15
ggc	G	0	0.15
gga	G	1	0.44
ggg	G	0	0.26
---	--	------	------
mPD		0.0099	0.67
		nPD:	0.01
		N. weight:	0.81
		Sc. PD:	-0.039
		Sc. rank:	-5545.1

HA

Pos. 18		obs:	exp:
tat	Y	0	1.04
tac	Y	2	0.96
cat	H	0	0.59
cac	H	1	0.41
aat	N	0	1.18
aac	N	2	0.82
gct	A	0	0.26
gcc	A	1	0.21
gca	A	0	0.47
gcg	A	0	0.06
gat	D	22	20950.00
gac	D	39739	18811.00
gaa	E	0	0.61
gag	E	1	0.39
ggt	G	0	0.30
ggc	G	2	0.30
gga	G	0	0.89
ggg	G	0	0.51
---	--	------	------
mPD		0.0016	0.50
		nPD:	0.
		N. weight:	0.8
		Sc. PD:	-0.048
		Sc. rank:	-22377.0

HA

Pos. 19		obs:	exp:
tct	S	0	0.18
tcc	S	0	0.12
tca	S	1	0.26
tcg	S	0	0.05
cgt	R	0	0.03
cgc	R	0	0.05
cga	R	0	0.10
cgg	R	0	0.07
att	I	0	12.74
atc	I	0	8.74
ata	I	35	13.52
act	T	215	10006.00
acc	T	5	8141.00
aca	T	39452	18158.00
acg	T	20	3387.00
aaa	K	41	23.39
aag	K	0	17.61
agt	S	0	0.19
agc	S	0	0.20
aga	R	1	0.50
agg	R	0	0.25
---	--	------	------
mPD		0.016	0.68
		nPD:	0.02
		N. weight:	0.81
		Sc. PD:	-0.032
		Sc. rank:	1471.8

HA

Pos. 20		obs:	exp:
tta	L	39534	4371.00
ttg	L	158	7486.00
ctt	L	1	7835.00
ctc	L	0	5616.00
cta	L	69	7655.00
ctg	L	0	6798.00
att	I	0	2.55
atc	I	0	1.75
ata	I	7	2.70
gtt	V	0	0.22
gtc	V	0	0.18
gta	V	1	0.27
gtg	V	0	0.33
---	--	------	------
mPD		0.012	1.1
		nPD:	0.01
		N. weight:	2.3
		Sc. PD:	-0.12
		Sc. rank:	-30895.0

HA

Pos. 21		obs:	exp:
tct	S	0	0.18
tcc	S	0	0.12
tca	S	0	0.26
tcg	S	0	0.05
tgt	C	39645	17948.00
tgc	C	120	21817.00
tgg	W	4	4.00
agt	S	1	0.19
agc	S	0	0.20
---	--	------	------
mPD		0.0063	0.50
		nPD:	0.01
		N. weight:	0.84
		Sc. PD:	-0.042
		Sc. rank:	-8523.9

HA

Pos. 22		obs:	exp:
att	I	1	14467.00
atc	I	5	9920.00
ata	I	39735	15354.00
atg	M	4	4.00
act	T	0	1.26
acc	T	0	1.03
aca	T	5	2.29
acg	T	0	0.43
gtt	V	0	4.42
gtc	V	0	3.63
gta	V	20	5.38
gtg	V	0	6.57
---	--	------	------
mPD		0.0018	0.66
		nPD:	0.
		N. weight:	1.
		Sc. PD:	-0.062
		Sc. rank:	-29380.0

HA

Pos. 23		obs:	exp:
tct	S	0	0.92
tcc	S	0	0.58
tca	S	0	1.30
tcg	S	0	0.27
agt	S	5	0.94
agc	S	0	0.99
ggt	G	39717	6030.00
ggc	G	28	5890.00
gga	G	13	17688.00
ggg	G	7	10157.00
---	--	------	------
mPD		0.0027	0.69
		nPD:	0.
		N. weight:	2.
		Sc. PD:	-0.12
		Sc. rank:	-52942.0

HA

Pos. 24		obs:	exp:
ttt	F	9	3.86
ttc	F	0	5.14
tct	S	1	0.18
tcc	S	0	0.12
tca	S	0	0.26
tcg	S	0	0.05
tat	Y	39488	20652.00
tac	Y	259	19095.00
cat	H	12	7.65
cac	H	1	5.35
agt	S	0	0.19
agc	S	0	0.20
---	--	------	------
mPD		0.014	0.50
		nPD:	0.03
		N. weight:	0.67
		Sc. PD:	-0.023
		Sc. rank:	3836.5

HA

Pos. 25		obs:	exp:
tat	Y	1	0.52
tac	Y	0	0.48
cat	H	39742	23385.00
cac	H	23	16380.00
caa	Q	1	0.53
cag	Q	0	0.47
cgt	R	3	0.08
cgc	R	0	0.16
cga	R	0	0.29
cgg	R	0	0.22
aga	R	0	1.51
agg	R	0	0.74
---	--	------	------
mPD		0.0014	0.48
		nPD:	0.
		N. weight:	0.57
		Sc. PD:	-0.034
		Sc. rank:	-16115.0

HA

Pos.	26	obs:	exp:
tct	S	0	0.18
tcc	S	0	0.12
tca	S	0	0.26
tcg	S	1	0.05
act	T	0	4.29
acc	T	0	3.49
aca	T	0	7.78
acg	T	17	1.45
agt	S	0	0.19
agc	S	0	0.20
gtt	V	0	0.44
gtc	V	0	0.36
gta	V	0	0.54
gtg	V	2	0.66
gct	A	29	10524.00
gcc	A	0	8228.00
gca	A	499	18669.00
gcg	A	39221	2329.00
ggt	G	0	0.15
ggc	G	0	0.15
gga	G	0	0.44
ggg	G	1	0.26
---	--	------	------
mPD		0.027	0.66
		nPD:	0.04
		N. weight:	3.
		Sc. PD:	-0.066
		Sc. rank:	37778.0

,

HA

Pos.	27	obs:	exp:
tct	S	0	0.37
tcc	S	0	0.23
tca	S	0	0.52
tcg	S	0	0.11
act	T	0	0.25
acc	T	1	0.21
aca	T	0	0.46
acg	T	0	0.09
aat	N	24	23519.00
aac	N	39741	16246.00
agt	S	0	0.38
agc	S	2	0.40
gat	D	0	1.05
gac	D	2	0.95
---	--	------	------
mPD		0.0015	0.48
		nPD:	0.
		N. weight:	0.96
		Sc. PD:	-0.057
		Sc. rank:	-26918.0

,

HA

Pos.	28	obs:	exp:
tct	S	0	0.18
tcc	S	0	0.12
tca	S	0	0.26
tcg	S	0	0.05
tat	Y	1	0.52
tac	Y	0	0.48
aat	N	39679	23517.00
aac	N	84	16246.00
agt	S	1	0.19
agc	S	0	0.20
gat	D	5	2.63
gac	D	0	2.37
---	--	------	------
mPD		0.0046	0.48
		nPD:	0.01
		N. weight:	0.55
		Sc. PD:	-0.029
		Sc. rank:	-8566.6

,

HA

Pos.	29	obs:	exp:
tta	L	1	0.11
ttg	L	0	0.19
tct	S	5	7318.00
tcc	S	9	4606.00
tca	S	39718	10349.00
tcg	S	33	2116.00
ctt	L	0	0.20
ctc	L	0	0.14
cta	L	0	0.19
ctg	L	0	0.17
cct	P	0	0.28
ccc	P	0	0.19
cca	P	1	0.43
ccg	P	0	0.11
act	T	0	0.25
acc	T	0	0.21
aca	T	1	0.46
acg	T	0	0.09
agt	S	0	7477.00
agc	S	0	7899.00
gct	A	0	0.53
gcc	A	0	0.41
gca	A	2	0.94
gcg	A	0	0.12
---	--	------	------
mPD		0.0026	1.6
		nPD:	0.
		N. weight:	1.4
		Sc. PD:	-0.088
		Sc. rank:	-44792.0

,

HA

Pos.	30	obs:	exp:
att	I	0	0.36
atc	I	0	0.25
ata	I	1	0.39
act	T	55	10025.00
acc	T	19	8157.00
aca	T	39624	18193.00
acg	T	70	3393.00
gct	A	0	0.26
gcc	A	0	0.21
gca	A	1	0.47
gcg	A	0	0.06
---	--	------	------
mPD		0.0073	0.68
		nPD:	0.01
		N. weight:	0.82
		Sc. PD:	-0.043
		Sc. rank:	-10180.0

,

HA

Pos.	31	obs:	exp:
tat	Y	0	2.08
tac	Y	4	1.92
aat	N	0	12.42
aac	N	21	8.58
gat	D	262	20899.00
gac	D	39403	18766.00
gaa	E	23	46.62
gag	E	54	30.38
ggt	G	0	0.45
ggc	G	3	0.44
gga	G	0	1.33
ggg	G	0	0.77
---	--	------	------
mPD		0.018	0.50
		nPD:	0.04
		N. weight:	0.76
		Sc. PD:	-0.02
		Sc. rank:	7837.1

,

HA

Pos.	32	obs:	exp:
tct	S	35	6.62
tcc	S	0	4.17
tca	S	0	9.37
tcg	S	0	1.92
att	I	26	9.46
atc	I	0	6.49
ata	I	0	10.05
atg	M	2	2.00
act	T	39645	10004.00
acc	T	28	8140.00
aca	T	11	18154.00
acg	T	0	3386.00
aat	N	7	4.14
aac	N	0	2.86
agt	S	1	6.77
agc	S	0	7.15
gct	A	15	3.97
gcc	A	0	3.10
gca	A	0	7.04
gcg	A	0	0.88
---	--	------	------
mPD		0.0064	0.68
		nPD:	0.01
		N. weight:	1.5
		Sc. PD:	-0.079
		Sc. rank:	-23049.0

,

HA

Pos.	33	obs:	exp:
tta	L	1	0.11
ttg	L	0	0.19
ctt	L	0	0.20
ctc	L	0	0.14
cta	L	0	0.19
ctg	L	0	0.17
att	I	0	6.55
atc	I	0	4.49
ata	I	18	6.95
gtt	V	22	8788.00
gtc	V	81	7223.00
gta	V	37487	10690.00
gtg	V	2160	13048.00
gaa	E	1	0.61
gag	E	0	0.39
---	--	------	------
mPD		0.11	0.74
		nPD:	0.15
		N. weight:	1.2
		Sc. PD:	0.095
		Sc. rank:	29325.0

,

HA

Pos.	34	obs:	exp:
tat	Y	0	0.52
tac	Y	1	0.48
aat	N	0	14.19
aac	N	24	9.81
gtt	V	0	0.22
gtc	V	1	0.18
gta	V	0	0.27
gtg	V	0	0.33
gct	A	0	0.26
gcc	A	1	0.21
gca	A	0	0.47
gcg	A	0	0.06
gat	D	1452	20939.00
gac	D	38289	18802.00
gaa	E	1	1.21
gag	E	1	0.79
---	--	------	------
mPD		0.072	0.50
		nPD:	0.14
		N. weight:	0.64
		Sc. PD:	0.05
		Sc. rank:	15821.0

,

HA

Pos.	35	obs:	exp:
tct	S	0	0.18
tcc	S	0	0.12
tca	S	1	0.26
tcg	S	0	0.05
act	T	8	10025.00
acc	T	16	8157.00
aca	T	39716	18193.00
acg	T	28	3393.00
agt	S	0	0.19
agc	S	0	0.20
gct	A	0	0.26
gcc	A	0	0.21
gca	A	1	0.47
gcg	A	0	0.06
---	--	------	------
mPD		0.0027	0.68
		nPD:	0.
		N. weight:	0.84
		Sc. PD:	-0.049
		Sc. rank:	-21609.0

,

HA

Pos.	36	obs:	exp:
tta	L	21	2.42
ttg	L	0	4.14
ctt	L	0	4.34
ctc	L	0	3.11
cta	L	1	4.24
ctg	L	0	3.76
att	I	0	70.26
atc	I	0	48.18
ata	I	193	74.57
gtt	V	2	8745.00
gtc	V	2	7188.00
gta	V	39189	10638.00
gtg	V	362	12984.00
---	--	------	------
mPD		0.029	0.75
		nPD:	0.04
		N. weight:	1.4
		Sc. PD:	-0.033
		Sc. rank:	15231.0

,

HA

Pos.	37	obs:	exp:
tta	L	153	4371.00
ttg	L	2	7486.00
ctt	L	6	7835.00
ctc	L	79	5616.00
cta	L	39468	7655.00
ctg	L	54	6798.00
att	I	0	2.91
atc	I	0	2.00
ata	I	8	3.09
---	--	------	------
mPD		0.015	1.1
		nPD:	0.01
		N. weight:	1.7
		Sc. PD:	-0.085
		Sc. rank:	-14389.0

,

HA

Pos.	38	obs:	exp:
aaa	K	10	5.70
aag	K	0	4.30
gaa	E	39394	24075.00
gag	E	366	15685.00
---	--	------	------
mPD		0.019	0.48
		nPD:	0.04
		N. weight:	0.49
		Sc. PD:	-0.012
		Sc. rank:	5636.0

,

HA

Pos.	39	obs:	exp:
cgt	R	0	1.99
cgc	R	0	3.82
cga	R	0	7.15
cgg	R	0	5.34
atg	M	1	1.00
act	T	0	0.25
acc	T	0	0.21
aca	T	0	0.46
acg	T	1	0.09
aaa	K	134	22643.00
aag	K	39560	17051.00
aga	R	1	36.65
agg	R	72	18.05
gaa	E	0	0.61
gag	E	1	0.39
---	--	------	------
mPD		0.011	0.49
		nPD:	0.02
		N. weight:	0.89
		Sc. PD:	-0.037
		Sc. rank:	262.4

,

HA

Pos.	40	obs:	exp:
tct	S	0	0.37
tcc	S	0	0.23
tca	S	0	0.52
tcg	S	0	0.11
aat	N	39698	23511.00
aac	N	55	16242.00
aaa	K	3	2.28
aag	K	1	1.72
agt	S	2	0.38
agc	S	0	0.40
gat	D	11	5.80
gac	D	0	5.20
---	--	------	------
mPD		0.0036	0.48
		nPD:	0.01
		N. weight:	0.56
		Sc. PD:	-0.031
		Sc. rank:	-10695.0

,

HA

Pos.	41	obs:	exp:
tta	L	2	0.44
ttg	L	1	0.75
ctt	L	0	0.79
ctc	L	0	0.56
cta	L	1	0.77
ctg	L	0	0.68
att	I	0	7.64
atc	I	0	5.24
ata	I	21	8.11
gtt	V	2	8786.00
gtc	V	17	7222.00
gta	V	39543	10688.00
gtg	V	180	13046.00
gct	A	0	0.53
gcc	A	0	0.41
gca	A	2	0.94
gcg	A	0	0.12
ggt	G	0	0.15
ggc	G	0	0.15
gga	G	1	0.44
ggg	G	0	0.26
---	--	------	------
mPD		0.011	0.74
		nPD:	0.02
		N. weight:	1.4
		Sc. PD:	-0.066
		Sc. rank:	-7700.9

,

HA

Pos.	42	obs:	exp:
cct	P	0	0.28
ccc	P	0	0.19
cca	P	1	0.43
ccg	P	0	0.11
att	I	0	2.18
atc	I	0	1.50
ata	I	6	2.32
act	T	14	10022.00
acc	T	23	8155.00
aca	T	39381	18188.00
acg	T	340	3392.00
aaa	K	1	0.57
aag	K	0	0.43
gct	A	0	1.06
gcc	A	0	0.83
gca	A	4	1.88
gcg	A	0	0.23
---	--	------	------
mPD		0.019	0.68
		nPD:	0.03
		N. weight:	0.8
		Sc. PD:	-0.028
		Sc. rank:	5275.9

,

HA

Pos.	43	obs:	exp:
att	I	0	5.46
atc	I	0	3.74
ata	I	15	5.80
gtt	V	4	8789.00
gtc	V	12	7223.00
gta	V	39541	10691.00
gtg	V	195	13049.00
gct	A	0	0.53
gcc	A	0	0.41
gca	A	2	0.94
gcg	A	0	0.12
ggt	G	0	0.15
ggc	G	0	0.15
gga	G	1	0.44
ggg	G	0	0.26
---	--	------	------
mPD		0.011	0.74
		nPD:	0.02
		N. weight:	1.4
		Sc. PD:	-0.065
		Sc. rank:	-7264.5

,

HA

Pos.	44	obs:	exp:
act	T	86	10025.00
acc	T	13	8157.00
aca	T	39626	18193.00
acg	T	44	3393.00
aaa	K	1	0.57
aag	K	0	0.43
---	--	------	------
mPD		0.0072	0.68
		nPD:	0.01
		N. weight:	0.82
		Sc. PD:	-0.043
		Sc. rank:	-10545.0

,

HA

Pos.	45	obs:	exp:
tat	Y	0	1.56
tac	Y	3	1.44
cct	P	0	0.28
ccc	P	1	0.19
cca	P	0	0.43
ccg	P	0	0.11
cat	H	168	23381.00
cac	H	39589	16376.00
caa	Q	5	3.17
cag	Q	1	2.83
cgt	R	0	0.08
cgc	R	3	0.16
cga	R	0	0.29
cgg	R	0	0.22
aga	R	0	1.51
agg	R	0	0.74
---	--	------	------
mPD		0.0091	0.49
		nPD:	0.02
		N. weight:	0.93
		Sc. PD:	-0.041
		Sc. rank:	-1938.4

,

HA

Pos.	46	obs:	exp:
ttt	F	1	0.43
ttc	F	0	0.57
tct	S	39667	7318.00
tcc	S	78	4606.00
tca	S	8	10349.00
tcg	S	11	2116.00
tat	Y	3	1.56
tac	Y	0	1.44
act	T	2	0.50
acc	T	0	0.41
aca	T	0	0.91
acg	T	0	0.17
agt	S	0	7477.00
agc	S	0	7899.00
---	--	------	------
mPD		0.0052	1.6
		nPD:	0.
		N. weight:	1.8
		Sc. PD:	-0.11
		Sc. rank:	-49675.0

,

HA

Pos.	47	obs:	exp:
ttt	F	3	1.29
ttc	F	0	1.71
att	I	21	12.74
atc	I	14	8.74
ata	I	0	13.52
gtt	V	29440	8770.00
gtc	V	10214	7208.00
gta	V	6	10668.00
gtg	V	6	13021.00
gct	A	49	17.47
gcc	A	17	13.66
gca	A	0	31.00
gcg	A	0	3.87
---	--	------	------
mPD		0.39	0.74
		nPD:	0.52
		N. weight:	1.1
		Sc. PD:	0.48
		Sc. rank:	32955.0

,

HA

Pos.	48	obs:	exp:
tct	S	0	0.74
tcc	S	0	0.46
tca	S	0	1.04
tcg	S	0	0.21
cat	H	0	0.59
cac	H	1	0.41
aat	N	27035	23470.00
aac	N	12648	16213.00
agt	S	3	0.75
agc	S	1	0.79
gat	D	15	43.21
gac	D	67	38.79
---	--	------	------
mPD		0.44	0.49
		nPD:	0.9
		N. weight:	0.019
		Sc. PD:	0.016
		Sc. rank:	618.9

,

HA

Pos.	49	obs:	exp:
ttt	F	4	1.72
ttc	F	0	2.28
tta	L	0	4332.00
ttg	L	0	7419.00
ctt	L	39276	7766.00
ctc	L	126	5566.00
cta	L	5	7587.00
ctg	L	1	6738.00
cct	P	2	0.55
ccc	P	0	0.37
cca	P	0	0.85
ccg	P	0	0.23
att	I	347	126.30
atc	I	0	86.62
ata	I	0	134.10
atg	M	1	1.00
gtt	V	1	1.77
gtc	V	0	1.45
gta	V	0	2.15
gtg	V	7	2.63
---	--	------	------
mPD		0.025	1.2
		nPD:	0.02
		N. weight:	1.7
		Sc. PD:	-0.071
		Sc. rank:	1061.2

,

HA

Pos.	50	obs:	exp:
tta	L	38	4371.00
ttg	L	31	7485.00
ctt	L	8	7834.00
ctc	L	2	5616.00
cta	L	12775	7654.00
ctg	L	26903	6797.00
atg	M	13	13.00
---	--	------	------
mPD		0.44	1.1
		nPD:	0.39
		N. weight:	1.2
		Sc. PD:	0.37
		Sc. rank:	34203.0

,

HA

Pos.	51	obs:	exp:
aaa	K	2	1.14
aag	K	0	0.86
gat	D	0	1.58
gac	D	3	1.42
gaa	E	39547	24077.00
gag	E	216	15686.00
ggt	G	0	0.30
ggc	G	0	0.30
gga	G	2	0.89
ggg	G	0	0.51
---	--	------	------
mPD		0.011	0.48
		nPD:	0.02
		N. weight:	0.51
		Sc. PD:	-0.02
		Sc. rank:	881.0

,

HA

Pos.	52	obs:	exp:
tct	S	0	0.18
tcc	S	0	0.12
tca	S	0	0.26
tcg	S	0	0.05
tat	Y	3	12.99
tac	Y	22	12.01
act	T	0	0.25
acc	T	1	0.21
aca	T	0	0.46
acg	T	0	0.09
aat	N	0	72.75
aac	N	123	50.25
agt	S	0	0.19
agc	S	1	0.20
gtt	V	0	0.88
gtc	V	4	0.73
gta	V	0	1.08
gtg	V	0	1.31
gct	A	0	0.26
gcc	A	1	0.21
gca	A	0	0.47
gcg	A	0	0.06
gat	D	99	20801.00
gac	D	39380	18678.00
gaa	E	22	13.32
gag	E	0	8.68
ggt	G	0	17.29
ggc	G	114	16.88
gga	G	0	50.71
ggg	G	0	29.12
---	--	------	------
mPD		0.020	0.51
		nPD:	0.04
		N. weight:	0.79
		Sc. PD:	-0.02
		Sc. rank:	8578.4

,

HA

Pos.	53	obs:	exp:
caa	Q	0	1.58
cag	Q	3	1.42
cgt	R	0	0.76
cgc	R	0	1.46
cga	R	0	2.74
cgg	R	0	2.05
att	I	1	0.36
atc	I	0	0.25
ata	I	0	0.39
atg	M	43	43.00
aat	N	40	23.66
aac	N	0	16.34
aaa	K	860	22600.00
aag	K	38759	17019.00
aga	R	1	14.06
agg	R	27	6.92
gaa	E	0	21.80
gag	E	36	14.20
---	--	------	------
mPD		0.050	0.50
		nPD:	0.1
		N. weight:	0.79
		Sc. PD:	0.028
		Sc. rank:	17907.0

,

HA

Pos.	54	obs:	exp:
tat	Y	5	2.60
tac	Y	0	2.40
cct	P	14	3.86
ccc	P	0	2.60
cca	P	0	5.96
ccg	P	0	1.59
cat	H	39251	23357.00
cac	H	465	16359.00
caa	Q	1	1.06
cag	Q	1	0.94
aat	N	33	19.52
aac	N	0	13.48
---	--	------	------
mPD		0.026	0.49
		nPD:	0.05
		N. weight:	0.51
		Sc. PD:	-0.0054
		Sc. rank:	7968.7

,

HA

Pos.	55	obs:	exp:
tct	S	0	2.58
tcc	S	0	1.62
tca	S	0	3.64
tcg	S	0	0.75
tat	Y	0	1.04
tac	Y	2	0.96
act	T	0	0.76
acc	T	3	0.62
aca	T	0	1.37
acg	T	0	0.26
aat	N	320	23408.00
aac	N	39258	16170.00
aaa	K	1	0.57
aag	K	0	0.43
agt	S	0	2.63
agc	S	14	2.78
gat	D	34	90.10
gac	D	137	80.90
gaa	E	1	0.61
gag	E	0	0.39
---	--	------	------
mPD		0.027	0.49
		nPD:	0.06
		N. weight:	0.91
		Sc. PD:	-0.0076
		Sc. rank:	14871.0

,

HA			
Pos.	56	obs:	exp:
tgg	W	3	3.00
cgt	R	0	1.64
cgc	R	0	3.14
cga	R	0	5.88
cgg	R	0	4.39
aga	R	41	30.12
agg	R	19	14.83
gtt	V	0	0.22
gtc	V	0	0.18
gta	V	0	0.27
gtg	V	1	0.33
gaa	E	31	36.94
gag	E	30	24.06
ggt	G	238	6011.00
ggc	G	1	5872.00
gga	G	27346	17635.00
ggg	G	12060	10127.00
---	--	------	------
mPD		0.44	0.70
		nPD:	0.63
		N. weight:	0.36
		Sc. PD:	0.2
		Sc. rank:	11375.0

,

HA			
Pos.	57	obs:	exp:
caa	Q	1	0.53
cag	Q	0	0.47
cgt	R	0	0.57
cgc	R	0	1.10
cga	R	0	2.06
cgg	R	0	1.54
act	T	0	0.25
acc	T	0	0.21
aca	T	1	0.46
acg	T	0	0.09
aat	N	0	2.96
aac	N	5	2.04
aaa	K	39673	22669.00
aag	K	67	17071.00
aga	R	21	10.54
agg	R	0	5.19
gaa	E	2	1.21
gag	E	0	0.79
---	--	------	------
mPD		0.0049	0.49
		nPD:	0.01
		N. weight:	0.59
		Sc. PD:	-0.031
		Sc. rank:	-8700.4

,

HA			
Pos.	58	obs:	exp:
tta	L	138	4371.00
ttg	L	0	7485.00
ctt	L	27	7835.00
ctc	L	8	5616.00
cta	L	39503	7654.00
ctg	L	83	6798.00
att	I	0	4.00
atc	I	0	2.75
ata	I	11	4.25
---	--	------	------
mPD		0.013	1.1
		nPD:	0.01
		N. weight:	1.7
		Sc. PD:	-0.089
		Sc. rank:	-18623.0

,

HA			
Pos.	59	obs:	exp:
tgt	C	61	17951.00
tgc	C	39709	21819.00
---	--	------	------
mPD		0.0031	0.50
		nPD:	0.01
		N. weight:	0.64
		Sc. PD:	-0.036
		Sc. rank:	-13772.0

,

HA			
Pos.	60	obs:	exp:
caa	Q	13	6.86
cag	Q	0	6.14
cgt	R	0	1.42
cgc	R	0	2.72
cga	R	0	5.09
cgg	R	0	3.81
att	I	0	0.73
atc	I	0	0.50
ata	I	2	0.77
act	T	0	5.04
acc	T	0	4.10
aca	T	20	9.15
acg	T	0	1.71
aat	N	1	4.14
aac	N	6	2.86
aaa	K	39609	22631.00
aag	K	64	17042.00
aga	R	52	26.11
agg	R	0	12.86
gaa	E	3	1.82
gag	E	0	1.18
---	--	------	------
mPD		0.0081	0.50
		nPD:	0.02
		N. weight:	0.59
		Sc. PD:	-0.028
		Sc. rank:	-2613.1

,

HA			
Pos.	61	obs:	exp:
tta	L	144	4370.00
ttg	L	0	7484.00
ctt	L	7	7834.00
ctc	L	0	5615.00
cta	L	39433	7653.00
ctg	L	170	6797.00
att	I	0	5.82
atc	I	0	3.99
ata	I	16	6.18
---	--	------	------
mPD		0.017	1.1
		nPD:	0.01
		N. weight:	1.7
		Sc. PD:	-0.083
		Sc. rank:	-11518.0

,

HA			
Pos.	62	obs:	exp:
tct	S	0	1.10
tcc	S	0	0.69
tca	S	0	1.56
tcg	S	0	0.32
caa	Q	9	4.75
cag	Q	0	4.25
cgt	R	0	1047.00
cgc	R	0	2006.00
cga	R	5	3757.00
cgg	R	0	2807.00
att	I	0	4.00
atc	I	0	2.75
ata	I	11	4.25
aaa	K	575	329.10
aag	K	2	247.90
agt	S	6	1.13
agc	S	0	1.19
aga	R	38148	19255.00
agg	R	201	9482.00
gaa	E	13	7.87
gag	E	0	5.13
ggt	G	0	121.30
ggc	G	0	118.50
gga	G	795	355.90
ggg	G	5	204.40
---	--	------	------
mPD		0.082	0.97
		nPD:	0.08
		N. weight:	0.71
		Sc. PD:	0.015
		Sc. rank:	14979.0

,

HA			
Pos.	63	obs:	exp:
gaa	E	0	5.45
gag	E	9	3.55
ggt	G	15	6029.00
ggc	G	0	5889.00
gga	G	912	17686.00
ggg	G	38834	10156.00
---	--	------	------
mPD		0.046	0.69
		nPD:	0.07
		N. weight:	1.3
		Sc. PD:	0.0034
		Sc. rank:	24895.0

,

HA			
Pos.	64	obs:	exp:
tta	L	133	15.17
ttg	L	0	25.98
ctt	L	0	27.19
ctc	L	0	19.49
cta	L	5	26.57
ctg	L	0	23.59
att	I	1	87.37
atc	I	0	59.91
ata	I	239	92.72
atg	M	3	3.00
gtt	V	60	8686.00
gtc	V	4	7139.00
gta	V	39102	10566.00
gtg	V	120	12896.00
gct	A	1	19.86
gcc	A	0	15.52
gca	A	74	35.22
gcg	A	0	4.39
gaa	E	20	12.11
gag	E	0	7.89
ggt	G	0	1.21
ggc	G	0	1.18
gga	G	8	3.56
ggg	G	0	2.04
---	--	------	------
mPD		0.034	0.76
		nPD:	0.04
		N. weight:	1.4
		Sc. PD:	-0.027
		Sc. rank:	18430.0

,

HA			
Pos.	65	obs:	exp:
tct	S	0	30.18
tcc	S	164	19.00
tca	S	0	42.68
tcg	S	0	8.73
cct	P	8	65.02
ccc	P	228	43.77
cca	P	0	100.50
ccg	P	0	26.72
act	T	0	17.90
acc	T	70	14.56
aca	T	1	32.48
acg	T	0	6.06
agt	S	0	30.84
agc	S	0	32.58
gtt	V	0	21.67
gtc	V	98	17.81
gta	V	0	26.36
gtg	V	0	32.17
gct	A	49	10377.00
gcc	A	39123	8113.00
gca	A	21	18408.00
gcg	A	0	2296.00
gat	D	0	3.69
gac	D	7	3.31
ggt	G	0	0.15
ggc	G	1	0.15
gga	G	0	0.44
ggg	G	0	0.26
---	--	------	------
mPD		0.033	0.70
		nPD:	0.05
		N. weight:	1.7
		Sc. PD:	-0.028
		Sc. rank:	24595.0

,

HA			
Pos.	66	obs:	exp:
tct	S	0	0.37
tcc	S	0	0.23
tca	S	2	0.52
tcg	S	0	0.11
cct	P	3	10956.00
ccc	P	32	7376.00
cca	P	39459	16933.00
ccg	P	273	4503.00
caa	Q	1	0.53
cag	Q	0	0.47
agt	S	0	0.38
agc	S	0	0.40
---	--	------	------
mPD		0.016	0.70
		nPD:	0.02
		N. weight:	0.88
		Sc. PD:	-0.036
		Sc. rank:	1161.7

,

HA			
Pos.	67	obs:	exp:
tta	L	158	4372.00
ttg	L	39535	7487.00
ctt	L	0	7837.00
ctc	L	0	5617.00
cta	L	0	7656.00
ctg	L	75	6799.00
atg	M	2	2.00
---	--	------	------
mPD		0.012	1.1
		nPD:	0.01
		N. weight:	1.8
		Sc. PD:	-0.093
		Sc. rank:	-24102.0

,

HA			
Pos.	68	obs:	exp:
tta	L	0	0.11
ttg	L	0	0.19
tat	Y	6	3.12
tac	Y	0	2.88
ctt	L	1	0.20
ctc	L	0	0.14
cta	L	0	0.19
ctg	L	0	0.17
cct	P	1	0.28
ccc	P	0	0.19
cca	P	0	0.43
ccg	P	0	0.11
cat	H	39607	23330.00
cac	H	64	16341.00
caa	Q	7	7.39
cag	Q	7	6.61
cgt	R	4	0.11
cgc	R	0	0.21
cga	R	0	0.39
cgg	R	0	0.29
aat	N	73	43.18
aac	N	0	29.82
aga	R	0	2.01
agg	R	0	0.99
---	--	------	------
mPD		0.0082	0.49
		nPD:	0.02
		N. weight:	0.56
		Sc. PD:	-0.026
		Sc. rank:	-2227.0

,

HA			
Pos.	69	obs:	exp:
tta	L	366	4372.00
ttg	L	39347	7487.00
ctt	L	0	7837.00
ctc	L	0	5617.00
cta	L	0	7656.00
ctg	L	56	6800.00
cct	P	0	0.28
ccc	P	0	0.19
cca	P	0	0.43
ccg	P	1	0.11
---	--	------	------
mPD		0.021	1.1
		nPD:	0.02
		N. weight:	1.7
		Sc. PD:	-0.077
		Sc. rank:	-4164.2

,

HA			
Pos.	70	obs:	exp:
tct	S	0	0.92
tcc	S	0	0.58
tca	S	0	1.30
tcg	S	0	0.27
agt	S	5	0.94
agc	S	0	0.99
gat	D	10	5.27
gac	D	0	4.73
ggt	G	39440	6028.00
ggc	G	290	5888.00
gga	G	19	17684.00
ggg	G	6	10155.00
---	--	------	------
mPD		0.016	0.69
		nPD:	0.02
		N. weight:	2.
		Sc. PD:	-0.078
		Sc. rank:	4637.8

,

HA			
Pos.	71	obs:	exp:
cat	H	2	1.18
cac	H	0	0.82
caa	Q	315	166.20
cag	Q	0	148.80
cgt	R	0	0.90
cgc	R	0	1.73
cga	R	0	3.23
cgg	R	0	2.42
att	I	0	1.82
atc	I	0	1.25
ata	I	5	1.93
act	T	0	0.50
acc	T	0	0.41
aca	T	2	0.91
acg	T	0	0.17
aat	N	4	32.53
aac	N	51	22.47
aaa	K	39298	22433.00
aag	K	28	16893.00
aga	R	33	16.57
agg	R	0	8.16
gaa	E	32	19.38
gag	E	0	12.62
---	--	------	------
mPD		0.024	0.51
		nPD:	0.05
		N. weight:	0.6
		Sc. PD:	-0.01
		Sc. rank:	8358.2

,

HA			
Pos.	72	obs:	exp:
tgt	C	38129	17950.00
tgc	C	1640	21819.00
cgt	R	1	0.03
cgc	R	0	0.05
cga	R	0	0.10
cgg	R	0	0.07
aga	R	0	0.50
agg	R	0	0.25
---	--	------	------
mPD		0.079	0.50
		nPD:	0.16
		N. weight:	0.67
		Sc. PD:	0.063
		Sc. rank:	17247.0

,

HA			
Pos.	73	obs:	exp:
tct	S	0	5.70
tcc	S	0	3.59
tca	S	0	8.07
tcg	S	0	1.65
act	T	0	1.01
acc	T	4	0.82
aca	T	0	1.83
acg	T	0	0.34
aat	N	131	23439.00
aac	N	39500	16192.00
aaa	K	1	0.57
aag	K	0	0.43
agt	S	0	5.83
agc	S	31	6.16
gat	D	0	54.27
gac	D	103	48.73
---	--	------	------
mPD		0.014	0.49
		nPD:	0.03
		N. weight:	0.94
		Sc. PD:	-0.034
		Sc. rank:	5041.8

,

HA			
Pos.	74	obs:	exp:
att	I	39564	14468.00
atc	I	135	9921.00
ata	I	46	15356.00
act	T	9	2.27
acc	T	0	1.85
aca	T	0	4.12
acg	T	0	0.77
gtt	V	16	3.54
gtc	V	0	2.91
gta	V	0	4.30
gtg	V	0	5.25
---	--	------	------
mPD		0.010	0.66
		nPD:	0.02
		N. weight:	1.1
		Sc. PD:	-0.05
		Sc. rank:	-5124.3

,

HA			
Pos.	75	obs:	exp:
gtt	V	1	0.22
gtc	V	0	0.18
gta	V	0	0.27
gtg	V	0	0.33
gct	A	39581	10529.00
gcc	A	49	8232.00
gca	A	25	18678.00
gcg	A	114	2330.00
---	--	------	------
mPD		0.0095	0.66
		nPD:	0.01
		N. weight:	1.4
		Sc. PD:	-0.068
		Sc. rank:	-9884.6

,

HA			
Pos.	76	obs:	exp:
tct	S	0	0.37
tcc	S	0	0.23
tca	S	0	0.52
tcg	S	0	0.11
agt	S	0	0.38
agc	S	2	0.40
ggt	G	52	6030.00
ggc	G	39592	5890.00
gga	G	119	17690.00
ggg	G	5	10158.00
---	--	------	------
mPD		0.0089	0.69
		nPD:	0.01
		N. weight:	2.
		Sc. PD:	-0.1
		Sc. rank:	-19124.0

,

HA			
Pos.	77	obs:	exp:
tta	L	0	0.22
ttg	L	2	0.38
tgg	W	39765	39765.00
ctt	L	0	0.39
ctc	L	0	0.28
cta	L	0	0.39
ctg	L	0	0.34
cgt	R	0	0.08
cgc	R	0	0.16
cga	R	0	0.29
cgg	R	0	0.22
aga	R	0	1.51
agg	R	3	0.74
---	--	------	------
mPD		0.00025	0.00049
		nPD:	0.51
		N. weight:	0.0002
		Sc. PD:	0.00009
		Sc. rank:	6.3

,

HA			
Pos.	78	obs:	exp:
ttt	F	0	0.43
ttc	F	1	0.57
tta	L	0	0.66
ttg	L	0	1.13
ctt	L	0	1.18
ctc	L	6	0.85
cta	L	0	1.16
ctg	L	0	1.03
att	I	158	14468.00
atc	I	39553	9921.00
ata	I	32	15355.00
act	T	0	0.76
acc	T	3	0.62
aca	T	0	1.37
acg	T	0	0.26
gtt	V	0	3.76
gtc	V	17	3.09
gta	V	0	4.57
gtg	V	0	5.58
---	--	------	------
mPD		0.011	0.66
		nPD:	0.02
		N. weight:	1.5
		Sc. PD:	-0.068
		Sc. rank:	-5892.0

,

HA			
Pos.	79	obs:	exp:
tta	L	2	4372.00
ttg	L	380	7487.00
ctt	L	22	7836.00
ctc	L	0	5617.00
cta	L	238	7656.00
ctg	L	39125	6799.00
cct	P	0	0.28
ccc	P	0	0.19
cca	P	0	0.43
ccg	P	1	0.11
atg	M	2	2.00
---	--	------	------
mPD		0.032	1.1
		nPD:	0.03
		N. weight:	1.8
		Sc. PD:	-0.063
		Sc. rank:	11074.0

,

HA			
Pos.	80	obs:	exp:
cgt	R	0	0.03
cgc	R	0	0.05
cga	R	0	0.10
cgg	R	0	0.07
aga	R	1	0.50
agg	R	0	0.25
gaa	E	1	0.61
gag	E	0	0.39
ggt	G	10	6030.00
ggc	G	2	5890.00
gga	G	39511	17690.00
ggg	G	245	10158.00
---	--	------	------
mPD		0.013	0.69
		nPD:	0.02
		N. weight:	0.84
		Sc. PD:	-0.037
		Sc. rank:	-1669.4

,

HA			
Pos.	81	obs:	exp:
tct	S	0	1.66
tcc	S	0	1.04
tca	S	0	2.34
tcg	S	0	0.48
tat	Y	1	0.52
tac	Y	0	0.48
act	T	2	0.50
acc	T	0	0.41
aca	T	0	0.91
acg	T	0	0.17
aat	N	39334	23506.00
aac	N	410	16238.00
agt	S	9	1.69
agc	S	0	1.79
gat	D	14	7.38
gac	D	0	6.62
---	--	------	------
mPD		0.022	0.48
		nPD:	0.04
		N. weight:	0.51
		Sc. PD:	-0.0095
		Sc. rank:	6831.8

,

HA			
Pos.	82	obs:	exp:
tta	L	0	0.22
ttg	L	0	0.38
tct	S	0	0.18
tcc	S	0	0.12
tca	S	1	0.26
tcg	S	0	0.05
ctt	L	0	0.39
ctc	L	0	0.28
cta	L	2	0.39
ctg	L	0	0.34
cct	P	10	10956.00
ccc	P	11	7376.00
cca	P	39720	16933.00
ccg	P	26	4503.00
agt	S	0	0.19
agc	S	0	0.20
---	--	------	------
mPD		0.0025	0.70
		nPD:	0.
		N. weight:	0.91
		Sc. PD:	-0.054
		Sc. rank:	-24602.0

,

HA			
Pos.	83	obs:	exp:
aaa	K	0	3.99
aag	K	7	3.01
gtt	V	0	0.22
gtc	V	0	0.18
gta	V	0	0.27
gtg	V	1	0.33
gct	A	0	0.53
gcc	A	0	0.41
gca	A	0	0.94
gcg	A	2	0.12
gat	D	14	7.90
gac	D	1	7.10
gaa	E	638	24064.00
gag	E	39104	15678.00
ggt	G	0	0.45
ggc	G	0	0.44
gga	G	0	1.33
ggg	G	3	0.77
---	--	------	------
mPD		0.033	0.48
		nPD:	0.07
		N. weight:	0.91
		Sc. PD:	0.0045
		Sc. rank:	17045.0

,

HA			
Pos.	84	obs:	exp:
tgt	C	39567	17950.00
tgc	C	201	21818.00
tgg	W	1	1.00
cgt	R	1	0.03
cgc	R	0	0.05
cga	R	0	0.10
cgg	R	0	0.07
aga	R	0	0.50
agg	R	0	0.25
---	--	------	------
mPD		0.010	0.50
		nPD:	0.02
		N. weight:	0.82
		Sc. PD:	-0.035
		Sc. rank:	-590.5

,

HA			
Pos.	85	obs:	exp:
aaa	K	3	1.71
aag	K	0	1.29
gtt	V	0	0.44
gtc	V	0	0.36
gta	V	2	0.54
gtg	V	0	0.66
gat	D	9	203.40
gac	D	377	182.60
gaa	E	39298	23829.00
gag	E	57	15526.00
ggt	G	0	3.64
ggc	G	0	3.55
gga	G	24	10.68
ggg	G	0	6.13
---	--	------	------
mPD		0.024	0.49
		nPD:	0.05
		N. weight:	0.53
		Sc. PD:	-0.0082
		Sc. rank:	8090.8

,

HA

Pos.	86	obs:	exp:
tta	L	45	4.95
ttg	L	0	8.47
tct	S	0	7242.00
tcc	S	4	4558.00
tca	S	39329	10242.00
tcg	S	20	2094.00
ctt	L	0	8.87
ctc	L	0	6.36
cta	L	0	8.66
ctg	L	0	7.69
cct	P	0	57.30
ccc	P	0	38.58
cca	P	208	88.57
ccg	P	0	23.55
act	T	0	40.84
acc	T	0	33.23
aca	T	161	74.11
acg	T	1	13.82
agt	S	0	7400.00
agc	S	0	7817.00
gct	A	0	0.53
gcc	A	0	0.41
gca	A	2	0.94
gcg	A	0	0.12
---	--	------	------
mPD		0.022	1.6
		nPD:	0.01
		N. weight:	1.4
		Sc. PD:	-0.071
		Sc. rank:	-11645.0

,

HA

Pos.	87	obs:	exp:
ttt	F	0	32.18
ttc	F	75	42.82
tta	L	2	4362.00
ttg	L	0	7470.00
ctt	L	48	7819.00
ctc	L	39587	5604.00
cta	L	40	7639.00
ctg	L	0	6784.00
cct	P	1	2.48
ccc	P	8	1.67
cca	P	0	3.83
ccg	P	0	1.02
att	I	0	2.55
atc	I	7	1.75
ata	I	0	2.70
gtt	V	0	0.44
gtc	V	2	0.36
gta	V	0	0.54
gtg	V	0	0.66
---	--	------	------
mPD		0.0093	1.1
		nPD:	0.01
		N. weight:	2.1
		Sc. PD:	-0.11
		Sc. rank:	-37988.0

,

HA

Pos.	88	obs:	exp:
ttt	F	0	11.59
ttc	F	27	15.41
tct	S	132	7304.00
tcc	S	39526	4597.00
tca	S	32	10330.00
tcg	S	0	2112.00
tat	Y	0	12.47
tac	Y	24	11.53
cct	P	0	3.86
ccc	P	14	2.60
cca	P	0	5.96
ccg	P	0	1.59
act	T	0	3.03
acc	T	12	2.46
aca	T	0	5.49
acg	T	0	1.02
agt	S	0	7463.00
agc	S	0	7884.00
gct	A	0	0.79
gcc	A	3	0.62
gca	A	0	1.41
gcg	A	0	0.18
---	--	------	------
mPD		0.012	1.6
		nPD:	0.01
		N. weight:	2.3
		Sc. PD:	-0.13
		Sc. rank:	-44400.0

,

HA

Pos.	89	obs:	exp:
tct	S	0	1.10
tcc	S	0	0.69
tca	S	6	1.56
tcg	S	0	0.32
cct	P	0	0.83
ccc	P	0	0.56
cca	P	3	1.28
ccg	P	0	0.34
cgt	R	0	0.03
cgc	R	0	0.05
cga	R	0	0.10
cgg	R	0	0.07
att	I	0	6.55
atc	I	0	4.49
ata	I	18	6.95
act	T	29	10007.00
acc	T	105	8142.00
aca	T	39284	18159.00
acg	T	277	3387.00
aaa	K	12	6.85
aag	K	0	5.15
agt	S	0	1.13
agc	S	0	1.19
aga	R	1	0.50
agg	R	0	0.25
gct	A	0	9.27
gcc	A	0	7.24
gca	A	35	16.44
gcg	A	0	2.05
---	--	------	------
mPD		0.024	0.68
		nPD:	0.04
		N. weight:	0.79
		Sc. PD:	-0.022
		Sc. rank:	7800.3

,

HA

Pos.	90	obs:	exp:
tct	S	0	13.07
tcc	S	0	8.22
tca	S	71	18.48
tcg	S	0	3.78
cct	P	0	0.28
ccc	P	0	0.19
cca	P	1	0.43
ccg	P	0	0.11
act	T	0	15.38
acc	T	0	12.51
aca	T	61	27.91
acg	T	0	5.20
agt	S	0	13.35
agc	S	0	14.10
gtt	V	0	9.73
gtc	V	0	8.00
gta	V	43	11.83
gtg	V	1	14.44
gct	A	6	10438.00
gcc	A	3	8161.00
gca	A	39385	18517.00
gcg	A	32	2310.00
gaa	E	167	101.10
gag	E	0	65.88
---	--	------	------
mPD		0.019	0.68
		nPD:	0.03
		N. weight:	0.81
		Sc. PD:	-0.028
		Sc. rank:	5045.5

,

HA

Pos.	91	obs:	exp:
tct	S	0	3371.00
tcc	S	0	2122.00
tca	S	0	4767.00
tcg	S	0	974.80
cat	H	1	0.59
cac	H	0	0.41
cgt	R	3	581.90
cgc	R	0	1115.00
cga	R	15	2087.00
cgg	R	0	1560.00
att	I	6	6.55
atc	I	7	4.49
ata	I	5	6.95
atg	M	1	1.00
act	T	0	0.25
acc	T	1	0.21
aca	T	0	0.46
acg	T	0	0.09
aat	N	16	20.11
aac	N	18	13.89
aaa	K	48	30.23
aag	K	5	22.77
agt	S	9886	3444.00
agc	S	8431	3638.00
aga	R	19973	10699.00
agg	R	1320	5268.00
ggt	G	6	5.16
ggc	G	7	5.04
gga	G	19	15.12
ggg	G	2	8.68
---	--	------	------
mPD		0.65	1.7
		nPD:	0.39
		N. weight:	0.76
		Sc. PD:	0.24
		Sc. rank:	22183.0

,

HA

Pos.	92	obs:	exp:
tta	L	2	0.22
ttg	L	0	0.38
tct	S	9	7317.00
tcc	S	14	4606.00
tca	S	39712	10348.00
tcg	S	27	2116.00
ctt	L	0	0.39
ctc	L	0	0.28
cta	L	0	0.39
ctg	L	0	0.34
cct	P	0	1.10
ccc	P	0	0.74
cca	P	4	1.70
ccg	P	0	0.45
act	T	0	0.25
acc	T	0	0.21
aca	T	1	0.46
acg	T	0	0.09
agt	S	0	7477.00
agc	S	0	7898.00
gct	A	0	0.26
gcc	A	0	0.21
gca	A	1	0.47
gcg	A	0	0.06
---	--	------	------
mPD		0.0029	1.6
		nPD:	0.
		N. weight:	1.4
		Sc. PD:	-0.088
		Sc. rank:	-44530.0

,

HA

Pos.	93	obs:	exp:
tgt	C	0	0.45
tgc	C	1	0.55
tgg	W	39769	39769.00
---	--	------	------
mPD		0.000050	0.000050
		nPD:	1.
		N. weight:	0.000016
		Sc. PD:	0.000015
		Sc. rank:	0.5

,

HA

Pos.	94	obs:	exp:
ttt	F	0	0.43
ttc	F	1	0.57
tct	S	71	7310.00
tcc	S	39620	4601.00
tca	S	34	10339.00
tcg	S	0	2114.00
cct	P	0	11.85
ccc	P	43	7.98
cca	P	0	18.31
ccg	P	0	4.87
act	T	0	0.25
acc	T	1	0.21
aca	T	0	0.46
acg	T	0	0.09
agt	S	0	7470.00
agc	S	0	7891.00
---	--	------	------
mPD		0.0075	1.6
		nPD:	0.
		N. weight:	2.3
		Sc. PD:	-0.13
		Sc. rank:	-57750.0

,

HA

Pos.	95	obs:	exp:
tat	Y	34	20664.00
tac	Y	39736	19106.00
---	--	------	------
mPD		0.0017	0.50
		nPD:	0.
		N. weight:	0.79
		Sc. PD:	-0.046
		Sc. rank:	-21308.0

HA

Pos.	96	obs:	exp:
tct	S	0	0.18
tcc	S	0	0.12
tca	S	0	0.26
tcg	S	0	0.05
att	I	39629	14474.00
atc	I	110	9925.00
ata	I	22	15362.00
act	T	3	0.76
acc	T	0	0.62
aca	T	0	1.37
acg	T	0	0.26
agt	S	1	0.19
agc	S	0	0.20
gtt	V	5	1.11
gtc	V	0	0.91
gta	V	0	1.34
gtg	V	0	1.64
---	--	------	------
mPD		0.0071	0.66
		nPD:	0.01
		N. weight:	1.1
		Sc. PD:	-0.056
		Sc. rank:	-13351.0

HA

Pos.	97	obs:	exp:
tta	L	0	0.11
ttg	L	1	0.19
ctt	L	0	0.20
ctc	L	0	0.14
cta	L	0	0.19
ctg	L	0	0.17
gtt	V	264	8791.00
gtc	V	0	7226.00
gta	V	106	10694.00
gtg	V	39394	13053.00
gct	A	0	1.32
gcc	A	0	1.03
gca	A	0	2.35
gcg	A	5	0.29
---	--	------	------
mPD		0.019	0.74
		nPD:	0.03
		N. weight:	1.1
		Sc. PD:	-0.043
		Sc. rank:	4394.4

HA

Pos.	98	obs:	exp:
aaa	K	1	0.57
aag	K	0	0.43
gaa	E	39678	24080.00
gag	E	90	15688.00
ggt	G	0	0.15
ggc	G	0	0.15
gga	G	1	0.44
ggg	G	0	0.26
---	--	------	------
mPD		0.0046	0.48
		nPD:	0.01
		N. weight:	0.53
		Sc. PD:	-0.028
		Sc. rank:	-8129.0

HA

Pos.	99	obs:	exp:
cct	P	0	1.10
ccc	P	0	0.74
cca	P	4	1.70
ccg	P	0	0.45
cgt	R	0	0.05
cgc	R	0	0.10
cga	R	0	0.20
cgg	R	0	0.15
att	I	0	2.18
atc	I	0	1.50
ata	I	6	2.32
atg	M	1	1.00
act	T	7	10012.00
acc	T	4	8147.00
aca	T	38314	18170.00
acg	T	1392	3389.00
aaa	K	13	7.99
aag	K	1	6.01
aga	R	2	1.00
agg	R	0	0.49
gct	A	0	6.88
gcc	A	0	5.38
gca	A	26	12.21
gcg	A	0	1.52
---	--	------	------
mPD		0.071	0.68
		nPD:	0.1
		N. weight:	0.74
		Sc. PD:	0.029
		Sc. rank:	16991.0

HA

Pos.	100	obs:	exp:
ttt	F	36	15.45
ttc	F	0	20.55
tta	L	0	0.11
ttg	L	0	0.19
tct	S	39431	7280.00
tcc	S	120	4582.00
tca	S	8	10296.00
tcg	S	2	2105.00
tat	Y	16	8.31
tac	Y	0	7.69
ctt	L	1	0.20
ctc	L	0	0.14
cta	L	0	0.19
ctg	L	0	0.17
cct	P	149	41.32
ccc	P	1	27.82
cca	P	0	63.87
ccg	P	0	16.98
act	T	5	1.26
acc	T	0	1.03
aca	T	0	2.29
acg	T	0	0.43
agt	S	0	7439.00
agc	S	0	7858.00
gct	A	1	0.26
gcc	A	0	0.21
gca	A	0	0.47
gcg	A	0	0.06
---	--	------	------
mPD		0.017	1.6
		nPD:	0.01
		N. weight:	1.8
		Sc. PD:	-0.094
		Sc. rank:	-24269.0

HA

Pos.	101	obs:	exp:
tct	S	0	2209.00
tcc	S	0	1390.00
tca	S	0	3124.00
tcg	S	0	638.80
cgt	R	0	0.03
cgc	R	0	0.05
cga	R	0	0.10
cgg	R	0	0.07
att	I	36	13.47
atc	I	1	9.24
ata	I	0	14.30
act	T	17	4.29
acc	T	0	3.49
aca	T	0	7.78
acg	T	0	1.45
aat	N	27484	16284.00
aac	N	48	11248.00
aaa	K	1	1.14
aag	K	1	0.86
agt	S	11984	2257.00
agc	S	19	2384.00
aga	R	0	0.50
agg	R	1	0.25
gat	D	21	11.06
gac	D	0	9.94
ggt	G	157	23.81
ggc	G	0	23.25
gga	G	0	69.84
ggg	G	0	40.10
---	--	------	------
mPD		0.44	1.4
		nPD:	0.32
		N. weight:	0.94
		Sc. PD:	0.24
		Sc. rank:	26262.0

HA

Pos.	102	obs:	exp:
tta	L	4	0.44
ttg	L	0	0.75
tct	S	13	7313.00
tcc	S	3	4603.00
tca	S	39683	10343.00
tcg	S	41	2115.00
ctt	L	0	0.79
ctc	L	0	0.56
cta	L	0	0.77
ctg	L	0	0.68
cct	P	0	5.51
ccc	P	0	3.71
cca	P	19	8.52
ccg	P	1	2.26
act	T	0	0.50
acc	T	0	0.41
aca	T	2	0.91
acg	T	0	0.17
agt	S	0	7472.00
agc	S	0	7894.00
gct	A	0	1.06
gcc	A	0	0.83
gca	A	4	1.88
gcg	A	0	0.23
---	--	------	------
mPD		0.0044	1.6
		nPD:	0.
		N. weight:	1.4
		Sc. PD:	-0.087
		Sc. rank:	-41099.0

HA

Pos.	103	obs:	exp:
tat	Y	0	2.08
tac	Y	4	1.92
aat	N	0	34.89
aac	N	59	24.11
gct	A	0	0.53
gcc	A	2	0.41
gca	A	0	0.94
gcg	A	0	0.12
gat	D	98	20849.00
gac	D	39471	18720.00
gaa	E	35	21.80
gag	E	1	14.20
ggt	G	0	15.16
ggc	G	100	14.81
gga	G	0	44.48
ggg	G	0	25.54
---	--	------	------
mPD		0.015	0.51
		nPD:	0.03
		N. weight:	0.79
		Sc. PD:	-0.027
		Sc. rank:	5425.9

HA

Pos.	104	obs:	exp:
tct	S	0	0.55
tcc	S	0	0.35
tca	S	0	0.78
tcg	S	0	0.16
tat	Y	1	0.52
tac	Y	0	0.48
aat	N	39732	23511.00
aac	N	21	16242.00
aaa	K	5	6.85
aag	K	7	5.15
agt	S	3	0.56
agc	S	0	0.60
gat	D	1	0.53
gac	D	0	0.47
---	--	------	------
mPD		0.0019	0.48
		nPD:	0.
		N. weight:	0.56
		Sc. PD:	-0.033
		Sc. rank:	-14623.0

HA

Pos.	105	obs:	exp:
ggt	G	7	6030.00
ggc	G	1	5890.00
gga	G	39345	17690.00
ggg	G	417	10159.00
---	--	------	------
mPD		0.021	0.69
		nPD:	0.03
		N. weight:	0.82
		Sc. PD:	-0.027
		Sc. rank:	6301.3

HA

Pos.	106	obs:	exp:
atg	M	11	11.00
act	T	73	10022.00
acc	T	1	8155.00
aca	T	1954	18188.00
acg	T	37730	3392.00
aaa	K	0	0.57
aag	K	1	0.43
---	--	------	------
mPD		0.098	0.68
		nPD:	0.14
		N. weight:	2.3
		Sc. PD:	0.18
		Sc. rank:	58902.0

HA			
Pos.	107	obs:	exp:
tgt	C	39640	17951.00
tgc	C	130	21819.00
---	--	------	------
mPD		0.0065	0.50
		nPD:	0.01
		N. weight:	0.84
		Sc. PD:	-0.041
		Sc. rank:	-7398.5

,

HA			
Pos.	108	obs:	exp:
ttt	F	0	0.43
ttc	F	1	0.57
tat	Y	142	20663.00
tac	Y	39627	19106.00
---	--	------	------
mPD		0.0072	0.50
		nPD:	0.01
		N. weight:	0.77
		Sc. PD:	-0.037
		Sc. rank:	-5331.3

,

HA			
Pos.	109	obs:	exp:
cct	P	71	10956.00
ccc	P	10	7376.00
cca	P	39633	16934.00
ccg	P	56	4503.00
---	--	------	------
mPD		0.0069	0.70
		nPD:	0.01
		N. weight:	0.9
		Sc. PD:	-0.047
		Sc. rank:	-13504.0

,

HA			
Pos.	110	obs:	exp:
ggt	G	15	6030.00
ggc	G	1	5890.00
gga	G	39557	17690.00
ggg	G	197	10159.00
---	--	------	------
mPD		0.011	0.69
		nPD:	0.02
		N. weight:	0.84
		Sc. PD:	-0.04
		Sc. rank:	-4816.1

,

HA			
Pos.	111	obs:	exp:
tat	Y	3	1.56
tac	Y	0	1.44
cat	H	1	0.59
cac	H	0	0.41
aat	N	165	97.59
aac	N	0	67.41
gtt	V	2	0.44
gtc	V	0	0.36
gta	V	0	0.54
gtg	V	0	0.66
gct	A	1	0.26
gcc	A	0	0.21
gca	A	0	0.47
gcg	A	0	0.06
gat	D	39298	20844.00
gac	D	261	18715.00
gaa	E	4	19.38
gag	E	28	12.62
ggt	G	7	1.06
ggc	G	0	1.04
gga	G	0	3.11
ggg	G	0	1.79
---	--	------	------
mPD		0.024	0.51
		nPD:	0.05
		N. weight:	0.65
		Sc. PD:	-0.011
		Sc. rank:	9159.1

,

HA			
Pos.	112	obs:	exp:
ttt	F	27	17066.00
ttc	F	39741	22702.00
tta	L	1	0.22
ttg	L	0	0.38
ctt	L	0	0.39
ctc	L	1	0.28
cta	L	0	0.39
ctg	L	0	0.34
---	--	------	------
mPD		0.0015	0.49
		nPD:	0.
		N. weight:	0.6
		Sc. PD:	-0.036
		Sc. rank:	-16860.0

,

HA			
Pos.	113	obs:	exp:
ttt	F	0	0.86
ttc	F	2	1.14
tta	L	0	0.44
ttg	L	0	0.75
tct	S	0	0.37
tcc	S	0	0.23
tca	S	0	0.52
tcg	S	0	0.11
tgt	C	0	0.45
tgc	C	1	0.55
ctt	L	0	0.79
ctc	L	4	0.56
cta	L	0	0.77
ctg	L	0	0.68
att	I	84	14433.00
atc	I	39500	9897.00
ata	I	65	15318.00
atg	M	1	1.00
act	T	0	7.56
acc	T	30	6.15
aca	T	0	13.72
acg	T	0	2.56
aat	N	0	5.32
aac	N	9	3.68
agt	S	0	0.38
agc	S	2	0.40
gtt	V	0	15.92
gtc	V	72	13.08
gta	V	0	19.36
gtg	V	0	23.63
---	--	------	------
mPD		0.014	0.66
		nPD:	0.02
		N. weight:	1.5
		Sc. PD:	-0.062
		Sc. rank:	-812.6

,

HA			
Pos.	114	obs:	exp:
tct	S	0	0.18
tcc	S	0	0.12
tca	S	0	0.26
tcg	S	0	0.05
tat	Y	1	0.52
tac	Y	0	0.48
cat	H	1	0.59
cac	H	0	0.41
caa	Q	0	0.53
cag	Q	1	0.47
aat	N	31937	19051.00
aac	N	274	13160.00
aaa	K	1	0.57
aag	K	0	0.43
agt	S	1	0.19
agc	S	0	0.20
gat	D	7547	3979.00
gac	D	5	3573.00
ggt	G	2	0.30
ggc	G	0	0.30
gga	G	0	0.89
ggg	G	0	0.51
---	--	------	------
mPD		0.32	0.80
		nPD:	0.4
		N. weight:	0.55
		Sc. PD:	0.18
		Sc. rank:	16135.0

,

HA			
Pos.	115	obs:	exp:
tct	S	1	0.18
tcc	S	0	0.12
tca	S	0	0.26
tcg	S	0	0.05
tat	Y	39747	20661.00
tac	Y	18	19104.00
tgt	C	1	0.45
tgc	C	0	0.55
cat	H	3	1.76
cac	H	0	1.24
agt	S	0	0.19
agc	S	0	0.20
---	--	------	------
mPD		0.0012	0.50
		nPD:	0.
		N. weight:	0.7
		Sc. PD:	-0.042
		Sc. rank:	-20929.0

,

HA			
Pos.	116	obs:	exp:
aaa	K	0	1.14
aag	K	2	0.86
gtt	V	0	0.44
gtc	V	0	0.36
gta	V	0	0.54
gtg	V	2	0.66
gat	D	1	0.53
gac	D	0	0.47
gaa	E	324	24075.00
gag	E	39436	15685.00
ggt	G	0	0.76
ggc	G	0	0.74
gga	G	1	2.22
ggg	G	4	1.28
---	--	------	------
mPD		0.017	0.48
		nPD:	0.03
		N. weight:	0.95
		Sc. PD:	-0.027
		Sc. rank:	9132.3

,

HA			
Pos.	117	obs:	exp:
gaa	E	1997	24080.00
gag	E	37771	15688.00
ggt	G	0	0.30
ggc	G	0	0.30
gga	G	0	0.89
ggg	G	2	0.51
---	--	------	------
mPD		0.095	0.48
		nPD:	0.2
		N. weight:	0.77
		Sc. PD:	0.1
		Sc. rank:	20491.0

,

HA			
Pos.	118	obs:	exp:
tta	L	48	4371.00
ttg	L	0	7486.00
ctt	L	2	7836.00
ctc	L	6	5616.00
cta	L	39516	7655.00
ctg	L	191	6799.00
caa	Q	1	0.53
cag	Q	0	0.47
att	I	0	0.73
atc	I	0	0.50
ata	I	2	0.77
gtt	V	0	0.88
gtc	V	0	0.73
gta	V	4	1.08
gtg	V	0	1.31
---	--	------	------
mPD		0.013	1.1
		nPD:	0.01
		N. weight:	1.7
		Sc. PD:	-0.09
		Sc. rank:	-20653.0

,

HA

Pos.	119	obs:	exp:
cgt	R	0	1086.00
cgc	R	0	2080.00
cga	R	11	3894.00
cgg	R	0	2910.00
att	I	0	0.36
atc	I	0	0.25
ata	I	1	0.39
aaa	K	11	6.27
aag	K	0	4.73
aga	R	38667	19959.00
agg	R	1079	9829.00
ggt	G	1	0.15
ggc	G	0	0.15
gga	G	0	0.44
ggg	G	0	0.26
---	--	------	------
mPD		0.054	0.91
		nPD:	0.06
		N. weight:	0.63
		Sc. PD:	-0.0027
		Sc. rank:	10725.0

,

HA

Pos.	120	obs:	exp:
gct	A	0	0.53
gcc	A	0	0.41
gca	A	0	0.94
gcg	A	2	0.12
gaa	E	132	24078.00
gag	E	39634	15688.00
ggt	G	0	0.30
ggc	G	0	0.30
gga	G	0	0.89
ggg	G	2	0.51
---	--	------	------
mPD		0.0068	0.48
		nPD:	0.01
		N. weight:	0.98
		Sc. PD:	-0.048
		Sc. rank:	-7051.7

,

HA

Pos.	121	obs:	exp:
tta	L	0	0.33
ttg	L	0	0.56
ctt	L	0	0.59
ctc	L	0	0.42
cta	L	3	0.58
ctg	L	0	0.51
cct	P	0	0.28
ccc	P	0	0.19
cca	P	1	0.43
ccg	P	0	0.11
cat	H	5	4.12
cac	H	2	2.88
caa	Q	38461	20975.00
cag	Q	1289	18775.00
cgt	R	0	0.22
cgc	R	0	0.42
cga	R	8	0.78
cgg	R	0	0.59
aaa	K	1	0.57
aag	K	0	0.43
aga	R	0	4.02
agg	R	0	1.98
---	--	------	------
mPD		0.064	0.50
		nPD:	0.13
		N. weight:	0.54
		Sc. PD:	0.034
		Sc. rank:	13139.0

,

HA

Pos.	122	obs:	exp:
tta	L	83	4371.00
ttg	L	37651	7486.00
ctt	L	0	7835.00
ctc	L	0	5616.00
cta	L	0	7655.00
ctg	L	2028	6798.00
atg	M	5	5.00
gtt	V	0	0.66
gtc	V	0	0.55
gta	V	0	0.81
gtg	V	3	0.98
---	--	------	------
mPD		0.10	1.1
		nPD:	0.09
		N. weight:	1.6
		Sc. PD:	0.039
		Sc. rank:	33835.0

,

HA

Pos.	123	obs:	exp:
tct	S	0	7315.00
tcc	S	0	4604.00
tca	S	0	10345.00
tcg	S	0	2116.00
att	I	0	0.73
atc	I	2	0.50
ata	I	0	0.77
act	T	0	0.50
acc	T	2	0.41
aca	T	0	0.91
acg	T	0	0.17
aat	N	0	8.28
aac	N	14	5.72
agt	S	684	7475.00
agc	S	39067	7896.00
ggt	G	0	0.15
ggc	G	1	0.15
gga	G	0	0.44
ggg	G	0	0.26
---	--	------	------
mPD		0.035	1.6
		nPD:	0.02
		N. weight:	1.7
		Sc. PD:	-0.069
		Sc. rank:	3.5

,

HA

Pos.	124	obs:	exp:
tta	L	2	0.22
ttg	L	0	0.38
tct	S	6	7318.00
tcc	S	5	4606.00
tca	S	39735	10350.00
tcg	S	22	2116.00
ctt	L	0	0.39
ctc	L	0	0.28
cta	L	0	0.39
ctg	L	0	0.34
agt	S	0	7478.00
agc	S	0	7899.00
---	--	------	------
mPD		0.0018	1.6
		nPD:	0.
		N. weight:	1.4
		Sc. PD:	-0.089
		Sc. rank:	-45558.0

,

HA

Pos.	125	obs:	exp:
tta	L	0	0.88
ttg	L	6	1.51
ctt	L	0	1.58
ctc	L	0	1.13
cta	L	0	1.54
ctg	L	2	1.37
atg	M	18	18.00
gtt	V	20	8786.00
gtc	V	10	7221.00
gta	V	288	10688.00
gtg	V	39423	13046.00
gct	A	0	0.79
gcc	A	0	0.62
gca	A	0	1.41
gcg	A	3	0.18
---	--	------	------
mPD		0.017	0.74
		nPD:	0.02
		N. weight:	1.2
		Sc. PD:	-0.046
		Sc. rank:	2247.4

,

HA

Pos.	126	obs:	exp:
tta	L	3	0.33
ttg	L	0	0.56
tct	S	11	7316.00
tcc	S	2	4605.00
tca	S	39551	10347.00
tcg	S	193	2116.00
ctt	L	0	0.59
ctc	L	0	0.42
cta	L	0	0.58
ctg	L	0	0.51
cct	P	0	0.83
ccc	P	0	0.56
cca	P	3	1.28
ccg	P	0	0.34
act	T	0	1.01
acc	T	0	0.82
aca	T	4	1.83
acg	T	0	0.34
agt	S	0	7476.00
agc	S	0	7897.00
gct	A	0	0.79
gcc	A	0	0.62
gca	A	3	1.41
gcg	A	0	0.18
---	--	------	------
mPD		0.011	1.6
		nPD:	0.01
		N. weight:	1.4
		Sc. PD:	-0.08
		Sc. rank:	-28992.0

,

HA

Pos.	127	obs:	exp:
tta	L	27	3.30
ttg	L	0	5.65
tct	S	16	7313.00
tcc	S	2	4603.00
tca	S	39262	10342.00
tcg	S	458	2115.00
ctt	L	0	5.91
ctc	L	0	4.24
cta	L	3	5.78
ctg	L	0	5.13
act	T	0	0.50
acc	T	0	0.41
aca	T	2	0.91
acg	T	0	0.17
agt	S	0	7472.00
agc	S	0	7893.00
---	--	------	------
mPD		0.025	1.6
		nPD:	0.02
		N. weight:	1.4
		Sc. PD:	-0.066
		Sc. rank:	-7596.1

,

HA

Pos.	128	obs:	exp:
ttt	F	39723	17060.00
ttc	F	31	22694.00
tta	L	1	1.54
ttg	L	2	2.64
ctt	L	11	2.76
ctc	L	0	1.98
cta	L	0	2.70
ctg	L	0	2.39
gtt	V	2	0.44
gtc	V	0	0.36
gta	V	0	0.54
gtg	V	0	0.66
---	--	------	------
mPD		0.0024	0.49
		nPD:	0.
		N. weight:	0.91
		Sc. PD:	-0.052
		Sc. rank:	-22145.0

,

HA

Pos.	129	obs:	exp:
aaa	K	49	28.52
aag	K	1	21.48
gtt	V	0	0.44
gtc	V	0	0.36
gta	V	2	0.54
gtg	V	0	0.66
gct	A	0	0.26
gcc	A	0	0.21
gca	A	1	0.47
gcg	A	0	0.06
gat	D	1	13.17
gac	D	24	11.83
gaa	E	38708	24029.00
gag	E	977	15656.00
ggt	G	0	1.06
ggc	G	0	1.04
gga	G	7	3.11
ggg	G	0	1.79
---	--	------	------
mPD		0.052	0.48
		nPD:	0.11
		N. weight:	0.43
		Sc. PD:	0.019
		Sc. rank:	9968.1

,

HA

Pos.	130	obs:	exp:
tct	S	0	1.29
tcc	S	0	0.81
tca	S	0	1.82
tcg	S	0	0.37
cgt	R	0	1079.00
cgc	R	0	2067.00
cga	R	0	3870.00
cgg	R	2	2892.00
atg	M	5	5.00
act	T	0	0.25
acc	T	0	0.21
aca	T	0	0.46
acg	T	1	0.09
aaa	K	28	135.80
aag	K	210	102.20
agt	S	4	1.32
agc	S	3	1.39
aga	R	2100	19837.00
agg	R	37412	9769.00
ggt	G	0	0.76
ggc	G	0	0.74
gga	G	0	2.22
ggg	G	5	1.28
---	--	------	------
mPD		0.11	0.92
		nPD:	0.12
		N. weight:	1.2
		Sc. PD:	0.073
		Sc. rank:	29927.0

,

HA			
Pos.	131	obs:	exp:
ttt	F	39733	17063.00
ttc	F	29	22699.00
tta	L	0	0.11
ttg	L	0	0.19
tat	Y	1	0.52
tac	Y	0	0.48
ctt	L	1	0.20
ctc	L	0	0.14
cta	L	0	0.19
ctg	L	0	0.17
att	I	2	0.73
atc	I	0	0.50
ata	I	0	0.77
gtt	V	4	0.88
gtc	V	0	0.73
gta	V	0	1.08
gtg	V	0	1.31
---	--	------	------
mPD		0.0019	0.49
		nPD:	0.
		N. weight:	0.91
		Sc. PD:	-0.053
		Sc. rank:	-23913.0

,

HA			
Pos.	132	obs:	exp:
aaa	K	1	0.57
aag	K	0	0.43
gaa	E	26721	24080.00
gag	E	13048	15689.00
---	--	------	------
mPD		0.44	0.48
		nPD:	0.92
		N. weight:	0.01
		Sc. PD:	0.0087
		Sc. rank:	329.6

,

HA			
Pos.	133	obs:	exp:
tta	L	3	0.33
ttg	L	0	0.56
ctt	L	0	0.59
ctc	L	0	0.42
cta	L	0	0.58
ctg	L	0	0.51
att	I	11	14411.00
atc	I	7	9882.00
ata	I	39570	15295.00
atg	M	159	159.00
gtt	V	0	4.42
gtc	V	0	3.63
gta	V	20	5.38
gtg	V	0	6.57
---	--	------	------
mPD		0.010	0.66
		nPD:	0.02
		N. weight:	1.
		Sc. PD:	-0.049
		Sc. rank:	-6316.1

,

HA			
Pos.	134	obs:	exp:
ttt	F	169	17065.00
ttc	F	39598	22702.00
tct	S	0	0.37
tcc	S	2	0.23
tca	S	0	0.52
tcg	S	0	0.11
agt	S	0	0.38
agc	S	0	0.40
gtt	V	0	0.22
gtc	V	1	0.18
gta	V	0	0.27
gtg	V	0	0.33
---	--	------	------
mPD		0.0086	0.49
		nPD:	0.02
		N. weight:	0.58
		Sc. PD:	-0.026
		Sc. rank:	-1770.4

,

HA			
Pos.	135	obs:	exp:
tct	S	0	3.68
tcc	S	20	2.32
tca	S	0	5.21
tcg	S	0	1.06
cct	P	176	10950.00
ccc	P	39002	7372.00
cca	P	570	16925.00
ccg	P	0	4501.00
cat	H	0	0.59
cac	H	1	0.41
act	T	0	0.25
acc	T	1	0.21
aca	T	0	0.46
acg	T	0	0.09
agt	S	0	3.76
agc	S	0	3.97
---	--	------	------
mPD		0.038	0.70
		nPD:	0.05
		N. weight:	1.7
		Sc. PD:	-0.015
		Sc. rank:	27249.0

,

HA			
Pos.	136	obs:	exp:
caa	Q	0	0.53
cag	Q	1	0.47
cgt	R	0	0.41
cgc	R	0	0.78
cga	R	0	1.47
cgg	R	0	1.10
atg	M	2	2.00
act	T	0	0.76
acc	T	0	0.62
aca	T	0	1.37
acg	T	3	0.26
aat	N	42	25.43
aac	N	1	17.57
aaa	K	942	22647.00
aag	K	38759	17054.00
aga	R	0	7.53
agg	R	15	3.71
gaa	E	0	3.03
gag	E	5	1.97
---	--	------	------
mPD		0.050	0.49
		nPD:	0.1
		N. weight:	0.79
		Sc. PD:	0.028
		Sc. rank:	17789.0

,

HA			
Pos.	137	obs:	exp:
tct	S	0	0.37
tcc	S	0	0.23
tca	S	2	0.52
tcg	S	0	0.11
cgt	R	0	0.08
cgc	R	0	0.16
cga	R	0	0.29
cgg	R	0	0.22
att	I	1	136.90
atc	I	0	93.86
ata	I	375	145.30
atg	M	2	2.00
act	T	18	9245.00
acc	T	1	7522.00
aca	T	36407	16777.00
acg	T	248	3129.00
aaa	K	9	5.13
aag	K	0	3.87
agt	S	0	0.38
agc	S	0	0.40
aga	R	3	1.51
agg	R	0	0.74
gtt	V	0	0.44
gtc	V	0	0.36
gta	V	2	0.54
gtg	V	0	0.66
gct	A	0	715.10
gcc	A	0	559.10
gca	A	2700	1269.00
gcg	A	1	158.20
gaa	E	1	0.61
gag	E	0	0.39
---	--	------	------
mPD		0.16	0.82
		nPD:	0.19
		N. weight:	0.81
		Sc. PD:	0.1
		Sc. rank:	21613.0

,

HA			
Pos.	138	obs:	exp:
tct	S	0	7218.00
tcc	S	0	4543.00
tca	S	0	10208.00
tcg	S	0	2087.00
cgt	R	0	0.30
cgc	R	0	0.58
cga	R	0	1.08
cgg	R	0	0.81
att	I	58	21.11
atc	I	0	14.48
ata	I	0	22.41
act	T	6	1.51
acc	T	0	1.23
aca	T	0	2.74
acg	T	0	0.51
aat	N	465	275.00
aac	N	0	190.00
agt	S	39150	7375.00
agc	S	72	7791.00
aga	R	1	5.52
agg	R	10	2.72
ggt	G	8	1.21
ggc	G	0	1.18
gga	G	0	3.56
ggg	G	0	2.04
---	--	------	------
mPD		0.031	1.7
		nPD:	0.02
		N. weight:	1.8
		Sc. PD:	-0.079
		Sc. rank:	-3894.3

,

HA			
Pos.	139	obs:	exp:
tct	S	10	7318.00
tcc	S	2	4606.00
tca	S	39696	10349.00
tcg	S	56	2116.00
cct	P	0	0.28
ccc	P	0	0.19
cca	P	1	0.43
ccg	P	0	0.11
act	T	0	0.76
acc	T	0	0.62
aca	T	3	1.37
acg	T	0	0.26
agt	S	0	7477.00
agc	S	0	7899.00
gct	A	0	0.53
gcc	A	0	0.41
gca	A	2	0.94
gcg	A	0	0.12
---	--	------	------
mPD		0.0037	1.6
		nPD:	0.
		N. weight:	1.4
		Sc. PD:	-0.087
		Sc. rank:	-43037.0

,

HA			
Pos.	140	obs:	exp:
tgg	W	39770	39770.00
---	--	------	------
mPD		0	0
		nPD:	1.
		N. weight:	0.
		Sc. PD:	0
		Sc. rank:	0

,

HA			
Pos.	141	obs:	exp:
tta	L	0	0.44
ttg	L	0	0.75
tct	S	2	0.55
tcc	S	1	0.35
tca	S	0	0.78
tcg	S	0	0.16
ctt	L	1	0.79
ctc	L	3	0.56
cta	L	0	0.77
ctg	L	0	0.68
cct	P	21098	10953.00
ccc	P	18583	7374.00
cca	P	76	16929.00
ccg	P	1	4502.00
cat	H	2	1.18
cac	H	0	0.82
act	T	1	0.76
acc	T	2	0.62
aca	T	0	1.37
acg	T	0	0.26
agt	S	0	0.56
agc	S	0	0.60
---	--	------	------
mPD		0.50	0.70
		nPD:	0.72
		N. weight:	0.83
		Sc. PD:	0.53
		Sc. rank:	26289.0

,

HA			
Pos.	142	obs:	exp:
tct	S	0	4.23
tcc	S	0	2.66
tca	S	0	5.99
tcg	S	0	1.22
tat	Y	1	0.52
tac	Y	0	0.48
cat	H	2	1.18
cac	H	0	0.82
att	I	1	0.36
atc	I	0	0.25
ata	I	0	0.39
act	T	1	0.25
acc	T	0	0.21
aca	T	0	0.46
acg	T	0	0.09
aat	N	39539	23434.00
aac	N	83	16188.00
aaa	K	5	2.85
aag	K	0	2.15
agt	S	23	4.32
agc	S	0	4.57
gat	D	114	60.59
gac	D	1	54.41
---	--	------	------
mPD		0.012	0.49
		nPD:	0.02
		N. weight:	0.55
		Sc. PD:	-0.022
		Sc. rank:	1113.4

,

HA

Pos.	143	obs:	exp:
tta	L	0	0.11
ttg	L	0	0.19
tat	Y	155	80.54
tac	Y	0	74.46
ctt	L	1	0.20
ctc	L	0	0.14
cta	L	0	0.19
ctg	L	0	0.17
cat	H	39568	23295.00
cac	H	44	16317.00
caa	Q	0	0.53
cag	Q	1	0.47
aat	N	1	0.59
aac	N	0	0.41
---	--	------	------
mPD		0.010	0.49
		nPD:	0.02
		N. weight:	0.57
		Sc. PD:	-0.024
		Sc. rank:	-277.2

,

HA

Pos.	144	obs:	exp:
aat	N	0	10.65
aac	N	18	7.35
gct	A	0	0.53
gcc	A	1	0.41
gca	A	1	0.94
gcg	A	0	0.12
gat	D	410	20895.00
gac	D	39246	18761.00
gaa	E	65	56.92
gag	E	29	37.08
---	--	------	------
mPD		0.026	0.50
		nPD:	0.05
		N. weight:	0.74
		Sc. PD:	-0.0086
		Sc. rank:	11543.0

,

HA

Pos.	145	obs:	exp:
tta	L	0	3.19
ttg	L	20	5.46
tct	S	69	7221.00
tcc	S	0	4545.00
tca	S	403	10212.00
tcg	S	38766	2088.00
ctt	L	0	5.71
ctc	L	0	4.10
cta	L	0	5.58
ctg	L	9	4.96
cct	P	0	123.40
ccc	P	0	83.09
cca	P	2	190.80
ccg	P	446	50.73
act	T	1	13.86
acc	T	0	11.28
aca	T	0	25.16
acg	T	54	4.69
agt	S	0	7378.00
agc	S	0	7794.00
---	--	------	------
mPD		0.051	1.7
		nPD:	0.03
		N. weight:	3.1
		Sc. PD:	-0.1
		Sc. rank:	25149.0

,

HA

Pos.	146	obs:	exp:
tct	S	0	3.31
tcc	S	0	2.08
tca	S	0	4.68
tcg	S	0	0.96
act	T	0	3.03
acc	T	12	2.46
aca	T	0	5.49
acg	T	0	1.02
aat	N	18	17172.00
aac	N	29016	11862.00
aaa	K	4	2.28
aag	K	0	1.72
agt	S	0	3.38
agc	S	18	3.58
gat	D	6	5634.00
gac	D	10686	5058.00
gaa	E	4	2.42
gag	E	0	1.58
ggt	G	0	0.91
ggc	G	6	0.89
gga	G	0	2.67
ggg	G	0	1.53
---	--	------	------
mPD		0.40	0.89
		nPD:	0.45
		N. weight:	0.92
		Sc. PD:	0.35
		Sc. rank:	27816.0

,

HA

Pos.	147	obs:	exp:
caa	Q	2	1.06
cag	Q	0	0.94
cgt	R	0	1.09
cgc	R	0	2.09
cga	R	0	3.92
cgg	R	0	2.93
att	I	2	0.73
atc	I	0	0.50
ata	I	0	0.77
aat	N	3402	2019.00
aac	N	12	1395.00
aaa	K	36261	20712.00
aag	K	49	15598.00
aga	R	40	20.08
agg	R	0	9.89
gaa	E	2	1.21
gag	E	0	0.79
---	--	------	------
mPD		0.16	0.57
		nPD:	0.28
		N. weight:	0.59
		Sc. PD:	0.13
		Sc. rank:	16140.0

,

HA

Pos.	148	obs:	exp:
tct	S	0	0.18
tcc	S	0	0.12
tca	S	0	0.26
tcg	S	0	0.05
tgt	C	0	0.45
tgc	C	1	0.55
agt	S	1	0.19
agc	S	0	0.20
ggt	G	39592	6030.00
ggc	G	157	5890.00
gga	G	8	17690.00
ggg	G	11	10158.00
---	--	------	------
mPD		0.0090	0.69
		nPD:	0.01
		N. weight:	2.
		Sc. PD:	-0.1
		Sc. rank:	-18312.0

,

HA

Pos.	149	obs:	exp:
tta	L	1	0.11
ttg	L	0	0.19
ctt	L	0	0.20
ctc	L	0	0.14
cta	L	0	0.19
ctg	L	0	0.17
att	I	1	12.01
atc	I	0	8.24
ata	I	32	12.75
gtt	V	14	8778.00
gtc	V	6	7214.00
gta	V	39411	10678.00
gtg	V	272	13033.00
gct	A	0	1.32
gcc	A	0	1.03
gca	A	5	2.35
gcg	A	0	0.29
gaa	E	28	16.95
gag	E	0	11.05
---	--	------	------
mPD		0.018	0.74
		nPD:	0.02
		N. weight:	1.4
		Sc. PD:	-0.053
		Sc. rank:	4221.2

,

HA

Pos.	150	obs:	exp:
tct	S	0	0.18
tcc	S	0	0.12
tca	S	0	0.26
tcg	S	1	0.05
cgt	R	0	0.05
cgc	R	0	0.10
cga	R	0	0.20
cgg	R	0	0.15
act	T	224	10025.00
acc	T	0	8157.00
aca	T	862	18192.00
acg	T	38681	3393.00
agt	S	0	0.19
agc	S	0	0.20
aga	R	0	1.00
agg	R	2	0.49
---	--	------	------
mPD		0.054	0.68
		nPD:	0.08
		N. weight:	2.5
		Sc. PD:	0.036
		Sc. rank:	49852.0

,

HA

Pos.	151	obs:	exp:
tct	S	0	0.37
tcc	S	0	0.23
tca	S	2	0.52
tcg	S	0	0.11
att	I	0	0.36
atc	I	0	0.25
ata	I	1	0.39
act	T	0	34.03
acc	T	0	27.69
aca	T	135	61.76
acg	T	0	11.52
agt	S	0	0.38
agc	S	0	0.40
gtt	V	0	2.21
gtc	V	0	1.82
gta	V	10	2.69
gtg	V	0	3.28
gct	A	86	10490.00
gcc	A	6	8202.00
gca	A	39519	18609.00
gcg	A	11	2321.00
---	--	------	------
mPD		0.013	0.67
		nPD:	0.02
		N. weight:	0.8
		Sc. PD:	-0.035
		Sc. rank:	-1444.8

,

HA

Pos.	152	obs:	exp:
tct	S	0	2.39
tcc	S	0	1.51
tca	S	13	3.38
tcg	S	0	0.69
cat	H	1	0.59
cac	H	0	0.41
agt	S	0	2.44
agc	S	0	2.58
gct	A	29	10526.00
gcc	A	3	8229.00
gca	A	39709	18672.00
gcg	A	15	2329.00
---	--	------	------
mPD		0.0032	0.66
		nPD:	0.
		N. weight:	0.81
		Sc. PD:	-0.047
		Sc. rank:	-19789.0

,

HA

Pos.	153	obs:	exp:
tgt	C	39456	17950.00
tgc	C	313	21819.00
gtt	V	0	0.22
gtc	V	1	0.18
gta	V	0	0.27
gtg	V	0	0.33
---	--	------	------
mPD		0.016	0.50
		nPD:	0.03
		N. weight:	0.81
		Sc. PD:	-0.025
		Sc. rank:	7052.3

,

HA

Pos.	154	obs:	exp:
tta	L	0	4.51
ttg	L	0	7.72
tct	S	1368	254.90
tcc	S	17	160.40
tca	S	0	360.50
tcg	S	0	73.71
ctt	L	41	8.08
ctc	L	0	5.79
cta	L	0	7.89
ctg	L	0	7.01
cct	P	38171	10560.00
ccc	P	140	7109.00
cca	P	16	16321.00
ccg	P	3	4340.00
cat	H	4	2.35
cac	H	0	1.65
act	T	9	2.27
acc	T	0	1.85
aca	T	0	4.12
acg	T	0	0.77
agt	S	0	260.40
agc	S	0	275.10
gct	A	1	0.26
gcc	A	0	0.21
gca	A	0	0.47
gcg	A	0	0.06
---	--	------	------
mPD		0.079	0.80
		nPD:	0.1
		N. weight:	1.4
		Sc. PD:	0.048
		Sc. rank:	30867.0

,

HA			
Pos.	155	obs:	exp:
tta	L	0	12.97
ttg	L	0	22.22
tat	Y	12	44.16
tac	Y	73	40.84
ctt	L	57	23.25
ctc	L	61	16.67
cta	L	0	22.72
ctg	L	0	20.18
cct	P	0	0.55
ccc	P	2	0.37
cca	P	0	0.85
ccg	P	0	0.23
cat	H	10244	23054.00
cac	H	28957	16147.00
caa	Q	212	121.40
cag	Q	18	108.60
cgt	R	102	3.28
cgc	R	18	6.28
cga	R	0	11.75
cgg	R	0	8.78
aat	N	6	8.28
aac	N	8	5.72
aga	R	0	60.24
agg	R	0	29.67
---	--	------	------
mPD		0.41	0.52
		nPD:	0.79
		N. weight:	0.25
		Sc. PD:	0.18
		Sc. rank:	7964.5

,

HA			
Pos.	156	obs:	exp:
tct	S	30	5.52
tcc	S	0	3.47
tca	S	0	7.81
tcg	S	0	1.60
act	T	41	10.34
acc	T	0	8.41
aca	T	0	18.76
acg	T	0	3.50
aat	N	2	1.18
aac	N	0	0.82
agt	S	0	5.64
agc	S	0	5.96
gtt	V	15	3.32
gtc	V	0	2.73
gta	V	0	4.03
gtg	V	0	4.92
gct	A	39491	10469.00
gcc	A	33	8185.00
gca	A	14	18571.00
gcg	A	4	2317.00
gat	D	140	73.77
gac	D	0	66.23
---	--	------	------
mPD		0.014	0.68
		nPD:	0.02
		N. weight:	1.4
		Sc. PD:	-0.06
		Sc. rank:	-349.6

,

HA			
Pos.	157	obs:	exp:
cgt	R	0	0.33
cgc	R	0	0.63
cga	R	0	1.18
cgg	R	0	0.88
aga	R	12	6.02
agg	R	0	2.97
gaa	E	16	9.69
gag	E	0	6.31
ggt	G	7	6026.00
ggc	G	0	5886.00
gga	G	38617	17678.00
ggg	G	1118	10152.00
---	--	------	------
mPD		0.056	0.69
		nPD:	0.08
		N. weight:	0.75
		Sc. PD:	0.013
		Sc. rank:	15374.0

,

HA			
Pos.	158	obs:	exp:
tct	S	0	8.28
tcc	S	0	5.21
tca	S	45	11.71
tcg	S	0	2.39
act	T	0	46.89
acc	T	0	38.15
aca	T	186	85.09
acg	T	0	15.87
agt	S	0	8.46
agc	S	0	8.94
gtt	V	0	5.97
gtc	V	0	4.91
gta	V	27	7.26
gtg	V	0	8.86
gct	A	8	10421.00
gcc	A	7	8148.00
gca	A	39336	18486.00
gcg	A	10	2306.00
gaa	E	151	91.43
gag	E	0	59.57
---	--	------	------
mPD		0.022	0.68
		nPD:	0.03
		N. weight:	0.81
		Sc. PD:	-0.026
		Sc. rank:	7006.1

,

HA			
Pos.	159	obs:	exp:
tct	S	0	0.37
tcc	S	0	0.23
tca	S	0	0.52
tcg	S	0	0.11
cgt	R	0	4.26
cgc	R	0	8.16
cga	R	0	15.28
cgg	R	0	11.42
aat	N	0	1.77
aac	N	3	1.23
aaa	K	39560	22594.00
aag	K	49	17015.00
agt	S	1	0.38
agc	S	1	0.40
aga	R	155	78.32
agg	R	1	38.57
---	--	------	------
mPD		0.011	0.50
		nPD:	0.02
		N. weight:	0.6
		Sc. PD:	-0.025
		Sc. rank:	117.3

,

HA			
Pos.	160	obs:	exp:
tct	S	0	7203.00
tcc	S	0	4534.00
tca	S	0	10187.00
tcg	S	0	2083.00
cgt	R	0	0.05
cgc	R	0	0.10
cga	R	0	0.20
cgg	R	0	0.15
aat	N	0	6.51
aac	N	11	4.49
agt	S	216	7360.00
agc	S	38925	7775.00
aga	R	2	1.00
agg	R	0	0.49
ggt	G	3	93.40
ggc	G	613	91.24
gga	G	0	274.00
ggg	G	0	157.30
---	--	------	------
mPD		0.042	1.7
		nPD:	0.03
		N. weight:	1.7
		Sc. PD:	-0.065
		Sc. rank:	6365.8

,

HA			
Pos.	161	obs:	exp:
ttt	F	63	17067.00
ttc	F	39707	22703.00
---	--	------	------
mPD		0.0032	0.49
		nPD:	0.01
		N. weight:	0.59
		Sc. PD:	-0.033
		Sc. rank:	-12496.0

,

HA			
Pos.	162	obs:	exp:
tct	S	0	0.18
tcc	S	1	0.12
tca	S	0	0.26
tcg	S	0	0.05
tat	Y	35	20663.00
tac	Y	39734	19106.00
agt	S	0	0.19
agc	S	0	0.20
---	--	------	------
mPD		0.0018	0.50
		nPD:	0.
		N. weight:	0.79
		Sc. PD:	-0.046
		Sc. rank:	-20908.0

,

HA			
Pos.	163	obs:	exp:
caa	Q	7	3.69
cag	Q	0	3.31
cgt	R	0	0.66
cgc	R	0	1.26
cga	R	0	2.35
cgg	R	0	1.76
att	I	0	0.36
atc	I	0	0.25
ata	I	1	0.39
act	T	0	1.76
acc	T	0	1.44
aca	T	7	3.20
acg	T	0	0.60
aat	N	1	11.24
aac	N	18	7.76
aaa	K	39649	22644.00
aag	K	47	17052.00
aga	R	24	12.05
agg	R	0	5.93
gaa	E	16	9.69
gag	E	0	6.31
---	--	------	------
mPD		0.0061	0.49
		nPD:	0.01
		N. weight:	0.6
		Sc. PD:	-0.03
		Sc. rank:	-6284.1

,

HA			
Pos.	164	obs:	exp:
tct	S	0	0.18
tcc	S	0	0.12
tca	S	0	0.26
tcg	S	0	0.05
act	T	2	2.52
acc	T	8	2.05
aca	T	0	4.57
acg	T	0	0.85
aat	N	14108	23514.00
aac	N	25650	16244.00
aaa	K	1	0.57
aag	K	0	0.43
agt	S	1	0.19
agc	S	0	0.20
---	--	------	------
mPD		0.46	0.48
		nPD:	0.95
		N. weight:	0.12
		Sc. PD:	0.11
		Sc. rank:	3913.5

,

HA			
Pos.	165	obs:	exp:
tta	L	12818	4372.00
ttg	L	26918	7487.00
ctt	L	0	7836.00
ctc	L	0	5617.00
cta	L	9	7656.00
ctg	L	22	6799.00
att	I	0	0.36
atc	I	0	0.25
ata	I	1	0.39
atg	M	1	1.00
gtt	V	0	0.22
gtc	V	0	0.18
gta	V	0	0.27
gtg	V	1	0.33
---	--	------	------
mPD		0.44	1.1
		nPD:	0.39
		N. weight:	1.3
		Sc. PD:	0.41
		Sc. rank:	37816.0

,

HA			
Pos.	166	obs:	exp:
tta	L	10	1.98
ttg	L	0	3.39
ctt	L	0	3.55
ctc	L	0	2.54
cta	L	8	3.47
ctg	L	0	3.08
att	I	80	14445.00
atc	I	7	9905.00
ata	I	39594	15331.00
atg	M	6	6.00
act	T	0	0.50
acc	T	0	0.41
aca	T	2	0.91
acg	T	0	0.17
gtt	V	0	13.93
gtc	V	0	11.45
gta	V	63	16.94
gtg	V	0	20.68
---	--	------	------
mPD		0.0088	0.66
		nPD:	0.01
		N. weight:	1.
		Sc. PD:	-0.05
		Sc. rank:	-8308.0

,

HA			
Pos.	167	obs:	exp:
tgt	C	0	0.90
tgc	C	2	1.10
tgg	W	39768	39768.00
---	--	------	------
mPD		0.00010	0.00010
		nPD:	1.
		N. weight:	0.000033
		Sc. PD:	0.00003
		Sc. rank:	1.0

,

HA			
Pos.	168	obs:	exp:
tta	L	29	4371.00
ttg	L	41	7487.00
ctt	L	447	7836.00
ctc	L	0	5617.00
cta	L	18943	7655.00
ctg	L	20305	6799.00
cct	P	0	0.28
ccc	P	0	0.19
cca	P	1	0.43
ccg	P	0	0.11
caa	Q	1	0.53
cag	Q	0	0.47
att	I	0	1.09
atc	I	0	0.75
ata	I	3	1.16
---	--	------	------
mPD		0.51	1.1
		nPD:	0.45
		N. weight:	1.
		Sc. PD:	0.39
		Sc. rank:	31369.0

,

HA			
Pos.	169	obs:	exp:
att	I	273	99.74
atc	I	1	68.40
ata	I	0	105.90
act	T	288	75.63
acc	T	1	61.53
aca	T	8	137.20
acg	T	3	25.60
gtt	V	39039	8666.00
gtc	V	157	7122.00
gta	V	0	10541.00
gtg	V	0	12867.00
---	--	------	------
mPD		0.052	0.78
		nPD:	0.07
		N. weight:	1.6
		Sc. PD:	0.004
		Sc. rank:	29449.0

,

HA			
Pos.	170	obs:	exp:
caa	Q	2	1.06
cag	Q	0	0.94
cgt	R	0	0.03
cgc	R	0	0.05
cga	R	0	0.10
cgg	R	0	0.07
aaa	K	39498	22675.00
aag	K	253	17076.00
aga	R	1	0.50
agg	R	0	0.25
gaa	E	16	9.69
gag	E	0	6.31
---	--	------	------
mPD		0.014	0.49
		nPD:	0.03
		N. weight:	0.57
		Sc. PD:	-0.02
		Sc. rank:	3168.3

,

HA			
Pos.	171	obs:	exp:
caa	Q	1	0.53
cag	Q	0	0.47
cgt	R	0	0.22
cgc	R	0	0.42
cga	R	0	0.78
cgg	R	0	0.59
act	T	0	0.76
acc	T	0	0.62
aca	T	3	1.37
acg	T	0	0.26
aat	N	2	1.77
aac	N	1	1.23
aaa	K	39565	22668.00
aag	K	174	17071.00
aga	R	8	4.02
agg	R	0	1.98
gaa	E	16	9.69
gag	E	0	6.31
---	--	------	------
mPD		0.010	0.49
		nPD:	0.02
		N. weight:	0.58
		Sc. PD:	-0.024
		Sc. rank:	-104.9

,

HA			
Pos.	172	obs:	exp:
cgt	R	0	1.23
cgc	R	0	2.35
cga	R	0	4.41
cgg	R	0	3.29
act	T	0	0.25
acc	T	0	0.21
aca	T	0	0.46
acg	T	1	0.09
aaa	K	1	0.57
aag	K	0	0.43
aga	R	45	22.59
agg	R	0	11.12
gtt	V	0	0.22
gtc	V	0	0.18
gta	V	1	0.27
gtg	V	0	0.33
gaa	E	227	138.70
gag	E	2	90.34
ggt	G	19	5988.00
ggc	G	4	5849.00
gga	G	37486	17567.00
ggg	G	1984	10088.00
---	--	------	------
mPD		0.11	0.71
		nPD:	0.16
		N. weight:	0.69
		Sc. PD:	0.062
		Sc. rank:	17648.0

,

HA			
Pos.	173	obs:	exp:
tct	S	0	6.26
tcc	S	0	3.94
tca	S	0	8.85
tcg	S	0	1.81
caa	Q	2	1.06
cag	Q	0	0.94
att	I	4	1.46
atc	I	0	1.00
ata	I	0	1.55
act	T	3	1.01
acc	T	1	0.82
aca	T	0	1.83
acg	T	0	0.34
aat	N	37167	22077.00
aac	N	161	15251.00
aaa	K	2329	1330.00
aag	K	2	1001.00
agt	S	34	6.39
agc	S	0	6.75
gat	D	63	33.19
gac	D	0	29.81
gaa	E	4	2.42
gag	E	0	1.58
---	--	------	------
mPD		0.12	0.55
		nPD:	0.23
		N. weight:	0.54
		Sc. PD:	0.087
		Sc. rank:	14649.0

,

HA			
Pos.	174	obs:	exp:
tta	L	41	4.62
ttg	L	0	7.91
tct	S	8	7304.00
tcc	S	19	4597.00
tca	S	39594	10330.00
tcg	S	71	2112.00
ctt	L	0	8.28
ctc	L	0	5.93
cta	L	1	8.09
ctg	L	0	7.18
cat	H	4	2.35
cac	H	0	1.65
act	T	0	6.81
acc	T	0	5.54
aca	T	27	12.35
acg	T	0	2.30
agt	S	0	7463.00
agc	S	0	7884.00
gct	A	0	1.32
gcc	A	0	1.03
gca	A	5	2.35
gcg	A	0	0.29
---	--	------	------
mPD		0.0093	1.6
		nPD:	0.01
		N. weight:	1.4
		Sc. PD:	-0.082
		Sc. rank:	-31934.0

,

HA			
Pos.	175	obs:	exp:
ttt	F	0	0.43
ttc	F	1	0.57
tat	Y	146	20661.00
tac	Y	39619	19104.00
act	T	0	1.01
acc	T	4	0.82
aca	T	0	1.83
acg	T	0	0.34
---	--	------	------
mPD		0.0078	0.50
		nPD:	0.02
		N. weight:	0.77
		Sc. PD:	-0.036
		Sc. rank:	-3794.2

,

HA			
Pos.	176	obs:	exp:
tta	L	0	0.22
ttg	L	0	0.38
tct	S	0	1.47
tcc	S	0	0.93
tca	S	8	2.08
tcg	S	0	0.43
ctt	L	0	0.39
ctc	L	0	0.28
cta	L	2	0.39
ctg	L	0	0.34
cct	P	46	10951.00
ccc	P	13	7373.00
cca	P	39667	16926.00
ccg	P	26	4501.00
caa	Q	5	2.64
cag	Q	0	2.36
act	T	0	0.76
acc	T	0	0.62
aca	T	3	1.37
acg	T	0	0.26
agt	S	0	1.50
agc	S	0	1.59
---	--	------	------
mPD		0.0052	0.70
		nPD:	0.01
		N. weight:	0.91
		Sc. PD:	-0.05
		Sc. rank:	-17768.0

,

HA			
Pos.	177	obs:	exp:
caa	Q	0	1.06
cag	Q	2	0.94
cgt	R	0	1.04
cgc	R	0	1.99
cga	R	0	3.72
cgg	R	0	2.78
att	I	0	0.73
atc	I	0	0.50
ata	I	2	0.77
atg	M	1314	1314.00
act	T	0	2.52
acc	T	0	2.05
aca	T	0	4.57
acg	T	10	0.85
aat	N	4	2.37
aac	N	0	1.63
aaa	K	202	21903.00
aag	K	38195	16494.00
aga	R	4	19.08
agg	R	34	9.39
gtt	V	0	0.22
gtc	V	0	0.18
gta	V	0	0.27
gtg	V	1	0.33
gaa	E	0	1.21
gag	E	2	0.79
---	--	------	------
mPD		0.077	0.56
		nPD:	0.14
		N. weight:	0.85
		Sc. PD:	0.061
		Sc. rank:	20876.0

,

HA			
Pos.	178	obs:	exp:
ttt	F	0	5.58
ttc	F	13	7.42
tta	L	0	4047.00
ttg	L	0	6931.00
tct	S	0	0.74
tcc	S	0	0.46
tca	S	4	1.04
tcg	S	0	0.21
ctt	L	88	7254.00
ctc	L	36676	5200.00
cta	L	49	7087.00
ctg	L	0	6294.00
att	I	0	1070.00
atc	I	2938	733.40
ata	I	0	1135.00
agt	S	0	0.75
agc	S	0	0.79
gtt	V	0	0.44
gtc	V	2	0.36
gta	V	0	0.54
gtg	V	0	0.66
---	--	------	------
mPD		0.14	1.2
		nPD:	0.12
		N. weight:	2.
		Sc. PD:	0.11
		Sc. rank:	48408.0

,

HA

Pos.	179	obs:	exp:
tct	S	0	2311.00
tcc	S	0	1454.00
tca	S	0	3268.00
tcg	S	0	668.20
cat	H	0	3.53
cac	H	6	2.47
cgt	R	0	1.06
cgc	R	28	2.04
cga	R	0	3.82
cgg	R	0	2.85
att	I	0	12.38
atc	I	34	8.49
ata	I	0	13.14
act	T	0	4.29
acc	T	17	3.49
aca	T	0	7.78
acg	T	0	1.45
aat	N	446	16035.00
aac	N	26665	11076.00
aaa	K	2	2.85
aag	K	3	2.15
agt	S	264	2361.00
agc	S	12292	2494.00
aga	R	11	19.58
agg	R	0	9.64
gat	D	0	1.05
gac	D	2	0.95
---	--	------	------
mPD		0.47	1.4
		nPD:	0.34
		N. weight:	1.1
		Sc. PD:	0.31
		Sc. rank:	31573.0

,

HA

Pos.	180	obs:	exp:
tta	L	0	0.66
ttg	L	0	1.13
ctt	L	0	1.18
ctc	L	0	0.85
cta	L	6	1.16
ctg	L	0	1.03
cat	H	0	0.59
cac	H	1	0.41
caa	Q	30493	16114.00
cag	Q	45	14424.00
cgt	R	0	0.85
cgc	R	0	1.62
cga	R	19	3.04
cgg	R	0	2.27
att	I	0	49.14
atc	I	0	33.70
ata	I	135	52.16
act	T	0	9.58
acc	T	0	7.79
aca	T	38	17.38
acg	T	0	3.24
aat	N	6	33.12
aac	N	50	22.88
aaa	K	8914	5102.00
aag	K	31	3843.00
aga	R	12	15.56
agg	R	0	7.66
gaa	E	20	12.11
gag	E	0	7.89
---	--	------	------
mPD		0.37	0.87
		nPD:	0.43
		N. weight:	0.66
		Sc. PD:	0.24
		Sc. rank:	19576.0

,

HA

Pos.	181	obs:	exp:
ttt	F	0	11.16
ttc	F	26	14.84
tct	S	42	3442.00
tcc	S	18618	2166.00
tca	S	39	4867.00
tcg	S	3	995.30
tat	Y	0	2.08
tac	Y	4	1.92
cct	P	4	1.38
ccc	P	1	0.93
cca	P	0	2.13
ccg	P	0	0.57
att	I	0	7.64
atc	I	21	5.24
ata	I	0	8.11
act	T	56	5294.00
acc	T	20924	4307.00
aca	T	20	9607.00
acg	T	0	1792.00
aat	N	0	1.18
aac	N	2	0.82
agt	S	0	3517.00
agc	S	0	3715.00
gct	A	0	2.65
gcc	A	10	2.07
gca	A	0	4.70
gcg	A	0	0.59
---	--	------	------
mPD		0.51	1.4
		nPD:	0.36
		N. weight:	2.
		Sc. PD:	0.57
		Sc. rank:	56191.0

,

HA

Pos.	182	obs:	exp:
ttt	F	0	0.86
ttc	F	2	1.14
tat	Y	113	20659.00
tac	Y	39648	19102.00
act	T	0	1.01
acc	T	0	0.82
aca	T	4	1.83
acg	T	0	0.34
gtt	V	0	0.66
gtc	V	3	0.55
gta	V	0	0.81
gtg	V	0	0.98
---	--	------	------
mPD		0.0067	0.50
		nPD:	0.01
		N. weight:	0.77
		Sc. PD:	-0.038
		Sc. rank:	-6763.6

,

HA

Pos.	183	obs:	exp:
ttt	F	37	15.88
ttc	F	0	21.12
tta	L	4	0.77
ttg	L	0	1.32
tct	S	0	1.84
tcc	S	0	1.16
tca	S	0	2.60
tcg	S	0	0.53
ctt	L	3	1.38
ctc	L	0	0.99
cta	L	0	1.35
ctg	L	0	1.20
att	I	39149	14332.00
atc	I	194	9828.00
ata	I	28	15211.00
atg	M	17	17.00
act	T	162	41.34
acc	T	2	33.64
aca	T	0	75.03
acg	T	0	13.99
agt	S	10	1.88
agc	S	0	1.99
gtt	V	164	36.26
gtc	V	0	29.80
gta	V	0	44.11
gtg	V	0	53.84
---	--	------	------
mPD		0.031	0.68
		nPD:	0.05
		N. weight:	1.1
		Sc. PD:	-0.018
		Sc. rank:	14822.0

,

HA

Pos.	184	obs:	exp:
tta	L	0	0.11
ttg	L	0	0.19
ctt	L	1	0.20
ctc	L	0	0.14
cta	L	0	0.19
ctg	L	0	0.17
cat	H	1	0.59
cac	H	0	0.41
atg	M	4	4.00
aat	N	39604	23517.00
aac	N	159	16246.00
gtt	V	1	0.22
gtc	V	0	0.18
gta	V	0	0.27
gtg	V	0	0.33
---	--	------	------
mPD		0.0086	0.48
		nPD:	0.02
		N. weight:	0.54
		Sc. PD:	-0.024
		Sc. rank:	-1541.4

,

HA

Pos.	185	obs:	exp:
tat	Y	1	0.52
tac	Y	0	0.48
att	I	0	1.46
atc	I	0	1.00
ata	I	4	1.55
aat	N	74	44.36
aac	N	1	30.64
gct	A	1	0.26
gcc	A	0	0.21
gca	A	0	0.47
gcg	A	0	0.06
gat	D	39074	20906.00
gac	D	603	18771.00
gaa	E	3	3.03
gag	E	2	1.97
ggt	G	7	1.06
ggc	G	0	1.04
gga	G	0	3.11
ggg	G	0	1.79
---	--	------	------
mPD		0.035	0.50
		nPD:	0.07
		N. weight:	0.61
		Sc. PD:	0.0034
		Sc. rank:	11468.0

,

HA

Pos.	186	obs:	exp:
caa	Q	1	0.53
cag	Q	0	0.47
cgt	R	0	0.85
cgc	R	0	1.62
cga	R	0	3.04
cgg	R	0	2.27
aat	N	1	1.18
aac	N	1	0.82
aaa	K	39399	22666.00
aag	K	336	17069.00
aga	R	31	15.56
agg	R	0	7.66
gaa	E	1	0.61
gag	E	0	0.39
---	--	------	------
mPD		0.019	0.49
		nPD:	0.04
		N. weight:	0.56
		Sc. PD:	-0.014
		Sc. rank:	5809.7

,

HA

Pos.	187	obs:	exp:
cgt	R	0	1.42
cgc	R	0	2.72
cga	R	0	5.09
cgg	R	0	3.81
aga	R	0	26.11
agg	R	52	12.86
gaa	E	0	26.64
gag	E	44	17.36
ggt	G	4	6016.00
ggc	G	2	5876.00
gga	G	398	17648.00
ggg	G	39270	10134.00
---	--	------	------
mPD		0.025	0.70
		nPD:	0.04
		N. weight:	1.4
		Sc. PD:	-0.039
		Sc. rank:	14111.0

,

HA

Pos.	188	obs:	exp:
cgt	R	0	1.58
cgc	R	0	3.03
cga	R	0	5.68
cgg	R	0	4.24
aat	N	1	0.59
aac	N	0	0.41
aaa	K	39688	22652.00
aag	K	23	17059.00
aga	R	57	29.12
agg	R	1	14.34
---	--	------	------
mPD		0.0042	0.49
		nPD:	0.01
		N. weight:	0.6
		Sc. PD:	-0.033
		Sc. rank:	-10687.0

,

HA

Pos.	189	obs:	exp:
caa	Q	1	0.53
cag	Q	0	0.47
cgt	R	0	0.03
cgc	R	0	0.05
cga	R	0	0.10
cgg	R	0	0.07
aat	N	4	2.37
aac	N	0	1.63
aaa	K	53	30.23
aag	K	0	22.77
aga	R	1	0.50
agg	R	0	0.25
gtt	V	0	0.44
gtc	V	0	0.36
gta	V	2	0.54
gtg	V	0	0.66
gat	D	44	28.98
gac	D	11	26.02
gaa	E	39514	24006.00
gag	E	133	15641.00
ggt	G	0	1.06
ggc	G	0	1.04
gga	G	7	3.11
ggg	G	0	1.79
---	--	------	------
mPD		0.013	0.48
		nPD:	0.03
		N. weight:	0.52
		Sc. PD:	-0.019
		Sc. rank:	2575.0

,

HA

Pos.	190	obs:	exp:
cct	P	4	1.10
ccc	P	0	0.74
cca	P	0	1.70
ccg	P	0	0.45
att	I	3	429.20
atc	I	1176	294.30
ata	I	0	455.50
aaa	K	0	0.57
aag	K	1	0.43
gtt	V	683	8531.00
gtc	V	37881	7011.00
gta	V	21	10377.00
gtg	V	0	12666.00
gct	A	0	0.26
gcc	A	1	0.21
gca	A	0	0.47
gcg	A	0	0.06
---	--	------	------
mPD		0.093	0.80
		nPD:	0.12
		N. weight:	1.7
		Sc. PD:	0.09
		Sc. rank:	40645.0

,

HA

Pos.	191	obs:	exp:
tta	L	2	4371.00
ttg	L	0	7485.00
ctt	L	161	7835.00
ctc	L	39379	5616.00
cta	L	215	7654.00
ctg	L	1	6798.00
cct	P	0	0.28
ccc	P	1	0.19
cca	P	0	0.43
ccg	P	0	0.11
cgt	R	4	0.11
cgc	R	0	0.21
cga	R	0	0.39
cgg	R	0	0.29
att	I	0	2.55
atc	I	7	1.75
ata	I	0	2.70
aga	R	0	2.01
agg	R	0	0.99
---	--	------	------
mPD		0.020	1.1
		nPD:	0.02
		N. weight:	2.
		Sc. PD:	-0.093
		Sc. rank:	-6862.8

,

HA

Pos.	192	obs:	exp:
tta	L	0	0.33
ttg	L	3	0.56
ctt	L	0	0.59
ctc	L	0	0.42
cta	L	0	0.58
ctg	L	0	0.51
atg	M	6	6.00
gtt	V	31	8790.00
gtc	V	1	7224.00
gta	V	120	10692.00
gtg	V	39605	13051.00
gct	A	4	1.06
gcc	A	0	0.83
gca	A	0	1.88
gcg	A	0	0.23
---	--	------	------
mPD		0.0085	0.74
		nPD:	0.01
		N. weight:	1.2
		Sc. PD:	-0.06
		Sc. rank:	-13345.0

,

HA

Pos.	193	obs:	exp:
tta	L	34	4370.00
ttg	L	68	7484.00
ctt	L	27	7834.00
ctc	L	0	5615.00
cta	L	8029	7653.00
ctg	L	31595	6797.00
att	I	0	1.09
atc	I	0	0.75
ata	I	3	1.16
atg	M	8	8.00
gtt	V	0	1.33
gtc	V	0	1.09
gta	V	1	1.61
gtg	V	5	1.97
---	--	------	------
mPD		0.33	1.1
		nPD:	0.29
		N. weight:	1.3
		Sc. PD:	0.29
		Sc. rank:	35999.0

,

HA

Pos.	194	obs:	exp:
tgg	W	39766	39766.00
---	-	4	4.00
---	--	------	------
mPD		0	0
		nPD:	1.
		N. weight:	0.
		Sc. PD:	0
		Sc. rank:	0

,

HA

Pos.	195	obs:	exp:
tct	S	0	0.55
tcc	S	0	0.35
tca	S	0	0.78
tcg	S	0	0.16
agt	S	0	0.56
agc	S	3	0.60
gct	A	0	6.09
gcc	A	23	4.76
gca	A	0	10.80
gcg	A	0	1.35
gat	D	0	0.53
gac	D	1	0.47
ggt	G	175	6026.00
ggc	G	39411	5886.00
gga	G	148	17678.00
ggg	G	9	10152.00
---	--	------	------
mPD		0.018	0.69
		nPD:	0.03
		N. weight:	2.
		Sc. PD:	-0.074
		Sc. rank:	8629.9

,

HA

Pos.	196	obs:	exp:
tta	L	0	0.44
ttg	L	0	0.75
ctt	L	4	0.79
ctc	L	0	0.56
cta	L	0	0.77
ctg	L	0	0.68
att	I	39450	14466.00
atc	I	210	9919.00
ata	I	78	15353.00
atg	M	3	3.00
gtt	V	25	5.53
gtc	V	0	4.54
gta	V	0	6.72
gtg	V	0	8.21
---	--	------	------
mPD		0.016	0.66
		nPD:	0.02
		N. weight:	1.
		Sc. PD:	-0.04
		Sc. rank:	3341.8

,

HA

Pos.	197	obs:	exp:
cat	H	294	23387.00
cac	H	39474	16381.00
caa	Q	0	1.06
cag	Q	2	0.94
---	--	------	------
mPD		0.015	0.48
		nPD:	0.03
		N. weight:	0.91
		Sc. PD:	-0.03
		Sc. rank:	6924.6

,

HA

Pos.	198	obs:	exp:
cat	H	39175	23384.00
cac	H	588	16379.00
caa	Q	1	0.53
cag	Q	0	0.47
aat	N	6	3.55
aac	N	0	2.45
---	--	------	------
mPD		0.029	0.48
		nPD:	0.06
		N. weight:	0.5
		Sc. PD:	-0.0015
		Sc. rank:	8692.4

,

HA			
Pos.	199	obs:	exp:
tct	S	0	0.74
tcc	S	0	0.46
tca	S	4	1.04
tcg	S	0	0.21
cct	P	29	10950.00
ccc	P	8	7372.00
cca	P	39666	16925.00
ccg	P	45	4501.00
caa	Q	18	9.50
cag	Q	0	8.50
agt	S	0	0.75
agc	S	0	0.79
---	--	------	------
mPD		0.0052	0.70
		nPD:	0.01
		N. weight:	0.91
		Sc. PD:	-0.05
		Sc. rank:	-17323.0

,

HA			
Pos.	200	obs:	exp:
ttt	F	1	0.43
ttc	F	0	0.57
tct	S	21594	4001.00
tcc	S	121	2518.00
tca	S	18	5658.00
tcg	S	6	1157.00
tat	Y	3	1.56
tac	Y	0	1.44
cct	P	17774	4962.00
ccc	P	231	3340.00
cca	P	3	7669.00
ccg	P	2	2039.00
act	T	2	0.50
acc	T	0	0.41
aca	T	0	0.91
acg	T	0	0.17
aat	N	2	1.18
aac	N	0	0.82
agt	S	0	4088.00
agc	S	0	4318.00
gct	A	13	3.44
gcc	A	0	2.69
gca	A	0	6.11
gcg	A	0	0.76
---	--	------	------
mPD		0.52	1.7
		nPD:	0.31
		N. weight:	1.6
		Sc. PD:	0.38
		Sc. rank:	43799.0

,

HA			
Pos.	201	obs:	exp:
tct	S	1	0.74
tcc	S	0	0.46
tca	S	0	1.04
tcg	S	0	0.21
att	I	2	0.73
atc	I	0	0.50
ata	I	0	0.77
act	T	39596	10015.00
acc	T	124	8149.00
aca	T	7	18174.00
acg	T	0	3390.00
aat	N	27	15.97
aac	N	0	11.03
agt	S	3	0.75
agc	S	0	0.79
gct	A	10	2.65
gcc	A	0	2.07
gca	A	0	4.70
gcg	A	0	0.59
---	--	------	------
mPD		0.0087	0.68
		nPD:	0.01
		N. weight:	1.5
		Sc. PD:	-0.073
		Sc. rank:	-14288.0

,

HA			
Pos.	202	obs:	exp:
ttt	F	3	1.29
ttc	F	0	1.71
tct	S	1	1328.00
tcc	S	0	836.00
tca	S	0	1879.00
tcg	S	0	384.10
cct	P	19	5.23
ccc	P	0	3.52
cca	P	0	8.09
ccg	P	0	2.15
att	I	11119	4049.00
atc	I	5	2777.00
ata	I	0	4298.00
atg	M	3	3.00
act	T	21107	5375.00
acc	T	198	4373.00
aca	T	12	9753.00
acg	T	3	1819.00
aat	N	47	27.80
aac	N	0	19.20
agt	S	7204	1357.00
agc	S	13	1434.00
gtt	V	8	1.77
gtc	V	0	1.45
gta	V	0	2.15
gtg	V	0	2.63
gct	A	27	7.41
gcc	A	1	5.80
gca	A	0	13.15
gcg	A	0	1.64
---	--	------	------
mPD		0.61	1.4
		nPD:	0.44
		N. weight:	1.4
		Sc. PD:	0.52
		Sc. rank:	42117.0

,

HA			
Pos.	203	obs:	exp:
tct	S	9	1.66
tcc	S	0	1.04
tca	S	0	2.34
tcg	S	0	0.48
cct	P	1	0.28
ccc	P	0	0.19
cca	P	0	0.43
ccg	P	0	0.11
act	T	1163	293.40
acc	T	1	238.80
aca	T	0	532.50
acg	T	0	99.32
aat	N	3	1.77
aac	N	0	1.23
agt	S	0	1.69
agc	S	0	1.79
gtt	V	22	4.86
gtc	V	0	4.00
gta	V	0	5.92
gtg	V	0	7.22
gct	A	38442	10210.00
gcc	A	63	7982.00
gca	A	56	18112.00
gcg	A	2	2259.00
gat	D	4	2.11
gac	D	0	1.89
ggt	G	3	0.61
ggc	G	0	0.59
gga	G	0	1.78
ggg	G	1	1.02
---	--	------	------
mPD		0.065	0.72
		nPD:	0.09
		N. weight:	1.4
		Sc. PD:	0.037
		Sc. rank:	30789.0

,

HA			
Pos.	204	obs:	exp:
tct	S	0	0.74
tcc	S	3	0.46
tca	S	0	1.04
tcg	S	0	0.21
tat	Y	0	2.60
tac	Y	5	2.40
act	T	1	2.27
acc	T	8	1.85
aca	T	0	4.12
acg	T	0	0.77
aat	N	0	8.87
aac	N	15	6.13
agt	S	0	0.75
agc	S	1	0.79
gtt	V	2	14.59
gtc	V	64	11.99
gta	V	0	17.75
gtg	V	0	21.67
gct	A	143	988.90
gcc	A	3590	773.10
gca	A	2	1754.00
gcg	A	0	218.80
gat	D	258	18918.00
gac	D	35646	16986.00
gaa	E	2	1.82
gag	E	1	1.18
ggt	G	1	4.40
ggc	G	28	4.30
gga	G	0	12.90
ggg	G	0	7.41
---	--	------	------
mPD		0.20	0.72
		nPD:	0.27
		N. weight:	0.84
		Sc. PD:	0.17
		Sc. rank:	22664.0

,

HA			
Pos.	205	obs:	exp:
caa	Q	39724	20980.00
cag	Q	35	18779.00
cgt	R	0	0.27
cgc	R	0	0.52
cga	R	10	0.98
cgg	R	0	0.73
aga	R	0	5.02
agg	R	0	2.47
gaa	E	1	0.61
gag	E	0	0.39
---	--	------	------
mPD		0.0023	0.50
		nPD:	0.
		N. weight:	0.68
		Sc. PD:	-0.04
		Sc. rank:	-16902.0

,

HA			
Pos.	206	obs:	exp:
tta	L	0	6.05
ttg	L	0	10.35
ctt	L	0	10.84
ctc	L	0	7.77
cta	L	55	10.59
ctg	L	0	9.40
cct	P	0	0.28
ccc	P	0	0.19
cca	P	1	0.43
ccg	P	0	0.11
cat	H	5	4.12
cac	H	2	2.88
caa	Q	35516	18797.00
cag	Q	107	16826.00
cgt	R	0	0.03
cgc	R	0	0.05
cga	R	1	0.10
cgg	R	0	0.07
aaa	K	2	1.14
aag	K	0	0.86
aga	R	0	0.50
agg	R	0	0.25
gaa	E	4077	2469.00
gag	E	1	1609.00
ggt	G	0	0.45
ggc	G	0	0.44
gga	G	3	1.33
ggg	G	0	0.77
---	--	------	------
mPD		0.19	0.69
		nPD:	0.28
		N. weight:	0.66
		Sc. PD:	0.14
		Sc. rank:	17889.0

,

HA			
Pos.	207	obs:	exp:
tct	S	0	7280.00
tcc	S	0	4582.00
tca	S	0	10296.00
tcg	S	0	2106.00
tgg	W	2	2.00
cgt	R	51	1.80
cgc	R	1	3.45
cga	R	0	6.46
cgg	R	0	4.83
att	I	59	21.84
atc	I	1	14.98
ata	I	0	23.18
act	T	4	1.01
acc	T	0	0.82
aca	T	0	1.83
acg	T	0	0.34
aat	N	48	28.98
aac	N	1	20.02
agt	S	38459	7439.00
agc	S	1103	7858.00
aga	R	6	33.13
agg	R	8	16.32
gtt	V	5	1.11
gtc	V	0	0.91
gta	V	0	1.34
gtg	V	0	1.64
ggt	G	21	3.34
ggc	G	1	3.26
gga	G	0	9.79
ggg	G	0	5.62
---	--	------	------
mPD		0.065	1.6
		nPD:	0.04
		N. weight:	1.7
		Sc. PD:	-0.04
		Sc. rank:	19801.0

,

HA			
Pos.	208	obs:	exp:
tta	L	0	4364.00
ttg	L	0	7474.00
ctt	L	65	7823.00
ctc	L	39626	5608.00
cta	L	9	7643.00
ctg	L	1	6788.00
cct	P	0	0.28
ccc	P	1	0.19
cca	P	0	0.43
ccg	P	0	0.11
att	I	0	24.75
atc	I	68	16.97
ata	I	0	26.27
---	--	------	------
mPD		0.0072	1.1
		nPD:	0.01
		N. weight:	2.1
		Sc. PD:	-0.12
		Sc. rank:	-44886.0

,

HA			
Pos.	209	obs:	exp:
tat	Y	39668	20663.00
tac	Y	100	19105.00
cat	H	1	0.59
cac	H	0	0.41
gat	D	1	0.53
gac	D	0	0.47
---	--	------	------
mPD		0.0051	0.50
		nPD:	0.01
		N. weight:	0.69
		Sc. PD:	-0.036
		Sc. rank:	-9587.7

,

HA			
Pos.	210	obs:	exp:
tta	L	0	0.55
ttg	L	0	0.94
ctt	L	0	0.99
ctc	L	0	0.71
cta	L	0	0.96
ctg	L	5	0.85
cct	P	0	0.28
ccc	P	0	0.19
cca	P	0	0.43
ccg	P	1	0.11
cat	H	6	3.53
cac	H	0	2.47
caa	Q	165	20976.00
cag	Q	39587	18776.00
aaa	K	0	3.42
aag	K	6	2.58
---	--	------	------
mPD		0.0092	0.50
		nPD:	0.02
		N. weight:	0.78
		Sc. PD:	-0.035
		Sc. rank:	-2018.6

,

HA			
Pos.	211	obs:	exp:
tct	S	0	0.92
tcc	S	0	0.58
tca	S	0	1.30
tcg	S	0	0.27
cat	H	5	2.94
cac	H	0	2.06
act	T	2	0.50
acc	T	0	0.41
aca	T	0	0.91
acg	T	0	0.17
aat	N	39721	23511.00
aac	N	32	16242.00
aaa	K	0	0.57
aag	K	1	0.43
agt	S	5	0.94
agc	S	0	0.99
gat	D	4	2.11
gac	D	0	1.89
---	--	------	------
mPD		0.0025	0.48
		nPD:	0.01
		N. weight:	0.56
		Sc. PD:	-0.032
		Sc. rank:	-13409.0

,

HA			
Pos.	212	obs:	exp:
tct	S	0	0.92
tcc	S	0	0.58
tca	S	5	1.30
tcg	S	0	0.27
act	T	0	20.17
acc	T	0	16.41
aca	T	80	36.60
acg	T	0	6.83
agt	S	0	0.94
agc	S	0	0.99
gtt	V	0	13.04
gtc	V	0	10.72
gta	V	59	15.87
gtg	V	0	19.37
gct	A	43	10478.00
gcc	A	4	8192.00
gca	A	39500	18587.00
gcg	A	29	2319.00
gaa	E	49	29.67
gag	E	0	19.33
ggt	G	0	0.15
ggc	G	0	0.15
gga	G	1	0.44
ggg	G	0	0.26
---	--	------	------
mPD		0.014	0.67
		nPD:	0.02
		N. weight:	0.8
		Sc. PD:	-0.034
		Sc. rank:	-727.4

,

HA			
Pos.	213	obs:	exp:
cat	H	2	1.18
cac	H	0	0.82
aat	N	12	7.10
aac	N	0	4.90
gat	D	39707	20944.00
gac	D	43	18806.00
gaa	E	4	3.03
gag	E	1	1.97
ggt	G	1	0.15
ggc	G	0	0.15
gga	G	0	0.44
ggg	G	0	0.26
---	--	------	------
mPD		0.0032	0.50
		nPD:	0.01
		N. weight:	0.68
		Sc. PD:	-0.039
		Sc. rank:	-14571.0

,

HA			
Pos.	214	obs:	exp:
tct	S	0	6.99
tcc	S	0	4.40
tca	S	38	9.89
tcg	S	0	2.02
cct	P	0	0.28
ccc	P	0	0.19
cca	P	1	0.43
ccg	P	0	0.11
act	T	0	177.20
acc	T	0	144.20
aca	T	703	321.60
acg	T	0	59.98
agt	S	0	7.15
agc	S	0	7.55
gct	A	10	10333.00
gcc	A	11	8078.00
gca	A	38940	18330.00
gcg	A	66	2286.00
gaa	E	1	0.61
gag	E	0	0.39
---	--	------	------
mPD		0.041	0.70
		nPD:	0.06
		N. weight:	0.8
		Sc. PD:	-0.0041
		Sc. rank:	13584.0

,

HA			
Pos.	215	obs:	exp:
ttt	F	4	1.72
ttc	F	0	2.28
tat	Y	39715	20652.00
tac	Y	32	19095.00
tgt	C	1	0.45
tgc	C	0	0.55
cat	H	13	7.65
cac	H	0	5.35
aat	N	3	1.77
aac	N	0	1.23
gat	D	2	1.05
gac	D	0	0.95
---	--	------	------
mPD		0.0028	0.50
		nPD:	0.01
		N. weight:	0.7
		Sc. PD:	-0.04
		Sc. rank:	-15669.0

,

HA			
Pos.	216	obs:	exp:
att	I	15	5.46
atc	I	0	3.74
ata	I	0	5.80
gtt	V	39348	8757.00
gtc	V	248	7197.00
gta	V	10	10652.00
gtg	V	1	13002.00
gct	A	145	38.39
gcc	A	0	30.01
gca	A	0	68.10
gcg	A	0	8.49
ggt	G	3	0.45
ggc	G	0	0.44
gga	G	0	1.33
ggg	G	0	0.77
---	--	------	------
mPD		0.021	0.75
		nPD:	0.03
		N. weight:	1.6
		Sc. PD:	-0.055
		Sc. rank:	9430.3

,

HA			
Pos.	217	obs:	exp:
ttt	F	39661	17061.00
ttc	F	97	22697.00
tta	L	1	0.55
ttg	L	2	0.94
tct	S	4	0.74
tcc	S	0	0.46
tca	S	0	1.04
tcg	S	0	0.21
tat	Y	2	1.04
tac	Y	0	0.96
ctt	L	2	0.99
ctc	L	0	0.71
cta	L	0	0.96
ctg	L	0	0.85
agt	S	0	0.75
agc	S	0	0.79
gtt	V	1	0.22
gtc	V	0	0.18
gta	V	0	0.27
gtg	V	0	0.33
---	--	------	------
mPD		0.0055	0.49
		nPD:	0.01
		N. weight:	0.9
		Sc. PD:	-0.046
		Sc. rank:	-10818.0

,

HA			
Pos.	218	obs:	exp:
atg	M	2	2.00
gtt	V	12	8792.00
gtc	V	1	7226.00
gta	V	63	10695.00
gtg	V	39691	13054.00
gct	A	0	0.26
gcc	A	0	0.21
gca	A	0	0.47
gcg	A	1	0.06
---	--	------	------
mPD		0.0040	0.74
		nPD:	0.01
		N. weight:	1.2
		Sc. PD:	-0.068
		Sc. rank:	-27016.0

,

HA			
Pos.	219	obs:	exp:
tct	S	0	0.18
tcc	S	0	0.12
tca	S	0	0.26
tcg	S	0	0.05
tgg	W	13	13.00
cgt	R	0	0.05
cgc	R	0	0.10
cga	R	0	0.20
cgg	R	0	0.15
agt	S	1	0.19
agc	S	0	0.20
aga	R	0	1.00
agg	R	2	0.49
gaa	E	0	33.30
gag	E	55	21.70
ggt	G	2	6020.00
ggc	G	1	5880.00
gga	G	342	17659.00
ggg	G	39354	10141.00
---	--	------	------
mPD		0.021	0.70
		nPD:	0.03
		N. weight:	1.4
		Sc. PD:	-0.047
		Sc. rank:	10288.0

,

HA			
Pos.	220	obs:	exp:
tta	L	1	0.11
ttg	L	0	0.19
tct	S	0	207.80
tcc	S	0	130.80
tca	S	1129	293.80
tcg	S	0	60.09
ctt	L	0	0.20
ctc	L	0	0.14
cta	L	0	0.19
ctg	L	0	0.17
act	T	24	9739.00
acc	T	16	7925.00
aca	T	38517	17675.00
acg	T	78	3296.00
agt	S	0	212.30
agc	S	0	224.30
gct	A	0	1.32
gcc	A	0	1.03
gca	A	5	2.35
gcg	A	0	0.29
---	--	------	------
mPD		0.061	0.74
		nPD:	0.08
		N. weight:	0.84
		Sc. PD:	0.016
		Sc. rank:	17626.0

,

HA			
Pos.	221	obs:	exp:
tct	S	6	7317.00
tcc	S	9	4606.00
tca	S	39703	10349.00
tcg	S	45	2116.00
cct	P	0	1.93
ccc	P	0	1.30
cca	P	7	2.98
ccg	P	0	0.79
agt	S	0	7477.00
agc	S	0	7898.00
---	--	------	------
mPD		0.0034	1.6
		nPD:	0.
		N. weight:	1.4
		Sc. PD:	-0.087
		Sc. rank:	-43756.0

,

HA			
Pos.	222	obs:	exp:
tct	S	0	1.10
tcc	S	0	0.69
tca	S	0	1.56
tcg	S	0	0.32
cgt	R	0	1056.00
cgc	R	0	2024.00
cga	R	6	3789.00
cgg	R	0	2831.00
att	I	0	1.09
atc	I	0	0.75
ata	I	3	1.16
act	T	0	0.50
acc	T	0	0.41
aca	T	2	0.91
acg	T	0	0.17
aat	N	2	1.18
aac	N	0	0.82
aaa	K	1038	593.20
aag	K	2	446.80
agt	S	2	1.13
agc	S	4	1.19
aga	R	38495	19423.00
agg	R	187	9564.00
ggt	G	0	4.40
ggc	G	0	4.30
gga	G	29	12.90
ggg	G	0	7.41
---	--	------	------
mPD		0.063	0.96
		nPD:	0.07
		N. weight:	0.71
		Sc. PD:	0.0014
		Sc. rank:	12983.0

,

HA			
Pos.	223	obs:	exp:
ttt	F	0	0.43
ttc	F	1	0.57
tat	Y	47	20661.00
tac	Y	39717	19103.00
tgt	C	0	0.45
tgc	C	1	0.55
cat	H	0	1.76
cac	H	3	1.24
gat	D	0	0.53
gac	D	1	0.47
---	--	------	------
mPD		0.0027	0.50
		nPD:	0.01
		N. weight:	0.78
		Sc. PD:	-0.045
		Sc. rank:	-17977.0

,

HA			
Pos.	224	obs:	exp:
tct	S	0	7315.00
tcc	S	0	4604.00
tca	S	0	10345.00
tcg	S	0	2116.00
tgt	C	0	0.45
tgc	C	1	0.55
att	I	0	0.73
atc	I	2	0.50
ata	I	0	0.77
aat	N	0	5.91
aac	N	10	4.09
agt	S	64	7475.00
agc	S	39687	7896.00
ggt	G	0	0.91
ggc	G	6	0.89
gga	G	0	2.67
ggg	G	0	1.53
---	--	------	------
mPD		0.0042	1.6
		nPD:	0.
		N. weight:	1.7
		Sc. PD:	-0.1
		Sc. rank:	-50353.0

,

HA			
Pos.	225	obs:	exp:
caa	Q	0	0.53
cag	Q	1	0.47
cgt	R	0	1.28
cgc	R	0	2.46
cga	R	0	4.60
cgg	R	0	3.44
aat	N	3	1.77
aac	N	0	1.23
aaa	K	267	22653.00
aag	K	39445	17059.00
aga	R	0	23.60
agg	R	47	11.62
gaa	E	0	4.24
gag	E	7	2.76
---	--	------	------
mPD		0.016	0.49
		nPD:	0.03
		N. weight:	0.87
		Sc. PD:	-0.026
		Sc. rank:	7878.1

,

HA			
Pos.	226	obs:	exp:
cgt	R	0	0.76
cgc	R	0	1.46
cga	R	0	2.74
cgg	R	0	2.05
att	I	0	0.73
atc	I	0	0.50
ata	I	2	0.77
atg	M	595	595.00
act	T	0	1.51
acc	T	0	1.23
aca	T	0	2.74
acg	T	6	0.51
aat	N	10	6.51
aac	N	1	4.49
aaa	K	319	22311.00
aag	K	38794	16802.00
aga	R	0	14.06
agg	R	28	6.92
gaa	E	1	9.08
gag	E	14	5.92
---	--	------	------
mPD		0.049	0.53
		nPD:	0.09
		N. weight:	0.85
		Sc. PD:	0.024
		Sc. rank:	18782.0

,

HA

Pos.	227	obs:	exp:
ttt	F	75	17053.00
ttc	F	39664	22686.00
tta	L	7	1.76
ttg	L	0	3.01
tct	S	0	1.29
tcc	S	7	0.81
tca	S	0	1.82
tcg	S	0	0.37
tat	Y	0	1.04
tac	Y	2	0.96
ctt	L	0	3.15
ctc	L	9	2.26
cta	L	0	3.08
ctg	L	0	2.74
agt	S	0	1.32
agc	S	0	1.39
gtt	V	0	1.33
gtc	V	6	1.09
gta	V	0	1.61
gtg	V	0	1.97
---	--	------	------
mPD		0.0053	0.49
		nPD:	0.01
		N. weight:	0.59
		Sc. PD:	-0.031
		Sc. rank:	-7316.4

,

HA

Pos.	228	obs:	exp:
caa	Q	0	1.06
cag	Q	2	0.94
cgt	R	0	2.84
cgc	R	0	5.44
cga	R	0	10.19
cgg	R	0	7.61
atg	M	1	1.00
act	T	0	1.01
acc	T	0	0.82
aca	T	0	1.83
acg	T	4	0.34
aat	N	15	9.46
aac	N	1	6.54
aaa	K	810	22606.00
aag	K	38819	17023.00
aga	R	72	52.21
agg	R	32	25.71
gtt	V	0	0.22
gtc	V	0	0.18
gta	V	0	0.27
gtg	V	1	0.33
gaa	E	0	7.87
gag	E	13	5.13
---	--	------	------
mPD		0.050	0.50
		nPD:	0.1
		N. weight:	0.8
		Sc. PD:	0.029
		Sc. rank:	18208.0

,

HA

Pos.	229	obs:	exp:
tta	L	0	0.11
ttg	L	0	0.19
tct	S	0	0.18
tcc	S	0	0.12
tca	S	0	0.26
tcg	S	1	0.05
ctt	L	0	0.20
ctc	L	0	0.14
cta	L	0	0.19
ctg	L	1	0.17
cct	P	21	10956.00
ccc	P	9	7376.00
cca	P	824	16933.00
ccg	P	38913	4503.00
cgt	R	0	0.03
cgc	R	0	0.05
cga	R	0	0.10
cgg	R	1	0.07
agt	S	0	0.19
agc	S	0	0.20
aga	R	0	0.50
agg	R	0	0.25
---	--	------	------
mPD		0.042	0.70
		nPD:	0.06
		N. weight:	2.2
		Sc. PD:	-0.0069
		Sc. rank:	38547.0

,

HA

Pos.	230	obs:	exp:
tta	L	2	0.22
ttg	L	0	0.38
ctt	L	0	0.39
ctc	L	0	0.28
cta	L	0	0.39
ctg	L	0	0.34
caa	Q	1	0.53
cag	Q	0	0.47
aaa	K	1	0.57
aag	K	0	0.43
gtt	V	0	0.22
gtc	V	0	0.18
gta	V	1	0.27
gtg	V	0	0.33
gat	D	0	1.05
gac	D	2	0.95
gaa	E	39629	24075.00
gag	E	132	15686.00
ggt	G	0	0.30
ggc	G	0	0.30
gga	G	2	0.89
ggg	G	0	0.51
---	--	------	------
mPD		0.0072	0.48
		nPD:	0.01
		N. weight:	0.52
		Sc. PD:	-0.025
		Sc. rank:	-3347.2

,

HA

Pos.	231	obs:	exp:
tta	L	0	0.22
ttg	L	0	0.38
ctt	L	0	0.39
ctc	L	0	0.28
cta	L	2	0.39
ctg	L	0	0.34
att	I	89	14467.00
atc	I	2	9920.00
ata	I	39650	15354.00
atg	M	5	5.00
act	T	0	1.51
acc	T	0	1.23
aca	T	6	2.74
acg	T	0	0.51
gtt	V	0	3.54
gtc	V	0	2.91
gta	V	15	4.30
gtg	V	1	5.25
---	--	------	------
mPD		0.0061	0.66
		nPD:	0.01
		N. weight:	1.
		Sc. PD:	-0.054
		Sc. rank:	-16053.0

,

HA

Pos.	232	obs:	exp:
tct	S	0	0.55
tcc	S	0	0.35
tca	S	3	0.78
tcg	S	0	0.16
act	T	2	6.81
acc	T	0	5.54
aca	T	25	12.35
acg	T	0	2.30
agt	S	0	0.56
agc	S	0	0.60
gtt	V	0	5.75
gtc	V	0	4.72
gta	V	26	6.99
gtg	V	0	8.53
gct	A	104	10396.00
gcc	A	6	8128.00
gca	A	39142	18443.00
gcg	A	16	2300.00
gaa	E	29	17.56
gag	E	0	11.44
ggt	G	0	63.23
ggc	G	0	61.76
gga	G	415	185.50
ggg	G	2	106.50
---	--	------	------
mPD		0.032	0.69
		nPD:	0.05
		N. weight:	0.8
		Sc. PD:	-0.014
		Sc. rank:	10948.0

,

HA

Pos.	233	obs:	exp:
tta	L	0	0.11
ttg	L	0	0.19
ctt	L	0	0.20
ctc	L	0	0.14
cta	L	1	0.19
ctg	L	0	0.17
cct	P	0	1.93
ccc	P	0	1.30
cca	P	7	2.98
ccg	P	0	0.79
cgt	R	0	0.19
cgc	R	0	0.37
cga	R	0	0.69
cgg	R	0	0.51
att	I	60	4574.00
atc	I	2	3136.00
ata	I	12502	4854.00
atg	M	11	11.00
act	T	12	6447.00
acc	T	3	5245.00
aca	T	25546	11699.00
acg	T	12	2182.00
aaa	K	1250	714.20
aag	K	2	537.80
aga	R	7	3.51
agg	R	0	1.73
gtt	V	1	32.72
gtc	V	0	26.89
gta	V	147	39.80
gtg	V	0	48.58
gct	A	0	54.80
gcc	A	0	42.85
gca	A	207	97.22
gcg	A	0	12.13
---	--	------	------
mPD		0.50	1.2
		nPD:	0.43
		N. weight:	0.88
		Sc. PD:	0.31
		Sc. rank:	26080.0

,

HA

Pos.	234	obs:	exp:
cgt	R	0	1086.00
cgc	R	0	2080.00
cga	R	1	3895.00
cgg	R	0	2911.00
aga	R	39664	19966.00
agg	R	105	9832.00
---	--	------	------
mPD		0.0053	0.91
		nPD:	0.01
		N. weight:	0.73
		Sc. PD:	-0.041
		Sc. rank:	-15923.0

,

HA			
Pos.	235	obs:	exp:
tta	L	0	0.88
ttg	L	0	1.51
tct	S	0	0.18
tcc	S	1	0.12
tca	S	0	0.26
tcg	S	0	0.05
ctt	L	0	1.58
ctc	L	8	1.13
cta	L	0	1.54
ctg	L	0	1.37
cct	P	60	10951.00
ccc	P	39663	7372.00
cca	P	26	16925.00
ccg	P	0	4501.00
cat	H	0	0.59
cac	H	1	0.41
act	T	0	0.25
acc	T	1	0.21
aca	T	0	0.46
acg	T	0	0.09
agt	S	0	0.19
agc	S	0	0.20
gct	A	0	2.65
gcc	A	9	2.07
gca	A	1	4.70
gcg	A	0	0.59
---	--	------	------
mPD		0.0054	0.70
		nPD:	0.01
		N. weight:	1.8
		Sc. PD:	-0.099
		Sc. rank:	-33633.0

,

HA			
Pos.	236	obs:	exp:
caa	Q	2	1.06
cag	Q	0	0.94
cgt	R	0	0.44
cgc	R	0	0.84
cga	R	0	1.57
cgg	R	0	1.17
att	I	0	0.36
atc	I	0	0.25
ata	I	1	0.39
aaa	K	39654	22675.00
aag	K	97	17076.00
aga	R	16	8.03
agg	R	0	3.96
---	--	------	------
mPD		0.0058	0.49
		nPD:	0.01
		N. weight:	0.59
		Sc. PD:	-0.03
		Sc. rank:	-6235.4

,

HA			
Pos.	237	obs:	exp:
tta	L	0	0.66
ttg	L	5	1.13
ctt	L	0	1.18
ctc	L	0	0.85
cta	L	0	1.16
ctg	L	1	1.03
att	I	0	0.36
atc	I	0	0.25
ata	I	1	0.39
atg	M	23	23.00
gtt	V	29	8785.00
gtc	V	3	7221.00
gta	V	159	10687.00
gtg	V	39547	13045.00
gct	A	0	0.26
gcc	A	0	0.21
gca	A	0	0.47
gcg	A	1	0.06
gaa	E	0	0.61
gag	E	1	0.39
---	--	------	------
mPD		0.011	0.74
		nPD:	0.02
		N. weight:	1.2
		Sc. PD:	-0.056
		Sc. rank:	-7375.1

,

HA			
Pos.	238	obs:	exp:
cgt	R	0	1085.00
cgc	R	0	2079.00
cga	R	0	3893.00
cgg	R	0	2909.00
atg	M	1	1.00
aaa	K	0	12.55
aag	K	22	9.45
aga	R	225	19954.00
agg	R	39521	9826.00
ggt	G	0	0.15
ggc	G	0	0.15
gga	G	0	0.44
ggg	G	1	0.26
---	--	------	------
mPD		0.012	0.91
		nPD:	0.01
		N. weight:	1.5
		Sc. PD:	-0.072
		Sc. rank:	-11585.0

,

HA			
Pos.	239	obs:	exp:
tat	Y	3	1.56
tac	Y	0	1.44
cat	H	1	0.59
cac	H	0	0.41
act	T	1	0.25
acc	T	0	0.21
aca	T	0	0.46
acg	T	0	0.09
aat	N	192	114.10
aac	N	1	78.85
gtt	V	1	0.22
gtc	V	0	0.18
gta	V	0	0.27
gtg	V	0	0.33
gct	A	3	0.79
gcc	A	0	0.62
gca	A	0	1.41
gcg	A	0	0.18
gat	D	38621	20406.00
gac	D	107	18322.00
gaa	E	492	299.70
gag	E	3	195.30
ggt	G	335	52.31
ggc	G	0	51.10
gga	G	9	153.50
ggg	G	1	88.13
---	--	------	------
mPD		0.058	0.54
		nPD:	0.11
		N. weight:	0.68
		Sc. PD:	0.028
		Sc. rank:	15758.0

,

HA			
Pos.	240	obs:	exp:
caa	Q	39391	20826.00
cag	Q	77	18642.00
cgt	R	0	8.22
cgc	R	0	15.75
cga	R	298	29.48
cgg	R	3	22.03
aaa	K	1	0.57
aag	K	0	0.43
aga	R	0	151.10
agg	R	0	74.41
---	--	------	------
mPD		0.019	0.53
		nPD:	0.04
		N. weight:	0.69
		Sc. PD:	-0.019
		Sc. rank:	7009.7

,

HA			
Pos.	241	obs:	exp:
caa	Q	1	0.53
cag	Q	0	0.47
aaa	K	35	19.97
aag	K	0	15.03
gtt	V	0	0.22
gtc	V	0	0.18
gta	V	1	0.27
gtg	V	0	0.33
gct	A	0	85.25
gcc	A	0	66.65
gca	A	322	151.20
gcg	A	0	18.86
gat	D	3	3.69
gac	D	4	3.31
gaa	E	39320	23850.00
gag	E	69	15539.00
ggt	G	0	2.27
ggc	G	0	2.22
gga	G	15	6.67
ggg	G	0	3.83
---	--	------	------
mPD		0.022	0.50
		nPD:	0.04
		N. weight:	0.53
		Sc. PD:	-0.0098
		Sc. rank:	7151.6

,

HA			
Pos.	242	obs:	exp:
cgt	R	0	0.05
cgc	R	0	0.10
cga	R	0	0.20
cgg	R	1	0.15
aga	R	0	1.00
agg	R	1	0.49
gct	A	0	1.06
gcc	A	0	0.83
gca	A	0	1.88
gcg	A	4	0.23
ggt	G	9	6029.00
ggc	G	0	5890.00
gga	G	228	17688.00
ggg	G	39527	10157.00
---	--	------	------
mPD		0.012	0.69
		nPD:	0.02
		N. weight:	1.4
		Sc. PD:	-0.065
		Sc. rank:	-4478.7

,

HA			
Pos.	243	obs:	exp:
cgt	R	0	1086.00
cgc	R	0	2080.00
cga	R	0	3895.00
cgg	R	0	2911.00
aga	R	39719	19966.00
agg	R	51	9832.00
---	--	------	------
mPD		0.0026	0.91
		nPD:	0.
		N. weight:	0.74
		Sc. PD:	-0.044
		Sc. rank:	-20867.0

,

HA			
Pos.	244	obs:	exp:
tta	L	0	0.11
ttg	L	0	0.19
ctt	L	0	0.20
ctc	L	0	0.14
cta	L	0	0.19
ctg	L	1	0.17
att	I	9	13.83
atc	I	1	9.49
ata	I	28	14.68
atg	M	39728	39728.00
gtt	V	0	0.66
gtc	V	0	0.55
gta	V	0	0.81
gtg	V	3	0.98
---	--	------	------
mPD		0.0021	0.0022
		nPD:	0.94
		N. weight:	0.00054
		Sc. PD:	0.00047
		Sc. rank:	17.2

,

HA			
Pos.	245	obs:	exp:
tct	S	0	3.86
tcc	S	0	2.43
tca	S	0	5.47
tcg	S	0	1.12
act	T	0	1.01
acc	T	4	0.82
aca	T	0	1.83
acg	T	0	0.34
aat	N	100	23488.00
aac	N	39613	16225.00
aaa	K	8	4.56
aag	K	0	3.44
agt	S	0	3.95
agc	S	21	4.17
gat	D	0	12.65
gac	D	24	11.35
---	--	------	------
mPD		0.0079	0.49
		nPD:	0.02
		N. weight:	0.95
		Sc. PD:	-0.044
		Sc. rank:	-4313.9

,

HA			
Pos.	246	obs:	exp:
tat	Y	39547	20664.00
tac	Y	223	19106.00
---	--	------	------
mPD		0.011	0.50
		nPD:	0.02
		N. weight:	0.67
		Sc. PD:	-0.027
		Sc. rank:	934.1

,

HA			
Pos.	247	obs:	exp:
tat	Y	48	20643.00
tac	Y	39682	19087.00
tgt	C	0	0.45
tgc	C	1	0.55
cat	H	0	22.35
cac	H	38	15.65
gat	D	0	0.53
gac	D	1	0.47
---	--	------	------
mPD		0.0044	0.50
		nPD:	0.01
		N. weight:	0.78
		Sc. PD:	-0.042
		Sc. rank:	-13105.0

HA			
Pos.	248	obs:	exp:
tgt	C	0	0.45
tgc	C	1	0.55
tgg	W	39769	39769.00
---	--	------	------
mPD		0.000050	0.000050
		nPD:	1.
		N. weight:	0.000016
		Sc. PD:	0.000015
		Sc. rank:	0.5

HA			
Pos.	249	obs:	exp:
att	I	0	9.46
atc	I	0	6.49
ata	I	26	10.05
act	T	8	10014.00
acc	T	5	8148.00
aca	T	39669	18173.00
acg	T	43	3389.00
gct	A	0	5.03
gcc	A	0	3.93
gca	A	19	8.92
gcg	A	0	1.11
---	--	------	------
mPD		0.0051	0.68
		nPD:	0.01
		N. weight:	0.84
		Sc. PD:	-0.046
		Sc. rank:	-16086.0

HA			
Pos.	250	obs:	exp:
tta	L	235	4302.00
ttg	L	0	7368.00
ctt	L	5	7712.00
ctc	L	5	5528.00
cta	L	38831	7534.00
ctg	L	58	6691.00
att	I	0	229.70
atc	I	0	157.50
ata	I	631	243.80
atg	M	1	1.00
gtt	V	0	0.88
gtc	V	0	0.73
gta	V	4	1.08
gtg	V	0	1.31
---	--	------	------
mPD		0.047	1.2
		nPD:	0.04
		N. weight:	1.7
		Sc. PD:	-0.04
		Sc. rank:	21249.0

HA			
Pos.	251	obs:	exp:
ttt	F	1	0.43
ttc	F	0	0.57
tta	L	47	5.39
ttg	L	0	9.23
ctt	L	0	9.66
ctc	L	0	6.92
cta	L	2	9.43
ctg	L	0	8.38
att	I	0	284.30
atc	I	0	195.00
ata	I	781	301.70
gtt	V	10	8608.00
gtc	V	1	7075.00
gta	V	38826	10472.00
gtg	V	100	12782.00
gct	A	0	0.26
gcc	A	0	0.21
gca	A	1	0.47
gcg	A	0	0.06
gaa	E	1	0.61
gag	E	0	0.39
---	--	------	------
mPD		0.047	0.78
		nPD:	0.06
		N. weight:	1.4
		Sc. PD:	-0.0055
		Sc. rank:	23933.0

HA			
Pos.	252	obs:	exp:
aat	N	0	0.59
aac	N	1	0.41
aaa	K	26	40.50
aag	K	45	30.50
gtt	V	0	0.22
gtc	V	0	0.18
gta	V	0	0.27
gtg	V	1	0.33
gct	A	0	0.53
gcc	A	0	0.41
gca	A	0	0.94
gcg	A	2	0.12
gat	D	65	997.40
gac	D	1828	895.60
gaa	E	10751	22876.00
gag	E	27029	14904.00
ggt	G	0	3.34
ggc	G	0	3.26
gga	G	7	9.79
ggg	G	15	5.62
---	--	------	------
mPD		0.47	0.53
		nPD:	0.88
		N. weight:	0.25
		Sc. PD:	0.2
		Sc. rank:	7887.5

HA			
Pos.	253	obs:	exp:
tta	L	0	0.11
ttg	L	0	0.19
tct	S	0	0.18
tcc	S	0	0.12
tca	S	1	0.26
tcg	S	0	0.05
ctt	L	0	0.20
ctc	L	0	0.14
cta	L	0	0.19
ctg	L	1	0.17
cct	P	52	10951.00
ccc	P	2	7373.00
cca	P	994	16926.00
ccg	P	38704	4501.00
caa	Q	0	1.58
cag	Q	3	1.42
agt	S	0	0.19
agc	S	0	0.20
gct	A	0	3.44
gcc	A	0	2.69
gca	A	1	6.11
gcg	A	12	0.76
---	--	------	------
mPD		0.052	0.70
		nPD:	0.08
		N. weight:	2.2
		Sc. PD:	0.024
		Sc. rank:	42551.0

HA			
Pos.	254	obs:	exp:
cgt	R	0	0.16
cgc	R	0	0.31
cga	R	0	0.59
cgg	R	0	0.44
aga	R	6	3.01
agg	R	0	1.48
gaa	E	6	4.24
gag	E	1	2.76
ggt	G	342	6028.00
ggc	G	2	5888.00
gga	G	39316	17685.00
ggg	G	97	10155.00
---	--	------	------
mPD		0.023	0.69
		nPD:	0.03
		N. weight:	0.82
		Sc. PD:	-0.025
		Sc. rank:	7311.6

HA			
Pos.	255	obs:	exp:
aat	N	0	2.37
aac	N	4	1.63
gat	D	276	20943.00
gac	D	39471	18804.00
gaa	E	8	4.84
gag	E	0	3.16
ggt	G	0	1.67
ggc	G	11	1.63
gga	G	0	4.89
ggg	G	0	2.81
---	--	------	------
mPD		0.015	0.50
		nPD:	0.03
		N. weight:	0.76
		Sc. PD:	-0.025
		Sc. rank:	5434.6

HA			
Pos.	256	obs:	exp:
caa	Q	2	1.06
cag	Q	0	0.94
cgt	R	0	0.90
cgc	R	0	1.73
cga	R	0	3.23
cgg	R	0	2.42
act	T	0	2.77
acc	T	0	2.26
aca	T	11	5.03
acg	T	0	0.94
aat	N	0	2.96
aac	N	5	2.04
aaa	K	39685	22655.00
aag	K	31	17061.00
aga	R	33	16.57
agg	R	0	8.16
gaa	E	3	1.82
gag	E	0	1.18
---	--	------	------
mPD		0.0043	0.49
		nPD:	0.01
		N. weight:	0.6
		Sc. PD:	-0.032
		Sc. rank:	-10369.0

HA			
Pos.	257	obs:	exp:
att	I	5	14393.00
atc	I	4	9869.00
ata	I	39529	15276.00
atg	M	2	2.00
gtt	V	0	50.85
gtc	V	0	41.79
gta	V	230	61.86
gtg	V	0	75.50
---	--	------	------
mPD		0.012	0.67
		nPD:	0.02
		N. weight:	1.
		Sc. PD:	-0.046
		Sc. rank:	-2744.2

HA			
Pos.	258	obs:	exp:
cct	P	0	0.55
ccc	P	0	0.37
cca	P	2	0.85
ccg	P	0	0.23
cgt	R	0	0.03
cgc	R	0	0.05
cga	R	0	0.10
cgg	R	0	0.07
att	I	0	13.47
atc	I	0	9.24
ata	I	37	14.30
act	T	17	10014.00
acc	T	8	8148.00
aca	T	39626	18173.00
acg	T	74	3389.00
aaa	K	4	2.28
aag	K	0	1.72
aga	R	1	0.50
agg	R	0	0.25
gct	A	0	0.26
gcc	A	0	0.21
gca	A	1	0.47
gcg	A	0	0.06
---	--	------	------
mPD		0.0072	0.68
		nPD:	0.01
		N. weight:	0.83
		Sc. PD:	-0.043
		Sc. rank:	-10733.0

HA			
Pos.	259	obs:	exp:
ttt	F	534	17062.00
ttc	F	39225	22697.00
tta	L	3	0.55
ttg	L	2	0.94
tat	Y	0	0.52
tac	Y	1	0.48
ctt	L	0	0.99
ctc	L	0	0.71
cta	L	0	0.96
ctg	L	0	0.85
att	I	0	1.82
atc	I	5	1.25
ata	I	0	1.93
---	--	------	------
mPD		0.027	0.49
		nPD:	0.06
		N. weight:	0.53
		Sc. PD:	-0.0046
		Sc. rank:	8653.0

,

HA			
Pos.	260	obs:	exp:
aaa	K	4	2.28
aag	K	0	1.72
gat	D	1	0.53
gac	D	0	0.47
gaa	E	39237	24077.00
gag	E	526	15686.00
ggt	G	0	0.30
ggc	G	0	0.30
gga	G	2	0.89
ggg	G	0	0.51
---	--	------	------
mPD		0.026	0.48
		nPD:	0.06
		N. weight:	0.47
		Sc. PD:	-0.0039
		Sc. rank:	7736.7

,

HA			
Pos.	261	obs:	exp:
tct	S	0	1.10
tcc	S	0	0.69
tca	S	6	1.56
tcg	S	0	0.32
act	T	0	1.76
acc	T	0	1.44
aca	T	7	3.20
acg	T	0	0.60
agt	S	0	1.13
agc	S	0	1.19
gct	A	28	10526.00
gcc	A	13	8230.00
gca	A	39704	18672.00
gcg	A	12	2329.00
---	--	------	------
mPD		0.0033	0.66
		nPD:	0.
		N. weight:	0.81
		Sc. PD:	-0.046
		Sc. rank:	-19311.0

,

HA			
Pos.	262	obs:	exp:
tct	S	3	0.55
tcc	S	0	0.35
tca	S	0	0.78
tcg	S	0	0.16
att	I	1	0.36
atc	I	0	0.25
ata	I	0	0.39
act	T	39738	10023.00
acc	T	10	8155.00
aca	T	7	18189.00
acg	T	5	3392.00
aat	N	6	3.55
aac	N	0	2.45
agt	S	0	0.56
agc	S	0	0.60
---	--	------	------
mPD		0.0016	0.68
		nPD:	0.
		N. weight:	1.5
		Sc. PD:	-0.089
		Sc. rank:	-43896.0

,

HA			
Pos.	263	obs:	exp:
ggt	G	2092	6030.00
ggc	G	2	5890.00
gga	G	37609	17690.00
ggg	G	67	10159.00
---	--	------	------
mPD		0.10	0.69
		nPD:	0.15
		N. weight:	0.7
		Sc. PD:	0.058
		Sc. rank:	17890.0

,

HA			
Pos.	264	obs:	exp:
cat	H	1	0.59
cac	H	0	0.41
aat	N	39648	23520.00
aac	N	120	16248.00
gat	D	1	0.53
gac	D	0	0.47
---	--	------	------
mPD		0.0061	0.48
		nPD:	0.01
		N. weight:	0.55
		Sc. PD:	-0.027
		Sc. rank:	-5535.0

,

HA			
Pos.	265	obs:	exp:
tta	L	37	4371.00
ttg	L	0	7486.00
ctt	L	11	7835.00
ctc	L	2	5616.00
cta	L	39568	7655.00
ctg	L	143	6798.00
att	I	0	3.28
atc	I	0	2.25
ata	I	9	3.48
---	--	------	------
mPD		0.010	1.1
		nPD:	0.01
		N. weight:	1.7
		Sc. PD:	-0.094
		Sc. rank:	-28783.0

,

HA			
Pos.	266	obs:	exp:
tta	L	0	30.34
ttg	L	273	51.96
tgg	W	1	1.00
ctt	L	0	54.39
ctc	L	0	38.98
cta	L	0	53.13
ctg	L	3	47.19
atg	M	15	15.00
gtt	V	9	8727.00
gtc	V	0	7173.00
gta	V	94	10616.00
gtg	V	39372	12958.00
gct	A	0	0.79
gcc	A	0	0.62
gca	A	0	1.41
gcg	A	3	0.18
---	--	------	------
mPD		0.020	0.75
		nPD:	0.03
		N. weight:	1.2
		Sc. PD:	-0.043
		Sc. rank:	5616.8

,

HA			
Pos.	267	obs:	exp:
tta	L	2	0.22
ttg	L	0	0.38
ctt	L	0	0.39
ctc	L	0	0.28
cta	L	0	0.39
ctg	L	0	0.34
att	I	0	9.83
atc	I	0	6.74
ata	I	27	10.43
act	T	0	0.25
acc	T	0	0.21
aca	T	1	0.46
acg	T	0	0.09
gtt	V	11	8263.00
gtc	V	1	6791.00
gta	V	37128	10052.00
gtg	V	235	12269.00
gct	A	1	626.10
gcc	A	1	489.50
gca	A	2356	1111.00
gcg	A	7	138.60
---	--	------	------
mPD		0.13	0.85
		nPD:	0.15
		N. weight:	1.3
		Sc. PD:	0.11
		Sc. rank:	34057.0

,

HA			
Pos.	268	obs:	exp:
tct	S	0	0.37
tcc	S	0	0.23
tca	S	0	0.52
tcg	S	2	0.11
cct	P	17	10956.00
ccc	P	0	7376.00
cca	P	650	16933.00
ccg	P	39101	4503.00
agt	S	0	0.38
agc	S	0	0.40
---	--	------	------
mPD		0.033	0.70
		nPD:	0.05
		N. weight:	2.2
		Sc. PD:	-0.036
		Sc. rank:	33632.0

,

HA			
Pos.	269	obs:	exp:
cgt	R	0	1085.00
cgc	R	0	2079.00
cga	R	1	3893.00
cgg	R	0	2909.00
att	I	0	0.36
atc	I	0	0.25
ata	I	1	0.39
aaa	K	16	9.13
aag	K	0	6.87
aga	R	38893	19956.00
agg	R	856	9827.00
ggt	G	0	0.45
ggc	G	0	0.44
gga	G	3	1.33
ggg	G	0	0.77
---	--	------	------
mPD		0.043	0.91
		nPD:	0.05
		N. weight:	0.65
		Sc. PD:	-0.01
		Sc. rank:	9595.1

,

HA			
Pos.	270	obs:	exp:
ttt	F	10	4.29
ttc	F	0	5.71
tct	S	1	0.18
tcc	S	0	0.12
tca	S	0	0.26
tcg	S	0	0.05
tat	Y	39663	20655.00
tac	Y	90	19098.00
tgt	C	1	0.45
tgc	C	0	0.55
cat	H	5	2.94
cac	H	0	2.06
agt	S	0	0.19
agc	S	0	0.20
---	--	------	------
mPD		0.0054	0.50
		nPD:	0.01
		N. weight:	0.69
		Sc. PD:	-0.036
		Sc. rank:	-8829.2

,

HA

Pos.	271	obs:	exp:
tct	S	0	0.18
tcc	S	0	0.12
tca	S	1	0.26
tcg	S	0	0.05
agt	S	0	0.19
agc	S	0	0.20
gtt	V	0	0.66
gtc	V	0	0.55
gta	V	3	0.81
gtg	V	0	0.98
gct	A	14	10528.00
gcc	A	3	8231.00
gca	A	39710	18677.00
gcg	A	39	2330.00
---	--	------	------
mPD		0.0030	0.66
		nPD:	0.
		N. weight:	0.81
		Sc. PD:	-0.047
		Sc. rank:	-20412.0

HA

Pos.	272	obs:	exp:
ttt	F	205	17065.00
ttc	F	39562	22702.00
tta	L	0	0.11
ttg	L	0	0.19
tat	Y	0	0.52
tac	Y	1	0.48
tgt	C	0	0.45
tgc	C	1	0.55
ctt	L	0	0.20
ctc	L	1	0.14
cta	L	0	0.19
ctg	L	0	0.17
---	--	------	------
mPD		0.010	0.49
		nPD:	0.02
		N. weight:	0.57
		Sc. PD:	-0.024
		Sc. rank:	37.7

HA

Pos.	273	obs:	exp:
tct	S	0	1.29
tcc	S	0	0.81
tca	S	7	1.82
tcg	S	0	0.37
cct	P	0	0.55
ccc	P	0	0.37
cca	P	2	0.85
ccg	P	0	0.23
att	I	0	71.35
atc	I	0	48.93
ata	I	196	75.73
atg	M	1	1.00
act	T	160	7646.00
acc	T	7	6221.00
aca	T	30143	13875.00
acg	T	19	2588.00
aaa	K	11	6.27
aag	K	0	4.73
agt	S	0	1.32
agc	S	0	1.39
gtt	V	0	2.87
gtc	V	0	2.36
gta	V	13	3.50
gtg	V	0	4.27
gct	A	1	2437.00
gcc	A	1	1905.00
gca	A	9197	4323.00
gcg	A	6	539.30
gaa	E	6	3.63
gag	E	0	2.37
---	--	------	------
mPD		0.38	1.0
		nPD:	0.36
		N. weight:	0.81
		Sc. PD:	0.24
		Sc. rank:	23161.0

HA

Pos.	274	obs:	exp:
tta	L	0	1.10
ttg	L	2	1.88
ctt	L	0	1.97
ctc	L	0	1.41
cta	L	0	1.93
ctg	L	8	1.71
att	I	76	59.34
atc	I	0	40.69
ata	I	87	62.98
atg	M	39557	39557.00
act	T	0	0.50
acc	T	0	0.41
aca	T	0	0.91
acg	T	2	0.17
gtt	V	0	8.40
gtc	V	0	6.90
gta	V	0	10.22
gtg	V	38	12.47
---	--	------	------
mPD		0.011	0.012
		nPD:	0.86
		N. weight:	0.003
		Sc. PD:	0.0024
		Sc. rank:	95.4

HA

Pos.	275	obs:	exp:
caa	Q	2	1.06
cag	Q	0	0.94
aaa	K	48	27.95
aag	K	1	21.05
gtt	V	0	1.99
gtc	V	0	1.64
gta	V	9	2.42
gtg	V	0	2.95
gct	A	0	0.26
gcc	A	1	0.21
gca	A	0	0.47
gcg	A	0	0.06
gat	D	1	5.80
gac	D	10	5.20
gaa	E	39214	24031.00
gag	E	473	15656.00
ggt	G	0	1.67
ggc	G	0	1.63
gga	G	11	4.89
ggg	G	0	2.81
---	--	------	------
mPD		0.028	0.48
		nPD:	0.06
		N. weight:	0.48
		Sc. PD:	-0.0029
		Sc. rank:	7966.4

HA

Pos.	276	obs:	exp:
tct	S	0	0.37
tcc	S	0	0.23
tca	S	0	0.52
tcg	S	0	0.11
cgt	R	0	1074.00
cgc	R	0	2058.00
cga	R	8	3854.00
cgg	R	0	2880.00
att	I	0	0.36
atc	I	0	0.25
ata	I	1	0.39
aaa	K	414	236.20
aag	K	0	177.80
agt	S	2	0.38
agc	S	0	0.40
aga	R	39259	19755.00
agg	R	83	9728.00
ggt	G	0	0.45
ggc	G	0	0.44
gga	G	3	1.33
ggg	G	0	0.77
---	--	------	------
mPD		0.025	0.93
		nPD:	0.03
		N. weight:	0.73
		Sc. PD:	-0.026
		Sc. rank:	3760.2

HA

Pos.	277	obs:	exp:
tct	S	0	6.07
tcc	S	0	3.82
tca	S	0	8.59
tcg	S	0	1.76
tat	Y	2	1.04
tac	Y	0	0.96
cat	H	8	5.29
cac	H	1	3.71
cgt	R	0	0.03
cgc	R	0	0.05
cga	R	0	0.10
cgg	R	0	0.07
att	I	2	0.73
atc	I	0	0.50
ata	I	0	0.77
act	T	65	16.89
acc	T	2	13.74
aca	T	0	30.65
acg	T	0	5.72
aat	N	26714	16103.00
aac	N	513	11124.00
aaa	K	11	9.13
aag	K	5	6.87
agt	S	33	6.21
agc	S	0	6.55
aga	R	1	0.50
agg	R	0	0.25
gct	A	1	0.26
gcc	A	0	0.21
gca	A	0	0.47
gcg	A	0	0.06
gat	D	12373	6530.00
gac	D	20	5863.00
gaa	E	14	9.69
gag	E	2	6.31
ggt	G	3	0.45
ggc	G	0	0.44
gga	G	0	1.33
ggg	G	0	0.77
---	--	------	------
mPD		0.46	0.93
		nPD:	0.5
		N. weight:	0.54
		Sc. PD:	0.23
		Sc. rank:	16611.0

HA

Pos.	278	obs:	exp:
tct	S	176	32.39
tcc	S	0	20.39
tca	S	0	45.80
tcg	S	0	9.37
act	T	119	30.00
acc	T	0	24.41
aca	T	0	54.44
acg	T	0	10.15
agt	S	0	33.09
agc	S	0	34.96
gtt	V	38	8.40
gtc	V	0	6.90
gta	V	0	10.22
gtg	V	0	12.47
gct	A	39251	10421.00
gcc	A	51	8147.00
gca	A	11	18486.00
gcg	A	47	2306.00
gat	D	74	38.99
gac	D	0	35.01
ggt	G	3	0.45
ggc	G	0	0.44
gga	G	0	1.33
ggg	G	0	0.77
---	--	------	------
mPD		0.026	0.69
		nPD:	0.04
		N. weight:	1.4
		Sc. PD:	-0.036
		Sc. rank:	15079.0

HA			
Pos.	279	obs:	exp:
cgt	R	0	0.11
cgc	R	0	0.21
cga	R	2	0.39
cgg	R	0	0.29
aga	R	2	2.01
agg	R	0	0.99
gct	A	0	0.53
gcc	A	0	0.41
gca	A	2	0.94
gcg	A	0	0.12
gaa	E	72	43.60
gag	E	0	28.40
ggt	G	11	6019.00
ggc	G	2	5879.00
gga	G	39421	17656.00
ggg	G	258	10139.00
---	--	------	------
mPD		0.017	0.70
		nPD:	0.03
		N. weight:	0.83
		Sc. PD:	-0.032
		Sc. rank:	2803.2

,

HA			
Pos.	280	obs:	exp:
ttt	F	10	4.29
ttc	F	0	5.71
tct	S	39650	7312.00
tcc	S	22	4602.00
tca	S	56	10341.00
tcg	S	3	2115.00
tat	Y	7	3.64
tac	Y	0	3.36
cct	P	4	1.10
ccc	P	0	0.74
cca	P	0	1.70
ccg	P	0	0.45
act	T	3	0.76
acc	T	0	0.62
aca	T	0	1.37
acg	T	0	0.26
agt	S	2	7471.00
agc	S	0	7892.00
gct	A	13	3.44
gcc	A	0	2.69
gca	A	0	6.11
gcg	A	0	0.76
---	--	------	------
mPD		0.0061	1.6
		nPD:	0.
		N. weight:	1.8
		Sc. PD:	-0.11
		Sc. rank:	-47884.0

,

HA			
Pos.	281	obs:	exp:
tct	S	0	1.47
tcc	S	0	0.93
tca	S	0	2.08
tcg	S	0	0.43
agt	S	8	1.50
agc	S	0	1.59
gct	A	2	0.53
gcc	A	0	0.41
gca	A	0	0.94
gcg	A	0	0.12
gat	D	3	1.58
gac	D	0	1.42
ggt	G	39555	6028.00
ggc	G	176	5888.00
gga	G	5	17685.00
ggg	G	21	10155.00
---	--	------	------
mPD		0.011	0.69
		nPD:	0.02
		N. weight:	2.
		Sc. PD:	-0.095
		Sc. rank:	-10084.0

,

HA			
Pos.	282	obs:	exp:
ttt	F	1	0.43
ttc	F	0	0.57
att	I	39637	14461.00
atc	I	87	9916.00
ata	I	1	15348.00
act	T	40	10.08
acc	T	0	8.20
aca	T	0	18.30
acg	T	0	3.41
aat	N	1	0.59
aac	N	0	0.41
gtt	V	3	0.66
gtc	V	0	0.55
gta	V	0	0.81
gtg	V	0	0.98
---	--	------	------
mPD		0.0067	0.66
		nPD:	0.01
		N. weight:	1.1
		Sc. PD:	-0.057
		Sc. rank:	-15301.0

,

HA			
Pos.	283	obs:	exp:
ttt	F	0	4.29
ttc	F	10	5.71
tta	L	0	1.21
ttg	L	0	2.07
ctt	L	0	2.17
ctc	L	11	1.55
cta	L	0	2.12
ctg	L	0	1.88
att	I	114	14416.00
atc	I	39466	9885.00
ata	I	20	15300.00
act	T	0	0.25
acc	T	1	0.21
aca	T	0	0.46
acg	T	0	0.09
gtt	V	0	32.72
gtc	V	148	26.89
gta	V	0	39.80
gtg	V	0	48.58
---	--	------	------
mPD		0.015	0.67
		nPD:	0.02
		N. weight:	1.5
		Sc. PD:	-0.059
		Sc. rank:	2471.1

,

HA			
Pos.	284	obs:	exp:
ttt	F	3	1.29
ttc	F	0	1.71
tta	L	0	0.55
ttg	L	0	0.94
tct	S	0	7.91
tcc	S	0	4.98
tca	S	0	11.19
tcg	S	0	2.29
ctt	L	5	0.99
ctc	L	0	0.71
cta	L	0	0.96
ctg	L	0	0.85
att	I	39359	14430.00
atc	I	275	9895.00
ata	I	6	15315.00
atg	M	2	2.00
act	T	64	16.13
acc	T	0	13.13
aca	T	0	29.28
acg	T	0	5.46
agt	S	43	8.09
agc	S	0	8.54
gtt	V	13	2.87
gtc	V	0	2.36
gta	V	0	3.50
gtg	V	0	4.27
---	--	------	------
mPD		0.021	0.66
		nPD:	0.03
		N. weight:	1.1
		Sc. PD:	-0.034
		Sc. rank:	8814.9

,

HA			
Pos.	285	obs:	exp:
tct	S	17	7316.00
tcc	S	10	4605.00
tca	S	39674	10346.00
tcg	S	54	2116.00
cct	P	0	0.55
ccc	P	0	0.37
cca	P	2	0.85
ccg	P	0	0.23
act	T	0	3.03
acc	T	0	2.46
aca	T	12	5.49
acg	T	0	1.02
agt	S	0	7475.00
agc	S	0	7897.00
gct	A	0	0.26
gcc	A	0	0.21
gca	A	1	0.47
gcg	A	0	0.06
---	--	------	------
mPD		0.0048	1.6
		nPD:	0.
		N. weight:	1.4
		Sc. PD:	-0.086
		Sc. rank:	-40545.0

,

HA			
Pos.	286	obs:	exp:
aat	N	58	34.89
aac	N	1	24.11
gat	D	39497	20912.00
gac	D	192	18777.00
gaa	E	9	6.66
gag	E	2	4.34
ggt	G	11	1.67
ggc	G	0	1.63
gga	G	0	4.89
ggg	G	0	2.81
---	--	------	------
mPD		0.014	0.50
		nPD:	0.03
		N. weight:	0.66
		Sc. PD:	-0.024
		Sc. rank:	3359.6

,

HA			
Pos.	287	obs:	exp:
tct	S	0	0.74
tcc	S	0	0.46
tca	S	4	1.04
tcg	S	0	0.21
att	I	0	4.00
atc	I	0	2.75
ata	I	11	4.25
atg	M	1	1.00
act	T	10	9976.00
acc	T	8	8117.00
aca	T	38995	18104.00
acg	T	560	3376.00
aaa	K	18	10.27
aag	K	0	7.73
agt	S	0	0.75
agc	S	0	0.79
gct	A	0	43.16
gcc	A	0	33.74
gca	A	163	76.56
gcg	A	0	9.55
---	--	------	------
mPD		0.039	0.69
		nPD:	0.06
		N. weight:	0.79
		Sc. PD:	-0.006
		Sc. rank:	13011.0

HA			
Pos.	288	obs:	exp:
tta	L	0	1.76
ttg	L	0	3.01
tct	S	0	41.96
tcc	S	0	26.41
tca	S	227	59.34
tcg	S	1	12.13
ctt	L	0	3.15
ctc	L	0	2.26
cta	L	16	3.08
ctg	L	0	2.74
cct	P	14	10874.00
ccc	P	2	7321.00
cca	P	39348	16806.00
ccg	P	106	4469.00
caa	Q	20	10.55
cag	Q	0	9.45
act	T	0	6.30
acc	T	0	5.13
aca	T	25	11.44
acg	T	0	2.13
agt	S	0	42.87
agc	S	0	45.29
gct	A	0	2.65
gcc	A	0	2.07
gca	A	10	4.70
gcg	A	0	0.59
ggt	G	0	0.15
ggc	G	0	0.15
gga	G	1	0.44
ggg	G	0	0.26
---	--	------	------
mPD		0.021	0.72
		nPD:	0.03
		N. weight:	0.91
		Sc. PD:	-0.03
		Sc. rank:	6370.9

HA			
Pos.	289	obs:	exp:
ttt	F	1	12.44
ttc	F	28	16.56
att	I	2	126.30
atc	I	345	86.62
ata	I	0	134.10
act	T	0	0.50
acc	T	2	0.41
aca	T	0	0.91
acg	T	0	0.17
gtt	V	478	8622.00
gtc	V	38499	7087.00
gta	V	21	10488.00
gtg	V	1	12802.00
gct	A	1	103.30
gcc	A	389	80.73
gca	A	0	183.20
gcg	A	0	22.85
ggt	G	0	0.45
ggc	G	3	0.44
gga	G	0	1.33
ggg	G	0	0.77
---	--	------	------
mPD		0.064	0.78
		nPD:	0.08
		N. weight:	1.8
		Sc. PD:	0.031
		Sc. rank:	36298.0

HA			
Pos.	290	obs:	exp:
tta	L	0	0.44
ttg	L	0	0.75
tat	Y	4	75.86
tac	Y	142	70.14
ctt	L	0	0.79
ctc	L	4	0.56
cta	L	0	0.77
ctg	L	0	0.68
cat	H	635	23173.00
cac	H	38768	16230.00
caa	Q	121	64.38
cag	Q	1	57.62
cgt	R	0	0.68
cgc	R	25	1.31
cga	R	0	2.45
cgg	R	0	1.83
aat	N	1	39.63
aac	N	66	27.37
aaa	K	1	0.57
aag	K	0	0.43
aga	R	0	12.55
agg	R	0	6.18
gat	D	0	1.05
gac	D	2	0.95
---	--	------	------
mPD		0.050	0.50
		nPD:	0.1
		N. weight:	0.86
		Sc. PD:	0.03
		Sc. rank:	19422.0

HA			
Pos.	291	obs:	exp:
tat	Y	9	4.68
tac	Y	0	4.32
cat	H	1	0.59
cac	H	0	0.41
aat	N	331	195.80
aac	N	0	135.20
gtt	V	1	0.22
gtc	V	0	0.18
gta	V	0	0.27
gtg	V	0	0.33
gat	D	38925	20755.00
gac	D	465	18635.00
gaa	E	8	14.53
gag	E	16	9.47
ggt	G	14	2.12
ggc	G	0	2.07
gga	G	0	6.23
ggg	G	0	3.58
---	--	------	------
mPD		0.042	0.52
		nPD:	0.08
		N. weight:	0.62
		Sc. PD:	0.011
		Sc. rank:	12710.0

HA			
Pos.	292	obs:	exp:
tgt	C	40	17951.00
tgc	C	39730	21819.00
---	--	------	------
mPD		0.0020	0.50
		nPD:	0.
		N. weight:	0.64
		Sc. PD:	-0.038
		Sc. rank:	-16531.0

HA			
Pos.	293	obs:	exp:
tct	S	0	2.94
tcc	S	0	1.85
tca	S	0	4.16
tcg	S	0	0.85
cat	H	1	0.59
cac	H	0	0.41
act	T	2	0.50
acc	T	0	0.41
aca	T	0	0.91
acg	T	0	0.17
aat	N	39322	23508.00
aac	N	425	16239.00
agt	S	16	3.01
agc	S	0	3.18
gat	D	4	2.11
gac	D	0	1.89
---	--	------	------
mPD		0.022	0.49
		nPD:	0.05
		N. weight:	0.51
		Sc. PD:	-0.0089
		Sc. rank:	7032.6

HA			
Pos.	294	obs:	exp:
tct	S	0	0.18
tcc	S	0	0.12
tca	S	1	0.26
tcg	S	0	0.05
act	T	8	10000.00
acc	T	5	8137.00
aca	T	39595	18148.00
acg	T	62	3385.00
agt	S	0	0.19
agc	S	0	0.20
gct	A	0	26.21
gcc	A	0	20.49
gca	A	99	46.50
gcg	A	0	5.80
---	--	------	------
mPD		0.0088	0.68
		nPD:	0.01
		N. weight:	0.83
		Sc. PD:	-0.042
		Sc. rank:	-8043.1

HA			
Pos.	295	obs:	exp:
tct	S	3	0.74
tcc	S	0	0.46
tca	S	0	1.04
tcg	S	0	0.21
att	I	17	6.19
atc	I	0	4.24
ata	I	0	6.57
act	T	39647	10015.00
acc	T	57	8149.00
aca	T	6	18174.00
acg	T	17	3390.00
aat	N	7	4.14
aac	N	0	2.86
agt	S	1	0.75
agc	S	0	0.79
gct	A	15	3.97
gcc	A	0	3.10
gca	A	0	7.04
gcg	A	0	0.88
---	--	------	------
mPD		0.0062	0.68
		nPD:	0.01
		N. weight:	1.5
		Sc. PD:	-0.079
		Sc. rank:	-23563.0

HA			
Pos.	296	obs:	exp:
tgt	C	39551	17950.00
tgc	C	218	21819.00
tgg	W	1	1.00
---	--	------	------
mPD		0.011	0.50
		nPD:	0.02
		N. weight:	0.82
		Sc. PD:	-0.034
		Sc. rank:	762.5

HA			
Pos.	297	obs:	exp:
caa	Q	980	20985.00
cag	Q	38789	18784.00
gaa	E	0	0.61
gag	E	1	0.39
---	--	------	------
mPD		0.048	0.50
		nPD:	0.1
		N. weight:	0.68
		Sc. PD:	0.022
		Sc. rank:	15188.0

HA			
Pos.	298	obs:	exp:
act	T	3	10025.00
acc	T	1590	8157.00
aca	T	38145	18192.00
acg	T	29	3393.00
gct	A	0	0.79
gcc	A	0	0.62
gca	A	3	1.41
gcg	A	0	0.18
---	--	------	------
mPD		0.078	0.68
		nPD:	0.12
		N. weight:	0.69
		Sc. PD:	0.035
		Sc. rank:	16137.0

HA			
Pos.	299	obs:	exp:
tta	L	0	1.87
ttg	L	0	3.20
tct	S	0	5.70
tcc	S	31	3.59
tca	S	0	8.07
tcg	S	0	1.65
ctt	L	0	3.35
ctc	L	17	2.40
cta	L	0	3.27
ctg	L	0	2.91
cct	P	2071	10734.00
ccc	P	36842	7227.00
cca	P	50	16591.00
ccg	P	1	4412.00
cat	H	0	0.59
cac	H	1	0.41
act	T	0	2.27
acc	T	9	1.85
aca	T	0	4.12
acg	T	0	0.77
agt	S	0	5.83
agc	S	0	6.16
gtt	V	0	0.88
gtc	V	4	0.73
gta	V	0	1.08
gtg	V	0	1.31
gct	A	0	197.00
gcc	A	743	154.00
gca	A	1	349.40
gcg	A	0	43.59
---	--	------	------
mPD		0.14	0.74
		nPD:	0.19
		N. weight :	1.6
		Sc. PD:	0.2
		Sc. rank:	41547.0

,

HA			
Pos.	300	obs:	exp:
caa	Q	0	1.58
cag	Q	3	1.42
cgt	R	0	0.63
cgc	R	0	1.20
cga	R	0	2.25
cgg	R	0	1.68
atg	M	1	1.00
act	T	0	0.25
acc	T	0	0.21
aca	T	0	0.46
acg	T	1	0.09
aat	N	47	28.98
aac	N	2	20.02
aaa	K	120	4802.00
aag	K	8299	3617.00
aga	R	0	11.55
agg	R	23	5.69
gtt	V	0	0.44
gtc	V	0	0.36
gta	V	0	0.54
gtg	V	2	0.66
gct	A	0	0.26
gcc	A	0	0.21
gca	A	0	0.47
gcg	A	1	0.06
gat	D	77	40.57
gac	D	0	36.43
gaa	E	409	18873.00
gag	E	30761	12297.00
ggt	G	0	3.64
ggc	G	0	3.55
gga	G	1	10.68
ggg	G	23	6.13
---	--	------	------
mPD		0.37	0.82
		nPD:	0.45
		N. weight :	0.9
		Sc. PD:	0.34
		Sc. rank:	27509.0

,

HA			
Pos.	301	obs:	exp:
tct	S	0	0.18
tcc	S	0	0.12
tca	S	0	0.26
tcg	S	0	0.05
agt	S	1	0.19
agc	S	0	0.20
gtt	V	2	0.44
gtc	V	0	0.36
gta	V	0	0.54
gtg	V	0	0.66
ggt	G	39652	6030.00
ggc	G	43	5890.00
gga	G	14	17689.00
ggg	G	58	10158.00
---	--	------	------
mPD		0.0059	0.69
		nPD:	0.01
		N. weight :	2.
		Sc. PD:	-0.11
		Sc. rank:	-34903.0

,

HA			
Pos.	302	obs:	exp:
tct	S	63	11.59
tcc	S	0	7.30
tca	S	0	16.40
tcg	S	0	3.35
act	T	2	0.50
acc	T	0	0.41
aca	T	0	0.91
acg	T	0	0.17
agt	S	0	11.85
agc	S	0	12.51
gct	A	39614	10512.00
gcc	A	63	8218.00
gca	A	6	18647.00
gcg	A	20	2326.00
gat	D	1	0.53
gac	D	0	0.47
ggt	G	1	0.15
ggc	G	0	0.15
gga	G	0	0.44
ggg	G	0	0.26
---	--	------	------
mPD		0.0078	0.67
		nPD:	0.01
		N. weight :	1.4
		Sc. PD:	-0.072
		Sc. rank:	-15517.0

,

HA			
Pos.	303	obs:	exp:
tta	L	14	30.01
ttg	L	0	51.40
ctt	L	0	53.80
ctc	L	0	38.56
cta	L	259	52.56
ctg	L	0	46.68
att	I	2	14248.00
atc	I	14	9770.00
ata	I	39124	15122.00
atg	M	26	26.00
act	T	0	0.50
acc	T	0	0.41
aca	T	2	0.91
acg	T	0	0.17
gtt	V	0	72.74
gtc	V	0	59.78
gta	V	329	88.48
gtg	V	0	108.00
---	--	------	------
mPD		0.032	0.69
		nPD:	0.05
		N. weight :	1.
		Sc. PD:	-0.017
		Sc. rank:	14815.0

,

HA			
Pos.	304	obs:	exp:
cat	H	0	0.59
cac	H	1	0.41
act	T	0	0.76
acc	T	3	0.62
aca	T	0	1.37
acg	T	0	0.26
aat	N	464	23510.00
aac	N	39287	16241.00
aaa	K	7	4.56
aag	K	1	3.44
gat	D	0	3.69
gac	D	7	3.31
---	--	------	------
mPD		0.024	0.48
		nPD:	0.05
		N. weight :	0.89
		Sc. PD:	-0.012
		Sc. rank:	13797.0

,

HA			
Pos.	305	obs:	exp:
act	T	0	0.25
acc	T	1	0.21
aca	T	0	0.46
acg	T	0	0.09
---	-	39769	39769.00
---	--	------	------
mPD		0	4.3×10^{-10}
		nPD:	0.
		N. weight :	0.
		Sc. PD:	0
		Sc. rank:	0

,

HA			
Pos.	306	obs:	exp:
tct	S	0	0.18
tcc	S	0	0.12
tca	S	0	0.26
tcg	S	0	0.05
agt	S	0	0.19
agc	S	1	0.20
---	-	39769	39769.00
---	--	------	------
mPD		0	1.0×10^{-9}
		nPD:	0.
		N. weight :	0.
		Sc. PD:	0
		Sc. rank:	0

,

HA

Pos.	307	obs:	exp:
tta	L	0	0.11
ttg	L	0	0.19
ctt	L	0	0.20
ctc	L	1	0.14
cta	L	0	0.19
ctg	L	0	0.17
---	-	39769	39769.00
---	--	------	------
mPD		0	7.2×10^{-10}
		nPD:	0.
		N. weight:	0.
		Sc. PD:	0
		Sc. rank:	0

,

HA

Pos.	308	obs:	exp:
tct	S	0	1.10
tcc	S	2	0.69
tca	S	0	1.56
tcg	S	0	0.32
cct	P	0	0.28
ccc	P	1	0.19
cca	P	0	0.43
ccg	P	0	0.11
att	I	0	0.36
atc	I	1	0.25
ata	I	0	0.39
act	T	60	10023.00
acc	T	39664	8155.00
aca	T	36	18189.00
acg	T	0	3392.00
aat	N	0	0.59
aac	N	1	0.41
agt	S	0	1.13
agc	S	4	1.19
gct	A	0	0.26
gcc	A	1	0.21
gca	A	0	0.47
gcg	A	0	0.06
---	--	------	------
mPD		0.0053	0.68
		nPD:	0.01
		N. weight:	1.7
		Sc. PD:	-0.093
		Sc. rank:	-31251.0

,

HA

Pos.	309	obs:	exp:
tct	S	0	7317.00
tcc	S	0	4606.00
tca	S	0	10349.00
tcg	S	0	2116.00
cgt	R	0	0.03
cgc	R	0	0.05
cga	R	0	0.10
cgg	R	0	0.07
att	I	0	0.36
atc	I	1	0.25
ata	I	0	0.39
aat	N	0	1.18
aac	N	2	0.82
agt	S	1540	7477.00
agc	S	38223	7898.00
aga	R	0	0.50
agg	R	1	0.25
ggt	G	0	0.45
ggc	G	3	0.44
gga	G	0	1.33
ggg	G	0	0.77
---	--	------	------
mPD		0.075	1.6
		nPD:	0.05
		N. weight:	1.6
		Sc. PD:	-0.028
		Sc. rank:	21509.0

,

HA

Pos.	310	obs:	exp:
ttt	F	2	5.58
ttc	F	11	7.42
tta	L	0	4370.00
ttg	L	0	7484.00
ctt	L	2010	7833.00
ctc	L	37262	5615.00
cta	L	478	7653.00
ctg	L	0	6796.00
cct	P	0	0.55
ccc	P	2	0.37
cca	P	0	0.85
ccg	P	0	0.23
cat	H	0	1.76
cac	H	3	1.24
cgt	R	1	0.05
cgc	R	0	0.10
cga	R	1	0.20
cgg	R	0	0.15
aga	R	0	1.00
agg	R	0	0.49
---	--	------	------
mPD		0.12	1.1
		nPD:	0.11
		N. weight:	1.8
		Sc. PD:	0.073
		Sc. rank:	41729.0

,

HA

Pos.	311	obs:	exp:
tct	S	0	0.37
tcc	S	0	0.23
tca	S	2	0.52
tcg	S	0	0.11
cct	P	7	10956.00
ccc	P	7	7376.00
cca	P	39734	16933.00
ccg	P	20	4503.00
agt	S	0	0.38
agc	S	0	0.40
---	--	------	------
mPD		0.0018	0.70
		nPD:	0.
		N. weight:	0.92
		Sc. PD:	-0.055
		Sc. rank:	-26495.0

,

HA

Pos.	312	obs:	exp:
ttt	F	39571	17064.00
ttc	F	192	22699.00
tct	S	1	0.18
tcc	S	0	0.12
tca	S	0	0.26
tcg	S	0	0.05
tgt	C	1	0.45
tgc	C	0	0.55
att	I	5	1.82
atc	I	0	1.25
ata	I	0	1.93
agt	S	0	0.19
agc	S	0	0.20
---	--	------	------
mPD		0.010	0.49
		nPD:	0.02
		N. weight:	0.88
		Sc. PD:	-0.038
		Sc. rank:	-710.8

,

HA

Pos.	313	obs:	exp:
cat	H	307	181.10
cac	H	1	126.90
caa	Q	244	20820.00
cag	Q	39212	18636.00
cgt	R	0	0.16
cgc	R	0	0.31
cga	R	0	0.59
cgg	R	6	0.44
aga	R	0	3.01
agg	R	0	1.48
---	--	------	------
mPD		0.028	0.51
		nPD:	0.05
		N. weight:	0.77
		Sc. PD:	-0.0068
		Sc. rank:	12413.0

,

HA

Pos.	314	obs:	exp:
tct	S	1	3.86
tcc	S	0	2.43
tca	S	0	5.47
tcg	S	0	1.12
tat	Y	1	1.04
tac	Y	1	0.96
cat	H	1	0.59
cac	H	0	0.41
act	T	2	0.50
acc	T	0	0.41
aca	T	0	0.91
acg	T	0	0.17
aat	N	39424	23500.00
aac	N	309	16233.00
aaa	K	4	3.99
aag	K	3	3.01
agt	S	17	3.95
agc	S	3	4.17
gat	D	4	2.11
gac	D	0	1.89
---	--	------	------
mPD		0.018	0.49
		nPD:	0.04
		N. weight:	0.52
		Sc. PD:	-0.014
		Sc. rank:	5267.5

,

HA

Pos.	315	obs:	exp:
tta	L	2	0.44
ttg	L	0	0.75
ctt	L	0	0.79
ctc	L	0	0.56
cta	L	2	0.77
ctg	L	0	0.68
att	I	5	6635.00
atc	I	2	4550.00
ata	I	18220	7042.00
atg	M	2	2.00
act	T	0	1.01
acc	T	0	0.82
aca	T	4	1.83
acg	T	0	0.34
aat	N	0	0.59
aac	N	1	0.41
gtt	V	2	4760.00
gtc	V	2	3912.00
gta	V	21429	5791.00
gtg	V	98	7068.00
gct	A	0	0.26
gcc	A	0	0.21
gca	A	1	0.47
gcg	A	0	0.06
---	--	------	------
mPD		0.50	1.2
		nPD:	0.41
		N. weight:	1.2
		Sc. PD:	0.41
		Sc. rank:	35923.0

,

HA

Pos.	316	obs:	exp:
tct	S	0	1.66
tcc	S	0	1.04
tca	S	0	2.34
tcg	S	0	0.48
tat	Y	8	4.16
tac	Y	0	3.84
cct	P	1	0.28
ccc	P	0	0.19
cca	P	0	0.43
ccg	P	0	0.11
cat	H	38317	22689.00
cac	H	264	15892.00
aat	N	1168	691.40
aac	N	1	477.60
agt	S	9	1.69
agc	S	0	1.79
gct	A	1	0.26
gcc	A	0	0.21
gca	A	0	0.47
gcg	A	0	0.06
gat	D	1	0.53
gac	D	0	0.47
---	--	------	------
mPD		0.072	0.54
		nPD:	0.13
		N. weight:	0.53
		Sc. PD:	0.036
		Sc. rank:	13027.0

,

HA

Pos.	317	obs:	exp:
tct	S	0	25.95
tcc	S	0	16.33
tca	S	0	36.70
tcg	S	141	7.50
cct	P	44	10914.00
ccc	P	0	7348.00
cca	P	888	16868.00
ccg	P	38683	4486.00
caa	Q	0	5.80
cag	Q	11	5.20
cgt	R	0	0.08
cgc	R	0	0.16
cga	R	0	0.29
cgg	R	3	0.22
agt	S	0	26.51
agc	S	0	28.01
aga	R	0	1.51
agg	R	0	0.74
---	--	------	------
mPD		0.054	0.71
		nPD:	0.08
		N. weight:	2.2
		Sc. PD:	0.026
		Sc. rank:	43466.0

,

HA

Pos.	318	obs:	exp:
ttt	F	0	1.29
ttc	F	3	1.71
tta	L	0	0.55
ttg	L	0	0.94
tct	S	0	0.55
tcc	S	1	0.35
tca	S	0	0.78
tcg	S	0	0.16
ctt	L	0	0.99
ctc	L	5	0.71
cta	L	0	0.96
ctg	L	0	0.85
att	I	3877	14189.00
atc	I	35066	9730.00
ata	I	35	15059.00
act	T	0	6.30
acc	T	25	5.13
aca	T	0	11.44
acg	T	0	2.13
aat	N	0	0.59
aac	N	1	0.41
agt	S	1	0.56
agc	S	1	0.60
gtt	V	86	166.90
gtc	V	669	137.20
gta	V	0	203.10
gtg	V	0	247.80
---	--	------	------
mPD		0.22	0.70
		nPD:	0.31
		N. weight:	1.1
		Sc. PD:	0.27
		Sc. rank:	31007.0

,

HA			
Pos.	319	obs:	exp:
tct	S	0	0.55
tcc	S	0	0.35
tca	S	2	0.78
tcg	S	1	0.16
cgt	R	0	0.03
cgc	R	0	0.05
cga	R	0	0.10
cgg	R	0	0.07
att	I	0	1.46
atc	I	0	1.00
ata	I	4	1.55
atg	M	1	1.00
act	T	15	10021.00
acc	T	23	8154.00
aca	T	38961	18186.00
acg	T	754	3392.00
aaa	K	3	1.71
aag	K	0	1.29
agt	S	0	0.56
agc	S	0	0.60
aga	R	1	0.50
agg	R	0	0.25
gct	A	0	1.32
gcc	A	0	1.03
gca	A	5	2.35
gcg	A	0	0.29
---	--	------	------
mPD		0.040	0.68
		nPD:	0.06
		N. weight:	0.77
		Sc. PD:	-0.0037
		Sc. rank:	13084.0

,

HA			
Pos.	320	obs:	exp:
tta	L	0	0.11
ttg	L	0	0.19
ctt	L	1	0.20
ctc	L	0	0.14
cta	L	0	0.19
ctg	L	0	0.17
att	I	39716	14468.00
atc	I	21	9921.00
ata	I	7	15355.00
atg	M	19	19.00
aat	N	1	0.59
aac	N	0	0.41
gtt	V	5	1.11
gtc	V	0	0.91
gta	V	0	1.34
gtg	V	0	1.64
---	--	------	------
mPD		0.0027	0.66
		nPD:	0.
		N. weight:	1.1
		Sc. PD:	-0.064
		Sc. rank:	-27707.0

,

HA			
Pos.	321	obs:	exp:
ggt	G	4	6030.00
ggc	G	1	5890.00
gga	G	12293	17690.00
ggg	G	27472	10159.00
---	--	------	------
mPD		0.43	0.69
		nPD:	0.62
		N. weight:	0.62
		Sc. PD:	0.34
		Sc. rank:	19408.0

,

HA			
Pos.	322	obs:	exp:
caa	Q	0	0.53
cag	Q	1	0.47
cgt	R	0	0.87
cgc	R	0	1.67
cga	R	0	3.13
cgg	R	0	2.34
att	I	0	0.36
atc	I	0	0.25
ata	I	1	0.39
act	T	0	338.30
acc	T	3	275.30
aca	T	1339	613.90
acg	T	0	114.50
aat	N	13	13.60
aac	N	10	9.40
aaa	K	38129	21834.00
aag	K	148	16443.00
aga	R	32	16.07
agg	R	0	7.91
gct	A	0	0.26
gcc	A	0	0.21
gca	A	1	0.47
gcg	A	0	0.06
gaa	E	93	56.31
gag	E	0	36.69
---	--	------	------
mPD		0.080	0.58
		nPD:	0.14
		N. weight:	0.59
		Sc. PD:	0.044
		Sc. rank:	14536.0

,

HA			
Pos.	323	obs:	exp:
tat	Y	1	0.52
tac	Y	0	0.48
tgt	C	39678	17950.00
tgc	C	91	21819.00
---	--	------	------
mPD		0.0046	0.50
		nPD:	0.01
		N. weight:	0.84
		Sc. PD:	-0.045
		Sc. rank:	-13298.0

,

HA			
Pos.	324	obs:	exp:
tct	S	0	10.49
tcc	S	0	6.60
tca	S	57	14.83
tcg	S	0	3.03
cct	P	8	10941.00
ccc	P	83	7366.00
cca	P	39610	16910.00
ccg	P	12	4497.00
agt	S	0	10.72
agc	S	0	11.32
---	--	------	------
mPD		0.0080	0.70
		nPD:	0.01
		N. weight:	0.91
		Sc. PD:	-0.046
		Sc. rank:	-10181.0

,

HA			
Pos.	325	obs:	exp:
cgt	R	0	0.08
cgc	R	0	0.16
cga	R	0	0.29
cgg	R	0	0.22
aat	N	1	0.59
aac	N	0	0.41
aaa	K	8671	22684.00
aag	K	31095	17082.00
aga	R	0	1.51
agg	R	3	0.74
---	--	------	------
mPD		0.34	0.49
		nPD:	0.7
		N. weight:	0.28
		Sc. PD:	0.17
		Sc. rank:	8780.8

,

HA			
Pos.	326	obs:	exp:
ttt	F	8	3.43
ttc	F	0	4.57
tat	Y	38867	20657.00
tac	Y	890	19100.00
cat	H	4	2.35
cac	H	0	1.65
gat	D	1	0.53
gac	D	0	0.47
---	--	------	------
mPD		0.044	0.50
		nPD:	0.09
		N. weight:	0.59
		Sc. PD:	0.015
		Sc. rank:	12633.0

,

HA			
Pos.	327	obs:	exp:
att	I	0	2.91
atc	I	0	2.00
ata	I	8	3.09
gtt	V	66	8790.00
gtc	V	6	7225.00
gta	V	38662	10693.00
gtg	V	1025	13051.00
gct	A	0	0.26
gcc	A	0	0.21
gca	A	1	0.47
gcg	A	0	0.06
ggt	G	0	0.30
ggc	G	0	0.30
gga	G	2	0.89
ggg	G	0	0.51
---	--	------	------
mPD		0.054	0.74
		nPD:	0.07
		N. weight:	1.3
		Sc. PD:	0.012
		Sc. rank:	24371.0

,

HA			
Pos.	328	obs:	exp:
cgt	R	0	11.52
cgc	R	0	22.07
cga	R	0	41.33
cgg	R	0	30.88
act	T	0	0.25
acc	T	0	0.21
aca	T	1	0.46
acg	T	0	0.09
aat	N	0	1.18
aac	N	2	0.82
aaa	K	39119	22435.00
aag	K	211	16895.00
aga	R	422	211.90
agg	R	0	104.30
gaa	E	15	9.08
gag	E	0	5.92
---	--	------	------
mPD		0.032	0.52
		nPD:	0.06
		N. weight:	0.57
		Sc. PD:	-0.00065
		Sc. rank:	10278.0

,

HA			
Pos.	329	obs:	exp:
tct	S	0	7317.00
tcc	S	0	4606.00
tca	S	0	10348.00
tcg	S	0	2116.00
cgt	R	0	0.03
cgc	R	0	0.05
cga	R	0	0.10
cgg	R	0	0.07
att	I	0	0.73
atc	I	2	0.50
ata	I	0	0.77
aat	N	0	0.59
aac	N	1	0.41
agt	S	52	7477.00
agc	S	39710	7898.00
aga	R	1	0.50
agg	R	0	0.25
ggt	G	0	0.61
ggc	G	4	0.59
gga	G	0	1.78
ggg	G	0	1.02
---	--	------	------
mPD		0.0030	1.6
		nPD:	0.
		N. weight:	1.7
		Sc. PD:	-0.11
		Sc. rank:	-53178.0

,

HA			
Pos.	330	obs:	exp:
tct	S	0	0.92
tcc	S	0	0.58
tca	S	5	1.30
tcg	S	0	0.27
cct	P	0	2.48
ccc	P	0	1.67
cca	P	9	3.83
ccg	P	0	1.02
att	I	0	10.92
atc	I	0	7.49
ata	I	30	11.59
atg	M	1	1.00
act	T	26	10006.00
acc	T	18	8141.00
aca	T	39550	18158.00
acg	T	97	3387.00
aaa	K	20	11.41
aag	K	0	8.59
agt	S	0	0.94
agc	S	0	0.99
gct	A	0	3.71
gcc	A	0	2.90
gca	A	14	6.58
gcg	A	0	0.82
---	--	------	------
mPD		0.011	0.68
		nPD:	0.02
		N. weight:	0.82
		Sc. PD:	-0.038
		Sc. rank:	-3666.6

,

HA			
Pos.	331	obs:	exp:
cgt	R	0	0.66
cgc	R	0	1.26
cga	R	0	2.35
cgg	R	0	1.76
act	T	0	11.09
acc	T	0	9.03
aca	T	44	20.13
acg	T	0	3.75
aat	N	14	9.46
aac	N	2	6.54
aaa	K	39507	22623.00
aag	K	153	17037.00
aga	R	24	12.05
agg	R	0	5.93
gaa	E	26	15.74
gag	E	0	10.26
---	--	------	------
mPD		0.013	0.50
		nPD:	0.03
		N. weight:	0.58
		Sc. PD:	-0.021
		Sc. rank:	2784.4

,

HA			
Pos.	332	obs:	exp:
tta	L	110	4371.00
ttg	L	39623	7486.00
ctt	L	0	7836.00
ctc	L	0	5617.00
cta	L	0	7655.00
ctg	L	31	6799.00
atg	M	5	5.00
gtt	V	0	0.22
gtc	V	0	0.18
gta	V	0	0.27
gtg	V	1	0.33
---	--	------	------
mPD		0.0074	1.1
		nPD:	0.01
		N. weight:	1.8
		Sc. PD:	-0.1
		Sc. rank:	-37090.0

,

HA			
Pos.	333	obs:	exp:
tct	S	0	0.55
tcc	S	0	0.35
tca	S	0	0.78
tcg	S	0	0.16
cgt	R	0	1085.00
cgc	R	0	2078.00
cga	R	4	3891.00
cgg	R	0	2907.00
att	I	0	2.91
atc	I	0	2.00
ata	I	8	3.09
aaa	K	34	19.97
aag	K	1	15.03
agt	S	2	0.56
agc	S	1	0.60
aga	R	37281	19942.00
agg	R	2438	9820.00
ggt	G	0	0.15
ggc	G	0	0.15
gga	G	1	0.44
ggg	G	0	0.26
---	--	------	------
mPD		0.12	0.91
		nPD:	0.13
		N. weight:	0.54
		Sc. PD:	0.035
		Sc. rank:	13197.0

,

HA			
Pos.	334	obs:	exp:
tta	L	0	4367.00
ttg	L	61	7478.00
ctt	L	13	7827.00
ctc	L	0	5611.00
cta	L	81	7647.00
ctg	L	39567	6792.00
atg	M	48	48.00
---	--	------	------
mPD		0.010	1.1
		nPD:	0.01
		N. weight:	1.9
		Sc. PD:	-0.1
		Sc. rank:	-30427.0

,

HA			
Pos.	335	obs:	exp:
tct	S	0	0.37
tcc	S	2	0.23
tca	S	0	0.52
tcg	S	0	0.11
act	T	0	0.25
acc	T	1	0.21
aca	T	0	0.46
acg	T	0	0.09
agt	S	0	0.38
agc	S	0	0.40
gtt	V	0	22.99
gtc	V	104	18.90
gta	V	0	27.97
gtg	V	0	34.14
gct	A	52	10501.00
gcc	A	39313	8210.00
gca	A	297	18628.00
gcg	A	0	2324.00
ggt	G	0	0.15
ggc	G	1	0.15
gga	G	0	0.44
ggg	G	0	0.26
---	--	------	------
mPD		0.023	0.67
		nPD:	0.03
		N. weight:	1.6
		Sc. PD:	-0.048
		Sc. rank:	15153.0

,

HA			
Pos.	336	obs:	exp:
tct	S	0	0.37
tcc	S	0	0.23
tca	S	2	0.52
tcg	S	0	0.11
cct	P	0	0.28
ccc	P	0	0.19
cca	P	1	0.43
ccg	P	0	0.11
act	T	57	10023.00
acc	T	10	8156.00
aca	T	39611	18190.00
acg	T	83	3393.00
aaa	K	1	0.57
aag	K	0	0.43
agt	S	0	0.38
agc	S	0	0.40
gct	A	0	1.32
gcc	A	0	1.03
gca	A	5	2.35
gcg	A	0	0.29
---	--	------	------
mPD		0.0080	0.68
		nPD:	0.01
		N. weight:	0.82
		Sc. PD:	-0.042
		Sc. rank:	-8732.6

,

HA			
Pos.	337	obs:	exp:
ggt	G	6	6030.00
ggc	G	17	5890.00
gga	G	39463	17690.00
ggg	G	284	10159.00
---	--	------	------
mPD		0.015	0.69
		nPD:	0.02
		N. weight:	0.83
		Sc. PD:	-0.034
		Sc. rank:	845.7

,

HA			
Pos.	338	obs:	exp:
tta	L	152	4371.00
ttg	L	39421	7486.00
tct	S	0	0.37
tcc	S	0	0.23
tca	S	1	0.52
tcg	S	1	0.11
tgg	W	1	1.00
ctt	L	0	7835.00
ctc	L	0	5616.00
cta	L	1	7655.00
ctg	L	187	6798.00
atg	M	3	3.00
agt	S	0	0.38
agc	S	0	0.40
gtt	V	0	0.44
gtc	V	0	0.36
gta	V	0	0.54
gtg	V	2	0.66
---	-	1	1.00
---	--	------	------
mPD		0.018	1.1
		nPD:	0.02
		N. weight:	1.7
		Sc. PD:	-0.083
		Sc. rank:	-10166.0

,

HA			
Pos.	339	obs:	exp:
cgt	R	0	1086.00
cgc	R	0	2080.00
cga	R	0	3895.00
cgg	R	1	2910.00
aaa	K	0	1.71
aag	K	3	1.29
aga	R	194	19964.00
agg	R	39571	9831.00
---	-	1	1.00
---	--	------	------
mPD		0.0099	0.91
		nPD:	0.01
		N. weight:	1.5
		Sc. PD:	-0.076
		Sc. rank:	-17945.0

,

HA			
Pos.	340	obs:	exp:
tct	S	0	0.74
tcc	S	0	0.46
tca	S	0	1.04
tcg	S	0	0.21
att	I	1	0.36
atc	I	0	0.25
ata	I	0	0.39
act	T	1	0.25
acc	T	0	0.21
aca	T	0	0.46
acg	T	0	0.09
aat	N	39712	23517.00
aac	N	51	16246.00
agt	S	4	0.75
agc	S	0	0.79
gat	D	1	0.53
gac	D	0	0.47
---	--	------	------
mPD		0.0029	0.48
		nPD:	0.01
		N. weight:	0.56
		Sc. PD:	-0.032
		Sc. rank:	-12102.0

,

HA			
Pos.	341	obs:	exp:
ttt	F	0	0.43
ttc	F	1	0.57
att	I	1	323.30
atc	I	843	221.70
ata	I	44	343.10
act	T	0	0.76
acc	T	2	0.62
aca	T	1	1.37
acg	T	0	0.26
gtt	V	339	8589.00
gtc	V	38497	7059.00
gta	V	13	10448.00
gtg	V	1	12753.00
gct	A	0	6.88
gcc	A	26	5.38
gca	A	0	12.21
gcg	A	0	1.52
gat	D	0	0.53
gac	D	1	0.47
gaa	E	1	0.61
gag	E	0	0.39
---	--	------	------
mPD		0.065	0.78
		nPD:	0.08
		N. weight:	1.8
		Sc. PD:	0.034
		Sc. rank:	36900.0

,

HA			
Pos.	342	obs:	exp:
tta	L	0	2.20
ttg	L	0	3.77
tct	S	0	0.37
tcc	S	0	0.23
tca	S	0	0.52
tcg	S	2	0.11
ctt	L	0	3.94
ctc	L	0	2.82
cta	L	0	3.85
ctg	L	20	3.42
cct	P	157	10948.00
ccc	P	1	7371.00
cca	P	2268	16921.00
ccg	P	37313	4500.00
caa	Q	0	3.69
cag	Q	7	3.31
cgt	R	0	0.03
cgc	R	0	0.05
cga	R	0	0.10
cgg	R	1	0.07
act	T	0	0.25
acc	T	0	0.21
aca	T	0	0.46
acg	T	1	0.09
agt	S	0	0.38
agc	S	0	0.40
aga	R	0	0.50
agg	R	0	0.25
---	--	------	------
mPD		0.12	0.70
		nPD:	0.17
		N. weight:	2.
		Sc. PD:	0.2
		Sc. rank:	52560.0

,

HA

Pos.	343	obs:	exp:
ttt	F	18	7.72
ttc	F	0	10.28
tct	S	39482	7314.00
tcc	S	232	4603.00
tca	S	29	10343.00
tcg	S	0	2115.00
tat	Y	1	0.52
tac	Y	0	0.48
tgt	C	1	0.45
tgc	C	0	0.55
cct	P	3	0.83
ccc	P	0	0.56
cca	P	0	1.28
ccg	P	0	0.34
act	T	3	0.76
acc	T	0	0.62
aca	T	0	1.37
acg	T	0	0.26
agt	S	0	7473.00
agc	S	0	7894.00
gct	A	1	0.26
gcc	A	0	0.21
gca	A	0	0.47
gcg	A	0	0.06
---	--	------	------
mPD		0.014	1.6
		nPD:	0.01
		N. weight:	1.8
		Sc. PD:	-0.096
		Sc. rank:	-30080.0

,

HA

Pos.	344	obs:	exp:
ttt	F	2	0.86
ttc	F	0	1.14
tta	L	0	0.11
ttg	L	0	0.19
tct	S	0	0.18
tcc	S	0	0.12
tca	S	0	0.26
tcg	S	0	0.05
ctt	L	1	0.20
ctc	L	0	0.14
cta	L	0	0.19
ctg	L	0	0.17
att	I	39246	14356.00
atc	I	181	9844.00
ata	I	10	15237.00
atg	M	6	6.00
act	T	3	0.76
acc	T	0	0.62
aca	T	0	1.37
acg	T	0	0.26
agt	S	1	0.19
agc	S	0	0.20
gtt	V	320	70.75
gtc	V	0	58.15
gta	V	0	86.06
gtg	V	0	105.00
---	--	------	------
mPD		0.026	0.67
		nPD:	0.04
		N. weight:	1.1
		Sc. PD:	-0.026
		Sc. rank:	12183.0

,

HA

Pos.	345	obs:	exp:
tta	L	0	0.44
ttg	L	0	0.75
ctt	L	0	0.79
ctc	L	0	0.56
cta	L	4	0.77
ctg	L	0	0.68
cat	H	1	0.59
cac	H	0	0.41
caa	Q	39672	20981.00
cag	Q	90	18781.00
cgt	R	0	0.08
cgc	R	0	0.16
cga	R	3	0.29
cgg	R	0	0.22
aga	R	0	1.51
agg	R	0	0.74
---	--	------	------
mPD		0.0049	0.50
		nPD:	0.01
		N. weight:	0.67
		Sc. PD:	-0.036
		Sc. rank:	-10317.0

,

HA

Pos.	346	obs:	exp:
ttt	F	2	0.86
ttc	F	0	1.14
tct	S	39491	7313.00
tcc	S	218	4603.00
tca	S	12	10343.00
tcg	S	19	2115.00
tat	Y	5	2.60
tac	Y	0	2.40
cct	P	18	5.23
ccc	P	1	3.52
cca	P	0	8.09
ccg	P	0	2.15
act	T	2	0.50
acc	T	0	0.41
aca	T	0	0.91
acg	T	0	0.17
agt	S	0	7472.00
agc	S	0	7894.00
gct	A	2	0.53
gcc	A	0	0.41
gca	A	0	0.94
gcg	A	0	0.12
---	--	------	------
mPD		0.014	1.6
		nPD:	0.01
		N. weight:	1.8
		Sc. PD:	-0.097
		Sc. rank:	-31402.0

,

HA

Pos.	347	obs:	exp:
cgt	R	0	1085.00
cgc	R	0	2079.00
cga	R	44	3893.00
cgg	R	0	2909.00
act	T	0	0.76
acc	T	0	0.62
aca	T	3	1.37
acg	T	0	0.26
aaa	K	18	10.27
aag	K	0	7.73
aga	R	39650	19955.00
agg	R	55	9827.00
---	--	------	------
mPD		0.0060	0.91
		nPD:	0.01
		N. weight:	0.73
		Sc. PD:	-0.041
		Sc. rank:	-14891.0

,

HA

Pos.	348	obs:	exp:
gct	A	0	0.26
gcc	A	1	0.21
gca	A	0	0.47
gcg	A	0	0.06
ggt	G	57	6030.00
ggc	G	39698	5890.00
gga	G	13	17690.00
ggg	G	1	10159.00
---	--	------	------
mPD		0.0036	0.69
		nPD:	0.01
		N. weight:	2.
		Sc. PD:	-0.12
		Sc. rank:	-48025.0

,

HA

Pos.	349	obs:	exp:
tta	L	43	4370.00
ttg	L	0	7485.00
ctt	L	9	7834.00
ctc	L	3	5615.00
cta	L	39615	7654.00
ctg	L	85	6797.00
att	I	0	5.10
atc	I	0	3.49
ata	I	14	5.41
gtt	V	0	0.22
gtc	V	0	0.18
gta	V	1	0.27
gtg	V	0	0.33
---	--	------	------
mPD		0.0078	1.1
		nPD:	0.01
		N. weight:	1.8
		Sc. PD:	-0.098
		Sc. rank:	-34920.0

,

HA

Pos.	350	obs:	exp:
ttt	F	8707	17055.00
ttc	F	31035	22687.00
tta	L	9	2.86
ttg	L	1	4.90
ctt	L	5	5.12
ctc	L	11	3.67
cta	L	0	5.01
ctg	L	0	4.45
att	I	0	0.36
atc	I	1	0.25
ata	I	0	0.39
gtt	V	0	0.22
gtc	V	1	0.18
gta	V	0	0.27
gtg	V	0	0.33
---	--	------	------
mPD		0.34	0.49
		nPD:	0.7
		N. weight:	0.11
		Sc. PD:	0.066
		Sc. rank:	3329.5

,

HA

Pos.	351	obs:	exp:
cgt	R	0	0.03
cgc	R	0	0.05
cga	R	0	0.10
cgg	R	1	0.07
aga	R	0	0.50
agg	R	0	0.25
ggt	G	10	6030.00
ggc	G	0	5890.00
gga	G	321	17690.00
ggg	G	39438	10159.00
---	--	------	------
mPD		0.017	0.69
		nPD:	0.02
		N. weight:	1.4
		Sc. PD:	-0.055
		Sc. rank:	3698.9

,

HA

Pos.	352	obs:	exp:
tct	S	0	0.18
tcc	S	1	0.12
tca	S	0	0.26
tcg	S	0	0.05
agt	S	0	0.19
agc	S	0	0.20
gct	A	29	10529.00
gcc	A	38818	8232.00
gca	A	45	18678.00
gcg	A	876	2330.00
ggt	G	0	0.15
ggc	G	1	0.15
gga	G	0	0.44
ggg	G	0	0.26
---	--	------	------
mPD		0.047	0.66
		nPD:	0.07
		N. weight:	1.6
		Sc. PD:	0.011
		Sc. rank:	30414.0

,

HA

Pos.	353	obs:	exp:
tta	L	0	0.22
ttg	L	0	0.38
ctt	L	2	0.39
ctc	L	0	0.28
cta	L	0	0.39
ctg	L	0	0.34
att	I	39708	14475.00
atc	I	53	9926.00
ata	I	3	15363.00
aat	N	1	0.59
aac	N	0	0.41
gtt	V	3	0.66
gtc	V	0	0.55
gta	V	0	0.81
gtg	V	0	0.98
---	--	------	------
mPD		0.0031	0.66
		nPD:	0.
		N. weight:	1.1
		Sc. PD:	-0.063
		Sc. rank:	-26587.0

,

HA

Pos.	354	obs:	exp:
cct	P	0	0.28
ccc	P	1	0.19
cca	P	0	0.43
ccg	P	0	0.11
act	T	0	0.76
acc	T	3	0.62
aca	T	0	1.37
acg	T	0	0.26
gct	A	10399	10528.00
gcc	A	29232	8231.00
gca	A	132	18677.00
gcg	A	3	2330.00
---	--	------	------
mPD		0.39	0.66
		nPD:	0.59
		N. weight:	0.99
		Sc. PD:	0.51
		Sc. rank:	30638.0

,

HA

Pos.	355	obs:	exp:
tct	S	0	0.18
tcc	S	0	0.12
tca	S	0	0.26
tcg	S	0	0.05
agt	S	0	0.19
agc	S	1	0.20
gct	A	1	0.26
gcc	A	0	0.21
gca	A	0	0.47
gcg	A	0	0.06
ggt	G	7310	6030.00
ggc	G	32415	5890.00
gga	G	42	17690.00
ggg	G	1	10158.00
---	--	------	------
mPD		0.30	0.69
		nPD:	0.44
		N. weight:	1.5
		Sc. PD:	0.56
		Sc. rank:	45797.0

,

HA

Pos.	356	obs:	exp:
ttt	F	99	17066.00
ttc	F	39670	22703.00
tta	L	1	0.11
ttg	L	0	0.19
ctt	L	0	0.20
ctc	L	0	0.14
cta	L	0	0.19
ctg	L	0	0.17
---	--	------	------
mPD		0.0050	0.49
		nPD:	0.01
		N. weight:	0.59
		Sc. PD:	-0.031
		Sc. rank:	-8227.6

,

HA

Pos.	357	obs:	exp:
tta	L	0	0.11
ttg	L	0	0.19
ctt	L	1	0.20
ctc	L	0	0.14
cta	L	0	0.19
ctg	L	0	0.17
att	I	37671	14477.00
atc	I	2087	9927.00
ata	I	10	15364.00
gtt	V	1	0.22
gtc	V	0	0.18
gta	V	0	0.27
gtg	V	0	0.33
---	--	------	------
mPD		0.10	0.66
		nPD:	0.15
		N. weight:	0.89
		Sc. PD:	0.077
		Sc. rank:	22761.0

,

HA

Pos.	358	obs:	exp:
aaa	K	1	0.57
aag	K	0	0.43
gat	D	1	0.53
gac	D	0	0.47
gaa	E	39765	24079.00
gag	E	2	15688.00
ggt	G	0	0.15
ggc	G	0	0.15
gga	G	1	0.44
ggg	G	0	0.26
---	--	------	------
mPD		0.00025	0.48
		nPD:	0.
		N. weight:	0.54
		Sc. PD:	-0.034
		Sc. rank:	-17178.0

,

HA

Pos.	359	obs:	exp:
cgt	R	0	0.08
cgc	R	0	0.16
cga	R	0	0.29
cgg	R	0	0.22
aga	R	0	1.51
agg	R	3	0.74
gaa	E	0	0.61
gag	E	1	0.39
ggt	G	5	6030.00
ggc	G	1	5890.00
gga	G	252	17689.00
ggg	G	39508	10158.00
---	--	------	------
mPD		0.013	0.69
		nPD:	0.02
		N. weight:	1.4
		Sc. PD:	-0.063
		Sc. rank:	-2218.5

,

HA

Pos.	360	obs:	exp:
ggt	G	22	6030.00
ggc	G	2	5890.00
gga	G	401	17690.00
ggg	G	39345	10159.00
---	--	------	------
mPD		0.021	0.69
		nPD:	0.03
		N. weight:	1.4
		Sc. PD:	-0.046
		Sc. rank:	11037.0

,

HA

Pos.	361	obs:	exp:
tta	L	0	0.11
ttg	L	1	0.19
tct	S	0	0.18
tcc	S	0	0.12
tca	S	0	0.26
tcg	S	0	0.05
tgg	W	39764	39764.00
ctt	L	0	0.20
ctc	L	0	0.14
cta	L	0	0.19
ctg	L	0	0.17
cgt	R	0	0.11
cgc	R	0	0.21
cga	R	0	0.39
cgg	R	0	0.29
agt	S	0	0.19
agc	S	1	0.20
aga	R	0	2.01
agg	R	4	0.99
---	--	------	------
mPD		0.00035	0.00055
		nPD:	0.64
		N. weight:	0.00024
		Sc. PD:	0.00014
		Sc. rank:	7.6

,

HA

Pos.	362	obs:	exp:
tct	S	0	0.18
tcc	S	0	0.12
tca	S	1	0.26
tcg	S	0	0.05
cct	P	0	0.28
ccc	P	0	0.19
cca	P	1	0.43
ccg	P	0	0.11
att	I	0	0.73
atc	I	0	0.50
ata	I	2	0.77
act	T	7	10024.00
acc	T	0	8156.00
aca	T	39755	18191.00
acg	T	1	3393.00
aaa	K	3	1.71
aag	K	0	1.29
agt	S	0	0.19
agc	S	0	0.20
---	--	------	------
mPD		0.00075	0.68
		nPD:	0.
		N. weight:	0.84
		Sc. PD:	-0.052
		Sc. rank:	-26452.0

,

HA

Pos.	363	obs:	exp:
cgt	R	0	0.03
cgc	R	0	0.05
cga	R	0	0.10
cgg	R	0	0.07
aga	R	0	0.50
agg	R	1	0.25
gaa	E	0	1.21
gag	E	2	0.79
ggt	G	15	6030.00
ggc	G	7	5890.00
gga	G	2023	17689.00
ggg	G	37722	10158.00
---	--	------	------
mPD		0.098	0.69
		nPD:	0.14
		N. weight:	1.2
		Sc. PD:	0.093
		Sc. rank:	30341.0

,

HA

Pos.	364	obs:	exp:
tta	L	0	0.22
ttg	L	2	0.38
ctt	L	0	0.39
ctc	L	0	0.28
cta	L	0	0.39
ctg	L	0	0.34
att	I	0	1.46
atc	I	1	1.00
ata	I	3	1.55
atg	M	39763	39763.00
act	T	0	0.25
acc	T	0	0.21
aca	T	0	0.46
acg	T	1	0.09
---	--	------	------
mPD		0.00035	0.00046
		nPD:	0.76
		N. weight:	0.00024
		Sc. PD:	0.00016
		Sc. rank:	7.5

,

HA

Pos.	365	obs:	exp:
tta	L	4	0.55
ttg	L	0	0.94
ctt	L	0	0.99
ctc	L	0	0.71
cta	L	1	0.96
ctg	L	0	0.85
att	I	0	8.74
atc	I	0	5.99
ata	I	24	9.27
gtt	V	47	8786.00
gtc	V	5	7221.00
gta	V	39614	10687.00
gtg	V	73	13045.00
gct	A	0	0.26
gcc	A	0	0.21
gca	A	1	0.47
gcg	A	0	0.06
gaa	E	1	0.61
gag	E	0	0.39
---	--	------	------
mPD		0.0078	0.74
		nPD:	0.01
		N. weight:	1.4
		Sc. PD:	-0.073
		Sc. rank:	-18178.0

,

HA

Pos.	366	obs:	exp:
tat	Y	2	1.04
tac	Y	0	0.96
aat	N	1	0.59
aac	N	0	0.41
gtt	V	0	0.22
gtc	V	0	0.18
gta	V	1	0.27
gtg	V	0	0.33
gct	A	1	0.26
gcc	A	0	0.21
gca	A	0	0.47
gcg	A	0	0.06
gat	D	39674	20943.00
gac	D	73	18804.00
gaa	E	5	10.90
gag	E	13	7.10
---	--	------	------
mPD		0.0049	0.50
		nPD:	0.01
		N. weight:	0.68
		Sc. PD:	-0.036
		Sc. rank:	-10429.0

,

HA

Pos.	367	obs:	exp:
ggt	G	14	6030.00
ggc	G	1	5890.00
gga	G	39691	17690.00
ggg	G	64	10159.00
---	--	------	------
mPD		0.0040	0.69
		nPD:	0.01
		N. weight:	0.86
		Sc. PD:	-0.049
		Sc. rank:	-19023.0

,

HA

Pos.	368	obs:	exp:
tgg	W	39770	39770.00
---	--	------	------
mPD		0	0
		nPD:	1.
		N. weight:	0.
		Sc. PD:	0
		Sc. rank:	0

,

HA

Pos.	369	obs:	exp:
ttt	F	0	0.86
ttc	F	2	1.14
tat	Y	1634	20662.00
tac	Y	38133	19105.00
cat	H	0	0.59
cac	H	1	0.41
---	--	------	------
mPD		0.079	0.50
		nPD:	0.16
		N. weight:	0.6
		Sc. PD:	0.056
		Sc. rank:	15601.0

,

HA

Pos.	370	obs:	exp:
ggt	G	39699	6030.00
ggc	G	33	5890.00
gga	G	1	17690.00
ggg	G	37	10159.00
---	--	------	------
mPD		0.0036	0.69
		nPD:	0.01
		N. weight:	2.
		Sc. PD:	-0.12
		Sc. rank:	-47879.0

,

HA			
Pos.	371	obs:	exp:
ttt	F	15	6.44
ttc	F	0	8.56
tat	Y	39495	20656.00
tac	Y	259	19098.00
cat	H	1	0.59
cac	H	0	0.41
---	--	------	------
mPD		0.014	0.50
		nPD:	0.03
		N. weight:	0.67
		Sc. PD:	-0.024
		Sc. rank:	3492.1

,

HA			
Pos.	372	obs:	exp:
tta	L	0	0.11
ttg	L	0	0.19
tat	Y	0	4.16
tac	Y	8	3.84
ctt	L	0	0.20
ctc	L	1	0.14
cta	L	0	0.19
ctg	L	0	0.17
cct	P	0	0.55
ccc	P	1	0.37
cca	P	1	0.85
ccg	P	0	0.23
cat	H	249	23375.00
cac	H	39498	16372.00
caa	Q	1	0.53
cag	Q	0	0.47
cgt	R	1	0.27
cgc	R	9	0.52
cga	R	0	0.98
cgg	R	0	0.73
aat	N	0	0.59
aac	N	1	0.41
aga	R	0	5.02
agg	R	0	2.47
---	--	------	------
mPD		0.014	0.49
		nPD:	0.03
		N. weight:	0.92
		Sc. PD:	-0.032
		Sc. rank:	5209.0

,

HA			
Pos.	373	obs:	exp:
tta	L	0	0.22
ttg	L	0	0.38
tat	Y	2	1.04
tac	Y	0	0.96
ctt	L	2	0.39
ctc	L	0	0.28
cta	L	0	0.39
ctg	L	0	0.34
cct	P	23	6.34
ccc	P	0	4.27
cca	P	0	9.79
ccg	P	0	2.60
cat	H	39650	23364.00
cac	H	78	16364.00
caa	Q	4	2.64
cag	Q	1	2.36
gat	D	9	4.74
gac	D	0	4.26
ggt	G	0	0.15
ggc	G	0	0.15
gga	G	0	0.44
ggg	G	1	0.26
---	--	------	------
mPD		0.0061	0.49
		nPD:	0.01
		N. weight:	0.56
		Sc. PD:	-0.028
		Sc. rank:	-5753.5

,

HA			
Pos.	374	obs:	exp:
tta	L	0	0.11
ttg	L	0	0.19
ctt	L	0	0.20
ctc	L	0	0.14
cta	L	1	0.19
ctg	L	0	0.17
cat	H	1	1.76
cac	H	2	1.24
caa	Q	39571	20976.00
cag	Q	182	18777.00
cgt	R	0	0.08
cgc	R	0	0.16
cga	R	3	0.29
cgg	R	0	0.22
aaa	K	7	3.99
aag	K	0	3.01
aga	R	0	1.51
agg	R	0	0.74
gaa	E	2	1.82
gag	E	1	1.18
---	--	------	------
mPD		0.010	0.50
		nPD:	0.02
		N. weight:	0.66
		Sc. PD:	-0.028
		Sc. rank:	-656.6

,

HA			
Pos.	375	obs:	exp:
cat	H	4	2.35
cac	H	0	1.65
caa	Q	4	2.11
cag	Q	0	1.89
act	T	2	0.50
acc	T	0	0.41
aca	T	0	0.91
acg	T	0	0.17
aat	N	39688	23514.00
aac	N	69	16243.00
aaa	K	3	1.71
aag	K	0	1.29
---	--	------	------
mPD		0.0043	0.48
		nPD:	0.01
		N. weight:	0.55
		Sc. PD:	-0.03
		Sc. rank:	-9116.7

,

HA			
Pos.	376	obs:	exp:
caa	Q	0	0.53
cag	Q	1	0.47
aaa	K	0	5.70
aag	K	10	4.30
gtt	V	0	0.22
gtc	V	0	0.18
gta	V	0	0.27
gtg	V	1	0.33
gct	A	0	12.44
gcc	A	0	9.73
gca	A	0	22.07
gcg	A	47	2.75
gat	D	28	15.28
gac	D	1	13.72
gaa	E	627	24005.00
gag	E	39018	15640.00
ggt	G	0	5.61
ggc	G	0	5.48
gga	G	0	16.46
ggg	G	37	9.45
---	--	------	------
mPD		0.037	0.48
		nPD:	0.08
		N. weight:	0.91
		Sc. PD:	0.012
		Sc. rank:	18140.0

,

HA			
Pos.	377	obs:	exp:
tta	L	0	0.22
ttg	L	0	0.38
ctt	L	0	0.39
ctc	L	0	0.28
cta	L	0	0.39
ctg	L	2	0.34
cat	H	2	1.18
cac	H	0	0.82
caa	Q	231	20980.00
cag	Q	39529	18780.00
cgt	R	0	0.08
cgc	R	0	0.16
cga	R	0	0.29
cgg	R	3	0.22
aaa	K	0	1.14
aag	K	2	0.86
aga	R	0	1.51
agg	R	0	0.74
gaa	E	0	0.61
gag	E	1	0.39
---	--	------	------
mPD		0.012	0.50
		nPD:	0.02
		N. weight:	0.77
		Sc. PD:	-0.03
		Sc. rank:	2146.2

,

HA			
Pos.	378	obs:	exp:
gaa	E	0	0.61
gag	E	1	0.39
ggt	G	135	6030.00
ggc	G	42	5890.00
gga	G	502	17690.00
ggg	G	39090	10159.00
---	--	------	------
mPD		0.034	0.69
		nPD:	0.05
		N. weight:	1.4
		Sc. PD:	-0.02
		Sc. rank:	20906.0

,

HA			
Pos.	379	obs:	exp:
tct	S	19	7317.00
tcc	S	4	4606.00
tca	S	39709	10348.00
tcg	S	30	2116.00
act	T	0	1.51
acc	T	0	1.23
aca	T	6	2.74
acg	T	0	0.51
agt	S	0	7477.00
agc	S	0	7898.00
gct	A	0	0.53
gcc	A	0	0.41
gca	A	2	0.94
gcg	A	0	0.12
---	--	------	------
mPD		0.0031	1.6
		nPD:	0.
		N. weight:	1.4
		Sc. PD:	-0.088
		Sc. rank:	-44003.0

,

HA			
Pos.	380	obs:	exp:
cgt	R	0	0.05
cgc	R	0	0.10
cga	R	0	0.20
cgg	R	0	0.15
aga	R	2	1.00
agg	R	0	0.49
gct	A	0	0.26
gcc	A	0	0.21
gca	A	1	0.47
gcg	A	0	0.06
ggt	G	0	6030.00
ggc	G	2	5890.00
gga	G	39315	17689.00
ggg	G	450	10158.00
---	--	------	------
mPD		0.023	0.69
		nPD:	0.03
		N. weight:	0.82
		Sc. PD:	-0.025
		Sc. rank:	7240.5

,

HA			
Pos.	381	obs:	exp:
ttt	F	8	3.43
ttc	F	0	4.57
tct	S	1	0.18
tcc	S	0	0.12
tca	S	0	0.26
tcg	S	0	0.05
tat	Y	39571	20649.00
tac	Y	171	19093.00
tgt	C	5	2.26
tgc	C	0	2.74
cat	H	11	6.47
cac	H	0	4.53
aat	N	2	1.18
aac	N	0	0.82
agt	S	0	0.19
agc	S	0	0.20
gat	D	1	0.53
gac	D	0	0.47
---	--	------	------
mPD		0.010	0.50
		nPD:	0.02
		N. weight:	0.68
		Sc. PD:	-0.029
		Sc. rank:	-767.8

,

HA			
Pos.	382	obs:	exp:
act	T	0	0.25
acc	T	0	0.21
aca	T	1	0.46
acg	T	0	0.09
gtt	V	0	0.22
gtc	V	0	0.18
gta	V	1	0.27
gtg	V	0	0.33
gct	A	23	10529.00
gcc	A	11	8232.00
gca	A	39698	18678.00
gcg	A	36	2330.00
---	--	------	------
mPD		0.0036	0.66
		nPD:	0.01
		N. weight:	0.8
		Sc. PD:	-0.046
		Sc. rank:	-18199.0

,

HA			
Pos.	383	obs:	exp:
tct	S	0	0.37
tcc	S	2	0.23
tca	S	0	0.52
tcg	S	0	0.11
cct	P	0	0.55
ccc	P	2	0.37
cca	P	0	0.85
ccg	P	0	0.23
agt	S	0	0.38
agc	S	0	0.40
gct	A	291	10528.00
gcc	A	39390	8231.00
gca	A	84	18676.00
gcg	A	0	2330.00
gat	D	0	0.53
gac	D	1	0.47
---	--	------	------
mPD		0.019	0.66
		nPD:	0.03
		N. weight:	1.6
		Sc. PD:	-0.057
		Sc. rank:	10643.0

,

HA			
Pos.	384	obs:	exp:
gat	D	21133	20954.00
gac	D	18636	18815.00
gaa	E	1	0.61
gag	E	0	0.39
---	--	------	------
mPD		0.50	0.50
		nPD:	1.
		N. weight:	0.00021
		Sc. PD:	0.00019
		Sc. rank:	6.6

,

HA			
Pos.	385	obs:	exp:
tta	L	0	4341.00
ttg	L	126	7434.00
ctt	L	370	7781.00
ctc	L	3	5578.00
cta	L	128	7602.00
ctg	L	38861	6751.00
caa	Q	0	125.60
cag	Q	238	112.40
cgt	R	0	0.76
cgc	R	0	1.46
cga	R	0	2.74
cgg	R	28	2.05
atg	M	15	15.00
aga	R	0	14.06
agg	R	0	6.92
gtt	V	0	0.22
gtc	V	0	0.18
gta	V	0	0.27
gtg	V	1	0.33
---	--	------	------
mPD		0.045	1.2
		nPD:	0.04
		N. weight:	1.8
		Sc. PD:	-0.043
		Sc. rank:	21014.0

,

HA			
Pos.	386	obs:	exp:
caa	Q	0	1.06
cag	Q	2	0.94
cgt	R	0	0.30
cgc	R	0	0.58
cga	R	0	1.08
cgg	R	0	0.81
atg	M	3	3.00
act	T	0	1.01
acc	T	0	0.82
aca	T	0	1.83
acg	T	4	0.34
aaa	K	547	22671.00
aag	K	39197	17073.00
aga	R	0	5.52
agg	R	11	2.72
gaa	E	0	3.63
gag	E	6	2.37
---	--	------	------
mPD		0.028	0.49
		nPD:	0.06
		N. weight:	0.83
		Sc. PD:	-0.0049
		Sc. rank:	13912.0

,

HA			
Pos.	387	obs:	exp:
tct	S	0	7318.00
tcc	S	0	4606.00
tca	S	0	10350.00
tcg	S	0	2116.00
cgt	R	0	0.03
cgc	R	1	0.05
cga	R	0	0.10
cgg	R	0	0.07
act	T	0	0.25
acc	T	1	0.21
aca	T	0	0.46
acg	T	0	0.09
agt	S	76	7478.00
agc	S	39691	7899.00
aga	R	0	0.50
agg	R	0	0.25
ggt	G	0	0.15
ggc	G	1	0.15
gga	G	0	0.44
ggg	G	0	0.26
---	--	------	------
mPD		0.0040	1.6
		nPD:	0.
		N. weight:	1.7
		Sc. PD:	-0.1
		Sc. rank:	-50911.0

,

HA			
Pos.	388	obs:	exp:
act	T	8	10025.00
acc	T	17	8157.00
aca	T	39708	18193.00
acg	T	36	3393.00
gct	A	0	0.26
gcc	A	0	0.21
gca	A	1	0.47
gcg	A	0	0.06
---	--	------	------
mPD		0.0031	0.68
		nPD:	0.
		N. weight:	0.83
		Sc. PD:	-0.048
		Sc. rank:	-20720.0

,

HA			
Pos.	389	obs:	exp:
caa	Q	31279	20985.00
cag	Q	8491	18785.00
---	--	------	------
mPD		0.34	0.50
		nPD:	0.67
		N. weight:	0.16
		Sc. PD:	0.093
		Sc. rank:	4895.9

,

HA			
Pos.	390	obs:	exp:
tct	S	0	24.48
tcc	S	0	15.41
tca	S	0	34.61
tcg	S	0	7.08
tat	Y	23	11.95
tac	Y	0	11.05
cat	H	154	90.57
cac	H	0	63.43
att	I	8	2.91
atc	I	0	2.00
ata	I	0	3.09
act	T	3	0.76
acc	T	0	0.62
aca	T	0	1.37
acg	T	0	0.26
aat	N	39343	23309.00
aac	N	67	16101.00
aaa	K	6	4.56
aag	K	2	3.44
agt	S	133	25.01
agc	S	0	26.42
gat	D	31	16.33
gac	D	0	14.67
---	--	------	------
mPD		0.021	0.51
		nPD:	0.04
		N. weight:	0.56
		Sc. PD:	-0.012
		Sc. rank:	7373.4

,

HA			
Pos.	391	obs:	exp:
tct	S	0	0.18
tcc	S	1	0.12
tca	S	0	0.26
tcg	S	0	0.05
cct	P	0	0.28
ccc	P	1	0.19
cca	P	0	0.43
ccg	P	0	0.11
act	T	0	1.01
acc	T	4	0.82
aca	T	0	1.83
acg	T	0	0.34
agt	S	0	0.19
agc	S	0	0.20
gtt	V	0	1.77
gtc	V	8	1.45
gta	V	0	2.15
gtg	V	0	2.63
gct	A	448	10526.00
gcc	A	39148	8229.00
gca	A	159	18672.00
gcg	A	1	2329.00
---	--	------	------
mPD		0.031	0.66
		nPD:	0.05
		N. weight:	1.6
		Sc. PD:	-0.027
		Sc. rank:	22829.0

,

HA			
Pos.	392	obs:	exp:
ttt	F	45	19.31
ttc	F	0	25.69
tta	L	0	4.18
ttg	L	0	7.15
ctt	L	38	7.49
ctc	L	0	5.37
cta	L	0	7.32
ctg	L	0	6.50
att	I	39261	14324.00
atc	I	85	9822.00
ata	I	2	15202.00
act	T	2	0.50
acc	T	0	0.41
aca	T	0	0.91
acg	T	0	0.17
aat	N	1	0.59
aac	N	0	0.41
gtt	V	336	74.28
gtc	V	0	61.05
gta	V	0	90.36
gtg	V	0	110.30
---	--	------	------
mPD		0.025	0.68
		nPD:	0.04
		N. weight:	1.1
		Sc. PD:	-0.028
		Sc. rank:	11212.0

,

HA			
Pos.	393	obs:	exp:
aat	N	4	3.55
aac	N	2	2.45
gat	D	21165	20952.00
gac	D	18599	18812.00
---	--	------	------
mPD		0.50	0.50
		nPD:	1.
		N. weight:	0.00024
		Sc. PD:	0.00022
		Sc. rank:	7.8

,

HA			
Pos.	394	obs:	exp:
caa	Q	33	20.58
cag	Q	6	18.42
cgt	R	0	1.06
cgc	R	0	2.04
cga	R	0	3.82
cgg	R	0	2.85
atg	M	2	2.00
aaa	K	2017	19343.00
aag	K	31892	14566.00
aga	R	1	19.58
agg	R	38	9.64
gaa	E	21	3471.00
gag	E	5712	2262.00
ggt	G	0	7.28
ggc	G	0	7.11
gga	G	1	21.35
ggg	G	47	12.26
---	--	------	------
mPD		0.35	0.74
		nPD:	0.47
		N. weight:	0.7
		Sc. PD:	0.28
		Sc. rank:	21444.0

,

HA			
Pos.	395	obs:	exp:
tct	S	0	0.18
tcc	S	0	0.12
tca	S	0	0.26
tcg	S	0	0.05
att	I	39457	14468.00
atc	I	283	9921.00
ata	I	4	15355.00
agt	S	1	0.19
agc	S	0	0.20
gtt	V	24	5.53
gtc	V	1	4.54
gta	V	0	6.72
gtg	V	0	8.21
---	--	------	------
mPD		0.016	0.66
		nPD:	0.02
		N. weight:	1.
		Sc. PD:	-0.041
		Sc. rank:	2571.8

,

HA			
Pos.	396	obs:	exp:
tct	S	2	0.37
tcc	S	0	0.23
tca	S	0	0.52
tcg	S	0	0.11
att	I	1	0.36
atc	I	0	0.25
ata	I	0	0.39
act	T	39124	10024.00
acc	T	593	8156.00
aca	T	45	18191.00
acg	T	3	3393.00
aat	N	1	0.59
aac	N	0	0.41
agt	S	0	0.38
agc	S	0	0.40
gct	A	1	0.26
gcc	A	0	0.21
gca	A	0	0.47
gcg	A	0	0.06
---	--	------	------
mPD		0.032	0.68
		nPD:	0.05
		N. weight:	1.4
		Sc. PD:	-0.023
		Sc. rank:	20576.0

,

HA			
Pos.	397	obs:	exp:
tct	S	0	0.92
tcc	S	0	0.58
tca	S	0	1.30
tcg	S	0	0.27
act	T	0	2.52
acc	T	10	2.05
aca	T	0	4.57
acg	T	0	0.85
aat	N	80	23511.00
aac	N	39673	16242.00
aaa	K	1	0.57
aag	K	0	0.43
agt	S	0	0.94
agc	S	5	0.99
gat	D	0	0.53
gac	D	1	0.47
---	--	------	------
mPD		0.0049	0.48
		nPD:	0.01
		N. weight:	0.95
		Sc. PD:	-0.05
		Sc. rank:	-13636.0

,

HA			
Pos.	398	obs:	exp:
cgt	R	0	0.19
cgc	R	0	0.37
cga	R	0	0.69
cgg	R	0	0.51
aat	N	0	0.59
aac	N	1	0.41
aaa	K	39656	22681.00
aag	K	105	17080.00
aga	R	7	3.51
agg	R	0	1.73
gaa	E	1	0.61
gag	E	0	0.39
---	--	------	------
mPD		0.0057	0.49
		nPD:	0.01
		N. weight:	0.59
		Sc. PD:	-0.03
		Sc. rank:	-6519.8

,

HA			
Pos.	399	obs:	exp:
att	I	0	5.46
atc	I	0	3.74
ata	I	15	5.80
gtt	V	9	8789.00
gtc	V	2	7223.00
gta	V	39674	10691.00
gtg	V	67	13049.00
gct	A	0	0.79
gcc	A	0	0.62
gca	A	3	1.41
gcg	A	0	0.18
---	--	------	------
mPD		0.0048	0.74
		nPD:	0.01
		N. weight:	1.4
		Sc. PD:	-0.079
		Sc. rank:	-29028.0

,

HA			
Pos.	400	obs:	exp:
tct	S	0	0.18
tcc	S	0	0.12
tca	S	0	0.26
tcg	S	0	0.05
aat	N	39330	23520.00
aac	N	438	16248.00
aaa	K	1	0.57
aag	K	0	0.43
agt	S	1	0.19
agc	S	0	0.20
---	--	------	------
mPD		0.022	0.48
		nPD:	0.05
		N. weight:	0.51
		Sc. PD:	-0.0092
		Sc. rank:	6843.2

,

HA			
Pos.	401	obs:	exp:
ttt	F	11	4.72
ttc	F	0	6.28
tct	S	39593	7316.00
tcc	S	53	4605.00
tca	S	101	10346.00
tcg	S	4	2116.00
tat	Y	1	0.52
tac	Y	0	0.48
cct	P	2	0.55
ccc	P	0	0.37
cca	P	0	0.85
ccg	P	0	0.23
agt	S	3	7475.00
agc	S	0	7897.00
gct	A	2	0.53
gcc	A	0	0.41
gca	A	0	0.94
gcg	A	0	0.12
---	--	------	------
mPD		0.0090	1.6
		nPD:	0.01
		N. weight:	1.8
		Sc. PD:	-0.1
		Sc. rank:	-40330.0

,

HA			
Pos.	402	obs:	exp:
ttt	F	1	0.43
ttc	F	0	0.57
att	I	26	9.46
atc	I	0	6.49
ata	I	0	10.05
gtt	V	39361	8786.00
gtc	V	365	7221.00
gta	V	12	10688.00
gtg	V	3	13046.00
gct	A	1	0.26
gcc	A	0	0.21
gca	A	0	0.47
gcg	A	0	0.06
gaa	E	0	0.61
gag	E	1	0.39
---	--	------	------
mPD		0.020	0.74
		nPD:	0.03
		N. weight:	1.6
		Sc. PD:	-0.056
		Sc. rank:	8686.3

,

HA			
Pos.	403	obs:	exp:
att	I	39548	14463.00
atc	I	128	9917.00
ata	I	54	15350.00
atg	M	2	2.00
act	T	2	0.50
acc	T	0	0.41
aca	T	0	0.91
acg	T	0	0.17
gtt	V	36	7.96
gtc	V	0	6.54
gta	V	0	9.68
gtg	V	0	11.82
---	--	------	------
mPD		0.011	0.66
		nPD:	0.02
		N. weight:	1.1
		Sc. PD:	-0.049
		Sc. rank:	-3905.4

,

HA			
Pos.	404	obs:	exp:
aaa	K	8	4.56
aag	K	0	3.44
gtt	V	0	0.66
gtc	V	0	0.55
gta	V	3	0.81
gtg	V	0	0.98
gct	A	0	0.79
gcc	A	0	0.62
gca	A	3	1.41
gcg	A	0	0.18
gat	D	1	4.22
gac	D	7	3.78
gaa	E	39696	24066.00
gag	E	49	15679.00
ggt	G	0	0.45
ggc	G	0	0.44
gga	G	3	1.33
ggg	G	0	0.77
---	--	------	------
mPD		0.0037	0.48
		nPD:	0.01
		N. weight:	0.53
		Sc. PD:	-0.029
		Sc. rank:	-10052.0

,

HA			
Pos.	405	obs:	exp:
cgt	R	0	0.22
cgc	R	0	0.42
cga	R	0	0.78
cgg	R	0	0.59
aaa	K	2427	22681.00
aag	K	37335	17081.00
aga	R	1	4.02
agg	R	7	1.98
---	--	------	------
mPD		0.12	0.49
		nPD:	0.23
		N. weight:	0.65
		Sc. PD:	0.11
		Sc. rank:	17439.0

,

HA			
Pos.	406	obs:	exp:
att	I	0	0.73
atc	I	1	0.50
ata	I	1	0.77
atg	M	39765	39765.00
act	T	0	0.25
acc	T	0	0.21
aca	T	0	0.46
acg	T	1	0.09
gtt	V	0	0.44
gtc	V	0	0.36
gta	V	0	0.54
gtg	V	2	0.66
---	--	------	------
mPD		0.00025	0.00036
		nPD:	0.69
		N. weight:	0.00017
		Sc. PD:	0.00011
		Sc. rank:	5.4

,

HA

Pos.	407	obs:	exp:
tct	S	0	0.18
tcc	S	0	0.12
tca	S	0	0.26
tcg	S	0	0.05
tat	Y	1	0.52
tac	Y	0	0.48
aat	N	39405	23520.00
aac	N	362	16247.00
agt	S	1	0.19
agc	S	0	0.20
gat	D	1	0.53
gac	D	0	0.47
---	--	------	------
mPD		0.018	0.48
		nPD:	0.04
		N. weight:	0.52
		Sc. PD:	-0.013
		Sc. rank:	5406.8

HA

Pos.	408	obs:	exp:
att	I	0	18.93
atc	I	0	12.98
ata	I	52	20.09
act	T	42	10009.00
acc	T	15	8144.00
aca	T	39616	18164.00
acg	T	31	3388.00
aaa	K	1	0.57
aag	K	0	0.43
gct	A	0	3.44
gcc	A	0	2.69
gca	A	13	6.11
gcg	A	0	0.76
---	--	------	------
mPD		0.0077	0.68
		nPD:	0.01
		N. weight:	0.83
		Sc. PD:	-0.043
		Sc. rank:	-9775.6

HA

Pos.	409	obs:	exp:
cat	H	9	5.88
cac	H	1	4.12
caa	Q	190	20976.00
cag	Q	39563	18777.00
cgt	R	0	0.14
cgc	R	0	0.26
cga	R	0	0.49
cgg	R	5	0.37
aaa	K	0	1.14
aag	K	2	0.86
aga	R	0	2.51
agg	R	0	1.24
---	--	------	------
mPD		0.010	0.50
		nPD:	0.02
		N. weight:	0.78
		Sc. PD:	-0.033
		Sc. rank:	-314.0

HA

Pos.	410	obs:	exp:
ttt	F	65	17065.00
ttc	F	39701	22701.00
tta	L	0	0.22
ttg	L	1	0.38
tct	S	0	0.18
tcc	S	1	0.12
tca	S	0	0.26
tcg	S	0	0.05
ctt	L	0	0.39
ctc	L	1	0.28
cta	L	0	0.39
ctg	L	0	0.34
att	I	0	0.36
atc	I	1	0.25
ata	I	0	0.39
agt	S	0	0.19
agc	S	0	0.20
---	--	------	------
mPD		0.0035	0.49
		nPD:	0.01
		N. weight:	0.59
		Sc. PD:	-0.033
		Sc. rank:	-11698.0

HA

Pos.	411	obs:	exp:
att	I	0	5.46
atc	I	0	3.74
ata	I	15	5.80
act	T	46	10020.00
acc	T	32	8153.00
aca	T	39652	18183.00
acg	T	17	3391.00
aaa	K	2	1.14
aag	K	0	0.86
gct	A	0	1.59
gcc	A	0	1.24
gca	A	6	2.82
gcg	A	0	0.35
---	--	------	------
mPD		0.0059	0.68
		nPD:	0.01
		N. weight:	0.83
		Sc. PD:	-0.045
		Sc. rank:	-14153.0

HA

Pos.	412	obs:	exp:
tct	S	0	11.96
tcc	S	0	7.53
tca	S	65	16.92
tcg	S	0	3.46
act	T	0	1.76
acc	T	0	1.44
aca	T	7	3.20
acg	T	0	0.60
agt	S	0	12.22
agc	S	0	12.91
gtt	V	0	0.22
gtc	V	0	0.18
gta	V	1	0.27
gtg	V	0	0.33
gct	A	6	10500.00
gcc	A	1	8209.00
gca	A	39603	18626.00
gcg	A	48	2323.00
gaa	E	3	1.82
gag	E	0	1.18
ggt	G	0	5.46
ggc	G	36	5.33
gga	G	0	16.01
ggg	G	0	9.20
---	--	------	------
mPD		0.010	0.67
		nPD:	0.02
		N. weight:	0.81
		Sc. PD:	-0.039
		Sc. rank:	-4944.4

HA

Pos.	413	obs:	exp:
tta	L	1	0.11
ttg	L	0	0.19
ctt	L	0	0.20
ctc	L	0	0.14
cta	L	0	0.19
ctg	L	0	0.17
caa	Q	0	19.00
cag	Q	36	17.00
att	I	0	3.64
atc	I	0	2.50
ata	I	10	3.86
atg	M	14	14.00
gtt	V	2096	8778.00
gtc	V	2	7214.00
gta	V	8438	10678.00
gtg	V	29167	13033.00
gct	A	0	1.32
gcc	A	0	1.03
gca	A	0	2.35
gcg	A	5	0.29
ggt	G	0	0.15
ggc	G	0	0.15
gga	G	1	0.44
ggg	G	0	0.26
---	--	------	------
mPD		0.42	0.74
		nPD:	0.56
		N. weight:	0.5
		Sc. PD:	0.25
		Sc. rank:	15624.0

HA

Pos.	414	obs:	exp:
tct	S	0	2.58
tcc	S	0	1.62
tca	S	0	3.64
tcg	S	0	0.75
tgt	C	36	16.25
tgc	C	0	19.75
agt	S	13	2.63
agc	S	1	2.78
gat	D	5	2.63
gac	D	0	2.37
ggt	G	39465	6022.00
ggc	G	238	5882.00
gga	G	7	17666.00
ggg	G	5	10145.00
---	--	------	------
mPD		0.015	0.70
		nPD:	0.02
		N. weight:	2.
		Sc. PD:	-0.082
		Sc. rank:	1668.4

HA

Pos.	415	obs:	exp:
cgt	R	0	0.08
cgc	R	0	0.16
cga	R	0	0.29
cgg	R	0	0.22
act	T	0	0.25
acc	T	0	0.21
aca	T	1	0.46
acg	T	0	0.09
aat	N	1	0.59
aac	N	0	0.41
aaa	K	39364	22681.00
aag	K	397	17080.00
aga	R	3	1.51
agg	R	0	0.74
gaa	E	4	2.42
gag	E	0	1.58
---	--	------	------
mPD		0.020	0.49
		nPD:	0.04
		N. weight:	0.55
		Sc. PD:	-0.012
		Sc. rank:	7051.6

HA

Pos.	416	obs:	exp:
aaa	K	0	1.14
aag	K	2	0.86
gat	D	1	0.53
gac	D	0	0.47
gaa	E	254	24078.00
gag	E	39511	15687.00
ggt	G	0	0.30
ggc	G	0	0.30
gga	G	0	0.89
ggg	G	2	0.51
---	--	------	------
mPD		0.013	0.48
		nPD:	0.03
		N. weight:	0.96
		Sc. PD:	-0.035
		Sc. rank:	4707.2

HA

Pos.	417	obs:	exp:
ttt	F	172	17065.00
ttc	F	39594	22701.00
tta	L	0	0.11
ttg	L	0	0.19
tct	S	0	0.37
tcc	S	2	0.23
tca	S	0	0.52
tcg	S	0	0.11
tat	Y	0	0.52
tac	Y	1	0.48
ctt	L	0	0.20
ctc	L	1	0.14
cta	L	0	0.19
ctg	L	0	0.17
agt	S	0	0.38
agc	S	0	0.40
---	--	------	------
mPD		0.0088	0.49
		nPD:	0.02
		N. weight:	0.58
		Sc. PD:	-0.026
		Sc. rank:	-1607.5

HA

Pos.	418	obs:	exp:
tct	S	0	3.68
tcc	S	0	2.32
tca	S	0	5.21
tcg	S	0	1.06
cat	H	0	0.59
cac	H	1	0.41
att	I	0	0.36
atc	I	1	0.25
ata	I	0	0.39
aat	N	207	23508.00
aac	N	39540	16239.00
agt	S	0	3.76
agc	S	20	3.97
gat	D	0	0.53
gac	D	1	0.47
---	--	------	------
mPD		0.012	0.49
		nPD:	0.02
		N. weight:	0.93
		Sc. PD:	-0.037
		Sc. rank:	1968.7

HA			
Pos.	419	obs:	exp:
tta	L	0	0.22
ttg	L	0	0.38
tat	Y	0	2.08
tac	Y	4	1.92
ctt	L	0	0.39
ctc	L	2	0.28
cta	L	0	0.39
ctg	L	0	0.34
cat	H	270	23306.00
cac	H	39360	16324.00
caa	Q	6	3.69
cag	Q	1	3.31
cgt	R	0	0.38
cgc	R	14	0.73
cga	R	0	1.37
cgg	R	0	1.02
aat	N	1	66.83
aac	N	112	46.17
aga	R	0	7.03
agg	R	0	3.46
---	--	------	------
mPD		0.021	0.49
		nPD:	0.04
		N. weight:	0.91
		Sc. PD:	-0.02
		Sc. rank:	11807.0

,

HA			
Pos.	420	obs:	exp:
ttt	F	7	3.00
ttc	F	0	4.00
tta	L	1	4371.00
ttg	L	1901	7486.00
ctt	L	20992	7835.00
ctc	L	10	5616.00
cta	L	43	7655.00
ctg	L	16814	6798.00
att	I	2	0.73
atc	I	0	0.50
ata	I	0	0.77
---	--	------	------
mPD		0.59	1.1
		nPD:	0.52
		N. weight:	0.9
		Sc. PD:	0.4
		Sc. rank:	27661.0

,

HA			
Pos.	421	obs:	exp:
gct	A	0	0.26
gcc	A	0	0.21
gca	A	1	0.47
gcg	A	0	0.06
gat	D	3	9.48
gac	D	15	8.52
gaa	E	38999	24066.00
gag	E	747	15680.00
ggt	G	0	0.76
ggc	G	0	0.74
gga	G	5	2.22
ggg	G	0	1.28
---	--	------	------
mPD		0.038	0.48
		nPD:	0.08
		N. weight:	0.45
		Sc. PD:	0.0069
		Sc. rank:	9131.8

,

HA			
Pos.	422	obs:	exp:
caa	Q	3	1.58
cag	Q	0	1.42
cgt	R	0	0.74
cgc	R	0	1.41
cga	R	0	2.64
cgg	R	0	1.98
att	I	0	0.73
atc	I	0	0.50
ata	I	2	0.77
act	T	0	1.76
acc	T	0	1.44
aca	T	7	3.20
acg	T	0	0.60
aat	N	2	1.18
aac	N	0	0.82
aaa	K	39561	22662.00
aag	K	167	17066.00
aga	R	27	13.55
agg	R	0	6.67
gaa	E	1	0.61
gag	E	0	0.39
---	--	------	------
mPD		0.010	0.49
		nPD:	0.02
		N. weight:	0.58
		Sc. PD:	-0.024
		Sc. rank:	75.3

,

HA			
Pos.	423	obs:	exp:
tct	S	0	1.10
tcc	S	0	0.69
tca	S	0	1.56
tcg	S	0	0.32
cgt	R	0	1085.00
cgc	R	0	2079.00
cga	R	1	3894.00
cgg	R	0	2909.00
aaa	K	4	2.28
aag	K	0	1.72
agt	S	4	1.13
agc	S	2	1.19
aga	R	39690	19957.00
agg	R	62	9828.00
ggt	G	0	1.06
ggc	G	0	1.04
gga	G	7	3.11
ggg	G	0	1.79
---	--	------	------
mPD		0.0040	0.91
		nPD:	0.
		N. weight:	0.73
		Sc. PD:	-0.043
		Sc. rank:	-18617.0

,

HA			
Pos.	424	obs:	exp:
tta	L	2	7.26
ttg	L	0	12.43
ctt	L	0	13.01
ctc	L	0	9.32
cta	L	63	12.71
ctg	L	1	11.28
att	I	30	13794.00
atc	I	1	9459.00
ata	I	37861	14640.00
atg	M	1790	1790.00
gtt	V	0	4.86
gtc	V	0	4.00
gta	V	22	5.92
gtg	V	0	7.22
---	--	------	------
mPD		0.092	0.69
		nPD:	0.13
		N. weight:	0.98
		Sc. PD:	0.067
		Sc. rank:	23937.0

,

HA			
Pos.	425	obs:	exp:
aaa	K	0	3.42
aag	K	6	2.58
gct	A	0	2.91
gcc	A	0	2.28
gca	A	0	5.17
gcg	A	11	0.64
gat	D	4	2.11
gac	D	0	1.89
gaa	E	1485	24050.00
gag	E	38234	15669.00
ggt	G	0	4.55
ggc	G	0	4.44
gga	G	1	13.34
ggg	G	29	7.66
---	--	------	------
mPD		0.074	0.48
		nPD:	0.16
		N. weight:	0.82
		Sc. PD:	0.073
		Sc. rank:	20986.0

,

HA			
Pos.	426	obs:	exp:
aat	N	39741	23522.00
aac	N	29	16248.00
---	--	------	------
mPD		0.0015	0.48
		nPD:	0.
		N. weight:	0.56
		Sc. PD:	-0.033
		Sc. rank:	-15680.0

,

HA			
Pos.	427	obs:	exp:
tta	L	12737	4372.00
ttg	L	20	7487.00
ctt	L	11	7836.00
ctc	L	0	5617.00
cta	L	26977	7656.00
ctg	L	21	6799.00
att	I	0	1.46
atc	I	0	1.00
ata	I	4	1.55
---	--	------	------
mPD		0.44	1.1
		nPD:	0.38
		N. weight:	1.3
		Sc. PD:	0.4
		Sc. rank:	37035.0

,

HA			
Pos.	428	obs:	exp:
tct	S	0	0.37
tcc	S	0	0.23
tca	S	0	0.52
tcg	S	0	0.11
aat	N	39506	23519.00
aac	N	259	16246.00
agt	S	2	0.38
agc	S	0	0.40
gat	D	3	1.58
gac	D	0	1.42
---	--	------	------
mPD		0.013	0.48
		nPD:	0.03
		N. weight:	0.53
		Sc. PD:	-0.019
		Sc. rank:	2648.3

,

HA			
Pos.	429	obs:	exp:
caa	Q	1	0.53
cag	Q	0	0.47
cgt	R	0	0.30
cgc	R	0	0.58
cga	R	0	1.08
cgg	R	0	0.81
act	T	0	0.25
acc	T	0	0.21
aca	T	1	0.46
acg	T	0	0.09
aat	N	0	0.59
aac	N	1	0.41
aaa	K	39725	22678.00
aag	K	31	17078.00
aga	R	11	5.52
agg	R	0	2.72
---	--	------	------
mPD		0.0023	0.49
		nPD:	0.
		N. weight:	0.6
		Sc. PD:	-0.035
		Sc. rank:	-14879.0

,

HA			
Pos.	430	obs:	exp:
cgt	R	0	0.05
cgc	R	0	0.10
cga	R	0	0.20
cgg	R	0	0.15
aaa	K	35763	22685.00
aag	K	4005	17083.00
aga	R	1	1.00
agg	R	1	0.49
---	--	------	------
mPD		0.18	0.49
		nPD:	0.37
		N. weight:	0.28
		Sc. PD:	0.085
		Sc. rank:	8161.9

,

HA

Pos.	431	obs:	exp:
tta	L	0	0.22
ttg	L	0	0.38
ctt	L	1	0.39
ctc	L	1	0.28
cta	L	0	0.39
ctg	L	0	0.34
att	I	154	56.06
atc	I	0	38.44
ata	I	0	59.50
gtt	V	39535	8758.00
gtc	V	13	7198.00
gta	V	57	10654.00
gtg	V	9	13004.00
---	--	------	------
mPD		0.012	0.75
		nPD:	0.02
		N. weight:	1.6
		Sc. PD:	-0.076
		Sc. rank:	-7505.1

,

HA

Pos.	432	obs:	exp:
aat	N	2	1.18
aac	N	0	0.82
gat	D	39390	20952.00
gac	D	374	18812.00
gaa	E	2	1.82
gag	E	1	1.18
ggt	G	0	0.15
ggc	G	0	0.15
gga	G	1	0.44
ggg	G	0	0.26
---	--	------	------
mPD		0.019	0.50
		nPD:	0.04
		N. weight:	0.64
		Sc. PD:	-0.016
		Sc. rank:	6818.5

,

HA

Pos.	433	obs:	exp:
gat	D	39118	20954.00
gac	D	651	18815.00
ggt	G	1	0.15
ggc	G	0	0.15
gga	G	0	0.44
ggg	G	0	0.26
---	--	------	------
mPD		0.032	0.50
		nPD:	0.06
		N. weight:	0.6
		Sc. PD:	0.00051
		Sc. rank:	10856.0

,

HA

Pos.	434	obs:	exp:
tct	S	0	0.18
tcc	S	0	0.12
tca	S	0	0.26
tcg	S	0	0.05
agt	S	1	0.19
agc	S	0	0.20
ggt	G	38850	6030.00
ggc	G	245	5890.00
gga	G	504	17690.00
ggg	G	170	10159.00
---	--	------	------
mPD		0.046	0.69
		nPD:	0.07
		N. weight:	1.9
		Sc. PD:	0.0036
		Sc. rank:	34139.0

,

HA

Pos.	435	obs:	exp:
ttt	F	332	17062.00
ttc	F	39427	22697.00
tta	L	6	1.10
ttg	L	0	1.88
ctt	L	0	1.97
ctc	L	4	1.41
cta	L	0	1.93
ctg	L	0	1.71
att	I	0	0.36
atc	I	1	0.25
ata	I	0	0.39
---	--	------	------
mPD		0.017	0.49
		nPD:	0.03
		N. weight:	0.56
		Sc. PD:	-0.016
		Sc. rank:	5290.0

,

HA

Pos.	436	obs:	exp:
tta	L	1	4370.00
ttg	L	240	7484.00
ctt	L	17	7833.00
ctc	L	1	5615.00
cta	L	163	7653.00
ctg	L	39329	6796.00
caa	Q	0	0.53
cag	Q	1	0.47
att	I	0	0.36
atc	I	1	0.25
ata	I	0	0.39
atg	M	17	17.00
---	--	------	------
mPD		0.022	1.1
		nPD:	0.02
		N. weight:	1.8
		Sc. PD:	-0.08
		Sc. rank:	-2214.5

,

HA

Pos.	437	obs:	exp:
aat	N	0	0.59
aac	N	1	0.41
gat	D	127	20954.00
gac	D	39641	18814.00
gaa	E	1	0.61
gag	E	0	0.39
---	--	------	------
mPD		0.0065	0.50
		nPD:	0.01
		N. weight:	0.79
		Sc. PD:	-0.039
		Sc. rank:	-7100.9

,

HA

Pos.	438	obs:	exp:
ttt	F	1	0.43
ttc	F	0	0.57
tta	L	0	0.55
ttg	L	0	0.94
ctt	L	5	0.99
ctc	L	0	0.71
cta	L	0	0.96
ctg	L	0	0.85
att	I	39244	14317.00
atc	I	63	9817.00
ata	I	22	15195.00
atg	M	1	1.00
act	T	0	0.25
acc	T	1	0.21
aca	T	0	0.46
acg	T	0	0.09
gtt	V	430	95.73
gtc	V	1	78.68
gta	V	2	116.50
gtg	V	0	142.10
---	--	------	------
mPD		0.026	0.68
		nPD:	0.04
		N. weight:	1.1
		Sc. PD:	-0.026
		Sc. rank:	12240.0

,

HA

Pos.	439	obs:	exp:
ttt	F	1	0.43
ttc	F	0	0.57
tct	S	0	0.18
tcc	S	0	0.12
tca	S	0	0.26
tcg	S	1	0.05
tgg	W	39767	39767.00
cct	P	0	0.28
ccc	P	0	0.19
cca	P	0	0.43
ccg	P	1	0.11
agt	S	0	0.19
agc	S	0	0.20
---	--	------	------
mPD		0.00025	0.00034
		nPD:	0.73
		N. weight:	0.00016
		Sc. PD:	0.00011
		Sc. rank:	5.1

,

HA

Pos.	440	obs:	exp:
act	T	39657	10025.00
acc	T	65	8157.00
aca	T	32	18193.00
acg	T	14	3393.00
gct	A	2	0.53
gcc	A	0	0.41
gca	A	0	0.94
gcg	A	0	0.12
---	--	------	------
mPD		0.0057	0.68
		nPD:	0.01
		N. weight:	1.5
		Sc. PD:	-0.08
		Sc. rank:	-26136.0

,

HA

Pos.	441	obs:	exp:
ttt	F	0	0.43
ttc	F	1	0.57
tat	Y	68	20663.00
tac	Y	39701	19106.00
---	--	------	------
mPD		0.0035	0.50
		nPD:	0.01
		N. weight:	0.78
		Sc. PD:	-0.043
		Sc. rank:	-15445.0

,

HA

Pos.	442	obs:	exp:
att	I	1	0.36
atc	I	0	0.25
ata	I	0	0.39
aat	N	39749	23521.00
aac	N	20	16248.00
---	--	------	------
mPD		0.0011	0.48
		nPD:	0.
		N. weight:	0.56
		Sc. PD:	-0.034
		Sc. rank:	-16890.0

,

HA

Pos.	443	obs:	exp:
tct	S	0	0.37
tcc	S	2	0.23
tca	S	0	0.52
tcg	S	0	0.11
act	T	0	1.01
acc	T	4	0.82
aca	T	0	1.83
acg	T	0	0.34
agt	S	0	0.38
agc	S	0	0.40
gtt	V	0	1.11
gtc	V	5	0.91
gta	V	0	1.34
gtg	V	0	1.64
gct	A	313	10526.00
gcc	A	39302	8230.00
gca	A	144	18673.00
gcg	A	0	2329.00
---	--	------	------
mPD		0.023	0.66
		nPD:	0.04
		N. weight:	1.6
		Sc. PD:	-0.046
		Sc. rank:	15873.0

,

HA

Pos.	444	obs:	exp:
caa	Q	1	0.53
cag	Q	0	0.47
aaa	K	6	3.42
aag	K	0	2.58
gaa	E	39635	24075.00
gag	E	126	15686.00
ggt	G	0	0.30
ggc	G	0	0.30
gga	G	2	0.89
ggg	G	0	0.51
---	--	------	------
mPD		0.0068	0.48
		nPD:	0.01
		N. weight:	0.52
		Sc. PD:	-0.025
		Sc. rank:	-3928.8

,

HA

Pos.	445	obs:	exp:
tta	L	0	4372.00
ttg	L	169	7487.00
ctt	L	21	7836.00
ctc	L	10	5617.00
cta	L	85	7656.00
ctg	L	39481	6799.00
atg	M	3	3.00
gtt	V	0	0.22
gtc	V	0	0.18
gta	V	0	0.27
gtg	V	1	0.33
---	--	------	------
mPD		0.014	1.1
		nPD:	0.01
		N. weight:	1.9
		Sc. PD:	-0.093
		Sc. rank:	-18395.0

,

HA

Pos.	446	obs:	exp:
ttt	F	2	1.29
ttc	F	1	1.71
tta	L	201	4371.00
ttg	L	37625	7486.00
tct	S	0	0.18
tcc	S	0	0.12
tca	S	0	0.26
tcg	S	1	0.05
ctt	L	1	7836.00
ctc	L	0	5616.00
cta	L	13	7655.00
ctg	L	1923	6799.00
atg	M	2	2.00
agt	S	0	0.19
agc	S	0	0.20
gct	A	1	0.26
gcc	A	0	0.21
gca	A	0	0.47
gcg	A	0	0.06
---	--	------	------
mPD		0.10	1.1
		nPD:	0.09
		N. weight:	1.6
		Sc. PD:	0.042
		Sc. rank:	34564.0

,

HA

Pos.	447	obs:	exp:
att	I	55	20.02
atc	I	0	13.73
ata	I	0	21.25
gtt	V	39586	8780.00
gtc	V	79	7216.00
gta	V	44	10680.00
gtg	V	3	13036.00
gct	A	3	0.79
gcc	A	0	0.62
gca	A	0	1.41
gcg	A	0	0.18
---	--	------	------
mPD		0.0092	0.74
		nPD:	0.01
		N. weight:	1.6
		Sc. PD:	-0.081
		Sc. rank:	-16595.0

,

HA

Pos.	448	obs:	exp:
ttt	F	1	0.43
ttc	F	0	0.57
tta	L	125	4371.00
ttg	L	0	7486.00
ctt	L	6	7836.00
ctc	L	1	5617.00
cta	L	38805	7655.00
ctg	L	827	6799.00
att	I	0	1.82
atc	I	0	1.25
ata	I	5	1.93
---	--	------	------
mPD		0.048	1.1
		nPD:	0.04
		N. weight:	1.7
		Sc. PD:	-0.036
		Sc. rank:	21663.0

,

HA

Pos.	449	obs:	exp:
ttt	F	1	0.86
ttc	F	1	1.14
tta	L	68	4371.00
ttg	L	18303	7487.00
tct	S	0	0.18
tcc	S	0	0.12
tca	S	0	0.26
tcg	S	1	0.05
ctt	L	6	7836.00
ctc	L	0	5617.00
cta	L	237	7655.00
ctg	L	21151	6799.00
atg	M	2	2.00
agt	S	0	0.19
agc	S	0	0.20
---	--	------	------
mPD		0.51	1.1
		nPD:	0.45
		N. weight:	1.1
		Sc. PD:	0.4
		Sc. rank:	32367.0

,

HA

Pos.	450	obs:	exp:
aaa	K	3	1.71
aag	K	0	1.29
gat	D	1	0.53
gac	D	0	0.47
gaa	E	39686	24078.00
gag	E	79	15687.00
ggt	G	0	0.15
ggc	G	0	0.15
gga	G	1	0.44
ggg	G	0	0.26
---	--	------	------
mPD		0.0042	0.48
		nPD:	0.01
		N. weight:	0.53
		Sc. PD:	-0.028
		Sc. rank:	-8809.0

,

HA

Pos.	451	obs:	exp:
aat	N	25727	23522.00
aac	N	14043	16248.00
---	--	------	------
mPD		0.46	0.48
		nPD:	0.95
		N. weight:	0.0071
		Sc. PD:	0.0062
		Sc. rank:	226.9

,

HA

Pos.	452	obs:	exp:
aaa	K	1	0.57
aag	K	0	0.43
gat	D	0	4.74
gac	D	9	4.26
gaa	E	39730	24072.00
gag	E	26	15684.00
ggt	G	0	0.61
ggc	G	0	0.59
gga	G	4	1.78
ggg	G	0	1.02
---	--	------	------
mPD		0.0020	0.48
		nPD:	0.
		N. weight:	0.54
		Sc. PD:	-0.031
		Sc. rank:	-13672.0

,

HA

Pos.	453	obs:	exp:
cgt	R	0	1085.00
cgc	R	0	2079.00
cga	R	5	3893.00
cgg	R	0	2909.00
att	I	0	1.82
atc	I	0	1.25
ata	I	5	1.93
aaa	K	14	8.56
aag	K	1	6.44
aga	R	39612	19955.00
agg	R	132	9827.00
ggt	G	0	0.15
ggc	G	0	0.15
gga	G	1	0.44
ggg	G	0	0.26
---	--	------	------
mPD		0.0080	0.91
		nPD:	0.01
		N. weight:	0.72
		Sc. PD:	-0.039
		Sc. rank:	-12302.0

,

HA

Pos.	454	obs:	exp:
att	I	18	6.55
atc	I	0	4.49
ata	I	0	6.95
act	T	39624	10011.00
acc	T	64	8146.00
aca	T	6	18168.00
acg	T	19	3388.00
aat	N	35	20.70
aac	N	0	14.30
gct	A	4	1.06
gcc	A	0	0.83
gca	A	0	1.88
gcg	A	0	0.23
---	--	------	------
mPD		0.0073	0.68
		nPD:	0.01
		N. weight:	1.5
		Sc. PD:	-0.076
		Sc. rank:	-18557.0

,

HA

Pos.	455	obs:	exp:
ttt	F	1	0.43
ttc	F	0	0.57
tta	L	82	4372.00
ttg	L	39651	7487.00
ctt	L	0	7837.00
ctc	L	0	5617.00
cta	L	0	7656.00
ctg	L	36	6800.00
---	--	------	------
mPD		0.0060	1.1
		nPD:	0.01
		N. weight:	1.8
		Sc. PD:	-0.1
		Sc. rank:	-41107.0

,

HA

Pos.	456	obs:	exp:
aat	N	0	3.55
aac	N	6	2.45
gtt	V	0	0.22
gtc	V	1	0.18
gta	V	0	0.27
gtg	V	0	0.33
gat	D	228	20948.00
gac	D	39529	18809.00
gaa	E	2	1.82
gag	E	1	1.18
ggt	G	0	0.45
ggc	G	3	0.44
gga	G	0	1.33
ggg	G	0	0.77
---	--	------	------
mPD		0.012	0.50
		nPD:	0.02
		N. weight:	0.77
		Sc. PD:	-0.03
		Sc. rank:	2076.3

,

HA

Pos.	457	obs:	exp:
tct	S	1	0.18
tcc	S	0	0.12
tca	S	0	0.26
tcg	S	0	0.05
tat	Y	27404	20658.00
tac	Y	12354	19100.00
cat	H	4	5.88
cac	H	6	4.12
aat	N	1	0.59
aac	N	0	0.41
agt	S	0	0.19
agc	S	0	0.20
---	--	------	------
mPD		0.43	0.50
		nPD:	0.86
		N. weight:	0.064
		Sc. PD:	0.05
		Sc. rank:	2045.2

,

HA

Pos.	458	obs:	exp:
cat	H	1198	23388.00
cac	H	38571	16381.00
caa	Q	1	0.53
cag	Q	0	0.47
---	--	------	------
mPD		0.058	0.48
		nPD:	0.12
		N. weight:	0.8
		Sc. PD:	0.045
		Sc. rank:	19067.0

,

HA

Pos.	459	obs:	exp:
tat	Y	1	0.52
tac	Y	0	0.48
cgt	R	1	0.03
cgc	R	0	0.05
cga	R	0	0.10
cgg	R	0	0.07
aat	N	25	15.38
aac	N	1	10.62
aga	R	0	0.50
agg	R	0	0.25
gtt	V	1	0.22
gtc	V	0	0.18
gta	V	0	0.27
gtg	V	0	0.33
gat	D	39448	20937.00
gac	D	289	18800.00
gaa	E	0	1.21
gag	E	2	0.79
ggt	G	2	0.30
ggc	G	0	0.30
gga	G	0	0.89
ggg	G	0	0.51
---	--	------	------
mPD		0.016	0.50
		nPD:	0.03
		N. weight:	0.65
		Sc. PD:	-0.02
		Sc. rank:	5689.7

,

HA

Pos.	460	obs:	exp:
tta	L	7	0.77
ttg	L	0	1.32
tct	S	24	7317.00
tcc	S	1	4605.00
tca	S	39690	10348.00
tcg	S	45	2116.00
ctt	L	0	1.38
ctc	L	0	0.99
cta	L	0	1.35
ctg	L	0	1.20
cct	P	0	0.55
ccc	P	0	0.37
cca	P	2	0.85
ccg	P	0	0.23
act	T	0	0.25
acc	T	0	0.21
aca	T	1	0.46
acg	T	0	0.09
agt	S	0	7476.00
agc	S	0	7898.00
---	--	------	------
mPD		0.0040	1.6
		nPD:	0.
		N. weight:	1.4
		Sc. PD:	-0.087
		Sc. rank:	-42153.0

,

HA

Pos.	461	obs:	exp:
tct	S	0	0.37
tcc	S	0	0.23
tca	S	0	0.52
tcg	S	0	0.11
tat	Y	1	0.52
tac	Y	0	0.48
cat	H	1	0.59
cac	H	0	0.41
att	I	1	0.36
atc	I	0	0.25
ata	I	0	0.39
aat	N	39687	23488.00
aac	N	27	16226.00
aaa	K	2	27.95
aag	K	47	21.05
agt	S	2	0.38
agc	S	0	0.40
gat	D	1	0.53
gac	D	0	0.47
ggt	G	1	0.15
ggc	G	0	0.15
gga	G	0	0.44
ggg	G	0	0.26
---	--	------	------
mPD		0.0042	0.49
		nPD:	0.01
		N. weight:	0.56
		Sc. PD:	-0.03
		Sc. rank:	-9674.4

,

HA

Pos.	462	obs:	exp:
atg	M	7	7.00
gtt	V	48	8791.00
gtc	V	0	7225.00
gta	V	265	10694.00
gtg	V	39450	13053.00
---	--	------	------
mPD		0.016	0.74
		nPD:	0.02
		N. weight:	1.2
		Sc. PD:	-0.048
		Sc. rank:	487.7

,

HA

Pos.	463	obs:	exp:
caa	Q	0	1.58
cag	Q	3	1.42
cgt	R	0	0.49
cgc	R	0	0.94
cga	R	0	1.76
cgg	R	0	1.32
atg	M	1	1.00
aat	N	2	1.18
aac	N	0	0.82
aaa	K	248	22668.00
aag	K	39490	17070.00
aga	R	0	9.04
agg	R	18	4.45
gaa	E	1	4.84
gag	E	7	3.16
---	--	------	------
mPD		0.014	0.49
		nPD:	0.03
		N. weight:	0.87
		Sc. PD:	-0.03
		Sc. rank:	5507.6

,

HA

Pos.	464	obs:	exp:
tct	S	0	2.58
tcc	S	0	1.62
tca	S	0	3.64
tcg	S	0	0.75
cat	H	0	1.18
cac	H	2	0.82
act	T	0	1.26
acc	T	5	1.03
aca	T	0	2.29
acg	T	0	0.43
aat	N	791	23462.00
aac	N	38879	16208.00
aaa	K	70	40.50
aag	K	1	30.50
agt	S	0	2.63
agc	S	14	2.78
gat	D	0	4.22
gac	D	8	3.78
---	--	------	------
mPD		0.044	0.49
		nPD:	0.09
		N. weight:	0.85
		Sc. PD:	0.022
		Sc. rank:	18471.0

,

HA

Pos.	465	obs:	exp:
tta	L	8065	4372.00
ttg	L	31562	7487.00
ctt	L	0	7837.00
ctc	L	0	5617.00
cta	L	3	7656.00
ctg	L	138	6799.00
atg	M	2	2.00
---	--	------	------
mPD		0.33	1.1
		nPD:	0.29
		N. weight:	1.4
		Sc. PD:	0.3
		Sc. rank:	37307.0

,

HA

Pos.	466	obs:	exp:
tat	Y	39711	20658.00
tac	Y	48	19101.00
tgt	C	2	0.90
tgc	C	0	1.10
cat	H	8	4.70
cac	H	0	3.30
aat	N	1	0.59
aac	N	0	0.41
---	--	------	------
mPD		0.0030	0.50
		nPD:	0.01
		N. weight:	0.7
		Sc. PD:	-0.04
		Sc. rank:	-15231.0

,

HA

Pos.	467	obs:	exp:
aaa	K	2	1.14
aag	K	0	0.86
gct	A	0	1.59
gcc	A	0	1.24
gca	A	6	2.82
gcg	A	0	0.35
gat	D	0	6.85
gac	D	13	6.15
gaa	E	39696	24064.00
gag	E	46	15678.00
ggt	G	0	1.06
ggc	G	0	1.04
gga	G	7	3.11
ggg	G	0	1.79
---	--	------	------
mPD		0.0037	0.48
		nPD:	0.01
		N. weight:	0.53
		Sc. PD:	-0.029
		Sc. rank:	-10094.0

,

HA

Pos.	468	obs:	exp:
cgt	R	0	0.93
cgc	R	0	1.78
cga	R	0	3.33
cgg	R	0	2.49
atg	M	1	1.00
act	T	0	0.25
acc	T	0	0.21
aca	T	1	0.46
acg	T	0	0.09
aat	N	1	1.18
aac	N	1	0.82
aaa	K	26713	22663.00
aag	K	13016	17066.00
aga	R	5	17.07
agg	R	29	8.41
gaa	E	0	1.82
gag	E	3	1.18
---	--	------	------
mPD		0.44	0.49
		nPD:	0.9
		N. weight:	0.025
		Sc. PD:	0.02
		Sc. rank:	783.3

,

HA

Pos.	469	obs:	exp:
tta	L	2	0.22
ttg	L	0	0.38
ctt	L	0	0.39
ctc	L	0	0.28
cta	L	0	0.39
ctg	L	0	0.34
att	I	0	36.77
atc	I	0	25.21
ata	I	101	39.02
gtt	V	61	8765.00
gtc	V	0	7204.00
gta	V	39410	10663.00
gtg	V	176	13015.00
gct	A	0	5.30
gcc	A	0	4.14
gca	A	20	9.39
gcg	A	0	1.17
---	--	------	------
mPD		0.018	0.74
		nPD:	0.02
		N. weight:	1.4
		Sc. PD:	-0.053
		Sc. rank:	4032.8

,

HA

Pos.	470	obs:	exp:
cgt	R	0	1083.00
cgc	R	0	2075.00
cga	R	3	3885.00
cgg	R	0	2902.00
att	I	0	0.73
atc	I	0	0.50
ata	I	2	0.77
aaa	K	97	55.90
aag	K	1	42.10
aga	R	38508	19910.00
agg	R	1148	9804.00
ggt	G	0	1.67
ggc	G	0	1.63
gga	G	3	4.89
ggg	G	8	2.81
---	--	------	------
mPD		0.062	0.91
		nPD:	0.07
		N. weight:	0.62
		Sc. PD:	0.0026
		Sc. rank:	11649.0

,

HA			
Pos.	471	obs:	exp:
tct	S	0	1327.00
tcc	S	0	835.30
tca	S	0	1877.00
tcg	S	0	383.80
cat	H	0	25.29
cac	H	43	17.71
cgt	R	0	0.16
cgc	R	2	0.31
cga	R	0	0.59
cgg	R	0	0.44
att	I	0	0.36
atc	I	1	0.25
ata	I	0	0.39
act	T	2	356.50
acc	T	1407	290.00
aca	T	5	646.90
acg	T	0	120.60
aat	N	142	18376.00
aac	N	30928	12694.00
aaa	K	12	6.85
aag	K	0	5.15
agt	S	16	1356.00
agc	S	7196	1433.00
aga	R	4	3.01
agg	R	0	1.48
gct	A	0	0.79
gcc	A	3	0.62
gca	A	0	1.41
gcg	A	0	0.18
gat	D	0	2.63
gac	D	5	2.37
ggt	G	0	0.61
ggc	G	4	0.59
gga	G	0	1.78
ggg	G	0	1.02
---	--	------	------
mPD		0.36	1.1
		nPD:	0.33
		N. weight:	1.1
		Sc. PD:	0.28
		Sc. rank:	31163.0

,

HA			
Pos.	472	obs:	exp:
cat	H	12	7.06
cac	H	0	4.94
caa	Q	1953	20978.00
cag	Q	37803	18778.00
cgt	R	0	0.03
cgc	R	0	0.05
cga	R	0	0.10
cgg	R	1	0.07
aaa	K	0	0.57
aag	K	1	0.43
aga	R	0	0.50
agg	R	0	0.25
---	--	------	------
mPD		0.094	0.50
		nPD:	0.19
		N. weight:	0.59
		Sc. PD:	0.073
		Sc. rank:	15642.0

,

HA			
Pos.	473	obs:	exp:
ttt	F	7	3.00
ttc	F	0	4.00
tta	L	38888	4371.00
ttg	L	182	7486.00
ctt	L	4	7836.00
ctc	L	0	5616.00
cta	L	689	7655.00
ctg	L	0	6799.00
---	--	------	------
mPD		0.044	1.1
		nPD:	0.04
		N. weight:	2.2
		Sc. PD:	-0.056
		Sc. rank:	24832.0

,

HA			
Pos.	474	obs:	exp:
caa	Q	4	2.11
cag	Q	0	1.89
cgt	R	0	1.69
cgc	R	0	3.24
cga	R	0	6.07
cgg	R	0	4.54
att	I	0	0.73
atc	I	0	0.50
ata	I	2	0.77
act	T	0	0.25
acc	T	0	0.21
aca	T	1	0.46
acg	T	0	0.09
aat	N	0	1.77
aac	N	3	1.23
aaa	K	39597	22640.00
aag	K	92	17049.00
aga	R	62	31.13
agg	R	0	15.33
gaa	E	9	5.45
gag	E	0	3.55
---	--	------	------
mPD		0.0087	0.49
		nPD:	0.02
		N. weight:	0.59
		Sc. PD:	-0.027
		Sc. rank:	-1940.0

,

HA			
Pos.	475	obs:	exp:
tct	S	0	25.58
tcc	S	0	16.10
tca	S	0	36.18
tcg	S	0	7.40
tat	Y	0	0.52
tac	Y	1	0.48
cat	H	0	0.59
cac	H	1	0.41
cgt	R	0	0.03
cgc	R	0	0.05
cga	R	0	0.10
cgg	R	0	0.07
att	I	0	0.36
atc	I	1	0.25
ata	I	0	0.39
act	T	0	86.72
acc	T	344	70.56
aca	T	0	157.40
acg	T	0	29.35
aat	N	74	23231.00
aac	N	39204	16047.00
agt	S	0	26.14
agc	S	139	27.61
aga	R	1	0.50
agg	R	0	0.25
gat	D	0	2.63
gac	D	5	2.37
---	--	------	------
mPD		0.028	0.52
		nPD:	0.05
		N. weight:	0.96
		Sc. PD:	-0.0087
		Sc. rank:	15274.0

,

HA			
Pos.	476	obs:	exp:
tct	S	0	0.55
tcc	S	0	0.35
tca	S	0	0.78
tcg	S	0	0.16
tat	Y	1	0.52
tac	Y	0	0.48
aat	N	39759	23519.00
aac	N	7	16247.00
agt	S	3	0.56
agc	S	0	0.60
---	--	------	------
mPD		0.00055	0.48
		nPD:	0.
		N. weight:	0.57
		Sc. PD:	-0.035
		Sc. rank:	-17718.0

,

HA			
Pos.	477	obs:	exp:
act	T	0	0.50
acc	T	2	0.41
aca	T	0	0.91
acg	T	0	0.17
gtt	V	0	0.66
gtc	V	3	0.55
gta	V	0	0.81
gtg	V	0	0.98
gct	A	192	10528.00
gcc	A	39371	8231.00
gca	A	201	18676.00
gcg	A	1	2330.00
---	--	------	------
mPD		0.020	0.66
		nPD:	0.03
		N. weight:	1.6
		Sc. PD:	-0.054
		Sc. rank:	12143.0

,

HA			
Pos.	478	obs:	exp:
caa	Q	3	3.17
cag	Q	3	2.83
cgt	R	0	2.02
cgc	R	0	3.87
cga	R	0	7.25
cgg	R	0	5.42
atg	M	1	1.00
aat	N	6	3.55
aac	N	0	2.45
aaa	K	619	22613.00
aag	K	39023	17029.00
aga	R	0	37.15
agg	R	74	18.29
gaa	E	0	24.83
gag	E	41	16.17
---	--	------	------
mPD		0.037	0.50
		nPD:	0.07
		N. weight:	0.82
		Sc. PD:	0.0089
		Sc. rank:	15865.0

,

HA

Pos.	479	obs:	exp:
aaa	K	1	0.57
aag	K	0	0.43
gat	D	0	1.05
gac	D	2	0.95
gaa	E	39681	24079.00
gag	E	86	15688.00
---	--	------	------
mPD		0.0045	0.48
		nPD:	0.01
		N. weight:	0.53
		Sc. PD:	-0.028
		Sc. rank:	-8288.2

,

HA

Pos.	480	obs:	exp:
tta	L	0	1.76
ttg	L	0	3.01
tct	S	0	0.18
tcc	S	0	0.12
tca	S	0	0.26
tcg	S	0	0.05
ctt	L	16	3.15
ctc	L	0	2.26
cta	L	0	3.08
ctg	L	0	2.74
att	I	39465	14394.00
atc	I	73	9870.00
ata	I	3	15277.00
act	T	39	9.83
acc	T	0	8.00
aca	T	0	17.84
acg	T	0	3.33
aat	N	1	0.59
aac	N	0	0.41
agt	S	1	0.19
agc	S	0	0.20
gtt	V	172	38.03
gtc	V	0	31.25
gta	V	0	46.26
gtg	V	0	56.46
---	--	------	------
mPD		0.015	0.67
		nPD:	0.02
		N. weight:	1.1
		Sc. PD:	-0.043
		Sc. rank:	1634.8

,

HA

Pos.	481	obs:	exp:
gct	A	0	0.26
gcc	A	0	0.21
gca	A	1	0.47
gcg	A	0	0.06
gaa	E	6	3.63
gag	E	0	2.37
ggt	G	5	6029.00
ggc	G	1	5889.00
gga	G	39611	17687.00
ggg	G	146	10157.00
---	--	------	------
mPD		0.0080	0.69
		nPD:	0.01
		N. weight:	0.85
		Sc. PD:	-0.044
		Sc. rank:	-9484.3

,

HA

Pos.	482	obs:	exp:
tct	S	0	0.18
tcc	S	0	0.12
tca	S	0	0.26
tcg	S	0	0.05
act	T	0	0.76
acc	T	3	0.62
aca	T	0	1.37
acg	T	0	0.26
aat	N	1604	23496.00
aac	N	38123	16231.00
aaa	K	19	11.98
aag	K	2	9.02
agt	S	0	0.19
agc	S	1	0.20
gat	D	0	9.48
gac	D	18	8.52
---	--	------	------
mPD		0.080	0.49
		nPD:	0.16
		N. weight:	0.77
		Sc. PD:	0.075
		Sc. rank:	19981.0

,

HA

Pos.	483	obs:	exp:
tct	S	0	0.55
tcc	S	0	0.35
tca	S	0	0.78
tcg	S	0	0.16
agt	S	0	0.56
agc	S	3	0.60
ggt	G	448	6030.00
ggc	G	38518	5890.00
gga	G	780	17689.00
ggg	G	21	10158.00
---	--	------	------
mPD		0.061	0.69
		nPD:	0.09
		N. weight:	1.9
		Sc. PD:	0.046
		Sc. rank:	39547.0

,

HA

Pos.	484	obs:	exp:
tgt	C	59	17951.00
tgc	C	39711	21819.00
---	--	------	------
mPD		0.0030	0.50
		nPD:	0.01
		N. weight:	0.64
		Sc. PD:	-0.036
		Sc. rank:	-13864.0

,

HA

Pos.	485	obs:	exp:
ttt	F	39585	17066.00
ttc	F	183	22702.00
tta	L	1	0.22
ttg	L	0	0.38
ctt	L	1	0.39
ctc	L	0	0.28
cta	L	0	0.39
ctg	L	0	0.34
---	--	------	------
mPD		0.0093	0.49
		nPD:	0.02
		N. weight:	0.88
		Sc. PD:	-0.039
		Sc. rank:	-1508.7

,

HA

Pos.	486	obs:	exp:
aaa	K	7	3.99
aag	K	0	3.01
gtt	V	0	0.88
gtc	V	0	0.73
gta	V	4	1.08
gtg	V	0	1.31
gat	D	0	0.53
gac	D	1	0.47
gaa	E	39064	24074.00
gag	E	694	15684.00
---	--	------	------
mPD		0.035	0.48
		nPD:	0.07
		N. weight:	0.46
		Sc. PD:	0.0041
		Sc. rank:	8675.4

,

HA

Pos.	487	obs:	exp:
ttt	F	39719	17064.00
ttc	F	45	22700.00
tta	L	3	0.55
ttg	L	1	0.94
ctt	L	1	0.99
ctc	L	0	0.71
cta	L	0	0.96
ctg	L	0	0.85
att	I	1	0.36
atc	I	0	0.25
ata	I	0	0.39
---	--	------	------
mPD		0.0026	0.49
		nPD:	0.01
		N. weight:	0.91
		Sc. PD:	-0.052
		Sc. rank:	-21116.0

,

HA

Pos.	488	obs:	exp:
ttt	F	0	3.86
ttc	F	9	5.14
tct	S	0	0.18
tcc	S	1	0.12
tca	S	0	0.26
tcg	S	0	0.05
tat	Y	164	20651.00
tac	Y	39581	19094.00
cat	H	0	7.65
cac	H	13	5.35
aat	N	0	1.18
aac	N	2	0.82
agt	S	0	0.19
agc	S	0	0.20
---	--	------	------
mPD		0.0095	0.50
		nPD:	0.02
		N. weight:	0.76
		Sc. PD:	-0.034
		Sc. rank:	-1120.8

,

HA

Pos.	489	obs:	exp:
cat	H	32	23388.00
cac	H	39737	16381.00
caa	Q	1	0.53
cag	Q	0	0.47
---	--	------	------
mPD		0.0017	0.48
		nPD:	0.
		N. weight:	0.95
		Sc. PD:	-0.056
		Sc. rank:	-25726.0

,

HA

Pos.	490	obs:	exp:
caa	Q	6	3.17
cag	Q	0	2.83
cgt	R	0	0.19
cgc	R	0	0.37
cga	R	0	0.69
cgg	R	0	0.51
att	I	0	0.36
atc	I	0	0.25
ata	I	1	0.39
act	T	0	0.25
acc	T	0	0.21
aca	T	1	0.46
acg	T	0	0.09
aat	N	1	0.59
aac	N	0	0.41
aaa	K	39574	22676.00
aag	K	179	17077.00
aga	R	7	3.51
agg	R	0	1.73
gaa	E	1	0.61
gag	E	0	0.39
---	--	------	------
mPD		0.0098	0.49
		nPD:	0.02
		N. weight:	0.58
		Sc. PD:	-0.025
		Sc. rank:	-616.4

,

HA

Pos.	491	obs:	exp:
tgt	C	305	17950.00
tgc	C	39464	21819.00
tgg	W	1	1.00
---	--	------	------
mPD		0.015	0.50
		nPD:	0.03
		N. weight:	0.6
		Sc. PD:	-0.019
		Sc. rank:	4980.1

,

HA

Pos.	492	obs:	exp:
aat	N	56	50.27
aac	N	29	34.73
gat	D	24952	20906.00
gac	D	14726	18772.00
gaa	E	1	0.61
gag	E	0	0.39
ggt	G	4	0.91
ggc	G	2	0.89
gga	G	0	2.67
ggg	G	0	1.53
---	--	------	------
mPD		0.47	0.50
		nPD:	0.94
		N. weight:	0.023
		Sc. PD:	0.02
		Sc. rank:	740.4

,

HA

Pos.	493	obs:	exp:
tct	S	0	0.55
tcc	S	0	0.35
tca	S	0	0.78
tcg	S	0	0.16
act	T	0	0.50
acc	T	1	0.41
aca	T	1	0.91
acg	T	0	0.17
aat	N	63	23461.00
aac	N	39605	16207.00
agt	S	0	0.56
agc	S	3	0.60
gat	D	0	51.11
gac	D	97	45.89
---	--	------	------
mPD		0.0083	0.49
		nPD:	0.02
		N. weight:	0.95
		Sc. PD:	-0.044
		Sc. rank:	-3414.9

,

HA

Pos.	494	obs:	exp:
tct	S	0	0.37
tcc	S	0	0.23
tca	S	1	0.52
tcg	S	1	0.11
cct	P	0	2.48
ccc	P	0	1.67
cca	P	2	3.83
ccg	P	7	1.02
cgt	R	0	0.03
cgc	R	0	0.05
cga	R	0	0.10
cgg	R	0	0.07
att	I	0	7.28
atc	I	0	4.99
ata	I	20	7.73
atg	M	266	266.00
act	T	42	9812.00
acc	T	11	7983.00
aca	T	21036	17806.00
acg	T	17833	3321.00
aaa	K	6	27.95
aag	K	43	21.05
agt	S	0	0.38
agc	S	0	0.40
aga	R	1	0.50
agg	R	0	0.25
gct	A	0	132.40
gcc	A	0	103.50
gca	A	447	234.80
gcg	A	53	29.29
gaa	E	1	0.61
gag	E	0	0.39
---	--	------	------
mPD		0.54	0.72
		nPD:	0.75
		N. weight:	0.91
		Sc. PD:	0.61
		Sc. rank:	28924.0

,

HA

Pos.	495	obs:	exp:
tct	S	0	0.37
tcc	S	0	0.23
tca	S	0	0.52
tcg	S	0	0.11
tgt	C	243	17950.00
tgc	C	39525	21818.00
agt	S	0	0.38
agc	S	2	0.40
---	--	------	------
mPD		0.012	0.50
		nPD:	0.02
		N. weight:	0.61
		Sc. PD:	-0.023
		Sc. rank:	1994.4

,

HA

Pos.	496	obs:	exp:
tta	L	0	0.44
ttg	L	3	0.75
ctt	L	0	0.79
ctc	L	0	0.56
cta	L	0	0.77
ctg	L	1	0.68
att	I	6	12.38
atc	I	0	8.49
ata	I	28	13.14
atg	M	39728	39728.00
act	T	0	0.25
acc	T	0	0.21
aca	T	0	0.46
acg	T	1	0.09
gtt	V	0	0.66
gtc	V	0	0.55
gta	V	0	0.81
gtg	V	3	0.98
---	--	------	------
mPD		0.0021	0.0024
		nPD:	0.88
		N. weight:	0.00081
		Sc. PD:	0.00065
		Sc. rank:	25.7

,

HA

Pos.	497	obs:	exp:
aaa	K	10	5.70
aag	K	0	4.30
gat	D	0	2.63
gac	D	5	2.37
gaa	E	39707	24067.00
gag	E	40	15680.00
ggt	G	0	1.21
ggc	G	0	1.18
gga	G	8	3.56
ggg	G	0	2.04
---	--	------	------
mPD		0.0032	0.48
		nPD:	0.01
		N. weight:	0.53
		Sc. PD:	-0.03
		Sc. rank:	-10984.0

,

HA

Pos.	498	obs:	exp:
tct	S	0	7307.00
tcc	S	0	4599.00
tca	S	0	10333.00
tcg	S	0	2113.00
aat	N	61	36.08
aac	N	0	24.92
agt	S	39664	7466.00
agc	S	40	7887.00
ggt	G	5	0.76
ggc	G	0	0.74
gga	G	0	2.22
ggg	G	0	1.28
---	--	------	------
mPD		0.0053	1.6
		nPD:	0.
		N. weight:	1.8
		Sc. PD:	-0.11
		Sc. rank:	-48907.0

,

HA

Pos.	499	obs:	exp:
att	I	0	47.32
atc	I	130	32.45
ata	I	0	50.23
gtt	V	57	8761.00
gtc	V	39438	7201.00
gta	V	133	10658.00
gtg	V	0	13008.00
gct	A	0	3.18
gcc	A	12	2.48
gca	A	0	5.64
gcg	A	0	0.70
---	--	------	------
mPD		0.017	0.75
		nPD:	0.02
		N. weight:	1.8
		Sc. PD:	-0.073
		Sc. rank:	2202.0

,

HA

Pos.	500	obs:	exp:
cgt	R	0	2.38
cgc	R	0	4.55
cga	R	0	8.52
cgg	R	0	6.37
act	T	0	0.25
acc	T	0	0.21
aca	T	1	0.46
acg	T	0	0.09
aat	N	0	0.59
aac	N	1	0.41
aaa	K	18534	22634.00
aag	K	21144	17044.00
aga	R	32	43.68
agg	R	55	21.51
gaa	E	2	1.82
gag	E	1	1.18
---	--	------	------
mPD		0.50	0.50
		nPD:	1.01
		N. weight:	0.025
		Sc. PD:	0.023
		Sc. rank:	782.5

,

HA

Pos.	501	obs:	exp:
tct	S	0	0.92
tcc	S	0	0.58
tca	S	0	1.30
tcg	S	0	0.27
cat	H	1	0.59
cac	H	0	0.41
att	I	3	1.09
atc	I	0	0.75
ata	I	0	1.16
act	T	1	0.25
acc	T	0	0.21
aca	T	0	0.46
acg	T	0	0.09
aat	N	39671	23511.00
aac	N	82	16242.00
aaa	K	1	1.14
aag	K	1	0.86
agt	S	5	0.94
agc	S	0	0.99
gat	D	5	2.63
gac	D	0	2.37
---	--	------	------
mPD		0.0050	0.48
		nPD:	0.01
		N. weight:	0.55
		Sc. PD:	-0.029
		Sc. rank:	-7658.6

,

HA

Pos.	502	obs:	exp:
tgg	W	3	3.00
cgt	R	0	0.08
cgc	R	0	0.16
cga	R	0	0.29
cgg	R	0	0.22
aga	R	0	1.51
agg	R	3	0.74
gaa	E	1	74.48
gag	E	122	48.52
ggt	G	6	6011.00
ggc	G	0	5871.00
gga	G	216	17633.00
ggg	G	39419	10126.00
---	--	------	------
mPD		0.018	0.70
		nPD:	0.03
		N. weight:	1.4
		Sc. PD:	-0.054
		Sc. rank:	5043.2

,

HA

Pos.	503	obs:	exp:
tct	S	2	1.66
tcc	S	0	1.04
tca	S	0	2.34
tcg	S	0	0.48
att	I	5	1.82
atc	I	0	1.25
ata	I	0	1.93
act	T	39636	10018.00
acc	T	98	8151.00
aca	T	1	18180.00
acg	T	4	3391.00
aat	N	5	2.96
aac	N	0	2.04
agt	S	7	1.69
agc	S	0	1.79
gct	A	12	3.18
gcc	A	0	2.48
gca	A	0	5.64
gcg	A	0	0.70
---	--	------	------
mPD		0.0067	0.68
		nPD:	0.01
		N. weight:	1.5
		Sc. PD:	-0.078
		Sc. rank:	-21866.0

HA

Pos.	504	obs:	exp:
tat	Y	39705	20664.00
tac	Y	65	19106.00
---	--	------	------
mPD		0.0033	0.50
		nPD:	0.01
		N. weight:	0.7
		Sc. PD:	-0.039
		Sc. rank:	-14323.0

HA

Pos.	505	obs:	exp:
tat	Y	0	2.08
tac	Y	4	1.92
aat	N	0	17.74
aac	N	30	12.26
gtt	V	0	0.22
gtc	V	1	0.18
gta	V	0	0.27
gtg	V	0	0.33
gat	D	302	20899.00
gac	D	39362	18765.00
gaa	E	65	41.17
gag	E	3	26.83
ggt	G	0	0.45
ggc	G	3	0.44
gga	G	0	1.33
ggg	G	0	0.77
---	--	------	------
mPD		0.020	0.50
		nPD:	0.04
		N. weight:	0.76
		Sc. PD:	-0.017
		Sc. rank:	9450.8

HA

Pos.	506	obs:	exp:
ttt	F	0	0.43
ttc	F	1	0.57
tat	Y	137	20661.00
tac	Y	39628	19104.00
tgt	C	0	0.45
tgc	C	1	0.55
cat	H	0	1.76
cac	H	3	1.24
---	--	------	------
mPD		0.0071	0.50
		nPD:	0.01
		N. weight:	0.77
		Sc. PD:	-0.037
		Sc. rank:	-5605.3

HA

Pos.	507	obs:	exp:
tta	L	0	0.11
ttg	L	0	0.19
tct	S	0	2.21
tcc	S	0	1.39
tca	S	12	3.12
tcg	S	0	0.64
ctt	L	0	0.20
ctc	L	0	0.14
cta	L	1	0.19
ctg	L	0	0.17
cct	P	15	10953.00
ccc	P	207	7374.00
cca	P	39508	16928.00
ccg	P	27	4502.00
agt	S	0	2.26
agc	S	0	2.38
---	--	------	------
mPD		0.013	0.70
		nPD:	0.02
		N. weight:	0.88
		Sc. PD:	-0.039
		Sc. rank:	-1678.4

HA

Pos.	508	obs:	exp:
caa	Q	1	0.53
cag	Q	0	0.47
cgt	R	0	5.46
cgc	R	0	10.46
cga	R	0	19.59
cgg	R	0	14.64
att	I	0	0.73
atc	I	0	0.50
ata	I	2	0.77
act	T	0	3.78
acc	T	0	3.08
aca	T	15	6.86
acg	T	0	1.28
aat	N	1	1.77
aac	N	2	1.23
aaa	K	39466	22556.00
aag	K	76	16986.00
aga	R	200	100.40
agg	R	0	49.44
gaa	E	7	4.24
gag	E	0	2.76
---	--	------	------
mPD		0.015	0.50
		nPD:	0.03
		N. weight:	0.59
		Sc. PD:	-0.02
		Sc. rank:	4460.5

HA

Pos.	509	obs:	exp:
ttt	F	0	3.00
ttc	F	7	4.00
tat	Y	121	20660.00
tac	Y	39641	19102.00
cat	H	0	0.59
cac	H	1	0.41
---	--	------	------
mPD		0.0065	0.50
		nPD:	0.01
		N. weight:	0.77
		Sc. PD:	-0.038
		Sc. rank:	-7199.0

HA

Pos.	510	obs:	exp:
tta	L	5	0.55
ttg	L	0	0.94
tct	S	12	7299.00
tcc	S	9	4594.00
tca	S	39610	10323.00
tcg	S	34	2111.00
ctt	L	0	0.99
ctc	L	0	0.71
cta	L	0	0.96
ctg	L	0	0.85
act	T	0	24.96
acc	T	0	20.31
aca	T	99	45.29
acg	T	0	8.45
agt	S	0	7458.00
agc	S	0	7879.00
gct	A	0	0.26
gcc	A	0	0.21
gca	A	1	0.47
gcg	A	0	0.06
---	--	------	------
mPD		0.0080	1.6
		nPD:	0.
		N. weight:	1.4
		Sc. PD:	-0.083
		Sc. rank:	-34974.0

HA

Pos.	511	obs:	exp:
cgt	R	0	0.03
cgc	R	0	0.05
cga	R	0	0.10
cgg	R	0	0.07
aaa	K	1	27.38
aag	K	47	20.62
aga	R	0	0.50
agg	R	1	0.25
gtt	V	0	0.22
gtc	V	0	0.18
gta	V	0	0.27
gtg	V	1	0.33
gct	A	0	0.26
gcc	A	0	0.21
gca	A	0	0.47
gcg	A	1	0.06
gat	D	7	3.69
gac	D	0	3.31
gaa	E	440	23791.00
gag	E	38851	15500.00
ggt	G	0	63.84
ggc	G	0	62.36
gga	G	1	187.30
ggg	G	420	107.50
---	--	------	------
mPD		0.046	0.50
		nPD:	0.09
		N. weight:	0.94
		Sc. PD:	0.025
		Sc. rank:	20566.0

HA

Pos.	512	obs:	exp:
gtt	V	0	0.66
gtc	V	0	0.55
gta	V	3	0.81
gtg	V	0	0.98
gct	A	0	3.44
gcc	A	0	2.69
gca	A	13	6.11
gcg	A	0	0.76
gaa	E	39588	24070.00
gag	E	164	15682.00
ggt	G	0	0.30
ggc	G	0	0.30
gga	G	2	0.89
ggg	G	0	0.51
---	--	------	------
mPD		0.0091	0.48
		nPD:	0.02
		N. weight:	0.52
		Sc. PD:	-0.023
		Sc. rank:	-727.5

HA

Pos.	513	obs:	exp:
tct	S	0	9.75
tcc	S	0	6.14
tca	S	53	13.79
tcg	S	0	2.82
act	T	0	2.52
acc	T	0	2.05
aca	T	9	4.57
acg	T	1	0.85
agt	S	0	9.97
agc	S	0	10.53
gtt	V	0	2.87
gtc	V	0	2.36
gta	V	13	3.50
gtg	V	0	4.27
gct	A	10	10509.00
gcc	A	36	8217.00
gca	A	39585	18643.00
gcg	A	63	2325.00
---	--	------	------
mPD		0.0093	0.67
		nPD:	0.01
		N. weight:	0.8
		Sc. PD:	-0.039
		Sc. rank:	-6237.7

HA

Pos.	514	obs:	exp:
caa	Q	3	1.58
cag	Q	0	1.42
cgt	R	0	0.85
cgc	R	0	1.62
cga	R	0	3.04
cgg	R	0	2.27
act	T	0	1.26
acc	T	0	1.03
aca	T	5	2.29
acg	T	0	0.43
aat	N	2	4.73
aac	N	6	3.27
aaa	K	39639	22656.00
aag	K	79	17062.00
aga	R	31	15.56
agg	R	0	7.66
gaa	E	5	3.03
gag	E	0	1.97
---	--	------	------
mPD		0.0066	0.49
		nPD:	0.01
		N. weight:	0.59
		Sc. PD:	-0.029
		Sc. rank:	-5153.2

HA			
Pos.	515	obs:	exp:
tta	L	39183	4370.00
ttg	L	234	7484.00
tct	S	0	0.18
tcc	S	0	0.12
tca	S	1	0.26
tcg	S	0	0.05
ctt	L	0	7834.00
ctc	L	0	5615.00
cta	L	337	7653.00
ctg	L	0	6797.00
att	I	0	4.73
atc	I	0	3.25
ata	I	13	5.02
agt	S	0	0.19
agc	S	0	0.20
gtt	V	0	0.44
gtc	V	0	0.36
gta	V	2	0.54
gtg	V	0	0.66
---	--	------	------
mPD		0.029	1.1
		nPD:	0.03
		N. weight:	2.3
		Sc. PD:	-0.085
		Sc. rank:	9354.4

,

HA			
Pos.	516	obs:	exp:
tct	S	0	279.50
tcc	S	0	175.90
tca	S	0	395.30
tcg	S	0	80.84
cgt	R	0	0.03
cgc	R	0	0.05
cga	R	0	0.10
cgg	R	0	0.07
att	I	0	0.73
atc	I	2	0.50
ata	I	0	0.77
act	T	0	3.78
acc	T	15	3.08
aca	T	0	6.86
acg	T	0	1.28
aat	N	33	22600.00
aac	N	38179	15612.00
aaa	K	15	8.56
aag	K	0	6.44
agt	S	3	285.60
agc	S	1516	301.70
aga	R	1	0.50
agg	R	0	0.25
gat	D	0	3.16
gac	D	6	2.84
---	--	------	------
mPD		0.077	0.62
		nPD:	0.13
		N. weight:	0.99
		Sc. PD:	0.059
		Sc. rank:	23979.0

,

HA			
Pos.	517	obs:	exp:
cgt	R	0	1085.00
cgc	R	0	2078.00
cga	R	9	3891.00
cgg	R	0	2908.00
att	I	0	0.36
atc	I	0	0.25
ata	I	1	0.39
act	T	0	0.25
acc	T	0	0.21
aca	T	1	0.46
acg	T	0	0.09
aaa	K	37	21.11
aag	K	0	15.89
aga	R	39675	19945.00
agg	R	45	9822.00
ggt	G	0	0.30
ggc	G	0	0.30
gga	G	2	0.89
ggg	G	0	0.51
---	--	------	------
mPD		0.0048	0.91
		nPD:	0.01
		N. weight:	0.73
		Sc. PD:	-0.042
		Sc. rank:	-17079.0

,

HA			
Pos.	518	obs:	exp:
aaa	K	17	9.70
aag	K	0	7.30
gtt	V	0	0.88
gtc	V	0	0.73
gta	V	4	1.08
gtg	V	0	1.31
gat	D	0	1.58
gac	D	3	1.42
gaa	E	39703	24062.00
gag	E	36	15677.00
ggt	G	0	1.06
ggc	G	0	1.04
gga	G	7	3.11
ggg	G	0	1.79
---	--	------	------
mPD		0.0034	0.48
		nPD:	0.01
		N. weight:	0.54
		Sc. PD:	-0.03
		Sc. rank:	-10582.0

,

HA			
Pos.	519	obs:	exp:
caa	Q	3	1.58
cag	Q	0	1.42
cgt	R	0	0.49
cgc	R	0	0.94
cga	R	0	1.76
cgg	R	0	1.32
att	I	0	3.28
atc	I	0	2.25
ata	I	9	3.48
act	T	0	1.26
acc	T	0	1.03
aca	T	5	2.29
acg	T	0	0.43
aat	N	0	1.18
aac	N	2	0.82
aaa	K	31337	17894.00
aag	K	33	13476.00
aga	R	18	9.04
agg	R	0	4.45
gaa	E	8347	5064.00
gag	E	16	3299.00
---	--	------	------
mPD		0.34	0.82
		nPD:	0.41
		N. weight:	0.58
		Sc. PD:	0.2
		Sc. rank:	17232.0

,

HA			
Pos.	520	obs:	exp:
tta	L	4	1.54
ttg	L	0	2.64
ctt	L	0	2.76
ctc	L	0	1.98
cta	L	10	2.70
ctg	L	0	2.39
att	I	37	14465.00
atc	I	0	9919.00
ata	I	39700	15352.00
gtt	V	0	4.20
gtc	V	0	3.45
gta	V	19	5.11
gtg	V	0	6.24
---	--	------	------
mPD		0.0035	0.66
		nPD:	0.01
		N. weight:	1.
		Sc. PD:	-0.058
		Sc. rank:	-23326.0

,

HA			
Pos.	521	obs:	exp:
tat	Y	21	10.91
tac	Y	0	10.09
aat	N	75	44.36
aac	N	0	30.64
gtt	V	0	0.22
gtc	V	0	0.18
gta	V	0	0.27
gtg	V	1	0.33
gct	A	1	0.26
gcc	A	0	0.21
gca	A	0	0.47
gcg	A	0	0.06
gat	D	39195	20670.00
gac	D	35	18560.00
gaa	E	428	264.00
gag	E	8	172.00
ggt	G	6	0.91
ggc	G	0	0.89
gga	G	0	2.67
ggg	G	0	1.53
---	--	------	------
mPD		0.029	0.51
		nPD:	0.06
		N. weight:	0.68
		Sc. PD:	-0.0054
		Sc. rank:	11228.0

,

HA			
Pos.	522	obs:	exp:
cgt	R	0	0.03
cgc	R	0	0.05
cga	R	0	0.10
cgg	R	0	0.07
aga	R	1	0.50
agg	R	0	0.25
gaa	E	2	3.03
gag	E	3	1.97
ggt	G	6	6029.00
ggc	G	3	5890.00
gga	G	21428	17688.00
ggg	G	18327	10157.00
---	--	------	------
mPD		0.50	0.69
		nPD:	0.72
		N. weight:	0.4
		Sc. PD:	0.26
		Sc. rank:	12736.0

,

HA			
Pos.	523	obs:	exp:
tta	L	0	0.22
ttg	L	0	0.38
ctt	L	0	0.39
ctc	L	0	0.28
cta	L	2	0.39
ctg	L	0	0.34
att	I	0	26.21
atc	I	0	17.97
ata	I	72	27.82
gtt	V	9	8776.00
gtc	V	3	7213.00
gta	V	39620	10675.00
gtg	V	62	13030.00
gct	A	0	0.53
gcc	A	0	0.41
gca	A	2	0.94
gcg	A	0	0.12
---	--	------	------
mPD		0.0075	0.74
		nPD:	0.01
		N. weight:	1.4
		Sc. PD:	-0.074
		Sc. rank:	-19722.0

,

HA			
Pos.	524	obs:	exp:
tct	S	0	0.18
tcc	S	0	0.12
tca	S	0	0.26
tcg	S	0	0.05
caa	Q	0	1.06
cag	Q	2	0.94
cgt	R	0	7.65
cgc	R	0	14.65
cga	R	0	27.43
cgg	R	0	20.49
atg	M	3	3.00
act	T	0	1.01
acc	T	0	0.82
aca	T	0	1.83
acg	T	4	0.34
aat	N	3	2.37
aac	N	1	1.63
aaa	K	606	22511.00
aag	K	38857	16952.00
agt	S	1	0.19
agc	S	0	0.20
aga	R	0	140.60
agg	R	280	69.22
gaa	E	0	7.87
gag	E	13	5.13
---	--	------	------
mPD		0.045	0.51
		nPD:	0.09
		N. weight:	0.83
		Sc. PD:	0.021
		Sc. rank:	17832.0

,

HA			
Pos.	525	obs:	exp:
tta	L	2	4372.00
ttg	L	248	7487.00
ctt	L	26	7837.00
ctc	L	0	5617.00
cta	L	95	7656.00
ctg	L	39399	6800.00
---	--	------	------
mPD		0.019	1.1
		nPD:	0.02
		N. weight:	1.8
		Sc. PD:	-0.086
		Sc. rank:	-7719.1

,

HA			
Pos.	526	obs:	exp:
aat	N	0	1.18
aac	N	2	0.82
gat	D	18	1878.00
gac	D	3546	1686.00
gaa	E	36116	21899.00
gag	E	51	14268.00
ggt	G	0	5.61
ggc	G	0	5.48
gga	G	37	16.46
ggg	G	0	9.45
---	--	------	------
mPD		0.17	0.57
		nPD:	0.3
		N. weight:	0.55
		Sc. PD:	0.13
		Sc. rank:	15295.0

HA

Pos.	527	obs:	exp:
tta	L	1	0.11
ttg	L	0	0.19
tct	S	23	7314.00
tcc	S	3	4603.00
tca	S	39637	10343.00
tcg	S	79	2115.00
ctt	L	0	0.20
ctc	L	0	0.14
cta	L	0	0.19
ctg	L	0	0.17
cct	P	0	4.96
ccc	P	0	3.34
cca	P	18	7.66
ccg	P	0	2.04
act	T	0	0.25
acc	T	0	0.21
aca	T	1	0.46
acg	T	0	0.09
agt	S	0	7473.00
agc	S	0	7894.00
gct	A	0	2.12
gcc	A	0	1.66
gca	A	8	3.76
gcg	A	0	0.47
---	--	------	------
mPD		0.0067	1.6
		nPD:	0.
		N. weight:	1.4
		Sc. PD:	-0.084
		Sc. rank:	-36851.0

,

HA

Pos.	528	obs:	exp:
att	I	0	3.28
atc	I	0	2.25
ata	I	9	3.48
act	T	1	10019.00
acc	T	4	8152.00
aca	T	39708	18181.00
acg	T	30	3391.00
aaa	K	6	3.42
aag	K	0	2.58
gct	A	0	3.18
gcc	A	0	2.48
gca	A	12	5.64
gcg	A	0	0.70
---	--	------	------
mPD		0.0031	0.68
		nPD:	0.
		N. weight:	0.84
		Sc. PD:	-0.049
		Sc. rank:	-20925.0

,

HA

Pos.	529	obs:	exp:
tct	S	0	0.18
tcc	S	0	0.12
tca	S	0	0.26
tcg	S	0	0.05
cgt	R	0	1081.00
cgc	R	0	2070.00
cga	R	0	3876.00
cgg	R	0	2896.00
atg	M	45	45.00
act	T	0	0.50
acc	T	0	0.41
aca	T	1	0.91
acg	T	1	0.17
aaa	K	6	71.87
aag	K	120	54.13
agt	S	1	0.19
agc	S	0	0.20
aga	R	794	19868.00
agg	R	38781	9784.00
ggt	G	0	3.18
ggc	G	0	3.11
gga	G	0	9.34
ggg	G	21	5.36
---	--	------	------
mPD		0.049	0.92
		nPD:	0.05
		N. weight:	1.4
		Sc. PD:	-0.014
		Sc. rank:	21862.0

,

HA

Pos.	530	obs:	exp:
tta	L	0	0.11
ttg	L	0	0.19
ctt	L	0	0.20
ctc	L	1	0.14
cta	L	0	0.19
ctg	L	0	0.17
att	I	13446	14250.00
atc	I	25687	9771.00
ata	I	12	15124.00
act	T	39	106.60
acc	T	384	86.76
aca	T	0	193.50
acg	T	0	36.09
gtt	V	108	44.44
gtc	V	93	36.52
gta	V	0	54.06
gtg	V	0	65.98
---	--	------	------
mPD		0.48	0.69
		nPD:	0.7
		N. weight:	0.67
		Sc. PD:	0.42
		Sc. rank:	21112.0

,

HA

Pos.	531	obs:	exp:
ttt	F	0	0.43
ttc	F	1	0.57
tat	Y	138	20662.00
tac	Y	39629	19105.00
cat	H	0	1.18
cac	H	2	0.82
---	--	------	------
mPD		0.0071	0.50
		nPD:	0.01
		N. weight:	0.77
		Sc. PD:	-0.037
		Sc. rank:	-5832.8

,

HA

Pos.	532	obs:	exp:
tta	L	0	0.44
ttg	L	0	0.75
ctt	L	0	0.79
ctc	L	0	0.56
cta	L	0	0.77
ctg	L	4	0.68
cct	P	0	0.28
ccc	P	0	0.19
cca	P	0	0.43
ccg	P	1	0.11
cat	H	5	2.94
cac	H	0	2.06
caa	Q	462	20977.00
cag	Q	39292	18777.00
cgt	R	0	0.08
cgc	R	0	0.16
cga	R	0	0.29
cgg	R	3	0.22
aaa	K	0	1.14
aag	K	2	0.86
aga	R	0	1.51
agg	R	0	0.74
gaa	E	0	0.61
gag	E	1	0.39
---	--	------	------
mPD		0.024	0.50
		nPD:	0.05
		N. weight:	0.74
		Sc. PD:	-0.012
		Sc. rank:	11208.0

,

HA

Pos.	533	obs:	exp:
tct	S	0	0.37
tcc	S	0	0.23
tca	S	0	0.52
tcg	S	0	0.11
att	I	39620	14472.00
atc	I	98	9923.00
ata	I	36	15359.00
act	T	1	0.25
acc	T	0	0.21
aca	T	0	0.46
acg	T	0	0.09
agt	S	2	0.38
agc	S	0	0.40
gtt	V	13	2.87
gtc	V	0	2.36
gta	V	0	3.50
gtg	V	0	4.27
---	--	------	------
mPD		0.0075	0.66
		nPD:	0.01
		N. weight:	1.1
		Sc. PD:	-0.055
		Sc. rank:	-12251.0

,

HA

Pos.	534	obs:	exp:
tta	L	18	4372.00
ttg	L	39710	7487.00
ctt	L	0	7837.00
ctc	L	0	5617.00
cta	L	0	7656.00
ctg	L	42	6800.00
---	--	------	------
mPD		0.0030	1.1
		nPD:	0.
		N. weight:	1.8
		Sc. PD:	-0.11
		Sc. rank:	-51503.0

,

HA

Pos.	535	obs:	exp:
tct	S	0	0.18
tcc	S	0	0.12
tca	S	0	0.26
tcg	S	1	0.05
act	T	0	4.03
acc	T	0	3.28
aca	T	0	7.32
acg	T	16	1.37
agt	S	0	0.19
agc	S	0	0.20
gtt	V	0	0.66
gtc	V	0	0.55
gta	V	0	0.81
gtg	V	3	0.98
gct	A	38	10524.00
gcc	A	0	8228.00
gca	A	781	18669.00
gcg	A	38931	2329.00
---	--	------	------
mPD		0.041	0.66
		nPD:	0.06
		N. weight:	2.9
		Sc. PD:	-0.0043
		Sc. rank:	51865.0

,

HA

Pos.	536	obs:	exp:
att	I	75	14474.00
atc	I	39641	9925.00
ata	I	45	15362.00
gtt	V	0	1.99
gtc	V	9	1.64
gta	V	0	2.42
gtg	V	0	2.95
---	--	------	------
mPD		0.0065	0.66
		nPD:	0.01
		N. weight:	1.5
		Sc. PD:	-0.078
		Sc. rank:	-22323.0

,

HA

Pos.	537	obs:	exp:
tat	Y	39180	20664.00
tac	Y	590	19106.00
---	--	------	------
mPD		0.029	0.50
		nPD:	0.06
		N. weight:	0.63
		Sc. PD:	-0.0032
		Sc. rank:	10517.0

,

HA

Pos.	538	obs:	exp:
tct	S	6	7318.00
tcc	S	0	4606.00
tca	S	39727	10350.00
tcg	S	35	2116.00
agt	S	0	7478.00
agc	S	0	7899.00
gct	A	0	0.53
gcc	A	0	0.41
gca	A	2	0.94
gcg	A	0	0.12
---	--	------	------
mPD		0.0022	1.6
		nPD:	0.
		N. weight:	1.4
		Sc. PD:	-0.089
		Sc. rank:	-45072.0

,

HA			
Pos.	539	obs:	exp:
tct	S	0	0.55
tcc	S	0	0.35
tca	S	0	0.78
tcg	S	0	0.16
act	T	39636	10021.00
acc	T	106	8154.00
aca	T	10	18186.00
acg	T	1	3392.00
agt	S	3	0.56
agc	S	0	0.60
gct	A	14	3.71
gcc	A	0	2.90
gca	A	0	6.58
gcg	A	0	0.82
---	--	------	------
mPD		0.0067	0.68
		nPD:	0.01
		N. weight:	1.5
		Sc. PD:	-0.078
		Sc. rank:	-21587.0

,

HA			
Pos.	540	obs:	exp:
att	I	3	6.55
atc	I	15	4.49
ata	I	0	6.95
gtt	V	9707	7986.00
gtc	V	26409	6564.00
gta	V	5	9714.00
gtg	V	0	11857.00
gct	A	781	961.10
gcc	A	2846	751.40
gca	A	0	1705.00
gcg	A	3	212.70
ggt	G	1	0.15
ggc	G	0	0.15
gga	G	0	0.44
ggg	G	0	0.26
---	--	------	------
mPD		0.56	0.91
		nPD:	0.61
		N. weight:	1.1
		Sc. PD:	0.62
		Sc. rank:	35863.0

,

HA			
Pos.	541	obs:	exp:
tct	S	0	0.55
tcc	S	3	0.35
tca	S	0	0.78
tcg	S	0	0.16
act	T	0	0.50
acc	T	2	0.41
aca	T	0	0.91
acg	T	0	0.17
agt	S	0	0.56
agc	S	0	0.60
gtt	V	0	0.44
gtc	V	2	0.36
gta	V	0	0.54
gtg	V	0	0.66
gct	A	161	10527.00
gcc	A	39593	8231.00
gca	A	8	18675.00
gcg	A	0	2329.00
ggt	G	0	0.15
ggc	G	1	0.15
gga	G	0	0.44
ggg	G	0	0.26
---	--	------	------
mPD		0.0089	0.66
		nPD:	0.01
		N. weight:	1.7
		Sc. PD:	-0.083
		Sc. rank:	-14233.0

,

HA			
Pos.	542	obs:	exp:
tct	S	0	7318.00
tcc	S	0	4606.00
tca	S	0	10350.00
tcg	S	0	2116.00
cgt	R	1	0.03
cgc	R	0	0.05
cga	R	0	0.10
cgg	R	0	0.07
att	I	1	0.36
atc	I	0	0.25
ata	I	0	0.39
agt	S	39667	7478.00
agc	S	100	7899.00
aga	R	0	0.50
agg	R	0	0.25
ggt	G	1	0.15
ggc	G	0	0.15
gga	G	0	0.44
ggg	G	0	0.26
---	--	------	------
mPD		0.0052	1.6
		nPD:	0.
		N. weight:	1.8
		Sc. PD:	-0.11
		Sc. rank:	-49390.0

,

HA			
Pos.	543	obs:	exp:
tct	S	18	7318.00
tcc	S	20	4606.00
tca	S	38725	10350.00
tcg	S	1004	2116.00
act	T	0	0.76
acc	T	0	0.62
aca	T	3	1.37
acg	T	0	0.26
agt	S	0	7478.00
agc	S	0	7899.00
---	--	------	------
mPD		0.051	1.6
		nPD:	0.03
		N. weight:	1.4
		Sc. PD:	-0.044
		Sc. rank:	11641.0

,

HA			
Pos.	544	obs:	exp:
ttt	F	29	12.44
ttc	F	0	16.56
tta	L	95	4367.00
ttg	L	39170	7480.00
tct	S	0	1.10
tcc	S	0	0.69
tca	S	0	1.56
tcg	S	6	0.32
ctt	L	0	7829.00
ctc	L	0	5611.00
cta	L	0	7648.00
ctg	L	463	6793.00
atg	M	4	4.00
agt	S	0	1.13
agc	S	0	1.19
gtt	V	0	0.66
gtc	V	0	0.55
gta	V	0	0.81
gtg	V	3	0.98
---	--	------	------
mPD		0.030	1.1
		nPD:	0.03
		N. weight:	1.7
		Sc. PD:	-0.063
		Sc. rank:	7751.3

,

HA			
Pos.	545	obs:	exp:
tta	L	3	0.33
ttg	L	0	0.56
ctt	L	0	0.59
ctc	L	0	0.42
cta	L	0	0.58
ctg	L	0	0.51
att	I	0	30.21
atc	I	0	20.72
ata	I	83	32.07
act	T	0	0.25
acc	T	0	0.21
aca	T	1	0.46
acg	T	0	0.09
gtt	V	3	8773.00
gtc	V	3	7210.00
gta	V	39561	10672.00
gtg	V	113	13025.00
gct	A	0	0.26
gcc	A	0	0.21
gca	A	1	0.47
gcg	A	0	0.06
gaa	E	2	1.21
gag	E	0	0.79
---	--	------	------
mPD		0.011	0.74
		nPD:	0.01
		N. weight:	1.4
		Sc. PD:	-0.068
		Sc. rank:	-10312.0

,

HA			
Pos.	546	obs:	exp:
tta	L	0	4367.00
ttg	L	62	7478.00
ctt	L	32	7827.00
ctc	L	0	5610.00
cta	L	83	7647.00
ctg	L	39543	6791.00
atg	M	48	48.00
aaa	K	0	0.57
aag	K	1	0.43
gtt	V	0	0.22
gtc	V	0	0.18
gta	V	0	0.27
gtg	V	1	0.33
---	--	------	------
mPD		0.011	1.1
		nPD:	0.01
		N. weight:	1.9
		Sc. PD:	-0.099
		Sc. rank:	-27009.0

,

HA			
Pos.	547	obs:	exp:
tta	L	14	2.09
ttg	L	0	3.58
ctt	L	0	3.74
ctc	L	0	2.68
cta	L	5	3.66
ctg	L	0	3.25
att	I	0	47.32
atc	I	0	32.45
ata	I	130	50.23
gtt	V	1	8751.00
gtc	V	3	7193.00
gta	V	39506	10645.00
gtg	V	73	12994.00
gct	A	0	10.06
gcc	A	0	7.87
gca	A	38	17.85
gcg	A	0	2.23
---	--	------	------
mPD		0.013	0.75
		nPD:	0.02
		N. weight:	1.4
		Sc. PD:	-0.063
		Sc. rank:	-4077.2

,

HA			
Pos.	548	obs:	exp:
ttt	F	0	0.43
ttc	F	1	0.57
att	I	0	33.13
atc	I	91	22.72
ata	I	0	35.16
gtt	V	1	8772.00
gtc	V	39675	7210.00
gta	V	1	10671.00
gtg	V	0	13025.00
gct	A	0	0.26
gcc	A	1	0.21
gca	A	0	0.47
gcg	A	0	0.06
---	--	------	------
mPD		0.0048	0.74
		nPD:	0.01
		N. weight:	1.8
		Sc. PD:	-0.1
		Sc. rank:	-38884.0

,

HA			
Pos.	549	obs:	exp:
ttt	F	0	1.72
ttc	F	4	2.28
tct	S	60	7317.00
tcc	S	39699	4605.00
tca	S	2	10348.00
tcg	S	0	2116.00
tat	Y	0	0.52
tac	Y	1	0.48
cct	P	0	0.28
ccc	P	1	0.19
cca	P	0	0.43
ccg	P	0	0.11
act	T	0	0.76
acc	T	3	0.62
aca	T	0	1.37
acg	T	0	0.26
agt	S	0	7476.00
agc	S	0	7898.00
---	--	------	------
mPD		0.0036	1.6
		nPD:	0.
		N. weight:	2.3
		Sc. PD:	-0.14
		Sc. rank:	-69599.0

,

HA			
Pos.	550	obs:	exp:
tta	L	0	4372.00
ttg	L	65	7487.00
ctt	L	2	7837.00
ctc	L	0	5617.00
cta	L	14	7656.00
ctg	L	39688	6800.00
gtt	V	0	0.22
gtc	V	0	0.18
gta	V	0	0.27
gtg	V	1	0.33
---	--	------	------
mPD		0.0041	1.1
		nPD:	0.
		N. weight:	1.9
		Sc. PD:	-0.11
		Sc. rank:	-50569.0

,

HA			
Pos.	551	obs:	exp:
cgt	R	0	0.03
cgc	R	0	0.05
cga	R	0	0.10
cgg	R	0	0.07
aga	R	0	0.50
agg	R	1	0.25
ggt	G	0	6030.00
ggc	G	0	5890.00
gga	G	2	17690.00
ggg	G	39767	10159.00
---	--	------	------
mPD		0.00015	0.69
		nPD:	0.
		N. weight:	1.5
		Sc. PD:	-0.092
		Sc. rank:	-46846.0

,

HA			
Pos.	552	obs:	exp:
gct	A	0	10529.00
gcc	A	2	8232.00
gca	A	39762	18678.00
gcg	A	5	2330.00
gaa	E	1	0.61
gag	E	0	0.39
---	--	------	------
mPD		0.00040	0.66
		nPD:	0.
		N. weight:	0.82
		Sc. PD:	-0.051
		Sc. rank:	-25779.0

,

HA			
Pos.	553	obs:	exp:
tta	L	0	0.55
ttg	L	0	0.94
ctt	L	0	0.99
ctc	L	5	0.71
cta	L	0	0.96
ctg	L	0	0.85
att	I	19	14445.00
atc	I	39662	9905.00
ata	I	0	15331.00
act	T	0	1.26
acc	T	5	1.03
aca	T	0	2.29
acg	T	0	0.43
gtt	V	0	17.47
gtc	V	79	14.36
gta	V	0	21.25
gtg	V	0	25.93
---	--	------	------
mPD		0.0054	0.66
		nPD:	0.01
		N. weight:	1.5
		Sc. PD:	-0.082
		Sc. rank:	-26913.0

,

HA			
Pos.	554	obs:	exp:
tct	S	0	7309.00
tcc	S	0	4600.00
tca	S	0	10336.00
tcg	S	0	2114.00
tgt	C	0	3.16
tgc	C	7	3.84
act	T	0	0.25
acc	T	1	0.21
aca	T	0	0.46
acg	T	0	0.09
aat	N	2	1.77
aac	N	1	1.23
agt	S	9486	7468.00
agc	S	30229	7889.00
ggt	G	4	6.67
ggc	G	40	6.52
gga	G	0	19.57
ggg	G	0	11.24
---	--	------	------
mPD		0.37	1.6
		nPD:	0.22
		N. weight:	1.2
		Sc. PD:	0.18
		Sc. rank:	31345.0

,

HA			
Pos.	555	obs:	exp:
ttt	F	551	17062.00
ttc	F	39208	22697.00
tta	L	8	1.10
ttg	L	0	1.88
tgt	C	0	0.45
tgc	C	1	0.55
ctt	L	0	1.97
ctc	L	2	1.41
cta	L	0	1.93
ctg	L	0	1.71
---	--	------	------
mPD		0.028	0.49
		nPD:	0.06
		N. weight:	0.53
		Sc. PD:	-0.0037
		Sc. rank:	8824.3

,

HA			
Pos.	556	obs:	exp:
tgg	W	39769	39769.00
cgt	R	0	0.03
cgc	R	0	0.05
cga	R	0	0.10
cgg	R	1	0.07
aga	R	0	0.50
agg	R	0	0.25
---	--	------	------
mPD		0.000050	0.000084
		nPD:	0.6
		N. weight:	0.000071
		Sc. PD:	0.000037
		Sc. rank:	2.2

,

HA			
Pos.	557	obs:	exp:
atg	M	39769	39769.00
gtt	V	0	0.22
gtc	V	0	0.18
gta	V	0	0.27
gtg	V	1	0.33
---	--	------	------
mPD		0.000050	0.000084
		nPD:	0.6
		N. weight:	0.00003
		Sc. PD:	0.000016
		Sc. rank:	0.9

,

HA			
Pos.	558	obs:	exp:
ttt	F	0	0.86
ttc	F	2	1.14
tgt	C	1018	17950.00
tgc	C	38750	21818.00
---	--	------	------
mPD		0.050	0.50
		nPD:	0.1
		N. weight:	0.53
		Sc. PD:	0.019
		Sc. rank:	11944.0

,

HA			
Pos.	559	obs:	exp:
ttt	F	1	0.43
ttc	F	0	0.57
tct	S	39393	7316.00
tcc	S	352	4604.00
tca	S	7	10346.00
tcg	S	1	2116.00
tat	Y	10	5.20
tac	Y	0	4.80
agt	S	0	7475.00
agc	S	0	7896.00
gct	A	6	1.59
gcc	A	0	1.24
gca	A	0	2.82
gcg	A	0	0.35
---	--	------	------
mPD		0.019	1.6
		nPD:	0.01
		N. weight:	1.8
		Sc. PD:	-0.091
		Sc. rank:	-20611.0

,

HA			
Pos.	560	obs:	exp:
tct	S	0	0.18
tcc	S	0	0.12
tca	S	0	0.26
tcg	S	0	0.05
tgg	W	5	5.00
cat	H	1	0.59
cac	H	0	0.41
aat	N	39668	23471.00
aac	N	16	16213.00
agt	S	1	0.19
agc	S	0	0.20
ggt	G	24	3.94
ggc	G	0	3.85
gga	G	2	11.57
ggg	G	0	6.64
---	-	53	53.00
---	--	------	------
mPD		0.0044	0.49
		nPD:	0.01
		N. weight:	0.56
		Sc. PD:	-0.03
		Sc. rank:	-9116.7

,

HA			
Pos.	561	obs:	exp:
tat	Y	1	0.52
tac	Y	0	0.48
atg	M	1	1.00
---	-	39768	39768.00
---	--	------	------
mPD		3.8×10^{-9}	4.1×10^{-9}
		nPD:	0.92
		N. weight:	0.
		Sc. PD:	0
		Sc. rank:	0

,

HA			
Pos.	562	obs:	exp:
cat	H	23	13.53
cac	H	0	9.47
atg	M	2	2.00
gtt	V	0	0.44
gtc	V	1	0.36
gta	V	0	0.54
gtg	V	1	0.66
ggt	G	59	6024.00
ggc	G	0	5884.00
gga	G	97	17671.00
ggg	G	39570	10148.00
---	-	17	17.00
---	--	------	------
mPD		0.012	0.69
		nPD:	0.02
		N. weight:	1.4
		Sc. PD:	-0.067
		Sc. rank:	-5488.4

,

HA

Pos.	563	obs:	exp:
tct	S	0	0.18
tcc	S	0	0.12
tca	S	0	0.26
tcg	S	0	0.05
agt	S	1	0.19
agc	S	0	0.20
---	-	39769	39769.00
---	--	------	------
mPD		0	1.0×10^{-9}
		nPD:	0.
		N. weight:	0.
		Sc. PD:	0
		Sc. rank:	0

,

HA

Pos.	564	obs:	exp:
tgg	W	10	10.00
cat	H	9	5.29
cac	H	0	3.71
ggt	G	0	0.30
ggc	G	0	0.30
gga	G	0	0.89
ggg	G	2	0.51
---	-	39749	39749.00
---	--	------	------
mPD		4.3×10^{-7}	4.8×10^{-7}
		nPD:	0.91
		N. weight:	0.
		Sc. PD:	0
		Sc. rank:	0

,

HA

Pos.	565	obs:	exp:
ttt	F	9	3.86
ttc	F	0	5.14
tta	L	0	0.22
ttg	L	0	0.38
tct	S	39645	7310.00
tcc	S	23	4601.00
tca	S	20	10338.00
tcg	S	3	2114.00
ctt	L	1	0.39
ctc	L	0	0.28
cta	L	0	0.39
ctg	L	1	0.34
cct	P	1	0.28
ccc	P	0	0.19
cca	P	0	0.43
ccg	P	0	0.11
cat	H	31	18.82
cac	H	1	13.18
agt	S	3	7469.00
agc	S	27	7890.00
ggt	G	2	0.45
ggc	G	0	0.44
gga	G	0	1.33
ggg	G	1	0.77
---	-	2	2.00
---	--	------	------
mPD		0.011	1.6
		nPD:	0.01
		N. weight:	1.8
		Sc. PD:	-0.1
		Sc. rank:	-36425.0

,

HA

Pos.	566	obs:	exp:
tta	L	0	0.11
ttg	L	0	0.19
tct	S	1	0.18
tcc	S	0	0.12
tca	S	0	0.26
tcg	S	0	0.05
tgt	C	1	0.45
tgc	C	0	0.55
ctt	L	1	0.20
ctc	L	0	0.14
cta	L	0	0.19
ctg	L	0	0.17
agt	S	0	0.19
agc	S	0	0.20
---	-	39767	39767.00
---	--	------	------
mPD		6.3×10^{-9}	1.1×10^{-8}
		nPD:	0.59
		N. weight:	0.
		Sc. PD:	0
		Sc. rank:	0

,

HA

Pos.	567	obs:	exp:
tta	L	505	4366.00
ttg	L	128	7477.00
ctt	L	3	7826.00
ctc	L	3	5609.00
cta	L	39060	7645.00
ctg	L	14	6790.00
cat	H	1	0.59
cac	H	0	0.41
---	-	56	56.00
---	--	------	------
mPD		0.039	1.1
		nPD:	0.03
		N. weight:	1.7
		Sc. PD:	-0.049
		Sc. rank:	15901.0

,

HA

Pos.	568	obs:	exp:
tta	L	1	0.33
ttg	L	0	0.56
tct	S	0	5.70
tcc	S	0	3.59
tca	S	0	8.07
tcg	S	0	1.65
ctt	L	0	0.59
ctc	L	0	0.42
cta	L	0	0.58
ctg	L	2	0.51
caa	Q	2687	20950.00
cag	Q	37016	18753.00
cgt	R	0	0.16
cgc	R	0	0.31
cga	R	0	0.59
cgg	R	6	0.44
aaa	K	0	0.57
aag	K	1	0.43
agt	S	0	5.83
agc	S	31	6.16
aga	R	0	3.01
agg	R	0	1.48
---	-	26	26.00
---	--	------	------
mPD		0.13	0.50
		nPD:	0.26
		N. weight:	0.54
		Sc. PD:	0.1
		Sc. rank:	14497.0

,

HA

Pos.	569	obs:	exp:
ttt	F	2	0.86
ttc	F	0	1.14
tct	S	0	0.18
tcc	S	0	0.12
tca	S	0	0.26
tcg	S	0	0.05
tat	Y	2	1.04
tac	Y	0	0.96
tgt	C	39510	17947.00
tgc	C	252	21815.00
tgg	W	1	1.00
agt	S	1	0.19
agc	S	0	0.20
ggt	G	0	0.30
ggc	G	0	0.30
gga	G	0	0.89
ggg	G	2	0.51
---	--	------	------
mPD		0.013	0.50
		nPD:	0.03
		N. weight:	0.82
		Sc. PD:	-0.03
		Sc. rank:	3755.8

,

HA

Pos.	570	obs:	exp:
tct	S	0	0.18
tcc	S	0	0.12
tca	S	0	0.26
tcg	S	0	0.05
cgt	R	0	1085.00
cgc	R	0	2079.00
cga	R	1	3893.00
cgg	R	0	2909.00
atg	M	1	1.00
act	T	0	0.25
acc	T	0	0.21
aca	T	1	0.46
acg	T	0	0.09
aaa	K	16	9.13
aag	K	0	6.87
agt	S	0	0.19
agc	S	1	0.20
aga	R	39702	19954.00
agg	R	44	9826.00
ggt	G	0	0.61
ggc	G	0	0.59
gga	G	2	1.78
ggg	G	2	1.02
---	--	------	------
mPD		0.0036	0.91
		nPD:	0.
		N. weight:	0.74
		Sc. PD:	-0.043
		Sc. rank:	-19163.0

,

HA

Pos.	571	obs:	exp:
tta	L	0	0.11
ttg	L	0	0.19
ctt	L	0	0.20
ctc	L	0	0.14
cta	L	1	0.19
ctg	L	0	0.17
cgt	R	0	0.08
cgc	R	0	0.16
cga	R	0	0.29
cgg	R	0	0.22
att	I	14	14448.00
atc	I	2	9907.00
ata	I	39673	15334.00
aaa	K	18	10.27
aag	K	0	7.73
aga	R	3	1.51
agg	R	0	0.74
gtt	V	0	12.60
gtc	V	0	10.36
gta	V	57	15.33
gtg	V	0	18.71
ggt	G	0	0.30
ggc	G	0	0.30
gga	G	1	0.89
ggg	G	1	0.51
---	--	------	------
mPD		0.0050	0.66
		nPD:	0.01
		N. weight:	1.
		Sc. PD:	-0.056
		Sc. rank:	-19448.0

,

HA

Pos.	572	obs:	exp:
tat	Y	1	0.52
tac	Y	0	0.48
tgt	C	39641	17950.00
tgc	C	127	21818.00
ggt	G	1	0.15
ggc	G	0	0.15
gga	G	0	0.44
ggg	G	0	0.26
---	--	------	------
mPD		0.0065	0.50
		nPD:	0.01
		N. weight:	0.84
		Sc. PD:	-0.042
		Sc. rank:	-7480.6

,

HA

Pos.	573	obs:	exp:
tta	L	0	0.11
ttg	L	0	0.19
ctt	L	1	0.20
ctc	L	0	0.14
cta	L	0	0.19
ctg	L	0	0.17
att	I	39696	14474.00
atc	I	64	9925.00
ata	I	1	15362.00
aaa	K	1	0.57
aag	K	0	0.43
gtt	V	7	1.55
gtc	V	0	1.27
gta	V	0	1.88
gtg	V	0	2.30
---	--	------	------
mPD		0.0038	0.66
		nPD:	0.01
		N. weight:	1.1
		Sc. PD:	-0.061
		Sc. rank:	-23743.0

,

HA

Pos.	574	obs:	exp:
taa	.	39642	18063.00
tag	.	13	10832.00
tga	.	115	10874.00
---	--	------	------
mPD		0.0064	0.79
		nPD:	0.01
		N. weight:	0.83
		Sc. PD:	-0.045
		Sc. rank:	-15268.0

}

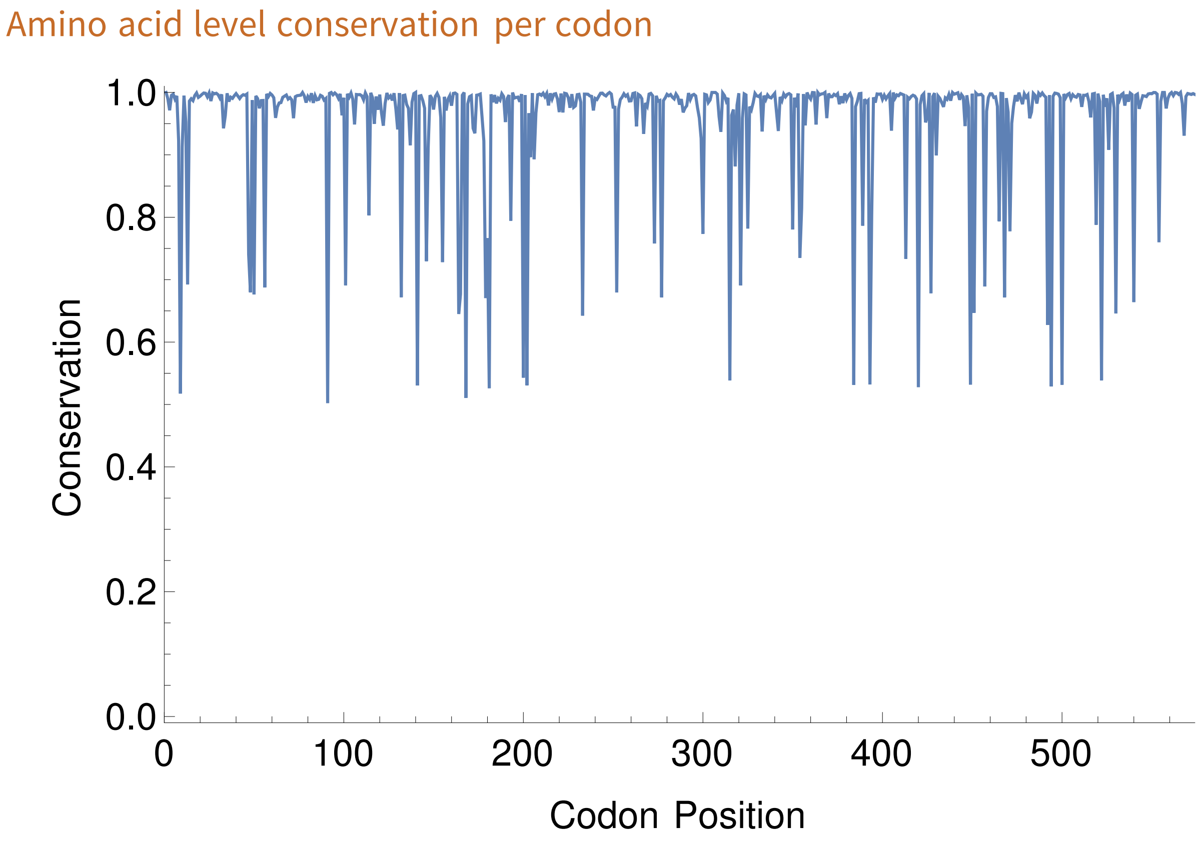

Amino acid level conservation per codon
Conservation
1.0
0.8
0.6
0.4
0.2
0.0
0
100
200
300
400
500
Codon Position

Conservation analysis plots

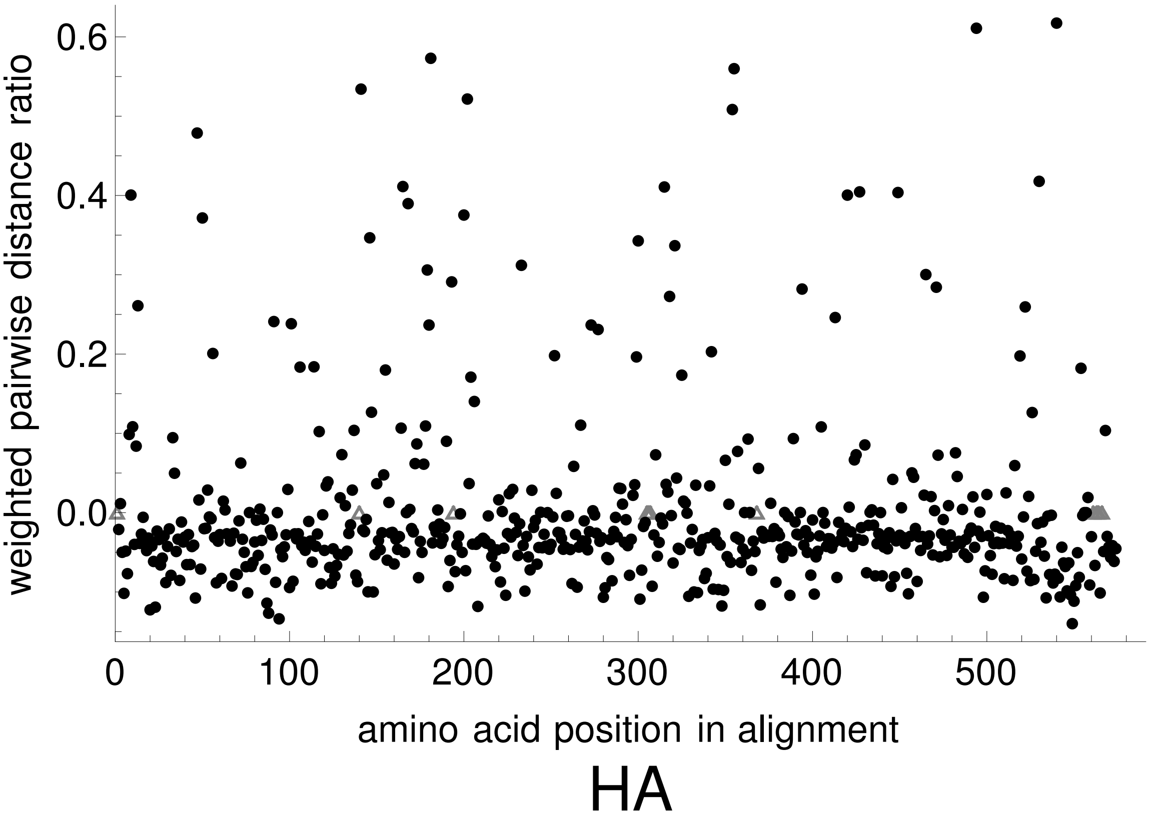

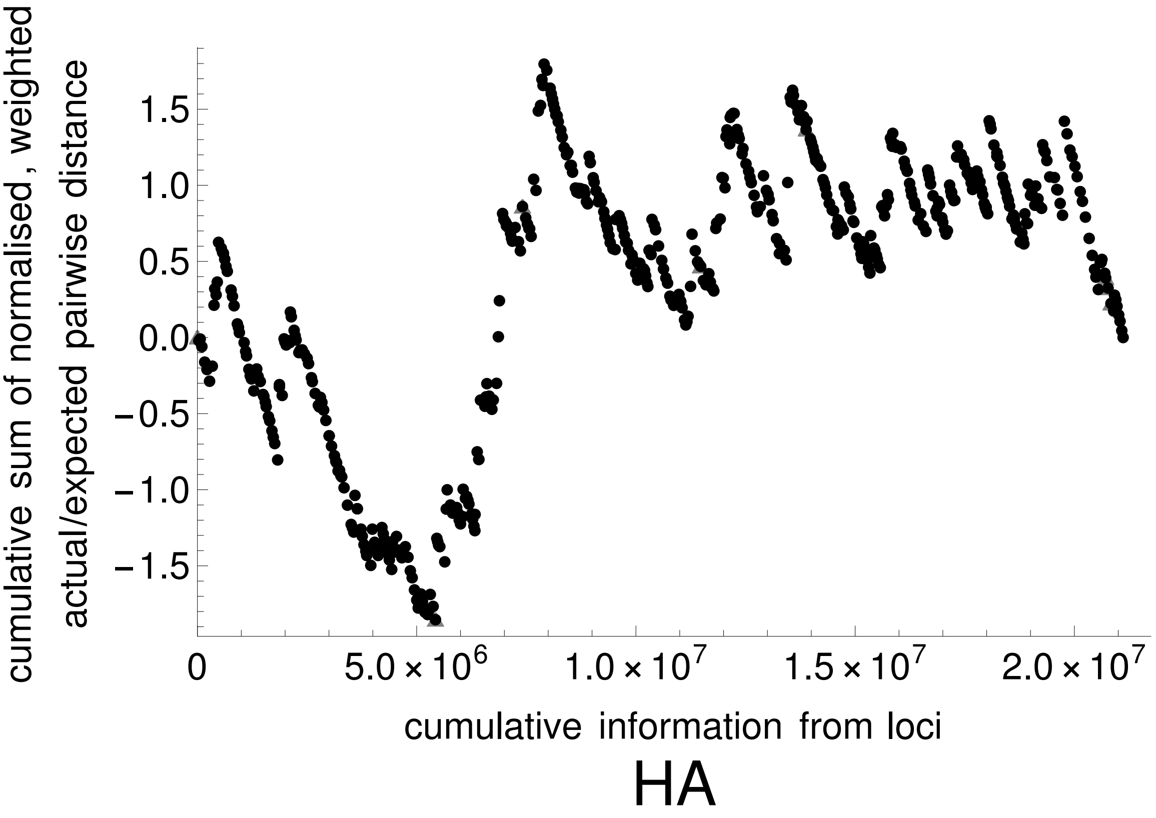

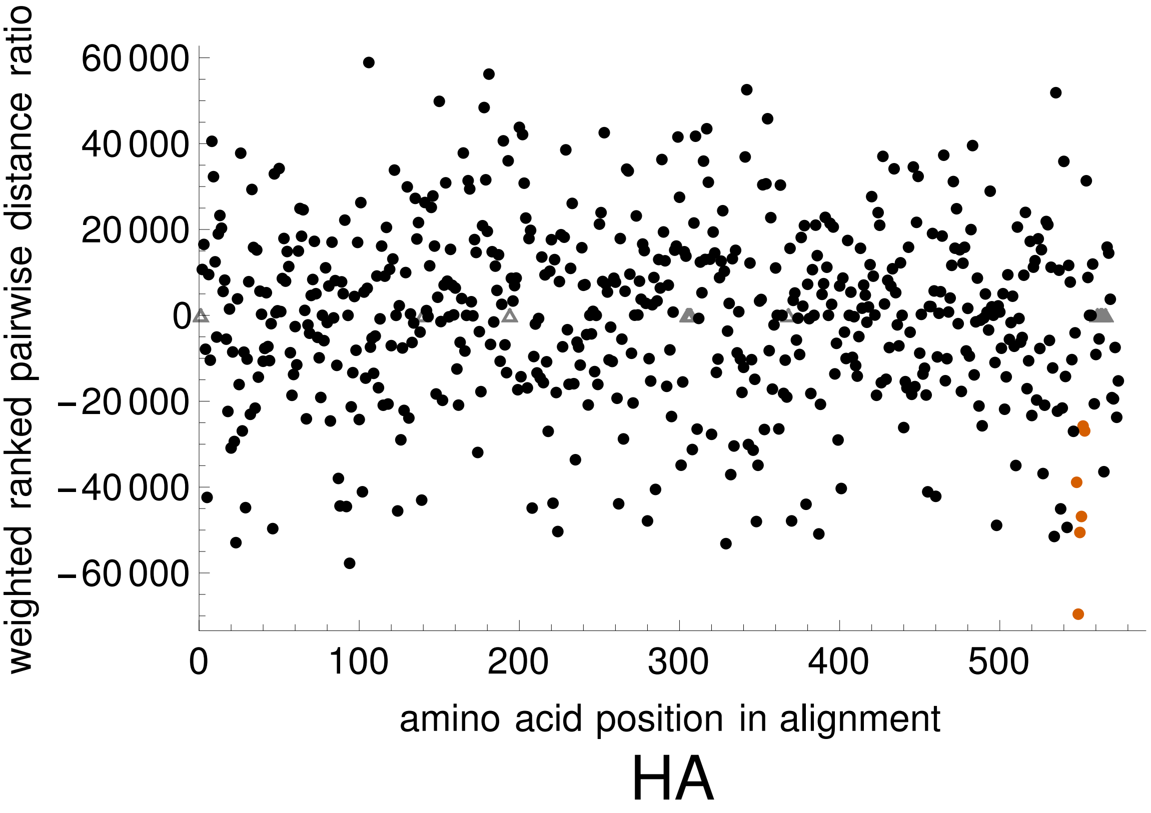
weighted ranked pairwise distance ratio
60 000
40 000
20 000
0
−20 000
−40 000
−60 000
0
100
200
300
400
500
amino acid position in alignment
HA

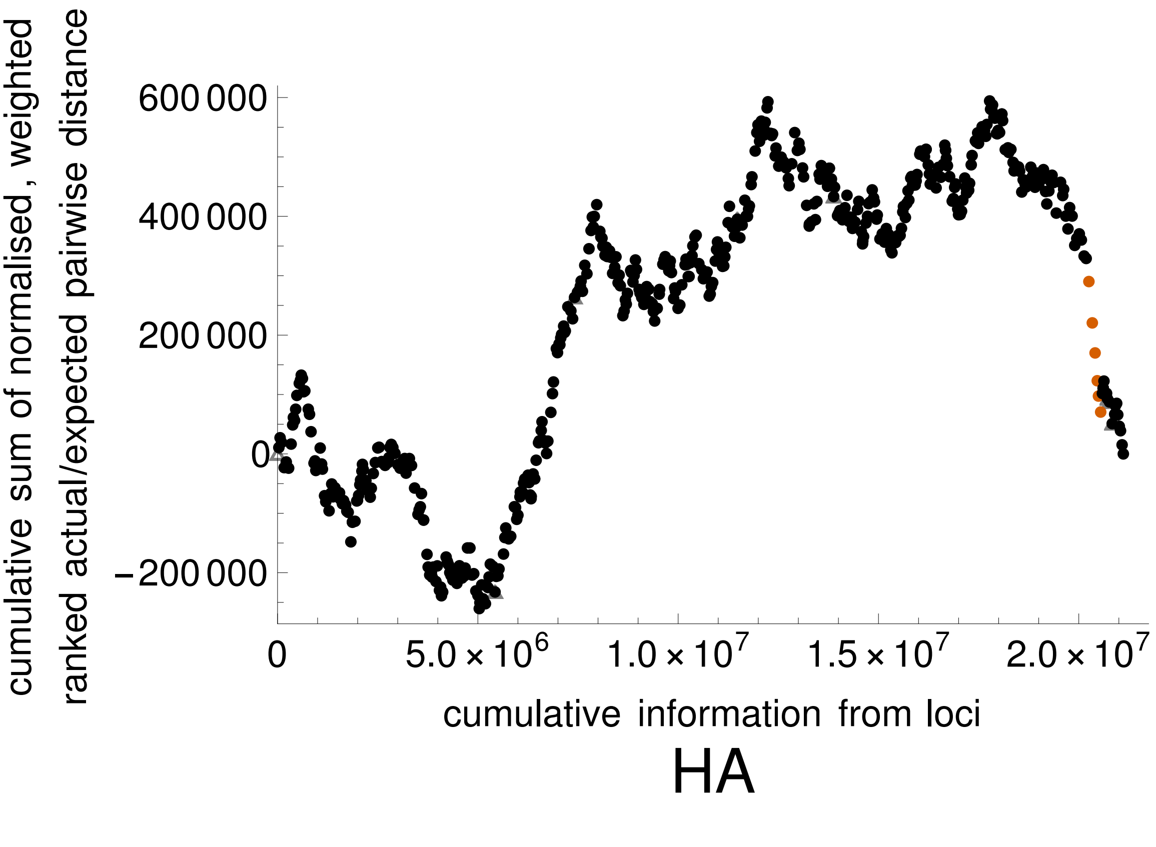
cumulative sum of normalised, weighted ranked actual/expected pairwise distance
600 000
400 000
200 000
0
−200 000
0
5.0 × 10^6
1.0 × 10^7
1.5 × 10^7
2.0 × 10^7
cumulative information from loci
HA

NP

Gene length histogram

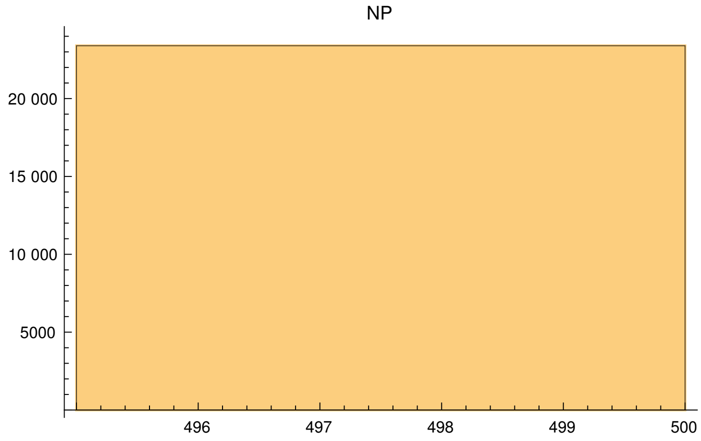

Information vs. nPD

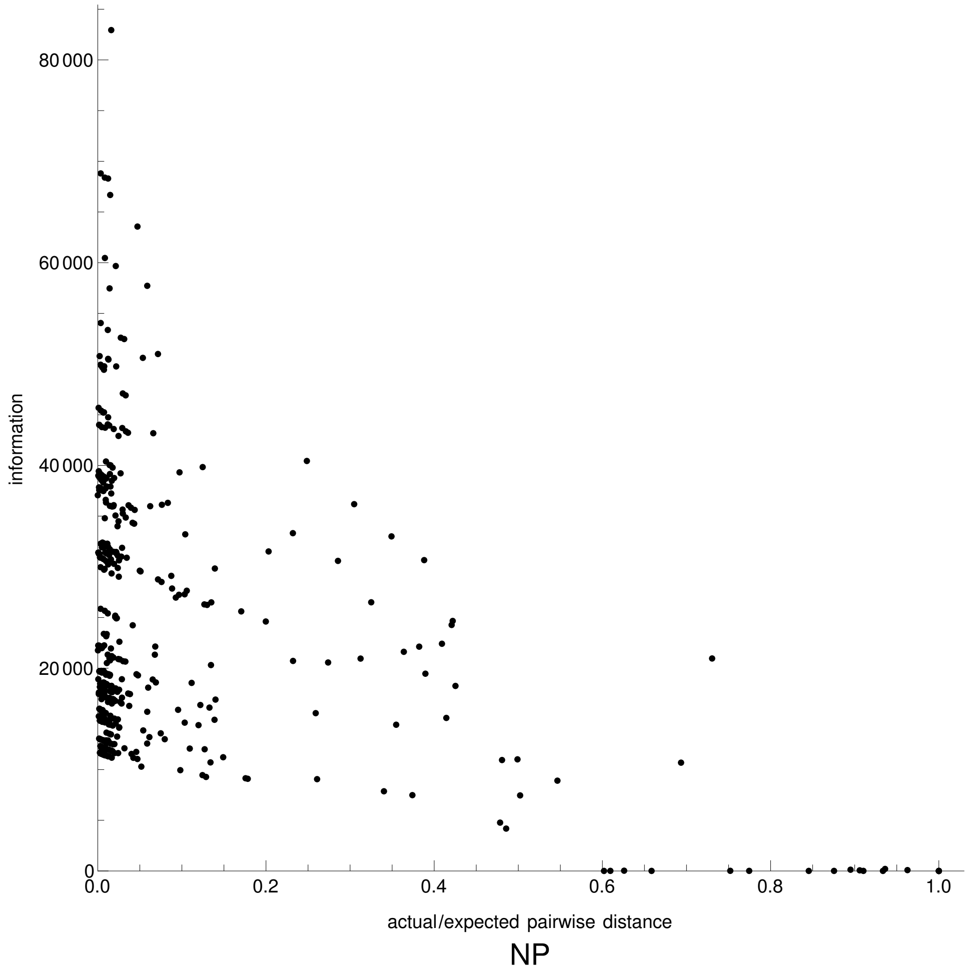

Example sequences highlighted by regions found in analyses to be conserved

```
Interesting points (by weighted raw PD) highlighted for gene NP:
-----------------ATGGCGTCTCAAGGCACCAAACGATCATATGAACAAATGGAGACTGGTGGGGAGCGCCA
    GGATACCACAGAAATCAGAGCATCTGTTGGAAGAATGATTGGTGGAATCGGGAGATTCTACATCCAAATGTGCAC
    TGAACTAAAACTCAGTGATTATGATGGACGACTAATCCAGAACAGCATAACAATAGAGAGGATGGTGCTTTCTTC
    TTTTGATGAGAGAAGAAATAAATACCTAGAAGAGCATCCAAGTGCTGGGAAGGACCCTAAGAAAACAGGAGGACC
    CATCTATAGAAGAATAGACGGAAAATGGACAAGAGAACTCATCCTTTATGACAAAGAAGAAATAAGAAGAGTTTG
    GCGCCAAGCAAACAATGGCGAAGATGCAACAGCAGGTCTTACTCATATCATGATTTGGCATTCCAATCTGAATGA
    TGCCACATATCAGAGGACAAGAGCACTTGTTCGCACTGGAATGGATCCCAGAATGTGCTCTCTAATGCAAGGTTC
    AACACTTCCCAGAAGGTCTGGTGCCGCAGGTGCTGCAGTAAAAGGAGTTGGAACAATAGCTATGGAGTTAATCAG
    AATGATAAAACGTGGAATCAATGACCGAAATTTCTGGAGGGGTGAAAATGGAAGAAGGACAAGAGTTGCTTATGA
    AAGAATGTGCAATATCCTCAAAGGAAAATTTCAAACAGCTGCCCAGAGGGCAATGATGGATCAAGTAAGAGAAAG
    TCGAAACCCAGGAAACGCTGAGATTGAAGACCTCATTTTCCTGGCACGGTCAGCACTCATTCTGAGAGGATCAGT
    TGCACATAAATCCTGCCTGCCTGCTTGTGTGTATGGGCTTGCAGTAGCAAGTGGCCATGACTTTGAAAGGGAAGG
    GTACTCACTGGTCGGGATAGACCCATTCAAATTACTCCAAAACAGTCAAGTGGTCAGCCTGATGAGACCAAATGA
    AAATCCAGCTCACAAGAGTCAATTGGTATGGATGGCATGCCACTCTGCTGCATTTGAAGATTTAAGAGTATCAAG
    TTTCATAAGAGGAAAGAAAGTGATCCCAAGAGGAAAGCTTTCCACAAGAGGGGTTCAGATTGCTTCAAATGAGAA
    TGTGGAAACCATGGACTCCAATACCCTGGAACTAAGAAGCAGATACTGGGCCATAAGAACCAGAAGTGGAGGAAA
    TACCAATCAACAGAAGGCATCTGCAGGCCAGATCAGTGTGCAGCCTACATTCTCAGTGCAGCGAAATCTCCCTTT
    TGAAAGAGCAACCATTATGGCAGCATTCAGCGGGAACAATGAAGGACGGACATCCGACATGCGAACAGAAATTAT
    AAGAATGATGGAAAGTGCAAAGCCAGAGGATTTGTCCTTCCAGGGGCGGGGAGTCTTCGAGCTCTCGGACGAAAA
    GGCAACGAACCCGATCGTGCCTTCCTTTGACATGAGTAATGAAGGGTCTTATTTCTTCGGAGACAATGCAGAGGA
    GTATGACAATTGA

Interesting points (by weighted ranked PD) highlighted for gene NP:
-----------------ATGGCGTCTCAAGGCACCAAACGATCATATGAACAAATGGAGACTGGTGGGGAGCGCCAGGA
    TACCACAGAAATCAGAGCATCTGTTGGAAGAATGATTGGTGGAATCGGGAGATTCTACATCCAAATGTGCACTGA
    ACTAAAACTCAGTGATTATGATGGACGACTAATCCAGAACAGCATAACAATAGAGAGGATGGTGCTTTCTTCTTT
    TGATGAGAGAAGAAATAAATACCTAGAAGAGCATCCAAGTGCTGGGAAGGACCCTAAGAAAACAGGAGGACCCAT
    CTATAGAAGAATAGACGGAAAATGGACAAGAGAACTCATCCTTTATGACAAAGAAGAAATAAGAAGAGTTTGGCG
    CCAAGCAAACAATGGCGAAGATGCAACAGCAGGTCTTACTCATATCATGATTTGGCATTCCAATCTGAATGATGC
    CACATATCAGAGGACAAGAGCACTTGTTCGCACTGGAATGGATCCCAGAATGTGCTCTCTAATGCAAGGTTCAAC
    ACTTCCCAGAAGGTCTGGTGCCGCAGGTGCTGCAGTAAAAGGAGTTGGAACAATAGCTATGGAGTTAATCAGAAT
    GATAAAACGTGGAATCAATGACCGAAATTTCTGGAGGGGTGAAAATGGAAGAAGGACAAGAGTTGCTTATGAAAG
    AATGTGCAATATCCTCAAAGGAAAATTTCAAACAGCTGCCCAGAGGGCAATGATGGATCAAGTAAGAGAAAGTCG
    AAACCCAGGAAACGCTGAGATTGAAGACCTCATTTTCCTGGCACGGTCAGCACTCATTCTGAGAGGATCAGTTGC
    ACATAAATCCTGCCTGCCTGCTTGTGTGTATGGGCTTGCAGTAGCAAGTGGCCATGACTTTGAAAGGGAAGGGTA
    CTCACTGGTCGGGATAGACCCATTCAAATTACTCCAAAACAGTCAAGTGGTCAGCCTGATGAGACCAAATGAAAA
    TCCAGCTCACAAGAGTCAATTGGTATGGATGGCATGCCACTCTGCTGCATTTGAAGATTTAAGAGTATCAAGTTT
    CATAAGAGGAAAGAAAGTGATCCCAAGAGGAAAGCTTTCCACAAGAGGGGTTCAGATTGCTTCAAATGAGAATGT
    GGAAACCATGGACTCCAATACCCTGGAACTAAGAAGCAGATACTGGGCCATAAGAACCAGAAGTGGAGGAAATAC
    CAATCAACAGAAGGCATCTGCAGGCCAGATCAGTGTGCAGCCTACATTCTCAGTGCAGCGAAATCTCCCTTTTGA
    AAGAGCAACCATTATGGCAGCATTCAGCGGGAACAATGAAGGACGGACATCCGACATGCGAACAGAAATTATAAG
    AATGATGGAAAGTGCAAAGCCAGAGGATTTGTCCTTCCAGGGGCGGGGAGTCTTCGAGCTCTCGGACGAAAAGGC
    AACGAACCCGATCGTGCCTTCCTTTGACATGAGTAATGAAGGGTCTTATTTCTTCGGAGACAATGCAGAGGAGTA
    TGACAATTGA
```

Per codon conservation report

NP

Pos.	1	obs:	exp:
atg	M	16542	16542.00
---	-	6852	6852.00
---	--	------	------
mPD		0	0
		nPD:	1.
		N. weight:	0.
		Sc. PD:	0
		Sc. rank:	0

NP

Pos.	2	obs:	exp:
tct	S	0	3036.00
tcc	S	0	1911.00
tca	S	0	4294.00
tcg	S	0	878.00
act	T	0	0.25
acc	T	0	0.21
aca	T	0	0.46
acg	T	1	0.09
aat	N	1	0.59
aac	N	0	0.41
agt	S	16496	3102.00
agc	S	2	3277.00
gct	A	0	10.85
gcc	A	0	8.49
gca	A	0	19.26
gcg	A	41	2.40
ggt	G	1	0.15
ggc	G	0	0.15
gga	G	0	0.44
ggg	G	0	0.26
---	-	6852	6852.00
---	--	------	------
mPD		0.0078	0.82
		nPD:	0.01
		N. weight:	0.
		Sc. PD:	0
		Sc. rank:	0

NP

Pos.	3	obs:	exp:
gat	D	1	8694.00
gac	D	16499	7806.00
---	-	6894	6894.00
---	--	------	------
mPD		0.000060	0.25
		nPD:	0.
		N. weight:	0.
		Sc. PD:	0
		Sc. rank:	0

NP

Pos.	4	obs:	exp:
att	I	67	6004.00
atc	I	16427	4117.00
ata	I	0	6372.00
act	T	0	1.26
acc	T	5	1.03
aca	T	0	2.29
acg	T	0	0.43
gtt	V	0	0.22
gtc	V	1	0.18
gta	V	0	0.27
gtg	V	0	0.33
---	-	6894	6894.00
---	--	------	------
mPD		0.0044	0.33
		nPD:	0.01
		N. weight:	0.
		Sc. PD:	0
		Sc. rank:	0

NP

Pos.	5	obs:	exp:
aaa	K	7	3.99
aag	K	0	3.01
gtt	V	0	0.22
gtc	V	0	0.18
gta	V	1	0.27
gtg	V	0	0.33
gct	A	0	0.53
gcc	A	0	0.41
gca	A	1	0.94
gcg	A	1	0.12
gat	D	1	0.53
gac	D	0	0.47
gaa	E	16486	9983.00
gag	E	1	6504.00
ggt	G	0	0.30
ggc	G	0	0.30
gga	G	2	0.89
ggg	G	0	0.51
---	-	6894	6894.00
---	--	------	------
mPD		0.00090	0.24
		nPD:	0.
		N. weight:	0.
		Sc. PD:	0
		Sc. rank:	0

NP

Pos.	6	obs:	exp:
tct	S	0	0.55
tcc	S	3	0.35
tca	S	0	0.78
tcg	S	0	0.16
att	I	0	0.36
atc	I	1	0.25
ata	I	0	0.39
act	T	0	31.01
acc	T	123	25.23
aca	T	0	56.27
acg	T	0	10.49
aat	N	0	0.59
aac	N	1	0.41
agt	S	0	0.56
agc	S	0	0.60
gtt	V	0	59.91
gtc	V	271	49.24
gta	V	0	72.88
gtg	V	0	88.96
gct	A	21	4263.00
gcc	A	16063	3333.00
gca	A	16	7562.00
gcg	A	0	943.20
gat	D	0	0.53
gac	D	1	0.47
---	-	6894	6894.00
---	--	------	------
mPD		0.026	0.36
		nPD:	0.07
		N. weight:	0.
		Sc. PD:	0
		Sc. rank:	0

NP

Pos.	7	obs:	exp:
atg	M	23352	23352.00
---	-	42	42.00
---	--	------	------
mPD		0	0
		nPD:	1.
		N. weight:	0.
		Sc. PD:	0
		Sc. rank:	0

NP

Pos.	8	obs:	exp:
gtt	V	0	3.10
gtc	V	0	2.54
gta	V	0	3.77
gtg	V	14	4.60
gct	A	30	6179.00
gcc	A	1	4831.00
gca	A	325	10961.00
gcg	A	22981	1367.00
gaa	E	0	0.61
gag	E	1	0.39
---	-	42	42.00
---	--	------	------
mPD		0.031	0.66
		nPD:	0.05
		N. weight:	2.8
		Sc. PD:	0.017
		Sc. rank:	36172.0

NP

Pos.	9	obs:	exp:
ttt	F	8	3.43
ttc	F	0	4.57
tct	S	23261	4301.00
tcc	S	32	2707.00
tca	S	2	6083.00
tcg	S	79	1244.00
tat	Y	3	1.56
tac	Y	0	1.44
cct	P	8	2.20
ccc	P	0	1.48
cca	P	0	3.41
ccg	P	0	0.91
act	T	1	0.25
acc	T	0	0.21
aca	T	0	0.46
acg	T	0	0.09
agt	S	0	4395.00
agc	S	0	4643.00
---	--	------	------
mPD		0.011	1.6
		nPD:	0.01
		N. weight:	1.7
		Sc. PD:	-0.056
		Sc. rank:	-12517.0

NP

Pos.	10	obs:	exp:
cct	P	0	0.28
ccc	P	0	0.19
cca	P	1	0.43
ccg	P	0	0.11
cat	H	1	1.76
cac	H	2	1.24
caa	Q	23317	12342.00
cag	Q	72	11047.00
aaa	K	1	0.57
aag	K	0	0.43
---	--	------	------
mPD		0.0066	0.50
		nPD:	0.01
		N. weight:	0.64
		Sc. PD:	-0.017
		Sc. rank:	492.5

NP

Pos.	11	obs:	exp:
ggt	G	95	3547.00
ggc	G	23298	3465.00
gga	G	1	10406.00
ggg	G	0	5976.00
---	--	------	------
mPD		0.0082	0.69
		nPD:	0.01
		N. weight:	1.9
		Sc. PD:	-0.054
		Sc. rank:	-1823.0

NP

Pos.	12	obs:	exp:
act	T	0	5897.00
acc	T	23394	4798.00
aca	T	0	10702.00
acg	T	0	1996.00
---	--	------	------
mPD		0	0.68
		nPD:	0.
		N. weight:	1.6
		Sc. PD:	-0.064
		Sc. rank:	-31964.0

NP

Pos.	13	obs:	exp:
aaa	K	23385	13345.00
aag	K	9	10049.00
---	--	------	------
mPD		0.00077	0.49
		nPD:	0.
		N. weight:	0.58
		Sc. PD:	-0.022
		Sc. rank:	-10245.0

NP

Pos.	14	obs:	exp:
cgt	R	1	638.80
cgc	R	0	1224.00
cga	R	23357	2291.00
cgg	R	26	1712.00
aga	R	10	11745.00
agg	R	0	5783.00
---	--	------	------
mPD		0.0032	0.91
		nPD:	0.
		N. weight:	2.4
		Sc. PD:	-0.086
		Sc. rank:	-31453.0

NP

Pos.	15	obs:	exp:
tct	S	4	4305.00
tcc	S	7	2709.00
tca	S	22929	6088.00
tcg	S	452	1245.00
cct	P	0	0.28
ccc	P	0	0.19
cca	P	1	0.43
ccg	P	0	0.11
act	T	0	0.25
acc	T	0	0.21
aca	T	1	0.46
acg	T	0	0.09
agt	S	0	4398.00
agc	S	0	4646.00
---	--	------	------
mPD		0.039	1.6
		nPD:	0.02
		N. weight:	1.3
		Sc. PD:	-0.022
		Sc. rank:	10776.0

NP			
Pos.	16	obs:	exp:
ttt	F	2	0.86
ttc	F	0	1.14
tat	Y	23385	12154.00
tac	Y	7	11238.00
---	--	------	------
mPD		0.00077	0.50
		nPD:	0.
		N. weight:	0.67
		Sc. PD:	-0.025
		Sc. rank:	-12072.0

,

NP			
Pos.	17	obs:	exp:
gaa	E	23365	14165.00
gag	E	29	9229.00
---	--	------	------
mPD		0.0025	0.48
		nPD:	0.01
		N. weight:	0.51
		Sc. PD:	-0.017
		Sc. rank:	-5277.2

,

NP			
Pos.	18	obs:	exp:
caa	Q	23379	12344.00
cag	Q	15	11050.00
---	--	------	------
mPD		0.0013	0.50
		nPD:	0.
		N. weight:	0.66
		Sc. PD:	-0.024
		Sc. rank:	-10431.0

,

NP			
Pos.	19	obs:	exp:
atg	M	23394	23394.00
---	--	------	------
mPD		0	0
		nPD:	1.
		N. weight:	0.
		Sc. PD:	0
		Sc. rank:	0

,

NP			
Pos.	20	obs:	exp:
gat	D	1475	777.70
gac	D	1	698.30
gaa	E	88	13271.00
gag	E	21830	8647.00
---	--	------	------
mPD		0.13	0.54
		nPD:	0.23
		N. weight:	0.92
		Sc. PD:	0.17
		Sc. rank:	16191.0

,

NP			
Pos.	21	obs:	exp:
att	I	7	2.55
atc	I	0	1.75
ata	I	0	2.70
act	T	23343	5896.00
acc	T	29	4797.00
aca	T	2	10699.00
acg	T	13	1995.00
---	--	------	------
mPD		0.0044	0.68
		nPD:	0.01
		N. weight:	1.4
		Sc. PD:	-0.047
		Sc. rank:	-12092.0

,

NP			
Pos.	22	obs:	exp:
tct	S	0	1.47
tcc	S	0	0.93
tca	S	0	2.08
tcg	S	0	0.43
agt	S	8	1.50
agc	S	0	1.59
gat	D	19	10.01
gac	D	0	8.99
ggt	G	23323	3543.00
ggc	G	18	3461.00
gga	G	11	10394.00
ggg	G	15	5969.00
---	--	------	------
mPD		0.0061	0.69
		nPD:	0.01
		N. weight:	1.9
		Sc. PD:	-0.06
		Sc. rank:	-7561.8

,

NP			
Pos.	23	obs:	exp:
cgt	R	0	0.05
cgc	R	0	0.10
cga	R	0	0.20
cgg	R	0	0.15
aga	R	1	1.00
agg	R	1	0.49
ggt	G	5	3547.00
ggc	G	0	3465.00
gga	G	882	10405.00
ggg	G	22505	5975.00
---	--	------	------
mPD		0.073	0.69
		nPD:	0.11
		N. weight:	1.2
		Sc. PD:	0.076
		Sc. rank:	19572.0

,

NP			
Pos.	24	obs:	exp:
gat	D	8	6.85
gac	D	5	6.15
gaa	E	79	14157.00
gag	E	23301	9223.00
ggt	G	0	0.15
ggc	G	0	0.15
gga	G	0	0.44
ggg	G	1	0.26
---	--	------	------
mPD		0.0079	0.48
		nPD:	0.02
		N. weight:	0.94
		Sc. PD:	-0.022
		Sc. rank:	4163.0

,

NP			
Pos.	25	obs:	exp:
tta	L	0	0.11
ttg	L	0	0.19
tct	S	0	0.18
tcc	S	0	0.12
tca	S	0	0.26
tcg	S	0	0.05
ctt	L	0	0.20
ctc	L	1	0.14
cta	L	0	0.19
ctg	L	0	0.17
cat	H	0	3.53
cac	H	6	2.47
cgt	R	28	638.50
cgc	R	23357	1223.00
cga	R	1	2291.00
cgg	R	0	1711.00
agt	S	0	0.19
agc	S	1	0.20
aga	R	0	11741.00
agg	R	0	5781.00
---	--	------	------
mPD		0.0032	0.91
		nPD:	0.
		N. weight:	3.
		Sc. PD:	-0.11
		Sc. rank:	-41100.0

,

NP			
Pos.	26	obs:	exp:
cct	P	0	1.10
ccc	P	0	0.74
cca	P	0	1.70
ccg	P	4	0.45
caa	Q	26	12342.00
cag	Q	23363	11047.00
cgt	R	0	0.03
cgc	R	0	0.05
cga	R	0	0.10
cgg	R	1	0.07
aga	R	0	0.50
agg	R	0	0.25
---	--	------	------
mPD		0.0026	0.50
		nPD:	0.01
		N. weight:	0.77
		Sc. PD:	-0.026
		Sc. rank:	-7571.5

,

NP			
Pos.	27	obs:	exp:
aat	N	2	1.18
aac	N	0	0.82
gat	D	23245	12324.00
gac	D	145	11066.00
gaa	E	1	0.61
gag	E	0	0.39
ggt	G	1	0.15
ggc	G	0	0.15
gga	G	0	0.44
ggg	G	0	0.26
---	--	------	------
mPD		0.013	0.50
		nPD:	0.03
		N. weight:	0.62
		Sc. PD:	-0.0093
		Sc. rank:	5624.0

,

NP			
Pos.	28	obs:	exp:
att	I	0	5.46
atc	I	15	3.74
ata	I	0	5.80
act	T	78	4900.00
acc	T	19330	3987.00
aca	T	28	8891.00
acg	T	0	1658.00
gtt	V	0	0.44
gtc	V	2	0.36
gta	V	0	0.54
gtg	V	0	0.66
gct	A	9	1043.00
gcc	A	3929	815.80
gca	A	3	1851.00
gcg	A	0	230.90
---	--	------	------
mPD		0.29	0.96
		nPD:	0.3
		N. weight:	1.6
		Sc. PD:	0.41
		Sc. rank:	29112.0

,

NP			
Pos.	29	obs:	exp:
tct	S	0	0.55
tcc	S	0	0.35
tca	S	3	0.78
tcg	S	0	0.16
act	T	14	5897.00
acc	T	1	4798.00
aca	T	23268	10701.00
acg	T	108	1996.00
agt	S	0	0.56
agc	S	0	0.60
---	--	------	------
mPD		0.011	0.68
		nPD:	0.02
		N. weight:	0.78
		Sc. PD:	-0.019
		Sc. rank:	2885.9

,

NP			
Pos.	30	obs:	exp:
gaa	E	23346	14165.00
gag	E	48	9229.00
---	--	------	------
mPD		0.0041	0.48
		nPD:	0.01
		N. weight:	0.5
		Sc. PD:	-0.016
		Sc. rank:	-2049.9

,

NP			
Pos.	31	obs:	exp:
att	I	264	8516.00
atc	I	23127	5840.00
ata	I	3	9038.00
---	--	------	------
mPD		0.023	0.66
		nPD:	0.03
		N. weight:	1.4
		Sc. PD:	-0.0084
		Sc. rank:	15600.0

,

NP			
Pos.	32	obs:	exp:
cgt	R	0	638.60
cgc	R	0	1223.00
cga	R	0	2291.00
cgg	R	0	1712.00
aaa	K	7	3.99
aag	K	0	3.01
aga	R	23371	11741.00
agg	R	16	5782.00
---	--	------	------
mPD		0.0020	0.91
		nPD:	0.
		N. weight:	0.71
		Sc. PD:	-0.026
		Sc. rank:	-11781.0

,

NP			
Pos.	33	obs:	exp:
tct	S	0	0.55
tcc	S	0	0.35
tca	S	3	0.78
tcg	S	0	0.16
act	T	0	1.26
acc	T	0	1.03
aca	T	5	2.29
acg	T	0	0.43
agt	S	0	0.56
agc	S	0	0.60
gtt	V	0	0.22
gtc	V	0	0.18
gta	V	1	0.27
gtg	V	0	0.33
gct	A	25	6191.00
gcc	A	0	4841.00
gca	A	23353	10983.00
gcg	A	7	1370.00
---	--	------	------
mPD		0.0035	0.66
		nPD:	0.01
		N. weight:	0.77
		Sc. PD:	-0.026
		Sc. rank:	-7944.1

,

NP			
Pos.	34	obs:	exp:
tct	S	23319	4305.00
tcc	S	54	2710.00
tca	S	17	6088.00
tcg	S	3	1245.00
cct	P	1	0.28
ccc	P	0	0.19
cca	P	0	0.43
ccg	P	0	0.11
agt	S	0	4399.00
agc	S	0	4647.00
---	--	------	------
mPD		0.0064	1.6
		nPD:	0.
		N. weight:	1.7
		Sc. PD:	-0.061
		Sc. rank:	-21440.0

,

NP			
Pos.	35	obs:	exp:
gtt	V	18883	5172.00
gtc	V	4503	4251.00
gta	V	5	6292.00
gtg	V	3	7679.00
---	--	------	------
mPD		0.31	0.74
		nPD:	0.42
		N. weight:	1.1
		Sc. PD:	0.4
		Sc. rank:	20929.0

,

NP

Pos.	36	obs:	exp:
ggt	G	8	3547.00
ggc	G	0	3465.00
gga	G	23332	10406.00
ggg	G	54	5976.00
---	--	------	------
mPD		0.0053	0.69
		nPD:	0.01
		N. weight:	0.82
		Sc. PD:	-0.026
		Sc. rank:	-4853.5

,

NP

Pos.	37	obs:	exp:
cgt	R	0	638.20
cgc	R	0	1223.00
cga	R	8	2289.00
cgg	R	0	1711.00
aaa	K	14	10.27
aag	K	4	7.73
aga	R	23141	11735.00
agg	R	225	5778.00
ggt	G	0	0.30
ggc	G	0	0.30
gga	G	2	0.89
ggg	G	0	0.51
---	--	------	------
mPD		0.022	0.91
		nPD:	0.02
		N. weight:	0.66
		Sc. PD:	-0.011
		Sc. rank:	5494.2

,

NP

Pos.	38	obs:	exp:
atg	M	23394	23394.00
---	--	------	------
mPD		0	0
		nPD:	1.
		N. weight:	0.
		Sc. PD:	0
		Sc. rank:	0

,

NP

Pos.	39	obs:	exp:
att	I	23195	8510.00
atc	I	168	5835.00
ata	I	13	9031.00
gtt	V	18	3.98
gtc	V	0	3.27
gta	V	0	4.84
gtg	V	0	5.91
---	--	------	------
mPD		0.017	0.66
		nPD:	0.03
		N. weight:	1.
		Sc. PD:	-0.015
		Sc. rank:	9126.8

,

NP

Pos.	40	obs:	exp:
tct	S	0	28.52
tcc	S	0	17.95
tca	S	0	40.34
tcg	S	0	8.25
aat	N	2	1.18
aac	N	0	0.82
agt	S	155	29.15
agc	S	0	30.79
gtt	V	4	0.88
gtc	V	0	0.73
gta	V	0	1.08
gtg	V	0	1.31
gct	A	9	2.38
gcc	A	0	1.86
gca	A	0	4.23
gcg	A	0	0.53
gat	D	348	183.90
gac	D	1	165.10
ggt	G	22812	3469.00
ggc	G	52	3388.00
gga	G	2	10175.00
ggg	G	9	5843.00
---	--	------	------
mPD		0.049	0.75
		nPD:	0.07
		N. weight:	1.9
		Sc. PD:	0.046
		Sc. rank:	26983.0

,

NP

Pos.	41	obs:	exp:
ggt	G	2	3547.00
ggc	G	6	3465.00
gga	G	23341	10406.00
ggg	G	45	5976.00
---	--	------	------
mPD		0.0045	0.69
		nPD:	0.01
		N. weight:	0.82
		Sc. PD:	-0.027
		Sc. rank:	-6944.1

,

NP

Pos.	42	obs:	exp:
att	I	110	8515.00
atc	I	23251	5839.00
ata	I	31	9038.00
gtt	V	0	0.44
gtc	V	2	0.36
gta	V	0	0.54
gtg	V	0	0.66
---	--	------	------
mPD		0.012	0.66
		nPD:	0.02
		N. weight:	1.4
		Sc. PD:	-0.03
		Sc. rank:	7893.8

,

NP

Pos.	43	obs:	exp:
ggt	G	17	3547.00
ggc	G	0	3465.00
gga	G	402	10406.00
ggg	G	22975	5976.00
---	--	------	------
mPD		0.035	0.69
		nPD:	0.05
		N. weight:	1.3
		Sc. PD:	0.013
		Sc. rank:	17167.0

,

NP

Pos.	44	obs:	exp:
tct	S	0	0.18
tcc	S	0	0.12
tca	S	0	0.26
tcg	S	0	0.05
cgt	R	0	638.10
cgc	R	0	1223.00
cga	R	0	2289.00
cgg	R	0	1710.00
aaa	K	22	12.55
aag	K	0	9.45
agt	S	1	0.19
agc	S	0	0.20
aga	R	23334	11733.00
agg	R	37	5778.00
---	--	------	------
mPD		0.0051	0.91
		nPD:	0.01
		N. weight:	0.7
		Sc. PD:	-0.024
		Sc. rank:	-6620.9

,

NP

Pos.	45	obs:	exp:
ttt	F	63	10038.00
ttc	F	23328	13353.00
tat	Y	0	1.56
tac	Y	3	1.44
---	--	------	------
mPD		0.0056	0.49
		nPD:	0.01
		N. weight:	0.56
		Sc. PD:	-0.016
		Sc. rank:	-675.9

,

NP

Pos.	46	obs:	exp:
tat	Y	26	12155.00
tac	Y	23368	11239.00
---	--	------	------
mPD		0.0022	0.50
		nPD:	0.
		N. weight:	0.75
		Sc. PD:	-0.026
		Sc. rank:	-8563.2

,

NP

Pos.	47	obs:	exp:
att	I	50	8515.00
atc	I	23333	5839.00
ata	I	8	9037.00
act	T	0	0.25
acc	T	1	0.21
aca	T	0	0.46
acg	T	0	0.09
aat	N	0	1.18
aac	N	2	0.82
---	--	------	------
mPD		0.0052	0.66
		nPD:	0.01
		N. weight:	1.4
		Sc. PD:	-0.045
		Sc. rank:	-7555.5

,

NP

Pos.	48	obs:	exp:
caa	Q	23363	12344.00
cag	Q	31	11050.00
---	--	------	------
mPD		0.0026	0.50
		nPD:	0.01
		N. weight:	0.65
		Sc. PD:	-0.022
		Sc. rank:	-6359.6

,

NP

Pos.	49	obs:	exp:
att	I	0	1.09
atc	I	0	0.75
ata	I	3	1.16
atg	M	23391	23391.00
---	--	------	------
mPD		0.00026	0.00026
		nPD:	1.
		N. weight:	0.00013
		Sc. PD:	0.00012
		Sc. rank:	2.5

,

NP

Pos.	50	obs:	exp:
tgt	C	91	10559.00
tgc	C	23303	12835.00
---	--	------	------
mPD		0.0077	0.50
		nPD:	0.02
		N. weight:	0.6
		Sc. PD:	-0.014
		Sc. rank:	2100.2

,

NP

Pos.	51	obs:	exp:
act	T	23355	5897.00
acc	T	24	4798.00
aca	T	13	10702.00
acg	T	2	1996.00
---	--	------	------
mPD		0.0033	0.68
		nPD:	0.
		N. weight:	1.4
		Sc. PD:	-0.049
		Sc. rank:	-14812.0

,

NP

Pos.	52	obs:	exp:
gaa	E	23363	14164.00
gag	E	30	9229.00
ggt	G	0	0.15
ggc	G	0	0.15
gga	G	1	0.44
ggg	G	0	0.26
---	--	------	------
mPD		0.0026	0.48
		nPD:	0.01
		N. weight:	0.51
		Sc. PD:	-0.017
		Sc. rank:	-4890.9

,

NP

Pos.	53	obs:	exp:
tta	L	395	2571.00
ttg	L	0	4403.00
ctt	L	12	4608.00
ctc	L	7787	3303.00
cta	L	15179	4502.00
ctg	L	12	3998.00
att	I	0	3.28
atc	I	1	2.25
ata	I	8	3.48
---	--	------	------
mPD		0.48	1.1
		nPD:	0.42
		N. weight:	1.1
		Sc. PD:	0.39
		Sc. rank:	20502.0

,

NP

Pos.	54	obs:	exp:
caa	Q	18	9.50
cag	Q	0	8.50
cgt	R	0	0.11
cgc	R	0	0.21
cga	R	0	0.39
cgg	R	0	0.29
aaa	K	23337	13332.00
aag	K	35	10040.00
aga	R	4	2.01
agg	R	0	0.99
---	--	------	------
mPD		0.0049	0.49
		nPD:	0.01
		N. weight:	0.57
		Sc. PD:	-0.017
		Sc. rank:	-1361.3

,

NP

Pos.	55	obs:	exp:
ttt	F	0	0.86
ttc	F	2	1.14
tta	L	0	2572.00
ttg	L	0	4404.00
ctt	L	5	4610.00
ctc	L	23381	3304.00
cta	L	6	4503.00
ctg	L	0	3999.00
---	--	------	------
mPD		0.0011	1.1
		nPD:	0.
		N. weight:	2.
		Sc. PD:	-0.077
		Sc. rank:	-37943.0

,

NP			
Pos.	56	obs:	exp:
tct	S	0	4302.00
tcc	S	0	2708.00
tca	S	0	6084.00
tcg	S	0	1244.00
aat	N	15	8.87
aac	N	0	6.13
agt	S	23355	4395.00
agc	S	21	4643.00
ggt	G	3	0.45
ggc	G	0	0.44
gga	G	0	1.33
ggg	G	0	0.77
---	--	------	------
mPD		0.0033	1.6
		nPD:	0.
		N. weight:	1.7
		Sc. PD:	-0.064
		Sc. rank:	-28937.0

,

NP			
Pos.	57	obs:	exp:
gat	D	23290	12326.00
gac	D	103	11067.00
gaa	E	0	0.61
gag	E	1	0.39
---	--	------	------
mPD		0.0089	0.50
		nPD:	0.02
		N. weight:	0.63
		Sc. PD:	-0.014
		Sc. rank:	3294.1

,

NP			
Pos.	58	obs:	exp:
tat	Y	23354	12153.00
tac	Y	35	11236.00
tgt	C	1	0.45
tgc	C	0	0.55
cat	H	4	2.35
cac	H	0	1.65
---	--	------	------
mPD		0.0034	0.50
		nPD:	0.01
		N. weight:	0.67
		Sc. PD:	-0.022
		Sc. rank:	-5021.5

,

NP			
Pos.	59	obs:	exp:
gat	D	23358	12316.00
gac	D	17	11059.00
gaa	E	8	11.50
gag	E	11	7.50
---	--	------	------
mPD		0.0031	0.50
		nPD:	0.01
		N. weight:	0.66
		Sc. PD:	-0.022
		Sc. rank:	-5913.6

,

NP			
Pos.	60	obs:	exp:
ggt	G	2	3547.00
ggc	G	4	3465.00
gga	G	23267	10406.00
ggg	G	121	5976.00
---	--	------	------
mPD		0.011	0.69
		nPD:	0.02
		N. weight:	0.8
		Sc. PD:	-0.02
		Sc. rank:	2742.3

,

NP			
Pos.	61	obs:	exp:
tta	L	0	6.38
ttg	L	0	10.92
ctt	L	0	11.43
ctc	L	0	8.19
cta	L	58	11.17
ctg	L	0	9.92
cgt	R	6	637.10
cgc	R	0	1221.00
cga	R	23100	2286.00
cgg	R	64	1708.00
att	I	0	0.36
atc	I	0	0.25
ata	I	1	0.39
aga	R	165	11715.00
agg	R	0	5769.00
---	--	------	------
mPD		0.025	0.92
		nPD:	0.03
		N. weight:	2.3
		Sc. PD:	-0.03
		Sc. rank:	22262.0

,

NP			
Pos.	62	obs:	exp:
tta	L	40	2572.00
ttg	L	4	4404.00
ctt	L	0	4610.00
ctc	L	0	3304.00
cta	L	23274	4504.00
ctg	L	76	4000.00
---	--	------	------
mPD		0.011	1.1
		nPD:	0.01
		N. weight:	1.7
		Sc. PD:	-0.051
		Sc. rank:	-5647.2

,

NP			
Pos.	63	obs:	exp:
tta	L	0	0.44
ttg	L	0	0.75
ctt	L	2	0.79
ctc	L	2	0.56
cta	L	0	0.77
ctg	L	0	0.68
att	I	678	8514.00
atc	I	22705	5838.00
ata	I	4	9036.00
act	T	0	0.25
acc	T	1	0.21
aca	T	0	0.46
acg	T	0	0.09
gtt	V	0	0.44
gtc	V	2	0.36
gta	V	0	0.54
gtg	V	0	0.66
---	--	------	------
mPD		0.057	0.66
		nPD:	0.09
		N. weight:	1.3
		Sc. PD:	0.058
		Sc. rank:	19507.0

,

NP			
Pos.	64	obs:	exp:
caa	Q	119	12344.00
cag	Q	23275	11050.00
---	--	------	------
mPD		0.010	0.50
		nPD:	0.02
		N. weight:	0.74
		Sc. PD:	-0.015
		Sc. rank:	4905.0

,

NP			
Pos.	65	obs:	exp:
aat	N	15307	13836.00
aac	N	8087	9558.00
---	--	------	------
mPD		0.45	0.48
		nPD:	0.94
		N. weight:	0.0088
		Sc. PD:	0.0076
		Sc. rank:	172.9

,

NP			
Pos.	66	obs:	exp:
tct	S	0	4305.00
tcc	S	0	2710.00
tca	S	0	6088.00
tcg	S	0	1245.00
agt	S	656	4399.00
agc	S	22738	4647.00
---	--	------	------
mPD		0.055	1.6
		nPD:	0.03
		N. weight:	1.5
		Sc. PD:	-0.011
		Sc. rank:	17210.0

,

NP			
Pos.	67	obs:	exp:
tta	L	3	0.77
ttg	L	3	1.32
ctt	L	0	1.38
ctc	L	0	0.99
cta	L	1	1.35
ctg	L	0	1.20
att	I	27	8484.00
atc	I	4	5818.00
ata	I	23276	9005.00
atg	M	69	69.00
act	T	0	0.76
acc	T	0	0.62
aca	T	3	1.37
acg	T	0	0.26
gtt	V	0	1.77
gtc	V	0	1.45
gta	V	8	2.15
gtg	V	0	2.63
---	--	------	------
mPD		0.010	0.66
		nPD:	0.02
		N. weight:	0.97
		Sc. PD:	-0.023
		Sc. rank:	3377.1

,

NP			
Pos.	68	obs:	exp:
act	T	3	5897.00
acc	T	1	4798.00
aca	T	23369	10702.00
acg	T	20	1996.00
gct	A	0	0.26
gcc	A	0	0.21
gca	A	1	0.47
gcg	A	0	0.06
---	--	------	------
mPD		0.0021	0.68
		nPD:	0.
		N. weight:	0.8
		Sc. PD:	-0.029
		Sc. rank:	-11654.0

,

NP			
Pos.	69	obs:	exp:
tta	L	0	0.33
ttg	L	0	0.56
ctt	L	0	0.59
ctc	L	0	0.42
cta	L	3	0.58
ctg	L	0	0.51
att	I	7	8502.00
atc	I	2	5830.00
ata	I	23346	9023.00
atg	M	24	24.00
gtt	V	0	2.65
gtc	V	0	2.18
gta	V	12	3.23
gtg	V	0	3.94
---	--	------	------
mPD		0.0041	0.66
		nPD:	0.01
		N. weight:	0.98
		Sc. PD:	-0.033
		Sc. rank:	-8697.2

,

NP			
Pos.	70	obs:	exp:
gaa	E	102	14165.00
gag	E	23292	9229.00
---	--	------	------
mPD		0.0087	0.48
		nPD:	0.02
		N. weight:	0.93
		Sc. PD:	-0.02
		Sc. rank:	4998.8

,

NP			
Pos.	71	obs:	exp:
cgt	R	0	638.70
cgc	R	0	1224.00
cga	R	0	2291.00
cgg	R	1	1712.00
aaa	K	3	1.71
aag	K	0	1.29
aga	R	1149	11743.00
agg	R	22241	5783.00
---	--	------	------
mPD		0.094	0.91
		nPD:	0.1
		N. weight:	1.2
		Sc. PD:	0.073
		Sc. rank:	19010.0

NP			
Pos.	72	obs:	exp:
atg	M	23394	23394.00
---	--	------	------
mPD		0	0
		nPD:	1.
		N. weight:	0.
		Sc. PD:	0
		Sc. rank:	0

NP			
Pos.	73	obs:	exp:
tta	L	0	0.55
ttg	L	3	0.94
ctt	L	0	0.99
ctc	L	0	0.71
cta	L	0	0.96
ctg	L	2	0.85
gtt	V	53	5169.00
gtc	V	2	4248.00
gta	V	127	6288.00
gtg	V	23198	7675.00
gct	A	0	2.12
gcc	A	0	1.66
gca	A	1	3.76
gcg	A	7	0.47
ggt	G	0	0.15
ggc	G	0	0.15
gga	G	0	0.44
ggg	G	1	0.26
---	--	------	------
mPD		0.017	0.74
		nPD:	0.02
		N. weight:	1.1
		Sc. PD:	-0.019
		Sc. rank:	8633.8

NP			
Pos.	74	obs:	exp:
tta	L	0	2572.00
ttg	L	0	4404.00
ctt	L	23340	4610.00
ctc	L	41	3304.00
cta	L	0	4504.00
ctg	L	12	4000.00
att	I	1	0.36
atc	I	0	0.25
ata	I	0	0.39
---	--	------	------
mPD		0.0046	1.1
		nPD:	0.
		N. weight:	1.7
		Sc. PD:	-0.059
		Sc. rank:	-20008.0

NP			
Pos.	75	obs:	exp:
tct	S	23372	4305.00
tcc	S	13	2709.00
tca	S	6	6088.00
tcg	S	1	1245.00
cct	P	2	0.55
ccc	P	0	0.37
cca	P	0	0.85
ccg	P	0	0.23
agt	S	0	4398.00
agc	S	0	4646.00
---	--	------	------
mPD		0.0019	1.6
		nPD:	0.
		N. weight:	1.7
		Sc. PD:	-0.067
		Sc. rank:	-32515.0

NP			
Pos.	76	obs:	exp:
tct	S	2315	436.90
tcc	S	59	275.00
tca	S	0	617.80
tcg	S	0	126.30
act	T	5	1.26
acc	T	0	1.03
aca	T	0	2.29
acg	T	0	0.43
agt	S	0	446.40
agc	S	0	471.60
gct	A	20990	5564.00
gcc	A	22	4350.00
gca	A	1	9870.00
gcg	A	2	1231.00
---	--	------	------
mPD		0.19	0.94
		nPD:	0.2
		N. weight:	1.4
		Sc. PD:	0.22
		Sc. rank:	24372.0

NP			
Pos.	77	obs:	exp:
ttt	F	23378	10036.00
ttc	F	9	13351.00
tct	S	1	0.18
tcc	S	0	0.12
tca	S	0	0.26
tcg	S	0	0.05
tat	Y	6	3.12
tac	Y	0	2.88
agt	S	0	0.19
agc	S	0	0.20
---	--	------	------
mPD		0.0014	0.49
		nPD:	0.
		N. weight:	0.87
		Sc. PD:	-0.032
		Sc. rank:	-13269.0

NP			
Pos.	78	obs:	exp:
gct	A	7	1.85
gcc	A	0	1.45
gca	A	0	3.29
gcg	A	0	0.41
gat	D	23360	12323.00
gac	D	27	11064.00
---	--	------	------
mPD		0.0029	0.50
		nPD:	0.01
		N. weight:	0.65
		Sc. PD:	-0.022
		Sc. rank:	-5990.1

NP			
Pos.	79	obs:	exp:
gat	D	11	5.80
gac	D	0	5.20
gaa	E	135	14158.00
gag	E	23247	9224.00
ggt	G	0	0.15
ggc	G	0	0.15
gga	G	0	0.44
ggg	G	1	0.26
---	--	------	------
mPD		0.012	0.48
		nPD:	0.03
		N. weight:	0.92
		Sc. PD:	-0.013
		Sc. rank:	8491.0

NP			
Pos.	80	obs:	exp:
cgt	R	0	638.70
cgc	R	0	1224.00
cga	R	4	2291.00
cgg	R	0	1712.00
aaa	K	1	0.57
aag	K	0	0.43
aga	R	23322	11744.00
agg	R	67	5783.00
---	--	------	------
mPD		0.0061	0.91
		nPD:	0.01
		N. weight:	0.69
		Sc. PD:	-0.023
		Sc. rank:	-5469.1

NP			
Pos.	81	obs:	exp:
cgt	R	0	638.80
cgc	R	0	1224.00
cga	R	4	2291.00
cgg	R	0	1712.00
aga	R	23299	11745.00
agg	R	91	5783.00
---	--	------	------
mPD		0.0081	0.91
		nPD:	0.01
		N. weight:	0.69
		Sc. PD:	-0.021
		Sc. rank:	-2513.3

NP			
Pos.	82	obs:	exp:
tct	S	0	0.18
tcc	S	0	0.12
tca	S	0	0.26
tcg	S	0	0.05
aat	N	23281	13835.00
aac	N	111	9557.00
agt	S	1	0.19
agc	S	0	0.20
gat	D	1	0.53
gac	D	0	0.47
---	--	------	------
mPD		0.0096	0.48
		nPD:	0.02
		N. weight:	0.51
		Sc. PD:	-0.01
		Sc. rank:	3306.0

NP			
Pos.	83	obs:	exp:
cgt	R	0	0.85
cgc	R	0	1.62
cga	R	0	3.04
cgg	R	0	2.27
aat	N	0	1.18
aac	N	2	0.82
aaa	K	23324	13326.00
aag	K	37	10035.00
aga	R	31	15.56
agg	R	0	7.66
---	--	------	------
mPD		0.0060	0.49
		nPD:	0.01
		N. weight:	0.57
		Sc. PD:	-0.016
		Sc. rank:	-354.0

NP			
Pos.	84	obs:	exp:
tat	Y	87	12149.00
tac	Y	23296	11234.00
cat	H	1	6.47
cac	H	10	4.53
---	--	------	------
mPD		0.0084	0.50
		nPD:	0.02
		N. weight:	0.73
		Sc. PD:	-0.017
		Sc. rank:	3463.5

NP			
Pos.	85	obs:	exp:
tta	L	73	2572.00
ttg	L	0	4404.00
ctt	L	5	4610.00
ctc	L	0	3304.00
cta	L	23180	4504.00
ctg	L	135	4000.00
caa	Q	1	0.53
cag	Q	0	0.47
---	--	------	------
mPD		0.018	1.1
		nPD:	0.02
		N. weight:	1.6
		Sc. PD:	-0.039
		Sc. rank:	6356.4

NP			
Pos.	86	obs:	exp:
aaa	K	1	0.57
aag	K	0	0.43
gaa	E	23325	14164.00
gag	E	68	9229.00
---	--	------	------
mPD		0.0059	0.48
		nPD:	0.01
		N. weight:	0.5
		Sc. PD:	-0.014
		Sc. rank:	89.6

NP			
Pos.	87	obs:	exp:
gat	D	9	4.74
gac	D	0	4.26
gaa	E	175	14159.00
gag	E	23209	9225.00
ggt	G	0	0.15
ggc	G	0	0.15
gga	G	0	0.44
ggg	G	1	0.26
---	--	------	------
mPD		0.016	0.48
		nPD:	0.03
		N. weight:	0.91
		Sc. PD:	-0.007
		Sc. rank:	9941.2

NP			
Pos.	88	obs:	exp:
cat	H	23256	13758.00
cac	H	138	9636.00
---	--	------	------
mPD		0.012	0.48
		nPD:	0.02
		N. weight:	0.51
		Sc. PD:	-0.0082
		Sc. rank:	4293.3

NP			
Pos.	89	obs:	exp:
tct	S	0	0.37
tcc	S	1	0.23
tca	S	1	0.52
tcg	S	0	0.11
cct	P	8	6444.00
ccc	P	4490	4339.00
cca	P	18548	9960.00
ccg	P	346	2649.00
agt	S	0	0.38
agc	S	0	0.40
---	--	------	------
mPD		0.33	0.70
		nPD:	0.48
		N. weight:	0.48
		Sc. PD:	0.2
		Sc. rank:	9342.5

NP			
Pos.	90	obs:	exp:
tct	S	0	4296.00
tcc	S	0	2704.00
tca	S	0	6076.00
tcg	S	0	1243.00
aat	N	47	27.80
aac	N	0	19.20
agt	S	23292	4390.00
agc	S	55	4638.00
---	--	------	------
mPD		0.0087	1.6
		nPD:	0.01
		N. weight:	1.7
		Sc. PD:	-0.058
		Sc. rank:	-17259.0

NP			
Pos.	91	obs:	exp:
tct	S	2	0.37
tcc	S	0	0.23
tca	S	0	0.52
tcg	S	0	0.11
act	T	65	16.39
acc	T	0	13.33
aca	T	0	29.74
acg	T	0	5.55
agt	S	0	0.38
agc	S	0	0.40
gtt	V	5	1.33
gtc	V	0	1.09
gta	V	0	1.61
gtg	V	1	1.97
gct	A	23272	6174.00
gcc	A	40	4827.00
gca	A	6	10953.00
gcg	A	3	1366.00
---	--	------	------
mPD		0.010	0.67
		nPD:	0.02
		N. weight:	1.4
		Sc. PD:	-0.033
		Sc. rank:	4934.6

,

NP			
Pos.	92	obs:	exp:
gaa	E	0	0.61
gag	E	1	0.39
ggt	G	2	3547.00
ggc	G	0	3465.00
gga	G	203	10406.00
ggg	G	23188	5975.00
---	--	------	------
mPD		0.017	0.69
		nPD:	0.03
		N. weight:	1.4
		Sc. PD:	-0.02
		Sc. rank:	12046.0

,

NP			
Pos.	93	obs:	exp:
cgt	R	0	0.08
cgc	R	0	0.16
cga	R	0	0.29
cgg	R	0	0.22
aaa	K	785	13343.00
aag	K	22606	10048.00
aga	R	0	1.51
agg	R	3	0.74
---	--	------	------
mPD		0.065	0.49
		nPD:	0.13
		N. weight:	0.71
		Sc. PD:	0.063
		Sc. rank:	11899.0

,

NP			
Pos.	94	obs:	exp:
aat	N	0	2.96
aac	N	5	2.04
gat	D	50	12324.00
gac	D	23339	11065.00
---	--	------	------
mPD		0.0047	0.50
		nPD:	0.01
		N. weight:	0.76
		Sc. PD:	-0.023
		Sc. rank:	-2521.9

,

NP			
Pos.	95	obs:	exp:
tct	S	3	0.55
tcc	S	0	0.35
tca	S	0	0.78
tcg	S	0	0.16
cct	P	23260	6444.00
ccc	P	116	4338.00
cca	P	11	9960.00
ccg	P	4	2649.00
agt	S	0	0.56
agc	S	0	0.60
---	--	------	------
mPD		0.011	0.70
		nPD:	0.02
		N. weight:	1.3
		Sc. PD:	-0.03
		Sc. rank:	5671.1

,

NP			
Pos.	96	obs:	exp:
cgt	R	0	0.44
cgc	R	0	0.84
cga	R	0	1.57
cgg	R	0	1.17
aat	N	1	0.59
aac	N	0	0.41
aaa	K	18	13334.00
aag	K	23358	10042.00
aga	R	0	8.03
agg	R	16	3.96
gaa	E	0	0.61
gag	E	1	0.39
---	--	------	------
mPD		0.0031	0.49
		nPD:	0.01
		N. weight:	0.87
		Sc. PD:	-0.029
		Sc. rank:	-7647.3

,

NP			
Pos.	97	obs:	exp:
cgt	R	0	0.08
cgc	R	0	0.16
cga	R	0	0.29
cgg	R	0	0.22
aaa	K	23353	13343.00
aag	K	38	10048.00
aga	R	3	1.51
agg	R	0	0.74
---	--	------	------
mPD		0.0035	0.49
		nPD:	0.01
		N. weight:	0.57
		Sc. PD:	-0.018
		Sc. rank:	-3991.0

,

NP			
Pos.	98	obs:	exp:
act	T	10	5896.00
acc	T	0	4797.00
aca	T	23368	10699.00
acg	T	9	1995.00
gct	A	0	1.85
gcc	A	0	1.45
gca	A	7	3.29
gcg	A	0	0.41
---	--	------	------
mPD		0.0022	0.68
		nPD:	0.
		N. weight:	0.8
		Sc. PD:	-0.029
		Sc. rank:	-11584.0

,

NP			
Pos.	99	obs:	exp:
ggt	G	9	3547.00
ggc	G	0	3465.00
gga	G	23271	10406.00
ggg	G	114	5976.00
---	--	------	------
mPD		0.010	0.69
		nPD:	0.02
		N. weight:	0.81
		Sc. PD:	-0.02
		Sc. rank:	2498.7

,

NP			
Pos.	100	obs:	exp:
ggt	G	0	3547.00
ggc	G	2	3465.00
gga	G	23312	10406.00
ggg	G	80	5976.00
---	--	------	------
mPD		0.0070	0.69
		nPD:	0.01
		N. weight:	0.82
		Sc. PD:	-0.024
		Sc. rank:	-1743.3

,

NP			
Pos.	101	obs:	exp:
tct	S	0	0.37
tcc	S	2	0.23
tca	S	0	0.52
tcg	S	0	0.11
cct	P	119	6444.00
ccc	P	23258	4339.00
cca	P	15	9960.00
ccg	P	0	2649.00
agt	S	0	0.38
agc	S	0	0.40
---	--	------	------
mPD		0.012	0.70
		nPD:	0.02
		N. weight:	1.7
		Sc. PD:	-0.04
		Sc. rank:	7851.1

,

NP			
Pos.	102	obs:	exp:
tta	L	0	0.11
ttg	L	0	0.19
ctt	L	0	0.20
ctc	L	1	0.14
cta	L	0	0.19
ctg	L	0	0.17
att	I	15	8507.00
atc	I	20152	5833.00
ata	I	3202	9029.00
gtt	V	0	5.31
gtc	V	24	4.36
gta	V	0	6.45
gtg	V	0	7.88
---	--	------	------
mPD		0.24	0.66
		nPD:	0.36
		N. weight:	0.96
		Sc. PD:	0.3
		Sc. rank:	17802.0

,

NP			
Pos.	103	obs:	exp:
tat	Y	23269	12155.00
tac	Y	125	11239.00
---	--	------	------
mPD		0.011	0.50
		nPD:	0.02
		N. weight:	0.64
		Sc. PD:	-0.012
		Sc. rank:	4547.7

,

NP			
Pos.	104	obs:	exp:
caa	Q	1	0.53
cag	Q	0	0.47
cgt	R	0	637.80
cgc	R	0	1222.00
cga	R	679	2288.00
cgg	R	3	1710.00
aaa	K	34	19.39
aag	K	0	14.61
aga	R	22579	11727.00
agg	R	98	5775.00
---	--	------	------
mPD		0.068	0.91
		nPD:	0.07
		N. weight:	0.6
		Sc. PD:	0.02
		Sc. rank:	8804.6

,

NP

Pos.	105	obs:	exp:
cgt	R	0	638.50
cgc	R	0	1223.00
cga	R	3	2291.00
cgg	R	0	1711.00
aaa	K	9	5.13
aag	K	0	3.87
aga	R	23234	11740.00
agg	R	148	5781.00
---	--	------	------
mPD		0.014	0.91
		nPD:	0.01
		N. weight:	0.68
		Sc. PD:	-0.017
		Sc. rank:	1934.5

,

NP

Pos.	106	obs:	exp:
tta	L	2	0.44
ttg	L	0	0.75
ctt	L	0	0.79
ctc	L	0	0.56
cta	L	2	0.77
ctg	L	0	0.68
cgt	R	0	0.03
cgc	R	0	0.05
cga	R	0	0.10
cgg	R	0	0.07
att	I	34	8320.00
atc	I	0	5705.00
ata	I	22822	8830.00
atg	M	34	34.00
act	T	0	2.77
acc	T	0	2.26
aca	T	11	5.03
acg	T	0	0.94
aga	R	1	0.50
agg	R	0	0.25
gtt	V	0	107.90
gtc	V	1	88.67
gta	V	487	131.20
gtg	V	0	160.20
---	--	------	------
mPD		0.048	0.70
		nPD:	0.07
		N. weight:	0.98
		Sc. PD:	0.026
		Sc. rank:	13979.0

,

NP

Pos.	107	obs:	exp:
aat	N	2	7.69
aac	N	11	5.31
gat	D	674	12303.00
gac	D	22675	11046.00
gaa	E	24	14.53
gag	E	0	9.47
ggt	G	2	1.21
ggc	G	6	1.18
gga	G	0	3.56
ggg	G	0	2.04
---	--	------	------
mPD		0.060	0.50
		nPD:	0.12
		N. weight:	0.64
		Sc. PD:	0.048
		Sc. rank:	10283.0

,

NP

Pos.	108	obs:	exp:
cgt	R	0	0.41
cgc	R	0	0.78
cga	R	0	1.47
cgg	R	0	1.10
aga	R	15	7.53
agg	R	0	3.71
ggt	G	6	3545.00
ggc	G	0	3463.00
gga	G	23261	10399.00
ggg	G	112	5972.00
---	--	------	------
mPD		0.011	0.69
		nPD:	0.02
		N. weight:	0.81
		Sc. PD:	-0.019
		Sc. rank:	3446.9

,

NP

Pos.	109	obs:	exp:
caa	Q	0	0.53
cag	Q	1	0.47
cgt	R	0	0.30
cgc	R	0	0.58
cga	R	0	1.08
cgg	R	0	0.81
act	T	0	0.25
acc	T	0	0.21
aca	T	1	0.46
acg	T	0	0.09
aaa	K	20218	13337.00
aag	K	3163	10044.00
aga	R	10	5.52
agg	R	1	2.72
---	--	------	------
mPD		0.24	0.49
		nPD:	0.48
		N. weight:	0.21
		Sc. PD:	0.089
		Sc. rank:	4063.6

,

NP

Pos.	110	obs:	exp:
tgt	C	1	0.45
tgc	C	0	0.55
tgg	W	23393	23393.00
---	--	------	------
mPD		0.000085	0.000085
		nPD:	1.
		N. weight:	0.000035
		Sc. PD:	0.000032
		Sc. rank:	0.7

,

NP

Pos.	111	obs:	exp:
tta	L	0	0.11
ttg	L	0	0.19
tct	S	0	0.18
tcc	S	0	0.12
tca	S	1	0.26
tcg	S	0	0.05
ctt	L	0	0.20
ctc	L	0	0.14
cta	L	0	0.19
ctg	L	1	0.17
att	I	0	0.36
atc	I	0	0.25
ata	I	1	0.39
atg	M	4004	4004.00
act	T	7	4877.00
acc	T	2	3969.00
aca	T	8032	8851.00
acg	T	11307	1651.00
agt	S	0	0.19
agc	S	0	0.20
gtt	V	0	0.88
gtc	V	0	0.73
gta	V	0	1.08
gtg	V	4	1.31
gct	A	0	9.27
gcc	A	0	7.24
gca	A	25	16.44
gcg	A	10	2.05
---	--	------	------
mPD		0.74	1.0
		nPD:	0.73
		N. weight:	0.93
		Sc. PD:	0.62
		Sc. rank:	18097.0

,

NP

Pos.	112	obs:	exp:
cgt	R	0	638.70
cgc	R	0	1224.00
cga	R	0	2291.00
cgg	R	0	1712.00
aaa	K	2	1.14
aag	K	0	0.86
aga	R	23375	11744.00
agg	R	17	5783.00
---	--	------	------
mPD		0.0016	0.91
		nPD:	0.
		N. weight:	0.71
		Sc. PD:	-0.027
		Sc. rank:	-12266.0

,

NP

Pos.	113	obs:	exp:
gat	D	0	0.53
gac	D	1	0.47
gaa	E	23369	14164.00
gag	E	23	9228.00
ggt	G	0	0.15
ggc	G	0	0.15
gga	G	1	0.44
ggg	G	0	0.26
---	--	------	------
mPD		0.0021	0.48
		nPD:	0.
		N. weight:	0.51
		Sc. PD:	-0.018
		Sc. rank:	-5824.3

,

NP

Pos.	114	obs:	exp:
ttt	F	0	0.43
ttc	F	1	0.57
tta	L	0	2570.00
ttg	L	0	4402.00
ctt	L	64	4607.00
ctc	L	23305	3303.00
cta	L	12	4501.00
ctg	L	0	3998.00
cct	P	0	0.28
ccc	P	1	0.19
cca	P	0	0.43
ccg	P	0	0.11
att	I	0	4.00
atc	I	11	2.75
ata	I	0	4.25
---	--	------	------
mPD		0.0076	1.1
		nPD:	0.01
		N. weight:	2.
		Sc. PD:	-0.066
		Sc. rank:	-16079.0

,

NP

Pos.	115	obs:	exp:
ttt	F	0	0.86
ttc	F	2	1.14
att	I	63	8507.00
atc	I	23300	5833.00
ata	I	5	9028.00
act	T	1	4.03
acc	T	15	3.28
aca	T	0	7.32
acg	T	0	1.37
aat	N	0	0.59
aac	N	1	0.41
gtt	V	0	1.55
gtc	V	7	1.27
gta	V	0	1.88
gtg	V	0	2.30
---	--	------	------
mPD		0.0081	0.66
		nPD:	0.01
		N. weight:	1.4
		Sc. PD:	-0.039
		Sc. rank:	408.1

,

NP

Pos.	116	obs:	exp:
tta	L	0	2572.00
ttg	L	0	4404.00
ctt	L	23375	4610.00
ctc	L	7	3304.00
cta	L	7	4504.00
ctg	L	4	4000.00
att	I	1	0.36
atc	I	0	0.25
ata	I	0	0.39
---	--	------	------
mPD		0.0016	1.1
		nPD:	0.
		N. weight:	1.7
		Sc. PD:	-0.063
		Sc. rank:	-30518.0

,

NP

Pos.	117	obs:	exp:
tat	Y	23347	12150.00
tac	Y	37	11234.00
tgt	C	4	1.81
tgc	C	0	2.19
cat	H	6	3.53
cac	H	0	2.47
---	--	------	------
mPD		0.0040	0.50
		nPD:	0.01
		N. weight:	0.66
		Sc. PD:	-0.021
		Sc. rank:	-3516.2

,

NP

Pos.	118	obs:	exp:
aat	N	0	0.59
aac	N	1	0.41
gat	D	50	12325.00
gac	D	23341	11066.00
gaa	E	2	1.21
gag	E	0	0.79
---	--	------	------
mPD		0.0045	0.50
		nPD:	0.01
		N. weight:	0.76
		Sc. PD:	-0.023
		Sc. rank:	-2695.4

,

NP

Pos.	119	obs:	exp:
aaa	K	23376	13345.00
aag	K	18	10049.00
---	--	------	------
mPD		0.0015	0.49
		nPD:	0.
		N. weight:	0.57
		Sc. PD:	-0.021
		Sc. rank:	-8498.6

,

NP

Pos.	120	obs:	exp:
gat	D	4	2.11
gac	D	0	1.89
gaa	E	23335	14163.00
gag	E	55	9227.00
---	--	------	------
mPD		0.0050	0.48
		nPD:	0.01
		N. weight:	0.5
		Sc. PD:	-0.015
		Sc. rank:	-823.7

,

NP

Pos.	121	obs:	exp:
gaa	E	23353	14165.00
gag	E	41	9229.00
---	--	------	------
mPD		0.0035	0.48
		nPD:	0.01
		N. weight:	0.51
		Sc. PD:	-0.016
		Sc. rank:	-3343.2

,

NP

Pos.	122	obs:	exp:
tta	L	0	0.11
ttg	L	0	0.19
ctt	L	0	0.20
ctc	L	0	0.14
cta	L	1	0.19
ctg	L	0	0.17
att	I	3	8513.00
atc	I	16	5838.00
ata	I	23367	9035.00
act	T	0	0.50
acc	T	0	0.41
aca	T	2	0.91
acg	T	0	0.17
gtt	V	0	1.11
gtc	V	0	0.91
gta	V	5	1.34
gtg	V	0	1.64
---	--	------	------
mPD		0.0023	0.66
		nPD:	0.
		N. weight:	0.98
		Sc. PD:	-0.035
		Sc. rank:	-12501.0

,

NP

Pos.	123	obs:	exp:
cgt	R	0	637.80
cgc	R	0	1222.00
cga	R	0	2288.00
cgg	R	1	1710.00
aaa	K	1	18.82
aag	K	32	14.18
aga	R	1439	11728.00
agg	R	21920	5775.00
ggt	G	0	0.15
ggc	G	0	0.15
gga	G	0	0.44
ggg	G	1	0.26
---	--	------	------
mPD		0.12	0.91
		nPD:	0.13
		N. weight:	1.2
		Sc. PD:	0.1
		Sc. rank:	19315.0

,

NP

Pos.	124	obs:	exp:
cgt	R	0	638.60
cgc	R	0	1223.00
cga	R	4	2291.00
cgg	R	0	1712.00
aaa	K	5	2.85
aag	K	0	2.15
aga	R	23357	11742.00
agg	R	28	5782.00
---	--	------	------
mPD		0.0032	0.91
		nPD:	0.
		N. weight:	0.7
		Sc. PD:	-0.025
		Sc. rank:	-9380.3

,

NP

Pos.	125	obs:	exp:
tta	L	0	0.33
ttg	L	0	0.56
ctt	L	3	0.59
ctc	L	0	0.42
cta	L	0	0.58
ctg	L	0	0.51
att	I	86	32.40
atc	I	3	22.22
ata	I	0	34.39
gtt	V	23181	5152.00
gtc	V	114	4234.00
gta	V	2	6267.00
gtg	V	5	7649.00
---	--	------	------
mPD		0.018	0.75
		nPD:	0.02
		N. weight:	1.5
		Sc. PD:	-0.024
		Sc. rank:	13251.0

,

NP

Pos.	126	obs:	exp:
tgg	W	23394	23394.00
---	--	------	------
mPD		0	0
		nPD:	1.
		N. weight:	0.
		Sc. PD:	0
		Sc. rank:	0

,

NP

Pos.	127	obs:	exp:
cat	H	0	0.59
cac	H	1	0.41
cgt	R	129	638.70
cgc	R	23263	1224.00
cga	R	1	2291.00
cgg	R	0	1712.00
aga	R	0	11744.00
agg	R	0	5783.00
---	--	------	------
mPD		0.011	0.91
		nPD:	0.01
		N. weight:	3.
		Sc. PD:	-0.083
		Sc. rank:	-874.6

,

NP

Pos.	128	obs:	exp:
caa	Q	23361	12344.00
cag	Q	33	11050.00
---	--	------	------
mPD		0.0028	0.50
		nPD:	0.01
		N. weight:	0.65
		Sc. PD:	-0.022
		Sc. rank:	-6069.2

,

NP

Pos.	129	obs:	exp:
tct	S	0	0.55
tcc	S	0	0.35
tca	S	3	0.78
tcg	S	0	0.16
agt	S	0	0.56
agc	S	0	0.60
gtt	V	0	0.22
gtc	V	0	0.18
gta	V	1	0.27
gtg	V	0	0.33
gct	A	4	6193.00
gcc	A	4	4842.00
gca	A	23375	10985.00
gcg	A	7	1370.00
---	--	------	------
mPD		0.0016	0.66
		nPD:	0.
		N. weight:	0.78
		Sc. PD:	-0.029
		Sc. rank:	-12560.0

,

NP

Pos.	130	obs:	exp:
aat	N	263	13836.00
aac	N	23131	9558.00
---	--	------	------
mPD		0.022	0.48
		nPD:	0.05
		N. weight:	0.86
		Sc. PD:	0.0043
		Sc. rank:	10824.0

,

NP

Pos.	131	obs:	exp:
tct	S	0	2.02
tcc	S	0	1.27
tca	S	0	2.86
tcg	S	0	0.59
aat	N	23347	13828.00
aac	N	33	9552.00
agt	S	11	2.07
agc	S	0	2.18
gat	D	3	1.58
gac	D	0	1.42
---	--	------	------
mPD		0.0040	0.49
		nPD:	0.01
		N. weight:	0.53
		Sc. PD:	-0.017
		Sc. rank:	-2603.6

,

NP

Pos.	132	obs:	exp:
tct	S	0	0.18
tcc	S	0	0.12
tca	S	0	0.26
tcg	S	0	0.05
agt	S	0	0.19
agc	S	1	0.20
ggt	G	97	3547.00
ggc	G	23240	3465.00
gga	G	55	10406.00
ggg	G	1	5975.00
---	--	------	------
mPD		0.013	0.69
		nPD:	0.02
		N. weight:	1.9
		Sc. PD:	-0.041
		Sc. rank:	11330.0

,

NP			
Pos.	133	obs:	exp:
act	T	0	0.25
acc	T	0	0.21
aca	T	1	0.46
acg	T	0	0.09
aaa	K	2	1.14
aag	K	0	0.86
gct	A	0	0.26
gcc	A	0	0.21
gca	A	1	0.47
gcg	A	0	0.06
gat	D	0	0.53
gac	D	1	0.47
gaa	E	23320	14159.00
gag	E	64	9225.00
ggt	G	0	0.76
ggc	G	0	0.74
gga	G	5	2.22
ggg	G	0	1.28
---	--	------	------
mPD		0.0064	0.48
		nPD :	0.01
		N. weight :	0.5
		Sc. PD:	-0.013
		Sc. rank :	429.6

,

NP			
Pos.	134	obs:	exp:
tat	Y	4	2.08
tac	Y	0	1.92
aat	N	11	6.51
aac	N	0	4.49
gat	D	23310	12287.00
gac	D	9	11032.00
gaa	E	40	32.09
gag	E	13	20.91
ggt	G	7	1.06
ggc	G	0	1.04
gga	G	0	3.11
ggg	G	0	1.79
---	--	------	------
mPD		0.0072	0.50
		nPD :	0.01
		N. weight :	0.66
		Sc. PD:	-0.017
		Sc. rank :	1284.0

,

NP			
Pos.	135	obs:	exp:
tct	S	0	27.60
tcc	S	0	17.37
tca	S	150	39.04
tcg	S	0	7.98
act	T	0	4.54
acc	T	0	3.69
aca	T	18	8.23
acg	T	0	1.54
agt	S	0	28.21
agc	S	0	29.80
gtt	V	0	1.11
gtc	V	0	0.91
gta	V	5	1.34
gtg	V	0	1.64
gct	A	6	6148.00
gcc	A	1	4806.00
gca	A	23207	10906.00
gcg	A	6	1360.00
ggt	G	0	0.15
ggc	G	0	0.15
gga	G	1	0.44
ggg	G	0	0.26
---	--	------	------
mPD		0.016	0.68
		nPD :	0.02
		N. weight :	0.78
		Sc. PD:	-0.013
		Sc. rank :	6200.1

,

NP			
Pos.	136	obs:	exp:
att	I	0	0.73
atc	I	0	0.50
ata	I	2	0.77
atg	M	1	1.00
act	T	15	5893.00
acc	T	5	4795.00
aca	T	23340	10695.00
acg	T	18	1995.00
aaa	K	3	1.71
aag	K	0	1.29
gct	A	0	2.65
gcc	A	0	2.07
gca	A	10	4.70
gcg	A	0	0.59
---	--	------	------
mPD		0.0047	0.68
		nPD :	0.01
		N. weight :	0.8
		Sc. PD:	-0.026
		Sc. rank :	-5748.5

,

NP			
Pos.	137	obs:	exp:
tct	S	3	1.10
tcc	S	0	0.69
tca	S	3	1.56
tcg	S	0	0.32
cct	P	0	0.55
ccc	P	0	0.37
cca	P	2	0.85
ccg	P	0	0.23
act	T	0	0.25
acc	T	0	0.21
aca	T	1	0.46
acg	T	0	0.09
agt	S	0	1.13
agc	S	0	1.19
gtt	V	0	0.22
gtc	V	0	0.18
gta	V	1	0.27
gtg	V	0	0.33
gct	A	18	6191.00
gcc	A	0	4840.00
gca	A	23353	10982.00
gcg	A	12	1370.00
ggt	G	0	0.15
ggc	G	0	0.15
gga	G	1	0.44
ggg	G	0	0.26
---	--	------	------
mPD		0.0038	0.66
		nPD :	0.01
		N. weight :	0.77
		Sc. PD:	-0.026
		Sc. rank :	-7161.5

,

NP			
Pos.	138	obs:	exp:
ggt	G	22337	3547.00
ggc	G	1055	3465.00
gga	G	0	10406.00
ggg	G	2	5976.00
---	--	------	------
mPD		0.086	0.69
		nPD :	0.12
		N. weight :	1.8
		Sc. PD:	0.14
		Sc. rank :	28874.0

,

NP			
Pos.	139	obs:	exp:
tta	L	0	2570.00
ttg	L	0	4402.00
ctt	L	23305	4607.00
ctc	L	67	3303.00
cta	L	7	4501.00
ctg	L	2	3998.00
att	I	13	4.73
atc	I	0	3.25
ata	I	0	5.02
---	--	------	------
mPD		0.0076	1.1
		nPD :	0.01
		N. weight :	1.7
		Sc. PD:	-0.054
		Sc. rank :	-13583.0

,

NP			
Pos.	140	obs:	exp:
tct	S	1	0.37
tcc	S	0	0.23
tca	S	0	0.52
tcg	S	0	0.11
act	T	23170	5895.00
acc	T	202	4797.00
aca	T	12	10698.00
acg	T	1	1995.00
agt	S	1	0.38
agc	S	0	0.40
gct	A	7	1.85
gcc	A	0	1.45
gca	A	0	3.29
gcg	A	0	0.41
---	--	------	------
mPD		0.019	0.68
		nPD :	0.03
		N. weight :	1.4
		Sc. PD:	-0.017
		Sc. rank :	13268.0

,

NP			
Pos.	141	obs:	exp:
cat	H	23124	13758.00
cac	H	270	9636.00
---	--	------	------
mPD		0.023	0.48
		nPD :	0.05
		N. weight :	0.49
		Sc. PD:	0.003
		Sc. rank :	6179.7

,

NP			
Pos.	142	obs:	exp:
tta	L	0	14.84
ttg	L	0	25.42
ctt	L	0	26.60
ctc	L	135	19.07
cta	L	0	25.99
ctg	L	0	23.08
att	I	20	8466.00
atc	I	23167	5805.00
ata	I	70	8985.00
gtt	V	0	0.44
gtc	V	2	0.36
gta	V	0	0.54
gtg	V	0	0.66
---	--	------	------
mPD		0.019	0.67
		nPD :	0.03
		N. weight :	1.4
		Sc. PD:	-0.016
		Sc. rank :	14048.0

,

NP			
Pos.	143	obs:	exp:
tta	L	0	0.11
ttg	L	1	0.19
ctt	L	0	0.20
ctc	L	0	0.14
cta	L	0	0.19
ctg	L	0	0.17
atg	M	23391	23391.00
gtt	V	0	0.44
gtc	V	0	0.36
gta	V	0	0.54
gtg	V	2	0.66
---	--	------	------
mPD		0.00026	0.00043
		nPD :	0.6
		N. weight :	0.00017
		Sc. PD:	0.000093
		Sc. rank :	3.3

,

NP			
Pos.	144	obs:	exp:
tta	L	0	0.11
ttg	L	0	0.19
ctt	L	1	0.20
ctc	L	0	0.14
cta	L	0	0.19
ctg	L	0	0.17
att	I	23317	8515.00
atc	I	71	5839.00
ata	I	4	9038.00
gtt	V	1	0.22
gtc	V	0	0.18
gta	V	0	0.27
gtg	V	0	0.33
---	--	------	------
mPD		0.0066	0.66
		nPD :	0.01
		N. weight :	1.
		Sc. PD:	-0.03
		Sc. rank :	-2252.5

,

NP

Pos.	145	obs:	exp:
tgg	W	23394	23394.00
---	--	------	------
mPD		0	0
		nPD:	1.
		N. weight:	0.
		Sc. PD:	0
		Sc. rank:	0

,

NP

Pos.	146	obs:	exp:
cat	H	23295	13757.00
cac	H	98	9636.00
caa	Q	1	0.53
cag	Q	0	0.47
---	--	------	------
mPD		0.0084	0.48
		nPD:	0.02
		N. weight:	0.52
		Sc. PD:	-0.012
		Sc. rank:	2618.4

,

NP

Pos.	147	obs:	exp:
tct	S	48	4305.00
tcc	S	23327	2710.00
tca	S	19	6088.00
tcg	S	0	1245.00
agt	S	0	4399.00
agc	S	0	4647.00
---	--	------	------
mPD		0.0057	1.6
		nPD:	0.
		N. weight:	2.2
		Sc. PD:	-0.079
		Sc. rank:	-28660.0

,

NP

Pos.	148	obs:	exp:
aat	N	14781	13836.00
aac	N	8613	9558.00
---	--	------	------
mPD		0.47	0.48
		nPD:	0.96
		N. weight:	0.0038
		Sc. PD:	0.0033
		Sc. rank:	73.5

,

NP

Pos.	149	obs:	exp:
tta	L	0	2572.00
ttg	L	75	4404.00
ctt	L	23	4610.00
ctc	L	0	3304.00
cta	L	89	4503.00
ctg	L	23205	3999.00
caa	Q	0	0.53
cag	Q	1	0.47
atg	M	1	1.00
---	--	------	------
mPD		0.016	1.1
		nPD:	0.01
		N. weight:	1.8
		Sc. PD:	-0.045
		Sc. rank:	3361.4

,

NP

Pos.	150	obs:	exp:
act	T	1	0.25
acc	T	0	0.21
aca	T	0	0.46
acg	T	0	0.09
aat	N	23369	13836.00
aac	N	24	9557.00
---	--	------	------
mPD		0.0021	0.48
		nPD:	0.
		N. weight:	0.54
		Sc. PD:	-0.019
		Sc. rank:	-6149.6

,

NP

Pos.	151	obs:	exp:
gat	D	23313	12326.00
gac	D	80	11067.00
ggt	G	1	0.15
ggc	G	0	0.15
gga	G	0	0.44
ggg	G	0	0.26
---	--	------	------
mPD		0.0069	0.50
		nPD:	0.01
		N. weight:	0.64
		Sc. PD:	-0.017
		Sc. rank:	838.3

,

NP

Pos.	152	obs:	exp:
tct	S	0	0.55
tcc	S	3	0.35
tca	S	0	0.78
tcg	S	0	0.16
act	T	0	2.77
acc	T	11	2.26
aca	T	0	5.03
acg	T	0	0.94
agt	S	0	0.56
agc	S	0	0.60
gct	A	32	6190.00
gcc	A	23318	4840.00
gca	A	29	10981.00
gcg	A	1	1370.00
---	--	------	------
mPD		0.0065	0.66
		nPD:	0.01
		N. weight:	1.6
		Sc. PD:	-0.048
		Sc. rank:	-4207.5

,

NP

Pos.	153	obs:	exp:
act	T	4	5897.00
acc	T	3	4798.00
aca	T	23279	10702.00
acg	T	108	1996.00
---	--	------	------
mPD		0.0098	0.68
		nPD:	0.01
		N. weight:	0.78
		Sc. PD:	-0.02
		Sc. rank:	1912.6

,

NP

Pos.	154	obs:	exp:
tat	Y	22627	12155.00
tac	Y	767	11239.00
---	--	------	------
mPD		0.063	0.50
		nPD:	0.13
		N. weight:	0.53
		Sc. PD:	0.044
		Sc. rank:	8771.2

,

NP

Pos.	155	obs:	exp:
cat	H	0	1.76
cac	H	3	1.24
caa	Q	111	12343.00
cag	Q	23280	11048.00
---	--	------	------
mPD		0.0097	0.50
		nPD:	0.02
		N. weight:	0.74
		Sc. PD:	-0.015
		Sc. rank:	4587.6

,

NP

Pos.	156	obs:	exp:
cgt	R	0	638.80
cgc	R	0	1224.00
cga	R	0	2291.00
cgg	R	0	1712.00
aga	R	3411	11745.00
agg	R	19983	5783.00
---	--	------	------
mPD		0.25	0.91
		nPD:	0.27
		N. weight:	0.91
		Sc. PD:	0.2
		Sc. rank:	16338.0

,

NP

Pos.	157	obs:	exp:
act	T	2	5897.00
acc	T	13	4798.00
aca	T	23352	10702.00
acg	T	26	1996.00
gct	A	0	0.26
gcc	A	0	0.21
gca	A	1	0.47
gcg	A	0	0.06
---	--	------	------
mPD		0.0036	0.68
		nPD:	0.01
		N. weight:	0.8
		Sc. PD:	-0.027
		Sc. rank:	-8152.7

,

NP

Pos.	158	obs:	exp:
cgt	R	0	638.80
cgc	R	0	1224.00
cga	R	2	2291.00
cgg	R	0	1712.00
aga	R	23357	11745.00
agg	R	35	5783.00
---	--	------	------
mPD		0.0032	0.91
		nPD:	0.
		N. weight:	0.7
		Sc. PD:	-0.025
		Sc. rank:	-9403.0

,

NP

Pos.	159	obs:	exp:
gtt	V	0	0.22
gtc	V	0	0.18
gta	V	0	0.27
gtg	V	1	0.33
gct	A	92	6193.00
gcc	A	3	4842.00
gca	A	20227	10987.00
gcg	A	3071	1370.00
---	--	------	------
mPD		0.24	0.66
		nPD:	0.35
		N. weight:	0.64
		Sc. PD:	0.19
		Sc. rank:	11830.0

,

NP

Pos.	160	obs:	exp:
tta	L	0	2570.00
ttg	L	0	4402.00
ctt	L	23352	4607.00
ctc	L	21	3302.00
cta	L	6	4501.00
ctg	L	1	3997.00
att	I	14	5.10
atc	I	0	3.49
ata	I	0	5.41
---	--	------	------
mPD		0.0036	1.1
		nPD:	0.
		N. weight:	1.7
		Sc. PD:	-0.061
		Sc. rank:	-24374.0

,

NP

Pos.	161	obs:	exp:
att	I	2	0.73
atc	I	0	0.50
ata	I	0	0.77
gtt	V	23322	5172.00
gtc	V	49	4251.00
gta	V	20	6291.00
gtg	V	1	7679.00
---	--	------	------
mPD		0.0061	0.74
		nPD:	0.01
		N. weight:	1.5
		Sc. PD:	-0.048
		Sc. rank:	-7189.6

,

NP

Pos.	162	obs:	exp:
cgt	R	51	638.80
cgc	R	23306	1224.00
cga	R	34	2291.00
cgg	R	2	1712.00
aga	R	0	11745.00
agg	R	1	5783.00
---	--	------	------
mPD		0.0076	0.91
		nPD:	0.01
		N. weight:	3.
		Sc. PD:	-0.094
		Sc. rank:	-13424.0

,

NP

Pos.	163	obs:	exp:
tct	S	1	0.55
tcc	S	1	0.35
tca	S	0	0.78
tcg	S	0	0.16
act	T	19507	5896.00
acc	T	3867	4798.00
aca	T	14	10700.00
acg	T	2	1996.00
agt	S	1	0.56
agc	S	0	0.60
gct	A	1	0.26
gcc	A	0	0.21
gca	A	0	0.47
gcg	A	0	0.06
---	--	------	------
mPD		0.28	0.68
		nPD:	0.41
		N. weight:	0.99
		Sc. PD:	0.35
		Sc. rank:	18802.0

,

NP

Pos.	164	obs:	exp:
ggt	G	2	3547.00
ggc	G	0	3465.00
gga	G	23187	10406.00
ggg	G	205	5976.00
---	--	------	------
mPD		0.018	0.69
		nPD:	0.03
		N. weight:	0.79
		Sc. PD:	-0.012
		Sc. rank:	7077.8

,

NP

Pos.	165	obs:	exp:
tta	L	0	0.77
ttg	L	1	1.32
ctt	L	0	1.38
ctc	L	0	0.99
cta	L	0	1.35
ctg	L	6	1.20
atg	M	23387	23387.00
---	--	------	------
mPD		0.00060	0.00098
		nPD:	0.61
		N. weight:	0.00046
		Sc. PD:	0.00025
		Sc. rank:	8.9

NP

Pos.	166	obs:	exp:
gat	D	23306	12326.00
gac	D	88	11068.00
---	--	------	------
mPD		0.0075	0.50
		nPD:	0.02
		N. weight:	0.64
		Sc. PD:	-0.016
		Sc. rank:	1854.3

NP

Pos.	167	obs:	exp:
cct	P	69	6445.00
ccc	P	23310	4339.00
cca	P	15	9961.00
ccg	P	0	2649.00
---	--	------	------
mPD		0.0072	0.70
		nPD:	0.01
		N. weight:	1.7
		Sc. PD:	-0.05
		Sc. rank:	-3178.3

NP

Pos.	168	obs:	exp:
cgt	R	0	638.80
cgc	R	0	1224.00
cga	R	13	2291.00
cgg	R	1	1712.00
aga	R	23353	11745.00
agg	R	27	5783.00
---	--	------	------
mPD		0.0036	0.91
		nPD:	0.
		N. weight:	0.7
		Sc. PD:	-0.025
		Sc. rank:	-8623.7

NP

Pos.	169	obs:	exp:
att	I	0	1.46
atc	I	0	1.00
ata	I	4	1.55
atg	M	23389	23389.00
act	T	0	0.25
acc	T	0	0.21
aca	T	0	0.46
acg	T	1	0.09
---	--	------	------
mPD		0.00043	0.00051
		nPD:	0.85
		N. weight:	0.00028
		Sc. PD:	0.00021
		Sc. rank:	5.4

NP

Pos.	170	obs:	exp:
tct	S	0	0.18
tcc	S	0	0.12
tca	S	0	0.26
tcg	S	0	0.05
tgt	C	79	10559.00
tgc	C	23314	12834.00
agt	S	0	0.19
agc	S	1	0.20
---	--	------	------
mPD		0.0068	0.50
		nPD:	0.01
		N. weight:	0.6
		Sc. PD:	-0.016
		Sc. rank:	754.5

NP

Pos.	171	obs:	exp:
tct	S	23262	4305.00
tcc	S	128	2710.00
tca	S	4	6088.00
tcg	S	0	1245.00
agt	S	0	4399.00
agc	S	0	4647.00
---	--	------	------
mPD		0.011	1.6
		nPD:	0.01
		N. weight:	1.7
		Sc. PD:	-0.056
		Sc. rank:	-12719.0

NP

Pos.	172	obs:	exp:
tta	L	49	2572.00
ttg	L	1	4404.00
ctt	L	0	4610.00
ctc	L	0	3304.00
cta	L	22899	4504.00
ctg	L	444	4000.00
cct	P	0	0.28
ccc	P	0	0.19
cca	P	1	0.43
ccg	P	0	0.11
---	--	------	------
mPD		0.042	1.1
		nPD:	0.04
		N. weight:	1.6
		Sc. PD:	-0.0065
		Sc. rank:	18742.0

NP

Pos.	173	obs:	exp:
att	I	0	0.73
atc	I	0	0.50
ata	I	2	0.77
atg	M	23392	23392.00
---	--	------	------
mPD		0.00017	0.00017
		nPD:	1.
		N. weight:	0.000084
		Sc. PD:	0.000078
		Sc. rank:	1.6

NP

Pos.	174	obs:	exp:
caa	Q	23317	12344.00
cag	Q	77	11050.00
---	--	------	------
mPD		0.0066	0.50
		nPD:	0.01
		N. weight:	0.64
		Sc. PD:	-0.017
		Sc. rank:	523.2

NP

Pos.	175	obs:	exp:
tct	S	0	0.18
tcc	S	0	0.12
tca	S	0	0.26
tcg	S	0	0.05
tgt	C	1	0.45
tgc	C	0	0.55
agt	S	1	0.19
agc	S	0	0.20
ggt	G	23193	3547.00
ggc	G	177	3465.00
gga	G	0	10405.00
ggg	G	22	5975.00
---	--	------	------
mPD		0.017	0.69
		nPD:	0.02
		N. weight:	1.9
		Sc. PD:	-0.029
		Sc. rank:	16261.0

NP

Pos.	176	obs:	exp:
tct	S	4	4305.00
tcc	S	13	2710.00
tca	S	23364	6088.00
tcg	S	13	1245.00
agt	S	0	4399.00
agc	S	0	4647.00
---	--	------	------
mPD		0.0026	1.6
		nPD:	0.
		N. weight:	1.4
		Sc. PD:	-0.052
		Sc. rank:	-24597.0

NP

Pos.	177	obs:	exp:
act	T	5	5897.00
acc	T	0	4798.00
aca	T	23374	10702.00
acg	T	15	1996.00
---	--	------	------
mPD		0.0017	0.68
		nPD:	0.
		N. weight:	0.8
		Sc. PD:	-0.03
		Sc. rank:	-12851.0

NP

Pos.	178	obs:	exp:
tta	L	0	2572.00
ttg	L	0	4404.00
ctt	L	23342	4610.00
ctc	L	45	3304.00
cta	L	3	4503.00
ctg	L	2	3999.00
att	I	2	0.73
atc	I	0	0.50
ata	I	0	0.77
---	--	------	------
mPD		0.0044	1.1
		nPD:	0.
		N. weight:	1.7
		Sc. PD:	-0.059
		Sc. rank:	-20875.0

NP

Pos.	179	obs:	exp:
cct	P	11	6445.00
ccc	P	23376	4339.00
cca	P	6	9961.00
ccg	P	1	2649.00
---	--	------	------
mPD		0.0015	0.70
		nPD:	0.
		N. weight:	1.7
		Sc. PD:	-0.064
		Sc. rank:	-28363.0

NP

Pos.	180	obs:	exp:
cgt	R	0	638.80
cgc	R	0	1224.00
cga	R	2	2291.00
cgg	R	1	1712.00
aga	R	10764	11745.00
agg	R	12627	5783.00
---	--	------	------
mPD		0.50	0.91
		nPD:	0.55
		N. weight:	0.39
		Sc. PD:	0.19
		Sc. rank:	7647.5

NP

Pos.	181	obs:	exp:
cgt	R	0	637.30
cgc	R	0	1221.00
cga	R	0	2286.00
cgg	R	5	1708.00
aaa	K	0	30.80
aag	K	54	23.20
aga	R	176	11718.00
agg	R	23159	5770.00
---	--	------	------
mPD		0.020	0.91
		nPD:	0.02
		N. weight:	1.4
		Sc. PD:	-0.025
		Sc. rank:	10269.0

,

NP

Pos.	182	obs:	exp:
tct	S	23306	4302.00
tcc	S	20	2708.00
tca	S	51	6084.00
tcg	S	1	1244.00
agt	S	0	4396.00
agc	S	0	4644.00
gct	A	16	4.24
gcc	A	0	3.31
gca	A	0	7.51
gcg	A	0	0.94
---	--	------	------
mPD		0.0075	1.6
		nPD:	0.
		N. weight:	1.7
		Sc. PD:	-0.06
		Sc. rank:	-19434.0

,

NP

Pos.	183	obs:	exp:
ggt	G	23355	3547.00
ggc	G	6	3465.00
gga	G	16	10406.00
ggg	G	17	5976.00
---	--	------	------
mPD		0.0033	0.69
		nPD:	0.
		N. weight:	1.9
		Sc. PD:	-0.067
		Sc. rank:	-20802.0

,

NP

Pos.	184	obs:	exp:
tct	S	0	0.18
tcc	S	1	0.12
tca	S	0	0.26
tcg	S	0	0.05
act	T	0	1.01
acc	T	4	0.82
aca	T	0	1.83
acg	T	0	0.34
agt	S	0	0.19
agc	S	0	0.20
gtt	V	0	0.22
gtc	V	1	0.18
gta	V	0	0.27
gtg	V	0	0.33
gct	A	93	6192.00
gcc	A	23262	4841.00
gca	A	33	10985.00
gcg	A	0	1370.00
---	--	------	------
mPD		0.011	0.66
		nPD:	0.02
		N. weight:	1.6
		Sc. PD:	-0.036
		Sc. rank:	7800.6

,

NP

Pos.	185	obs:	exp:
gct	A	13	6194.00
gcc	A	2	4842.00
gca	A	23370	10987.00
gcg	A	9	1371.00
---	--	------	------
mPD		0.0021	0.66
		nPD:	0.
		N. weight:	0.77
		Sc. PD:	-0.028
		Sc. rank:	-11585.0

,

NP

Pos.	186	obs:	exp:
ggt	G	23379	3547.00
ggc	G	8	3465.00
gga	G	4	10406.00
ggg	G	3	5976.00
---	--	------	------
mPD		0.0013	0.69
		nPD:	0.
		N. weight:	1.9
		Sc. PD:	-0.073
		Sc. rank:	-33439.0

,

NP

Pos.	187	obs:	exp:
tct	S	6	1.10
tcc	S	0	0.69
tca	S	0	1.56
tcg	S	0	0.32
act	T	1	0.25
acc	T	0	0.21
aca	T	0	0.46
acg	T	0	0.09
agt	S	0	1.13
agc	S	0	1.19
gct	A	23318	6191.00
gcc	A	16	4841.00
gca	A	43	10983.00
gcg	A	8	1370.00
ggt	G	2	0.30
ggc	G	0	0.30
gga	G	0	0.89
ggg	G	0	0.51
---	--	------	------
mPD		0.0065	0.66
		nPD:	0.01
		N. weight:	1.4
		Sc. PD:	-0.04
		Sc. rank:	-3397.4

,

NP

Pos.	188	obs:	exp:
tct	S	0	0.37
tcc	S	0	0.23
tca	S	2	0.52
tcg	S	0	0.11
act	T	0	0.25
acc	T	0	0.21
aca	T	1	0.46
acg	T	0	0.09
agt	S	0	0.38
agc	S	0	0.40
gct	A	13	6193.00
gcc	A	4	4842.00
gca	A	19551	10986.00
gcg	A	3823	1370.00
---	--	------	------
mPD		0.27	0.66
		nPD:	0.41
		N. weight:	0.67
		Sc. PD:	0.24
		Sc. rank:	12699.0

,

NP

Pos.	189	obs:	exp:
att	I	0	6.92
atc	I	0	4.74
ata	I	19	7.34
gtt	V	7	5167.00
gtc	V	2	4247.00
gta	V	18835	6285.00
gtg	V	4526	7672.00
gct	A	0	1.32
gcc	A	0	1.03
gca	A	5	2.35
gcg	A	0	0.29
---	--	------	------
mPD		0.31	0.74
		nPD:	0.43
		N. weight:	0.81
		Sc. PD:	0.3
		Sc. rank:	15540.0

,

NP

Pos.	190	obs:	exp:
aaa	K	23354	13345.00
aag	K	40	10049.00
---	--	------	------
mPD		0.0034	0.49
		nPD:	0.01
		N. weight:	0.57
		Sc. PD:	-0.019
		Sc. rank:	-4074.0

,

NP

Pos.	191	obs:	exp:
ggt	G	6	3547.00
ggc	G	0	3465.00
gga	G	23222	10406.00
ggg	G	166	5976.00
---	--	------	------
mPD		0.015	0.69
		nPD:	0.02
		N. weight:	0.8
		Sc. PD:	-0.015
		Sc. rank:	5589.9

,

NP

Pos.	192	obs:	exp:
tta	L	0	0.11
ttg	L	0	0.19
ctt	L	1	0.20
ctc	L	0	0.14
cta	L	0	0.19
ctg	L	0	0.17
att	I	253	92.83
atc	I	2	63.65
ata	I	0	98.52
gtt	V	23025	5115.00
gtc	V	33	4204.00
gta	V	10	6223.00
gtg	V	70	7595.00
---	--	------	------
mPD		0.031	0.76
		nPD:	0.04
		N. weight:	1.5
		Sc. PD:	0.00083
		Sc. rank:	18445.0

,

NP			
Pos.	193	obs:	exp:
ggt	G	3	3547.00
ggc	G	0	3465.00
gga	G	22594	10406.00
ggg	G	797	5976.00
---	--	------	------
mPD		0.066	0.69
		nPD:	0.1
		N. weight:	0.7
		Sc. PD:	0.037
		Sc. rank:	10823.0

,

NP			
Pos.	194	obs:	exp:
act	T	209	5897.00
acc	T	2	4798.00
aca	T	23164	10702.00
acg	T	19	1996.00
---	--	------	------
mPD		0.019	0.68
		nPD:	0.03
		N. weight:	0.76
		Sc. PD:	-0.0088
		Sc. rank:	7450.4

,

NP			
Pos.	195	obs:	exp:
tta	L	2	0.22
ttg	L	0	0.38
ctt	L	0	0.39
ctc	L	0	0.28
cta	L	0	0.39
ctg	L	0	0.34
att	I	0	8495.00
atc	I	0	5825.00
ata	I	23335	9016.00
atg	M	13	13.00
act	T	0	0.76
acc	T	0	0.62
aca	T	3	1.37
acg	T	0	0.26
gtt	V	0	9.06
gtc	V	0	7.45
gta	V	41	11.03
gtg	V	0	13.46
---	--	------	------
mPD		0.0050	0.66
		nPD:	0.01
		N. weight:	0.98
		Sc. PD:	-0.031
		Sc. rank:	-5887.5

,

NP			
Pos.	196	obs:	exp:
tct	S	3	0.55
tcc	S	0	0.35
tca	S	0	0.78
tcg	S	0	0.16
att	I	1	0.36
atc	I	0	0.25
ata	I	0	0.39
act	T	131	33.78
acc	T	0	27.49
aca	T	3	61.30
acg	T	0	11.43
agt	S	0	0.56
agc	S	0	0.60
gtt	V	203	45.76
gtc	V	0	37.61
gta	V	2	55.67
gtg	V	2	67.95
gct	A	19798	6102.00
gcc	A	112	4771.00
gca	A	3137	10825.00
gcg	A	2	1350.00
---	--	------	------
mPD		0.27	0.69
		nPD:	0.39
		N. weight:	0.86
		Sc. PD:	0.29
		Sc. rank:	16259.0

,

NP			
Pos.	197	obs:	exp:
atg	M	23394	23394.00
---	--	------	------
mPD		0	0
		nPD:	1.
		N. weight:	0.
		Sc. PD:	0
		Sc. rank:	0

,

NP			
Pos.	198	obs:	exp:
gaa	E	641	14165.00
gag	E	22753	9229.00
---	--	------	------
mPD		0.053	0.48
		nPD:	0.11
		N. weight:	0.82
		Sc. PD:	0.056
		Sc. rank:	13221.0

,

NP			
Pos.	199	obs:	exp:
tta	L	23103	2572.00
ttg	L	219	4404.00
tct	S	0	0.18
tcc	S	0	0.12
tca	S	1	0.26
tcg	S	0	0.05
ctt	L	0	4610.00
ctc	L	0	3304.00
cta	L	68	4504.00
ctg	L	3	4000.00
agt	S	0	0.19
agc	S	0	0.20
---	--	------	------
mPD		0.025	1.1
		nPD:	0.02
		N. weight:	2.2
		Sc. PD:	-0.04
		Sc. rank:	16616.0

,

NP			
Pos.	200	obs:	exp:
att	I	15	8515.00
atc	I	23369	5839.00
ata	I	6	9037.00
act	T	1	0.25
acc	T	0	0.21
aca	T	0	0.46
acg	T	0	0.09
gtt	V	0	0.66
gtc	V	3	0.55
gta	V	0	0.81
gtg	V	0	0.98
---	--	------	------
mPD		0.0022	0.66
		nPD:	0.
		N. weight:	1.4
		Sc. PD:	-0.051
		Sc. rank:	-19862.0

,

NP			
Pos.	201	obs:	exp:
cgt	R	0	638.80
cgc	R	0	1224.00
cga	R	2	2291.00
cgg	R	0	1712.00
aga	R	23334	11745.00
agg	R	58	5783.00
---	--	------	------
mPD		0.0051	0.91
		nPD:	0.01
		N. weight:	0.7
		Sc. PD:	-0.024
		Sc. rank:	-6529.3

,

NP			
Pos.	202	obs:	exp:
atg	M	23394	23394.00
---	--	------	------
mPD		0	0
		nPD:	1.
		N. weight:	0.
		Sc. PD:	0
		Sc. rank:	0

,

NP			
Pos.	203	obs:	exp:
cat	H	0	0.59
cac	H	1	0.41
att	I	4	8490.00
atc	I	8093	5822.00
ata	I	15226	9011.00
atg	M	6	6.00
act	T	0	1.76
acc	T	3	1.44
aca	T	4	3.20
acg	T	0	0.60
gtt	V	0	12.60
gtc	V	17	10.36
gta	V	40	15.33
gtg	V	0	18.71
---	--	------	------
mPD		0.46	0.66
		nPD:	0.69
		N. weight:	0.47
		Sc. PD:	0.3
		Sc. rank:	9189.7

,

NP			
Pos.	204	obs:	exp:
cgt	R	0	0.08
cgc	R	0	0.16
cga	R	0	0.29
cgg	R	0	0.22
aaa	K	22339	13343.00
aag	K	1052	10048.00
aga	R	3	1.51
agg	R	0	0.74
---	--	------	------
mPD		0.086	0.49
		nPD:	0.18
		N. weight:	0.4
		Sc. PD:	0.053
		Sc. rank:	6981.7

,

NP			
Pos.	205	obs:	exp:
cgt	R	23223	638.80
cgc	R	106	1224.00
cga	R	18	2291.00
cgg	R	47	1712.00
aga	R	0	11745.00
agg	R	0	5783.00
---	--	------	------
mPD		0.015	0.91
		nPD:	0.02
		N. weight:	3.7
		Sc. PD:	-0.088
		Sc. rank:	15430.0

,

NP			
Pos.	206	obs:	exp:
gaa	E	1	0.61
gag	E	0	0.39
ggt	G	2	3547.00
ggc	G	3	3465.00
gga	G	23336	10406.00
ggg	G	52	5975.00
---	--	------	------
mPD		0.0049	0.69
		nPD:	0.01
		N. weight:	0.82
		Sc. PD:	-0.027
		Sc. rank:	-5643.2

,

NP			
Pos.	207	obs:	exp:
att	I	20	8493.00
atc	I	23308	5824.00
ata	I	3	9014.00
act	T	0	1.01
acc	T	4	0.82
aca	T	0	1.83
acg	T	0	0.34
gtt	V	2	13.04
gtc	V	57	10.72
gta	V	0	15.87
gtg	V	0	19.37
---	--	------	------
mPD		0.0075	0.66
		nPD:	0.01
		N. weight:	1.4
		Sc. PD:	-0.04
		Sc. rank:	-1780.0

,

NP			
Pos.	208	obs:	exp:
tct	S	0	3.31
tcc	S	0	2.08
tca	S	0	4.68
tcg	S	0	0.96
act	T	1	0.25
acc	T	0	0.21
aca	T	0	0.46
acg	T	0	0.09
aat	N	23325	13825.00
aac	N	50	9550.00
agt	S	18	3.38
agc	S	0	3.58
---	--	------	------
mPD		0.0059	0.49
		nPD:	0.01
		N. weight:	0.53
		Sc. PD:	-0.015
		Sc. rank:	-307.5

,

NP			
Pos.	209	obs:	exp:
gat	D	51	12326.00
gac	D	23343	11068.00
---	--	------	------
mPD		0.0044	0.50
		nPD:	0.01
		N. weight:	0.76
		Sc. PD:	-0.023
		Sc. rank:	-2920.3

NP			
Pos.	210	obs:	exp:
cgt	R	2	638.80
cgc	R	0	1224.00
cga	R	22812	2291.00
cgg	R	29	1712.00
aga	R	550	11745.00
agg	R	1	5783.00
---	--	------	------
mPD		0.049	0.91
		nPD:	0.05
		N. weight:	2.2
		Sc. PD:	0.028
		Sc. rank:	29871.0

NP			
Pos.	211	obs:	exp:
aat	N	23342	13836.00
aac	N	52	9558.00
---	--	------	------
mPD		0.0044	0.48
		nPD:	0.01
		N. weight:	0.53
		Sc. PD:	-0.016
		Sc. rank:	-1854.9

NP			
Pos.	212	obs:	exp:
ttt	F	88	10039.00
ttc	F	23306	13355.00
---	--	------	------
mPD		0.0075	0.49
		nPD:	0.02
		N. weight:	0.56
		Sc. PD:	-0.014
		Sc. rank:	1822.2

NP			
Pos.	213	obs:	exp:
tgg	W	23394	23394.00
---	--	------	------
mPD		0	0
		nPD:	1.
		N. weight:	0.
		Sc. PD:	0
		Sc. rank:	0

NP			
Pos.	214	obs:	exp:
cgt	R	0	638.80
cgc	R	0	1224.00
cga	R	0	2291.00
cgg	R	3	1712.00
aga	R	254	11745.00
agg	R	23137	5783.00
---	--	------	------
mPD		0.022	0.91
		nPD:	0.02
		N. weight:	1.4
		Sc. PD:	-0.022
		Sc. rank:	11356.0

NP			
Pos.	215	obs:	exp:
ggt	G	23381	3547.00
ggc	G	9	3465.00
gga	G	3	10406.00
ggg	G	1	5976.00
---	--	------	------
mPD		0.0011	0.69
		nPD:	0.
		N. weight:	1.9
		Sc. PD:	-0.073
		Sc. rank:	-34237.0

NP			
Pos.	216	obs:	exp:
gat	D	0	2.63
gac	D	5	2.37
gaa	E	23360	14162.00
gag	E	29	9227.00
---	--	------	------
mPD		0.0029	0.48
		nPD:	0.01
		N. weight:	0.51
		Sc. PD:	-0.017
		Sc. rank:	-4619.1

NP			
Pos.	217	obs:	exp:
tct	S	0	2.94
tcc	S	0	1.85
tca	S	0	4.16
tcg	S	0	0.85
cat	H	1	0.59
cac	H	0	0.41
att	I	1	0.36
atc	I	0	0.25
ata	I	0	0.39
aat	N	23365	13824.00
aac	N	8	9549.00
agt	S	16	3.01
agc	S	0	3.18
gat	D	3	1.58
gac	D	0	1.42
---	--	------	------
mPD		0.0025	0.49
		nPD:	0.01
		N. weight:	0.54
		Sc. PD:	-0.019
		Sc. rank:	-5623.6

NP			
Pos.	218	obs:	exp:
ggt	G	0	3547.00
ggc	G	40	3465.00
gga	G	23277	10406.00
ggg	G	77	5976.00
---	--	------	------
mPD		0.010	0.69
		nPD:	0.01
		N. weight:	0.81
		Sc. PD:	-0.02
		Sc. rank:	1830.1

NP			
Pos.	219	obs:	exp:
cgt	R	1	638.80
cgc	R	0	1224.00
cga	R	20488	2291.00
cgg	R	474	1712.00
aga	R	2430	11745.00
agg	R	1	5783.00
---	--	------	------
mPD		0.23	0.91
		nPD:	0.25
		N. weight:	1.8
		Sc. PD:	0.36
		Sc. rank:	31854.0

NP			
Pos.	220	obs:	exp:
cgt	R	0	638.10
cgc	R	0	1223.00
cga	R	0	2289.00
cgg	R	8	1710.00
aaa	K	2	13.12
aag	K	21	9.88
aga	R	209	11733.00
agg	R	23154	5778.00
---	--	------	------
mPD		0.021	0.91
		nPD:	0.02
		N. weight:	1.4
		Sc. PD:	-0.024
		Sc. rank:	10709.0

NP			
Pos.	221	obs:	exp:
act	T	5	5896.00
acc	T	5	4797.00
aca	T	23364	10699.00
acg	T	14	1996.00
gct	A	0	1.59
gcc	A	0	1.24
gca	A	6	2.82
gcg	A	0	0.35
---	--	------	------
mPD		0.0026	0.68
		nPD:	0.
		N. weight:	0.8
		Sc. PD:	-0.029
		Sc. rank:	-10152.0

NP			
Pos.	222	obs:	exp:
cgt	R	0	638.00
cgc	R	0	1222.00
cga	R	1	2289.00
cgg	R	1	1710.00
aaa	K	25	14.83
aag	K	1	11.17
aga	R	20182	11732.00
agg	R	3184	5777.00
---	--	------	------
mPD		0.24	0.91
		nPD:	0.26
		N. weight:	0.4
		Sc. PD:	0.085
		Sc. rank:	7166.3

NP			
Pos.	223	obs:	exp:
tta	L	0	0.11
ttg	L	0	0.19
tct	S	0	0.37
tcc	S	0	0.23
tca	S	0	0.52
tcg	S	0	0.11
ctt	L	1	0.20
ctc	L	0	0.14
cta	L	0	0.19
ctg	L	0	0.17
att	I	136	49.51
atc	I	0	33.95
ata	I	0	52.54
agt	S	2	0.38
agc	S	0	0.40
gtt	V	23210	5136.00
gtc	V	2	4221.00
gta	V	19	6248.00
gtg	V	0	7626.00
gct	A	24	6.35
gcc	A	0	4.97
gca	A	0	11.27
gcg	A	0	1.41
---	--	------	------
mPD		0.016	0.75
		nPD:	0.02
		N. weight:	1.5
		Sc. PD:	-0.029
		Sc. rank:	10785.0

NP			
Pos.	224	obs:	exp:
gct	A	23373	6194.00
gcc	A	16	4842.00
gca	A	4	10987.00
gcg	A	1	1371.00
---	--	------	------
mPD		0.0018	0.66
		nPD:	0.
		N. weight:	1.4
		Sc. PD:	-0.05
		Sc. rank:	-21074.0

NP			
Pos.	225	obs:	exp:
ttt	F	27	11.59
ttc	F	0	15.41
tat	Y	23354	12141.00
tac	Y	13	11226.00
---	--	------	------
mPD		0.0034	0.50
		nPD:	0.01
		N. weight:	0.67
		Sc. PD:	-0.022
		Sc. rank:	-5179.0

,

NP			
Pos.	226	obs:	exp:
gat	D	0	6.85
gac	D	13	6.15
gaa	E	23352	14157.00
gag	E	29	9224.00
---	--	------	------
mPD		0.0036	0.48
		nPD:	0.01
		N. weight:	0.51
		Sc. PD:	-0.016
		Sc. rank:	-3120.7

,

NP			
Pos.	227	obs:	exp:
cgt	R	0	638.70
cgc	R	0	1224.00
cga	R	19	2291.00
cgg	R	0	1712.00
aaa	K	1	0.57
aag	K	0	0.43
aga	R	23182	11744.00
agg	R	191	5783.00
ggt	G	0	0.15
ggc	G	0	0.15
gga	G	1	0.44
ggg	G	0	0.26
---	--	------	------
mPD		0.018	0.91
		nPD:	0.02
		N. weight:	0.66
		Sc. PD:	-0.013
		Sc. rank:	4247.0

,

NP			
Pos.	228	obs:	exp:
tta	L	0	0.55
ttg	L	2	0.94
ctt	L	0	0.99
ctc	L	0	0.71
cta	L	0	0.96
ctg	L	3	0.85
att	I	1	2.18
atc	I	0	1.50
ata	I	5	2.32
atg	M	23383	23383.00
---	--	------	------
mPD		0.00094	0.0012
		nPD:	0.77
		N. weight:	0.00046
		Sc. PD:	0.00032
		Sc. rank:	8.9

,

NP			
Pos.	229	obs:	exp:
tct	S	0	0.18
tcc	S	0	0.12
tca	S	0	0.26
tcg	S	0	0.05
tgt	C	133	10559.00
tgc	C	23260	12834.00
agt	S	0	0.19
agc	S	1	0.20
---	--	------	------
mPD		0.011	0.50
		nPD:	0.02
		N. weight:	0.59
		Sc. PD:	-0.01
		Sc. rank:	4614.8

,

NP			
Pos.	230	obs:	exp:
tct	S	0	0.18
tcc	S	0	0.12
tca	S	0	0.26
tcg	S	0	0.05
aat	N	23354	13835.00
aac	N	38	9557.00
agt	S	1	0.19
agc	S	0	0.20
gat	D	1	0.53
gac	D	0	0.47
---	--	------	------
mPD		0.0034	0.48
		nPD:	0.01
		N. weight:	0.53
		Sc. PD:	-0.017
		Sc. rank:	-3753.2

,

NP			
Pos.	231	obs:	exp:
att	I	91	8514.00
atc	I	23283	5838.00
ata	I	14	9036.00
act	T	0	0.25
acc	T	1	0.21
aca	T	0	0.46
acg	T	0	0.09
gtt	V	0	1.11
gtc	V	5	0.91
gta	V	0	1.34
gtg	V	0	1.64
---	--	------	------
mPD		0.0095	0.66
		nPD:	0.01
		N. weight:	1.4
		Sc. PD:	-0.036
		Sc. rank:	3336.4

,

NP			
Pos.	232	obs:	exp:
tta	L	0	2571.00
ttg	L	0	4404.00
ctt	L	154	4609.00
ctc	L	23229	3304.00
cta	L	7	4503.00
ctg	L	0	3999.00
att	I	0	1.46
atc	I	4	1.00
ata	I	0	1.55
---	--	------	------
mPD		0.014	1.1
		nPD:	0.01
		N. weight:	2.
		Sc. PD:	-0.054
		Sc. rank:	72.9

,

NP			
Pos.	233	obs:	exp:
att	I	0	0.36
atc	I	0	0.25
ata	I	1	0.39
aaa	K	23374	13344.00
aag	K	19	10049.00
---	--	------	------
mPD		0.0017	0.49
		nPD:	0.
		N. weight:	0.57
		Sc. PD:	-0.021
		Sc. rank:	-7433.6

,

NP			
Pos.	234	obs:	exp:
gtt	V	0	0.22
gtc	V	0	0.18
gta	V	1	0.27
gtg	V	0	0.33
ggt	G	2	3547.00
ggc	G	1	3465.00
gga	G	22211	10406.00
ggg	G	1179	5975.00
---	--	------	------
mPD		0.096	0.69
		nPD:	0.14
		N. weight:	0.66
		Sc. PD:	0.062
		Sc. rank:	11176.0

,

NP			
Pos.	235	obs:	exp:
aaa	K	22323	13345.00
aag	K	1071	10049.00
---	--	------	------
mPD		0.087	0.49
		nPD:	0.18
		N. weight:	0.4
		Sc. PD:	0.053
		Sc. rank:	6953.7

,

NP			
Pos.	236	obs:	exp:
ttt	F	23313	10038.00
ttc	F	78	13353.00
tta	L	0	0.33
ttg	L	0	0.56
ctt	L	3	0.59
ctc	L	0	0.42
cta	L	0	0.58
ctg	L	0	0.51
---	--	------	------
mPD		0.0069	0.49
		nPD:	0.01
		N. weight:	0.85
		Sc. PD:	-0.022
		Sc. rank:	1499.7

,

NP			
Pos.	237	obs:	exp:
caa	Q	23379	12344.00
cag	Q	15	11050.00
---	--	------	------
mPD		0.0013	0.50
		nPD:	0.
		N. weight:	0.66
		Sc. PD:	-0.024
		Sc. rank:	-10431.0

,

NP			
Pos.	238	obs:	exp:
act	T	10	5897.00
acc	T	3	4798.00
aca	T	23373	10701.00
acg	T	6	1996.00
gct	A	0	0.53
gcc	A	0	0.41
gca	A	2	0.94
gcg	A	0	0.12
---	--	------	------
mPD		0.0018	0.68
		nPD:	0.
		N. weight:	0.8
		Sc. PD:	-0.029
		Sc. rank:	-12587.0

,

NP			
Pos.	239	obs:	exp:
act	T	13	3.28
acc	T	0	2.67
aca	T	0	5.95
acg	T	0	1.11
gct	A	23341	6190.00
gcc	A	12	4840.00
gca	A	25	10981.00
gcg	A	3	1370.00
---	--	------	------
mPD		0.0045	0.66
		nPD:	0.01
		N. weight:	1.4
		Sc. PD:	-0.044
		Sc. rank:	-10327.0

,

NP			
Pos.	240	obs:	exp:
tct	S	0	1.47
tcc	S	8	0.93
tca	S	0	2.08
tcg	S	0	0.43
agt	S	0	1.50
agc	S	0	1.59
gtt	V	0	0.44
gtc	V	2	0.36
gta	V	0	0.54
gtg	V	0	0.66
gct	A	125	6191.00
gcc	A	23250	4840.00
gca	A	8	10982.00
gcg	A	0	1370.00
ggt	G	0	0.15
ggc	G	1	0.15
gga	G	0	0.44
ggg	G	0	0.26
---	--	------	------
mPD		0.012	0.66
		nPD:	0.02
		N. weight:	1.6
		Sc. PD:	-0.034
		Sc. rank:	8732.6

,

NP			
Pos.	241	obs:	exp:
caa	Q	220	12344.00
cag	Q	23174	11050.00
---	--	------	------
mPD		0.019	0.50
		nPD:	0.04
		N. weight:	0.72
		Sc. PD:	-0.0024
		Sc. rank:	8499.9

,

NP			
Pos.	242	obs:	exp:
cgt	R	0	638.30
cgc	R	0	1223.00
cga	R	0	2290.00
cgg	R	1	1711.00
aaa	K	0	8.56
aag	K	15	6.44
aga	R	90	11737.00
agg	R	23288	5780.00
---	--	------	------
mPD		0.0090	0.91
		nPD:	0.01
		N. weight:	1.4
		Sc. PD:	-0.042
		Sc. rank:	-3228.3

,

NP			
Pos.	243	obs:	exp:
tct	S	0	0.74
tcc	S	0	0.46
tca	S	4	1.04
tcg	S	0	0.21
act	T	0	0.50
acc	T	0	0.41
aca	T	2	0.91
acg	T	0	0.17
agt	S	0	0.75
agc	S	0	0.79
gct	A	3	6192.00
gcc	A	2	4841.00
gca	A	23366	10985.00
gcg	A	17	1370.00
---	--	------	------
mPD		0.0024	0.66
		nPD:	0.
		N. weight:	0.77
		Sc. PD:	-0.028
		Sc. rank:	-9873.3

,

NP			
Pos.	244	obs:	exp:
tta	L	0	0.11
ttg	L	0	0.19
ctt	L	0	0.20
ctc	L	0	0.14
cta	L	0	0.19
ctg	L	1	0.17
att	I	2	2.91
atc	I	0	2.00
ata	I	6	3.09
atg	M	23385	23385.00
---	--	------	------
mPD		0.00077	0.00082
		nPD:	0.93
		N. weight:	0.00027
		Sc. PD:	0.00023
		Sc. rank:	5.3

,

NP			
Pos.	245	obs:	exp:
att	I	2	1.46
atc	I	0	1.00
ata	I	2	1.55
atg	M	23374	23374.00
act	T	0	1.26
acc	T	0	1.03
aca	T	0	2.29
acg	T	5	0.43
gtt	V	0	2.43
gtc	V	0	2.00
gta	V	0	2.96
gtg	V	11	3.61
---	--	------	------
mPD		0.0017	0.0027
		nPD:	0.63
		N. weight:	0.0012
		Sc. PD:	0.00066
		Sc. rank:	22.9

,

NP			
Pos.	246	obs:	exp:
gat	D	23344	12325.00
gac	D	48	11067.00
gaa	E	2	1.21
gag	E	0	0.79
---	--	------	------
mPD		0.0043	0.50
		nPD:	0.01
		N. weight:	0.65
		Sc. PD:	-0.02
		Sc. rank:	-2681.9

,

NP			
Pos.	247	obs:	exp:
caa	Q	23379	12344.00
cag	Q	15	11050.00
---	--	------	------
mPD		0.0013	0.50
		nPD:	0.
		N. weight:	0.66
		Sc. PD:	-0.024
		Sc. rank:	-10431.0

,

NP			
Pos.	248	obs:	exp:
att	I	0	1.82
atc	I	0	1.25
ata	I	5	1.93
gtt	V	6	5170.00
gtc	V	2	4249.00
gta	V	22564	6289.00
gtg	V	811	7676.00
gct	A	0	1.59
gcc	A	0	1.24
gca	A	6	2.82
gcg	A	0	0.35
---	--	------	------
mPD		0.069	0.74
		nPD:	0.09
		N. weight:	1.2
		Sc. PD:	0.06
		Sc. rank:	18295.0

,

NP			
Pos.	249	obs:	exp:
cgt	R	0	638.50
cgc	R	0	1223.00
cga	R	2	2291.00
cgg	R	0	1711.00
aaa	K	6	4.56
aag	K	2	3.44
aga	R	21685	11741.00
agg	R	1699	5781.00
---	--	------	------
mPD		0.14	0.91
		nPD:	0.15
		N. weight:	0.5
		Sc. PD:	0.052
		Sc. rank:	8505.6

,

NP			
Pos.	250	obs:	exp:
gaa	E	23301	14165.00
gag	E	93	9229.00
---	--	------	------
mPD		0.0079	0.48
		nPD:	0.02
		N. weight:	0.49
		Sc. PD:	-0.012
		Sc. rank:	2213.9

,

NP			
Pos.	251	obs:	exp:
tct	S	0	4305.00
tcc	S	0	2710.00
tca	S	0	6088.00
tcg	S	0	1245.00
aat	N	1	0.59
aac	N	0	0.41
agt	S	23271	4399.00
agc	S	122	4647.00
---	--	------	------
mPD		0.010	1.6
		nPD:	0.01
		N. weight:	1.7
		Sc. PD:	-0.056
		Sc. rank:	-14712.0

,

NP			
Pos.	252	obs:	exp:
cgt	R	1	638.80
cgc	R	23	1224.00
cga	R	23056	2291.00
cgg	R	287	1712.00
aga	R	27	11745.00
agg	R	0	5783.00
---	--	------	------
mPD		0.029	0.91
		nPD:	0.03
		N. weight:	2.3
		Sc. PD:	-0.021
		Sc. rank:	24991.0

,

NP			
Pos.	253	obs:	exp:
tct	S	0	136.90
tcc	S	0	86.18
tca	S	0	193.60
tcg	S	0	39.60
act	T	0	6.81
acc	T	27	5.54
aca	T	0	12.35
acg	T	0	2.30
aat	N	194	13380.00
aac	N	22428	9242.00
agt	S	0	139.90
agc	S	744	147.80
gat	D	0	0.53
gac	D	1	0.47
---	--	------	------
mPD		0.080	0.60
		nPD:	0.13
		N. weight:	0.9
		Sc. PD:	0.081
		Sc. rank:	15094.0

,

NP			
Pos.	254	obs:	exp:
cct	P	8	6445.00
ccc	P	13	4339.00
cca	P	23317	9961.00
ccg	P	55	2649.00
act	T	0	0.25
acc	T	0	0.21
aca	T	1	0.46
acg	T	0	0.09
---	--	------	------
mPD		0.0066	0.70
		nPD:	0.01
		N. weight:	0.86
		Sc. PD:	-0.026
		Sc. rank:	-2701.3

,

NP			
Pos.	255	obs:	exp:
ggt	G	3	3547.00
ggc	G	18	3465.00
gga	G	23264	10406.00
ggg	G	109	5976.00
---	--	------	------
mPD		0.011	0.69
		nPD:	0.02
		N. weight:	0.8
		Sc. PD:	-0.019
		Sc. rank:	3151.9

,

NP			
Pos.	256	obs:	exp:
aat	N	375	13836.00
aac	N	23019	9558.00
---	--	------	------
mPD		0.032	0.48
		nPD:	0.07
		N. weight:	0.83
		Sc. PD:	0.02
		Sc. rank:	11677.0

,

NP			
Pos.	257	obs:	exp:
gtt	V	19	4.20
gtc	V	0	3.45
gta	V	0	5.11
gtg	V	0	6.24
gct	A	22620	6189.00
gcc	A	11	4839.00
gca	A	728	10978.00
gcg	A	16	1369.00
---	--	------	------
mPD		0.064	0.66
		nPD:	0.1
		N. weight:	1.2
		Sc. PD:	0.065
		Sc. rank:	18661.0

,

NP			
Pos.	258	obs:	exp:
gaa	E	169	14164.00
gag	E	23224	9229.00
ggt	G	0	0.15
ggc	G	0	0.15
gga	G	0	0.44
ggg	G	1	0.26
---	--	------	------
mPD		0.014	0.48
		nPD:	0.03
		N. weight:	0.91
		Sc. PD:	-0.0093
		Sc. rank:	9690.6

,

NP			
Pos.	259	obs:	exp:
att	I	23339	8509.00
atc	I	33	5835.00
ata	I	3	9031.00
act	T	9	2.27
acc	T	0	1.85
aca	T	0	4.12
acg	T	0	0.77
gtt	V	10	2.21
gtc	V	0	1.82
gta	V	0	2.69
gtg	V	0	3.28
---	--	------	------
mPD		0.0047	0.66
		nPD:	0.01
		N. weight:	1.
		Sc. PD:	-0.033
		Sc. rank:	-7172.1

,

NP			
Pos.	260	obs:	exp:
gaa	E	23370	14165.00
gag	E	24	9229.00
---	--	------	------
mPD		0.0020	0.48
		nPD:	0.
		N. weight:	0.51
		Sc. PD:	-0.018
		Sc. rank:	-5940.9

,

NP			
Pos.	261	obs:	exp:
gat	D	118	12326.00
gac	D	23276	11068.00
---	--	------	------
mPD		0.010	0.50
		nPD:	0.02
		N. weight:	0.74
		Sc. PD:	-0.015
		Sc. rank:	4852.9

,

NP			
Pos.	262	obs:	exp:
ttt	F	0	0.43
ttc	F	1	0.57
tta	L	0	2566.00
ttg	L	0	4394.00
ctt	L	236	4599.00
ctc	L	23001	3296.00
cta	L	100	4493.00
ctg	L	0	3990.00
att	I	0	20.39
atc	I	56	13.98
ata	I	0	21.64
---	--	------	------
mPD		0.033	1.1
		nPD:	0.03
		N. weight:	1.9
		Sc. PD:	-0.022
		Sc. rank:	19545.0

,

NP			
Pos.	263	obs:	exp:
tta	L	0	4.18
ttg	L	0	7.15
ctt	L	38	7.49
ctc	L	0	5.37
cta	L	0	7.32
ctg	L	0	6.50
att	I	23312	8502.00
atc	I	42	5830.00
ata	I	1	9023.00
gtt	V	1	0.22
gtc	V	0	0.18
gta	V	0	0.27
gtg	V	0	0.33
---	--	------	------
mPD		0.0070	0.66
		nPD:	0.01
		N. weight:	1.
		Sc. PD:	-0.03
		Sc. rank:	-1609.1

,

NP			
Pos.	264	obs:	exp:
ttt	F	183	10039.00
ttc	F	23211	13355.00
---	--	------	------
mPD		0.016	0.49
		nPD:	0.03
		N. weight:	0.53
		Sc. PD:	-0.0047
		Sc. rank:	5786.0

,

NP			
Pos.	265	obs:	exp:
tta	L	1	2571.00
ttg	L	28	4404.00
ctt	L	15	4609.00
ctc	L	0	3304.00
cta	L	155	4503.00
ctg	L	23192	3999.00
caa	Q	0	0.53
cag	Q	1	0.47
atg	M	2	2.00
---	--	------	------
mPD		0.017	1.1
		nPD:	0.02
		N. weight:	1.8
		Sc. PD:	-0.044
		Sc. rank:	5727.8

,

NP			
Pos.	266	obs:	exp:
tct	S	0	0.18
tcc	S	0	0.12
tca	S	1	0.26
tcg	S	0	0.05
act	T	0	7.81
acc	T	0	6.36
aca	T	31	14.18
acg	T	0	2.65
agt	S	0	0.19
agc	S	0	0.20
gtt	V	0	0.88
gtc	V	0	0.73
gta	V	4	1.08
gtg	V	0	1.31
gct	A	3	6184.00
gcc	A	1	4835.00
gca	A	23335	10969.00
gcg	A	17	1368.00
ggt	G	0	0.30
ggc	G	0	0.30
gga	G	2	0.89
ggg	G	0	0.51
---	--	------	------
mPD		0.0050	0.67
		nPD:	0.01
		N. weight:	0.78
		Sc. PD:	-0.025
		Sc. rank:	-4701.4

,

NP			
Pos.	267	obs:	exp:
cgt	R	14	638.80
cgc	R	71	1224.00
cga	R	531	2291.00
cgg	R	22755	1712.00
aga	R	1	11745.00
agg	R	22	5783.00
---	--	------	------
mPD		0.053	0.91
		nPD:	0.06
		N. weight:	2.6
		Sc. PD:	0.044
		Sc. rank:	34939.0

,

NP			
Pos.	268	obs:	exp:
tct	S	9	4305.00
tcc	S	2	2710.00
tca	S	23370	6088.00
tcg	S	13	1245.00
agt	S	0	4399.00
agc	S	0	4647.00
---	--	------	------
mPD		0.0021	1.6
		nPD:	0.
		N. weight:	1.4
		Sc. PD:	-0.053
		Sc. rank:	-25516.0

,

NP			
Pos.	269	obs:	exp:
tct	S	0	0.55
tcc	S	0	0.35
tca	S	3	0.78
tcg	S	0	0.16
act	T	0	0.50
acc	T	0	0.41
aca	T	2	0.91
acg	T	0	0.17
agt	S	0	0.56
agc	S	0	0.60
gct	A	4	6192.00
gcc	A	91	4841.00
gca	A	23286	10985.00
gcg	A	8	1370.00
---	--	------	------
mPD		0.0092	0.66
		nPD:	0.01
		N. weight:	0.75
		Sc. PD:	-0.02
		Sc. rank:	1131.7

,

NP			
Pos.	270	obs:	exp:
tta	L	0	2572.00
ttg	L	0	4404.00
ctt	L	446	4610.00
ctc	L	22940	3304.00
cta	L	7	4504.00
ctg	L	1	4000.00
---	--	------	------
mPD		0.038	1.1
		nPD:	0.03
		N. weight:	1.9
		Sc. PD:	-0.014
		Sc. rank:	21692.0

,

NP			
Pos.	271	obs:	exp:
tct	S	0	0.18
tcc	S	0	0.12
tca	S	0	0.26
tcg	S	0	0.05
att	I	22861	8512.00
atc	I	518	5837.00
ata	I	3	9034.00
agt	S	1	0.19
agc	S	0	0.20
gtt	V	11	2.43
gtc	V	0	2.00
gta	V	0	2.96
gtg	V	0	3.61
---	--	------	------
mPD		0.045	0.66
		nPD:	0.07
		N. weight:	0.94
		Sc. PD:	0.025
		Sc. rank:	13400.0

,

NP			
Pos.	272	obs:	exp:
tta	L	1	2571.00
ttg	L	280	4403.00
ctt	L	4	4609.00
ctc	L	0	3303.00
cta	L	73	4503.00
ctg	L	23030	3999.00
atg	M	6	6.00
---	--	------	------
mPD		0.031	1.1
		nPD:	0.03
		N. weight:	1.7
		Sc. PD:	-0.023
		Sc. rank:	16288.0

,

NP			
Pos.	273	obs:	exp:
cgt	R	0	638.80
cgc	R	0	1224.00
cga	R	0	2291.00
cgg	R	0	1712.00
aga	R	18309	11745.00
agg	R	5085	5783.00
---	--	------	------
mPD		0.34	0.91
		nPD:	0.37
		N. weight:	0.33
		Sc. PD:	0.11
		Sc. rank:	6167.9

,

NP			
Pos.	274	obs:	exp:
ggt	G	9	3547.00
ggc	G	1	3465.00
gga	G	23334	10406.00
ggg	G	50	5976.00
---	--	------	------
mPD		0.0051	0.69
		nPD:	0.01
		N. weight:	0.82
		Sc. PD:	-0.026
		Sc. rank:	-5311.9

,

NP

Pos.	275	obs:	exp:
tct	S	6	4305.00
tcc	S	0	2710.00
tca	S	23355	6088.00
tcg	S	33	1245.00
agt	S	0	4399.00
agc	S	0	4647.00
---	--	------	------
mPD		0.0033	1.6
		nPD:	0.
		N. weight:	1.4
		Sc. PD:	-0.052
		Sc. rank:	-23420.0

NP

Pos.	276	obs:	exp:
ttt	F	1	0.43
ttc	F	0	0.57
att	I	6	2.18
atc	I	0	1.50
ata	I	0	2.32
gtt	V	23190	5170.00
gtc	V	38	4249.00
gta	V	6	6289.00
gtg	V	152	7677.00
gct	A	1	0.26
gcc	A	0	0.21
gca	A	0	0.47
gcg	A	0	0.06
---	--	------	------
mPD		0.017	0.74
		nPD:	0.02
		N. weight:	1.5
		Sc. PD:	-0.025
		Sc. rank:	12106.0

NP

Pos.	277	obs:	exp:
gct	A	7	6194.00
gcc	A	5	4842.00
gca	A	23373	10987.00
gcg	A	9	1371.00
---	--	------	------
mPD		0.0018	0.66
		nPD:	0.
		N. weight:	0.77
		Sc. PD:	-0.028
		Sc. rank:	-11897.0

NP

Pos.	278	obs:	exp:
cat	H	23152	13758.00
cac	H	242	9636.00
---	--	------	------
mPD		0.020	0.48
		nPD:	0.04
		N. weight:	0.49
		Sc. PD:	0.0007
		Sc. rank:	6055.1

NP

Pos.	279	obs:	exp:
aaa	K	23290	13345.00
aag	K	104	10049.00
---	--	------	------
mPD		0.0089	0.49
		nPD:	0.02
		N. weight:	0.55
		Sc. PD:	-0.012
		Sc. rank:	2926.3

NP

Pos.	280	obs:	exp:
tct	S	40	4305.00
tcc	S	22810	2710.00
tca	S	544	6088.00
tcg	S	0	1245.00
agt	S	0	4399.00
agc	S	0	4647.00
---	--	------	------
mPD		0.049	1.6
		nPD:	0.03
		N. weight:	2.1
		Sc. PD:	-0.022
		Sc. rank:	21661.0

NP

Pos.	281	obs:	exp:
tct	S	0	0.18
tcc	S	0	0.12
tca	S	0	0.26
tcg	S	0	0.05
tgt	C	803	10559.00
tgc	C	22590	12834.00
agt	S	0	0.19
agc	S	1	0.20
---	--	------	------
mPD		0.066	0.50
		nPD:	0.13
		N. weight:	0.47
		Sc. PD:	0.042
		Sc. rank:	7925.3

NP

Pos.	282	obs:	exp:
tta	L	0	2572.00
ttg	L	89	4404.00
ctt	L	11	4610.00
ctc	L	0	3304.00
cta	L	136	4504.00
ctg	L	23158	4000.00
---	--	------	------
mPD		0.020	1.1
		nPD:	0.02
		N. weight:	1.8
		Sc. PD:	-0.039
		Sc. rank:	9051.3

NP

Pos.	283	obs:	exp:
cct	P	23333	6444.00
ccc	P	35	4339.00
cca	P	20	9960.00
ccg	P	4	2649.00
cgt	R	1	0.03
cgc	R	0	0.05
cga	R	0	0.10
cgg	R	0	0.07
aga	R	0	0.50
agg	R	0	0.25
gct	A	1	0.26
gcc	A	0	0.21
gca	A	0	0.47
gcg	A	0	0.06
---	--	------	------
mPD		0.0052	0.70
		nPD:	0.01
		N. weight:	1.3
		Sc. PD:	-0.042
		Sc. rank:	-8093.0

NP

Pos.	284	obs:	exp:
tct	S	3	0.55
tcc	S	0	0.35
tca	S	0	0.78
tcg	S	0	0.16
act	T	5	1.26
acc	T	0	1.03
aca	T	0	2.29
acg	T	0	0.43
agt	S	0	0.56
agc	S	0	0.60
gct	A	23357	6192.00
gcc	A	24	4841.00
gca	A	2	10984.00
gcg	A	3	1370.00
---	--	------	------
mPD		0.0032	0.66
		nPD:	0.
		N. weight:	1.4
		Sc. PD:	-0.047
		Sc. rank:	-14870.0

NP

Pos.	285	obs:	exp:
tgt	C	23378	10559.00
tgc	C	16	12835.00
---	--	------	------
mPD		0.0014	0.50
		nPD:	0.
		N. weight:	0.82
		Sc. PD:	-0.03
		Sc. rank:	-12494.0

NP

Pos.	286	obs:	exp:
atg	M	1	1.00
gtt	V	20	5171.00
gtc	V	10	4250.00
gta	V	158	6291.00
gtg	V	23203	7678.00
gct	A	0	0.53
gcc	A	0	0.41
gca	A	1	0.94
gcg	A	1	0.12
---	--	------	------
mPD		0.016	0.74
		nPD:	0.02
		N. weight:	1.1
		Sc. PD:	-0.02
		Sc. rank:	8413.4

NP

Pos.	287	obs:	exp:
tat	Y	23382	12155.00
tac	Y	12	11239.00
---	--	------	------
mPD		0.0010	0.50
		nPD:	0.
		N. weight:	0.67
		Sc. PD:	-0.025
		Sc. rank:	-11281.0

NP

Pos.	288	obs:	exp:
ggt	G	3	3547.00
ggc	G	1	3465.00
gga	G	105	10406.00
ggg	G	23285	5976.00
---	--	------	------
mPD		0.0093	0.69
		nPD:	0.01
		N. weight:	1.4
		Sc. PD:	-0.036
		Sc. rank:	1329.0

NP

Pos.	289	obs:	exp:
tta	L	0	2570.00
ttg	L	0	4402.00
ctt	L	23348	4608.00
ctc	L	22	3303.00
cta	L	5	4501.00
ctg	L	7	3998.00
cct	P	1	0.28
ccc	P	0	0.19
cca	P	0	0.43
ccg	P	0	0.11
att	I	11	4.00
atc	I	0	2.75
ata	I	0	4.25
---	--	------	------
mPD		0.0039	1.1
		nPD:	0.
		N. weight:	1.7
		Sc. PD:	-0.06
		Sc. rank:	-22925.0

NP

Pos.	290	obs:	exp:
tct	S	0	0.37
tcc	S	0	0.23
tca	S	2	0.52
tcg	S	0	0.11
agt	S	0	0.38
agc	S	0	0.40
gtt	V	0	0.22
gtc	V	0	0.18
gta	V	1	0.27
gtg	V	0	0.33
gct	A	21	6193.00
gcc	A	2	4842.00
gca	A	23337	10985.00
gcg	A	30	1370.00
ggt	G	0	0.15
ggc	G	0	0.15
gga	G	1	0.44
ggg	G	0	0.26
---	--	------	------
mPD		0.0049	0.66
		nPD:	0.01
		N. weight:	0.77
		Sc. PD:	-0.025
		Sc. rank:	-5016.3

NP

Pos.	291	obs:	exp:
tta	L	1	0.11
ttg	L	0	0.19
ctt	L	0	0.20
ctc	L	0	0.14
cta	L	0	0.19
ctg	L	0	0.17
att	I	1	88.09
atc	I	0	60.41
ata	I	241	93.50
gtt	V	10	5118.00
gtc	V	0	4207.00
gta	V	22595	6226.00
gtg	V	545	7599.00
gct	A	0	0.26
gcc	A	0	0.21
gca	A	1	0.47
gcg	A	0	0.06
---	--	------	------
mPD		0.067	0.76
		nPD:	0.09
		N. weight:	1.2
		Sc. PD:	0.056
		Sc. rank:	18788.0

NP			
Pos.	292	obs:	exp:
tct	S	0	0.18
tcc	S	1	0.12
tca	S	0	0.26
tcg	S	0	0.05
agt	S	0	0.19
agc	S	0	0.20
gct	A	5	6193.00
gcc	A	3	4842.00
gca	A	23374	10987.00
gcg	A	11	1370.00
---	--	------	------
mPD		0.0018	0.66
		nPD:	0.
		N. weight:	0.78
		Sc. PD:	-0.028
		Sc. rank:	-12040.0

,

NP			
Pos.	293	obs:	exp:
tct	S	0	4299.00
tcc	S	0	2706.00
tca	S	0	6080.00
tcg	S	0	1243.00
att	I	1	0.36
atc	I	0	0.25
ata	I	0	0.39
act	T	1	0.25
acc	T	0	0.21
aca	T	0	0.46
acg	T	0	0.09
aat	N	30	17.74
aac	N	0	12.26
agt	S	23334	4393.00
agc	S	28	4641.00
---	--	------	------
mPD		0.0051	1.6
		nPD:	0.
		N. weight:	1.7
		Sc. PD:	-0.062
		Sc. rank:	-25484.0

,

NP			
Pos.	294	obs:	exp:
ggt	G	48	3547.00
ggc	G	20121	3465.00
gga	G	34	10406.00
ggg	G	3191	5976.00
---	--	------	------
mPD		0.24	0.69
		nPD:	0.35
		N. weight:	1.5
		Sc. PD:	0.43
		Sc. rank:	26996.0

,

NP			
Pos.	295	obs:	exp:
tat	Y	2	1.56
tac	Y	1	1.44
cat	H	23378	13755.00
cac	H	12	9635.00
aat	N	1	0.59
aac	N	0	0.41
---	--	------	------
mPD		0.0015	0.48
		nPD:	0.
		N. weight:	0.54
		Sc. PD:	-0.02
		Sc. rank:	-8272.5

,

NP			
Pos.	296	obs:	exp:
aat	N	1	8.87
aac	N	14	6.13
gat	D	82	12315.00
gac	D	23290	11057.00
gaa	E	7	4.24
gag	E	0	2.76
---	--	------	------
mPD		0.0089	0.50
		nPD:	0.02
		N. weight:	0.75
		Sc. PD:	-0.017
		Sc. rank:	3937.7

,

NP			
Pos.	297	obs:	exp:
ttt	F	23366	10039.00
ttc	F	28	13355.00
---	--	------	------
mPD		0.0024	0.49
		nPD:	0.
		N. weight:	0.86
		Sc. PD:	-0.03
		Sc. rank:	-9175.0

,

NP			
Pos.	298	obs:	exp:
gaa	E	23380	14162.00
gag	E	9	9227.00
ggt	G	0	0.76
ggc	G	0	0.74
gga	G	5	2.22
ggg	G	0	1.28
---	--	------	------
mPD		0.0012	0.48
		nPD:	0.
		N. weight:	0.52
		Sc. PD:	-0.019
		Sc. rank:	-8288.8

,

NP			
Pos.	299	obs:	exp:
cgt	R	0	638.70
cgc	R	0	1224.00
cga	R	0	2291.00
cgg	R	1	1712.00
aaa	K	1	1.71
aag	K	2	1.29
aga	R	831	11743.00
agg	R	22559	5783.00
---	--	------	------
mPD		0.069	0.91
		nPD:	0.08
		N. weight:	1.3
		Sc. PD:	0.042
		Sc. rank:	18609.0

,

NP			
Pos.	300	obs:	exp:
gtt	V	0	0.22
gtc	V	0	0.18
gta	V	1	0.27
gtg	V	0	0.33
gat	D	1	2.63
gac	D	4	2.37
gaa	E	23357	14161.00
gag	E	30	9226.00
ggt	G	0	0.15
ggc	G	0	0.15
gga	G	1	0.44
ggg	G	0	0.26
---	--	------	------
mPD		0.0032	0.48
		nPD:	0.01
		N. weight:	0.51
		Sc. PD:	-0.017
		Sc. rank:	-4284.7

,

NP			
Pos.	301	obs:	exp:
ggt	G	4	3547.00
ggc	G	0	3465.00
gga	G	407	10406.00
ggg	G	22983	5976.00
---	--	------	------
mPD		0.035	0.69
		nPD:	0.05
		N. weight:	1.3
		Sc. PD:	0.011
		Sc. rank:	17075.0

,

NP			
Pos.	302	obs:	exp:
tat	Y	70	12155.00
tac	Y	23323	11238.00
cat	H	0	0.59
cac	H	1	0.41
---	--	------	------
mPD		0.0061	0.50
		nPD:	0.01
		N. weight:	0.74
		Sc. PD:	-0.02
		Sc. rank:	-386.0

,

NP			
Pos.	303	obs:	exp:
tct	S	2	4305.00
tcc	S	1	2710.00
tca	S	23382	6088.00
tcg	S	8	1245.00
act	T	0	0.25
acc	T	0	0.21
aca	T	1	0.46
acg	T	0	0.09
agt	S	0	4399.00
agc	S	0	4647.00
---	--	------	------
mPD		0.0010	1.6
		nPD:	0.
		N. weight:	1.4
		Sc. PD:	-0.054
		Sc. rank:	-26452.0

,

NP			
Pos.	304	obs:	exp:
tta	L	1	2572.00
ttg	L	1091	4404.00
ctt	L	8	4610.00
ctc	L	0	3304.00
cta	L	60	4504.00
ctg	L	22234	4000.00
---	--	------	------
mPD		0.095	1.1
		nPD:	0.08
		N. weight:	1.6
		Sc. PD:	0.066
		Sc. rank:	24173.0

,

NP			
Pos.	305	obs:	exp:
gtt	V	128	5172.00
gtc	V	23225	4251.00
gta	V	40	6292.00
gtg	V	1	7679.00
---	--	------	------
mPD		0.014	0.74
		nPD:	0.02
		N. weight:	1.7
		Sc. PD:	-0.035
		Sc. rank:	10791.0

,

NP			
Pos.	306	obs:	exp:
ggt	G	2	3547.00
ggc	G	0	3465.00
gga	G	1471	10406.00
ggg	G	21921	5976.00
---	--	------	------
mPD		0.12	0.69
		nPD:	0.17
		N. weight:	1.1
		Sc. PD:	0.14
		Sc. rank:	19513.0

,

NP			
Pos.	307	obs:	exp:
tta	L	3	0.44
ttg	L	0	0.75
ctt	L	0	0.79
ctc	L	0	0.56
cta	L	1	0.77
ctg	L	0	0.68
att	I	11	8511.00
atc	I	1	5836.00
ata	I	23367	9033.00
gtt	V	0	2.43
gtc	V	0	2.00
gta	V	11	2.96
gtg	V	0	3.61
---	--	------	------
mPD		0.0023	0.66
		nPD:	0.
		N. weight:	0.98
		Sc. PD:	-0.035
		Sc. rank:	-12606.0

,

NP			
Pos.	308	obs:	exp:
gat	D	164	12325.00
gac	D	23228	11067.00
gaa	E	2	1.21
gag	E	0	0.79
---	--	------	------
mPD		0.014	0.50
		nPD:	0.03
		N. weight:	0.73
		Sc. PD:	-0.0088
		Sc. rank:	7110.1

NP			
Pos.	309	obs:	exp:
tct	S	0	0.55
tcc	S	0	0.35
tca	S	3	0.78
tcg	S	0	0.16
cct	P	12	6444.00
ccc	P	5	4338.00
cca	P	23327	9960.00
ccg	P	47	2649.00
agt	S	0	0.56
agc	S	0	0.60
---	--	------	------
mPD		0.0057	0.70
		nPD:	0.01
		N. weight:	0.86
		Sc. PD:	-0.027
		Sc. rank:	-4257.8

NP			
Pos.	310	obs:	exp:
ttt	F	75	10039.00
ttc	F	23319	13355.00
---	--	------	------
mPD		0.0064	0.49
		nPD:	0.01
		N. weight:	0.56
		Sc. PD:	-0.015
		Sc. rank:	402.0

NP			
Pos.	311	obs:	exp:
cgt	R	0	0.46
cgc	R	0	0.89
cga	R	1	1.67
cgg	R	0	1.24
aaa	K	23321	13335.00
aag	K	56	10042.00
aga	R	16	8.53
agg	R	0	4.20
---	--	------	------
mPD		0.0063	0.49
		nPD:	0.01
		N. weight:	0.56
		Sc. PD:	-0.015
		Sc. rank:	381.6

NP			
Pos.	312	obs:	exp:
tta	L	23224	2572.00
ttg	L	80	4404.00
ctt	L	0	4610.00
ctc	L	0	3304.00
cta	L	89	4504.00
ctg	L	0	4000.00
att	I	0	0.36
atc	I	0	0.25
ata	I	1	0.39
---	--	------	------
mPD		0.014	1.1
		nPD:	0.01
		N. weight:	2.2
		Sc. PD:	-0.06
		Sc. rank:	1262.7

NP			
Pos.	313	obs:	exp:
tta	L	0	2572.00
ttg	L	0	4404.00
ctt	L	480	4610.00
ctc	L	22905	3304.00
cta	L	6	4503.00
ctg	L	1	3999.00
att	I	0	0.73
atc	I	2	0.50
ata	I	0	0.77
---	--	------	------
mPD		0.041	1.1
		nPD:	0.04
		N. weight:	1.9
		Sc. PD:	-0.0089
		Sc. rank:	22109.0

NP			
Pos.	314	obs:	exp:
caa	Q	23379	12344.00
cag	Q	15	11050.00
---	--	------	------
mPD		0.0013	0.50
		nPD:	0.
		N. weight:	0.66
		Sc. PD:	-0.024
		Sc. rank:	-10431.0

NP			
Pos.	315	obs:	exp:
tct	S	0	0.18
tcc	S	0	0.12
tca	S	0	0.26
tcg	S	0	0.05
tat	Y	0	0.52
tac	Y	1	0.48
aat	N	59	13835.00
aac	N	23333	9557.00
agt	S	0	0.19
agc	S	1	0.20
---	--	------	------
mPD		0.0052	0.48
		nPD:	0.01
		N. weight:	0.91
		Sc. PD:	-0.026
		Sc. rank:	-1291.5

NP			
Pos.	316	obs:	exp:
tct	S	0	4305.00
tcc	S	0	2709.00
tca	S	0	6088.00
tcg	S	0	1245.00
att	I	1	0.36
atc	I	0	0.25
ata	I	0	0.39
agt	S	20320	4398.00
agc	S	3072	4646.00
ggt	G	0	0.15
ggc	G	1	0.15
gga	G	0	0.44
ggg	G	0	0.26
---	--	------	------
mPD		0.23	1.6
		nPD:	0.14
		N. weight:	1.3
		Sc. PD:	0.12
		Sc. rank:	22492.0

NP			
Pos.	317	obs:	exp:
caa	Q	23376	12344.00
cag	Q	18	11050.00
---	--	------	------
mPD		0.0015	0.50
		nPD:	0.
		N. weight:	0.65
		Sc. PD:	-0.024
		Sc. rank:	-9844.5

NP			
Pos.	318	obs:	exp:
att	I	0	0.36
atc	I	0	0.25
ata	I	1	0.39
atg	M	3	3.00
gtt	V	10	5171.00
gtc	V	0	4250.00
gta	V	349	6291.00
gtg	V	23031	7678.00
---	--	------	------
mPD		0.031	0.74
		nPD:	0.04
		N. weight:	1.1
		Sc. PD:	0.00076
		Sc. rank:	13105.0

NP			
Pos.	319	obs:	exp:
ttt	F	0	3.00
ttc	F	7	4.00
tta	L	0	0.11
ttg	L	0	0.19
tat	Y	0	0.52
tac	Y	1	0.48
ctt	L	0	0.20
ctc	L	1	0.14
cta	L	0	0.19
ctg	L	0	0.17
att	I	0	325.80
atc	I	894	223.40
ata	I	1	345.80
act	T	0	3.03
acc	T	12	2.46
aca	T	0	5.49
acg	T	0	1.02
gtt	V	20	4969.00
gtc	V	22444	4084.00
gta	V	13	6045.00
gtg	V	0	7378.00
gct	A	0	0.26
gcc	A	1	0.21
gca	A	0	0.47
gcg	A	0	0.06
---	--	------	------
mPD		0.079	0.82
		nPD:	0.1
		N. weight:	1.7
		Sc. PD:	0.094
		Sc. rank:	27167.0

NP			
Pos.	320	obs:	exp:
tct	S	0	4299.00
tcc	S	0	2706.00
tca	S	0	6080.00
tcg	S	0	1243.00
tgt	C	0	15.35
tgc	C	34	18.65
agt	S	826	4392.00
agc	S	22534	4640.00
---	--	------	------
mPD		0.071	1.6
		nPD:	0.04
		N. weight:	1.5
		Sc. PD:	0.0036
		Sc. rank:	18760.0

NP			
Pos.	321	obs:	exp:
tta	L	1	2571.00
ttg	L	63	4403.00
ctt	L	730	4609.00
ctc	L	0	3304.00
cta	L	249	4503.00
ctg	L	22346	3999.00
atg	M	4	4.00
gtt	V	0	0.22
gtc	V	0	0.18
gta	V	0	0.27
gtg	V	1	0.33
---	--	------	------
mPD		0.087	1.1
		nPD:	0.08
		N. weight:	1.6
		Sc. PD:	0.054
		Sc. rank:	23777.0

NP			
Pos.	322	obs:	exp:
tta	L	0	0.11
ttg	L	1	0.19
ctt	L	0	0.20
ctc	L	0	0.14
cta	L	0	0.19
ctg	L	0	0.17
att	I	4	24.75
atc	I	1	16.97
ata	I	63	26.27
atg	M	23314	23314.00
act	T	0	0.25
acc	T	0	0.21
aca	T	0	0.46
acg	T	1	0.09
gtt	V	0	2.21
gtc	V	0	1.82
gta	V	0	2.69
gtg	V	10	3.28
---	--	------	------
mPD		0.0068	0.0075
		nPD:	0.91
		N. weight:	0.0028
		Sc. PD:	0.0023
		Sc. rank:	54.3

NP			
Pos.	323	obs:	exp:
cgt	R	0	638.70
cgc	R	0	1224.00
cga	R	1	2291.00
cgg	R	0	1712.00
act	T	0	0.25
acc	T	0	0.21
aca	T	1	0.46
acg	T	0	0.09
aga	R	23328	11744.00
agg	R	64	5783.00
---	--	------	------
mPD		0.0056	0.91
		nPD:	0.01
		N. weight:	0.69
		Sc. PD:	-0.023
		Sc. rank:	-6224.4

NP			
Pos.	324	obs:	exp:
tta	L	0	0.22
ttg	L	0	0.38
tct	S	0	1.47
tcc	S	0	0.93
tca	S	8	2.08
tcg	S	0	0.43
ctt	L	0	0.39
ctc	L	0	0.28
cta	L	2	0.39
ctg	L	0	0.34
cct	P	2	6439.00
ccc	P	19	4335.00
cca	P	23337	9951.00
ccg	P	13	2646.00
caa	Q	10	5.28
cag	Q	0	4.72
act	T	0	0.50
acc	T	0	0.41
aca	T	2	0.91
acg	T	0	0.17
agt	S	0	1.50
agc	S	0	1.59
gct	A	0	0.26
gcc	A	0	0.21
gca	A	1	0.47
gcg	A	0	0.06
---	--	------	------
mPD		0.0049	0.70
		nPD:	0.01
		N. weight:	0.87
		Sc. PD:	-0.028
		Sc. rank:	-6195.7

NP			
Pos.	325	obs:	exp:
tct	S	0	0.55
tcc	S	0	0.35
tca	S	0	0.78
tcg	S	0	0.16
act	T	1	0.25
acc	T	0	0.21
aca	T	0	0.46
acg	T	0	0.09
aat	N	23369	13833.00
aac	N	20	9556.00
aaa	K	1	0.57
aag	K	0	0.43
agt	S	3	0.56
agc	S	0	0.60
---	--	------	------
mPD		0.0021	0.48
		nPD:	0.
		N. weight:	0.54
		Sc. PD:	-0.019
		Sc. rank:	-6187.0

NP			
Pos.	326	obs:	exp:
gaa	E	23345	14165.00
gag	E	49	9229.00
---	--	------	------
mPD		0.0042	0.48
		nPD:	0.01
		N. weight:	0.5
		Sc. PD:	-0.016
		Sc. rank:	-1869.5

NP			
Pos.	327	obs:	exp:
tct	S	0	0.74
tcc	S	0	0.46
tca	S	0	1.04
tcg	S	0	0.21
aat	N	20215	13834.00
aac	N	3175	9556.00
agt	S	4	0.75
agc	S	0	0.79
---	--	------	------
mPD		0.23	0.48
		nPD:	0.49
		N. weight:	0.18
		Sc. PD:	0.079
		Sc. rank:	3572.3

NP			
Pos.	328	obs:	exp:
cct	P	6	6445.00
ccc	P	3	4339.00
cca	P	23360	9961.00
ccg	P	25	2649.00
---	--	------	------
mPD		0.0029	0.70
		nPD:	0.
		N. weight:	0.87
		Sc. PD:	-0.031
		Sc. rank:	-10347.0

NP			
Pos.	329	obs:	exp:
tct	S	6	1.10
tcc	S	0	0.69
tca	S	0	1.56
tcg	S	0	0.32
act	T	11	2.77
acc	T	0	2.26
aca	T	0	5.03
acg	T	0	0.94
agt	S	0	1.13
agc	S	0	1.19
gtt	V	5	1.11
gtc	V	0	0.91
gta	V	0	1.34
gtg	V	0	1.64
gct	A	23243	6188.00
gcc	A	120	4838.00
gca	A	8	10977.00
gcg	A	1	1369.00
---	--	------	------
mPD		0.013	0.67
		nPD:	0.02
		N. weight:	1.3
		Sc. PD:	-0.028
		Sc. rank:	8177.4

NP			
Pos.	330	obs:	exp:
cat	H	821	13757.00
cac	H	22572	9636.00
aat	N	0	0.59
aac	N	1	0.41
---	--	------	------
mPD		0.068	0.48
		nPD:	0.14
		N. weight:	0.75
		Sc. PD:	0.071
		Sc. rank:	12788.0

NP			
Pos.	331	obs:	exp:
aaa	K	56	13345.00
aag	K	23338	10049.00
---	--	------	------
mPD		0.0048	0.49
		nPD:	0.01
		N. weight:	0.86
		Sc. PD:	-0.026
		Sc. rank:	-2347.8

NP			
Pos.	332	obs:	exp:
tct	S	0	4305.00
tcc	S	0	2710.00
tca	S	0	6088.00
tcg	S	0	1245.00
agt	S	23192	4399.00
agc	S	202	4647.00
---	--	------	------
mPD		0.017	1.6
		nPD:	0.01
		N. weight:	1.7
		Sc. PD:	-0.049
		Sc. rank:	-2811.5

NP			
Pos.	333	obs:	exp:
tta	L	0	0.11
ttg	L	0	0.19
ctt	L	0	0.20
ctc	L	0	0.14
cta	L	1	0.19
ctg	L	0	0.17
caa	Q	23363	12344.00
cag	Q	30	11049.00
---	--	------	------
mPD		0.0026	0.50
		nPD:	0.01
		N. weight:	0.65
		Sc. PD:	-0.022
		Sc. rank:	-6395.7

NP			
Pos.	334	obs:	exp:
tta	L	168	2572.00
ttg	L	23192	4404.00
ctt	L	0	4610.00
ctc	L	0	3304.00
cta	L	0	4504.00
ctg	L	33	4000.00
gtt	V	0	0.22
gtc	V	0	0.18
gta	V	0	0.27
gtg	V	1	0.33
---	--	------	------
mPD		0.017	1.1
		nPD:	0.02
		N. weight:	1.7
		Sc. PD:	-0.042
		Sc. rank:	5092.7

NP			
Pos.	335	obs:	exp:
att	I	0	9.83
atc	I	0	6.74
ata	I	27	10.43
gtt	V	4	5166.00
gtc	V	1	4246.00
gta	V	20282	6284.00
gtg	V	3080	7671.00
---	--	------	------
mPD		0.23	0.74
		nPD:	0.31
		N. weight:	0.93
		Sc. PD:	0.24
		Sc. rank:	16923.0

NP			
Pos.	336	obs:	exp:
tgg	W	23394	23394.00
---	--	------	------
mPD		0	0
		nPD:	1.
		N. weight:	0.
		Sc. PD:	0
		Sc. rank:	0

NP			
Pos.	337	obs:	exp:
tta	L	0	0.22
ttg	L	2	0.38
ctt	L	0	0.39
ctc	L	0	0.28
cta	L	0	0.39
ctg	L	0	0.34
att	I	6	4.00
atc	I	0	2.75
ata	I	5	4.25
atg	M	23381	23381.00
---	--	------	------
mPD		0.0011	0.0012
		nPD:	0.91
		N. weight:	0.00035
		Sc. PD:	0.0003
		Sc. rank:	6.9

NP			
Pos.	338	obs:	exp:
gct	A	64	6194.00
gcc	A	8	4842.00
gca	A	23288	10987.00
gcg	A	34	1371.00
---	--	------	------
mPD		0.0090	0.66
		nPD:	0.01
		N. weight:	0.75
		Sc. PD:	-0.02
		Sc. rank:	801.1

,

NP			
Pos.	339	obs:	exp:
tat	Y	0	0.52
tac	Y	1	0.48
tgt	C	59	10559.00
tgc	C	23334	12834.00
---	--	------	------
mPD		0.0051	0.50
		nPD:	0.01
		N. weight:	0.6
		Sc. PD:	-0.018
		Sc. rank:	-1090.1

,

NP			
Pos.	340	obs:	exp:
cat	H	398	13758.00
cac	H	22996	9636.00
---	--	------	------
mPD		0.033	0.48
		nPD:	0.07
		N. weight:	0.82
		Sc. PD:	0.022
		Sc. rank:	11796.0

,

NP			
Pos.	341	obs:	exp:
ttt	F	40	17.17
ttc	F	0	22.83
tct	S	23210	4297.00
tcc	S	121	2705.00
tca	S	21	6077.00
tcg	S	0	1243.00
agt	S	0	4391.00
agc	S	0	4639.00
gct	A	2	0.53
gcc	A	0	0.41
gca	A	0	0.94
gcg	A	0	0.12
---	--	------	------
mPD		0.016	1.6
		nPD:	0.01
		N. weight:	1.7
		Sc. PD:	-0.051
		Sc. rank:	-5133.8

,

NP			
Pos.	342	obs:	exp:
aat	N	1	0.59
aac	N	0	0.41
gtt	V	1	0.22
gtc	V	0	0.18
gta	V	0	0.27
gtg	V	0	0.33
gct	A	23374	6193.00
gcc	A	15	4842.00
gca	A	2	10986.00
gcg	A	1	1370.00
---	--	------	------
mPD		0.0018	0.66
		nPD:	0.
		N. weight:	1.4
		Sc. PD:	-0.05
		Sc. rank:	-21318.0

,

NP			
Pos.	343	obs:	exp:
act	T	0	0.25
acc	T	0	0.21
aca	T	1	0.46
acg	T	0	0.09
gct	A	12	6193.00
gcc	A	1	4842.00
gca	A	23349	10987.00
gcg	A	31	1370.00
---	--	------	------
mPD		0.0038	0.66
		nPD:	0.01
		N. weight:	0.77
		Sc. PD:	-0.026
		Sc. rank:	-7089.1

,

NP			
Pos.	344	obs:	exp:
ttt	F	23376	10039.00
ttc	F	18	13355.00
---	--	------	------
mPD		0.0015	0.49
		nPD:	0.
		N. weight:	0.87
		Sc. PD:	-0.031
		Sc. rank:	-12789.0

,

NP			
Pos.	345	obs:	exp:
gaa	E	23377	14165.00
gag	E	17	9229.00
---	--	------	------
mPD		0.0015	0.48
		nPD:	0.
		N. weight:	0.51
		Sc. PD:	-0.019
		Sc. rank:	-7739.7

,

NP			
Pos.	346	obs:	exp:
gat	D	22738	12326.00
gac	D	656	11068.00
---	--	------	------
mPD		0.055	0.50
		nPD:	0.11
		N. weight:	0.53
		Sc. PD:	0.035
		Sc. rank:	8570.3

,

NP			
Pos.	347	obs:	exp:
tta	L	23231	2572.00
ttg	L	127	4404.00
ctt	L	0	4610.00
ctc	L	0	3304.00
cta	L	34	4504.00
ctg	L	1	4000.00
gtt	V	0	0.22
gtc	V	0	0.18
gta	V	1	0.27
gtg	V	0	0.33
---	--	------	------
mPD		0.014	1.1
		nPD:	0.01
		N. weight:	2.2
		Sc. PD:	-0.061
		Sc. rank:	-260.2

,

NP			
Pos.	348	obs:	exp:
cgt	R	0	638.50
cgc	R	0	1223.00
cga	R	3	2291.00
cgg	R	0	1711.00
att	I	0	0.36
atc	I	0	0.25
ata	I	1	0.39
aaa	K	8	4.56
aag	K	0	3.44
aga	R	23350	11740.00
agg	R	32	5781.00
---	--	------	------
mPD		0.0038	0.91
		nPD:	0.
		N. weight:	0.7
		Sc. PD:	-0.025
		Sc. rank:	-8371.7

,

NP			
Pos.	349	obs:	exp:
tta	L	0	0.33
ttg	L	1	0.56
ctt	L	0	0.59
ctc	L	0	0.42
cta	L	2	0.58
ctg	L	0	0.51
att	I	0	13.47
atc	I	0	9.24
ata	I	37	14.30
gtt	V	1	5163.00
gtc	V	4	4243.00
gta	V	23288	6281.00
gtg	V	60	7666.00
gct	A	0	0.26
gcc	A	0	0.21
gca	A	1	0.47
gcg	A	0	0.06
---	--	------	------
mPD		0.0091	0.74
		nPD:	0.01
		N. weight:	1.3
		Sc. PD:	-0.037
		Sc. rank:	187.8

,

NP			
Pos.	350	obs:	exp:
tta	L	2	0.22
ttg	L	0	0.38
tct	S	1	4305.00
tcc	S	0	2709.00
tca	S	23379	6088.00
tcg	S	12	1245.00
ctt	L	0	0.39
ctc	L	0	0.28
cta	L	0	0.39
ctg	L	0	0.34
agt	S	0	4398.00
agc	S	0	4646.00
---	--	------	------
mPD		0.0013	1.6
		nPD:	0.
		N. weight:	1.4
		Sc. PD:	-0.053
		Sc. rank:	-26299.0

,

NP			
Pos.	351	obs:	exp:
tct	S	0	4305.00
tcc	S	0	2709.00
tca	S	0	6088.00
tcg	S	0	1245.00
aat	N	1	0.59
aac	N	0	0.41
agt	S	23313	4398.00
agc	S	78	4646.00
ggt	G	2	0.30
ggc	G	0	0.30
gga	G	0	0.89
ggg	G	0	0.51
---	--	------	------
mPD		0.0069	1.6
		nPD:	0.
		N. weight:	1.7
		Sc. PD:	-0.06
		Sc. rank:	-19995.0

,

NP			
Pos.	352	obs:	exp:
ttt	F	30	10036.00
ttc	F	23356	13350.00
tta	L	1	0.44
ttg	L	0	0.75
tat	Y	0	1.56
tac	Y	3	1.44
ctt	L	0	0.79
ctc	L	3	0.56
cta	L	0	0.77
ctg	L	0	0.68
gtt	V	0	0.22
gtc	V	1	0.18
gta	V	0	0.27
gtg	V	0	0.33
---	--	------	------
mPD		0.0032	0.49
		nPD:	0.01
		N. weight:	0.57
		Sc. PD:	-0.019
		Sc. rank:	-4749.8

,

NP			
Pos.	353	obs:	exp:
att	I	0	8513.00
atc	I	2	5837.00
ata	I	23383	9035.00
atg	M	1	1.00
gtt	V	0	1.77
gtc	V	0	1.45
gta	V	8	2.15
gtg	V	0	2.63
---	--	------	------
mPD		0.00094	0.66
		nPD:	0.
		N. weight:	0.98
		Sc. PD:	-0.037
		Sc. rank:	-17855.0

,

NP			
Pos.	354	obs:	exp:
cgt	R	0	638.70
cgc	R	0	1224.00
cga	R	1	2291.00
cgg	R	0	1712.00
aaa	K	1	0.57
aag	K	0	0.43
aga	R	22514	11744.00
agg	R	877	5783.00
ggt	G	0	0.15
ggc	G	0	0.15
gga	G	1	0.44
ggg	G	0	0.26
---	--	------	------
mPD		0.072	0.91
		nPD:	0.08
		N. weight:	0.57
		Sc. PD:	0.021
		Sc. rank:	8581.5

,

NP			
Pos.	355	obs:	exp:
ggt	G	1	3547.00
ggc	G	1	3465.00
gga	G	23268	10406.00
ggg	G	124	5976.00
---	--	------	------
mPD		0.011	0.69
		nPD:	0.02
		N. weight:	0.81
		Sc. PD:	-0.02
		Sc. rank:	2694.7

,

NP			
Pos.	356	obs:	exp:
cgt	R	0	0.19
cgc	R	0	0.37
cga	R	0	0.69
cgg	R	0	0.51
act	T	0	0.25
acc	T	0	0.21
aca	T	1	0.46
acg	T	0	0.09
aaa	K	53	13340.00
aag	K	23333	10046.00
aga	R	0	3.51
agg	R	7	1.73
---	--	------	------
mPD		0.0053	0.49
		nPD:	0.01
		N. weight:	0.86
		Sc. PD:	-0.025
		Sc. rank:	-1163.6

,

NP			
Pos.	357	obs:	exp:
cgt	R	0	0.35
cgc	R	0	0.68
cga	R	0	1.27
cgg	R	0	0.95
aaa	K	23280	13337.00
aag	K	101	10044.00
aga	R	13	6.53
agg	R	0	3.21
---	--	------	------
mPD		0.0097	0.49
		nPD:	0.02
		N. weight:	0.55
		Sc. PD:	-0.011
		Sc. rank:	3507.9

,

NP			
Pos.	358	obs:	exp:
tta	L	0	1.10
ttg	L	10	1.88
ctt	L	0	1.97
ctc	L	0	1.41
cta	L	0	1.93
ctg	L	0	1.71
atg	M	26	26.00
gtt	V	3	5164.00
gtc	V	0	4244.00
gta	V	141	6282.00
gtg	V	23213	7667.00
gct	A	0	0.26
gcc	A	0	0.21
gca	A	0	0.47
gcg	A	1	0.06
---	--	------	------
mPD		0.015	0.74
		nPD:	0.02
		N. weight:	1.1
		Sc. PD:	-0.021
		Sc. rank:	7611.9

,

NP			
Pos.	359	obs:	exp:
tta	L	0	0.22
ttg	L	0	0.38
tct	S	1	0.37
tcc	S	0	0.23
tca	S	1	0.52
tcg	S	0	0.11
ctt	L	1	0.39
ctc	L	1	0.28
cta	L	0	0.39
ctg	L	0	0.34
att	I	3075	8386.00
atc	I	19955	5750.00
ata	I	7	8900.00
agt	S	0	0.38
agc	S	0	0.40
gtt	V	67	78.04
gtc	V	286	64.14
gta	V	0	94.94
gtg	V	0	115.90
---	--	------	------
mPD		0.26	0.69
		nPD:	0.38
		N. weight:	0.98
		Sc. PD:	0.32
		Sc. rank:	18319.0

,

NP			
Pos.	360	obs:	exp:
tct	S	0	0.55
tcc	S	0	0.35
tca	S	3	0.78
tcg	S	0	0.16
cct	P	14	6444.00
ccc	P	1	4338.00
cca	P	23290	9959.00
ccg	P	84	2648.00
act	T	0	0.50
acc	T	0	0.41
aca	T	2	0.91
acg	T	0	0.17
agt	S	0	0.56
agc	S	0	0.60
---	--	------	------
mPD		0.0089	0.70
		nPD:	0.01
		N. weight:	0.86
		Sc. PD:	-0.023
		Sc. rank:	543.1

,

NP			
Pos.	361	obs:	exp:
cgt	R	0	638.80
cgc	R	0	1224.00
cga	R	0	2291.00
cgg	R	1	1712.00
aga	R	23349	11745.00
agg	R	44	5783.00
---	--	------	------
mPD		0.0039	0.91
		nPD:	0.
		N. weight:	0.7
		Sc. PD:	-0.025
		Sc. rank:	-8095.2

,

NP			
Pos.	362	obs:	exp:
ggt	G	2	3547.00
ggc	G	4	3465.00
gga	G	23098	10406.00
ggg	G	290	5976.00
---	--	------	------
mPD		0.025	0.69
		nPD:	0.04
		N. weight:	0.77
		Sc. PD:	-0.0035
		Sc. rank:	8999.8

,

NP			
Pos.	363	obs:	exp:
cgt	R	0	0.82
cgc	R	0	1.57
cga	R	0	2.94
cgg	R	0	2.20
aaa	K	136	13328.00
aag	K	23228	10036.00
aga	R	0	15.06
agg	R	30	7.42
---	--	------	------
mPD		0.014	0.49
		nPD:	0.03
		N. weight:	0.84
		Sc. PD:	-0.0098
		Sc. rank:	8195.5

,

NP			
Pos.	364	obs:	exp:
ttt	F	1	0.43
ttc	F	0	0.57
tta	L	0	2572.00
ttg	L	0	4404.00
ctt	L	23322	4610.00
ctc	L	62	3304.00
cta	L	1	4503.00
ctg	L	7	3999.00
att	I	1	0.36
atc	I	0	0.25
ata	I	0	0.39
---	--	------	------
mPD		0.0061	1.1
		nPD:	0.01
		N. weight:	1.7
		Sc. PD:	-0.057
		Sc. rank:	-15991.0

,

NP			
Pos.	365	obs:	exp:
ttt	F	0	30.47
ttc	F	71	40.53
tct	S	45	4290.00
tcc	S	23246	2700.00
tca	S	22	6067.00
tcg	S	0	1241.00
tat	Y	0	0.52
tac	Y	1	0.48
act	T	0	2.27
acc	T	9	1.85
aca	T	0	4.12
acg	T	0	0.77
agt	S	0	4384.00
agc	S	0	4631.00
---	--	------	------
mPD		0.013	1.6
		nPD:	0.01
		N. weight:	2.2
		Sc. PD:	-0.07
		Sc. rank:	-12654.0

,

NP			
Pos.	366	obs:	exp:
act	T	4	5897.00
acc	T	7	4798.00
aca	T	23258	10702.00
acg	T	125	1996.00
---	--	------	------
mPD		0.012	0.68
		nPD:	0.02
		N. weight:	0.78
		Sc. PD:	-0.018
		Sc. rank:	3874.8

,

NP			
Pos.	367	obs:	exp:
cgt	R	0	638.80
cgc	R	0	1224.00
cga	R	0	2291.00
cgg	R	0	1712.00
aga	R	23357	11745.00
agg	R	37	5783.00
---	--	------	------
mPD		0.0032	0.91
		nPD:	0.
		N. weight:	0.7
		Sc. PD:	-0.025
		Sc. rank:	-9443.9

,

NP			
Pos.	368	obs:	exp:
ggt	G	26	3547.00
ggc	G	0	3465.00
gga	G	568	10406.00
ggg	G	22800	5976.00
---	--	------	------
mPD		0.050	0.69
		nPD:	0.07
		N. weight:	1.3
		Sc. PD:	0.038
		Sc. rank:	18590.0

,

NP			
Pos.	369	obs:	exp:
tta	L	0	0.22
ttg	L	0	0.38
ctt	L	0	0.39
ctc	L	2	0.28
cta	L	0	0.39
ctg	L	0	0.34
att	I	72	27.30
atc	I	3	18.72
ata	I	0	28.98
gtt	V	20134	5155.00
gtc	V	3170	4237.00
gta	V	10	6271.00
gtg	V	3	7654.00
---	--	------	------
mPD		0.24	0.74
		nPD:	0.33
		N. weight:	1.2
		Sc. PD:	0.32
		Sc. rank:	21515.0

,

NP

Pos.	370	obs:	exp:
cat	H	1	0.59
cac	H	0	0.41
caa	Q	120	12344.00
cag	Q	23273	11049.00
---	--	------	------
mPD		0.010	0.50
		nPD:	0.02
		N. weight:	0.74
		Sc. PD:	-0.014
		Sc. rank:	4946.1

,

NP

Pos.	371	obs:	exp:
att	I	23318	8511.00
atc	I	17	5836.00
ata	I	45	9033.00
act	T	1	0.25
acc	T	0	0.21
aca	T	0	0.46
acg	T	0	0.09
gtt	V	13	2.87
gtc	V	0	2.36
gta	V	0	3.50
gtg	V	0	4.27
---	--	------	------
mPD		0.0065	0.66
		nPD:	0.01
		N. weight:	1.
		Sc. PD:	-0.031
		Sc. rank:	-2495.5

,

NP

Pos.	372	obs:	exp:
tct	S	1	0.18
tcc	S	0	0.12
tca	S	0	0.26
tcg	S	0	0.05
agt	S	0	0.19
agc	S	0	0.20
gtt	V	1	0.22
gtc	V	0	0.18
gta	V	0	0.27
gtg	V	0	0.33
gct	A	23298	6193.00
gcc	A	84	4842.00
gca	A	2	10986.00
gcg	A	8	1370.00
---	--	------	------
mPD		0.0082	0.66
		nPD:	0.01
		N. weight:	1.3
		Sc. PD:	-0.037
		Sc. rank:	529.9

,

NP

Pos.	373	obs:	exp:
tct	S	6	4305.00
tcc	S	2	2709.00
tca	S	23365	6088.00
tcg	S	18	1245.00
agt	S	0	4398.00
agc	S	0	4646.00
gct	A	0	0.79
gcc	A	0	0.62
gca	A	3	1.41
gcg	A	0	0.18
---	--	------	------
mPD		0.0025	1.6
		nPD:	0.
		N. weight:	1.4
		Sc. PD:	-0.052
		Sc. rank:	-24844.0

,

NP

Pos.	374	obs:	exp:
tct	S	0	1.10
tcc	S	0	0.69
tca	S	0	1.56
tcg	S	0	0.32
att	I	1	0.36
atc	I	0	0.25
ata	I	0	0.39
act	T	1	0.25
acc	T	0	0.21
aca	T	0	0.46
acg	T	0	0.09
aat	N	22641	13831.00
aac	N	745	9555.00
agt	S	6	1.13
agc	S	0	1.19
---	--	------	------
mPD		0.062	0.48
		nPD:	0.13
		N. weight:	0.41
		Sc. PD:	0.035
		Sc. rank:	6788.6

,

NP

Pos.	375	obs:	exp:
gaa	E	104	14165.00
gag	E	23290	9229.00
---	--	------	------
mPD		0.0089	0.48
		nPD:	0.02
		N. weight:	0.93
		Sc. PD:	-0.02
		Sc. rank:	5177.9

,

NP

Pos.	376	obs:	exp:
tct	S	0	64.96
tcc	S	0	40.89
tca	S	0	91.87
tcg	S	0	18.79
act	T	3	0.76
acc	T	0	0.62
aca	T	0	1.37
acg	T	0	0.26
aat	N	23020	13624.00
aac	N	16	9412.00
aaa	K	1	0.57
aag	K	0	0.43
agt	S	353	66.38
agc	S	0	70.12
gat	D	1	0.53
gac	D	0	0.47
---	--	------	------
mPD		0.032	0.54
		nPD:	0.06
		N. weight:	0.56
		Sc. PD:	0.0096
		Sc. rank:	7468.9

,

NP

Pos.	377	obs:	exp:
tta	L	0	0.22
ttg	L	1	0.38
ctt	L	0	0.39
ctc	L	0	0.28
cta	L	0	0.39
ctg	L	1	0.34
att	I	1	0.36
atc	I	0	0.25
ata	I	0	0.39
atg	M	11	11.00
gtt	V	4	5169.00
gtc	V	1	4248.00
gta	V	50	6287.00
gtg	V	23323	7674.00
gct	A	0	0.53
gcc	A	0	0.41
gca	A	0	0.94
gcg	A	2	0.12
---	--	------	------
mPD		0.0061	0.74
		nPD:	0.01
		N. weight:	1.1
		Sc. PD:	-0.035
		Sc. rank:	-5438.0

,

NP

Pos.	378	obs:	exp:
aaa	K	1	0.57
aag	K	0	0.43
gat	D	1	2.11
gac	D	3	1.89
gaa	E	23376	14162.00
gag	E	13	9227.00
---	--	------	------
mPD		0.0015	0.48
		nPD:	0.
		N. weight:	0.51
		Sc. PD:	-0.019
		Sc. rank:	-7455.8

,

NP

Pos.	379	obs:	exp:
tct	S	0	1.10
tcc	S	1	0.69
tca	S	0	1.56
tcg	S	0	0.32
cct	P	0	2.20
ccc	P	8	1.48
cca	P	0	3.41
ccg	P	0	0.91
att	I	1	85.18
atc	I	233	58.41
ata	I	0	90.41
act	T	21	5801.00
acc	T	22875	4720.00
aca	T	115	10527.00
acg	T	0	1963.00
aat	N	2	31.94
aac	N	52	22.06
agt	S	0	1.13
agc	S	5	1.19
gct	A	0	21.45
gcc	A	81	16.77
gca	A	0	38.04
gcg	A	0	4.75
---	--	------	------
mPD		0.044	0.71
		nPD:	0.06
		N. weight:	1.6
		Sc. PD:	0.033
		Sc. rank:	22146.0

,

NP

Pos.	380	obs:	exp:
tta	L	0	0.33
ttg	L	1	0.56
ctt	L	0	0.59
ctc	L	0	0.42
cta	L	0	0.58
ctg	L	2	0.51
att	I	0	0.36
atc	I	0	0.25
ata	I	1	0.39
atg	M	23389	23389.00
gtt	V	0	0.22
gtc	V	0	0.18
gta	V	0	0.27
gtg	V	1	0.33
---	--	------	------
mPD		0.00043	0.00065
		nPD:	0.66
		N. weight:	0.00027
		Sc. PD:	0.00016
		Sc. rank:	5.3

,

NP

Pos.	381	obs:	exp:
aat	N	1	5.91
aac	N	9	4.09
gct	A	0	0.26
gcc	A	1	0.21
gca	A	0	0.47
gcg	A	0	0.06
gat	D	88	12316.00
gac	D	23286	11058.00
gaa	E	5	3.03
gag	E	0	1.97
ggt	G	0	0.61
ggc	G	3	0.59
gga	G	1	1.78
ggg	G	0	1.02
---	--	------	------
mPD		0.0094	0.50
		nPD:	0.02
		N. weight:	0.75
		Sc. PD:	-0.016
		Sc. rank:	4287.0

,

NP

Pos.	382	obs:	exp:
tct	S	42	4305.00
tcc	S	23330	2709.00
tca	S	19	6088.00
tcg	S	0	1245.00
cct	P	0	0.28
ccc	P	1	0.19
cca	P	0	0.43
ccg	P	0	0.11
act	T	0	0.25
acc	T	1	0.21
aca	T	0	0.46
acg	T	0	0.09
agt	S	0	4398.00
agc	S	0	4646.00
gct	A	0	0.26
gcc	A	1	0.21
gca	A	0	0.47
gcg	A	0	0.06
---	--	------	------
mPD		0.0055	1.6
		nPD:	0.
		N. weight:	2.2
		Sc. PD:	-0.08
		Sc. rank:	-31163.0

,

NP

Pos.	383	obs:	exp:
tct	S	0	6.07
tcc	S	0	3.82
tca	S	0	8.59
tcg	S	0	1.76
tat	Y	2	1.04
tac	Y	0	0.96
aat	N	22666	13815.00
aac	N	693	9544.00
agt	S	29	6.21
agc	S	4	6.55
---	--	------	------
mPD		0.061	0.49
		nPD:	0.12
		N. weight:	0.42
		Sc. PD:	0.034
		Sc. rank:	6795.2

,

NP

Pos.	384	obs:	exp:
act	T	225	5897.00
acc	T	23158	4798.00
aca	T	11	10702.00
acg	T	0	1996.00
---	--	------	------
mPD		0.020	0.68
		nPD:	0.03
		N. weight:	1.6
		Sc. PD:	-0.017
		Sc. rank:	16140.0

,

NP

Pos.	385	obs:	exp:
tta	L	1	2572.00
ttg	L	60	4404.00
ctt	L	19	4610.00
ctc	L	0	3304.00
cta	L	47	4503.00
ctg	L	23265	3999.00
atg	M	2	2.00
---	--	------	------
mPD		0.011	1.1
		nPD:	0.01
		N. weight:	1.8
		Sc. PD:	-0.053
		Sc. rank:	-5016.4

,

NP

Pos.	386	obs:	exp:
gaa	E	23346	14164.00
gag	E	47	9229.00
ggt	G	0	0.15
ggc	G	0	0.15
gga	G	1	0.44
ggg	G	0	0.26
---	--	------	------
mPD		0.0041	0.48
		nPD:	0.01
		N. weight:	0.5
		Sc. PD:	-0.016
		Sc. rank:	-2070.7

,

NP

Pos.	387	obs:	exp:
tta	L	126	2571.00
ttg	L	0	4404.00
ctt	L	3	4609.00
ctc	L	0	3304.00
cta	L	22860	4503.00
ctg	L	401	3999.00
att	I	0	1.09
atc	I	0	0.75
ata	I	3	1.16
gtt	V	0	0.22
gtc	V	0	0.18
gta	V	1	0.27
gtg	V	0	0.33
---	--	------	------
mPD		0.045	1.1
		nPD:	0.04
		N. weight:	1.6
		Sc. PD:	-0.002
		Sc. rank:	18994.0

,

NP

Pos.	388	obs:	exp:
cgt	R	0	638.70
cgc	R	0	1224.00
cga	R	3	2291.00
cgg	R	0	1712.00
aaa	K	3	1.71
aag	K	0	1.29
aga	R	23367	11743.00
agg	R	21	5783.00
---	--	------	------
mPD		0.0023	0.91
		nPD:	0.
		N. weight:	0.7
		Sc. PD:	-0.026
		Sc. rank:	-11249.0

,

NP

Pos.	389	obs:	exp:
tct	S	0	4305.00
tcc	S	0	2710.00
tca	S	0	6088.00
tcg	S	0	1245.00
agt	S	33	4399.00
agc	S	23361	4647.00
---	--	------	------
mPD		0.0028	1.6
		nPD:	0.
		N. weight:	1.7
		Sc. PD:	-0.062
		Sc. rank:	-29011.0

,

NP

Pos.	390	obs:	exp:
cgt	R	0	638.60
cgc	R	0	1223.00
cga	R	0	2291.00
cgg	R	0	1712.00
aaa	K	5	2.85
aag	K	0	2.15
aga	R	23293	11741.00
agg	R	94	5782.00
ggt	G	0	0.30
ggc	G	0	0.30
gga	G	1	0.89
ggg	G	1	0.51
---	--	------	------
mPD		0.0087	0.91
		nPD:	0.01
		N. weight:	0.69
		Sc. PD:	-0.021
		Sc. rank:	-2030.0

,

NP

Pos.	391	obs:	exp:
tat	Y	97	12155.00
tac	Y	23296	11238.00
cat	H	0	0.59
cac	H	1	0.41
---	--	------	------
mPD		0.0083	0.50
		nPD:	0.02
		N. weight:	0.73
		Sc. PD:	-0.017
		Sc. rank:	3410.5

,

NP

Pos.	392	obs:	exp:
tgg	W	23394	23394.00
---	--	------	------
mPD		0	0
		nPD:	1.
		N. weight:	0.
		Sc. PD:	0
		Sc. rank:	0

,

NP

Pos.	393	obs:	exp:
tct	S	0	0.18
tcc	S	1	0.12
tca	S	0	0.26
tcg	S	0	0.05
agt	S	0	0.19
agc	S	0	0.20
gct	A	51	6193.00
gcc	A	23282	4842.00
gca	A	60	10987.00
gcg	A	0	1370.00
---	--	------	------
mPD		0.0095	0.66
		nPD:	0.01
		N. weight:	1.6
		Sc. PD:	-0.04
		Sc. rank:	3301.5

,

NP

Pos.	394	obs:	exp:
att	I	5	8516.00
atc	I	31	5839.00
ata	I	23357	9038.00
gtt	V	0	0.22
gtc	V	0	0.18
gta	V	1	0.27
gtg	V	0	0.33
---	--	------	------
mPD		0.0032	0.66
		nPD:	0.
		N. weight:	0.97
		Sc. PD:	-0.034
		Sc. rank:	-10372.0

,

NP

Pos.	395	obs:	exp:
cgt	R	0	638.60
cgc	R	0	1224.00
cga	R	2	2291.00
cgg	R	1	1712.00
aaa	K	2	2.28
aag	K	2	1.72
aga	R	18923	11743.00
agg	R	4464	5782.00
---	--	------	------
mPD		0.31	0.91
		nPD:	0.34
		N. weight:	0.35
		Sc. PD:	0.1
		Sc. rank:	6390.9

,

NP

Pos.	396	obs:	exp:
act	T	141	5897.00
acc	T	23243	4798.00
aca	T	10	10702.00
acg	T	0	1996.00
---	--	------	------
mPD		0.013	0.68
		nPD:	0.02
		N. weight:	1.6
		Sc. PD:	-0.034
		Sc. rank:	9574.8

,

NP

Pos.	397	obs:	exp:
cgt	R	0	638.60
cgc	R	0	1223.00
cga	R	0	2291.00
cgg	R	2	1712.00
aaa	K	1	3.99
aag	K	6	3.01
aga	R	1426	11741.00
agg	R	21959	5782.00
---	--	------	------
mPD		0.12	0.91
		nPD:	0.13
		N. weight:	1.2
		Sc. PD:	0.096
		Sc. rank:	19163.0

,

NP

Pos.	398	obs:	exp:
tct	S	0	4305.00
tcc	S	0	2710.00
tca	S	0	6088.00
tcg	S	0	1245.00
agt	S	23382	4399.00
agc	S	12	4647.00
---	--	------	------
mPD		0.0010	1.6
		nPD:	0.
		N. weight:	1.7
		Sc. PD:	-0.067
		Sc. rank:	-33029.0

NP

Pos.	399	obs:	exp:
gtt	V	0	0.22
gtc	V	0	0.18
gta	V	1	0.27
gtg	V	0	0.33
ggt	G	27	3547.00
ggc	G	30	3465.00
gga	G	23231	10406.00
ggg	G	105	5975.00
---	--	------	------
mPD		0.014	0.69
		nPD:	0.02
		N. weight:	0.79
		Sc. PD:	-0.016
		Sc. rank:	5158.0

NP

Pos.	400	obs:	exp:
gct	A	0	0.26
gcc	A	0	0.21
gca	A	1	0.47
gcg	A	0	0.06
ggt	G	0	3547.00
ggc	G	1	3465.00
gga	G	23335	10406.00
ggg	G	57	5975.00
---	--	------	------
mPD		0.0050	0.69
		nPD:	0.01
		N. weight:	0.82
		Sc. PD:	-0.027
		Sc. rank:	-5592.7

NP

Pos.	401	obs:	exp:
tct	S	0	4.42
tcc	S	0	2.78
tca	S	0	6.25
tcg	S	0	1.28
cat	H	2	1.18
cac	H	0	0.82
act	T	3	0.76
acc	T	0	0.62
aca	T	0	1.37
acg	T	0	0.26
aat	N	22822	13819.00
aac	N	543	9546.00
agt	S	24	4.51
agc	S	0	4.77
---	--	------	------
mPD		0.048	0.49
		nPD:	0.1
		N. weight:	0.44
		Sc. PD:	0.024
		Sc. rank:	6884.6

NP

Pos.	402	obs:	exp:
tct	S	0	0.37
tcc	S	2	0.23
tca	S	0	0.52
tcg	S	0	0.11
act	T	33	5893.00
acc	T	23319	4795.00
aca	T	23	10693.00
acg	T	0	1994.00
aat	N	0	1.77
aac	N	3	1.23
agt	S	0	0.38
agc	S	0	0.40
gct	A	0	3.71
gcc	A	14	2.90
gca	A	0	6.58
gcg	A	0	0.82
---	--	------	------
mPD		0.0064	0.68
		nPD:	0.01
		N. weight:	1.6
		Sc. PD:	-0.049
		Sc. rank:	-5179.9

NP

Pos.	403	obs:	exp:
tct	S	0	3.86
tcc	S	0	2.43
tca	S	0	5.47
tcg	S	0	1.12
tat	Y	1	0.52
tac	Y	0	0.48
act	T	1	0.25
acc	T	0	0.21
aca	T	0	0.46
acg	T	0	0.09
aat	N	23317	13823.00
aac	N	54	9548.00
agt	S	21	3.95
agc	S	0	4.17
---	--	------	------
mPD		0.0066	0.49
		nPD:	0.01
		N. weight:	0.53
		Sc. PD:	-0.014
		Sc. rank:	531.4

NP

Pos.	404	obs:	exp:
tta	L	0	0.33
ttg	L	0	0.56
ctt	L	0	0.59
ctc	L	0	0.42
cta	L	3	0.58
ctg	L	0	0.51
cct	P	0	0.28
ccc	P	0	0.19
cca	P	1	0.43
ccg	P	0	0.11
cat	H	0	2.94
cac	H	5	2.06
caa	Q	23030	12335.00
cag	Q	346	11041.00
cgt	R	0	0.05
cgc	R	0	0.10
cga	R	2	0.20
cgg	R	0	0.15
aaa	K	7	3.99
aag	K	0	3.01
aga	R	0	1.00
agg	R	0	0.49
---	--	------	------
mPD		0.031	0.50
		nPD:	0.06
		N. weight:	0.58
		Sc. PD:	0.012
		Sc. rank:	8055.3

NP

Pos.	405	obs:	exp:
tta	L	0	0.22
ttg	L	0	0.38
ctt	L	0	0.39
ctc	L	0	0.28
cta	L	0	0.39
ctg	L	2	0.34
cat	H	19	11.76
cac	H	1	8.24
caa	Q	3413	12332.00
cag	Q	19958	11039.00
cgt	R	0	0.03
cgc	R	0	0.05
cga	R	0	0.10
cgg	R	1	0.07
aga	R	0	0.50
agg	R	0	0.25
---	--	------	------
mPD		0.25	0.50
		nPD:	0.5
		N. weight:	0.33
		Sc. PD:	0.15
		Sc. rank:	6389.8

NP

Pos.	406	obs:	exp:
caa	Q	0	0.53
cag	Q	1	0.47
cgt	R	0	5.38
cgc	R	0	10.30
cga	R	0	19.30
cgg	R	0	14.42
aaa	K	74	13232.00
aag	K	23122	9964.00
aga	R	16	98.90
agg	R	181	48.70
---	--	------	------
mPD		0.024	0.51
		nPD:	0.05
		N. weight:	0.85
		Sc. PD:	0.0058
		Sc. rank:	11036.0

NP

Pos.	407	obs:	exp:
act	T	0	5.29
acc	T	0	4.31
aca	T	21	9.61
acg	T	0	1.79
gtt	V	0	0.22
gtc	V	0	0.18
gta	V	1	0.27
gtg	V	0	0.33
gct	A	9	6188.00
gcc	A	4	4838.00
gca	A	23355	10977.00
gcg	A	4	1369.00
---	--	------	------
mPD		0.0033	0.66
		nPD:	0.01
		N. weight:	0.78
		Sc. PD:	-0.027
		Sc. rank:	-8094.7

NP

Pos.	408	obs:	exp:
ttt	F	1	0.86
ttc	F	1	1.14
tct	S	8550	4304.00
tcc	S	14761	2709.00
tca	S	75	6086.00
tcg	S	0	1245.00
tat	Y	1	0.52
tac	Y	0	0.48
cct	P	0	0.28
ccc	P	1	0.19
cca	P	0	0.43
ccg	P	0	0.11
act	T	0	0.76
acc	T	3	0.62
aca	T	0	1.37
acg	T	0	0.26
agt	S	0	4397.00
agc	S	0	4645.00
gct	A	0	0.26
gcc	A	1	0.21
gca	A	0	0.47
gcg	A	0	0.06
---	--	------	------
mPD		0.47	1.6
		nPD:	0.29
		N. weight:	1.4
		Sc. PD:	0.32
		Sc. rank:	24442.0

NP

Pos.	409	obs:	exp:
tct	S	0	0.74
tcc	S	0	0.46
tca	S	4	1.04
tcg	S	0	0.21
act	T	0	4.03
acc	T	0	3.28
aca	T	16	7.32
acg	T	0	1.37
agt	S	0	0.75
agc	S	0	0.79
gct	A	13	6188.00
gcc	A	7	4838.00
gca	A	23334	10978.00
gcg	A	20	1369.00
---	--	------	------
mPD		0.0051	0.66
		nPD:	0.01
		N. weight:	0.77
		Sc. PD:	-0.025
		Sc. rank:	-4379.8

NP			
Pos.	410	obs:	exp:
ggt	G	104	3547.00
ggc	G	23283	3465.00
gga	G	5	10406.00
ggg	G	2	5976.00
---	--	------	------
mPD		0.0094	0.69
		nPD:	0.01
		N. weight:	1.9
		Sc. PD:	-0.051
		Sc. rank:	2359.4

NP			
Pos.	411	obs:	exp:
caa	Q	618	12344.00
cag	Q	22775	11049.00
cgt	R	0	0.03
cgc	R	0	0.05
cga	R	0	0.10
cgg	R	1	0.07
aga	R	0	0.50
agg	R	0	0.25
---	--	------	------
mPD		0.052	0.50
		nPD:	0.1
		N. weight:	0.65
		Sc. PD:	0.039
		Sc. rank:	10223.0

NP			
Pos.	412	obs:	exp:
att	I	17	8502.00
atc	I	23334	5830.00
ata	I	4	9023.00
act	T	0	1.26
acc	T	5	1.03
aca	T	0	2.29
acg	T	0	0.43
gtt	V	0	7.52
gtc	V	34	6.18
gta	V	0	9.14
gtg	V	0	11.16
---	--	------	------
mPD		0.0051	0.66
		nPD:	0.01
		N. weight:	1.4
		Sc. PD:	-0.045
		Sc. rank:	-7967.2

NP			
Pos.	413	obs:	exp:
tct	S	0	4305.00
tcc	S	0	2710.00
tca	S	0	6088.00
tcg	S	0	1245.00
agt	S	23358	4399.00
agc	S	36	4647.00
---	--	------	------
mPD		0.0031	1.6
		nPD:	0.
		N. weight:	1.7
		Sc. PD:	-0.064
		Sc. rank:	-29287.0

NP			
Pos.	414	obs:	exp:
att	I	0	0.36
atc	I	0	0.25
ata	I	1	0.39
atg	M	10	10.00
gtt	V	14	5170.00
gtc	V	0	4249.00
gta	V	154	6289.00
gtg	V	23215	7676.00
---	--	------	------
mPD		0.015	0.74
		nPD:	0.02
		N. weight:	1.1
		Sc. PD:	-0.021
		Sc. rank:	7475.3

NP			
Pos.	415	obs:	exp:
caa	Q	99	12344.00
cag	Q	23295	11050.00
---	--	------	------
mPD		0.0084	0.50
		nPD:	0.02
		N. weight:	0.75
		Sc. PD:	-0.017
		Sc. rank:	3575.2

NP			
Pos.	416	obs:	exp:
cct	P	23189	6445.00
ccc	P	196	4339.00
cca	P	9	9961.00
ccg	P	0	2649.00
---	--	------	------
mPD		0.017	0.70
		nPD:	0.02
		N. weight:	1.3
		Sc. PD:	-0.02
		Sc. rank:	11279.0

NP			
Pos.	417	obs:	exp:
act	T	9	5895.00
acc	T	3	4797.00
aca	T	23360	10699.00
acg	T	14	1995.00
gct	A	0	2.12
gcc	A	0	1.66
gca	A	8	3.76
gcg	A	0	0.47
---	--	------	------
mPD		0.0029	0.68
		nPD:	0.
		N. weight:	0.8
		Sc. PD:	-0.028
		Sc. rank:	-9335.5

NP			
Pos.	418	obs:	exp:
ttt	F	66	10039.00
ttc	F	23328	13355.00
---	--	------	------
mPD		0.0056	0.49
		nPD:	0.01
		N. weight:	0.56
		Sc. PD:	-0.016
		Sc. rank:	-650.6

NP			
Pos.	419	obs:	exp:
tct	S	5	4305.00
tcc	S	0	2710.00
tca	S	23366	6088.00
tcg	S	23	1245.00
agt	S	0	4399.00
agc	S	0	4647.00
---	--	------	------
mPD		0.0024	1.6
		nPD:	0.
		N. weight:	1.4
		Sc. PD:	-0.052
		Sc. rank:	-24993.0

NP			
Pos.	420	obs:	exp:
tta	L	0	0.22
ttg	L	0	0.38
ctt	L	0	0.39
ctc	L	0	0.28
cta	L	0	0.39
ctg	L	2	0.34
gtt	V	9	5172.00
gtc	V	0	4251.00
gta	V	92	6291.00
gtg	V	23291	7679.00
---	--	------	------
mPD		0.0088	0.74
		nPD:	0.01
		N. weight:	1.1
		Sc. PD:	-0.031
		Sc. rank:	-953.6

NP			
Pos.	421	obs:	exp:
caa	Q	348	12344.00
cag	Q	23046	11050.00
---	--	------	------
mPD		0.029	0.50
		nPD:	0.06
		N. weight:	0.69
		Sc. PD:	0.012
		Sc. rank:	9368.1

NP			
Pos.	422	obs:	exp:
cgt	R	0	638.70
cgc	R	0	1224.00
cga	R	22608	2291.00
cgg	R	759	1712.00
aga	R	25	11744.00
agg	R	0	5783.00
ggt	G	0	0.30
ggc	G	0	0.30
gga	G	2	0.89
ggg	G	0	0.51
---	--	------	------
mPD		0.065	0.91
		nPD:	0.07
		N. weight:	2.3
		Sc. PD:	0.067
		Sc. rank:	32740.0

NP			
Pos.	423	obs:	exp:
tct	S	0	3.31
tcc	S	0	2.08
tca	S	0	4.68
tcg	S	0	0.96
cat	H	1	0.59
cac	H	0	0.41
act	T	5	1.26
acc	T	0	1.03
aca	T	0	2.29
acg	T	0	0.43
aat	N	23303	13822.00
aac	N	67	9548.00
agt	S	18	3.38
agc	S	0	3.58
---	--	------	------
mPD		0.0078	0.49
		nPD:	0.02
		N. weight:	0.53
		Sc. PD:	-0.013
		Sc. rank:	1961.0

NP			
Pos.	424	obs:	exp:
tta	L	0	2571.00
ttg	L	0	4404.00
ctt	L	43	4609.00
ctc	L	23340	3304.00
cta	L	7	4503.00
ctg	L	0	3999.00
att	I	0	1.46
atc	I	4	1.00
ata	I	0	1.55
---	--	------	------
mPD		0.0046	1.1
		nPD:	0.
		N. weight:	2.
		Sc. PD:	-0.071
		Sc. rank:	-24407.0

NP			
Pos.	425	obs:	exp:
cct	P	21637	6445.00
ccc	P	1755	4339.00
cca	P	1	9961.00
ccg	P	1	2649.00
---	--	------	------
mPD		0.14	0.70
		nPD:	0.2
		N. weight:	1.1
		Sc. PD:	0.17
		Sc. rank:	18911.0

NP			
Pos.	426	obs:	exp:
ttt	F	22673	10039.00
ttc	F	721	13355.00
---	--	------	------
mPD		0.060	0.49
		nPD:	0.12
		N. weight:	0.72
		Sc. PD:	0.057
		Sc. rank:	11744.0

,

NP			
Pos.	427	obs:	exp:
gaa	E	23375	14165.00
gag	E	19	9229.00
---	--	------	------
mPD		0.0016	0.48
		nPD:	0.
		N. weight:	0.51
		Sc. PD:	-0.018
		Sc. rank:	-7112.2

,

NP			
Pos.	428	obs:	exp:
cgt	R	0	638.70
cgc	R	0	1224.00
cga	R	7	2291.00
cgg	R	0	1712.00
aaa	K	1	1.14
aag	K	1	0.86
aga	R	23323	11743.00
agg	R	61	5783.00
ggt	G	0	0.15
ggc	G	0	0.15
gga	G	1	0.44
ggg	G	0	0.26
---	--	------	------
mPD		0.0061	0.91
		nPD:	0.01
		N. weight:	0.69
		Sc. PD:	-0.023
		Sc. rank:	-5435.5

,

NP			
Pos.	429	obs:	exp:
tct	S	0	2.76
tcc	S	0	1.74
tca	S	15	3.90
tcg	S	0	0.80
act	T	0	9.33
acc	T	0	7.59
aca	T	37	16.93
acg	T	0	3.16
agt	S	0	2.82
agc	S	0	2.98
gtt	V	0	0.22
gtc	V	0	0.18
gta	V	1	0.27
gtg	V	0	0.33
gct	A	0	6180.00
gcc	A	0	4832.00
gca	A	23336	10962.00
gcg	A	5	1367.00
---	--	------	------
mPD		0.0050	0.67
		nPD:	0.01
		N. weight:	0.78
		Sc. PD:	-0.025
		Sc. rank:	-4888.0

,

NP			
Pos.	430	obs:	exp:
tct	S	0	0.18
tcc	S	1	0.12
tca	S	0	0.26
tcg	S	0	0.05
att	I	0	2.18
atc	I	6	1.50
ata	I	0	2.32
act	T	837	5895.00
acc	T	22539	4796.00
aca	T	8	10698.00
acg	T	0	1995.00
aat	N	0	0.59
aac	N	1	0.41
agt	S	0	0.19
agc	S	0	0.20
gct	A	1	0.53
gcc	A	1	0.41
gca	A	0	0.94
gcg	A	0	0.12
---	--	------	------
mPD		0.071	0.68
		nPD:	0.1
		N. weight:	1.5
		Sc. PD:	0.089
		Sc. rank:	23371.0

,

NP			
Pos.	431	obs:	exp:
att	I	8518	3104.00
atc	I	10	2129.00
ata	I	0	3295.00
gtt	V	14824	3287.00
gtc	V	25	2701.00
gta	V	4	3998.00
gtg	V	13	4880.00
---	--	------	------
mPD		0.47	1.2
		nPD:	0.39
		N. weight:	1.4
		Sc. PD:	0.45
		Sc. rank:	25529.0

,

NP			
Pos.	432	obs:	exp:
tta	L	1	0.88
ttg	L	5	1.51
ctt	L	0	1.58
ctc	L	0	1.13
cta	L	0	1.54
ctg	L	2	1.37
att	I	2	2.18
atc	I	0	1.50
ata	I	4	2.32
atg	M	23377	23377.00
aaa	K	0	1.14
aag	K	2	0.86
gtt	V	0	0.22
gtc	V	0	0.18
gta	V	0	0.27
gtg	V	1	0.33
---	--	------	------
mPD		0.0015	0.0020
		nPD:	0.75
		N. weight:	0.00066
		Sc. PD:	0.00045
		Sc. rank:	12.9

,

NP			
Pos.	433	obs:	exp:
tct	S	0	0.18
tcc	S	0	0.12
tca	S	1	0.26
tcg	S	0	0.05
agt	S	0	0.19
agc	S	0	0.20
gct	A	2	6193.00
gcc	A	4	4842.00
gca	A	23384	10987.00
gcg	A	3	1370.00
---	--	------	------
mPD		0.00085	0.66
		nPD:	0.
		N. weight:	0.78
		Sc. PD:	-0.03
		Sc. rank:	-14301.0

,

NP			
Pos.	434	obs:	exp:
tct	S	0	0.92
tcc	S	0	0.58
tca	S	5	1.30
tcg	S	0	0.27
cct	P	0	0.55
ccc	P	0	0.37
cca	P	2	0.85
ccg	P	0	0.23
agt	S	0	0.94
agc	S	0	0.99
gct	A	6	6192.00
gcc	A	1	4841.00
gca	A	23358	10984.00
gcg	A	22	1370.00
---	--	------	------
mPD		0.0031	0.66
		nPD:	0.
		N. weight:	0.77
		Sc. PD:	-0.027
		Sc. rank:	-8613.5

,

NP			
Pos.	435	obs:	exp:
ttt	F	37	10038.00
ttc	F	23355	13354.00
tta	L	0	0.11
ttg	L	1	0.19
tat	Y	0	0.52
tac	Y	1	0.48
ctt	L	0	0.20
ctc	L	0	0.14
cta	L	0	0.19
ctg	L	0	0.17
---	--	------	------
mPD		0.0033	0.49
		nPD:	0.01
		N. weight:	0.57
		Sc. PD:	-0.019
		Sc. rank:	-4405.0

,

NP			
Pos.	436	obs:	exp:
tct	S	0	4297.00
tcc	S	0	2704.00
tca	S	0	6077.00
tcg	S	0	1243.00
tgt	C	0	0.45
tgc	C	1	0.55
cgt	R	0	0.03
cgc	R	0	0.05
cga	R	0	0.10
cgg	R	0	0.07
att	I	0	0.73
atc	I	2	0.50
ata	I	0	0.77
act	T	1	0.25
acc	T	0	0.21
aca	T	0	0.46
acg	T	0	0.09
aat	N	3	22.47
aac	N	35	15.53
agt	S	535	4390.00
agc	S	22814	4638.00
aga	R	1	0.50
agg	R	0	0.25
ggt	G	0	0.30
ggc	G	2	0.30
gga	G	0	0.89
ggg	G	0	0.51
---	--	------	------
mPD		0.049	1.6
		nPD:	0.03
		N. weight:	1.6
		Sc. PD:	-0.017
		Sc. rank:	16403.0

,

NP			
Pos.	437	obs:	exp:
cgt	R	0	0.03
cgc	R	0	0.05
cga	R	0	0.10
cgg	R	1	0.07
aga	R	0	0.50
agg	R	0	0.25
gaa	E	0	1.82
gag	E	3	1.18
ggt	G	122	3547.00
ggc	G	0	3464.00
gga	G	1017	10404.00
ggg	G	22251	5975.00
---	--	------	------
mPD		0.093	0.69
		nPD:	0.13
		N. weight:	1.2
		Sc. PD:	0.11
		Sc. rank:	19795.0

,

NP

Pos.	438	obs:	exp:
tct	S	0	1.66
tcc	S	0	1.04
tca	S	0	2.34
tcg	S	0	0.48
tat	Y	0	1.04
tac	Y	2	0.96
act	T	0	11.34
acc	T	45	9.23
aca	T	0	20.59
acg	T	0	3.84
aat	N	25	13801.00
aac	N	23310	9534.00
aaa	K	3	1.71
aag	K	0	1.29
agt	S	0	1.69
agc	S	9	1.79
---	--	------	------
mPD		0.0072	0.49
		nPD:	0.01
		N. weight:	0.92
		Sc. PD:	-0.023
		Sc. rank:	2367.2

NP

Pos.	439	obs:	exp:
tct	S	0	2.76
tcc	S	0	1.74
tca	S	0	3.90
tcg	S	0	0.80
tat	Y	1	0.52
tac	Y	0	0.48
att	I	1	0.36
atc	I	0	0.25
ata	I	0	0.39
act	T	1	0.50
acc	T	0	0.41
aca	T	0	0.91
acg	T	1	0.17
aat	N	23163	13779.00
aac	N	134	9518.00
aaa	K	0	1.71
aag	K	3	1.29
agt	S	15	2.82
agc	S	0	2.98
gat	D	75	39.52
gac	D	0	35.48
---	--	------	------
mPD		0.020	0.49
		nPD:	0.04
		N. weight:	0.51
		Sc. PD:	-0.00034
		Sc. rank:	6140.3

NP

Pos.	440	obs:	exp:
gat	D	1	1.05
gac	D	1	0.95
gaa	E	23344	14164.00
gag	E	48	9228.00
---	--	------	------
mPD		0.0043	0.48
		nPD:	0.01
		N. weight:	0.5
		Sc. PD:	-0.015
		Sc. rank:	-1823.8

NP

Pos.	441	obs:	exp:
ggt	G	3	3547.00
ggc	G	0	3465.00
gga	G	23080	10406.00
ggg	G	311	5976.00
---	--	------	------
mPD		0.026	0.69
		nPD:	0.04
		N. weight:	0.77
		Sc. PD:	-0.0019
		Sc. rank:	9156.3

NP

Pos.	442	obs:	exp:
cgt	R	1	638.80
cgc	R	0	1224.00
cga	R	184	2291.00
cgg	R	23166	1712.00
aga	R	1	11745.00
agg	R	42	5783.00
---	--	------	------
mPD		0.019	0.91
		nPD:	0.02
		N. weight:	2.6
		Sc. PD:	-0.049
		Sc. rank:	19188.0

NP

Pos.	443	obs:	exp:
tct	S	0	0.92
tcc	S	0	0.58
tca	S	5	1.30
tcg	S	0	0.27
att	I	0	4.73
atc	I	0	3.25
ata	I	13	5.02
act	T	13	5892.00
acc	T	18	4794.00
aca	T	23330	10693.00
acg	T	13	1994.00
agt	S	0	0.94
agc	S	0	0.99
gct	A	0	0.53
gcc	A	0	0.41
gca	A	2	0.94
gcg	A	0	0.12
---	--	------	------
mPD		0.0055	0.68
		nPD:	0.01
		N. weight:	0.79
		Sc. PD:	-0.025
		Sc. rank:	-4153.4

NP

Pos.	444	obs:	exp:
tct	S	118	4305.00
tcc	S	23253	2710.00
tca	S	23	6088.00
tcg	S	0	1245.00
agt	S	0	4399.00
agc	S	0	4647.00
---	--	------	------
mPD		0.012	1.6
		nPD:	0.01
		N. weight:	2.2
		Sc. PD:	-0.07
		Sc. rank:	-14513.0

NP

Pos.	445	obs:	exp:
gat	D	37	12326.00
gac	D	23356	11067.00
ggt	G	0	0.15
ggc	G	1	0.15
gga	G	0	0.44
ggg	G	0	0.26
---	--	------	------
mPD		0.0032	0.50
		nPD:	0.01
		N. weight:	0.76
		Sc. PD:	-0.025
		Sc. rank:	-6486.6

NP

Pos.	446	obs:	exp:
atg	M	23394	23394.00
---	--	------	------
mPD		0	0
		nPD:	1.
		N. weight:	0.
		Sc. PD:	0
		Sc. rank:	0

NP

Pos.	447	obs:	exp:
cgt	R	0	638.80
cgc	R	0	1224.00
cga	R	23268	2291.00
cgg	R	40	1712.00
aga	R	85	11745.00
agg	R	1	5783.00
---	--	------	------
mPD		0.011	0.91
		nPD:	0.01
		N. weight:	2.4
		Sc. PD:	-0.066
		Sc. rank:	-1571.4

NP

Pos.	448	obs:	exp:
tct	S	0	0.18
tcc	S	0	0.12
tca	S	1	0.26
tcg	S	0	0.05
act	T	5	5897.00
acc	T	0	4798.00
aca	T	23370	10701.00
acg	T	16	1996.00
agt	S	0	0.19
agc	S	0	0.20
gct	A	0	0.53
gcc	A	0	0.41
gca	A	2	0.94
gcg	A	0	0.12
---	--	------	------
mPD		0.0021	0.68
		nPD:	0.
		N. weight:	0.8
		Sc. PD:	-0.029
		Sc. rank:	-12135.0

NP

Pos.	449	obs:	exp:
gaa	E	23101	14165.00
gag	E	293	9229.00
---	--	------	------
mPD		0.025	0.48
		nPD:	0.05
		N. weight:	0.45
		Sc. PD:	0.0048
		Sc. rank:	5995.1

NP			
Pos.	450	obs:	exp:
ttt	F	3	1.29
ttc	F	0	1.71
att	I	2859	1045.00
atc	I	13	716.90
ata	I	0	1110.00
gtt	V	20451	4536.00
gtc	V	57	3729.00
gta	V	1	5518.00
gtg	V	10	6736.00
---	--	------	------
mPD		0.22	0.96
		nPD:	0.23
		N. weight:	1.5
		Sc. PD:	0.27
		Sc. rank:	25936.0

,

NP			
Pos.	451	obs:	exp:
att	I	1	8515.00
atc	I	0	5839.00
ata	I	23390	9037.00
act	T	0	0.25
acc	T	0	0.21
aca	T	1	0.46
acg	T	0	0.09
gtt	V	0	0.44
gtc	V	0	0.36
gta	V	2	0.54
gtg	V	0	0.66
---	--	------	------
mPD		0.00034	0.66
		nPD:	0.
		N. weight:	0.98
		Sc. PD:	-0.038
		Sc. rank:	-19032.0

,

NP			
Pos.	452	obs:	exp:
cgt	R	0	637.50
cgc	R	0	1221.00
cga	R	18	2287.00
cgg	R	0	1709.00
att	I	0	0.73
atc	I	0	0.50
ata	I	2	0.77
act	T	0	0.25
acc	T	0	0.21
aca	T	1	0.46
acg	T	0	0.09
aaa	K	40	23.96
aag	K	2	18.04
aga	R	22807	11722.00
agg	R	524	5772.00
---	--	------	------
mPD		0.049	0.91
		nPD:	0.05
		N. weight:	0.61
		Sc. PD:	0.0078
		Sc. rank:	8210.7

,

NP			
Pos.	453	obs:	exp:
tta	L	0	0.22
ttg	L	0	0.38
ctt	L	0	0.39
ctc	L	0	0.28
cta	L	0	0.39
ctg	L	2	0.34
att	I	2	2.55
atc	I	0	1.75
ata	I	5	2.70
atg	M	23385	23385.00
---	--	------	------
mPD		0.00077	0.00088
		nPD:	0.88
		N. weight:	0.00032
		Sc. PD:	0.00026
		Sc. rank:	6.3

,

NP			
Pos.	454	obs:	exp:
att	I	0	0.36
atc	I	0	0.25
ata	I	1	0.39
atg	M	23393	23393.00
---	--	------	------
mPD		0.000085	0.000085
		nPD:	1.
		N. weight:	0.000042
		Sc. PD:	0.000039
		Sc. rank:	0.8

,

NP			
Pos.	455	obs:	exp:
gaa	E	23356	14165.00
gag	E	38	9229.00
---	--	------	------
mPD		0.0032	0.48
		nPD:	0.01
		N. weight:	0.51
		Sc. PD:	-0.017
		Sc. rank:	-3954.4

,

NP			
Pos.	456	obs:	exp:
tct	S	0	4284.00
tcc	S	0	2696.00
tca	S	0	6058.00
tcg	S	0	1239.00
cgt	R	0	0.08
cgc	R	0	0.16
cga	R	0	0.29
cgg	R	0	0.22
att	I	4	1.82
atc	I	1	1.25
ata	I	0	1.93
aat	N	102	60.33
aac	N	0	41.67
agt	S	22526	4377.00
agc	S	751	4624.00
aga	R	2	1.51
agg	R	1	0.74
ggt	G	6	1.06
ggc	G	1	1.04
gga	G	0	3.11
ggg	G	0	1.79
---	--	------	------
mPD		0.072	1.6
		nPD:	0.04
		N. weight:	1.6
		Sc. PD:	0.0047
		Sc. rank:	19697.0

,

NP			
Pos.	457	obs:	exp:
tct	S	0	0.18
tcc	S	0	0.12
tca	S	1	0.26
tcg	S	0	0.05
act	T	0	1.26
acc	T	0	1.03
aca	T	5	2.29
acg	T	0	0.43
agt	S	0	0.19
agc	S	0	0.20
gtt	V	0	0.44
gtc	V	0	0.36
gta	V	2	0.54
gtg	V	0	0.66
gct	A	4	6192.00
gcc	A	8	4841.00
gca	A	23348	10984.00
gcg	A	26	1370.00
---	--	------	------
mPD		0.0039	0.66
		nPD:	0.01
		N. weight:	0.77
		Sc. PD:	-0.026
		Sc. rank:	-7016.1

,

NP			
Pos.	458	obs:	exp:
cgt	R	0	1.26
cgc	R	0	2.41
cga	R	0	4.51
cgg	R	0	3.37
aaa	K	305	13318.00
aag	K	23043	10030.00
aga	R	0	23.09
agg	R	46	11.37
---	--	------	------
mPD		0.030	0.50
		nPD:	0.06
		N. weight:	0.8
		Sc. PD:	0.015
		Sc. rank:	10996.0

,

NP			
Pos.	459	obs:	exp:
tta	L	0	0.22
ttg	L	0	0.38
tct	S	0	1.29
tcc	S	0	0.81
tca	S	7	1.82
tcg	S	0	0.37
ctt	L	0	0.39
ctc	L	0	0.28
cta	L	2	0.39
ctg	L	0	0.34
cct	P	64	6439.00
ccc	P	4	4335.00
cca	P	23275	9952.00
ccg	P	29	2646.00
caa	Q	3	1.58
cag	Q	0	1.42
act	T	0	2.02
acc	T	0	1.64
aca	T	8	3.66
acg	T	0	0.68
agt	S	0	1.32
agc	S	0	1.39
gct	A	0	0.53
gcc	A	0	0.41
gca	A	2	0.94
gcg	A	0	0.12
---	--	------	------
mPD		0.010	0.70
		nPD:	0.01
		N. weight:	0.86
		Sc. PD:	-0.022
		Sc. rank:	2144.1

,

NP			
Pos.	460	obs:	exp:
aaa	K	4	3.42
aag	K	2	2.58
gat	D	7	4.22
gac	D	1	3.78
gaa	E	3218	14154.00
gag	E	20158	9222.00
ggt	G	0	0.61
ggc	G	0	0.59
gga	G	1	1.78
ggg	G	3	1.02
---	--	------	------
mPD		0.24	0.48
		nPD:	0.5
		N. weight:	0.49
		Sc. PD:	0.21
		Sc. rank:	9425.9

,

NP			
Pos.	461	obs:	exp:
aat	N	4	2.37
aac	N	0	1.63
gtt	V	1	0.22
gtc	V	0	0.18
gta	V	0	0.27
gtg	V	0	0.33
gat	D	23244	12318.00
gac	D	135	11061.00
gaa	E	5	6.06
gag	E	5	3.94
---	--	------	------
mPD		0.013	0.50
		nPD:	0.03
		N. weight:	0.63
		Sc. PD:	-0.0092
		Sc. rank:	5672.3

,

NP			
Pos.	462	obs:	exp:
tta	L	20	2571.00
ttg	L	23332	4404.00
ctt	L	0	4609.00
ctc	L	0	3304.00
cta	L	0	4503.00
ctg	L	38	3999.00
gtt	V	0	0.88
gtc	V	0	0.73
gta	V	0	1.08
gtg	V	4	1.31
---	--	------	------
mPD		0.0053	1.1
		nPD:	0.
		N. weight:	1.7
		Sc. PD:	-0.059
		Sc. rank:	-18796.0

,

NP			
Pos.	463	obs:	exp:
tct	S	81	4305.00
tcc	S	23292	2710.00
tca	S	20	6088.00
tcg	S	0	1245.00
act	T	0	0.25
acc	T	1	0.21
aca	T	0	0.46
acg	T	0	0.09
agt	S	0	4399.00
agc	S	0	4647.00
---	--	------	------
mPD		0.0087	1.6
		nPD:	0.01
		N. weight:	2.2
		Sc. PD:	-0.075
		Sc. rank:	-21809.0

,

NP			
Pos.	464	obs:	exp:
ttt	F	265	10039.00
ttc	F	23129	13355.00
---	--	------	------
mPD		0.022	0.49
		nPD:	0.05
		N. weight:	0.52
		Sc. PD:	0.0025
		Sc. rank:	6515.5

,

NP			
Pos.	465	obs:	exp:
cat	H	8	4.70
cac	H	0	3.30
caa	Q	142	12337.00
cag	Q	23238	11043.00
cgt	R	0	0.11
cgc	R	0	0.21
cga	R	0	0.39
cgg	R	4	0.29
aaa	K	0	1.14
aag	K	2	0.86
aga	R	0	2.01
agg	R	0	0.99
---	--	------	------
mPD		0.013	0.50
		nPD:	0.03
		N. weight:	0.74
		Sc. PD:	-0.01
		Sc. rank:	6825.6

,

NP			
Pos.	466	obs:	exp:
cgt	R	0	0.11
cgc	R	0	0.21
cga	R	0	0.39
cgg	R	0	0.29
aga	R	0	2.01
agg	R	4	0.99
ggt	G	2	3547.00
ggc	G	5	3464.00
gga	G	60	10404.00
ggg	G	23323	5975.00
---	--	------	------
mPD		0.0061	0.69
		nPD:	0.01
		N. weight:	1.4
		Sc. PD:	-0.043
		Sc. rank:	-5202.5

,

NP			
Pos.	467	obs:	exp:
cgt	R	1	638.80
cgc	R	0	1224.00
cga	R	52	2291.00
cgg	R	23304	1712.00
aga	R	0	11745.00
agg	R	37	5783.00
---	--	------	------
mPD		0.0077	0.91
		nPD:	0.01
		N. weight:	2.7
		Sc. PD:	-0.083
		Sc. rank:	-11313.0

,

NP			
Pos.	468	obs:	exp:
ggt	G	3	3547.00
ggc	G	1	3465.00
gga	G	23307	10406.00
ggg	G	83	5976.00
---	--	------	------
mPD		0.0074	0.69
		nPD:	0.01
		N. weight:	0.81
		Sc. PD:	-0.024
		Sc. rank:	-1216.8

,

NP			
Pos.	469	obs:	exp:
att	I	0	1.09
atc	I	3	0.75
ata	I	0	1.16
gtt	V	64	5167.00
gtc	V	23269	4247.00
gta	V	37	6285.00
gtg	V	0	7672.00
gct	A	0	5.56
gcc	A	21	4.35
gca	A	0	9.86
gcg	A	0	1.23
---	--	------	------
mPD		0.011	0.74
		nPD:	0.01
		N. weight:	1.7
		Sc. PD:	-0.044
		Sc. rank:	3821.9

,

NP			
Pos.	470	obs:	exp:
ttt	F	19	10039.00
ttc	F	23375	13355.00
---	--	------	------
mPD		0.0016	0.49
		nPD:	0.
		N. weight:	0.57
		Sc. PD:	-0.021
		Sc. rank:	-8249.9

,

NP			
Pos.	471	obs:	exp:
caa	Q	0	0.53
cag	Q	1	0.47
gaa	E	0	14164.00
gag	E	23393	9229.00
---	--	------	------
mPD		0.000085	0.48
		nPD:	0.
		N. weight:	0.96
		Sc. PD:	-0.038
		Sc. rank:	-18695.0

,

NP			
Pos.	472	obs:	exp:
ttt	F	0	8.15
ttc	F	19	10.85
tta	L	0	2568.00
ttg	L	0	4398.00
ctt	L	26	4603.00
ctc	L	23295	3300.00
cta	L	40	4497.00
ctg	L	0	3994.00
cct	P	0	0.55
ccc	P	2	0.37
cca	P	0	0.85
ccg	P	0	0.23
att	I	0	4.37
atc	I	12	3.00
ata	I	0	4.64
---	--	------	------
mPD		0.0084	1.1
		nPD:	0.01
		N. weight:	2.
		Sc. PD:	-0.064
		Sc. rank:	-12646.0

,

NP			
Pos.	473	obs:	exp:
tta	L	0	1.76
ttg	L	16	3.01
tct	S	20	4301.00
tcc	S	0	2707.00
tca	S	244	6083.00
tcg	S	23108	1244.00
ctt	L	0	3.15
ctc	L	0	2.26
cta	L	0	3.08
ctg	L	0	2.74
act	T	0	0.50
acc	T	0	0.41
aca	T	0	0.91
acg	T	2	0.17
agt	S	0	4395.00
agc	S	0	4643.00
gct	A	0	1.06
gcc	A	0	0.83
gca	A	0	1.88
gcg	A	4	0.23
---	--	------	------
mPD		0.024	1.6
		nPD:	0.01
		N. weight:	2.9
		Sc. PD:	-0.074
		Sc. rank:	8271.7

,

NP			
Pos.	474	obs:	exp:
gat	D	81	12326.00
gac	D	23313	11068.00
---	--	------	------
mPD		0.0069	0.50
		nPD:	0.01
		N. weight:	0.75
		Sc. PD:	-0.019
		Sc. rank:	1026.5

,

NP			
Pos.	475	obs:	exp:
gaa	E	23372	14164.00
gag	E	21	9229.00
ggt	G	0	0.15
ggc	G	0	0.15
gga	G	1	0.44
ggg	G	0	0.26
---	--	------	------
mPD		0.0019	0.48
		nPD:	0.
		N. weight:	0.51
		Sc. PD:	-0.018
		Sc. rank:	-6333.4

,

NP			
Pos.	476	obs:	exp:
cgt	R	0	0.05
cgc	R	0	0.10
cga	R	0	0.20
cgg	R	0	0.15
aaa	K	8	13344.00
aag	K	23384	10048.00
aga	R	0	1.00
agg	R	2	0.49
---	--	------	------
mPD		0.00085	0.49
		nPD:	0.
		N. weight:	0.87
		Sc. PD:	-0.033
		Sc. rank:	-15152.0

,

NP			
Pos.	477	obs:	exp:
gct	A	2	6194.00
gcc	A	0	4842.00
gca	A	23378	10987.00
gcg	A	14	1371.00
---	--	------	------
mPD		0.0014	0.66
		nPD:	0.
		N. weight:	0.78
		Sc. PD:	-0.029
		Sc. rank:	-12983.0

,

NP

Pos.	478	obs:	exp:
tct	S	0	0.18
tcc	S	0	0.12
tca	S	0	0.26
tcg	S	1	0.05
act	T	5	5873.00
acc	T	0	4778.00
aca	T	10	10657.00
acg	T	23281	1988.00
agt	S	0	0.19
agc	S	0	0.20
gct	A	0	25.68
gcc	A	0	20.08
gca	A	0	45.56
gcg	A	97	5.68
---	--	------	------
mPD		0.0096	0.69
		nPD:	0.01
		N. weight:	2.5
		Sc. PD:	-0.065
		Sc. rank:	4308.9

,

NP

Pos.	479	obs:	exp:
tct	S	0	2.76
tcc	S	0	1.74
tca	S	0	3.90
tcg	S	0	0.80
cat	H	0	1.18
cac	H	2	0.82
act	T	0	13.86
acc	T	55	11.28
aca	T	0	25.16
acg	T	0	4.69
aat	N	1	13789.00
aac	N	23314	9526.00
aaa	K	1	0.57
aag	K	0	0.43
agt	S	0	2.82
agc	S	15	2.98
gat	D	0	3.16
gac	D	6	2.84
---	--	------	------
mPD		0.0068	0.49
		nPD:	0.01
		N. weight:	0.93
		Sc. PD:	-0.024
		Sc. rank:	1336.0

,

NP

Pos.	480	obs:	exp:
cct	P	6	6445.00
ccc	P	0	4339.00
cca	P	12	9961.00
ccg	P	23376	2649.00
---	--	------	------
mPD		0.0015	0.70
		nPD:	0.
		N. weight:	2.2
		Sc. PD:	-0.083
		Sc. rank:	-37000.0

,

NP

Pos.	481	obs:	exp:
att	I	5	8504.00
atc	I	23351	5831.00
ata	I	4	9025.00
act	T	0	0.25
acc	T	1	0.21
aca	T	0	0.46
acg	T	0	0.09
gtt	V	0	7.30
gtc	V	33	6.00
gta	V	0	8.88
gtg	V	0	10.83
---	--	------	------
mPD		0.0037	0.66
		nPD:	0.01
		N. weight:	1.4
		Sc. PD:	-0.049
		Sc. rank:	-13590.0

,

NP

Pos.	482	obs:	exp:
tta	L	0	0.33
ttg	L	3	0.56
ctt	L	0	0.59
ctc	L	0	0.42
cta	L	0	0.58
ctg	L	0	0.51
atg	M	1	1.00
gtt	V	12	5171.00
gtc	V	0	4250.00
gta	V	13	6291.00
gtg	V	23365	7678.00
---	--	------	------
mPD		0.0025	0.74
		nPD:	0.
		N. weight:	1.1
		Sc. PD:	-0.041
		Sc. rank:	-16036.0

,

NP

Pos.	483	obs:	exp:
cct	P	23367	6445.00
ccc	P	27	4339.00
cca	P	0	9961.00
ccg	P	0	2649.00
---	--	------	------
mPD		0.0023	0.70
		nPD:	0.
		N. weight:	1.3
		Sc. PD:	-0.048
		Sc. rank:	-18925.0

,

NP

Pos.	484	obs:	exp:
ttt	F	0	3.00
ttc	F	7	4.00
tct	S	569	4304.00
tcc	S	22742	2709.00
tca	S	76	6087.00
tcg	S	0	1245.00
agt	S	0	4398.00
agc	S	0	4645.00
---	--	------	------
mPD		0.054	1.6
		nPD:	0.03
		N. weight:	2.1
		Sc. PD:	-0.015
		Sc. rank:	22913.0

,

NP

Pos.	485	obs:	exp:
ttt	F	23380	10039.00
ttc	F	14	13355.00
---	--	------	------
mPD		0.0012	0.49
		nPD:	0.
		N. weight:	0.87
		Sc. PD:	-0.032
		Sc. rank:	-14167.0

,

NP

Pos.	486	obs:	exp:
aat	N	0	2.37
aac	N	4	1.63
gat	D	18	12322.00
gac	D	23367	11063.00
gaa	E	2	1.82
gag	E	1	1.18
ggt	G	0	0.30
ggc	G	2	0.30
gga	G	0	0.89
ggg	G	0	0.51
---	--	------	------
mPD		0.0023	0.50
		nPD:	0.
		N. weight:	0.77
		Sc. PD:	-0.027
		Sc. rank:	-8594.5

,

NP

Pos.	487	obs:	exp:
tta	L	0	2.86
ttg	L	17	4.90
ctt	L	0	5.12
ctc	L	0	3.67
cta	L	0	5.01
ctg	L	9	4.45
att	I	4	42.59
atc	I	0	29.21
ata	I	113	45.20
atg	M	23251	23251.00
---	--	------	------
mPD		0.012	0.014
		nPD:	0.9
		N. weight:	0.0055
		Sc. PD:	0.0045
		Sc. rank:	107.9

,

NP

Pos.	488	obs:	exp:
tct	S	0	4285.00
tcc	S	0	2697.00
tca	S	0	6060.00
tcg	S	0	1239.00
cgt	R	0	0.03
cgc	R	0	0.05
cga	R	0	0.10
cgg	R	0	0.07
att	I	5	1.82
atc	I	0	1.25
ata	I	0	1.93
aat	N	94	55.60
aac	N	0	38.40
agt	S	23238	4378.00
agc	S	47	4625.00
aga	R	1	0.50
agg	R	0	0.25
ggt	G	9	1.36
ggc	G	0	1.33
gga	G	0	4.00
ggg	G	0	2.30
---	--	------	------
mPD		0.013	1.6
		nPD:	0.01
		N. weight:	1.7
		Sc. PD:	-0.054
		Sc. rank:	-8699.5

,

NP

Pos.	489	obs:	exp:
tct	S	0	0.55
tcc	S	0	0.35
tca	S	0	0.78
tcg	S	0	0.16
act	T	1	0.25
acc	T	0	0.21
aca	T	0	0.46
acg	T	0	0.09
aat	N	23356	13832.00
aac	N	31	9555.00
aaa	K	1	0.57
aag	K	0	0.43
agt	S	3	0.56
agc	S	0	0.60
gat	D	2	1.05
gac	D	0	0.95
---	--	------	------
mPD		0.0032	0.48
		nPD:	0.01
		N. weight:	0.53
		Sc. PD:	-0.018
		Sc. rank:	-4237.9

,

NP

Pos.	490	obs:	exp:
gaa	E	23357	14165.00
gag	E	37	9229.00
---	--	------	------
mPD		0.0032	0.48
		nPD:	0.01
		N. weight:	0.51
		Sc. PD:	-0.017
		Sc. rank:	-4248.6

,

NP

Pos.	491	obs:	exp:
tgg	W	1	1.00
cgt	R	0	0.03
cgc	R	0	0.05
cga	R	0	0.10
cgg	R	0	0.07
aga	R	0	0.50
agg	R	1	0.25
ggt	G	9	3547.00
ggc	G	1	3465.00
gga	G	71	10405.00
ggg	G	23311	5975.00
---	--	------	------
mPD		0.0071	0.69
		nPD:	0.01
		N. weight:	1.4
		Sc. PD:	-0.041
		Sc. rank:	-2811.5

,

NP

Pos.	492	obs:	exp:
tct	S	23244	4304.00
tcc	S	141	2709.00
tca	S	0	6087.00
tcg	S	3	1245.00
tat	Y	5	2.60
tac	Y	0	2.40
act	T	1	0.25
acc	T	0	0.21
aca	T	0	0.46
acg	T	0	0.09
agt	S	0	4398.00
agc	S	0	4646.00
---	--	------	------
mPD		0.013	1.6
		nPD:	0.01
		N. weight:	1.7
		Sc. PD:	-0.054
		Sc. rank:	-9337.9

,

NP

Pos.	493	obs:	exp:
tat	Y	23387	12155.00
tac	Y	7	11239.00
---	--	------	------
mPD		0.00060	0.50
		nPD:	0.
		N. weight:	0.67
		Sc. PD:	-0.026
		Sc. rank:	-12545.0

,

NP			
Pos.	494	obs:	exp:
ttt	F	14	10039.00
ttc	F	23380	13355.00
---	--	------	------
mPD		0.0012	0.49
		nPD:	0.
		N. weight:	0.57
		Sc. PD:	-0.021
		Sc. rank:	-9364.3

,

NP			
Pos.	495	obs:	exp:
ttt	F	58	10039.00
ttc	F	23335	13354.00
tta	L	1	0.11
ttg	L	0	0.19
ctt	L	0	0.20
ctc	L	0	0.14
cta	L	0	0.19
ctg	L	0	0.17
---	--	------	------
mPD		0.0050	0.49
		nPD:	0.01
		N. weight:	0.56
		Sc. PD:	-0.017
		Sc. rank:	-1118.0

,

NP			
Pos.	496	obs:	exp:
cgt	R	0	0.03
cgc	R	0	0.05
cga	R	0	0.10
cgg	R	0	0.07
aga	R	1	0.50
agg	R	0	0.25
ggt	G	2	3547.00
ggc	G	0	3465.00
gga	G	23389	10406.00
ggg	G	2	5975.00
---	--	------	------
mPD		0.00043	0.69
		nPD:	0.
		N. weight:	0.84
		Sc. PD:	-0.032
		Sc. rank:	-16134.0

,

NP			
Pos.	497	obs:	exp:
gtt	V	0	0.22
gtc	V	1	0.18
gta	V	0	0.27
gtg	V	0	0.33
gat	D	6	12326.00
gac	D	23387	11067.00
---	--	------	------
mPD		0.00060	0.50
		nPD:	0.
		N. weight:	0.77
		Sc. PD:	-0.029
		Sc. rank:	-14308.0

,

NP			
Pos.	498	obs:	exp:
tct	S	0	7.18
tcc	S	0	4.52
tca	S	0	10.15
tcg	S	0	2.08
aat	N	23345	13813.00
aac	N	10	9542.00
agt	S	39	7.33
agc	S	0	7.75
---	--	------	------
mPD		0.0042	0.49
		nPD:	0.01
		N. weight:	0.54
		Sc. PD:	-0.017
		Sc. rank:	-2269.3

,

NP			
Pos.	499	obs:	exp:
tct	S	0	0.18
tcc	S	0	0.12
tca	S	1	0.26
tcg	S	0	0.05
cct	P	0	0.28
ccc	P	0	0.19
cca	P	1	0.43
ccg	P	0	0.11
agt	S	0	0.19
agc	S	0	0.20
gct	A	12	6193.00
gcc	A	1	4842.00
gca	A	23375	10986.00
gcg	A	4	1370.00
---	--	------	------
mPD		0.0016	0.66
		nPD:	0.
		N. weight:	0.78
		Sc. PD:	-0.029
		Sc. rank:	-12501.0

,

NP			
Pos.	500	obs:	exp:
gat	D	8	4.22
gac	D	0	3.78
gaa	E	129	14160.00
gag	E	23257	9226.00
---	--	------	------
mPD		0.012	0.48
		nPD:	0.02
		N. weight:	0.92
		Sc. PD:	-0.015
		Sc. rank:	7786.6

,

NP			
Pos.	501	obs:	exp:
aaa	K	0	0.57
aag	K	1	0.43
gat	D	7	3.69
gac	D	0	3.31
gaa	E	57	14160.00
gag	E	23328	9225.00
ggt	G	0	0.15
ggc	G	0	0.15
gga	G	0	0.44
ggg	G	1	0.26
---	--	------	------
mPD		0.0056	0.48
		nPD:	0.01
		N. weight:	0.94
		Sc. PD:	-0.026
		Sc. rank:	-1025.4

,

NP			
Pos.	502	obs:	exp:
ttt	F	2	0.86
ttc	F	0	1.14
tat	Y	23370	12154.00
tac	Y	21	11237.00
tgt	C	1	0.45
tgc	C	0	0.55
---	--	------	------
mPD		0.0021	0.50
		nPD:	0.
		N. weight:	0.67
		Sc. PD:	-0.024
		Sc. rank:	-8025.6

,

NP			
Pos.	503	obs:	exp:
tat	Y	0	0.52
tac	Y	1	0.48
aat	N	0	5.32
aac	N	9	3.68
gat	D	16	12314.00
gac	D	23355	11057.00
gaa	E	2	4.24
gag	E	5	2.76
ggt	G	0	0.91
ggc	G	6	0.89
gga	G	0	2.67
ggg	G	0	1.53
---	--	------	------
mPD		0.0033	0.50
		nPD:	0.01
		N. weight:	0.77
		Sc. PD:	-0.025
		Sc. rank:	-6135.7

,

NP			
Pos.	504	obs:	exp:
tct	S	0	583.20
tcc	S	0	367.10
tca	S	0	824.70
tcg	S	0	168.70
aat	N	20171	11962.00
aac	N	54	8263.00
agt	S	3169	595.90
agc	S	0	629.50
---	--	------	------
mPD		0.24	0.92
		nPD:	0.26
		N. weight:	0.69
		Sc. PD:	0.14
		Sc. rank:	12298.0

,

NP			
Pos.	505	obs:	exp:
taa	.	76	10625.00
tag	.	0	6372.00
tga	.	23318	6397.00
---	--	------	------
mPD		0.0065	0.79
		nPD:	0.01
		N. weight:	1.3
		Sc. PD:	-0.041
		Sc. rank:	-6571.0

}

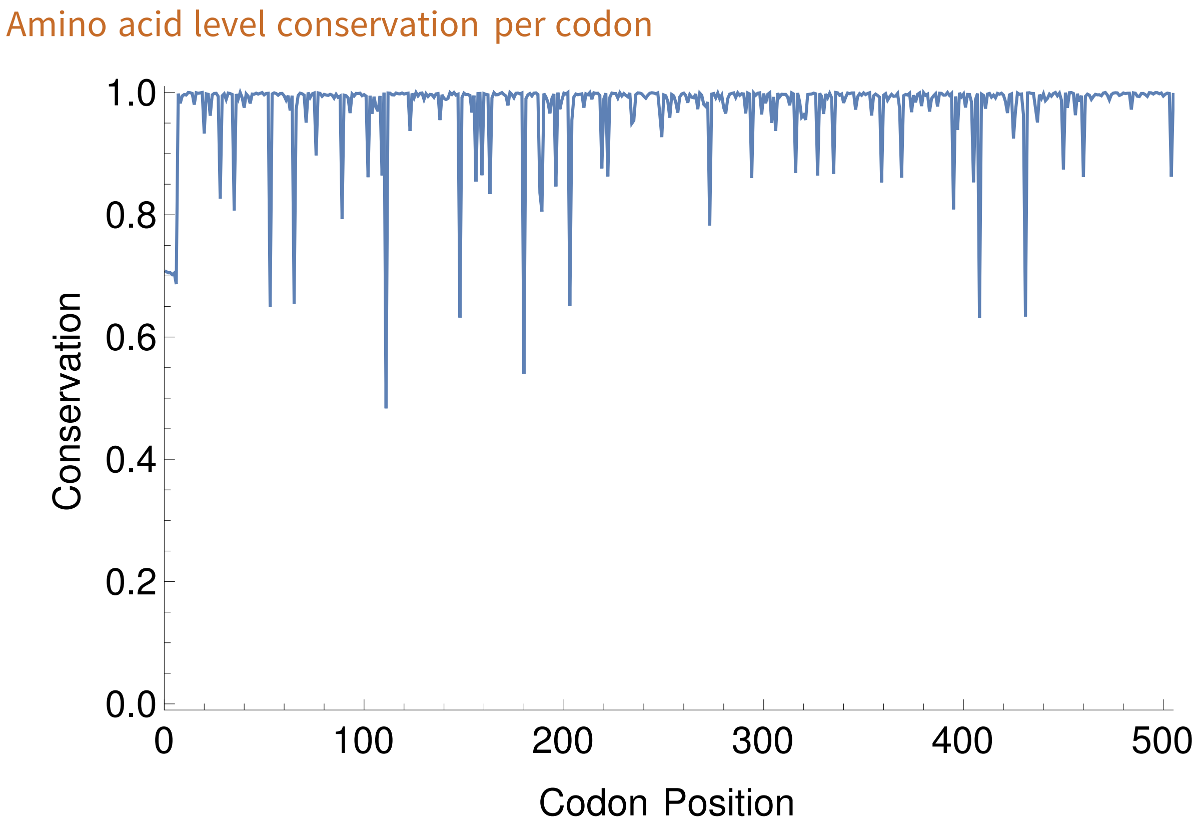
Amino acid level conservation per codon
Conservation
1.0
0.8
0.6
0.4
0.2
0.0
0
100
200
300
400
500
Codon Position

Conservation analysis plots

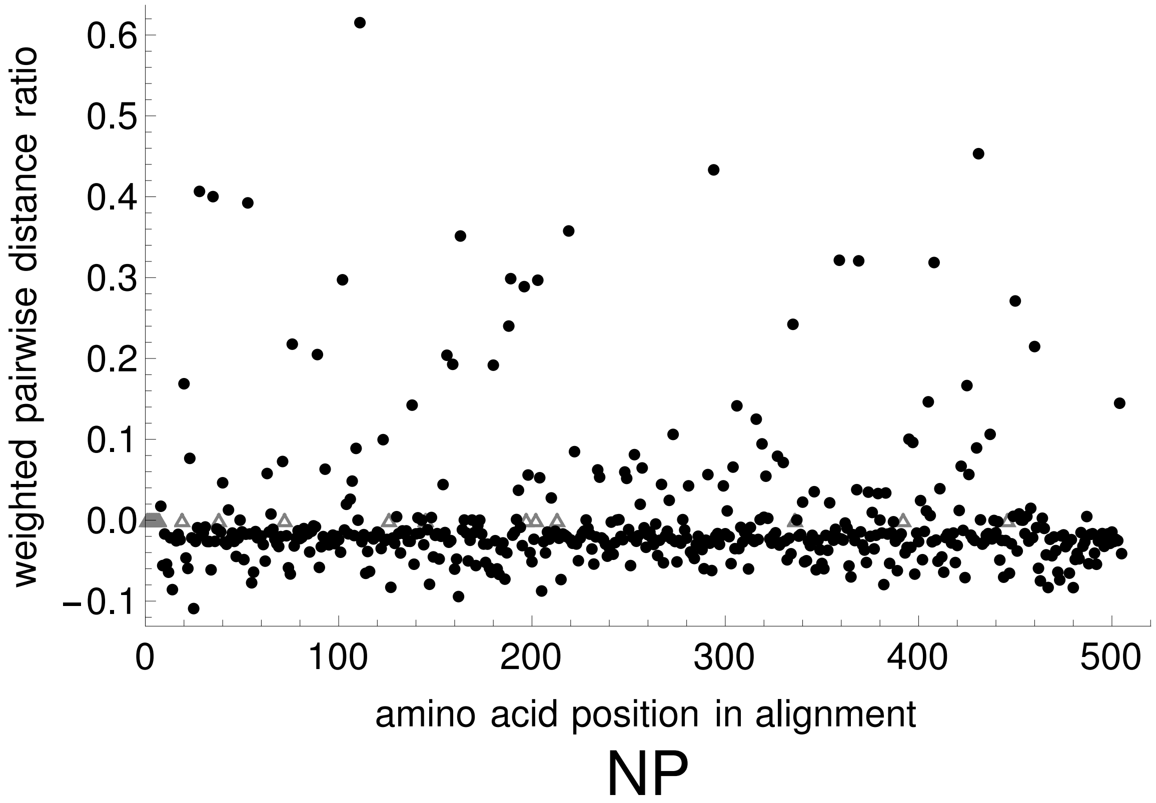

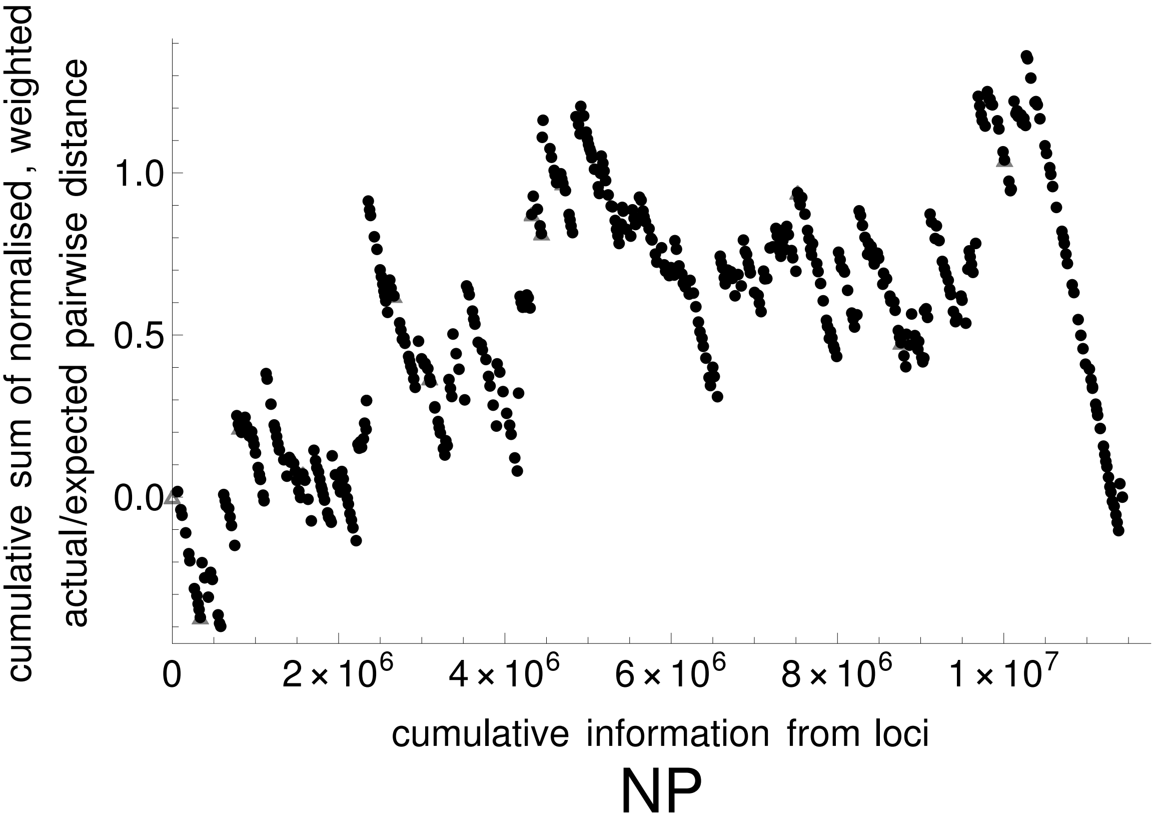

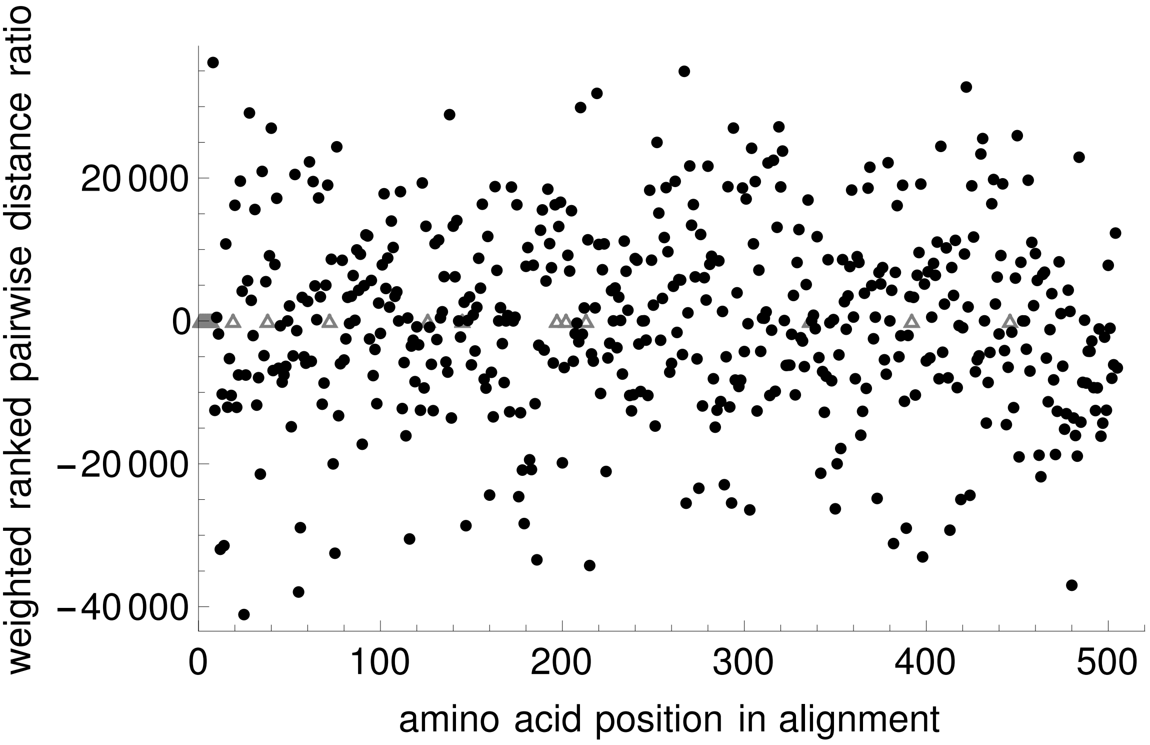
weighted ranked pairwise distance ratio
20 000
0
−20 000
−40 000
0
100
200
300
400
500
amino acid position in alignment

NP

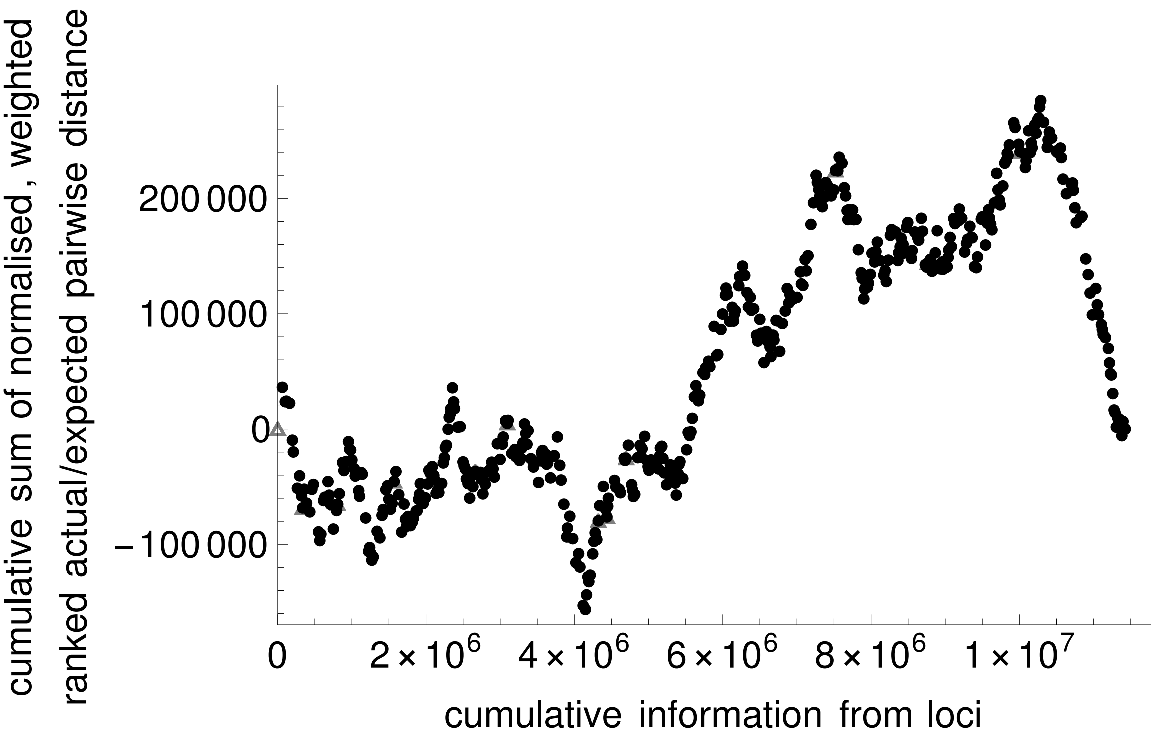
cumulative sum of normalised, weighted ranked actual/expected pairwise distance
200 000
100 000
0
−100 000
0
2 × 10^6
4 × 10^6
6 × 10^6
8 × 10^6
1 × 10^7
cumulative information from loci

NP

NA

Gene length histogram

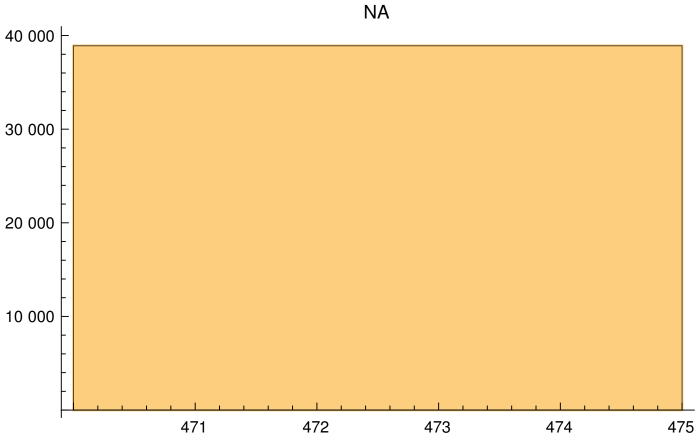

Information vs. nPD

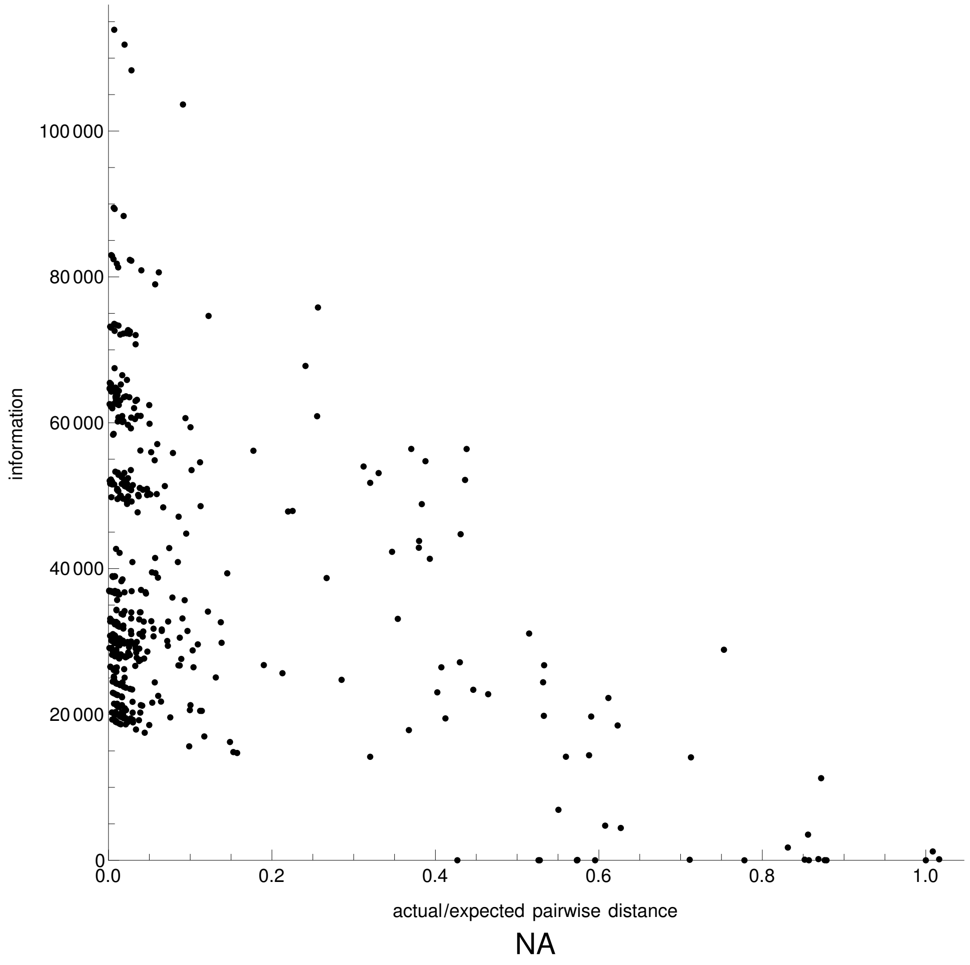

Example sequences highlighted by regions found in analyses to be conserved

Interesting points (by weighted raw PD) highlighted for gene NA:

ATGAATCCAAACCAAAAG ---------------ATAATAACCATTGGTTCTATCTGTATGGCAATTGGAACGGCTAA
CTTAATATTGCAAATTGGAAACATAATCTCAATATGGGTTAGCCACTCAATTCAAATTGGAAATCAAAGTCAGAT
TGAAACATGCAACAAAAGCGTCATTACTTATGAAAACAACACTTGGGTAAATCAGACATTTGTTAACATCAGCAA
CACTAACTCTGCTGCTAGACAGTCAGTGGCTTCCGTGAAATTAGCGGGCAATTCCTCTCTCTGCCCTGTTAGTGG
ATGGGCTATATACAGTAAAGACAACAGTGTAAGAATCGGTTCCAAGGGGGATGTGTTTGTCATAAGGGAACCATT
CATATCATGCTCTCCCTTGGAATGCAGAACCTTCTTCTTGACTCAAGGGGCTTTGCTAAATGACAAACATTCCAA
TGGAACCATTAAAGACAGAAGCCCATACCGAACCCTAATGAGCTGTCCTATTGGTGAAGTTCCCTCTCCATACAA
CTCAAGATTTGAGTCAGTCGCTTGGTCAGCAAGTGCTTGTCATGATGGCACCAATTGGCTAACAATTGGAATTTC
TGGCCCAGACAGTGGGGCAGTGGCTGTGTTAAAATACAATGGCATAATAACAGACACTATCAAGAGTTGGAGGAA
CAAGATATTGAGAACACAAGAGTCTGAATGTGCATGTGTAAATGGTTCTTGCTTTACCATAATGACCGATGGACC
AAGTGATGGACAGGCCTCATACAAAATCTTCAGAATAGAAAAGGGAAAGATAATCAAATCAGTCGAAATGAAAGC
CCCTAATTACCACTATGAAGAATGCTCCTGTTACCCTGATTCTAGTGAAATCACATGTGTGTGCAGGGATAACTG
GCATGGCTCGAATCGACCGTGGGTGTCTTTCAACCAGAATCTGGAATATCAGATGGGATACATATGCAGTGGGGT
TTTCGGAGACAATCCACGCCCTAATGATAAGACAGGCAGTTGTGGTCCAGTATCGTCTAATGGAGCAAATGGGGT
AAAAGGATTTTCATTCAAATACGGCAATGGTGTTTGGATAGGGAGAACTAAGAGCATTAGTTCAAGAAAAGGTTT
TGAGATGATTTGGGATCCGAATGGATGGACTGGGACTGACAATAAATTCTCAAAAAAGCAAGATATCGTAGGAAT
AAATGAGTGGTCAGGGTATAGCGGGAGTTTTGTTCAGCATCCAGAACTAACAGGGCTGAATTGTATAAGACCTTG
CTTCTGGGTTGAACTAATAAGAGGACGACCCGAAGAGAACACAATCTGGACTAGCGGGAGCAGCATATCCTTTTG
TGGTGTAGACAGTGACATTGTGGGTTGGTCTTGGCCAGACGGTGCTGAGTTG ------CCATTTACCATTGACAAG
---------TAA

Interesting points (by weighted ranked PD) highlighted for gene NA:

ATGAATCCAAACCAAAAG ---------------ATAATAACCATTGGTTCTATCTGTATGGCAATTGGAACGGCTAACT
TAATATTGCAAATTGGAAACATAATCTCAATATGGGTTAGCCACTCAATTCAAATTGGAAATCAAAGTCAGATTG
AAACATGCAACAAAAGCGTCATTACTTATGAAAACAACACTTGGGTAAATCAGACATTTGTTAACATCAGCAACA
CTAACTCTGCTGCTAGACAGTCAGTGGCTTCCGTGAAATTAGCGGGCAATTCCTCTCTCTGCCCTGTTAGTGGAT
GGGCTATATACAGTAAAGACAACAGTGTAAGAATCGGTTCCAAGGGGGATGTGTTTGTCATAAGGGAACCATTCA
TATCATGCTCTCCCTTGGAATGCAGAACCTTCTTCTTGACTCAAGGGGCTTTGCTAAATGACAAACATTCCAATG
GAACCATTAAAGACAGAAGCCCATACCGAACCCTAATGAGCTGTCCTATTGGTGAAGTTCCCTCTCCATACAACT
CAAGATTTGAGTCAGTCGCTTGGTCAGCAAGTGCTTGTCATGATGGCACCAATTGGCTAACAATTGGAATTTCTG
GCCCAGACAGTGGGGCAGTGGCTGTGTTAAAATACAATGGCATAATAACAGACACTATCAAGAGTTGGAGGAACA
AGATATTGAGAACACAAGAGTCTGAATGTGCATGTGTAAATGGTTCTTGCTTTACCATAATGACCGATGGACCAA
GTGATGGACAGGCCTCATACAAAATCTTCAGAATAGAAAAGGGAAAGATAATCAAATCAGTCGAAATGAAAGCCC
CTAATTACCACTATGAAGAATGCTCCTGTTACCCTGATTCTAGTGAAATCACATGTGTGTGCAGGGATAACTGGC
ATGGCTCGAATCGACCGTGGGTGTCTTTCAACCAGAATCTGGAATATCAGATGGGATACATATGCAGTGGGGTTT
TCGGAGACAATCCACGCCCTAATGATAAGACAGGCAGTTGTGGTCCAGTATCGTCTAATGGAGCAAATGGGGTAA
AAGGATTTTCATTCAAATACGGCAATGGTGTTTGGATAGGGAGAACTAAGAGCATTAGTTCAAGAAAAGGTTTTG
AGATGATTTGGGATCCGAATGGATGGACTGGGACTGACAATAAATTCTCAAAAAAGCAAGATATCGTAGGAATAA
ATGAGTGGTCAGGGTATAGCGGGAGTTTTGTTCAGCATCCAGAACTAACAGGGCTGAATTGTATAAGACCTTGCT
TCTGGGTTGAACTAATAAGAGGACGACCCGAAGAGAACACAATCTGGACTAGCGGGAGCAGCATATCCTTTTGTG
GTGTAGACAGTGACATTGTGGGTTGGTCTTGGCCA
GACGGTGCTGAGTTG------CCATTTACCATTGACAAG ---------TAA

Per codon conservation report

NA

Pos.	1	obs:	exp:
atg	M	38907	38907.00
---	--	------	------
mPD		0	0
		nPD:	1.
		N. weight:	0.
		Sc. PD:	0
		Sc. rank:	0

NA

Pos.	2	obs:	exp:
tct	S	0	0.18
tcc	S	0	0.12
tca	S	0	0.26
tcg	S	0	0.05
cat	H	2	1.18
cac	H	0	0.82
cgt	R	0	0.03
cgc	R	0	0.05
cga	R	0	0.10
cgg	R	0	0.07
att	I	3	1.09
atc	I	0	0.75
ata	I	0	1.16
aat	N	38868	23000.00
aac	N	20	15888.00
aaa	K	10	5.70
aag	K	0	4.30
agt	S	1	0.19
agc	S	0	0.20
aga	R	0	0.50
agg	R	1	0.25
gat	D	2	1.05
gac	D	0	0.95
---	--	------	------
mPD		0.0021	0.48
		nPD:	0.
		N. weight:	0.54
		Sc. PD:	-0.027
		Sc. rank:	-13489.0

NA

Pos.	3	obs:	exp:
tta	L	0	0.11
ttg	L	0	0.19
tct	S	0	2.21
tcc	S	0	1.39
tca	S	12	3.12
tcg	S	0	0.64
ctt	L	0	0.20
ctc	L	0	0.14
cta	L	1	0.19
ctg	L	0	0.17
cct	P	2	10714.00
ccc	P	4	7213.00
cca	P	38878	16559.00
ccg	P	5	4404.00
act	T	0	1.26
acc	T	0	1.03
aca	T	5	2.29
acg	T	0	0.43
agt	S	0	2.26
agc	S	0	2.38
---	--	------	------
mPD		0.0015	0.70
		nPD:	0.
		N. weight:	0.88
		Sc. PD:	-0.047
		Sc. rank:	-26197.0

NA

Pos.	4	obs:	exp:
tct	S	0	0.18
tcc	S	0	0.12
tca	S	0	0.26
tcg	S	0	0.05
att	I	0	1.09
atc	I	3	0.75
ata	I	0	1.16
act	T	0	12.10
acc	T	48	9.85
aca	T	0	21.96
acg	T	0	4.10
aat	N	351	22975.00
aac	N	38495	15871.00
aaa	K	6	5.13
aag	K	3	3.87
agt	S	0	0.19
agc	S	1	0.20
---	--	------	------
mPD		0.021	0.49
		nPD:	0.04
		N. weight:	0.87
		Sc. PD:	-0.012
		Sc. rank:	14428.0

NA

Pos.	5	obs:	exp:
cct	P	0	1.93
ccc	P	0	1.30
cca	P	7	2.98
ccg	P	0	0.79
cat	H	1	1.76
cac	H	2	1.24
caa	Q	38623	20512.00
cag	Q	249	18360.00
cgt	R	0	0.41
cgc	R	0	0.78
cga	R	15	1.47
cgg	R	0	1.10
aaa	K	10	5.70
aag	K	0	4.30
aga	R	0	7.53
agg	R	0	3.71
---	--	------	------
mPD		0.015	0.50
		nPD:	0.03
		N. weight:	0.62
		Sc. PD:	-0.017
		Sc. rank:	6535.3

NA

Pos.	6	obs:	exp:
caa	Q	0	0.53
cag	Q	1	0.47
cgt	R	0	0.27
cgc	R	0	0.52
cga	R	0	0.98
cgg	R	0	0.73
act	T	0	0.25
acc	T	0	0.21
aca	T	0	0.46
acg	T	1	0.09
aat	N	7	4.14
aac	N	0	2.86
aaa	K	61	22181.00
aag	K	38824	16704.00
aga	R	0	5.02
agg	R	10	2.47
gaa	E	0	1.82
gag	E	3	1.18
---	--	------	------
mPD		0.0043	0.49
		nPD:	0.01
		N. weight:	0.86
		Sc. PD:	-0.04
		Sc. rank:	-12903.0

NA

Pos.	7	obs:	exp:
gat	D	1	0.53
gac	D	0	0.47
---	-	38906	38906.00
---	--	------	------
mPD		0	3.3×10^{-10}
		nPD:	0.
		N. weight:	0.
		Sc. PD:	0
		Sc. rank:	0

NA

Pos.	8	obs:	exp:
aat	N	1	0.59
aac	N	0	0.41
---	-	38906	38906.00
---	--	------	------
mPD		0	3.2×10^{-10}
		nPD:	0.
		N. weight:	0.
		Sc. PD:	0
		Sc. rank:	0

NA

Pos.	9	obs:	exp:
aat	N	0	0.59
aac	N	1	0.41
---	-	38906	38906.00
---	--	------	------
mPD		0	3.2×10^{-10}
		nPD:	0.
		N. weight:	0.
		Sc. PD:	0
		Sc. rank:	0

NA

Pos.	10	obs:	exp:
cat	H	1	0.59
cac	H	0	0.41
---	-	38906	38906.00
---	--	------	------
mPD		0	3.2×10^{-10}
		nPD:	0.
		N. weight:	0.
		Sc. PD:	0
		Sc. rank:	0

NA

Pos.	11	obs:	exp:
tgg	W	1	1.00
---	-	38906	38906.00
---	--	------	------
mPD		0	0
		nPD:	1.
		N. weight:	0.
		Sc. PD:	0
		Sc. rank:	0

NA

Pos.	12	obs:	exp:
att	I	2	14161.00
atc	I	0	9710.00
ata	I	38899	15030.00
atg	M	2	2.00
act	T	0	0.25
acc	T	0	0.21
aca	T	1	0.46
acg	T	0	0.09
gtt	V	1	0.66
gtc	V	1	0.55
gta	V	1	0.81
gtg	V	0	0.98
---	--	------	------
mPD		0.00051	0.66
		nPD:	0.
		N. weight:	0.98
		Sc. PD:	-0.053
		Sc. rank:	-31976.0

NA

Pos.	13	obs:	exp:
tta	L	2	0.33
ttg	L	0	0.56
ctt	L	0	0.59
ctc	L	0	0.42
cta	L	1	0.58
ctg	L	0	0.51
cgt	R	0	0.03
cgc	R	0	0.05
cga	R	1	0.10
cgg	R	0	0.07
att	I	8	14152.00
atc	I	3	9704.00
ata	I	38865	15020.00
atg	M	5	5.00
act	T	0	4.29
acc	T	0	3.49
aca	T	17	7.78
acg	T	0	1.45
aga	R	0	0.50
agg	R	0	0.25
gtt	V	0	1.11
gtc	V	0	0.91
gta	V	5	1.34
gtg	V	0	1.64
---	--	------	------
mPD		0.0022	0.66
		nPD:	0.
		N. weight:	0.98
		Sc. PD:	-0.051
		Sc. rank:	-26542.0

NA

Pos.	14	obs:	exp:
tct	S	1	0.37
tcc	S	0	0.23
tca	S	0	0.52
tcg	S	0	0.11
att	I	0	18.57
atc	I	51	12.73
ata	I	0	19.70
act	T	84	9788.00
acc	T	38692	7964.00
aca	T	51	17763.00
acg	T	2	3313.00
aat	N	0	0.59
aac	N	1	0.41
aaa	K	1	0.57
aag	K	0	0.43
agt	S	0	0.38
agc	S	1	0.40
gct	A	0	6.09
gcc	A	23	4.76
gca	A	0	10.80
gcg	A	0	1.35
---	--	------	------
mPD		0.011	0.68
		nPD:	0.02
		N. weight:	1.6
		Sc. PD:	-0.063
		Sc. rank:	-4755.8

,

NA

Pos.	15	obs:	exp:
ttt	F	3	1.29
ttc	F	0	1.71
tta	L	0	0.22
ttg	L	0	0.38
ctt	L	2	0.39
ctc	L	0	0.28
cta	L	0	0.39
ctg	L	0	0.34
att	I	38845	14158.00
atc	I	44	9708.00
ata	I	4	15026.00
act	T	2	0.50
acc	T	0	0.41
aca	T	0	0.91
acg	T	0	0.17
gtt	V	7	1.55
gtc	V	0	1.27
gta	V	0	1.88
gtg	V	0	2.30
---	--	------	------
mPD		0.0032	0.66
		nPD:	0.
		N. weight:	1.
		Sc. PD:	-0.052
		Sc. rank:	-24396.0

,

NA

Pos.	16	obs:	exp:
tct	S	0	15.64
tcc	S	0	9.85
tca	S	0	22.12
tcg	S	0	4.52
tgt	C	2	0.90
tgc	C	0	1.10
agt	S	69	15.98
agc	S	16	16.88
gtt	V	16	3.54
gtc	V	0	2.91
gta	V	0	4.30
gtg	V	0	5.25
gct	A	2	0.53
gcc	A	0	0.41
gca	A	0	0.94
gcg	A	0	0.12
gat	D	3	1.58
gac	D	0	1.42
ggt	G	35090	5883.00
ggc	G	3667	5746.00
gga	G	30	17258.00
ggg	G	11	9910.00
---	-	1	1.00
---	--	------	------
mPD		0.18	0.70
		nPD:	0.26
		N. weight:	1.6
		Sc. PD:	0.31
		Sc. rank:	45260.0

,

NA

Pos.	17	obs:	exp:
ttt	F	38	16.31
ttc	F	0	21.69
tta	L	0	8.14
ttg	L	74	13.93
tct	S	11130	7132.00
tcc	S	51	4489.00
tca	S	80	10087.00
tcg	S	27496	2063.00
tat	Y	6	3.12
tac	Y	0	2.88
tgg	W	1	1.00
ctt	L	0	14.58
ctc	L	0	10.45
cta	L	0	14.25
ctg	L	0	12.65
act	T	0	0.76
acc	T	0	0.62
aca	T	0	1.37
acg	T	3	0.26
agt	S	0	7288.00
agc	S	0	7699.00
gct	A	0	7.15
gcc	A	0	5.59
gca	A	0	12.68
gcg	A	27	1.58
---	-	1	1.00
---	--	------	------
mPD		0.42	1.6
		nPD:	0.26
		N. weight:	2.
		Sc. PD:	0.39
		Sc. rank:	56894.0

,

NA

Pos.	18	obs:	exp:
ttt	F	0	0.43
ttc	F	1	0.57
att	I	101	10179.00
atc	I	27850	6980.00
ata	I	11	10803.00
act	T	0	2.52
acc	T	10	2.05
aca	T	0	4.57
acg	T	0	0.85
gtt	V	30	2416.00
gtc	V	10891	1985.00
gta	V	5	2938.00
gtg	V	0	3587.00
gct	A	0	1.32
gcc	A	5	1.03
gca	A	0	2.35
gcg	A	0	0.29
ggt	G	0	0.30
ggc	G	2	0.30
gga	G	0	0.89
ggg	G	0	0.51
---	-	1	1.00
---	--	------	------
mPD		0.41	1.1
		nPD:	0.37
		N. weight:	1.5
		Sc. PD:	0.45
		Sc. rank:	44419.0

,

NA

Pos.	19	obs:	exp:
tct	S	4	1.84
tcc	S	0	1.16
tca	S	0	2.60
tcg	S	0	0.53
tgt	C	38779	17552.00
tgc	C	107	21334.00
tgg	W	1	1.00
agt	S	6	1.88
agc	S	0	1.99
ggt	G	9	1.36
ggc	G	0	1.33
gga	G	0	4.00
ggg	G	0	2.30
---	-	1	1.00
---	--	------	------
mPD		0.0065	0.50
		nPD:	0.01
		N. weight:	0.8
		Sc. PD:	-0.034
		Sc. rank:	-5240.4

,

NA

Pos.	20	obs:	exp:
tta	L	0	0.99
ttg	L	6	1.69
ctt	L	0	1.77
ctc	L	0	1.27
cta	L	0	1.73
ctg	L	3	1.54
att	I	62	78.63
atc	I	0	53.92
ata	I	154	83.45
atg	M	38631	38631.00
act	T	0	3.03
acc	T	0	2.46
aca	T	0	5.49
acg	T	12	1.02
gtt	V	0	8.62
gtc	V	0	7.09
gta	V	1	10.49
gtg	V	38	12.80
---	--	------	------
mPD		0.014	0.016
		nPD:	0.87
		N. weight:	0.0043
		Sc. PD:	0.0034
		Sc. rank:	140.4

,

NA

Pos.	21	obs:	exp:
tct	S	0	0.92
tcc	S	0	0.58
tca	S	5	1.30
tcg	S	0	0.27
att	I	0	14.93
atc	I	0	10.23
ata	I	41	15.84
act	T	16	9558.00
acc	T	4	7777.00
aca	T	37868	17345.00
acg	T	26	3235.00
aaa	K	1	0.57
aag	K	0	0.43
agt	S	0	0.94
agc	S	0	0.99
gct	A	0	250.20
gcc	A	1	195.60
gca	A	944	443.80
gcg	A	0	55.36
ggt	G	0	0.15
ggc	G	0	0.15
gga	G	1	0.44
ggg	G	0	0.26
---	--	------	------
mPD		0.052	0.73
		nPD:	0.07
		N. weight:	0.8
		Sc. PD:	0.011
		Sc. rank:	17298.0

,

NA

Pos.	22	obs:	exp:
ttt	F	2	0.86
ttc	F	0	1.14
tta	L	0	0.33
ttg	L	0	0.56
tct	S	0	0.18
tcc	S	0	0.12
tca	S	0	0.26
tcg	S	0	0.05
ctt	L	3	0.59
ctc	L	0	0.42
cta	L	0	0.58
ctg	L	0	0.51
att	I	38805	14145.00
atc	I	44	9699.00
ata	I	8	15013.00
atg	M	4	4.00
act	T	12	3.28
acc	T	1	2.67
aca	T	0	5.95
acg	T	0	1.11
aat	N	1	0.59
aac	N	0	0.41
agt	S	1	0.19
agc	S	0	0.20
gtt	V	26	5.75
gtc	V	0	4.72
gta	V	0	6.99
gtg	V	0	8.53
---	--	------	------
mPD		0.0053	0.66
		nPD:	0.01
		N. weight:	1.
		Sc. PD:	-0.049
		Sc. rank:	-16825.0

,

NA

Pos.	23	obs:	exp:
gaa	E	7	4.24
gag	E	0	2.76
ggt	G	104	5898.00
ggc	G	3	5762.00
gga	G	38669	17303.00
ggg	G	124	9937.00
---	--	------	------
mPD		0.012	0.69
		nPD:	0.02
		N. weight:	0.8
		Sc. PD:	-0.03
		Sc. rank:	-1015.6

,

NA			
Pos.	24	obs:	exp:
tta	L	0	9.45
ttg	L	13	16.19
ctt	L	0	16.95
ctc	L	0	12.15
cta	L	0	16.56
ctg	L	73	14.70
cgt	R	0	0.03
cgc	R	0	0.05
cga	R	0	0.10
cgg	R	0	0.07
att	I	44	92.83
atc	I	0	63.65
ata	I	211	98.52
atg	M	35722	35722.00
act	T	1	688.20
acc	T	0	560.00
aca	T	22	1249.00
acg	T	2707	232.90
aga	R	0	0.50
agg	R	1	0.25
gtt	V	0	24.76
gtc	V	0	20.35
gta	V	0	30.12
gtg	V	112	36.77
gct	A	0	0.26
gcc	A	0	0.21
gca	A	0	0.47
gcg	A	1	0.06
---	--	------	------
mPD		0.15	0.28
		nPD:	0.55
		N. weight:	0.18
		Sc. PD:	0.087
		Sc. rank:	5873.4

,

NA			
Pos.	25	obs:	exp:
tct	S	17	3.13
tcc	S	0	1.97
tca	S	0	4.42
tcg	S	0	0.90
act	T	118	29.75
acc	T	0	24.20
aca	T	0	53.98
acg	T	0	10.07
agt	S	0	3.20
agc	S	0	3.38
gtt	V	66	15.03
gtc	V	2	12.36
gta	V	0	18.29
gtg	V	0	22.32
gct	A	38018	10246.00
gcc	A	672	8011.00
gca	A	9	18176.00
gcg	A	2	2267.00
gat	D	1	0.53
gac	D	0	0.47
ggt	G	2	0.30
ggc	G	0	0.30
gga	G	0	0.89
ggg	G	0	0.51
---	--	------	------
mPD		0.045	0.67
		nPD:	0.07
		N. weight:	1.3
		Sc. PD:	0.012
		Sc. rank:	27463.0

,

NA			
Pos.	26	obs:	exp:
tct	S	0	1.47
tcc	S	0	0.93
tca	S	0	2.08
tcg	S	0	0.43
aat	N	613	23005.00
aac	N	38284	15892.00
aaa	K	1	0.57
aag	K	0	0.43
agt	S	1	1.50
agc	S	7	1.59
gat	D	0	0.53
gac	D	1	0.47
---	--	------	------
mPD		0.032	0.48
		nPD:	0.07
		N. weight:	0.83
		Sc. PD:	0.0066
		Sc. rank:	17703.0

,

NA			
Pos.	27	obs:	exp:
ttt	F	5	4.29
ttc	F	5	5.71
tta	L	38321	4276.00
ttg	L	472	7322.00
tct	S	0	0.55
tcc	S	0	0.35
tca	S	3	0.78
tcg	S	0	0.16
ctt	L	0	7664.00
ctc	L	0	5493.00
cta	L	99	7487.00
ctg	L	0	6650.00
att	I	0	0.73
atc	I	0	0.50
ata	I	2	0.77
agt	S	0	0.56
agc	S	0	0.60
---	--	------	------
mPD		0.030	1.1
		nPD:	0.03
		N. weight:	2.2
		Sc. PD:	-0.065
		Sc. rank:	15901.0

,

NA			
Pos.	28	obs:	exp:
tta	L	0	1.10
ttg	L	0	1.88
ctt	L	0	1.97
ctc	L	0	1.41
cta	L	10	1.93
ctg	L	0	1.71
att	I	8	14117.00
atc	I	7	9680.00
ata	I	38764	14982.00
atg	M	65	65.00
act	T	0	7.56
acc	T	0	6.15
aca	T	30	13.72
acg	T	0	2.56
gtt	V	0	5.08
gtc	V	0	4.18
gta	V	23	6.19
gtg	V	0	7.55
---	--	------	------
mPD		0.0073	0.66
		nPD:	0.01
		N. weight:	0.98
		Sc. PD:	-0.043
		Sc. rank:	-9682.5

,

NA			
Pos.	29	obs:	exp:
tta	L	37997	4276.00
ttg	L	843	7324.00
ctt	L	0	7665.00
ctc	L	0	5495.00
cta	L	60	7489.00
ctg	L	0	6651.00
att	I	0	2.18
atc	I	0	1.50
ata	I	6	2.32
gtt	V	0	0.22
gtc	V	0	0.18
gta	V	1	0.27
gtg	V	0	0.33
---	--	------	------
mPD		0.046	1.1
		nPD:	0.04
		N. weight:	2.1
		Sc. PD:	-0.035
		Sc. rank:	34374.0

,

NA			
Pos.	30	obs:	exp:
cat	H	1	0.59
cac	H	0	0.41
caa	Q	38817	20529.00
cag	Q	88	18376.00
cgt	R	0	0.03
cgc	R	0	0.05
cga	R	1	0.10
cgg	R	0	0.07
aga	R	0	0.50
agg	R	0	0.25
---	--	------	------
mPD		0.0046	0.50
		nPD:	0.01
		N. weight:	0.64
		Sc. PD:	-0.03
		Sc. rank:	-8837.9

,

NA			
Pos.	31	obs:	exp:
tta	L	0	0.33
ttg	L	0	0.56
tct	S	0	0.18
tcc	S	0	0.12
tca	S	0	0.26
tcg	S	0	0.05
ctt	L	3	0.59
ctc	L	0	0.42
cta	L	0	0.58
ctg	L	0	0.51
att	I	38812	14146.00
atc	I	40	9700.00
ata	I	8	15014.00
atg	M	5	5.00
act	T	15	3.78
acc	T	0	3.08
aca	T	0	6.86
acg	T	0	1.28
agt	S	1	0.19
agc	S	0	0.20
gtt	V	23	5.08
gtc	V	0	4.18
gta	V	0	6.19
gtg	V	0	7.55
---	--	------	------
mPD		0.0049	0.66
		nPD:	0.01
		N. weight:	1.
		Sc. PD:	-0.049
		Sc. rank:	-19045.0

,

NA			
Pos.	32	obs:	exp:
cgt	R	0	0.03
cgc	R	0	0.05
cga	R	0	0.10
cgg	R	0	0.07
aga	R	0	0.50
agg	R	1	0.25
gaa	E	1	0.61
gag	E	0	0.39
ggt	G	5	5899.00
ggc	G	0	5762.00
gga	G	37052	17306.00
ggg	G	1848	9938.00
---	--	------	------
mPD		0.091	0.69
		nPD:	0.13
		N. weight:	0.66
		Sc. PD:	0.048
		Sc. rank:	17481.0

,

NA			
Pos.	33	obs:	exp:
tct	S	0	3.31
tcc	S	0	2.08
tca	S	0	4.68
tcg	S	0	0.96
tat	Y	0	1.04
tac	Y	2	0.96
cat	H	0	1.76
cac	H	3	1.24
act	T	0	1.01
acc	T	4	0.82
aca	T	0	1.83
acg	T	0	0.34
aat	N	63	22994.00
aac	N	38815	15884.00
aaa	K	1	0.57
aag	K	0	0.43
agt	S	0	3.38
agc	S	18	3.58
gat	D	0	0.53
gac	D	1	0.47
---	--	------	------
mPD		0.0047	0.49
		nPD:	0.01
		N. weight:	0.91
		Sc. PD:	-0.042
		Sc. rank:	-11668.0

,

NA

Pos. 34		obs:	exp:
tta	L	4	0.44
ttg	L	0	0.75
ctt	L	0	0.79
ctc	L	0	0.56
cta	L	0	0.77
ctg	L	0	0.68
att	I	5	14074.00
atc	I	2	9651.00
ata	I	38655	14937.00
atg	M	190	190.00
act	T	0	10.84
acc	T	0	8.82
aca	T	41	19.67
acg	T	2	3.67
gtt	V	0	1.77
gtc	V	0	1.45
gta	V	8	2.15
gtg	V	0	2.63
---	--	------	------
mPD		0.013	0.66
		nPD:	0.02
		N. weight:	0.97
		Sc. PD:	-0.035
		Sc. rank:	1294.7

,

NA

Pos. 35		obs:	exp:
ttt	F	0	0.43
ttc	F	1	0.57
tta	L	0	0.77
ttg	L	0	1.32
ctt	L	0	1.38
ctc	L	7	0.99
cta	L	0	1.35
ctg	L	0	1.20
att	I	384	14120.00
atc	I	38295	9682.00
ata	I	110	14986.00
act	T	0	15.13
acc	T	60	12.31
aca	T	0	27.45
acg	T	0	5.12
gtt	V	0	11.05
gtc	V	50	9.09
gta	V	0	13.45
gtg	V	0	16.41
---	--	------	------
mPD		0.031	0.66
		nPD:	0.05
		N. weight:	1.4
		Sc. PD:	-0.013
		Sc. rank:	23279.0

,

NA

Pos. 36		obs:	exp:
tta	L	13	1.54
ttg	L	1	2.64
tct	S	526	7156.00
tcc	S	3	4504.00
tca	S	38335	10121.00
tcg	S	23	2070.00
ctt	L	0	2.76
ctc	L	0	1.98
cta	L	0	2.70
ctg	L	0	2.39
act	T	0	1.26
acc	T	0	1.03
aca	T	5	2.29
acg	T	0	0.43
agt	S	0	7312.00
agc	S	0	7724.00
gct	A	0	0.26
gcc	A	0	0.21
gca	A	1	0.47
gcg	A	0	0.06
---	--	------	------
mPD		0.029	1.6
		nPD:	0.02
		N. weight:	1.3
		Sc. PD:	-0.05
		Sc. rank:	-1437.0

,

NA

Pos. 37		obs:	exp:
tta	L	1	0.22
ttg	L	0	0.38
ctt	L	0	0.39
ctc	L	0	0.28
cta	L	1	0.39
ctg	L	0	0.34
cgt	R	0	0.03
cgc	R	0	0.05
cga	R	0	0.10
cgg	R	0	0.07
att	I	21	14143.00
atc	I	25	9698.00
ata	I	38806	15011.00
atg	M	12	12.00
act	T	0	5.55
acc	T	0	4.51
aca	T	22	10.06
acg	T	0	1.88
aga	R	1	0.50
agg	R	0	0.25
gtt	V	0	3.98
gtc	V	0	3.27
gta	V	18	4.84
gtg	V	0	5.91
---	--	------	------
mPD		0.0052	0.66
		nPD:	0.01
		N. weight:	0.97
		Sc. PD:	-0.046
		Sc. rank:	-16191.0

,

NA

Pos. 38		obs:	exp:
tta	L	0	0.22
ttg	L	2	0.38
tgg	W	38905	38905.00
ctt	L	0	0.39
ctc	L	0	0.28
cta	L	0	0.39
ctg	L	0	0.34
---	--	------	------
mPD		0.00010	0.00024
		nPD:	0.43
		N. weight:	0.000089
		Sc. PD:	0.000032
		Sc. rank:	2.7

,

NA

Pos. 39		obs:	exp:
ttt	F	4	1.72
ttc	F	0	2.28
tta	L	2	5.50
ttg	L	0	9.41
ctt	L	48	9.85
ctc	L	0	7.06
cta	L	0	9.63
ctg	L	0	8.55
cct	P	0	0.55
ccc	P	0	0.37
cca	P	2	0.85
ccg	P	0	0.23
att	I	8796	3206.00
atc	I	10	2198.00
ata	I	0	3402.00
atg	M	1	1.00
act	T	3	1.01
acc	T	1	0.82
aca	T	0	1.83
acg	T	0	0.34
gtt	V	29858	6638.00
gtc	V	103	5456.00
gta	V	63	8075.00
gtg	V	2	9856.00
gct	A	9	2.91
gcc	A	2	2.28
gca	A	0	5.17
gcg	A	0	0.64
ggt	G	3	0.45
ggc	G	0	0.44
gga	G	0	1.33
ggg	G	0	0.77
---	--	------	------
mPD		0.36	1.1
		nPD:	0.33
		N. weight:	1.4
		Sc. PD:	0.37
		Sc. rank:	41052.0

,

NA

Pos. 40		obs:	exp:
tta	L	0	0.11
ttg	L	0	0.19
tct	S	0	7144.00
tcc	S	0	4496.00
tca	S	0	10103.00
tcg	S	0	2066.00
tgt	C	0	0.90
tgc	C	2	1.10
ctt	L	0	0.20
ctc	L	1	0.14
cta	L	0	0.19
ctg	L	0	0.17
cgt	R	0	0.25
cgc	R	0	0.47
cga	R	0	0.88
cgg	R	0	0.66
att	I	0	7.64
atc	I	21	5.24
ata	I	0	8.11
act	T	0	0.50
acc	T	2	0.41
aca	T	0	0.91
acg	T	0	0.17
aat	N	0	15.97
aac	N	27	11.03
agt	S	502	7299.00
agc	S	38317	7711.00
aga	R	9	4.52
agg	R	0	2.22
gtt	V	0	0.66
gtc	V	3	0.55
gta	V	0	0.81
gtg	V	0	0.98
ggt	G	0	3.49
ggc	G	23	3.41
gga	G	0	10.23
ggg	G	0	5.88
---	--	------	------
mPD		0.030	1.6
		nPD:	0.02
		N. weight:	1.6
		Sc. PD:	-0.06
		Sc. rank:	-303.9

,

NA

Pos. 41		obs:	exp:
tta	L	0	0.11
ttg	L	0	0.19
tat	Y	0	6.75
tac	Y	13	6.25
ctt	L	0	0.20
ctc	L	1	0.14
cta	L	0	0.19
ctg	L	0	0.17
cct	P	0	1.10
ccc	P	4	0.74
cca	P	0	1.70
ccg	P	0	0.45
cat	H	104	22853.00
cac	H	38755	16006.00
caa	Q	8	4.22
cag	Q	0	3.78
cgt	R	0	0.49
cgc	R	18	0.94
cga	R	0	1.76
cgg	R	0	1.32
aat	N	0	2.37
aac	N	4	1.63
aga	R	0	9.04
agg	R	0	4.45
---	--	------	------
mPD		0.0078	0.49
		nPD:	0.02
		N. weight:	0.9
		Sc. PD:	-0.035
		Sc. rank:	-3157.5

,

NA

Pos. 42		obs:	exp:
tta	L	13	1.43
ttg	L	0	2.45
tct	S	8	7145.00
tcc	S	0	4497.00
tca	S	38811	10105.00
tcg	S	8	2066.00
ctt	L	0	2.56
ctc	L	0	1.84
cta	L	0	2.50
ctg	L	0	2.22
cct	P	0	6.61
ccc	P	0	4.45
cca	P	24	10.22
ccg	P	0	2.72
act	T	0	10.84
acc	T	0	8.82
aca	T	43	19.67
acg	T	0	3.67
agt	S	0	7301.00
agc	S	0	7712.00
---	--	------	------
mPD		0.0049	1.6
		nPD:	0.
		N. weight:	1.4
		Sc. PD:	-0.072
		Sc. rank:	-39443.0

,

NA

Pos. 43		obs:	exp:
ttt	F	2	0.86
ttc	F	0	1.14
tta	L	0	0.77
ttg	L	0	1.32
tct	S	0	0.74
tcc	S	0	0.46
tca	S	0	1.04
tcg	S	0	0.21
ctt	L	7	1.38
ctc	L	0	0.99
cta	L	0	1.35
ctg	L	0	1.20
att	I	38704	14148.00
atc	I	136	9701.00
ata	I	25	15016.00
act	T	15	3.78
acc	T	0	3.08
aca	T	0	6.86
acg	T	0	1.28
aat	N	6	3.55
aac	N	0	2.45
agt	S	4	0.75
agc	S	0	0.79
gtt	V	8	1.77
gtc	V	0	1.45
gta	V	0	2.15
gtg	V	0	2.63
---	--	------	------
mPD		0.010	0.66
		nPD:	0.02
		N. weight:	1.
		Sc. PD:	-0.04
		Sc. rank:	-3699.8

,

NA

Pos.	44	obs:	exp:
cct	P	0	0.28
ccc	P	0	0.19
cca	P	1	0.43
ccg	P	0	0.11
cat	H	1	6.47
cac	H	10	4.53
caa	Q	38834	20513.00
cag	Q	41	18362.00
cgt	R	0	0.30
cgc	R	0	0.58
cga	R	11	1.08
cgg	R	0	0.81
aaa	K	8	4.56
aag	K	0	3.44
aga	R	0	5.52
agg	R	0	2.72
gaa	E	1	0.61
gag	E	0	0.39
---	--	------	------
mPD		0.0037	0.50
		nPD:	0.01
		N. weight:	0.65
		Sc. PD:	-0.031
		Sc. rank:	-11627.0

,

NA

Pos.	45	obs:	exp:
ttt	F	16	6.87
ttc	F	0	9.13
tta	L	0	986.80
ttg	L	0	1690.00
tct	S	0	0.37
tcc	S	0	0.23
tca	S	0	0.52
tcg	S	0	0.11
ctt	L	8962	1769.00
ctc	L	11	1268.00
cta	L	1	1728.00
ctg	L	2	1535.00
cct	P	4	1.10
ccc	P	0	0.74
cca	P	0	1.70
ccg	P	0	0.45
cgt	R	2	0.05
cgc	R	0	0.10
cga	R	0	0.20
cgg	R	0	0.15
att	I	28220	10281.00
atc	I	20	7050.00
ata	I	3	10912.00
atg	M	2	2.00
act	T	262	381.40
acc	T	1249	310.30
aca	T	1	692.20
acg	T	1	129.10
aat	N	2	1.77
aac	N	1	1.23
agt	S	2	0.38
agc	S	0	0.40
aga	R	0	1.00
agg	R	0	0.49
gtt	V	146	32.28
gtc	V	0	26.53
gta	V	0	39.27
gtg	V	0	47.93
---	--	------	------
mPD		0.50	1.2
		nPD:	0.43
		N. weight:	1.2
		Sc. PD:	0.43
		Sc. rank:	36804.0

,

NA

Pos.	46	obs:	exp:
tgg	W	1	1.00
cgt	R	0	6.94
cgc	R	0	13.29
cga	R	0	24.88
cgg	R	0	18.59
atg	M	3	3.00
aat	N	1	0.59
aac	N	0	0.41
aaa	K	0	1.71
aag	K	3	1.29
aga	R	39	127.50
agg	R	215	62.79
gtt	V	0	8.18
gtc	V	0	6.72
gta	V	10	9.95
gtg	V	27	12.15
gat	D	18	9.48
gac	D	0	8.52
gaa	E	46	49.65
gag	E	36	32.35
ggt	G	259	5839.00
ggc	G	4	5703.00
gga	G	29115	17129.00
ggg	G	9130	9836.00
---	--	------	------
mPD		0.40	0.71
		nPD:	0.56
		N. weight:	0.38
		Sc. PD:	0.18
		Sc. rank:	12066.0

,

NA

Pos.	47	obs:	exp:
tct	S	0	1.66
tcc	S	0	1.04
tca	S	0	2.34
tcg	S	0	0.48
tat	Y	3	1.56
tac	Y	0	1.44
cat	H	3	1.76
cac	H	0	1.24
act	T	4	1.01
acc	T	0	0.82
aca	T	0	1.83
acg	T	0	0.34
aat	N	37407	22973.00
aac	N	1435	15869.00
aaa	K	4	3.99
aag	K	3	3.01
agt	S	8	1.69
agc	S	1	1.79
gat	D	38	20.02
gac	D	0	17.98
---	-	1	1.00
---	--	------	------
mPD		0.074	0.49
		nPD:	0.15
		N. weight:	0.39
		Sc. PD:	0.036
		Sc. rank:	10537.0

,

NA

Pos.	48	obs:	exp:
tta	L	0	0.33
ttg	L	0	0.56
ctt	L	0	0.59
ctc	L	0	0.42
cta	L	3	0.58
ctg	L	0	0.51
cct	P	0	0.55
ccc	P	0	0.37
cca	P	2	0.85
ccg	P	0	0.23
cat	H	18	15.29
cac	H	8	10.71
caa	Q	38737	20478.00
cag	Q	72	18331.00
cgt	R	0	0.87
cgc	R	0	1.67
cga	R	32	3.13
cgg	R	0	2.34
aaa	K	34	19.39
aag	K	0	14.61
aga	R	0	16.07
agg	R	0	7.91
---	-	1	1.00
---	--	------	------
mPD		0.0087	0.50
		nPD:	0.02
		N. weight:	0.65
		Sc. PD:	-0.025
		Sc. rank:	-893.0

,

NA

Pos.	49	obs:	exp:
tct	S	0	5954.00
tcc	S	0	3747.00
tca	S	0	8420.00
tcg	S	0	1722.00
tgt	C	0	0.45
tgc	C	1	0.55
cat	H	2	1.18
cac	H	0	0.82
cgt	R	0	0.05
cgc	R	1	0.10
cga	R	0	0.20
cgg	R	0	0.15
att	I	0	1.46
atc	I	4	1.00
ata	I	0	1.55
act	T	3	1.01
acc	T	1	0.82
aca	T	0	1.83
acg	T	0	0.34
aat	N	6445	3858.00
aac	N	78	2665.00
aaa	K	0	1.14
aag	K	2	0.86
agt	S	5228	6084.00
agc	S	27126	6427.00
aga	R	1	1.00
agg	R	0	0.49
gat	D	4	2.11
gac	D	0	1.89
ggt	G	5	1.52
ggc	G	5	1.48
gga	G	0	4.45
ggg	G	0	2.55
---	-	1	1.00
---	--	------	------
mPD		0.70	1.8
		nPD:	0.39
		N. weight:	1.1
		Sc. PD:	0.36
		Sc. rank:	33471.0

,

NA

Pos.	50	obs:	exp:
tta	L	0	1.54
ttg	L	0	2.64
ctt	L	0	2.76
ctc	L	0	1.98
cta	L	0	2.70
ctg	L	14	2.39
cct	P	2	1.65
ccc	P	0	1.11
cca	P	0	2.55
ccg	P	4	0.68
cat	H	94	55.28
cac	H	0	38.72
caa	Q	260	20441.00
cag	Q	38478	18297.00
cgt	R	0	1.04
cgc	R	0	1.99
cga	R	1	3.72
cgg	R	37	2.78
aat	N	1	0.59
aac	N	0	0.41
aaa	K	0	9.13
aag	K	16	6.87
aga	R	0	19.08
agg	R	0	9.39
---	--	------	------
mPD		0.022	0.51
		nPD:	0.04
		N. weight:	0.73
		Sc. PD:	-0.0095
		Sc. rank:	12269.0

,

NA

Pos.	51	obs:	exp:
ttt	F	13	5.58
ttc	F	0	7.42
tta	L	0	0.11
ttg	L	0	0.19
tct	S	1	0.18
tcc	S	0	0.12
tca	S	0	0.26
tcg	S	0	0.05
ctt	L	1	0.20
ctc	L	0	0.14
cta	L	0	0.19
ctg	L	0	0.17
att	I	37278	14053.00
atc	I	1320	9636.00
ata	I	6	14915.00
atg	M	2	2.00
act	T	101	27.23
acc	T	7	22.15
aca	T	0	49.41
acg	T	0	9.21
aat	N	1	0.59
aac	N	0	0.41
agt	S	0	0.19
agc	S	0	0.20
gtt	V	175	39.13
gtc	V	2	32.16
gta	V	0	47.60
gtg	V	0	58.10
---	--	------	------
mPD		0.082	0.67
		nPD:	0.12
		N. weight:	0.9
		Sc. PD:	0.056
		Sc. rank:	23453.0

,

NA

Pos.	52	obs:	exp:
aaa	K	48	27.38
aag	K	0	20.62
gtt	V	0	0.66
gtc	V	0	0.55
gta	V	3	0.81
gtg	V	0	0.98
gat	D	2	6.85
gac	D	11	6.15
gaa	E	38645	23425.00
gag	E	42	15262.00
ggt	G	0	23.65
ggc	G	0	23.11
gga	G	156	69.39
ggg	G	0	39.85
---	--	------	------
mPD		0.013	0.49
		nPD:	0.03
		N. weight:	0.51
		Sc. PD:	-0.015
		Sc. rank:	4119.2

,

NA

Pos.	53	obs:	exp:
tct	S	0	0.37
tcc	S	0	0.23
tca	S	2	0.52
tcg	S	0	0.11
cct	P	0	8.82
ccc	P	0	5.94
cca	P	32	13.63
ccg	P	0	3.62
cgt	R	0	0.03
cgc	R	0	0.05
cga	R	0	0.10
cgg	R	0	0.07
att	I	0	45.50
atc	I	0	31.20
ata	I	125	48.29
act	T	12	9708.00
acc	T	5	7899.00
aca	T	38435	17618.00
acg	T	59	3286.00
aaa	K	64	36.51
aag	K	0	27.49
agt	S	0	0.38
agc	S	0	0.40
aga	R	1	0.50
agg	R	0	0.25
gct	A	0	45.54
gcc	A	0	35.60
gca	A	172	80.78
gcg	A	0	10.08
---	--	------	------
mPD		0.024	0.70
		nPD:	0.03
		N. weight:	0.79
		Sc. PD:	-0.017
		Sc. rank:	10334.0

,

NA			
Pos.	54	obs:	exp:
ttt	F	0	1.29
ttc	F	3	1.71
tat	Y	0	5.20
tac	Y	10	4.80
tgt	C	140	17554.00
tgc	C	38751	21337.00
cgt	R	0	0.05
cgc	R	2	0.10
cga	R	0	0.20
cgg	R	0	0.15
aga	R	0	1.00
agg	R	0	0.49
ggt	G	0	0.15
ggc	G	1	0.15
gga	G	0	0.44
ggg	G	0	0.26
---	--	------	------
mPD		0.0080	0.50
		nPD:	0.02
		N. weight:	0.6
		Sc. PD:	-0.024
		Sc. rank:	-2051.0

,

NA			
Pos.	55	obs:	exp:
tct	S	0	7.91
tcc	S	0	4.98
tca	S	0	11.19
tcg	S	0	2.29
tat	Y	5	2.60
tac	Y	0	2.40
cat	H	1	0.59
cac	H	0	0.41
att	I	23	8.37
atc	I	0	5.74
ata	I	0	8.89
act	T	15	3.78
acc	T	0	3.08
aca	T	0	6.86
acg	T	0	1.28
aat	N	38427	22947.00
aac	N	371	15851.00
aaa	K	9	9.13
aag	K	7	6.87
agt	S	43	8.09
agc	S	0	8.54
gat	D	6	3.16
gac	D	0	2.84
---	--	------	------
mPD		0.024	0.49
		nPD:	0.05
		N. weight:	0.49
		Sc. PD:	-0.0034
		Sc. rank:	8850.3

,

NA			
Pos.	56	obs:	exp:
cct	P	0	0.28
ccc	P	0	0.19
cca	P	1	0.43
ccg	P	0	0.11
cat	H	1	3.53
cac	H	5	2.47
caa	Q	27660	14665.00
cag	Q	132	13127.00
cgt	R	0	1.99
cgc	R	0	3.82
cga	R	36	7.15
cgg	R	0	5.34
aat	N	1	1.77
aac	N	2	1.23
aaa	K	10956	6293.00
aag	K	76	4739.00
aga	R	37	36.65
agg	R	0	18.05
---	--	------	------
mPD		0.42	0.91
		nPD:	0.46
		N. weight:	0.6
		Sc. PD:	0.24
		Sc. rank:	19084.0

,

NA			
Pos.	57	obs:	exp:
tct	S	0	6509.00
tcc	S	0	4097.00
tca	S	0	9205.00
tcg	S	0	1882.00
tgt	C	0	0.90
tgc	C	2	1.10
cgt	R	0	1.45
cgc	R	2	2.77
cga	R	0	5.19
cgg	R	0	3.88
att	I	1	0.36
atc	I	0	0.25
ata	I	0	0.39
act	T	0	12.60
acc	T	50	10.26
aca	T	0	22.87
acg	T	0	4.27
aat	N	25	2028.00
aac	N	3404	1401.00
aaa	K	3	1.71
aag	K	0	1.29
agt	S	124	6650.00
agc	S	35244	7025.00
aga	R	51	26.61
agg	R	0	13.10
ggt	G	0	0.15
ggc	G	1	0.15
gga	G	0	0.44
ggg	G	0	0.26
---	--	------	------
mPD		0.17	1.7
		nPD:	0.1
		N. weight:	1.6
		Sc. PD:	0.066
		Sc. rank:	38945.0

,

NA			
Pos.	58	obs:	exp:
ttt	F	0	16.31
ttc	F	38	21.69
tta	L	0	0.33
ttg	L	0	0.56
ctt	L	0	0.59
ctc	L	3	0.42
cta	L	0	0.58
ctg	L	0	0.51
att	I	0	83.36
atc	I	229	57.16
ata	I	0	88.47
gtt	V	18	8537.00
gtc	V	38574	7017.00
gta	V	23	10385.00
gtg	V	0	12676.00
gct	A	0	5.82
gcc	A	22	4.55
gca	A	0	10.33
gcg	A	0	1.29
---	--	------	------
mPD		0.017	0.75
		nPD:	0.02
		N. weight:	1.7
		Sc. PD:	-0.058
		Sc. rank:	7423.3

,

NA			
Pos.	59	obs:	exp:
ttt	F	2	0.86
ttc	F	0	1.14
att	I	37068	14155.00
atc	I	1810	9706.00
ata	I	5	15023.00
atg	M	1	1.00
act	T	2	1.01
acc	T	2	0.82
aca	T	0	1.83
acg	T	0	0.34
gtt	V	16	3.76
gtc	V	0	3.09
gta	V	1	4.57
gtg	V	0	5.58
---	--	------	------
mPD		0.090	0.66
		nPD:	0.14
		N. weight:	0.87
		Sc. PD:	0.067
		Sc. rank:	22862.0

,

NA			
Pos.	60	obs:	exp:
tct	S	2	0.55
tcc	S	0	0.35
tca	S	0	0.78
tcg	S	0	0.16
cct	P	2	0.55
ccc	P	0	0.37
cca	P	0	0.85
ccg	P	0	0.23
att	I	112	41.14
atc	I	1	28.21
ata	I	0	43.66
act	T	38589	9772.00
acc	T	151	7951.00
aca	T	6	17734.00
acg	T	18	3307.00
aat	N	25	14.79
aac	N	0	10.21
agt	S	1	0.56
agc	S	0	0.60
---	--	------	------
mPD		0.016	0.69
		nPD:	0.02
		N. weight:	1.4
		Sc. PD:	-0.044
		Sc. rank:	7718.5

,

NA			
Pos.	61	obs:	exp:
ttt	F	16	6.87
ttc	F	0	9.13
tat	Y	38843	20201.00
tac	Y	36	18678.00
tgt	C	2	0.90
tgc	C	0	1.10
cat	H	10	5.88
cac	H	0	4.12
---	--	------	------
mPD		0.0033	0.50
		nPD:	0.01
		N. weight:	0.67
		Sc. PD:	-0.033
		Sc. rank:	-14031.0

,

NA			
Pos.	62	obs:	exp:
caa	Q	2	1.06
cag	Q	0	0.94
aaa	K	17	9.70
aag	K	0	7.30
gtt	V	0	0.66
gtc	V	0	0.55
gta	V	3	0.81
gtg	V	0	0.98
gaa	E	38591	23544.00
gag	E	292	15339.00
ggt	G	0	0.30
ggc	G	0	0.30
gga	G	2	0.89
ggg	G	0	0.51
---	--	------	------
mPD		0.016	0.48
		nPD:	0.03
		N. weight:	0.48
		Sc. PD:	-0.011
		Sc. rank:	6039.7

,

NA			
Pos.	63	obs:	exp:
tct	S	0	1.29
tcc	S	0	0.81
tca	S	0	1.82
tcg	S	0	0.37
cat	H	0	4.12
cac	H	7	2.88
act	T	0	1.01
acc	T	4	0.82
aca	T	0	1.83
acg	T	0	0.34
aat	N	72	22999.00
aac	N	38814	15887.00
aaa	K	1	0.57
aag	K	0	0.43
agt	S	0	1.32
agc	S	7	1.39
gat	D	0	1.05
gac	D	2	0.95
---	--	------	------
mPD		0.0048	0.48
		nPD:	0.01
		N. weight:	0.91
		Sc. PD:	-0.041
		Sc. rank:	-11458.0

,

NA			
Pos.	64	obs:	exp:
tct	S	0	0.37
tcc	S	0	0.23
tca	S	0	0.52
tcg	S	0	0.11
tat	Y	0	1.04
tac	Y	2	0.96
cat	H	0	5.29
cac	H	9	3.71
cgt	R	0	0.05
cgc	R	0	0.10
cga	R	0	0.20
cgg	R	0	0.15
att	I	0	0.73
atc	I	2	0.50
ata	I	0	0.77
aat	N	1825	22979.00
aac	N	37027	15873.00
aaa	K	30	21.11
aag	K	7	15.89
agt	S	0	0.38
agc	S	2	0.40
aga	R	2	1.00
agg	R	0	0.49
gat	D	0	0.53
gac	D	1	0.47
---	--	------	------
mPD		0.092	0.49
		nPD:	0.19
		N. weight:	0.71
		Sc. PD:	0.091
		Sc. rank:	19255.0

,

NA			
Pos.	65	obs:	exp:
att	I	8	2.91
atc	I	0	2.00
ata	I	0	3.09
act	T	38343	9805.00
acc	T	549	7978.00
aca	T	3	17793.00
acg	T	0	3319.00
aat	N	2	1.18
aac	N	0	0.82
gct	A	2	0.53
gcc	A	0	0.41
gca	A	0	0.94
gcg	A	0	0.12
---	--	------	------
mPD		0.029	0.68
		nPD:	0.04
		N. weight:	1.3
		Sc. PD:	-0.019
		Sc. rank:	22087.0

,

NA			
Pos.	66	obs:	exp:
tta	L	0	0.11
ttg	L	1	0.19
tgg	W	38904	38904.00
ctt	L	0	0.20
ctc	L	0	0.14
cta	L	0	0.19
ctg	L	0	0.17
cgt	R	0	0.05
cgc	R	0	0.10
cga	R	0	0.20
cgg	R	2	0.15
aga	R	0	1.00
agg	R	0	0.49
---	--	------	------
mPD		0.00015	0.00029
		nPD:	0.53
		N. weight:	0.00018
		Sc. PD:	0.000083
		Sc. rank:	5.8

,

NA			
Pos.	67	obs:	exp:
tta	L	7	0.77
ttg	L	0	1.32
ctt	L	0	1.38
ctc	L	0	0.99
cta	L	0	1.35
ctg	L	0	1.20
att	I	0	217.70
atc	I	0	149.30
ata	I	598	231.00
gtt	V	2	8468.00
gtc	V	1	6960.00
gta	V	38189	10301.00
gtg	V	109	12573.00
gct	A	0	0.26
gcc	A	0	0.21
gca	A	1	0.47
gcg	A	0	0.06
---	--	------	------
mPD		0.036	0.77
		nPD:	0.05
		N. weight:	1.3
		Sc. PD:	-0.012
		Sc. rank:	23133.0

,

NA			
Pos.	68	obs:	exp:
tct	S	0	0.37
tcc	S	0	0.23
tca	S	0	0.52
tcg	S	0	0.11
cat	H	2	1.18
cac	H	0	0.82
act	T	2	0.50
acc	T	0	0.41
aca	T	0	0.91
acg	T	0	0.17
aat	N	38623	23005.00
aac	N	274	15892.00
aaa	K	2	1.71
aag	K	1	1.29
agt	S	2	0.38
agc	S	0	0.40
gat	D	1	0.53
gac	D	0	0.47
---	--	------	------
mPD		0.014	0.48
		nPD:	0.03
		N. weight:	0.5
		Sc. PD:	-0.013
		Sc. rank:	5588.5

,

NA

Pos.	69	obs:	exp:
tta	L	0	0.77
ttg	L	0	1.32
ctt	L	0	1.38
ctc	L	0	0.99
cta	L	0	1.35
ctg	L	7	1.20
cct	P	0	0.28
ccc	P	0	0.19
cca	P	0	0.43
ccg	P	1	0.11
cat	H	33	19.41
cac	H	0	13.59
caa	Q	318	20504.00
cag	Q	38540	18354.00
cgt	R	0	0.08
cgc	R	0	0.16
cga	R	0	0.29
cgg	R	3	0.22
aaa	K	0	2.85
aag	K	5	2.15
aga	R	0	1.51
agg	R	0	0.74
---	--	------	------
mPD		0.019	0.50
		nPD:	0.04
		N. weight:	0.72
		Sc. PD:	-0.014
		Sc. rank:	10256.0

,

NA

Pos.	70	obs:	exp:
att	I	0	0.36
atc	I	0	0.25
ata	I	1	0.39
act	T	13	9807.00
acc	T	12	7980.00
aca	T	38758	17798.00
acg	T	121	3319.00
aaa	K	1	0.57
aag	K	0	0.43
gct	A	0	0.26
gcc	A	0	0.21
gca	A	1	0.47
gcg	A	0	0.06
---	--	------	------
mPD		0.0076	0.68
		nPD:	0.01
		N. weight:	0.79
		Sc. PD:	-0.035
		Sc. rank:	-7543.2

,

NA

Pos.	71	obs:	exp:
ttt	F	2752	1181.00
ttc	F	0	1571.00
tta	L	0	0.11
ttg	L	0	0.19
tat	Y	36097	18779.00
tac	Y	46	17364.00
tgt	C	2	0.90
tgc	C	0	1.10
ctt	L	1	0.20
ctc	L	0	0.14
cta	L	0	0.19
ctg	L	0	0.17
cat	H	7	4.12
cac	H	0	2.88
aat	N	2	1.18
aac	N	0	0.82
---	--	------	------
mPD		0.13	0.63
		nPD:	0.21
		N. weight:	0.68
		Sc. PD:	0.1
		Sc. rank:	18516.0

,

NA

Pos.	72	obs:	exp:
ttt	F	3	1.29
ttc	F	0	1.71
tta	L	0	0.11
ttg	L	0	0.19
ctt	L	1	0.20
ctc	L	0	0.14
cta	L	0	0.19
ctg	L	0	0.17
att	I	742	271.60
atc	I	4	186.20
ata	I	0	288.20
gtt	V	37699	8435.00
gtc	V	438	6933.00
gta	V	3	10261.00
gtg	V	15	12525.00
gat	D	1	0.53
gac	D	0	0.47
ggt	G	1	0.15
ggc	G	0	0.15
gga	G	0	0.44
ggg	G	0	0.26
---	--	------	------
mPD		0.061	0.78
		nPD:	0.08
		N. weight:	1.5
		Sc. PD:	0.031
		Sc. rank:	33286.0

,

NA

Pos.	73	obs:	exp:
tct	S	0	2.76
tcc	S	0	1.74
tca	S	0	3.90
tcg	S	0	0.80
tat	Y	0	1.04
tac	Y	2	0.96
act	T	1	5.29
acc	T	20	4.31
aca	T	0	9.61
acg	T	0	1.79
aat	N	289	22970.00
aac	N	38549	15868.00
aaa	K	25	14.26
aag	K	0	10.74
agt	S	0	2.82
agc	S	15	2.98
gat	D	0	3.16
gac	D	6	2.84
---	--	------	------
mPD		0.018	0.49
		nPD:	0.04
		N. weight:	0.88
		Sc. PD:	-0.016
		Sc. rank:	12507.0

,

NA

Pos.	74	obs:	exp:
ttt	F	0	0.43
ttc	F	1	0.57
tta	L	0	0.22
ttg	L	0	0.38
tct	S	0	0.18
tcc	S	0	0.12
tca	S	0	0.26
tcg	S	0	0.05
ctt	L	0	0.39
ctc	L	2	0.28
cta	L	0	0.39
ctg	L	0	0.34
att	I	113	14150.00
atc	I	38696	9703.00
ata	I	62	15018.00
act	T	0	1.01
acc	T	4	0.82
aca	T	0	1.83
acg	T	0	0.34
aat	N	0	0.59
aac	N	1	0.41
agt	S	0	0.19
agc	S	1	0.20
gtt	V	0	5.97
gtc	V	27	4.91
gta	V	0	7.26
gtg	V	0	8.86
---	--	------	------
mPD		0.011	0.66
		nPD:	0.02
		N. weight:	1.4
		Sc. PD:	-0.055
		Sc. rank:	-3612.8

,

NA

Pos.	75	obs:	exp:
tct	S	0	7118.00
tcc	S	0	4480.00
tca	S	0	10066.00
tcg	S	0	2058.00
tgt	C	0	1.35
tgc	C	3	1.65
cgt	R	0	0.25
cgc	R	0	0.47
cga	R	0	0.88
cgg	R	0	0.66
att	I	0	2.18
atc	I	6	1.50
ata	I	0	2.32
act	T	0	0.25
acc	T	1	0.21
aca	T	0	0.46
acg	T	0	0.09
aat	N	2	122.40
aac	N	205	84.57
agt	S	312	7273.00
agc	S	38366	7683.00
aga	R	9	4.52
agg	R	0	2.22
ggt	G	0	0.45
ggc	G	3	0.44
gga	G	0	1.33
ggg	G	0	0.77
---	--	------	------
mPD		0.028	1.6
		nPD:	0.02
		N. weight:	1.6
		Sc. PD:	-0.063
		Sc. rank:	-3451.7

,

NA

Pos.	76	obs:	exp:
tct	S	0	5.89
tcc	S	0	3.71
tca	S	0	8.33
tcg	S	0	1.70
tat	Y	0	4.16
tac	Y	8	3.84
cat	H	0	1.76
cac	H	3	1.24
att	I	0	2.91
atc	I	8	2.00
ata	I	0	3.09
act	T	0	6.30
acc	T	25	5.13
aca	T	0	11.44
acg	T	0	2.13
aat	N	94	22956.00
aac	N	38719	15857.00
aaa	K	3	2.28
aag	K	1	1.72
agt	S	0	6.02
agc	S	32	6.36
gat	D	0	7.38
gac	D	14	6.62
---	--	------	------
mPD		0.0096	0.49
		nPD:	0.02
		N. weight:	0.91
		Sc. PD:	-0.033
		Sc. rank:	1681.3

,

NA

Pos.	77	obs:	exp:
ttt	F	0	0.43
ttc	F	1	0.57
tta	L	0	0.22
ttg	L	0	0.38
tct	S	0	1.10
tcc	S	0	0.69
tca	S	0	1.56
tcg	S	0	0.32
ctt	L	0	0.39
ctc	L	2	0.28
cta	L	0	0.39
ctg	L	0	0.34
cct	P	0	1.10
ccc	P	4	0.74
cca	P	0	1.70
ccg	P	0	0.45
att	I	397	918.40
atc	I	2124	629.80
ata	I	2	974.80
act	T	1754	8935.00
acc	T	33674	7270.00
aca	T	15	16214.00
acg	T	0	3024.00
aat	N	2	537.60
aac	N	907	371.40
agt	S	0	1.13
agc	S	6	1.19
gtt	V	1	2.21
gtc	V	9	1.82
gta	V	0	2.69
gtg	V	0	3.28
gct	A	0	2.38
gcc	A	9	1.86
gca	A	0	4.23
gcg	A	0	0.53
---	--	------	------
mPD		0.27	0.85
		nPD:	0.32
		N. weight:	1.4
		Sc. PD:	0.35
		Sc. rank:	39754.0

,

NA			
Pos.	78	obs:	exp:
tct	S	0	0.37
tcc	S	0	0.23
tca	S	0	0.52
tcg	S	0	0.11
tat	Y	0	1.56
tac	Y	3	1.44
cat	H	0	3.53
cac	H	6	2.47
act	T	0	0.25
acc	T	1	0.21
aca	T	0	0.46
acg	T	0	0.09
aat	N	567	22977.00
aac	N	38282	15872.00
aaa	K	16	9.13
aag	K	0	6.87
agt	S	0	0.38
agc	S	2	0.40
gat	D	0	15.81
gac	D	30	14.19
---	--	------	------
mPD		0.032	0.49
		nPD:	0.07
		N. weight:	0.84
		Sc. PD:	0.0066
		Sc. rank:	17720.0

,

NA			
Pos.	79	obs:	exp:
ttt	F	26362	11318.00
ttc	F	13	15057.00
tta	L	15	175.30
ttg	L	1532	300.30
tct	S	10825	1996.00
tcc	S	3	1256.00
tca	S	12	2822.00
tcg	S	4	577.10
tat	Y	55	28.58
tac	Y	0	26.42
tgt	C	1	0.45
tgc	C	0	0.55
tgg	W	1	1.00
ctt	L	47	314.30
ctc	L	1	225.30
cta	L	0	307.10
ctg	L	0	272.70
cct	P	9	2.48
ccc	P	0	1.67
cca	P	0	3.83
ccg	P	0	1.02
att	I	5	1.82
atc	I	0	1.25
ata	I	0	1.93
act	T	1	0.25
acc	T	0	0.21
aca	T	0	0.46
acg	T	0	0.09
agt	S	0	2039.00
agc	S	0	2154.00
gtt	V	19	4.42
gtc	V	0	3.63
gta	V	0	5.38
gtg	V	1	6.57
gct	A	1	0.26
gcc	A	0	0.21
gca	A	0	0.47
gcg	A	0	0.06
---	--	------	------
mPD		0.49	1.3
		nPD:	0.38
		N. weight:	1.1
		Sc. PD:	0.35
		Sc. rank:	33925.0

,

NA			
Pos.	80	obs:	exp:
tct	S	2	0.37
tcc	S	0	0.23
tca	S	0	0.52
tcg	S	0	0.11
att	I	5	1.82
atc	I	0	1.25
ata	I	0	1.93
act	T	16	4.03
acc	T	0	3.28
aca	T	0	7.32
acg	T	0	1.37
agt	S	0	0.38
agc	S	0	0.40
gtt	V	195	43.11
gtc	V	0	35.43
gta	V	0	52.44
gtg	V	0	64.01
gct	A	38617	10238.00
gcc	A	18	8005.00
gca	A	35	18162.00
gcg	A	0	2265.00
gat	D	18	9.48
gac	D	0	8.52
ggt	G	1	0.15
ggc	G	0	0.15
gga	G	0	0.44
ggg	G	0	0.26
---	--	------	------
mPD		0.015	0.68
		nPD:	0.02
		N. weight:	1.4
		Sc. PD:	-0.046
		Sc. rank:	5101.2

,

NA			
Pos.	81	obs:	exp:
tct	S	27	4.97
tcc	S	0	3.13
tca	S	0	7.03
tcg	S	0	1.44
cct	P	1	0.28
ccc	P	0	0.19
cca	P	0	0.43
ccg	P	0	0.11
act	T	73	18.65
acc	T	0	15.18
aca	T	1	33.85
acg	T	0	6.31
agt	S	0	5.08
agc	S	0	5.36
gtt	V	89	19.68
gtc	V	0	16.17
gta	V	0	23.94
gtg	V	0	29.22
gct	A	38545	10235.00
gcc	A	98	8002.00
gca	A	12	18156.00
gcg	A	3	2265.00
gat	D	55	28.98
gac	D	0	26.02
ggt	G	3	0.45
ggc	G	0	0.44
gga	G	0	1.33
ggg	G	0	0.77
---	--	------	------
mPD		0.019	0.68
		nPD:	0.03
		N. weight:	1.3
		Sc. PD:	-0.038
		Sc. rank:	11420.0

,

NA			
Pos.	82	obs:	exp:
tct	S	0	0.37
tcc	S	0	0.23
tca	S	0	0.52
tcg	S	0	0.11
cgt	R	0	575.10
cgc	R	0	1102.00
cga	R	1	2063.00
cgg	R	0	1541.00
att	I	0	14.93
atc	I	0	10.23
ata	I	41	15.84
act	T	0	1.51
acc	T	0	1.23
aca	T	6	2.74
acg	T	0	0.51
aaa	K	276	158.00
aag	K	1	119.00
agt	S	2	0.38
agc	S	0	0.40
aga	R	21022	10574.00
agg	R	40	5207.00
gtt	V	0	5.08
gtc	V	0	4.18
gta	V	23	6.19
gtg	V	0	7.55
gaa	E	90	55.10
gag	E	1	35.90
ggt	G	1	2639.00
ggc	G	1	2578.00
gga	G	17073	7742.00
ggg	G	329	4446.00
---	--	------	------
mPD		0.54	1.2
		nPD:	0.43
		N. weight:	0.72
		Sc. PD:	0.26
		Sc. rank:	22231.0

,

NA			
Pos.	83	obs:	exp:
tta	L	0	0.99
ttg	L	1	1.69
ctt	L	0	1.77
ctc	L	0	1.27
cta	L	0	1.73
ctg	L	8	1.54
cct	P	0	3.03
ccc	P	0	2.04
cca	P	0	4.68
ccg	P	11	1.25
cat	H	55	32.34
cac	H	0	22.66
caa	Q	162	20480.00
cag	Q	38651	18333.00
cgt	R	0	0.30
cgc	R	0	0.58
cga	R	0	1.08
cgg	R	11	0.81
aaa	K	0	2.85
aag	K	5	2.15
aga	R	0	5.52
agg	R	0	2.72
gaa	E	0	1.82
gag	E	3	1.18
---	--	------	------
mPD		0.013	0.50
		nPD:	0.03
		N. weight:	0.75
		Sc. PD:	-0.022
		Sc. rank:	5512.6

,

NA			
Pos.	84	obs:	exp:
tta	L	54	5.94
ttg	L	0	10.17
tct	S	4	7051.00
tcc	S	6	4438.00
tca	S	37700	9972.00
tcg	S	605	2039.00
ctt	L	0	10.64
ctc	L	0	7.63
cta	L	0	10.40
ctg	L	0	9.23
cct	P	0	130.30
ccc	P	0	87.73
cca	P	473	201.40
ccg	P	0	53.56
act	T	0	15.88
acc	T	0	12.92
aca	T	63	28.82
acg	T	0	5.38
agt	S	0	7205.00
agc	S	0	7611.00
gct	A	0	0.53
gcc	A	0	0.41
gca	A	1	0.94
gcg	A	1	0.12
---	--	------	------
mPD		0.061	1.7
		nPD:	0.04
		N. weight:	1.3
		Sc. PD:	-0.026
		Sc. rank:	18521.0

,

NA			
Pos.	85	obs:	exp:
tta	L	1	1.65
ttg	L	13	2.82
tct	S	0	0.18
tcc	S	0	0.12
tca	S	1	0.26
tcg	S	0	0.05
ctt	L	0	2.96
ctc	L	0	2.12
cta	L	0	2.89
ctg	L	1	2.56
cgt	R	0	0.03
cgc	R	0	0.05
cga	R	0	0.10
cgg	R	0	0.07
att	I	0	0.73
atc	I	0	0.50
ata	I	2	0.77
atg	M	934	934.00
act	T	0	0.25
acc	T	0	0.21
aca	T	0	0.46
acg	T	1	0.09
agt	S	0	0.19
agc	S	0	0.20
aga	R	0	0.50
agg	R	1	0.25
gtt	V	30	8362.00
gtc	V	0	6873.00
gta	V	205	10172.00
gtg	V	37587	12416.00
gct	A	1	10.33
gcc	A	0	8.07
gca	A	0	18.32
gcg	A	38	2.28
gaa	E	0	49.65
gag	E	82	32.35
ggt	G	0	1.52
ggc	G	0	1.48
gga	G	0	4.45
ggg	G	10	2.55
---	--	------	------
mPD		0.067	0.79
		nPD:	0.08
		N. weight:	1.1
		Sc. PD:	0.029
		Sc. rank:	24531.0

,

NA			
Pos.	86	obs:	exp:
tct	S	12	2.21
tcc	S	0	1.39
tca	S	0	3.12
tcg	S	0	0.64
cct	P	1	0.28
ccc	P	0	0.19
cca	P	0	0.43
ccg	P	0	0.11
att	I	117	42.59
atc	I	0	29.21
ata	I	0	45.20
act	T	153	38.57
acc	T	0	31.38
aca	T	0	69.99
acg	T	0	13.05
agt	S	0	2.26
agc	S	0	2.38
gtt	V	17695	3939.00
gtc	V	114	3238.00
gta	V	5	4792.00
gtg	V	3	5849.00
gct	A	20333	5497.00
gcc	A	428	4298.00
gca	A	0	9751.00
gcg	A	1	1216.00
gat	D	30	15.81
gac	D	0	14.19
ggt	G	15	2.27
ggc	G	0	2.22
gga	G	0	6.67
ggg	G	0	3.83
---	--	------	------
mPD		0.54	1.2
		nPD:	0.44
		N. weight:	1.4
		Sc. PD:	0.51
		Sc. rank:	43190.0

,

NA

Pos.	87	obs:	exp:
ttt	F	0	6.01
ttc	F	14	7.99
tct	S	616	6946.00
tcc	S	37020	4372.00
tca	S	107	9823.00
tcg	S	0	2009.00
tat	Y	0	4.16
tac	Y	8	3.84
cct	P	10	314.30
ccc	P	1129	211.60
cca	P	2	485.80
ccg	P	0	129.20
act	T	0	0.25
acc	T	1	0.21
aca	T	0	0.46
acg	T	0	0.09
agt	S	0	7097.00
agc	S	0	7497.00
mPD		0.095	1.7
		nPD:	0.06
		N. weight:	2.1
		Sc. PD:	0.00029
		Sc. rank:	41630.0

NA

Pos.	88	obs:	exp:
tta	L	0	10.11
ttg	L	91	17.32
ctt	L	0	18.13
ctc	L	0	12.99
cta	L	0	17.71
ctg	L	1	15.73
att	I	0	0.36
atc	I	0	0.25
ata	I	1	0.39
atg	M	358	358.00
gtt	V	14	8477.00
gtc	V	0	6968.00
gta	V	277	10312.00
gtg	V	38053	12587.00
gct	A	0	27.53
gcc	A	0	21.53
gca	A	2	48.85
gcg	A	102	6.09
gaa	E	0	1.21
gag	E	2	0.79
ggt	G	0	0.91
ggc	G	0	0.89
gga	G	0	2.67
ggg	G	6	1.53
mPD		0.044	0.77
		nPD:	0.06
		N. weight:	1.1
		Sc. PD:	0.000089
		Sc. rank:	21538.0

NA

Pos.	89	obs:	exp:
caa	Q	1	0.53
cag	Q	0	0.47
cgt	R	0	0.76
cgc	R	0	1.46
cga	R	0	2.74
cgg	R	0	2.05
att	I	0	0.73
atc	I	0	0.50
ata	I	2	0.77
act	T	0	1.26
acc	T	0	1.03
aca	T	5	2.29
acg	T	0	0.43
aat	N	13	12.42
aac	N	8	8.58
aaa	K	38748	22149.00
aag	K	81	16680.00
aga	R	28	14.06
agg	R	0	6.92
gaa	E	21	12.72
gag	E	0	8.28
mPD		0.0082	0.49
		nPD:	0.02
		N. weight:	0.56
		Sc. PD:	-0.022
		Sc. rank:	-1415.3

NA

Pos.	90	obs:	exp:
ttt	F	33	14.59
ttc	F	1	19.41
tta	L	37494	4200.00
ttg	L	445	7194.00
tct	S	0	0.92
tcc	S	0	0.58
tca	S	5	1.30
tcg	S	0	0.27
ctt	L	0	7529.00
ctc	L	0	5397.00
cta	L	270	7356.00
ctg	L	0	6533.00
cct	P	0	0.28
ccc	P	0	0.19
cca	P	1	0.43
ccg	P	0	0.11
att	I	0	225.70
atc	I	0	154.80
ata	I	620	239.50
agt	S	0	0.94
agc	S	0	0.99
gtt	V	0	8.40
gtc	V	0	6.90
gta	V	38	10.22
gtg	V	0	12.47
mPD		0.072	1.2
		nPD:	0.06
		N. weight:	2.1
		Sc. PD:	0.0095
		Sc. rank:	44722.0

NA

Pos.	91	obs:	exp:
tct	S	0	2.94
tcc	S	0	1.85
tca	S	0	4.16
tcg	S	16	0.85
atg	M	2	2.00
act	T	0	31.01
acc	T	0	25.23
aca	T	1	56.27
acg	T	122	10.49
agt	S	0	3.01
agc	S	0	3.18
gtt	V	0	41.12
gtc	V	0	33.80
gta	V	0	50.02
gtg	V	186	61.06
gct	A	103	10213.00
gcc	A	0	7985.00
gca	A	791	18118.00
gcg	A	37682	2260.00
gaa	E	0	2.42
gag	E	4	1.58
mPD		0.062	0.68
		nPD:	0.09
		N. weight:	2.7
		Sc. PD:	0.091
		Sc. rank:	65101.0

NA

Pos.	92	obs:	exp:
tct	S	0	2.94
tcc	S	0	1.85
tca	S	0	4.16
tcg	S	0	0.85
agt	S	0	3.01
agc	S	16	3.18
gtt	V	0	0.22
gtc	V	1	0.18
gta	V	0	0.27
gtg	V	0	0.33
gct	A	0	2.38
gcc	A	9	1.86
gca	A	0	4.23
gcg	A	0	0.53
ggt	G	257	5896.00
ggc	G	38558	5759.00
gga	G	66	17295.00
ggg	G	0	9932.00
mPD		0.018	0.69
		nPD:	0.03
		N. weight:	1.9
		Sc. PD:	-0.058
		Sc. rank:	13373.0

NA

Pos.	93	obs:	exp:
tct	S	0	10.49
tcc	S	0	6.60
tca	S	0	14.83
tcg	S	0	3.03
tat	Y	1	0.52
tac	Y	0	0.48
att	I	3	1.09
atc	I	0	0.75
ata	I	0	1.16
act	T	2	0.50
acc	T	0	0.41
aca	T	0	0.91
acg	T	0	0.17
aat	N	38622	22969.00
aac	N	214	15867.00
aaa	K	3	3.99
aag	K	4	3.01
agt	S	57	10.72
agc	S	0	11.32
gat	D	1	0.53
gac	D	0	0.47
mPD		0.015	0.49
		nPD:	0.03
		N. weight:	0.51
		Sc. PD:	-0.013
		Sc. rank:	5655.7

NA

Pos.	94	obs:	exp:
ttt	F	0	12.87
ttc	F	30	17.13
tta	L	0	0.11
ttg	L	0	0.19
tct	S	235	7153.00
tcc	S	38580	4502.00
tca	S	55	10116.00
tcg	S	1	2069.00
tat	Y	0	1.04
tac	Y	2	0.96
ctt	L	0	0.20
ctc	L	1	0.14
cta	L	0	0.19
ctg	L	0	0.17
cct	P	0	0.28
ccc	P	1	0.19
cca	P	0	0.43
ccg	P	0	0.11
act	T	0	0.25
acc	T	1	0.21
aca	T	0	0.46
acg	T	0	0.09
agt	S	0	7309.00
agc	S	0	7721.00
gct	A	0	0.26
gcc	A	1	0.21
gca	A	0	0.47
gcg	A	0	0.06
mPD		0.017	1.6
		nPD:	0.01
		N. weight:	2.2
		Sc. PD:	-0.098
		Sc. rank:	-25263.0

NA

Pos.	95	obs:	exp:
ttt	F	4	1.72
ttc	F	0	2.28
tct	S	37627	7149.00
tcc	S	1214	4500.00
tca	S	5	10110.00
tcg	S	1	2067.00
tat	Y	1	0.52
tac	Y	0	0.48
cct	P	33	9.37
ccc	P	1	6.31
cca	P	0	14.48
ccg	P	0	3.85
act	T	6	1.51
acc	T	0	1.23
aca	T	0	2.74
acg	T	0	0.51
agt	S	0	7305.00
agc	S	0	7716.00
gct	A	0	3.97
gcc	A	15	3.10
gca	A	0	7.04
gcg	A	0	0.88
mPD		0.065	1.6
		nPD:	0.04
		N. weight:	1.6
		Sc. PD:	-0.028
		Sc. rank:	24919.0

NA

Pos.	96	obs:	exp:
tta	L	0	4277.00
ttg	L	0	7324.00
ctt	L	689	7666.00
ctc	L	38158	5495.00
cta	L	53	7489.00
ctg	L	2	6651.00
att	I	0	1.82
atc	I	4	1.25
ata	I	1	1.93
mPD		0.038	1.1
		nPD:	0.03
		N. weight:	1.9
		Sc. PD:	-0.044
		Sc. rank:	23932.0

NA

Pos.	97	obs:	exp:
tgt	C	402	17561.00
tgc	C	38505	21346.00
---	--	------	------
mPD		0.020	0.50
		nPD:	0.04
		N. weight:	0.56
		Sc. PD:	-0.0085
		Sc. rank:	9050.8

,

NA

Pos.	98	obs:	exp:
tta	L	0	2.75
ttg	L	0	4.71
tct	S	232	42.69
tcc	S	0	26.87
tca	S	0	60.38
tcg	S	0	12.35
tat	Y	2	1.04
tac	Y	0	0.96
ctt	L	24	4.93
ctc	L	0	3.53
cta	L	0	4.81
ctg	L	1	4.27
cct	P	37121	10248.00
ccc	P	48	6899.00
cca	P	25	15839.00
ccg	P	4	4212.00
cat	H	1446	850.40
cac	H	0	595.60
act	T	4	1.01
acc	T	0	0.82
aca	T	0	1.83
acg	T	0	0.34
agt	S	0	43.62
agc	S	0	46.08
---	--	------	------
mPD		0.089	0.79
		nPD:	0.11
		N. weight:	1.3
		Sc. PD:	0.069
		Sc. rank:	33066.0

,

NA

Pos.	99	obs:	exp:
att	I	39	14.93
atc	I	2	10.23
ata	I	0	15.84
gtt	V	35442	8580.00
gtc	V	3358	7052.00
gta	V	7	10437.00
gtg	V	2	12740.00
gct	A	56	15.09
gcc	A	1	11.80
gca	A	0	26.77
gcg	A	0	3.34
---	--	------	------
mPD		0.16	0.74
		nPD:	0.22
		N. weight:	1.3
		Sc. PD:	0.2
		Sc. rank:	34740.0

,

NA

Pos.	100	obs:	exp:
tct	S	0	7092.00
tcc	S	0	4464.00
tca	S	0	10029.00
tcg	S	0	2051.00
cgt	R	2	0.14
cgc	R	0	0.26
cga	R	0	0.49
cgg	R	0	0.37
att	I	11	4.00
atc	I	0	2.75
ata	I	0	4.25
act	T	1	0.25
acc	T	0	0.21
aca	T	0	0.46
acg	T	0	0.09
aat	N	288	170.30
aac	N	0	117.70
agt	S	38496	7246.00
agc	S	41	7655.00
aga	R	1	2.51
agg	R	2	1.24
ggt	G	65	9.86
ggc	G	0	9.63
gga	G	0	28.91
ggg	G	0	16.60
---	--	------	------
mPD		0.021	1.7
		nPD:	0.01
		N. weight:	1.7
		Sc. PD:	-0.073
		Sc. rank:	-11606.0

,

NA

Pos.	101	obs:	exp:
tct	S	0	6.62
tcc	S	0	4.17
tca	S	0	9.37
tcg	S	0	1.92
cgt	R	0	0.03
cgc	R	0	0.05
cga	R	1	0.10
cgg	R	0	0.07
aat	N	0	0.59
aac	N	1	0.41
agt	S	0	6.77
agc	S	36	7.15
aga	R	0	0.50
agg	R	0	0.25
ggt	G	7	5894.00
ggc	G	294	5757.00
gga	G	38294	17290.00
ggg	G	274	9929.00
---	--	------	------
mPD		0.033	0.70
		nPD:	0.05
		N. weight:	0.76
		Sc. PD:	-0.0069
		Sc. rank:	13295.0

,

NA

Pos.	102	obs:	exp:
tgg	W	38907	38907.00
---	--	------	------
mPD		0	0
		nPD:	1.
		N. weight:	0.
		Sc. PD:	0
		Sc. rank:	0

,

NA

Pos.	103	obs:	exp:
tct	S	1	0.18
tcc	S	0	0.12
tca	S	0	0.26
tcg	S	0	0.05
cct	P	1	0.28
ccc	P	0	0.19
cca	P	0	0.43
ccg	P	0	0.11
act	T	10	2.52
acc	T	0	2.05
aca	T	0	4.57
acg	T	0	0.85
agt	S	0	0.19
agc	S	0	0.20
gtt	V	19	4.20
gtc	V	0	3.45
gta	V	0	5.11
gtg	V	0	6.24
gct	A	38768	10293.00
gcc	A	84	8047.00
gca	A	10	18259.00
gcg	A	14	2278.00
---	--	------	------
mPD		0.0071	0.66
		nPD:	0.01
		N. weight:	1.4
		Sc. PD:	-0.06
		Sc. rank:	-14337.0

,

NA

Pos.	104	obs:	exp:
tta	L	1	0.11
ttg	L	0	0.19
ctt	L	0	0.20
ctc	L	0	0.14
cta	L	0	0.19
ctg	L	0	0.17
att	I	9	14035.00
atc	I	12	9624.00
ata	I	38535	14896.00
atg	M	18	18.00
act	T	0	0.25
acc	T	0	0.21
aca	T	1	0.46
acg	T	0	0.09
gtt	V	0	73.18
gtc	V	0	60.15
gta	V	328	89.02
gtg	V	3	108.70
---	--	------	------
mPD		0.019	0.68
		nPD:	0.03
		N. weight:	0.98
		Sc. PD:	-0.027
		Sc. rank:	10214.0

,

NA

Pos.	105	obs:	exp:
tat	Y	50	20195.00
tac	Y	38817	18672.00
cat	H	0	21.76
cac	H	37	15.24
aat	N	0	1.77
aac	N	3	1.23
---	--	------	------
mPD		0.0046	0.50
		nPD:	0.01
		N. weight:	0.75
		Sc. PD:	-0.034
		Sc. rank:	-10515.0

,

NA

Pos.	106	obs:	exp:
tct	S	0	7158.00
tcc	S	0	4506.00
tca	S	0	10124.00
tcg	S	0	2070.00
tgt	C	1	0.45
tgc	C	0	0.55
cgt	R	0	0.03
cgc	R	0	0.05
cga	R	0	0.10
cgg	R	0	0.07
act	T	5	1.26
acc	T	0	1.03
aca	T	0	2.29
acg	T	0	0.43
agt	S	38622	7314.00
agc	S	277	7727.00
aga	R	1	0.50
agg	R	0	0.25
ggt	G	1	0.15
ggc	G	0	0.15
gga	G	0	0.44
ggg	G	0	0.26
---	--	------	------
mPD		0.015	1.6
		nPD:	0.01
		N. weight:	1.7
		Sc. PD:	-0.078
		Sc. rank:	-24725.0

,

NA

Pos.	107	obs:	exp:
cgt	R	0	0.08
cgc	R	0	0.16
cga	R	0	0.29
cgg	R	0	0.22
aaa	K	38809	22192.00
aag	K	95	16712.00
aga	R	3	1.51
agg	R	0	0.74
---	--	------	------
mPD		0.0050	0.49
		nPD:	0.01
		N. weight:	0.56
		Sc. PD:	-0.025
		Sc. rank:	-6505.2

,

NA

Pos.	108	obs:	exp:
aat	N	0	0.59
aac	N	1	0.41
gat	D	47	20500.00
gac	D	38859	18406.00
---	--	------	------
mPD		0.0025	0.50
		nPD:	0.
		N. weight:	0.76
		Sc. PD:	-0.038
		Sc. rank:	-17843.0

,

NA

Pos.	109	obs:	exp:
aat	N	167	23011.00
aac	N	38739	15895.00
aaa	K	0	0.57
aag	K	1	0.43
---	--	------	------
mPD		0.0086	0.48
		nPD:	0.02
		N. weight:	0.89
		Sc. PD:	-0.034
		Sc. rank:	-867.6

,

NA

Pos.	110	obs:	exp:
tct	S	0	7117.00
tcc	S	0	4480.00
tca	S	0	10066.00
tcg	S	0	2058.00
att	I	2	0.73
atc	I	0	0.50
ata	I	0	0.77
aat	N	179	105.90
aac	N	0	73.13
agt	S	38637	7272.00
agc	S	39	7682.00
ggt	G	50	7.58
ggc	G	0	7.41
gga	G	0	22.24
ggg	G	0	12.77
---	--	------	------
mPD		0.014	1.6
		nPD:	0.01
		N. weight:	1.7
		Sc. PD:	-0.08
		Sc. rank:	-26965.0

,

NA

Pos.	111	obs:	exp:
tta	L	9	0.99
ttg	L	0	1.69
ctt	L	0	1.77
ctc	L	0	1.27
cta	L	0	1.73
ctg	L	0	1.54
att	I	0	2409.00
atc	I	0	1652.00
ata	I	6617	2556.00
atg	M	1	1.00
gtt	V	2	7136.00
gtc	V	13	5865.00
gta	V	30788	8680.00
gtg	V	1473	10595.00
gct	A	0	1.06
gcc	A	0	0.83
gca	A	4	1.88
gcg	A	0	0.23
---	--	------	------
mPD		0.36	1.0
		nPD:	0.35
		N. weight:	1.1
		Sc. PD:	0.31
		Sc. rank:	32875.0

,

NA

Pos.	112	obs:	exp:
cgt	R	0	1062.00
cgc	R	0	2035.00
cga	R	32	3811.00
cgg	R	0	2847.00
aaa	K	1	0.57
aag	K	0	0.43
aga	R	38793	19532.00
agg	R	80	9618.00
ggt	G	0	0.15
ggc	G	0	0.15
gga	G	1	0.44
ggg	G	0	0.26
---	--	------	------
mPD		0.0059	0.91
		nPD:	0.01
		N. weight:	0.69
		Sc. PD:	-0.034
		Sc. rank:	-14817.0

,

NA

Pos.	113	obs:	exp:
tta	L	0	1.10
ttg	L	0	1.88
ctt	L	0	1.97
ctc	L	10	1.41
cta	L	0	1.93
ctg	L	0	1.71
att	I	646	14127.00
atc	I	38137	9687.00
ata	I	24	14993.00
atg	M	44	44.00
act	T	0	0.50
acc	T	2	0.41
aca	T	0	0.91
acg	T	0	0.17
gtt	V	0	9.73
gtc	V	44	8.00
gta	V	0	11.83
gtg	V	0	14.44
---	--	------	------
mPD		0.039	0.66
		nPD:	0.06
		N. weight:	1.3
		Sc. PD:	0.0026
		Sc. rank:	26901.0

,

NA

Pos.	114	obs:	exp:
tct	S	0	0.74
tcc	S	0	0.46
tca	S	0	1.04
tcg	S	0	0.21
agt	S	4	0.75
agc	S	0	0.79
ggt	G	38455	5899.00
ggc	G	403	5762.00
gga	G	12	17305.00
ggg	G	33	9937.00
---	--	------	------
mPD		0.023	0.69
		nPD:	0.03
		N. weight:	1.9
		Sc. PD:	-0.043
		Sc. rank:	23025.0

,

NA

Pos.	115	obs:	exp:
ttt	F	0	1.72
ttc	F	4	2.28
tct	S	118	7158.00
tcc	S	38767	4505.00
tca	S	13	10123.00
tcg	S	0	2070.00
tgt	C	0	0.45
tgc	C	1	0.55
act	T	0	0.76
acc	T	3	0.62
aca	T	0	1.37
acg	T	0	0.26
agt	S	0	7314.00
agc	S	0	7727.00
gct	A	0	0.26
gcc	A	1	0.21
gca	A	0	0.47
gcg	A	0	0.06
---	--	------	------
mPD		0.0072	1.6
		nPD:	0.
		N. weight:	2.2
		Sc. PD:	-0.11
		Sc. rank:	-54069.0

,

NA

Pos.	116	obs:	exp:
cgt	R	0	0.93
cgc	R	0	1.78
cga	R	0	3.33
cgg	R	0	2.49
aat	N	4	2.37
aac	N	0	1.63
aaa	K	226	22171.00
aag	K	38642	16697.00
aga	R	0	17.07
agg	R	34	8.41
gaa	E	0	0.61
gag	E	1	0.39
---	--	------	------
mPD		0.014	0.49
		nPD:	0.03
		N. weight:	0.83
		Sc. PD:	-0.024
		Sc. rank:	7149.5

,

NA

Pos.	117	obs:	exp:
ggt	G	1	5899.00
ggc	G	0	5763.00
gga	G	335	17307.00
ggg	G	38571	9938.00
---	--	------	------
mPD		0.017	0.69
		nPD:	0.02
		N. weight:	1.4
		Sc. PD:	-0.042
		Sc. rank:	8495.5

,

NA

Pos.	118	obs:	exp:
cat	H	1	0.59
cac	H	0	0.41
aat	N	13	7.69
aac	N	0	5.31
gtt	V	1	0.22
gtc	V	0	0.18
gta	V	0	0.27
gtg	V	0	0.33
gat	D	38769	20486.00
gac	D	112	18395.00
gaa	E	7	6.66
gag	E	4	4.34
---	--	------	------
mPD		0.0071	0.50
		nPD:	0.01
		N. weight:	0.64
		Sc. PD:	-0.027
		Sc. rank:	-3399.3

,

NA

Pos.	119	obs:	exp:
att	I	0	1.46
atc	I	0	1.00
ata	I	4	1.55
gtt	V	47	8599.00
gtc	V	2	7067.00
gta	V	129	10460.00
gtg	V	38715	12767.00
gct	A	0	2.65
gcc	A	0	2.07
gca	A	0	4.70
gcg	A	10	0.59
---	--	------	------
mPD		0.010	0.74
		nPD:	0.01
		N. weight:	1.1
		Sc. PD:	-0.047
		Sc. rank:	-6561.6

,

NA

Pos.	120	obs:	exp:
ttt	F	38851	16682.00
ttc	F	22	22191.00
tta	L	2	2.31
ttg	L	3	3.95
tat	Y	13	6.75
tac	Y	0	6.25
ctt	L	16	4.14
ctc	L	0	2.97
cta	L	0	4.04
ctg	L	0	3.59
---	--	------	------
mPD		0.0029	0.49
		nPD:	0.01
		N. weight:	0.87
		Sc. PD:	-0.043
		Sc. rank:	-19274.0

,

NA

Pos.	121	obs:	exp:
tta	L	0	0.11
ttg	L	0	0.19
ctt	L	0	0.20
ctc	L	1	0.14
cta	L	0	0.19
ctg	L	0	0.17
att	I	1	24.75
atc	I	67	16.97
ata	I	0	26.27
gtt	V	111	8586.00
gtc	V	38689	7057.00
gta	V	35	10444.00
gtg	V	0	12748.00
gct	A	0	0.79
gcc	A	3	0.62
gca	A	0	1.41
gcg	A	0	0.18
---	--	------	------
mPD		0.011	0.74
		nPD:	0.02
		N. weight:	1.7
		Sc. PD:	-0.07
		Sc. rank:	-6723.2

,

NA

Pos.	122	obs:	exp:
tta	L	1	0.11
ttg	L	0	0.19
ctt	L	0	0.20
ctc	L	0	0.14
cta	L	0	0.19
ctg	L	0	0.17
cgt	R	0	0.08
cgc	R	0	0.16
cga	R	0	0.29
cgg	R	0	0.22
att	I	10	13708.00
atc	I	8	9400.00
ata	I	37639	14549.00
atg	M	1235	1235.00
act	T	0	1.76
acc	T	0	1.44
aca	T	7	3.20
acg	T	0	0.60
aaa	K	2	1.14
aag	K	0	0.86
aga	R	3	1.51
agg	R	0	0.74
gtt	V	0	0.44
gtc	V	0	0.36
gta	V	2	0.54
gtg	V	0	0.66
---	--	------	------
mPD		0.063	0.68
		nPD:	0.09
		N. weight:	0.95
		Sc. PD:	0.033
		Sc. rank:	22524.0

,

NA

Pos.	123	obs:	exp:
cgt	R	0	1062.00
cgc	R	0	2035.00
cga	R	0	3811.00
cgg	R	17	2847.00
aga	R	328	19533.00
agg	R	38562	9619.00
---	--	------	------
mPD		0.018	0.91
		nPD:	0.02
		N. weight:	1.4
		Sc. PD:	-0.05
		Sc. rank:	1100.6

,

NA

Pos.	124	obs:	exp:
aaa	K	37	21.11
aag	K	0	15.89
gct	A	0	0.26
gcc	A	0	0.21
gca	A	1	0.47
gcg	A	0	0.06
gaa	E	37964	23533.00
gag	E	902	15333.00
ggt	G	0	0.45
ggc	G	0	0.44
gga	G	3	1.33
ggg	G	0	0.77
---	--	------	------
mPD		0.047	0.48
		nPD:	0.1
		N. weight:	0.41
		Sc. PD:	0.017
		Sc. rank:	10098.0

,

NA

Pos.	125	obs:	exp:
tta	L	0	0.33
ttg	L	0	0.56
ctt	L	0	0.59
ctc	L	0	0.42
cta	L	3	0.58
ctg	L	0	0.51
cct	P	35	10717.00
ccc	P	2	7215.00
cca	P	38809	16564.00
ccg	P	54	4405.00
act	T	0	0.76
acc	T	0	0.62
aca	T	3	1.37
acg	T	0	0.26
gct	A	0	0.26
gcc	A	0	0.21
gca	A	1	0.47
gcg	A	0	0.06
---	--	------	------
mPD		0.0050	0.70
		nPD:	0.01
		N. weight:	0.86
		Sc. PD:	-0.042
		Sc. rank:	-16452.0

,

NA

Pos.	126	obs:	exp:
ttt	F	200	16695.00
ttc	F	38705	22210.00
tta	L	1	0.11
ttg	L	0	0.19
ctt	L	0	0.20
ctc	L	0	0.14
cta	L	0	0.19
ctg	L	0	0.17
gtt	V	0	0.22
gtc	V	1	0.18
gta	V	0	0.27
gtg	V	0	0.33
---	--	------	------
mPD		0.010	0.49
		nPD:	0.02
		N. weight:	0.55
		Sc. PD:	-0.019
		Sc. rank:	1443.2

,

NA

Pos.	127	obs:	exp:
tta	L	0	0.11
ttg	L	0	0.19
ctt	L	0	0.20
ctc	L	0	0.14
cta	L	1	0.19
ctg	L	0	0.17
att	I	10	14148.00
atc	I	7	9702.00
ata	I	38849	15016.00
atg	M	12	12.00
act	T	0	1.76
acc	T	0	1.44
aca	T	7	3.20
acg	T	0	0.60
gtt	V	0	4.64
gtc	V	0	3.82
gta	V	21	5.65
gtg	V	0	6.89
---	--	------	------
mPD		0.0030	0.66
		nPD:	0.
		N. weight:	0.98
		Sc. PD:	-0.049
		Sc. rank:	-23625.0

,

NA

Pos.	128	obs:	exp:
tct	S	9	7159.00
tcc	S	4	4506.00
tca	S	38797	10124.00
tcg	S	91	2070.00
agt	S	0	7315.00
agc	S	0	7727.00
gct	A	0	1.59
gcc	A	0	1.24
gca	A	6	2.82
gcg	A	0	0.35
---	--	------	------
mPD		0.0056	1.6
		nPD:	0.
		N. weight:	1.4
		Sc. PD:	-0.071
		Sc. rank:	-36978.0

,

NA

Pos.	129	obs:	exp:
tct	S	0	0.18
tcc	S	0	0.12
tca	S	0	0.26
tcg	S	0	0.05
tgt	C	105	17560.00
tgc	C	38800	21345.00
cgt	R	0	0.03
cgc	R	1	0.05
cga	R	0	0.10
cgg	R	0	0.07
agt	S	0	0.19
agc	S	1	0.20
aga	R	0	0.50
agg	R	0	0.25
---	--	------	------
mPD		0.0055	0.50
		nPD:	0.01
		N. weight:	0.6
		Sc. PD:	-0.027
		Sc. rank:	-6039.1

,

NA

Pos.	130	obs:	exp:
tct	S	29446	7160.00
tcc	S	9336	4506.00
tca	S	121	10125.00
tcg	S	2	2071.00
cct	P	0	0.28
ccc	P	1	0.19
cca	P	0	0.43
ccg	P	0	0.11
agt	S	0	7315.00
agc	S	0	7728.00
gct	A	1	0.26
gcc	A	0	0.21
gca	A	0	0.47
gcg	A	0	0.06
---	--	------	------
mPD		0.37	1.6
		nPD:	0.23
		N. weight:	1.3
		Sc. PD:	0.21
		Sc. rank:	35015.0

,

NA

Pos.	131	obs:	exp:
tta	L	0	5.83
ttg	L	0	9.98
tct	S	1	6.07
tcc	S	32	3.82
tca	S	0	8.59
tcg	S	0	1.76
ctt	L	0	10.44
ctc	L	53	7.49
cta	L	0	10.20
ctg	L	0	9.06
cct	P	255	10685.00
ccc	P	38429	7193.00
cca	P	100	16514.00
ccg	P	0	4392.00
cat	H	1	15.88
cac	H	26	11.12
act	T	0	2.52
acc	T	10	2.05
aca	T	0	4.57
acg	T	0	0.85
agt	S	0	6.21
agc	S	0	6.55
---	--	------	------
mPD		0.025	0.70
		nPD:	0.03
		N. weight:	1.7
		Sc. PD:	-0.036
		Sc. rank:	22168.0

,

NA

Pos.	132	obs:	exp:
ttt	F	427	183.20
ttc	F	0	243.80
tta	L	190	4224.00
ttg	L	38158	7234.00
tct	S	0	5.15
tcc	S	0	3.24
tca	S	0	7.29
tcg	S	28	1.49
tgg	W	10	10.00
ctt	L	0	7572.00
ctc	L	0	5427.00
cta	L	0	7397.00
ctg	L	76	6570.00
atg	M	9	9.00
agt	S	0	5.26
agc	S	0	5.56
gtt	V	0	1.99
gtc	V	0	1.64
gta	V	0	2.42
gtg	V	9	2.95
---	--	------	------
mPD		0.038	1.2
		nPD:	0.03
		N. weight:	1.7
		Sc. PD:	-0.039
		Sc. rank:	20069.0

,

NA			
Pos.	133	obs:	exp:
aaa	K	1	0.57
aag	K	0	0.43
gat	D	2	1.58
gac	D	1	1.42
gaa	E	38709	23527.00
gag	E	146	15328.00
ggt	G	0	7.28
ggc	G	0	7.11
gga	G	48	21.35
ggg	G	0	12.26
---	--	------	------
mPD		0.010	0.48
		nPD:	0.02
		N. weight:	0.49
		Sc. PD:	-0.017
		Sc. rank:	1337.8

,

NA			
Pos.	134	obs:	exp:
tgt	C	52	17561.00
tgc	C	38855	21346.00
---	--	------	------
mPD		0.0027	0.50
		nPD:	0.01
		N. weight:	0.61
		Sc. PD:	-0.03
		Sc. rank:	-14151.0

,

NA			
Pos.	135	obs:	exp:
tct	S	0	0.37
tcc	S	0	0.23
tca	S	0	0.52
tcg	S	0	0.11
cgt	R	0	1062.00
cgc	R	0	2035.00
cga	R	2	3810.00
cgg	R	0	2847.00
act	T	0	0.25
acc	T	0	0.21
aca	T	1	0.46
acg	T	0	0.09
aaa	K	3	1.71
aag	K	0	1.29
agt	S	2	0.38
agc	S	0	0.40
aga	R	38736	19530.00
agg	R	163	9617.00
---	--	------	------
mPD		0.0088	0.91
		nPD:	0.01
		N. weight:	0.69
		Sc. PD:	-0.031
		Sc. rank:	-9026.7

,

NA			
Pos.	136	obs:	exp:
att	I	0	2.18
atc	I	6	1.50
ata	I	0	2.32
atg	M	1	1.00
act	T	1435	9802.00
acc	T	37372	7976.00
aca	T	76	17788.00
acg	T	1	3318.00
aat	N	0	6.51
aac	N	11	4.49
gct	A	0	1.32
gcc	A	5	1.03
gca	A	0	2.35
gcg	A	0	0.29
---	--	------	------
mPD		0.076	0.68
		nPD:	0.11
		N. weight:	1.4
		Sc. PD:	0.077
		Sc. rank:	36800.0

,

NA			
Pos.	137	obs:	exp:
ttt	F	206	16696.00
ttc	F	38701	22211.00
---	--	------	------
mPD		0.011	0.49
		nPD:	0.02
		N. weight:	0.55
		Sc. PD:	-0.019
		Sc. rank:	1791.1

,

NA			
Pos.	138	obs:	exp:
ttt	F	115	16696.00
ttc	F	38792	22211.00
---	--	------	------
mPD		0.0059	0.49
		nPD:	0.01
		N. weight:	0.56
		Sc. PD:	-0.024
		Sc. rank:	-4587.5

,

NA			
Pos.	139	obs:	exp:
tta	L	72	4277.00
ttg	L	38710	7324.00
ctt	L	1	7666.00
ctc	L	0	5495.00
cta	L	0	7490.00
ctg	L	120	6651.00
atg	M	4	4.00
---	--	------	------
mPD		0.010	1.1
		nPD:	0.01
		N. weight:	1.7
		Sc. PD:	-0.078
		Sc. rank:	-24459.0

,

NA			
Pos.	140	obs:	exp:
tct	S	1	0.37
tcc	S	0	0.23
tca	S	0	0.52
tcg	S	0	0.11
act	T	38510	9806.00
acc	T	377	7979.00
aca	T	10	17796.00
acg	T	3	3319.00
aat	N	1	0.59
aac	N	0	0.41
agt	S	1	0.38
agc	S	0	0.40
gct	A	4	1.06
gcc	A	0	0.83
gca	A	0	1.88
gcg	A	0	0.23
---	--	------	------
mPD		0.020	0.68
		nPD:	0.03
		N. weight:	1.4
		Sc. PD:	-0.036
		Sc. rank:	15015.0

,

NA			
Pos.	141	obs:	exp:
cat	H	1	0.59
cac	H	0	0.41
caa	Q	38833	20522.00
cag	Q	58	18369.00
cgt	R	0	0.25
cgc	R	0	0.47
cga	R	9	0.88
cgg	R	0	0.66
aaa	K	6	3.42
aag	K	0	2.58
aga	R	0	4.52
agg	R	0	2.22
---	--	------	------
mPD		0.0038	0.50
		nPD:	0.01
		N. weight:	0.65
		Sc. PD:	-0.031
		Sc. rank:	-11183.0

,

NA			
Pos.	142	obs:	exp:
ggt	G	8	5899.00
ggc	G	1	5763.00
gga	G	261	17307.00
ggg	G	38637	9938.00
---	--	------	------
mPD		0.014	0.69
		nPD:	0.02
		N. weight:	1.4
		Sc. PD:	-0.049
		Sc. rank:	2899.4

,

NA			
Pos.	143	obs:	exp:
tct	S	1	11.96
tcc	S	64	7.53
tca	S	0	16.92
tcg	S	0	3.46
act	T	13	19.16
acc	T	63	15.59
aca	T	0	34.77
acg	T	0	6.48
agt	S	0	12.22
agc	S	0	12.91
gtt	V	0	0.66
gtc	V	3	0.55
gta	V	0	0.81
gtg	V	0	0.98
gct	A	20963	10263.00
gcc	A	17730	8024.00
gca	A	69	18206.00
gcg	A	1	2271.00
---	--	------	------
mPD		0.51	0.67
		nPD:	0.75
		N. weight:	0.77
		Sc. PD:	0.51
		Sc. rank:	24883.0

,

NA			
Pos.	144	obs:	exp:
tta	L	945	4277.00
ttg	L	37778	7325.00
ctt	L	0	7667.00
ctc	L	0	5495.00
cta	L	9	7490.00
ctg	L	174	6652.00
atg	M	1	1.00
---	--	------	------
mPD		0.057	1.1
		nPD:	0.05
		N. weight:	1.6
		Sc. PD:	-0.01
		Sc. rank:	28906.0

,

NA			
Pos.	145	obs:	exp:
tta	L	84	4272.00
ttg	L	0	7317.00
ctt	L	14	7658.00
ctc	L	7	5489.00
cta	L	38706	7482.00
ctg	L	52	6645.00
att	I	0	16.02
atc	I	0	10.98
ata	I	44	17.00
---	--	------	------
mPD		0.010	1.1
		nPD:	0.01
		N. weight:	1.7
		Sc. PD:	-0.077
		Sc. rank:	-23802.0

,

NA			
Pos.	146	obs:	exp:
tct	S	0	3.13
tcc	S	0	1.97
tca	S	0	4.42
tcg	S	0	0.90
act	T	3	0.76
acc	T	0	0.62
aca	T	0	1.37
acg	T	0	0.26
aat	N	38748	22992.00
aac	N	126	15882.00
aaa	K	0	1.14
aag	K	2	0.86
agt	S	17	3.20
agc	S	0	3.38
gat	D	11	5.80
gac	D	0	5.20
---	--	------	------
mPD		0.0082	0.49
		nPD:	0.02
		N. weight:	0.52
		Sc. PD:	-0.02
		Sc. rank:	-1228.5

,

NA			
Pos.	147	obs:	exp:
tat	Y	0	0.52
tac	Y	1	0.48
aat	N	0	2.37
aac	N	4	1.63
gat	D	86	20497.00
gac	D	38816	18405.00
---	--	------	------
mPD		0.0047	0.50
		nPD:	0.01
		N. weight:	0.76
		Sc. PD:	-0.035
		Sc. rank:	-10188.0

,

NA			
Pos.	148	obs:	exp:
caa	Q	5	2.64
cag	Q	0	2.36
cgt	R	0	2.10
cgc	R	0	4.03
cga	R	0	7.54
cgg	R	0	5.64
att	I	0	3.28
atc	I	0	2.25
ata	I	9	3.48
aaa	K	38530	22141.00
aag	K	285	16674.00
aga	R	76	38.66
agg	R	1	19.04
gaa	E	1	0.61
gag	E	0	0.39
---	--	------	------
mPD		0.019	0.50
		nPD:	0.04
		N. weight:	0.54
		Sc. PD:	-0.0094
		Sc. rank:	7988.5

,

NA			
Pos.	149	obs:	exp:
cat	H	37362	22880.00
cac	H	1543	16025.00
caa	Q	1	1.06
cag	Q	1	0.94
---	--	------	------
mPD		0.076	0.48
		nPD:	0.16
		N. weight:	0.39
		Sc. PD:	0.038
		Sc. rank:	10469.0

,

NA			
Pos.	150	obs:	exp:
ttt	F	0	0.43
ttc	F	1	0.57
tct	S	68	7160.00
tcc	S	38796	4506.00
tca	S	41	10125.00
tcg	S	0	2071.00
cct	P	0	0.28
ccc	P	1	0.19
cca	P	0	0.43
ccg	P	0	0.11
agt	S	0	7315.00
agc	S	0	7728.00
---	--	------	------
mPD		0.0057	1.6
		nPD:	0.
		N. weight:	2.2
		Sc. PD:	-0.11
		Sc. rank:	-58118.0

,

NA			
Pos.	151	obs:	exp:
aat	N	38835	23011.00
aac	N	72	15896.00
---	--	------	------
mPD		0.0037	0.48
		nPD:	0.01
		N. weight:	0.53
		Sc. PD:	-0.025
		Sc. rank:	-9003.4

,

NA			
Pos.	152	obs:	exp:
cgt	R	0	0.46
cgc	R	0	0.89
cga	R	0	1.67
cgg	R	0	1.24
aga	R	17	8.53
agg	R	0	4.20
gtt	V	0	0.22
gtc	V	0	0.18
gta	V	1	0.27
gtg	V	0	0.33
gaa	E	14	8.48
gag	E	0	5.52
ggt	G	11	5895.00
ggc	G	3	5758.00
gga	G	38589	17292.00
ggg	G	272	9930.00
---	--	------	------
mPD		0.016	0.69
		nPD:	0.02
		N. weight:	0.79
		Sc. PD:	-0.026
		Sc. rank:	4088.3

,

NA			
Pos.	153	obs:	exp:
tct	S	0	0.74
tcc	S	2	0.46
tca	S	0	1.04
tcg	S	0	0.21
att	I	0	0.73
atc	I	2	0.50
ata	I	0	0.77
act	T	30	9806.00
acc	T	38542	7979.00
aca	T	328	17796.00
acg	T	0	3319.00
aat	N	0	0.59
aac	N	1	0.41
agt	S	0	0.75
agc	S	2	0.79
---	--	------	------
mPD		0.019	0.68
		nPD:	0.03
		N. weight:	1.6
		Sc. PD:	-0.045
		Sc. rank:	13002.0

,

NA			
Pos.	154	obs:	exp:
ttt	F	4	1.72
ttc	F	0	2.28
tta	L	0	3.08
ttg	L	0	5.27
tct	S	0	0.92
tcc	S	0	0.58
tca	S	0	1.30
tcg	S	0	0.27
ctt	L	28	5.52
ctc	L	0	3.95
cta	L	0	5.39
ctg	L	0	4.79
att	I	37869	14057.00
atc	I	745	9639.00
ata	I	2	14919.00
atg	M	8	8.00
act	T	2	0.76
acc	T	1	0.62
aca	T	0	1.37
acg	T	0	0.26
aat	N	5	2.96
aac	N	0	2.04
agt	S	5	0.94
agc	S	0	0.99
gtt	V	238	52.62
gtc	V	0	43.25
gta	V	0	64.01
gtg	V	0	78.13
---	--	------	------
mPD		0.053	0.67
		nPD:	0.08
		N. weight:	0.96
		Sc. PD:	0.02
		Sc. rank:	21280.0

,

NA			
Pos.	155	obs:	exp:
cgt	R	0	0.49
cgc	R	0	0.94
cga	R	0	1.76
cgg	R	0	1.32
act	T	0	0.25
acc	T	0	0.21
aca	T	1	0.46
acg	T	0	0.09
aat	N	3	2.96
aac	N	2	2.04
aaa	K	38715	22177.00
aag	K	162	16700.00
aga	R	18	9.04
agg	R	0	4.45
gaa	E	6	3.63
gag	E	0	2.37
---	--	------	------
mPD		0.0098	0.49
		nPD:	0.02
		N. weight:	0.55
		Sc. PD:	-0.02
		Sc. rank:	1219.2

,

NA			
Pos.	156	obs:	exp:
aat	N	0	2.37
aac	N	4	1.63
gat	D	176	20475.00
gac	D	38684	18385.00
gaa	E	42	26.04
gag	E	1	16.96
---	--	------	------
mPD		0.011	0.50
		nPD:	0.02
		N. weight:	0.74
		Sc. PD:	-0.024
		Sc. rank:	3495.1

,

NA			
Pos.	157	obs:	exp:
cgt	R	0	1062.00
cgc	R	0	2035.00
cga	R	0	3811.00
cgg	R	3	2847.00
aaa	K	3	1.71
aag	K	0	1.29
aga	R	11004	19531.00
agg	R	27897	9618.00
---	--	------	------
mPD		0.41	0.91
		nPD:	0.45
		N. weight:	0.62
		Sc. PD:	0.23
		Sc. rank:	19540.0

,

NA			
Pos.	158	obs:	exp:
tct	S	0	7157.00
tcc	S	0	4505.00
tca	S	0	10122.00
tcg	S	0	2070.00
att	I	0	0.36
atc	I	1	0.25
ata	I	0	0.39
aat	N	0	5.91
aac	N	10	4.09
agt	S	136	7313.00
agc	S	38757	7726.00
ggt	G	0	0.45
ggc	G	3	0.44
gga	G	0	1.33
ggg	G	0	0.77
---	--	------	------
mPD		0.0077	1.6
		nPD:	0.
		N. weight:	1.6
		Sc. PD:	-0.083
		Sc. rank:	-39263.0

,

NA			
Pos.	159	obs:	exp:
tct	S	0	9.20
tcc	S	0	5.79
tca	S	50	13.01
tcg	S	0	2.66
cct	P	39	10705.00
ccc	P	4	7207.00
cca	P	38778	16545.00
ccg	P	35	4400.00
caa	Q	1	0.53
cag	Q	0	0.47
agt	S	0	9.40
agc	S	0	9.93
---	--	------	------
mPD		0.0066	0.70
		nPD:	0.01
		N. weight:	0.87
		Sc. PD:	-0.04
		Sc. rank:	-11582.0

,

NA			
Pos.	160	obs:	exp:
ttt	F	10	4.72
ttc	F	1	6.28
tat	Y	37902	20159.00
tac	Y	896	18639.00
cat	H	98	57.63
cac	H	0	40.37
---	--	------	------
mPD		0.051	0.50
		nPD:	0.1
		N. weight:	0.56
		Sc. PD:	0.024
		Sc. rank:	13830.0

,

NA			
Pos.	161	obs:	exp:
cgt	R	4	1062.00
cgc	R	0	2035.00
cga	R	38793	3811.00
cgg	R	64	2847.00
aga	R	46	19533.00
agg	R	0	9619.00
---	--	------	------
mPD		0.0059	0.91
		nPD:	0.01
		N. weight:	2.4
		Sc. PD:	-0.12
		Sc. rank:	-50047.0

,

NA			
Pos.	162	obs:	exp:
tct	S	0	0.37
tcc	S	0	0.23
tca	S	0	0.52
tcg	S	0	0.11
att	I	0	4.37
atc	I	12	3.00
ata	I	0	4.64
act	T	245	9802.00
acc	T	38592	7975.00
aca	T	44	17788.00
acg	T	1	3318.00
aat	N	0	2.37
aac	N	4	1.63
agt	S	1	0.38
agc	S	1	0.40
gct	A	0	1.85
gcc	A	7	1.45
gca	A	0	3.29
gcg	A	0	0.41
---	--	------	------
mPD		0.016	0.68
		nPD:	0.02
		N. weight:	1.6
		Sc. PD:	-0.051
		Sc. rank:	8496.9

,

NA			
Pos.	163	obs:	exp:
tta	L	624	4277.00
ttg	L	0	7325.00
ctt	L	11	7667.00
ctc	L	2	5496.00
cta	L	38178	7490.00
ctg	L	92	6652.00
---	--	------	------
mPD		0.037	1.1
		nPD:	0.03
		N. weight:	1.6
		Sc. PD:	-0.038
		Sc. rank:	18590.0

,

NA			
Pos.	164	obs:	exp:
atg	M	38907	38907.00
---	--	------	------
mPD		0	0
		nPD:	1.
		N. weight:	0.
		Sc. PD:	0
		Sc. rank:	0

,

NA			
Pos.	165	obs:	exp:
tct	S	0	7160.00
tcc	S	0	4506.00
tca	S	0	10126.00
tcg	S	0	2071.00
agt	S	92	7316.00
agc	S	38815	7728.00
---	--	------	------
mPD		0.0047	1.6
		nPD:	0.
		N. weight:	1.7
		Sc. PD:	-0.086
		Sc. rank:	-47330.0

,

NA			
Pos.	166	obs:	exp:
tct	S	0	1.47
tcc	S	0	0.93
tca	S	0	2.08
tcg	S	0	0.43
tat	Y	1	0.52
tac	Y	0	0.48
tgt	C	38048	17556.00
tgc	C	849	21341.00
cgt	R	1	0.03
cgc	R	0	0.05
cga	R	0	0.10
cgg	R	0	0.07
agt	S	8	1.50
agc	S	0	1.59
aga	R	0	0.50
agg	R	0	0.25
---	--	------	------
mPD		0.043	0.50
		nPD:	0.09
		N. weight:	0.71
		Sc. PD:	0.021
		Sc. rank:	16280.0

,

NA			
Pos.	167	obs:	exp:
tta	L	0	0.11
ttg	L	0	0.19
tct	S	12	2.21
tcc	S	0	1.39
tca	S	0	3.12
tcg	S	0	0.64
ctt	L	1	0.20
ctc	L	0	0.14
cta	L	0	0.19
ctg	L	0	0.17
cct	P	38418	10715.00
ccc	P	461	7214.00
cca	P	11	16561.00
ccg	P	3	4404.00
act	T	1	0.25
acc	T	0	0.21
aca	T	0	0.46
acg	T	0	0.09
agt	S	0	2.26
agc	S	0	2.38
---	--	------	------
mPD		0.025	0.70
		nPD:	0.04
		N. weight:	1.3
		Sc. PD:	-0.026
		Sc. rank:	17248.0

,

NA			
Pos.	168	obs:	exp:
tta	L	0	2.20
ttg	L	0	3.77
tct	S	0	0.18
tcc	S	0	0.12
tca	S	0	0.26
tcg	S	0	0.05
ctt	L	20	3.94
ctc	L	0	2.82
cta	L	0	3.85
ctg	L	0	3.42
att	I	38686	14131.00
atc	I	131	9690.00
ata	I	1	14997.00
atg	M	2	2.00
act	T	37	9.33
acc	T	0	7.59
aca	T	0	16.93
acg	T	0	3.16
agt	S	1	0.19
agc	S	0	0.20
gtt	V	29	6.41
gtc	V	0	5.27
gta	V	0	7.80
gtg	V	0	9.52
---	--	------	------
mPD		0.011	0.66
		nPD:	0.02
		N. weight:	1.
		Sc. PD:	-0.039
		Sc. rank:	-1499.8

,

NA

Pos.	169	obs:	exp:
tct	S	0	1.29
tcc	S	0	0.81
tca	S	0	1.82
tcg	S	0	0.37
agt	S	7	1.32
agc	S	0	1.39
gat	D	1	0.53
gac	D	0	0.47
ggt	G	38863	5898.00
ggc	G	19	5761.00
gga	G	14	17303.00
ggg	G	3	9936.00
---	--	------	------
mPD		0.0023	0.69
		nPD:	0.
		N. weight:	1.9
		Sc. PD:	-0.1
		Sc. rank:	-52813.0

NA

Pos.	170	obs:	exp:
aaa	K	5	2.85
aag	K	0	2.15
gtt	V	0	0.88
gtc	V	0	0.73
gta	V	4	1.08
gtg	V	0	1.31
gct	A	0	0.26
gcc	A	0	0.21
gca	A	1	0.47
gcg	A	0	0.06
gat	D	1	1.58
gac	D	2	1.42
gaa	E	38864	23550.00
gag	E	29	15343.00
ggt	G	0	0.15
ggc	G	0	0.15
gga	G	1	0.44
ggg	G	0	0.26
---	--	------	------
mPD		0.0022	0.48
		nPD:	0.
		N. weight:	0.51
		Sc. PD:	-0.026
		Sc. rank:	-12340.0

NA

Pos.	171	obs:	exp:
ttt	F	1	0.43
ttc	F	0	0.57
att	I	163	59.70
atc	I	0	40.94
ata	I	1	63.36
gtt	V	38339	8563.00
gtc	V	268	7038.00
gta	V	120	10417.00
gtg	V	6	12715.00
gct	A	7	2.38
gcc	A	2	1.86
gca	A	0	4.23
gcg	A	0	0.53
---	--	------	------
mPD		0.029	0.75
		nPD:	0.04
		N. weight:	1.5
		Sc. PD:	-0.026
		Sc. rank:	22468.0

NA

Pos.	172	obs:	exp:
tct	S	0	0.55
tcc	S	3	0.35
tca	S	0	0.78
tcg	S	0	0.16
cct	P	253	10718.00
ccc	P	38615	7215.00
cca	P	35	16565.00
ccg	P	0	4405.00
act	T	0	0.25
acc	T	1	0.21
aca	T	0	0.46
acg	T	0	0.09
agt	S	0	0.56
agc	S	0	0.60
---	--	------	------
mPD		0.015	0.70
		nPD:	0.02
		N. weight:	1.7
		Sc. PD:	-0.058
		Sc. rank:	5097.8

NA

Pos.	173	obs:	exp:
tct	S	38670	7158.00
tcc	S	135	4506.00
tca	S	86	10124.00
tcg	S	8	2070.00
cct	P	1	0.28
ccc	P	0	0.19
cca	P	0	0.43
ccg	P	0	0.11
act	T	7	1.76
acc	T	0	1.44
aca	T	0	3.20
acg	T	0	0.60
agt	S	0	7314.00
agc	S	0	7727.00
---	--	------	------
mPD		0.012	1.6
		nPD:	0.01
		N. weight:	1.7
		Sc. PD:	-0.082
		Sc. rank:	-31682.0

NA

Pos.	174	obs:	exp:
tta	L	0	0.55
ttg	L	0	0.94
tct	S	0	0.18
tcc	S	0	0.12
tca	S	1	0.26
tcg	S	0	0.05
ctt	L	0	0.99
ctc	L	0	0.71
cta	L	5	0.96
ctg	L	0	0.85
cct	P	27	10717.00
ccc	P	61	7215.00
cca	P	38717	16564.00
ccg	P	96	4405.00
agt	S	0	0.19
agc	S	0	0.20
---	--	------	------
mPD		0.0097	0.70
		nPD:	0.01
		N. weight:	0.85
		Sc. PD:	-0.035
		Sc. rank:	-4677.4

NA

Pos.	175	obs:	exp:
tct	S	0	0.74
tcc	S	4	0.46
tca	S	0	1.04
tcg	S	0	0.21
tat	Y	108	20208.00
tac	Y	38785	18685.00
tgt	C	0	0.45
tgc	C	1	0.55
cat	H	0	5.29
cac	H	9	3.71
agt	S	0	0.75
agc	S	0	0.79
---	--	------	------
mPD		0.0063	0.50
		nPD:	0.01
		N. weight:	0.74
		Sc. PD:	-0.032
		Sc. rank:	-5345.6

NA

Pos.	176	obs:	exp:
aat	N	261	23010.00
aac	N	38644	15895.00
aaa	K	1	1.14
aag	K	1	0.86
---	--	------	------
mPD		0.013	0.48
		nPD:	0.03
		N. weight:	0.88
		Sc. PD:	-0.025
		Sc. rank:	7902.6

NA

Pos.	177	obs:	exp:
tta	L	1	0.11
ttg	L	0	0.19
tct	S	14	7159.00
tcc	S	13	4506.00
tca	S	38795	10125.00
tcg	S	82	2071.00
ctt	L	0	0.20
ctc	L	0	0.14
cta	L	0	0.19
ctg	L	0	0.17
caa	Q	1	0.53
cag	Q	0	0.47
act	T	0	0.25
acc	T	0	0.21
aca	T	1	0.46
acg	T	0	0.09
agt	S	0	7315.00
agc	S	0	7728.00
---	--	------	------
mPD		0.0058	1.6
		nPD:	0.
		N. weight:	1.4
		Sc. PD:	-0.071
		Sc. rank:	-35692.0

NA

Pos.	178	obs:	exp:
cgt	R	0	1059.00
cgc	R	0	2028.00
cga	R	2	3798.00
cgg	R	0	2838.00
att	I	0	0.36
atc	I	0	0.25
ata	I	1	0.39
aaa	K	129	73.59
aag	K	0	55.41
aga	R	38727	19467.00
agg	R	47	9586.00
gat	D	1	0.53
gac	D	0	0.47
---	--	------	------
mPD		0.0093	0.92
		nPD:	0.01
		N. weight:	0.7
		Sc. PD:	-0.032
		Sc. rank:	-8374.9

NA

Pos.	179	obs:	exp:
ttt	F	38733	16695.00
ttc	F	170	22208.00
tta	L	0	0.11
ttg	L	1	0.19
tct	S	1	0.18
tcc	S	0	0.12
tca	S	0	0.26
tcg	S	0	0.05
tat	Y	1	0.52
tac	Y	0	0.48
ctt	L	0	0.20
ctc	L	0	0.14
cta	L	0	0.19
ctg	L	0	0.17
agt	S	0	0.19
agc	S	0	0.20
gtt	V	1	0.22
gtc	V	0	0.18
gta	V	0	0.27
gtg	V	0	0.33
---	--	------	------
mPD		0.0089	0.49
		nPD:	0.02
		N. weight:	0.84
		Sc. PD:	-0.032
		Sc. rank:	-441.3

NA

Pos.	180	obs:	exp:
tct	S	0	0.18
tcc	S	0	0.12
tca	S	0	0.26
tcg	S	0	0.05
agt	S	1	0.19
agc	S	0	0.20
gaa	E	358	23558.00
gag	E	38548	15348.00
---	--	------	------
mPD		0.018	0.48
		nPD:	0.04
		N. weight:	0.9
		Sc. PD:	-0.016
		Sc. rank:	13348.0

NA			
Pos.	181	obs:	exp:
tct	S	55	7158.00
tcc	S	3	4506.00
tca	S	37986	10124.00
tcg	S	855	2070.00
cct	P	0	0.28
ccc	P	0	0.19
cca	P	1	0.43
ccg	P	0	0.11
cat	H	1	0.59
cac	H	0	0.41
act	T	0	1.01
acc	T	0	0.82
aca	T	4	1.83
acg	T	0	0.34
agt	S	0	7314.00
agc	S	0	7727.00
gct	A	0	0.53
gcc	A	0	0.41
gca	A	2	0.94
gcg	A	0	0.12
---	--	------	------
mPD		0.046	1.6
		nPD:	0.03
		N. weight:	1.3
		Sc. PD:	-0.036
		Sc. rank:	13443.0

,

NA			
Pos.	182	obs:	exp:
tct	S	1	0.18
tcc	S	0	0.12
tca	S	0	0.26
tcg	S	0	0.05
att	I	0	1.46
atc	I	4	1.00
ata	I	0	1.55
agt	S	0	0.19
agc	S	0	0.20
gtt	V	669	8601.00
gtc	V	38180	7069.00
gta	V	53	10462.00
gtg	V	0	12770.00
---	--	------	------
mPD		0.037	0.74
		nPD:	0.05
		N. weight:	1.7
		Sc. PD:	-0.011
		Sc. rank:	29673.0

,

NA			
Pos.	183	obs:	exp:
gct	A	38751	10301.00
gcc	A	71	8054.00
gca	A	21	18273.00
gcg	A	64	2279.00
---	--	------	------
mPD		0.0080	0.66
		nPD:	0.01
		N. weight:	1.3
		Sc. PD:	-0.058
		Sc. rank:	-10785.0

,

NA			
Pos.	184	obs:	exp:
tgg	W	38906	38906.00
ggt	G	0	0.15
ggc	G	0	0.15
gga	G	0	0.44
ggg	G	1	0.26
---	--	------	------
mPD		0.000051	0.000090
		nPD:	0.57
		N. weight:	0.000036
		Sc. PD:	0.000018
		Sc. rank:	1.2

,

NA			
Pos.	185	obs:	exp:
tct	S	30	7160.00
tcc	S	1	4506.00
tca	S	38763	10125.00
tcg	S	112	2071.00
cct	P	0	0.28
ccc	P	0	0.19
cca	P	1	0.43
ccg	P	0	0.11
agt	S	0	7316.00
agc	S	0	7728.00
---	--	------	------
mPD		0.0074	1.6
		nPD:	0.
		N. weight:	1.4
		Sc. PD:	-0.069
		Sc. rank:	-33232.0

,

NA			
Pos.	186	obs:	exp:
tct	S	0	0.55
tcc	S	0	0.35
tca	S	3	0.78
tcg	S	0	0.16
agt	S	0	0.56
agc	S	0	0.60
gct	A	16	10300.00
gcc	A	4	8053.00
gca	A	38456	18272.00
gcg	A	428	2279.00
---	--	------	------
mPD		0.023	0.66
		nPD:	0.03
		N. weight:	0.74
		Sc. PD:	-0.016
		Sc. rank:	9505.2

,

NA			
Pos.	187	obs:	exp:
tct	S	0	7158.00
tcc	S	0	4505.00
tca	S	0	10123.00
tcg	S	0	2070.00
act	T	11	2.77
acc	T	0	2.26
aca	T	0	5.03
acg	T	0	0.94
agt	S	38830	7314.00
agc	S	65	7726.00
ggt	G	1	0.15
ggc	G	0	0.15
gga	G	0	0.44
ggg	G	0	0.26
---	--	------	------
mPD		0.0040	1.6
		nPD:	0.
		N. weight:	1.7
		Sc. PD:	-0.09
		Sc. rank:	-50071.0

,

NA			
Pos.	188	obs:	exp:
gtt	V	1	0.22
gtc	V	0	0.18
gta	V	0	0.27
gtg	V	0	0.33
gct	A	38757	10301.00
gcc	A	119	8053.00
gca	A	28	18273.00
gcg	A	2	2279.00
---	--	------	------
mPD		0.0077	0.66
		nPD:	0.01
		N. weight:	1.3
		Sc. PD:	-0.059
		Sc. rank:	-11781.0

,

NA			
Pos.	189	obs:	exp:
tgt	C	38719	17561.00
tgc	C	188	21346.00
---	--	------	------
mPD		0.0096	0.50
		nPD:	0.02
		N. weight:	0.79
		Sc. PD:	-0.029
		Sc. rank:	863.3

,

NA			
Pos.	190	obs:	exp:
cat	H	38785	22880.00
cac	H	121	16026.00
aat	N	1	0.59
aac	N	0	0.41
---	--	------	------
mPD		0.0063	0.48
		nPD:	0.01
		N. weight:	0.53
		Sc. PD:	-0.022
		Sc. rank:	-3546.6

,

NA			
Pos.	191	obs:	exp:
gat	D	38701	20500.00
gac	D	206	18407.00
---	--	------	------
mPD		0.011	0.50
		nPD:	0.02
		N. weight:	0.63
		Sc. PD:	-0.022
		Sc. rank:	1752.5

,

NA			
Pos.	192	obs:	exp:
ggt	G	299	5899.00
ggc	G	38597	5763.00
gga	G	11	17307.00
ggg	G	0	9938.00
---	--	------	------
mPD		0.016	0.69
		nPD:	0.02
		N. weight:	1.9
		Sc. PD:	-0.063
		Sc. rank:	9555.1

,

NA			
Pos.	193	obs:	exp:
ttt	F	0	15.45
ttc	F	36	20.55
tta	L	0	0.11
ttg	L	0	0.19
tct	S	0	1.66
tcc	S	7	1.04
tca	S	0	2.34
tcg	S	0	0.48
ctt	L	0	0.20
ctc	L	1	0.14
cta	L	0	0.19
ctg	L	0	0.17
att	I	35	6568.00
atc	I	17993	4504.00
ata	I	15	6971.00
act	T	190	5232.00
acc	T	20547	4257.00
aca	T	16	9494.00
acg	T	0	1771.00
aat	N	0	10.65
aac	N	18	7.35
agt	S	0	1.69
agc	S	2	1.79
gtt	V	0	7.52
gtc	V	34	6.18
gta	V	0	9.14
gtg	V	0	11.16
gct	A	0	3.44
gcc	A	13	2.69
gca	A	0	6.11
gcg	A	0	0.76
---	--	------	------
mPD		0.52	1.2
		nPD:	0.44
		N. weight:	1.5
		Sc. PD:	0.55
		Sc. rank:	47008.0

,

NA			
Pos.	194	obs:	exp:
tct	S	0	17.30
tcc	S	0	10.89
tca	S	0	24.46
tcg	S	0	5.00
tat	Y	4	2.08
tac	Y	0	1.92
cat	H	1	0.59
cac	H	0	0.41
aat	N	38744	22949.00
aac	N	58	15853.00
aaa	K	0	0.57
aag	K	1	0.43
agt	S	94	17.68
agc	S	0	18.67
gat	D	5	2.63
gac	D	0	2.37
---	--	------	------
mPD		0.0084	0.49
		nPD:	0.02
		N. weight:	0.53
		Sc. PD:	-0.021
		Sc. rank:	-973.8

,

NA			
Pos.	195	obs:	exp:
tgg	W	38907	38907.00
---	--	------	------
mPD		0	0
		nPD:	1.
		N. weight:	0.
		Sc. PD:	0
		Sc. rank:	0

,

NA			
Pos.	196	obs:	exp:
tta	L	107	4277.00
ttg	L	0	7325.00
ctt	L	8	7667.00
ctc	L	9	5495.00
cta	L	38675	7490.00
ctg	L	107	6652.00
att	I	0	0.36
atc	I	0	0.25
ata	I	1	0.39
---	--	------	------
mPD		0.012	1.1
		nPD:	0.01
		N. weight:	1.7
		Sc. PD:	-0.075
		Sc. rank:	-18702.0

,

NA			
Pos.	197	obs:	exp:
cct	P	0	0.28
ccc	P	0	0.19
cca	P	1	0.43
ccg	P	0	0.11
att	I	0	1.09
atc	I	0	0.75
ata	I	3	1.16
act	T	6	9807.00
acc	T	10	7980.00
aca	T	38856	17797.00
acg	T	31	3319.00
---	--	------	------
mPD		0.0026	0.68
		nPD:	0.
		N. weight:	0.8
		Sc. PD:	-0.041
		Sc. rank:	-20377.0

,

NA			
Pos.	198	obs:	exp:
att	I	38836	14160.00
atc	I	54	9710.00
ata	I	9	15029.00
gtt	V	8	1.77
gtc	V	0	1.45
gta	V	0	2.15
gtg	V	0	2.63
---	--	------	------
mPD		0.0036	0.66
		nPD:	0.01
		N. weight:	1.
		Sc. PD:	-0.051
		Sc. rank:	-23439.0

,

NA			
Pos.	199	obs:	exp:
gct	A	0	0.26
gcc	A	0	0.21
gca	A	1	0.47
gcg	A	0	0.06
ggt	G	10	5899.00
ggc	G	1	5762.00
gga	G	38808	17306.00
ggg	G	87	9938.00
---	--	------	------
mPD		0.0051	0.69
		nPD:	0.01
		N. weight:	0.82
		Sc. PD:	-0.039
		Sc. rank:	-15392.0

,

NA			
Pos.	200	obs:	exp:
att	I	38319	14137.00
atc	I	509	9694.00
ata	I	7	15004.00
gtt	V	72	15.92
gtc	V	0	13.08
gta	V	0	19.36
gtg	V	0	23.63
---	--	------	------
mPD		0.030	0.66
		nPD:	0.05
		N. weight:	0.98
		Sc. PD:	-0.011
		Sc. rank:	16498.0

,

NA			
Pos.	201	obs:	exp:
tct	S	38856	7159.00
tcc	S	26	4506.00
tca	S	9	10125.00
tcg	S	13	2071.00
cct	P	0	0.28
ccc	P	0	0.19
cca	P	0	0.43
ccg	P	1	0.11
act	T	2	0.50
acc	T	0	0.41
aca	T	0	0.91
acg	T	0	0.17
agt	S	0	7315.00
agc	S	0	7728.00
---	--	------	------
mPD		0.0027	1.6
		nPD:	0.
		N. weight:	1.7
		Sc. PD:	-0.093
		Sc. rank:	-54032.0

,

NA			
Pos.	202	obs:	exp:
ggt	G	225	5899.00
ggc	G	38604	5763.00
gga	G	17	17307.00
ggg	G	61	9938.00
---	--	------	------
mPD		0.015	0.69
		nPD:	0.02
		N. weight:	1.9
		Sc. PD:	-0.064
		Sc. rank:	7688.5

,

NA			
Pos.	203	obs:	exp:
tta	L	0	0.11
ttg	L	0	0.19
tct	S	0	7.73
tcc	S	0	4.86
tca	S	42	10.93
tcg	S	0	2.24
ctt	L	0	0.20
ctc	L	0	0.14
cta	L	1	0.19
ctg	L	0	0.17
cct	P	81	10705.00
ccc	P	12	7207.00
cca	P	38755	16546.00
ccg	P	11	4400.00
caa	Q	5	2.64
cag	Q	0	2.36
agt	S	0	7.90
agc	S	0	8.34
---	--	------	------
mPD		0.0078	0.70
		nPD:	0.01
		N. weight:	0.86
		Sc. PD:	-0.038
		Sc. rank:	-8450.9

,

NA			
Pos.	204	obs:	exp:
tat	Y	0	3.12
tac	Y	6	2.88
aat	N	0	79.84
aac	N	135	55.16
gat	D	91	20422.00
gac	D	38668	18337.00
gaa	E	0	0.61
gag	E	1	0.39
ggt	G	0	0.91
ggc	G	6	0.89
gga	G	0	2.67
ggg	G	0	1.53
---	--	------	------
mPD		0.012	0.51
		nPD:	0.02
		N. weight:	0.76
		Sc. PD:	-0.024
		Sc. rank:	4610.0

,

NA

Pos.	205	obs:	exp:
tct	S	0	5748.00
tcc	S	0	3618.00
tca	S	0	8128.00
tcg	S	0	1662.00
tat	Y	4	2.08
tac	Y	0	1.92
cat	H	6	3.53
cac	H	0	2.47
cgt	R	3	0.22
cgc	R	0	0.42
cga	R	0	0.78
cgg	R	0	0.59
att	I	6	2.18
atc	I	0	1.50
ata	I	0	2.32
act	T	1	0.25
acc	T	0	0.21
aca	T	0	0.46
acg	T	0	0.09
aat	N	7641	4521.00
aac	N	3	3123.00
aaa	K	0	0.57
aag	K	1	0.43
agt	S	31226	5873.00
agc	S	6	6204.00
aga	R	4	4.02
agg	R	1	1.98
ggt	G	5	0.76
ggc	G	0	0.74
gga	G	0	2.22
ggg	G	0	1.28
---	--	------	------
mPD		0.32	1.8
		nPD:	0.18
		N. weight:	1.5
		Sc. PD:	0.17
		Sc. rank:	40254.0

,

NA

Pos.	206	obs:	exp:
cgt	R	0	0.52
cgc	R	0	0.99
cga	R	0	1.86
cgg	R	0	1.39
aga	R	0	9.54
agg	R	19	4.70
gaa	E	0	50.86
gag	E	84	33.14
ggt	G	7	5884.00
ggc	G	0	5747.00
gga	G	200	17261.00
ggg	G	38597	9912.00
---	--	------	------
mPD		0.016	0.70
		nPD:	0.02
		N. weight:	1.4
		Sc. PD:	-0.045
		Sc. rank:	6316.0

,

NA

Pos.	207	obs:	exp:
tct	S	0	0.37
tcc	S	0	0.23
tca	S	2	0.52
tcg	S	0	0.11
agt	S	0	0.38
agc	S	0	0.40
gct	A	21	10298.00
gcc	A	4	8052.00
gca	A	38860	18269.00
gcg	A	13	2279.00
gaa	E	1	0.61
gag	E	0	0.39
ggt	G	0	0.91
ggc	G	0	0.89
gga	G	6	2.67
ggg	G	0	1.53
---	--	------	------
mPD		0.0024	0.66
		nPD:	0.
		N. weight:	0.77
		Sc. PD:	-0.04
		Sc. rank:	-19799.0

,

NA

Pos.	208	obs:	exp:
tta	L	0	0.88
ttg	L	7	1.51
ctt	L	0	1.58
ctc	L	0	1.13
cta	L	0	1.54
ctg	L	1	1.37
att	I	0	0.36
atc	I	0	0.25
ata	I	1	0.39
atg	M	40	40.00
gtt	V	10	8591.00
gtc	V	0	7061.00
gta	V	73	10450.00
gtg	V	38774	12755.00
gct	A	0	0.26
gcc	A	0	0.21
gca	A	0	0.47
gcg	A	1	0.06
---	--	------	------
mPD		0.0069	0.74
		nPD:	0.01
		N. weight:	1.1
		Sc. PD:	-0.052
		Sc. rank:	-15379.0

,

NA

Pos.	209	obs:	exp:
act	T	3	0.76
acc	T	0	0.62
aca	T	0	1.37
acg	T	0	0.26
gtt	V	1	0.22
gtc	V	0	0.18
gta	V	0	0.27
gtg	V	0	0.33
gct	A	38771	10299.00
gcc	A	111	8052.00
gca	A	12	18270.00
gcg	A	7	2279.00
gat	D	1	0.53
gac	D	0	0.47
ggt	G	1	0.15
ggc	G	0	0.15
gga	G	0	0.44
ggg	G	0	0.26
---	--	------	------
mPD		0.0070	0.66
		nPD:	0.01
		N. weight:	1.3
		Sc. PD:	-0.06
		Sc. rank:	-14943.0

,

NA

Pos.	210	obs:	exp:
att	I	0	0.36
atc	I	0	0.25
ata	I	1	0.39
gtt	V	15	8601.00
gtc	V	1	7069.00
gta	V	824	10463.00
gtg	V	38065	12771.00
ggt	G	0	0.15
ggc	G	0	0.15
gga	G	0	0.44
ggg	G	1	0.26
---	--	------	------
mPD		0.042	0.74
		nPD:	0.06
		N. weight:	1.
		Sc. PD:	0.00046
		Sc. rank:	20907.0

,

NA

Pos.	211	obs:	exp:
ttt	F	5	2.15
ttc	F	0	2.85
tta	L	38281	4275.00
ttg	L	533	7321.00
tct	S	0	1.84
tcc	S	0	1.16
tca	S	10	2.60
tcg	S	0	0.53
ctt	L	0	7663.00
ctc	L	0	5493.00
cta	L	72	7486.00
ctg	L	0	6649.00
att	I	0	0.73
atc	I	1	0.50
ata	I	1	0.77
agt	S	0	1.88
agc	S	0	1.99
gtt	V	0	0.88
gtc	V	0	0.73
gta	V	4	1.08
gtg	V	0	1.31
---	--	------	------
mPD		0.032	1.1
		nPD:	0.03
		N. weight:	2.2
		Sc. PD:	-0.061
		Sc. rank:	21227.0

,

NA

Pos.	212	obs:	exp:
cgt	R	0	0.19
cgc	R	0	0.37
cga	R	0	0.69
cgg	R	0	0.51
aaa	K	20620	22190.00
aag	K	18280	16710.00
aga	R	4	3.51
agg	R	3	1.73
---	--	------	------
mPD		0.50	0.49
		nPD:	1.02
		N. weight:	0.0036
		Sc. PD:	0.0034
		Sc. rank:	118.6

,

NA			
Pos.	213	obs:	exp:
tat	Y	76	20215.00
tac	Y	38830	18691.00
cat	H	0	0.59
cac	H	1	0.41
---	--	------	------
mPD		0.0040	0.50
		nPD:	0.01
		N. weight:	0.74
		Sc. PD:	-0.035
		Sc. rank:	-12291.0

,

NA			
Pos.	214	obs:	exp:
tct	S	0	0.55
tcc	S	0	0.35
tca	S	0	0.78
tcg	S	0	0.16
aat	N	31664	23006.00
aac	N	7235	15893.00
aaa	K	3	1.71
aag	K	0	1.29
agt	S	1	0.56
agc	S	2	0.60
gat	D	2	1.05
gac	D	0	0.95
---	--	------	------
mPD		0.30	0.48
		nPD:	0.63
		N. weight:	0.12
		Sc. PD:	0.065
		Sc. rank:	3801.7

,

NA			
Pos.	215	obs:	exp:
tct	S	0	0.92
tcc	S	0	0.58
tca	S	0	1.30
tcg	S	0	0.27
aat	N	0	0.59
aac	N	1	0.41
agt	S	0	0.94
agc	S	5	0.99
gat	D	0	4.74
gac	D	9	4.26
ggt	G	67	5897.00
ggc	G	38783	5760.00
gga	G	40	17300.00
ggg	G	2	9934.00
---	--	------	------
mPD		0.0064	0.69
		nPD:	0.01
		N. weight:	1.9
		Sc. PD:	-0.09
		Sc. rank:	-26914.0

,

NA			
Pos.	216	obs:	exp:
tta	L	1	0.33
ttg	L	0	0.56
ctt	L	0	0.59
ctc	L	0	0.42
cta	L	2	0.58
ctg	L	0	0.51
att	I	6	14129.00
atc	I	1	9688.00
ata	I	38806	14996.00
atg	M	24	24.00
act	T	0	0.25
acc	T	0	0.21
aca	T	1	0.46
acg	T	0	0.09
gtt	V	0	14.37
gtc	V	0	11.81
gta	V	65	17.48
gtg	V	0	21.34
gct	A	0	0.26
gcc	A	0	0.21
gca	A	1	0.47
gcg	A	0	0.06
---	--	------	------
mPD		0.0052	0.66
		nPD:	0.01
		N. weight:	0.98
		Sc. PD:	-0.046
		Sc. rank:	-16100.0

,

NA			
Pos.	217	obs:	exp:
tta	L	1	0.11
ttg	L	0	0.19
ctt	L	0	0.20
ctc	L	0	0.14
cta	L	0	0.19
ctg	L	0	0.17
att	I	8	14162.00
atc	I	3	9711.00
ata	I	38893	15031.00
atg	M	1	1.00
act	T	0	0.25
acc	T	0	0.21
aca	T	1	0.46
acg	T	0	0.09
---	--	------	------
mPD		0.00072	0.66
		nPD:	0.
		N. weight:	0.98
		Sc. PD:	-0.053
		Sc. rank:	-31777.0

,

NA			
Pos.	218	obs:	exp:
tct	S	0	0.18
tcc	S	0	0.12
tca	S	1	0.26
tcg	S	0	0.05
act	T	21	9807.00
acc	T	11	7980.00
aca	T	38805	17798.00
acg	T	68	3319.00
aaa	K	1	0.57
aag	K	0	0.43
agt	S	0	0.19
agc	S	0	0.20
---	--	------	------
mPD		0.0052	0.68
		nPD:	0.01
		N. weight:	0.79
		Sc. PD:	-0.038
		Sc. rank:	-13367.0

,

NA			
Pos.	219	obs:	exp:
aat	N	0	1.77
aac	N	3	1.23
gat	D	181	20495.00
gac	D	38717	18403.00
ggt	G	0	0.91
ggc	G	6	0.89
gga	G	0	2.67
ggg	G	0	1.53
---	--	------	------
mPD		0.0097	0.50
		nPD:	0.02
		N. weight:	0.74
		Sc. PD:	-0.027
		Sc. rank:	886.7

,

NA			
Pos.	220	obs:	exp:
tct	S	2	0.74
tcc	S	0	0.46
tca	S	0	1.04
tcg	S	0	0.21
cct	P	1	0.28
ccc	P	0	0.19
cca	P	0	0.43
ccg	P	0	0.11
att	I	53	19.29
atc	I	0	13.23
ata	I	0	20.48
act	T	38692	9793.00
acc	T	138	7968.00
aca	T	16	17772.00
acg	T	2	3315.00
agt	S	2	0.75
agc	S	0	0.79
gct	A	1	0.26
gcc	A	0	0.21
gca	A	0	0.47
gcg	A	0	0.06
---	--	------	------
mPD		0.011	0.68
		nPD:	0.02
		N. weight:	1.4
		Sc. PD:	-0.055
		Sc. rank:	-4532.5

,

NA			
Pos.	221	obs:	exp:
ttt	F	0	0.43
ttc	F	1	0.57
tta	L	0	0.22
ttg	L	0	0.38
ctt	L	0	0.39
ctc	L	2	0.28
cta	L	0	0.39
ctg	L	0	0.34
att	I	78	14114.00
atc	I	38656	9678.00
ata	I	39	14980.00
act	T	0	0.76
acc	T	3	0.62
aca	T	0	1.37
acg	T	0	0.26
gtt	V	0	28.30
gtc	V	128	23.26
gta	V	0	34.42
gtg	V	0	42.02
---	--	------	------
mPD		0.013	0.66
		nPD:	0.02
		N. weight:	1.4
		Sc. PD:	-0.051
		Sc. rank:	1391.7

,

NA			
Pos.	222	obs:	exp:
caa	Q	0	0.53
cag	Q	1	0.47
cgt	R	0	1.50
cgc	R	0	2.88
cga	R	0	5.39
cgg	R	0	4.03
atg	M	1	1.00
act	T	0	0.76
acc	T	0	0.62
aca	T	0	1.37
acg	T	3	0.26
aaa	K	305	22158.00
aag	K	38540	16687.00
aga	R	0	27.61
agg	R	55	13.60
gaa	E	0	1.21
gag	E	2	0.79
---	--	------	------
mPD		0.019	0.49
		nPD:	0.04
		N. weight:	0.82
		Sc. PD:	-0.015
		Sc. rank:	11934.0

,

NA			
Pos.	223	obs:	exp:
tct	S	0	7159.00
tcc	S	0	4506.00
tca	S	0	10125.00
tcg	S	0	2071.00
act	T	3	0.76
acc	T	0	0.62
aca	T	0	1.37
acg	T	0	0.26
agt	S	38796	7315.00
agc	S	108	7728.00
---	--	------	------
mPD		0.0057	1.6
		nPD:	0.
		N. weight:	1.7
		Sc. PD:	-0.088
		Sc. rank:	-45521.0

,

NA			
Pos.	224	obs:	exp:
tgg	W	38906	38906.00
ggt	G	0	0.15
ggc	G	0	0.15
gga	G	0	0.44
ggg	G	1	0.26
---	--	------	------
mPD		0.000051	0.000090
		nPD:	0.57
		N. weight:	0.000036
		Sc. PD:	0.000018
		Sc. rank:	1.2

,

NA

Pos.	225	obs:	exp:
tct	S	0	1.10
tcc	S	0	0.69
tca	S	0	1.56
tcg	S	0	0.32
cgt	R	0	1056.00
cgc	R	0	2023.00
cga	R	0	3789.00
cgg	R	2	2831.00
atg	M	2	2.00
aaa	K	126	112.40
aag	K	71	84.63
agt	S	6	1.13
agc	S	0	1.19
aga	R	9130	19420.00
agg	R	29550	9563.00
gaa	E	1	0.61
gag	E	0	0.39
ggt	G	0	2.88
ggc	G	0	2.81
gga	G	11	8.45
ggg	G	8	4.85
---	--	------	------
mPD		0.37	0.92
		nPD:	0.41
		N. weight:	0.7
		Sc. PD:	0.24
		Sc. rank:	21550.0

,

NA

Pos.	226	obs:	exp:
tct	S	0	24.66
tcc	S	0	15.52
tca	S	0	34.87
tcg	S	0	7.13
tat	Y	0	1.56
tac	Y	3	1.44
cat	H	0	2.35
cac	H	4	1.65
act	T	0	0.25
acc	T	1	0.21
aca	T	0	0.46
acg	T	0	0.09
aat	N	351	22918.00
aac	N	38398	15831.00
aaa	K	6	4.56
aag	K	2	3.44
agt	S	0	25.20
agc	S	134	26.62
gat	D	1	4.22
gac	D	7	3.78
---	--	------	------
mPD		0.026	0.50
		nPD:	0.05
		N. weight:	0.87
		Sc. PD:	-0.0039
		Sc. rank:	16268.0

,

NA

Pos.	227	obs:	exp:
tct	S	0	6.62
tcc	S	0	4.17
tca	S	0	9.37
tcg	S	0	1.92
tat	Y	11	6.24
tac	Y	1	5.76
cat	H	9	5.29
cac	H	0	3.71
cgt	R	0	0.03
cgc	R	0	0.05
cga	R	0	0.10
cgg	R	0	0.07
act	T	1	0.25
acc	T	0	0.21
aca	T	0	0.46
acg	T	0	0.09
aat	N	34265	20726.00
aac	N	778	14317.00
aaa	K	32	1573.00
aag	K	2725	1184.00
agt	S	36	6.77
agc	S	0	7.15
aga	R	0	0.50
agg	R	1	0.25
gat	D	1046	551.70
gac	D	1	495.30
gaa	E	0	0.61
gag	E	1	0.39
---	--	------	------
mPD		0.22	0.61
		nPD:	0.37
		N. weight:	0.47
		Sc. PD:	0.14
		Sc. rank:	13961.0

,

NA

Pos.	228	obs:	exp:
tta	L	1	0.11
ttg	L	0	0.19
ctt	L	0	0.20
ctc	L	0	0.14
cta	L	0	0.19
ctg	L	0	0.17
cgt	R	0	0.41
cgc	R	0	0.78
cga	R	0	1.47
cgg	R	0	1.10
att	I	10	14129.00
atc	I	66	9688.00
ata	I	38736	14995.00
atg	M	3	3.00
act	T	0	5.80
acc	T	0	4.72
aca	T	23	10.52
acg	T	0	1.96
aaa	K	11	6.27
aag	K	0	4.73
aga	R	15	7.53
agg	R	0	3.71
gtt	V	0	9.29
gtc	V	0	7.63
gta	V	42	11.30
gtg	V	0	13.79
---	--	------	------
mPD		0.0088	0.66
		nPD:	0.01
		N. weight:	0.97
		Sc. PD:	-0.041
		Sc. rank:	-5989.3

,

NA

Pos.	229	obs:	exp:
tta	L	187	4276.00
ttg	L	38586	7323.00
ctt	L	0	7664.00
ctc	L	0	5494.00
cta	L	0	7488.00
ctg	L	121	6650.00
atg	M	13	13.00
---	--	------	------
mPD		0.016	1.1
		nPD:	0.01
		N. weight:	1.7
		Sc. PD:	-0.069
		Sc. rank:	-7424.2

,

NA

Pos.	230	obs:	exp:
cgt	R	0	1062.00
cgc	R	0	2035.00
cga	R	3	3811.00
cgg	R	0	2847.00
aaa	K	1	0.57
aag	K	0	0.43
aga	R	38554	19532.00
agg	R	349	9618.00
---	--	------	------
mPD		0.018	0.91
		nPD:	0.02
		N. weight:	0.66
		Sc. PD:	-0.024
		Sc. rank:	1292.2

,

NA

Pos.	231	obs:	exp:
act	T	6	9805.00
acc	T	7	7978.00
aca	T	38859	17793.00
acg	T	23	3319.00
gct	A	0	3.18
gcc	A	0	2.48
gca	A	12	5.64
gcg	A	0	0.70
---	--	------	------
mPD		0.0025	0.68
		nPD:	0.
		N. weight:	0.8
		Sc. PD:	-0.041
		Sc. rank:	-20593.0

,

NA

Pos.	232	obs:	exp:
cct	P	0	0.28
ccc	P	0	0.19
cca	P	1	0.43
ccg	P	0	0.11
caa	Q	38857	20530.00
cag	Q	49	18376.00
---	--	------	------
mPD		0.0026	0.50
		nPD:	0.01
		N. weight:	0.65
		Sc. PD:	-0.033
		Sc. rank:	-15148.0

,

NA

Pos.	233	obs:	exp:
aaa	K	0	1.14
aag	K	2	0.86
gaa	E	1330	23557.00
gag	E	37575	15348.00
---	--	------	------
mPD		0.066	0.48
		nPD:	0.14
		N. weight:	0.79
		Sc. PD:	0.062
		Sc. rank:	20975.0

,

NA

Pos.	234	obs:	exp:
tct	S	38840	7160.00
tcc	S	51	4506.00
tca	S	6	10126.00
tcg	S	10	2071.00
agt	S	0	7316.00
agc	S	0	7728.00
---	--	------	------
mPD		0.0034	1.6
		nPD:	0.
		N. weight:	1.7
		Sc. PD:	-0.092
		Sc. rank:	-52325.0

,

NA

Pos.	235	obs:	exp:
gtt	V	0	0.22
gtc	V	0	0.18
gta	V	1	0.27
gtg	V	0	0.33
gat	D	0	0.53
gac	D	1	0.47
gaa	E	38825	23557.00
gag	E	80	15348.00
---	--	------	------
mPD		0.0042	0.48
		nPD:	0.01
		N. weight:	0.5
		Sc. PD:	-0.023
		Sc. rank:	-7522.9

,

NA

Pos.	236	obs:	exp:
tgt	C	38860	17561.00
tgc	C	46	21345.00
tgg	W	1	1.00
---	--	------	------
mPD		0.0024	0.50
		nPD:	0.
		N. weight:	0.81
		Sc. PD:	-0.041
		Sc. rank:	-19064.0

,

NA

Pos.	237	obs:	exp:
tct	S	0	0.37
tcc	S	0	0.23
tca	S	2	0.52
tcg	S	0	0.11
act	T	0	5.80
acc	T	0	4.72
aca	T	23	10.52
acg	T	0	1.96
agt	S	0	0.38
agc	S	0	0.40
gtt	V	0	58.37
gtc	V	0	47.97
gta	V	264	71.00
gtg	V	0	86.66
gct	A	15	10224.00
gcc	A	3	7994.00
gca	A	38572	18138.00
gcg	A	28	2262.00
---	--	------	------
mPD		0.017	0.68
		nPD:	0.03
		N. weight:	0.78
		Sc. PD:	-0.024
		Sc. rank:	5036.0

,

NA

Pos.	238	obs:	exp:
tct	S	0	0.18
tcc	S	0	0.12
tca	S	0	0.26
tcg	S	0	0.05
tgt	C	38792	17561.00
tgc	C	114	21345.00
agt	S	1	0.19
agc	S	0	0.20
---	--	------	------
mPD		0.0059	0.50
		nPD:	0.01
		N. weight:	0.8
		Sc. PD:	-0.035
		Sc. rank:	-6689.1

,

NA

Pos.	239	obs:	exp:
tta	L	17	2.09
ttg	L	1	3.58
ctt	L	0	3.74
ctc	L	0	2.68
cta	L	1	3.66
ctg	L	0	3.25
att	I	0	41.14
atc	I	0	28.21
ata	I	113	43.66
atg	M	2	2.00
gtt	V	4	8561.00
gtc	V	10	7036.00
gta	V	38559	10414.00
gtg	V	150	12711.00
gct	A	0	13.24
gcc	A	0	10.35
gca	A	50	23.48
gcg	A	0	2.93
---	--	------	------
mPD		0.018	0.75
		nPD:	0.02
		N. weight:	1.3
		Sc. PD:	-0.042
		Sc. rank:	7935.9

,

NA

Pos.	240	obs:	exp:
tat	Y	1	0.52
tac	Y	0	0.48
aat	N	38730	23001.00
aac	N	160	15889.00
aaa	K	1	1.14
aag	K	1	0.86
gat	D	14	7.38
gac	D	0	6.62
---	--	------	------
mPD		0.0091	0.48
		nPD:	0.02
		N. weight:	0.52
		Sc. PD:	-0.019
		Sc. rank:	146.8

,

NA			
Pos.	241	obs:	exp:
tct	S	0	0.74
tcc	S	0	0.46
tca	S	0	1.04
tcg	S	0	0.21
agt	S	4	0.75
agc	S	0	0.79
ggt	G	38712	5899.00
ggc	G	163	5762.00
gga	G	3	17305.00
ggg	G	25	9937.00
---	--	------	------
mPD		0.010	0.69
		nPD:	0.01
		N. weight:	1.9
		Sc. PD:	-0.079
		Sc. rank:	-8921.7

,

NA			
Pos.	242	obs:	exp:
tct	S	38555	7160.00
tcc	S	284	4506.00
tca	S	57	10125.00
tcg	S	9	2071.00
agt	S	0	7315.00
agc	S	0	7728.00
gct	A	2	0.53
gcc	A	0	0.41
gca	A	0	0.94
gcg	A	0	0.12
---	--	------	------
mPD		0.018	1.6
		nPD:	0.01
		N. weight:	1.7
		Sc. PD:	-0.075
		Sc. rank:	-17444.0

,

NA			
Pos.	243	obs:	exp:
tat	Y	0	1.04
tac	Y	2	0.96
tgt	C	159	17560.00
tgc	C	38746	21345.00
---	--	------	------
mPD		0.0082	0.50
		nPD:	0.02
		N. weight:	0.59
		Sc. PD:	-0.023
		Sc. rank:	-1439.2

,

NA			
Pos.	244	obs:	exp:
ttt	F	38781	16696.00
ttc	F	126	22211.00
---	--	------	------
mPD		0.0065	0.49
		nPD:	0.01
		N. weight:	0.85
		Sc. PD:	-0.036
		Sc. rank:	-5374.4

,

NA			
Pos.	245	obs:	exp:
tct	S	0	0.18
tcc	S	0	0.12
tca	S	0	0.26
tcg	S	0	0.05
cct	P	0	0.28
ccc	P	1	0.19
cca	P	0	0.43
ccg	P	0	0.11
att	I	5	2.91
atc	I	3	2.00
ata	I	0	3.09
act	T	5876	9805.00
acc	T	33006	7978.00
aca	T	12	17793.00
acg	T	1	3319.00
aat	N	0	0.59
aac	N	1	0.41
agt	S	0	0.19
agc	S	1	0.20
gct	A	1	0.26
gcc	A	0	0.21
gca	A	0	0.47
gcg	A	0	0.06
---	--	------	------
mPD		0.26	0.68
		nPD:	0.38
		N. weight:	1.2
		Sc. PD:	0.36
		Sc. rank:	34840.0

,

NA			
Pos.	246	obs:	exp:
tta	L	10	1.21
ttg	L	0	2.07
ctt	L	0	2.17
ctc	L	0	1.55
cta	L	1	2.12
ctg	L	0	1.88
att	I	3	12129.00
atc	I	4	8317.00
ata	I	33313	12873.00
atg	M	3	3.00
act	T	0	0.25
acc	T	0	0.21
aca	T	1	0.46
acg	T	0	0.09
gtt	V	0	1232.00
gtc	V	0	1012.00
gta	V	5529	1499.00
gtg	V	43	1829.00
---	--	------	------
mPD		0.25	0.93
		nPD:	0.27
		N. weight:	1.
		Sc. PD:	0.21
		Sc. rank:	29190.0

,

NA			
Pos.	247	obs:	exp:
tta	L	0	4.07
ttg	L	1	6.97
ctt	L	0	7.29
ctc	L	0	5.23
cta	L	0	7.12
ctg	L	36	6.33
att	I	12	8.01
atc	I	0	5.49
ata	I	10	8.50
atg	M	38847	38847.00
gtt	V	0	0.22
gtc	V	0	0.18
gta	V	0	0.27
gtg	V	1	0.33
---	--	------	------
mPD		0.0031	0.0043
		nPD:	0.71
		N. weight:	0.0019
		Sc. PD:	0.0012
		Sc. rank:	60.9

,

NA			
Pos.	248	obs:	exp:
act	T	197	9808.00
acc	T	38680	7980.00
aca	T	28	17799.00
acg	T	1	3320.00
gct	A	0	0.26
gcc	A	1	0.21
gca	A	0	0.47
gcg	A	0	0.06
---	--	------	------
mPD		0.012	0.68
		nPD:	0.02
		N. weight:	1.6
		Sc. PD:	-0.061
		Sc. rank:	-2561.6

,

NA			
Pos.	249	obs:	exp:
gat	D	38725	20500.00
gac	D	182	18407.00
---	--	------	------
mPD		0.0093	0.50
		nPD:	0.02
		N. weight:	0.63
		Sc. PD:	-0.023
		Sc. rank:	135.2

,

NA			
Pos.	250	obs:	exp:
gaa	E	2	1.21
gag	E	0	0.79
ggt	G	12	5899.00
ggc	G	10	5762.00
gga	G	38797	17306.00
ggg	G	86	9938.00
---	--	------	------
mPD		0.0056	0.69
		nPD:	0.01
		N. weight:	0.82
		Sc. PD:	-0.038
		Sc. rank:	-13213.0

,

NA			
Pos.	251	obs:	exp:
tta	L	0	0.11
ttg	L	0	0.19
tct	S	0	0.18
tcc	S	0	0.12
tca	S	1	0.26
tcg	S	0	0.05
ctt	L	0	0.20
ctc	L	0	0.14
cta	L	1	0.19
ctg	L	0	0.17
cct	P	5	10718.00
ccc	P	15	7215.00
cca	P	38864	16565.00
ccg	P	19	4405.00
act	T	0	0.25
acc	T	0	0.21
aca	T	1	0.46
acg	T	0	0.09
aaa	K	1	0.57
aag	K	0	0.43
agt	S	0	0.19
agc	S	0	0.20
---	--	------	------
mPD		0.0023	0.70
		nPD:	0.
		N. weight:	0.87
		Sc. PD:	-0.045
		Sc. rank:	-24131.0

,

NA			
Pos.	252	obs:	exp:
tct	S	0	7125.00
tcc	S	0	4485.00
tca	S	0	10077.00
tcg	S	0	2061.00
cgt	R	2	0.11
cgc	R	0	0.21
cga	R	0	0.39
cgg	R	0	0.29
att	I	5	1.82
atc	I	0	1.25
ata	I	0	1.93
aat	N	174	102.90
aac	N	0	71.09
agt	S	38692	7280.00
agc	S	26	7691.00
aga	R	1	2.01
agg	R	1	0.99
ggt	G	6	0.91
ggc	G	0	0.89
gga	G	0	2.67
ggg	G	0	1.53
---	--	------	------
mPD		0.011	1.6
		nPD:	0.01
		N. weight:	1.7
		Sc. PD:	-0.083
		Sc. rank:	-35579.0

,

NA			
Pos.	253	obs:	exp:
cat	H	2	1.18
cac	H	0	0.82
aat	N	505	298.70
aac	N	0	206.30
gat	D	38322	20228.00
gac	D	68	18162.00
ggt	G	10	1.52
ggc	G	0	1.48
gga	G	0	4.45
ggg	G	0	2.55
---	--	------	------
mPD		0.030	0.52
		nPD:	0.06
		N. weight:	0.65
		Sc. PD:	-0.00023
		Sc. rank:	12577.0

,

NA			
Pos.	254	obs:	exp:
cgt	R	0	1.20
cgc	R	0	2.30
cga	R	1	4.31
cgg	R	0	3.22
aga	R	42	22.09
agg	R	1	10.88
gaa	E	63	38.75
gag	E	1	25.25
ggt	G	5	5883.00
ggc	G	4	5747.00
gga	G	38532	17259.00
ggg	G	258	9911.00
---	--	------	------
mPD		0.019	0.70
		nPD:	0.03
		N. weight:	0.8
		Sc. PD:	-0.023
		Sc. rank:	6908.0

,

NA			
Pos.	255	obs:	exp:
tta	L	0	0.11
ttg	L	0	0.19
ctt	L	0	0.20
ctc	L	0	0.14
cta	L	0	0.19
ctg	L	1	0.17
caa	Q	143	20527.00
cag	Q	38759	18375.00
cgt	R	0	0.05
cgc	R	0	0.10
cga	R	0	0.20
cgg	R	2	0.15
aaa	K	0	1.14
aag	K	2	0.86
aga	R	0	1.00
agg	R	0	0.49
---	--	------	------
mPD		0.0076	0.50
		nPD:	0.02
		N. weight:	0.75
		Sc. PD:	-0.03
		Sc. rank:	-2832.6

,

NA			
Pos.	256	obs:	exp:
tct	S	0	0.55
tcc	S	3	0.35
tca	S	0	0.78
tcg	S	0	0.16
cct	P	0	0.28
ccc	P	1	0.19
cca	P	0	0.43
ccg	P	0	0.11
agt	S	0	0.56
agc	S	0	0.60
gct	A	736	10300.00
gcc	A	38122	8053.00
gca	A	45	18271.00
gcg	A	0	2279.00
---	--	------	------
mPD		0.040	0.66
		nPD:	0.06
		N. weight:	1.5
		Sc. PD:	0.0039
		Sc. rank:	30884.0

,

NA			
Pos.	257	obs:	exp:
tct	S	12	7160.00
tcc	S	1	4506.00
tca	S	38859	10125.00
tcg	S	34	2071.00
act	T	0	0.25
acc	T	0	0.21
aca	T	1	0.46
acg	T	0	0.09
agt	S	0	7316.00
agc	S	0	7728.00
---	--	------	------
mPD		0.0025	1.6
		nPD:	0.
		N. weight:	1.4
		Sc. PD:	-0.074
		Sc. rank:	-43598.0

,

NA			
Pos.	258	obs:	exp:
ttt	F	0	0.43
ttc	F	1	0.57
tct	S	0	0.18
tcc	S	1	0.12
tca	S	0	0.26
tcg	S	0	0.05
tat	Y	125	20214.00
tac	Y	38779	18690.00
cat	H	0	0.59
cac	H	1	0.41
agt	S	0	0.19
agc	S	0	0.20
---	--	------	------
mPD		0.0066	0.50
		nPD:	0.01
		N. weight:	0.73
		Sc. PD:	-0.031
		Sc. rank:	-4728.0

,

NA			
Pos.	259	obs:	exp:
cgt	R	0	0.68
cgc	R	0	1.31
cga	R	0	2.45
cgg	R	0	1.83
atg	M	1	1.00
aaa	K	27803	22179.00
aag	K	11078	16702.00
aga	R	21	12.55
agg	R	4	6.18
---	--	------	------
mPD		0.41	0.49
		nPD:	0.83
		N. weight:	0.046
		Sc. PD:	0.035
		Sc. rank:	1509.6

,

NA			
Pos.	260	obs:	exp:
tta	L	0	0.44
ttg	L	0	0.75
ctt	L	0	0.79
ctc	L	4	0.56
cta	L	0	0.77
ctg	L	0	0.68
att	I	95	14161.00
atc	I	38751	9710.00
ata	I	54	15029.00
gtt	V	0	0.66
gtc	V	3	0.55
gta	V	0	0.81
gtg	V	0	0.98
---	--	------	------
mPD		0.0080	0.66
		nPD:	0.01
		N. weight:	1.4
		Sc. PD:	-0.061
		Sc. rank:	-10685.0

,

NA			
Pos.	261	obs:	exp:
ttt	F	108	16685.00
ttc	F	38773	22196.00
tta	L	2	2.64
ttg	L	0	4.52
tat	Y	0	0.52
tac	Y	1	0.48
ctt	L	0	4.73
ctc	L	22	3.39
cta	L	0	4.62
ctg	L	0	4.10
att	I	0	0.36
atc	I	1	0.25
ata	I	0	0.39
---	--	------	------
mPD		0.0069	0.49
		nPD:	0.01
		N. weight:	0.56
		Sc. PD:	-0.023
		Sc. rank:	-3146.7

,

NA			
Pos.	262	obs:	exp:
cgt	R	0	1055.00
cgc	R	0	2022.00
cga	R	30	3785.00
cgg	R	0	2828.00
aaa	K	261	148.90
aag	K	0	112.10
aga	R	38561	19402.00
agg	R	55	9554.00
---	--	------	------
mPD		0.018	0.92
		nPD:	0.02
		N. weight:	0.69
		Sc. PD:	-0.025
		Sc. rank:	422.7

,

NA			
Pos.	263	obs:	exp:
att	I	5	14156.00
atc	I	2	9707.00
ata	I	38880	15024.00
atg	M	4	4.00
act	T	0	0.25
acc	T	0	0.21
aca	T	1	0.46
acg	T	0	0.09
gtt	V	0	3.32
gtc	V	0	2.73
gta	V	15	4.03
gtg	V	0	4.92
---	--	------	------
mPD		0.0014	0.66
		nPD:	0.
		N. weight:	0.98
		Sc. PD:	-0.052
		Sc. rank:	-29324.0

,

NA			
Pos.	264	obs:	exp:
aaa	K	8	4.56
aag	K	0	3.44
gtt	V	0	0.44
gtc	V	0	0.36
gta	V	2	0.54
gtg	V	0	0.66
gat	D	2	4.74
gac	D	7	4.26
gaa	E	38490	23544.00
gag	E	394	15340.00
ggt	G	0	0.61
ggc	G	0	0.59
gga	G	4	1.78
ggg	G	0	1.02
---	--	------	------
mPD		0.021	0.48
		nPD:	0.04
		N. weight:	0.46
		Sc. PD:	-0.0057
		Sc. rank:	7787.1

,

NA

Pos.	265	obs:	exp:
caa	Q	0	3.17
cag	Q	6	2.83
cgt	R	0	4.53
cgc	R	0	8.68
cga	R	0	16.26
cgg	R	0	12.15
act	T	0	0.25
acc	T	0	0.21
aca	T	0	0.46
acg	T	1	0.09
aat	N	3	1.77
aac	N	0	1.23
aaa	K	814	22074.00
aag	K	37883	16623.00
aga	R	2	83.34
agg	R	164	41.04
gaa	E	0	20.59
gag	E	34	13.41
---	--	------	------
mPD		0.052	0.50
		nPD:	0.1
		N. weight:	0.76
		Sc. PD:	0.034
		Sc. rank:	19103.0

,

NA

Pos.	266	obs:	exp:
ggt	G	6	5899.00
ggc	G	17	5763.00
gga	G	38613	17307.00
ggg	G	271	9938.00
---	--	------	------
mPD		0.015	0.69
		nPD:	0.02
		N. weight:	0.79
		Sc. PD:	-0.027
		Sc. rank:	2684.7

,

NA

Pos.	267	obs:	exp:
cgt	R	0	1.50
cgc	R	0	2.88
cga	R	0	5.39
cgg	R	0	4.03
act	T	0	0.25
acc	T	0	0.21
aca	T	0	0.46
acg	T	1	0.09
aaa	K	651	22162.00
aag	K	38200	16689.00
aga	R	2	27.61
agg	R	53	13.60
---	--	------	------
mPD		0.036	0.49
		nPD:	0.07
		N. weight:	0.78
		Sc. PD:	0.012
		Sc. rank:	17001.0

,

NA

Pos.	268	obs:	exp:
tta	L	1	0.22
ttg	L	0	0.38
ctt	L	0	0.39
ctc	L	0	0.28
cta	L	1	0.39
ctg	L	0	0.34
att	I	8	13972.00
atc	I	2	9581.00
ata	I	38371	14829.00
atg	M	13	13.00
act	T	0	1.26
acc	T	0	1.03
aca	T	5	2.29
acg	T	0	0.43
gtt	V	1	111.60
gtc	V	0	91.76
gta	V	504	135.80
gtg	V	0	165.80
gct	A	0	0.26
gcc	A	0	0.21
gca	A	1	0.47
gcg	A	0	0.06
---	--	------	------
mPD		0.027	0.69
		nPD:	0.04
		N. weight:	0.98
		Sc. PD:	-0.016
		Sc. rank:	15465.0

,

NA

Pos.	269	obs:	exp:
tta	L	0	0.11
ttg	L	0	0.19
tct	S	0	0.37
tcc	S	0	0.23
tca	S	0	0.52
tcg	S	0	0.11
ctt	L	0	0.20
ctc	L	1	0.14
cta	L	0	0.19
ctg	L	0	0.17
att	I	147	10214.00
atc	I	27683	7004.00
ata	I	228	10840.00
act	T	0	100.60
acc	T	399	81.84
aca	T	0	182.50
acg	T	0	34.04
agt	S	0	0.38
agc	S	2	0.40
gtt	V	38	2309.00
gtc	V	10405	1898.00
gta	V	2	2809.00
gtg	V	0	3429.00
gct	A	0	0.53
gcc	A	2	0.41
gca	A	0	0.94
gcg	A	0	0.12
---	--	------	------
mPD		0.43	1.1
		nPD:	0.39
		N. weight:	1.5
		Sc. PD:	0.46
		Sc. rank:	44091.0

,

NA

Pos.	270	obs:	exp:
cgt	R	0	0.30
cgc	R	0	0.58
cga	R	0	1.08
cgg	R	0	0.81
aat	N	2	1.77
aac	N	1	1.23
aaa	K	38842	22186.00
aag	K	51	16707.00
aga	R	11	5.52
agg	R	0	2.72
---	--	------	------
mPD		0.0033	0.49
		nPD:	0.01
		N. weight:	0.57
		Sc. PD:	-0.028
		Sc. rank:	-11677.0

,

NA

Pos.	271	obs:	exp:
tct	S	7	7159.00
tcc	S	6	4506.00
tca	S	38780	10124.00
tcg	S	109	2070.00
act	T	0	0.25
acc	T	0	0.21
aca	T	1	0.46
acg	T	0	0.09
agt	S	0	7315.00
agc	S	0	7727.00
gct	A	0	1.06
gcc	A	0	0.83
gca	A	4	1.88
gcg	A	0	0.23
---	--	------	------
mPD		0.0065	1.6
		nPD:	0.
		N. weight:	1.4
		Sc. PD:	-0.07
		Sc. rank:	-34738.0

,

NA

Pos.	272	obs:	exp:
att	I	4	21.48
atc	I	55	14.73
ata	I	0	22.79
gtt	V	1155	8570.00
gtc	V	37583	7044.00
gta	V	25	10425.00
gtg	V	0	12724.00
gct	A	78	22.50
gcc	A	7	17.59
gca	A	0	39.92
gcg	A	0	4.98
---	--	------	------
mPD		0.070	0.75
		nPD:	0.09
		N. weight:	1.6
		Sc. PD:	0.058
		Sc. rank:	38643.0

,

NA

Pos.	273	obs:	exp:
aaa	K	1	0.57
aag	K	0	0.43
gat	D	0	1.05
gac	D	2	0.95
gaa	E	38810	23556.00
gag	E	94	15348.00
---	--	------	------
mPD		0.0050	0.48
		nPD:	0.01
		N. weight:	0.5
		Sc. PD:	-0.023
		Sc. rank:	-5764.8

NA

Pos.	274	obs:	exp:
tta	L	0	1.32
ttg	L	4	2.26
ctt	L	0	2.36
ctc	L	0	1.69
cta	L	0	2.31
ctg	L	8	2.05
att	I	8	28.39
atc	I	0	19.47
ata	I	70	30.14
atg	M	38801	38801.00
gtt	V	0	3.54
gtc	V	0	2.91
gta	V	0	4.30
gtg	V	16	5.25
---	--	------	------
mPD		0.0054	0.0064
		nPD:	0.85
		N. weight:	0.0022
		Sc. PD:	0.0017
		Sc. rank:	71.7

NA

Pos.	275	obs:	exp:
tct	S	0	0.37
tcc	S	0	0.23
tca	S	0	0.52
tcg	S	0	0.11
cgt	R	0	0.74
cgc	R	0	1.41
cga	R	0	2.64
cgg	R	0	1.98
att	I	4	1.46
atc	I	0	1.00
ata	I	0	1.55
act	T	1	0.25
acc	T	0	0.21
aca	T	0	0.46
acg	T	0	0.09
aat	N	10490	6208.00
aac	N	7	4289.00
aaa	K	28015	16169.00
aag	K	331	12177.00
agt	S	2	0.38
agc	S	0	0.40
aga	R	27	13.55
agg	R	0	6.67
gat	D	12	6.32
gac	D	0	5.68
gaa	E	18	10.90
gag	E	0	7.10
---	--	------	------
mPD		0.41	0.69
		nPD:	0.59
		N. weight:	0.52
		Sc. PD:	0.27
		Sc. rank:	16812.0

NA

Pos.	276	obs:	exp:
tct	S	0	0.18
tcc	S	1	0.12
tca	S	0	0.26
tcg	S	0	0.05
act	T	0	0.50
acc	T	2	0.41
aca	T	0	0.91
acg	T	0	0.17
agt	S	0	0.19
agc	S	0	0.20
gct	A	89	10300.00
gcc	A	38760	8053.00
gca	A	55	18272.00
gcg	A	0	2279.00
---	--	------	------
mPD		0.0075	0.66
		nPD:	0.01
		N. weight:	1.6
		Sc. PD:	-0.07
		Sc. rank:	-14588.0

NA

Pos.	277	obs:	exp:
tta	L	0	4.62
ttg	L	0	7.91
tct	S	31	5.70
tcc	S	0	3.59
tca	S	0	8.07
tcg	S	0	1.65
ctt	L	42	8.28
ctc	L	0	5.93
cta	L	0	8.09
ctg	L	0	7.18
cct	P	38595	10693.00
ccc	P	210	7199.00
cca	P	8	16527.00
ccg	P	1	4395.00
cat	H	16	9.41
cac	H	0	6.59
act	T	4	1.01
acc	T	0	0.82
aca	T	0	1.83
acg	T	0	0.34
agt	S	0	5.83
agc	S	0	6.16
---	--	------	------
mPD		0.016	0.70
		nPD:	0.02
		N. weight:	1.3
		Sc. PD:	-0.043
		Sc. rank:	5735.3

NA

Pos.	278	obs:	exp:
tct	S	0	1.84
tcc	S	0	1.16
tca	S	0	2.60
tcg	S	0	0.53
cat	H	1	0.59
cac	H	0	0.41
act	T	1	0.25
acc	T	0	0.21
aca	T	0	0.46
acg	T	0	0.09
aat	N	38850	22996.00
aac	N	32	15886.00
aaa	K	1	1.71
aag	K	2	1.29
agt	S	10	1.88
agc	S	0	1.99
gat	D	8	4.22
gac	D	0	3.78
ggt	G	2	0.30
ggc	G	0	0.30
gga	G	0	0.89
ggg	G	0	0.51
---	--	------	------
mPD		0.0030	0.49
		nPD:	0.01
		N. weight:	0.54
		Sc. PD:	-0.026
		Sc. rank:	-11637.0

NA

Pos.	279	obs:	exp:
ttt	F	1	0.43
ttc	F	0	0.57
tat	Y	37763	20203.00
tac	Y	1120	18680.00
tgt	C	2	0.90
tgc	C	0	1.10
cat	H	21	12.35
cac	H	0	8.65
---	--	------	------
mPD		0.057	0.50
		nPD:	0.11
		N. weight:	0.54
		Sc. PD:	0.03
		Sc. rank:	13989.0

NA

Pos.	280	obs:	exp:
tat	Y	3	390.70
tac	Y	749	361.30
cat	H	181	22439.00
cac	H	37974	15716.00
---	--	------	------
mPD		0.047	0.52
		nPD:	0.09
		N. weight:	0.88
		Sc. PD:	0.028
		Sc. rank:	20503.0

NA

Pos.	281	obs:	exp:
ttt	F	6	2.57
ttc	F	0	3.43
tat	Y	38840	20211.00
tac	Y	59	18688.00
tgt	C	2	0.90
tgc	C	0	1.10
---	--	------	------
mPD		0.0034	0.50
		nPD:	0.01
		N. weight:	0.66
		Sc. PD:	-0.032
		Sc. rank:	-13559.0

NA

Pos.	282	obs:	exp:
gaa	E	2861	23558.00
gag	E	36046	15349.00
---	--	------	------
mPD		0.14	0.48
		nPD:	0.29
		N. weight:	0.66
		Sc. PD:	0.14
		Sc. rank:	18723.0

NA

Pos.	283	obs:	exp:
gaa	E	38766	23558.00
gag	E	141	15349.00
---	--	------	------
mPD		0.0072	0.48
		nPD:	0.02
		N. weight:	0.49
		Sc. PD:	-0.02
		Sc. rank:	-2073.1

NA

Pos.	284	obs:	exp:
tgt	C	95	17561.00
tgc	C	38812	21346.00
---	--	------	------
mPD		0.0049	0.50
		nPD:	0.01
		N. weight:	0.6
		Sc. PD:	-0.027
		Sc. rank:	-7664.7

NA

Pos.	285	obs:	exp:
tct	S	159	7160.00
tcc	S	38709	4506.00
tca	S	38	10126.00
tcg	S	1	2071.00
agt	S	0	7316.00
agc	S	0	7728.00
---	--	------	------
mPD		0.010	1.6
		nPD:	0.01
		N. weight:	2.2
		Sc. PD:	-0.11
		Sc. rank:	-47635.0

NA

Pos.	286	obs:	exp:
tgt	C	38851	17561.00
tgc	C	56	21346.00
---	--	------	------
mPD		0.0029	0.50
		nPD:	0.01
		N. weight:	0.81
		Sc. PD:	-0.04
		Sc. rank:	-18069.0

NA

Pos.	287	obs:	exp:
tat	Y	12035	20215.00
tac	Y	26871	18691.00
tgt	C	0	0.45
tgc	C	1	0.55
---	--	------	------
mPD		0.43	0.50
		nPD:	0.86
		N. weight:	0.093
		Sc. PD:	0.072
		Sc. rank:	3034.5

NA

Pos.	288	obs:	exp:
cct	P	37575	10719.00
ccc	P	1318	7216.00
cca	P	10	16567.00
ccg	P	4	4406.00
---	--	------	------
mPD		0.066	0.70
		nPD:	0.1
		N. weight:	1.2
		Sc. PD:	0.044
		Sc. rank:	28743.0

NA			
Pos.	289	obs:	exp:
tct	S	0	0.37
tcc	S	0	0.23
tca	S	0	0.52
tcg	S	0	0.11
tat	Y	2	1.04
tac	Y	0	0.96
aat	N	1	0.59
aac	N	0	0.41
agt	S	2	0.38
agc	S	0	0.40
gtt	V	1	0.22
gtc	V	0	0.18
gta	V	0	0.27
gtg	V	0	0.33
gct	A	3	0.79
gcc	A	0	0.62
gca	A	0	1.41
gcg	A	0	0.18
gat	D	37916	20476.00
gac	D	946	18386.00
gaa	E	2	20.59
gag	E	32	13.41
ggt	G	2	0.30
ggc	G	0	0.30
gga	G	0	0.89
ggg	G	0	0.51
---	--	------	------
mPD		0.050	0.50
		nPD:	0.1
		N. weight:	0.55
		Sc. PD:	0.022
		Sc. rank:	13349.0

,

NA			
Pos.	290	obs:	exp:
ttt	F	5	2.57
ttc	F	1	3.43
tct	S	38584	7157.00
tcc	S	297	4505.00
tca	S	11	10122.00
tcg	S	0	2070.00
tat	Y	3	1.56
tac	Y	0	1.44
act	T	4	1.01
acc	T	0	0.82
aca	T	0	1.83
acg	T	0	0.34
agt	S	0	7313.00
agc	S	0	7725.00
gct	A	2	0.53
gcc	A	0	0.41
gca	A	0	0.94
gcg	A	0	0.12
---	--	------	------
mPD		0.017	1.6
		nPD:	0.01
		N. weight:	1.7
		Sc. PD:	-0.077
		Sc. rank:	-20645.0

,

NA			
Pos.	291	obs:	exp:
tct	S	0	7094.00
tcc	S	0	4465.00
tca	S	0	10032.00
tcg	S	0	2052.00
aat	N	41	24.25
aac	N	0	16.75
agt	S	38295	7248.00
agc	S	252	7657.00
ggt	G	318	48.37
ggc	G	1	47.25
gga	G	0	141.90
ggg	G	0	81.48
---	--	------	------
mPD		0.031	1.7
		nPD:	0.02
		N. weight:	1.7
		Sc. PD:	-0.062
		Sc. rank:	864.9

,

NA			
Pos.	292	obs:	exp:
aaa	K	115	65.60
aag	K	0	49.40
gtt	V	0	0.44
gtc	V	0	0.36
gta	V	2	0.54
gtg	V	0	0.66
gat	D	1	1.58
gac	D	2	1.42
gaa	E	38689	23484.00
gag	E	95	15300.00
ggt	G	0	0.45
ggc	G	0	0.44
gga	G	3	1.33
ggg	G	0	0.77
---	--	------	------
mPD		0.011	0.48
		nPD:	0.02
		N. weight:	0.5
		Sc. PD:	-0.016
		Sc. rank:	2528.1

,

NA			
Pos.	293	obs:	exp:
att	I	49	14058.00
atc	I	38547	9640.00
ata	I	21	14920.00
act	T	0	0.76
acc	T	3	0.62
aca	T	0	1.37
acg	T	0	0.26
gtt	V	0	63.45
gtc	V	287	52.15
gta	V	0	77.19
gtg	V	0	94.21
---	--	------	------
mPD		0.018	0.67
		nPD:	0.03
		N. weight:	1.4
		Sc. PD:	-0.041
		Sc. rank:	11343.0

,

NA			
Pos.	294	obs:	exp:
att	I	0	29.49
atc	I	1	20.22
ata	I	80	31.29
act	T	19	9787.00
acc	T	319	7964.00
aca	T	38454	17761.00
acg	T	33	3313.00
gct	A	0	0.26
gcc	A	0	0.21
gca	A	1	0.47
gcg	A	0	0.06
---	--	------	------
mPD		0.023	0.68
		nPD:	0.03
		N. weight:	0.76
		Sc. PD:	-0.017
		Sc. rank:	9672.2

,

NA			
Pos.	295	obs:	exp:
tgt	C	38888	17561.00
tgc	C	19	21346.00
---	--	------	------
mPD		0.00098	0.50
		nPD:	0.
		N. weight:	0.82
		Sc. PD:	-0.043
		Sc. rank:	-25207.0

,

NA			
Pos.	296	obs:	exp:
gtt	V	396	8602.00
gtc	V	3	7070.00
gta	V	485	10464.00
gtg	V	38023	12772.00
---	--	------	------
mPD		0.045	0.74
		nPD:	0.06
		N. weight:	1.
		Sc. PD:	0.0035
		Sc. rank:	21117.0

,

NA			
Pos.	297	obs:	exp:
tgt	C	68	17561.00
tgc	C	38839	21346.00
---	--	------	------
mPD		0.0035	0.50
		nPD:	0.01
		N. weight:	0.61
		Sc. PD:	-0.029
		Sc. rank:	-11927.0

,

NA			
Pos.	298	obs:	exp:
cgt	R	0	1062.00
cgc	R	0	2035.00
cga	R	0	3811.00
cgg	R	4	2847.00
aga	R	144	19532.00
agg	R	38758	9618.00
ggt	G	0	0.15
ggc	G	0	0.15
gga	G	0	0.44
ggg	G	1	0.26
---	--	------	------
mPD		0.0076	0.91
		nPD:	0.01
		N. weight:	1.4
		Sc. PD:	-0.066
		Sc. rank:	-22602.0

,

NA			
Pos.	299	obs:	exp:
gat	D	38768	20500.00
gac	D	139	18407.00
---	--	------	------
mPD		0.0071	0.50
		nPD:	0.01
		N. weight:	0.64
		Sc. PD:	-0.026
		Sc. rank:	-3208.8

,

NA			
Pos.	300	obs:	exp:
tct	S	0	0.74
tcc	S	0	0.46
tca	S	0	1.04
tcg	S	0	0.21
cat	H	0	0.59
cac	H	1	0.41
aat	N	1052	23008.00
aac	N	37850	15894.00
agt	S	0	0.75
agc	S	4	0.79
---	--	------	------
mPD		0.053	0.48
		nPD:	0.11
		N. weight:	0.79
		Sc. PD:	0.04
		Sc. rank:	19810.0

,

NA			
Pos.	301	obs:	exp:
tgg	W	38907	38907.00
---	--	------	------
mPD		0	0
		nPD:	1.
		N. weight:	0.
		Sc. PD:	0
		Sc. rank:	0

,

NA			
Pos.	302	obs:	exp:
cat	H	38804	22881.00
cac	H	103	16026.00
---	--	------	------
mPD		0.0053	0.48
		nPD:	0.01
		N. weight:	0.53
		Sc. PD:	-0.024
		Sc. rank:	-5585.5

,

NA			
Pos.	303	obs:	exp:
tct	S	0	0.74
tcc	S	0	0.46
tca	S	0	1.04
tcg	S	0	0.21
agt	S	0	0.75
agc	S	4	0.79
gct	A	0	29.39
gcc	A	111	22.98
gca	A	0	52.13
gcg	A	0	6.50
ggt	G	129	5882.00
ggc	G	38580	5746.00
gga	G	76	17255.00
ggg	G	7	9909.00
---	--	------	------
mPD		0.017	0.70
		nPD:	0.02
		N. weight:	1.9
		Sc. PD:	-0.061
		Sc. rank:	11215.0

,

NA			
Pos.	304	obs:	exp:
tct	S	32	7156.00
tcc	S	0	4504.00
tca	S	862	10119.00
tcg	S	37989	2069.00
agt	S	0	7311.00
agc	S	0	7724.00
gct	A	0	6.35
gcc	A	0	4.97
gca	A	0	11.27
gcg	A	24	1.41
---	--	------	------
mPD		0.046	1.6
		nPD:	0.03
		N. weight:	2.9
		Sc. PD:	-0.08
		Sc. rank:	29089.0

,

NA			
Pos.	305	obs:	exp:
aat	N	38836	23011.00
aac	N	71	15896.00
---	--	------	------
mPD		0.0036	0.48
		nPD:	0.01
		N. weight:	0.53
		Sc. PD:	-0.025
		Sc. rank:	-9335.5

,

NA			
Pos.	306	obs:	exp:
cgt	R	1	1062.00
cgc	R	1	2035.00
cga	R	38773	3811.00
cgg	R	67	2847.00
aga	R	65	19533.00
agg	R	0	9619.00
---	--	------	------
mPD		0.0069	0.91
		nPD:	0.01
		N. weight:	2.4
		Sc. PD:	-0.11
		Sc. rank:	-41045.0

,

NA			
Pos.	307	obs:	exp:
cct	P	423	10719.00
ccc	P	3	7216.00
cca	P	1293	16567.00
ccg	P	37188	4406.00
---	--	------	------
mPD		0.085	0.70
		nPD:	0.12
		N. weight:	2.
		Sc. PD:	0.13
		Sc. rank:	51871.0

,

NA			
Pos.	308	obs:	exp:
tgg	W	38906	38906.00
ggt	G	0	0.15
ggc	G	0	0.15
gga	G	0	0.44
ggg	G	1	0.26
---	--	------	------
mPD		0.000051	0.000090
		nPD:	0.57
		N. weight:	0.000036
		Sc. PD:	0.000018
		Sc. rank:	1.2

,

NA			
Pos.	309	obs:	exp:
tta	L	0	0.11
ttg	L	1	0.19
ctt	L	0	0.20
ctc	L	0	0.14
cta	L	0	0.19
ctg	L	0	0.17
att	I	0	0.73
atc	I	0	0.50
ata	I	2	0.77
gtt	V	82	8601.00
gtc	V	7	7069.00
gta	V	330	10463.00
gtg	V	38485	12771.00
---	--	------	------
mPD		0.022	0.74
		nPD:	0.03
		N. weight:	1.1
		Sc. PD:	-0.029
		Sc. rank:	11570.0

,

NA			
Pos.	310	obs:	exp:
tct	S	38811	7156.00
tcc	S	49	4504.00
tca	S	7	10119.00
tcg	S	16	2069.00
tat	Y	1	0.52
tac	Y	0	0.48
act	T	20	5.04
acc	T	0	4.10
aca	T	0	9.15
acg	T	0	1.71
agt	S	0	7311.00
agc	S	0	7724.00
gtt	V	1	0.22
gtc	V	0	0.18
gta	V	0	0.27
gtg	V	0	0.33
gct	A	2	0.53
gcc	A	0	0.41
gca	A	0	0.94
gcg	A	0	0.12
---	--	------	------
mPD		0.0050	1.6
		nPD:	0.
		N. weight:	1.7
		Sc. PD:	-0.09
		Sc. rank:	-48928.0

,

NA			
Pos.	311	obs:	exp:
ttt	F	123	16696.00
ttc	F	38783	22210.00
att	I	0	0.36
atc	I	1	0.25
ata	I	0	0.39
---	--	------	------
mPD		0.0064	0.49
		nPD:	0.01
		N. weight:	0.56
		Sc. PD:	-0.024
		Sc. rank:	-3707.3

,

NA			
Pos.	312	obs:	exp:
tct	S	0	0.37
tcc	S	0	0.23
tca	S	0	0.52
tcg	S	0	0.11
act	T	0	0.50
acc	T	2	0.41
aca	T	0	0.91
acg	T	0	0.17
aat	N	827	23004.00
aac	N	38068	15891.00
aaa	K	0	0.57
aag	K	1	0.43
agt	S	0	0.38
agc	S	2	0.40
gat	D	0	3.69
gac	D	7	3.31
---	--	------	------
mPD		0.042	0.48
		nPD:	0.09
		N. weight:	0.81
		Sc. PD:	0.024
		Sc. rank:	18693.0

,

NA			
Pos.	313	obs:	exp:
tta	L	0	0.99
ttg	L	0	1.69
ctt	L	0	1.77
ctc	L	0	1.27
cta	L	0	1.73
ctg	L	9	1.54
cct	P	0	1.65
ccc	P	0	1.11
cca	P	0	2.55
ccg	P	6	0.68
cat	H	43	25.29
cac	H	0	17.71
caa	Q	295	20480.00
cag	Q	38518	18333.00
cgt	R	0	0.82
cgc	R	0	1.57
cga	R	0	2.94
cgg	R	30	2.20
aaa	K	0	3.42
aag	K	6	2.58
aga	R	0	15.06
agg	R	0	7.42
---	--	------	------
mPD		0.020	0.50
		nPD:	0.04
		N. weight:	0.73
		Sc. PD:	-0.012
		Sc. rank:	11312.0

,

NA			
Pos.	314	obs:	exp:
tct	S	0	0.37
tcc	S	0	0.23
tca	S	0	0.52
tcg	S	0	0.11
cat	H	1	0.59
cac	H	0	0.41
aat	N	38648	22955.00
aac	N	164	15857.00
agt	S	2	0.38
agc	S	0	0.40
gat	D	92	48.47
gac	D	0	43.53
---	--	------	------
mPD		0.013	0.49
		nPD:	0.03
		N. weight:	0.52
		Sc. PD:	-0.015
		Sc. rank:	4023.7

,

NA			
Pos.	315	obs:	exp:
tta	L	0	4277.00
ttg	L	66	7325.00
ctt	L	25	7667.00
ctc	L	0	5495.00
cta	L	73	7490.00
ctg	L	38742	6652.00
cct	P	0	0.28
ccc	P	0	0.19
cca	P	0	0.43
ccg	P	1	0.11
---	--	------	------
mPD		0.0085	1.1
		nPD:	0.01
		N. weight:	1.8
		Sc. PD:	-0.086
		Sc. rank:	-32553.0

,

NA			
Pos.	316	obs:	exp:
caa	Q	1	0.53
cag	Q	0	0.47
aaa	K	11	6.27
aag	K	0	4.73
gat	D	2	7.38
gac	D	12	6.62
gaa	E	38822	23542.00
gag	E	58	15338.00
ggt	G	0	0.15
ggc	G	0	0.15
gga	G	1	0.44
ggg	G	0	0.26
---	--	------	------
mPD		0.0044	0.48
		nPD:	0.01
		N. weight:	0.51
		Sc. PD:	-0.023
		Sc. rank:	-7191.6

,

NA			
Pos.	317	obs:	exp:
tat	Y	35499	20216.00
tac	Y	3408	18691.00
---	--	------	------
mPD		0.16	0.50
		nPD:	0.32
		N. weight:	0.38
		Sc. PD:	0.096
		Sc. rank:	10830.0

,

NA			
Pos.	318	obs:	exp:
tta	L	0	0.22
ttg	L	0	0.38
ctt	L	0	0.39
ctc	L	0	0.28
cta	L	0	0.39
ctg	L	2	0.34
cat	H	63	37.05
cac	H	0	25.95
caa	Q	374	20232.00
cag	Q	37969	18111.00
cgt	R	0	8.41
cgc	R	0	16.11
cga	R	1	30.17
cgg	R	307	22.54
aaa	K	1	109.00
aag	K	190	82.05
aga	R	0	154.60
agg	R	0	76.14
---	--	------	------
mPD		0.048	0.54
		nPD:	0.09
		N. weight:	0.73
		Sc. PD:	0.023
		Sc. rank:	16982.0

,

NA			
Pos.	319	obs:	exp:
tta	L	9	4.07
ttg	L	0	6.97
ctt	L	0	7.29
ctc	L	0	5.23
cta	L	0	7.12
ctg	L	28	6.33
att	I	46	4399.00
atc	I	3	3016.00
ata	I	12035	4669.00
atg	M	26751	26751.00
act	T	0	3.53
acc	T	0	2.87
aca	T	2	6.40
acg	T	12	1.19
aaa	K	3	2.85
aag	K	2	2.15
gtt	V	0	3.54
gtc	V	0	2.91
gta	V	5	4.30
gtg	V	11	5.25
---	--	------	------
mPD		0.43	0.50
		nPD:	0.87
		N. weight:	0.3
		Sc. PD:	0.23
		Sc. rank:	9721.4

,

NA			
Pos.	320	obs:	exp:
ggt	G	7	5899.00
ggc	G	4	5763.00
gga	G	38393	17307.00
ggg	G	503	9938.00
---	--	------	------
mPD		0.026	0.69
		nPD:	0.04
		N. weight:	0.77
		Sc. PD:	-0.014
		Sc. rank:	11070.0

,

NA			
Pos.	321	obs:	exp:
tat	Y	43	20216.00
tac	Y	38864	18691.00
---	--	------	------
mPD		0.0022	0.50
		nPD:	0.
		N. weight:	0.75
		Sc. PD:	-0.038
		Sc. rank:	-18312.0

,

NA			
Pos.	322	obs:	exp:
tta	L	1	0.11
ttg	L	0	0.19
ctt	L	0	0.20
ctc	L	0	0.14
cta	L	0	0.19
ctg	L	0	0.17
att	I	4	14160.00
atc	I	6	9710.00
ata	I	38889	15029.00
atg	M	1	1.00
gtt	V	0	1.33
gtc	V	0	1.09
gta	V	6	1.61
gtg	V	0	1.97
---	--	------	------
mPD		0.00093	0.66
		nPD:	0.
		N. weight:	0.98
		Sc. PD:	-0.053
		Sc. rank:	-31105.0

,

NA			
Pos.	323	obs:	exp:
tgt	C	285	17561.00
tgc	C	38621	21345.00
cgt	R	0	0.03
cgc	R	1	0.05
cga	R	0	0.10
cgg	R	0	0.07
aga	R	0	0.50
agg	R	0	0.25
---	--	------	------
mPD		0.015	0.50
		nPD:	0.03
		N. weight:	0.58
		Sc. PD:	-0.015
		Sc. rank:	6193.9

,

NA			
Pos.	324	obs:	exp:
tct	S	0	7160.00
tcc	S	0	4506.00
tca	S	0	10125.00
tcg	S	0	2071.00
cgt	R	0	0.03
cgc	R	0	0.05
cga	R	0	0.10
cgg	R	0	0.07
agt	S	38863	7316.00
agc	S	43	7728.00
aga	R	0	0.50
agg	R	1	0.25
---	--	------	------
mPD		0.0023	1.6
		nPD:	0.
		N. weight:	1.7
		Sc. PD:	-0.092
		Sc. rank:	-55131.0

,

NA			
Pos.	325	obs:	exp:
ggt	G	21	5899.00
ggc	G	0	5763.00
gga	G	474	17307.00
ggg	G	38412	9938.00
---	--	------	------
mPD		0.025	0.69
		nPD:	0.04
		N. weight:	1.3
		Sc. PD:	-0.027
		Sc. rank:	18355.0

,

NA			
Pos.	326	obs:	exp:
tta	L	0	0.11
ttg	L	0	0.19
ctt	L	1	0.20
ctc	L	0	0.14
cta	L	0	0.19
ctg	L	0	0.17
att	I	8128	2965.00
atc	I	16	2033.00
ata	I	2	3147.00
act	T	2	0.50
acc	T	0	0.41
aca	T	0	0.91
acg	T	0	0.17
gtt	V	30684	6799.00
gtc	V	56	5589.00
gta	V	1	8271.00
gtg	V	14	10096.00
gct	A	3	0.79
gcc	A	0	0.62
gca	A	0	1.41
gcg	A	0	0.18
---	--	------	------
mPD		0.34	1.1
		nPD:	0.31
		N. weight:	1.4
		Sc. PD:	0.35
		Sc. rank:	41134.0

,

NA			
Pos.	327	obs:	exp:
ttt	F	110	16513.00
ttc	F	38370	21967.00
tta	L	12	46.28
ttg	L	1	79.26
tct	S	0	0.18
tcc	S	1	0.12
tca	S	0	0.26
tcg	S	0	0.05
tat	Y	0	2.60
tac	Y	5	2.40
ctt	L	2	82.96
ctc	L	406	59.47
cta	L	0	81.05
ctg	L	0	71.98
agt	S	0	0.19
agc	S	0	0.20
---	--	------	------
mPD		0.027	0.51
		nPD:	0.05
		N. weight:	0.57
		Sc. PD:	-0.0019
		Sc. rank:	10851.0

,

NA			
Pos.	328	obs:	exp:
gaa	E	1	0.61
gag	E	0	0.39
ggt	G	6	5899.00
ggc	G	2	5762.00
gga	G	38834	17306.00
ggg	G	64	9938.00
---	--	------	------
mPD		0.0037	0.69
		nPD:	0.01
		N. weight:	0.82
		Sc. PD:	-0.041
		Sc. rank:	-19011.0

,

NA

Pos.	329	obs:	exp:
aaa	K	1	0.57
aag	K	0	0.43
gat	D	202	20500.00
gac	D	38704	18406.00
---	--	------	------
mPD		0.010	0.50
		nPD:	0.02
		N. weight:	0.74
		Sc. PD:	-0.026
		Sc. rank:	1841.5

,

NA

Pos.	330	obs:	exp:
tct	S	0	2.02
tcc	S	0	1.27
tca	S	0	2.86
tcg	S	0	0.59
att	I	1	0.36
atc	I	0	0.25
ata	I	0	0.39
act	T	4	1.01
acc	T	0	0.82
aca	T	0	1.83
acg	T	0	0.34
aat	N	38764	23000.00
aac	N	125	15889.00
aaa	K	1	0.57
aag	K	0	0.43
agt	S	11	2.07
agc	S	0	2.18
gat	D	1	0.53
gac	D	0	0.47
---	--	------	------
mPD		0.0073	0.48
		nPD:	0.02
		N. weight:	0.52
		Sc. PD:	-0.021
		Sc. rank:	-2148.1

,

NA

Pos.	331	obs:	exp:
cct	P	8	10719.00
ccc	P	4	7216.00
cca	P	38813	16567.00
ccg	P	82	4406.00
---	--	------	------
mPD		0.0048	0.70
		nPD:	0.01
		N. weight:	0.87
		Sc. PD:	-0.042
		Sc. rank:	-17570.0

,

NA

Pos.	332	obs:	exp:
cgt	R	81	1062.00
cgc	R	38782	2035.00
cga	R	36	3811.00
cgg	R	8	2847.00
aga	R	0	19533.00
agg	R	0	9619.00
---	--	------	------
mPD		0.0064	0.91
		nPD:	0.01
		N. weight:	3.
		Sc. PD:	-0.15
		Sc. rank:	-58148.0

,

NA

Pos.	333	obs:	exp:
tct	S	54	9.94
tcc	S	0	6.25
tca	S	0	14.05
tcg	S	0	2.87
cct	P	38757	10703.00
ccc	P	90	7206.00
cca	P	4	16543.00
ccg	P	0	4399.00
act	T	2	0.50
acc	T	0	0.41
aca	T	0	0.91
acg	T	0	0.17
agt	S	0	10.15
agc	S	0	10.73
---	--	------	------
mPD		0.0077	0.70
		nPD:	0.01
		N. weight:	1.3
		Sc. PD:	-0.058
		Sc. rank:	-13312.0

,

NA

Pos.	334	obs:	exp:
tct	S	0	12.33
tcc	S	0	7.76
tca	S	0	17.44
tcg	S	0	3.57
tat	Y	1	0.52
tac	Y	0	0.48
cat	H	9	5.29
cac	H	0	3.71
att	I	22	8.01
atc	I	0	5.49
ata	I	0	8.50
act	T	7	1.76
acc	T	0	1.44
aca	T	0	3.20
acg	T	0	0.60
aat	N	38751	22935.00
aac	N	27	15843.00
aaa	K	2	1.71
aag	K	1	1.29
agt	S	67	12.60
agc	S	0	13.31
gat	D	20	10.54
gac	D	0	9.46
---	--	------	------
mPD		0.0080	0.49
		nPD:	0.02
		N. weight:	0.54
		Sc. PD:	-0.021
		Sc. rank:	-1713.2

,

NA

Pos.	335	obs:	exp:
gat	D	38273	20500.00
gac	D	634	18407.00
---	--	------	------
mPD		0.032	0.50
		nPD:	0.06
		N. weight:	0.58
		Sc. PD:	0.0041
		Sc. rank:	12117.0

,

NA

Pos.	336	obs:	exp:
cgt	R	0	3.30
cgc	R	0	6.33
cga	R	0	11.85
cgg	R	0	8.86
atg	M	1	1.00
aat	N	25	14.79
aac	N	0	10.21
aaa	K	378	22102.00
aag	K	38369	16645.00
aga	R	0	60.75
agg	R	121	29.91
gaa	E	0	6.66
gag	E	11	4.34
ggt	G	0	0.30
ggc	G	0	0.30
gga	G	0	0.89
ggg	G	2	0.51
---	--	------	------
mPD		0.028	0.50
		nPD:	0.06
		N. weight:	0.81
		Sc. PD:	-0.0015
		Sc. rank:	15505.0

,

NA

Pos.	337	obs:	exp:
tct	S	0	0.74
tcc	S	0	0.46
tca	S	4	1.04
tcg	S	0	0.21
att	I	0	10.56
atc	I	0	7.24
ata	I	29	11.20
act	T	7	9763.00
acc	T	6	7944.00
aca	T	38570	17718.00
acg	T	147	3305.00
aaa	K	106	60.47
aag	K	0	45.53
agt	S	0	0.75
agc	S	0	0.79
gct	A	0	10.06
gcc	A	0	7.87
gca	A	38	17.85
gcg	A	0	2.23
---	--	------	------
mPD		0.017	0.69
		nPD:	0.03
		N. weight:	0.78
		Sc. PD:	-0.024
		Sc. rank:	5013.0

,

NA

Pos.	338	obs:	exp:
ggt	G	72	5899.00
ggc	G	38812	5763.00
gga	G	22	17307.00
ggg	G	1	9938.00
---	--	------	------
mPD		0.0049	0.69
		nPD:	0.01
		N. weight:	2.
		Sc. PD:	-0.094
		Sc. rank:	-38520.0

,

NA

Pos.	339	obs:	exp:
tct	S	0	7152.00
tcc	S	0	4501.00
tca	S	0	10114.00
tcg	S	0	2068.00
cgt	R	2	0.08
cgc	R	0	0.16
cga	R	0	0.29
cgg	R	0	0.22
att	I	1	0.36
atc	I	0	0.25
ata	I	0	0.39
aat	N	40	23.66
aac	N	0	16.34
agt	S	38631	7308.00
agc	S	232	7720.00
aga	R	0	1.51
agg	R	1	0.74
---	--	------	------
mPD		0.014	1.6
		nPD:	0.01
		N. weight:	1.7
		Sc. PD:	-0.079
		Sc. rank:	-25619.0

,

NA

Pos.	340	obs:	exp:
tct	S	0	0.18
tcc	S	0	0.12
tca	S	0	0.26
tcg	S	0	0.05
tgt	C	38064	17561.00
tgc	C	842	21345.00
agt	S	1	0.19
agc	S	0	0.20
---	--	------	------
mPD		0.042	0.50
		nPD:	0.09
		N. weight:	0.71
		Sc. PD:	0.02
		Sc. rank:	16103.0

NA

Pos.	341	obs:	exp:
tct	S	0	3.13
tcc	S	0	1.97
tca	S	0	4.42
tcg	S	0	0.90
agt	S	17	3.20
agc	S	0	3.38
gat	D	66	34.78
gac	D	0	31.22
ggt	G	38660	5887.00
ggc	G	159	5750.00
gga	G	5	17270.00
ggg	G	0	9917.00
---	--	------	------
mPD		0.013	0.70
		nPD:	0.02
		N. weight:	1.9
		Sc. PD:	-0.072
		Sc. rank:	-1222.5

,

NA

Pos.	342	obs:	exp:
cct	P	43	10719.00
ccc	P	4	7216.00
cca	P	38825	16567.00
ccg	P	35	4406.00
---	--	------	------
mPD		0.0042	0.70
		nPD:	0.01
		N. weight:	0.87
		Sc. PD:	-0.043
		Sc. rank:	-19119.0

,

NA

Pos.	343	obs:	exp:
tta	L	6	0.77
ttg	L	1	1.32
ctt	L	0	1.38
ctc	L	0	0.99
cta	L	0	1.35
ctg	L	0	1.20
att	I	0	7.64
atc	I	0	5.24
ata	I	21	8.11
gtt	V	17	8593.00
gtc	V	1	7063.00
gta	V	38702	10453.00
gtg	V	148	12759.00
gct	A	0	2.38
gcc	A	0	1.86
gca	A	9	4.23
gcg	A	0	0.53
gaa	E	2	1.21
gag	E	0	0.79
---	--	------	------
mPD		0.011	0.74
		nPD:	0.01
		N. weight:	1.3
		Sc. PD:	-0.055
		Sc. rank:	-6783.7

,

NA

Pos.	344	obs:	exp:
tta	L	0	26.27
ttg	L	238	45.00
tct	S	45	7085.00
tcc	S	0	4459.00
tca	S	184	10020.00
tcg	S	38272	2049.00
tat	Y	0	0.52
tac	Y	1	0.48
ctt	L	0	47.10
ctc	L	0	33.76
cta	L	0	46.01
ctg	L	1	40.86
cct	P	0	37.19
ccc	P	0	25.04
cca	P	0	57.48
ccg	P	135	15.29
act	T	0	3.53
acc	T	0	2.87
aca	T	0	6.40
acg	T	14	1.19
agt	S	0	7239.00
agc	S	0	7648.00
gct	A	0	4.50
gcc	A	0	3.52
gca	A	0	7.98
gcg	A	17	1.00
---	--	------	------
mPD		0.033	1.7
		nPD:	0.02
		N. weight:	3.
		Sc. PD:	-0.11
		Sc. rank:	5137.4

,

NA

Pos.	345	obs:	exp:
ttt	F	83	35.62
ttc	F	0	47.38
tct	S	38588	7136.00
tcc	S	175	4491.00
tca	S	3	10091.00
tcg	S	9	2064.00
tat	Y	9	4.68
tac	Y	0	4.32
tgt	C	2	0.90
tgc	C	0	1.10
cct	P	25	7.16
ccc	P	1	4.82
cca	P	0	11.07
ccg	P	0	2.94
act	T	7	1.76
acc	T	0	1.44
aca	T	0	3.20
acg	T	0	0.60
agt	S	0	7291.00
agc	S	0	7702.00
gct	A	5	1.32
gcc	A	0	1.03
gca	A	0	2.35
gcg	A	0	0.29
---	--	------	------
mPD		0.016	1.6
		nPD:	0.01
		N. weight:	1.7
		Sc. PD:	-0.078
		Sc. rank:	-21197.0

,

NA

Pos.	346	obs:	exp:
tct	S	0	13.80
tcc	S	0	8.69
tca	S	0	19.52
tcg	S	0	3.99
tat	Y	5	2.60
tac	Y	0	2.40
att	I	1	0.36
atc	I	0	0.25
ata	I	0	0.39
aat	N	38736	22953.00
aac	N	73	15856.00
aaa	K	2	2.85
aag	K	3	2.15
agt	S	75	14.10
agc	S	0	14.90
gat	D	12	6.32
gac	D	0	5.68
---	--	------	------
mPD		0.0088	0.49
		nPD:	0.02
		N. weight:	0.53
		Sc. PD:	-0.02
		Sc. rank:	-477.2

,

NA

Pos.	347	obs:	exp:
gaa	E	1	0.61
gag	E	0	0.39
ggt	G	63	5899.00
ggc	G	12	5762.00
gga	G	38716	17306.00
ggg	G	115	9938.00
---	--	------	------
mPD		0.0098	0.69
		nPD:	0.01
		N. weight:	0.8
		Sc. PD:	-0.033
		Sc. rank:	-4332.6

,

NA

Pos.	348	obs:	exp:
tta	L	1	0.11
ttg	L	0	0.19
tct	S	0	1.84
tcc	S	0	1.16
tca	S	10	2.60
tcg	S	0	0.53
ctt	L	0	0.20
ctc	L	0	0.14
cta	L	0	0.19
ctg	L	0	0.17
cct	P	0	0.28
ccc	P	0	0.19
cca	P	1	0.43
ccg	P	0	0.11
act	T	0	1.76
acc	T	0	1.44
aca	T	7	3.20
acg	T	0	0.60
agt	S	0	1.88
agc	S	0	1.99
gtt	V	0	1.33
gtc	V	0	1.09
gta	V	6	1.61
gtg	V	0	1.97
gct	A	47	10289.00
gcc	A	7	8045.00
gca	A	38784	18253.00
gcg	A	26	2277.00
gaa	E	13	7.87
gag	E	0	5.13
ggt	G	0	0.76
ggc	G	0	0.74
gga	G	5	2.22
ggg	G	0	1.28
---	--	------	------
mPD		0.0064	0.67
		nPD:	0.01
		N. weight:	0.77
		Sc. PD:	-0.035
		Sc. rank:	-10146.0

,

NA

Pos.	349	obs:	exp:
tct	S	0	0.37
tcc	S	0	0.23
tca	S	0	0.52
tcg	S	0	0.11
tat	Y	1	0.52
tac	Y	0	0.48
cat	H	1	0.59
cac	H	0	0.41
act	T	1	0.25
acc	T	0	0.21
aca	T	0	0.46
acg	T	0	0.09
aat	N	38843	22994.00
aac	N	35	15884.00
aaa	K	5	3.42
aag	K	1	2.58
agt	S	2	0.38
agc	S	0	0.40
gat	D	18	9.48
gac	D	0	8.52
---	--	------	------
mPD		0.0033	0.48
		nPD:	0.01
		N. weight:	0.53
		Sc. PD:	-0.026
		Sc. rank:	-11013.0

,

NA

Pos.	350	obs:	exp:
cgt	R	0	0.03
cgc	R	0	0.05
cga	R	0	0.10
cgg	R	0	0.07
aga	R	1	0.50
agg	R	0	0.25
ggt	G	4	5899.00
ggc	G	0	5762.00
gga	G	27852	17306.00
ggg	G	11050	9938.00
---	--	------	------
mPD		0.41	0.69
		nPD:	0.59
		N. weight:	0.38
		Sc. PD:	0.2
		Sc. rank:	12258.0

,

NA

Pos.	351	obs:	exp:
tta	L	1	0.22
ttg	L	0	0.38
ctt	L	0	0.39
ctc	L	0	0.28
cta	L	1	0.39
ctg	L	0	0.34
att	I	0	11.28
atc	I	0	7.74
ata	I	31	11.98
gtt	V	15	8594.00
gtc	V	1	7064.00
gta	V	38584	10455.00
gtg	V	273	12761.00
gct	A	0	0.26
gcc	A	0	0.21
gca	A	1	0.47
gcg	A	0	0.06
---	--	------	------
mPD		0.016	0.74
		nPD:	0.02
		N. weight:	1.3
		Sc. PD:	-0.044
		Sc. rank:	4659.3

,

NA

Pos.	352	obs:	exp:
aaa	K	37755	22194.00
aag	K	1152	16713.00
---	--	------	------
mPD		0.057	0.49
		nPD:	0.12
		N. weight:	0.45
		Sc. PD:	0.026
		Sc. rank:	11624.0

,

NA

Pos.	353	obs:	exp:
ggt	G	7	5899.00
ggc	G	1	5763.00
gga	G	38723	17307.00
ggg	G	176	9938.00
---	--	------	------
mPD		0.0094	0.69
		nPD:	0.01
		N. weight:	0.81
		Sc. PD:	-0.034
		Sc. rank:	-4646.8

,

NA

Pos.	354	obs:	exp:
ttt	F	38799	16696.00
ttc	F	108	22211.00
---	--	------	------
mPD		0.0055	0.49
		nPD:	0.01
		N. weight:	0.85
		Sc. PD:	-0.038
		Sc. rank:	-7977.7

,

NA

Pos.	355	obs:	exp:
tct	S	6	7150.00
tcc	S	14	4500.00
tca	S	38779	10112.00
tcg	S	54	2068.00
agt	S	0	7306.00
agc	S	0	7718.00
gct	A	0	14.30
gcc	A	0	11.18
gca	A	54	25.36
gcg	A	0	3.16
---	--	------	------
mPD		0.0066	1.6
		nPD:	0.
		N. weight:	1.4
		Sc. PD:	-0.07
		Sc. rank:	-34597.0

,

NA

Pos.	356	obs:	exp:
ttt	F	1394	16659.00
ttc	F	37427	22162.00
tat	Y	0	44.68
tac	Y	86	41.32
---	--	------	------
mPD		0.074	0.49
		nPD:	0.15
		N. weight:	0.43
		Sc. PD:	0.038
		Sc. rank:	11494.0

,

NA

Pos.	357	obs:	exp:
caa	Q	2	1.06
cag	Q	0	0.94
cgt	R	0	1.31
cgc	R	0	2.51
cga	R	0	4.70
cgg	R	0	3.51
aaa	K	38805	22165.00
aag	K	52	16692.00
aga	R	48	24.10
agg	R	0	11.87
---	--	------	------
mPD		0.0052	0.49
		nPD:	0.01
		N. weight:	0.57
		Sc. PD:	-0.025
		Sc. rank:	-6147.3

,

NA

Pos.	358	obs:	exp:
ttt	F	0	0.86
ttc	F	2	1.14
tat	Y	318	20214.00
tac	Y	38586	18690.00
tgt	C	0	0.45
tgc	C	1	0.55
---	--	------	------
mPD		0.016	0.50
		nPD:	0.03
		N. weight:	0.71
		Sc. PD:	-0.017
		Sc. rank:	8332.9

,

NA

Pos.	359	obs:	exp:
tct	S	0	0.55
tcc	S	0	0.35
tca	S	0	0.78
tcg	S	0	0.16
aat	N	0	0.59
aac	N	1	0.41
agt	S	0	0.56
agc	S	3	0.60
gat	D	0	16.86
gac	D	32	15.14
ggt	G	109	5894.00
ggc	G	38742	5757.00
gga	G	17	17291.00
ggg	G	3	9929.00
---	--	------	------
mPD		0.0085	0.69
		nPD:	0.01
		N. weight:	1.9
		Sc. PD:	-0.084
		Sc. rank:	-14303.0

,

NA

Pos.	360	obs:	exp:
tct	S	0	4.05
tcc	S	0	2.55
tca	S	0	5.73
tcg	S	0	1.17
tat	Y	1	0.52
tac	Y	0	0.48
cat	H	3	1.76
cac	H	0	1.24
act	T	4	1.01
acc	T	0	0.82
aca	T	0	1.83
acg	T	0	0.34
aat	N	38825	22973.00
aac	N	17	15869.00
aaa	K	1	3.42
aag	K	5	2.58
agt	S	22	4.14
agc	S	0	4.37
gat	D	29	15.28
gac	D	0	13.72
---	--	------	------
mPD		0.0042	0.49
		nPD:	0.01
		N. weight:	0.54
		Sc. PD:	-0.025
		Sc. rank:	-8155.0

,

NA

Pos.	361	obs:	exp:
tct	S	0	0.37
tcc	S	0	0.23
tca	S	0	0.52
tcg	S	0	0.11
agt	S	2	0.38
agc	S	0	0.40
gat	D	1	0.53
gac	D	0	0.47
ggt	G	38864	5899.00
ggc	G	30	5762.00
gga	G	6	17305.00
ggg	G	4	9938.00
---	--	------	------
mPD		0.0022	0.69
		nPD:	0.
		N. weight:	1.9
		Sc. PD:	-0.1
		Sc. rank:	-53773.0

NA

Pos.	362	obs:	exp:
att	I	24	8.74
atc	I	0	5.99
ata	I	0	9.27
gtt	V	38827	8594.00
gtc	V	38	7063.00
gta	V	2	10454.00
gtg	V	3	12760.00
gct	A	12	3.44
gcc	A	1	2.69
gca	A	0	6.11
gcg	A	0	0.76
---	--	------	------
mPD		0.0042	0.74
		nPD:	0.01
		N. weight:	1.5
		Sc. PD:	-0.077
		Sc. rank:	-34722.0

NA

Pos.	363	obs:	exp:
tgg	W	38905	38905.00
cgt	R	0	0.05
cgc	R	0	0.10
cga	R	0	0.20
cgg	R	1	0.15
aga	R	0	1.00
agg	R	1	0.49
---	--	------	------
mPD		0.00010	0.00017
		nPD:	0.6
		N. weight:	0.000088
		Sc. PD:	0.000046
		Sc. rank:	2.8

NA

Pos.	364	obs:	exp:
tta	L	5	0.55
ttg	L	0	0.94
ctt	L	0	0.99
ctc	L	0	0.71
cta	L	0	0.96
ctg	L	0	0.85
att	I	8	14151.00
atc	I	18	9703.00
ata	I	38846	15018.00
atg	M	12	12.00
act	T	0	0.25
acc	T	0	0.21
aca	T	1	0.46
acg	T	0	0.09
aaa	K	1	0.57
aag	K	0	0.43
gtt	V	0	3.54
gtc	V	0	2.91
gta	V	16	4.30
gtg	V	0	5.25
---	--	------	------
mPD		0.0031	0.66
		nPD:	0.
		N. weight:	0.98
		Sc. PD:	-0.049
		Sc. rank:	-23169.0

NA

Pos.	365	obs:	exp:
ggt	G	1	5899.00
ggc	G	1	5763.00
gga	G	375	17307.00
ggg	G	38530	9938.00
---	--	------	------
mPD		0.019	0.69
		nPD:	0.03
		N. weight:	1.3
		Sc. PD:	-0.038
		Sc. rank:	11934.0

NA

Pos.	366	obs:	exp:
cgt	R	0	1062.00
cgc	R	0	2035.00
cga	R	1	3811.00
cgg	R	0	2847.00
aga	R	38864	19533.00
agg	R	42	9619.00
---	--	------	------
mPD		0.0022	0.91
		nPD:	0.
		N. weight:	0.7
		Sc. PD:	-0.037
		Sc. rank:	-20497.0

NA

Pos.	367	obs:	exp:
tct	S	1	0.18
tcc	S	0	0.12
tca	S	0	0.26
tcg	S	0	0.05
att	I	31	11.28
atc	I	0	7.74
ata	I	0	11.98
act	T	38632	9800.00
acc	T	231	7974.00
aca	T	5	17784.00
acg	T	6	3317.00
agt	S	0	0.19
agc	S	0	0.20
gct	A	1	0.26
gcc	A	0	0.21
gca	A	0	0.47
gcg	A	0	0.06
---	--	------	------
mPD		0.014	0.68
		nPD:	0.02
		N. weight:	1.4
		Sc. PD:	-0.048
		Sc. rank:	3310.1

NA

Pos.	368	obs:	exp:
caa	Q	2	1.06
cag	Q	0	0.94
cgt	R	0	0.11
cgc	R	0	0.21
cga	R	0	0.39
cgg	R	0	0.29
aaa	K	17372	22190.00
aag	K	21529	16711.00
aga	R	4	2.01
agg	R	0	0.99
---	--	------	------
mPD		0.49	0.49
		nPD:	1.01
		N. weight:	0.032
		Sc. PD:	0.03
		Sc. rank:	1046.1

NA

Pos.	369	obs:	exp:
tct	S	0	7153.00
tcc	S	0	4502.00
tca	S	0	10115.00
tcg	S	0	2069.00
cgt	R	0	0.05
cgc	R	0	0.10
cga	R	0	0.20
cgg	R	0	0.15
att	I	0	0.36
atc	I	1	0.25
ata	I	0	0.39
act	T	0	0.25
acc	T	1	0.21
aca	T	0	0.46
acg	T	0	0.09
aat	N	1	18.33
aac	N	30	12.67
agt	S	57	7308.00
agc	S	38810	7720.00
aga	R	2	1.00
agg	R	0	0.49
ggt	G	0	0.76
ggc	G	5	0.74
gga	G	0	2.22
ggg	G	0	1.28
---	--	------	------
mPD		0.0050	1.6
		nPD:	0.
		N. weight:	1.7
		Sc. PD:	-0.086
		Sc. rank:	-46400.0

NA

Pos.	370	obs:	exp:
tct	S	0	1.29
tcc	S	0	0.81
tca	S	0	1.82
tcg	S	0	0.37
att	I	36636	13377.00
atc	I	107	9173.00
ata	I	3	14197.00
atg	M	1	1.00
act	T	2134	539.50
acc	T	6	438.90
aca	T	0	979.00
acg	T	0	182.60
aat	N	5	2.96
aac	N	0	2.04
agt	S	7	1.32
agc	S	0	1.39
gtt	V	7	1.55
gtc	V	0	1.27
gta	V	0	1.88
gtg	V	0	2.30
gct	A	1	0.26
gcc	A	0	0.21
gca	A	0	0.47
gcg	A	0	0.06
---	--	------	------
mPD		0.11	0.76
		nPD:	0.15
		N. weight:	1.
		Sc. PD:	0.089
		Sc. rank:	27822.0

NA

Pos.	371	obs:	exp:
tct	S	0	7054.00
tcc	S	0	4440.00
tca	S	0	9976.00
tcg	S	0	2040.00
cgt	R	1	0.16
cgc	R	0	0.31
cga	R	0	0.59
cgg	R	0	0.44
att	I	16	5.82
atc	I	0	3.99
ata	I	0	6.18
aat	N	524	311.10
aac	N	2	214.90
agt	S	37905	7208.00
agc	S	427	7614.00
aga	R	3	3.01
agg	R	2	1.48
gat	D	20	10.54
gac	D	0	9.46
ggt	G	7	1.06
ggc	G	0	1.04
gga	G	0	3.11
ggg	G	0	1.79
---	--	------	------
mPD		0.052	1.7
		nPD:	0.03
		N. weight:	1.6
		Sc. PD:	-0.041
		Sc. rank:	18691.0

NA

Pos.	372	obs:	exp:
tta	L	49	5.39
ttg	L	0	9.23
tct	S	4	7150.00
tcc	S	2	4500.00
tca	S	38683	10112.00
tcg	S	166	2068.00
ctt	L	0	9.66
ctc	L	0	6.92
cta	L	0	9.43
ctg	L	0	8.38
cct	P	0	0.28
ccc	P	0	0.19
cca	P	1	0.43
ccg	P	0	0.11
agt	S	0	7306.00
agc	S	0	7718.00
gct	A	0	0.53
gcc	A	0	0.41
gca	A	2	0.94
gcg	A	0	0.12
---	--	------	------
mPD		0.011	1.6
		nPD:	0.01
		N. weight:	1.4
		Sc. PD:	-0.066
		Sc. rank:	-27500.0

NA

Pos.	373	obs:	exp:
cgt	R	0	1062.00
cgc	R	0	2035.00
cga	R	6	3810.00
cgg	R	0	2847.00
aaa	K	3	1.71
aag	K	0	1.29
aga	R	37800	19531.00
agg	R	1097	9618.00
gct	A	0	0.26
gcc	A	0	0.21
gca	A	1	0.47
gcg	A	0	0.06
---	--	------	------
mPD		0.055	0.91
		nPD:	0.06
		N. weight:	0.6
		Sc. PD:	0.0022
		Sc. rank:	12334.0

,

NA

Pos.	374	obs:	exp:
tct	S	0	3.86
tcc	S	0	2.43
tca	S	0	5.47
tcg	S	0	1.12
cat	H	0	1.76
cac	H	3	1.24
caa	Q	5	2.64
cag	Q	0	2.36
cgt	R	0	0.19
cgc	R	0	0.37
cga	R	0	0.69
cgg	R	0	0.51
att	I	0	0.36
atc	I	0	0.25
ata	I	1	0.39
act	T	0	4.29
acc	T	17	3.49
aca	T	0	7.78
acg	T	0	1.45
aat	N	61	3235.00
aac	N	5408	2234.00
aaa	K	33340	19043.00
aag	K	44	14341.00
agt	S	1	3.95
agc	S	20	4.17
aga	R	7	3.51
agg	R	0	1.73
---	--	------	------
mPD		0.25	0.62
		nPD:	0.4
		N. weight:	0.61
		Sc. PD:	0.2
		Sc. rank:	18696.0

,

NA

Pos.	375	obs:	exp:
ggt	G	38806	5899.00
ggc	G	50	5763.00
gga	G	0	17307.00
ggg	G	51	9938.00
---	--	------	------
mPD		0.0052	0.69
		nPD:	0.01
		N. weight:	1.9
		Sc. PD:	-0.092
		Sc. rank:	-34517.0

,

NA

Pos.	376	obs:	exp:
ttt	F	38887	16695.00
ttc	F	18	22210.00
tta	L	0	0.11
ttg	L	0	0.19
tat	Y	1	0.52
tac	Y	0	0.48
ctt	L	1	0.20
ctc	L	0	0.14
cta	L	0	0.19
ctg	L	0	0.17
---	--	------	------
mPD		0.0010	0.49
		nPD:	0.
		N. weight:	0.87
		Sc. PD:	-0.046
		Sc. rank:	-26123.0

,

NA

Pos.	377	obs:	exp:
gaa	E	920	23558.00
gag	E	37987	15349.00
---	--	------	------
mPD		0.046	0.48
		nPD:	0.1
		N. weight:	0.83
		Sc. PD:	0.032
		Sc. rank:	20271.0

,

NA

Pos.	378	obs:	exp:
tct	S	0	0.18
tcc	S	0	0.12
tca	S	0	0.26
tcg	S	0	0.05
att	I	5	6.19
atc	I	0	4.24
ata	I	12	6.57
atg	M	38885	38885.00
agt	S	1	0.19
agc	S	0	0.20
gtt	V	0	0.88
gtc	V	0	0.73
gta	V	0	1.08
gtg	V	4	1.31
---	--	------	------
mPD		0.0012	0.0013
		nPD:	0.88
		N. weight:	0.00037
		Sc. PD:	0.00029
		Sc. rank:	12.0

,

NA

Pos.	379	obs:	exp:
att	I	38810	14148.00
atc	I	56	9702.00
ata	I	0	15016.00
aaa	K	1	0.57
aag	K	0	0.43
gtt	V	40	8.84
gtc	V	0	7.27
gta	V	0	10.76
gtg	V	0	13.13
---	--	------	------
mPD		0.0050	0.66
		nPD:	0.01
		N. weight:	1.
		Sc. PD:	-0.049
		Sc. rank:	-17657.0

,

NA

Pos.	380	obs:	exp:
tgt	C	1	0.45
tgc	C	0	0.55
tgg	W	38905	38905.00
atg	M	1	1.00
---	--	------	------
mPD		0.00015	0.00015
		nPD:	1.
		N. weight:	0.000021
		Sc. PD:	0.000019
		Sc. rank:	0.7

,

NA

Pos.	381	obs:	exp:
aat	N	2	1.18
aac	N	0	0.82
gat	D	38798	20499.00
gac	D	107	18406.00
---	--	------	------
mPD		0.0056	0.50
		nPD:	0.01
		N. weight:	0.64
		Sc. PD:	-0.028
		Sc. rank:	-6235.3

,

NA

Pos.	382	obs:	exp:
tta	L	0	0.11
ttg	L	0	0.19
tct	S	0	2.58
tcc	S	0	1.62
tca	S	1	3.64
tcg	S	13	0.75
ctt	L	0	0.20
ctc	L	0	0.14
cta	L	0	0.19
ctg	L	1	0.17
cct	P	65	10714.00
ccc	P	3	7213.00
cca	P	3502	16559.00
ccg	P	35319	4404.00
caa	Q	0	1.58
cag	Q	3	1.42
agt	S	0	2.63
agc	S	0	2.78
---	--	------	------
mPD		0.17	0.70
		nPD:	0.24
		N. weight:	1.8
		Sc. PD:	0.32
		Sc. rank:	49989.0

,

NA

Pos.	383	obs:	exp:
aat	N	31948	23006.00
aac	N	6950	15892.00
gat	D	7	4.74
gac	D	2	4.26
---	--	------	------
mPD		0.29	0.48
		nPD:	0.61
		N. weight:	0.13
		Sc. PD:	0.067
		Sc. rank:	4053.5

,

NA

Pos.	384	obs:	exp:
cgt	R	0	0.03
cgc	R	0	0.05
cga	R	0	0.10
cgg	R	0	0.07
aga	R	1	0.50
agg	R	0	0.25
ggt	G	58	5899.00
ggc	G	1	5762.00
gga	G	38713	17306.00
ggg	G	134	9938.00
---	--	------	------
mPD		0.0099	0.69
		nPD:	0.01
		N. weight:	0.8
		Sc. PD:	-0.033
		Sc. rank:	-3961.8

,

NA

Pos.	385	obs:	exp:
tgg	W	38906	38906.00
aaa	K	0	0.57
aag	K	1	0.43
---	--	------	------
mPD		0.00010	0.00013
		nPD:	0.78
		N. weight:	0.000022
		Sc. PD:	0.000016
		Sc. rank:	0.7

,

NA

Pos.	386	obs:	exp:
ttt	F	2	0.86
ttc	F	0	1.14
tct	S	1	0.37
tcc	S	0	0.23
tca	S	0	0.52
tcg	S	0	0.11
att	I	549	200.20
atc	I	0	137.30
ata	I	1	212.50
act	T	37948	9657.00
acc	T	336	7857.00
aca	T	18	17524.00
acg	T	5	3268.00
aat	N	32	18.93
aac	N	0	13.07
agt	S	1	0.38
agc	S	0	0.40
gct	A	14	3.71
gcc	A	0	2.90
gca	A	0	6.58
gcg	A	0	0.82
---	--	------	------
mPD		0.049	0.71
		nPD:	0.07
		N. weight:	1.4
		Sc. PD:	0.016
		Sc. rank:	29371.0

,

NA

Pos.	387	obs:	exp:
cgt	R	0	1.06
cgc	R	0	2.04
cga	R	0	3.82
cgg	R	0	2.85
atg	M	1	1.00
aga	R	0	19.58
agg	R	39	9.64
gct	A	0	0.53
gcc	A	0	0.41
gca	A	0	0.94
gcg	A	2	0.12
gaa	E	3	109.60
gag	E	178	71.40
ggt	G	8	5866.00
ggc	G	1	5730.00
gga	G	291	17207.00
ggg	G	38384	9881.00
---	--	------	------
mPD		0.027	0.70
		nPD:	0.04
		N. weight:	1.4
		Sc. PD:	-0.024
		Sc. rank:	19876.0

,

NA

Pos.	388	obs:	exp:
tct	S	2	0.37
tcc	S	0	0.23
tca	S	0	0.52
tcg	S	0	0.11
att	I	15	6.19
atc	I	0	4.24
ata	I	2	6.57
act	T	29717	9800.00
acc	T	24	7974.00
aca	T	9133	17785.00
acg	T	3	3317.00
aat	N	3	1.77
aac	N	0	1.23
aaa	K	3	1.71
aag	K	0	1.29
agt	S	0	0.38
agc	S	0	0.40
gct	A	1	1.32
gcc	A	0	1.03
gca	A	4	2.35
gcg	A	0	0.29
---	--	------	------
mPD		0.36	0.68
		nPD:	0.53
		N. weight:	0.71
		Sc. PD:	0.33
		Sc. rank:	22673.0

,

NA

Pos.	389	obs:	exp:
att	I	1	0.36
atc	I	0	0.25
ata	I	0	0.39
aat	N	0	0.59
aac	N	1	0.41
gat	D	94	20498.00
gac	D	38810	18406.00
ggt	G	0	0.15
ggc	G	1	0.15
gga	G	0	0.44
ggg	G	0	0.26
---	--	------	------
mPD		0.0051	0.50
		nPD:	0.01
		N. weight:	0.75
		Sc. PD:	-0.034
		Sc. rank:	-9081.3

,

NA

Pos.	390	obs:	exp:
tct	S	0	13.07
tcc	S	0	8.22
tca	S	0	18.48
tcg	S	0	3.78
tat	Y	3	1.56
tac	Y	0	1.44
cat	H	2	1.18
cac	H	0	0.82
att	I	1	0.73
atc	I	0	0.50
ata	I	1	0.77
act	T	24	6.30
acc	T	0	5.13
aca	T	1	11.44
acg	T	0	2.13
aat	N	38550	22937.00
aac	N	231	15844.00
aaa	K	18	10.27
aag	K	0	7.73
agt	S	71	13.35
agc	S	0	14.10
gat	D	5	2.63
gac	D	0	2.37
---	--	------	------
mPD		0.018	0.49
		nPD:	0.04
		N. weight:	0.51
		Sc. PD:	-0.0097
		Sc. rank:	7162.9

,

NA

Pos.	391	obs:	exp:
tct	S	0	54.10
tcc	S	0	34.05
tca	S	0	76.51
tcg	S	0	15.65
tat	Y	1	7.79
tac	Y	14	7.21
cat	H	0	1.18
cac	H	2	0.82
caa	Q	1	0.53
cag	Q	0	0.47
cgt	R	0	0.16
cgc	R	0	0.31
cga	R	0	0.59
cgg	R	0	0.44
att	I	0	0.73
atc	I	2	0.50
ata	I	0	0.77
act	T	3	0.76
acc	T	0	0.62
aca	T	0	1.37
acg	T	0	0.26
aat	N	39	4925.00
aac	N	8288	3402.00
aaa	K	30149	17219.00
aag	K	37	12967.00
agt	S	1	55.28
agc	S	293	58.40
aga	R	6	3.01
agg	R	0	1.48
gat	D	0	31.61
gac	D	60	28.39
gaa	E	10	6.06
gag	E	0	3.94
ggt	G	0	0.15
ggc	G	1	0.15
gga	G	0	0.44
ggg	G	0	0.26
---	--	------	------
mPD		0.37	0.69
		nPD:	0.53
		N. weight:	0.65
		Sc. PD:	0.3
		Sc. rank:	20582.0

,

NA

Pos.	392	obs:	exp:
ttt	F	278	16694.00
ttc	F	38623	22207.00
tta	L	0	0.11
ttg	L	1	0.19
tct	S	0	0.37
tcc	S	2	0.23
tca	S	0	0.52
tcg	S	0	0.11
tat	Y	0	1.04
tac	Y	2	0.96
ctt	L	0	0.20
ctc	L	0	0.14
cta	L	0	0.19
ctg	L	0	0.17
att	I	0	0.36
atc	I	1	0.25
ata	I	0	0.39
agt	S	0	0.38
agc	S	0	0.40
---	--	------	------
mPD		0.014	0.49
		nPD:	0.03
		N. weight:	0.54
		Sc. PD:	-0.014
		Sc. rank:	5810.2

,

NA

Pos.	393	obs:	exp:
tta	L	23	2.53
ttg	L	0	4.33
tct	S	47	7152.00
tcc	S	6	4502.00
tca	S	38796	10115.00
tcg	S	16	2068.00
ctt	L	0	4.53
ctc	L	0	3.25
cta	L	0	4.43
ctg	L	0	3.93
cct	P	0	0.55
ccc	P	0	0.37
cca	P	2	0.85
ccg	P	0	0.23
act	T	0	2.77
acc	T	0	2.26
aca	T	11	5.03
acg	T	0	0.94
agt	S	0	7308.00
agc	S	0	7720.00
gct	A	0	1.59
gcc	A	0	1.24
gca	A	6	2.82
gcg	A	0	0.35
---	--	------	------
mPD		0.0057	1.6
		nPD:	0.
		N. weight:	1.4
		Sc. PD:	-0.071
		Sc. rank:	-36071.0

,

NA

Pos.	394	obs:	exp:
tta	L	0	0.44
ttg	L	0	0.75
ctt	L	0	0.79
ctc	L	0	0.56
cta	L	4	0.77
ctg	L	0	0.68
cgt	R	0	0.66
cgc	R	0	1.26
cga	R	0	2.35
cgg	R	0	1.76
att	I	10	10684.00
atc	I	1	7326.00
ata	I	29339	11339.00
atg	M	69	69.00
act	T	0	24.45
acc	T	0	19.90
aca	T	97	44.38
acg	T	0	8.28
aaa	K	9283	5304.00
aag	K	16	3995.00
aga	R	24	12.05
agg	R	0	5.93
gtt	V	0	14.15
gtc	V	0	11.63
gta	V	64	17.21
gtg	V	0	21.01
---	--	------	------
mPD		0.38	1.1
		nPD:	0.35
		N. weight:	0.88
		Sc. PD:	0.25
		Sc. rank:	25835.0

,

NA

Pos.	395	obs:	exp:
caa	Q	0	1.06
cag	Q	2	0.94
cgt	R	0	4.45
cgc	R	0	8.53
cga	R	0	15.97
cgg	R	0	11.93
aaa	K	252	22100.00
aag	K	38490	16642.00
aga	R	0	81.83
agg	R	163	40.30
---	--	------	------
mPD		0.021	0.50
		nPD:	0.04
		N. weight:	0.83
		Sc. PD:	-0.012
		Sc. rank:	13734.0

,

NA

Pos.	396	obs:	exp:
tta	L	0	0.11
ttg	L	0	0.19
ctt	L	0	0.20
ctc	L	0	0.14
cta	L	1	0.19
ctg	L	0	0.17
cct	P	0	0.55
ccc	P	0	0.37
cca	P	2	0.85
ccg	P	0	0.23
cat	H	1	1.18
cac	H	1	0.82
caa	Q	38804	20527.00
cag	Q	98	18375.00
---	--	------	------
mPD		0.0053	0.50
		nPD:	0.01
		N. weight:	0.64
		Sc. PD:	-0.029
		Sc. rank:	-7062.6

,

NA

Pos.	397	obs:	exp:
cat	H	1	0.59
cac	H	0	0.41
aat	N	10	5.91
aac	N	0	4.09
gat	D	38649	20489.00
gac	D	237	18397.00
gaa	E	6	5.45
gag	E	3	3.55
ggt	G	1	0.15
ggc	G	0	0.15
gga	G	0	0.44
ggg	G	0	0.26
---	--	------	------
mPD		0.013	0.50
		nPD:	0.03
		N. weight:	0.62
		Sc. PD:	-0.018
		Sc. rank:	4819.5

,

NA

Pos.	398	obs:	exp:
att	I	526	14156.00
atc	I	38243	9707.00
ata	I	118	15024.00
atg	M	1	1.00
gtt	V	0	4.20
gtc	V	19	3.45
gta	V	0	5.11
gtg	V	0	6.24
---	--	------	------
mPD		0.034	0.66
		nPD:	0.05
		N. weight:	1.3
		Sc. PD:	-0.0074
		Sc. rank:	24467.0

,

NA

Pos.	399	obs:	exp:
tta	L	81	8.90
ttg	L	0	15.25
ctt	L	0	15.96
ctc	L	0	11.44
cta	L	0	15.59
ctg	L	0	13.85
att	I	0	210.80
atc	I	0	144.50
ata	I	579	223.70
gtt	V	11	8454.00
gtc	V	2	6949.00
gta	V	38168	10284.00
gtg	V	59	12553.00
gct	A	0	1.85
gcc	A	0	1.45
gca	A	7	3.29
gcg	A	0	0.41
---	--	------	------
mPD		0.037	0.77
		nPD:	0.05
		N. weight:	1.3
		Sc. PD:	-0.011
		Sc. rank:	23652.0

,

NA

Pos.	400	obs:	exp:
cgt	R	0	0.46
cgc	R	0	0.89
cga	R	0	1.67
cgg	R	0	1.24
aga	R	17	8.53
agg	R	0	4.20
gaa	E	33	19.98
gag	E	0	13.02
ggt	G	4	5892.00
ggc	G	0	5755.00
gga	G	38464	17284.00
ggg	G	389	9926.00
---	--	------	------
mPD		0.023	0.69
		nPD:	0.03
		N. weight:	0.78
		Sc. PD:	-0.019
		Sc. rank:	9150.6

,

NA

Pos.	401	obs:	exp:
tta	L	34	7.81
ttg	L	0	13.37
ctt	L	0	13.99
ctc	L	0	10.03
cta	L	37	13.67
ctg	L	0	12.14
cct	P	0	1.38
ccc	P	0	0.93
cca	P	5	2.13
ccg	P	0	0.57
cgt	R	0	0.05
cgc	R	0	0.10
cga	R	0	0.20
cgg	R	0	0.15
att	I	28	13955.00
atc	I	3	9569.00
ata	I	38304	14811.00
atg	M	205	205.00
act	T	0	41.34
acc	T	0	33.64
aca	T	164	75.03
acg	T	0	13.99
aaa	K	3	1.71
aag	K	0	1.29
aga	R	2	1.00
agg	R	0	0.49
gtt	V	0	26.75
gtc	V	0	21.99
gta	V	120	32.54
gtg	V	1	39.72
gct	A	0	0.26
gcc	A	0	0.21
gca	A	1	0.47
gcg	A	0	0.06
---	--	------	------
mPD		0.031	0.68
		nPD:	0.05
		N. weight:	0.97
		Sc. PD:	-0.01
		Sc. rank:	16541.0

,

NA

Pos.	402	obs:	exp:
tct	S	0	5.70
tcc	S	0	3.59
tca	S	0	8.07
tcg	S	0	1.65
tat	Y	1	0.52
tac	Y	0	0.48
cat	H	2	1.18
cac	H	0	0.82
cgt	R	0	0.08
cgc	R	0	0.16
cga	R	0	0.29
cgg	R	0	0.22
act	T	6	1.51
acc	T	0	1.23
aca	T	0	2.74
acg	T	0	0.51
aat	N	38161	22644.00
aac	N	125	15642.00
aaa	K	530	320.60
aag	K	32	241.40
agt	S	31	5.83
agc	S	0	6.16
aga	R	3	1.51
agg	R	0	0.74
gat	D	16	8.43
gac	D	0	7.57
---	--	------	------
mPD		0.038	0.50
		nPD:	0.08
		N. weight:	0.52
		Sc. PD:	0.0093
		Sc. rank:	11506.0

,

NA

Pos.	403	obs:	exp:
caa	Q	0	0.53
cag	Q	1	0.47
aaa	K	3	8.56
aag	K	12	6.44
gtt	V	0	0.22
gtc	V	0	0.18
gta	V	0	0.27
gtg	V	1	0.33
gat	D	49	25.82
gac	D	0	23.18
gaa	E	617	23514.00
gag	E	38217	15320.00
ggt	G	0	1.06
ggc	G	0	1.04
gga	G	0	3.11
ggg	G	7	1.79
---	--	------	------
mPD		0.035	0.48
		nPD:	0.07
		N. weight:	0.87
		Sc. PD:	0.013
		Sc. rank:	19029.0

,

NA

Pos.	404	obs:	exp:
tta	L	0	0.55
ttg	L	5	0.94
tct	S	0	0.18
tcc	S	0	0.12
tca	S	0	0.26
tcg	S	1	0.05
tgt	C	1	0.90
tgc	C	1	1.10
tgg	W	38886	38886.00
ctt	L	0	0.99
ctc	L	0	0.71
cta	L	0	0.96
ctg	L	0	0.85
caa	Q	0	0.53
cag	Q	1	0.47
cgt	R	0	0.33
cgc	R	0	0.63
cga	R	0	1.18
cgg	R	12	0.88
agt	S	0	0.19
agc	S	0	0.20
aga	R	0	6.02
agg	R	0	2.97
---	--	------	------
mPD		0.0011	0.0020
		nPD:	0.57
		N. weight:	0.0012
		Sc. PD:	0.00058
		Sc. rank:	37.5

,

NA

Pos.	405	obs:	exp:
tct	S	26	7144.00
tcc	S	1	4496.00
tca	S	38768	10103.00
tcg	S	24	2066.00
act	T	0	22.18
acc	T	0	18.05
aca	T	88	40.26
acg	T	0	7.51
agt	S	0	7299.00
agc	S	0	7711.00
---	--	------	------
mPD		0.0071	1.6
		nPD:	0.
		N. weight:	1.4
		Sc. PD:	-0.07
		Sc. rank:	-34301.0

,

NA

Pos.	406	obs:	exp:
ggt	G	17	5899.00
ggc	G	77	5763.00
gga	G	11665	17307.00
ggg	G	27148	9938.00
---	--	------	------
mPD		0.42	0.69
		nPD:	0.61
		N. weight:	0.59
		Sc. PD:	0.32
		Sc. rank:	19042.0

,

NA

Pos.	407	obs:	exp:
tat	Y	37785	20214.00
tac	Y	1119	18690.00
cat	H	1	0.59
cac	H	0	0.41
aat	N	1	0.59
aac	N	0	0.41
gat	D	1	0.53
gac	D	0	0.47
---	--	------	------
mPD		0.056	0.50
		nPD:	0.11
		N. weight:	0.54
		Sc. PD:	0.029
		Sc. rank:	13857.0

,

NA

Pos.	408	obs:	exp:
tct	S	0	7160.00
tcc	S	0	4506.00
tca	S	0	10125.00
tcg	S	0	2071.00
cgt	R	0	0.03
cgc	R	0	0.05
cga	R	0	0.10
cgg	R	0	0.07
agt	S	388	7315.00
agc	S	38517	7728.00
aga	R	1	0.50
agg	R	0	0.25
ggt	G	0	0.15
ggc	G	1	0.15
gga	G	0	0.44
ggg	G	0	0.26
---	--	------	------
mPD		0.020	1.6
		nPD:	0.01
		N. weight:	1.6
		Sc. PD:	-0.07
		Sc. rank:	-12581.0

,

NA

Pos.	409	obs:	exp:
cgt	R	0	0.08
cgc	R	0	0.16
cga	R	0	0.29
cgg	R	1	0.22
aga	R	0	1.51
agg	R	2	0.74
ggt	G	6	5899.00
ggc	G	0	5762.00
gga	G	212	17305.00
ggg	G	38686	9938.00
---	--	------	------
mPD		0.011	0.69
		nPD:	0.02
		N. weight:	1.4
		Sc. PD:	-0.054
		Sc. rank:	-3798.2

,

NA

Pos.	410	obs:	exp:
tct	S	0	7156.00
tcc	S	0	4504.00
tca	S	0	10121.00
tcg	S	0	2070.00
tgt	C	2	0.90
tgc	C	0	1.10
aat	N	15	8.87
aac	N	0	6.13
agt	S	38831	7312.00
agc	S	57	7725.00
ggt	G	2	0.30
ggc	G	0	0.30
gga	G	0	0.89
ggg	G	0	0.51
---	--	------	------
mPD		0.0039	1.6
		nPD:	0.
		N. weight:	1.7
		Sc. PD:	-0.09
		Sc. rank:	-50498.0

,

NA

Pos.	411	obs:	exp:
ttt	F	38832	16696.00
ttc	F	74	22210.00
tct	S	1	0.18
tcc	S	0	0.12
tca	S	0	0.26
tcg	S	0	0.05
agt	S	0	0.19
agc	S	0	0.20
---	--	------	------
mPD		0.0038	0.49
		nPD:	0.01
		N. weight:	0.86
		Sc. PD:	-0.041
		Sc. rank:	-14420.0

,

NA

Pos.	412	obs:	exp:
att	I	2	0.73
atc	I	0	0.50
ata	I	0	0.77
gtt	V	38078	8601.00
gtc	V	823	7069.00
gta	V	3	10463.00
gtg	V	1	12771.00
---	--	------	------
mPD		0.042	0.74
		nPD:	0.06
		N. weight:	1.5
		Sc. PD:	-0.00069
		Sc. rank:	28153.0

,

NA

Pos.	413	obs:	exp:
caa	Q	67	20530.00
cag	Q	38839	18376.00
aaa	K	0	0.57
aag	K	1	0.43
---	--	------	------
mPD		0.0035	0.50
		nPD:	0.01
		N. weight:	0.76
		Sc. PD:	-0.037
		Sc. rank:	-15223.0

,

NA

Pos.	414	obs:	exp:
cat	H	38708	22879.00
cac	H	196	16025.00
caa	Q	0	0.53
cag	Q	1	0.47
cgt	R	1	0.03
cgc	R	0	0.05
cga	R	0	0.10
cgg	R	0	0.07
aat	N	1	0.59
aac	N	0	0.41
aga	R	0	0.50
agg	R	0	0.25
---	--	------	------
mPD		0.010	0.48
		nPD:	0.02
		N. weight:	0.52
		Sc. PD:	-0.018
		Sc. rank:	1326.2

,

NA

Pos.	415	obs:	exp:
tct	S	0	0.37
tcc	S	0	0.23
tca	S	2	0.52
tcg	S	0	0.11
cct	P	114	10718.00
ccc	P	162	7216.00
cca	P	38527	16565.00
ccg	P	101	4405.00
act	T	0	0.25
acc	T	0	0.21
aca	T	1	0.46
acg	T	0	0.09
agt	S	0	0.38
agc	S	0	0.40
---	--	------	------
mPD		0.019	0.70
		nPD:	0.03
		N. weight:	0.82
		Sc. PD:	-0.023
		Sc. rank:	7662.9

,

NA

Pos.	416	obs:	exp:
caa	Q	1	0.53
cag	Q	0	0.47
gat	D	0	0.53
gac	D	1	0.47
gaa	E	38778	23557.00
gag	E	127	15348.00
---	--	------	------
mPD		0.0066	0.48
		nPD:	0.01
		N. weight:	0.5
		Sc. PD:	-0.021
		Sc. rank:	-2826.1

,

NA

Pos.	417	obs:	exp:
tta	L	178	4275.00
ttg	L	0	7321.00
ctt	L	25	7663.00
ctc	L	1	5493.00
cta	L	38627	7486.00
ctg	L	55	6649.00
cct	P	0	0.55
ccc	P	0	0.37
cca	P	2	0.85
ccg	P	0	0.23
caa	Q	13	6.86
cag	Q	0	6.14
att	I	0	1.82
atc	I	0	1.25
ata	I	5	1.93
atg	M	1	1.00
---	--	------	------
mPD		0.014	1.1
		nPD:	0.01
		N. weight:	1.7
		Sc. PD:	-0.071
		Sc. rank:	-11639.0

,

NA

Pos.	418	obs:	exp:
tct	S	0	0.18
tcc	S	0	0.12
tca	S	1	0.26
tcg	S	0	0.05
cct	P	0	0.28
ccc	P	0	0.19
cca	P	1	0.43
ccg	P	0	0.11
act	T	63	9807.00
acc	T	4	7980.00
aca	T	38813	17798.00
acg	T	25	3319.00
agt	S	0	0.19
agc	S	0	0.20
---	--	------	------
mPD		0.0048	0.68
		nPD:	0.01
		N. weight:	0.79
		Sc. PD:	-0.038
		Sc. rank:	-15130.0

,

NA

Pos.	419	obs:	exp:
cgt	R	0	0.25
cgc	R	0	0.47
cga	R	0	0.88
cgg	R	1	0.66
aga	R	0	4.52
agg	R	8	2.22
gaa	E	0	0.61
gag	E	1	0.39
ggt	G	12	5898.00
ggc	G	3	5761.00
gga	G	266	17302.00
ggg	G	38616	9936.00
---	--	------	------
mPD		0.015	0.69
		nPD:	0.02
		N. weight:	1.4
		Sc. PD:	-0.047
		Sc. rank:	4356.2

,

NA

Pos.	420	obs:	exp:
tta	L	2	4268.00
ttg	L	70	7309.00
ctt	L	113	7650.00
ctc	L	2	5483.00
cta	L	101	7474.00
ctg	L	38533	6637.00
caa	Q	0	0.53
cag	Q	1	0.47
cgt	R	0	0.03
cgc	R	0	0.05
cga	R	0	0.10
cgg	R	1	0.07
atg	M	82	82.00
aga	R	0	0.50
agg	R	0	0.25
gtt	V	0	0.44
gtc	V	0	0.36
gta	V	0	0.54
gtg	V	2	0.66
---	--	------	------
mPD		0.019	1.1
		nPD:	0.02
		N. weight:	1.8
		Sc. PD:	-0.068
		Sc. rank:	-3345.8

,

NA

Pos.	421	obs:	exp:
tct	S	0	1.66
tcc	S	0	1.04
tca	S	0	2.34
tcg	S	0	0.48
cat	H	1	0.59
cac	H	0	0.41
aat	N	14047	8317.00
aac	N	16	5746.00
aaa	K	1	78.15
aag	K	136	58.85
agt	S	9	1.69
agc	S	0	1.79
gat	D	23858	12994.00
gac	D	804	11668.00
gaa	E	0	1.82
gag	E	3	1.18
ggt	G	32	4.85
ggc	G	0	4.74
gga	G	0	14.23
ggg	G	0	8.17
---	--	------	------
mPD		0.51	0.97
		nPD:	0.53
		N. weight:	0.53
		Sc. PD:	0.24
		Sc. rank:	16740.0

,

NA

Pos.	422	obs:	exp:
tgt	C	38837	17561.00
tgc	C	69	21345.00
ggt	G	1	0.15
ggc	G	0	0.15
gga	G	0	0.44
ggg	G	0	0.26
---	--	------	------
mPD		0.0036	0.50
		nPD:	0.01
		N. weight:	0.81
		Sc. PD:	-0.039
		Sc. rank:	-15269.0

,

NA

Pos.	423	obs:	exp:
att	I	3	14160.00
atc	I	11	9709.00
ata	I	38883	15028.00
atg	M	4	4.00
gtt	V	0	1.33
gtc	V	0	1.09
gta	V	6	1.61
gtg	V	0	1.97
---	--	------	------
mPD		0.0012	0.66
		nPD:	0.
		N. weight:	0.98
		Sc. PD:	-0.052
		Sc. rank:	-30302.0

,

NA

Pos.	424	obs:	exp:
tct	S	0	0.37
tcc	S	0	0.23
tca	S	0	0.52
tcg	S	0	0.11
cgt	R	0	1060.00
cgc	R	0	2031.00
cga	R	3	3802.00
cgg	R	0	2841.00
att	I	0	2.55
atc	I	0	1.75
ata	I	7	2.70
act	T	0	0.25
acc	T	0	0.21
aca	T	1	0.46
acg	T	0	0.09
aaa	K	77	43.92
aag	K	0	33.08
agt	S	0	0.38
agc	S	2	0.40
aga	R	38757	19489.00
agg	R	60	9597.00
---	--	------	------
mPD		0.0077	0.91
		nPD:	0.01
		N. weight:	0.7
		Sc. PD:	-0.033
		Sc. rank:	-10958.0

,

NA

Pos.	425	obs:	exp:
tta	L	0	0.11
ttg	L	0	0.19
ctt	L	1	0.20
ctc	L	0	0.14
cta	L	0	0.19
ctg	L	0	0.17
cct	P	38859	10718.00
ccc	P	36	7216.00
cca	P	7	16566.00
ccg	P	3	4405.00
cat	H	1	0.59
cac	H	0	0.41
---	--	------	------
mPD		0.0025	0.70
		nPD:	0.
		N. weight:	1.3
		Sc. PD:	-0.068
		Sc. rank:	-34140.0

,

NA

Pos.	426	obs:	exp:
tgt	C	385	17561.00
tgc	C	38522	21346.00
---	--	------	------
mPD		0.020	0.50
		nPD:	0.04
		N. weight:	0.56
		Sc. PD:	-0.0095
		Sc. rank:	8799.3

,

NA

Pos.	427	obs:	exp:
ttt	F	111	16695.00
ttc	F	38793	22209.00
tta	L	1	0.11
ttg	L	0	0.19
tct	S	0	0.37
tcc	S	2	0.23
tca	S	0	0.52
tcg	S	0	0.11
ctt	L	0	0.20
ctc	L	0	0.14
cta	L	0	0.19
ctg	L	0	0.17
agt	S	0	0.38
agc	S	0	0.40
---	--	------	------
mPD		0.0058	0.49
		nPD:	0.01
		N. weight:	0.56
		Sc. PD:	-0.024
		Sc. rank:	-4635.9

,

NA

Pos.	428	obs:	exp:
tta	L	0	0.22
ttg	L	2	0.38
tgt	C	1	0.45
tgc	C	0	0.55
tgg	W	38904	38904.00
ctt	L	0	0.39
ctc	L	0	0.28
cta	L	0	0.39
ctg	L	0	0.34
---	--	------	------
mPD		0.00015	0.00029
		nPD:	0.53
		N. weight:	0.00011
		Sc. PD:	0.00005
		Sc. rank:	3.5

,

NA

Pos.	429	obs:	exp:
att	I	67	24.39
atc	I	0	16.72
ata	I	0	25.89
gtt	V	38815	8587.00
gtc	V	22	7058.00
gta	V	1	10446.00
gtg	V	2	12750.00
---	--	------	------
mPD		0.0047	0.74
		nPD:	0.01
		N. weight:	1.6
		Sc. PD:	-0.076
		Sc. rank:	-33352.0

,

NA

Pos.	430	obs:	exp:
gaa	E	38783	23558.00
gag	E	123	15348.00
ggt	G	0	0.15
ggc	G	0	0.15
gga	G	1	0.44
ggg	G	0	0.26
---	--	------	------
mPD		0.0064	0.48
		nPD:	0.01
		N. weight:	0.5
		Sc. PD:	-0.021
		Sc. rank:	-3045.9

,

NA

Pos.	431	obs:	exp:
tta	L	206	4276.00
ttg	L	1	7323.00
ctt	L	1	7665.00
ctc	L	3	5494.00
cta	L	38286	7488.00
ctg	L	398	6650.00
caa	Q	3	1.58
cag	Q	0	1.42
att	I	0	3.28
atc	I	0	2.25
ata	I	9	3.48
---	--	------	------
mPD		0.032	1.1
		nPD:	0.03
		N. weight:	1.6
		Sc. PD:	-0.045
		Sc. rank:	14818.0

,

NA

Pos.	432	obs:	exp:
att	I	12	14160.00
atc	I	11104	9710.00
ata	I	27782	15028.00
atg	M	1	1.00
act	T	0	0.50
acc	T	1	0.41
aca	T	1	0.91
acg	T	0	0.17
aaa	K	1	0.57
aag	K	0	0.43
gtt	V	0	1.11
gtc	V	1	0.91
gta	V	2	1.34
gtg	V	2	1.64
---	--	------	------
mPD		0.41	0.66
		nPD:	0.62
		N. weight:	0.49
		Sc. PD:	0.27
		Sc. rank:	15850.0

,

NA

Pos.	433	obs:	exp:
tct	S	0	0.18
tcc	S	0	0.12
tca	S	0	0.26
tcg	S	0	0.05
cgt	R	0	1062.00
cgc	R	0	2035.00
cga	R	7	3810.00
cgg	R	0	2847.00
att	I	0	0.73
atc	I	0	0.50
ata	I	2	0.77
aaa	K	0	0.57
aag	K	1	0.43
agt	S	1	0.19
agc	S	0	0.20
aga	R	38867	19530.00
agg	R	28	9617.00
ggt	G	0	0.15
ggc	G	0	0.15
gga	G	1	0.44
ggg	G	0	0.26
---	--	------	------
mPD		0.0021	0.91
		nPD:	0.
		N. weight:	0.7
		Sc. PD:	-0.037
		Sc. rank:	-20927.0

,

NA

Pos.	434	obs:	exp:
cgt	R	0	0.03
cgc	R	0	0.05
cga	R	0	0.10
cgg	R	1	0.07
aga	R	0	0.50
agg	R	0	0.25
gct	A	0	0.26
gcc	A	0	0.21
gca	A	0	0.47
gcg	A	1	0.06
ggt	G	7	5899.00
ggc	G	21	5762.00
gga	G	21842	17306.00
ggg	G	17035	9938.00
---	--	------	------
mPD		0.49	0.69
		nPD:	0.71
		N. weight:	0.37
		Sc. PD:	0.24
		Sc. rank:	12126.0

,

NA

Pos.	435	obs:	exp:
tta	L	0	1.43
ttg	L	0	2.45
ctt	L	0	2.56
ctc	L	0	1.84
cta	L	13	2.50
ctg	L	0	2.22
caa	Q	28	14.77
cag	Q	0	13.23
cgt	R	3	1061.00
cgc	R	53	2033.00
cga	R	38576	3807.00
cgg	R	178	2844.00
aga	R	56	19512.00
agg	R	0	9608.00
---	--	------	------
mPD		0.017	0.91
		nPD:	0.02
		N. weight:	2.3
		Sc. PD:	-0.087
		Sc. rank:	300.9

,

NA

Pos.	436	obs:	exp:
tta	L	0	1.54
ttg	L	0	2.64
tct	S	0	5.89
tcc	S	32	3.71
tca	S	0	8.33
tcg	S	0	1.70
ctt	L	0	2.76
ctc	L	14	1.98
cta	L	0	2.70
ctg	L	0	2.39
cct	P	206	10704.00
ccc	P	38558	7206.00
cca	P	90	16544.00
ccg	P	0	4400.00
cat	H	0	2.35
cac	H	4	1.65
act	T	0	0.76
acc	T	3	0.62
aca	T	0	1.37
acg	T	0	0.26
agt	S	0	6.02
agc	S	0	6.36
---	--	------	------
mPD		0.018	0.70
		nPD:	0.03
		N. weight:	1.7
		Sc. PD:	-0.051
		Sc. rank:	11324.0

,

NA

Pos.	437	obs:	exp:
caa	Q	1	0.53
cag	Q	0	0.47
cgt	R	0	0.05
cgc	R	0	0.10
cga	R	0	0.20
cgg	R	0	0.15
aat	N	0	1.18
aac	N	2	0.82
aaa	K	8028	4594.00
aag	K	26	3460.00
aga	R	2	1.00
agg	R	0	0.49
gct	A	0	0.26
gcc	A	0	0.21
gca	A	1	0.47
gcg	A	0	0.06
gat	D	3	3.69
gac	D	4	3.31
gaa	E	30767	18663.00
gag	E	56	12160.00
ggt	G	0	2.58
ggc	G	0	2.52
gga	G	17	7.56
ggg	G	0	4.34
---	--	------	------
mPD		0.33	0.81
		nPD:	0.41
		N. weight:	0.52
		Sc. PD:	0.18
		Sc. rank:	15881.0

,

NA

Pos.	438	obs:	exp:
caa	Q	0	1.06
cag	Q	2	0.94
cgt	R	0	0.03
cgc	R	0	0.05
cga	R	0	0.10
cgg	R	0	0.07
aaa	K	0	0.57
aag	K	1	0.43
aga	R	0	0.50
agg	R	1	0.25
gct	A	0	0.26
gcc	A	0	0.21
gca	A	0	0.47
gcg	A	1	0.06
gat	D	5	2.63
gac	D	0	2.37
gaa	E	356	23552.00
gag	E	38540	15344.00
ggt	G	0	0.15
ggc	G	0	0.15
gga	G	0	0.44
ggg	G	1	0.26
---	--	------	------
mPD		0.019	0.48
		nPD:	0.04
		N. weight:	0.9
		Sc. PD:	-0.016
		Sc. rank:	13712.0

,

NA

Pos.	439	obs:	exp:
tct	S	0	11.96
tcc	S	0	7.53
tca	S	0	16.92
tcg	S	0	3.46
act	T	0	2.27
acc	T	9	1.85
aca	T	0	4.12
acg	T	0	0.77
aat	N	118	22922.00
aac	N	38639	15835.00
aaa	K	67	42.21
aag	K	7	31.79
agt	S	0	12.22
agc	S	65	12.91
gat	D	0	1.05
gac	D	2	0.95
---	--	------	------
mPD		0.014	0.49
		nPD:	0.03
		N. weight:	0.9
		Sc. PD:	-0.025
		Sc. rank:	8508.5

,

NA

Pos.	440	obs:	exp:
tct	S	0	1.10
tcc	S	0	0.69
tca	S	6	1.56
tcg	S	0	0.32
att	I	0	0.36
atc	I	0	0.25
ata	I	1	0.39
act	T	17	9804.00
acc	T	6	7977.00
aca	T	38818	17792.00
acg	T	50	3318.00
agt	S	0	1.13
agc	S	0	1.19
gct	A	0	2.38
gcc	A	0	1.86
gca	A	9	4.23
gcg	A	0	0.53
---	--	------	------
mPD		0.0046	0.68
		nPD:	0.01
		N. weight:	0.79
		Sc. PD:	-0.039
		Sc. rank:	-16406.0

,

NA

Pos.	441	obs:	exp:
ttt	F	0	0.43
ttc	F	1	0.57
tta	L	0	0.77
ttg	L	0	1.32
tct	S	0	0.18
tcc	S	0	0.12
tca	S	0	0.26
tcg	S	0	0.05
ctt	L	0	1.38
ctc	L	7	0.99
cta	L	0	1.35
ctg	L	0	1.20
att	I	76	13663.00
atc	I	37419	9369.00
ata	I	37	14501.00
act	T	0	3.28
acc	T	13	2.67
aca	T	0	5.95
acg	T	0	1.11
agt	S	0	0.19
agc	S	1	0.20
gtt	V	2	299.10
gtc	V	1346	245.90
gta	V	5	363.90
gtg	V	0	444.10
---	--	------	------
mPD		0.074	0.73
		nPD:	0.1
		N. weight:	1.4
		Sc. PD:	0.061
		Sc. rank:	35363.0

,

NA

Pos.	442	obs:	exp:
tgg	W	38907	38907.00
---	--	------	------
mPD		0	0
		nPD:	1.
		N. weight:	0.
		Sc. PD:	0
		Sc. rank:	0

,

NA

Pos.	443	obs:	exp:
tct	S	1	0.37
tcc	S	0	0.23
tca	S	0	0.52
tcg	S	0	0.11
att	I	2	0.73
atc	I	0	0.50
ata	I	0	0.77
act	T	38755	9785.00
acc	T	44	7961.00
aca	T	3	17756.00
acg	T	12	3312.00
aat	N	1	0.59
aac	N	0	0.41
aaa	K	1	0.57
aag	K	0	0.43
agt	S	1	0.38
agc	S	0	0.40
gct	A	87	23.03
gcc	A	0	18.01
gca	A	0	40.86
gcg	A	0	5.10
---	--	------	------
mPD		0.0079	0.68
		nPD:	0.01
		N. weight:	1.4
		Sc. PD:	-0.062
		Sc. rank:	-12615.0

,

NA

Pos.	444	obs:	exp:
tct	S	0	7154.00
tcc	S	0	4503.00
tca	S	0	10118.00
tcg	S	0	2069.00
tgt	C	0	0.90
tgc	C	2	1.10
cgt	R	0	0.03
cgc	R	0	0.05
cga	R	0	0.10
cgg	R	0	0.07
agt	S	1714	7310.00
agc	S	37163	7722.00
aga	R	1	0.50
agg	R	0	0.25
ggt	G	0	4.09
ggc	G	27	4.00
gga	G	0	12.01
ggg	G	0	6.90
---	--	------	------
mPD		0.086	1.6
		nPD:	0.05
		N. weight:	1.5
		Sc. PD:	-0.0068
		Sc. rank:	27591.0

,

NA

Pos.	445	obs:	exp:
gtt	V	0	1.33
gtc	V	0	1.09
gta	V	1	1.61
gtg	V	5	1.97
gct	A	0	0.26
gcc	A	0	0.21
gca	A	0	0.47
gcg	A	1	0.06
gaa	E	0	0.61
gag	E	1	0.39
ggt	G	5	5898.00
ggc	G	1	5761.00
gga	G	1177	17303.00
ggg	G	37716	9936.00
---	--	------	------
mPD		0.059	0.69
		nPD:	0.09
		N. weight:	1.2
		Sc. PD:	0.035
		Sc. rank:	28597.0

,

NA

Pos.	446	obs:	exp:
tct	S	0	7160.00
tcc	S	0	4506.00
tca	S	0	10125.00
tcg	S	0	2071.00
cgt	R	0	0.03
cgc	R	0	0.05
cga	R	0	0.10
cgg	R	0	0.07
agt	S	43	7316.00
agc	S	38863	7728.00
aga	R	1	0.50
agg	R	0	0.25
---	--	------	------
mPD		0.0023	1.6
		nPD:	0.
		N. weight:	1.7
[truncated: 391,095 more chars]
